# Development and Genome-Wide Analysis of a Blast-Resistant *japonica* Rice Variety

**DOI:** 10.3390/plants12203536

**Published:** 2023-10-11

**Authors:** Glòria Escolà, Víctor M. González-Miguel, Sonia Campo, Mar Catala-Forner, Concha Domingo, Luis Marqués, Blanca San Segundo

**Affiliations:** 1 Centre for Research in Agricultural Genomics (CRAG) CSIC-IRTA-UAB-UB, Campus Universitat Autònoma de Barcelona (UAB), Bellaterra (Cerdanyola del Vallés), C/de la Vall Moronta, CRAG Building, 08193 Barcelona, Spain; gloriaescolaoliva@gmail.com (G.E.); victor.gonzalez@cragenomica.es (V.M.G.-M.); soniacampo@fundaciomiquelagusti.cat (S.C.); 2 Institute of Agrifood Research and Technology (IRTA), Field Crops, Ctra. Balada km. 1, 43870 Tarragona, Spain; mar.catala@irta.cat; 3 Instituto Valenciano de Investigaciones Agrarias (IVIA), Departamento del Arroz and Centro de Genómica. Ctra Moncada-Náquera km 10.7, 46113 Moncada, Spain; domingo_concar@gva.es; 4 Cooperativa de Productores de Semillas de Arroz, S.C.L. (COPSEMAR) Avda del Mar 1, 46410 Sueca, Spain; luismarques@copsemar.com; 5 Consejo Superior de Investigaciones Científicas (CSIC), 08193 Barcelona, Spain

**Keywords:** breeding, defense, genome mapping, *japonica*, *Magnaporthe oryzae*, microRNA (miRNA), *Oryza sativa*, resistance

## Abstract

Rice is one of the most important crops in the world, and its production is severely affected by the rice blast disease caused by the fungus *Magnaporthe oryzae*. Several major blast resistance genes and QTLs associated with blast resistance have been described and mostly identified in *indica* rice varieties. In this work, we report the obtention of a blast-resistant rice breeding line derived from crosses between the resistant *indica* variety CT13432 and the *japonica* elite cultivar JSendra (highly susceptible to blast). The breeding line, named COPSEMAR9, was found to exhibit resistance to leaf blast and panicle blast, as demonstrated by disease assays under controlled and field conditions. Furthermore, a high-quality genome sequence of the blast-resistant breeding line was obtained using a strategy that combines short-read sequencing (Illumina sequencing) and long-read sequencing (Pacbio sequencing). The use of a whole-genome approach allowed the fine mapping of DNA regions of *indica* and *japonica* origin present in the COPSEMAR9 genome and the identification of parental gene regions potentially contributing to blast resistance in the breeding line. Rice blast resistance genes (including *Pi33* derived from the resistant parent) and defense-related genes in the genome of COPSEMAR9 were identified. Whole-genome analyses also revealed the presence of microRNAs (miRNAs) with a known function in the rice response to *M. oryzae* infection in COPSEMAR9, which might also contribute to its phenotype of blast resistance. From this study, the genomic information and analysis methods provide valuable knowledge that will be useful in breeding programs for blast resistance in *japonica* rice cultivars.

## 1. Introduction

Rice is a staple food for more than half of the world’s population. It belongs to the genus *Oryza* which includes two independently domesticated species, *Oryza sativa* (Asian rice) and *Oryza glaberrima* (African rice). Most of the cultivated rice varieties belong to the *O. sativa* group which comprises *indica* and *japonica* subspecies [1]. Whereas *indica* varieties are mainly cultivated in tropical environments, *japonica* subspecies are grown in temperate regions. *O. sativa japonica* is further differentiated into temperate *japonica* (*japonica*) and tropical *japonica* (*javanica*) varieties [1]. It is generally assumed that the two major groups of cultivated rice, *indica* and *japonica*, originated from two different ancestral wild rice populations, *O. rufipogon* and *O. nivara* [2], whereas the African rice *O. glaberrima* is thought to have been domesticated from the wild ancestor *Oryza barthii* [3]. During domestication, both *indica* and *japonica* rice have undergone significant changes related to their morphological characteristics, agronomic traits, yield and grain quality, and stress resistance. Despite major progress being made during recent years, the evolutionary and domestication history of cultivated rice is still a matter of debate.

One of the major factors limiting rice production is the rice blast disease caused by the fungal pathogen *Magnaporthe oryzae* (syn. *Pyricularia grisea* B. C. Couch) [4]. *M. oryzae* infections can occur on leaves, collars, necks and panicles, and at different stages of plant growth, from seedling in nursery to heading in the field. Agrochemicals are used to control rice blast and frequent use of these chemicals pose health risks and environmental damage in rice growing areas. Breeding for blast resistance has become a key issue among rice breeders.

During the last decade, great progress has been achieved in understanding how rice plants defend themselves against *M. oryzae*. Rice employs a two-layered innate immune system to fight *M. oryzae* invasion which are defined by the type of molecules that are recognized by the plant. The first layer of immunity is activated after recognition of pathogen-derived molecules (or PAMPs, Pathogen-Associated Molecular Patterns) by receptors on the host cell membrane, referred to as PAMP-triggered immunity (PTI) [5,6]. To subvert PTI, virulent *M. oryzae* isolates secrete effectors (also known as Avirulence, or AVR effectors) that function as invasion weapons to successfully invade and propagate in the host plant [7]. In turn, rice plants evolved another immune response that relies on the recognition of the microbial effectors by proteins encoded by resistance (*R*) genes. The R proteins interact directly with microbial effector proteins (or host proteins modified by effectors) and this interaction triggers a strong immune response referred to as effector-triggered immunity (ETI) [8]. ETI and PTI both involve massive transcriptional reprogramming in the plant for the activation of defense-related genes and production of antimicrobial compounds. Unlike PTI, disease resistance mediated by ETI is generally specific to the race of a pathogen and the host cultivar. Increasing evidence also supports the critical role of microRNAs (miRNAs) in rice innate immunity [9,10,11,12,13,14]. MiRNAs are small non-coding RNAs (encoded by *MIR* genes) that direct gene silencing at the post-transcriptional level through cleavage or translational inhibition of target transcripts [15].

Currently, 146 blast resistance (*Pi*) genes have been identified in rice and over 37 of them have been molecularly characterized [16,17,18]. Most blast resistance genes are found in tandem in distinct regions of the genome, exist as allelic series, and have pseudogenes. This genetic structure makes it challenging to clone individual *R* genes and to distinguish allelic variants from pseudogenes. The existence of allelic variants of *R* genes indicates that they most probably arose through coevolution of the rice/*M. oryzae* interactions [7]. On the other hand, the introgression of blast resistance genes has proven to be a good strategy to obtain blast resistance into elite rice varieties [18]. However, not all *R* genes are equally effective in providing resistance to different isolates of *M. oryzae* in natural pathogen populations and, accordingly, resistance conferred by specific *R* genes/alleles needs to be verified in geographic areas in which those cultivars are grown. It is also well known that resistance conferred by the introduction of a single *R* gene often breaks down in a few years due to the high genetic variability and fast-evolving populations of the fungus. Pyramiding multiple *R* genes into a rice variety offers the possibility of broad and durable blast resistance [19]. The development of blast-resistant varieties through sequential introduction of single *R* genes is, however, a long and time-consuming process. On the other hand, in pyramiding strategies using a donor parent harboring multiple *R* genes, different introgression patterns can be found in the progeny plants in which *R* genes are integrated either separately or in combinations that would determine the level of blast resistance. 

In addition to *R* genes, more than 500 QTLs associated with blast resistance have been reported, suggesting that effective pyramided major *R* genes must coexist with appropriate QTLs in the recipient genome. Along with this, not only the type of introgressed *R* gene(s), but also interactions of these genes with the recipient genetic background might affect the rice response to *M. oryzae* infection. As an additional complexity, there are examples in which the function of a given *R* gene (e.g., the *Pik* locus) relies on the function of two alleles (e.g., *Pikm-1* and *Pikm-2*), both of them being required to confer *Pik* resistance [20]. On this basis, breeding programs involving the introduction of *R* genes into locally adapted rice cultivars require to evaluate blast resistance of individuals among the progeny in that particular geographical area. 

Many efforts are being made by rice breeders to develop *japonica* cultivars adapted to temperate rice growing areas with improved resistance to blast. However, most blast *R* genes for blast resistance have been identified in *indica* accessions (Asian cultivated rice), and less is known about *R* genes effective against *M. oryzae* isolates in *japonica* rice cultivars. It should be mentioned here that because of reproductive barriers, hybrids between *indica* and *japonica* cultivars are usually sterile [21]. The production of blast-resistant *japonica* lines derived from *japonica* × *indica* crosses well adapted to temperate conditions remains challenging, not only because of the spectrum of resistance against *M. oryzae* races and the risk of resistance breakdown, but also because of the risk of the sterility of hybrids. Even so, the introgression of *indica R* genes (*Piz*, *Pib*, *Pita and Pik*) into a susceptible *japonica* variety has proven to be effective in conferring resistance against the blast population in Northern Italy [22]. 

In this study, we report the obtention of a blast-resistant variety derived from crosses between the *japonica* elite cultivar JSendra (highly susceptible to *M. oryzae* infection) and the *indica* rice variety CT13432-34 (harboring the *Pi1*, *Pi2* and *Pi33* blast resistance genes) [23]. The breeding line generated in this study, which has been named as COPSEMAR9, exhibited resistance to leaf blast and panicle blast under controlled conditions, as well as under field conditions. By combining Illumina and Pac-Bio sequencing, we generated a high-quality genome sequence of the blast-resistant breeding line COPSEMAR9. This information allowed us to identify *indica* DNA regions introgressed into the *japonica* background at the genome-wide level, and to infer *indica R* genes present in the COPSEMAR9 genome. Of the various *R* genes that are present in the COPSEMAR9 genome, *Pi33* was identified in a large chromosomal region (chromosome 8) highly enriched in *indica* loci, suggesting that *Pi33* in COPSEMAR9 derives from CT13432. The high-quality genome annotations for this breeding line also allowed us to search for the presence/absence of microRNAs (miRNAs) for which a function in blast resistance has been demonstrated. The information gained in this study will eventually help in breeding programs in *japonica* rice varieties addressing blast resistance. 

## 2. Results

### 2.1. Resistance to Infection by the Rice Blast Fungus Magnaporthe oryzae in the Breeding Line COPSEMAR9

In this study, the *indica* cultivar CT13432-34 (henceforth CT13432) was used as the resistant parent to improve blast resistance in the *japonica* cultivar JSendra. The CT13432 variety contains three blast *R* genes in its genome, namely *Pi-1*, *Pi-2*, and *Pi-33*, and derives from the cross between the *indica* varieties C101 LAC (carrying *Pi-1* and *Pi-33*), and C101A51 (carrying *Pi-2* in the genetic background of CO39). CT13432 was developed at the International Center for Tropical Agriculture (CIAT, Colombia) [23]. The *japonica* cultivar JSendra is a medium-grain commercial rice cultivar well adapted to European climate conditions (temperate regions), that shows susceptibility to infection by the rice blast fungus *M. oryzae*. JSendra was obtained by the Valencian Institute for Agricultural Research (IVIA) and has good levels of resistance to lodging. 

Performance of the breeding lines obtained from the initial crosses between CT13432 (male donor parent) and JSendra was monitored in the field through successive selfing generations (up to 4 generations). Visual inspection of blast symptoms of breeding lines through the successive generations was compared with that of the susceptible parent JSendra and commercial, locally adapted *japonica* varieties. A breeding line named as COPSEMAR9 consistently showed increased resistance against *M. oryzae* isolates in the testing fields (e.g., the rice-growing region of Valencia, Spain). This line was examined in more detail for blast resistance. 

**Figure 1:**
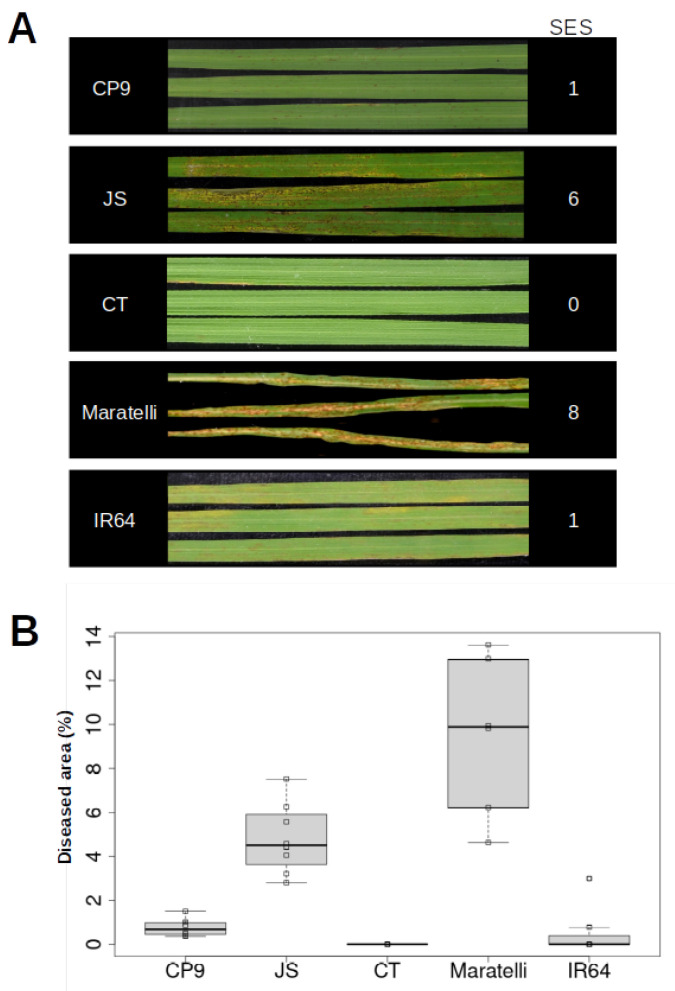
Evaluation of leaf blast resistance of COPSEMAR9 plants under controlled conditions. Rice seedlings at the 3–4 leaf stage were inoculated with *M. oryzae* spores (5 × 105 spores/mL). Parents used for the generation of COPSEMAR9 were included JSendra (JS, susceptible parent) and CT13432 (CT, resistant parent). Reference cultivars were Maratelli (highly susceptible) and IR64 (resistant). Three independent experiments were carried out with similar results (12 plants each experiment). (**A**). Representative images of disease symptoms at 7 days post-inoculation with *M. oryzae* spores. Disease scoring was performed using the 0–9 SES scale (Standard Evaluation System) of IRRI: highly resistant (SES, 0), resistant (SES, 1), moderately resistant (SES, 2–3), moderately susceptible (SES, 4–5), susceptible (SES, 6–7), and highly susceptible (SES, 8–9). SES values are indicated on the right side. (**B**). Percentage of the leaf area affected by blast lesions as determined by image analysis. Values obtained for each biological replicate were plotted. The horizontal line within the box represents the median value. Outliers are included.

Initially, we evaluated blast resistance of COPSEMAR9 plants at the seedling stage. For this, greenhouse-grown plants at the 3–4 leaf stage were inoculated with a spore suspension of *M. oryzae* (strain Guy11), or mock-inoculated, and allowed to continue growth under controlled conditions. Previous studies indicated that the *M. oryzae* strain Guy11 shows a wide spectrum of virulence in both *japonica* and *indica* varieties [24]. The development of disease symptoms in *M.oryzae*-inoculated COPSEMAR9 seedlings was compared with that of the two parental lines (CT13432 and JSendra). As the resistant check we used IR64 (*indica*-type genotype containing *Pi33*, among other *R* genes) [25], whereas Maratelli served as the susceptible check. The Standard Evaluation System (SES) from IRRI was used to evaluate blast disease. As shown in Figure 1A, COPSEMAR9 showed a SES score of 1 (resistant), while its parent varieties showed scores of 6 (JSendra, susceptible) and 0 (CT13432, highly resistant). As expected, Maratelli plants were severely affected by *M. oryzae* infection (SES score of 8, highly susceptible) whereas IR64 (harboring *Pi33*) showed resistance to leaf blast disease, with a SES score of 1 (resistant) (Figure 1A). Consistent with visual inspection of disease symptoms (SES scores), quantification of the leaf area covered with blast lesions revealed a significant reduction in diseased leaf area in leaves of COPSEMAR9 compared with its susceptible parent JSendra (Figure 1B). As expected, the susceptible check Maratelli showed the highest level of diseased leaf area. 

Tests were performed for confirmation of *M. oryzae* as the causal agent of the disease ssymptoms observed in our blast infection assays, following the Koch’s postulates. They included (i) identification of *M. oryzae* from diseased rice leaves. For this, leaves showing disease symptoms were cut into small pieces and placed on CMA medium to allow fungal growth; (ii) obtention of pure *M. oryzae* cultures (subculturing) and preparation of spore suspensions followed by microscopic examination; and (iii) inoculation of healthy seedlings and evaluation of disease symptoms (controlled conditions). Disease symptoms caused by the re-isolated fungus were the same as the originally inoculated pathogen indicating that disease symptoms observed in our experiments could be positively linked to the fungus *M. oryzae*. Overall, results obtained in infection assays under controlled conditions revealed blast resistance in COPSEMAR9 plants at the seedling stage.

**Figure 2:**
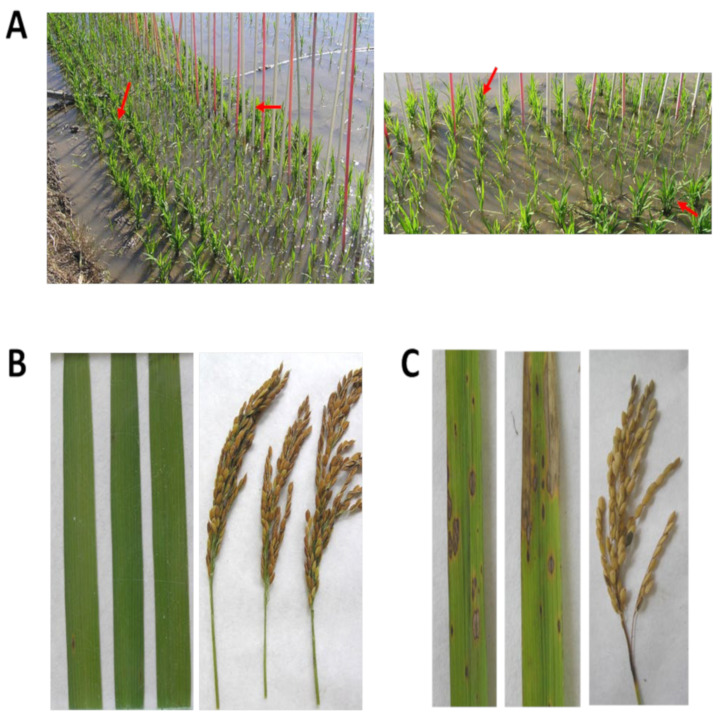
Evaluation of leaf and panicle blast resistance of COPSEMAR9 plants under natural infection conditions. (**A**). Experimental design used in this study for field trials. Plants were transplanted in rows of 5 plants with a 10 × 10 cm spacing. The plot was surrounded by Baixet plants (highly susceptible to blast (red arrows). A row of Baixet plants was also included every two rows (red labels). (**B**,**C**), Representative images of leaves and panicles of COPSEMA9 (**B**) and Baixet (**C**) plants at 20–25 days after heading. Additional images of blast symptoms in panicles of COPSEMAR9 and Baixet plants are shown in Figure S1.

Field trials were carried out to determine blast resistance of COPSEMAR9 under natural infection conditions. Blast resistance was assessed in adult plants (20–25 days after heading), focusing on leaf and panicle blast symptoms. Checks in field trials were Baixet and Cormoran (*japonica*-type), these cultivars showing susceptibility and resistance, respectively, to natural blast infection in the same geographical region. Baixet also served as natural spreader of the infection in field assays (Figure 2A). COPSEMAR9 plants showed a resistant phenotype, with a SES score of 3 (moderately resistant, in both year 1 and year 2), whereas its susceptible parent JSendra and the susceptible check Baixet were rated as susceptible to *M. oryzae* (SES score of 6 and 5, year 1 and year 2, respectively) (Table 1). Finally, infection experiments were carried out to confirm that *M. oryzae* was responsible for causing disease symptoms observed in COPSEMAR9 plants. For this, the fungus was recovered from the infected leaves from field trials and used for inoculation of healthy plants (under controlled conditions). Symptoms were the same as those observed in the field under natural infection conditions. 

As for panicle blast, COPSEMAR9 was found to be resistant against panicle blast under natural infection conditions (7.8% and 23% of panicles showed lesions in more than 75% of the panicle in years 1 and 2, respectively), whereas JSendra panicles were severely damaged (90.0 and 100% of the panicles with more than 75% of lesions in years 1 and 2, respectively) (Table 1; Figure S1). Representative images of leaf blast and panicle blast in field-grown COPSEMAR9 plants are shown in Figure 2B and Figure S1. For a comparison, blast symptoms in leaves and panicles of Baixet plants are shown in Figure 2C and Figure S1. Together, infection assays under controlled and natural infection conditions demonstrated that COPSEMAR9 plants exhibit resistance to leaf blast and panicle blast.

### 2.2. Sequencing, Assembly and Annotation of the COPSEMAR9 Genome

To obtain insights into the genetic nature of blast resistance in COPSEMAR9, we obtained its genome sequence using a strategy that combines short-read sequencing with Illumina short reads (NovaSeq, 2 × 150 bp reads), and long-read sequencing with Pacbio (RSII reads). Details for assembly of the COPSEMAR9 genome are described in the Methods section. The cleaned PacBio sequences had an average length of 12 Kb, the longest sequence being 166 Kb. A total of two million PacBio long reads (62.5× in coverage) were assembled into contigs (Table 2). Then, 160 million Illumina reads were mapped on the PacBio assembly, using the software Pilon to correct misassemblies and errors. 

This assembly, which consists of 335 scaffolds, was then anchored to the 12 rice chromosomes using *O. sativa japonica* genome as reference (GCA_000005425.2). The final sequence contains 393.86 Mb, has an N50 of 31.3 Mb, and consists of 23 scaffolds (the 12 rice nuclear chromosomes, the 2 organellar chromosomes, and 9 unanchored scaffolds) (Table 2). The completeness of the genome assembly was estimated to be of ca. 98%, based on collocated sets of ubiquitous and single-copy genes within a phylogenetic lineage (Figure S2). The heterozygosity of the assembled genome was found to be 0.068%, as revealed by the Genoscope software applied to the Illumina reads, meaning that the genome is highly homozygous. The average GC content of the COPSEMAR9 genome is 43.52% (Table 2). A total of 28,258 protein coding genes could be annotated in COPSEMAR9 (Table 2).

As the sequenced DNA is a cross between a *japonica* (JSendra, susceptible) and an *indica* (CT13432, resistant) variety, we reasoned that blast resistance in COPSEMAR9 would be associated with the presence of *indica* genes conferring that resistance. Accordingly, a genome-wide mapping was carried out to assign DNA regions in the COPSEMAR9 genome as *indica* or *japonica* regions, and to identify genes that could be clearly associated with the *indica* parent. This approach has been successfully used for precise genotyping of a salt tolerant introgression line derived from *japonica* × *indica* crosses [26]. To accomplish this goal, we first compared the genome sequence of COPSEMAR9 with that of *indica* and *japonica* fully sequenced genomes using the software MASH [27]. Principal Component Analysis (PCA) based on the distance of the *indica*, *japonica* and COPSEMAR9 genomes is presented in Figure S3. The distance matrix is found in Table S1. 

**Figure 3:**
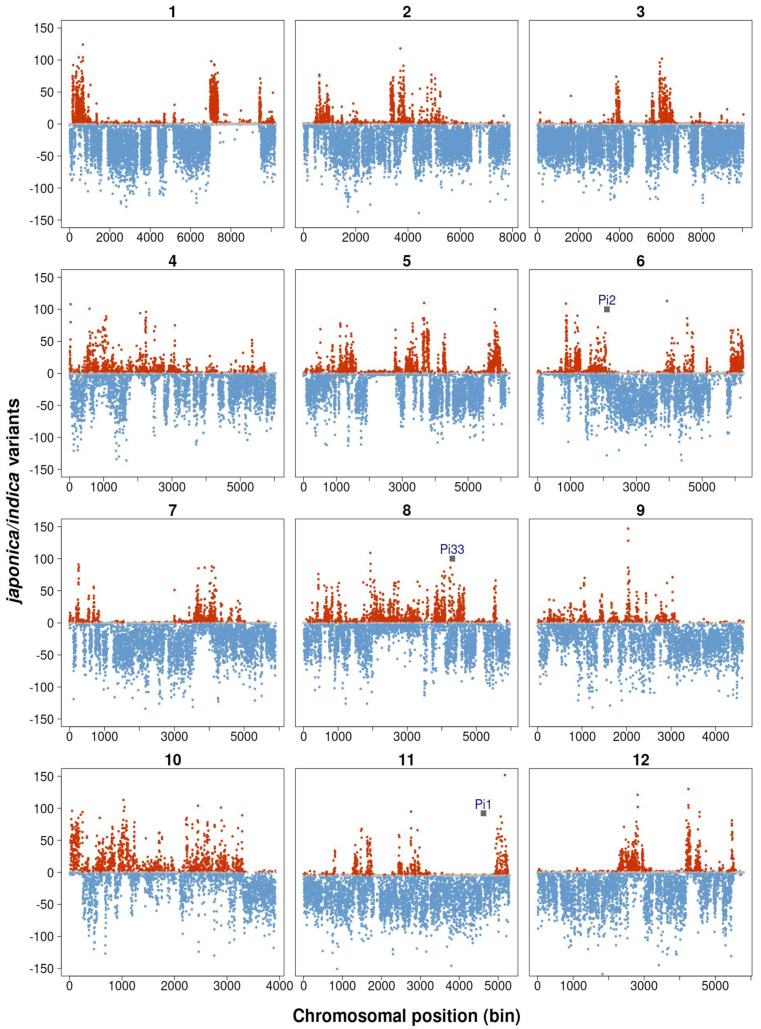
Identification of indica DNA regions introgressed into the genome of COPSEMAR9. Scatter plots showing the classification of genomic sequences (bins) as *indica*, *japonica* or not assigned sequences (red, blue and grey dots). Details on the procedure used for classification of genomic sequences as *indica* or *japonica* sequences, and data processing can be found in Materials and Methods. For each chromosome, the *x*-axis shows the bin/window number. The *y*-axis shows the difference of the counts of variants between *japonica* and *indica* (e.g., subtracting *japonica* from *indica* reads). The chromosomal location of *Pi1*, *Pi2* and *Pi33* is indicated (Chromosome 11, 6 and 8, respectively).

We then further investigated genes associated with *indica* or *japonica* regions in the COPSEMAR9 genome. For this, two independent methods were used. Firstly, the COPSEMAR predicted proteins were aligned against the *japonica* and *indica* protein sequences and the relative differences in bit scores were used to determine the matching parent (variant calling analysis) (Table S2). As an independent approach, simulated datasets of 150-bp paired-end reads from the *indica* and *japonica* reference genomes were mapped against the assembled genome. Then, differences in *indica* and *japonica* polymorphisms were calculated from non-overlapping 5 Kb windows of the COPSEMAR genome (bit score difference analysis) (Table S2). Combining both analyses, a total of 5713 predicted proteins distributed among the various rice chromosomes could be classified with certainty as of either *indica* or else *japonica* origin (Table S3). A representation of the *indica* regions introgressed into the *japonica* genetic background of COPSEMAR9 is presented in Figure 3. The length and distribution of *indica* segments showed a great difference among the 12 rice chromosomes. Importantly, this analysis allowed us to obtain fine mapping of *indica* DNA regions in the genome of COPSEMAR9. 

### 2.3. Resistance Genes in COPSEMAR9

As previously mentioned CT-13432 (resistant parent of COPSEMAR 9) contains the *Pi1* (an allele of the *Pik* locus), *Pi2* and *Pi33* resistance genes, which were previously mapped on chromosome 11 (*Pi1*), Chromosome 6 (*Pi2*), and Chromosome 8 (*Pi33*) [25,28,29]. In this study, we assessed the presence/absence of these *R* genes in the *indica* regions identified in the genome of COPSEMAR9. Interestingly, *Pi33* was identified in a large region at chromosome 8 highly enriched in *indica* loci in the COPSEMAR9 genome, whereas *Pi2* was found at the boundary of a large *indica* region in Chromosome 6 (Figure 3). These observations suggest that *Pi33* in COPSEMAR9, most probably, derives from the *indica* parent CT13432. The presence of this *R* gene in COPSEMAR9 might then contribute to blast resistance in the breeding line of COPSEMAR9. Regarding *Pi2*, however, it cannot be concluded whether this *R* gene derives from the *indica* parent, or not. Finally, no *indica* regions were identified at the *Pi1* location in Chromosome 11 (Figure 3). Likely, *Pi1* (and perhaps *Pi2*) might derive from the parent JSendra, but this gene either is not functional, or is not effective in conferring blast resistance in the testing fields. 

Next, we explored the spectrum of *R* genes in the genome of COPSEMAR9. To date, 146 blast resistance genes/alleles against *M. oryzae* have been identified in rice [17]. Except for a few *R* genes, most of the rice blast resistance genes encode proteins that have a nucleotide-binding site (NBS) and a leucine-rich repeat (LRR) domain [18]. Other blast *R* genes are known to encode a B-lectin-kinase domain protein (*Pi-d2*) [30], a proline-rich protein with a heavy metal domain (*Pi21*) [31], or an atypical protein with an armadillo repeat (*Ptr*) [32]. COPSEMAR9 was found to contain a large number of known blast resistance (*Pi*) genes, either full-length or fragmented versions of these genes (Table S4; Table S5). *Pi* genes identified in COPSEMAR 9 included several *Pik* alleles (*Piks-1*, *Piks-2*, *Pikm2*), *Pi63*, *Pita*, *Pi54*, several *Pib* genes, *PID3*, *PiBP1* and *Ptr.* The COPSEMAR9 genome also contains an important number of genes encoding Resistance Gene Analogs (RGAs) (Table S4). Among them, we identified *Pi63*, also named as *Pikahei-1(t)* and previously known as RGA3 [33]. As R proteins, RGAs have conserved domains that play specific roles in pathogen resistance. Well-known RGAs are nucleotide-binding site–leucine-rich repeat proteins, receptor-like kinases and receptor-like proteins [34]. Further investigation is needed to determine whether the various *R* and *RGA* genes identified in COPSEMAR9 are functional, and whether they play a role in resistance to *M. oryzae* infection in this breeding line.

During the past years, a diversity of genes encoding different groups of proteins with different biochemical functions have been shown to confer blast resistance [20]. Contrary to pathogen-specific *R* genes, defense regulator genes often confer partial resistance to a broad spectrum of *M. oryzae* isolates or various pathogens. These defense regulator genes encode transcription factors, kinases, E3 ubiquitin ligases and proteins involved in protection against oxidative stress, among others [35]. Defense regulator genes identified in the COPSEMAR9 genome are shown in Table S4. These genes might as well contribute to blast resistance in COPSEMAR9.

### 2.4. Mining of Blast-Associated miRNAs in the Genome of COPSEMAR9

Increasing evidence supports that miRNAs modulate rice immunity against *M. oryzae* by regulating the expression of target genes [9,10,11,12,36]. The availability of a high-quality genome sequence of COPSEMAR9 opened the possibility of performing a genome-wide analysis of *MIR* genes with a known function in blast resistance (hereinafter blast-associated miRNAs).

MiRNAs are transcribed as long single-stranded primary transcripts, or miRNA precursors of variable length which are then processed to produce mature miRNAs (21–24 nucleotides in length). In this study, a BLAST search was carried out to identify miRNA precursors of blast-associated miRNAs in the COPSEMAR9 genome. The nucleotide sequence of the miRNA precursors was retrieved from miRBase (https://www.mirbase.org/, accessed on 1 January 2023). Blast-associated miRNAs identified in the COPSEMAR9 genome are listed in (Table 3). They were: miR156fhl-3p, miR160a, miR162a, miR164a, miR166k-5p, miR167d, miR168a, miR169a, miR319b, miR396 (a, c, d, h), miR398b, miR399f, miR439a, miR444b.2, miR812w, miR1432, miR1871, miR1873, miR7695, and miR9664 (of them, miR812w and miR9664 have not been annotated in miRBase yet) (Table 3). Most blast-associated miRNAs were found to be represented by 1–3 loci in the COPSEMAR9 genome (Table 3). In contrast, miR439a, miR812w and miR7695 were encoded by multiple loci in the genome of this breeding line (e.g., 16, 132 and 181 loci, respectively.

## 3. Discussion

In this work, we report the development of a blast-resistant rice variety derived from crosses between the blast-resistant *indica* cultivar (CT13432) and the *japonica* cultivar JSendra well adapted to temperate rice growing areas. JSendra was selected for improvement because of its wide adaptability to temperate climate; however, it is susceptible to *M. oryzae* infection. It is well known that breeding lines carrying different resistance genes might exhibit different responses which depend on combinations of *R* genes/alleles and/or interactions of *R* genes with the host genome. With so many blast *R* genes/alleles and QTLs (more than 500 QTLs have been described), actual interactions among them and with the host genome of *R* genes are difficult to predict, and their effectiveness inevitably also depends on the virulence spectra of these genes on the pathogen population. Accordingly, blast resistance was monitored in the breeding lines over successive generations of selfing in the field.

One breeding line consistently exhibited blast resistance through the successive generations compared to other commercial varieties. One critical limitation of this study was that, in our hands, diagnostic molecular markers for target *R* genes (e.g., SSR markers for *Pi1*, *Pi2* and *Pi33*) were not useful to track the presence/absence of these genes in the progeny plants. Most probably, the heterogeneous genetic backgrounds in the parent varieties due to breeding processes made by rice farmers for the obtention of these varieties (JSendra and CT13432) would explain failure in the use of SSR markers in genotyping progeny plants derived from JSendra × CT13432 crosses. Because of this drawback, we decided to build a high-quality genome sequence of COPSEMAR9. Here, it should be mentioned that previous research in molecular plant breeding focused on genotyping progeny plants using molecular markers and/or marker-assisted selection (MAS), and more recently genotyping-by-sequencing (GBS). Results obtained in this work demonstrated the usefulness of combining phenotyping for blast resistance with whole-genome sequencing for the generation and analysis of blast-resistant breeding lines in rice. 

An interesting finding in this study was that, in the testing field, adult COPSEMAR9 plants exhibit resistance to both leaf and panicle blast. Disease evaluation under controlled conditions and artificial inoculation also revealed a clear phenotype of blast resistance in COPSEMAR9 plants at the seedling stage. Based on the results obtained in these studies, it can be concluded that blast resistance mechanisms operating in COPSEMAR9 are effective at different developmental stages. In the literature, correlation between leaf blast resistance and panicle blast resistance is not frequently found, with varieties showing high level of resistance to leaf blast but susceptibility to panicle blast or vice versa. It is also true that most blast resistance genes so far characterized confer resistance against leaf blast, while only a few *R* genes are effective for resistance to both seedling and panicle [18]. Compared with leaf blast resistance, our understanding of panicle blast resistance is, however, limited. Collectively, results presented here support that COPSEMAR9 contains an appropriate combination of resistance and defense-related genes to confer resistance to leaf and panicle blast.

Evidence in the literature support that the introduction of distinct blast *R* genes into a susceptible variety through conventional breeding methods, either alone or in combinations, is effective to confer resistance against *M. oryzae* infection. They include *Pi1*, *Pi2*, *Pi9*, *Pi20(t)*, *Pi33*, *Pi39* and *Pi40(t)*, *Pi47*, *Pi48*, *Pi54*, *Pi56*, *PiZ*, *Pizt* and *Pig*, among others [16,18,22,28,55]. In particular, the *Pigm* gene has been shown to be effective to control both leaf and panicle [56]. By mapping *indica* regions introgressed into the *japonica* genetic background of COPSEMAR9, we were able to identify the *Pi33* gene within a large DNA region highly enriched in *indica* genes in chromosome 8, suggesting that *Pi33* gene derives from the donor parent CT13432. *Pi33* is considered to confer broad-spectrum resistance gene, as it has been shown to be effective against over 2000 isolates originating from 55 [25]. The COPSEMAR9 genome also contains several other *R* genes, including several alleles of the *Pik* gene (e.g., *Pi1*, *Pikm-1 and Pikm-2*, *Pik-s and Pik-p*), and *Pi63*, among others. Regarding *Pik*, this locus has a complex structure of tandem repeats of genes with a high homology, and *Pikm*-specific resistance is conferred by the cooperation of two adjacent genes (*Pikm1* and *Pikm-2*; both alleles being present in COPSEMAR9) [20]. Although *Pik* was originally described as a broad-spectrum blast resistance gene [28], more recently it was described that protection conferred by *Pik* genes varies depending on the rice-growing region [57]. Then, it will be of interest to verify whether *Pik* alleles identified in the COPSEMAR9 genome are functional and responsible for blast resistance COPSEMAR9.

Several possibilities, which are not mutually exclusive, can be envisaged to explain blast resistance in COPSEMAR9. At the genotype level, resistance can be governed by the number and type of *R* genes identified in its genome, and the specific combinations of genes derived from one or another parent. The introduction of *R* genes not yet functionally characterized in the resistant parent CT13432 into COPSEMAR9 should also be considered. Although CT13432 has long been recognized to carry *Pi1*, *Pi2* and *Pi33*, more recently, it was described that CT13432 carries *Pi63*, also known as *Pikahei-1(t)* (or RGA3, Resistance Gene Analogue 3) which was originally identified in the rice cultivar Kahei [33,58]. To note, *Pi63* was identified in the COPSEMAR9 genome. Still, COPSEMAR9 might carry unidentified blast resistance genes (other than *Pi1*, *Pi2*, *Pi33*, *Pi63*) contributing to blast resistance. Furthermore, novel combinations of the *Pi* genes and/or interactions of *indica R* genes with genes in the *japonica* background might underlie resistance in COPSEMAR9. Clearly, additional investigation is still required to understand the actual levels of blast resistances conferred by individual and/or combinations of *R* genes, and the interactions occurring between the introgressed *indica* loci and the *japonica* genomic landscape in COPSEMAR9 plants. From the perspective of practical application, the fine mapping of *indica* genetic introgressions into the genome of COPSEMAR9 (*japonica* background) provides a new avenue for future research on blast resistance in *japonica* varieties, including breeding lines derived from crosses between *indica* and *japonica* rice cultivars.

A previously mentioned, evidence supports an important role of miRNAs in rice immunity, both ETI and PTI [9,10,11,12]. Links between miRNA regulation and *R* gene expression have also been demonstrated, as illustrated by the existence of several miRNAs playing a role in *Pi54*-mediated blast resistance [59]. Here, it should be mentioned that miRNA precursors are not usually detected in transcriptome analysis aiming the identification of transcripts for protein coding genes, probably because miRNA precursors are rapidly processed to mature miRNAs. The identification of pathogen-regulated miRNAs requires sequencing of small RNA libraries. Using this strategy, a large number of miRNAs were reported to be regulated during *M. oryzae* infection, or treatment with elicitors prepared from this fungus, in rice tissues [36,38,60,61]. The availability of a high-quality genome sequence of COPSEMAR9 allowed the identification of blast-associated miRNAs in this breeding line. The COPSEMAR9 genome was found to contain 24 blast-associated miRNAs belonging to 20 different miRNA families. These miRNAs might well mediate blast resistance in COPSEMAR9. 

The development of COPSEMAR9 represents a new resource for developing blast resistance in *japonica* varieties. Although varietal resistance is recognized as a good strategy to control blast, more efforts are needed for the development of *japonica* rice varieties adapted to temperate regions. In particular, the introduction of genes from an *indica* variety into a *japonica* genetic background is a need in current breeding programes. Breeding for blast resistance could also be approached by taking advantage of heterosis, a phenomenon in which the phenotypes of the hybrid progeny surpass those of their parents. Indeed, the utilization of heterosis has been useful to obtain important increases in yield in modern rice varieties [62], and heterosis-related QTLs for agronomic traits (e.g., grain traits, plant height) have been described in rice populations derived from *indica* × *japonica* crosses [63,64]. However, the utilization of heterosis in *indica* × *japonica* crosses for blast resistance still requires uncovering heterosis-related QTLs for blast resistance, which is an interesting aspect for both rice breeders and researchers. The information gained in this study should be taken into account when considering genetic improvement for blast resistance in rice by crossing *indica* and *japonica* varieties. 

An important requisite for improvement of blast resistance is to understand which *R* genes (and defense-related genes) are functional in modern rice varieties and to know the *M. oryzae* isolates against which those *R* genes are effective. Additionally, to breed new rice varieties resistant to rice blast, it is important to know the allelic state of the donor genes transferred into the hybrid progeny, that is to identify functional and non-functional alleles. With the increase in genetic research onto blast resistance and the important advances that have been made in recent years in the elucidation of rice genome sequences, both *japonica* and *indica* accessions, understanding resistance controlled by multiple loci has become a very important goal in rice blast resistance. The availability of the COPSEMAR9 genome represents an important step in this direction as it offers the possibility of exploring molecular mechanisms involved in blast resistance in *japonica* rice varieties. Clearly, a better understanding of mechanisms involved in immunity against the rice blast fungus in COPSEMAR9 might allow the development of strategies for effective management of the blast disease in temperate *japonica* rice. At present, the control of the rice blast disease relies on the use of chemical treatments, also as preventive management. Chemical activators of natural plant defenses, such as probenazole, are also used for blast control [65]. As the indiscriminate use of agrochemicals has adverse effects on the environment and human health, it is critical to have alternative methods to control the rice blast disease in an environmentally friendly way.

## 4. Materials and Methods 

### 4.1. Plant Material and DNA Extraction

Temperate *japonica* rice (*Oryza sativa*) cultivars and *indica* cultivars were grown under controlled conditions at 28 °C ± 2 °C under a 16 h/8 h photoperiod. They were: JSendra (provided by COPSEMAR, Sueca, Valencia), Maratelli (provided by the Council for Agricultural Research and Economics-CREA, Vercelli, Italy), and CT13432 (provided by the Center for Tropical Agriculture-CIAT, Colombia). IR64 harboring the *Pi33* gene was obtained from the International Rice Research Institute (IRRI, Los Banos, Philippines). 

Genomic DNA was obtained from leaves of three-week-old plants using MATAB (100 mM of TRIS-HCl pH 8.0, 1.4 M NaCl, 20 mM EDTA, 2% MATAB, containing 1% PEG 6000 and 0.5% sodium sulfite) as the extraction buffer (6 mL MATAB/gr fresh weight) with modifications [66]. Briefly, the mix was maintained at 65 °C for 30 min with gentle mixing by inversion every 15 min. Then, chloroform:IAA (24:1; 6 mL) was added to the mixture followed by centrifugation (4400 rpm, 15 min). The supernatant was recovered and incubated with RNAse A (50 µL, 10 µg/µL) for 30 min at 37 °C. A second round of chloroform:IAA (24:1) extraction was carried out using 10 mL of Chloroform:IAA. The gDNA was precipitated by adding cold isopropanol (6 mL). The genomic DNA was rescued with a pasteur glass pipette, transferred to an Eppendorf tube containing H_2_O (30 μL). The quantity and quality of DNA samples were determined by using a Nanodrop 2000 spectrophotometer (Nanodrop technologies, Wilmington, DE) and agarose gel electrophoresis.

### 4.2. Phenotyping for Resistance to Leaf Blast in Rice Seedlings under Controlled Conditions

Blast resistance of COPSEMAR9 was evaluated at the seedling stage under controlled conditions. Infections were carried out using the *M. oryzae* strain Guy-11. The fungus was grown in Complete Media Agar (CMA, containing 30 mg/L chloramphenicol) for 15 days at 28 °C (16 h/8 h light/dark). *M. oryzae* spores were prepared as described [36]. Soil-grown plants at the 3–4 leaf stage were infected by spraying a *M. oryzae* spore suspension (10^5^ spores/mL; 0.3 mL/plant) using an aerograph (pressure, 2 atmospheres). The *M. oryzae*-inoculated and mock-inoculated seedlings were maintained overnight in the dark under high humidity and then allowed to continue growth under controlled conditions. Scoring of leaf blast disease was performed using the 0–9 scale of the International Rice Research Institute (IRRI, Standard Evaluation System, SES) [67]. The SES rates symptoms of infection according to the lesion type and size, and percentage of the leaf area with lesions (lower scores indicate blast resistance while high scores indicate susceptibility. The percentage of leaf area affected by blast lesions was determined by image analysis (APS Assess 2.0 program). Resistant IR64 (harboring *Pi33*) and susceptible (Maratelli) checks were included in this study. To assess that the blast fungus causes disease symptoms in our disease assays, the fungus recovered from infected leaves was found to cause the same disease symptoms when used for inoculation of healthy seedlings, satisfying Koch’s postulates. 

### 4.3. Field Experiments 

The resistance of Copsemar-9 plants was evaluated in a field trial at the IVIA (Valencian Institute of Agricultural Research; 39°17′50.4″ N, 0°19′24.7″ W) from May to September in two years (2018 and 2021), under favorable conditions of infection by blast. The commercial varieties Baixet and Cormorán varieties were used as highly susceptible and highly resistant control varieties, respectively. Seeds were sown in pots and transplanted to the field when the plants reached the 2–3 leaf stage. Each field trial consisted of three replications (plots), each one containing COPSEMAR9, JSendra, Baixet and Cormorán plants. In each plot, the rice varieties were distributed in lines (3 lines, 5 plants/line, with a 10 × 10 cm spacing between plants) (Figure 2A and Figure S4). As Baixet is a highly susceptible variety, a row of Baixet plants was grown every second row (Figure 2A and Figure S4). Additionally, each plot was surrounded by Baixet plant to maintain a high level of inoculum in the field and to achieve a sufficient level of inoculum pressure and infection. This also served to ensure equal conditions in all the varieties in the trial. The cultivation was carried out under sprinklers and sprayed with water every 60 min to maintain high humidity, thus, favoring the incidence and intensity of the disease. Scoring of leaf blast and panicle blast was carried out in adult plants at 20 days after heading. On leaves, the size of the lesions and the degree of affectation was assessed based on the Standard Evaluation System for rice (SES) of the International Rice Research Institute [67]. Assessment of panicle blast infection was carried out on 20 randomly selected panicles for each variety, based on the number of panicles with more than 75% of lesions covering the node, neck or lower part of the panicle axis (symptom type 9 in the SES scale from IRRI [67].

### 4.4. Whole-Genome Sequencing on the PacBio and Illumina Platforms, De Novo Assembly and Reference-Based Annotation

Illumina Novaseq 2 × 150 bp reads and Pacbio RSII sequencing libraries were produced using standard protocols (Sequentia Biotech SL, Spain). The quality of the Illumina raw reads was assessed with the FASTQC software (https://www.bioinformatics.babraham.ac.uk/projects/fastqc/, accessed on 1 June 2022). Then, low-quality bases and adapter sequences were removed with the software BBDuk (minimum Phred quality 35 and minimum length 35 bp) (https://jgi.doe.gov/data-and-tools/software-tools/bbtools/bb-tools-user-guide/bbduk-guide/, accessed on 1 June 2022) to produce a total of 160,036,468 trimmed reads. The GenomeScope software was used to provide an estimation of the genome size based on 21 K-mer distribution (http://qb.cshl.edu/genomescope/, accessed on 1 June 2022).

The pipeline for the de novo genome assembly consisted in the following steps: first, a de novo assembly was created only with the Pacbio reads using Canu [68]. Second, a de novo assembly was created only with the Pacbio reads using wtdbg2 [69]. Finally, a consensus assembly was generated with the outputs of Canu and wtdbg2 using quickmerge [70].

The Illumina reads were then mapped on the obtained assembly and the software pilon was used to correct mis-assemblies and errors performing 11 iterations of correction [71]. The obtained assembly was composed of 335 scaffolds which were then anchored to the rice chromosomes using the software DGENIES [72] and the *Oryza sativa* (ssp. *japonica*) genome as reference (GCA_000005425.2). Scaffolds that could not be anchored to the reference genome were left as “unanchored” sequences. The final assembly is composed by the 12 expected rice chromosomes, the mitochondrial and chloroplast genomes and additional 9 “unanchored” scaffolds, totaling 393,859,641 bp, being only 1% larger than estimated using k-mer distributions. The completeness of the assembled genome was evaluated using BUSCO [73].

The COPSEMAR9 genome was annotated using the Maker pipeline (https://www.yandell-lab.org/software/maker.html, accessed on 1 June 2022) [74] by combining ab initio predictions using Augustus [75] with the genome annotations of *japonica* (Nipponbare, GCA_000005425.2) and *indica* (R498, http://mbkbase.org/R498/, accessed on 1 June 2022) reference genomes. The functional annotation, including descriptions, Gene Ontology and KEGG was obtained using the Pannzer2 pipeline (http://ekhidna2.biocenter.helsinki.fi/sanspanz/, accessed on 1 June 2022) [76]. 

### 4.5. Mapping of Indica Regions

The software MASH [27] was used to generate a distance matrix between the hybrid and 16 *indica* and 13 *japonica* genomes which allowed to position the assembled genome relative to those rice genomes in a PCA-based bluster analysis. To classify the genome as “*japonica*” or “*indica*” regions, and to identify genes that could be clearly associated with one of the parentals, two independent methods were used. First, the predicted protein sequences of the genes identified in the genome were aligned against the *japonica* (Nipponbare) and *indica* (R498) protein sequences. Only proteins found in all three genomes (Nipponbare, R498 and COPSEMAR 9) and located in the same chromosome were considered. For each gene, the difference in the bitscore was used to determine the matching parent. Finally, if the ratio between the difference of the Bitscores and the maximum Bitscore was higher or equal than 0.1 then the gene was classified as of *indica* or *japonica* origin.

For the second approach, the reference genomes of *indica* and *japonica* were used to generate a dataset of simulated paired-end reads with the software wgsim (https://github.com/lh3/wgsim/, accessed on 1 June 2022) (17.5 millions of paired-end 150 bp reads were simulated from each genome). The genome sequence of Copsemar was indexed with minimap2 (v2.17-r941) and then the simulated reads of *indica* and *japonica* were mapped using minimap2 with the option ‘-x sr’ [77]. The resulting BAM files were sorted using samtools (v1.10) [78] A 97.89% of the *indica* reads and 98.89% of the *japonica* reads mapped on the genome. The variant caller Platypus (v0.8.1.1) [79] was then used to identify the variants between the references and the sample using default parameters, with the exception of: ‘trimReadFlank’: 0, ‘trimSoftClipped’: 1, ‘minReads’: 6, ‘maxSize’: 1500, ‘trimAdapter’: 1, ‘minPosterior’: 10, ‘trimOverlapping’: 1, ‘filterDuplicates’: 1, ‘minFlank’: 10, ‘filterReadsWithUnmappedMates’: 1, ‘filterReadsWithDistantMates’: 1, ‘minMapQual’: 30, and ‘minBaseQual’: 30. Approximately 1.8 million variants were detected with *indica* and approximately 572,000 variants were detected with *japonica*, highlighting that the hybrid is more similar to *japonica* than *indica*. Finally, the genome was divided into not overlapping 5 Kb windows and for each window the number of *indica* and *japonica* polymorphisms were calculated. The difference in the number of variants per window between *japonica* and *indica* was finally obtained. In each window, if the number of variants was at least 5 but the difference between *japonica* and *indica* was less than 30%, the window remained unclassified. If the difference in the number of variants was higher than 30% and more variants were identified against *japonica*, the window was classified as *indica*, and vice versa.

### 4.6. Mining of Blast Disease Resistance and Blast-Associated MIR Genes

The list of rice genes with a known function in blast resistance was retrieved from the RAPDB database (https://www.ncbi.nlm.nih.gov/pmc/articles/PMC3583025/, accessed on 1 June 2022) [80]. Additionally, the DRAGO2 pipeline [81] was applied to the predicted proteome of COPSEMAR9 to identify and classify genes involved in resistance. The nucleotide sequences of precursors of miRNAs with a known function in blast resistance in rice were retrieved from the miRbase (https://www.mirbase.org/, accessed on 1 June 2022, release 22.1) [82].

## Figures and Tables

**Table 1:** Resistance of COPSEMAR9 to leaf and panicle blast under natural infection conditions. Disease symptoms were recorded in field-grown plants at 20–25 days after heading. Scoring of leaf blast was performed using the 0–9 scale of IRRI. Incidence of panicle blast was determined on severely affected panicles (>75% of lesions) with lesions covering the node, neck or lower part of the panicle axis (20 panicles were randomly harvested from the experimental field). The percentage of panicles showing blast symptoms in each year (2018, 2021) is shown.

	Leaf Blast		Panicle Blast (>75% of Lesions)	
	2018	2021	2018	2021
COPSEMAR9	3	3	7.8	23.0
JSendra	6	5	90.0	100
Baixet	5	8	42.0	76.6
Cormoran	4	5	2.5	13.0

**Table 2:** Summary of the COPSEMAR9 genome assembly and annotation.

Genome Coverage	61.5× (Illumina)/62.5× (Pacbio)
Genome size (bp)	393,859,641
Number of scaffolds	23
Scaffold N50 (bp) *	31,322,768
L50	5
Largest contig (Mb)	51.11
GC content	43.52%
BUSCO completeness (%) **	98% (Eukaryotic genes); 97% (Viridiplantae genes)
Transposable elements	Retrotransposons, 24.81%; DNA transposons, 16.67; Unclassified, 3.98%
Number of protein-coding genes	28,258

* N50, minimum sequence length needed to cover 50% of the genome. ** BUSCO, Benchmarking Universal Single-Copy Orthologs score.

**Table 3:** miRNAs identified in COPSEMAR9 with a known function in blast resistance in rice. The number of loci encoding each miRNA precursor and their chromosomal location is indicated. (-), the nucleotide sequence of the miR9664 precursor is missing from the present miRBase catalogue.

Name	Loci (Ner)	Chromosome	Reference
miR156h	1	8	[37]
miR156l	1	5	[37]
miR160a	3	2, 6	[38]
miR162a	2	2, 4	[39]
miR164a	1	7	[40]
miR166k	2	2, 9	[41]
miR167d	1	7	[42]
miR168a	1	2	[43]
miR169a	1	1	[44]
miR319b	1	1	[45]
miR396a	1	2	[46]
miR396c	1	2	[46]
miR396d	2	2, 4	[46]
miR396h	3	2, 6	[46]
miR398b	1	7	[38]
miR399f	1	6	[47]
miR439a	16	1, 3, 4, 5, 6, 7, 8, 9, 11	[48]
miR444b	3	2, 4	[49]
miR812w	132	1, 2, 3, 4, 5, 6, 7, 8, 9, 10, 11, 12	[50]
miR1432	1	7	[51]
miR1871	1	6	[52]
miR1873	1	7	[53]
miR7695	181	1, 2, 3, 4, 5, 6, 7, 8, 9, 10, 11, 12	[36]
miR9664	(-)	(-)	[54]

## Data Availability

The genome sequence of the breeding line developed in this study can be found at the “European Nucleotide Archive” (ENA) under the accession number GCA_951799355 (https://urldefense.com/v3/__http://www.ebi.ac.uk/ena/browser/view/__;!!D9dNQwwGXtA!UBsZoQ6hdqcLPnRnnfGHxQLOa98lID5PNadIE8rfSgjaJrqtSTWIXB-Pxw0ngN4zbjAun77KeC9Dh-RsLjE2Y6u0nX-cGvKY8fg78r7l$ (accessed on 1 January 2023)).

## Supplementary Materials

The following supporting information can be downloaded at: https://www.mdpi.com/article/10.3390/plants12203536/s1. Figure S1. Evaluation of panicle blast resistance of COPSEMAR9 under natural infection conditions. Figure S2: Graphical representation of the results of the BUSCO analysis using the Eukaryote (top) and Viridiplantae (bottom) databases. Figure S3: PCA analysis based on the distance of the *indica*, *japonica* and COPSEMAR 9 genomes. Figure S4: Experimental design used to assess blast resistance of COPSEMAR9 under field conditions. Table S1: Matrix of distances between the COPSEMAR9 assembly and *indica* and *japonica* fully sequenced genomes. Table S2: Genes identified in the genome of COPSEMAR9. Table S3: Homologues in *japonica* and *indica*. Table S4: *R* and defense-related genes. Table S5: Chromosomal coordinates and nucleotide sequences of *Pi1*, *Pi2* and *Pi33.*
