# Supplementary material for: Development and Genome-Wide Analysis of a Blast-Resistant japonica Rice Variety
Source: Plants (Basel). 2023 Oct 11;12(20):3536. doi: 10.3390/plants12203536 (PMC10667994; doi:10.3390/plants12203536)
Supplement: Supplementary file 1 [file plants-12-03536-s001.zip › Supplementary Material Escol_Suppl Fig S1 Leaves Panicles Field.pdf]

A

B

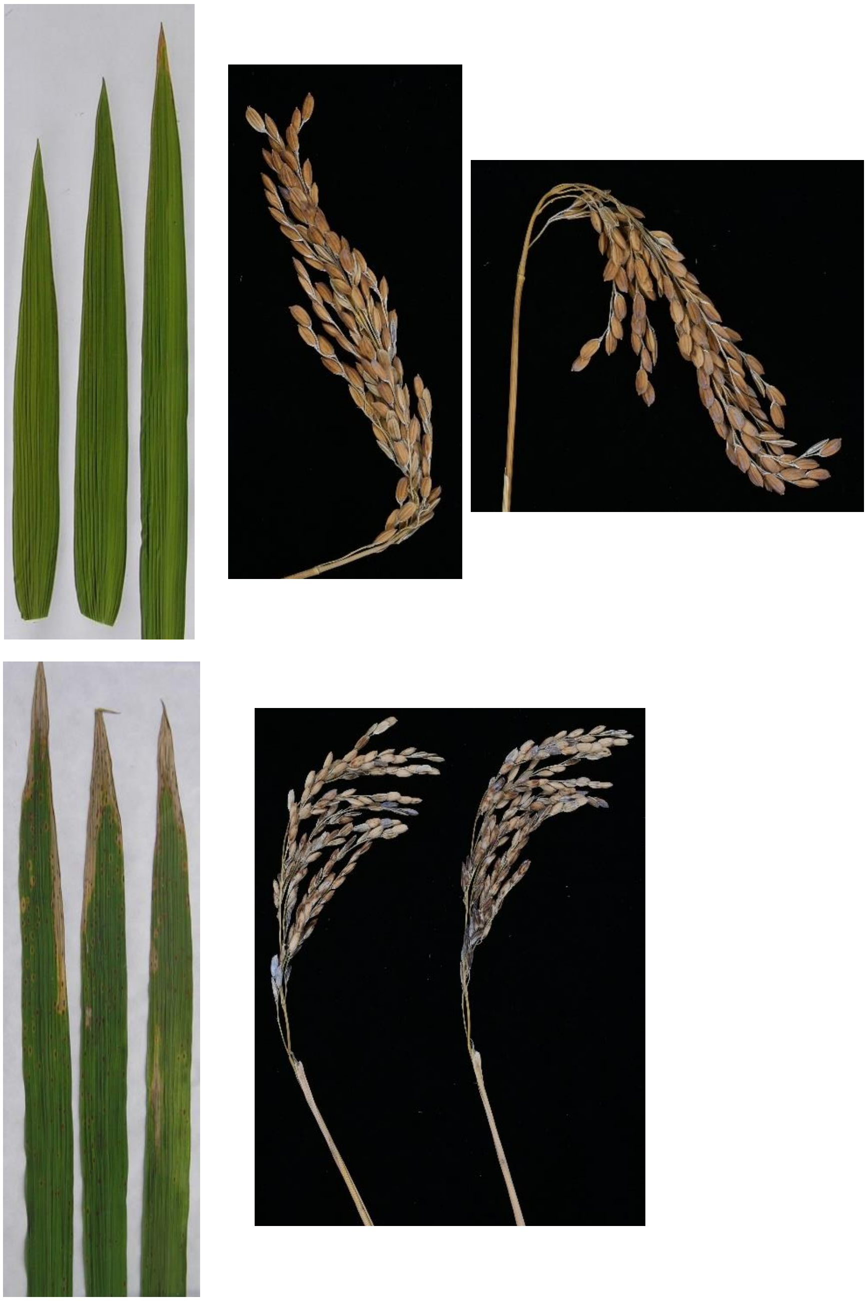

Figure S1. Evaluation of leaf and panicle blast resistance of COPSEMAR9 plants under natural infection conditions (field trial carried out in year 2). Representative images of leaves and panicles of COPSEMAR9 (A) and Baixet (B) plants.
