# Supplementary material for: Development and Genome-Wide Analysis of a Blast-Resistant japonica Rice Variety
Source: Plants (Basel). 2023 Oct 11;12(20):3536. doi: 10.3390/plants12203536 (PMC10667994; doi:10.3390/plants12203536)
Supplement: Supplementary file 1 [file plants-12-03536-s001.zip › Supplementary Material Escol_Suppl Fig S2 BUSCO analysis.pdf]

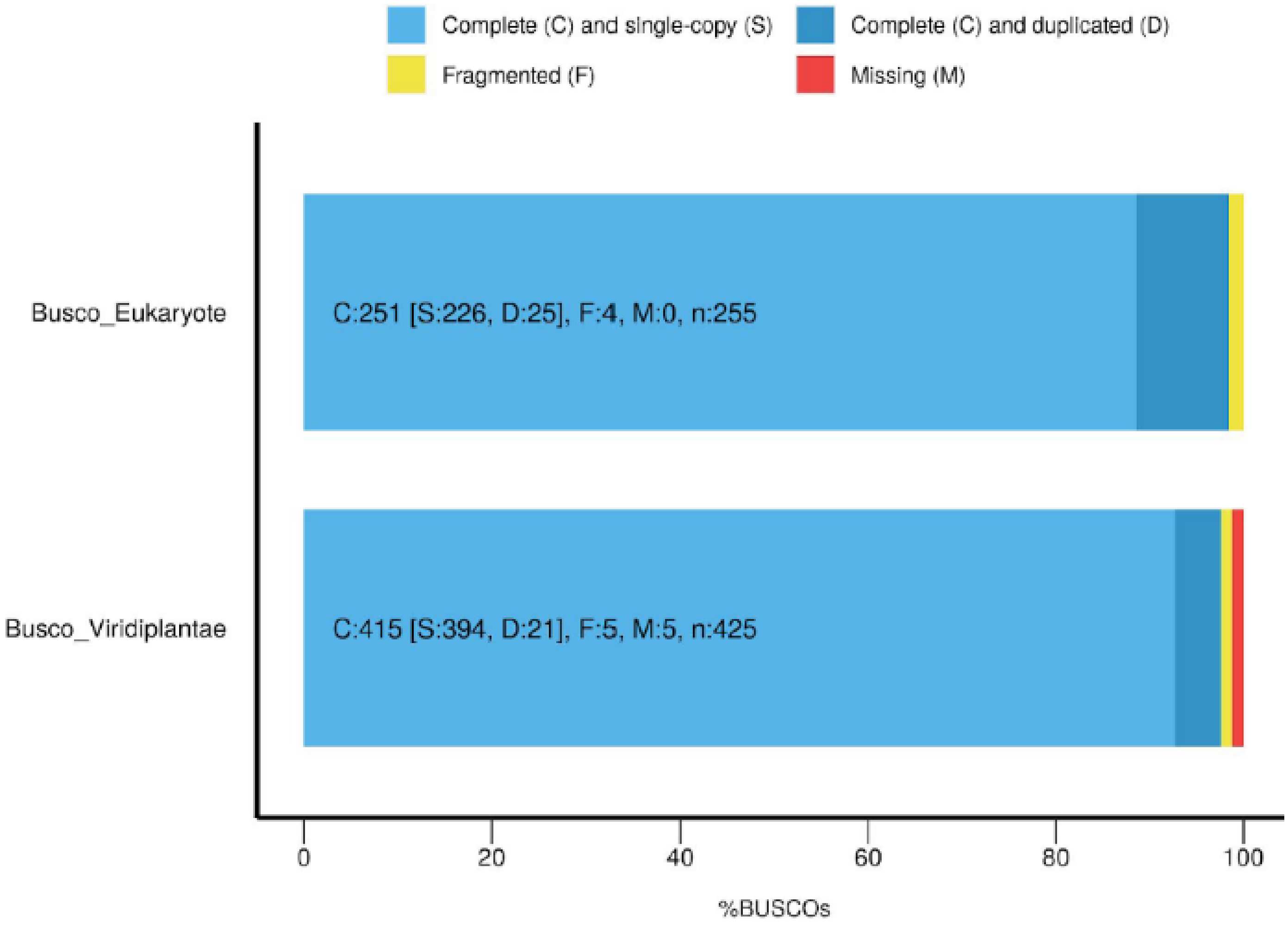

Figure S2. Graphical representation of results obtained on the BUSCO analysis using the Eukaryote (top) and Viridiplantae (bottom) databases. The percentage of completeness was 98,4% and 97,6%, respectively. C, complete; S, single copy; D, duplicated; F, fragmented; M, missing.
