# Supplementary material for: Development and Genome-Wide Analysis of a Blast-Resistant japonica Rice Variety
Source: Plants (Basel). 2023 Oct 11;12(20):3536. doi: 10.3390/plants12203536 (PMC10667994; doi:10.3390/plants12203536)
Supplement: Supplementary file 1 [file plants-12-03536-s001.zip › Supplementary Material Escol_Suppl Fig S3 PCA Jap Ind Hybrid.pdf]

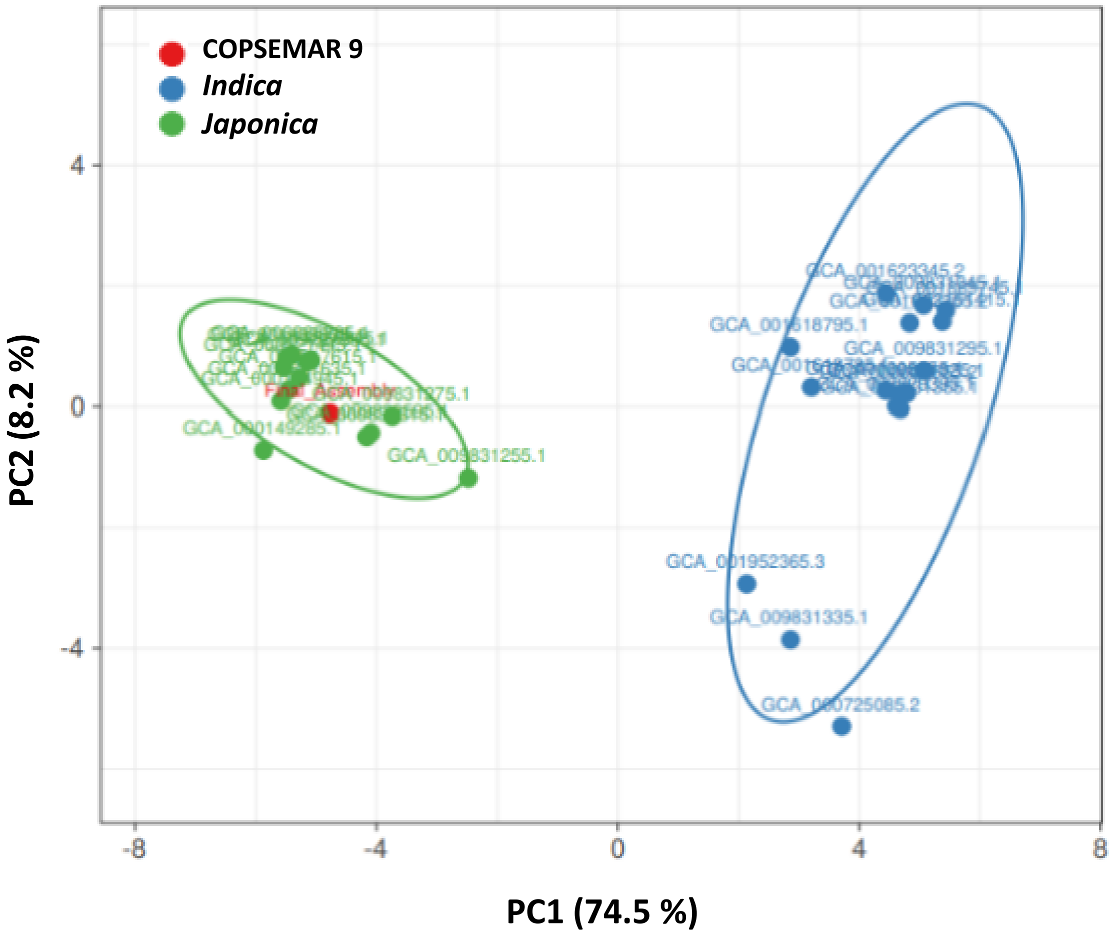

Figure S3. PCA analysis based on the distance of the *indica*, *japonica* and COPSEMAR 9 genomes. Each point represents a genome which is labeled as *indica*, *japonica* or COPSEMAR 9 (blue, green and red, respectively). Genome sequences were retrieved from the NCBI Genomes database. The rice varieties (and accessions) used for this analysis were the following. For *japonica* varieties: Nipponbare (GCA_000149285.1; GCA_003865235.1; and GCF_001433935.1); Kitaake (GCA_009797565.1); Zhonghua 11 (GCA_014526345.1); Azucena (GCA_009830595.1); Koshihikari (GCA_000164945.1); Hitomebore (GCA_000321445.1); HEG4 (GCA_000817615.1); A123 (GCA_000817635.1); ARC 10497::IRGC 12485-1 (GCA_009831255.1); KETAN NANGKA::IRGC 19961-2 (GCA_009831275.1); and CHAO MEO::IRGC 80273-1 (GCA_009831315.1). For *indica* varieties: R498/Shuhui498 (GCA_002151415.1); LIMA::IRGC 81487-1 (GCA_009829395.1); IR8 (GCA_001889745.1); Minghui63 (GCA_001618785.1; GCA_001623365.2); Zhenshan97 (GCA_001618795.1; GCA_001623345.2); PR 106::IRGC 53418-1 (GCA_009831045.1); HR-12 (GCA_000725085.2); N 22::IRGC 19379-1 (IRGC 117534, GCA_001952365.3); GOBOL SAIL (BALAM)::IRGC 26624-2 (GCA_009831025.1); RP Bio-226 (GCA_001305255.1); 93-11 (GCA_000004655.2); KHAO YAI GUANG::IRGC 65972-1 (GCA_009831295.1); NATEL BORO::IRGC 34749-1 (GCA_009831335.1); and LARHA MUGAD::IRGC 52339-1 (GCA_009831355.1).
