# Supplementary material for: Development and Genome-Wide Analysis of a Blast-Resistant japonica Rice Variety
Source: Plants (Basel). 2023 Oct 11;12(20):3536. doi: 10.3390/plants12203536 (PMC10667994; doi:10.3390/plants12203536)
Supplement: Supplementary file 1 [file plants-12-03536-s001.zip › Supplementary Material Escol_Suppl Fig S4 Design field experiments.pdf]

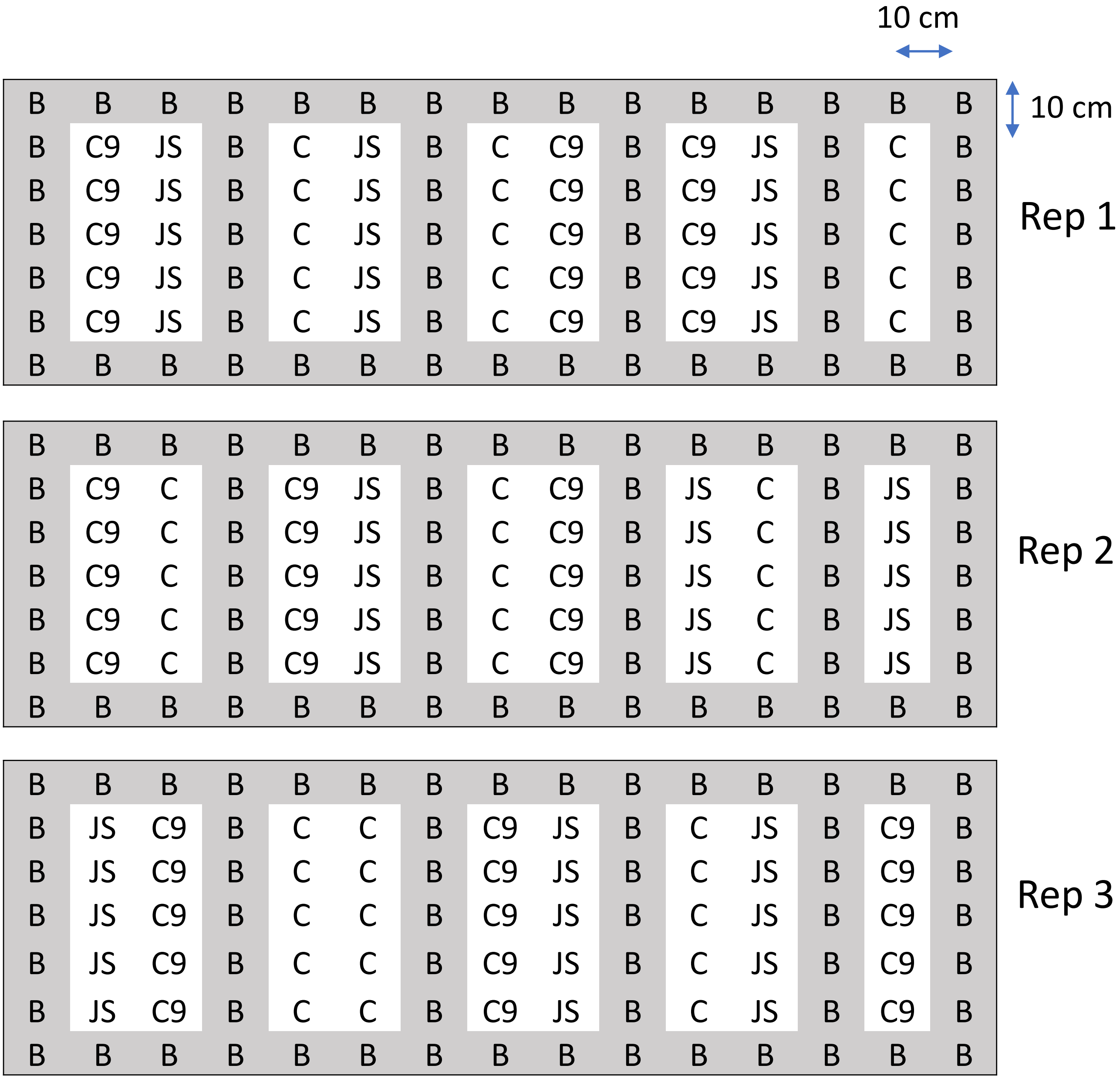

Figure S4. Experimental design used to assess blast resistance of COPSEMAR9 under field conditions. Three plots (Rep 1, Rep2 and Rep3) were made, each one consisting of COPSEMAR9 (C9), JSendra (JS), Baixet (B) and Cormoran (C) plants (each plot containing 15 plants of each variety**,** with a 10 cm spacing between plants). In each plot, a row of Baixet plants was included every two rows. Baixet plants were also grown surrounding each experimental plot. The same experimental design was used in field assays carried out in 2018 and 2021.
