# Supplementary material for: Development and Genome-Wide Analysis of a Blast-Resistant japonica Rice Variety
Source: Plants (Basel). 2023 Oct 11;12(20):3536. doi: 10.3390/plants12203536 (PMC10667994; doi:10.3390/plants12203536)
Supplement: Supplementary file 1 [file plants-12-03536-s001.zip › Supplementary Material Escol_Suppl Table S1 Distance Matrix.pdf]

Supplemental Table S1. Matrix of distances between the COPSEMAR9 assembly and *indica* and *japonica* fully sequenced genomes. Genome sequences were retrieved from the NCBI Genemes database. Lower values correspond to higher similarities. The matrix was produced using the softare MASH.

**Distance**	**Genotype**	**Final_Assembly**	**GCA_000004655.2**	**GCA_000149285.1**	**GCA_000164945.1**	**GCA_000321445.1**	**GCA_000725085.2**	**GCA_000817615.1**
Final_Assembly	Hybrid	0	0,0132573	0,00574147	0,00508714	0,00445566	0,0167251	0,00492718
GCA_000004655.2	Indica	0,0132573	0	0,0147665	0,0140996	0,0135016	0,0123533	0,0135016
GCA_000149285.1	Japonica	0,00574147	0,0147665	0	0,00314086	0,00251903	0,0175939	0,00334352
GCA_000164945.1	Japonica	0,00508714	0,0140996	0,00314086	0	0,00113443	0,0166117	0,00249129
GCA_000321445.1	Japonica	0,00445566	0,0135016	0,00251903	0,00113443	0	0,0162749	0,00199971
GCA_000725085.2	Indica	0,0167251	0,0123533	0,0175939	0,0166117	0,0162749	0	0,0158882
GCA_000817615.1	Japonica	0,00492718	0,0135016	0,00334352	0,00249129	0,00199971	0,0158882	0
GCA_000817635.1	Japonica	0,00458019	0,0137986	0,00337267	0,00251903	0,00202665	0,0162749	0,00113443
GCA_001305255.1	Indica	0,0129677	0,00628226	0,0146626	0,0132573	0,0128243	0,0121217	0,0129677
GCA_001618785.1	Indica	0,0124	0,0100207	0,0132087	0,0125405	0,0122141	0,0130157	0,011984
GCA_001618795.1	Indica	0,0122141	0,00932572	0,0124467	0,0116661	0,0110882	0,0129677	0,0112643
GCA_001623345.2	Indica	0,0118471	0,00669844	0,0129198	0,0119383	0,0115313	0,0111321	0,011621
GCA_001623365.2	Indica	0,0124	0,00752278	0,0139486	0,0130157	0,0124935	0,0114419	0,0122604
GCA_001889745.1	Indica	0,0124935	0,004991	0,0138985	0,012872	0,0123068	0,0104835	0,0124
GCA_001952365.3	Indica	0,0118927	0,0112201	0,013112	0,011984	0,0118017	0,0121679	0,0117564
GCA_002151415.1	Indica	0,0122141	0,00577481	0,0139988	0,013112	0,0125875	0,0105688	0,0124467
GCA_003865235.1	Japonica	0,00411762	0,0132087	0,00186568	0,00149601	0,000931334	0,0161637	0,00202665
GCA_009797565.1	Japonica	0,00439372	0,0132573	0,0034604	0,0026025	0,00197282	0,0160531	0,00213483
GCA_009829395.1	Indica	0,0132573	0,00789262	0,0145592	0,0133059	0,013112	0,0111761	0,0130638
GCA_009830595.1	Japonica	0,00414809	0,0132573	0,00734051	0,0064892	0,00590877	0,015998	0,00587519
GCA_009831025.1	Indica	0,0124935	0,00804257	0,0141502	0,0136991	0,0130638	0,0109136	0,0130157
GCA_009831045.1	Indica	0,0121679	0,00515153	0,0135016	0,0124935	0,0120298	0,0106116	0,0118927
GCA_009831255.1	Japonica	0,00800497	0,0127767	0,00969064	0,00877107	0,00804257	0,0152404	0,00834599
GCA_009831275.1	Japonica	0,00557568	0,012872	0,00759618	0,00673355	0,00604372	0,0158882	0,00638544
GCA_009831295.1	Indica	0,0130638	0,00737682	0,0142518	0,0134036	0,0129198	0,0106545	0,0128243
GCA_009831315.1	Japonica	0,00550978	0,0132087	0,00712406	0,006248	0,00557568	0,0154006	0,00594242
GCA_009831335.1	Indica	0,0134525	0,011621	0,0149759	0,0138985	0,0136002	0,0135508	0,0134036
GCA_009831355.1	Indica	0,0124935	0,00834599	0,0142009	0,0135508	0,012872	0,0101878	0,0130157
GCA_014526345.1	Japonica	0,00473708	0,0129677	0,00325637	0,00232584	0,00178577	0,0158882	0,00173271
GCF_001433935.1	Japonica	0,00411762	0,0133059	0,00191916	0,00136596	0,000805673	0,0162749	0,00210772

GCA_000817635.1	GCA_001305255.1	GCA_001618785.1	GCA_001618795.1	GCA_001623345.2	GCA_001623365.2	GCA_001889745.1	GCA_001952365.3	GCA_002151415.1
0,00458019	0,0129677	0,0124	0,0122141	0,0118471	0,0124	0,0124935	0,0118927	0,0122141
0,0137986	0,00628226	0,0100207	0,00932572	0,00669844	0,00752278	0,004991	0,0112201	0,00577481
0,00337267	0,0146626	0,0132087	0,0124467	0,0129198	0,0139486	0,0138985	0,013112	0,0139988
0,00251903	0,0132573	0,0125405	0,0116661	0,0119383	0,0130157	0,012872	0,011984	0,013112
0,00202665	0,0128243	0,0122141	0,0110882	0,0115313	0,0124935	0,0123068	0,0118017	0,0125875
0,0162749	0,0121217	0,0130157	0,0129677	0,0111321	0,0114419	0,0104835	0,0121679	0,0105688
0,00113443	0,0129677	0,011984	0,0112643	0,011621	0,0122604	0,0124	0,0117564	0,0124467
0	0,0129677	0,0123533	0,0113529	0,0120298	0,012872	0,0128243	0,0122604	0,0127293
0,0129677	0	0,00944662	0,0090861	0,00691005	0,00741321	0,00584167	0,0107836	0,00662843
0,0123533	0,00944662	0	0,00932572	0,00920555	0,00396608	0,00838426	0,0113974	0,00673355
0,0113529	0,0090861	0,00932572	0	0,00378594	0,00904645	0,00734051	0,0108269	0,00826969
0,0120298	0,00691005	0,00920555	0,00378594	0	0,0064892	0,00454897	0,00964976	0,00550978
0,012872	0,00741321	0,00396608	0,00904645	0,0064892	0	0,00524853	0,0101459	0,00354859
0,0128243	0,00584167	0,00838426	0,00734051	0,00454897	0,00524853	0	0,00916565	0,0037263
0,0122604	0,0107836	0,0113974	0,0108269	0,00964976	0,0101459	0,00916565	0	0,0090861
0,0127293	0,00662843	0,00673355	0,00826969	0,00550978	0,00354859	0,0037263	0,0090861	0
0,00194597	0,0127767	0,0117112	0,0106116	0,0113974	0,0123068	0,0121679	0,0114419	0,0124935
0,00227104	0,0126347	0,0120298	0,0109571	0,0110007	0,0120298	0,0118927	0,0113086	0,0123068
0,0134525	0,00785531	0,00900687	0,00952763	0,00712406	0,00673355	0,00614558	0,0103986	0,00631659
0,00628226	0,0132087	0,0124467	0,0121679	0,0113529	0,0115313	0,0124467	0,0112201	0,0121217
0,013112	0,00774383	0,00924552	0,00900687	0,00611156	0,0064892	0,00590877	0,00960897	0,00580821
0,0123068	0,00577481	0,00808024	0,00785531	0,00524853	0,00495906	0,00191916	0,00904645	0,00322742
0,00865422	0,0124	0,0124935	0,0118927	0,0110882	0,0120757	0,0115761	0,0106974	0,0115761
0,00666341	0,0125875	0,0120757	0,0113974	0,0108269	0,0114419	0,0118017	0,0114419	0,0118017
0,013112	0,00737682	0,00826969	0,00892796	0,00617965	0,00557568	0,00524853	0,00989629	0,00587519
0,00641996	0,0132087	0,0126819	0,0122141	0,0113974	0,0122604	0,011984	0,0113974	0,0125875
0,0136991	0,0111321	0,0121679	0,0114419	0,0100207	0,0106974	0,00944662	0,00604372	0,00960897
0,0131603	0,00811798	0,00916565	0,00846101	0,00604372	0,00687462	0,00607761	0,00892796	0,00607761
0,0018924	0,0126347	0,0118017	0,0110007	0,0113974	0,0119383	0,0121217	0,011621	0,0120298
0,00202665	0,0128243	0,0118017	0,0106974	0,0114865	0,0124	0,0122604	0,0115313	0,0125875

GCA_003865235.1	GCA_009797565.1	GCA_009829395.1	GCA_009830595.1	GCA_009831025.1	GCA_009831045.1	GCA_009831255.1	GCA_009831275.1	GCA_009831295.1
0,00411762	0,00439372	0,0132573	0,00414809	0,0124935	0,0121679	0,00800497	0,00557568	0,0130638
0,0132087	0,0132573	0,00789262	0,0132573	0,00804257	0,00515153	0,0127767	0,012872	0,00737682
0,00186568	0,0034604	0,0145592	0,00734051	0,0141502	0,0135016	0,00969064	0,00759618	0,0142518
0,00149601	0,0026025	0,0133059	0,0064892	0,0136991	0,0124935	0,00877107	0,00673355	0,0134036
0,000931334	0,00197282	0,013112	0,00590877	0,0130638	0,0120298	0,00804257	0,00604372	0,0129198
0,0161637	0,0160531	0,0111761	0,015998	0,0109136	0,0106116	0,0152404	0,0158882	0,0106545
0,00202665	0,00213483	0,0130638	0,00587519	0,0130157	0,0118927	0,00834599	0,00638544	0,0128243
0,00194597	0,00227104	0,0134525	0,00628226	0,013112	0,0123068	0,00865422	0,00666341	0,013112
0,0127767	0,0126347	0,00785531	0,0132087	0,00774383	0,00577481	0,0124	0,0125875	0,00737682
0,0117112	0,0120298	0,00900687	0,0124467	0,00924552	0,00808024	0,0124935	0,0120757	0,00826969
0,0106116	0,0109571	0,00952763	0,0121679	0,00900687	0,00785531	0,0118927	0,0113974	0,00892796
0,0113974	0,0110007	0,00712406	0,0113529	0,00611156	0,00524853	0,0110882	0,0108269	0,00617965
0,0123068	0,0120298	0,00673355	0,0115313	0,0064892	0,00495906	0,0120757	0,0114419	0,00557568
0,0121679	0,0118927	0,00614558	0,0124467	0,00590877	0,00191916	0,0115761	0,0118017	0,00524853
0,0114419	0,0113086	0,0103986	0,0112201	0,00960897	0,00904645	0,0106974	0,0114419	0,00989629
0,0124935	0,0123068	0,00631659	0,0121217	0,00580821	0,00322742	0,0115761	0,0118017	0,00587519
0	0,00167981	0,0128243	0,00580821	0,0127293	0,0118017	0,00800497	0,00614558	0,0125405
0,00167981	0	0,0130157	0,00544411	0,0127293	0,0115313	0,00763299	0,00611156	0,0124935
0,0128243	0,0130157	0	0,0126819	0,00607761	0,00680395	0,0114419	0,0123068	0,00528099
0,00580821	0,00544411	0,0126819	0	0,0123068	0,0118927	0,00755945	0,00518381	0,0124467
0,0127293	0,0127293	0,00607761	0,0123068	0	0,00641996	0,0115313	0,0123068	0,00518381
0,0118017	0,0115313	0,00680395	0,0118927	0,00641996	0	0,0110882	0,0113974	0,00567497
0,00800497	0,00763299	0,0114419	0,00755945	0,0115313	0,0110882	0	0,00759618	0,0121679
0,00614558	0,00611156	0,0123068	0,00518381	0,0123068	0,0113974	0,00759618	0	0,0128243
0,0125405	0,0124935	0,00528099	0,0124467	0,00518381	0,00567497	0,0121679	0,0128243	0
0,00557568	0,00550978	0,0124	0,00467416	0,0124467	0,0117112	0,00723198	0,00531349	0,0123533
0,0132087	0,0133547	0,0100623	0,0125875	0,0097316	0,00977265	0,0117112	0,0133547	0,0100623
0,0126819	0,0123068	0,00759618	0,0120757	0,00641996	0,00628226	0,0115313	0,0124935	0,00621379
0,00167981	0,00199971	0,0127293	0,00614558	0,0125405	0,0115761	0,0081558	0,00628226	0,0124935
0,000119496	0,00170624	0,0129198	0,00580821	0,0128243	0,0118927	0,00804257	0,00607761	0,0126347

GCA_009831315.1	GCA_009831335.1	GCA_009831355.1	GCA_014526345.1	GCF_001433935.1
0,00550978	0,0134525	0,0124935	0,00473708	0,00411762
0,0132087	0,011621	0,00834599	0,0129677	0,0133059
0,00712406	0,0149759	0,0142009	0,00325637	0,00191916
0,006248	0,0138985	0,0135508	0,00232584	0,00136596
0,00557568	0,0136002	0,012872	0,00178577	0,000805673
0,0154006	0,0135508	0,0101878	0,0158882	0,0162749
0,00594242	0,0134036	0,0130157	0,00173271	0,00210772
0,00641996	0,0136991	0,0131603	0,0018924	0,00202665
0,0132087	0,0111321	0,00811798	0,0126347	0,0128243
0,0126819	0,0121679	0,00916565	0,0118017	0,0118017
0,0122141	0,0114419	0,00846101	0,0110007	0,0106974
0,0113974	0,0100207	0,00604372	0,0113974	0,0114865
0,0122604	0,0106974	0,00687462	0,0119383	0,0124
0,011984	0,00944662	0,00607761	0,0121217	0,0122604
0,0113974	0,00604372	0,00892796	0,011621	0,0115313
0,0125875	0,00960897	0,00607761	0,0120298	0,0125875
0,00557568	0,0132087	0,0126819	0,00167981	0,000119496
0,00550978	0,0133547	0,0123068	0,00199971	0,00170624
0,0124	0,0100623	0,00759618	0,0127293	0,0129198
0,00467416	0,0125875	0,0120757	0,00614558	0,00580821
0,0124467	0,0097316	0,00641996	0,0125405	0,0128243
0,0117112	0,00977265	0,00628226	0,0115761	0,0118927
0,00723198	0,0117112	0,0115313	0,0081558	0,00804257
0,00531349	0,0133547	0,0124935	0,00628226	0,00607761
0,0123533	0,0100623	0,00621379	0,0124935	0,0126347
0	0,0132573	0,0127767	0,00604372	0,00550978
0,0132573	0	0,00956826	0,0136991	0,0133059
0,0127767	0,00956826	0	0,0125405	0,0127767
0,00604372	0,0136991	0,0125405	0	0,00170624
0,00550978	0,0133059	0,0127767	0,00170624	0
