# Supplementary material for: Development and Genome-Wide Analysis of a Blast-Resistant japonica Rice Variety
Source: Plants (Basel). 2023 Oct 11;12(20):3536. doi: 10.3390/plants12203536 (PMC10667994; doi:10.3390/plants12203536)
Supplement: Supplementary file 1 [file plants-12-03536-s001.zip › Supplementary Material Escol_Suppl Table S2 List of genes in COPSEMAR9.pdf]

**Supplemental Table S2. Genes identified in the genome of COPSEMAR9**. A. Genes identified by the variant calling analysis (Assignment Variants) or bit score difference (Assignment Similarity) are listed and assigned as *indica* or *japonica* genes.

| Locus | Assignment Variants | Assignment Similarity | Resistance Gene Class | Japonica Locus | Indica Locus | Description |
|---|---|---|---|---|---|---|
| augustus-1-processed-gene-15.2-mRNA-1 | Indica | Japonica | | Os01g0127600 | OsR498G0100091300.01.T01 | |
| augustus-1-processed-gene-15.3-mRNA-1 | Indica | NA | Kinase | | OsR498G0100091500.01.T01 | G-type lectin S-receptor-like serine/threonine-protein kinase |
| augustus-1-processed-gene-17.20-mRNA-1 | Indica | NA | | Os01g0131300 | OsR498G0100108700.01.T01 | PORR domain-containing protein |
| augustus-1-processed-gene-17.23-mRNA-1 | Indica | NA | | Os01g0131600 | OsR498G0100109900.01.T06 | Pathogenesis-related transcriptional activator PTI6 |
| augustus-1-processed-gene-18.41-mRNA-1 | Indica | NA | | Os02g0452800 | OsR498G0714367900.01.T01 | Epimerase domain-containing protein |
| augustus-1-processed-gene-19.26-mRNA-1 | Indica | NA | | Os01g0135800 | OsR498G0100121300.01.T01 | 16.9 kDa class I heat shock protein 3 |
| augustus-1-processed-gene-19.37-mRNA-1 | Indica | Indica | | Os01g0134800 | OsR498G0100119800.01.T01 | Endo-1,4-beta-xylanase |
| augustus-1-processed-gene-19.38-mRNA-1 | Indica | Indica | | Os01g0134900 | OsR498G0100120000.01.T01 | Endo-1,4-beta-xylanase |
| augustus-1-processed-gene-19.42-mRNA-1 | Indica | NA | | Os01g0136200 | OsR498G0100124300.01.T01 | Class I heat shock protein 1 |
| augustus-1-processed-gene-21.29-mRNA-1 | Indica | NA | | | | WAK1-OsWAK receptor-like cytoplasmic kinase (OsWAK-RLCK) |
| augustus-1-processed-gene-21.32-mRNA-1 | Indica | NA | | Os01g0138300 | OsR498G0100133900.01.T01 | Putative serine/threonine-protein kinase |
| augustus-1-processed-gene-21.48-mRNA-1 | Indica | NA | | Os01g0139600 | OsR498G0100138300.01.T01 | Lipid phosphate phosphatase |
| augustus-1-processed-gene-222.16-mRNA-1 | Indica | Japonica | | Os01g0564300 | OsR498G0101347500.01.T01 | Peptidylprolyl isomerase |
| augustus-1-processed-gene-23.56-mRNA-1 | Indica | Japonica | | Os01g0141700 | OsR498G0100150400.01.T02 | Protein DAMAGED DNA-BINDING 2 isoform A |
| augustus-1-processed-gene-23.61-mRNA-1 | Indica | Indica | | Os02g0547100 | OsR498G0101158000.01.T01 | Transposon protein, putative, Mutator sub-class, expressed |
| augustus-1-processed-gene-234.11-mRNA-1 | Indica | NA | | Os01g0585200 | OsR498G0101417500.01.T01 | Glycerophosphocholine acyltransferase 1 |
| augustus-1-processed-gene-260.34-mRNA-1 | Indica | NA | | Os01g0633200 | OsR498G0101598900.01.T01 | Factor of DNA methylation 1 |
| augustus-1-processed-gene-28.37-mRNA-1 | Indica | NA | | Os01g0151100 | OsR498G0100183100.01.T06 | Putative translation initiation factor IF-2 |
| augustus-1-processed-gene-29.20-mRNA-1 | Indica | NA | | Os01g0153800 | OsR498G0100193100.01.T01 | Putative carboxylesterase 15 |
| augustus-1-processed-gene-29.21-mRNA-1 | Indica | NA | | Os01g0154300 | OsR498G0100194100.01.T01 | Ubiquitin-like domain-containing protein |
| augustus-1-processed-gene-30.34-mRNA-1 | Indica | NA | Kinase-TM | Os01g0155500 | OsR498G0100197900.01.T01 | Receptor-like protein kinase HERK 1 |
| augustus-1-processed-gene-30.53-mRNA-1 | Indica | NA | | Os01g0157200 | | |
| augustus-1-processed-gene-30.54-mRNA-1 | Indica | NA | | | OsR498G0100202500.01.T01 | |
| augustus-1-processed-gene-31.35-mRNA-1 | Indica | Indica | | Os05g0163100 | OsR498G0100208400.01.T01 | PHD finger protein ALFIN-LIKE 1-like |
| augustus-1-processed-gene-33.59-mRNA-1 | Indica | NA | | Os01g0165000 | OsR498G0100216100.01.T03 | Dehydration-responsive element-binding protein 2 |
| augustus-1-processed-gene-34.49-mRNA-1 | Indica | Indica | | Os01g0166700 | OsR498G0100220900.01.T02 | Saposin-like type B, 1 |
| augustus-1-processed-gene-34.53-mRNA-1 | Indica | NA | | Os01g0167400 | OsR498G0100222500.01.T01 | rRNA N-glycosidase |
| augustus-1-processed-gene-351.41-mRNA-1 | Indica | NA | | | OsR498G0102186300.01.T01 | |
| augustus-1-processed-gene-352.13-mRNA-1 | Indica | NA | | Os01g0811200 | OsR498G0102193100.01.T01 | Serine-type endopeptidase inhibitor |
| augustus-1-processed-gene-352.14-mRNA-1 | Indica | NA | | Os01g0811300 | OsR498G0102193500.01.T01 | Histone-lysine N-methyltransferase H3 lysine-9 specific SUVH3 |
| augustus-1-processed-gene-353.52-mRNA-1 | Indica | NA | | Os01g0812200 | OsR498G0102197300.01.T01 | CMP/dCMP-type deaminase domain-containing protein (Fragment) |
| augustus-1-processed-gene-353.68-mRNA-1 | Indica | NA | | Os01g0813400 | OsR498G0102200300.01.T05 | ADP-ribosylation factor |
| augustus-1-processed-gene-353.71-mRNA-1 | Indica | Indica | | Os01g0814100 | OsR498G0102202300.01.T01 | AAI domain-containing protein |
| augustus-1-processed-gene-354.59-mRNA-1 | Indica | NA | | Os01g0816000 | OsR498G0102207500.01.T01 | Pentatricopeptide repeat-containing protein mitochondrial |
| augustus-1-processed-gene-355.34-mRNA-1 | Indica | NA | | Os01g0816700 | OsR498G0102211100.01.T01 | L-ascorbate oxidase like |
| augustus-1-processed-gene-359.17-mRNA-1 | Indica | NA | | | OsR498G0100075100.01.T01 | Epstein-Barr virus EBNA-1-like |
| augustus-1-processed-gene-360.26-mRNA-1 | Indica | NA | | | OsR498G0102241700.01.T01 | Epidermal patterning factor-like protein 6-like |
| augustus-1-processed-gene-361.11-mRNA-1 | Indica | NA | | Os01g0827500 | OsR498G0102253700.01.T05 | Exocyst complex component EXO70B1 |
| augustus-1-processed-gene-361.12-mRNA-1 | Indica | NA | | Os01g0827600 | | Exocyst subunit Exo70 family protein (Fragment) |
| augustus-1-processed-gene-361.13-mRNA-1 | Indica | NA | | Os05g0473000 | OsR498G0511028200.01.T01 | Abscisic acid receptor 6 |
| augustus-1-processed-gene-362.10-mRNA-1 | Indica | Indica | | Os01g0829800 | OsR498G0102260500.01.T01 | Malate dehydrogenase |
| augustus-1-processed-gene-362.11-mRNA-1 | Indica | NA | | Os01g0830100 | OsR498G0102261300.01.T03 | Internal alternative NAD(P)H-ubiquinone oxidoreductase |
| augustus-1-processed-gene-364.40-mRNA-1 | Indica | NA | | Os01g0832900 | OsR498G0102273300.01.T03 | Dual specificity tyrosine-phosphorylation-regulated kinase mbk-1 |
| augustus-1-processed-gene-364.41-mRNA-1 | Indica | NA | | Os01g0833000 | | Pentatricopeptide repeat-containing protein chloroplastic |
| augustus-1-processed-gene-365.39-mRNA-1 | Indica | NA | | Os01g0835900 | OsR498G0102286500.01.T01 | Multifunctional fusion protein |
| augustus-1-processed-gene-366.1-mRNA-1 | Indica | NA | | Os01g0836600 | OsR498G0102288300.01.T01 | ABC transporter domain-containing protein (Fragment) |
| augustus-1-processed-gene-366.10-mRNA-1 | Indica | NA | | Os01g0836900 | | probable membrane-associated kinase regulator 4 |
| augustus-1-processed-gene-367.49-mRNA-1 | Indica | NA | | Os01g0839400 | OsR498G0102299500.01.T01 | Pentatricopeptide repeat-containing protein mitochondrial |
| augustus-1-processed-gene-45.5-mRNA-1 | Indica | NA | | | OsR498G0100283300.01.T01 | Alkane hydroxylase MAH1 |
| augustus-1-processed-gene-46.17-mRNA-1 | Indica | NA | | Os01g0186600 | OsR498G0100291700.01.T01 | |
| augustus-1-processed-gene-46.8-mRNA-1 | Indica | NA | | Os01g0658300 | | Glutaminyl-tRNA synthetase |
| augustus-1-processed-gene-470.17-mRNA-1 | Indica | NA | | Os01g0905700 | OsR498G0102537700.01.T01 | peroxisome biogenesis factor 10-like |
| augustus-1-processed-gene-472.32-mRNA-1 | Indica | NA | | Os01g0908800 | OsR498G0102549700.01.T02 | Fertility restorer homologue A-like |
| augustus-1-processed-gene-474.14-mRNA-1 | Indica | NA | | | | Transposon protein, putative, Mutator sub-class, expressed |
| augustus-1-processed-gene-494.2-mRNA-1 | Indica | NA | | | OsR498G0204046800.01.T01 | Circumsporozoite protein-like |
| augustus-1-processed-gene-7.38-mRNA-1 | Indica | NA | | Os01g0112600 | | |
| augustus-1-processed-gene-8.0-mRNA-1 | Indica | Indica | CC-Kinase-TM | Os01g0113350 | OsR498G0100044500.01.T01 | Putative receptor serine/threonine kinase PR5K |
| augustus-1-processed-gene-8.3-mRNA-1 | Indica | Japonica | Kinase-TM | Os01g0113650 | OsR498G0100044500.01.T01 | Putative receptor serine/threonine kinase PR5K |
| augustus-10-processed-gene-1.21-mRNA-1 | Indica | NA | Kinase-TM | Os10g0101000 | OsR498G1017996700.01.T01 | Non-specific serine/threonine protein kinase |

| | | | | | | |
|---|---|---|---|---|---|---|
| augustus-10-processed-gene-11.10-mRNA-1 | Indica | NA | | | | Hydroxyproline-rich glycoprotein-like |
| augustus-10-processed-gene-111.13-mRNA-1 | Indica | NA | | Os10g0442700 | OsR498G1018800100.01.T02 | Pentatricopeptide repeat-containing protein, chloroplastic |
| augustus-10-processed-gene-12.15-mRNA-1 | Indica | NA | | Os10g0113000 | OsR498G1018038100.01.T01 | Deoxymugineic acid synthase 1-B |
| augustus-10-processed-gene-126.13-mRNA-1 | Indica | NA | | | OsR498G0407539000.01.T01 | |
| augustus-10-processed-gene-128.0-mRNA-1 | Indica | NA | | Os04g0137400 | OsR498G0407530000.01.T01 | |
| augustus-10-processed-gene-132.21-mRNA-1 | Indica | NA | | Os05g0471800 | | Far-red impaired response protein-like |
| augustus-10-processed-gene-138.36-mRNA-1 | Indica | NA | Kinase-LRR-TM | | OsR498G0407494600.01.T01 | Putative LRR receptor-like serine/threonine-protein kinase |
| augustus-10-processed-gene-142.24-mRNA-1 | Indica | NA | | | OsR498G0815762600.01.T01 | |
| augustus-10-processed-gene-145.25-mRNA-1 | Indica | NA | | Os04g0110500 | OsR498G0407447400.01.T01 | Pentatricopeptide repeat-containing protein ELI1, chloroplastic |
| augustus-10-processed-gene-145.35-mRNA-1 | Indica | NA | | Os04g0110100 | OsR498G0407446000.01.T01 | Legume lectins beta domain containing protein |
| augustus-10-processed-gene-147.14-mRNA-1 | Indica | NA | | Os04g0106000 | OsR498G0407429800.01.T02 | PMEI domain-containing protein (Fragment) |
| augustus-10-processed-gene-147.4-mRNA-1 | Indica | NA | | Os04g0106500 | | VARLMGL domain-containing protein |
| augustus-10-processed-gene-148.12-mRNA-1 | Indica | NA | | Os04g0103900 | OsR498G0407422800.01.T01 | Chalcone synthase G |
| augustus-10-processed-gene-159.29-mRNA-1 | Indica | NA | | Os10g0459600 | OsR498G1018847300.01.T01 | Xyloglucan galactosyltransferase KATAMARI1 |
| augustus-10-processed-gene-16.14-mRNA-1 | Indica | NA | | | | Transposase, putative, expressed |
| augustus-10-processed-gene-29.3-mRNA-1 | Indica | NA | Kinase | Os10g0142600 | OsR498G1018125300.01.T01 | Receptor-like protein kinase FERONIA |
| augustus-10-processed-gene-3.2-mRNA-1 | Indica | Indica | | Os01g0524500 | OsR498G1018003900.01.T01 | Transcription factor MYBS1 |
| augustus-10-processed-gene-31.29-mRNA-1 | Indica | NA | | | OsR498G1018144500.01.T01 | Cellulase |
| augustus-10-processed-gene-31.30-mRNA-1 | Indica | NA | | Os10g0148000 | OsR498G1018144900.01.T01 | Protease inhibitor/seed storage/LTP family protein, expressed |
| augustus-10-processed-gene-34.19-mRNA-1 | Indica | NA | | Os10g0150200 | OsR498G1018151700.01.T01 | IGR protein motif |
| augustus-10-processed-gene-37.10-mRNA-1 | Indica | NA | | Os10g0155300 | OsR498G1018168100.01.T01 | F-box domain containing protein, expressed |
| augustus-10-processed-gene-4.40-mRNA-1 | Indica | NA | | | OsR498G1018011100.01.T01 | Putative Mitochondrial editing factor 22 |
| augustus-10-processed-gene-47.0-mRNA-1 | Indica | NA | | Os10g0164600 | OsR498G1018230300.01.T01 | Geraniol 8-hydroxylase-like |
| augustus-10-processed-gene-47.2-mRNA-1 | Indica | NA | | Os10g0165000 | OsR498G1018231300.01.T01 | Core-2/I-branching beta-16-N-acetylglucosaminyltransferase family protein |
| augustus-10-processed-gene-49.26-mRNA-1 | Indica | Indica | | Os10g0166600 | OsR498G1018238900.01.T02 | Cytochrome P450 family protein, expressed |
| augustus-10-processed-gene-49.41-mRNA-1 | Indica | NA | | | OsR498G1018240900.01.T01 | |
| augustus-10-processed-gene-50.23-mRNA-1 | Indica | NA | | Os10g0169200 | OsR498G1018246900.01.T01 | |
| augustus-10-processed-gene-51.30-mRNA-1 | Indica | Indica | | Os12g0435200 | OsR498G1018251900.01.T01 | Nine-cis-epoxycarotenoid dioxygenase 4 |
| augustus-10-processed-gene-51.42-mRNA-1 | Indica | Japonica | | Os10g0171400 | OsR498G1018303700.01.T01 | |
| augustus-10-processed-gene-53.2-mRNA-1 | Indica | Indica | | Os10g0173000 | OsR498G1018256500.01.T02 | |
| augustus-10-processed-gene-55.14-mRNA-1 | Indica | NA | | Os10g0176300 | | |
| augustus-10-processed-gene-56.1-mRNA-1 | Indica | NA | | | OsR498G1018268500.01.T01 | AAI domain-containing protein (Fragment) |
| augustus-10-processed-gene-60.15-mRNA-1 | Indica | NA | | Os03g0732200 | | Dynamin GTPase |
| augustus-10-processed-gene-60.39-mRNA-1 | Indica | NA | | | OsR498G0306743300.01.T01 | Hydroxyproline-rich glycoprotein-like |
| augustus-10-processed-gene-61.0-mRNA-1 | Indica | NA | | Os10g0186200 | | PMEI domain-containing protein (Fragment) |
| augustus-10-processed-gene-7.10-mRNA-1 | Indica | Indica | | Os10g0108700 | OsR498G1018024500.01.T01 | Methanol O-anthraniloyltransferase |
| augustus-11-processed-gene-146.34-mRNA-1 | Indica | Japonica | | Os11g0486000 | OsR498G0409297100.01.T04 | Truncated Sr21 |
| augustus-11-processed-gene-147.1-mRNA-1 | Indica | NA | | | OsR498G1120161500.01.T01 | Protease inhibitor/seed storage/LTP family protein |
| augustus-11-processed-gene-250.11-mRNA-1 | Indica | NA | | Os11g0560000 | OsR498G1018527700.01.T01 | Disease resistance protein RPM1 |
| augustus-11-processed-gene-252.13-mRNA-1 | Indica | NA | CC-NBS-TM | Os11g0684200 | OsR498G1120731300.01.T01 | NB-ARC domain containing protein, expressed |
| augustus-11-processed-gene-255.16-mRNA-1 | Indica | NA | | | OsR498G1120738700.01.T03 | |
| augustus-11-processed-gene-261.22-mRNA-1 | Indica | Japonica | | Os11g0703000 | OsR498G1221905100.01.T01 | Pathogenesis-related protein 5 |
| augustus-11-processed-gene-261.26-mRNA-1 | Indica | NA | | Os11g0703600 | OsR498G1120766900.01.T02 | Protein UPSTREAM OF FLC |
| augustus-11-processed-gene-73.31-mRNA-1 | Indica | NA | | Os11g0211800 | OsR498G1119608900.01.T02 | |
| augustus-12-processed-gene-114.41-mRNA-1 | Indica | NA | | | OsR498G1221348100.01.T02 | Helicase and polymerase-containing protein TEBICHI |
| augustus-12-processed-gene-115.12-mRNA-1 | Indica | NA | | | | Transposon protein, putative, Mutator sub-class, expressed |
| augustus-12-processed-gene-126.23-mRNA-1 | Indica | NA | | Os12g0407800 | OsR498G1221372700.01.T03 | Pentatricopeptide repeat-containing protein |
| augustus-12-processed-gene-133.13-mRNA-1 | Indica | NA | | | OsR498G1221406900.01.T01 | |
| augustus-12-processed-gene-134.14-mRNA-1 | Indica | NA | | | OsR498G1221411100.01.T01 | NAC domain-containing protein (Fragment) |
| augustus-12-processed-gene-137.11-mRNA-1 | Indica | NA | | Os12g0422200 | OsR498G1221421300.01.T01 | DUF295 domain-containing protein |
| augustus-12-processed-gene-137.12-mRNA-1 | Indica | NA | | Os12g0424600 | OsR498G1221421500.01.T01 | DUF295 domain-containing protein |
| augustus-12-processed-gene-140.30-mRNA-1 | Indica | NA | | Os12g0428200 | | Putative chloroplast nucleoid DNA binding protein |
| augustus-12-processed-gene-143.10-mRNA-1 | Indica | NA | | Os12g0432600 | OsR498G1221448500.01.T01 | RING finger domain-containing |
| augustus-12-processed-gene-209.6-mRNA-1 | Indica | NA | | Os12g0515500 | OsR498G1221715900.01.T02 | Zinc finger CCCH domain-containing protein 67 |
| augustus-12-processed-gene-209.8-mRNA-1 | Indica | Indica | | Os12g0516900 | OsR498G1221718100.01.T02 | F-box domain containing protein, expressed |
| augustus-12-processed-gene-210.14-mRNA-1 | Indica | NA | | Os12g0517000 | OsR498G1221720100.01.T01 | F-box domain-containing protein (Fragment) |
| augustus-12-processed-gene-210.15-mRNA-1 | Indica | NA | | Os12g0517100 | OsR498G1221720500.01.T01 | F-box domain containing protein, expressed |
| augustus-12-processed-gene-210.16-mRNA-1 | Indica | NA | | Os12g0517200 | OsR498G1221720900.01.T02 | Ribosomal protein S10, mitochondrial |
| augustus-12-processed-gene-211.34-mRNA-1 | Indica | NA | | | | Transposon protein, putative, Mutator sub-class, expressed |
| augustus-12-processed-gene-212.13-mRNA-1 | Indica | Indica | | Os12g0529900 | OsR498G1221728500.01.T01 | Flagellar attachment zone protein 1 |
| augustus-12-processed-gene-215.2-mRNA-1 | Indica | NA | | | OsR498G1221744300.01.T01 | Epstein-Barr virus EBNA-1-like |

| | | | | | | |
|---|---|---|---|---|---|---|
| augustus-12-processed-gene-227.14-mRNA-1 | Indica | NA | | | OsR498G1221852500.01.T01 | Protein DETOXIFICATION |
| augustus-2-processed-gene-151.22-mRNA-1 | Indica | NA | | | OsR498G1221513100.01.T01 | Hydroxyproline-rich glycoprotein-like |
| augustus-2-processed-gene-166.21-mRNA-1 | Indica | NA | LRR-TM | | OsR498G0203803900.01.T01 | NB-ARC domain-containing protein |
| augustus-2-processed-gene-167.54-mRNA-1 | Indica | Japonica | | Os02g0478900 | OsR498G0202966300.01.T03 | Protein kinase domain-containing protein |
| augustus-2-processed-gene-167.55-mRNA-1 | Indica | NA | | Os02g0478900 | OsR498G0203805900.01.T02 | Splicing factor |
| augustus-2-processed-gene-167.67-mRNA-1 | Indica | NA | | | OsR498G0203809700.01.T01 | Calcineurin b-like protein 3-like |
| augustus-2-processed-gene-167.68-mRNA-1 | Indica | NA | | | OsR498G0203810500.01.T01 | |
| augustus-2-processed-gene-167.81-mRNA-1 | Indica | NA | | Os02g0480100 | | ATP-dependent DNA helicase |
| augustus-2-processed-gene-169.3-mRNA-1 | Indica | Japonica | | Os02g0482400 | OsR498G0917892000.01.T01 | Diaminopimelate decarboxylase 2 chloroplastic |
| augustus-2-processed-gene-170.0-mRNA-1 | Indica | NA | | Os02g0484600 | OsR498G0203823300.01.T01 | Putative quercetin 3-O-glucoside-6''-O-malonyltransferase |
| augustus-2-processed-gene-170.23-mRNA-1 | Indica | NA | | Os02g0485800 | OsR498G0203826500.01.T01 | Putative quercetin 3-O-glucoside-6''-O-malonyltransferase |
| augustus-2-processed-gene-172.16-mRNA-1 | Indica | Indica | | Os02g0225200 | OsR498G0203832900.01.T01 | F-box family protein-like |
| augustus-2-processed-gene-172.35-mRNA-1 | Indica | NA | | Os01g0967000 | OsR498G0305701300.01.T01 | |
| augustus-2-processed-gene-181.13-mRNA-1 | Indica | Japonica | | Os02g0503300 | OsR498G0203881700.01.T01 | X8 domain-containing protein (Fragment) |
| augustus-2-processed-gene-182.13-mRNA-1 | Indica | NA | | | OsR498G0203883900.01.T02 | UDP-N-acetylmuramoyl-L-alanyl-D-glutamate--2, 6-diaminopimelate ligase |
| augustus-2-processed-gene-185.36-mRNA-1 | Indica | NA | | Os02g0510000 | OsR498G0203902900.01.T01 | J domain-containing protein (Fragment) |
| augustus-2-processed-gene-185.37-mRNA-1 | Indica | Japonica | | Os02g0510200 | OsR498G0203903500.01.T02 | Acetolactate synthase |
| augustus-2-processed-gene-185.38-mRNA-1 | Indica | NA | | Os02g0510600 | OsR498G0203903900.01.T01 | Farnesylated protein 3 |
| augustus-2-processed-gene-187.10-mRNA-1 | Indica | Japonica | | Os02g0511400 | OsR498G0203905300.01.T04 | Cis-2,3-dihydrobiphenyl-2,3-diol dehydrogenase |
| augustus-2-processed-gene-187.9-mRNA-1 | Indica | NA | | Os02g0511400 | OsR498G0203905300.01.T03 | 11-beta-hydroxysteroid dehydrogenase-like 6 |
| augustus-2-processed-gene-190.44-mRNA-1 | Indica | NA | | Os02g0516400 | OsR498G0203926000.01.T02 | Major ampullate spidroin 2-2 |
| augustus-2-processed-gene-191.26-mRNA-1 | Indica | NA | | | OsR498G0203930900.01.T01 | |
| augustus-2-processed-gene-192.13-mRNA-1 | Indica | Indica | | Os02g0302500 | OsR498G0203943500.01.T02 | zf-RVT domain-containing protein |
| augustus-2-processed-gene-192.20-mRNA-1 | Indica | NA | | Os02g0517700 | OsR498G0203938000.01.T01 | |
| augustus-2-processed-gene-192.23-mRNA-1 | Indica | NA | | Os02g0518000 | OsR498G0203939200.01.T01 | |
| augustus-2-processed-gene-208.21-mRNA-1 | Indica | NA | | | OsR498G0612206300.01.T01 | Beta-hexosaminidase |
| augustus-2-processed-gene-223.26-mRNA-1 | Indica | NA | | | OsR498G0204155200.01.T01 | RING-H2 finger protein ATL65 |
| augustus-2-processed-gene-234.36-mRNA-1 | Indica | NA | | Os02g0594300 | | Ethylene-responsive transcription factor LEP |
| augustus-2-processed-gene-24.8-mRNA-1 | Indica | NA | | Os02g0143400 | OsR498G0202954000.01.T01 | Auxin-induced protein 6B |
| augustus-2-processed-gene-245.29-mRNA-1 | Indica | NA | | Os06g0272601 | OsR498G0204300200.01.T01 | dNK domain-containing protein |
| augustus-2-processed-gene-247.15-mRNA-1 | Indica | Indica | LRR-TM | Os02g0615300 | OsR498G0204309700.01.T01 | Putative LRR receptor-like serine/threonine-protein kinase (Fragment) |
| augustus-2-processed-gene-247.42-mRNA-1 | Indica | NA | | | OsR498G0204310000.01.T01 | |
| augustus-2-processed-gene-25.12-mRNA-1 | Indica | NA | | | OsR498G0202962100.01.T01 | |
| augustus-2-processed-gene-251.11-mRNA-1 | Indica | NA | LRR-TM | | OsR498G0204320700.01.T01 | Leucine-rich repeat-like protein |
| augustus-2-processed-gene-254.22-mRNA-1 | Indica | NA | | | OsR498G0204330800.01.T01 | |
| augustus-2-processed-gene-26.11-mRNA-1 | Indica | Indica | | Os02g0147300 | OsR498G0202966300.01.T03 | TOG domain-containing protein (Fragment) |
| augustus-2-processed-gene-26.25-mRNA-1 | Indica | NA | | Os02g0147600 | OsR498G0202967600.01.T01 | |
| augustus-2-processed-gene-262.50-mRNA-1 | Indica | NA | | Os02g0633200 | | Membrane-associated salt-inducible protein like |
| augustus-2-processed-gene-267.39-mRNA-1 | Indica | NA | | | OsR498G0204414300.01.T05 | Pentatricopeptide repeat-containing protein mitochondrial |
| augustus-2-processed-gene-30.34-mRNA-1 | Indica | NA | | Os02g0154600 | OsR498G0202991900.01.T04 | TPT domain-containing protein |
| augustus-2-processed-gene-30.39-mRNA-1 | Indica | NA | Kinase-LRR-TM | Os02g0153100 | OsR498G0202989000.01.T01 | Putative Phytosulfokine receptor |
| augustus-2-processed-gene-30.40-mRNA-1 | Indica | NA | Kinase-LRR-TM | Os02g0153200 | OsR498G0202989400.01.T01 | Putative Phytosulfokine receptor |
| augustus-2-processed-gene-30.41-mRNA-1 | Indica | NA | Kinase-LRR-TM | | | Putative leucine-rich repeat receptor-like kinase |
| augustus-2-processed-gene-30.42-mRNA-1 | Indica | Japonica | Kinase-LRR-TM | Os02g0153700 | OsR498G0202991600.01.T01 | Putative leucine-rich repeat receptor-like kinase |
| augustus-2-processed-gene-30.44-mRNA-1 | Indica | NA | Kinase-LRR-TM | Os02g0153900 | OsR498G0202990800.01.T01 | Putative leucine-rich repeat receptor-like kinase |
| augustus-2-processed-gene-31.11-mRNA-1 | Indica | NA | LRR-TM | Os02g0155700 | | Tyrosine-sulfated glycopeptide receptor 1 |
| augustus-2-processed-gene-31.8-mRNA-1 | Indica | Indica | | Os05g0523100 | OsR498G0202992400.01.T01 | |
| augustus-2-processed-gene-32.19-mRNA-1 | Indica | NA | | Os02g0158800 | OsR498G0203005900.01.T01 | Alpha-galactosidase |
| augustus-2-processed-gene-32.4-mRNA-1 | Indica | Indica | | Os08g0114000 | OsR498G0203003900.01.T01 | T-complex protein 1 subunit eta |
| augustus-2-processed-gene-33.34-mRNA-1 | Indica | NA | Kinase-TM | Os02g0161000 | OsR498G0203015000.01.T02 | Non-specific serine/threonine protein kinase |
| augustus-2-processed-gene-34.2-mRNA-1 | Indica | Japonica | LRR-TM | Os02g0161700 | OsR498G0203018700.01.T01 | LRR receptor-like serine/threonine-protein kinase FLS2 |
| augustus-2-processed-gene-37.63-mRNA-1 | Indica | NA | | Os02g0168500 | OsR498G0203045700.01.T03 | O-fucosyltransferase family protein (Fragment) |
| augustus-2-processed-gene-45.1-mRNA-1 | Indica | Indica | | Os02g0180200 | OsR498G0203091300.01.T03 | Nucleoid-associated protein chloroplastic |
| augustus-2-processed-gene-46.17-mRNA-1 | Indica | NA | | | OsR498G0203101000.01.T01 | Zinc finger protein 2 |
| augustus-2-processed-gene-49.25-mRNA-1 | Indica | NA | | Os02g0188600 | | |
| augustus-2-processed-gene-49.4-mRNA-1 | Indica | Indica | | Os02g0187100 | OsR498G0203117500.01.T02 | cyclase-like protein 3 |
| augustus-2-processed-gene-50.9-mRNA-1 | Indica | NA | | Os02g0189100 | OsR498G0203126600.01.T04 | Mediator of RNA polymerase II transcription subunit 11 |
| augustus-2-processed-gene-55.14-mRNA-1 | Indica | Indica | | Os06g0609700 | OsR498G0203157500.01.T04 | Alpha/beta-Hydrolases superfamily protein |
| augustus-2-processed-gene-55.22-mRNA-1 | Indica | NA | | | OsR498G0203154800.01.T01 | GRAS domain-containing protein (Fragment) |
| augustus-2-processed-gene-55.24-mRNA-1 | Indica | NA | | | OsR498G0203156600.01.T01 | Chlorophyll a-b binding protein, chloroplastic |
| augustus-2-processed-gene-95.22-mRNA-1 | Indica | NA | | | | Transposon protein, putative, CACTA, En/Spm sub-class |

| | | | | | | |
|---|---|---|---|---|---|---|
| augustus-2-processed-gene-98.19-mRNA-1 | Indica | Indica | | Os02g0270933 | OsR498G0203388600.01.T02 | Subtilisin-like protease |
| augustus-3-processed-gene-324.15-mRNA-1 | Indica | NA | | Os10g0379100 | OsR498G1018470900.01.T01 | Tryptamine hydroxycinnamoyl transferase |
| augustus-3-processed-gene-328.20-mRNA-1 | Indica | NA | | Os08g0381000 | OsR498G0916869000.01.T01 | Cell wall protein-like |
| augustus-4-processed-gene-1.21-mRNA-1 | Indica | Japonica | | Os04g0171600 | OsR498G0407629800.01.T01 | Tyrosine N-monooxygenase |
| augustus-4-processed-gene-104.14-mRNA-1 | Indica | Indica | | Os04g0341500 | OsR498G0408172900.01.T01 | (+)-delta-cadinene synthase isozyme A |
| augustus-4-processed-gene-111.34-mRNA-1 | Indica | NA | | Os04g0348600 | OsR498G0408222700.01.T01 | Response regulatory domain-containing protein (Fragment) |
| augustus-4-processed-gene-112.0-mRNA-1 | Indica | Indica | | Os04g0351333 | OsR498G0408226900.01.T02 | |
| augustus-4-processed-gene-118.13-mRNA-1 | Indica | Indica | | Os04g0361500 | OsR498G0408287300.01.T01 | L-gulonolactone oxidase |
| augustus-4-processed-gene-125.16-mRNA-1 | Indica | NA | | Os04g0370500 | OsR498G0408346500.01.T01 | F-box domain-containing protein |
| augustus-4-processed-gene-131.48-mRNA-1 | Indica | NA | | | OsR498G0408386900.01.T01 | |
| augustus-4-processed-gene-134.17-mRNA-1 | Indica | NA | | Os04g0384100 | | |
| augustus-4-processed-gene-134.2-mRNA-1 | Indica | NA | | Os04g0383700 | OsR498G0408405300.01.T01 | |
| augustus-4-processed-gene-134.4-mRNA-1 | Indica | NA | | | OsR498G0408405500.01.T01 | |
| augustus-4-processed-gene-135.0-mRNA-1 | Indica | NA | | | OsR498G0408414500.01.T01 | Branchpoint-bridging |
| augustus-4-processed-gene-135.3-mRNA-1 | Indica | Japonica | | Os04g0386300 | OsR498G0203898100.01.T01 | Protein SHOOT GRAVITROPISM 5 |
| augustus-4-processed-gene-136.6-mRNA-1 | Indica | Indica | | Os04g0387400 | OsR498G0408424300.01.T04 | MADS-box transcription factor 6 |
| augustus-4-processed-gene-137.2-mRNA-1 | Indica | Indica | | Os04g0388800 | OsR498G0408429700.01.T01 | J domain-containing protein (Fragment) |
| augustus-4-processed-gene-145.29-mRNA-1 | Indica | Japonica | | Os04g0402700 | OsR498G0407604900.01.T01 | |
| augustus-4-processed-gene-148.27-mRNA-1 | Indica | Indica | | Os04g0407500 | OsR498G0408513300.01.T01 | |
| augustus-4-processed-gene-148.28-mRNA-1 | Indica | NA | | Os04g0407800 | | Gibberellin 2-beta-dioxygenase 8 |
| augustus-4-processed-gene-148.59-mRNA-1 | Indica | NA | | Os04g0409500 | OsR498G0408518400.01.T04 | Metallophos domain-containing protein |
| augustus-4-processed-gene-149.46-mRNA-1 | Indica | NA | | Os04g0411200 | OsR498G0408521900.01.T01 | Protein LITTLE ZIPPER 4 isoform A |
| augustus-4-processed-gene-149.49-mRNA-1 | Indica | NA | | Os04g0411800 | OsR498G0408524100.01.T01 | AB hydrolase-1 domain-containing protein |
| augustus-4-processed-gene-149.70-mRNA-1 | Indica | NA | | Os04g0411400 | OsR498G0408522400.01.T01 | Centroradialis |
| augustus-4-processed-gene-150.1-mRNA-1 | Indica | NA | | Os04g0412100 | OsR498G0408525300.01.T01 | Ferredoxin (Fragment) |
| augustus-4-processed-gene-151.11-mRNA-1 | Indica | NA | | Os04g0414800 | OsR498G0408537500.01.T03 | Pentatricopeptide repeat-containing protein, mitochondrial |
| augustus-4-processed-gene-154.22-mRNA-1 | Indica | NA | Kinase-TM | Os04g0420600 | OsR498G0408563300.01.T01 | Non-specific serine/threonine protein kinase (Fragment) |
| augustus-4-processed-gene-159.17-mRNA-1 | Indica | NA | | Os04g0429800 | OsR498G0408584900.01.T04 | Nicotinate phosphoribosyltransferase |
| augustus-4-processed-gene-201.1-mRNA-1 | Indica | NA | | Os04g0508600 | OsR498G0408868100.01.T01 | Putative lung seven transmembrane receptor 1 |
| augustus-4-processed-gene-201.2-mRNA-1 | Indica | NA | | | OsR498G0408868500.01.T01 | |
| augustus-4-processed-gene-244.32-mRNA-1 | Indica | NA | | | | Poly(A) polymerase |
| augustus-4-processed-gene-254.28-mRNA-1 | Indica | Indica | | Os08g0224700 | OsR498G0409250600.01.T01 | 26S proteasome non-ATPase regulatory subunit 1 homolog |
| augustus-4-processed-gene-284.34-mRNA-1 | Indica | NA | | Os04g0663100 | OsR498G0409437500.01.T05 | Orphans transcription factor |
| augustus-4-processed-gene-285.29-mRNA-1 | Indica | Indica | | Os04g0664500 | OsR498G0409443100.01.T01 | Agmatine hydroxycinnamoyltransferase 1 |
| augustus-4-processed-gene-34.22-mRNA-1 | Indica | NA | | Os04g0225700 | OsR498G0407767500.01.T01 | BURP domain-containing protein 5 |
| augustus-4-processed-gene-37.12-mRNA-1 | Indica | NA | | | OsR498G0510283100.01.T01 | Splicing coactivator subunit-like |
| augustus-4-processed-gene-86.16-mRNA-1 | Indica | NA | | | OsR498G0408109200.01.T01 | SRF-type transcription factor family protein |
| augustus-5-processed-gene-141.59-mRNA-1 | Indica | NA | | Os05g0305700 | OsR498G0510414000.01.T01 | Homeobox protein SIX3-like isoform X2 |
| augustus-5-processed-gene-155.44-mRNA-1 | Indica | NA | | Os03g0110200 | OsR498G0101158000.01.T01 | Transposon protein, putative, Mutator sub-class, expressed |
| augustus-5-processed-gene-160.34-mRNA-1 | Indica | NA | | Os12g0468900 | OsR498G0713957300.01.T01 | Cell wall protein-like |
| augustus-5-processed-gene-161.11-mRNA-1 | Indica | NA | | | OsR498G0510526300.01.T01 | |
| augustus-5-processed-gene-161.19-mRNA-1 | Indica | NA | | Os05g0332000 | OsR498G0510526000.01.T01 | Putaive prolamin 7 |
| augustus-5-processed-gene-161.27-mRNA-1 | Indica | Indica | | Os05g0332600 | OsR498G0510530600.01.T01 | Adenine/guanine permease AZG1 |
| augustus-5-processed-gene-161.5-mRNA-1 | Indica | NA | | Os05g0332000 | OsR498G0510526000.01.T01 | Putaive prolamin 7 |
| augustus-5-processed-gene-162.23-mRNA-1 | Indica | Japonica | | Os05g0333000 | OsR498G1019239600.01.T06 | 60S ribosomal export protein NMD3 |
| augustus-5-processed-gene-164.6-mRNA-1 | Indica | Indica | | Os05g0335800 | OsR498G0510544200.01.T01 | Peptide transporter PTR3-A |
| augustus-5-processed-gene-173.12-mRNA-1 | Indica | NA | | | OsR498G0510603400.01.T01 | |
| augustus-5-processed-gene-173.13-mRNA-1 | Indica | Japonica | | Os05g0350900 | OsR498G0510603600.01.T01 | Tuber-specific and sucrose-responsive element binding factor |
| augustus-5-processed-gene-181.27-mRNA-1 | Indica | NA | | | OsR498G0510664200.01.T02 | |
| augustus-5-processed-gene-181.28-mRNA-1 | Indica | NA | | Os05g0363600 | OsR498G0510666200.01.T01 | Catalytic/ oxidoreductase, acting on NADH or NADPH |
| augustus-5-processed-gene-183.31-mRNA-1 | Indica | NA | | | | |
| augustus-5-processed-gene-183.53-mRNA-1 | Indica | NA | | Os12g0635700 | OsR498G1222170400.01.T01 | Elongation of fatty acids protein 3-like |
| augustus-5-processed-gene-187.11-mRNA-1 | Indica | NA | | Os05g0370000 | OsR498G0510689400.01.T01 | Pentatricopeptide repeat |
| augustus-5-processed-gene-187.34-mRNA-1 | Indica | Japonica | | Os05g0370300 | OsR498G0714930700.01.T01 | C2 domain-containing protein (Fragment) |
| augustus-5-processed-gene-190.25-mRNA-1 | Indica | NA | | | OsR498G0510708400.01.T01 | ABC-type lipoprotein export system, ATPase component |
| augustus-5-processed-gene-190.32-mRNA-1 | Indica | NA | | Os05g0375400 | OsR498G0510710800.01.T02 | Putative O-Glycosyl hydrolase superfamily protein |
| augustus-5-processed-gene-212.28-mRNA-1 | Indica | NA | | Os05g0412300 | | |
| augustus-5-processed-gene-223.35-mRNA-1 | Indica | NA | | Os05g0433000 | | Serine/threonine-protein kinase SAPK8 |
| augustus-5-processed-gene-25.28-mRNA-1 | Indica | NA | | Os05g0145100 | OsR498G0509725400.01.T01 | |
| augustus-5-processed-gene-281.27-mRNA-1 | Indica | NA | | | OsR498G0815745200.01.T01 | SWIM-type domain-containing protein |
| augustus-5-processed-gene-282.38-mRNA-1 | Indica | NA | | | OsR498G0917328500.01.T01 | Ribonuclease H |

| | | | | | | |
|---|---|---|---|---|---|---|
| augustus-5-processed-gene-286.59-mRNA-1 | Indica | NA | | Os05g0554100 | OsR498G0511312600.01.T01 | Ribosomal protein L7/L12 oligomerization |
| augustus-5-processed-gene-287.4-mRNA-1 | Indica | Indica | | Os01g0681900 | OsR498G0511318100.01.T02 | NADH-dependent glutamate synthase |
| augustus-5-processed-gene-288.20-mRNA-1 | Indica | NA | | Os05g0558000 | OsR498G0511325800.01.T01 | Ribosomal L1 domain-containing protein 1 |
| augustus-5-processed-gene-288.25-mRNA-1 | Indica | Indica | | Os10g0549850 | OsR498G0511327600.01.T01 | |
| augustus-5-processed-gene-292.53-mRNA-1 | Indica | NA | | | OsR498G0511350600.01.T01 | Ethylene-responsive transcription factor |
| augustus-5-processed-gene-292.54-mRNA-1 | Indica | NA | | Os05g0565500 | OsR498G0511352200.01.T01 | Putative 3-hydroxyisobutyrate dehydrogenase-like 3 mitochondrial |
| augustus-5-processed-gene-293.32-mRNA-1 | Indica | NA | | Os05g0567500 | OsR498G0511359200.01.T06 | ENDO3c domain-containing protein (Fragment) |
| augustus-5-processed-gene-293.4-mRNA-1 | Indica | NA | Kinase-TM | Os05g0566400 | OsR498G0511355000.01.T05 | Mitogen-activated protein kinase |
| augustus-5-processed-gene-293.5-mRNA-1 | Indica | NA | | Os05g0566500 | OsR498G0511356000.01.T01 | Eukaryotic translation initiation factor 3 subunit D |
| augustus-5-processed-gene-294.30-mRNA-1 | Indica | NA | | Os05g0568300 | OsR498G0511363600.01.T01 | Large subunit ribosomal protein L7/L12 |
| augustus-5-processed-gene-294.34-mRNA-1 | Indica | NA | | | OsR498G0511368000.01.T01 | CTP synthase (glutamine hydrolyzing) |
| augustus-5-processed-gene-295.46-mRNA-1 | Indica | NA | | Os05g0570300 | OsR498G0511370200.01.T01 | DUF295 domain-containing protein |
| augustus-5-processed-gene-296.27-mRNA-1 | Indica | NA | | Os05g0572900 | OsR498G0511376600.01.T01 | Pentatricopeptide repeat |
| augustus-5-processed-gene-298.25-mRNA-1 | Indica | NA | | Os05g0577500 | OsR498G0511394400.01.T01 | Probable calcium-binding protein CML14 |
| augustus-5-processed-gene-298.4-mRNA-1 | Indica | NA | | | OsR498G0511390200.01.T01 | Non-specific serine/threonine protein kinase |
| augustus-5-processed-gene-298.6-mRNA-1 | Indica | NA | | Os05g0576600 | OsR498G0511391600.01.T01 | J domain-containing protein |
| augustus-5-processed-gene-307.45-mRNA-1 | Indica | NA | | Os05g0594200 | OsR498G0511449400.01.T01 | Vacuolar cation/proton exchanger |
| augustus-5-processed-gene-46.28-mRNA-1 | Indica | NA | | Os05g0178400 | OsR498G0509853800.01.T01 | Serine/threonine protein phosphatase 2A regulatory subunit |
| augustus-5-processed-gene-56.19-mRNA-1 | Indica | Indica | | Os05g0192100 | OsR498G0509914200.01.T01 | Stem 28 kDa glycoprotein |
| augustus-5-processed-gene-66.22-mRNA-1 | Indica | Japonica | Kinase-TM | Os05g0208100 | OsR498G0816176500.01.T01 | Non-specific serine/threonine protein kinase |
| augustus-5-processed-gene-67.3-mRNA-1 | Indica | Indica | | Os01g0209700 | OsR498G0509994400.01.T02 | Gibberellin 2-beta-dioxygenase |
| augustus-5-processed-gene-71.21-mRNA-1 | Indica | NA | | Os05g0215700 | | |
| augustus-5-processed-gene-74.24-mRNA-1 | Indica | NA | | | OsR498G0510045400.01.T01 | |
| augustus-5-processed-gene-75.27-mRNA-1 | Indica | NA | | Os05g0223000 | OsR498G0510053600.01.T01 | Probable calcium-binding protein CML18 |
| augustus-5-processed-gene-75.5-mRNA-1 | Indica | Indica | | Os05g0217800 | OsR498G0510051200.01.T01 | BURP domain-containing protein 8 |
| augustus-6-processed-gene-299.7-mRNA-1 | Indica | NA | | Os06g0704400 | | Transposon protein, putative, mutator sub-class |
| augustus-6-processed-gene-309.53-mRNA-1 | Indica | NA | | | OsR498G0511427100.01.T01 | Epstein-Barr virus EBNA-1-like |
| augustus-6-processed-gene-44.67-mRNA-1 | Indica | NA | | Os06g0186400 | OsR498G0611742200.01.T02 | Carboxypeptidase |
| augustus-7-processed-gene-1.0-mRNA-1 | Indica | NA | | Os07g0101200 | OsR498G0713357100.01.T04 | BHLH domain-containing protein (Fragment) |
| augustus-7-processed-gene-10.16-mRNA-1 | Indica | NA | | Os07g0127500 | OsR498G0713445900.01.T01 | pathogenesis-related protein PR-1 |
| augustus-7-processed-gene-10.20-mRNA-1 | Indica | Indica | | Os01g0382400 | OsR498G0713447700.01.T01 | SCP domain-containing protein |
| augustus-7-processed-gene-10.4-mRNA-1 | Indica | NA | | | OsR498G1220887700.01.T01 | Hydroxyproline-rich glycoprotein-like |
| augustus-7-processed-gene-11.6-mRNA-1 | Indica | Indica | | Os07g0129200 | OsR498G0713449300.01.T01 | Pathogenesis-related protein class I (Fragment) |
| augustus-7-processed-gene-12.18-mRNA-1 | Indica | NA | Kinase-TM | Os07g0130100 | OsR498G0713460400.01.T01 | L-type lectin-domain containing receptor kinase IV.2 |
| augustus-7-processed-gene-12.19-mRNA-1 | Indica | NA | Kinase-TM | Os07g0130200 | OsR498G0713461200.01.T01 | Putative receptor kinase Lecrk |
| augustus-7-processed-gene-12.20-mRNA-1 | Indica | Japonica | Kinase-TM | Os07g0130300 | OsR498G0713999000.01.T01 | L-type lectin-domain containing receptor kinase IV.1 |
| augustus-7-processed-gene-12.25-mRNA-1 | Indica | NA | Kinase-TM | Os07g0130800 | OsR498G0713462000.01.T01 | Putative receptor kinase Lecrk |
| augustus-7-processed-gene-12.31-mRNA-1 | Indica | NA | Kinase-TM | Os07g0132150 | OsR498G0713464000.01.T01 | L-type lectin-domain containing receptor kinase IV.2 |
| augustus-7-processed-gene-13.14-mRNA-1 | Indica | NA | Kinase-TM | | OsR498G0713464300.01.T06 | Putative receptor-type protein kinase LRK1 |
| augustus-7-processed-gene-13.27-mRNA-1 | Indica | Indica | Kinase-TM | Os07g0130200 | OsR498G0713465300.01.T01 | Putative receptor-type protein kinase LRK1 |
| augustus-7-processed-gene-13.37-mRNA-1 | Indica | Indica | Kinase-TM | Os07g0133100 | OsR498G0713465800.01.T01 | Non-specific serine/threonine protein kinase |
| augustus-7-processed-gene-158.9-mRNA-1 | Indica | Japonica | | Os07g0460100 | OsR498G0509914200.01.T01 | Stem 28 kDa glycoprotein |
| augustus-7-processed-gene-181.23-mRNA-1 | Indica | NA | Kinase-TM | Os07g0493800 | OsR498G0714347500.01.T01 | Wall-associated protein kinase |
| augustus-7-processed-gene-182.53-mRNA-1 | Indica | NA | | Os07g0496300 | | protein FAR-RED IMPAIRED RESPONSE 1-like |
| augustus-7-processed-gene-184.26-mRNA-1 | Indica | Indica | | Os02g0452800 | OsR498G0714367900.01.T01 | DUF295 domain-containing protein |
| augustus-7-processed-gene-184.42-mRNA-1 | Indica | NA | | Os07g0499900 | | DUF295 domain-containing protein |
| augustus-7-processed-gene-184.5-mRNA-1 | Indica | NA | | | | |
| augustus-7-processed-gene-193.58-mRNA-1 | Indica | Japonica | | Os07g0514801 | OsR498G0306608800.01.T06 | |
| augustus-7-processed-gene-198.23-mRNA-1 | Indica | NA | CC-NBS-TM | Os07g0521800 | OsR498G0714451300.01.T01 | Putative resistance complex protein I2C-2 |
| augustus-7-processed-gene-200.18-mRNA-1 | Indica | NA | | Os07g0526600 | OsR498G0714472500.01.T01 | Putative carboxylesterase 2 |
| augustus-7-processed-gene-201.21-mRNA-1 | Indica | NA | | | OsR498G0714474500.01.T01 | |
| augustus-7-processed-gene-204.10-mRNA-1 | Indica | NA | | | OsR498G0204046800.01.T01 | Epstein-Barr virus EBNA-1-like |
| augustus-7-processed-gene-205.42-mRNA-1 | Indica | NA | | Os07g0533800 | OsR498G0714494400.01.T01 | Putative nucleoid DNA-binding protein cnd41 |
| augustus-7-processed-gene-206.31-mRNA-1 | Indica | NA | | | OsR498G0714501300.01.T02 | Gnk2-homologous domain-containing protein (Fragment) |
| augustus-7-processed-gene-208.7-mRNA-1 | Indica | NA | Kinase-TM | | | Receptor-like serine/threonine-protein kinase |
| augustus-7-processed-gene-216.24-mRNA-1 | Indica | NA | | | OsR498G0714552500.01.T01 | F-box domain-containing protein |
| augustus-7-processed-gene-216.35-mRNA-1 | Indica | Japonica | | Os07g0554200 | OsR498G0714554100.01.T01 | F-box domain-containing protein |
| augustus-7-processed-gene-216.36-mRNA-1 | Indica | NA | | | | NAM-associated domain-containing protein |
| augustus-7-processed-gene-231.4-mRNA-1 | Indica | NA | | Os07g0577300 | OsR498G0714622900.01.T06 | Glucan endo-1,3-beta-D-glucosidase |
| augustus-7-processed-gene-232.24-mRNA-1 | Indica | NA | | Os10g0460000 | OsR498G0204348500.01.T01 | |
| augustus-7-processed-gene-241.42-mRNA-1 | Indica | Indica | | Os12g0230600 | OsR498G0714697900.01.T01 | |

| | | | | | | |
|---|---|---|---|---|---|---|
| augustus-7-processed-gene-34.14-mRNA-1 | Indica | NA | | Os07g0172600 | OsR498G0713602400.01.T01 | Pentatricopeptide repeat-containing protein103 |
| augustus-7-processed-gene-35.1-mRNA-1 | Indica | Indica | | Os07g0174700 | OsR498G0713607100.01.T02 | Constitutive photomorphogenesis protein 10 |
| augustus-7-processed-gene-5.16-mRNA-1 | Indica | NA | | | OsR498G0713409700.01.T01 | DUF4220 domain-containing protein (Fragment) |
| augustus-8-processed-gene-102.30-mRNA-1 | Indica | NA | | | OsR498G0306743300.01.T01 | Transposon protein, putative, CACTA, En/Spm sub-class |
| augustus-8-processed-gene-103.47-mRNA-1 | Indica | NA | LRR-TM | Os08g0266400 | | Putative somatic embryogenesis protein kinase 1 |
| augustus-8-processed-gene-104.16-mRNA-1 | Indica | NA | | Os08g0267000 | OsR498G0815588200.01.T01 | FBD domain-containing protein |
| augustus-8-processed-gene-122.25-mRNA-1 | Indica | NA | | | | |
| augustus-8-processed-gene-131.7-mRNA-1 | Indica | NA | | Os08g0297500 | | |
| augustus-8-processed-gene-156.20-mRNA-1 | Indica | NA | | | OsR498G0102174700.01.T01 | Transposon protein, putative, unclassified |
| augustus-8-processed-gene-164.30-mRNA-1 | Indica | NA | | | | |
| augustus-8-processed-gene-165.22-mRNA-1 | Indica | NA | | Os08g0351300 | | |
| augustus-8-processed-gene-165.3-mRNA-1 | Indica | NA | | | OsR498G0815866000.01.T01 | |
| augustus-8-processed-gene-178.22-mRNA-1 | Indica | NA | | | OsR498G0713579700.01.T01 | Hydroxyproline-rich glycoprotein-like |
| augustus-8-processed-gene-189.5-mRNA-1 | Indica | NA | NBS-TM | Os08g0373400 | | CC-NBS-LRR type disease resistance protein NLR1-V |
| augustus-8-processed-gene-190.23-mRNA-1 | Indica | NA | | | OsR498G0815948900.01.T01 | Transposon protein, putative, CACTA, En/Spm sub-class |
| augustus-8-processed-gene-192.14-mRNA-1 | Indica | NA | | Os08g0378800 | OsR498G0815961000.01.T01 | SAM domain-containing protein (Fragment) |
| augustus-8-processed-gene-192.16-mRNA-1 | Indica | NA | | Os08g0379000 | OsR498G0815961400.01.T05 | Nuclear factor of activated T-cells 5 isoform X2 |
| augustus-8-processed-gene-194.33-mRNA-1 | Indica | NA | | | | Transposon protein, putative, Mutator sub-class, expressed |
| augustus-8-processed-gene-215.20-mRNA-1 | Indica | NA | | Os08g0421850 | OsR498G0816100800.01.T01 | RING-type E3 ubiquitin transferase |
| augustus-8-processed-gene-221.59-mRNA-1 | Indica | NA | | Os04g0184800 | | Transposon protein, putative, Mutator sub-class |
| augustus-8-processed-gene-223.31-mRNA-1 | Indica | Indica | | Os08g0434300 | OsR498G0816151600.01.T02 | Malate dehydrogenase |
| augustus-8-processed-gene-231.5-mRNA-1 | Indica | NA | Kinase-LRR-TM | Os08g0446200 | OsR498G0816198200.01.T01 | Leucine-rich repeat receptor-like protein kinase PEPR1 |
| augustus-8-processed-gene-276.62-mRNA-1 | Indica | NA | | Os07g0513300 | OsR498G0102262600.01.T01 | Transposable element protein, putative, MuDR, expressed |
| augustus-8-processed-gene-32.23-mRNA-1 | Indica | NA | | Os08g0152600 | OsR498G0815203800.01.T01 | Protein enhanced disease resistance 2 |
| augustus-8-processed-gene-33.7-mRNA-1 | Indica | Indica | | Os08g0153600 | OsR498G0815207400.01.T04 | Pentatricopeptide repeat-containing protein mitochondrial |
| augustus-8-processed-gene-33.8-mRNA-1 | Indica | NA | | Os08g0153800 | OsR498G0815207800.01.T01 | TRP-like ion channel pkd2 |
| augustus-8-processed-gene-36.22-mRNA-1 | Indica | NA | | Os08g0158200 | OsR498G0815235000.01.T01 | Reticuline oxidase |
| augustus-8-processed-gene-41.17-mRNA-1 | Indica | NA | | | OsR498G0204368800.01.T01 | N-acetylmuramoyl-L-alanine amidase |
| augustus-8-processed-gene-62.12-mRNA-1 | Indica | NA | | Os08g0203900 | OsR498G0815388600.01.T01 | GDP-mannose transporter GONST4 |
| augustus-9-processed-gene-100.5-mRNA-1 | Indica | NA | | | OsR498G0917167800.01.T01 | |
| augustus-9-processed-gene-103.24-mRNA-1 | Indica | Japonica | | Os09g0333600 | OsR498G0917178000.01.T05 | Pleiotropic drug resistance protein 4 |
| augustus-9-processed-gene-132.23-mRNA-1 | Indica | NA | | Os09g0384900 | OsR498G0917335000.01.T01 | DUF295 domain-containing protein (Fragment) |
| augustus-9-processed-gene-133.2-mRNA-1 | Indica | NA | | | | Zinc finger transcription factor |
| augustus-9-processed-gene-139.32-mRNA-1 | Indica | NA | | Os09g0395400 | | |
| augustus-9-processed-gene-141.32-mRNA-1 | Indica | NA | Kinase-LRR-TM | Os09g0400500 | OsR498G0917406000.01.T01 | Atypical receptor-like kinase MARK |
| augustus-9-processed-gene-144.32-mRNA-1 | Indica | NA | | Os09g0407800 | OsR498G0917422800.01.T01 | Pentatricopeptide repeat-containing protein chloroplastic |
| augustus-9-processed-gene-70.14-mRNA-1 | Indica | NA | | | | Transposon protein, putative, Mutator sub-class, expressed |
| augustus-9-processed-gene-71.15-mRNA-1 | Indica | NA | | | OsR498G1018968000.01.T01 | ATP-dependent DNA helicase |
| augustus-9-processed-gene-72.22-mRNA-1 | Indica | NA | | | OsR498G0204768400.01.T01 | Epstein-Barr virus EBNA-1-like |
| augustus_masked-1-processed-gene-16.17-mRNA-1 | Indica | NA | | Os01g0130900 | OsR498G0100106000.01.T01 | Mediator of RNA polymerase II transcription subunit 25 (Fragment) |
| augustus_masked-1-processed-gene-17.43-mRNA-1 | Indica | NA | | Os01g0132200 | OsR498G0100112800.01.T01 | Translation initiation factor IF-2-like |
| augustus_masked-1-processed-gene-17.46-mRNA-1 | Indica | NA | | Os01g0132766 | | Yin/yang transcription factor |
| augustus_masked-1-processed-gene-19.51-mRNA-1 | Indica | NA | | Os01g0136300 | OsR498G0100124800.01.T01 | |
| augustus_masked-1-processed-gene-19.53-mRNA-1 | Indica | NA | | | OsR498G0100125400.01.T01 | Protein kinase domain-containing protein |
| augustus_masked-1-processed-gene-19.55-mRNA-1 | Indica | NA | | | | Protein kinase domain-containing protein |
| augustus_masked-1-processed-gene-20.51-mRNA-1 | Indica | NA | | | | DDE Tnp4 domain-containing protein (Fragment) |
| augustus_masked-1-processed-gene-21.41-mRNA-1 | Indica | NA | | | OsR498G0100133400.01.T01 | |
| augustus_masked-1-processed-gene-26.22-mRNA-1 | Indica | NA | | Os01g0148500 | OsR498G0100175200.01.T01 | |
| augustus_masked-1-processed-gene-29.44-mRNA-1 | Indica | Japonica | | Os01g0154900 | OsR498G0100193100.01.T01 | Putative carboxylesterase 15 |
| augustus_masked-1-processed-gene-29.45-mRNA-1 | Indica | NA | | Os01g0155000 | OsR498G0100195800.01.T01 | Putative carboxylesterase 15 |
| augustus_masked-1-processed-gene-30.82-mRNA-1 | Indica | NA | | | OsR498G0100202400.01.T01 | |
| augustus_masked-1-processed-gene-30.85-mRNA-1 | Indica | NA | | Os01g0158100 | OsR498G0100204400.01.T01 | protein THYLAKOID ASSEMBLY 8, chloroplastic-like |
| augustus_masked-1-processed-gene-31.28-mRNA-1 | Indica | Indica | | Os01g0159200 | OsR498G0100209000.01.T03 | FAS1 domain-containing protein (Fragment) |
| augustus_masked-1-processed-gene-35.34-mRNA-1 | Indica | NA | | Os01g0168100 | OsR498G0100225000.01.T04 | NAB domain-containing protein (Fragment) |
| augustus_masked-1-processed-gene-35.53-mRNA-1 | Indica | NA | | Os01g0169400 | OsR498G0100232000.01.T02 | Ramosa 2 |
| augustus_masked-1-processed-gene-350.13-mRNA-1 | Indica | NA | | | OsR498G0102184300.01.T01 | Anaphase-promoting complex subunit 5 |
| augustus_masked-1-processed-gene-353.62-mRNA-1 | Indica | NA | | | | 60S ribosomal protein L30 |
| augustus_masked-1-processed-gene-353.78-mRNA-1 | Indica | NA | | Os01g0812900 | OsR498G0102199000.01.T01 | Protein RETICULATA-RELATED 3 chloroplastic |
| augustus_masked-1-processed-gene-354.66-mRNA-1 | Indica | NA | | Os01g0815900 | OsR498G0102207400.01.T02 | Pentatricopeptide repeat-containing protein mitochondrial |
| augustus_masked-1-processed-gene-356.51-mRNA-1 | Indica | NA | | | OsR498G0102215800.01.T02 | Pentatricopeptide repeat-containing protein |
| augustus_masked-1-processed-gene-359.33-mRNA-1 | Indica | NA | | Os01g0823700 | OsR498G0102239000.01.T04 | DUF641 domain-containing protein |

| | | | | | | |
|---|---|---|---|---|---|---|
| augustus_masked-1-processed-gene-359.34-mRNA-1 | Indica | NA | | Os01g0823900 | OsR498G0102239800.01.T01 | RING-type E3 ubiquitin transferase |
| augustus_masked-1-processed-gene-359.9-mRNA-1 | Indica | NA | | Os01g0822900 | OsR498G0102234200.01.T02 | Non-specific lipid-transfer protein (Fragment) |
| augustus_masked-1-processed-gene-362.22-mRNA-1 | Indica | NA | | Os01g0829600 | | |
| augustus_masked-1-processed-gene-362.25-mRNA-1 | Indica | NA | | Os01g0830200 | OsR498G0102261800.01.T03 | E3 ubiquitin-protein ligase RNF5 |
| augustus_masked-1-processed-gene-362.28-mRNA-1 | Indica | NA | | | OsR498G0102265800.01.T01 | Ring finger protein 13 (Fragment) |
| augustus_masked-1-processed-gene-365.30-mRNA-1 | Indica | NA | | | OsR498G0102280600.01.T01 | |
| augustus_masked-1-processed-gene-366.27-mRNA-1 | Indica | NA | | Os01g0836800 | OsR498G0102289000.01.T01 | Lung seven transmembrane receptor family protein |
| augustus_masked-1-processed-gene-366.31-mRNA-1 | Indica | NA | | Os01g0837800 | OsR498G0102292400.01.T02 | Metal tolerance protein 5 |
| augustus_masked-1-processed-gene-366.5-mRNA-1 | Indica | NA | | | OsR498G0102287000.01.T01 | |
| augustus_masked-1-processed-gene-367.40-mRNA-1 | Indica | NA | | Os01g0838200 | OsR498G0102294600.01.T01 | Protein kinase domain-containing protein |
| augustus_masked-1-processed-gene-367.41-mRNA-1 | Indica | NA | | Os01g0838600 | OsR498G0102296000.01.T01 | Zinc finger transcription factor ZFP19 |
| augustus_masked-1-processed-gene-367.56-mRNA-1 | Indica | NA | | Os07g0433200 | OsR498G0510328900.01.T01 | Retrotransposon protein, putative, Ty1-copia subclass |
| augustus_masked-1-processed-gene-367.57-mRNA-1 | Indica | NA | | | OsR498G0510328900.01.T01 | |
| augustus_masked-1-processed-gene-367.60-mRNA-1 | Indica | Japonica | | Os01g0839500 | OsR498G0305687300.01.T01 | |
| augustus_masked-1-processed-gene-368.24-mRNA-1 | Indica | NA | | Os01g0840200 | OsR498G0102302600.01.T01 | Putative translation initiation factor IF-2 |
| augustus_masked-1-processed-gene-470.25-mRNA-1 | Indica | NA | | Os01g0905800 | OsR498G0102538200.01.T01 | Fructose-bisphosphate aldolase |
| augustus_masked-1-processed-gene-470.30-mRNA-1 | Indica | NA | | Os01g0906425 | OsR498G0102541200.01.T01 | |
| augustus_masked-1-processed-gene-472.64-mRNA-1 | Indica | NA | | Os01g0909400 | OsR498G0102552000.01.T01 | DUF868 domain-containing protein |
| augustus_masked-1-processed-gene-473.25-mRNA-1 | Indica | NA | | | | Transcription factor BTF3 |
| augustus_masked-1-processed-gene-474.24-mRNA-1 | Indica | NA | | | OsR498G0102558800.01.T01 | |
| augustus_masked-1-processed-gene-491.62-mRNA-1 | Indica | NA | | Os01g0941200 | OsR498G0102675100.01.T01 | Glucan endo-1,3-beta-glucosidase isoenzyme I |
| augustus_masked-1-processed-gene-7.51-mRNA-1 | Indica | NA | | Os01g0112100 | OsR498G0100039200.01.T02 | |
| augustus_masked-1-processed-gene-7.53-mRNA-1 | Indica | NA | | Os01g0112400 | OsR498G0100040400.01.T02 | probable aquaporin NIP-type |
| augustus_masked-1-processed-gene-8.12-mRNA-1 | Indica | NA | Kinase-TM | Os01g0113400 | OsR498G0100045000.01.T04 | Cysteine-rich receptor-like protein kinase 37 |
| augustus_masked-1-processed-gene-81.28-mRNA-1 | Indica | NA | | Os01g0246100 | OsR498G0100539200.01.T05 | Histone acetyltransferase |
| augustus_masked-1-processed-gene-9.31-mRNA-1 | Indica | Indica | Kinase-TM | Os01g0117400 | OsR498G0100056000.01.T03 | Receptor-like kinase |
| augustus_masked-1-processed-gene-9.4-mRNA-1 | Indica | NA | Kinase-TM | Os01g0115700 | OsR498G0100051200.01.T01 | Receptor-like kinase |
| augustus_masked-10-processed-gene-1.26-mRNA-1 | Indica | Japonica | | Os10g0100700 | OsR498G0713353000.01.T01 | Pyridoxal 5'-phosphate synthase subunit PDX1 |
| augustus_masked-10-processed-gene-112.18-mRNA-1 | Indica | NA | | Os10g0444700 | OsR498G1018804000.01.T01 | H(+)/Pi cotransporter |
| augustus_masked-10-processed-gene-122.15-mRNA-1 | Indica | NA | | Os04g0156200 | OsR498G0407574100.01.T01 | |
| augustus_masked-10-processed-gene-122.33-mRNA-1 | Indica | NA | | | OsR498G0407569700.01.T01 | DUF4220 domain-containing protein (Fragment) |
| augustus_masked-10-processed-gene-128.17-mRNA-1 | Indica | NA | | Os09g0543400 | OsR498G0407525900.01.T01 | Diaminopimelate decarboxylase 2 chloroplastic |
| augustus_masked-10-processed-gene-131.8-mRNA-1 | Indica | NA | | Os04g0125700 | OsR498G0407519500.01.T01 | Putative receptor-type protein kinase LRK1 |
| augustus_masked-10-processed-gene-142.16-mRNA-1 | Indica | NA | | | | |
| augustus_masked-10-processed-gene-148.16-mRNA-1 | Indica | NA | Kinase-TM | Os04g0103500 | OsR498G0407421200.01.T01 | G-type lectin S-receptor-like serine/threonine-protein kinase |
| augustus_masked-10-processed-gene-155.25-mRNA-1 | Indica | NA | | Os10g0451700 | OsR498G1018827600.01.T01 | Transposon protein, putative, CACTA, En/Spm sub-class, expressed |
| augustus_masked-10-processed-gene-156.11-mRNA-1 | Indica | NA | | Os10g0453200 | | Putative retrotransposon protein ty3-gypsy subclass |
| augustus_masked-10-processed-gene-156.8-mRNA-1 | Indica | NA | | Os10g0452900 | OsR498G1018830200.01.T01 | Glycine-rich cell wall structural protein 2 |
| augustus_masked-10-processed-gene-159.40-mRNA-1 | Indica | NA | | Os10g0459700 | OsR498G1018848000.01.T01 | Xyloglucan galactosyltransferase KATAMARI1 |
| augustus_masked-10-processed-gene-161.6-mRNA-1 | Indica | NA | | | OsR498G1018855600.01.T01 | |
| augustus_masked-10-processed-gene-25.16-mRNA-1 | Indica | NA | LRR-NBS-TM | Os10g0136100 | | NB-ARC domain containing protein, expressed |
| augustus_masked-10-processed-gene-27.39-mRNA-1 | Indica | NA | | Os10g0139700 | OsR498G1018117600.01.T01 | Cytochrome P450 family 87 subfamily A polypeptide 6 |
| augustus_masked-10-processed-gene-28.25-mRNA-1 | Indica | NA | | Os10g0140700 | OsR498G1018118600.01.T01 | |
| augustus_masked-10-processed-gene-28.27-mRNA-1 | Indica | NA | | Os10g0140300 | OsR498G1018119400.01.T01 | GDSL esterase/lipase |
| augustus_masked-10-processed-gene-31.37-mRNA-1 | Indica | NA | | Os10g0147900 | OsR498G1018144800.01.T02 | Armadillo/beta-catenin-like repeat family protein, expressed |
| augustus_masked-10-processed-gene-38.31-mRNA-1 | Indica | Japonica | Kinase | Os10g0157400 | OsR498G1018162400.01.T01 | Putative cyclin-dependent kinase F-2 |
| augustus_masked-10-processed-gene-39.19-mRNA-1 | Indica | NA | | Os10g0158100 | OsR498G1018179200.01.T01 | Very-long-chain 3-oxoacyl-CoA synthase |
| augustus_masked-10-processed-gene-41.11-mRNA-1 | Indica | NA | | Os10g0160100 | OsR498G1018192000.01.T01 | Glucan endo-1,3-beta-glucosidase 7 |
| augustus_masked-10-processed-gene-5.20-mRNA-1 | Indica | NA | Kinase-TM | Os10g0104800 | OsR498G1018012400.01.T01 | Proline-rich receptor-like protein kinase PERK4 |
| augustus_masked-10-processed-gene-50.39-mRNA-1 | Indica | NA | | Os12g0484600 | OsR498G0307220700.01.T01 | Nodulin-like domain-containing protein (Fragment) |
| augustus_masked-10-processed-gene-51.21-mRNA-1 | Indica | Indica | | Os11g0708500 | OsR498G1018251600.01.T01 | Pyridoxal biosynthesis lyase pdxS |
| augustus_masked-10-processed-gene-54.31-mRNA-1 | Indica | Japonica | Kinase | Os10g0174800 | OsR498G1018205800.01.T02 | Wall-associated receptor kinase 2 |
| augustus_masked-10-processed-gene-66.35-mRNA-1 | Indica | NA | | | OsR498G1018333000.01.T01 | PMD domain-containing protein |
| augustus_masked-10-processed-gene-68.13-mRNA-1 | Indica | NA | | | OsR498G0510213600.01.T01 | |
| augustus_masked-10-processed-gene-87.19-mRNA-1 | Indica | Indica | | Os10g0213700 | OsR498G1018399800.01.T01 | Acidic leucine-rich nuclear phosphoprotein 32-related protein |
| augustus_masked-10-processed-gene-92.4-mRNA-1 | Indica | NA | | | OsR498G0203809100.01.T01 | DUF834 domain-containing protein |
| augustus_masked-11-processed-gene-252.24-mRNA-1 | Indica | Indica | | Os11g0683800 | OsR498G1120730400.01.T01 | Pectinesterase |
| augustus_masked-11-processed-gene-253.37-mRNA-1 | Indica | NA | NBS-TM | Os11g0686900 | OsR498G1120736800.01.T01 | NBS-LRR disease resistance protein homologue-like |
| augustus_masked-11-processed-gene-255.23-mRNA-1 | Indica | NA | | | OsR498G0511458200.01.T01 | |
| augustus_masked-11-processed-gene-259.30-mRNA-1 | Indica | NA | | Os10g0192300 | OsR498G0306363500.01.T01 | ATP-dependent DNA helicase |
| augustus_masked-11-processed-gene-69.1-mRNA-1 | Indica | NA | | | OsR498G0305447100.01.T01 | |

| | | | | | | |
|---|---|---|---|---|---|---|
| augustus_masked-11-processed-gene-83.18-mRNA-1 | Indica | NA | CC-LRR-NBS-TM | | | NBS-LRR type protein (Fragment) |
| augustus_masked-11-processed-gene-84.45-mRNA-1 | Indica | Indica | | Os11g0231400 | OsR498G1119654600.01.T01 | F-box domain containing protein-like |
| augustus_masked-12-processed-gene-113.3-mRNA-1 | Indica | NA | | Os12g0288400 | OsR498G1221338800.01.T02 | E3 ubiquitin-protein ligase RGLG1 |
| augustus_masked-12-processed-gene-117.13-mRNA-1 | Indica | NA | | Os12g0295600 | | Thioredoxin M-type |
| augustus_masked-12-processed-gene-148.19-mRNA-1 | Indica | NA | | | OsR498G1221454800.01.T01 | |
| augustus_masked-12-processed-gene-150.5-mRNA-1 | Indica | NA | | | OsR498G0511427100.01.T01 | Epstein-Barr virus EBNA-1-like |
| augustus_masked-2-processed-gene-167.87-mRNA-1 | Indica | NA | | | OsR498G0203808600.01.T01 | |
| augustus_masked-3-processed-gene-162.61-mRNA-1 | Indica | NA | | | OsR498G0203810500.01.T01 | |
| augustus_masked-3-processed-gene-180.60-mRNA-1 | Indica | NA | | Os03g0370250 | OsR498G0306020800.01.T01 | GeBP transcription factor |
| augustus_masked-3-processed-gene-185.22-mRNA-1 | Indica | NA | | Os03g0379500 | OsR498G0305248900.01.T01 | 30S ribosomal protein S4, chloroplastic |
| augustus_masked-3-processed-gene-192.19-mRNA-1 | Indica | NA | | Os03g0392300 | OsR498G0306092300.01.T01 | GTP-binding protein 1 isoform 2 |
| augustus_masked-3-processed-gene-192.29-mRNA-1 | Indica | Indica | | Os04g0465100 | OsR498G0306091600.01.T01 | Peroxidase |
| augustus_masked-3-processed-gene-192.30-mRNA-1 | Indica | NA | | Os03g0392400 | OsR498G0306092800.01.T02 | J domain-containing protein |
| augustus_masked-3-processed-gene-197.1-mRNA-1 | Indica | NA | | Os03g0397500 | OsR498G0306113900.01.T02 | Pre-mRNA-processing factor |
| augustus_masked-3-processed-gene-198.17-mRNA-1 | Indica | Japonica | | Os03g0399800 | OsR498G0306125100.01.T01 | Mannose-binding lectin |
| augustus_masked-3-processed-gene-199.47-mRNA-1 | Indica | NA | | Os03g0401200 | OsR498G0306131600.01.T01 | J domain-containing protein |
| augustus_masked-3-processed-gene-265.45-mRNA-1 | Indica | NA | | | OsR498G1018686900.01.T02 | Exonuclease family protein, expressed |
| augustus_masked-3-processed-gene-283.22-mRNA-1 | Indica | NA | | Os10g0397800 | OsR498G1018652000.01.T02 | Solute carrier family 25 member 44 |
| augustus_masked-3-processed-gene-299.2-mRNA-1 | Indica | NA | | Os10g0374200 | OsR498G1018572600.01.T01 | |
| augustus_masked-3-processed-gene-302.15-mRNA-1 | Indica | Indica | | Os03g0193000 | OsR498G1018558000.01.T02 | GRAS domain-containing protein (Fragment) |
| augustus_masked-3-processed-gene-302.25-mRNA-1 | Indica | NA | | Os10g0369500 | OsR498G1018557800.01.T01 | Yippee domain-containing protein |
| augustus_masked-3-processed-gene-302.27-mRNA-1 | Indica | NA | | Os10g0369000 | OsR498G1018557000.01.T01 | Glutamyl-tRNA synthetase |
| augustus_masked-3-processed-gene-304.34-mRNA-1 | Indica | NA | | Os10g0363600 | OsR498G1018549300.01.T04 | F-box/kelch-repeat protein At1g22040 |
| augustus_masked-3-processed-gene-306.0-mRNA-1 | Indica | NA | | Os10g0362300 | OsR498G1018542200.01.T02 | Histidine kinase |
| augustus_masked-3-processed-gene-306.14-mRNA-1 | Indica | NA | | Os10g0361350 | OsR498G1018537100.01.T01 | PLAT domain-containing protein (Fragment) |
| augustus_masked-3-processed-gene-306.15-mRNA-1 | Indica | NA | | | OsR498G1018537500.01.T01 | |
| augustus_masked-3-processed-gene-306.16-mRNA-1 | Indica | NA | | Os10g0361000 | OsR498G1018537100.01.T01 | PLAT domain-containing protein (Fragment) |
| augustus_masked-3-processed-gene-308.31-mRNA-1 | Indica | Indica | | Os03g0199100 | OsR498G1018531100.01.T01 | |
| augustus_masked-3-processed-gene-311.17-mRNA-1 | Indica | NA | | | | Nucleoside phosphatase |
| augustus_masked-3-processed-gene-312.16-mRNA-1 | Indica | NA | | Os10g0348600 | OsR498G1018516200.01.T01 | DUF4220 domain-containing protein (Fragment) |
| augustus_masked-3-processed-gene-326.32-mRNA-1 | Indica | NA | | Os10g0327800 | OsR498G1018453300.01.T03 | Tyrosine-sulfated glycopeptide receptor 1 |
| augustus_masked-3-processed-gene-327.12-mRNA-1 | Indica | NA | | Os10g0326400 | OsR498G1018446200.01.T01 | F-box domain containing protein, expressed |
| augustus_masked-3-processed-gene-392.5-mRNA-1 | Indica | NA | | | OsR498G0306695700.01.T01 | |
| augustus_masked-3-processed-gene-462.38-mRNA-1 | Indica | NA | | | OsR498G0307158400.01.T01 | Pentatricopeptide repeat-containing protein (Fragment) |
| augustus_masked-4-processed-gene-0.19-mRNA-1 | Indica | NA | | Os04g0169500 | OsR498G0407624000.01.T02 | Putative oxaloacetate decarboxylase gamma chain |
| augustus_masked-4-processed-gene-1.39-mRNA-1 | Indica | Japonica | | Os04g0172400 | OsR498G0407698300.01.T01 | Acyl transferase 15 |
| augustus_masked-4-processed-gene-111.43-mRNA-1 | Indica | NA | | | | U11/U12 small nuclear ribonucleoprotein 48 kDa protein |
| augustus_masked-4-processed-gene-112.15-mRNA-1 | Indica | Indica | | Os04g0350100 | OsR498G0408228000.01.T01 | Cysteine proteinase inhibitor B |
| augustus_masked-4-processed-gene-126.20-mRNA-1 | Indica | NA | | | OsR498G0714707400.01.T01 | |
| augustus_masked-4-processed-gene-133.17-mRNA-1 | Indica | NA | | Os04g0382900 | OsR498G0408404100.01.T01 | |
| augustus_masked-4-processed-gene-136.25-mRNA-1 | Indica | NA | | Os04g0387600 | OsR498G0408425200.01.T01 | |
| augustus_masked-4-processed-gene-148.20-mRNA-1 | Indica | NA | | | OsR498G0408512800.01.T01 | IQ calmodulin-binding motif family protein, expressed |
| augustus_masked-4-processed-gene-150.28-mRNA-1 | Indica | NA | | Os04g0412400 | | |
| augustus_masked-4-processed-gene-153.9-mRNA-1 | Indica | NA | | | OsR498G0408553000.01.T01 | Protein kinase domain-containing protein |
| augustus_masked-4-processed-gene-154.33-mRNA-1 | Indica | Japonica | Kinase-TM | Os04g0420900 | OsR498G0408563300.01.T01 | Non-specific serine/threonine protein kinase (Fragment) |
| augustus_masked-4-processed-gene-173.34-mRNA-1 | Indica | NA | | | OsR498G0408681600.01.T02 | Sugar transport protein 5 |
| augustus_masked-4-processed-gene-176.60-mRNA-1 | Indica | NA | | | OsR498G0408694600.01.T01 | |
| augustus_masked-4-processed-gene-204.64-mRNA-1 | Indica | NA | | Os04g0516200 | OsR498G0408895000.01.T03 | ALOG domain transcriptional regulator family protein 4-2A |
| augustus_masked-4-processed-gene-209.41-mRNA-1 | Indica | NA | | Os04g0523600 | OsR498G0408924200.01.T01 | Glycosyltransferase |
| augustus_masked-4-processed-gene-209.42-mRNA-1 | Indica | NA | | Os04g0523700 | OsR498G0408924600.01.T01 | Glycosyltransferase |
| augustus_masked-4-processed-gene-22.27-mRNA-1 | Indica | Indica | Kinase-TM | Os04g0202350 | OsR498G0407718200.01.T01 | Non-specific serine/threonine protein kinase |
| augustus_masked-4-processed-gene-267.34-mRNA-1 | Indica | NA | | | OsR498G0511458200.01.T01 | |
| augustus_masked-4-processed-gene-285.35-mRNA-1 | Indica | NA | | Os04g0664600 | OsR498G0409443400.01.T01 | Agmatine hydroxycinnamoyltransferase 1 |
| augustus_masked-4-processed-gene-29.12-mRNA-1 | Indica | Japonica | CC-NBS-TM | Os04g0219600 | OsR498G1119645400.01.T01 | NBS-LRR type protein (Fragment) |
| augustus_masked-4-processed-gene-30.25-mRNA-1 | Indica | Indica | | Os04g0221300 | OsR498G0407755600.01.T06 | Pentatricopeptide repeat-containing protein |
| augustus_masked-4-processed-gene-34.26-mRNA-1 | Indica | NA | | Os04g0225100 | OsR498G0407765200.01.T01 | Werner syndrome ATP-dependent helicase |
| augustus_masked-4-processed-gene-51.9-mRNA-1 | Indica | Japonica | | Os04g0258900 | OsR498G0407867000.01.T01 | F-box domain-containing protein |
| augustus_masked-4-processed-gene-87.12-mRNA-1 | Indica | NA | | Os04g0324100 | OsR498G0408099900.01.T01 | Glycosyltransferase |
| augustus_masked-4-processed-gene-89.16-mRNA-1 | Indica | NA | | Os04g0317500 | OsR498G0408137500.01.T01 | Expansin-like CBD domain-containing protein |
| augustus_masked-4-processed-gene-98.1-mRNA-1 | Indica | NA | | | OsR498G0713442500.01.T01 | |
| augustus_masked-5-processed-gene-139.21-mRNA-1 | Indica | NA | | Os05g0302600 | OsR498G0510398900.01.T01 | TLC domain-containing protein (Fragment) |

| | | | | | | |
|---|---|---|---|---|---|---|
| augustus_masked-5-processed-gene-140.24-mRNA-1 | Indica | NA | | Os05g0304800 | OsR498G0510409700.01.T01 | Nuclear factor Y transcription factor |
| augustus_masked-5-processed-gene-150.18-mRNA-1 | Indica | NA | | Os12g0468900 | OsR498G0917373300.01.T01 | Cell wall protein-like |
| augustus_masked-5-processed-gene-155.32-mRNA-1 | Indica | Indica | | Os12g0468900 | OsR498G0510503700.01.T01 | Cell wall protein-like |
| augustus_masked-5-processed-gene-155.34-mRNA-1 | Indica | NA | | Os05g0326400 | OsR498G0510504100.01.T02 | |
| augustus_masked-5-processed-gene-160.12-mRNA-1 | Indica | NA | | Os05g0330600 | OsR498G0510523500.01.T01 | Putaive prolamin 7 |
| augustus_masked-5-processed-gene-161.28-mRNA-1 | Indica | NA | | Os05g0331550 | OsR498G0510526000.01.T01 | Putaive prolamin 7 |
| augustus_masked-5-processed-gene-161.9-mRNA-1 | Indica | NA | Kinase-TM | Os05g0332300 | OsR498G0510529300.01.T01 | Non-specific serine/threonine protein kinase |
| augustus_masked-5-processed-gene-169.12-mRNA-1 | Indica | Japonica | | Os07g0433200 | OsR498G0510328900.01.T01 | Retrotransposon protein, putative, Ty1-copia subclass |
| augustus_masked-5-processed-gene-183.26-mRNA-1 | Indica | Indica | | Os05g0365000 | OsR498G0510671900.01.T01 | phytochrome A-associated F-box protein isoform X1 |
| augustus_masked-5-processed-gene-183.36-mRNA-1 | Indica | NA | | Os05g0365500 | OsR498G0510674100.01.T01 | Pentatricopeptide repeat |
| augustus_masked-5-processed-gene-187.21-mRNA-1 | Indica | NA | | Os05g0370600 | OsR498G0510692500.01.T06 | FT-interacting protein 1-like |
| augustus_masked-5-processed-gene-189.55-mRNA-1 | Indica | NA | | Os05g0373900 | OsR498G0510704700.01.T02 | Eukaryotic peptide chain release factor subunit |
| augustus_masked-5-processed-gene-191.20-mRNA-1 | Indica | NA | | Os04g0499100 | OsR498G0611743600.01.T01 | Folate-biopterin transporter |
| augustus_masked-5-processed-gene-213.18-mRNA-1 | Indica | NA | | Os05g0413000 | OsR498G0510836900.01.T02 | |
| augustus_masked-5-processed-gene-213.32-mRNA-1 | Indica | NA | CC-NBS-TM | | OsR498G0510842500.01.T01 | Putative disease resistance RPP13-like protein 1 |
| augustus_masked-5-processed-gene-266.30-mRNA-1 | Indica | NA | | Os05g0514500 | OsR498G0511177100.01.T01 | Interactor of constitutive active ROPs 1 |
| augustus_masked-5-processed-gene-280.46-mRNA-1 | Indica | NA | | Os05g0541400 | OsR498G0511268300.01.T01 | Transcription factor bHLH87 |
| augustus_masked-5-processed-gene-282.8-mRNA-1 | Indica | Japonica | | Os05g0546300 | OsR498G0511285700.01.T01 | |
| augustus_masked-5-processed-gene-283.54-mRNA-1 | Indica | Japonica | | Os05g0548600 | OsR498G0511288100.01.T01 | NOP5NT domain-containing protein |
| augustus_masked-5-processed-gene-286.37-mRNA-1 | Indica | NA | | | | t-SNARE coiled-coil homology domain-containing protein (Fragment) |
| augustus_masked-5-processed-gene-286.47-mRNA-1 | Indica | NA | | | | CDP-diacylglycerol--serine O-phosphatidyltransferase 1-like |
| augustus_masked-5-processed-gene-292.28-mRNA-1 | Indica | NA | | Os05g0564200 | OsR498G0511348300.01.T03 | Splicing factor U2af small subunit B |
| augustus_masked-5-processed-gene-293.0-mRNA-1 | Indica | NA | | | OsR498G0511354700.01.T05 | FCP1 homology domain-containing protein |
| augustus_masked-5-processed-gene-294.11-mRNA-1 | Indica | NA | | | OsR498G0511363900.01.T01 | |
| augustus_masked-5-processed-gene-294.12-mRNA-1 | Indica | NA | | Os05g0568600 | | |
| augustus_masked-5-processed-gene-294.14-mRNA-1 | Indica | NA | | Os05g0568800 | OsR498G0511364900.01.T01 | |
| augustus_masked-5-processed-gene-294.15-mRNA-1 | Indica | NA | | Os05g0569100 | | Reverse transcriptase Ty1/copia-type domain-containing protein |
| augustus_masked-5-processed-gene-295.29-mRNA-1 | Indica | NA | | | OsR498G0511371100.01.T01 | Reverse transcriptase Ty1/copia-type domain-containing protein |
| augustus_masked-5-processed-gene-295.30-mRNA-1 | Indica | NA | | Os05g0571100 | OsR498G0511372100.01.T01 | PORR domain-containing protein |
| augustus_masked-5-processed-gene-299.37-mRNA-1 | Indica | NA | | Os05g0578500 | OsR498G0511397100.01.T02 | Cinnamoyl-CoA reductase-like SNL6 |
| augustus_masked-5-processed-gene-65.3-mRNA-1 | Indica | NA | | Os05g0205400 | | |
| augustus_masked-5-processed-gene-65.8-mRNA-1 | Indica | NA | | Os05g0206000 | | |
| augustus_masked-5-processed-gene-65.9-mRNA-1 | Indica | NA | | Os05g0206100 | | |
| augustus_masked-5-processed-gene-71.9-mRNA-1 | Indica | NA | | Os05g0215600 | OsR498G0510025900.01.T02 | |
| augustus_masked-6-processed-gene-102.20-mRNA-1 | Indica | NA | | Os06g0288200 | OsR498G0612167300.01.T01 | C-glycosyltransferase |
| augustus_masked-6-processed-gene-102.3-mRNA-1 | Indica | NA | NBS-TM | | OsR498G0612165700.01.T01 | Disease resistance protein RPM1 |
| augustus_masked-6-processed-gene-102.31-mRNA-1 | Indica | NA | Kinase-TM | Os06g0288100 | OsR498G0612167200.01.T01 | Leucine-rich repeat receptor-like serine/threonine/tyrosine-protein kinase S |
| augustus_masked-6-processed-gene-103.24-mRNA-1 | Indica | Indica | | Os06g0289900 | OsR498G0612168500.01.T01 | C-glycosyltransferase |
| augustus_masked-6-processed-gene-103.3-mRNA-1 | Indica | NA | | Os06g0289200 | OsR498G0612167500.01.T01 | C-glycosyltransferase |
| augustus_masked-6-processed-gene-206.15-mRNA-1 | Indica | NA | | Os06g0548100 | OsR498G0612721800.01.T01 | Berberine bridge enzyme-like 28 |
| augustus_masked-6-processed-gene-236.10-mRNA-1 | Indica | NA | | Os06g0597500 | OsR498G0612883400.01.T01 | Pentatricopeptide repeat-containing protein mitochondrial |
| augustus_masked-6-processed-gene-236.15-mRNA-1 | Indica | Japonica | | Os06g0599200 | OsR498G0204145300.01.T01 | Oryzalexin D synthase |
| augustus_masked-6-processed-gene-236.3-mRNA-1 | Indica | NA | | Os06g0597800 | | |
| augustus_masked-6-processed-gene-257.23-mRNA-1 | Indica | NA | | Os12g0265500 | OsR498G1221250300.01.T01 | HAT family dimerisation domain containing protein, expressed |
| augustus_masked-6-processed-gene-26.27-mRNA-1 | Indica | NA | | | OsR498G0511427100.01.T01 | Epstein-Barr virus EBNA-1-like |
| augustus_masked-6-processed-gene-28.1-mRNA-1 | Indica | NA | | | OsR498G0611640900.01.T01 | |
| augustus_masked-6-processed-gene-293.18-mRNA-1 | Indica | NA | | Os06g0697500 | OsR498G0613242700.01.T01 | Adenosinetriphosphatase |
| augustus_masked-6-processed-gene-294.12-mRNA-1 | Indica | NA | | Os06g0697600 | OsR498G0613243200.01.T01 | Adenosinetriphosphatase |
| augustus_masked-6-processed-gene-294.22-mRNA-1 | Indica | NA | | Os06g0698600 | OsR498G0613245900.01.T01 | Exocyst subunit Exo70 family protein (Fragment) |
| augustus_masked-6-processed-gene-295.37-mRNA-1 | Indica | NA | Kinase-TM | Os06g0699400 | OsR498G0613249300.01.T03 | Mitogen-activated protein kinase |
| augustus_masked-6-processed-gene-295.52-mRNA-1 | Indica | NA | | Os06g0700000 | OsR498G0613251700.01.T01 | Subtilisin-like serine protease |
| augustus_masked-6-processed-gene-295.53-mRNA-1 | Indica | NA | | Os06g0700100 | OsR498G0613251900.01.T01 | Pentatricopeptide repeat-containing protein |
| augustus_masked-6-processed-gene-296.7-mRNA-1 | Indica | Indica | | Os01g0532700 | OsR498G0613255900.01.T01 | |
| augustus_masked-6-processed-gene-297.26-mRNA-1 | Indica | NA | | Os06g0702400 | OsR498G0613262800.01.T01 | |
| augustus_masked-6-processed-gene-299.1-mRNA-1 | Indica | NA | | Os06g0704100 | OsR498G0613270100.01.T01 | Ternary complex factor MIP1, leucine-zipper |
| augustus_masked-6-processed-gene-300.24-mRNA-1 | Indica | Indica | Kinase | Os02g0623600 | OsR498G0613277600.01.T01 | Putative tyrosine-protein kinase, insulin-like receptor |
| augustus_masked-6-processed-gene-301.13-mRNA-1 | Indica | NA | | Os06g0709000 | OsR498G0613284300.01.T01 | Arogenate dehydrogenase alpha |
| augustus_masked-6-processed-gene-302.26-mRNA-1 | Indica | NA | | Os06g0711800 | OsR498G0613289800.01.T02 | PMEI domain-containing protein (Fragment) |
| augustus_masked-6-processed-gene-302.6-mRNA-1 | Indica | Indica | | Os06g0710900 | OsR498G0613287200.01.T01 | Translation initiation factor IF-2-like |
| augustus_masked-6-processed-gene-302.7-mRNA-1 | Indica | NA | | Os06g0711000 | OsR498G0613287400.01.T01 | |
| augustus_masked-6-processed-gene-302.8-mRNA-1 | Indica | NA | | Os06g0711700 | OsR498G0613288400.01.T01 | F-box/kelch-repeat protein SKIP25 |

| | | | | | | |
|---|---|---|---|---|---|---|
| augustus_masked-6-processed-gene-303.39-mRNA-1 | Indica | Indica | | Os06g0713000 | OsR498G0613296500.01.T01 | Zinc finger protein CONSTANS |
| augustus_masked-6-processed-gene-304.44-mRNA-1 | Indica | NA | | Os06g0714300 | OsR498G0613300200.01.T02 | Auxin-responsive protein SAUR32 |
| augustus_masked-6-processed-gene-304.50-mRNA-1 | Indica | NA | | Os06g0715200 | | DNA ligase 3 (Fragment) |
| augustus_masked-6-processed-gene-304.60-mRNA-1 | Indica | Japonica | | Os06g0714600 | OsR498G0917833300.01.T01 | Ras-related protein RABA3 |
| augustus_masked-6-processed-gene-304.61-mRNA-1 | Indica | NA | | Os06g0714700 | OsR498G0613301800.01.T01 | |
| augustus_masked-6-processed-gene-304.62-mRNA-1 | Indica | NA | | | OsR498G0613302000.01.T05 | DNA-directed RNA polymerase II subunit RPB2 |
| augustus_masked-6-processed-gene-305.1-mRNA-1 | Indica | NA | | Os06g0716100 | OsR498G0613306500.01.T01 | J domain-containing protein (Fragment) |
| augustus_masked-6-processed-gene-305.23-mRNA-1 | Indica | Indica | | Os06g0716800 | OsR498G0613307800.01.T01 | Basic-leucine zipper transcription factor |
| augustus_masked-6-processed-gene-305.28-mRNA-1 | Indica | NA | | | OsR498G0613312200.01.T01 | |
| augustus_masked-6-processed-gene-305.6-mRNA-1 | Indica | NA | | Os07g0543700 | OsR498G1120733700.01.T01 | 4-diphosphocytidyl-2C-methyl-D-erythritol synthase |
| augustus_masked-6-processed-gene-308.25-mRNA-1 | Indica | NA | | | | Non-specific serine/threonine protein kinase |
| augustus_masked-6-processed-gene-308.35-mRNA-1 | Indica | Indica | | Os06g0725700 | OsR498G0613330300.01.T01 | |
| augustus_masked-6-processed-gene-309.47-mRNA-1 | Indica | Indica | | Os06g0727700 | OsR498G0613335700.01.T03 | Storage protein-like |
| augustus_masked-6-processed-gene-309.56-mRNA-1 | Indica | NA | | Os06g0727000 | OsR498G0613333800.01.T01 | Zinc finger protein ZAT3-like |
| augustus_masked-6-processed-gene-310.34-mRNA-1 | Indica | Japonica | | Os06g0727900 | OsR498G0613336100.01.T01 | Glycosyltransferase family 92 protein (Fragment) |
| augustus_masked-6-processed-gene-310.37-mRNA-1 | Indica | NA | | Os06g0728500 | OsR498G0613337700.01.T04 | Translation initiation factor IF-2-like |
| augustus_masked-6-processed-gene-43.10-mRNA-1 | Indica | Indica | | Os06g0185500 | OsR498G0611739300.01.T01 | Putrescine hydroxycinnamoyltransferase 2 |
| augustus_masked-6-processed-gene-43.21-mRNA-1 | Indica | NA | | Os06g0184866 | OsR498G0611738000.01.T01 | Pentatricopeptide repeat-containing protein mitochondrial |
| augustus_masked-6-processed-gene-43.23-mRNA-1 | Indica | NA | | | | |
| augustus_masked-6-processed-gene-43.8-mRNA-1 | Indica | NA | | Os06g0184900 | OsR498G0611738500.01.T01 | Hydroxycinnamoyl-CoA quinate hydroxycinnamoyltransferase |
| augustus_masked-6-processed-gene-44.42-mRNA-1 | Indica | NA | | Os06g0185700 | OsR498G0611740100.01.T01 | Pentatricopeptide repeat-containing protein mitochondrial |
| augustus_masked-6-processed-gene-44.48-mRNA-1 | Indica | Japonica | | Os06g0185800 | OsR498G0611740600.01.T01 | Pentatricopeptide repeat-containing protein isoform X1 |
| augustus_masked-6-processed-gene-44.56-mRNA-1 | Indica | NA | | | OsR498G0611745700.01.T01 | Putative RING-H2 finger protein ATL49 |
| augustus_masked-6-processed-gene-44.65-mRNA-1 | Indica | NA | | | OsR498G0611744000.01.T01 | RING-type domain-containing protein |
| augustus_masked-6-processed-gene-49.31-mRNA-1 | Indica | Indica | | Os07g0110200 | OsR498G0611778200.01.T02 | Nucleolar GTP-binding protein 1 |
| augustus_masked-6-processed-gene-49.32-mRNA-1 | Indica | NA | | Os06g0196200 | OsR498G0611779600.01.T01 | |
| augustus_masked-6-processed-gene-56.40-mRNA-1 | Indica | Indica | | Os02g0768700 | OsR498G0611835500.01.T02 | GeBP transcription factor (Fragment) |
| augustus_masked-6-processed-gene-57.30-mRNA-1 | Indica | NA | | Os06g0210300 | | Ethylene-responsive transcription factor ERF014 |
| augustus_masked-6-processed-gene-57.31-mRNA-1 | Indica | NA | | Os06g0210500 | OsR498G0611837900.01.T01 | Mitochondrial phosphate carrier protein like |
| augustus_masked-6-processed-gene-57.46-mRNA-1 | Indica | Japonica | Kinase-TM | Os06g0210400 | OsR498G1019094500.01.T01 | Mitogen-activated protein kinase kinase kinase |
| augustus_masked-6-processed-gene-57.47-mRNA-1 | Indica | NA | | | OsR498G0611837800.01.T01 | RING-type E3 ubiquitin transferase |
| augustus_masked-6-processed-gene-58.29-mRNA-1 | Indica | Japonica | | Os06g0212600 | OsR498G0204823700.01.T01 | Fucosyltransferase |
| augustus_masked-6-processed-gene-58.3-mRNA-1 | Indica | NA | | | OsR498G0611842100.01.T01 | Scarecrow-like protein 3 |
| augustus_masked-6-processed-gene-59.18-mRNA-1 | Indica | NA | | | OsR498G0611846900.01.T01 | Putative 41 kD chloroplast nucleoid DNA binding protein |
| augustus_masked-6-processed-gene-59.23-mRNA-1 | Indica | Indica | | Os02g0762900 | OsR498G0611848400.01.T01 | Polcalcin Syr v 3-like |
| augustus_masked-6-processed-gene-59.28-mRNA-1 | Indica | NA | | Os06g0213900 | OsR498G0611849700.01.T01 | |
| augustus_masked-6-processed-gene-60.36-mRNA-1 | Indica | NA | | Os06g0215100 | OsR498G0611853300.01.T01 | |
| augustus_masked-6-processed-gene-60.49-mRNA-1 | Indica | Indica | | Os10g0122000 | OsR498G0611856800.01.T01 | Glycosyltransferase |
| augustus_masked-6-processed-gene-60.53-mRNA-1 | Indica | NA | | Os06g0216400 | OsR498G0611857800.01.T01 | Transcription factor EMB1444 |
| augustus_masked-6-processed-gene-61.17-mRNA-1 | Indica | Indica | | Os06g0217500 | OsR498G0611860600.01.T05 | RNA polymerase II transcriptional regulation mediator |
| augustus_masked-6-processed-gene-62.41-mRNA-1 | Indica | NA | | Os06g0218800 | | |
| augustus_masked-6-processed-gene-63.45-mRNA-1 | Indica | NA | | Os06g0219800 | OsR498G0611870800.01.T02 | Serine/threonine-protein phosphatase 2A activator |
| augustus_masked-6-processed-gene-63.46-mRNA-1 | Indica | NA | | Os06g0219900 | OsR498G0611871000.01.T01 | EXORDIUM like 2 |
| augustus_masked-6-processed-gene-64.15-mRNA-1 | Indica | NA | | Os06g0220900 | OsR498G0611875500.01.T01 | |
| augustus_masked-6-processed-gene-78.17-mRNA-1 | Indica | NA | | Os06g0247800 | OsR498G0611996700.01.T05 | Dynamin GTPase |
| augustus_masked-6-processed-gene-89.31-mRNA-1 | Indica | NA | | Os06g0265900 | OsR498G0612062300.01.T01 | PORR domain-containing protein (Fragment) |
| augustus_masked-6-processed-gene-91.11-mRNA-1 | Indica | NA | | | OsR498G1221332900.01.T01 | Retrotransposon protein, putative, unclassified |
| augustus_masked-6-processed-gene-92.22-mRNA-1 | Indica | NA | | Os06g0271000 | OsR498G0612075700.01.T01 | UDP-glycosyltransferase 90A1 |
| augustus_masked-6-processed-gene-94.23-mRNA-1 | Indica | NA | | Os06g0275500 | OsR498G0612092700.01.T01 | Histone-lysine N-methyltransferase EZ1 |
| augustus_masked-8-processed-gene-153.21-mRNA-1 | Indica | NA | | Os08g0334900 | OsR498G0815812500.01.T01 | Fucosyltransferase |
| augustus_masked-8-processed-gene-161.8-mRNA-1 | Indica | NA | | Os08g0346400 | OsR498G0815852300.01.T01 | HTH myb-type domain-containing protein |
| augustus_masked-8-processed-gene-165.0-mRNA-1 | Indica | Indica | | Os08g0351200 | OsR498G0815866300.01.T03 | |
| augustus_masked-8-processed-gene-191.12-mRNA-1 | Indica | NA | | Os08g0375700 | OsR498G0815952700.01.T01 | O-fucosyltransferase family protein |
| augustus_masked-8-processed-gene-193.0-mRNA-1 | Indica | NA | | | | Phospholipid-transporting ATPase |
| augustus_masked-8-processed-gene-194.11-mRNA-1 | Indica | Indica | | Os08g0381600 | OsR498G0815968100.01.T01 | thaumatin-like protein isoform X1 |
| augustus_masked-8-processed-gene-195.42-mRNA-1 | Indica | Indica | | Os05g0510100 | OsR498G0815976100.01.T01 | |
| augustus_masked-8-processed-gene-200.9-mRNA-1 | Indica | NA | | | OsR498G0816003100.01.T01 | |
| augustus_masked-8-processed-gene-212.0-mRNA-1 | Indica | NA | | | OsR498G0816082900.01.T03 | NHL domain-containing protein |
| augustus_masked-8-processed-gene-215.10-mRNA-1 | Indica | Japonica | | Os08g0421700 | OsR498G0816100100.01.T01 | CCHC-type domain-containing protein |
| augustus_masked-8-processed-gene-223.4-mRNA-1 | Indica | NA | | Os08g0433300 | OsR498G0816148900.01.T01 | Glycosyl hydrolase, five-bladed beta-propellor domain-containing protein |
| augustus_masked-8-processed-gene-225.11-mRNA-1 | Indica | NA | | | OsR498G0816160100.01.T01 | Zinc finger A20 and AN1 domain-containing stress-associated protein 9 |

| | | | | | | |
|---|---|---|---|---|---|---|
| augustus_masked-8-processed-gene-227.32-mRNA-1 | Indica | NA | | Os08g0439600 | OsR498G0816169900.01.T01 | |
| augustus_masked-8-processed-gene-227.33-mRNA-1 | Indica | NA | | Os08g0439900 | OsR498G0816170300.01.T01 | Mitochondrial glycoprotein family protein, putative |
| augustus_masked-8-processed-gene-228.11-mRNA-1 | Indica | Indica | Kinase-TM | Os08g0441100 | OsR498G0816176500.01.T01 | Non-specific serine/threonine protein kinase |
| augustus_masked-8-processed-gene-230.11-mRNA-1 | Indica | Indica | | Os07g0597625 | OsR498G0816195700.01.T01 | Glyoxylate/hydroxypyruvate reductase HPR3 |
| augustus_masked-8-processed-gene-232.17-mRNA-1 | Indica | NA | | Os08g0450100 | OsR498G0816215500.01.T01 | Pectinesterase |
| augustus_masked-8-processed-gene-232.18-mRNA-1 | Indica | NA | | Os08g0450100 | OsR498G0816215500.01.T01 | Pectinesterase |
| augustus_masked-8-processed-gene-260.36-mRNA-1 | Indica | NA | | Os08g0499800 | OsR498G0816397900.01.T02 | Pentatricopeptide repeat-containing protein mitochondrial |
| augustus_masked-8-processed-gene-260.48-mRNA-1 | Indica | NA | | Os08g0500500 | OsR498G0816401500.01.T01 | DNA-binding protein-like |
| augustus_masked-8-processed-gene-276.75-mRNA-1 | Indica | NA | | Os08g0529400 | | F-box domain-containing protein |
| augustus_masked-8-processed-gene-277.11-mRNA-1 | Indica | NA | | Os08g0530300 | OsR498G0816515500.01.T01 | Exocyst subunit Exo70 family protein (Fragment) |
| augustus_masked-8-processed-gene-30.14-mRNA-1 | Indica | NA | | | | |
| augustus_masked-8-processed-gene-31.12-mRNA-1 | Indica | NA | | Os08g0150800 | OsR498G0815197900.01.T01 | Tyrosyl-tRNA synthetase |
| augustus_masked-8-processed-gene-33.2-mRNA-1 | Indica | NA | CC-NBS-TM | | OsR498G0815208900.01.T01 | NB-ARC domain containing protein, expressed |
| augustus_masked-8-processed-gene-36.1-mRNA-1 | Indica | NA | | | OsR498G0815232500.01.T01 | TF-B3 domain-containing protein (Fragment) |
| augustus_masked-8-processed-gene-36.9-mRNA-1 | Indica | NA | | Os08g0159000 | OsR498G0815236700.01.T01 | DnaJ homolog subfamily C member 2 |
| augustus_masked-8-processed-gene-41.20-mRNA-1 | Indica | NA | | | OsR498G0815268900.01.T01 | Serine-rich protein-related |
| augustus_masked-8-processed-gene-42.1-mRNA-1 | Indica | NA | | Os08g0168600 | OsR498G0815272900.01.T01 | Glycosyltransferase |
| augustus_masked-9-processed-gene-102.1-mRNA-1 | Indica | NA | | Os09g0332100 | OsR498G0917174700.01.T01 | |
| augustus_masked-9-processed-gene-110.29-mRNA-1 | Indica | NA | | Os09g0342000 | OsR498G0917198400.01.T01 | CRIB domain-containing protein |
| augustus_masked-9-processed-gene-143.9-mRNA-1 | Indica | NA | | | | |
| augustus_masked-9-processed-gene-73.12-mRNA-1 | Indica | NA | | Os09g0292900 | OsR498G0916991700.01.T01 | Kelch repeat-containing F-box-like protein |
| augustus_masked-9-processed-gene-8.3-mRNA-1 | Indica | NA | | Os02g0212975 | OsR498G0202873200.01.T01 | |
| maker-1-augustus-gene-10.28-mRNA-1 | Indica | NA | | Os01g0117900 | OsR498G0100057100.01.T04 | |
| maker-1-augustus-gene-10.31-mRNA-1 | Indica | Japonica | | Os01g0118000 | OsR498G1119486400.01.T02 | Fructose-bisphosphate aldolase |
| maker-1-augustus-gene-10.34-mRNA-1 | Indica | NA | | | OsR498G0100057700.01.T05 | DExH-box ATP-dependent RNA helicase DExH3 |
| maker-1-augustus-gene-10.40-mRNA-1 | Indica | NA | | | | CDP-diacylglycerol--serine O-phosphatidyltransferase 1 isoform X1 |
| maker-1-augustus-gene-14.60-mRNA-1 | Indica | Indica | | Os01g0127000 | OsR498G0100088600.01.T01 | Multicopper oxidase LPR1 |
| maker-1-augustus-gene-14.63-mRNA-1 | Indica | Indica | | Os01g0127200 | OsR498G0100089400.01.T01 | Multicopper oxidase LPR1 |
| maker-1-augustus-gene-14.66-mRNA-1 | Indica | NA | | Os01g0127300 | OsR498G0100089600.01.T01 | Protein ABCI7 chloroplastic |
| maker-1-augustus-gene-15.29-mRNA-1 | Indica | Indica | | Os01g0127400 | OsR498G0100089700.01.T04 | HTH myb-type domain-containing protein |
| maker-1-augustus-gene-15.31-mRNA-1 | Indica | NA | | Os01g0127500 | OsR498G0100089900.01.T01 | Tetraketide alpha-pyrone reductase |
| maker-1-augustus-gene-15.35-mRNA-1 | Indica | NA | | Os01g0127900 | OsR498G0100091900.01.T02 | Mannose-6-phosphate isomerase |
| maker-1-augustus-gene-16.30-mRNA-1 | Indica | NA | | Os01g0129950 | | |
| maker-1-augustus-gene-16.32-mRNA-1 | Indica | NA | | Os01g0130400 | OsR498G0100104900.01.T01 | Alpha-xylosidase 1 |
| maker-1-augustus-gene-16.36-mRNA-1 | Indica | NA | | Os01g0130000 | OsR498G0100102600.01.T02 | Metal tolerance protein 5 |
| maker-1-augustus-gene-17.0-mRNA-1 | Indica | NA | | | OsR498G0100109900.01.T01 | AP2/ERF domain-containing protein |
| maker-1-augustus-gene-17.11-mRNA-1 | Indica | Japonica | | Os01g0132000 | OsR498G0100111200.01.T01 | Wound-induced serine protease inhibitor |
| maker-1-augustus-gene-17.13-mRNA-1 | Indica | NA | | Os01g0132400 | OsR498G0100113200.01.T01 | |
| maker-1-augustus-gene-17.15-mRNA-1 | Indica | NA | | Os01g0132700 | OsR498G0100113600.01.T05 | Mediator of RNA polymerase II transcription subunit 22b like |
| maker-1-augustus-gene-17.18-mRNA-1 | Indica | NA | | Os01g0132800 | | Chloroplastic group IIB intron splicing facilitator CRS2-B, chloroplastic |
| maker-1-augustus-gene-17.2-mRNA-1 | Indica | NA | | Os01g0131800 | OsR498G0100110900.01.T01 | Signal peptidase subunit |
| maker-1-augustus-gene-17.7-mRNA-1 | Indica | Indica | | Os01g0132500 | OsR498G0100113300.01.T01 | RNA polymerase-associated protein (Fragment) |
| maker-1-augustus-gene-17.9-mRNA-1 | Indica | NA | | Os01g0131900 | OsR498G0100111200.01.T01 | Wound-induced serine protease inhibitor |
| maker-1-augustus-gene-18.0-mRNA-1 | Indica | Indica | | Os01g0133100 | OsR498G0100115900.01.T01 | VARLMGL domain-containing protein |
| maker-1-augustus-gene-18.13-mRNA-1 | Indica | Indica | | Os01g0133700 | OsR498G0100117100.01.T01 | Core-2/I-branching beta-16-N-acetylglucosaminyltransferase family protein |
| maker-1-augustus-gene-18.15-mRNA-1 | Indica | Japonica | | Os01g0133700 | OsR498G0100117500.01.T01 | Core-2/I-branching beta-16-N-acetylglucosaminyltransferase family protein |
| maker-1-augustus-gene-18.17-mRNA-1 | Indica | NA | Kinase-LRR-TM | Os01g0133900 | OsR498G0100117900.01.T02 | probable leucine-rich repeat receptor-like protein kinase At1g68400 |
| maker-1-augustus-gene-18.20-mRNA-1 | Indica | NA | | Os06g0313260 | | Epimerase domain-containing protein |
| maker-1-augustus-gene-18.22-mRNA-1 | Indica | NA | | Os01g0134500 | OsR498G0100119100.01.T01 | Aldehyde oxygenase (deformylating) |
| maker-1-augustus-gene-18.3-mRNA-1 | Indica | NA | | Os01g0133400 | OsR498G0100116700.01.T02 | Plastidic glucose transporter like |
| maker-1-augustus-gene-18.9-mRNA-1 | Indica | NA | | Os01g0133500 | OsR498G0100116900.01.T07 | Metallo-hydrolase/oxidoreductase superfamily protein |
| maker-1-augustus-gene-19.0-mRNA-1 | Indica | NA | | Os01g0134700 | OsR498G0100119500.01.T01 | Protein SAR DEFICIENT 1 isoform B |
| maker-1-augustus-gene-19.10-mRNA-1 | Indica | NA | | Os01g0136400 | OsR498G0100125200.01.T03 | Putative receptor serine/threonine kinase PR5K |
| maker-1-augustus-gene-19.21-mRNA-1 | Indica | NA | Kinase-TM | Os01g0136800 | OsR498G0100126800.01.T01 | WAK2-OsWAK receptor-like cytoplasmic kinase (OsWAK-RLCK) |
| maker-1-augustus-gene-19.3-mRNA-1 | Indica | Indica | | Os01g0137100 | OsR498G0100127300.01.T01 | Putative receptor serine/threonine kinase PR5K |
| maker-1-augustus-gene-19.9-mRNA-1 | Indica | NA | Kinase-TM | | OsR498G0100125200.01.T02 | Putative serine/threonine-protein kinase |
| maker-1-augustus-gene-20.0-mRNA-1 | Indica | NA | Kinase-TM | | | Protein kinase domain-containing protein (Fragment) |
| maker-1-augustus-gene-20.11-mRNA-1 | Indica | NA | Kinase-TM | | | Putative receptor serine/threonine kinase PR5K |
| maker-1-augustus-gene-20.13-mRNA-1 | Indica | NA | Kinase-TM | | OsR498G0100130500.01.T01 | Putative receptor serine/threonine kinase PR5K |
| maker-1-augustus-gene-20.18-mRNA-1 | Indica | NA | Kinase-TM | | | Protein kinase domain-containing protein (Fragment) |
| maker-1-augustus-gene-20.20-mRNA-1 | Indica | Indica | | Os06g0586600 | OsR498G0100130200.01.T01 | Transposon protein, putative, CACTA, En/Spm sub-class |

| | | | | | | |
|---|---|---|---|---|---|---|
| maker-1-augustus-gene-20.22-mRNA-1 | Indica | NA | | | OsR498G0100131400.01.T03 | |
| maker-1-augustus-gene-20.4-mRNA-1 | Indica | NA | | | OsR498G0100127900.01.T03 | Putative receptor serine/threonine kinase PR5K |
| maker-1-augustus-gene-20.6-mRNA-1 | Indica | NA | | | | DUF223 domain-containing protein |
| maker-1-augustus-gene-20.9-mRNA-1 | Indica | NA | Kinase-TM | | | Protein kinase domain-containing protein (Fragment) |
| maker-1-augustus-gene-21.10-mRNA-1 | Indica | NA | | Os01g0139000 | OsR498G0100136300.01.T01 | Reticulon domain-containing protein |
| maker-1-augustus-gene-21.16-mRNA-1 | Indica | NA | | Os01g0138600 | OsR498G0100135400.01.T03 | Cysteine proteinase inhibitor |
| maker-1-augustus-gene-21.2-mRNA-1 | Indica | NA | Kinase-TM | Os01g0138400 | OsR498G0100134300.01.T01 | Stress-induced receptor-like kinase 2 |
| maker-1-augustus-gene-21.23-mRNA-1 | Indica | NA | | Os01g0138900 | OsR498G0100136000.01.T03 | Dipeptide epimerase |
| maker-1-augustus-gene-21.27-mRNA-1 | Indica | NA | | Os01g0139200 | OsR498G0100136800.01.T05 | PB1 domain-containing protein (Fragment) |
| maker-1-augustus-gene-21.4-mRNA-1 | Indica | NA | | Os01g0138500 | OsR498G0100135100.01.T03 | Coagulation factor VIII |
| maker-1-augustus-gene-21.7-mRNA-1 | Indica | NA | | Os01g0138800 | OsR498G0100135700.01.T01 | Class I glutamine amidotransferase |
| maker-1-augustus-gene-23.0-mRNA-1 | Indica | Indica | | Os01g0141600 | OsR498G0100150500.01.T02 | Root UVB sensitive family |
| maker-1-augustus-gene-23.10-mRNA-1 | Indica | Indica | | Os01g0141900 | OsR498G0100151100.01.T03 | Putative proteophosphoglycan |
| maker-1-augustus-gene-23.14-mRNA-1 | Indica | NA | | Os01g0142200 | OsR498G0100151500.01.T05 | EKC/KEOPS complex subunit Tprkb like |
| maker-1-augustus-gene-23.18-mRNA-1 | Indica | NA | | Os01g0142300 | OsR498G0100151700.01.T01 | Sulfoquinovosyl transferase SQD2 |
| maker-1-augustus-gene-23.23-mRNA-1 | Indica | NA | | Os01g0142800 | OsR498G0100152900.01.T04 | Peptide transporter |
| maker-1-augustus-gene-23.31-mRNA-1 | Indica | NA | | Os01g0142000 | | ATP binding protein |
| maker-1-augustus-gene-23.33-mRNA-1 | Indica | NA | | Os01g0142100 | OsR498G0100151400.01.T01 | Probable zinc metalloprotease EGY2, chloroplastic |
| maker-1-augustus-gene-23.41-mRNA-1 | Indica | NA | | Os01g0142500 | OsR498G0100152200.01.T02 | Transcription factor DIVARICATA |
| maker-1-augustus-gene-234.41-mRNA-1 | Indica | NA | | Os01g0584900 | OsR498G0101416500.01.T01 | WRKY transcription factor |
| maker-1-augustus-gene-234.43-mRNA-1 | Indica | NA | | Os01g0585100 | OsR498G0101417300.01.T03 | Glycerophosphocholine acyltransferase 1 |
| maker-1-augustus-gene-234.47-mRNA-1 | Indica | NA | | | | DNA damage-binding protein 1 |
| maker-1-augustus-gene-234.60-mRNA-1 | Indica | NA | | Os01g0585300 | OsR498G0101418800.01.T01 | DNA-directed RNA polymerase, phage-type |
| maker-1-augustus-gene-24.60-mRNA-1 | Indica | NA | | Os01g0143100 | OsR498G0100159200.01.T05 | Mitochondrial deoxynucleotide carrier, putative |
| maker-1-augustus-gene-24.66-mRNA-1 | Indica | Japonica | | Os01g0143700 | OsR498G0100161600.01.T01 | Mitochondrial glycoprotein-like |
| maker-1-augustus-gene-24.69-mRNA-1 | Indica | NA | | Os01g0143800 | OsR498G0100162200.01.T01 | Mitochondrial glycoprotein family protein |
| maker-1-augustus-gene-26.30-mRNA-1 | Indica | NA | | Os01g0148400 | OsR498G0100174700.01.T01 | Brix domain-containing protein |
| maker-1-augustus-gene-26.40-mRNA-1 | Indica | NA | | Os01g0147700 | OsR498G0100172800.01.T01 | Factor of DNA methylation 1 |
| maker-1-augustus-gene-26.43-mRNA-1 | Indica | NA | | | OsR498G0100173000.01.T01 | DUF547 domain-containing protein |
| maker-1-augustus-gene-26.48-mRNA-1 | Indica | NA | | Os01g0147900 | OsR498G0100173200.01.T01 | Triose phosphate isomerase cytosolic isoform |
| maker-1-augustus-gene-260.2-mRNA-1 | Indica | NA | | Os01g0632700 | OsR498G0101597500.01.T03 | |
| maker-1-augustus-gene-260.3-mRNA-1 | Indica | NA | | | | ATP-dependent DNA helicase |
| maker-1-augustus-gene-260.7-mRNA-1 | Indica | NA | | Os01g0633100 | OsR498G0101598700.01.T09 | Glucose-1-phosphate adenylyltransferase large subunit, chloroplastic/amylo |
| maker-1-augustus-gene-27.40-mRNA-1 | Indica | Indica | | Os01g0150100 | OsR498G0100180100.01.T02 | Geranylgeranyl transferase type I subunit beta |
| maker-1-augustus-gene-27.45-mRNA-1 | Indica | NA | | Os01g0150200 | OsR498G0100180300.01.T03 | Very-long-chain (3R)-3-hydroxyacyl-CoA dehydratase PASTICCINO 2 |
| maker-1-augustus-gene-27.62-mRNA-1 | Indica | Indica | Kinase-LRR-TM | Os01g0149700 | OsR498G0100178400.01.T01 | Putative LRR receptor-like serine/threonine-protein kinase |
| maker-1-augustus-gene-27.65-mRNA-1 | Indica | NA | | Os01g0149800 | OsR498G0100176800.01.T01 | Metallothionein-like protein type 2 |
| maker-1-augustus-gene-27.67-mRNA-1 | Indica | NA | | Os01g0149900 | OsR498G0100179000.01.T01 | |
| maker-1-augustus-gene-27.71-mRNA-1 | Indica | NA | | Os01g0150000 | OsR498G0100179800.01.T01 | very-long-chain enoyl-CoA reductase isoform X1 |
| maker-1-augustus-gene-28.0-mRNA-1 | Indica | NA | | | | Very-long-chain (3R)-3-hydroxyacyl-CoA dehydratase |
| maker-1-augustus-gene-28.11-mRNA-1 | Indica | NA | | Os01g0151200 | OsR498G0100183300.01.T03 | Inner membrane protein translocase involved in respiratory chain assembly |
| maker-1-augustus-gene-28.25-mRNA-1 | Indica | NA | | Os01g0151700 | OsR498G0100184500.01.T02 | Short-chain dehydrogenase TIC 32, chloroplastic |
| maker-1-augustus-gene-28.4-mRNA-1 | Indica | NA | | Os01g0150800 | | Very-long-chain (3R)-3-hydroxyacyl-CoA dehydratase |
| maker-1-augustus-gene-29.77-mRNA-1 | Indica | NA | | | OsR498G0100191900.01.T01 | Aspartate--tRNA ligase, chloroplastic/mitochondrial |
| maker-1-augustus-gene-29.88-mRNA-1 | Indica | NA | | Os01g0154500 | OsR498G0100194300.01.T01 | |
| maker-1-augustus-gene-29.90-mRNA-1 | Indica | NA | | Os01g0154600 | OsR498G0100194700.01.T02 | |
| maker-1-augustus-gene-29.94-mRNA-1 | Indica | NA | | | | Tyramine N-feruloyltransferase 4/11-like |
| maker-1-augustus-gene-30.0-mRNA-1 | Indica | Indica | | Os01g0155800 | OsR498G0100198300.01.T01 | GPRP |
| maker-1-augustus-gene-30.13-mRNA-1 | Indica | NA | | Os01g0155400 | OsR498G0100197800.01.T04 | |
| maker-1-augustus-gene-30.14-mRNA-1 | Indica | NA | | | OsR498G0100197800.01.T01 | GRIP and coiled-coil domain-containing protein 2 isoform X1 |
| maker-1-augustus-gene-30.2-mRNA-1 | Indica | NA | | | OsR498G0100203300.01.T01 | Glycosyltransferases |
| maker-1-augustus-gene-30.27-mRNA-1 | Indica | Japonica | | Os01g0155600 | OsR498G0509618800.01.T01 | Serine/arginine-rich splicing factor RS2Z33 like |
| maker-1-augustus-gene-30.29-mRNA-1 | Indica | Indica | | Os01g0156000 | OsR498G0100198600.01.T01 | Protein REVEILLE 8 isoform A |
| maker-1-augustus-gene-30.31-mRNA-1 | Indica | NA | | Os01g0158400 | OsR498G0100205600.01.T02 | Lectin |
| maker-1-augustus-gene-30.4-mRNA-1 | Indica | NA | | Os01g0158000 | OsR498G0100204100.01.T01 | Importin subunit alpha |
| maker-1-augustus-gene-30.8-mRNA-1 | Indica | NA | | Os01g0158200 | OsR498G0100204500.01.T01 | Carboxypeptidase |
| maker-1-augustus-gene-31.44-mRNA-1 | Indica | NA | | Os01g0159800 | OsR498G0100210300.01.T02 | Transcription factor bHLH51 |
| maker-1-augustus-gene-31.46-mRNA-1 | Indica | NA | | Os01g0158900 | OsR498G0100208600.01.T06 | NF-X1-type zinc finger protein NFXL2 |
| maker-1-augustus-gene-31.51-mRNA-1 | Indica | NA | | Os01g0159000 | OsR498G0100208800.01.T01 | DOG1 domain-containing protein (Fragment) |
| maker-1-augustus-gene-31.53-mRNA-1 | Indica | NA | | Os01g0159300 | OsR498G0100209200.01.T02 | E3 ubiquitin-protein ligase MBR1 |
| maker-1-augustus-gene-31.59-mRNA-1 | Indica | NA | | Os01g0159400 | OsR498G0100209800.01.T01 | Acyl-coenzyme A oxidase 4 peroxisomal |

| | | | | | | |
|---|---|---|---|---|---|---|
| maker-1-augustus-gene-31.63-mRNA-1 | Indica | NA | | Os01g0160100 | OsR498G0100211200.01.T01 | Pyruvate decarboxylase |
| maker-1-augustus-gene-32.51-mRNA-1 | Indica | NA | | Os01g0160800 | OsR498G0100211800.01.T01 | rRNA N-glycosidase |
| maker-1-augustus-gene-32.52-mRNA-1 | Indica | Indica | | Os01g0160800 | OsR498G0100212800.01.T01 | rRNA N-glycosidase |
| maker-1-augustus-gene-32.54-mRNA-1 | Indica | NA | | | OsR498G0204301500.01.T06 | ATP-dependent DNA helicase |
| maker-1-augustus-gene-33.0-mRNA-1 | Indica | NA | | | OsR498G0100215100.01.T05 | Transcription initiation factor TFIID subunit 15b |
| maker-1-augustus-gene-33.11-mRNA-1 | Indica | NA | | Os01g0164500 | OsR498G0100215400.01.T01 | DEAD-box ATP-dependent RNA helicase |
| maker-1-augustus-gene-33.16-mRNA-1 | Indica | NA | | Os01g0164600 | OsR498G0100215600.01.T01 | PPM-type phosphatase domain-containing protein |
| maker-1-augustus-gene-33.22-mRNA-1 | Indica | NA | | | OsR498G0100216000.01.T02 | Tower domain-containing protein (Fragment) |
| maker-1-augustus-gene-33.29-mRNA-1 | Indica | Indica | | Os01g0164900 | OsR498G0100216000.01.T05 | Tower domain-containing protein |
| maker-1-augustus-gene-33.3-mRNA-1 | Indica | NA | | | OsR498G0100215700.01.T02 | Golgi to ER traffic protein 4 |
| maker-1-augustus-gene-33.31-mRNA-1 | Indica | NA | | Os01g0165100 | OsR498G0100216400.01.T01 | WD repeat-containing 63 |
| maker-1-augustus-gene-33.33-mRNA-1 | Indica | NA | | Os01g0165200 | OsR498G0100216600.01.T03 | DCD (Development and Cell Death) domain protein |
| maker-1-augustus-gene-34.0-mRNA-1 | Indica | NA | | Os01g0166100 | OsR498G0100219100.01.T01 | Staphylococcal-like nuclease CAN2 isoform A |
| maker-1-augustus-gene-34.13-mRNA-1 | Indica | Indica | | Os01g0167100 | OsR498G0100221500.01.T06 | |
| maker-1-augustus-gene-34.15-mRNA-1 | Indica | NA | | | OsR498G0100223300.01.T02 | |
| maker-1-augustus-gene-34.18-mRNA-1 | Indica | NA | | | OsR498G0100218400.01.T06 | HORMA domain-containing protein |
| maker-1-augustus-gene-34.20-mRNA-1 | Indica | NA | | Os01g0166800 | OsR498G0100221200.01.T01 | E2F target protein 1 |
| maker-1-augustus-gene-34.24-mRNA-1 | Indica | NA | | Os01g0167500 | OsR498G0100222800.01.T01 | TPT domain-containing protein |
| maker-1-augustus-gene-34.3-mRNA-1 | Indica | NA | | | OsR498G0100219300.01.T03 | Staphylococcal-like nuclease CAN2 isoform A |
| maker-1-augustus-gene-34.30-mRNA-1 | Indica | NA | | | OsR498G0100223800.01.T05 | |
| maker-1-augustus-gene-348.24-mRNA-1 | Indica | NA | | Os01g0805800 | OsR498G0102167600.01.T01 | DNA-directed RNA polymerases II 24 kDa polypeptide |
| maker-1-augustus-gene-348.28-mRNA-1 | Indica | Indica | | Os01g0268800 | OsR498G0102168400.01.T03 | Ubiquitin-associated domain-containing protein 2-like isoform X1 |
| maker-1-augustus-gene-348.31-mRNA-1 | Indica | NA | | Os01g0806200 | OsR498G0102168600.01.T01 | F-box family protein-like |
| maker-1-augustus-gene-348.6-mRNA-1 | Indica | Indica | | Os01g0805900 | OsR498G0102167900.01.T01 | Tubulin beta chain |
| maker-1-augustus-gene-349.41-mRNA-1 | Indica | NA | | | OsR498G0102174700.01.T01 | Putative zinc finger, CCHC-type |
| maker-1-augustus-gene-349.42-mRNA-1 | Indica | NA | | | OsR498G0102176300.01.T02 | |
| maker-1-augustus-gene-349.44-mRNA-1 | Indica | NA | | Os01g0807900 | OsR498G0102176900.01.T03 | Pyrimidine 2 isoform 1 |
| maker-1-augustus-gene-349.48-mRNA-1 | Indica | Indica | | Os01g0279900 | OsR498G0102177400.01.T02 | Transcription factor TGAL1 |
| maker-1-augustus-gene-35.0-mRNA-1 | Indica | NA | | Os01g0168400 | OsR498G0100226300.01.T01 | RING finger and transmembrane domain-containing protein 2 |
| maker-1-augustus-gene-35.13-mRNA-1 | Indica | NA | | | OsR498G0100229500.01.T01 | Transcription factor MYB3 |
| maker-1-augustus-gene-35.15-mRNA-1 | Indica | NA | | Os01g0167800 | | PMD domain-containing protein (Fragment) |
| maker-1-augustus-gene-35.18-mRNA-1 | Indica | NA | | Os01g0167900 | OsR498G0100224600.01.T02 | NAB domain-containing protein |
| maker-1-augustus-gene-35.20-mRNA-1 | Indica | NA | | Os01g0168200 | OsR498G0100225200.01.T02 | Thioredoxin H-type like |
| maker-1-augustus-gene-35.23-mRNA-1 | Indica | NA | | Os01g0168300 | OsR498G0100225600.01.T01 | Import inner membrane translocase subunit |
| maker-1-augustus-gene-35.25-mRNA-1 | Indica | NA | | Os01g0168800 | OsR498G0100227000.01.T02 | AB hydrolase-1 domain-containing protein |
| maker-1-augustus-gene-35.28-mRNA-1 | Indica | NA | | | OsR498G0100227600.01.T01 | Transcription factor TT2 |
| maker-1-augustus-gene-35.6-mRNA-1 | Indica | NA | | Os01g0168500 | OsR498G0100226500.01.T03 | Homolog of yeast autophagy 18C |
| maker-1-augustus-gene-35.8-mRNA-1 | Indica | Indica | | Os01g0168600 | OsR498G0100226700.01.T02 | O-fucosyltransferase family protein (Fragment) |
| maker-1-augustus-gene-350.22-mRNA-1 | Indica | NA | LRR-TM | | OsR498G0102182300.01.T02 | BRASSINOSTEROID INSENSITIVE 1-associated receptor kinase 1 |
| maker-1-augustus-gene-350.26-mRNA-1 | Indica | NA | | | OsR498G0102183300.01.T01 | |
| maker-1-augustus-gene-350.28-mRNA-1 | Indica | NA | | | OsR498G0102183700.01.T01 | C2H2-type domain-containing protein |
| maker-1-augustus-gene-351.12-mRNA-1 | Indica | NA | | Os01g0810000 | OsR498G0102186800.01.T02 | U3 small nucleolar RNA-associated protein 11 |
| maker-1-augustus-gene-351.17-mRNA-1 | Indica | NA | | Os01g0810100 | OsR498G0102187200.01.T01 | RNase III domain-containing protein (Fragment) |
| maker-1-augustus-gene-351.2-mRNA-1 | Indica | Indica | | Os01g0809900 | OsR498G0102186500.01.T01 | DAO domain-containing protein (Fragment) |
| maker-1-augustus-gene-351.20-mRNA-1 | Indica | Indica | Kinase-LRR-TM | Os01g0810533 | OsR498G0102188400.01.T04 | Receptor-like protein kinase |
| maker-1-augustus-gene-351.26-mRNA-1 | Indica | NA | Kinase-TM | Os01g0810600 | | Putative light repressible receptor protein kinase |
| maker-1-augustus-gene-351.6-mRNA-1 | Indica | NA | | Os01g0810200 | OsR498G0102187300.01.T02 | RmlC-like jelly roll |
| maker-1-augustus-gene-351.8-mRNA-1 | Indica | NA | | Os01g0810300 | OsR498G0102187500.01.T02 | Calmodulin |
| maker-1-augustus-gene-352.35-mRNA-1 | Indica | NA | | Os01g0811100 | OsR498G0102192900.01.T01 | Proteasome endopeptidase complex |
| maker-1-augustus-gene-352.41-mRNA-1 | Indica | NA | | Os01g0812000 | OsR498G0102195500.01.T02 | Transcription factor GAMYB |
| maker-1-augustus-gene-352.45-mRNA-1 | Indica | NA | | Os01g0812050 | OsR498G0102195900.01.T01 | Ulp1 protease family C-terminal catalytic domain containing protein express |
| maker-1-augustus-gene-352.53-mRNA-1 | Indica | NA | Kinase-LRR-TM | | OsR498G0102191000.01.T01 | Putative light repressible receptor protein kinase |
| maker-1-augustus-gene-352.57-mRNA-1 | Indica | NA | Kinase-LRR-TM | Os01g0810900 | OsR498G0102192000.01.T04 | Putative light repressible receptor protein kinase |
| maker-1-augustus-gene-352.63-mRNA-1 | Indica | NA | | Os01g0811400 | OsR498G0102193800.01.T02 | Exostosin domain-containing protein |
| maker-1-augustus-gene-353.0-mRNA-1 | Indica | NA | | | OsR498G0102200100.01.T02 | Ethylene-responsive transcription factor WRI1 |
| maker-1-augustus-gene-353.15-mRNA-1 | Indica | NA | | Os01g0814000 | OsR498G0102201900.01.T01 | NUFIP1 domain-containing protein (Fragment) |
| maker-1-augustus-gene-353.22-mRNA-1 | Indica | NA | | Os01g0812600 | OsR498G0102198200.01.T01 | |
| maker-1-augustus-gene-353.26-mRNA-1 | Indica | Indica | | Os03g0322500 | OsR498G0102199200.01.T02 | 14 kDa zinc-binding protein, putative, expressed |
| maker-1-augustus-gene-353.31-mRNA-1 | Indica | NA | | Os01g0813100 | OsR498G0102199400.01.T02 | ABSCISIC ACID-INSENSITIVE 5-like protein 2 |
| maker-1-augustus-gene-353.35-mRNA-1 | Indica | NA | | Os01g0813500 | OsR498G0102200600.01.T01 | GLUE N-terminal domain-containing protein |
| maker-1-augustus-gene-353.40-mRNA-1 | Indica | Indica | | Os01g0930800 | OsR498G0102200800.01.T01 | Non-cyanogenic beta-glucosidase |

| | | | | | | |
|---|---|---|---|---|---|---|
| maker-1-augustus-gene-353.41-mRNA-1 | Indica | Indica | | Os01g0930800 | OsR498G0102200800.01.T01 | Beta-glucosidase 2 |
| maker-1-augustus-gene-353.44-mRNA-1 | Indica | Indica | | Os01g0930800 | OsR498G0102201400.01.T03 | 4-hydroxy-7-methoxy-3-oxo-3,4-dihydro-2H-1,4-benzoxazin-2-yl glucosidebe |
| maker-1-augustus-gene-353.8-mRNA-1 | Indica | NA | | Os01g0813900 | OsR498G0102201700.01.T06 | probable ADP-ribosylation factor GTPase-activating protein AGD14 |
| maker-1-augustus-gene-354.0-mRNA-1 | Indica | NA | | Os01g0814150 | | |
| maker-1-augustus-gene-354.10-mRNA-1 | Indica | Indica | | Os05g0489000 | OsR498G0102202600.01.T02 | IPPc domain-containing protein (Fragment) |
| maker-1-augustus-gene-354.14-mRNA-1 | Indica | NA | | Os01g0814400 | OsR498G0102203000.01.T01 | Dentin sialophosphoprotein |
| maker-1-augustus-gene-354.16-mRNA-1 | Indica | Indica | | Os01g0814700 | OsR498G0102203400.01.T05 | F-box protein SKIP24 |
| maker-1-augustus-gene-354.2-mRNA-1 | Indica | NA | | Os01g0814300 | OsR498G0102202700.01.T01 | Pentatricopeptide repeat-containing protein chloroplastic/mitochondrial |
| maker-1-augustus-gene-354.20-mRNA-1 | Indica | NA | | Os01g0814800 | OsR498G0102203600.01.T01 | Cysteine synthase |
| maker-1-augustus-gene-354.23-mRNA-1 | Indica | NA | | Os01g0814900 | OsR498G0102204200.01.T01 | NADH-cytochrome b5 reductase |
| maker-1-augustus-gene-354.27-mRNA-1 | Indica | Japonica | | Os01g0815400 | OsR498G0511077900.01.T01 | Laminin subunit gamma-3 |
| maker-1-augustus-gene-354.30-mRNA-1 | Indica | NA | | Os01g0816100 | OsR498G0102207800.01.T02 | Stress-induced transcription factor NAC1 |
| maker-1-augustus-gene-354.32-mRNA-1 | Indica | NA | | Os01g0816400 | OsR498G0102209000.01.T04 | VHS domain-containing protein |
| maker-1-augustus-gene-354.4-mRNA-1 | Indica | NA | | Os01g0815700 | OsR498G0102206700.01.T01 | Zinc finger protein VAR3 chloroplastic |
| maker-1-augustus-gene-354.8-mRNA-1 | Indica | NA | | Os01g0815800 | OsR498G0102206900.01.T03 | TRASH domain-containing protein |
| maker-1-augustus-gene-355.0-mRNA-1 | Indica | NA | Kinase-LRR-TM | | OsR498G0102210700.01.T02 | Leucine-rich repeat protein kinase family protein |
| maker-1-augustus-gene-355.13-mRNA-1 | Indica | NA | | Os01g0817700 | OsR498G0102213900.01.T04 | 2,3-bisphosphoglycerate-independent phosphoglycerate mutase |
| maker-1-augustus-gene-355.18-mRNA-1 | Indica | Indica | | Os01g0816400 | OsR498G0102210200.01.T01 | TOG domain-containing protein |
| maker-1-augustus-gene-355.21-mRNA-1 | Indica | NA | | Os01g0817000 | OsR498G0102211400.01.T01 | Calcium uniporter protein (Fragment) |
| maker-1-augustus-gene-355.24-mRNA-1 | Indica | NA | | Os01g0817100 | OsR498G0102211800.01.T05 | TLC domain-containing protein (Fragment) |
| maker-1-augustus-gene-355.26-mRNA-1 | Indica | NA | | Os01g0817800 | OsR498G0102214200.01.T01 | Transducin/WD40 repeat-like superfamily protein isoform 1 |
| maker-1-augustus-gene-355.9-mRNA-1 | Indica | Indica | | Os01g0817400 | OsR498G0102212100.01.T01 | Cation/H(+) antiporter 15 |
| maker-1-augustus-gene-356.0-mRNA-1 | Indica | NA | | Os01g0818000 | OsR498G0102215500.01.T06 | PIN-formed protein14 |
| maker-1-augustus-gene-356.11-mRNA-1 | Indica | NA | | Os01g0818400 | OsR498G0102216700.01.T02 | Homeobox domain-containing protein (Fragment) |
| maker-1-augustus-gene-356.13-mRNA-1 | Indica | NA | | Os01g0819000 | OsR498G0102218300.01.T01 | Proline-rich family protein |
| maker-1-augustus-gene-356.16-mRNA-1 | Indica | Indica | Kinase-LRR-TM | Os05g0480400 | OsR498G0102218900.01.T03 | Inactive receptor kinase |
| maker-1-augustus-gene-356.23-mRNA-1 | Indica | NA | Kinase-LRR-TM | | OsR498G0102217000.01.T01 | Non-specific serine/threonine protein kinase (Fragment) |
| maker-1-augustus-gene-356.25-mRNA-1 | Indica | Indica | | Os09g0538800 | OsR498G0102219200.01.T01 | Calcium lipid binding protein-like |
| maker-1-augustus-gene-356.37-mRNA-1 | Indica | Indica | | Os09g0538800 | OsR498G0102219400.01.T03 | Calcium lipid binding protein-like |
| maker-1-augustus-gene-356.4-mRNA-1 | Indica | NA | | | OsR498G0102215900.01.T01 | C2 calcium-dependent membrane targeting |
| maker-1-augustus-gene-356.7-mRNA-1 | Indica | NA | | Os01g0818300 | OsR498G0102216100.01.T01 | KH domain-containing protein SPIN1 |
| maker-1-augustus-gene-357.0-mRNA-1 | Indica | NA | | Os01g0819400 | OsR498G0102220900.01.T02 | Ubiquitin-conjugating enzyme E2 |
| maker-1-augustus-gene-357.1-mRNA-1 | Indica | NA | | Os01g0819400 | OsR498G0102221700.01.T02 | UBC core domain-containing protein (Fragment) |
| maker-1-augustus-gene-357.10-mRNA-1 | Indica | NA | | | OsR498G0102224700.01.T01 | WRKY transcription factor |
| maker-1-augustus-gene-357.12-mRNA-1 | Indica | NA | | | OsR498G0102225700.01.T01 | WRKY transcription factor 18 |
| maker-1-augustus-gene-357.14-mRNA-1 | Indica | NA | | | OsR498G0102226300.01.T01 | WRKY transcription factor 20 |
| maker-1-augustus-gene-357.16-mRNA-1 | Indica | NA | | Os01g0819900 | OsR498G0102222600.01.T01 | Inactive serine/threonine-protein kinase scy1 |
| maker-1-augustus-gene-357.20-mRNA-1 | Indica | Japonica | | Os01g0820000 | OsR498G0510486800.01.T01 | Trans-cinnamate 4-monooxygenase |
| maker-1-augustus-gene-357.6-mRNA-1 | Indica | NA | | Os01g0819400 | OsR498G0102220900.01.T02 | UBC core domain-containing protein (Fragment) |
| maker-1-augustus-gene-358.0-mRNA-1 | Indica | NA | | Os01g0821600 | OsR498G0102228300.01.T01 | WRKY transcription factor 1 |
| maker-1-augustus-gene-358.13-mRNA-1 | Indica | NA | | Os01g0821800 | OsR498G0102228800.01.T01 | tRNA-binding domain-containing protein (Fragment) |
| maker-1-augustus-gene-358.18-mRNA-1 | Indica | Indica | Kinase-LRR-TM | Os01g0821900 | OsR498G0102230200.01.T01 | Serine/threonine kinase |
| maker-1-augustus-gene-358.2-mRNA-1 | Indica | NA | | | | Glutathione S-transferase T2-like |
| maker-1-augustus-gene-358.20-mRNA-1 | Indica | NA | Kinase-TM | Os01g0822200 | OsR498G0102231000.01.T04 | Serine/threonine-protein kinase NAK |
| maker-1-augustus-gene-358.4-mRNA-1 | Indica | NA | | Os01g0821300 | OsR498G0102227200.01.T01 | WRKY55-superfamily of TFs having WRKY and zinc finger domains |
| maker-1-augustus-gene-358.8-mRNA-1 | Indica | NA | | | OsR498G0102228600.01.T02 | Protein FATTY ACID EXPORT 3 chloroplastic |
| maker-1-augustus-gene-359.42-mRNA-1 | Indica | NA | | Os01g0823100 | OsR498G0102236100.01.T01 | Expansin |
| maker-1-augustus-gene-359.44-mRNA-1 | Indica | NA | | | | TPT domain-containing protein |
| maker-1-augustus-gene-359.49-mRNA-1 | Indica | NA | | Os01g0823300 | OsR498G0102236500.01.T01 | 40S ribosomal protein S26 |
| maker-1-augustus-gene-359.51-mRNA-1 | Indica | NA | | Os01g0823400 | OsR498G0102236900.01.T02 | 2-oxoglutarate (2OG) and Fe(II)-dependent oxygenase superfamily protein |
| maker-1-augustus-gene-359.54-mRNA-1 | Indica | NA | | Os01g0823600 | OsR498G0102238700.01.T01 | GATA-N domain-containing protein |
| maker-1-augustus-gene-359.56-mRNA-1 | Indica | NA | | | OsR498G0102239900.01.T01 | |
| maker-1-augustus-gene-36.36-mRNA-1 | Indica | Indica | Kinase-LRR-TM | Os01g0170300 | OsR498G0100236300.01.T02 | Mitogen-activated protein kinase kinase kinase |
| maker-1-augustus-gene-36.46-mRNA-1 | Indica | NA | | Os01g0169500 | OsR498G0100232800.01.T02 | |
| maker-1-augustus-gene-36.55-mRNA-1 | Indica | NA | | | OsR498G0100235000.01.T02 | Galactinol--sucrose galactosyltransferase |
| maker-1-augustus-gene-360.10-mRNA-1 | Indica | NA | | | OsR498G0102242400.01.T01 | |
| maker-1-augustus-gene-360.12-mRNA-1 | Indica | NA | | Os01g0825500 | OsR498G0102245400.01.T03 | Nodulin-like domain-containing protein (Fragment) |
| maker-1-augustus-gene-360.14-mRNA-1 | Indica | NA | | Os01g0825700 | OsR498G0102246600.01.T01 | Target of Myb protein 1-like |
| maker-1-augustus-gene-360.20-mRNA-1 | Indica | Indica | | Os01g0825800 | OsR498G0102247400.01.T01 | Amino acid-polyamine transporter |
| maker-1-augustus-gene-360.21-mRNA-1 | Indica | NA | | Os01g0825800 | OsR498G0102247400.01.T02 | Amino acid-polyamine transporter |
| maker-1-augustus-gene-360.3-mRNA-1 | Indica | NA | Kinase-TM | Os01g0824600 | OsR498G0102242100.01.T06 | Non-specific serine/threonine protein kinase |

| | | | | | | |
|---|---|---|---|---|---|---|
| maker-1-augustus-gene-360.4-mRNA-1 | Indica | NA | | Os01g0824800 | OsR498G0102242700.01.T02 | |
| maker-1-augustus-gene-360.6-mRNA-1 | Indica | NA | | Os01g0825000 | OsR498G0102243500.01.T01 | LOB domain-containing protein 15 |
| maker-1-augustus-gene-360.8-mRNA-1 | Indica | NA | | Os01g0826000 | OsR498G0102247900.01.T01 | HMA domain-containing protein |
| maker-1-augustus-gene-361.38-mRNA-1 | Indica | NA | | Os01g0826400 | OsR498G0102249300.01.T01 | Transcription factor WRKY33 |
| maker-1-augustus-gene-361.40-mRNA-1 | Indica | NA | | | OsR498G0102249900.01.T01 | |
| maker-1-augustus-gene-361.43-mRNA-1 | Indica | NA | | Os01g0827300 | OsR498G0102253300.01.T01 | Laccase |
| maker-1-augustus-gene-361.45-mRNA-1 | Indica | NA | | | OsR498G0102255300.01.T01 | Cinnamoyl-CoA reductase |
| maker-1-augustus-gene-361.48-mRNA-1 | Indica | NA | | | OsR498G0102255500.01.T01 | Gamma interferon inducible lysosomal thiol reductase GILT |
| maker-1-augustus-gene-361.51-mRNA-1 | Indica | NA | | Os01g0828300 | OsR498G0102255700.01.T04 | Methyltransferase |
| maker-1-augustus-gene-361.57-mRNA-1 | Indica | NA | | | | |
| maker-1-augustus-gene-361.60-mRNA-1 | Indica | NA | | Os01g0826900 | OsR498G0102252200.01.T01 | Protein RETICULATA-RELATED 6 chloroplastic |
| maker-1-augustus-gene-361.64-mRNA-1 | Indica | NA | | Os01g0827200 | OsR498G0102252800.01.T01 | PX domain-containing protein (Fragment) |
| maker-1-augustus-gene-361.68-mRNA-1 | Indica | Indica | | Os01g0827700 | OsR498G0102254000.01.T01 | GDSL esterase/lipase |
| maker-1-augustus-gene-362.37-mRNA-1 | Indica | NA | | | OsR498G0611768000.01.T01 | |
| maker-1-augustus-gene-362.39-mRNA-1 | Indica | NA | | Os01g0829000 | OsR498G0102257700.01.T02 | Thioredoxin-like fold domain-containing protein MRL7, chloroplastic |
| maker-1-augustus-gene-362.48-mRNA-1 | Indica | NA | | Os01g0830700 | OsR498G0102265100.01.T04 | Protein trichome birefringence-like |
| maker-1-augustus-gene-362.53-mRNA-1 | Indica | NA | | Os01g0829100 | OsR498G0102258000.01.T01 | Cyclin dependent kinase inhibitor 2D |
| maker-1-augustus-gene-362.55-mRNA-1 | Indica | NA | | Os01g0829300 | | |
| maker-1-augustus-gene-362.57-mRNA-1 | Indica | NA | | Os01g0829900 | OsR498G0102261000.01.T01 | Probable metal-nicotianamine transporter YSL18 |
| maker-1-augustus-gene-362.59-mRNA-1 | Indica | NA | | Os01g0830000 | OsR498G0102261200.01.T01 | UPF0051 protein ABCI8, chloroplastic |
| maker-1-augustus-gene-362.61-mRNA-1 | Indica | NA | | Os01g0830500 | OsR498G0102262400.01.T01 | Fe2OG dioxygenase domain-containing protein (Fragment) |
| maker-1-augustus-gene-363.14-mRNA-1 | Indica | NA | | Os01g0832600 | OsR498G0102271500.01.T02 | Jasmonate-regulated gene 21 |
| maker-1-augustus-gene-363.16-mRNA-1 | Indica | Indica | | Os01g0831900 | OsR498G0102268800.01.T01 | Ammonium transporter |
| maker-1-augustus-gene-363.18-mRNA-1 | Indica | NA | | Os01g0831900 | OsR498G0102269200.01.T01 | Ammonium transporter |
| maker-1-augustus-gene-363.20-mRNA-1 | Indica | NA | | | OsR498G0102269400.01.T02 | Phytol kinase |
| maker-1-augustus-gene-363.25-mRNA-1 | Indica | Indica | | Os05g0467300 | OsR498G0102270000.01.T01 | |
| maker-1-augustus-gene-363.6-mRNA-1 | Indica | NA | | Os01g0832100 | OsR498G0102269700.01.T01 | Gdsl esteraselipase |
| maker-1-augustus-gene-363.8-mRNA-1 | Indica | Indica | | Os05g0467000 | OsR498G0102270900.01.T05 | Calcium-dependent protein kinase |
| maker-1-augustus-gene-364.11-mRNA-1 | Indica | NA | | Os01g0834200 | OsR498G0102276700.01.T05 | V-type proton ATPase subunit a |
| maker-1-augustus-gene-364.18-mRNA-1 | Indica | NA | | Os01g0833100 | OsR498G0102273800.01.T01 | FCP1 homology domain-containing protein |
| maker-1-augustus-gene-364.22-mRNA-1 | Indica | NA | | Os01g0833500 | OsR498G0102274800.01.T01 | Carboxypeptidase |
| maker-1-augustus-gene-364.25-mRNA-1 | Indica | NA | | Os01g0833600 | OsR498G0102275000.01.T01 | ATP-dependent Clp protease adapter protein ClpS |
| maker-1-augustus-gene-364.27-mRNA-1 | Indica | NA | | Os01g0833700 | OsR498G0102275200.01.T03 | Coatomer subunit delta |
| maker-1-augustus-gene-364.32-mRNA-1 | Indica | NA | | Os01g0833800 | OsR498G0102275400.01.T05 | IQ-domain 3 |
| maker-1-augustus-gene-364.35-mRNA-1 | Indica | Indica | | Os01g0834100 | OsR498G0102275800.01.T02 | Interactor of constitutive active ROPs 3 |
| maker-1-augustus-gene-364.38-mRNA-1 | Indica | NA | | | OsR498G0102275800.01.T05 | |
| maker-1-augustus-gene-364.6-mRNA-1 | Indica | NA | | Os01g0833200 | OsR498G0102274100.01.T02 | Putative rmlC-like cupin domain-containing protein |
| maker-1-augustus-gene-364.9-mRNA-1 | Indica | NA | | Os01g0833400 | OsR498G0102274300.01.T02 | Chlorophyll A-B binding family protein |
| maker-1-augustus-gene-365.0-mRNA-1 | Indica | NA | | Os01g0834700 | OsR498G0102279700.01.T01 | Zinc finger CCCH domain-containing protein 11 |
| maker-1-augustus-gene-365.11-mRNA-1 | Indica | NA | | Os01g0834500 | OsR498G0102279400.01.T04 | 40S ribosomal protein S23 |
| maker-1-augustus-gene-365.13-mRNA-1 | Indica | NA | | Os01g0834601 | OsR498G0102279400.01.T04 | 40S ribosomal protein S23 |
| maker-1-augustus-gene-365.15-mRNA-1 | Indica | Indica | | Os05g0465100 | OsR498G0102281200.01.T01 | Rab-GAP TBC domain-containing protein |
| maker-1-augustus-gene-365.20-mRNA-1 | Indica | NA | | Os01g0835700 | OsR498G0102285600.01.T01 | CCT domain |
| maker-1-augustus-gene-365.22-mRNA-1 | Indica | NA | | Os01g0835800 | OsR498G0102286000.01.T02 | NAB domain-containing protein |
| maker-1-augustus-gene-365.3-mRNA-1 | Indica | NA | | Os01g0835500 | OsR498G0102283700.01.T01 | Respiratory burst oxidase like protein H |
| maker-1-augustus-gene-365.9-mRNA-1 | Indica | Indica | | Os05g0463800 | OsR498G0102279000.01.T01 | Nuclear transcription factor Y subunit B |
| maker-1-augustus-gene-366.40-mRNA-1 | Indica | Indica | | Os01g0836400 | OsR498G0102287100.01.T04 | PCI domain-containing protein |
| maker-1-augustus-gene-366.45-mRNA-1 | Indica | NA | | | OsR498G0102290700.01.T01 | caffeoylshikimate esterase-like |
| maker-1-augustus-gene-366.49-mRNA-1 | Indica | NA | | Os01g0837300 | OsR498G0102291100.01.T01 | UDP-glucuronic acid decarboxylase |
| maker-1-augustus-gene-366.53-mRNA-1 | Indica | NA | | Os01g0837500 | OsR498G0102291300.01.T04 | DNA helicase |
| maker-1-augustus-gene-366.59-mRNA-1 | Indica | Indica | | Os01g0837600 | OsR498G0102291500.01.T01 | Plant-specific domain TIGR01589 family protein expressed |
| maker-1-augustus-gene-366.63-mRNA-1 | Indica | Indica | | Os01g0837000 | OsR498G0102290000.01.T02 | Ankyrin repeat-containing protein NPR4 |
| maker-1-augustus-gene-366.73-mRNA-1 | Indica | NA | | Os01g0837900 | OsR498G0102292600.01.T02 | Serinethreonine-protein kinase afc1 |
| maker-1-augustus-gene-367.0-mRNA-1 | Indica | NA | | | OsR498G0102293500.01.T02 | Transcription repressor OFP4 |
| maker-1-augustus-gene-367.1-mRNA-1 | Indica | NA | | Os01g0838100 | OsR498G0102294100.01.T02 | Replication protein A 70 kDa DNA-binding subunit B-like |
| maker-1-augustus-gene-367.14-mRNA-1 | Indica | NA | | Os04g0132900 | | |
| maker-1-augustus-gene-367.16-mRNA-1 | Indica | NA | | Os01g0838800 | OsR498G0102297100.01.T01 | Coatomer subunit zeta |
| maker-1-augustus-gene-367.19-mRNA-1 | Indica | NA | | | OsR498G0204529200.01.T01 | Putative non-LTR retroelement reverse transcriptase |
| maker-1-augustus-gene-367.20-mRNA-1 | Indica | NA | | | OsR498G0102299300.01.T04 | RplQ |
| maker-1-augustus-gene-367.26-mRNA-1 | Indica | NA | | Os01g0838900 | | DDE Tnp4 domain-containing protein (Fragment) |
| maker-1-augustus-gene-367.28-mRNA-1 | Indica | NA | | Os01g0839200 | OsR498G0102299000.01.T01 | UPSTREAM OF FLC protein (DUF966) |

| | | | | | | |
|---|---|---|---|---|---|---|
| maker-1-augustus-gene-367.30-mRNA-1 | Indica | NA | | Os01g0839700 | OsR498G0102300200.01.T01 | Ubiquitin-conjugating enzyme |
| maker-1-augustus-gene-368.0-mRNA-1 | Indica | NA | | Os01g0840100 | OsR498G0102302100.01.T02 | Heat shock cognate 70 kDa protein 2 |
| maker-1-augustus-gene-368.7-mRNA-1 | Indica | NA | | Os01g0840300 | OsR498G0102304200.01.T01 | Homeobox domain |
| maker-1-augustus-gene-37.33-mRNA-1 | Indica | NA | | Os01g0172200 | OsR498G0100242400.01.T02 | DEAD-box ATP-dependent RNA helicase 14 |
| maker-1-augustus-gene-37.36-mRNA-1 | Indica | NA | | Os01g0172300 | OsR498G0100242600.01.T02 | DUF4050 domain-containing protein (Fragment) |
| maker-1-augustus-gene-37.39-mRNA-1 | Indica | NA | | Os01g0172400 | OsR498G0100242800.01.T02 | Phospholipase D |
| maker-1-augustus-gene-37.6-mRNA-1 | Indica | NA | | | OsR498G0100242900.01.T04 | Peroxidase |
| maker-1-augustus-gene-383.1-mRNA-1 | Indica | NA | | Os01g0869200 | OsR498G0102410700.01.T01 | Magnesium transporter |
| maker-1-augustus-gene-383.32-mRNA-1 | Indica | Indica | | Os07g0232900 | OsR498G0102410400.01.T01 | Cadmium/zinc-transporting ATPase HMA2 |
| maker-1-augustus-gene-39.47-mRNA-1 | Indica | NA | | Os01g0176500 | OsR498G0100254600.01.T01 | Myosin-10 like |
| maker-1-augustus-gene-39.9-mRNA-1 | Indica | Indica | | Os05g0178000 | OsR498G0100254300.01.T01 | NPH3 domain-containing protein (Fragment) |
| maker-1-augustus-gene-422.62-mRNA-1 | Indica | Indica | | Os03g0855700 | OsR498G0102324800.01.T02 | DUF223 domain-containing protein |
| maker-1-augustus-gene-422.66-mRNA-1 | Indica | Indica | | Os01g0630700 | OsR498G1221780100.01.T02 | Putative transcription factor DP, helitron helicase-like domain-containing pro |
| maker-1-augustus-gene-425.0-mRNA-1 | Indica | NA | | Os01g0850800 | OsR498G0102340700.01.T01 | Laccase |
| maker-1-augustus-gene-44.5-mRNA-1 | Indica | NA | | | OsR498G0100281900.01.T04 | Spatacsin |
| maker-1-augustus-gene-445.9-mRNA-1 | Indica | Indica | Kinase-TM | Os01g0890600 | OsR498G0102491100.01.T02 | Receptor-like serine/threonine-protein kinase |
| maker-1-augustus-gene-448.23-mRNA-1 | Indica | NA | Kinase | | OsR498G0102507600.01.T01 | Cell division control protein 2 |
| maker-1-augustus-gene-45.72-mRNA-1 | Indica | NA | | Os01g0183700 | | Histone-lysine N-methyltransferase ATX1 |
| maker-1-augustus-gene-46.38-mRNA-1 | Indica | NA | | | OsR498G0100288300.01.T02 | Cysteine dioxygenase |
| maker-1-augustus-gene-47.22-mRNA-1 | Indica | NA | | Os01g0187000 | OsR498G0100294100.01.T01 | Skp1 domain-containing protein |
| maker-1-augustus-gene-47.42-mRNA-1 | Indica | NA | Kinase-TM | Os01g0186700 | OsR498G0100292200.01.T03 | AGC (cAMP-dependent cGMP-dependent and protein kinase C) kinase family |
| maker-1-augustus-gene-470.39-mRNA-1 | Indica | NA | | Os01g0905200 | OsR498G0102535900.01.T01 | Transposon protein, putative, unclassified |
| maker-1-augustus-gene-470.41-mRNA-1 | Indica | Indica | | Os01g0905300 | OsR498G0102536100.01.T01 | Exocyst complex component EXO70A1 |
| maker-1-augustus-gene-470.47-mRNA-1 | Indica | NA | | Os01g0905400 | OsR498G0102536400.01.T01 | B3 domain-containing protein |
| maker-1-augustus-gene-470.50-mRNA-1 | Indica | NA | | | OsR498G0102539200.01.T02 | Ceramide glucosyltransferase |
| maker-1-augustus-gene-470.58-mRNA-1 | Indica | NA | | | OsR498G0102540800.01.T02 | Vacuolar protein sorting-associated protein 53 A like |
| maker-1-augustus-gene-471.10-mRNA-1 | Indica | NA | | Os01g0908200 | OsR498G0102547300.01.T01 | BTB/POZ and TAZ domain-containing protein 1 |
| maker-1-augustus-gene-471.13-mRNA-1 | Indica | NA | | | OsR498G0102548500.01.T02 | Proline transporter 2 |
| maker-1-augustus-gene-471.14-mRNA-1 | Indica | NA | | | OsR498G0102548500.01.T01 | Proline transporter 1 |
| maker-1-augustus-gene-471.2-mRNA-1 | Indica | NA | | Os01g0907300 | OsR498G0102542500.01.T02 | GPI transamidase component PIG-S |
| maker-1-augustus-gene-471.27-mRNA-1 | Indica | NA | | | | Ypt/Rab-GAP domain of gyp1p superfamily protein isoform 2 |
| maker-1-augustus-gene-471.32-mRNA-1 | Indica | Indica | | Os01g0908500 | OsR498G0102547800.01.T01 | Magnesium transporter |
| maker-1-augustus-gene-471.37-mRNA-1 | Indica | Indica | | Os01g0908700 | OsR498G0102548800.01.T01 | E3 ubiquitin-protein ligase RGLG2 |
| maker-1-augustus-gene-471.6-mRNA-1 | Indica | NA | | Os01g0907400 | | Lysine-specific demethylase |
| maker-1-augustus-gene-471.8-mRNA-1 | Indica | NA | | Os01g0907900 | OsR498G0102544500.01.T01 | RRM domain-containing protein (Fragment) |
| maker-1-augustus-gene-472.0-mRNA-1 | Indica | NA | | | OsR498G0102550900.01.T02 | NPL domain-containing protein |
| maker-1-augustus-gene-472.11-mRNA-1 | Indica | NA | | Os01g0910400 | OsR498G0102553900.01.T09 | Lysine ketoglutarate reductase trans-splicing related 1 |
| maker-1-augustus-gene-472.17-mRNA-1 | Indica | NA | | Os01g0909200 | OsR498G0102551400.01.T02 | Endoribonuclease Dicer a |
| maker-1-augustus-gene-472.30-mRNA-1 | Indica | NA | | Os01g0909500 | | NPL domain-containing protein |
| maker-1-augustus-gene-472.6-mRNA-1 | Indica | NA | | Os01g0909300 | | Wiskott-Aldrich syndrome protein family member 2 like |
| maker-1-augustus-gene-473.0-mRNA-1 | Indica | NA | | Os01g0911200 | OsR498G0102555700.01.T01 | Dolichyl-diphosphooligosaccharide--protein glycosyltransferase subunit 2 |
| maker-1-augustus-gene-473.13-mRNA-1 | Indica | NA | | | OsR498G1119986200.01.T02 | 4F5 domain-containing protein (Fragment) |
| maker-1-augustus-gene-473.15-mRNA-1 | Indica | NA | NBS-TM | Os01g0911300 | OsR498G0102556000.01.T01 | ABC transporter B family member 26, chloroplastic |
| maker-1-augustus-gene-473.6-mRNA-1 | Indica | NA | | Os01g0911000 | | Neuroguidin |
| maker-1-augustus-gene-473.8-mRNA-1 | Indica | NA | | | OsR498G0102555000.01.T06 | DEAD-box ATP-dependent RNA helicase |
| maker-1-augustus-gene-474.33-mRNA-1 | Indica | NA | | Os01g0911800 | OsR498G0102558100.01.T01 | BLISTER |
| maker-1-augustus-gene-474.40-mRNA-1 | Indica | NA | | | OsR498G0102559900.01.T01 | snRNA-activating protein complex subunit |
| maker-1-augustus-gene-474.44-mRNA-1 | Indica | NA | | Os01g0912700 | OsR498G0102560100.01.T03 | Thioredoxin F-type chloroplastic |
| maker-1-augustus-gene-474.48-mRNA-1 | Indica | NA | | Os01g0911700 | OsR498G0102558000.01.T02 | Regulatory protein viviparous-1 |
| maker-1-augustus-gene-474.51-mRNA-1 | Indica | NA | | | OsR498G0102558400.01.T05 | Protein arginine methyltransferase NDUFAF7 |
| maker-1-augustus-gene-491.15-mRNA-1 | Indica | NA | | Os07g0513300 | OsR498G1120703200.01.T01 | PMD domain-containing protein (Fragment) |
| maker-1-augustus-gene-495.0-mRNA-1 | Indica | NA | | Os01g0949700 | OsR498G0102701900.01.T03 | Glutathione S-transferase, N-terminal domain containing protein, expressed |
| maker-1-augustus-gene-497.64-mRNA-1 | Indica | NA | | Os01g0953600 | OsR498G0102713200.01.T01 | NAD(P)H dehydrogenase (quinone) |
| maker-1-augustus-gene-499.21-mRNA-1 | Indica | NA | | Os01g0956700 | OsR498G0102729800.01.T01 | |
| maker-1-augustus-gene-500.16-mRNA-1 | Indica | NA | | Os01g0958500 | OsR498G0102735500.01.T01 | RNA-binding family protein isoform 1 |
| maker-1-augustus-gene-501.12-mRNA-1 | Indica | NA | | | | |
| maker-1-augustus-gene-501.50-mRNA-1 | Indica | NA | | Os01g0961600 | OsR498G0102743800.01.T01 | |
| maker-1-augustus-gene-501.55-mRNA-1 | Indica | Indica | | Os01g0962200 | OsR498G0102744800.01.T01 | Senescence associated proteingene 18 isoform 1 |
| maker-1-augustus-gene-501.8-mRNA-1 | Indica | Indica | | Os07g0651500 | OsR498G0102743500.01.T03 | ATP-dependent DNA helicase |
| maker-1-augustus-gene-504.60-mRNA-1 | Indica | NA | | Os01g0968300 | OsR498G0102762700.01.T01 | Late embryogenesis abundant protein-related / LEA protein-related |
| maker-1-augustus-gene-504.78-mRNA-1 | Indica | Indica | | Os01g0968300 | OsR498G0102762600.01.T01 | Late embryogenesis abundant protein-related / LEA protein-related |

| | | | | | | |
|---|---|---|---|---|---|---|
| maker-1-augustus-gene-505.22-mRNA-1 | Indica | NA | | | OsR498G0102769200.01.T02 | |
| maker-1-augustus-gene-52.29-mRNA-1 | Indica | NA | | Os01g0195500 | OsR498G0100335800.01.T01 | Translation machinery-associated protein 22 |
| maker-1-augustus-gene-67.0-mRNA-1 | Indica | Indica | | Os05g0215600 | OsR498G0100439900.01.T01 | Delta(8)-fatty-acid desaturase-like |
| maker-1-augustus-gene-67.11-mRNA-1 | Indica | NA | | | OsR498G0100441000.01.T02 | Peptidase C45, acyl-coenzyme A/6-aminopenicillanic acid acyl-transferase |
| maker-1-augustus-gene-67.2-mRNA-1 | Indica | NA | | Os01g0221600 | OsR498G0100440500.01.T08 | Aluminum-activated malate transporter like |
| maker-1-augustus-gene-67.6-mRNA-1 | Indica | NA | | | OsR498G0100437400.01.T01 | Jasmonic acid-amino acid-conjugating enzyme |
| maker-1-augustus-gene-67.9-mRNA-1 | Indica | NA | | Os01g0221300 | OsR498G0100439000.01.T01 | Chromodomain-helicase-DNA-binding protein 1-like |
| maker-1-augustus-gene-7.14-mRNA-1 | Indica | Indica | | Os05g0103700 | OsR498G0100040000.01.T01 | |
| maker-1-augustus-gene-7.22-mRNA-1 | Indica | NA | | | OsR498G0100040600.01.T01 | U-box domain-containing protein 4 |
| maker-1-augustus-gene-7.25-mRNA-1 | Indica | NA | Kinase-TM | Os01g0113200 | OsR498G0100043400.01.T02 | Receptor serine/threonine kinase |
| maker-1-augustus-gene-7.9-mRNA-1 | Indica | NA | Kinase-TM | Os01g0113300 | OsR498G0100043700.01.T01 | Iron-deficiency induced receptor-like kinase |
| maker-1-augustus-gene-8.130-mRNA-1 | Indica | Japonica | | Os01g0115200 | OsR498G1119343800.01.T03 | Amidase signature enzyme |
| maker-1-augustus-gene-8.133-mRNA-1 | Indica | Indica | Kinase-TM | Os01g0113400 | OsR498G0100045600.01.T01 | Serine/threonine protein kinase |
| maker-1-augustus-gene-8.134-mRNA-1 | Indica | Japonica | Kinase-TM | Os01g0114100 | OsR498G0100046400.01.T01 | Putative rust resistance kinase Lr10 |
| maker-1-augustus-gene-8.139-mRNA-1 | Indica | Indica | Kinase-TM | Os01g0116200 | OsR498G0100046400.01.T07 | Putative rust resistance kinase Lr10 |
| maker-1-augustus-gene-8.141-mRNA-1 | Indica | Japonica | Kinase-TM | Os01g0114600 | OsR498G0100047600.01.T01 | Receptor-like kinase |
| maker-1-augustus-gene-8.144-mRNA-1 | Indica | Indica | | Os01g0115750 | OsR498G0100050600.01.T01 | Putative rust resistance kinase Lr10 |
| maker-1-augustus-gene-8.47-mRNA-1 | Indica | Indica | Kinase-TM | Os01g0113800 | OsR498G0100045100.01.T01 | Putative rust resistance kinase Lr10 |
| maker-1-augustus-gene-8.48-mRNA-1 | Indica | NA | Kinase-TM | Os01g0113800 | OsR498G0100045700.01.T01 | Resistance-related receptor-like kinase |
| maker-1-augustus-gene-8.49-mRNA-1 | Indica | NA | Kinase-TM | Os01g0115900 | OsR498G0100046700.01.T01 | Receptor-like protein kinase (Fragment) |
| maker-1-augustus-gene-8.50-mRNA-1 | Indica | Japonica | Kinase-TM | Os01g0114500 | OsR498G0100047300.01.T01 | Receptor serine/threonine kinase |
| maker-1-augustus-gene-8.51-mRNA-1 | Indica | NA | Kinase-TM | Os01g0114700 | OsR498G0100047900.01.T01 | Resistance-related receptor-like kinase |
| maker-1-augustus-gene-80.44-mRNA-1 | Indica | NA | | Os02g0140500 | OsR498G0101278400.01.T01 | Transposon protein, putative, Mutator sub-class, expressed |
| maker-1-augustus-gene-80.55-mRNA-1 | Indica | NA | | Os01g0244400 | OsR498G0100533600.01.T01 | RNA-binding glycine-rich protein-like |
| maker-1-augustus-gene-81.4-mRNA-1 | Indica | Indica | | Os01g0246700 | OsR498G0100541900.01.T01 | WRKY transcription factor |
| maker-1-augustus-gene-9.101-mRNA-1 | Indica | NA | Kinase-TM | Os01g0116400 | OsR498G0100053600.01.T01 | Receptor-like kinase |
| maker-1-augustus-gene-9.102-mRNA-1 | Indica | Indica | | Os01g0116600 | OsR498G0100054400.01.T01 | Tr-type G domain-containing protein |
| maker-1-augustus-gene-9.103-mRNA-1 | Indica | NA | | Os01g0116400 | OsR498G0100053600.01.T01 | Receptor kinase |
| maker-1-augustus-gene-9.105-mRNA-1 | Indica | Indica | Kinase-TM | Os01g0117400 | OsR498G0100056600.01.T01 | Putative rust resistance kinase Lr10 |
| maker-1-augustus-gene-9.181-mRNA-1 | Indica | NA | | Os01g0117850 | OsR498G0100057000.01.T04 | |
| maker-1-augustus-gene-9.48-mRNA-1 | Indica | NA | | Os01g0115600 | OsR498G0100050900.01.T02 | Ser/Thr receptor-like kinase |
| maker-1-augustus-gene-9.51-mRNA-1 | Indica | NA | Kinase-TM | Os01g0115900 | OsR498G0100052100.01.T01 | Receptor-like protein kinase (Fragment) |
| maker-1-augustus-gene-9.52-mRNA-1 | Indica | Japonica | Kinase-TM | Os01g0114500 | OsR498G0100045700.01.T01 | Protein kinase domain-containing protein |
| maker-1-augustus-gene-9.53-mRNA-1 | Indica | Japonica | | Os01g0116500 | OsR498G0100054700.01.T03 | Receptor kinase |
| maker-1-augustus-gene-9.75-mRNA-1 | Indica | Indica | Kinase-TM | Os01g0117500 | OsR498G0100056100.01.T02 | Putative rust resistance kinase Lr10 |
| maker-1-augustus-gene-9.81-mRNA-1 | Indica | Japonica | Kinase-TM | Os01g0117100 | OsR498G0100056700.01.T02 | Receptor kinase |
| maker-1-augustus-gene-9.86-mRNA-1 | Indica | NA | Kinase-TM | Os01g0115750 | OsR498G0100051800.01.T01 | Putative rust resistance kinase Lr10 |
| maker-1-augustus-gene-9.99-mRNA-1 | Indica | Indica | Kinase-TM | Os01g0116000 | OsR498G0100052000.01.T01 | Serine/threonine protein kinase |
| maker-1-augustus-gene-94.39-mRNA-1 | Indica | NA | CC-NBS-TM | Os01g0269500 | OsR498G0100629900.01.T03 | Putative NBS-LRR type disease resistance protein |
| maker-1-augustus-gene-96.8-mRNA-1 | Indica | NA | | | OsR498G0509629200.01.T01 | |
| maker-10-augustus-gene-1.0-mRNA-1 | Indica | Indica | CC-Kinase-TM | Os10g0100500 | OsR498G1017994900.01.T04 | E3 ubiquitin ligase |
| maker-10-augustus-gene-1.7-mRNA-1 | Indica | NA | | | OsR498G1017994000.01.T01 | Protein TIC 62, chloroplastic |
| maker-10-augustus-gene-111.39-mRNA-1 | Indica | Indica | | Os03g0151800 | OsR498G1018799900.01.T01 | Cell division control protein 48 E |
| maker-10-augustus-gene-111.45-mRNA-1 | Indica | NA | | Os10g0442900 | OsR498G1018800500.01.T01 | putative white-brown complex homolog protein 30 isoform X1 |
| maker-10-augustus-gene-111.52-mRNA-1 | Indica | NA | | | OsR498G1018798600.01.T02 | Phosphoglycerate kinase |
| maker-10-augustus-gene-111.57-mRNA-1 | Indica | Indica | | Os10g0442300 | OsR498G1018798800.01.T01 | Glycosyltransferase |
| maker-10-augustus-gene-111.62-mRNA-1 | Indica | NA | Kinase | | OsR498G1018800400.01.T01 | Putative serine/threonine-protein kinase NAK |
| maker-10-augustus-gene-111.64-mRNA-1 | Indica | NA | | Os03g0307400 | OsR498G0305792900.01.T01 | Six-bladed beta-propeller |
| maker-10-augustus-gene-112.34-mRNA-1 | Indica | Japonica | | Os10g0443200 | OsR498G1018801300.01.T01 | Translin like |
| maker-10-augustus-gene-112.37-mRNA-1 | Indica | NA | | Os10g0443800 | OsR498G1018801700.01.T01 | HTH myb-type domain-containing protein (Fragment) |
| maker-10-augustus-gene-112.38-mRNA-1 | Indica | NA | | Os10g0444400 | OsR498G1018802500.01.T02 | Macro domain-containing protein |
| maker-10-augustus-gene-115.0-mRNA-1 | Indica | NA | | Os04g0167900 | OsR498G0407615000.01.T02 | Protein-serine/threonine phosphatase |
| maker-10-augustus-gene-117.44-mRNA-1 | Indica | NA | | Os04g0165000 | OsR498G0407602000.01.T01 | Thaumatin-like protein 1a |
| maker-10-augustus-gene-117.47-mRNA-1 | Indica | NA | | | OsR498G0407601700.01.T02 | Limit dextrinase type starch debranching enzyme |
| maker-10-augustus-gene-118.0-mRNA-1 | Indica | NA | | Os04g0162100 | OsR498G0407596300.01.T01 | VEFS-Box domain-containing protein |
| maker-10-augustus-gene-12.38-mRNA-1 | Indica | Indica | | Os10g0370700 | OsR498G1018036700.01.T02 | Putative LeOPT1-oligopeptide transporter |
| maker-10-augustus-gene-12.44-mRNA-1 | Indica | Indica | | Os10g0112100 | OsR498G1018036600.01.T02 | Plant cadmium resistance 2 |
| maker-10-augustus-gene-12.48-mRNA-1 | Indica | NA | | Os10g0112600 | OsR498G1018037200.01.T01 | Transmembrane 9 superfamily member |
| maker-10-augustus-gene-122.0-mRNA-1 | Indica | NA | | Os04g0155000 | OsR498G0407571100.01.T01 | |
| maker-10-augustus-gene-122.2-mRNA-1 | Indica | NA | | Os04g0155200 | OsR498G0407574100.01.T01 | |
| maker-10-augustus-gene-122.4-mRNA-1 | Indica | NA | | Os04g0155000 | OsR498G0407569300.01.T01 | |

| | | | | | | |
|---|---|---|---|---|---|---|
| maker-10-augustus-gene-122.5-mRNA-1 | Indica | NA | | | OsR498G0407568700.01.T01 | DUF4220 domain-containing protein (Fragment) |
| maker-10-augustus-gene-126.25-mRNA-1 | Indica | NA | | Os04g0142400 | OsR498G0407539400.01.T02 | |
| maker-10-augustus-gene-127.46-mRNA-1 | Indica | NA | Kinase-TM | Os04g0141400 | OsR498G0407537100.01.T01 | Putative receptor-type protein kinase LRK1 |
| maker-10-augustus-gene-127.48-mRNA-1 | Indica | NA | Kinase-TM | Os04g0141200 | OsR498G0407536900.01.T01 | Receptor-like serine/threonine-protein kinase |
| maker-10-augustus-gene-127.52-mRNA-1 | Indica | NA | | Os02g0824700 | OsR498G0407530700.01.T04 | V-type proton ATPase subunit F |
| maker-10-augustus-gene-128.23-mRNA-1 | Indica | NA | | Os04g0136700 | OsR498G0407526200.01.T01 | CBS domain-containing protein CBSX1, chloroplastic |
| maker-10-augustus-gene-128.30-mRNA-1 | Indica | NA | | Os04g0136900 | OsR498G0407526500.01.T01 | Serine-rich 25 kDa antigen protein |
| maker-10-augustus-gene-129.0-mRNA-1 | Indica | NA | | Os04g0476100 | OsR498G0407524800.01.T01 | Amine oxidase |
| maker-10-augustus-gene-131.28-mRNA-1 | Indica | NA | Kinase-TM | Os04g0125700 | OsR498G0407519500.01.T01 | Lectin-domain containing receptor kinase A4.3 |
| maker-10-augustus-gene-131.30-mRNA-1 | Indica | NA | | | OsR498G0407523100.01.T01 | Histidine decarboxylase |
| maker-10-augustus-gene-132.26-mRNA-1 | Indica | NA | | | | |
| maker-10-augustus-gene-132.30-mRNA-1 | Indica | NA | | Os04g0134800 | OsR498G0407518700.01.T01 | Peroxidase |
| maker-10-augustus-gene-133.0-mRNA-1 | Indica | NA | | Os04g0131900 | OsR498G0407513000.01.T01 | Sterol 3-beta-glucosyltransferase |
| maker-10-augustus-gene-133.8-mRNA-1 | Indica | NA | | Os04g0132300 | OsR498G0407513700.01.T03 | A1 cistron-splicing factor, AAR2 |
| maker-10-augustus-gene-136.28-mRNA-1 | Indica | NA | | Os04g0127200 | OsR498G0407499700.01.T01 | Subtilisin-like protease SBT3.3 |
| maker-10-augustus-gene-136.29-mRNA-1 | Indica | NA | | Os04g0127300 | OsR498G0407499700.01.T01 | Subtilisin-like protease SBT3.3 |
| maker-10-augustus-gene-136.44-mRNA-1 | Indica | NA | | Os04g0121100 | OsR498G0407492700.01.T01 | Subtilisin-like serine endopeptidase family protein |
| maker-10-augustus-gene-138.14-mRNA-1 | Indica | NA | | Os04g0121300 | OsR498G0407492700.01.T01 | Subtilisin-like protein protease SBT3.7 |
| maker-10-augustus-gene-138.18-mRNA-1 | Indica | NA | | | | Subtilisin-like protease |
| maker-10-augustus-gene-139.33-mRNA-1 | Indica | NA | | Os04g0118400 | OsR498G0407485900.01.T07 | Tr-type G domain-containing protein |
| maker-10-augustus-gene-141.0-mRNA-1 | Indica | NA | | | OsR498G0407475000.01.T01 | Putative leucine rich repeat |
| maker-10-augustus-gene-141.2-mRNA-1 | Indica | NA | | | | Transposon protein, putative, Mutator sub-class |
| maker-10-augustus-gene-142.3-mRNA-1 | Indica | NA | Kinase-TM | Os01g0136400 | OsR498G0100125200.01.T04 | Putative serine/threonine-protein kinase |
| maker-10-augustus-gene-144.32-mRNA-1 | Indica | NA | CC-LRR-NBS-TM | Os04g0111900 | OsR498G0407452000.01.T01 | Putative NB-ARC domain-containing protein (Fragment) |
| maker-10-augustus-gene-144.34-mRNA-1 | Indica | NA | | Os03g0743900 | OsR498G0407449600.01.T01 | Sulfate adenylyltransferase |
| maker-10-augustus-gene-145.10-mRNA-1 | Indica | NA | Kinase-TM | Os04g0109100 | OsR498G0407442600.01.T01 | Non-specific serine/threonine protein kinase |
| maker-10-augustus-gene-145.12-mRNA-1 | Indica | NA | | Os04g0110400 | OsR498G0407447100.01.T01 | SASA domain-containing protein |
| maker-10-augustus-gene-145.15-mRNA-1 | Indica | NA | | Os04g0110300 | OsR498G0407446700.01.T01 | SWI/SNF complex subunit SWI3D |
| maker-10-augustus-gene-145.2-mRNA-1 | Indica | NA | | | OsR498G0407447600.01.T01 | Zn-finger, RanBP-type, containing protein |
| maker-10-augustus-gene-145.8-mRNA-1 | Indica | NA | | | OsR498G0407445800.01.T01 | |
| maker-10-augustus-gene-146.42-mRNA-1 | Indica | NA | | Os09g0482610 | OsR498G0407437300.01.T01 | Heat shock protein 2 |
| maker-10-augustus-gene-147.42-mRNA-1 | Indica | NA | | Os04g0105700 | OsR498G0407429000.01.T10 | Dimethylallyl, adenosine tRNA methylthiotransferase |
| maker-10-augustus-gene-147.45-mRNA-1 | Indica | NA | | | OsR498G0407429000.01.T05 | Adenylate kinase |
| maker-10-augustus-gene-147.49-mRNA-1 | Indica | NA | | Os04g0105400 | OsR498G0407428600.01.T01 | Ion channel regulatory protein, UNC-93 |
| maker-10-augustus-gene-147.53-mRNA-1 | Indica | NA | | Os04g0106400 | OsR498G0407430900.01.T02 | Glyoxylate/hydroxypyruvate reductase HPR3 |
| maker-10-augustus-gene-147.56-mRNA-1 | Indica | NA | | Os04g0106300 | OsR498G0407430700.01.T01 | Mitochondrion arginase |
| maker-10-augustus-gene-147.62-mRNA-1 | Indica | NA | | Os04g0105300 | OsR498G0407428300.01.T01 | Oxidoreductase |
| maker-10-augustus-gene-148.27-mRNA-1 | Indica | NA | | Os04g0105100 | OsR498G0407426700.01.T01 | RING-CH-type domain-containing protein (Fragment) |
| maker-10-augustus-gene-148.33-mRNA-1 | Indica | NA | | | | Zinc finger CCCH domain-containing protein 24 |
| maker-10-augustus-gene-158.39-mRNA-1 | Indica | NA | | Os10g0457700 | OsR498G1018842700.01.T02 | ATP-dependent helicase ATRX |
| maker-10-augustus-gene-159.9-mRNA-1 | Indica | NA | | | | |
| maker-10-augustus-gene-161.35-mRNA-1 | Indica | NA | | | OsR498G1018856300.01.T01 | Plastid transcriptionally active 3 |
| maker-10-augustus-gene-161.40-mRNA-1 | Indica | NA | | Os10g0462900 | OsR498G1018856700.01.T01 | Mitochondrial chaperonin-60 |
| maker-10-augustus-gene-162.78-mRNA-1 | Indica | Indica | | Os10g0465000 | OsR498G1018863400.01.T04 | WD repeat-containing protein 26 |
| maker-10-augustus-gene-163.13-mRNA-1 | Indica | NA | | Os10g0467600 | OsR498G1018869700.01.T01 | mRNA cap-binding protein (Fragment) |
| maker-10-augustus-gene-163.16-mRNA-1 | Indica | NA | | Os10g0467800 | OsR498G1018869900.01.T01 | Cellulose synthase |
| maker-10-augustus-gene-163.28-mRNA-1 | Indica | Indica | Kinase-LRR-TM | Os10g0467900 | OsR498G1018870200.01.T01 | Receptor protein kinase |
| maker-10-augustus-gene-164.27-mRNA-1 | Indica | NA | | Os10g0469900 | OsR498G1018875100.01.T01 | Peptide transporter PTR3-A |
| maker-10-augustus-gene-164.29-mRNA-1 | Indica | Japonica | LRR-TM | Os10g0469600 | OsR498G1018872900.01.T01 | MDIS1-interacting receptor like kinase 2 |
| maker-10-augustus-gene-2.0-mRNA-1 | Indica | NA | | Os10g0101100 | OsR498G1017997000.01.T05 | Serine carboxypeptidase-like 18 |
| maker-10-augustus-gene-2.4-mRNA-1 | Indica | NA | | Os10g0101200 | OsR498G1017997800.01.T02 | Serine carboxypeptidase |
| maker-10-augustus-gene-26.3-mRNA-1 | Indica | NA | | Os10g0138300 | OsR498G1018113300.01.T01 | F-box domain-containing protein (Fragment) |
| maker-10-augustus-gene-27.0-mRNA-1 | Indica | NA | | Os10g0138600 | OsR498G1018113700.01.T01 | F-box domain containing protein, expressed |
| maker-10-augustus-gene-27.2-mRNA-1 | Indica | NA | | | OsR498G1018116900.01.T01 | F-box domain containing protein, expressed |
| maker-10-augustus-gene-28.0-mRNA-1 | Indica | NA | | Os10g0141900 | OsR498G1018123300.01.T02 | Queuine tRNA-ribosyltransferase accessory subunit 2 |
| maker-10-augustus-gene-28.13-mRNA-1 | Indica | NA | | | | Putative alpha-mannosidase |
| maker-10-augustus-gene-28.14-mRNA-1 | Indica | NA | | | | Putative alpha-mannosidase |
| maker-10-augustus-gene-29.0-mRNA-1 | Indica | NA | | | OsR498G1018125700.01.T01 | DUF4220 domain-containing protein (Fragment) |
| maker-10-augustus-gene-3.24-mRNA-1 | Indica | Indica | | Os07g0248800 | OsR498G1018004100.01.T01 | |
| maker-10-augustus-gene-30.0-mRNA-1 | Indica | Japonica | | Os10g0144700 | OsR498G1018131800.01.T01 | Cytochrome P450 family protein, expressed |
| maker-10-augustus-gene-30.11-mRNA-1 | Indica | NA | | Os10g0146200 | OsR498G1018137200.01.T02 | Thaumatin-like protein 1 |

| | | | | | | |
|---|---|---|---|---|---|---|
| maker-10-augustus-gene-30.2-mRNA-1 | Indica | NA | | Os10g0144800 | | F-box domain-containing protein |
| maker-10-augustus-gene-31.0-mRNA-1 | Indica | Indica | | Os03g0244200 | OsR498G1018140100.01.T01 | Thaumatin-like protein 1 |
| maker-10-augustus-gene-31.4-mRNA-1 | Indica | NA | | Os10g0148100 | OsR498G1018145800.01.T01 | Proline-rich protein 4 |
| maker-10-augustus-gene-32.0-mRNA-1 | Indica | NA | | Os10g0148400 | OsR498G1018147200.01.T01 | Peptide transporter PTR5 |
| maker-10-augustus-gene-32.10-mRNA-1 | Indica | Indica | | Os10g0149200 | OsR498G1018149800.01.T01 | Proline-rich protein, putative, expressed |
| maker-10-augustus-gene-32.2-mRNA-1 | Indica | NA | | Os10g0148700 | OsR498G1018147400.01.T01 | |
| maker-10-augustus-gene-32.4-mRNA-1 | Indica | NA | | | | F-box domain-containing protein |
| maker-10-augustus-gene-32.9-mRNA-1 | Indica | Indica | | Os10g0149200 | OsR498G1018149000.01.T01 | Proline-rich protein, putative, expressed |
| maker-10-augustus-gene-33.23-mRNA-1 | Indica | Japonica | | Os10g0149400 | OsR498G1018150600.01.T01 | Proline-rich protein, putative, expressed |
| maker-10-augustus-gene-34.14-mRNA-1 | Indica | NA | | Os10g0150300 | OsR498G1018152000.01.T01 | Proline-rich protein (Fragment) |
| maker-10-augustus-gene-35.0-mRNA-1 | Indica | NA | Kinase-TM | | | Wall-associated receptor kinase 4 |
| maker-10-augustus-gene-36.47-mRNA-1 | Indica | NA | | Os10g0151800 | OsR498G1018157200.01.T02 | Heterogeneous nuclear ribonucleoprotein like |
| maker-10-augustus-gene-37.38-mRNA-1 | Indica | Japonica | | Os10g0155400 | OsR498G1018168500.01.T04 | Amidase family protein, expressed |
| maker-10-augustus-gene-37.42-mRNA-1 | Indica | Japonica | Kinase-LRR-TM | Os10g0155733 | OsR498G1018170700.01.T01 | Leucine-rich repeat receptor-like protein kinase PEPR1 |
| maker-10-augustus-gene-37.44-mRNA-1 | Indica | NA | | Os10g0155500 | OsR498G1018169000.01.T01 | Aldose 1-epimerase |
| maker-10-augustus-gene-4.11-mRNA-1 | Indica | NA | | Os10g0104500 | OsR498G1018011400.01.T01 | Notchless protein isogeny |
| maker-10-augustus-gene-4.12-mRNA-1 | Indica | NA | | Os10g0104500 | OsR498G1018011400.01.T01 | Notchless protein like |
| maker-10-augustus-gene-4.3-mRNA-1 | Indica | Indica | | Os10g0561800 | OsR498G1018006400.01.T01 | Homeobox-leucine zipper protein HOX15 |
| maker-10-augustus-gene-4.6-mRNA-1 | Indica | NA | | Os10g0103800 | OsR498G1018008200.01.T01 | Inositol-tetrakisphosphate 1-kinase |
| maker-10-augustus-gene-40.0-mRNA-1 | Indica | Indica | | Os10g0159600 | OsR498G1018187400.01.T01 | SHSP domain-containing protein |
| maker-10-augustus-gene-40.3-mRNA-1 | Indica | NA | | Os10g0159700 | OsR498G1018188000.01.T01 | SHSP domain-containing protein |
| maker-10-augustus-gene-40.8-mRNA-1 | Indica | NA | | Os10g0159800 | OsR498G1018188600.01.T01 | Alcohol dehydrogenase |
| maker-10-augustus-gene-41.20-mRNA-1 | Indica | Japonica | | Os10g0141400 | OsR498G0305555000.01.T04 | Putative 26S proteasome regulatory subunit S5A |
| maker-10-augustus-gene-41.22-mRNA-1 | Indica | NA | | | | Ubiquitin carboxyl-terminal hydrolase |
| maker-10-augustus-gene-41.28-mRNA-1 | Indica | NA | | Os10g0160700 | OsR498G1018196400.01.T01 | |
| maker-10-augustus-gene-42.42-mRNA-1 | Indica | NA | | Os10g0161700 | OsR498G1018198900.01.T02 | DUF295 domain-containing protein |
| maker-10-augustus-gene-42.43-mRNA-1 | Indica | Indica | | Os10g0162400 | OsR498G1018201700.01.T05 | Filament-like plant protein 4 |
| maker-10-augustus-gene-42.47-mRNA-1 | Indica | NA | | | OsR498G1018197000.01.T02 | |
| maker-10-augustus-gene-42.49-mRNA-1 | Indica | NA | | | OsR498G1018199600.01.T01 | Fork head domain transcription factor slp2 like |
| maker-10-augustus-gene-43.32-mRNA-1 | Indica | NA | | Os10g0162600 | OsR498G1018202600.01.T01 | LOB domain-containing protein 18 |
| maker-10-augustus-gene-43.39-mRNA-1 | Indica | Indica | Kinase-TM | Os10g0174800 | OsR498G1018204400.01.T01 | Wall associated kinase |
| maker-10-augustus-gene-47.26-mRNA-1 | Indica | Indica | | Os10g0166600 | OsR498G1018232300.01.T01 | Geraniol 8-hydroxylase |
| maker-10-augustus-gene-48.36-mRNA-1 | Indica | NA | | Os10g0166600 | OsR498G1018238100.01.T01 | 7-ethoxycoumarin O-deethylase |
| maker-10-augustus-gene-49.10-mRNA-1 | Indica | Indica | | Os10g0167900 | OsR498G1018243700.01.T01 | Putative chalcone synthase |
| maker-10-augustus-gene-49.2-mRNA-1 | Indica | Japonica | | Os10g0167500 | OsR498G1018239500.01.T02 | RNA recognition motif family protein, expressed |
| maker-10-augustus-gene-49.20-mRNA-1 | Indica | NA | | | OsR498G1018239800.01.T03 | DNA photolyase |
| maker-10-augustus-gene-49.7-mRNA-1 | Indica | NA | | Os10g0167900 | OsR498G1018241300.01.T01 | Putative chalcone synthase |
| maker-10-augustus-gene-5.0-mRNA-1 | Indica | NA | | | OsR498G1018014500.01.T02 | Serine decarboxylase 2 |
| maker-10-augustus-gene-5.14-mRNA-1 | Indica | NA | | Os10g0105900 | OsR498G1018015000.01.T01 | Acyl transferase 1 |
| maker-10-augustus-gene-5.4-mRNA-1 | Indica | NA | | | OsR498G1018012600.01.T02 | DNA methyl transferase2 |
| maker-10-augustus-gene-5.9-mRNA-1 | Indica | NA | | Os10g0105400 | OsR498G1018013200.01.T01 | |
| maker-10-augustus-gene-50.11-mRNA-1 | Indica | NA | | | OsR498G1018247800.01.T01 | |
| maker-10-augustus-gene-50.2-mRNA-1 | Indica | NA | | Os10g0169800 | OsR498G1018248100.01.T06 | Maspardin |
| maker-10-augustus-gene-51.0-mRNA-1 | Indica | NA | | Os10g0170200 | OsR498G1018250900.01.T02 | Ribosomal protein S10 |
| maker-10-augustus-gene-51.3-mRNA-1 | Indica | NA | | Os10g0170300 | OsR498G1018251300.01.T01 | GPI ethanolamine phosphate transferase 3 |
| maker-10-augustus-gene-51.9-mRNA-1 | Indica | NA | | Os10g0171300 | | Cytochrome P450 family protein, expressed |
| maker-10-augustus-gene-52.0-mRNA-1 | Indica | NA | | | OsR498G1018256100.01.T01 | 5-epiaristolochene 1,3-dihydroxylase |
| maker-10-augustus-gene-52.2-mRNA-1 | Indica | NA | | | OsR498G1018254200.01.T01 | Geraniol 8-hydroxylase |
| maker-10-augustus-gene-54.0-mRNA-1 | Indica | NA | | | | Wall-associated receptor kinase 1 |
| maker-10-augustus-gene-54.2-mRNA-1 | Indica | NA | | Os10g0174800 | | Wall-associated receptor kinase 1 |
| maker-10-augustus-gene-55.0-mRNA-1 | Indica | NA | | | OsR498G1018264800.01.T01 | No apical meristem protein, expressed |
| maker-10-augustus-gene-55.1-mRNA-1 | Indica | NA | | Os10g0177200 | OsR498G1018265600.01.T01 | 39 kDa EF-Hand containing protein, putative, expressed |
| maker-10-augustus-gene-56.25-mRNA-1 | Indica | Japonica | | Os10g0177300 | OsR498G1018222500.01.T01 | Chalcone and stilbene synthases, N-terminal domain containing protein |
| maker-10-augustus-gene-56.28-mRNA-1 | Indica | NA | | | OsR498G1018267400.01.T04 | TCP domain-containing protein |
| maker-10-augustus-gene-58.41-mRNA-1 | Indica | NA | | Os10g0182000 | OsR498G1018282900.01.T01 | Alanine--tRNA ligase |
| maker-10-augustus-gene-58.52-mRNA-1 | Indica | NA | | Os10g0181700 | OsR498G1018282200.01.T01 | Protein MICRORCHIDIA 1 |
| maker-10-augustus-gene-6.0-mRNA-1 | Indica | NA | | Os10g0106701 | | Acyl transferase 1 |
| maker-10-augustus-gene-6.1-mRNA-1 | Indica | Japonica | | Os10g0106701 | OsR498G1018015700.01.T01 | Benzyl alcohol O-benzoyltransferase |
| maker-10-augustus-gene-6.2-mRNA-1 | Indica | NA | | | OsR498G1018019400.01.T01 | ATP-dependent DNA helicase |
| maker-10-augustus-gene-61.19-mRNA-1 | Indica | NA | CC-Kinase-TM | Os03g0251700 | OsR498G0305588100.01.T01 | Receptor-like serine/threonine-protein kinase |
| maker-10-augustus-gene-62.11-mRNA-1 | Indica | NA | | Os10g0188100 | OsR498G1018308400.01.T02 | Transcription initiation factor IIF subunit alpha |

| | | | | | | |
|---|---|---|---|---|---|---|
| maker-10-augustus-gene-64.18-mRNA-1 | Indica | NA | | Os10g0190500 | OsR498G1018321600.01.T03 | DUF4220 domain-containing protein (Fragment) |
| maker-10-augustus-gene-68.35-mRNA-1 | Indica | NA | | | OsR498G1221332900.01.T01 | Retrotransposon protein, putative, unclassified |
| maker-10-augustus-gene-7.33-mRNA-1 | Indica | NA | | Os10g0108700 | OsR498G1018023900.01.T01 | Acyl transferase 15 |
| maker-10-augustus-gene-7.34-mRNA-1 | Indica | NA | | Os12g0468900 | OsR498G0305961100.01.T01 | Cell wall protein-like |
| maker-10-augustus-gene-73.43-mRNA-1 | Indica | NA | | Os10g0197132 | | |
| maker-10-augustus-gene-78.7-mRNA-1 | Indica | NA | | Os10g0204400 | OsR498G1018371000.01.T02 | Phosphoenolpyruvate carboxykinase (ATP) |
| maker-10-augustus-gene-8.0-mRNA-1 | Indica | NA | | Os10g0109300 | OsR498G1018027200.01.T02 | Peroxidase |
| maker-10-augustus-gene-8.3-mRNA-1 | Indica | NA | | | OsR498G1018029600.01.T01 | POT family protein, expressed |
| maker-10-augustus-gene-8.5-mRNA-1 | Indica | NA | | Os10g0110600 | OsR498G1018031200.01.T01 | Putative proton-dependent oligopeptide transport |
| maker-10-augustus-gene-80.3-mRNA-1 | Indica | NA | | Os10g0206800 | OsR498G1018380100.01.T08 | Protein DETOXIFICATION |
| maker-10-augustus-gene-81.0-mRNA-1 | Indica | Indica | | Os10g0207500 | OsR498G1018384500.01.T01 | Protein TAPETUM DETERMINANT 1 |
| maker-10-augustus-gene-81.2-mRNA-1 | Indica | NA | | Os10g0207700 | OsR498G1018383500.01.T01 | protein PYRICULARIA ORYZAE RESISTANCE 21-like |
| maker-10-augustus-gene-81.5-mRNA-1 | Indica | Indica | | Os02g0586400 | OsR498G1018385400.01.T01 | GTP-binding 2 |
| maker-10-augustus-gene-82.0-mRNA-1 | Indica | NA | | | OsR498G1018387300.01.T05 | Oxoglutarate/iron-dependent dioxygenase |
| maker-10-augustus-gene-82.2-mRNA-1 | Indica | Japonica | | Os10g0208600 | OsR498G1018387600.01.T01 | Pollen Ole e 1 allergen and extensin family protein |
| maker-10-augustus-gene-83.3-mRNA-1 | Indica | NA | | Os10g0464400 | OsR498G1018862400.01.T01 | Riboflavin kinase |
| maker-10-augustus-gene-9.27-mRNA-1 | Indica | Indica | | Os10g0370700 | OsR498G1018031600.01.T06 | Major Facilitator Superfamily protein, expressed |
| maker-10-augustus-gene-9.32-mRNA-1 | Indica | Indica | | Os10g0370700 | OsR498G1018032200.01.T01 | Putative LeOPT1-oligopeptide transporter |
| maker-10-augustus-gene-9.40-mRNA-1 | Indica | Indica | | Os10g0111700 | OsR498G1018034200.01.T01 | Putative LeOPT1-oligopeptide transporter |
| maker-10-augustus-gene-91.0-mRNA-1 | Indica | NA | | | OsR498G1018396800.01.T02 | Arginine and glutamate-rich protein 1 |
| maker-10-augustus-gene-92.18-mRNA-1 | Indica | NA | | Os10g0213100 | | 4-aminobutyrate--pyruvate transaminase |
| maker-11-augustus-gene-122.25-mRNA-1 | Indica | Indica | | Os11g0446500 | OsR498G1120055700.01.T02 | Phospholipid-transporting ATPase |
| maker-11-augustus-gene-123.41-mRNA-1 | Indica | Japonica | Kinase-LRR-TM | Os11g0448000 | OsR498G1120059500.01.T01 | Receptor-like kinase TMK4 isoform A |
| maker-11-augustus-gene-133.30-mRNA-1 | Indica | Japonica | | Os11g0465200 | OsR498G1120113400.01.T01 | Indole-2-monooxygenase |
| maker-11-augustus-gene-137.48-mRNA-1 | Indica | NA | | | OsR498G1120128100.01.T01 | Aromatic amino acid decarboxylase 2 |
| maker-11-augustus-gene-138.0-mRNA-1 | Indica | Indica | | Os11g0474800 | OsR498G1120128800.01.T01 | Ent-isokaurene synthase |
| maker-11-augustus-gene-14.71-mRNA-1 | Indica | Japonica | | Os11g0146100 | OsR498G1119406000.01.T01 | Phosphatidylinositol 4-phosphate 5-kinase |
| maker-11-augustus-gene-14.72-mRNA-1 | Indica | NA | | | | 1-phosphatidylinositol-4-phosphate 5-kinase |
| maker-11-augustus-gene-142.50-mRNA-1 | Indica | NA | NBS-TM | | | Pollen signalling protein with adenylyl cyclase activity (Fragment) |
| maker-11-augustus-gene-143.25-mRNA-1 | Indica | NA | | Os11g0481200 | OsR498G1120145600.01.T01 | O-fucosyltransferase family protein (Fragment) |
| maker-11-augustus-gene-147.38-mRNA-1 | Indica | NA | | Os11g0485200 | OsR498G1120160600.01.T05 | ATPase 2 plasma membrane-type |
| maker-11-augustus-gene-149.39-mRNA-1 | Indica | NA | CC-NBS-TM | | | Putative NBS-LRR type disease resistance protein |
| maker-11-augustus-gene-246.17-mRNA-1 | Indica | NA | | Os11g0671000 | OsR498G1120700800.01.T01 | Auxin-repressed 12.5 kDa protein (Fragment) |
| maker-11-augustus-gene-247.0-mRNA-1 | Indica | NA | CC-Kinase-TM | | | S-receptor kinase-like protein 1-like |
| maker-11-augustus-gene-248.27-mRNA-1 | Indica | NA | CC-LRR-NBS-TM | Os11g0677101 | OsR498G1120709900.01.T01 | Putative nucleotide-binding leucine-rich-repeat protein 1 |
| maker-11-augustus-gene-248.28-mRNA-1 | Indica | NA | | | OsR498G1120713900.01.T01 | Retrotransposon protein, putative, unclassified |
| maker-11-augustus-gene-248.45-mRNA-1 | Indica | Indica | | Os11g0673200 | OsR498G1120707800.01.T01 | Beta-xylosidase 2 |
| maker-11-augustus-gene-254.0-mRNA-1 | Indica | NA | | Os11g0687100 | OsR498G1120737300.01.T01 | von Willebrand factor type A domain-like |
| maker-11-augustus-gene-254.2-mRNA-1 | Indica | NA | | Os11g0687200 | OsR498G1120737500.01.T01 | von Willebrand factor type A domain-like |
| maker-11-augustus-gene-255.12-mRNA-1 | Indica | NA | | Os11g0689300 | OsR498G1120738400.01.T01 | Calmodulin-binding protein MPCBP |
| maker-11-augustus-gene-255.2-mRNA-1 | Indica | NA | | Os11g0689400 | OsR498G1120738500.01.T02 | DEAD-box ATP-dependent RNA helicase 40 |
| maker-11-augustus-gene-256.34-mRNA-1 | Indica | NA | Kinase-TM | Os11g0695850 | OsR498G1120743300.01.T02 | Wall-associated receptor kinase |
| maker-11-augustus-gene-256.35-mRNA-1 | Indica | NA | Kinase-TM | | | Putative inactive receptor kinase RLK902 |
| maker-11-augustus-gene-259.37-mRNA-1 | Indica | Indica | | Os12g0566300 | OsR498G1120756900.01.T01 | ATP citrate synthase |
| maker-11-augustus-gene-259.44-mRNA-1 | Indica | NA | | Os11g0696400 | OsR498G1120757100.01.T01 | Beta-D-xylosidase |
| maker-11-augustus-gene-259.46-mRNA-1 | Indica | NA | | Os11g0695900 | OsR498G1120756600.01.T02 | ARM repeat superfamily protein |
| maker-11-augustus-gene-259.52-mRNA-1 | Indica | NA | | Os10g0186800 | OsR498G1221417300.01.T01 | |
| maker-11-augustus-gene-260.2-mRNA-1 | Indica | NA | | | OsR498G0815996300.01.T01 | Transposon protein, putative, CACTA, En/Spm sub-class |
| maker-11-augustus-gene-261.65-mRNA-1 | Indica | NA | | Os11g0703400 | OsR498G1120766200.01.T01 | Charged multivesicular body protein 2a |
| maker-11-augustus-gene-262.39-mRNA-1 | Indica | NA | | Os11g0703900 | OsR498G1120767700.01.T01 | Non-cell-autonomous heat shock cognate protein 70 |
| maker-11-augustus-gene-262.42-mRNA-1 | Indica | NA | | Os11g0704000 | OsR498G1120768100.01.T01 | Selenoprotein H |
| maker-11-augustus-gene-262.44-mRNA-1 | Indica | NA | | Os11g0704300 | OsR498G1120769100.01.T01 | Probable GTP-binding protein OBGM, mitochondrial |
| maker-11-augustus-gene-38.28-mRNA-1 | Indica | NA | | Os11g0186200 | OsR498G1119534500.01.T01 | Aldehyde dehydrogenase |
| maker-11-augustus-gene-38.32-mRNA-1 | Indica | NA | | Os11g0186300 | OsR498G1119534700.01.T01 | Methyltransferase |
| maker-11-augustus-gene-40.1-mRNA-1 | Indica | NA | | | | Terpene cyclase/mutase family member |
| maker-11-augustus-gene-40.6-mRNA-1 | Indica | NA | | | OsR498G1119542100.01.T01 | |
| maker-11-augustus-gene-40.9-mRNA-1 | Indica | NA | | Os11g0189900 | | NAD kinase 2 chloroplastic |
| maker-11-augustus-gene-41.0-mRNA-1 | Indica | NA | | | OsR498G1119543500.01.T01 | |
| maker-11-augustus-gene-65.38-mRNA-1 | Indica | NA | | Os11g0199700 | | Target of Myb protein 1 |
| maker-11-augustus-gene-66.39-mRNA-1 | Indica | NA | | Os11g0200600 | OsR498G1119570700.01.T03 | F-box domain containing protein, expressed |
| maker-11-augustus-gene-66.44-mRNA-1 | Indica | NA | | | OsR498G1119570600.01.T04 | F-box domain containing protein, expressed |

| | | | | | | |
|---|---|---|---|---|---|---|
| maker-11-augustus-gene-66.48-mRNA-1 | Indica | NA | | | | F-box domain-containing protein (Fragment) |
| maker-11-augustus-gene-67.46-mRNA-1 | Indica | NA | | Os11g0202600 | | zf-HYPF domain-containing protein |
| maker-11-augustus-gene-67.59-mRNA-1 | Indica | NA | | | | Glutamic acid-rich protein, putative (Fragment) |
| maker-11-augustus-gene-68.0-mRNA-1 | Indica | NA | | Os05g0580000 | OsR498G0511403400.01.T04 | Glucose-1-phosphate adenylyltransferase large subunit, chloroplastic/amylo |
| maker-11-augustus-gene-69.35-mRNA-1 | Indica | NA | | Os11g0205500 | OsR498G1119581200.01.T01 | |
| maker-11-augustus-gene-69.39-mRNA-1 | Indica | Japonica | | Os11g0205900 | OsR498G1119582800.01.T01 | F-box domain containing protein, expressed |
| maker-11-augustus-gene-73.0-mRNA-1 | Indica | Indica | | Os06g0614300 | OsR498G1119608300.01.T06 | TBCC domain-containing protein 1 |
| maker-11-augustus-gene-73.12-mRNA-1 | Indica | NA | | Os11g0210600 | OsR498G1119607600.01.T02 | Cinnamyl-alcohol dehydrogenase |
| maker-11-augustus-gene-73.13-mRNA-1 | Indica | Indica | | Os11g0210600 | OsR498G1119607600.01.T03 | Cinnamyl-alcohol dehydrogenase |
| maker-11-augustus-gene-74.11-mRNA-1 | Indica | NA | Kinase-TM | | OsR498G1119612600.01.T01 | Protein kinase domain containing protein, expressed |
| maker-11-augustus-gene-74.2-mRNA-1 | Indica | NA | LRR-TM | | OsR498G1119610600.01.T03 | NBS-LRR disease resistance protein family-4 |
| maker-11-augustus-gene-75.0-mRNA-1 | Indica | NA | | | | |
| maker-11-augustus-gene-82.0-mRNA-1 | Indica | Japonica | CC-NBS-TM RGA5, Pia | Os11g0225300 | OsR498G1119642700.01.T03 | NBS-LRR type protein (Fragment) |
| maker-11-augustus-gene-82.2-mRNA-1 | Indica | NA | Kinase | | | Retrotransposon protein, putative, LINE subclass |
| maker-11-augustus-gene-83.3-mRNA-1 | Indica | Japonica | CC-LRR-NBS-TM | Os11g0227100 | OsR498G1119648000.01.T02 | NBS-LRR type resistance protein (Fragment) |
| maker-11-augustus-gene-84.6-mRNA-1 | Indica | NA | | Os11g0231600 | OsR498G1119654700.01.T01 | F-box domain containing protein, expressed |
| maker-11-augustus-gene-86.11-mRNA-1 | Indica | NA | | | OsR498G1119665400.01.T02 | Glycerophosphodiester phosphodiesterase |
| maker-11-augustus-gene-86.3-mRNA-1 | Indica | NA | | Os11g0234200 | OsR498G1119661800.01.T01 | PHD domain-containing protein (Fragment) |
| maker-11-augustus-gene-86.6-mRNA-1 | Indica | NA | | Os11g0235200 | OsR498G1119662400.01.T01 | Proton-dependent oligopeptide transporter family |
| maker-11-augustus-gene-87.44-mRNA-1 | Indica | NA | NBS | Os12g0263800 | | Cyprosin |
| maker-12-augustus-gene-114.22-mRNA-1 | Indica | NA | | | | SGF29 C-terminal domain-containing protein |
| maker-12-augustus-gene-126.0-mRNA-1 | Indica | NA | | | | |
| maker-12-augustus-gene-126.2-mRNA-1 | Indica | NA | | | | Phosphatidylinositol 3,4,5-trisphosphate 3-phosphatase and protein-tyrosin |
| maker-12-augustus-gene-129.27-mRNA-1 | Indica | NA | | Os12g0411700 | OsR498G1221382800.01.T01 | ABC transporter domain-containing protein |
| maker-12-augustus-gene-131.20-mRNA-1 | Indica | NA | | Os12g0414500 | OsR498G1221394200.01.T01 | Asparagine--tRNA ligase |
| maker-12-augustus-gene-133.0-mRNA-1 | Indica | NA | | | | |
| maker-12-augustus-gene-134.26-mRNA-1 | Indica | NA | | Os02g0701833 | OsR498G0203665800.01.T01 | ATP-dependent DNA helicase |
| maker-12-augustus-gene-134.28-mRNA-1 | Indica | NA | | Os02g0701833 | OsR498G0916979800.01.T01 | ATP-dependent DNA helicase |
| maker-12-augustus-gene-135.11-mRNA-1 | Indica | NA | | Os12g0420400 | OsR498G1221413300.01.T03 | PSI subunit V |
| maker-12-augustus-gene-135.13-mRNA-1 | Indica | Japonica | | Os12g0420000 | OsR498G0408810600.01.T03 | 2-phosphoglycolate phosphatase, eukaryotic |
| maker-12-augustus-gene-135.2-mRNA-1 | Indica | NA | | Os12g0420200 | OsR498G1221412700.01.T01 | Chloroplast stem-loop binding protein like |
| maker-12-augustus-gene-135.7-mRNA-1 | Indica | NA | | | OsR498G1221413100.01.T02 | Tyrosine-protein phosphatase DSP1 |
| maker-12-augustus-gene-138.36-mRNA-1 | Indica | NA | | Os01g0284200 | OsR498G0100683700.01.T01 | |
| maker-12-augustus-gene-139.0-mRNA-1 | Indica | NA | | Os12g0425600 | OsR498G1221425300.01.T01 | O-fucosyltransferase family protein (Fragment) |
| maker-12-augustus-gene-140.4-mRNA-1 | Indica | NA | | | | Autophagy-related protein 101 |
| maker-12-augustus-gene-147.2-mRNA-1 | Indica | NA | | | OsR498G1221451000.01.T01 | PHD-type domain-containing protein |
| maker-12-augustus-gene-147.6-mRNA-1 | Indica | NA | Kinase-TM | Os12g0433500 | OsR498G1221451200.01.T03 | serine/threonine-protein kinase TIO |
| maker-12-augustus-gene-148.23-mRNA-1 | Indica | NA | | Os12g0434400 | OsR498G1221453900.01.T01 | Putative aminopeptidase of the M17 family |
| maker-12-augustus-gene-2.56-mRNA-1 | Indica | NA | | Os11g0104200 | OsR498G1119282100.01.T01 | IBR domain-containing protein |
| maker-12-augustus-gene-209.21-mRNA-1 | Indica | NA | | Os12g0515400 | OsR498G1221715500.01.T01 | Dicarboxylate transporter 1 chloroplastic |
| maker-12-augustus-gene-209.24-mRNA-1 | Indica | NA | | Os12g0516000 | | |
| maker-12-augustus-gene-209.28-mRNA-1 | Indica | NA | | Os12g0515300 | OsR498G1221715000.01.T01 | Transcription factor like |
| maker-12-augustus-gene-209.30-mRNA-1 | Indica | NA | | Os12g0515600 | OsR498G1221716600.01.T01 | Guanylate kinase |
| maker-12-augustus-gene-211.37-mRNA-1 | Indica | NA | | Os12g0527500 | OsR498G1221723300.01.T01 | F-box domain containing protein, expressed |
| maker-12-augustus-gene-211.39-mRNA-1 | Indica | NA | | | OsR498G1221724500.01.T02 | Non-specific serine/threonine protein kinase |
| maker-12-augustus-gene-211.42-mRNA-1 | Indica | Indica | | Os12g0527850 | OsR498G1221725100.01.T01 | Pentatricopeptide repeat-containing protein mitochondrial |
| maker-12-augustus-gene-211.44-mRNA-1 | Indica | NA | | | | PHD finger family protein isoform 1 |
| maker-12-augustus-gene-211.49-mRNA-1 | Indica | Indica | | Os12g0528000 | OsR498G1221725400.01.T02 | DNA-binding protein PD2 |
| maker-12-augustus-gene-212.40-mRNA-1 | Indica | Indica | | Os12g0528300 | OsR498G1221726300.01.T01 | MHD domain-containing death-inducing |
| maker-12-augustus-gene-212.44-mRNA-1 | Indica | NA | | | OsR498G1221726500.01.T02 | Glutathione synthetase, chloroplastic |
| maker-12-augustus-gene-212.49-mRNA-1 | Indica | NA | | Os12g0529400 | OsR498G1221728300.01.T02 | Auxin-binding protein 4 |
| maker-12-augustus-gene-212.52-mRNA-1 | Indica | NA | | | | zinc finger MYM-type protein 1-like |
| maker-12-augustus-gene-212.54-mRNA-1 | Indica | NA | | Os12g0530000 | OsR498G1221729000.01.T01 | |
| maker-12-augustus-gene-212.56-mRNA-1 | Indica | NA | | Os12g0530100 | OsR498G1221729200.01.T02 | Peroxidase |
| maker-12-augustus-gene-213.0-mRNA-1 | Indica | NA | | | | Ribosomal protein-like |
| maker-12-augustus-gene-216.65-mRNA-1 | Indica | NA | | Os12g0534200 | OsR498G1221759000.01.T01 | Acyl carrier protein 1, chloroplastic |
| maker-12-augustus-gene-217.50-mRNA-1 | Indica | Japonica | | Os12g0535600 | OsR498G1221764600.01.T01 | rRNA N-glycosidase |
| maker-12-augustus-gene-221.0-mRNA-1 | Indica | NA | | | OsR498G0407881700.01.T01 | Transposon protein, putative, Mutator sub-class, expressed |
| maker-12-augustus-gene-223.29-mRNA-1 | Indica | NA | | | | NAM-associated domain-containing protein |
| maker-12-augustus-gene-225.11-mRNA-1 | Indica | NA | | Os12g0548100 | OsR498G1221838400.01.T02 | Cnd1 domain-containing protein (Fragment) |
| maker-12-augustus-gene-226.10-mRNA-1 | Indica | Indica | | Os12g0551200 | OsR498G1221848400.01.T01 | Patatin |

| | | | | | | |
|---|---|---|---|---|---|---|
| maker-12-augustus-gene-226.4-mRNA-1 | Indica | NA | | Os12g0548200 | OsR498G1221839000.01.T01 | Auxilin-related protein 1 |
| maker-12-augustus-gene-228.29-mRNA-1 | Indica | Indica | | Os12g0552700 | OsR498G1221853700.01.T01 | F-box/LRR-repeat protein 3-like isoform X2 |
| maker-12-augustus-gene-228.61-mRNA-1 | Indica | Japonica | | Os12g0554500 | OsR498G1221858800.01.T02 | Mono-/di-acylglycerol lipase N-terminal |
| maker-12-augustus-gene-237.38-mRNA-1 | Indica | Indica | | Os12g0571100 | OsR498G1221902600.01.T01 | Metallothionein-like protein 1, putative, expressed |
| maker-12-augustus-gene-256.0-mRNA-1 | Indica | Japonica | | Os12g0601000 | OsR498G1222049900.01.T02 | F-box/LRR-repeat protein 17-like isoform X1 |
| maker-12-augustus-gene-256.18-mRNA-1 | Indica | NA | | Os12g0601300 | OsR498G1222050600.01.T01 | Auxin-responsive protein IAA30 |
| maker-12-augustus-gene-256.3-mRNA-1 | Indica | NA | NBS-TM | Os12g0601200 | OsR498G1222050300.01.T04 | PRK domain-containing protein (Fragment) |
| maker-12-augustus-gene-259.14-mRNA-1 | Indica | NA | | Os12g0606600 | OsR498G1222072300.01.T02 | HORMA domain containing protein, expressed |
| maker-12-augustus-gene-273.36-mRNA-1 | Indica | NA | | Os12g0631100 | OsR498G1222155700.01.T01 | Ras-related protein RHN1 |
| maker-12-augustus-gene-273.45-mRNA-1 | Indica | NA | | Os12g0631600 | OsR498G1222159300.01.T01 | |
| maker-12-augustus-gene-273.47-mRNA-1 | Indica | NA | | Os12g0630700 | OsR498G1222154600.01.T04 | Haloacid dehalogenase-like hydrolase domain-containing protein |
| maker-12-augustus-gene-273.52-mRNA-1 | Indica | NA | | | OsR498G1222155200.01.T01 | Transcription factor JUNGBRUNNEN 1 |
| maker-12-augustus-gene-273.56-mRNA-1 | Indica | Japonica | | Os12g0631800 | OsR498G1222159600.01.T01 | Putative amine oxidase, FAD/NAD(P)-binding domain-containing protein |
| maker-12-augustus-gene-274.0-mRNA-1 | Indica | NA | | Os12g0632000 | OsR498G1222159700.01.T01 | Glycine rich RNA binding protein |
| maker-12-augustus-gene-275.13-mRNA-1 | Indica | NA | | Os07g0105000 | OsR498G0713368100.01.T01 | Phytocyanin domain-containing protein (Fragment) |
| maker-12-augustus-gene-275.3-mRNA-1 | Indica | NA | | Os08g0395500 | OsR498G0102642600.01.T02 | |
| maker-12-augustus-gene-278.12-mRNA-1 | Indica | NA | | | OsR498G0713384500.01.T01 | |
| maker-12-augustus-gene-279.59-mRNA-1 | Indica | NA | | Os07g0110800 | OsR498G0713388200.01.T01 | |
| maker-2-augustus-gene-111.39-mRNA-1 | Indica | NA | | Os02g0292100 | | GDSL-motif lipase/hydrolase-like |
| maker-2-augustus-gene-167.0-mRNA-1 | Indica | Indica | | Os02g0478550 | OsR498G0203804900.01.T01 | 30S ribosomal protein S8, chloroplastic |
| maker-2-augustus-gene-167.13-mRNA-1 | Indica | NA | | | | Calcineurin B |
| maker-2-augustus-gene-167.15-mRNA-1 | Indica | NA | | Os02g0478525 | | Arogenate dehydratase |
| maker-2-augustus-gene-167.18-mRNA-1 | Indica | NA | | Os02g0479300 | OsR498G0203807200.01.T01 | dCTP pyrophosphatase 1 |
| maker-2-augustus-gene-167.21-mRNA-1 | Indica | NA | | | | WH1 domain-containing protein |
| maker-2-augustus-gene-167.3-mRNA-1 | Indica | NA | | Os02g0478600 | OsR498G0203805100.01.T02 | 30S ribosomal protein S8, chloroplastic |
| maker-2-augustus-gene-167.35-mRNA-1 | Indica | Indica | | Os02g0480100 | OsR498G0203808800.01.T04 | ATP-dependent DNA helicase |
| maker-2-augustus-gene-167.5-mRNA-1 | Indica | NA | | Os02g0478700 | OsR498G0203805300.01.T02 | 40S ribosomal protein S27 |
| maker-2-augustus-gene-167.9-mRNA-1 | Indica | NA | | | OsR498G0203807700.01.T02 | Calcineurin B-like protein 3 |
| maker-2-augustus-gene-168.16-mRNA-1 | Indica | NA | | Os02g0480900 | OsR498G0203811600.01.T02 | Polyadenylate-binding protein-interacting protein 3 |
| maker-2-augustus-gene-168.21-mRNA-1 | Indica | NA | | Os02g0481000 | OsR498G0203812000.01.T02 | 28S ribosomal protein S34, mitochondrial (Fragment) |
| maker-2-augustus-gene-168.24-mRNA-1 | Indica | NA | | | OsR498G0203816400.01.T04 | Putative P-loop containing nucleoside triphosphate hydrolase, alpha/Beta h[illegible] |
| maker-2-augustus-gene-168.3-mRNA-1 | Indica | Indica | | Os02g0481900 | OsR498G0203816900.01.T01 | 5'-3' exoribonuclease |
| maker-2-augustus-gene-168.30-mRNA-1 | Indica | NA | | Os10g0199200 | OsR498G1018346900.01.T03 | Hydroxyproline-rich glycoprotein-like |
| maker-2-augustus-gene-169.37-mRNA-1 | Indica | NA | | Os02g0482400 | OsR498G0203817800.01.T01 | Ornithine decarboxylase |
| maker-2-augustus-gene-171.20-mRNA-1 | Indica | NA | | | | |
| maker-2-augustus-gene-172.41-mRNA-1 | Indica | NA | | | OsR498G0203833700.01.T01 | WRC domain-containing protein |
| maker-2-augustus-gene-172.43-mRNA-1 | Indica | NA | | Os02g0488701 | OsR498G0203835200.01.T03 | RING-type E3 ubiquitin transferase |
| maker-2-augustus-gene-181.2-mRNA-1 | Indica | Indica | | Os02g0503300 | OsR498G0203881700.01.T01 | X8 domain-containing protein |
| maker-2-augustus-gene-181.7-mRNA-1 | Indica | NA | | | OsR498G0203882200.01.T01 | 3-oxoacyl-[acyl-carrier-protein] reductase |
| maker-2-augustus-gene-182.36-mRNA-1 | Indica | NA | | Os02g0503900 | OsR498G0203883100.01.T02 | Isoflavone 2'-hydroxylase |
| maker-2-augustus-gene-182.43-mRNA-1 | Indica | Indica | | Os02g0503900 | OsR498G0203883500.01.T01 | Isoflavone 2'-hydroxylase |
| maker-2-augustus-gene-182.49-mRNA-1 | Indica | NA | NBS-TM | | | Disease resistance protein RGA2 |
| maker-2-augustus-gene-183.0-mRNA-1 | Indica | NA | | Os02g0504500 | OsR498G0203886100.01.T01 | UBA domain-containing protein/PUB domain-containing protein |
| maker-2-augustus-gene-183.13-mRNA-1 | Indica | NA | | Os02g0504900 | OsR498G0203887600.01.T02 | F-box/kelch-repeat protein At1g67480 |
| maker-2-augustus-gene-183.9-mRNA-1 | Indica | NA | | | OsR498G0203891300.01.T01 | |
| maker-2-augustus-gene-185.10-mRNA-1 | Indica | NA | | | OsR498G0203901200.01.T01 | Gamma carbonic anhydrase-like 2 mitochondrial |
| maker-2-augustus-gene-185.12-mRNA-1 | Indica | Indica | | Os02g0510100 | OsR498G0203903200.01.T01 | Sm domain-containing protein |
| maker-2-augustus-gene-185.15-mRNA-1 | Indica | NA | | Os02g0510300 | OsR498G0203903800.01.T02 | Proline-rich protein HaeIII subfamily 1 |
| maker-2-augustus-gene-185.5-mRNA-1 | Indica | NA | | Os02g0509600 | OsR498G0203902500.01.T01 | |
| maker-2-augustus-gene-187.27-mRNA-1 | Indica | Indica | | Os02g0511400 | OsR498G0203904700.01.T01 | Cis-2,3-dihydrobiphenyl-2,3-diol dehydrogenase |
| maker-2-augustus-gene-187.29-mRNA-1 | Indica | NA | | | | HTH myb-type domain-containing protein |
| maker-2-augustus-gene-187.35-mRNA-1 | Indica | NA | | Os02g0511600 | OsR498G0203906100.01.T01 | Beta-13-N-Acetylglucosaminyltransferase family protein |
| maker-2-augustus-gene-187.42-mRNA-1 | Indica | Indica | | Os02g0511800 | OsR498G0203906400.01.T02 | NOP5NT domain-containing protein |
| maker-2-augustus-gene-188.0-mRNA-1 | Indica | NA | | Os02g0511900 | | DNA polymerase epsilon catalytic subunit A |
| maker-2-augustus-gene-188.7-mRNA-1 | Indica | NA | | | OsR498G0203908300.01.T02 | Ribosomal pseudouridine synthase |
| maker-2-augustus-gene-189.0-mRNA-1 | Indica | NA | Kinase-TM | Os02g0513000 | OsR498G0203912300.01.T01 | Serine/threonine-protein kinase PBS1 |
| maker-2-augustus-gene-189.3-mRNA-1 | Indica | NA | | | OsR498G0203917700.01.T01 | Glycerophosphodiester phosphodiesterase |
| maker-2-augustus-gene-189.8-mRNA-1 | Indica | NA | | Os02g0513100 | OsR498G0203912800.01.T01 | Bidirectional sugar transporter SWEET |
| maker-2-augustus-gene-190.16-mRNA-1 | Indica | NA | | Os02g0515600 | OsR498G0203923000.01.T01 | GSH-induced LITAF domain-containing protein |
| maker-2-augustus-gene-191.0-mRNA-1 | Indica | NA | | Os02g0517300 | OsR498G0203929500.01.T02 | Topoisomerase II-associated protein PAT1 |
| maker-2-augustus-gene-191.13-mRNA-1 | Indica | NA | | Os02g0516800 | OsR498G0203927400.01.T01 | Trihelix transcription factor ASR3 |

| | | | | | | |
|---|---|---|---|---|---|---|
| maker-2-augustus-gene-191.7-mRNA-1 | Indica | NA | | | | |
| maker-2-augustus-gene-191.9-mRNA-1 | Indica | NA | | Os02g0516600 | OsR498G0203926800.01.T01 | RING finger protein 10 |
| maker-2-augustus-gene-192.35-mRNA-1 | Indica | NA | | Os02g0517531 | OsR498G0203934500.01.T01 | RNA recognition motif domain |
| maker-2-augustus-gene-192.43-mRNA-1 | Indica | NA | | | OsR498G0203940900.01.T01 | |
| maker-2-augustus-gene-192.45-mRNA-1 | Indica | NA | | Os02g0517600 | | Ubiquitinyl hydrolase 1 |
| maker-2-augustus-gene-192.50-mRNA-1 | Indica | NA | | Os02g0518100 | OsR498G0203939400.01.T01 | Probable magnesium transporter |
| maker-2-augustus-gene-192.56-mRNA-1 | Indica | NA | | Os02g0518600 | OsR498G0203941800.01.T02 | Potassium transporter |
| maker-2-augustus-gene-193.0-mRNA-1 | Indica | NA | | Os02g0519900 | OsR498G0203948900.01.T06 | Tr-type G domain-containing protein |
| maker-2-augustus-gene-193.1-mRNA-1 | Indica | NA | | Os02g0519100 | OsR498G0203945200.01.T01 | Potassium transporter |
| maker-2-augustus-gene-193.10-mRNA-1 | Indica | NA | | Os02g0520100 | OsR498G0203951000.01.T02 | Nudix hydrolase 13 mitochondrial |
| maker-2-augustus-gene-193.3-mRNA-1 | Indica | Indica | | Os04g0661300 | OsR498G0203946800.01.T01 | Myosin-H heavy chain isoform 1 |
| maker-2-augustus-gene-196.18-mRNA-1 | Indica | Indica | | Os02g0526000 | OsR498G0203972200.01.T01 | Heparan-alpha-glucosaminide N-acetyltransferase-like isoform X1 |
| maker-2-augustus-gene-196.5-mRNA-1 | Indica | NA | | Os02g0525900 | OsR498G0203971900.01.T01 | Acetyl-coenzyme A synthetase |
| maker-2-augustus-gene-203.0-mRNA-1 | Indica | NA | | | OsR498G0204016900.01.T01 | Chaperone protein ClpB1-like |
| maker-2-augustus-gene-203.4-mRNA-1 | Indica | NA | | Os02g0537700 | OsR498G0204017500.01.T01 | Thioredoxin peroxidase |
| maker-2-augustus-gene-203.8-mRNA-1 | Indica | Japonica | | Os02g0537800 | OsR498G0204018500.01.T01 | Butenolide signaling repressing protein-like |
| maker-2-augustus-gene-204.22-mRNA-1 | Indica | NA | | Os02g0538900 | OsR498G0204022400.01.T01 | Basic leucine zipper 6 |
| maker-2-augustus-gene-208.0-mRNA-1 | Indica | Indica | | Os02g0816900 | OsR498G0204065700.01.T01 | Myosin motor domain-containing protein |
| maker-2-augustus-gene-208.6-mRNA-1 | Indica | NA | | Os02g0545300 | | ATP synthase subunit-like protein |
| maker-2-augustus-gene-230.62-mRNA-1 | Indica | NA | | Os02g0587300 | | |
| maker-2-augustus-gene-230.64-mRNA-1 | Indica | NA | | Os02g0587600 | OsR498G0204199700.01.T01 | Xylan 14-beta-xylosidase |
| maker-2-augustus-gene-237.126-mRNA-1 | Indica | NA | | Os02g0600400 | OsR498G0204248100.01.T05 | Glucose-6-phosphate dehydrogenase (NADP(+)) |
| maker-2-augustus-gene-237.139-mRNA-1 | Indica | NA | | Os02g0600200 | OsR498G0204248000.01.T03 | Ribosome-recycling factor, mitochondrial (Fragment) |
| maker-2-augustus-gene-237.48-mRNA-1 | Indica | NA | | | | Protein-serine/threonine phosphatase |
| maker-2-augustus-gene-237.49-mRNA-1 | Indica | NA | | | | Protein-serine/threonine phosphatase |
| maker-2-augustus-gene-24.37-mRNA-1 | Indica | NA | | Os02g0143200 | OsR498G0202953500.01.T02 | ARM repeat superfamily protein |
| maker-2-augustus-gene-244.34-mRNA-1 | Indica | NA | | Os02g0614100 | OsR498G0204296700.01.T01 | TPT domain-containing protein (Fragment) |
| maker-2-augustus-gene-244.39-mRNA-1 | Indica | NA | | | OsR498G0204297900.01.T02 | AP2-like ethylene-responsive transcription factor BBM2 |
| maker-2-augustus-gene-244.43-mRNA-1 | Indica | NA | | Os02g0614400 | OsR498G0204298500.01.T02 | Calcium-dependent protein kinase 20 |
| maker-2-augustus-gene-244.54-mRNA-1 | Indica | NA | | Os02g0614200 | OsR498G0204297200.01.T01 | Lysine-specific demethylase 2A-like |
| maker-2-augustus-gene-245.0-mRNA-1 | Indica | NA | | Os02g0614600 | OsR498G0204299100.01.T02 | Filament-like plant protein 7 |
| maker-2-augustus-gene-245.16-mRNA-1 | Indica | NA | | Os02g0614500 | OsR498G0204298800.01.T01 | Nucleotide sugar transporter-KT 1 |
| maker-2-augustus-gene-245.21-mRNA-1 | Indica | Japonica | | Os02g0614700 | OsR498G0204299400.01.T01 | SPRY domain-containing protein |
| maker-2-augustus-gene-247.10-mRNA-1 | Indica | NA | | | OsR498G0204310000.01.T03 | |
| maker-2-augustus-gene-247.3-mRNA-1 | Indica | NA | LRR-TM | | OsR498G0204309100.01.T01 | LRR receptor-like serine/threonine-protein kinase EFR |
| maker-2-augustus-gene-251.19-mRNA-1 | Indica | NA | | | | |
| maker-2-augustus-gene-252.27-mRNA-1 | Indica | NA | | Os02g0616501 | | PAZ domain-containing protein/Piwi domain-containing protein/DUF1785 d |
| maker-2-augustus-gene-253.8-mRNA-1 | Indica | NA | | Os02g0617900 | OsR498G0204327100.01.T01 | 39S ribosomal protein L41-A, mitochondrial |
| maker-2-augustus-gene-255.57-mRNA-1 | Indica | Indica | | Os04g0515900 | OsR498G0204341900.01.T01 | NAC transcription factor |
| maker-2-augustus-gene-258.5-mRNA-1 | Indica | NA | | Os01g0281200 | OsR498G0100675400.01.T01 | CYCLIN domain-containing protein (Fragment) |
| maker-2-augustus-gene-259.54-mRNA-1 | Indica | NA | | Os02g0629800 | OsR498G0204365900.01.T01 | Knot1 domain-containing protein (Fragment) |
| maker-2-augustus-gene-26.37-mRNA-1 | Indica | NA | | Os02g0145600 | OsR498G0202963300.01.T06 | NB-ARC domain-containing protein |
| maker-2-augustus-gene-26.40-mRNA-1 | Indica | NA | | | OsR498G0202966500.01.T04 | Dolichol kinase |
| maker-2-augustus-gene-26.47-mRNA-1 | Indica | NA | | Os02g0146600 | OsR498G0202966700.01.T02 | Translation initiation factor |
| maker-2-augustus-gene-26.49-mRNA-1 | Indica | NA | | Os02g0146700 | OsR498G0202967300.01.T01 | 26S proteasome non-ATPase regulatory subunit 2 A |
| maker-2-augustus-gene-26.56-mRNA-1 | Indica | NA | | Os02g0147700 | OsR498G0202969700.01.T02 | DNA-binding protein BIN4-like isoform X1 |
| maker-2-augustus-gene-26.65-mRNA-1 | Indica | NA | | | OsR498G0202966200.01.T01 | |
| maker-2-augustus-gene-26.67-mRNA-1 | Indica | NA | | | OsR498G0202966500.01.T04 | Dolichol kinase |
| maker-2-augustus-gene-26.74-mRNA-1 | Indica | Japonica | | Os02g0147300 | OsR498G0202968200.01.T02 | TOG domain-containing protein |
| maker-2-augustus-gene-260.6-mRNA-1 | Indica | Japonica | Kinase-TM | Os02g0632100 | OsR498G0509684800.01.T02 | Wall-associated receptor kinase 3 |
| maker-2-augustus-gene-262.0-mRNA-1 | Indica | Indica | | Os04g0524600 | OsR498G0204380300.01.T01 | Metal-nicotianamine transporter YSL12 |
| maker-2-augustus-gene-262.30-mRNA-1 | Indica | NA | Kinase-TM | | | Wall-associated receptor kinase 4 |
| maker-2-augustus-gene-262.38-mRNA-1 | Indica | NA | | | | B-block binding subunit of TFIIIC |
| maker-2-augustus-gene-27.0-mRNA-1 | Indica | NA | | Os02g0147500 | | Resistance protein-like |
| maker-2-augustus-gene-30.0-mRNA-1 | Indica | NA | | | | DUF547 domain-containing protein |
| maker-2-augustus-gene-30.16-mRNA-1 | Indica | NA | | Os02g0152900 | OsR498G0202988400.01.T01 | Putative WAS/WASL-interacting protein family member 1-like |
| maker-2-augustus-gene-30.20-mRNA-1 | Indica | Indica | Kinase-LRR-TM | Os02g0154000 | OsR498G0202991600.01.T01 | Putative leucine-rich repeat receptor-like kinase |
| maker-2-augustus-gene-31.54-mRNA-1 | Indica | NA | Kinase-TM | | OsR498G0202996100.01.T01 | L-type lectin-domain containing receptor kinase IX.2 |
| maker-2-augustus-gene-32.53-mRNA-1 | Indica | NA | | Os02g0158300 | OsR498G0203004900.01.T01 | Arabinose 5-phosphate isomerase KdsD |
| maker-2-augustus-gene-32.58-mRNA-1 | Indica | NA | | Os02g0158900 | OsR498G0203006100.01.T01 | 5'-AMP-activated protein kinase subunit gamma-3 |
| maker-2-augustus-gene-32.60-mRNA-1 | Indica | NA | | Os02g0159200 | OsR498G0203007700.01.T01 | Regulator of Vps4 activity in the MVB pathway protein |

| | | | | | | |
|---|---|---|---|---|---|---|
| maker-2-augustus-gene-32.64-mRNA-1 | Indica | NA | | | OsR498G0203008100.01.T01 | translation machinery-associated protein 7-like |
| maker-2-augustus-gene-32.66-mRNA-1 | Indica | Indica | | Os01g0141100 | OsR498G0203008900.01.T01 | Protein SUPPRESSOR OF K(+) TRANSPORT GROWTH DEFECT 1 |
| maker-2-augustus-gene-32.73-mRNA-1 | Indica | NA | | Os02g0158100 | OsR498G0203003800.01.T01 | EH domain-containing protein 1 |
| maker-2-augustus-gene-32.77-mRNA-1 | Indica | NA | | | | single-stranded DNA-binding protein WHY2, mitochondrial |
| maker-2-augustus-gene-32.82-mRNA-1 | Indica | Indica | | Os02g0158500 | OsR498G0203005400.01.T01 | Plant-specific domain TIGR01627 family protein |
| maker-2-augustus-gene-32.86-mRNA-1 | Indica | NA | | | OsR498G0203008400.01.T01 | |
| maker-2-augustus-gene-32.91-mRNA-1 | Indica | Indica | | Os02g0159700 | OsR498G0203008600.01.T04 | Protein SCO1 homolog 1 mitochondrial |
| maker-2-augustus-gene-33.0-mRNA-1 | Indica | Japonica | | Os02g0160100 | OsR498G0203010500.01.T01 | |
| maker-2-augustus-gene-33.3-mRNA-1 | Indica | NA | | Os02g0160200 | OsR498G0203010900.01.T01 | FBD domain-containing protein |
| maker-2-augustus-gene-33.5-mRNA-1 | Indica | NA | | Os02g0161100 | OsR498G0203015300.01.T01 | Formin-like protein 10 |
| maker-2-augustus-gene-33.7-mRNA-1 | Indica | NA | | | OsR498G0203009200.01.T03 | DExH-box ATP-dependent RNA helicase DExH11 |
| maker-2-augustus-gene-34.35-mRNA-1 | Indica | NA | | | | Zinc finger CCCH domain-containing protein 13 |
| maker-2-augustus-gene-34.40-mRNA-1 | Indica | NA | | | OsR498G0203016300.01.T01 | TATA-binding protein-associated factor BTAF1 |
| maker-2-augustus-gene-37.13-mRNA-1 | Indica | NA | | Os02g0168300 | OsR498G0203044700.01.T01 | Glutathione transferase |
| maker-2-augustus-gene-37.15-mRNA-1 | Indica | NA | | | OsR498G0203044900.01.T01 | Putative E3 ubiquitin-protein ligase LIN |
| maker-2-augustus-gene-37.41-mRNA-1 | Indica | NA | | Os02g0168200 | OsR498G0203044600.01.T01 | probable transcription factor KAN3 |
| maker-2-augustus-gene-38.0-mRNA-1 | Indica | NA | | Os02g0168800 | OsR498G0203046700.01.T01 | Hydroxymethylbilane synthase |
| maker-2-augustus-gene-38.10-mRNA-1 | Indica | NA | | | OsR498G0203052900.01.T02 | Glycine cleavage system protein GcvH |
| maker-2-augustus-gene-38.14-mRNA-1 | Indica | NA | | Os02g0167700 | OsR498G0203043400.01.T03 | Cullin-associated NEDD8-dissociated protein 1 |
| maker-2-augustus-gene-38.23-mRNA-1 | Indica | Indica | | Os02g0170000 | OsR498G0203053700.01.T02 | Pentatricopeptide repeat |
| maker-2-augustus-gene-38.28-mRNA-1 | Indica | NA | | Os02g0168600 | OsR498G0203046000.01.T03 | OTU domain-containing protein |
| maker-2-augustus-gene-38.31-mRNA-1 | Indica | NA | | Os02g0168700 | OsR498G0203046400.01.T01 | Peptidylprolyl isomerase |
| maker-2-augustus-gene-38.33-mRNA-1 | Indica | NA | | Os02g0168900 | OsR498G0203047200.01.T01 | Ribonuclease H2 subunit C |
| maker-2-augustus-gene-38.39-mRNA-1 | Indica | NA | | | OsR498G0203051200.01.T03 | Piwi domain-containing protein |
| maker-2-augustus-gene-38.4-mRNA-1 | Indica | NA | | Os02g0169300 | OsR498G0203048100.01.T02 | Phosphoglycerate kinase |
| maker-2-augustus-gene-38.45-mRNA-1 | Indica | NA | | Os02g0169900 | OsR498G0203053600.01.T04 | Pentatricopeptide repeat-containing protein |
| maker-2-augustus-gene-38.8-mRNA-1 | Indica | NA | | | OsR498G0203051500.01.T01 | |
| maker-2-augustus-gene-41.11-mRNA-1 | Indica | NA | | Os02g0174300 | OsR498G0203068700.01.T01 | Trihelix transcription factor ASR3 |
| maker-2-augustus-gene-41.14-mRNA-1 | Indica | NA | Kinase-LRR-TM | | OsR498G0203068900.01.T01 | Pollen receptor-like kinase |
| maker-2-augustus-gene-41.37-mRNA-1 | Indica | NA | Kinase-TM | Os02g0174200 | OsR498G0203068200.01.T01 | serine/threonine-protein kinase STY13 |
| maker-2-augustus-gene-41.7-mRNA-1 | Indica | NA | | Os02g0174100 | OsR498G0203067900.01.T02 | SBP-type domain-containing protein |
| maker-2-augustus-gene-44.13-mRNA-1 | Indica | NA | | Os02g0179500 | OsR498G0203089700.01.T03 | Heptahelical transmembrane protein 2 |
| maker-2-augustus-gene-44.18-mRNA-1 | Indica | NA | | Os02g0179200 | OsR498G0203088200.01.T01 | Gamma-glutamyl peptidase |
| maker-2-augustus-gene-44.2-mRNA-1 | Indica | NA | CC-Kinase | | OsR498G0203087100.01.T01 | Mitogen-activated protein kinase kinase kinase ANP1 |
| maker-2-augustus-gene-44.20-mRNA-1 | Indica | NA | | | OsR498G0203089400.01.T01 | TUB transcription factor |
| maker-2-augustus-gene-44.24-mRNA-1 | Indica | Indica | | Os02g0179800 | OsR498G0203090200.01.T01 | ATG11 domain-containing protein |
| maker-2-augustus-gene-44.5-mRNA-1 | Indica | NA | | Os02g0179100 | OsR498G0203087700.01.T04 | 5'-deoxynucleotidase |
| maker-2-augustus-gene-45.21-mRNA-1 | Indica | NA | | | OsR498G0203090500.01.T01 | U2 snRNP-associated SURP motif-containing protein |
| maker-2-augustus-gene-45.28-mRNA-1 | Indica | NA | | Os02g0180000 | OsR498G0203090900.01.T02 | Protein-serine/threonine phosphatase |
| maker-2-augustus-gene-45.37-mRNA-1 | Indica | NA | | | OsR498G0203092100.01.T01 | probable N-acetyltransferase HLS1 |
| maker-2-augustus-gene-45.39-mRNA-1 | Indica | NA | | Os02g0180700 | OsR498G0203093300.01.T01 | Nucleoside-diphosphate-sugar epimerase |
| maker-2-augustus-gene-45.44-mRNA-1 | Indica | NA | | Os02g0180100 | OsR498G0203091200.01.T03 | Ubiquitin carboxyl-terminal hydrolase |
| maker-2-augustus-gene-45.48-mRNA-1 | Indica | NA | | Os02g0180500 | OsR498G0203092800.01.T01 | Chorismate mutase |
| maker-2-augustus-gene-46.34-mRNA-1 | Indica | NA | | Os02g0182700 | OsR498G0203102700.01.T01 | |
| maker-2-augustus-gene-46.38-mRNA-1 | Indica | Indica | | Os02g0182800 | OsR498G0203103100.01.T02 | Knotted 7 |
| maker-2-augustus-gene-46.44-mRNA-1 | Indica | NA | | Os02g0182100 | OsR498G0203099000.01.T01 | Two-component response regulator |
| maker-2-augustus-gene-46.47-mRNA-1 | Indica | Indica | | Os06g0643100 | OsR498G0203101400.01.T01 | Proteasome endopeptidase complex |
| maker-2-augustus-gene-46.50-mRNA-1 | Indica | NA | Kinase-TM | | OsR498G0203101600.01.T02 | Phytosulfokine receptor |
| maker-2-augustus-gene-47.36-mRNA-1 | Indica | NA | | Os02g0184100 | OsR498G0203106300.01.T01 | Putative prostatic spermine-binding protein (Fragment) |
| maker-2-augustus-gene-47.38-mRNA-1 | Indica | NA | | Os02g0184400 | OsR498G0203107300.01.T02 | UDP-glucose-fructose-phosphate glucosyltransferase |
| maker-2-augustus-gene-47.44-mRNA-1 | Indica | Indica | | Os02g0184500 | OsR498G0203107900.01.T01 | Ubiquitin system component Cue protein |
| maker-2-augustus-gene-47.47-mRNA-1 | Indica | NA | | Os02g0183000 | OsR498G0203104200.01.T01 | PLATZ transcription factor (Fragment) |
| maker-2-augustus-gene-47.49-mRNA-1 | Indica | NA | | Os02g0183700 | | Isoleucyl-tRNA synthetase |
| maker-2-augustus-gene-47.58-mRNA-1 | Indica | Indica | | Os02g0183900 | OsR498G0203106000.01.T01 | Isoleucyl-tRNA synthetase |
| maker-2-augustus-gene-47.61-mRNA-1 | Indica | NA | | Os02g0184000 | OsR498G0203106200.01.T03 | S-acyltransferase |
| maker-2-augustus-gene-47.66-mRNA-1 | Indica | Indica | | Os06g0644200 | OsR498G0203106600.01.T02 | H(+)-exporting diphosphatase |
| maker-2-augustus-gene-47.72-mRNA-1 | Indica | Japonica | | Os02g0184600 | OsR498G0203112700.01.T01 | Premnaspirodiene oxygenase |
| maker-2-augustus-gene-47.74-mRNA-1 | Indica | NA | | Os02g0184700 | OsR498G0203108600.01.T01 | Premnaspirodiene oxygenase |
| maker-2-augustus-gene-48.49-mRNA-1 | Indica | NA | | Os02g0185200 | OsR498G0203111900.01.T01 | Premnaspirodiene oxygenase |
| maker-2-augustus-gene-48.54-mRNA-1 | Indica | Indica | | Os02g0184900 | OsR498G0203112900.01.T02 | Premnaspirodiene oxygenase-like |
| maker-2-augustus-gene-48.57-mRNA-1 | Indica | Indica | | Os02g0185500 | OsR498G0203113300.01.T01 | Premnaspirodiene oxygenase |

| | | | | | | |
|---|---|---|---|---|---|---|
| maker-2-augustus-gene-48.59-mRNA-1 | Indica | Indica | | Os02g0186100 | OsR498G0203113700.01.T01 | Premnaspirodiene oxygenase |
| maker-2-augustus-gene-48.62-mRNA-1 | Indica | Indica | | Os02g0184700 | OsR498G0203113900.01.T01 | Premnaspirodiene oxygenase |
| maker-2-augustus-gene-49.32-mRNA-1 | Indica | Indica | | Os02g0186700 | OsR498G0203116700.01.T02 | |
| maker-2-augustus-gene-49.35-mRNA-1 | Indica | NA | | Os02g0186800 | OsR498G0203116900.01.T01 | Premnaspirodiene oxygenase |
| maker-2-augustus-gene-49.37-mRNA-1 | Indica | Indica | | Os02g0186900 | OsR498G0203117100.01.T01 | Premnaspirodiene oxygenase |
| maker-2-augustus-gene-49.39-mRNA-1 | Indica | NA | | Os02g0187000 | OsR498G0203117300.01.T01 | Premnaspirodiene oxygenase |
| maker-2-augustus-gene-49.45-mRNA-1 | Indica | NA | | Os02g0187600 | OsR498G0203119500.01.T01 | Protein TIC 21, chloroplastic isoform A |
| maker-2-augustus-gene-49.48-mRNA-1 | Indica | NA | | Os02g0187200 | OsR498G0203118200.01.T01 | Exostosin domain-containing protein (Fragment) |
| maker-2-augustus-gene-49.53-mRNA-1 | Indica | NA | | | OsR498G0203118400.01.T03 | SNARE associated Golgi protein family |
| maker-2-augustus-gene-49.60-mRNA-1 | Indica | NA | | Os02g0187500 | OsR498G0203119400.01.T01 | Putative thiamine phosphate synthase |
| maker-2-augustus-gene-49.74-mRNA-1 | Indica | NA | | Os02g0188500 | OsR498G0203124200.01.T01 | UDPGT domain-containing protein |
| maker-2-augustus-gene-50.36-mRNA-1 | Indica | NA | | Os02g0188900 | | F-box domain containing protein, expressed |
| maker-2-augustus-gene-50.38-mRNA-1 | Indica | NA | | Os02g0189300 | OsR498G0203127100.01.T02 | GDSL esterase/lipase |
| maker-2-augustus-gene-50.43-mRNA-1 | Indica | NA | | Os02g0189000 | OsR498G0203126400.01.T01 | 30S ribosomal protein S21, chloroplastic |
| maker-2-augustus-gene-55.37-mRNA-1 | Indica | Indica | | Os02g0197200 | OsR498G0203154300.01.T02 | Endoglucanase |
| maker-2-augustus-gene-55.50-mRNA-1 | Indica | Indica | | Os02g0198200 | OsR498G0203158500.01.T02 | probable calcium-binding protein CML21 |
| maker-2-augustus-gene-55.54-mRNA-1 | Indica | NA | | Os02g0198300 | OsR498G0203158700.01.T04 | ADP-ribosylation factor GTPase-activating protein AGD12 |
| maker-2-augustus-gene-55.62-mRNA-1 | Indica | NA | | Os02g0196900 | OsR498G0203153400.01.T01 | 3-oxoacyl-acyl-carrier-protein synthase |
| maker-2-augustus-gene-55.77-mRNA-1 | Indica | NA | | Os02g0197700 | OsR498G0203156800.01.T01 | Ditrans,polycis-polyprenyl diphosphate synthase ((2E,6E)-farnesyldiphospha |
| maker-2-augustus-gene-55.81-mRNA-1 | Indica | Indica | | Os02g0197800 | OsR498G0203157400.01.T02 | DUF971 domain-containing protein (Fragment) |
| maker-2-augustus-gene-56.0-mRNA-1 | Indica | NA | | Os02g0198600 | OsR498G0203160100.01.T05 | DNA damage-inducible protein 1 |
| maker-2-augustus-gene-56.16-mRNA-1 | Indica | NA | | | OsR498G0203159400.01.T02 | |
| maker-2-augustus-gene-56.19-mRNA-1 | Indica | NA | | Os02g0198700 | OsR498G0203160400.01.T01 | Subtilisin-like serine protease |
| maker-2-augustus-gene-66.67-mRNA-1 | Indica | Indica | | Os03g0233200 | OsR498G0202837900.01.T01 | Putative lecithin-cholesterol acyl transferase |
| maker-2-augustus-gene-73.19-mRNA-1 | Indica | NA | | Os01g0265300 | | Transposable element protein, putative, MuDR |
| maker-2-augustus-gene-98.13-mRNA-1 | Indica | NA | | Os04g0120000 | | Hydroxyproline-rich glycoprotein-like |
| maker-3-augustus-gene-173.41-mRNA-1 | Indica | Indica | | Os03g0359000 | OsR498G0305987400.01.T01 | Alpha/beta-Hydrolases superfamily protein |
| maker-3-augustus-gene-180.13-mRNA-1 | Indica | NA | | | OsR498G0306020300.01.T01 | NYN domain-containing protein (Fragment) |
| maker-3-augustus-gene-186.0-mRNA-1 | Indica | NA | | Os03g0380600 | | Transposon protein, putative, CACTA, En/Spm sub-class |
| maker-3-augustus-gene-192.7-mRNA-1 | Indica | Indica | | Os07g0620800 | OsR498G0306091200.01.T02 | Cyclin dependent kinase regulator |
| maker-3-augustus-gene-195.10-mRNA-1 | Indica | NA | | Os03g0395300 | OsR498G0306105600.01.T01 | Protein WEAK CHLOROPLAST MOVEMENT UNDER BLUE LIGHT 1 |
| maker-3-augustus-gene-196.33-mRNA-1 | Indica | NA | | | OsR498G0306106700.01.T02 | Switch-associated protein 70 |
| maker-3-augustus-gene-196.39-mRNA-1 | Indica | NA | | | OsR498G0306108700.01.T05 | Suppressor of white apricot N-terminal domain |
| maker-3-augustus-gene-196.44-mRNA-1 | Indica | Japonica | | Os03g0396900 | OsR498G0306112500.01.T01 | Trimethylguanosine synthase |
| maker-3-augustus-gene-196.49-mRNA-1 | Indica | Indica | | Os03g0397300 | OsR498G0306113300.01.T02 | Metallophos domain-containing protein (Fragment) |
| maker-3-augustus-gene-196.54-mRNA-1 | Indica | NA | | Os03g0396500 | OsR498G0306111400.01.T01 | Tify domain-containing protein |
| maker-3-augustus-gene-197.30-mRNA-1 | Indica | NA | | Os03g0397400 | OsR498G0306113700.01.T04 | Vacuolar cation/proton exchanger |
| maker-3-augustus-gene-197.33-mRNA-1 | Indica | Indica | Kinase-LRR-TM | Os07g0618400 | OsR498G0306114400.01.T02 | Protein STRUBBELIG-RECEPTOR FAMILY 6 |
| maker-3-augustus-gene-198.4-mRNA-1 | Indica | NA | | | OsR498G0306123200.01.T01 | 1-phosphatidylinositol-3-phosphate 5-kinase |
| maker-3-augustus-gene-199.0-mRNA-1 | Indica | NA | Kinase | | OsR498G0306131300.01.T02 | Serine/threonine-protein kinase CTR1 |
| maker-3-augustus-gene-199.10-mRNA-1 | Indica | NA | LRR-TM | Os03g0400850 | OsR498G0306129200.01.T01 | Putative LRR receptor-like serine/threonine-protein kinase |
| maker-3-augustus-gene-199.12-mRNA-1 | Indica | NA | | Os03g0401300 | OsR498G0306132000.01.T03 | Sucrose synthase |
| maker-3-augustus-gene-199.5-mRNA-1 | Indica | NA | | Os03g0400700 | | Late embryogenesis abundant protein |
| maker-3-augustus-gene-279.10-mRNA-1 | Indica | NA | | | OsR498G1018671500.01.T01 | BAH domain-containing protein |
| maker-3-augustus-gene-279.2-mRNA-1 | Indica | NA | | Os10g0404900 | OsR498G1018674900.01.T01 | Homeobox-leucine zipper protein ATHB-23 |
| maker-3-augustus-gene-279.4-mRNA-1 | Indica | NA | | Os02g0452800 | | Epimerase domain-containing protein |
| maker-3-augustus-gene-279.5-mRNA-1 | Indica | NA | | Os10g0404500 | OsR498G1018673700.01.T01 | Sucrose transporter |
| maker-3-augustus-gene-279.7-mRNA-1 | Indica | Indica | | Os03g0171300 | OsR498G1018672900.01.T01 | Transcription factor ILI7 |
| maker-3-augustus-gene-279.9-mRNA-1 | Indica | NA | | | OsR498G1018671700.01.T01 | BAH domain-containing protein |
| maker-3-augustus-gene-280.23-mRNA-1 | Indica | Indica | | Os03g0171700 | OsR498G1018669400.01.T01 | Transcription factor ILI3 |
| maker-3-augustus-gene-280.25-mRNA-1 | Indica | NA | | Os10g0403000 | OsR498G1018666000.01.T01 | Cytochrome P450, family 78, subfamily A, polypeptide 7 |
| maker-3-augustus-gene-280.27-mRNA-1 | Indica | NA | | | OsR498G1018668900.01.T02 | Glucose-fructose oxidoreductase, bacterial |
| maker-3-augustus-gene-281.20-mRNA-1 | Indica | NA | | Os10g0400900 | OsR498G1018659900.01.T02 | Mic1 domain-containing protein (Fragment) |
| maker-3-augustus-gene-281.4-mRNA-1 | Indica | NA | | Os10g0400800 | OsR498G1018659800.01.T01 | Phenylalanine--tRNA ligase alpha subunit, cytoplasmic |
| maker-3-augustus-gene-282.33-mRNA-1 | Indica | NA | | Os10g0400200 | OsR498G1018658200.01.T01 | Glutelin type-A 2 |
| maker-3-augustus-gene-282.35-mRNA-1 | Indica | NA | | Os10g0399200 | OsR498G1018655600.01.T01 | Putative O-succinylhomoserine (Thiol)-lyase |
| maker-3-augustus-gene-282.37-mRNA-1 | Indica | NA | | Os10g0400100 | OsR498G1018657900.01.T01 | Methionyl-tRNA synthetase |
| maker-3-augustus-gene-282.42-mRNA-1 | Indica | NA | | Os10g0399700 | OsR498G1018656500.01.T01 | Putative O-succinylhomoserine (Thiol)-lyase |
| maker-3-augustus-gene-283.5-mRNA-1 | Indica | NA | | | OsR498G1018652100.01.T01 | CCAAT-binding transcription factor B |
| maker-3-augustus-gene-297.11-mRNA-1 | Indica | NA | | | OsR498G1018579600.01.T01 | |
| maker-3-augustus-gene-297.15-mRNA-1 | Indica | NA | | | | |

| | | | | | | |
|---|---|---|---|---|---|---|
| maker-3-augustus-gene-297.17-mRNA-1 | Indica | NA | | Os10g0376900 | OsR498G1018581100.01.T01 | Transcription factor bHLH96 |
| maker-3-augustus-gene-298.34-mRNA-1 | Indica | NA | LRR-TM | Os10g0376200 | OsR498G1018578500.01.T02 | Dynein assembly factor 1, axonemal |
| maker-3-augustus-gene-298.38-mRNA-1 | Indica | Indica | | Os03g0388800 | OsR498G1018576700.01.T01 | 2-hydroxy-6-oxononadienedioate/2-hydroxy-6-oxononatrienedioate hydrola |
| maker-3-augustus-gene-298.41-mRNA-1 | Indica | NA | | Os10g0375600 | OsR498G1018576500.01.T03 | Calmodulin-binding transcription activator 2 |
| maker-3-augustus-gene-298.46-mRNA-1 | Indica | NA | Kinase-LRR-TM | Os10g0375000 | OsR498G1018574700.01.T03 | Putative receptor like protein kinase |
| maker-3-augustus-gene-299.35-mRNA-1 | Indica | NA | | | | |
| maker-3-augustus-gene-299.40-mRNA-1 | Indica | NA | | Os10g0372800 | | NAC domain-containing protein |
| maker-3-augustus-gene-299.43-mRNA-1 | Indica | NA | Kinase-LRR-TM | Os10g0374666 | OsR498G1018573900.01.T01 | LRR receptor-like serine/threonine-protein kinase EFR isoform B |
| maker-3-augustus-gene-299.46-mRNA-1 | Indica | NA | | Os10g0373400 | | |
| maker-3-augustus-gene-299.48-mRNA-1 | Indica | NA | | | OsR498G1018569700.01.T01 | |
| maker-3-augustus-gene-299.50-mRNA-1 | Indica | NA | | | OsR498G1018568500.01.T01 | |
| maker-3-augustus-gene-301.25-mRNA-1 | Indica | NA | NBS-TM | Os10g0370100 | | calmodulin-binding transcription activator 3 |
| maker-3-augustus-gene-303.26-mRNA-1 | Indica | NA | | Os10g0368400 | OsR498G1018555200.01.T01 | AP complex subunit sigma |
| maker-3-augustus-gene-303.30-mRNA-1 | Indica | NA | | Os10g0528300 | OsR498G1018554900.01.T02 | Glutathione S-transferase |
| maker-3-augustus-gene-304.0-mRNA-1 | Indica | NA | | | OsR498G1018550000.01.T01 | C2 NT-type domain-containing protein (Fragment) |
| maker-3-augustus-gene-304.12-mRNA-1 | Indica | NA | | | OsR498G1018551500.01.T01 | Protein translocase/ protein transporter |
| maker-3-augustus-gene-304.14-mRNA-1 | Indica | NA | NBS-TM | | OsR498G1018551100.01.T01 | NB-ARC domain containing protein, expressed |
| maker-3-augustus-gene-305.24-mRNA-1 | Indica | NA | | Os10g0363500 | OsR498G1018548900.01.T02 | Pentatricopeptide repeat-containing protein mitochondrial |
| maker-3-augustus-gene-305.25-mRNA-1 | Indica | NA | | | OsR498G1018548100.01.T01 | Acetyl-CoA carboxylase |
| maker-3-augustus-gene-305.32-mRNA-1 | Indica | NA | | Os10g0363100 | OsR498G1018546700.01.T07 | Hexosyltransferase |
| maker-3-augustus-gene-307.0-mRNA-1 | Indica | NA | | Os10g0360900 | OsR498G1018535600.01.T03 | |
| maker-3-augustus-gene-307.8-mRNA-1 | Indica | NA | Kinase-LRR-TM | Os10g0360933 | OsR498G1018534900.01.T01 | Putative LRR receptor-like serine/threonine-protein kinase |
| maker-3-augustus-gene-308.0-mRNA-1 | Indica | NA | | Os10g0359500 | OsR498G1018531800.01.T03 | NAC domain-containing protein 68 |
| maker-3-augustus-gene-309.0-mRNA-1 | Indica | NA | | Os10g0357800 | OsR498G1018526700.01.T05 | N(2),N(2)-dimethylguanosine tRNA methyltransferase 1 |
| maker-3-augustus-gene-310.24-mRNA-1 | Indica | NA | | Os10g0351600 | OsR498G1018523300.01.T01 | GH10 domain-containing protein (Fragment) |
| maker-3-augustus-gene-311.0-mRNA-1 | Indica | NA | | Os10g0351300 | OsR498G1018520800.01.T01 | Cytochrome P450 family 76 subfamily C polypeptide 7 |
| maker-3-augustus-gene-311.4-mRNA-1 | Indica | NA | | Os10g0350400 | | Ribosomal protein S12 containing protein |
| maker-3-augustus-gene-311.9-mRNA-1 | Indica | NA | | | OsR498G1018520500.01.T02 | |
| maker-3-augustus-gene-313.30-mRNA-1 | Indica | NA | | Os10g0347800 | OsR498G1018515600.01.T01 | Formin-like protein 4 |
| maker-3-augustus-gene-315.0-mRNA-1 | Indica | NA | | Os10g0347000 | OsR498G1018513200.01.T01 | (1->3)-beta-glucan endohydrolase |
| maker-3-augustus-gene-315.10-mRNA-1 | Indica | NA | | Os10g0346200 | OsR498G1018510900.01.T01 | Zinc finger, RING/FYVE/PHD-type |
| maker-3-augustus-gene-315.2-mRNA-1 | Indica | NA | | Os10g0346600 | OsR498G1018512600.01.T03 | Vacuolar-sorting receptor |
| maker-3-augustus-gene-315.8-mRNA-1 | Indica | NA | | Os10g0346300 | OsR498G1018511200.01.T01 | Laccase |
| maker-3-augustus-gene-316.21-mRNA-1 | Indica | NA | | Os10g0345100 | OsR498G1018505600.01.T02 | Protein DETOXIFICATION |
| maker-3-augustus-gene-317.42-mRNA-1 | Indica | NA | | Os10g0343500 | | |
| maker-3-augustus-gene-322.9-mRNA-1 | Indica | NA | | Os10g0333700 | OsR498G1018475500.01.T01 | |
| maker-3-augustus-gene-324.45-mRNA-1 | Indica | NA | | Os10g0330600 | OsR498G0612997600.01.T01 | Beta-galactosidase 14 |
| maker-3-augustus-gene-328.13-mRNA-1 | Indica | NA | | Os10g0324600 | OsR498G1018442500.01.T01 | zinc finger protein 346 |
| maker-3-augustus-gene-328.2-mRNA-1 | Indica | NA | | Os10g0490400 | OsR498G1018442800.01.T03 | Transposon protein, putative, unclassified, expressed |
| maker-3-augustus-gene-330.12-mRNA-1 | Indica | NA | | Os10g0322250 | | Inorganic phosphate transporter 6 |
| maker-3-augustus-gene-393.7-mRNA-1 | Indica | NA | | Os03g0656800 | OsR498G0306703500.01.T01 | Glucan endo-1,3-beta-D-glucosidase |
| maker-3-augustus-gene-462.59-mRNA-1 | Indica | NA | | | | Transcription factor PIF5 |
| maker-3-augustus-gene-463.0-mRNA-1 | Indica | NA | | | OsR498G0307158500.01.T05 | Actin-related protein 7 isoform 1 |
| maker-3-augustus-gene-6.21-mRNA-1 | Indica | Japonica | | Os03g0115300 | OsR498G0305117000.01.T04 | Pentatricopeptide repeat-containing protein (Fragment) |
| maker-3-augustus-gene-82.3-mRNA-1 | Indica | NA | | Os03g0259300 | OsR498G0305611700.01.T03 | ERAD-associated E3 ubiquitin-protein ligase component HRD3A like |
| maker-4-augustus-gene-0.3-mRNA-1 | Indica | NA | | | OsR498G0816152000.01.T01 | |
| maker-4-augustus-gene-103.29-mRNA-1 | Indica | Indica | | Os02g0474700 | OsR498G0408169100.01.T06 | Importin-beta |
| maker-4-augustus-gene-103.36-mRNA-1 | Indica | NA | | Os04g0337500 | OsR498G0408169700.01.T01 | Probable aldo-keto reductase 1 |
| maker-4-augustus-gene-103.39-mRNA-1 | Indica | Japonica | | Os04g0337700 | OsR498G0408170900.01.T04 | Probable aldo-keto reductase 2 |
| maker-4-augustus-gene-103.41-mRNA-1 | Indica | NA | | Os04g0338000 | OsR498G0408170900.01.T04 | Aldo-keto reductase 2 |
| maker-4-augustus-gene-103.45-mRNA-1 | Indica | Indica | | Os04g0338000 | OsR498G0408171100.01.T04 | Aldo-keto reductase 2 |
| maker-4-augustus-gene-105.6-mRNA-1 | Indica | NA | | Os04g0341100 | | Putative aldo/keto reductase AKR (Fragment) |
| maker-4-augustus-gene-105.9-mRNA-1 | Indica | NA | | Os04g0340300 | OsR498G0408179000.01.T02 | (+)-delta-cadinene synthase isozyme A |
| maker-4-augustus-gene-108.29-mRNA-1 | Indica | NA | | | | Transposon protein, putative, CACTA, En/Spm sub-class |
| maker-4-augustus-gene-108.32-mRNA-1 | Indica | Indica | | Os04g0341800 | OsR498G0408202600.01.T03 | Terpene synthase TPS38 |
| maker-4-augustus-gene-110.38-mRNA-1 | Indica | NA | | | OsR498G0408219900.01.T01 | MAU2 chromatid cohesion factor like |
| maker-4-augustus-gene-110.44-mRNA-1 | Indica | NA | | | OsR498G0408220300.01.T01 | |
| maker-4-augustus-gene-110.59-mRNA-1 | Indica | NA | | | OsR498G0408217400.01.T01 | Prolyl 4-hydroxylase 1 |
| maker-4-augustus-gene-110.61-mRNA-1 | Indica | Indica | | Os04g0347200 | OsR498G0408218400.01.T01 | Acidic endochitinase |
| maker-4-augustus-gene-111.0-mRNA-1 | Indica | Indica | | Os04g0348300 | OsR498G0408222300.01.T01 | Cell division cycle 5-related protein |
| maker-4-augustus-gene-111.15-mRNA-1 | Indica | NA | | Os04g0348400 | OsR498G0408222600.01.T01 | RING-type E3 ubiquitin transferase |

| | | | | | | |
|---|---|---|---|---|---|---|
| maker-4-augustus-gene-111.22-mRNA-1 | Indica | NA | | Os04g0349500 | OsR498G0408225600.01.T01 | 40S ribosomal protein S8 |
| maker-4-augustus-gene-111.5-mRNA-1 | Indica | NA | | | | Response regulatory domain-containing protein |
| maker-4-augustus-gene-112.37-mRNA-1 | Indica | NA | | Os04g0350300 | OsR498G0408228100.01.T01 | Phragmoplast-associated kinesin-related protein |
| maker-4-augustus-gene-112.45-mRNA-1 | Indica | Indica | LRR-TM | Os04g0349700 | OsR498G0408227200.01.T02 | Putative verticillium wilt disease resistance protein |
| maker-4-augustus-gene-112.47-mRNA-1 | Indica | NA | | Os04g0350700 | OsR498G0408230800.01.T01 | Transcription factor bHLH77 |
| maker-4-augustus-gene-113.36-mRNA-1 | Indica | NA | | Os04g0352400 | OsR498G0408236200.01.T01 | Peptidylprolyl isomerase |
| maker-4-augustus-gene-113.40-mRNA-1 | Indica | NA | | | OsR498G0408239000.01.T03 | Phospholipid-transporting ATPase |
| maker-4-augustus-gene-124.31-mRNA-1 | Indica | NA | | Os04g0367400 | OsR498G0408336300.01.T01 | Reticulon-like protein (Fragment) |
| maker-4-augustus-gene-124.33-mRNA-1 | Indica | NA | | | | Putative WAK family receptor-like protein kinase |
| maker-4-augustus-gene-125.39-mRNA-1 | Indica | NA | CC-Kinase-TM | | OsR498G0408339000.01.T01 | Jacalin-like lectin domain containing protein |
| maker-4-augustus-gene-126.0-mRNA-1 | Indica | NA | Kinase-TM | | | Protein kinase domain-containing protein (Fragment) |
| maker-4-augustus-gene-127.31-mRNA-1 | Indica | NA | | | | |
| maker-4-augustus-gene-130.31-mRNA-1 | Indica | NA | | Os03g0598600 | | |
| maker-4-augustus-gene-130.4-mRNA-1 | Indica | Indica | | Os04g0377600 | OsR498G0408380900.01.T01 | Rab-GAP TBC domain-containing protein (Fragment) |
| maker-4-augustus-gene-131.22-mRNA-1 | Indica | NA | | Os04g0379900 | OsR498G0408386800.01.T01 | Stearoyl-acyl-carrier protein desaturase |
| maker-4-augustus-gene-132.14-mRNA-1 | Indica | NA | | Os04g0381000 | OsR498G0408395400.01.T05 | Dynamin-related protein 3A isoform 2 |
| maker-4-augustus-gene-133.31-mRNA-1 | Indica | NA | | Os04g0382700 | OsR498G0408403100.01.T01 | Vacuolar protein sorting-associated protein 11 isogeny |
| maker-4-augustus-gene-133.33-mRNA-1 | Indica | NA | | Os04g0382800 | OsR498G0408403700.01.T02 | |
| maker-4-augustus-gene-134.43-mRNA-1 | Indica | NA | | | | |
| maker-4-augustus-gene-137.34-mRNA-1 | Indica | Indica | | Os08g0532400 | OsR498G0408429300.01.T01 | Nodulin-like domain-containing protein |
| maker-4-augustus-gene-138.0-mRNA-1 | Indica | NA | | | OsR498G0408437300.01.T01 | Probable metal-nicotianamine transporter YSL6 |
| maker-4-augustus-gene-138.12-mRNA-1 | Indica | NA | | Os04g0390700 | OsR498G0408437700.01.T01 | Hydroxysteroid dehydrogenase 1 |
| maker-4-augustus-gene-138.14-mRNA-1 | Indica | NA | | Os04g0390800 | OsR498G0408438300.01.T01 | 11-beta-hydroxysteroid dehydrogenase 1B |
| maker-4-augustus-gene-138.15-mRNA-1 | Indica | NA | | Os04g0390800 | OsR498G0408438300.01.T01 | 11-beta-hydroxysteroid dehydrogenase 1B |
| maker-4-augustus-gene-138.27-mRNA-1 | Indica | NA | | Os04g0391000 | OsR498G0408438600.01.T03 | RecQ-mediated genome instability protein 1 |
| maker-4-augustus-gene-138.6-mRNA-1 | Indica | NA | | | OsR498G0408437500.01.T01 | Probable metal-nicotianamine transporter YSL6 |
| maker-4-augustus-gene-147.38-mRNA-1 | Indica | Indica | | Os02g0527200 | OsR498G0408507000.01.T03 | |
| maker-4-augustus-gene-148.0-mRNA-1 | Indica | NA | | Os04g0408600 | OsR498G0408516600.01.T01 | RAB6-interacting golgin |
| maker-4-augustus-gene-148.6-mRNA-1 | Indica | Indica | | Os02g0528200 | OsR498G0408518200.01.T01 | 1,4-alpha-glucan branching enzyme |
| maker-4-augustus-gene-149.11-mRNA-1 | Indica | NA | | Os04g0410300 | OsR498G0408520300.01.T03 | Late embryogenesis abundant protein-related / LEA protein-related |
| maker-4-augustus-gene-149.15-mRNA-1 | Indica | NA | | Os04g0410400 | OsR498G0408520500.01.T01 | Late embryogenesis abundant protein-related / LEA protein-related |
| maker-4-augustus-gene-149.20-mRNA-1 | Indica | NA | | Os04g0410200 | OsR498G0408519000.01.T02 | Serine/threonine-protein kinase KIPK |
| maker-4-augustus-gene-149.21-mRNA-1 | Indica | NA | | Os04g0410250 | | |
| maker-4-augustus-gene-149.28-mRNA-1 | Indica | NA | | Os04g0410600 | OsR498G0408520800.01.T01 | Purple acid phosphatase |
| maker-4-augustus-gene-150.38-mRNA-1 | Indica | NA | | Os04g0412300 | OsR498G0408526500.01.T02 | Glucan endo-13-beta-glucosidase 14 |
| maker-4-augustus-gene-150.40-mRNA-1 | Indica | NA | | | OsR498G0408527700.01.T04 | Bifunctional monodehydroascorbate reductase and carbonic anhydrase nect |
| maker-4-augustus-gene-151.36-mRNA-1 | Indica | NA | | Os04g0414000 | | Mitochondrial fission protein ELM1 |
| maker-4-augustus-gene-152.37-mRNA-1 | Indica | NA | | Os04g0415900 | OsR498G0408543100.01.T01 | AAI domain-containing protein (Fragment) |
| maker-4-augustus-gene-152.39-mRNA-1 | Indica | NA | | Os04g0416000 | OsR498G0408543300.01.T01 | DUF1279 domain-containing protein (Fragment) |
| maker-4-augustus-gene-152.50-mRNA-1 | Indica | Indica | | Os02g0537500 | OsR498G0408543600.01.T04 | Transcription factor E2FA |
| maker-4-augustus-gene-153.37-mRNA-1 | Indica | Japonica | | Os04g0417900 | OsR498G0408552100.01.T01 | |
| maker-4-augustus-gene-153.39-mRNA-1 | Indica | NA | | Os04g0418000 | OsR498G0408552500.01.T01 | Succinate dehydrogenase subunit 5 like |
| maker-4-augustus-gene-153.41-mRNA-1 | Indica | NA | | Os04g0417800 | OsR498G0408551400.01.T02 | Protein phosphatase type 2A regulator/ signal transducer |
| maker-4-augustus-gene-172.40-mRNA-1 | Indica | NA | | Os04g0451100 | | Polyadenylate-binding protein 2 |
| maker-4-augustus-gene-172.44-mRNA-1 | Indica | NA | | Os04g0451200 | OsR498G0408673700.01.T01 | Glycosyltransferase |
| maker-4-augustus-gene-200.40-mRNA-1 | Indica | NA | | Os04g0508100 | | Isocitrate dehydrogenase [NADP] |
| maker-4-augustus-gene-200.42-mRNA-1 | Indica | NA | | | OsR498G0408865700.01.T01 | Isocitrate dehydrogenase |
| maker-4-augustus-gene-204.6-mRNA-1 | Indica | NA | | Os04g0515900 | OsR498G0408893100.01.T01 | NAC transcription factor |
| maker-4-augustus-gene-205.15-mRNA-1 | Indica | NA | | Os04g0517100 | OsR498G0408899800.01.T01 | Transcription factor MYB4 |
| maker-4-augustus-gene-205.17-mRNA-1 | Indica | NA | | Os04g0517300 | OsR498G0408901000.01.T01 | Root UVB sensitive family |
| maker-4-augustus-gene-205.3-mRNA-1 | Indica | Indica | | Os04g0517400 | OsR498G0408901100.01.T01 | Hexosyltransferase |
| maker-4-augustus-gene-205.9-mRNA-1 | Indica | NA | Kinase-TM | Os04g0517500 | OsR498G0408901300.01.T01 | Phosphoenolpyruvate carboxylase kinase |
| maker-4-augustus-gene-208.49-mRNA-1 | Indica | Indica | | Os04g0521200 | OsR498G0408917900.01.T01 | V-type proton ATPase subunit G1 |
| maker-4-augustus-gene-209.2-mRNA-1 | Indica | NA | | Os04g0524300 | OsR498G0408926300.01.T01 | Two-component response regulator ORR5 |
| maker-4-augustus-gene-213.61-mRNA-1 | Indica | NA | | Os04g0534000 | OsR498G0408964300.01.T02 | CAP10 domain-containing protein (Fragment) |
| maker-4-augustus-gene-214.0-mRNA-1 | Indica | Indica | | Os02g0642700 | OsR498G0408964500.01.T05 | Downstream target of AGL15 2 |
| maker-4-augustus-gene-216.39-mRNA-1 | Indica | NA | | Os04g0538000 | OsR498G0408982500.01.T02 | Hsp organizing protein/stress-inducible protein |
| maker-4-augustus-gene-216.57-mRNA-1 | Indica | Indica | | Os04g0538100 | OsR498G0408982800.01.T03 | Elongation factor G, chloroplastic |
| maker-4-augustus-gene-22.46-mRNA-1 | Indica | Indica | | Os02g0101000 | OsR498G0407717000.01.T02 | Vacuolar amino acid transporter 1 |
| maker-4-augustus-gene-241.68-mRNA-1 | Indica | NA | | | | RecBCD enzyme subunit RecB, P-loop containing nucleoside triphosphate hy |
| maker-4-augustus-gene-244.0-mRNA-1 | Indica | NA | | | | DNA replication ATP-dependent helicase/nuclease |

| | | | | | | |
|---|---|---|---|---|---|---|
| maker-4-augustus-gene-249.38-mRNA-1 | Indica | NA | | Os04g0600400 | OsR498G0409221000.01.T01 | Baculoviral IAP repeat-containing protein 1a |
| maker-4-augustus-gene-249.41-mRNA-1 | Indica | Indica | | Os02g0700700 | OsR498G0409221200.01.T03 | Protein BREVIS RADIX |
| maker-4-augustus-gene-249.7-mRNA-1 | Indica | NA | | | | UvrD-helicase domain-containing protein (Fragment) |
| maker-4-augustus-gene-254.30-mRNA-1 | Indica | NA | | Os04g0608400 | OsR498G0409250300.01.T01 | Nuclear pore complex protein NUP88 |
| maker-4-augustus-gene-254.34-mRNA-1 | Indica | NA | | Os04g0608800 | OsR498G0409252300.01.T01 | Evolutionarily conserved C-terminal region 10 |
| maker-4-augustus-gene-254.37-mRNA-1 | Indica | NA | | Os04g0607600 | OsR498G0409246400.01.T03 | Probable cation transporter HKT7 |
| maker-4-augustus-gene-254.53-mRNA-1 | Indica | NA | | | OsR498G0409251200.01.T01 | probable nucleoredoxin 3 |
| maker-4-augustus-gene-255.17-mRNA-1 | Indica | NA | | Os04g0609700 | OsR498G0409256800.01.T04 | PHD-type domain-containing protein |
| maker-4-augustus-gene-255.7-mRNA-1 | Indica | NA | Kinase-TM | Os04g0608900 | OsR498G0409252600.01.T01 | serine/threonine-protein kinase STY13 |
| maker-4-augustus-gene-26.27-mRNA-1 | Indica | Indica | Kinase-TM | Os11g0692100 | OsR498G0407758000.01.T01 | Bacterial blight resistance protein XA26 |
| maker-4-augustus-gene-263.39-mRNA-1 | Indica | NA | | | OsR498G0407881700.01.T01 | Transposon protein, putative, Mutator sub-class |
| maker-4-augustus-gene-267.54-mRNA-1 | Indica | Indica | | Os04g0630800 | OsR498G0409329100.01.T02 | Epimerase domain-containing protein (Fragment) |
| maker-4-augustus-gene-267.55-mRNA-1 | Indica | Indica | | Os04g0631000 | OsR498G0409329500.01.T01 | Epimerase domain-containing protein |
| maker-4-augustus-gene-267.58-mRNA-1 | Indica | NA | | | | Epimerase domain-containing protein (Fragment) |
| maker-4-augustus-gene-268.3-mRNA-1 | Indica | NA | Kinase-TM | Os04g0631800 | OsR498G0409332600.01.T02 | Serine/threonine-protein kinase |
| maker-4-augustus-gene-268.39-mRNA-1 | Indica | Japonica | Kinase-TM | Os04g0634000 | OsR498G0409338400.01.T01 | G-type lectin S-receptor-like serine/threonine-protein kinase |
| maker-4-augustus-gene-268.7-mRNA-1 | Indica | NA | Kinase-TM | Os04g0632100 | OsR498G0409332800.01.T04 | Non-specific serine/threonine protein kinase |
| maker-4-augustus-gene-278.31-mRNA-1 | Indica | NA | | Os04g0652900 | OsR498G0409400600.01.T04 | Aminoacyl-tRNA hydrolase |
| maker-4-augustus-gene-278.7-mRNA-1 | Indica | NA | | | | Aspergillus nuclease S(1) |
| maker-4-augustus-gene-28.36-mRNA-1 | Indica | NA | | | OsR498G1019263000.01.T04 | Serrate RNA effector molecule (Fragment) |
| maker-4-augustus-gene-28.42-mRNA-1 | Indica | NA | | Os04g0218900 | OsR498G0407752500.01.T03 | TRAF-type domain-containing protein |
| maker-4-augustus-gene-28.48-mRNA-1 | Indica | NA | | | | |
| maker-4-augustus-gene-280.15-mRNA-1 | Indica | NA | | Os04g0656500 | OsR498G0409415100.01.T01 | Squamosa promoter-binding-like protein 8 |
| maker-4-augustus-gene-284.21-mRNA-1 | Indica | NA | | | OsR498G0409438800.01.T01 | Homeobox domain-containing protein (Fragment) |
| maker-4-augustus-gene-284.23-mRNA-1 | Indica | NA | | Os04g0663700 | OsR498G0409439800.01.T03 | TLDc domain-containing protein (Fragment) |
| maker-4-augustus-gene-284.27-mRNA-1 | Indica | NA | | | OsR498G0409440000.01.T01 | Peptidyl-prolyl cis-trans isomerase |
| maker-4-augustus-gene-285.0-mRNA-1 | Indica | NA | | Os04g0665000 | OsR498G0409445100.01.T01 | cytochrome c oxidase-assembly factor COX23, mitochondrial |
| maker-4-augustus-gene-285.17-mRNA-1 | Indica | NA | | | OsR498G0409445000.01.T04 | Beta-fructofuranosidase, insoluble isoenzyme 6 |
| maker-4-augustus-gene-285.21-mRNA-1 | Indica | NA | | Os04g0665200 | OsR498G0409445400.01.T02 | Indole-3-acetate O-methyltransferase 1 |
| maker-4-augustus-gene-285.7-mRNA-1 | Indica | NA | | Os04g0665700 | OsR498G0409449300.01.T02 | Putative plastid-lipid-associated protein 13 chloroplastic |
| maker-4-augustus-gene-286.22-mRNA-1 | Indica | NA | | Os04g0666900 | OsR498G0409453600.01.T03 | Kinesin-like calmodulin-binding protein (ZWICHEL) isoform 4 |
| maker-4-augustus-gene-29.0-mRNA-1 | Indica | NA | | Os04g0220300 | | Wall-associated receptor kinase-like 16 |
| maker-4-augustus-gene-30.0-mRNA-1 | Indica | NA | | Os04g0221000 | OsR498G0407755400.01.T01 | |
| maker-4-augustus-gene-30.4-mRNA-1 | Indica | NA | | Os04g0221600 | OsR498G0407756000.01.T02 | PHD domain-containing protein |
| maker-4-augustus-gene-31.39-mRNA-1 | Indica | Indica | Kinase-TM | Os11g0692100 | OsR498G0407758000.01.T01 | Receptor kinase MRKa |
| maker-4-augustus-gene-33.0-mRNA-1 | Indica | NA | | | | Vacuolar protein sorting-associated protein 33 like |
| maker-4-augustus-gene-33.11-mRNA-1 | Indica | NA | | Os04g0223901 | OsR498G0407762400.01.T01 | Flavin-containing monooxygenase |
| maker-4-augustus-gene-33.6-mRNA-1 | Indica | NA | | Os04g0223300 | OsR498G0407761800.01.T01 | L-ascorbate peroxidase |
| maker-4-augustus-gene-34.0-mRNA-1 | Indica | NA | | Os04g0224600 | OsR498G0407764000.01.T02 | SEC1 family transport protein SLY1-like |
| maker-4-augustus-gene-34.4-mRNA-1 | Indica | Indica | | Os04g0224900 | OsR498G0407764800.01.T05 | Glycerol-3-phosphate dehydrogenase |
| maker-4-augustus-gene-62.50-mRNA-1 | Indica | NA | | | OsR498G0407924400.01.T01 | FMN-linked oxidoreductase superfamily protein |
| maker-4-augustus-gene-63.3-mRNA-1 | Indica | NA | | | | CYTH domain-containing protein |
| maker-4-augustus-gene-63.7-mRNA-1 | Indica | Japonica | | Os04g0280500 | OsR498G0407926900.01.T01 | F-box domain-containing protein |
| maker-4-augustus-gene-64.0-mRNA-1 | Indica | NA | | Os04g0282200 | OsR498G0407930900.01.T03 | 1-phosphatidylinositol-4,5-bisphosphate phosphodiesterase epsilon-1 |
| maker-4-augustus-gene-64.2-mRNA-1 | Indica | NA | | Os04g0282400 | OsR498G0407932400.01.T02 | Flowering-promoting factor 1 |
| maker-4-augustus-gene-68.40-mRNA-1 | Indica | Japonica | | Os04g0288100 | OsR498G0816248400.01.T01 | Germin-like protein 8-14 |
| maker-4-augustus-gene-86.32-mRNA-1 | Indica | NA | CC-NBS-TM | | OsR498G0408112900.01.T01 | Putative NBS-LRR class RGA |
| maker-4-augustus-gene-89.4-mRNA-1 | Indica | NA | | Os10g0343500 | | |
| maker-4-augustus-gene-92.18-mRNA-1 | Indica | NA | | Os04g0321800 | OsR498G0408083400.01.T01 | PPM-type phosphatase domain-containing protein |
| maker-4-augustus-gene-97.3-mRNA-1 | Indica | NA | | Os04g0326300 | OsR498G0408118000.01.T02 | tRNA (adenine(58)-N(1))-methyltransferase |
| maker-5-augustus-gene-12.3-mRNA-1 | Indica | NA | | | OsR498G0714022200.01.T01 | |
| maker-5-augustus-gene-12.5-mRNA-1 | Indica | NA | | | | Ulp1 protease family, C-terminal catalytic domain containing protein, expres |
| maker-5-augustus-gene-138.19-mRNA-1 | Indica | NA | | Os05g0302300 | OsR498G0510395600.01.T01 | Lysine-specific demethylase |
| maker-5-augustus-gene-139.10-mRNA-1 | Indica | NA | | | OsR498G0510403200.01.T01 | RRM domain-containing protein |
| maker-5-augustus-gene-139.6-mRNA-1 | Indica | NA | | | OsR498G0510402900.01.T01 | RRM domain-containing protein (Fragment) |
| maker-5-augustus-gene-139.8-mRNA-1 | Indica | NA | | Os05g0302700 | OsR498G0510399200.01.T02 | ADP ATP carrier protein 3 mitochondrial |
| maker-5-augustus-gene-140.0-mRNA-1 | Indica | NA | | Os05g0304400 | OsR498G0510406900.01.T01 | Guanosine nucleotide diphosphate dissociation inhibitor |
| maker-5-augustus-gene-140.10-mRNA-1 | Indica | Japonica | | Os05g0304200 | OsR498G1120445500.01.T02 | Plastid-lipid-associated protein 6, chloroplastic |
| maker-5-augustus-gene-140.7-mRNA-1 | Indica | NA | | Os05g0304600 | OsR498G0510408500.01.T01 | Lipoxygenase |
| maker-5-augustus-gene-140.9-mRNA-1 | Indica | NA | | Os05g0319900 | OsR498G0510483800.01.T01 | Endo-1,4-beta-xylanase |
| maker-5-augustus-gene-141.0-mRNA-1 | Indica | NA | | Os05g0305100 | OsR498G0510411900.01.T02 | Transcription factor IIIB subunit |

| | | | | | | |
|---|---|---|---|---|---|---|
| maker-5-augustus-gene-141.16-mRNA-1 | Indica | Indica | | Os05g0304900 | OsR498G0510410400.01.T02 | GH10 domain-containing protein (Fragment) |
| maker-5-augustus-gene-146.0-mRNA-1 | Indica | NA | | Os08g0299000 | OsR498G0815695900.01.T05 | UPF0103/Mediator of ErbB2-driven cell motility (Memo-related) |
| maker-5-augustus-gene-156.0-mRNA-1 | Indica | NA | | Os05g0326650 | | DUF659 domain-containing protein |
| maker-5-augustus-gene-156.1-mRNA-1 | Indica | NA | | Os05g0326650 | | DUF659 domain-containing protein |
| maker-5-augustus-gene-156.4-mRNA-1 | Indica | Japonica | | Os05g0327100 | OsR498G1221921500.01.T05 | Pumilio/PUF RNA binding protein 6 |
| maker-5-augustus-gene-161.0-mRNA-1 | Indica | NA | | Os05g0331200 | OsR498G0510525000.01.T01 | External alternative NAD(P)H-ubiquinone oxidoreductase B3, mitochondrial |
| maker-5-augustus-gene-162.1-mRNA-1 | Indica | NA | | Os07g0212400 | | HGWP repeat containing protein-like |
| maker-5-augustus-gene-162.17-mRNA-1 | Indica | NA | | | OsR498G0510534010.01.T01 | YTH domain-containing protein |
| maker-5-augustus-gene-162.19-mRNA-1 | Indica | NA | | Os05g0334400 | OsR498G0510534000.01.T01 | Heat shock protein DnaJ |
| maker-5-augustus-gene-162.8-mRNA-1 | Indica | NA | | Os05g0333200 | OsR498G0510532800.01.T01 | Guanine nucleotide-binding protein alpha-1 subunit |
| maker-5-augustus-gene-163.0-mRNA-1 | Indica | NA | | | | Saposin B-type domain-containing protein |
| maker-5-augustus-gene-163.1-mRNA-1 | Indica | NA | | Os05g0334400 | OsR498G0510534000.01.T01 | Heat shock protein DnaJ |
| maker-5-augustus-gene-163.15-mRNA-1 | Indica | NA | | | OsR498G0510541400.01.T08 | Phospholipid-transporting ATPase |
| maker-5-augustus-gene-164.35-mRNA-1 | Indica | NA | | | | Retrotransposon protein, putative, unclassified |
| maker-5-augustus-gene-164.37-mRNA-1 | Indica | NA | | | | Putative peptide/nitrate transporter |
| maker-5-augustus-gene-164.41-mRNA-1 | Indica | NA | | Os05g0337200 | OsR498G0510547400.01.T01 | Transcription factor bHLH61 like |
| maker-5-augustus-gene-164.43-mRNA-1 | Indica | NA | | | | Polynucleotidyl transferase, ribonuclease H-like superfamily protein, putativ |
| maker-5-augustus-gene-165.31-mRNA-1 | Indica | Indica | | Os05g0338933 | OsR498G0510554500.01.T01 | Nitrate excretion transporter 1 |
| maker-5-augustus-gene-165.35-mRNA-1 | Indica | Japonica | | Os05g0339200 | OsR498G0510555700.01.T02 | SAM domain-containing protein |
| maker-5-augustus-gene-165.37-mRNA-1 | Indica | NA | | | OsR498G0510554800.01.T02 | GAT domain-containing protein |
| maker-5-augustus-gene-166.36-mRNA-1 | Indica | NA | | Os05g0341600 | OsR498G0510561200.01.T01 | Ras-related protein Rab-21 |
| maker-5-augustus-gene-167.32-mRNA-1 | Indica | NA | | Os12g0275500 | OsR498G1221283500.01.T01 | |
| maker-5-augustus-gene-169.33-mRNA-1 | Indica | NA | | | OsR498G0510592600.01.T01 | NADH dehydrogenase-like complex L |
| maker-5-augustus-gene-171.2-mRNA-1 | Indica | NA | | | OsR498G0510583100.01.T01 | Cellular retinaldehyde binding/alpha-tocopherol transport |
| maker-5-augustus-gene-172.0-mRNA-1 | Indica | NA | | Os05g0349700 | OsR498G0510598100.01.T06 | Chlorophyll synthase chloroplastic |
| maker-5-augustus-gene-172.19-mRNA-1 | Indica | NA | | Os05g0349500 | OsR498G0510597400.01.T02 | Nuclear pore complex protein NUP50A |
| maker-5-augustus-gene-172.20-mRNA-1 | Indica | NA | | Os05g0349800 | OsR498G0510598400.01.T02 | Em protein H2 |
| maker-5-augustus-gene-172.9-mRNA-1 | Indica | Indica | | Os07g0693500 | OsR498G0510595800.01.T01 | AMP deaminase |
| maker-5-augustus-gene-173.21-mRNA-1 | Indica | NA | | Os05g0350500 | OsR498G0510601700.01.T01 | ERBB-3 BINDING PROTEIN 1 |
| maker-5-augustus-gene-173.26-mRNA-1 | Indica | NA | | Os05g0350600 | OsR498G0510602300.01.T01 | Ran-binding protein c |
| maker-5-augustus-gene-173.29-mRNA-1 | Indica | NA | | Os05g0350700 | OsR498G0510602900.01.T02 | Serine/arginine repetitive matrix protein 2 isoform 4 |
| maker-5-augustus-gene-180.36-mRNA-1 | Indica | NA | | Os05g0361500 | OsR498G0510656300.01.T01 | Pectinesterase |
| maker-5-augustus-gene-181.6-mRNA-1 | Indica | NA | | | | Putative E3 ubiquitin-protein ligase LIN-1 |
| maker-5-augustus-gene-182.18-mRNA-1 | Indica | NA | | Os05g0364600 | OsR498G0510668900.01.T03 | Arginine/serine-rich splicing factor SR30 transcript VI |
| maker-5-augustus-gene-182.23-mRNA-1 | Indica | Indica | | Os01g0586600 | OsR498G0510669100.01.T01 | SNF1-related protein kinase regulatory subunit gamma 1 |
| maker-5-augustus-gene-182.25-mRNA-1 | Indica | NA | | Os01g0597400 | OsR498G0101422900.01.T01 | Amino acid/polyamine transporter II |
| maker-5-augustus-gene-183.21-mRNA-1 | Indica | NA | | Os05g0366000 | OsR498G0510676300.01.T03 | Sinigrinase |
| maker-5-augustus-gene-183.3-mRNA-1 | Indica | NA | | | | Beta-glucosidase 20-like isoform X1 |
| maker-5-augustus-gene-183.6-mRNA-1 | Indica | Indica | | Os05g0366000 | OsR498G0510674700.01.T01 | 4-hydroxy-7-methoxy-3-oxo-3,4-dihydro-2H-1,4-benzoxazin-2-yl glucosidebe |
| maker-5-augustus-gene-184.0-mRNA-1 | Indica | Indica | | Os05g0366000 | OsR498G0510676700.01.T01 | Beta-glucosidase 20 |
| maker-5-augustus-gene-184.2-mRNA-1 | Indica | NA | | Os05g0366300 | OsR498G0510677300.01.T03 | C2 domain-containing protein / GRAM domain-containing protein isoform 1 |
| maker-5-augustus-gene-184.6-mRNA-1 | Indica | NA | | Os05g0366600 | OsR498G0510677900.01.T01 | Non-cyanogenic beta-glucosidase |
| maker-5-augustus-gene-185.12-mRNA-1 | Indica | NA | | Os05g0367400 | OsR498G0510680800.01.T01 | Thiamine pyrophosphokinase |
| maker-5-augustus-gene-186.0-mRNA-1 | Indica | NA | | Os05g0368300 | OsR498G0510685100.01.T01 | S4 RNA-binding domain-containing protein (Fragment) |
| maker-5-augustus-gene-188.24-mRNA-1 | Indica | NA | | Os05g0371200 | OsR498G0510696700.01.T01 | 26S proteasome regulatory subunit RPN11 |
| maker-5-augustus-gene-188.30-mRNA-1 | Indica | Indica | Kinase | Os01g0936100 | OsR498G0510698900.01.T01 | Serine/threonine-protein kinase PBL27 |
| maker-5-augustus-gene-188.34-mRNA-1 | Indica | NA | | Os05g0370700 | OsR498G0510694200.01.T01 | AB hydrolase-1 domain-containing protein (Fragment) |
| maker-5-augustus-gene-188.36-mRNA-1 | Indica | Indica | | Os05g0370700 | OsR498G0510694600.01.T01 | Salicylic acid-binding protein 2 |
| maker-5-augustus-gene-188.38-mRNA-1 | Indica | Japonica | | Os05g0371100 | OsR498G0510695800.01.T02 | CW-type domain-containing protein |
| maker-5-augustus-gene-188.44-mRNA-1 | Indica | NA | Kinase-TM | | OsR498G0510697600.01.T02 | Receptor-like protein kinase ANXUR1 |
| maker-5-augustus-gene-188.47-mRNA-1 | Indica | NA | | Os05g0372000 | OsR498G0510698400.01.T01 | Succinate dehydrogenase subunit 4 mitochondrial |
| maker-5-augustus-gene-189.0-mRNA-1 | Indica | NA | | Os05g0372300 | OsR498G0510699300.01.T01 | Cytochrome P450 family 72 subfamily A polypeptide 8 |
| maker-5-augustus-gene-189.12-mRNA-1 | Indica | NA | | Os05g0373400 | OsR498G0510703100.01.T01 | RNA-binding protein BRN1 |
| maker-5-augustus-gene-189.17-mRNA-1 | Indica | NA | | Os05g0373700 | OsR498G0510704100.01.T01 | NAC-A/B domain-containing protein (Fragment) |
| maker-5-augustus-gene-189.2-mRNA-1 | Indica | NA | | Os05g0372800 | OsR498G0510700100.01.T01 | F-box domain-containing protein (Fragment) |
| maker-5-augustus-gene-189.20-mRNA-1 | Indica | NA | | Os05g0372400 | OsR498G0510699600.01.T02 | probable feruloyl esterase A |
| maker-5-augustus-gene-189.25-mRNA-1 | Indica | NA | | | OsR498G0510701600.01.T01 | Enolase-phosphatase E1-like |
| maker-5-augustus-gene-189.27-mRNA-1 | Indica | NA | | Os05g0373000 | OsR498G0510701800.01.T01 | Vesicle-associated protein 2-2 |
| maker-5-augustus-gene-189.32-mRNA-1 | Indica | Indica | | Os01g0938600 | OsR498G0510703600.01.T01 | Tyrosine-protein phosphatase non-receptor type 23-like |
| maker-5-augustus-gene-189.5-mRNA-1 | Indica | NA | | Os05g0373300 | OsR498G0510702700.01.T03 | VWFA domain-containing protein |
| maker-5-augustus-gene-19.2-mRNA-1 | Indica | NA | | Os08g0448900 | | ATP-dependent DNA helicase |

| | | | | | | |
|---|---|---|---|---|---|---|
| maker-5-augustus-gene-190.0-mRNA-1 | Indica | NA | | Os05g0374200 | OsR498G0510705500.01.T01 | Cytokinin dehydrogenase |
| maker-5-augustus-gene-190.3-mRNA-1 | Indica | NA | | | OsR498G0510706700.01.T01 | Heat shock protein DnaJ with tetratricopeptide repeat |
| maker-5-augustus-gene-190.8-mRNA-1 | Indica | Indica | | Os01g0940100 | OsR498G0510709000.01.T01 | Phosphotransferase |
| maker-5-augustus-gene-191.54-mRNA-1 | Indica | Japonica | | Os05g0376501 | OsR498G0408835700.01.T02 | Integral membrane transporter-like protein |
| maker-5-augustus-gene-20.9-mRNA-1 | Indica | NA | | Os05g0134800 | OsR498G0509684100.01.T01 | Peroxidase |
| maker-5-augustus-gene-204.20-mRNA-1 | Indica | NA | | | OsR498G0510795700.01.T01 | Gdsl esteraselipase |
| maker-5-augustus-gene-204.24-mRNA-1 | Indica | NA | | | OsR498G0510796500.01.T04 | 23 kDa subunit of oxygen evolving system of photosystem II |
| maker-5-augustus-gene-204.32-mRNA-1 | Indica | NA | | Os05g0401100 | OsR498G0510796000.01.T01 | UPF0603 protein, chloroplastic |
| maker-5-augustus-gene-204.34-mRNA-1 | Indica | NA | | | OsR498G0510796200.01.T02 | Guanine nucleotide-binding protein-like 3-like protein |
| maker-5-augustus-gene-205.0-mRNA-1 | Indica | NA | | | | Putative alpha-ketoglutarate-dependent dioxygenase ABH5 (Fragment) |
| maker-5-augustus-gene-212.34-mRNA-1 | Indica | Indica | | Os05g0410900 | OsR498G0510828700.01.T01 | Putative proton-dependent oligopeptide transporter (POT) |
| maker-5-augustus-gene-212.36-mRNA-1 | Indica | NA | | | OsR498G0510830700.01.T01 | SUZ domain-containing protein |
| maker-5-augustus-gene-212.42-mRNA-1 | Indica | Indica | | Os05g0411200 | OsR498G0510829000.01.T03 | Putative endonuclease or glycosyl hydrolase |
| maker-5-augustus-gene-212.51-mRNA-1 | Indica | Indica | | Os05g0411300 | OsR498G0510829200.01.T01 | BZIP transcription factor |
| maker-5-augustus-gene-213.0-mRNA-1 | Indica | NA | | Os05g0413200 | OsR498G0510837700.01.T02 | Tubulin beta chain |
| maker-5-augustus-gene-213.15-mRNA-1 | Indica | NA | | Os05g0414400 | | DUF659 domain-containing protein |
| maker-5-augustus-gene-213.3-mRNA-1 | Indica | NA | | Os05g0413400 | OsR498G0510838300.01.T01 | Isopentenyl-diphosphate Delta-isomerase |
| maker-5-augustus-gene-213.7-mRNA-1 | Indica | NA | | | OsR498G0510836400.01.T01 | Glutathione S-transferase GST 41 |
| maker-5-augustus-gene-213.9-mRNA-1 | Indica | NA | | | OsR498G0510840800.01.T01 | TFIIS N-terminal domain-containing protein |
| maker-5-augustus-gene-214.30-mRNA-1 | Indica | NA | | | OsR498G0510844100.01.T01 | Glutathione gamma-glutamylcysteinyltransferase |
| maker-5-augustus-gene-214.34-mRNA-1 | Indica | NA | | Os05g0415700 | OsR498G0510845100.01.T03 | Beta-N-acetylhexosaminidase |
| maker-5-augustus-gene-214.38-mRNA-1 | Indica | NA | | | OsR498G0510845300.01.T01 | Obtusifoliol 14-alpha demethylase |
| maker-5-augustus-gene-214.40-mRNA-1 | Indica | NA | Kinase-LRR-TM | Os05g0414700 | OsR498G0510843600.01.T02 | Serine/threonine protein kinase |
| maker-5-augustus-gene-214.45-mRNA-1 | Indica | NA | | | OsR498G0510845000.01.T04 | |
| maker-5-augustus-gene-215.38-mRNA-1 | Indica | NA | | Os05g0417300 | OsR498G0510850300.01.T01 | RPA-interacting protein B |
| maker-5-augustus-gene-215.41-mRNA-1 | Indica | NA | | Os05g0417000 | OsR498G0510848800.01.T01 | LOB domain-containing protein 6 |
| maker-5-augustus-gene-216.28-mRNA-1 | Indica | NA | | Os05g0418000 | OsR498G0510853700.01.T01 | Guanosine nucleotide diphosphate dissociation inhibitor |
| maker-5-augustus-gene-216.40-mRNA-1 | Indica | Indica | | Os05g0418100 | OsR498G0510854000.01.T02 | |
| maker-5-augustus-gene-23.30-mRNA-1 | Indica | NA | | Os05g0140100 | OsR498G0509709100.01.T03 | MYB18 transcription factor |
| maker-5-augustus-gene-23.33-mRNA-1 | Indica | NA | | Os05g0140333 | OsR498G0509709900.01.T01 | Bromodomain adjacent to zinc finger domain protein 2B-like |
| maker-5-augustus-gene-247.14-mRNA-1 | Indica | NA | Kinase-LRR-TM | Os05g0481100 | OsR498G0511055300.01.T09 | Putative LRR receptor-like serine/threonine-protein kinase-like |
| maker-5-augustus-gene-247.29-mRNA-1 | Indica | NA | Kinase-LRR-TM | Os05g0480400 | OsR498G0511051800.01.T01 | Putative inactive receptor kinase |
| maker-5-augustus-gene-25.84-mRNA-1 | Indica | NA | | Os05g0145000 | OsR498G0509725000.01.T01 | E3 ubiquitin-protein ligase SIS3 |
| maker-5-augustus-gene-26.18-mRNA-1 | Indica | NA | | Os05g0146900 | | PDZ domain-containing protein |
| maker-5-augustus-gene-266.6-mRNA-1 | Indica | Indica | | Os05g0514500 | OsR498G0511177100.01.T01 | |
| maker-5-augustus-gene-27.65-mRNA-1 | Indica | NA | | | OsR498G0509732600.01.T01 | BRCT domain-containing DNA repair protein |
| maker-5-augustus-gene-274.13-mRNA-1 | Indica | NA | | Os05g0531200 | OsR498G0511234900.01.T01 | Non-classical arabinogalactan protein 31 |
| maker-5-augustus-gene-280.0-mRNA-1 | Indica | NA | | | OsR498G0511269300.01.T01 | Transcription factor bHLH87 |
| maker-5-augustus-gene-280.2-mRNA-1 | Indica | Indica | | Os05g0541800 | OsR498G0511270300.01.T02 | Jacalin-type lectin domain-containing protein (Fragment) |
| maker-5-augustus-gene-280.25-mRNA-1 | Indica | Indica | | Os01g0706500 | OsR498G0511270600.01.T01 | 60S ribosomal protein l28 |
| maker-5-augustus-gene-281.0-mRNA-1 | Indica | NA | | Os05g0543600 | OsR498G0511275900.01.T01 | Leucine-rich repeat and WD repeat-containing protein 1 (Fragment) |
| maker-5-augustus-gene-281.18-mRNA-1 | Indica | NA | | | | J domain-containing protein |
| maker-5-augustus-gene-281.2-mRNA-1 | Indica | NA | | Os05g0545000 | OsR498G0511280900.01.T06 | CRAL-TRIO domain-containing protein |
| maker-5-augustus-gene-281.21-mRNA-1 | Indica | Indica | | Os01g0700200 | OsR498G0511281600.01.T02 | Brevis radix-like domain-containing protein |
| maker-5-augustus-gene-281.8-mRNA-1 | Indica | NA | | Os05g0543700 | OsR498G0511276200.01.T01 | J domain-containing protein |
| maker-5-augustus-gene-282.58-mRNA-1 | Indica | Japonica | | Os05g0545900 | OsR498G0511285100.01.T01 | |
| maker-5-augustus-gene-282.60-mRNA-1 | Indica | NA | | | OsR498G0511285500.01.T01 | |
| maker-5-augustus-gene-282.62-mRNA-1 | Indica | NA | | Os07g0513300 | OsR498G0100612800.01.T01 | PMD domain-containing protein (Fragment) |
| maker-5-augustus-gene-282.65-mRNA-1 | Indica | NA | | Os05g0546600 | OsR498G0511284300.01.T01 | FHA domain-containing protein DDL |
| maker-5-augustus-gene-282.67-mRNA-1 | Indica | Japonica | | Os05g0546800 | OsR498G0511285100.01.T01 | |
| maker-5-augustus-gene-282.72-mRNA-1 | Indica | Japonica | | Os05g0546500 | OsR498G0511286400.01.T02 | Double-stranded DNA-binding family protein (Fragment) |
| maker-5-augustus-gene-282.74-mRNA-1 | Indica | NA | | Os05g0547850 | OsR498G0511286400.01.T01 | Double-stranded DNA-binding family protein |
| maker-5-augustus-gene-283.0-mRNA-1 | Indica | NA | | | OsR498G0511287500.01.T01 | ABC transporter B family member 9 |
| maker-5-augustus-gene-283.30-mRNA-1 | Indica | NA | | Os05g0549000 | OsR498G0511289200.01.T02 | ENTH domain-containing protein |
| maker-5-augustus-gene-283.37-mRNA-1 | Indica | NA | Kinase-TM | Os05g0549100 | OsR498G0511289600.01.T01 | Serine/threonine-protein kinase SNT7 |
| maker-5-augustus-gene-283.4-mRNA-1 | Indica | NA | | | OsR498G0511287900.01.T01 | ABC transporter B family member 9 |
| maker-5-augustus-gene-284.29-mRNA-1 | Indica | NA | Kinase-TM | | OsR498G0511296500.01.T02 | Wall-associated kinase |
| maker-5-augustus-gene-284.33-mRNA-1 | Indica | NA | | | | RING finger and CHY zinc finger domain-containing protein 1 |
| maker-5-augustus-gene-284.53-mRNA-1 | Indica | NA | | Os05g0551350 | | Formin-like protein 5 |
| maker-5-augustus-gene-284.55-mRNA-1 | Indica | NA | | Os05g0551500 | OsR498G0511300200.01.T01 | |
| maker-5-augustus-gene-285.33-mRNA-1 | Indica | NA | | Os05g0551600 | OsR498G0511300500.01.T01 | Formin-like protein 18 |

| | | | | | | |
|---|---|---|---|---|---|---|
| maker-5-augustus-gene-285.36-mRNA-1 | Indica | NA | | Os05g0552400 | OsR498G0511303700.01.T02 | RING-CH-type domain-containing protein (Fragment) |
| maker-5-augustus-gene-286.17-mRNA-1 | Indica | NA | | Os05g0554500 | OsR498G0511313100.01.T02 | CDP-diacylglycerol--serine O-phosphatidyltransferase 2 |
| maker-5-augustus-gene-286.26-mRNA-1 | Indica | NA | | Os05g0555000 | OsR498G0511315400.01.T01 | |
| maker-5-augustus-gene-286.7-mRNA-1 | Indica | Indica | | Os01g0684900 | OsR498G0511311900.01.T01 | Protein DETOXIFICATION |
| maker-5-augustus-gene-287.38-mRNA-1 | Indica | NA | | Os05g0556000 | OsR498G0511319100.01.T01 | Oxoglutarate/iron-dependent dioxygenase |
| maker-5-augustus-gene-287.48-mRNA-1 | Indica | Japonica | | Os05g0556500 | OsR498G0511320500.01.T02 | Mediator of RNA polymerase II transcription subunit 1-like |
| maker-5-augustus-gene-287.65-mRNA-1 | Indica | NA | | | OsR498G0511323500.01.T03 | ARM repeat superfamily protein (Fragment) |
| maker-5-augustus-gene-287.68-mRNA-1 | Indica | NA | | Os05g0556100 | OsR498G0511319400.01.T01 | Dynamin-related protein 5A |
| maker-5-augustus-gene-288.38-mRNA-1 | Indica | NA | | Os05g0558400 | OsR498G0511327300.01.T04 | Branched-chain-amino-acid aminotransferase |
| maker-5-augustus-gene-288.69-mRNA-1 | Indica | NA | | Os05g0558900 | OsR498G0511329100.01.T01 | Phenylalanine--tRNA ligase beta subunit cytoplasmic |
| maker-5-augustus-gene-288.74-mRNA-1 | Indica | NA | | Os05g0557600 | OsR498G0511324400.01.T01 | Ubiquitin-conjugating enzyme |
| maker-5-augustus-gene-288.79-mRNA-1 | Indica | NA | | | OsR498G0511325000.01.T01 | probable ubiquitin-conjugating enzyme E2 24 isoform X3 |
| maker-5-augustus-gene-288.81-mRNA-1 | Indica | NA | | Os05g0557800 | OsR498G0511325400.01.T01 | Large subunit ribosomal protein L21 |
| maker-5-augustus-gene-288.85-mRNA-1 | Indica | NA | | Os05g0557900 | OsR498G0511325600.01.T03 | Alpha/beta-Hydrolases superfamily protein (Fragment) |
| maker-5-augustus-gene-289.31-mRNA-1 | Indica | NA | | Os05g0559600 | OsR498G0511331900.01.T02 | Glycosyltransferases |
| maker-5-augustus-gene-289.34-mRNA-1 | Indica | NA | | Os05g0560000 | OsR498G0511332700.01.T01 | Photosystem I reaction center subunit VI like |
| maker-5-augustus-gene-289.50-mRNA-1 | Indica | NA | | Os05g0559900 | OsR498G0511332400.01.T05 | Golgin candidate 5 |
| maker-5-augustus-gene-289.56-mRNA-1 | Indica | NA | | Os05g0560100 | OsR498G0511333400.01.T01 | PRONE domain |
| maker-5-augustus-gene-289.59-mRNA-1 | Indica | NA | Kinase-TM | Os05g0560300 | OsR498G0511334000.01.T02 | Protein MALE DISCOVERER 1 |
| maker-5-augustus-gene-290.0-mRNA-1 | Indica | Indica | | Os05g0561600 | OsR498G0511339900.01.T01 | Hydroxyproline-rich glycoprotein family protein |
| maker-5-augustus-gene-290.13-mRNA-1 | Indica | NA | | Os05g0562200 | OsR498G0511341400.01.T01 | Drought-induced 19 |
| maker-5-augustus-gene-290.17-mRNA-1 | Indica | NA | | Os05g0562300 | OsR498G0511341800.01.T01 | Chaperone protein dnaJ 3 |
| maker-5-augustus-gene-290.19-mRNA-1 | Indica | Indica | | Os05g0562400 | OsR498G0511342000.01.T01 | Putative homeodomain-like transcription factor superfamily protein (Fragme |
| maker-5-augustus-gene-290.2-mRNA-1 | Indica | NA | | Os05g0560900 | OsR498G0511337200.01.T01 | Gibberellin 2-beta-dioxygenase |
| maker-5-augustus-gene-290.4-mRNA-1 | Indica | NA | | | | 3-deoxy-manno-octulosonate cytidylyltransferase, mitochondrial |
| maker-5-augustus-gene-290.7-mRNA-1 | Indica | NA | | | OsR498G0511340800.01.T01 | |
| maker-5-augustus-gene-290.9-mRNA-1 | Indica | NA | | | OsR498G0511341200.01.T03 | zf-Di19 domain-containing protein |
| maker-5-augustus-gene-291.0-mRNA-1 | Indica | NA | | Os05g0563000 | OsR498G0511343100.01.T01 | NAC domain-containing protein 8 |
| maker-5-augustus-gene-291.13-mRNA-1 | Indica | NA | | | OsR498G0511346000.01.T01 | E3 SUMO-protein like |
| maker-5-augustus-gene-291.17-mRNA-1 | Indica | NA | | | OsR498G0511348000.01.T06 | |
| maker-5-augustus-gene-291.2-mRNA-1 | Indica | NA | | Os05g0563400 | OsR498G0511345700.01.T09 | Auxin response factor |
| maker-5-augustus-gene-291.9-mRNA-1 | Indica | NA | | | OsR498G0511343400.01.T01 | GB1/RHD3-type G domain-containing protein |
| maker-5-augustus-gene-292.0-mRNA-1 | Indica | NA | | Os05g0564300 | OsR498G0511348700.01.T04 | E3 ubiquitin-protein ligase arkadia isoform X2 |
| maker-5-augustus-gene-292.10-mRNA-1 | Indica | NA | | Os05g0565000 | OsR498G0511350900.01.T01 | Urease accessory protein G |
| maker-5-augustus-gene-292.13-mRNA-1 | Indica | NA | | Os05g0565100 | OsR498G0511351300.01.T01 | Cytochrome b561 domain-containing protein |
| maker-5-augustus-gene-292.15-mRNA-1 | Indica | NA | | Os05g0565200 | OsR498G0511351500.01.T01 | cobW domain-containing protein (Fragment) |
| maker-5-augustus-gene-292.18-mRNA-1 | Indica | Japonica | | Os05g0565900 | OsR498G0511354100.01.T01 | WRKY transcription factor |
| maker-5-augustus-gene-292.21-mRNA-1 | Indica | NA | | Os05g0564400 | OsR498G0511349200.01.T01 | Ran GTPase |
| maker-5-augustus-gene-292.4-mRNA-1 | Indica | Indica | | Os05g0564500 | OsR498G0511349700.01.T01 | Homeobox domain |
| maker-5-augustus-gene-292.7-mRNA-1 | Indica | NA | | | OsR498G0511350700.01.T01 | Hydroxyacylglutathione hydrolase 3, mitochondrial |
| maker-5-augustus-gene-293.41-mRNA-1 | Indica | NA | | | OsR498G0511354700.01.T01 | Ribosome maturation factor rimM |
| maker-5-augustus-gene-293.45-mRNA-1 | Indica | NA | | Os02g0102850 | OsR498G0612917800.01.T09 | |
| maker-5-augustus-gene-293.47-mRNA-1 | Indica | NA | | Os05g0566900 | OsR498G0511357700.01.T03 | GBF-interacting protein 1 |
| maker-5-augustus-gene-293.53-mRNA-1 | Indica | NA | | Os05g0567100 | | Aspartic acid protease |
| maker-5-augustus-gene-293.55-mRNA-1 | Indica | NA | | | OsR498G0511358500.01.T01 | WRKY transcription factor |
| maker-5-augustus-gene-293.57-mRNA-1 | Indica | NA | | Os05g0567400 | OsR498G0511358900.01.T01 | Putative rRNA methyltransferase |
| maker-5-augustus-gene-293.62-mRNA-1 | Indica | NA | | | OsR498G0511359700.01.T04 | MFS domain-containing protein (Fragment) |
| maker-5-augustus-gene-293.67-mRNA-1 | Indica | NA | | Os05g0567800 | OsR498G0511360100.01.T04 | Sugar transporter protein ERD6-S |
| maker-5-augustus-gene-293.78-mRNA-1 | Indica | Indica | | Os05g0566600 | OsR498G0511356200.01.T03 | Endosulphine |
| maker-5-augustus-gene-293.80-mRNA-1 | Indica | NA | | Os05g0566800 | OsR498G0511356600.01.T02 | Cold-regulated inner membrane protein 2, chloroplastic |
| maker-5-augustus-gene-293.83-mRNA-1 | Indica | NA | | | OsR498G0511358800.01.T01 | GTP-binding protein era, putative |
| maker-5-augustus-gene-294.44-mRNA-1 | Indica | NA | | Os05g0568100 | OsR498G0511362900.01.T01 | Iron-sulfur cluster assembly protein 1 |
| maker-5-augustus-gene-294.48-mRNA-1 | Indica | NA | | Os05g0568200 | OsR498G0511363300.01.T01 | MYB-related transcription factor |
| maker-5-augustus-gene-294.50-mRNA-1 | Indica | NA | | Os05g0569200 | OsR498G0511366100.01.T01 | Multidrug resistance-associated protein 14 isoform 1 |
| maker-5-augustus-gene-294.52-mRNA-1 | Indica | NA | | Os05g0569300 | OsR498G0511366500.01.T01 | G-box binding factor |
| maker-5-augustus-gene-294.58-mRNA-1 | Indica | Indica | | Os01g0658700 | OsR498G0511367100.01.T01 | Glucose-6-phosphate 1-epimerase |
| maker-5-augustus-gene-294.62-mRNA-1 | Indica | NA | | Os05g0569500 | OsR498G0511367500.01.T01 | Oil body-associated protein like |
| maker-5-augustus-gene-294.64-mRNA-1 | Indica | Indica | | Os01g0662800 | OsR498G0511360600.01.T01 | GATA transcription factor 19 |
| maker-5-augustus-gene-294.66-mRNA-1 | Indica | NA | | Os05g0568900 | OsR498G0511365200.01.T02 | DegP protease 1 isoform 1 |
| maker-5-augustus-gene-294.71-mRNA-1 | Indica | NA | | | | |
| maker-5-augustus-gene-294.74-mRNA-1 | Indica | Indica | | Os05g0569700 | OsR498G0511370300.01.T01 | CTP synthase (glutamine hydrolyzing) |

| | | | | | | |
|---|---|---|---|---|---|---|
| maker-5-augustus-gene-295.11-mRNA-1 | Indica | NA | | Os05g0570900 | OsR498G0511371400.01.T01 | Phytocyanin domain-containing protein (Fragment) |
| maker-5-augustus-gene-295.13-mRNA-1 | Indica | NA | | Os05g0571000 | OsR498G0511371600.01.T02 | Protein SUPPRESSOR OF PHYA-105 1 |
| maker-5-augustus-gene-295.2-mRNA-1 | Indica | NA | | | | B-block binding subunit of TFIIIC |
| maker-5-augustus-gene-295.4-mRNA-1 | Indica | NA | | Os05g0569800 | OsR498G0511368600.01.T02 | THO complex subunit 7 |
| maker-5-augustus-gene-295.6-mRNA-1 | Indica | Indica | | Os05g0573300 | OsR498G0511370300.01.T01 | CTP synthase |
| maker-5-augustus-gene-296.37-mRNA-1 | Indica | NA | | Os05g0573200 | OsR498G0511377300.01.T04 | Isocitrate dehydrogenase [NADP] |
| maker-5-augustus-gene-296.53-mRNA-1 | Indica | NA | | Os05g0572700 | OsR498G0511375800.01.T01 | Protein-serine/threonine phosphatase |
| maker-5-augustus-gene-296.56-mRNA-1 | Indica | Indica | | Os05g0573000 | OsR498G0511376800.01.T01 | |
| maker-5-augustus-gene-296.60-mRNA-1 | Indica | NA | | Os05g0573300 | OsR498G0511377600.01.T02 | CTP synthase |
| maker-5-augustus-gene-297.46-mRNA-1 | Indica | NA | | Os05g0573500 | OsR498G0511379100.01.T02 | CCAAT-binding transcription factor A |
| maker-5-augustus-gene-297.51-mRNA-1 | Indica | Indica | | Os05g0574000 | OsR498G0511381100.01.T01 | Phospholipase |
| maker-5-augustus-gene-297.53-mRNA-1 | Indica | Japonica | | Os05g0574800 | OsR498G0511385100.01.T01 | Pentatricopeptide repeat-containing protein chloroplastic |
| maker-5-augustus-gene-297.54-mRNA-1 | Indica | Indica | | Os05g0575300 | OsR498G0511387900.01.T01 | Tr-type G domain-containing protein |
| maker-5-augustus-gene-297.65-mRNA-1 | Indica | NA | | Os05g0573700 | OsR498G0511380000.01.T01 | Acetohydroxy-acid reductoisomerase |
| maker-5-augustus-gene-297.70-mRNA-1 | Indica | NA | | | OsR498G0511380600.01.T02 | Tetratricopeptide repeat-like superfamily protein isoform 2 |
| maker-5-augustus-gene-297.74-mRNA-1 | Indica | NA | | Os05g0574100 | OsR498G0511381600.01.T01 | Phospholipase A1-IIgamma |
| maker-5-augustus-gene-297.76-mRNA-1 | Indica | NA | | Os05g0574400 | OsR498G0511383800.01.T01 | Malate dehydrogenase |
| maker-5-augustus-gene-297.79-mRNA-1 | Indica | NA | | Os05g0574500 | OsR498G0511384000.01.T01 | GTP-binding nuclear protein Ran-2 |
| maker-5-augustus-gene-297.82-mRNA-1 | Indica | NA | | Os05g0574700 | OsR498G0511384800.01.T04 | Very-long-chain 3-oxoacyl-CoA synthase |
| maker-5-augustus-gene-298.40-mRNA-1 | Indica | NA | Kinase-TM | Os05g0576800 | OsR498G0511392300.01.T01 | Mitogen-activated protein kinase |
| maker-5-augustus-gene-298.45-mRNA-1 | Indica | NA | | Os05g0578000 | OsR498G0511395300.01.T01 | Peroxin-14 |
| maker-5-augustus-gene-298.51-mRNA-1 | Indica | NA | | | OsR498G0511391200.01.T01 | Fe2OG dioxygenase domain-containing protein (Fragment) |
| maker-5-augustus-gene-298.53-mRNA-1 | Indica | Indica | | Os01g0643300 | OsR498G0511393400.01.T01 | Auxin efflux carrier component |
| maker-5-augustus-gene-298.55-mRNA-1 | Indica | Indica | | Os05g0577200 | OsR498G0511394200.01.T05 | Isoprenylcysteine alpha-carbonyl methylesterase ICME |
| maker-5-augustus-gene-298.59-mRNA-1 | Indica | NA | Kinase-TM | Os05g0577700 | OsR498G0511394600.01.T02 | MAP kinase kinase kinase55 |
| maker-5-augustus-gene-298.68-mRNA-1 | Indica | NA | | Os05g0577900 | OsR498G0511395200.01.T01 | Protein kinase domain-containing protein |
| maker-5-augustus-gene-299.16-mRNA-1 | Indica | Indica | | Os05g0578600 | OsR498G0511397800.01.T02 | Polygalacturonase |
| maker-5-augustus-gene-299.20-mRNA-1 | Indica | NA | | Os05g0579000 | OsR498G0511399000.01.T02 | Sugar transporter protein ERD6-S |
| maker-5-augustus-gene-299.3-mRNA-1 | Indica | NA | | | OsR498G0511397900.01.T05 | |
| maker-5-augustus-gene-36.46-mRNA-1 | Indica | NA | | Os05g0161800 | OsR498G0509794300.01.T01 | GTP diphosphokinase |
| maker-5-augustus-gene-36.65-mRNA-1 | Indica | NA | | | OsR498G0509796800.01.T01 | HTH myb-type domain-containing protein |
| maker-5-augustus-gene-37.3-mRNA-1 | Indica | NA | | Os05g0164100 | OsR498G0509801900.01.T06 | 6-phosphofructo-2-kinase/fructose-26-bisphosphatase |
| maker-5-augustus-gene-45.13-mRNA-1 | Indica | Indica | | Os05g0173500 | OsR498G0509840800.01.T04 | |
| maker-5-augustus-gene-45.25-mRNA-1 | Indica | NA | | Os05g0176500 | OsR498G0509848400.01.T02 | Mitochondria isoform 2 |
| maker-5-augustus-gene-45.7-mRNA-1 | Indica | NA | | | | |
| maker-5-augustus-gene-46.49-mRNA-1 | Indica | NA | | Os05g0178600 | OsR498G0509854200.01.T01 | Auxin-responsive protein (Fragment) |
| maker-5-augustus-gene-55.0-mRNA-1 | Indica | NA | | Os09g0258800 | | Ulp1 protease family, C-terminal catalytic domain containing protein |
| maker-5-augustus-gene-57.0-mRNA-1 | Indica | Indica | | Os05g0194900 | OsR498G0509918900.01.T01 | ATP-dependent 6-phosphofructokinase 3 |
| maker-5-augustus-gene-57.6-mRNA-1 | Indica | NA | | | | Probable tRNA N6-adenosine threonylcarbamoyltransferase |
| maker-5-augustus-gene-57.8-mRNA-1 | Indica | NA | | Os05g0195700 | OsR498G0509922600.01.T02 | Transcription factor DIVARICATA |
| maker-5-augustus-gene-63.31-mRNA-1 | Indica | NA | | | | |
| maker-5-augustus-gene-64.34-mRNA-1 | Indica | NA | | Os05g0204600 | OsR498G0509981100.01.T01 | B-box zinc finger protein 22 |
| maker-5-augustus-gene-64.36-mRNA-1 | Indica | NA | | Os05g0204900 | OsR498G0509982500.01.T03 | Serine/threonine-protein phosphatase 5 |
| maker-5-augustus-gene-64.46-mRNA-1 | Indica | Indica | | Os02g0232900 | OsR498G0509982800.01.T01 | Aquaporin NIP-type |
| maker-5-augustus-gene-66.35-mRNA-1 | Indica | NA | | Os05g0207900 | OsR498G0509992300.01.T03 | Sentrin-specific protease 6 |
| maker-5-augustus-gene-66.41-mRNA-1 | Indica | NA | | Os05g0208000 | OsR498G0509992900.01.T01 | Mitochondrial dicarboxylate/tricarboxylate transporter DTC |
| maker-5-augustus-gene-66.46-mRNA-1 | Indica | NA | | | | NAM-associated domain-containing protein |
| maker-5-augustus-gene-66.49-mRNA-1 | Indica | NA | | Os05g0207400 | OsR498G0509990800.01.T01 | RING-CH-type domain-containing protein (Fragment) |
| maker-5-augustus-gene-66.51-mRNA-1 | Indica | NA | Kinase-TM | Os05g0207500 | OsR498G0509991200.01.T01 | Non-specific serine/threonine protein kinase |
| maker-5-augustus-gene-66.56-mRNA-1 | Indica | NA | Kinase-TM | Os05g0207700 | OsR498G0509991800.01.T03 | Serine/threonine-protein kinase CDL1 |
| maker-5-augustus-gene-70.0-mRNA-1 | Indica | NA | | Os05g0213500 | OsR498G0510012300.01.T01 | Abscisic acid receptor 8 |
| maker-5-augustus-gene-71.26-mRNA-1 | Indica | Indica | | Os05g0214900 | OsR498G0510020300.01.T01 | Protein trichome birefringence-like 25 |
| maker-5-augustus-gene-71.29-mRNA-1 | Indica | Indica | | Os05g0215000 | OsR498G0510022000.01.T01 | BURP domain-containing protein 2 |
| maker-5-augustus-gene-72.0-mRNA-1 | Indica | NA | | Os05g0217800 | OsR498G0510033900.01.T01 | BURP domain-containing protein 2 |
| maker-5-augustus-gene-72.2-mRNA-1 | Indica | NA | | Os05g0215800 | OsR498G0510026400.01.T02 | Nucleotide sugar transporter-KT 1 |
| maker-5-augustus-gene-72.7-mRNA-1 | Indica | Japonica | | Os05g0217000 | OsR498G0612586700.01.T02 | Arabinogalactan peptide |
| maker-5-augustus-gene-73.26-mRNA-1 | Indica | Indica | Kinase-TM | Os01g0227200 | OsR498G0510036200.01.T03 | Receptor protein kinase PERK1 |
| maker-5-augustus-gene-73.31-mRNA-1 | Indica | Indica | | Os01g0303800 | OsR498G0510635800.01.T01 | Usp domain-containing protein |
| maker-5-augustus-gene-74.40-mRNA-1 | Indica | NA | | Os05g0220900 | OsR498G0510046700.01.T01 | |
| maker-5-augustus-gene-74.44-mRNA-1 | Indica | NA | | | OsR498G0510043000.01.T02 | DNA helicase |
| maker-5-augustus-gene-74.48-mRNA-1 | Indica | NA | | Os05g0219900 | OsR498G0510043600.01.T01 | S-type anion channel SLAH3 |

| | | | | | | |
|---|---|---|---|---|---|---|
| maker-5-augustus-gene-74.50-mRNA-1 | Indica | NA | | Os05g0220100 | OsR498G0510044200.01.T04 | Post-GPI attachment to proteins factor 3 |
| maker-5-augustus-gene-74.58-mRNA-1 | Indica | Indica | | Os01g0750500 | OsR498G0510048200.01.T01 | Coiled-coil domain-containing protein 25 |
| maker-5-augustus-gene-75.36-mRNA-1 | Indica | Indica | | Os05g0223200 | OsR498G0510053900.01.T01 | Glycine-rich RNA-binding protein GRP1A |
| maker-5-augustus-gene-75.42-mRNA-1 | Indica | NA | NBS-TM | Os05g0222200 | OsR498G0510052200.01.T01 | Eceriferum 5 (Fragment) |
| maker-5-augustus-gene-79.41-mRNA-1 | Indica | Japonica | | Os05g0229300 | OsR498G0815323400.01.T01 | |
| maker-6-augustus-gene-100.8-mRNA-1 | Indica | NA | | Os06g0286146 | OsR498G0612156000.01.T01 | PGG domain-containing protein (Fragment) |
| maker-6-augustus-gene-101.19-mRNA-1 | Indica | Indica | | Os06g0286310 | OsR498G0612158300.01.T01 | DNA oxidative demethylase |
| maker-6-augustus-gene-101.30-mRNA-1 | Indica | NA | | Os10g0531750 | OsR498G0408675500.01.T01 | Retrotransposon protein, putative, Ty3-gypsy sub-class |
| maker-6-augustus-gene-102.41-mRNA-1 | Indica | NA | CC-NBS-TM | | | NBS-LRR |
| maker-6-augustus-gene-102.42-mRNA-1 | Indica | NA | NBS-TM | | | Disease resistance protein RPM1 |
| maker-6-augustus-gene-105.5-mRNA-1 | Indica | Japonica | | Os06g0293500 | OsR498G0612176400.01.T01 | |
| maker-6-augustus-gene-110.30-mRNA-1 | Indica | NA | | Os06g0301300 | OsR498G0612205500.01.T05 | Proteasome activator subunit 4 |
| maker-6-augustus-gene-195.22-mRNA-1 | Indica | NA | | Os06g0527800 | | Bet v I allergen-like |
| maker-6-augustus-gene-197.0-mRNA-1 | Indica | Indica | | Os06g0530300 | OsR498G0612674500.01.T01 | Putative membrane protein YuiD |
| maker-6-augustus-gene-202.48-mRNA-1 | Indica | Indica | | Os02g0244600 | OsR498G0612706500.01.T01 | Polyadenylate-binding protein-interacting protein 8-like |
| maker-6-augustus-gene-204.31-mRNA-1 | Indica | NA | | Os06g0545400 | OsR498G0612717800.01.T01 | Group 13 grass pollen allergen (Fragment) |
| maker-6-augustus-gene-205.52-mRNA-1 | Indica | NA | | Os06g0547100 | OsR498G0612720200.01.T01 | Peroxidase |
| maker-6-augustus-gene-227.0-mRNA-1 | Indica | Indica | Kinase-LRR-TM | Os06g0586150 | OsR498G0612844500.01.T01 | Putative LRR receptor-like serine/threonine-protein kinase |
| maker-6-augustus-gene-227.3-mRNA-1 | Indica | Indica | Kinase-LRR-TM | Os06g0587500 | OsR498G0612845300.01.T01 | Putative LRR receptor-like serine/threonine-protein kinase |
| maker-6-augustus-gene-228.1-mRNA-1 | Indica | NA | Kinase-LRR-TM | | OsR498G0612847900.01.T01 | Putative LRR receptor-like serine/threonine-protein kinase |
| maker-6-augustus-gene-231.7-mRNA-1 | Indica | Indica | Kinase-LRR-TM | Os06g0587900 | OsR498G0612839000.01.T02 | Putative LRR receptor-like serine/threonine-protein kinase |
| maker-6-augustus-gene-234.11-mRNA-1 | Indica | Indica | | Os06g0594600 | OsR498G0612877000.01.T03 | 3'-N-debenzoyl-2'-deoxytaxol N-benzoyltransferase |
| maker-6-augustus-gene-236.36-mRNA-1 | Indica | Indica | | Os06g0597400 | OsR498G0612883100.01.T02 | Exonuclease/helicase-like |
| maker-6-augustus-gene-236.44-mRNA-1 | Indica | NA | | Os06g0597600 | OsR498G0612883500.01.T01 | FscO |
| maker-6-augustus-gene-236.48-mRNA-1 | Indica | NA | | Os06g0597900 | OsR498G0612883900.01.T04 | Heme-binding-like protein, chloroplastic |
| maker-6-augustus-gene-236.51-mRNA-1 | Indica | NA | | Os06g0598900 | OsR498G0612886000.01.T01 | serine-threonine kinase receptor-associated protein isoform X1 |
| maker-6-augustus-gene-257.3-mRNA-1 | Indica | Indica | | Os06g0632200 | OsR498G0613011200.01.T02 | Tryptophan synthase |
| maker-6-augustus-gene-258.44-mRNA-1 | Indica | NA | | | OsR498G0613013000.01.T01 | Putative B3 domain-containing protein (Fragment) |
| maker-6-augustus-gene-293.47-mRNA-1 | Indica | Indica | | Os02g0150100 | OsR498G0613241100.01.T01 | Putative RNA helicase transcription factor interactor and regulator CCHC(Zn) |
| maker-6-augustus-gene-293.49-mRNA-1 | Indica | NA | | Os06g0697300 | OsR498G0613241500.01.T01 | |
| maker-6-augustus-gene-293.54-mRNA-1 | Indica | Indica | | Os03g0845700 | OsR498G0613241800.01.T01 | DNA-directed RNA polymerases II, IV and V subunit 8B |
| maker-6-augustus-gene-294.50-mRNA-1 | Indica | NA | | | OsR498G0613245200.01.T02 | Protein ENHANCED DISEASE RESISTANCE like |
| maker-6-augustus-gene-295.19-mRNA-1 | Indica | NA | | Os06g0699500 | OsR498G0613249800.01.T01 | Macrophage migration inhibitory factor isogeny |
| maker-6-augustus-gene-296.41-mRNA-1 | Indica | NA | | Os06g0701400 | OsR498G0613255600.01.T01 | 60s acidic ribosomal protein-domain-containing protein |
| maker-6-augustus-gene-296.46-mRNA-1 | Indica | NA | | Os06g0701700 | OsR498G0613256600.01.T01 | HKT24 transporter |
| maker-6-augustus-gene-297.40-mRNA-1 | Indica | NA | | | OsR498G0613262500.01.T01 | |
| maker-6-augustus-gene-297.42-mRNA-1 | Indica | Indica | | Os06g0701700 | OsR498G0613258400.01.T02 | High-affinity potassium uptake transporter |
| maker-6-augustus-gene-297.46-mRNA-1 | Indica | NA | | Os11g0620500 | OsR498G0510972100.01.T01 | Plant disease resistance polyprotein-like |
| maker-6-augustus-gene-298.0-mRNA-1 | Indica | NA | | Os06g0702500 | OsR498G0613263100.01.T06 | L-arabinokinase |
| maker-6-augustus-gene-298.12-mRNA-1 | Indica | NA | | Os06g0702800 | OsR498G0613265100.01.T03 | 3-phosphoinositide-dependent protein kinase-1 |
| maker-6-augustus-gene-298.22-mRNA-1 | Indica | NA | | Os06g0702700 | OsR498G0613265000.01.T01 | GGACT domain-containing protein (Fragment) |
| maker-6-augustus-gene-298.25-mRNA-1 | Indica | NA | Kinase-TM | Os06g0703000 | OsR498G0613265800.01.T01 | Putative serine/threonine-protein kinase PBL17 isoform B |
| maker-6-augustus-gene-298.32-mRNA-1 | Indica | NA | | Os06g0703500 | OsR498G0613267000.01.T02 | Squamosa-promoter binding protein-like protein |
| maker-6-augustus-gene-298.4-mRNA-1 | Indica | Indica | | Os06g0702600 | OsR498G0613264700.01.T02 | Auxin response factor |
| maker-6-augustus-gene-299.36-mRNA-1 | Indica | Japonica | | Os06g0703900 | OsR498G0202933000.01.T03 | Protein PHOSPHATE STARVATION RESPONSE 3 |
| maker-6-augustus-gene-300.44-mRNA-1 | Indica | Indica | | Os06g0705600 | OsR498G0613275100.01.T02 | Peptide transporter PTR2 |
| maker-6-augustus-gene-300.45-mRNA-1 | Indica | NA | | Os06g0705600 | OsR498G0613275100.01.T03 | Peptide transporter PTR2 |
| maker-6-augustus-gene-300.52-mRNA-1 | Indica | NA | | Os06g0705700 | OsR498G0613275500.01.T01 | Peptide transporter PTR2 |
| maker-6-augustus-gene-300.54-mRNA-1 | Indica | NA | | Os06g0705900 | OsR498G0613276300.01.T01 | Peptide transporter PTR2 |
| maker-6-augustus-gene-300.56-mRNA-1 | Indica | Indica | | Os06g0705700 | OsR498G0613276500.01.T06 | Peptide transporter PTR2 |
| maker-6-augustus-gene-300.61-mRNA-1 | Indica | NA | | Os06g0707100 | OsR498G0613278300.01.T09 | Protein DETOXIFICATION |
| maker-6-augustus-gene-301.39-mRNA-1 | Indica | NA | Kinase | Os06g0708000 | OsR498G0613281300.01.T01 | Mitogen-activated protein kinase |
| maker-6-augustus-gene-301.44-mRNA-1 | Indica | NA | | | OsR498G0613282100.01.T01 | Type 2 DNA topoisomerase 6 subunit B-like |
| maker-6-augustus-gene-301.51-mRNA-1 | Indica | Indica | | Os01g0106200 | OsR498G0613282300.01.T01 | Peptidylprolyl isomerase |
| maker-6-augustus-gene-301.53-mRNA-1 | Indica | Indica | | Os06g0708500 | OsR498G0613282500.01.T01 | Peptidyl-prolyl cis-trans isomerase |
| maker-6-augustus-gene-301.55-mRNA-1 | Indica | NA | | Os06g0708500 | OsR498G0613282700.01.T01 | Peptidyl-prolyl cis-trans isomerase |
| maker-6-augustus-gene-301.61-mRNA-1 | Indica | Indica | | Os06g0709100 | OsR498G0613284500.01.T03 | F-box protein SKIP31 |
| maker-6-augustus-gene-301.67-mRNA-1 | Indica | NA | | | | Putative isoprenylcysteine alpha-carbonyl methylesterase ICME |
| maker-6-augustus-gene-301.71-mRNA-1 | Indica | Indica | | Os06g0708700 | OsR498G0613283200.01.T02 | Conserved oligomeric Golgi complex subunit 6 |
| maker-6-augustus-gene-301.75-mRNA-1 | Indica | NA | | | OsR498G0613284200.01.T04 | Zinc finger CCHC domain-containing protein |
| maker-6-augustus-gene-301.81-mRNA-1 | Indica | NA | | Os06g0710300 | OsR498G0613285800.01.T02 | ATP synthase gamma chain |

| | | | | | | |
|---|---|---|---|---|---|---|
| maker-6-augustus-gene-302.38-mRNA-1 | Indica | NA | | Os06g0711100 | OsR498G0613287700.01.T04 | WAS/WASL-interacting protein family member 1-like |
| maker-6-augustus-gene-302.41-mRNA-1 | Indica | NA | | Os06g0711200 | OsR498G0613287900.01.T01 | Pre-mRNA-splicing factor syf2 like |
| maker-6-augustus-gene-302.43-mRNA-1 | Indica | NA | | Os06g0711600 | OsR498G0613288100.01.T02 | GCIP-interacting family protein isoform 1 |
| maker-6-augustus-gene-302.52-mRNA-1 | Indica | NA | | Os06g0712400 | OsR498G0613292600.01.T02 | NF-E2 inducible protein-like |
| maker-6-augustus-gene-303.0-mRNA-1 | Indica | Indica | | Os09g0531900 | OsR498G0613293100.01.T01 | Hexosyltransferase |
| maker-6-augustus-gene-303.13-mRNA-1 | Indica | NA | | Os06g0713100 | OsR498G0613297400.01.T01 | Mitochondrion protein |
| maker-6-augustus-gene-303.17-mRNA-1 | Indica | Indica | | Os06g0713900 | OsR498G0613298200.01.T02 | |
| maker-6-augustus-gene-303.21-mRNA-1 | Indica | NA | | Os06g0714000 | OsR498G0613298600.01.T04 | Transmembrane protein |
| maker-6-augustus-gene-303.3-mRNA-1 | Indica | Indica | | Os06g0712600 | OsR498G0613294100.01.T01 | Lateral root primordium (LRP) protein-related |
| maker-6-augustus-gene-303.8-mRNA-1 | Indica | NA | | Os06g0713800 | OsR498G0613297500.01.T02 | Alpha-amylase type A isozyme |
| maker-6-augustus-gene-304.0-mRNA-1 | Indica | Indica | | Os10g0510700 | OsR498G0613299500.01.T02 | Calcium-dependent protein kinase |
| maker-6-augustus-gene-304.13-mRNA-1 | Indica | NA | | Os06g0715400 | OsR498G0613303900.01.T05 | Golgin subfamily A member like |
| maker-6-augustus-gene-304.15-mRNA-1 | Indica | NA | | Os06g0715500 | OsR498G0613303900.01.T06 | translocon-associated protein subunit alpha-like |
| maker-6-augustus-gene-304.20-mRNA-1 | Indica | NA | Kinase | Os06g0714900 | OsR498G0613302400.01.T04 | probable serine/threonine-protein kinase PBL19 |
| maker-6-augustus-gene-304.23-mRNA-1 | Indica | NA | | | OsR498G0613303400.01.T02 | Target SNARE coiled-coil domain |
| maker-6-augustus-gene-304.28-mRNA-1 | Indica | NA | | Os06g0715300 | OsR498G0613303600.01.T02 | Endoglucanase |
| maker-6-augustus-gene-304.30-mRNA-1 | Indica | NA | | Os06g0715600 | OsR498G0613304200.01.T01 | BRCA1-A complex subunit BRE |
| maker-6-augustus-gene-304.35-mRNA-1 | Indica | NA | | Os06g0715700 | OsR498G0613304400.01.T01 | Probable magnesium transporter |
| maker-6-augustus-gene-304.4-mRNA-1 | Indica | NA | TM | Os06g0714500 | OsR498G0613301300.01.T01 | ATPase family AAA domain-containing protein 1-A |
| maker-6-augustus-gene-304.6-mRNA-1 | Indica | NA | | Os06g0714800 | OsR498G0613302100.01.T01 | FLZ-type domain-containing protein (Fragment) |
| maker-6-augustus-gene-304.8-mRNA-1 | Indica | NA | | Os06g0715000 | OsR498G0613303100.01.T02 | UV radiation resistance-associated |
| maker-6-augustus-gene-305.35-mRNA-1 | Indica | NA | | Os06g0716000 | OsR498G0613305900.01.T01 | DUF668 domain-containing protein (Fragment) |
| maker-6-augustus-gene-305.40-mRNA-1 | Indica | NA | | Os06g0716200 | OsR498G0613306700.01.T01 | Thaumatin-like protein 1a |
| maker-6-augustus-gene-305.43-mRNA-1 | Indica | Japonica | | Os06g0717300 | OsR498G0613308900.01.T01 | |
| maker-6-augustus-gene-305.48-mRNA-1 | Indica | NA | | Os06g0717900 | | Chloroplast nucleoid DNA-binding protein-like protein |
| maker-6-augustus-gene-305.50-mRNA-1 | Indica | NA | | Os06g0718100 | OsR498G0613311900.01.T01 | Expansin |
| maker-6-augustus-gene-305.52-mRNA-1 | Indica | NA | | Os06g0716700 | OsR498G0613307600.01.T01 | Endoplasmin like |
| maker-6-augustus-gene-305.58-mRNA-1 | Indica | NA | | Os06g0717100 | OsR498G0613308200.01.T01 | Senescence domain-containing protein (Fragment) |
| maker-6-augustus-gene-305.61-mRNA-1 | Indica | NA | Kinase-LRR-TM | Os06g0717200 | OsR498G0613308600.01.T01 | Leucine-rich repeat receptor-like kinase protein THICK TASSEL DWARF1 |
| maker-6-augustus-gene-305.63-mRNA-1 | Indica | NA | | Os06g0717400 | OsR498G0613309200.01.T01 | RNA pseudourine synthase 2, chloroplastic |
| maker-6-augustus-gene-305.67-mRNA-1 | Indica | NA | | Os06g0717800 | OsR498G0613310200.01.T01 | Protein-serine/threonine phosphatase |
| maker-6-augustus-gene-305.68-mRNA-1 | Indica | NA | | | OsR498G0613310200.01.T02 | |
| maker-6-augustus-gene-306.33-mRNA-1 | Indica | Indica | | Os06g0721800 | OsR498G0613312700.01.T01 | Blue copper binding protein-like |
| maker-6-augustus-gene-306.35-mRNA-1 | Indica | NA | | | | NF-E2 inducible protein-like |
| maker-6-augustus-gene-306.63-mRNA-1 | Indica | NA | | Os06g0722100 | OsR498G0613323500.01.T01 | DUF1618 domain-containing protein (Fragment) |
| maker-6-augustus-gene-307.42-mRNA-1 | Indica | NA | | Os06g0724100 | OsR498G0613324700.01.T01 | FYVE-type domain-containing protein (Fragment) |
| maker-6-augustus-gene-307.48-mRNA-1 | Indica | NA | | Os06g0724200 | OsR498G0613324900.01.T02 | Lateral signaling target protein 2-like protein |
| maker-6-augustus-gene-307.59-mRNA-1 | Indica | NA | | Os06g0722100 | OsR498G0613323500.01.T01 | DUF1618 domain-containing protein (Fragment) |
| maker-6-augustus-gene-307.63-mRNA-1 | Indica | NA | | | | Putative retroelement |
| maker-6-augustus-gene-307.66-mRNA-1 | Indica | Indica | | Os06g0724000 | OsR498G0613324000.01.T01 | BZIP transcription factor |
| maker-6-augustus-gene-307.73-mRNA-1 | Indica | NA | | Os06g0724300 | OsR498G0613325200.01.T02 | DUF616 domain-containing protein |
| maker-6-augustus-gene-308.21-mRNA-1 | Indica | NA | | Os06g0725900 | OsR498G0613331200.01.T01 | AAA domain-containing protein (Fragment) |
| maker-6-augustus-gene-309.0-mRNA-1 | Indica | Japonica | | Os06g0726900 | OsR498G0816324600.01.T01 | RING-type E3 ubiquitin transferase |
| maker-6-augustus-gene-309.14-mRNA-1 | Indica | NA | | Os06g0726400 | OsR498G0613332800.01.T02 | 1,4-alpha-glucan-branching enzyme, chloroplastic/amyloplastic |
| maker-6-augustus-gene-309.19-mRNA-1 | Indica | NA | | Os06g0726600 | OsR498G0613333000.01.T01 | Lazarus |
| maker-6-augustus-gene-309.2-mRNA-1 | Indica | NA | | Os06g0727200 | OsR498G0613333900.01.T01 | Catalase isozyme b |
| maker-6-augustus-gene-309.24-mRNA-1 | Indica | NA | | Os06g0726800 | OsR498G0613333200.01.T03 | Cyclin N-terminal domain-containing protein |
| maker-6-augustus-gene-309.29-mRNA-1 | Indica | NA | | Os06g0727300 | OsR498G0613334200.01.T02 | Hexosyltransferase |
| maker-6-augustus-gene-309.34-mRNA-1 | Indica | NA | Kinase | Os06g0727400 | OsR498G0613334400.01.T01 | probable serine/threonine-protein kinase PIX13 |
| maker-6-augustus-gene-309.7-mRNA-1 | Indica | Indica | | Os06g0727700 | OsR498G0613334900.01.T01 | Storage protein-like |
| maker-6-augustus-gene-309.9-mRNA-1 | Indica | NA | | Os06g0727700 | OsR498G0613335300.01.T01 | Transmembrane protein, putative (DUF707) |
| maker-6-augustus-gene-310.0-mRNA-1 | Indica | Indica | | Os06g0728000 | OsR498G0613336700.01.T02 | DNA-binding protein MNB1B |
| maker-6-augustus-gene-310.10-mRNA-1 | Indica | NA | | Os06g0728600 | OsR498G0613337900.01.T01 | Calcium-binding EF hand protein-like |
| maker-6-augustus-gene-310.15-mRNA-1 | Indica | NA | | | | Piwi domain-containing protein |
| maker-6-augustus-gene-310.21-mRNA-1 | Indica | NA | | Os06g0728700 | OsR498G0613338800.01.T01 | HTH myb-type domain-containing protein |
| maker-6-augustus-gene-310.23-mRNA-1 | Indica | NA | | | OsR498G0613339400.01.T02 | Callose synthase 3 |
| maker-6-augustus-gene-310.30-mRNA-1 | Indica | NA | | Os06g0729000 | OsR498G0613340000.01.T02 | Phytoene synthase 1, chloroplastic |
| maker-6-augustus-gene-310.7-mRNA-1 | Indica | Japonica | | Os06g0728200 | OsR498G0613337300.01.T01 | |
| maker-6-augustus-gene-311.0-mRNA-1 | Indica | NA | | Os06g0729400 | OsR498G0613342300.01.T03 | Gibberellin-regulated protein 3 |
| maker-6-augustus-gene-311.10-mRNA-1 | Indica | NA | | Os06g0730500 | OsR498G0613347500.01.T01 | Gamma-tubulin complex component |
| maker-6-augustus-gene-311.14-mRNA-1 | Indica | NA | | Os06g0730800 | OsR498G0613347900.01.T01 | Coiled-coil domain-containing protein 12 |

| | | | | | | |
|---|---|---|---|---|---|---|
| maker-6-augustus-gene-311.18-mRNA-1 | Indica | NA | | | OsR498G0613342600.01.T05 | |
| maker-6-augustus-gene-311.2-mRNA-1 | Indica | Indica | | Os02g0634700 | OsR498G0613345100.01.T01 | Carboxypeptidase |
| maker-6-augustus-gene-311.23-mRNA-1 | Indica | NA | | | OsR498G0613342800.01.T03 | Photosystem II stability/assembly factor like |
| maker-6-augustus-gene-311.27-mRNA-1 | Indica | NA | | Os06g0730600 | OsR498G0613347800.01.T05 | Oligopeptidase B |
| maker-6-augustus-gene-311.4-mRNA-1 | Indica | NA | | Os07g0172200 | OsR498G0613346900.01.T02 | DNA-binding protein RHL1 |
| maker-6-augustus-gene-311.7-mRNA-1 | Indica | NA | | Os06g0730300 | OsR498G0613347100.01.T03 | Transmembrane protein 53 |
| maker-6-augustus-gene-312.44-mRNA-1 | Indica | NA | | | | PA domain-containing protein |
| maker-6-augustus-gene-312.50-mRNA-1 | Indica | NA | | Os06g0731000 | | No-mechanoreceptor potential A, putative |
| maker-6-augustus-gene-312.51-mRNA-1 | Indica | NA | | Os06g0731400 | OsR498G0613349700.01.T01 | Tetraspanin-18 |
| maker-6-augustus-gene-312.55-mRNA-1 | Indica | NA | | Os06g0731900 | OsR498G0613351300.01.T01 | Putative lysine ketoglutarate reductase trans-splicing related 1 |
| maker-6-augustus-gene-312.62-mRNA-1 | Indica | NA | | | | Acyl-CoA N-acyltransferase with RING/FYVE/PHD-type zinc finger domain |
| maker-6-augustus-gene-312.68-mRNA-1 | Indica | NA | | Os06g0731200 | OsR498G0613349200.01.T01 | White-brown complex like protein 30 |
| maker-6-augustus-gene-312.73-mRNA-1 | Indica | Indica | | Os06g0731300 | OsR498G0613349400.01.T01 | Chaperone protein like |
| maker-6-augustus-gene-312.75-mRNA-1 | Indica | NA | | Os06g0731500 | | JmjC domain-containing protein |
| maker-6-augustus-gene-312.77-mRNA-1 | Indica | NA | | | OsR498G0613350200.01.T07 | Lysine-specific demethylase |
| maker-6-augustus-gene-312.79-mRNA-1 | Indica | NA | | Os06g0731700 | OsR498G0613350600.01.T01 | Clathrin light chain 2 |
| maker-6-augustus-gene-312.83-mRNA-1 | Indica | NA | | Os06g0731800 | OsR498G0613351200.01.T01 | Clathrin light chain |
| maker-6-augustus-gene-32.0-mRNA-1 | Indica | NA | | | OsR498G0611667500.01.T02 | FBD domain-containing protein |
| maker-6-augustus-gene-35.49-mRNA-1 | Indica | NA | | | OsR498G0611688900.01.T01 | X8 domain-containing protein |
| maker-6-augustus-gene-36.2-mRNA-1 | Indica | NA | | Os06g0169900 | OsR498G0611689700.01.T01 | Jacalin-like lectin domain containing protein, expressed |
| maker-6-augustus-gene-37.16-mRNA-1 | Indica | NA | | Os06g0171700 | OsR498G0611697700.01.T01 | CDK-activating kinase assembly factor MAT1 |
| maker-6-augustus-gene-37.32-mRNA-1 | Indica | NA | | | | Fanconi-associated nuclease |
| maker-6-augustus-gene-42.10-mRNA-1 | Indica | NA | | | OsR498G0611733400.01.T04 | CHD3-type chromatin-remodeling factor PICKLE |
| maker-6-augustus-gene-42.18-mRNA-1 | Indica | NA | | Os01g0258600 | OsR498G0100585300.01.T03 | FYVE-type domain-containing protein |
| maker-6-augustus-gene-42.20-mRNA-1 | Indica | NA | | Os06g0184200 | OsR498G0611735400.01.T01 | Protein POLAR LOCALIZATION DURING ASYMMETRIC DIVISION AND REDISTR |
| maker-6-augustus-gene-43.37-mRNA-1 | Indica | NA | | | OsR498G0611737500.01.T03 | ABC transporter C family member 13 isoform A |
| maker-6-augustus-gene-43.46-mRNA-1 | Indica | Indica | | Os06g0184800 | OsR498G0611737800.01.T01 | Stress-induced hydrophobic peptide 1 |
| maker-6-augustus-gene-43.48-mRNA-1 | Indica | NA | | | OsR498G0611738400.01.T01 | BSD domain-containing protein |
| maker-6-augustus-gene-44.0-mRNA-1 | Indica | Indica | | Os02g0664000 | OsR498G0611740700.01.T01 | Phospholipid hydroperoxide glutathione peroxidase 1, chloroplastic |
| maker-6-augustus-gene-44.14-mRNA-1 | Indica | NA | | Os06g0187000 | OsR498G0611744100.01.T03 | Origin of replication complex subunit 1 |
| maker-6-augustus-gene-44.19-mRNA-1 | Indica | NA | | | OsR498G0611744500.01.T01 | Trimethylguanosine synthase |
| maker-6-augustus-gene-44.21-mRNA-1 | Indica | Indica | Kinase-LRR-TM | Os06g0186300 | OsR498G0611741800.01.T01 | Serine/threonine protein kinase |
| maker-6-augustus-gene-44.23-mRNA-1 | Indica | Japonica | Kinase-LRR-TM | Os06g0186300 | OsR498G0611742000.01.T01 | Serine/threonine protein kinase |
| maker-6-augustus-gene-44.31-mRNA-1 | Indica | NA | | Os06g0186600 | OsR498G0611743000.01.T01 | 50S ribosomal protein L35 isoform 1 |
| maker-6-augustus-gene-44.35-mRNA-1 | Indica | NA | | | | Folate-biopterin transporter |
| maker-6-augustus-gene-44.38-mRNA-1 | Indica | NA | | Os06g0186900 | OsR498G0611743800.01.T01 | RuvB-like helicase |
| maker-6-augustus-gene-44.40-mRNA-1 | Indica | Indica | | Os06g0187200 | OsR498G0611744800.01.T01 | UDP-glucuronate 4-epimerase 1 |
| maker-6-augustus-gene-44.5-mRNA-1 | Indica | NA | Kinase-LRR-TM | | | LRR receptor-like serine/threonine-protein kinase EFR |
| maker-6-augustus-gene-44.8-mRNA-1 | Indica | NA | | | OsR498G0611742500.01.T02 | HAUS-augmin3 domain-containing protein |
| maker-6-augustus-gene-45.11-mRNA-1 | Indica | NA | | Os06g0188100 | OsR498G0611747800.01.T02 | UDP-galactose/UDP-glucose transporter 7 |
| maker-6-augustus-gene-45.4-mRNA-1 | Indica | Indica | | Os06g0188000 | OsR498G0611747500.01.T01 | Cyclic nucleotide-gated ion channel 17 |
| maker-6-augustus-gene-46.4-mRNA-1 | Indica | NA | | | OsR498G0611748600.01.T01 | PBPe domain-containing protein (Fragment) |
| maker-6-augustus-gene-46.5-mRNA-1 | Indica | NA | | Os06g0190700 | OsR498G0611753200.01.T01 | PBPe domain-containing protein |
| maker-6-augustus-gene-49.0-mRNA-1 | Indica | Indica | | Os06g0195800 | OsR498G0611777900.01.T01 | J domain-containing protein (Fragment) |
| maker-6-augustus-gene-49.7-mRNA-1 | Indica | NA | | Os06g0195600 | OsR498G0611776200.01.T02 | Phosphoinositide phosphatase SAC2 |
| maker-6-augustus-gene-50.45-mRNA-1 | Indica | NA | | | OsR498G0611782500.01.T05 | Auxin response factor |
| maker-6-augustus-gene-50.59-mRNA-1 | Indica | Indica | | Os02g0781400 | OsR498G0611782900.01.T06 | Chaperonin, chloroplastic |
| maker-6-augustus-gene-50.68-mRNA-1 | Indica | Indica | | Os06g0196300 | OsR498G0611780000.01.T01 | Peroxiredoxin Q chloroplastic |
| maker-6-augustus-gene-50.71-mRNA-1 | Indica | Japonica | | Os06g0196500 | OsR498G0611780600.01.T01 | NTF2-domain-containing protein |
| maker-6-augustus-gene-55.38-mRNA-1 | Indica | NA | | Os06g0207000 | OsR498G0611822700.01.T01 | Pantothenate kinase 1 |
| maker-6-augustus-gene-57.0-mRNA-1 | Indica | NA | | | OsR498G0611835900.01.T01 | |
| maker-6-augustus-gene-57.14-mRNA-1 | Indica | NA | | Os06g0210900 | OsR498G0611839300.01.T01 | Fungal lipase-like domain containing protein |
| maker-6-augustus-gene-57.2-mRNA-1 | Indica | Indica | | Os02g0768300 | OsR498G0611836500.01.T01 | |
| maker-6-augustus-gene-57.5-mRNA-1 | Indica | NA | | Os06g0210100 | OsR498G0611836900.01.T01 | Transmembrane emp24 domain-containing protein 10 |
| maker-6-augustus-gene-57.8-mRNA-1 | Indica | NA | | Os06g0210200 | OsR498G0611837100.01.T01 | N-acyl-L-amino-acid amidohydrolase |
| maker-6-augustus-gene-58.40-mRNA-1 | Indica | NA | | Os06g0211200 | OsR498G0611841300.01.T05 | ABA responsive element binding factor 1 |
| maker-6-augustus-gene-58.45-mRNA-1 | Indica | NA | | Os06g0211600 | OsR498G0611842900.01.T01 | Fucosyltransferase |
| maker-6-augustus-gene-58.49-mRNA-1 | Indica | NA | | Os06g0211700 | OsR498G0611843200.01.T01 | Fucosyltransferase |
| maker-6-augustus-gene-59.0-mRNA-1 | Indica | NA | | Os06g0214100 | OsR498G0611849900.01.T01 | Apple domain-containing protein |
| maker-6-augustus-gene-59.5-mRNA-1 | Indica | NA | | | OsR498G0611846800.01.T01 | |
| maker-6-augustus-gene-59.7-mRNA-1 | Indica | NA | | | OsR498G0611848000.01.T01 | Protein TIC 22, chloroplastic isoform A |

| | | | | | | | |
|---|---|---|---|---|---|---|---|
| maker-6-augustus-gene-60.0-mRNA-1 | Indica | Indica | | Os06g0214900 | OsR498G0611853000.01.T01 | Alternative NAD(P)H-ubiquinone oxidoreductase |
| maker-6-augustus-gene-60.15-mRNA-1 | Indica | NA | | Os06g0215400 | OsR498G0611854400.01.T08 | Dipeptidyl peptidase family member 6 |
| maker-6-augustus-gene-60.25-mRNA-1 | Indica | NA | | Os06g0215900 | OsR498G0611856400.01.T01 | 12-oxo-phytodienoic acid reductase |
| maker-6-augustus-gene-60.27-mRNA-1 | Indica | Indica | | Os06g0216300 | OsR498G0611856600.01.T01 | 12-oxo-phytodienoic acid reductase |
| maker-6-augustus-gene-60.29-mRNA-1 | Indica | Indica | | Os06g0216200 | OsR498G0611857200.01.T01 | 12-oxo-phytodienoic acid reductase |
| maker-6-augustus-gene-60.32-mRNA-1 | Indica | NA | | Os06g0216300 | OsR498G0611857600.01.T01 | 12-oxo-phytodienoic acid reductase |
| maker-6-augustus-gene-60.34-mRNA-1 | Indica | NA | | Os06g0216700 | OsR498G0611858400.01.T01 | Phytocyanin domain-containing protein |
| maker-6-augustus-gene-60.7-mRNA-1 | Indica | NA | | Os06g0215200 | OsR498G0611854200.01.T01 | Zinc finger protein ZOP1 |
| maker-6-augustus-gene-60.9-mRNA-1 | Indica | NA | | Os06g0215300 | OsR498G0611854400.01.T01 | Acylaminoacyl-peptidase1 |
| maker-6-augustus-gene-61.27-mRNA-1 | Indica | NA | | Os06g0216800 | OsR498G0611858600.01.T02 | Peptidyl-prolyl cis-trans isomerase |
| maker-6-augustus-gene-61.32-mRNA-1 | Indica | NA | | Os06g0217300 | OsR498G0611859800.01.T01 | MADS-box transcription factor 6 |
| maker-6-augustus-gene-61.37-mRNA-1 | Indica | NA | | | | Mediator of RNA polymerase II transcription subunit 6 |
| maker-6-augustus-gene-61.43-mRNA-1 | Indica | NA | | | OsR498G0611861000.01.T05 | Kinesin-like protein KCA2 |
| maker-6-augustus-gene-61.50-mRNA-1 | Indica | NA | | Os06g0217700 | OsR498G0611862200.01.T01 | CAAD domain-containing protein (Fragment) |
| maker-6-augustus-gene-62.0-mRNA-1 | Indica | NA | | | | Cyclin dependent kinase regulator |
| maker-6-augustus-gene-62.16-mRNA-1 | Indica | NA | | Os06g0218500 | OsR498G0611867000.01.T01 | DNA helicase |
| maker-6-augustus-gene-62.21-mRNA-1 | Indica | NA | | Os06g0218900 | OsR498G0611867800.01.T04 | LMBR1 domain-containing protein 2 isogeny A |
| maker-6-augustus-gene-62.4-mRNA-1 | Indica | NA | | | OsR498G0611864700.01.T02 | GLABRA2 expression modulator |
| maker-6-augustus-gene-62.7-mRNA-1 | Indica | NA | | Os06g0218600 | OsR498G0611867100.01.T01 | Phytocyanin domain-containing protein (Fragment) |
| maker-6-augustus-gene-62.9-mRNA-1 | Indica | NA | | | OsR498G0611865000.01.T03 | DnaJ-class molecular chaperone |
| maker-6-augustus-gene-63.0-mRNA-1 | Indica | NA | | Os06g0219400 | OsR498G0611869700.01.T02 | O-fucosyltransferase family protein |
| maker-6-augustus-gene-63.13-mRNA-1 | Indica | NA | | Os06g0220800 | OsR498G0611874100.01.T02 | 5'-adenylylsulfate reductase-like 3 |
| maker-6-augustus-gene-63.16-mRNA-1 | Indica | NA | | Os06g0219500 | OsR498G0611870000.01.T01 | Heat shock protein, mitochondrial |
| maker-6-augustus-gene-63.20-mRNA-1 | Indica | NA | | | OsR498G0611873600.01.T01 | RRM domain-containing protein |
| maker-6-augustus-gene-64.0-mRNA-1 | Indica | NA | | Os06g0221000 | OsR498G0611876100.01.T01 | Myb domain protein 93 |
| maker-6-augustus-gene-64.11-mRNA-1 | Indica | Indica | | Os06g0222100 | OsR498G0611878400.01.T02 | Trehalose 6-phosphate phosphatase |
| maker-6-augustus-gene-64.3-mRNA-1 | Indica | NA | | Os06g0221200 | OsR498G0611877100.01.T01 | Annexin |
| maker-6-augustus-gene-64.5-mRNA-1 | Indica | Indica | | Os05g0477800 | OsR498G0611877700.01.T01 | Sulfate transporter-like |
| maker-6-augustus-gene-64.7-mRNA-1 | Indica | NA | | Os06g0221100 | OsR498G0611876600.01.T01 | TLDc domain-containing protein |
| maker-6-augustus-gene-65.3-mRNA-1 | Indica | NA | | Os06g0222800 | OsR498G0611882600.01.T01 | Metallophosphoesterase 1 |
| maker-6-augustus-gene-69.30-mRNA-1 | Indica | Indica | | Os06g0229066 | OsR498G0611932200.01.T01 | ATP-dependent zinc metalloprotease FTSH 6 chloroplastic |
| maker-6-augustus-gene-78.46-mRNA-1 | Indica | NA | | Os06g0246700 | | SWIM-type domain-containing protein (Fragment) |
| maker-6-augustus-gene-80.15-mRNA-1 | Indica | Indica | | Os06g0125200 | OsR498G0611641600.01.T03 | FLZ-type domain-containing protein (Fragment) |
| maker-6-augustus-gene-80.17-mRNA-1 | Indica | NA | | Os06g0251700 | OsR498G0612011400.01.T01 | Microtubule-associated protein 70 |
| maker-6-augustus-gene-80.8-mRNA-1 | Indica | NA | | Os06g0251100 | OsR498G0612010300.01.T01 | MACPF domain-containing protein |
| maker-6-augustus-gene-89.12-mRNA-1 | Indica | Indica | | Os02g0731400 | OsR498G0612063600.01.T01 | Chemocyanin |
| maker-6-augustus-gene-89.14-mRNA-1 | Indica | Indica | | Os06g0266800 | OsR498G0612064200.01.T01 | gibberellin-regulated protein 6-like |
| maker-6-augustus-gene-89.8-mRNA-1 | Indica | Indica | | Os06g0265400 | OsR498G0612061600.01.T03 | BZIP transcription factor family protein isoform 1 |
| maker-6-augustus-gene-90.36-mRNA-1 | Indica | NA | | | OsR498G0612070200.01.T02 | Aluminum-activated malate transporter 9 |
| maker-6-augustus-gene-92.0-mRNA-1 | Indica | NA | | Os06g0270900 | OsR498G0612075300.01.T02 | T-cytoplasm male sterility restorer factor 2 |
| maker-6-augustus-gene-92.6-mRNA-1 | Indica | NA | | | OsR498G0407658300.01.T01 | Amidase domain-containing protein |
| maker-6-augustus-gene-94.6-mRNA-1 | Indica | NA | | Os06g0275600 | OsR498G0612093100.01.T03 | Transcription factor bHLH62 isoform A |
| maker-6-augustus-gene-97.2-mRNA-1 | Indica | NA | | Os06g0281300 | OsR498G0612134400.01.T01 | O-methyltransferase ZRP4 |
| maker-7-augustus-gene-1.34-mRNA-1 | Indica | NA | | Os07g0101300 | | BHLH domain-containing protein |
| maker-7-augustus-gene-1.56-mRNA-1 | Indica | NA | | Os07g0102500 | OsR498G0713361200.01.T01 | HTH La-type RNA-binding domain-containing protein (Fragment) |
| maker-7-augustus-gene-11.0-mRNA-1 | Indica | NA | | | | |
| maker-7-augustus-gene-11.2-mRNA-1 | Indica | NA | | Os07g0128800 | OsR498G0713450900.01.T01 | Pathogenesis-related protein class 1 |
| maker-7-augustus-gene-12.46-mRNA-1 | Indica | NA | | Os07g0129700 | OsR498G0713458300.01.T01 | Homeobox protein rough sheath 1 |
| maker-7-augustus-gene-12.47-mRNA-1 | Indica | NA | | Os07g0129700 | OsR498G0713458300.01.T01 | Homeobox protein rough sheath 1 |
| maker-7-augustus-gene-12.50-mRNA-1 | Indica | NA | | Os07g0131600 | OsR498G0713463100.01.T01 | Sugar transport protein 3 |
| maker-7-augustus-gene-12.52-mRNA-1 | Indica | NA | Kinase-TM | Os07g0129800 | OsR498G0713458800.01.T01 | Putative receptor kinase Lecrk |
| maker-7-augustus-gene-12.54-mRNA-1 | Indica | NA | Kinase-TM | Os07g0131000 | | L-type lectin-domain containing receptor kinase IV.1 |
| maker-7-augustus-gene-13.0-mRNA-1 | Indica | NA | | Os07g0132300 | OsR498G0713464300.01.T05 | EPIDERMAL PATTERNING FACTOR-like protein 2 |
| maker-7-augustus-gene-13.7-mRNA-1 | Indica | NA | | Os07g0133500 | OsR498G0713466600.01.T01 | Formin-like protein 18 |
| maker-7-augustus-gene-13.9-mRNA-1 | Indica | NA | | Os07g0134000 | OsR498G0713468000.01.T01 | Amino acid transporter |
| maker-7-augustus-gene-150.33-mRNA-1 | Indica | Indica | | Os07g0448100 | OsR498G0714192500.01.T01 | Aquaporin |
| maker-7-augustus-gene-181.0-mRNA-1 | Indica | NA | | Os07g0495000 | OsR498G0714350300.01.T06 | Ureidoglycine aminohydrolase |
| maker-7-augustus-gene-181.16-mRNA-1 | Indica | NA | | Os07g0495200 | OsR498G0714351000.01.T01 | ATPase, F1 complex, delta/epsilon subunit |
| maker-7-augustus-gene-181.19-mRNA-1 | Indica | Indica | | Os07g0495300 | OsR498G0714351200.01.T03 | H+-transporting two-sector ATPase family protein |
| maker-7-augustus-gene-181.4-mRNA-1 | Indica | NA | | Os07g0495100 | OsR498G0714350500.01.T01 | Casein kinase II subunit beta |
| maker-7-augustus-gene-181.8-mRNA-1 | Indica | NA | Kinase | | | Receptor-like serine/threonine-protein kinase |

| | | | | | | |
|---|---|---|---|---|---|---|
| maker-7-augustus-gene-182.18-mRNA-1 | Indica | NA | | Os07g0496600 | | SWIM-type domain-containing protein |
| maker-7-augustus-gene-182.20-mRNA-1 | Indica | NA | | Os07g0496900 | OsR498G0714356900.01.T02 | 2-oxoglutarate and FeII-dependent oxygenase superfamily protein isoform 2 |
| maker-7-augustus-gene-182.24-mRNA-1 | Indica | NA | | Os07g0496000 | OsR498G0714353000.01.T01 | GTP-binding protein Rab6 |
| maker-7-augustus-gene-182.8-mRNA-1 | Indica | NA | | Os07g0496200 | OsR498G0714353900.01.T01 | Malic enzyme |
| maker-7-augustus-gene-183.0-mRNA-1 | Indica | NA | | | OsR498G0714357500.01.T01 | peptide-N(4)-(N-acetyl-beta- glucosaminyl)asparagine amidase |
| maker-7-augustus-gene-183.12-mRNA-1 | Indica | NA | | Os07g0498900 | OsR498G0714363100.01.T01 | Protein trichome birefringence-like protein 13 |
| maker-7-augustus-gene-183.14-mRNA-1 | Indica | NA | | | OsR498G0714357200.01.T02 | CHD3-type chromatin remodeling factor D |
| maker-7-augustus-gene-183.20-mRNA-1 | Indica | NA | | Os07g0497500 | OsR498G0714358000.01.T01 | Transcription factor like |
| maker-7-augustus-gene-183.22-mRNA-1 | Indica | NA | Kinase-LRR-TM | Os07g0498400 | OsR498G0714359600.01.T01 | LRR receptor-like serine/threonine-protein kinase |
| maker-7-augustus-gene-183.24-mRNA-1 | Indica | NA | | Os07g0498800 | OsR498G0714362600.01.T04 | Ubiquitin-like domain-containing protein |
| maker-7-augustus-gene-183.8-mRNA-1 | Indica | NA | | | OsR498G0714359300.01.T02 | Excitatory amino acid transporter |
| maker-7-augustus-gene-184.0-mRNA-1 | Indica | NA | | Os07g0499800 | OsR498G0714366700.01.T02 | RING/U-box superfamily protein |
| maker-7-augustus-gene-185.37-mRNA-1 | Indica | NA | | Os07g0500300 | OsR498G0714368300.01.T01 | Synaptotagmin-4 |
| maker-7-augustus-gene-185.41-mRNA-1 | Indica | NA | | Os07g0501100 | OsR498G0714371600.01.T01 | Chalcone synthase 2 |
| maker-7-augustus-gene-186.28-mRNA-1 | Indica | NA | | | OsR498G0714374000.01.T05 | Transmembrane E3 ubiquitin-protein ligase 1 |
| maker-7-augustus-gene-186.34-mRNA-1 | Indica | Indica | | Os07g0502200 | OsR498G0714374800.01.T01 | Protein DETOXIFICATION |
| maker-7-augustus-gene-188.32-mRNA-1 | Indica | NA | | Os07g0506000 | OsR498G0714387600.01.T01 | Organic solute transporter subunit alpha/Transmembrane protein |
| maker-7-augustus-gene-189.27-mRNA-1 | Indica | NA | | Os07g0507700 | OsR498G0714393300.01.T02 | Bromo domain-containing protein |
| maker-7-augustus-gene-189.43-mRNA-1 | Indica | NA | | | OsR498G0714392400.01.T02 | Targeting protein for Xklp2 |
| maker-7-augustus-gene-189.51-mRNA-1 | Indica | NA | Kinase-TM | Os07g0507300 | OsR498G0714392600.01.T04 | germinal center kinase 1 isoform X3 |
| maker-7-augustus-gene-189.63-mRNA-1 | Indica | NA | | Os07g0507600 | OsR498G0714393200.01.T03 | PMD domain-containing protein (Fragment) |
| maker-7-augustus-gene-193.13-mRNA-1 | Indica | NA | | | OsR498G0714420400.01.T01 | DUF629 domain-containing protein |
| maker-7-augustus-gene-195.56-mRNA-1 | Indica | NA | | Os07g0516500 | | mediator of RNA polymerase II transcription subunit 15a-like |
| maker-7-augustus-gene-201.0-mRNA-1 | Indica | NA | | Os07g0527000 | OsR498G0714474100.01.T01 | |
| maker-7-augustus-gene-201.7-mRNA-1 | Indica | Japonica | | Os07g0527600 | OsR498G0714476000.01.T01 | Putative carboxylesterase 13 |
| maker-7-augustus-gene-202.39-mRNA-1 | Indica | NA | | | OsR498G0714478300.01.T01 | GDSL esterase/lipase |
| maker-7-augustus-gene-202.41-mRNA-1 | Indica | NA | | | OsR498G0714478700.01.T01 | GDSL esterase/lipase |
| maker-7-augustus-gene-202.43-mRNA-1 | Indica | Japonica | | Os07g0528900 | OsR498G0714480300.01.T01 | t-SNARE coiled-coil homology domain-containing protein (Fragment) |
| maker-7-augustus-gene-202.46-mRNA-1 | Indica | NA | | Os07g0529600 | OsR498G0714482300.01.T01 | Thiamine thiazole synthase, chloroplastic |
| maker-7-augustus-gene-203.16-mRNA-1 | Indica | NA | | Os07g0530100 | OsR498G0714483400.01.T01 | Tyrosyl-DNA phosphodiesterase |
| maker-7-augustus-gene-203.23-mRNA-1 | Indica | NA | | Os07g0530400 | | F-box domain containing protein-like |
| maker-7-augustus-gene-203.6-mRNA-1 | Indica | NA | | | OsR498G0714486700.01.T01 | Peroxidase |
| maker-7-augustus-gene-203.8-mRNA-1 | Indica | NA | | Os07g0531400 | OsR498G0714487100.01.T01 | Peroxidase |
| maker-7-augustus-gene-205.0-mRNA-1 | Indica | NA | | | OsR498G0714492700.01.T01 | Homeobox domain-containing protein (Fragment) |
| maker-7-augustus-gene-205.14-mRNA-1 | Indica | NA | Kinase-TM | Os07g0534700 | OsR498G0714497100.01.T02 | Cysteine-rich receptor-like protein kinase 25 |
| maker-7-augustus-gene-205.2-mRNA-1 | Indica | NA | | | OsR498G0714494500.01.T01 | Thylakoid lumenal 15.0 kDa protein 2 chloroplastic |
| maker-7-augustus-gene-205.9-mRNA-1 | Indica | NA | Kinase-TM | | OsR498G0714496300.01.T01 | Receptor-like serine/threonine-protein kinase |
| maker-7-augustus-gene-206.10-mRNA-1 | Indica | Indica | Kinase-TM | Os07g0628700 | OsR498G0714502100.01.T01 | Cysteine-rich receptor-like protein kinase 8 |
| maker-7-augustus-gene-207.46-mRNA-1 | Indica | NA | Kinase-TM | | OsR498G0714502300.01.T01 | Cysteine-rich receptor-like protein kinase 8 |
| maker-7-augustus-gene-207.58-mRNA-1 | Indica | Indica | Kinase-TM | Os07g0537900 | OsR498G0714507500.01.T01 | Cysteine-rich receptor-like protein kinase 8 |
| maker-7-augustus-gene-207.71-mRNA-1 | Indica | Indica | Kinase-TM | Os07g0537900 | OsR498G0714504400.01.T04 | Cysteine-rich receptor-like protein kinase 8 |
| maker-7-augustus-gene-207.76-mRNA-1 | Indica | NA | | Os07g0539100 | OsR498G0714508400.01.T02 | Glucan endo-1,3-beta-D-glucosidase |
| maker-7-augustus-gene-207.81-mRNA-1 | Indica | NA | | | | Glucan endo-1,3-beta-D-glucosidase |
| maker-7-augustus-gene-208.36-mRNA-1 | Indica | NA | Kinase-TM | Os07g0541000 | OsR498G0714516300.01.T01 | Receptor-like serine-threonine protein kinase |
| maker-7-augustus-gene-208.38-mRNA-1 | Indica | Japonica | | Os07g0539900 | OsR498G0714510800.01.T02 | Glucan endo-1,3-beta-D-glucosidase |
| maker-7-augustus-gene-208.47-mRNA-1 | Indica | NA | | | OsR498G0714514000.01.T02 | DCD domain-containing protein (Fragment) |
| maker-7-augustus-gene-209.0-mRNA-1 | Indica | NA | Kinase-TM | Os07g0541400 | OsR498G0714516900.01.T02 | Cysteine-rich receptor kinase |
| maker-7-augustus-gene-209.11-mRNA-1 | Indica | NA | Kinase-TM | Os07g0540800 | OsR498G0714515800.01.T01 | Receptor-like serine-threonine protein kinase |
| maker-7-augustus-gene-209.12-mRNA-1 | Indica | NA | Kinase-TM | Os07g0541000 | OsR498G0714516800.01.T01 | Cysteine-rich receptor-like protein kinase 10 |
| maker-7-augustus-gene-209.21-mRNA-1 | Indica | Indica | Kinase-TM | Os07g0541500 | OsR498G0714517200.01.T02 | Cysteine-rich receptor kinase |
| maker-7-augustus-gene-210.47-mRNA-1 | Indica | NA | Kinase-TM | Os07g0542400 | OsR498G0714519200.01.T05 | Putative Zea mays retrotransposon Opie-2 |
| maker-7-augustus-gene-217.0-mRNA-1 | Indica | NA | | | OsR498G0714556100.01.T03 | Eukaryotic translation initiation factor 4G |
| maker-7-augustus-gene-218.16-mRNA-1 | Indica | Indica | | Os07g0557200 | OsR498G0714563000.01.T01 | Equilibrative nucleotide transporter like |
| maker-7-augustus-gene-218.17-mRNA-1 | Indica | NA | | Os07g0557400 | OsR498G0714563000.01.T01 | Equilibrative nucleoside transporter |
| maker-7-augustus-gene-229.47-mRNA-1 | Indica | Indica | | Os07g0573300 | OsR498G0714613700.01.T01 | FYVE-type domain-containing protein (Fragment) |
| maker-7-augustus-gene-23.32-mRNA-1 | Indica | Japonica | | Os07g0151901 | OsR498G0713527800.01.T01 | |
| maker-7-augustus-gene-231.60-mRNA-1 | Indica | Indica | | Os07g0576500 | OsR498G0714622800.01.T01 | Indole-3-acetic acid amido synthetase |
| maker-7-augustus-gene-241.2-mRNA-1 | Indica | NA | | Os07g0598000 | OsR498G0714699100.01.T01 | Dihydroflavonol-4-reductase |
| maker-7-augustus-gene-241.6-mRNA-1 | Indica | NA | | | OsR498G0714699300.01.T04 | 21S rRNA (GM2251-2'-O)-methyltransferase |
| maker-7-augustus-gene-242.49-mRNA-1 | Indica | NA | | Os07g0598700 | OsR498G0714702000.01.T02 | Golgin subfamily A member 4 isoform X1 |
| maker-7-augustus-gene-243.39-mRNA-1 | Indica | NA | | Os07g0602200 | OsR498G0714712900.01.T04 | Histone deacetylase |

| | | | | | | |
|---|---|---|---|---|---|---|
| maker-7-augustus-gene-26.3-mRNA-1 | Indica | NA | | Os07g0155600 | OsR498G0713546500.01.T02 | Ethylene insensitive |
| maker-7-augustus-gene-27.31-mRNA-1 | Indica | NA | | Os07g0158900 | OsR498G0713555000.01.T04 | FBD domain-containing protein |
| maker-7-augustus-gene-27.35-mRNA-1 | Indica | NA | | Os07g0159500 | OsR498G0713558200.01.T02 | Putative arylformamidase |
| maker-7-augustus-gene-28.39-mRNA-1 | Indica | NA | | | OsR498G0713563700.01.T04 | F-box domain-containing protein |
| maker-7-augustus-gene-28.41-mRNA-1 | Indica | NA | | | OsR498G0713563700.01.T06 | FBD domain-containing protein |
| maker-7-augustus-gene-28.45-mRNA-1 | Indica | Indica | Kinase-TM | Os07g0159700 | OsR498G0713558800.01.T02 | Protein kinase domain-containing protein (Fragment) |
| maker-7-augustus-gene-28.49-mRNA-1 | Indica | NA | | Os07g0160400 | OsR498G0713563400.01.T01 | VOC domain-containing protein (Fragment) |
| maker-7-augustus-gene-33.76-mRNA-1 | Indica | NA | | | | 2-oxoisovalerate dehydrogenase subunit beta |
| maker-7-augustus-gene-33.86-mRNA-1 | Indica | Indica | | Os07g0171200 | OsR498G0713599800.01.T01 | ADP-glucose phosphorylase |
| maker-7-augustus-gene-34.53-mRNA-1 | Indica | NA | | | OsR498G0713603300.01.T01 | |
| maker-7-augustus-gene-34.54-mRNA-1 | Indica | NA | | Os07g0172900 | OsR498G0713603500.01.T05 | Folate-biopterin transporter 1 chloroplastic |
| maker-7-augustus-gene-34.65-mRNA-1 | Indica | NA | | | OsR498G0713605500.01.T05 | Acyl-CoA N-acyltransferase with RING/FYVE/PHD-type zinc finger protein |
| maker-7-augustus-gene-34.70-mRNA-1 | Indica | NA | | Os07g0173700 | OsR498G0713605700.01.T01 | 40S ribosomal protein S18 isoform B |
| maker-7-augustus-gene-34.73-mRNA-1 | Indica | Indica | | Os07g0174400 | OsR498G0713606300.01.T01 | AAI domain-containing protein (Fragment) |
| maker-7-augustus-gene-34.75-mRNA-1 | Indica | NA | | Os07g0172500 | OsR498G0713602200.01.T01 | DNA-directed RNA polymerases II IV and V subunit 11 |
| maker-7-augustus-gene-34.79-mRNA-1 | Indica | Indica | | Os07g0173200 | OsR498G0713604200.01.T03 | 5-methyltetrahydropteroyltriglutamate--homocysteine S-methyltransferase |
| maker-7-augustus-gene-35.44-mRNA-1 | Indica | Japonica | | Os07g0175000 | OsR498G0713608300.01.T01 | AAI domain-containing protein |
| maker-7-augustus-gene-35.46-mRNA-1 | Indica | NA | | Os07g0175400 | OsR498G0713610100.01.T01 | Inwardly rectifying potassium channel subunit |
| maker-7-augustus-gene-35.49-mRNA-1 | Indica | NA | | Os07g0175500 | OsR498G0713610300.01.T01 | AAI domain-containing protein (Fragment) |
| maker-7-augustus-gene-35.51-mRNA-1 | Indica | NA | | Os07g0175600 | OsR498G0713610900.01.T01 | non-specific lipid-transfer protein-like protein At2g13820 |
| maker-7-augustus-gene-35.58-mRNA-1 | Indica | NA | | Os07g0174900 | OsR498G0713608000.01.T01 | AAI domain-containing protein |
| maker-7-augustus-gene-35.60-mRNA-1 | Indica | NA | | Os07g0175100 | | protein ANTAGONIST OF LIKE HETEROCHROMATIN PROTEIN 1 |
| maker-7-augustus-gene-35.62-mRNA-1 | Indica | Indica | | Os07g0175300 | OsR498G0713609400.01.T01 | FLZ-type domain-containing protein (Fragment) |
| maker-7-augustus-gene-38.0-mRNA-1 | Indica | Indica | | Os11g0135900 | OsR498G0713631300.01.T04 | MFS domain-containing protein |
| maker-7-augustus-gene-40.44-mRNA-1 | Indica | NA | | | OsR498G0713637100.01.T03 | DNA (cytosine-5)-methyltransferase |
| maker-7-augustus-gene-41.14-mRNA-1 | Indica | NA | | Os07g0186000 | OsR498G0713649800.01.T01 | Thioredoxin H-type like |
| maker-7-augustus-gene-9.19-mRNA-1 | Indica | Indica | | Os03g0770800 | OsR498G0713440600.01.T01 | Probable xyloglucan glycosyltransferase 1 |
| maker-7-augustus-gene-9.22-mRNA-1 | Indica | NA | | Os07g0124800 | OsR498G0713441000.01.T02 | J domain-containing protein (Fragment) |
| maker-7-augustus-gene-9.5-mRNA-1 | Indica | Indica | | Os07g0124700 | OsR498G0713440300.01.T02 | AP2/ERF domain-containing transcription factor |
| maker-8-augustus-gene-100.32-mRNA-1 | Indica | NA | | Os08g0260600 | OsR498G0815571400.01.T01 | Tetraspanin-8 |
| maker-8-augustus-gene-102.41-mRNA-1 | Indica | NA | | | OsR498G0408029600.01.T01 | Transposon protein, putative, CACTA, En/Spm sub-class |
| maker-8-augustus-gene-103.0-mRNA-1 | Indica | NA | | | OsR498G0815582300.01.T01 | Lactation elevated protein 1 |
| maker-8-augustus-gene-103.14-mRNA-1 | Indica | NA | | Os08g0266700 | OsR498G0815585700.01.T01 | Sister chromatid cohesion 1 protein 3 |
| maker-8-augustus-gene-104.0-mRNA-1 | Indica | NA | | | | F-box domain-containing protein |
| maker-8-augustus-gene-104.10-mRNA-1 | Indica | NA | | Os08g0267800 | OsR498G0815589400.01.T01 | F-box domain-containing protein |
| maker-8-augustus-gene-104.2-mRNA-1 | Indica | NA | | | OsR498G0815591300.01.T02 | DNA-directed RNA polymerase III subunit RPC5 |
| maker-8-augustus-gene-104.6-mRNA-1 | Indica | NA | | Os08g0267300 | | Peptidase A1 domain-containing protein (Fragment) |
| maker-8-augustus-gene-104.8-mRNA-1 | Indica | Indica | | Os08g0267800 | OsR498G0815588800.01.T01 | F-box domain-containing protein |
| maker-8-augustus-gene-105.29-mRNA-1 | Indica | NA | | Os08g0270200 | OsR498G0815595900.01.T01 | Nuclear nucleic acid-binding protein C1D |
| maker-8-augustus-gene-105.33-mRNA-1 | Indica | Indica | | Os08g0269600 | OsR498G0815593400.01.T01 | |
| maker-8-augustus-gene-105.35-mRNA-1 | Indica | NA | | Os08g0269700 | OsR498G0815593600.01.T01 | |
| maker-8-augustus-gene-105.37-mRNA-1 | Indica | Indica | | Os08g0269800 | OsR498G0815593800.01.T02 | F-box protein SKIP1 |
| maker-8-augustus-gene-106.31-mRNA-1 | Indica | Indica | | Os08g0270466 | OsR498G0815597600.01.T01 | Cinnamyl-alcohol dehydrogenase |
| maker-8-augustus-gene-110.33-mRNA-1 | Indica | NA | | Os08g0277200 | OsR498G0815620900.01.T01 | Epimerase domain-containing protein (Fragment) |
| maker-8-augustus-gene-110.35-mRNA-1 | Indica | Indica | | Os08g0240000 | OsR498G0815621600.01.T01 | Sulfotransferase |
| maker-8-augustus-gene-113.32-mRNA-1 | Indica | NA | | | OsR498G0815633900.01.T03 | Formin-like protein 6 |
| maker-8-augustus-gene-114.0-mRNA-1 | Indica | Indica | | Os02g0116300 | OsR498G0815634100.01.T01 | Probable metal-nicotianamine transporter YSL17 |
| maker-8-augustus-gene-115.41-mRNA-1 | Indica | NA | | Os08g0282000 | OsR498G0815638800.01.T01 | Tobamovirus multiplication protein like |
| maker-8-augustus-gene-119.0-mRNA-1 | Indica | NA | | Os08g0288200 | OsR498G0815658200.01.T02 | Adenylate kinase |
| maker-8-augustus-gene-124.27-mRNA-1 | Indica | NA | | | | ubiquitin-like domain-containing protein CIP73 isoform X1 |
| maker-8-augustus-gene-125.24-mRNA-1 | Indica | NA | | Os08g0307400 | OsR498G0815726600.01.T05 | Phosphatidylinositol 3-kinase |
| maker-8-augustus-gene-125.31-mRNA-1 | Indica | NA | | Os08g0295300 | OsR498G0815686000.01.T01 | Threonyl-tRNA synthetase |
| maker-8-augustus-gene-125.37-mRNA-1 | Indica | NA | | Os08g0307500 | | PHD finger protein ING |
| maker-8-augustus-gene-126.0-mRNA-1 | Indica | NA | | Os08g0307000 | OsR498G0815725100.01.T05 | AAA domain-containing protein |
| maker-8-augustus-gene-128.32-mRNA-1 | Indica | NA | | | OsR498G0100616300.01.T02 | ATP-dependent DNA helicase |
| maker-8-augustus-gene-129.47-mRNA-1 | Indica | NA | | | OsR498G0815694100.01.T01 | Fatty acyl-CoA reductase |
| maker-8-augustus-gene-13.37-mRNA-1 | Indica | NA | | Os08g0121800 | OsR498G0815093500.01.T01 | |
| maker-8-augustus-gene-149.39-mRNA-1 | Indica | NA | | Os08g0327400 | OsR498G0815789900.01.T05 | Enoyl-ACP reductase |
| maker-8-augustus-gene-149.44-mRNA-1 | Indica | NA | | Os08g0327200 | OsR498G0815788600.01.T01 | Exopolygalacturonase |
| maker-8-augustus-gene-152.35-mRNA-1 | Indica | Indica | | Os09g0281900 | OsR498G0815804700.01.T04 | Mediator of RNA polymerase II transcription subunit 14 |
| maker-8-augustus-gene-153.0-mRNA-1 | Indica | NA | | | OsR498G0815813000.01.T01 | Pre-mRNA-splicing factor ATP-dependent RNA helicase DEAH1 |

| | | | | | | |
|---|---|---|---|---|---|---|
| maker-8-augustus-gene-156.31-mRNA-1 | Indica | NA | | | | general transcription and DNA repair factor IIH subunit TFB1-1-like isoform X |
| maker-8-augustus-gene-159.13-mRNA-1 | Indica | Indica | | Os08g0344300 | OsR498G0815843700.01.T03 | Mitochondrial pyruvate carrier |
| maker-8-augustus-gene-159.16-mRNA-1 | Indica | NA | | Os08g0344600 | OsR498G0815845300.01.T01 | Phosphoenolpyruvate/phosphate translocator like |
| maker-8-augustus-gene-160.28-mRNA-1 | Indica | Indica | | Os08g0345400 | OsR498G0815848100.01.T02 | Rho GTPase-activating protein 21-A (Fragment) |
| maker-8-augustus-gene-160.39-mRNA-1 | Indica | Indica | | Os08g0345800 | OsR498G0815850400.01.T01 | Glucose-1-phosphate adenylyltransferase small subunit, chloroplastic/amylo |
| maker-8-augustus-gene-161.4-mRNA-1 | Indica | NA | | | OsR498G0511410300.01.T03 | Solanesyl diphosphate synthase 2 chloroplastic |
| maker-8-augustus-gene-167.31-mRNA-1 | Indica | NA | | Os08g0349100 | OsR498G0815862900.01.T01 | Dirigent protein (Fragment) |
| maker-8-augustus-gene-168.20-mRNA-1 | Indica | NA | | Os08g0349100 | OsR498G0815862900.01.T01 | Dirigent protein (Fragment) |
| maker-8-augustus-gene-17.36-mRNA-1 | Indica | NA | | Os08g0129500 | OsR498G0815110600.01.T01 | Electron carrier/ iron ion binding protein |
| maker-8-augustus-gene-17.39-mRNA-1 | Indica | NA | | Os08g0129600 | OsR498G0815110800.01.T02 | Protein CHUP1, chloroplastic isoform A |
| maker-8-augustus-gene-180.24-mRNA-1 | Indica | NA | | Os08g0363000 | OsR498G0815846800.01.T01 | F-box domain containing protein, expressed |
| maker-8-augustus-gene-189.23-mRNA-1 | Indica | NA | | Os08g0374100 | OsR498G0815943800.01.T01 | UBC core domain-containing protein (Fragment) |
| maker-8-augustus-gene-189.26-mRNA-1 | Indica | Indica | | Os08g0374200 | OsR498G0815944200.01.T01 | UBC core domain-containing protein |
| maker-8-augustus-gene-19.9-mRNA-1 | Indica | NA | | Os08g0133000 | OsR498G0815124300.01.T02 | Cysteine dioxygenase |
| maker-8-augustus-gene-190.0-mRNA-1 | Indica | Indica | Kinase-TM | Os08g0374600 | OsR498G0815946800.01.T03 | Cytokinin-regulated kinase-like |
| maker-8-augustus-gene-190.2-mRNA-1 | Indica | NA | | Os08g0374800 | OsR498G0815947600.01.T01 | UDP-glucose 4-epimerase 4 |
| maker-8-augustus-gene-191.10-mRNA-1 | Indica | NA | Kinase-LRR-TM | Os08g0376300 | OsR498G0815955000.01.T01 | Receptor-like protein kinase 5 |
| maker-8-augustus-gene-192.33-mRNA-1 | Indica | NA | | Os08g0377400 | OsR498G0815960600.01.T05 | RNA exonuclease 4 |
| maker-8-augustus-gene-192.35-mRNA-1 | Indica | NA | | Os08g0378900 | OsR498G0815961200.01.T02 | Thioredoxin-like protein CITRX, chloroplastic |
| maker-8-augustus-gene-193.36-mRNA-1 | Indica | NA | | Os08g0379300 | OsR498G0815961900.01.T06 | Phosphoglucan phosphatase LSF |
| maker-8-augustus-gene-193.41-mRNA-1 | Indica | NA | | Os08g0380100 | OsR498G0815964500.01.T03 | BURP domain-containing protein 12 |
| maker-8-augustus-gene-193.43-mRNA-1 | Indica | NA | | Os08g0379400 | OsR498G0815962200.01.T01 | 2-methylene-furan-3-one reductase |
| maker-8-augustus-gene-194.8-mRNA-1 | Indica | NA | | | | |
| maker-8-augustus-gene-195.13-mRNA-1 | Indica | NA | | Os08g0384100 | OsR498G0815974800.01.T01 | double-stranded RNA-binding protein 3-like |
| maker-8-augustus-gene-195.17-mRNA-1 | Indica | Indica | | Os08g0384500 | OsR498G0815975800.01.T02 | ABC transporter domain-containing protein |
| maker-8-augustus-gene-195.4-mRNA-1 | Indica | Indica | | Os08g0384000 | OsR498G0815974500.01.T01 | probable transmembrane ascorbate ferrireductase 2 |
| maker-8-augustus-gene-20.35-mRNA-1 | Indica | NA | | | | Skp1 domain-containing protein |
| maker-8-augustus-gene-20.38-mRNA-1 | Indica | NA | | | OsR498G0307051300.01.T01 | Cysteine dioxygenase |
| maker-8-augustus-gene-203.30-mRNA-1 | Indica | NA | | Os08g0397900 | OsR498G0816026100.01.T01 | Putative S-acyltransferase |
| maker-8-augustus-gene-203.33-mRNA-1 | Indica | NA | | Os08g0398300 | OsR498G0816026500.01.T01 | ABC transporter A family member 6 |
| maker-8-augustus-gene-203.36-mRNA-1 | Indica | Indica | | Os08g0398350 | OsR498G0816026900.01.T01 | ABC transporter domain-containing protein (Fragment) |
| maker-8-augustus-gene-203.41-mRNA-1 | Indica | NA | | Os08g0398400 | OsR498G0816027300.01.T06 | Hypersensitive-induced response protein 1 |
| maker-8-augustus-gene-203.45-mRNA-1 | Indica | NA | | Os08g0398700 | OsR498G0816027700.01.T01 | Aminopeptidase |
| maker-8-augustus-gene-203.51-mRNA-1 | Indica | NA | | Os08g0398000 | OsR498G0816026400.01.T01 | ABC transporter domain-containing protein (Fragment) |
| maker-8-augustus-gene-203.55-mRNA-1 | Indica | NA | | Os08g0398500 | OsR498G0816027600.01.T01 | H/ACA ribonucleoprotein complex subunit CBF5 |
| maker-8-augustus-gene-204.2-mRNA-1 | Indica | NA | | Os08g0400300 | OsR498G0816031900.01.T04 | DnaJ subfamily C member 2, N-terminally processed like |
| maker-8-augustus-gene-204.6-mRNA-1 | Indica | Indica | | Os08g0400800 | OsR498G0816032500.01.T02 | F-box domain containing protein, expressed |
| maker-8-augustus-gene-204.9-mRNA-1 | Indica | NA | | Os08g0401100 | OsR498G0816033100.01.T01 | PGG domain-containing protein |
| maker-8-augustus-gene-205.4-mRNA-1 | Indica | NA | | Os08g0401500 | OsR498G0816035400.01.T02 | |
| maker-8-augustus-gene-205.7-mRNA-1 | Indica | NA | | Os08g0401800 | OsR498G0816036000.01.T01 | Phospholipase D |
| maker-8-augustus-gene-21.38-mRNA-1 | Indica | NA | | Os08g0135400 | OsR498G0815132500.01.T01 | E3 ubiquitin-protein like |
| maker-8-augustus-gene-21.42-mRNA-1 | Indica | NA | | Os08g0135800 | OsR498G0815133100.01.T02 | Zinc finger CCCH domain-containing protein 55 |
| maker-8-augustus-gene-21.44-mRNA-1 | Indica | NA | | Os08g0135900 | OsR498G0815133300.01.T01 | Tryptophan synthase beta chain 2, chloroplastic |
| maker-8-augustus-gene-21.56-mRNA-1 | Indica | NA | | | OsR498G0815133000.01.T01 | Mediator of RNA polymerase II transcription subunit 21 |
| maker-8-augustus-gene-21.58-mRNA-1 | Indica | Indica | | Os04g0627000 | OsR498G0815133600.01.T01 | Homeobox-leucine zipper protein ROC7 |
| maker-8-augustus-gene-210.50-mRNA-1 | Indica | NA | CC-LRR-NBS-TM | | OsR498G0816075400.01.T01 | Disease resistance protein RPM1 |
| maker-8-augustus-gene-210.59-mRNA-1 | Indica | NA | | Os08g0412500 | OsR498G0816077400.01.T03 | Mitochondrial pyruvate carrier |
| maker-8-augustus-gene-210.62-mRNA-1 | Indica | NA | | | OsR498G0816077600.01.T01 | Ecotropic viral integration site 5 protein isogeny |
| maker-8-augustus-gene-211.53-mRNA-1 | Indica | NA | | Os08g0414200 | OsR498G0816080700.01.T01 | RING-type E3 ubiquitin transferase BRCA1 |
| maker-8-augustus-gene-211.55-mRNA-1 | Indica | NA | | Os08g0414300 | OsR498G0816080900.01.T01 | Cyclic AMP-responsive element-binding protein like |
| maker-8-augustus-gene-211.59-mRNA-1 | Indica | NA | | Os08g0413000 | OsR498G0816078200.01.T01 | Cell division control protein 48 |
| maker-8-augustus-gene-212.49-mRNA-1 | Indica | NA | | Os08g0414700 | OsR498G0816083200.01.T01 | Trehalose-phosphatase/synthase 9 |
| maker-8-augustus-gene-212.56-mRNA-1 | Indica | NA | | Os08g0416100 | OsR498G0816086800.01.T01 | DEAD-box ATP-dependent RNA helicase 29 |
| maker-8-augustus-gene-215.49-mRNA-1 | Indica | NA | | | OsR498G0816099200.01.T01 | Cyclin N-terminal domain-containing protein |
| maker-8-augustus-gene-215.52-mRNA-1 | Indica | NA | CC-Kinase-TM Pi33 | Os08g0421800 | OsR498G0816100400.01.T01 | Pyricularia oryzae resistance-33(t) *Pi33* R gene |
| maker-8-augustus-gene-222.21-mRNA-1 | Indica | NA | | Os08g0432500 | OsR498G0816144000.01.T01 | ATP-dependent Clp protease adaptor protein ClpS containing protein |
| maker-8-augustus-gene-222.25-mRNA-1 | Indica | NA | | Os08g0432800 | OsR498G0816145600.01.T01 | Transcription factor bHLH30 like |
| maker-8-augustus-gene-223.39-mRNA-1 | Indica | NA | | Os08g0433200 | OsR498G0816148700.01.T03 | Cytosolic fe-s cluster assembly factor nar1 |
| maker-8-augustus-gene-223.41-mRNA-1 | Indica | NA | | Os08g0434000 | OsR498G0816150900.01.T05 | Selenium-binding protein-like |
| maker-8-augustus-gene-223.42-mRNA-1 | Indica | NA | | Os08g0434100 | OsR498G0816151100.01.T01 | Wounding-induced ribonuclease |
| maker-8-augustus-gene-223.50-mRNA-1 | Indica | NA | | Os08g0433400 | OsR498G0816149200.01.T05 | MIXTA1 transcription factor |

| | | | | | | |
|---|---|---|---|---|---|---|
| maker-8-augustus-gene-224.0-mRNA-1 | Indica | NA | | | OsR498G0816153900.01.T02 | Putative mannan synthase 7 |
| maker-8-augustus-gene-224.5-mRNA-1 | Indica | NA | | | | Myb-related protein 2 |
| maker-8-augustus-gene-225.31-mRNA-1 | Indica | Japonica | | Os08g0435700 | OsR498G0816157500.01.T01 | Transcription factor MYB105 |
| maker-8-augustus-gene-225.34-mRNA-1 | Indica | NA | | Os08g0436100 | OsR498G0816159500.01.T04 | Apyrase 6 |
| maker-8-augustus-gene-225.45-mRNA-1 | Indica | NA | | Os08g0435800 | OsR498G0816158800.01.T02 | Biotinyl protein ligase (BPL) and lipoyl protein ligase (LPL), catalytic domain c |
| maker-8-augustus-gene-225.48-mRNA-1 | Indica | NA | | Os08g0435900 | OsR498G0816159000.01.T01 | Chlorophyll a-b binding protein, chloroplastic |
| maker-8-augustus-gene-225.51-mRNA-1 | Indica | NA | | Os08g0436000 | OsR498G0816159200.01.T01 | Polypyrimidine tract-binding protein isogeny 1 |
| maker-8-augustus-gene-226.24-mRNA-1 | Indica | NA | | Os08g0438600 | OsR498G0816166700.01.T02 | Exostosin domain-containing protein (Fragment) |
| maker-8-augustus-gene-227.12-mRNA-1 | Indica | NA | | | | Temperature-induced lipocalin-2 |
| maker-8-augustus-gene-227.14-mRNA-1 | Indica | NA | | | | Poly(A)-specific ribonuclease PARN-like |
| maker-8-augustus-gene-227.20-mRNA-1 | Indica | NA | | Os08g0440000 | OsR498G0816170600.01.T03 | Golgi SNAP receptor complex member 1 |
| maker-8-augustus-gene-228.34-mRNA-1 | Indica | NA | | Os08g0440800 | OsR498G0816174900.01.T01 | Aldedh domain-containing protein |
| maker-8-augustus-gene-228.42-mRNA-1 | Indica | NA | | | | Glycosyltransferase 39 like protein (Fragment) |
| maker-8-augustus-gene-228.46-mRNA-1 | Indica | NA | | | OsR498G0816175400.01.T01 | Omega-6 fatty acid desaturase chloroplastic |
| maker-8-augustus-gene-230.0-mRNA-1 | Indica | NA | | Os08g0445700 | OsR498G0816196900.01.T01 | Trehalose-phosphatase |
| maker-8-augustus-gene-231.37-mRNA-1 | Indica | NA | Kinase-LRR-TM | | OsR498G0816198400.01.T04 | Receptor-like protein kinase |
| maker-8-augustus-gene-231.39-mRNA-1 | Indica | Indica | | Os06g0655100 | OsR498G0816202800.01.T01 | D-3-phosphoglycerate dehydrogenase |
| maker-8-augustus-gene-231.42-mRNA-1 | Indica | NA | | Os08g0447200 | OsR498G0816204200.01.T01 | Protein ecdysoneless like |
| maker-8-augustus-gene-232.51-mRNA-1 | Indica | NA | | Os08g0447900 | OsR498G0816205500.01.T02 | Adenylyl cyclase-associated protein |
| maker-8-augustus-gene-232.53-mRNA-1 | Indica | Japonica | | Os08g0447300 | OsR498G0816204600.01.T04 | DUF676 domain-containing protein |
| maker-8-augustus-gene-232.60-mRNA-1 | Indica | NA | | | OsR498G0816205000.01.T02 | Dentin sialophosphoprotein-related |
| maker-8-augustus-gene-232.62-mRNA-1 | Indica | NA | | Os08g0448000 | OsR498G0816205800.01.T02 | 4-coumarate--CoA ligase |
| maker-8-augustus-gene-253.23-mRNA-1 | Indica | NA | | | | |
| maker-8-augustus-gene-260.0-mRNA-1 | Indica | Indica | LRR-TM | Os08g0499900 | OsR498G0816398100.01.T01 | F-box protein At-B |
| maker-8-augustus-gene-260.26-mRNA-1 | Indica | NA | | Os08g0500300 | OsR498G0816399200.01.T02 | PPM-type phosphatase domain-containing protein (Fragment) |
| maker-8-augustus-gene-275.32-mRNA-1 | Indica | NA | | Os08g0526500 | OsR498G0816500100.01.T02 | Ankyrin repeat and SAM domain-containing protein 6 |
| maker-8-augustus-gene-275.35-mRNA-1 | Indica | NA | | | | UBA domain-containing protein (Fragment) |
| maker-8-augustus-gene-275.39-mRNA-1 | Indica | NA | | | OsR498G0816499400.01.T01 | E3 ubiquitin ligase RNF4 |
| maker-8-augustus-gene-275.42-mRNA-1 | Indica | Indica | | Os08g0526400 | OsR498G0816499600.01.T02 | RPM1-interacting protein 4 isoform B |
| maker-8-augustus-gene-275.44-mRNA-1 | Indica | NA | | Os08g0527100 | OsR498G0816502800.01.T01 | Ubiquitinyl hydrolase 1 |
| maker-8-augustus-gene-275.52-mRNA-1 | Indica | NA | | | | Ubiquitinyl hydrolase 1 |
| maker-8-augustus-gene-276.0-mRNA-1 | Indica | NA | | Os08g0528900 | OsR498G0816508900.01.T02 | FHA domain-containing protein FHA2 |
| maker-8-augustus-gene-276.19-mRNA-1 | Indica | NA | | Os08g0528100 | | Ubiquitinyl hydrolase 1 |
| maker-8-augustus-gene-276.2-mRNA-1 | Indica | NA | | Os08g0529100 | OsR498G0816510300.01.T01 | Proteasome endopeptidase complex |
| maker-8-augustus-gene-276.22-mRNA-1 | Indica | NA | | Os08g0528500 | OsR498G0816507800.01.T02 | Probable magnesium transporter |
| maker-8-augustus-gene-276.26-mRNA-1 | Indica | NA | | Os08g0528601 | OsR498G0816508200.01.T02 | GRIP/coiled-coil protein |
| maker-8-augustus-gene-276.29-mRNA-1 | Indica | NA | | | OsR498G0816508400.01.T01 | Mitochondrial transcription termination factor-like family-4 |
| maker-8-augustus-gene-276.30-mRNA-1 | Indica | NA | | Os08g0528800 | OsR498G0816508600.01.T01 | Peptidase S1, PA clan |
| maker-8-augustus-gene-276.32-mRNA-1 | Indica | NA | | Os08g0529000 | OsR498G0816509400.01.T01 | Auxin efflux carrier component |
| maker-8-augustus-gene-276.5-mRNA-1 | Indica | NA | | Os04g0691400 | OsR498G0409539100.01.T01 | Peptide transporter PTR1 |
| maker-8-augustus-gene-277.37-mRNA-1 | Indica | Indica | | Os07g0294800 | OsR498G0816513900.01.T01 | Regulatory protein-like |
| maker-8-augustus-gene-277.39-mRNA-1 | Indica | NA | | Os08g0530400 | OsR498G0816515900.01.T01 | Sulfite oxidase |
| maker-8-augustus-gene-277.46-mRNA-1 | Indica | NA | | Os08g0531000 | OsR498G0816517100.01.T02 | Purple acid phosphatase |
| maker-8-augustus-gene-277.54-mRNA-1 | Indica | NA | | Os08g0531100 | OsR498G0816517500.01.T06 | 65-kDa microtubule-associated protein 8 |
| maker-8-augustus-gene-277.60-mRNA-1 | Indica | NA | | Os08g0529800 | OsR498G0816512800.01.T01 | Triacylglycerol lipase 2 |
| maker-8-augustus-gene-277.63-mRNA-1 | Indica | NA | | Os08g0530200 | OsR498G0816514600.01.T01 | Ribosomal protein L17c |
| maker-8-augustus-gene-277.67-mRNA-1 | Indica | NA | | Os08g0530500 | OsR498G0816516200.01.T01 | COMM domain-containing protein 9 |
| maker-8-augustus-gene-277.69-mRNA-1 | Indica | NA | | | | Auxin response factor 12 |
| maker-8-augustus-gene-278.0-mRNA-1 | Indica | NA | | Os08g0531300 | OsR498G0816518100.01.T01 | YrdC-like domain-containing protein (Fragment) |
| maker-8-augustus-gene-30.34-mRNA-1 | Indica | NA | | Os08g0148500 | OsR498G0815189100.01.T01 | |
| maker-8-augustus-gene-30.65-mRNA-1 | Indica | NA | | | | F-box domain-containing protein (Fragment) |
| maker-8-augustus-gene-31.29-mRNA-1 | Indica | NA | | Os08g0150700 | OsR498G0815197100.01.T01 | Phloem-specific lectin |
| maker-8-augustus-gene-31.39-mRNA-1 | Indica | NA | | | OsR498G0815198500.01.T03 | CCA-type MYB transcription factor 1h (Fragment) |
| maker-8-augustus-gene-31.62-mRNA-1 | Indica | NA | | | OsR498G0815196000.01.T02 | FBD domain-containing protein |
| maker-8-augustus-gene-31.65-mRNA-1 | Indica | NA | | Os08g0151300 | OsR498G0815199200.01.T01 | Transcription factor MYB26 |
| maker-8-augustus-gene-32.36-mRNA-1 | Indica | NA | | | OsR498G0815202500.01.T01 | Aquaporin NIP3-3 |
| maker-8-augustus-gene-32.38-mRNA-1 | Indica | NA | | Os08g0152700 | OsR498G0815204100.01.T01 | Sphingoid long-chain bases kinase 1 |
| maker-8-augustus-gene-32.42-mRNA-1 | Indica | NA | | | OsR498G0815205300.01.T01 | Sulfonylurea receptor, P-loop containing nucleoside triphosphate hydrolase |
| maker-8-augustus-gene-32.45-mRNA-1 | Indica | NA | | | | Multidrug/pheromone exporter, MDR family, ABC transporter family |
| maker-8-augustus-gene-32.46-mRNA-1 | Indica | NA | | Os08g0152500 | OsR498G0815203600.01.T04 | Protein ENHANCED DISEASE RESISTANCE 2-like |
| maker-8-augustus-gene-32.50-mRNA-1 | Indica | Indica | | Os02g0106800 | OsR498G0815204400.01.T02 | Transmembrane protein 120 |

| | | | | | | |
|---|---|---|---|---|---|---|
| maker-8-augustus-gene-32.54-mRNA-1 | Indica | NA | | | OsR498G0815205000.01.T01 | HEAT repeat-containing protein 6 |
| maker-8-augustus-gene-33.40-mRNA-1 | Indica | NA | | Os08g0154000 | OsR498G0815208700.01.T02 | Tricin synthase 1 |
| maker-8-augustus-gene-33.44-mRNA-1 | Indica | NA | | Os08g0154600 | | DNA topoisomerase I |
| maker-8-augustus-gene-33.46-mRNA-1 | Indica | Indica | | Os08g0154700 | OsR498G0815210500.01.T01 | Sm domain-containing protein (Fragment) |
| maker-8-augustus-gene-33.61-mRNA-1 | Indica | Indica | | Os08g0159900 | OsR498G0815209400.01.T01 | DEAD-box ATP-dependent RNA helicase |
| maker-8-augustus-gene-33.64-mRNA-1 | Indica | Indica | | Os08g0154250 | OsR498G0815209800.01.T01 | Monocopper oxidase |
| maker-8-augustus-gene-33.66-mRNA-1 | Indica | NA | | Os08g0154300 | OsR498G0815210200.01.T01 | Transaldolase |
| maker-8-augustus-gene-35.30-mRNA-1 | Indica | NA | | Os08g0156700 | OsR498G0815224800.01.T01 | MSP domain-containing protein (Fragment) |
| maker-8-augustus-gene-35.43-mRNA-1 | Indica | NA | | Os08g0157500 | OsR498G0815228600.01.T01 | Caffeic acid-O-methyltransferase |
| maker-8-augustus-gene-36.34-mRNA-1 | Indica | Indica | | Os08g0157600 | OsR498G0815231500.01.T07 | Circadian clock associated protein 1 |
| maker-8-augustus-gene-36.39-mRNA-1 | Indica | NA | | Os08g0158500 | OsR498G0815235900.01.T03 | PHB domain-containing protein (Fragment) |
| maker-8-augustus-gene-36.48-mRNA-1 | Indica | NA | | Os08g0159100 | OsR498G0815236900.01.T04 | Tobamovirus multiplication protein 2A |
| maker-8-augustus-gene-36.51-mRNA-1 | Indica | NA | | Os08g0157800 | OsR498G0815232800.01.T01 | |
| maker-8-augustus-gene-36.58-mRNA-1 | Indica | NA | | Os08g0158900 | OsR498G0815236600.01.T02 | Nucleolar GTP-binding protein 1 |
| maker-8-augustus-gene-37.19-mRNA-1 | Indica | NA | | Os08g0159500 | OsR498G0815239000.01.T04 | LOL1 (LSD ONE LIKE 1) |
| maker-8-augustus-gene-4.60-mRNA-1 | Indica | NA | | Os08g0106900 | | DDE Tnp4 domain-containing protein |
| maker-8-augustus-gene-40.56-mRNA-1 | Indica | NA | | | | Deoxyribonuclease TATDN1 |
| maker-8-augustus-gene-40.61-mRNA-1 | Indica | Japonica | | Os08g0163900 | OsR498G0815262100.01.T01 | DUF569 domain-containing protein |
| maker-8-augustus-gene-41.0-mRNA-1 | Indica | NA | | Os08g0166450 | | SWIM-type domain-containing protein |
| maker-8-augustus-gene-41.11-mRNA-1 | Indica | NA | | Os08g0167400 | OsR498G0815269800.01.T01 | Pentatricopeptide (PPR) repeat-containing protein-like |
| maker-8-augustus-gene-41.14-mRNA-1 | Indica | Japonica | | Os08g0167600 | OsR498G0815270400.01.T01 | |
| maker-8-augustus-gene-41.2-mRNA-1 | Indica | NA | | | OsR498G0815268500.01.T01 | ABC transporter domain-containing protein |
| maker-8-augustus-gene-41.4-mRNA-1 | Indica | NA | | Os08g0167500 | OsR498G0815270100.01.T02 | Chlororespiratory reduction 6 |
| maker-8-augustus-gene-41.7-mRNA-1 | Indica | Japonica | | Os08g0168000 | OsR498G0815271100.01.T02 | Sesquiterpene synthase |
| maker-8-augustus-gene-41.9-mRNA-1 | Indica | Japonica | | Os08g0168150 | OsR498G0917743600.01.T01 | EIN3-binding F-box protein 1 |
| maker-8-augustus-gene-42.33-mRNA-1 | Indica | NA | | Os08g0168700 | OsR498G0815272900.01.T02 | Glycosyltransferase |
| maker-8-augustus-gene-52.14-mRNA-1 | Indica | NA | | Os08g0187700 | OsR498G0815333200.01.T01 | Polyadenylation and cleavage factor homolog 5 |
| maker-8-augustus-gene-53.35-mRNA-1 | Indica | NA | | Os08g0189200 | OsR498G0815337700.01.T01 | Germin-like protein 8-3 |
| maker-8-augustus-gene-53.49-mRNA-1 | Indica | Japonica | | Os08g0190700 | OsR498G0815344500.01.T02 | CAP-Gly domain-containing linker protein 1-like |
| maker-8-augustus-gene-53.54-mRNA-1 | Indica | Japonica | CC-LRR-NBS-TM | Os08g0190300 | OsR498G0815343400.01.T01 | |
| maker-8-augustus-gene-54.2-mRNA-1 | Indica | Indica | | Os04g0624600 | OsR498G0815345700.01.T01 | Starch synthase IIIa |
| maker-8-augustus-gene-54.6-mRNA-1 | Indica | NA | | Os08g0191600 | OsR498G0815346100.01.T04 | Autophagy-related protein 8B |
| maker-8-augustus-gene-57.61-mRNA-1 | Indica | Indica | | Os08g0197500 | OsR498G0815366900.01.T01 | F-box domain-containing protein (Fragment) |
| maker-8-augustus-gene-60.12-mRNA-1 | Indica | NA | NBS-TM | Os08g0202400 | OsR498G0815386000.01.T02 | NB-ARC domain-containing protein |
| maker-8-augustus-gene-60.8-mRNA-1 | Indica | Indica | CC-Kinase-LRR-TM | Os08g0201700 | OsR498G0815385700.01.T13 | Non-specific serine/threonine protein kinase |
| maker-8-augustus-gene-85.14-mRNA-1 | Indica | Indica | | Os08g0236900 | OsR498G0815500400.01.T02 | |
| maker-8-augustus-gene-87.11-mRNA-1 | Indica | NA | | | | Complex 1 family protein, putative, expressed |
| maker-8-augustus-gene-87.21-mRNA-1 | Indica | NA | | | | |
| maker-8-augustus-gene-88.38-mRNA-1 | Indica | Indica | | Os08g0239100 | OsR498G0815510300.01.T01 | Endo-1,31,4-beta-D-glucanase, putative, expressed |
| maker-8-augustus-gene-91.5-mRNA-1 | Indica | Japonica | | Os08g0244500 | OsR498G0714509400.01.T03 | Glucan endo-1,3-beta-D-glucosidase |
| maker-8-augustus-gene-91.6-mRNA-1 | Indica | NA | | Os08g0244500 | OsR498G0714509400.01.T01 | Glucan endo-1,3-beta-D-glucosidase |
| maker-8-augustus-gene-96.10-mRNA-1 | Indica | NA | | Os08g0254600 | OsR498G0815559000.01.T01 | Cyclic nucleotide-gated ion channel 17 |
| maker-8-augustus-gene-96.5-mRNA-1 | Indica | NA | | Os08g0254500 | OsR498G0815558700.01.T05 | Preprotein translocase subunit SECY, chloroplastic |
| maker-8-augustus-gene-97.0-mRNA-1 | Indica | NA | | Os08g0254900 | OsR498G0815559700.01.T01 | Glutamyl-tRNA reductase-binding protein chloroplastic |
| maker-9-augustus-gene-100.40-mRNA-1 | Indica | Japonica | | Os09g0330700 | OsR498G0917172500.01.T01 | |
| maker-9-augustus-gene-101.0-mRNA-1 | Indica | NA | | | | Protein disulfide-isomerase |
| maker-9-augustus-gene-102.26-mRNA-1 | Indica | NA | | Os09g0331900 | OsR498G0917174600.01.T01 | |
| maker-9-augustus-gene-102.29-mRNA-1 | Indica | NA | | Os09g0332360 | | ABC transporter domain-containing protein |
| maker-9-augustus-gene-102.30-mRNA-1 | Indica | Indica | | Os01g0609066 | OsR498G0917178000.01.T01 | Pleiotropic drug resistance protein 4 |
| maker-9-augustus-gene-103.30-mRNA-1 | Indica | NA | | Os09g0333600 | OsR498G0917179000.01.T01 | ABC transporter domain containing protein,expressed |
| maker-9-augustus-gene-104.2-mRNA-1 | Indica | NA | | Os09g0353700 | OsR498G0917230700.01.T01 | Iron/ascorbate-dependent oxidoreductase-like |
| maker-9-augustus-gene-109.7-mRNA-1 | Indica | NA | | Os09g0346600 | OsR498G0917207600.01.T03 | Short-chain dehydrogenase TIC 32, chloroplastic |
| maker-9-augustus-gene-112.25-mRNA-1 | Indica | NA | | | | Glycerophosphodiester phosphodiesterase |
| maker-9-augustus-gene-113.29-mRNA-1 | Indica | NA | | Os02g0545300 | | Putative ATP synthase subunit |
| maker-9-augustus-gene-115.2-mRNA-1 | Indica | NA | Kinase-TM | | | Serine/threonine-specific receptor protein kinase-like |
| maker-9-augustus-gene-117.3-mRNA-1 | Indica | Indica | Kinase-TM | Os09g0356800 | OsR498G0917240400.01.T05 | Protein kinase domain-containing protein |
| maker-9-augustus-gene-122.2-mRNA-1 | Indica | NA | | | OsR498G0917264000.01.T01 | |
| maker-9-augustus-gene-122.4-mRNA-1 | Indica | NA | | | OsR498G0917264600.01.T01 | |
| maker-9-augustus-gene-13.31-mRNA-1 | Indica | Indica | | Os09g0115900 | OsR498G0916693700.01.T01 | DOG1 domain-containing protein |
| maker-9-augustus-gene-136.28-mRNA-1 | Indica | NA | | | | Pyruvate kinase |
| maker-9-augustus-gene-139.0-mRNA-1 | Indica | NA | | Os09g0395300 | OsR498G0917383500.01.T01 | Homeodomain-like superfamily protein, putative isoform 1 |

| | | | | | | |
|---|---|---|---|---|---|---|
| maker-9-augustus-gene-139.12-mRNA-1 | Indica | Indica | | Os09g0395600 | OsR498G0917385200.01.T01 | Xyloglucan endotransglucosylase/hydrolase |
| maker-9-augustus-gene-141.42-mRNA-1 | Indica | Indica | | Os09g0400000 | OsR498G0917404400.01.T01 | Cinnamyl alcohol dehydrogenase 3 |
| maker-9-augustus-gene-141.44-mRNA-1 | Indica | NA | | Os09g0400400 | OsR498G0917405100.01.T01 | Cinnamyl alcohol dehydrogenase 3 |
| maker-9-augustus-gene-141.46-mRNA-1 | Indica | NA | | Os09g0401000 | OsR498G0917407900.01.T01 | MYB transcriptional factor |
| maker-9-augustus-gene-141.48-mRNA-1 | Indica | NA | | Os09g0399800 | OsR498G0917404000.01.T01 | Cinnamyl alcohol dehydrogenase |
| maker-9-augustus-gene-141.50-mRNA-1 | Indica | Indica | | Os09g0400000 | OsR498G0917404400.01.T01 | Cinnamyl alcohol dehydrogenase 3 |
| maker-9-augustus-gene-142.33-mRNA-1 | Indica | Indica | | Os09g0401100 | OsR498G0917409300.01.T01 | Half-size ABCG transporter |
| maker-9-augustus-gene-142.44-mRNA-1 | Indica | NA | | Os09g0401200 | OsR498G0917409600.01.T06 | TPR repeat-containing thioredoxin TDX |
| maker-9-augustus-gene-142.49-mRNA-1 | Indica | NA | | | | DEAD/DEAH box helicase |
| maker-9-augustus-gene-142.50-mRNA-1 | Indica | NA | | Os09g0401300 | OsR498G0917410000.01.T01 | Tify domain-containing protein |
| maker-9-augustus-gene-144.42-mRNA-1 | Indica | NA | | | OsR498G0917420300.01.T01 | |
| maker-9-augustus-gene-144.54-mRNA-1 | Indica | NA | | Os09g0406100 | OsR498G0917419000.01.T01 | |
| maker-9-augustus-gene-145.39-mRNA-1 | Indica | NA | Kinase-TM | Os09g0408800 | OsR498G0917427900.01.T01 | Cysteine-rich receptor-like protein kinase 10 |
| maker-9-augustus-gene-15.2-mRNA-1 | Indica | NA | | Os04g0274700 | OsR498G0613115000.01.T04 | |
| maker-9-augustus-gene-151.27-mRNA-1 | Indica | NA | | | OsR498G0917466700.01.T02 | |
| maker-9-augustus-gene-151.32-mRNA-1 | Indica | NA | | Os09g0419200 | OsR498G0917467800.01.T01 | Cinnamoyl CoA reductase |
| maker-9-augustus-gene-154.28-mRNA-1 | Indica | NA | | | | FAR1 domain-containing protein |
| maker-9-augustus-gene-154.43-mRNA-1 | Indica | NA | | | OsR498G0407890700.01.T01 | |
| maker-9-augustus-gene-25.35-mRNA-1 | Indica | NA | | | | 4-diphosphocytidyl-2C-methyl-D-erythritol synthase |
| maker-9-augustus-gene-25.43-mRNA-1 | Indica | NA | | | | |
| maker-9-augustus-gene-41.6-mRNA-1 | Indica | NA | | Os09g0248100 | OsR498G0916818100.01.T01 | Fasciclin-like arabinogalactan protein 2 |
| maker-9-augustus-gene-52.39-mRNA-1 | Indica | Indica | | Os09g0264400 | OsR498G0916863800.01.T01 | Flavonoid 3'-monooxygenase |
| maker-9-augustus-gene-53.34-mRNA-1 | Indica | Indica | | Os05g0524500 | OsR498G0916869900.01.T01 | Putative serine/threonine-specific protein kinase |
| maker-9-augustus-gene-54.0-mRNA-1 | Indica | NA | | Os09g0266400 | OsR498G0916872700.01.T01 | Hydrolase acting on ester bonds |
| maker-9-augustus-gene-71.22-mRNA-1 | Indica | NA | | Os04g0316800 | | Transposon protein, putative, unclassified |
| maker-9-augustus-gene-72.0-mRNA-1 | Indica | NA | | | | DDE Tnp4 domain-containing protein (Fragment) |
| maker-9-augustus-gene-84.12-mRNA-1 | Indica | NA | | | | |
| maker-9-augustus-gene-88.25-mRNA-1 | Indica | NA | NBS-TM | | | NBS-LRR protein (Fragment) |
| maker-9-augustus-gene-90.13-mRNA-1 | Indica | NA | | Os09g0318600 | OsR498G0917118200.01.T02 | NAM-associated domain-containing protein |
| maker-9-augustus-gene-99.0-mRNA-1 | Indica | NA | LRR-NBS-TM | Os09g0327800 | | NBS-LRR disease resistance protein-like |
| augustus-1-processed-gene-1.67-mRNA-1 | Japonica | NA | | | OsR498G0100004300.01.T04 | Ste24 endopeptidase (Fragment) |
| augustus-1-processed-gene-1.72-mRNA-1 | Japonica | NA | | Os01g0101600 | OsR498G0100006300.01.T06 | Nuclear protein-like |
| augustus-1-processed-gene-100.51-mRNA-1 | Japonica | Japonica | | Os01g0862500 | OsR498G0509898500.01.T01 | IQ-domain 25 |
| augustus-1-processed-gene-102.35-mRNA-1 | Japonica | NA | | Os01g0283400 | OsR498G0100680700.01.T01 | |
| augustus-1-processed-gene-105.7-mRNA-1 | Japonica | NA | | | OsR498G0100702900.01.T01 | MADS-box domain-containing protein |
| augustus-1-processed-gene-106.19-mRNA-1 | Japonica | Japonica | | Os01g0290600 | OsR498G0100711500.01.T01 | Putative L-cysteine desulfhydrase chloroplastic |
| augustus-1-processed-gene-107.17-mRNA-1 | Japonica | NA | | Os01g0292700 | OsR498G0100721100.01.T03 | Myosin heavy chain, cardiac protein |
| augustus-1-processed-gene-108.20-mRNA-1 | Japonica | NA | | Os01g0293200 | OsR498G0100723300.01.T03 | PORR domain-containing protein (Fragment) |
| augustus-1-processed-gene-110.23-mRNA-1 | Japonica | NA | | Os01g0298400 | OsR498G0100734500.01.T01 | Transcription factor MYB108 |
| augustus-1-processed-gene-111.52-mRNA-1 | Japonica | NA | | Os01g0300400 | | ATP-dependent DNA helicase |
| augustus-1-processed-gene-113.11-mRNA-1 | Japonica | Japonica | | Os01g0302900 | OsR498G0100517100.01.T01 | |
| augustus-1-processed-gene-113.13-mRNA-1 | Japonica | NA | | Os01g0303100 | OsR498G0100750300.01.T01 | Acidic endochitinase |
| augustus-1-processed-gene-114.34-mRNA-1 | Japonica | NA | | | | Transposable element protein, putative, MuDR |
| augustus-1-processed-gene-115.16-mRNA-1 | Japonica | NA | | Os01g0306400 | OsR498G0100760300.01.T01 | DOG1 domain-containing protein (Fragment) |
| augustus-1-processed-gene-117.14-mRNA-1 | Japonica | NA | | Os01g0309100 | OsR498G0100773300.01.T01 | Actin cytoskeleton-regulatory complex protein pan-1-like |
| augustus-1-processed-gene-118.39-mRNA-1 | Japonica | NA | | Os01g0311400 | OsR498G0100778900.01.T01 | RING-H2 finger protein ATL3F |
| augustus-1-processed-gene-119.52-mRNA-1 | Japonica | NA | | Os03g0110200 | | Transposon protein, putative, Mutator sub-class |
| augustus-1-processed-gene-120.1-mRNA-1 | Japonica | Indica | | Os03g0341300 | OsR498G0100787900.01.T01 | Bidirectional sugar transporter SWEET |
| augustus-1-processed-gene-120.2-mRNA-1 | Japonica | NA | NBS-TM | | OsR498G0100788500.01.T01 | NB-ARC domain-containing protein |
| augustus-1-processed-gene-121.18-mRNA-1 | Japonica | NA | | | OsR498G0100073600.01.T01 | |
| augustus-1-processed-gene-122.10-mRNA-1 | Japonica | NA | | Os01g0317800 | OsR498G0100798100.01.T01 | Caffeoylshikimate esterase |
| augustus-1-processed-gene-123.7-mRNA-1 | Japonica | NA | | | OsR498G0100809200.01.T01 | |
| augustus-1-processed-gene-125.19-mRNA-1 | Japonica | NA | | | | |
| augustus-1-processed-gene-125.21-mRNA-1 | Japonica | NA | | | | Putative mudrA protein-maize transposon MuDR |
| augustus-1-processed-gene-125.23-mRNA-1 | Japonica | NA | | | OsR498G0100824700.01.T01 | Carboxymethylenebutenolidase homolog |
| augustus-1-processed-gene-127.18-mRNA-1 | Japonica | Japonica | | Os01g0327000 | OsR498G0100832700.01.T01 | Peroxidase |
| augustus-1-processed-gene-129.4-mRNA-1 | Japonica | NA | | Os01g0330500 | OsR498G0100844100.01.T01 | Organic cation/carnitine transporter 3 |
| augustus-1-processed-gene-13.21-mRNA-1 | Japonica | NA | | Os01g0125600 | OsR498G0100084700.01.T01 | Putative heavy metal-associated domain, HMA |
| augustus-1-processed-gene-130.3-mRNA-1 | Japonica | NA | | Os10g0181100 | OsR498G0509574000.01.T01 | |
| augustus-1-processed-gene-133.14-mRNA-1 | Japonica | NA | | | OsR498G0100073600.01.T01 | RNA binding protein |
| augustus-1-processed-gene-134.35-mRNA-1 | Japonica | NA | | Os01g0337500 | OsR498G0100861300.01.T01 | H(+)-exporting diphosphatase |

| | | | | | | |
|---|---|---|---|---|---|---|
| augustus-1-processed-gene-134.47-mRNA-1 | Japonica | NA | | Os01g0338200 | OsR498G0100863300.01.T01 | AMSH-like ubiquitin thiolesterase 3 |
| augustus-1-processed-gene-135.19-mRNA-1 | Japonica | NA | | Os01g0339400 | | |
| augustus-1-processed-gene-135.33-mRNA-1 | Japonica | NA | | | OsR498G0100867100.01.T01 | MADS-box domain-containing protein |
| augustus-1-processed-gene-136.18-mRNA-1 | Japonica | NA | | Os01g0342200 | | Cysteine-rich receptor-like protein kinase 15 |
| augustus-1-processed-gene-137.0-mRNA-1 | Japonica | NA | | Os01g0342700 | OsR498G0100880900.01.T01 | Pleiotropic drug resistance protein 4 |
| augustus-1-processed-gene-137.12-mRNA-1 | Japonica | NA | | Os01g0343200 | OsR498G0100883700.01.T06 | Importin subunit alpha |
| augustus-1-processed-gene-139.30-mRNA-1 | Japonica | Japonica | | Os01g0347300 | OsR498G0100897900.01.T02 | |
| augustus-1-processed-gene-14.19-mRNA-1 | Japonica | NA | | | OsR498G0511427100.01.T01 | Epstein-Barr virus EBNA-1-like |
| augustus-1-processed-gene-140.15-mRNA-1 | Japonica | NA | | Os01g0348700 | OsR498G0100901500.01.T01 | 60S ribosomal protein like |
| augustus-1-processed-gene-141.8-mRNA-1 | Japonica | NA | | Os01g0350000 | OsR498G0100905900.01.T01 | (Z)-3-hexen-1-ol acetyltransferase |
| augustus-1-processed-gene-142.10-mRNA-1 | Japonica | NA | | Os01g0351300 | OsR498G0100909500.01.T03 | Exocyst complex component |
| augustus-1-processed-gene-142.12-mRNA-1 | Japonica | NA | | Os01g0351800 | OsR498G0100911700.01.T01 | Fe2OG dioxygenase domain-containing protein (Fragment) |
| augustus-1-processed-gene-144.31-mRNA-1 | Japonica | NA | | Os01g0355000 | OsR498G0100917100.01.T04 | Pentatricopeptide repeat-containing protein, chloroplastic |
| augustus-1-processed-gene-144.33-mRNA-1 | Japonica | NA | | Os01g0355400 | | MATH domain-containing protein |
| augustus-1-processed-gene-145.20-mRNA-1 | Japonica | NA | | | OsR498G0100919100.01.T05 | probable phosphoinositide phosphatase SAC9 |
| augustus-1-processed-gene-146.10-mRNA-1 | Japonica | Indica | | Os01g0357100 | OsR498G0100931900.01.T01 | Ferredoxin--nitrite reductase chloroplastic |
| augustus-1-processed-gene-146.13-mRNA-1 | Japonica | NA | | Os01g0357800 | OsR498G0100933100.01.T05 | Pentatricopeptide (PPR) repeat-containing protein-like |
| augustus-1-processed-gene-151.21-mRNA-1 | Japonica | Indica | CC-Kinase-TM | Os01g0364400 | OsR498G0100957400.01.T01 | Wall-associated receptor kinase 3 |
| augustus-1-processed-gene-152.17-mRNA-1 | Japonica | NA | CC-Kinase | Os01g0367700 | OsR498G0100962700.01.T05 | probable serine/threonine-protein kinase At1g54610 |
| augustus-1-processed-gene-153.3-mRNA-1 | Japonica | NA | | Os01g0368700 | OsR498G0100966100.01.T01 | DUF679 domain membrane protein 7 |
| augustus-1-processed-gene-154.5-mRNA-1 | Japonica | NA | | | OsR498G0511458200.01.T01 | |
| augustus-1-processed-gene-154.7-mRNA-1 | Japonica | NA | | Os01g0370600 | OsR498G0100971500.01.T01 | Glutathione transferase |
| augustus-1-processed-gene-155.25-mRNA-1 | Japonica | NA | | | OsR498G0100979300.01.T01 | DUF4220 domain-containing protein (Fragment) |
| augustus-1-processed-gene-159.16-mRNA-1 | Japonica | NA | | Os03g0412050 | | Transposon protein, putative, Mutator sub-class, expressed |
| augustus-1-processed-gene-160.8-mRNA-1 | Japonica | NA | | Os01g0380800 | OsR498G0100999600.01.T01 | F-box domain-containing protein |
| augustus-1-processed-gene-162.13-mRNA-1 | Japonica | NA | | | OsR498G0612612900.01.T01 | DUF834 domain-containing protein |
| augustus-1-processed-gene-162.22-mRNA-1 | Japonica | NA | | | | |
| augustus-1-processed-gene-162.23-mRNA-1 | Japonica | NA | | Os07g0543700 | OsR498G0408675500.01.T01 | 4-diphosphocytidyl-2C-methyl-D-erythritol synthase |
| augustus-1-processed-gene-162.24-mRNA-1 | Japonica | NA | | Os01g0383900 | OsR498G0101004300.01.T01 | Nuclear pore complex protein NUP98A |
| augustus-1-processed-gene-162.25-mRNA-1 | Japonica | Indica | Kinase-TM | Os01g0384300 | OsR498G0101005100.01.T01 | LRR receptor-like serine/threonine-protein kinase RKF3 |
| augustus-1-processed-gene-166.18-mRNA-1 | Japonica | NA | | Os01g0389700 | OsR498G0101026700.01.T01 | DUF679 domain membrane protein 7 |
| augustus-1-processed-gene-166.23-mRNA-1 | Japonica | NA | | Os01g0390600 | OsR498G0101029900.01.T05 | tRNA (guanine(37)-N1)-methyltransferase |
| augustus-1-processed-gene-166.3-mRNA-1 | Japonica | NA | | Os01g0389200 | OsR498G0101025500.01.T01 | DUF679 domain membrane protein 7 |
| augustus-1-processed-gene-179.10-mRNA-1 | Japonica | NA | | Os03g0154300 | OsR498G0203061400.01.T01 | |
| augustus-1-processed-gene-180.6-mRNA-1 | Japonica | NA | | Os01g0500900 | OsR498G0101059100.01.T01 | Tyrosine--tRNA ligase |
| augustus-1-processed-gene-181.31-mRNA-1 | Japonica | NA | | | OsR498G0101068100.01.T01 | |
| augustus-1-processed-gene-183.20-mRNA-1 | Japonica | NA | | | OsR498G0101077500.01.T01 | Retrotransposon protein, putative, unclassified |
| augustus-1-processed-gene-184.10-mRNA-1 | Japonica | NA | | Os01g0507500 | OsR498G0101082100.01.T01 | Ell-associated factor Eaf like |
| augustus-1-processed-gene-185.0-mRNA-1 | Japonica | NA | | Os01g0508100 | OsR498G0101085900.01.T01 | |
| augustus-1-processed-gene-186.0-mRNA-1 | Japonica | NA | | | OsR498G0510283100.01.T01 | Retrotransposon protein, putative, unclassified |
| augustus-1-processed-gene-187.14-mRNA-1 | Japonica | NA | | | OsR498G0101105700.01.T01 | AP-3 complex subunit delta |
| augustus-1-processed-gene-188.1-mRNA-1 | Japonica | Japonica | | Os01g0512700 | OsR498G0101106700.01.T01 | Yin/yang transcription factor |
| augustus-1-processed-gene-191.13-mRNA-1 | Japonica | NA | | | OsR498G0101121100.01.T01 | Luminal-binding protein 4 |
| augustus-1-processed-gene-194.22-mRNA-1 | Japonica | NA | | | OsR498G0101140500.01.T01 | |
| augustus-1-processed-gene-198.17-mRNA-1 | Japonica | NA | | | OsR498G0713993600.01.T01 | Putative zinc finger, CCHC-type |
| augustus-1-processed-gene-198.5-mRNA-1 | Japonica | NA | | Os01g0530400 | OsR498G0101163500.01.T07 | Monothiol glutaredoxin |
| augustus-1-processed-gene-199.19-mRNA-1 | Japonica | NA | | Os01g0532100 | OsR498G0101168500.01.T01 | |
| augustus-1-processed-gene-199.31-mRNA-1 | Japonica | NA | | | | Condensin complex subunit 2 |
| augustus-1-processed-gene-2.43-mRNA-1 | Japonica | Indica | | Os01g0102400 | OsR498G0100011300.01.T01 | Histone domain-containing protein |
| augustus-1-processed-gene-2.48-mRNA-1 | Japonica | NA | | Os01g0103100 | OsR498G0100013300.01.T01 | Major facilitator superfamily protein isoform 1 |
| augustus-1-processed-gene-221.13-mRNA-1 | Japonica | NA | | Os01g0563000 | OsR498G0101346000.01.T07 | Peptidylprolyl isomerase |
| augustus-1-processed-gene-223.13-mRNA-1 | Japonica | NA | | | OsR498G0101356600.01.T01 | |
| augustus-1-processed-gene-226.45-mRNA-1 | Japonica | NA | | | OsR498G0101371100.01.T01 | |
| augustus-1-processed-gene-226.46-mRNA-1 | Japonica | NA | | | OsR498G0101371500.01.T01 | |
| augustus-1-processed-gene-230.28-mRNA-1 | Japonica | NA | | | OsR498G0101393500.01.T01 | Regulatory protein B-Peru-like |
| augustus-1-processed-gene-231.38-mRNA-1 | Japonica | NA | | Os01g0578800 | | |
| augustus-1-processed-gene-231.54-mRNA-1 | Japonica | NA | | Os01g0579800 | OsR498G0101399700.01.T01 | DEAD-box ATP-dependent RNA helicase 20 |
| augustus-1-processed-gene-232.42-mRNA-1 | Japonica | NA | | | OsR498G0101403900.01.T01 | |
| augustus-1-processed-gene-233.12-mRNA-1 | Japonica | NA | | | | Translation initiation factor |
| augustus-1-processed-gene-233.25-mRNA-1 | Japonica | NA | | | | Zinc ion binding |
| augustus-1-processed-gene-235.12-mRNA-1 | Japonica | Indica | | Os01g0597400 | OsR498G0101422900.01.T01 | Vacuolar amino acid transporter like |

| | | | | | | |
|---|---|---|---|---|---|---|
| augustus-1-processed-gene-237.19-mRNA-1 | Japonica | NA | | Os01g0589300 | OsR498G0101436500.01.T01 | MYB transcription factor |
| augustus-1-processed-gene-238.17-mRNA-1 | Japonica | NA | | Os02g0547100 | | Transposon protein, putative, Mutator sub-class, expressed |
| augustus-1-processed-gene-239.8-mRNA-1 | Japonica | NA | | Os01g0593600 | OsR498G0101455300.01.T01 | |
| augustus-1-processed-gene-241.11-mRNA-1 | Japonica | NA | | Os07g0433200 | OsR498G0510328900.01.T01 | Retrotransposon protein, putative, Ty1-copia subclass |
| augustus-1-processed-gene-258.26-mRNA-1 | Japonica | Japonica | | Os01g0629600 | OsR498G0511411500.01.T01 | Carboxypeptidase |
| augustus-1-processed-gene-263.18-mRNA-1 | Japonica | NA | | Os01g0638000 | OsR498G0101614100.01.T01 | Glycosyltransferase |
| augustus-1-processed-gene-263.38-mRNA-1 | Japonica | NA | | Os01g0638600 | OsR498G0101615700.01.T01 | Glycosyltransferase |
| augustus-1-processed-gene-266.23-mRNA-1 | Japonica | Japonica | | Os01g0643900 | OsR498G0511392000.01.T01 | |
| augustus-1-processed-gene-266.30-mRNA-1 | Japonica | NA | | Os01g0644900 | OsR498G0101640300.01.T01 | |
| augustus-1-processed-gene-267.31-mRNA-1 | Japonica | NA | | | OsR498G0101644900.01.T01 | Pentatricopeptide repeat |
| augustus-1-processed-gene-267.50-mRNA-1 | Japonica | NA | | Os01g0646300 | OsR498G0101645600.01.T01 | DELLA transcription factor |
| augustus-1-processed-gene-269.39-mRNA-1 | Japonica | Japonica | | Os01g0649300 | OsR498G0101656100.01.T02 | GDSL esterase/lipase |
| augustus-1-processed-gene-269.56-mRNA-1 | Japonica | NA | | | OsR498G1119312200.01.T01 | Non-specific serine/threonine protein kinase |
| augustus-1-processed-gene-269.58-mRNA-1 | Japonica | NA | | Os01g0650200 | OsR498G0101658300.01.T01 | GDSL esterase/lipase |
| augustus-1-processed-gene-269.60-mRNA-1 | Japonica | NA | | | OsR498G0204046800.01.T01 | Epstein-Barr virus EBNA-1-like |
| augustus-1-processed-gene-270.28-mRNA-1 | Japonica | Indica | | Os01g0651100 | OsR498G0101660900.01.T05 | Pentatricopeptide repeat-containing protein |
| augustus-1-processed-gene-270.42-mRNA-1 | Japonica | NA | | Os01g0651500 | OsR498G0101661700.01.T01 | Wax synthase isoform 3 |
| augustus-1-processed-gene-271.11-mRNA-1 | Japonica | NA | | Os01g0653200 | OsR498G0101668900.01.T01 | UDP-Glycosyltransferase superfamily protein |
| augustus-1-processed-gene-271.2-mRNA-1 | Japonica | NA | | Os01g0652700 | OsR498G0101665900.01.T01 | Calcineurin B-like protein 1 |
| augustus-1-processed-gene-273.41-mRNA-1 | Japonica | NA | | | OsR498G0101679300.01.T01 | |
| augustus-1-processed-gene-273.66-mRNA-1 | Japonica | NA | | | | Eukaryotic translation initiation factor 3 subunit 10 |
| augustus-1-processed-gene-275.15-mRNA-1 | Japonica | Indica | | Os01g0659800 | OsR498G0101693700.01.T01 | C2 domain-containing protein |
| augustus-1-processed-gene-275.17-mRNA-1 | Japonica | NA | | Os01g0660200 | OsR498G0101694700.01.T03 | Acidic endochitinase |
| augustus-1-processed-gene-275.8-mRNA-1 | Japonica | NA | | Os01g0658300 | | Glutaminyl-tRNA synthetase |
| augustus-1-processed-gene-277.26-mRNA-1 | Japonica | NA | | Os01g0664100 | OsR498G0101705900.01.T02 | zinc transport protein ZntB-like isoform X2 |
| augustus-1-processed-gene-278.1-mRNA-1 | Japonica | NA | | | OsR498G0101707500.01.T01 | |
| augustus-1-processed-gene-278.5-mRNA-1 | Japonica | NA | | Os02g0733600 | OsR498G1018053500.01.T01 | |
| augustus-1-processed-gene-28.49-mRNA-1 | Japonica | NA | | Os01g0151900 | OsR498G0100184700.01.T01 | Pentatricopeptide repeat |
| augustus-1-processed-gene-280.9-mRNA-1 | Japonica | NA | | | OsR498G0101725500.01.T01 | |
| augustus-1-processed-gene-281.14-mRNA-1 | Japonica | NA | | | OsR498G0612788100.01.T01 | |
| augustus-1-processed-gene-283.12-mRNA-1 | Japonica | NA | | | OsR498G0100324300.01.T01 | Cyclic dof factor like |
| augustus-1-processed-gene-285.22-mRNA-1 | Japonica | Indica | | Os01g0677900 | OsR498G0101757700.01.T01 | CCHC-type domain-containing protein |
| augustus-1-processed-gene-285.23-mRNA-1 | Japonica | Japonica | | Os01g0678000 | OsR498G0101758100.01.T01 | CCHC-type domain-containing protein |
| augustus-1-processed-gene-285.24-mRNA-1 | Japonica | NA | | Os01g0678100 | | |
| augustus-1-processed-gene-286.15-mRNA-1 | Japonica | NA | | Os01g0680200 | OsR498G0101765100.01.T01 | Purine permease |
| augustus-1-processed-gene-286.9-mRNA-1 | Japonica | NA | | Os01g0679500 | | Peptidase A1 domain-containing protein (Fragment) |
| augustus-1-processed-gene-287.1-mRNA-1 | Japonica | NA | | Os01g0680600 | OsR498G0101767700.01.T01 | TAFII28 domain-containing protein |
| augustus-1-processed-gene-289.45-mRNA-1 | Japonica | NA | | Os01g0685000 | | USP domain-containing protein |
| augustus-1-processed-gene-289.49-mRNA-1 | Japonica | Japonica | | Os01g0686000 | OsR498G0101785300.01.T01 | DUF4228 domain-containing protein (Fragment) |
| augustus-1-processed-gene-289.51-mRNA-1 | Japonica | NA | | Os01g0686200 | OsR498G0101785900.01.T01 | Glycosyltransferase |
| augustus-1-processed-gene-290.33-mRNA-1 | Japonica | NA | | Os01g0686300 | OsR498G0101786500.01.T01 | UDP-glucosyltransferase |
| augustus-1-processed-gene-290.35-mRNA-1 | Japonica | Indica | | Os01g0686500 | OsR498G0101787300.01.T01 | |
| augustus-1-processed-gene-290.55-mRNA-1 | Japonica | Indica | | Os05g0551700 | OsR498G0101792300.01.T01 | Adenylate isopentenyltransferase |
| augustus-1-processed-gene-291.37-mRNA-1 | Japonica | NA | | Os05g0269800 | OsR498G0409509300.01.T01 | Transposon protein, putative, CACTA, En/Spm sub-class |
| augustus-1-processed-gene-292.59-mRNA-1 | Japonica | Indica | | Os01g0691600 | OsR498G0101801900.01.T03 | DNA helicase |
| augustus-1-processed-gene-295.15-mRNA-1 | Japonica | Indica | | Os01g0696800 | OsR498G0101823100.01.T01 | Peptidase A1 domain-containing protein (Fragment) |
| augustus-1-processed-gene-296.25-mRNA-1 | Japonica | Japonica | Kinase-TM | Os01g0699400 | OsR498G0101831600.01.T01 | Mitogen-activated protein kinase kinase kinase A |
| augustus-1-processed-gene-298.58-mRNA-1 | Japonica | Indica | | Os01g0704100 | OsR498G0101845100.01.T01 | Nitrate transporter |
| augustus-1-processed-gene-299.40-mRNA-1 | Japonica | NA | | Os01g0705700 | OsR498G0101853300.01.T01 | BHLH transcription factor |
| augustus-1-processed-gene-3.52-mRNA-1 | Japonica | NA | | Os01g0104400 | OsR498G0100016300.01.T01 | Stress responsive protein 1 |
| augustus-1-processed-gene-300.27-mRNA-1 | Japonica | NA | | Os01g0706600 | OsR498G0101856700.01.T01 | |
| augustus-1-processed-gene-300.54-mRNA-1 | Japonica | NA | | Os07g0513300 | | Transposon protein, putative, Mutator sub-class, expressed |
| augustus-1-processed-gene-301.49-mRNA-1 | Japonica | NA | | Os01g0708100 | OsR498G0101861900.01.T01 | Glycylpeptide N-tetradecanoyltransferase |
| augustus-1-processed-gene-301.61-mRNA-1 | Japonica | NA | | | OsR498G0307184000.01.T01 | DH domain-containing protein |
| augustus-1-processed-gene-302.67-mRNA-1 | Japonica | NA | | | OsR498G0101872100.01.T01 | |
| augustus-1-processed-gene-304.43-mRNA-1 | Japonica | Indica | | Os01g0715200 | OsR498G0101882900.01.T01 | |
| augustus-1-processed-gene-304.48-mRNA-1 | Japonica | Japonica | | Os01g0715700 | OsR498G0101885900.01.T01 | Pentatricopeptide repeat |
| augustus-1-processed-gene-307.31-mRNA-1 | Japonica | NA | | | OsR498G0101907500.01.T03 | GDSL esterase/lipase |
| augustus-1-processed-gene-307.41-mRNA-1 | Japonica | Japonica | | Os01g0720500 | OsR498G0101476300.01.T01 | Chlorophyll a-b binding protein, chloroplastic |
| augustus-1-processed-gene-307.44-mRNA-1 | Japonica | NA | | Os01g0721000 | OsR498G0101910700.01.T05 | Fusarium resistance protein I2C-5-like |
| augustus-1-processed-gene-307.47-mRNA-1 | Japonica | Indica | | Os01g0721500 | OsR498G0101911900.01.T01 | Fusarium resistance protein I2C-5-like |

| | | | | | | |
|---|---|---|---|---|---|---|
| augustus-1-processed-gene-308.60-mRNA-1 | Japonica | NA | | Os01g0722800 | OsR498G0101916100.01.T01 | 4-hydroxy-4-methyl-2-oxoglutarate aldolase |
| augustus-1-processed-gene-308.66-mRNA-1 | Japonica | Indica | | Os01g0723400 | OsR498G0101918500.01.T03 | Malic enzyme |
| augustus-1-processed-gene-309.10-mRNA-1 | Japonica | NA | | Os07g0121500 | | |
| augustus-1-processed-gene-309.9-mRNA-1 | Japonica | Japonica | | Os01g0724700 | OsR498G0101922900.01.T01 | Lachrymatory-factor synthase |
| augustus-1-processed-gene-310.17-mRNA-1 | Japonica | NA | | | OsR498G0101932500.01.T04 | |
| augustus-1-processed-gene-311.1-mRNA-1 | Japonica | NA | LRR-TM | Os01g0729400 | OsR498G0101933900.01.T01 | Receptor like protein 29 |
| augustus-1-processed-gene-312.11-mRNA-1 | Japonica | NA | | | OsR498G0101940700.01.T01 | Ubiquitin-60S ribosomal protein L40 |
| augustus-1-processed-gene-312.15-mRNA-1 | Japonica | NA | | Os01g0732300 | OsR498G0101942300.01.T01 | Transcription repressor |
| augustus-1-processed-gene-313.24-mRNA-1 | Japonica | NA | | Os01g0733500 | OsR498G0101946900.01.T03 | BURP domain-containing protein 3 |
| augustus-1-processed-gene-313.38-mRNA-1 | Japonica | NA | | Os01g0734501 | | |
| augustus-1-processed-gene-313.39-mRNA-1 | Japonica | NA | | Os01g0734600 | OsR498G0101949900.01.T03 | Glycosyltransferase |
| augustus-1-processed-gene-313.43-mRNA-1 | Japonica | NA | | Os01g0735300 | OsR498G0101951500.01.T01 | Glycosyltransferase |
| augustus-1-processed-gene-314.51-mRNA-1 | Japonica | NA | | Os02g0212975 | OsR498G0305421900.01.T01 | |
| augustus-1-processed-gene-314.54-mRNA-1 | Japonica | NA | | Os01g0737600 | OsR498G0101957500.01.T01 | Werner syndrome ATP-dependent helicase |
| augustus-1-processed-gene-314.55-mRNA-1 | Japonica | NA | | Os01g0737700 | OsR498G0101957700.01.T01 | 3'-5' exonuclease domain-containing protein (Fragment) |
| augustus-1-processed-gene-315.5-mRNA-1 | Japonica | NA | | Os01g0738400 | OsR498G0101960500.01.T01 | Zinc finger CCCH domain-containing protein 2 |
| augustus-1-processed-gene-316.39-mRNA-1 | Japonica | NA | | Os01g0740800 | | |
| augustus-1-processed-gene-322.37-mRNA-1 | Japonica | NA | | | OsR498G0102004100.01.T01 | F-box domain-containing protein (Fragment) |
| augustus-1-processed-gene-322.48-mRNA-1 | Japonica | Japonica | | Os01g0751400 | OsR498G0511182600.01.T01 | Inorganic diphosphatase |
| augustus-1-processed-gene-322.54-mRNA-1 | Japonica | NA | | Os01g0752500 | | Pathogenesis-related transcriptional activator like |
| augustus-1-processed-gene-323.15-mRNA-1 | Japonica | NA | | Os01g0753800 | OsR498G0102010900.01.T02 | Translation initiation factor IF-2-like |
| augustus-1-processed-gene-324.26-mRNA-1 | Japonica | NA | | Os01g0755700 | OsR498G0102015300.01.T01 | RING-H2 finger protein ATL2K |
| augustus-1-processed-gene-325.45-mRNA-1 | Japonica | NA | | | OsR498G0204046800.01.T01 | Circumsporozoite protein-like |
| augustus-1-processed-gene-327.0-mRNA-1 | Japonica | Indica | | Os01g0760000 | OsR498G0102038900.01.T02 | Dynein light chain |
| augustus-1-processed-gene-327.1-mRNA-1 | Japonica | Indica | | Os01g0760300 | OsR498G0102039300.01.T02 | PRONE domain-containing protein |
| augustus-1-processed-gene-327.10-mRNA-1 | Japonica | NA | | Os01g0760600 | OsR498G0102040200.01.T01 | Aspartate aminotransferase |
| augustus-1-processed-gene-327.12-mRNA-1 | Japonica | NA | | Os01g0761000 | OsR498G0102040900.01.T03 | Cyanobacterial aminoacyl-tRNA synthetase, CAAD domain containing protei |
| augustus-1-processed-gene-328.52-mRNA-1 | Japonica | Japonica | | Os01g0763650 | OsR498G0102047500.01.T02 | Chlororespiratory reduction 7 |
| augustus-1-processed-gene-328.53-mRNA-1 | Japonica | NA | | Os01g0763700 | OsR498G0102047500.01.T03 | Exocyst subunit Exo70 family protein (Fragment) |
| augustus-1-processed-gene-329.51-mRNA-1 | Japonica | NA | | | OsR498G0102053700.01.T01 | Zinc finger A20 and AN1 domain-containing stress-associated protein 3 |
| augustus-1-processed-gene-331.36-mRNA-1 | Japonica | NA | | Os01g0768333 | | |
| augustus-1-processed-gene-331.38-mRNA-1 | Japonica | NA | | | OsR498G0204046800.01.T01 | Circumsporozoite protein-like |
| augustus-1-processed-gene-331.40-mRNA-1 | Japonica | NA | | Os02g0547100 | OsR498G0510938300.01.T01 | Transposon mutator sub-class |
| augustus-1-processed-gene-331.48-mRNA-1 | Japonica | NA | | Os01g0769000 | OsR498G0102062300.01.T06 | Topoisomerase II-associated protein PAT1 |
| augustus-1-processed-gene-331.52-mRNA-1 | Japonica | NA | Kinase-TM | Os01g0769700 | OsR498G0102064500.01.T01 | Receptor-like protein kinase FERONIA |
| augustus-1-processed-gene-334.41-mRNA-1 | Japonica | NA | | Os01g0774300 | | DUF295 domain-containing protein |
| augustus-1-processed-gene-334.43-mRNA-1 | Japonica | NA | | | OsR498G0511427100.01.T01 | Epstein-Barr virus EBNA-1-like |
| augustus-1-processed-gene-335.9-mRNA-1 | Japonica | NA | | Os01g0776300 | | Gnk2-homologous domain-containing protein |
| augustus-1-processed-gene-336.57-mRNA-1 | Japonica | Indica | | Os01g0778100 | OsR498G0102093700.01.T01 | Protein UPSTREAM OF FLC isoform B |
| augustus-1-processed-gene-337.1-mRNA-1 | Japonica | NA | Kinase-TM | Os01g0779300 | OsR498G0102097100.01.T01 | Non-specific serine/threonine protein kinase |
| augustus-1-processed-gene-338.41-mRNA-1 | Japonica | NA | | Os01g0783100 | OsR498G0102106700.01.T04 | Pentatricopeptide repeat-containing protein |
| augustus-1-processed-gene-339.2-mRNA-1 | Japonica | Japonica | Kinase-TM | Os01g0784200 | OsR498G0102109300.01.T01 | Receptor-like serine/threonine-protein kinase |
| augustus-1-processed-gene-339.20-mRNA-1 | Japonica | NA | | Os01g0784800 | OsR498G0102111100.01.T02 | NAD(P)H dehydrogenase (quinone) |
| augustus-1-processed-gene-339.26-mRNA-1 | Japonica | NA | | Os01g0785700 | OsR498G0102115700.01.T05 | Pentatricopeptide repeat-containing protein mitochondrial |
| augustus-1-processed-gene-339.4-mRNA-1 | Japonica | NA | Kinase-TM | Os01g0784200 | | Non-specific serine/threonine protein kinase |
| augustus-1-processed-gene-341.6-mRNA-1 | Japonica | NA | | Os03g0110200 | | Transposon protein, putative, Mutator sub-class |
| augustus-1-processed-gene-342.1-mRNA-1 | Japonica | Indica | | Os01g0793200 | OsR498G0102128300.01.T02 | Pentatricopeptide repeat-containing protein mitochondrial |
| augustus-1-processed-gene-343.10-mRNA-1 | Japonica | NA | | | | Inositol-1,4,5-trisphosphate 5-phosphatase 4 isoform 1 |
| augustus-1-processed-gene-343.19-mRNA-1 | Japonica | NA | | | OsR498G0100073600.01.T01 | RNA binding protein |
| augustus-1-processed-gene-343.25-mRNA-1 | Japonica | NA | | Os01g0795600 | OsR498G0102135900.01.T01 | |
| augustus-1-processed-gene-343.28-mRNA-1 | Japonica | Japonica | | Os01g0796001 | OsR498G0102135900.01.T01 | Type II secretion system protein D |
| augustus-1-processed-gene-344.34-mRNA-1 | Japonica | NA | | Os01g0797600 | OsR498G0102141700.01.T01 | Ethylene responsive element binding factor3 |
| augustus-1-processed-gene-345.19-mRNA-1 | Japonica | NA | | Os01g0800400 | OsR498G0102184800.01.T02 | Spo11/DNA topoisomerase VI, subunit A protein |
| augustus-1-processed-gene-345.2-mRNA-1 | Japonica | NA | NBS-TM | Os01g0799100 | OsR498G0102144900.01.T02 | NB-ARC domain-containing protein |
| augustus-1-processed-gene-346.23-mRNA-1 | Japonica | NA | | | | AAI domain-containing protein |
| augustus-1-processed-gene-346.33-mRNA-1 | Japonica | Indica | | Os05g0495200 | OsR498G0102154100.01.T04 | Basic leucine zipper like |
| augustus-1-processed-gene-346.34-mRNA-1 | Japonica | NA | | Os01g0802000 | OsR498G0102154700.01.T01 | RING-type E3 ubiquitin transferase |
| augustus-1-processed-gene-348.38-mRNA-1 | Japonica | Indica | | Os01g0805100 | OsR498G0102165700.01.T01 | Glycoside hydrolase starch-binding domain-containing family protein |
| augustus-1-processed-gene-38.5-mRNA-1 | Japonica | NA | | | OsR498G0511311000.01.T01 | |
| augustus-1-processed-gene-39.50-mRNA-1 | Japonica | NA | | Os01g0175500 | OsR498G0100251500.01.T01 | DUF1618 domain-containing protein (Fragment) |
| augustus-1-processed-gene-39.73-mRNA-1 | Japonica | NA | | Os01g0176800 | OsR498G0100256900.01.T01 | |

| | | | | | | |
|---|---|---|---|---|---|---|
| augustus-1-processed-gene-4.42-mRNA-1 | Japonica | NA | | Os01g0105500 | | |
| augustus-1-processed-gene-4.45-mRNA-1 | Japonica | NA | | Os01g0106200 | OsR498G0100022900.01.T01 | Peptidylprolyl isomerase |
| augustus-1-processed-gene-40.0-mRNA-1 | Japonica | NA | | Os01g0177100 | OsR498G0100258100.01.T04 | Transcriptional corepressor LEUNIG |
| augustus-1-processed-gene-40.17-mRNA-1 | Japonica | NA | | | OsR498G0100261400.01.T01 | Retrotransposon protein, putative, unclassified, expressed |
| augustus-1-processed-gene-41.38-mRNA-1 | Japonica | NA | | Os01g0178600 | OsR498G0100265100.01.T01 | Peptidase A1 domain-containing protein |
| augustus-1-processed-gene-41.46-mRNA-1 | Japonica | NA | | Os01g0178700 | OsR498G0100266100.01.T05 | RING-type domain-containing protein (Fragment) |
| augustus-1-processed-gene-45.13-mRNA-1 | Japonica | NA | | Os01g0183800 | OsR498G0100284500.01.T01 | DUF295 domain-containing protein |
| augustus-1-processed-gene-45.20-mRNA-1 | Japonica | Japonica | | Os01g0184700 | OsR498G0100286300.01.T01 | FACT complex subunit SSRP1 |
| augustus-1-processed-gene-475.12-mRNA-1 | Japonica | NA | | Os01g0913800 | OsR498G0102563100.01.T01 | Fertility restorer homologue-like |
| augustus-1-processed-gene-476.50-mRNA-1 | Japonica | NA | | Os09g0552200 | OsR498G0306910900.01.T01 | Zinc finger (C3HC4 type RING finger) protein, putative |
| augustus-1-processed-gene-478.3-mRNA-1 | Japonica | Japonica | | Os01g0919300 | OsR498G0102583100.01.T01 | Sad1/UNC domain-containing protein, putative |
| augustus-1-processed-gene-479.17-mRNA-1 | Japonica | Japonica | | Os01g0921100 | OsR498G0102592100.01.T01 | Regulatory protein-like |
| augustus-1-processed-gene-479.20-mRNA-1 | Japonica | Indica | | Os01g0921400 | OsR498G0102593100.01.T01 | Exocyst subunit Exo70 family protein (Fragment) |
| augustus-1-processed-gene-480.10-mRNA-1 | Japonica | NA | | Os08g0561800 | OsR498G1120733700.01.T01 | Retrotransposon protein, putative, Ty3-gypsy sub-class |
| augustus-1-processed-gene-481.12-mRNA-1 | Japonica | NA | | Os01g0923800 | OsR498G0102610500.01.T01 | J domain-containing protein |
| augustus-1-processed-gene-481.14-mRNA-1 | Japonica | NA | | Os01g0924200 | OsR498G0102612100.01.T01 | B-cell lymphoma leukemia 11A isoform X1 |
| augustus-1-processed-gene-482.0-mRNA-1 | Japonica | NA | | Os01g0924400 | OsR498G0102613700.01.T03 | TCP transcription factor |
| augustus-1-processed-gene-482.2-mRNA-1 | Japonica | NA | | Os01g0924900 | OsR498G0102614900.01.T01 | glutamic acid-rich protein isoform X2 |
| augustus-1-processed-gene-484.34-mRNA-1 | Japonica | NA | Kinase-TM | Os01g0927500 | OsR498G0102625300.01.T03 | Inactive leucine-rich repeat receptor-like protein kinase CORYNE |
| augustus-1-processed-gene-488.1-mRNA-1 | Japonica | Japonica | | Os01g0936000 | OsR498G0102656100.01.T01 | Glutaredoxin domain-containing protein (Fragment) |
| augustus-1-processed-gene-489.1-mRNA-1 | Japonica | NA | | Os01g0937600 | OsR498G0102663900.01.T01 | Basic 7S globulin 2 |
| augustus-1-processed-gene-489.2-mRNA-1 | Japonica | NA | | Os01g0937800 | OsR498G0102664300.01.T01 | Basic 7S globulin 2 |
| augustus-1-processed-gene-489.22-mRNA-1 | Japonica | NA | | Os01g0939500 | OsR498G0102668500.01.T01 | Eukaryotic peptide chain release factor subunit |
| augustus-1-processed-gene-489.3-mRNA-1 | Japonica | NA | | Os01g0937900 | OsR498G0102664700.01.T01 | Classical arabinogalactan protein 26-like |
| augustus-1-processed-gene-489.4-mRNA-1 | Japonica | NA | | | OsR498G0102665100.01.T01 | PPR-repeat protein-like |
| augustus-1-processed-gene-492.12-mRNA-1 | Japonica | NA | | | OsR498G0102679100.01.T01 | |
| augustus-1-processed-gene-492.24-mRNA-1 | Japonica | NA | | | OsR498G0102681100.01.T07 | Retrotransposon protein, putative, unclassified |
| augustus-1-processed-gene-493.14-mRNA-1 | Japonica | NA | | | | ENTH domain-containing protein |
| augustus-1-processed-gene-493.15-mRNA-1 | Japonica | NA | | | OsR498G0102687100.01.T01 | |
| augustus-1-processed-gene-493.17-mRNA-1 | Japonica | NA | | | OsR498G0102687300.01.T01 | |
| augustus-1-processed-gene-493.2-mRNA-1 | Japonica | NA | | Os01g0941400 | OsR498G0102675700.01.T01 | Truncated pathogenesis-related protein 6 |
| augustus-1-processed-gene-496.59-mRNA-1 | Japonica | NA | | Os01g0950300 | OsR498G0102702900.01.T01 | Glutathione S-transferase GST 29 |
| augustus-1-processed-gene-497.8-mRNA-1 | Japonica | NA | | Os01g0953100 | OsR498G0102710700.01.T01 | 1-phosphatidylinositol 4,5-bisphosphate phosphodiesterase beta-1 |
| augustus-1-processed-gene-498.4-mRNA-1 | Japonica | Indica | | Os01g0955100 | OsR498G0102722100.01.T01 | Putative calcium-binding protein CML19 |
| augustus-1-processed-gene-499.37-mRNA-1 | Japonica | NA | | | OsR498G0102729500.01.T01 | |
| augustus-1-processed-gene-499.43-mRNA-1 | Japonica | NA | | | OsR498G0102733100.01.T01 | 5-epiaristolochene 13-dihydroxylase |
| augustus-1-processed-gene-5.14-mRNA-1 | Japonica | Japonica | | Os01g0108500 | OsR498G0101358900.01.T01 | BHLH domain-containing protein |
| augustus-1-processed-gene-500.93-mRNA-1 | Japonica | NA | | Os01g0960000 | OsR498G0102739900.01.T01 | probable gamma-secretase subunit PEN-2 |
| augustus-1-processed-gene-501.73-mRNA-1 | Japonica | NA | | Os01g0961000 | | |
| augustus-1-processed-gene-503.48-mRNA-1 | Japonica | Indica | | Os01g0964800 | OsR498G0102752500.01.T01 | E3 ubiquitin-protein ligase HERC2 |
| augustus-1-processed-gene-503.49-mRNA-1 | Japonica | NA | | Os01g0964900 | OsR498G0102752700.01.T01 | Protein MITOFERRINLIKE 1 chloroplastic |
| augustus-1-processed-gene-503.61-mRNA-1 | Japonica | NA | | | | Acyl-CoA N-acyltransferase with RING/FYVE/PHD-type zinc finger protein |
| augustus-1-processed-gene-503.63-mRNA-1 | Japonica | NA | | Os01g0966300 | OsR498G0102757100.01.T05 | Mitochondrial processing peptidase |
| augustus-1-processed-gene-504.23-mRNA-1 | Japonica | NA | | Os01g0968800 | OsR498G0102764300.01.T01 | Dehydration-responsive element-binding protein 1D |
| augustus-1-processed-gene-506.28-mRNA-1 | Japonica | NA | | Os01g0972000 | OsR498G0102775500.01.T01 | RING-type E3 ubiquitin transferase |
| augustus-1-processed-gene-507.75-mRNA-1 | Japonica | Japonica | | Os01g0974900 | OsR498G0204247700.01.T03 | 26S proteasome non-ATPase regulatory subunit 6 |
| augustus-1-processed-gene-508.13-mRNA-1 | Japonica | NA | | Os01g0976300 | | Protein SODIUM POTASSIUM ROOT DEFECTIVE 1 |
| augustus-1-processed-gene-508.32-mRNA-1 | Japonica | NA | | | | Retrotransposon protein, putative, unclassified, expressed |
| augustus-1-processed-gene-509.40-mRNA-1 | Japonica | NA | | | OsR498G0102792700.01.T01 | Extra-large GTP-binding protein 3 |
| augustus-1-processed-gene-509.44-mRNA-1 | Japonica | NA | | Os01g0978400 | | Cinnamoyl-CoA reductase |
| augustus-1-processed-gene-52.11-mRNA-1 | Japonica | NA | | Os01g0196600 | OsR498G0100340100.01.T01 | Venom phosphodiesterase 1 |
| augustus-1-processed-gene-53.40-mRNA-1 | Japonica | NA | | Os01g0197350 | OsR498G0100343500.01.T03 | |
| augustus-1-processed-gene-57.25-mRNA-1 | Japonica | Japonica | | Os01g0203400 | OsR498G0100368300.01.T01 | |
| augustus-1-processed-gene-6.45-mRNA-1 | Japonica | NA | | Os01g0109500 | OsR498G0100031500.01.T03 | |
| augustus-1-processed-gene-63.39-mRNA-1 | Japonica | NA | | | OsR498G0100414300.01.T01 | GDSL esterase/lipase |
| augustus-1-processed-gene-66.29-mRNA-1 | Japonica | NA | | Os01g0219300 | OsR498G0100429300.01.T01 | |
| augustus-1-processed-gene-66.31-mRNA-1 | Japonica | NA | | | | Transposable element protein, putative, MuDR |
| augustus-1-processed-gene-68.14-mRNA-1 | Japonica | NA | Kinase-TM | Os01g0223900 | OsR498G0100448100.01.T01 | Non-specific serine/threonine protein kinase (Fragment) |
| augustus-1-processed-gene-68.16-mRNA-1 | Japonica | NA | | Os01g0224100 | OsR498G0100451500.01.T01 | Ethylene-responsive transcription factor CRF2 |
| augustus-1-processed-gene-70.29-mRNA-1 | Japonica | NA | | | OsR498G0100464900.01.T01 | Transcription repressor |
| augustus-1-processed-gene-72.51-mRNA-1 | Japonica | NA | | Os01g0229300 | OsR498G0100474900.01.T01 | |

| | | | | | | |
|---|---|---|---|---|---|---|
| augustus-1-processed-gene-73.17-mRNA-1 | Japonica | NA | Kinase | Os01g0231500 | OsR498G0100481100.01.T06 | Casein kinase (Serine/threonine/tyrosine protein kinase) |
| augustus-1-processed-gene-73.19-mRNA-1 | Japonica | NA | | Os01g0231800 | OsR498G0100481700.01.T05 | Putative RNA ligase/cyclic nucleotide phosphodiesterase |
| augustus-1-processed-gene-74.23-mRNA-1 | Japonica | NA | | Os01g0233100 | | Cyclin N-terminal domain-containing protein (Fragment) |
| augustus-1-processed-gene-75.1-mRNA-1 | Japonica | Japonica | | Os01g0234300 | OsR498G0100490700.01.T01 | Pectinesterase |
| augustus-1-processed-gene-75.18-mRNA-1 | Japonica | Japonica | | Os01g0235500 | OsR498G0100494900.01.T01 | |
| augustus-1-processed-gene-75.2-mRNA-1 | Japonica | NA | | Os01g0234300 | OsR498G0100490700.01.T01 | Pectinesterase |
| augustus-1-processed-gene-75.20-mRNA-1 | Japonica | NA | | Os01g0235900 | OsR498G0100497300.01.T01 | Glutaredoxin domain-containing cysteine-rich protein 1 |
| augustus-1-processed-gene-75.21-mRNA-1 | Japonica | NA | | Os01g0236000 | OsR498G0100497500.01.T02 | Phytolongin Phyl1.1 |
| augustus-1-processed-gene-77.55-mRNA-1 | Japonica | NA | | Os01g0239100 | OsR498G0100510100.01.T01 | Heat-shock protein DnaJ |
| augustus-1-processed-gene-78.1-mRNA-1 | Japonica | NA | Kinase-LRR-TM | Os01g0239700 | OsR498G0100512300.01.T01 | Receptor-like protein kinase HSL1 |
| augustus-1-processed-gene-94.11-mRNA-1 | Japonica | NA | | Os01g0270700 | OsR498G0100633700.01.T01 | Inositol or phosphatidylinositol kinase |
| augustus-1-processed-gene-95.16-mRNA-1 | Japonica | NA | | Os01g0273300 | OsR498G0100640100.01.T01 | BSD domain-containing protein 1 |
| augustus-1-processed-gene-96.22-mRNA-1 | Japonica | NA | | Os01g0274500 | OsR498G0100644500.01.T01 | Expansin |
| augustus-1-processed-gene-97.6-mRNA-1 | Japonica | NA | | | OsR498G0100650800.01.T02 | ArgoN domain-containing protein |
| augustus-1-processed-gene-98.12-mRNA-1 | Japonica | NA | | Os01g0277500 | OsR498G0100658300.01.T01 | Dof-type domain-containing protein |
| augustus-10-processed-gene-104.20-mRNA-1 | Japonica | Indica | | Os10g0428500 | OsR498G1018762300.01.T01 | BTB/POZ domain containing protein, expressed |
| augustus-10-processed-gene-105.59-mRNA-1 | Japonica | NA | | | OsR498G0612113800.01.T01 | RNase H domain-containing protein (Fragment) |
| augustus-10-processed-gene-105.63-mRNA-1 | Japonica | NA | | | OsR498G0204046800.01.T01 | Circumsporozoite protein-like |
| augustus-10-processed-gene-105.68-mRNA-1 | Japonica | NA | | Os10g0430950 | OsR498G1018764700.01.T01 | |
| augustus-10-processed-gene-106.25-mRNA-1 | Japonica | NA | | | OsR498G1018767100.01.T01 | Transcription repressor |
| augustus-10-processed-gene-107.3-mRNA-1 | Japonica | Indica | | Os10g0435400 | OsR498G1018774700.01.T01 | BTB/POZ domain containing protein |
| augustus-10-processed-gene-108.15-mRNA-1 | Japonica | Indica | | Os03g0748500 | OsR498G1018781100.01.T02 | NAD(P)H dehydrogenase (quinone) |
| augustus-10-processed-gene-108.16-mRNA-1 | Japonica | NA | | | OsR498G1018781300.01.T01 | NAD(P)H dehydrogenase (quinone) |
| augustus-10-processed-gene-108.17-mRNA-1 | Japonica | NA | | Os10g0436900 | OsR498G1018781700.01.T01 | Cationcalcium exchanger 1 |
| augustus-10-processed-gene-108.19-mRNA-1 | Japonica | NA | | | OsR498G1018783900.01.T01 | Retrotransposon protein, putative, unclassified |
| augustus-10-processed-gene-109.50-mRNA-1 | Japonica | NA | | Os10g0438800 | OsR498G1018789500.01.T01 | RING finger protein 165 |
| augustus-10-processed-gene-111.2-mRNA-1 | Japonica | NA | Kinase-TM | Os10g0442000 | OsR498G1018798100.01.T01 | Putative receptor-type protein kinase LRK1 |
| augustus-10-processed-gene-113.19-mRNA-1 | Japonica | NA | | | OsR498G1018812900.01.T01 | ARID domain-containing protein |
| augustus-10-processed-gene-116.20-mRNA-1 | Japonica | NA | | Os04g0165900 | | Cysteine-rich receptor-like protein kinase 41 |
| augustus-10-processed-gene-118.10-mRNA-1 | Japonica | NA | | Os04g0162600 | OsR498G0407598800.01.T01 | DUF295 domain-containing protein (Fragment) |
| augustus-10-processed-gene-134.27-mRNA-1 | Japonica | NA | | Os04g0129900 | OsR498G0407509800.01.T01 | Dynamin family protein, putative, expressed |
| augustus-10-processed-gene-139.47-mRNA-1 | Japonica | NA | | Os04g0118900 | OsR498G0407487200.01.T03 | Serine/arginine-rich splicing factor like |
| augustus-10-processed-gene-15.21-mRNA-1 | Japonica | NA | | | | |
| augustus-10-processed-gene-153.26-mRNA-1 | Japonica | NA | | | OsR498G1018823300.01.T01 | |
| augustus-10-processed-gene-153.3-mRNA-1 | Japonica | NA | | | OsR498G0917193500.01.T01 | |
| augustus-10-processed-gene-155.0-mRNA-1 | Japonica | NA | | | OsR498G1018826300.01.T01 | RRM domain-containing protein (Fragment) |
| augustus-10-processed-gene-156.21-mRNA-1 | Japonica | NA | | | OsR498G1018831700.01.T01 | Eukaryotic translation initiation factor 4 gamma 2 putative |
| augustus-10-processed-gene-157.44-mRNA-1 | Japonica | NA | | | OsR498G1018836700.01.T01 | Enhanced disease resistance 2 isoform 1 |
| augustus-10-processed-gene-157.47-mRNA-1 | Japonica | NA | | Os10g0456400 | OsR498G1018837500.01.T01 | Calcium ion binding |
| augustus-10-processed-gene-16.14-mRNA-1 | Japonica | NA | | | | Transposase, putative, expressed |
| augustus-10-processed-gene-168.11-mRNA-1 | Japonica | NA | | Os10g0475900 | OsR498G1018898300.01.T01 | Polyubiquitin-like |
| augustus-10-processed-gene-168.12-mRNA-1 | Japonica | NA | | Os10g0476000 | OsR498G1018898500.01.T03 | Non-intrinsic ABC protein 4 |
| augustus-10-processed-gene-172.0-mRNA-1 | Japonica | Indica | | Os10g0479900 | OsR498G1018914700.01.T01 | Auxin response factor |
| augustus-10-processed-gene-172.5-mRNA-1 | Japonica | Indica | | Os07g0693100 | OsR498G1018918700.01.T01 | Pyruvate decarboxylase |
| augustus-10-processed-gene-173.21-mRNA-1 | Japonica | NA | | | OsR498G1018930300.01.T01 | RNase H domain-containing protein (Fragment) |
| augustus-10-processed-gene-175.49-mRNA-1 | Japonica | NA | | | | Tetratricopeptide repeat protein 1 |
| augustus-10-processed-gene-176.14-mRNA-1 | Japonica | NA | | | OsR498G1018947100.01.T05 | SMAP domain-containing protein (Fragment) |
| augustus-10-processed-gene-177.26-mRNA-1 | Japonica | Indica | | Os10g0488900 | OsR498G1018950500.01.T02 | Pentatricopeptide repeat-containing protein chloroplastic |
| augustus-10-processed-gene-178.18-mRNA-1 | Japonica | NA | Kinase-LRR-TM | | OsR498G1018962700.01.T01 | Tyrosine-protein kinase |
| augustus-10-processed-gene-179.42-mRNA-1 | Japonica | NA | | Os10g0493600 | OsR498G1018966500.01.T04 | Alpha-galactosidase |
| augustus-10-processed-gene-18.26-mRNA-1 | Japonica | NA | | Os10g0124500 | | F-box domain containing protein, expressed |
| augustus-10-processed-gene-181.75-mRNA-1 | Japonica | NA | | Os10g0495000 | OsR498G1018975500.01.T02 | KH domain containing protein, expressed |
| augustus-10-processed-gene-182.45-mRNA-1 | Japonica | NA | | Os10g0498500 | OsR498G1018987100.01.T01 | AB hydrolase superfamily protein yfhM |
| augustus-10-processed-gene-182.57-mRNA-1 | Japonica | NA | | | OsR498G1018988900.01.T01 | |
| augustus-10-processed-gene-182.59-mRNA-1 | Japonica | NA | | Os10g0499500 | OsR498G1018991500.01.T01 | Rf1 protein, mitochondrial, putative, expressed |
| augustus-10-processed-gene-182.61-mRNA-1 | Japonica | Japonica | | Os10g0499800 | OsR498G1018978200.01.T01 | Rf1 protein, mitochondrial, putative, expressed |
| augustus-10-processed-gene-183.9-mRNA-1 | Japonica | NA | | Os10g0502100 | OsR498G1018999100.01.T01 | Putative alkaline/neutral invertase F |
| augustus-10-processed-gene-184.39-mRNA-1 | Japonica | NA | | Os10g0503200 | OsR498G1019001800.01.T04 | Folylpoly-gamma-glutamate synthetase |
| augustus-10-processed-gene-185.13-mRNA-1 | Japonica | Japonica | | Os10g0506000 | OsR498G1019008700.01.T01 | Pentatricopeptide repeat |
| augustus-10-processed-gene-185.16-mRNA-1 | Japonica | NA | | Os04g0384000 | | |
| augustus-10-processed-gene-186.1-mRNA-1 | Japonica | Japonica | | Os10g0507500 | OsR498G1019011500.01.T02 | leukocyte receptor cluster member 1 |

| | | | | | | |
|---|---|---|---|---|---|---|
| augustus-10-processed-gene-186.15-mRNA-1 | Japonica | NA | | Os10g0508100 | OsR498G1019014300.01.T02 | IRK-interacting protein-like |
| augustus-10-processed-gene-188.30-mRNA-1 | Japonica | NA | | Os10g0510400 | OsR498G1019023100.01.T04 | Methyltransferase |
| augustus-10-processed-gene-188.39-mRNA-1 | Japonica | NA | | Os10g0511400 | OsR498G1019027100.01.T02 | Prolyl carboxypeptidase like protein, putative, expressed |
| augustus-10-processed-gene-189.23-mRNA-1 | Japonica | NA | | Os10g0513300 | OsR498G1019032900.01.T03 | Ubiquinone biosynthesis protein COQ4-like, mitochondrial (Fragment) |
| augustus-10-processed-gene-189.24-mRNA-1 | Japonica | Indica | | Os10g0513550 | OsR498G1019033100.01.T01 | Cytochrome P450 family 87 subfamily A polypeptide 6 |
| augustus-10-processed-gene-190.0-mRNA-1 | Japonica | Japonica | | Os10g0513900 | OsR498G1019033100.01.T01 | Cytochrome P450 family 87 subfamily A polypeptide 6 |
| augustus-10-processed-gene-190.2-mRNA-1 | Japonica | Indica | | Os10g0513900 | OsR498G1019033500.01.T01 | Cytochrome P450 family 87 subfamily A polypeptide 6 |
| augustus-10-processed-gene-190.27-mRNA-1 | Japonica | NA | | Os10g0515200 | OsR498G1019034900.01.T01 | Cytochrome P450 family 87 subfamily A polypeptide 6 |
| augustus-10-processed-gene-190.3-mRNA-1 | Japonica | Indica | | Os10g0513900 | OsR498G1019033500.01.T01 | Cytochrome P450 family 87 subfamily A polypeptide 6 |
| augustus-10-processed-gene-190.30-mRNA-1 | Japonica | NA | | Os10g0515900 | OsR498G1019035500.01.T01 | Cytochrome P450 family 87 subfamily A polypeptide 6 |
| augustus-10-processed-gene-190.4-mRNA-1 | Japonica | NA | | Os10g0514400 | | |
| augustus-10-processed-gene-192.22-mRNA-1 | Japonica | NA | | Os10g0519800 | OsR498G1019053500.01.T01 | DUF295 domain-containing protein |
| augustus-10-processed-gene-193.32-mRNA-1 | Japonica | Japonica | | Os10g0520100 | OsR498G1019053700.01.T01 | DUF295 domain-containing protein |
| augustus-10-processed-gene-193.33-mRNA-1 | Japonica | Japonica | | Os10g0520400 | OsR498G1019054500.01.T01 | DUF295 domain-containing protein |
| augustus-10-processed-gene-195.38-mRNA-1 | Japonica | NA | | Os10g0524300 | OsR498G1019069700.01.T01 | Peptidoglycan-binding LysM domain-containing protein, putative |
| augustus-10-processed-gene-20.32-mRNA-1 | Japonica | NA | | Os10g0128400 | OsR498G1018085500.01.T04 | |
| augustus-10-processed-gene-21.31-mRNA-1 | Japonica | Japonica | | Os10g0130200 | OsR498G0203391400.01.T01 | F-box domain containing protein, expressed |
| augustus-10-processed-gene-22.0-mRNA-1 | Japonica | Japonica | | Os10g0131000 | OsR498G1018101100.01.T01 | Disease resistance protein RPM1 |
| augustus-10-processed-gene-23.0-mRNA-1 | Japonica | NA | | Os01g0726700 | OsR498G0101927700.01.T01 | Protodermal factor 1 |
| augustus-10-processed-gene-23.14-mRNA-1 | Japonica | Indica | | Os10g0133500 | OsR498G1018102300.01.T01 | Benzyl alcohol O-benzoyltransferase |
| augustus-10-processed-gene-24.12-mRNA-1 | Japonica | Indica | | Os10g0134400 | OsR498G1018104100.01.T01 | |
| augustus-10-processed-gene-24.14-mRNA-1 | Japonica | Japonica | | Os10g0134700 | OsR498G1018104900.01.T01 | F-box domain-containing protein |
| augustus-10-processed-gene-24.15-mRNA-1 | Japonica | Indica | | Os10g0135100 | OsR498G1018105500.01.T01 | Putative disease resistance protein RGA4 |
| augustus-10-processed-gene-24.16-mRNA-1 | Japonica | Indica | | Os10g0135100 | OsR498G1018105900.01.T01 | NB-ARC domain containing protein |
| augustus-10-processed-gene-24.19-mRNA-1 | Japonica | Japonica | | Os10g0135300 | OsR498G1018107100.01.T03 | F-box domain containing protein, expressed |
| augustus-10-processed-gene-25.0-mRNA-1 | Japonica | NA | | Os10g0135400 | OsR498G1018107700.01.T05 | F-box domain containing protein, expressed |
| augustus-10-processed-gene-25.2-mRNA-1 | Japonica | NA | | Os10g0135600 | OsR498G1018108500.01.T01 | caffeoylshikimate esterase |
| augustus-10-processed-gene-26.10-mRNA-1 | Japonica | NA | | Os10g0137100 | | |
| augustus-10-processed-gene-34.19-mRNA-1 | Japonica | NA | | Os10g0150200 | OsR498G1018151700.01.T01 | IGR protein motif |
| augustus-10-processed-gene-35.7-mRNA-1 | Japonica | Indica | Kinase-TM | Os10g0151100 | OsR498G1018156300.01.T01 | Wall associated kinase |
| augustus-10-processed-gene-44.0-mRNA-1 | Japonica | NA | LRR-NBS-TM | | OsR498G1018206100.01.T04 | NB-ARC domain containing protein, expressed |
| augustus-10-processed-gene-45.26-mRNA-1 | Japonica | NA | | Os05g0527100 | OsR498G0511219800.01.T01 | Glycosyltransferase |
| augustus-10-processed-gene-46.22-mRNA-1 | Japonica | Indica | | Os10g0163201 | OsR498G1018228800.01.T01 | Lecithin-cholesterol acyltransferase-like 1 |
| augustus-10-processed-gene-58.33-mRNA-1 | Japonica | NA | | Os11g0205200 | OsR498G0101869200.01.T01 | Putative copia-type pol polyprotein |
| augustus-10-processed-gene-59.16-mRNA-1 | Japonica | NA | | | | |
| augustus-10-processed-gene-77.12-mRNA-1 | Japonica | Indica | | Os03g0845100 | OsR498G1018361500.01.T01 | Transposable element protein, putative, MuDR |
| augustus-10-processed-gene-94.21-mRNA-1 | Japonica | Indica | | Os05g0267732 | OsR498G1018411500.01.T01 | |
| augustus-11-processed-gene-1.13-mRNA-1 | Japonica | Japonica | | Os11g0119200 | OsR498G1220832400.01.T01 | RING-type E3 ubiquitin transferase |
| augustus-11-processed-gene-100.22-mRNA-1 | Japonica | NA | | | | Transposon protein, putative, mutator sub-class |
| augustus-11-processed-gene-11.17-mRNA-1 | Japonica | NA | | Os11g0138700 | OsR498G1119390700.01.T01 | Kelch motif family protein, expressed |
| augustus-11-processed-gene-11.19-mRNA-1 | Japonica | NA | | Os11g0138900 | OsR498G1119391100.01.T01 | Cell death associated protein, putative, expressed |
| augustus-11-processed-gene-11.2-mRNA-1 | Japonica | NA | | | OsR498G1119385200.01.T01 | putative RING-H2 finger protein ATL61 |
| augustus-11-processed-gene-11.20-mRNA-1 | Japonica | NA | | Os11g0139000 | OsR498G1119391300.01.T01 | Cell death associated protein, putative, expressed |
| augustus-11-processed-gene-11.22-mRNA-1 | Japonica | NA | | Os11g0139400 | OsR498G1119391900.01.T01 | TPT domain-containing protein |
| augustus-11-processed-gene-11.3-mRNA-1 | Japonica | NA | | Os11g0138300 | OsR498G1119389500.01.T01 | Putative cytochrome P450-dependent fatty acid hydroxylase |
| augustus-11-processed-gene-110.13-mRNA-1 | Japonica | NA | | Os11g0427700 | OsR498G1119995500.01.T01 | Adenineguanine permease azg2 |
| augustus-11-processed-gene-110.14-mRNA-1 | Japonica | NA | | Os11g0427800 | OsR498G1119996500.01.T01 | Non-specific lipid-transfer protein (Fragment) |
| augustus-11-processed-gene-111.16-mRNA-1 | Japonica | NA | | | OsR498G0407753100.01.T01 | HGWP repeat containing protein-like |
| augustus-11-processed-gene-113.23-mRNA-1 | Japonica | NA | | | OsR498G1120012500.01.T01 | |
| augustus-11-processed-gene-113.24-mRNA-1 | Japonica | NA | | Os11g0432400 | OsR498G1120014500.01.T02 | Mitochondrial dicarboxylatetricarboxylate transporter dtc |
| augustus-11-processed-gene-113.27-mRNA-1 | Japonica | NA | | Os11g0433100 | OsR498G1120016500.01.T01 | Pentatricopeptide repeat-containing protein mitochondrial |
| augustus-11-processed-gene-114.1-mRNA-1 | Japonica | NA | | Os11g0433300 | | BTB domain-containing protein (Fragment) |
| augustus-11-processed-gene-114.4-mRNA-1 | Japonica | Indica | | Os11g0433600 | OsR498G1120018500.01.T01 | Pentatricopeptide repeat-containing protein, mitochondrial |
| augustus-11-processed-gene-116.16-mRNA-1 | Japonica | NA | | | OsR498G1120023700.01.T01 | BHLH domain-containing protein |
| augustus-11-processed-gene-116.17-mRNA-1 | Japonica | NA | | | | |
| augustus-11-processed-gene-119.2-mRNA-1 | Japonica | NA | Kinase-TM | Os11g0441900 | OsR498G1120029400.01.T01 | G-type lectin S-receptor-like serine/threonine-protein kinase |
| augustus-11-processed-gene-12.14-mRNA-1 | Japonica | NA | | | OsR498G0511427100.01.T01 | Epstein-Barr virus EBNA-1-like |
| augustus-11-processed-gene-121.11-mRNA-1 | Japonica | NA | Kinase-TM | Os11g0445300 | OsR498G1120050700.01.T01 | Brassinosteroid insensitive 1-associated receptor kinase 1 |
| augustus-11-processed-gene-123.17-mRNA-1 | Japonica | NA | | | OsR498G1120061700.01.T01 | |
| augustus-11-processed-gene-129.11-mRNA-1 | Japonica | NA | | | OsR498G1120090300.01.T01 | MATH domain-containing protein |
| augustus-11-processed-gene-13.26-mRNA-1 | Japonica | Indica | | Os11g0142300 | OsR498G1220908700.01.T01 | Strictosidine synthase |

| | | | | | | |
|---|---|---|---|---|---|---|
| augustus-11-processed-gene-13.44-mRNA-1 | Japonica | NA | | | OsR498G1119403900.01.T01 | |
| augustus-11-processed-gene-13.45-mRNA-1 | Japonica | NA | | Os12g0140200 | OsR498G1220912300.01.T01 | Regulator of gene silencing |
| augustus-11-processed-gene-13.53-mRNA-1 | Japonica | NA | | Os11g0144900 | OsR498G1119405700.01.T01 | Embryogenesis transmembrane protein-like |
| augustus-11-processed-gene-130.0-mRNA-1 | Japonica | NA | | Os11g0459300 | OsR498G1120090900.01.T01 | |
| augustus-11-processed-gene-130.11-mRNA-1 | Japonica | NA | | | | Pentatricopeptide, putative, expressed |
| augustus-11-processed-gene-131.15-mRNA-1 | Japonica | Indica | | Os12g0575000 | OsR498G1120094300.01.T01 | Endopolygalacturonase AN8327 (Fragment) |
| augustus-11-processed-gene-131.26-mRNA-1 | Japonica | Indica | | Os11g0457300 | OsR498G1120095300.01.T01 | Transposon protein, putative, Mariner sub-class, expressed |
| augustus-11-processed-gene-131.27-mRNA-1 | Japonica | NA | | Os11g0462100 | OsR498G1120097100.01.T01 | class V chitinase-like |
| augustus-11-processed-gene-133.6-mRNA-1 | Japonica | Indica | | Os11g0180000 | OsR498G1120104500.01.T01 | Dirigent protein (Fragment) |
| augustus-11-processed-gene-135.10-mRNA-1 | Japonica | NA | | | OsR498G1120114700.01.T01 | |
| augustus-11-processed-gene-137.11-mRNA-1 | Japonica | NA | | Os11g0473000 | OsR498G1120125900.01.T01 | ER lumen protein-retaining receptor |
| augustus-11-processed-gene-14.0-mRNA-1 | Japonica | Indica | | Os12g0143150 | OsR498G1119406500.01.T02 | Ubiquitin-like protein 5 |
| augustus-11-processed-gene-14.2-mRNA-1 | Japonica | NA | | Os11g0145600 | | SPRY domain-containing protein |
| augustus-11-processed-gene-14.24-mRNA-1 | Japonica | Indica | | Os12g0143750 | OsR498G1119407500.01.T01 | E3 ubiquitin-protein ligase rnf12-B |
| augustus-11-processed-gene-144.6-mRNA-1 | Japonica | NA | | Os11g0482400 | OsR498G1120148300.01.T01 | Pentatricopeptide repeat-containing protein chloroplastic |
| augustus-11-processed-gene-144.9-mRNA-1 | Japonica | NA | | Os11g0483000 | | Cytochrome P450 family 96 subfamily A polypeptide 1 |
| augustus-11-processed-gene-145.40-mRNA-1 | Japonica | NA | | Os11g0484500 | OsR498G1120152900.01.T01 | 6-phosphogluconate dehydrogenase, decarboxylating |
| augustus-11-processed-gene-146.14-mRNA-1 | Japonica | NA | | | OsR498G1120155900.01.T01 | |
| augustus-11-processed-gene-146.15-mRNA-1 | Japonica | NA | | Os11g0487700 | | |
| augustus-11-processed-gene-154.13-mRNA-1 | Japonica | NA | | | OsR498G1120197300.01.T02 | |
| augustus-11-processed-gene-154.17-mRNA-1 | Japonica | Indica | | Os11g0505200 | OsR498G1120198900.01.T01 | Sulfotransferase |
| augustus-11-processed-gene-155.4-mRNA-1 | Japonica | NA | | Os11g0505200 | OsR498G1120199500.01.T01 | Sulfotransferase |
| augustus-11-processed-gene-157.0-mRNA-1 | Japonica | NA | | Os07g0513300 | | Transposon protein, putative, Mutator sub-class, expressed |
| augustus-11-processed-gene-157.14-mRNA-1 | Japonica | NA | | | OsR498G0408287200.01.T01 | RNase H domain-containing protein |
| augustus-11-processed-gene-158.17-mRNA-1 | Japonica | Indica | | Os11g0512200 | OsR498G1120212500.01.T01 | No apical meristem protein, expressed |
| augustus-11-processed-gene-159.48-mRNA-1 | Japonica | NA | | Os12g0550700 | | Transposon protein, putative, Pong sub-class, expressed |
| augustus-11-processed-gene-16.32-mRNA-1 | Japonica | NA | | | | Transposon protein, putative, Mutator sub-class, expressed |
| augustus-11-processed-gene-16.33-mRNA-1 | Japonica | NA | | | OsR498G1119418900.01.T01 | Zinc finger, C3HC4 type family protein, expressed |
| augustus-11-processed-gene-16.34-mRNA-1 | Japonica | NA | | Os11g0149900 | OsR498G1119419500.01.T01 | PAN domain-containing protein |
| augustus-11-processed-gene-16.36-mRNA-1 | Japonica | NA | | Os03g0793100 | OsR498G0307191800.01.T02 | Glycosyltransferases |
| augustus-11-processed-gene-165.25-mRNA-1 | Japonica | NA | | Os11g0525200 | OsR498G1120251900.01.T01 | Obtusifoliol 14-alpha demethylase |
| augustus-11-processed-gene-166.38-mRNA-1 | Japonica | NA | | Os11g0527000 | | |
| augustus-11-processed-gene-169.13-mRNA-1 | Japonica | NA | | Os11g0531900 | OsR498G1120275200.01.T01 | |
| augustus-11-processed-gene-169.14-mRNA-1 | Japonica | NA | | Os11g0533500 | OsR498G1120278500.01.T06 | Retinoblastoma-related protein 2 |
| augustus-11-processed-gene-169.15-mRNA-1 | Japonica | NA | | | OsR498G1120279100.01.T01 | |
| augustus-11-processed-gene-17.15-mRNA-1 | Japonica | Indica | | Os12g0151000 | OsR498G1119427900.01.T01 | Purple acid phosphatase |
| augustus-11-processed-gene-17.18-mRNA-1 | Japonica | NA | | | OsR498G1119429500.01.T01 | DUF295 domain-containing protein |
| augustus-11-processed-gene-170.12-mRNA-1 | Japonica | NA | | Os11g0535600 | OsR498G1120283900.01.T04 | C2 domain containing protein, expressed |
| augustus-11-processed-gene-171.26-mRNA-1 | Japonica | NA | | Os11g0537300 | OsR498G1120288100.01.T01 | Germin-like protein 11-1 |
| augustus-11-processed-gene-171.32-mRNA-1 | Japonica | NA | | | OsR498G1120288600.01.T01 | Respiratory burst oxidase like protein C |
| augustus-11-processed-gene-171.36-mRNA-1 | Japonica | NA | | Os11g0538400 | | FBD domain-containing protein |
| augustus-11-processed-gene-171.37-mRNA-1 | Japonica | Japonica | | Os11g0538600 | OsR498G1120290900.01.T01 | FBD domain-containing protein |
| augustus-11-processed-gene-173.1-mRNA-1 | Japonica | NA | | Os11g0540900 | OsR498G1120300300.01.T01 | |
| augustus-11-processed-gene-173.2-mRNA-1 | Japonica | Indica | | Os11g0541600 | OsR498G1120301100.01.T01 | |
| augustus-11-processed-gene-175.0-mRNA-1 | Japonica | Indica | | Os11g0544000 | OsR498G1120311700.01.T06 | Pentatricopeptide repeat superfamily protein, putative isoform 1 |
| augustus-11-processed-gene-178.46-mRNA-1 | Japonica | NA | | Os11g0549635 | OsR498G1120330300.01.T02 | ABC1 domain-containing protein (Fragment) |
| augustus-11-processed-gene-180.33-mRNA-1 | Japonica | Japonica | CC-LRR-NBS-TM | Os11g0551300 | OsR498G1120334900.01.T01 | Disease resistance protein RPM1 |
| augustus-11-processed-gene-181.0-mRNA-1 | Japonica | NA | | | OsR498G0509784600.01.T01 | SANT domain-containing protein |
| augustus-11-processed-gene-182.37-mRNA-1 | Japonica | NA | Kinase-TM | Os11g0557500 | OsR498G1120348700.01.T01 | Proline-rich receptor-like protein kinase PERK14 |
| augustus-11-processed-gene-182.41-mRNA-1 | Japonica | NA | | Os11g0558300 | OsR498G1120351100.01.T01 | AMP-binding enzyme family protein, expressed |
| augustus-11-processed-gene-184.34-mRNA-1 | Japonica | NA | | Os11g0562100 | OsR498G1120361100.01.T02 | Terpene cyclase/mutase family member |
| augustus-11-processed-gene-185.22-mRNA-1 | Japonica | NA | LRR-TM | Os11g0565000 | OsR498G1120370300.01.T01 | Leucine Rich Repeat family protein, expressed |
| augustus-11-processed-gene-187.24-mRNA-1 | Japonica | NA | | Os11g0568500 | OsR498G1120379500.01.T02 | Prefoldin subunit |
| augustus-11-processed-gene-189.16-mRNA-1 | Japonica | NA | | Os11g0571900 | OsR498G1120390900.01.T01 | TPX2 domain-containing protein (Fragment) |
| augustus-11-processed-gene-19.24-mRNA-1 | Japonica | NA | | Os11g0156300 | OsR498G1119444100.01.T01 | Transcription repressor |
| augustus-11-processed-gene-193.13-mRNA-1 | Japonica | Japonica | | Os11g0577800 | OsR498G0307190300.01.T02 | Glucan endo-1,3-beta-D-glucosidase |
| augustus-11-processed-gene-193.14-mRNA-1 | Japonica | Indica | | Os03g0804800 | OsR498G1120402700.01.T01 | CCT-theta |
| augustus-11-processed-gene-193.22-mRNA-1 | Japonica | NA | | Os11g0578100 | OsR498G1120403100.01.T01 | J domain-containing protein (Fragment) |
| augustus-11-processed-gene-193.30-mRNA-1 | Japonica | NA | | Os11g0578700 | OsR498G1120405100.01.T01 | F-box only protein 7 |
| augustus-11-processed-gene-194.1-mRNA-1 | Japonica | Japonica | | Os11g0579600 | OsR498G1120405500.01.T01 | F-box domain containing protein, expressed |
| augustus-11-processed-gene-196.0-mRNA-1 | Japonica | NA | | Os11g0584100 | OsR498G1120415500.01.T01 | F-box only protein 7 |

| | | | | | | |
|---|---|---|---|---|---|---|
| augustus-11-processed-gene-197.15-mRNA-1 | Japonica | NA | | Os11g0586900 | OsR498G1120421300.01.T02 | ADP-ribosylation factor-like protein 5 |
| augustus-11-processed-gene-2.36-mRNA-1 | Japonica | NA | | Os11g0119700 | OsR498G1220832200.01.T01 | Secologanin synthase |
| augustus-11-processed-gene-2.54-mRNA-1 | Japonica | NA | | Os12g0120900 | OsR498G1220825200.01.T01 | High density lipoprotein binding protein |
| augustus-11-processed-gene-200.40-mRNA-1 | Japonica | NA | | Os11g0591600 | OsR498G1120433100.01.T01 | DJ-1 protein-like protein E |
| augustus-11-processed-gene-200.53-mRNA-1 | Japonica | NA | | Os11g0592200 | OsR498G1120434500.01.T01 | Barwin domain-containing protein |
| augustus-11-processed-gene-201.32-mRNA-1 | Japonica | NA | | Os11g0593100 | OsR498G1120437900.01.T01 | DUF295 domain-containing protein (Fragment) |
| augustus-11-processed-gene-201.44-mRNA-1 | Japonica | Indica | | Os11g0593500 | OsR498G1120438300.01.T01 | DUF295 domain-containing protein (Fragment) |
| augustus-11-processed-gene-201.45-mRNA-1 | Japonica | Japonica | | Os11g0593500 | OsR498G1120439100.01.T01 | DUF295 domain-containing protein (Fragment) |
| augustus-11-processed-gene-201.48-mRNA-1 | Japonica | NA | | Os11g0594700 | OsR498G1120444100.01.T01 | F-box domain-containing protein |
| augustus-11-processed-gene-201.49-mRNA-1 | Japonica | NA | | Os11g0594800 | OsR498G1120444500.01.T01 | F-box domain-containing protein |
| augustus-11-processed-gene-201.50-mRNA-1 | Japonica | NA | | Os11g0595000 | OsR498G1120445100.01.T01 | F-box domain-containing protein |
| augustus-11-processed-gene-204.32-mRNA-1 | Japonica | Japonica | CC-NBS | Os11g0598500 | OsR498G1120448400.01.T01 | Leucine Rich Repeat family protein, expressed |
| augustus-11-processed-gene-204.33-mRNA-1 | Japonica | NA | | Os04g0576300 | OsR498G0409126100.01.T01 | PUA domain-containing protein (Fragment) |
| augustus-11-processed-gene-205.0-mRNA-1 | Japonica | Indica | | Os09g0434900 | OsR498G1120456900.01.T01 | IBR domain-containing protein |
| augustus-11-processed-gene-206.1-mRNA-1 | Japonica | NA | | Os11g0600700 | OsR498G1120466900.01.T02 | RING-CH-type domain-containing protein |
| augustus-11-processed-gene-206.17-mRNA-1 | Japonica | Japonica | Kinase-TM | Os11g0601500 | OsR498G1120470500.01.T01 | Putative serine/threonine-specific protein kinase |
| augustus-11-processed-gene-206.20-mRNA-1 | Japonica | NA | | Os11g0601600 | OsR498G1120470700.01.T01 | Methyltransferase |
| augustus-11-processed-gene-207.20-mRNA-1 | Japonica | NA | | Os11g0602200 | OsR498G1120473300.01.T05 | Histone-lysine N-methyltransferase H3 lysine-9 specific SUVH3 |
| augustus-11-processed-gene-207.23-mRNA-1 | Japonica | Japonica | | Os11g0602400 | OsR498G0612676800.01.T03 | tRNA-intron lyase |
| augustus-11-processed-gene-207.24-mRNA-1 | Japonica | NA | | Os11g0602600 | OsR498G1120474500.01.T03 | 30S ribosomal protein S4, chloroplastic |
| augustus-11-processed-gene-208.16-mRNA-1 | Japonica | NA | | | OsR498G1120479500.01.T01 | |
| augustus-11-processed-gene-209.35-mRNA-1 | Japonica | Japonica | NBS-TM | Os11g0606500 | OsR498G1120485700.01.T01 | Nucleotide binding-leucine rich repeat protein |
| augustus-11-processed-gene-209.38-mRNA-1 | Japonica | NA | | Os11g0607100 | OsR498G1120486300.01.T02 | Pentatricopeptide repeat-containing protein isoform A |
| augustus-11-processed-gene-211.13-mRNA-1 | Japonica | NA | | Os11g0610600 | OsR498G1120490700.01.T02 | RING-H2 finger protein ATL54 |
| augustus-11-processed-gene-211.18-mRNA-1 | Japonica | NA | | | OsR498G1120492100.01.T01 | |
| augustus-11-processed-gene-214.19-mRNA-1 | Japonica | NA | | Os11g0615600 | OsR498G1120508300.01.T01 | |
| augustus-11-processed-gene-215.12-mRNA-1 | Japonica | NA | | Os11g0616200 | OsR498G1120511100.01.T01 | Peptidylprolyl isomerase |
| augustus-11-processed-gene-217.16-mRNA-1 | Japonica | NA | | | OsR498G1120524700.01.T02 | Monosaccharide-sensing protein 2 |
| augustus-11-processed-gene-217.18-mRNA-1 | Japonica | NA | | Os11g0620800 | | |
| augustus-11-processed-gene-218.12-mRNA-1 | Japonica | NA | | Os11g0622800 | OsR498G1120532700.01.T01 | Cinnamyl alcohol dehydrogenase |
| augustus-11-processed-gene-219.22-mRNA-1 | Japonica | NA | | Os07g0543700 | OsR498G0408675500.01.T01 | |
| augustus-11-processed-gene-219.23-mRNA-1 | Japonica | Indica | | Os11g0624500 | OsR498G1120542900.01.T01 | DUF295 domain-containing protein (Fragment) |
| augustus-11-processed-gene-22.10-mRNA-1 | Japonica | NA | | | OsR498G1119463500.01.T01 | Nodulation-signaling pathway 2 protein-like |
| augustus-11-processed-gene-22.11-mRNA-1 | Japonica | NA | | | | Fusarium resistance protein I2C-5-like |
| augustus-11-processed-gene-22.12-mRNA-1 | Japonica | NA | | Os11g0160800 | OsR498G1119464700.01.T01 | NB-ARC domain, putative |
| augustus-11-processed-gene-22.6-mRNA-1 | Japonica | Indica | | Os11g0160000 | OsR498G1119460700.01.T01 | |
| augustus-11-processed-gene-22.7-mRNA-1 | Japonica | Indica | | Os11g0160000 | OsR498G1119461100.01.T01 | |
| augustus-11-processed-gene-22.8-mRNA-1 | Japonica | NA | | Os11g0160400 | | DD-transpeptidase |
| augustus-11-processed-gene-22.9-mRNA-1 | Japonica | Indica | | Os11g0160500 | OsR498G1119463100.01.T01 | Transcription factor VIP1 |
| augustus-11-processed-gene-221.22-mRNA-1 | Japonica | NA | | Os06g0489700 | OsR498G0510061500.01.T01 | DUF295 domain-containing protein |
| augustus-11-processed-gene-221.23-mRNA-1 | Japonica | NA | | Os11g0628800 | | HAT family dimerisation domain containing protein |
| augustus-11-processed-gene-222.14-mRNA-1 | Japonica | NA | | Os11g0630600 | | MATH domain-containing protein |
| augustus-11-processed-gene-223.15-mRNA-1 | Japonica | NA | | Os11g0631600 | OsR498G1120574300.01.T01 | MATH domain-containing protein (Fragment) |
| augustus-11-processed-gene-223.18-mRNA-1 | Japonica | NA | | Os11g0631900 | | Epstein-Barr virus EBNA-1-like protein |
| augustus-11-processed-gene-224.16-mRNA-1 | Japonica | NA | | | OsR498G0917372400.01.T01 | BKRF1 encodes EBNA-1 protein-like |
| augustus-11-processed-gene-228.19-mRNA-1 | Japonica | Japonica | LRR-NBS-TM | Os11g0640300 | OsR498G1120612100.01.T01 | Leucine Rich Repeat family protein, expressed |
| augustus-11-processed-gene-228.9-mRNA-1 | Japonica | NA | | Os11g0639300 | OsR498G1120609300.01.T01 | DUF4220 domain-containing protein (Fragment) |
| augustus-11-processed-gene-23.0-mRNA-1 | Japonica | NA | | Os11g0161000 | OsR498G1119465100.01.T03 | NB-ARC domain, putative |
| augustus-11-processed-gene-23.11-mRNA-1 | Japonica | Indica | | Os04g0538400 | OsR498G1119466100.01.T01 | Vacuolar iron transporter homolog 4 |
| augustus-11-processed-gene-231.20-mRNA-1 | Japonica | NA | | | OsR498G1120631500.01.T01 | |
| augustus-11-processed-gene-231.21-mRNA-1 | Japonica | Indica | NBS-LRR | Os11g0644700 | OsR498G1120631700.01.T01 | Disease Resistance protein *Pi1* (allele at the *Pik* locus) |
| augustus-11-processed-gene-235.0-mRNA-1 | Japonica | Japonica | | Os11g0650100 | OsR498G0306323800.01.T01 | |
| augustus-11-processed-gene-235.26-mRNA-1 | Japonica | NA | CC-NBS-TM | Os11g0652100 | OsR498G1120654000.01.T01 | Disease resistance protein RPM1 |
| augustus-11-processed-gene-236.18-mRNA-1 | Japonica | NA | NBS-TM | Os11g0654100 | | NB-ARC domain containing protein, expressed |
| augustus-11-processed-gene-237.18-mRNA-1 | Japonica | NA | CC-NBS-TM | Os11g0655300 | OsR498G1120667300.01.T01 | NB-ARC domain containing protein, expressed |
| augustus-11-processed-gene-24.1-mRNA-1 | Japonica | Japonica | | Os11g0163500 | OsR498G1119469700.01.T03 | Trihelix transcription factor |
| augustus-11-processed-gene-24.10-mRNA-1 | Japonica | NA | | Os11g0163800 | OsR498G1119470500.01.T01 | |
| augustus-11-processed-gene-241.31-mRNA-1 | Japonica | NA | | | OsR498G1120687300.01.T02 | Auxilin-related protein 1 |
| augustus-11-processed-gene-245.2-mRNA-1 | Japonica | NA | | | OsR498G1120693500.01.T01 | |
| augustus-11-processed-gene-246.20-mRNA-1 | Japonica | NA | | | OsR498G1120697900.01.T02 | Calmodulin-binding protein-like |
| augustus-11-processed-gene-246.34-mRNA-1 | Japonica | NA | | | OsR498G1120698700.01.T01 | Serine/threonine kinase-like |

| | | | | | | |
|---|---|---|---|---|---|---|
| augustus-11-processed-gene-246.40-mRNA-1 | Japonica | NA | | Os11g0670900 | | Phorbol-ester/DAG-type domain-containing protein |
| augustus-11-processed-gene-247.35-mRNA-1 | Japonica | NA | | Os11g0672900 | OsR498G1120706700.01.T01 | Calmodulin binding protein-like |
| augustus-11-processed-gene-248.0-mRNA-1 | Japonica | NA | | | | Transposon protein, putative, CACTA, En/Spm sub-class |
| augustus-11-processed-gene-25.0-mRNA-1 | Japonica | NA | | Os11g0166100 | OsR498G1119471900.01.T01 | F-box domain-containing protein (Fragment) |
| augustus-11-processed-gene-251.13-mRNA-1 | Japonica | NA | | | OsR498G0612612900.01.T01 | DUF834 domain-containing protein |
| augustus-11-processed-gene-252.15-mRNA-1 | Japonica | NA | | | OsR498G0408884400.01.T03 | Epstein-Barr virus EBNA-1-like |
| augustus-11-processed-gene-252.2-mRNA-1 | Japonica | NA | | Os11g0683600 | | |
| augustus-11-processed-gene-261.0-mRNA-1 | Japonica | NA | | Os11g0701100 | OsR498G1120762300.01.T01 | Xylanase inhibitor |
| augustus-11-processed-gene-261.1-mRNA-1 | Japonica | NA | | Os11g0701200 | OsR498G1120762500.01.T01 | Xylanase inhibitor XIP-III |
| augustus-11-processed-gene-261.18-mRNA-1 | Japonica | NA | | Os11g0701800 | OsR498G1120763500.01.T01 | Xylanase inhibitor |
| augustus-11-processed-gene-262.22-mRNA-1 | Japonica | NA | | Os11g0705200 | OsR498G1120771100.01.T01 | GRAS transcription factor |
| augustus-11-processed-gene-263.49-mRNA-1 | Japonica | Indica | | Os11g0705200 | OsR498G1120772300.01.T01 | GRAS family transcription factor containing protein, expressed |
| augustus-11-processed-gene-263.50-mRNA-1 | Japonica | Japonica | | Os11g0705900 | OsR498G1120772300.01.T01 | GRAS transcription factor |
| augustus-11-processed-gene-263.51-mRNA-1 | Japonica | NA | | Os11g0706000 | | GRAS transcription factor |
| augustus-11-processed-gene-263.52-mRNA-1 | Japonica | NA | | Os11g0706200 | | GRAS transcription factor |
| augustus-11-processed-gene-263.65-mRNA-1 | Japonica | Japonica | | Os11g0708500 | OsR498G1120778700.01.T01 | probable pyridoxal 5'-phosphate synthase subunit PDX1 |
| augustus-11-processed-gene-27.38-mRNA-1 | Japonica | NA | | | OsR498G1119475700.01.T01 | DUF676 domain-containing protein |
| augustus-11-processed-gene-27.39-mRNA-1 | Japonica | Japonica | | Os11g0167600 | OsR498G1119475900.01.T01 | Exocyst subunit Exo70 family protein (Fragment) |
| augustus-11-processed-gene-29.29-mRNA-1 | Japonica | NA | | Os11g0171300 | OsR498G1119486400.01.T02 | Fructose-bisphosphate aldolase |
| augustus-11-processed-gene-30.12-mRNA-1 | Japonica | Japonica | Kinase-LRR-TM | Os11g0172800 | OsR498G1119491700.01.T01 | Protein kinase domain-containing protein |
| augustus-11-processed-gene-31.0-mRNA-1 | Japonica | Indica | Kinase-LRR-TM | Os11g0171800 | OsR498G1119492700.01.T01 | Leucine-rich repeat receptor kinase (LRR-RK) |
| augustus-11-processed-gene-31.11-mRNA-1 | Japonica | NA | | | OsR498G0511427100.01.T01 | Epstein-Barr virus EBNA-1-like |
| augustus-11-processed-gene-32.20-mRNA-1 | Japonica | NA | | | OsR498G0101775500.01.T01 | Putative YVTN beta-propeller repeat-containing protein |
| augustus-11-processed-gene-33.42-mRNA-1 | Japonica | NA | | | OsR498G1119510100.01.T01 | Serine/arginine repetitive matrix protein 1-like |
| augustus-11-processed-gene-34.15-mRNA-1 | Japonica | Indica | | Os11g0179700 | OsR498G1119517500.01.T01 | Dirigent protein 2 |
| augustus-11-processed-gene-34.18-mRNA-1 | Japonica | NA | | Os11g0180000 | OsR498G1119518700.01.T02 | |
| augustus-11-processed-gene-36.0-mRNA-1 | Japonica | NA | | Os11g0181800 | OsR498G1119522900.01.T07 | Glucose/ribitol dehydrogenase |
| augustus-11-processed-gene-37.29-mRNA-1 | Japonica | NA | | Os11g0184100 | OsR498G1119530100.01.T04 | Steroid 5-alpha-reductase DET2 |
| augustus-11-processed-gene-37.32-mRNA-1 | Japonica | NA | | Os09g0491100 | OsR498G0917727000.01.T01 | 4-hydroxy-7-methoxy-3-oxo-3,4-dihydro-2H-1,4-benzoxazin-2-yl glucosidebe |
| augustus-11-processed-gene-38.0-mRNA-1 | Japonica | NA | | Os11g0185100 | | |
| augustus-11-processed-gene-39.2-mRNA-1 | Japonica | NA | | Os11g0186800 | OsR498G1119536300.01.T02 | Anion transporter 5 like |
| augustus-11-processed-gene-40.14-mRNA-1 | Japonica | NA | | Os05g0267732 | OsR498G0510209200.01.T01 | |
| augustus-11-processed-gene-40.15-mRNA-1 | Japonica | NA | | Os05g0267732 | OsR498G0510209200.01.T01 | |
| augustus-11-processed-gene-41.20-mRNA-1 | Japonica | NA | | Os11g0194600 | OsR498G1119545900.01.T01 | PMEI domain-containing protein |
| augustus-11-processed-gene-42.15-mRNA-1 | Japonica | NA | | | | |
| augustus-11-processed-gene-43.12-mRNA-1 | Japonica | NA | | Os11g0261900 | OsR498G1119742100.01.T04 | Shewanella-like protein phosphatase 2 |
| augustus-11-processed-gene-43.45-mRNA-1 | Japonica | Japonica | NBS-TM | Os11g0263000 | OsR498G1119746200.01.T02 | Similar to NBS-LRR disease resistance protein homologue |
| augustus-11-processed-gene-46.39-mRNA-1 | Japonica | NA | | Os11g0267000 | OsR498G1119770300.01.T01 | tetrapyrrole-binding protein, chloroplastic-like |
| augustus-11-processed-gene-47.25-mRNA-1 | Japonica | NA | | Os07g0415700 | OsR498G1018346700.01.T01 | Hydroxyproline-rich glycoprotein-like |
| augustus-11-processed-gene-49.12-mRNA-1 | Japonica | NA | | | | Disease resistance protein RPM1 |
| augustus-11-processed-gene-49.13-mRNA-1 | Japonica | NA | CC-LRR-NBS-TM | Os11g0270500 | OsR498G1119789300.01.T01 | NB-ARC domain, putative |
| augustus-11-processed-gene-54.21-mRNA-1 | Japonica | NA | | | OsR498G1119729300.01.T01 | |
| augustus-11-processed-gene-55.15-mRNA-1 | Japonica | NA | | | OsR498G0510294600.01.T01 | Zinc knuckle containing protein-like |
| augustus-11-processed-gene-6.102-mRNA-1 | Japonica | NA | | Os11g0128400 | OsR498G1119357300.01.T01 | Cell division control protein 45 |
| augustus-11-processed-gene-6.103-mRNA-1 | Japonica | NA | | Os11g0128600 | | |
| augustus-11-processed-gene-61.5-mRNA-1 | Japonica | NA | | | | Exopolygalacturonase clone GBGE184 |
| augustus-11-processed-gene-61.7-mRNA-1 | Japonica | Indica | Kinase-LRR-TM | Os11g0249900 | OsR498G1119716500.01.T01 | Probably inactive leucine-rich repeat receptor-like protein kinase |
| augustus-11-processed-gene-69.18-mRNA-1 | Japonica | NA | | | OsR498G0307081900.01.T01 | |
| augustus-11-processed-gene-7.15-mRNA-1 | Japonica | NA | | Os11g0131100 | OsR498G1119366100.01.T01 | VQ motif family protein, expressed |
| augustus-11-processed-gene-7.17-mRNA-1 | Japonica | NA | | Os12g0128300 | OsR498G1220873500.01.T01 | Pentatricopeptide, putative, expressed |
| augustus-11-processed-gene-70.2-mRNA-1 | Japonica | NA | | Os11g0207100 | OsR498G1119587900.01.T03 | Transcriptional corepressor SEUSS |
| augustus-11-processed-gene-75.39-mRNA-1 | Japonica | NA | LRR-TM | Os11g0213500 | OsR498G1119614900.01.T01 | Pentatricopeptide repeat-containing protein |
| augustus-11-processed-gene-75.43-mRNA-1 | Japonica | Japonica | | Os11g0214400 | OsR498G1119617700.01.T01 | Dirigent protein (Fragment) |
| augustus-11-processed-gene-76.18-mRNA-1 | Japonica | NA | | Os11g0215100 | OsR498G1119617900.01.T01 | Dirigent protein (Fragment) |
| augustus-11-processed-gene-76.4-mRNA-1 | Japonica | NA | | Os11g0214700 | OsR498G1119617700.01.T01 | Dirigent protein (Fragment) |
| augustus-11-processed-gene-79.18-mRNA-1 | Japonica | NA | | Os11g0221300 | OsR498G1119635700.01.T01 | Putative auxin-responsive protein IAA29 |
| augustus-11-processed-gene-8.78-mRNA-1 | Japonica | NA | | | OsR498G1119368700.01.T03 | Non-reducing end alpha-L-arabinofuranosidase |
| augustus-11-processed-gene-8.93-mRNA-1 | Japonica | NA | | | OsR498G0203751900.01.T06 | PORR domain-containing protein (Fragment) |
| augustus-11-processed-gene-8.94-mRNA-1 | Japonica | NA | CC-Kinase-TM | Os11g0132900 | OsR498G1119371300.01.T01 | Non-specific serine/threonine protein kinase |
| augustus-11-processed-gene-8.96-mRNA-1 | Japonica | Indica | Kinase-TM | Os12g0130300 | OsR498G1119373100.01.T01 | Receptor-like serine/threonine-protein kinase |
| augustus-11-processed-gene-8.97-mRNA-1 | Japonica | NA | | Os11g0133600 | OsR498G1119374500.01.T01 | Heterogeneous nuclear ribonucleoprotein F |

| | | | | | | |
|---|---|---|---|---|---|---|
| augustus-11-processed-gene-81.10-mRNA-1 | Japonica | NA | | | OsR498G1019116800.01.T01 | |
| augustus-11-processed-gene-81.11-mRNA-1 | Japonica | Japonica | | Os07g0543700 | OsR498G1120733700.01.T01 | Retrotransposon protein, putative, Ty3-gypsy sub-class |
| augustus-11-processed-gene-81.30-mRNA-1 | Japonica | NA | | | | |
| augustus-11-processed-gene-81.9-mRNA-1 | Japonica | NA | | | OsR498G1119639100.01.T01 | |
| augustus-11-processed-gene-83.15-mRNA-1 | Japonica | NA | LRR-NBS-TM | Os11g0229400 | OsR498G1119649900.01.T03 | NBS-LRR type protein (Fragment) |
| augustus-11-processed-gene-84.35-mRNA-1 | Japonica | NA | | | OsR498G1119653300.01.T01 | |
| augustus-11-processed-gene-89.15-mRNA-1 | Japonica | NA | | Os11g0241000 | OsR498G1119684100.01.T01 | |
| augustus-11-processed-gene-94.0-mRNA-1 | Japonica | NA | | | OsR498G1119917900.01.T01 | F-box/LRR-repeat protein 4 |
| augustus-12-processed-gene-0.3-mRNA-1 | Japonica | NA | | Os11g0100150 | | |
| augustus-12-processed-gene-0.4-mRNA-1 | Japonica | NA | | Os11g0100200 | OsR498G1220782900.01.T03 | THO complex subunit 4-A |
| augustus-12-processed-gene-10.1-mRNA-1 | Japonica | NA | | | OsR498G1220828000.01.T03 | Bis(5'-adenosyl)-triphosphatase |
| augustus-12-processed-gene-10.19-mRNA-1 | Japonica | Japonica | | Os12g0120900 | OsR498G1220825200.01.T01 | Pentatricopeptide repeat-containing protein |
| augustus-12-processed-gene-10.21-mRNA-1 | Japonica | NA | | Os12g0121600 | OsR498G1220848300.01.T01 | Cation/H(+) antiporter 15 |
| augustus-12-processed-gene-100.11-mRNA-1 | Japonica | NA | | | OsR498G1221264300.01.T02 | Pentatricopeptide repeat |
| augustus-12-processed-gene-103.15-mRNA-1 | Japonica | NA | | | | |
| augustus-12-processed-gene-106.25-mRNA-1 | Japonica | NA | | | OsR498G1221301600.01.T01 | |
| augustus-12-processed-gene-109.1-mRNA-1 | Japonica | NA | | Os06g0114366 | OsR498G0613198100.01.T01 | Selenium-binding protein-like |
| augustus-12-processed-gene-11.44-mRNA-1 | Japonica | NA | | | OsR498G1220852300.01.T01 | Retrotransposon protein, putative, unclassified |
| augustus-12-processed-gene-110.0-mRNA-1 | Japonica | NA | | Os12g0283400 | OsR498G1221320300.01.T01 | Pectinesterase inhibitor 28 |
| augustus-12-processed-gene-110.9-mRNA-1 | Japonica | NA | | | | protein ACCELERATED CELL DEATH 6-like |
| augustus-12-processed-gene-114.41-mRNA-1 | Japonica | NA | | | OsR498G1221348100.01.T02 | Helicase and polymerase-containing protein TEBICHI |
| augustus-12-processed-gene-115.14-mRNA-1 | Japonica | NA | | | | RNA-directed DNA polymerase |
| augustus-12-processed-gene-124.16-mRNA-1 | Japonica | NA | | Os12g0405100 | OsR498G1221366300.01.T01 | Zinc finger CCCH domain-containing protein 66 |
| augustus-12-processed-gene-126.23-mRNA-1 | Japonica | NA | | Os12g0407800 | OsR498G1221372700.01.T03 | Pentatricopeptide repeat-containing protein |
| augustus-12-processed-gene-127.10-mRNA-1 | Japonica | NA | | | OsR498G1221377300.01.T04 | |
| augustus-12-processed-gene-137.1-mRNA-1 | Japonica | Indica | | Os01g0389200 | OsR498G1221417500.01.T01 | |
| augustus-12-processed-gene-14.55-mRNA-1 | Japonica | NA | | Os12g0128300 | OsR498G1220873500.01.T01 | Pentatricopeptide, putative, expressed |
| augustus-12-processed-gene-14.68-mRNA-1 | Japonica | NA | | Os11g0132100 | OsR498G1119369400.01.T01 | PMEI domain-containing protein (Fragment) |
| augustus-12-processed-gene-14.82-mRNA-1 | Japonica | NA | | | | Aladin isoform A |
| augustus-12-processed-gene-15.1-mRNA-1 | Japonica | NA | | | OsR498G1220878500.01.T02 | |
| augustus-12-processed-gene-15.14-mRNA-1 | Japonica | NA | | Os12g0131000 | OsR498G1220884900.01.T01 | Heterogeneous nuclear ribonucleoprotein F |
| augustus-12-processed-gene-15.2-mRNA-1 | Japonica | Japonica | | Os12g0129700 | OsR498G1220879500.01.T01 | F-box domain containing protein, expressed |
| augustus-12-processed-gene-151.7-mRNA-1 | Japonica | NA | NBS-TM | Os12g0438300 | OsR498G1221468900.01.T01 | Disease resistance protein RGA2 |
| augustus-12-processed-gene-156.16-mRNA-1 | Japonica | NA | | Os12g0443500 | OsR498G1221501200.01.T01 | UDP-glucose 6-dehydrogenase 5 |
| augustus-12-processed-gene-156.17-mRNA-1 | Japonica | NA | | Os12g0443600 | OsR498G1221500400.01.T03 | UDP-glucose 6-dehydrogenase 3 |
| augustus-12-processed-gene-167.10-mRNA-1 | Japonica | NA | | Os12g0455000 | OsR498G1221537900.01.T01 | Kazal-like domain-containing protein |
| augustus-12-processed-gene-167.22-mRNA-1 | Japonica | Japonica | | Os12g0456100 | OsR498G1221540900.01.T05 | Putative pentatricopeptide repeat-containing protein mitochondrial |
| augustus-12-processed-gene-167.8-mRNA-1 | Japonica | Japonica | Kinase-TM | Os12g0454800 | OsR498G1221536100.01.T01 | Histidine kinase2 |
| augustus-12-processed-gene-170.13-mRNA-1 | Japonica | NA | | Os12g0460000 | OsR498G1221549300.01.T01 | |
| augustus-12-processed-gene-170.7-mRNA-1 | Japonica | NA | | | | AT hook motif-containing protein, putative, expressed |
| augustus-12-processed-gene-171.6-mRNA-1 | Japonica | NA | | | OsR498G1221550500.01.T01 | |
| augustus-12-processed-gene-173.17-mRNA-1 | Japonica | Indica | | Os12g0465100 | OsR498G1221564100.01.T01 | |
| augustus-12-processed-gene-173.6-mRNA-1 | Japonica | NA | | Os12g0464200 | OsR498G1221563300.01.T01 | F-box domain containing protein, expressed |
| augustus-12-processed-gene-175.1-mRNA-1 | Japonica | Indica | | Os12g0467200 | OsR498G1221571300.01.T01 | |
| augustus-12-processed-gene-178.12-mRNA-1 | Japonica | NA | | Os12g0471100 | OsR498G1221576100.01.T01 | Adenosinetriphosphatase |
| augustus-12-processed-gene-179.10-mRNA-1 | Japonica | NA | | Os12g0472500 | OsR498G1221579700.01.T01 | TPD1 protein homolog 1 |
| augustus-12-processed-gene-180.1-mRNA-1 | Japonica | NA | | | OsR498G1221583500.01.T01 | Protease inhibitor/seed storage/LTP family protein |
| augustus-12-processed-gene-182.1-mRNA-1 | Japonica | NA | | | OsR498G1221585700.01.T01 | Retrotransposon protein, putative, Ty3-gypsy subclass |
| augustus-12-processed-gene-182.13-mRNA-1 | Japonica | Indica | CC-LRR-NBS-TM | Os12g0477100 | OsR498G1221588100.01.T01 | NB-ARC domain containing protein, expressed |
| augustus-12-processed-gene-184.1-mRNA-1 | Japonica | NA | | Os12g0478400 | | Wall-associated receptor kinase 3 |
| augustus-12-processed-gene-184.2-mRNA-1 | Japonica | NA | | | OsR498G1120619900.01.T01 | UvrD-like helicase C-terminal domain/Helix-hairpin-helix containing domain/ |
| augustus-12-processed-gene-186.9-mRNA-1 | Japonica | NA | | | OsR498G0815948900.01.T01 | Hydroxyproline-rich glycoprotein-like |
| augustus-12-processed-gene-187.0-mRNA-1 | Japonica | Indica | | Os12g0482600 | OsR498G1221614700.01.T01 | Protein CONTINUOUS VASCULAR RING like |
| augustus-12-processed-gene-187.33-mRNA-1 | Japonica | NA | | | OsR498G0510294600.01.T01 | Zinc knuckle domain-like |
| augustus-12-processed-gene-188.25-mRNA-1 | Japonica | Indica | | Os12g0484900 | OsR498G1221621100.01.T02 | Growth-regulating factor like |
| augustus-12-processed-gene-19.11-mRNA-1 | Japonica | NA | | Os12g0134900 | OsR498G1119389500.01.T01 | Putative cytochrome P450-dependent fatty acid hydroxylase |
| augustus-12-processed-gene-19.12-mRNA-1 | Japonica | NA | | Os12g0135200 | OsR498G1119390700.01.T01 | Kelch motif family protein, expressed |
| augustus-12-processed-gene-19.18-mRNA-1 | Japonica | Japonica | | Os12g0136100 | OsR498G1220899500.01.T01 | TPT domain-containing protein |
| augustus-12-processed-gene-19.20-mRNA-1 | Japonica | Indica | | Os05g0495200 | OsR498G1220899900.01.T02 | Basic-leucine zipper transcription factor |
| augustus-12-processed-gene-190.2-mRNA-1 | Japonica | NA | | Os12g0486800 | | |
| augustus-12-processed-gene-191.1-mRNA-1 | Japonica | NA | | | OsR498G0511427100.01.T01 | Epstein-Barr virus EBNA-1-like |

| | | | | | | |
|---|---|---|---|---|---|---|
| augustus-12-processed-gene-193.0-mRNA-1 | Japonica | NA | | | | Transcription termination factor MTERF15, mitochondrial |
| augustus-12-processed-gene-198.43-mRNA-1 | Japonica | Japonica | Kinase-LRR-TM | Os12g0498650 | OsR498G1221664100.01.T01 | Protein kinase domain-containing protein (Fragment) |
| augustus-12-processed-gene-198.45-mRNA-1 | Japonica | NA | | Os12g0498900 | OsR498G1221666100.01.T01 | Transmembrane protein |
| augustus-12-processed-gene-199.19-mRNA-1 | Japonica | NA | | | OsR498G0611633900.01.T01 | |
| augustus-12-processed-gene-200.10-mRNA-1 | Japonica | NA | | Os12g0502200 | OsR498G1221670300.01.T04 | Glycine-rich protein2 |
| augustus-12-processed-gene-202.12-mRNA-1 | Japonica | NA | | Os05g0128900 | OsR498G0816083200.01.T02 | Trehalose-phosphatase |
| augustus-12-processed-gene-204.10-mRNA-1 | Japonica | NA | | Os12g0508400 | OsR498G1221692700.01.T02 | |
| augustus-12-processed-gene-205.31-mRNA-1 | Japonica | NA | | | | Putative retroelement |
| augustus-12-processed-gene-205.35-mRNA-1 | Japonica | NA | NBS-TM | | OsR498G1221700900.01.T01 | NB-ARC domain containing protein, expressed |
| augustus-12-processed-gene-207.6-mRNA-1 | Japonica | NA | | Os12g0512100 | OsR498G1221705000.01.T01 | MFS domain-containing protein (Fragment) |
| augustus-12-processed-gene-21.14-mRNA-1 | Japonica | NA | | Os12g0139400 | OsR498G1220910900.01.T01 | Two-component response regulator ORR10 |
| augustus-12-processed-gene-21.15-mRNA-1 | Japonica | NA | | | OsR498G1119403900.01.T01 | |
| augustus-12-processed-gene-21.18-mRNA-1 | Japonica | Japonica | | Os12g0140200 | OsR498G1119404100.01.T01 | probable calcium-binding protein CML25 |
| augustus-12-processed-gene-21.24-mRNA-1 | Japonica | NA | | Os12g0140600 | OsR498G1220914900.01.T04 | RING finger protein 38 |
| augustus-12-processed-gene-219.15-mRNA-1 | Japonica | NA | | Os12g0540000 | OsR498G1221781100.01.T01 | |
| augustus-12-processed-gene-22.0-mRNA-1 | Japonica | Indica | | Os11g0144900 | OsR498G1220915700.01.T01 | PGG domain-containing protein |
| augustus-12-processed-gene-22.1-mRNA-1 | Japonica | NA | | Os12g0141000 | OsR498G1119405700.01.T01 | Embryogenesis transmembrane protein-like |
| augustus-12-processed-gene-220.0-mRNA-1 | Japonica | NA | | | | Cell wall protein-like |
| augustus-12-processed-gene-227.9-mRNA-1 | Japonica | Indica | | Os12g0261300 | OsR498G1221851600.01.T01 | Patatin |
| augustus-12-processed-gene-229.35-mRNA-1 | Japonica | Indica | | Os12g0556100 | OsR498G1221864300.01.T01 | Protein DEHYDRATION-INDUCED 19 homolog 3 |
| augustus-12-processed-gene-229.43-mRNA-1 | Japonica | NA | | Os12g0556200 | OsR498G1221864900.01.T02 | Calmodulin-binding protein, putative, expressed |
| augustus-12-processed-gene-229.48-mRNA-1 | Japonica | Japonica | | Os12g0556900 | OsR498G1221867300.01.T01 | Calmodulin-binding protein-like |
| augustus-12-processed-gene-231.28-mRNA-1 | Japonica | NA | | Os12g0559400 | | Disease resistance protein RPM1 |
| augustus-12-processed-gene-232.11-mRNA-1 | Japonica | NA | | | OsR498G1221878700.01.T02 | BZIP transcription factor |
| augustus-12-processed-gene-234.3-mRNA-1 | Japonica | Indica | | Os12g0563600 | OsR498G1221888700.01.T01 | 26S proteasome non-ATPase regulatory subunit like |
| augustus-12-processed-gene-237.22-mRNA-1 | Japonica | Indica | | Os11g0703900 | OsR498G1221905900.01.T03 | Putative mediator of RNA polymerase II transcription subunit 37c |
| augustus-12-processed-gene-237.23-mRNA-1 | Japonica | NA | | Os12g0569900 | OsR498G1221906500.01.T01 | Dof-type zinc finger protein 28 |
| augustus-12-processed-gene-237.4-mRNA-1 | Japonica | NA | | Os12g0569000 | | |
| augustus-12-processed-gene-238.40-mRNA-1 | Japonica | Japonica | | Os12g0571700 | OsR498G1221912500.01.T01 | |
| augustus-12-processed-gene-239.1-mRNA-1 | Japonica | Japonica | | Os12g0572700 | OsR498G1018848000.01.T01 | Exostosin family protein, expressed |
| augustus-12-processed-gene-239.13-mRNA-1 | Japonica | NA | | Os12g0573200 | OsR498G1221918700.01.T01 | GRAS family transcription factor containing protein, expressed |
| augustus-12-processed-gene-24.33-mRNA-1 | Japonica | NA | | Os12g0144100 | | Jacalin-type lectin domain-containing protein (Fragment) |
| augustus-12-processed-gene-243.22-mRNA-1 | Japonica | Indica | | Os12g0580400 | OsR498G1221944300.01.T01 | Polyamine transporter like |
| augustus-12-processed-gene-243.28-mRNA-1 | Japonica | NA | | Os12g0580900 | OsR498G1221945900.01.T01 | Protein-serine/threonine phosphatase |
| augustus-12-processed-gene-248.16-mRNA-1 | Japonica | NA | | Os12g0587200 | OsR498G1221992910.01.T02 | PMD domain-containing protein |
| augustus-12-processed-gene-25.23-mRNA-1 | Japonica | NA | | Os12g0147200 | OsR498G1220936500.01.T01 | RING-type domain-containing protein |
| augustus-12-processed-gene-250.31-mRNA-1 | Japonica | NA | | Os12g0590900 | OsR498G1222008300.01.T01 | Pyridoxal phosphate (PLP)-dependent transferases superfamily protein |
| augustus-12-processed-gene-250.39-mRNA-1 | Japonica | Indica | | Os12g0592300 | OsR498G1222010700.01.T01 | B3 DNA binding domain containing protein |
| augustus-12-processed-gene-251.13-mRNA-1 | Japonica | NA | | | OsR498G1222021700.01.T01 | |
| augustus-12-processed-gene-251.7-mRNA-1 | Japonica | Indica | | Os12g0591600 | OsR498G1222011000.01.T01 | probable transcription factor KAN4 |
| augustus-12-processed-gene-253.57-mRNA-1 | Japonica | Indica | | Os12g0595800 | OsR498G1222032500.01.T01 | Protein kinase domain-containing protein |
| augustus-12-processed-gene-253.59-mRNA-1 | Japonica | Japonica | | Os12g0596300 | OsR498G1222033300.01.T01 | Phorbol-ester/DAG-type domain-containing protein (Fragment) |
| augustus-12-processed-gene-254.0-mRNA-1 | Japonica | Indica | | Os12g0597600 | OsR498G1222036300.01.T03 | Nucleic acid binding protein |
| augustus-12-processed-gene-255.23-mRNA-1 | Japonica | NA | | Os12g0599800 | OsR498G1222046500.01.T01 | PMEI domain-containing protein (Fragment) |
| augustus-12-processed-gene-26.12-mRNA-1 | Japonica | NA | | Os12g0149000 | OsR498G1220942300.01.T03 | |
| augustus-12-processed-gene-260.18-mRNA-1 | Japonica | Japonica | Kinase-TM | Os12g0608500 | OsR498G1222080600.01.T01 | Lectin protein kinase, putative, expressed |
| augustus-12-processed-gene-260.19-mRNA-1 | Japonica | Japonica | | Os12g0609200 | OsR498G0816121400.01.T01 | Tify domain-containing protein |
| augustus-12-processed-gene-260.23-mRNA-1 | Japonica | NA | | | OsR498G0305810200.01.T01 | |
| augustus-12-processed-gene-260.4-mRNA-1 | Japonica | Indica | | Os12g0607800 | OsR498G1222075700.01.T01 | DUF295 domain-containing protein |
| augustus-12-processed-gene-261.30-mRNA-1 | Japonica | NA | | Os12g0610100 | | |
| augustus-12-processed-gene-261.31-mRNA-1 | Japonica | NA | | Os12g0610200 | OsR498G1222083900.01.T01 | BHLH domain-containing protein (Fragment) |
| augustus-12-processed-gene-262.26-mRNA-1 | Japonica | NA | | Os03g0110200 | | Transposon protein, putative, Mutator sub-class, expressed |
| augustus-12-processed-gene-262.8-mRNA-1 | Japonica | NA | | Os12g0611900 | OsR498G1222092500.01.T02 | Beta-1,4-mannosyl-glycoprotein 4-beta-N-acetylglucosaminyltransferase |
| augustus-12-processed-gene-264.32-mRNA-1 | Japonica | NA | Kinase-TM | Os12g0614600 | OsR498G1222101900.01.T01 | Protein kinase PINOID |
| augustus-12-processed-gene-264.34-mRNA-1 | Japonica | Indica | Kinase-TM | Os12g0615300 | OsR498G1222102900.01.T01 | Wall-associated receptor kinase 5 |
| augustus-12-processed-gene-264.46-mRNA-1 | Japonica | NA | Kinase-TM | Os12g0615300 | OsR498G1222104500.01.T04 | Wall-associated receptor kinase 5 |
| augustus-12-processed-gene-264.49-mRNA-1 | Japonica | Indica | | Os12g0615900 | OsR498G1222106300.01.T01 | p21-activated protein kinase-interacting protein 1-like isoform X1 |
| augustus-12-processed-gene-266.21-mRNA-1 | Japonica | NA | | Os12g0617400 | OsR498G1222114300.01.T01 | Plastid 9-cis-epoxycarotenoid dioxygenase |
| augustus-12-processed-gene-268.23-mRNA-1 | Japonica | NA | | Os12g0621000 | OsR498G1222128900.01.T02 | Ubiquitin carboxyl-terminal hydrolase |
| augustus-12-processed-gene-269.45-mRNA-1 | Japonica | Japonica | | Os12g0622500 | OsR498G1222134700.01.T01 | |
| augustus-12-processed-gene-27.0-mRNA-1 | Japonica | Japonica | | Os12g0150500 | OsR498G0815138700.01.T01 | Phytocyanin domain-containing protein (Fragment) |

| | | | | | | |
|---|---|---|---|---|---|---|
| augustus-12-processed-gene-27.15-mRNA-1 | Japonica | NA | | Os12g0151500 | OsR498G1220949700.01.T01 | sialyltransferase-like protein 1 isoform X1 |
| augustus-12-processed-gene-270.2-mRNA-1 | Japonica | NA | | Os12g0624200 | OsR498G1222139900.01.T01 | Sodium/calcium exchanger protein, expressed |
| augustus-12-processed-gene-270.3-mRNA-1 | Japonica | NA | | | OsR498G1222140300.01.T01 | Aminoacyl-tRNA hydrolase |
| augustus-12-processed-gene-271.4-mRNA-1 | Japonica | NA | | | OsR498G1222145800.01.T01 | |
| augustus-12-processed-gene-272.25-mRNA-1 | Japonica | Japonica | | Os12g0628200 | OsR498G1222149500.01.T01 | |
| augustus-12-processed-gene-272.27-mRNA-1 | Japonica | NA | | Os12g0630200 | OsR498G1222152700.01.T01 | Zeamatin |
| augustus-12-processed-gene-34.26-mRNA-1 | Japonica | NA | | Os12g0162800 | | |
| augustus-12-processed-gene-35.38-mRNA-1 | Japonica | Japonica | | Os12g0164600 | OsR498G1119470500.01.T01 | |
| augustus-12-processed-gene-37.39-mRNA-1 | Japonica | NA | | Os12g0168800 | OsR498G1221019100.01.T03 | GATA transcription factor 5 |
| augustus-12-processed-gene-38.0-mRNA-1 | Japonica | NA | | Os12g0170400 | | Sensitive to proton rhizotoxicity1 |
| augustus-12-processed-gene-39.32-mRNA-1 | Japonica | NA | | Os12g0173333 | OsR498G1221028300.01.T01 | TCP transcription factor |
| augustus-12-processed-gene-4.20-mRNA-1 | Japonica | NA | | Os12g0107600 | OsR498G1119296700.01.T01 | Protein DETOXIFICATION |
| augustus-12-processed-gene-40.23-mRNA-1 | Japonica | NA | | Os12g0174300 | OsR498G1221031900.01.T01 | Serine/arginine repetitive matrix protein 1-like |
| augustus-12-processed-gene-41.13-mRNA-1 | Japonica | NA | Kinase-TM | | OsR498G1221041100.01.T01 | Non-specific serine/threonine protein kinase |
| augustus-12-processed-gene-41.14-mRNA-1 | Japonica | Indica | | Os11g0186200 | OsR498G1221041300.01.T01 | Aldehyde dehydrogenase NADP-dependent |
| augustus-12-processed-gene-41.15-mRNA-1 | Japonica | NA | | | OsR498G1221042100.01.T01 | probable methyltransferase PMT26 |
| augustus-12-processed-gene-43.43-mRNA-1 | Japonica | Japonica | | Os12g0181900 | OsR498G1221049100.01.T04 | Pentatricopeptide repeat |
| augustus-12-processed-gene-44.36-mRNA-1 | Japonica | NA | | Os12g0182500 | OsR498G1221050900.01.T01 | |
| augustus-12-processed-gene-44.55-mRNA-1 | Japonica | NA | | Os02g0806500 | OsR498G0204973200.01.T01 | Poly (A) polymerase |
| augustus-12-processed-gene-44.57-mRNA-1 | Japonica | NA | | | OsR498G0815170600.01.T01 | DUF834 domain-containing protein |
| augustus-12-processed-gene-47.30-mRNA-1 | Japonica | NA | | Os12g0189300 | | Carboxyvinyl-carboxyphosphonate phosphorylmutase, chloroplastic |
| augustus-12-processed-gene-5.47-mRNA-1 | Japonica | NA | | Os12g0109600 | OsR498G1220812100.01.T01 | |
| augustus-12-processed-gene-5.48-mRNA-1 | Japonica | NA | | Os12g0109800 | OsR498G1220813700.01.T01 | Pentatricopeptide, putative |
| augustus-12-processed-gene-53.1-mRNA-1 | Japonica | NA | | | OsR498G1221088300.01.T01 | Serine/threonine-protein kinase OXI1 |
| augustus-12-processed-gene-53.29-mRNA-1 | Japonica | NA | | Os12g0198700 | OsR498G1221091800.01.T01 | Dirigent protein (Fragment) |
| augustus-12-processed-gene-53.30-mRNA-1 | Japonica | NA | | Os12g0199000 | OsR498G1221093800.01.T01 | inactive protein RESTRICTED TEV MOVEMENT 1-like |
| augustus-12-processed-gene-56.15-mRNA-1 | Japonica | NA | | | OsR498G1221095700.01.T05 | Putative serine/threonine-protein kinase |
| augustus-12-processed-gene-57.3-mRNA-1 | Japonica | NA | CC-NBS-TM | Os12g0204700 | OsR498G1221097900.01.T01 | NB-ARC domain containing protein, expressed |
| augustus-12-processed-gene-58.0-mRNA-1 | Japonica | NA | | Os12g0206700 | OsR498G1221102500.01.T03 | |
| augustus-12-processed-gene-6.89-mRNA-1 | Japonica | NA | | Os12g0111000 | | Retrotransposon protein, putative, Ty3-gypsy subclass |
| augustus-12-processed-gene-6.95-mRNA-1 | Japonica | NA | | Os12g0112250 | OsR498G1220818300.01.T05 | Surfactant protein B containing protein |
| augustus-12-processed-gene-65.13-mRNA-1 | Japonica | NA | | | OsR498G0408300400.01.T01 | Hydroxyproline-rich glycoprotein-like |
| augustus-12-processed-gene-65.15-mRNA-1 | Japonica | NA | | Os12g0218300 | | |
| augustus-12-processed-gene-66.2-mRNA-1 | Japonica | NA | | Os12g0218800 | | |
| augustus-12-processed-gene-66.21-mRNA-1 | Japonica | NA | | | OsR498G1221133700.01.T01 | |
| augustus-12-processed-gene-66.22-mRNA-1 | Japonica | Indica | LRR-TM | Os12g0218500 | OsR498G1221134900.01.T01 | Leucine Rich Repeat family protein, expressed |
| augustus-12-processed-gene-7.39-mRNA-1 | Japonica | NA | | Os12g0113900 | OsR498G1119317100.01.T03 | DUF641 domain-containing protein (Fragment) |
| augustus-12-processed-gene-7.44-mRNA-1 | Japonica | NA | | Os11g0115466 | OsR498G1220845600.01.T01 | Non-specific lipid-transfer protein (Fragment) |
| augustus-12-processed-gene-7.46-mRNA-1 | Japonica | NA | | Os12g0115100 | OsR498G1220845200.01.T01 | Non-specific lipid-transfer protein (Fragment) |
| augustus-12-processed-gene-71.43-mRNA-1 | Japonica | Indica | | Os12g0226900 | OsR498G1221147500.01.T01 | NADP-dependent oxidoreductase P1, putative, expressed |
| augustus-12-processed-gene-75.37-mRNA-1 | Japonica | NA | | Os12g0233900 | OsR498G1221166700.01.T01 | FAS1 domain-containing protein (Fragment) |
| augustus-12-processed-gene-76.11-mRNA-1 | Japonica | NA | | | | ATP-dependent DNA helicase |
| augustus-12-processed-gene-79.5-mRNA-1 | Japonica | NA | | Os07g0543700 | OsR498G1120733700.01.T01 | Retrotransposon protein, putative, Ty3-gypsy sub-class |
| augustus-12-processed-gene-8.0-mRNA-1 | Japonica | NA | | Os12g0115300 | OsR498G1220844800.01.T01 | Non-specific lipid-transfer protein (Fragment) |
| augustus-12-processed-gene-8.2-mRNA-1 | Japonica | NA | | | OsR498G1220843600.01.T04 | RING-H2 finger protein ATL44 |
| augustus-12-processed-gene-81.16-mRNA-1 | Japonica | NA | | Os12g0243100 | OsR498G1221200500.01.T01 | |
| augustus-12-processed-gene-9.82-mRNA-1 | Japonica | NA | | Os12g0118800 | OsR498G1220833300.01.T06 | RNase H domain-containing protein |
| augustus-12-processed-gene-91.12-mRNA-1 | Japonica | NA | | Os12g0257500 | OsR498G1221223100.01.T02 | Reverse transcriptase domain-containing protein |
| augustus-12-processed-gene-94.10-mRNA-1 | Japonica | NA | | Os12g0262000 | | HAT family dimerisation domain containing protein, expressed |
| augustus-12-processed-gene-94.9-mRNA-1 | Japonica | NA | | | | Hydroxyproline-rich glycoprotein-like |
| augustus-12-processed-gene-96.25-mRNA-1 | Japonica | NA | | Os12g0264500 | OsR498G1221246300.01.T06 | Beta-hydroxyisobutyryl-CoA hydrolase 1 |
| augustus-12-processed-gene-97.14-mRNA-1 | Japonica | NA | | Os12g0265500 | OsR498G0306920300.01.T01 | HAT family dimerisation domain containing protein, expressed |
| augustus-12-processed-gene-98.13-mRNA-1 | Japonica | NA | | Os12g0267500 | | |
| augustus-2-processed-gene-1.25-mRNA-1 | Japonica | NA | | Os02g0102000 | | Glutaredoxin |
| augustus-2-processed-gene-101.2-mRNA-1 | Japonica | Indica | | Os02g0274700 | OsR498G0203400900.01.T01 | RanBP2-type domain-containing protein (Fragment) |
| augustus-2-processed-gene-102.26-mRNA-1 | Japonica | NA | | | OsR498G0203403500.01.T01 | PMD domain-containing protein |
| augustus-2-processed-gene-104.36-mRNA-1 | Japonica | NA | | Os02g0280300 | OsR498G0203416300.01.T01 | Xyloglucan endotransglucosylase/hydrolase |
| augustus-2-processed-gene-112.40-mRNA-1 | Japonica | NA | | Os02g0294000 | | MFAP1 domain-containing protein |
| augustus-2-processed-gene-112.52-mRNA-1 | Japonica | NA | | Os02g0294100 | OsR498G0203464000.01.T02 | F-box domain-containing protein (Fragment) |
| augustus-2-processed-gene-114.14-mRNA-1 | Japonica | Indica | Kinase-TM | Os02g0298200 | OsR498G0203485500.01.T01 | Putative receptor-type protein kinase LRK1 |
| augustus-2-processed-gene-115.18-mRNA-1 | Japonica | Indica | Kinase-TM | Os02g0298200 | OsR498G0203485500.01.T01 | Putative receptor-type protein kinase LRK1 |

| | | | | | | | |
|---|---|---|---|---|---|---|---|
| augustus-2-processed-gene-115.36-mRNA-1 | Japonica | Indica | | Os02g0297200 | OsR498G0203489200.01.T01 | DUF4220 domain-containing protein (Fragment) |
| augustus-2-processed-gene-117.13-mRNA-1 | Japonica | Japonica | | Os02g0302400 | OsR498G0203521400.01.T03 | Nicotianamine aminotransferase A |
| augustus-2-processed-gene-118.30-mRNA-1 | Japonica | NA | | Os02g0303500 | OsR498G0203504400.01.T01 | glycine-rich cell wall structural protein-like |
| augustus-2-processed-gene-119.16-mRNA-1 | Japonica | NA | | Os02g0304800 | OsR498G0203508300.01.T04 | Pentatricopeptide repeat-containing protein |
| augustus-2-processed-gene-121.20-mRNA-1 | Japonica | Indica | | Os02g0308400 | OsR498G0203527800.01.T01 | FAS1 domain-containing protein (Fragment) |
| augustus-2-processed-gene-121.21-mRNA-1 | Japonica | NA | | | OsR498G0203529000.01.T01 | Speckle-type POZ protein B |
| augustus-2-processed-gene-121.7-mRNA-1 | Japonica | NA | | Os02g0307900 | | Chromatin remodeling protein EBS |
| augustus-2-processed-gene-121.9-mRNA-1 | Japonica | NA | | Os02g0308400 | OsR498G0203527200.01.T01 | Fasciclin-like arabinogalactan protein 6 |
| augustus-2-processed-gene-124.18-mRNA-1 | Japonica | NA | | | OsR498G0203542800.01.T01 | putative glycine-rich cell wall structural protein 1 |
| augustus-2-processed-gene-124.9-mRNA-1 | Japonica | NA | | Os02g0314100 | OsR498G0203543900.01.T09 | Vacuolar cation/proton exchanger |
| augustus-2-processed-gene-125.1-mRNA-1 | Japonica | NA | | Os02g0315400 | OsR498G0203547100.01.T01 | Oligosaccharyltransferase complex subunit ostc-A |
| augustus-2-processed-gene-126.12-mRNA-1 | Japonica | NA | | | OsR498G0203555300.01.T01 | |
| augustus-2-processed-gene-126.2-mRNA-1 | Japonica | NA | | Os02g0317200 | OsR498G0203551700.01.T01 | DUF295 domain-containing protein |
| augustus-2-processed-gene-126.23-mRNA-1 | Japonica | NA | | Os02g0318200 | OsR498G0203556600.01.T01 | DUF295 domain-containing protein |
| augustus-2-processed-gene-126.24-mRNA-1 | Japonica | NA | | | OsR498G0203557200.01.T01 | |
| augustus-2-processed-gene-126.25-mRNA-1 | Japonica | NA | | Os02g0318400 | OsR498G0203558200.01.T01 | F-box domain-containing protein |
| augustus-2-processed-gene-126.8-mRNA-1 | Japonica | NA | | Os02g0317300 | OsR498G0203552200.01.T01 | Putative F-box/LRR-repeat protein 23 |
| augustus-2-processed-gene-128.15-mRNA-1 | Japonica | NA | | Os02g0321600 | OsR498G0203567500.01.T01 | CRAL-TRIO domain-containing protein |
| augustus-2-processed-gene-130.24-mRNA-1 | Japonica | NA | | | | |
| augustus-2-processed-gene-130.25-mRNA-1 | Japonica | NA | | | OsR498G1018240500.01.T04 | |
| augustus-2-processed-gene-130.30-mRNA-1 | Japonica | NA | | Os02g0324400 | OsR498G0203578500.01.T01 | Inactive protein FON2 SPARE1 |
| augustus-2-processed-gene-130.43-mRNA-1 | Japonica | Japonica | | Os02g0324500 | OsR498G0203579600.01.T01 | 40S ribosomal protein S7 |
| augustus-2-processed-gene-132.17-mRNA-1 | Japonica | NA | | Os02g0327500 | OsR498G0203595100.01.T01 | Core-2/I-branching beta-1,6-N-acetylglucosaminyltransferase family protein |
| augustus-2-processed-gene-134.23-mRNA-1 | Japonica | NA | | | OsR498G0203612200.01.T01 | |
| augustus-2-processed-gene-136.12-mRNA-1 | Japonica | Indica | | Os02g0332700 | OsR498G0714227900.01.T02 | |
| augustus-2-processed-gene-141.15-mRNA-1 | Japonica | NA | | | | |
| augustus-2-processed-gene-141.18-mRNA-1 | Japonica | NA | | | OsR498G0203639100.01.T01 | |
| augustus-2-processed-gene-142.27-mRNA-1 | Japonica | NA | | | OsR498G1018048100.01.T01 | ATP-dependent DNA helicase |
| augustus-2-processed-gene-143.19-mRNA-1 | Japonica | NA | | Os02g0439700 | OsR498G0203652100.01.T01 | Translation initiation factor |
| augustus-2-processed-gene-143.24-mRNA-1 | Japonica | NA | | | | Transposon protein, putative, Mutator sub-class, expressed |
| augustus-2-processed-gene-146.33-mRNA-1 | Japonica | Japonica | | Os02g0701833 | OsR498G1018968000.01.T01 | ATP-dependent DNA helicase |
| augustus-2-processed-gene-146.9-mRNA-1 | Japonica | NA | | Os12g0114500 | OsR498G1220846600.01.T01 | Non-specific lipid-transfer protein (Fragment) |
| augustus-2-processed-gene-147.12-mRNA-1 | Japonica | NA | | Os02g0448000 | OsR498G0203675100.01.T01 | AT-hook motif nuclear-localized protein 22 |
| augustus-2-processed-gene-147.2-mRNA-1 | Japonica | NA | | | | Retrotransposon protein, putative, unclassified |
| augustus-2-processed-gene-148.16-mRNA-1 | Japonica | NA | | Os04g0574150 | | Exostosin domain-containing protein |
| augustus-2-processed-gene-149.12-mRNA-1 | Japonica | NA | | Os02g0450000 | OsR498G0203686900.01.T01 | DUF659 domain-containing protein |
| augustus-2-processed-gene-151.19-mRNA-1 | Japonica | NA | | | OsR498G0305137000.01.T01 | |
| augustus-2-processed-gene-151.22-mRNA-1 | Japonica | NA | | | OsR498G1221513100.01.T01 | Hydroxyproline-rich glycoprotein-like |
| augustus-2-processed-gene-152.24-mRNA-1 | Japonica | NA | | Os02g0452700 | | |
| augustus-2-processed-gene-152.26-mRNA-1 | Japonica | NA | | | OsR498G0203710000.01.T06 | |
| augustus-2-processed-gene-153.1-mRNA-1 | Japonica | NA | | Os02g0454500 | OsR498G0203714300.01.T01 | Putative long-chain-alcohol O-fatty-acyltransferase 4 |
| augustus-2-processed-gene-153.20-mRNA-1 | Japonica | NA | | Os02g0455400 | OsR498G0203719700.01.T01 | |
| augustus-2-processed-gene-154.0-mRNA-1 | Japonica | NA | | Os02g0455800 | OsR498G0203720900.01.T01 | TF-B3 domain-containing protein |
| augustus-2-processed-gene-154.1-mRNA-1 | Japonica | NA | | | OsR498G0203721700.01.T01 | TF-B3 domain-containing protein |
| augustus-2-processed-gene-154.19-mRNA-1 | Japonica | NA | CC-LRR-NBS-TM | Os02g0456800 | OsR498G0203724300.01.T01 | Putative NB-ARC domain-containing protein (Fragment) |
| augustus-2-processed-gene-155.18-mRNA-1 | Japonica | Japonica | | Os02g0458900 | OsR498G0613026600.01.T01 | GDSL esterase/lipase |
| augustus-2-processed-gene-155.27-mRNA-1 | Japonica | NA | | | | |
| augustus-2-processed-gene-156.10-mRNA-1 | Japonica | NA | Kinase-TM | Os02g0459600 | OsR498G0203737300.01.T03 | Non-specific serine/threonine protein kinase |
| augustus-2-processed-gene-156.30-mRNA-1 | Japonica | NA | | | OsR498G0203737600.01.T01 | Putative Glutamate receptor 2 plant |
| augustus-2-processed-gene-156.32-mRNA-1 | Japonica | NA | | Os02g0460200 | OsR498G0203738600.01.T01 | Flowering-promoting factor 1 |
| augustus-2-processed-gene-157.13-mRNA-1 | Japonica | NA | | Os02g0461200 | | Glucan endo-1,3-beta-D-glucosidase |
| augustus-2-processed-gene-157.18-mRNA-1 | Japonica | NA | | Os02g0461500 | OsR498G0203744600.01.T01 | FAS1 domain-containing protein (Fragment) |
| augustus-2-processed-gene-157.23-mRNA-1 | Japonica | NA | | | | |
| augustus-2-processed-gene-157.35-mRNA-1 | Japonica | NA | | Os02g0462200 | OsR498G0203746200.01.T01 | |
| augustus-2-processed-gene-161.48-mRNA-1 | Japonica | NA | | Os02g0469900 | OsR498G0203776900.01.T02 | High mobility group B protein 15 |
| augustus-2-processed-gene-161.55-mRNA-1 | Japonica | Indica | | Os02g0470000 | OsR498G0203777200.01.T01 | Pentatricopeptide repeat-containing protein |
| augustus-2-processed-gene-164.28-mRNA-1 | Japonica | NA | | Os10g0489650 | | |
| augustus-2-processed-gene-164.41-mRNA-1 | Japonica | NA | | | OsR498G0203790200.01.T01 | BHLH domain-containing protein |
| augustus-2-processed-gene-165.15-mRNA-1 | Japonica | NA | | | OsR498G0203796800.01.T01 | |
| augustus-2-processed-gene-166.8-mRNA-1 | Japonica | Japonica | | Os02g0477401 | OsR498G0816166700.01.T02 | Exostosin domain-containing protein (Fragment) |
| augustus-2-processed-gene-174.15-mRNA-1 | Japonica | NA | | Os02g0491300 | OsR498G0203846900.01.T01 | Fasciclin-like arabinogalactan protein 21 |

| | | | | | | |
|---|---|---|---|---|---|---|
| augustus-2-processed-gene-174.17-mRNA-1 | Japonica | NA | | Os02g0491800 | OsR498G0203848300.01.T01 | |
| augustus-2-processed-gene-175.0-mRNA-1 | Japonica | NA | | Os06g0342750 | OsR498G1221118000.01.T01 | Putative transcription factor |
| augustus-2-processed-gene-175.11-mRNA-1 | Japonica | NA | | Os02g0492500 | | DUF1618 domain-containing protein (Fragment) |
| augustus-2-processed-gene-176.40-mRNA-1 | Japonica | NA | | Os05g0225100 | OsR498G0510061500.01.T01 | DUF295 domain-containing protein |
| augustus-2-processed-gene-178.20-mRNA-1 | Japonica | NA | | | OsR498G0203863300.01.T01 | Transcriptional activator DEMETER |
| augustus-2-processed-gene-179.17-mRNA-1 | Japonica | NA | | | | Aldehyde oxygenase (deformylating) |
| augustus-2-processed-gene-180.12-mRNA-1 | Japonica | NA | | Os02g0502500 | OsR498G0203880700.01.T01 | Calcium and calcium/calmodulin-dependent serine/threonine-protein kinase |
| augustus-2-processed-gene-184.15-mRNA-1 | Japonica | Japonica | | Os02g0506600 | OsR498G0203894500.01.T02 | Chaperone protein dnaJ-related |
| augustus-2-processed-gene-184.17-mRNA-1 | Japonica | NA | | | OsR498G0203898100.01.T01 | Protein indeterminate-domain 16 |
| augustus-2-processed-gene-185.18-mRNA-1 | Japonica | NA | | Os02g0507600 | OsR498G0203900100.01.T01 | Argininosuccinate synthase (Fragment) |
| augustus-2-processed-gene-185.21-mRNA-1 | Japonica | NA | | | OsR498G0203900500.01.T01 | |
| augustus-2-processed-gene-185.55-mRNA-1 | Japonica | Indica | | Os02g0212975 | OsR498G1019116500.01.T01 | |
| augustus-2-processed-gene-192.23-mRNA-1 | Japonica | NA | | Os02g0518000 | OsR498G0203939200.01.T01 | |
| augustus-2-processed-gene-194.41-mRNA-1 | Japonica | NA | | Os02g0521800 | | |
| augustus-2-processed-gene-196.24-mRNA-1 | Japonica | NA | | Os02g0524500 | | Inhibitor of apoptosis-like protein |
| augustus-2-processed-gene-2.18-mRNA-1 | Japonica | Indica | | Os10g0536100 | OsR498G0202808500.01.T03 | MADS box transcription factor 5 |
| augustus-2-processed-gene-2.4-mRNA-1 | Japonica | Indica | | Os02g0103500 | OsR498G0202807000.01.T01 | Auxin-induced protein 5NG4 |
| augustus-2-processed-gene-20.27-mRNA-1 | Japonica | NA | LRR-TM | Os02g0138000 | OsR498G0202928200.01.T01 | Leucine-rich repeat extensin-like protein 1 |
| augustus-2-processed-gene-21.0-mRNA-1 | Japonica | NA | | Os02g0138600 | OsR498G0202932100.01.T01 | |
| augustus-2-processed-gene-21.8-mRNA-1 | Japonica | NA | | Os02g0139100 | OsR498G0202933600.01.T01 | Chaperone binding ATPase activator |
| augustus-2-processed-gene-21.9-mRNA-1 | Japonica | NA | | Os02g0139200 | OsR498G0202933800.01.T02 | Protein arginine N-methyltransferase |
| augustus-2-processed-gene-210.25-mRNA-1 | Japonica | NA | | Os02g0548700 | OsR498G0204080800.01.T01 | RING-type E3 ubiquitin transferase |
| augustus-2-processed-gene-212.41-mRNA-1 | Japonica | NA | | Os02g0551600 | OsR498G0204094500.01.T01 | |
| augustus-2-processed-gene-213.14-mRNA-1 | Japonica | NA | | | OsR498G0204103100.01.T05 | Histone-lysine N-methyltransferase ASHH2 |
| augustus-2-processed-gene-213.25-mRNA-1 | Japonica | Indica | | Os02g0554300 | OsR498G0204104000.01.T02 | SAC domain-containing protein |
| augustus-2-processed-gene-213.6-mRNA-1 | Japonica | Indica | Kinase-LRR-TM | Os01g0296000 | OsR498G0204100400.01.T01 | Putative LRR receptor-like serine/threonine-protein kinase |
| augustus-2-processed-gene-214.15-mRNA-1 | Japonica | NA | | Os02g0555100 | | Pentatricopeptide repeat-containing protein |
| augustus-2-processed-gene-214.3-mRNA-1 | Japonica | NA | | Os02g0554800 | OsR498G0204106300.01.T01 | |
| augustus-2-processed-gene-215.16-mRNA-1 | Japonica | Indica | | Os02g0557000 | OsR498G0204111500.01.T01 | Tubulin-folding cofactor C |
| augustus-2-processed-gene-216.37-mRNA-1 | Japonica | Indica | | Os04g0549700 | OsR498G0204118400.01.T01 | Ethylene-responsive transcription factor ERF021 |
| augustus-2-processed-gene-217.25-mRNA-1 | Japonica | NA | | Os02g0559800 | OsR498G0204121600.01.T01 | E3 ubiquitin-protein ligase EL5 |
| augustus-2-processed-gene-217.31-mRNA-1 | Japonica | NA | | Os02g0562100 | OsR498G0204123300.01.T02 | DNA repair protein XRCC |
| augustus-2-processed-gene-217.37-mRNA-1 | Japonica | NA | | | OsR498G0305104500.01.T01 | |
| augustus-2-processed-gene-217.51-mRNA-1 | Japonica | NA | | Os02g0563301 | OsR498G0204127000.01.T01 | |
| augustus-2-processed-gene-218.50-mRNA-1 | Japonica | NA | | Os02g0564300 | | Copper ion transmembrane transporters |
| augustus-2-processed-gene-218.52-mRNA-1 | Japonica | NA | | | OsR498G0204131200.01.T01 | Transcription factor bHLH82 |
| augustus-2-processed-gene-218.55-mRNA-1 | Japonica | NA | | Os02g0565000 | OsR498G0204131600.01.T01 | Trihelix transcription factor |
| augustus-2-processed-gene-218.57-mRNA-1 | Japonica | NA | | Os02g0565150 | | |
| augustus-2-processed-gene-218.61-mRNA-1 | Japonica | NA | Kinase-TM | Os02g0565500 | OsR498G0204133200.01.T01 | Tyrosine-protein kinase |
| augustus-2-processed-gene-22.0-mRNA-1 | Japonica | NA | | Os02g0140300 | OsR498G0202938500.01.T01 | Molybdopterin synthase catalytic subunit |
| augustus-2-processed-gene-22.18-mRNA-1 | Japonica | Japonica | | Os02g0141400 | OsR498G0202944000.01.T01 | Arginyl-tRNA synthetase |
| augustus-2-processed-gene-22.6-mRNA-1 | Japonica | NA | | Os02g0140800 | OsR498G0202940300.01.T01 | Mitochondrial transcription termination factor family protein |
| augustus-2-processed-gene-220.14-mRNA-1 | Japonica | NA | | Os02g0569000 | OsR498G0204143300.01.T01 | Oryzalexin E synthase |
| augustus-2-processed-gene-221.21-mRNA-1 | Japonica | NA | | Os02g0569900 | OsR498G0204146300.01.T01 | Oryzalexin D synthase |
| augustus-2-processed-gene-221.8-mRNA-1 | Japonica | NA | | Os02g0569400 | OsR498G0204145300.01.T01 | Oryzalexin D synthase |
| augustus-2-processed-gene-223.24-mRNA-1 | Japonica | NA | | Os02g0571900 | OsR498G0204154400.01.T01 | Oryzalexin E synthase |
| augustus-2-processed-gene-223.38-mRNA-1 | Japonica | Indica | | Os02g0572350 | OsR498G0204155600.01.T01 | RING-H2 finger protein ATL51 |
| augustus-2-processed-gene-224.14-mRNA-1 | Japonica | NA | | | OsR498G0204164700.01.T01 | Putative ETHYLENE INSENSITIVE 3-like 4 protein isoform X1 |
| augustus-2-processed-gene-224.15-mRNA-1 | Japonica | NA | | Os02g0574900 | OsR498G0204164900.01.T01 | F-box/kelch-repeat protein At1g22040 |
| augustus-2-processed-gene-224.24-mRNA-1 | Japonica | NA | | | OsR498G0204164600.01.T01 | |
| augustus-2-processed-gene-225.20-mRNA-1 | Japonica | NA | | Os02g0575700 | OsR498G0204166900.01.T01 | Putative WRKY transcription factor 19 |
| augustus-2-processed-gene-225.22-mRNA-1 | Japonica | NA | | Os02g0575900 | OsR498G0204167700.01.T01 | |
| augustus-2-processed-gene-225.26-mRNA-1 | Japonica | NA | | | | |
| augustus-2-processed-gene-225.36-mRNA-1 | Japonica | NA | | Os10g0531750 | OsR498G0408675500.01.T01 | Retrotransposon protein, putative, Ty3-gypsy sub-class |
| augustus-2-processed-gene-227.26-mRNA-1 | Japonica | NA | | | OsR498G0204046800.01.T01 | Circumsporozoite protein-like |
| augustus-2-processed-gene-227.49-mRNA-1 | Japonica | Japonica | | Os02g0580500 | OsR498G0408710600.01.T03 | Prohibitin |
| augustus-2-processed-gene-229.20-mRNA-1 | Japonica | NA | | Os11g0519100 | | |
| augustus-2-processed-gene-229.23-mRNA-1 | Japonica | NA | | Os02g0582800 | OsR498G0204189200.01.T01 | |
| augustus-2-processed-gene-229.26-mRNA-1 | Japonica | NA | | Os02g0583400 | | |
| augustus-2-processed-gene-229.38-mRNA-1 | Japonica | NA | | Os03g0204201 | OsR498G0408675500.01.T01 | Retrotransposon protein, putative, Ty3-gypsy sub-class |
| augustus-2-processed-gene-229.53-mRNA-1 | Japonica | NA | | Os02g0585200 | OsR498G0204193200.01.T01 | HMA domain-containing protein |

| | | | | | | |
|---|---|---|---|---|---|---|
| augustus-2-processed-gene-23.24-mRNA-1 | Japonica | Indica | | Os02g0144800 | OsR498G0202945800.01.T01 | |
| augustus-2-processed-gene-230.0-mRNA-1 | Japonica | NA | | Os02g0586400 | OsR498G0204196300.01.T01 | Ras-related protein Rab-2-B |
| augustus-2-processed-gene-235.0-mRNA-1 | Japonica | NA | | Os02g0595200 | | |
| augustus-2-processed-gene-235.10-mRNA-1 | Japonica | NA | | Os02g0595700 | OsR498G0204234900.01.T01 | Elongation factor Tu |
| augustus-2-processed-gene-235.12-mRNA-1 | Japonica | NA | | Os02g0596200 | OsR498G0204236900.01.T01 | Mannan endo-1,4-beta-mannosidase |
| augustus-2-processed-gene-235.13-mRNA-1 | Japonica | Indica | | Os02g0596200 | OsR498G0204236900.01.T01 | Mannan endo-1,4-beta-mannosidase |
| augustus-2-processed-gene-235.4-mRNA-1 | Japonica | NA | | Os02g0595400 | OsR498G0204234200.01.T01 | Coiled-coil domain-containing protein 86 |
| augustus-2-processed-gene-238.18-mRNA-1 | Japonica | NA | | Os02g0604100 | OsR498G0204259500.01.T01 | |
| augustus-2-processed-gene-238.2-mRNA-1 | Japonica | NA | | | OsR498G0204253500.01.T01 | Transcription termination factor like |
| augustus-2-processed-gene-238.26-mRNA-1 | Japonica | NA | Kinase-TM | Os02g0603000 | OsR498G0204255800.01.T01 | Serine/threonine-protein kinase OXI1 |
| augustus-2-processed-gene-238.27-mRNA-1 | Japonica | NA | LRR-TM | Os02g0603100 | OsR498G0204256200.01.T01 | Leucine-rich repeat receptor-like protein CLAVATA2 |
| augustus-2-processed-gene-238.28-mRNA-1 | Japonica | NA | | | OsR498G0203722500.01.T01 | Cytokinin inducible protein-like |
| augustus-2-processed-gene-238.31-mRNA-1 | Japonica | Japonica | | Os02g0604000 | OsR498G0204259400.01.T01 | SGNH hydrolase-type esterase superfamily protein, putative |
| augustus-2-processed-gene-238.9-mRNA-1 | Japonica | Indica | | Os02g0602500 | OsR498G0204253800.01.T01 | Pre-mRNA-splicing factor ISY1 |
| augustus-2-processed-gene-239.45-mRNA-1 | Japonica | NA | | | OsR498G0204263100.01.T01 | Protein-serine/threonine phosphatase |
| augustus-2-processed-gene-239.59-mRNA-1 | Japonica | Japonica | | Os02g0605000 | OsR498G0204264300.01.T01 | Cyclin N-terminal domain-containing protein (Fragment) |
| augustus-2-processed-gene-239.61-mRNA-1 | Japonica | Japonica | | Os02g0605400 | OsR498G0204265100.01.T04 | VASt domain-containing protein |
| augustus-2-processed-gene-240.35-mRNA-1 | Japonica | Indica | | Os02g0606900 | OsR498G0204270600.01.T03 | Protein-serine/threonine phosphatase |
| augustus-2-processed-gene-240.36-mRNA-1 | Japonica | NA | | Os02g0605000 | OsR498G0204270800.01.T01 | Cyclin N-terminal domain-containing protein (Fragment) |
| augustus-2-processed-gene-241.31-mRNA-1 | Japonica | Indica | | Os02g0608600 | OsR498G0204275900.01.T01 | Acetohydroxy-acid synthase small subunit |
| augustus-2-processed-gene-241.38-mRNA-1 | Japonica | NA | | Os02g0608801 | OsR498G0204276600.01.T01 | Acetylajmalan esterase-like |
| augustus-2-processed-gene-241.48-mRNA-1 | Japonica | NA | LRR-TM | Os02g0609900 | | Receptor-like protein kinase |
| augustus-2-processed-gene-242.0-mRNA-1 | Japonica | NA | LRR-TM | | OsR498G0204282100.01.T01 | Protein kinase domain-containing protein (Fragment) |
| augustus-2-processed-gene-243.34-mRNA-1 | Japonica | NA | | | OsR498G0204288300.01.T01 | GrpE protein homolog |
| augustus-2-processed-gene-243.41-mRNA-1 | Japonica | NA | | Os02g0612200 | | RRM domain-containing protein |
| augustus-2-processed-gene-244.8-mRNA-1 | Japonica | Indica | | Os02g0613900 | OsR498G0204296100.01.T03 | Adenine phosphoribosyltransferase |
| augustus-2-processed-gene-254.39-mRNA-1 | Japonica | NA | | Os02g0620500 | OsR498G0204334300.01.T01 | Ammonium transporter |
| augustus-2-processed-gene-254.41-mRNA-1 | Japonica | NA | | Os02g0620600 | OsR498G0204334900.01.T01 | Ammonium transporter |
| augustus-2-processed-gene-254.52-mRNA-1 | Japonica | NA | | | OsR498G0204332400.01.T01 | |
| augustus-2-processed-gene-254.53-mRNA-1 | Japonica | NA | | Os02g0620800 | OsR498G0204335200.01.T04 | Pentatricopeptide repeat-containing protein |
| augustus-2-processed-gene-255.5-mRNA-1 | Japonica | Indica | | Os02g0621800 | OsR498G0204338000.01.T02 | Long-chain-alcohol oxidase |
| augustus-2-processed-gene-256.29-mRNA-1 | Japonica | Indica | | Os10g0460000 | OsR498G0204348500.01.T01 | |
| augustus-2-processed-gene-256.35-mRNA-1 | Japonica | Japonica | | Os02g0625100 | OsR498G0204350100.01.T01 | Pentatricopeptide repeat-containing protein, chloroplastic |
| augustus-2-processed-gene-257.1-mRNA-1 | Japonica | Japonica | CC-Kinase-TM | Os02g0625300 | OsR498G0204350900.01.T01 | Phosphoenolpyruvate carboxylase kinase |
| augustus-2-processed-gene-257.31-mRNA-1 | Japonica | Japonica | | Os02g0626600 | OsR498G0204355400.01.T01 | Phenylalanine ammonia-lyase |
| augustus-2-processed-gene-258.32-mRNA-1 | Japonica | NA | | | OsR498G0204357400.01.T01 | |
| augustus-2-processed-gene-259.0-mRNA-1 | Japonica | NA | | | | UDP-glucuronic acid/UDP-N-acetylgalactosamine transporter |
| augustus-2-processed-gene-259.22-mRNA-1 | Japonica | NA | | Os02g0629000 | OsR498G0204363000.01.T01 | Senescence regulator |
| augustus-2-processed-gene-259.25-mRNA-1 | Japonica | NA | Kinase-LRR-TM | Os02g0629400 | OsR498G0204364800.01.T01 | Phytosulfokine receptor 1 |
| augustus-2-processed-gene-259.26-mRNA-1 | Japonica | NA | | Os08g0114000 | OsR498G0204365600.01.T01 | T-complex protein 1 subunit eta |
| augustus-2-processed-gene-260.21-mRNA-1 | Japonica | NA | | Os02g0631100 | OsR498G0204370100.01.T03 | |
| augustus-2-processed-gene-262.64-mRNA-1 | Japonica | NA | | Os02g0634500 | OsR498G0204382100.01.T04 | ATP-dependent Clp protease proteolytic subunit |
| augustus-2-processed-gene-262.79-mRNA-1 | Japonica | NA | | | OsR498G0204381000.01.T01 | Putative protein phosphatase 2C 23 |
| augustus-2-processed-gene-262.80-mRNA-1 | Japonica | NA | | | OsR498G0204381600.01.T01 | Putative protein phosphatase 2C 24 |
| augustus-2-processed-gene-263.17-mRNA-1 | Japonica | NA | | Os02g0636300 | OsR498G0204386000.01.T01 | DEAD-box ATP-dependent RNA helicase |
| augustus-2-processed-gene-264.29-mRNA-1 | Japonica | NA | | Os02g0639800 | OsR498G0204397200.01.T01 | E3 ubiquitin-protein ligase RMA1 |
| augustus-2-processed-gene-264.4-mRNA-1 | Japonica | NA | | Os02g0638300 | OsR498G0204392200.01.T03 | Ferredoxin-thioredoxin reductase, variable chain |
| augustus-2-processed-gene-264.6-mRNA-1 | Japonica | NA | | Os02g0638650 | OsR498G0204392800.01.T01 | Ethylene-responsive transcription factor ERF053 |
| augustus-2-processed-gene-265.32-mRNA-1 | Japonica | NA | | Os02g0639900 | OsR498G0204397600.01.T01 | Chloroplast thioredoxin M-type 2 |
| augustus-2-processed-gene-265.40-mRNA-1 | Japonica | NA | | Os02g0640700 | OsR498G0204400100.01.T01 | Plant UBX domain-containing protein 7 |
| augustus-2-processed-gene-265.58-mRNA-1 | Japonica | NA | Kinase-TM | Os02g0640500 | OsR498G0204399600.01.T01 | L-type lectin-domain containing receptor kinase IV.2 |
| augustus-2-processed-gene-266.17-mRNA-1 | Japonica | Japonica | | Os02g0643800 | OsR498G0204410500.01.T01 | Auxin-responsive protein SAUR36 |
| augustus-2-processed-gene-267.39-mRNA-1 | Japonica | NA | | | OsR498G0204414300.01.T05 | Pentatricopeptide repeat-containing protein mitochondrial |
| augustus-2-processed-gene-267.45-mRNA-1 | Japonica | Indica | | Os02g0644200 | OsR498G0204412400.01.T01 | Vacuolar iron transporter like |
| augustus-2-processed-gene-268.23-mRNA-1 | Japonica | NA | | | OsR498G0204417500.01.T01 | Nucleolin 2 (Fragment) |
| augustus-2-processed-gene-268.30-mRNA-1 | Japonica | Indica | | Os02g0646900 | OsR498G0204419300.01.T01 | Polyamine oxidase 1 |
| augustus-2-processed-gene-268.32-mRNA-1 | Japonica | Indica | | Os01g0346700 | OsR498G0204419500.01.T01 | Ccr4-not complex subunit mot2 |
| augustus-2-processed-gene-27.57-mRNA-1 | Japonica | NA | | Os02g0148600 | OsR498G0202973900.01.T01 | |
| augustus-2-processed-gene-271.53-mRNA-1 | Japonica | NA | | | | |
| augustus-2-processed-gene-272.16-mRNA-1 | Japonica | NA | | Os02g0655200 | OsR498G0204444300.01.T01 | Ethylene-responsive transcription factor 5 |
| augustus-2-processed-gene-272.26-mRNA-1 | Japonica | NA | | Os02g0655300 | OsR498G0204445200.01.T02 | LeuD |

| | | | | | | |
|---|---|---|---|---|---|---|
| augustus-2-processed-gene-272.8-mRNA-1 | Japonica | NA | | Os02g0654700 | OsR498G0204443200.01.T01 | Ethylene-responsive factor |
| augustus-2-processed-gene-273.34-mRNA-1 | Japonica | NA | | Os02g0656600 | OsR498G0204448900.01.T01 | Ethylene-responsive transcription factor TINY-like |
| augustus-2-processed-gene-273.35-mRNA-1 | Japonica | NA | | Os02g0657000 | OsR498G0204450900.01.T01 | Ethylene-responsive transcription factor ERF034 |
| augustus-2-processed-gene-274.15-mRNA-1 | Japonica | NA | | Os02g0659100 | OsR498G0204458500.01.T01 | Zinc finger protein ZAT2 |
| augustus-2-processed-gene-274.18-mRNA-1 | Japonica | NA | | Os02g0659500 | OsR498G0204460300.01.T01 | Zinc finger protein ZAT4 |
| augustus-2-processed-gene-274.9-mRNA-1 | Japonica | NA | | | | Remorin, C-terminal |
| augustus-2-processed-gene-275.17-mRNA-1 | Japonica | NA | | Os02g0659600 | OsR498G0204461300.01.T01 | |
| augustus-2-processed-gene-275.45-mRNA-1 | Japonica | NA | | Os02g0661300 | OsR498G0204465000.01.T03 | DOG1 domain-containing protein (Fragment) |
| augustus-2-processed-gene-276.22-mRNA-1 | Japonica | NA | | Os02g0662000 | OsR498G0204467700.01.T01 | Proline-rich protein DC2.15 |
| augustus-2-processed-gene-276.23-mRNA-1 | Japonica | NA | | Os02g0662100 | OsR498G0204468300.01.T01 | 14 kDa polypeptide |
| augustus-2-processed-gene-276.30-mRNA-1 | Japonica | Indica | | Os02g0662700 | OsR498G0204470900.01.T01 | Scarecrow-like protein 6 |
| augustus-2-processed-gene-276.32-mRNA-1 | Japonica | NA | | Os02g0663100 | OsR498G0204471300.01.T01 | GRAS transcription factor |
| augustus-2-processed-gene-277.46-mRNA-1 | Japonica | NA | | | OsR498G0509838600.01.T01 | HGWP repeat containing protein-like |
| augustus-2-processed-gene-277.48-mRNA-1 | Japonica | NA | | | OsR498G0204474000.01.T01 | C2 domain-containing protein |
| augustus-2-processed-gene-277.54-mRNA-1 | Japonica | NA | | Os02g0664000 | OsR498G0204474300.01.T01 | Glutathione peroxidase |
| augustus-2-processed-gene-278.30-mRNA-1 | Japonica | NA | | Os02g0665300 | OsR498G0204477900.01.T01 | Subtilisin-like serine protease |
| augustus-2-processed-gene-278.42-mRNA-1 | Japonica | NA | | Os02g0666700 | | Purine-cytosine permease |
| augustus-2-processed-gene-278.52-mRNA-1 | Japonica | NA | | Os02g0666500 | OsR498G0204480600.01.T02 | Cytochrome P450, E-class, group I |
| augustus-2-processed-gene-279.23-mRNA-1 | Japonica | Japonica | | Os02g0667701 | OsR498G0204484100.01.T01 | |
| augustus-2-processed-gene-279.35-mRNA-1 | Japonica | NA | | Os02g0668100 | OsR498G0204485000.01.T02 | Chloroplast geranyl diphosphate synthase small subunit II |
| augustus-2-processed-gene-279.40-mRNA-1 | Japonica | NA | | Os02g0668200 | | |
| augustus-2-processed-gene-279.44-mRNA-1 | Japonica | Indica | | Os04g0562500 | OsR498G0204490700.01.T01 | |
| augustus-2-processed-gene-279.45-mRNA-1 | Japonica | NA | | | OsR498G0204490900.01.T01 | |
| augustus-2-processed-gene-279.54-mRNA-1 | Japonica | Indica | | Os02g0111900 | OsR498G0204487800.01.T01 | DUF295 domain-containing protein |
| augustus-2-processed-gene-28.25-mRNA-1 | Japonica | Indica | | Os02g0149500 | OsR498G0202976900.01.T01 | Peptidase C32 domain-containing protein |
| augustus-2-processed-gene-28.33-mRNA-1 | Japonica | Indica | | Os02g0149700 | OsR498G0202977600.01.T03 | Exocyst subunit Exo70 family protein (Fragment) |
| augustus-2-processed-gene-28.42-mRNA-1 | Japonica | NA | Kinase-LRR-NBS | | | Disease resistance protein RGA2 |
| augustus-2-processed-gene-28.53-mRNA-1 | Japonica | NA | | Os02g0150000 | OsR498G0202979600.01.T01 | |
| augustus-2-processed-gene-280.27-mRNA-1 | Japonica | NA | | Os02g0670900 | OsR498G0204494100.01.T03 | Lysine histidine transporter-like 3 |
| augustus-2-processed-gene-280.39-mRNA-1 | Japonica | NA | | | OsR498G0204046800.01.T01 | Epstein-Barr virus EBNA-1-like |
| augustus-2-processed-gene-281.52-mRNA-1 | Japonica | NA | | Os02g0672500 | OsR498G0204501000.01.T01 | Mitochondrial inner membrane translocase subunit |
| augustus-2-processed-gene-282.9-mRNA-1 | Japonica | Indica | | Os02g0673700 | OsR498G0204506800.01.T01 | Dof zinc finger protein 2 |
| augustus-2-processed-gene-283.14-mRNA-1 | Japonica | Japonica | | Os02g0676400 | OsR498G0204516700.01.T01 | Protein DETOXIFICATION |
| augustus-2-processed-gene-283.6-mRNA-1 | Japonica | NA | | | OsR498G0204046800.01.T01 | Epstein-Barr virus EBNA-1-like |
| augustus-2-processed-gene-284.1-mRNA-1 | Japonica | NA | | Os02g0676800 | OsR498G0204519700.01.T01 | Ethylene-responsive transcription factor ERF025 |
| augustus-2-processed-gene-284.11-mRNA-1 | Japonica | NA | | Os02g0677300 | OsR498G0204522100.01.T01 | C-repeats binding factor 4 |
| augustus-2-processed-gene-285.12-mRNA-1 | Japonica | NA | | Os02g0679700 | OsR498G0204528100.01.T01 | Transcription repressor |
| augustus-2-processed-gene-285.30-mRNA-1 | Japonica | Indica | | Os02g0559800 | OsR498G0204527400.01.T01 | E3 ubiquitin-protein ligase EL5 |
| augustus-2-processed-gene-285.31-mRNA-1 | Japonica | NA | | | | Transposon protein CACTA, En/Spm sub-class |
| augustus-2-processed-gene-286.35-mRNA-1 | Japonica | NA | | Os02g0681632 | OsR498G0204533500.01.T01 | Serine/threonine receptor-like kinase NFP |
| augustus-2-processed-gene-286.46-mRNA-1 | Japonica | NA | | Os02g0681900 | OsR498G0204535200.01.T01 | GRAS domain-containing protein (Fragment) |
| augustus-2-processed-gene-287.15-mRNA-1 | Japonica | NA | | Os02g0684000 | OsR498G0204544300.01.T02 | Protein SLOW GREEN 1, chloroplastic |
| augustus-2-processed-gene-287.23-mRNA-1 | Japonica | NA | | Os02g0683500 | OsR498G0204540600.01.T02 | ABI3VP1 transcription factor |
| augustus-2-processed-gene-287.24-mRNA-1 | Japonica | NA | | Os02g0684100 | | Sulfotransferase |
| augustus-2-processed-gene-287.25-mRNA-1 | Japonica | NA | | | OsR498G0204544800.01.T01 | tRNA-splicing endonuclease positive effector-like |
| augustus-2-processed-gene-287.4-mRNA-1 | Japonica | NA | | Os02g0682500 | OsR498G0204537800.01.T02 | Transparent testa |
| augustus-2-processed-gene-288.25-mRNA-1 | Japonica | NA | | Os02g0684500 | OsR498G0204546200.01.T01 | Multifunctional fusion protein |
| augustus-2-processed-gene-288.49-mRNA-1 | Japonica | NA | | Os02g0686100 | OsR498G0204552000.01.T01 | RING-H2 finger protein ATL1R |
| augustus-2-processed-gene-289.33-mRNA-1 | Japonica | NA | | | OsR498G0204553600.01.T01 | |
| augustus-2-processed-gene-290.24-mRNA-1 | Japonica | NA | | Os02g0688200 | | PMEI domain-containing protein (Fragment) |
| augustus-2-processed-gene-290.34-mRNA-1 | Japonica | NA | | | | |
| augustus-2-processed-gene-290.41-mRNA-1 | Japonica | NA | | Os02g0688800 | OsR498G0204564700.01.T01 | E3 ubiquitin-protein ligase ATL4 |
| augustus-2-processed-gene-290.53-mRNA-1 | Japonica | NA | | | | AAA domain-containing protein |
| augustus-2-processed-gene-290.58-mRNA-1 | Japonica | NA | | Os02g0689500 | OsR498G0204567600.01.T02 | |
| augustus-2-processed-gene-291.32-mRNA-1 | Japonica | NA | | Os02g0690200 | OsR498G0204571100.01.T02 | Proton-dependent oligopeptide transport family protein |
| augustus-2-processed-gene-291.33-mRNA-1 | Japonica | NA | | Os02g0690500 | OsR498G0204571900.01.T01 | PPM-type phosphatase domain-containing protein (Fragment) |
| augustus-2-processed-gene-291.34-mRNA-1 | Japonica | NA | | Os02g0690600 | OsR498G0204572100.01.T01 | RING-type E3 ubiquitin transferase |
| augustus-2-processed-gene-292.16-mRNA-1 | Japonica | NA | | Os02g0693000 | | |
| augustus-2-processed-gene-292.9-mRNA-1 | Japonica | NA | | Os02g0692000 | OsR498G0204575600.01.T01 | Putative RING-H2 finger protein ATL53 |
| augustus-2-processed-gene-293.65-mRNA-1 | Japonica | Japonica | | Os02g0695700 | OsR498G0409312500.01.T01 | DNA repair protein like |
| augustus-2-processed-gene-295.63-mRNA-1 | Japonica | NA | | Os02g0699600 | OsR498G0204598300.01.T02 | 60S ribosomal protein L12 |

| | | | | | | |
|---|---|---|---|---|---|---|
| augustus-2-processed-gene-296.27-mRNA-1 | Japonica | NA | | Os02g0700500 | OsR498G0204600800.01.T01 | Polyamine transporter |
| augustus-2-processed-gene-297.11-mRNA-1 | Japonica | NA | | | OsR498G1018968000.01.T01 | ATP-dependent DNA helicase |
| augustus-2-processed-gene-297.12-mRNA-1 | Japonica | NA | | Os02g0702100 | | Transcription factor |
| augustus-2-processed-gene-297.26-mRNA-1 | Japonica | NA | | Os02g0703300 | OsR498G0204609700.01.T01 | |
| augustus-2-processed-gene-298.32-mRNA-1 | Japonica | NA | | | | Ribosomal protein (Fragment) |
| augustus-2-processed-gene-298.33-mRNA-1 | Japonica | NA | | Os02g0703800 | OsR498G0204612000.01.T01 | DUF295 domain-containing protein |
| augustus-2-processed-gene-298.47-mRNA-1 | Japonica | Japonica | | Os02g0704000 | OsR498G1221463200.01.T01 | Carotenoid cleavage dioxygenase |
| augustus-2-processed-gene-3.48-mRNA-1 | Japonica | NA | | Os02g0105200 | OsR498G0202813500.01.T05 | Dihydrolipoamide acetyltransferase component of pyruvate dehydrogenase |
| augustus-2-processed-gene-3.67-mRNA-1 | Japonica | NA | | Os02g0105800 | OsR498G0202815200.01.T04 | FBD domain-containing protein |
| augustus-2-processed-gene-3.71-mRNA-1 | Japonica | NA | | Os02g0106300 | OsR498G0202817800.01.T05 | Putative tetratricopeptide-like helical domain, DYW domain-containing prot |
| augustus-2-processed-gene-300.0-mRNA-1 | Japonica | NA | | Os02g0706400 | OsR498G0204619500.01.T03 | Transcription factor RADIALIS |
| augustus-2-processed-gene-300.10-mRNA-1 | Japonica | NA | | Os02g0708000 | | BED-type domain-containing protein |
| augustus-2-processed-gene-300.6-mRNA-1 | Japonica | NA | | Os02g0706600 | OsR498G0204620600.01.T04 | Zinc-finger homeodomain protein 7 |
| augustus-2-processed-gene-300.9-mRNA-1 | Japonica | NA | | | OsR498G0204624500.01.T01 | |
| augustus-2-processed-gene-301.16-mRNA-1 | Japonica | NA | | Os02g0709300 | OsR498G0204629800.01.T01 | Histidinol-phosphate aminotransferase, chloroplastic |
| augustus-2-processed-gene-302.24-mRNA-1 | Japonica | NA | | Os02g0711100 | | Pentatricopeptide repeat-containing protein, mitochondrial |
| augustus-2-processed-gene-303.37-mRNA-1 | Japonica | NA | | Os05g0346800 | OsR498G0102644000.01.T01 | LOB domain-containing protein 12 |
| augustus-2-processed-gene-303.38-mRNA-1 | Japonica | NA | | | OsR498G0204642900.01.T01 | Cytochrome b561/ferric reductase transmembrane |
| augustus-2-processed-gene-304.10-mRNA-1 | Japonica | NA | | | OsR498G0204648100.01.T02 | Trafficking protein particle complex subunit 11 |
| augustus-2-processed-gene-305.27-mRNA-1 | Japonica | NA | | Os02g0717200 | OsR498G0204654900.01.T03 | Rho GTPase-activating gacO-like protein |
| augustus-2-processed-gene-306.38-mRNA-1 | Japonica | NA | | Os02g0717700 | OsR498G0204657300.01.T02 | Glucosamine 6-phosphate N-acetyltransferase |
| augustus-2-processed-gene-306.39-mRNA-1 | Japonica | NA | | Os02g0718000 | | LIM domain-containing serine/threonine-protein kinase |
| augustus-2-processed-gene-306.62-mRNA-1 | Japonica | NA | | Os02g0719800 | OsR498G0204662500.01.T03 | Homogentisate 1,2-dioxygenase |
| augustus-2-processed-gene-307.43-mRNA-1 | Japonica | NA | | Os02g0720400 | OsR498G0204663700.01.T01 | |
| augustus-2-processed-gene-307.53-mRNA-1 | Japonica | NA | | Os02g0789700 | OsR498G1120753400.01.T01 | |
| augustus-2-processed-gene-308.12-mRNA-1 | Japonica | Japonica | | Os02g0723200 | OsR498G1120322800.01.T01 | Galactomannan galactosyltransferase |
| augustus-2-processed-gene-309.50-mRNA-1 | Japonica | Japonica | | Os02g0724100 | OsR498G0204681800.01.T01 | Myb-like transcription factor-like |
| augustus-2-processed-gene-309.58-mRNA-1 | Japonica | NA | | Os02g0725200 | OsR498G0204683700.01.T02 | Ribulose-1,5 bisphosphate carboxylase/oxygenase large subunit N-methyltra |
| augustus-2-processed-gene-31.36-mRNA-1 | Japonica | NA | LRR-TM | Os02g0156400 | OsR498G0202998000.01.T01 | Putative Phytosulfokine receptor |
| augustus-2-processed-gene-310.3-mRNA-1 | Japonica | NA | | Os02g0726000 | OsR498G0204687000.01.T01 | Fasciclin-like arabinogalactan protein 2 |
| augustus-2-processed-gene-310.4-mRNA-1 | Japonica | NA | | Os02g0726300 | OsR498G0204687800.01.T01 | Dof zinc finger protein like |
| augustus-2-processed-gene-311.20-mRNA-1 | Japonica | Indica | | Os06g0271600 | OsR498G0204702700.01.T01 | E3 ubiquitin-protein ligase ATL15 |
| augustus-2-processed-gene-311.21-mRNA-1 | Japonica | NA | | Os02g0729900 | OsR498G0204702900.01.T01 | RING-H2 finger protein ATL1M |
| augustus-2-processed-gene-311.8-mRNA-1 | Japonica | Indica | | Os03g0351300 | OsR498G0204693600.01.T01 | Beta-amylase |
| augustus-2-processed-gene-312.0-mRNA-1 | Japonica | NA | | Os02g0730300 | OsR498G0204704700.01.T04 | Potassium transporter |
| augustus-2-processed-gene-312.18-mRNA-1 | Japonica | NA | | Os02g0731900 | OsR498G0204710500.01.T01 | Very-long-chain 3-oxoacyl-CoA synthase |
| augustus-2-processed-gene-312.26-mRNA-1 | Japonica | NA | | Os02g0730900 | | Pentatricopeptide repeat-containing protein chloroplastic |
| augustus-2-processed-gene-313.10-mRNA-1 | Japonica | NA | | Os02g0732500 | OsR498G0204713000.01.T03 | ADP-ribosylation factor-like protein 8c isoform A |
| augustus-2-processed-gene-313.26-mRNA-1 | Japonica | NA | | Os02g0733500 | OsR498G0204717000.01.T02 | Probable calcium-binding protein CML20 |
| augustus-2-processed-gene-314.13-mRNA-1 | Japonica | Indica | | Os02g0734900 | OsR498G0204722100.01.T01 | Endoplasmic reticulum transmembrane protein (Fragment) |
| augustus-2-processed-gene-314.16-mRNA-1 | Japonica | Indica | | Os03g0589100 | OsR498G0204724500.01.T01 | Retrotransposon protein, putative, Ty3-gypsy sub-class |
| augustus-2-processed-gene-316.47-mRNA-1 | Japonica | NA | | Os02g0739400 | OsR498G0204738600.01.T01 | Hexosyltransferase |
| augustus-2-processed-gene-317.49-mRNA-1 | Japonica | NA | | Os02g0740700 | OsR498G0204744400.01.T01 | Metalloendoproteinase 2-MMP |
| augustus-2-processed-gene-318.5-mRNA-1 | Japonica | Indica | | Os02g0742000 | OsR498G0204748700.01.T01 | BAH and coiled-coil domain-containing protein 1 |
| augustus-2-processed-gene-319.0-mRNA-1 | Japonica | NA | | Os02g0742700 | OsR498G0204750500.01.T01 | protein RICE SALT SENSITIVE 3 |
| augustus-2-processed-gene-319.4-mRNA-1 | Japonica | NA | | | OsR498G0204752800.01.T01 | Retrotransposon protein, putative, unclassified |
| augustus-2-processed-gene-319.8-mRNA-1 | Japonica | Japonica | | Os02g0743100 | OsR498G0204753500.01.T01 | RING-H2 finger protein ATL5I |
| augustus-2-processed-gene-320.59-mRNA-1 | Japonica | NA | | | OsR498G0204765500.01.T01 | |
| augustus-2-processed-gene-321.24-mRNA-1 | Japonica | NA | | | OsR498G0511427100.01.T01 | Epstein-Barr virus EBNA-1-like |
| augustus-2-processed-gene-335.26-mRNA-1 | Japonica | NA | | | OsR498G0613106300.01.T01 | |
| augustus-2-processed-gene-337.13-mRNA-1 | Japonica | Indica | | Os01g0740700 | OsR498G0204773100.01.T01 | Phytocyanin domain-containing protein |
| augustus-2-processed-gene-337.2-mRNA-1 | Japonica | NA | | Os02g0747400 | OsR498G0204771300.01.T01 | Transcription factor TCP subgroup |
| augustus-2-processed-gene-337.29-mRNA-1 | Japonica | NA | | | OsR498G0204776600.01.T02 | F-box/kelch-repeat protein SKIP11 |
| augustus-2-processed-gene-337.30-mRNA-1 | Japonica | NA | | Os02g0748800 | | Glutaredoxin domain-containing protein (Fragment) |
| augustus-2-processed-gene-338.1-mRNA-1 | Japonica | NA | | | OsR498G0204779900.01.T01 | |
| augustus-2-processed-gene-338.10-mRNA-1 | Japonica | NA | | Os02g0749900 | OsR498G0204782500.01.T01 | Mitochondrial transcription termination factor-like |
| augustus-2-processed-gene-338.9-mRNA-1 | Japonica | NA | | | OsR498G0204782300.01.T02 | mTERF transcription factor |
| augustus-2-processed-gene-34.13-mRNA-1 | Japonica | NA | | Os02g0162600 | OsR498G0203023100.01.T01 | |
| augustus-2-processed-gene-34.2-mRNA-1 | Japonica | Japonica | LRR-TM | Os02g0161700 | OsR498G0203018700.01.T01 | LRR receptor-like serine/threonine-protein kinase FLS2 |
| augustus-2-processed-gene-35.50-mRNA-1 | Japonica | Indica | | Os06g0679500 | OsR498G0203023900.01.T03 | Hexosyltransferase |
| augustus-2-processed-gene-35.52-mRNA-1 | Japonica | NA | | | | Agamous-like MADS-box protein AGL61 |

| | | | | | | |
|---|---|---|---|---|---|---|
| augustus-2-processed-gene-35.56-mRNA-1 | Japonica | NA | | Os02g0164600 | OsR498G0203026500.01.T06 | Pentatricopeptide repeat (PPR) superfamily protein isoform 1 |
| augustus-2-processed-gene-355.36-mRNA-1 | Japonica | NA | | Os02g0757700 | OsR498G0204807100.01.T06 | F-box protein FBW2 |
| augustus-2-processed-gene-355.39-mRNA-1 | Japonica | NA | | | OsR498G0204808900.01.T01 | |
| augustus-2-processed-gene-355.47-mRNA-1 | Japonica | NA | | Os02g0758200 | | |
| augustus-2-processed-gene-356.1-mRNA-1 | Japonica | NA | | Os02g0759400 | OsR498G0204812100.01.T01 | E3 ubiquitin-protein ligase ATL6 |
| augustus-2-processed-gene-356.17-mRNA-1 | Japonica | NA | | Os02g0760400 | | |
| augustus-2-processed-gene-356.19-mRNA-1 | Japonica | NA | | Os02g0760700 | | |
| augustus-2-processed-gene-356.2-mRNA-1 | Japonica | NA | | Os02g0759500 | OsR498G0204812500.01.T01 | Pentatricopeptide repeat-containing protein mitochondrial |
| augustus-2-processed-gene-356.28-mRNA-1 | Japonica | NA | | Os02g0759800 | OsR498G0204814200.01.T01 | SNW/SKI-interacting protein-like |
| augustus-2-processed-gene-357.24-mRNA-1 | Japonica | NA | | Os02g0762100 | OsR498G0204819800.01.T01 | 4-hydroxy-4-methyl-2-oxoglutarate aldolase |
| augustus-2-processed-gene-357.5-mRNA-1 | Japonica | NA | | Os02g0761300 | | |
| augustus-2-processed-gene-358.22-mRNA-1 | Japonica | NA | | | OsR498G0204046800.01.T01 | Epstein-Barr virus EBNA-1-like |
| augustus-2-processed-gene-358.40-mRNA-1 | Japonica | NA | | | OsR498G0204046800.01.T01 | Circumsporozoite protein-like |
| augustus-2-processed-gene-358.55-mRNA-1 | Japonica | NA | | Os02g0764700 | OsR498G0204826400.01.T01 | Ethylene-responsive element binding protein 2 |
| augustus-2-processed-gene-359.43-mRNA-1 | Japonica | NA | | Os02g0766600 | OsR498G0204829100.01.T01 | HTH-type transcriptional repressor YcgE |
| augustus-2-processed-gene-359.50-mRNA-1 | Japonica | NA | | Os01g0357100 | OsR498G0100930500.01.T02 | Ferredoxin--nitrite reductase chloroplastic |
| augustus-2-processed-gene-359.51-mRNA-1 | Japonica | NA | | | OsR498G0204827800.01.T02 | Protein DCL, chloroplastic |
| augustus-2-processed-gene-359.60-mRNA-1 | Japonica | NA | | | OsR498G0204832700.01.T01 | RING-type E3 ubiquitin transferase |
| augustus-2-processed-gene-359.68-mRNA-1 | Japonica | NA | Kinase-TM | Os02g0767400 | OsR498G0204832400.01.T01 | Non-specific serine/threonine protein kinase |
| augustus-2-processed-gene-359.70-mRNA-1 | Japonica | NA | | Os02g0767700 | OsR498G0204833000.01.T03 | Prolyl-tRNA synthetase associated domain-containing protein 1 |
| augustus-2-processed-gene-360.31-mRNA-1 | Japonica | NA | | Os02g0768700 | OsR498G0204836200.01.T01 | GeBP transcription factor (Fragment) |
| augustus-2-processed-gene-361.17-mRNA-1 | Japonica | NA | | Os02g0770600 | OsR498G0204844300.01.T02 | G-protein coupled receptor 15 like |
| augustus-2-processed-gene-361.28-mRNA-1 | Japonica | NA | | Os02g0771200 | OsR498G0204846200.01.T01 | Putative tyrosine-protein phosphatase |
| augustus-2-processed-gene-361.8-mRNA-1 | Japonica | Indica | | Os02g0769900 | OsR498G0204841800.01.T01 | Chloroplast biogenesis 19 (Fragment) |
| augustus-2-processed-gene-362.38-mRNA-1 | Japonica | NA | | Os01g0703000 | OsR498G0101842900.01.T01 | |
| augustus-2-processed-gene-363.60-mRNA-1 | Japonica | NA | | Os02g0773600 | OsR498G0204854400.01.T01 | WRC domain-containing protein (Fragment) |
| augustus-2-processed-gene-363.83-mRNA-1 | Japonica | NA | | Os02g0775300 | OsR498G0204858200.01.T02 | |
| augustus-2-processed-gene-364.17-mRNA-1 | Japonica | NA | | Os02g0775600 | OsR498G0204860400.01.T01 | Zinc finger protein 6 |
| augustus-2-processed-gene-364.42-mRNA-1 | Japonica | NA | | | OsR498G0204863800.01.T02 | CCAAT-binding transcription factor B |
| augustus-2-processed-gene-366.26-mRNA-1 | Japonica | NA | | Os02g0779200 | OsR498G0204874800.01.T01 | Senescence-associated subtilisin protease |
| augustus-2-processed-gene-366.8-mRNA-1 | Japonica | Japonica | | Os02g0778900 | OsR498G0204874500.01.T01 | Subtilisin-like protease |
| augustus-2-processed-gene-367.69-mRNA-1 | Japonica | NA | | Os07g0527600 | OsR498G0917525300.01.T01 | Putative cell death associated protein |
| augustus-2-processed-gene-367.78-mRNA-1 | Japonica | Indica | | Os02g0781500 | OsR498G0204884700.01.T01 | GPI ethanolamine phosphate transferase 2 |
| augustus-2-processed-gene-367.82-mRNA-1 | Japonica | NA | | | OsR498G0204881400.01.T01 | ATP-dependent DNA helicase |
| augustus-2-processed-gene-368.18-mRNA-1 | Japonica | NA | | Os02g0783100 | OsR498G0204892600.01.T01 | Mitochondrial transcription termination factor |
| augustus-2-processed-gene-369.26-mRNA-1 | Japonica | NA | | | OsR498G0204897800.01.T01 | DUF295 domain-containing protein |
| augustus-2-processed-gene-369.28-mRNA-1 | Japonica | NA | | | OsR498G0204898400.01.T01 | Plastid transcriptionally active 18 |
| augustus-2-processed-gene-369.5-mRNA-1 | Japonica | NA | | | OsR498G0204894500.01.T01 | F-box domain-containing protein (Fragment) |
| augustus-2-processed-gene-370.38-mRNA-1 | Japonica | NA | | Os02g0785800 | OsR498G0204900900.01.T02 | 60S ribosomal protein l33-b |
| augustus-2-processed-gene-370.47-mRNA-1 | Japonica | Indica | | Os02g0785000 | OsR498G0204900600.01.T01 | Hexosyltransferase |
| augustus-2-processed-gene-370.81-mRNA-1 | Japonica | NA | | Os02g0787000 | | SCP domain-containing protein (Fragment) |
| augustus-2-processed-gene-371.37-mRNA-1 | Japonica | NA | Kinase | Os02g0787300 | OsR498G0204906600.01.T01 | MAP kinase kinase |
| augustus-2-processed-gene-372.27-mRNA-1 | Japonica | NA | | Os02g0791200 | OsR498G0204917500.01.T02 | Probable E3 ubiquitin-protein ligase XBOS32 |
| augustus-2-processed-gene-372.29-mRNA-1 | Japonica | NA | | Os02g0791500 | OsR498G0204919300.01.T01 | UDP-glucuronate 4-epimerase 1 |
| augustus-2-processed-gene-372.39-mRNA-1 | Japonica | NA | | Os02g0790600 | OsR498G0204916600.01.T01 | Putative RING-H2 finger protein ATL49 |
| augustus-2-processed-gene-373.44-mRNA-1 | Japonica | NA | | Os02g0792800 | | Protein TIC 55 chloroplastic |
| augustus-2-processed-gene-373.46-mRNA-1 | Japonica | Indica | | Os02g0793200 | OsR498G0204924100.01.T02 | Zinc finger (CCCH-type) protein-like |
| augustus-2-processed-gene-375.1-mRNA-1 | Japonica | NA | | | OsR498G0204932500.01.T01 | RNA polymerase II C-terminal domain phosphatase-like |
| augustus-2-processed-gene-375.4-mRNA-1 | Japonica | NA | | Os02g0796300 | | Poly(A)-specific ribonuclease |
| augustus-2-processed-gene-376.0-mRNA-1 | Japonica | NA | | Os02g0797300 | OsR498G0204938900.01.T01 | Plasma membrane ATPase |
| augustus-2-processed-gene-376.19-mRNA-1 | Japonica | NA | | Os02g0798450 | OsR498G0204942600.01.T01 | |
| augustus-2-processed-gene-377.26-mRNA-1 | Japonica | NA | | Os02g0799000 | OsR498G0204944700.01.T02 | Protein-serine/threonine phosphatase |
| augustus-2-processed-gene-377.29-mRNA-1 | Japonica | Indica | | Os02g0799600 | OsR498G0204947300.01.T01 | Serine-rich protein-related |
| augustus-2-processed-gene-377.42-mRNA-1 | Japonica | NA | | Os02g0800300 | OsR498G0204954900.01.T01 | |
| augustus-2-processed-gene-377.52-mRNA-1 | Japonica | NA | | | | NADH dehydrogenase [ubiquinone] 1 alpha subcomplex subunit 9 mitochon |
| augustus-2-processed-gene-378.13-mRNA-1 | Japonica | NA | | | OsR498G0204960100.01.T01 | |
| augustus-2-processed-gene-378.15-mRNA-1 | Japonica | NA | | | OsR498G0204961100.01.T02 | |
| augustus-2-processed-gene-378.29-mRNA-1 | Japonica | NA | | | OsR498G1018994800.01.T01 | |
| augustus-2-processed-gene-379.15-mRNA-1 | Japonica | NA | | Os02g0805400 | | influenza virus NS1A-binding protein homolog A |
| augustus-2-processed-gene-379.16-mRNA-1 | Japonica | NA | | Os02g0805600 | OsR498G0204968900.01.T01 | Quinone oxidoreductase PIG3 |
| augustus-2-processed-gene-379.30-mRNA-1 | Japonica | NA | | Os02g0805500 | OsR498G0204968800.01.T01 | 3'(2'),5'-bisphosphate nucleotidase |

| | | | | | | |
|---|---|---|---|---|---|---|
| augustus-2-processed-gene-381.26-mRNA-1 | Japonica | NA | | Os02g0809100 | OsR498G0204980200.01.T01 | PB1 domain-containing protein (Fragment) |
| augustus-2-processed-gene-381.28-mRNA-1 | Japonica | NA | | | OsR498G0511427100.01.T01 | Circumsporozoite protein-like |
| augustus-2-processed-gene-382.2-mRNA-1 | Japonica | NA | | Os02g0809800 | OsR498G0204983100.01.T04 | Phosphate transporter PHO1-2 |
| augustus-2-processed-gene-382.3-mRNA-1 | Japonica | NA | | Os02g0809800 | OsR498G0204983100.01.T04 | Phosphate transporter PHO1-2 |
| augustus-2-processed-gene-383.42-mRNA-1 | Japonica | Japonica | | Os02g0812700 | OsR498G0204994700.01.T01 | Phloem-specific lectin |
| augustus-2-processed-gene-383.44-mRNA-1 | Japonica | Indica | | Os02g0813300 | OsR498G0204994900.01.T01 | F-box family protein-like |
| augustus-2-processed-gene-384.80-mRNA-1 | Japonica | NA | | Os02g0814700 | OsR498G0205000500.01.T01 | Ribosomal protein L17 |
| augustus-2-processed-gene-385.76-mRNA-1 | Japonica | NA | | Os02g0816000 | OsR498G0205003700.01.T01 | FT-interacting protein 1 |
| augustus-2-processed-gene-385.80-mRNA-1 | Japonica | NA | | Os02g0816800 | OsR498G0205007100.01.T01 | Epimerase domain-containing protein |
| augustus-2-processed-gene-386.43-mRNA-1 | Japonica | NA | | Os02g0817000 | OsR498G0205007900.01.T02 | Myb-like domain-containing protein (Fragment) |
| augustus-2-processed-gene-386.56-mRNA-1 | Japonica | NA | | Os02g0817700 | OsR498G0205010200.01.T01 | Acetyl-CoA C-acyltransferase-like protein |
| augustus-2-processed-gene-386.60-mRNA-1 | Japonica | NA | | | OsR498G0205011000.01.T02 | Putative NB-ARC domain-containing protein (Fragment) |
| augustus-2-processed-gene-386.62-mRNA-1 | Japonica | NA | | Os02g0818500 | | |
| augustus-2-processed-gene-387.38-mRNA-1 | Japonica | NA | | Os02g0818800 | | Selenium-binding protein-like |
| augustus-2-processed-gene-387.52-mRNA-1 | Japonica | NA | | Os02g0820200 | OsR498G0205020500.01.T01 | RING-H2 finger protein ATL1 |
| augustus-2-processed-gene-388.51-mRNA-1 | Japonica | NA | | Os02g0820800 | OsR498G0205022500.01.T01 | PPC domain-containing protein (Fragment) |
| augustus-2-processed-gene-388.56-mRNA-1 | Japonica | NA | | Os02g0820400 | OsR498G0205021000.01.T01 | Anthocyanin 5-aromatic acyltransferase |
| augustus-2-processed-gene-389.43-mRNA-1 | Japonica | NA | | Os02g0822700 | | 4-coumarate--CoA ligase-like 5 |
| augustus-2-processed-gene-389.59-mRNA-1 | Japonica | Japonica | | Os02g0824100 | OsR498G0100001000.01.T01 | Cytokinin trans-hydroxylase |
| augustus-2-processed-gene-389.67-mRNA-1 | Japonica | NA | | | | Zinc finger protein 1 |
| augustus-2-processed-gene-39.27-mRNA-1 | Japonica | NA | | Os02g0170701 | | SLT domain-containing protein |
| augustus-2-processed-gene-39.9-mRNA-1 | Japonica | NA | | Os02g0170500 | OsR498G0203056200.01.T03 | CCAAT-binding transcription factor C |
| augustus-2-processed-gene-390.14-mRNA-1 | Japonica | Indica | | Os02g0825100 | OsR498G0205034900.01.T01 | F-box domain-containing protein |
| augustus-2-processed-gene-390.33-mRNA-1 | Japonica | NA | | Os02g0826300 | | |
| augustus-2-processed-gene-390.4-mRNA-1 | Japonica | Japonica | | Os02g0825500 | OsR498G0205034900.01.T01 | F-box domain-containing protein |
| augustus-2-processed-gene-390.45-mRNA-1 | Japonica | NA | | Os02g0826400 | OsR498G0205038000.01.T03 | U-box domain-containing protein 30 |
| augustus-2-processed-gene-391.79-mRNA-1 | Japonica | Indica | | Os02g0828500 | OsR498G0205043500.01.T03 | E3 ubiquitin-protein ligase RNF25 |
| augustus-2-processed-gene-391.80-mRNA-1 | Japonica | NA | | Os02g0828500 | OsR498G0205044100.01.T01 | Tetratricopeptide-like helical |
| augustus-2-processed-gene-392.11-mRNA-1 | Japonica | NA | | Os02g0830000 | | Pentatricopeptide repeat |
| augustus-2-processed-gene-392.20-mRNA-1 | Japonica | NA | | Os02g0829800 | OsR498G0205048000.01.T01 | Membrane-associated salt-inducible protein-like |
| augustus-2-processed-gene-392.5-mRNA-1 | Japonica | NA | | Os09g0562400 | OsR498G0101823900.01.T01 | Transposon protein, putative, Mutator sub-class, expressed |
| augustus-2-processed-gene-393.107-mRNA-1 | Japonica | NA | | Os02g0831800 | | Homoserine kinase |
| augustus-2-processed-gene-393.111-mRNA-1 | Japonica | NA | | Os02g0832600 | OsR498G0205054500.01.T02 | Large tegument protein like |
| augustus-2-processed-gene-393.118-mRNA-1 | Japonica | NA | | Os02g0833500 | OsR498G0205056900.01.T01 | Cation/H(+) antiporter 1 |
| augustus-2-processed-gene-393.149-mRNA-1 | Japonica | NA | | Os02g0832150 | | RING-H2 finger protein ATL44 |
| augustus-2-processed-gene-393.152-mRNA-1 | Japonica | NA | | Os02g0833300 | OsR498G0205056400.01.T05 | ESCRT-I complex subunit TSG101 |
| augustus-2-processed-gene-4.37-mRNA-1 | Japonica | NA | Kinase-LRR-TM | Os02g0211200 | | Putative LRR receptor-like serine/threonine-protein kinase |
| augustus-2-processed-gene-4.49-mRNA-1 | Japonica | NA | | Os02g0107600 | OsR498G0202821800.01.T04 | SEC63 domain-containing protein |
| augustus-2-processed-gene-43.21-mRNA-1 | Japonica | Japonica | | Os02g0178300 | OsR498G0203084800.01.T01 | Protein GLUTAMINE DUMPER 5 |
| augustus-2-processed-gene-48.1-mRNA-1 | Japonica | Indica | | Os02g0184900 | OsR498G0203109500.01.T01 | Premnaspirodiene oxygenase |
| augustus-2-processed-gene-49.23-mRNA-1 | Japonica | Indica | | Os02g0188000 | OsR498G0203122000.01.T02 | Glycosyltransferase |
| augustus-2-processed-gene-5.21-mRNA-1 | Japonica | NA | | Os02g0108400 | OsR498G0202827500.01.T01 | SEC63 domain-containing protein |
| augustus-2-processed-gene-51.25-mRNA-1 | Japonica | Indica | | Os02g0190600 | OsR498G0203132400.01.T02 | Lycopene beta cyclase chloroplastic |
| augustus-2-processed-gene-51.31-mRNA-1 | Japonica | Japonica | | Os02g0191200 | OsR498G0613274200.01.T01 | Pentatricopeptide repeat-containing protein chloroplastic/mitochondrial |
| augustus-2-processed-gene-51.36-mRNA-1 | Japonica | Japonica | CC-LRR-NBS-TM | Os02g0191000 | OsR498G0203022300.01.T01 | Disease resistance protein RPM1 |
| augustus-2-processed-gene-51.39-mRNA-1 | Japonica | NA | | Os02g0191600 | | BZIP domain-containing protein (Fragment) |
| augustus-2-processed-gene-52.12-mRNA-1 | Japonica | NA | Kinase-TM | | OsR498G0203139900.01.T01 | Proline-rich receptor-like protein kinase PERK9 (Fragment) |
| augustus-2-processed-gene-52.13-mRNA-1 | Japonica | NA | | | OsR498G0203142100.01.T02 | Subtilisin-like protease-like isoform X1 |
| augustus-2-processed-gene-52.19-mRNA-1 | Japonica | NA | | Os02g0193100 | OsR498G0203140400.01.T01 | TMV response protein |
| augustus-2-processed-gene-52.20-mRNA-1 | Japonica | NA | | Os02g0193200 | OsR498G0203141200.01.T01 | |
| augustus-2-processed-gene-53.2-mRNA-1 | Japonica | NA | | Os02g0193900 | OsR498G0203143300.01.T01 | Mediator of RNA polymerase II transcription subunit like |
| augustus-2-processed-gene-57.16-mRNA-1 | Japonica | NA | | Os02g0202000 | OsR498G0203172500.01.T01 | AP domain containing transcription factor |
| augustus-2-processed-gene-57.24-mRNA-1 | Japonica | NA | | | OsR498G0203169800.01.T02 | |
| augustus-2-processed-gene-58.4-mRNA-1 | Japonica | NA | | Os02g0203300 | OsR498G0203177300.01.T01 | Glycosyltransferase |
| augustus-2-processed-gene-59.5-mRNA-1 | Japonica | NA | | Os02g0204100 | | Tetratricopeptide repeat (TPR)-like superfamily protein |
| augustus-2-processed-gene-6.0-mRNA-1 | Japonica | NA | | Os02g0110400 | OsR498G0202833200.01.T02 | Pentatricopeptide repeat |
| augustus-2-processed-gene-6.11-mRNA-1 | Japonica | NA | | Os02g0111900 | OsR498G0202836100.01.T01 | Nucleic acid binding,ATP-dependent helicase |
| augustus-2-processed-gene-6.12-mRNA-1 | Japonica | NA | | Os02g0112600 | OsR498G0202837400.01.T01 | High affinity nitrate transporter |
| augustus-2-processed-gene-6.27-mRNA-1 | Japonica | NA | Kinase-LRR-TM | Os02g0111800 | OsR498G0202835600.01.T01 | Leucine-rich repeat receptor-like protein kinase PXL2 |
| augustus-2-processed-gene-6.8-mRNA-1 | Japonica | NA | | | OsR498G0202834300.01.T01 | Peptidyl-prolyl cis-trans isomerase |
| augustus-2-processed-gene-60.18-mRNA-1 | Japonica | NA | | | OsR498G0611960200.01.T01 | |

| | | | | | | |
|---|---|---|---|---|---|---|
| augustus-2-processed-gene-61.12-mRNA-1 | Japonica | NA | | | OsR498G0203195100.01.T04 | RRM domain-containing protein (Fragment) |
| augustus-2-processed-gene-61.21-mRNA-1 | Japonica | NA | | Os02g0207900 | OsR498G0203194400.01.T01 | Caffeoylshikimate esterase |
| augustus-2-processed-gene-61.22-mRNA-1 | Japonica | Indica | | Os02g0208700 | OsR498G0203197400.01.T01 | Kelch repeat type 1 |
| augustus-2-processed-gene-62.56-mRNA-1 | Japonica | NA | | Os02g0212975 | OsR498G1019116500.01.T01 | |
| augustus-2-processed-gene-62.71-mRNA-1 | Japonica | Japonica | | Os02g0209400 | OsR498G0203200800.01.T02 | |
| augustus-2-processed-gene-63.19-mRNA-1 | Japonica | NA | | Os02g0211000 | OsR498G0203203700.01.T01 | ATP-binding cassette transporter subfamily A member |
| augustus-2-processed-gene-64.18-mRNA-1 | Japonica | NA | | | | |
| augustus-2-processed-gene-64.19-mRNA-1 | Japonica | NA | | Os02g0213800 | | |
| augustus-2-processed-gene-65.10-mRNA-1 | Japonica | NA | | Os02g0214600 | | Retrotransposon protein, putative, Ty3-gypsy subclass |
| augustus-2-processed-gene-65.14-mRNA-1 | Japonica | Japonica | | Os02g0215100 | OsR498G0203218300.01.T01 | |
| augustus-2-processed-gene-67.10-mRNA-1 | Japonica | NA | | Os02g0218700 | OsR498G0203227900.01.T01 | Allene oxide synthase, chloroplastic |
| augustus-2-processed-gene-67.11-mRNA-1 | Japonica | NA | | Os02g0218800 | OsR498G0203228700.01.T01 | Allene oxide synthase, chloroplastic |
| augustus-2-processed-gene-67.28-mRNA-1 | Japonica | NA | | Os02g0219000 | OsR498G0203229000.01.T01 | Interferon-related developmental regulator family protein / IFRD protein far |
| augustus-2-processed-gene-67.29-mRNA-1 | Japonica | Japonica | | Os02g0219050 | OsR498G0816363900.01.T01 | Interferon-related developmental regulator family protein / IFRD protein far |
| augustus-2-processed-gene-69.10-mRNA-1 | Japonica | Indica | | Os06g0564500 | OsR498G0203238300.01.T03 | PALP domain-containing protein |
| augustus-2-processed-gene-69.18-mRNA-1 | Japonica | NA | | Os02g0221500 | OsR498G0203237200.01.T01 | Heterogeneous nuclear ribonucleoprotein 1 |
| augustus-2-processed-gene-70.32-mRNA-1 | Japonica | NA | | Os02g0223200 | | GTP cyclohydrolase II |
| augustus-2-processed-gene-72.46-mRNA-1 | Japonica | NA | | | | Hydroxyproline-rich glycoprotein-like |
| augustus-2-processed-gene-74.10-mRNA-1 | Japonica | NA | | Os02g0229400 | OsR498G0203272500.01.T04 | Monosaccharide-sensing protein 2 |
| augustus-2-processed-gene-75.0-mRNA-1 | Japonica | NA | | Os02g0230200 | OsR498G0203275100.01.T05 | Secretion-regulating guanine nucleotide exchange factor |
| augustus-2-processed-gene-76.28-mRNA-1 | Japonica | NA | | Os02g0231600 | | DDE Tnp4 domain-containing protein (Fragment) |
| augustus-2-processed-gene-76.43-mRNA-1 | Japonica | NA | | | OsR498G0713376400.01.T01 | |
| augustus-2-processed-gene-77.9-mRNA-1 | Japonica | Indica | | Os02g0234300 | OsR498G0203292500.01.T01 | RING-type E3 ubiquitin transferase |
| augustus-2-processed-gene-78.37-mRNA-1 | Japonica | NA | | Os02g0234500 | OsR498G0203293100.01.T04 | NAD(P)-binding rossmann-fold protein |
| augustus-2-processed-gene-79.23-mRNA-1 | Japonica | NA | | Os02g0236500 | | TTF-type domain-containing protein |
| augustus-2-processed-gene-79.29-mRNA-1 | Japonica | Japonica | | Os02g0237000 | OsR498G0203303300.01.T01 | Peroxidase |
| augustus-2-processed-gene-81.50-mRNA-1 | Japonica | Japonica | | Os02g0241700 | OsR498G0203313200.01.T01 | Glycosyltransferase |
| augustus-2-processed-gene-82.10-mRNA-1 | Japonica | NA | | Os02g0242100 | OsR498G0203313200.01.T01 | Glycosyltransferase |
| augustus-2-processed-gene-82.27-mRNA-1 | Japonica | NA | | Os02g0242900 | OsR498G0203314800.01.T01 | Glycosyltransferase |
| augustus-2-processed-gene-82.31-mRNA-1 | Japonica | NA | | Os02g0243300 | OsR498G0203315000.01.T02 | Glycosyltransferase |
| augustus-2-processed-gene-83.9-mRNA-1 | Japonica | NA | | Os02g0245100 | OsR498G0203320200.01.T01 | Peroxisome bioproteinsis protein 7 |
| augustus-2-processed-gene-84.13-mRNA-1 | Japonica | NA | | | OsR498G0713983500.01.T01 | |
| augustus-2-processed-gene-84.20-mRNA-1 | Japonica | NA | | | OsR498G0203321400.01.T01 | |
| augustus-2-processed-gene-84.27-mRNA-1 | Japonica | NA | | Os02g0245900 | | |
| augustus-2-processed-gene-85.17-mRNA-1 | Japonica | NA | | | OsR498G0203327800.01.T01 | E3 ubiquitin-protein ligase EL5-like |
| augustus-2-processed-gene-85.20-mRNA-1 | Japonica | Indica | | Os02g0248320 | OsR498G0203329000.01.T01 | RING-H2 finger protein ATL5H |
| augustus-2-processed-gene-85.24-mRNA-1 | Japonica | NA | | | | RING-H2 finger protein ATL5H |
| augustus-2-processed-gene-85.4-mRNA-1 | Japonica | Japonica | | Os02g0247200 | OsR498G0203326000.01.T01 | T-complex protein 1 subunit gamma |
| augustus-2-processed-gene-85.9-mRNA-1 | Japonica | NA | | | OsR498G0203327100.01.T01 | |
| augustus-2-processed-gene-86.0-mRNA-1 | Japonica | NA | | Os02g0249300 | OsR498G0203331500.01.T01 | RING-H2 finger protein ATL1N |
| augustus-2-processed-gene-86.2-mRNA-1 | Japonica | NA | | Os02g0249500 | | Cupin type-1 domain-containing protein |
| augustus-2-processed-gene-86.20-mRNA-1 | Japonica | NA | | Os01g0265300 | OsR498G0100612800.01.T01 | Transposable element protein, putative, MuDR |
| augustus-2-processed-gene-86.23-mRNA-1 | Japonica | NA | | Os02g0250600 | | Seed biotin-containing protein SBP65 isoform X1 |
| augustus-2-processed-gene-86.24-mRNA-1 | Japonica | NA | | | OsR498G0203337800.01.T01 | |
| augustus-2-processed-gene-87.0-mRNA-1 | Japonica | NA | | Os02g0251700 | OsR498G0203339500.01.T02 | Dual specificity phosphatase, catalytic domain containing protein, expressed |
| augustus-2-processed-gene-87.20-mRNA-1 | Japonica | NA | | Os02g0252400 | OsR498G0203342200.01.T01 | Dof zinc finger protein like |
| augustus-2-processed-gene-88.44-mRNA-1 | Japonica | NA | | Os02g0255100 | OsR498G0203347900.01.T01 | PPM-type phosphatase domain-containing protein |
| augustus-2-processed-gene-90.22-mRNA-1 | Japonica | NA | | Os02g0258800 | OsR498G0203360700.01.T03 | |
| augustus-2-processed-gene-90.30-mRNA-1 | Japonica | Indica | | Os06g0523100 | OsR498G0203361400.01.T03 | |
| augustus-2-processed-gene-93.14-mRNA-1 | Japonica | Japonica | NBS-TM | Os02g0262800 | OsR498G0203369300.01.T01 | Powdery mildew resistance protein |
| augustus-2-processed-gene-95.1-mRNA-1 | Japonica | Japonica | | Os02g0265300 | OsR498G0203374300.01.T01 | |
| augustus-2-processed-gene-99.35-mRNA-1 | Japonica | Indica | | Os10g0130200 | OsR498G0203391400.01.T01 | F-box domain containing protein, expressed |
| augustus-3-processed-gene-100.38-mRNA-1 | Japonica | NA | | | OsR498G0305744400.01.T01 | |
| augustus-3-processed-gene-101.76-mRNA-1 | Japonica | NA | | | OsR498G0305753500.01.T05 | |
| augustus-3-processed-gene-102.33-mRNA-1 | Japonica | NA | | | OsR498G0305757300.01.T02 | Pentatricopeptide repeat |
| augustus-3-processed-gene-102.65-mRNA-1 | Japonica | Japonica | | Os03g0299600 | OsR498G0305763200.01.T01 | Senescence associated gene 20 |
| augustus-3-processed-gene-110.47-mRNA-1 | Japonica | NA | | Os03g0314500 | OsR498G0305816300.01.T01 | Tol-Pal system beta propeller repeat-containing protein |
| augustus-3-processed-gene-118.23-mRNA-1 | Japonica | Japonica | | Os03g0103400 | OsR498G0305078700.01.T01 | GRAS domain-containing protein (Fragment) |
| augustus-3-processed-gene-144.3-mRNA-1 | Japonica | NA | | Os10g0550000 | OsR498G1019144500.01.T01 | Phosphatidylinositol N-acetyglucosaminlytransferase subunit P-related |
| augustus-3-processed-gene-144.4-mRNA-1 | Japonica | NA | | | OsR498G1019145300.01.T01 | Mediator of RNA polymerase II transcription subunit 12 |
| augustus-3-processed-gene-149.22-mRNA-1 | Japonica | NA | | Os03g0325700 | OsR498G0305859100.01.T01 | QXW lectin repeat family protein |

| | | | | | | |
|---|---|---|---|---|---|---|
| augustus-3-processed-gene-151.33-mRNA-1 | Japonica | NA | | | | DDE Tnp4 domain-containing protein |
| augustus-3-processed-gene-151.34-mRNA-1 | Japonica | Japonica | | Os03g0328200 | OsR498G0510886900.01.T01 | Transposon protein, putative, CACTA, En/Spm sub-class |
| augustus-3-processed-gene-178.51-mRNA-1 | Japonica | Indica | | Os07g0640900 | OsR498G0306009600.01.T01 | Heat shock transcription factor, other eukaryote |
| augustus-3-processed-gene-215.19-mRNA-1 | Japonica | NA | | Os03g0425600 | OsR498G0306243700.01.T01 | UDP-glucose 6-dehydrogenase 5 |
| augustus-3-processed-gene-22.3-mRNA-1 | Japonica | NA | | Os03g0143600 | OsR498G0305218500.01.T01 | PHD finger protein ING |
| augustus-3-processed-gene-232.24-mRNA-1 | Japonica | NA | | | OsR498G0100933000.01.T01 | Retrotransposon protein, putative, Ty3-gypsy sub-class |
| augustus-3-processed-gene-232.3-mRNA-1 | Japonica | NA | | | OsR498G0510213600.01.T01 | |
| augustus-3-processed-gene-26.23-mRNA-1 | Japonica | NA | | | OsR498G0100724200.01.T01 | Transposon protein, putative, Mutator sub-class, expressed |
| augustus-3-processed-gene-281.57-mRNA-1 | Japonica | NA | | | OsR498G1018661500.01.T01 | |
| augustus-3-processed-gene-29.11-mRNA-1 | Japonica | NA | | Os03g0158600 | OsR498G0305257100.01.T02 | RNase H domain-containing protein (Fragment) |
| augustus-3-processed-gene-290.10-mRNA-1 | Japonica | NA | | | | Aminotransferase-like |
| augustus-3-processed-gene-294.25-mRNA-1 | Japonica | NA | | Os10g0381700 | | F-box domain containing protein, expressed |
| augustus-3-processed-gene-3.69-mRNA-1 | Japonica | NA | | Os03g0110400 | OsR498G0305103900.01.T02 | THO complex, subunit THOC |
| augustus-3-processed-gene-303.4-mRNA-1 | Japonica | NA | | | OsR498G0204301500.01.T06 | ATP-dependent DNA helicase |
| augustus-3-processed-gene-322.36-mRNA-1 | Japonica | NA | | Os12g0468900 | OsR498G0407549800.01.T01 | Cell wall protein-like |
| augustus-3-processed-gene-335.16-mRNA-1 | Japonica | NA | | Os03g0558000 | | |
| augustus-3-processed-gene-388.39-mRNA-1 | Japonica | NA | | Os03g0648400 | OsR498G0306670300.01.T02 | Chaperone protein dnaJ 3 |
| augustus-3-processed-gene-392.3-mRNA-1 | Japonica | NA | | | | Transposable element protein, putative, MuDR |
| augustus-3-processed-gene-402.30-mRNA-1 | Japonica | NA | | | OsR498G1120483500.01.T01 | Transposon protein, putative, CACTA, En/Spm sub-class |
| augustus-3-processed-gene-406.41-mRNA-1 | Japonica | NA | | | | Vacuolar protein sorting-associated protein 26 |
| augustus-3-processed-gene-41.35-mRNA-1 | Japonica | NA | | | OsR498G0305342800.01.T01 | Plasma membrane ATPase |
| augustus-3-processed-gene-410.74-mRNA-1 | Japonica | NA | | | | Tetratricopeptide repeat (TPR)-like superfamily protein |
| augustus-3-processed-gene-412.43-mRNA-1 | Japonica | NA | | Os03g0689300 | OsR498G0306830100.01.T03 | Plasma membrane ATPase |
| augustus-3-processed-gene-414.55-mRNA-1 | Japonica | NA | | | | CW14 protein isoform 2 |
| augustus-3-processed-gene-416.20-mRNA-1 | Japonica | NA | | | OsR498G0306743300.01.T01 | Hydroxyproline-rich glycoprotein-like |
| augustus-3-processed-gene-416.21-mRNA-1 | Japonica | NA | | Os03g0697200 | OsR498G0306865100.01.T01 | Cytokinin riboside 5'-monophosphate phosphoribohydrolase |
| augustus-3-processed-gene-417.2-mRNA-1 | Japonica | Japonica | | Os03g0110200 | OsR498G0101158000.01.T01 | Transposon protein, putative, Mutator sub-class, expressed |
| augustus-3-processed-gene-419.75-mRNA-1 | Japonica | NA | | | | Transposon protein, putative, Mutator sub-class |
| augustus-3-processed-gene-426.27-mRNA-1 | Japonica | NA | | | | Endonuclease |
| augustus-3-processed-gene-43.53-mRNA-1 | Japonica | NA | | | OsR498G0305352900.01.T04 | Dentin sialophosphoprotein-related |
| augustus-3-processed-gene-430.30-mRNA-1 | Japonica | NA | | Os03g0721700 | OsR498G0306955400.01.T01 | DUF569 domain-containing protein |
| augustus-3-processed-gene-438.73-mRNA-1 | Japonica | NA | | Os03g0735000 | OsR498G0307002800.01.T02 | Glucose-1-phosphate adenylyltransferase |
| augustus-3-processed-gene-440.40-mRNA-1 | Japonica | NA | | Os03g0738100 | OsR498G0307011300.01.T09 | Phosphatidylinositol 3-and 4-kinase family protein, expressed |
| augustus-3-processed-gene-444.35-mRNA-1 | Japonica | NA | | | OsR498G0307040600.01.T01 | |
| augustus-3-processed-gene-450.67-mRNA-1 | Japonica | NA | | | OsR498G0307071000.01.T03 | |
| augustus-3-processed-gene-451.11-mRNA-1 | Japonica | NA | | | OsR498G0307071200.01.T02 | |
| augustus-3-processed-gene-459.2-mRNA-1 | Japonica | NA | | Os03g0773600 | OsR498G0307130100.01.T04 | Kinesin-like protein KIN-8B |
| augustus-3-processed-gene-46.67-mRNA-1 | Japonica | Indica | | Os09g0368500 | OsR498G0305377500.01.T02 | Flavin containing polyamine oxidase |
| augustus-3-processed-gene-465.45-mRNA-1 | Japonica | NA | | Os03g0786500 | OsR498G0307172400.01.T03 | DNA topoisomerase |
| augustus-3-processed-gene-465.54-mRNA-1 | Japonica | NA | | Os03g0787300 | OsR498G0307174300.01.T01 | Chaperone protein dnaJ 3 |
| augustus-3-processed-gene-469.45-mRNA-1 | Japonica | NA | | Os03g0795100 | OsR498G0307197400.01.T01 | |
| augustus-3-processed-gene-47.35-mRNA-1 | Japonica | NA | | | OsR498G0101349400.01.T01 | Splicing coactivator subunit-like |
| augustus-3-processed-gene-472.68-mRNA-1 | Japonica | NA | | | OsR498G0307222300.01.T01 | |
| augustus-3-processed-gene-478.14-mRNA-1 | Japonica | NA | | Os03g0812200 | OsR498G0307259300.01.T02 | RING-type E3 ubiquitin transferase |
| augustus-3-processed-gene-479.34-mRNA-1 | Japonica | NA | | Os08g0338000 | | DDE Tnp4 domain-containing protein (Fragment) |
| augustus-3-processed-gene-490.28-mRNA-1 | Japonica | NA | | Os03g0110200 | | MuDR family transposase domain containing protein |
| augustus-3-processed-gene-491.56-mRNA-1 | Japonica | NA | Kinase-TM | | | probable receptor-like serine/threonine-protein kinase At5g57670 |
| augustus-3-processed-gene-493.101-mRNA-1 | Japonica | NA | | Os03g0844900 | OsR498G0307357600.01.T02 | Nucleic acid-binding proteins superfamily |
| augustus-3-processed-gene-493.104-mRNA-1 | Japonica | Indica | | Os03g0845300 | OsR498G0307358600.01.T01 | Nuclear RNA binding protein A |
| augustus-3-processed-gene-493.97-mRNA-1 | Japonica | NA | Kinase | Os03g0844100 | OsR498G0307355200.01.T01 | Tyrosine-protein kinase |
| augustus-3-processed-gene-496.5-mRNA-1 | Japonica | NA | | Os03g0849900 | OsR498G0307369900.01.T01 | Glycosyltransferase |
| augustus-3-processed-gene-77.33-mRNA-1 | Japonica | NA | | Os03g0251000 | OsR498G0305585800.01.T01 | AAI domain-containing protein |
| augustus-3-processed-gene-80.65-mRNA-1 | Japonica | NA | | Os03g0255500 | OsR498G0305600800.01.T07 | Phosphoenolpyruvate carboxykinase (ATP) |
| augustus-4-processed-gene-109.27-mRNA-1 | Japonica | NA | | Os04g0346000 | OsR498G0408213500.01.T05 | Prolyl 4-hydroxylase alpha subunit |
| augustus-4-processed-gene-11.2-mRNA-1 | Japonica | NA | | Os04g0186400 | OsR498G0407666500.01.T01 | H(+)/Pi cotransporter |
| augustus-4-processed-gene-118.0-mRNA-1 | Japonica | NA | | | OsR498G0408285100.01.T01 | |
| augustus-4-processed-gene-12.15-mRNA-1 | Japonica | Indica | | Os04g0188500 | OsR498G0407674500.01.T02 | |
| augustus-4-processed-gene-12.17-mRNA-1 | Japonica | NA | | | OsR498G0509838600.01.T01 | HGWP repeat containing protein-like |
| augustus-4-processed-gene-120.22-mRNA-1 | Japonica | Japonica | | Os04g0364800 | OsR498G0204388300.01.T01 | Kiwellin |
| augustus-4-processed-gene-131.46-mRNA-1 | Japonica | NA | | Os04g0379600 | OsR498G0408386100.01.T01 | Nitrate-induced NOI protein |
| augustus-4-processed-gene-138.55-mRNA-1 | Japonica | NA | | | OsR498G0408442900.01.T01 | GLTSCR1 domain-containing protein (Fragment) |

| | | | | | | |
|---|---|---|---|---|---|---|
| augustus-4-processed-gene-139.19-mRNA-1 | Japonica | NA | | Os04g0394200 | OsR498G0408449100.01.T04 | Dihydrolipoyllysine-residue succinyltransferase |
| augustus-4-processed-gene-153.23-mRNA-1 | Japonica | NA | | Os04g0419100 | | |
| augustus-4-processed-gene-154.8-mRNA-1 | Japonica | NA | | | | RING-H2 finger protein ATL78 |
| augustus-4-processed-gene-158.17-mRNA-1 | Japonica | NA | | Os04g0428300 | | |
| augustus-4-processed-gene-159.17-mRNA-1 | Japonica | NA | | Os04g0429800 | OsR498G0408584900.01.T04 | Nicotinate phosphoribosyltransferase |
| augustus-4-processed-gene-159.18-mRNA-1 | Japonica | NA | | Os04g0429900 | | Nicotinate phosphoribosyltransferase |
| augustus-4-processed-gene-159.22-mRNA-1 | Japonica | NA | | Os04g0430500 | | |
| augustus-4-processed-gene-160.37-mRNA-1 | Japonica | NA | | Os04g0430700 | OsR498G0408588300.01.T01 | Subtilisin-like protease |
| augustus-4-processed-gene-160.53-mRNA-1 | Japonica | NA | | Os04g0432366 | OsR498G0408597500.01.T01 | Alpha-1,4-fucosyltransferase |
| augustus-4-processed-gene-161.32-mRNA-1 | Japonica | Indica | | Os04g0433300 | OsR498G0408601300.01.T01 | |
| augustus-4-processed-gene-162.10-mRNA-1 | Japonica | NA | | | OsR498G0408604500.01.T01 | PsbP domain-containing protein 2, chloroplastic |
| augustus-4-processed-gene-168.12-mRNA-1 | Japonica | NA | | Os04g0444100 | OsR498G0408646700.01.T01 | C2H2 zinc finger malformed spikelet |
| augustus-4-processed-gene-169.39-mRNA-1 | Japonica | NA | | Os04g0444300 | OsR498G0408647300.01.T03 | Auxin regulated gene involved in organ size 2 |
| augustus-4-processed-gene-169.41-mRNA-1 | Japonica | NA | | Os04g0444600 | OsR498G0408648100.01.T02 | Proteasome component (PCI) domain |
| augustus-4-processed-gene-169.57-mRNA-1 | Japonica | NA | | Os04g0445400 | OsR498G0408650500.01.T01 | kinetochore protein SPC24 homolog |
| augustus-4-processed-gene-169.58-mRNA-1 | Japonica | NA | | Os04g0445600 | OsR498G0408651100.01.T01 | Trihelix transcription factor |
| augustus-4-processed-gene-169.61-mRNA-1 | Japonica | Indica | | Os04g0446200 | OsR498G0408653100.01.T01 | OBP3-responsive protein 4 isoform 1 |
| augustus-4-processed-gene-171.51-mRNA-1 | Japonica | NA | | Os04g0448500 | | Peptidase A1 domain-containing protein |
| augustus-4-processed-gene-171.69-mRNA-1 | Japonica | Japonica | | Os04g0449800 | OsR498G0101579700.01.T01 | Gibberellin receptor GID1L2 |
| augustus-4-processed-gene-171.70-mRNA-1 | Japonica | Indica | | Os04g0449900 | OsR498G0408667100.01.T02 | Polyadenylate-binding protein RBP47B |
| augustus-4-processed-gene-175.2-mRNA-1 | Japonica | NA | | Os04g0456200 | OsR498G0408687100.01.T01 | PLAT domain-containing protein 2 |
| augustus-4-processed-gene-175.21-mRNA-1 | Japonica | Indica | | Os04g0457300 | OsR498G0408689100.01.T02 | Nascent polypeptide-associated complex subunit beta |
| augustus-4-processed-gene-175.23-mRNA-1 | Japonica | NA | | Os04g0458000 | | |
| augustus-4-processed-gene-175.25-mRNA-1 | Japonica | NA | | Os04g0458200 | OsR498G0408691500.01.T02 | Putative WRKY transcription factor 19 |
| augustus-4-processed-gene-182.0-mRNA-1 | Japonica | Japonica | CC-NBS-TM | Os04g0470500 | OsR498G0408741100.01.T01 | Disease resistance protein RPS2 |
| augustus-4-processed-gene-183.27-mRNA-1 | Japonica | NA | | Os04g0472900 | OsR498G0408750100.01.T01 | FT-interacting protein 1 |
| augustus-4-processed-gene-185.46-mRNA-1 | Japonica | Japonica | | Os04g0475900 | OsR498G0408760900.01.T01 | DUF295 domain-containing protein |
| augustus-4-processed-gene-185.47-mRNA-1 | Japonica | NA | | | OsR498G0408761100.01.T01 | DUF295 domain-containing protein |
| augustus-4-processed-gene-185.61-mRNA-1 | Japonica | NA | | Os04g0476500 | | |
| augustus-4-processed-gene-186.0-mRNA-1 | Japonica | Japonica | | Os04g0477900 | OsR498G0511078900.01.T01 | AAA domain-containing protein |
| augustus-4-processed-gene-19.0-mRNA-1 | Japonica | NA | | Os04g0197100 | | Cysteine-rich receptor-like protein kinase 6 |
| augustus-4-processed-gene-19.9-mRNA-1 | Japonica | NA | | | OsR498G0407708700.01.T01 | |
| augustus-4-processed-gene-192.20-mRNA-1 | Japonica | NA | CC-LRR-NBS-TM | Os04g0491100 | OsR498G0408811900.01.T02 | Disease resistance RPP13-like protein 4 |
| augustus-4-processed-gene-192.23-mRNA-1 | Japonica | NA | | Os04g0492100 | OsR498G0408815100.01.T01 | probable E3 ubiquitin-protein ligase ARI8 |
| augustus-4-processed-gene-192.3-mRNA-1 | Japonica | NA | | Os04g0490700 | OsR498G0408810300.01.T01 | Chaperone protein ClpB 2 |
| augustus-4-processed-gene-193.26-mRNA-1 | Japonica | NA | | Os04g0492500 | OsR498G0408817300.01.T05 | Non-specific serine/threonine protein kinase |
| augustus-4-processed-gene-193.34-mRNA-1 | Japonica | NA | | Os04g0492600 | OsR498G0408817300.01.T06 | Non-specific serine/threonine protein kinase |
| augustus-4-processed-gene-193.35-mRNA-1 | Japonica | NA | | Os04g0492800 | OsR498G0408817700.01.T01 | Probable calcium-binding protein CML28 |
| augustus-4-processed-gene-194.48-mRNA-1 | Japonica | NA | | Os04g0494800 | OsR498G0408823900.01.T01 | Putative Galactose-binding domain-containing protein |
| augustus-4-processed-gene-195.60-mRNA-1 | Japonica | Japonica | | Os04g0497900 | OsR498G0408832900.01.T01 | |
| augustus-4-processed-gene-196.2-mRNA-1 | Japonica | NA | | Os04g0498600 | OsR498G0408834300.01.T04 | S-adenosylmethionine decarboxylase proenzyme |
| augustus-4-processed-gene-197.34-mRNA-1 | Japonica | NA | | Os04g0502300 | OsR498G0408845600.01.T01 | 40S ribosomal protein S11 |
| augustus-4-processed-gene-20.3-mRNA-1 | Japonica | NA | | Os05g0233900 | | TTF-type domain-containing protein |
| augustus-4-processed-gene-209.57-mRNA-1 | Japonica | NA | | Os04g0525600 | OsR498G0408931100.01.T02 | ATP-dependent Clp protease proteolytic subunit |
| augustus-4-processed-gene-210.1-mRNA-1 | Japonica | NA | | Os04g0526000 | OsR498G0408933100.01.T01 | TCP transcription factor |
| augustus-4-processed-gene-210.3-mRNA-1 | Japonica | NA | | Os04g0526300 | OsR498G0408933900.01.T01 | Sulfotransferase |
| augustus-4-processed-gene-218.28-mRNA-1 | Japonica | NA | | Os04g0541500 | OsR498G0409002300.01.T01 | Sphingoid long-chain bases kinase 1 |
| augustus-4-processed-gene-218.39-mRNA-1 | Japonica | NA | | | OsR498G0409004500.01.T01 | Senescence regulator S40 |
| augustus-4-processed-gene-219.1-mRNA-1 | Japonica | NA | | Os04g0543500 | OsR498G0409009300.01.T01 | FRIGIDA interacting protein 2 |
| augustus-4-processed-gene-219.16-mRNA-1 | Japonica | NA | | | OsR498G0409011100.01.T01 | Keratin, type II cytoskeletal 79 (Fragment) |
| augustus-4-processed-gene-220.42-mRNA-1 | Japonica | NA | | | OsR498G0409018100.01.T05 | PORR domain-containing protein (Fragment) |
| augustus-4-processed-gene-221.0-mRNA-1 | Japonica | NA | | Os04g0547600 | OsR498G0409022300.01.T01 | Ethylene-responsive transcription factor 5 |
| augustus-4-processed-gene-222.20-mRNA-1 | Japonica | NA | | Os04g0549700 | OsR498G0409030100.01.T02 | Ethylene-responsive transcription factor TINY-like |
| augustus-4-processed-gene-222.21-mRNA-1 | Japonica | NA | | | OsR498G0409030700.01.T01 | Ethylene-responsive transcription factor TINY-like |
| augustus-4-processed-gene-222.27-mRNA-1 | Japonica | NA | | Os04g0550200 | OsR498G0409032100.01.T01 | Ethylene-responsive transcription factor ERF034 |
| augustus-4-processed-gene-222.28-mRNA-1 | Japonica | NA | | Os04g0550500 | OsR498G0409033700.01.T01 | Acetylglutamate kinase chloroplastic |
| augustus-4-processed-gene-223.11-mRNA-1 | Japonica | NA | | Os04g0552300 | OsR498G0409041500.01.T02 | Cyclin |
| augustus-4-processed-gene-223.12-mRNA-1 | Japonica | NA | | Os04g0552400 | OsR498G0409042500.01.T01 | Cys2/His2 zinc-finger transcription factor |
| augustus-4-processed-gene-223.13-mRNA-1 | Japonica | NA | | Os04g0552700 | OsR498G0409043500.01.T01 | Cys2/His2 zinc-finger transcription factor |
| augustus-4-processed-gene-223.17-mRNA-1 | Japonica | NA | | Os04g0553000 | OsR498G0409045300.01.T02 | Protein TRIGALACTOSYLDIACYLGLYCEROL 1 chloroplastic |
| augustus-4-processed-gene-223.8-mRNA-1 | Japonica | NA | | Os04g0551800 | OsR498G0409039700.01.T01 | CCT-alpha |

| | | | | | | |
|---|---|---|---|---|---|---|
| augustus-4-processed-gene-224.13-mRNA-1 | Japonica | Japonica | | Os04g0554600 | OsR498G0409049300.01.T01 | Proline-rich protein DC2.15 |
| augustus-4-processed-gene-224.15-mRNA-1 | Japonica | NA | | Os04g0555000 | OsR498G0409052300.01.T01 | GRAS transcription factor |
| augustus-4-processed-gene-225.42-mRNA-1 | Japonica | NA | | Os04g0557000 | OsR498G0409059700.01.T03 | Genetic modifier |
| augustus-4-processed-gene-226.33-mRNA-1 | Japonica | NA | | | | R-type basic helix-loop-helix protein |
| augustus-4-processed-gene-234.18-mRNA-1 | Japonica | NA | | Os04g0571600 | OsR498G0409109900.01.T01 | Protein DETOXIFICATION |
| augustus-4-processed-gene-235.41-mRNA-1 | Japonica | Indica | | Os02g0676800 | OsR498G0409113500.01.T01 | Ethylene-responsive transcription factor ERF025 |
| augustus-4-processed-gene-235.42-mRNA-1 | Japonica | Japonica | | Os04g0572400 | OsR498G0204522100.01.T01 | Dehydration-responsive element-binding protein 1G |
| augustus-4-processed-gene-236.10-mRNA-1 | Japonica | Japonica | | Os04g0574100 | OsR498G0612527500.01.T01 | Exostosin domain-containing protein (Fragment) |
| augustus-4-processed-gene-236.15-mRNA-1 | Japonica | NA | | Os04g0574700 | OsR498G0409122500.01.T01 | Guard cell S-type anion channel like |
| augustus-4-processed-gene-237.18-mRNA-1 | Japonica | Indica | Kinase-LRR-TM | Os04g0576900 | OsR498G0409129100.01.T01 | Probable inactive leucine-rich repeat receptor kinase XIAO |
| augustus-4-processed-gene-237.19-mRNA-1 | Japonica | NA | | Os04g0577000 | OsR498G0409129500.01.T02 | Ubiquitin fusion degradation protein UFD |
| augustus-4-processed-gene-237.4-mRNA-1 | Japonica | Indica | | Os10g0531750 | OsR498G0408675500.01.T01 | Retrotransposon protein, putative, Ty3-gypsy sub-class |
| augustus-4-processed-gene-238.39-mRNA-1 | Japonica | NA | | Os04g0577700 | OsR498G0409132300.01.T04 | Transcription repressor |
| augustus-4-processed-gene-238.56-mRNA-1 | Japonica | NA | | Os04g0578400 | OsR498G0409134900.01.T01 | Aldehyde oxygenase (deformylating) |
| augustus-4-processed-gene-239.17-mRNA-1 | Japonica | NA | | Os04g0578900 | | MULE domain-containing protein |
| augustus-4-processed-gene-241.16-mRNA-1 | Japonica | NA | | Os04g0583150 | OsR498G0409160700.01.T06 | Pentatricopeptide repeat-containing protein chloroplastic |
| augustus-4-processed-gene-243.14-mRNA-1 | Japonica | NA | | Os04g0586500 | OsR498G0409175700.01.T01 | Receptor-like protein kinase FERONIA |
| augustus-4-processed-gene-243.15-mRNA-1 | Japonica | NA | | Os04g0586700 | OsR498G0409176100.01.T01 | E3 ubiquitin-protein ligase ATL4 |
| augustus-4-processed-gene-243.19-mRNA-1 | Japonica | NA | | Os04g0587100 | OsR498G0409177300.01.T01 | PMEI domain-containing protein |
| augustus-4-processed-gene-243.20-mRNA-1 | Japonica | NA | | Os04g0587200 | OsR498G0409177500.01.T01 | Invertase inhibitor 1 |
| augustus-4-processed-gene-243.24-mRNA-1 | Japonica | Indica | | Os04g0587400 | OsR498G0409178500.01.T02 | Purine nucleobase transmembrane transport |
| augustus-4-processed-gene-244.33-mRNA-1 | Japonica | NA | | Os10g0460000 | OsR498G1019116500.01.T01 | |
| augustus-4-processed-gene-244.45-mRNA-1 | Japonica | NA | | Os04g0589401 | | |
| augustus-4-processed-gene-244.46-mRNA-1 | Japonica | Indica | | Os04g0589500 | OsR498G0409185300.01.T01 | Proton-dependent oligopeptide transport family protein |
| augustus-4-processed-gene-244.48-mRNA-1 | Japonica | NA | | Os04g0589700 | OsR498G0409185900.01.T01 | RING-type E3 ubiquitin transferase |
| augustus-4-processed-gene-245.12-mRNA-1 | Japonica | NA | | Os04g0590400 | OsR498G0409189100.01.T01 | scarecrow-like protein 9 |
| augustus-4-processed-gene-247.8-mRNA-1 | Japonica | NA | | Os04g0594800 | OsR498G0409204100.01.T01 | Oligopeptide transporter like |
| augustus-4-processed-gene-247.9-mRNA-1 | Japonica | NA | | Os04g0595000 | OsR498G0409204700.01.T01 | Peptidase A1 domain-containing protein (Fragment) |
| augustus-4-processed-gene-248.10-mRNA-1 | Japonica | Indica | | Os04g0597900 | OsR498G0409211100.01.T01 | |
| augustus-4-processed-gene-248.3-mRNA-1 | Japonica | NA | | Os04g0597600 | OsR498G0409209900.01.T01 | |
| augustus-4-processed-gene-248.8-mRNA-1 | Japonica | NA | | Os02g0212975 | OsR498G1019086300.01.T01 | |
| augustus-4-processed-gene-248.9-mRNA-1 | Japonica | NA | | Os04g0597800 | OsR498G0409210900.01.T01 | |
| augustus-4-processed-gene-249.67-mRNA-1 | Japonica | NA | | | OsR498G0409219500.01.T01 | |
| augustus-4-processed-gene-251.19-mRNA-1 | Japonica | NA | | | OsR498G0409231700.01.T01 | |
| augustus-4-processed-gene-253.53-mRNA-1 | Japonica | NA | | | | HTH myb-type domain-containing protein |
| augustus-4-processed-gene-256.1-mRNA-1 | Japonica | NA | | Os04g0610400 | OsR498G0409258900.01.T01 | Ethylene-responsive element binding protein homolog |
| augustus-4-processed-gene-256.16-mRNA-1 | Japonica | NA | | Os04g0611700 | OsR498G0409264300.01.T01 | Ent-kaurene synthase B |
| augustus-4-processed-gene-257.0-mRNA-1 | Japonica | NA | | Os04g0611800 | OsR498G0409264900.01.T05 | Ent-kaurene synthase I (Fragment) |
| augustus-4-processed-gene-257.10-mRNA-1 | Japonica | Indica | | Os04g0612300 | OsR498G0409266300.01.T01 | AAI domain-containing protein (Fragment) |
| augustus-4-processed-gene-258.13-mRNA-1 | Japonica | NA | | | | Adenosylmethionine aminotransferase1 |
| augustus-4-processed-gene-260.31-mRNA-1 | Japonica | NA | | Os04g0616800 | OsR498G0409282900.01.T01 | |
| augustus-4-processed-gene-260.32-mRNA-1 | Japonica | NA | | Os04g0616900 | OsR498G0409283300.01.T01 | |
| augustus-4-processed-gene-260.47-mRNA-1 | Japonica | NA | | Os04g0618400 | | |
| augustus-4-processed-gene-261.28-mRNA-1 | Japonica | NA | | Os04g0619300 | OsR498G0409292300.01.T01 | F-box/kelch-repeat protein At5g60570 |
| augustus-4-processed-gene-261.33-mRNA-1 | Japonica | NA | | Os04g0619900 | OsR498G0409294900.01.T01 | BAG family molecular chaperone regulator like |
| augustus-4-processed-gene-262.14-mRNA-1 | Japonica | Japonica | CC-NBS-TM | Os04g0621750 | OsR498G0409301900.01.T01 | Disease resistance protein RGA2 |
| augustus-4-processed-gene-262.16-mRNA-1 | Japonica | Japonica | | Os04g0621900 | OsR498G0409303900.01.T01 | BED-type domain-containing protein (Fragment) |
| augustus-4-processed-gene-262.8-mRNA-1 | Japonica | Japonica | | Os04g0621300 | OsR498G0409297100.01.T04 | Disease Resistance RGA protein fragment (Pi63) |
| augustus-4-processed-gene-264.34-mRNA-1 | Japonica | NA | | Os04g0624400 | OsR498G0409310700.01.T01 | Polyphenol oxidase, chloroplastic |
| augustus-4-processed-gene-265.33-mRNA-1 | Japonica | NA | | Os04g0625600 | OsR498G0409315900.01.T01 | Speckle-type POZ protein B |
| augustus-4-processed-gene-265.35-mRNA-1 | Japonica | NA | | Os04g0625700 | | Putative chromatin remodeling & transcription regulator BTB-POZ-MATH far |
| augustus-4-processed-gene-265.36-mRNA-1 | Japonica | NA | | Os04g0625800 | OsR498G0409316900.01.T03 | Polyadenylate-binding protein RBP45C |
| augustus-4-processed-gene-266.66-mRNA-1 | Japonica | NA | | Os04g0628700 | OsR498G0409325300.01.T03 | Peroxisome biogenesis protein 22 |
| augustus-4-processed-gene-266.67-mRNA-1 | Japonica | Japonica | | Os04g0628800 | OsR498G0409325300.01.T01 | Cyclin |
| augustus-4-processed-gene-268.79-mRNA-1 | Japonica | Japonica | Kinase-TM | Os04g0633800 | OsR498G0409334600.01.T02 | Non-specific serine/threonine protein kinase |
| augustus-4-processed-gene-271.17-mRNA-1 | Japonica | NA | | Os04g0640900 | OsR498G0409355300.01.T01 | Putative WRKY transcription factor 19 |
| augustus-4-processed-gene-271.20-mRNA-1 | Japonica | NA | | Os04g0641300 | | Glutaredoxin |
| augustus-4-processed-gene-273.58-mRNA-1 | Japonica | NA | | Os04g0643700 | OsR498G0409364700.01.T04 | Pentatricopeptide repeat-containing protein mitochondrial |
| augustus-4-processed-gene-274.2-mRNA-1 | Japonica | NA | | Os04g0645500 | | Methyltransferase-16 |
| augustus-4-processed-gene-275.33-mRNA-1 | Japonica | NA | LRR-TM | | | Phytosulfokine receptor 1 |
| augustus-4-processed-gene-275.39-mRNA-1 | Japonica | NA | | Os04g0648600 | OsR498G0409380300.01.T01 | |

| | | | | | | |
|---|---|---|---|---|---|---|
| augustus-4-processed-gene-276.11-mRNA-1 | Japonica | NA | | Os04g0649900 | OsR498G0409389300.01.T01 | Glucuronoxylan 4-O-methyltransferase 3 |
| augustus-4-processed-gene-277.63-mRNA-1 | Japonica | Indica | | Os04g0650700 | OsR498G0409393500.01.T01 | Isoaspartyl peptidase/L-asparaginase |
| augustus-4-processed-gene-277.65-mRNA-1 | Japonica | NA | | | OsR498G0409395100.01.T01 | Wall-associated receptor kinase 2 |
| augustus-4-processed-gene-279.43-mRNA-1 | Japonica | Japonica | Kinase-TM | Os04g0655000 | OsR498G0409408100.01.T01 | Serine/threonine-protein kinase |
| augustus-4-processed-gene-281.13-mRNA-1 | Japonica | NA | | Os04g0658100 | OsR498G0409419300.01.T06 | Transcriptional regulator of RNA polII, SAGA, subunit |
| augustus-4-processed-gene-282.9-mRNA-1 | Japonica | NA | | | OsR498G0409426700.01.T05 | |
| augustus-4-processed-gene-284.33-mRNA-1 | Japonica | NA | | Os04g0662800 | OsR498G0409436100.01.T01 | E3 ubiquitin-protein ligase HERC2 |
| augustus-4-processed-gene-289.0-mRNA-1 | Japonica | NA | | Os04g0672800 | OsR498G0409471300.01.T02 | Peroxisome biogenesis protein 22 |
| augustus-4-processed-gene-290.57-mRNA-1 | Japonica | NA | | Os04g0674400 | OsR498G0409505400.01.T01 | Anamorsin homolog |
| augustus-4-processed-gene-291.12-mRNA-1 | Japonica | NA | | Os04g0677600 | | |
| augustus-4-processed-gene-293.51-mRNA-1 | Japonica | NA | | | OsR498G0409501500.01.T01 | Allantoinase |
| augustus-4-processed-gene-293.70-mRNA-1 | Japonica | NA | | Os04g0681800 | | |
| augustus-4-processed-gene-295.1-mRNA-1 | Japonica | Indica | Kinase-TM | Os04g0684200 | OsR498G0409514300.01.T06 | CASP-like protein 1 |
| augustus-4-processed-gene-295.13-mRNA-1 | Japonica | Indica | | Os04g0684900 | OsR498G0409516900.01.T01 | Poly(A)-specific ribonuclease |
| augustus-4-processed-gene-295.9-mRNA-1 | Japonica | Indica | | Os04g0684400 | OsR498G0409515700.01.T01 | |
| augustus-4-processed-gene-296.36-mRNA-1 | Japonica | NA | | Os04g0686800 | OsR498G0409525900.01.T01 | Vacuolar iron transporter |
| augustus-4-processed-gene-297.16-mRNA-1 | Japonica | NA | | Os04g0687800 | OsR498G0409528100.01.T01 | Plant-drug/metabolite exporter |
| augustus-4-processed-gene-297.18-mRNA-1 | Japonica | NA | | Os04g0688000 | OsR498G0409529300.01.T01 | DUF793 domain-containing protein |
| augustus-4-processed-gene-299.19-mRNA-1 | Japonica | NA | | Os04g0691800 | OsR498G0409540700.01.T01 | FT-interacting protein 1 |
| augustus-4-processed-gene-299.29-mRNA-1 | Japonica | NA | | | OsR498G0409538800.01.T02 | DDB1-and CUL4-associated factor-like protein 1 |
| augustus-4-processed-gene-299.4-mRNA-1 | Japonica | NA | | Os04g0691000 | | F-box domain-containing protein |
| augustus-4-processed-gene-3.15-mRNA-1 | Japonica | NA | | Os04g0175400 | OsR498G0407633900.01.T01 | |
| augustus-4-processed-gene-35.36-mRNA-1 | Japonica | NA | Kinase-LRR-TM | | | Leucine-rich repeat protein kinase family protein |
| augustus-4-processed-gene-5.20-mRNA-1 | Japonica | NA | | Os04g0178300 | OsR498G0407643700.01.T01 | Syn-copalyl diphosphate synthase |
| augustus-4-processed-gene-59.5-mRNA-1 | Japonica | NA | | Os04g0272700 | OsR498G0407907100.01.T01 | Glycosyltransferase |
| augustus-4-processed-gene-65.0-mRNA-1 | Japonica | Indica | | Os02g0461500 | OsR498G0407938700.01.T01 | FAS1 domain-containing protein |
| augustus-4-processed-gene-66.2-mRNA-1 | Japonica | NA | | Os05g0234700 | | Retrotransposon protein, putative, unclassified |
| augustus-4-processed-gene-7.13-mRNA-1 | Japonica | NA | | Os04g0180900 | | |
| augustus-4-processed-gene-72.16-mRNA-1 | Japonica | NA | | Os04g0295000 | OsR498G0407959000.01.T02 | Type 1 phosphatases regulator like |
| augustus-4-processed-gene-74.12-mRNA-1 | Japonica | NA | | Os04g0297800 | OsR498G0407971100.01.T01 | Bifunctional hemolysin/adenylate cyclase |
| augustus-4-processed-gene-76.18-mRNA-1 | Japonica | NA | | Os04g0300175 | | ATP-dependent DNA helicase |
| augustus-4-processed-gene-8.6-mRNA-1 | Japonica | NA | | | | |
| augustus-4-processed-gene-80.14-mRNA-1 | Japonica | NA | | Os04g0305700 | OsR498G0408009100.01.T01 | Glycosyltransferase |
| augustus-4-processed-gene-81.17-mRNA-1 | Japonica | NA | | Os04g0306400 | OsR498G0408010700.01.T01 | Ribose-5-phosphate isomerase |
| augustus-4-processed-gene-82.4-mRNA-1 | Japonica | Japonica | Kinase-TM | Os04g0307900 | OsR498G0408016900.01.T01 | Wall-associated receptor kinase 5 |
| augustus-4-processed-gene-82.5-mRNA-1 | Japonica | NA | Kinase-TM | | | Wall-associated receptor kinase 3 |
| augustus-4-processed-gene-83.5-mRNA-1 | Japonica | NA | | | | |
| augustus-5-processed-gene-10.31-mRNA-1 | Japonica | Japonica | | Os05g0120000 | OsR498G0509618600.01.T01 | ABC transporter G family member 6 |
| augustus-5-processed-gene-10.34-mRNA-1 | Japonica | NA | | Os10g0460000 | OsR498G1019116500.01.T01 | |
| augustus-5-processed-gene-11.16-mRNA-1 | Japonica | NA | | Os05g0120900 | OsR498G0509622800.01.T01 | probable membrane-associated kinase regulator 4 |
| augustus-5-processed-gene-12.45-mRNA-1 | Japonica | NA | | Os05g0122500 | OsR498G0509629600.01.T01 | PWWP domain-containing protein (Fragment) |
| augustus-5-processed-gene-13.33-mRNA-1 | Japonica | NA | | Os05g0124000 | OsR498G0509638000.01.T02 | |
| augustus-5-processed-gene-14.7-mRNA-1 | Japonica | Indica | | Os05g0124000 | OsR498G0509641800.01.T05 | |
| augustus-5-processed-gene-141.37-mRNA-1 | Japonica | NA | | Os05g0305300 | OsR498G0510413000.01.T01 | Pentatricopeptide repeat |
| augustus-5-processed-gene-141.59-mRNA-1 | Japonica | NA | | Os05g0305700 | OsR498G0510414000.01.T01 | Homeobox protein SIX3-like isoform X2 |
| augustus-5-processed-gene-145.12-mRNA-1 | Japonica | NA | | | OsR498G0510430800.01.T01 | Hydroxyproline-rich glycoprotein-like protein |
| augustus-5-processed-gene-147.9-mRNA-1 | Japonica | Indica | | Os03g0356900 | OsR498G0510442200.01.T01 | Adenylate isopentenyltransferase |
| augustus-5-processed-gene-148.47-mRNA-1 | Japonica | NA | | Os05g0313700 | OsR498G0510453400.01.T01 | NET domain-containing protein |
| augustus-5-processed-gene-149.15-mRNA-1 | Japonica | NA | | Os05g0315401 | | Transposon protein, putative, Mutator sub-class, expressed |
| augustus-5-processed-gene-149.16-mRNA-1 | Japonica | NA | | Os05g0314700 | OsR498G0510463400.01.T01 | Fanconi anemia group I protein isoform X1 |
| augustus-5-processed-gene-15.32-mRNA-1 | Japonica | Indica | | Os01g0881800 | OsR498G0509652400.01.T01 | |
| augustus-5-processed-gene-151.23-mRNA-1 | Japonica | NA | | Os05g0317200 | OsR498G0510474100.01.T03 | Long-chain-fatty-acid--CoA ligase 3 |
| augustus-5-processed-gene-152.17-mRNA-1 | Japonica | NA | Kinase-TM | | OsR498G0510479400.01.T04 | Receptor-like protein kinase FERONIA |
| augustus-5-processed-gene-152.19-mRNA-1 | Japonica | NA | Kinase-TM | Os05g0318700 | OsR498G0510480600.01.T01 | Receptor-like protein kinase FERONIA |
| augustus-5-processed-gene-153.24-mRNA-1 | Japonica | Japonica | | Os05g0319800 | OsR498G0510483600.01.T01 | Plasma membrane ATPase |
| augustus-5-processed-gene-153.25-mRNA-1 | Japonica | NA | | Os05g0319900 | OsR498G0510483800.01.T01 | GH10 domain-containing protein |
| augustus-5-processed-gene-153.26-mRNA-1 | Japonica | NA | | Os05g0320100 | OsR498G0510484400.01.T03 | F-box domain containing protein, expressed |
| augustus-5-processed-gene-16.30-mRNA-1 | Japonica | NA | | Os05g0128400 | OsR498G0509662200.01.T03 | Metal tolerance protein 1 |
| augustus-5-processed-gene-17.8-mRNA-1 | Japonica | NA | | Os05g0129000 | OsR498G0509662700.01.T04 | Glutamate--cysteine ligase A, chloroplastic |
| augustus-5-processed-gene-18.35-mRNA-1 | Japonica | NA | | | OsR498G0509669200.01.T01 | Zinc finger, RanBP2-type |
| augustus-5-processed-gene-18.60-mRNA-1 | Japonica | NA | | | OsR498G0408652000.01.T01 | CIA30 domain-containing protein |

| | | | | | | |
|---|---|---|---|---|---|---|
| augustus-5-processed-gene-194.45-mRNA-1 | Japonica | Japonica | | Os05g0382550 | OsR498G0612562800.01.T01 | |
| augustus-5-processed-gene-195.28-mRNA-1 | Japonica | NA | | Os05g0384300 | OsR498G0510745600.01.T01 | Peptidase A1 domain-containing protein |
| augustus-5-processed-gene-195.29-mRNA-1 | Japonica | NA | NBS-TM | Os05g0384600 | OsR498G0510747200.01.T01 | ATPase, coupled to transmembrane movement of substances |
| augustus-5-processed-gene-199.0-mRNA-1 | Japonica | NA | | Os05g0390600 | | probable membrane-associated kinase regulator 1 |
| augustus-5-processed-gene-199.1-mRNA-1 | Japonica | NA | | Os05g0390800 | OsR498G0510767800.01.T01 | VQ domain-containing protein (Fragment) |
| augustus-5-processed-gene-199.7-mRNA-1 | Japonica | NA | | | | RMUA |
| augustus-5-processed-gene-201.32-mRNA-1 | Japonica | NA | | Os05g0395000 | OsR498G0510782000.01.T03 | Peptide-N4-(N-acetyl-beta-glucosaminyl)asparagine amidase A |
| augustus-5-processed-gene-202.19-mRNA-1 | Japonica | NA | | | | |
| augustus-5-processed-gene-202.25-mRNA-1 | Japonica | NA | | Os05g0396900 | OsR498G0510787600.01.T01 | Gelation factor like |
| augustus-5-processed-gene-204.46-mRNA-1 | Japonica | NA | LRR-TM | Os05g0399800 | OsR498G0510794200.01.T01 | Inactive LRR receptor-like serine/threonine-protein kinase BIR2 |
| augustus-5-processed-gene-205.29-mRNA-1 | Japonica | NA | | | OsR498G0510801000.01.T01 | |
| augustus-5-processed-gene-206.10-mRNA-1 | Japonica | NA | | Os05g0403400 | OsR498G0510803600.01.T01 | mTERF transcription factor (Fragment) |
| augustus-5-processed-gene-206.11-mRNA-1 | Japonica | NA | | Os05g0403600 | OsR498G0510804000.01.T01 | mTERF transcription factor (Fragment) |
| augustus-5-processed-gene-208.36-mRNA-1 | Japonica | NA | | Os05g0404600 | OsR498G0510805800.01.T01 | Methyl-CpG-binding domain-containing protein 4 |
| augustus-5-processed-gene-208.37-mRNA-1 | Japonica | NA | | Os05g0404700 | OsR498G0510806000.01.T01 | Methyl-CpG-binding domain-containing protein 1 |
| augustus-5-processed-gene-208.51-mRNA-1 | Japonica | NA | | Os12g0501100 | OsR498G0613150400.01.T01 | Protein-serine/threonine phosphatase |
| augustus-5-processed-gene-209.36-mRNA-1 | Japonica | NA | | Os05g0406600 | OsR498G0510812600.01.T01 | |
| augustus-5-processed-gene-210.25-mRNA-1 | Japonica | NA | | Os05g0409000 | OsR498G0510821800.01.T01 | UBA domain-containing protein (Fragment) |
| augustus-5-processed-gene-210.5-mRNA-1 | Japonica | NA | | Os05g0407900 | OsR498G0510818600.01.T04 | Pentatricopeptide repeat |
| augustus-5-processed-gene-217.37-mRNA-1 | Japonica | NA | | | OsR498G0510862200.01.T01 | Ethylene-responsive transcription factor ERF020 |
| augustus-5-processed-gene-217.56-mRNA-1 | Japonica | Japonica | | Os05g0420900 | OsR498G0203891300.01.T01 | |
| augustus-5-processed-gene-218.24-mRNA-1 | Japonica | NA | | | OsR498G0306294300.01.T01 | Oxidoreductase, 2OG-Fe oxygenase family protein, expressed |
| augustus-5-processed-gene-219.17-mRNA-1 | Japonica | NA | | Os05g0424300 | OsR498G0510875200.01.T01 | Flavonoid 3'-monooxygenase |
| augustus-5-processed-gene-22.36-mRNA-1 | Japonica | Japonica | | Os05g0139200 | OsR498G0509702800.01.T01 | Putative receptor kinase |
| augustus-5-processed-gene-220.45-mRNA-1 | Japonica | Japonica | | Os05g0427400 | OsR498G0204355400.01.T01 | Phenylalanine ammonia-lyase |
| augustus-5-processed-gene-221.26-mRNA-1 | Japonica | Japonica | | Os05g0428800 | OsR498G0510892400.01.T01 | Glycosyltransferase |
| augustus-5-processed-gene-222.3-mRNA-1 | Japonica | NA | | Os05g0429700 | OsR498G0510895200.01.T01 | FT-interacting protein 1 |
| augustus-5-processed-gene-227.10-mRNA-1 | Japonica | Indica | | Os05g0440100 | OsR498G0510928400.01.T02 | Histone deacetylase |
| augustus-5-processed-gene-227.18-mRNA-1 | Japonica | Indica | | Os10g0326400 | OsR498G0509612600.01.T01 | F-box domain containing protein, expressed |
| augustus-5-processed-gene-227.20-mRNA-1 | Japonica | Indica | | Os05g0441400 | OsR498G0510930400.01.T01 | Transcription repressor |
| augustus-5-processed-gene-23.16-mRNA-1 | Japonica | Indica | | Os05g0141200 | OsR498G0509711600.01.T02 | VQ domain-containing protein |
| augustus-5-processed-gene-23.18-mRNA-1 | Japonica | Japonica | | Os05g0141300 | OsR498G0713871400.01.T01 | (Z)-3-hexen-1-ol acetyltransferase |
| augustus-5-processed-gene-23.20-mRNA-1 | Japonica | NA | | Os05g0141500 | OsR498G0509713200.01.T03 | E3 ubiquitin-protein like |
| augustus-5-processed-gene-230.35-mRNA-1 | Japonica | NA | | Os05g0447000 | OsR498G0510949800.01.T01 | Pleckstrin likey domain-containing protein 1 |
| augustus-5-processed-gene-230.37-mRNA-1 | Japonica | NA | | Os05g0447200 | OsR498G0510950000.01.T01 | Auxin transporter protein 1 |
| augustus-5-processed-gene-231.43-mRNA-1 | Japonica | NA | | Os05g0449200 | OsR498G0510954600.01.T01 | BAHD acyltransferase DCR |
| augustus-5-processed-gene-234.2-mRNA-1 | Japonica | NA | | | | Retrotransposon protein, putative, unclassified, expressed |
| augustus-5-processed-gene-238.4-mRNA-1 | Japonica | NA | | Os05g0462700 | OsR498G0510997800.01.T01 | Multifunctional fusion protein |
| augustus-5-processed-gene-24.41-mRNA-1 | Japonica | NA | | Os05g0142600 | | |
| augustus-5-processed-gene-242.44-mRNA-1 | Japonica | NA | | Os05g0471100 | OsR498G0511022200.01.T01 | Glutaredoxin domain-containing cysteine-rich protein 1 |
| augustus-5-processed-gene-243.31-mRNA-1 | Japonica | NA | | Os05g0473500 | OsR498G0511029000.01.T02 | Exocyst subunit exo70 family protein B2 |
| augustus-5-processed-gene-244.42-mRNA-1 | Japonica | Indica | | Os05g0475400 | OsR498G0511033800.01.T01 | Alanine--glyoxylate aminotransferase 2 |
| augustus-5-processed-gene-245.27-mRNA-1 | Japonica | NA | | | OsR498G0511043200.01.T01 | RING-H2 finger protein ATL1N |
| augustus-5-processed-gene-246.7-mRNA-1 | Japonica | NA | | | OsR498G0511046600.01.T01 | |
| augustus-5-processed-gene-247.79-mRNA-1 | Japonica | NA | | | OsR498G0511056000.01.T01 | |
| augustus-5-processed-gene-248.2-mRNA-1 | Japonica | Japonica | | Os05g0481900 | OsR498G0511058000.01.T02 | PIN-formed protein14 |
| augustus-5-processed-gene-248.35-mRNA-1 | Japonica | NA | | | OsR498G1119312200.01.T01 | |
| augustus-5-processed-gene-250.30-mRNA-1 | Japonica | NA | | Os05g0486200 | OsR498G0511076000.01.T01 | Dolichyl-diphosphooligosaccharide--protein glycosyltransferase subunit KCP |
| augustus-5-processed-gene-251.63-mRNA-1 | Japonica | NA | | Os05g0488800 | OsR498G0511080200.01.T03 | RING-type E3 ubiquitin transferase |
| augustus-5-processed-gene-252.18-mRNA-1 | Japonica | NA | | Os05g0490400 | OsR498G0511085200.01.T01 | |
| augustus-5-processed-gene-252.21-mRNA-1 | Japonica | NA | | Os05g0490700 | OsR498G0511085800.01.T02 | Histone-lysine N-methyltransferase H3 lysine-9 specific SUVH3 |
| augustus-5-processed-gene-252.8-mRNA-1 | Japonica | NA | | Os05g0490000 | | KRR1-interacting protein involved in 40S ribosome biogenesis |
| augustus-5-processed-gene-254.54-mRNA-1 | Japonica | NA | | | OsR498G0511098600.01.T03 | RING-type E3 ubiquitin transferase |
| augustus-5-processed-gene-254.55-mRNA-1 | Japonica | NA | | Os05g0495200 | OsR498G0511099200.01.T03 | BZIP transcription factor |
| augustus-5-processed-gene-255.29-mRNA-1 | Japonica | NA | | | OsR498G0511103200.01.T01 | |
| augustus-5-processed-gene-255.34-mRNA-1 | Japonica | NA | | Os05g0497300 | OsR498G0511105200.01.T01 | Ethylene-responsive transcription factor 4 |
| augustus-5-processed-gene-256.29-mRNA-1 | Japonica | NA | | Os05g0498200 | OsR498G0511109000.01.T01 | Sonic hedgehog protein A |
| augustus-5-processed-gene-256.34-mRNA-1 | Japonica | NA | | | OsR498G0511110000.01.T01 | |
| augustus-5-processed-gene-256.37-mRNA-1 | Japonica | NA | | | OsR498G0917893100.01.T01 | Trypsin |
| augustus-5-processed-gene-257.14-mRNA-1 | Japonica | NA | | Os10g0460000 | OsR498G0305532000.01.T01 | Kinesin heavy chain-like |
| augustus-5-processed-gene-260.3-mRNA-1 | Japonica | NA | | | OsR498G0511135400.01.T01 | DUF834 domain-containing protein |

| | | | | | | |
|---|---|---|---|---|---|---|
| augustus-5-processed-gene-264.47-mRNA-1 | Japonica | NA | | Os05g0510600 | OsR498G0511162200.01.T01 | Tyrosine decarboxylase |
| augustus-5-processed-gene-264.67-mRNA-1 | Japonica | NA | | Os05g0511300 | OsR498G0511165400.01.T02 | Carnosine N-methyltransferase |
| augustus-5-processed-gene-266.40-mRNA-1 | Japonica | Indica | | Os05g0514600 | OsR498G0511177800.01.T03 | Gibberellin 2-beta-dioxygenase |
| augustus-5-processed-gene-266.41-mRNA-1 | Japonica | Indica | | Os05g0514600 | OsR498G0511177800.01.T03 | Gibberellin 2-beta-dioxygenase |
| augustus-5-processed-gene-267.54-mRNA-1 | Japonica | NA | | Os05g0515600 | OsR498G0511181400.01.T01 | O-methyltransferase ZRP4 |
| augustus-5-processed-gene-269.56-mRNA-1 | Japonica | NA | | Os05g0519800 | | probable transcriptional regulatory protein At2g25830 |
| augustus-5-processed-gene-27.10-mRNA-1 | Japonica | NA | | Os05g0147800 | | BED-type domain-containing protein |
| augustus-5-processed-gene-27.9-mRNA-1 | Japonica | NA | | Os05g0147700 | OsR498G0509734200.01.T01 | |
| augustus-5-processed-gene-271.36-mRNA-1 | Japonica | NA | | Os05g0522700 | OsR498G0511207200.01.T01 | |
| augustus-5-processed-gene-271.37-mRNA-1 | Japonica | NA | | Os10g0531750 | OsR498G1120733700.01.T01 | Retrotransposon protein, putative, Ty3-gypsy sub-class |
| augustus-5-processed-gene-272.36-mRNA-1 | Japonica | NA | | Os05g0525900 | OsR498G0511216000.01.T01 | C3H transcription factor |
| augustus-5-processed-gene-273.35-mRNA-1 | Japonica | NA | | Os05g0527000 | OsR498G0511218600.01.T01 | Glycosyltransferase |
| augustus-5-processed-gene-273.36-mRNA-1 | Japonica | NA | | Os05g0527100 | OsR498G0511219800.01.T01 | Glycosyltransferase |
| augustus-5-processed-gene-273.52-mRNA-1 | Japonica | Japonica | | Os05g0527400 | OsR498G0511221000.01.T01 | Glycosyltransferase |
| augustus-5-processed-gene-273.55-mRNA-1 | Japonica | Japonica | | Os05g0527700 | OsR498G0511221000.01.T01 | Glycosyltransferase |
| augustus-5-processed-gene-273.58-mRNA-1 | Japonica | NA | | | | Respiratory burst oxidase protein B variant beta |
| augustus-5-processed-gene-28.60-mRNA-1 | Japonica | NA | | Os05g0149300 | OsR498G0509738700.01.T01 | 1-aminocyclopropane-1-carboxylate oxidase |
| augustus-5-processed-gene-285.14-mRNA-1 | Japonica | NA | | Os05g0552200 | OsR498G0511303400.01.T01 | Hexosyltransferase |
| augustus-5-processed-gene-285.2-mRNA-1 | Japonica | Japonica | | Os05g0551700 | OsR498G0101792300.01.T01 | Adenylate isopentenyltransferase |
| augustus-5-processed-gene-299.66-mRNA-1 | Japonica | NA | | Os05g0579300 | OsR498G0511400300.01.T01 | Zinc-finger homeodomain protein 6 |
| augustus-5-processed-gene-300.24-mRNA-1 | Japonica | NA | | Os05g0582000 | OsR498G0511409400.01.T01 | Plant basic secretory protein (BSP) family protein |
| augustus-5-processed-gene-300.9-mRNA-1 | Japonica | NA | | Os08g0106900 | OsR498G0511404600.01.T01 | Transposon protein, putative, CACTA, En/Spm sub-class |
| augustus-5-processed-gene-305.4-mRNA-1 | Japonica | Japonica | | Os05g0590000 | OsR498G0101471900.01.T01 | Peptidase A1 domain-containing protein (Fragment) |
| augustus-5-processed-gene-306.77-mRNA-1 | Japonica | NA | | Os05g0593900 | OsR498G0511448600.01.T01 | Vacuolar-processing enzyme gamma-isozyme |
| augustus-5-processed-gene-307.45-mRNA-1 | Japonica | NA | | Os05g0594200 | OsR498G0511449400.01.T01 | Vacuolar cation/proton exchanger |
| augustus-5-processed-gene-31.10-mRNA-1 | Japonica | NA | | Os05g0154600 | OsR498G0509766800.01.T01 | E3 ubiquitin-protein ligase RFI2 |
| augustus-5-processed-gene-32.51-mRNA-1 | Japonica | Japonica | | Os05g0155400 | OsR498G0100884100.01.T02 | GATA-type domain-containing protein |
| augustus-5-processed-gene-33.67-mRNA-1 | Japonica | NA | | | OsR498G0204960100.01.T01 | |
| augustus-5-processed-gene-35.37-mRNA-1 | Japonica | NA | | Os05g0160300 | OsR498G0509788400.01.T01 | AAI domain-containing protein (Fragment) |
| augustus-5-processed-gene-36.27-mRNA-1 | Japonica | NA | | Os05g0163300 | OsR498G0509798800.01.T02 | FAS1 domain-containing protein (Fragment) |
| augustus-5-processed-gene-39.57-mRNA-1 | Japonica | NA | | Os05g0168500 | OsR498G0509820600.01.T01 | Transmembrane 9 superfamily member |
| augustus-5-processed-gene-46.7-mRNA-1 | Japonica | NA | | Os05g0177500 | OsR498G0509850800.01.T01 | Glycosyltransferase |
| augustus-5-processed-gene-47.20-mRNA-1 | Japonica | NA | LRR-TM | Os05g0180300 | OsR498G0509866000.01.T01 | Leucine-rich repeat extensin-like protein 3 |
| augustus-5-processed-gene-49.36-mRNA-1 | Japonica | NA | | | OsR498G0306050700.01.T01 | Transposon protein, putative, Mutator sub-class |
| augustus-5-processed-gene-5.23-mRNA-1 | Japonica | Japonica | | Os05g0109100 | OsR498G0509581600.01.T01 | |
| augustus-5-processed-gene-5.3-mRNA-1 | Japonica | NA | CC-Kinase | Os05g0108300 | OsR498G0509579900.01.T01 | Non-specific serine/threonine protein kinase |
| augustus-5-processed-gene-6.47-mRNA-1 | Japonica | NA | | Os05g0110400 | | Scarecrow-like protein 9 isoform A |
| augustus-5-processed-gene-67.2-mRNA-1 | Japonica | NA | | Os05g0209000 | | |
| augustus-5-processed-gene-67.20-mRNA-1 | Japonica | NA | | Os05g0209300 | OsR498G0509996000.01.T01 | E3 ubiquitin-protein ligase ATL9 |
| augustus-5-processed-gene-68.23-mRNA-1 | Japonica | NA | | Os02g0711400 | OsR498G1220919400.01.T01 | RING-type domain-containing protein |
| augustus-5-processed-gene-69.16-mRNA-1 | Japonica | NA | | Os05g0212900 | OsR498G0510009000.01.T01 | Chalcone and stilbene synthases, N-terminal domain containing protein |
| augustus-5-processed-gene-7.13-mRNA-1 | Japonica | NA | | Os05g0112750 | OsR498G0509596200.01.T01 | Embryo defective |
| augustus-5-processed-gene-7.14-mRNA-1 | Japonica | NA | | Os05g0112800 | OsR498G0509596400.01.T01 | cysteine-rich repeat secretory protein 55 |
| augustus-5-processed-gene-75.3-mRNA-1 | Japonica | NA | | Os03g0110200 | | Transposon protein, putative, Mutator sub-class |
| augustus-5-processed-gene-82.16-mRNA-1 | Japonica | NA | | Os05g0233900 | | |
| augustus-5-processed-gene-85.45-mRNA-1 | Japonica | NA | | Os05g0238400 | OsR498G0510112800.01.T01 | Glycine-rich protein family |
| augustus-5-processed-gene-85.47-mRNA-1 | Japonica | NA | | Os05g0239150 | | Zinc finger BED domain-containing protein DAYSLEEPER |
| augustus-5-processed-gene-86.37-mRNA-1 | Japonica | NA | | | OsR498G0203809100.01.T01 | DUF834 domain-containing protein |
| augustus-5-processed-gene-87.38-mRNA-1 | Japonica | NA | | Os05g0242600 | OsR498G0510126400.01.T01 | |
| augustus-5-processed-gene-87.40-mRNA-1 | Japonica | NA | | | | |
| augustus-5-processed-gene-89.36-mRNA-1 | Japonica | NA | | | OsR498G0510294600.01.T01 | Zinc knuckle containing protein-like |
| augustus-5-processed-gene-89.48-mRNA-1 | Japonica | NA | | Os05g0247100 | OsR498G0510133000.01.T02 | Xylanase inhibitor protein XIP |
| augustus-5-processed-gene-9.33-mRNA-1 | Japonica | Indica | | Os10g0326400 | OsR498G0509612600.01.T01 | F-box domain containing protein, expressed |
| augustus-5-processed-gene-9.9-mRNA-1 | Japonica | NA | | Os05g0116600 | OsR498G0509609000.01.T01 | F-box protein interaction domain containing protein |
| augustus-5-processed-gene-92.27-mRNA-1 | Japonica | Japonica | | Os05g0251200 | OsR498G0510143400.01.T01 | DUF1618 domain-containing protein (Fragment) |
| augustus-5-processed-gene-92.6-mRNA-1 | Japonica | NA | | | OsR498G0510148400.01.T01 | |
| augustus-5-processed-gene-94.24-mRNA-1 | Japonica | NA | | | OsR498G0510162000.01.T01 | |
| augustus-5-processed-gene-95.48-mRNA-1 | Japonica | NA | | Os05g0256033 | | |
| augustus-5-processed-gene-96.25-mRNA-1 | Japonica | Indica | | Os05g0360900 | OsR498G0510171400.01.T01 | |
| augustus-6-processed-gene-106.26-mRNA-1 | Japonica | NA | | | OsR498G0612182100.01.T01 | Embryogenesis transmembrane protein-like |
| augustus-6-processed-gene-112.27-mRNA-1 | Japonica | NA | | Os06g0305200 | OsR498G0612219700.01.T01 | Nuclear pore complex protein NUP155 |

| | | | | | | |
|---|---|---|---|---|---|---|
| augustus-6-processed-gene-120.40-mRNA-1 | Japonica | NA | | Os06g0318200 | OsR498G0612270500.01.T03 | BTB/POZ domain-containing protein At2g04740 |
| augustus-6-processed-gene-124.5-mRNA-1 | Japonica | Indica | | Os02g0716800 | OsR498G0612283100.01.T01 | Peptide transporter PTR1 |
| augustus-6-processed-gene-134.19-mRNA-1 | Japonica | NA | | | OsR498G0612526300.01.T01 | Putative K domain-containing protein |
| augustus-6-processed-gene-155.34-mRNA-1 | Japonica | NA | | | OsR498G0612423700.01.T01 | |
| augustus-6-processed-gene-159.6-mRNA-1 | Japonica | NA | | Os06g0475800 | | |
| augustus-6-processed-gene-164.11-mRNA-1 | Japonica | NA | | | | Retrotransposon protein, putative, LINE subclass |
| augustus-6-processed-gene-171.32-mRNA-1 | Japonica | Indica | | Os06g0494400 | OsR498G0612327800.01.T02 | Protein DETOXIFICATION |
| augustus-6-processed-gene-172.17-mRNA-1 | Japonica | NA | | | OsR498G0612323800.01.T01 | Transcription factor bHLH83 |
| augustus-6-processed-gene-174.0-mRNA-1 | Japonica | NA | | | OsR498G0713993600.01.T01 | Putative zinc finger, CCHC-type |
| augustus-6-processed-gene-189.9-mRNA-1 | Japonica | NA | | Os01g0387333 | OsR498G0815569700.01.T02 | |
| augustus-6-processed-gene-222.28-mRNA-1 | Japonica | NA | | | | DDE Tnp4 domain-containing protein (Fragment) |
| augustus-6-processed-gene-245.23-mRNA-1 | Japonica | NA | | | | Retrotransposon protein, putative, unclassified, expressed |
| augustus-6-processed-gene-255.29-mRNA-1 | Japonica | NA | | Os12g0468900 | OsR498G0916869000.01.T01 | Cell wall protein-like |
| augustus-6-processed-gene-256.35-mRNA-1 | Japonica | NA | | Os03g0110200 | | Transposon protein, putative, Mutator sub-class, expressed |
| augustus-6-processed-gene-260.26-mRNA-1 | Japonica | NA | | Os06g0636300 | OsR498G0613025400.01.T02 | Ubiquitin-specific protease family C19-related protein |
| augustus-6-processed-gene-288.73-mRNA-1 | Japonica | NA | | Os06g0687000 | OsR498G0613209600.01.T01 | Regulator of Vps4 activity in the MVB pathway protein |
| augustus-6-processed-gene-291.18-mRNA-1 | Japonica | NA | Kinase-TM | Os06g0693200 | OsR498G0613227500.01.T01 | probable receptor-like serine/threonine-protein kinase At5g57670 |
| augustus-6-processed-gene-4.32-mRNA-1 | Japonica | NA | | Os06g0106100 | OsR498G0611480000.01.T01 | Pre-mRNA-splicing factor Cwf15/Cwc15 |
| augustus-6-processed-gene-5.13-mRNA-1 | Japonica | NA | | | | |
| augustus-6-processed-gene-76.24-mRNA-1 | Japonica | Indica | | Os06g0244000 | OsR498G0611978800.01.T01 | Anthranilate O-methyltransferase 2 |
| augustus-6-processed-gene-86.24-mRNA-1 | Japonica | NA | | | OsR498G0306743300.01.T01 | Transposon protein, putative, CACTA, En/Spm sub-class |
| augustus-6-processed-gene-93.17-mRNA-1 | Japonica | NA | Kinase-LRR-TM | Os06g0274500 | OsR498G0612087700.01.T01 | Putative somatic embryogenesis protein kinase 1 |
| augustus-7-processed-gene-100.22-mRNA-1 | Japonica | Japonica | | Os07g0277400 | OsR498G1221660800.01.T01 | FBD domain-containing protein (Fragment) |
| augustus-7-processed-gene-103.25-mRNA-1 | Japonica | NA | | Os07g0281200 | OsR498G0713993200.01.T01 | |
| augustus-7-processed-gene-104.13-mRNA-1 | Japonica | NA | Kinase-TM | Os07g0283050 | OsR498G0713999000.01.T01 | Putative receptor-type protein kinase LRK1 |
| augustus-7-processed-gene-108.12-mRNA-1 | Japonica | NA | | | | ABC transporter domain-containing protein |
| augustus-7-processed-gene-109.15-mRNA-1 | Japonica | Indica | | Os07g0290200 | OsR498G0714023700.01.T01 | AAI domain-containing protein |
| augustus-7-processed-gene-109.21-mRNA-1 | Japonica | Indica | | Os01g0860500 | OsR498G0714025000.01.T01 | Endo-beta-N-acetylglucosaminidase |
| augustus-7-processed-gene-109.35-mRNA-1 | Japonica | NA | | Os07g0291100 | OsR498G0714026000.01.T01 | Transport protein particle (TRAPP) complex subunit |
| augustus-7-processed-gene-113.19-mRNA-1 | Japonica | NA | | Os07g0297300 | OsR498G0714043200.01.T03 | Methyltransferase-like protein 17, mitochondrial |
| augustus-7-processed-gene-114.19-mRNA-1 | Japonica | NA | | Os03g0110200 | | Transposon protein, putative, Mutator sub-class, expressed |
| augustus-7-processed-gene-115.10-mRNA-1 | Japonica | NA | | | | Cell wall protein-like |
| augustus-7-processed-gene-116.5-mRNA-1 | Japonica | NA | | Os07g0302100 | OsR498G0714058400.01.T01 | Non-specific serine/threonine protein kinase (Fragment) |
| augustus-7-processed-gene-123.1-mRNA-1 | Japonica | NA | | Os07g0407900 | OsR498G0714077500.01.T01 | DUF679 domain membrane protein 7 |
| augustus-7-processed-gene-124.34-mRNA-1 | Japonica | NA | | Os07g0409400 | OsR498G0714085000.01.T05 | Transcription termination factor MTEF18, mitochondrial |
| augustus-7-processed-gene-124.37-mRNA-1 | Japonica | NA | | Os07g0409500 | OsR498G0714085700.01.T01 | Skp1 domain-containing protein |
| augustus-7-processed-gene-125.1-mRNA-1 | Japonica | NA | | Os07g0410700 | OsR498G0714091600.01.T01 | Ethylene-responsive transcription factor 15 |
| augustus-7-processed-gene-127.1-mRNA-1 | Japonica | NA | | Os07g0414000 | OsR498G0714098100.01.T01 | Anthocyanin 5-aromatic acyltransferase |
| augustus-7-processed-gene-127.20-mRNA-1 | Japonica | NA | | Os07g0415200 | OsR498G0714100900.01.T01 | Putative mitochondrial ribosomal protein L23 |
| augustus-7-processed-gene-128.21-mRNA-1 | Japonica | NA | | Os07g0417200 | OsR498G0714108300.01.T01 | Omega-6 fatty acid desaturase, endoplasmic reticulum isozyme 2 |
| augustus-7-processed-gene-128.31-mRNA-1 | Japonica | NA | | Os07g0416900 | OsR498G0714107400.01.T02 | Omega-6 fatty acid desaturase, endoplasmic reticulum isozyme 2 |
| augustus-7-processed-gene-129.18-mRNA-1 | Japonica | NA | | Os07g0417600 | OsR498G0714110200.01.T01 | Thaumatin-like protein 1 isoform B |
| augustus-7-processed-gene-130.12-mRNA-1 | Japonica | NA | | Os07g0418600 | OsR498G0714114600.01.T01 | Proline and lysine rich protein |
| augustus-7-processed-gene-130.17-mRNA-1 | Japonica | Indica | | Os04g0131900 | OsR498G0714118700.01.T07 | Sterol 3-beta-glucosyltransferase 1 |
| augustus-7-processed-gene-130.25-mRNA-1 | Japonica | Japonica | | Os07g0418500 | OsR498G0714820100.01.T01 | Secologanin synthase |
| augustus-7-processed-gene-130.28-mRNA-1 | Japonica | Indica | | Os07g0419800 | OsR498G0714121200.01.T03 | C2H2-type domain-containing protein |
| augustus-7-processed-gene-130.6-mRNA-1 | Japonica | Japonica | | Os07g0418700 | OsR498G0714114600.01.T01 | Early nodulin 75 protein |
| augustus-7-processed-gene-131.27-mRNA-1 | Japonica | NA | | Os07g0421000 | OsR498G0714126600.01.T02 | Monogalactosyldiacylglycerol synthase |
| augustus-7-processed-gene-131.8-mRNA-1 | Japonica | Indica | | Os11g0701800 | OsR498G0714121900.01.T01 | Acidic endochitinase |
| augustus-7-processed-gene-132.14-mRNA-1 | Japonica | Japonica | | Os07g0421300 | OsR498G0714125300.01.T05 | Heteroglycan glucosidase 1 |
| augustus-7-processed-gene-133.14-mRNA-1 | Japonica | NA | | Os07g0423000 | OsR498G0714131800.01.T03 | Polyubiquitin |
| augustus-7-processed-gene-133.20-mRNA-1 | Japonica | NA | | | OsR498G0510760300.01.T01 | |
| augustus-7-processed-gene-134.22-mRNA-1 | Japonica | NA | | | OsR498G0714140800.01.T01 | |
| augustus-7-processed-gene-141.26-mRNA-1 | Japonica | NA | | | OsR498G0714153600.01.T01 | Tetratricopeptide repeat (TPR)-like superfamily protein |
| augustus-7-processed-gene-142.18-mRNA-1 | Japonica | Japonica | | Os07g0436600 | OsR498G0714158500.01.T05 | Selenium-binding protein-like |
| augustus-7-processed-gene-142.19-mRNA-1 | Japonica | NA | | Os07g0436700 | | |
| augustus-7-processed-gene-142.27-mRNA-1 | Japonica | NA | | Os07g0437000 | OsR498G0714159000.01.T02 | Flavin-containing monooxygenase |
| augustus-7-processed-gene-142.9-mRNA-1 | Japonica | NA | | Os07g0435900 | OsR498G0714156900.01.T02 | Histone-lysine N-methyltransferase family member SUVH9 |
| augustus-7-processed-gene-143.0-mRNA-1 | Japonica | NA | | Os01g0293600 | | zinc finger MYM-type protein 1-like |
| augustus-7-processed-gene-143.5-mRNA-1 | Japonica | NA | | Os07g0437500 | OsR498G0714162100.01.T05 | Aromatic-L-amino-acid/L-histidine decarboxylase |
| augustus-7-processed-gene-144.23-mRNA-1 | Japonica | NA | | | OsR498G0714169700.01.T01 | DUF295 domain-containing protein |

| | | | | | | |
|---|---|---|---|---|---|---|
| augustus-7-processed-gene-144.41-mRNA-1 | Japonica | NA | | Os07g0440100 | OsR498G0714170000.01.T03 | glycine-rich cell wall structural protein 1 |
| augustus-7-processed-gene-146.14-mRNA-1 | Japonica | Indica | | Os07g0443500 | OsR498G0714178700.01.T01 | SANT domain-containing protein |
| augustus-7-processed-gene-149.7-mRNA-1 | Japonica | Japonica | | Os07g0446800 | OsR498G0509895600.01.T01 | Phosphotransferase |
| augustus-7-processed-gene-152.4-mRNA-1 | Japonica | NA | | | OsR498G0713980300.01.T01 | |
| augustus-7-processed-gene-152.6-mRNA-1 | Japonica | Japonica | | Os07g0451300 | OsR498G0714197900.01.T01 | |
| augustus-7-processed-gene-155.2-mRNA-1 | Japonica | NA | | | OsR498G0100075100.01.T01 | Epstein-Barr virus EBNA-1-like |
| augustus-7-processed-gene-155.23-mRNA-1 | Japonica | NA | | Os07g0456900 | OsR498G0714218600.01.T01 | NAC domain-containing protein (Fragment) |
| augustus-7-processed-gene-156.20-mRNA-1 | Japonica | Indica | | Os07g0457400 | OsR498G0714219900.01.T01 | |
| augustus-7-processed-gene-158.9-mRNA-1 | Japonica | Japonica | | Os07g0460100 | OsR498G0509914200.01.T01 | Stem 28 kDa glycoprotein |
| augustus-7-processed-gene-159.35-mRNA-1 | Japonica | NA | | Os05g0129000 | OsR498G0714240400.01.T04 | Glutamate--cysteine ligase B, chloroplastic |
| augustus-7-processed-gene-165.21-mRNA-1 | Japonica | NA | | Os07g0251301 | | 60s acidic ribosomal protein domain-containing protein |
| augustus-7-processed-gene-165.32-mRNA-1 | Japonica | NA | | Os07g0470400 | OsR498G0714265800.01.T02 | |
| augustus-7-processed-gene-169.15-mRNA-1 | Japonica | NA | | Os07g0475700 | OsR498G0714287500.01.T01 | Auxin-responsive protein SAUR61 |
| augustus-7-processed-gene-170.35-mRNA-1 | Japonica | NA | | Os07g0477500 | OsR498G0714295500.01.T01 | zinc finger CCCH domain-containing protein 13-like isoform X2 |
| augustus-7-processed-gene-171.13-mRNA-1 | Japonica | NA | | Os03g0204201 | OsR498G0408675500.01.T01 | Retrotransposon protein, putative, Ty3-gypsy sub-class |
| augustus-7-processed-gene-174.27-mRNA-1 | Japonica | NA | | Os07g0483500 | OsR498G0714312400.01.T04 | FT-interacting protein 1 |
| augustus-7-processed-gene-175.13-mRNA-1 | Japonica | NA | | Os07g0485000 | OsR498G0714317500.01.T02 | Trehalose 6-phosphate phosphatase |
| augustus-7-processed-gene-175.9-mRNA-1 | Japonica | NA | | Os07g0484500 | OsR498G0714315500.01.T02 | Spindle and kinetochore-associated protein 1 |
| augustus-7-processed-gene-177.15-mRNA-1 | Japonica | Japonica | | Os07g0487100 | OsR498G0714323900.01.T01 | Glycosyltransferase |
| augustus-7-processed-gene-191.1-mRNA-1 | Japonica | NA | | | OsR498G0511427100.01.T01 | Epstein-Barr virus EBNA-1-like |
| augustus-7-processed-gene-193.35-mRNA-1 | Japonica | NA | | Os07g0513000 | | ATP synthase gamma chain like |
| augustus-7-processed-gene-193.51-mRNA-1 | Japonica | NA | | Os07g0513200 | OsR498G0714418200.01.T02 | Pentatricopeptide repeat-containing protein mitochondrial |
| augustus-7-processed-gene-201.21-mRNA-1 | Japonica | NA | | | OsR498G0714474500.01.T01 | |
| augustus-7-processed-gene-204.30-mRNA-1 | Japonica | NA | | Os07g0532800 | | Peptidase A1 domain-containing protein (Fragment) |
| augustus-7-processed-gene-204.8-mRNA-1 | Japonica | NA | | | | Putative ankyrin repeat-containing domain, PGG domain-containing protein |
| augustus-7-processed-gene-209.52-mRNA-1 | Japonica | NA | | | | Transposon protein, putative, Mutator sub-class, expressed |
| augustus-7-processed-gene-21.26-mRNA-1 | Japonica | Japonica | | Os07g0148600 | OsR498G1221267000.01.T01 | Sulfotransferase |
| augustus-7-processed-gene-21.29-mRNA-1 | Japonica | NA | | | OsR498G0511146000.01.T01 | |
| augustus-7-processed-gene-21.6-mRNA-1 | Japonica | NA | Kinase-TM | Os07g0147600 | OsR498G0713511000.01.T02 | Mitogen-activated protein kinase kinase kinase |
| augustus-7-processed-gene-210.26-mRNA-1 | Japonica | Indica | | Os07g0542400 | OsR498G0714520000.01.T02 | Cysteine-rich receptor-like protein kinase 15 |
| augustus-7-processed-gene-211.28-mRNA-1 | Japonica | Japonica | | Os07g0543600 | OsR498G0714523900.01.T01 | Strictosidine synthase 1 |
| augustus-7-processed-gene-212.0-mRNA-1 | Japonica | NA | | Os07g0545800 | OsR498G0714527300.01.T01 | Chitin-inducible gibberellin-responsive protein 1 |
| augustus-7-processed-gene-212.12-mRNA-1 | Japonica | Indica | | Os07g0546400 | OsR498G0714529100.01.T04 | NPH3 domain-containing protein (Fragment) |
| augustus-7-processed-gene-212.25-mRNA-1 | Japonica | NA | | Os11g0433800 | | Ribosomal protein-like |
| augustus-7-processed-gene-213.14-mRNA-1 | Japonica | Indica | | Os07g0548500 | OsR498G0714534100.01.T01 | |
| augustus-7-processed-gene-213.24-mRNA-1 | Japonica | Japonica | | Os07g0548000 | OsR498G0714534100.01.T01 | FBD domain-containing protein |
| augustus-7-processed-gene-214.34-mRNA-1 | Japonica | NA | | | OsR498G0714536900.01.T01 | Vacuolar ATP synthase subunit h |
| augustus-7-processed-gene-214.36-mRNA-1 | Japonica | NA | | Os07g0549900 | OsR498G0714537700.01.T01 | Multifunctional fusion protein |
| augustus-7-processed-gene-214.43-mRNA-1 | Japonica | NA | | Os07g0549100 | | Transmembrane protein, putative |
| augustus-7-processed-gene-214.51-mRNA-1 | Japonica | NA | | | OsR498G0714537900.01.T01 | |
| augustus-7-processed-gene-216.21-mRNA-1 | Japonica | NA | | | OsR498G0714551300.01.T01 | RNA helicase |
| augustus-7-processed-gene-216.24-mRNA-1 | Japonica | NA | | | OsR498G0714552500.01.T01 | F-box domain-containing protein |
| augustus-7-processed-gene-216.33-mRNA-1 | Japonica | NA | Kinase-TM | Os07g0553633 | OsR498G0714550000.01.T01 | Non-specific serine/threonine protein kinase (Fragment) |
| augustus-7-processed-gene-219.36-mRNA-1 | Japonica | Indica | | Os07g0559100 | OsR498G0714569500.01.T01 | |
| augustus-7-processed-gene-220.17-mRNA-1 | Japonica | NA | | Os07g0562400 | OsR498G0714577900.01.T01 | Organic cation/carnitine transporter 2 |
| augustus-7-processed-gene-220.6-mRNA-1 | Japonica | NA | | | OsR498G0714573400.01.T01 | WAS/WASL-interacting protein family member 3-like |
| augustus-7-processed-gene-222.3-mRNA-1 | Japonica | NA | | | | Glycine-rich rna-binding protein |
| augustus-7-processed-gene-222.32-mRNA-1 | Japonica | Indica | | Os07g0564200 | OsR498G0714585000.01.T01 | Tyrosine-protein kinase receptor UFO like |
| augustus-7-processed-gene-223.11-mRNA-1 | Japonica | NA | | | OsR498G0714591300.01.T01 | F-box domain-containing protein |
| augustus-7-processed-gene-223.21-mRNA-1 | Japonica | NA | | Os07g0565800 | OsR498G0714589800.01.T01 | |
| augustus-7-processed-gene-223.6-mRNA-1 | Japonica | NA | | Os04g0613400 | OsR498G0714587700.01.T03 | Cell differentiation protein rcd1 |
| augustus-7-processed-gene-224.10-mRNA-1 | Japonica | NA | | Os07g0567000 | OsR498G0714593500.01.T01 | Exostosin domain-containing protein (Fragment) |
| augustus-7-processed-gene-224.15-mRNA-1 | Japonica | NA | | | OsR498G0714594900.01.T02 | Scarecrow-like protein 23 |
| augustus-7-processed-gene-224.26-mRNA-1 | Japonica | NA | | Os07g0566500 | OsR498G0714592800.01.T01 | NAC transcription factor |
| augustus-7-processed-gene-225.12-mRNA-1 | Japonica | NA | | Os07g0568450 | | |
| augustus-7-processed-gene-225.2-mRNA-1 | Japonica | NA | | Os07g0568300 | OsR498G0714596900.01.T03 | Zinc finger CCCH domain-containing protein 50 |
| augustus-7-processed-gene-225.23-mRNA-1 | Japonica | NA | | Os07g0568800 | | |
| augustus-7-processed-gene-226.0-mRNA-1 | Japonica | NA | | Os07g0569500 | OsR498G0714601500.01.T01 | Protein-lysine N-methyltransferase rrg1 (Fragment) |
| augustus-7-processed-gene-226.3-mRNA-1 | Japonica | Japonica | Kinase-TM | Os07g0569800 | OsR498G0714602900.01.T01 | L-type lectin-domain containing receptor kinase IX.1 |
| augustus-7-processed-gene-229.3-mRNA-1 | Japonica | NA | | Os07g0573200 | OsR498G0714613300.01.T01 | Adenylyl-sulfate kinase |
| augustus-7-processed-gene-23.2-mRNA-1 | Japonica | Indica | | Os04g0393500 | OsR498G0713525100.01.T01 | Glutaredoxin |

| | | | | | | |
|---|---|---|---|---|---|---|
| augustus-7-processed-gene-230.20-mRNA-1 | Japonica | NA | | Os07g0575401 | | |
| augustus-7-processed-gene-230.22-mRNA-1 | Japonica | NA | | | OsR498G0714619600.01.T01 | Ethylene-responsive transcription factor |
| augustus-7-processed-gene-230.27-mRNA-1 | Japonica | NA | Kinase-TM | Os07g0575600 | OsR498G0714620600.01.T01 | Putative receptor-type protein kinase LRK1 |
| augustus-7-processed-gene-230.37-mRNA-1 | Japonica | NA | | Os07g0576100 | OsR498G0714622300.01.T01 | Indole-3-acetic acid amido synthetase |
| augustus-7-processed-gene-230.47-mRNA-1 | Japonica | NA | Kinase-TM | Os07g0575700 | OsR498G0714621000.01.T01 | Lectin receptor-type kinase |
| augustus-7-processed-gene-230.48-mRNA-1 | Japonica | Indica | Kinase-TM | Os07g0575600 | OsR498G0714621200.01.T01 | Lectin receptor-type kinase |
| augustus-7-processed-gene-231.28-mRNA-1 | Japonica | NA | | Os07g0578300 | | Subtilisin-like protease |
| augustus-7-processed-gene-232.34-mRNA-1 | Japonica | NA | | Os07g0578700 | | |
| augustus-7-processed-gene-233.32-mRNA-1 | Japonica | NA | | Os07g0583000 | OsR498G0714642400.01.T01 | Stress-associated endoplasmic reticulum protein 2 |
| augustus-7-processed-gene-234.40-mRNA-1 | Japonica | NA | | Os07g0583600 | OsR498G0714645900.01.T01 | Chitin-inducible gibberellin-responsive protein 2 |
| augustus-7-processed-gene-235.37-mRNA-1 | Japonica | NA | | Os07g0585100 | OsR498G0714651700.01.T02 | Proteinase inhibitor I25, cystatin |
| augustus-7-processed-gene-235.43-mRNA-1 | Japonica | NA | | Os07g0585700 | OsR498G0714655100.01.T01 | |
| augustus-7-processed-gene-235.56-mRNA-1 | Japonica | Indica | | Os03g0431200 | OsR498G0714654200.01.T02 | Amino-acid N-acetyltransferase |
| augustus-7-processed-gene-236.17-mRNA-1 | Japonica | NA | | Os07g0587000 | | F-box domain-containing protein |
| augustus-7-processed-gene-236.18-mRNA-1 | Japonica | Indica | | Os07g0587500 | OsR498G0714665300.01.T01 | U-box domain-containing protein 4 |
| augustus-7-processed-gene-236.25-mRNA-1 | Japonica | NA | | Os07g0587300 | | |
| augustus-7-processed-gene-236.26-mRNA-1 | Japonica | NA | | Os07g0587400 | OsR498G0714664800.01.T03 | Eukaryotic peptide chain release factor subunit 1 |
| augustus-7-processed-gene-236.9-mRNA-1 | Japonica | NA | | Os07g0586600 | OsR498G0714658200.01.T01 | GIY-YIG domain-containing protein |
| augustus-7-processed-gene-237.10-mRNA-1 | Japonica | Japonica | | Os07g0588600 | OsR498G0714669700.01.T01 | Zinc finger protein 1 |
| augustus-7-processed-gene-237.12-mRNA-1 | Japonica | Japonica | | Os07g0588700 | OsR498G0714669700.01.T01 | C2H2-type zinc finger transcription factor |
| augustus-7-processed-gene-237.13-mRNA-1 | Japonica | NA | | Os07g0588800 | OsR498G0714670300.01.T04 | Queuosine salvage protein (Fragment) |
| augustus-7-processed-gene-237.17-mRNA-1 | Japonica | NA | | Os07g0589200 | OsR498G0714672500.01.T01 | Scarecrow transcription factor family protein |
| augustus-7-processed-gene-238.25-mRNA-1 | Japonica | NA | | Os07g0589600 | OsR498G0714673400.01.T01 | Zinc finger CCCH domain-containing protein 18 |
| augustus-7-processed-gene-238.31-mRNA-1 | Japonica | NA | | Os07g0590100 | OsR498G0714674100.01.T01 | Zinc finger protein 1 |
| augustus-7-processed-gene-238.32-mRNA-1 | Japonica | Japonica | | Os07g0590600 | OsR498G0714675900.01.T01 | Pentatricopeptide repeat-containing protein (Fragment) |
| augustus-7-processed-gene-238.48-mRNA-1 | Japonica | NA | | | OsR498G0714676400.01.T01 | Transcriptional regulatory protein algP |
| augustus-7-processed-gene-239.59-mRNA-1 | Japonica | NA | | | OsR498G0714685600.01.T01 | RING-type E3 ubiquitin transferase |
| augustus-7-processed-gene-239.62-mRNA-1 | Japonica | NA | | | | tRNA/rRNA methyltransferase (SpoU) family protein |
| augustus-7-processed-gene-240.2-mRNA-1 | Japonica | NA | | | OsR498G0714690900.01.T01 | Son of sevenless |
| augustus-7-processed-gene-243.15-mRNA-1 | Japonica | NA | | Os07g0601600 | OsR498G0714710500.01.T01 | 3-oxo-Delta(45)-steroid 5-beta-reductase |
| augustus-7-processed-gene-243.4-mRNA-1 | Japonica | NA | | | OsR498G0100073600.01.T01 | RNA binding protein |
| augustus-7-processed-gene-245.12-mRNA-1 | Japonica | NA | | Os07g0604700 | OsR498G0714724300.01.T01 | NADH-ubiquinone reductase complex 1 MLRQ subunit |
| augustus-7-processed-gene-245.24-mRNA-1 | Japonica | NA | | Os07g0604500 | OsR498G0714723800.01.T02 | Mitochondrial inner membrane translocase complex, subunit Tim |
| augustus-7-processed-gene-245.3-mRNA-1 | Japonica | NA | | Os07g0603900 | OsR498G0714721300.01.T03 | Pentatricopeptide repeat |
| augustus-7-processed-gene-246.18-mRNA-1 | Japonica | NA | | | OsR498G0511427100.01.T01 | Epstein-Barr virus EBNA-1-like |
| augustus-7-processed-gene-246.22-mRNA-1 | Japonica | NA | | Os07g0605800 | OsR498G0714728400.01.T01 | Sulfotransferase |
| augustus-7-processed-gene-246.5-mRNA-1 | Japonica | NA | | Os07g0605200 | OsR498G0714725500.01.T04 | MADS-box transcription factor CDM41 |
| augustus-7-processed-gene-246.7-mRNA-1 | Japonica | Indica | | Os07g0605900 | OsR498G0714726500.01.T01 | Protein EGG APPARATUS-1 |
| augustus-7-processed-gene-247.10-mRNA-1 | Japonica | NA | | Os07g0606600 | OsR498G0714730000.01.T02 | Nuclear transcription factor Y subunit B |
| augustus-7-processed-gene-247.18-mRNA-1 | Japonica | NA | | | OsR498G0714733100.01.T01 | BAH domain-containing protein |
| augustus-7-processed-gene-247.2-mRNA-1 | Japonica | NA | | Os07g0606800 | OsR498G0714731100.01.T01 | Gibberellin receptor GID1L2 |
| augustus-7-processed-gene-247.23-mRNA-1 | Japonica | NA | | Os07g0608100 | OsR498G0714734900.01.T01 | Putative 6,7-dimethyl-8-ribityllumazine synthase-like |
| augustus-7-processed-gene-247.33-mRNA-1 | Japonica | NA | | Os07g0607200 | OsR498G0714733000.01.T01 | |
| augustus-7-processed-gene-247.6-mRNA-1 | Japonica | Indica | | Os10g0190100 | OsR498G0714729200.01.T01 | Sulfotransferase |
| augustus-7-processed-gene-248.17-mRNA-1 | Japonica | Japonica | | Os07g0609000 | OsR498G0714737500.01.T05 | Stress-response A/B barrel domain-containing protein UP3 |
| augustus-7-processed-gene-249.33-mRNA-1 | Japonica | NA | | Os07g0611500 | OsR498G0714740700.01.T01 | CHP-rich zinc finger protein-like |
| augustus-7-processed-gene-250.37-mRNA-1 | Japonica | Japonica | | Os07g0611700 | OsR498G0714741500.01.T02 | |
| augustus-7-processed-gene-252.0-mRNA-1 | Japonica | Indica | | Os07g0616500 | OsR498G0714756300.01.T02 | (S)-2-hydroxy-acid oxidase |
| augustus-7-processed-gene-252.16-mRNA-1 | Japonica | NA | | | OsR498G0510294600.01.T01 | Zinc knuckle containing protein-like |
| augustus-7-processed-gene-252.20-mRNA-1 | Japonica | NA | | | OsR498G0714763000.01.T01 | RING-H2 finger protein ATL3 |
| augustus-7-processed-gene-252.7-mRNA-1 | Japonica | NA | | Os07g0617100 | OsR498G0714759700.01.T01 | |
| augustus-7-processed-gene-253.19-mRNA-1 | Japonica | NA | | Os07g0619500 | OsR498G0714766900.01.T01 | Hormone receptor 4 like |
| augustus-7-processed-gene-254.35-mRNA-1 | Japonica | NA | | | OsR498G0714769900.01.T01 | Putative caltractin |
| augustus-7-processed-gene-254.36-mRNA-1 | Japonica | NA | | Os07g0620200 | OsR498G0714770300.01.T01 | AT hook-containing MAR binding 1-like protein (Fragment) |
| augustus-7-processed-gene-254.45-mRNA-1 | Japonica | NA | | Os07g0620600 | | AP-3 complex subunit mu like |
| augustus-7-processed-gene-254.46-mRNA-1 | Japonica | Japonica | | Os07g0621100 | OsR498G0714772900.01.T01 | Pentatricopeptide repeat-containing protein |
| augustus-7-processed-gene-254.61-mRNA-1 | Japonica | NA | | Os07g0621800 | OsR498G0714774200.01.T01 | Alcohol dehydrogenase superfamily, zinc-type |
| augustus-7-processed-gene-255.1-mRNA-1 | Japonica | NA | | Os07g0622100 | OsR498G0714775300.01.T01 | 40S ribosomal protein S6 |
| augustus-7-processed-gene-255.14-mRNA-1 | Japonica | NA | | Os07g0622700 | OsR498G0714778400.01.T01 | 2-hydroxy-6-oxononadienedioate/2-hydroxy-6-oxononatrienedioate hydrola |
| augustus-7-processed-gene-255.15-mRNA-1 | Japonica | NA | | Os07g0622900 | OsR498G0714778600.01.T01 | Peptidase |
| augustus-7-processed-gene-256.33-mRNA-1 | Japonica | Indica | | Os07g0625500 | OsR498G0714786700.01.T01 | AIF-MLS domain-containing protein |

| | | | | | | |
|---|---|---|---|---|---|---|
| augustus-7-processed-gene-256.48-mRNA-1 | Japonica | NA | | | OsR498G0714782600.01.T01 | VQ motif family protein, expressed |
| augustus-7-processed-gene-257.19-mRNA-1 | Japonica | NA | | | OsR498G0714792900.01.T01 | 4-alpha-glucanotransferase |
| augustus-7-processed-gene-257.36-mRNA-1 | Japonica | Indica | | Os07g0626800 | OsR498G0714792000.01.T01 | 15-cis-phytoene desaturase, chloroplastic/chromoplastic |
| augustus-7-processed-gene-259.23-mRNA-1 | Japonica | NA | | Os07g0631700 | OsR498G0714808300.01.T02 | Putative calcium-binding protein CML45 |
| augustus-7-processed-gene-259.31-mRNA-1 | Japonica | NA | | Os07g0630800 | OsR498G0714804800.01.T02 | Malate dehydrogenase |
| augustus-7-processed-gene-259.36-mRNA-1 | Japonica | NA | | Os02g0212975 | OsR498G0305421900.01.T01 | |
| augustus-7-processed-gene-260.3-mRNA-1 | Japonica | NA | | Os07g0632600 | OsR498G0714810500.01.T03 | J domain-containing protein |
| augustus-7-processed-gene-260.35-mRNA-1 | Japonica | NA | | Os07g0633600 | OsR498G0714814400.01.T01 | O-fucosyltransferase family protein |
| augustus-7-processed-gene-261.41-mRNA-1 | Japonica | NA | | Os07g0635300 | OsR498G0714820500.01.T01 | Secologanin synthase |
| augustus-7-processed-gene-261.57-mRNA-1 | Japonica | NA | | Os07g0636000 | OsR498G0714822200.01.T01 | PUA domain-containing protein (Fragment) |
| augustus-7-processed-gene-262.45-mRNA-1 | Japonica | NA | | Os07g0636400 | OsR498G0714824300.01.T01 | 50S ribosomal protein like |
| augustus-7-processed-gene-263.0-mRNA-1 | Japonica | Japonica | | Os07g0637700 | OsR498G0714825200.01.T01 | Zinc finger Ran-binding domain-containing protein 3 |
| augustus-7-processed-gene-263.10-mRNA-1 | Japonica | NA | | Os07g0638300 | OsR498G0714830900.01.T01 | Peroxiredoxin |
| augustus-7-processed-gene-263.33-mRNA-1 | Japonica | Japonica | | Os07g0638600 | OsR498G0714833300.01.T01 | Peroxidase |
| augustus-7-processed-gene-263.8-mRNA-1 | Japonica | NA | | Os07g0638100 | OsR498G0714830500.01.T01 | TolB protein-related |
| augustus-7-processed-gene-264.74-mRNA-1 | Japonica | NA | | Os07g0639400 | OsR498G0714834500.01.T01 | Peroxidase |
| augustus-7-processed-gene-264.97-mRNA-1 | Japonica | NA | | Os07g0641700 | OsR498G0714840300.01.T01 | Chaperonin, mitochondrial |
| augustus-7-processed-gene-265.14-mRNA-1 | Japonica | Japonica | | Os07g0643000 | OsR498G0714846000.01.T01 | probable carboxylesterase 18 |
| augustus-7-processed-gene-265.15-mRNA-1 | Japonica | Japonica | | Os07g0643800 | OsR498G0714825200.01.T01 | Zinc finger Ran-binding domain-containing protein 3 |
| augustus-7-processed-gene-265.23-mRNA-1 | Japonica | NA | | Os07g0643100 | OsR498G0714846000.01.T01 | Putative carboxylesterase 18 |
| augustus-7-processed-gene-265.27-mRNA-1 | Japonica | NA | | Os07g0643400 | OsR498G0714846800.01.T01 | Putative carboxylesterase 18 |
| augustus-7-processed-gene-265.6-mRNA-1 | Japonica | NA | | | | ATP-dependent DNA helicase |
| augustus-7-processed-gene-266.21-mRNA-1 | Japonica | NA | | Os01g0304500 | OsR498G0100876000.01.T01 | Transposon protein, putative, Mutator sub-class |
| augustus-7-processed-gene-267.21-mRNA-1 | Japonica | Indica | | Os07g0646300 | OsR498G0714855400.01.T01 | |
| augustus-7-processed-gene-267.5-mRNA-1 | Japonica | Indica | | Os07g0645300 | OsR498G0714853500.01.T01 | DUF679 domain membrane protein 2 |
| augustus-7-processed-gene-268.75-mRNA-1 | Japonica | NA | | Os07g0647400 | OsR498G0714863900.01.T01 | BSD domain-containing protein 1 |
| augustus-7-processed-gene-269.14-mRNA-1 | Japonica | Japonica | | Os07g0649300 | OsR498G0714870700.01.T01 | |
| augustus-7-processed-gene-269.29-mRNA-1 | Japonica | NA | | Os05g0412300 | | |
| augustus-7-processed-gene-274.58-mRNA-1 | Japonica | NA | | Os07g0656400 | OsR498G0714881300.01.T02 | Putative glycosyltransferase STELLO1 |
| augustus-7-processed-gene-274.70-mRNA-1 | Japonica | NA | | | OsR498G0714881200.01.T01 | Glycosyl hydrolase superfamily protein |
| augustus-7-processed-gene-275.51-mRNA-1 | Japonica | NA | | Os07g0657400 | OsR498G0714885700.01.T01 | EGF domain-specific O-linked N-acetylglucosamine transferase |
| augustus-7-processed-gene-275.60-mRNA-1 | Japonica | NA | | Os07g0658300 | OsR498G0714887700.01.T04 | Pleckstrin homology (PH) domain-containing protein-like |
| augustus-7-processed-gene-275.66-mRNA-1 | Japonica | NA | | | OsR498G0714887200.01.T03 | Signal recognition particle SEC65 subunit |
| augustus-7-processed-gene-275.71-mRNA-1 | Japonica | NA | | Os07g0658700 | OsR498G0714888800.01.T01 | Phosphatidylinositol 4-phosphate 5-kinase |
| augustus-7-processed-gene-277.8-mRNA-1 | Japonica | NA | | Os07g0661400 | OsR498G0714897400.01.T03 | Protein EARLY RESPONSIVE TO DEHYDRATION 15 |
| augustus-7-processed-gene-28.12-mRNA-1 | Japonica | NA | | Os07g0160600 | OsR498G0713564300.01.T01 | Fasciclin-like arabinogalactan protein 16 |
| augustus-7-processed-gene-280.75-mRNA-1 | Japonica | NA | | | OsR498G0714920800.01.T01 | F-box domain-containing protein |
| augustus-7-processed-gene-281.53-mRNA-1 | Japonica | NA | | | OsR498G0714929500.01.T02 | Cysteine-tryptophan domain-containing zinc finger protein 7 |
| augustus-7-processed-gene-281.55-mRNA-1 | Japonica | NA | | Os07g0670200 | OsR498G0714930300.01.T01 | C2 domain-containing protein (Fragment) |
| augustus-7-processed-gene-281.56-mRNA-1 | Japonica | NA | | Os07g0670300 | OsR498G0714930700.01.T01 | C2 domain-containing protein (Fragment) |
| augustus-7-processed-gene-281.71-mRNA-1 | Japonica | NA | | Os07g0670000 | | Pentatricopeptide repeat-containing protein mitochondrial |
| augustus-7-processed-gene-282.50-mRNA-1 | Japonica | NA | | Os07g0671000 | OsR498G0714931700.01.T03 | Flowering-promoting factor 1-like protein 5 |
| augustus-7-processed-gene-282.56-mRNA-1 | Japonica | Japonica | | Os07g0671200 | OsR498G0714932400.01.T01 | Pentatricopeptide repeat-containing protein |
| augustus-7-processed-gene-283.29-mRNA-1 | Japonica | NA | | Os07g0673700 | OsR498G0714939900.01.T02 | F-box/kelch-repeat protein SKIP6 |
| augustus-7-processed-gene-283.41-mRNA-1 | Japonica | Japonica | | Os07g0673801 | OsR498G0305924500.01.T01 | |
| augustus-7-processed-gene-283.58-mRNA-1 | Japonica | NA | | Os07g0674700 | OsR498G0714944100.01.T01 | Cytoplasmic ribosomal protein L18 |
| augustus-7-processed-gene-283.66-mRNA-1 | Japonica | NA | | Os07g0674300 | OsR498G0714942400.01.T01 | Pentatricopeptide repeat |
| augustus-7-processed-gene-284.1-mRNA-1 | Japonica | Japonica | | Os07g0675250 | OsR498G0714945700.01.T01 | IBB domain-containing protein |
| augustus-7-processed-gene-284.21-mRNA-1 | Japonica | NA | | Os07g0676700 | | Endonuclease/Exonuclease/phosphatase family protein |
| augustus-7-processed-gene-286.0-mRNA-1 | Japonica | NA | | Os07g0679200 | OsR498G0714958700.01.T01 | Transcription repressor |
| augustus-7-processed-gene-287.10-mRNA-1 | Japonica | NA | | Os07g0681400 | OsR498G0714966300.01.T03 | Probable calcium-binding protein CML32 |
| augustus-7-processed-gene-288.49-mRNA-1 | Japonica | NA | | | OsR498G0204046800.01.T01 | Circumsporozoite protein-like |
| augustus-7-processed-gene-288.63-mRNA-1 | Japonica | NA | | | OsR498G0511427100.01.T01 | Epstein-Barr virus EBNA-1-like |
| augustus-7-processed-gene-289.31-mRNA-1 | Japonica | NA | | Os07g0685900 | OsR498G0714985300.01.T01 | Subtilisin-like protease |
| augustus-7-processed-gene-289.41-mRNA-1 | Japonica | NA | | Os07g0685700 | OsR498G0714984400.01.T02 | Ethylene signal transcription factor |
| augustus-7-processed-gene-29.24-mRNA-1 | Japonica | Indica | | Os07g0161900 | OsR498G0713566700.01.T01 | VQ motif-containing protein 8, chloroplastic |
| augustus-7-processed-gene-29.25-mRNA-1 | Japonica | Japonica | | Os07g0161900 | OsR498G0713566700.01.T01 | VQ domain-containing protein |
| augustus-7-processed-gene-29.44-mRNA-1 | Japonica | NA | | Os07g0162600 | OsR498G0713570600.01.T01 | Putative carboxylesterase 15 |
| augustus-7-processed-gene-29.45-mRNA-1 | Japonica | NA | | Os07g0162700 | OsR498G0713571400.01.T01 | probable carboxylesterase 15 |
| augustus-7-processed-gene-290.2-mRNA-1 | Japonica | NA | | Os07g0686600 | OsR498G0714990300.01.T01 | VQ domain-containing protein |
| augustus-7-processed-gene-290.20-mRNA-1 | Japonica | NA | | Os07g0688000 | OsR498G0714994900.01.T04 | Manganese-dependent ADP-ribose/CDP-alcohol diphosphatase |

| | | | | | | |
|---|---|---|---|---|---|---|
| augustus-7-processed-gene-291.43-mRNA-1 | Japonica | Indica | | Os03g0311600 | OsR498G0714996600.01.T01 | Transcription factor bHLH149 like |
| augustus-7-processed-gene-292.16-mRNA-1 | Japonica | NA | | | | D-alanine--D-alanine ligase family |
| augustus-7-processed-gene-292.9-mRNA-1 | Japonica | NA | | Os07g0690900 | OsR498G0715005400.01.T01 | COBRA-like protein 7 |
| augustus-7-processed-gene-293.46-mRNA-1 | Japonica | NA | | Os07g0692401 | OsR498G0715011300.01.T01 | Putative methyltransferases-like |
| augustus-7-processed-gene-293.48-mRNA-1 | Japonica | NA | | Os07g0692900 | OsR498G0715011900.01.T01 | E1 ubiquitin-activating enzyme |
| augustus-7-processed-gene-294.43-mRNA-1 | Japonica | Indica | | Os07g0694500 | OsR498G0715018100.01.T02 | PWWP domain-containing protein (Fragment) |
| augustus-7-processed-gene-294.52-mRNA-1 | Japonica | NA | | Os07g0694600 | OsR498G0715018400.01.T01 | Arogenate dehydratase |
| augustus-7-processed-gene-294.64-mRNA-1 | Japonica | NA | Kinase | Os07g0695300 | OsR498G0715020300.01.T01 | Putative serine/threonine-protein kinase PBL7 |
| augustus-7-processed-gene-30.1-mRNA-1 | Japonica | NA | | | | Ubiquitinyl hydrolase 1 |
| augustus-7-processed-gene-30.9-mRNA-1 | Japonica | NA | | Os07g0164800 | OsR498G0713577500.01.T01 | PIN2/TERF1-interacting telomerase inhibitor 1 |
| augustus-7-processed-gene-31.25-mRNA-1 | Japonica | Indica | | Os12g0468900 | OsR498G0713579700.01.T01 | Cell wall protein-like |
| augustus-7-processed-gene-31.28-mRNA-1 | Japonica | NA | | Os07g0165100 | OsR498G0713578600.01.T01 | FT-interacting protein 1-like protein |
| augustus-7-processed-gene-36.18-mRNA-1 | Japonica | NA | | | OsR498G0713622400.01.T01 | |
| augustus-7-processed-gene-37.14-mRNA-1 | Japonica | Indica | | Os07g0180300 | OsR498G0713629900.01.T01 | DUF4220 domain-containing protein (Fragment) |
| augustus-7-processed-gene-37.4-mRNA-1 | Japonica | NA | | Os07g0179000 | OsR498G0713626300.01.T05 | Pentatricopeptide repeat-containing protein, putative |
| augustus-7-processed-gene-41.21-mRNA-1 | Japonica | NA | | Os07g0185300 | OsR498G0713645400.01.T01 | |
| augustus-7-processed-gene-41.40-mRNA-1 | Japonica | Japonica | Kinase-TM | Os07g0186200 | OsR498G0713651200.01.T01 | Non-specific serine/threonine protein kinase (Fragment) |
| augustus-7-processed-gene-42.1-mRNA-1 | Japonica | NA | LRR-NBS-TM | Os07g0186500 | OsR498G0713652100.01.T01 | Disease resistance protein RPM1 |
| augustus-7-processed-gene-43.0-mRNA-1 | Japonica | NA | | Os12g0484600 | OsR498G0307220700.01.T01 | Nodulin-like domain-containing protein (Fragment) |
| augustus-7-processed-gene-43.29-mRNA-1 | Japonica | Japonica | | Os07g0189400 | OsR498G0306754100.01.T02 | F-box domain-containing protein (Fragment) |
| augustus-7-processed-gene-44.14-mRNA-1 | Japonica | NA | | | OsR498G0713663800.01.T01 | |
| augustus-7-processed-gene-47.19-mRNA-1 | Japonica | NA | | Os07g0195300 | OsR498G0713687000.01.T01 | F-box domain-containing protein |
| augustus-7-processed-gene-48.12-mRNA-1 | Japonica | NA | | Os07g0196300 | OsR498G0713690500.01.T01 | Retrovirus-related Pol polyprotein LINE-1 |
| augustus-7-processed-gene-48.26-mRNA-1 | Japonica | Indica | | Os07g0242400 | OsR498G0713692900.01.T02 | F-box domain-containing protein (Fragment) |
| augustus-7-processed-gene-48.37-mRNA-1 | Japonica | Japonica | | Os07g0196700 | OsR498G0713691600.01.T01 | NAC domain-containing protein |
| augustus-7-processed-gene-5.27-mRNA-1 | Japonica | Indica | | Os07g0117000 | OsR498G0713412200.01.T02 | Fusarium resistance protein I2C-5-like |
| augustus-7-processed-gene-50.35-mRNA-1 | Japonica | NA | | Os07g0201100 | OsR498G0713703300.01.T01 | Coatomer subunit gamma |
| augustus-7-processed-gene-51.1-mRNA-1 | Japonica | Indica | | Os07g0201500 | OsR498G0713705500.01.T01 | Glycosyltransferase |
| augustus-7-processed-gene-51.3-mRNA-1 | Japonica | NA | | Os07g0202000 | OsR498G0713706700.01.T01 | Glycosyltransferase |
| augustus-7-processed-gene-52.62-mRNA-1 | Japonica | NA | | Os07g0204500 | OsR498G0713712600.01.T03 | 5'-nucleotidase |
| augustus-7-processed-gene-6.22-mRNA-1 | Japonica | NA | | | | Fusarium resistance protein I2C-5-like |
| augustus-7-processed-gene-6.32-mRNA-1 | Japonica | Japonica | | Os07g0118200 | OsR498G0713415100.01.T01 | NB-ARC domain-containing protein (Fragment) |
| augustus-7-processed-gene-6.33-mRNA-1 | Japonica | Japonica | | Os07g0118250 | OsR498G0713423300.01.T01 | NB-ARC domain-containing protein (Fragment) |
| augustus-7-processed-gene-60.2-mRNA-1 | Japonica | NA | | | OsR498G0511427100.01.T01 | Epstein-Barr virus EBNA-1-like |
| augustus-7-processed-gene-60.3-mRNA-1 | Japonica | NA | | | OsR498G0511427100.01.T01 | Epstein-Barr virus EBNA-1-like |
| augustus-7-processed-gene-61.0-mRNA-1 | Japonica | NA | | Os07g0217600 | OsR498G0713782100.01.T01 | Ent-cassadiene C2-hydroxylase |
| augustus-7-processed-gene-64.39-mRNA-1 | Japonica | NA | | Os07g0223700 | OsR498G0713794900.01.T02 | RING-type E3 ubiquitin transferase |
| augustus-7-processed-gene-64.42-mRNA-1 | Japonica | NA | | Os07g0224000 | OsR498G0713795700.01.T01 | TRASH domain-containing protein |
| augustus-7-processed-gene-64.51-mRNA-1 | Japonica | NA | | Os07g0223832 | | RING-type domain-containing protein |
| augustus-7-processed-gene-65.9-mRNA-1 | Japonica | Indica | | Os07g0224900 | OsR498G0713804500.01.T01 | Small nucleolar ribonucleoprotein complex subunit |
| augustus-7-processed-gene-66.11-mRNA-1 | Japonica | NA | | Os10g0531750 | OsR498G0408675500.01.T01 | Retrotransposon protein, putative, Ty3-gypsy sub-class |
| augustus-7-processed-gene-67.20-mRNA-1 | Japonica | NA | | Os07g0227200 | OsR498G0713819100.01.T01 | |
| augustus-7-processed-gene-67.31-mRNA-1 | Japonica | NA | | Os07g0227600 | | Ethylene-responsive transcription factor CRF3 |
| augustus-7-processed-gene-67.34-mRNA-1 | Japonica | NA | | | | FBD domain-containing protein |
| augustus-7-processed-gene-68.11-mRNA-1 | Japonica | NA | | Os07g0230600 | | Fe2OG dioxygenase domain-containing protein |
| augustus-7-processed-gene-68.18-mRNA-1 | Japonica | NA | | Os07g0229900 | OsR498G0713825000.01.T01 | 40S ribosomal protein S12 |
| augustus-7-processed-gene-68.3-mRNA-1 | Japonica | Indica | | Os07g0229200 | OsR498G0713822600.01.T01 | FBD domain-containing protein |
| augustus-7-processed-gene-69.27-mRNA-1 | Japonica | NA | | Os07g0231500 | OsR498G0713829700.01.T01 | |
| augustus-7-processed-gene-7.55-mRNA-1 | Japonica | Japonica | | Os07g0121000 | OsR498G0713426300.01.T04 | |
| augustus-7-processed-gene-7.56-mRNA-1 | Japonica | NA | | Os07g0120900 | OsR498G0713426300.01.T04 | WW domain-containing protein (Fragment) |
| augustus-7-processed-gene-7.57-mRNA-1 | Japonica | NA | | Os07g0121100 | OsR498G0713426900.01.T02 | |
| augustus-7-processed-gene-7.68-mRNA-1 | Japonica | Japonica | | Os07g0120000 | OsR498G0713423300.01.T01 | NB-ARC domain-containing protein (Fragment) |
| augustus-7-processed-gene-7.71-mRNA-1 | Japonica | NA | | Os07g0120500 | OsR498G0713415100.01.T01 | NB-ARC domain-containing protein (Fragment) |
| augustus-7-processed-gene-7.73-mRNA-1 | Japonica | NA | | Os07g0120500 | | F-box domain-containing protein |
| augustus-7-processed-gene-7.75-mRNA-1 | Japonica | Japonica | | Os07g0120600 | OsR498G0713423300.01.T01 | NB-ARC domain-containing protein (Fragment) |
| augustus-7-processed-gene-7.76-mRNA-1 | Japonica | NA | | | | Enoyl-CoA hydratase 2, peroxisomal |
| augustus-7-processed-gene-70.11-mRNA-1 | Japonica | NA | | Os09g0258800 | | Ulp1 protease family, C-terminal catalytic domain containing protein |
| augustus-7-processed-gene-72.2-mRNA-1 | Japonica | NA | | Os04g0617200 | OsR498G0409285000.01.T01 | |
| augustus-7-processed-gene-72.24-mRNA-1 | Japonica | NA | | Os07g0236700 | OsR498G0713848400.01.T02 | Zinc finger, Dof-type |
| augustus-7-processed-gene-72.28-mRNA-1 | Japonica | Japonica | | Os07g0237900 | OsR498G0613330300.01.T01 | |
| augustus-7-processed-gene-73.34-mRNA-1 | Japonica | NA | | Os07g0239600 | OsR498G0713856800.01.T03 | Pentatricopeptide repeat-containing protein mitochondrial |

| | | | | | | |
|---|---|---|---|---|---|---|
| augustus-7-processed-gene-75.12-mRNA-1 | Japonica | NA | | Os07g0242000 | | |
| augustus-7-processed-gene-75.37-mRNA-1 | Japonica | NA | | Os07g0243000 | OsR498G0713868400.01.T01 | UDP-glycosyltransferase 76E4 |
| augustus-7-processed-gene-76.2-mRNA-1 | Japonica | NA | | Os05g0225100 | OsR498G0510061500.01.T01 | DUF295 domain-containing protein |
| augustus-7-processed-gene-76.42-mRNA-1 | Japonica | NA | | Os07g0244400 | OsR498G0713872200.01.T01 | Pentatricopeptide repeat-containing protein, putative |
| augustus-7-processed-gene-77.14-mRNA-1 | Japonica | NA | | Os07g0246200 | OsR498G0713877300.01.T02 | Calreticulin |
| augustus-7-processed-gene-77.23-mRNA-1 | Japonica | NA | | Os07g0246300 | OsR498G0713877600.01.T02 | Protein transport protein SEC13 B like |
| augustus-7-processed-gene-78.1-mRNA-1 | Japonica | NA | | | OsR498G0713878900.01.T01 | Serine/threonine kinase (Fragment) |
| augustus-7-processed-gene-78.2-mRNA-1 | Japonica | NA | | Os07g0247000 | OsR498G0713879300.01.T01 | PMEI domain-containing protein (Fragment) |
| augustus-7-processed-gene-79.29-mRNA-1 | Japonica | Japonica | | Os07g0250133 | OsR498G0714323900.01.T01 | Glycosyltransferase |
| augustus-7-processed-gene-79.3-mRNA-1 | Japonica | NA | | Os07g0248900 | | Putative mutator-like transposase |
| augustus-7-processed-gene-8.0-mRNA-1 | Japonica | Japonica | | Os07g0121600 | OsR498G0511374000.01.T01 | |
| augustus-7-processed-gene-8.4-mRNA-1 | Japonica | NA | | | | |
| augustus-7-processed-gene-8.5-mRNA-1 | Japonica | NA | | Os07g0122200 | OsR498G0713429500.01.T03 | |
| augustus-7-processed-gene-8.6-mRNA-1 | Japonica | NA | | Os07g0122300 | OsR498G0713429700.01.T01 | |
| augustus-7-processed-gene-80.14-mRNA-1 | Japonica | Japonica | | Os07g0251200 | OsR498G0713892000.01.T01 | |
| augustus-7-processed-gene-80.15-mRNA-1 | Japonica | NA | | Os07g0251301 | | 60S acidic ribosomal protein P2-B |
| augustus-7-processed-gene-81.29-mRNA-1 | Japonica | NA | | | OsR498G0510294600.01.T01 | HGWP repeat containing protein-like |
| augustus-7-processed-gene-83.15-mRNA-1 | Japonica | NA | | Os07g0255200 | OsR498G0713577000.01.T01 | ATP-dependent DNA helicase |
| augustus-7-processed-gene-85.12-mRNA-1 | Japonica | NA | | Os07g0257200 | OsR498G0713916200.01.T02 | Metal transporter |
| augustus-7-processed-gene-86.10-mRNA-1 | Japonica | NA | | Os07g0259400 | | Pentatricopeptide repeat-containing protein mitochondrial |
| augustus-7-processed-gene-88.17-mRNA-1 | Japonica | NA | | | OsR498G0713939900.01.T01 | L-type lectin-domain containing receptor kinase IV.1 |
| augustus-7-processed-gene-9.29-mRNA-1 | Japonica | NA | | Os07g0124000 | | |
| augustus-7-processed-gene-9.32-mRNA-1 | Japonica | NA | | Os07g0123800 | OsR498G0713435600.01.T01 | |
| augustus-7-processed-gene-9.33-mRNA-1 | Japonica | NA | | Os07g0123900 | OsR498G0713436000.01.T01 | Eggshell protein 2A-like |
| augustus-7-processed-gene-9.41-mRNA-1 | Japonica | NA | | | OsR498G0713437300.01.T01 | BZIP transcription factor family protein expressed |
| augustus-7-processed-gene-90.2-mRNA-1 | Japonica | NA | | Os09g0265800 | OsR498G0407753100.01.T01 | HGWP repeat containing protein-like |
| augustus-7-processed-gene-93.17-mRNA-1 | Japonica | Japonica | | Os07g0268800 | OsR498G0713959500.01.T02 | DUF4220 domain-containing protein (Fragment) |
| augustus-7-processed-gene-94.12-mRNA-1 | Japonica | Indica | | Os07g0268800 | OsR498G0713961500.01.T01 | DUF4220 domain-containing protein (Fragment) |
| augustus-7-processed-gene-95.20-mRNA-1 | Japonica | NA | | | OsR498G0713965300.01.T01 | |
| augustus-7-processed-gene-96.0-mRNA-1 | Japonica | NA | | | OsR498G0713967500.01.T01 | DUF4220 domain-containing protein (Fragment) |
| augustus-7-processed-gene-96.6-mRNA-1 | Japonica | NA | | | OsR498G0100073600.01.T01 | RNA binding protein |
| augustus-7-processed-gene-97.10-mRNA-1 | Japonica | NA | | Os07g0272400 | OsR498G0713971700.01.T01 | FYVE-type domain-containing protein |
| augustus-7-processed-gene-97.23-mRNA-1 | Japonica | NA | | Os07g0272700 | OsR498G0713975100.01.T01 | NAC transcription factor 69 |
| augustus-7-processed-gene-99.16-mRNA-1 | Japonica | NA | | | OsR498G0713983500.01.T01 | |
| augustus-8-processed-gene-1.19-mRNA-1 | Japonica | NA | | Os08g0101700 | OsR498G0815026400.01.T01 | Cytochrome b561 domain-containing protein (Fragment) |
| augustus-8-processed-gene-1.20-mRNA-1 | Japonica | NA | | Os08g0102000 | | Putative N-acetyltransferase ycf52 |
| augustus-8-processed-gene-11.47-mRNA-1 | Japonica | NA | | Os08g0119800 | OsR498G0815087200.01.T06 | Photosystem II core complex proteins psbY |
| augustus-8-processed-gene-122.25-mRNA-1 | Japonica | NA | | | | |
| augustus-8-processed-gene-132.21-mRNA-1 | Japonica | NA | | Os03g0114600 | OsR498G0612855600.01.T01 | Calcineurin-like phosphoesterase family protein, putative |
| augustus-8-processed-gene-14.24-mRNA-1 | Japonica | Japonica | Kinase-TM | Os08g0124100 | OsR498G0815098200.01.T02 | Putative receptor-type protein kinase LRK1 |
| augustus-8-processed-gene-140.6-mRNA-1 | Japonica | NA | | Os03g0110200 | | Transposon protein, putative, Mutator sub-class, expressed |
| augustus-8-processed-gene-153.14-mRNA-1 | Japonica | NA | Kinase-TM | | OsR498G0815808800.01.T01 | Putative glycerophosphodiester phosphodiesterase, protein kinase RLK-Pelle |
| augustus-8-processed-gene-155.17-mRNA-1 | Japonica | NA | | | OsR498G0815821700.01.T01 | |
| augustus-8-processed-gene-158.35-mRNA-1 | Japonica | NA | Kinase-LRR-TM | Os08g0342300 | OsR498G0815837200.01.T01 | Brassinosteroid LRR receptor kinase BRL3 |
| augustus-8-processed-gene-16.47-mRNA-1 | Japonica | Japonica | | Os08g0126800 | OsR498G0815103200.01.T01 | DUF295 domain-containing protein (Fragment) |
| augustus-8-processed-gene-161.15-mRNA-1 | Japonica | NA | | | OsR498G0815762600.01.T01 | |
| augustus-8-processed-gene-162.16-mRNA-1 | Japonica | Indica | | Os10g0461100 | OsR498G0815858000.01.T02 | PsbP family protein, expressed |
| augustus-8-processed-gene-17.50-mRNA-1 | Japonica | NA | | Os08g0127800 | OsR498G0815105800.01.T01 | ATP-dependent zinc metalloprotease FTSH protein |
| augustus-8-processed-gene-174.21-mRNA-1 | Japonica | NA | | Os08g0356700 | OsR498G0815875600.01.T01 | |
| augustus-8-processed-gene-176.30-mRNA-1 | Japonica | NA | | | OsR498G0815882100.01.T01 | |
| augustus-8-processed-gene-191.39-mRNA-1 | Japonica | NA | | | OsR498G0815957800.01.T01 | |
| augustus-8-processed-gene-197.37-mRNA-1 | Japonica | Indica | | Os08g0387100 | OsR498G0815983300.01.T01 | |
| augustus-8-processed-gene-206.35-mRNA-1 | Japonica | NA | | | OsR498G0816048600.01.T01 | Phorbol-ester/DAG-type domain-containing protein (Fragment) |
| augustus-8-processed-gene-207.40-mRNA-1 | Japonica | NA | | Os08g0406500 | OsR498G0816055400.01.T01 | BTB/POZ and MATH domain-containing protein 2 |
| augustus-8-processed-gene-208.19-mRNA-1 | Japonica | NA | | Os08g0406600 | OsR498G0816056200.01.T01 | Zinc finger POZ domain protein-like |
| augustus-8-processed-gene-208.37-mRNA-1 | Japonica | NA | | | OsR498G0204046800.01.T01 | Circumsporozoite protein-like |
| augustus-8-processed-gene-208.38-mRNA-1 | Japonica | NA | | | OsR498G0816060800.01.T01 | |
| augustus-8-processed-gene-208.39-mRNA-1 | Japonica | Japonica | | Os08g0408300 | OsR498G0816061400.01.T01 | |
| augustus-8-processed-gene-21.7-mRNA-1 | Japonica | NA | | | | Retrotransposon protein, putative, unclassified, expressed |
| augustus-8-processed-gene-210.11-mRNA-1 | Japonica | NA | | | OsR498G0816071400.01.T02 | Polynucleotide adenylyltransferase |
| augustus-8-processed-gene-210.7-mRNA-1 | Japonica | Japonica | | Os08g0410266 | OsR498G0816070200.01.T01 | DUF295 domain-containing protein (Fragment) |

| | | | | | | |
|---|---|---|---|---|---|---|
| augustus-8-processed-gene-212.31-mRNA-1 | Japonica | NA | | Os08g0416000 | OsR498G0816085600.01.T01 | Class I homeodomain-leucine zipper protein 5 |
| augustus-8-processed-gene-213.7-mRNA-1 | Japonica | NA | | Os08g0417100 | OsR498G0816090200.01.T01 | Putative 2-oxoglutarate-dependent dioxygenase |
| augustus-8-processed-gene-228.28-mRNA-1 | Japonica | Indica | | Os08g0441900 | OsR498G0816183200.01.T02 | Matrin-type domain-containing protein |
| augustus-8-processed-gene-229.25-mRNA-1 | Japonica | NA | | Os08g0442900 | OsR498G0816186400.01.T01 | glycine-rich cell wall structural protein-like |
| augustus-8-processed-gene-23.24-mRNA-1 | Japonica | NA | | | OsR498G0815141200.01.T01 | RING-type E3 ubiquitin transferase |
| augustus-8-processed-gene-230.5-mRNA-1 | Japonica | NA | | Os08g0444500 | OsR498G0816191600.01.T01 | |
| augustus-8-processed-gene-233.23-mRNA-1 | Japonica | Japonica | | Os08g0451700 | OsR498G0816219600.01.T02 | AT-rich interactive domain-containing protein 4 |
| augustus-8-processed-gene-236.27-mRNA-1 | Japonica | NA | | Os08g0455800 | OsR498G0816234300.01.T01 | Liver carboxylesterase (Fragment) |
| augustus-8-processed-gene-236.30-mRNA-1 | Japonica | NA | | | OsR498G0816232200.01.T01 | T-complex protein 1 subunit delta |
| augustus-8-processed-gene-236.33-mRNA-1 | Japonica | NA | | Os08g0456200 | OsR498G0816235600.01.T01 | Flavonoid 3'-monooxygenase |
| augustus-8-processed-gene-238.17-mRNA-1 | Japonica | NA | | Os08g0459300 | OsR498G0816246800.01.T01 | F-box domain-containing protein |
| augustus-8-processed-gene-24.24-mRNA-1 | Japonica | NA | | Os08g0140500 | OsR498G0815146000.01.T01 | Aromatic-L-amino-acid decarboxylase-like |
| augustus-8-processed-gene-24.34-mRNA-1 | Japonica | Japonica | | Os08g0141000 | OsR498G0815146600.01.T01 | |
| augustus-8-processed-gene-24.40-mRNA-1 | Japonica | Japonica | | Os08g0140700 | OsR498G0815146800.01.T01 | RING-type domain-containing protein |
| augustus-8-processed-gene-24.44-mRNA-1 | Japonica | NA | | Os08g0141700 | OsR498G0815149600.01.T01 | |
| augustus-8-processed-gene-240.18-mRNA-1 | Japonica | NA | | Os08g0462800 | OsR498G0816257400.01.T01 | Plant viral-response family protein-like |
| augustus-8-processed-gene-243.45-mRNA-1 | Japonica | NA | | Os08g0468700 | OsR498G0816276200.01.T01 | Nitrate reductase |
| augustus-8-processed-gene-244.22-mRNA-1 | Japonica | Japonica | | Os08g0471100 | OsR498G0102262600.01.T01 | Transposable element protein, putative, MuDR, expressed |
| augustus-8-processed-gene-244.24-mRNA-1 | Japonica | NA | | | OsR498G0816287000.01.T01 | Basic helix-loop-helix transcription factor |
| augustus-8-processed-gene-245.21-mRNA-1 | Japonica | NA | | Os08g0472400 | OsR498G0816290400.01.T01 | Putative zeaxanthin epoxidase |
| augustus-8-processed-gene-246.49-mRNA-1 | Japonica | Japonica | | Os08g0474500 | OsR498G0816298400.01.T01 | BRI1 kinase inhibitor 1 |
| augustus-8-processed-gene-247.10-mRNA-1 | Japonica | NA | | Os08g0475000 | OsR498G0816299200.01.T01 | Tuliposide A-converting enzyme 1, chloroplastic |
| augustus-8-processed-gene-248.5-mRNA-1 | Japonica | NA | | | OsR498G0204046800.01.T01 | Epstein-Barr virus EBNA-1-like |
| augustus-8-processed-gene-249.4-mRNA-1 | Japonica | NA | | Os08g0478466 | OsR498G0816310200.01.T01 | PPC domain-containing protein (Fragment) |
| augustus-8-processed-gene-25.12-mRNA-1 | Japonica | Japonica | | Os08g0142900 | OsR498G0815150800.01.T01 | PMEI domain-containing protein |
| augustus-8-processed-gene-254.17-mRNA-1 | Japonica | NA | | Os08g0487100 | | BZIP domain-containing protein (Fragment) |
| augustus-8-processed-gene-262.30-mRNA-1 | Japonica | NA | | | | Putative transparent testa |
| augustus-8-processed-gene-263.22-mRNA-1 | Japonica | NA | LRR-TM | Os08g0505900 | OsR498G0816425400.01.T01 | DNA-damage-repair/toleration protein DRT100 |
| augustus-8-processed-gene-263.23-mRNA-1 | Japonica | NA | | | OsR498G0408884400.01.T03 | Epstein-Barr virus EBNA-1-like |
| augustus-8-processed-gene-264.18-mRNA-1 | Japonica | NA | | Os08g0507301 | | |
| augustus-8-processed-gene-264.24-mRNA-1 | Japonica | NA | | Os08g0507100 | OsR498G0816430400.01.T01 | |
| augustus-8-processed-gene-264.29-mRNA-1 | Japonica | NA | | | | Kinesin motor domain-containing protein |
| augustus-8-processed-gene-266.22-mRNA-1 | Japonica | NA | | Os08g0510800 | OsR498G0816446400.01.T01 | Cyclic nucleotide-gated ion channel 2 |
| augustus-8-processed-gene-267.70-mRNA-1 | Japonica | NA | | Os08g0513300 | | Ribosomal protein S24/S35 mitochondrial |
| augustus-8-processed-gene-269.10-mRNA-1 | Japonica | NA | | Os08g0515800 | | Mitochondrial transcription termination factor |
| augustus-8-processed-gene-269.28-mRNA-1 | Japonica | NA | | Os08g0516600 | OsR498G0816464400.01.T01 | Cyclin-dependent protein kinase inhibitor SMR3 |
| augustus-8-processed-gene-270.36-mRNA-1 | Japonica | NA | | Os08g0517600 | OsR498G0816467200.01.T04 | S-adenosyl-L-methionine-dependent methyltransferases superfamily protei |
| augustus-8-processed-gene-270.56-mRNA-1 | Japonica | Japonica | | Os08g0518200 | OsR498G0816469400.01.T01 | Mitochondrial transcription termination factor |
| augustus-8-processed-gene-270.61-mRNA-1 | Japonica | Indica | | Os08g0519100 | OsR498G0816473200.01.T01 | L-gulonolactone oxidase |
| augustus-8-processed-gene-272.41-mRNA-1 | Japonica | NA | | Os08g0520600 | OsR498G0816481000.01.T01 | |
| augustus-8-processed-gene-273.21-mRNA-1 | Japonica | NA | | | OsR498G0816486800.01.T01 | TD and POZ domain-containing protein 3 (Fragment) |
| augustus-8-processed-gene-274.53-mRNA-1 | Japonica | Japonica | | Os08g0526100 | OsR498G0917763200.01.T01 | UDP-D-glucuronate 4-epimerase 6 |
| augustus-8-processed-gene-283.64-mRNA-1 | Japonica | NA | | | OsR498G0816542800.01.T01 | |
| augustus-8-processed-gene-285.16-mRNA-1 | Japonica | NA | | Os04g0184800 | | Transposon protein, putative, Mutator sub-class, expressed |
| augustus-8-processed-gene-285.39-mRNA-1 | Japonica | NA | | Os02g0778800 | | |
| augustus-8-processed-gene-286.21-mRNA-1 | Japonica | NA | CC-NBS-TM | Os08g0543100 | | Putative NBS-LRR resistance protein RGH1 |
| augustus-8-processed-gene-286.42-mRNA-1 | Japonica | NA | | Os09g0519100 | OsR498G0917808800.01.T01 | BHLH transcription factor |
| augustus-8-processed-gene-287.15-mRNA-1 | Japonica | NA | | Os08g0544800 | OsR498G0816564400.01.T02 | Transcription factor PCF2 |
| augustus-8-processed-gene-287.16-mRNA-1 | Japonica | NA | | Os08g0545000 | | Pyridoxal phosphate (PLP)-dependent transferase superfamily protein |
| augustus-8-processed-gene-287.17-mRNA-1 | Japonica | NA | | Os08g0545400 | OsR498G0816566200.01.T01 | Dehydration-responsive element-binding protein 1J |
| augustus-8-processed-gene-287.18-mRNA-1 | Japonica | Japonica | | Os08g0545500 | OsR498G0917817800.01.T01 | Dehydration-responsive element-binding protein 1A |
| augustus-8-processed-gene-287.21-mRNA-1 | Japonica | NA | | Os08g0545900 | OsR498G0816568200.01.T01 | Protein DETOXIFICATION |
| augustus-8-processed-gene-288.48-mRNA-1 | Japonica | NA | | Os08g0547200 | OsR498G0816572100.01.T01 | small G protein signaling modulator 1 isoform X1 |
| augustus-8-processed-gene-289.1-mRNA-1 | Japonica | NA | | Os08g0547900 | OsR498G0816576400.01.T01 | Geraniol 8-hydroxylase |
| augustus-8-processed-gene-290.76-mRNA-1 | Japonica | NA | | Os08g0550700 | OsR498G0816586000.01.T02 | Auxin-responsive protein SAUR36-like |
| augustus-8-processed-gene-292.28-mRNA-1 | Japonica | NA | | | | Skp1 domain-containing protein |
| augustus-8-processed-gene-293.57-mRNA-1 | Japonica | NA | | Os08g0557200 | OsR498G0816600400.01.T01 | Manganese-dependent ADP-ribose/CDP-alcohol diphosphatase-like |
| augustus-8-processed-gene-295.24-mRNA-1 | Japonica | NA | | Os08g0562600 | OsR498G0816619400.01.T06 | C2 domain-containing protein (Fragment) |
| augustus-8-processed-gene-296.13-mRNA-1 | Japonica | Japonica | | Os08g0563200 | OsR498G0816620000.01.T01 | AT-hook motif nuclear-localized protein 20 |
| augustus-8-processed-gene-296.16-mRNA-1 | Japonica | NA | | | OsR498G0816621000.01.T01 | RING-H2 finger protein ATL5K |
| augustus-8-processed-gene-296.37-mRNA-1 | Japonica | NA | | Os08g0564500 | OsR498G0816624200.01.T01 | zinc finger protein 593 homolog |

| | | | | | | |
|---|---|---|---|---|---|---|
| augustus-8-processed-gene-296.38-mRNA-1 | Japonica | NA | Kinase-LRR-TM | Os08g0564700 | OsR498G0816627000.01.T01 | Serine/threonine protein kinase |
| augustus-8-processed-gene-297.53-mRNA-1 | Japonica | NA | | Os08g0565200 | OsR498G0816629600.01.T01 | Dehydration-responsive element-binding protein 2C |
| augustus-8-processed-gene-297.80-mRNA-1 | Japonica | Japonica | | Os08g0565700 | OsR498G0816630800.01.T05 | Histone-lysine N-methyltransferase family member SUVH2 |
| augustus-8-processed-gene-3.20-mRNA-1 | Japonica | NA | | Os08g0105400 | OsR498G0815038200.01.T01 | Indole-2-monooxygenase |
| augustus-8-processed-gene-3.21-mRNA-1 | Japonica | Japonica | | Os08g0105600 | OsR498G0815038400.01.T01 | 3-hydroxyindolin-2-one monooxygenase |
| augustus-8-processed-gene-3.23-mRNA-1 | Japonica | Indica | | Os08g0105400 | OsR498G0815038600.01.T01 | Indole-2-monooxygenase |
| augustus-8-processed-gene-3.3-mRNA-1 | Japonica | NA | | Os08g0104400 | OsR498G0815034200.01.T01 | Seed maturation protein |
| augustus-8-processed-gene-37.9-mRNA-1 | Japonica | NA | | Os08g0159900 | OsR498G0815240800.01.T04 | DEAD-box ATP-dependent RNA helicase 42 |
| augustus-8-processed-gene-39.47-mRNA-1 | Japonica | NA | | Os08g0162400 | OsR498G0815252800.01.T01 | RING finger protein 5 |
| augustus-8-processed-gene-4.12-mRNA-1 | Japonica | NA | | Os08g0106100 | | |
| augustus-8-processed-gene-4.32-mRNA-1 | Japonica | NA | | Os08g0107100 | OsR498G0815041800.01.T04 | Polyadenylate-binding protein 1-B-binding protein |
| augustus-8-processed-gene-4.36-mRNA-1 | Japonica | NA | | Os08g0108100 | OsR498G0815044600.01.T01 | Pectinesterase inhibitor 28 |
| augustus-8-processed-gene-4.37-mRNA-1 | Japonica | NA | | Os08g0108300 | OsR498G0815045600.01.T01 | Nuclear receptor subfamily 4 group A member 3 |
| augustus-8-processed-gene-40.39-mRNA-1 | Japonica | NA | | Os08g0165300 | | |
| augustus-8-processed-gene-45.24-mRNA-1 | Japonica | Japonica | | Os08g0174500 | OsR498G0815298200.01.T02 | Truncated days to heading 8 |
| augustus-8-processed-gene-45.6-mRNA-1 | Japonica | NA | | Os08g0173700 | OsR498G0815297000.01.T01 | Ethylene-responsive transcription factor ERF088 |
| augustus-8-processed-gene-47.15-mRNA-1 | Japonica | NA | | Os08g0178100 | OsR498G0815307700.01.T01 | vacuolar protein sorting-associated protein 18 homolog |
| augustus-8-processed-gene-47.22-mRNA-1 | Japonica | NA | | | | Similarity to RNase H |
| augustus-8-processed-gene-5.39-mRNA-1 | Japonica | NA | | Os08g0108500 | OsR498G0815046800.01.T01 | Basic helix-loop-helix transcription factor |
| augustus-8-processed-gene-59.34-mRNA-1 | Japonica | NA | | Os08g0201100 | OsR498G0815382600.01.T01 | |
| augustus-8-processed-gene-6.21-mRNA-1 | Japonica | NA | | Os08g0111300 | OsR498G0815058200.01.T01 | Putative acetyltransferase |
| augustus-8-processed-gene-6.6-mRNA-1 | Japonica | NA | | Os08g0110300 | OsR498G0815055200.01.T01 | Longin domain-containing protein |
| augustus-8-processed-gene-64.20-mRNA-1 | Japonica | NA | | | OsR498G0815402700.01.T01 | GTP-binding protein ERG |
| augustus-8-processed-gene-64.27-mRNA-1 | Japonica | NA | | Os08g0207800 | | Peptidase A1 domain-containing protein |
| augustus-8-processed-gene-64.34-mRNA-1 | Japonica | Japonica | | Os08g0208300 | OsR498G1018122200.01.T01 | |
| augustus-8-processed-gene-65.8-mRNA-1 | Japonica | NA | | | OsR498G1120154400.01.T01 | Endonuclease |
| augustus-8-processed-gene-66.14-mRNA-1 | Japonica | NA | | | | Cell wall protein-like |
| augustus-8-processed-gene-7.22-mRNA-1 | Japonica | NA | | Os08g0112200 | OsR498G0815060200.01.T01 | Putative acetyltransferase |
| augustus-8-processed-gene-7.23-mRNA-1 | Japonica | NA | | Os08g0112300 | OsR498G0815060400.01.T01 | Putative acetyltransferase |
| augustus-8-processed-gene-7.24-mRNA-1 | Japonica | NA | | Os08g0112400 | OsR498G0815060600.01.T01 | Pentatricopeptide repeat-containing protein |
| augustus-8-processed-gene-7.5-mRNA-1 | Japonica | NA | | Os08g0111800 | OsR498G0815058600.01.T01 | Putative acetyltransferase |
| augustus-8-processed-gene-75.17-mRNA-1 | Japonica | NA | | Os03g0110200 | | Transposon protein, putative, Mutator sub-class, expressed |
| augustus-8-processed-gene-75.19-mRNA-1 | Japonica | NA | | Os08g0220400 | OsR498G0815445900.01.T01 | Pectinesterase |
| augustus-8-processed-gene-78.29-mRNA-1 | Japonica | NA | | | OsR498G0815456200.01.T01 | |
| augustus-8-processed-gene-79.25-mRNA-1 | Japonica | Japonica | | Os08g0225100 | OsR498G0815469000.01.T03 | Reticulon domain-containing protein (Fragment) |
| augustus-8-processed-gene-8.33-mRNA-1 | Japonica | Japonica | | Os08g0113500 | OsR498G0815064300.01.T01 | NAC domain containing protein 35 |
| augustus-8-processed-gene-8.35-mRNA-1 | Japonica | NA | | Os08g0113400 | | Putative methyltransferase PMT21 |
| augustus-8-processed-gene-80.21-mRNA-1 | Japonica | NA | | Os04g0286500 | OsR498G0916692600.01.T01 | Transposon protein, putative, Pong sub-class, expressed |
| augustus-8-processed-gene-80.22-mRNA-1 | Japonica | Japonica | | Os08g0226800 | OsR498G0815473500.01.T01 | TD and POZ domain-containing protein 4 |
| augustus-8-processed-gene-80.23-mRNA-1 | Japonica | Indica | | Os08g0226800 | OsR498G0815471800.01.T01 | TD and POZ domain-containing protein 4 |
| augustus-8-processed-gene-81.7-mRNA-1 | Japonica | NA | | Os08g0227100 | OsR498G0815475400.01.T02 | BTB domain-containing protein (Fragment) |
| augustus-8-processed-gene-81.8-mRNA-1 | Japonica | NA | | Os08g0227200 | OsR498G0815475600.01.T01 | Speckle-type POZ protein |
| augustus-8-processed-gene-82.8-mRNA-1 | Japonica | NA | | | OsR498G0815482600.01.T01 | |
| augustus-8-processed-gene-83.24-mRNA-1 | Japonica | NA | | Os08g0230500 | OsR498G0815486600.01.T02 | Endonuclease or glycosyl hydrolase |
| augustus-8-processed-gene-86.13-mRNA-1 | Japonica | NA | | Os08g0235800 | OsR498G0815502400.01.T01 | WRKY transcription factor |
| augustus-8-processed-gene-86.28-mRNA-1 | Japonica | Japonica | CC-Kinase-TM | Os08g0230800 | OsR498G0815488100.01.T01 | Non-specific serine/threonine protein kinase |
| augustus-8-processed-gene-89.42-mRNA-1 | Japonica | NA | | | OsR498G0306506500.01.T01 | |
| augustus-8-processed-gene-90.27-mRNA-1 | Japonica | Indica | | Os02g0797300 | OsR498G0815517200.01.T01 | Plasma membrane ATPase |
| augustus-8-processed-gene-91.51-mRNA-1 | Japonica | NA | | Os08g0243900 | OsR498G0815525200.01.T04 | MHD domain-containing protein (Fragment) |
| augustus-8-processed-gene-93.30-mRNA-1 | Japonica | NA | | Os08g0246300 | OsR498G0815530100.01.T01 | Disease resistance protein RPM1 |
| augustus-8-processed-gene-99.15-mRNA-1 | Japonica | NA | | Os10g0460000 | OsR498G0305532000.01.T01 | Kinesin heavy chain-like |
| augustus-8-processed-gene-99.30-mRNA-1 | Japonica | NA | NBS-TM | Os08g0258700 | OsR498G0815569000.01.T01 | Nucleotide-binding leucine-rich-repeat protein 1 |
| augustus-9-processed-gene-104.52-mRNA-1 | Japonica | Indica | | Os09g0353400 | OsR498G0917228700.01.T01 | Oxoglutarate-dependent dioxygenase 2 |
| augustus-9-processed-gene-105.19-mRNA-1 | Japonica | NA | | | | NAM-associated domain-containing protein (Fragment) |
| augustus-9-processed-gene-108.28-mRNA-1 | Japonica | NA | | Os09g0347300 | OsR498G0917209900.01.T01 | Exocyst subunit Exo70 family protein (Fragment) |
| augustus-9-processed-gene-112.18-mRNA-1 | Japonica | NA | | Os09g0338400 | OsR498G0917192300.01.T01 | Cysteine desulfurase |
| augustus-9-processed-gene-114.38-mRNA-1 | Japonica | NA | | | | Transposon protein, putative, Mutator sub-class, expressed |
| augustus-9-processed-gene-119.47-mRNA-1 | Japonica | NA | | Os09g0361400 | OsR498G0917246900.01.T01 | Mitochondrial outer membrane porin |
| augustus-9-processed-gene-12.26-mRNA-1 | Japonica | NA | | | OsR498G0916689600.01.T01 | WIYLD domain-containing protein |
| augustus-9-processed-gene-121.26-mRNA-1 | Japonica | Japonica | LRR-NBS-TM | Os09g0365000 | OsR498G0917259000.01.T01 | PPC domain-containing protein |
| augustus-9-processed-gene-125.49-mRNA-1 | Japonica | NA | | Os07g0693100 | OsR498G1018918700.01.T01 | Pyruvate decarboxylase |

| | | | | | | |
|---|---|---|---|---|---|---|
| augustus-9-processed-gene-125.71-mRNA-1 | Japonica | NA | | Os09g0372900 | OsR498G0917292200.01.T02 | Protein kinase domain-containing protein |
| augustus-9-processed-gene-127.29-mRNA-1 | Japonica | Indica | | Os09g0376400 | OsR498G0917299200.01.T01 | BHLH domain-containing protein |
| augustus-9-processed-gene-127.4-mRNA-1 | Japonica | NA | | | OsR498G0917294800.01.T01 | Pentatricopeptide repeat-containing protein mitochondrial |
| augustus-9-processed-gene-128.30-mRNA-1 | Japonica | NA | | Os02g0651200 | | |
| augustus-9-processed-gene-129.12-mRNA-1 | Japonica | Japonica | | Os09g0379300 | OsR498G0917311200.01.T01 | Glycosyltransferase |
| augustus-9-processed-gene-129.34-mRNA-1 | Japonica | NA | | Os09g0379900 | OsR498G0917313200.01.T01 | Endo-1,3(4)-beta-glucanase |
| augustus-9-processed-gene-131.20-mRNA-1 | Japonica | NA | | | | BED-type domain-containing protein |
| augustus-9-processed-gene-133.28-mRNA-1 | Japonica | Indica | | Os09g0387150 | OsR498G0917343200.01.T03 | |
| augustus-9-processed-gene-133.3-mRNA-1 | Japonica | NA | | Os09g0386200 | OsR498G0917340000.01.T01 | RING-type E3 ubiquitin transferase |
| augustus-9-processed-gene-133.6-mRNA-1 | Japonica | NA | | | OsR498G0917342000.01.T01 | AUGMIN subunit like |
| augustus-9-processed-gene-138.2-mRNA-1 | Japonica | NA | | Os09g0394300 | OsR498G0917376000.01.T02 | Endoglucanase |
| augustus-9-processed-gene-147.41-mRNA-1 | Japonica | NA | | Os09g0411600 | OsR498G0917435800.01.T03 | Tetratricopeptide repeat (TPR)-like superfamily protein, putative isoform 1 |
| augustus-9-processed-gene-147.68-mRNA-1 | Japonica | NA | | | OsR498G0917439000.01.T01 | S-adenosylmethionine decarboxylase proenzyme |
| augustus-9-processed-gene-147.71-mRNA-1 | Japonica | Japonica | | Os09g0413300 | OsR498G0917441800.01.T03 | Pentatricopeptide repeat-containing protein chloroplastic |
| augustus-9-processed-gene-149.17-mRNA-1 | Japonica | NA | | Os09g0415300 | OsR498G0917452000.01.T01 | |
| augustus-9-processed-gene-149.4-mRNA-1 | Japonica | NA | | | OsR498G0917449000.01.T03 | Zinc-finger homeodomain protein 9 |
| augustus-9-processed-gene-154.23-mRNA-1 | Japonica | NA | | Os09g0424300 | OsR498G0917484800.01.T01 | S-adenosylmethionine decarboxylase proenzyme |
| augustus-9-processed-gene-155.13-mRNA-1 | Japonica | NA | | Os09g0425200 | OsR498G0917486200.01.T01 | Putative cell-wall protein (Fragment) |
| augustus-9-processed-gene-156.32-mRNA-1 | Japonica | NA | | | OsR498G0408432600.01.T01 | |
| augustus-9-processed-gene-158.20-mRNA-1 | Japonica | NA | | | | CBS domain-containing protein |
| augustus-9-processed-gene-159.1-mRNA-1 | Japonica | Indica | | Os09g0431900 | OsR498G0917513200.01.T01 | Zinc finger protein KNUCKLES |
| augustus-9-processed-gene-159.21-mRNA-1 | Japonica | NA | | Os09g0433000 | OsR498G0917516800.01.T01 | Hexosyltransferase |
| augustus-9-processed-gene-160.31-mRNA-1 | Japonica | NA | | Os09g0433700 | OsR498G0917518800.01.T01 | Pectinesterase |
| augustus-9-processed-gene-160.32-mRNA-1 | Japonica | NA | | Os09g0433700 | OsR498G0917518800.01.T01 | Pectinesterase |
| augustus-9-processed-gene-160.54-mRNA-1 | Japonica | NA | | Os09g0434900 | OsR498G0917523000.01.T01 | IBR domain-containing protein |
| augustus-9-processed-gene-162.28-mRNA-1 | Japonica | NA | | Os09g0438100 | OsR498G0917532800.01.T01 | |
| augustus-9-processed-gene-165.3-mRNA-1 | Japonica | Indica | | Os08g0499400 | OsR498G0917547600.01.T01 | |
| augustus-9-processed-gene-166.41-mRNA-1 | Japonica | Indica | | Os05g0567400 | OsR498G0917558800.01.T01 | Putative rRNA methyltransferase |
| augustus-9-processed-gene-168.25-mRNA-1 | Japonica | NA | | Os09g0449000 | OsR498G0917576300.01.T01 | PHD finger protein MALE STERILITY like |
| augustus-9-processed-gene-168.30-mRNA-1 | Japonica | Japonica | | Os09g0448100 | OsR498G0917580500.01.T06 | F-box protein interaction domain containing protein, expressed |
| augustus-9-processed-gene-168.33-mRNA-1 | Japonica | NA | | Os09g0449100 | | Transposon protein, putative, CACTA, En/Spm sub-class |
| augustus-9-processed-gene-168.7-mRNA-1 | Japonica | Indica | | Os01g0587300 | OsR498G0917572600.01.T01 | Phosphoribosylanthranilate transferase-like protein |
| augustus-9-processed-gene-17.17-mRNA-1 | Japonica | NA | | | | |
| augustus-9-processed-gene-172.34-mRNA-1 | Japonica | NA | | Os09g0455500 | OsR498G0917604800.01.T01 | Tuliposide A-converting enzyme 2, chloroplastic |
| augustus-9-processed-gene-172.41-mRNA-1 | Japonica | NA | | Os09g0455900 | OsR498G0917605400.01.T01 | Tuliposide A-converting enzyme b1, amyloplastic |
| augustus-9-processed-gene-173.1-mRNA-1 | Japonica | NA | | Os09g0456800 | OsR498G0917608700.01.T01 | Heat shock transcription factor |
| augustus-9-processed-gene-173.17-mRNA-1 | Japonica | NA | | Os07g0418100 | | ATP-dependent DNA helicase |
| augustus-9-processed-gene-173.22-mRNA-1 | Japonica | NA | | Os09g0336500 | OsR498G1220975100.01.T01 | Zinc knuckle family protein |
| augustus-9-processed-gene-173.3-mRNA-1 | Japonica | NA | | Os09g0456700 | OsR498G0917608000.01.T01 | |
| augustus-9-processed-gene-174.37-mRNA-1 | Japonica | NA | | Os09g0459600 | OsR498G0917618800.01.T01 | PPM-type phosphatase domain-containing protein |
| augustus-9-processed-gene-175.34-mRNA-1 | Japonica | NA | | Os09g0462200 | OsR498G0917625800.01.T01 | Tuliposide A-converting enzyme 2, chloroplastic |
| augustus-9-processed-gene-176.33-mRNA-1 | Japonica | NA | | Os09g0464100 | | |
| augustus-9-processed-gene-176.35-mRNA-1 | Japonica | Indica | | Os08g0478466 | OsR498G0917637200.01.T01 | PPC domain-containing protein (Fragment) |
| augustus-9-processed-gene-178.20-mRNA-1 | Japonica | Japonica | | Os09g0467500 | OsR498G0917649000.01.T01 | Purine transporter |
| augustus-9-processed-gene-178.23-mRNA-1 | Japonica | NA | | Os09g0468150 | OsR498G0917650800.01.T01 | Clathrin assembly protein |
| augustus-9-processed-gene-178.25-mRNA-1 | Japonica | NA | | Os09g0468300 | OsR498G0917651200.01.T01 | E3 ubiquitin-protein like |
| augustus-9-processed-gene-178.6-mRNA-1 | Japonica | NA | | Os09g0466400 | OsR498G0917645800.01.T01 | Zinc-finger homeodomain protein 1 |
| augustus-9-processed-gene-179.27-mRNA-1 | Japonica | NA | | | OsR498G0917654200.01.T04 | RING-H2 finger protein ATL52 |
| augustus-9-processed-gene-182.29-mRNA-1 | Japonica | NA | | Os09g0474000 | | BZIP transcription factor |
| augustus-9-processed-gene-182.42-mRNA-1 | Japonica | NA | | | OsR498G0917671300.01.T01 | Pentatricopeptide repeat-containing protein, chloroplastic |
| augustus-9-processed-gene-182.56-mRNA-1 | Japonica | NA | | Os09g0474800 | OsR498G0917674800.01.T01 | Phosphatidylinositol 4-kinase type 2-alpha |
| augustus-9-processed-gene-187.13-mRNA-1 | Japonica | Indica | | Os09g0482780 | OsR498G0917706600.01.T02 | Fasciclin-like arabinogalactan protein 7 |
| augustus-9-processed-gene-187.38-mRNA-1 | Japonica | NA | | Os09g0482860 | OsR498G0917707800.01.T01 | Glycosyltransferase |
| augustus-9-processed-gene-187.40-mRNA-1 | Japonica | NA | | Os09g0482900 | OsR498G0917708600.01.T01 | Glycosyltransferase |
| augustus-9-processed-gene-187.42-mRNA-1 | Japonica | NA | | Os09g0483600 | OsR498G0917709600.01.T03 | JmjC domain-containing protein 7 |
| augustus-9-processed-gene-187.45-mRNA-1 | Japonica | NA | | Os08g0165300 | | |
| augustus-9-processed-gene-189.20-mRNA-1 | Japonica | NA | | Os09g0490200 | OsR498G0917725400.01.T02 | Ethylene signal transcription factor |
| augustus-9-processed-gene-190.20-mRNA-1 | Japonica | NA | | Os09g0491676 | OsR498G0917731000.01.T01 | Cyclic nucleotide-gated ion channel 2 |
| augustus-9-processed-gene-192.7-mRNA-1 | Japonica | Indica | | Os04g0577000 | OsR498G0917738000.01.T04 | Ubiquitin fusion degradation protein 1 |
| augustus-9-processed-gene-195.32-mRNA-1 | Japonica | NA | | | OsR498G0917759200.01.T04 | |
| augustus-9-processed-gene-195.38-mRNA-1 | Japonica | NA | | | | FBD domain-containing protein |

| | | | | | | |
|---|---|---|---|---|---|---|
| augustus-9-processed-gene-196.50-mRNA-1 | Japonica | NA | | Os09g0504000 | OsR498G0917763200.01.T01 | UDP-D-glucuronate 4-epimerase 6 |
| augustus-9-processed-gene-197.66-mRNA-1 | Japonica | NA | | | OsR498G0917769800.01.T01 | |
| augustus-9-processed-gene-198.7-mRNA-1 | Japonica | NA | | Os09g0507900 | | Calmodulin binding protein |
| augustus-9-processed-gene-201.109-mRNA-1 | Japonica | NA | | Os09g0514200 | OsR498G0917793800.01.T02 | Calcium-dependent protein kinase |
| augustus-9-processed-gene-203.53-mRNA-1 | Japonica | NA | | | OsR498G0917809200.01.T01 | |
| augustus-9-processed-gene-205.6-mRNA-1 | Japonica | NA | | | OsR498G0917816200.01.T01 | SPX domain-containing membrane protein isoform A |
| augustus-9-processed-gene-205.9-mRNA-1 | Japonica | NA | | Os09g0522100 | OsR498G0917817400.01.T01 | C-repeat binding factor 3 |
| augustus-9-processed-gene-212.14-mRNA-1 | Japonica | NA | | Os09g0535500 | OsR498G0917860600.01.T01 | RING-H2 finger protein ATL2A |
| augustus-9-processed-gene-214.39-mRNA-1 | Japonica | NA | | Os09g0540500 | OsR498G0917882200.01.T01 | Mediator of RNA polymerase II transcription subunit 4 |
| augustus-9-processed-gene-214.52-mRNA-1 | Japonica | Japonica | | Os09g0540800 | OsR498G0917882700.01.T01 | Abscisic acid-insensitive 5-like protein 2-like |
| augustus-9-processed-gene-214.59-mRNA-1 | Japonica | Japonica | | Os09g0541100 | OsR498G0917884600.01.T01 | Phytocyanin domain-containing protein |
| augustus-9-processed-gene-215.37-mRNA-1 | Japonica | NA | | Os09g0543400 | OsR498G0917892000.01.T01 | Diaminopimelate decarboxylase 2 chloroplastic |
| augustus-9-processed-gene-218.78-mRNA-1 | Japonica | NA | | Os09g0549500 | OsR498G0917917200.01.T02 | U11/U12 small nuclear ribonucleoprotein 31 kDa protein (Fragment) |
| augustus-9-processed-gene-218.79-mRNA-1 | Japonica | Japonica | | Os09g0549700 | OsR498G0917917600.01.T02 | Ribosomal protein L18/L5 |
| augustus-9-processed-gene-22.29-mRNA-1 | Japonica | NA | | | OsR498G0408153000.01.T01 | Splicing coactivator subunit-like |
| augustus-9-processed-gene-220.73-mRNA-1 | Japonica | NA | | Os09g0553100 | OsR498G0917927000.01.T01 | Multifunctional fusion protein |
| augustus-9-processed-gene-221.46-mRNA-1 | Japonica | NA | | Os09g0554200 | OsR498G0917930200.01.T01 | probable E3 ubiquitin-protein ligase ATL44 |
| augustus-9-processed-gene-222.40-mRNA-1 | Japonica | Indica | | Os02g0184100 | OsR498G0917939700.01.T01 | Zinc finger protein hangover |
| augustus-9-processed-gene-223.32-mRNA-1 | Japonica | NA | | Os09g0559000 | OsR498G0917945400.01.T01 | Putative E3 ubiquitin-protein ligase ARI5 |
| augustus-9-processed-gene-223.33-mRNA-1 | Japonica | NA | | Os09g0559100 | OsR498G0917945800.01.T01 | Putative E3 ubiquitin-protein ligase ARI5 |
| augustus-9-processed-gene-223.35-mRNA-1 | Japonica | Indica | | Os09g0559400 | OsR498G0917946400.01.T01 | DUF4220 domain-containing protein (Fragment) |
| augustus-9-processed-gene-223.8-mRNA-1 | Japonica | NA | | | OsR498G0917941800.01.T01 | DOMON domain-containing protein |
| augustus-9-processed-gene-224.58-mRNA-1 | Japonica | NA | | Os09g0560300 | OsR498G0917948400.01.T01 | Malic enzyme |
| augustus-9-processed-gene-225.20-mRNA-1 | Japonica | NA | | Os09g0563950 | | DUF295 domain-containing protein |
| augustus-9-processed-gene-225.22-mRNA-1 | Japonica | NA | | | OsR498G1120619900.01.T01 | Splicing factor 3B subunit 4 |
| augustus-9-processed-gene-226.9-mRNA-1 | Japonica | Indica | | Os09g0564000 | OsR498G0917959000.01.T01 | Cathepsin K |
| augustus-9-processed-gene-227.41-mRNA-1 | Japonica | NA | | Os09g0566400 | OsR498G0917963800.01.T01 | Protein kinase domain-containing protein |
| augustus-9-processed-gene-227.42-mRNA-1 | Japonica | NA | | Os09g0566400 | OsR498G0917963800.01.T01 | Protein kinase domain-containing protein |
| augustus-9-processed-gene-227.49-mRNA-1 | Japonica | Indica | | Os08g0557300 | OsR498G0917966400.01.T01 | |
| augustus-9-processed-gene-228.93-mRNA-1 | Japonica | Indica | | Os09g0568700 | OsR498G0917970600.01.T01 | Germin-like protein 9-3 |
| augustus-9-processed-gene-228.94-mRNA-1 | Japonica | NA | | Os09g0568700 | OsR498G0917971000.01.T01 | Germin-like protein 9-3 |
| augustus-9-processed-gene-229.83-mRNA-1 | Japonica | Japonica | | Os09g0571200 | OsR498G0816619400.01.T06 | C2 calcium-dependent membrane targeting |
| augustus-9-processed-gene-229.86-mRNA-1 | Japonica | NA | | | OsR498G0917979800.01.T01 | Ethylene-responsive transcription factor |
| augustus-9-processed-gene-23.22-mRNA-1 | Japonica | NA | | Os09g0127700 | OsR498G0916739000.01.T01 | |
| augustus-9-processed-gene-230.3-mRNA-1 | Japonica | NA | Kinase-TM | Os09g0572100 | | lysM domain receptor-like kinase 3 |
| augustus-9-processed-gene-25.26-mRNA-1 | Japonica | NA | | | OsR498G0916743600.01.T02 | |
| augustus-9-processed-gene-30.29-mRNA-1 | Japonica | NA | | Os09g0135800 | OsR498G0916770000.01.T01 | protein CELLULOSE SYNTHASE INTERACTIVE 1-like |
| augustus-9-processed-gene-30.32-mRNA-1 | Japonica | NA | | Os05g0267732 | OsR498G0510213400.01.T01 | |
| augustus-9-processed-gene-4.39-mRNA-1 | Japonica | Japonica | | Os09g0103400 | OsR498G0916647000.01.T01 | |
| augustus-9-processed-gene-41.25-mRNA-1 | Japonica | NA | | | | Auxin transport protein BIG |
| augustus-9-processed-gene-41.42-mRNA-1 | Japonica | NA | | | OsR498G0916819000.01.T01 | Annexin Max4 |
| augustus-9-processed-gene-42.34-mRNA-1 | Japonica | NA | | | | |
| augustus-9-processed-gene-44.19-mRNA-1 | Japonica | Japonica | Kinase-TM | Os09g0251250 | OsR498G0916836300.01.T01 | Serine/threonine protein kinase |
| augustus-9-processed-gene-44.26-mRNA-1 | Japonica | NA | | Os09g0251500 | OsR498G0100917100.01.T04 | Pentatricopeptide repeat-containing protein, chloroplastic |
| augustus-9-processed-gene-57.20-mRNA-1 | Japonica | Japonica | | Os09g0270900 | OsR498G0916883200.01.T02 | Cysteine desulfuration |
| augustus-9-processed-gene-57.22-mRNA-1 | Japonica | Indica | | Os09g0271100 | OsR498G0916884000.01.T01 | HMA domain-containing protein |
| augustus-9-processed-gene-58.13-mRNA-1 | Japonica | NA | | | | Transposon protein, putative, Mutator sub-class |
| augustus-9-processed-gene-59.32-mRNA-1 | Japonica | NA | | Os09g0273800 | OsR498G0916892000.01.T01 | SCF ubiquitin ligase complex protein SKP1a |
| augustus-9-processed-gene-59.9-mRNA-1 | Japonica | Japonica | | Os09g0272900 | OsR498G0916894000.01.T01 | Skp1 domain-containing protein (Fragment) |
| augustus-9-processed-gene-60.6-mRNA-1 | Japonica | NA | | Os09g0273800 | OsR498G0916892000.01.T01 | Skp1 domain-containing protein |
| augustus-9-processed-gene-62.10-mRNA-1 | Japonica | NA | | Os08g0561800 | OsR498G0408675500.01.T01 | 4-diphosphocytidyl-2C-methyl-D-erythritol synthase |
| augustus-9-processed-gene-65.2-mRNA-1 | Japonica | NA | | Os09g0282300 | | Zinc finger protein WIP2 |
| augustus-9-processed-gene-65.24-mRNA-1 | Japonica | NA | | | OsR498G0916922600.01.T01 | Putative disease resistance protein RGA3 |
| augustus-9-processed-gene-65.6-mRNA-1 | Japonica | NA | | | OsR498G0916921200.01.T01 | |
| augustus-9-processed-gene-7.4-mRNA-1 | Japonica | NA | | Os09g0106700 | OsR498G0916657400.01.T01 | Tuber-specific and sucrose-responsive element binding factor |
| augustus-9-processed-gene-74.22-mRNA-1 | Japonica | NA | Kinase-LRR-TM | Os09g0293500 | OsR498G0917002400.01.T01 | Brassinosteroid LRR receptor kinase BRL1 |
| augustus-9-processed-gene-76.0-mRNA-1 | Japonica | NA | | Os09g0295000 | OsR498G0917006600.01.T01 | NAC domain-containing protein (Fragment) |
| augustus-9-processed-gene-76.3-mRNA-1 | Japonica | Japonica | | Os09g0295700 | OsR498G0917007400.01.T01 | Ankyrin repeat and SOCS box protein 3 |
| augustus-9-processed-gene-84.43-mRNA-1 | Japonica | NA | | | OsR498G0917046500.01.T02 | Mediator of RNA polymerase II transcription subunit 25 |
| augustus-9-processed-gene-9.33-mRNA-1 | Japonica | NA | | | OsR498G0916670900.01.T04 | CitMHS domain-containing protein (Fragment) |
| augustus-9-processed-gene-94.8-mRNA-1 | Japonica | NA | | | | |

| | | | | | | |
|---|---|---|---|---|---|---|
| augustus-9-processed-gene-96.31-mRNA-1 | Japonica | NA | | Os09g0325000 | | F-box domain-containing protein |
| augustus-9-processed-gene-97.7-mRNA-1 | Japonica | NA | | | | |
| augustus-9-processed-gene-99.31-mRNA-1 | Japonica | NA | | Os09g0329200 | OsR498G0917166200.01.T01 | Glycosyltransferase |
| augustus_masked-1-processed-gene-10.16-mRNA-1 | Japonica | Indica | | Os01g0119500 | OsR498G0100063000.01.T01 | |
| augustus_masked-1-processed-gene-101.40-mRNA-1 | Japonica | NA | | | OsR498G0100676800.01.T01 | Temperature sensing protein-related |
| augustus_masked-1-processed-gene-103.29-mRNA-1 | Japonica | NA | | Os07g0543700 | OsR498G1120733700.01.T01 | Retrotransposon protein, putative, Ty3-gypsy sub-class |
| augustus_masked-1-processed-gene-108.23-mRNA-1 | Japonica | NA | | Os01g0293000 | OsR498G0100722000.01.T02 | S-adenosylmethionine synthase |
| augustus_masked-1-processed-gene-109.50-mRNA-1 | Japonica | NA | | | | N(6)-L-threonylcarbamoyladenine synthase |
| augustus_masked-1-processed-gene-110.37-mRNA-1 | Japonica | Indica | | Os05g0539300 | OsR498G0100732200.01.T01 | DUF295 domain-containing protein |
| augustus_masked-1-processed-gene-111.61-mRNA-1 | Japonica | Japonica | | Os01g0299700 | OsR498G0100738800.01.T01 | 3'-5' exonuclease domain-containing protein (Fragment) |
| augustus_masked-1-processed-gene-111.62-mRNA-1 | Japonica | NA | | | OsR498G0100738600.01.T01 | Alpha/beta hydrolase related protein |
| augustus_masked-1-processed-gene-112.19-mRNA-1 | Japonica | NA | | Os01g0301500 | OsR498G0100745200.01.T01 | Pentatricopeptide repeat-containing protein mitochondrial |
| augustus_masked-1-processed-gene-118.52-mRNA-1 | Japonica | NA | | Os01g0311600 | OsR498G0100779400.01.T01 | Sulfotransferase |
| augustus_masked-1-processed-gene-123.20-mRNA-1 | Japonica | NA | | | | |
| augustus_masked-1-processed-gene-124.41-mRNA-1 | Japonica | NA | | | OsR498G0100817800.01.T01 | Delta(1)-pyrroline-2-carboxylate reductase |
| augustus_masked-1-processed-gene-125.33-mRNA-1 | Japonica | Indica | | Os01g0293000 | OsR498G0100820800.01.T01 | S-adenosylmethionine synthase |
| augustus_masked-1-processed-gene-125.39-mRNA-1 | Japonica | Indica | | Os08g0545400 | OsR498G0100826200.01.T01 | Dehydration-responsive element-binding protein 1J |
| augustus_masked-1-processed-gene-128.24-mRNA-1 | Japonica | NA | | | | Endonuclease |
| augustus_masked-1-processed-gene-13.36-mRNA-1 | Japonica | NA | | Os01g0125500 | OsR498G0100084600.01.T04 | RING-type E3 ubiquitin transferase |
| augustus_masked-1-processed-gene-130.14-mRNA-1 | Japonica | NA | | | | Gibberellin 2-beta-dioxygenase 2 |
| augustus_masked-1-processed-gene-135.45-mRNA-1 | Japonica | Japonica | | Os01g0340600 | OsR498G0100874800.01.T01 | |
| augustus_masked-1-processed-gene-135.47-mRNA-1 | Japonica | NA | | Os01g0341000 | | |
| augustus_masked-1-processed-gene-136.12-mRNA-1 | Japonica | Japonica | | Os01g0341200 | OsR498G0100874800.01.T01 | |
| augustus_masked-1-processed-gene-136.22-mRNA-1 | Japonica | Indica | | Os06g0489700 | OsR498G0100876900.01.T01 | DUF295 domain-containing protein |
| augustus_masked-1-processed-gene-136.28-mRNA-1 | Japonica | NA | | Os01g0342500 | | |
| augustus_masked-1-processed-gene-137.5-mRNA-1 | Japonica | Japonica | | Os01g0343100 | OsR498G0100882700.01.T01 | DUF4220 domain-containing protein (Fragment) |
| augustus_masked-1-processed-gene-138.26-mRNA-1 | Japonica | NA | | | OsR498G0100887200.01.T01 | |
| augustus_masked-1-processed-gene-139.38-mRNA-1 | Japonica | NA | | Os01g0346700 | OsR498G0100895200.01.T01 | Putative glycine-rich cell wall structural protein 1.0 |
| augustus_masked-1-processed-gene-139.41-mRNA-1 | Japonica | Japonica | | Os01g0347200 | OsR498G0100897800.01.T01 | |
| augustus_masked-1-processed-gene-146.27-mRNA-1 | Japonica | Japonica | | Os01g0357900 | OsR498G0204493300.01.T02 | Rapid ALkalinization Factor |
| augustus_masked-1-processed-gene-147.20-mRNA-1 | Japonica | NA | NBS-TM | Os01g0359400 | OsR498G0100940200.01.T05 | Truncated powdery mildew resistance protein Pm3 |
| augustus_masked-1-processed-gene-147.22-mRNA-1 | Japonica | Japonica | LRR-NBS-TM | Os01g0359800 | OsR498G0100941400.01.T02 | Powdery mildew resistance protein PM3 variant |
| augustus_masked-1-processed-gene-149.26-mRNA-1 | Japonica | NA | | Os01g0362100 | OsR498G0100949800.01.T01 | Acyltransferase-like protein chloroplastic |
| augustus_masked-1-processed-gene-150.19-mRNA-1 | Japonica | NA | | Os01g0364400 | OsR498G0100955800.01.T01 | Wall-associated receptor kinase 3 |
| augustus_masked-1-processed-gene-151.19-mRNA-1 | Japonica | NA | | | OsR498G0100957400.01.T01 | Wall-associated receptor kinase 3 |
| augustus_masked-1-processed-gene-155.37-mRNA-1 | Japonica | Indica | | Os01g0373400 | OsR498G0714828400.01.T01 | NAM-associated domain-containing protein (Fragment) |
| augustus_masked-1-processed-gene-156.37-mRNA-1 | Japonica | NA | | Os01g0375100 | OsR498G0100985200.01.T03 | J domain-containing protein (Fragment) |
| augustus_masked-1-processed-gene-158.23-mRNA-1 | Japonica | NA | | Os07g0691100 | OsR498G0715007300.01.T01 | Pectinesterase |
| augustus_masked-1-processed-gene-161.25-mRNA-1 | Japonica | NA | | Os01g0382400 | OsR498G0100996100.01.T01 | Pathogenesis-related protein 1-2 |
| augustus_masked-1-processed-gene-178.20-mRNA-1 | Japonica | NA | | Os01g0498300 | OsR498G0101052000.01.T01 | Protein O-linked-mannose beta-1,4-N-acetylglucosaminyltransferase 2 (Frag |
| augustus_masked-1-processed-gene-181.37-mRNA-1 | Japonica | NA | | Os05g0574300 | OsR498G0305687300.01.T01 | Histone domain-containing protein |
| augustus_masked-1-processed-gene-182.10-mRNA-1 | Japonica | NA | | Os01g0504100 | OsR498G0101070200.01.T01 | Purine permease 4 |
| augustus_masked-1-processed-gene-182.25-mRNA-1 | Japonica | NA | | | OsR498G0101073300.01.T01 | RING-type domain-containing protein |
| augustus_masked-1-processed-gene-183.15-mRNA-1 | Japonica | NA | | Os01g0505500 | OsR498G0101075200.01.T01 | Pentatricopeptide repeat-containing protein, mitochondrial |
| augustus_masked-1-processed-gene-186.29-mRNA-1 | Japonica | NA | | Os01g0510200 | OsR498G0101096000.01.T01 | |
| augustus_masked-1-processed-gene-187.21-mRNA-1 | Japonica | NA | | Os01g0511200 | OsR498G0101102600.01.T01 | Copper ion binding |
| augustus_masked-1-processed-gene-187.3-mRNA-1 | Japonica | Indica | | Os01g0511000 | OsR498G0101099600.01.T01 | LOB transcription factor |
| augustus_masked-1-processed-gene-192.4-mRNA-1 | Japonica | NA | | Os12g0468900 | OsR498G0407549800.01.T01 | Cell wall protein-like |
| augustus_masked-1-processed-gene-198.24-mRNA-1 | Japonica | Indica | | Os01g0530500 | OsR498G0101164000.01.T01 | DUF295 domain-containing protein |
| augustus_masked-1-processed-gene-199.34-mRNA-1 | Japonica | NA | | Os01g0532300 | OsR498G0101169200.01.T01 | |
| augustus_masked-1-processed-gene-2.38-mRNA-1 | Japonica | NA | | Os01g0102000 | | Non-hemolytic phospholipase C |
| augustus_masked-1-processed-gene-200.17-mRNA-1 | Japonica | Indica | | Os02g0822300 | OsR498G0101174600.01.T01 | 1,3-beta-glucan synthase |
| augustus_masked-1-processed-gene-220.39-mRNA-1 | Japonica | NA | | Os02g0102850 | OsR498G0307365000.01.T04 | |
| augustus_masked-1-processed-gene-220.40-mRNA-1 | Japonica | NA | | Os01g0561600 | OsR498G0101341800.01.T01 | Oryzalexin D synthase |
| augustus_masked-1-processed-gene-222.25-mRNA-1 | Japonica | NA | | Os01g0564600 | | Cell wall protein-like |
| augustus_masked-1-processed-gene-224.11-mRNA-1 | Japonica | NA | | Os01g0566800 | OsR498G0101357600.01.T01 | Transcription factor bHLH87 |
| augustus_masked-1-processed-gene-225.24-mRNA-1 | Japonica | NA | | Os01g0568400 | | Cysteine-rich receptor-like protein kinase 15 |
| augustus_masked-1-processed-gene-226.38-mRNA-1 | Japonica | NA | | | OsR498G0101368000.01.T01 | |
| augustus_masked-1-processed-gene-228.7-mRNA-1 | Japonica | NA | | Os11g0708500 | OsR498G1018251600.01.T01 | Pyridoxal biosynthesis lyase pdxS |
| augustus_masked-1-processed-gene-229.0-mRNA-1 | Japonica | Japonica | | Os01g0575500 | OsR498G0101383200.01.T01 | DENN domain and WD repeat-containing protein SCD1 |
| augustus_masked-1-processed-gene-229.21-mRNA-1 | Japonica | NA | | | OsR498G0101387800.01.T01 | |

| | | | | | | |
|---|---|---|---|---|---|---|
| augustus_masked-1-processed-gene-231.44-mRNA-1 | Japonica | NA | | | | SP-RING-type domain-containing protein |
| augustus_masked-1-processed-gene-231.69-mRNA-1 | Japonica | Japonica | | Os01g0580300 | OsR498G0307172900.01.T05 | Cullin 4 |
| augustus_masked-1-processed-gene-236.30-mRNA-1 | Japonica | NA | | Os01g0587500 | | SHSP domain-containing protein |
| augustus_masked-1-processed-gene-25.36-mRNA-1 | Japonica | NA | | | | Factor of DNA methylation 4 |
| augustus_masked-1-processed-gene-260.26-mRNA-1 | Japonica | NA | | Os01g0632000 | | Putative GPI-anchored protein 58 |
| augustus_masked-1-processed-gene-261.33-mRNA-1 | Japonica | NA | | Os01g0635550 | OsR498G0101603800.01.T02 | Zinc-finger homeodomain protein 5 |
| augustus_masked-1-processed-gene-266.41-mRNA-1 | Japonica | NA | | | OsR498G0101637600.01.T01 | F-box domain-containing protein (Fragment) |
| augustus_masked-1-processed-gene-266.42-mRNA-1 | Japonica | NA | | Os01g0644200 | OsR498G0101637800.01.T01 | Little protein 1 |
| augustus_masked-1-processed-gene-267.44-mRNA-1 | Japonica | NA | | Os01g0645900 | OsR498G0101644400.01.T01 | Molybdate transporter like |
| augustus_masked-1-processed-gene-268.45-mRNA-1 | Japonica | NA | | Os01g0648700 | OsR498G0101654400.01.T01 | Transcription factor GTE4 |
| augustus_masked-1-processed-gene-269.75-mRNA-1 | Japonica | NA | | | OsR498G0101656600.01.T01 | |
| augustus_masked-1-processed-gene-275.26-mRNA-1 | Japonica | Japonica | | Os01g0660000 | OsR498G0101694200.01.T01 | |
| augustus_masked-1-processed-gene-276.12-mRNA-1 | Japonica | NA | | Os01g0660700 | OsR498G0101696800.01.T01 | DUF295 domain-containing protein |
| augustus_masked-1-processed-gene-277.15-mRNA-1 | Japonica | Indica | | Os07g0443500 | OsR498G0101702400.01.T01 | SANT domain-containing protein |
| augustus_masked-1-processed-gene-28.60-mRNA-1 | Japonica | NA | | Os01g0152300 | OsR498G0100191200.01.T01 | Histone domain-containing protein |
| augustus_masked-1-processed-gene-280.18-mRNA-1 | Japonica | Japonica | Kinase-TM | Os01g0668600 | OsR498G0101726500.01.T01 | Serine/threonine-protein kinase |
| augustus_masked-1-processed-gene-280.20-mRNA-1 | Japonica | NA | Kinase-TM | Os01g0669100 | OsR498G0101727800.01.T01 | Non-specific serine/threonine protein kinase |
| augustus_masked-1-processed-gene-281.23-mRNA-1 | Japonica | NA | Kinase-TM | Os01g0670100 | OsR498G0101730000.01.T01 | Non-specific serine/threonine protein kinase |
| augustus_masked-1-processed-gene-281.25-mRNA-1 | Japonica | NA | | Os01g0670500 | | |
| augustus_masked-1-processed-gene-282.15-mRNA-1 | Japonica | NA | | | OsR498G0101735400.01.T01 | Pentatricopeptide repeat-containing protein |
| augustus_masked-1-processed-gene-282.16-mRNA-1 | Japonica | NA | | | OsR498G0101737000.01.T02 | 1,3-beta-glucan synthase |
| augustus_masked-1-processed-gene-283.22-mRNA-1 | Japonica | NA | | Os01g0674000 | OsR498G0101745200.01.T01 | 6b-interacting protein 1 |
| augustus_masked-1-processed-gene-283.24-mRNA-1 | Japonica | Indica | | Os05g0560400 | OsR498G0101747200.01.T01 | Tubby-like F-box protein 1 |
| augustus_masked-1-processed-gene-284.23-mRNA-1 | Japonica | NA | | Os01g0675800 | OsR498G0101751200.01.T01 | NAC domain-containing protein 72 |
| augustus_masked-1-processed-gene-285.32-mRNA-1 | Japonica | NA | | Os01g0677100 | OsR498G0101756200.01.T02 | |
| augustus_masked-1-processed-gene-288.5-mRNA-1 | Japonica | NA | | | OsR498G0101774600.01.T01 | GPI transamidase component Gaa |
| augustus_masked-1-processed-gene-290.62-mRNA-1 | Japonica | NA | | Os01g0687400 | OsR498G0101789800.01.T01 | Acidic endochitinase |
| augustus_masked-1-processed-gene-291.42-mRNA-1 | Japonica | NA | | | OsR498G0101792600.01.T01 | |
| augustus_masked-1-processed-gene-291.60-mRNA-1 | Japonica | Japonica | | Os01g0689900 | OsR498G0101799400.01.T02 | Receptor-like protein kinase-like |
| augustus_masked-1-processed-gene-291.61-mRNA-1 | Japonica | NA | | Os01g0690200 | OsR498G0101800200.01.T01 | Protein kinase domain-containing protein |
| augustus_masked-1-processed-gene-292.73-mRNA-1 | Japonica | NA | | Os01g0691300 | OsR498G0101801400.01.T01 | AAI domain-containing protein |
| augustus_masked-1-processed-gene-292.76-mRNA-1 | Japonica | NA | | | | |
| augustus_masked-1-processed-gene-293.29-mRNA-1 | Japonica | Japonica | | Os01g0693400 | OsR498G0101809000.01.T03 | AP2/ERF and B3 domain-containing transcription repressor TEM1 |
| augustus_masked-1-processed-gene-293.42-mRNA-1 | Japonica | NA | | Os01g0693800 | OsR498G0101813200.01.T01 | Threonine synthase |
| augustus_masked-1-processed-gene-298.71-mRNA-1 | Japonica | Indica | | Os01g0704300 | OsR498G0101846200.01.T01 | Kelch repeat-containing F-box-like protein |
| augustus_masked-1-processed-gene-299.35-mRNA-1 | Japonica | NA | | Os03g0395700 | | |
| augustus_masked-1-processed-gene-300.49-mRNA-1 | Japonica | NA | | Os01g0707500 | OsR498G0101859800.01.T02 | Transcription factor bHLH87 |
| augustus_masked-1-processed-gene-301.72-mRNA-1 | Japonica | NA | | | OsR498G0101865200.01.T01 | |
| augustus_masked-1-processed-gene-302.56-mRNA-1 | Japonica | NA | | Os01g0710200 | OsR498G0101867800.01.T01 | Polyamine oxidase |
| augustus_masked-1-processed-gene-302.57-mRNA-1 | Japonica | NA | | Os01g0710700 | OsR498G0101869400.01.T04 | Phospholipase A1-Igamma2, chloroplastic |
| augustus_masked-1-processed-gene-302.81-mRNA-1 | Japonica | NA | | | OsR498G0101873200.01.T01 | |
| augustus_masked-1-processed-gene-303.6-mRNA-1 | Japonica | NA | | Os01g0712700 | OsR498G0101875000.01.T01 | Cytidine deaminase |
| augustus_masked-1-processed-gene-304.65-mRNA-1 | Japonica | NA | | Os01g0715900 | OsR498G0101886400.01.T01 | Pentatricopeptide repeat-containing protein, chloroplastic |
| augustus_masked-1-processed-gene-305.22-mRNA-1 | Japonica | NA | | | OsR498G0101897800.01.T01 | |
| augustus_masked-1-processed-gene-306.22-mRNA-1 | Japonica | NA | Kinase-LRR-TM | Os01g0718300 | OsR498G0101902000.01.T01 | Brassinosteroid-insensitive 1 |
| augustus_masked-1-processed-gene-306.23-mRNA-1 | Japonica | NA | | Os01g0718900 | OsR498G0101903800.01.T02 | Trihelix transcription factor ASIL1 |
| augustus_masked-1-processed-gene-307.62-mRNA-1 | Japonica | Indica | | Os10g0160600 | OsR498G0101912800.01.T01 | |
| augustus_masked-1-processed-gene-309.26-mRNA-1 | Japonica | NA | | | OsR498G0101927200.01.T01 | |
| augustus_masked-1-processed-gene-310.26-mRNA-1 | Japonica | NA | | Os01g0727600 | | |
| augustus_masked-1-processed-gene-310.27-mRNA-1 | Japonica | NA | | Os01g0727700 | | Thiamine thiazole synthase (Fragment) |
| augustus_masked-1-processed-gene-311.29-mRNA-1 | Japonica | Indica | | Os01g0730200 | OsR498G0101936400.01.T01 | FBD domain-containing protein |
| augustus_masked-1-processed-gene-311.30-mRNA-1 | Japonica | NA | | Os01g0730400 | | |
| augustus_masked-1-processed-gene-311.31-mRNA-1 | Japonica | Indica | | Os01g0730600 | OsR498G0101938000.01.T01 | |
| augustus_masked-1-processed-gene-314.35-mRNA-1 | Japonica | NA | | Os01g0735900 | OsR498G0101953400.01.T01 | Glycosyltransferase |
| augustus_masked-1-processed-gene-314.37-mRNA-1 | Japonica | NA | | Os01g0736100 | OsR498G0101953800.01.T01 | Glycosyltransferase |
| augustus_masked-1-processed-gene-314.38-mRNA-1 | Japonica | Japonica | | Os01g0736300 | OsR498G0101954200.01.T01 | Glycosyltransferase |
| augustus_masked-1-processed-gene-314.69-mRNA-1 | Japonica | NA | | Os01g0736600 | OsR498G0101955400.01.T01 | RING-H2 finger protein ATL68 |
| augustus_masked-1-processed-gene-314.70-mRNA-1 | Japonica | Indica | | Os01g0737100 | OsR498G0101956800.01.T01 | |
| augustus_masked-1-processed-gene-316.55-mRNA-1 | Japonica | Japonica | | Os01g0740700 | OsR498G0204773100.01.T01 | TIR domain-containing adapter molecule 1 |
| augustus_masked-1-processed-gene-317.4-mRNA-1 | Japonica | NA | | | OsR498G0101972400.01.T02 | rRNA methyltransferase 1, mitochondrial |
| augustus_masked-1-processed-gene-318.57-mRNA-1 | Japonica | NA | | | OsR498G0101981600.01.T01 | |

| | | | | | | |
|---|---|---|---|---|---|---|
| augustus_masked-1-processed-gene-320.46-mRNA-1 | Japonica | NA | | Os01g0747600 | | Pentatricopeptide repeat-containing protein mitochondrial |
| augustus_masked-1-processed-gene-320.69-mRNA-1 | Japonica | NA | | Os01g0748200 | OsR498G0101990600.01.T01 | Protodermal factor 1-like |
| augustus_masked-1-processed-gene-320.70-mRNA-1 | Japonica | NA | | Os01g0748300 | OsR498G0101991000.01.T01 | |
| augustus_masked-1-processed-gene-323.30-mRNA-1 | Japonica | NA | | Os01g0754701 | OsR498G0102012800.01.T01 | Pentatricopeptide repeat-containing protein |
| augustus_masked-1-processed-gene-324.53-mRNA-1 | Japonica | Indica | | Os04g0208500 | OsR498G0102018800.01.T01 | F-box domain-containing protein |
| augustus_masked-1-processed-gene-325.60-mRNA-1 | Japonica | Indica | | Os01g0757600 | OsR498G0102023400.01.T01 | Myosin heavy chain 1 (Fragment) |
| augustus_masked-1-processed-gene-325.61-mRNA-1 | Japonica | NA | | | | Pentatricopeptide repeat |
| augustus_masked-1-processed-gene-326.19-mRNA-1 | Japonica | NA | | | OsR498G0511427100.01.T01 | Epstein-Barr virus EBNA-1-like |
| augustus_masked-1-processed-gene-326.20-mRNA-1 | Japonica | NA | Kinase-TM | Os01g0759200 | OsR498G0102036200.01.T01 | Non-specific serine/threonine protein kinase |
| augustus_masked-1-processed-gene-326.21-mRNA-1 | Japonica | NA | Kinase-TM | Os01g0759400 | OsR498G0102036600.01.T01 | Non-specific serine/threonine protein kinase |
| augustus_masked-1-processed-gene-327.27-mRNA-1 | Japonica | NA | | | OsR498G0306953600.01.T01 | probable isoaspartyl peptidase/L-asparaginase 2 |
| augustus_masked-1-processed-gene-327.6-mRNA-1 | Japonica | NA | TIR-TM | Os01g0760400 | OsR498G0102039800.01.T01 | ADP-ribosyl cyclase/cyclic ADP-ribose hydrolase |
| augustus_masked-1-processed-gene-328.45-mRNA-1 | Japonica | NA | | Os01g0763200 | OsR498G0102046000.01.T04 | TCP transcription factor |
| augustus_masked-1-processed-gene-328.65-mRNA-1 | Japonica | NA | | Os11g0631900 | | |
| augustus_masked-1-processed-gene-329.58-mRNA-1 | Japonica | NA | | Os01g0764950 | OsR498G0102052200.01.T05 | Pentatricopeptide repeat-containing protein |
| augustus_masked-1-processed-gene-329.67-mRNA-1 | Japonica | NA | | Os01g0766200 | OsR498G0102054200.01.T02 | E3 ubiquitin-protein ligase RMA1H1 |
| augustus_masked-1-processed-gene-330.37-mRNA-1 | Japonica | NA | | Os01g0766300 | OsR498G0102055400.01.T01 | |
| augustus_masked-1-processed-gene-332.20-mRNA-1 | Japonica | NA | | Os01g0771000 | OsR498G0102067800.01.T01 | Translation initiation factor eIF-2B subunit alpha |
| augustus_masked-1-processed-gene-332.4-mRNA-1 | Japonica | NA | | Os01g0770200 | OsR498G0102066200.01.T01 | Tyrosine decarboxylase |
| augustus_masked-1-processed-gene-332.5-mRNA-1 | Japonica | NA | | Os01g0770400 | OsR498G0102066400.01.T02 | F-box family protein-like |
| augustus_masked-1-processed-gene-334.32-mRNA-1 | Japonica | NA | | Os01g0773500 | | |
| augustus_masked-1-processed-gene-334.53-mRNA-1 | Japonica | NA | | Os01g0774100 | | Gnk2-homologous domain-containing protein |
| augustus_masked-1-processed-gene-336.77-mRNA-1 | Japonica | NA | | Os01g0778900 | OsR498G0102096000.01.T01 | |
| augustus_masked-1-processed-gene-337.19-mRNA-1 | Japonica | NA | | Os01g0779800 | OsR498G0102098200.01.T01 | Serine/arginine repetitive matrix protein 1 |
| augustus_masked-1-processed-gene-338.56-mRNA-1 | Japonica | NA | | Os01g0783700 | OsR498G0102108600.01.T01 | Calcium-binding protein PBP1 |
| augustus_masked-1-processed-gene-339.36-mRNA-1 | Japonica | Japonica | | Os01g0785600 | OsR498G0511115700.01.T02 | S-adenosyl-L-methionine-dependent methyltransferase superfamily protein |
| augustus_masked-1-processed-gene-341.24-mRNA-1 | Japonica | NA | CC-NBS-TM | Os01g0788500 | OsR498G0102125800.01.T03 | Disease resistance protein RPS2 |
| augustus_masked-1-processed-gene-341.25-mRNA-1 | Japonica | NA | | Os01g0788900 | OsR498G0102126800.01.T01 | Pentatricopeptide repeat |
| augustus_masked-1-processed-gene-342.7-mRNA-1 | Japonica | NA | | Os01g0793300 | | Catechol oxidase |
| augustus_masked-1-processed-gene-343.4-mRNA-1 | Japonica | NA | | Os01g0793800 | OsR498G0102129800.01.T01 | Protein odd-skipped |
| augustus_masked-1-processed-gene-343.41-mRNA-1 | Japonica | Indica | | Os10g0402400 | OsR498G0102133400.01.T01 | |
| augustus_masked-1-processed-gene-343.46-mRNA-1 | Japonica | NA | | | OsR498G0102136800.01.T01 | |
| augustus_masked-1-processed-gene-343.5-mRNA-1 | Japonica | NA | | | OsR498G0204046800.01.T01 | Epstein-Barr virus EBNA-1-like |
| augustus_masked-1-processed-gene-343.6-mRNA-1 | Japonica | NA | | Os01g0793900 | OsR498G0102130200.01.T01 | Protein odd-skipped |
| augustus_masked-1-processed-gene-343.7-mRNA-1 | Japonica | NA | | Os01g0794100 | OsR498G0102130600.01.T01 | Protein odd-skipped |
| augustus_masked-1-processed-gene-345.30-mRNA-1 | Japonica | NA | | Os01g0800300 | OsR498G0102148800.01.T04 | Polyadenylate-binding protein 1-B-binding protein |
| augustus_masked-1-processed-gene-346.48-mRNA-1 | Japonica | NA | | Os01g0802200 | | WAS/WASL-interacting protein family member 3-like |
| augustus_masked-1-processed-gene-347.43-mRNA-1 | Japonica | NA | | | OsR498G0102163600.01.T01 | |
| augustus_masked-1-processed-gene-347.7-mRNA-1 | Japonica | Japonica | | Os01g0802850 | OsR498G0511097100.01.T04 | Plastidic phosphate translocator-like protein1 |
| augustus_masked-1-processed-gene-38.21-mRNA-1 | Japonica | NA | | Os01g0174100 | OsR498G0100247800.01.T01 | MAPK kinase substrate protein |
| augustus_masked-1-processed-gene-4.64-mRNA-1 | Japonica | NA | | Os01g0106500 | OsR498G0100023600.01.T01 | |
| augustus_masked-1-processed-gene-4.65-mRNA-1 | Japonica | Japonica | | Os01g0106600 | OsR498G0100023800.01.T01 | |
| augustus_masked-1-processed-gene-40.8-mRNA-1 | Japonica | NA | | Os01g0177400 | OsR498G0100258600.01.T01 | GA 3beta-hydroxylase |
| augustus_masked-1-processed-gene-42.29-mRNA-1 | Japonica | NA | LRR-TM | Os01g0180000 | OsR498G0100270600.01.T01 | Leucine-rich repeat extensin-like protein 3 |
| augustus_masked-1-processed-gene-44.32-mRNA-1 | Japonica | NA | | Os01g0182400 | OsR498G0100278600.01.T01 | TSL-kinase interacting protein 1 |
| augustus_masked-1-processed-gene-475.28-mRNA-1 | Japonica | NA | | | OsR498G0102562800.01.T03 | glycine-rich RNA-binding protein 4, mitochondrial |
| augustus_masked-1-processed-gene-475.29-mRNA-1 | Japonica | NA | | Os01g0913400 | OsR498G0102562800.01.T01 | RRM domain-containing protein |
| augustus_masked-1-processed-gene-475.35-mRNA-1 | Japonica | NA | | Os01g0914600 | OsR498G0102565000.01.T05 | Pentatricopeptide repeat-containing protein chloroplastic |
| augustus_masked-1-processed-gene-475.7-mRNA-1 | Japonica | NA | | Os01g0912900 | OsR498G0102561600.01.T02 | Pentatricopeptide repeat-containing protein, mitochondrial |
| augustus_masked-1-processed-gene-475.8-mRNA-1 | Japonica | NA | | Os01g0913100 | OsR498G0102562000.01.T02 | Nuclear transition protein like |
| augustus_masked-1-processed-gene-476.60-mRNA-1 | Japonica | Japonica | | Os01g0915900 | OsR498G0102571400.01.T03 | NB-ARC domain-containing protein |
| augustus_masked-1-processed-gene-476.61-mRNA-1 | Japonica | NA | | Os01g0916000 | OsR498G0102571600.01.T02 | Putative clathrin assembly protein |
| augustus_masked-1-processed-gene-476.62-mRNA-1 | Japonica | Indica | | Os01g0916100 | OsR498G0102571800.01.T01 | Loricrin |
| augustus_masked-1-processed-gene-477.46-mRNA-1 | Japonica | NA | | Os01g0917900 | OsR498G0102578800.01.T01 | E3 ubiquitin-protein ligase LUL4 |
| augustus_masked-1-processed-gene-478.7-mRNA-1 | Japonica | NA | | | OsR498G0917172900.01.T01 | DUF834 domain-containing protein |
| augustus_masked-1-processed-gene-479.12-mRNA-1 | Japonica | NA | | | OsR498G0204046800.01.T01 | Circumsporozoite protein-like |
| augustus_masked-1-processed-gene-480.13-mRNA-1 | Japonica | NA | | Os01g0922100 | OsR498G0102598800.01.T01 | vitellogenin-2 |
| augustus_masked-1-processed-gene-482.15-mRNA-1 | Japonica | NA | | Os01g0924966 | OsR498G0102616400.01.T01 | Auxin-responsive protein SAUR71 |
| augustus_masked-1-processed-gene-485.70-mRNA-1 | Japonica | NA | | Os10g0531750 | OsR498G0408675500.01.T01 | Retrotransposon protein, putative, Ty3-gypsy sub-class |
| augustus_masked-1-processed-gene-487.45-mRNA-1 | Japonica | NA | | Os01g0934100 | OsR498G0102649000.01.T02 | C2 calcium-dependent membrane targeting |
| augustus_masked-1-processed-gene-487.62-mRNA-1 | Japonica | Indica | | Os01g0935200 | OsR498G0102652200.01.T01 | CCAAT-binding transcription factor A |

| | | | | | | |
|---|---|---|---|---|---|---|
| augustus_masked-1-processed-gene-488.16-mRNA-1 | Japonica | Japonica | | Os01g0936900 | OsR498G0102660600.01.T01 | Xylanase inhibitor TAXI-IV |
| augustus_masked-1-processed-gene-488.17-mRNA-1 | Japonica | Indica | | Os01g0936900 | OsR498G0102660200.01.T01 | Peptidase A1 domain-containing protein (Fragment) |
| augustus_masked-1-processed-gene-488.18-mRNA-1 | Japonica | Indica | | Os01g0936900 | OsR498G0102660600.01.T01 | Putative xylanase inhibitor |
| augustus_masked-1-processed-gene-488.20-mRNA-1 | Japonica | Japonica | | Os01g0937000 | OsR498G0102660800.01.T01 | Xylanase inhibitor TAXI-IV |
| augustus_masked-1-processed-gene-488.22-mRNA-1 | Japonica | NA | | Os01g0937100 | OsR498G0102661800.01.T01 | Xylanase inhibitor TAXI-IV |
| augustus_masked-1-processed-gene-488.23-mRNA-1 | Japonica | Japonica | CC-NBS-TM | Os01g0937300 | OsR498G0102662000.01.T03 | Disease resistance protein RGA2 |
| augustus_masked-1-processed-gene-489.31-mRNA-1 | Japonica | NA | | Os01g0938400 | OsR498G0102666600.01.T01 | VWFA domain-containing protein |
| augustus_masked-1-processed-gene-490.8-mRNA-1 | Japonica | NA | | Os01g0940100 | OsR498G0102672200.01.T02 | Phosphotransferase |
| augustus_masked-1-processed-gene-493.39-mRNA-1 | Japonica | NA | | Os01g0946550 | | NAC domain-containing protein |
| augustus_masked-1-processed-gene-494.27-mRNA-1 | Japonica | NA | | Os01g0948500 | OsR498G0102694000.01.T01 | U-box domain-containing protein 43 |
| augustus_masked-1-processed-gene-496.71-mRNA-1 | Japonica | NA | | | | BTB/POZ domain and ankyrin repeat-containing protein NH5.1 |
| augustus_masked-1-processed-gene-498.14-mRNA-1 | Japonica | NA | | Os01g0954900 | OsR498G0102720400.01.T01 | Nucleoid DNA-binding-like protein |
| augustus_masked-1-processed-gene-498.16-mRNA-1 | Japonica | NA | | Os01g0955100 | OsR498G0102721000.01.T01 | Probable calcium-binding protein CML31 |
| augustus_masked-1-processed-gene-498.17-mRNA-1 | Japonica | Indica | | Os01g0955100 | OsR498G0102721800.01.T01 | Putative calcium-binding protein CML23 |
| augustus_masked-1-processed-gene-5.23-mRNA-1 | Japonica | NA | | | OsR498G0100073600.01.T01 | |
| augustus_masked-1-processed-gene-503.71-mRNA-1 | Japonica | NA | | Os01g0965800 | | |
| augustus_masked-1-processed-gene-503.73-mRNA-1 | Japonica | NA | | | OsR498G0102756200.01.T05 | Oxoprolinase 1 |
| augustus_masked-1-processed-gene-506.35-mRNA-1 | Japonica | NA | | Os01g0971800 | OsR498G0102775000.01.T01 | PHYTOCLOCK 1 |
| augustus_masked-1-processed-gene-508.23-mRNA-1 | Japonica | NA | | Os01g0976200 | OsR498G0102788800.01.T01 | Glutelin type-A 1 |
| augustus_masked-1-processed-gene-509.35-mRNA-1 | Japonica | NA | | Os01g0977300 | | Transcription factor MYB44 |
| augustus_masked-1-processed-gene-52.19-mRNA-1 | Japonica | NA | | Os01g0196500 | OsR498G0100339800.01.T01 | |
| augustus_masked-1-processed-gene-52.2-mRNA-1 | Japonica | NA | | | OsR498G0100335400.01.T01 | Polyamine oxidase 5 |
| augustus_masked-1-processed-gene-53.47-mRNA-1 | Japonica | NA | | Os01g0197500 | OsR498G0100343800.01.T01 | Pentatricopeptide repeat-containing protein (Fragment) |
| augustus_masked-1-processed-gene-55.41-mRNA-1 | Japonica | NA | | | OsR498G0100359200.01.T01 | VQ motif-containing protein 4 |
| augustus_masked-1-processed-gene-56.27-mRNA-1 | Japonica | NA | | | OsR498G0204046800.01.T01 | Epstein-Barr virus EBNA-1-like |
| augustus_masked-1-processed-gene-57.34-mRNA-1 | Japonica | NA | | Os01g0203300 | OsR498G0100367600.01.T05 | Glycosyltransferase |
| augustus_masked-1-processed-gene-57.38-mRNA-1 | Japonica | NA | | Os01g0203800 | OsR498G0100369400.01.T02 | DUF641 domain-containing protein (Fragment) |
| augustus_masked-1-processed-gene-60.23-mRNA-1 | Japonica | NA | | Os01g0209500 | OsR498G0100387600.01.T03 | RRM domain-containing protein |
| augustus_masked-1-processed-gene-60.38-mRNA-1 | Japonica | NA | | Os02g0212975 | OsR498G0305421900.01.T01 | Kinesin heavy chain-like |
| augustus_masked-1-processed-gene-62.30-mRNA-1 | Japonica | NA | | | OsR498G0100075100.01.T01 | Epstein-Barr virus EBNA-1-like |
| augustus_masked-1-processed-gene-62.34-mRNA-1 | Japonica | NA | | Os01g0213400 | | RING-H2 finger protein ATL2L |
| augustus_masked-1-processed-gene-66.53-mRNA-1 | Japonica | NA | | | | Protein kinase domain-containing protein |
| augustus_masked-1-processed-gene-68.24-mRNA-1 | Japonica | Indica | Kinase-TM | Os01g0223700 | OsR498G0100448000.01.T02 | Non-specific serine/threonine protein kinase (Fragment) |
| augustus_masked-1-processed-gene-68.25-mRNA-1 | Japonica | Japonica | Kinase-TM | Os01g0224000 | OsR498G0100448100.01.T01 | Non-specific serine/threonine protein kinase (Fragment) |
| augustus_masked-1-processed-gene-7.32-mRNA-1 | Japonica | NA | | Os01g0111700 | OsR498G0100037600.01.T01 | |
| augustus_masked-1-processed-gene-70.37-mRNA-1 | Japonica | NA | | Os01g0226400 | OsR498G0100462600.01.T01 | AAA domain-containing protein |
| augustus_masked-1-processed-gene-72.63-mRNA-1 | Japonica | Indica | | Os01g0229800 | OsR498G0100476800.01.T01 | Bark storage protein A |
| augustus_masked-1-processed-gene-75.31-mRNA-1 | Japonica | Indica | | Os01g0235400 | OsR498G0100494600.01.T01 | Importin-beta |
| augustus_masked-1-processed-gene-79.58-mRNA-1 | Japonica | NA | | | OsR498G0100526000.01.T01 | Transcription factor bHLH148 |
| augustus_masked-1-processed-gene-85.2-mRNA-1 | Japonica | Indica | | Os01g0254300 | OsR498G0100568000.01.T01 | Pectinesterase |
| augustus_masked-1-processed-gene-97.17-mRNA-1 | Japonica | Indica | | Os01g0276400 | OsR498G0100655000.01.T01 | Presenilin |
| augustus_masked-1-processed-gene-98.24-mRNA-1 | Japonica | NA | | Os01g0278000 | OsR498G0100660800.01.T01 | Calmodulin-binding protein 25 |
| augustus_masked-1-processed-gene-99.5-mRNA-1 | Japonica | NA | | | OsR498G0100663000.01.T02 | Importin N-terminal domain-containing protein |
| augustus_masked-10-processed-gene-106.46-mRNA-1 | Japonica | NA | | | OsR498G1018770200.01.T01 | |
| augustus_masked-10-processed-gene-107.8-mRNA-1 | Japonica | Japonica | | Os10g0434200 | OsR498G1018774700.01.T01 | BTB/POZ domain containing protein |
| augustus_masked-10-processed-gene-116.11-mRNA-1 | Japonica | NA | | Os04g0166900 | | F-box domain-containing protein (Fragment) |
| augustus_masked-10-processed-gene-117.32-mRNA-1 | Japonica | NA | | Os04g0163800 | OsR498G0407600700.01.T01 | Disease resistance protein |
| augustus_masked-10-processed-gene-13.17-mRNA-1 | Japonica | NA | Kinase-LRR-TM | Os10g0114400 | | Mitogen-activated protein kinase kinase kinase |
| augustus_masked-10-processed-gene-13.18-mRNA-1 | Japonica | NA | | Os10g0114500 | OsR498G1018044000.01.T01 | Barley B recombinant-like protein A |
| augustus_masked-10-processed-gene-13.19-mRNA-1 | Japonica | NA | | Os10g0114500 | OsR498G1018044000.01.T01 | Barley B recombinant-like protein A |
| augustus_masked-10-processed-gene-139.35-mRNA-1 | Japonica | NA | | Os04g0120300 | OsR498G0407492700.01.T01 | subtilisin-like protease SDD1 |
| augustus_masked-10-processed-gene-14.23-mRNA-1 | Japonica | Japonica | | Os10g0115500 | OsR498G1018044000.01.T01 | Barley B recombinant-like protein C |
| augustus_masked-10-processed-gene-144.26-mRNA-1 | Japonica | NA | CC-NBS-TM | | | NBS-LRR resistant protein (Fragment) |
| augustus_masked-10-processed-gene-146.3-mRNA-1 | Japonica | NA | | | OsR498G0204768400.01.T01 | Epstein-Barr virus EBNA-1-like |
| augustus_masked-10-processed-gene-155.29-mRNA-1 | Japonica | NA | | Os10g0452300 | OsR498G1018828200.01.T01 | |
| augustus_masked-10-processed-gene-155.30-mRNA-1 | Japonica | NA | | Os10g0452201 | OsR498G1018828400.01.T01 | |
| augustus_masked-10-processed-gene-156.31-mRNA-1 | Japonica | NA | | Os10g0453900 | OsR498G1018832400.01.T01 | Glycine-rich cell wall structural protein 2 |
| augustus_masked-10-processed-gene-156.35-mRNA-1 | Japonica | NA | | Os10g0454500 | OsR498G1018834800.01.T01 | Root cap-specific glycine-rich protein |
| augustus_masked-10-processed-gene-156.5-mRNA-1 | Japonica | NA | | Os10g0452700 | OsR498G1018829600.01.T01 | Glycine-rich cell wall structural protein 2 (Fragment) |
| augustus_masked-10-processed-gene-157.36-mRNA-1 | Japonica | Japonica | | Os10g0455000 | OsR498G1018835400.01.T01 | Glycine-rich cell wall structural protein 2 |
| augustus_masked-10-processed-gene-157.37-mRNA-1 | Japonica | Indica | | Os10g0455000 | OsR498G1018836000.01.T01 | Glycine-rich cell wall structural protein 2-like |

| | | | | | | |
|---|---|---|---|---|---|---|
| augustus_masked-10-processed-gene-157.38-mRNA-1 | Japonica | NA | | Os10g0455100 | OsR498G1018836200.01.T01 | Glycine-rich cell wall structural protein 2 |
| augustus_masked-10-processed-gene-167.49-mRNA-1 | Japonica | NA | | Os10g0474000 | | |
| augustus_masked-10-processed-gene-168.21-mRNA-1 | Japonica | Indica | | Os10g0474966 | OsR498G1018896800.01.T01 | Long-chain-alcohol oxidase |
| augustus_masked-10-processed-gene-168.25-mRNA-1 | Japonica | NA | | Os10g0476200 | | |
| augustus_masked-10-processed-gene-169.20-mRNA-1 | Japonica | NA | | Os10g0476900 | OsR498G1018901200.01.T01 | Pentatricopeptide, putative, expressed |
| augustus_masked-10-processed-gene-169.21-mRNA-1 | Japonica | Indica | | Os10g0477200 | OsR498G1018901800.01.T01 | Pentatricopeptide repeat-containing protein mitochondrial |
| augustus_masked-10-processed-gene-170.36-mRNA-1 | Japonica | NA | | Os10g0478400 | OsR498G1018908200.01.T01 | |
| augustus_masked-10-processed-gene-173.8-mRNA-1 | Japonica | NA | | Os10g0481300 | OsR498G1018923400.01.T01 | GST C-terminal domain-containing protein |
| augustus_masked-10-processed-gene-174.59-mRNA-1 | Japonica | NA | | Os10g0484300 | OsR498G1018936200.01.T02 | Pentatricopeptide repeat-containing protein |
| augustus_masked-10-processed-gene-176.10-mRNA-1 | Japonica | Indica | | Os10g0487400 | OsR498G1018946600.01.T01 | RING-type E3 ubiquitin transferase |
| augustus_masked-10-processed-gene-177.35-mRNA-1 | Japonica | NA | | | | Putative ripening-related protein 7 |
| augustus_masked-10-processed-gene-177.58-mRNA-1 | Japonica | Indica | | Os10g0489800 | OsR498G1018955800.01.T02 | DUF1308 domain-containing protein (Fragment) |
| augustus_masked-10-processed-gene-178.7-mRNA-1 | Japonica | Indica | | Os10g0490900 | OsR498G1018957600.01.T01 | Basic secretory protease |
| augustus_masked-10-processed-gene-179.66-mRNA-1 | Japonica | NA | | Os10g0494100 | OsR498G1018971000.01.T01 | |
| augustus_masked-10-processed-gene-18.40-mRNA-1 | Japonica | Japonica | CC-NBS | Os10g0124300 | OsR498G1018071200.01.T02 | Disease resistance protein RPM1 |
| augustus_masked-10-processed-gene-180.3-mRNA-1 | Japonica | NA | | Os10g0495200 | OsR498G1018978200.01.T01 | Rf1 protein, mitochondrial, putative, expressed |
| augustus_masked-10-processed-gene-182.53-mRNA-1 | Japonica | NA | | | OsR498G1018987600.01.T02 | PsbB mRNA maturation factor Mbb1, chloroplastic |
| augustus_masked-10-processed-gene-185.4-mRNA-1 | Japonica | Japonica | | Os10g0505500 | OsR498G1019007500.01.T01 | AAI domain-containing protein (Fragment) |
| augustus_masked-10-processed-gene-187.32-mRNA-1 | Japonica | NA | | Os10g0508700 | OsR498G1019016200.01.T01 | PMEI domain-containing protein |
| augustus_masked-10-processed-gene-187.33-mRNA-1 | Japonica | Japonica | | Os10g0508900 | OsR498G1019017000.01.T01 | |
| augustus_masked-10-processed-gene-187.59-mRNA-1 | Japonica | NA | | Os10g0510300 | OsR498G1019022800.01.T01 | PORR domain-containing protein (Fragment) |
| augustus_masked-10-processed-gene-188.32-mRNA-1 | Japonica | NA | | Os10g0510500 | OsR498G1019024000.01.T01 | Auxin-responsive protein SAUR32 |
| augustus_masked-10-processed-gene-189.16-mRNA-1 | Japonica | NA | | Os10g0512500 | OsR498G1019030200.01.T01 | RNA-binding |
| augustus_masked-10-processed-gene-190.20-mRNA-1 | Japonica | NA | | Os10g0514600 | OsR498G1019034400.01.T01 | T-complex protein 1 subunit delta |
| augustus_masked-10-processed-gene-192.10-mRNA-1 | Japonica | NA | | | OsR498G0511427100.01.T01 | Epstein-Barr virus EBNA-1-like |
| augustus_masked-10-processed-gene-195.52-mRNA-1 | Japonica | NA | | Os10g0523700 | OsR498G1019068000.01.T01 | Arogenate dehydratase |
| augustus_masked-10-processed-gene-195.55-mRNA-1 | Japonica | NA | | Os10g0524600 | OsR498G1019071400.01.T01 | Subtilisin-like serine protease |
| augustus_masked-10-processed-gene-21.43-mRNA-1 | Japonica | NA | NBS-TM | | | Powdery mildew resistance protein PM17 |
| augustus_masked-10-processed-gene-23.22-mRNA-1 | Japonica | NA | | Os10g0133100 | | F-box domain containing protein, expressed |
| augustus_masked-10-processed-gene-24.9-mRNA-1 | Japonica | NA | | | OsR498G1018103600.01.T01 | |
| augustus_masked-10-processed-gene-25.18-mRNA-1 | Japonica | Indica | | Os10g0134500 | OsR498G1018108800.01.T01 | F-box domain-containing protein |
| augustus_masked-10-processed-gene-25.20-mRNA-1 | Japonica | NA | | Os10g0136400 | OsR498G1018109000.01.T02 | Cysteine-rich receptor-like protein kinase 10 |
| augustus_masked-10-processed-gene-26.16-mRNA-1 | Japonica | NA | | Os10g0136900 | OsR498G1018109800.01.T01 | F-box domain containing protein, expressed |
| augustus_masked-10-processed-gene-26.17-mRNA-1 | Japonica | NA | | Os10g0137000 | OsR498G1018110000.01.T01 | F-box domain-containing protein (Fragment) |
| augustus_masked-10-processed-gene-30.28-mRNA-1 | Japonica | NA | | Os10g0145100 | OsR498G1018134000.01.T02 | F-box domain containing protein, expressed |
| augustus_masked-10-processed-gene-36.6-mRNA-1 | Japonica | Indica | Kinase-TM | Os10g0152000 | OsR498G1018157400.01.T01 | Wall-associated receptor kinase 2 |
| augustus_masked-10-processed-gene-45.15-mRNA-1 | Japonica | Indica | LRR-TM | Os02g0156400 | OsR498G1018221800.01.T01 | Putative Phytosulfokine receptor |
| augustus_masked-10-processed-gene-46.19-mRNA-1 | Japonica | Indica | | Os10g0163201 | OsR498G1018230000.01.T01 | Retrotransposon protein, putative, Ty3-gypsy subclass |
| augustus_masked-10-processed-gene-58.25-mRNA-1 | Japonica | NA | | | OsR498G0409006400.01.T04 | |
| augustus_masked-10-processed-gene-59.23-mRNA-1 | Japonica | NA | | | OsR498G1018292000.01.T01 | F-box domain-containing protein (Fragment) |
| augustus_masked-10-processed-gene-63.36-mRNA-1 | Japonica | NA | | Os10g0188800 | OsR498G1018312800.01.T01 | Retrotransposon protein, putative, Ty3-gypsy subclass, expressed |
| augustus_masked-10-processed-gene-69.21-mRNA-1 | Japonica | NA | | Os08g0381000 | OsR498G0917373300.01.T01 | Retrotransposon protein, putative, Ty3-gypsy subclass |
| augustus_masked-10-processed-gene-73.21-mRNA-1 | Japonica | NA | | Os12g0518100 | | |
| augustus_masked-10-processed-gene-73.5-mRNA-1 | Japonica | NA | | | OsR498G1018341400.01.T01 | F-box domain-containing protein (Fragment) |
| augustus_masked-10-processed-gene-75.28-mRNA-1 | Japonica | NA | | Os10g0200700 | OsR498G1018354200.01.T01 | OVATE domain-containing protein |
| augustus_masked-11-processed-gene-0.43-mRNA-1 | Japonica | NA | | | OsR498G0511427100.01.T01 | Epstein-Barr virus EBNA-1-like |
| augustus_masked-11-processed-gene-10.103-mRNA-1 | Japonica | NA | | | OsR498G1119385200.01.T01 | putative RING-H2 finger protein ATL61 |
| augustus_masked-11-processed-gene-10.82-mRNA-1 | Japonica | NA | | Os11g0135900 | OsR498G1119380200.01.T01 | Transporter, putative, expressed |
| augustus_masked-11-processed-gene-109.23-mRNA-1 | Japonica | NA | | | OsR498G0203809100.01.T01 | DUF834 domain-containing protein |
| augustus_masked-11-processed-gene-11.10-mRNA-1 | Japonica | Indica | | Os11g0138300 | OsR498G1119390400.01.T01 | Cytochrome P450 family protein, expressed |
| augustus_masked-11-processed-gene-111.22-mRNA-1 | Japonica | Japonica | NBS-TM | Os11g0429100 | OsR498G1120005800.01.T01 | NBS-LRR type resistance protein (Fragment) |
| augustus_masked-11-processed-gene-111.9-mRNA-1 | Japonica | NA | | Os11g0428500 | | |
| augustus_masked-11-processed-gene-114.21-mRNA-1 | Japonica | NA | | | OsR498G1120019600.01.T01 | PGG domain-containing protein |
| augustus_masked-11-processed-gene-115.3-mRNA-1 | Japonica | NA | | | | |
| augustus_masked-11-processed-gene-119.8-mRNA-1 | Japonica | NA | | | OsR498G0305447100.01.T01 | |
| augustus_masked-11-processed-gene-12.25-mRNA-1 | Japonica | NA | | Os12g0137900 | OsR498G1220905400.01.T01 | Alpha-galactosidase |
| augustus_masked-11-processed-gene-12.26-mRNA-1 | Japonica | Japonica | | Os12g0137900 | OsR498G1119397800.01.T01 | Sulfotransferase |
| augustus_masked-11-processed-gene-12.28-mRNA-1 | Japonica | NA | | | OsR498G1119398600.01.T01 | GRAS domain-containing protein (Fragment) |
| augustus_masked-11-processed-gene-12.30-mRNA-1 | Japonica | NA | | | OsR498G1119399600.01.T01 | GRAS domain-containing protein (Fragment) |
| augustus_masked-11-processed-gene-125.1-mRNA-1 | Japonica | NA | | Os11g0450300 | OsR498G1120065200.01.T01 | Sulfotransferase |
| augustus_masked-11-processed-gene-128.18-mRNA-1 | Japonica | NA | | Os11g0456000 | OsR498G1120082000.01.T03 | Rapid alkalinization factor 1 |

| | | | | | | |
|---|---|---|---|---|---|---|
| augustus_masked-11-processed-gene-129.4-mRNA-1 | Japonica | NA | | Os11g0457300 | OsR498G1120085200.01.T01 | Glycosyltransferase |
| augustus_masked-11-processed-gene-14.10-mRNA-1 | Japonica | NA | | Os11g0145200 | OsR498G1119406400.01.T01 | Glycosyltransferase |
| augustus_masked-11-processed-gene-14.9-mRNA-1 | Japonica | NA | | Os11g0145100 | OsR498G1119406200.01.T03 | tRNA-uridine aminocarboxypropyltransferase |
| augustus_masked-11-processed-gene-149.6-mRNA-1 | Japonica | NA | | | | Adenosinetriphosphatase |
| augustus_masked-11-processed-gene-151.6-mRNA-1 | Japonica | Japonica | | Os11g0495950 | OsR498G0612724200.01.T01 | Inactive tetrahydrocannabinolic acid synthase |
| augustus_masked-11-processed-gene-153.19-mRNA-1 | Japonica | NA | | Os11g0500100 | | HAT dimerisation domain-containing protein-like |
| augustus_masked-11-processed-gene-153.30-mRNA-1 | Japonica | NA | | Os11g0501000 | | |
| augustus_masked-11-processed-gene-155.25-mRNA-1 | Japonica | NA | | | | Transposon protein, putative, Pong sub-class |
| augustus_masked-11-processed-gene-156.22-mRNA-1 | Japonica | NA | | | OsR498G0407665800.01.T01 | Retrotransposon protein, putative, unclassified |
| augustus_masked-11-processed-gene-156.4-mRNA-1 | Japonica | NA | | | OsR498G1120205800.01.T01 | GRAS transcription factor |
| augustus_masked-11-processed-gene-159.51-mRNA-1 | Japonica | NA | | Os11g0514100 | OsR498G1120215400.01.T01 | ATP/DNA-binding protein (Fragment) |
| augustus_masked-11-processed-gene-159.56-mRNA-1 | Japonica | Indica | | Os11g0515000 | OsR498G1120218800.01.T01 | Armadillo/beta-catenin-like repeat family protein, expressed |
| augustus_masked-11-processed-gene-167.45-mRNA-1 | Japonica | Indica | | Os07g0501100 | OsR498G1120265000.01.T01 | Chalcone and stilbene synthases, N-terminal domain containing protein |
| augustus_masked-11-processed-gene-169.20-mRNA-1 | Japonica | NA | | Os11g0533100 | OsR498G1120278000.01.T02 | CarD-like transcriptional regulator family protein, expressed |
| augustus_masked-11-processed-gene-169.21-mRNA-1 | Japonica | NA | | Os11g0533400 | OsR498G1120278400.01.T01 | NADH-ubiquinone reductase complex 1 MLRQ subunit |
| augustus_masked-11-processed-gene-17.31-mRNA-1 | Japonica | Indica | | Os10g0326400 | OsR498G1119430200.01.T01 | F-box domain containing protein, expressed |
| augustus_masked-11-processed-gene-17.6-mRNA-1 | Japonica | NA | | Os11g0151300 | OsR498G1119422800.01.T02 | KOW domain-containing protein |
| augustus_masked-11-processed-gene-17.7-mRNA-1 | Japonica | NA | | Os11g0151400 | OsR498G1119425200.01.T01 | Cytochrome P450 family protein, expressed |
| augustus_masked-11-processed-gene-171.47-mRNA-1 | Japonica | Japonica | | Os11g0537800 | OsR498G0407604900.01.T01 | |
| augustus_masked-11-processed-gene-175.4-mRNA-1 | Japonica | NA | | Os11g0543900 | | |
| augustus_masked-11-processed-gene-175.7-mRNA-1 | Japonica | NA | | Os11g0544300 | OsR498G1120313000.01.T03 | PORR domain-containing protein (Fragment) |
| augustus_masked-11-processed-gene-176.62-mRNA-1 | Japonica | NA | | Os11g0546500 | OsR498G1120322800.01.T01 | Glycosyltransferase 6 |
| augustus_masked-11-processed-gene-177.15-mRNA-1 | Japonica | NA | | Os11g0547000 | OsR498G1120324400.01.T01 | Adagio-like protein 3 |
| augustus_masked-11-processed-gene-18.46-mRNA-1 | Japonica | NA | | Os11g0152900 | OsR498G1119432200.01.T01 | |
| augustus_masked-11-processed-gene-18.79-mRNA-1 | Japonica | NA | | Os11g0155100 | | F-box domain containing protein, expressed |
| augustus_masked-11-processed-gene-180.64-mRNA-1 | Japonica | Japonica | | Os11g0552500 | OsR498G1120340000.01.T01 | Agenet-like domain-containing protein |
| augustus_masked-11-processed-gene-181.6-mRNA-1 | Japonica | Indica | | Os10g0445600 | OsR498G1120341200.01.T01 | Rubisco accumulation factor 1, chloroplastic-like |
| augustus_masked-11-processed-gene-181.7-mRNA-1 | Japonica | NA | Kinase-TM | Os11g0553500 | OsR498G1120341600.01.T01 | Wall-associated receptor kinase 3 |
| augustus_masked-11-processed-gene-182.29-mRNA-1 | Japonica | NA | Kinase-TM | Os11g0556400 | | Wall-associated receptor kinase 1 |
| augustus_masked-11-processed-gene-182.53-mRNA-1 | Japonica | NA | | Os11g0557300 | OsR498G1120348600.01.T01 | BSD domain containing protein, expressed |
| augustus_masked-11-processed-gene-182.55-mRNA-1 | Japonica | NA | | Os11g0557300 | OsR498G1120348600.01.T02 | BSD domain containing protein, expressed |
| augustus_masked-11-processed-gene-182.58-mRNA-1 | Japonica | Japonica | LRR-TM | Os11g0558400 | OsR498G1120351600.01.T01 | Leucine Rich Repeat family protein, expressed |
| augustus_masked-11-processed-gene-183.18-mRNA-1 | Japonica | NA | LRR-TM | | | Leucine-rich repeat receptor protein kinase MSL1 |
| augustus_masked-11-processed-gene-183.19-mRNA-1 | Japonica | NA | LRR-NBS-TM | Os11g0560000 | OsR498G1120357400.01.T01 | Disease resistance protein RPM1 |
| augustus_masked-11-processed-gene-183.4-mRNA-1 | Japonica | NA | LRR-TM | Os11g0558900 | OsR498G1120352700.01.T01 | Leucine Rich Repeat family protein, expressed |
| augustus_masked-11-processed-gene-185.13-mRNA-1 | Japonica | NA | | Os08g0564800 | OsR498G0816627600.01.T04 | |
| augustus_masked-11-processed-gene-19.28-mRNA-1 | Japonica | Japonica | | Os11g0155900 | OsR498G0611661000.01.T01 | Histone domain-containing protein (Fragment) |
| augustus_masked-11-processed-gene-192.34-mRNA-1 | Japonica | Japonica | | Os11g0576700 | OsR498G0815097600.01.T01 | |
| augustus_masked-11-processed-gene-193.38-mRNA-1 | Japonica | Indica | | Os11g0578500 | OsR498G1120404900.01.T02 | J domain-containing protein |
| augustus_masked-11-processed-gene-197.22-mRNA-1 | Japonica | NA | | Os06g0272800 | OsR498G0612082400.01.T01 | Leaf senescence protein-like |
| augustus_masked-11-processed-gene-197.24-mRNA-1 | Japonica | NA | | Os11g0587100 | OsR498G1120421800.01.T05 | Pentatricopeptide repeat-containing protein |
| augustus_masked-11-processed-gene-198.19-mRNA-1 | Japonica | NA | | | OsR498G1120424600.01.T01 | Late embryogenesis abundant (LEA) hydroxyproline-rich glycoprotein family |
| augustus_masked-11-processed-gene-2.45-mRNA-1 | Japonica | Japonica | | Os11g0120000 | OsR498G0612633600.01.T01 | Harpin-induced protein 1 containing protein, expressed |
| augustus_masked-11-processed-gene-2.47-mRNA-1 | Japonica | NA | | Os12g0120100 | OsR498G1119335000.01.T01 | Eukaryotic translation initiation factor 2B family protein, putative, expressed |
| augustus_masked-11-processed-gene-2.65-mRNA-1 | Japonica | NA | Kinase | Os11g0121800 | | Serine/threonine-protein kinase At5g01020 |
| augustus_masked-11-processed-gene-20.42-mRNA-1 | Japonica | NA | | Os11g0157300 | OsR498G1119449800.01.T01 | Harpin-induced protein 1 containing protein, expressed |
| augustus_masked-11-processed-gene-200.69-mRNA-1 | Japonica | NA | | Os11g0592650 | | |
| augustus_masked-11-processed-gene-201.35-mRNA-1 | Japonica | NA | | Os11g0592900 | OsR498G1120437600.01.T01 | Thioredoxin |
| augustus_masked-11-processed-gene-201.62-mRNA-1 | Japonica | NA | | Os11g0593600 | | DUF295 domain-containing protein (Fragment) |
| augustus_masked-11-processed-gene-201.63-mRNA-1 | Japonica | NA | | Os11g0593700 | | DUF295 domain-containing protein (Fragment) |
| augustus_masked-11-processed-gene-201.65-mRNA-1 | Japonica | NA | | | OsR498G1120440400.01.T03 | DUF629 domain-containing protein |
| augustus_masked-11-processed-gene-201.67-mRNA-1 | Japonica | NA | | Os11g0594400 | OsR498G1120441400.01.T01 | DUF295 domain-containing protein |
| augustus_masked-11-processed-gene-203.21-mRNA-1 | Japonica | NA | CC-NBS-TM | Os11g0598300 | OsR498G1120448400.01.T01 | Leucine Rich Repeat family protein, expressed |
| augustus_masked-11-processed-gene-204.54-mRNA-1 | Japonica | NA | | Os11g0599200 | OsR498G1120454800.01.T01 | Glycosyltransferase |
| augustus_masked-11-processed-gene-206.12-mRNA-1 | Japonica | NA | | Os11g0600500 | OsR498G1120466600.01.T01 | Putative calcium-binding protein CML45 |
| augustus_masked-11-processed-gene-207.40-mRNA-1 | Japonica | NA | | Os11g0602800 | OsR498G1120475600.01.T01 | F-boxkelch-repeat protein skip11 |
| augustus_masked-11-processed-gene-207.43-mRNA-1 | Japonica | NA | | | OsR498G1120478400.01.T01 | RNA recognition motif domain containing protein |
| augustus_masked-11-processed-gene-208.22-mRNA-1 | Japonica | Japonica | | Os11g0604500 | OsR498G1019153500.01.T01 | |
| augustus_masked-11-processed-gene-21.25-mRNA-1 | Japonica | NA | | Os11g0157900 | | |
| augustus_masked-11-processed-gene-211.2-mRNA-1 | Japonica | NA | LRR | Os11g0609700 | | Disease resistance protein RPM1 |
| augustus_masked-11-processed-gene-211.3-mRNA-1 | Japonica | NA | CC-NBS-TM | Os11g0609700 | OsR498G1120489600.01.T01 | Disease resistance protein RPM1 |

| | | | | | | |
|---|---|---|---|---|---|---|
| augustus_masked-11-processed-gene-216.22-mRNA-1 | Japonica | NA | | | OsR498G1120521200.01.T01 | USP domain-containing protein |
| augustus_masked-11-processed-gene-217.29-mRNA-1 | Japonica | NA | | Os11g0621000 | OsR498G1120525600.01.T03 | C globular stage isoform 1 |
| augustus_masked-11-processed-gene-218.4-mRNA-1 | Japonica | NA | | Os11g0622600 | OsR498G1120530800.01.T01 | BTB/POZ domain containing protein, expressed |
| augustus_masked-11-processed-gene-219.12-mRNA-1 | Japonica | NA | CC-LRR-NBS-TM | Os11g0623800 | OsR498G1120539400.01.T03 | Putative nucleotide-binding leucine-rich-repeat protein 1 |
| augustus_masked-11-processed-gene-219.32-mRNA-1 | Japonica | NA | Kinase-LRR-TM | Os11g0624600 | OsR498G1120543200.01.T03 | GPCR kinase |
| augustus_masked-11-processed-gene-227.3-mRNA-1 | Japonica | NA | | Os11g0637200 | OsR498G1120603000.01.T01 | Polyol transporter 5 |
| augustus_masked-11-processed-gene-228.26-mRNA-1 | Japonica | NA | LRR-NBS-TM | Os11g0639600 | OsR498G1120610200.01.T02 | NB-ARC domain containing protein, expressed |
| augustus_masked-11-processed-gene-228.29-mRNA-1 | Japonica | NA | CC-LRR-NBS-TM | Os11g0640000 | OsR498G1120611600.01.T01 | Leucine Rich Repeat family protein, expressed |
| augustus_masked-11-processed-gene-23.33-mRNA-1 | Japonica | NA | | Os11g0162900 | | |
| augustus_masked-11-processed-gene-23.6-mRNA-1 | Japonica | Japonica | | Os11g0161033 | OsR498G0611509300.01.T02 | Ozone-responsive stress related protein |
| augustus_masked-11-processed-gene-234.34-mRNA-1 | Japonica | NA | | Os11g0649500 | OsR498G1120645800.01.T01 | |
| augustus_masked-11-processed-gene-236.22-mRNA-1 | Japonica | NA | CC-NBS-TM | Os11g0653300 | | NBS-LRR type resistance protein-like |
| augustus_masked-11-processed-gene-237.27-mRNA-1 | Japonica | NA | CC-NBS-TM | Os11g0654800 | OsR498G1120666400.01.T01 | Disease resistance protein RPM1 |
| augustus_masked-11-processed-gene-239.33-mRNA-1 | Japonica | NA | CC-NBS-TM | | OsR498G1120676400.01.T02 | Putative disease resistance RPP8-like protein 2 |
| augustus_masked-11-processed-gene-240.39-mRNA-1 | Japonica | Japonica | | Os11g0659600 | OsR498G1120730000.01.T01 | Pectinesterase |
| augustus_masked-11-processed-gene-240.41-mRNA-1 | Japonica | NA | | Os11g0659700 | | Pectinesterase |
| augustus_masked-11-processed-gene-244.4-mRNA-1 | Japonica | NA | CC-Kinase-TM | Os11g0664000 | OsR498G1120691000.01.T01 | L-type lectin-domain containing receptor kinase IX.1 |
| augustus_masked-11-processed-gene-245.11-mRNA-1 | Japonica | NA | CC-NBS | Os11g0668000 | OsR498G1120693800.01.T01 | Putative NBS-LRR class RGA |
| augustus_masked-11-processed-gene-245.12-mRNA-1 | Japonica | NA | | Os11g0668000 | OsR498G1120693800.01.T01 | Putative NBS-LRR class RGA |
| augustus_masked-11-processed-gene-245.30-mRNA-1 | Japonica | NA | | Os07g0543700 | OsR498G0408675500.01.T01 | Retrotransposon protein, putative, Ty3-gypsy sub-class |
| augustus_masked-11-processed-gene-247.40-mRNA-1 | Japonica | NA | | Os11g0673000 | OsR498G1120707000.01.T03 | Pentatricopeptide repeat |
| augustus_masked-11-processed-gene-25.8-mRNA-1 | Japonica | NA | | | OsR498G1119472200.01.T01 | DNA-binding factor of bZIP class |
| augustus_masked-11-processed-gene-25.9-mRNA-1 | Japonica | Japonica | | Os11g0166201 | OsR498G1220787900.01.T02 | Enoyl-[acyl-carrier-protein] reductase, mitochondrial |
| augustus_masked-11-processed-gene-252.23-mRNA-1 | Japonica | NA | | Os11g0683700 | OsR498G1120730000.01.T01 | Pectinesterase |
| augustus_masked-11-processed-gene-253.37-mRNA-1 | Japonica | NA | NBS-TM | Os11g0686900 | OsR498G1120736800.01.T01 | NBS-LRR disease resistance protein homologue-like |
| augustus_masked-11-processed-gene-259.11-mRNA-1 | Japonica | NA | | | OsR498G0511427100.01.T01 | Epstein-Barr virus EBNA-1-like |
| augustus_masked-11-processed-gene-260.33-mRNA-1 | Japonica | Japonica | | Os11g0701000 | OsR498G1120761400.01.T01 | Xylanase inhibitor protein I |
| augustus_masked-11-processed-gene-261.41-mRNA-1 | Japonica | NA | | Os11g0701900 | OsR498G1120763800.01.T02 | Xylanase inhibitor |
| augustus_masked-11-processed-gene-261.42-mRNA-1 | Japonica | NA | | Os11g0702100 | OsR498G1120764000.01.T01 | Xylanase inhibitor |
| augustus_masked-11-processed-gene-261.43-mRNA-1 | Japonica | NA | | Os11g0702200 | OsR498G1120764200.01.T01 | Xylanase inhibitor protein 1 |
| augustus_masked-11-processed-gene-261.44-mRNA-1 | Japonica | NA | | | | Zinc finger protein ZAT5 |
| augustus_masked-11-processed-gene-261.9-mRNA-1 | Japonica | Japonica | | Os11g0701000 | OsR498G1120761400.01.T01 | Xylanase inhibitor protein I |
| augustus_masked-11-processed-gene-263.100-mRNA-1 | Japonica | NA | | Os11g0707900 | OsR498G1120777600.01.T01 | UPF0503 protein, chloroplastic |
| augustus_masked-11-processed-gene-263.91-mRNA-1 | Japonica | NA | | Os11g0707100 | OsR498G1120775000.01.T01 | Chloroplast ribulose bisphosphate carboxylase/oxygenase activase |
| augustus_masked-11-processed-gene-263.95-mRNA-1 | Japonica | NA | | Os11g0707300 | | Putative transcriptional regulator RABBIT EARS |
| augustus_masked-11-processed-gene-27.55-mRNA-1 | Japonica | NA | | Os01g0647800 | OsR498G0101650800.01.T02 | |
| augustus_masked-11-processed-gene-27.58-mRNA-1 | Japonica | NA | | Os11g0168000 | OsR498G1119478400.01.T01 | Egg cell-secreted protein 1.4 |
| augustus_masked-11-processed-gene-28.24-mRNA-1 | Japonica | NA | | Os11g0169600 | | Hyccin |
| augustus_masked-11-processed-gene-28.25-mRNA-1 | Japonica | NA | | Os11g0169700 | OsR498G1119484000.01.T03 | Aldehyde oxidase glox |
| augustus_masked-11-processed-gene-29.21-mRNA-1 | Japonica | Indica | | Os11g0171400 | OsR498G1119486600.01.T01 | Integrator complex subunit 3 isogeny |
| augustus_masked-11-processed-gene-3.21-mRNA-1 | Japonica | NA | | Os11g0123033 | OsR498G1119343800.01.T03 | Amidase signature enzyme |
| augustus_masked-11-processed-gene-32.50-mRNA-1 | Japonica | NA | | Os11g0175700 | OsR498G1119504600.01.T02 | TCP transcription factor |
| augustus_masked-11-processed-gene-34.21-mRNA-1 | Japonica | Indica | | Os11g0179100 | OsR498G1119516000.01.T01 | Pterocarpan synthase 1 |
| augustus_masked-11-processed-gene-34.5-mRNA-1 | Japonica | NA | | Os11g0178800 | OsR498G1119514600.01.T02 | Dirigent protein 15 |
| augustus_masked-11-processed-gene-34.6-mRNA-1 | Japonica | NA | | Os11g0179000 | OsR498G1119515000.01.T01 | Dirigent protein (Fragment) |
| augustus_masked-11-processed-gene-36.6-mRNA-1 | Japonica | Japonica | | Os11g0182100 | OsR498G1119524400.01.T01 | keratin, type II cytoskeletal 2 epidermal |
| augustus_masked-11-processed-gene-37.45-mRNA-1 | Japonica | NA | | | OsR498G0511427100.01.T01 | Epstein-Barr virus EBNA-1-like |
| augustus_masked-11-processed-gene-40.22-mRNA-1 | Japonica | NA | | Os07g0433200 | OsR498G0510328900.01.T01 | Retrotransposon protein, putative, Ty1-copia subclass |
| augustus_masked-11-processed-gene-40.23-mRNA-1 | Japonica | NA | | Os07g0433200 | OsR498G0510328900.01.T01 | Retrotransposon protein, putative, Ty1-copia subclass |
| augustus_masked-11-processed-gene-45.46-mRNA-1 | Japonica | NA | | Os11g0264900 | | Peptidylprolyl isomerase |
| augustus_masked-11-processed-gene-49.6-mRNA-1 | Japonica | NA | | Os11g0270000 | OsR498G1119787000.01.T01 | |
| augustus_masked-11-processed-gene-52.26-mRNA-1 | Japonica | NA | | Os05g0269800 | OsR498G0409509300.01.T01 | Transposon protein, putative, CACTA, En/Spm sub-class |
| augustus_masked-11-processed-gene-52.5-mRNA-1 | Japonica | NA | | | OsR498G0204046800.01.T01 | Epstein-Barr virus EBNA-1-like |
| augustus_masked-11-processed-gene-54.29-mRNA-1 | Japonica | Japonica | | Os11g0256100 | OsR498G1119726400.01.T03 | Acetohydroxy-acid synthase small subunit |
| augustus_masked-11-processed-gene-54.33-mRNA-1 | Japonica | NA | | | OsR498G1119728000.01.T01 | |
| augustus_masked-11-processed-gene-56.8-mRNA-1 | Japonica | NA | | Os11g0258900 | | |
| augustus_masked-11-processed-gene-57.10-mRNA-1 | Japonica | NA | CC-LRR-NBS-TM | | OsR498G1119693000.01.T02 | Putative nucleotide-binding leucine-rich-repeat protein 1 |
| augustus_masked-11-processed-gene-57.11-mRNA-1 | Japonica | Indica | | Os06g0103200 | OsR498G1119695000.01.T01 | N-hydroxycinnamoyl benzoyltransferase-like protein |
| augustus_masked-11-processed-gene-58.3-mRNA-1 | Japonica | NA | | Os11g0244600 | OsR498G1119697000.01.T01 | Clathrin assembly protein |
| augustus_masked-11-processed-gene-58.5-mRNA-1 | Japonica | NA | | Os11g0244900 | | FAR1 domain-containing protein |
| augustus_masked-11-processed-gene-59.18-mRNA-1 | Japonica | NA | | Os11g0247000 | OsR498G1119707600.01.T01 | |

| | | | | | | |
|---|---|---|---|---|---|---|
| augustus_masked-11-processed-gene-59.4-mRNA-1 | Japonica | NA | | | OsR498G1119701200.01.T01 | UDP-glycosyltransferase-like |
| augustus_masked-11-processed-gene-59.6-mRNA-1 | Japonica | NA | | Os11g0246200 | OsR498G1119702200.01.T01 | F-box/kelch-repeat protein SKIP20 |
| augustus_masked-11-processed-gene-6.97-mRNA-1 | Japonica | NA | | Os11g0127900 | OsR498G1119354800.01.T01 | Small subunit ribosomal protein S16e |
| augustus_masked-11-processed-gene-60.24-mRNA-1 | Japonica | NA | | Os11g0247500 | | FtsJ domain-containing protein |
| augustus_masked-11-processed-gene-63.31-mRNA-1 | Japonica | NA | | Os11g0195700 | OsR498G1119554600.01.T01 | Dihydrolipoamide acetyltransferase component of pyruvate dehydrogenase |
| augustus_masked-11-processed-gene-65.23-mRNA-1 | Japonica | NA | | Os11g0199600 | OsR498G1119568600.01.T01 | Zinc finger CCHC domain-containing protein 10 |
| augustus_masked-11-processed-gene-7.27-mRNA-1 | Japonica | NA | | | | |
| augustus_masked-11-processed-gene-7.31-mRNA-1 | Japonica | NA | | | OsR498G1119367200.01.T01 | Histone-lysine N-methyltransferase H3 lysine-9 specific SUVH3 |
| augustus_masked-11-processed-gene-7.32-mRNA-1 | Japonica | NA | | Os11g0132100 | OsR498G1119369000.01.T01 | PMEI domain-containing protein (Fragment) |
| augustus_masked-11-processed-gene-7.7-mRNA-1 | Japonica | NA | | Os11g0130400 | OsR498G1119364000.01.T01 | Harpin-induced protein 1 containing protein, expressed |
| augustus_masked-11-processed-gene-70.20-mRNA-1 | Japonica | Japonica | | Os11g0207300 | OsR498G1119592200.01.T01 | Ribosome-recycling factor |
| augustus_masked-11-processed-gene-70.21-mRNA-1 | Japonica | NA | NBS-TM | Os11g0207400 | | NB-ARC domain-containing protein |
| augustus_masked-11-processed-gene-71.36-mRNA-1 | Japonica | Indica | Kinase-TM | Os04g0103700 | OsR498G1119598200.01.T01 | Non-specific serine/threonine protein kinase (Fragment) |
| augustus_masked-11-processed-gene-73.23-mRNA-1 | Japonica | NA | | Os11g0210201 | OsR498G1119605600.01.T01 | |
| augustus_masked-11-processed-gene-74.40-mRNA-1 | Japonica | NA | Kinase-LRR-TM | | | Putative LRR receptor-like serine/threonine-protein kinase (Fragment) |
| augustus_masked-11-processed-gene-76.32-mRNA-1 | Japonica | Japonica | | Os11g0215400 | OsR498G1119618400.01.T01 | Eukaryotic aspartyl protease family protein, expressed |
| augustus_masked-11-processed-gene-76.33-mRNA-1 | Japonica | NA | | | OsR498G1119618800.01.T01 | Carboxyl-terminal peptidase, putative, expressed |
| augustus_masked-11-processed-gene-77.16-mRNA-1 | Japonica | NA | | Os11g0216400 | OsR498G1119621400.01.T01 | Multiple organellar RNA editing factor 1 mitochondrial |
| augustus_masked-11-processed-gene-8.106-mRNA-1 | Japonica | NA | Kinase-TM | Os11g0133100 | OsR498G1119372000.01.T01 | Receptor-like serine/threonine-protein kinase |
| augustus_masked-11-processed-gene-8.107-mRNA-1 | Japonica | Japonica | | Os11g0133200 | OsR498G0306499400.01.T02 | PPR domain-containing protein |
| augustus_masked-11-processed-gene-8.108-mRNA-1 | Japonica | NA | Kinase-TM | Os11g0133500 | OsR498G1220884400.01.T01 | Receptor-like serine/threonine-protein kinase |
| augustus_masked-11-processed-gene-8.82-mRNA-1 | Japonica | Indica | | Os11g0132100 | OsR498G1119369400.01.T01 | PMEI domain-containing protein |
| augustus_masked-11-processed-gene-80.5-mRNA-1 | Japonica | NA | Kinase-TM | Os11g0222000 | OsR498G1119637400.01.T01 | Serine/threonine-protein kinase-like protein CCR4 |
| augustus_masked-11-processed-gene-81.41-mRNA-1 | Japonica | NA | | Os11g0224400 | OsR498G1119640600.01.T01 | |
| augustus_masked-11-processed-gene-81.43-mRNA-1 | Japonica | NA | | Os11g0224600 | OsR498G1119641200.01.T01 | |
| augustus_masked-11-processed-gene-81.45-mRNA-1 | Japonica | Japonica | LRR-TM | Os11g0224900 | OsR498G1119642200.01.T01 | NBS-LRR type protein (Fragment) |
| augustus_masked-11-processed-gene-82.26-mRNA-1 | Japonica | Indica | CC-NBS-TM | Os11g0226933 | OsR498G1119644200.01.T01 | NBS-LRR type protein (Fragment) |
| augustus_masked-11-processed-gene-83.18-mRNA-1 | Japonica | NA | CC-LRR-NBS-TM | | | NBS-LRR type protein (Fragment) |
| augustus_masked-11-processed-gene-85.7-mRNA-1 | Japonica | Indica | LRR-TM | Os11g0232100 | OsR498G1119658200.01.T01 | Receptor protein-tyrosine kinase CEPR2 |
| augustus_masked-11-processed-gene-88.41-mRNA-1 | Japonica | NA | | | OsR498G1119676200.01.T01 | Serpin (Serine protease inhibitor) |
| augustus_masked-11-processed-gene-88.46-mRNA-1 | Japonica | Japonica | | Os11g0240000 | OsR498G1119681000.01.T01 | Gibberellin receptor GID1L2 |
| augustus_masked-11-processed-gene-88.49-mRNA-1 | Japonica | NA | | Os11g0240600 | OsR498G1119681000.01.T01 | Gibberellin receptor GID1L2 |
| augustus_masked-12-processed-gene-1.54-mRNA-1 | Japonica | NA | | Os11g0100800 | OsR498G1220784400.01.T01 | |
| augustus_masked-12-processed-gene-1.78-mRNA-1 | Japonica | NA | | Os12g0102300 | OsR498G1220788600.01.T03 | NEDD8-specific protease 1 |
| augustus_masked-12-processed-gene-10.8-mRNA-1 | Japonica | NA | | Os12g0120100 | OsR498G1119335000.01.T01 | Eukaryotic translation initiation factor 2B family protein, putative, expressed |
| augustus_masked-12-processed-gene-100.19-mRNA-1 | Japonica | NA | | | | Retrotransposon protein, putative, Ty1-copia subclass |
| augustus_masked-12-processed-gene-100.23-mRNA-1 | Japonica | NA | CC-NBS-TM | | | NBS-LRR disease resistance protein, putative |
| augustus_masked-12-processed-gene-101.18-mRNA-1 | Japonica | NA | | Os04g0563600 | OsR498G0204553600.01.T01 | |
| augustus_masked-12-processed-gene-102.18-mRNA-1 | Japonica | NA | CC-NBS-TM | Os12g0273000 | OsR498G1221277200.01.T02 | Disease resistance protein RPM1 |
| augustus_masked-12-processed-gene-103.25-mRNA-1 | Japonica | NA | | Os12g0275201 | | |
| augustus_masked-12-processed-gene-104.26-mRNA-1 | Japonica | NA | | | OsR498G1221290600.01.T01 | Polyubiquitin 9 |
| augustus_masked-12-processed-gene-108.24-mRNA-1 | Japonica | NA | NBS-TM PITA | Os12g0281300 | OsR498G1221311800.01.T01 | Blast resistance protein Pi-ta |
| augustus_masked-12-processed-gene-109.7-mRNA-1 | Japonica | Japonica | | Os12g0282000 | OsR498G1221316000.01.T02 | |
| augustus_masked-12-processed-gene-111.17-mRNA-1 | Japonica | NA | | Os12g0286200 | OsR498G1221327800.01.T01 | |
| augustus_masked-12-processed-gene-123.23-mRNA-1 | Japonica | NA | | Os02g0319400 | | GST C-terminal domain-containing protein |
| augustus_masked-12-processed-gene-126.16-mRNA-1 | Japonica | Indica | | Os12g0407400 | OsR498G1221371000.01.T01 | MADS-box domain-containing protein (Fragment) |
| augustus_masked-12-processed-gene-126.33-mRNA-1 | Japonica | NA | | Os12g0407300 | | Mitochondrial protein ymf11 |
| augustus_masked-12-processed-gene-126.35-mRNA-1 | Japonica | Japonica | | Os12g0407400 | OsR498G1221371000.01.T01 | MADS-box domain-containing protein (Fragment) |
| augustus_masked-12-processed-gene-127.3-mRNA-1 | Japonica | NA | | | OsR498G1221373700.01.T01 | |
| augustus_masked-12-processed-gene-14.78-mRNA-1 | Japonica | Indica | | Os11g0132100 | OsR498G1220875000.01.T01 | PMEI domain-containing protein (Fragment) |
| augustus_masked-12-processed-gene-149.15-mRNA-1 | Japonica | NA | | Os12g0435200 | OsR498G1221463200.01.T01 | Carotenoid cleavage dioxygenase |
| augustus_masked-12-processed-gene-15.24-mRNA-1 | Japonica | NA | Kinase-TM | Os12g0130300 | OsR498G1119372000.01.T01 | Receptor-like serine/threonine-protein kinase |
| augustus_masked-12-processed-gene-15.26-mRNA-1 | Japonica | NA | Kinase-TM | Os11g0133100 | OsR498G1119373100.01.T01 | Receptor-like serine/threonine-protein kinase |
| augustus_masked-12-processed-gene-15.29-mRNA-1 | Japonica | NA | Kinase-TM | Os11g0133500 | OsR498G1220884400.01.T01 | Receptor-like serine/threonine-protein kinase |
| augustus_masked-12-processed-gene-152.21-mRNA-1 | Japonica | NA | | Os12g0438900 | OsR498G1221472800.01.T01 | Signal peptidase complex subunit like |
| augustus_masked-12-processed-gene-155.9-mRNA-1 | Japonica | NA | | Os12g0442700 | OsR498G1221505800.01.T01 | |
| augustus_masked-12-processed-gene-161.13-mRNA-1 | Japonica | NA | | | OsR498G1221521800.01.T01 | |
| augustus_masked-12-processed-gene-161.18-mRNA-1 | Japonica | NA | | | OsR498G1221525000.01.T01 | Peptidase A1 domain-containing protein |
| augustus_masked-12-processed-gene-162.11-mRNA-1 | Japonica | Indica | | Os12g0453500 | OsR498G1221528600.01.T01 | |
| augustus_masked-12-processed-gene-178.3-mRNA-1 | Japonica | NA | | Os12g0470700 | OsR498G1221574600.01.T01 | Adenosinetriphosphatase |
| augustus_masked-12-processed-gene-182.6-mRNA-1 | Japonica | NA | | Os03g0126900 | | |

| | | | | | | |
|---|---|---|---|---|---|---|
| augustus_masked-12-processed-gene-189.7-mRNA-1 | Japonica | NA | | | OsR498G0101192000.01.T01 | Zinc knuckle containing protein-like |
| augustus_masked-12-processed-gene-189.9-mRNA-1 | Japonica | NA | | | OsR498G0307015300.01.T01 | Retrotransposon protein, putative, unclassified, expressed |
| augustus_masked-12-processed-gene-19.38-mRNA-1 | Japonica | Japonica | | Os12g0134600 | OsR498G1220896200.01.T01 | Tryptamine hydroxycinnamoyltransferase 2 |
| augustus_masked-12-processed-gene-19.44-mRNA-1 | Japonica | NA | | Os06g0688500 | OsR498G0510924300.01.T03 | |
| augustus_masked-12-processed-gene-191.5-mRNA-1 | Japonica | NA | | | OsR498G1221637400.01.T01 | |
| augustus_masked-12-processed-gene-192.21-mRNA-1 | Japonica | NA | NBS-TM | Os12g0489800 | OsR498G1221649100.01.T01 | NB-ARC domain-containing protein |
| augustus_masked-12-processed-gene-192.6-mRNA-1 | Japonica | NA | | Os12g0488900 | OsR498G1221645800.01.T01 | Pumilio 12 like |
| augustus_masked-12-processed-gene-20.10-mRNA-1 | Japonica | NA | | | OsR498G1220902200.01.T01 | NAC domain-containing protein |
| augustus_masked-12-processed-gene-20.28-mRNA-1 | Japonica | Indica | | Os12g0137600 | OsR498G1220904600.01.T01 | Alpha-galactosidase |
| augustus_masked-12-processed-gene-20.29-mRNA-1 | Japonica | NA | | Os12g0137700 | OsR498G1220904600.01.T02 | Alpha-galactosidase |
| augustus_masked-12-processed-gene-20.32-mRNA-1 | Japonica | NA | | | OsR498G1119398600.01.T01 | Scarecrow-like protein 3 |
| augustus_masked-12-processed-gene-20.33-mRNA-1 | Japonica | NA | | | OsR498G1220906800.01.T01 | GRAS domain-containing protein (Fragment) |
| augustus_masked-12-processed-gene-203.27-mRNA-1 | Japonica | Japonica | | Os12g0506100 | OsR498G1221682400.01.T01 | |
| augustus_masked-12-processed-gene-203.42-mRNA-1 | Japonica | NA | | | OsR498G1221685900.01.T04 | Autophagy 10 variant 1 |
| augustus_masked-12-processed-gene-203.47-mRNA-1 | Japonica | NA | | Os12g0506500 | | Calmodulin-binding protein |
| augustus_masked-12-processed-gene-203.49-mRNA-1 | Japonica | NA | | Os12g0507400 | OsR498G1221689000.01.T01 | |
| augustus_masked-12-processed-gene-204.23-mRNA-1 | Japonica | NA | | | OsR498G0100073600.01.T01 | RNA binding protein |
| augustus_masked-12-processed-gene-205.48-mRNA-1 | Japonica | Indica | | Os12g0510750 | OsR498G1221701800.01.T01 | |
| augustus_masked-12-processed-gene-207.17-mRNA-1 | Japonica | Indica | | Os12g0512700 | OsR498G1221706000.01.T05 | Pleiotropic drug resistance protein 3 |
| augustus_masked-12-processed-gene-21.40-mRNA-1 | Japonica | NA | | | OsR498G1220912200.01.T01 | |
| augustus_masked-12-processed-gene-21.42-mRNA-1 | Japonica | NA | | Os12g0140700 | OsR498G1220912600.01.T01 | RING finger protein 44 |
| augustus_masked-12-processed-gene-23.34-mRNA-1 | Japonica | NA | | Os12g0142600 | OsR498G1220919200.01.T02 | tRNA-uridine aminocarboxypropyltransferase |
| augustus_masked-12-processed-gene-23.36-mRNA-1 | Japonica | Indica | | Os02g0711400 | OsR498G1220919400.01.T01 | RING-type domain-containing protein |
| augustus_masked-12-processed-gene-23.54-mRNA-1 | Japonica | NA | | Os12g0143300 | OsR498G1220920800.01.T05 | Excinuclease ABC C subunit N-terminal |
| augustus_masked-12-processed-gene-230.38-mRNA-1 | Japonica | NA | | Os12g0557800 | OsR498G1221868800.01.T03 | Putative pentatricopeptide repeat-containing protein, mitochondrial |
| augustus_masked-12-processed-gene-233.33-mRNA-1 | Japonica | Indica | | Os10g0122000 | OsR498G1221881400.01.T01 | Glycosyltransferase |
| augustus_masked-12-processed-gene-238.53-mRNA-1 | Japonica | NA | | Os12g0571300 | OsR498G1221911600.01.T01 | Microneme/rhoptry antigen |
| augustus_masked-12-processed-gene-240.15-mRNA-1 | Japonica | NA | Kinase-TM | | OsR498G1221922000.01.T01 | Mitogen-activated protein kinase kinase kinase |
| augustus_masked-12-processed-gene-240.16-mRNA-1 | Japonica | Japonica | | Os12g0574800 | OsR498G1221924200.01.T03 | Transcription initiation factor TFIID subunit 8 |
| augustus_masked-12-processed-gene-240.4-mRNA-1 | Japonica | NA | | | OsR498G1221920600.01.T01 | rRNA N-glycosidase |
| augustus_masked-12-processed-gene-241.20-mRNA-1 | Japonica | NA | | Os10g0186900 | OsR498G0101463500.01.T01 | FBD domain-containing protein |
| augustus_masked-12-processed-gene-250.25-mRNA-1 | Japonica | NA | | Os12g0590400 | OsR498G1222005000.01.T01 | DUF1618 domain-containing protein (Fragment) |
| augustus_masked-12-processed-gene-253.75-mRNA-1 | Japonica | NA | | | OsR498G1222033000.01.T01 | Ring finger protein, putative |
| augustus_masked-12-processed-gene-255.40-mRNA-1 | Japonica | NA | | | OsR498G1222046400.01.T01 | Pectinesterase inhibitor domain containing protein, expressed |
| augustus_masked-12-processed-gene-259.67-mRNA-1 | Japonica | NA | | Os12g0606000 | | Gnk2-homologous domain-containing protein (Fragment) |
| augustus_masked-12-processed-gene-26.21-mRNA-1 | Japonica | NA | | Os12g0149300 | OsR498G1220943800.01.T01 | Putative xyloglucan 6-xylosyltransferase |
| augustus_masked-12-processed-gene-26.24-mRNA-1 | Japonica | NA | | Os12g0150100 | OsR498G1220945800.01.T01 | KOW domain-containing protein |
| augustus_masked-12-processed-gene-26.25-mRNA-1 | Japonica | NA | | Os12g0150200 | OsR498G1220946600.01.T01 | Cytochrome P450 family protein, expressed |
| augustus_masked-12-processed-gene-260.34-mRNA-1 | Japonica | NA | | Os12g0166700 | OsR498G1120733700.01.T01 | Retrotransposon protein, putative, Ty3-gypsy sub-class |
| augustus_masked-12-processed-gene-260.37-mRNA-1 | Japonica | NA | Kinase-TM | Os12g0608900 | OsR498G1222080600.01.T01 | Lectin protein kinase, putative, expressed |
| augustus_masked-12-processed-gene-260.42-mRNA-1 | Japonica | NA | | Os12g0609600 | OsR498G1222082800.01.T01 | Small auxin up regulated protein |
| augustus_masked-12-processed-gene-261.35-mRNA-1 | Japonica | Japonica | | Os12g0609900 | OsR498G1222083400.01.T01 | F-box domain containing protein expressed |
| augustus_masked-12-processed-gene-261.37-mRNA-1 | Japonica | NA | | Os12g0610300 | | Formin-like protein 5 |
| augustus_masked-12-processed-gene-264.61-mRNA-1 | Japonica | NA | | Os12g0615800 | OsR498G1222106200.01.T03 | Septin and tuftelin-interacting protein 1 |
| augustus_masked-12-processed-gene-265.12-mRNA-1 | Japonica | NA | | Os12g0616800 | OsR498G1222111000.01.T01 | 101 kDa malaria antigen-like |
| augustus_masked-12-processed-gene-265.14-mRNA-1 | Japonica | NA | | Os12g0617001 | | C2H2-type zinc-finger transcription factor (Fragment) |
| augustus_masked-12-processed-gene-27.26-mRNA-1 | Japonica | Japonica | | Os12g0150750 | OsR498G1220948000.01.T01 | Purple acid phosphatase |
| augustus_masked-12-processed-gene-27.29-mRNA-1 | Japonica | NA | | Os12g0152200 | | DUF295 domain-containing protein |
| augustus_masked-12-processed-gene-272.42-mRNA-1 | Japonica | Japonica | | Os12g0628600 | OsR498G1222150400.01.T01 | Thaumatin-like protein (Fragment) |
| augustus_masked-12-processed-gene-3.8-mRNA-1 | Japonica | NA | | Os12g0104900 | OsR498G1220796000.01.T01 | Probable calcium-binding protein CML31 |
| augustus_masked-12-processed-gene-34.46-mRNA-1 | Japonica | NA | | Os12g0163500 | OsR498G1221003600.01.T02 | GTgamma-3 |
| augustus_masked-12-processed-gene-34.47-mRNA-1 | Japonica | NA | | Os12g0163600 | OsR498G1221004000.01.T01 | Pentatricopeptide repeat |
| augustus_masked-12-processed-gene-36.32-mRNA-1 | Japonica | NA | | Os04g0429450 | OsR498G0408675500.01.T01 | Retrotransposon protein, putative, Ty3-gypsy sub-class |
| augustus_masked-12-processed-gene-42.49-mRNA-1 | Japonica | NA | | | OsR498G1221045400.01.T01 | |
| augustus_masked-12-processed-gene-44.69-mRNA-1 | Japonica | NA | | | OsR498G1221053400.01.T01 | |
| augustus_masked-12-processed-gene-50.21-mRNA-1 | Japonica | NA | CC-NBS-TM | | OsR498G1221076200.01.T01 | Disease resistance protein RPM1 |
| augustus_masked-12-processed-gene-51.11-mRNA-1 | Japonica | Japonica | | Os12g0195100 | OsR498G1221047500.01.T01 | Amino acid permease 2 |
| augustus_masked-12-processed-gene-51.5-mRNA-1 | Japonica | NA | | Os12g0194800 | | tRNA pseudouridine synthase |
| augustus_masked-12-processed-gene-51.9-mRNA-1 | Japonica | NA | | Os07g0561900 | OsR498G0100572500.01.T01 | Thiamine diphosphokinase |
| augustus_masked-12-processed-gene-57.32-mRNA-1 | Japonica | Japonica | NBS-TM | Os12g0205500 | OsR498G1221100000.01.T02 | NB-ARC domain containing protein, expressed |
| augustus_masked-12-processed-gene-59.18-mRNA-1 | Japonica | NA | CC-NBS-TM | | | Disease resistance protein RGA2 |

| | | | | | | |
|---|---|---|---|---|---|---|
| augustus_masked-12-processed-gene-60.19-mRNA-1 | Japonica | Japonica | LRR-TM | Os12g0211500 | OsR498G1221115800.01.T01 | Leucine Rich Repeat family protein, expressed |
| augustus_masked-12-processed-gene-60.2-mRNA-1 | Japonica | NA | | Os12g0210500 | OsR498G1221110600.01.T01 | |
| augustus_masked-12-processed-gene-69.26-mRNA-1 | Japonica | NA | | | OsR498G0100073600.01.T01 | RNA binding protein |
| augustus_masked-12-processed-gene-7.58-mRNA-1 | Japonica | NA | | | OsR498G1220822400.01.T01 | Protein kinase domain-containing protein |
| augustus_masked-12-processed-gene-7.59-mRNA-1 | Japonica | NA | Kinase-TM | Os12g0113500 | OsR498G1220822800.01.T01 | Non-specific serine/threonine protein kinase |
| augustus_masked-12-processed-gene-7.62-mRNA-1 | Japonica | NA | | | OsR498G1119316400.01.T01 | Laminin subunit alpha-5 (Fragment) |
| augustus_masked-12-processed-gene-70.36-mRNA-1 | Japonica | NA | | Os12g0225300 | | FAR1 domain-containing protein |
| augustus_masked-12-processed-gene-73.22-mRNA-1 | Japonica | NA | | Os12g0230133 | OsR498G1221156200.01.T01 | UVR domain-containing protein |
| augustus_masked-12-processed-gene-75.47-mRNA-1 | Japonica | NA | | Os12g0233200 | OsR498G1221165600.01.T05 | Selenium-binding protein-like protein |
| augustus_masked-12-processed-gene-78.39-mRNA-1 | Japonica | Indica | | Os09g0552200 | OsR498G1221184800.01.T01 | |
| augustus_masked-12-processed-gene-8.23-mRNA-1 | Japonica | NA | | Os11g0116500 | OsR498G1119324200.01.T02 | |
| augustus_masked-12-processed-gene-80.15-mRNA-1 | Japonica | NA | | | OsR498G1221193800.01.T01 | |
| augustus_masked-12-processed-gene-81.3-mRNA-1 | Japonica | NA | | Os12g0242100 | OsR498G1221196400.01.T01 | |
| augustus_masked-12-processed-gene-9.51-mRNA-1 | Japonica | NA | | Os12g0117400 | OsR498G1220835700.01.T01 | Root phototropism protein 2 |
| augustus_masked-12-processed-gene-96.38-mRNA-1 | Japonica | NA | | | OsR498G1221247400.01.T01 | |
| augustus_masked-2-processed-gene-120.57-mRNA-1 | Japonica | Indica | | Os08g0543050 | OsR498G0203525000.01.T01 | ADP-ribosyl cyclase/cyclic ADP-ribose hydrolase |
| augustus_masked-2-processed-gene-128.31-mRNA-1 | Japonica | NA | | Os02g0321000 | OsR498G0203567200.01.T02 | Fertility restorer |
| augustus_masked-2-processed-gene-155.20-mRNA-1 | Japonica | NA | | | OsR498G0100463800.01.T01 | HGWP repeat containing protein-like |
| augustus_masked-2-processed-gene-162.2-mRNA-1 | Japonica | NA | | | OsR498G0203777900.01.T01 | |
| augustus_masked-2-processed-gene-184.28-mRNA-1 | Japonica | NA | | | OsR498G0203894800.01.T01 | |
| augustus_masked-2-processed-gene-186.16-mRNA-1 | Japonica | NA | | | OsR498G1120154400.01.T01 | |
| augustus_masked-2-processed-gene-195.15-mRNA-1 | Japonica | NA | | Os02g0523800 | OsR498G0203962500.01.T02 | Inositol polyphosphate multikinase |
| augustus_masked-2-processed-gene-220.18-mRNA-1 | Japonica | NA | | | OsR498G0204142300.01.T01 | Hepatoma-derived growth factor-related protein 2-like |
| augustus_masked-2-processed-gene-258.41-mRNA-1 | Japonica | Indica | | Os01g0721800 | OsR498G0204358400.01.T01 | Fusarium resistance protein I2C-5-like |
| augustus_masked-2-processed-gene-307.71-mRNA-1 | Japonica | NA | | Os02g0721200 | | Ubiquitin-conjugating enzyme E2 S |
| augustus_masked-2-processed-gene-367.74-mRNA-1 | Japonica | NA | | Os02g0780200 | OsR498G0204877800.01.T01 | Subtilisin-like protease |
| augustus_masked-2-processed-gene-376.26-mRNA-1 | Japonica | NA | | Os02g0798450 | OsR498G0204942600.01.T01 | |
| augustus_masked-2-processed-gene-393.133-mRNA-1 | Japonica | Indica | | Os02g0832700 | OsR498G0205054700.01.T03 | Metal tolerance protein 5 |
| augustus_masked-2-processed-gene-54.4-mRNA-1 | Japonica | Indica | | Os02g0194900 | OsR498G0203147100.01.T01 | Transcription factor TGA1 |
| augustus_masked-2-processed-gene-66.43-mRNA-1 | Japonica | NA | | | OsR498G1221141800.01.T01 | Retrotransposon protein, putative, Ty3-gypsy sub-class |
| augustus_masked-2-processed-gene-78.49-mRNA-1 | Japonica | NA | | Os02g0235600 | OsR498G0203297700.01.T02 | Ribosomal L5P family protein isoform 1 |
| augustus_masked-2-processed-gene-87.31-mRNA-1 | Japonica | NA | | Os02g0254550 | | BED-type domain-containing protein |
| augustus_masked-2-processed-gene-88.59-mRNA-1 | Japonica | NA | | Os02g0255000 | OsR498G0203347800.01.T01 | Cyclic nucleotide-gated ion channel 1 |
| augustus_masked-3-processed-gene-1.13-mRNA-1 | Japonica | NA | | Os03g0106900 | OsR498G0305087100.01.T01 | Group I pollen allergen |
| augustus_masked-3-processed-gene-1.25-mRNA-1 | Japonica | NA | | Os03g0106800 | OsR498G0305087800.01.T01 | Group I pollen allergen |
| augustus_masked-3-processed-gene-100.35-mRNA-1 | Japonica | NA | | | | |
| augustus_masked-3-processed-gene-100.44-mRNA-1 | Japonica | NA | | Os03g0294500 | | Trihelix transcription factor ASIL2 |
| augustus_masked-3-processed-gene-100.58-mRNA-1 | Japonica | Indica | | Os03g0295300 | OsR498G0305748800.01.T01 | |
| augustus_masked-3-processed-gene-101.64-mRNA-1 | Japonica | NA | | | OsR498G0305753100.01.T01 | (1->3)-beta-glucan endohydrolase |
| augustus_masked-3-processed-gene-101.65-mRNA-1 | Japonica | NA | | Os03g0296600 | OsR498G0305753300.01.T01 | Egg cell-secreted protein 1.1 |
| augustus_masked-3-processed-gene-101.81-mRNA-1 | Japonica | NA | | Os03g0295800 | OsR498G0305751000.01.T04 | Gamma interferon inducible lysosomal thiol reductase GILT |
| augustus_masked-3-processed-gene-102.36-mRNA-1 | Japonica | NA | | Os03g0297600 | OsR498G0305756400.01.T01 | Abscisic acid receptor 7 |
| augustus_masked-3-processed-gene-103.14-mRNA-1 | Japonica | Indica | | Os07g0690900 | OsR498G0305770700.01.T01 | COBRA-like protein 7 |
| augustus_masked-3-processed-gene-103.2-mRNA-1 | Japonica | NA | | Os03g0300100 | | |
| augustus_masked-3-processed-gene-103.23-mRNA-1 | Japonica | NA | | Os03g0300500 | OsR498G0305767200.01.T01 | Pectinesterase |
| augustus_masked-3-processed-gene-103.25-mRNA-1 | Japonica | NA | | Os03g0300700 | | |
| augustus_masked-3-processed-gene-103.6-mRNA-1 | Japonica | NA | | Os03g0299800 | OsR498G0305764800.01.T01 | Protein PLANT CADMIUM RESISTANCE 7 |
| augustus_masked-3-processed-gene-103.7-mRNA-1 | Japonica | NA | | | OsR498G0714024600.01.T01 | |
| augustus_masked-3-processed-gene-103.8-mRNA-1 | Japonica | Indica | | Os03g0300000 | OsR498G0305766000.01.T01 | Xyloglucan xylosyltransferase |
| augustus_masked-3-processed-gene-104.16-mRNA-1 | Japonica | NA | | Os03g0302800 | OsR498G0305774900.01.T01 | Beta-fructofuranosidase, insoluble isoenzyme CWINV6 (Fragment) |
| augustus_masked-3-processed-gene-104.18-mRNA-1 | Japonica | NA | | Os03g0303100 | OsR498G0305776300.01.T01 | Calmodulin-binding protein-like |
| augustus_masked-3-processed-gene-104.2-mRNA-1 | Japonica | NA | | Os03g0301950 | OsR498G0305773500.01.T01 | Peroxisomal membrane protein like |
| augustus_masked-3-processed-gene-104.3-mRNA-1 | Japonica | NA | | Os03g0302000 | OsR498G0305773700.01.T02 | Peroxisomal membrane protein like |
| augustus_masked-3-processed-gene-106.38-mRNA-1 | Japonica | NA | | | OsR498G0305786100.01.T01 | Argininosuccinate lyase |
| augustus_masked-3-processed-gene-106.47-mRNA-1 | Japonica | NA | | Os03g0305200 | | Multidrug resistance protein ABC transporter family protein |
| augustus_masked-3-processed-gene-106.55-mRNA-1 | Japonica | Japonica | | Os03g0306100 | OsR498G0305787900.01.T01 | Xyloglucan 6-xylosyltransferase 2 |
| augustus_masked-3-processed-gene-106.57-mRNA-1 | Japonica | Japonica | | Os03g0306800 | OsR498G0100750200.01.T01 | CP12 domain-containing protein (Fragment) |
| augustus_masked-3-processed-gene-106.65-mRNA-1 | Japonica | Japonica | | Os03g0305800 | OsR498G0305787900.01.T01 | Xyloglucan 6-xylosyltransferase 2 |
| augustus_masked-3-processed-gene-107.1-mRNA-1 | Japonica | NA | | Os03g0307200 | OsR498G0305791800.01.T01 | Nicotianamine synthase |
| augustus_masked-3-processed-gene-107.11-mRNA-1 | Japonica | NA | | Os03g0307900 | | |
| augustus_masked-3-processed-gene-107.12-mRNA-1 | Japonica | NA | | Os03g0308000 | OsR498G0305793900.01.T03 | Ubiquitin-conjugating enzyme like |

| | | | | | | |
|---|---|---|---|---|---|---|
| augustus_masked-3-processed-gene-108.42-mRNA-1 | Japonica | NA | | Os03g0309800 | OsR498G0305799900.01.T04 | Pentatricopeptide repeat-containing protein, chloroplastic |
| augustus_masked-3-processed-gene-109.2-mRNA-1 | Japonica | NA | | Os03g0311600 | OsR498G0305805500.01.T01 | Transcription factor bHLH148 |
| augustus_masked-3-processed-gene-110.41-mRNA-1 | Japonica | NA | | Os03g0314400 | OsR498G0305815500.01.T05 | Pentatricopeptide repeat-containing protein mitochondrial |
| augustus_masked-3-processed-gene-111.43-mRNA-1 | Japonica | NA | | | OsR498G0100075100.01.T01 | Epstein-Barr virus EBNA-1-like |
| augustus_masked-3-processed-gene-112.0-mRNA-1 | Japonica | NA | | Os03g0317100 | OsR498G0305825700.01.T04 | Pentatricopeptide repeat-containing protein, mitochondrial |
| augustus_masked-3-processed-gene-112.1-mRNA-1 | Japonica | NA | | Os03g0317300 | OsR498G0305826500.01.T01 | Peptidase A1 domain-containing protein (Fragment) |
| augustus_masked-3-processed-gene-112.15-mRNA-1 | Japonica | Japonica | | Os03g0318400 | OsR498G0305828500.01.T01 | Peptidase A1 domain-containing protein (Fragment) |
| augustus_masked-3-processed-gene-112.16-mRNA-1 | Japonica | NA | | | OsR498G0305830700.01.T01 | VQ motif family protein expressed |
| augustus_masked-3-processed-gene-113.14-mRNA-1 | Japonica | NA | | Os03g0320900 | OsR498G0305839500.01.T03 | Guanylate kinase |
| augustus_masked-3-processed-gene-113.22-mRNA-1 | Japonica | NA | | Os03g0320500 | OsR498G0305836200.01.T01 | VQ domain-containing protein |
| augustus_masked-3-processed-gene-114.35-mRNA-1 | Japonica | NA | | Os03g0321300 | OsR498G0305840900.01.T01 | F-box domain containing protein, expressed |
| augustus_masked-3-processed-gene-114.60-mRNA-1 | Japonica | NA | | Os03g0321800 | OsR498G0305845000.01.T04 | Lipoyl(octanoyl) transferase |
| augustus_masked-3-processed-gene-118.15-mRNA-1 | Japonica | NA | | Os03g0103300 | OsR498G0305077300.01.T01 | AAI domain-containing protein (Fragment) |
| augustus_masked-3-processed-gene-119.28-mRNA-1 | Japonica | NA | | Os10g0557900 | OsR498G1019184200.01.T01 | Metalloendoproteinase |
| augustus_masked-3-processed-gene-119.31-mRNA-1 | Japonica | NA | | Os10g0557900 | OsR498G1019184200.01.T01 | ZnMc domain-containing protein (Fragment) |
| augustus_masked-3-processed-gene-119.9-mRNA-1 | Japonica | Japonica | | Os03g0104400 | OsR498G0305082800.01.T01 | PB1 domain-containing protein |
| augustus_masked-3-processed-gene-12.55-mRNA-1 | Japonica | NA | Kinase-TM | Os03g0124200 | OsR498G0305151700.01.T01 | Receptor-like protein kinase HERK 1 isoform A |
| augustus_masked-3-processed-gene-12.56-mRNA-1 | Japonica | Indica | | Os03g0124300 | OsR498G0305151900.01.T04 | Malectin-like carbohydrate-binding domain |
| augustus_masked-3-processed-gene-121.47-mRNA-1 | Japonica | NA | | Os10g0560700 | OsR498G1019194600.01.T01 | Ethylene-responsive transcription factor TINY-like |
| augustus_masked-3-processed-gene-125.65-mRNA-1 | Japonica | NA | | Os04g0559000 | OsR498G1019224100.01.T01 | Subtilisin-like protease |
| augustus_masked-3-processed-gene-125.67-mRNA-1 | Japonica | NA | | Os10g0568900 | OsR498G1019225500.01.T04 | Haloacid dehalogenase-like hydrolase domain-containing protein Sgpp |
| augustus_masked-3-processed-gene-125.81-mRNA-1 | Japonica | NA | | Os10g0568000 | OsR498G1019222800.01.T01 | |
| augustus_masked-3-processed-gene-125.82-mRNA-1 | Japonica | NA | | | OsR498G1019222800.01.T01 | F-box domain containing protein, expressed |
| augustus_masked-3-processed-gene-125.83-mRNA-1 | Japonica | NA | | Os10g0568100 | OsR498G1019223000.01.T04 | VAN3-binding protein-like |
| augustus_masked-3-processed-gene-126.14-mRNA-1 | Japonica | NA | | Os11g0145100 | OsR498G0917727700.01.T01 | tRNA-uridine aminocarboxypropyltransferase |
| augustus_masked-3-processed-gene-126.20-mRNA-1 | Japonica | NA | | Os10g0570700 | OsR498G1019231300.01.T01 | rRNA N-glycosidase |
| augustus_masked-3-processed-gene-127.0-mRNA-1 | Japonica | NA | | Os10g0571600 | OsR498G1019234100.01.T01 | NAC domain-containing protein 86 |
| augustus_masked-3-processed-gene-127.23-mRNA-1 | Japonica | NA | | Os10g0573200 | | RPM1 interacting protein 13 |
| augustus_masked-3-processed-gene-127.9-mRNA-1 | Japonica | NA | | | OsR498G0306492600.01.T01 | protein FAR-RED IMPAIRED RESPONSE 1-like |
| augustus_masked-3-processed-gene-128.15-mRNA-1 | Japonica | NA | | Os10g0573900 | OsR498G1019239600.01.T06 | 60S ribosomal export protein NMD3 |
| augustus_masked-3-processed-gene-128.23-mRNA-1 | Japonica | NA | | Os10g0574400 | | E3 ubiquitin-protein ligase ATL6 |
| augustus_masked-3-processed-gene-128.26-mRNA-1 | Japonica | NA | | Os10g0575000 | OsR498G1019243900.01.T01 | Transcription factor like |
| augustus_masked-3-processed-gene-129.20-mRNA-1 | Japonica | NA | | Os10g0576100 | OsR498G1019247600.01.T01 | Inositol-tetrakisphosphate 1-kinase |
| augustus_masked-3-processed-gene-130.1-mRNA-1 | Japonica | NA | | | OsR498G1019250500.01.T01 | Putative WEB family protein chloroplastic |
| augustus_masked-3-processed-gene-130.5-mRNA-1 | Japonica | NA | | Os10g0577200 | OsR498G1019250800.01.T02 | VQ domain-containing protein |
| augustus_masked-3-processed-gene-131.1-mRNA-1 | Japonica | NA | | Os10g0578600 | OsR498G1019255200.01.T01 | Amelogenin, X like |
| augustus_masked-3-processed-gene-134.34-mRNA-1 | Japonica | NA | | Os10g0530700 | OsR498G1019083900.01.T01 | Glutathione transferase |
| augustus_masked-3-processed-gene-134.35-mRNA-1 | Japonica | NA | | Os10g0530900 | OsR498G1019083900.01.T01 | Glutathione S-transferase GSTU6 |
| augustus_masked-3-processed-gene-134.36-mRNA-1 | Japonica | NA | | Os10g0531400 | OsR498G1019074700.01.T01 | Glutathione transferase |
| augustus_masked-3-processed-gene-134.38-mRNA-1 | Japonica | NA | | | OsR498G1019086300.01.T01 | Kinesin heavy chain-like |
| augustus_masked-3-processed-gene-134.57-mRNA-1 | Japonica | NA | CC-Kinase-LRR-TM | Os10g0531700 | OsR498G1019085800.01.T01 | LRR receptor-like serine/threonine-protein kinase |
| augustus_masked-3-processed-gene-135.10-mRNA-1 | Japonica | NA | | Os10g0533200 | OsR498G1019092300.01.T01 | Mu-crystallin |
| augustus_masked-3-processed-gene-135.13-mRNA-1 | Japonica | NA | Kinase-TM | Os10g0533800 | OsR498G1019094500.01.T01 | L-type lectin-domain containing receptor kinase IV.1 |
| augustus_masked-3-processed-gene-135.20-mRNA-1 | Japonica | NA | | Os10g0532300 | OsR498G1019088600.01.T01 | HMA domain-containing protein |
| augustus_masked-3-processed-gene-135.22-mRNA-1 | Japonica | NA | | Os10g0533100 | OsR498G1019091800.01.T01 | ABC-type Co2+ transport system, permease component |
| augustus_masked-3-processed-gene-135.8-mRNA-1 | Japonica | NA | | Os10g0532600 | OsR498G1019090300.01.T01 | Transcription repressor |
| augustus_masked-3-processed-gene-136.7-mRNA-1 | Japonica | NA | Kinase-TM | Os10g0534500 | OsR498G1019095800.01.T03 | Receptor-like protein kinase THESEUS 1 |
| augustus_masked-3-processed-gene-137.12-mRNA-1 | Japonica | NA | | Os10g0537800 | OsR498G1019107900.01.T01 | Putative chloroplast nucleoid DNA binding protein |
| augustus_masked-3-processed-gene-137.26-mRNA-1 | Japonica | NA | | | OsR498G0204768400.01.T01 | Epstein-Barr virus EBNA-1-like |
| augustus_masked-3-processed-gene-138.13-mRNA-1 | Japonica | NA | | Os10g0538200 | OsR498G1019109300.01.T01 | Putative chloroplast nucleoid DNA binding protein |
| augustus_masked-3-processed-gene-138.23-mRNA-1 | Japonica | NA | | Os10g0538200 | OsR498G1019109700.01.T01 | Putative chloroplast nucleoid DNA binding protein |
| augustus_masked-3-processed-gene-138.24-mRNA-1 | Japonica | NA | | Os10g0538200 | OsR498G1019110300.01.T01 | Putative chloroplast nucleoid DNA binding protein |
| augustus_masked-3-processed-gene-138.28-mRNA-1 | Japonica | NA | | Os10g0539700 | OsR498G1019112900.01.T01 | C2 NT-type domain-containing protein (Fragment) |
| augustus_masked-3-processed-gene-138.38-mRNA-1 | Japonica | NA | | | OsR498G1019111000.01.T01 | N-acetylneuraminate 7-O(Or 9-O)-acetyltransferase |
| augustus_masked-3-processed-gene-138.39-mRNA-1 | Japonica | NA | | Os10g0539500 | OsR498G1019111200.01.T01 | Multifunctional fusion protein |
| augustus_masked-3-processed-gene-138.42-mRNA-1 | Japonica | NA | | Os10g0540100 | OsR498G1019115200.01.T01 | Pentatricopeptide repeat |
| augustus_masked-3-processed-gene-138.6-mRNA-1 | Japonica | NA | | Os10g0538200 | OsR498G1019107900.01.T01 | Putative chloroplast nucleoid DNA binding protein |
| augustus_masked-3-processed-gene-138.8-mRNA-1 | Japonica | NA | | Os10g0538200 | OsR498G1019108300.01.T01 | Putative chloroplast nucleoid DNA binding protein |
| augustus_masked-3-processed-gene-139.10-mRNA-1 | Japonica | NA | | Os07g0543700 | OsR498G0408675500.01.T01 | Retrotransposon protein, putative, Ty3-gypsy sub-class |
| augustus_masked-3-processed-gene-139.11-mRNA-1 | Japonica | NA | | | OsR498G1019116800.01.T01 | |
| augustus_masked-3-processed-gene-139.14-mRNA-1 | Japonica | NA | | Os10g0540900 | OsR498G1019118300.01.T03 | |

| | | | | | | |
|---|---|---|---|---|---|---|
| augustus_masked-3-processed-gene-139.3-mRNA-1 | Japonica | NA | | Os10g0540800 | OsR498G1019117300.01.T01 | |
| augustus_masked-3-processed-gene-14.40-mRNA-1 | Japonica | NA | | | OsR498G0305165100.01.T01 | Monosaccharide-sensing protein 3 |
| augustus_masked-3-processed-gene-14.50-mRNA-1 | Japonica | NA | | Os03g0129300 | OsR498G0305166900.01.T02 | Glyceraldehyde-3-phosphate dehydrogenase B, chloroplastic |
| augustus_masked-3-processed-gene-140.42-mRNA-1 | Japonica | NA | | Os10g0542900 | OsR498G1019125300.01.T01 | Class II chitinase |
| augustus_masked-3-processed-gene-140.55-mRNA-1 | Japonica | NA | | Os10g0543150 | | |
| augustus_masked-3-processed-gene-142.52-mRNA-1 | Japonica | NA | | Os10g0548400 | | Mediator of RNA polymerase II transcription subunit like |
| augustus_masked-3-processed-gene-142.63-mRNA-1 | Japonica | NA | | Os10g0548100 | OsR498G1019140200.01.T01 | 3-oxoacyl-[acyl-carrier-protein] synthase III, chloroplastic |
| augustus_masked-3-processed-gene-142.65-mRNA-1 | Japonica | NA | CC-Kinase-TM | Os10g0548300 | OsR498G1019140600.01.T06 | E3 ubiquitin ligase |
| augustus_masked-3-processed-gene-143.4-mRNA-1 | Japonica | NA | | Os10g0548800 | OsR498G1019141200.01.T01 | Translocase of chloroplast 120 chloroplastic |
| augustus_masked-3-processed-gene-144.14-mRNA-1 | Japonica | NA | | Os10g0550900 | OsR498G1019148500.01.T01 | Proline dehydrogenase |
| augustus_masked-3-processed-gene-144.30-mRNA-1 | Japonica | NA | | Os10g0551200 | OsR498G1019149600.01.T01 | GRAS transcription factor |
| augustus_masked-3-processed-gene-145.2-mRNA-1 | Japonica | NA | | Os10g0551900 | OsR498G1019153400.01.T01 | Cortical cell-delineating protein-like |
| augustus_masked-3-processed-gene-145.5-mRNA-1 | Japonica | NA | | Os10g0552300 | OsR498G1019154800.01.T01 | Cortical cell delineating protein, putative, expressed |
| augustus_masked-3-processed-gene-145.7-mRNA-1 | Japonica | NA | | Os10g0552600 | OsR498G1019156200.01.T01 | Cortical cell-delineating protein-like |
| augustus_masked-3-processed-gene-146.27-mRNA-1 | Japonica | NA | | Os10g0554800 | OsR498G1019164500.01.T01 | AAI domain-containing protein (Fragment) |
| augustus_masked-3-processed-gene-146.34-mRNA-1 | Japonica | NA | | Os10g0554900 | OsR498G1019164800.01.T02 | QWRF motif-containing protein 7 |
| augustus_masked-3-processed-gene-148.37-mRNA-1 | Japonica | NA | | Os03g0324700 | OsR498G0305854900.01.T01 | Arabinosyltransferase ARAD1 |
| augustus_masked-3-processed-gene-148.50-mRNA-1 | Japonica | NA | CC-LRR-NBS-TM | Os03g0324600 | OsR498G0305854800.01.T01 | NB-ARC domain containing protein, expressed |
| augustus_masked-3-processed-gene-149.0-mRNA-1 | Japonica | NA | | Os03g0324900 | OsR498G0305855900.01.T02 | RING-type E3 ubiquitin transferase |
| augustus_masked-3-processed-gene-149.24-mRNA-1 | Japonica | NA | | Os03g0325600 | OsR498G0305857800.01.T02 | Homeobox domain containing protein, expressed |
| augustus_masked-3-processed-gene-15.54-mRNA-1 | Japonica | NA | Kinase-TM | | | Serine/threonine-protein kinase Cx32, chloroplast, putative, expressed |
| augustus_masked-3-processed-gene-152.37-mRNA-1 | Japonica | NA | | | OsR498G0305877200.01.T01 | |
| augustus_masked-3-processed-gene-153.48-mRNA-1 | Japonica | NA | Kinase-LRR-TM | Os03g0332900 | OsR498G0305887500.01.T03 | Putative inactive receptor kinase |
| augustus_masked-3-processed-gene-154.30-mRNA-1 | Japonica | Japonica | Kinase-TM | Os03g0333200 | OsR498G0305888100.01.T01 | Receptor-like protein kinase FERONIA |
| augustus_masked-3-processed-gene-154.31-mRNA-1 | Japonica | NA | | Os03g0333300 | OsR498G0305888500.01.T01 | Eukaryotic translation initiation factor 2 subunit beta |
| augustus_masked-3-processed-gene-155.25-mRNA-1 | Japonica | NA | | Os03g0336400 | OsR498G0305898600.01.T01 | Expansin |
| augustus_masked-3-processed-gene-155.3-mRNA-1 | Japonica | NA | | | OsR498G0305897100.01.T01 | |
| augustus_masked-3-processed-gene-156.55-mRNA-1 | Japonica | NA | | Os03g0336900 | OsR498G0305902200.01.T01 | Transcription repressor |
| augustus_masked-3-processed-gene-156.60-mRNA-1 | Japonica | NA | | Os03g0339100 | OsR498G0305907700.01.T06 | PP1/PP2A phosphatases pleiotropic regulator PRL1 |
| augustus_masked-3-processed-gene-156.69-mRNA-1 | Japonica | NA | | | OsR498G0305907400.01.T01 | |
| augustus_masked-3-processed-gene-157.0-mRNA-1 | Japonica | NA | | | OsR498G0305908100.01.T01 | Papain fold toxin 1 (Glutamine deamidase) of polymorphic toxin system |
| augustus_masked-3-processed-gene-160.5-mRNA-1 | Japonica | Indica | | Os03g0346200 | OsR498G0305934000.01.T01 | Flowering-promoting factor 1-like protein 5 |
| augustus_masked-3-processed-gene-162.64-mRNA-1 | Japonica | NA | | Os03g0351800 | OsR498G0305953600.01.T03 | RING-type E3 ubiquitin transferase |
| augustus_masked-3-processed-gene-169.13-mRNA-1 | Japonica | NA | | Os03g0356400 | OsR498G0305969100.01.T01 | Glutaredoxin family protein, expressed |
| augustus_masked-3-processed-gene-169.6-mRNA-1 | Japonica | NA | | Os03g0355100 | OsR498G0305965400.01.T02 | |
| augustus_masked-3-processed-gene-171.26-mRNA-1 | Japonica | NA | | Os03g0356900 | OsR498G0305981500.01.T01 | Isopentenyl transferase IPT2 |
| augustus_masked-3-processed-gene-171.33-mRNA-1 | Japonica | NA | | | OsR498G0305979400.01.T01 | |
| augustus_masked-3-processed-gene-172.18-mRNA-1 | Japonica | NA | | | OsR498G0305982900.01.T01 | Non-specific serine/threonine protein kinase |
| augustus_masked-3-processed-gene-172.42-mRNA-1 | Japonica | Indica | | Os03g0356900 | OsR498G0305987200.01.T01 | Isopentenyl transferase IPT2 |
| augustus_masked-3-processed-gene-175.39-mRNA-1 | Japonica | NA | | Os03g0360600 | OsR498G0305990700.01.T01 | Transcription termination factor MTEF1, chloroplastic |
| augustus_masked-3-processed-gene-177.41-mRNA-1 | Japonica | NA | | Os03g0363800 | OsR498G0305999700.01.T01 | Serine/arginine rich splicing factor |
| augustus_masked-3-processed-gene-177.48-mRNA-1 | Japonica | NA | | Os03g0363700 | OsR498G0305999600.01.T05 | Transferase, transferring glycosyl groups |
| augustus_masked-3-processed-gene-178.42-mRNA-1 | Japonica | NA | | Os03g0366100 | OsR498G0306007200.01.T01 | |
| augustus_masked-3-processed-gene-178.43-mRNA-1 | Japonica | Japonica | | Os03g0366800 | OsR498G0306009000.01.T01 | F-box domain containing protein |
| augustus_masked-3-processed-gene-178.46-mRNA-1 | Japonica | NA | | | | OB domain-containing protein (Fragment) |
| augustus_masked-3-processed-gene-179.28-mRNA-1 | Japonica | NA | | | OsR498G0306012100.01.T01 | F-box domain-containing protein |
| augustus_masked-3-processed-gene-18.0-mRNA-1 | Japonica | Indica | | Os03g0136500 | OsR498G0305190100.01.T01 | Peptide methionine sulfoxide reductase MrsB |
| augustus_masked-3-processed-gene-18.13-mRNA-1 | Japonica | NA | | Os03g0137400 | OsR498G0305193900.01.T01 | Calcium-dependent channel |
| augustus_masked-3-processed-gene-18.4-mRNA-1 | Japonica | NA | | Os03g0136400 | OsR498G0305190000.01.T01 | H(+)/Pi cotransporter |
| augustus_masked-3-processed-gene-180.37-mRNA-1 | Japonica | NA | | Os03g0369700 | | Pentatricopeptide repeat-containing protein mitochondrial |
| augustus_masked-3-processed-gene-180.38-mRNA-1 | Japonica | NA | | Os03g0370000 | OsR498G0306019700.01.T02 | Protein-serine/threonine kinase |
| augustus_masked-3-processed-gene-180.41-mRNA-1 | Japonica | NA | | Os03g0370500 | OsR498G0306021700.01.T01 | PUA domain-containing protein (Fragment) |
| augustus_masked-3-processed-gene-180.42-mRNA-1 | Japonica | Japonica | | Os03g0370800 | OsR498G0408541500.01.T01 | Copper transporter |
| augustus_masked-3-processed-gene-181.2-mRNA-1 | Japonica | NA | | Os03g0371600 | | |
| augustus_masked-3-processed-gene-182.28-mRNA-1 | Japonica | NA | | Os03g0372900 | OsR498G0306031300.01.T01 | F-box domain containing protein, expressed |
| augustus_masked-3-processed-gene-185.16-mRNA-1 | Japonica | NA | | | | |
| augustus_masked-3-processed-gene-185.17-mRNA-1 | Japonica | NA | | | OsR498G0204046800.01.T01 | Epstein-Barr virus EBNA-1-like |
| augustus_masked-3-processed-gene-188.16-mRNA-1 | Japonica | NA | | Os03g0386000 | OsR498G0306062300.01.T03 | Putative WD repeat-containing protein C2A9.03 |
| augustus_masked-3-processed-gene-189.10-mRNA-1 | Japonica | Indica | | Os03g0387300 | OsR498G0306070300.01.T01 | VQ motif family protein, expressed |
| augustus_masked-3-processed-gene-189.11-mRNA-1 | Japonica | NA | | | OsR498G0306071900.01.T01 | |
| augustus_masked-3-processed-gene-19.0-mRNA-1 | Japonica | NA | | Os03g0138200 | OsR498G0305198900.01.T01 | Cytochrome P450 family 96 subfamily A polypeptide 1 |

| | | | | | | |
|---|---|---|---|---|---|---|
| augustus_masked-3-processed-gene-19.1-mRNA-1 | Japonica | NA | | Os03g0138400 | OsR498G0305199100.01.T02 | |
| augustus_masked-3-processed-gene-19.14-mRNA-1 | Japonica | NA | | Os03g0139000 | OsR498G0305201100.01.T01 | |
| augustus_masked-3-processed-gene-19.16-mRNA-1 | Japonica | NA | | Os03g0139300 | OsR498G0305202300.01.T01 | |
| augustus_masked-3-processed-gene-19.17-mRNA-1 | Japonica | Indica | | Os03g0140100 | OsR498G0305203100.01.T01 | Cytochrome P450 family 96 subfamily A polypeptide 1 |
| augustus_masked-3-processed-gene-19.19-mRNA-1 | Japonica | Indica | | Os03g0140100 | OsR498G0305203500.01.T01 | Cytochrome P450 family 96 subfamily A polypeptide 1 |
| augustus_masked-3-processed-gene-19.22-mRNA-1 | Japonica | NA | | Os03g0140100 | OsR498G0305205100.01.T01 | Cytochrome P450 family 96 subfamily A polypeptide 1 |
| augustus_masked-3-processed-gene-192.16-mRNA-1 | Japonica | NA | | Os03g0391700 | OsR498G0306087800.01.T02 | Putative HLH DNA-binding domain superfamily protein isoform 1 |
| augustus_masked-3-processed-gene-197.14-mRNA-1 | Japonica | NA | | Os03g0398600 | OsR498G0306118900.01.T01 | RING-H2 finger protein ATL3 |
| augustus_masked-3-processed-gene-197.15-mRNA-1 | Japonica | NA | | Os03g0399000 | OsR498G0306119900.01.T01 | Pectinesterase |
| augustus_masked-3-processed-gene-197.16-mRNA-1 | Japonica | NA | | Os03g0399000 | OsR498G0306119900.01.T01 | Pectinesterase |
| augustus_masked-3-processed-gene-198.9-mRNA-1 | Japonica | NA | | Os03g0399400 | OsR498G0306121800.01.T01 | F-box only protein 13 |
| augustus_masked-3-processed-gene-2.58-mRNA-1 | Japonica | NA | | | | Skp1 family, dimerisation domain containing protein, expressed |
| augustus_masked-3-processed-gene-2.66-mRNA-1 | Japonica | NA | | Os03g0107700 | | Cyclin-dependent protein kinase inhibitor EL2 |
| augustus_masked-3-processed-gene-20.20-mRNA-1 | Japonica | Indica | | Os03g0140100 | OsR498G0305205900.01.T01 | Cytochrome P450 family 96 subfamily A polypeptide 1 |
| augustus_masked-3-processed-gene-20.21-mRNA-1 | Japonica | Indica | | Os03g0140100 | OsR498G0305207300.01.T01 | Cytochrome P450 family 96 subfamily A polypeptide 1 |
| augustus_masked-3-processed-gene-20.24-mRNA-1 | Japonica | NA | | Os03g0140500 | OsR498G0305207800.01.T01 | Cysteine-rich repeat secretory protein 3 |
| augustus_masked-3-processed-gene-201.3-mRNA-1 | Japonica | NA | | | OsR498G0306141500.01.T01 | |
| augustus_masked-3-processed-gene-201.5-mRNA-1 | Japonica | NA | | | OsR498G0306142300.01.T01 | FLZ-type domain-containing protein |
| augustus_masked-3-processed-gene-202.2-mRNA-1 | Japonica | Indica | | Os03g0403600 | OsR498G0306146500.01.T01 | VWFA domain-containing protein (Fragment) |
| augustus_masked-3-processed-gene-203.15-mRNA-1 | Japonica | NA | | | OsR498G0815762600.01.T01 | |
| augustus_masked-3-processed-gene-209.28-mRNA-1 | Japonica | NA | | | OsR498G0306205600.01.T01 | Zinc finger protein WIP2 (Fragment) |
| augustus_masked-3-processed-gene-209.29-mRNA-1 | Japonica | NA | | Os12g0135200 | OsR498G1220897500.01.T01 | F-box/kelch-repeat protein At5g26960 |
| augustus_masked-3-processed-gene-21.38-mRNA-1 | Japonica | NA | | | OsR498G0305216300.01.T01 | Subtilisin-like protease |
| augustus_masked-3-processed-gene-210.0-mRNA-1 | Japonica | NA | | Os03g0415500 | OsR498G0306207300.01.T02 | Puative inner mitochondrial membrane translocase |
| augustus_masked-3-processed-gene-211.19-mRNA-1 | Japonica | Japonica | | Os03g0417700 | OsR498G0306216600.01.T01 | Cytochrome P450, family 78, subfamily A, polypeptide 10 (Fragment) |
| augustus_masked-3-processed-gene-211.25-mRNA-1 | Japonica | NA | | Os03g0418000 | OsR498G0306219500.01.T01 | Chitinase |
| augustus_masked-3-processed-gene-211.33-mRNA-1 | Japonica | NA | | | OsR498G0306220400.01.T01 | |
| augustus_masked-3-processed-gene-212.33-mRNA-1 | Japonica | NA | | | OsR498G0306226600.01.T01 | |
| augustus_masked-3-processed-gene-213.12-mRNA-1 | Japonica | NA | | Os03g0423000 | OsR498G0306236100.01.T02 | F-box domain containing protein, expressed |
| augustus_masked-3-processed-gene-213.29-mRNA-1 | Japonica | Indica | | Os03g0423300 | OsR498G0306236800.01.T02 | Acyl-[acyl-carrier-protein] desaturase 4, chloroplastic |
| augustus_masked-3-processed-gene-213.6-mRNA-1 | Japonica | Japonica | | Os03g0422300 | OsR498G1120568600.01.T01 | |
| augustus_masked-3-processed-gene-215.23-mRNA-1 | Japonica | NA | | Os03g0425900 | | Zinc finger protein ZAT4 |
| augustus_masked-3-processed-gene-215.25-mRNA-1 | Japonica | Indica | Kinase-TM | Os03g0426300 | OsR498G0306245800.01.T01 | G-type lectin S-receptor-like serine/threonine-protein kinase |
| augustus_masked-3-processed-gene-216.18-mRNA-1 | Japonica | NA | | Os03g0426800 | OsR498G0306246300.01.T03 | Membrane-anchored ubiquitin-fold protein 1 |
| augustus_masked-3-processed-gene-216.22-mRNA-1 | Japonica | NA | | Os03g0427000 | OsR498G0306247200.01.T03 | EID1-like F-box protein 2 isoform C |
| augustus_masked-3-processed-gene-216.23-mRNA-1 | Japonica | NA | | Os03g0427100 | OsR498G0306247800.01.T02 | RING/U-box superfamily protein isoform 1 |
| augustus_masked-3-processed-gene-217.19-mRNA-1 | Japonica | NA | | Os03g0428700 | OsR498G0306252300.01.T01 | Expansin |
| augustus_masked-3-processed-gene-217.21-mRNA-1 | Japonica | NA | | Os03g0429000 | OsR498G0306253900.01.T01 | Cystatin domain-containing protein (Fragment) |
| augustus_masked-3-processed-gene-218.21-mRNA-1 | Japonica | NA | | Os02g0212975 | OsR498G0305421900.01.T01 | Kinesin heavy chain-like |
| augustus_masked-3-processed-gene-218.24-mRNA-1 | Japonica | NA | | Os10g0460000 | OsR498G0204348500.01.T01 | |
| augustus_masked-3-processed-gene-218.4-mRNA-1 | Japonica | NA | | Os03g0430500 | OsR498G0306258700.01.T01 | Subtilisin-like protease |
| augustus_masked-3-processed-gene-22.19-mRNA-1 | Japonica | NA | | | OsR498G1120638300.01.T05 | |
| augustus_masked-3-processed-gene-22.21-mRNA-1 | Japonica | NA | | Os03g0144300 | OsR498G0305219500.01.T02 | Xyloglucan galactosyltransferase KATAMARI |
| augustus_masked-3-processed-gene-22.22-mRNA-1 | Japonica | Indica | | Os03g0144500 | OsR498G0305220100.01.T01 | Exostosin domain-containing protein (Fragment) |
| augustus_masked-3-processed-gene-22.25-mRNA-1 | Japonica | NA | | Os03g0144800 | OsR498G0305220900.01.T03 | Xyloglucan galactosyltransferase KATAMARI |
| augustus_masked-3-processed-gene-22.39-mRNA-1 | Japonica | NA | Kinase-LRR-TM | Os03g0145000 | OsR498G0305221600.01.T01 | Leucine-rich repeat receptor-like protein kinase PXL2 |
| augustus_masked-3-processed-gene-220.10-mRNA-1 | Japonica | NA | | Os03g0433200 | OsR498G0306272800.01.T01 | GRAS transcription factor |
| augustus_masked-3-processed-gene-222.22-mRNA-1 | Japonica | NA | | Os03g0437100 | OsR498G0306284800.01.T01 | Zinc finger protein 1 |
| augustus_masked-3-processed-gene-222.23-mRNA-1 | Japonica | Japonica | | Os03g0437200 | OsR498G0306284800.01.T01 | Zinc finger protein 1 |
| augustus_masked-3-processed-gene-222.8-mRNA-1 | Japonica | NA | | Os03g0435900 | OsR498G0306281600.01.T01 | Mitotic-spindle organizing protein 1A |
| augustus_masked-3-processed-gene-222.9-mRNA-1 | Japonica | Japonica | | Os03g0436400 | OsR498G0714710500.01.T01 | 3-oxo-Delta(45)-steroid 5-beta-reductase |
| augustus_masked-3-processed-gene-223.16-mRNA-1 | Japonica | NA | | Os03g0439000 | OsR498G0306291900.01.T01 | Zinc finger CCCH domain-containing protein 18 |
| augustus_masked-3-processed-gene-224.44-mRNA-1 | Japonica | NA | | | | |
| augustus_masked-3-processed-gene-224.45-mRNA-1 | Japonica | NA | | | | |
| augustus_masked-3-processed-gene-225.5-mRNA-1 | Japonica | NA | | Os03g0441400 | OsR498G0306299200.01.T05 | Pentatricopeptide repeat-containing protein, chloroplastic |
| augustus_masked-3-processed-gene-225.6-mRNA-1 | Japonica | NA | | Os03g0441500 | OsR498G0306299600.01.T03 | ABC transporter family protein, expressed |
| augustus_masked-3-processed-gene-226.0-mRNA-1 | Japonica | NA | | | OsR498G0204046800.01.T01 | Epstein-Barr virus EBNA-1-like |
| augustus_masked-3-processed-gene-227.7-mRNA-1 | Japonica | Indica | | Os03g0444900 | OsR498G0306310300.01.T02 | WRKY DNA binding domain containing protein, expressed |
| augustus_masked-3-processed-gene-23.1-mRNA-1 | Japonica | NA | | | OsR498G0305222300.01.T01 | Beta-glucuronosyltransferase GlcAT14A |
| augustus_masked-3-processed-gene-23.8-mRNA-1 | Japonica | NA | | Os03g0145600 | | Zinc finger CCCH domain-containing protein 48 |
| augustus_masked-3-processed-gene-232.9-mRNA-1 | Japonica | NA | | | OsR498G0306330400.01.T01 | |

| | | | | | | |
|---|---|---|---|---|---|---|
| augustus_masked-3-processed-gene-24.47-mRNA-1 | Japonica | Japonica | | Os03g0148400 | OsR498G0305233000.01.T02 | Leucine-rich repeat-containing protein 16B-like |
| augustus_masked-3-processed-gene-25.1-mRNA-1 | Japonica | NA | | Os03g0149000 | OsR498G0305234100.01.T01 | LOB transcription factor |
| augustus_masked-3-processed-gene-25.19-mRNA-1 | Japonica | Japonica | | Os03g0150500 | OsR498G0305239100.01.T01 | H(+)/Pi cotransporter |
| augustus_masked-3-processed-gene-25.2-mRNA-1 | Japonica | NA | | Os03g0149000 | OsR498G0305234100.01.T01 | LOB domain-containing protein 29 |
| augustus_masked-3-processed-gene-25.30-mRNA-1 | Japonica | NA | | Os03g0149700 | OsR498G0305237600.01.T01 | Ring-h2 finger protein atl51 |
| augustus_masked-3-processed-gene-25.31-mRNA-1 | Japonica | NA | | Os03g0149800 | OsR498G0305237800.01.T01 | RING-H2 finger protein ATL16 |
| augustus_masked-3-processed-gene-26.8-mRNA-1 | Japonica | Indica | | Os03g0150600 | OsR498G0305239400.01.T01 | H(+)/Pi cotransporter |
| augustus_masked-3-processed-gene-26.9-mRNA-1 | Japonica | NA | | Os03g0150800 | OsR498G0305240200.01.T01 | H(+)/Pi cotransporter |
| augustus_masked-3-processed-gene-265.19-mRNA-1 | Japonica | NA | | Os10g0427000 | OsR498G1018762100.01.T01 | BTB/POZ domain containing protein, expressed |
| augustus_masked-3-processed-gene-265.30-mRNA-1 | Japonica | NA | | Os10g0426600 | OsR498G1018761700.01.T01 | BTB/POZ domain containing protein, expressed |
| augustus_masked-3-processed-gene-265.31-mRNA-1 | Japonica | NA | | Os10g0426500 | OsR498G1018758400.01.T01 | BTB/POZ and MATH domain-containing protein 1 |
| augustus_masked-3-processed-gene-265.45-mRNA-1 | Japonica | NA | | | OsR498G1018686900.01.T02 | Exonuclease family protein, expressed |
| augustus_masked-3-processed-gene-265.50-mRNA-1 | Japonica | NA | | Os10g0425700 | | BTB/POZ and MATH domain-containing protein 2 |
| augustus_masked-3-processed-gene-266.16-mRNA-1 | Japonica | NA | | Os10g0423800 | OsR498G1018750800.01.T01 | BTB/POZ and MATH domain-containing protein 2 |
| augustus_masked-3-processed-gene-266.25-mRNA-1 | Japonica | NA | | Os10g0424500 | OsR498G1018753900.01.T01 | BTB domain-containing protein |
| augustus_masked-3-processed-gene-266.26-mRNA-1 | Japonica | NA | | Os10g0424100 | OsR498G1018753500.01.T01 | BTB domain-containing protein (Fragment) |
| augustus_masked-3-processed-gene-266.27-mRNA-1 | Japonica | NA | | Os10g0424100 | OsR498G1018753500.01.T01 | BTB/POZ and MATH domain-containing protein 2 |
| augustus_masked-3-processed-gene-266.28-mRNA-1 | Japonica | NA | | Os10g0423900 | | BTB/POZ domain containing protein, expressed |
| augustus_masked-3-processed-gene-266.4-mRNA-1 | Japonica | NA | | Os10g0425400 | OsR498G1018754900.01.T01 | BTB domain-containing protein |
| augustus_masked-3-processed-gene-267.17-mRNA-1 | Japonica | NA | | Os10g0423000 | OsR498G1018750400.01.T03 | Ribosomal N-lysine methyltransferase 5 |
| augustus_masked-3-processed-gene-267.25-mRNA-1 | Japonica | NA | | Os10g0423300 | OsR498G1018750800.01.T01 | BTB/POZ and MATH domain-containing protein 1 |
| augustus_masked-3-processed-gene-267.27-mRNA-1 | Japonica | Japonica | | Os10g0422800 | OsR498G0307042100.01.T01 | |
| augustus_masked-3-processed-gene-267.31-mRNA-1 | Japonica | NA | | Os10g0422600 | OsR498G1018748200.01.T01 | Zf-FLZ domain containing protein |
| augustus_masked-3-processed-gene-268.1-mRNA-1 | Japonica | NA | | Os10g0421800 | OsR498G1018744600.01.T05 | Pentatricopeptide repeat-containing protein, chloroplastic |
| augustus_masked-3-processed-gene-269.38-mRNA-1 | Japonica | NA | | | OsR498G1018738400.01.T01 | EGF-like domain-containing protein |
| augustus_masked-3-processed-gene-27.13-mRNA-1 | Japonica | NA | | Os03g0154000 | OsR498G0305247900.01.T01 | FAD binding domain containing protein, expressed |
| augustus_masked-3-processed-gene-27.15-mRNA-1 | Japonica | Indica | | Os11g0602400 | OsR498G0305248300.01.T01 | tRNA-intron lyase |
| augustus_masked-3-processed-gene-27.35-mRNA-1 | Japonica | Japonica | | Os03g0153400 | OsR498G0916883200.01.T02 | SufE domain-containing protein (Fragment) |
| augustus_masked-3-processed-gene-27.36-mRNA-1 | Japonica | NA | | | OsR498G0305247600.01.T01 | |
| augustus_masked-3-processed-gene-270.0-mRNA-1 | Japonica | NA | | | | Ribonuclease 3 |
| augustus_masked-3-processed-gene-270.1-mRNA-1 | Japonica | NA | | | OsR498G1018732200.01.T01 | Calcium-transporting ATPase |
| augustus_masked-3-processed-gene-271.1-mRNA-1 | Japonica | NA | | | OsR498G1018724200.01.T01 | Leucine Rich Repeat family protein |
| augustus_masked-3-processed-gene-271.10-mRNA-1 | Japonica | NA | | Os10g0416500 | OsR498G1018723700.01.T01 | Class III chitinase |
| augustus_masked-3-processed-gene-271.17-mRNA-1 | Japonica | NA | | Os10g0416200 | OsR498G1018721400.01.T01 | Very-long-chain 3-oxoacyl-CoA synthase |
| augustus_masked-3-processed-gene-271.8-mRNA-1 | Japonica | NA | | Os10g0416800 | OsR498G1018725300.01.T01 | Class III chitinase |
| augustus_masked-3-processed-gene-272.25-mRNA-1 | Japonica | NA | | Os10g0415200 | OsR498G1018718400.01.T02 | Exocyst complex component |
| augustus_masked-3-processed-gene-272.58-mRNA-1 | Japonica | NA | | Os10g0460000 | OsR498G0305421900.01.T01 | |
| augustus_masked-3-processed-gene-273.1-mRNA-1 | Japonica | NA | | | | |
| augustus_masked-3-processed-gene-273.7-mRNA-1 | Japonica | NA | | Os10g0412800 | OsR498G1018709700.01.T05 | |
| augustus_masked-3-processed-gene-278.41-mRNA-1 | Japonica | NA | | | | Inorganic diphosphatase |
| augustus_masked-3-processed-gene-279.21-mRNA-1 | Japonica | NA | | | | |
| augustus_masked-3-processed-gene-284.46-mRNA-1 | Japonica | NA | | Os10g0396100 | OsR498G1018645800.01.T01 | F-box domain containing protein, expressed |
| augustus_masked-3-processed-gene-288.33-mRNA-1 | Japonica | NA | | Os10g0390000 | OsR498G1018626000.01.T01 | |
| augustus_masked-3-processed-gene-288.39-mRNA-1 | Japonica | NA | | Os10g0389500 | OsR498G1018624000.01.T04 | |
| augustus_masked-3-processed-gene-289.25-mRNA-1 | Japonica | NA | | Os10g0388200 | OsR498G1018620600.01.T01 | F-box/kelch-repeat protein SKIP30 |
| augustus_masked-3-processed-gene-289.48-mRNA-1 | Japonica | NA | Kinase | Os09g0376600 | OsR498G1018618100.01.T01 | Putative leucine-rich repeat receptor-like protein kinase |
| augustus_masked-3-processed-gene-29.12-mRNA-1 | Japonica | NA | | Os03g0158000 | OsR498G1221887800.01.T01 | |
| augustus_masked-3-processed-gene-29.18-mRNA-1 | Japonica | Japonica | | Os03g0158900 | OsR498G0307003200.01.T01 | Pentatricopeptide repeat-containing protein |
| augustus_masked-3-processed-gene-29.19-mRNA-1 | Japonica | NA | Kinase-TM | Os03g0159100 | OsR498G0305257900.01.T01 | probable serine/threonine-protein kinase PBL16 |
| augustus_masked-3-processed-gene-29.23-mRNA-1 | Japonica | NA | | Os03g0160200 | OsR498G0305260700.01.T02 | Ribosomal N-lysine methyltransferase 5 |
| augustus_masked-3-processed-gene-29.3-mRNA-1 | Japonica | NA | | | OsR498G0307316800.01.T01 | Putative Anter-specific proline-rich protein APG |
| augustus_masked-3-processed-gene-29.33-mRNA-1 | Japonica | NA | | Os03g0159400 | OsR498G0305258600.01.T01 | Transcription repressor |
| augustus_masked-3-processed-gene-29.36-mRNA-1 | Japonica | Indica | | Os03g0159900 | OsR498G0305260000.01.T02 | |
| augustus_masked-3-processed-gene-290.8-mRNA-1 | Japonica | NA | | Os10g0386300 | | |
| augustus_masked-3-processed-gene-293.17-mRNA-1 | Japonica | NA | | | OsR498G1221194600.01.T01 | BKRF1 encodes EBNA-1 protein-like |
| augustus_masked-3-processed-gene-294.27-mRNA-1 | Japonica | NA | | Os10g0380800 | OsR498G1018593600.01.T01 | Tyrosine decarboxylase |
| augustus_masked-3-processed-gene-295.7-mRNA-1 | Japonica | NA | | | OsR498G0305652000.01.T01 | SR-rich pre-mRNA splicing activator |
| augustus_masked-3-processed-gene-296.8-mRNA-1 | Japonica | NA | | Os10g0378400 | OsR498G1018586600.01.T01 | IRK-interacting protein-like |
| augustus_masked-3-processed-gene-297.22-mRNA-1 | Japonica | NA | | Os10g0377400 | OsR498G1018583800.01.T03 | Ras-related protein RGP1 |
| augustus_masked-3-processed-gene-30.19-mRNA-1 | Japonica | Japonica | | Os03g0160300 | OsR498G0307042100.01.T01 | Major facilitator superfamily protein isoform 1 |
| augustus_masked-3-processed-gene-30.22-mRNA-1 | Japonica | Indica | | Os03g0160900 | OsR498G0305261800.01.T04 | |

| | | | | | | |
|---|---|---|---|---|---|---|
| augustus_masked-3-processed-gene-309.6-mRNA-1 | Japonica | NA | NBS-TM | | | Disease resistance protein RPM1 |
| augustus_masked-3-processed-gene-31.40-mRNA-1 | Japonica | NA | | Os03g0162500 | OsR498G0305274100.01.T01 | Plant-specific domain TIGR01615 family protein |
| augustus_masked-3-processed-gene-312.2-mRNA-1 | Japonica | NA | | Os10g0349300 | OsR498G1018517600.01.T01 | Cortical cell-delineating protein-like |
| augustus_masked-3-processed-gene-312.3-mRNA-1 | Japonica | NA | | Os10g0349300 | OsR498G1018517000.01.T01 | Cortical cell delineating protein, putative, expressed |
| augustus_masked-3-processed-gene-312.4-mRNA-1 | Japonica | NA | | Os10g0349300 | OsR498G1018516600.01.T01 | Cortical cell delineating protein, putative, expressed |
| augustus_masked-3-processed-gene-32.2-mRNA-1 | Japonica | NA | | Os03g0163500 | OsR498G0305276900.01.T02 | Exocyst complex component |
| augustus_masked-3-processed-gene-32.8-mRNA-1 | Japonica | NA | | Os03g0164300 | OsR498G0305279700.01.T01 | TF-B3 domain-containing protein |
| augustus_masked-3-processed-gene-321.20-mRNA-1 | Japonica | NA | | Os10g0337700 | OsR498G1018481900.01.T01 | WPP domain-containing protein |
| augustus_masked-3-processed-gene-321.7-mRNA-1 | Japonica | NA | | Os10g0338500 | | E3 ubiquitin-protein ligase SPL11 |
| augustus_masked-3-processed-gene-325.8-mRNA-1 | Japonica | NA | | Os10g0327000 | OsR498G1018449400.01.T03 | cysteine-rich repeat secretory protein 38-like isoform X3 |
| augustus_masked-3-processed-gene-326.30-mRNA-1 | Japonica | NA | | | OsR498G1018455500.01.T01 | WRKY transcription factor 18 |
| augustus_masked-3-processed-gene-326.31-mRNA-1 | Japonica | NA | | Os10g0328100 | OsR498G0101190400.01.T03 | HDOD domain protein |
| augustus_masked-3-processed-gene-33.21-mRNA-1 | Japonica | NA | | Os03g0166000 | OsR498G0305285000.01.T04 | Alba domain-containing protein (Fragment) |
| augustus_masked-3-processed-gene-332.10-mRNA-1 | Japonica | NA | | | OsR498G0306342800.01.T01 | Embryo defective 2016 |
| augustus_masked-3-processed-gene-334.0-mRNA-1 | Japonica | NA | Kinase-TM | Os03g0556600 | OsR498G0306350500.01.T01 | Non-specific serine/threonine protein kinase |
| augustus_masked-3-processed-gene-334.22-mRNA-1 | Japonica | NA | | | | |
| augustus_masked-3-processed-gene-336.16-mRNA-1 | Japonica | NA | | Os03g0559700 | OsR498G0306366400.01.T01 | HTH myb-type domain-containing protein |
| augustus_masked-3-processed-gene-336.3-mRNA-1 | Japonica | NA | | Os03g0558700 | | HTH myb-type domain-containing protein |
| augustus_masked-3-processed-gene-338.27-mRNA-1 | Japonica | NA | | Os03g0562200 | OsR498G0306377600.01.T01 | F-box domain containing protein expressed |
| augustus_masked-3-processed-gene-34.24-mRNA-1 | Japonica | NA | | Os03g0168400 | OsR498G0305292300.01.T06 | Pentatricopeptide repeat-containing protein |
| augustus_masked-3-processed-gene-34.39-mRNA-1 | Japonica | NA | | | OsR498G0305293000.01.T01 | Pentatricopeptide repeat |
| augustus_masked-3-processed-gene-34.41-mRNA-1 | Japonica | NA | | Os03g0168700 | OsR498G0305293400.01.T01 | SET and MYND domain-containing protein 3 |
| augustus_masked-3-processed-gene-340.16-mRNA-1 | Japonica | Japonica | | Os03g0564600 | OsR498G0306388200.01.T01 | Cysteine-rich repeat secretory protein 38 |
| augustus_masked-3-processed-gene-341.23-mRNA-1 | Japonica | NA | LRR-NBS-TM | Os03g0566700 | | NBS-LRR protein (Fragment) |
| augustus_masked-3-processed-gene-341.24-mRNA-1 | Japonica | NA | | Os03g0566800 | OsR498G0306397400.01.T01 | Eukaryotic translation initiation factor |
| augustus_masked-3-processed-gene-341.26-mRNA-1 | Japonica | NA | | Os03g0567100 | OsR498G0306398000.01.T01 | |
| augustus_masked-3-processed-gene-342.28-mRNA-1 | Japonica | NA | | Os03g0568433 | | 4-hydroxyphenylacetaldehyde oxime monooxygenase |
| augustus_masked-3-processed-gene-342.29-mRNA-1 | Japonica | Japonica | | Os03g0568466 | OsR498G0306401700.01.T01 | 4-hydroxyphenylacetaldehyde oxime monooxygenase |
| augustus_masked-3-processed-gene-342.30-mRNA-1 | Japonica | NA | | Os03g0568600 | OsR498G0306402100.01.T01 | tRNA-uridine aminocarboxypropyltransferase |
| augustus_masked-3-processed-gene-343.29-mRNA-1 | Japonica | NA | | Os03g0569900 | OsR498G0306409400.01.T02 | polyadenylate-binding protein RBP45-like isoform X4 |
| augustus_masked-3-processed-gene-343.30-mRNA-1 | Japonica | Indica | | Os03g0570100 | OsR498G0306410000.01.T01 | Tyrosine N-monooxygenase |
| augustus_masked-3-processed-gene-343.8-mRNA-1 | Japonica | NA | | Os03g0569800 | OsR498G0306408900.01.T01 | Putative membrane-associated salt-inducible protein |
| augustus_masked-3-processed-gene-348.16-mRNA-1 | Japonica | Indica | | Os03g0578500 | OsR498G0306438900.01.T01 | Myosin-2 |
| augustus_masked-3-processed-gene-348.27-mRNA-1 | Japonica | NA | | | OsR498G0100073600.01.T01 | |
| augustus_masked-3-processed-gene-35.11-mRNA-1 | Japonica | Indica | | Os04g0286500 | OsR498G0305295200.01.T01 | Transposon protein, putative, Pong sub-class, expressed |
| augustus_masked-3-processed-gene-35.2-mRNA-1 | Japonica | NA | | Os03g0169300 | OsR498G0305295300.01.T01 | Pentatricopeptide repeat-containing protein chloroplastic |
| augustus_masked-3-processed-gene-35.26-mRNA-1 | Japonica | Indica | Kinase-TM | Os10g0405100 | OsR498G0305298200.01.T01 | probable serine/threonine-protein kinase PBL3 |
| augustus_masked-3-processed-gene-35.27-mRNA-1 | Japonica | NA | | Os03g0170500 | OsR498G0305299000.01.T01 | NAD(P)-linked oxidoreductase superfamily protein isoform 1 |
| augustus_masked-3-processed-gene-36.18-mRNA-1 | Japonica | NA | | Os03g0171600 | OsR498G0305304300.01.T05 | F-box/kelch-repeat protein SKIP11 |
| augustus_masked-3-processed-gene-36.42-mRNA-1 | Japonica | Indica | | Os03g0172400 | OsR498G0305306800.01.T01 | Cysteine proteinase inhibitor |
| augustus_masked-3-processed-gene-36.43-mRNA-1 | Japonica | Japonica | | Os03g0172400 | OsR498G0305307000.01.T01 | Cysteine proteinase inhibitor |
| augustus_masked-3-processed-gene-374.10-mRNA-1 | Japonica | NA | | Os03g0624300 | OsR498G0306587700.01.T01 | |
| augustus_masked-3-processed-gene-374.21-mRNA-1 | Japonica | NA | | Os03g0624800 | OsR498G0306589900.01.T06 | Putative methyltransferase PMT9 |
| augustus_masked-3-processed-gene-376.15-mRNA-1 | Japonica | NA | | Os03g0627500 | OsR498G0306600500.01.T02 | Nucleic acid binding protein |
| augustus_masked-3-processed-gene-376.9-mRNA-1 | Japonica | NA | | Os06g0114366 | OsR498G0612361200.01.T01 | Selenium-binding protein-like |
| augustus_masked-3-processed-gene-38.39-mRNA-1 | Japonica | NA | | | OsR498G0305810200.01.T01 | |
| augustus_masked-3-processed-gene-38.45-mRNA-1 | Japonica | NA | | | OsR498G0305314600.01.T01 | |
| augustus_masked-3-processed-gene-38.46-mRNA-1 | Japonica | NA | | Os03g0175800 | | |
| augustus_masked-3-processed-gene-38.66-mRNA-1 | Japonica | NA | | Os03g0176900 | OsR498G0305319400.01.T01 | Phosphatidylserine decarboxylase beta chain like |
| augustus_masked-3-processed-gene-38.68-mRNA-1 | Japonica | NA | | | OsR498G0305320600.01.T01 | |
| augustus_masked-3-processed-gene-381.22-mRNA-1 | Japonica | NA | LRR-TM | Os03g0637600 | OsR498G0306623400.01.T01 | Leucine-rich repeat extensin-like protein 6 |
| augustus_masked-3-processed-gene-381.26-mRNA-1 | Japonica | NA | | Os03g0637800 | OsR498G0306624300.01.T01 | Serine/threonine-protein kinase-like protein CR4 |
| augustus_masked-3-processed-gene-381.27-mRNA-1 | Japonica | NA | Kinase-TM | Os03g0637800 | OsR498G0306624300.01.T01 | Receptor protein kinase CRINKLY4 |
| augustus_masked-3-processed-gene-382.18-mRNA-1 | Japonica | NA | | Os03g0638900 | OsR498G0306629400.01.T01 | F-box domain containing protein expressed |
| augustus_masked-3-processed-gene-382.26-mRNA-1 | Japonica | Japonica | | Os03g0639400 | OsR498G0306635200.01.T01 | PMEI domain-containing protein |
| augustus_masked-3-processed-gene-386.29-mRNA-1 | Japonica | Japonica | | Os03g0645900 | OsR498G1222114300.01.T01 | Plastid 9-cis-epoxycarotenoid dioxygenase |
| augustus_masked-3-processed-gene-387.30-mRNA-1 | Japonica | NA | | Os03g0646300 | OsR498G0306664500.01.T01 | Cyclic nucleotide-gated ion channel 8 |
| augustus_masked-3-processed-gene-387.36-mRNA-1 | Japonica | NA | | Os03g0646200 | | PORR domain-containing protein (Fragment) |
| augustus_masked-3-processed-gene-388.27-mRNA-1 | Japonica | NA | | Os03g0648100 | OsR498G0306669800.01.T03 | Core-2/I-branching beta-16-N-acetylglucosaminyltransferase family protein |
| augustus_masked-3-processed-gene-388.48-mRNA-1 | Japonica | Japonica | | Os03g0648800 | OsR498G0306671600.01.T01 | Glutaredoxin domain-containing cysteine-rich protein 1 |
| augustus_masked-3-processed-gene-39.56-mRNA-1 | Japonica | NA | | | | Germin-like protein 3-1 |

| | | | | | | | |
|---|---|---|---|---|---|---|---|
| augustus_masked-3-processed-gene-39.57-mRNA-1 | Japonica | NA | | Os03g0179200 | OsR498G0305324900.01.T01 | Cupin type-1 domain-containing protein |
| augustus_masked-3-processed-gene-39.75-mRNA-1 | Japonica | NA | | Os03g0180000 | OsR498G0305327800.01.T01 | Sodium/calcium exchanger membrane region |
| augustus_masked-3-processed-gene-391.1-mRNA-1 | Japonica | NA | | | OsR498G0306689300.01.T01 | F-box domain-containing protein (Fragment) |
| augustus_masked-3-processed-gene-392.2-mRNA-1 | Japonica | NA | | Os03g0654400 | OsR498G0306693800.01.T01 | Amino acid permease family protein, expressed |
| augustus_masked-3-processed-gene-4.59-mRNA-1 | Japonica | NA | | | OsR498G0305104500.01.T01 | Modular polyketide synthase (Fragment) |
| augustus_masked-3-processed-gene-4.66-mRNA-1 | Japonica | NA | | Os03g0111000 | | |
| augustus_masked-3-processed-gene-40.12-mRNA-1 | Japonica | NA | | Os03g0181100 | OsR498G0305331700.01.T01 | Tify domain-containing protein (Fragment) |
| augustus_masked-3-processed-gene-40.14-mRNA-1 | Japonica | NA | | Os03g0181400 | OsR498G0305333100.01.T01 | PORR domain-containing protein (Fragment) |
| augustus_masked-3-processed-gene-40.28-mRNA-1 | Japonica | NA | | | OsR498G0511427100.01.T01 | Epstein-Barr virus EBNA-1-like |
| augustus_masked-3-processed-gene-403.24-mRNA-1 | Japonica | Indica | | Os03g0673100 | OsR498G0306772900.01.T01 | |
| augustus_masked-3-processed-gene-403.30-mRNA-1 | Japonica | NA | | | | |
| augustus_masked-3-processed-gene-404.32-mRNA-1 | Japonica | NA | | Os03g0676400 | OsR498G0306781800.01.T01 | VQ domain-containing protein (Fragment) |
| augustus_masked-3-processed-gene-404.6-mRNA-1 | Japonica | NA | | Os02g0770700 | OsR498G0612483600.01.T01 | Separase |
| augustus_masked-3-processed-gene-405.19-mRNA-1 | Japonica | NA | CC-Kinase-TM | Os03g0678100 | OsR498G0306787500.01.T01 | Serinethreonine-protein kinase blus1 |
| augustus_masked-3-processed-gene-406.25-mRNA-1 | Japonica | Japonica | | Os03g0678800 | OsR498G0305988900.01.T01 | Hexosyltransferase |
| augustus_masked-3-processed-gene-408.19-mRNA-1 | Japonica | NA | | | OsR498G0306808200.01.T01 | PAZ domain-containing protein/Piwi domain-containing protein/DUF1785 d |
| augustus_masked-3-processed-gene-409.26-mRNA-1 | Japonica | Japonica | | Os03g0683300 | OsR498G1019231300.01.T01 | rRNA N-glycosidase |
| augustus_masked-3-processed-gene-409.27-mRNA-1 | Japonica | NA | | Os01g0240700 | OsR498G0100514900.01.T01 | DUF1618 domain-containing protein (Fragment) |
| augustus_masked-3-processed-gene-409.7-mRNA-1 | Japonica | NA | | | OsR498G0306810100.01.T01 | Agmatine coumaroyltransferase-2 |
| augustus_masked-3-processed-gene-41.18-mRNA-1 | Japonica | NA | Kinase-LRR-TM | Os03g0183800 | OsR498G0305342500.01.T01 | Strubbelig-receptor family 6 |
| augustus_masked-3-processed-gene-41.19-mRNA-1 | Japonica | NA | | Os03g0184000 | OsR498G0305342900.01.T03 | 15-cis-phytoene desaturase, chloroplastic/chromoplastic |
| augustus_masked-3-processed-gene-411.5-mRNA-1 | Japonica | Indica | | Os06g0488200 | OsR498G0306819800.01.T02 | Myosin family protein with Dil domain isoform 1 |
| augustus_masked-3-processed-gene-412.41-mRNA-1 | Japonica | Indica | | Os03g0689800 | OsR498G0306832100.01.T01 | NB-ARC domain containing protein |
| augustus_masked-3-processed-gene-413.0-mRNA-1 | Japonica | NA | | Os03g0690500 | OsR498G0306837400.01.T01 | 2-oxoglutarate-dependent oxygenase |
| augustus_masked-3-processed-gene-414.46-mRNA-1 | Japonica | NA | | | OsR498G0306849600.01.T02 | DUF538 domain-containing protein |
| augustus_masked-3-processed-gene-414.47-mRNA-1 | Japonica | NA | | | OsR498G0306849600.01.T01 | |
| augustus_masked-3-processed-gene-415.15-mRNA-1 | Japonica | NA | | Os03g0694800 | OsR498G0306856500.01.T03 | 50S ribosomal protein L18, cyanelle |
| augustus_masked-3-processed-gene-415.2-mRNA-1 | Japonica | NA | | Os03g0693400 | OsR498G0306851600.01.T02 | |
| augustus_masked-3-processed-gene-415.3-mRNA-1 | Japonica | NA | | Os03g0693700 | OsR498G0306853200.01.T01 | Oxalate oxidase 2 |
| augustus_masked-3-processed-gene-415.31-mRNA-1 | Japonica | NA | LRR-TM | | OsR498G0306857800.01.T01 | Putative LRR receptor-like protein kinase |
| augustus_masked-3-processed-gene-415.5-mRNA-1 | Japonica | NA | | Os03g0693900 | OsR498G0306854600.01.T01 | Oxalate oxidase 2 |
| augustus_masked-3-processed-gene-417.12-mRNA-1 | Japonica | NA | | Os03g0698800 | OsR498G0306869300.01.T05 | Zinc finger CCCH domain-containing protein 24 |
| augustus_masked-3-processed-gene-418.28-mRNA-1 | Japonica | Indica | | Os07g0196700 | OsR498G0306874200.01.T01 | NAC domain-containing protein |
| augustus_masked-3-processed-gene-419.57-mRNA-1 | Japonica | NA | | Os03g0702500 | OsR498G0306882500.01.T01 | Glycosyltransferase |
| augustus_masked-3-processed-gene-419.59-mRNA-1 | Japonica | NA | | Os03g0702800 | OsR498G0306884100.01.T02 | Eukaryotic peptide chain release factor subunit 1-3 |
| augustus_masked-3-processed-gene-419.67-mRNA-1 | Japonica | NA | | | OsR498G0306883000.01.T01 | Histone acetyltransferase of the MYST family 1 isoform 1 |
| augustus_masked-3-processed-gene-419.73-mRNA-1 | Japonica | NA | | | OsR498G0306885000.01.T06 | Protein kinase domain-containing protein |
| augustus_masked-3-processed-gene-42.16-mRNA-1 | Japonica | Japonica | | Os03g0185000 | OsR498G0305393200.01.T01 | Serine O-acetyltransferase |
| augustus_masked-3-processed-gene-42.32-mRNA-1 | Japonica | NA | | Os03g0185200 | OsR498G0305348400.01.T05 | Pentatricopeptide repeat-containing protein DOT4, chloroplastic |
| augustus_masked-3-processed-gene-42.34-mRNA-1 | Japonica | NA | | Os03g0185700 | OsR498G0305349200.01.T01 | Agmatine coumaroyltransferase-2 |
| augustus_masked-3-processed-gene-421.0-mRNA-1 | Japonica | Japonica | | Os03g0705433 | OsR498G0306891900.01.T01 | Putative transcription factor RL9 |
| augustus_masked-3-processed-gene-421.1-mRNA-1 | Japonica | NA | | | | ATP-dependent helicase |
| augustus_masked-3-processed-gene-421.12-mRNA-1 | Japonica | NA | | Os03g0706500 | OsR498G0306897300.01.T01 | Transcription factor TEOSINTE BRANCHED 1 |
| augustus_masked-3-processed-gene-422.40-mRNA-1 | Japonica | NA | | Os03g0707600 | OsR498G0306900900.01.T01 | DELLA protein RHT-1 |
| augustus_masked-3-processed-gene-423.36-mRNA-1 | Japonica | NA | | Os03g0708200 | OsR498G0306904300.01.T01 | DUF295 domain-containing protein (Fragment) |
| augustus_masked-3-processed-gene-423.41-mRNA-1 | Japonica | NA | | Os03g0708600 | OsR498G0306905200.01.T03 | DEAD-box ATP-dependent RNA helicase |
| augustus_masked-3-processed-gene-423.53-mRNA-1 | Japonica | Indica | | Os09g0552200 | OsR498G0306910900.01.T01 | |
| augustus_masked-3-processed-gene-423.73-mRNA-1 | Japonica | NA | | Os03g0710550 | | |
| augustus_masked-3-processed-gene-424.48-mRNA-1 | Japonica | NA | | | OsR498G0306916100.01.T01 | DNA-directed RNA polymerase I subunit H |
| augustus_masked-3-processed-gene-424.55-mRNA-1 | Japonica | NA | | | OsR498G0306915600.01.T02 | H15 domain-containing protein (Fragment) |
| augustus_masked-3-processed-gene-425.21-mRNA-1 | Japonica | NA | | | OsR498G1221284200.01.T02 | |
| augustus_masked-3-processed-gene-426.1-mRNA-1 | Japonica | Indica | Kinase-TM | Os03g0225100 | OsR498G0306923500.01.T01 | Mitogen-activated protein kinase kinase 7 |
| augustus_masked-3-processed-gene-427.8-mRNA-1 | Japonica | NA | | | OsR498G0306930000.01.T01 | |
| augustus_masked-3-processed-gene-428.46-mRNA-1 | Japonica | NA | | Os03g0719100 | OsR498G0306943300.01.T02 | E3 SUMO-protein ligase SIZ2 |
| augustus_masked-3-processed-gene-429.9-mRNA-1 | Japonica | NA | | Os03g0720400 | OsR498G0306947300.01.T01 | F-box only protein 7 |
| augustus_masked-3-processed-gene-43.37-mRNA-1 | Japonica | NA | | Os03g0186900 | OsR498G0305351700.01.T01 | Peptidase A1 domain-containing protein (Fragment) |
| augustus_masked-3-processed-gene-43.61-mRNA-1 | Japonica | NA | | Os03g0187800 | OsR498G0305357000.01.T01 | Purine permease |
| augustus_masked-3-processed-gene-43.62-mRNA-1 | Japonica | NA | | Os03g0188200 | | RING-H2 finger protein ATL3B |
| augustus_masked-3-processed-gene-430.12-mRNA-1 | Japonica | NA | | Os03g0722500 | OsR498G0306957100.01.T02 | Glucan endo-1,3-beta-D-glucosidase |
| augustus_masked-3-processed-gene-430.27-mRNA-1 | Japonica | NA | | Os03g0722000 | | Hyccin domain-containing protein (Fragment) |
| augustus_masked-3-processed-gene-431.1-mRNA-1 | Japonica | NA | | Os03g0723000 | OsR498G0306958900.01.T01 | Scarecrow-like protein 4 |

| | | | | | | |
|---|---|---|---|---|---|---|
| augustus_masked-3-processed-gene-432.0-mRNA-1 | Japonica | NA | | Os03g0724700 | OsR498G0306965500.01.T05 | Maf-like protein, expressed |
| augustus_masked-3-processed-gene-433.0-mRNA-1 | Japonica | NA | | Os03g0726400 | OsR498G0306972700.01.T01 | Transcription initiation factor TFIID subunit 8 |
| augustus_masked-3-processed-gene-433.13-mRNA-1 | Japonica | NA | | | OsR498G0306975400.01.T02 | KNOTTED1 homeodomain protein (Fragment) |
| augustus_masked-3-processed-gene-434.24-mRNA-1 | Japonica | NA | | Os03g0727800 | OsR498G0306978500.01.T01 | Protein kinase domain-containing protein |
| augustus_masked-3-processed-gene-434.27-mRNA-1 | Japonica | NA | | Os03g0728200 | OsR498G0306979100.01.T02 | Pentatricopeptide repeat |
| augustus_masked-3-processed-gene-434.28-mRNA-1 | Japonica | NA | | Os03g0728500 | OsR498G0306979300.01.T01 | FHA domain containing protein, expressed |
| augustus_masked-3-processed-gene-435.43-mRNA-1 | Japonica | NA | | Os03g0728700 | | |
| augustus_masked-3-processed-gene-437.47-mRNA-1 | Japonica | Indica | | Os03g0732100 | OsR498G0306994100.01.T01 | Homeodomain protein JUBEL1 |
| augustus_masked-3-processed-gene-437.59-mRNA-1 | Japonica | NA | | Os03g0733400 | | Zinc finger BED domain-containing protein DAYSLEEPER |
| augustus_masked-3-processed-gene-438.50-mRNA-1 | Japonica | Indica | | Os03g0734100 | OsR498G0307000500.01.T02 | |
| augustus_masked-3-processed-gene-438.62-mRNA-1 | Japonica | NA | | Os03g0734400 | OsR498G0307001400.01.T01 | Mitochondrial ribosomal protein L7/L12 |
| augustus_masked-3-processed-gene-439.13-mRNA-1 | Japonica | NA | | Os03g0736200 | OsR498G0307006100.01.T01 | Pentatricopeptide repeat-containing protein |
| augustus_masked-3-processed-gene-439.14-mRNA-1 | Japonica | Indica | OsSYP121 | Os03g0787000 | OsR498G0307007300.01.T01 | Syntaxin-related protein KNOLLE |
| augustus_masked-3-processed-gene-439.15-mRNA-1 | Japonica | NA | | Os03g0736600 | OsR498G0307007700.01.T01 | ATP synthase subunit delta, chloroplastic |
| augustus_masked-3-processed-gene-44.10-mRNA-1 | Japonica | NA | | | OsR498G0305363300.01.T01 | |
| augustus_masked-3-processed-gene-44.14-mRNA-1 | Japonica | Indica | | Os03g0189900 | OsR498G0305365900.01.T01 | VQ motif-containing protein 9 |
| augustus_masked-3-processed-gene-44.24-mRNA-1 | Japonica | NA | | Os03g0189300 | OsR498G0305364200.01.T01 | Myosin heavy chain-related protein |
| augustus_masked-3-processed-gene-441.29-mRNA-1 | Japonica | NA | | | OsR498G0307016800.01.T01 | |
| augustus_masked-3-processed-gene-441.40-mRNA-1 | Japonica | NA | | Os03g0741600 | OsR498G0307023100.01.T02 | |
| augustus_masked-3-processed-gene-442.43-mRNA-1 | Japonica | NA | | | OsR498G0307028900.01.T04 | Methyltransferase small domain |
| augustus_masked-3-processed-gene-442.45-mRNA-1 | Japonica | NA | | | OsR498G0202820500.01.T01 | U5 small nuclear ribonucleoprotein 200 kDa helicase |
| augustus_masked-3-processed-gene-442.52-mRNA-1 | Japonica | NA | | Os03g0743650 | OsR498G0307030000.01.T01 | Retrotransposon protein, putative, Ty3-gypsy sub-class |
| augustus_masked-3-processed-gene-444.32-mRNA-1 | Japonica | NA | | Os03g0746700 | OsR498G0307041400.01.T01 | Zinc finger MYM-type protein 6 |
| augustus_masked-3-processed-gene-445.12-mRNA-1 | Japonica | NA | | Os03g0748000 | OsR498G0307045700.01.T01 | Evolutionarily conserved C-terminal region 2 |
| augustus_masked-3-processed-gene-446.39-mRNA-1 | Japonica | NA | | Os03g0749400 | OsR498G0307051300.01.T01 | Circumsporozoite protein-like protein |
| augustus_masked-3-processed-gene-446.45-mRNA-1 | Japonica | NA | | Os03g0750500 | OsR498G0307053300.01.T02 | Atrophin-1 like |
| augustus_masked-3-processed-gene-448.17-mRNA-1 | Japonica | NA | | Os03g0753500 | OsR498G0307062200.01.T01 | |
| augustus_masked-3-processed-gene-45.19-mRNA-1 | Japonica | NA | | Os03g0191700 | OsR498G0305370400.01.T03 | PUM-HD domain-containing protein (Fragment) |
| augustus_masked-3-processed-gene-45.20-mRNA-1 | Japonica | NA | | Os03g0191900 | | DRE binding factor |
| augustus_masked-3-processed-gene-450.56-mRNA-1 | Japonica | NA | Kinase-LRR-TM | Os03g0756200 | OsR498G0307070000.01.T03 | Lrr receptor-like serinethreonine-protein kinase rpk2 |
| augustus_masked-3-processed-gene-451.17-mRNA-1 | Japonica | NA | | Os03g0757600 | OsR498G0307074100.01.T01 | UDP-glycosyltransferase 83A1 |
| augustus_masked-3-processed-gene-451.2-mRNA-1 | Japonica | NA | | | | DUF591 domain-containing protein |
| augustus_masked-3-processed-gene-451.28-mRNA-1 | Japonica | NA | | Os03g0757100 | OsR498G0307073200.01.T01 | UDP-glycosyltransferase 83A1 |
| augustus_masked-3-processed-gene-452.58-mRNA-1 | Japonica | NA | | | OsR498G0307081900.01.T01 | |
| augustus_masked-3-processed-gene-452.60-mRNA-1 | Japonica | Indica | | Os03g0759700 | OsR498G0307082900.01.T01 | BHLH transcription factor (Fragment) |
| augustus_masked-3-processed-gene-452.62-mRNA-1 | Japonica | NA | | Os03g0760000 | OsR498G0307083700.01.T02 | Isoflavone 2'-hydroxylase |
| augustus_masked-3-processed-gene-452.65-mRNA-1 | Japonica | NA | | Os03g0760100 | OsR498G0307084100.01.T01 | |
| augustus_masked-3-processed-gene-453.47-mRNA-1 | Japonica | NA | | Os03g0760900 | | |
| augustus_masked-3-processed-gene-453.48-mRNA-1 | Japonica | NA | | Os03g0761300 | OsR498G0307086900.01.T01 | Pentatricopeptide repeat-containing protein, mitochondrial |
| augustus_masked-3-processed-gene-453.78-mRNA-1 | Japonica | NA | | Os03g0761500 | OsR498G0307087600.01.T01 | Subtilisin-like protease |
| augustus_masked-3-processed-gene-453.81-mRNA-1 | Japonica | NA | | Os03g0762700 | OsR498G0307092400.01.T01 | Transcriptional regulator of RNA polII SAGA subunit |
| augustus_masked-3-processed-gene-454.49-mRNA-1 | Japonica | NA | | Os03g0764100 | | Zinc finger transcription factor ZF1 |
| augustus_masked-3-processed-gene-454.52-mRNA-1 | Japonica | NA | | | OsR498G0511427100.01.T01 | Epstein-Barr virus EBNA-1-like |
| augustus_masked-3-processed-gene-455.35-mRNA-1 | Japonica | NA | | Os03g0764900 | OsR498G0307100600.01.T01 | Dof zinc finger protein like |
| augustus_masked-3-processed-gene-455.39-mRNA-1 | Japonica | NA | | Os03g0765800 | OsR498G0307104500.01.T01 | Molybdenum cofactor sulfurase |
| augustus_masked-3-processed-gene-456.26-mRNA-1 | Japonica | NA | | Os03g0766900 | OsR498G0307107500.01.T04 | Proteasome assembly chaperone 2 |
| augustus_masked-3-processed-gene-456.33-mRNA-1 | Japonica | NA | | Os03g0767000 | OsR498G0307107800.01.T01 | Allene oxide synthase chloroplastic |
| augustus_masked-3-processed-gene-456.40-mRNA-1 | Japonica | NA | | Os03g0768900 | | |
| augustus_masked-3-processed-gene-456.47-mRNA-1 | Japonica | NA | | Os03g0767700 | | Pentatricopeptide repeat-containing protein, mitochondrial |
| augustus_masked-3-processed-gene-457.23-mRNA-1 | Japonica | NA | | Os03g0769900 | OsR498G0307115300.01.T01 | BSD domain-containing protein |
| augustus_masked-3-processed-gene-458.45-mRNA-1 | Japonica | Indica | Kinase-TM | Os03g0772600 | OsR498G0307124500.01.T01 | Lectin protein kinase, putative, expressed |
| augustus_masked-3-processed-gene-458.51-mRNA-1 | Japonica | Indica | Kinase | Os04g0531500 | OsR498G0307125400.01.T01 | Non-specific serine/threonine protein kinase |
| augustus_masked-3-processed-gene-458.53-mRNA-1 | Japonica | NA | | Os03g0772900 | OsR498G0307126600.01.T01 | DUF862 domain-containing protein |
| augustus_masked-3-processed-gene-46.58-mRNA-1 | Japonica | NA | | Os03g0193000 | OsR498G0305375300.01.T05 | GRAS domain-containing protein (Fragment) |
| augustus_masked-3-processed-gene-460.12-mRNA-1 | Japonica | NA | | Os03g0775400 | | Pentatricopeptide repeat |
| augustus_masked-3-processed-gene-460.35-mRNA-1 | Japonica | NA | | Os03g0776400 | OsR498G0307139600.01.T01 | |
| augustus_masked-3-processed-gene-461.14-mRNA-1 | Japonica | NA | | Os03g0778400 | OsR498G0307149500.01.T01 | Keratinocyte proline-rich protein |
| augustus_masked-3-processed-gene-461.26-mRNA-1 | Japonica | NA | | | OsR498G0307150200.01.T01 | DUF295 domain-containing protein |
| augustus_masked-3-processed-gene-462.9-mRNA-1 | Japonica | NA | | | OsR498G0511427100.01.T01 | Epstein-Barr virus EBNA-1-like |
| augustus_masked-3-processed-gene-463.16-mRNA-1 | Japonica | NA | | Os03g0783100 | | |
| augustus_masked-3-processed-gene-463.19-mRNA-1 | Japonica | Indica | | Os03g0783100 | OsR498G0307161100.01.T01 | Sentrin-specific protease 7 (Fragment) |

| | | | | | | |
|---|---|---|---|---|---|---|
| augustus_masked-3-processed-gene-463.29-mRNA-1 | Japonica | NA | | Os03g0784000 | OsR498G0307165900.01.T08 | Amine oxidase, putative, expressed |
| augustus_masked-3-processed-gene-464.23-mRNA-1 | Japonica | NA | | | | |
| augustus_masked-3-processed-gene-465.44-mRNA-1 | Japonica | NA | | Os03g0786600 | OsR498G0307172600.01.T01 | PORR domain-containing protein (Fragment) |
| augustus_masked-3-processed-gene-465.50-mRNA-1 | Japonica | NA | | Os03g0788100 | OsR498G0307176100.01.T01 | RING-H2 finger protein ATL71 |
| augustus_masked-3-processed-gene-466.1-mRNA-1 | Japonica | NA | | Os03g0788300 | OsR498G0307176700.01.T02 | Nuclear pore complex protein NUP1 |
| augustus_masked-3-processed-gene-466.8-mRNA-1 | Japonica | NA | | Os03g0788600 | OsR498G0307177200.01.T01 | Fasciclin-like arabinogalactan protein 16 |
| augustus_masked-3-processed-gene-467.46-mRNA-1 | Japonica | NA | | Os03g0790500 | OsR498G0307182700.01.T01 | probable carboxylesterase 15 |
| augustus_masked-3-processed-gene-467.64-mRNA-1 | Japonica | NA | | Os03g0791600 | | |
| augustus_masked-3-processed-gene-468.20-mRNA-1 | Japonica | NA | | Os03g0794000 | OsR498G0307194900.01.T01 | AAI domain-containing protein (Fragment) |
| augustus_masked-3-processed-gene-468.30-mRNA-1 | Japonica | NA | | Os03g0793000 | OsR498G0307191600.01.T01 | Zinc finger AN1 domain-containing stress-associated protein 14 |
| augustus_masked-3-processed-gene-468.9-mRNA-1 | Japonica | NA | | | OsR498G0307189200.01.T01 | DUF295 domain-containing protein (Fragment) |
| augustus_masked-3-processed-gene-469.46-mRNA-1 | Japonica | NA | | Os03g0795200 | OsR498G0307197700.01.T01 | Pentatricopeptide repeat-containing protein chloroplastic |
| augustus_masked-3-processed-gene-469.62-mRNA-1 | Japonica | NA | | | OsR498G0307200200.01.T01 | F-box domain-containing protein |
| augustus_masked-3-processed-gene-469.63-mRNA-1 | Japonica | Japonica | | Os03g0796100 | OsR498G0511251800.01.T01 | F-box domain-containing protein (Fragment) |
| augustus_masked-3-processed-gene-47.0-mRNA-1 | Japonica | NA | | Os05g0548200 | | TFIIB-type domain-containing protein |
| augustus_masked-3-processed-gene-47.1-mRNA-1 | Japonica | NA | | Os03g0193700 | | Geranylgeranyl transferase type-2 subunit beta |
| augustus_masked-3-processed-gene-47.15-mRNA-1 | Japonica | NA | | | OsR498G0511460800.01.T01 | |
| augustus_masked-3-processed-gene-47.16-mRNA-1 | Japonica | NA | | Os03g0194100 | OsR498G0305379000.01.T04 | C2 calcium-dependent membrane targeting |
| augustus_masked-3-processed-gene-47.17-mRNA-1 | Japonica | NA | | Os03g0194300 | OsR498G0305379200.01.T01 | Cytochrome b561 and DOMON domain-containing protein |
| augustus_masked-3-processed-gene-47.6-mRNA-1 | Japonica | NA | | Os03g0194600 | OsR498G0305379700.01.T02 | DOMON domain-containing protein (Fragment) |
| augustus_masked-3-processed-gene-470.39-mRNA-1 | Japonica | NA | | Os03g0797600 | OsR498G0307212100.01.T04 | Transcription factor UNE12 |
| augustus_masked-3-processed-gene-470.48-mRNA-1 | Japonica | NA | | Os03g0797000 | OsR498G0307209000.01.T03 | Tryptophan synthase |
| augustus_masked-3-processed-gene-471.32-mRNA-1 | Japonica | Japonica | | Os03g0798400 | OsR498G0307023100.01.T02 | |
| augustus_masked-3-processed-gene-472.55-mRNA-1 | Japonica | Indica | | Os03g0800000 | OsR498G0307220700.01.T01 | Nodulin-like domain-containing protein (Fragment) |
| augustus_masked-3-processed-gene-472.78-mRNA-1 | Japonica | NA | | Os03g0801350 | OsR498G0307223000.01.T01 | |
| augustus_masked-3-processed-gene-473.14-mRNA-1 | Japonica | Japonica | | Os03g0802500 | OsR498G0713673300.01.T01 | Adenosinetriphosphatase |
| augustus_masked-3-processed-gene-473.17-mRNA-1 | Japonica | Indica | | Os03g0802600 | OsR498G0307226500.01.T01 | Adenosinetriphosphatase |
| augustus_masked-3-processed-gene-473.3-mRNA-1 | Japonica | NA | | Os03g0802150 | OsR498G0307225100.01.T01 | DUF295 domain-containing protein (Fragment) |
| augustus_masked-3-processed-gene-473.4-mRNA-1 | Japonica | Japonica | | Os03g0802200 | OsR498G0102093100.01.T01 | Cysteine-rich transmembrane CYSTM domain containing protein |
| augustus_masked-3-processed-gene-476.34-mRNA-1 | Japonica | NA | | Os03g0809000 | OsR498G0307249200.01.T01 | Dirigent protein 15 |
| augustus_masked-3-processed-gene-477.48-mRNA-1 | Japonica | NA | | Os03g0810100 | OsR498G0307251700.01.T01 | Adenylate isopentenyltransferase |
| augustus_masked-3-processed-gene-477.57-mRNA-1 | Japonica | NA | | | OsR498G0307253600.01.T01 | |
| augustus_masked-3-processed-gene-478.0-mRNA-1 | Japonica | NA | | | | DUF591 domain-containing protein |
| augustus_masked-3-processed-gene-478.19-mRNA-1 | Japonica | NA | | Os03g0811800 | OsR498G0307258000.01.T01 | Ribosomal protein (Fragment) |
| augustus_masked-3-processed-gene-478.8-mRNA-1 | Japonica | NA | | Os03g0811700 | OsR498G0307257700.01.T01 | RRM domain-containing protein (Fragment) |
| augustus_masked-3-processed-gene-48.14-mRNA-1 | Japonica | NA | | Os03g0196250 | OsR498G0305387400.01.T01 | Putative metal ion binding protein |
| augustus_masked-3-processed-gene-48.15-mRNA-1 | Japonica | Indica | | Os02g0316200 | OsR498G0305390400.01.T01 | F-box/kelch-repeat protein OR23 |
| augustus_masked-3-processed-gene-48.16-mRNA-1 | Japonica | NA | | Os03g0196500 | OsR498G0305391600.01.T01 | F-box protein SNE |
| augustus_masked-3-processed-gene-48.17-mRNA-1 | Japonica | NA | | Os03g0196600 | OsR498G0305393200.01.T01 | Serine O-acetyltransferase |
| augustus_masked-3-processed-gene-48.9-mRNA-1 | Japonica | NA | | | OsR498G0305389700.01.T02 | X8 domain-containing protein |
| augustus_masked-3-processed-gene-480.66-mRNA-1 | Japonica | NA | | Os03g0815800 | OsR498G0307268600.01.T01 | Ethylene-responsive transcription factor CRF3 |
| augustus_masked-3-processed-gene-481.1-mRNA-1 | Japonica | NA | | Os03g0817200 | OsR498G0307275500.01.T01 | Lysine histidine transporter 1 |
| augustus_masked-3-processed-gene-482.11-mRNA-1 | Japonica | NA | | Os03g0820100 | OsR498G0307285100.01.T04 | NEDD8-activating enzyme E1 regulatory subunit |
| augustus_masked-3-processed-gene-482.15-mRNA-1 | Japonica | NA | | Os03g0820600 | OsR498G0307286500.01.T01 | Actin-depolymerizing factor |
| augustus_masked-3-processed-gene-483.47-mRNA-1 | Japonica | NA | Kinase-TM | Os03g0821900 | OsR498G0307292500.01.T01 | Protein kinase APK1A chloroplastic |
| augustus_masked-3-processed-gene-483.53-mRNA-1 | Japonica | NA | | Os03g0822900 | | HAT family dimerisation domain containing protein, expressed |
| augustus_masked-3-processed-gene-483.63-mRNA-1 | Japonica | NA | | Os03g0821700 | OsR498G0307291800.01.T01 | Putative tetratricopeptide-like helical domain, DYW domain-containing prot |
| augustus_masked-3-processed-gene-484.15-mRNA-1 | Japonica | Japonica | | Os03g0824600 | OsR498G0307299700.01.T01 | DIMBOA UDP-glucosyltransferase BX8 |
| augustus_masked-3-processed-gene-484.2-mRNA-1 | Japonica | Indica | | Os03g0823400 | OsR498G0307296200.01.T01 | Bowman-Birk type trypsin inhibitor |
| augustus_masked-3-processed-gene-484.21-mRNA-1 | Japonica | NA | | Os03g0823700 | OsR498G0307297400.01.T01 | Ras-related protein Rab-11A |
| augustus_masked-3-processed-gene-484.25-mRNA-1 | Japonica | Indica | | Os03g0824000 | OsR498G0307298400.01.T02 | Mitotic-spindle organizing protein 1A |
| augustus_masked-3-processed-gene-485.64-mRNA-1 | Japonica | NA | | Os03g0826500 | OsR498G0307305400.01.T01 | Anthranilate synthase alpha subunit 1, chloroplastic |
| augustus_masked-3-processed-gene-486.14-mRNA-1 | Japonica | NA | Kinase-TM | Os03g0828800 | OsR498G0307310600.01.T02 | Dual specificity testis-specific protein kinase 2 |
| augustus_masked-3-processed-gene-486.29-mRNA-1 | Japonica | NA | | Os03g0829600 | OsR498G0307312600.01.T01 | PMEI domain-containing protein (Fragment) |
| augustus_masked-3-processed-gene-490.33-mRNA-1 | Japonica | NA | Kinase-TM | Os03g0838100 | OsR498G0307339000.01.T02 | Non-specific serine/threonine protein kinase (Fragment) |
| augustus_masked-3-processed-gene-491.47-mRNA-1 | Japonica | NA | | Os03g0839400 | | |
| augustus_masked-3-processed-gene-491.50-mRNA-1 | Japonica | NA | | Os03g0839800 | | |
| augustus_masked-3-processed-gene-492.101-mRNA-1 | Japonica | NA | | Os03g0843300 | OsR498G0307353000.01.T03 | Salt tolerance protein I |
| augustus_masked-3-processed-gene-492.63-mRNA-1 | Japonica | NA | | | OsR498G0307346400.01.T01 | SANT domain-containing protein |
| augustus_masked-3-processed-gene-493.61-mRNA-1 | Japonica | NA | | Os03g0843700 | | |
| augustus_masked-3-processed-gene-493.78-mRNA-1 | Japonica | NA | | Os03g0845100 | | Transposon protein, putative, Mutator sub-class |

| | | | | | | |
|---|---|---|---|---|---|---|
| augustus_masked-3-processed-gene-494.16-mRNA-1 | Japonica | NA | | | OsR498G0307363000.01.T01 | |
| augustus_masked-3-processed-gene-494.5-mRNA-1 | Japonica | NA | | Os03g0845600 | OsR498G0307359400.01.T01 | Glucan endo-1,3-beta-D-glucosidase |
| augustus_masked-3-processed-gene-496.24-mRNA-1 | Japonica | NA | | Os03g0850300 | OsR498G0307370800.01.T02 | SAM domain-containing protein (Fragment) |
| augustus_masked-3-processed-gene-496.26-mRNA-1 | Japonica | NA | | Os03g0850700 | OsR498G0307373200.01.T01 | Polyphosphoinositide binding protein Ssh2p |
| augustus_masked-3-processed-gene-496.32-mRNA-1 | Japonica | NA | | Os03g0851300 | OsR498G0307375200.01.T01 | 26S proteasome non-ATPase regulatory subunit A |
| augustus_masked-3-processed-gene-497.11-mRNA-1 | Japonica | NA | | Os03g0852800 | OsR498G0307378500.01.T01 | Non-specific phospholipase |
| augustus_masked-3-processed-gene-497.22-mRNA-1 | Japonica | NA | | | OsR498G0307376800.01.T01 | Pentatricopeptide repeat |
| augustus_masked-3-processed-gene-497.24-mRNA-1 | Japonica | NA | | Os03g0852400 | OsR498G0307377400.01.T01 | Amino acid adenylation domain-containing protein |
| augustus_masked-3-processed-gene-497.26-mRNA-1 | Japonica | NA | | Os03g0852700 | OsR498G0307378000.01.T01 | Pentatricopeptide repeat-containing protein, chloroplastic |
| augustus_masked-3-processed-gene-498.10-mRNA-1 | Japonica | NA | | Os03g0854000 | | Gamma-glutamylcyclotransferase family protein (Fragment) |
| augustus_masked-3-processed-gene-498.22-mRNA-1 | Japonica | NA | | Os03g0854400 | | RNase III domain-containing protein (Fragment) |
| augustus_masked-3-processed-gene-498.5-mRNA-1 | Japonica | NA | | Os03g0853700 | OsR498G0307382200.01.T02 | GCFC domain-containing protein (Fragment) |
| augustus_masked-3-processed-gene-499.41-mRNA-1 | Japonica | NA | | Os03g0856000 | OsR498G0307391300.01.T01 | S-norcoclaurine synthase 1 |
| augustus_masked-3-processed-gene-499.42-mRNA-1 | Japonica | Indica | | Os03g0856100 | OsR498G0307391500.01.T01 | Smr domain-containing protein (Fragment) |
| augustus_masked-3-processed-gene-50.6-mRNA-1 | Japonica | NA | | Os03g0199100 | OsR498G0305405700.01.T01 | |
| augustus_masked-3-processed-gene-50.7-mRNA-1 | Japonica | NA | | Os03g0199300 | | |
| augustus_masked-3-processed-gene-500.0-mRNA-1 | Japonica | NA | | Os03g0856700 | OsR498G0307394300.01.T01 | Gibberellin 20-oxidase |
| augustus_masked-3-processed-gene-500.10-mRNA-1 | Japonica | NA | | | | |
| augustus_masked-3-processed-gene-500.11-mRNA-1 | Japonica | NA | | Os03g0857600 | OsR498G0307396700.01.T01 | SASA domain-containing protein (Fragment) |
| augustus_masked-3-processed-gene-500.21-mRNA-1 | Japonica | NA | | Os03g0857750 | OsR498G0307397000.01.T01 | PREDICTED |
| augustus_masked-3-processed-gene-500.26-mRNA-1 | Japonica | NA | | Os03g0858600 | OsR498G0307399000.01.T01 | Myelin-associated oligodendrocyte basic protein |
| augustus_masked-3-processed-gene-500.9-mRNA-1 | Japonica | Japonica | | Os03g0857100 | OsR498G0307395900.01.T01 | Outer membrane autotransporter |
| augustus_masked-3-processed-gene-501.21-mRNA-1 | Japonica | NA | | Os03g0860600 | OsR498G0307403900.01.T01 | Oxidoreductase, 2OG-Fe oxygenase family protein, expressed |
| augustus_masked-3-processed-gene-501.3-mRNA-1 | Japonica | NA | | Os03g0858800 | OsR498G0307399200.01.T05 | Protein DETOXIFICATION |
| augustus_masked-3-processed-gene-501.32-mRNA-1 | Japonica | NA | | Os03g0860100 | OsR498G0307401600.01.T01 | Ethylene-responsive transcription factor |
| augustus_masked-3-processed-gene-501.33-mRNA-1 | Japonica | NA | | | OsR498G0408884400.01.T03 | Epstein-Barr virus EBNA-1-like |
| augustus_masked-3-processed-gene-501.34-mRNA-1 | Japonica | NA | | | OsR498G0204046800.01.T01 | Epstein-Barr virus EBNA-1-like |
| augustus_masked-3-processed-gene-501.36-mRNA-1 | Japonica | NA | | | OsR498G0307403800.01.T02 | Dilute domain-containing protein |
| augustus_masked-3-processed-gene-502.11-mRNA-1 | Japonica | NA | | Os10g0531750 | OsR498G0408675500.01.T01 | Retrotransposon protein, putative, Ty3-gypsy sub-class |
| augustus_masked-3-processed-gene-502.4-mRNA-1 | Japonica | NA | | Os03g0861000 | | |
| augustus_masked-3-processed-gene-51.76-mRNA-1 | Japonica | NA | | | OsR498G0305416200.01.T01 | Retrotransposon protein, putative, LINE subclass |
| augustus_masked-3-processed-gene-52.21-mRNA-1 | Japonica | Japonica | | Os03g0204201 | OsR498G0408675500.01.T01 | Retrotransposon protein, putative, Ty3-gypsy sub-class |
| augustus_masked-3-processed-gene-53.17-mRNA-1 | Japonica | NA | | Os03g0206100 | OsR498G0305428000.01.T03 | Protein SNOWY COTYLEDON 3 |
| augustus_masked-3-processed-gene-54.18-mRNA-1 | Japonica | NA | | Os03g0208600 | OsR498G0305434900.01.T01 | Pentatricopeptide repeat |
| augustus_masked-3-processed-gene-55.10-mRNA-1 | Japonica | NA | | Os03g0209600 | OsR498G0305438500.01.T05 | Methionyl-tRNA synthetase |
| augustus_masked-3-processed-gene-55.12-mRNA-1 | Japonica | Indica | | Os03g0209700 | OsR498G0305438700.01.T04 | Rho GTPase-activating protein REN1-like |
| augustus_masked-3-processed-gene-55.24-mRNA-1 | Japonica | Indica | | Os03g0210200 | OsR498G0305439800.01.T01 | Cysteine proteinase inhibitor |
| augustus_masked-3-processed-gene-55.26-mRNA-1 | Japonica | NA | | | OsR498G0305440400.01.T01 | Myosin heavy chain |
| augustus_masked-3-processed-gene-55.28-mRNA-1 | Japonica | Indica | | Os03g0210600 | OsR498G0305441000.01.T01 | Nodulin-related protein 1 |
| augustus_masked-3-processed-gene-56.11-mRNA-1 | Japonica | NA | | | OsR498G0305443800.01.T01 | |
| augustus_masked-3-processed-gene-56.17-mRNA-1 | Japonica | Japonica | | Os03g0212000 | OsR498G0305445700.01.T01 | Glycosyltransferase |
| augustus_masked-3-processed-gene-56.2-mRNA-1 | Japonica | NA | | Os03g0211400 | OsR498G0305443500.01.T02 | Plant calmodulin-binding protein-related |
| augustus_masked-3-processed-gene-56.9-mRNA-1 | Japonica | NA | | Os03g0211200 | | |
| augustus_masked-3-processed-gene-57.51-mRNA-1 | Japonica | Indica | | Os03g0213200 | OsR498G0305448900.01.T03 | Stellacyanin |
| augustus_masked-3-processed-gene-57.72-mRNA-1 | Japonica | NA | | | OsR498G0305450200.01.T07 | |
| augustus_masked-3-processed-gene-57.73-mRNA-1 | Japonica | NA | | Os03g0213800 | OsR498G0305450400.01.T01 | mitochondrial substrate carrier family protein E |
| augustus_masked-3-processed-gene-58.35-mRNA-1 | Japonica | NA | | Os03g0215000 | OsR498G0305452800.01.T01 | TPT domain-containing protein |
| augustus_masked-3-processed-gene-59.34-mRNA-1 | Japonica | NA | | Os03g0215900 | OsR498G0305457400.01.T01 | Putative Smr domain, tetratricopeptide-like helical domain, pentacotripeptic |
| augustus_masked-3-processed-gene-59.35-mRNA-1 | Japonica | NA | | Os03g0216000 | OsR498G0305457800.01.T01 | Zinc finger protein GIS |
| augustus_masked-3-processed-gene-59.55-mRNA-1 | Japonica | NA | | Os03g0216500 | OsR498G0305459600.01.T01 | Delta-latroinsectotoxin-Lt1a |
| augustus_masked-3-processed-gene-59.58-mRNA-1 | Japonica | NA | | Os03g0217000 | | Recombination protein like |
| augustus_masked-3-processed-gene-60.14-mRNA-1 | Japonica | Japonica | | Os03g0219500 | OsR498G0204385500.01.T01 | Uv-induced protein uvi31 |
| augustus_masked-3-processed-gene-60.21-mRNA-1 | Japonica | NA | | Os03g0218500 | OsR498G0305464200.01.T01 | Heat shock protein 8 |
| augustus_masked-3-processed-gene-61.13-mRNA-1 | Japonica | NA | Kinase-TM | Os03g0221700 | | G-type lectin S-receptor-like serine/threonine-protein kinase |
| augustus_masked-3-processed-gene-61.4-mRNA-1 | Japonica | NA | | Os03g0219700 | OsR498G0305467400.01.T01 | DEAD-box ATP-dependent RNA helicase |
| augustus_masked-3-processed-gene-62.20-mRNA-1 | Japonica | NA | | | OsR498G0305476500.01.T01 | Caleosin |
| augustus_masked-3-processed-gene-62.32-mRNA-1 | Japonica | NA | | Os03g0223100 | OsR498G0305478400.01.T01 | Cytochrome P450 family 94 subfamily D polypeptide 2 |
| augustus_masked-3-processed-gene-62.4-mRNA-1 | Japonica | NA | | Os03g0222500 | | F-box domain-containing protein |
| augustus_masked-3-processed-gene-63.50-mRNA-1 | Japonica | NA | | Os03g0225300 | OsR498G0305487200.01.T01 | Pentatricopeptide repeat-containing protein |
| augustus_masked-3-processed-gene-64.37-mRNA-1 | Japonica | NA | | Os03g0225900 | OsR498G0305488900.01.T01 | Allene oxide synthase, chloroplastic |
| augustus_masked-3-processed-gene-64.45-mRNA-1 | Japonica | NA | | | OsR498G0204046800.01.T01 | Epstein-Barr virus EBNA-1-like |

| | | | | | | |
|---|---|---|---|---|---|---|
| augustus_masked-3-processed-gene-64.51-mRNA-1 | Japonica | NA | | | OsR498G0305491700.01.T01 | DNA methyl transferase5 |
| augustus_masked-3-processed-gene-65.40-mRNA-1 | Japonica | NA | | | OsR498G0511427100.01.T01 | Circumsporozoite protein-like |
| augustus_masked-3-processed-gene-68.20-mRNA-1 | Japonica | NA | | Os03g0232000 | OsR498G0305516600.01.T08 | Helix-loop-helix DNA-binding domain containing protein, expressed |
| augustus_masked-3-processed-gene-68.43-mRNA-1 | Japonica | NA | | Os03g0232600 | OsR498G0305520600.01.T01 | RING-type E3 ubiquitin transferase |
| augustus_masked-3-processed-gene-69.20-mRNA-1 | Japonica | Japonica | | Os03g0235200 | OsR498G0305528900.01.T01 | Pentatricopeptide repeat |
| augustus_masked-3-processed-gene-7.58-mRNA-1 | Japonica | NA | | Os03g0116900 | OsR498G0305123700.01.T05 | Armadillo/beta-catenin-like repeat family protein, expressed |
| augustus_masked-3-processed-gene-70.1-mRNA-1 | Japonica | NA | | | OsR498G0305531300.01.T01 | transcription factor MYB3R-3-like isoform X2 |
| augustus_masked-3-processed-gene-71.34-mRNA-1 | Japonica | NA | | | OsR498G0305536700.01.T03 | DUF775 domain-containing protein |
| augustus_masked-3-processed-gene-71.40-mRNA-1 | Japonica | Indica | | Os03g0237950 | OsR498G0305538800.01.T01 | FAS1 domain-containing protein (Fragment) |
| augustus_masked-3-processed-gene-71.55-mRNA-1 | Japonica | NA | | Os10g0531750 | OsR498G0408675500.01.T01 | Retrotransposon protein, putative, Ty3-gypsy sub-class |
| augustus_masked-3-processed-gene-71.61-mRNA-1 | Japonica | NA | | Os03g0239300 | OsR498G0305543200.01.T02 | Zinc finger protein 1 |
| augustus_masked-3-processed-gene-72.40-mRNA-1 | Japonica | NA | | Os03g0240400 | OsR498G0305545900.01.T01 | |
| augustus_masked-3-processed-gene-72.41-mRNA-1 | Japonica | NA | | Os03g0240800 | OsR498G0305546700.01.T01 | Small nuclear ribonucleoprotein Sm D3 |
| augustus_masked-3-processed-gene-72.47-mRNA-1 | Japonica | NA | | Os03g0240600 | OsR498G0305546400.01.T01 | RING-type E3 ubiquitin transferase |
| augustus_masked-3-processed-gene-73.67-mRNA-1 | Japonica | NA | | Os03g0242300 | OsR498G0305550600.01.T01 | |
| augustus_masked-3-processed-gene-73.69-mRNA-1 | Japonica | NA | | Os03g0242900 | OsR498G0305553400.01.T01 | Subtilisin-like serine protease 2 |
| augustus_masked-3-processed-gene-74.55-mRNA-1 | Japonica | Japonica | | Os03g0243400 | OsR498G0305555400.01.T01 | |
| augustus_masked-3-processed-gene-74.62-mRNA-1 | Japonica | NA | | Os03g0244200 | OsR498G0305557100.01.T01 | Thaumatin-like protein 1 |
| augustus_masked-3-processed-gene-74.64-mRNA-1 | Japonica | NA | | | OsR498G0204960100.01.T01 | |
| augustus_masked-3-processed-gene-74.75-mRNA-1 | Japonica | Indica | | Os03g0244950 | OsR498G0305559400.01.T01 | J domain-containing protein (Fragment) |
| augustus_masked-3-processed-gene-75.42-mRNA-1 | Japonica | Indica | | Os06g0236300 | OsR498G0305563100.01.T01 | Protein-serine/threonine phosphatase |
| augustus_masked-3-processed-gene-75.51-mRNA-1 | Japonica | NA | | Os03g0246100 | OsR498G0305562600.01.T01 | Glucan endo-1,3-beta-glucosidase 7 |
| augustus_masked-3-processed-gene-76.30-mRNA-1 | Japonica | NA | | Os03g0247100 | OsR498G0305567700.01.T01 | ABC transporter C family member 9 |
| augustus_masked-3-processed-gene-77.19-mRNA-1 | Japonica | NA | | Os03g0251100 | | |
| augustus_masked-3-processed-gene-77.26-mRNA-1 | Japonica | NA | | Os03g0250400 | OsR498G0305584400.01.T01 | |
| augustus_masked-3-processed-gene-77.4-mRNA-1 | Japonica | NA | | Os03g0249500 | OsR498G0305578700.01.T01 | UDP-D-glucuronate 4-epimerase 2 |
| augustus_masked-3-processed-gene-78.0-mRNA-1 | Japonica | NA | | | | V-type proton ATPase subunit a |
| augustus_masked-3-processed-gene-78.13-mRNA-1 | Japonica | NA | | | OsR498G0305591300.01.T02 | Piwi domain containing protein, expressed |
| augustus_masked-3-processed-gene-78.2-mRNA-1 | Japonica | NA | Kinase-TM | Os03g0251700 | OsR498G0305588100.01.T01 | Serine/threonine-protein kinase PBS1 |
| augustus_masked-3-processed-gene-78.6-mRNA-1 | Japonica | NA | | Os03g0251350 | OsR498G0305586800.01.T04 | CCAAT-binding transcription factor C |
| augustus_masked-3-processed-gene-79.1-mRNA-1 | Japonica | NA | Kinase-TM | Os03g0253200 | OsR498G0305593900.01.T01 | Serine/threonine-protein kinase WAG2 |
| augustus_masked-3-processed-gene-79.14-mRNA-1 | Japonica | NA | | Os03g0254400 | OsR498G0305597100.01.T01 | Patatin |
| augustus_masked-3-processed-gene-79.8-mRNA-1 | Japonica | NA | | Os03g0253500 | OsR498G0305594600.01.T10 | G-patch domain containing protein, expressed |
| augustus_masked-3-processed-gene-8.48-mRNA-1 | Japonica | NA | | Os03g0119700 | OsR498G0305132500.01.T01 | Pentatricopeptide repeat-containing protein mitochondrial |
| augustus_masked-3-processed-gene-8.49-mRNA-1 | Japonica | NA | | Os03g0119900 | OsR498G0305133100.01.T01 | Multifunctional fusion protein |
| augustus_masked-3-processed-gene-8.57-mRNA-1 | Japonica | NA | | Os03g0119300 | OsR498G0305130200.01.T01 | Serine protease |
| augustus_masked-3-processed-gene-81.35-mRNA-1 | Japonica | NA | | | | |
| augustus_masked-3-processed-gene-82.29-mRNA-1 | Japonica | NA | | Os03g0258900 | OsR498G0305610500.01.T01 | Galactose oxidase |
| augustus_masked-3-processed-gene-82.60-mRNA-1 | Japonica | NA | | | OsR498G0305615000.01.T01 | |
| augustus_masked-3-processed-gene-83.29-mRNA-1 | Japonica | NA | | Os03g0261800 | OsR498G0305617800.01.T01 | The fantastic four family |
| augustus_masked-3-processed-gene-84.12-mRNA-1 | Japonica | NA | | Os03g0263000 | OsR498G0305623600.01.T01 | AP2 domain class transcription factor |
| augustus_masked-3-processed-gene-84.2-mRNA-1 | Japonica | Indica | | Os03g0262900 | OsR498G0305623100.01.T01 | Vacuolar protein sorting-associated protein VPS9 |
| augustus_masked-3-processed-gene-84.22-mRNA-1 | Japonica | NA | | Os03g0263900 | OsR498G0305627100.01.T01 | ATP binding microtubule motor family protein, putative isoform 1 |
| augustus_masked-3-processed-gene-84.37-mRNA-1 | Japonica | NA | | Os03g0264600 | OsR498G0305631000.01.T01 | Zinc finger, C2H2 type family protein, expressed |
| augustus_masked-3-processed-gene-84.38-mRNA-1 | Japonica | NA | | Os03g0264700 | OsR498G0305631200.01.T01 | BTB/POZ domain-containing protein At3g09030 |
| augustus_masked-3-processed-gene-85.15-mRNA-1 | Japonica | Indica | | Os03g0266000 | OsR498G0305635900.01.T06 | Ubiquitinyl hydrolase 1 |
| augustus_masked-3-processed-gene-86.70-mRNA-1 | Japonica | NA | | Os03g0267200 | OsR498G0305639000.01.T01 | SHSP domain-containing protein |
| augustus_masked-3-processed-gene-87.32-mRNA-1 | Japonica | NA | | | OsR498G0305649500.01.T01 | Fringe protein, putative, expressed |
| augustus_masked-3-processed-gene-88.1-mRNA-1 | Japonica | NA | | Os03g0270300 | | |
| augustus_masked-3-processed-gene-88.22-mRNA-1 | Japonica | NA | | Os03g0271600 | OsR498G0305655100.01.T01 | RING-type E3 ubiquitin transferase |
| augustus_masked-3-processed-gene-9.46-mRNA-1 | Japonica | NA | | Os03g0120900 | OsR498G0305138300.01.T02 | ABI3VP1 transcription factor |
| augustus_masked-3-processed-gene-90.1-mRNA-1 | Japonica | NA | | | OsR498G0305667700.01.T06 | Initiator binding protein1 |
| augustus_masked-3-processed-gene-90.20-mRNA-1 | Japonica | NA | | Os03g0275500 | OsR498G0305672800.01.T02 | Epsin N-terminal domain-containing protein / clathrin assembly protein-rela |
| augustus_masked-3-processed-gene-90.9-mRNA-1 | Japonica | NA | | Os03g0274700 | OsR498G0305669100.01.T01 | Centrosome-associated protein CEP250 |
| augustus_masked-3-processed-gene-97.8-mRNA-1 | Japonica | NA | | Os03g0288000 | OsR498G0305724500.01.T01 | Metallothionein-like protein 1A |
| augustus_masked-3-processed-gene-99.11-mRNA-1 | Japonica | NA | Kinase-TM | | OsR498G0305741500.01.T01 | Mitogen-activated protein kinase kinase kinase 2 |
| augustus_masked-3-processed-gene-99.9-mRNA-1 | Japonica | Indica | | Os03g0292200 | OsR498G0305739900.01.T03 | Mitochondrial succinate-fumarate transporter like |
| augustus_masked-4-processed-gene-11.23-mRNA-1 | Japonica | NA | | | OsR498G0407671900.01.T01 | Histone-glutamine methyltransferase |
| augustus_masked-4-processed-gene-118.4-mRNA-1 | Japonica | NA | | Os07g0561900 | OsR498G0102180600.01.T01 | DUF295 domain-containing protein |
| augustus_masked-4-processed-gene-120.10-mRNA-1 | Japonica | NA | | | OsR498G0408305400.01.T05 | Single-stranded DNA-binding protein, mitochondrial |
| augustus_masked-4-processed-gene-140.52-mRNA-1 | Japonica | Japonica | | Os04g0395700 | OsR498G0408454800.01.T02 | Histone H2A.Z-specific chaperone CHZ1 (Fragment) |

| | | | | | | |
|---|---|---|---|---|---|---|
| augustus_masked-4-processed-gene-142.8-mRNA-1 | Japonica | NA | | | | HTH myb-type domain-containing protein |
| augustus_masked-4-processed-gene-143.28-mRNA-1 | Japonica | NA | | Os04g0398900 | | |
| augustus_masked-4-processed-gene-15.18-mRNA-1 | Japonica | Indica | | Os04g0191800 | OsR498G0407683800.01.T05 | F-box domain-containing protein |
| augustus_masked-4-processed-gene-15.6-mRNA-1 | Japonica | NA | | Os04g0191400 | OsR498G0407682200.01.T02 | |
| augustus_masked-4-processed-gene-15.7-mRNA-1 | Japonica | NA | | Os04g0191500 | | |
| augustus_masked-4-processed-gene-157.1-mRNA-1 | Japonica | NA | | | | Transposon protein, putative, unclassified |
| augustus_masked-4-processed-gene-158.31-mRNA-1 | Japonica | NA | | | OsR498G0408582600.01.T01 | Alba domain-containing protein |
| augustus_masked-4-processed-gene-16.33-mRNA-1 | Japonica | NA | | Os04g0193300 | | F-box domain-containing protein |
| augustus_masked-4-processed-gene-161.23-mRNA-1 | Japonica | NA | | | OsR498G0408599200.01.T02 | Pentatricopeptide repeat-containing protein mitochondrial |
| augustus_masked-4-processed-gene-165.41-mRNA-1 | Japonica | NA | Kinase-TM | Os04g0439600 | OsR498G0408622600.01.T01 | Serine/threonine-protein kinase-like protein CCR4 |
| augustus_masked-4-processed-gene-166.28-mRNA-1 | Japonica | NA | | Os04g0442300 | OsR498G0408633800.01.T01 | Two-component response regulator ORR1 |
| augustus_masked-4-processed-gene-169.50-mRNA-1 | Japonica | NA | | Os04g0444700 | OsR498G0408648400.01.T01 | Putative methyltransferase PMT21 |
| augustus_masked-4-processed-gene-169.76-mRNA-1 | Japonica | NA | | Os04g0446100 | OsR498G0408652800.01.T02 | Pentatricopeptide repeat-containing protein |
| augustus_masked-4-processed-gene-17.37-mRNA-1 | Japonica | NA | | Os04g0194000 | OsR498G0407697400.01.T01 | F-box domain-containing protein |
| augustus_masked-4-processed-gene-17.39-mRNA-1 | Japonica | NA | | Os04g0194600 | OsR498G0407699600.01.T01 | TCP transcription factor |
| augustus_masked-4-processed-gene-173.34-mRNA-1 | Japonica | NA | | | OsR498G0408681600.01.T02 | Sugar transport protein 5 |
| augustus_masked-4-processed-gene-175.36-mRNA-1 | Japonica | Japonica | | Os04g0457400 | OsR498G0204165200.01.T01 | |
| augustus_masked-4-processed-gene-18.10-mRNA-1 | Japonica | NA | | Os04g0195100 | OsR498G0407700600.01.T01 | F-box domain-containing protein |
| augustus_masked-4-processed-gene-182.30-mRNA-1 | Japonica | NA | | Os04g0472200 | OsR498G0408747800.01.T01 | FAS1 domain-containing protein |
| augustus_masked-4-processed-gene-184.25-mRNA-1 | Japonica | NA | | Os04g0474800 | OsR498G0408756800.01.T01 | Beta-glucosidase 13 |
| augustus_masked-4-processed-gene-184.27-mRNA-1 | Japonica | NA | | Os04g0475300 | OsR498G0408759400.01.T01 | Glycogen phosphorylase 1-like |
| augustus_masked-4-processed-gene-185.54-mRNA-1 | Japonica | NA | | Os04g0475800 | | Pentatricopeptide repeat-containing protein |
| augustus_masked-4-processed-gene-185.72-mRNA-1 | Japonica | NA | | | OsR498G0408765500.01.T01 | |
| augustus_masked-4-processed-gene-185.74-mRNA-1 | Japonica | NA | | Os04g0476800 | OsR498G0408764200.01.T01 | ZZ-type zinc finger-containing protein |
| augustus_masked-4-processed-gene-188.46-mRNA-1 | Japonica | NA | | Os04g0481800 | OsR498G0408776400.01.T01 | Wax synthase isoform 1 |
| augustus_masked-4-processed-gene-188.47-mRNA-1 | Japonica | Japonica | | Os04g0481900 | OsR498G0408776400.01.T01 | Wax synthase isoform 1 |
| augustus_masked-4-processed-gene-19.21-mRNA-1 | Japonica | Japonica | | Os04g0197500 | OsR498G0407707200.01.T01 | Glycosyltransferase |
| augustus_masked-4-processed-gene-193.51-mRNA-1 | Japonica | NA | | Os04g0493600 | OsR498G0408821800.01.T03 | Chitinase |
| augustus_masked-4-processed-gene-194.43-mRNA-1 | Japonica | NA | | | OsR498G0307361200.01.T01 | |
| augustus_masked-4-processed-gene-195.46-mRNA-1 | Japonica | NA | | | OsR498G0917172900.01.T01 | DUF834 domain-containing protein |
| augustus_masked-4-processed-gene-196.21-mRNA-1 | Japonica | NA | | Os05g0376300 | OsR498G0510716000.01.T03 | N-acetyltransferase ESCO1 |
| augustus_masked-4-processed-gene-196.30-mRNA-1 | Japonica | NA | | Os04g0500600 | | |
| augustus_masked-4-processed-gene-199.27-mRNA-1 | Japonica | NA | | Os04g0505300 | | |
| augustus_masked-4-processed-gene-199.35-mRNA-1 | Japonica | NA | | Os04g0505400 | OsR498G0408855200.01.T01 | Transmembrane protein, putative (DUF247) |
| augustus_masked-4-processed-gene-199.37-mRNA-1 | Japonica | Indica | | Os04g0505700 | OsR498G0408856000.01.T01 | Putative F-box/LRR-repeat protein 8 |
| augustus_masked-4-processed-gene-199.39-mRNA-1 | Japonica | NA | Kinase-LRR-TM | | | probable leucine-rich repeat receptor-like protein kinase At2g33170 |
| augustus_masked-4-processed-gene-2.9-mRNA-1 | Japonica | NA | | Os04g0174100 | OsR498G0407629800.01.T01 | Tyrosine N-monooxygenase |
| augustus_masked-4-processed-gene-200.23-mRNA-1 | Japonica | NA | | | OsR498G0408862600.01.T07 | Protein LOW PSII ACCUMULATION 1, chloroplastic isoform A |
| augustus_masked-4-processed-gene-201.8-mRNA-1 | Japonica | NA | | Os04g0508400 | | NAC transcription factor |
| augustus_masked-4-processed-gene-202.26-mRNA-1 | Japonica | NA | | Os04g0510400 | OsR498G0408876800.01.T01 | DEAD-box ATP-dependent RNA helicase |
| augustus_masked-4-processed-gene-203.42-mRNA-1 | Japonica | NA | | Os04g0512500 | OsR498G0408882000.01.T01 | Embryo sac development arrest 6 |
| augustus_masked-4-processed-gene-208.18-mRNA-1 | Japonica | Japonica | | Os04g0521900 | OsR498G0713495500.01.T01 | Vegetative cell wall protein gp1 |
| augustus_masked-4-processed-gene-209.69-mRNA-1 | Japonica | NA | | | OsR498G0408932000.01.T01 | Isochorismatase |
| augustus_masked-4-processed-gene-210.14-mRNA-1 | Japonica | NA | | Os04g0526600 | OsR498G0408934200.01.T01 | Alpha-amylasesubtilisin inhibitor |
| augustus_masked-4-processed-gene-219.18-mRNA-1 | Japonica | NA | | Os04g0544100 | OsR498G0409011400.01.T01 | Histone-lysine N-methyltransferase, H3 lysine-9 specific SUVH5 |
| augustus_masked-4-processed-gene-220.30-mRNA-1 | Japonica | NA | | Os04g0545300 | | Streptogrisin D (Fragment) |
| augustus_masked-4-processed-gene-220.56-mRNA-1 | Japonica | NA | | Os04g0546800 | OsR498G0409019800.01.T01 | Ethylene-responsive factor |
| augustus_masked-4-processed-gene-221.10-mRNA-1 | Japonica | NA | | | | Putative Anter-specific proline-rich protein APG |
| augustus_masked-4-processed-gene-221.11-mRNA-1 | Japonica | Indica | LRR-NBS-TM | Os04g0548100 | OsR498G0409024200.01.T01 | Disease resistance protein RPM1 |
| augustus_masked-4-processed-gene-221.29-mRNA-1 | Japonica | NA | | | OsR498G0409024800.01.T01 | Glycoprotein homolog |
| augustus_masked-4-processed-gene-225.45-mRNA-1 | Japonica | NA | | Os04g0556100 | | |
| augustus_masked-4-processed-gene-225.47-mRNA-1 | Japonica | NA | | Os04g0556400 | OsR498G0409056600.01.T01 | Putative cis-zeatin O-glucosyltransferase |
| augustus_masked-4-processed-gene-225.48-mRNA-1 | Japonica | NA | | Os04g0556500 | OsR498G0409058200.01.T01 | Glycosyltransferase |
| augustus_masked-4-processed-gene-225.49-mRNA-1 | Japonica | NA | | Os04g0556600 | OsR498G0409058600.01.T01 | Glycosyltransferase |
| augustus_masked-4-processed-gene-235.67-mRNA-1 | Japonica | NA | | Os04g0573300 | | Subtilisin-like serine protease |
| augustus_masked-4-processed-gene-239.16-mRNA-1 | Japonica | NA | | Os04g0579200 | OsR498G0409138800.01.T01 | E3 ubiquitin-protein ligase RHA2B |
| augustus_masked-4-processed-gene-239.31-mRNA-1 | Japonica | NA | | Os04g0580300 | OsR498G0409142800.01.T01 | GRAS domain-containing protein (Fragment) |
| augustus_masked-4-processed-gene-239.34-mRNA-1 | Japonica | NA | | Os04g0580500 | OsR498G0409144800.01.T01 | Transposon protein, putative, unclassified |
| augustus_masked-4-processed-gene-240.22-mRNA-1 | Japonica | NA | | | OsR498G0409146200.01.T01 | RING-H2 finger protein ATL34 |
| augustus_masked-4-processed-gene-240.33-mRNA-1 | Japonica | Indica | | Os04g0581400 | OsR498G0409149800.01.T02 | TF-B3 domain-containing protein (Fragment) |
| augustus_masked-4-processed-gene-241.31-mRNA-1 | Japonica | NA | | Os04g0583600 | OsR498G0409163000.01.T01 | Multifunctional fusion protein |

| | | | | | | |
|---|---|---|---|---|---|---|
| augustus_masked-4-processed-gene-242.57-mRNA-1 | Japonica | NA | | | OsR498G0409165000.01.T01 | L-type lectin-domain containing receptor kinase S.5 |
| augustus_masked-4-processed-gene-242.67-mRNA-1 | Japonica | NA | | | OsR498G0409168400.01.T01 | RING-H2 finger protein ATL1R |
| augustus_masked-4-processed-gene-245.15-mRNA-1 | Japonica | NA | | Os04g0590200 | OsR498G0409187800.01.T01 | AT-hook motif nuclear-localized protein 23 |
| augustus_masked-4-processed-gene-245.30-mRNA-1 | Japonica | NA | | Os04g0590900 | OsR498G0409190600.01.T01 | Ring-h2 finger protein atl52 |
| augustus_masked-4-processed-gene-246.37-mRNA-1 | Japonica | Indica | | Os01g0235900 | OsR498G0409198000.01.T01 | Glutaredoxin domain-containing protein |
| augustus_masked-4-processed-gene-246.8-mRNA-1 | Japonica | NA | | Os04g0591900 | | F-box domain-containing protein (Fragment) |
| augustus_masked-4-processed-gene-247.20-mRNA-1 | Japonica | NA | | Os12g0582600 | OsR498G0816427900.01.T01 | Putative mediator of RNA polymerase II transcription subunit 26c |
| augustus_masked-4-processed-gene-252.41-mRNA-1 | Japonica | NA | | Os04g0606000 | OsR498G0409239000.01.T01 | Putative quercetin 3-O-glucoside-6''-O-malonyltransferase |
| augustus_masked-4-processed-gene-257.23-mRNA-1 | Japonica | NA | | Os04g0612800 | | Putative Smr domain, pentacotripeptide-repeat region of PRORP |
| augustus_masked-4-processed-gene-258.8-mRNA-1 | Japonica | NA | | Os04g0613900 | OsR498G0409271000.01.T03 | Potassium transporter |
| augustus_masked-4-processed-gene-260.62-mRNA-1 | Japonica | NA | | Os04g0618500 | OsR498G0409288800.01.T03 | gamma-soluble NSF attachment protein-like |
| augustus_masked-4-processed-gene-261.39-mRNA-1 | Japonica | NA | Kinase-TM | Os04g0619600 | OsR498G0409294000.01.T01 | Mitogen-activated protein kinase kinase kinase |
| augustus_masked-4-processed-gene-261.40-mRNA-1 | Japonica | NA | | Os04g0619700 | OsR498G0409294200.01.T01 | F-box/WD-40 repeat-containing protein At5g21040 |
| augustus_masked-4-processed-gene-264.46-mRNA-1 | Japonica | NA | | | OsR498G0409312200.01.T01 | |
| augustus_masked-4-processed-gene-265.58-mRNA-1 | Japonica | NA | | Os04g0626100 | OsR498G0409317600.01.T01 | AT hook motif family protein |
| augustus_masked-4-processed-gene-266.79-mRNA-1 | Japonica | NA | | Os04g0627900 | OsR498G0409323200.01.T01 | Eukaryotic translation initiation factor SUI1 |
| augustus_masked-4-processed-gene-267.10-mRNA-1 | Japonica | NA | | Os04g0629400 | | Thioredoxin-like protein CXXS1 |
| augustus_masked-4-processed-gene-269.30-mRNA-1 | Japonica | NA | | Os04g0635800 | OsR498G0409343400.01.T01 | N-terminal acetyltransferase complex ARD1 subunit A |
| augustus_masked-4-processed-gene-269.6-mRNA-1 | Japonica | NA | | Os04g0635000 | OsR498G0409340800.01.T01 | Wound-induced protein |
| augustus_masked-4-processed-gene-270.6-mRNA-1 | Japonica | NA | | Os04g0636900 | OsR498G0409346400.01.T03 | UBP1-associated protein 2A |
| augustus_masked-4-processed-gene-270.8-mRNA-1 | Japonica | NA | | Os04g0637500 | | |
| augustus_masked-4-processed-gene-273.88-mRNA-1 | Japonica | Japonica | | Os04g0644200 | OsR498G0409366400.01.T01 | DDE Tnp4 domain-containing protein (Fragment) |
| augustus_masked-4-processed-gene-275.25-mRNA-1 | Japonica | NA | | Os04g0647300 | OsR498G0409376000.01.T01 | Ubiquitinyl hydrolase 1 |
| augustus_masked-4-processed-gene-276.20-mRNA-1 | Japonica | NA | | Os04g0649600 | OsR498G0409388800.01.T01 | |
| augustus_masked-4-processed-gene-279.52-mRNA-1 | Japonica | NA | | Os04g0654400 | OsR498G0409406000.01.T02 | Pre-mRNA-splicing helicase BRR2, putative |
| augustus_masked-4-processed-gene-279.53-mRNA-1 | Japonica | NA | | Os04g0654500 | OsR498G0409406200.01.T01 | Related to large-conductance mechanosensitive channel |
| augustus_masked-4-processed-gene-28.4-mRNA-1 | Japonica | NA | | Os04g0218100 | OsR498G0407751100.01.T01 | Pentatricopeptide repeat |
| augustus_masked-4-processed-gene-281.28-mRNA-1 | Japonica | NA | | Os04g0658600 | OsR498G0409421400.01.T01 | Ribosomal RNA processing protein A |
| augustus_masked-4-processed-gene-281.29-mRNA-1 | Japonica | NA | | Os04g0658800 | | |
| augustus_masked-4-processed-gene-283.84-mRNA-1 | Japonica | NA | | Os04g0662200 | OsR498G0409433600.01.T01 | Indole-3-acetic acid-induced protein ARG7 |
| augustus_masked-4-processed-gene-288.13-mRNA-1 | Japonica | NA | | Os04g0671800 | OsR498G0409467000.01.T01 | Zinc finger CCCH domain-containing protein 32 |
| augustus_masked-4-processed-gene-288.15-mRNA-1 | Japonica | NA | Kinase-LRR-TM | Os04g0672100 | OsR498G0409468600.01.T01 | Phytosulfokine receptor |
| augustus_masked-4-processed-gene-289.5-mRNA-1 | Japonica | Japonica | LRR-TM | Os04g0672600 | OsR498G0409468600.01.T01 | Phytosulfokine receptor 2 |
| augustus_masked-4-processed-gene-289.6-mRNA-1 | Japonica | NA | | Os04g0672700 | OsR498G0409471000.01.T04 | Pentatricopeptide repeat-containing protein |
| augustus_masked-4-processed-gene-290.76-mRNA-1 | Japonica | NA | | Os04g0675600 | OsR498G0409484400.01.T05 | Pentatricopeptide repeat-containing protein |
| augustus_masked-4-processed-gene-290.77-mRNA-1 | Japonica | NA | | Os04g0675700 | | DEAD/DEAH box helicase (Fragment) |
| augustus_masked-4-processed-gene-291.21-mRNA-1 | Japonica | Indica | | Os04g0677033 | OsR498G0409489200.01.T01 | |
| augustus_masked-4-processed-gene-296.29-mRNA-1 | Japonica | Indica | | Os04g0685700 | OsR498G0409520600.01.T01 | Putative S-receptor kinase |
| augustus_masked-4-processed-gene-296.39-mRNA-1 | Japonica | Indica | | Os04g0686000 | OsR498G0409522400.01.T01 | RING-type E3 ubiquitin transferase |
| augustus_masked-4-processed-gene-296.42-mRNA-1 | Japonica | NA | | Os04g0686500 | OsR498G0409524600.01.T01 | PPR domain-containing protein/PPR_2 domain-containing protein/DYW_dea |
| augustus_masked-4-processed-gene-296.45-mRNA-1 | Japonica | NA | | Os04g0686700 | OsR498G0409525000.01.T02 | SKP1-interacting partner 15 |
| augustus_masked-4-processed-gene-299.27-mRNA-1 | Japonica | Indica | | Os08g0103900 | OsR498G0409539000.01.T01 | Secondary wall NAC master switch |
| augustus_masked-4-processed-gene-3.22-mRNA-1 | Japonica | Indica | | Os04g0174800 | OsR498G0407632800.01.T01 | Peptidase S10, serine carboxypeptidase, Alpha/Beta hydrolase fold, DYW do |
| augustus_masked-4-processed-gene-300.96-mRNA-1 | Japonica | NA | NBS-TM | | OsR498G0409545600.01.T01 | White-brown-complex ABC transporter family |
| augustus_masked-4-processed-gene-4.8-mRNA-1 | Japonica | NA | | Os04g0175500 | OsR498G0407635400.01.T01 | Acyl transferase 15 |
| augustus_masked-4-processed-gene-43.23-mRNA-1 | Japonica | NA | | Os04g0309500 | | |
| augustus_masked-4-processed-gene-5.24-mRNA-1 | Japonica | NA | | | OsR498G0407642900.01.T01 | |
| augustus_masked-4-processed-gene-57.27-mRNA-1 | Japonica | NA | | Os04g0270900 | OsR498G0407902000.01.T01 | UDP-glucosyltransferase |
| augustus_masked-4-processed-gene-58.26-mRNA-1 | Japonica | Japonica | | Os04g0271700 | OsR498G0407905000.01.T01 | Glycosyltransferase |
| augustus_masked-4-processed-gene-58.30-mRNA-1 | Japonica | NA | | Os04g0272400 | OsR498G0407905600.01.T01 | Peroxiredoxin-5, mitochondrial |
| augustus_masked-4-processed-gene-6.13-mRNA-1 | Japonica | NA | | Os04g0179100 | OsR498G0407645000.01.T01 | Momilactone synthase like |
| augustus_masked-4-processed-gene-60.12-mRNA-1 | Japonica | NA | Kinase-TM | Os04g0275100 | OsR498G0407914000.01.T01 | Wall-associated receptor kinase 3 |
| augustus_masked-4-processed-gene-73.4-mRNA-1 | Japonica | NA | | | OsR498G0407965000.01.T01 | |
| augustus_masked-4-processed-gene-77.21-mRNA-1 | Japonica | NA | | | OsR498G0100073600.01.T01 | |
| augustus_masked-4-processed-gene-78.23-mRNA-1 | Japonica | Japonica | Kinase-TM | Os04g0303300 | OsR498G0407999200.01.T01 | Receptor-like serine/threonine-protein kinase |
| augustus_masked-4-processed-gene-79.42-mRNA-1 | Japonica | Indica | | Os11g0566800 | OsR498G0408006600.01.T01 | Chalcone and stilbene synthases, N-terminal domain containing protein, exp |
| augustus_masked-4-processed-gene-8.29-mRNA-1 | Japonica | NA | | Os04g0182300 | | Protease |
| augustus_masked-4-processed-gene-81.20-mRNA-1 | Japonica | NA | | | | thaumatin-like protein isoform X1 |
| augustus_masked-4-processed-gene-81.23-mRNA-1 | Japonica | NA | | | OsR498G1120155900.01.T01 | Skp1 domain-containing protein |
| augustus_masked-4-processed-gene-81.46-mRNA-1 | Japonica | NA | Kinase-TM | Os04g0307500 | OsR498G0408014500.01.T02 | Wall-associated receptor kinase 1 |
| augustus_masked-4-processed-gene-88.4-mRNA-1 | Japonica | NA | | | OsR498G0408093700.01.T01 | Poly [ADP-ribose] polymerase |

| | | | | | | |
|---|---|---|---|---|---|---|
| augustus_masked-4-processed-gene-89.32-mRNA-1 | Japonica | Indica | | Os04g0317800 | OsR498G0408135500.01.T01 | Expansin-like CBD domain-containing protein |
| augustus_masked-5-processed-gene-10.1-mRNA-1 | Japonica | NA | | Os05g0119000 | OsR498G0509614300.01.T01 | Aluminum sensitive 3 |
| augustus_masked-5-processed-gene-10.16-mRNA-1 | Japonica | NA | | Os05g0120200 | OsR498G0509618900.01.T01 | ABC transporter G family member 6 |
| augustus_masked-5-processed-gene-10.17-mRNA-1 | Japonica | NA | | Os05g0120300 | OsR498G0509620300.01.T01 | |
| augustus_masked-5-processed-gene-13.16-mRNA-1 | Japonica | Indica | | Os05g0123200 | OsR498G0509634700.01.T01 | IQ-domain 3 |
| augustus_masked-5-processed-gene-14.15-mRNA-1 | Japonica | NA | Kinase-TM | Os05g0125200 | OsR498G0509646500.01.T01 | Receptor-like kinase LIP2 |
| augustus_masked-5-processed-gene-141.41-mRNA-1 | Japonica | NA | | | OsR498G0510413500.01.T01 | Putative disease resistance protein RGA3 |
| augustus_masked-5-processed-gene-141.42-mRNA-1 | Japonica | Japonica | LRR-NBS | Os05g0305600 | OsR498G0510413500.01.T01 | NB-ARC domain containing protein, expressed |
| augustus_masked-5-processed-gene-145.19-mRNA-1 | Japonica | NA | | | OsR498G0510435500.01.T01 | Retrotransposon protein, putative, LINE subclass |
| augustus_masked-5-processed-gene-15.0-mRNA-1 | Japonica | Japonica | | Os05g0125600 | OsR498G0509648100.01.T01 | |
| augustus_masked-5-processed-gene-15.21-mRNA-1 | Japonica | NA | Kinase-TM | | OsR498G0509654700.01.T01 | Serine/threonine-protein kinase-like protein CCR3 |
| augustus_masked-5-processed-gene-151.20-mRNA-1 | Japonica | Japonica | CC-Kinase-TM | Os05g0317900 | OsR498G0510480600.01.T01 | Receptor-like protein kinase FERONIA |
| augustus_masked-5-processed-gene-151.7-mRNA-1 | Japonica | NA | | | OsR498G0510472300.01.T01 | |
| augustus_masked-5-processed-gene-153.32-mRNA-1 | Japonica | NA | | Os05g0320800 | | |
| augustus_masked-5-processed-gene-168.18-mRNA-1 | Japonica | NA | CC-Kinase-TM | | OsR498G0510577100.01.T01 | Receptor-like protein kinase FERONIA |
| augustus_masked-5-processed-gene-168.7-mRNA-1 | Japonica | NA | | | OsR498G0510594200.01.T01 | RNase H domain-containing protein (Fragment) |
| augustus_masked-5-processed-gene-17.1-mRNA-1 | Japonica | NA | | Os05g0128600 | | Transcription factor bHLH148 |
| augustus_masked-5-processed-gene-17.20-mRNA-1 | Japonica | Japonica | | Os05g0129400 | OsR498G0100416900.01.T01 | RRM domain-containing protein |
| augustus_masked-5-processed-gene-170.1-mRNA-1 | Japonica | NA | | | OsR498G0510587600.01.T01 | |
| augustus_masked-5-processed-gene-170.3-mRNA-1 | Japonica | NA | | Os05g0346300 | OsR498G0510586600.01.T01 | 40S ribosomal protein S7 |
| augustus_masked-5-processed-gene-178.3-mRNA-1 | Japonica | NA | | | OsR498G0510643100.01.T01 | SPX domain-containing protein |
| augustus_masked-5-processed-gene-179.0-mRNA-1 | Japonica | NA | | Os05g0359700 | | |
| augustus_masked-5-processed-gene-179.11-mRNA-1 | Japonica | Indica | | Os05g0360400 | OsR498G0510650500.01.T01 | RING-H2 finger protein ATL3F |
| augustus_masked-5-processed-gene-179.17-mRNA-1 | Japonica | Japonica | | Os05g0361000 | OsR498G0510653900.01.T01 | Putative ferulate 5-hydroxylase |
| augustus_masked-5-processed-gene-18.43-mRNA-1 | Japonica | NA | | | OsR498G0509671900.01.T03 | Fe2OG dioxygenase domain-containing protein (Fragment) |
| augustus_masked-5-processed-gene-19.28-mRNA-1 | Japonica | NA | | | | |
| augustus_masked-5-processed-gene-19.31-mRNA-1 | Japonica | NA | | Os05g0133900 | OsR498G0509678900.01.T01 | SAM-dependent MTase DRM-type domain-containing protein |
| augustus_masked-5-processed-gene-193.19-mRNA-1 | Japonica | Japonica | NBS-TM | Os05g0380800 | OsR498G0510731100.01.T01 | Disease resistance protein RPM1 |
| augustus_masked-5-processed-gene-194.37-mRNA-1 | Japonica | Indica | | Os01g0951500 | OsR498G0510739700.01.T01 | Cytochrome P450 family 94 subfamily D polypeptide 2 |
| augustus_masked-5-processed-gene-195.20-mRNA-1 | Japonica | NA | | Os05g0385400 | OsR498G0510749100.01.T01 | |
| augustus_masked-5-processed-gene-196.18-mRNA-1 | Japonica | NA | | Os05g0386000 | OsR498G0510752300.01.T01 | Hydroxyproline O-arabinosyltransferase |
| augustus_masked-5-processed-gene-197.22-mRNA-1 | Japonica | NA | | | OsR498G0510760300.01.T01 | |
| augustus_masked-5-processed-gene-197.3-mRNA-1 | Japonica | NA | CC-Kinase-TM | Os05g0387600 | | serine/threonine-protein kinase-like protein At3g51990 |
| augustus_masked-5-processed-gene-200.42-mRNA-1 | Japonica | NA | | | OsR498G0510774100.01.T04 | SNF2 domain-containing protein CLASSY 3 |
| augustus_masked-5-processed-gene-200.46-mRNA-1 | Japonica | NA | | Os05g0393200 | OsR498G0510775900.01.T01 | Putative ribosomal protein L34e superfamily protein-like |
| augustus_masked-5-processed-gene-201.18-mRNA-1 | Japonica | NA | | | OsR498G0510782300.01.T01 | BRO1 domain-containing protein |
| augustus_masked-5-processed-gene-203.36-mRNA-1 | Japonica | NA | | | OsR498G0510789700.01.T06 | |
| augustus_masked-5-processed-gene-203.37-mRNA-1 | Japonica | Indica | | Os05g0397800 | OsR498G0510790300.01.T04 | J domain-containing protein (Fragment) |
| augustus_masked-5-processed-gene-203.56-mRNA-1 | Japonica | NA | | Os05g0399300 | OsR498G0510793300.01.T01 | Chitinase |
| augustus_masked-5-processed-gene-206.0-mRNA-1 | Japonica | NA | | Os05g0402900 | OsR498G0510802100.01.T01 | Peptidase A1 domain-containing protein (Fragment) |
| augustus_masked-5-processed-gene-206.1-mRNA-1 | Japonica | NA | | Os05g0403000 | OsR498G0510802300.01.T01 | Peptidase A1 domain-containing protein |
| augustus_masked-5-processed-gene-206.15-mRNA-1 | Japonica | NA | | Os05g0404000 | OsR498G0510804300.01.T01 | Mitochodrial transcription termination factor |
| augustus_masked-5-processed-gene-206.3-mRNA-1 | Japonica | NA | | Os05g0403300 | OsR498G0510803300.01.T01 | Peptidase A1 domain-containing protein (Fragment) |
| augustus_masked-5-processed-gene-210.15-mRNA-1 | Japonica | Japonica | | Os05g0409100 | OsR498G0510822100.01.T01 | Wall-associated receptor kinase galacturonan-binding domain-containing pr |
| augustus_masked-5-processed-gene-211.21-mRNA-1 | Japonica | NA | | Os05g0409800 | OsR498G0510825100.01.T01 | Pentatricopeptide repeat-containing protein |
| augustus_masked-5-processed-gene-212.0-mRNA-1 | Japonica | NA | | | OsR498G0510828300.01.T01 | |
| augustus_masked-5-processed-gene-218.0-mRNA-1 | Japonica | Japonica | | Os05g0422200 | OsR498G0612271200.01.T01 | TLDc domain-containing protein (Fragment) |
| augustus_masked-5-processed-gene-218.9-mRNA-1 | Japonica | NA | | | OsR498G0510871500.01.T05 | |
| augustus_masked-5-processed-gene-219.23-mRNA-1 | Japonica | Indica | | Os06g0261300 | OsR498G0510877500.01.T01 | |
| augustus_masked-5-processed-gene-22.10-mRNA-1 | Japonica | NA | | Os05g0137600 | OsR498G0509697700.01.T05 | PAB-dependent poly(A)-specific ribonuclease subunit PAN3 (Fragment) |
| augustus_masked-5-processed-gene-22.13-mRNA-1 | Japonica | Japonica | | Os05g0138200 | OsR498G0100702400.01.T01 | Class II chitinase |
| augustus_masked-5-processed-gene-220.31-mRNA-1 | Japonica | NA | | Os05g0426900 | OsR498G0510884500.01.T01 | |
| augustus_masked-5-processed-gene-220.34-mRNA-1 | Japonica | NA | | Os05g0427100 | OsR498G0510884900.01.T02 | PB1 domain-containing protein |
| augustus_masked-5-processed-gene-220.35-mRNA-1 | Japonica | NA | | | | Protein ACCELERATED CELL DEATH 6-like |
| augustus_masked-5-processed-gene-226.22-mRNA-1 | Japonica | NA | | Os05g0440000 | OsR498G0510927500.01.T01 | Dof-type domain-containing protein (Fragment) |
| augustus_masked-5-processed-gene-227.13-mRNA-1 | Japonica | NA | | | | |
| augustus_masked-5-processed-gene-227.2-mRNA-1 | Japonica | NA | | Os05g0440900 | OsR498G0510929500.01.T01 | Transcription repressor |
| augustus_masked-5-processed-gene-229.13-mRNA-1 | Japonica | NA | | Os05g0445100 | OsR498G0510943100.01.T01 | |
| augustus_masked-5-processed-gene-23.6-mRNA-1 | Japonica | Indica | | Os05g0140500 | OsR498G0509710100.01.T01 | RNA-binding protein 8A-B |
| augustus_masked-5-processed-gene-232.3-mRNA-1 | Japonica | Indica | | Os05g0450600 | OsR498G0510960100.01.T01 | UPF0503 protein chloroplastic |
| augustus_masked-5-processed-gene-233.26-mRNA-1 | Japonica | NA | | Os05g0452800 | OsR498G0510966100.01.T01 | RING-CH-type domain-containing protein (Fragment) |

| | | | | | | |
|---|---|---|---|---|---|---|
| augustus_masked-5-processed-gene-234.11-mRNA-1 | Japonica | NA | | Os05g0455900 | OsR498G0510977300.01.T03 | Pentatricopeptide repeat |
| augustus_masked-5-processed-gene-236.8-mRNA-1 | Japonica | NA | | Os05g0459300 | OsR498G0510985100.01.T02 | Glucan endo-1,3-beta-glucosidase isoform 1 |
| augustus_masked-5-processed-gene-238.0-mRNA-1 | Japonica | NA | | Os05g0462500 | OsR498G0510997100.01.T02 | Transmembrane receptor |
| augustus_masked-5-processed-gene-239.31-mRNA-1 | Japonica | NA | | Os05g0466600 | OsR498G0510997800.01.T01 | Multifunctional fusion protein |
| augustus_masked-5-processed-gene-240.55-mRNA-1 | Japonica | Japonica | LRR-TM | Os05g0468200 | OsR498G0511013500.01.T01 | DUF1618 domain-containing protein (Fragment) |
| augustus_masked-5-processed-gene-241.37-mRNA-1 | Japonica | NA | | Os05g0470700 | OsR498G0511020500.01.T01 | E3 ubiquitin-protein ligase RNF5 |
| augustus_masked-5-processed-gene-242.21-mRNA-1 | Japonica | NA | | Os05g0470900 | | |
| augustus_masked-5-processed-gene-242.39-mRNA-1 | Japonica | NA | | | OsR498G0511025500.01.T01 | |
| augustus_masked-5-processed-gene-243.16-mRNA-1 | Japonica | NA | | Os05g0474400 | OsR498G0511029900.01.T01 | |
| augustus_masked-5-processed-gene-244.18-mRNA-1 | Japonica | NA | | Os05g0474700 | OsR498G0511031100.01.T01 | |
| augustus_masked-5-processed-gene-244.29-mRNA-1 | Japonica | Indica | | Os04g0544200 | OsR498G0511033100.01.T01 | Cyclin |
| augustus_masked-5-processed-gene-245.14-mRNA-1 | Japonica | NA | | Os05g0476700 | OsR498G0511042100.01.T01 | RING-type E3 ubiquitin transferase |
| augustus_masked-5-processed-gene-245.15-mRNA-1 | Japonica | NA | | Os05g0477200 | OsR498G0511043500.01.T01 | Transcription repressor |
| augustus_masked-5-processed-gene-245.17-mRNA-1 | Japonica | NA | | Os05g0477900 | OsR498G0511045900.01.T01 | Non-specific lipid-transfer protein (Fragment) |
| augustus_masked-5-processed-gene-246.1-mRNA-1 | Japonica | NA | | | OsR498G0511046900.01.T01 | |
| augustus_masked-5-processed-gene-248.14-mRNA-1 | Japonica | NA | | Os05g0482400 | OsR498G0511060710.01.T02 | Secologanin synthase |
| augustus_masked-5-processed-gene-249.40-mRNA-1 | Japonica | NA | | | OsR498G0511072900.01.T01 | GRAS domain-containing protein (Fragment) |
| augustus_masked-5-processed-gene-251.49-mRNA-1 | Japonica | NA | | Os05g0489100 | OsR498G0511080900.01.T03 | Procollagen-proline 4-dioxygenase |
| augustus_masked-5-processed-gene-252.1-mRNA-1 | Japonica | NA | | Os05g0490100 | OsR498G0511083100.01.T01 | 60S ribosomal protein L30 |
| augustus_masked-5-processed-gene-253.1-mRNA-1 | Japonica | NA | CC-LRR-NBS-TM | | OsR498G0511090900.01.T01 | NBS-LRR type resistance protein (Fragment) |
| augustus_masked-5-processed-gene-253.16-mRNA-1 | Japonica | Japonica | CC-LRR-NBS-TM | Os05g0492600 | OsR498G0511090900.01.T01 | NBS-LRR type resistance protein (Fragment) |
| augustus_masked-5-processed-gene-254.30-mRNA-1 | Japonica | NA | | Os05g0494500 | OsR498G0511097100.01.T04 | Plastidic phosphate translocator-like protein1 |
| augustus_masked-5-processed-gene-254.31-mRNA-1 | Japonica | NA | | | OsR498G0511098100.01.T01 | WAS/WASL-interacting protein family member 3-like |
| augustus_masked-5-processed-gene-254.42-mRNA-1 | Japonica | NA | | Os05g0495100 | OsR498G0511098700.01.T01 | Coiled-coil domain-containing protein 16 |
| augustus_masked-5-processed-gene-254.45-mRNA-1 | Japonica | NA | | | OsR498G0408669000.01.T01 | Leucine Rich Repeat family protein, expressed |
| augustus_masked-5-processed-gene-254.47-mRNA-1 | Japonica | NA | | Os05g0496000 | OsR498G0511102100.01.T01 | Putative glucan endo-13-beta-glucosidase BG1 |
| augustus_masked-5-processed-gene-255.23-mRNA-1 | Japonica | NA | | | OsR498G0511103500.01.T01 | SMC hinge domain-containing protein |
| augustus_masked-5-processed-gene-255.40-mRNA-1 | Japonica | Indica | | Os05g0497500 | OsR498G0511105800.01.T06 | Zinc finger CCCH domain-containing protein 36 |
| augustus_masked-5-processed-gene-257.22-mRNA-1 | Japonica | NA | | Os05g0500000 | OsR498G0511113900.01.T01 | UDP-glucosyltransferase |
| augustus_masked-5-processed-gene-257.25-mRNA-1 | Japonica | NA | | Os05g0500600 | OsR498G0511115500.01.T01 | Scarecrow-like protein 32 |
| augustus_masked-5-processed-gene-257.26-mRNA-1 | Japonica | NA | | Os05g0500700 | OsR498G0511115700.01.T01 | S-adenosyl-L-methionine-dependent methyltransferase superfamily protein |
| augustus_masked-5-processed-gene-257.3-mRNA-1 | Japonica | NA | | Os05g0499600 | OsR498G0511113100.01.T01 | UDP-glucosyltransferase |
| augustus_masked-5-processed-gene-257.5-mRNA-1 | Japonica | NA | | Os05g0499800 | OsR498G0511113500.01.T01 | UDP-glucosyltransferase |
| augustus_masked-5-processed-gene-259.3-mRNA-1 | Japonica | Indica | | Os01g0778400 | OsR498G0511123500.01.T01 | Arabinogalactan peptide |
| augustus_masked-5-processed-gene-261.38-mRNA-1 | Japonica | NA | | Os05g0506000 | OsR498G0511140700.01.T01 | Protein SULFUR DEFICIENCY-INDUCED like |
| augustus_masked-5-processed-gene-261.52-mRNA-1 | Japonica | Japonica | | Os05g0507000 | OsR498G0511143300.01.T01 | Putative mutator-like transposase |
| augustus_masked-5-processed-gene-261.53-mRNA-1 | Japonica | Indica | | Os05g0507300 | OsR498G0511143700.01.T02 | Calreticulin |
| augustus_masked-5-processed-gene-261.54-mRNA-1 | Japonica | NA | | Os05g0507400 | | |
| augustus_masked-5-processed-gene-262.19-mRNA-1 | Japonica | NA | | | OsR498G0511154300.01.T01 | |
| augustus_masked-5-processed-gene-263.12-mRNA-1 | Japonica | NA | | | OsR498G0511160300.01.T01 | Major facilitator superfamily protein isoform 1 |
| augustus_masked-5-processed-gene-263.13-mRNA-1 | Japonica | NA | | Os05g0510100 | OsR498G0511160900.01.T02 | |
| augustus_masked-5-processed-gene-264.42-mRNA-1 | Japonica | NA | | Os05g0510400 | OsR498G0511161700.01.T02 | F-box family protein-like |
| augustus_masked-5-processed-gene-264.52-mRNA-1 | Japonica | NA | | Os05g0511050 | | Peptidase A1 domain-containing protein (Fragment) |
| augustus_masked-5-processed-gene-265.1-mRNA-1 | Japonica | NA | | Os05g0512200 | OsR498G0511168700.01.T02 | Lysine-tRNA ligase |
| augustus_masked-5-processed-gene-265.3-mRNA-1 | Japonica | NA | | Os05g0512600 | OsR498G0511170100.01.T01 | X8 domain-containing protein |
| augustus_masked-5-processed-gene-266.19-mRNA-1 | Japonica | NA | Kinase-TM | Os05g0514200 | OsR498G0511175300.01.T02 | Non-specific serine/threonine protein kinase |
| augustus_masked-5-processed-gene-266.30-mRNA-1 | Japonica | NA | | Os05g0514500 | OsR498G0511177100.01.T01 | Interactor of constitutive active ROPs 1 |
| augustus_masked-5-processed-gene-267.47-mRNA-1 | Japonica | NA | | Os05g0515700 | OsR498G0511181500.01.T01 | Transcription factor VOZ1 |
| augustus_masked-5-processed-gene-267.60-mRNA-1 | Japonica | NA | | Os05g0516300 | OsR498G0511182700.01.T04 | Glucosamine-phosphate N-acetyltransferase |
| augustus_masked-5-processed-gene-268.21-mRNA-1 | Japonica | NA | | Os05g0519000 | OsR498G0511193300.01.T01 | VQ motif-containing protein 4 |
| augustus_masked-5-processed-gene-269.37-mRNA-1 | Japonica | NA | Kinase-TM | Os05g0519200 | OsR498G0511194100.01.T05 | Serine/threonine protein kinase |
| augustus_masked-5-processed-gene-269.38-mRNA-1 | Japonica | NA | | | | |
| augustus_masked-5-processed-gene-269.52-mRNA-1 | Japonica | Indica | | Os02g0563800 | OsR498G0511196700.01.T01 | F-box domain-containing protein |
| augustus_masked-5-processed-gene-27.1-mRNA-1 | Japonica | NA | | | OsR498G0509733700.01.T01 | Protease Do-like protein 2, chloroplastic |
| augustus_masked-5-processed-gene-273.24-mRNA-1 | Japonica | NA | | Os05g0526600 | OsR498G0511217700.01.T01 | RING-H2 finger protein ATL68 |
| augustus_masked-5-processed-gene-273.26-mRNA-1 | Japonica | Japonica | | Os05g0526900 | OsR498G0101954200.01.T01 | Glycosyltransferase |
| augustus_masked-5-processed-gene-28.69-mRNA-1 | Japonica | NA | | Os05g0150100 | OsR498G0509740900.01.T01 | DUF295 domain-containing protein |
| augustus_masked-5-processed-gene-282.4-mRNA-1 | Japonica | NA | | Os05g0545700 | | |
| augustus_masked-5-processed-gene-282.5-mRNA-1 | Japonica | NA | | | OsR498G0511284900.01.T01 | |
| augustus_masked-5-processed-gene-299.52-mRNA-1 | Japonica | NA | | Os05g0579200 | OsR498G0511399700.01.T01 | Long-chain-fatty-acid--AMP ligase FadD29 |
| augustus_masked-5-processed-gene-299.59-mRNA-1 | Japonica | Indica | | Os05g0579900 | OsR498G0511403100.01.T01 | Auxilin-like protein 1 |

| | | | | | | |
|---|---|---|---|---|---|---|
| augustus_masked-5-processed-gene-30.20-mRNA-1 | Japonica | Japonica | | Os05g0152900 | OsR498G0509751100.01.T01 | RING-type E3 ubiquitin transferase |
| augustus_masked-5-processed-gene-30.27-mRNA-1 | Japonica | NA | | Os05g0153200 | OsR498G0509751900.01.T03 | Factor of DNA methylation 1 |
| augustus_masked-5-processed-gene-30.28-mRNA-1 | Japonica | NA | | Os05g0153400 | OsR498G0509752700.01.T01 | Vegetative storage protein |
| augustus_masked-5-processed-gene-30.29-mRNA-1 | Japonica | NA | | Os05g0128900 | OsR498G0816083200.01.T02 | Trehalose phosphate synthase |
| augustus_masked-5-processed-gene-300.15-mRNA-1 | Japonica | NA | | Os05g0581300 | | Pentatricopeptide repeat-containing protein |
| augustus_masked-5-processed-gene-300.16-mRNA-1 | Japonica | NA | | Os05g0581700 | | Putative GPI-anchored protein 58 |
| augustus_masked-5-processed-gene-300.17-mRNA-1 | Japonica | NA | | Os05g0581800 | OsR498G0511407900.01.T03 | GBF-interacting protein 1 |
| augustus_masked-5-processed-gene-300.2-mRNA-1 | Japonica | NA | | Os05g0580800 | | |
| augustus_masked-5-processed-gene-301.25-mRNA-1 | Japonica | NA | | Os05g0582500 | OsR498G0511410700.01.T01 | Carboxypeptidase |
| augustus_masked-5-processed-gene-301.26-mRNA-1 | Japonica | NA | | Os05g0582600 | OsR498G0511410900.01.T01 | Carboxypeptidase |
| augustus_masked-5-processed-gene-301.27-mRNA-1 | Japonica | NA | | Os05g0582800 | OsR498G0511411500.01.T01 | Carboxypeptidase |
| augustus_masked-5-processed-gene-301.39-mRNA-1 | Japonica | NA | | Os03g0733400 | | Zinc finger BED domain-containing protein DAYSLEEPER |
| augustus_masked-5-processed-gene-301.42-mRNA-1 | Japonica | NA | | Os05g0583200 | | BED-type domain-containing protein |
| augustus_masked-5-processed-gene-301.45-mRNA-1 | Japonica | NA | | Os05g0583400 | OsR498G0511413700.01.T01 | Mitochondrial import receptor subunit TOM7 |
| augustus_masked-5-processed-gene-301.47-mRNA-1 | Japonica | Indica | | Os05g0583600 | OsR498G0511414500.01.T01 | Transcription repressor |
| augustus_masked-5-processed-gene-302.32-mRNA-1 | Japonica | Japonica | Kinase-TM | Os05g0585800 | OsR498G0101562800.01.T03 | mitogen-activated protein kinase kinase kinase 7 |
| augustus_masked-5-processed-gene-303.47-mRNA-1 | Japonica | NA | | | OsR498G0511427500.01.T01 | Pentatricopeptide repeat-containing protein mitochondrial |
| augustus_masked-5-processed-gene-304.14-mRNA-1 | Japonica | NA | Kinase-LRR-TM | Os05g0588250 | OsR498G0511432300.01.T02 | Putative inactive receptor kinase |
| augustus_masked-5-processed-gene-304.15-mRNA-1 | Japonica | NA | Kinase | Os05g0588300 | OsR498G0511432300.01.T01 | Receptor-like protein kinase 1-like |
| augustus_masked-5-processed-gene-305.12-mRNA-1 | Japonica | Japonica | | Os05g0591300 | OsR498G0511439500.01.T01 | Peptidase A1 domain-containing protein (Fragment) |
| augustus_masked-5-processed-gene-305.14-mRNA-1 | Japonica | Japonica | | Os05g0591400 | OsR498G0511440100.01.T01 | Luminal-binding protein-like |
| augustus_masked-5-processed-gene-31.3-mRNA-1 | Japonica | Indica | | Os05g0154700 | OsR498G0509767100.01.T01 | Kinesin-like protein KIN-13A |
| augustus_masked-5-processed-gene-32.55-mRNA-1 | Japonica | NA | | Os07g0550600 | OsR498G0714539100.01.T01 | Benzyl alcohol O-benzoyltransferase |
| augustus_masked-5-processed-gene-32.59-mRNA-1 | Japonica | NA | | | OsR498G0509773100.01.T01 | J domain-containing protein (Fragment) |
| augustus_masked-5-processed-gene-33.40-mRNA-1 | Japonica | NA | | Os05g0157100 | OsR498G0509774500.01.T02 | DNA-directed RNA polymerase V subunit 7 |
| augustus_masked-5-processed-gene-36.15-mRNA-1 | Japonica | NA | Kinase-TM | | OsR498G0509796100.01.T01 | Receptor-like protein kinase HERK 1 |
| augustus_masked-5-processed-gene-37.37-mRNA-1 | Japonica | NA | | Os05g0164200 | OsR498G0509802100.01.T01 | RING-H2 finger protein ATL3F |
| augustus_masked-5-processed-gene-39.47-mRNA-1 | Japonica | NA | | Os05g0457000 | OsR498G1019116500.01.T01 | Kinesin heavy chain-like |
| augustus_masked-5-processed-gene-4.11-mRNA-1 | Japonica | NA | | Os05g0107300 | OsR498G0509576500.01.T05 | |
| augustus_masked-5-processed-gene-42.0-mRNA-1 | Japonica | NA | | | OsR498G0510294600.01.T01 | Zinc knuckle containing protein-like |
| augustus_masked-5-processed-gene-45.45-mRNA-1 | Japonica | NA | | | OsR498G1221765900.01.T01 | Interactor of constitutive active ROPs 2, chloroplastic |
| augustus_masked-5-processed-gene-47.0-mRNA-1 | Japonica | NA | | Os05g0179100 | OsR498G0509858900.01.T01 | |
| augustus_masked-5-processed-gene-48.24-mRNA-1 | Japonica | NA | | Os05g0181200 | | Alkane hydroxylase MAH1 |
| augustus_masked-5-processed-gene-48.26-mRNA-1 | Japonica | NA | | Os05g0181800 | | |
| augustus_masked-5-processed-gene-5.13-mRNA-1 | Japonica | Indica | | Os05g0110000 | OsR498G0509583500.01.T01 | RING-type E3 ubiquitin transferase |
| augustus_masked-5-processed-gene-53.39-mRNA-1 | Japonica | NA | | Os05g0188100 | OsR498G0509901500.01.T01 | |
| augustus_masked-5-processed-gene-54.10-mRNA-1 | Japonica | NA | | | OsR498G0509904900.01.T01 | |
| augustus_masked-5-processed-gene-6.59-mRNA-1 | Japonica | NA | | Os05g0111200 | OsR498G0509588500.01.T01 | Outer envelope pore protein 16 chloroplastic |
| augustus_masked-5-processed-gene-67.8-mRNA-1 | Japonica | Japonica | | Os05g0208900 | OsR498G0509995200.01.T01 | |
| augustus_masked-5-processed-gene-69.1-mRNA-1 | Japonica | Japonica | | Os05g0212100 | OsR498G0510003100.01.T01 | Pentatricopeptide repeat-containing protein chloroplastic |
| augustus_masked-5-processed-gene-7.0-mRNA-1 | Japonica | NA | | | OsR498G0509595100.01.T01 | |
| augustus_masked-5-processed-gene-7.3-mRNA-1 | Japonica | NA | | Os05g0112900 | OsR498G0509596900.01.T01 | Pentatricopeptide repeat-containing protein mitochondrial |
| augustus_masked-5-processed-gene-8.19-mRNA-1 | Japonica | NA | | | OsR498G0509598900.01.T01 | |
| augustus_masked-5-processed-gene-86.26-mRNA-1 | Japonica | NA | | Os09g0503400 | OsR498G0917762300.01.T01 | Leucyl-tRNA synthetase |
| augustus_masked-5-processed-gene-87.12-mRNA-1 | Japonica | Japonica | | Os05g0242000 | OsR498G0101343800.01.T01 | 60S ribosomal protein L21-1 |
| augustus_masked-5-processed-gene-9.14-mRNA-1 | Japonica | NA | | | OsR498G0509609300.01.T01 | |
| augustus_masked-5-processed-gene-9.20-mRNA-1 | Japonica | NA | | Os05g0118000 | OsR498G0509612100.01.T01 | SRR1 domain-containing protein |
| augustus_masked-5-processed-gene-90.19-mRNA-1 | Japonica | NA | | Os05g0248200 | OsR498G0510139500.01.T01 | Xylanase inhibitor protein XIP |
| augustus_masked-6-processed-gene-100.11-mRNA-1 | Japonica | NA | | Os06g0285200 | OsR498G0612149700.01.T01 | HAP3 transcriptional-activator |
| augustus_masked-6-processed-gene-100.19-mRNA-1 | Japonica | NA | | | OsR498G0204046800.01.T01 | Circumsporozoite protein-like |
| augustus_masked-6-processed-gene-104.34-mRNA-1 | Japonica | NA | | Os06g0292100 | OsR498G0612172400.01.T01 | Embryogenesis transmembrane protein |
| augustus_masked-6-processed-gene-104.35-mRNA-1 | Japonica | Indica | | Os06g0292400 | OsR498G0612172800.01.T02 | PGG domain-containing protein |
| augustus_masked-6-processed-gene-106.30-mRNA-1 | Japonica | NA | | | OsR498G0612181600.01.T01 | |
| augustus_masked-6-processed-gene-106.32-mRNA-1 | Japonica | NA | | Os03g0122400 | OsR498G0100876000.01.T01 | Transposon protein, putative, Mutator sub-class |
| augustus_masked-6-processed-gene-107.37-mRNA-1 | Japonica | NA | | Os06g0297500 | OsR498G0612189500.01.T01 | Embryogenesis transmembrane protein-like |
| augustus_masked-6-processed-gene-107.49-mRNA-1 | Japonica | NA | | | OsR498G0612188800.01.T01 | Embryogenesis transmembrane protein-like |
| augustus_masked-6-processed-gene-108.11-mRNA-1 | Japonica | Japonica | | Os06g0298700 | OsR498G0612194000.01.T01 | Hydroxysteroid dehydrogenase-like protein 1 |
| augustus_masked-6-processed-gene-108.6-mRNA-1 | Japonica | NA | | | OsR498G0612193600.01.T01 | |
| augustus_masked-6-processed-gene-109.10-mRNA-1 | Japonica | NA | | Os06g0300700 | OsR498G0612202300.01.T02 | Serine/threonine-protein phosphatase 4 regulatory subunit like |
| augustus_masked-6-processed-gene-110.19-mRNA-1 | Japonica | NA | | Os03g0124100 | OsR498G0305151500.01.T02 | Beta-1,3-N-acetylglucosaminyltransferase lunatic fringe |
| augustus_masked-6-processed-gene-110.6-mRNA-1 | Japonica | NA | | | OsR498G0612206300.01.T01 | Amino acid transporter-like |

| | | | | | | |
|---|---|---|---|---|---|---|
| augustus_masked-6-processed-gene-110.8-mRNA-1 | Japonica | Indica | | Os06g0302000 | OsR498G0612208900.01.T01 | BURP domain-containing protein 11 |
| augustus_masked-6-processed-gene-111.21-mRNA-1 | Japonica | NA | | | OsR498G0612212800.01.T01 | Chemotaxis protein CheA |
| augustus_masked-6-processed-gene-112.0-mRNA-1 | Japonica | NA | | Os06g0303700 | OsR498G0612215700.01.T04 | MYB transcription factor-like |
| augustus_masked-6-processed-gene-112.1-mRNA-1 | Japonica | NA | | Os06g0303700 | OsR498G0612215700.01.T01 | Putative transcriptional regulator ATRX-like |
| augustus_masked-6-processed-gene-112.3-mRNA-1 | Japonica | NA | | | OsR498G0612217500.01.T01 | C2H2-type domain-containing protein |
| augustus_masked-6-processed-gene-113.17-mRNA-1 | Japonica | NA | | Os06g0306600 | OsR498G0612221700.01.T01 | probable carboxylesterase 18 |
| augustus_masked-6-processed-gene-118.1-mRNA-1 | Japonica | Japonica | | Os06g0314100 | OsR498G0612258000.01.T01 | Pentatricopeptide repeat |
| augustus_masked-6-processed-gene-118.3-mRNA-1 | Japonica | Indica | | Os06g0314501 | OsR498G0612260000.01.T01 | |
| augustus_masked-6-processed-gene-120.19-mRNA-1 | Japonica | Indica | | Os06g0317200 | OsR498G0612267300.01.T01 | glycine-rich cell wall structural protein 2-like |
| augustus_masked-6-processed-gene-120.45-mRNA-1 | Japonica | Japonica | | Os05g0422200 | OsR498G0612271200.01.T01 | TLDc domain-containing protein (Fragment) |
| augustus_masked-6-processed-gene-121.41-mRNA-1 | Japonica | NA | | | OsR498G0612274700.01.T01 | |
| augustus_masked-6-processed-gene-122.12-mRNA-1 | Japonica | NA | | Os06g0321700 | | |
| augustus_masked-6-processed-gene-122.2-mRNA-1 | Japonica | NA | | Os06g0320300 | OsR498G0612275800.01.T01 | Linoleate 13S-lipoxygenase 2-1 |
| augustus_masked-6-processed-gene-123.1-mRNA-1 | Japonica | Japonica | | Os06g0322300 | OsR498G0612281500.01.T01 | Putative jasmonate O-methyltransferase |
| augustus_masked-6-processed-gene-124.0-mRNA-1 | Japonica | NA | | | OsR498G0612383800.01.T01 | |
| augustus_masked-6-processed-gene-124.16-mRNA-1 | Japonica | NA | | | OsR498G0612286300.01.T01 | |
| augustus_masked-6-processed-gene-124.27-mRNA-1 | Japonica | NA | | Os06g0325700 | | |
| augustus_masked-6-processed-gene-125.0-mRNA-1 | Japonica | Japonica | | Os06g0325900 | OsR498G0815038200.01.T01 | Putative ferulate 5-hydroxylase |
| augustus_masked-6-processed-gene-125.10-mRNA-1 | Japonica | NA | | Os06g0326900 | OsR498G0612296100.01.T02 | PPC domain-containing protein (Fragment) |
| augustus_masked-6-processed-gene-125.3-mRNA-1 | Japonica | NA | | Os08g0381000 | OsR498G0713957300.01.T01 | |
| augustus_masked-6-processed-gene-127.24-mRNA-1 | Japonica | NA | | Os08g0152400 | OsR498G0815203000.01.T01 | Cytochrome P450 family 87 subfamily A polypeptide 6 |
| augustus_masked-6-processed-gene-128.14-mRNA-1 | Japonica | NA | | | OsR498G0612309300.01.T01 | |
| augustus_masked-6-processed-gene-128.25-mRNA-1 | Japonica | NA | | Os06g0331900 | OsR498G0612561200.01.T01 | Aluminum-activated malate transporter 10 |
| augustus_masked-6-processed-gene-129.38-mRNA-1 | Japonica | NA | | Os06g0332800 | OsR498G0612558900.01.T01 | Protein BUNDLE SHEATH DEFECTIVE 2, chloroplastic |
| augustus_masked-6-processed-gene-131.41-mRNA-1 | Japonica | NA | | | OsR498G0917449000.01.T03 | Zinc-finger homeodomain protein 9 |
| augustus_masked-6-processed-gene-131.42-mRNA-1 | Japonica | NA | | | OsR498G0612544300.01.T01 | AP2/ERF domain-containing protein |
| augustus_masked-6-processed-gene-132.28-mRNA-1 | Japonica | NA | | Os05g0457000 | OsR498G0305421900.01.T01 | |
| augustus_masked-6-processed-gene-133.14-mRNA-1 | Japonica | Japonica | | Os06g0339800 | OsR498G0815054500.01.T01 | Structural constituent of ribosome |
| augustus_masked-6-processed-gene-134.16-mRNA-1 | Japonica | NA | | Os06g0343100 | OsR498G0612524900.01.T01 | RNA helicase |
| augustus_masked-6-processed-gene-134.17-mRNA-1 | Japonica | NA | | Os06g0343600 | OsR498G0612523700.01.T01 | Glycosyltransferase |
| augustus_masked-6-processed-gene-134.3-mRNA-1 | Japonica | NA | | Os06g0342000 | OsR498G0612527500.01.T01 | Exostosin domain-containing protein (Fragment) |
| augustus_masked-6-processed-gene-139.11-mRNA-1 | Japonica | NA | | | OsR498G0815996300.01.T02 | Hydroxyproline-rich glycoprotein-like |
| augustus_masked-6-processed-gene-139.6-mRNA-1 | Japonica | NA | | Os06g0349800 | OsR498G0612504300.01.T01 | |
| augustus_masked-6-processed-gene-140.31-mRNA-1 | Japonica | NA | | Os06g0352200 | OsR498G0612495900.01.T01 | DUF679 domain membrane protein 7 |
| augustus_masked-6-processed-gene-141.10-mRNA-1 | Japonica | NA | | Os06g0352900 | OsR498G0612492500.01.T02 | Chloroplast protein HCF243 |
| augustus_masked-6-processed-gene-142.11-mRNA-1 | Japonica | NA | | Os06g0354601 | OsR498G0612489400.01.T01 | |
| augustus_masked-6-processed-gene-142.12-mRNA-1 | Japonica | NA | | Os06g0354700 | OsR498G0612488000.01.T03 | Pheophytinase, chloroplastic |
| augustus_masked-6-processed-gene-144.0-mRNA-1 | Japonica | NA | | Os12g0468900 | OsR498G0713579700.01.T01 | Cell wall protein-like |
| augustus_masked-6-processed-gene-147.12-mRNA-1 | Japonica | NA | | Os06g0362100 | | |
| augustus_masked-6-processed-gene-154.8-mRNA-1 | Japonica | NA | | Os06g0468300 | OsR498G0612438500.01.T06 | Putative RING zinc finger domain superfamily protein isoform 1 |
| augustus_masked-6-processed-gene-155.23-mRNA-1 | Japonica | NA | | | | |
| augustus_masked-6-processed-gene-156.17-mRNA-1 | Japonica | NA | | Os06g0472700 | OsR498G0612396600.01.T01 | WRKY transcription factor |
| augustus_masked-6-processed-gene-156.9-mRNA-1 | Japonica | Indica | | Os06g0472300 | OsR498G0612388200.01.T02 | Pentatricopeptide repeat-containing protein mitochondrial |
| augustus_masked-6-processed-gene-157.11-mRNA-1 | Japonica | NA | | Os06g0473800 | OsR498G0612402300.01.T01 | Oleosin Zm-II |
| augustus_masked-6-processed-gene-158.0-mRNA-1 | Japonica | NA | | Os06g0474300 | OsR498G0612405300.01.T01 | S-adenosyl-L-methionine-dependent methyltransferase superfamily protein |
| augustus_masked-6-processed-gene-159.18-mRNA-1 | Japonica | NA | | Os06g0476800 | | |
| augustus_masked-6-processed-gene-159.19-mRNA-1 | Japonica | Japonica | | Os06g0476900 | OsR498G0714121500.01.T06 | |
| augustus_masked-6-processed-gene-161.0-mRNA-1 | Japonica | NA | | Os06g0478100 | | |
| augustus_masked-6-processed-gene-161.13-mRNA-1 | Japonica | NA | LRR-TM | Os06g0478600 | OsR498G0612420500.01.T01 | Leucine-rich repeat receptor-like serine/threonine-protein kinase BAM2 |
| augustus_masked-6-processed-gene-161.23-mRNA-1 | Japonica | NA | | Os06g0479233 | | |
| augustus_masked-6-processed-gene-166.12-mRNA-1 | Japonica | Indica | | Os06g0114366 | OsR498G0612361200.01.T01 | Selenium-binding protein-like |
| augustus_masked-6-processed-gene-167.37-mRNA-1 | Japonica | NA | | Os07g0561900 | OsR498G0102180600.01.T01 | Peptide transporter PTR1 |
| augustus_masked-6-processed-gene-168.19-mRNA-1 | Japonica | NA | | | OsR498G0408540300.01.T01 | |
| augustus_masked-6-processed-gene-168.7-mRNA-1 | Japonica | NA | | Os06g0490000 | OsR498G0612352500.01.T02 | rRNA adenine N(6)-methyltransferase |
| augustus_masked-6-processed-gene-169.22-mRNA-1 | Japonica | NA | | | OsR498G0612342000.01.T01 | |
| augustus_masked-6-processed-gene-170.19-mRNA-1 | Japonica | NA | | Os06g0493800 | OsR498G0612334700.01.T04 | Pentatricopeptide repeat-containing protein |
| augustus_masked-6-processed-gene-171.20-mRNA-1 | Japonica | NA | | Os06g0494700 | OsR498G0612329400.01.T01 | Protein DETOXIFICATION |
| augustus_masked-6-processed-gene-171.7-mRNA-1 | Japonica | NA | | Os06g0494000 | OsR498G0612334000.01.T02 | Paired amphipathic helix repeat-containing family protein |
| augustus_masked-6-processed-gene-174.1-mRNA-1 | Japonica | NA | | Os06g0498150 | | |
| augustus_masked-6-processed-gene-174.2-mRNA-1 | Japonica | NA | | Os01g0726000 | OsR498G0917309200.01.T01 | |
| augustus_masked-6-processed-gene-176.24-mRNA-1 | Japonica | NA | | Os06g0501900 | OsR498G0612571700.01.T01 | |

| | | | | | | |
|---|---|---|---|---|---|---|
| augustus_masked-6-processed-gene-177.4-mRNA-1 | Japonica | NA | | Os06g0502900 | OsR498G0612566000.01.T02 | Putative WRKY transcription factor |
| augustus_masked-6-processed-gene-178.10-mRNA-1 | Japonica | NA | | Os06g0504100 | | |
| augustus_masked-6-processed-gene-179.1-mRNA-1 | Japonica | NA | | | | Arabinogalactan peptide |
| augustus_masked-6-processed-gene-179.11-mRNA-1 | Japonica | NA | | Os06g0506100 | OsR498G0612587700.01.T03 | Pentatricopeptide repeat-containing protein, mitochondrial |
| augustus_masked-6-processed-gene-180.0-mRNA-1 | Japonica | NA | | Os06g0507101 | OsR498G0612592000.01.T01 | Prolamin |
| augustus_masked-6-processed-gene-180.1-mRNA-1 | Japonica | NA | | Os06g0507300 | OsR498G0612592400.01.T01 | BTB domain-containing protein |
| augustus_masked-6-processed-gene-180.20-mRNA-1 | Japonica | NA | | | OsR498G0612594200.01.T01 | Intracellular protein transport protein USO1-related |
| augustus_masked-6-processed-gene-189.11-mRNA-1 | Japonica | NA | | | OsR498G1221584400.01.T01 | |
| augustus_masked-6-processed-gene-190.9-mRNA-1 | Japonica | NA | | | | Eukaryotic translation initiation factor isoform 4G-2 |
| augustus_masked-6-processed-gene-191.10-mRNA-1 | Japonica | NA | | | | Plant cadmium resistance protein |
| augustus_masked-6-processed-gene-191.31-mRNA-1 | Japonica | Indica | | Os12g0119800 | OsR498G0612633600.01.T01 | Harpin-induced protein 1 containing protein, expressed |
| augustus_masked-6-processed-gene-192.18-mRNA-1 | Japonica | Indica | | Os06g0523100 | OsR498G0612638500.01.T03 | WRKY transcription factor |
| augustus_masked-6-processed-gene-193.7-mRNA-1 | Japonica | NA | | Os06g0523300 | OsR498G0612640000.01.T01 | Nucleotide-sugar transporter |
| augustus_masked-6-processed-gene-193.8-mRNA-1 | Japonica | NA | | Os06g0523400 | OsR498G0612640400.01.T06 | Nucleotide-sugar transporter |
| augustus_masked-6-processed-gene-194.3-mRNA-1 | Japonica | NA | | | OsR498G0612647700.01.T01 | |
| augustus_masked-6-processed-gene-196.4-mRNA-1 | Japonica | Japonica | | Os06g0528700 | OsR498G0100964300.01.T01 | Flavin-containing monooxygenase |
| augustus_masked-6-processed-gene-199.12-mRNA-1 | Japonica | Japonica | | Os06g0534900 | OsR498G0612688700.01.T01 | RING-H2 finger protein ATL22 |
| augustus_masked-6-processed-gene-199.22-mRNA-1 | Japonica | NA | | Os12g0550700 | | Transposon protein, putative, Pong sub-class, expressed |
| augustus_masked-6-processed-gene-199.9-mRNA-1 | Japonica | NA | | Os06g0534800 | OsR498G0612688300.01.T01 | E3 ubiquitin-protein ligase ATL6 |
| augustus_masked-6-processed-gene-2.13-mRNA-1 | Japonica | NA | | | OsR498G0611471700.01.T01 | CCT domain-containing protein |
| augustus_masked-6-processed-gene-2.14-mRNA-1 | Japonica | Japonica | | Os06g0103200 | OsR498G1119695000.01.T01 | N-hydroxycinnamoyl benzoyltransferase-like protein |
| augustus_masked-6-processed-gene-200.10-mRNA-1 | Japonica | NA | | | OsR498G0612691300.01.T01 | Zinc finger, RING/FYVE/PHD-type |
| augustus_masked-6-processed-gene-200.12-mRNA-1 | Japonica | NA | | | OsR498G0612692100.01.T01 | Poly(A)-specific ribonuclease |
| augustus_masked-6-processed-gene-208.2-mRNA-1 | Japonica | Indica | | Os02g0232900 | OsR498G0612734500.01.T01 | Aquaporin NIP-type like |
| augustus_masked-6-processed-gene-209.17-mRNA-1 | Japonica | NA | | | OsR498G0612740100.01.T01 | Dehydration-responsive element-binding protein 3 |
| augustus_masked-6-processed-gene-209.35-mRNA-1 | Japonica | NA | | Os06g0554300 | OsR498G0612743600.01.T03 | Dentin sialophosphoprotein-like |
| augustus_masked-6-processed-gene-209.36-mRNA-1 | Japonica | NA | | Os06g0554600 | OsR498G0612745200.01.T01 | |
| augustus_masked-6-processed-gene-210.24-mRNA-1 | Japonica | Indica | | Os06g0556200 | OsR498G0612751900.01.T01 | Amino acid permease 1 |
| augustus_masked-6-processed-gene-211.22-mRNA-1 | Japonica | Japonica | LRR-TM | Os06g0557400 | OsR498G0612758000.01.T01 | LRR receptor-like serine/threonine-protein kinase HSL2 |
| augustus_masked-6-processed-gene-211.24-mRNA-1 | Japonica | NA | | | OsR498G0612760200.01.T01 | Protein DETOXIFICATION |
| augustus_masked-6-processed-gene-214.4-mRNA-1 | Japonica | NA | | Os06g0562200 | OsR498G0612770700.01.T01 | Abscisic acid receptor PYL 2 |
| augustus_masked-6-processed-gene-215.35-mRNA-1 | Japonica | NA | | Os06g0564000 | | |
| augustus_masked-6-processed-gene-216.0-mRNA-1 | Japonica | Japonica | | Os06g0565000 | OsR498G0612787500.01.T02 | Putative fertility restorer homologue |
| augustus_masked-6-processed-gene-217.12-mRNA-1 | Japonica | NA | | Os06g0567000 | OsR498G0612796100.01.T01 | Dolichyl-diphosphooligosaccharide-protein glycosyltransferase subunit OST5 |
| augustus_masked-6-processed-gene-217.26-mRNA-1 | Japonica | NA | | | OsR498G0100560400.01.T01 | |
| augustus_masked-6-processed-gene-218.16-mRNA-1 | Japonica | Japonica | | Os06g0568800 | OsR498G0612797900.01.T01 | SWIM-type domain-containing protein |
| augustus_masked-6-processed-gene-218.17-mRNA-1 | Japonica | Japonica | | Os06g0569300 | OsR498G0816542100.01.T01 | |
| augustus_masked-6-processed-gene-218.31-mRNA-1 | Japonica | NA | | Os06g0568600 | OsR498G0612797400.01.T01 | Ent-kaurene oxidase |
| augustus_masked-6-processed-gene-220.23-mRNA-1 | Japonica | Japonica | | Os06g0572000 | OsR498G0916743100.01.T07 | |
| augustus_masked-6-processed-gene-221.22-mRNA-1 | Japonica | NA | | Os06g0573700 | OsR498G0612810500.01.T02 | |
| augustus_masked-6-processed-gene-222.36-mRNA-1 | Japonica | Indica | | Os04g0205200 | OsR498G0612819600.01.T01 | Disease resistance protein RPM1 |
| augustus_masked-6-processed-gene-226.0-mRNA-1 | Japonica | NA | Kinase-LRR-TM | | | U-box domain-containing protein 33-like isoform X1 |
| augustus_masked-6-processed-gene-227.33-mRNA-1 | Japonica | NA | | | OsR498G0204046800.01.T01 | Epstein-Barr virus EBNA-1-like |
| augustus_masked-6-processed-gene-231.29-mRNA-1 | Japonica | Indica | Kinase-LRR-TM | Os06g0587500 | OsR498G0612856100.01.T01 | Leucine-rich repeat protein kinase family protein |
| augustus_masked-6-processed-gene-231.42-mRNA-1 | Japonica | NA | | Os06g0588900 | OsR498G0612856600.01.T02 | CTLH domain-containing protein |
| augustus_masked-6-processed-gene-233.1-mRNA-1 | Japonica | NA | | Os06g0591701 | | |
| augustus_masked-6-processed-gene-233.37-mRNA-1 | Japonica | NA | | Os06g0593800 | OsR498G0612872400.01.T01 | Glycosyltransferase |
| augustus_masked-6-processed-gene-234.17-mRNA-1 | Japonica | Japonica | CC-NBS-TM | Os06g0594200 | OsR498G0612874800.01.T01 | Putative disease resistance RPP13-like protein 1 |
| augustus_masked-6-processed-gene-236.14-mRNA-1 | Japonica | NA | | Os06g0598800 | OsR498G0612885700.01.T02 | Very-long-chain 3-oxoacyl-CoA synthase |
| augustus_masked-6-processed-gene-237.8-mRNA-1 | Japonica | NA | | Os06g0600400 | OsR498G0612888300.01.T01 | Secologanin synthase |
| augustus_masked-6-processed-gene-238.11-mRNA-1 | Japonica | NA | | Os06g0602400 | OsR498G0612892900.01.T01 | DEAD-box ATP-dependent RNA helicase 52 |
| augustus_masked-6-processed-gene-238.19-mRNA-1 | Japonica | NA | Kinase-TM | Os06g0602500 | OsR498G0612893400.01.T01 | Non-specific serine/threonine protein kinase |
| augustus_masked-6-processed-gene-239.21-mRNA-1 | Japonica | NA | | | OsR498G0612897600.01.T03 | RNA polymerase Rpc34 subunit family protein |
| augustus_masked-6-processed-gene-241.41-mRNA-1 | Japonica | NA | | Os06g0607100 | OsR498G0612914100.01.T01 | Phosphatidic acid phosphatase-related /PAP2-related |
| augustus_masked-6-processed-gene-241.49-mRNA-1 | Japonica | NA | | Os06g0606800 | OsR498G0612911400.01.T03 | TPX2 domain-containing protein |
| augustus_masked-6-processed-gene-243.13-mRNA-1 | Japonica | NA | | | OsR498G0612922300.01.T01 | Lateral suppressor |
| augustus_masked-6-processed-gene-244.40-mRNA-1 | Japonica | Japonica | | Os06g0611200 | OsR498G0612925400.01.T01 | Pentatricopeptide (PPR) repeat-containing protein-like |
| augustus_masked-6-processed-gene-245.16-mRNA-1 | Japonica | NA | | Os06g0612800 | OsR498G0612933300.01.T05 | Stress-associated protein 8 |
| augustus_masked-6-processed-gene-246.2-mRNA-1 | Japonica | Japonica | | Os06g0614400 | OsR498G1019138600.01.T01 | |
| augustus_masked-6-processed-gene-247.13-mRNA-1 | Japonica | Japonica | | Os06g0616900 | OsR498G0101162300.01.T01 | |
| augustus_masked-6-processed-gene-247.6-mRNA-1 | Japonica | NA | | | | |

| | | | | | | |
|---|---|---|---|---|---|---|
| augustus_masked-6-processed-gene-248.39-mRNA-1 | Japonica | NA | | Os06g0618700 | OsR498G0612961000.01.T01 | VQ motif-containing protein 25 |
| augustus_masked-6-processed-gene-250.13-mRNA-1 | Japonica | NA | | | OsR498G0612968300.01.T01 | |
| augustus_masked-6-processed-gene-251.1-mRNA-1 | Japonica | Indica | | Os07g0543600 | OsR498G0612972300.01.T01 | Putative strictosidine synthase |
| augustus_masked-6-processed-gene-251.7-mRNA-1 | Japonica | Indica | | Os07g0543600 | OsR498G0612972200.01.T01 | Strictosidine synthase 1 |
| augustus_masked-6-processed-gene-252.18-mRNA-1 | Japonica | NA | | Os06g0624900 | OsR498G0612977500.01.T03 | PLATZ transcription factor family protein isoform 2 |
| augustus_masked-6-processed-gene-252.19-mRNA-1 | Japonica | Indica | | Os06g0625000 | OsR498G0612978300.01.T01 | |
| augustus_masked-6-processed-gene-253.26-mRNA-1 | Japonica | NA | | Os06g0626600 | OsR498G0612988400.01.T01 | Sulfotransferase |
| augustus_masked-6-processed-gene-253.28-mRNA-1 | Japonica | NA | | | OsR498G0612989000.01.T01 | |
| augustus_masked-6-processed-gene-254.49-mRNA-1 | Japonica | NA | | Os06g0627400 | OsR498G0612992400.01.T01 | |
| augustus_masked-6-processed-gene-255.13-mRNA-1 | Japonica | NA | LRR-TM | Os06g0627500 | OsR498G0612993000.01.T01 | piriformospora indica-insensitive protein 2 |
| augustus_masked-6-processed-gene-258.14-mRNA-1 | Japonica | NA | | Os06g0633900 | OsR498G0613018300.01.T02 | Alpha/beta hydrolase fold |
| augustus_masked-6-processed-gene-258.27-mRNA-1 | Japonica | NA | | | OsR498G0613017200.01.T01 | RING-type E3 ubiquitin transferase |
| augustus_masked-6-processed-gene-259.35-mRNA-1 | Japonica | NA | | Os06g0634300 | OsR498G0613019800.01.T03 | MutT domain protein-like |
| augustus_masked-6-processed-gene-260.2-mRNA-1 | Japonica | NA | | | OsR498G0917172900.01.T01 | DUF834 domain-containing protein |
| augustus_masked-6-processed-gene-261.16-mRNA-1 | Japonica | NA | | Os06g0639300 | OsR498G0613035300.01.T01 | Methyltransferase-like protein 22 |
| augustus_masked-6-processed-gene-261.17-mRNA-1 | Japonica | NA | Kinase-TM | Os06g0639500 | OsR498G0613036700.01.T01 | E3 ubiquitin-protein ligase KEG |
| augustus_masked-6-processed-gene-288.54-mRNA-1 | Japonica | NA | | Os06g0687200 | OsR498G0613210300.01.T01 | Peptidoglycan glycosyltransferase FtsW |
| augustus_masked-6-processed-gene-289.25-mRNA-1 | Japonica | NA | | Os06g0690100 | | |
| augustus_masked-6-processed-gene-289.27-mRNA-1 | Japonica | NA | | | OsR498G0613217300.01.T01 | |
| augustus_masked-6-processed-gene-289.7-mRNA-1 | Japonica | Indica | | Os09g0472900 | OsR498G0613213400.01.T01 | Expansin-like EG45 domain-containing protein (Fragment) |
| augustus_masked-6-processed-gene-289.8-mRNA-1 | Japonica | Indica | | Os09g0472900 | OsR498G0613213600.01.T01 | Expansin-like EG45 domain-containing protein (Fragment) |
| augustus_masked-6-processed-gene-290.36-mRNA-1 | Japonica | Indica | Kinase-LRR-TM | Os06g0692600 | OsR498G0613224200.01.T01 | Tyrosine-sulfated glycopeptide receptor 1 |
| augustus_masked-6-processed-gene-290.37-mRNA-1 | Japonica | NA | Kinase-LRR-TM | Os06g0692600 | OsR498G0613225000.01.T01 | Tyrosine-sulfated glycopeptide receptor 1 |
| augustus_masked-6-processed-gene-290.7-mRNA-1 | Japonica | Japonica | | Os06g0690900 | OsR498G0613218100.01.T02 | Pentatricopeptide repeat-containing protein |
| augustus_masked-6-processed-gene-290.8-mRNA-1 | Japonica | NA | | Os06g0691100 | OsR498G0613218700.01.T01 | Ethylene-responsive transcription factor 3 |
| augustus_masked-6-processed-gene-290.9-mRNA-1 | Japonica | NA | | Os06g0691200 | OsR498G0613219300.01.T02 | thaumatin-like protein isoform X1 |
| augustus_masked-6-processed-gene-291.11-mRNA-1 | Japonica | NA | | Os06g0693700 | OsR498G0613228500.01.T01 | C2H2-type domain-containing protein (Fragment) |
| augustus_masked-6-processed-gene-291.33-mRNA-1 | Japonica | Japonica | | Os06g0694600 | OsR498G0613230800.01.T01 | Pentatricopeptide repeat |
| augustus_masked-6-processed-gene-292.26-mRNA-1 | Japonica | NA | | Os06g0695700 | OsR498G0613235300.01.T01 | Homeobox protein ARX-like |
| augustus_masked-6-processed-gene-292.34-mRNA-1 | Japonica | NA | | Os06g0695900 | OsR498G0613236200.01.T01 | Anaphase-promoting complex subunit 11 RING-H2 finger protein |
| augustus_masked-6-processed-gene-296.16-mRNA-1 | Japonica | NA | | | OsR498G0203878000.01.T01 | Cell wall protein-like |
| augustus_masked-6-processed-gene-297.9-mRNA-1 | Japonica | NA | | Os06g0702100 | OsR498G0613261700.01.T03 | Methyl-CpG-binding domain-containing protein 2 |
| augustus_masked-6-processed-gene-3.1-mRNA-1 | Japonica | NA | | Os06g0104100 | OsR498G0611474500.01.T01 | NADH dehydrogenasecomplex I, assembly factor 6 like |
| augustus_masked-6-processed-gene-3.22-mRNA-1 | Japonica | NA | | Os06g0104900 | OsR498G0611476400.01.T01 | L-lactate dehydrogenase A |
| augustus_masked-6-processed-gene-308.31-mRNA-1 | Japonica | NA | | | OsR498G0511427100.01.T01 | Epstein-Barr virus EBNA-1-like |
| augustus_masked-6-processed-gene-308.41-mRNA-1 | Japonica | NA | | | OsR498G0511427100.01.T01 | Epstein-Barr virus EBNA-1-like |
| augustus_masked-6-processed-gene-4.0-mRNA-1 | Japonica | Indica | | Os06g0105200 | OsR498G0611477700.01.T01 | GRAS transcription factor |
| augustus_masked-6-processed-gene-4.29-mRNA-1 | Japonica | NA | | Os06g0106900 | OsR498G0611482600.01.T02 | MADS-box transcription factor 8 |
| augustus_masked-6-processed-gene-4.4-mRNA-1 | Japonica | NA | Kinase-TM | | OsR498G0611477400.01.T01 | Receptor-like serine/threonine-protein kinase |
| augustus_masked-6-processed-gene-4.5-mRNA-1 | Japonica | NA | | Os06g0105400 | OsR498G0611478000.01.T02 | Dihydrolipoamide acetyltransferase component of pyruvate dehydrogenase |
| augustus_masked-6-processed-gene-43.0-mRNA-1 | Japonica | NA | | Os06g0184400 | OsR498G0611736100.01.T01 | C2 tensin-type domain-containing protein |
| augustus_masked-6-processed-gene-46.60-mRNA-1 | Japonica | NA | | | OsR498G0611752600.01.T01 | |
| augustus_masked-6-processed-gene-47.12-mRNA-1 | Japonica | Japonica | Kinase-TM | Os06g0191300 | OsR498G0204906600.01.T01 | MAP kinase kinase |
| augustus_masked-6-processed-gene-47.20-mRNA-1 | Japonica | NA | | | OsR498G0611760900.01.T01 | |
| augustus_masked-6-processed-gene-47.21-mRNA-1 | Japonica | NA | | Os06g0192800 | OsR498G0611761500.01.T01 | RING-H2 finger protein ATL60 |
| augustus_masked-6-processed-gene-47.6-mRNA-1 | Japonica | NA | | Os04g0471100 | OsR498G0510159400.01.T01 | Transposon protein, putative, CACTA, En/Spm sub-class, expressed |
| augustus_masked-6-processed-gene-48.23-mRNA-1 | Japonica | NA | | | | |
| augustus_masked-6-processed-gene-48.3-mRNA-1 | Japonica | NA | | Os06g0193200 | OsR498G0611762700.01.T02 | Pectinesterase |
| augustus_masked-6-processed-gene-49.13-mRNA-1 | Japonica | NA | | | OsR498G0511427100.01.T01 | Epstein-Barr virus EBNA-1-like |
| augustus_masked-6-processed-gene-5.39-mRNA-1 | Japonica | NA | | Os06g0109000 | OsR498G0611488200.01.T03 | Phosphoglycerate mutase-like protein AT74 |
| augustus_masked-6-processed-gene-5.41-mRNA-1 | Japonica | Japonica | | Os06g0109500 | OsR498G0611490800.01.T01 | Haloacid dehalogenase-like hydrolase domain-containing protein Sgpp |
| augustus_masked-6-processed-gene-6.1-mRNA-1 | Japonica | NA | | Os02g0540000 | OsR498G1019218100.01.T01 | RING-type domain-containing protein |
| augustus_masked-6-processed-gene-6.12-mRNA-1 | Japonica | NA | | Os06g0111200 | OsR498G0611495300.01.T01 | DUF295 domain-containing protein |
| augustus_masked-6-processed-gene-6.13-mRNA-1 | Japonica | NA | | Os06g0111500 | OsR498G0611496300.01.T02 | 6-phosphogluconate dehydrogenase, decarboxylating |
| augustus_masked-6-processed-gene-6.27-mRNA-1 | Japonica | NA | | Os06g0111100 | OsR498G0611495200.01.T01 | DUF295 domain-containing protein |
| augustus_masked-6-processed-gene-6.8-mRNA-1 | Japonica | NA | | Os06g0110200 | OsR498G0611494100.01.T01 | 18 kDa seed maturation protein-like |
| augustus_masked-6-processed-gene-65.20-mRNA-1 | Japonica | Japonica | | Os06g0223000 | OsR498G0611882900.01.T01 | t-SNARE coiled-coil homology domain-containing protein (Fragment) |
| augustus_masked-6-processed-gene-65.35-mRNA-1 | Japonica | Indica | | Os02g0751800 | OsR498G0611883600.01.T01 | Phosphoglycerate mutase |
| augustus_masked-6-processed-gene-7.27-mRNA-1 | Japonica | NA | | Os06g0112000 | OsR498G0611498500.01.T01 | Pentatricopeptide repeat-containing protein chloroplastic |
| augustus_masked-6-processed-gene-74.22-mRNA-1 | Japonica | NA | | Os06g0238000 | OsR498G0611967700.01.T01 | RING-type E3 ubiquitin transferase |
| augustus_masked-6-processed-gene-74.39-mRNA-1 | Japonica | NA | | Os06g0239100 | OsR498G0611970300.01.T01 | Metalloendoproteinase 2-MMP |

| | | | | | | |
|---|---|---|---|---|---|---|
| augustus_masked-6-processed-gene-74.44-mRNA-1 | Japonica | NA | | Os10g0460000 | OsR498G0204348500.01.T01 | |
| augustus_masked-6-processed-gene-74.55-mRNA-1 | Japonica | NA | | Os06g0238900 | OsR498G0611970000.01.T02 | TMV response protein |
| augustus_masked-6-processed-gene-75.5-mRNA-1 | Japonica | Indica | | Os06g0240800 | OsR498G0611972800.01.T01 | Acetylserotonin O-methyltransferase 1 |
| augustus_masked-6-processed-gene-79.7-mRNA-1 | Japonica | NA | | | OsR498G0611999800.01.T01 | F-box domain-containing protein (Fragment) |
| augustus_masked-6-processed-gene-85.4-mRNA-1 | Japonica | NA | | | OsR498G1220782800.01.T03 | |
| augustus_masked-6-processed-gene-86.15-mRNA-1 | Japonica | Japonica | | Os06g0262200 | OsR498G0612046000.01.T01 | 3-ketoacyl-CoA synthase |
| augustus_masked-6-processed-gene-87.22-mRNA-1 | Japonica | NA | | | | |
| augustus_masked-6-processed-gene-87.24-mRNA-1 | Japonica | NA | | Os06g0263400 | OsR498G0612054600.01.T01 | Very-long-chain 3-oxoacyl-CoA synthase |
| augustus_masked-6-processed-gene-87.3-mRNA-1 | Japonica | Japonica | | Os06g0262800 | OsR498G0612046000.01.T01 | Very-long-chain 3-oxoacyl-CoA synthase |
| augustus_masked-6-processed-gene-88.8-mRNA-1 | Japonica | Japonica | | Os06g0264200 | OsR498G0612055300.01.T01 | NADH2 dehydrogenase family protein |
| augustus_masked-6-processed-gene-98.31-mRNA-1 | Japonica | NA | | Os06g0283400 | OsR498G0612143000.01.T01 | Phosphatidylinositol 4-kinase type 2-beta |
| augustus_masked-7-processed-gene-105.9-mRNA-1 | Japonica | NA | | | | |
| augustus_masked-7-processed-gene-124.33-mRNA-1 | Japonica | NA | | | OsR498G0714084500.01.T06 | D-ribulose kinase |
| augustus_masked-7-processed-gene-133.32-mRNA-1 | Japonica | NA | | | OsR498G0714134600.01.T01 | |
| augustus_masked-7-processed-gene-155.25-mRNA-1 | Japonica | NA | | | OsR498G0714217800.01.T01 | Phosphatidylinositol/phosphatidylcholine transfer protein SFH5 |
| augustus_masked-7-processed-gene-168.25-mRNA-1 | Japonica | NA | | | | PMD domain-containing protein (Fragment) |
| augustus_masked-7-processed-gene-253.24-mRNA-1 | Japonica | NA | | Os07g0619200 | OsR498G0714765300.01.T03 | Polcalcin Syr v 3-like |
| augustus_masked-7-processed-gene-262.50-mRNA-1 | Japonica | NA | | Os08g0487300 | OsR498G0102102900.01.T01 | Transposon protein, putative, Mutator sub-class, expressed |
| augustus_masked-7-processed-gene-269.9-mRNA-1 | Japonica | NA | | | OsR498G0714866500.01.T01 | |
| augustus_masked-7-processed-gene-6.31-mRNA-1 | Japonica | NA | | | | Fusarium resistance protein I2C-5-like |
| augustus_masked-7-processed-gene-74.35-mRNA-1 | Japonica | NA | | Os07g0240600 | OsR498G0713860700.01.T01 | DIMBOA UDP-glucosyltransferase BX9 |
| augustus_masked-7-processed-gene-89.22-mRNA-1 | Japonica | NA | | Os07g0264000 | OsR498G0713942500.01.T04 | |
| augustus_masked-7-processed-gene-96.9-mRNA-1 | Japonica | NA | | | OsR498G0713968800.01.T03 | GlcNAc-PI synthesis protein (Fragment) |
| augustus_masked-8-processed-gene-1.7-mRNA-1 | Japonica | NA | | Os08g0101500 | OsR498G0815025500.01.T01 | Molybdate transporter like |
| augustus_masked-8-processed-gene-12.1-mRNA-1 | Japonica | NA | | | OsR498G0305908100.01.T01 | |
| augustus_masked-8-processed-gene-12.13-mRNA-1 | Japonica | NA | | Os08g0120900 | | |
| augustus_masked-8-processed-gene-151.29-mRNA-1 | Japonica | NA | Kinase-LRR-TM | | OsR498G0815800700.01.T01 | LRR receptor-like serine/threonine-protein kinase EFR |
| augustus_masked-8-processed-gene-153.20-mRNA-1 | Japonica | NA | | | OsR498G0815809900.01.T01 | |
| augustus_masked-8-processed-gene-17.55-mRNA-1 | Japonica | NA | | Os08g0128000 | OsR498G0815106700.01.T03 | Galactose oxidase/kelch repeat superfamily protein |
| augustus_masked-8-processed-gene-17.59-mRNA-1 | Japonica | NA | | Os08g0128700 | OsR498G0815108100.01.T01 | BTB/POZ domain-containing protein At1g55760 |
| augustus_masked-8-processed-gene-18.47-mRNA-1 | Japonica | Indica | | Os08g0131200 | OsR498G0815118100.01.T01 | AAI domain-containing protein |
| augustus_masked-8-processed-gene-2.1-mRNA-1 | Japonica | NA | | | OsR498G0815028100.01.T01 | Transcription repressor |
| augustus_masked-8-processed-gene-2.8-mRNA-1 | Japonica | Japonica | | Os08g0102800 | OsR498G0713661400.01.T01 | |
| augustus_masked-8-processed-gene-205.21-mRNA-1 | Japonica | NA | | Os08g0402100 | OsR498G0816036700.01.T01 | LOB domain-containing protein 22 |
| augustus_masked-8-processed-gene-209.19-mRNA-1 | Japonica | NA | | Os08g0409500 | OsR498G0816066900.01.T01 | VQ domain-containing protein (Fragment) |
| augustus_masked-8-processed-gene-209.5-mRNA-1 | Japonica | NA | | Os08g0408500 | OsR498G0816061900.01.T01 | AP domain DRE binding factor |
| augustus_masked-8-processed-gene-210.0-mRNA-1 | Japonica | Indica | | Os08g0410300 | OsR498G0816070500.01.T01 | Carbohydrate transporter/ sugar porter/ transporter |
| augustus_masked-8-processed-gene-211.17-mRNA-1 | Japonica | NA | | Os07g0579000 | OsR498G0714629300.01.T01 | AP2/ERF transcription factor |
| augustus_masked-8-processed-gene-211.3-mRNA-1 | Japonica | Indica | | Os08g0413100 | OsR498G0816078500.01.T01 | |
| augustus_masked-8-processed-gene-212.16-mRNA-1 | Japonica | NA | | Os08g0415600 | OsR498G0816084700.01.T01 | RING-type E3 ubiquitin transferase |
| augustus_masked-8-processed-gene-214.23-mRNA-1 | Japonica | Japonica | | Os08g0419100 | OsR498G0816093100.01.T01 | |
| augustus_masked-8-processed-gene-214.34-mRNA-1 | Japonica | NA | | | OsR498G0816095500.01.T01 | |
| augustus_masked-8-processed-gene-217.0-mRNA-1 | Japonica | NA | NBS-TM | Os08g0424700 | | NB-ARC domain-containing protein |
| augustus_masked-8-processed-gene-22.53-mRNA-1 | Japonica | Japonica | | Os08g0137900 | OsR498G0815137300.01.T01 | Phytocyanin domain-containing protein (Fragment) |
| augustus_masked-8-processed-gene-220.17-mRNA-1 | Japonica | NA | | | OsR498G0816130300.01.T01 | |
| augustus_masked-8-processed-gene-229.14-mRNA-1 | Japonica | NA | | Os08g0443800 | OsR498G0816189900.01.T01 | Putative senescence-associated protein 5 |
| augustus_masked-8-processed-gene-23.0-mRNA-1 | Japonica | NA | | Os08g0138200 | OsR498G0815138300.01.T01 | Phytocyanin domain-containing protein (Fragment) |
| augustus_masked-8-processed-gene-233.2-mRNA-1 | Japonica | Indica | | Os05g0350900 | OsR498G0816216500.01.T01 | R2R3-MYB transcription factor MYB7 |
| augustus_masked-8-processed-gene-234.10-mRNA-1 | Japonica | NA | | Os08g0452100 | OsR498G0816220900.01.T02 | Subtilisin-like serine protease |
| augustus_masked-8-processed-gene-235.25-mRNA-1 | Japonica | Japonica | | Os08g0453700 | OsR498G0816228700.01.T06 | Respiratory burst oxidase E |
| augustus_masked-8-processed-gene-235.31-mRNA-1 | Japonica | NA | | Os08g0454400 | OsR498G0816230500.01.T02 | Peroxidase |
| augustus_masked-8-processed-gene-236.19-mRNA-1 | Japonica | NA | | Os08g0455700 | OsR498G0816233900.01.T01 | Exocyst subunit Exo70 family protein (Fragment) |
| augustus_masked-8-processed-gene-237.14-mRNA-1 | Japonica | Indica | | Os08g0457100 | OsR498G0816237900.01.T01 | |
| augustus_masked-8-processed-gene-238.9-mRNA-1 | Japonica | NA | | Os08g0459400 | | DDE Tnp4 domain-containing protein (Fragment) |
| augustus_masked-8-processed-gene-240.26-mRNA-1 | Japonica | NA | | Os08g0463500 | OsR498G0816258900.01.T01 | Zinc finger protein GIS |
| augustus_masked-8-processed-gene-241.10-mRNA-1 | Japonica | Indica | | Os08g0464400 | OsR498G0816261100.01.T01 | RING-type E3 ubiquitin transferase |
| augustus_masked-8-processed-gene-241.21-mRNA-1 | Japonica | NA | | Os08g0465700 | OsR498G0816264700.01.T02 | |
| augustus_masked-8-processed-gene-242.18-mRNA-1 | Japonica | NA | | Os08g0466200 | OsR498G0816265900.01.T01 | Potassium transporter |
| augustus_masked-8-processed-gene-242.37-mRNA-1 | Japonica | NA | | Os08g0467300 | OsR498G0816271500.01.T01 | ENTH domain-containing protein |
| augustus_masked-8-processed-gene-243.13-mRNA-1 | Japonica | Japonica | | Os08g0467600 | OsR498G0816272300.01.T01 | GLABROUS1 enhancer-binding protein-like |
| augustus_masked-8-processed-gene-243.20-mRNA-1 | Japonica | NA | | Os08g0468300 | | |

| | | | | | | |
|---|---|---|---|---|---|---|
| augustus_masked-8-processed-gene-243.22-mRNA-1 | Japonica | Japonica | | Os08g0469000 | OsR498G0816277700.01.T01 | Aspartic proteinase nepenthesin-1 |
| augustus_masked-8-processed-gene-243.26-mRNA-1 | Japonica | NA | | | OsR498G0816279300.01.T01 | |
| augustus_masked-8-processed-gene-245.8-mRNA-1 | Japonica | NA | | Os08g0472000 | OsR498G0816288900.01.T01 | ABSCISIC ACID-INSENSITIVE 5-like protein 5 |
| augustus_masked-8-processed-gene-246.32-mRNA-1 | Japonica | Indica | | Os08g0474000 | OsR498G0816296700.01.T01 | Ethylene-responsive transcription factor ERF109 |
| augustus_masked-8-processed-gene-246.35-mRNA-1 | Japonica | Japonica | | Os08g0474400 | OsR498G0816363600.01.T01 | Protein transport protein SEC23 |
| augustus_masked-8-processed-gene-247.16-mRNA-1 | Japonica | Japonica | | Os08g0475300 | OsR498G0611850100.01.T01 | Putative carboxylesterase 2 |
| augustus_masked-8-processed-gene-247.20-mRNA-1 | Japonica | NA | | Os10g0460000 | OsR498G1019116500.01.T01 | |
| augustus_masked-8-processed-gene-247.23-mRNA-1 | Japonica | NA | | Os08g0476600 | OsR498G0816303700.01.T01 | Plant-specific domain TIGR01570 family protein |
| augustus_masked-8-processed-gene-248.1-mRNA-1 | Japonica | NA | | | OsR498G0511427100.01.T01 | Epstein-Barr virus EBNA-1-like |
| augustus_masked-8-processed-gene-248.18-mRNA-1 | Japonica | NA | | Os02g0212975 | OsR498G1019116500.01.T01 | Kinesin heavy chain-like |
| augustus_masked-8-processed-gene-248.19-mRNA-1 | Japonica | NA | | Os08g0477700 | OsR498G0816307300.01.T01 | J domain-containing protein (Fragment) |
| augustus_masked-8-processed-gene-249.0-mRNA-1 | Japonica | NA | | | | |
| augustus_masked-8-processed-gene-25.15-mRNA-1 | Japonica | NA | | Os08g0142500 | OsR498G0815150600.01.T01 | PMEI domain-containing protein |
| augustus_masked-8-processed-gene-25.22-mRNA-1 | Japonica | Japonica | | Os08g0143600 | OsR498G0815152700.01.T01 | Putative DEFECTIVE IN ANTHER DEHISCENCE1 |
| augustus_masked-8-processed-gene-260.51-mRNA-1 | Japonica | NA | | Os08g0500800 | OsR498G0816402300.01.T01 | Ubiquitin and WLM domain-containing metalloprotease |
| augustus_masked-8-processed-gene-262.23-mRNA-1 | Japonica | NA | | Os08g0504500 | OsR498G0816418300.01.T02 | Thylakoid lumenal protein, chloroplastic |
| augustus_masked-8-processed-gene-262.27-mRNA-1 | Japonica | Japonica | Kinase-TM | Os08g0504800 | OsR498G0917718500.01.T01 | Serine/threonine-protein kinase UCNL |
| augustus_masked-8-processed-gene-262.29-mRNA-1 | Japonica | Indica | | Os08g0505001 | OsR498G0816421500.01.T02 | Complex I subunit |
| augustus_masked-8-processed-gene-263.16-mRNA-1 | Japonica | Japonica | LRR-TM | Os08g0506100 | OsR498G0816425900.01.T01 | RNase H domain-containing protein (Fragment) |
| augustus_masked-8-processed-gene-264.15-mRNA-1 | Japonica | NA | | Os08g0507400 | OsR498G0816432100.01.T01 | Oryzalexin E synthase |
| augustus_masked-8-processed-gene-264.16-mRNA-1 | Japonica | Japonica | | Os08g0508000 | OsR498G0816434700.01.T01 | |
| augustus_masked-8-processed-gene-265.12-mRNA-1 | Japonica | NA | | Os08g0508700 | OsR498G0816439300.01.T02 | Ethylene signal transcription factor |
| augustus_masked-8-processed-gene-266.11-mRNA-1 | Japonica | Japonica | | Os08g0511000 | OsR498G0816446900.01.T01 | Pentatricopeptide repeat-containing protein |
| augustus_masked-8-processed-gene-266.12-mRNA-1 | Japonica | Indica | | Os08g0511500 | OsR498G0816449300.01.T03 | TFIIS N-terminal domain-containing protein |
| augustus_masked-8-processed-gene-267.63-mRNA-1 | Japonica | NA | | Os08g0513400 | | Calvin cycle protein CP12-3 chloroplastic |
| augustus_masked-8-processed-gene-269.21-mRNA-1 | Japonica | NA | | Os08g0517100 | OsR498G0816465900.01.T01 | LOB domain-containing protein 23 |
| augustus_masked-8-processed-gene-270.44-mRNA-1 | Japonica | NA | | Os08g0518100 | | Rab-GAP TBC domain-containing protein |
| augustus_masked-8-processed-gene-270.46-mRNA-1 | Japonica | NA | Kinase-LRR-TM | Os08g0518400 | | Serine/threonine-protein kinase |
| augustus_masked-8-processed-gene-270.47-mRNA-1 | Japonica | NA | | Os08g0518900 | OsR498G0816472500.01.T01 | Xylanase inhibitor protein 1 |
| augustus_masked-8-processed-gene-271.11-mRNA-1 | Japonica | Japonica | | Os08g0519800 | OsR498G0816477900.01.T01 | Pumilio homolog 5 |
| augustus_masked-8-processed-gene-271.12-mRNA-1 | Japonica | NA | | Os08g0520100 | | RNA pseudourine synthase 3, mitochondrial |
| augustus_masked-8-processed-gene-272.52-mRNA-1 | Japonica | NA | | | OsR498G0816484100.01.T01 | |
| augustus_masked-8-processed-gene-273.10-mRNA-1 | Japonica | NA | | | OsR498G0816485800.01.T01 | Zinc finger POZ domain protein-like |
| augustus_masked-8-processed-gene-274.24-mRNA-1 | Japonica | NA | | Os08g0524400 | OsR498G0816491700.01.T01 | DOMON domain-containing protein (Fragment) |
| augustus_masked-8-processed-gene-278.41-mRNA-1 | Japonica | NA | | | OsR498G0816520700.01.T05 | Plant-specific TFIIB-related protein PTF2 |
| augustus_masked-8-processed-gene-282.14-mRNA-1 | Japonica | Japonica | | Os08g0537001 | OsR498G0816537900.01.T03 | |
| augustus_masked-8-processed-gene-282.24-mRNA-1 | Japonica | NA | | | OsR498G0816539700.01.T01 | |
| augustus_masked-8-processed-gene-283.51-mRNA-1 | Japonica | Indica | | Os08g0538100 | OsR498G0816542100.01.T01 | Glycine--tRNA ligase, mitochondrial 1 (Fragment) |
| augustus_masked-8-processed-gene-285.24-mRNA-1 | Japonica | NA | | Os08g0541700 | | PMEI domain-containing protein |
| augustus_masked-8-processed-gene-285.26-mRNA-1 | Japonica | NA | | Os08g0542600 | OsR498G0816556100.01.T02 | p-Nitrophenyl phosphatase |
| augustus_masked-8-processed-gene-286.27-mRNA-1 | Japonica | Japonica | | Os08g0543200 | OsR498G0816559700.01.T02 | Anthranilate N-benzoyltransferase protein 3 |
| augustus_masked-8-processed-gene-289.12-mRNA-1 | Japonica | NA | | Os08g0548500 | OsR498G0816579100.01.T01 | Armadillo repeat only 1 |
| augustus_masked-8-processed-gene-289.16-mRNA-1 | Japonica | NA | | Os08g0548900 | OsR498G0816580100.01.T01 | Proteasome endopeptidase complex |
| augustus_masked-8-processed-gene-290.32-mRNA-1 | Japonica | NA | | Os08g0549900 | OsR498G0816583500.01.T01 | |
| augustus_masked-8-processed-gene-290.33-mRNA-1 | Japonica | NA | | Os08g0550000 | | OBG-type G domain-containing protein |
| augustus_masked-8-processed-gene-290.48-mRNA-1 | Japonica | NA | | Os08g0550900 | | |
| augustus_masked-8-processed-gene-290.51-mRNA-1 | Japonica | Japonica | | Os08g0551300 | OsR498G0816589700.01.T01 | |
| augustus_masked-8-processed-gene-290.53-mRNA-1 | Japonica | Japonica | | Os08g0551500 | OsR498G0816590300.01.T01 | |
| augustus_masked-8-processed-gene-290.57-mRNA-1 | Japonica | Japonica | | Os08g0552300 | OsR498G0816589300.01.T01 | |
| augustus_masked-8-processed-gene-291.32-mRNA-1 | Japonica | NA | | Os08g0553000 | OsR498G0816589300.01.T01 | |
| augustus_masked-8-processed-gene-291.33-mRNA-1 | Japonica | NA | | Os08g0553000 | OsR498G0816589300.01.T01 | |
| augustus_masked-8-processed-gene-291.36-mRNA-1 | Japonica | Japonica | | Os08g0553050 | OsR498G0816588900.01.T01 | |
| augustus_masked-8-processed-gene-291.37-mRNA-1 | Japonica | Indica | | Os08g0553200 | OsR498G0816589300.01.T01 | |
| augustus_masked-8-processed-gene-291.39-mRNA-1 | Japonica | Japonica | | Os08g0553400 | OsR498G0816590300.01.T01 | |
| augustus_masked-8-processed-gene-291.55-mRNA-1 | Japonica | Japonica | | Os08g0553600 | OsR498G0816589700.01.T01 | |
| augustus_masked-8-processed-gene-291.57-mRNA-1 | Japonica | Indica | | Os08g0552300 | OsR498G0816590300.01.T01 | |
| augustus_masked-8-processed-gene-291.62-mRNA-1 | Japonica | Japonica | | Os08g0554300 | OsR498G0816591500.01.T01 | Rhamnogalacturonan endolyase |
| augustus_masked-8-processed-gene-292.19-mRNA-1 | Japonica | NA | | Os08g0556400 | OsR498G0816598100.01.T01 | S-acyltransferase |
| augustus_masked-8-processed-gene-292.20-mRNA-1 | Japonica | NA | | | OsR498G0816598300.01.T01 | Peroxin-1 |
| augustus_masked-8-processed-gene-293.69-mRNA-1 | Japonica | NA | | Os08g0558300 | OsR498G0816603700.01.T01 | WHy domain-containing protein (Fragment) |
| augustus_masked-8-processed-gene-295.10-mRNA-1 | Japonica | NA | | Os08g0561700 | OsR498G0816616100.01.T01 | Superoxide dismutase |

| | | | | | | |
|---|---|---|---|---|---|---|
| augustus_masked-8-processed-gene-295.11-mRNA-1 | Japonica | NA | | Os08g0561800 | OsR498G0408675500.01.T01 | Retrotransposon protein, putative, Ty3-gypsy sub-class |
| augustus_masked-8-processed-gene-296.10-mRNA-1 | Japonica | Indica | | Os05g0437133 | OsR498G0816621300.01.T01 | Ethylene-responsive transcription factor ERF098 |
| augustus_masked-8-processed-gene-296.23-mRNA-1 | Japonica | NA | | Os08g0564000 | OsR498G0816622500.01.T01 | H(+)/Pi cotransporter |
| augustus_masked-8-processed-gene-296.25-mRNA-1 | Japonica | Indica | | Os08g0564300 | OsR498G0816623900.01.T03 | p-glycoprotein 1 |
| augustus_masked-8-processed-gene-296.28-mRNA-1 | Japonica | Indica | | Os08g0564750 | OsR498G0816627100.01.T01 | Small acidic protein 2 |
| augustus_masked-8-processed-gene-39.34-mRNA-1 | Japonica | NA | | Os08g0162200 | OsR498G0815251700.01.T02 | Pentatricopeptide repeat |
| augustus_masked-8-processed-gene-39.39-mRNA-1 | Japonica | NA | | Os08g0162700 | OsR498G0815253700.01.T01 | PCI domain-containing protein |
| augustus_masked-8-processed-gene-4.18-mRNA-1 | Japonica | Japonica | CC-NBS-TM | Os08g0107000 | OsR498G0815041300.01.T01 | Disease resistance protein RPP13 |
| augustus_masked-8-processed-gene-40.0-mRNA-1 | Japonica | NA | | Os08g0164300 | OsR498G0815258700.01.T01 | DUF569 domain-containing protein |
| augustus_masked-8-processed-gene-40.19-mRNA-1 | Japonica | Japonica | | Os08g0165200 | OsR498G0815259700.01.T01 | Protein kinase domain-containing protein |
| augustus_masked-8-processed-gene-42.11-mRNA-1 | Japonica | NA | | Os08g0168932 | | |
| augustus_masked-8-processed-gene-44.35-mRNA-1 | Japonica | NA | | Os08g0173300 | OsR498G0815295300.01.T01 | |
| augustus_masked-8-processed-gene-47.11-mRNA-1 | Japonica | NA | | Os08g0178300 | OsR498G0815308500.01.T02 | Pre-mRNA-splicing factor of RES complex protein |
| augustus_masked-8-processed-gene-47.12-mRNA-1 | Japonica | Indica | | Os08g0178500 | OsR498G0815309100.01.T01 | Wound induced protein |
| augustus_masked-8-processed-gene-47.6-mRNA-1 | Japonica | NA | | | OsR498G0815306300.01.T01 | |
| augustus_masked-8-processed-gene-5.49-mRNA-1 | Japonica | NA | Kinase-TM | Os08g0109800 | OsR498G0815051500.01.T01 | Serine/threonine-protein kinase-like protein CCR4 |
| augustus_masked-8-processed-gene-53.22-mRNA-1 | Japonica | NA | | Os08g0191000 | OsR498G0815344900.01.T01 | Auxin efflux carrier |
| augustus_masked-8-processed-gene-56.11-mRNA-1 | Japonica | Indica | | Os11g0638801 | OsR498G0815358900.01.T01 | DUF4220 domain-containing protein (Fragment) |
| augustus_masked-8-processed-gene-57.39-mRNA-1 | Japonica | NA | | | | Zinc finger BED domain-containing protein DAYSLEEPER |
| augustus_masked-8-processed-gene-6.12-mRNA-1 | Japonica | NA | | | OsR498G0203043500.01.T01 | |
| augustus_masked-8-processed-gene-62.18-mRNA-1 | Japonica | NA | | | OsR498G0815389900.01.T01 | |
| augustus_masked-8-processed-gene-63.34-mRNA-1 | Japonica | Indica | | Os08g0205300 | OsR498G0815393500.01.T02 | Histone-lysine N-methyltransferase ASHR2 |
| augustus_masked-8-processed-gene-64.2-mRNA-1 | Japonica | NA | | Os08g0207300 | | |
| augustus_masked-8-processed-gene-66.11-mRNA-1 | Japonica | NA | | Os05g0267732 | OsR498G0510213400.01.T01 | |
| augustus_masked-8-processed-gene-75.29-mRNA-1 | Japonica | NA | | | | |
| augustus_masked-8-processed-gene-75.7-mRNA-1 | Japonica | Japonica | | Os08g0221000 | OsR498G0815443700.01.T01 | |
| augustus_masked-8-processed-gene-76.10-mRNA-1 | Japonica | NA | | Os08g0219100 | OsR498G0815449300.01.T01 | PMEI domain-containing protein |
| augustus_masked-8-processed-gene-77.2-mRNA-1 | Japonica | NA | | Os08g0218700 | OsR498G0815450300.01.T01 | PMEI domain-containing protein |
| augustus_masked-8-processed-gene-80.9-mRNA-1 | Japonica | NA | | | OsR498G0815473300.01.T01 | BTB/POZ and MATH domain-containing protein 2 |
| augustus_masked-8-processed-gene-81.26-mRNA-1 | Japonica | Japonica | | Os08g0227400 | OsR498G0815478400.01.T01 | BTB/POZ and MATH domain-containing protein 1 |
| augustus_masked-8-processed-gene-83.0-mRNA-1 | Japonica | NA | | Os08g0230200 | OsR498G0815484900.01.T02 | Ubiquitin-specific protease family C19-related protein |
| augustus_masked-8-processed-gene-83.1-mRNA-1 | Japonica | NA | | | OsR498G0815485700.01.T01 | |
| augustus_masked-8-processed-gene-87.33-mRNA-1 | Japonica | Japonica | | Os08g0238100 | OsR498G1119809300.01.T02 | Pentatricopeptide repeat-containing protein (Fragment) |
| augustus_masked-8-processed-gene-88.1-mRNA-1 | Japonica | Japonica | | Os08g0239300 | OsR498G0815510700.01.T03 | DLH domain-containing protein |
| augustus_masked-8-processed-gene-88.13-mRNA-1 | Japonica | Indica | | Os08g0240533 | OsR498G0815512300.01.T01 | Xyloglucan endotransglucosylase/hydrolase |
| augustus_masked-8-processed-gene-90.38-mRNA-1 | Japonica | NA | | | OsR498G0815522700.01.T01 | rRNA N-glycosidase |
| augustus_masked-8-processed-gene-93.26-mRNA-1 | Japonica | NA | LRR-NBS-TM | Os08g0246300 | OsR498G0815530100.01.T01 | Disease resistance protein RPM1 |
| augustus_masked-8-processed-gene-94.1-mRNA-1 | Japonica | Indica | | Os10g0495200 | OsR498G0815537100.01.T01 | Rf1 protein, mitochondrial, putative, expressed |
| augustus_masked-8-processed-gene-97.14-mRNA-1 | Japonica | NA | | Os08g0256700 | | Protein kinase domain-containing protein |
| augustus_masked-8-processed-gene-97.16-mRNA-1 | Japonica | NA | | | | |
| augustus_masked-9-processed-gene-106.11-mRNA-1 | Japonica | Indica | | Os04g0461800 | OsR498G0917216400.01.T01 | Formin-like protein 2 |
| augustus_masked-9-processed-gene-11.0-mRNA-1 | Japonica | NA | | | OsR498G0916682700.01.T01 | VIER F-box protein 1 |
| augustus_masked-9-processed-gene-110.25-mRNA-1 | Japonica | NA | | | OsR498G1119549200.01.T02 | |
| augustus_masked-9-processed-gene-110.29-mRNA-1 | Japonica | NA | | Os09g0342000 | OsR498G0917198400.01.T01 | CRIB domain-containing protein |
| augustus_masked-9-processed-gene-110.30-mRNA-1 | Japonica | NA | | Os09g0341600 | OsR498G0917198200.01.T01 | F-box domain-containing protein (Fragment) |
| augustus_masked-9-processed-gene-111.0-mRNA-1 | Japonica | Indica | | Os09g0341500 | OsR498G0917198000.01.T01 | F-box domain-containing protein (Fragment) |
| augustus_masked-9-processed-gene-114.24-mRNA-1 | Japonica | Japonica | | Os09g0336500 | OsR498G1220975100.01.T01 | Zinc knuckle family protein |
| augustus_masked-9-processed-gene-118.25-mRNA-1 | Japonica | Indica | Kinase-LRR-TM | Os09g0352000 | OsR498G0917242100.01.T03 | Putative leucine-rich repeat receptor-like serine/threonine-protein kinase |
| augustus_masked-9-processed-gene-119.44-mRNA-1 | Japonica | NA | | Os09g0362500 | OsR498G0917249500.01.T02 | Aminopeptidase |
| augustus_masked-9-processed-gene-120.13-mRNA-1 | Japonica | NA | | Os09g0364000 | OsR498G0917253500.01.T01 | Zinc finger CCCH domain-containing protein 59 |
| augustus_masked-9-processed-gene-121.10-mRNA-1 | Japonica | Indica | | Os09g0364800 | OsR498G0917256500.01.T01 | HMA domain-containing protein (Fragment) |
| augustus_masked-9-processed-gene-121.19-mRNA-1 | Japonica | Japonica | LRR-NBS-TM | Os09g0365300 | OsR498G0917259200.01.T01 | Protein kinase domain-containing protein |
| augustus_masked-9-processed-gene-123.14-mRNA-1 | Japonica | NA | | Os09g0369000 | OsR498G0917276700.01.T01 | AP domain DRE binding factor |
| augustus_masked-9-processed-gene-124.0-mRNA-1 | Japonica | Japonica | | Os09g0369400 | OsR498G0917279900.01.T04 | Trehalose 6-phosphate phosphatase |
| augustus_masked-9-processed-gene-124.6-mRNA-1 | Japonica | NA | | Os09g0370000 | | |
| augustus_masked-9-processed-gene-125.36-mRNA-1 | Japonica | NA | | | OsR498G0917285500.01.T01 | MFS transporter |
| augustus_masked-9-processed-gene-126.1-mRNA-1 | Japonica | NA | | Os09g0373200 | OsR498G0917293100.01.T01 | Strictosidine synthase 3 |
| augustus_masked-9-processed-gene-127.1-mRNA-1 | Japonica | NA | | Os03g0154300 | OsR498G1119747800.01.T01 | RNA-directed RNA polymerase |
| augustus_masked-9-processed-gene-129.0-mRNA-1 | Japonica | NA | | Os09g0378700 | OsR498G0917310300.01.T01 | RING-type E3 ubiquitin transferase |
| augustus_masked-9-processed-gene-129.20-mRNA-1 | Japonica | NA | | | OsR498G0917313700.01.T05 | Acyl-activating enzyme 17 |
| augustus_masked-9-processed-gene-130.19-mRNA-1 | Japonica | NA | | Os09g0380300 | | GRAS domain-containing protein (Fragment) |

| | | | | | | |
|---|---|---|---|---|---|---|
| augustus_masked-9-processed-gene-136.0-mRNA-1 | Japonica | NA | | | | |
| augustus_masked-9-processed-gene-142.26-mRNA-1 | Japonica | NA | | | | HAT dimerisation domain-containing protein, putative |
| augustus_masked-9-processed-gene-146.33-mRNA-1 | Japonica | Indica | | Os09g0410500 | OsR498G0917433500.01.T02 | Retarded palea 1 protein (Fragment) |
| augustus_masked-9-processed-gene-147.54-mRNA-1 | Japonica | NA | | Os09g0412900 | OsR498G0917439900.01.T01 | Pentatricopeptide repeat |
| augustus_masked-9-processed-gene-150.22-mRNA-1 | Japonica | Indica | | Os09g0416600 | OsR498G0917456300.01.T01 | |
| augustus_masked-9-processed-gene-150.23-mRNA-1 | Japonica | Japonica | | Os09g0416800 | OsR498G0917457300.01.T01 | Poly(A)-specific ribonuclease |
| augustus_masked-9-processed-gene-150.28-mRNA-1 | Japonica | NA | | Os09g0417500 | OsR498G0917462100.01.T01 | Pentatricopeptide repeat-containing protein |
| augustus_masked-9-processed-gene-155.16-mRNA-1 | Japonica | NA | | | OsR498G0917487700.01.T01 | Glycylpeptide N-tetradecanoyltransferase |
| augustus_masked-9-processed-gene-155.4-mRNA-1 | Japonica | NA | | Os09g0425300 | | Voltage-dependent T-type calcium channel subunit alpha |
| augustus_masked-9-processed-gene-157.20-mRNA-1 | Japonica | NA | | | OsR498G0917503500.01.T01 | Zinc finger protein 2 |
| augustus_masked-9-processed-gene-160.21-mRNA-1 | Japonica | NA | | Os09g0433600 | OsR498G0917518300.01.T01 | Multifunctional fusion protein |
| augustus_masked-9-processed-gene-160.38-mRNA-1 | Japonica | NA | | Os09g0434500 | OsR498G0917520500.01.T01 | EREBP transcription factor |
| augustus_masked-9-processed-gene-160.39-mRNA-1 | Japonica | NA | | | OsR498G0917522700.01.T01 | RNA helicase |
| augustus_masked-9-processed-gene-161.0-mRNA-1 | Japonica | NA | | | OsR498G0917524700.01.T01 | Putative cell death associated protein |
| augustus_masked-9-processed-gene-161.11-mRNA-1 | Japonica | NA | | | | |
| augustus_masked-9-processed-gene-163.13-mRNA-1 | Japonica | NA | | Os09g0439600 | OsR498G0917538300.01.T01 | Exocyst subunit Exo70 family protein (Fragment) |
| augustus_masked-9-processed-gene-165.9-mRNA-1 | Japonica | NA | | Os09g0442400 | OsR498G0917550500.01.T02 | Protein GAMETE EXPRESSED 1 |
| augustus_masked-9-processed-gene-166.15-mRNA-1 | Japonica | NA | | | OsR498G0917553100.01.T01 | PABS domain-containing protein |
| augustus_masked-9-processed-gene-167.14-mRNA-1 | Japonica | Indica | | Os09g0445500 | OsR498G0917561300.01.T01 | Zinc finger protein GIS |
| augustus_masked-9-processed-gene-167.17-mRNA-1 | Japonica | Japonica | | Os09g0445700 | OsR498G0917563300.01.T01 | Adenosinetriphosphatase |
| augustus_masked-9-processed-gene-170.23-mRNA-1 | Japonica | NA | | Os09g0451500 | OsR498G0917589100.01.T01 | Protein disulfide-isomerase |
| augustus_masked-9-processed-gene-170.35-mRNA-1 | Japonica | NA | | Os09g0452400 | OsR498G0917591700.01.T01 | Aspartic proteinase nepenthesin |
| augustus_masked-9-processed-gene-170.37-mRNA-1 | Japonica | NA | | | OsR498G0714603300.01.T01 | |
| augustus_masked-9-processed-gene-172.10-mRNA-1 | Japonica | NA | | | OsR498G1119312200.01.T01 | |
| augustus_masked-9-processed-gene-172.11-mRNA-1 | Japonica | NA | | Os09g0455300 | OsR498G0917602900.01.T02 | Transcription factor HEC1 |
| augustus_masked-9-processed-gene-173.0-mRNA-1 | Japonica | NA | | Os09g0456800 | OsR498G0917608700.01.T01 | Heat shock transcription factor |
| augustus_masked-9-processed-gene-174.20-mRNA-1 | Japonica | Japonica | | Os09g0458800 | OsR498G0917615900.01.T02 | Polcalcin Phl p 7-like |
| augustus_masked-9-processed-gene-174.25-mRNA-1 | Japonica | NA | | Os09g0460000 | OsR498G0917620100.01.T03 | Diphthamide biosynthesis protein 4 |
| augustus_masked-9-processed-gene-174.6-mRNA-1 | Japonica | NA | | Os09g0458700 | | Calmodulin-like protein 1 |
| augustus_masked-9-processed-gene-175.15-mRNA-1 | Japonica | Japonica | | Os09g0461700 | OsR498G0917605400.01.T01 | Tuliposide A-converting enzyme 2, chloroplastic |
| augustus_masked-9-processed-gene-175.17-mRNA-1 | Japonica | NA | | Os09g0462100 | OsR498G0917625500.01.T01 | Tuliposide A-converting enzyme 1, chloroplastic |
| augustus_masked-9-processed-gene-175.18-mRNA-1 | Japonica | Japonica | | Os09g0462300 | OsR498G0816299500.01.T01 | Tuliposide A-converting enzyme 2, chloroplastic |
| augustus_masked-9-processed-gene-175.2-mRNA-1 | Japonica | NA | | Os09g0460300 | OsR498G0917621300.01.T01 | Tuliposide A-converting enzyme b1, amyloplastic |
| augustus_masked-9-processed-gene-175.3-mRNA-1 | Japonica | Japonica | | Os09g0460400 | OsR498G0917621300.01.T01 | Tuliposide A-converting enzyme b1, amyloplastic |
| augustus_masked-9-processed-gene-175.5-mRNA-1 | Japonica | NA | | Os09g0460700 | OsR498G0917622300.01.T01 | Tuliposide A-converting enzyme 1, chloroplastic |
| augustus_masked-9-processed-gene-175.6-mRNA-1 | Japonica | NA | | Os09g0460800 | OsR498G0917622500.01.T01 | Tuliposide A-converting enzyme b1, amyloplastic |
| augustus_masked-9-processed-gene-176.18-mRNA-1 | Japonica | NA | | Os09g0463600 | OsR498G0917633900.01.T01 | Plant-specific domain TIGR01570 family protein |
| augustus_masked-9-processed-gene-176.19-mRNA-1 | Japonica | NA | | Os09g0463700 | OsR498G0917634300.01.T02 | J domain-containing protein (Fragment) |
| augustus_masked-9-processed-gene-176.21-mRNA-1 | Japonica | NA | | | OsR498G0917635900.01.T01 | |
| augustus_masked-9-processed-gene-179.1-mRNA-1 | Japonica | NA | | | OsR498G0917653300.01.T01 | |
| augustus_masked-9-processed-gene-181.10-mRNA-1 | Japonica | NA | | Os09g0473400 | OsR498G0917668500.01.T01 | PORR domain-containing protein (Fragment) |
| augustus_masked-9-processed-gene-181.6-mRNA-1 | Japonica | NA | | Os09g0472700 | OsR498G0917666300.01.T01 | Blight-associated protein p12 |
| augustus_masked-9-processed-gene-181.7-mRNA-1 | Japonica | NA | | Os09g0472900 | OsR498G0917667700.01.T01 | Expansin-like EG45 domain-containing protein (Fragment) |
| augustus_masked-9-processed-gene-182.22-mRNA-1 | Japonica | Indica | | Os09g0473600 | OsR498G0917669300.01.T01 | probable serine/threonine-protein kinase fhkB |
| augustus_masked-9-processed-gene-182.37-mRNA-1 | Japonica | NA | | | OsR498G0917673500.01.T01 | |
| augustus_masked-9-processed-gene-183.24-mRNA-1 | Japonica | NA | | Os09g0475800 | OsR498G0917678300.01.T01 | DNA-binding with one finger protein 22 |
| augustus_masked-9-processed-gene-184.10-mRNA-1 | Japonica | Indica | | Os09g0517800 | OsR498G0917689100.01.T06 | F-box domain-containing protein |
| augustus_masked-9-processed-gene-185.10-mRNA-1 | Japonica | Japonica | | Os09g0480200 | OsR498G0917693300.01.T01 | Putative chalcone synthase |
| augustus_masked-9-processed-gene-185.15-mRNA-1 | Japonica | NA | | | OsR498G0917695700.01.T01 | BURP domain-containing protein 15 |
| augustus_masked-9-processed-gene-186.43-mRNA-1 | Japonica | Indica | | Os09g0481600 | OsR498G0917698300.01.T01 | AAA domain-containing protein (Fragment) |
| augustus_masked-9-processed-gene-186.44-mRNA-1 | Japonica | NA | | | | Polyubiquitin 9 |
| augustus_masked-9-processed-gene-186.58-mRNA-1 | Japonica | NA | | Os09g0482700 | | Heat shock protein 81-3 |
| augustus_masked-9-processed-gene-187.20-mRNA-1 | Japonica | NA | | Os09g0483300 | OsR498G0917709300.01.T01 | Calcium-binding EF hand family protein |
| augustus_masked-9-processed-gene-188.10-mRNA-1 | Japonica | NA | Kinase-TM | Os09g0486700 | OsR498G0917718500.01.T01 | Serine/threonine-protein kinase UCNL |
| augustus_masked-9-processed-gene-188.5-mRNA-1 | Japonica | NA | | Os06g0612300 | | Zinc finger protein WIP2 |
| augustus_masked-9-processed-gene-190.13-mRNA-1 | Japonica | NA | | Os09g0491756 | OsR498G0917732900.01.T04 | Splicing factor U2af small subunit A |
| augustus_masked-9-processed-gene-193.57-mRNA-1 | Japonica | Indica | | Os09g0498100 | OsR498G0917746500.01.T10 | 30S ribosomal protein S16-2 chloroplastic/mitochondrial |
| augustus_masked-9-processed-gene-194.40-mRNA-1 | Japonica | Indica | | Os02g0179600 | OsR498G0917753100.01.T01 | |
| augustus_masked-9-processed-gene-195.0-mRNA-1 | Japonica | Indica | | Os08g0524400 | OsR498G0917755900.01.T01 | DOMON domain-containing protein (Fragment) |
| augustus_masked-9-processed-gene-195.16-mRNA-1 | Japonica | NA | | | OsR498G0917761100.01.T01 | Chloroplastic quinone-oxidoreductase |
| augustus_masked-9-processed-gene-195.19-mRNA-1 | Japonica | NA | | Os09g0503400 | OsR498G0917762300.01.T01 | Leucyl-tRNA synthetase |

| | | | | | | |
|---|---|---|---|---|---|---|
| augustus_masked-9-processed-gene-197.76-mRNA-1 | Japonica | NA | | Os09g0507100 | OsR498G0917772100.01.T04 | SBP-box family member protein (Fragment) |
| augustus_masked-9-processed-gene-199.17-mRNA-1 | Japonica | NA | | | OsR498G0917786700.01.T01 | |
| augustus_masked-9-processed-gene-199.18-mRNA-1 | Japonica | NA | | | OsR498G0917787300.01.T01 | |
| augustus_masked-9-processed-gene-20.15-mRNA-1 | Japonica | NA | | | | Ankyrin repeat protein-like |
| augustus_masked-9-processed-gene-203.27-mRNA-1 | Japonica | Indica | | Os09g0517800 | OsR498G0917804100.01.T01 | F-box domain-containing protein |
| augustus_masked-9-processed-gene-203.37-mRNA-1 | Japonica | Japonica | | Os09g0518000 | OsR498G0917804500.01.T01 | Glycosyltransferase |
| augustus_masked-9-processed-gene-205.19-mRNA-1 | Japonica | NA | | Os09g0522800 | OsR498G0917819100.01.T01 | |
| augustus_masked-9-processed-gene-208.35-mRNA-1 | Japonica | NA | | | OsR498G0917840100.01.T02 | SH3 and multiple ankyrin repeat domains protein 1 |
| augustus_masked-9-processed-gene-209.0-mRNA-1 | Japonica | NA | | Os09g0529900 | OsR498G0917840500.01.T01 | Rhodanese-like domain-containing protein 14, chloroplastic |
| augustus_masked-9-processed-gene-21.2-mRNA-1 | Japonica | NA | | Os02g0212975 | OsR498G0305421900.01.T01 | Kinesin-like protein KIN-14G |
| augustus_masked-9-processed-gene-211.43-mRNA-1 | Japonica | Indica | | Os11g0138300 | OsR498G0917856700.01.T01 | Cytochrome P450 family protein, expressed |
| augustus_masked-9-processed-gene-212.1-mRNA-1 | Japonica | NA | | Os09g0535400 | OsR498G0917860300.01.T01 | Collagen, type IV, alpha 5 |
| augustus_masked-9-processed-gene-212.2-mRNA-1 | Japonica | Indica | | Os07g0593400 | OsR498G0917860900.01.T02 | WPP domain-containing protein |
| augustus_masked-9-processed-gene-212.5-mRNA-1 | Japonica | NA | | Os09g0536200 | OsR498G0917863900.01.T01 | Armadillo repeat only 1 |
| augustus_masked-9-processed-gene-213.15-mRNA-1 | Japonica | NA | | Os09g0538750 | OsR498G0917877500.01.T01 | DUF632 domain-containing protein (Fragment) |
| augustus_masked-9-processed-gene-213.19-mRNA-1 | Japonica | NA | | | OsR498G0203810500.01.T01 | |
| augustus_masked-9-processed-gene-214.48-mRNA-1 | Japonica | NA | | | OsR498G0917884900.01.T01 | |
| augustus_masked-9-processed-gene-215.28-mRNA-1 | Japonica | NA | | | OsR498G0917893100.01.T01 | |
| augustus_masked-9-processed-gene-215.29-mRNA-1 | Japonica | NA | | Os09g0543900 | OsR498G0917894100.01.T01 | Putrescine hydroxycinnamoyltransferase |
| augustus_masked-9-processed-gene-218.34-mRNA-1 | Japonica | NA | | | OsR498G0917910900.01.T01 | F-box domain-containing protein |
| augustus_masked-9-processed-gene-218.56-mRNA-1 | Japonica | NA | | | OsR498G0917916500.01.T03 | BED-type domain-containing protein |
| augustus_masked-9-processed-gene-22.21-mRNA-1 | Japonica | Japonica | | Os09g0126600 | OsR498G0916735500.01.T01 | Tyrosine specific protein phosphatase-like |
| augustus_masked-9-processed-gene-220.58-mRNA-1 | Japonica | Indica | | Os09g0553800 | OsR498G0917928900.01.T01 | Ecotropic viral integration site protein |
| augustus_masked-9-processed-gene-220.59-mRNA-1 | Japonica | NA | | Os09g0553900 | | Plastid movement impaired protein |
| augustus_masked-9-processed-gene-221.53-mRNA-1 | Japonica | NA | | Os09g0555150 | OsR498G0917933500.01.T01 | Sulfotransferase |
| augustus_masked-9-processed-gene-221.55-mRNA-1 | Japonica | NA | | Os09g0555300 | OsR498G0917934300.01.T01 | F-box/kelch-repeat protein At5g42360 |
| augustus_masked-9-processed-gene-221.56-mRNA-1 | Japonica | NA | | | OsR498G0917934700.01.T01 | |
| augustus_masked-9-processed-gene-223.14-mRNA-1 | Japonica | NA | | Os09g0558200 | OsR498G0917943500.01.T01 | Transcription factor |
| augustus_masked-9-processed-gene-223.18-mRNA-1 | Japonica | Indica | | Os09g0559600 | OsR498G0917946500.01.T02 | Thioredoxin domain-containing protein |
| augustus_masked-9-processed-gene-225.0-mRNA-1 | Japonica | NA | Kinase-TM WAK92 | Os09g0562600 | OsR498G0917953300.01.T02 | Wall-associated receptor kinase 3 |
| augustus_masked-9-processed-gene-225.13-mRNA-1 | Japonica | NA | | Os05g0116600 | OsR498G0509609000.01.T01 | F-box protein interaction domain containing protein |
| augustus_masked-9-processed-gene-227.15-mRNA-1 | Japonica | Japonica | | Os09g0565700 | OsR498G0917961300.01.T03 | Arogenate dehydratase |
| augustus_masked-9-processed-gene-227.17-mRNA-1 | Japonica | Japonica | | Os09g0566000 | OsR498G0917961300.01.T03 | Arogenate dehydratase |
| augustus_masked-9-processed-gene-228.76-mRNA-1 | Japonica | NA | | Os09g0569100 | OsR498G0917971500.01.T01 | Genetic modifier |
| augustus_masked-9-processed-gene-229.63-mRNA-1 | Japonica | Japonica | | Os09g0570800 | OsR498G0816609100.01.T01 | feruloyl CoA ortho-hydroxylase 2 |
| augustus_masked-9-processed-gene-229.64-mRNA-1 | Japonica | NA | | Os09g0571100 | OsR498G0917977900.01.T01 | Pectinesterase |
| augustus_masked-9-processed-gene-229.68-mRNA-1 | Japonica | NA | | Os09g0572000 | OsR498G0917980300.01.T01 | Ethylene-responsive transcription factor 15 |
| augustus_masked-9-processed-gene-230.0-mRNA-1 | Japonica | NA | | Os09g0572200 | OsR498G0917980900.01.T03 | P-loop NTPase domain-containing protein LPA1 |
| augustus_masked-9-processed-gene-230.10-mRNA-1 | Japonica | NA | | Os09g0572700 | OsR498G0917982900.01.T01 | Phytocyanin domain-containing protein |
| augustus_masked-9-processed-gene-24.35-mRNA-1 | Japonica | NA | | | OsR498G0917242300.01.T01 | |
| augustus_masked-9-processed-gene-26.17-mRNA-1 | Japonica | NA | LRR-TM | Os09g0131200 | | Receptor like protein 44 isoform 2 |
| augustus_masked-9-processed-gene-26.21-mRNA-1 | Japonica | NA | | Os07g0562400 | OsR498G0714577900.01.T01 | MFS domain-containing protein (Fragment) |
| augustus_masked-9-processed-gene-26.5-mRNA-1 | Japonica | NA | | | OsR498G0916747900.01.T01 | |
| augustus_masked-9-processed-gene-28.17-mRNA-1 | Japonica | NA | | Os09g0132200 | OsR498G0916754500.01.T01 | GDSL-motif lipase/hydrolase-like |
| augustus_masked-9-processed-gene-30.9-mRNA-1 | Japonica | Indica | | Os09g0135400 | OsR498G0916767700.01.T01 | Receptor kinase |
| augustus_masked-9-processed-gene-35.25-mRNA-1 | Japonica | NA | | Os09g0241550 | OsR498G0916787100.01.T01 | F-box domain containing protein, expressed |
| augustus_masked-9-processed-gene-37.10-mRNA-1 | Japonica | Japonica | | Os09g0244600 | OsR498G1019219100.01.T02 | |
| augustus_masked-9-processed-gene-4.14-mRNA-1 | Japonica | NA | | Os09g0102300 | | DNA-binding storekeeper protein-related |
| augustus_masked-9-processed-gene-45.19-mRNA-1 | Japonica | NA | | | OsR498G0916838100.01.T05 | Exosome complex component RRP41 |
| augustus_masked-9-processed-gene-46.10-mRNA-1 | Japonica | Indica | | Os09g0258700 | OsR498G0916851500.01.T01 | Bidirectional sugar transporter SWEET |
| augustus_masked-9-processed-gene-47.13-mRNA-1 | Japonica | NA | | Os09g0255900 | OsR498G0916844700.01.T01 | |
| augustus_masked-9-processed-gene-47.9-mRNA-1 | Japonica | NA | | | OsR498G0511259700.01.T01 | Transposon protein, putative, Mutator sub-class, expressed |
| augustus_masked-9-processed-gene-48.0-mRNA-1 | Japonica | NA | | Os09g0256100 | OsR498G0916845900.01.T01 | Sulfotransferase |
| augustus_masked-9-processed-gene-5.28-mRNA-1 | Japonica | NA | | Os09g0103700 | OsR498G0916647700.01.T01 | CAX-interacting protein 4 |
| augustus_masked-9-processed-gene-55.25-mRNA-1 | Japonica | Indica | Kinase-TM | Os07g0283125 | OsR498G0916878900.01.T01 | Lectin-domain containing receptor kinase A4.2 |
| augustus_masked-9-processed-gene-56.0-mRNA-1 | Japonica | NA | | | OsR498G0916880300.01.T01 | |
| augustus_masked-9-processed-gene-56.17-mRNA-1 | Japonica | NA | | | OsR498G0407871400.01.T01 | Retrotransposon protein, putative, Ty3-gypsy sub-class |
| augustus_masked-9-processed-gene-62.16-mRNA-1 | Japonica | NA | | | OsR498G0916902900.01.T01 | |
| augustus_masked-9-processed-gene-63.28-mRNA-1 | Japonica | NA | | | OsR498G0916907500.01.T01 | |
| augustus_masked-9-processed-gene-77.12-mRNA-1 | Japonica | Indica | | Os07g0559700 | OsR498G0917012900.01.T01 | Sugar carrier protein C |
| augustus_masked-9-processed-gene-82.11-mRNA-1 | Japonica | NA | | Os09g0304500 | OsR498G0917038900.01.T01 | |

| | | | | | | |
|---|---|---|---|---|---|---|
| augustus_masked-9-processed-gene-92.16-mRNA-1 | Japonica | NA | | | OsR498G0917242300.01.T01 | |
| augustus_masked-9-processed-gene-95.31-mRNA-1 | Japonica | NA | | | | |
| augustus_masked-9-processed-gene-96.18-mRNA-1 | Japonica | NA | | Os09g0324300 | OsR498G0917155300.01.T01 | F-box domain-containing protein (Fragment) |
| augustus_masked-9-processed-gene-96.19-mRNA-1 | Japonica | NA | | Os09g0324400 | OsR498G0917155900.01.T01 | F-box domain-containing protein (Fragment) |
| augustus_masked-9-processed-gene-96.2-mRNA-1 | Japonica | NA | | Os09g0324200 | OsR498G0917154900.01.T01 | F-box domain-containing protein (Fragment) |
| augustus_masked-9-processed-gene-96.23-mRNA-1 | Japonica | NA | | Os09g0325700 | OsR498G0917159900.01.T01 | Protein-serine/threonine phosphatase |
| augustus_masked-9-processed-gene-97.17-mRNA-1 | Japonica | NA | | Os09g0326800 | OsR498G0917161300.01.T01 | 26S proteasome non-ATPase regulatory subunit 2 A |
| augustus_masked-9-processed-gene-98.4-mRNA-1 | Japonica | NA | | Os09g0326900 | OsR498G0917161700.01.T06 | W2 domain-containing protein (Fragment) |
| augustus_masked-9-processed-gene-99.19-mRNA-1 | Japonica | NA | | Os09g0329000 | OsR498G0917165300.01.T01 | BURP domain-containing protein 14 |
| maker-1-augustus-gene-1.12-mRNA-1 | Japonica | NA | | Os01g0100500 | OsR498G0100001700.01.T05 | Monocopper oxidase-like protein SKU5 |
| maker-1-augustus-gene-1.16-mRNA-1 | Japonica | NA | | Os01g0100600 | OsR498G0100002100.01.T01 | SUZ domain-containing protein (Fragment) |
| maker-1-augustus-gene-1.20-mRNA-1 | Japonica | NA | | Os01g0100700 | OsR498G0100002300.01.T01 | 40S ribosomal protein like |
| maker-1-augustus-gene-1.22-mRNA-1 | Japonica | NA | | | | Putative 1-phosphatidylinositol-3-phosphate 5-kinase FAB1C |
| maker-1-augustus-gene-1.29-mRNA-1 | Japonica | NA | | Os01g0100900 | OsR498G0100003100.01.T01 | Sphingosine-1-phosphate lyase |
| maker-1-augustus-gene-1.3-mRNA-1 | Japonica | Indica | | Os01g0100100 | OsR498G0100000700.01.T01 | Rab-GAP TBC domain-containing protein |
| maker-1-augustus-gene-1.34-mRNA-1 | Japonica | NA | | Os01g0101200 | | probable bifunctional methylthioribulose-1-phosphate dehydratase/enolase |
| maker-1-augustus-gene-1.47-mRNA-1 | Japonica | NA | | Os01g0101300 | OsR498G0100004600.01.T01 | Snurportin-1 |
| maker-1-augustus-gene-1.8-mRNA-1 | Japonica | Indica | | Os01g0100400 | OsR498G0100001500.01.T01 | Monocopper oxidase-like protein SKU5 |
| maker-1-augustus-gene-10.45-mRNA-1 | Japonica | NA | | Os01g0119500 | OsR498G0100063300.01.T02 | |
| maker-1-augustus-gene-10.47-mRNA-1 | Japonica | Indica | | Os01g0118400 | OsR498G0100059400.01.T01 | Glycosyltransferase family 61 protein |
| maker-1-augustus-gene-10.50-mRNA-1 | Japonica | NA | | Os01g0118600 | OsR498G0100060400.01.T01 | Putative glycosyltransferase AGO61 |
| maker-1-augustus-gene-10.53-mRNA-1 | Japonica | NA | | Os01g0118700 | OsR498G0100060600.01.T01 | Glycosyltransferase, HGA-like, expressed |
| maker-1-augustus-gene-10.57-mRNA-1 | Japonica | NA | | Os01g0119000 | OsR498G0100061000.01.T01 | Putative glycosyltransferase AGO61 |
| maker-1-augustus-gene-10.59-mRNA-1 | Japonica | Indica | | Os01g0119100 | OsR498G0100062400.01.T02 | protein O-linked-mannose beta-1,4-N-acetylglucosaminyltransferase 2-like |
| maker-1-augustus-gene-100.0-mRNA-1 | Japonica | NA | | Os01g0279300 | OsR498G0100668900.01.T01 | Calmodulin |
| maker-1-augustus-gene-100.10-mRNA-1 | Japonica | NA | | Os12g0170100 | OsR498G0100672300.01.T01 | Pentatricopeptide repeat-containing protein mitochondrial |
| maker-1-augustus-gene-100.13-mRNA-1 | Japonica | NA | | Os01g0279400 | OsR498G0100669200.01.T01 | MFS domain-containing protein |
| maker-1-augustus-gene-100.2-mRNA-1 | Japonica | NA | | Os01g0279700 | OsR498G0100670100.01.T03 | Probable anion transporter 1, chloroplastic |
| maker-1-augustus-gene-100.20-mRNA-1 | Japonica | NA | LRR-TM | Os01g0279800 | OsR498G0100670400.01.T01 | Somatic embryoproteinsis receptor kinase 5 |
| maker-1-augustus-gene-100.23-mRNA-1 | Japonica | NA | | Os01g0280000 | OsR498G0100671400.01.T02 | Cell death-related nuclease 4 |
| maker-1-augustus-gene-100.27-mRNA-1 | Japonica | NA | | Os01g0280500 | OsR498G0100672600.01.T01 | Eukaryotic translation initiation factor 6 |
| maker-1-augustus-gene-100.6-mRNA-1 | Japonica | NA | | Os01g0279900 | OsR498G0100671100.01.T04 | Transcription factor TGAL1 |
| maker-1-augustus-gene-101.0-mRNA-1 | Japonica | NA | | Os01g0281000 | OsR498G0100674900.01.T01 | F-box family protein isoform 1 |
| maker-1-augustus-gene-101.13-mRNA-1 | Japonica | NA | | Os01g0281600 | OsR498G0100676600.01.T01 | Phytocyanin domain-containing protein (Fragment) |
| maker-1-augustus-gene-101.3-mRNA-1 | Japonica | Indica | | Os01g0281100 | OsR498G0100675200.01.T01 | |
| maker-1-augustus-gene-101.7-mRNA-1 | Japonica | Indica | | Os01g0281200 | OsR498G0100675400.01.T01 | Cyclin N-terminal domain-containing protein |
| maker-1-augustus-gene-101.9-mRNA-1 | Japonica | Indica | | Os11g0174000 | OsR498G0100676000.01.T02 | Coatomer subunit beta |
| maker-1-augustus-gene-102.0-mRNA-1 | Japonica | NA | | Os01g0283100 | OsR498G0100679900.01.T01 | Gamma carbonic anhydrase 1 mitochondrial |
| maker-1-augustus-gene-102.10-mRNA-1 | Japonica | NA | | Os01g0282800 | OsR498G0100678600.01.T01 | Tubulin beta chain |
| maker-1-augustus-gene-102.12-mRNA-1 | Japonica | NA | | Os01g0283000 | OsR498G0100679800.01.T02 | Nucleotide-diphospho-sugar transferase |
| maker-1-augustus-gene-102.14-mRNA-1 | Japonica | NA | | Os01g0283500 | OsR498G0100681000.01.T01 | Rhomboid-like protein 14, mitochondrial |
| maker-1-augustus-gene-102.16-mRNA-1 | Japonica | NA | | Os01g0284500 | OsR498G0100684800.01.T01 | Germin-like protein 1-1 |
| maker-1-augustus-gene-102.5-mRNA-1 | Japonica | NA | | Os01g0283600 | OsR498G0100681500.01.T01 | Epimerase domain-containing protein (Fragment) |
| maker-1-augustus-gene-102.8-mRNA-1 | Japonica | NA | | Os01g0283700 | OsR498G0100683300.01.T01 | Epimerase domain-containing protein (Fragment) |
| maker-1-augustus-gene-103.44-mRNA-1 | Japonica | Indica | | Os05g0103200 | OsR498G0100687900.01.T04 | Peptidyl-prolyl cis-trans isomerase |
| maker-1-augustus-gene-103.47-mRNA-1 | Japonica | NA | | | | |
| maker-1-augustus-gene-103.51-mRNA-1 | Japonica | NA | | Os01g0284900 | OsR498G0100688400.01.T01 | Bromodomain adjacent to zinc finger domain protein 2B-like |
| maker-1-augustus-gene-103.53-mRNA-1 | Japonica | NA | | | OsR498G0100689400.01.T01 | Berberine bridge enzyme-like 27 |
| maker-1-augustus-gene-103.55-mRNA-1 | Japonica | NA | | Os01g0285300 | OsR498G0100690200.01.T01 | Transcription factor MYB61 |
| maker-1-augustus-gene-103.58-mRNA-1 | Japonica | NA | | Os01g0286100 | OsR498G0100694000.01.T01 | Transcription factor APG |
| maker-1-augustus-gene-104.0-mRNA-1 | Japonica | NA | | Os01g0286600 | OsR498G0100695700.01.T01 | Chloroplast protoporphyrinogen IX oxidase |
| maker-1-augustus-gene-104.10-mRNA-1 | Japonica | NA | | Os01g0287600 | OsR498G0100702400.01.T01 | Class II chitinase |
| maker-1-augustus-gene-104.12-mRNA-1 | Japonica | NA | | | OsR498G0100702600.01.T08 | |
| maker-1-augustus-gene-104.6-mRNA-1 | Japonica | Indica | | Os01g0286900 | OsR498G0100698500.01.T01 | Auxin-responsive protein IAA4 |
| maker-1-augustus-gene-104.8-mRNA-1 | Japonica | Indica | | Os06g0620000 | OsR498G0100695400.01.T01 | |
| maker-1-augustus-gene-105.0-mRNA-1 | Japonica | NA | | Os01g0288200 | | Phosphatase methylesterase 1 |
| maker-1-augustus-gene-105.3-mRNA-1 | Japonica | NA | | Os03g0114600 | OsR498G0612855600.01.T01 | Transposable element protein, putative, MuDR |
| maker-1-augustus-gene-106.0-mRNA-1 | Japonica | NA | | | OsR498G0100708900.01.T01 | WRKY transcription factor |
| maker-1-augustus-gene-106.1-mRNA-1 | Japonica | NA | | Os01g0289900 | | Anthocyanin 5-aromatic acyltransferase |
| maker-1-augustus-gene-106.3-mRNA-1 | Japonica | NA | | Os01g0290000 | | Retrotransposon protein SINE subclass |
| maker-1-augustus-gene-106.5-mRNA-1 | Japonica | NA | | Os01g0290300 | | Putative HAT, C-terminal dimerization domain-containing protein |

| | | | | | | |
|---|---|---|---|---|---|---|
| maker-1-augustus-gene-106.7-mRNA-1 | Japonica | NA | | | OsR498G0100712200.01.T02 | ABC transporter B family member 4 |
| maker-1-augustus-gene-107.29-mRNA-1 | Japonica | NA | Kinase-TM | Os01g0292200 | OsR498G0100720500.01.T01 | Non-specific serine/threonine protein kinase |
| maker-1-augustus-gene-107.39-mRNA-1 | Japonica | NA | | Os01g0291500 | OsR498G0100715000.01.T01 | Acyl transferase 4 |
| maker-1-augustus-gene-108.0-mRNA-1 | Japonica | NA | | Os01g0292900 | OsR498G0100721700.01.T06 | SBP-type domain-containing protein |
| maker-1-augustus-gene-108.11-mRNA-1 | Japonica | Japonica | | Os01g0293900 | OsR498G0100724000.01.T01 | Peroxidase |
| maker-1-augustus-gene-108.13-mRNA-1 | Japonica | Indica | | Os01g0294700 | OsR498G0100725400.01.T01 | Peroxidase |
| maker-1-augustus-gene-108.15-mRNA-1 | Japonica | Indica | | Os05g0134800 | OsR498G0100725400.01.T01 | Peroxidase |
| maker-1-augustus-gene-108.17-mRNA-1 | Japonica | NA | | Os01g0294700 | OsR498G0100726000.01.T01 | Peroxidase |
| maker-1-augustus-gene-108.5-mRNA-1 | Japonica | NA | | Os01g0293100 | OsR498G0100722600.01.T01 | Transcription factor TIP2 |
| maker-1-augustus-gene-108.7-mRNA-1 | Japonica | NA | | Os07g0157000 | OsR498G0100723800.01.T01 | Peroxidase |
| maker-1-augustus-gene-109.10-mRNA-1 | Japonica | Japonica | | Os01g0296200 | OsR498G0100728900.01.T01 | Polygalacturonase |
| maker-1-augustus-gene-109.14-mRNA-1 | Japonica | NA | | | OsR498G0100727400.01.T01 | Protein kinase domain-containing protein |
| maker-1-augustus-gene-109.17-mRNA-1 | Japonica | NA | | Os01g0295700 | OsR498G0100727600.01.T01 | Protein-serine/threonine phosphatase |
| maker-1-augustus-gene-109.26-mRNA-1 | Japonica | NA | Kinase-TM | Os01g0296000 | OsR498G0100728600.01.T01 | Putative LRR receptor-like serine/threonine-protein kinase |
| maker-1-augustus-gene-109.5-mRNA-1 | Japonica | NA | Kinase-TM | Os01g0296100 | OsR498G0100728700.01.T04 | Non-specific serine/threonine protein kinase |
| maker-1-augustus-gene-11.42-mRNA-1 | Japonica | NA | | | OsR498G0100063700.01.T01 | Ubiquitin-like domain-containing protein |
| maker-1-augustus-gene-11.44-mRNA-1 | Japonica | NA | | | OsR498G0100063900.01.T01 | Ubiquitin-like domain-containing protein |
| maker-1-augustus-gene-110.50-mRNA-1 | Japonica | Indica | | Os01g0296700 | OsR498G0100730500.01.T01 | Beta-xylosidasealpha-l-arabinofuranosidase 1 |
| maker-1-augustus-gene-110.52-mRNA-1 | Japonica | Japonica | | Os01g0296900 | OsR498G0100730900.01.T01 | Adenosinetriphosphatase |
| maker-1-augustus-gene-110.55-mRNA-1 | Japonica | NA | | Os01g0297200 | OsR498G0100731500.01.T01 | Adenosinetriphosphatase |
| maker-1-augustus-gene-110.57-mRNA-1 | Japonica | NA | | Os01g0297700 | OsR498G0100733500.01.T02 | |
| maker-1-augustus-gene-110.59-mRNA-1 | Japonica | NA | | | OsR498G1222106000.01.T03 | |
| maker-1-augustus-gene-110.60-mRNA-1 | Japonica | NA | | Os04g0564200 | OsR498G1222106000.01.T02 | TPX2 domain-containing protein |
| maker-1-augustus-gene-111.0-mRNA-1 | Japonica | NA | | | OsR498G0100736700.01.T01 | Pentatricopeptide repeat-containing protein |
| maker-1-augustus-gene-111.13-mRNA-1 | Japonica | Japonica | | Os01g0299100 | OsR498G0202947900.01.T01 | Transposon protein, putative, CACTA, En/Spm sub-class |
| maker-1-augustus-gene-111.16-mRNA-1 | Japonica | Japonica | | Os01g0299300 | OsR498G0100737000.01.T01 | Acyltransferase family protein, expressed |
| maker-1-augustus-gene-111.18-mRNA-1 | Japonica | Indica | | Os01g0299500 | OsR498G0100737600.01.T03 | PAB-dependent poly(A)-specific ribonuclease |
| maker-1-augustus-gene-111.2-mRNA-1 | Japonica | NA | | Os01g0299400 | OsR498G0100737300.01.T01 | IGR protein motif |
| maker-1-augustus-gene-111.21-mRNA-1 | Japonica | NA | | Os01g0300200 | OsR498G0100740400.01.T01 | ATP citrate synthase |
| maker-1-augustus-gene-111.28-mRNA-1 | Japonica | NA | | | OsR498G0100742000.01.T01 | Carbohydrate-binding-like fold |
| maker-1-augustus-gene-112.30-mRNA-1 | Japonica | NA | | Os01g0300900 | OsR498G0100743700.01.T01 | Rab9 effector protein with kelch motifs |
| maker-1-augustus-gene-112.33-mRNA-1 | Japonica | NA | | Os01g0301000 | OsR498G0100744000.01.T01 | Pentatricopeptide repeat-containing protein mitochondrial |
| maker-1-augustus-gene-112.35-mRNA-1 | Japonica | Japonica | | Os01g0301500 | OsR498G0100745200.01.T01 | Pentatricopeptide repeat-containing protein mitochondrial |
| maker-1-augustus-gene-112.37-mRNA-1 | Japonica | NA | | Os01g0301700 | OsR498G0100745600.01.T02 | Pentatricopeptide repeat-containing protein |
| maker-1-augustus-gene-112.39-mRNA-1 | Japonica | NA | | Os01g0301900 | | |
| maker-1-augustus-gene-113.37-mRNA-1 | Japonica | NA | | | | Knotted1-like homeodomain protein liguleless3 (Fragment) |
| maker-1-augustus-gene-113.39-mRNA-1 | Japonica | NA | | Os01g0303200 | OsR498G0100750500.01.T05 | |
| maker-1-augustus-gene-113.45-mRNA-1 | Japonica | NA | | Os01g0303800 | OsR498G0100752500.01.T04 | USP-like protein isoform 3 |
| maker-1-augustus-gene-113.48-mRNA-1 | Japonica | Indica | | Os03g0305600 | OsR498G0100750800.01.T02 | Outer envelope pore protein like |
| maker-1-augustus-gene-113.53-mRNA-1 | Japonica | NA | | Os01g0303600 | OsR498G0100751600.01.T01 | RING-CH-type domain-containing protein |
| maker-1-augustus-gene-114.0-mRNA-1 | Japonica | Indica | | Os05g0531500 | OsR498G0100753700.01.T01 | Inner membrane protein oxaA |
| maker-1-augustus-gene-114.3-mRNA-1 | Japonica | NA | | | | Endosulphine |
| maker-1-augustus-gene-114.8-mRNA-1 | Japonica | NA | | | OsR498G0100753400.01.T02 | Cation-chloride cotransporter 1 |
| maker-1-augustus-gene-115.3-mRNA-1 | Japonica | NA | | Os01g0306800 | OsR498G0100761500.01.T03 | 4-hydroxy-3-methylbut-2-enyl diphosphatereductase |
| maker-1-augustus-gene-115.9-mRNA-1 | Japonica | NA | | Os01g0306900 | OsR498G0100762200.01.T01 | |
| maker-1-augustus-gene-116.31-mRNA-1 | Japonica | NA | LRR-NBS-TM | Os01g0308300 | OsR498G0100770300.01.T01 | Putative NB-ARC domain-containing protein (Fragment) |
| maker-1-augustus-gene-116.33-mRNA-1 | Japonica | NA | | Os01g0307500 | OsR498G0100765000.01.T01 | High affinity potassium transporter 2 |
| maker-1-augustus-gene-116.35-mRNA-1 | Japonica | Indica | | Os05g0357800 | OsR498G0100767800.01.T04 | Lysophospholipid acyltransferase LPEAT1 |
| maker-1-augustus-gene-116.40-mRNA-1 | Japonica | NA | | | OsR498G0100768200.01.T01 | Embryonic abundant protein-like |
| maker-1-augustus-gene-117.0-mRNA-1 | Japonica | NA | | Os01g0309800 | OsR498G0100775700.01.T02 | HMA domain-containing protein |
| maker-1-augustus-gene-117.2-mRNA-1 | Japonica | NA | | Os01g0309900 | OsR498G0100776100.01.T01 | Mono-/di-acylglycerol lipase N-terminal |
| maker-1-augustus-gene-118.0-mRNA-1 | Japonica | Japonica | Kinase-TM | Os01g0310400 | OsR498G0100776900.01.T03 | Wall-associated receptor kinase 5 |
| maker-1-augustus-gene-118.12-mRNA-1 | Japonica | Japonica | | Os01g0311500 | OsR498G0100779100.01.T02 | Dual specificity protein phosphatase PHS1 |
| maker-1-augustus-gene-118.18-mRNA-1 | Japonica | NA | | Os01g0311700 | OsR498G0100779700.01.T01 | Pectin methyl esterase inhibitor |
| maker-1-augustus-gene-118.20-mRNA-1 | Japonica | NA | | | OsR498G0100776800.01.T04 | Phospholipase D |
| maker-1-augustus-gene-118.27-mRNA-1 | Japonica | NA | | Os01g0311800 | OsR498G0100780000.01.T01 | Pectinesterase |
| maker-1-augustus-gene-118.4-mRNA-1 | Japonica | Japonica | | Os01g0310500 | OsR498G0409269800.01.T05 | Protein kinase domain-containing protein |
| maker-1-augustus-gene-118.6-mRNA-1 | Japonica | Indica | Kinase-TM | Os01g0310400 | OsR498G0100778100.01.T02 | PTI1-like tyrosine-protein kinase 3 |
| maker-1-augustus-gene-119.0-mRNA-1 | Japonica | Indica | | Os01g0312500 | OsR498G0100781700.01.T01 | Pectinesterase |
| maker-1-augustus-gene-119.10-mRNA-1 | Japonica | NA | | | | Peripheral subunit-binding (PSBD) domain-containing protein (Fragment) |
| maker-1-augustus-gene-119.14-mRNA-1 | Japonica | NA | | Os01g0314300 | OsR498G0100786800.01.T01 | MCT-1 like PUA RNA binding-domain containing protein |

| | | | | | | |
|---|---|---|---|---|---|---|
| maker-1-augustus-gene-119.4-mRNA-1 | Japonica | NA | | Os01g0312800 | OsR498G0100782500.01.T01 | Endoglucanase |
| maker-1-augustus-gene-119.6-mRNA-1 | Japonica | NA | | Os01g0313300 | OsR498G0100785100.01.T01 | Ethylene-responsive transcription factor 1 |
| maker-1-augustus-gene-119.8-mRNA-1 | Japonica | NA | | | OsR498G0100786200.01.T01 | Dihydrolipoamide acetyltransferase component of pyruvate dehydrogenase |
| maker-1-augustus-gene-120.24-mRNA-1 | Japonica | Indica | | Os05g0363100 | OsR498G0100791300.01.T02 | Caffeoylshikimate esterase |
| maker-1-augustus-gene-120.31-mRNA-1 | Japonica | Indica | | Os01g0314800 | OsR498G0100789400.01.T01 | Indole-3-acetic acid-induced protein ARG2-like |
| maker-1-augustus-gene-120.33-mRNA-1 | Japonica | NA | | Os01g0315800 | OsR498G0100791600.01.T01 | UDP-glucuronic acid decarboxylase |
| maker-1-augustus-gene-121.0-mRNA-1 | Japonica | NA | | Os01g0316600 | OsR498G0100794100.01.T01 | Serine/arginine-rich-splicing factor SR34 |
| maker-1-augustus-gene-121.16-mRNA-1 | Japonica | NA | | Os01g0316500 | OsR498G0100793800.01.T02 | LYR motif-containing protein 7 isoform 2 |
| maker-1-augustus-gene-121.9-mRNA-1 | Japonica | Indica | | Os01g0316900 | OsR498G0100795100.01.T03 | |
| maker-1-augustus-gene-122.35-mRNA-1 | Japonica | NA | | | OsR498G0100800300.01.T03 | Protein ABC transporter 1 mitochondrial |
| maker-1-augustus-gene-122.40-mRNA-1 | Japonica | NA | | Os01g0319000 | OsR498G0100800900.01.T01 | Pectin acetylesterase |
| maker-1-augustus-gene-122.45-mRNA-1 | Japonica | NA | | | OsR498G0100801300.01.T01 | |
| maker-1-augustus-gene-122.47-mRNA-1 | Japonica | NA | | Os01g0319200 | OsR498G0100803600.01.T01 | |
| maker-1-augustus-gene-123.24-mRNA-1 | Japonica | NA | | Os01g0320400 | OsR498G0100809600.01.T01 | |
| maker-1-augustus-gene-124.0-mRNA-1 | Japonica | NA | | Os01g0321800 | OsR498G0100814500.01.T01 | NEFA-interacting nuclear protein NIP30 |
| maker-1-augustus-gene-124.14-mRNA-1 | Japonica | NA | | Os01g0321700 | OsR498G0100814400.01.T01 | Protein transport protein SEC23 |
| maker-1-augustus-gene-124.17-mRNA-1 | Japonica | NA | | Os01g0322800 | OsR498G0100817200.01.T01 | Suppressor of mec-8 and unc-52 protein 1 |
| maker-1-augustus-gene-124.2-mRNA-1 | Japonica | NA | Kinase-TM | Os01g0323000 | OsR498G0100818500.01.T02 | Serine/threonine protein kinase |
| maker-1-augustus-gene-124.7-mRNA-1 | Japonica | NA | | | | Chloroplast protein-transporting ATPase |
| maker-1-augustus-gene-125.0-mRNA-1 | Japonica | NA | | Os01g0323300 | | CRS2-associated factor 2, chloroplastic |
| maker-1-augustus-gene-125.5-mRNA-1 | Japonica | NA | | | | Sulfotransferase |
| maker-1-augustus-gene-125.7-mRNA-1 | Japonica | NA | Kinase-TM | Os01g0323100 | OsR498G0100818800.01.T01 | Pto-interacting protein 1 |
| maker-1-augustus-gene-125.9-mRNA-1 | Japonica | NA | | | OsR498G0100824600.01.T01 | |
| maker-1-augustus-gene-126.0-mRNA-1 | Japonica | NA | | Os01g0326000 | OsR498G0100830700.01.T01 | Peroxidase |
| maker-1-augustus-gene-126.2-mRNA-1 | Japonica | NA | | Os01g0326100 | OsR498G0100832100.01.T01 | Peroxidase |
| maker-1-augustus-gene-126.3-mRNA-1 | Japonica | Indica | | Os01g0326000 | OsR498G0100832100.01.T01 | Peroxidase |
| maker-1-augustus-gene-127.0-mRNA-1 | Japonica | Japonica | | Os01g0327000 | OsR498G0100832700.01.T01 | Peroxidase |
| maker-1-augustus-gene-127.10-mRNA-1 | Japonica | NA | | | OsR498G0100834200.01.T07 | Putative inactive serine/threonine-protein kinase lvsG |
| maker-1-augustus-gene-127.14-mRNA-1 | Japonica | Japonica | | Os01g0327900 | OsR498G0100834400.01.T01 | DUF295 domain-containing protein |
| maker-1-augustus-gene-127.3-mRNA-1 | Japonica | NA | | Os01g0327100 | OsR498G0100833100.01.T01 | Peroxidase |
| maker-1-augustus-gene-127.5-mRNA-1 | Japonica | NA | | Os01g0327400 | OsR498G0100833700.01.T03 | Peroxidase |
| maker-1-augustus-gene-127.7-mRNA-1 | Japonica | NA | | Os01g0328200 | | |
| maker-1-augustus-gene-128.33-mRNA-1 | Japonica | NA | | Os01g0328500 | OsR498G0100834900.01.T03 | BCNT-C domain-containing protein (Fragment) |
| maker-1-augustus-gene-128.35-mRNA-1 | Japonica | NA | | Os01g0328600 | OsR498G0100835100.01.T06 | Polyketide cyclase/dehydrase and lipid transport superfamily protein |
| maker-1-augustus-gene-128.41-mRNA-1 | Japonica | NA | | Os01g0329300 | OsR498G0100840300.01.T01 | Polygalacturonase |
| maker-1-augustus-gene-128.43-mRNA-1 | Japonica | NA | | Os01g0329800 | OsR498G0100841100.01.T01 | Evolutionarily conserved C-terminal region 7 |
| maker-1-augustus-gene-128.45-mRNA-1 | Japonica | NA | | Os01g0328700 | OsR498G0100835400.01.T01 | Dihydrolipoyl dehydrogenase |
| maker-1-augustus-gene-128.47-mRNA-1 | Japonica | NA | | Os01g0328900 | OsR498G0100836400.01.T01 | Hydroxyproline O-galactosyltransferase GALT3 |
| maker-1-augustus-gene-128.52-mRNA-1 | Japonica | NA | | Os01g0329000 | OsR498G0100836600.01.T01 | PlsC domain-containing protein (Fragment) |
| maker-1-augustus-gene-128.54-mRNA-1 | Japonica | NA | | Os01g0329400 | OsR498G0100840600.01.T01 | Solute carrier family 25 member like |
| maker-1-augustus-gene-129.37-mRNA-1 | Japonica | NA | | | OsR498G0100842700.01.T03 | GDSL esterase/lipase |
| maker-1-augustus-gene-129.40-mRNA-1 | Japonica | NA | | Os01g0330300 | OsR498G0100843300.01.T01 | Putative cysteine protease |
| maker-1-augustus-gene-129.42-mRNA-1 | Japonica | NA | | Os01g0330500 | OsR498G0714577900.01.T01 | Organic cation/carnitine transporter 3 |
| maker-1-augustus-gene-129.44-mRNA-1 | Japonica | Indica | | Os01g0329900 | OsR498G0100845700.01.T01 | GDSL esterase/lipase |
| maker-1-augustus-gene-129.46-mRNA-1 | Japonica | NA | | Os01g0330200 | OsR498G0100843200.01.T01 | Cysteine protease 1 |
| maker-1-augustus-gene-13.50-mRNA-1 | Japonica | NA | | | OsR498G0100083100.01.T01 | RING-type E3 ubiquitin transferase |
| maker-1-augustus-gene-13.60-mRNA-1 | Japonica | NA | | Os01g0124601 | OsR498G0100082800.01.T01 | Putative Bowman Birk trypsin inhibitor |
| maker-1-augustus-gene-130.26-mRNA-1 | Japonica | NA | | Os01g0331900 | OsR498G0100847500.01.T01 | Golgi apparatus membrane protein TVP23 |
| maker-1-augustus-gene-130.30-mRNA-1 | Japonica | NA | | Os01g0332100 | OsR498G0100848700.01.T01 | Beta-fructofuranosidase |
| maker-1-augustus-gene-130.36-mRNA-1 | Japonica | NA | | Os01g0332500 | OsR498G0100849700.01.T01 | Carboxypeptidase |
| maker-1-augustus-gene-130.42-mRNA-1 | Japonica | NA | | Os01g0332800 | OsR498G0100850400.01.T01 | Carboxypeptidase |
| maker-1-augustus-gene-130.45-mRNA-1 | Japonica | Indica | | Os01g0332900 | OsR498G0100851000.01.T01 | Immediate-early protein RSP40 |
| maker-1-augustus-gene-133.35-mRNA-1 | Japonica | NA | | Os01g0336300 | | DUF4220 domain-containing protein (Fragment) |
| maker-1-augustus-gene-133.39-mRNA-1 | Japonica | NA | | Os01g0337100 | | Sesquithujene synthase A |
| maker-1-augustus-gene-133.42-mRNA-1 | Japonica | NA | CC-LRR-NBS-TM | Os01g0335700 | OsR498G0100857600.01.T01 | Putative NB-ARC domain-containing protein (Fragment) |
| maker-1-augustus-gene-134.0-mRNA-1 | Japonica | NA | | Os01g0337500 | OsR498G0100861300.01.T01 | H(+)-exporting diphosphatase |
| maker-1-augustus-gene-134.10-mRNA-1 | Japonica | NA | | Os01g0338000 | OsR498G0100862700.01.T01 | GTP-binding protein SAR1A |
| maker-1-augustus-gene-134.13-mRNA-1 | Japonica | NA | | | OsR498G0100863100.01.T05 | Transcription initiation factor TFIID subunit like |
| maker-1-augustus-gene-134.21-mRNA-1 | Japonica | NA | | Os01g0337600 | OsR498G0100861600.01.T01 | SMK-1 domain-containing protein |
| maker-1-augustus-gene-134.26-mRNA-1 | Japonica | NA | | Os01g0338600 | OsR498G0100864800.01.T03 | Cysteine sulfinate desulfinase/cysteine desulfurase and related enzymes |
| maker-1-augustus-gene-134.4-mRNA-1 | Japonica | NA | | Os01g0337900 | OsR498G0100862300.01.T01 | Putative lipoamide dehydrogenase |

| | | | | | | |
|---|---|---|---|---|---|---|
| maker-1-augustus-gene-135.1-mRNA-1 | Japonica | Indica | | Os01g0339600 | OsR498G0100866300.01.T03 | Proteasome assembly chaperone 3 |
| maker-1-augustus-gene-135.11-mRNA-1 | Japonica | Indica | | Os01g0339500 | OsR498G0100866200.01.T01 | No apical meristem protein, expressed |
| maker-1-augustus-gene-135.4-mRNA-1 | Japonica | NA | | Os01g0339900 | OsR498G0100866500.01.T01 | Protein disulfide-isomerase |
| maker-1-augustus-gene-137.38-mRNA-1 | Japonica | NA | | Os01g0342900 | OsR498G0100882300.01.T01 | Acyl-activating enzyme 9 |
| maker-1-augustus-gene-137.54-mRNA-1 | Japonica | NA | | Os01g0342800 | OsR498G0100881200.01.T01 | LYR motif-containing protein (Fragment) |
| maker-1-augustus-gene-137.56-mRNA-1 | Japonica | NA | | | OsR498G0100885800.01.T04 | Lipoprotein NlpI |
| maker-1-augustus-gene-139.1-mRNA-1 | Japonica | NA | | Os01g0346501 | | Transposon protein, putative, CACTA, En/Spm sub-class |
| maker-1-augustus-gene-139.11-mRNA-1 | Japonica | Indica | | Os01g0347100 | OsR498G0100897400.01.T02 | Glycine-rich domain-containing protein 1 |
| maker-1-augustus-gene-139.4-mRNA-1 | Japonica | Japonica | | Os01g0346600 | OsR498G0100894900.01.T01 | |
| maker-1-augustus-gene-139.5-mRNA-1 | Japonica | NA | | Os01g0347000 | OsR498G0100897100.01.T01 | Dual specificity protein phosphatase 4 |
| maker-1-augustus-gene-14.41-mRNA-1 | Japonica | NA | | Os01g0125800 | OsR498G0100085300.01.T01 | Actin-interacting protein 1-2 |
| maker-1-augustus-gene-14.45-mRNA-1 | Japonica | Indica | | Os01g0125900 | OsR498G0100085500.01.T03 | UBC core domain-containing protein (Fragment) |
| maker-1-augustus-gene-14.49-mRNA-1 | Japonica | NA | | Os01g0126100 | OsR498G0100086500.01.T01 | Multicopper oxidase, type 1 |
| maker-1-augustus-gene-14.51-mRNA-1 | Japonica | NA | | Os01g0126200 | OsR498G0100086900.01.T05 | Cupredoxin superfamily protein isoform 1 |
| maker-1-augustus-gene-14.53-mRNA-1 | Japonica | NA | | | | Factor of DNA methylation 1 |
| maker-1-augustus-gene-14.55-mRNA-1 | Japonica | NA | | Os01g0126900 | OsR498G0100087500.01.T01 | Multicopper oxidase LPR1 |
| maker-1-augustus-gene-14.58-mRNA-1 | Japonica | NA | | Os01g0125700 | OsR498G0100085200.01.T02 | Glutamyl-tRNA (Gln) amidotransferase subunit C, mitochondrial |
| maker-1-augustus-gene-140.41-mRNA-1 | Japonica | Indica | | Os01g0330200 | OsR498G0100899700.01.T01 | Cysteine proteinase Cathepsin L |
| maker-1-augustus-gene-140.43-mRNA-1 | Japonica | NA | | Os01g0348000 | OsR498G0100899600.01.T01 | Putative cysteine protease |
| maker-1-augustus-gene-140.45-mRNA-1 | Japonica | NA | | Os01g0348600 | OsR498G0100901300.01.T01 | 3-hydroxyacyl-CoA dehydrogenase |
| maker-1-augustus-gene-140.53-mRNA-1 | Japonica | NA | | Os01g0347500 | OsR498G0100899600.01.T01 | Putative cysteine protease |
| maker-1-augustus-gene-140.55-mRNA-1 | Japonica | Indica | | Os01g0330200 | OsR498G0100900600.01.T01 | Cysteine proteinase Cathepsin L |
| maker-1-augustus-gene-140.57-mRNA-1 | Japonica | NA | | Os01g0348900 | OsR498G0100902200.01.T02 | Jacalin-like lectin domain containing protein, expressed |
| maker-1-augustus-gene-141.26-mRNA-1 | Japonica | NA | | Os01g0349800 | OsR498G0100904900.01.T01 | Secologanin synthase |
| maker-1-augustus-gene-141.29-mRNA-1 | Japonica | Indica | | Os05g0154600 | OsR498G0100907900.01.T03 | E3 ubiquitin-protein ligase RFI2 isoform X1 |
| maker-1-augustus-gene-141.34-mRNA-1 | Japonica | NA | | Os01g0349400 | OsR498G0100904600.01.T01 | Serine/threonine-protein phosphatase |
| maker-1-augustus-gene-141.37-mRNA-1 | Japonica | NA | NBS-TM | | OsR498G0100907000.01.T01 | NB-ARC domain-containing protein |
| maker-1-augustus-gene-141.39-mRNA-1 | Japonica | NA | | Os01g0350500 | OsR498G0100907200.01.T01 | |
| maker-1-augustus-gene-142.27-mRNA-1 | Japonica | NA | | Os01g0351100 | OsR498G0100908700.01.T01 | Poly [ADP-ribose] polymerase |
| maker-1-augustus-gene-142.31-mRNA-1 | Japonica | NA | | | OsR498G0100909100.01.T04 | Poly [ADP-ribose] polymerase |
| maker-1-augustus-gene-142.40-mRNA-1 | Japonica | NA | | Os01g0352100 | OsR498G0100913700.01.T01 | Fe2OG dioxygenase domain-containing protein (Fragment) |
| maker-1-augustus-gene-143.37-mRNA-1 | Japonica | NA | | Os01g0353400 | OsR498G0100914900.01.T01 | Glutathione S-transferase IV |
| maker-1-augustus-gene-143.39-mRNA-1 | Japonica | NA | | Os03g0228400 | OsR498G0305498900.01.T01 | AP complex subunit sigma |
| maker-1-augustus-gene-143.49-mRNA-1 | Japonica | NA | | | OsR498G0100914600.01.T01 | |
| maker-1-augustus-gene-143.51-mRNA-1 | Japonica | NA | | | OsR498G0100916200.01.T01 | Jacalin-type lectin domain-containing protein |
| maker-1-augustus-gene-144.0-mRNA-1 | Japonica | NA | | | OsR498G0100916700.01.T02 | Polynucleotide 5'-hydroxyl-kinase NOL9 |
| maker-1-augustus-gene-144.14-mRNA-1 | Japonica | NA | | Os01g0355500 | OsR498G0100918400.01.T02 | Auxilin-related protein 1 |
| maker-1-augustus-gene-144.8-mRNA-1 | Japonica | NA | | | OsR498G0100916600.01.T04 | Sterol C-14 reductase |
| maker-1-augustus-gene-145.10-mRNA-1 | Japonica | NA | | | OsR498G0100920800.01.T01 | Multidrug resistance-associated protein/mitoxantrone resistance protein, Al |
| maker-1-augustus-gene-145.14-mRNA-1 | Japonica | Indica | | Os01g0356500 | OsR498G0100923200.01.T02 | Thiamine pyrophosphokinase |
| maker-1-augustus-gene-145.5-mRNA-1 | Japonica | NA | | Os01g0355800 | OsR498G0100920300.01.T01 | Methylesterase 7 |
| maker-1-augustus-gene-145.7-mRNA-1 | Japonica | NA | | Os01g0355900 | OsR498G0100920600.01.T01 | Ubiquitin-like-specific protease ESD4 |
| maker-1-augustus-gene-146.36-mRNA-1 | Japonica | NA | | Os01g0356800 | | AIG1-type G domain-containing protein |
| maker-1-augustus-gene-146.38-mRNA-1 | Japonica | Indica | | Os01g0357100 | OsR498G0100930500.01.T02 | Ferredoxin--nitrite reductase chloroplastic |
| maker-1-augustus-gene-146.39-mRNA-1 | Japonica | Indica | | Os02g0765800 | OsR498G0100930700.01.T02 | SCP2 sterol-binding domain |
| maker-1-augustus-gene-146.53-mRNA-1 | Japonica | Indica | | Os02g0765600 | OsR498G0100931500.01.T01 | 1,4-alpha-D-glucan glucanohydrolase |
| maker-1-augustus-gene-146.54-mRNA-1 | Japonica | NA | | Os01g0357400 | OsR498G0100931500.01.T01 | Alpha-amylase 2 (Fragment) |
| maker-1-augustus-gene-146.60-mRNA-1 | Japonica | NA | | Os01g0358400 | OsR498G0100935300.01.T01 | S4 RNA-binding domain-containing protein (Fragment) |
| maker-1-augustus-gene-146.63-mRNA-1 | Japonica | NA | | Os01g0358300 | OsR498G0100935200.01.T02 | Putative tetratricopeptide-like helical domain, protein LOW PSII ACCUMULA |
| maker-1-augustus-gene-147.35-mRNA-1 | Japonica | NA | CC-NBS-TM | | OsR498G0100940800.01.T01 | Powdery mildew resistance protein PM3 variant |
| maker-1-augustus-gene-148.26-mRNA-1 | Japonica | Indica | NBS-TM | Os01g0359800 | OsR498G0100942600.01.T01 | Powdery mildew resistance protein PM3 variant |
| maker-1-augustus-gene-148.29-mRNA-1 | Japonica | NA | | Os01g0360200 | OsR498G0100943000.01.T04 | Respiratory burst oxidase B |
| maker-1-augustus-gene-148.34-mRNA-1 | Japonica | NA | | Os01g0360600 | OsR498G0100944800.01.T01 | Dephospho-CoA kinase |
| maker-1-augustus-gene-148.37-mRNA-1 | Japonica | NA | | | OsR498G0408211800.01.T01 | |
| maker-1-augustus-gene-149.40-mRNA-1 | Japonica | NA | | | OsR498G0100949500.01.T02 | rRNA adenine N(6)-methyltransferase |
| maker-1-augustus-gene-149.44-mRNA-1 | Japonica | NA | | Os01g0363300 | OsR498G0100952300.01.T01 | |
| maker-1-augustus-gene-149.47-mRNA-1 | Japonica | NA | | Os01g0361500 | OsR498G0100948400.01.T04 | Acyltransferase |
| maker-1-augustus-gene-149.54-mRNA-1 | Japonica | NA | | | OsR498G0100949000.01.T02 | Acyltransferase-like protein chloroplastic |
| maker-1-augustus-gene-150.31-mRNA-1 | Japonica | Indica | | Os01g0363900 | OsR498G0100952700.01.T02 | Xpo1 domain-containing protein |
| maker-1-augustus-gene-150.40-mRNA-1 | Japonica | NA | Kinase-TM | Os01g0364800 | OsR498G0100956900.01.T01 | Wall-associated receptor kinase |
| maker-1-augustus-gene-151.1-mRNA-1 | Japonica | NA | | | OsR498G0100957900.01.T01 | Neprosin domain-containing protein (Fragment) |

| | | | | | | |
|---|---|---|---|---|---|---|
| maker-1-augustus-gene-151.2-mRNA-1 | Japonica | NA | | | OsR498G0100958900.01.T02 | |
| maker-1-augustus-gene-151.6-mRNA-1 | Japonica | NA | | Os01g0367100 | OsR498G0100959800.01.T07 | NAD(P)-binding Rossmann-fold superfamily protein |
| maker-1-augustus-gene-152.40-mRNA-1 | Japonica | Japonica | | Os01g0368000 | OsR498G0100964300.01.T01 | Flavin-containing monooxygenase |
| maker-1-augustus-gene-152.42-mRNA-1 | Japonica | NA | | | | RRM domain-containing protein |
| maker-1-augustus-gene-152.44-mRNA-1 | Japonica | NA | | Os01g0367300 | OsR498G0100960200.01.T07 | RRM domain-containing protein |
| maker-1-augustus-gene-152.46-mRNA-1 | Japonica | Indica | | Os01g0367400 | OsR498G0100961200.01.T04 | NAD(P)-binding Rossmann-fold superfamily protein |
| maker-1-augustus-gene-152.52-mRNA-1 | Japonica | NA | | Os01g0367900 | OsR498G0100964000.01.T02 | Chromatin-remodeling complex ATPase chain |
| maker-1-augustus-gene-153.34-mRNA-1 | Japonica | NA | | Os01g0369200 | OsR498G0100968500.01.T01 | Cullin-1 |
| maker-1-augustus-gene-153.36-mRNA-1 | Japonica | NA | | Os01g0369000 | OsR498G0100968000.01.T03 | Cullin-1 |
| maker-1-augustus-gene-153.41-mRNA-1 | Japonica | NA | | | OsR498G0100968800.01.T01 | Potassium transporter |
| maker-1-augustus-gene-153.43-mRNA-1 | Japonica | NA | | Os01g0369700 | OsR498G0100969800.01.T01 | Glutathione S-transferase IV |
| maker-1-augustus-gene-153.45-mRNA-1 | Japonica | Indica | | Os01g0369900 | OsR498G0100970600.01.T01 | 12-oxo-phytodienoic acid reductase |
| maker-1-augustus-gene-153.48-mRNA-1 | Japonica | NA | | | OsR498G0100971200.01.T01 | N-ethylmaleimide reductase |
| maker-1-augustus-gene-154.28-mRNA-1 | Japonica | NA | | Os01g0371400 | | Glutathione transferase |
| maker-1-augustus-gene-154.30-mRNA-1 | Japonica | NA | | Os01g0371500 | OsR498G0100975500.01.T01 | Glutathione transferase |
| maker-1-augustus-gene-154.32-mRNA-1 | Japonica | Japonica | | Os01g0370200 | OsR498G0100971400.01.T01 | Glutathione transferase |
| maker-1-augustus-gene-154.35-mRNA-1 | Japonica | Indica | | Os01g0371200 | OsR498G0100972400.01.T01 | Glutathione transferase |
| maker-1-augustus-gene-154.38-mRNA-1 | Japonica | NA | | Os01g0371200 | OsR498G0100974400.01.T01 | Glutathione transferase |
| maker-1-augustus-gene-155.0-mRNA-1 | Japonica | NA | | | | Glutathione transferase |
| maker-1-augustus-gene-155.2-mRNA-1 | Japonica | NA | | Os01g0372400 | OsR498G0100978100.01.T01 | Glutathione transferase |
| maker-1-augustus-gene-155.4-mRNA-1 | Japonica | NA | | Os01g0372700 | OsR498G0100979000.01.T01 | Asparagine--tRNA ligase |
| maker-1-augustus-gene-155.7-mRNA-1 | Japonica | NA | | Os06g0247200 | OsR498G0408464800.01.T01 | Fe2OG dioxygenase domain-containing protein |
| maker-1-augustus-gene-156.0-mRNA-1 | Japonica | NA | | Os01g0374000 | OsR498G0100980700.01.T01 | Glutathione transferase |
| maker-1-augustus-gene-156.12-mRNA-1 | Japonica | NA | | Os01g0375000 | OsR498G0100984800.01.T01 | Guanine nucleotide-binding protein-like 3 |
| maker-1-augustus-gene-156.2-mRNA-1 | Japonica | Indica | | Os01g0374600 | OsR498G0100983700.01.T01 | Laccase |
| maker-1-augustus-gene-156.5-mRNA-1 | Japonica | NA | | Os01g0374900 | OsR498G0100984500.01.T01 | Putative E3 ubiquitin-protein ligase LUL2 |
| maker-1-augustus-gene-156.7-mRNA-1 | Japonica | Japonica | | Os01g0375200 | OsR498G0100985300.01.T01 | Shikimate biosynthesis protein aroDE |
| maker-1-augustus-gene-157.0-mRNA-1 | Japonica | Indica | | Os01g0375200 | OsR498G0100985700.01.T01 | Shikimate biosynthesis protein aroDE |
| maker-1-augustus-gene-157.4-mRNA-1 | Japonica | NA | | Os01g0376700 | OsR498G0100989200.01.T02 | Sucrose-phosphate phosphatase |
| maker-1-augustus-gene-158.0-mRNA-1 | Japonica | NA | | Os01g0377700 | OsR498G0100990900.01.T01 | Nuclear pore localization protein Npl |
| maker-1-augustus-gene-158.2-mRNA-1 | Japonica | NA | | Os01g0377500 | OsR498G0100990800.01.T01 | CYCLIN domain-containing protein |
| maker-1-augustus-gene-158.5-mRNA-1 | Japonica | Indica | | Os12g0530100 | OsR498G0100992000.01.T01 | Peroxidase |
| maker-1-augustus-gene-160.28-mRNA-1 | Japonica | NA | | Os01g0722550 | | HAT dimerization domain-containing protein / transposase-related |
| maker-1-augustus-gene-161.34-mRNA-1 | Japonica | NA | | Os01g0382200 | OsR498G0100996400.01.T01 | Putative hydroxyanthranilate hydroxycinnamoyltransferase 2 |
| maker-1-augustus-gene-161.36-mRNA-1 | Japonica | NA | | Os01g0382700 | OsR498G0101000100.01.T02 | Stress regulated protein isoform 3 |
| maker-1-augustus-gene-161.41-mRNA-1 | Japonica | NA | | Os05g0267732 | OsR498G0510209200.01.T01 | |
| maker-1-augustus-gene-161.45-mRNA-1 | Japonica | NA | | | | |
| maker-1-augustus-gene-162.0-mRNA-1 | Japonica | Indica | | Os05g0144100 | OsR498G0101004100.01.T01 | Transducin family protein / WD-40 repeat family protein |
| maker-1-augustus-gene-162.10-mRNA-1 | Japonica | NA | | | OsR498G0101003000.01.T01 | |
| maker-1-augustus-gene-163.26-mRNA-1 | Japonica | NA | | Os01g0385000 | OsR498G0101009400.01.T01 | |
| maker-1-augustus-gene-163.28-mRNA-1 | Japonica | NA | | Os01g0385400 | OsR498G0101010400.01.T02 | S-type anion channel SLAH3 |
| maker-1-augustus-gene-163.31-mRNA-1 | Japonica | Indica | | Os01g0385600 | OsR498G0101011200.01.T01 | Ribosomal protein-like |
| maker-1-augustus-gene-165.11-mRNA-1 | Japonica | Indica | | Os02g0204700 | OsR498G0101021000.01.T02 | |
| maker-1-augustus-gene-165.5-mRNA-1 | Japonica | NA | | | | Piezo-type mechanosensitive ion channel like |
| maker-1-augustus-gene-166.38-mRNA-1 | Japonica | NA | | | OsR498G0101029700.01.T05 | tRNA (guanine(37)-N1)-methyltransferase |
| maker-1-augustus-gene-166.39-mRNA-1 | Japonica | NA | | | OsR498G0101029700.01.T01 | tRNA (guanine(37)-N1)-methyltransferase |
| maker-1-augustus-gene-166.43-mRNA-1 | Japonica | NA | | | OsR498G0101029600.01.T03 | |
| maker-1-augustus-gene-167.0-mRNA-1 | Japonica | NA | | Os01g0390900 | OsR498G0101031000.01.T01 | Dual specificity phosphatase |
| maker-1-augustus-gene-168.40-mRNA-1 | Japonica | NA | | Os01g0392100 | OsR498G0101039100.01.T02 | |
| maker-1-augustus-gene-169.0-mRNA-1 | Japonica | NA | | | OsR498G0101041900.01.T02 | |
| maker-1-augustus-gene-169.11-mRNA-1 | Japonica | NA | | Os01g0393100 | OsR498G0101043400.01.T01 | NAC domain class transcription factor |
| maker-1-augustus-gene-169.14-mRNA-1 | Japonica | NA | | Os04g0495900 | OsR498G0408827700.01.T01 | Glycerol-3-phosphatase 1 |
| maker-1-augustus-gene-169.2-mRNA-1 | Japonica | Indica | | Os01g0104600 | OsR498G0101041900.01.T05 | Light-mediated development protein DET1 |
| maker-1-augustus-gene-169.4-mRNA-1 | Japonica | NA | | | | Transposon protein, putative, CACTA, En/Spm sub-class |
| maker-1-augustus-gene-169.9-mRNA-1 | Japonica | Japonica | | Os01g0393400 | OsR498G0101043700.01.T03 | ABC transporter B family member 4 |
| maker-1-augustus-gene-177.36-mRNA-1 | Japonica | NA | | Os01g0495900 | OsR498G0101046700.01.T01 | RNA-binding |
| maker-1-augustus-gene-177.42-mRNA-1 | Japonica | NA | | Os01g0496900 | OsR498G0101049400.01.T01 | Sterol O-acyltransferase |
| maker-1-augustus-gene-178.0-mRNA-1 | Japonica | NA | | | OsR498G0101051700.01.T01 | Expressed protein (Fragment) |
| maker-1-augustus-gene-178.6-mRNA-1 | Japonica | NA | | | OsR498G0101050600.01.T01 | Putative calcium-binding protein annexin 6 |
| maker-1-augustus-gene-179.25-mRNA-1 | Japonica | NA | | Os01g0499300 | OsR498G0101054600.01.T01 | AMSH-like ubiquitin thioesterase 3 |
| maker-1-augustus-gene-179.31-mRNA-1 | Japonica | Japonica | | Os01g0499600 | OsR498G1119310800.01.T01 | |

| | | | | | | |
|---|---|---|---|---|---|---|
| maker-1-augustus-gene-180.31-mRNA-1 | Japonica | NA | | Os01g0501800 | OsR498G0101060700.01.T01 | Oxygen-evolving enhancer protein like |
| maker-1-augustus-gene-180.33-mRNA-1 | Japonica | NA | | | OsR498G0101058200.01.T01 | DUF632 domain-containing protein |
| maker-1-augustus-gene-180.35-mRNA-1 | Japonica | NA | | | | Mediator of RNA polymerase II transcription subunit like |
| maker-1-augustus-gene-180.38-mRNA-1 | Japonica | NA | | Os01g0501700 | OsR498G0101060600.01.T01 | Sec-independent protein translocase protein TATC chloroplastic |
| maker-1-augustus-gene-181.0-mRNA-1 | Japonica | NA | | | OsR498G0101063500.01.T01 | Acyl-[acyl-carrier-protein] hydrolase |
| maker-1-augustus-gene-181.10-mRNA-1 | Japonica | NA | NRAMP6 | Os01g0503400 | OsR498G0101068400.01.T03 | Metal transporter |
| maker-1-augustus-gene-181.2-mRNA-1 | Japonica | NA | | Os01g0502400 | OsR498G0101064300.01.T02 | Oxoglutarate/iron-dependent dioxygenase |
| maker-1-augustus-gene-181.8-mRNA-1 | Japonica | NA | | Os01g0502700 | OsR498G0101065300.01.T01 | |
| maker-1-augustus-gene-182.32-mRNA-1 | Japonica | NA | | | OsR498G0101074900.01.T01 | 2-hydroxyacyl-CoA lyase-like |
| maker-1-augustus-gene-182.34-mRNA-1 | Japonica | NA | | Os01g0504500 | OsR498G0101071600.01.T03 | Protein DETOXIFICATION |
| maker-1-augustus-gene-183.0-mRNA-1 | Japonica | NA | | | OsR498G0101075900.01.T02 | Ribosome quality control complex subunit 2 |
| maker-1-augustus-gene-183.3-mRNA-1 | Japonica | NA | | Os01g0506100 | OsR498G0101078100.01.T03 | Pentatricopeptide repeat-containing protein mitochondrial |
| maker-1-augustus-gene-183.4-mRNA-1 | Japonica | NA | | Os01g0506200 | OsR498G0101079200.01.T04 | Tetratricopeptide repeat protein 5 |
| maker-1-augustus-gene-184.27-mRNA-1 | Japonica | NA | | Os01g0507700 | OsR498G0101084100.01.T03 | Farnesylated protein 2 |
| maker-1-augustus-gene-184.29-mRNA-1 | Japonica | NA | | Os01g0507900 | OsR498G0101084700.01.T02 | ATP-dependent Clp protease proteolytic subunit |
| maker-1-augustus-gene-184.34-mRNA-1 | Japonica | NA | | Os01g0508000 | OsR498G0101085300.01.T01 | Beta-glucosidase 44 |
| maker-1-augustus-gene-185.37-mRNA-1 | Japonica | NA | | | OsR498G0101090900.01.T01 | |
| maker-1-augustus-gene-186.36-mRNA-1 | Japonica | Japonica | | Os01g0509900 | OsR498G0101094500.01.T01 | BED-type domain-containing protein |
| maker-1-augustus-gene-186.38-mRNA-1 | Japonica | NA | Kinase | | | MAPKK |
| maker-1-augustus-gene-186.42-mRNA-1 | Japonica | NA | | | OsR498G0101097500.01.T01 | Protein ENHANCED DOWNY MILDEW 2 |
| maker-1-augustus-gene-186.49-mRNA-1 | Japonica | NA | | | OsR498G0101098100.01.T01 | |
| maker-1-augustus-gene-186.51-mRNA-1 | Japonica | NA | | Os01g0510600 | OsR498G0101097800.01.T01 | Protein FLUORESCENT IN BLUE LIGHT, chloroplastic |
| maker-1-augustus-gene-187.33-mRNA-1 | Japonica | NA | | Os01g0512100 | OsR498G0101105500.01.T01 | J domain-containing protein (Fragment) |
| maker-1-augustus-gene-187.39-mRNA-1 | Japonica | NA | | | | TAF domain-containing protein (Fragment) |
| maker-1-augustus-gene-187.45-mRNA-1 | Japonica | NA | | Os01g0511100 | OsR498G0101102400.01.T01 | Usp domain-containing protein |
| maker-1-augustus-gene-187.48-mRNA-1 | Japonica | NA | | Os01g0511300 | OsR498G0101102800.01.T01 | 26S proteasome non-ATPase regulatory subunit B |
| maker-1-augustus-gene-187.53-mRNA-1 | Japonica | Indica | | Os01g0511600 | OsR498G0101103600.01.T02 | Plastidal glycolate/glycerate translocator 1 chloroplastic |
| maker-1-augustus-gene-188.26-mRNA-1 | Japonica | NA | | Os01g0513400 | OsR498G0101110500.01.T01 | |
| maker-1-augustus-gene-188.29-mRNA-1 | Japonica | NA | | Os01g0513900 | OsR498G0101111700.01.T03 | Kinesin-like protein KIN-7A |
| maker-1-augustus-gene-188.34-mRNA-1 | Japonica | NA | | | OsR498G0101108400.01.T01 | PPM-type phosphatase domain-containing protein (Fragment) |
| maker-1-augustus-gene-188.39-mRNA-1 | Japonica | NA | | | OsR498G0101109600.01.T01 | Nicotinamide adenine dinucleotide transporter like |
| maker-1-augustus-gene-188.46-mRNA-1 | Japonica | NA | | Os01g0513700 | OsR498G0101111200.01.T01 | Trafficking protein particle complex subunit |
| maker-1-augustus-gene-188.49-mRNA-1 | Japonica | NA | | Os01g0513800 | OsR498G0101111400.01.T01 | Brix domain-containing protein 1 |
| maker-1-augustus-gene-189.11-mRNA-1 | Japonica | NA | Kinase-LRR-TM | Os01g0514700 | OsR498G0101114500.01.T01 | Receptor protein kinase-like protein ZAR1 |
| maker-1-augustus-gene-189.13-mRNA-1 | Japonica | NA | Kinase-LRR-TM | | | Protein kinase domain-containing protein |
| maker-1-augustus-gene-189.18-mRNA-1 | Japonica | NA | | Os08g0381000 | OsR498G0407574900.01.T01 | Cell wall protein-like |
| maker-1-augustus-gene-189.5-mRNA-1 | Japonica | NA | | Os01g0514600 | OsR498G0101113500.01.T01 | Fimbrin-2 |
| maker-1-augustus-gene-190.0-mRNA-1 | Japonica | NA | | Os01g0516400 | OsR498G0101116900.01.T01 | Stress responsive alpha-beta barrel |
| maker-1-augustus-gene-190.18-mRNA-1 | Japonica | NA | | | OsR498G0101119400.01.T01 | F-box domain-containing protein |
| maker-1-augustus-gene-190.20-mRNA-1 | Japonica | NA | | Os01g0517500 | OsR498G0101120600.01.T01 | Exopolygalacturonase |
| maker-1-augustus-gene-190.4-mRNA-1 | Japonica | NA | | Os01g0516600 | OsR498G0101117500.01.T01 | Stress responsive alpha-beta barrel |
| maker-1-augustus-gene-190.6-mRNA-1 | Japonica | Indica | | Os01g0516700 | OsR498G0101118000.01.T03 | UDP-N-acetylglucosamine--N-acetylmuramyl- (Pentapeptide) pyrophosphor |
| maker-1-augustus-gene-190.7-mRNA-1 | Japonica | NA | | | | Plant PDR ABC transporter associated |
| maker-1-augustus-gene-191.10-mRNA-1 | Japonica | NA | | | | |
| maker-1-augustus-gene-191.2-mRNA-1 | Japonica | Japonica | | Os01g0517800 | OsR498G0101120800.01.T01 | PLATZ transcription factor (Fragment) |
| maker-1-augustus-gene-192.30-mRNA-1 | Japonica | NA | | | | |
| maker-1-augustus-gene-192.32-mRNA-1 | Japonica | NA | CC-NBS-TM | | OsR498G0101131600.01.T02 | Blight resistance protein RGA1-like protein |
| maker-1-augustus-gene-193.0-mRNA-1 | Japonica | Indica | | Os01g0521400 | OsR498G0101133900.01.T01 | Gastric triacylglycerol lipase |
| maker-1-augustus-gene-193.10-mRNA-1 | Japonica | Japonica | | Os01g0522433 | OsR498G0101136300.01.T01 | |
| maker-1-augustus-gene-193.3-mRNA-1 | Japonica | NA | | Os01g0521500 | OsR498G0101134300.01.T01 | J domain-containing protein (Fragment) |
| maker-1-augustus-gene-193.9-mRNA-1 | Japonica | NA | | Os01g0522100 | OsR498G0101136300.01.T01 | VQ domain-containing protein |
| maker-1-augustus-gene-194.0-mRNA-1 | Japonica | NA | | | | Protein kinase domain-containing protein |
| maker-1-augustus-gene-194.2-mRNA-1 | Japonica | NA | | Os11g0205200 | OsR498G0407482700.01.T01 | |
| maker-1-augustus-gene-194.6-mRNA-1 | Japonica | Indica | | Os01g0524500 | OsR498G0101142900.01.T01 | Transcription factor MYBS1 |
| maker-1-augustus-gene-194.8-mRNA-1 | Japonica | NA | | | | Callose synthase 5 |
| maker-1-augustus-gene-195.0-mRNA-1 | Japonica | NA | | Os01g0524700 | OsR498G0101143700.01.T02 | ARM repeat superfamily protein isoform 1 |
| maker-1-augustus-gene-195.4-mRNA-1 | Japonica | NA | | | OsR498G0101148900.01.T01 | DNA-damage-repair/toleration protein, chloroplastic |
| maker-1-augustus-gene-195.6-mRNA-1 | Japonica | NA | | Os01g0526200 | OsR498G0101149100.01.T01 | rRNA processing protein-related |
| maker-1-augustus-gene-196.2-mRNA-1 | Japonica | Japonica | | Os01g0527400 | OsR498G0101154900.01.T04 | Bromo adjacent homology (BAH) domain |
| maker-1-augustus-gene-196.7-mRNA-1 | Japonica | NA | | | OsR498G0101155200.01.T01 | RNA-dependent RNA polymerase |
| maker-1-augustus-gene-197.0-mRNA-1 | Japonica | NA | | | | Tau95 domain-containing protein |

| | | | | | | |
|---|---|---|---|---|---|---|
| maker-1-augustus-gene-197.12-mRNA-1 | Japonica | NA | | | | GRF-type domain-containing protein |
| maker-1-augustus-gene-197.14-mRNA-1 | Japonica | NA | | Os01g0527700 | OsR498G0101155400.01.T01 | Heme O synthase |
| maker-1-augustus-gene-197.18-mRNA-1 | Japonica | Indica | | Os06g0521000 | OsR498G0101155600.01.T01 | Transposon protein, putative, ping/pong/SNOOPY sub-class |
| maker-1-augustus-gene-197.5-mRNA-1 | Japonica | NA | | | | Arogenate dehydratase |
| maker-1-augustus-gene-197.7-mRNA-1 | Japonica | NA | | Os01g0528800 | OsR498G0101158700.01.T01 | Cinnamoyl CoA reductase-like 2a |
| maker-1-augustus-gene-198.37-mRNA-1 | Japonica | Indica | | Os01g0530300 | OsR498G0101163100.01.T01 | SANTA domain-containing protein |
| maker-1-augustus-gene-198.42-mRNA-1 | Japonica | NA | | | OsR498G0101163300.01.T01 | RNA polymerase archaeal subunit P/eukaryotic subunit RPABC |
| maker-1-augustus-gene-198.48-mRNA-1 | Japonica | NA | | Os01g0531500 | OsR498G0101166100.01.T01 | DLH domain-containing protein (Fragment) |
| maker-1-augustus-gene-198.52-mRNA-1 | Japonica | NA | | Os01g0541600 | OsR498G0101165600.01.T01 | BAH domain-containing protein |
| maker-1-augustus-gene-198.55-mRNA-1 | Japonica | NA | | Os01g0531200 | OsR498G0101960700.01.T03 | PMD domain-containing protein |
| maker-1-augustus-gene-198.57-mRNA-1 | Japonica | NA | | | | Katanin p80 WD40 repeat-containing subunit B1 homolog |
| maker-1-augustus-gene-198.60-mRNA-1 | Japonica | NA | | Os01g0756600 | OsR498G0102017700.01.T01 | Putative Galactose-binding domain-containing protein |
| maker-1-augustus-gene-199.0-mRNA-1 | Japonica | Japonica | | Os01g0532600 | OsR498G0613256400.01.T02 | Probable cation transporter HKT9 |
| maker-1-augustus-gene-199.13-mRNA-1 | Japonica | NA | | | | |
| maker-1-augustus-gene-199.6-mRNA-1 | Japonica | NA | | | OsR498G0101168800.01.T02 | Pre-mRNA-processing protein 40A |
| maker-1-augustus-gene-2.0-mRNA-1 | Japonica | Indica | | Os01g0101700 | OsR498G0100006700.01.T02 | J domain-containing protein (Fragment) |
| maker-1-augustus-gene-2.14-mRNA-1 | Japonica | NA | | Os01g0102700 | OsR498G0100011900.01.T01 | Translocon-associated protein subunit beta |
| maker-1-augustus-gene-2.17-mRNA-1 | Japonica | NA | | Os01g0102800 | OsR498G0100012100.01.T01 | Protein CHROMATIN REMODELING like |
| maker-1-augustus-gene-2.19-mRNA-1 | Japonica | NA | | Os01g0102900 | OsR498G0100012800.01.T01 | Light regulated Lir |
| maker-1-augustus-gene-2.2-mRNA-1 | Japonica | NA | | Os01g0101800 | OsR498G0100006900.01.T01 | Putative aldehyde dehydrogenase family protein |
| maker-1-augustus-gene-2.22-mRNA-1 | Japonica | NA | | Os01g0102950 | OsR498G0100013000.01.T02 | Vacuolar protein sorting-associated protein 60.2 |
| maker-1-augustus-gene-2.26-mRNA-1 | Japonica | NA | | Os01g0103600 | OsR498G0100014200.01.T01 | 3-beta-hydroxysteroid-delta-isomerase |
| maker-1-augustus-gene-2.27-mRNA-1 | Japonica | NA | | Os01g0103600 | OsR498G0100014200.01.T03 | EXPERA domain-containing protein (Fragment) |
| maker-1-augustus-gene-2.4-mRNA-1 | Japonica | NA | | Os01g0102300 | OsR498G0100010900.01.T01 | Thylakoid lumenal 17.9 kDa protein, chloroplastic |
| maker-1-augustus-gene-2.6-mRNA-1 | Japonica | NA | | Os01g0102500 | OsR498G0100011500.01.T01 | Gap junction beta-4 protein isoform 1 |
| maker-1-augustus-gene-2.9-mRNA-1 | Japonica | NA | | Os01g0102600 | OsR498G0100011700.01.T01 | Shikimate kinase, chloroplastic |
| maker-1-augustus-gene-200.26-mRNA-1 | Japonica | Indica | | Os01g0533900 | OsR498G0101176700.01.T02 | ABC transporter B family member 9 |
| maker-1-augustus-gene-200.32-mRNA-1 | Japonica | NA | | | | 1,3-beta-glucan synthase |
| maker-1-augustus-gene-200.39-mRNA-1 | Japonica | NA | | Os01g0533400 | OsR498G0101174400.01.T01 | Beta-galactosidase 13 |
| maker-1-augustus-gene-200.41-mRNA-1 | Japonica | NA | | Os01g0533500 | OsR498G0101174800.01.T01 | 1,3-beta-glucan synthase |
| maker-1-augustus-gene-200.44-mRNA-1 | Japonica | NA | | | | Beta-galactosidase |
| maker-1-augustus-gene-218.51-mRNA-1 | Japonica | NA | | Os01g0559000 | OsR498G0101334100.01.T01 | PITH domain-containing protein 1 |
| maker-1-augustus-gene-219.11-mRNA-1 | Japonica | NA | | Os01g0559300 | OsR498G0101335100.01.T01 | Histone-lysine N-methyltransferase EHMT2 |
| maker-1-augustus-gene-219.14-mRNA-1 | Japonica | Japonica | | Os01g0559500 | OsR498G0101336300.01.T05 | Pentatricopeptide repeat-containing protein chloroplastic |
| maker-1-augustus-gene-219.18-mRNA-1 | Japonica | NA | | Os01g0559600 | OsR498G0101337500.01.T01 | Vacuolar-processing enzyme |
| maker-1-augustus-gene-219.22-mRNA-1 | Japonica | NA | | Os01g0560000 | OsR498G0101339800.01.T01 | IAA-amino acid hydrolase |
| maker-1-augustus-gene-219.7-mRNA-1 | Japonica | NA | | Os01g0559200 | OsR498G0101334900.01.T01 | Phosphorylated adapter RNA export protein (Fragment) |
| maker-1-augustus-gene-220.11-mRNA-1 | Japonica | NA | | | | |
| maker-1-augustus-gene-220.3-mRNA-1 | Japonica | NA | | Os01g0560350 | | V-SNARE domain-containing protein (Fragment) |
| maker-1-augustus-gene-220.5-mRNA-1 | Japonica | NA | | | | Peptidylprolyl isomerase |
| maker-1-augustus-gene-221.30-mRNA-1 | Japonica | Japonica | | Os01g0562400 | OsR498G0101342100.01.T02 | Peptidylprolyl isomerase |
| maker-1-augustus-gene-221.35-mRNA-1 | Japonica | Japonica | | Os01g0563000 | OsR498G0101346000.01.T04 | Peptidylprolyl isomerase |
| maker-1-augustus-gene-222.1-mRNA-1 | Japonica | NA | | Os01g0564300 | | Peptidylprolyl isomerase |
| maker-1-augustus-gene-223.34-mRNA-1 | Japonica | NA | | Os01g0565600 | OsR498G0101352200.01.T02 | Ribonuclease MRP protein subunit SNM1 |
| maker-1-augustus-gene-223.37-mRNA-1 | Japonica | NA | | Os01g0565800 | OsR498G0101352600.01.T01 | DUF538 domain-containing protein |
| maker-1-augustus-gene-223.39-mRNA-1 | Japonica | Indica | | Os01g0566100 | OsR498G0101353600.01.T04 | Early flowering protein 3 |
| maker-1-augustus-gene-223.42-mRNA-1 | Japonica | NA | | Os01g0566500 | OsR498G0101357200.01.T02 | Carotenoid cleavage dioxygenase |
| maker-1-augustus-gene-224.0-mRNA-1 | Japonica | NA | | Os01g0567500 | OsR498G0101359700.01.T01 | Sugar carrier protein C |
| maker-1-augustus-gene-224.2-mRNA-1 | Japonica | NA | | Os01g0566900 | OsR498G0101358200.01.T04 | PiRNA biogenesis protein EXD1 |
| maker-1-augustus-gene-224.8-mRNA-1 | Japonica | NA | | Os01g0567600 | OsR498G0101360200.01.T01 | Sugar carrier protein C |
| maker-1-augustus-gene-225.1-mRNA-1 | Japonica | NA | | Os01g0569200 | | |
| maker-1-augustus-gene-225.2-mRNA-1 | Japonica | NA | | Os01g0568400 | | Cysteine-rich receptor-like protein kinase 25 |
| maker-1-augustus-gene-225.4-mRNA-1 | Japonica | NA | Kinase-TM | Os01g0568400 | | receptor-like kinase TMK3 |
| maker-1-augustus-gene-226.0-mRNA-1 | Japonica | NA | | Os01g0570500 | OsR498G0101367300.01.T01 | Casein kinase I |
| maker-1-augustus-gene-226.13-mRNA-1 | Japonica | NA | | Os01g0571200 | OsR498G0101370700.01.T01 | 54S ribosomal protein L34, mitochondrial |
| maker-1-augustus-gene-226.17-mRNA-1 | Japonica | NA | | Os01g0571300 | OsR498G0101370900.01.T01 | Heat stress transcription factor A-2 |
| maker-1-augustus-gene-226.19-mRNA-1 | Japonica | NA | | | OsR498G0101372900.01.T02 | C3H-type transcription factor |
| maker-1-augustus-gene-226.23-mRNA-1 | Japonica | NA | | Os01g0570800 | OsR498G0101369200.01.T01 | IQ domain-containing protein IQM1 |
| maker-1-augustus-gene-226.28-mRNA-1 | Japonica | Indica | | Os01g0571100 | OsR498G0101369800.01.T03 | |
| maker-1-augustus-gene-226.5-mRNA-1 | Japonica | NA | | | OsR498G0101368900.01.T01 | Carbamoyl-phosphate synthase (glutamine-hydrolyzing) |
| maker-1-augustus-gene-226.7-mRNA-1 | Japonica | Indica | | Os01g0571000 | OsR498G0101369500.01.T01 | Graves disease carrier |

| | | | | | | |
|---|---|---|---|---|---|---|
| maker-1-augustus-gene-227.30-mRNA-1 | Japonica | NA | | Os01g0572300 | | Protein indeterminate-domain 9 |
| maker-1-augustus-gene-227.32-mRNA-1 | Japonica | NA | | Os05g0112000 | OsR498G1018864100.01.T01 | E3 ubiquitin-protein like |
| maker-1-augustus-gene-227.34-mRNA-1 | Japonica | NA | | | OsR498G0101376000.01.T03 | Calcium-binding EF hand family protein |
| maker-1-augustus-gene-228.33-mRNA-1 | Japonica | NA | NBS-TM | Os01g0574400 | OsR498G0101378500.01.T03 | ATP-dependent zinc metalloprotease FTSH 4 mitochondrial |
| maker-1-augustus-gene-228.36-mRNA-1 | Japonica | NA | NBS-TM | Os01g0574500 | OsR498G0101378700.01.T01 | ATP-dependent zinc metalloprotease FTSH 4 mitochondrial |
| maker-1-augustus-gene-228.39-mRNA-1 | Japonica | NA | | Os01g0575000 | OsR498G0101381500.01.T01 | Protein ROOT HAIR DEFECTIVE 3 homolog |
| maker-1-augustus-gene-228.46-mRNA-1 | Japonica | Indica | | Os01g0575200 | OsR498G0101382500.01.T01 | BHLH domain-containing protein |
| maker-1-augustus-gene-228.47-mRNA-1 | Japonica | NA | | Os01g0575200 | OsR498G0101382500.01.T01 | BHLH transcription factor |
| maker-1-augustus-gene-228.52-mRNA-1 | Japonica | NA | | Os01g0574600 | OsR498G0101379000.01.T01 | Glyoxylate/succinic semialdehyde reductase |
| maker-1-augustus-gene-229.29-mRNA-1 | Japonica | Indica | | Os04g0557200 | OsR498G0101393500.01.T01 | Anthocyanin regulatory R-S protein-like |
| maker-1-augustus-gene-229.30-mRNA-1 | Japonica | NA | | Os01g0576100 | OsR498G0101387300.01.T01 | Anthocyanin regulatory R-S protein-like |
| maker-1-augustus-gene-229.43-mRNA-1 | Japonica | NA | | | | |
| maker-1-augustus-gene-230.10-mRNA-1 | Japonica | NA | Kinase-LRR-TM | Os01g0577600 | OsR498G0101394600.01.T01 | Leucine-rich repeat receptor protein kinase EXS |
| maker-1-augustus-gene-230.13-mRNA-1 | Japonica | NA | | Os01g0578000 | OsR498G0101395400.01.T01 | Recombination protein51 gene c |
| maker-1-augustus-gene-230.18-mRNA-1 | Japonica | Indica | | Os03g0300200 | OsR498G0101395800.01.T03 | Programmed cell death protein 2 |
| maker-1-augustus-gene-230.8-mRNA-1 | Japonica | NA | | Os01g0578500 | | FBD domain-containing protein |
| maker-1-augustus-gene-231.15-mRNA-1 | Japonica | Japonica | | Os01g0580100 | OsR498G0101400300.01.T03 | Mannosyltransferase |
| maker-1-augustus-gene-231.21-mRNA-1 | Japonica | NA | | Os01g0580200 | OsR498G0101400900.01.T02 | Beta-galactosidase |
| maker-1-augustus-gene-231.28-mRNA-1 | Japonica | NA | | Os01g0579900 | OsR498G0101400000.01.T01 | Alpha/beta-Hydrolases superfamily protein |
| maker-1-augustus-gene-231.33-mRNA-1 | Japonica | NA | | Os01g0580000 | OsR498G0101400200.01.T02 | Alpha/beta-Hydrolases superfamily protein |
| maker-1-augustus-gene-231.6-mRNA-1 | Japonica | Indica | | Os01g0711500 | OsR498G0101399300.01.T01 | Calcineurin B-like protein 9 |
| maker-1-augustus-gene-232.0-mRNA-1 | Japonica | NA | | Os01g0580500 | | 1-aminocyclopropane-1-carboxylic acid oxidase |
| maker-1-augustus-gene-232.12-mRNA-1 | Japonica | NA | | | OsR498G0101402200.01.T01 | Actin binding protein family |
| maker-1-augustus-gene-232.13-mRNA-1 | Japonica | Indica | | Os01g0580800 | OsR498G0101402200.01.T01 | Alpha-mannosidase-like |
| maker-1-augustus-gene-232.18-mRNA-1 | Japonica | Japonica | | Os01g0581800 | OsR498G0101404800.01.T01 | NB-ARC domain-containing protein |
| maker-1-augustus-gene-232.2-mRNA-1 | Japonica | NA | | Os01g0581300 | OsR498G0101403500.01.T04 | Lycopene epsilon-cyclase |
| maker-1-augustus-gene-232.7-mRNA-1 | Japonica | NA | CC-Kinase-TM | Os01g0581400 | OsR498G0101403700.01.T01 | Lycopene epsilon cyclase, chloroplastic |
| maker-1-augustus-gene-232.9-mRNA-1 | Japonica | NA | | Os01g0580400 | OsR498G0101401800.01.T01 | Histone domain-containing protein |
| maker-1-augustus-gene-233.29-mRNA-1 | Japonica | Indica | | Os05g0592800 | OsR498G0101409500.01.T01 | Protein-serine/threonine phosphatase |
| maker-1-augustus-gene-233.37-mRNA-1 | Japonica | NA | | Os01g0582400 | OsR498G0101407000.01.T02 | Peptidyl-prolyl cis-trans isomerase |
| maker-1-augustus-gene-233.42-mRNA-1 | Japonica | NA | | Os01g0583200 | | UBP1-associated proteins 1C isoform A |
| maker-1-augustus-gene-234.49-mRNA-1 | Japonica | NA | | Os01g0585600 | OsR498G0101420100.01.T02 | DUF632 domain-containing protein |
| maker-1-augustus-gene-235.35-mRNA-1 | Japonica | NA | | Os01g0586600 | OsR498G0101423200.01.T04 | 5'-AMP-activated protein kinase subunit gamma-3 |
| maker-1-augustus-gene-235.38-mRNA-1 | Japonica | NA | | | OsR498G0101423600.01.T01 | WRKY transcription factor |
| maker-1-augustus-gene-235.40-mRNA-1 | Japonica | NA | | Os01g0587000 | OsR498G0101425200.01.T03 | V-type proton ATPase subunit |
| maker-1-augustus-gene-236.0-mRNA-1 | Japonica | NA | | Os01g0588700 | OsR498G0101430700.01.T01 | YJU2 splicing factor homolog isoform X1 |
| maker-1-augustus-gene-236.13-mRNA-1 | Japonica | NA | | Os01g0588200 | OsR498G0101428400.01.T02 | Outer plastidial membrane protein porin |
| maker-1-augustus-gene-236.17-mRNA-1 | Japonica | NA | | Os01g0588400 | OsR498G0101428600.01.T01 | Hypersensitive-induced response protein 1 |
| maker-1-augustus-gene-236.19-mRNA-1 | Japonica | NA | Kinase | Os01g0588500 | OsR498G0101429400.01.T01 | Putative serine/threonine-protein kinase PIX7 |
| maker-1-augustus-gene-236.22-mRNA-1 | Japonica | NA | | Os01g0588900 | OsR498G0101433200.01.T01 | Cytokinin riboside 5'-monophosphate phosphoribohydrolase LOG |
| maker-1-augustus-gene-236.4-mRNA-1 | Japonica | NA | | Os01g0588800 | OsR498G0101432900.01.T06 | Type II protein arginine methyltransferase |
| maker-1-augustus-gene-236.9-mRNA-1 | Japonica | NA | Kinase-TM | Os01g0587400 | OsR498G0101426800.01.T01 | Receptor-like serine/threonine-protein kinase |
| maker-1-augustus-gene-237.0-mRNA-1 | Japonica | Japonica | | Os01g0589100 | OsR498G0511248800.01.T01 | S-adenosyl-L-methionine-dependent methyltransferase superfamily protein |
| maker-1-augustus-gene-237.11-mRNA-1 | Japonica | NA | | Os01g0589000 | OsR498G0101435800.01.T03 | Ribosomal protein S1 |
| maker-1-augustus-gene-237.12-mRNA-1 | Japonica | Japonica | | Os01g0589500 | OsR498G0101437000.01.T01 | Holocarboxylase synthetase |
| maker-1-augustus-gene-237.14-mRNA-1 | Japonica | NA | | Os01g0590200 | OsR498G0101439000.01.T01 | |
| maker-1-augustus-gene-237.2-mRNA-1 | Japonica | NA | | Os01g0589200 | | Trans-aconitate 3-methyltransferase |
| maker-1-augustus-gene-237.9-mRNA-1 | Japonica | NA | | Os01g0589800 | OsR498G0101437300.01.T01 | Photosystem II 22 kDa, chloroplastic |
| maker-1-augustus-gene-238.39-mRNA-1 | Japonica | NA | | | OsR498G0101442100.01.T02 | ATP-dependent zinc metalloprotease FtsH |
| maker-1-augustus-gene-238.42-mRNA-1 | Japonica | NA | | Os01g0590900 | OsR498G0101443500.01.T01 | Serine/threonine-protein kinase AFC3 |
| maker-1-augustus-gene-238.48-mRNA-1 | Japonica | NA | | Os01g0591000 | OsR498G0101443900.01.T01 | Aldehyde dehydrogenase3 |
| maker-1-augustus-gene-238.55-mRNA-1 | Japonica | NA | | Os01g0591300 | OsR498G0101445100.01.T02 | Aldedh domain-containing protein (Fragment) |
| maker-1-augustus-gene-238.58-mRNA-1 | Japonica | NA | | | | Bidirectional sugar transporter SWEET |
| maker-1-augustus-gene-238.60-mRNA-1 | Japonica | Indica | | Os01g0590800 | OsR498G0101443000.01.T01 | |
| maker-1-augustus-gene-238.63-mRNA-1 | Japonica | NA | | Os01g0590800 | OsR498G0101443400.01.T01 | |
| maker-1-augustus-gene-239.28-mRNA-1 | Japonica | NA | | Os01g0593000 | OsR498G0101451500.01.T05 | Secretory carrier-associated membrane protein (Fragment) |
| maker-1-augustus-gene-239.36-mRNA-1 | Japonica | NA | | Os01g0593200 | OsR498G0101453300.01.T05 | FLZ-type domain-containing protein |
| maker-1-augustus-gene-239.40-mRNA-1 | Japonica | NA | | Os01g0593700 | OsR498G0101455700.01.T01 | Sulfate transporter |
| maker-1-augustus-gene-239.42-mRNA-1 | Japonica | NA | | Os01g0592900 | OsR498G0101450600.01.T01 | Regulator of telomere elongation helicase |
| maker-1-augustus-gene-239.47-mRNA-1 | Japonica | Japonica | | Os01g0593100 | OsR498G0714656200.01.T02 | A-kinase anchor protein 17A |
| maker-1-augustus-gene-239.49-mRNA-1 | Japonica | NA | | Os01g0593500 | OsR498G0101455200.01.T01 | Large subunit ribosomal protein L25 |

| | | | | | | |
|---|---|---|---|---|---|---|
| maker-1-augustus-gene-24.50-mRNA-1 | Japonica | NA | | Os01g0144200 | OsR498G0306795400.01.T03 | Glucose-induced degradation protein 4 |
| maker-1-augustus-gene-24.64-mRNA-1 | Japonica | NA | | Os01g0143300 | OsR498G0100159600.01.T01 | Mitochondrial glycoprotein family protein, putative isoform 1 |
| maker-1-augustus-gene-24.78-mRNA-1 | Japonica | NA | | Os01g0144100 | OsR498G0100162800.01.T01 | Thylakoid lumenal protein 1, chloroplastic |
| maker-1-augustus-gene-247.28-mRNA-1 | Japonica | NA | | Os01g0609066 | OsR498G0101514000.01.T02 | ABC transporter G family member 34 |
| maker-1-augustus-gene-25.13-mRNA-1 | Japonica | NA | | | OsR498G0100172200.01.T02 | Putative E3 ubiquitin-protein ligase |
| maker-1-augustus-gene-25.2-mRNA-1 | Japonica | NA | | | | TFIIS N-terminal domain-containing protein (Fragment) |
| maker-1-augustus-gene-25.6-mRNA-1 | Japonica | Indica | | Os11g0704800 | OsR498G0100171500.01.T01 | RHOMBOID-like protein (Fragment) |
| maker-1-augustus-gene-256.64-mRNA-1 | Japonica | NA | | | OsR498G0101575300.01.T01 | Kinesin-like protein KIN-13B |
| maker-1-augustus-gene-257.52-mRNA-1 | Japonica | Japonica | | Os01g0627933 | OsR498G0101582500.01.T01 | Secologanin synthase |
| maker-1-augustus-gene-257.53-mRNA-1 | Japonica | Japonica | | Os01g0627966 | OsR498G0101582500.01.T01 | Cytochrome P450 monooxygenase |
| maker-1-augustus-gene-257.60-mRNA-1 | Japonica | Indica | | Os01g0627900 | OsR498G0101583500.01.T01 | Secologanin synthase |
| maker-1-augustus-gene-258.10-mRNA-1 | Japonica | NA | Kinase-TM | Os01g0629900 | OsR498G0101587800.01.T02 | Mitogen-activated protein kinase |
| maker-1-augustus-gene-258.7-mRNA-1 | Japonica | NA | | | OsR498G0101586100.01.T07 | FCP1 homology domain-containing protein |
| maker-1-augustus-gene-259.1-mRNA-1 | Japonica | NA | | Os03g0855700 | | DUF223 domain-containing protein |
| maker-1-augustus-gene-259.2-mRNA-1 | Japonica | Indica | | Os01g0630700 | OsR498G0101590100.01.T01 | AT hook motif-containing protein, putative, expressed |
| maker-1-augustus-gene-259.20-mRNA-1 | Japonica | NA | | Os01g0631100 | OsR498G0101592900.01.T01 | Protein REDUCED WALL ACETYLATION like |
| maker-1-augustus-gene-259.26-mRNA-1 | Japonica | Indica | | Os01g0631200 | OsR498G0101593100.01.T03 | Urophorphyrin methylase 1 |
| maker-1-augustus-gene-259.29-mRNA-1 | Japonica | Indica | | Os01g0631400 | OsR498G0101593500.01.T01 | Glycerol-3-phosphate 1-O-acyltransferase |
| maker-1-augustus-gene-259.3-mRNA-1 | Japonica | Japonica | | Os01g0630700 | OsR498G0102324800.01.T02 | DUF223 domain-containing protein |
| maker-1-augustus-gene-259.31-mRNA-1 | Japonica | NA | | Os01g0631500 | OsR498G0101593900.01.T01 | Carbohydrate-binding X8 domain superfamily protein, putative |
| maker-1-augustus-gene-259.33-mRNA-1 | Japonica | NA | Kinase-TM | Os01g0631700 | OsR498G0101594500.01.T03 | Receptor-like protein kinase |
| maker-1-augustus-gene-259.38-mRNA-1 | Japonica | NA | | | | Transposable element protein, putative, MuDR |
| maker-1-augustus-gene-259.42-mRNA-1 | Japonica | Indica | NBS | Os12g0539800 | OsR498G0101591800.01.T01 | |
| maker-1-augustus-gene-26.33-mRNA-1 | Japonica | Indica | | Os01g0147600 | OsR498G0100172400.01.T01 | Centromere protein B |
| maker-1-augustus-gene-26.36-mRNA-1 | Japonica | NA | | | OsR498G0100172600.01.T02 | Factor of DNA methylation 1 |
| maker-1-augustus-gene-260.0-mRNA-1 | Japonica | NA | | Os01g0631900 | | Aspartic proteinase |
| maker-1-augustus-gene-261.45-mRNA-1 | Japonica | NA | | Os01g0635400 | OsR498G0101603500.01.T02 | Coatomer beta subunit |
| maker-1-augustus-gene-261.51-mRNA-1 | Japonica | NA | | Os01g0634300 | OsR498G0101600400.01.T03 | Auxilin-like protein 1 |
| maker-1-augustus-gene-261.55-mRNA-1 | Japonica | Indica | | Os01g0842500 | OsR498G0101601600.01.T01 | Laccase |
| maker-1-augustus-gene-261.57-mRNA-1 | Japonica | NA | | Os01g0634600 | OsR498G0101601800.01.T01 | Pectinesterase |
| maker-1-augustus-gene-262.11-mRNA-1 | Japonica | NA | | Os01g0636700 | | SWI/SNF-related matrix-associated actin-dependent regulator 1 of chromati |
| maker-1-augustus-gene-262.4-mRNA-1 | Japonica | Indica | | Os02g0706900 | OsR498G0101606000.01.T01 | AB hydrolase-1 domain-containing protein |
| maker-1-augustus-gene-262.7-mRNA-1 | Japonica | NA | | Os01g0637600 | OsR498G0101612800.01.T01 | Peptide deformylase |
| maker-1-augustus-gene-263.0-mRNA-1 | Japonica | NA | | Os01g0637800 | OsR498G0101613100.01.T01 | Myb-like transcription factor EOBI (Fragment) |
| maker-1-augustus-gene-263.10-mRNA-1 | Japonica | NA | | Os01g0637600 | OsR498G0101612800.01.T01 | Peptide deformylase |
| maker-1-augustus-gene-263.14-mRNA-1 | Japonica | NA | | Os01g0639200 | OsR498G0101616800.01.T01 | Cinnamoyl-CoA reductase 2 |
| maker-1-augustus-gene-263.2-mRNA-1 | Japonica | NA | | Os01g0639100 | OsR498G0101616500.01.T03 | Eukaryotic translation initiation factor |
| maker-1-augustus-gene-263.7-mRNA-1 | Japonica | NA | | | | |
| maker-1-augustus-gene-264.35-mRNA-1 | Japonica | NA | | Os01g0639900 | OsR498G0101621700.01.T03 | Carbonic anhydrase, chloroplastic |
| maker-1-augustus-gene-264.49-mRNA-1 | Japonica | NA | Kinase-TM | Os01g0641000 | OsR498G0101624900.01.T01 | Serine/threonine-protein kinase STY8 |
| maker-1-augustus-gene-264.51-mRNA-1 | Japonica | NA | | | OsR498G0101625100.01.T01 | 5'-deoxynucleotidase |
| maker-1-augustus-gene-264.57-mRNA-1 | Japonica | NA | | Os01g0640800 | OsR498G0101624600.01.T04 | Ribosomal RNA large subunit methyltransferase RlmN |
| maker-1-augustus-gene-265.39-mRNA-1 | Japonica | Indica | | Os01g0643300 | OsR498G0101632900.01.T01 | Auxin efflux carrier component |
| maker-1-augustus-gene-266.0-mRNA-1 | Japonica | NA | | Os01g0644000 | OsR498G0101637100.01.T01 | |
| maker-1-augustus-gene-266.10-mRNA-1 | Japonica | NA | | | | MHD2 domain-containing protein |
| maker-1-augustus-gene-266.12-mRNA-1 | Japonica | NA | | Os01g0645000 | OsR498G0101640600.01.T01 | Butyrate response factor 1 |
| maker-1-augustus-gene-266.2-mRNA-1 | Japonica | NA | | Os01g0643600 | OsR498G0101634400.01.T01 | Homeobox-leucine zipper protein ATHB-4 |
| maker-1-augustus-gene-267.0-mRNA-1 | Japonica | NA | | Os01g0645200 | OsR498G0101641700.01.T02 | Sodium/pyruvate cotransporter BASS2, chloroplastic |
| maker-1-augustus-gene-267.7-mRNA-1 | Japonica | NA | | | OsR498G0101643100.01.T01 | Flavin-containing monooxygenase |
| maker-1-augustus-gene-267.9-mRNA-1 | Japonica | NA | | Os01g0647000 | OsR498G0101647300.01.T01 | F-box domain |
| maker-1-augustus-gene-268.0-mRNA-1 | Japonica | NA | | Os01g0647700 | OsR498G0101650500.01.T04 | AB hydrolase-1 domain-containing protein |
| maker-1-augustus-gene-268.12-mRNA-1 | Japonica | NA | | Os01g0648000 | OsR498G0101651000.01.T01 | Potassium channel |
| maker-1-augustus-gene-268.7-mRNA-1 | Japonica | NA | | Os01g0647100 | OsR498G0101647600.01.T01 | ADP,ATP carrier protein (Fragment) |
| maker-1-augustus-gene-268.9-mRNA-1 | Japonica | NA | | | OsR498G1119498700.01.T01 | |
| maker-1-augustus-gene-269.0-mRNA-1 | Japonica | NA | | | | Small-subunit processome, Utp12 |
| maker-1-augustus-gene-269.10-mRNA-1 | Japonica | Indica | | Os01g0649900 | OsR498G0101655500.01.T01 | GDSL esterase/lipase |
| maker-1-augustus-gene-269.16-mRNA-1 | Japonica | NA | | Os01g0649400 | OsR498G0101655700.01.T01 | GDSL esterase/lipase |
| maker-1-augustus-gene-269.19-mRNA-1 | Japonica | NA | | Os01g0649900 | OsR498G0101656100.01.T01 | GDSL esterase/lipase |
| maker-1-augustus-gene-269.22-mRNA-1 | Japonica | Japonica | | Os01g0650000 | OsR498G0101656300.01.T02 | |
| maker-1-augustus-gene-269.6-mRNA-1 | Japonica | NA | | Os01g0649100 | OsR498G0101655100.01.T01 | Malate dehydrogenase |
| maker-1-augustus-gene-27.71-mRNA-1 | Japonica | NA | | Os01g0150000 | OsR498G0100179800.01.T01 | very-long-chain enoyl-CoA reductase isoform X1 |

| | | | | | | |
|---|---|---|---|---|---|---|
| maker-1-augustus-gene-270.0-mRNA-1 | Japonica | Japonica | | Os01g0650900 | OsR498G0101660700.01.T01 | GDSL esterase/lipase |
| maker-1-augustus-gene-270.11-mRNA-1 | Japonica | NA | | Os01g0651800 | OsR498G0101662900.01.T01 | Phospholipase A1-IIgamma |
| maker-1-augustus-gene-270.13-mRNA-1 | Japonica | NA | | | OsR498G0101664300.01.T01 | Tetratricopeptide repeat-like superfamily protein isoform 1 |
| maker-1-augustus-gene-270.2-mRNA-1 | Japonica | NA | | | OsR498G0101660700.01.T01 | GDSL esterase/lipase |
| maker-1-augustus-gene-270.21-mRNA-1 | Japonica | NA | | Os01g0652100 | OsR498G0101664600.01.T01 | Protein trichome birefringence-like protein 38 |
| maker-1-augustus-gene-270.5-mRNA-1 | Japonica | NA | | Os01g0651200 | OsR498G0101661100.01.T03 | Phospholipase A1-IIgamma |
| maker-1-augustus-gene-270.7-mRNA-1 | Japonica | NA | | | OsR498G0101661500.01.T02 | SGNH hydrolase-type esterase superfamily protein, putative |
| maker-1-augustus-gene-270.8-mRNA-1 | Japonica | NA | | Os01g0651400 | OsR498G0101661500.01.T02 | |
| maker-1-augustus-gene-271.26-mRNA-1 | Japonica | NA | | Os01g0652600 | OsR498G0101665700.01.T03 | Acetohydroxy-acid reductoisomerase |
| maker-1-augustus-gene-271.32-mRNA-1 | Japonica | Indica | | Os05g0574100 | OsR498G0101665200.01.T02 | Phospholipase A1-IIgamma |
| maker-1-augustus-gene-271.34-mRNA-1 | Japonica | NA | | Os01g0652800 | OsR498G0101666400.01.T01 | Leaf senescence protein-like |
| maker-1-augustus-gene-271.36-mRNA-1 | Japonica | NA | | Os01g0653100 | OsR498G0101668200.01.T01 | Leaf senescence protein-like |
| maker-1-augustus-gene-271.38-mRNA-1 | Japonica | NA | | | OsR498G0101669800.01.T01 | Small subunit rRNA processing factor, putative |
| maker-1-augustus-gene-271.40-mRNA-1 | Japonica | NA | | Os01g0653800 | OsR498G0101671400.01.T03 | Transcription initiation factor TFIID subunit 5 |
| maker-1-augustus-gene-272.20-mRNA-1 | Japonica | NA | | Os01g0654500 | OsR498G0101677000.01.T01 | Isocitrate dehydrogenase [NADP] |
| maker-1-augustus-gene-273.0-mRNA-1 | Japonica | NA | | Os01g0654966 | | |
| maker-1-augustus-gene-273.19-mRNA-1 | Japonica | NA | | Os01g0655500 | OsR498G0101681300.01.T01 | Serine/threonine-protein kinase STN7, chloroplastic |
| maker-1-augustus-gene-273.25-mRNA-1 | Japonica | NA | | Os01g0655800 | OsR498G0101682500.01.T08 | Fatty acyl-CoA synthetase A |
| maker-1-augustus-gene-273.34-mRNA-1 | Japonica | NA | | | OsR498G0101679000.01.T01 | |
| maker-1-augustus-gene-273.8-mRNA-1 | Japonica | NA | | | OsR498G0101680700.01.T01 | histone-lysine N-methyltransferase ATX4-like isoform X1 |
| maker-1-augustus-gene-274.0-mRNA-1 | Japonica | Japonica | | Os01g0656900 | OsR498G0101687300.01.T02 | |
| maker-1-augustus-gene-274.3-mRNA-1 | Japonica | Japonica | | Os01g0657000 | OsR498G0203372600.01.T01 | Arabinogalactan peptide, AGP |
| maker-1-augustus-gene-274.7-mRNA-1 | Japonica | NA | | | OsR498G0101686000.01.T01 | Myosin heavy chain-like |
| maker-1-augustus-gene-274.9-mRNA-1 | Japonica | NA | | Os01g0657100 | OsR498G0101687800.01.T01 | H(+)/Pi cotransporter |
| maker-1-augustus-gene-275.29-mRNA-1 | Japonica | Indica | | Os06g0330400 | OsR498G0101691100.01.T01 | Aldehyde oxygenase (deformylating) |
| maker-1-augustus-gene-275.31-mRNA-1 | Japonica | NA | | Os01g0659200 | OsR498G0101691900.01.T02 | V-type proton ATPase subunit E like |
| maker-1-augustus-gene-275.36-mRNA-1 | Japonica | NA | | Os01g0659900 | OsR498G0101693900.01.T01 | F-box/kelch-repeat protein At3g61590 |
| maker-1-augustus-gene-275.39-mRNA-1 | Japonica | NA | | Os01g0658400 | OsR498G0101690600.01.T03 | UBC core domain-containing protein (Fragment) |
| maker-1-augustus-gene-275.41-mRNA-1 | Japonica | NA | | | OsR498G0101690800.01.T01 | Vacuolar protein sorting-associated protein 25 |
| maker-1-augustus-gene-275.45-mRNA-1 | Japonica | NA | | Os01g0658700 | OsR498G0101691400.01.T01 | Glucose-6-phosphate 1-epimerase |
| maker-1-augustus-gene-275.49-mRNA-1 | Japonica | NA | | Os01g0658900 | OsR498G0101691600.01.T01 | G-box binding factor |
| maker-1-augustus-gene-276.26-mRNA-1 | Japonica | NA | | Os01g0660900 | OsR498G0101697900.01.T02 | DUF588 domain-containing protein |
| maker-1-augustus-gene-276.30-mRNA-1 | Japonica | NA | | Os01g0661000 | OsR498G0101698100.01.T03 | DUF588 domain-containing protein |
| maker-1-augustus-gene-276.32-mRNA-1 | Japonica | NA | | Os01g0661400 | OsR498G0101698300.01.T04 | Ribosomal RNA-processing protein 40 |
| maker-1-augustus-gene-276.37-mRNA-1 | Japonica | NA | | Os01g0661500 | OsR498G0101698300.01.T05 | AMSH-like ubiquitin thioesterase 2 |
| maker-1-augustus-gene-276.39-mRNA-1 | Japonica | Indica | | Os10g0571200 | OsR498G0101695000.01.T01 | Pyruvate kinase |
| maker-1-augustus-gene-277.46-mRNA-1 | Japonica | Indica | | Os01g0663300 | OsR498G0101703900.01.T01 | Mannan endo-1,4-beta-mannosidase |
| maker-1-augustus-gene-277.50-mRNA-1 | Japonica | NA | | Os01g0663500 | OsR498G0101704500.01.T04 | Pterin-4-alpha-carbinolamine dehydratase 2, mitochondrial |
| maker-1-augustus-gene-277.56-mRNA-1 | Japonica | NA | | Os01g0664000 | OsR498G0101705300.01.T02 | PDZ domain-containing protein |
| maker-1-augustus-gene-277.65-mRNA-1 | Japonica | NA | | Os01g0662600 | OsR498G0101701400.01.T01 | Iron-sulfur cluster assembly protein 1 |
| maker-1-augustus-gene-277.67-mRNA-1 | Japonica | NA | | Os01g0662700 | OsR498G0101701600.01.T01 | 1,4-dihydroxy-2-naphthoyl-CoA synthase, peroxisomal like |
| maker-1-augustus-gene-277.71-mRNA-1 | Japonica | Japonica | | Os01g0662800 | OsR498G0101701800.01.T01 | GATA transcription factor 19 |
| maker-1-augustus-gene-277.73-mRNA-1 | Japonica | NA | | Os01g0663400 | | Aspartic proteinase |
| maker-1-augustus-gene-277.75-mRNA-1 | Japonica | NA | | Os01g0663800 | OsR498G0101704800.01.T03 | GBF-interacting protein 1 |
| maker-1-augustus-gene-277.80-mRNA-1 | Japonica | NA | Kinase-TM | Os01g0664200 | OsR498G0101706400.01.T01 | Putative serine/threonine-protein kinase |
| maker-1-augustus-gene-278.40-mRNA-1 | Japonica | NA | Kinase-TM | Os01g0665200 | OsR498G0101709700.01.T01 | Mitogen-activated protein kinase |
| maker-1-augustus-gene-278.45-mRNA-1 | Japonica | NA | | Os01g0665400 | OsR498G0101710100.01.T02 | Ribokinase |
| maker-1-augustus-gene-278.47-mRNA-1 | Japonica | NA | | | | High mobility group box domain |
| maker-1-augustus-gene-278.50-mRNA-1 | Japonica | NA | | Os01g0665300 | OsR498G0101710000.01.T02 | FCP1 homology domain-containing protein |
| maker-1-augustus-gene-278.55-mRNA-1 | Japonica | Japonica | | Os01g0665500 | OsR498G0101711000.01.T01 | WRKY transcription factor |
| maker-1-augustus-gene-278.58-mRNA-1 | Japonica | NA | | Os01g0666000 | OsR498G0101714600.01.T02 | acidPPc domain-containing protein |
| maker-1-augustus-gene-279.30-mRNA-1 | Japonica | NA | | Os01g0666600 | OsR498G0101715500.01.T01 | Alpha/beta-Hydrolases superfamily protein |
| maker-1-augustus-gene-279.32-mRNA-1 | Japonica | NA | | | OsR498G0101716900.01.T02 | |
| maker-1-augustus-gene-279.35-mRNA-1 | Japonica | NA | | | OsR498G0101718500.01.T01 | Peroxidase |
| maker-1-augustus-gene-279.37-mRNA-1 | Japonica | NA | | Os01g0667600 | OsR498G0101720100.01.T01 | |
| maker-1-augustus-gene-279.39-mRNA-1 | Japonica | NA | | Os01g0666400 | OsR498G0101715200.01.T01 | Lipase class 3-like |
| maker-1-augustus-gene-279.41-mRNA-1 | Japonica | NA | | Os01g0666500 | OsR498G0101715400.01.T02 | Cytochrome b561/ferric reductase transmembrane protein family |
| maker-1-augustus-gene-279.43-mRNA-1 | Japonica | NA | | Os01g0666900 | OsR498G0101717400.01.T01 | Urease accessory protein G |
| maker-1-augustus-gene-279.46-mRNA-1 | Japonica | Indica | | Os01g0667100 | OsR498G0101718000.01.T01 | Hydroxyacylglutathione hydrolase 3, mitochondrial |
| maker-1-augustus-gene-279.48-mRNA-1 | Japonica | NA | | Os01g0667200 | OsR498G0101718200.01.T01 | Hydroxyacylglutathione hydrolase 3, mitochondrial |
| maker-1-augustus-gene-279.53-mRNA-1 | Japonica | NA | | | OsR498G0101719200.01.T01 | Homeobox domain-containing protein (Fragment) |

| | | | | | | |
|---|---|---|---|---|---|---|
| maker-1-augustus-gene-279.55-mRNA-1 | Japonica | NA | | Os01g0667700 | OsR498G0101720400.01.T03 | E3 ubiquitin-protein ligase arkadia isoform X2 |
| maker-1-augustus-gene-28.15-mRNA-1 | Japonica | Indica | | Os01g0151400 | OsR498G0100183500.01.T01 | Gamma-glutamyltranspeptidase 3 |
| maker-1-augustus-gene-28.30-mRNA-1 | Japonica | NA | Kinase-LRR-TM | Os01g0152000 | OsR498G0100185300.01.T01 | LRR receptor-like serine/threonine-protein kinase EFR |
| maker-1-augustus-gene-280.33-mRNA-1 | Japonica | NA | | Os01g0668000 | OsR498G0101723500.01.T01 | Nuclear distribution protein NUDC |
| maker-1-augustus-gene-280.36-mRNA-1 | Japonica | NA | | | OsR498G0101724300.01.T03 | PHD-type domain-containing protein (Fragment) |
| maker-1-augustus-gene-281.1-mRNA-1 | Japonica | NA | | Os01g0670800 | OsR498G0101731800.01.T01 | Auxin response factor |
| maker-1-augustus-gene-282.23-mRNA-1 | Japonica | Indica | | Os05g0562400 | OsR498G0101736100.01.T02 | Homeobox-DDT domain protein RLT1 |
| maker-1-augustus-gene-282.35-mRNA-1 | Japonica | NA | | Os01g0672400 | OsR498G0101736500.01.T01 | Drought-induced 19 |
| maker-1-augustus-gene-282.40-mRNA-1 | Japonica | NA | | | OsR498G0101734600.01.T03 | NAC transcriptional factor |
| maker-1-augustus-gene-283.34-mRNA-1 | Japonica | NA | | Os01g0672700 | OsR498G0101737900.01.T01 | Non-canonical poly(A) RNA polymerase |
| maker-1-augustus-gene-283.40-mRNA-1 | Japonica | Indica | | Os01g0672800 | OsR498G0101738400.01.T01 | DUF1421 domain-containing protein (Fragment) |
| maker-1-augustus-gene-283.43-mRNA-1 | Japonica | NA | NBS-TM | | OsR498G0101739800.01.T04 | Katanin p60 ATPase-containing subunit |
| maker-1-augustus-gene-283.48-mRNA-1 | Japonica | NA | | Os01g0673600 | OsR498G0101740200.01.T01 | UBC core domain-containing protein (Fragment) |
| maker-1-augustus-gene-283.52-mRNA-1 | Japonica | Indica | | Os01g0673800 | OsR498G0101741200.01.T06 | ATP synthase protein I |
| maker-1-augustus-gene-283.55-mRNA-1 | Japonica | NA | | | OsR498G0101744400.01.T03 | RING/U-box superfamily protein |
| maker-1-augustus-gene-283.61-mRNA-1 | Japonica | NA | | | OsR498G0101745400.01.T01 | Serine/threonine-protein kinase EDR1 |
| maker-1-augustus-gene-284.34-mRNA-1 | Japonica | NA | | | OsR498G0101747500.01.T04 | Pentatricopeptide repeat-containing protein chloroplastic |
| maker-1-augustus-gene-284.39-mRNA-1 | Japonica | NA | Kinase-LRR-TM | | OsR498G0101747700.01.T01 | Protein MALE DISCOVERER 1 |
| maker-1-augustus-gene-284.46-mRNA-1 | Japonica | NA | | Os01g0675000 | OsR498G0101748500.01.T01 | PRONE domain-containing protein (Fragment) |
| maker-1-augustus-gene-284.48-mRNA-1 | Japonica | NA | | Os01g0675700 | OsR498G0101750900.01.T03 | Auxin-responsive protein IAA5 |
| maker-1-augustus-gene-284.51-mRNA-1 | Japonica | NA | | Os01g0675100 | OsR498G0101748800.01.T01 | Glutaredoxin-dependent peroxiredoxin |
| maker-1-augustus-gene-284.54-mRNA-1 | Japonica | NA | | Os01g0675500 | OsR498G0101749600.01.T01 | Glycosyltransferases |
| maker-1-augustus-gene-285.0-mRNA-1 | Japonica | Japonica | | Os01g0676900 | OsR498G0101755300.01.T02 | SPOROCYTELESS-like EAR-containing protein 3 |
| maker-1-augustus-gene-285.10-mRNA-1 | Japonica | NA | | | | Ubiquitin-conjugating enzyme 25 |
| maker-1-augustus-gene-285.12-mRNA-1 | Japonica | NA | | | | USP domain-containing protein |
| maker-1-augustus-gene-285.4-mRNA-1 | Japonica | NA | | Os01g0677000 | OsR498G0101755700.01.T01 | Glyoxal oxidase |
| maker-1-augustus-gene-286.30-mRNA-1 | Japonica | NA | | Os01g0678600 | OsR498G0101760500.01.T01 | 30S ribosomal protein S20 chloroplastic |
| maker-1-augustus-gene-286.32-mRNA-1 | Japonica | NA | | Os01g0679000 | OsR498G0101761900.01.T03 | RNA polymerase III subunit C3 |
| maker-1-augustus-gene-286.36-mRNA-1 | Japonica | NA | | Os01g0679600 | OsR498G0101763500.01.T01 | UPF0678 fatty acid-binding protein-like protein isoform A |
| maker-1-augustus-gene-286.38-mRNA-1 | Japonica | NA | | Os01g0679900 | OsR498G0101764500.01.T01 | Evolutionarily conserved C-terminal region 8 |
| maker-1-augustus-gene-286.42-mRNA-1 | Japonica | NA | | | | Two-pore calcium channel |
| maker-1-augustus-gene-286.46-mRNA-1 | Japonica | NA | | Os01g0678700 | OsR498G0101760800.01.T01 | Transcription factor-like protein DPB |
| maker-1-augustus-gene-286.50-mRNA-1 | Japonica | NA | | Os01g0678800 | OsR498G0101761000.01.T01 | HMA domain-containing protein |
| maker-1-augustus-gene-286.52-mRNA-1 | Japonica | NA | | Os01g0679700 | OsR498G0101763800.01.T01 | Ribosomal protein L37ae |
| maker-1-augustus-gene-287.31-mRNA-1 | Japonica | NA | | Os01g0680400 | OsR498G0101767300.01.T01 | TAFII28 domain-containing protein (Fragment) |
| maker-1-augustus-gene-287.34-mRNA-1 | Japonica | Indica | | Os05g0556100 | OsR498G0101770300.01.T01 | Dynamin-related protein 5A |
| maker-1-augustus-gene-287.38-mRNA-1 | Japonica | NA | | Os01g0681600 | OsR498G0101771700.01.T01 | Autophagy 6 isoform 1 |
| maker-1-augustus-gene-287.40-mRNA-1 | Japonica | NA | | Os01g0680700 | OsR498G0101768200.01.T01 | Linker for activation of T-cells family member 2 |
| maker-1-augustus-gene-287.45-mRNA-1 | Japonica | Indica | | Os01g0680900 | OsR498G0101769600.01.T01 | Cytochrome b561 and DOMON domain-containing protein |
| maker-1-augustus-gene-287.48-mRNA-1 | Japonica | NA | | Os01g0681000 | OsR498G0101770000.01.T01 | Diacylglycerol O-acyltransferase |
| maker-1-augustus-gene-287.51-mRNA-1 | Japonica | NA | | Os01g0681200 | OsR498G0101770600.01.T03 | Long chain acyl-CoA synthetase 4 |
| maker-1-augustus-gene-287.57-mRNA-1 | Japonica | NA | | Os01g0681400 | OsR498G0101771000.01.T03 | beclin-1-like protein isoform X1 |
| maker-1-augustus-gene-287.60-mRNA-1 | Japonica | NA | | Os01g0681900 | OsR498G0101772400.01.T03 | NADH-dependent glutamate synthase |
| maker-1-augustus-gene-288.28-mRNA-1 | Japonica | NA | | Os01g0682500 | OsR498G0101775100.01.T01 | D-glycerate 3-kinase chloroplastic |
| maker-1-augustus-gene-288.33-mRNA-1 | Japonica | NA | | Os01g0683100 | OsR498G0101775900.01.T01 | Katanin p60 ATPase-containing subunit A1 |
| maker-1-augustus-gene-288.35-mRNA-1 | Japonica | NA | | Os01g0683400 | OsR498G0101778300.01.T06 | Origin of replication complex subunit 4 |
| maker-1-augustus-gene-288.42-mRNA-1 | Japonica | NA | | | OsR498G0101778700.01.T02 | ER membrane protein complex subunit 6 |
| maker-1-augustus-gene-288.47-mRNA-1 | Japonica | NA | | Os01g0683700 | OsR498G0101778900.01.T02 | |
| maker-1-augustus-gene-288.49-mRNA-1 | Japonica | NA | | Os01g0684200 | OsR498G0101779900.01.T08 | Protein prenylyltransferase superfamily protein |
| maker-1-augustus-gene-288.59-mRNA-1 | Japonica | Indica | | Os05g0554400 | OsR498G0101778600.01.T03 | CDP-diacylglycerol--serine O-phosphatidyltransferase 1 isoform X1 |
| maker-1-augustus-gene-289.0-mRNA-1 | Japonica | Indica | | Os01g0684200 | OsR498G0101781300.01.T02 | protein prenyltransferase alpha subunit repeat-containing protein 1-B |
| maker-1-augustus-gene-289.10-mRNA-1 | Japonica | NA | | Os01g0685800 | OsR498G0101784700.01.T01 | ATP synthase subunit beta |
| maker-1-augustus-gene-289.16-mRNA-1 | Japonica | NA | | Os01g0685900 | OsR498G0101784900.01.T01 | 65-kDa microtubule-associated protein 3 |
| maker-1-augustus-gene-289.20-mRNA-1 | Japonica | NA | | Os01g0684900 | OsR498G0101781600.01.T03 | Protein DETOXIFICATION |
| maker-1-augustus-gene-289.24-mRNA-1 | Japonica | NA | | | OsR498G0101782000.01.T01 | t-SNARE coiled-coil homology domain-containing protein (Fragment) |
| maker-1-augustus-gene-289.27-mRNA-1 | Japonica | NA | | | OsR498G0101784200.01.T01 | DNA ligase |
| maker-1-augustus-gene-289.8-mRNA-1 | Japonica | Indica | | Os01g0685400 | OsR498G0101782700.01.T01 | Transcription factor RAX3 |
| maker-1-augustus-gene-29.59-mRNA-1 | Japonica | Japonica | Kinase-LRR-TM | Os01g0152600 | OsR498G0100187100.01.T01 | Putative LRR receptor-like serine/threonine-protein kinase |
| maker-1-augustus-gene-29.60-mRNA-1 | Japonica | Japonica | Kinase-LRR-TM | Os01g0152800 | OsR498G0100189500.01.T01 | LRR receptor-like serine/threonine-protein kinase EFR |
| maker-1-augustus-gene-29.83-mRNA-1 | Japonica | Indica | | Os01g0153500 | OsR498G0100192100.01.T01 | |
| maker-1-augustus-gene-29.92-mRNA-1 | Japonica | NA | | | | DUF674 domain-containing protein |

| | | | | | | |
|---|---|---|---|---|---|---|
| maker-1-augustus-gene-290.0-mRNA-1 | Japonica | NA | | | OsR498G0101787100.01.T01 | Protein-lysine N-methyltransferase |
| maker-1-augustus-gene-290.11-mRNA-1 | Japonica | NA | | Os01g0687500 | OsR498G0101790100.01.T05 | Methionyl-tRNA formyltransferase, mitochondrial |
| maker-1-augustus-gene-290.16-mRNA-1 | Japonica | Indica | | Os01g0686700 | OsR498G0101788000.01.T01 | RING-CH-type domain-containing protein |
| maker-1-augustus-gene-290.19-mRNA-1 | Japonica | NA | | Os01g0687600 | OsR498G0101790400.01.T02 | serine/arginine-rich splicing factor 4-like isoform X1 |
| maker-1-augustus-gene-290.21-mRNA-1 | Japonica | Japonica | | Os01g0687700 | OsR498G0101790400.01.T04 | Regulator of Vps4 activity in the MVB pathway protein |
| maker-1-augustus-gene-290.26-mRNA-1 | Japonica | NA | | Os01g0687800 | OsR498G0101791000.01.T01 | L-gulonolactone oxidase |
| maker-1-augustus-gene-290.28-mRNA-1 | Japonica | NA | | Os01g0688200 | OsR498G0101791800.01.T02 | Caffeoylshikimate esterase |
| maker-1-augustus-gene-290.5-mRNA-1 | Japonica | NA | | Os01g0686800 | OsR498G0101788300.01.T02 | Guanine nucleotide-binding protein subunit beta-like protein A |
| maker-1-augustus-gene-290.7-mRNA-1 | Japonica | NA | | Os01g0687300 | OsR498G0101789300.01.T05 | |
| maker-1-augustus-gene-291.11-mRNA-1 | Japonica | Indica | | Os01g0688600 | OsR498G0101794400.01.T01 | Formin-like protein 18 |
| maker-1-augustus-gene-291.13-mRNA-1 | Japonica | Indica | | Os05g0460000 | OsR498G0101795400.01.T02 | Heat shock protein cognate 70 |
| maker-1-augustus-gene-291.15-mRNA-1 | Japonica | Indica | | Os05g0551000 | OsR498G0101796800.01.T03 | Zinc finger protein BRUTUS (Fragment) |
| maker-1-augustus-gene-291.20-mRNA-1 | Japonica | NA | | Os01g0689600 | OsR498G0101798600.01.T05 | Retrovirus-related Pol polyprotein LINE-1 |
| maker-1-augustus-gene-291.26-mRNA-1 | Japonica | Indica | Kinase-TM | Os01g0689900 | OsR498G0101799400.01.T01 | Wall-associated receptor-like kinase 2 |
| maker-1-augustus-gene-291.4-mRNA-1 | Japonica | NA | | Os01g0689000 | OsR498G0101795900.01.T01 | Formin-like protein 5 |
| maker-1-augustus-gene-291.6-mRNA-1 | Japonica | NA | | Os01g0689800 | OsR498G0101799100.01.T01 | Alpha/beta hydrolase domain-containing protein 17C-like |
| maker-1-augustus-gene-292.0-mRNA-1 | Japonica | NA | | | | Protein kinase domain-containing protein |
| maker-1-augustus-gene-292.1-mRNA-1 | Japonica | NA | Kinase-TM | Os01g0690800 | OsR498G0101800500.01.T01 | Protein kinase domain-containing protein (Fragment) |
| maker-1-augustus-gene-292.17-mRNA-1 | Japonica | NA | | Os01g0691700 | OsR498G0101802300.01.T01 | Phytochrome-associated serine/threonine-protein phosphatase |
| maker-1-augustus-gene-292.22-mRNA-1 | Japonica | Indica | | Os01g0692300 | OsR498G0101803900.01.T01 | Protein PLASTID MOVEMENT IMPAIRED 2 |
| maker-1-augustus-gene-292.25-mRNA-1 | Japonica | NA | | Os01g0692600 | OsR498G0101804100.01.T01 | Abhydrolase domain-containing protein 1 (Fragment) |
| maker-1-augustus-gene-292.29-mRNA-1 | Japonica | Indica | | Os01g0691500 | OsR498G0101801800.01.T01 | Cytidylyltransferase domain protein |
| maker-1-augustus-gene-292.4-mRNA-1 | Japonica | NA | Kinase-TM | | OsR498G0101800900.01.T01 | Acidic endochitinase |
| maker-1-augustus-gene-292.40-mRNA-1 | Japonica | NA | | Os01g0692100 | OsR498G0101803400.01.T01 | Glutathione S-transferase GSTU6 |
| maker-1-augustus-gene-292.42-mRNA-1 | Japonica | Indica | | Os01g0692700 | OsR498G0101804400.01.T01 | E3 ubiquitin ligase BIG BROTHER |
| maker-1-augustus-gene-292.7-mRNA-1 | Japonica | Indica | | Os01g0691400 | OsR498G0101801500.01.T01 | Reticulon-like protein (Fragment) |
| maker-1-augustus-gene-293.0-mRNA-1 | Japonica | NA | | Os01g0693300 | OsR498G0101806500.01.T03 | Lipid phosphate phosphatase 2 |
| maker-1-augustus-gene-293.15-mRNA-1 | Japonica | NA | | Os01g0694200 | OsR498G0101815200.01.T03 | |
| maker-1-augustus-gene-293.18-mRNA-1 | Japonica | NA | | | OsR498G0101815600.01.T01 | Symplekin |
| maker-1-augustus-gene-293.3-mRNA-1 | Japonica | NA | Kinase-LRR-TM | | | Receptor kinase MRKa |
| maker-1-augustus-gene-293.5-mRNA-1 | Japonica | NA | Kinase-LRR-TM | | | Leucine-rich repeat receptor kinase-like protein |
| maker-1-augustus-gene-293.8-mRNA-1 | Japonica | NA | | Os01g0693900 | OsR498G0101813400.01.T03 | Peptidyl-tRNA hydrolase chloroplastic |
| maker-1-augustus-gene-294.0-mRNA-1 | Japonica | NA | | Os01g0694900 | OsR498G0101817300.01.T05 | ENTH domain-containing protein (Fragment) |
| maker-1-augustus-gene-294.10-mRNA-1 | Japonica | Indica | | Os01g0695200 | OsR498G0101818100.01.T01 | Core-2/I-branching beta-1,6-N-acetylglucosaminyltransferase family protein |
| maker-1-augustus-gene-294.16-mRNA-1 | Japonica | NA | | Os01g0695700 | OsR498G0101819100.01.T06 | ABC transporter B family member 9 |
| maker-1-augustus-gene-294.22-mRNA-1 | Japonica | NA | | Os01g0695900 | OsR498G0101820100.01.T01 | Transcription repressor MYB5 |
| maker-1-augustus-gene-294.24-mRNA-1 | Japonica | NA | | | OsR498G0101820500.01.T05 | ABC transporter B family member 4-like |
| maker-1-augustus-gene-294.27-mRNA-1 | Japonica | Indica | | Os01g0695300 | OsR498G0101818600.01.T03 | Farnesyl pyrophosphate synthetase |
| maker-1-augustus-gene-294.28-mRNA-1 | Japonica | NA | | | OsR498G0101818600.01.T05 | D-cysteine desulfhydrase 2 mitochondrial |
| maker-1-augustus-gene-294.43-mRNA-1 | Japonica | NA | | | | p-glycoprotein-like |
| maker-1-augustus-gene-294.48-mRNA-1 | Japonica | NA | | Os01g0696100 | OsR498G0101820800.01.T02 | Two-pore potassium channel 2 |
| maker-1-augustus-gene-294.5-mRNA-1 | Japonica | NA | | Os01g0695100 | OsR498G0101817900.01.T01 | Phosphomethylethanolamine N-methyltransferase-like |
| maker-1-augustus-gene-295.0-mRNA-1 | Japonica | Indica | | Os01g0695700 | OsR498G0101822500.01.T01 | ABC transporter B family member 4 |
| maker-1-augustus-gene-295.10-mRNA-1 | Japonica | NA | | Os01g0698300 | | Zinc finger BED domain-containing protein DAYSLEEPER |
| maker-1-augustus-gene-295.5-mRNA-1 | Japonica | NA | | Os01g0698100 | OsR498G0101826700.01.T03 | SWAP (Suppressor-of-White-APricot)/surp domain-containing protein |
| maker-1-augustus-gene-296.0-mRNA-1 | Japonica | NA | | Os01g0698900 | OsR498G0101828700.01.T01 | Nascent polypeptide associated complex alpha subunit |
| maker-1-augustus-gene-296.11-mRNA-1 | Japonica | NA | | Os01g0700200 | OsR498G0101835300.01.T04 | E3 ubiquitin-protein ligase HERC2 |
| maker-1-augustus-gene-296.16-mRNA-1 | Japonica | Japonica | | Os01g0700500 | OsR498G0101835700.01.T01 | Cytochrome P450 family 706 subfamily A polypeptide 5 |
| maker-1-augustus-gene-296.18-mRNA-1 | Japonica | Indica | | Os01g0698800 | OsR498G0101828000.01.T01 | |
| maker-1-augustus-gene-296.20-mRNA-1 | Japonica | NA | Kinase | Os01g0699500 | OsR498G0101831600.01.T01 | Putative MAPKKK family protein kinase |
| maker-1-augustus-gene-296.21-mRNA-1 | Japonica | Indica | | Os01g0701700 | OsR498G0101835600.01.T01 | SABATH methyltransferase 5 |
| maker-1-augustus-gene-296.4-mRNA-1 | Japonica | NA | | Os01g0699900 | OsR498G0101833700.01.T01 | Plasma membrane associated protein-like protein (Fragment) |
| maker-1-augustus-gene-296.6-mRNA-1 | Japonica | NA | | Os01g0700000 | OsR498G0101834700.01.T02 | |
| maker-1-augustus-gene-296.8-mRNA-1 | Japonica | NA | | Os01g0700100 | OsR498G0101835100.01.T01 | Bidirectional sugar transporter SWEET |
| maker-1-augustus-gene-297.1-mRNA-1 | Japonica | Japonica | | Os01g0701300 | OsR498G0101836700.01.T01 | Thromboxane-A synthase |
| maker-1-augustus-gene-297.13-mRNA-1 | Japonica | NA | | Os01g0702000 | OsR498G0101838600.01.T03 | BFN domain-containing protein (Fragment) |
| maker-1-augustus-gene-297.4-mRNA-1 | Japonica | Indica | | Os01g0701500 | OsR498G0101836900.01.T01 | Thromboxane-A synthase |
| maker-1-augustus-gene-297.8-mRNA-1 | Japonica | Indica | | Os01g0701900 | OsR498G0101838400.01.T03 | Putative CRAL/TRIO domain containing, Sec14p-like phosphatidylinositol tra |
| maker-1-augustus-gene-298.0-mRNA-1 | Japonica | NA | | Os01g0702300 | OsR498G0101839700.01.T01 | Subtilisin-like serine protease |
| maker-1-augustus-gene-298.14-mRNA-1 | Japonica | NA | | Os01g0702500 | OsR498G0101840500.01.T01 | Dehydrin Xero 1 |
| maker-1-augustus-gene-298.16-mRNA-1 | Japonica | NA | | | OsR498G0101843700.01.T01 | RING/U-box domain-containing protein isoform 2 |

| | | | | | | |
|---|---|---|---|---|---|---|
| maker-1-augustus-gene-298.18-mRNA-1 | Japonica | NA | | Os01g0703400 | OsR498G0101844100.01.T01 | Farnesyl pyrophosphate synthase mRNA |
| maker-1-augustus-gene-298.23-mRNA-1 | Japonica | NA | | Os01g0703600 | OsR498G0101844300.01.T01 | MHD domain-containing protein |
| maker-1-augustus-gene-298.3-mRNA-1 | Japonica | NA | | Os01g0702400 | OsR498G0101839900.01.T01 | J domain-containing protein |
| maker-1-augustus-gene-298.30-mRNA-1 | Japonica | NA | | | OsR498G0101845300.01.T04 | Transmembrane protein, putative |
| maker-1-augustus-gene-298.35-mRNA-1 | Japonica | NA | | Os01g0702700 | OsR498G0101841800.01.T01 | MYB transcription factor |
| maker-1-augustus-gene-298.38-mRNA-1 | Japonica | NA | | Os01g0704000 | OsR498G0101844800.01.T01 | PMEI domain-containing protein |
| maker-1-augustus-gene-298.9-mRNA-1 | Japonica | Indica | | Os01g0702450 | OsR498G0101840300.01.T01 | J domain-containing protein |
| maker-1-augustus-gene-299.0-mRNA-1 | Japonica | NA | | | OsR498G0101847100.01.T05 | Chloride channel protein CLC-e |
| maker-1-augustus-gene-299.11-mRNA-1 | Japonica | NA | | | OsR498G0101855500.01.T01 | Ribosomal RNA apurinic site specific lyase-like |
| maker-1-augustus-gene-299.13-mRNA-1 | Japonica | NA | | Os01g0704400 | | zf-RVT domain-containing protein |
| maker-1-augustus-gene-299.16-mRNA-1 | Japonica | NA | | Os01g0705200 | OsR498G0101849200.01.T01 | Late embryogenesis abundant protein, group 3 |
| maker-1-augustus-gene-299.18-mRNA-1 | Japonica | NA | | Os01g0705300 | OsR498G0101850000.01.T01 | Plastid transcriptionally active7 |
| maker-1-augustus-gene-299.20-mRNA-1 | Japonica | NA | | Os01g0706000 | OsR498G0101854200.01.T01 | RNA polymerase II transcriptional coactivator KELP |
| maker-1-augustus-gene-299.6-mRNA-1 | Japonica | NA | | Os01g0706100 | OsR498G0101854900.01.T01 | |
| maker-1-augustus-gene-299.9-mRNA-1 | Japonica | NA | | | OsR498G0101855100.01.T01 | Cullin domain-containing protein |
| maker-1-augustus-gene-3.0-mRNA-1 | Japonica | NA | | | | |
| maker-1-augustus-gene-3.15-mRNA-1 | Japonica | NA | | Os01g0104100 | OsR498G0100015500.01.T03 | E3 ubiquitin-protein ligase CHFR |
| maker-1-augustus-gene-3.17-mRNA-1 | Japonica | Indica | | Os01g0104200 | OsR498G0100015900.01.T01 | NAC transcription factor |
| maker-1-augustus-gene-3.21-mRNA-1 | Japonica | NA | | Os01g0104800 | OsR498G0100017900.01.T04 | Sas10 domain-containing protein |
| maker-1-augustus-gene-3.26-mRNA-1 | Japonica | NA | Kinase-TM | Os01g0104000 | OsR498G0100015400.01.T01 | C-type lectin receptor-like tyrosine-protein kinase |
| maker-1-augustus-gene-3.29-mRNA-1 | Japonica | NA | | Os01g0104600 | OsR498G0100017400.01.T04 | Light-mediated development protein DET1 |
| maker-1-augustus-gene-3.33-mRNA-1 | Japonica | Japonica | | Os01g0104900 | OsR498G0100018400.01.T01 | Omega-hydroxypalmitate O-feruloyl transferase |
| maker-1-augustus-gene-3.36-mRNA-1 | Japonica | Indica | | Os01g0104900 | OsR498G0100018800.01.T01 | Omega-hydroxypalmitate O-feruloyl transferase |
| maker-1-augustus-gene-3.4-mRNA-1 | Japonica | NA | | Os01g0103800 | OsR498G0100014700.01.T02 | Hydroxyproline-rich glycoprotein family protein |
| maker-1-augustus-gene-3.8-mRNA-1 | Japonica | Indica | | Os01g0103900 | OsR498G0100015100.01.T01 | |
| maker-1-augustus-gene-300.0-mRNA-1 | Japonica | NA | | Os01g0706400 | OsR498G0101856100.01.T01 | Regulator of Vps4 activity in the MVB pathway-like protein isoform 1 |
| maker-1-augustus-gene-300.11-mRNA-1 | Japonica | NA | | Os01g0706700 | OsR498G0101856600.01.T04 | |
| maker-1-augustus-gene-300.17-mRNA-1 | Japonica | NA | | Os01g0706800 | OsR498G0101857000.01.T01 | Jacalin-type lectin domain-containing protein (Fragment) |
| maker-1-augustus-gene-300.19-mRNA-1 | Japonica | NA | | Os01g0706900 | OsR498G0101857400.01.T01 | IAA-amino acid hydrolase |
| maker-1-augustus-gene-300.4-mRNA-1 | Japonica | NA | | Os01g0706500 | OsR498G0101856300.01.T02 | 60S ribosomal protein l28 |
| maker-1-augustus-gene-300.7-mRNA-1 | Japonica | NA | | Os01g0707300 | OsR498G0101858100.01.T01 | Vesicle transport v-SNARE 12 |
| maker-1-augustus-gene-301.0-mRNA-1 | Japonica | NA | | Os01g0708000 | OsR498G0101861700.01.T02 | Homeodomain-like/winged-helix DNA-binding family protein (Fragment) |
| maker-1-augustus-gene-301.12-mRNA-1 | Japonica | Indica | Kinase | Os05g0593800 | OsR498G0101862300.01.T01 | Serine/threonine protein kinase |
| maker-1-augustus-gene-301.17-mRNA-1 | Japonica | NA | | | OsR498G0101862900.01.T03 | Trafficking protein particle complex subunit |
| maker-1-augustus-gene-301.21-mRNA-1 | Japonica | NA | | Os01g0708900 | OsR498G0101864100.01.T01 | Graves disease carrier |
| maker-1-augustus-gene-301.26-mRNA-1 | Japonica | NA | | Os01g0709000 | OsR498G0101864900.01.T01 | Transcription factor MYB26 |
| maker-1-augustus-gene-301.29-mRNA-1 | Japonica | NA | | Os01g0709400 | OsR498G0101865300.01.T01 | 5'-nucleotidase SurE |
| maker-1-augustus-gene-301.32-mRNA-1 | Japonica | NA | | Os01g0708500 | OsR498G0101862600.01.T04 | Cytokinin riboside 5'-monophosphate phosphoribohydrolase |
| maker-1-augustus-gene-301.38-mRNA-1 | Japonica | NA | | Os01g0708700 | OsR498G0101863200.01.T02 | IQ-domain 6 |
| maker-1-augustus-gene-301.40-mRNA-1 | Japonica | NA | Kinase-TM | Os01g0709500 | OsR498G0101865600.01.T04 | Receptor-like serine/threonine-protein kinase ALE2 |
| maker-1-augustus-gene-301.6-mRNA-1 | Japonica | NA | | | OsR498G0101862100.01.T02 | Probable magnesium transporter |
| maker-1-augustus-gene-302.0-mRNA-1 | Japonica | NA | | | | WD-40 repeat-containing protein MSI4 |
| maker-1-augustus-gene-302.11-mRNA-1 | Japonica | Indica | | Os01g0711100 | OsR498G0101869900.01.T01 | Mitochondrial-processing peptidase subunit beta |
| maker-1-augustus-gene-302.17-mRNA-1 | Japonica | NA | Kinase-LRR-TM | Os01g0711200 | OsR498G0101870300.01.T01 | Leucine-rich repeat transmembrane protein kinase |
| maker-1-augustus-gene-302.19-mRNA-1 | Japonica | NA | | Os01g0711400 | OsR498G0101870900.01.T01 | Glycine dehydrogenase (aminomethyl-transferring) |
| maker-1-augustus-gene-302.29-mRNA-1 | Japonica | NA | | Os01g0711500 | OsR498G0101871100.01.T02 | Calcineurin B-like protein 9 |
| maker-1-augustus-gene-302.35-mRNA-1 | Japonica | NA | | Os01g0711600 | OsR498G0101871300.01.T01 | Reversion-to-ethylene sensitivity1 |
| maker-1-augustus-gene-302.37-mRNA-1 | Japonica | Indica | | Os01g0924700 | OsR498G0101868400.01.T02 | Protein kinase domain-containing protein |
| maker-1-augustus-gene-302.39-mRNA-1 | Japonica | NA | | Os01g0711800 | OsR498G0101872000.01.T01 | Nucleosome assembly protein like |
| maker-1-augustus-gene-302.41-mRNA-1 | Japonica | Japonica | | Os01g0712500 | OsR498G0101874800.01.T02 | Cytidine deaminase-like |
| maker-1-augustus-gene-302.5-mRNA-1 | Japonica | NA | | Os01g0711000 | OsR498G0101869700.01.T02 | Vacuolar proton pump subunit B |
| maker-1-augustus-gene-303.30-mRNA-1 | Japonica | NA | | | OsR498G0101876100.01.T01 | Peroxidase |
| maker-1-augustus-gene-303.32-mRNA-1 | Japonica | NA | | Os01g0713200 | OsR498G0101877100.01.T01 | Glucan endo-1,3-beta-glucosidase GII |
| maker-1-augustus-gene-303.34-mRNA-1 | Japonica | Indica | | Os01g0713600 | OsR498G0101878300.01.T01 | B3 domain transcription factor |
| maker-1-augustus-gene-303.36-mRNA-1 | Japonica | Japonica | | Os01g0713700 | OsR498G0101878900.01.T01 | KRR-R motif-containing protein 1 |
| maker-1-augustus-gene-303.39-mRNA-1 | Japonica | NA | | Os03g0237200 | | Zinc finger, C2H2 type family protein, expressed |
| maker-1-augustus-gene-303.41-mRNA-1 | Japonica | NA | | | | Myosin-17 |
| maker-1-augustus-gene-303.47-mRNA-1 | Japonica | NA | | | OsR498G0101880200.01.T01 | Protein PLASTID MOVEMENT IMPAIRED 1-RELATED like |
| maker-1-augustus-gene-304.1-mRNA-1 | Japonica | NA | | | OsR498G0101882900.01.T01 | Signal recognition particle 54 kDa protein, chloroplastic-like |
| maker-1-augustus-gene-304.11-mRNA-1 | Japonica | NA | | Os01g0714900 | OsR498G0101881600.01.T01 | Ras-related protein Rab7 |
| maker-1-augustus-gene-304.15-mRNA-1 | Japonica | Indica | | Os01g0715000 | OsR498G0101881800.01.T03 | Cellular nucleic acid-binding protein |

| | | | | | | |
|---|---|---|---|---|---|---|
| maker-1-augustus-gene-304.18-mRNA-1 | Japonica | NA | | | OsR498G0101881800.01.T05 | Zinc finger CCHC domain-containing protein 9 isoform X1 |
| maker-1-augustus-gene-304.20-mRNA-1 | Japonica | NA | | Os01g0715500 | OsR498G0101884600.01.T01 | Outer mitochondrial membrane protein porin |
| maker-1-augustus-gene-304.23-mRNA-1 | Japonica | NA | | | OsR498G0101885400.01.T03 | Auxin efflux carrier component |
| maker-1-augustus-gene-304.26-mRNA-1 | Japonica | NA | | Os01g0715800 | OsR498G0101886200.01.T02 | Eukaryotic translation initiation factor 3 subunit C (Fragment) |
| maker-1-augustus-gene-304.3-mRNA-1 | Japonica | Indica | | Os01g0715400 | OsR498G0101884300.01.T03 | Alpha-amylase |
| maker-1-augustus-gene-304.9-mRNA-1 | Japonica | Japonica | | Os01g0714800 | OsR498G0101881000.01.T01 | WRKY transcription factor |
| maker-1-augustus-gene-305.27-mRNA-1 | Japonica | NA | | Os01g0716400 | OsR498G0101888500.01.T04 | VDE domain-containing protein (Fragment) |
| maker-1-augustus-gene-305.30-mRNA-1 | Japonica | NA | | Os01g0716500 | OsR498G0101888500.01.T01 | Embryonic abundant protein-like |
| maker-1-augustus-gene-305.32-mRNA-1 | Japonica | NA | | | | Type IV inositol polyphosphate 5-phosphatase 3 |
| maker-1-augustus-gene-305.35-mRNA-1 | Japonica | NA | | Os01g0717400 | OsR498G0101896900.01.T01 | Alliin lyase |
| maker-1-augustus-gene-305.38-mRNA-1 | Japonica | NA | | Os01g0716200 | OsR498G0101888000.01.T05 | Orphans transcription factor |
| maker-1-augustus-gene-305.42-mRNA-1 | Japonica | Japonica | | Os01g0716300 | OsR498G0101888400.01.T01 | MRN complex-interacting protein |
| maker-1-augustus-gene-305.45-mRNA-1 | Japonica | NA | | Os01g0717000 | OsR498G0101891000.01.T01 | Choline/ethanolamine kinase |
| maker-1-augustus-gene-305.49-mRNA-1 | Japonica | Indica | | Os01g0718000 | OsR498G0101897400.01.T01 | Zinc finger A20 and AN1 domain-containing stress-associated protein 2 |
| maker-1-augustus-gene-306.30-mRNA-1 | Japonica | NA | | Os01g0717700 | OsR498G0101898100.01.T01 | Tryptophan aminotransferase-related protein 4 |
| maker-1-augustus-gene-306.33-mRNA-1 | Japonica | NA | | | OsR498G0101903500.01.T01 | Potassium uptake channel |
| maker-1-augustus-gene-306.35-mRNA-1 | Japonica | NA | | Os01g0719000 | OsR498G0101904300.01.T01 | FLZ-type domain-containing protein |
| maker-1-augustus-gene-306.37-mRNA-1 | Japonica | Indica | | Os01g0719250 | OsR498G0101904900.01.T02 | |
| maker-1-augustus-gene-306.39-mRNA-1 | Japonica | NA | | Os01g0719300 | OsR498G0101906500.01.T02 | STAS domain-containing protein (Fragment) |
| maker-1-augustus-gene-306.40-mRNA-1 | Japonica | NA | | Os01g0719300 | OsR498G0101906500.01.T01 | Sulfate transporter 3.5 like |
| maker-1-augustus-gene-306.49-mRNA-1 | Japonica | NA | | Os01g0719100 | OsR498G0101904800.01.T06 | E3 ubiquitin-protein like |
| maker-1-augustus-gene-307.0-mRNA-1 | Japonica | Indica | | Os01g0719400 | OsR498G0101906700.01.T02 | Btz domain-containing protein (Fragment) |
| maker-1-augustus-gene-307.11-mRNA-1 | Japonica | Indica | | Os01g0719700 | OsR498G0101907300.01.T01 | Fe-S cluster assembly factor HCF101 chloroplastic |
| maker-1-augustus-gene-307.16-mRNA-1 | Japonica | NA | | Os01g0720800 | OsR498G0101910100.01.T02 | Fusarium resistance protein I2C-5-like |
| maker-1-augustus-gene-307.18-mRNA-1 | Japonica | Indica | | Os05g0533800 | OsR498G0101908000.01.T01 | Hydrogen-transporting ATP synthase, rotational mechanism |
| maker-1-augustus-gene-307.20-mRNA-1 | Japonica | Indica | | Os05g0533700 | OsR498G0101908200.01.T01 | NADH dehydrogenase2 |
| maker-1-augustus-gene-307.22-mRNA-1 | Japonica | NA | | Os01g0720600 | OsR498G0101909600.01.T03 | Starch synthase, chloroplastic/amyloplastic |
| maker-1-augustus-gene-307.8-mRNA-1 | Japonica | NA | | Os01g0719600 | OsR498G0101907100.01.T01 | HMA domain-containing protein |
| maker-1-augustus-gene-308.0-mRNA-1 | Japonica | NA | | Os01g0722100 | OsR498G0101914500.01.T07 | Putative acyl-acyl-carrier-proteinUDP-N-acetylglucosamine O-acyltransferas |
| maker-1-augustus-gene-308.12-mRNA-1 | Japonica | Indica | | Os01g0722700 | OsR498G0101915900.01.T01 | Phosphotransferase |
| maker-1-augustus-gene-308.18-mRNA-1 | Japonica | NA | | | OsR498G0101916300.01.T01 | BED-type domain-containing protein |
| maker-1-augustus-gene-308.21-mRNA-1 | Japonica | NA | | Os01g0723100 | OsR498G0101917500.01.T02 | Senescence domain-containing protein (Fragment) |
| maker-1-augustus-gene-308.29-mRNA-1 | Japonica | NA | | Os01g0723500 | OsR498G0101918700.01.T01 | B3 domain-containing protein (Fragment) |
| maker-1-augustus-gene-308.32-mRNA-1 | Japonica | NA | | Os01g0723600 | OsR498G0101918900.01.T01 | Ribose-phosphate pyrophosphokinase |
| maker-1-augustus-gene-308.35-mRNA-1 | Japonica | NA | | Os01g0723700 | OsR498G0101919300.01.T03 | TF-B3 domain-containing protein (Fragment) |
| maker-1-augustus-gene-308.37-mRNA-1 | Japonica | NA | | Os01g0721900 | OsR498G0101914200.01.T01 | RGG repeats nuclear RNA binding protein A |
| maker-1-augustus-gene-308.41-mRNA-1 | Japonica | NA | | Os01g0723000 | OsR498G0101917000.01.T03 | Tr-type G domain-containing protein |
| maker-1-augustus-gene-308.43-mRNA-1 | Japonica | NA | | Os01g0723200 | OsR498G0101917800.01.T01 | 40S ribosomal protein S24 |
| maker-1-augustus-gene-308.6-mRNA-1 | Japonica | NA | | Os01g0722300 | OsR498G0101914900.01.T01 | transcription factor RAX2 |
| maker-1-augustus-gene-309.37-mRNA-1 | Japonica | NA | | | OsR498G0101922300.01.T01 | Pleiotropic drug resistance transporter 5 |
| maker-1-augustus-gene-309.44-mRNA-1 | Japonica | NA | | Os01g0725400 | OsR498G0101925700.01.T03 | |
| maker-1-augustus-gene-309.45-mRNA-1 | Japonica | NA | | Os01g0725600 | OsR498G0101926100.01.T03 | Ultraviolet-B receptor like |
| maker-1-augustus-gene-309.56-mRNA-1 | Japonica | Indica | CC-Kinase-TM | Os01g0725800 | OsR498G0101926500.01.T05 | Protein kinase-like domain, Coatomer beta' subunit (COPB2), WD40 repeat, |
| maker-1-augustus-gene-309.60-mRNA-1 | Japonica | NA | | Os01g0723800 | OsR498G0101919800.01.T01 | Multidrug/pheromone exporter, MDR family, ABC transporter family |
| maker-1-augustus-gene-309.62-mRNA-1 | Japonica | NA | | Os01g0725900 | OsR498G0101926800.01.T01 | Non-classical arabinogalactan protein 31-like |
| maker-1-augustus-gene-309.64-mRNA-1 | Japonica | NA | | Os01g0726100 | OsR498G0101925400.01.T02 | Non-classical arabinogalactan protein 31-like |
| maker-1-augustus-gene-309.66-mRNA-1 | Japonica | NA | | Os01g0726400 | OsR498G0101927400.01.T02 | MADS-box transcription factor 32 |
| maker-1-augustus-gene-310.44-mRNA-1 | Japonica | NA | | Os01g0726700 | OsR498G0101927700.01.T01 | Protodermal factor 1 like |
| maker-1-augustus-gene-310.47-mRNA-1 | Japonica | NA | | Os01g0727100 | OsR498G0101929300.01.T01 | Hexosyltransferase |
| maker-1-augustus-gene-310.50-mRNA-1 | Japonica | NA | | Os01g0727400 | OsR498G0101929500.01.T05 | Complex I intermediate-associated protein 30 |
| maker-1-augustus-gene-310.56-mRNA-1 | Japonica | NA | | Os01g0727900 | OsR498G0101931300.01.T03 | DNA primase small subunit |
| maker-1-augustus-gene-310.58-mRNA-1 | Japonica | Indica | | Os05g0518300 | OsR498G0101931500.01.T02 | GDSL esterase/lipase |
| maker-1-augustus-gene-310.60-mRNA-1 | Japonica | NA | | | OsR498G0101928600.01.T01 | |
| maker-1-augustus-gene-310.62-mRNA-1 | Japonica | Indica | | Os01g0727800 | OsR498G0101931000.01.T01 | Subtilisin-like protease |
| maker-1-augustus-gene-310.66-mRNA-1 | Japonica | NA | | | OsR498G0101932000.01.T02 | |
| maker-1-augustus-gene-310.69-mRNA-1 | Japonica | NA | | Os01g0728300 | OsR498G0101932200.01.T01 | Secologanin synthase |
| maker-1-augustus-gene-311.42-mRNA-1 | Japonica | NA | | Os01g0729100 | OsR498G0101933500.01.T02 | DUF92 domain-containing protein (Fragment) |
| maker-1-augustus-gene-311.45-mRNA-1 | Japonica | NA | | Os01g0729900 | OsR498G0101935700.01.T05 | 6-hydroxymethyl-7,8-dihydropterin pyrophosphokinase |
| maker-1-augustus-gene-311.48-mRNA-1 | Japonica | NA | | | OsR498G0101935900.01.T01 | Inhibitor I9 domain-containing protein |
| maker-1-augustus-gene-311.50-mRNA-1 | Japonica | NA | | Os01g0730300 | OsR498G0101936700.01.T01 | Trehalose-phosphatase |
| maker-1-augustus-gene-311.53-mRNA-1 | Japonica | NA | | Os01g0730500 | OsR498G0101937700.01.T02 | Ferredoxin-1 (Fragment) |

| | | | | | | |
|---|---|---|---|---|---|---|
| maker-1-augustus-gene-311.57-mRNA-1 | Japonica | NA | | Os01g0728700 | OsR498G0101932800.01.T01 | Oil body-associated protein 2B |
| maker-1-augustus-gene-311.59-mRNA-1 | Japonica | NA | | Os01g0729600 | OsR498G0101934800.01.T01 | Putative pyridoxal phosphate-dependent transferase |
| maker-1-augustus-gene-311.61-mRNA-1 | Japonica | NA | | Os01g0730100 | OsR498G0101936200.01.T01 | FBD domain-containing protein (Fragment) |
| maker-1-augustus-gene-311.63-mRNA-1 | Japonica | NA | | Os01g0730700 | OsR498G0101938600.01.T01 | WRKY transcription factor 5 |
| maker-1-augustus-gene-312.38-mRNA-1 | Japonica | NA | | Os01g0730800 | OsR498G0101939300.01.T04 | Peroxisomal membrane protein 2 |
| maker-1-augustus-gene-312.42-mRNA-1 | Japonica | NA | | Os01g0732200 | OsR498G0101942100.01.T02 | EngB-type G domain-containing protein (Fragment) |
| maker-1-augustus-gene-312.44-mRNA-1 | Japonica | Indica | | Os01g0732600 | OsR498G0101944100.01.T01 | Flavin-containing monooxygenase |
| maker-1-augustus-gene-312.46-mRNA-1 | Japonica | NA | | Os01g0733200 | OsR498G0101946100.01.T01 | Heat shock transcription factor class C |
| maker-1-augustus-gene-312.48-mRNA-1 | Japonica | Indica | | Os01g0730900 | OsR498G0101939600.01.T03 | DNA polymerase III subunit gamma/tau |
| maker-1-augustus-gene-312.52-mRNA-1 | Japonica | NA | | Os01g0731000 | OsR498G0101940000.01.T01 | UBD domain-containing protein |
| maker-1-augustus-gene-312.56-mRNA-1 | Japonica | NA | | Os01g0731100 | OsR498G0101940200.01.T01 | Pathogenesis-related family protein isoform 1 |
| maker-1-augustus-gene-312.58-mRNA-1 | Japonica | Indica | | Os01g0731100 | OsR498G0101941200.01.T01 | Pathogenesis-related family protein isoform 1 |
| maker-1-augustus-gene-312.60-mRNA-1 | Japonica | NA | | Os01g0731800 | OsR498G0101941400.01.T01 | RING-CH-type domain-containing protein (Fragment) |
| maker-1-augustus-gene-313.13-mRNA-1 | Japonica | Japonica | | Os01g0735500 | OsR498G0101951500.01.T01 | Glycosyltransferase |
| maker-1-augustus-gene-313.14-mRNA-1 | Japonica | NA | | Os01g0733600 | OsR498G0101947200.01.T02 | NADPH-dependent diflavin oxidoreductase 1 |
| maker-1-augustus-gene-313.2-mRNA-1 | Japonica | NA | | Os01g0734000 | OsR498G0101947500.01.T01 | WRKY transcription factor 24 |
| maker-1-augustus-gene-313.21-mRNA-1 | Japonica | Indica | | Os06g0279950 | OsR498G0101949000.01.T01 | |
| maker-1-augustus-gene-313.4-mRNA-1 | Japonica | NA | | Os01g0734200 | OsR498G0101948700.01.T02 | Respiratory burst oxidase protein B |
| maker-1-augustus-gene-314.0-mRNA-1 | Japonica | Indica | | Os10g0560900 | OsR498G0101954300.01.T01 | Serine C-palmitoyltransferase |
| maker-1-augustus-gene-314.11-mRNA-1 | Japonica | NA | | | OsR498G0101957100.01.T01 | Bifunctional polynucleotide phosphatase/kinase |
| maker-1-augustus-gene-314.18-mRNA-1 | Japonica | Indica | | Os01g0737500 | OsR498G0101957300.01.T01 | Aluminum-activated malate transporter |
| maker-1-augustus-gene-314.21-mRNA-1 | Japonica | NA | | Os01g0737900 | OsR498G0101958100.01.T01 | Pentatricopeptide repeat-containing protein, mitochondrial |
| maker-1-augustus-gene-314.23-mRNA-1 | Japonica | NA | | Os01g0737800 | OsR498G0101958000.01.T02 | CAAX farnesyltransferase subunit beta |
| maker-1-augustus-gene-314.8-mRNA-1 | Japonica | NA | | Os01g0736900 | OsR498G0101956100.01.T03 | Reticulon-like protein (Fragment) |
| maker-1-augustus-gene-315.34-mRNA-1 | Japonica | NA | | Os01g0738000 | OsR498G0101958300.01.T01 | Pentatricopeptide repeat-containing protein |
| maker-1-augustus-gene-315.36-mRNA-1 | Japonica | NA | Kinase-TM | Os01g0738300 | OsR498G0101960100.01.T01 | Proline-rich receptor-like protein kinase PERK10 isoform A |
| maker-1-augustus-gene-315.40-mRNA-1 | Japonica | NA | | Os01g0531200 | OsR498G0101960700.01.T03 | PMD domain-containing protein |
| maker-1-augustus-gene-315.42-mRNA-1 | Japonica | NA | | Os01g0738600 | OsR498G0101960900.01.T03 | Clathrin interactor EPSIN 1 |
| maker-1-augustus-gene-315.48-mRNA-1 | Japonica | NA | | Os01g0739200 | OsR498G0101963300.01.T01 | Dual specificity protein phosphatase DSP8 |
| maker-1-augustus-gene-315.52-mRNA-1 | Japonica | NA | | | OsR498G0101958800.01.T01 | Ubiquitin-like-specific protease 1D |
| maker-1-augustus-gene-315.55-mRNA-1 | Japonica | Indica | | Os05g0524400 | OsR498G0101961400.01.T03 | ATP-dependent 6-phosphofructokinase |
| maker-1-augustus-gene-315.61-mRNA-1 | Japonica | NA | | Os01g0289900 | | Rosmarinic acid synthase |
| maker-1-augustus-gene-315.63-mRNA-1 | Japonica | NA | | Os01g0739000 | OsR498G0101962600.01.T01 | Mitochondrial-processing peptidase subunit alpha |
| maker-1-augustus-gene-316.0-mRNA-1 | Japonica | NA | | | OsR498G0101964500.01.T01 | Haloacid dehalogenase-like hydrolase-like |
| maker-1-augustus-gene-316.13-mRNA-1 | Japonica | NA | | Os01g0739500 | OsR498G0101964800.01.T01 | Oxidative stress 3 |
| maker-1-augustus-gene-316.15-mRNA-1 | Japonica | NA | | Os01g0740500 | | Glutamate carboxypeptidase 2 |
| maker-1-augustus-gene-316.19-mRNA-1 | Japonica | Indica | | Os01g0743300 | OsR498G0101968800.01.T01 | Glutamate carboxypeptidase 2 |
| maker-1-augustus-gene-316.24-mRNA-1 | Japonica | NA | Kinase-TM | Os01g0741200 | OsR498G0101969600.01.T01 | Putative serine/threonine-protein kinase RLCKVII |
| maker-1-augustus-gene-316.5-mRNA-1 | Japonica | NA | | Os01g0739700 | OsR498G0101966500.01.T01 | Glucan endo-1,3-beta-D-glucosidase |
| maker-1-augustus-gene-316.7-mRNA-1 | Japonica | Indica | | Os01g0740400 | OsR498G0101967900.01.T01 | Formin-like protein 18 |
| maker-1-augustus-gene-316.9-mRNA-1 | Japonica | NA | | Os01g0741900 | OsR498G0101971900.01.T04 | Auxin-responsive protein IAA6 |
| maker-1-augustus-gene-317.33-mRNA-1 | Japonica | NA | | Os01g0742200 | OsR498G0101972900.01.T02 | Tr-type G domain-containing protein |
| maker-1-augustus-gene-317.35-mRNA-1 | Japonica | NA | Kinase-LRR-TM | Os01g0742400 | OsR498G0101973900.01.T01 | Protein kinase family protein with leucine-rich repeat domain |
| maker-1-augustus-gene-317.37-mRNA-1 | Japonica | Indica | | Os01g0743100 | OsR498G0101975300.01.T01 | IQ-domain 25 |
| maker-1-augustus-gene-317.39-mRNA-1 | Japonica | NA | | Os01g0743300 | OsR498G0101976900.01.T03 | Protease-associated domain |
| maker-1-augustus-gene-317.44-mRNA-1 | Japonica | NA | | Os01g0743500 | OsR498G0101977300.01.T01 | NADP-dependent malic enzyme |
| maker-1-augustus-gene-317.48-mRNA-1 | Japonica | NA | | Os01g0742500 | OsR498G0101974200.01.T01 | Phosphotransferase |
| maker-1-augustus-gene-317.53-mRNA-1 | Japonica | NA | | Os01g0743200 | OsR498G0101976000.01.T01 | Pectinesterase |
| maker-1-augustus-gene-317.57-mRNA-1 | Japonica | NA | | Os01g0743400 | OsR498G0101977200.01.T03 | Tryptophanyl-tRNA synthetase |
| maker-1-augustus-gene-318.0-mRNA-1 | Japonica | Japonica | | Os01g0743800 | OsR498G0101978300.01.T01 | Pseudo histidine-containing phosphotransfer protein 1 |
| maker-1-augustus-gene-318.10-mRNA-1 | Japonica | NA | | Os01g0744200 | OsR498G0101979100.01.T01 | Downstream target of AGL15-4 |
| maker-1-augustus-gene-318.14-mRNA-1 | Japonica | NA | | Os01g0743600 | OsR498G0101977600.01.T03 | Lon N-terminal domain-containing protein |
| maker-1-augustus-gene-318.19-mRNA-1 | Japonica | NA | | Os01g0744300 | OsR498G0101979400.01.T04 | Casein kinase I-like protein 2 |
| maker-1-augustus-gene-318.26-mRNA-1 | Japonica | NA | | Os01g0744400 | OsR498G0101979800.01.T03 | Golgin candidate 1 |
| maker-1-augustus-gene-318.3-mRNA-1 | Japonica | NA | | Os01g0744000 | OsR498G0101978700.01.T04 | p-loop nucleoside triphosphate hydrolase superfamily protein with CH (Calp |
| maker-1-augustus-gene-318.33-mRNA-1 | Japonica | Indica | | Os01g0200200 | OsR498G0101980400.01.T01 | |
| maker-1-augustus-gene-318.35-mRNA-1 | Japonica | NA | | Os01g0745700 | OsR498G0101982800.01.T02 | GATA transcription factor |
| maker-1-augustus-gene-319.32-mRNA-1 | Japonica | NA | | | | Nuclear pore complex protein Nup85 |
| maker-1-augustus-gene-319.37-mRNA-1 | Japonica | Indica | | Os01g0747300 | OsR498G0101986700.01.T01 | Plant-specific domain TIGR01615 family protein expressed |
| maker-1-augustus-gene-319.40-mRNA-1 | Japonica | NA | | Os01g0746400 | OsR498G0101984600.01.T01 | Carotenoid cleavage dioxygenase |
| maker-1-augustus-gene-319.42-mRNA-1 | Japonica | NA | | Os01g0746700 | OsR498G0101985200.01.T01 | Mannan endo-1,4-beta-mannosidase |

| | | | | | | |
|---|---|---|---|---|---|---|
| maker-1-augustus-gene-319.46-mRNA-1 | Japonica | NA | Kinase-TM | Os01g0747400 | OsR498G0101987200.01.T01 | Serine/threonine-protein kinase HT1 |
| maker-1-augustus-gene-320.0-mRNA-1 | Japonica | NA | | Os01g0747500 | OsR498G0101987900.01.T06 | Dihydroorotase |
| maker-1-augustus-gene-320.10-mRNA-1 | Japonica | NA | | Os01g0748900 | OsR498G0101993100.01.T02 | MACPF domain-containing protein CAD1 |
| maker-1-augustus-gene-320.14-mRNA-1 | Japonica | NA | | | OsR498G0101993300.01.T01 | Proton-dependent oligopeptide transporter |
| maker-1-augustus-gene-320.16-mRNA-1 | Japonica | Indica | | Os01g0749000 | OsR498G0101993500.01.T01 | Oil body-associated protein 2A |
| maker-1-augustus-gene-320.18-mRNA-1 | Japonica | NA | | | OsR498G0101993700.01.T01 | Forkhead box protein like |
| maker-1-augustus-gene-320.22-mRNA-1 | Japonica | NA | | Os01g0749200 | OsR498G0101994100.01.T01 | 50S ribosomal protein L13 chloroplastic |
| maker-1-augustus-gene-320.26-mRNA-1 | Japonica | NA | | Os01g0749300 | OsR498G0101994300.01.T01 | Heat shock responsive transcription factor |
| maker-1-augustus-gene-320.29-mRNA-1 | Japonica | NA | | | | Dynamin-related protein 5A isoform A |
| maker-1-augustus-gene-320.3-mRNA-1 | Japonica | NA | | Os01g0747700 | | S4 RNA-binding domain-containing protein (Fragment) |
| maker-1-augustus-gene-320.32-mRNA-1 | Japonica | NA | | Os01g0748150 | OsR498G0101990200.01.T01 | Phytocyanin domain-containing protein |
| maker-1-augustus-gene-320.34-mRNA-1 | Japonica | NA | | | OsR498G0101992000.01.T02 | Dual specificity protein kinase splA |
| maker-1-augustus-gene-320.40-mRNA-1 | Japonica | NA | | Os01g0749400 | OsR498G0101994600.01.T02 | Trehalose-phosphatase |
| maker-1-augustus-gene-320.5-mRNA-1 | Japonica | Indica | | Os05g0518300 | OsR498G0101991700.01.T01 | GDSL esterase/lipase |
| maker-1-augustus-gene-320.8-mRNA-1 | Japonica | NA | | Os01g0748800 | OsR498G0101992300.01.T01 | Phosphatidylethanolamine-binding protein |
| maker-1-augustus-gene-321.0-mRNA-1 | Japonica | NA | | Os01g0749900 | OsR498G0101996500.01.T01 | Putative sugar phosphate/phosphate translocator |
| maker-1-augustus-gene-321.10-mRNA-1 | Japonica | NA | | Os01g0750300 | OsR498G0101999100.01.T02 | Cellulose synthase A catalytic subunit 4 |
| maker-1-augustus-gene-321.14-mRNA-1 | Japonica | NA | | | OsR498G0101997600.01.T01 | WRKY transcription factor 5 |
| maker-1-augustus-gene-321.16-mRNA-1 | Japonica | NA | | Os01g0750500 | OsR498G0102001400.01.T01 | Coiled-coil domain-containing protein 25 |
| maker-1-augustus-gene-321.19-mRNA-1 | Japonica | NA | Kinase-TM | Os01g0750600 | OsR498G0102002200.01.T01 | proline-rich receptor-like protein kinase PERK5 |
| maker-1-augustus-gene-321.8-mRNA-1 | Japonica | NA | | Os01g0750000 | OsR498G0101997100.01.T01 | Ras-related protein RIC2 |
| maker-1-augustus-gene-322.0-mRNA-1 | Japonica | NA | | Os01g0751300 | OsR498G0102004500.01.T02 | Tobamovirus multiplication protein 1 |
| maker-1-augustus-gene-322.11-mRNA-1 | Japonica | NA | | Os01g0752700 | OsR498G0102008100.01.T01 | Elongation factor family protein isoform 1 |
| maker-1-augustus-gene-322.17-mRNA-1 | Japonica | NA | | Os01g0751600 | OsR498G0102005200.01.T01 | Fungal lipase-like domain containing protein |
| maker-1-augustus-gene-322.19-mRNA-1 | Japonica | NA | | Os01g0752200 | OsR498G0102006800.01.T01 | 3-hydroxyisobutyryl-CoA hydrolase-like protein 5 |
| maker-1-augustus-gene-322.23-mRNA-1 | Japonica | NA | | Os01g0752300 | OsR498G0102007200.01.T01 | Urease accessory protein G |
| maker-1-augustus-gene-322.26-mRNA-1 | Japonica | NA | | Os01g0752400 | OsR498G0102007400.01.T02 | PRKCSH-like domain-containing protein |
| maker-1-augustus-gene-322.30-mRNA-1 | Japonica | NA | | Os01g0752600 | OsR498G0102008000.01.T04 | Lipid-A-disaccharide synthase |
| maker-1-augustus-gene-322.8-mRNA-1 | Japonica | NA | | Os01g0752100 | OsR498G0102006500.01.T01 | FBD domain-containing protein |
| maker-1-augustus-gene-323.40-mRNA-1 | Japonica | NA | | Os01g0753100 | OsR498G0102009100.01.T01 | reticulon-4-interacting protein 1, mitochondrial isoform X1 |
| maker-1-augustus-gene-323.44-mRNA-1 | Japonica | NA | | | OsR498G0102009900.01.T01 | trans-resveratrol di-O-methyltransferase-like isoform X2 |
| maker-1-augustus-gene-323.46-mRNA-1 | Japonica | NA | | | OsR498G0102010300.01.T01 | Protein kinase domain-containing protein |
| maker-1-augustus-gene-323.48-mRNA-1 | Japonica | NA | | Os01g0754000 | OsR498G0102011100.01.T06 | Protein NUCLEAR FUSION DEFECTIVE 6 chloroplastic/mitochondrial |
| maker-1-augustus-gene-323.50-mRNA-1 | Japonica | NA | | Os01g0752800 | OsR498G0102008400.01.T02 | Casein kinase substrate, phosphoprotein PP28 |
| maker-1-augustus-gene-323.54-mRNA-1 | Japonica | Indica | | Os05g0515700 | OsR498G0102009000.01.T03 | Transcription factor VOZ1 |
| maker-1-augustus-gene-323.56-mRNA-1 | Japonica | NA | | Os01g0753150 | OsR498G0102009600.01.T01 | optic atrophy 3 protein homolog |
| maker-1-augustus-gene-323.58-mRNA-1 | Japonica | Indica | | Os02g0788500 | OsR498G0102009800.01.T01 | F-box protein SKIP8 |
| maker-1-augustus-gene-323.60-mRNA-1 | Japonica | Indica | | Os01g0753500 | OsR498G0102010200.01.T02 | Auxin response factor |
| maker-1-augustus-gene-323.66-mRNA-1 | Japonica | NA | | Os01g0754100 | OsR498G0102011400.01.T01 | Integrator complex subunit 9-like protein |
| maker-1-augustus-gene-323.68-mRNA-1 | Japonica | NA | | Os01g0754500 | OsR498G0102012200.01.T02 | Structural constituent of cell wall protein, putative |
| maker-1-augustus-gene-324.0-mRNA-1 | Japonica | NA | | Os01g0755500 | OsR498G0102014300.01.T01 | transcription factor PCF5 |
| maker-1-augustus-gene-324.10-mRNA-1 | Japonica | NA | | Os01g0756700 | OsR498G0102018500.01.T01 | Voltage dependent potassium channel |
| maker-1-augustus-gene-324.12-mRNA-1 | Japonica | NA | | Os01g0755100 | OsR498G0102013800.01.T03 | |
| maker-1-augustus-gene-324.18-mRNA-1 | Japonica | NA | | | OsR498G0102017200.01.T01 | HTH La-type RNA-binding domain-containing protein |
| maker-1-augustus-gene-324.2-mRNA-1 | Japonica | NA | | Os01g0756200 | OsR498G0102016300.01.T01 | Basic leucine zipper 6 |
| maker-1-augustus-gene-324.6-mRNA-1 | Japonica | Japonica | | Os01g0531400 | OsR498G0102017700.01.T01 | Putative Galactose-binding domain-containing protein |
| maker-1-augustus-gene-325.0-mRNA-1 | Japonica | NA | | Os01g0757200 | OsR498G0102021500.01.T01 | Gibberellin 2-beta-dioxygenase |
| maker-1-augustus-gene-325.15-mRNA-1 | Japonica | NA | | Os01g0757500 | OsR498G0102022800.01.T01 | Calcyclin-binding protein (Fragment) |
| maker-1-augustus-gene-325.2-mRNA-1 | Japonica | NA | NBS | Os01g0757400 | OsR498G0102022500.01.T02 | AAA domain-containing protein (Fragment) |
| maker-1-augustus-gene-325.26-mRNA-1 | Japonica | NA | | | OsR498G0102025400.01.T04 | Bifunctional riboflavin kinasefmn phosphatase |
| maker-1-augustus-gene-325.32-mRNA-1 | Japonica | NA | | Os01g0758000 | OsR498G0102025600.01.T01 | Heavy metal-associated isoprenylated plant protein 26 |
| maker-1-augustus-gene-325.34-mRNA-1 | Japonica | Indica | | Os01g0758300 | OsR498G0102028800.01.T01 | Phosphoenolpyruvate carboxylase, housekeeping isozyme |
| maker-1-augustus-gene-325.38-mRNA-1 | Japonica | Indica | | Os01g0758400 | OsR498G0102029400.01.T05 | Phosphatidate cytidylyltransferase |
| maker-1-augustus-gene-325.9-mRNA-1 | Japonica | Indica | | Os01g0757800 | OsR498G0102025100.01.T01 | UmuC domain-containing protein |
| maker-1-augustus-gene-326.32-mRNA-1 | Japonica | NA | | Os01g0758900 | OsR498G0102034300.01.T01 | F-box domain-containing protein |
| maker-1-augustus-gene-326.34-mRNA-1 | Japonica | NA | | Os01g0759000 | OsR498G0102034900.01.T01 | |
| maker-1-augustus-gene-326.36-mRNA-1 | Japonica | Indica | | Os01g0759700 | OsR498G0102038300.01.T02 | Transcriptional regulator family protein-like |
| maker-1-augustus-gene-326.40-mRNA-1 | Japonica | Indica | | Os01g0759900 | OsR498G0102038700.01.T02 | Nucleobase-ascorbate transporter like |
| maker-1-augustus-gene-326.48-mRNA-1 | Japonica | Japonica | | Os01g0759100 | OsR498G0102035200.01.T02 | Tubby-like F-box protein 1 |
| maker-1-augustus-gene-327.48-mRNA-1 | Japonica | NA | | | | Putative acyl-activating enzyme |
| maker-1-augustus-gene-327.52-mRNA-1 | Japonica | NA | | Os01g0761900 | OsR498G0102043900.01.T01 | RING-type E3 ubiquitin transferase |

| | | | | | | |
|---|---|---|---|---|---|---|
| maker-1-augustus-gene-327.54-mRNA-1 | Japonica | NA | | Os01g0762500 | OsR498G0102044500.01.T01 | Glutelin type-A 1 |
| maker-1-augustus-gene-327.62-mRNA-1 | Japonica | NA | | | OsR498G0102040600.01.T01 | Homeobox-leucine zipper protein ROC9 |
| maker-1-augustus-gene-327.65-mRNA-1 | Japonica | NA | | Os01g0760900 | OsR498G0102040800.01.T01 | Probable protein ABIL5 |
| maker-1-augustus-gene-327.69-mRNA-1 | Japonica | NA | | Os01g0761100 | OsR498G0102041200.01.T01 | CRC domain |
| maker-1-augustus-gene-327.72-mRNA-1 | Japonica | NA | | Os01g0761400 | OsR498G0102042000.01.T02 | Peptide/nitrate transporter plant |
| maker-1-augustus-gene-327.75-mRNA-1 | Japonica | NA | | Os01g0761500 | OsR498G0102042600.01.T01 | Putative Nitrate transporter |
| maker-1-augustus-gene-327.77-mRNA-1 | Japonica | NA | | Os01g0762000 | OsR498G0102044200.01.T01 | Sugar-dependent1 |
| maker-1-augustus-gene-327.79-mRNA-1 | Japonica | NA | | Os01g0762300 | OsR498G0102044400.01.T01 | Putative 54S ribosomal protein L32, mitochondrial (Fragment) |
| maker-1-augustus-gene-328.0-mRNA-1 | Japonica | Japonica | | Os01g0763100 | OsR498G0102045300.01.T04 | RHOMBOID-like protein 12 mitochondrial |
| maker-1-augustus-gene-328.13-mRNA-1 | Japonica | Indica | | Os07g0694800 | OsR498G0102049700.01.T01 | DUF155 domain-containing protein |
| maker-1-augustus-gene-328.18-mRNA-1 | Japonica | Japonica | | Os01g0762600 | OsR498G0102044400.01.T06 | FCP1 homology domain-containing protein (Fragment) |
| maker-1-augustus-gene-328.21-mRNA-1 | Japonica | NA | | Os01g0763000 | OsR498G0102045200.01.T01 | |
| maker-1-augustus-gene-328.25-mRNA-1 | Japonica | NA | | | OsR498G0102047800.01.T01 | X8 domain-containing protein |
| maker-1-augustus-gene-328.27-mRNA-1 | Japonica | NA | | Os01g0763900 | OsR498G0102048000.01.T02 | X8 domain-containing protein |
| maker-1-augustus-gene-328.30-mRNA-1 | Japonica | NA | | Os01g0764000 | OsR498G0102048200.01.T01 | Glutathione transferase |
| maker-1-augustus-gene-328.32-mRNA-1 | Japonica | NA | | Os01g0764400 | OsR498G0102050000.01.T01 | Chorismate mutase |
| maker-1-augustus-gene-328.6-mRNA-1 | Japonica | NA | | Os01g0763600 | OsR498G0102047300.01.T01 | Glycerophosphodiester phosphodiesterase |
| maker-1-augustus-gene-329.0-mRNA-1 | Japonica | NA | | Os01g0764700 | OsR498G0102050900.01.T05 | YqgFc domain-containing protein |
| maker-1-augustus-gene-329.11-mRNA-1 | Japonica | NA | | Os08g0172200 | OsR498G0815291700.01.T04 | UvrB/uvrC motif-containing protein |
| maker-1-augustus-gene-329.16-mRNA-1 | Japonica | NA | | Os01g0764600 | OsR498G0102050800.01.T02 | Isopentenyl phosphate kinase |
| maker-1-augustus-gene-329.22-mRNA-1 | Japonica | NA | | Os01g0764900 | | Pentatricopeptide (PPR) repeat protein-like |
| maker-1-augustus-gene-329.24-mRNA-1 | Japonica | NA | | | OsR498G0102052400.01.T03 | Deoxycytidylate deaminase |
| maker-1-augustus-gene-329.25-mRNA-1 | Japonica | NA | | Os01g0765000 | OsR498G0102052400.01.T01 | Deoxycytidylate deaminase |
| maker-1-augustus-gene-329.3-mRNA-1 | Japonica | NA | | Os01g0764800 | OsR498G0102051900.01.T01 | Indole-3-acetic acid-amido synthetase |
| maker-1-augustus-gene-329.32-mRNA-1 | Japonica | NA | | Os01g0765400 | OsR498G0102053200.01.T01 | Serpin (Serine protease inhibitor) |
| maker-1-augustus-gene-329.34-mRNA-1 | Japonica | NA | | Os01g0765500 | OsR498G0102053400.01.T01 | |
| maker-1-augustus-gene-329.36-mRNA-1 | Japonica | Japonica | | Os01g0766000 | OsR498G0102054000.01.T01 | Protein DETOXIFICATION |
| maker-1-augustus-gene-329.6-mRNA-1 | Japonica | NA | | | OsR498G0102052700.01.T02 | Nucleic acid binding |
| maker-1-augustus-gene-330.0-mRNA-1 | Japonica | NA | | Os01g0766400 | OsR498G0102055700.01.T01 | |
| maker-1-augustus-gene-330.12-mRNA-1 | Japonica | NA | | Os01g0767900 | OsR498G0102060100.01.T03 | Regulatory protein NPR3 |
| maker-1-augustus-gene-330.16-mRNA-1 | Japonica | Indica | | Os03g0770800 | OsR498G0102057400.01.T01 | Probable xyloglucan glycosyltransferase 1 |
| maker-1-augustus-gene-330.19-mRNA-1 | Japonica | NA | | Os01g0767000 | OsR498G0102058400.01.T01 | Branched-chain-amino-acid aminotransferase 5 chloroplastic |
| maker-1-augustus-gene-330.23-mRNA-1 | Japonica | NA | | Os01g0767100 | OsR498G0102058600.01.T02 | Lysosomal Pro-X carboxypeptidase |
| maker-1-augustus-gene-330.3-mRNA-1 | Japonica | NA | | Os01g0766600 | OsR498G0102055900.01.T01 | BSD domain-containing protein |
| maker-1-augustus-gene-330.5-mRNA-1 | Japonica | NA | | Os01g0767600 | OsR498G0102058900.01.T01 | DNA-directed RNA polymerase subunit beta |
| maker-1-augustus-gene-330.7-mRNA-1 | Japonica | NA | | Os01g0767700 | OsR498G0102059100.01.T04 | RNA helicase |
| maker-1-augustus-gene-331.0-mRNA-1 | Japonica | NA | | | OsR498G0102061700.01.T02 | Maternal effect embryo arrest 22 |
| maker-1-augustus-gene-331.15-mRNA-1 | Japonica | NA | | Os01g0769100 | OsR498G0102062700.01.T02 | Lipoyl synthase, chloroplastic |
| maker-1-augustus-gene-331.20-mRNA-1 | Japonica | NA | | Os01g0769200 | OsR498G0102064100.01.T01 | Subtilisin-like protease |
| maker-1-augustus-gene-331.22-mRNA-1 | Japonica | NA | | Os01g0769900 | OsR498G0102064700.01.T04 | Plastid transcriptionally active 12 |
| maker-1-augustus-gene-331.26-mRNA-1 | Japonica | Indica | | Os01g0768200 | OsR498G0102060800.01.T03 | TLC domain-containing protein |
| maker-1-augustus-gene-331.31-mRNA-1 | Japonica | NA | | | OsR498G0102061000.01.T01 | TLC domain-containing protein |
| maker-1-augustus-gene-331.34-mRNA-1 | Japonica | NA | | Os01g0768400 | | HGWP repeat containing protein-like |
| maker-1-augustus-gene-332.32-mRNA-1 | Japonica | NA | | Os01g0771200 | OsR498G0102068700.01.T01 | Mal d 1-associated protein |
| maker-1-augustus-gene-332.34-mRNA-1 | Japonica | NA | | Os01g0770000 | OsR498G0102065000.01.T01 | Diacylglycerol O-acyltransferase |
| maker-1-augustus-gene-332.35-mRNA-1 | Japonica | NA | | Os01g0770100 | | Diacylglycerol O-acyltransferase |
| maker-1-augustus-gene-332.48-mRNA-1 | Japonica | NA | | Os01g0770500 | OsR498G0102066600.01.T01 | ATP-binding ABC transporter |
| maker-1-augustus-gene-332.50-mRNA-1 | Japonica | NA | | Os01g0771100 | OsR498G0102068600.01.T01 | Mitochondrial acidic protein mam33 |
| maker-1-augustus-gene-332.52-mRNA-1 | Japonica | NA | | | OsR498G0102070400.01.T02 | Ubiquitinyl hydrolase 1 |
| maker-1-augustus-gene-333.32-mRNA-1 | Japonica | NA | | Os01g0772600 | OsR498G0102073700.01.T02 | Casein kinase |
| maker-1-augustus-gene-333.40-mRNA-1 | Japonica | NA | | | OsR498G0102074100.01.T03 | ARM repeat superfamily protein |
| maker-1-augustus-gene-333.46-mRNA-1 | Japonica | NA | | Os01g0772800 | OsR498G0102074300.01.T01 | Signal recognition particle 54 kDa protein |
| maker-1-augustus-gene-333.51-mRNA-1 | Japonica | NA | | Os01g0773100 | OsR498G0102075300.01.T02 | Dymeclin |
| maker-1-augustus-gene-333.56-mRNA-1 | Japonica | NA | | Os01g0771900 | OsR498G0102070800.01.T06 | lysosomal beta glucosidase-like isoform X2 |
| maker-1-augustus-gene-333.61-mRNA-1 | Japonica | NA | | | OsR498G0102071200.01.T01 | Double-stranded RNA-binding protein 1 |
| maker-1-augustus-gene-333.64-mRNA-1 | Japonica | Indica | | Os01g0772100 | OsR498G0102071600.01.T01 | LOB domain-containing protein 4 |
| maker-1-augustus-gene-333.67-mRNA-1 | Japonica | Indica | | Os01g0772150 | OsR498G0102071800.01.T01 | Histone-lysine N-methyltransferase SUVR3 |
| maker-1-augustus-gene-333.71-mRNA-1 | Japonica | NA | | Os01g0772500 | OsR498G0102073000.01.T01 | Beta-glucuronosyltransferase GlcAT14A |
| maker-1-augustus-gene-333.73-mRNA-1 | Japonica | NA | | Os01g0772900 | OsR498G0102074800.01.T02 | Homocysteine S-methyltransferase 4 |
| maker-1-augustus-gene-333.80-mRNA-1 | Japonica | NA | | | OsR498G0102075000.01.T01 | TRUD domain-containing protein (Fragment) |
| maker-1-augustus-gene-333.82-mRNA-1 | Japonica | NA | | | | jmjC domain-containing protein C isoform X2 |

| | | | | | | |
|---|---|---|---|---|---|---|
| maker-1-augustus-gene-334.0-mRNA-1 | Japonica | NA | | | OsR498G0102076700.01.T01 | |
| maker-1-augustus-gene-334.10-mRNA-1 | Japonica | NA | | | | FBD domain-containing protein |
| maker-1-augustus-gene-334.12-mRNA-1 | Japonica | NA | | Os01g0775200 | OsR498G0102082700.01.T01 | GTD-binding domain-containing protein (Fragment) |
| maker-1-augustus-gene-334.15-mRNA-1 | Japonica | NA | | Os01g0773700 | OsR498G0102077400.01.T01 | Photosystem II reaction center W protein, chloroplastic |
| maker-1-augustus-gene-334.17-mRNA-1 | Japonica | Japonica | | Os01g0774500 | OsR498G0102081600.01.T01 | p-loop containing nucleoside triphosphate hydrolase superfamily protein |
| maker-1-augustus-gene-334.2-mRNA-1 | Japonica | NA | | Os01g0773600 | OsR498G0102077100.01.T02 | alpha-1,2-Mannosidase |
| maker-1-augustus-gene-334.20-mRNA-1 | Japonica | Indica | | Os01g0775100 | OsR498G0102081800.01.T02 | Plus3 domain-containing protein |
| maker-1-augustus-gene-334.8-mRNA-1 | Japonica | NA | | Os01g0773800 | OsR498G0102078100.01.T01 | BHLH domain-containing protein (Fragment) |
| maker-1-augustus-gene-335.29-mRNA-1 | Japonica | NA | | | OsR498G0102087900.01.T01 | |
| maker-1-augustus-gene-335.31-mRNA-1 | Japonica | Indica | | Os01g0776500 | OsR498G0102088500.01.T02 | Superoxide dismutase |
| maker-1-augustus-gene-335.37-mRNA-1 | Japonica | NA | | Os01g0776800 | OsR498G0102089900.01.T01 | Nucleotide binding (Fragment) |
| maker-1-augustus-gene-335.40-mRNA-1 | Japonica | NA | | Os01g0777000 | OsR498G0102090300.01.T03 | Conserved oligomeric Golgi complex subunit 1 |
| maker-1-augustus-gene-335.45-mRNA-1 | Japonica | NA | | Os01g0775300 | OsR498G0102083400.01.T02 | Math domain containing protein (Fragment) |
| maker-1-augustus-gene-335.50-mRNA-1 | Japonica | NA | | Os01g0775400 | OsR498G0102083800.01.T01 | Cytokinin dehydrogenase |
| maker-1-augustus-gene-335.53-mRNA-1 | Japonica | Indica | | Os01g0775500 | OsR498G0102085600.01.T01 | Mannose-binding lectin |
| maker-1-augustus-gene-335.56-mRNA-1 | Japonica | NA | | Os01g0775600 | OsR498G0102085800.01.T03 | LisH dimerization motif |
| maker-1-augustus-gene-335.60-mRNA-1 | Japonica | NA | | | OsR498G0102088400.01.T03 | WD repeat-containing protein 13 |
| maker-1-augustus-gene-335.65-mRNA-1 | Japonica | NA | | Os01g0776600 | OsR498G0102089000.01.T01 | Purple acid phosphatase |
| maker-1-augustus-gene-335.68-mRNA-1 | Japonica | Indica | | Os01g0776700 | OsR498G0102089400.01.T01 | |
| maker-1-augustus-gene-336.0-mRNA-1 | Japonica | Indica | | Os01g0776900 | OsR498G0102091500.01.T01 | Aspartic proteinase nepenthesin-2 |
| maker-1-augustus-gene-336.11-mRNA-1 | Japonica | NA | | | OsR498G0102091900.01.T01 | RRM domain-containing protein (Fragment) |
| maker-1-augustus-gene-336.13-mRNA-1 | Japonica | NA | | Os01g0777900 | OsR498G0102093100.01.T01 | Cysteine-rich transmembrane CYSTM domain containing protein |
| maker-1-augustus-gene-336.18-mRNA-1 | Japonica | NA | | Os01g0778700 | OsR498G0102095500.01.T01 | Vacuole membrane protein KMS1 |
| maker-1-augustus-gene-336.21-mRNA-1 | Japonica | NA | | | | Insulin-degrading enzyme-like 1 peroxisomal |
| maker-1-augustus-gene-336.31-mRNA-1 | Japonica | Indica | | Os03g0689100 | OsR498G0102092400.01.T02 | Inositol hexakisphosphate and diphosphoinositol-pentakisphosphate kinase |
| maker-1-augustus-gene-336.37-mRNA-1 | Japonica | NA | | | OsR498G0102092800.01.T02 | Ultraviolet-B receptor like |
| maker-1-augustus-gene-336.42-mRNA-1 | Japonica | Indica | | Os01g0778500 | OsR498G0102095000.01.T01 | Putative serine/threonine-protein kinase yrzF |
| maker-1-augustus-gene-336.6-mRNA-1 | Japonica | NA | | Os01g0777300 | OsR498G0102091700.01.T01 | Exonuclease 1 |
| maker-1-augustus-gene-337.36-mRNA-1 | Japonica | NA | | | | Zinc-metallopeptidase, peroxisomal |
| maker-1-augustus-gene-337.38-mRNA-1 | Japonica | NA | | | | Katanin p80 WD40 repeat-containing subunit B1 homolog |
| maker-1-augustus-gene-337.44-mRNA-1 | Japonica | NA | | Os01g0780500 | OsR498G0102099700.01.T01 | Secretory carrier-associated membrane protein 2 |
| maker-1-augustus-gene-337.50-mRNA-1 | Japonica | Indica | | Os01g0779400 | OsR498G0102097400.01.T02 | SNF2 domain-containing protein / helicase domain-containing protein / zinc |
| maker-1-augustus-gene-337.55-mRNA-1 | Japonica | Indica | | Os01g0780600 | OsR498G0102100000.01.T01 | Non-phototropic hypocotyl |
| maker-1-augustus-gene-337.58-mRNA-1 | Japonica | NA | | Os01g0780800 | OsR498G0102100200.01.T01 | SAND domain-containing protein |
| maker-1-augustus-gene-338.0-mRNA-1 | Japonica | NA | | | OsR498G0102108100.01.T01 | Ferroxidase |
| maker-1-augustus-gene-338.11-mRNA-1 | Japonica | NA | | Os01g0782700 | OsR498G0102105000.01.T01 | Cyclic nucleotide-gated ion channel 4 |
| maker-1-augustus-gene-338.12-mRNA-1 | Japonica | Japonica | | Os01g0782700 | OsR498G0511120900.01.T03 | Cyclic nucleotide-gated ion channel 4 |
| maker-1-augustus-gene-338.17-mRNA-1 | Japonica | NA | | Os03g0207200 | OsR498G0305431600.01.T04 | |
| maker-1-augustus-gene-338.19-mRNA-1 | Japonica | NA | | | OsR498G0102105600.01.T01 | LysM domain-containing protein |
| maker-1-augustus-gene-338.21-mRNA-1 | Japonica | NA | | Os01g0783200 | OsR498G0102107000.01.T01 | diacylglycerol kinase |
| maker-1-augustus-gene-338.27-mRNA-1 | Japonica | NA | | | OsR498G0102107400.01.T01 | DRMBL domain-containing protein (Fragment) |
| maker-1-augustus-gene-338.30-mRNA-1 | Japonica | NA | | Os01g0783500 | OsR498G0102107800.01.T01 | Usp domain-containing protein (Fragment) |
| maker-1-augustus-gene-338.4-mRNA-1 | Japonica | NA | | Os01g0782200 | OsR498G0102103600.01.T02 | Sphingosine kinase |
| maker-1-augustus-gene-338.9-mRNA-1 | Japonica | NA | | Os01g0782500 | OsR498G0102103800.01.T03 | 1-acylglycerol-3-phosphate O-acyltransferase |
| maker-1-augustus-gene-339.39-mRNA-1 | Japonica | Japonica | Kinase-TM | Os01g0783800 | OsR498G0102108700.01.T06 | G-type lectin S-receptor-like serine/threonine-protein kinase |
| maker-1-augustus-gene-339.42-mRNA-1 | Japonica | NA | Kinase-TM | | | Serine/threonine-protein kinase |
| maker-1-augustus-gene-339.51-mRNA-1 | Japonica | Indica | | Os01g0784600 | OsR498G0102109900.01.T01 | Nodulation protein H |
| maker-1-augustus-gene-339.58-mRNA-1 | Japonica | NA | | Os01g0785400 | OsR498G0102114500.01.T01 | Indole-3-acetic acid amido synthetase |
| maker-1-augustus-gene-339.61-mRNA-1 | Japonica | NA | Kinase-TM | Os01g0784700 | OsR498G0102110200.01.T02 | Serine/threonine-protein kinase |
| maker-1-augustus-gene-339.64-mRNA-1 | Japonica | NA | | Os01g0784900 | OsR498G0102111800.01.T01 | BHLH domain-containing protein (Fragment) |
| maker-1-augustus-gene-340.0-mRNA-1 | Japonica | NA | | Os01g0787400 | OsR498G0102121700.01.T01 | Armadillo repeat-containing X-linked protein 2 (Fragment) |
| maker-1-augustus-gene-340.10-mRNA-1 | Japonica | Indica | | Os01g0786700 | OsR498G0102119200.01.T01 | Membrane protein-like |
| maker-1-augustus-gene-340.13-mRNA-1 | Japonica | NA | | Os01g0786800 | OsR498G0102119400.01.T01 | Membrane protein-like |
| maker-1-augustus-gene-340.16-mRNA-1 | Japonica | NA | | Os01g0786900 | OsR498G0102119600.01.T03 | BCAS3 domain-containing protein |
| maker-1-augustus-gene-340.21-mRNA-1 | Japonica | NA | | Os01g0787000 | OsR498G0102120400.01.T01 | Peroxidase |
| maker-1-augustus-gene-340.4-mRNA-1 | Japonica | NA | | Os01g0787600 | OsR498G0102122700.01.T01 | AB hydrolase-1 domain-containing protein (Fragment) |
| maker-1-augustus-gene-340.6-mRNA-1 | Japonica | NA | | Os01g0785900 | OsR498G0102116800.01.T01 | C2H2-type domain-containing protein (Fragment) |
| maker-1-augustus-gene-340.8-mRNA-1 | Japonica | Indica | | Os01g0786500 | OsR498G0102118600.01.T01 | Phytocyanin domain-containing protein (Fragment) |
| maker-1-augustus-gene-341.36-mRNA-1 | Japonica | NA | | Os01g0788200 | OsR498G0102124700.01.T01 | NTF2 domain-containing protein (Fragment) |
| maker-1-augustus-gene-341.38-mRNA-1 | Japonica | NA | | Os01g0788400 | OsR498G0102125500.01.T01 | Pectinesterase |
| maker-1-augustus-gene-341.40-mRNA-1 | Japonica | NA | | Os01g0788800 | OsR498G0102126500.01.T01 | Homeodomain leucine zipper family IV protein |

| | | | | | | | |
|---|---|---|---|---|---|---|---|
| maker-1-augustus-gene-341.44-mRNA-1 | Japonica | NA | Kinase | | | probable serine/threonine-protein kinase PBL23 |
| maker-1-augustus-gene-341.46-mRNA-1 | Japonica | Indica | | Os03g0115200 | OsR498G0102127000.01.T01 | KH domain-containing protein (Fragment) |
| maker-1-augustus-gene-341.48-mRNA-1 | Japonica | NA | | Os01g0789000 | OsR498G0102127200.01.T01 | F-box domain-containing protein (Fragment) |
| maker-1-augustus-gene-341.51-mRNA-1 | Japonica | NA | | Os01g0789100 | OsR498G0102127400.01.T01 | CDP-diacylglycerol--glycerol-3-phosphate 3-phosphatidyltransferase 1, chlor |
| maker-1-augustus-gene-342.12-mRNA-1 | Japonica | NA | | | | |
| maker-1-augustus-gene-343.104-mRNA-1 | Japonica | NA | | Os01g0794500 | OsR498G0102132600.01.T02 | SUI1 domain-containing protein (Fragment) |
| maker-1-augustus-gene-343.59-mRNA-1 | Japonica | NA | | Os01g0794400 | OsR498G0102132300.01.T02 | Probable nucleoredoxin 2 |
| maker-1-augustus-gene-343.64-mRNA-1 | Japonica | NA | | Os01g0795000 | OsR498G0102133700.01.T02 | Subtilisin-like protease |
| maker-1-augustus-gene-343.75-mRNA-1 | Japonica | NA | | Os01g0795100 | | Subtilisin-like serine proteinase-like protein |
| maker-1-augustus-gene-343.84-mRNA-1 | Japonica | Indica | | Os01g0795200 | OsR498G0102134900.01.T06 | Subtilisin-like protease |
| maker-1-augustus-gene-343.93-mRNA-1 | Japonica | NA | | | OsR498G0102129600.01.T02 | Inositol-1,4,5-trisphosphate 5-phosphatase 4 isoform 1 |
| maker-1-augustus-gene-344.0-mRNA-1 | Japonica | NA | | Os01g0796400 | OsR498G0102139500.01.T01 | 3-hydroxybutyryl-CoA dehydrogenase |
| maker-1-augustus-gene-344.10-mRNA-1 | Japonica | NA | | Os01g0796700 | OsR498G0102140600.01.T05 | Putative E3 ubiquitin-protein ligase RING1a |
| maker-1-augustus-gene-344.11-mRNA-1 | Japonica | NA | | Os01g0796900 | OsR498G0102140600.01.T06 | RING-type domain-containing protein |
| maker-1-augustus-gene-344.20-mRNA-1 | Japonica | NA | | Os01g0798200 | | |
| maker-1-augustus-gene-344.21-mRNA-1 | Japonica | NA | | | | |
| maker-1-augustus-gene-344.3-mRNA-1 | Japonica | NA | | Os01g0798500 | OsR498G0102143900.01.T01 | Protein CHLOROPLAST ENHANCING STRESS TOLERANCE, chloroplastic |
| maker-1-augustus-gene-344.6-mRNA-1 | Japonica | NA | | | OsR498G0102139800.01.T01 | Haloacid dehalogenase superfamily protein |
| maker-1-augustus-gene-345.35-mRNA-1 | Japonica | NA | | Os01g0799500 | OsR498G0102146300.01.T01 | probable GMP synthase [glutamine-hydrolyzing] |
| maker-1-augustus-gene-345.39-mRNA-1 | Japonica | NA | | Os01g0799900 | OsR498G0102147300.01.T01 | Metacaspase-5 |
| maker-1-augustus-gene-345.41-mRNA-1 | Japonica | Japonica | | Os01g0800266 | OsR498G0511102000.01.T04 | Phosphoglycerate kinase |
| maker-1-augustus-gene-345.43-mRNA-1 | Japonica | NA | | Os01g0800500 | OsR498G0102149100.01.T01 | Purple acid phosphatase |
| maker-1-augustus-gene-345.47-mRNA-1 | Japonica | NA | | Os01g0798800 | OsR498G0102144600.01.T01 | 1-phosphatidylinositol 4,5-bisphosphate phosphodiesterase beta-1 |
| maker-1-augustus-gene-346.0-mRNA-1 | Japonica | NA | | Os01g0801000 | OsR498G0102151700.01.T05 | DNA-(Apurinic or apyrimidinic site) lyase chloroplastic |
| maker-1-augustus-gene-346.12-mRNA-1 | Japonica | NA | | Os01g0801200 | OsR498G0102152400.01.T01 | DNA repair protein recA-like protein (Fragment) |
| maker-1-augustus-gene-346.14-mRNA-1 | Japonica | NA | | Os01g0801500 | OsR498G0102153200.01.T01 | Glucan endo-1,3-beta-glucosidase GVI |
| maker-1-augustus-gene-346.16-mRNA-1 | Japonica | NA | | Os01g0801600 | OsR498G0102153400.01.T03 | Glycerol-3-phosphate dehydrogenase |
| maker-1-augustus-gene-346.19-mRNA-1 | Japonica | NA | | Os01g0801700 | OsR498G0102153800.01.T02 | Protein GAMETE CELL DEFECTIVE 1, mitochondrial |
| maker-1-augustus-gene-346.21-mRNA-1 | Japonica | NA | | Os01g0802700 | OsR498G0102156000.01.T01 | Auxin efflux carrier component |
| maker-1-augustus-gene-346.5-mRNA-1 | Japonica | NA | | Os01g0801100 | OsR498G0102152100.01.T03 | DNA-(apurinic or apyrimidinic site) endonuclease, chloroplastic |
| maker-1-augustus-gene-346.7-mRNA-1 | Japonica | NA | | Os01g0802100 | OsR498G0102154900.01.T05 | 4-(cytidine-5'-diphospho)-2-C-methyl-D-erythritol kinase |
| maker-1-augustus-gene-347.50-mRNA-1 | Japonica | NA | | Os01g0803200 | OsR498G0102157700.01.T05 | Cysteine proteinase inhibitor |
| maker-1-augustus-gene-347.52-mRNA-1 | Japonica | Indica | | Os01g0803300 | OsR498G0102159600.01.T01 | Auxin-induced protein 5NG4 (Fragment) |
| maker-1-augustus-gene-348.0-mRNA-1 | Japonica | NA | | Os01g0805300 | OsR498G0102166300.01.T01 | PsbP domain-containing protein 6 chloroplastic |
| maker-1-augustus-gene-348.10-mRNA-1 | Japonica | Indica | | Os01g0805000 | OsR498G0102165600.01.T01 | 50S ribosomal protein L34 chloroplastic |
| maker-1-augustus-gene-348.12-mRNA-1 | Japonica | NA | | Os01g0805200 | OsR498G0102166000.01.T01 | MAR-binding filament-like protein 1-1 isoform 2 |
| maker-1-augustus-gene-348.14-mRNA-1 | Japonica | Indica | | Os01g0805600 | OsR498G0102167000.01.T01 | Cyclin IaZm |
| maker-1-augustus-gene-348.17-mRNA-1 | Japonica | NA | | Os01g0805700 | OsR498G0102167200.01.T01 | Stomatal closure-related actin-binding protein 1 |
| maker-1-augustus-gene-348.2-mRNA-1 | Japonica | NA | | Os01g0805400 | OsR498G0102166500.01.T01 | UDP-glucosyltransferase |
| maker-1-augustus-gene-348.24-mRNA-1 | Japonica | NA | | Os01g0805800 | OsR498G0102167600.01.T01 | DNA-directed RNA polymerases II 24 kDa polypeptide |
| maker-1-augustus-gene-348.4-mRNA-1 | Japonica | Japonica | | Os01g0805500 | OsR498G0102166700.01.T02 | Indole-3-acetate beta-glucosyltransferase |
| maker-1-augustus-gene-36.40-mRNA-1 | Japonica | NA | | Os01g0170600 | | Usp domain-containing protein (Fragment) |
| maker-1-augustus-gene-36.42-mRNA-1 | Japonica | NA | | Os01g0170700 | OsR498G0100237500.01.T01 | |
| maker-1-augustus-gene-37.2-mRNA-1 | Japonica | NA | | Os01g0172100 | OsR498G0100242100.01.T02 | Phosphoenolpyruvate/phosphate translocator 2 chloroplastic |
| maker-1-augustus-gene-37.33-mRNA-1 | Japonica | NA | | Os01g0172200 | OsR498G0100242400.01.T02 | DEAD-box ATP-dependent RNA helicase 14 |
| maker-1-augustus-gene-37.36-mRNA-1 | Japonica | NA | | Os01g0172300 | OsR498G0100242600.01.T02 | DUF4050 domain-containing protein (Fragment) |
| maker-1-augustus-gene-37.39-mRNA-1 | Japonica | NA | | Os01g0172400 | OsR498G0100242800.01.T02 | Phospholipase D |
| maker-1-augustus-gene-37.42-mRNA-1 | Japonica | NA | | Os01g0173000 | OsR498G0100243400.01.T05 | Thiol-disulphide oxidoreductase DCC |
| maker-1-augustus-gene-37.46-mRNA-1 | Japonica | NA | | Os01g0173100 | OsR498G0100243800.01.T01 | Alba domain-containing protein (Fragment) |
| maker-1-augustus-gene-37.8-mRNA-1 | Japonica | NA | | | | Polygalacturonase |
| maker-1-augustus-gene-373.15-mRNA-1 | Japonica | NA | | Os01g0850800 | OsR498G0102340700.01.T01 | Laccase |
| maker-1-augustus-gene-38.31-mRNA-1 | Japonica | NA | | Os01g0173600 | OsR498G0100245300.01.T01 | VOC domain-containing protein (Fragment) |
| maker-1-augustus-gene-38.35-mRNA-1 | Japonica | NA | | Os01g0173900 | OsR498G0100246500.01.T02 | ABC transporter C family member 3 |
| maker-1-augustus-gene-38.42-mRNA-1 | Japonica | NA | | Os01g0174000 | OsR498G0100246800.01.T01 | BZIP domain-containing protein (Fragment) |
| maker-1-augustus-gene-38.44-mRNA-1 | Japonica | NA | | Os01g0174300 | OsR498G0100248000.01.T01 | NADH-cytochrome b5 reductase |
| maker-1-augustus-gene-38.52-mRNA-1 | Japonica | NA | | Os01g0174600 | OsR498G0100248800.01.T01 | Zinc finger CCCH domain-containing protein 1 |
| maker-1-augustus-gene-38.54-mRNA-1 | Japonica | NA | | Os01g0175000 | OsR498G0100250000.01.T02 | Acyl-protein thioesterase 2 |
| maker-1-augustus-gene-39.0-mRNA-1 | Japonica | Indica | | Os01g0175300 | OsR498G0510826100.01.T01 | ATP-dependent DNA helicase |
| maker-1-augustus-gene-39.12-mRNA-1 | Japonica | NA | | Os01g0176700 | OsR498G0100256100.01.T02 | HTH myb-type domain-containing protein |
| maker-1-augustus-gene-39.15-mRNA-1 | Japonica | NA | | Os01g0175200 | | Ankyrin-3 |
| maker-1-augustus-gene-39.16-mRNA-1 | Japonica | NA | | Os01g0175400 | OsR498G0100251400.01.T05 | Boron transporter-like protein 2 |

| | | | | | | |
|---|---|---|---|---|---|---|
| maker-1-augustus-gene-39.3-mRNA-1 | Japonica | NA | | Os01g0176000 | OsR498G0100253300.01.T01 | Glycosyltransferase |
| maker-1-augustus-gene-39.41-mRNA-1 | Japonica | NA | | Os01g0175600 | OsR498G0100252000.01.T02 | Boron transporter |
| maker-1-augustus-gene-39.43-mRNA-1 | Japonica | Indica | | Os01g0175600 | OsR498G0100253000.01.T01 | Boron transporter |
| maker-1-augustus-gene-39.45-mRNA-1 | Japonica | NA | | Os01g0175700 | | Glycosyltransferase |
| maker-1-augustus-gene-39.5-mRNA-1 | Japonica | NA | | Os01g0176100 | OsR498G0100253700.01.T01 | Glycosyltransferase |
| maker-1-augustus-gene-39.7-mRNA-1 | Japonica | NA | | Os01g0176200 | OsR498G0100254100.01.T01 | Glycosyltransferase |
| maker-1-augustus-gene-39.9-mRNA-1 | Japonica | Indica | | Os05g0178000 | OsR498G0100254300.01.T01 | NPH3 domain-containing protein (Fragment) |
| maker-1-augustus-gene-4.0-mRNA-1 | Japonica | Indica | | Os01g0513900 | OsR498G0100018900.01.T04 | Kinesin-related protein2 |
| maker-1-augustus-gene-4.10-mRNA-1 | Japonica | NA | | | | Non-specific serine/threonine protein kinase |
| maker-1-augustus-gene-4.19-mRNA-1 | Japonica | NA | | Os01g0106800 | OsR498G0100024900.01.T01 | RING-box protein 1a |
| maker-1-augustus-gene-4.21-mRNA-1 | Japonica | NA | | Os01g0105800 | OsR498G0100021200.01.T03 | Iron-sulfur assembly protein IscA-like 1 mitochondrial |
| maker-1-augustus-gene-4.23-mRNA-1 | Japonica | NA | | Os01g0105900 | OsR498G0100021400.01.T01 | PfkB family carbohydrate kinase |
| maker-1-augustus-gene-4.28-mRNA-1 | Japonica | NA | | Os01g0106300 | OsR498G0100023200.01.T01 | NmrA domain-containing protein (Fragment) |
| maker-1-augustus-gene-4.30-mRNA-1 | Japonica | NA | | Os01g0106400 | OsR498G0100023400.01.T01 | NmrA domain-containing protein (Fragment) |
| maker-1-augustus-gene-4.32-mRNA-1 | Japonica | NA | | Os01g0106900 | OsR498G0100024800.01.T01 | 1-deoxy-D-xylulose-5-phosphate reductoisomerase |
| maker-1-augustus-gene-4.37-mRNA-1 | Japonica | NA | | Os01g0107000 | OsR498G0100025600.01.T02 | Peroxin-14 |
| maker-1-augustus-gene-4.4-mRNA-1 | Japonica | NA | | Os01g0105700 | OsR498G0100020700.01.T01 | BHLH domain-containing protein (Fragment) |
| maker-1-augustus-gene-40.26-mRNA-1 | Japonica | NA | | Os01g0177200 | OsR498G0100258300.01.T01 | Ubiquitin carboxyl-terminal hydrolase |
| maker-1-augustus-gene-40.34-mRNA-1 | Japonica | NA | | | OsR498G0100261800.01.T01 | ABC transporter |
| maker-1-augustus-gene-40.42-mRNA-1 | Japonica | NA | | Os01g0178000 | OsR498G0100262600.01.T06 | Aromatic aminotransferase ISS1 |
| maker-1-augustus-gene-41.0-mRNA-1 | Japonica | NA | | Os01g0178100 | | Tudor domain-containing protein 3 |
| maker-1-augustus-gene-41.14-mRNA-1 | Japonica | NA | | | OsR498G0100267100.01.T02 | |
| maker-1-augustus-gene-41.16-mRNA-1 | Japonica | NA | | Os01g0179200 | OsR498G0100268100.01.T01 | t-SNARE coiled-coil homology domain-containing protein |
| maker-1-augustus-gene-41.20-mRNA-1 | Japonica | NA | | Os01g0179300 | OsR498G0100268300.01.T01 | Nuclear ubiquitous casein and cyclin-dependent kinase substrate 1-like prot |
| maker-1-augustus-gene-41.22-mRNA-1 | Japonica | NA | | | | Embryo defective 2410 |
| maker-1-augustus-gene-41.29-mRNA-1 | Japonica | NA | | Os01g0179500 | OsR498G0100268900.01.T02 | Enhancer of polycomb-like transcription factor protein |
| maker-1-augustus-gene-41.31-mRNA-1 | Japonica | Indica | | Os05g0178400 | OsR498G0100264200.01.T01 | Serine/threonine protein phosphatase 2A regulatory subunit |
| maker-1-augustus-gene-41.33-mRNA-1 | Japonica | NA | | Os01g0178500 | OsR498G0100264600.01.T01 | Auxin-responsive protein (Fragment) |
| maker-1-augustus-gene-41.6-mRNA-1 | Japonica | NA | | Os01g0178200 | OsR498G0100263500.01.T05 | Tudor domain-containing protein 3 isoform X1 |
| maker-1-augustus-gene-415.40-mRNA-1 | Japonica | Japonica | | Os01g0602400 | OsR498G0101582500.01.T01 | Secologanin synthase |
| maker-1-augustus-gene-42.0-mRNA-1 | Japonica | Indica | | Os01g0179700 | OsR498G0100269800.01.T05 | GTP-binding protein YPTM2 |
| maker-1-augustus-gene-42.11-mRNA-1 | Japonica | Indica | | Os01g0179901 | OsR498G0100270400.01.T02 | |
| maker-1-augustus-gene-42.17-mRNA-1 | Japonica | NA | | | OsR498G0100271800.01.T03 | FLZ-type domain-containing protein (Fragment) |
| maker-1-augustus-gene-42.19-mRNA-1 | Japonica | NA | | | | ARF guanine-nucleotide exchange factor GNL2 |
| maker-1-augustus-gene-42.23-mRNA-1 | Japonica | NA | | | OsR498G0100274000.01.T03 | DDT domain-containing protein DDR4 |
| maker-1-augustus-gene-42.5-mRNA-1 | Japonica | NA | | Os01g0179800 | OsR498G0100270200.01.T01 | Serine incorporator |
| maker-1-augustus-gene-43.0-mRNA-1 | Japonica | NA | | Os01g0180800 | OsR498G0100274100.01.T04 | Heat shock protein 4 |
| maker-1-augustus-gene-43.10-mRNA-1 | Japonica | NA | | Os01g0181000 | OsR498G0100275400.01.T01 | |
| maker-1-augustus-gene-43.13-mRNA-1 | Japonica | NA | | | OsR498G0100276000.01.T02 | |
| maker-1-augustus-gene-43.15-mRNA-1 | Japonica | NA | | | | Ubiquitin-like domain-containing protein |
| maker-1-augustus-gene-43.4-mRNA-1 | Japonica | Japonica | | Os01g0180900 | OsR498G0100274900.01.T01 | PKHD-type hydroxylase |
| maker-1-augustus-gene-43.6-mRNA-1 | Japonica | NA | | Os01g0181200 | | |
| maker-1-augustus-gene-44.1-mRNA-1 | Japonica | NA | | Os01g0182500 | | |
| maker-1-augustus-gene-44.12-mRNA-1 | Japonica | NA | | Os01g0182200 | OsR498G0100277600.01.T02 | Aquaporin SIP |
| maker-1-augustus-gene-44.18-mRNA-1 | Japonica | NA | | Os01g0182600 | OsR498G0100279000.01.T05 | Gigantea-like protein (Fragment) |
| maker-1-augustus-gene-44.23-mRNA-1 | Japonica | NA | | | | WRKY transcription factor 21 |
| maker-1-augustus-gene-45.47-mRNA-1 | Japonica | NA | | | | Transposon protein, putative, Mutator sub-class, expressed |
| maker-1-augustus-gene-45.51-mRNA-1 | Japonica | NA | | Os01g0183100 | OsR498G0100282100.01.T01 | probable U3 small nucleolar RNA-associated protein 7 |
| maker-1-augustus-gene-45.56-mRNA-1 | Japonica | NA | | Os01g0184000 | OsR498G0100285700.01.T01 | Polynucleotide 5'-hydroxyl-kinase NOL9 |
| maker-1-augustus-gene-45.64-mRNA-1 | Japonica | NA | | Os01g0184900 | OsR498G0100286700.01.T01 | FACT complex subunit SSRP1-A |
| maker-1-augustus-gene-45.70-mRNA-1 | Japonica | NA | | Os01g0183300 | OsR498G0100282600.01.T01 | Type IV inositol polyphosphate 5-phosphatase 3 |
| maker-1-augustus-gene-45.72-mRNA-1 | Japonica | NA | | Os01g0183700 | | Histone-lysine N-methyltransferase ATX1 |
| maker-1-augustus-gene-45.76-mRNA-1 | Japonica | NA | | Os01g0184500 | OsR498G0100286200.01.T02 | DEAD-box ATP-dependent RNA helicase |
| maker-1-augustus-gene-45.81-mRNA-1 | Japonica | NA | | Os01g0184800 | OsR498G0100286600.01.T01 | Phosducin domain-containing protein (Fragment) |
| maker-1-augustus-gene-46.30-mRNA-1 | Japonica | NA | | | OsR498G0100286900.01.T01 | DNA double-strand break repair Rad50 ATPase (Fragment) |
| maker-1-augustus-gene-46.32-mRNA-1 | Japonica | NA | | Os01g0185300 | OsR498G0100287700.01.T02 | Acyl transferase 9 |
| maker-1-augustus-gene-46.34-mRNA-1 | Japonica | Indica | | Os01g0185400 | OsR498G0100287900.01.T03 | Pleiotropic regulator 1 (Fragment) |
| maker-1-augustus-gene-46.42-mRNA-1 | Japonica | NA | | Os01g0185900 | OsR498G0100288900.01.T01 | DNA-binding WRKY |
| maker-1-augustus-gene-46.50-mRNA-1 | Japonica | NA | | Os01g0186200 | OsR498G0100290000.01.T02 | Putative LOV domain-containing protein |
| maker-1-augustus-gene-470.50-mRNA-1 | Japonica | NA | | | OsR498G0102539200.01.T02 | Ceramide glucosyltransferase |
| maker-1-augustus-gene-471.0-mRNA-1 | Japonica | NA | | Os01g0907200 | OsR498G0102542100.01.T01 | BCL-2 binding anthanogene-1 |

| | | | | | | |
|---|---|---|---|---|---|---|
| maker-1-augustus-gene-471.2-mRNA-1 | Japonica | NA | | Os01g0907300 | OsR498G0102542500.01.T02 | GPI transamidase component PIG-S |
| maker-1-augustus-gene-474.44-mRNA-1 | Japonica | NA | | Os01g0912700 | OsR498G0102560100.01.T03 | Thioredoxin F-type chloroplastic |
| maker-1-augustus-gene-475.46-mRNA-1 | Japonica | NA | | | OsR498G0102560700.01.T01 | Phorbol-ester/DAG-type domain-containing protein |
| maker-1-augustus-gene-475.48-mRNA-1 | Japonica | NA | | Os01g0913000 | OsR498G0102561700.01.T01 | Thioredoxin F-type like |
| maker-1-augustus-gene-475.51-mRNA-1 | Japonica | NA | | Os01g0913300 | | Putative proton-dependent oligopeptide or low-affinity nitrate transporter ( |
| maker-1-augustus-gene-475.53-mRNA-1 | Japonica | NA | | Os01g0914400 | OsR498G0102565100.01.T01 | EPIDERMAL PATTERNING FACTOR-like protein 9 |
| maker-1-augustus-gene-475.56-mRNA-1 | Japonica | NA | | Os01g0914700 | OsR498G0102565500.01.T01 | Putative PA domain, presenilin/signal peptide peptidase |
| maker-1-augustus-gene-475.58-mRNA-1 | Japonica | NA | | Os01g0914800 | OsR498G0102565700.01.T01 | Trichome birefringence-like 18 isoform 1 |
| maker-1-augustus-gene-475.62-mRNA-1 | Japonica | NA | | | OsR498G0102565900.01.T02 | |
| maker-1-augustus-gene-475.64-mRNA-1 | Japonica | NA | | | OsR498G0102560400.01.T01 | Phorbol-ester/DAG-type domain-containing protein |
| maker-1-augustus-gene-475.69-mRNA-1 | Japonica | NA | | Os01g0913600 | OsR498G0102563000.01.T01 | RHO protein GDP dissociation inhibitor |
| maker-1-augustus-gene-475.71-mRNA-1 | Japonica | NA | | Os01g0914000 | OsR498G0102563800.01.T02 | ACT domain-containing protein ACR4 |
| maker-1-augustus-gene-476.0-mRNA-1 | Japonica | NA | | Os01g0916400 | OsR498G0102572700.01.T01 | Methanethiol oxidase |
| maker-1-augustus-gene-476.11-mRNA-1 | Japonica | NA | | | OsR498G0102568000.01.T01 | Protein O-GlcNAc transferase |
| maker-1-augustus-gene-476.17-mRNA-1 | Japonica | NA | | Os01g0915600 | OsR498G0102568600.01.T04 | Transcription factor bHLH62 |
| maker-1-augustus-gene-476.20-mRNA-1 | Japonica | NA | | Os01g0915800 | OsR498G0102571000.01.T01 | Peptidylprolyl isomerase |
| maker-1-augustus-gene-476.25-mRNA-1 | Japonica | NA | | Os01g0916200 | OsR498G0102572000.01.T01 | AP-4 complex subunit epsilon |
| maker-1-augustus-gene-476.31-mRNA-1 | Japonica | NA | | Os01g0916300 | OsR498G0102572200.01.T01 | Polyglutamine tract-binding protein 1 |
| maker-1-augustus-gene-476.37-mRNA-1 | Japonica | Japonica | | Os01g0916800 | OsR498G0102573800.01.T02 | Tho2 domain-containing protein |
| maker-1-augustus-gene-476.6-mRNA-1 | Japonica | NA | | Os01g0916600 | OsR498G0102573300.01.T05 | Putative glycine-rich RNA-binding protein 2 |
| maker-1-augustus-gene-476.9-mRNA-1 | Japonica | NA | | Os01g0916700 | OsR498G0102573500.01.T01 | SKI family transcriptional corepressor 2-like |
| maker-1-augustus-gene-477.0-mRNA-1 | Japonica | NA | | Os01g0917100 | | Jas domain-containing protein |
| maker-1-augustus-gene-477.11-mRNA-1 | Japonica | NA | | Os01g0917400 | OsR498G0102576200.01.T01 | Zinc finger CCCH domain-containing protein 33 |
| maker-1-augustus-gene-477.15-mRNA-1 | Japonica | NA | | Os01g0917700 | OsR498G0102577800.01.T02 | ROP-INTERACTIVE CRIB MOTIF-CONTAINING protein 6 |
| maker-1-augustus-gene-477.17-mRNA-1 | Japonica | NA | | Os01g0918100 | OsR498G0102580200.01.T01 | |
| maker-1-augustus-gene-477.2-mRNA-1 | Japonica | Indica | | Os01g0917200 | OsR498G0102575500.01.T01 | Peptidase S1, PA clan |
| maker-1-augustus-gene-477.20-mRNA-1 | Japonica | NA | | Os01g0918200 | OsR498G0102580600.01.T01 | Small ubiquitin-related modifier |
| maker-1-augustus-gene-477.22-mRNA-1 | Japonica | NA | | Os01g0918300 | OsR498G0102580600.01.T02 | Small ubiquitin-related modifier |
| maker-1-augustus-gene-477.25-mRNA-1 | Japonica | NA | | Os01g0918500 | OsR498G0102581400.01.T01 | |
| maker-1-augustus-gene-477.5-mRNA-1 | Japonica | NA | Kinase-LRR-TM | Os01g0917500 | OsR498G0102576500.01.T05 | Leucine-rich repeat receptor protein kinase MSP1 |
| maker-1-augustus-gene-477.6-mRNA-1 | Japonica | NA | | | OsR498G0102581100.01.T01 | |
| maker-1-augustus-gene-477.8-mRNA-1 | Japonica | NA | | | OsR498G0102575800.01.T01 | CRP7-Cysteine-rich family protein expressed |
| maker-1-augustus-gene-478.31-mRNA-1 | Japonica | NA | | | OsR498G0102582900.01.T01 | Protein DETOXIFICATION |
| maker-1-augustus-gene-478.33-mRNA-1 | Japonica | NA | | Os01g0919500 | OsR498G0102585300.01.T01 | E3 ubiquitin-protein ligase RNF4 |
| maker-1-augustus-gene-478.35-mRNA-1 | Japonica | NA | | Os01g0919600 | OsR498G0102586700.01.T02 | Lysine ketoglutarate reductase trans-splicing related 1-like |
| maker-1-augustus-gene-478.38-mRNA-1 | Japonica | NA | | Os01g0919700 | OsR498G0102586900.01.T02 | Alpha/beta hydrolase-1 |
| maker-1-augustus-gene-478.45-mRNA-1 | Japonica | NA | | Os01g0920100 | OsR498G0102589300.01.T01 | |
| maker-1-augustus-gene-478.48-mRNA-1 | Japonica | NA | | | OsR498G0102584400.01.T01 | UDP-glucose-fructose-phosphate glucosyltransferase |
| maker-1-augustus-gene-478.50-mRNA-1 | Japonica | NA | | Os01g0919800 | OsR498G0102587200.01.T02 | Auxin efflux carrier component |
| maker-1-augustus-gene-478.52-mRNA-1 | Japonica | NA | | Os01g0919900 | OsR498G0102588000.01.T01 | Acyl-(Acyl-carrier-protein) desaturase, chloroplastic |
| maker-1-augustus-gene-478.54-mRNA-1 | Japonica | Indica | | Os01g0920000 | OsR498G0102588400.01.T01 | Cystathionine beta-synthase, core |
| maker-1-augustus-gene-478.56-mRNA-1 | Japonica | Indica | | Os01g0920200 | OsR498G0102589600.01.T01 | Transcription and mRNA export factor ENY2 |
| maker-1-augustus-gene-479.36-mRNA-1 | Japonica | NA | | | OsR498G0102589900.01.T03 | MTS domain-containing protein |
| maker-1-augustus-gene-479.43-mRNA-1 | Japonica | Indica | | Os03g0849900 | OsR498G0102590500.01.T01 | Nucleotide-diphospho-sugar transferase family protein |
| maker-1-augustus-gene-479.45-mRNA-1 | Japonica | Japonica | | Os01g0920700 | OsR498G0102591100.01.T01 | Glycosyltransferase |
| maker-1-augustus-gene-479.48-mRNA-1 | Japonica | NA | | Os01g0921000 | OsR498G0102591900.01.T01 | |
| maker-1-augustus-gene-479.52-mRNA-1 | Japonica | NA | | Os01g0921200 | OsR498G0102592700.01.T01 | Mannosyl-oligosaccharide glucosidase |
| maker-1-augustus-gene-479.53-mRNA-1 | Japonica | NA | | Os01g0921300 | OsR498G0102592900.01.T01 | Exostosin domain-containing protein (Fragment) |
| maker-1-augustus-gene-479.70-mRNA-1 | Japonica | NA | | Os01g0921600 | OsR498G0102593500.01.T01 | Mitochondrial import receptor subunit like |
| maker-1-augustus-gene-479.76-mRNA-1 | Japonica | NA | | Os01g0920400 | OsR498G0102590200.01.T01 | Dynamin-related protein 3A isoform 2 |
| maker-1-augustus-gene-479.81-mRNA-1 | Japonica | NA | | Os01g0921500 | OsR498G0102593400.01.T01 | AMP-activated protein kinase, gamma regulatory subunit |
| maker-1-augustus-gene-479.83-mRNA-1 | Japonica | NA | | Os01g0921800 | OsR498G0102595400.01.T01 | MYND-type domain-containing protein |
| maker-1-augustus-gene-480.0-mRNA-1 | Japonica | Indica | | Os01g0922600 | OsR498G0102601900.01.T01 | SBP-box family member protein (Fragment) |
| maker-1-augustus-gene-480.2-mRNA-1 | Japonica | NA | | | OsR498G0102603900.01.T02 | MADS-box transcription factor |
| maker-1-augustus-gene-480.8-mRNA-1 | Japonica | NA | | | OsR498G0102604200.01.T01 | |
| maker-1-augustus-gene-481.22-mRNA-1 | Japonica | NA | | | OsR498G0102606300.01.T02 | PB1 domain-containing protein/CBS domain-containing protein |
| maker-1-augustus-gene-481.28-mRNA-1 | Japonica | NA | | Os01g0923600 | OsR498G0102607100.01.T06 | Calmodulin-binding transcription activator 4 |
| maker-1-augustus-gene-481.33-mRNA-1 | Japonica | NA | | Os01g0924000 | OsR498G0102611700.01.T01 | Mitochondrial ribosomal protein MRP7 (L2) |
| maker-1-augustus-gene-481.35-mRNA-1 | Japonica | NA | | | OsR498G0102607600.01.T04 | Histidine kinase |
| maker-1-augustus-gene-481.41-mRNA-1 | Japonica | NA | | Os01g0924300 | OsR498G0102612600.01.T02 | Putative WD repeat-containing protein C17D11.16 |
| maker-1-augustus-gene-482.27-mRNA-1 | Japonica | NA | | Os01g0924700 | OsR498G0102614300.01.T01 | Protein kinase domain-containing protein |

| | | | | | | |
|---|---|---|---|---|---|---|
| maker-1-augustus-gene-482.35-mRNA-1 | Japonica | NA | | Os01g0925200 | OsR498G0102617500.01.T01 | Delta(3,5)-Delta(2,4)-dienoyl-CoA isomerase, mitochondrial |
| maker-1-augustus-gene-482.37-mRNA-1 | Japonica | NA | | | OsR498G0102614000.01.T03 | PERQ amino acid-rich with GYF domain-containing protein 2 |
| maker-1-augustus-gene-482.42-mRNA-1 | Japonica | Japonica | | Os01g0924800 | OsR498G0102614600.01.T01 | |
| maker-1-augustus-gene-482.45-mRNA-1 | Japonica | NA | | Os01g0925000 | OsR498G0102617200.01.T02 | CID domain-containing protein |
| maker-1-augustus-gene-482.51-mRNA-1 | Japonica | NA | CC-NBS-TM | | OsR498G0102617400.01.T01 | Disease resistance protein RPP13 |
| maker-1-augustus-gene-482.53-mRNA-1 | Japonica | NA | | Os01g0925300 | OsR498G0102617800.01.T01 | S-acyltransferase |
| maker-1-augustus-gene-483.34-mRNA-1 | Japonica | Indica | | Os01g0925600 | OsR498G0102618900.01.T01 | |
| maker-1-augustus-gene-483.37-mRNA-1 | Japonica | NA | | | OsR498G0102620300.01.T01 | Tify domain-containing protein (Fragment) |
| maker-1-augustus-gene-483.39-mRNA-1 | Japonica | NA | | Os01g0926200 | OsR498G0102620500.01.T03 | E3 ubiquitin-protein ligase |
| maker-1-augustus-gene-483.41-mRNA-1 | Japonica | NA | | Os01g0926800 | OsR498G0102622700.01.T01 | CRAL-TRIO domain-containing protein (Fragment) |
| maker-1-augustus-gene-483.44-mRNA-1 | Japonica | NA | | Os01g0927000 | OsR498G0102623300.01.T01 | Histone-lysine N-methyltransferase H3 lysine-9 specific SUVH4 |
| maker-1-augustus-gene-483.49-mRNA-1 | Japonica | Japonica | | Os01g0927200 | OsR498G0102623900.01.T01 | Trihelix transcription factor |
| maker-1-augustus-gene-483.51-mRNA-1 | Japonica | Indica | Kinase-TM | Os03g0349200 | OsR498G0102619200.01.T01 | probable serine/threonine-protein kinase At1g54610 |
| maker-1-augustus-gene-483.55-mRNA-1 | Japonica | NA | | Os01g0925800 | OsR498G0102620000.01.T01 | E2 ubiquitin-conjugating enzyme |
| maker-1-augustus-gene-483.58-mRNA-1 | Japonica | NA | | Os01g0926300 | OsR498G0102620800.01.T01 | Transaldolase |
| maker-1-augustus-gene-483.60-mRNA-1 | Japonica | NA | | Os01g0926400 | OsR498G0102621000.01.T01 | Exostosin domain-containing protein |
| maker-1-augustus-gene-483.62-mRNA-1 | Japonica | NA | | Os01g0926600 | OsR498G0102622000.01.T02 | Secondary cell wall glycosyltransferase family 47 |
| maker-1-augustus-gene-483.65-mRNA-1 | Japonica | Indica | | Os01g0926600 | OsR498G0102622400.01.T02 | Beta-1,4-xylosyltransferase IRX10L |
| maker-1-augustus-gene-484.0-mRNA-1 | Japonica | NA | | Os01g0927600 | OsR498G0102626700.01.T03 | Auxin response factor |
| maker-1-augustus-gene-484.11-mRNA-1 | Japonica | NA | | Os01g0927300 | OsR498G0102624400.01.T03 | HMA domain-containing protein |
| maker-1-augustus-gene-484.14-mRNA-1 | Japonica | NA | | Os01g0927900 | OsR498G0102627600.01.T02 | Aspartate kinase |
| maker-1-augustus-gene-484.19-mRNA-1 | Japonica | NA | | Os01g0928000 | OsR498G0102627800.01.T02 | Inducer of CBF expression |
| maker-1-augustus-gene-484.22-mRNA-1 | Japonica | NA | | Os01g0928100 | OsR498G0102629600.01.T01 | Coiled-coil domain-containing protein SCD2 |
| maker-1-augustus-gene-484.28-mRNA-1 | Japonica | Japonica | | Os01g0928400 | OsR498G0102630800.01.T01 | Serine C-palmitoyltransferase |
| maker-1-augustus-gene-484.30-mRNA-1 | Japonica | Indica | | Os01g0928700 | OsR498G0102631800.01.T01 | Serine C-palmitoyltransferase |
| maker-1-augustus-gene-484.32-mRNA-1 | Japonica | NA | | Os01g0928700 | OsR498G0102632000.01.T01 | Serine C-palmitoyltransferase |
| maker-1-augustus-gene-484.4-mRNA-1 | Japonica | NA | | | OsR498G0102630300.01.T02 | RNA polymerase II subunit 5-mediating protein homolog |
| maker-1-augustus-gene-485.0-mRNA-1 | Japonica | NA | | Os01g0929000 | OsR498G0102632900.01.T04 | Aminopeptidase |
| maker-1-augustus-gene-485.11-mRNA-1 | Japonica | NA | | Os01g0930400 | OsR498G0102636900.01.T01 | Potassium transporter |
| maker-1-augustus-gene-485.13-mRNA-1 | Japonica | NA | | Os01g0930800 | OsR498G0102638700.01.T06 | Non-cyanogenic beta-glucosidase |
| maker-1-augustus-gene-485.16-mRNA-1 | Japonica | NA | | Os01g0930900 | OsR498G0102638900.01.T01 | Tropinone reductase |
| maker-1-augustus-gene-485.18-mRNA-1 | Japonica | NA | | Os01g0931000 | OsR498G0102639100.01.T01 | Tropinone reductase |
| maker-1-augustus-gene-485.20-mRNA-1 | Japonica | NA | | Os01g0928800 | OsR498G0102632600.01.T01 | Serine C-palmitoyltransferase |
| maker-1-augustus-gene-485.26-mRNA-1 | Japonica | NA | Kinase-TM | | OsR498G0102633200.01.T02 | proline-rich receptor-like protein kinase PERK3 isoform X2 |
| maker-1-augustus-gene-485.29-mRNA-1 | Japonica | NA | | Os01g0931100 | OsR498G0102639400.01.T05 | Heparan-alpha-glucosaminide N-acetyltransferase isoform A |
| maker-1-augustus-gene-485.35-mRNA-1 | Japonica | NA | | Os01g0931200 | OsR498G0102639800.01.T01 | TOG array regulator of axonemal microtubules protein 1 |
| maker-1-augustus-gene-485.37-mRNA-1 | Japonica | NA | | Os01g0931300 | OsR498G0102640000.01.T01 | PlsC domain-containing protein |
| maker-1-augustus-gene-485.4-mRNA-1 | Japonica | NA | | Os01g0929100 | | Photosynthetic NDH subunit of subcomplex B 5 chloroplastic |
| maker-1-augustus-gene-485.41-mRNA-1 | Japonica | NA | | Os01g0931400 | OsR498G0102640200.01.T04 | Thiamine pyrophosphokinase |
| maker-1-augustus-gene-485.6-mRNA-1 | Japonica | NA | | Os01g0930000 | OsR498G0102635300.01.T03 | protein disulfide-isomerase LQY1, chloroplastic isoform X2 |
| maker-1-augustus-gene-485.9-mRNA-1 | Japonica | NA | | Os01g0930200 | OsR498G0102635500.01.T01 | |
| maker-1-augustus-gene-486.0-mRNA-1 | Japonica | NA | | | OsR498G0102641100.01.T01 | LURP1-related protein domain containing protein |
| maker-1-augustus-gene-486.11-mRNA-1 | Japonica | Indica | | Os01g0932825 | OsR498G0102645300.01.T01 | Knot1 domain-containing protein (Fragment) |
| maker-1-augustus-gene-486.13-mRNA-1 | Japonica | NA | | | OsR498G0102640800.01.T01 | |
| maker-1-augustus-gene-486.15-mRNA-1 | Japonica | NA | | Os01g0932500 | OsR498G0102644600.01.T01 | Potassium transporter |
| maker-1-augustus-gene-486.17-mRNA-1 | Japonica | NA | | Os01g0933200 | OsR498G0102646200.01.T02 | HMA domain-containing protein |
| maker-1-augustus-gene-486.2-mRNA-1 | Japonica | NA | | | | |
| maker-1-augustus-gene-486.20-mRNA-1 | Japonica | NA | | Os01g0933600 | OsR498G0102647600.01.T01 | NPH3 domain-containing protein |
| maker-1-augustus-gene-486.23-mRNA-1 | Japonica | NA | | Os01g0933900 | OsR498G0102648200.01.T01 | Glutathione transferase |
| maker-1-augustus-gene-486.25-mRNA-1 | Japonica | NA | | Os01g0934000 | OsR498G0102648800.01.T01 | Autophagy-related protein 18a |
| maker-1-augustus-gene-486.6-mRNA-1 | Japonica | NA | | Os01g0932600 | OsR498G0102645100.01.T02 | BTB domain-containing protein |
| maker-1-augustus-gene-487.0-mRNA-1 | Japonica | NA | | Os01g0934200 | OsR498G0102649300.01.T07 | Mitochondrial thiamine pyrophosphate carrier like |
| maker-1-augustus-gene-487.12-mRNA-1 | Japonica | Indica | | Os01g0934400 | OsR498G0102649700.01.T01 | PsbP domain-containing protein 7 chloroplastic |
| maker-1-augustus-gene-487.15-mRNA-1 | Japonica | NA | | Os01g0935300 | OsR498G0102653900.01.T06 | Cullin domain-containing protein |
| maker-1-augustus-gene-487.19-mRNA-1 | Japonica | NA | | Os01g0935500 | OsR498G0102654700.01.T01 | Potassium transporter |
| maker-1-augustus-gene-487.22-mRNA-1 | Japonica | Japonica | | Os01g0934600 | OsR498G0102650600.01.T01 | Methyl jasmonate esterase |
| maker-1-augustus-gene-487.25-mRNA-1 | Japonica | NA | PIR7B | Os01g0934800 | OsR498G0102650800.01.T01 | AB hydrolase-1 domain-containing protein |
| maker-1-augustus-gene-487.26-mRNA-1 | Japonica | NA | PIR7A | Os01g0934900 | OsR498G0102650800.01.T03 | AB hydrolase-1 domain-containing protein (Fragment) |
| maker-1-augustus-gene-487.35-mRNA-1 | Japonica | NA | | Os01g0935000 | | Protein indeterminate-domain 12 |
| maker-1-augustus-gene-487.37-mRNA-1 | Japonica | NA | | Os01g0935400 | OsR498G0102654200.01.T01 | Oxoglutarate/iron-dependent dioxygenase |
| maker-1-augustus-gene-487.8-mRNA-1 | Japonica | NA | | Os01g0934300 | OsR498G0102649500.01.T04 | Homeobox protein LUMINIDEPENDENS |

| | | | | | | |
|---|---|---|---|---|---|---|
| maker-1-augustus-gene-488.36-mRNA-1 | Japonica | NA | | Os01g0935700 | OsR498G0102655100.01.T05 | Cytochrome c1 1 heme protein mitochondrial |
| maker-1-augustus-gene-488.40-mRNA-1 | Japonica | NA | Kinase | Os01g0936100 | OsR498G0102657500.01.T01 | Serine/threonine-protein kinase PBL27 |
| maker-1-augustus-gene-488.44-mRNA-1 | Japonica | NA | | Os01g0936500 | | |
| maker-1-augustus-gene-488.46-mRNA-1 | Japonica | NA | | | OsR498G0102655000.01.T04 | spermatogenesis-associated protein 20-like |
| maker-1-augustus-gene-488.53-mRNA-1 | Japonica | Indica | Kinase-TM | Os01g0935800 | OsR498G0102655400.01.T01 | Receptor-like protein kinase ANXUR1 |
| maker-1-augustus-gene-488.56-mRNA-1 | Japonica | NA | | Os01g0935900 | OsR498G0102655800.01.T01 | Succinate dehydrogenase subunit 4 mitochondrial |
| maker-1-augustus-gene-488.58-mRNA-1 | Japonica | Indica | | Os01g0936200 | OsR498G0102657800.01.T02 | probable feruloyl esterase A |
| maker-1-augustus-gene-488.63-mRNA-1 | Japonica | NA | | Os01g0936700 | OsR498G0102658400.01.T01 | |
| maker-1-augustus-gene-488.65-mRNA-1 | Japonica | NA | | Os01g0936800 | OsR498G0102658800.01.T02 | MSP domain-containing protein (Fragment) |
| maker-1-augustus-gene-488.70-mRNA-1 | Japonica | NA | CC-NBS-TM | Os01g0937400 | OsR498G0102663200.01.T06 | Disease resistance protein RGA2 |
| maker-1-augustus-gene-489.37-mRNA-1 | Japonica | Japonica | | Os01g0937500 | OsR498G0102664300.01.T01 | Putative xylanase inhibitor |
| maker-1-augustus-gene-489.39-mRNA-1 | Japonica | NA | | Os01g0938200 | OsR498G0102666300.01.T01 | RNA-binding protein BRN1 |
| maker-1-augustus-gene-489.44-mRNA-1 | Japonica | NA | | Os01g0938900 | OsR498G0102667300.01.T02 | NAC-A/B domain-containing protein |
| maker-1-augustus-gene-489.48-mRNA-1 | Japonica | NA | | Os01g0939100 | OsR498G0102667700.01.T01 | Calcium-transporting ATPase |
| maker-1-augustus-gene-489.53-mRNA-1 | Japonica | NA | | Os01g0939600 | OsR498G0102668700.01.T03 | Glycerol-3-phosphate dehydrogenase [NAD(+)] |
| maker-1-augustus-gene-489.56-mRNA-1 | Japonica | NA | | Os01g0938600 | OsR498G0102666800.01.T01 | Tyrosine-protein phosphatase non-receptor type 23-like |
| maker-1-augustus-gene-489.58-mRNA-1 | Japonica | Indica | | Os01g0939200 | OsR498G0102668000.01.T02 | F-box domain containing protein, expressed |
| maker-1-augustus-gene-489.60-mRNA-1 | Japonica | NA | | Os01g0939300 | OsR498G0102668200.01.T01 | BRCT domain-containing DNA repair protein |
| maker-1-augustus-gene-489.62-mRNA-1 | Japonica | NA | | Os01g0939400 | OsR498G0102668400.01.T01 | Vegetative cell wall protein gp1 |
| maker-1-augustus-gene-490.13-mRNA-1 | Japonica | Japonica | | Os01g0939700 | OsR498G0102668910.01.T07 | S-formylglutathione hydrolase |
| maker-1-augustus-gene-490.20-mRNA-1 | Japonica | NA | | Os01g0940000 | OsR498G0102671700.01.T03 | Cytokinin dehydrogenase |
| maker-1-augustus-gene-491.0-mRNA-1 | Japonica | NA | | Os01g0940600 | OsR498G0102673700.01.T01 | High chlorophyll fluorescence 153 |
| maker-1-augustus-gene-491.1-mRNA-1 | Japonica | Indica | | Os01g0941400 | OsR498G0102674100.01.T01 | Truncated pathogenesis-related protein 6 |
| maker-1-augustus-gene-491.12-mRNA-1 | Japonica | NA | | Os01g0941400 | OsR498G0102675700.01.T01 | Truncated pathogenesis-related protein 6 |
| maker-1-augustus-gene-492.10-mRNA-1 | Japonica | NA | | Os01g0944000 | | |
| maker-1-augustus-gene-492.6-mRNA-1 | Japonica | Indica | | Os05g0375400 | OsR498G0102679000.01.T01 | Licheninase |
| maker-1-augustus-gene-493.101-mRNA-1 | Japonica | Indica | | Os01g0945766 | OsR498G0102687600.01.T01 | Amino-acid permease BAT1 like |
| maker-1-augustus-gene-493.102-mRNA-1 | Japonica | NA | | Os01g0945766 | OsR498G0102688000.01.T03 | Putative GABA-specific permease |
| maker-1-augustus-gene-493.115-mRNA-1 | Japonica | NA | | Os01g0945800 | OsR498G0102688200.01.T02 | RNA-binding protein Musashi homolog 1-like isoform X2 |
| maker-1-augustus-gene-493.54-mRNA-1 | Japonica | Japonica | | Os01g0944200 | OsR498G0102683900.01.T04 | RNase H domain-containing protein |
| maker-1-augustus-gene-493.58-mRNA-1 | Japonica | NA | | Os01g0946300 | OsR498G0102688700.01.T01 | Lecithin-cholesterol acyltransferase-like 1 |
| maker-1-augustus-gene-493.60-mRNA-1 | Japonica | NA | | Os01g0944700 | OsR498G0102685400.01.T01 | Endo-1,3-beta-glucanase |
| maker-1-augustus-gene-493.61-mRNA-1 | Japonica | NA | | Os01g0944800 | | Putative beta 1,3-glucanase |
| maker-1-augustus-gene-493.66-mRNA-1 | Japonica | NA | | | OsR498G0102686200.01.T02 | DNA repair protein recA, mitochondrial |
| maker-1-augustus-gene-493.70-mRNA-1 | Japonica | Indica | | Os01g0945200 | OsR498G0102686800.01.T01 | Putative GABA-specific permease |
| maker-1-augustus-gene-493.74-mRNA-1 | Japonica | Japonica | | Os01g0945200 | OsR498G0102687000.01.T04 | Putative GABA-specific permease |
| maker-1-augustus-gene-493.75-mRNA-1 | Japonica | NA | | Os01g0945300 | OsR498G0102687000.01.T06 | Putative GABA-specific permease |
| maker-1-augustus-gene-493.76-mRNA-1 | Japonica | NA | | Os01g0945300 | OsR498G0102687000.01.T08 | Amino-acid permease BAT1 |
| maker-1-augustus-gene-494.35-mRNA-1 | Japonica | NA | | | | Glucan endo-1,3-beta-glucosidase GII |
| maker-1-augustus-gene-494.38-mRNA-1 | Japonica | NA | | Os01g0947000 | OsR498G0102690700.01.T01 | Glucan endo-1,3-beta-glucosidase GIII |
| maker-1-augustus-gene-494.40-mRNA-1 | Japonica | NA | | Os01g0948100 | OsR498G0102690900.01.T02 | ERCC4 domain-containing protein (Fragment) |
| maker-1-augustus-gene-494.46-mRNA-1 | Japonica | NA | | Os01g0948300 | OsR498G0102693300.01.T01 | Cellular retinaldehyde-binding/triple function, C-terminal |
| maker-1-augustus-gene-494.50-mRNA-1 | Japonica | NA | | Os01g0948400 | OsR498G0102693800.01.T02 | Pyrroline-5-carboxylate reductase |
| maker-1-augustus-gene-495.4-mRNA-1 | Japonica | Indica | | Os01g0948900 | OsR498G0102696200.01.T01 | BTB/POZ domain and ankyrin repeat-containing protein NPR5 |
| maker-1-augustus-gene-495.8-mRNA-1 | Japonica | NA | | | | Protein OSB3, chloroplastic/mitochondrial |
| maker-1-augustus-gene-496.0-mRNA-1 | Japonica | NA | | Os01g0950000 | OsR498G0102702700.01.T01 | Glutathione S-transferase GST 29 |
| maker-1-augustus-gene-496.11-mRNA-1 | Japonica | NA | | Os01g0951100 | OsR498G0102704700.01.T01 | probable ADP-ribosylation factor GTPase-activating protein AGD11 |
| maker-1-augustus-gene-496.25-mRNA-1 | Japonica | NA | | Os01g0952000 | OsR498G0102706900.01.T02 | Germin-like protein 1-3 |
| maker-1-augustus-gene-496.27-mRNA-1 | Japonica | NA | | Os01g0952100 | OsR498G0102707100.01.T01 | Germin-like protein 1-4 |
| maker-1-augustus-gene-496.29-mRNA-1 | Japonica | NA | | Os01g0952200 | OsR498G0102707300.01.T01 | Helicase C-terminal domain-containing protein |
| maker-1-augustus-gene-496.41-mRNA-1 | Japonica | NA | | | | Flowering time control protein FY |
| maker-1-augustus-gene-496.47-mRNA-1 | Japonica | NA | | Os01g0951800 | OsR498G0102706400.01.T01 | Mitochondrial fission 1 protein A (Fragment) |
| maker-1-augustus-gene-496.49-mRNA-1 | Japonica | NA | | Os01g0952300 | OsR498G0102707600.01.T01 | Regulator of chromosome condensation (RCC1) family with FYVE zinc finger |
| maker-1-augustus-gene-497.55-mRNA-1 | Japonica | Japonica | LRR-NBS-TM | Os01g0953400 | OsR498G0102712500.01.T01 | Leucine Rich Repeat family protein, expressed |
| maker-1-augustus-gene-497.56-mRNA-1 | Japonica | NA | | Os01g0954000 | OsR498G0102714500.01.T01 | NAD(P)H dehydrogenase (quinone) |
| maker-1-augustus-gene-497.58-mRNA-1 | Japonica | NA | | | OsR498G0102716500.01.T01 | E3 ubiquitin-protein ligase RHF2A isoform X1 |
| maker-1-augustus-gene-497.62-mRNA-1 | Japonica | NA | | Os01g0952800 | OsR498G0102710200.01.T03 | Transcription factor ORG2 |
| maker-1-augustus-gene-498.28-mRNA-1 | Japonica | NA | | | OsR498G0102722700.01.T01 | |
| maker-1-augustus-gene-498.31-mRNA-1 | Japonica | NA | | | OsR498G0102723100.01.T03 | Chloroquine-resistance transporter-like |
| maker-1-augustus-gene-498.36-mRNA-1 | Japonica | NA | | Os01g0954500 | OsR498G0102717800.01.T04 | Zinc finger, lateral root primordium type 1 |
| maker-1-augustus-gene-498.39-mRNA-1 | Japonica | NA | | Os01g0955000 | OsR498G0102720600.01.T01 | Non-specific phospholipase |

| | | | | | | |
|---|---|---|---|---|---|---|
| maker-1-augustus-gene-499.0-mRNA-1 | Japonica | Indica | | Os01g0956500 | OsR498G0102727900.01.T02 | |
| maker-1-augustus-gene-499.14-mRNA-1 | Japonica | NA | Kinase-LRR-TM | Os01g0957100 | OsR498G0102731100.01.T01 | Inactive leucine-rich repeat receptor-like protein kinase-like |
| maker-1-augustus-gene-499.16-mRNA-1 | Japonica | NA | | Os01g0957200 | OsR498G0102731500.01.T01 | Protein RETICULATA-RELATED 4, chloroplastic isoform A |
| maker-1-augustus-gene-499.19-mRNA-1 | Japonica | NA | | | OsR498G0102733500.01.T01 | Germacrene A oxidase |
| maker-1-augustus-gene-499.21-mRNA-1 | Japonica | NA | | Os01g0956700 | OsR498G0102729800.01.T01 | |
| maker-1-augustus-gene-499.6-mRNA-1 | Japonica | NA | | Os01g0956600 | OsR498G0102728900.01.T01 | RRM domain-containing protein |
| maker-1-augustus-gene-499.9-mRNA-1 | Japonica | NA | | | OsR498G0102730700.01.T03 | NAD kinase 1 |
| maker-1-augustus-gene-5.29-mRNA-1 | Japonica | NA | | Os01g0107700 | OsR498G0100027300.01.T01 | Exostosin domain-containing protein |
| maker-1-augustus-gene-5.32-mRNA-1 | Japonica | Japonica | | Os01g0108000 | OsR498G0100027900.01.T01 | |
| maker-1-augustus-gene-5.36-mRNA-1 | Japonica | NA | | Os01g0108200 | OsR498G0100028100.01.T01 | Serine-type endopeptidase |
| maker-1-augustus-gene-5.40-mRNA-1 | Japonica | Japonica | | Os01g0108400 | OsR498G0100028700.01.T02 | BHLH domain-containing protein (Fragment) |
| maker-1-augustus-gene-5.43-mRNA-1 | Japonica | Indica | | Os01g0108600 | OsR498G0100029700.01.T01 | BHLH transcription factor |
| maker-1-augustus-gene-5.45-mRNA-1 | Japonica | NA | | | OsR498G0100030100.01.T02 | |
| maker-1-augustus-gene-5.48-mRNA-1 | Japonica | NA | Kinase-TM | | OsR498G0100026400.01.T01 | Serine/threonine protein kinase |
| maker-1-augustus-gene-5.52-mRNA-1 | Japonica | NA | | | OsR498G0100027600.01.T03 | Executer 1 |
| maker-1-augustus-gene-500.1-mRNA-1 | Japonica | NA | | | OsR498G0102733900.01.T03 | NUC173 domain-containing protein |
| maker-1-augustus-gene-500.16-mRNA-1 | Japonica | NA | | Os01g0958500 | OsR498G0102735500.01.T01 | RNA-binding family protein isoform 1 |
| maker-1-augustus-gene-500.20-mRNA-1 | Japonica | NA | | Os01g0958700 | OsR498G0102736100.01.T01 | MACPF domain-containing protein isoform X1 |
| maker-1-augustus-gene-500.25-mRNA-1 | Japonica | NA | | | | |
| maker-1-augustus-gene-500.26-mRNA-1 | Japonica | NA | | Os01g0958900 | OsR498G0102736500.01.T03 | DNA mismatch repair protein MLH1 |
| maker-1-augustus-gene-500.39-mRNA-1 | Japonica | NA | | Os01g0959000 | OsR498G0102736700.01.T03 | Serine/arginine-rich splicing factor 33 |
| maker-1-augustus-gene-500.42-mRNA-1 | Japonica | NA | | Os01g0959200 | OsR498G0102737100.01.T01 | Abscisic stress-ripening protein 1 |
| maker-1-augustus-gene-500.46-mRNA-1 | Japonica | NA | | Os01g0959900 | OsR498G0102739700.01.T01 | NADH dehydrogenase-like complex O |
| maker-1-augustus-gene-500.60-mRNA-1 | Japonica | Indica | | Os01g0958800 | OsR498G0102736400.01.T02 | Lipid droplet-associated hydrolase |
| maker-1-augustus-gene-500.66-mRNA-1 | Japonica | NA | | Os01g0959100 | OsR498G0102737000.01.T01 | Abscisic stress-ripening protein 1 |
| maker-1-augustus-gene-500.68-mRNA-1 | Japonica | NA | | | OsR498G0102739200.01.T02 | Phosphatidylserine decarboxylase |
| maker-1-augustus-gene-501.0-mRNA-1 | Japonica | NA | | Os01g0961200 | OsR498G0102743100.01.T01 | CBS-PB1 domain contining protein |
| maker-1-augustus-gene-501.16-mRNA-1 | Japonica | NA | | Os01g0962300 | OsR498G0102745100.01.T01 | V-type proton ATPase proteolipid subunit |
| maker-1-augustus-gene-501.18-mRNA-1 | Japonica | NA | | Os01g0960300 | OsR498G0102740600.01.T01 | Glucose-inhibited division family A protein isoform 1 |
| maker-1-augustus-gene-501.23-mRNA-1 | Japonica | NA | Kinase-LRR-TM | Os01g0960400 | OsR498G0102740800.01.T02 | Putative leucine-rich repeat receptor-like serine/threonine-protein kinase |
| maker-1-augustus-gene-501.38-mRNA-1 | Japonica | NA | | Os01g0960500 | OsR498G0102742000.01.T01 | E3 ubiquitin-protein ligase RGLG3 |
| maker-1-augustus-gene-501.43-mRNA-1 | Japonica | Indica | | Os01g0956500 | OsR498G0102742200.01.T02 | Protein kinase domain-containing protein |
| maker-1-augustus-gene-501.46-mRNA-1 | Japonica | NA | | Os01g0960800 | OsR498G0102742400.01.T01 | Mitochondrial import inner membrane translocase |
| maker-1-augustus-gene-501.48-mRNA-1 | Japonica | NA | | | OsR498G0102742800.01.T01 | Nitrate excretion transporter 1 |
| maker-1-augustus-gene-501.55-mRNA-1 | Japonica | Indica | | Os01g0962200 | OsR498G0102744800.01.T01 | Senescence associated proteingene 18 isoform 1 |
| maker-1-augustus-gene-501.58-mRNA-1 | Japonica | NA | | Os01g0962400 | OsR498G0102745400.01.T01 | Ubiquitin-fold modifier 1 |
| maker-1-augustus-gene-501.6-mRNA-1 | Japonica | NA | | Os01g0961300 | OsR498G0102743300.01.T03 | |
| maker-1-augustus-gene-501.61-mRNA-1 | Japonica | NA | | Os01g0962500 | OsR498G0102745600.01.T01 | HIT-type domain-containing protein |
| maker-1-augustus-gene-501.64-mRNA-1 | Japonica | NA | | Os01g0962600 | OsR498G0102745800.01.T01 | Small subunit ribosomal protein S10e |
| maker-1-augustus-gene-502.0-mRNA-1 | Japonica | NA | | Os01g0962700 | OsR498G0102746100.01.T01 | Peroxidase |
| maker-1-augustus-gene-502.10-mRNA-1 | Japonica | NA | | | OsR498G0102748900.01.T01 | |
| maker-1-augustus-gene-502.12-mRNA-1 | Japonica | NA | | Os01g0963600 | OsR498G0102749300.01.T01 | Abscisic stress-ripening protein 2 |
| maker-1-augustus-gene-502.14-mRNA-1 | Japonica | NA | | | OsR498G0102749900.01.T02 | Bile salt-activated lipase-like |
| maker-1-augustus-gene-502.16-mRNA-1 | Japonica | NA | | Os01g0963300 | OsR498G0102748600.01.T01 | t-SNARE coiled-coil homology domain-containing protein (Fragment) |
| maker-1-augustus-gene-502.2-mRNA-1 | Japonica | NA | | Os01g0962900 | OsR498G0102746700.01.T01 | Peroxidase |
| maker-1-augustus-gene-502.21-mRNA-1 | Japonica | NA | | Os01g0964000 | OsR498G0102750400.01.T01 | Synaptobrevin-like protein YKT6 |
| maker-1-augustus-gene-502.24-mRNA-1 | Japonica | Indica | | Os05g0106600 | OsR498G0102750600.01.T01 | Beta-actin |
| maker-1-augustus-gene-502.4-mRNA-1 | Japonica | Indica | | Os01g0963200 | OsR498G0102748300.01.T01 | Peroxidase |
| maker-1-augustus-gene-502.6-mRNA-1 | Japonica | Indica | | Os01g0963400 | OsR498G0102748700.01.T02 | Thioredoxin Y, chloroplastic |
| maker-1-augustus-gene-503.14-mRNA-1 | Japonica | NA | | | OsR498G0102756700.01.T05 | ABC transporter D family member 1 |
| maker-1-augustus-gene-503.3-mRNA-1 | Japonica | NA | Kinase-TM | | OsR498G0102753900.01.T01 | Putative serine-threonine/tyrosine-protein kinase catalytic domain-containi |
| maker-1-augustus-gene-503.31-mRNA-1 | Japonica | NA | | | | DNA-directed RNA polymerase subunit |
| maker-1-augustus-gene-503.34-mRNA-1 | Japonica | NA | | Os01g0965600 | OsR498G0102755000.01.T01 | Coiled-coil domain-containing protein R3HCC1L |
| maker-1-augustus-gene-503.37-mRNA-1 | Japonica | NA | | Os01g0965900 | OsR498G0102755800.01.T01 | Transcription initiation factor TFIID subunit like |
| maker-1-augustus-gene-503.45-mRNA-1 | Japonica | NA | LRR-TM | Os01g0966400 | OsR498G0102757400.01.T01 | Protein phosphatase 1 regulatory subunit like |
| maker-1-augustus-gene-503.5-mRNA-1 | Japonica | NA | | Os01g0965400 | OsR498G0102754100.01.T01 | Uridine monophosphate kinase |
| maker-1-augustus-gene-503.9-mRNA-1 | Japonica | NA | | | OsR498G0102754300.01.T01 | Histone-lysine N-methyltransferase ATXR6 |
| maker-1-augustus-gene-504.53-mRNA-1 | Japonica | NA | | Os01g0967800 | OsR498G0102761500.01.T01 | |
| maker-1-augustus-gene-504.55-mRNA-1 | Japonica | NA | | Os01g0967900 | OsR498G0102761700.01.T01 | Aspartyl aminopeptidase |
| maker-1-augustus-gene-504.64-mRNA-1 | Japonica | NA | | | | BACK domain-containing protein |
| maker-1-augustus-gene-504.74-mRNA-1 | Japonica | NA | | Os01g0968000 | OsR498G0102762000.01.T01 | Ribonuclease P |

| | | | | | | |
|---|---|---|---|---|---|---|
| maker-1-augustus-gene-504.76-mRNA-1 | Japonica | NA | | Os01g0968100 | OsR498G0102762200.01.T01 | Late embryogenesis abundant protein-related / LEA protein-related |
| maker-1-augustus-gene-504.78-mRNA-1 | Japonica | Indica | | Os01g0968300 | OsR498G0102762600.01.T01 | Late embryogenesis abundant protein-related / LEA protein-related |
| maker-1-augustus-gene-504.80-mRNA-1 | Japonica | NA | | Os01g0969000 | OsR498G0102764600.01.T01 | Chloroplast outer envelope 24 kD protein |
| maker-1-augustus-gene-504.82-mRNA-1 | Japonica | NA | | Os01g0969100 | OsR498G0102764800.01.T01 | UDP-D-apiose/UDP-D-xylose synthase |
| maker-1-augustus-gene-506.16-mRNA-1 | Japonica | Indica | | Os01g0971700 | OsR498G0102774800.01.T03 | Ribosome association toxin RatA (Fragment) |
| maker-1-augustus-gene-506.18-mRNA-1 | Japonica | NA | | Os01g0971900 | OsR498G0102775200.01.T02 | Pumilio homolog 3 |
| maker-1-augustus-gene-506.5-mRNA-1 | Japonica | NA | | Os01g0972800 | OsR498G0102778700.01.T01 | DNA-binding WRKY |
| maker-1-augustus-gene-506.7-mRNA-1 | Japonica | NA | | Os01g0972900 | OsR498G0102778900.01.T01 | B-type cell cycle switch protein ccs52B |
| maker-1-augustus-gene-507.0-mRNA-1 | Japonica | NA | | Os01g0973000 | OsR498G0102779100.01.T03 | Putative pyridoxal phosphatase |
| maker-1-augustus-gene-507.12-mRNA-1 | Japonica | Indica | | Os01g0973300 | OsR498G0102779700.01.T01 | AP-3 complex subunit beta |
| maker-1-augustus-gene-507.18-mRNA-1 | Japonica | NA | | Os01g0973600 | OsR498G0102780500.01.T01 | Plant-specific domain TIGR01615 family protein (Fragment) |
| maker-1-augustus-gene-507.20-mRNA-1 | Japonica | NA | | Os01g0974000 | OsR498G0102780900.01.T01 | Protein TRIGALACTOSYLDIACYLGLYCEROL 2 chloroplastic |
| maker-1-augustus-gene-507.23-mRNA-1 | Japonica | Indica | | Os05g0111200 | OsR498G0102781100.01.T02 | Amino acid selective channel protein |
| maker-1-augustus-gene-507.28-mRNA-1 | Japonica | NA | | Os01g0974600 | OsR498G0102782900.01.T04 | RRM domain-containing protein |
| maker-1-augustus-gene-507.31-mRNA-1 | Japonica | NA | | Os01g0973200 | OsR498G0102779600.01.T01 | Kinetochore protein spc25 |
| maker-1-augustus-gene-507.36-mRNA-1 | Japonica | NA | | Os01g0973400 | OsR498G0102780000.01.T01 | Elp3 domain-containing protein (Fragment) |
| maker-1-augustus-gene-507.39-mRNA-1 | Japonica | NA | Kinase-TM | Os01g0973500 | OsR498G0102780200.01.T01 | receptor-like cytoplasmic kinase 176 |
| maker-1-augustus-gene-507.4-mRNA-1 | Japonica | Indica | | Os01g0550800 | OsR498G0102779300.01.T02 | Carboxyl-terminal peptidase-like |
| maker-1-augustus-gene-507.43-mRNA-1 | Japonica | NA | | | | |
| maker-1-augustus-gene-507.48-mRNA-1 | Japonica | NA | | Os01g0974500 | OsR498G0102782800.01.T03 | Pectin acetylesterase |
| maker-1-augustus-gene-507.51-mRNA-1 | Japonica | Indica | | Os05g0114000 | OsR498G0102783200.01.T04 | Inorganic diphosphatase |
| maker-1-augustus-gene-507.55-mRNA-1 | Japonica | NA | | Os01g0975000 | OsR498G0102784200.01.T01 | UPSTREAM OF FLC protein (DUF966) |
| maker-1-augustus-gene-508.34-mRNA-1 | Japonica | Indica | | Os01g0975300 | OsR498G0102785500.01.T01 | Myb-related protein MYBAS2 |
| maker-1-augustus-gene-508.37-mRNA-1 | Japonica | NA | | | | fructose-bisphosphate aldolase-lysine N-methyltransferase, chloroplastic isc |
| maker-1-augustus-gene-508.42-mRNA-1 | Japonica | NA | | Os01g0976500 | OsR498G0102789100.01.T03 | NAB domain-containing protein (Fragment) |
| maker-1-augustus-gene-508.44-mRNA-1 | Japonica | NA | | Os01g0975900 | OsR498G0102787800.01.T01 | Aquaporin TIP3 |
| maker-1-augustus-gene-508.46-mRNA-1 | Japonica | NA | | Os01g0976000 | OsR498G0102788400.01.T03 | Vacuolar fusion protein MON1 homolog |
| maker-1-augustus-gene-508.49-mRNA-1 | Japonica | Indica | | Os03g0280000 | OsR498G0102788600.01.T03 | ABC transporter |
| maker-1-augustus-gene-508.54-mRNA-1 | Japonica | NA | | Os01g0976600 | OsR498G0102789400.01.T01 | 2-methoxy-6-polyprenyl-1,4-benzoquinol methylase, mitochondrial |
| maker-1-augustus-gene-508.57-mRNA-1 | Japonica | NA | | Os01g0976800 | OsR498G0102790200.01.T01 | GATA-type domain-containing protein |
| maker-1-augustus-gene-509.16-mRNA-1 | Japonica | Indica | Kinase-LRR-TM | Os01g0976900 | OsR498G0102790800.01.T02 | Mitogen-activated protein kinase kinase kinase |
| maker-1-augustus-gene-509.2-mRNA-1 | Japonica | NA | | | OsR498G0102791300.01.T01 | DnaJ protein P58IPK homolog B |
| maker-1-augustus-gene-509.21-mRNA-1 | Japonica | NA | | Os01g0977600 | OsR498G0102792600.01.T01 | E3 ubiquitin-protein ligase listerin |
| maker-1-augustus-gene-509.26-mRNA-1 | Japonica | Indica | | Os01g0978100 | OsR498G0102794600.01.T01 | Cysteine synthase |
| maker-1-augustus-gene-509.7-mRNA-1 | Japonica | NA | | | OsR498G0102794100.01.T05 | Mitochondrial uncoupling protein 1 |
| maker-1-augustus-gene-52.26-mRNA-1 | Japonica | NA | | Os01g0195700 | OsR498G0100335900.01.T01 | BHLH transcription factor |
| maker-1-augustus-gene-53.0-mRNA-1 | Japonica | NA | | Os01g0197100 | OsR498G0100341300.01.T01 | 3-epi-6-deoxocathasterone 23-monooxygenase isoform X2 |
| maker-1-augustus-gene-53.10-mRNA-1 | Japonica | NA | | | OsR498G0100341800.01.T04 | Dead box ATP-dependent RNA helicase |
| maker-1-augustus-gene-53.14-mRNA-1 | Japonica | NA | | Os01g0197500 | OsR498G0100344000.01.T02 | Ceramidase |
| maker-1-augustus-gene-53.17-mRNA-1 | Japonica | NA | | | OsR498G0100344200.01.T01 | Cytokinin dehydrogenase |
| maker-1-augustus-gene-53.19-mRNA-1 | Japonica | Japonica | | Os01g0197900 | OsR498G0100345200.01.T02 | RNA-dependent RNA polymerase |
| maker-1-augustus-gene-53.21-mRNA-1 | Japonica | Indica | | Os01g0198100 | OsR498G0100345000.01.T05 | RNA-dependent RNA polymerase |
| maker-1-augustus-gene-53.22-mRNA-1 | Japonica | Indica | | Os01g0198100 | OsR498G0100345000.01.T05 | RNA-dependent RNA polymerase |
| maker-1-augustus-gene-53.25-mRNA-1 | Japonica | NA | | | OsR498G0100345200.01.T01 | RNA-dependent RNA polymerase |
| maker-1-augustus-gene-53.32-mRNA-1 | Japonica | NA | | Os01g0198200 | OsR498G0100345600.01.T01 | Casein kinase I hhp1 |
| maker-1-augustus-gene-53.4-mRNA-1 | Japonica | NA | | Os01g0197400 | OsR498G0100343500.01.T04 | CA-responsive protein |
| maker-1-augustus-gene-55.18-mRNA-1 | Japonica | NA | Kinase-TM | Os01g0201200 | OsR498G0100357200.01.T04 | Serine/threonine-protein kinase |
| maker-1-augustus-gene-55.7-mRNA-1 | Japonica | Indica | | Os03g0692000 | OsR498G0100356900.01.T01 | beta-glucuronosyltransferase GlcAT14B-like |
| maker-1-augustus-gene-56.0-mRNA-1 | Japonica | NA | | Os01g0201600 | OsR498G0100361000.01.T01 | Meiosis 5 |
| maker-1-augustus-gene-56.2-mRNA-1 | Japonica | Indica | | Os01g0201700 | OsR498G0100361800.01.T03 | MADS-box transcription factor |
| maker-1-augustus-gene-56.6-mRNA-1 | Japonica | Indica | | Os01g0201900 | OsR498G0100362800.01.T07 | Nodulin-26 like intrinsic protein |
| maker-1-augustus-gene-57.0-mRNA-1 | Japonica | NA | | Os01g0202500 | OsR498G0100364900.01.T01 | B-box zinc finger protein 22 |
| maker-1-augustus-gene-57.16-mRNA-1 | Japonica | NA | | Os01g0204000 | OsR498G0100370600.01.T01 | DNA polymerase delta subunit 3 |
| maker-1-augustus-gene-57.2-mRNA-1 | Japonica | Indica | | Os06g0157500 | OsR498G0100365100.01.T01 | Protein TWIN SISTER of FT (Fragment) |
| maker-1-augustus-gene-57.4-mRNA-1 | Japonica | NA | | Os01g0202800 | OsR498G0100366800.01.T02 | Nodulin-26 like intrinsic protein |
| maker-1-augustus-gene-57.5-mRNA-1 | Japonica | NA | | | OsR498G0100366800.01.T02 | Aquaporin NIP-type |
| maker-1-augustus-gene-57.6-mRNA-1 | Japonica | NA | | Os01g0203000 | OsR498G0100367000.01.T01 | BES transcription factor |
| maker-1-augustus-gene-58.0-mRNA-1 | Japonica | NA | | Os01g0205100 | OsR498G0100372900.01.T01 | Leucine-rich repeat and WD repeat-containing protein 1 |
| maker-1-augustus-gene-59.12-mRNA-1 | Japonica | NA | | Os01g0208700 | OsR498G0100384500.01.T01 | Phosphoenolpyruvate carboxylase |
| maker-1-augustus-gene-59.16-mRNA-1 | Japonica | NA | | Os01g0209000 | OsR498G0100385100.01.T03 | Mannosyltransferase |
| maker-1-augustus-gene-6.0-mRNA-1 | Japonica | NA | | | OsR498G0100031100.01.T02 | Deoxynucleoside triphosphate triphosphohydrolase SAMHD1 |

| | | | | | | |
|---|---|---|---|---|---|---|
| maker-1-augustus-gene-6.10-mRNA-1 | Japonica | NA | | Os01g0110400 | OsR498G0100033900.01.T03 | Acetyl-CoA acetyltransferase, cytosolic 2 |
| maker-1-augustus-gene-6.15-mRNA-1 | Japonica | NA | | Os01g0111200 | OsR498G0100035700.01.T01 | Protein decapping like |
| maker-1-augustus-gene-6.19-mRNA-1 | Japonica | NA | | Os01g0109700 | OsR498G0100031800.01.T01 | Paired amphipathic helix protein Sin3 |
| maker-1-augustus-gene-6.26-mRNA-1 | Japonica | NA | | Os01g0109750 | OsR498G0100032000.01.T04 | Paired amphipathic helix protein Sin3-like 4 (Fragment) |
| maker-1-augustus-gene-6.29-mRNA-1 | Japonica | NA | | Os01g0110200 | OsR498G0100033600.01.T01 | Protein O-GlcNAc transferase (Fragment) |
| maker-1-augustus-gene-6.31-mRNA-1 | Japonica | NA | Kinase-TM | Os01g0110500 | OsR498G0100034200.01.T01 | Proline-rich receptor-like protein kinase PERK8 |
| maker-1-augustus-gene-6.35-mRNA-1 | Japonica | NA | | Os01g0110700 | OsR498G0100034600.01.T01 | Phosphoenolpyruvate carboxylase |
| maker-1-augustus-gene-6.37-mRNA-1 | Japonica | Indica | LRR-TM | Os01g0110800 | OsR498G0100035200.01.T01 | Piriformospora indica-insensitive protein 2 |
| maker-1-augustus-gene-6.40-mRNA-1 | Japonica | NA | | Os01g0111100 | OsR498G0100035600.01.T01 | Peptidyl-prolyl cis-trans isomerase |
| maker-1-augustus-gene-6.6-mRNA-1 | Japonica | Indica | | Os06g0493600 | OsR498G0100033300.01.T01 | Phosphate transporter PHO1 like 1 |
| maker-1-augustus-gene-60.0-mRNA-1 | Japonica | NA | | Os01g0209200 | | Tyrosine 3-monooxygenase/tryptophan 5-monooxygenase activation protei |
| maker-1-augustus-gene-60.12-mRNA-1 | Japonica | NA | | Os01g0209700 | OsR498G0100389000.01.T01 | Gibberellin 2-beta-dioxygenase |
| maker-1-augustus-gene-60.14-mRNA-1 | Japonica | NA | | Os01g0209800 | OsR498G0100389600.01.T01 | Cationic amino acid transporter like |
| maker-1-augustus-gene-60.16-mRNA-1 | Japonica | Japonica | | Os01g0210400 | OsR498G0100391800.01.T01 | PIK-related kinase, FAT |
| maker-1-augustus-gene-60.18-mRNA-1 | Japonica | NA | | Os01g0210500 | OsR498G0100392000.01.T01 | Heme-binding protein 2 |
| maker-1-augustus-gene-60.3-mRNA-1 | Japonica | Japonica | | Os01g0210300 | OsR498G0100391500.01.T01 | |
| maker-1-augustus-gene-60.6-mRNA-1 | Japonica | NA | | Os01g0209400 | OsR498G0100386600.01.T01 | Polyadenylate-binding protein-interacting protein 9 |
| maker-1-augustus-gene-61.24-mRNA-1 | Japonica | Indica | | Os01g0210800 | OsR498G0100394100.01.T01 | phospho-N-acetylmuramoyl-pentapeptide- transferase homolog isoform X1 |
| maker-1-augustus-gene-61.29-mRNA-1 | Japonica | NA | | Os01g0210700 | OsR498G0100393600.01.T03 | K+-channel ERG |
| maker-1-augustus-gene-62.5-mRNA-1 | Japonica | NA | | Os01g0212400 | OsR498G0100402300.01.T01 | sodium/calcium exchanger NCL |
| maker-1-augustus-gene-62.8-mRNA-1 | Japonica | NA | | | OsR498G0100402600.01.T01 | SPOROCYTELESS-like EAR-containing protein 2 |
| maker-1-augustus-gene-63.0-mRNA-1 | Japonica | NA | | | OsR498G0100410300.01.T01 | Transcription factor like |
| maker-1-augustus-gene-63.11-mRNA-1 | Japonica | Indica | | Os01g0214600 | OsR498G0100411100.01.T01 | GDSL esterase/lipase |
| maker-1-augustus-gene-63.13-mRNA-1 | Japonica | NA | | Os01g0214800 | OsR498G0100411900.01.T01 | GDSL esterase/lipase |
| maker-1-augustus-gene-63.15-mRNA-1 | Japonica | Japonica | | Os01g0215000 | OsR498G0100444200.01.T01 | ABC transporter D family member 2, chloroplastic (Fragment) |
| maker-1-augustus-gene-63.2-mRNA-1 | Japonica | NA | | | OsR498G0100410500.01.T04 | Ferritin-like superfamily |
| maker-1-augustus-gene-63.20-mRNA-1 | Japonica | NA | | Os01g0214200 | OsR498G0100410000.01.T01 | Isoamyl acetate-hydrolyzing esterase |
| maker-1-augustus-gene-63.7-mRNA-1 | Japonica | NA | | Os01g0214500 | OsR498G0100410700.01.T01 | |
| maker-1-augustus-gene-63.9-mRNA-1 | Japonica | NA | | | OsR498G1119638400.01.T01 | Transposon protein, putative, unclassified |
| maker-1-augustus-gene-64.0-mRNA-1 | Japonica | NA | | Os01g0215700 | OsR498G0100415100.01.T01 | Gdsl esteraselipase |
| maker-1-augustus-gene-64.2-mRNA-1 | Japonica | NA | | Os01g0216000 | OsR498G0100416300.01.T01 | GDSL esterase/lipase |
| maker-1-augustus-gene-64.4-mRNA-1 | Japonica | NA | | Os01g0216300 | OsR498G0100416500.01.T01 | GDSL esterase/lipase |
| maker-1-augustus-gene-65.12-mRNA-1 | Japonica | NA | | Os01g0218100 | OsR498G0100424900.01.T01 | Transcription factor bHLH30 like |
| maker-1-augustus-gene-65.14-mRNA-1 | Japonica | NA | | Os01g0218200 | OsR498G0100426100.01.T01 | Inactive TPR repeat-containing thioredoxin TTL3 |
| maker-1-augustus-gene-65.19-mRNA-1 | Japonica | NA | | Os01g0218500 | OsR498G0100427300.01.T01 | Phosphatidylethanolamine-binding protein PEBP |
| maker-1-augustus-gene-65.23-mRNA-1 | Japonica | NA | | Os01g0218700 | OsR498G0100427600.01.T02 | Peroxisomal membrane ABC transporter family isoform 1 |
| maker-1-augustus-gene-65.29-mRNA-1 | Japonica | NA | | | | histone-lysine N-methyltransferase ATX4 |
| maker-1-augustus-gene-65.34-mRNA-1 | Japonica | NA | | Os01g0218900 | OsR498G0100428600.01.T03 | Histone-lysine N-methyltransferase ATX4 |
| maker-1-augustus-gene-65.6-mRNA-1 | Japonica | NA | | | OsR498G0100423700.01.T04 | Transcriptional activator DEMETER |
| maker-1-augustus-gene-66.0-mRNA-1 | Japonica | NA | | Os01g0219100 | OsR498G0100428900.01.T03 | Vesicle transport protein |
| maker-1-augustus-gene-66.10-mRNA-1 | Japonica | NA | | Os01g0219600 | OsR498G0100430000.01.T01 | Endoglucanase |
| maker-1-augustus-gene-66.12-mRNA-1 | Japonica | NA | | Os01g0220100 | OsR498G0100430400.01.T02 | Endoglucanase |
| maker-1-augustus-gene-66.14-mRNA-1 | Japonica | Indica | | Os05g0212400 | OsR498G0100431000.01.T02 | Ectonucleotide pyrophosphatase/phosphodiesterase family member 3 |
| maker-1-augustus-gene-66.19-mRNA-1 | Japonica | NA | | Os01g0220500 | OsR498G0100433200.01.T01 | Inhibitor I9 domain-containing protein (Fragment) |
| maker-1-augustus-gene-66.21-mRNA-1 | Japonica | NA | | Os01g0220700 | OsR498G0100435800.01.T03 | Bidirectional sugar transporter SWEET |
| maker-1-augustus-gene-66.4-mRNA-1 | Japonica | NA | | | | Reduced male fertility |
| maker-1-augustus-gene-66.6-mRNA-1 | Japonica | NA | | Os01g0219500 | OsR498G0100429700.01.T01 | AAI domain-containing protein |
| maker-1-augustus-gene-66.8-mRNA-1 | Japonica | NA | | | | Translation elongation factor EF1B/ribosomal protein S6 family protein isofo |
| maker-1-augustus-gene-69.0-mRNA-1 | Japonica | NA | | Os01g0224300 | OsR498G0100453300.01.T01 | Cysteine alpha-hairpin motif superfamily |
| maker-1-augustus-gene-69.15-mRNA-1 | Japonica | NA | | | OsR498G0100458500.01.T01 | GTP-binding protein EngA |
| maker-1-augustus-gene-69.2-mRNA-1 | Japonica | NA | | | OsR498G0100453700.01.T01 | Mitochondrial carrier protein CGI-69 |
| maker-1-augustus-gene-69.20-mRNA-1 | Japonica | NA | | | OsR498G0100458700.01.T02 | GTP binding |
| maker-1-augustus-gene-69.26-mRNA-1 | Japonica | NA | | Os01g0224200 | OsR498G0100453200.01.T01 | Plastid division protein PDV1 |
| maker-1-augustus-gene-69.28-mRNA-1 | Japonica | NA | | Os01g0224500 | OsR498G0100454000.01.T01 | Phosphatidylinositol N-acetylglucosaminyltransferase (Fragment) |
| maker-1-augustus-gene-69.31-mRNA-1 | Japonica | NA | | Os01g0225000 | OsR498G0100456800.01.T01 | Mitochondrial arginine transporter like |
| maker-1-augustus-gene-69.34-mRNA-1 | Japonica | NA | | | OsR498G0100459000.01.T01 | 3-methyl-2-oxobutanoate hydroxymethyltransferase |
| maker-1-augustus-gene-69.37-mRNA-1 | Japonica | NA | | | OsR498G0100459200.01.T01 | 3-methyl-2-oxobutanoate hydroxymethyltransferase |
| maker-1-augustus-gene-69.7-mRNA-1 | Japonica | NA | | Os01g0224700 | OsR498G0100455300.01.T01 | Flavin-containing monooxygenase |
| maker-1-augustus-gene-69.9-mRNA-1 | Japonica | NA | | | OsR498G0100458300.01.T04 | Smr domain-containing protein |
| maker-1-augustus-gene-7.0-mRNA-1 | Japonica | NA | | Os01g0111250 | OsR498G0100035900.01.T03 | Usp domain-containing protein |
| maker-1-augustus-gene-7.11-mRNA-1 | Japonica | NA | | Os01g0111500 | OsR498G0100036600.01.T01 | Transcription factor bHLH83 |

| | | | | | | |
|---|---|---|---|---|---|---|
| maker-1-augustus-gene-7.5-mRNA-1 | Japonica | NA | | Os01g0111400 | | Zinc finger BED domain-containing protein DAYSLEEPER |
| maker-1-augustus-gene-7.7-mRNA-1 | Japonica | NA | | Os01g0111600 | OsR498G0100037100.01.T01 | Protein MOTHER of FT and TF 1 |
| maker-1-augustus-gene-70.0-mRNA-1 | Japonica | Indica | | Os01g0226300 | OsR498G0100462300.01.T01 | Reticulon-like protein (Fragment) |
| maker-1-augustus-gene-70.4-mRNA-1 | Japonica | NA | | Os01g0226500 | OsR498G0100462700.01.T05 | U11/U12 small nuclear ribonucleoprotein 59 kDa protein isoform A |
| maker-1-augustus-gene-70.7-mRNA-1 | Japonica | NA | | Os01g0226600 | OsR498G0100463300.01.T03 | S-type anion channel SLAH3 |
| maker-1-augustus-gene-70.9-mRNA-1 | Japonica | NA | | Os01g0226200 | OsR498G0100461600.01.T01 | probable membrane-associated kinase regulator 2 |
| maker-1-augustus-gene-71.28-mRNA-1 | Japonica | NA | | Os01g0227100 | OsR498G0100466700.01.T02 | Putative chlorophyll(Ide) b reductase NYC1 |
| maker-1-augustus-gene-71.33-mRNA-1 | Japonica | NA | Kinase-TM | Os01g0227200 | OsR498G0100467900.01.T01 | Receptor protein kinase PERK1 |
| maker-1-augustus-gene-71.37-mRNA-1 | Japonica | NA | | Os01g0227300 | OsR498G0100468100.01.T01 | Ras-related protein Rab7 |
| maker-1-augustus-gene-71.41-mRNA-1 | Japonica | Indica | | Os01g0227500 | OsR498G0100468300.01.T01 | Putative ferulate 5-hydroxylase |
| maker-1-augustus-gene-71.43-mRNA-1 | Japonica | NA | | Os01g0227500 | OsR498G0100468500.01.T01 | Putative ferulate 5-hydroxylase |
| maker-1-augustus-gene-71.45-mRNA-1 | Japonica | Indica | | Os01g0227800 | OsR498G0100468900.01.T01 | |
| maker-1-augustus-gene-71.47-mRNA-1 | Japonica | NA | | Os01g0227800 | OsR498G0100469700.01.T01 | Putative ferulate 5-hydroxylase |
| maker-1-augustus-gene-71.50-mRNA-1 | Japonica | Japonica | Kinase-LRR-TM | Os01g0228200 | OsR498G0100470100.01.T01 | Putative receptor kinase TRKc |
| maker-1-augustus-gene-71.53-mRNA-1 | Japonica | NA | | Os01g0228400 | OsR498G0100471100.01.T02 | Pentatricopeptide repeat-containing protein isoform X1 |
| maker-1-augustus-gene-71.55-mRNA-1 | Japonica | NA | | Os01g0228300 | OsR498G0100470400.01.T01 | Peroxisomal membrane protein PMP22 |
| maker-1-augustus-gene-71.57-mRNA-1 | Japonica | NA | | Os01g0228500 | OsR498G0100471400.01.T04 | Late embryogenesis abundant protein group 2 |
| maker-1-augustus-gene-71.59-mRNA-1 | Japonica | Indica | | Os01g0228600 | OsR498G0100471600.01.T02 | Hydroxyacid dehydrogenase |
| maker-1-augustus-gene-71.62-mRNA-1 | Japonica | NA | | Os01g0228800 | OsR498G0100472000.01.T02 | VARLMGL domain-containing protein |
| maker-1-augustus-gene-72.0-mRNA-1 | Japonica | Indica | | Os12g0238000 | OsR498G0100472700.01.T01 | Transcription factor myb |
| maker-1-augustus-gene-72.14-mRNA-1 | Japonica | NA | | Os01g0229500 | OsR498G0100476300.01.T01 | 4HBT domain-containing protein (Fragment) |
| maker-1-augustus-gene-72.16-mRNA-1 | Japonica | NA | | | | 4HBT domain-containing protein (Fragment) |
| maker-1-augustus-gene-72.18-mRNA-1 | Japonica | Indica | | Os01g0233900 | OsR498G0100477300.01.T01 | Ubiquitin-conjugating enzyme family protein, expressed |
| maker-1-augustus-gene-72.27-mRNA-1 | Japonica | NA | | Os01g0229100 | OsR498G0100473000.01.T01 | Eukaryotic translation initiation factor 3 subunit L |
| maker-1-augustus-gene-72.31-mRNA-1 | Japonica | NA | | Os01g0229400 | OsR498G0100475200.01.T01 | Rac-like GTP-binding protein ARAC7 |
| maker-1-augustus-gene-72.34-mRNA-1 | Japonica | NA | | Os01g0229700 | OsR498G0100476600.01.T01 | RING-type E3 ubiquitin transferase |
| maker-1-augustus-gene-72.7-mRNA-1 | Japonica | NA | | Os01g0229200 | OsR498G0100473100.01.T04 | Putative VHS/GAT domain containing family protein |
| maker-1-augustus-gene-73.38-mRNA-1 | Japonica | NA | | Os01g0231000 | OsR498G0100479700.01.T01 | Auxin-responsive protein IAA3 |
| maker-1-augustus-gene-73.41-mRNA-1 | Japonica | NA | | Os01g0861400 | | VACUOLAR SORTING RECEPTOR 2 |
| maker-1-augustus-gene-73.50-mRNA-1 | Japonica | NA | | | | KH domain-containing protein (Fragment) |
| maker-1-augustus-gene-73.55-mRNA-1 | Japonica | NA | | Os01g0232000 | OsR498G0100482300.01.T01 | Aquaporin TIP4-4 |
| maker-1-augustus-gene-73.57-mRNA-1 | Japonica | NA | | Os01g0232100 | OsR498G0100482700.01.T03 | Aquaporin TIP4-3 |
| maker-1-augustus-gene-73.59-mRNA-1 | Japonica | NA | | Os01g0230200 | | Transcription factor bHLH68 isoform B |
| maker-1-augustus-gene-73.60-mRNA-1 | Japonica | NA | | Os01g0230200 | OsR498G0100477800.01.T05 | BHLH domain-containing protein |
| maker-1-augustus-gene-73.71-mRNA-1 | Japonica | NA | | Os01g0231700 | OsR498G0100481600.01.T02 | 60s acidic ribosomal protein-domain-containing protein |
| maker-1-augustus-gene-74.28-mRNA-1 | Japonica | NA | | Os01g0232300 | OsR498G0100484700.01.T02 | Zinc phosphodiesterase |
| maker-1-augustus-gene-74.34-mRNA-1 | Japonica | NA | | Os01g0232400 | OsR498G0100484900.01.T01 | VHS domain-containing protein |
| maker-1-augustus-gene-74.36-mRNA-1 | Japonica | NA | | Os01g0232500 | OsR498G0100485700.01.T02 | Putative ubiquitin-onjugating enzyme E2 |
| maker-1-augustus-gene-74.39-mRNA-1 | Japonica | NA | | Os01g0232700 | OsR498G0100486100.01.T02 | Histidinol dehydrogenase |
| maker-1-augustus-gene-74.47-mRNA-1 | Japonica | NA | Kinase-TM | Os01g0233800 | OsR498G0100488900.01.T01 | Serine/threonine-protein kinase |
| maker-1-augustus-gene-74.49-mRNA-1 | Japonica | NA | | | OsR498G0100486400.01.T01 | Glycosyltransferase family 92 protein (Fragment) |
| maker-1-augustus-gene-74.55-mRNA-1 | Japonica | NA | | Os01g0233000 | OsR498G0100487200.01.T02 | Salt stress root protein RS1 |
| maker-1-augustus-gene-74.58-mRNA-1 | Japonica | NA | | Os01g0233500 | OsR498G0100488600.01.T01 | Cyclin N-terminal domain-containing protein (Fragment) |
| maker-1-augustus-gene-74.73-mRNA-1 | Japonica | NA | | Os01g0233900 | OsR498G0100489200.01.T05 | Ubiquitin-conjugating enzyme-like |
| maker-1-augustus-gene-75.42-mRNA-1 | Japonica | NA | | Os01g0234100 | OsR498G0100489900.01.T01 | TF-B3 domain-containing protein |
| maker-1-augustus-gene-75.46-mRNA-1 | Japonica | NA | | Os01g0235100 | OsR498G0100493500.01.T01 | Transcriptional coactivator p15 (PC4) family protein (KELP) |
| maker-1-augustus-gene-75.49-mRNA-1 | Japonica | NA | | Os01g0235300 | OsR498G0100494100.01.T01 | SOUL heme-binding |
| maker-1-augustus-gene-75.51-mRNA-1 | Japonica | NA | | | OsR498G0100496700.01.T05 | RNA-binding KH domain-containing protein RCF3 |
| maker-1-augustus-gene-75.56-mRNA-1 | Japonica | NA | | Os02g0699700 | OsR498G0204598600.01.T05 | DNA topoisomerase 2 |
| maker-1-augustus-gene-75.58-mRNA-1 | Japonica | NA | | | OsR498G0100492200.01.T04 | SEC22 vesicle trafficking protein-like protein B |
| maker-1-augustus-gene-75.63-mRNA-1 | Japonica | NA | | Os01g0234850 | OsR498G0100492600.01.T01 | Phosphoinositide 4-kinase gamma 4, gamma 4,ubdk gamma 4,pi4k gamma 4 |
| maker-1-augustus-gene-75.66-mRNA-1 | Japonica | NA | | Os01g0234900 | OsR498G0100493200.01.T02 | RING-type E3 ubiquitin transferase |
| maker-1-augustus-gene-75.68-mRNA-1 | Japonica | NA | | Os01g0235200 | OsR498G0100493800.01.T02 | Defective in meristem silencing 3 |
| maker-1-augustus-gene-75.73-mRNA-1 | Japonica | NA | | | OsR498G0100494400.01.T01 | WPP domain-interacting tail-anchored protein 1 |
| maker-1-augustus-gene-75.76-mRNA-1 | Japonica | NA | | Os01g0235700 | OsR498G0100495800.01.T01 | Transcription factor bHLH13 |
| maker-1-augustus-gene-76.32-mRNA-1 | Japonica | NA | | Os01g0236300 | OsR498G0100499700.01.T01 | Auxin response factor |
| maker-1-augustus-gene-76.38-mRNA-1 | Japonica | NA | | Os01g0236400 | OsR498G0100500300.01.T01 | Probable protein ABIL3 |
| maker-1-augustus-gene-76.42-mRNA-1 | Japonica | NA | | Os01g0236700 | OsR498G0100502100.01.T04 | NIN like protein 7 |
| maker-1-augustus-gene-76.47-mRNA-1 | Japonica | NA | | Os01g0237000 | OsR498G0100503300.01.T01 | |
| maker-1-augustus-gene-76.49-mRNA-1 | Japonica | NA | | Os01g0236800 | OsR498G0100502400.01.T01 | Beta-amylase |
| maker-1-augustus-gene-76.54-mRNA-1 | Japonica | NA | | Os01g0237100 | OsR498G0100503600.01.T01 | probable 2-carboxy-D-arabinitol-1-phosphatase |

| | | | | | | |
|---|---|---|---|---|---|---|
| maker-1-augustus-gene-76.56-mRNA-1 | Japonica | NA | | | OsR498G0100504200.01.T01 | Phenylcoumaran benzylic ether reductase (Fragment) |
| maker-1-augustus-gene-77.0-mRNA-1 | Japonica | NA | | Os01g0238500 | OsR498G0100507700.01.T01 | Branched-chain-amino-acid aminotransferase-like protein chloroplastic-like |
| maker-1-augustus-gene-77.18-mRNA-1 | Japonica | Indica | | Os01g0237500 | OsR498G0100505800.01.T02 | NmrA domain-containing protein (Fragment) |
| maker-1-augustus-gene-77.19-mRNA-1 | Japonica | Indica | | Os01g0237366 | OsR498G0100505800.01.T02 | NmrA domain-containing protein (Fragment) |
| maker-1-augustus-gene-77.24-mRNA-1 | Japonica | NA | | Os01g0238800 | | Protein NUCLEAR FUSION DEFECTIVE 6 chloroplastic/mitochondrial |
| maker-1-augustus-gene-77.26-mRNA-1 | Japonica | NA | | Os01g0239000 | OsR498G0100509200.01.T02 | Transcription activator GLK1 isoform X2 |
| maker-1-augustus-gene-77.5-mRNA-1 | Japonica | NA | | Os01g0238600 | OsR498G0100507900.01.T01 | Putative metal-nicotianamine transporter YSL1 |
| maker-1-augustus-gene-77.7-mRNA-1 | Japonica | NA | | Os01g0238700 | OsR498G0100508300.01.T01 | Probable metal-nicotianamine transporter YSL1 |
| maker-1-augustus-gene-77.9-mRNA-1 | Japonica | NA | | | OsR498G0100508900.01.T01 | DDB1- and CUL4-associated factor 13 |
| maker-1-augustus-gene-78.38-mRNA-1 | Japonica | NA | | | OsR498G0100514500.01.T02 | |
| maker-1-augustus-gene-78.41-mRNA-1 | Japonica | Indica | | Os01g0240700 | OsR498G0100514900.01.T01 | Reverse transcriptase domain-containing protein |
| maker-1-augustus-gene-78.43-mRNA-1 | Japonica | Japonica | | Os01g0240700 | OsR498G0100515300.01.T01 | DUF1618 domain-containing protein (Fragment) |
| maker-1-augustus-gene-78.46-mRNA-1 | Japonica | Indica | | Os01g0241000 | OsR498G0100519100.01.T01 | |
| maker-1-augustus-gene-78.48-mRNA-1 | Japonica | NA | | Os01g0239200 | OsR498G0100510600.01.T02 | Triose phosphate/phosphate translocator TPT chloroplastic |
| maker-1-augustus-gene-78.54-mRNA-1 | Japonica | NA | | | OsR498G0100519400.01.T02 | Lecithin-cholesterol acyltransferase-like 1 |
| maker-1-augustus-gene-79.0-mRNA-1 | Japonica | NA | | | | Zinc finger protein MAGPIE |
| maker-1-augustus-gene-79.12-mRNA-1 | Japonica | Indica | | Os01g0242500 | OsR498G0100522400.01.T02 | D-aminoacyl-tRNA deacylase |
| maker-1-augustus-gene-79.16-mRNA-1 | Japonica | NA | | Os01g0242600 | OsR498G0100522600.01.T02 | C2 domain-containing protein (Fragment) |
| maker-1-augustus-gene-79.2-mRNA-1 | Japonica | NA | | Os01g0242300 | OsR498G0100521900.01.T03 | Optic atrophy 3 protein |
| maker-1-augustus-gene-79.22-mRNA-1 | Japonica | Japonica | | Os01g0243000 | OsR498G0100524200.01.T01 | GDSL esterase/lipase |
| maker-1-augustus-gene-79.24-mRNA-1 | Japonica | Indica | | Os01g0243200 | OsR498G0100525200.01.T01 | Folate-biopterin transporter like |
| maker-1-augustus-gene-79.5-mRNA-1 | Japonica | NA | | Os01g0242900 | OsR498G0100523900.01.T01 | Ribosomal protein L18ae family |
| maker-1-augustus-gene-79.8-mRNA-1 | Japonica | NA | | | | P-loop nucleoside triphosphate hydrolases superfamily protein with Calponi |
| maker-1-augustus-gene-80.50-mRNA-1 | Japonica | Japonica | | Os01g0243900 | OsR498G0100529200.01.T01 | Zinc finger CCCH domain-containing protein 4 |
| maker-1-augustus-gene-94.43-mRNA-1 | Japonica | NA | | Os01g0270300 | OsR498G0100632700.01.T01 | Peroxidase |
| maker-1-augustus-gene-94.45-mRNA-1 | Japonica | NA | | Os01g0271000 | OsR498G0100633900.01.T01 | Mitochondrial inner membrane translocase subunit Tim17/Tim22/Tim23/pe |
| maker-1-augustus-gene-94.55-mRNA-1 | Japonica | NA | | Os01g0270100 | OsR498G0100632000.01.T01 | Cysteine proteinase inhibitor |
| maker-1-augustus-gene-94.57-mRNA-1 | Japonica | NA | | Os01g0271200 | OsR498G0100634200.01.T01 | Glutamyl-tRNA synthetase |
| maker-1-augustus-gene-94.59-mRNA-1 | Japonica | NA | | Os01g0271400 | OsR498G0100634400.01.T03 | ATP-dependent Clp protease proteolytic subunit |
| maker-1-augustus-gene-95.33-mRNA-1 | Japonica | NA | | Os01g0271500 | OsR498G0100634700.01.T03 | Ubiquitin-activating enzyme repeat |
| maker-1-augustus-gene-95.37-mRNA-1 | Japonica | NA | | Os01g0272600 | OsR498G0100638100.01.T01 | Hydroxyproline O-arabinosyltransferase |
| maker-1-augustus-gene-95.42-mRNA-1 | Japonica | NA | | | OsR498G0100640700.01.T05 | DnaJ homolog subfamily C member 7 |
| maker-1-augustus-gene-95.48-mRNA-1 | Japonica | NA | | | OsR498G0100635600.01.T01 | ARID domain-containing protein C08B11.3 |
| maker-1-augustus-gene-95.51-mRNA-1 | Japonica | NA | | | OsR498G0100638400.01.T01 | Phytocyanin domain-containing protein (Fragment) |
| maker-1-augustus-gene-95.53-mRNA-1 | Japonica | NA | | | | Transglutaminase family protein |
| maker-1-augustus-gene-95.58-mRNA-1 | Japonica | NA | | Os01g0273000 | OsR498G0100639400.01.T01 | Deleted in split hand/splt foot protein 1 |
| maker-1-augustus-gene-95.60-mRNA-1 | Japonica | NA | | | | |
| maker-1-augustus-gene-96.0-mRNA-1 | Japonica | NA | | Os01g0273800 | OsR498G0100642500.01.T01 | Flavin-containing monooxygenase |
| maker-1-augustus-gene-96.10-mRNA-1 | Japonica | NA | | | OsR498G0100647800.01.T01 | Transcription factor MYB105 |
| maker-1-augustus-gene-96.2-mRNA-1 | Japonica | NA | | | OsR498G0714022200.01.T01 | |
| maker-1-augustus-gene-96.4-mRNA-1 | Japonica | NA | | | | Ulp1 protease family, C-terminal catalytic domain containing protein, expres |
| maker-1-augustus-gene-96.6-mRNA-1 | Japonica | NA | | Os01g0274100 | OsR498G0100643300.01.T01 | Flavin-containing monooxygenase |
| maker-1-augustus-gene-97.24-mRNA-1 | Japonica | NA | | Os01g0276100 | OsR498G0100653900.01.T05 | Isocitrate dehydrogenase [NAD] catalytic subunit 5, mitochondrial |
| maker-1-augustus-gene-97.29-mRNA-1 | Japonica | NA | | Os01g0276300 | OsR498G0100654700.01.T01 | Embryonic protein DC-8 |
| maker-1-augustus-gene-97.41-mRNA-1 | Japonica | NA | | Os01g0275600 | OsR498G0100651200.01.T03 | Argonaute |
| maker-1-augustus-gene-97.48-mRNA-1 | Japonica | NA | | | | |
| maker-1-augustus-gene-97.55-mRNA-1 | Japonica | NA | | Os01g0276000 | OsR498G0100653000.01.T01 | 60S ribosomal protein L30 |
| maker-1-augustus-gene-97.60-mRNA-1 | Japonica | NA | | Os01g0276200 | OsR498G0100654200.01.T01 | Mitochondrial import receptor subunit TOM40-1, N-terminally processed lik |
| maker-1-augustus-gene-98.28-mRNA-1 | Japonica | NA | | Os01g0277700 | OsR498G0100658900.01.T01 | Fe(3+) dicitrate transport system permease fecC |
| maker-1-augustus-gene-98.31-mRNA-1 | Japonica | NA | | Os01g0277900 | OsR498G0100660500.01.T01 | J domain-containing protein (Fragment) |
| maker-1-augustus-gene-98.34-mRNA-1 | Japonica | NA | | Os01g0276500 | OsR498G0100655200.01.T01 | Phosphoribosyl-AMP cyclohydrolase |
| maker-1-augustus-gene-98.38-mRNA-1 | Japonica | NA | | Os01g0276700 | OsR498G0100655600.01.T01 | Pyruvate kinase |
| maker-1-augustus-gene-98.41-mRNA-1 | Japonica | NA | | Os01g0276800 | OsR498G0100656000.01.T01 | Glucosidase 2 subunit beta |
| maker-1-augustus-gene-98.47-mRNA-1 | Japonica | NA | | Os01g0277600 | OsR498G0100658600.01.T01 | Phospholipid-transporting ATPase |
| maker-1-augustus-gene-99.28-mRNA-1 | Japonica | NA | | | | |
| maker-1-augustus-gene-99.33-mRNA-1 | Japonica | NA | | Os01g0278900 | OsR498G0100667700.01.T02 | 50S ribosomal protein 5 chloroplastic |
| maker-1-augustus-gene-99.35-mRNA-1 | Japonica | NA | | | OsR498G0100667900.01.T01 | CO(2)-response secreted protease |
| maker-1-augustus-gene-99.37-mRNA-1 | Japonica | NA | | Os01g0279000 | OsR498G0100668100.01.T01 | S-acyltransferase |
| maker-1-augustus-gene-99.40-mRNA-1 | Japonica | NA | | Os01g0279100 | OsR498G0100668500.01.T01 | Magnesium-protoporphyrin IX monomethyl ester (oxidative) cyclase |
| maker-1-augustus-gene-99.44-mRNA-1 | Japonica | NA | | Os01g0279200 | OsR498G0100668700.01.T02 | COP9 signalosome complex subunit |
| maker-10-augustus-gene-105.10-mRNA-1 | Japonica | NA | | Os10g0430700 | OsR498G1018763700.01.T01 | |

| | | | | | | |
|---|---|---|---|---|---|---|
| maker-10-augustus-gene-105.21-mRNA-1 | Japonica | NA | | Os10g0430800 | | |
| maker-10-augustus-gene-105.27-mRNA-1 | Japonica | NA | | | OsR498G1018763200.01.T03 | Cinnamyl alcohol dehydrogenase 7 |
| maker-10-augustus-gene-105.30-mRNA-1 | Japonica | NA | Kinase-TM | Os10g0430900 | OsR498G1018764400.01.T03 | Serine/threonine-protein kinase EDR1 |
| maker-10-augustus-gene-105.4-mRNA-1 | Japonica | NA | | Os10g0430600 | OsR498G1018763500.01.T01 | |
| maker-10-augustus-gene-105.9-mRNA-1 | Japonica | NA | | Os10g0430700 | OsR498G1018763700.01.T03 | |
| maker-10-augustus-gene-106.0-mRNA-1 | Japonica | Indica | | Os10g0431000 | OsR498G1018765300.01.T01 | DWNN domain a CCHC-type zinc finger |
| maker-10-augustus-gene-106.13-mRNA-1 | Japonica | NA | Kinase | Os10g0431900 | OsR498G1018768400.01.T02 | Probable serine/threonine-protein kinase PBL16 |
| maker-10-augustus-gene-106.16-mRNA-1 | Japonica | NA | | Os10g0432300 | OsR498G1018769200.01.T03 | TATA-box-binding protein isoform A |
| maker-10-augustus-gene-106.7-mRNA-1 | Japonica | Indica | | Os03g0159600 | OsR498G1018765500.01.T01 | Late embryogenesis abundant |
| maker-10-augustus-gene-106.9-mRNA-1 | Japonica | NA | | | | RNAse l inhibitor protein 2 |
| maker-10-augustus-gene-108.44-mRNA-1 | Japonica | NA | | Os10g0437000 | OsR498G1018782300.01.T01 | Beta-glucuronosyltransferase GlcAT14A |
| maker-10-augustus-gene-108.48-mRNA-1 | Japonica | NA | | Os10g0437100 | OsR498G1018782800.01.T02 | Amino acid permease family protein, expressed |
| maker-10-augustus-gene-108.52-mRNA-1 | Japonica | NA | | | OsR498G1018783200.01.T04 | HRDC domain-containing protein |
| maker-10-augustus-gene-109.0-mRNA-1 | Japonica | NA | | Os10g0437500 | OsR498G1018785700.01.T01 | Usp domain-containing protein (Fragment) |
| maker-10-augustus-gene-109.16-mRNA-1 | Japonica | NA | | Os10g0438000 | OsR498G1018787000.01.T01 | FH protein interacting protein FIP2 |
| maker-10-augustus-gene-109.19-mRNA-1 | Japonica | Japonica | | Os10g0438300 | OsR498G1018787600.01.T01 | |
| maker-10-augustus-gene-109.2-mRNA-1 | Japonica | NA | | Os10g0437600 | OsR498G1018786300.01.T05 | Starch synthase, chloroplastic/amyloplastic |
| maker-10-augustus-gene-109.8-mRNA-1 | Japonica | NA | | Os10g0438100 | OsR498G1018787300.01.T01 | Skp1 family, dimerization domain-containing protein (Fragment) |
| maker-10-augustus-gene-110.38-mRNA-1 | Japonica | NA | | | OsR498G1018792300.01.T04 | Splicing factor, CC1-like family protein, expressed |
| maker-10-augustus-gene-110.45-mRNA-1 | Japonica | NA | | | OsR498G1018794500.01.T01 | HMA domain-containing protein (Fragment) |
| maker-10-augustus-gene-111.36-mRNA-1 | Japonica | NA | | Os10g0441800 | OsR498G1018797700.01.T01 | Ras-related protein RABF1 |
| maker-10-augustus-gene-113.39-mRNA-1 | Japonica | NA | | | OsR498G1018810900.01.T01 | WRC domain-containing protein |
| maker-10-augustus-gene-113.41-mRNA-1 | Japonica | NA | | | | Transmembrane protein like |
| maker-10-augustus-gene-113.43-mRNA-1 | Japonica | NA | | | | Transmembrane protein 8B |
| maker-10-augustus-gene-113.51-mRNA-1 | Japonica | NA | | Os10g0445400 | OsR498G1018809400.01.T02 | E3 ubiquitin-protein ligase AIRP2 |
| maker-10-augustus-gene-113.55-mRNA-1 | Japonica | Japonica | | Os10g0446100 | OsR498G1018811400.01.T01 | WRC domain-containing protein |
| maker-10-augustus-gene-116.37-mRNA-1 | Japonica | NA | | Os04g0166400 | OsR498G0407609800.01.T01 | ELM2 domain-containing protein |
| maker-10-augustus-gene-116.39-mRNA-1 | Japonica | NA | | Os04g0165700 | OsR498G0407604600.01.T01 | Beta-cyanoalanine synthase |
| maker-10-augustus-gene-116.46-mRNA-1 | Japonica | NA | | Os04g0166900 | OsR498G0407612300.01.T01 | F-box domain-containing protein |
| maker-10-augustus-gene-116.54-mRNA-1 | Japonica | NA | | Os04g0165600 | OsR498G0407604300.01.T04 | Mitochondrial inner membrane protease subunit 2 |
| maker-10-augustus-gene-117.46-mRNA-1 | Japonica | NA | | Os04g0165300 | OsR498G0407603300.01.T02 | ATG8-interacting protein 1 |
| maker-10-augustus-gene-117.53-mRNA-1 | Japonica | NA | | | | |
| maker-10-augustus-gene-12.42-mRNA-1 | Japonica | NA | | Os10g0113100 | OsR498G1018038500.01.T01 | Non-functional NADPH-dependent codeinone reductase 2-like |
| maker-10-augustus-gene-123.31-mRNA-1 | Japonica | NA | | Os05g0499400 | OsR498G0407563000.01.T01 | Peroxidase |
| maker-10-augustus-gene-123.33-mRNA-1 | Japonica | NA | | | OsR498G0407560200.01.T01 | DUF4220 domain-containing protein (Fragment) |
| maker-10-augustus-gene-123.34-mRNA-1 | Japonica | NA | | Os04g0151900 | OsR498G0407559800.01.T01 | Cysteine dioxygenase |
| maker-10-augustus-gene-127.41-mRNA-1 | Japonica | NA | Kinase-TM | Os04g0125700 | OsR498G0407534800.01.T01 | Non-specific serine/threonine protein kinase |
| maker-10-augustus-gene-127.44-mRNA-1 | Japonica | NA | | Os03g0114600 | OsR498G0407534000.01.T01 | Calcineurin-like phosphoesterase family protein, putative |
| maker-10-augustus-gene-127.50-mRNA-1 | Japonica | NA | | | OsR498G0407531300.01.T01 | DUF4220 domain-containing protein (Fragment) |
| maker-10-augustus-gene-128.26-mRNA-1 | Japonica | NA | | Os04g0137100 | OsR498G0407528100.01.T01 | Pectate lyase |
| maker-10-augustus-gene-129.0-mRNA-1 | Japonica | NA | | Os04g0476100 | OsR498G0407524800.01.T01 | Amine oxidase |
| maker-10-augustus-gene-13.28-mRNA-1 | Japonica | NA | | Os10g0113900 | OsR498G1018041500.01.T01 | Deoxymugineic acid synthase 1-D |
| maker-10-augustus-gene-13.31-mRNA-1 | Japonica | NA | | Os10g0114300 | OsR498G1018042100.01.T03 | Non-functional NADPH-dependent codeinone reductase 2 |
| maker-10-augustus-gene-133.8-mRNA-1 | Japonica | NA | | Os04g0132300 | OsR498G0407513700.01.T03 | A1 cistron-splicing factor, AAR2 |
| maker-10-augustus-gene-134.2-mRNA-1 | Japonica | NA | | Os04g0129900 | OsR498G0407510000.01.T01 | Dynamin-related protein 4C |
| maker-10-augustus-gene-134.4-mRNA-1 | Japonica | NA | | Os04g0129600 | OsR498G0407508700.01.T01 | Lipoyl synthase |
| maker-10-augustus-gene-134.6-mRNA-1 | Japonica | NA | | Os04g0129500 | OsR498G0407508500.01.T02 | Sec23/Sec24 protein transport family protein isoform 1 |
| maker-10-augustus-gene-136.28-mRNA-1 | Japonica | NA | | Os04g0127200 | OsR498G0407499700.01.T01 | Subtilisin-like protease SBT3.3 |
| maker-10-augustus-gene-136.29-mRNA-1 | Japonica | NA | | Os04g0127300 | OsR498G0407499700.01.T01 | Subtilisin-like protease SBT3.3 |
| maker-10-augustus-gene-138.12-mRNA-1 | Japonica | NA | | | OsR498G0407495100.01.T01 | Jacalin-type lectin domain-containing protein (Fragment) |
| maker-10-augustus-gene-138.3-mRNA-1 | Japonica | NA | | Os04g0127200 | OsR498G0407495300.01.T02 | Subtilisin-like protease |
| maker-10-augustus-gene-138.4-mRNA-1 | Japonica | NA | | | | Subtilisin-like protein protease SBT3.3 |
| maker-10-augustus-gene-139.0-mRNA-1 | Japonica | NA | | Os04g0119500 | OsR498G0407488200.01.T01 | START domain-containing protein |
| maker-10-augustus-gene-139.17-mRNA-1 | Japonica | NA | | Os04g0120100 | OsR498G0407490700.01.T01 | Subtilase family protein, expressed |
| maker-10-augustus-gene-139.19-mRNA-1 | Japonica | NA | | Os04g0120000 | OsR498G0407490300.01.T02 | Subtilisin-like protein protease SBT3.7 |
| maker-10-augustus-gene-139.21-mRNA-1 | Japonica | NA | LRR-TM | | OsR498G0407494400.01.T01 | Protein kinase domain-containing protein |
| maker-10-augustus-gene-139.24-mRNA-1 | Japonica | NA | | Os04g0119400 | OsR498G0407487700.01.T01 | Pyruvate dehydrogenase E1 component subunit alpha |
| maker-10-augustus-gene-139.26-mRNA-1 | Japonica | NA | | Os04g0119300 | OsR498G0305093300.01.T01 | DUF1990 domain-containing protein (Fragment) |
| maker-10-augustus-gene-139.28-mRNA-1 | Japonica | NA | | Os04g0119000 | | |
| maker-10-augustus-gene-139.30-mRNA-1 | Japonica | NA | CC-NBS-TM | Os12g0246700 | OsR498G0407486900.01.T01 | NB-ARC domain containing protein, expressed |
| maker-10-augustus-gene-139.5-mRNA-1 | Japonica | NA | | | OsR498G0306740100.01.T01 | |

| | | | | | | |
|---|---|---|---|---|---|---|
| maker-10-augustus-gene-14.0-mRNA-1 | Japonica | NA | | Os10g0115550 | OsR498G1018044900.01.T01 | Gibberellin-regulated protein 12 |
| maker-10-augustus-gene-14.13-mRNA-1 | Japonica | NA | | | | |
| maker-10-augustus-gene-14.16-mRNA-1 | Japonica | NA | | | OsR498G1018049000.01.T03 | |
| maker-10-augustus-gene-14.3-mRNA-1 | Japonica | NA | | | OsR498G1018048500.01.T01 | Troponin T |
| maker-10-augustus-gene-14.7-mRNA-1 | Japonica | NA | | Os10g0115600 | OsR498G1018045600.01.T03 | U1 small nuclear ribonucleoprotein 70 kDa |
| maker-10-augustus-gene-140.45-mRNA-1 | Japonica | NA | | Os04g0117200 | OsR498G0407483200.01.T03 | MAR-binding filament-like protein 1-1 |
| maker-10-augustus-gene-140.48-mRNA-1 | Japonica | NA | | | OsR498G0407483000.01.T01 | ATP synthase subunit e, mitochondrial |
| maker-10-augustus-gene-140.50-mRNA-1 | Japonica | NA | | Os04g0116900 | OsR498G0407482000.01.T01 | WEB family protein isoform A |
| maker-10-augustus-gene-140.52-mRNA-1 | Japonica | NA | | Os04g0116200 | OsR498G0407478600.01.T01 | |
| maker-10-augustus-gene-140.60-mRNA-1 | Japonica | NA | | Os04g0117800 | OsR498G0407484500.01.T01 | Amidase domain-containing protein (Fragment) |
| maker-10-augustus-gene-140.63-mRNA-1 | Japonica | NA | | Os04g0117600 | OsR498G0407484100.01.T01 | tRNA-dihydrouridine synthase |
| maker-10-augustus-gene-142.0-mRNA-1 | Japonica | NA | | Os08g0230450 | OsR498G1221360100.01.T03 | |
| maker-10-augustus-gene-142.3-mRNA-1 | Japonica | NA | Kinase-TM | Os01g0136400 | OsR498G0100125200.01.T04 | Putative serine/threonine-protein kinase |
| maker-10-augustus-gene-143.0-mRNA-1 | Japonica | NA | | Os04g0112300 | OsR498G0407453400.01.T02 | tRNA (adenine(58)-N(1))-methyltransferase non-catalytic subunit TRM6 |
| maker-10-augustus-gene-143.6-mRNA-1 | Japonica | NA | | Os04g0112200 | | E2F-DP transcription factor |
| maker-10-augustus-gene-145.0-mRNA-1 | Japonica | NA | | | OsR498G0407447800.01.T03 | |
| maker-10-augustus-gene-146.37-mRNA-1 | Japonica | NA | | | OsR498G0407441900.01.T01 | Alpha-humulene synthase |
| maker-10-augustus-gene-15.10-mRNA-1 | Japonica | NA | | Os10g0116900 | OsR498G1018051200.01.T01 | Hydroxyproline-rich glycoprotein family protein, putative, expressed |
| maker-10-augustus-gene-15.19-mRNA-1 | Japonica | NA | | Os10g0118200 | OsR498G1018056400.01.T01 | Acetylserotonin O-methyltransferase 2 |
| maker-10-augustus-gene-15.5-mRNA-1 | Japonica | NA | | Os10g0117800 | OsR498G1018054500.01.T02 | G-protein alpha subunit, putative, expressed |
| maker-10-augustus-gene-156.4-mRNA-1 | Japonica | NA | | | | Myozenin 1 |
| maker-10-augustus-gene-157.10-mRNA-1 | Japonica | NA | | Os10g0455800 | | |
| maker-10-augustus-gene-157.12-mRNA-1 | Japonica | NA | LRR | Os10g0456200 | OsR498G1018837200.01.T01 | LRR repeat and ubiquitin-like domain-containing protein isoform X2 |
| maker-10-augustus-gene-157.13-mRNA-1 | Japonica | NA | | | | LRR repeats and ubiquitin-like domain-containing protein |
| maker-10-augustus-gene-157.24-mRNA-1 | Japonica | NA | | Os10g0456600 | OsR498G1018838600.01.T01 | GTP-binding protein yptV3 |
| maker-10-augustus-gene-157.4-mRNA-1 | Japonica | NA | | | | Glutamate-ammonia ligase |
| maker-10-augustus-gene-158.24-mRNA-1 | Japonica | Indica | | Os10g0457000 | OsR498G1018839900.01.T02 | Splicing factor 3B subunit like |
| maker-10-augustus-gene-158.29-mRNA-1 | Japonica | NA | | Os10g0457400 | OsR498G1018841300.01.T01 | Shugoshin, C-terminal |
| maker-10-augustus-gene-158.46-mRNA-1 | Japonica | NA | | Os10g0456800 | OsR498G1018839000.01.T03 | RING finger and CHY zinc finger domain-containing protein 1 |
| maker-10-augustus-gene-158.52-mRNA-1 | Japonica | NA | | | OsR498G1018839200.01.T01 | Midasin |
| maker-10-augustus-gene-158.55-mRNA-1 | Japonica | NA | | | OsR498G1018839400.01.T05 | Midasin |
| maker-10-augustus-gene-158.69-mRNA-1 | Japonica | NA | | Os10g0457500 | OsR498G1018841600.01.T01 | Inosine triphosphate pyrophosphatase |
| maker-10-augustus-gene-159.7-mRNA-1 | Japonica | NA | | | | ATP-dependent DNA helicase |
| maker-10-augustus-gene-16.34-mRNA-1 | Japonica | NA | | | OsR498G1018061500.01.T02 | Formin-like protein 14 |
| maker-10-augustus-gene-16.43-mRNA-1 | Japonica | NA | | Os10g0118800 | | Cytochrome b reductase 1 |
| maker-10-augustus-gene-16.45-mRNA-1 | Japonica | NA | | | OsR498G1018057400.01.T08 | p-loop containing nucleoside triphosphate hydrolases superfamily protein is |
| maker-10-augustus-gene-16.50-mRNA-1 | Japonica | NA | Kinase-LRR-TM | Os10g0119200 | OsR498G1018061000.01.T01 | Putative LRR receptor-like serine/threonine-protein kinase |
| maker-10-augustus-gene-160.6-mRNA-1 | Japonica | Indica | | Os10g0461100 | OsR498G1018850800.01.T01 | PsbP family protein, expressed |
| maker-10-augustus-gene-160.9-mRNA-1 | Japonica | NA | | Os10g0461100 | OsR498G1018853400.01.T01 | Photosystem II PsbP, oxygen evolving complex |
| maker-10-augustus-gene-162.53-mRNA-1 | Japonica | Indica | | Os10g0465900 | OsR498G1018865700.01.T02 | PfkB domain-containing protein |
| maker-10-augustus-gene-162.65-mRNA-1 | Japonica | NA | | Os10g0464100 | OsR498G1018862000.01.T04 | WD repeat-containing protein 70 |
| maker-10-augustus-gene-162.69-mRNA-1 | Japonica | NA | | Os10g0464400 | OsR498G1018862400.01.T05 | Riboflavin kinase |
| maker-10-augustus-gene-162.86-mRNA-1 | Japonica | Indica | | Os10g0466000 | OsR498G1018866000.01.T03 | RPM1 interacting protein 13 |
| maker-10-augustus-gene-163.24-mRNA-1 | Japonica | NA | | Os10g0466700 | OsR498G1018867600.01.T01 | Ribosomal protein L17 |
| maker-10-augustus-gene-164.25-mRNA-1 | Japonica | NA | Kinase-LRR-TM | Os10g0468500 | OsR498G1018872900.01.T01 | MDIS1-interacting receptor like kinase 2 |
| maker-10-augustus-gene-164.29-mRNA-1 | Japonica | Japonica | LRR-TM | Os10g0469600 | OsR498G1018872900.01.T01 | MDIS1-interacting receptor like kinase 2 |
| maker-10-augustus-gene-165.0-mRNA-1 | Japonica | NA | | Os10g0470700 | OsR498G1018878300.01.T02 | Putative proton-dependent oligopeptide or low-affinity nitrate transporter |
| maker-10-augustus-gene-165.3-mRNA-1 | Japonica | NA | | | OsR498G1018878700.01.T04 | |
| maker-10-augustus-gene-166.0-mRNA-1 | Japonica | NA | | | | Cyanate hydratase |
| maker-10-augustus-gene-166.15-mRNA-1 | Japonica | NA | | Os10g0471400 | OsR498G1018883200.01.T03 | No pollen germination related 2 |
| maker-10-augustus-gene-166.17-mRNA-1 | Japonica | NA | | Os10g0471700 | OsR498G1018884800.01.T01 | Cyclin-dependent kinase inhibitor |
| maker-10-augustus-gene-166.4-mRNA-1 | Japonica | NA | | Os10g0471100 | OsR498G1018881800.01.T01 | Aldehyde oxygenase (deformylating) |
| maker-10-augustus-gene-166.9-mRNA-1 | Japonica | NA | | | OsR498G1018883000.01.T02 | RNA helicase |
| maker-10-augustus-gene-167.0-mRNA-1 | Japonica | NA | | Os10g0472400 | OsR498G1018887500.01.T01 | DUF862 domain-containing protein (Fragment) |
| maker-10-augustus-gene-167.13-mRNA-1 | Japonica | NA | | Os10g0473400 | OsR498G1018890800.01.T03 | Non-lysosomal glucosylceramidase |
| maker-10-augustus-gene-167.19-mRNA-1 | Japonica | NA | | Os10g0474800 | OsR498G1018893600.01.T01 | Long-chain-alcohol oxidase |
| maker-10-augustus-gene-167.20-mRNA-1 | Japonica | Japonica | | Os10g0474900 | OsR498G1018893600.01.T01 | Long-chain-alcohol oxidase |
| maker-10-augustus-gene-167.3-mRNA-1 | Japonica | NA | | Os10g0472900 | OsR498G1018889900.01.T01 | Very-long-chain 3-oxoacyl-CoA synthase |
| maker-10-augustus-gene-167.5-mRNA-1 | Japonica | NA | CC-LRR-NBS-TM | Os10g0474500 | OsR498G1018892300.01.T01 | Putative disease resistance protein RGA3 |
| maker-10-augustus-gene-167.6-mRNA-1 | Japonica | Japonica | | Os10g0474600 | OsR498G1018892700.01.T01 | Putative disease resistance RPP13-like protein 1 |
| maker-10-augustus-gene-168.34-mRNA-1 | Japonica | NA | | | | |

| | | | | | | |
|---|---|---|---|---|---|---|
| maker-10-augustus-gene-168.35-mRNA-1 | Japonica | Japonica | | Os10g0474933 | OsR498G1018894000.01.T01 | Putative polyprotein from transposon TNT |
| maker-10-augustus-gene-168.44-mRNA-1 | Japonica | NA | Kinase-TM | Os10g0476100 | OsR498G1018898700.01.T01 | serine/threonine-protein kinase tricorner isoform X1 |
| maker-10-augustus-gene-168.50-mRNA-1 | Japonica | NA | | Os10g0474300 | OsR498G1018894000.01.T01 | Putative serine protease |
| maker-10-augustus-gene-168.53-mRNA-1 | Japonica | NA | | Os10g0474966 | OsR498G1018896600.01.T01 | Long-chain-alcohol oxidase |
| maker-10-augustus-gene-168.55-mRNA-1 | Japonica | NA | | Os10g0475000 | | Long-chain-alcohol oxidase |
| maker-10-augustus-gene-169.27-mRNA-1 | Japonica | NA | Kinase | Os10g0476300 | OsR498G1018899100.01.T01 | Casein kinase I isoform epsilon |
| maker-10-augustus-gene-169.30-mRNA-1 | Japonica | NA | | Os10g0476600 | OsR498G1018900500.01.T03 | Serine/threonine-protein phosphatase 2A regulatory subunit B'' subunit alpl |
| maker-10-augustus-gene-169.35-mRNA-1 | Japonica | NA | | Os10g0477000 | OsR498G1018901300.01.T01 | Exportin-4 |
| maker-10-augustus-gene-169.38-mRNA-1 | Japonica | NA | | Os10g0477100 | OsR498G1018901500.01.T05 | Putative methyltransferase PMT8 |
| maker-10-augustus-gene-169.43-mRNA-1 | Japonica | NA | | Os10g0476400 | OsR498G1018899600.01.T02 | Vacuolar protein sorting-associated protein 2 |
| maker-10-augustus-gene-169.48-mRNA-1 | Japonica | NA | | Os09g0348800 | OsR498G1221562500.01.T02 | Proline rich 12b |
| maker-10-augustus-gene-17.37-mRNA-1 | Japonica | NA | | Os10g0123200 | OsR498G1018069300.01.T01 | F-box domain containing protein, expressed |
| maker-10-augustus-gene-170.0-mRNA-1 | Japonica | NA | | Os10g0477600 | OsR498G1018903500.01.T01 | NAC transcription factor |
| maker-10-augustus-gene-170.10-mRNA-1 | Japonica | NA | | Os10g0478200 | OsR498G1018906500.01.T01 | Malate dehydrogenase |
| maker-10-augustus-gene-170.15-mRNA-1 | Japonica | Indica | | Os04g0630100 | OsR498G1018904200.01.T01 | Epimerase domain-containing protein (Fragment) |
| maker-10-augustus-gene-170.17-mRNA-1 | Japonica | NA | | Os10g0478000 | OsR498G1018905000.01.T01 | ALOG domain transcriptional regulator family protein 4-2B |
| maker-10-augustus-gene-170.18-mRNA-1 | Japonica | NA | | Os10g0478100 | OsR498G1018906000.01.T05 | Chloroplast J-like domain 1 |
| maker-10-augustus-gene-170.2-mRNA-1 | Japonica | NA | | | | Nuclear transportin |
| maker-10-augustus-gene-170.24-mRNA-1 | Japonica | NA | | Os10g0478300 | OsR498G1018907600.01.T01 | Transcription factor MYB4 |
| maker-10-augustus-gene-171.0-mRNA-1 | Japonica | Indica | | Os10g0478900 | OsR498G1018910500.01.T01 | |
| maker-10-augustus-gene-171.10-mRNA-1 | Japonica | NA | | Os10g0479700 | OsR498G1018913100.01.T03 | TPT domain-containing protein |
| maker-10-augustus-gene-171.12-mRNA-1 | Japonica | NA | | | OsR498G1018914100.01.T03 | START domain-containing protein |
| maker-10-augustus-gene-171.19-mRNA-1 | Japonica | NA | | Os10g0478500 | | MATH domain-containing protein (Fragment) |
| maker-10-augustus-gene-171.22-mRNA-1 | Japonica | NA | | Os10g0478600 | OsR498G1018910000.01.T05 | |
| maker-10-augustus-gene-171.3-mRNA-1 | Japonica | Japonica | | Os10g0479200 | OsR498G1018911300.01.T01 | Putative membrane-associated salt-inducible protein |
| maker-10-augustus-gene-171.7-mRNA-1 | Japonica | NA | | Os10g0479500 | OsR498G1018912700.01.T01 | Cytokinin riboside 5'-monophosphate phosphoribohydrolase |
| maker-10-augustus-gene-172.21-mRNA-1 | Japonica | NA | | Os10g0480200 | OsR498G1018916700.01.T06 | Rolled leaf 2 |
| maker-10-augustus-gene-172.27-mRNA-1 | Japonica | NA | | Os10g0480500 | OsR498G1018917600.01.T02 | Double-stranded RNA-binding protein 6 |
| maker-10-augustus-gene-173.42-mRNA-1 | Japonica | NA | | Os10g0481100 | OsR498G1018923100.01.T01 | Aquaporin PIP2-1 |
| maker-10-augustus-gene-173.44-mRNA-1 | Japonica | NA | | Os10g0481500 | OsR498G1018924900.01.T01 | Flotillin-like protein 1 |
| maker-10-augustus-gene-173.46-mRNA-1 | Japonica | Indica | | Os10g0481400 | OsR498G1018923600.01.T02 | Histone acetyltransferase |
| maker-10-augustus-gene-173.48-mRNA-1 | Japonica | NA | | | OsR498G1018923800.01.T04 | RING-CH-type domain-containing protein |
| maker-10-augustus-gene-173.52-mRNA-1 | Japonica | NA | | | | |
| maker-10-augustus-gene-173.54-mRNA-1 | Japonica | Indica | | Os10g0481500 | OsR498G1018930000.01.T01 | Flotillin-like protein 3 |
| maker-10-augustus-gene-173.56-mRNA-1 | Japonica | Indica | | Os10g0481500 | OsR498G1018931000.01.T01 | Flotillin-like protein 3 |
| maker-10-augustus-gene-173.58-mRNA-1 | Japonica | NA | | Os10g0482900 | OsR498G1018931200.01.T01 | Dishevelled |
| maker-10-augustus-gene-174.0-mRNA-1 | Japonica | Indica | Kinase-TM | Os10g0483400 | OsR498G1120115000.01.T01 | Protein kinase domain containing protein, expressed |
| maker-10-augustus-gene-174.12-mRNA-1 | Japonica | NA | | | OsR498G1018931800.01.T04 | Nascent polypeptide-associated complex subunit beta |
| maker-10-augustus-gene-174.14-mRNA-1 | Japonica | NA | | Os10g0483100 | OsR498G1018932000.01.T01 | Nascent polypeptide-associated complex subunit beta |
| maker-10-augustus-gene-174.16-mRNA-1 | Japonica | NA | | Os10g0484400 | OsR498G1018936400.01.T01 | Ribosomal protein L18/L5 |
| maker-10-augustus-gene-174.18-mRNA-1 | Japonica | NA | | Os10g0484700 | OsR498G1018937800.01.T01 | Kynurenine--oxoglutarate transaminase |
| maker-10-augustus-gene-174.23-mRNA-1 | Japonica | NA | | Os10g0485000 | OsR498G1018938800.01.T03 | Pre-mRNA-processing protein 40C |
| maker-10-augustus-gene-174.27-mRNA-1 | Japonica | Indica | | Os10g0485100 | OsR498G1018939200.01.T03 | Thioredoxin domain-containing protein |
| maker-10-augustus-gene-174.3-mRNA-1 | Japonica | NA | | Os10g0483500 | OsR498G1018934100.01.T02 | Cytokinin dehydrogenase |
| maker-10-augustus-gene-174.6-mRNA-1 | Japonica | Indica | | Os10g0484800 | OsR498G1018938300.01.T01 | Chalcone and stilbene synthases, N-terminal domain containing protein, exp |
| maker-10-augustus-gene-174.9-mRNA-1 | Japonica | NA | | Os10g0483000 | OsR498G1018931800.01.T03 | Nascent polypeptide-associated complex subunit beta |
| maker-10-augustus-gene-175.0-mRNA-1 | Japonica | NA | | | OsR498G1018939300.01.T02 | Protein EXECUTER 1 chloroplastic |
| maker-10-augustus-gene-175.14-mRNA-1 | Japonica | NA | | Os10g0486000 | OsR498G1018941500.01.T02 | HD domain containing protein, expressed |
| maker-10-augustus-gene-175.17-mRNA-1 | Japonica | NA | | | | Mitosis protein YLS8 |
| maker-10-augustus-gene-175.19-mRNA-1 | Japonica | NA | TPR1 | Os10g0486900 | OsR498G1018945500.01.T01 | Tetratricopeptide repeat protein 1 |
| maker-10-augustus-gene-175.22-mRNA-1 | Japonica | Indica | | Os10g0487200 | OsR498G1018945900.01.T01 | |
| maker-10-augustus-gene-175.24-mRNA-1 | Japonica | NA | | Os10g0487300 | OsR498G1018946300.01.T01 | nijmegen breakage syndrome 1 protein isoform X1 |
| maker-10-augustus-gene-175.28-mRNA-1 | Japonica | NA | | | OsR498G1018940800.01.T02 | |
| maker-10-augustus-gene-175.30-mRNA-1 | Japonica | NA | | Os10g0485900 | OsR498G1018941400.01.T01 | |
| maker-10-augustus-gene-175.32-mRNA-1 | Japonica | NA | | Os10g0486100 | OsR498G1018941800.01.T01 | Cytochrome P450 family protein, expressed |
| maker-10-augustus-gene-175.34-mRNA-1 | Japonica | Japonica | | Os10g0486300 | OsR498G1018943600.01.T02 | C2H2-type domain-containing protein |
| maker-10-augustus-gene-175.6-mRNA-1 | Japonica | NA | | Os10g0485500 | OsR498G1018940100.01.T01 | LysM domain containing protein, expressed |
| maker-10-augustus-gene-175.8-mRNA-1 | Japonica | NA | | | | Endoribonuclease Dicer b |
| maker-10-augustus-gene-176.30-mRNA-1 | Japonica | NA | | Os10g0487500 | OsR498G1018946700.01.T02 | SMAP domain-containing protein |
| maker-10-augustus-gene-176.33-mRNA-1 | Japonica | NA | | Os10g0487600 | OsR498G1018946900.01.T04 | SMAP domain-containing protein |
| maker-10-augustus-gene-176.40-mRNA-1 | Japonica | NA | | Os10g0488100 | | Prefoldin alpha subunit |

| | | | | | | |
|---|---|---|---|---|---|---|
| maker-10-augustus-gene-176.44-mRNA-1 | Japonica | NA | | Os10g0488300 | OsR498G1018947300.01.T04 | Vacuolar protein sorting-associated protein 52 A |
| maker-10-augustus-gene-176.47-mRNA-1 | Japonica | NA | | | | Myosin motor domain-containing protein |
| maker-10-augustus-gene-177.0-mRNA-1 | Japonica | NA | | Os10g0489100 | OsR498G1018951100.01.T01 | PrmA domain-containing protein |
| maker-10-augustus-gene-177.11-mRNA-1 | Japonica | NA | | Os10g0489200 | OsR498G1018951400.01.T02 | DNA ligase |
| maker-10-augustus-gene-177.18-mRNA-1 | Japonica | NA | | Os10g0489500 | OsR498G1018952600.01.T01 | Terpene synthase family, metal binding domain containing protein, expresse |
| maker-10-augustus-gene-177.6-mRNA-1 | Japonica | NA | | | OsR498G1018955900.01.T01 | CDT1 domain-containing protein (Fragment) |
| maker-10-augustus-gene-177.8-mRNA-1 | Japonica | NA | | Os10g0489000 | OsR498G1018950800.01.T03 | GRAM domain containing protein, expressed |
| maker-10-augustus-gene-178.40-mRNA-1 | Japonica | NA | | Os10g0491000 | OsR498G1018957900.01.T01 | Basic secretory protease |
| maker-10-augustus-gene-178.42-mRNA-1 | Japonica | NA | | Os10g0491900 | OsR498G1018960700.01.T01 | G patch domain and ankyrin repeat-containing protein 1 |
| maker-10-augustus-gene-178.46-mRNA-1 | Japonica | NA | | Os10g0492600 | OsR498G1018963100.01.T01 | Aquaporin TIP |
| maker-10-augustus-gene-178.48-mRNA-1 | Japonica | NA | | | OsR498G1018963700.01.T01 | Alpha-galactosidase |
| maker-10-augustus-gene-178.58-mRNA-1 | Japonica | NA | | | OsR498G1018961200.01.T01 | Chloroplast inner envelope protein, putative, expressed |
| maker-10-augustus-gene-178.64-mRNA-1 | Japonica | NA | | Os10g0492200 | OsR498G1018962000.01.T03 | Glycosyltransferase, putative, expressed |
| maker-10-augustus-gene-178.66-mRNA-1 | Japonica | NA | | Os10g0492300 | OsR498G1018962600.01.T01 | Protein, chloroplastic |
| maker-10-augustus-gene-178.72-mRNA-1 | Japonica | Indica | | Os10g0492800 | OsR498G1018963400.01.T02 | Metallophos domain-containing protein (Fragment) |
| maker-10-augustus-gene-179.11-mRNA-1 | Japonica | NA | | | OsR498G1018968700.01.T02 | WAT1-related protein, chloroplastic isoform A |
| maker-10-augustus-gene-179.15-mRNA-1 | Japonica | Japonica | | Os10g0494200 | OsR498G0306568200.01.T03 | Probable N-acetyl-gamma-glutamyl-phosphate reductase, chloroplastic |
| maker-10-augustus-gene-179.18-mRNA-1 | Japonica | NA | | Os10g0494300 | OsR498G1018972100.01.T02 | ABC-2 type transporter family protein, expressed |
| maker-10-augustus-gene-179.21-mRNA-1 | Japonica | NA | | Os10g0494500 | OsR498G1018972500.01.T01 | RING-type E3 ubiquitin transferase |
| maker-10-augustus-gene-179.24-mRNA-1 | Japonica | NA | | Os10g0495000 | OsR498G1018975500.01.T02 | KH domain containing protein, expressed |
| maker-10-augustus-gene-179.27-mRNA-1 | Japonica | NA | | Os10g0494000 | OsR498G1018969000.01.T01 | (RS)-norcoclaurine 6-O-methyltransferase-like isoform 1 |
| maker-10-augustus-gene-179.33-mRNA-1 | Japonica | NA | | Os10g0494800 | OsR498G1018972800.01.T04 | Katanin p80 WD40 repeat-containing subunit B1 homolog |
| maker-10-augustus-gene-179.7-mRNA-1 | Japonica | NA | | Os10g0493800 | OsR498G1018968500.01.T04 | Small nuclear RNA activating complex (SNAPc), subunit SNAP |
| maker-10-augustus-gene-18.0-mRNA-1 | Japonica | Japonica | | Os10g0124000 | OsR498G1018070300.01.T01 | U4/U6 small nuclear ribonucleoprotein SNU13 |
| maker-10-augustus-gene-18.3-mRNA-1 | Japonica | Japonica | | Os10g0124100 | OsR498G1018070700.01.T01 | |
| maker-10-augustus-gene-18.5-mRNA-1 | Japonica | NA | | | OsR498G1018071200.01.T01 | NB-ARC domain containing protein |
| maker-10-augustus-gene-18.8-mRNA-1 | Japonica | NA | | Os10g0124700 | OsR498G1018071800.01.T01 | F-box domain containing protein, expressed |
| maker-10-augustus-gene-180.43-mRNA-1 | Japonica | NA | | Os10g0494950 | OsR498G1018972900.01.T01 | Sox C-terminal domain-containing protein |
| maker-10-augustus-gene-180.46-mRNA-1 | Japonica | NA | | Os10g0495000 | OsR498G1018975500.01.T01 | Nucleic acid binding protein |
| maker-10-augustus-gene-180.51-mRNA-1 | Japonica | Indica | | Os04g0678200 | OsR498G1018979500.01.T01 | Fiber protein Fb34 |
| maker-10-augustus-gene-180.53-mRNA-1 | Japonica | Indica | | Os10g0496900 | OsR498G1018982300.01.T01 | NADPH-protochlorophyllide oxidoreductase |
| maker-10-augustus-gene-180.57-mRNA-1 | Japonica | NA | | Os10g0495300 | OsR498G1018978600.01.T01 | ALG-2 interacting protein like |
| maker-10-augustus-gene-180.58-mRNA-1 | Japonica | NA | | Os10g0495400 | OsR498G1018978800.01.T02 | Rf1 protein, mitochondrial, putative, expressed |
| maker-10-augustus-gene-180.67-mRNA-1 | Japonica | NA | | | OsR498G1018979200.01.T01 | Nucleolar complex protein 2 |
| maker-10-augustus-gene-180.74-mRNA-1 | Japonica | NA | | | OsR498G1018979400.01.T01 | DNA-directed RNA polymerase subunit beta |
| maker-10-augustus-gene-180.82-mRNA-1 | Japonica | NA | | | OsR498G1018980800.01.T01 | |
| maker-10-augustus-gene-180.84-mRNA-1 | Japonica | Indica | | Os10g0493600 | OsR498G1018981000.01.T01 | Glycosyl hydrolase, family 13, all-beta |
| maker-10-augustus-gene-181.0-mRNA-1 | Japonica | NA | | Os10g0497000 | OsR498G1018982700.01.T05 | WAPL (Wings apart-like protein regulation of heterochromatin) protein |
| maker-10-augustus-gene-181.1-mRNA-1 | Japonica | NA | | Os10g0497100 | OsR498G1018982900.01.T01 | 1-acyl-sn-glycerol-3-phosphate acyltransferase |
| maker-10-augustus-gene-181.2-mRNA-1 | Japonica | NA | | Os10g0497300 | OsR498G1018983100.01.T03 | Rf1 protein, mitochondrial, putative, expressed |
| maker-10-augustus-gene-181.28-mRNA-1 | Japonica | NA | | Os10g0495000 | OsR498G1018975500.01.T01 | Nucleic acid binding protein |
| maker-10-augustus-gene-181.3-mRNA-1 | Japonica | NA | | | OsR498G1018984100.01.T06 | Rf1 protein, mitochondrial, putative, expressed |
| maker-10-augustus-gene-181.34-mRNA-1 | Japonica | NA | Kinase-TM | Os10g0497600 | OsR498G1018985300.01.T01 | Putative serine/threonine-protein kinase |
| maker-10-augustus-gene-181.39-mRNA-1 | Japonica | NA | | Os10g0497700 | OsR498G1018985500.01.T04 | COBRA-like protein 3 |
| maker-10-augustus-gene-181.43-mRNA-1 | Japonica | NA | | Os10g0497800 | OsR498G1018985700.01.T02 | Prolyl 4-hydroxylase alpha subunit |
| maker-10-augustus-gene-181.47-mRNA-1 | Japonica | Japonica | | Os10g0497900 | OsR498G1018985900.01.T02 | LanC-like protein 2 |
| maker-10-augustus-gene-181.50-mRNA-1 | Japonica | NA | | Os10g0498200 | OsR498G1018986700.01.T01 | Hydrolase, alpha/beta fold family protein, expressed |
| maker-10-augustus-gene-181.53-mRNA-1 | Japonica | NA | | Os10g0498300 | OsR498G1018986900.01.T01 | AB hydrolase-1 domain-containing protein (Fragment) |
| maker-10-augustus-gene-181.56-mRNA-1 | Japonica | NA | | Os10g0498000 | OsR498G1018986200.01.T01 | Soluble epoxide hydrolase / lipid-phosphate phosphatase |
| maker-10-augustus-gene-182.10-mRNA-1 | Japonica | NA | | | OsR498G1018988500.01.T05 | Mediator of RNA polymerase II transcription subunit 16 isoform X2 |
| maker-10-augustus-gene-182.16-mRNA-1 | Japonica | Indica | | Os10g0499400 | OsR498G1018991100.01.T01 | CBS domain-containing protein CBSX5 |
| maker-10-augustus-gene-182.19-mRNA-1 | Japonica | NA | | Os10g0499600 | | Protein NEOXANTHIN-DEFICIENT 1 |
| maker-10-augustus-gene-182.22-mRNA-1 | Japonica | NA | | Os10g0500000 | OsR498G1018993300.01.T01 | E3 ubiquitin-protein ligase SHPRH |
| maker-10-augustus-gene-182.27-mRNA-1 | Japonica | NA | | Os10g0500500 | OsR498G1018993700.01.T01 | Structural constituent of ribosome, putative |
| maker-10-augustus-gene-182.29-mRNA-1 | Japonica | NA | | Os10g0500600 | OsR498G1018994500.01.T03 | Monothiol glutaredoxin-S11 |
| maker-10-augustus-gene-182.3-mRNA-1 | Japonica | NA | | Os10g0498700 | OsR498G1018988100.01.T04 | Mediator of RNA polymerase II transcription subunit |
| maker-10-augustus-gene-182.33-mRNA-1 | Japonica | Indica | | Os10g0500700 | OsR498G1018994500.01.T06 | Monothiol glutaredoxin |
| maker-10-augustus-gene-182.35-mRNA-1 | Japonica | Japonica | | Os10g0501000 | OsR498G1018995300.01.T01 | Pentatricopeptide, putative, expressed |
| maker-10-augustus-gene-182.37-mRNA-1 | Japonica | NA | | Os10g0498900 | OsR498G1018988800.01.T01 | Microtubule-associated protein RP/EB family member 3 |
| maker-10-augustus-gene-182.39-mRNA-1 | Japonica | NA | | | | Transcription factor RAX3 |
| maker-10-augustus-gene-182.41-mRNA-1 | Japonica | Indica | | Os08g0248700 | OsR498G1018992400.01.T01 | Transcription factor MYB36 |

| | | | | | | |
|---|---|---|---|---|---|---|
| maker-10-augustus-gene-182.43-mRNA-1 | Japonica | NA | | Os10g0500400 | OsR498G1018993600.01.T01 | Acyl-CoA N-acyltransferases (NAT) superfamily protein |
| maker-10-augustus-gene-183.40-mRNA-1 | Japonica | Indica | | Os10g0501300 | OsR498G1018995300.01.T01 | Pentatricopeptide, putative, expressed |
| maker-10-augustus-gene-183.42-mRNA-1 | Japonica | NA | | Os10g0501300 | OsR498G1018995500.01.T01 | Pentatricopeptide repeat-containing protein mitochondrial |
| maker-10-augustus-gene-183.44-mRNA-1 | Japonica | NA | | Os10g0501500 | OsR498G1018997100.01.T01 | Calcium uniporter protein (Fragment) |
| maker-10-augustus-gene-183.47-mRNA-1 | Japonica | NA | | Os10g0501900 | OsR498G1018998500.01.T01 | Tetratricopeptide repeat (TPR)-like superfamily protein |
| maker-10-augustus-gene-183.49-mRNA-1 | Japonica | NA | | Os10g0502000 | OsR498G1018998800.01.T01 | Thylakoid lumenal 17 protein, chloroplastic |
| maker-10-augustus-gene-183.51-mRNA-1 | Japonica | NA | | Os10g0502400 | OsR498G1019000200.01.T01 | Glutamyl-tRNA reductase |
| maker-10-augustus-gene-183.53-mRNA-1 | Japonica | NA | | Os10g0502500 | OsR498G1019000800.01.T01 | Cytochrome b5 heme-binding domain-containing protein (Fragment) |
| maker-10-augustus-gene-183.56-mRNA-1 | Japonica | NA | | Os10g0502600 | OsR498G1019001200.01.T04 | Membrane steroid-binding protein 1 |
| maker-10-augustus-gene-183.58-mRNA-1 | Japonica | NA | | Os10g0502900 | OsR498G1019001400.01.T02 | F-box/LRR-repeat protein At3g48880 |
| maker-10-augustus-gene-183.61-mRNA-1 | Japonica | Japonica | | Os10g0503100 | OsR498G0204401000.01.T01 | LIM domain-containing protein PLIM2b |
| maker-10-augustus-gene-184.0-mRNA-1 | Japonica | NA | | Os10g0503500 | OsR498G1019003300.01.T01 | Malic enzyme |
| maker-10-augustus-gene-184.15-mRNA-1 | Japonica | NA | | Os10g0503600 | OsR498G1019004000.01.T01 | Tetraspanin-10 |
| maker-10-augustus-gene-184.19-mRNA-1 | Japonica | NA | | Os10g0503700 | OsR498G1019004200.01.T01 | DEAD-box ATP-dependent RNA helicase |
| maker-10-augustus-gene-184.23-mRNA-1 | Japonica | Indica | | Os02g0824500 | OsR498G1019004800.01.T01 | Remorin |
| maker-10-augustus-gene-184.25-mRNA-1 | Japonica | NA | | Os10g0504200 | OsR498G1019005800.01.T01 | Ascorbate-specific transmembrane electron transporter 2 |
| maker-10-augustus-gene-184.7-mRNA-1 | Japonica | NA | | Os10g0504600 | OsR498G1019006300.01.T01 | C2 NT-type domain-containing protein (Fragment) |
| maker-10-augustus-gene-185.37-mRNA-1 | Japonica | NA | | Os10g0505900 | OsR498G1019008300.01.T01 | Low-temperature-induced 65 kDa protein |
| maker-10-augustus-gene-185.39-mRNA-1 | Japonica | NA | | | | Valyl-tRNA synthetase |
| maker-10-augustus-gene-185.42-mRNA-1 | Japonica | NA | | | | COP9 signalosome complex subunit 6 |
| maker-10-augustus-gene-185.43-mRNA-1 | Japonica | NA | | | | COP9 signalosome complex subunit 6 |
| maker-10-augustus-gene-185.50-mRNA-1 | Japonica | NA | | | OsR498G1019011400.01.T01 | |
| maker-10-augustus-gene-185.52-mRNA-1 | Japonica | NA | | Os10g0506100 | OsR498G1019009000.01.T02 | HMA domain-containing protein (Fragment) |
| maker-10-augustus-gene-185.54-mRNA-1 | Japonica | NA | | Os10g0506800 | OsR498G1019010000.01.T02 | SET and MYND domain-containing protein 4 |
| maker-10-augustus-gene-186.25-mRNA-1 | Japonica | Indica | | Os01g0100400 | OsR498G1019013500.01.T01 | Monocopper oxidase-like protein SKU5 |
| maker-10-augustus-gene-186.28-mRNA-1 | Japonica | NA | | Os10g0508400 | OsR498G1019015500.01.T01 | Methionine aminopeptidase |
| maker-10-augustus-gene-186.33-mRNA-1 | Japonica | Indica | | Os10g0507600 | OsR498G1019011800.01.T01 | MSP domain containing protein, expressed |
| maker-10-augustus-gene-186.37-mRNA-1 | Japonica | NA | | Os10g0507800 | OsR498G1019013400.01.T01 | Chaperone protein like |
| maker-10-augustus-gene-186.43-mRNA-1 | Japonica | Japonica | | Os10g0508300 | OsR498G1019015000.01.T01 | C2C2-YABBY-transcription factor 12 |
| maker-10-augustus-gene-187.0-mRNA-1 | Japonica | Japonica | | Os10g0508600 | OsR498G1019015900.01.T01 | Protein jagunal like |
| maker-10-augustus-gene-187.10-mRNA-1 | Japonica | NA | | | OsR498G1019018100.01.T01 | Plastid-lipid-associated protein 10 |
| maker-10-augustus-gene-187.14-mRNA-1 | Japonica | NA | | Os10g0510000 | OsR498G1019021500.01.T02 | Actin |
| maker-10-augustus-gene-187.16-mRNA-1 | Japonica | Japonica | | Os10g0508500 | OsR498G1019015800.01.T02 | Tubulin-specific chaperone D |
| maker-10-augustus-gene-187.22-mRNA-1 | Japonica | NA | | Os10g0509800 | | |
| maker-10-augustus-gene-187.3-mRNA-1 | Japonica | Indica | | Os10g0509000 | OsR498G1019017300.01.T07 | Endonuclease MutS2 isoform X1 |
| maker-10-augustus-gene-187.9-mRNA-1 | Japonica | NA | | Os10g0509100 | OsR498G1019017900.01.T05 | Cyclic AMP-responsive element-binding 3 |
| maker-10-augustus-gene-188.13-mRNA-1 | Japonica | NA | | | OsR498G1019028300.01.T01 | Zinc finger CCCH domain-containing protein 44 |
| maker-10-augustus-gene-188.19-mRNA-1 | Japonica | Japonica | | Os10g0510700 | OsR498G1019025000.01.T01 | Calcium protein ( calmodulin-dependent protein kinase homolog\|CaM kinas |
| maker-10-augustus-gene-188.25-mRNA-1 | Japonica | Japonica | | Os10g0512100 | OsR498G1019028800.01.T02 | Putative DUF26 domain receptor-like protein kinase |
| maker-10-augustus-gene-188.8-mRNA-1 | Japonica | Indica | | Os10g0511400 | OsR498G1019027700.01.T03 | Prolyl carboxypeptidase like protein, putative, expressed |
| maker-10-augustus-gene-189.0-mRNA-1 | Japonica | NA | | Os10g0512400 | OsR498G1019030000.01.T01 | Ferulate 5-hydroxylase |
| maker-10-augustus-gene-189.12-mRNA-1 | Japonica | NA | | | OsR498G1019031400.01.T01 | Aquaporin NIP |
| maker-10-augustus-gene-189.2-mRNA-1 | Japonica | NA | | Os10g0512700 | OsR498G1019030400.01.T01 | SH3 domain-containing protein 2 |
| maker-10-augustus-gene-189.7-mRNA-1 | Japonica | NA | | | OsR498G1019030600.01.T03 | Kinesin-like protein KIN-7D mitochondrial |
| maker-10-augustus-gene-19.32-mRNA-1 | Japonica | NA | | | OsR498G1018073600.01.T01 | F-box domain containing protein, expressed |
| maker-10-augustus-gene-19.34-mRNA-1 | Japonica | NA | | | OsR498G1018073800.01.T01 | F-box domain containing protein, expressed |
| maker-10-augustus-gene-19.36-mRNA-1 | Japonica | Japonica | | Os10g0126500 | OsR498G1018080600.01.T01 | F-box domain containing protein, expressed |
| maker-10-augustus-gene-19.38-mRNA-1 | Japonica | NA | | Os10g0126600 | OsR498G1018081000.01.T01 | F-box domain containing protein, expressed |
| maker-10-augustus-gene-19.40-mRNA-1 | Japonica | NA | | Os10g0126700 | OsR498G1018081200.01.T01 | F-box domain containing protein, expressed |
| maker-10-augustus-gene-190.57-mRNA-1 | Japonica | NA | | Os10g0516100 | OsR498G1019036300.01.T01 | Glycine cleavage system H protein, mitochondrial |
| maker-10-augustus-gene-190.59-mRNA-1 | Japonica | Indica | | Os10g0516300 | OsR498G1019038500.01.T07 | Electron transfer flavoprotein-ubiquinone oxidoreductase |
| maker-10-augustus-gene-190.66-mRNA-1 | Japonica | Indica | Kinase | Os03g0113000 | OsR498G1019036800.01.T01 | Putative receptor protein kinase |
| maker-10-augustus-gene-191.0-mRNA-1 | Japonica | NA | | Os10g0516800 | OsR498G1019043100.01.T01 | Sphingoid long-chain bases kinase 1 |
| maker-10-augustus-gene-191.13-mRNA-1 | Japonica | NA | | Os10g0516600 | OsR498G1019042200.01.T01 | Beta-1,3-N-acetylglucosaminyltransferase lunatic fringe |
| maker-10-augustus-gene-191.16-mRNA-1 | Japonica | NA | | Os10g0516900 | OsR498G1019043400.01.T01 | |
| maker-10-augustus-gene-191.18-mRNA-1 | Japonica | NA | | | | |
| maker-10-augustus-gene-191.20-mRNA-1 | Japonica | NA | | Os10g0517500 | OsR498G1019047400.01.T01 | Cys/Met metabolism PLP-dependent enzyme family protein, expressed |
| maker-10-augustus-gene-191.6-mRNA-1 | Japonica | NA | | Os10g0517400 | OsR498G1019046900.01.T05 | Pyridoxal reductase, chloroplastic |
| maker-10-augustus-gene-191.9-mRNA-1 | Japonica | NA | | | OsR498G1019040200.01.T03 | GAMYB transcription factor |
| maker-10-augustus-gene-192.39-mRNA-1 | Japonica | NA | | Os10g0518100 | OsR498G1019048900.01.T02 | Rab-GAP TBC domain-containing protein (Fragment) |
| maker-10-augustus-gene-192.42-mRNA-1 | Japonica | NA | | Os10g0518300 | OsR498G1019049300.01.T03 | Hop-interacting protein THI002 |

| | | | | | | |
|---|---|---|---|---|---|---|
| maker-10-augustus-gene-192.47-mRNA-1 | Japonica | NA | Kinase-TM | Os10g0518800 | OsR498G1019051100.01.T01 | Protein kinase domain containing protein, expressed |
| maker-10-augustus-gene-192.53-mRNA-1 | Japonica | NA | | Os10g0519600 | OsR498G1019053100.01.T01 | Major facilitator superfamily domain-containing protein 5 |
| maker-10-augustus-gene-192.59-mRNA-1 | Japonica | NA | | | | Cytochrome b5 heme-binding domain-containing protein (Fragment) |
| maker-10-augustus-gene-192.62-mRNA-1 | Japonica | NA | | Os10g0518900 | OsR498G1019051400.01.T01 | DNA excision repair protein like |
| maker-10-augustus-gene-193.0-mRNA-1 | Japonica | NA | | | OsR498G1019056900.01.T01 | Impaired sucrose induction protein, putative |
| maker-10-augustus-gene-193.11-mRNA-1 | Japonica | NA | | | | Alpha,alpha-trehalase |
| maker-10-augustus-gene-193.16-mRNA-1 | Japonica | NA | | Os10g0521100 | OsR498G1019058200.01.T01 | Actin-depolymerizing factor |
| maker-10-augustus-gene-193.19-mRNA-1 | Japonica | NA | | | | E3 ubiquitin-protein ligase XBAT33 |
| maker-10-augustus-gene-193.2-mRNA-1 | Japonica | NA | | | OsR498G1019058300.01.T02 | Inner membrane protein translocase involved in respiratory chain assembly |
| maker-10-augustus-gene-193.26-mRNA-1 | Japonica | NA | Kinase-TM | Os10g0521700 | OsR498G1019060000.01.T01 | Non-specific serine/threonine protein kinase |
| maker-10-augustus-gene-193.5-mRNA-1 | Japonica | NA | | Os10g0521500 | OsR498G1019058900.01.T01 | AB hydrolase-1 domain-containing protein |
| maker-10-augustus-gene-193.7-mRNA-1 | Japonica | Indica | | Os10g0520600 | OsR498G1019056600.01.T01 | Plant UBX domain-containing protein 10 |
| maker-10-augustus-gene-193.9-mRNA-1 | Japonica | NA | | Os10g0520700 | OsR498G1019056800.01.T01 | Zinc finger HIT domain-containing protein 2 |
| maker-10-augustus-gene-194.0-mRNA-1 | Japonica | NA | | Os10g0521900 | OsR498G1019060300.01.T01 | RHOMBOID-like protein (Fragment) |
| maker-10-augustus-gene-194.20-mRNA-1 | Japonica | NA | | | | 2-oxoglutarate-Fe(II) type oxidoreductase hxnY isoform B |
| maker-10-augustus-gene-194.34-mRNA-1 | Japonica | NA | | Os10g0523100 | OsR498G1019066700.01.T02 | Fe2OG dioxygenase domain-containing protein (Fragment) |
| maker-10-augustus-gene-194.38-mRNA-1 | Japonica | NA | | | OsR498G1019060800.01.T01 | Methyltransferase |
| maker-10-augustus-gene-194.5-mRNA-1 | Japonica | Indica | | Os10g0522400 | OsR498G1019063700.01.T01 | F-box family protein, putative, expressed |
| maker-10-augustus-gene-194.8-mRNA-1 | Japonica | NA | | | OsR498G1019064100.01.T01 | Protein COFACTOR ASSEMBLY OF COMPLEX C SUBUNIT B like |
| maker-10-augustus-gene-195.0-mRNA-1 | Japonica | NA | | Os10g0524000 | OsR498G1019068700.01.T01 | |
| maker-10-augustus-gene-195.15-mRNA-1 | Japonica | Indica | | Os10g0525000 | OsR498G1019071800.01.T01 | Cytochrome P450 family 704 subfamily A polypeptide 2 |
| maker-10-augustus-gene-195.18-mRNA-1 | Japonica | Japonica | | Os10g0525000 | OsR498G1019073200.01.T01 | Cytochrome P450 family 704 subfamily A polypeptide 2 |
| maker-10-augustus-gene-195.2-mRNA-1 | Japonica | NA | | Os10g0524500 | OsR498G1019070300.01.T01 | Mandelonitrile lyase |
| maker-10-augustus-gene-195.21-mRNA-1 | Japonica | NA | | Os10g0525200 | OsR498G1019073800.01.T01 | Cytochrome P450 family 704 subfamily A polypeptide 2 |
| maker-10-augustus-gene-195.23-mRNA-1 | Japonica | NA | | Os10g0525400 | OsR498G1019074000.01.T01 | Glutathione S-transferase U22 |
| maker-10-augustus-gene-195.25-mRNA-1 | Japonica | Indica | | Os10g0525800 | OsR498G1019074400.01.T01 | Glutathione transferase31 |
| maker-10-augustus-gene-195.27-mRNA-1 | Japonica | NA | | Os10g0525800 | OsR498G1019075200.01.T01 | Glutathione S-transferase, N-terminal domain containing protein, expressed |
| maker-10-augustus-gene-195.4-mRNA-1 | Japonica | NA | | Os10g0525600 | OsR498G1019074700.01.T01 | Glutathione S-transferase, N-terminal domain containing protein, expressed |
| maker-10-augustus-gene-195.6-mRNA-1 | Japonica | NA | | Os10g0524100 | OsR498G1019069000.01.T01 | Post-GPI attachment to proteins factor 3 |
| maker-10-augustus-gene-195.9-mRNA-1 | Japonica | NA | | Os10g0524400 | OsR498G1019070200.01.T02 | Phospholipase D |
| maker-10-augustus-gene-20.0-mRNA-1 | Japonica | Indica | | Os10g0126600 | OsR498G1018082400.01.T02 | F-box domain containing protein, expressed |
| maker-10-augustus-gene-20.3-mRNA-1 | Japonica | NA | | Os10g0127700 | OsR498G1018082800.01.T01 | DUF639 domain-containing protein |
| maker-10-augustus-gene-21.0-mRNA-1 | Japonica | Japonica | | Os10g0130500 | OsR498G1018089500.01.T01 | |
| maker-10-augustus-gene-21.12-mRNA-1 | Japonica | Indica | NBS-TM | Os10g0130600 | OsR498G1018091000.01.T01 | Disease resistance protein RGA4 |
| maker-10-augustus-gene-21.3-mRNA-1 | Japonica | NA | | Os05g0269800 | | DDE Tnp4 domain-containing protein |
| maker-10-augustus-gene-22.26-mRNA-1 | Japonica | Indica | NBS-TM PIK, PIKM | Os11g0689100 | OsR498G1018095600.01.T01 | Disease resistance protein Piks-2 |
| maker-10-augustus-gene-22.27-mRNA-1 | Japonica | NA | CC-NBS-TM | Os10g0131901 | OsR498G1018095600.01.T01 | Disease resistance protein Pikm2-TS |
| maker-10-augustus-gene-23.27-mRNA-1 | Japonica | NA | LRR-NBS-TM | Os10g0132500 | OsR498G1018099300.01.T02 | Putative retrotransposable elements TNP2 |
| maker-10-augustus-gene-23.34-mRNA-1 | Japonica | Indica | CC-NBS-TM | Os10g0131000 | OsR498G1018101100.01.T01 | Disease resistance protein RPM1 |
| maker-10-augustus-gene-23.37-mRNA-1 | Japonica | NA | | Os10g0133500 | OsR498G1018102900.01.T01 | Acyl transferase 1 |
| maker-10-augustus-gene-23.41-mRNA-1 | Japonica | NA | | | OsR498G1018098600.01.T01 | Beta-galactosidase |
| maker-10-augustus-gene-24.0-mRNA-1 | Japonica | NA | | Os10g0134500 | OsR498G1018104900.01.T01 | F-box domain-containing protein |
| maker-10-augustus-gene-24.2-mRNA-1 | Japonica | NA | | Os10g0135200 | OsR498G1018106600.01.T02 | Ras-related small GTP-binding family protein |
| maker-10-augustus-gene-25.33-mRNA-1 | Japonica | Japonica | Kinase-TM | Os10g0136500 | OsR498G1018109100.01.T04 | Protein kinase domain containing protein, expressed |
| maker-10-augustus-gene-25.38-mRNA-1 | Japonica | Japonica | | Os10g0136200 | OsR498G1018108800.01.T03 | F-box domain containing protein, expressed |
| maker-10-augustus-gene-25.40-mRNA-1 | Japonica | NA | | | | F-box domain containing protein, expressed |
| maker-10-augustus-gene-26.0-mRNA-1 | Japonica | NA | | Os10g0138100 | OsR498G1018112500.01.T01 | Aldehyde oxidase1 |
| maker-10-augustus-gene-26.6-mRNA-1 | Japonica | NA | | Os10g0138000 | | F-box domain containing protein |
| maker-10-augustus-gene-29.0-mRNA-1 | Japonica | NA | | | OsR498G1018125700.01.T01 | DUF4220 domain-containing protein (Fragment) |
| maker-10-augustus-gene-34.0-mRNA-1 | Japonica | NA | | Os10g0149900 | OsR498G1018151500.01.T01 | Proline-rich protein, putative, expressed |
| maker-10-augustus-gene-34.11-mRNA-1 | Japonica | Japonica | Kinase-TM | Os10g0151100 | OsR498G1018157400.01.T01 | Wall-associated receptor kinase 2 |
| maker-10-augustus-gene-34.14-mRNA-1 | Japonica | NA | | Os10g0150300 | OsR498G1018152000.01.T01 | Proline-rich protein (Fragment) |
| maker-10-augustus-gene-34.16-mRNA-1 | Japonica | Japonica | | Os10g0150400 | OsR498G1018152200.01.T01 | Proline-rich protein (Fragment) |
| maker-10-augustus-gene-34.5-mRNA-1 | Japonica | Japonica | | Os10g0150600 | OsR498G1018152300.01.T01 | Proline-rich protein 4 |
| maker-10-augustus-gene-34.7-mRNA-1 | Japonica | NA | | Os10g0150700 | OsR498G1018152700.01.T01 | Proline-rich protein 4 |
| maker-10-augustus-gene-34.9-mRNA-1 | Japonica | Japonica | | Os10g0150800 | OsR498G1018152900.01.T01 | Proline-rich protein 4 |
| maker-10-augustus-gene-35.0-mRNA-1 | Japonica | NA | Kinase-TM | | | Wall-associated receptor kinase 4 |
| maker-10-augustus-gene-37.30-mRNA-1 | Japonica | NA | | | OsR498G1018165700.01.T03 | Nuclear pore complex protein Nup188a |
| maker-10-augustus-gene-37.35-mRNA-1 | Japonica | NA | | | OsR498G1018166100.01.T01 | Nucleoporin NUP188 |
| maker-10-augustus-gene-39.27-mRNA-1 | Japonica | NA | | | OsR498G1018182100.01.T01 | TF-B3 domain-containing protein |
| maker-10-augustus-gene-43.34-mRNA-1 | Japonica | NA | | | | Amino-acid permease BAT1 |

| | | | | | | |
|---|---|---|---|---|---|---|
| maker-10-augustus-gene-43.37-mRNA-1 | Japonica | NA | | | OsR498G1018204200.01.T02 | NPH3 domain-containing protein |
| maker-10-augustus-gene-43.39-mRNA-1 | Japonica | Indica | Kinase-TM | Os10g0174800 | OsR498G1018204400.01.T01 | Wall associated kinase |
| maker-10-augustus-gene-43.42-mRNA-1 | Japonica | NA | Kinase-TM | | OsR498G1018205800.01.T01 | Wall associated kinase |
| maker-10-augustus-gene-44.23-mRNA-1 | Japonica | Indica | | Os01g0946300 | OsR498G1018221300.01.T01 | Lecithin-cholesterol acyltransferase-like 1 |
| maker-10-augustus-gene-45.0-mRNA-1 | Japonica | NA | | Os10g0158400 | OsR498G1018222500.01.T01 | Putative chalcone synthase |
| maker-10-augustus-gene-45.2-mRNA-1 | Japonica | Indica | | Os10g0158400 | OsR498G1018222000.01.T01 | Putative chalcone synthase |
| maker-10-augustus-gene-45.4-mRNA-1 | Japonica | NA | | | | Autophagy-related protein 9 |
| maker-10-augustus-gene-45.6-mRNA-1 | Japonica | NA | | | OsR498G1018226400.01.T01 | |
| maker-10-augustus-gene-46.31-mRNA-1 | Japonica | NA | | Os08g0459400 | OsR498G0305568900.01.T01 | DDE Tnp4 domain-containing protein |
| maker-10-augustus-gene-48.33-mRNA-1 | Japonica | NA | | | | |
| maker-10-augustus-gene-58.47-mRNA-1 | Japonica | Japonica | | Os10g0181200 | OsR498G1018281600.01.T07 | Pentatricopeptide repeat |
| maker-10-augustus-gene-59.35-mRNA-1 | Japonica | NA | | Os10g0183900 | OsR498G1018292300.01.T01 | rRNA adenine N(6)-methyltransferase |
| maker-10-augustus-gene-59.37-mRNA-1 | Japonica | NA | NBS-TM | Os10g0183000 | OsR498G1018288800.01.T04 | NB-ARC domain containing protein, expressed |
| maker-10-augustus-gene-60.0-mRNA-1 | Japonica | Indica | | Os03g0251500 | OsR498G1018295100.01.T01 | V-type proton ATPase subunit a |
| maker-10-augustus-gene-62.0-mRNA-1 | Japonica | NA | | | | PAP-associated domain-containing protein |
| maker-10-augustus-gene-62.5-mRNA-1 | Japonica | NA | | | OsR498G1018311100.01.T01 | RAB6-interacting golgin |
| maker-10-augustus-gene-63.0-mRNA-1 | Japonica | NA | | | OsR498G1018313100.01.T01 | Oxidoreductase/ zinc ion binding protein |
| maker-10-augustus-gene-63.22-mRNA-1 | Japonica | NA | | Os10g0189100 | OsR498G1018314600.01.T01 | Phosphoglucomutase (alpha-D-glucose-1,6-bisphosphate-dependent) |
| maker-10-augustus-gene-64.14-mRNA-1 | Japonica | NA | | Os08g0271600 | | |
| maker-10-augustus-gene-64.20-mRNA-1 | Japonica | NA | | | OsR498G1018323800.01.T01 | Putative proline-rich cell wall protein |
| maker-10-augustus-gene-64.3-mRNA-1 | Japonica | NA | | | OsR498G1018322500.01.T06 | Nitroreductase |
| maker-10-augustus-gene-64.6-mRNA-1 | Japonica | Japonica | | Os10g0190900 | OsR498G0408361700.01.T01 | Protein DETOXIFICATION |
| maker-10-augustus-gene-64.9-mRNA-1 | Japonica | NA | | Os10g0189600 | OsR498G1018317800.01.T02 | Long chain base biosynthesis protein 1 |
| maker-10-augustus-gene-66.0-mRNA-1 | Japonica | Japonica | | Os10g0193100 | OsR498G1018327500.01.T01 | AAI domain-containing protein |
| maker-10-augustus-gene-66.2-mRNA-1 | Japonica | NA | | Os10g0193500 | OsR498G1018329300.01.T01 | AAI domain-containing protein |
| maker-10-augustus-gene-72.39-mRNA-1 | Japonica | Indica | | Os10g0196100 | OsR498G1018339300.01.T01 | Coumaroyl shikimate 3-hydroxylase 2 |
| maker-10-augustus-gene-72.41-mRNA-1 | Japonica | NA | | | OsR498G1018339700.01.T04 | |
| maker-10-augustus-gene-73.33-mRNA-1 | Japonica | NA | | Os10g0198600 | OsR498G1018343700.01.T03 | IENR2 domain-containing protein (Fragment) |
| maker-10-augustus-gene-73.45-mRNA-1 | Japonica | NA | | Os10g0322250 | | HAT family dimerisation domain containing protein, expressed |
| maker-10-augustus-gene-73.49-mRNA-1 | Japonica | NA | | Os10g0198100 | OsR498G1018341600.01.T01 | |
| maker-10-augustus-gene-73.51-mRNA-1 | Japonica | NA | | | | Mevalonate kinase |
| maker-10-augustus-gene-74.0-mRNA-1 | Japonica | NA | | | | |
| maker-10-augustus-gene-74.5-mRNA-1 | Japonica | Indica | | Os10g0199500 | OsR498G1018348300.01.T03 | |
| maker-10-augustus-gene-75.0-mRNA-1 | Japonica | NA | Kinase | | OsR498G1018352600.01.T02 | Putative receptor-like protein kinase |
| maker-10-augustus-gene-75.3-mRNA-1 | Japonica | NA | | Os03g0129000 | OsR498G1018354600.01.T02 | Phosphatidylinositol-glycan biosynthesis class F |
| maker-10-augustus-gene-77.25-mRNA-1 | Japonica | Japonica | | Os10g0202900 | OsR498G1018362000.01.T01 | |
| maker-10-augustus-gene-77.27-mRNA-1 | Japonica | NA | | Os10g0203000 | OsR498G1018362600.01.T01 | Calcium-binding endonuclease/exonuclease/phosphatase family |
| maker-10-augustus-gene-78.5-mRNA-1 | Japonica | NA | | Os10g0204100 | OsR498G1018368400.01.T01 | probable E3 ubiquitin-protein ligase LOG2 |
| maker-10-augustus-gene-79.33-mRNA-1 | Japonica | NA | | | OsR498G0100876000.01.T01 | Transposon protein, putative, Mutator sub-class |
| maker-10-augustus-gene-84.0-mRNA-1 | Japonica | NA | | Os10g0209700 | OsR498G1018391500.01.T01 | Heavy metal-associated isoprenylated plant protein 8 |
| maker-11-augustus-gene-0.17-mRNA-1 | Japonica | Japonica | | Os11g0116900 | OsR498G1119325200.01.T01 | WRKY DNA binding domain containing protein, expressed |
| maker-11-augustus-gene-1.39-mRNA-1 | Japonica | NA | | Os12g0117100 | OsR498G1119329500.01.T03 | Methylesterase 17 |
| maker-11-augustus-gene-1.43-mRNA-1 | Japonica | NA | | | | Methylesterase 17 |
| maker-11-augustus-gene-1.47-mRNA-1 | Japonica | NA | | Os11g0119100 | OsR498G1119332100.01.T03 | bifunctional epoxide hydrolase 2 isoform X1 |
| maker-11-augustus-gene-1.51-mRNA-1 | Japonica | Indica | | Os12g0118900 | OsR498G1119332500.01.T01 | Secologanin synthase |
| maker-11-augustus-gene-1.55-mRNA-1 | Japonica | NA | | Os11g0117400 | OsR498G1119327000.01.T01 | WRKY DNA binding domain containing protein, expressed |
| maker-11-augustus-gene-1.57-mRNA-1 | Japonica | NA | | Os11g0117500 | OsR498G1119327400.01.T01 | WRKY transcription factor 64 |
| maker-11-augustus-gene-1.60-mRNA-1 | Japonica | NA | | Os11g0117600 | OsR498G1119327800.01.T01 | WRKY DNA binding domain containing protein, expressed |
| maker-11-augustus-gene-1.62-mRNA-1 | Japonica | NA | | Os11g0117700 | OsR498G1119328600.01.T01 | |
| maker-11-augustus-gene-1.64-mRNA-1 | Japonica | Indica | | Os12g0117250 | OsR498G1119330000.01.T01 | Mannan endo-1,4-beta-mannosidase |
| maker-11-augustus-gene-1.67-mRNA-1 | Japonica | NA | | Os11g0118300 | OsR498G1119330200.01.T02 | Root phototropism protein 2 |
| maker-11-augustus-gene-1.70-mRNA-1 | Japonica | NA | | Os12g0117600 | OsR498G1119330600.01.T01 | BTB/POZ domain-containing protein At3g08570 |
| maker-11-augustus-gene-1.72-mRNA-1 | Japonica | NA | | Os12g0117800 | OsR498G1119331000.01.T02 | NAM-associated domain-containing protein |
| maker-11-augustus-gene-1.73-mRNA-1 | Japonica | NA | | Os11g0118800 | OsR498G1119331600.01.T01 | Mannan endo-1,4-beta-mannosidase |
| maker-11-augustus-gene-1.79-mRNA-1 | Japonica | NA | | | OsR498G1120154400.01.T01 | Endonuclease |
| maker-11-augustus-gene-10.0-mRNA-1 | Japonica | NA | | Os12g0518200 | OsR498G1220892100.01.T01 | |
| maker-11-augustus-gene-10.15-mRNA-1 | Japonica | NA | | | OsR498G1119384700.01.T01 | Transcription initiation factor IIE subunit alpha |
| maker-11-augustus-gene-10.18-mRNA-1 | Japonica | NA | | | OsR498G1119384900.01.T03 | Transcription initiation factor IIE subunit alpha |
| maker-11-augustus-gene-10.23-mRNA-1 | Japonica | NA | | | | Transporter, putative, expressed |
| maker-11-augustus-gene-10.3-mRNA-1 | Japonica | NA | | Os11g0136500 | OsR498G1119382100.01.T03 | ACPS domain-containing protein (Fragment) |
| maker-11-augustus-gene-10.40-mRNA-1 | Japonica | NA | | Os11g0136400 | OsR498G1119382000.01.T03 | MFS domain-containing protein (Fragment) |

| | | | | | | |
|---|---|---|---|---|---|---|
| maker-11-augustus-gene-10.49-mRNA-1 | Japonica | NA | | Os11g0136701 | OsR498G1119382600.01.T02 | Guanine nucleotide-binding protein G(T) subunit alpha-3 like |
| maker-11-augustus-gene-10.53-mRNA-1 | Japonica | NA | | Os11g0136800 | OsR498G1119382800.01.T02 | Protein-tyrosine-phosphatase |
| maker-11-augustus-gene-10.58-mRNA-1 | Japonica | NA | | Os11g0137000 | OsR498G1119383800.01.T01 | Auxin efflux carrier component |
| maker-11-augustus-gene-10.6-mRNA-1 | Japonica | NA | Kinase-TM | Os11g0136600 | OsR498G1119382300.01.T01 | Calcium-dependent protein kinase |
| maker-11-augustus-gene-10.60-mRNA-1 | Japonica | NA | | Os12g0133900 | OsR498G1119384000.01.T01 | U3 small nucleolar ribonucleoprotein protein MPP10 |
| maker-11-augustus-gene-10.65-mRNA-1 | Japonica | Indica | | Os11g0137300 | OsR498G1119384600.01.T01 | Proteasome stabiliser ECM29 |
| maker-11-augustus-gene-10.9-mRNA-1 | Japonica | Indica | | Os12g0134000 | OsR498G1119384300.01.T04 | Pyruvate carboxyltransferase domain-containing protein (Fragment) |
| maker-11-augustus-gene-101.37-mRNA-1 | Japonica | NA | | Os02g0530900 | OsR498G0203991600.01.T01 | |
| maker-11-augustus-gene-101.39-mRNA-1 | Japonica | NA | | Os11g0416900 | OsR498G1119927600.01.T02 | White-brown complex like protein 30 |
| maker-11-augustus-gene-102.0-mRNA-1 | Japonica | NA | | Os11g0417400 | OsR498G1119930100.01.T01 | Pyruvate dehydrogenase phosphatase |
| maker-11-augustus-gene-105.33-mRNA-1 | Japonica | NA | | Os11g0421800 | OsR498G1119966200.01.T01 | |
| maker-11-augustus-gene-106.0-mRNA-1 | Japonica | Indica | | Os11g0423800 | OsR498G1119975500.01.T07 | Mitochondrial arginine transporter like |
| maker-11-augustus-gene-107.0-mRNA-1 | Japonica | NA | | Os11g0424400 | OsR498G1119977800.01.T01 | Myosin family protein, putative, expressed |
| maker-11-augustus-gene-108.35-mRNA-1 | Japonica | Japonica | | Os11g0425100 | OsR498G1119982900.01.T01 | Fruit bromelain |
| maker-11-augustus-gene-108.38-mRNA-1 | Japonica | NA | | Os11g0425300 | OsR498G1119984600.01.T03 | Protein PEP-RELATED DEVELOPMENT ARRESTED 1 homolog, chloroplastic |
| maker-11-augustus-gene-109.0-mRNA-1 | Japonica | NA | | Os11g0425800 | OsR498G1119987500.01.T02 | Ribosomal protein L13a |
| maker-11-augustus-gene-109.2-mRNA-1 | Japonica | Indica | | Os11g0426400 | OsR498G1119990700.01.T01 | Phytocyanin domain-containing protein (Fragment) |
| maker-11-augustus-gene-11.49-mRNA-1 | Japonica | NA | | Os12g0136200 | OsR498G1119392100.01.T01 | Heterogeneous nuclear ribonucleoprotein Q |
| maker-11-augustus-gene-11.55-mRNA-1 | Japonica | NA | | Os12g0136900 | OsR498G1119394100.01.T05 | Calcium-transporting ATPase |
| maker-11-augustus-gene-11.61-mRNA-1 | Japonica | NA | | | | Phosphoglycerate mutase family protein, expressed |
| maker-11-augustus-gene-11.66-mRNA-1 | Japonica | NA | | Os11g0138600 | OsR498G1119390600.01.T03 | Phosphoglycerate mutase gpmB |
| maker-11-augustus-gene-11.71-mRNA-1 | Japonica | NA | | Os11g0139100 | OsR498G1119391800.01.T01 | KOW domain-containing protein |
| maker-11-augustus-gene-11.76-mRNA-1 | Japonica | NA | | Os11g0139850 | OsR498G1119393400.01.T04 | Gamma-glutamylcyclotransferase family protein (Fragment) |
| maker-11-augustus-gene-11.79-mRNA-1 | Japonica | NA | | Os11g0140100 | OsR498G1119393800.01.T04 | Mitochondrial acidic protein MAM33 |
| maker-11-augustus-gene-110.0-mRNA-1 | Japonica | NA | | Os08g0230450 | | |
| maker-11-augustus-gene-111.32-mRNA-1 | Japonica | NA | | Os11g0428800 | OsR498G1120003500.01.T01 | Phytocyanin domain-containing protein (Fragment) |
| maker-11-augustus-gene-111.33-mRNA-1 | Japonica | Indica | | Os08g0560300 | OsR498G1120003100.01.T01 | PLATZ transcription factor family protein |
| maker-11-augustus-gene-111.40-mRNA-1 | Japonica | Indica | | Os11g0429600 | OsR498G1120007200.01.T01 | Serine carboxypeptidase-like 19 |
| maker-11-augustus-gene-111.43-mRNA-1 | Japonica | NA | | Os11g0430000 | OsR498G1120008800.01.T02 | Serine carboxypeptidase-like 19 |
| maker-11-augustus-gene-112.25-mRNA-1 | Japonica | NA | | Os08g0230450 | | |
| maker-11-augustus-gene-112.27-mRNA-1 | Japonica | Indica | | Os11g0432900 | OsR498G1120010000.01.T02 | Serine carboxypeptidase-like 19 |
| maker-11-augustus-gene-112.30-mRNA-1 | Japonica | NA | | Os11g0431400 | | Serine carboxypeptidase-like 11 |
| maker-11-augustus-gene-112.31-mRNA-1 | Japonica | NA | | | OsR498G1120010600.01.T04 | Serine carboxypeptidase-like 19 |
| maker-11-augustus-gene-113.3-mRNA-1 | Japonica | NA | | Os11g0432600 | OsR498G1120015100.01.T01 | Very-long-chain 3-oxoacyl-CoA reductase 1 |
| maker-11-augustus-gene-113.5-mRNA-1 | Japonica | Indica | | Os11g0432900 | OsR498G1120011800.01.T03 | Serine carboxypeptidase-like 19 |
| maker-11-augustus-gene-113.7-mRNA-1 | Japonica | NA | | Os11g0432800 | OsR498G1120015800.01.T02 | Protein RETICULATA, chloroplastic |
| maker-11-augustus-gene-113.8-mRNA-1 | Japonica | NA | | | | Serine carboxypeptidase-like 11 |
| maker-11-augustus-gene-114.31-mRNA-1 | Japonica | Indica | | Os11g0433200 | OsR498G1120016900.01.T02 | Signal peptide peptidase family protein, expressed isoform 1 |
| maker-11-augustus-gene-114.35-mRNA-1 | Japonica | NA | | | OsR498G1120018300.01.T03 | Protein transport protein SEC23 |
| maker-11-augustus-gene-114.42-mRNA-1 | Japonica | Indica | | Os07g0546700 | OsR498G1120018900.01.T01 | 60S ribosomal protein L38 |
| maker-11-augustus-gene-114.46-mRNA-1 | Japonica | NA | | Os11g0434000 | OsR498G1120019200.01.T01 | Magnesium-dependent phosphatase 1 |
| maker-11-augustus-gene-115.34-mRNA-1 | Japonica | NA | | | OsR498G1120020200.01.T01 | SWI/SNF complex subunit SWI3C-like |
| maker-11-augustus-gene-115.36-mRNA-1 | Japonica | NA | | Os11g0435500 | | Galactinol--sucrose galactosyltransferase |
| maker-11-augustus-gene-116.34-mRNA-1 | Japonica | NA | | | OsR498G1120025500.01.T01 | probable 2-oxoglutarate-dependent dioxygenase At3g111800 |
| maker-11-augustus-gene-117.0-mRNA-1 | Japonica | Indica | | Os11g0438700 | OsR498G1120043200.01.T01 | Noroxomaritidine/norcraugsodine reductase |
| maker-11-augustus-gene-117.3-mRNA-1 | Japonica | NA | | Os11g0438700 | OsR498G1120041800.01.T03 | Tropinone reductase I |
| maker-11-augustus-gene-117.8-mRNA-1 | Japonica | NA | | Os11g0439100 | OsR498G1120039600.01.T01 | Tropinone reductase like |
| maker-11-augustus-gene-118.28-mRNA-1 | Japonica | NA | | | OsR498G0100876000.01.T01 | Transposon protein, putative, Mutator sub-class, expressed |
| maker-11-augustus-gene-118.30-mRNA-1 | Japonica | NA | | Os11g0440200 | OsR498G1120037500.01.T01 | GDA1/CD39 family protein, expressed |
| maker-11-augustus-gene-118.36-mRNA-1 | Japonica | NA | | Os11g0440200 | OsR498G1120037500.01.T01 | GDA1/CD39 family protein, expressed |
| maker-11-augustus-gene-119.31-mRNA-1 | Japonica | NA | | Os11g0441500 | OsR498G1120030500.01.T01 | Glycosyltransferase |
| maker-11-augustus-gene-119.33-mRNA-1 | Japonica | NA | | Os04g0691000 | | F-box domain-containing protein |
| maker-11-augustus-gene-119.34-mRNA-1 | Japonica | NA | | Os11g0442650 | OsR498G1120026900.01.T01 | BHLH transcription factor |
| maker-11-augustus-gene-12.41-mRNA-1 | Japonica | NA | | | OsR498G1119395500.01.T08 | Kallikrein-2 like |
| maker-11-augustus-gene-12.50-mRNA-1 | Japonica | NA | | Os11g0140900 | OsR498G1119395900.01.T01 | |
| maker-11-augustus-gene-12.52-mRNA-1 | Japonica | NA | | Os12g0137500 | OsR498G1220904300.01.T01 | PP2A regulatory subunit like |
| maker-11-augustus-gene-12.61-mRNA-1 | Japonica | NA | | Os01g0861400 | OsR498G0100480900.01.T01 | Vacuolar-sorting receptor 1 |
| maker-11-augustus-gene-12.63-mRNA-1 | Japonica | NA | | Os11g0141900 | OsR498G1119400100.01.T02 | BTB/POZ domain and ankyrin repeat-containing protein NH5.1 |
| maker-11-augustus-gene-12.66-mRNA-1 | Japonica | NA | | Os11g0140600 | OsR498G1119394800.01.T02 | probable calcium-binding protein CML49 |
| maker-11-augustus-gene-120.32-mRNA-1 | Japonica | NA | | Os11g0442900 | | |
| maker-11-augustus-gene-120.33-mRNA-1 | Japonica | NA | | Os11g0443700 | OsR498G1120046000.01.T01 | Tropinone reductase I |

| | | | | | | |
|---|---|---|---|---|---|---|
| maker-11-augustus-gene-120.35-mRNA-1 | Japonica | NA | | Os11g0444000 | OsR498G1120044700.01.T01 | DIMBOA UDP-glucosyltransferase BX8 |
| maker-11-augustus-gene-121.22-mRNA-1 | Japonica | NA | | Os11g0444700 | OsR498G1120048900.01.T01 | PB1 domain-containing protein (Fragment) |
| maker-11-augustus-gene-121.24-mRNA-1 | Japonica | Indica | | Os11g0444700 | OsR498G1120049700.01.T01 | PB1 domain-containing protein (Fragment) |
| maker-11-augustus-gene-121.26-mRNA-1 | Japonica | Indica | | Os11g0446000 | OsR498G1120054300.01.T01 | Serine/threonine-protein kinase WNK (With No Lysine)-related |
| maker-11-augustus-gene-123.36-mRNA-1 | Japonica | NA | | | | Short integuments 2 mitochondrial |
| maker-11-augustus-gene-123.43-mRNA-1 | Japonica | NA | Kinase-TM | | OsR498G1120059900.01.T01 | probable serine/threonine-protein kinase PBL28 |
| maker-11-augustus-gene-123.45-mRNA-1 | Japonica | NA | | Os11g0448400 | OsR498G1120060300.01.T02 | RNA polymerase sigma factor |
| maker-11-augustus-gene-123.48-mRNA-1 | Japonica | NA | Kinase-TM | | | Putative serine/threonine-protein kinase WNK9 (Fragment) |
| maker-11-augustus-gene-126.0-mRNA-1 | Japonica | NA | | Os11g0451700 | OsR498G1120068200.01.T02 | Dehydrin |
| maker-11-augustus-gene-127.29-mRNA-1 | Japonica | NA | | | | |
| maker-11-augustus-gene-127.31-mRNA-1 | Japonica | NA | | Os11g0453900 | OsR498G1120073200.01.T01 | Dehydrin family protein, expressed |
| maker-11-augustus-gene-127.33-mRNA-1 | Japonica | NA | | Os11g0454000 | OsR498G1120074000.01.T01 | Dehydrin |
| maker-11-augustus-gene-127.35-mRNA-1 | Japonica | NA | | Os11g0454200 | OsR498G1120074800.01.T01 | Dehydrin family protein, expressed |
| maker-11-augustus-gene-127.37-mRNA-1 | Japonica | Japonica | | Os11g0454300 | OsR498G1120075200.01.T01 | Deyhydrin 4 |
| maker-11-augustus-gene-128.30-mRNA-1 | Japonica | NA | | Os11g0455800 | OsR498G1120081300.01.T01 | Serine hydroxymethyltransferase |
| maker-11-augustus-gene-128.33-mRNA-1 | Japonica | NA | | Os11g0456300 | OsR498G1120082900.01.T01 | |
| maker-11-augustus-gene-128.36-mRNA-1 | Japonica | NA | | | OsR498G1120078800.01.T01 | Protein MICRORCHIDIA 6 |
| maker-11-augustus-gene-128.38-mRNA-1 | Japonica | NA | | Os11g0455500 | OsR498G1120079200.01.T01 | Adenosylhomocysteinase |
| maker-11-augustus-gene-128.40-mRNA-1 | Japonica | NA | | Os11g0456100 | OsR498G1120082200.01.T01 | UV-B-induced protein, chloroplastic-like protein |
| maker-11-augustus-gene-128.43-mRNA-1 | Japonica | NA | | Os11g0456200 | OsR498G1120082600.01.T01 | |
| maker-11-augustus-gene-128.45-mRNA-1 | Japonica | Japonica | | Os11g0457000 | OsR498G1120085200.01.T01 | Transposon protein, putative, Mariner sub-class, expressed |
| maker-11-augustus-gene-129.0-mRNA-1 | Japonica | NA | | | OsR498G1120089900.01.T01 | DUF629 domain-containing protein |
| maker-11-augustus-gene-13.0-mRNA-1 | Japonica | NA | | | OsR498G1119401900.01.T02 | Strictosidine synthase 3 |
| maker-11-augustus-gene-13.13-mRNA-1 | Japonica | NA | | Os11g0143200 | OsR498G1119403200.01.T02 | CPD brassinosteroid C-23 hydroxylase |
| maker-11-augustus-gene-13.16-mRNA-1 | Japonica | NA | | | | RNA polymerase Rpb5, N-terminal domain containing protein, expressed |
| maker-11-augustus-gene-13.19-mRNA-1 | Japonica | NA | | Os11g0143500 | OsR498G1119403800.01.T01 | L-galactono-1,4-lactone dehydrogenase, mitochondrial |
| maker-11-augustus-gene-13.24-mRNA-1 | Japonica | NA | | | | FBD domain-containing protein (Fragment) |
| maker-11-augustus-gene-13.3-mRNA-1 | Japonica | NA | | Os12g0138900 | OsR498G1220909100.01.T02 | 2-isopropylmalate synthase 1 chloroplastic |
| maker-11-augustus-gene-13.9-mRNA-1 | Japonica | NA | | Os11g0143300 | OsR498G1220910900.01.T01 | Two-component response regulator ORR10 |
| maker-11-augustus-gene-130.29-mRNA-1 | Japonica | NA | | Os11g0461000 | | Serine carboxypeptidase-like |
| maker-11-augustus-gene-130.32-mRNA-1 | Japonica | Indica | | Os11g0460800 | OsR498G1120091900.01.T04 | Serine carboxypeptidase-like protein (Fragment) |
| maker-11-augustus-gene-131.2-mRNA-1 | Japonica | NA | | Os11g0461000 | | Serine carboxypeptidase-like 7 |
| maker-11-augustus-gene-131.6-mRNA-1 | Japonica | NA | | | | Serine carboxypeptidase-like 6 |
| maker-11-augustus-gene-131.7-mRNA-1 | Japonica | Indica | | Os11g0460800 | OsR498G1120091900.01.T04 | Serine carboxypeptidase 1 |
| maker-11-augustus-gene-132.28-mRNA-1 | Japonica | NA | NBS-TM | | | F-box protein FBX14 |
| maker-11-augustus-gene-132.30-mRNA-1 | Japonica | Indica | | Os11g0463600 | OsR498G1120101100.01.T02 | FHA domain containing protein, expressed |
| maker-11-augustus-gene-132.33-mRNA-1 | Japonica | NA | | | OsR498G1120099600.01.T01 | |
| maker-11-augustus-gene-132.35-mRNA-1 | Japonica | NA | | Os11g0463700 | OsR498G1120101600.01.T03 | CRP7-Cysteine-rich family protein expressed |
| maker-11-augustus-gene-133.28-mRNA-1 | Japonica | Indica | | Os08g0381000 | OsR498G1119949600.01.T01 | Cell wall protein-like |
| maker-11-augustus-gene-134.3-mRNA-1 | Japonica | NA | | | OsR498G1120106600.01.T03 | Putative Pyridoxal phosphate (PLP)-dependent transferases superfamily pro |
| maker-11-augustus-gene-135.25-mRNA-1 | Japonica | NA | | | OsR498G0100828800.01.T01 | Hydroxyproline-rich glycoprotein-like |
| maker-11-augustus-gene-135.29-mRNA-1 | Japonica | Indica | | Os11g0469900 | OsR498G1120113400.01.T01 | Indole-2-monooxygenase |
| maker-11-augustus-gene-135.30-mRNA-1 | Japonica | Indica | | Os11g0465200 | OsR498G1120113400.01.T01 | Indole-2-monooxygenase |
| maker-11-augustus-gene-135.35-mRNA-1 | Japonica | Indica | Kinase-TM | Os11g0470200 | OsR498G1120115000.01.T01 | Protein kinase domain containing protein, expressed |
| maker-11-augustus-gene-136.24-mRNA-1 | Japonica | NA | | Os11g0471200 | | |
| maker-11-augustus-gene-136.26-mRNA-1 | Japonica | NA | | Os11g0472000 | OsR498G1120119800.01.T02 | Zinc finger CCCH domain-containing protein 63 |
| maker-11-augustus-gene-137.33-mRNA-1 | Japonica | Indica | | Os11g0472500 | OsR498G1120121900.01.T06 | Probable magnesium transporter |
| maker-11-augustus-gene-137.38-mRNA-1 | Japonica | NA | | | OsR498G1120122500.01.T01 | ROP-INTERACTIVE CRIB MOTIF-CONTAINING protein 2 |
| maker-11-augustus-gene-137.43-mRNA-1 | Japonica | NA | | | OsR498G1120126500.01.T01 | Ubiquitinyl hydrolase 1 |
| maker-11-augustus-gene-137.50-mRNA-1 | Japonica | NA | | Os11g0473100 | OsR498G1120126200.01.T01 | CDK-activating kinase assembly factor MAT1 |
| maker-11-augustus-gene-138.2-mRNA-1 | Japonica | Japonica | | Os11g0474800 | OsR498G0204151800.01.T05 | Ent-isokaurene synthase |
| maker-11-augustus-gene-14.50-mRNA-1 | Japonica | NA | | Os11g0146500 | OsR498G1119406700.01.T01 | Set1/Ash2 histone methyltransferase complex subunit ASH2 |
| maker-11-augustus-gene-14.60-mRNA-1 | Japonica | NA | | | | NAC domain-containing protein |
| maker-11-augustus-gene-14.62-mRNA-1 | Japonica | NA | | Os11g0147100 | OsR498G1119408900.01.T01 | R3H-associated N-terminal domain-containing protein |
| maker-11-augustus-gene-14.66-mRNA-1 | Japonica | NA | | Os11g0147500 | OsR498G1119411100.01.T02 | HMA domain-containing protein (Fragment) |
| maker-11-augustus-gene-14.68-mRNA-1 | Japonica | NA | | Os12g0141100 | OsR498G1119406000.01.T01 | Phosphatidylinositol 4-phosphate 5-kinase |
| maker-11-augustus-gene-14.74-mRNA-1 | Japonica | NA | | Os11g0146700 | OsR498G1119407400.01.T02 | Suppressor of mec-8 and unc-52 protein 2 |
| maker-11-augustus-gene-14.81-mRNA-1 | Japonica | Indica | | Os11g0146800 | OsR498G1119407800.01.T03 | DNA meiotic recombinase 1 |
| maker-11-augustus-gene-14.86-mRNA-1 | Japonica | NA | | Os11g0147000 | OsR498G1119408600.01.T02 | AMP-binding enzyme family protein, expressed |
| maker-11-augustus-gene-14.96-mRNA-1 | Japonica | NA | | | OsR498G1119410000.01.T03 | |
| maker-11-augustus-gene-142.31-mRNA-1 | Japonica | NA | | | | |

| | | | | | | |
|---|---|---|---|---|---|---|
| maker-11-augustus-gene-142.34-mRNA-1 | Japonica | NA | | Os11g0479000 | OsR498G1120142300.01.T01 | Acetylglucosaminyltransferase family protein |
| maker-11-augustus-gene-142.36-mRNA-1 | Japonica | Indica | | Os11g0479232 | OsR498G1120143900.01.T01 | GPI transamidase component PIG-T |
| maker-11-augustus-gene-142.39-mRNA-1 | Japonica | NA | | | | Rab-GAP TBC domain-containing protein |
| maker-11-augustus-gene-142.46-mRNA-1 | Japonica | NA | CC-NBS-TM | Os11g0479100 | OsR498G1120143800.01.T02 | NB-ARC domain, putative |
| maker-11-augustus-gene-144.30-mRNA-1 | Japonica | NA | | Os11g0482100 | OsR498G1120147500.01.T01 | Retrotransposon protein, putative, unclassified, expressed |
| maker-11-augustus-gene-144.36-mRNA-1 | Japonica | NA | | Os11g0482000 | OsR498G1120146800.01.T01 | 40S ribosomal protein S5 isoform A |
| maker-11-augustus-gene-144.38-mRNA-1 | Japonica | Indica | | Os11g0482200 | OsR498G1120147800.01.T01 | AvrRpt-cleavage domain-containing protein |
| maker-11-augustus-gene-144.41-mRNA-1 | Japonica | Indica | | Os11g0482500 | OsR498G1120148800.01.T01 | PHD domain-containing protein |
| maker-11-augustus-gene-145.0-mRNA-1 | Japonica | NA | | Os11g0483600 | OsR498G1120151100.01.T04 | DET1- and DDB1-associated protein 1 |
| maker-11-augustus-gene-145.11-mRNA-1 | Japonica | NA | | Os11g0484300 | OsR498G1120152300.01.T01 | DNA replication licensing factor MCM2 |
| maker-11-augustus-gene-145.13-mRNA-1 | Japonica | NA | | | OsR498G1120152500.01.T02 | Bifunctional dihydrofolate reductase-thymidylate synthase |
| maker-11-augustus-gene-145.18-mRNA-1 | Japonica | NA | | Os11g0487600 | OsR498G1120153700.01.T02 | Cytochrome P450 family 78 subfamily A polypeptide 8 |
| maker-11-augustus-gene-145.21-mRNA-1 | Japonica | NA | | Os11g0484000 | OsR498G1120151800.01.T02 | Probable bifunctional methylthioribulose-1-phosphate dehydratase/enolase |
| maker-11-augustus-gene-145.5-mRNA-1 | Japonica | Japonica | | Os11g0483900 | OsR498G1120151500.01.T04 | ELMO domain-containing protein A |
| maker-11-augustus-gene-145.9-mRNA-1 | Japonica | NA | | | | DNA replication licensing factor MCM2 |
| maker-11-augustus-gene-146.0-mRNA-1 | Japonica | NA | | Os11g0488600 | OsR498G1120154700.01.T02 | ABC transporter domain-containing protein |
| maker-11-augustus-gene-146.10-mRNA-1 | Japonica | NA | | Os11g0488500 | OsR498G1120155000.01.T01 | Alkylated DNA repair protein alkB isogeny |
| maker-11-augustus-gene-148.29-mRNA-1 | Japonica | NA | | Os11g0490600 | OsR498G1120166100.01.T01 | |
| maker-11-augustus-gene-148.31-mRNA-1 | Japonica | NA | | | OsR498G1120167100.01.T01 | Non-intrinsic ABC protein 14 |
| maker-11-augustus-gene-148.34-mRNA-1 | Japonica | NA | | Os11g0490900 | OsR498G1120167900.01.T01 | WRKY transcription factor |
| maker-11-augustus-gene-148.36-mRNA-1 | Japonica | NA | | Os11g0491400 | OsR498G1120168100.01.T03 | Lipase/lipooxygenase, putative isoform 3 |
| maker-11-augustus-gene-149.35-mRNA-1 | Japonica | Indica | | Os11g0492700 | OsR498G1120174100.01.T01 | AB hydrolase-1 domain-containing protein (Fragment) |
| maker-11-augustus-gene-149.40-mRNA-1 | Japonica | NA | CC-NBS-TM | Os11g0493700 | | NB-ARC domain containing protein, expressed |
| maker-11-augustus-gene-149.41-mRNA-1 | Japonica | Indica | | Os11g0492100 | OsR498G1120172400.01.T01 | NB-ARC domain containing protein, expressed |
| maker-11-augustus-gene-15.28-mRNA-1 | Japonica | Indica | | Os12g0145400 | OsR498G1119414500.01.T01 | Protein trichome birefringence-like 10 |
| maker-11-augustus-gene-15.30-mRNA-1 | Japonica | Indica | | Os12g0145500 | OsR498G1119414700.01.T01 | Peptidylprolyl isomerase |
| maker-11-augustus-gene-15.35-mRNA-1 | Japonica | NA | | Os11g0148400 | OsR498G1119414900.01.T02 | Peptidylprolyl isomerase |
| maker-11-augustus-gene-15.41-mRNA-1 | Japonica | NA | | Os11g0147800 | OsR498G1119413400.01.T03 | Stem-specific protein TSJT1 |
| maker-11-augustus-gene-15.47-mRNA-1 | Japonica | NA | | Os11g0148500 | OsR498G1119415200.01.T01 | Pyruvate kinase |
| maker-11-augustus-gene-15.56-mRNA-1 | Japonica | NA | | Os11g0148600 | OsR498G1119415400.01.T01 | |
| maker-11-augustus-gene-15.58-mRNA-1 | Japonica | Indica | | Os11g0148700 | OsR498G1119415600.01.T01 | DDT domain-containing protein PTM |
| maker-11-augustus-gene-15.62-mRNA-1 | Japonica | Indica | | Os11g0148800 | OsR498G1119415800.01.T04 | DDT domain-containing protein PTM |
| maker-11-augustus-gene-150.0-mRNA-1 | Japonica | NA | CC-NBS-TM | | OsR498G1120176700.01.T01 | NBS-LRR type resistance protein (Fragment) |
| maker-11-augustus-gene-150.2-mRNA-1 | Japonica | NA | CC-NBS-TM | Os11g0495032 | | NB-ARC domain containing protein, expressed |
| maker-11-augustus-gene-151.31-mRNA-1 | Japonica | NA | | Os11g0497000 | OsR498G1120184700.01.T01 | Ubiquitin-activating enzyme E |
| maker-11-augustus-gene-151.37-mRNA-1 | Japonica | NA | | | OsR498G1120182000.01.T01 | CBS-PB1 domain contining protein |
| maker-11-augustus-gene-151.40-mRNA-1 | Japonica | NA | | | OsR498G1120183000.01.T03 | Squamosa promoter-binding protein 15 |
| maker-11-augustus-gene-152.33-mRNA-1 | Japonica | NA | | Os11g0498400 | OsR498G1120186500.01.T01 | Zinc finger protein 511 |
| maker-11-augustus-gene-152.36-mRNA-1 | Japonica | NA | | | | |
| maker-11-augustus-gene-153.0-mRNA-1 | Japonica | NA | | Os11g0499600 | OsR498G1120188500.01.T01 | Cis-2,3-dihydrobiphenyl-2,3-diol dehydrogenase |
| maker-11-augustus-gene-153.10-mRNA-1 | Japonica | Indica | | Os11g0501500 | OsR498G1120193400.01.T01 | |
| maker-11-augustus-gene-154.41-mRNA-1 | Japonica | Indica | | Os11g0502700 | OsR498G1120196500.01.T01 | Acetylglucosaminyltransferase family protein |
| maker-11-augustus-gene-154.43-mRNA-1 | Japonica | NA | | Os11g0503400 | OsR498G1120197300.01.T02 | |
| maker-11-augustus-gene-154.45-mRNA-1 | Japonica | NA | | Os11g0503900 | OsR498G1120197700.01.T01 | Sulfotransferase |
| maker-11-augustus-gene-154.47-mRNA-1 | Japonica | NA | | Os11g0504000 | | Putative transposable element |
| maker-11-augustus-gene-155.2-mRNA-1 | Japonica | NA | | | OsR498G1120204600.01.T01 | |
| maker-11-augustus-gene-156.31-mRNA-1 | Japonica | NA | | Os11g0507200 | OsR498G1120205300.01.T01 | Omega-hydroxypalmitate O-feruloyl transferase |
| maker-11-augustus-gene-156.35-mRNA-1 | Japonica | Japonica | | Os11g0507400 | OsR498G1120206200.01.T01 | Rhamnogalacturonate lyase family protein |
| maker-11-augustus-gene-156.38-mRNA-1 | Japonica | NA | | Os11g0508600 | OsR498G1120210200.01.T01 | Bidirectional sugar transporter SWEET |
| maker-11-augustus-gene-158.0-mRNA-1 | Japonica | NA | | Os11g0512000 | OsR498G1120212100.01.T01 | NAC domain-containing protein 78 |
| maker-11-augustus-gene-158.2-mRNA-1 | Japonica | NA | | | OsR498G1120212300.01.T01 | No apical meristem protein, expressed |
| maker-11-augustus-gene-158.6-mRNA-1 | Japonica | NA | | | OsR498G1120213100.01.T01 | NAC domain-containing protein |
| maker-11-augustus-gene-159.0-mRNA-1 | Japonica | NA | | | | |
| maker-11-augustus-gene-159.18-mRNA-1 | Japonica | NA | LRR-TM | Os11g0514400 | OsR498G1120215800.01.T01 | Somatic embryogenesis receptor kinase 1 |
| maker-11-augustus-gene-159.21-mRNA-1 | Japonica | Indica | LRR-TM | Os11g0514500 | OsR498G1120216000.01.T01 | BRASSINOSTEROID INSENSITIVE 1-associated receptor kinase 1, putative, exp |
| maker-11-augustus-gene-159.26-mRNA-1 | Japonica | Indica | LRR-TM | Os11g0514600 | OsR498G1120216600.01.T01 | Somatic embryogenesis receptor kinase 1 |
| maker-11-augustus-gene-159.29-mRNA-1 | Japonica | Japonica | LRR-TM | Os11g0514700 | OsR498G1120218000.01.T01 | Somatic embryogenesis receptor kinase 1 |
| maker-11-augustus-gene-159.32-mRNA-1 | Japonica | NA | | Os11g0514800 | OsR498G1120218600.01.T02 | BRO1 domain-containing protein BROX |
| maker-11-augustus-gene-159.4-mRNA-1 | Japonica | NA | | Os11g0514000 | OsR498G1120216800.01.T02 | Putative histidine kinase-like ATPase domain-containing protein |
| maker-11-augustus-gene-16.0-mRNA-1 | Japonica | NA | | Os11g0149100 | OsR498G1119416900.01.T01 | AT-hook motif nuclear-localized protein (Fragment) |
| maker-11-augustus-gene-16.12-mRNA-1 | Japonica | Indica | | Os03g0793100 | OsR498G1119421100.01.T02 | Glycosyltransferases |

| | | | | | | |
|---|---|---|---|---|---|---|
| maker-11-augustus-gene-16.16-mRNA-1 | Japonica | Japonica | Kinase-TM | Os11g0150700 | OsR498G1119421300.01.T01 | Serine/threonine-protein kinase D6PKL2 |
| maker-11-augustus-gene-16.18-mRNA-1 | Japonica | NA | | Os11g0150400 | OsR498G1119420600.01.T01 | Stress-response A/B barrel domain-containing protein (Fragment) |
| maker-11-augustus-gene-16.20-mRNA-1 | Japonica | Indica | | Os11g0150450 | OsR498G1119420800.01.T02 | RING-H2 finger protein ATL38 |
| maker-11-augustus-gene-16.3-mRNA-1 | Japonica | NA | | Os11g0149400 | OsR498G1119418100.01.T01 | Phytosulfokine-alpha |
| maker-11-augustus-gene-16.6-mRNA-1 | Japonica | NA | | Os11g0150100 | OsR498G1119419700.01.T04 | probable 2-carboxy-D-arabinitol-1-phosphatase |
| maker-11-augustus-gene-16.9-mRNA-1 | Japonica | NA | | Os11g0150200 | OsR498G1119420100.01.T04 | |
| maker-11-augustus-gene-160.8-mRNA-1 | Japonica | NA | | Os11g0515500 | OsR498G1120221400.01.T02 | Auxin signaling F-box protein 2 |
| maker-11-augustus-gene-163.3-mRNA-1 | Japonica | Japonica | | Os11g0520500 | OsR498G1120235900.01.T02 | Coiled-coil domain-containing protein SCD2 |
| maker-11-augustus-gene-163.8-mRNA-1 | Japonica | NA | | Os11g0522900 | OsR498G1120238700.01.T01 | Carboxypeptidase |
| maker-11-augustus-gene-164.0-mRNA-1 | Japonica | NA | | Os11g0523110 | OsR498G1120240900.01.T01 | zerumbone synthase |
| maker-11-augustus-gene-164.2-mRNA-1 | Japonica | NA | | | OsR498G1120242700.01.T01 | |
| maker-11-augustus-gene-164.4-mRNA-1 | Japonica | NA | | Os11g0523700 | OsR498G1120244700.01.T01 | Inducer of CBF expression |
| maker-11-augustus-gene-164.6-mRNA-1 | Japonica | Indica | | Os03g0193700 | OsR498G1120243200.01.T01 | Geranylgeranyl transferase type-2 subunit beta |
| maker-11-augustus-gene-164.9-mRNA-1 | Japonica | NA | | Os11g0523800 | OsR498G1120245800.01.T02 | Auxin response factor |
| maker-11-augustus-gene-165.0-mRNA-1 | Japonica | NA | | Os11g0524300 | OsR498G1120247700.01.T01 | Chromophore lyase CRL chloroplastic |
| maker-11-augustus-gene-165.14-mRNA-1 | Japonica | NA | CC-NBS-TM | | OsR498G1120249800.01.T02 | NBS-LRR-like resistance protein (Fragment) |
| maker-11-augustus-gene-165.6-mRNA-1 | Japonica | NA | | Os11g0525600 | OsR498G1120252900.01.T01 | Alpha-mannosidase |
| maker-11-augustus-gene-166.0-mRNA-1 | Japonica | NA | | Os11g0525700 | OsR498G1120253100.01.T01 | DCD domain-containing protein (Fragment) |
| maker-11-augustus-gene-166.11-mRNA-1 | Japonica | NA | | Os11g0525800 | OsR498G1120253400.01.T01 | N-acetyltransferase MAK3 isogeny |
| maker-11-augustus-gene-166.14-mRNA-1 | Japonica | NA | | | OsR498G1120259000.01.T02 | FBD domain-containing protein |
| maker-11-augustus-gene-166.4-mRNA-1 | Japonica | Indica | | Os11g0526200 | OsR498G1120254900.01.T01 | Cofactor assembly of complex C subunit B |
| maker-11-augustus-gene-166.9-mRNA-1 | Japonica | Indica | | Os07g0278866 | OsR498G1120261100.01.T02 | ATPase inhibitor |
| maker-11-augustus-gene-167.0-mRNA-1 | Japonica | NA | | | | No exine formation 1 |
| maker-11-augustus-gene-167.13-mRNA-1 | Japonica | NA | | Os11g0528500 | OsR498G1120263000.01.T01 | Rubredoxin-like domain-containing protein (Fragment) |
| maker-11-augustus-gene-167.16-mRNA-1 | Japonica | Indica | | Os11g0528700 | OsR498G1120263200.01.T01 | Indole-3-acetic acid-amido synthetase |
| maker-11-augustus-gene-167.2-mRNA-1 | Japonica | NA | | Os11g0528300 | OsR498G1120262500.01.T01 | Succinate dehydrogenase assembly factor 2 mitochondrial |
| maker-11-augustus-gene-167.20-mRNA-1 | Japonica | NA | | Os11g0529500 | OsR498G1120265600.01.T01 | Bisdemethoxycurcumin synthase |
| maker-11-augustus-gene-167.22-mRNA-1 | Japonica | Japonica | | Os11g0529900 | OsR498G1120266400.01.T01 | Bisdemethoxycurcumin synthase |
| maker-11-augustus-gene-167.4-mRNA-1 | Japonica | NA | | Os12g0512200 | | Transposon protein, putative, Mariner sub-class |
| maker-11-augustus-gene-167.7-mRNA-1 | Japonica | Japonica | | Os11g0529800 | OsR498G1120266100.01.T01 | Bisdemethoxycurcumin synthase |
| maker-11-augustus-gene-167.9-mRNA-1 | Japonica | NA | | Os11g0528400 | OsR498G1120262800.01.T02 | Aminopeptidase |
| maker-11-augustus-gene-168.23-mRNA-1 | Japonica | NA | | | OsR498G1018146800.01.T01 | |
| maker-11-augustus-gene-168.27-mRNA-1 | Japonica | NA | | Os11g0531300 | OsR498G1120272700.01.T01 | CCHC-type domain-containing protein |
| maker-11-augustus-gene-168.29-mRNA-1 | Japonica | NA | | Os11g0531700 | OsR498G1120274700.01.T02 | Nudix hydrolase |
| maker-11-augustus-gene-168.32-mRNA-1 | Japonica | NA | | Os11g0530600 | OsR498G1120269200.01.T01 | Chalcone synthase |
| maker-11-augustus-gene-169.30-mRNA-1 | Japonica | Japonica | | Os11g0532600 | OsR498G1120276200.01.T01 | FBD domain-containing protein |
| maker-11-augustus-gene-169.35-mRNA-1 | Japonica | NA | | | OsR498G1120277500.01.T03 | |
| maker-11-augustus-gene-169.45-mRNA-1 | Japonica | NA | | Os11g0533100 | OsR498G1120278000.01.T01 | CarD-like transcriptional regulator family protein, expressed |
| maker-11-augustus-gene-169.54-mRNA-1 | Japonica | NA | | Os11g0533800 | OsR498G1120279400.01.T07 | FBD domain-containing protein |
| maker-11-augustus-gene-17.42-mRNA-1 | Japonica | Indica | | Os11g0151100 | OsR498G1119422500.01.T01 | Root cap periphery protein 2 |
| maker-11-augustus-gene-17.45-mRNA-1 | Japonica | Indica | | Os11g0151800 | OsR498G1119427900.01.T01 | Purple acid phosphatase |
| maker-11-augustus-gene-17.48-mRNA-1 | Japonica | NA | | | OsR498G1119428100.01.T01 | |
| maker-11-augustus-gene-17.50-mRNA-1 | Japonica | Indica | | Os12g0149900 | OsR498G1119421800.01.T01 | IQ domain-containing protein IQM3 |
| maker-11-augustus-gene-17.54-mRNA-1 | Japonica | NA | | Os11g0151500 | OsR498G1119426800.01.T02 | Hippocampus abundant transcript-like protein 1 |
| maker-11-augustus-gene-17.60-mRNA-1 | Japonica | NA | | Os12g0256300 | OsR498G1221220300.01.T01 | MI domain-containing protein |
| maker-11-augustus-gene-17.65-mRNA-1 | Japonica | Indica | | Os11g0151700 | OsR498G1119427200.01.T01 | Purple acid phosphatase |
| maker-11-augustus-gene-17.67-mRNA-1 | Japonica | NA | | Os11g0152500 | OsR498G1119431200.01.T01 | Terminal flower 1 |
| maker-11-augustus-gene-17.70-mRNA-1 | Japonica | NA | | Os11g0152700 | OsR498G1119432000.01.T01 | Transcription factor TGAL11 |
| maker-11-augustus-gene-170.27-mRNA-1 | Japonica | NA | | Os11g0534300 | OsR498G1120281000.01.T01 | S-acyltransferase |
| maker-11-augustus-gene-171.0-mRNA-1 | Japonica | NA | | | OsR498G1120287900.01.T01 | SNARE associated Golgi protein family |
| maker-11-augustus-gene-171.12-mRNA-1 | Japonica | NA | | Os11g0536800 | OsR498G1120287000.01.T02 | Amidase domain-containing protein (Fragment) |
| maker-11-augustus-gene-171.2-mRNA-1 | Japonica | NA | | Os11g0120700 | OsR498G1119336000.01.T01 | |
| maker-11-augustus-gene-171.4-mRNA-1 | Japonica | NA | | Os11g0538900 | OsR498G1120292700.01.T01 | Citrate synthase |
| maker-11-augustus-gene-172.0-mRNA-1 | Japonica | NA | | Os11g0539200 | OsR498G1120293500.01.T01 | Xyloglucan endotransglucosylase/hydrolase |
| maker-11-augustus-gene-172.13-mRNA-1 | Japonica | NA | | | | Xaa-Pro aminopeptidase P |
| maker-11-augustus-gene-172.18-mRNA-1 | Japonica | Indica | | Os11g0540100 | OsR498G1120296000.01.T02 | Xaa-Pro aminopeptidase |
| maker-11-augustus-gene-172.2-mRNA-1 | Japonica | Indica | | Os11g0539700 | OsR498G1120295700.01.T01 | F-box domain containing protein, expressed |
| maker-11-augustus-gene-172.5-mRNA-1 | Japonica | NA | | Os11g0540600 | OsR498G1120297100.01.T01 | |
| maker-11-augustus-gene-172.7-mRNA-1 | Japonica | Japonica | | Os11g0539500 | OsR498G1120294800.01.T01 | 5-pentadecatrienyl resorcinol O-methyltransferase |
| maker-11-augustus-gene-172.9-mRNA-1 | Japonica | Indica | | Os11g0539600 | OsR498G1120295400.01.T01 | F-box domain containing protein, expressed |
| maker-11-augustus-gene-173.44-mRNA-1 | Japonica | NA | | Os11g0540900 | | |

| | | | | | | |
|---|---|---|---|---|---|---|
| maker-11-augustus-gene-173.46-mRNA-1 | Japonica | NA | | | OsR498G1119817100.01.T01 | Transposon protein, putative, CACTA, En/Spm sub-class |
| maker-11-augustus-gene-173.49-mRNA-1 | Japonica | Indica | | Os11g0540900 | OsR498G1120304700.01.T02 | |
| maker-11-augustus-gene-173.50-mRNA-1 | Japonica | Japonica | | Os11g0541600 | OsR498G1120304700.01.T01 | |
| maker-11-augustus-gene-173.54-mRNA-1 | Japonica | NA | | | | |
| maker-11-augustus-gene-174.0-mRNA-1 | Japonica | NA | | | OsR498G1120154400.01.T01 | Endonuclease |
| maker-11-augustus-gene-174.3-mRNA-1 | Japonica | Japonica | | Os11g0543300 | OsR498G1120311100.01.T01 | |
| maker-11-augustus-gene-174.5-mRNA-1 | Japonica | NA | | Os11g0543500 | OsR498G1120311400.01.T01 | Heparan-alpha-glucosaminide N-acetyltransferase-like isoform X1 |
| maker-11-augustus-gene-175.50-mRNA-1 | Japonica | NA | | | OsR498G1120311600.01.T06 | Xpo1 domain-containing protein (Fragment) |
| maker-11-augustus-gene-175.54-mRNA-1 | Japonica | Japonica | | Os11g0544100 | OsR498G1120312000.01.T01 | Protein AUXIN RESPONSE 4 |
| maker-11-augustus-gene-175.56-mRNA-1 | Japonica | NA | | Os11g0544200 | OsR498G1120312200.01.T02 | Arginine/serine-rich coiled coil |
| maker-11-augustus-gene-175.61-mRNA-1 | Japonica | NA | | | | GDT1-like protein 2, chloroplastic |
| maker-11-augustus-gene-175.66-mRNA-1 | Japonica | NA | | Os11g0544800 | OsR498G1120315000.01.T02 | Glutamyl-tRNA(Gln) amidotransferase subunit B, chloroplastic/mitochondria |
| maker-11-augustus-gene-175.71-mRNA-1 | Japonica | Japonica | | Os11g0545000 | OsR498G1120315800.01.T02 | NOP5NT domain-containing protein |
| maker-11-augustus-gene-176.0-mRNA-1 | Japonica | NA | | | | Ubiquitin carboxyl-terminal hydrolase family protein, expressed |
| maker-11-augustus-gene-176.11-mRNA-1 | Japonica | NA | | Os04g0101300 | OsR498G0407412900.01.T05 | MRG domain-containing protein |
| maker-11-augustus-gene-176.18-mRNA-1 | Japonica | NA | | | OsR498G1120320600.01.T02 | RZZ complex |
| maker-11-augustus-gene-176.2-mRNA-1 | Japonica | NA | | Os11g0546000 | OsR498G1120321500.01.T01 | 68 kDa protein HP68 |
| maker-11-augustus-gene-176.25-mRNA-1 | Japonica | NA | | Os11g0545800 | OsR498G1120321000.01.T01 | Ultraviolet-B receptor like |
| maker-11-augustus-gene-176.28-mRNA-1 | Japonica | NA | NBS-TM | | OsR498G1120322000.01.T02 | Armadillo repeat-containing protein 7 |
| maker-11-augustus-gene-176.32-mRNA-1 | Japonica | NA | | Os11g0546300 | OsR498G1120322200.01.T03 | Patatin |
| maker-11-augustus-gene-176.6-mRNA-1 | Japonica | NA | | Os11g0546100 | OsR498G1120321700.01.T01 | Transmembrane receptor, eukaryota |
| maker-11-augustus-gene-177.0-mRNA-1 | Japonica | NA | | Os11g0546900 | OsR498G1120324100.01.T01 | G-box binding factor |
| maker-11-augustus-gene-177.4-mRNA-1 | Japonica | Indica | | Os03g0708000 | OsR498G1120323400.01.T01 | Secretory phospholipase A2 |
| maker-11-augustus-gene-178.0-mRNA-1 | Japonica | NA | | Os07g0514400 | | DUF629 domain-containing protein |
| maker-11-augustus-gene-178.24-mRNA-1 | Japonica | NA | | | OsR498G1120327200.01.T01 | Binding partner of ACD11 1 |
| maker-11-augustus-gene-178.27-mRNA-1 | Japonica | Japonica | | Os11g0549615 | OsR498G0305541500.01.T01 | Purple acid phosphatase |
| maker-11-augustus-gene-178.29-mRNA-1 | Japonica | NA | | Os11g0549620 | OsR498G1120328800.01.T01 | Purple acid phosphatase |
| maker-11-augustus-gene-178.3-mRNA-1 | Japonica | NA | | Os11g0549610 | | DUF629 domain-containing protein |
| maker-11-augustus-gene-178.31-mRNA-1 | Japonica | NA | | | OsR498G1120329400.01.T01 | |
| maker-11-augustus-gene-178.9-mRNA-1 | Japonica | NA | | | OsR498G1120330300.01.T02 | ABC1 domain-containing protein (Fragment) |
| maker-11-augustus-gene-179.38-mRNA-1 | Japonica | Indica | | Os11g0549700 | OsR498G1120331100.01.T01 | S-acyltransferase |
| maker-11-augustus-gene-179.42-mRNA-1 | Japonica | NA | | | OsR498G1120330600.01.T01 | CDP-diacylglycerol synthase |
| maker-11-augustus-gene-179.50-mRNA-1 | Japonica | NA | | | OsR498G1120330600.01.T01 | CDP-diacylglycerol synthase |
| maker-11-augustus-gene-179.54-mRNA-1 | Japonica | Indica | | Os11g0549600 | OsR498G1120331000.01.T01 | Maf-like protein isoform B |
| maker-11-augustus-gene-18.0-mRNA-1 | Japonica | NA | | Os11g0153400 | OsR498G1119433900.01.T02 | Rho GTPase-activating protein gacA |
| maker-11-augustus-gene-18.15-mRNA-1 | Japonica | NA | | Os11g0153800 | OsR498G1119435300.01.T01 | 40S ribosomal protein S25 |
| maker-11-augustus-gene-18.16-mRNA-1 | Japonica | NA | | | | 40S ribosomal protein S25 |
| maker-11-augustus-gene-18.23-mRNA-1 | Japonica | NA | | Os11g0153900 | OsR498G1119435900.01.T01 | Ribonuclease |
| maker-11-augustus-gene-18.28-mRNA-1 | Japonica | NA | NAC17 | Os11g0154500 | OsR498G1119437300.01.T01 | NAC domain-containing protein 90 |
| maker-11-augustus-gene-18.3-mRNA-1 | Japonica | Indica | | Os11g0153700 | OsR498G1119434700.01.T02 | SRP54 domain-containing protein (Fragment) |
| maker-11-augustus-gene-18.30-mRNA-1 | Japonica | NA | | | OsR498G1119437500.01.T01 | DNA-binding factor of bZIP class |
| maker-11-augustus-gene-18.34-mRNA-1 | Japonica | Indica | | Os12g0156400 | OsR498G1119438100.01.T01 | mRNA-decapping enzyme-like protein isoform X1 |
| maker-11-augustus-gene-18.9-mRNA-1 | Japonica | NA | | Os11g0153700 | OsR498G1119435100.01.T02 | SRP54 domain-containing protein (Fragment) |
| maker-11-augustus-gene-180.11-mRNA-1 | Japonica | Japonica | | Os11g0550900 | OsR498G1120334400.01.T01 | |
| maker-11-augustus-gene-180.15-mRNA-1 | Japonica | Indica | | Os11g0551350 | OsR498G1120335400.01.T03 | |
| maker-11-augustus-gene-180.19-mRNA-1 | Japonica | NA | | Os11g0551800 | OsR498G1120336600.01.T05 | Yippee domain-containing protein |
| maker-11-augustus-gene-180.2-mRNA-1 | Japonica | NA | CC-NBS-TM | Os11g0551700 | | Disease resistance protein RPM1 |
| maker-11-augustus-gene-180.22-mRNA-1 | Japonica | NA | | Os11g0552000 | | probable aminotransferase TAT2 |
| maker-11-augustus-gene-180.26-mRNA-1 | Japonica | NA | | Os11g0552100 | OsR498G1120337800.01.T01 | DUF724 domain-containing protein 2 |
| maker-11-augustus-gene-180.4-mRNA-1 | Japonica | NA | | Os11g0551900 | OsR498G1120336900.01.T01 | Growth-regulating factor like |
| maker-11-augustus-gene-180.8-mRNA-1 | Japonica | NA | | | OsR498G1120337900.01.T02 | DUF724 domain-containing protein 2 |
| maker-11-augustus-gene-181.30-mRNA-1 | Japonica | NA | CC-NBS-TM | | | Rust resistance protein RP1-D homolog |
| maker-11-augustus-gene-181.32-mRNA-1 | Japonica | Indica | | Os11g0552600 | OsR498G1120340200.01.T01 | Kinesin-like protein KIN-7L |
| maker-11-augustus-gene-182.0-mRNA-1 | Japonica | NA | | Os11g0557000 | OsR498G1120348100.01.T01 | Phytosulfokine-alpha |
| maker-11-augustus-gene-182.14-mRNA-1 | Japonica | NA | | | OsR498G1120351700.01.T01 | rRNA N-glycosidase |
| maker-11-augustus-gene-182.18-mRNA-1 | Japonica | NA | | Os12g0512200 | | Transposon protein, putative, Mariner sub-class |
| maker-11-augustus-gene-182.2-mRNA-1 | Japonica | NA | | Os11g0557700 | OsR498G1120349100.01.T01 | S-adenosylmethionine-dependent methyltransferase/ catalytic |
| maker-11-augustus-gene-182.21-mRNA-1 | Japonica | NA | | Os11g0558200 | OsR498G1120350400.01.T01 | Transcription factor MYB3 |
| maker-11-augustus-gene-182.24-mRNA-1 | Japonica | NA | | | OsR498G1120357900.01.T01 | |
| maker-11-augustus-gene-182.6-mRNA-1 | Japonica | NA | | Os11g0557900 | OsR498G1120349900.01.T01 | Actin-related protein 2/3 complex subunit 5 |
| maker-11-augustus-gene-183.28-mRNA-1 | Japonica | NA | Kinase-LRR-TM | Os11g0559200 | OsR498G1120353500.01.T01 | Receptor kinase, putative, expressed |

| | | | | | | |
|---|---|---|---|---|---|---|
| maker-11-augustus-gene-184.0-mRNA-1 | Japonica | NA | LRR-TM | | | |
| maker-11-augustus-gene-184.2-mRNA-1 | Japonica | NA | | | | Terpene cyclase/mutase family member |
| maker-11-augustus-gene-185.0-mRNA-1 | Japonica | NA | Kinase-TM | Os11g0565300 | OsR498G1120370600.01.T01 | Wall-associated receptor kinase 2 |
| maker-11-augustus-gene-185.3-mRNA-1 | Japonica | NA | | | OsR498G1120370800.01.T02 | E3 ubiquitin-protein like |
| maker-11-augustus-gene-186.38-mRNA-1 | Japonica | Japonica | | Os11g0566800 | OsR498G1120376700.01.T01 | Chalcone and stilbene synthases, N-terminal domain containing protein, exp |
| maker-11-augustus-gene-187.0-mRNA-1 | Japonica | NA | | Os11g0568600 | OsR498G1120379700.01.T04 | THUMP domain-containing protein 1 |
| maker-11-augustus-gene-187.12-mRNA-1 | Japonica | Japonica | Kinase | Os11g0568800 | OsR498G1120381200.01.T01 | Protein kinase domain-containing protein |
| maker-11-augustus-gene-187.15-mRNA-1 | Japonica | Japonica | Kinase-LRR-TM | Os11g0569300 | OsR498G1120383600.01.T02 | Leucin-rich receptor-like kinase |
| maker-11-augustus-gene-187.20-mRNA-1 | Japonica | Japonica | Kinase-LRR-TM | Os11g0569500 | OsR498G1120381200.01.T01 | Putative LRR receptor-like serine/threonine-protein kinase |
| maker-11-augustus-gene-187.5-mRNA-1 | Japonica | NA | | Os11g0568700 | OsR498G1120380300.01.T05 | Formin-E like |
| maker-11-augustus-gene-187.9-mRNA-1 | Japonica | NA | | Os11g0568300 | OsR498G1120379000.01.T01 | Cell division cycle-associated protein 7 |
| maker-11-augustus-gene-188.40-mRNA-1 | Japonica | Indica | Kinase-LRR-TM | Os11g0569600 | OsR498G1120386200.01.T01 | Receptor kinase-like protein (Fragment) |
| maker-11-augustus-gene-188.45-mRNA-1 | Japonica | NA | | Os11g0570400 | OsR498G1120387200.01.T01 | |
| maker-11-augustus-gene-189.4-mRNA-1 | Japonica | NA | NBS-TM | Os11g0572100 | OsR498G1120391500.01.T01 | Replication factor C subunit 1 |
| maker-11-augustus-gene-19.0-mRNA-1 | Japonica | NA | | Os11g0155500 | OsR498G1119440500.01.T01 | Cationic amino acid transporter |
| maker-11-augustus-gene-19.11-mRNA-1 | Japonica | NA | | Os11g0156000 | OsR498G1119443000.01.T01 | TF-B3 domain-containing protein (Fragment) |
| maker-11-augustus-gene-19.14-mRNA-1 | Japonica | NA | | Os11g0156200 | OsR498G1119443600.01.T01 | Prolylcarboxypeptidase |
| maker-11-augustus-gene-19.3-mRNA-1 | Japonica | NA | | | OsR498G1119440800.01.T01 | Multidrug resistance-associated protein3 |
| maker-11-augustus-gene-190.0-mRNA-1 | Japonica | NA | | Os11g0572400 | OsR498G1120393700.01.T01 | Sialyltransferase-like protein 1 |
| maker-11-augustus-gene-190.12-mRNA-1 | Japonica | NA | | | | Zinc knuckle (CCHC-type) family protein |
| maker-11-augustus-gene-190.15-mRNA-1 | Japonica | NA | | | OsR498G1120393600.01.T01 | NB-ARC domain-containing protein |
| maker-11-augustus-gene-190.17-mRNA-1 | Japonica | NA | | | OsR498G1120394000.01.T01 | E3 ubiquitin-protein ligase RHF2A |
| maker-11-augustus-gene-190.22-mRNA-1 | Japonica | NA | | Os11g0572800 | OsR498G1120394400.01.T01 | Bifunctional arginine demethylase andlysyl-hydroxylase JMJD6 |
| maker-11-augustus-gene-190.24-mRNA-1 | Japonica | NA | | | OsR498G1120395000.01.T01 | GATA transcription factor |
| maker-11-augustus-gene-190.26-mRNA-1 | Japonica | NA | | | OsR498G1120395200.01.T01 | Ubiquitinyl hydrolase 1 |
| maker-11-augustus-gene-190.5-mRNA-1 | Japonica | Indica | | Os11g0572700 | OsR498G1120394100.01.T01 | Prolycopene isomerase, chloroplastic |
| maker-11-augustus-gene-191.35-mRNA-1 | Japonica | NA | | | | J domain-containing protein (Fragment) |
| maker-11-augustus-gene-191.36-mRNA-1 | Japonica | Indica | | Os11g0574200 | OsR498G1120403500.01.T01 | J domain-containing protein |
| maker-11-augustus-gene-192.2-mRNA-1 | Japonica | NA | | | OsR498G1120396500.01.T01 | Myosin-binding protein 7 |
| maker-11-augustus-gene-192.4-mRNA-1 | Japonica | NA | | | | DUF295 domain-containing protein (Fragment) |
| maker-11-augustus-gene-192.5-mRNA-1 | Japonica | NA | | | OsR498G1120399100.01.T01 | |
| maker-11-augustus-gene-193.0-mRNA-1 | Japonica | NA | | Os04g0590366 | OsR498G0407482700.01.T01 | DDE Tnp4 domain-containing protein |
| maker-11-augustus-gene-193.3-mRNA-1 | Japonica | NA | | | OsR498G1120402100.01.T01 | C2H2-type domain-containing protein |
| maker-11-augustus-gene-193.7-mRNA-1 | Japonica | NA | | Os11g0574200 | | J domain-containing protein |
| maker-11-augustus-gene-194.34-mRNA-1 | Japonica | NA | | Os11g0579800 | OsR498G1120405900.01.T02 | H/ACA ribonucleoprotein complex subunit |
| maker-11-augustus-gene-194.36-mRNA-1 | Japonica | NA | | Os11g0580000 | OsR498G1120406500.01.T01 | Survival motor neuron interacting protein 1 |
| maker-11-augustus-gene-194.38-mRNA-1 | Japonica | Indica | | Os11g0581700 | OsR498G1120410300.01.T01 | CHALLAH-LIKE 2, EPIDERMAL PATTERNING FACTOR-LIKE protein 4 |
| maker-11-augustus-gene-194.40-mRNA-1 | Japonica | NA | | | OsR498G1120405800.01.T01 | XS domain containing protein expressed |
| maker-11-augustus-gene-194.42-mRNA-1 | Japonica | NA | | Os11g0579900 | OsR498G1120406200.01.T01 | Pumilio domain-containing protein |
| maker-11-augustus-gene-194.44-mRNA-1 | Japonica | NA | | Os11g0580100 | | SERPIN domain-containing protein |
| maker-11-augustus-gene-194.46-mRNA-1 | Japonica | Indica | | Os11g0545000 | OsR498G1120407000.01.T01 | NOP5NT domain-containing protein |
| maker-11-augustus-gene-194.48-mRNA-1 | Japonica | NA | | Os11g0580800 | OsR498G1120407400.01.T01 | High chlorophyll fluorescence106c |
| maker-11-augustus-gene-195.0-mRNA-1 | Japonica | NA | | Os11g0581900 | OsR498G1120411100.01.T01 | Transmembrane BAX inhibitor motif-containing protein 4 |
| maker-11-augustus-gene-195.10-mRNA-1 | Japonica | NA | | | OsR498G1120412700.01.T03 | F-box domain-containing protein |
| maker-11-augustus-gene-195.12-mRNA-1 | Japonica | NA | | Os11g0583200 | OsR498G1120413700.01.T03 | Pentatricopeptide repeat |
| maker-11-augustus-gene-195.16-mRNA-1 | Japonica | NA | | | OsR498G1120411400.01.T01 | EGF-like domain-containing protein |
| maker-11-augustus-gene-195.2-mRNA-1 | Japonica | NA | NBS-TM | Os11g0582300 | OsR498G1120411700.01.T01 | Protein ROOT HAIR DEFECTIVE 3 homolog |
| maker-11-augustus-gene-195.20-mRNA-1 | Japonica | NA | | Os11g0582100 | OsR498G1120411600.01.T01 | E3 ubiquitin-protein ligase RING1 |
| maker-11-augustus-gene-195.22-mRNA-1 | Japonica | NA | | | OsR498G1120413600.01.T01 | AAI domain-containing protein (Fragment) |
| maker-11-augustus-gene-195.8-mRNA-1 | Japonica | NA | | Os11g0582500 | OsR498G1120412300.01.T01 | AAI domain-containing protein |
| maker-11-augustus-gene-196.36-mRNA-1 | Japonica | NA | | | OsR498G1120416500.01.T03 | PHD finger protein rhinoceros-like |
| maker-11-augustus-gene-196.39-mRNA-1 | Japonica | NA | | | | Ribosomal protein L4/L1 family isoform 1 |
| maker-11-augustus-gene-196.42-mRNA-1 | Japonica | NA | | Os11g0585900 | OsR498G1120418500.01.T02 | Tetratricopeptide TPR-1 |
| maker-11-augustus-gene-197.35-mRNA-1 | Japonica | NA | | | | Protein-serine/threonine phosphatase |
| maker-11-augustus-gene-197.44-mRNA-1 | Japonica | NA | | | OsR498G1120421500.01.T01 | Beta-carotene isomerase D27 chloroplastic |
| maker-11-augustus-gene-197.46-mRNA-1 | Japonica | NA | | | OsR498G1120419600.01.T01 | Calmodulin like 5 |
| maker-11-augustus-gene-197.48-mRNA-1 | Japonica | Indica | | Os11g0586300 | OsR498G1120419800.01.T02 | Protein RNA-directed DNA methylation like |
| maker-11-augustus-gene-197.50-mRNA-1 | Japonica | NA | | | | |
| maker-11-augustus-gene-198.0-mRNA-1 | Japonica | Indica | | Os07g0485500 | OsR498G1120426500.01.T01 | |
| maker-11-augustus-gene-198.10-mRNA-1 | Japonica | Indica | | Os07g0525400 | OsR498G1120424800.01.T01 | Rab-GAP TBC domain-containing protein |
| maker-11-augustus-gene-198.15-mRNA-1 | Japonica | NA | | | OsR498G1120425000.01.T01 | Pleiotropic drug resistance protein 5 |

| | | | | | | | |
|---|---|---|---|---|---|---|---|
| maker-11-augustus-gene-198.2-mRNA-1 | Japonica | NA | | Os11g0588300 | OsR498G1120426900.01.T01 | Theta class glutathione S-transferase |
| maker-11-augustus-gene-198.7-mRNA-1 | Japonica | NA | | Os11g0588300 | OsR498G1120426900.01.T01 | Glutathione S-transferase, N-terminal domain containing protein, expressed |
| maker-11-augustus-gene-199.32-mRNA-1 | Japonica | Japonica | NBS-TM | Os11g0590400 | OsR498G1120429100.01.T01 | Putative CC-NBS-LRR resistance protein MLA13 |
| maker-11-augustus-gene-199.35-mRNA-1 | Japonica | Japonica | CC-NBS-TM | Os11g0590700 | OsR498G1120430700.01.T02 | Putative CC-NBS-LRR resistance protein MLA13 |
| maker-11-augustus-gene-199.37-mRNA-1 | Japonica | NA | CC-NBS-TM | Os11g0590900 | OsR498G1120430300.01.T01 | NB-ARC domain containing protein, expressed |
| maker-11-augustus-gene-199.40-mRNA-1 | Japonica | Indica | CC-LRR-NBS-TM | Os11g0590900 | OsR498G1120430700.01.T02 | Putative CC-NBS-LRR resistance protein MLA13 |
| maker-11-augustus-gene-2.0-mRNA-1 | Japonica | NA | | Os12g0119000 | OsR498G1220832200.01.T01 | Secologanin synthase |
| maker-11-augustus-gene-2.11-mRNA-1 | Japonica | NA | | Os11g0120700 | OsR498G1119336000.01.T01 | Putative tubby C-terminal-like domain-containing protein |
| maker-11-augustus-gene-2.14-mRNA-1 | Japonica | NA | | Os12g0120500 | OsR498G1119336800.01.T03 | |
| maker-11-augustus-gene-2.17-mRNA-1 | Japonica | NA | | Os11g0121300 | OsR498G1119338600.01.T02 | Protein kinase APK1B chloroplast |
| maker-11-augustus-gene-2.19-mRNA-1 | Japonica | NA | Kinase | Os11g0121400 | OsR498G1119338800.01.T01 | Serine/threonine-protein kinase NAK |
| maker-11-augustus-gene-2.2-mRNA-1 | Japonica | Indica | | Os12g0119700 | OsR498G1119333300.01.T02 | Retrotransposon protein, putative, Ty1-copia subclass |
| maker-11-augustus-gene-2.20-mRNA-1 | Japonica | NA | Kinase | | | Putative serine/threonine-protein kinase PBL8 |
| maker-11-augustus-gene-2.4-mRNA-1 | Japonica | Indica | | Os11g0120600 | OsR498G1119335700.01.T01 | Bis(5'-adenosyl)-triphosphatase |
| maker-11-augustus-gene-2.7-mRNA-1 | Japonica | NA | | Os12g0120400 | OsR498G1220828000.01.T02 | Bis(5'-adenosyl)-triphosphatase |
| maker-11-augustus-gene-2.9-mRNA-1 | Japonica | NA | | Os11g0120200 | OsR498G1119333800.01.T01 | Serine/arginine repetitive matrix protein |
| maker-11-augustus-gene-20.0-mRNA-1 | Japonica | NA | | Os11g0156600 | OsR498G1119445300.01.T01 | |
| maker-11-augustus-gene-20.11-mRNA-1 | Japonica | Japonica | | Os11g0157000 | OsR498G1119447400.01.T01 | 5'-3' exoribonuclease |
| maker-11-augustus-gene-20.13-mRNA-1 | Japonica | Indica | | Os11g0157000 | OsR498G1119448000.01.T01 | 5'-3' exoribonuclease |
| maker-11-augustus-gene-20.15-mRNA-1 | Japonica | NA | | | OsR498G1119448400.01.T01 | 5'-3' exoribonuclease |
| maker-11-augustus-gene-20.17-mRNA-1 | Japonica | NA | | Os11g0157400 | OsR498G1119450600.01.T03 | |
| maker-11-augustus-gene-20.3-mRNA-1 | Japonica | Indica | | Os11g0156800 | OsR498G1119446100.01.T01 | Butenolide signaling repressing protein-like |
| maker-11-augustus-gene-20.5-mRNA-1 | Japonica | NA | | | OsR498G1119448900.01.T01 | Cyclin-T1-5 like |
| maker-11-augustus-gene-200.10-mRNA-1 | Japonica | NA | NBS-TM | | | Protein-serine/threonine phosphatase |
| maker-11-augustus-gene-200.12-mRNA-1 | Japonica | NA | | Os11g0592500 | | PPM-type phosphatase domain-containing protein |
| maker-11-augustus-gene-200.14-mRNA-1 | Japonica | NA | | Os11g0592600 | OsR498G1120436500.01.T01 | TPX2 domain-containing protein |
| maker-11-augustus-gene-200.16-mRNA-1 | Japonica | NA | | | OsR498G1120436900.01.T01 | Hflx-type G domain-containing protein |
| maker-11-augustus-gene-200.19-mRNA-1 | Japonica | NA | | Os11g0591100 | OsR498G1120431000.01.T02 | GDP-mannose 3,5-epimerase 1 |
| maker-11-augustus-gene-200.2-mRNA-1 | Japonica | NA | | Os11g0591800 | OsR498G1120433700.01.T01 | Barwin domain-containing protein (Fragment) |
| maker-11-augustus-gene-200.24-mRNA-1 | Japonica | Indica | | Os11g0591200 | OsR498G1120432000.01.T01 | Very-long-chain 3-oxoacyl-CoA synthase |
| maker-11-augustus-gene-200.26-mRNA-1 | Japonica | NA | | | | |
| maker-11-augustus-gene-200.28-mRNA-1 | Japonica | NA | | Os11g0592300 | OsR498G1120434800.01.T05 | Hexosyltransferase |
| maker-11-augustus-gene-200.31-mRNA-1 | Japonica | NA | | | OsR498G1120435200.01.T01 | CRM-domain containing factor CFM3, chloroplastic/mitochondrial |
| maker-11-augustus-gene-200.4-mRNA-1 | Japonica | NA | | Os11g0592000 | OsR498G1120433900.01.T01 | Barwin domain-containing protein |
| maker-11-augustus-gene-200.6-mRNA-1 | Japonica | NA | | Os11g0592100 | OsR498G1120434100.01.T01 | Barwin domain-containing protein |
| maker-11-augustus-gene-201.0-mRNA-1 | Japonica | Indica | | Os06g0141000 | OsR498G1120440500.01.T01 | MFS domain-containing protein (Fragment) |
| maker-11-augustus-gene-201.10-mRNA-1 | Japonica | NA | | Os11g0595200 | OsR498G1120445500.01.T02 | Harpin binding protein 1 |
| maker-11-augustus-gene-201.12-mRNA-1 | Japonica | NA | | Os11g0592800 | OsR498G1120437200.01.T01 | TRAM domain |
| maker-11-augustus-gene-201.14-mRNA-1 | Japonica | Indica | | Os11g0593000 | OsR498G1120437800.01.T01 | Non-hemolytic phospholipase C |
| maker-11-augustus-gene-201.16-mRNA-1 | Japonica | NA | | | OsR498G1120440400.01.T02 | DUF629 domain-containing protein |
| maker-11-augustus-gene-201.17-mRNA-1 | Japonica | NA | | | OsR498G1120442000.01.T01 | DUF295 domain-containing protein |
| maker-11-augustus-gene-201.2-mRNA-1 | Japonica | NA | | Os11g0594200 | OsR498G1120441100.01.T01 | Notchless protein like |
| maker-11-augustus-gene-201.30-mRNA-1 | Japonica | NA | | Os11g0595100 | OsR498G1120445400.01.T01 | DUF295 domain-containing protein |
| maker-11-augustus-gene-202.0-mRNA-1 | Japonica | NA | | | | DUF629 domain-containing protein |
| maker-11-augustus-gene-202.1-mRNA-1 | Japonica | NA | | | | DUF629 domain-containing protein |
| maker-11-augustus-gene-202.8-mRNA-1 | Japonica | Japonica | | Os11g0596400 | OsR498G1120452900.01.T05 | rRNA N-glycosidase |
| maker-11-augustus-gene-203.30-mRNA-1 | Japonica | Japonica | | Os11g0597700 | OsR498G1120452300.01.T01 | Disease resistance protein RPM1 |
| maker-11-augustus-gene-203.32-mRNA-1 | Japonica | NA | | Os11g0597500 | | |
| maker-11-augustus-gene-203.34-mRNA-1 | Japonica | NA | | Os11g0579600 | OsR498G1120448000.01.T01 | F-box only protein 7 |
| maker-11-augustus-gene-204.0-mRNA-1 | Japonica | NA | | Os11g0598800 | OsR498G1120452900.01.T01 | rRNA N-glycosidase |
| maker-11-augustus-gene-204.19-mRNA-1 | Japonica | NA | | | | DUF629 domain-containing protein |
| maker-11-augustus-gene-204.24-mRNA-1 | Japonica | Indica | | Os11g0599101 | OsR498G1120454300.01.T02 | DUF629 domain-containing protein |
| maker-11-augustus-gene-204.28-mRNA-1 | Japonica | NA | | Os11g0599500 | OsR498G1120456100.01.T01 | Dead box ATP-dependent RNA helicase, putative isoform 1 |
| maker-11-augustus-gene-204.30-mRNA-1 | Japonica | NA | | | OsR498G0612819100.01.T01 | L10-interacting MYB domain-containing protein |
| maker-11-augustus-gene-204.6-mRNA-1 | Japonica | NA | | Os11g0598900 | | DUF629 domain-containing protein |
| maker-11-augustus-gene-206.35-mRNA-1 | Japonica | NA | | | OsR498G1120466700.01.T03 | |
| maker-11-augustus-gene-206.38-mRNA-1 | Japonica | NA | | | | RING-CH-type domain-containing protein |
| maker-11-augustus-gene-206.49-mRNA-1 | Japonica | Indica | | Os01g0127900 | OsR498G1120467700.01.T01 | Mannose-6-phosphate isomerase |
| maker-11-augustus-gene-206.57-mRNA-1 | Japonica | NA | | Os11g0601700 | OsR498G1120471000.01.T01 | Transcription factor bHLH47 |
| maker-11-augustus-gene-207.0-mRNA-1 | Japonica | NA | | Os11g0602300 | OsR498G1120474100.01.T02 | tRNA-intron lyase |
| maker-11-augustus-gene-207.11-mRNA-1 | Japonica | NA | | Os11g0603000 | OsR498G1120477300.01.T01 | BHLH domain-containing protein |

| | | | | | | |
|---|---|---|---|---|---|---|
| maker-11-augustus-gene-207.13-mRNA-1 | Japonica | NA | | Os11g0603200 | OsR498G1120478100.01.T02 | ABC transporter family protein, expressed |
| maker-11-augustus-gene-207.18-mRNA-1 | Japonica | NA | | Os11g0603100 | OsR498G1120477800.01.T01 | Elongation factor 1-delta 1-like |
| maker-11-augustus-gene-207.5-mRNA-1 | Japonica | NA | | Os11g0602600 | OsR498G1120475100.01.T01 | 30S ribosomal protein S4, chloroplastic |
| maker-11-augustus-gene-208.0-mRNA-1 | Japonica | NA | | Os11g0604600 | OsR498G1120482500.01.T01 | RING-CH-type domain-containing protein (Fragment) |
| maker-11-augustus-gene-208.11-mRNA-1 | Japonica | NA | | | OsR498G1120483200.01.T02 | Acyl-coenzyme A oxidase |
| maker-11-augustus-gene-208.5-mRNA-1 | Japonica | NA | | | | Thioredoxin domain-containing protein 9 like |
| maker-11-augustus-gene-209.1-mRNA-1 | Japonica | NA | NBS-TM | Os11g0606900 | OsR498G1120485700.01.T01 | Disease resistance protein RPM1 |
| maker-11-augustus-gene-209.14-mRNA-1 | Japonica | NA | Kinase-LRR-TM | Os11g0607200 | OsR498G1120487000.01.T01 | BRASSINOSTEROID INSENSITIVE 1-associated receptor kinase 1 |
| maker-11-augustus-gene-209.8-mRNA-1 | Japonica | Japonica | LRR-NBS-TM | Os11g0606400 | OsR498G1120484800.01.T01 | NB-ARC domain containing protein, expressed |
| maker-11-augustus-gene-209.9-mRNA-1 | Japonica | Indica | LRR-NBS-TM | Os11g0606400 | OsR498G1120485600.01.T01 | NB-ARC domain containing protein, expressed |
| maker-11-augustus-gene-21.37-mRNA-1 | Japonica | NA | | Os11g0157600 | OsR498G1119451500.01.T01 | Two-component response regulator-like APRR9 |
| maker-11-augustus-gene-21.43-mRNA-1 | Japonica | NA | | Os11g0158200 | OsR498G1119453100.01.T01 | Apoptosis-inducing factor B |
| maker-11-augustus-gene-21.45-mRNA-1 | Japonica | NA | | Os11g0158400 | OsR498G1119453700.01.T02 | Digalactosyldiacylglycerol synthase |
| maker-11-augustus-gene-21.54-mRNA-1 | Japonica | NA | | | OsR498G1119452200.01.T01 | |
| maker-11-augustus-gene-210.24-mRNA-1 | Japonica | NA | | | OsR498G1120489000.01.T02 | 14-3-3 protein zeta (inferred by orthology to a D. melanogaster protein) |
| maker-11-augustus-gene-211.54-mRNA-1 | Japonica | NA | | Os11g0610900 | OsR498G1120491500.01.T01 | Seryl-tRNA synthetase |
| maker-11-augustus-gene-211.55-mRNA-1 | Japonica | NA | | Os11g0610900 | OsR498G1120491500.01.T03 | Seryl-tRNA synthetase |
| maker-11-augustus-gene-211.69-mRNA-1 | Japonica | NA | | Os11g0610700 | OsR498G1120491200.01.T01 | Putative Prolactin regulatory element-binding protein |
| maker-11-augustus-gene-213.27-mRNA-1 | Japonica | NA | | Os11g0613800 | | Transposon protein, putative, CACTA, En/Spm sub-class |
| maker-11-augustus-gene-214.40-mRNA-1 | Japonica | NA | | Os11g0614400 | OsR498G1120503300.01.T01 | Patatin |
| maker-11-augustus-gene-214.42-mRNA-1 | Japonica | NA | | Os11g0614800 | OsR498G1120504700.01.T01 | Cyclin-dependent kinase inhibitor |
| maker-11-augustus-gene-214.45-mRNA-1 | Japonica | Indica | | Os11g0614900 | OsR498G1120505100.01.T05 | Transmembrane protein like |
| maker-11-augustus-gene-214.50-mRNA-1 | Japonica | NA | | | OsR498G1120505900.01.T02 | Phosphatidate phosphatase |
| maker-11-augustus-gene-214.55-mRNA-1 | Japonica | NA | | Os11g0615100 | OsR498G1120506100.01.T01 | Phosphatidate phosphatase |
| maker-11-augustus-gene-214.58-mRNA-1 | Japonica | NA | | Os11g0615800 | OsR498G1120508900.01.T01 | DNA repair protein RAD51 homolog |
| maker-11-augustus-gene-214.60-mRNA-1 | Japonica | NA | | | OsR498G1120509500.01.T01 | NB-ARC domain-containing protein |
| maker-11-augustus-gene-214.62-mRNA-1 | Japonica | NA | | Os11g0615200 | OsR498G1120506400.01.T01 | GRF1-interacting factor 3 isoform A |
| maker-11-augustus-gene-214.64-mRNA-1 | Japonica | NA | | Os11g0615700 | OsR498G1120508800.01.T01 | Proteasome endopeptidase complex |
| maker-11-augustus-gene-214.70-mRNA-1 | Japonica | NA | | | OsR498G1120509800.01.T01 | |
| maker-11-augustus-gene-215.0-mRNA-1 | Japonica | NA | Kinase-TM | Os11g0616100 | OsR498G1120510900.01.T01 | Leucine-rich repeat receptor protein kinase EXS |
| maker-11-augustus-gene-215.5-mRNA-1 | Japonica | NA | | Os11g0616300 | OsR498G1120512200.01.T01 | Remorin, C-terminal |
| maker-11-augustus-gene-215.8-mRNA-1 | Japonica | NA | | | OsR498G1120513600.01.T01 | |
| maker-11-augustus-gene-216.34-mRNA-1 | Japonica | NA | Kinase-TM | | OsR498G1120517600.01.T01 | Protein kinase domain-containing protein |
| maker-11-augustus-gene-216.36-mRNA-1 | Japonica | NA | Kinase-TM | | OsR498G1120517800.01.T01 | Protein kinase domain containing protein, expressed |
| maker-11-augustus-gene-216.38-mRNA-1 | Japonica | Indica | | Os11g0618600 | OsR498G1120518800.01.T01 | Protein kinase domain-containing protein (Fragment) |
| maker-11-augustus-gene-216.43-mRNA-1 | Japonica | NA | | | | USP domain-containing protein |
| maker-11-augustus-gene-216.45-mRNA-1 | Japonica | NA | | | OsR498G1120520600.01.T01 | USP domain-containing protein |
| maker-11-augustus-gene-217.42-mRNA-1 | Japonica | NA | Kinase-LRR-TM | Os11g0620500 | OsR498G1120524900.01.T01 | Leucine-rich repeat receptor protein kinase EXS, putative |
| maker-11-augustus-gene-217.44-mRNA-1 | Japonica | Indica | | Os11g0620000 | OsR498G1120523200.01.T03 | Mitochondrial inner membrane protease |
| maker-11-augustus-gene-217.48-mRNA-1 | Japonica | NA | | Os11g0620100 | OsR498G1120523800.01.T02 | oligouridylate-binding protein 1-like isoform X1 |
| maker-11-augustus-gene-217.55-mRNA-1 | Japonica | NA | | | OsR498G1120526000.01.T01 | Polyubiquitin |
| maker-11-augustus-gene-217.57-mRNA-1 | Japonica | Japonica | | Os11g0621400 | OsR498G1120526200.01.T02 | |
| maker-11-augustus-gene-218.23-mRNA-1 | Japonica | Japonica | | Os11g0622150 | OsR498G1120530800.01.T01 | BTB/POZ domain containing protein, expressed |
| maker-11-augustus-gene-219.36-mRNA-1 | Japonica | NA | | | OsR498G1120544700.01.T01 | |
| maker-11-augustus-gene-22.27-mRNA-1 | Japonica | NA | | Os11g0159500 | | Large tegument protein |
| maker-11-augustus-gene-22.29-mRNA-1 | Japonica | NA | | Os11g0160000 | OsR498G1119460300.01.T01 | |
| maker-11-augustus-gene-22.35-mRNA-1 | Japonica | Indica | | Os11g0160100 | OsR498G1119461400.01.T01 | PHD-finger family protein, expressed |
| maker-11-augustus-gene-22.37-mRNA-1 | Japonica | NA | CC-Kinase-TM | Os11g0160300 | OsR498G1119462200.01.T01 | Probable serine/threonine-protein kinase WNK6 |
| maker-11-augustus-gene-22.40-mRNA-1 | Japonica | NA | | Os11g0160700 | OsR498G1119464000.01.T02 | PHD domain-containing protein |
| maker-11-augustus-gene-220.21-mRNA-1 | Japonica | NA | | Os07g0651500 | | ATP-dependent DNA helicase |
| maker-11-augustus-gene-220.23-mRNA-1 | Japonica | Japonica | Kinase-LRR-TM | Os11g0625900 | OsR498G1120550600.01.T01 | Putative LRR receptor-like serine/threonine-protein kinase |
| maker-11-augustus-gene-221.0-mRNA-1 | Japonica | Japonica | Kinase-LRR-TM | Os11g0628000 | OsR498G1120550600.01.T01 | Putative LRR receptor-like serine/threonine-protein kinase |
| maker-11-augustus-gene-221.12-mRNA-1 | Japonica | NA | | Os11g0629300 | OsR498G1120561500.01.T01 | RING-type domain-containing protein |
| maker-11-augustus-gene-221.4-mRNA-1 | Japonica | NA | | Os11g0629100 | OsR498G1120561100.01.T02 | Protein PYRICULARIA ORYZAE RESISTANCE 21 |
| maker-11-augustus-gene-221.6-mRNA-1 | Japonica | NA | | Os11g0629200 | OsR498G1120561300.01.T01 | Vacuolar protein sorting-associated protein 26 |
| maker-11-augustus-gene-222.39-mRNA-1 | Japonica | NA | CC-NBS-TM | | OsR498G1120562500.01.T05 | Disease resistance protein RPM1 |
| maker-11-augustus-gene-222.42-mRNA-1 | Japonica | NA | NBS-TM | | OsR498G1120563300.01.T01 | NB-ARC domain containing protein, expressed |
| maker-11-augustus-gene-222.45-mRNA-1 | Japonica | NA | | Os11g0629600 | | BTB/POZ and MATH domain-containing protein 2 |
| maker-11-augustus-gene-222.46-mRNA-1 | Japonica | NA | | Os11g0629400 | OsR498G1120562000.01.T01 | Methylesterase 11 |
| maker-11-augustus-gene-222.48-mRNA-1 | Japonica | NA | | Os11g0629500 | OsR498G1120562200.01.T03 | O-phosphoserine phosphohydrolase |
| maker-11-augustus-gene-223.0-mRNA-1 | Japonica | NA | | | OsR498G1120575900.01.T01 | |

| | | | | | | |
|---|---|---|---|---|---|---|
| maker-11-augustus-gene-223.2-mRNA-1 | Japonica | Indica | | Os02g0701833 | OsR498G1120576600.01.T02 | ATP-dependent DNA helicase |
| maker-11-augustus-gene-224.0-mRNA-1 | Japonica | NA | LRR-NBS-TM | Os11g0633500 | OsR498G1120581500.01.T03 | Disease resistance protein RPP8 |
| maker-11-augustus-gene-224.3-mRNA-1 | Japonica | NA | | Os11g0633800 | OsR498G1120584100.01.T01 | F-box domain containing protein, expressed |
| maker-11-augustus-gene-224.5-mRNA-1 | Japonica | NA | | Os11g0634200 | OsR498G1120584800.01.T01 | |
| maker-11-augustus-gene-225.0-mRNA-1 | Japonica | Indica | | Os11g0634500 | OsR498G1120587100.01.T02 | Ribosomal protein S14p/S29e containing protein, expressed |
| maker-11-augustus-gene-225.2-mRNA-1 | Japonica | NA | | | OsR498G1120587900.01.T01 | BHLH domain-containing protein (Fragment) |
| maker-11-augustus-gene-225.3-mRNA-1 | Japonica | Indica | NBS-TM | Os07g0573100 | OsR498G1120588100.01.T01 | Adenylyl-sulfate kinase |
| maker-11-augustus-gene-225.6-mRNA-1 | Japonica | Indica | | Os11g0635500 | OsR498G1120590900.01.T01 | Ent-cassadiene C2-hydroxylase |
| maker-11-augustus-gene-225.8-mRNA-1 | Japonica | NA | | Os11g0635500 | OsR498G1120591500.01.T01 | Ent-cassadiene C2-hydroxylase-like |
| maker-11-augustus-gene-226.0-mRNA-1 | Japonica | NA | | Os09g0244000 | | |
| maker-11-augustus-gene-226.2-mRNA-1 | Japonica | Japonica | | Os11g0636200 | OsR498G1120596000.01.T03 | CPSF (Cleavage and polyadenylation specific factor), subunit A, putative |
| maker-11-augustus-gene-226.7-mRNA-1 | Japonica | NA | | Os11g0636900 | OsR498G1120601800.01.T01 | U2 snRNP auxiliary factor large subunit |
| maker-11-augustus-gene-227.36-mRNA-1 | Japonica | Japonica | | Os11g0637300 | OsR498G1120603300.01.T01 | F-box domain containing protein, expressed |
| maker-11-augustus-gene-227.38-mRNA-1 | Japonica | NA | | Os11g0637700 | OsR498G1120604700.01.T02 | Heterogeneous nuclear ribonucleoprotein 1 |
| maker-11-augustus-gene-227.40-mRNA-1 | Japonica | NA | | | OsR498G1120605300.01.T06 | GTP-binding protein EngA |
| maker-11-augustus-gene-227.45-mRNA-1 | Japonica | NA | | Os11g0638200 | | |
| maker-11-augustus-gene-227.48-mRNA-1 | Japonica | Indica | | Os11g0637200 | OsR498G1120602400.01.T01 | Polyol transporter 5 |
| maker-11-augustus-gene-227.53-mRNA-1 | Japonica | Indica | | Os11g0637200 | OsR498G1120604000.01.T02 | Polyol transporter 5 |
| maker-11-augustus-gene-227.55-mRNA-1 | Japonica | Indica | | Os11g0637400 | OsR498G1120604200.01.T01 | Stress-associated endoplasmic reticulum protein 2 |
| maker-11-augustus-gene-227.57-mRNA-1 | Japonica | NA | | | OsR498G1120604600.01.T01 | Potyviral VPg interacting protein 1 (Fragment) |
| maker-11-augustus-gene-227.59-mRNA-1 | Japonica | NA | | | OsR498G1120605000.01.T01 | 1-acylglycerol-3-phosphate O-acyltransferase |
| maker-11-augustus-gene-227.63-mRNA-1 | Japonica | NA | | | OsR498G1120608000.01.T03 | Armadillo repeat-containing protein 8 |
| maker-11-augustus-gene-228.0-mRNA-1 | Japonica | NA | | | OsR498G1120608700.01.T01 | Folate-sensitive fragile site protein Fra10Ac1 |
| maker-11-augustus-gene-228.6-mRNA-1 | Japonica | NA | CC-LRR-NBS | Os11g0640600 | OsR498G1120613000.01.T01 | Blast resistance protein Pi54 variant (Fragment) |
| maker-11-augustus-gene-23.40-mRNA-1 | Japonica | NA | | Os11g0161000 | OsR498G1119465100.01.T02 | NB-ARC domain, putative |
| maker-11-augustus-gene-23.42-mRNA-1 | Japonica | Japonica | | Os11g0161650 | OsR498G1119465700.01.T05 | NB-ARC domain, putative |
| maker-11-augustus-gene-23.45-mRNA-1 | Japonica | NA | | | OsR498G1119465700.01.T04 | NB-ARC domain, putative |
| maker-11-augustus-gene-23.48-mRNA-1 | Japonica | NA | | Os11g0162200 | OsR498G1119466700.01.T01 | Cytochrome b-c1 complex subunit 6-1, mitochondrial |
| maker-11-augustus-gene-23.54-mRNA-1 | Japonica | NA | | | OsR498G0100693500.01.T01 | Transposon protein, putative, Mutator sub-class |
| maker-11-augustus-gene-23.57-mRNA-1 | Japonica | NA | | | OsR498G1119466400.01.T01 | ATG13 domain-containing protein |
| maker-11-augustus-gene-23.60-mRNA-1 | Japonica | NA | | Os11g0162300 | | |
| maker-11-augustus-gene-231.0-mRNA-1 | Japonica | NA | | | OsR498G1120633100.01.T01 | Oxidoreductase, aldo/keto reductase family protein, expressed |
| maker-11-augustus-gene-231.11-mRNA-1 | Japonica | NA | | | OsR498G1120632000.01.T02 | Nicotianamine aminotransferase A |
| maker-11-augustus-gene-231.7-mRNA-1 | Japonica | Japonica | | Os11g0645700 | OsR498G1120634500.01.T01 | |
| maker-11-augustus-gene-233.24-mRNA-1 | Japonica | NA | | | OsR498G1120642100.01.T01 | |
| maker-11-augustus-gene-233.29-mRNA-1 | Japonica | NA | | Os11g0648000 | OsR498G1120635000.01.T01 | Sodium-hydrogen exchanger 2 |
| maker-11-augustus-gene-233.32-mRNA-1 | Japonica | NA | | | OsR498G1120635400.01.T03 | kinesin-like protein klp-3 isoform X2 |
| maker-11-augustus-gene-233.38-mRNA-1 | Japonica | Indica | | Os11g0648400 | OsR498G1120641000.01.T02 | |
| maker-11-augustus-gene-233.45-mRNA-1 | Japonica | NA | | | OsR498G1120641400.01.T01 | |
| maker-11-augustus-gene-233.47-mRNA-1 | Japonica | NA | | | OsR498G1120642600.01.T01 | |
| maker-11-augustus-gene-233.51-mRNA-1 | Japonica | NA | | | | |
| maker-11-augustus-gene-234.0-mRNA-1 | Japonica | NA | | | OsR498G1120643700.01.T01 | |
| maker-11-augustus-gene-234.11-mRNA-1 | Japonica | NA | | Os11g0649600 | OsR498G1120646000.01.T01 | |
| maker-11-augustus-gene-234.13-mRNA-1 | Japonica | NA | | Os11g0649700 | OsR498G1120646200.01.T01 | |
| maker-11-augustus-gene-234.2-mRNA-1 | Japonica | NA | | | OsR498G1120650900.01.T01 | von Willebrand factor type A domain containing protein, expressed |
| maker-11-augustus-gene-234.4-mRNA-1 | Japonica | NA | | | OsR498G1120644200.01.T01 | |
| maker-11-augustus-gene-234.9-mRNA-1 | Japonica | NA | | | OsR498G1120645000.01.T01 | |
| maker-11-augustus-gene-236.36-mRNA-1 | Japonica | Indica | | Os11g0652600 | OsR498G1120655200.01.T01 | DUF4220 domain-containing protein (Fragment) |
| maker-11-augustus-gene-236.39-mRNA-1 | Japonica | NA | | Os11g0652900 | OsR498G1120655800.01.T02 | Tropinone reductase |
| maker-11-augustus-gene-237.33-mRNA-1 | Japonica | Japonica | | Os11g0655000 | OsR498G1120661900.01.T01 | |
| maker-11-augustus-gene-237.37-mRNA-1 | Japonica | NA | | Os11g0654400 | OsR498G1120665000.01.T01 | Tropinone reductase |
| maker-11-augustus-gene-238.30-mRNA-1 | Japonica | NA | | Os11g0655800 | OsR498G1120669300.01.T05 | probable feruloyl esterase A |
| maker-11-augustus-gene-238.37-mRNA-1 | Japonica | Indica | | Os11g0657000 | OsR498G1120670900.01.T02 | Kelch motif family protein, expressed |
| maker-11-augustus-gene-238.40-mRNA-1 | Japonica | NA | | Os11g0657100 | OsR498G1120671100.01.T01 | RF-1 domain-containing protein/PCRF domain-containing protein |
| maker-11-augustus-gene-238.46-mRNA-1 | Japonica | NA | | Os11g0657300 | OsR498G1120671500.01.T02 | Sm protein F |
| maker-11-augustus-gene-238.50-mRNA-1 | Japonica | NA | | Os11g0657200 | OsR498G1120671400.01.T01 | alkylated DNA repair protein alkB homolog 8 |
| maker-11-augustus-gene-239.0-mRNA-1 | Japonica | NA | | Os11g0658700 | OsR498G1120679700.01.T02 | Agamous-like MADS-box protein AGL65 |
| maker-11-augustus-gene-239.13-mRNA-1 | Japonica | NA | | Os11g0658800 | OsR498G1120680000.01.T02 | Glycoside hydrolase family 28 protein, putative, expressed |
| maker-11-augustus-gene-239.3-mRNA-1 | Japonica | NA | | | OsR498G1120681300.01.T01 | Mono-/di-acylglycerol lipase N-terminal |
| maker-11-augustus-gene-24.35-mRNA-1 | Japonica | NA | | Os11g0163100 | OsR498G1119468300.01.T04 | Beta-actin |
| maker-11-augustus-gene-24.40-mRNA-1 | Japonica | Indica | | Os11g0163600 | OsR498G1119469900.01.T05 | Tubby-like F-box protein 13 |

| | | | | | | |
|---|---|---|---|---|---|---|
| maker-11-augustus-gene-24.44-mRNA-1 | Japonica | NA | | Os11g0165700 | OsR498G1119471500.01.T01 | Transposon protein, putative, CACTA, En/Spm sub-class, expressed |
| maker-11-augustus-gene-240.0-mRNA-1 | Japonica | NA | | Os11g0659200 | OsR498G1120681700.01.T01 | FLZ-type domain-containing protein |
| maker-11-augustus-gene-240.11-mRNA-1 | Japonica | NA | | Os11g0660000 | OsR498G1120683400.01.T02 | Magnesiumproton exchanger |
| maker-11-augustus-gene-240.18-mRNA-1 | Japonica | NA | | Os11g0660500 | OsR498G1120685800.01.T01 | TCTP domain-containing protein (Fragment) |
| maker-11-augustus-gene-240.2-mRNA-1 | Japonica | NA | | Os11g0660300 | OsR498G1120683700.01.T01 | WD repeat-containing protein 61 |
| maker-11-augustus-gene-240.4-mRNA-1 | Japonica | NA | | Os11g0659500 | OsR498G1120682400.01.T03 | Acyl-[acyl-carrier-protein] hydrolase |
| maker-11-augustus-gene-240.8-mRNA-1 | Japonica | NA | | | | Pectinesterase |
| maker-11-augustus-gene-241.10-mRNA-1 | Japonica | Indica | | Os07g0672500 | OsR498G1120687800.01.T02 | AAA-type ATPase family protein |
| maker-11-augustus-gene-241.18-mRNA-1 | Japonica | NA | | Os11g0661800 | | |
| maker-11-augustus-gene-241.20-mRNA-1 | Japonica | NA | | Os08g0522100 | | ATP-dependent DNA helicase |
| maker-11-augustus-gene-241.8-mRNA-1 | Japonica | NA | | Os11g0661600 | OsR498G1120688300.01.T01 | Peroxidase |
| maker-11-augustus-gene-243.4-mRNA-1 | Japonica | Japonica | | Os11g0664800 | OsR498G1120688900.01.T01 | Calmodulin-binding protein 60 F |
| maker-11-augustus-gene-243.6-mRNA-1 | Japonica | Indica | | Os11g0665600 | OsR498G1120688800.01.T01 | |
| maker-11-augustus-gene-244.49-mRNA-1 | Japonica | NA | Kinase-TM | | OsR498G1120691300.01.T01 | Cytokinin-regulated kinase-like |
| maker-11-augustus-gene-244.53-mRNA-1 | Japonica | Japonica | CC-Kinase-TM | Os11g0666200 | OsR498G1120691200.01.T01 | Protein kinase domain-containing protein |
| maker-11-augustus-gene-245.38-mRNA-1 | Japonica | NA | | | OsR498G1120694900.01.T03 | |
| maker-11-augustus-gene-245.39-mRNA-1 | Japonica | NA | | | | Calmodulin-binding protein-like |
| maker-11-augustus-gene-245.48-mRNA-1 | Japonica | NA | Kinase-TM | | OsR498G1120696900.01.T05 | Protein kinase domain containing protein, expressed |
| maker-11-augustus-gene-245.50-mRNA-1 | Japonica | NA | | Os11g0669100 | OsR498G1120697100.01.T01 | Calmodulin-binding protein-like |
| maker-11-augustus-gene-245.56-mRNA-1 | Japonica | NA | Kinase-TM | | OsR498G1120696900.01.T05 | G-type lectin S-receptor-like serine/threonine-protein kinase |
| maker-11-augustus-gene-245.58-mRNA-1 | Japonica | NA | | | | |
| maker-11-augustus-gene-246.7-mRNA-1 | Japonica | Indica | | Os09g0529700 | OsR498G1120699900.01.T01 | Vacuolar protein sorting-associated protein |
| maker-11-augustus-gene-246.8-mRNA-1 | Japonica | NA | | Os09g0529400 | OsR498G0917840000.01.T04 | Vacuolar protein sorting-associated protein |
| maker-11-augustus-gene-247.3-mRNA-1 | Japonica | NA | | Os11g0672300 | OsR498G1120704100.01.T05 | Protein MID1-COMPLEMENTING ACTIVITY 1 |
| maker-11-augustus-gene-247.5-mRNA-1 | Japonica | NA | | | OsR498G1120704400.01.T01 | p-loop nucleoside triphosphate hydrolase superfamily protein with CH (Calp |
| maker-11-augustus-gene-248.27-mRNA-1 | Japonica | NA | CC-LRR-NBS-TM | Os11g0677101 | OsR498G1120709900.01.T01 | Putative nucleotide-binding leucine-rich-repeat protein 1 |
| maker-11-augustus-gene-248.32-mRNA-1 | Japonica | Japonica | LRR-NBS | Os11g0673900 | OsR498G1120709100.01.T03 | Putative nucleotide-binding leucine-rich-repeat protein 1 |
| maker-11-augustus-gene-248.45-mRNA-1 | Japonica | Indica | | Os11g0673200 | OsR498G1120707800.01.T01 | Beta-xylosidase 2 |
| maker-11-augustus-gene-25.37-mRNA-1 | Japonica | NA | | Os11g0166300 | | 60 kDa jasmonate-induced protein, putative |
| maker-11-augustus-gene-250.32-mRNA-1 | Japonica | Indica | CC-Kinase-TM | Os12g0562500 | OsR498G1120719100.01.T01 | Serine/threonine-protein kinase CTR1 |
| maker-11-augustus-gene-250.37-mRNA-1 | Japonica | NA | | Os11g0679700 | OsR498G1120723300.01.T01 | PlsC domain-containing protein (Fragment) |
| maker-11-augustus-gene-250.39-mRNA-1 | Japonica | NA | | | OsR498G1120721800.01.T01 | VWFA domain-containing protein (Fragment) |
| maker-11-augustus-gene-251.40-mRNA-1 | Japonica | NA | Kinase-TM | | OsR498G1120726800.01.T02 | Receptor protein kinase-like |
| maker-11-augustus-gene-252.38-mRNA-1 | Japonica | NA | | Os11g0682300 | OsR498G1120728700.01.T06 | U2 snRNP auxiliary factor large subunit |
| maker-11-augustus-gene-252.48-mRNA-1 | Japonica | NA | | Os11g0683500 | OsR498G1120729800.01.T02 | Beta-glucosidase 5 |
| maker-11-augustus-gene-252.53-mRNA-1 | Japonica | NA | | Os11g0684000 | OsR498G1120730600.01.T01 | Transcription factor MYB108 |
| maker-11-augustus-gene-252.57-mRNA-1 | Japonica | Japonica | NBS | Os11g0684600 | OsR498G1120731800.01.T01 | NBS-LRR disease resistance protein homologue-like |
| maker-11-augustus-gene-253.1-mRNA-1 | Japonica | Indica | NBS-TM | Os11g0684100 | OsR498G1120735400.01.T01 | NBS-LRR disease resistance protein homologue-like |
| maker-11-augustus-gene-254.0-mRNA-1 | Japonica | NA | | Os11g0687100 | OsR498G1120737300.01.T01 | von Willebrand factor type A domain-like |
| maker-11-augustus-gene-254.11-mRNA-1 | Japonica | NA | LRR-NBS-TM | | OsR498G1120737800.01.T01 | Disease resistance protein Piks-1 |
| maker-11-augustus-gene-254.4-mRNA-1 | Japonica | NA | | Os11g0687100 | OsR498G1120737300.01.T01 | von Willebrand factor type A domain-like |
| maker-11-augustus-gene-254.7-mRNA-1 | Japonica | Indica | | Os11g0687200 | OsR498G1120737500.01.T01 | von Willebrand factor type A domain-like |
| maker-11-augustus-gene-255.0-mRNA-1 | Japonica | Indica | NBS-TM PIK, PIKM | Os11g0689100 | OsR498G1120737900.01.T02 | Disease resistance protein Piks-2 |
| maker-11-augustus-gene-256.34-mRNA-1 | Japonica | NA | Kinase-TM | Os11g0695850 | OsR498G1120743300.01.T02 | Wall-associated receptor kinase |
| maker-11-augustus-gene-256.35-mRNA-1 | Japonica | NA | Kinase-TM | | | Putative inactive receptor kinase RLK902 |
| maker-11-augustus-gene-256.36-mRNA-1 | Japonica | NA | Kinase | | | Major disease resistance protein Xa4 |
| maker-11-augustus-gene-257.26-mRNA-1 | Japonica | Indica | Kinase-LRR-TM | Os11g0695700 | OsR498G1120747200.01.T01 | Putative receptor kinase TRKc |
| maker-11-augustus-gene-257.28-mRNA-1 | Japonica | NA | Kinase-LRR-TM | | OsR498G1120751000.01.T01 | Protein kinase domain containing protein, expressed |
| maker-11-augustus-gene-258.22-mRNA-1 | Japonica | NA | Kinase-LRR-TM | Os11g0695600 | OsR498G1120747600.01.T01 | Serine-threonine protein kinase plant-type |
| maker-11-augustus-gene-260.0-mRNA-1 | Japonica | Japonica | CC-NBS-TM | Os11g0699100 | OsR498G1120757300.01.T01 | Disease resistance protein RPP13 |
| maker-11-augustus-gene-260.5-mRNA-1 | Japonica | NA | | Os11g0700500 | OsR498G1120760800.01.T01 | MYB transcription factor MYBAS1 |
| maker-11-augustus-gene-262.53-mRNA-1 | Japonica | NA | | Os11g0704700 | OsR498G1120770400.01.T02 | RRM domain-containing protein (Fragment) |
| maker-11-augustus-gene-262.58-mRNA-1 | Japonica | NA | | Os11g0704800 | OsR498G1120770600.01.T01 | RHOMBOID-like protein (Fragment) |
| maker-11-augustus-gene-263.0-mRNA-1 | Japonica | NA | | | OsR498G1120777300.01.T02 | Mitochondrial uncoupling protein 2 |
| maker-11-augustus-gene-263.16-mRNA-1 | Japonica | Japonica | | Os11g0706100 | OsR498G0203365400.01.T04 | |
| maker-11-augustus-gene-263.21-mRNA-1 | Japonica | Indica | | Os11g0706600 | OsR498G1120773800.01.T02 | thaumatin-like protein isoform X2 |
| maker-11-augustus-gene-263.23-mRNA-1 | Japonica | NA | | | OsR498G1120774000.01.T01 | Cyclin N-terminal domain-containing protein |
| maker-11-augustus-gene-263.25-mRNA-1 | Japonica | Indica | | Os11g0707000 | OsR498G1120774600.01.T03 | Ribulose bisphosphate carboxylase/oxygenase activase |
| maker-11-augustus-gene-263.30-mRNA-1 | Japonica | Japonica | | Os11g0707100 | OsR498G1120774600.01.T02 | Rubisco activase small subunit |
| maker-11-augustus-gene-263.32-mRNA-1 | Japonica | NA | | Os11g0707600 | OsR498G1120776600.01.T01 | Aldehyde oxygenase (deformylating) |
| maker-11-augustus-gene-263.37-mRNA-1 | Japonica | NA | | Os11g0707700 | OsR498G1120776800.01.T01 | Elongin-C |

| | | | | | | |
|---|---|---|---|---|---|---|
| maker-11-augustus-gene-263.39-mRNA-1 | Japonica | Indica | | Os11g0708600 | OsR498G1120779000.01.T02 | mediator of RNA polymerase II transcription subunit 34 isoform X1 |
| maker-11-augustus-gene-263.4-mRNA-1 | Japonica | NA | | | OsR498G1120778300.01.T01 | Laccase |
| maker-11-augustus-gene-263.6-mRNA-1 | Japonica | NA | | Os11g0708400 | OsR498G1120778500.01.T01 | GDSL esterase/lipase |
| maker-11-augustus-gene-263.9-mRNA-1 | Japonica | Japonica | | Os11g0708900 | OsR498G0407560600.01.T01 | Phenylalanine ammonia-lyase |
| maker-11-augustus-gene-27.0-mRNA-1 | Japonica | NA | | | OsR498G1119475100.01.T01 | SEC14 cytosolic factor family protein / phosphoglyceride transfer family prot |
| maker-11-augustus-gene-27.12-mRNA-1 | Japonica | NA | | Os11g0168200 | OsR498G1119478900.01.T01 | Ribosomal protein L3 |
| maker-11-augustus-gene-27.16-mRNA-1 | Japonica | Indica | | Os11g0166800 | OsR498G1119474600.01.T02 | probable mediator of RNA polymerase II transcription subunit 26c |
| maker-11-augustus-gene-27.20-mRNA-1 | Japonica | NA | | Os11g0167300 | OsR498G1119475600.01.T01 | HTH OST-type domain-containing protein (Fragment) |
| maker-11-augustus-gene-27.22-mRNA-1 | Japonica | NA | | Os11g0167800 | OsR498G1119476600.01.T01 | Abcissic acid stress ripening |
| maker-11-augustus-gene-27.8-mRNA-1 | Japonica | Indica | | Os01g0963300 | OsR498G1119478500.01.T02 | t-SNARE coiled-coil homology domain-containing protein |
| maker-11-augustus-gene-28.36-mRNA-1 | Japonica | Japonica | | Os11g0168300 | OsR498G0408582600.01.T02 | Alba domain-containing protein (Fragment) |
| maker-11-augustus-gene-28.37-mRNA-1 | Japonica | NA | | Os11g0168300 | | |
| maker-11-augustus-gene-28.42-mRNA-1 | Japonica | Indica | Kinase-TM | Os02g0194400 | OsR498G1119480300.01.T01 | Leucine-rich repeat receptor protein kinase MSL1 |
| maker-11-augustus-gene-28.44-mRNA-1 | Japonica | NA | | | | Transposon protein, putative, CACTA, En/Spm sub-class |
| maker-11-augustus-gene-28.48-mRNA-1 | Japonica | NA | | Os11g0169100 | OsR498G1119481900.01.T01 | Sec1 family domain-containing protein 2 |
| maker-11-augustus-gene-28.50-mRNA-1 | Japonica | NA | | Os11g0169400 | OsR498G1119483300.01.T01 | Zinc finger, C2H2 type family protein, expressed |
| maker-11-augustus-gene-28.52-mRNA-1 | Japonica | Indica | | Os11g0169800 | OsR498G1119484100.01.T01 | Long-chain-fatty-acid--CoA ligase 3 |
| maker-11-augustus-gene-28.57-mRNA-1 | Japonica | NA | | Os11g0168500 | OsR498G1119479600.01.T01 | Ethylene response factor 9 |
| maker-11-augustus-gene-28.59-mRNA-1 | Japonica | NA | | Os11g0169200 | OsR498G1119482600.01.T01 | AUX/LAX auxin transporter |
| maker-11-augustus-gene-28.61-mRNA-1 | Japonica | NA | | Os09g0545700 | OsR498G0815439200.01.T01 | Auxin-responsive protein SAUR36-like |
| maker-11-augustus-gene-28.63-mRNA-1 | Japonica | NA | | Os11g0169900 | OsR498G1119484400.01.T01 | V-type proton ATPase proteolipid subunit |
| maker-11-augustus-gene-28.66-mRNA-1 | Japonica | Japonica | | Os11g0170000 | OsR498G1221019600.01.T03 | Amidase family protein, expressed |
| maker-11-augustus-gene-28.68-mRNA-1 | Japonica | NA | | Os11g0170200 | OsR498G1119484800.01.T07 | Filament-like plant protein 3 |
| maker-11-augustus-gene-29.32-mRNA-1 | Japonica | Indica | | Os12g0169400 | OsR498G1119485200.01.T02 | CBS domain containing protein CBSCBSPB4 |
| maker-11-augustus-gene-29.41-mRNA-1 | Japonica | NA | | Os11g0171500 | OsR498G1119487000.01.T02 | Calcium-dependent protein kinase SK5 |
| maker-11-augustus-gene-3.0-mRNA-1 | Japonica | NA | | | | Auxin efflux carrier component |
| maker-11-augustus-gene-3.2-mRNA-1 | Japonica | NA | | | OsR498G1119344700.01.T01 | |
| maker-11-augustus-gene-30.26-mRNA-1 | Japonica | NA | Kinase-LRR-TM | Os11g0171800 | OsR498G1119488300.01.T02 | Putative LRR receptor-like serine/threonine-protein kinase |
| maker-11-augustus-gene-30.27-mRNA-1 | Japonica | Indica | | Os01g0788400 | OsR498G1119488700.01.T01 | Pectinesterase |
| maker-11-augustus-gene-30.29-mRNA-1 | Japonica | NA | LRR | Os11g0172300 | | Protein kinase domain-containing protein |
| maker-11-augustus-gene-30.62-mRNA-1 | Japonica | Japonica | Kinase-LRR-TM | Os11g0172700 | OsR498G1119488300.01.T02 | Putative LRR receptor-like serine/threonine-protein kinase |
| maker-11-augustus-gene-30.66-mRNA-1 | Japonica | NA | | Os11g0171700 | OsR498G1119488200.01.T01 | FHA domain containing protein, expressed |
| maker-11-augustus-gene-31.34-mRNA-1 | Japonica | Indica | Kinase-LRR-TM | Os11g0173700 | OsR498G1119493500.01.T01 | Putative LRR receptor-like serine/threonine-protein kinase |
| maker-11-augustus-gene-31.36-mRNA-1 | Japonica | Indica | Kinase-LRR-TM | Os11g0173500 | OsR498G1119494900.01.T07 | Putative LRR receptor-like serine/threonine-protein kinase |
| maker-11-augustus-gene-31.38-mRNA-1 | Japonica | Japonica | Kinase-LRR-TM | Os11g0173700 | OsR498G1119493500.01.T01 | Putative LRR receptor-like serine/threonine-protein kinase |
| maker-11-augustus-gene-31.40-mRNA-1 | Japonica | NA | Kinase-LRR-TM | Os11g0173800 | OsR498G1119496700.01.T02 | Leucine Rich Repeat family protein, expressed |
| maker-11-augustus-gene-31.44-mRNA-1 | Japonica | NA | Kinase-LRR-TM | Os11g0173900 | OsR498G1119496900.01.T01 | Leucine-rich repeat receptor kinase (LRR-RK) |
| maker-11-augustus-gene-31.54-mRNA-1 | Japonica | NA | | Os11g0174000 | OsR498G1119497200.01.T02 | Coatomer subunit beta |
| maker-11-augustus-gene-33.12-mRNA-1 | Japonica | NA | | | OsR498G0102010300.01.T01 | Protein kinase domain-containing protein |
| maker-11-augustus-gene-33.20-mRNA-1 | Japonica | Indica | | Os11g0177400 | OsR498G1119510400.01.T02 | ABC transporter domain-containing protein (Fragment) |
| maker-11-augustus-gene-34.38-mRNA-1 | Japonica | Indica | | Os11g0180100 | OsR498G1119519000.01.T01 | |
| maker-11-augustus-gene-35.26-mRNA-1 | Japonica | NA | | | OsR498G1119520500.01.T03 | DEAD-box ATP-dependent RNA helicase FANCM |
| maker-11-augustus-gene-35.30-mRNA-1 | Japonica | NA | | Os11g0180900 | | R2R3-Myb transcription factor |
| maker-11-augustus-gene-35.33-mRNA-1 | Japonica | NA | | Os11g0181100 | OsR498G1119521900.01.T01 | Transmembrane 9 superfamily member |
| maker-11-augustus-gene-35.37-mRNA-1 | Japonica | NA | | | OsR498G1119522500.01.T01 | Mlo like protein 4 (Fragment) |
| maker-11-augustus-gene-35.42-mRNA-1 | Japonica | NA | | Os11g0181500 | OsR498G1119522700.01.T01 | NifU-like protein 2 chloroplastic |
| maker-11-augustus-gene-35.44-mRNA-1 | Japonica | Indica | | Os12g0174800 | OsR498G1119519800.01.T04 | Protein-tyrosine-phosphatase PTP1 |
| maker-11-augustus-gene-36.43-mRNA-1 | Japonica | NA | | Os11g0181900 | OsR498G1119523700.01.T02 | |
| maker-11-augustus-gene-36.46-mRNA-1 | Japonica | Japonica | | Os11g0182200 | OsR498G0816559700.01.T01 | Putrescine hydroxycinnamoyltransferase 1 |
| maker-11-augustus-gene-36.48-mRNA-1 | Japonica | NA | | Os11g0182400 | OsR498G1119525400.01.T01 | F-box/WD-40 repeat-containing protein (Fragment) |
| maker-11-augustus-gene-36.54-mRNA-1 | Japonica | Japonica | | Os11g0182500 | OsR498G1119526200.01.T01 | SWI/SNF complex subunit SWI3C-like |
| maker-11-augustus-gene-36.56-mRNA-1 | Japonica | NA | | Os11g0182900 | OsR498G1119527200.01.T02 | Ankyrin repeat family protein, putative, expressed |
| maker-11-augustus-gene-36.60-mRNA-1 | Japonica | NA | | | OsR498G1119527800.01.T01 | SWI/SNF complex subunit SWI3C-like |
| maker-11-augustus-gene-37.0-mRNA-1 | Japonica | NA | | Os11g0183700 | OsR498G1119529300.01.T01 | SWI/SNF complex subunit SWI3C |
| maker-11-augustus-gene-37.10-mRNA-1 | Japonica | NA | | Os11g0183900 | | Eukaryotic aspartyl protease family protein, expressed |
| maker-11-augustus-gene-37.12-mRNA-1 | Japonica | Japonica | | Os11g0184200 | OsR498G0917727000.01.T01 | Beta-glucosidase 11 isoform F |
| maker-11-augustus-gene-37.15-mRNA-1 | Japonica | NA | | Os11g0184800 | | Aspartic proteinase |
| maker-11-augustus-gene-37.17-mRNA-1 | Japonica | NA | NBS-TM | Os11g0184900 | OsR498G1119531400.01.T01 | NAC domain transcription factor |
| maker-11-augustus-gene-37.5-mRNA-1 | Japonica | NA | | | OsR498G1119529700.01.T03 | Protein CELLULOSE SYNTHASE INTERACTIVE 3 isoform C |
| maker-11-augustus-gene-38.36-mRNA-1 | Japonica | Indica | | Os01g0221100 | OsR498G1119535300.01.T01 | Jasmonoyl--L-amino acid synthetase GH3.3 |
| maker-11-augustus-gene-39.45-mRNA-1 | Japonica | NA | | Os11g0186900 | OsR498G1119536600.01.T01 | 1-aminocyclopropane-1-carboxylate oxidase |

| | | | | | | |
|---|---|---|---|---|---|---|
| maker-11-augustus-gene-39.53-mRNA-1 | Japonica | NA | | Os11g0187500 | OsR498G1119541000.01.T01 | Heat shock 70 kDa protein 3 |
| maker-11-augustus-gene-4.52-mRNA-1 | Japonica | NA | | Os12g0121600 | OsR498G1119346300.01.T01 | Cation/H(+) antiporter 15 |
| maker-11-augustus-gene-4.54-mRNA-1 | Japonica | NA | | Os11g0124300 | OsR498G1119347500.01.T01 | Transcription factor GRAS |
| maker-11-augustus-gene-4.56-mRNA-1 | Japonica | Indica | | Os11g0124500 | OsR498G1119348100.01.T01 | Calcium uniporter protein (Fragment) |
| maker-11-augustus-gene-4.58-mRNA-1 | Japonica | Japonica | | Os11g0125600 | OsR498G1220855500.01.T04 | Glutathione transferase GST 23 |
| maker-11-augustus-gene-4.60-mRNA-1 | Japonica | NA | | | | Ethanolamine-phosphate cytidylyltransferase |
| maker-11-augustus-gene-4.65-mRNA-1 | Japonica | NA | | Os11g0123500 | OsR498G1119346000.01.T02 | Vacuolar protein sorting-associated protein 32 |
| maker-11-augustus-gene-4.70-mRNA-1 | Japonica | NA | | Os11g0124900 | OsR498G1119348600.01.T01 | Hexosyltransferase |
| maker-11-augustus-gene-4.74-mRNA-1 | Japonica | NA | | | | Pentatricopeptide repeat-containing protein (Fragment) |
| maker-11-augustus-gene-41.0-mRNA-1 | Japonica | NA | | | OsR498G1119543500.01.T01 | |
| maker-11-augustus-gene-41.6-mRNA-1 | Japonica | NA | | | | DNA topoisomerase 2-binding protein 1 |
| maker-11-augustus-gene-41.9-mRNA-1 | Japonica | NA | | Os11g0191400 | OsR498G1119545300.01.T02 | NAD kinase 2 chloroplastic |
| maker-11-augustus-gene-42.0-mRNA-1 | Japonica | NA | CC-NBS-TM | | | NB-ARC domain, putative |
| maker-11-augustus-gene-43.0-mRNA-1 | Japonica | NA | | | | F-box domain-containing protein (Fragment) |
| maker-11-augustus-gene-43.2-mRNA-1 | Japonica | NA | | | OsR498G1119741400.01.T01 | Subtilisin-like serine endopeptidase family protein |
| maker-11-augustus-gene-43.3-mRNA-1 | Japonica | NA | | | OsR498G1119741400.01.T03 | Subtilisin-like serine endopeptidase family protein |
| maker-11-augustus-gene-44.31-mRNA-1 | Japonica | NA | | Os10g0199200 | | Transposon protein, putative, CACTA, En/Spm sub-class, expressed |
| maker-11-augustus-gene-44.36-mRNA-1 | Japonica | Indica | | Os11g0264200 | OsR498G1119754700.01.T02 | F-box/LRR-repeat protein 12 |
| maker-11-augustus-gene-44.38-mRNA-1 | Japonica | Indica | | Os11g0641500 | OsR498G1119753600.01.T01 | Laccase |
| maker-11-augustus-gene-44.40-mRNA-1 | Japonica | NA | | | OsR498G1119755000.01.T01 | NLP transcription factor (Fragment) |
| maker-11-augustus-gene-45.0-mRNA-1 | Japonica | NA | | Os11g0264500 | OsR498G1119761300.01.T01 | |
| maker-11-augustus-gene-45.10-mRNA-1 | Japonica | NA | | Os11g0265000 | OsR498G1119763500.01.T01 | Uridine kinase |
| maker-11-augustus-gene-45.17-mRNA-1 | Japonica | NA | | Os11g0265400 | OsR498G1119764700.01.T01 | Dehydrogenase/reductase SDR family member 7 like |
| maker-11-augustus-gene-45.24-mRNA-1 | Japonica | Indica | | Os11g0264700 | OsR498G1119762000.01.T01 | ApaG domain-containing protein |
| maker-11-augustus-gene-45.28-mRNA-1 | Japonica | NA | | Os11g0265200 | OsR498G1119764400.01.T02 | F-box protein SKIP16 |
| maker-11-augustus-gene-45.32-mRNA-1 | Japonica | NA | | | OsR498G1119764600.01.T01 | |
| maker-11-augustus-gene-45.4-mRNA-1 | Japonica | NA | | | OsR498G1119761700.01.T01 | ER membrane protein complex subunit 10 like |
| maker-11-augustus-gene-46.0-mRNA-1 | Japonica | NA | | Os11g0265600 | | Diphthamide synthesis DHP2, eukaryotic |
| maker-11-augustus-gene-46.11-mRNA-1 | Japonica | NA | | | OsR498G1119765000.01.T02 | RNA-binding ASCH domain protein |
| maker-11-augustus-gene-46.18-mRNA-1 | Japonica | NA | | Os11g0266000 | OsR498G1119767600.01.T01 | |
| maker-11-augustus-gene-46.22-mRNA-1 | Japonica | Indica | NBS-TM | Os11g0265900 | OsR498G1119768400.01.T01 | NB-ARC domain, putative |
| maker-11-augustus-gene-46.24-mRNA-1 | Japonica | NA | | | | Tetratricopeptide repeat-containing domain |
| maker-11-augustus-gene-46.6-mRNA-1 | Japonica | Japonica | | Os11g0265800 | OsR498G1119766900.01.T01 | 2-oxoglutarate-dependent dioxygenase, putative |
| maker-11-augustus-gene-46.8-mRNA-1 | Japonica | Japonica | NBS-TM | Os11g0266500 | OsR498G1119768700.01.T02 | Disease resistance protein RPM1 |
| maker-11-augustus-gene-47.27-mRNA-1 | Japonica | NA | | | OsR498G1119770900.01.T01 | Endonuclease III 1, chloroplastic |
| maker-11-augustus-gene-47.33-mRNA-1 | Japonica | NA | | Os11g0267100 | OsR498G1119770200.01.T04 | Alpha-1,6-mannosyl-glycoprotein 6-beta-N-acetylglucosaminyltransferase |
| maker-11-augustus-gene-47.42-mRNA-1 | Japonica | NA | | Os11g0267400 | OsR498G1119771200.01.T01 | Chaperone protein ClpC, chloroplastic |
| maker-11-augustus-gene-48.25-mRNA-1 | Japonica | Indica | | Os11g0267400 | OsR498G1119779800.01.T01 | chaperone protein ClpC, chloroplastic |
| maker-11-augustus-gene-49.33-mRNA-1 | Japonica | NA | Kinase-TM | Os11g0271100 | OsR498G1119793700.01.T06 | Mitogen-activated protein kinase |
| maker-11-augustus-gene-49.37-mRNA-1 | Japonica | NA | | Os11g0269900 | OsR498G1119786800.01.T01 | Enoyl-CoA hydratase/isomerase family protein, expressed |
| maker-11-augustus-gene-5.32-mRNA-1 | Japonica | NA | | Os12g0123500 | OsR498G1220856100.01.T03 | GDA1/CD39 family protein, expressed |
| maker-11-augustus-gene-5.47-mRNA-1 | Japonica | Japonica | | Os11g0126400 | OsR498G1119350900.01.T01 | GDA1/CD39 family protein, expressed |
| maker-11-augustus-gene-5.50-mRNA-1 | Japonica | Indica | | Os11g0126400 | OsR498G1119351300.01.T01 | GDA1/CD39 family protein, expressed |
| maker-11-augustus-gene-5.54-mRNA-1 | Japonica | NA | | | OsR498G1119352100.01.T01 | NAC domain transcription factor |
| maker-11-augustus-gene-5.57-mRNA-1 | Japonica | NA | | Os11g0127000 | OsR498G1119352900.01.T02 | NAC domain-containing protein 7 |
| maker-11-augustus-gene-5.62-mRNA-1 | Japonica | NA | | Os11g0127600 | OsR498G1119353500.01.T01 | NAC transcription factor 8 |
| maker-11-augustus-gene-52.38-mRNA-1 | Japonica | Japonica | | Os11g0252900 | OsR498G1119717500.01.T01 | DDE Tnp4 domain-containing protein |
| maker-11-augustus-gene-52.53-mRNA-1 | Japonica | NA | CC-Kinase-TM | Os11g0274300 | OsR498G1119797800.01.T03 | Protein kinase domain containing protein, expressed |
| maker-11-augustus-gene-52.59-mRNA-1 | Japonica | Indica | | Os11g0251200 | OsR498G1119719010.01.T01 | Protein ACCELERATED CELL DEATH 6-like isoform X1 |
| maker-11-augustus-gene-54.41-mRNA-1 | Japonica | Indica | | Os11g0257200 | OsR498G1119727500.01.T01 | Anthranilate O-methyltransferase 2-like |
| maker-11-augustus-gene-54.43-mRNA-1 | Japonica | Indica | | Os11g0257200 | OsR498G1119728500.01.T01 | SAM dependent carboxyl methyltransferase |
| maker-11-augustus-gene-54.47-mRNA-1 | Japonica | NA | | Os11g0257200 | OsR498G1119729900.01.T01 | Anthranilate O-methyltransferase 2 |
| maker-11-augustus-gene-54.49-mRNA-1 | Japonica | NA | | Os11g0255300 | OsR498G1119723500.01.T01 | Thiol protease |
| maker-11-augustus-gene-54.52-mRNA-1 | Japonica | NA | | Os11g0255500 | OsR498G1119723100.01.T01 | NADP-dependent oxidoreductase P1, putative, expressed |
| maker-11-augustus-gene-54.54-mRNA-1 | Japonica | Indica | | Os02g0608600 | OsR498G1119725800.01.T01 | Acetohydroxy-acid synthase small subunit |
| maker-11-augustus-gene-54.58-mRNA-1 | Japonica | NA | | Os11g0256200 | OsR498G1119726600.01.T01 | Calcineurin-like metallo-phosphoesterase superfamily protein |
| maker-11-augustus-gene-55.0-mRNA-1 | Japonica | Indica | | Os11g0257200 | OsR498G1119733300.01.T01 | Anthranilate O-methyltransferase 2 |
| maker-11-augustus-gene-55.2-mRNA-1 | Japonica | NA | | | | Transposable element protein, putative, MuDR |
| maker-11-augustus-gene-55.6-mRNA-1 | Japonica | Indica | | Os11g0257200 | OsR498G1119733300.01.T01 | Anthranilate O-methyltransferase 2 |
| maker-11-augustus-gene-55.8-mRNA-1 | Japonica | Japonica | CC-LRR-NBS-TM | Os11g0258500 | OsR498G1119734000.01.T01 | NB-ARC domain, putative |
| maker-11-augustus-gene-56.36-mRNA-1 | Japonica | Indica | | Os04g0557800 | OsR498G1119735000.01.T01 | Anthocyanin regulatory R-S protein-like |

| | | | | | | |
|---|---|---|---|---|---|---|
| maker-11-augustus-gene-56.37-mRNA-1 | Japonica | NA | | Os11g0258700 | OsR498G1119735000.01.T01 | Anthocyanin regulatory R-S protein-like |
| maker-11-augustus-gene-58.25-mRNA-1 | Japonica | NA | | | OsR498G1119698500.01.T02 | Glutathione S-transferase zeta class |
| maker-11-augustus-gene-58.29-mRNA-1 | Japonica | Indica | Kinase-LRR-TM | Os12g0210400 | OsR498G1119698900.01.T01 | probable leucine-rich repeat receptor-like protein kinase At5g49770 |
| maker-11-augustus-gene-58.33-mRNA-1 | Japonica | Indica | | Os12g0209300 | OsR498G1119695800.01.T01 | ATPase, AAA family protein, expressed |
| maker-11-augustus-gene-58.36-mRNA-1 | Japonica | Japonica | | Os11g0244800 | OsR498G1119697600.01.T01 | PHD finger protein Alfin1 |
| maker-11-augustus-gene-58.40-mRNA-1 | Japonica | NA | | | OsR498G1119698400.01.T01 | CW-type domain-containing protein |
| maker-11-augustus-gene-58.42-mRNA-1 | Japonica | NA | | Os11g0245500 | OsR498G1119700200.01.T01 | START domain-containing protein 10 |
| maker-11-augustus-gene-58.44-mRNA-1 | Japonica | NA | | | OsR498G1119700800.01.T01 | Heparan-alpha-glucosaminide N-acetyltransferase |
| maker-11-augustus-gene-59.22-mRNA-1 | Japonica | NA | NBS-TM | | OsR498G1119700900.01.T04 | Leucine Rich Repeat family protein, expressed |
| maker-11-augustus-gene-59.25-mRNA-1 | Japonica | NA | | Os11g0246100 | OsR498G1119701700.01.T01 | Mitochondrial transcription termination factor-like family-1 |
| maker-11-augustus-gene-59.28-mRNA-1 | Japonica | NA | | Os11g0246600 | | Trypsin family protein with PDZ domain |
| maker-11-augustus-gene-59.32-mRNA-1 | Japonica | NA | | Os11g0246900 | OsR498G1119707300.01.T01 | F-box/ankyrin repeat protein SKIP35 |
| maker-11-augustus-gene-59.35-mRNA-1 | Japonica | NA | | | OsR498G0203537600.01.T01 | Transposon protein, putative, ping/pong/SNOOPY sub-class |
| maker-11-augustus-gene-6.0-mRNA-1 | Japonica | NA | | Os11g0127800 | OsR498G1119354300.01.T01 | FHA domain containing protein, expressed |
| maker-11-augustus-gene-6.11-mRNA-1 | Japonica | NA | | Os11g0127700 | OsR498G1119354000.01.T01 | TNF receptor-associated factor 5 |
| maker-11-augustus-gene-6.19-mRNA-1 | Japonica | NA | | Os11g0128500 | OsR498G1119358600.01.T01 | Myb-like DNA-binding domain containing protein, expressed |
| maker-11-augustus-gene-6.21-mRNA-1 | Japonica | NA | | Os12g0125300 | OsR498G1220865000.01.T01 | BUB1 N-terminal domain-containing protein (Fragment) |
| maker-11-augustus-gene-6.24-mRNA-1 | Japonica | NA | | Os11g0128800 | OsR498G1119359200.01.T02 | Homoserine dehydrogenase |
| maker-11-augustus-gene-6.31-mRNA-1 | Japonica | NA | | Os11g0126100 | OsR498G1119360200.01.T01 | MatE family protein, expressed |
| maker-11-augustus-gene-6.35-mRNA-1 | Japonica | NA | | Os11g0128900 | OsR498G1119360400.01.T03 | MatE family protein, expressed |
| maker-11-augustus-gene-6.4-mRNA-1 | Japonica | NA | | Os12g0126200 | OsR498G1119361700.01.T02 | ELMO domain-containing protein A |
| maker-11-augustus-gene-6.80-mRNA-1 | Japonica | NA | | | OsR498G1119361400.01.T01 | GDSL-like Lipase/Acylhydrolase family protein, expressed |
| maker-11-augustus-gene-6.84-mRNA-1 | Japonica | Japonica | | Os12g0126300 | OsR498G1119362000.01.T01 | Ethylene-responsive transcription factor WRI1 |
| maker-11-augustus-gene-60.0-mRNA-1 | Japonica | NA | | Os11g0247300 | OsR498G1119708900.01.T01 | Tubulin alpha chain |
| maker-11-augustus-gene-60.11-mRNA-1 | Japonica | NA | | Os11g0247800 | | HAT family dimerisation domain containing protein |
| maker-11-augustus-gene-60.13-mRNA-1 | Japonica | Indica | | Os11g0248200 | OsR498G1119712400.01.T01 | Transposon protein, putative, Mutator sub-class, expressed |
| maker-11-augustus-gene-60.4-mRNA-1 | Japonica | NA | | Os11g0252400 | OsR498G1119717400.01.T04 | Protein ACCELERATED CELL DEATH 6-like isoform X1 |
| maker-11-augustus-gene-60.6-mRNA-1 | Japonica | NA | | | | TTF-type domain-containing protein (Fragment) |
| maker-11-augustus-gene-60.8-mRNA-1 | Japonica | NA | | Os11g0247400 | OsR498G1119709200.01.T02 | S4 RNA-binding domain-containing protein (Fragment) |
| maker-11-augustus-gene-61.35-mRNA-1 | Japonica | Japonica | CC-NBS-TM | Os11g0249000 | OsR498G1119714500.01.T01 | Disease resistance protein RPM1 |
| maker-11-augustus-gene-61.47-mRNA-1 | Japonica | NA | | | OsR498G1119715800.01.T01 | Polygalacturonase, putative, expressed |
| maker-11-augustus-gene-61.49-mRNA-1 | Japonica | Indica | | Os10g0151800 | OsR498G1119717000.01.T01 | RNA recognition motif family protein, expressed |
| maker-11-augustus-gene-62.0-mRNA-1 | Japonica | NA | | Os11g0194800 | OsR498G1119550500.01.T01 | DNA-directed RNA polymerase II subunit 10 |
| maker-11-augustus-gene-62.2-mRNA-1 | Japonica | NA | | Os11g0251400 | OsR498G1119717200.01.T01 | Protein ACCELERATED CELL DEATH 6-like isoform X1 |
| maker-11-augustus-gene-62.4-mRNA-1 | Japonica | NA | | Os11g0252400 | OsR498G1119717400.01.T03 | Protein ACCELERATED CELL DEATH 6-like isoform X2 |
| maker-11-augustus-gene-62.7-mRNA-1 | Japonica | NA | Kinase-TM | Os11g0194900 | OsR498G1119551000.01.T02 | Protein kinase domain-containing protein |
| maker-11-augustus-gene-63.0-mRNA-1 | Japonica | Japonica | | Os11g0195100 | OsR498G1119553300.01.T05 | Protein translocase subunit SecA |
| maker-11-augustus-gene-63.4-mRNA-1 | Japonica | Indica | | Os11g0195500 | OsR498G1119553700.01.T03 | Lipase family protein, expressed |
| maker-11-augustus-gene-63.8-mRNA-1 | Japonica | NA | | Os11g0195600 | OsR498G1119554100.01.T01 | Amino acid permease |
| maker-11-augustus-gene-64.3-mRNA-1 | Japonica | Indica | | Os01g0882300 | OsR498G1119559800.01.T02 | Probable magnesium transporter |
| maker-11-augustus-gene-65.38-mRNA-1 | Japonica | NA | | Os11g0199700 | | Target of Myb protein 1 |
| maker-11-augustus-gene-66.50-mRNA-1 | Japonica | NA | | Os11g0201332 | OsR498G1119571800.01.T02 | F-box family-2 |
| maker-11-augustus-gene-67.43-mRNA-1 | Japonica | NA | | Os06g0272601 | OsR498G0204300200.01.T01 | dNK domain-containing protein |
| maker-11-augustus-gene-67.48-mRNA-1 | Japonica | NA | | | | F-box domain, putative |
| maker-11-augustus-gene-67.51-mRNA-1 | Japonica | Japonica | | Os11g0201800 | OsR498G1119574200.01.T01 | F-box domain, putative |
| maker-11-augustus-gene-67.55-mRNA-1 | Japonica | Indica | | Os11g0201800 | OsR498G1119574400.01.T01 | F-box family-4 |
| maker-11-augustus-gene-67.57-mRNA-1 | Japonica | NA | | Os11g0202000 | OsR498G1119574800.01.T01 | F-box family-2 |
| maker-11-augustus-gene-69.50-mRNA-1 | Japonica | NA | | Os11g0206700 | OsR498G1119585800.01.T01 | Extra-large guanine nucleotide-binding protein like |
| maker-11-augustus-gene-7.42-mRNA-1 | Japonica | NA | | Os11g0130300 | OsR498G1119363500.01.T01 | RING-type E3 ubiquitin transferase |
| maker-11-augustus-gene-7.46-mRNA-1 | Japonica | NA | | Os11g0130600 | OsR498G1119364700.01.T01 | |
| maker-11-augustus-gene-7.53-mRNA-1 | Japonica | NA | | Os11g0131900 | OsR498G1119368100.01.T04 | Non-reducing end alpha-L-arabinofuranosidase |
| maker-11-augustus-gene-7.54-mRNA-1 | Japonica | Indica | | Os11g0129800 | OsR498G1119362400.01.T01 | |
| maker-11-augustus-gene-7.59-mRNA-1 | Japonica | NA | | | | CID domain-containing protein |
| maker-11-augustus-gene-7.65-mRNA-1 | Japonica | NA | | Os11g0130200 | OsR498G1119363200.01.T01 | CID domain-containing protein |
| maker-11-augustus-gene-7.68-mRNA-1 | Japonica | NA | | | OsR498G1220870800.01.T01 | FBD domain-containing protein |
| maker-11-augustus-gene-7.73-mRNA-1 | Japonica | Indica | NBS-TM | Os08g0170200 | OsR498G1119365800.01.T01 | Disease resistance protein RPM1 |
| maker-11-augustus-gene-7.75-mRNA-1 | Japonica | Indica | | Os11g0131200 | OsR498G1119366400.01.T02 | Peroxisomal membrane 22 kDa family protein isoform 1 |
| maker-11-augustus-gene-7.80-mRNA-1 | Japonica | NA | | Os11g0130900 | OsR498G1220874000.01.T02 | Mitochondrial import inner membrane translocase subunit |
| maker-11-augustus-gene-70.29-mRNA-1 | Japonica | NA | | | | Transcriptional corepressor SEUSS |
| maker-11-augustus-gene-70.39-mRNA-1 | Japonica | Indica | Kinase-TM | Os11g0207200 | OsR498G1119591700.01.T01 | Mitogen-activated protein kinase kinase kinase 3 |
| maker-11-augustus-gene-70.45-mRNA-1 | Japonica | NA | | Os11g0207600 | OsR498G1119593300.01.T01 | transcription factor MYB52 |

| | | | | | | |
|---|---|---|---|---|---|---|
| maker-11-augustus-gene-70.47-mRNA-1 | Japonica | NA | | | OsR498G0203391700.01.T01 | Flavin-containing monooxygenase |
| maker-11-augustus-gene-71.0-mRNA-1 | Japonica | NA | | Os11g0208000 | OsR498G1119594500.01.T03 | F-box domain containing protein, expressed |
| maker-11-augustus-gene-71.10-mRNA-1 | Japonica | Japonica | Kinase-LRR-TM | Os11g0208900 | OsR498G1119599000.01.T01 | Serine-threonine protein kinase plant-type |
| maker-11-augustus-gene-71.3-mRNA-1 | Japonica | NA | | Os11g0208100 | OsR498G1119595800.01.T02 | F-box family-2 |
| maker-11-augustus-gene-71.5-mRNA-1 | Japonica | NA | | | OsR498G1119596600.01.T02 | F-box family-2 |
| maker-11-augustus-gene-71.7-mRNA-1 | Japonica | NA | | | OsR498G1119597400.01.T01 | F-box domain containing protein, expressed |
| maker-11-augustus-gene-71.9-mRNA-1 | Japonica | Indica | Kinase-TM | Os07g0186200 | OsR498G1119598200.01.T01 | Serine/threonine-protein kinase |
| maker-11-augustus-gene-72.24-mRNA-1 | Japonica | NA | | Os11g0209100 | OsR498G1119599800.01.T01 | F-box domain containing protein |
| maker-11-augustus-gene-72.26-mRNA-1 | Japonica | NA | | Os11g0209200 | OsR498G1119600000.01.T01 | F-box family-6 |
| maker-11-augustus-gene-72.29-mRNA-1 | Japonica | NA | | | OsR498G1119601000.01.T02 | F-box domain containing protein, expressed |
| maker-11-augustus-gene-72.31-mRNA-1 | Japonica | Indica | | Os11g0209500 | OsR498G1119601400.01.T02 | F-box domain containing protein, expressed |
| maker-11-augustus-gene-72.34-mRNA-1 | Japonica | NA | | | OsR498G1119603400.01.T01 | Phosphatidylinositol kinase |
| maker-11-augustus-gene-72.41-mRNA-1 | Japonica | Japonica | | Os11g0209900 | OsR498G1119604000.01.T01 | Cysteine desulfurylase |
| maker-11-augustus-gene-72.43-mRNA-1 | Japonica | Indica | | Os12g0191500 | OsR498G1119605000.01.T01 | Peroxidase |
| maker-11-augustus-gene-73.13-mRNA-1 | Japonica | Indica | | Os11g0210600 | OsR498G1119607600.01.T03 | Cinnamyl-alcohol dehydrogenase |
| maker-11-augustus-gene-73.3-mRNA-1 | Japonica | NA | | Os11g0210300 | OsR498G1119605800.01.T01 | Alcohol dehydrogenase ADH1 |
| maker-11-augustus-gene-73.9-mRNA-1 | Japonica | NA | | Os11g0210500 | OsR498G1119606600.01.T01 | Alcohol dehydrogenase ADH2 |
| maker-11-augustus-gene-74.0-mRNA-1 | Japonica | NA | | | | DUF4220 domain-containing protein (Fragment) |
| maker-11-augustus-gene-74.2-mRNA-1 | Japonica | NA | LRR-TM | | OsR498G1119610600.01.T03 | NBS-LRR disease resistance protein family-4 |
| maker-11-augustus-gene-74.5-mRNA-1 | Japonica | NA | CC-LRR-NBS-TM | | OsR498G1119611800.01.T01 | NBS-LRR disease resistance protein family-4 |
| maker-11-augustus-gene-75.13-mRNA-1 | Japonica | NA | | Os11g0213600 | OsR498G1119615200.01.T01 | Carboxypeptidase |
| maker-11-augustus-gene-75.17-mRNA-1 | Japonica | Indica | LRR-TM | Os11g0213700 | OsR498G1119615600.01.T02 | Disease resistance protein RGA2 |
| maker-11-augustus-gene-75.2-mRNA-1 | Japonica | NA | Kinase-TM | | OsR498G1119613600.01.T01 | Coatomer beta subunit, putative |
| maker-11-augustus-gene-75.20-mRNA-1 | Japonica | NA | CC-NBS-TM | Os11g0213800 | OsR498G1119615600.01.T05 | NBS-LRR type protein (Fragment) |
| maker-11-augustus-gene-75.29-mRNA-1 | Japonica | NA | | | OsR498G1119616200.01.T02 | Ubiquitin-like-specific protease 2 |
| maker-11-augustus-gene-75.8-mRNA-1 | Japonica | NA | Kinase-TM | | OsR498G1119614000.01.T02 | Putative mitogen-activated protein (MAP) kinase kinase kinase Ste11, Crypto |
| maker-11-augustus-gene-76.0-mRNA-1 | Japonica | Japonica | | Os11g0215300 | OsR498G1119618200.01.T01 | |
| maker-11-augustus-gene-77.35-mRNA-1 | Japonica | NA | | Os11g0216000 | OsR498G1119619100.01.T03 | Pyruvate kinase |
| maker-11-augustus-gene-77.39-mRNA-1 | Japonica | NA | | Os11g0216900 | OsR498G1119622100.01.T02 | Methylthioribose-1-phosphate isomerase |
| maker-11-augustus-gene-77.44-mRNA-1 | Japonica | NA | | Os11g0217300 | OsR498G1119622300.01.T03 | Glucuronokinase G |
| maker-11-augustus-gene-77.48-mRNA-1 | Japonica | NA | | Os11g0218000 | OsR498G1119624300.01.T01 | BZIP type transcription factor |
| maker-11-augustus-gene-77.51-mRNA-1 | Japonica | NA | | Os11g0216300 | OsR498G1119619600.01.T01 | Protein ACTIVITY OF BC1 COMPLEX KINASE 1, chloroplastic isoform A |
| maker-11-augustus-gene-77.60-mRNA-1 | Japonica | Indica | | Os12g0193200 | OsR498G1119622600.01.T01 | GTP--RNA guanylyltransferase |
| maker-11-augustus-gene-78.0-mRNA-1 | Japonica | NA | | | OsR498G1119629900.01.T02 | RNase H domain-containing protein |
| maker-11-augustus-gene-78.2-mRNA-1 | Japonica | NA | | | OsR498G1119628600.01.T02 | Lon protease |
| maker-11-augustus-gene-79.33-mRNA-1 | Japonica | NA | | | OsR498G1119632900.01.T01 | Calmodulin-interacting protein 111 |
| maker-11-augustus-gene-79.35-mRNA-1 | Japonica | NA | | | | Reverse transcriptase domain-containing protein |
| maker-11-augustus-gene-79.40-mRNA-1 | Japonica | Indica | | Os11g0221000 | OsR498G1119634900.01.T01 | Auxin-responsive protein IAA27 |
| maker-11-augustus-gene-8.2-mRNA-1 | Japonica | NA | | Os11g0132600 | OsR498G1119370100.01.T06 | Non-reducing end alpha-L-arabinofuranosidase |
| maker-11-augustus-gene-8.45-mRNA-1 | Japonica | NA | | Os11g0133800 | OsR498G1119375500.01.T01 | S-acyltransferase |
| maker-11-augustus-gene-8.49-mRNA-1 | Japonica | Indica | | Os12g0129000 | OsR498G1119369600.01.T02 | Transmembrane protein 230 |
| maker-11-augustus-gene-8.55-mRNA-1 | Japonica | NA | | Os11g0132700 | OsR498G1119370400.01.T05 | Aladin |
| maker-11-augustus-gene-8.67-mRNA-1 | Japonica | NA | | | OsR498G1119374800.01.T02 | Glutamine--fructose-6-phosphate transaminase (isomerizing) |
| maker-11-augustus-gene-8.76-mRNA-1 | Japonica | Japonica | | Os11g0133900 | OsR498G1119375800.01.T02 | Glucosamine fructose-6-phosphate aminotransferase1 |
| maker-11-augustus-gene-80.32-mRNA-1 | Japonica | Indica | | Os07g0179600 | OsR498G1119638500.01.T01 | Neprosin domain-containing protein (Fragment) |
| maker-11-augustus-gene-80.34-mRNA-1 | Japonica | NA | | Os11g0222225 | OsR498G1119638200.01.T01 | SERPIN domain-containing protein (Fragment) |
| maker-11-augustus-gene-81.0-mRNA-1 | Japonica | Indica | | Os07g0179600 | OsR498G1119638500.01.T01 | Neprosin domain-containing protein (Fragment) |
| maker-11-augustus-gene-82.2-mRNA-1 | Japonica | NA | Kinase | | | Retrotransposon protein, putative, LINE subclass |
| maker-11-augustus-gene-82.6-mRNA-1 | Japonica | Indica | CC-NBS-TM | Os11g0226700 | OsR498G1119646100.01.T02 | NBS-LRR type protein (Fragment) |
| maker-11-augustus-gene-82.8-mRNA-1 | Japonica | NA | CC-NBS-TM | | | Protein kinase domain-containing protein |
| maker-11-augustus-gene-83.0-mRNA-1 | Japonica | NA | | | | |
| maker-11-augustus-gene-83.3-mRNA-1 | Japonica | Japonica | CC-LRR-NBS-TM | Os11g0227100 | OsR498G1119648000.01.T02 | NBS-LRR type resistance protein (Fragment) |
| maker-11-augustus-gene-84.0-mRNA-1 | Japonica | Indica | | Os11g0230400 | OsR498G1119652500.01.T01 | SERPIN domain-containing protein (Fragment) |
| maker-11-augustus-gene-84.4-mRNA-1 | Japonica | Indica | | Os11g0230700 | OsR498G1119653900.01.T01 | SERPIN domain-containing protein (Fragment) |
| maker-11-augustus-gene-85.28-mRNA-1 | Japonica | NA | Kinase-LRR-TM | | OsR498G1119660500.01.T01 | Leucine-rich repeat receptor-like serine/threonine-protein kinase BAM3 |
| maker-11-augustus-gene-86.16-mRNA-1 | Japonica | NA | | | OsR498G1119665600.01.T03 | UDP-glucose-fructose-phosphate glucosyltransferase |
| maker-11-augustus-gene-86.17-mRNA-1 | Japonica | NA | | | | UDP-glucose-fructose-phosphate glucosyltransferase |
| maker-11-augustus-gene-86.6-mRNA-1 | Japonica | NA | | Os11g0235200 | OsR498G1119662400.01.T01 | Proton-dependent oligopeptide transporter family |
| maker-11-augustus-gene-87.36-mRNA-1 | Japonica | NA | CC-LRR-NBS-TM | Os11g0237900 | OsR498G1119671300.01.T01 | Putative disease resistance RPP13-like protein 3 |
| maker-11-augustus-gene-87.38-mRNA-1 | Japonica | Indica | | Os05g0318300 | OsR498G1119671000.01.T02 | RNase III domain-containing protein (Fragment) |
| maker-11-augustus-gene-88.0-mRNA-1 | Japonica | NA | | | OsR498G1119676200.01.T01 | Serpin (Serine protease inhibitor) |

| | | | | | | |
|---|---|---|---|---|---|---|
| maker-11-augustus-gene-88.2-mRNA-1 | Japonica | NA | | | OsR498G1119677200.01.T01 | Serpin (Serine protease inhibitor) |
| maker-11-augustus-gene-88.3-mRNA-1 | Japonica | NA | | Os03g0193900 | OsR498G0101213400.01.T05 | SERPIN domain-containing protein |
| maker-11-augustus-gene-89.0-mRNA-1 | Japonica | NA | | Os11g0240900 | OsR498G1119683100.01.T04 | CCR4-NOT transcription complex subunit 11 |
| maker-11-augustus-gene-89.5-mRNA-1 | Japonica | NA | | | | PH domain-containing protein |
| maker-11-augustus-gene-89.7-mRNA-1 | Japonica | NA | | Os11g0241900 | OsR498G1119686000.01.T01 | Protein trichome birefringence-like protein 14 |
| maker-11-augustus-gene-9.0-mRNA-1 | Japonica | NA | Kinase-TM | Os12g0132200 | OsR498G1119377100.01.T07 | Non-specific serine/threonine protein kinase |
| maker-11-augustus-gene-9.17-mRNA-1 | Japonica | NA | | Os12g0132300 | OsR498G1119377400.01.T01 | Calmodulin-4 |
| maker-11-augustus-gene-9.18-mRNA-1 | Japonica | NA | | Os11g0134500 | OsR498G1119377600.01.T01 | Glutamate-rich WD repeat-containing protein 1 |
| maker-11-augustus-gene-9.25-mRNA-1 | Japonica | NA | | | | Major facilitator superfamily antiporter, putative, expressed |
| maker-11-augustus-gene-9.40-mRNA-1 | Japonica | NA | | Os11g0135000 | OsR498G1119378200.01.T02 | Major facilitator superfamily antiporter |
| maker-11-augustus-gene-9.46-mRNA-1 | Japonica | NA | | Os12g0133000 | OsR498G1220891200.01.T01 | Major facilitator superfamily antiporter |
| maker-11-augustus-gene-9.50-mRNA-1 | Japonica | Indica | | Os08g0130500 | OsR498G1119380000.01.T01 | 60S acidic ribosomal protein P0 |
| maker-11-augustus-gene-9.6-mRNA-1 | Japonica | Indica | | Os12g0131900 | OsR498G1119376200.01.T01 | Rhamnogalacturonan endolyase |
| maker-11-augustus-gene-90.0-mRNA-1 | Japonica | NA | | Os11g0242100 | OsR498G1119686500.01.T06 | Non-lysosomal glucosylceramidase |
| maker-11-augustus-gene-90.12-mRNA-1 | Japonica | Japonica | | Os11g0242700 | OsR498G1119689500.01.T01 | fibroin heavy chain isoform X1 |
| maker-11-augustus-gene-90.15-mRNA-1 | Japonica | NA | | Os11g0242800 | OsR498G1119689900.01.T02 | Chlorophyll a-b binding protein, chloroplastic |
| maker-11-augustus-gene-90.20-mRNA-1 | Japonica | NA | | Os11g0242400 | OsR498G1119688800.01.T03 | Rieske domain-containing protein (Fragment) |
| maker-11-augustus-gene-90.7-mRNA-1 | Japonica | NA | | Os11g0242200 | OsR498G1119687300.01.T01 | PPM-type phosphatase domain-containing protein |
| maker-11-augustus-gene-90.9-mRNA-1 | Japonica | NA | Kinase-TM | Os11g0242500 | OsR498G1119688900.01.T03 | Cyclin-dependent kinase C |
| maker-11-augustus-gene-99.0-mRNA-1 | Japonica | NA | | | OsR498G0917954800.01.T01 | Transposon protein, putative, CACTA, En/Spm sub-class |
| maker-11-augustus-gene-99.3-mRNA-1 | Japonica | NA | | Os07g0165500 | OsR498G0203722500.01.T01 | Cytokinin inducible protein-like |
| maker-11-augustus-gene-99.5-mRNA-1 | Japonica | NA | | Os11g0414000 | OsR498G1119939800.01.T02 | Basic leucine zipper and W2 domain-containing protein 2 |
| maker-12-augustus-gene-0.34-mRNA-1 | Japonica | NA | | | | Pleiotropic drug resistance protein 3 |
| maker-12-augustus-gene-0.41-mRNA-1 | Japonica | NA | | | OsR498G1220782800.01.T02 | Autophagy-related protein 8D |
| maker-12-augustus-gene-0.43-mRNA-1 | Japonica | Japonica | | Os11g0100300 | OsR498G1220783200.01.T01 | |
| maker-12-augustus-gene-0.45-mRNA-1 | Japonica | NA | | Os12g0100500 | OsR498G1220783400.01.T01 | Caffeoylshikimate esterase |
| maker-12-augustus-gene-1.0-mRNA-1 | Japonica | Japonica | | Os12g0101000 | OsR498G1220784700.01.T03 | CCT domain-containing protein |
| maker-12-augustus-gene-1.22-mRNA-1 | Japonica | NA | | Os12g0102400 | OsR498G1220788900.01.T02 | RNA binding (RRM/RBD/RNP motifs) family protein |
| maker-12-augustus-gene-1.23-mRNA-1 | Japonica | NA | | Os12g0102400 | OsR498G1220788900.01.T03 | RNA binding (RRM/RBD/RNP motifs) family protein |
| maker-12-augustus-gene-1.34-mRNA-1 | Japonica | NA | Kinase-LRR-TM | | OsR498G1220789200.01.T06 | Leucine-rich repeat protein kinase family protein |
| maker-12-augustus-gene-1.5-mRNA-1 | Japonica | NA | Kinase-TM | | | Nonphototropic hypocotyl 1 |
| maker-12-augustus-gene-10.42-mRNA-1 | Japonica | Japonica | | Os12g0119700 | OsR498G1119333300.01.T02 | Retrotransposon protein, putative, Ty1-copia subclass |
| maker-12-augustus-gene-10.47-mRNA-1 | Japonica | NA | | Os11g0120600 | OsR498G1119336300.01.T01 | Bis(5'-adenosyl)-triphosphatase |
| maker-12-augustus-gene-10.52-mRNA-1 | Japonica | NA | | Os12g0120000 | OsR498G1220829700.01.T01 | Serine/arginine repetitive matrix protein |
| maker-12-augustus-gene-10.54-mRNA-1 | Japonica | NA | | Os11g0120700 | OsR498G1220827300.01.T01 | |
| maker-12-augustus-gene-10.57-mRNA-1 | Japonica | NA | | Os12g0120500 | OsR498G1119336800.01.T03 | |
| maker-12-augustus-gene-10.60-mRNA-1 | Japonica | NA | | Os12g0121000 | OsR498G1220824900.01.T02 | Protein kinase APK1B chloroplast |
| maker-12-augustus-gene-10.62-mRNA-1 | Japonica | NA | Kinase | Os11g0121400 | OsR498G1220824700.01.T01 | Serine-threonine kinase STK11 |
| maker-12-augustus-gene-10.68-mRNA-1 | Japonica | NA | | | | Ethanolamine-phosphate cytidylyltransferase |
| maker-12-augustus-gene-10.73-mRNA-1 | Japonica | NA | | Os12g0121400 | OsR498G1220823700.01.T03 | Vacuolar protein sorting-associated protein 32 |
| maker-12-augustus-gene-100.37-mRNA-1 | Japonica | NA | | Os12g0270100 | OsR498G1221264000.01.T03 | Putative heme chaperone |
| maker-12-augustus-gene-100.40-mRNA-1 | Japonica | Indica | | Os12g0270900 | OsR498G1221267000.01.T01 | Sulfotransferase |
| maker-12-augustus-gene-101.37-mRNA-1 | Japonica | NA | | Os12g0271600 | OsR498G1221270500.01.T02 | |
| maker-12-augustus-gene-101.39-mRNA-1 | Japonica | NA | | Os12g0271700 | OsR498G1221270800.01.T01 | Solanesyl-diphosphate synthase 2, chloroplastic |
| maker-12-augustus-gene-102.0-mRNA-1 | Japonica | Japonica | CC-NBS-TM | Os12g0273266 | OsR498G1221272600.01.T01 | Disease resistance protein RPM1, putative, expressed |
| maker-12-augustus-gene-102.3-mRNA-1 | Japonica | NA | CC-NBS-TM | | | NB-ARC domain containing protein, expressed |
| maker-12-augustus-gene-103.40-mRNA-1 | Japonica | Indica | | Os12g0273700 | OsR498G1221278700.01.T03 | TPX2 (Targeting protein for Xklp2) protein family |
| maker-12-augustus-gene-103.44-mRNA-1 | Japonica | Indica | | Os12g0273900 | OsR498G1221278900.01.T01 | Senescence-specific cysteine protease SAG39 |
| maker-12-augustus-gene-103.49-mRNA-1 | Japonica | Indica | | Os12g0274200 | OsR498G1221280200.01.T01 | GDSL-motif lipase/hydrolase, putative |
| maker-12-augustus-gene-103.51-mRNA-1 | Japonica | NA | | Os12g0274700 | OsR498G1221281600.01.T01 | Ribulose bisphosphate carboxylase small chain |
| maker-12-augustus-gene-104.35-mRNA-1 | Japonica | Japonica | | Os12g0275500 | OsR498G1221283500.01.T01 | |
| maker-12-augustus-gene-104.37-mRNA-1 | Japonica | NA | | | OsR498G0714211300.01.T01 | |
| maker-12-augustus-gene-104.40-mRNA-1 | Japonica | NA | | | OsR498G1221284200.01.T02 | Cation/H(+) antiporter 15 |
| maker-12-augustus-gene-104.42-mRNA-1 | Japonica | NA | | Os12g0276100 | OsR498G1221290200.01.T02 | Rho GTPase-activating protein like |
| maker-12-augustus-gene-105.23-mRNA-1 | Japonica | NA | | Os12g0277500 | OsR498G1221297100.01.T01 | ruBisCO large subunit-binding protein subunit alpha |
| maker-12-augustus-gene-105.27-mRNA-1 | Japonica | NA | | | OsR498G1221293000.01.T01 | |
| maker-12-augustus-gene-105.29-mRNA-1 | Japonica | NA | | Os12g0277000 | OsR498G1221295000.01.T01 | RING-type E3 ubiquitin transferase |
| maker-12-augustus-gene-105.31-mRNA-1 | Japonica | NA | | Os12g0277400 | OsR498G1221297000.01.T01 | RING-type E3 ubiquitin transferase |
| maker-12-augustus-gene-105.34-mRNA-1 | Japonica | NA | | | OsR498G1221297800.01.T01 | |
| maker-12-augustus-gene-105.36-mRNA-1 | Japonica | NA | | | OsR498G1221298800.01.T01 | |
| maker-12-augustus-gene-106.29-mRNA-1 | Japonica | NA | | | OsR498G1120154400.01.T01 | |

| | | | | | | |
|---|---|---|---|---|---|---|
| maker-12-augustus-gene-106.34-mRNA-1 | Japonica | NA | | Os01g0650700 | | Transposon protein, putative, CACTA, En/Spm sub-class |
| maker-12-augustus-gene-106.36-mRNA-1 | Japonica | NA | | Os12g0278700 | OsR498G1221303800.01.T03 | Cystinosin like |
| maker-12-augustus-gene-107.15-mRNA-1 | Japonica | NA | | | OsR498G1221305700.01.T02 | RNA helicase, ATP-dependent, SK12/DOB1 protein |
| maker-12-augustus-gene-107.19-mRNA-1 | Japonica | NA | | Os12g0278800 | OsR498G1221304600.01.T03 | Zinc finger CCCH domain-containing protein 65 |
| maker-12-augustus-gene-107.23-mRNA-1 | Japonica | NA | | Os12g0278900 | OsR498G1221305600.01.T01 | |
| maker-12-augustus-gene-107.25-mRNA-1 | Japonica | NA | | Os12g0279100 | | Lysine-specific demethylase REF6 |
| maker-12-augustus-gene-107.27-mRNA-1 | Japonica | NA | | Os12g0279600 | OsR498G1221307200.01.T02 | DNA-(apurinic or apyrimidinic site) endonuclease |
| maker-12-augustus-gene-108.36-mRNA-1 | Japonica | NA | | | OsR498G1221308400.01.T01 | Cysteine proteinase EP-B 1 |
| maker-12-augustus-gene-109.40-mRNA-1 | Japonica | NA | CC-NBS-TM Pi42 | Os12g0281600 | OsR498G1221312700.01.T02 | NB-ARC domain containing protein, expressed |
| maker-12-augustus-gene-109.42-mRNA-1 | Japonica | NA | | Os12g0282400 | | ATP synthase subunit-like protein |
| maker-12-augustus-gene-109.46-mRNA-1 | Japonica | NA | | | | |
| maker-12-augustus-gene-109.48-mRNA-1 | Japonica | NA | | Os12g0283100 | OsR498G1221319200.01.T01 | PAP/OAS1 substrate-binding domain superfamily |
| maker-12-augustus-gene-11.0-mRNA-1 | Japonica | NA | | Os12g0122000 | OsR498G1220851500.01.T01 | Transcription factor GRAS |
| maker-12-augustus-gene-11.2-mRNA-1 | Japonica | Indica | | Os12g0122100 | OsR498G1220852100.01.T01 | Calcium uniporter protein (Fragment) |
| maker-12-augustus-gene-11.32-mRNA-1 | Japonica | NA | | Os11g0124900 | OsR498G1220854200.01.T01 | Hexosyltransferase |
| maker-12-augustus-gene-110.39-mRNA-1 | Japonica | NA | | Os12g0283900 | OsR498G1221321500.01.T01 | Pentatricopeptide repeat |
| maker-12-augustus-gene-110.41-mRNA-1 | Japonica | Japonica | | Os12g0284000 | OsR498G1221322100.01.T01 | Ultraviolet-B receptor UVR8 |
| maker-12-augustus-gene-110.45-mRNA-1 | Japonica | Indica | PTR | Os12g0285100 | OsR498G1221322300.01.T01 | Disease resistance protein |
| maker-12-augustus-gene-110.47-mRNA-1 | Japonica | NA | | | OsR498G1221322700.01.T01 | Disease resistance protein (Fragment) |
| maker-12-augustus-gene-110.52-mRNA-1 | Japonica | NA | | | OsR498G1221321400.01.T01 | Protein RETICULATA-RELATED 3 chloroplastic |
| maker-12-augustus-gene-111.26-mRNA-1 | Japonica | Japonica | PTR | Os12g0285100 | OsR498G1221325200.01.T02 | Disease resistance protein (Fragment) |
| maker-12-augustus-gene-111.31-mRNA-1 | Japonica | NA | | | OsR498G1221325900.01.T02 | Ufm1-specific protease |
| maker-12-augustus-gene-111.37-mRNA-1 | Japonica | NA | | Os12g0285600 | OsR498G1221326600.01.T03 | Oxysterol-binding protein-related protein 1D |
| maker-12-augustus-gene-112.28-mRNA-1 | Japonica | Indica | | Os12g0287200 | OsR498G1221331700.01.T03 | Mago nashi |
| maker-12-augustus-gene-112.30-mRNA-1 | Japonica | NA | | Os12g0286600 | OsR498G1221330000.01.T01 | Abhydrolase domain-containing protein FAM108C1 family |
| maker-12-augustus-gene-112.36-mRNA-1 | Japonica | Indica | | Os12g0287300 | OsR498G1221332000.01.T03 | Cysteine desulfurase |
| maker-12-augustus-gene-113.42-mRNA-1 | Japonica | NA | | | OsR498G1221342900.01.T03 | AAA+ ATPase domain |
| maker-12-augustus-gene-113.58-mRNA-1 | Japonica | NA | | Os12g0288900 | OsR498G1221340400.01.T01 | Mitochondrial inner membrane protease ATP23 |
| maker-12-augustus-gene-114.0-mRNA-1 | Japonica | Indica | Kinase | Os05g0511400 | OsR498G1221346100.01.T01 | Serine/threonine-protein kinase CBK1 |
| maker-12-augustus-gene-114.12-mRNA-1 | Japonica | NA | | | OsR498G1221346400.01.T07 | Quinolinate synthase, chloroplastic |
| maker-12-augustus-gene-115.19-mRNA-1 | Japonica | Japonica | | Os12g0291200 | OsR498G1221348500.01.T01 | Ribulose bisphosphate carboxylase small chain |
| maker-12-augustus-gene-115.21-mRNA-1 | Japonica | NA | | Os12g0291400 | OsR498G1221349500.01.T01 | Ribulose bisphosphate carboxylase small chain |
| maker-12-augustus-gene-115.23-mRNA-1 | Japonica | NA | | Os12g0292400 | OsR498G1221350300.01.T01 | Ribulose bisphosphate carboxylase small chain |
| maker-12-augustus-gene-116.14-mRNA-1 | Japonica | NA | | | OsR498G1221351500.01.T04 | Chloroplast sensor kinase chloroplastic |
| maker-12-augustus-gene-116.19-mRNA-1 | Japonica | NA | | | OsR498G1221356100.01.T05 | WD repeat-containing protein 89 homolog |
| maker-12-augustus-gene-116.24-mRNA-1 | Japonica | NA | | Os12g0293100 | OsR498G1221351800.01.T01 | Telomerase reverse transcriptase |
| maker-12-augustus-gene-118.0-mRNA-1 | Japonica | NA | | | OsR498G0714235700.01.T01 | HGWP repeat containing protein-like |
| maker-12-augustus-gene-118.2-mRNA-1 | Japonica | Japonica | | Os12g0297500 | OsR498G1221360600.01.T01 | Phosphoglucan water dikinase chloroplastic |
| maker-12-augustus-gene-122.10-mRNA-1 | Japonica | NA | | Os12g0403800 | OsR498G1221362900.01.T03 | 3'-5'-exoribonuclease family protein isoform 2 |
| maker-12-augustus-gene-125.29-mRNA-1 | Japonica | NA | | Os12g0405200 | OsR498G1221366900.01.T02 | 15-cis-zeta-carotene isomerase |
| maker-12-augustus-gene-125.32-mRNA-1 | Japonica | NA | | Os12g0405700 | | Wound-inducible basic |
| maker-12-augustus-gene-125.34-mRNA-1 | Japonica | NA | | Os12g0406100 | OsR498G1221368800.01.T03 | Beta-glucanase, putative |
| maker-12-augustus-gene-125.38-mRNA-1 | Japonica | NA | | Os12g0406200 | OsR498G1221369000.01.T01 | |
| maker-12-augustus-gene-126.0-mRNA-1 | Japonica | NA | | | | |
| maker-12-augustus-gene-126.4-mRNA-1 | Japonica | NA | | Os12g0407500 | OsR498G1221372600.01.T03 | Phosphatidylinositol 3 4 5-trisphosphate 3-phosphatase and dual-specificity |
| maker-12-augustus-gene-127.37-mRNA-1 | Japonica | NA | | | OsR498G1221377100.01.T01 | |
| maker-12-augustus-gene-127.39-mRNA-1 | Japonica | Indica | | Os12g0409000 | OsR498G1221374800.01.T01 | Serine hydroxymethyltransferase |
| maker-12-augustus-gene-127.42-mRNA-1 | Japonica | NA | | Os12g0409700 | OsR498G1221377600.01.T01 | ABC transporter domain-containing protein (Fragment) |
| maker-12-augustus-gene-132.38-mRNA-1 | Japonica | NA | | Os12g0414900 | | Tyrosyl-DNA phosphodiesterase |
| maker-12-augustus-gene-132.40-mRNA-1 | Japonica | Indica | | Os12g0416500 | OsR498G1221400300.01.T05 | Biogenesis of lysosome-related organelles complex 1 subunit 1 |
| maker-12-augustus-gene-135.0-mRNA-1 | Japonica | NA | | Os12g0420100 | OsR498G1221412500.01.T01 | B-S glucosidase 44 |
| maker-12-augustus-gene-135.2-mRNA-1 | Japonica | NA | | Os12g0420200 | OsR498G1221412700.01.T01 | Chloroplast stem-loop binding protein like |
| maker-12-augustus-gene-138.36-mRNA-1 | Japonica | NA | | Os01g0284200 | OsR498G0100683700.01.T01 | |
| maker-12-augustus-gene-14.0-mRNA-1 | Japonica | NA | | Os12g0128700 | OsR498G1220874100.01.T06 | Non-reducing end alpha-L-arabinofuranosidase |
| maker-12-augustus-gene-14.14-mRNA-1 | Japonica | NA | | Os11g0132600 | OsR498G1119370100.01.T06 | Non-reducing end alpha-L-arabinofuranosidase |
| maker-12-augustus-gene-14.18-mRNA-1 | Japonica | NA | | | OsR498G1220876500.01.T01 | Zinc finger, CCCH-type |
| maker-12-augustus-gene-14.21-mRNA-1 | Japonica | NA | | | OsR498G1220873000.01.T04 | Peroxisomal membrane 22 kDa family protein isoform 2 |
| maker-12-augustus-gene-14.26-mRNA-1 | Japonica | NA | | Os12g0128600 | OsR498G1220874000.01.T02 | Mitochondrial import inner membrane translocase subunit |
| maker-12-augustus-gene-14.28-mRNA-1 | Japonica | Japonica | | Os12g0129000 | OsR498G1220874600.01.T01 | Transmembrane protein 230 |
| maker-12-augustus-gene-14.6-mRNA-1 | Japonica | NA | | Os12g0128800 | OsR498G1220874300.01.T01 | Non-reducing end alpha-L-arabinofuranosidase |
| maker-12-augustus-gene-15.46-mRNA-1 | Japonica | NA | Kinase-TM | Os12g0130200 | OsR498G1220880500.01.T01 | Receptor-like serine/threonine-protein kinase |

| | | | | | | |
|---|---|---|---|---|---|---|
| maker-12-augustus-gene-15.49-mRNA-1 | Japonica | NA | | Os12g0131200 | OsR498G1220885500.01.T01 | S-acyltransferase |
| maker-12-augustus-gene-15.54-mRNA-1 | Japonica | Japonica | | Os12g0131300 | OsR498G1119370400.01.T05 | Aladin |
| maker-12-augustus-gene-15.56-mRNA-1 | Japonica | NA | | Os12g0131500 | | Putative resistance protein (Fragment) |
| maker-12-augustus-gene-15.58-mRNA-1 | Japonica | NA | | | | |
| maker-12-augustus-gene-15.61-mRNA-1 | Japonica | NA | | Os12g0130900 | OsR498G1220884800.01.T01 | X8 domain-containing protein (Fragment) |
| maker-12-augustus-gene-15.62-mRNA-1 | Japonica | NA | | | OsR498G1220885200.01.T01 | Glutamine--fructose-6-phosphate transaminase (isomerizing) |
| maker-12-augustus-gene-15.71-mRNA-1 | Japonica | Japonica | | Os12g0131400 | OsR498G1220885800.01.T01 | Glucosamine fructose-6-phosphate aminotransferase1 |
| maker-12-augustus-gene-15.73-mRNA-1 | Japonica | Indica | | Os12g0131600 | OsR498G1220879500.01.T01 | Phloem protein 2-6 |
| maker-12-augustus-gene-151.23-mRNA-1 | Japonica | NA | | | OsR498G1221469100.01.T04 | Acyl-[acyl-carrier-protein]--UDP-N-acetylglucosamine O-acyltransferase |
| maker-12-augustus-gene-151.27-mRNA-1 | Japonica | NA | | | | Serine proteinase inhibitor |
| maker-12-augustus-gene-151.29-mRNA-1 | Japonica | NA | | Os12g0438000 | OsR498G1221468200.01.T01 | |
| maker-12-augustus-gene-151.31-mRNA-1 | Japonica | NA | | Os12g0438100 | | |
| maker-12-augustus-gene-152.31-mRNA-1 | Japonica | NA | | Os12g0438600 | OsR498G1221471600.01.T01 | Chloride channel protein CLC-a |
| maker-12-augustus-gene-152.32-mRNA-1 | Japonica | Indica | | Os12g0438600 | OsR498G1221471600.01.T02 | Chloride channel protein CLC-a |
| maker-12-augustus-gene-152.37-mRNA-1 | Japonica | NA | | | OsR498G1221475600.01.T01 | |
| maker-12-augustus-gene-154.0-mRNA-1 | Japonica | Indica | | Os12g0445200 | OsR498G1221486600.01.T01 | O-methyltransferase ZRP4 |
| maker-12-augustus-gene-154.2-mRNA-1 | Japonica | Indica | | Os12g0444800 | OsR498G1221488200.01.T01 | O-methyltransferase ZRP4-like |
| maker-12-augustus-gene-155.18-mRNA-1 | Japonica | NA | | Os12g0442800 | OsR498G1221505500.01.T01 | Sulfite oxidase |
| maker-12-augustus-gene-155.22-mRNA-1 | Japonica | NA | | Os12g0442900 | OsR498G1221505100.01.T03 | DET1- and DDB1-associated protein 1 |
| maker-12-augustus-gene-156.0-mRNA-1 | Japonica | NA | | | | KDEL-tailed cysteine endopeptidase CEP3 |
| maker-12-augustus-gene-156.10-mRNA-1 | Japonica | NA | | | OsR498G1221499700.01.T01 | ATPase family AAA domain-containing protein FIGL1 |
| maker-12-augustus-gene-156.2-mRNA-1 | Japonica | NA | | Os12g0443700 | OsR498G1221500100.01.T01 | Prolyl-tRNA synthetase |
| maker-12-augustus-gene-157.0-mRNA-1 | Japonica | Japonica | | Os12g0444800 | OsR498G1221494600.01.T01 | O-methyltransferase ZRP4-like |
| maker-12-augustus-gene-157.2-mRNA-1 | Japonica | Japonica | | Os12g0445200 | OsR498G1221486600.01.T01 | O-methyltransferase ZRP4 |
| maker-12-augustus-gene-158.28-mRNA-1 | Japonica | NA | | | OsR498G1221506900.01.T03 | Bifunctional dihydrofolate reductase-thymidylate synthase |
| maker-12-augustus-gene-158.33-mRNA-1 | Japonica | NA | | Os12g0446700 | OsR498G1221506800.01.T02 | Autophagy-related protein 101 |
| maker-12-augustus-gene-16.0-mRNA-1 | Japonica | NA | Kinase-TM | Os12g0132200 | OsR498G1119377100.01.T07 | Non-specific serine/threonine protein kinase |
| maker-12-augustus-gene-16.11-mRNA-1 | Japonica | NA | | Os12g0132300 | OsR498G1220890000.01.T01 | Putative calmodulin-like protein 2 |
| maker-12-augustus-gene-16.12-mRNA-1 | Japonica | NA | | Os11g0134500 | OsR498G1220890200.01.T02 | Glutamate-rich WD repeat-containing protein 1 |
| maker-12-augustus-gene-16.19-mRNA-1 | Japonica | NA | | | | MFS domain-containing protein |
| maker-12-augustus-gene-16.28-mRNA-1 | Japonica | NA | | Os12g0132800 | OsR498G1220890400.01.T05 | Major facilitator superfamily antiporter |
| maker-12-augustus-gene-16.42-mRNA-1 | Japonica | NA | | Os12g0133000 | OsR498G1220891200.01.T01 | Major facilitator superfamily antiporter |
| maker-12-augustus-gene-16.47-mRNA-1 | Japonica | Indica | | Os08g0130500 | OsR498G1220891400.01.T01 | 60S acidic ribosomal protein P0 |
| maker-12-augustus-gene-16.53-mRNA-1 | Japonica | NA | | Os11g0135900 | OsR498G1220891600.01.T02 | Transporter, putative, expressed |
| maker-12-augustus-gene-16.6-mRNA-1 | Japonica | NA | | Os12g0131900 | OsR498G1220889000.01.T01 | Rhamnogalacturonan endolyase |
| maker-12-augustus-gene-160.0-mRNA-1 | Japonica | NA | | Os12g0448900 | OsR498G1221518200.01.T03 | Alpha-dioxygenase, putative, expressed |
| maker-12-augustus-gene-161.29-mRNA-1 | Japonica | NA | | | OsR498G1221520300.01.T01 | Dirigent protein (Fragment) |
| maker-12-augustus-gene-161.32-mRNA-1 | Japonica | NA | | | | Retrotransposon protein, putative, unclassified |
| maker-12-augustus-gene-166.0-mRNA-1 | Japonica | NA | | Os12g0454600 | OsR498G1221535900.01.T01 | Phytocyanin domain-containing protein (Fragment) |
| maker-12-augustus-gene-167.0-mRNA-1 | Japonica | NA | | | OsR498G1221542300.01.T06 | Nuclear pore complex protein NUP1 |
| maker-12-augustus-gene-167.3-mRNA-1 | Japonica | NA | | Os12g0456200 | OsR498G1221541200.01.T01 | Iron-sulfur cluster co-chaperone protein HscB, mitochondrial |
| maker-12-augustus-gene-169.0-mRNA-1 | Japonica | Indica | | Os12g0458100 | OsR498G1221544200.01.T01 | Acyl transferase 1 |
| maker-12-augustus-gene-169.2-mRNA-1 | Japonica | NA | | Os12g0458100 | OsR498G1221546000.01.T01 | Acyl transferase 1 |
| maker-12-augustus-gene-169.4-mRNA-1 | Japonica | NA | | Os12g0458100 | OsR498G1221546000.01.T01 | Benzyl alcohol O-benzoyltransferase |
| maker-12-augustus-gene-169.5-mRNA-1 | Japonica | NA | | | | Acyl transferase 1 |
| maker-12-augustus-gene-17.27-mRNA-1 | Japonica | Japonica | | Os12g0518200 | OsR498G1220892100.01.T01 | |
| maker-12-augustus-gene-17.30-mRNA-1 | Japonica | NA | | Os12g0133400 | OsR498G1220892700.01.T02 | L-aminoadipate-semialdehyde dehydrogenase-phosphopantetheinyl transfe |
| maker-12-augustus-gene-17.34-mRNA-1 | Japonica | Japonica | Kinase-TM | Os12g0133500 | OsR498G1119382300.01.T01 | Calcium-dependent protein kinase |
| maker-12-augustus-gene-17.55-mRNA-1 | Japonica | NA | | | | Carbohydrate transporter/ sugar porter/ transporter |
| maker-12-augustus-gene-17.58-mRNA-1 | Japonica | NA | | Os11g0136701 | | Guanine nucleotide-binding protein G(T) subunit alpha-3 like |
| maker-12-augustus-gene-17.62-mRNA-1 | Japonica | NA | | Os12g0133700 | OsR498G1220893400.01.T01 | Protein-tyrosine-phosphatase |
| maker-12-augustus-gene-17.67-mRNA-1 | Japonica | NA | | Os11g0137000 | OsR498G1220894000.01.T01 | Auxin efflux carrier component |
| maker-12-augustus-gene-17.69-mRNA-1 | Japonica | NA | | Os12g0133900 | OsR498G1220894600.01.T01 | U3 small nucleolar ribonucleoprotein protein MPP10 |
| maker-12-augustus-gene-170.4-mRNA-1 | Japonica | NA | | Os07g0485900 | OsR498G0100084400.01.T01 | Transposon protein, putative, Mutator sub-class |
| maker-12-augustus-gene-171.0-mRNA-1 | Japonica | Indica | | Os01g0837900 | OsR498G1221553400.01.T03 | Serinethreonine-protein kinase afc2 |
| maker-12-augustus-gene-173.36-mRNA-1 | Japonica | NA | | Os09g0348800 | | Transposon protein, putative, CACTA, En/Spm sub-class, expressed |
| maker-12-augustus-gene-173.40-mRNA-1 | Japonica | Japonica | | Os12g0464400 | OsR498G1221563700.01.T01 | 11-beta-hydroxysteroid dehydrogenase-like 5 isoform X1 |
| maker-12-augustus-gene-173.42-mRNA-1 | Japonica | NA | | Os05g0269800 | OsR498G0409509300.01.T01 | DDE Tnp4 domain-containing protein |
| maker-12-augustus-gene-174.0-mRNA-1 | Japonica | Indica | | Os12g0465100 | OsR498G1221566500.01.T01 | |
| maker-12-augustus-gene-174.2-mRNA-1 | Japonica | NA | | Os12g0465100 | OsR498G1221566900.01.T01 | Acetylglucosaminyltransferase family protein |
| maker-12-augustus-gene-174.4-mRNA-1 | Japonica | NA | | | OsR498G1221568200.01.T01 | GLTSCR1 domain-containing protein (Fragment) |

| | | | | | | |
|---|---|---|---|---|---|---|
| maker-12-augustus-gene-176.28-mRNA-1 | Japonica | Indica | | Os03g0646300 | OsR498G1221573200.01.T01 | Cyclic nucleotide-gated ion channel 9 |
| maker-12-augustus-gene-176.31-mRNA-1 | Japonica | NA | | Os12g0468600 | OsR498G1221572900.01.T01 | 5-methylthioadenosine/S-adenosylhomocysteine deaminase-like isoform X1 |
| maker-12-augustus-gene-179.0-mRNA-1 | Japonica | NA | | Os12g0471100 | OsR498G1221576100.01.T01 | Adenosinetriphosphatase |
| maker-12-augustus-gene-179.3-mRNA-1 | Japonica | NA | | Os12g0472800 | OsR498G1221580600.01.T01 | Pollen-specific protein-like |
| maker-12-augustus-gene-182.24-mRNA-1 | Japonica | Indica | | Os11g0508600 | OsR498G1221587200.01.T01 | Bidirectional sugar transporter SWEET |
| maker-12-augustus-gene-183.27-mRNA-1 | Japonica | NA | | Os12g0477400 | OsR498G1221589500.01.T01 | NAC domain-containing protein 83 |
| maker-12-augustus-gene-183.29-mRNA-1 | Japonica | NA | | | OsR498G1221591100.01.T01 | Neprosin domain-containing protein |
| maker-12-augustus-gene-183.31-mRNA-1 | Japonica | NA | | Os12g0477600 | | Putative ribosomal protein L31 |
| maker-12-augustus-gene-183.33-mRNA-1 | Japonica | NA | | | OsR498G1221590800.01.T02 | DNA binding,ATP binding protein |
| maker-12-augustus-gene-184.25-mRNA-1 | Japonica | Indica | | Os09g0466300 | OsR498G1221591800.01.T01 | GRAM domain-containing protein / ABA-responsive |
| maker-12-augustus-gene-184.27-mRNA-1 | Japonica | NA | | Os12g0479100 | OsR498G1221596400.01.T01 | Extensin isoform X1 |
| maker-12-augustus-gene-185.18-mRNA-1 | Japonica | NA | | Os12g0479900 | OsR498G1221601700.01.T01 | Aminopeptidase |
| maker-12-augustus-gene-185.24-mRNA-1 | Japonica | NA | | | | S-acyltransferase |
| maker-12-augustus-gene-185.29-mRNA-1 | Japonica | NA | | Os12g0480100 | OsR498G1221602100.01.T02 | High-light-induced protein chloroplastic |
| maker-12-augustus-gene-185.32-mRNA-1 | Japonica | NA | | Os11g0523800 | OsR498G1120245800.01.T03 | Auxin response factor |
| maker-12-augustus-gene-185.37-mRNA-1 | Japonica | Indica | Kinase | Os12g0480200 | OsR498G1221602400.01.T01 | Serine/threonine-protein kinase KIPK |
| maker-12-augustus-gene-186.37-mRNA-1 | Japonica | Indica | | Os12g0481200 | OsR498G1221605700.01.T01 | Putative Histone-lysine N-methyltransferase SUV420H14-20 |
| maker-12-augustus-gene-186.42-mRNA-1 | Japonica | Indica | | Os11g0543300 | OsR498G1221605200.01.T01 | |
| maker-12-augustus-gene-186.44-mRNA-1 | Japonica | NA | | Os12g0481100 | OsR498G1221605600.01.T01 | DEAD-box ATP-dependent RNA helicase |
| maker-12-augustus-gene-186.49-mRNA-1 | Japonica | Japonica | | Os12g0482500 | OsR498G1221614600.01.T01 | GTD-binding domain-containing protein (Fragment) |
| maker-12-augustus-gene-187.37-mRNA-1 | Japonica | NA | | Os12g0482700 | OsR498G1221615000.01.T01 | L-galactose dehydrogenase |
| maker-12-augustus-gene-188.13-mRNA-1 | Japonica | NA | | Os12g0485000 | OsR498G1221621600.01.T02 | Probable tRNA N6-adenosine threonylcarbamoyltransferase, mitochondrial |
| maker-12-augustus-gene-188.18-mRNA-1 | Japonica | NA | | Os12g0485500 | OsR498G1221623000.01.T01 | Iron-sulfur assembly protein IscA-like 1 mitochondrial |
| maker-12-augustus-gene-188.2-mRNA-1 | Japonica | NA | | Os12g0485400 | OsR498G1221622500.01.T02 | Cyclin-T1-5 like |
| maker-12-augustus-gene-188.20-mRNA-1 | Japonica | NA | | Os12g0485600 | OsR498G1221624200.01.T01 | Lysine histidine transporter-like protein 8 |
| maker-12-augustus-gene-188.7-mRNA-1 | Japonica | NA | | Os12g0484700 | OsR498G1221620800.01.T01 | Yippee domain-containing protein |
| maker-12-augustus-gene-189.12-mRNA-1 | Japonica | Japonica | CC-NBS-TM | Os12g0485900 | OsR498G1221625000.01.T01 | NB-ARC domain, putative |
| maker-12-augustus-gene-19.52-mRNA-1 | Japonica | NA | | Os08g0343300 | OsR498G1119384700.01.T01 | Transcription initiation factor IIE subunit alpha |
| maker-12-augustus-gene-19.53-mRNA-1 | Japonica | NA | | | OsR498G1119384700.01.T01 | Transcription initiation factor IIE subunit alpha |
| maker-12-augustus-gene-19.56-mRNA-1 | Japonica | NA | | Os12g0136200 | OsR498G1220899700.01.T01 | Heterogeneous nuclear ribonucleoprotein Q |
| maker-12-augustus-gene-19.62-mRNA-1 | Japonica | Indica | | Os11g0137300 | OsR498G1220895400.01.T01 | proteasome-associated protein ECM29 homolog |
| maker-12-augustus-gene-19.73-mRNA-1 | Japonica | NA | | | | Phosphoglycerate mutase family protein, expressed |
| maker-12-augustus-gene-19.78-mRNA-1 | Japonica | NA | | Os11g0138600 | OsR498G1220897400.01.T02 | Phosphoglycerate mutase gpmB |
| maker-12-augustus-gene-19.83-mRNA-1 | Japonica | NA | | Os11g0139100 | OsR498G1220899400.01.T01 | KOW domain-containing protein |
| maker-12-augustus-gene-190.28-mRNA-1 | Japonica | NA | | Os12g0486600 | OsR498G1221633300.01.T01 | Nuclear pore complex protein NUP1-like |
| maker-12-augustus-gene-190.29-mRNA-1 | Japonica | NA | | Os12g0486600 | OsR498G1221633300.01.T01 | Calcium-dependent calmodulin-independent protein kinase |
| maker-12-augustus-gene-190.35-mRNA-1 | Japonica | Japonica | Kinase-TM | Os12g0486900 | OsR498G1221633900.01.T01 | receptor-like serine/threonine-protein kinase ALE2 isoform X2 |
| maker-12-augustus-gene-192.30-mRNA-1 | Japonica | Japonica | | Os12g0488600 | OsR498G1120417100.01.T01 | 50S ribosomal protein L4 |
| maker-12-augustus-gene-192.32-mRNA-1 | Japonica | NA | | Os12g0488700 | OsR498G1221645100.01.T01 | Myosin-binding protein 7 |
| maker-12-augustus-gene-192.34-mRNA-1 | Japonica | NA | | | OsR498G1221646900.01.T05 | Ubiquitinyl hydrolase 1 |
| maker-12-augustus-gene-192.41-mRNA-1 | Japonica | NA | | | OsR498G1119369600.01.T06 | PMEI domain-containing protein |
| maker-12-augustus-gene-192.43-mRNA-1 | Japonica | NA | | | | DNA-directed RNA polymerase III subunit RPC9 |
| maker-12-augustus-gene-192.50-mRNA-1 | Japonica | NA | | Os12g0489300 | OsR498G1221647600.01.T01 | Putative DNA/RNA-binding protein Alba |
| maker-12-augustus-gene-194.20-mRNA-1 | Japonica | NA | | Os12g0492900 | | F-box domain containing protein, expressed |
| maker-12-augustus-gene-194.22-mRNA-1 | Japonica | Indica | | Os12g0491800 | OsR498G1221651000.01.T01 | Kaurene synthase3 |
| maker-12-augustus-gene-194.24-mRNA-1 | Japonica | NA | | Os12g0491800 | OsR498G1221651400.01.T02 | Ent-kaurene synthase like 4 |
| maker-12-augustus-gene-195.41-mRNA-1 | Japonica | NA | | Os12g0493900 | OsR498G1221652500.01.T01 | Maternal protein pumilio |
| maker-12-augustus-gene-195.44-mRNA-1 | Japonica | NA | | Os10g0199200 | OsR498G1018346900.01.T03 | |
| maker-12-augustus-gene-196.0-mRNA-1 | Japonica | NA | CC-NBS-TM | Os12g0495600 | OsR498G1221658200.01.T01 | Putative CC-NBS-LRR resistance protein MLA13 |
| maker-12-augustus-gene-197.0-mRNA-1 | Japonica | NA | NBS-TM | | OsR498G1221658300.01.T01 | NB-ARC domain containing protein, expressed |
| maker-12-augustus-gene-197.2-mRNA-1 | Japonica | NA | | | | Phototropic-responsive NPH3 family protein |
| maker-12-augustus-gene-197.3-mRNA-1 | Japonica | NA | | | OsR498G1221660800.01.T01 | FBD domain-containing protein (Fragment) |
| maker-12-augustus-gene-197.5-mRNA-1 | Japonica | NA | | Os12g0496900 | OsR498G1221661000.01.T01 | GRF1-interacting factor 3 isoform B |
| maker-12-augustus-gene-197.8-mRNA-1 | Japonica | Indica | | Os12g0497400 | OsR498G1221661600.01.T01 | eIF-2B GDP-GTP exchange factor subunit alpha |
| maker-12-augustus-gene-198.0-mRNA-1 | Japonica | NA | | | | BHLH domain-containing protein (Fragment) |
| maker-12-augustus-gene-198.11-mRNA-1 | Japonica | NA | | Os12g0498700 | OsR498G1221664300.01.T01 | GPI mannosyltransferase 2 |
| maker-12-augustus-gene-198.18-mRNA-1 | Japonica | NA | | Os12g0498400 | OsR498G1221663000.01.T02 | Plastid division regulator MinE |
| maker-12-augustus-gene-198.2-mRNA-1 | Japonica | Indica | | Os05g0482600 | OsR498G1221662700.01.T01 | |
| maker-12-augustus-gene-198.20-mRNA-1 | Japonica | NA | | | OsR498G1221663400.01.T01 | NADPH-quinone oxidoreductase subunit U, chloroplastic |
| maker-12-augustus-gene-198.24-mRNA-1 | Japonica | NA | | Os12g0498800 | OsR498G1221665000.01.T01 | |
| maker-12-augustus-gene-199.0-mRNA-1 | Japonica | NA | | Os12g0500700 | OsR498G1221667300.01.T05 | Vacuolar protein sorting-associated protein 26 |

| | | | | | | |
|---|---|---|---|---|---|---|
| maker-12-augustus-gene-2.66-mRNA-1 | Japonica | NA | | Os12g0104300 | OsR498G1220794300.01.T01 | Double CLP-N motif-containing P-loop nucleoside triphosphate hydrolases su |
| maker-12-augustus-gene-20.40-mRNA-1 | Japonica | NA | | Os12g0136900 | OsR498G1220901700.01.T01 | Calcium-transporting ATPase |
| maker-12-augustus-gene-20.46-mRNA-1 | Japonica | NA | | | OsR498G1220903100.01.T07 | Kallikrein-2 like |
| maker-12-augustus-gene-20.55-mRNA-1 | Japonica | Indica | | Os11g0140900 | OsR498G1220903700.01.T01 | Bromodomain-containing protein |
| maker-12-augustus-gene-20.57-mRNA-1 | Japonica | NA | | Os12g0137500 | OsR498G1220904300.01.T01 | PP2A regulatory subunit like |
| maker-12-augustus-gene-20.65-mRNA-1 | Japonica | NA | | Os11g0141900 | OsR498G1220907100.01.T01 | BTB/POZ domain and ankyrin repeat-containing protein NH5.2 |
| maker-12-augustus-gene-20.68-mRNA-1 | Japonica | Indica | | Os11g0140100 | OsR498G1220901400.01.T01 | Mitochondrial acidic protein mam33 |
| maker-12-augustus-gene-20.71-mRNA-1 | Japonica | Indica | | Os12g0137100 | OsR498G1220902400.01.T01 | probable calcium-binding protein CML49 |
| maker-12-augustus-gene-20.76-mRNA-1 | Japonica | NA | | | OsR498G1220903400.01.T01 | Cytochrome c-type biogenesis ccda chloroplastic protein isoform 1 |
| maker-12-augustus-gene-200.27-mRNA-1 | Japonica | NA | | Os08g0402700 | | ZF-HD dimerization-type domain-containing protein |
| maker-12-augustus-gene-200.32-mRNA-1 | Japonica | NA | | Os12g0502500 | OsR498G1221671900.01.T01 | Nitrilase-like protein 2 isoform A |
| maker-12-augustus-gene-200.35-mRNA-1 | Japonica | NA | | Os12g0502700 | OsR498G1221672300.01.T02 | DnaJ domain containing protein, expressed |
| maker-12-augustus-gene-200.38-mRNA-1 | Japonica | Japonica | | Os12g0501400 | OsR498G1221669000.01.T02 | Usp domain-containing protein (Fragment) |
| maker-12-augustus-gene-200.41-mRNA-1 | Japonica | Japonica | | Os12g0501700 | OsR498G1221669400.01.T01 | Agamous-like MADS-box protein AGL8 |
| maker-12-augustus-gene-200.45-mRNA-1 | Japonica | NA | | Os12g0502000 | OsR498G1221670000.01.T02 | Non-structural polyprotein 1AB |
| maker-12-augustus-gene-200.47-mRNA-1 | Japonica | NA | | Os12g0502100 | | SKIP interacting protein 15 |
| maker-12-augustus-gene-200.50-mRNA-1 | Japonica | NA | | Os12g0502300 | OsR498G1221671000.01.T01 | Cyclin N-terminal domain-containing protein |
| maker-12-augustus-gene-200.54-mRNA-1 | Japonica | NA | | Os12g0502400 | OsR498G1221671600.01.T01 | O-phosphoserine phosphohydrolase |
| maker-12-augustus-gene-200.62-mRNA-1 | Japonica | NA | | Os12g0502800 | OsR498G1221672600.01.T01 | Ureide permease |
| maker-12-augustus-gene-201.28-mRNA-1 | Japonica | NA | | | OsR498G1221673100.01.T04 | Translation initiation factor |
| maker-12-augustus-gene-201.31-mRNA-1 | Japonica | NA | | Os12g0503000 | OsR498G1221672800.01.T02 | Ureide permease |
| maker-12-augustus-gene-201.38-mRNA-1 | Japonica | NA | | Os12g0503300 | | Ureide permease |
| maker-12-augustus-gene-203.0-mRNA-1 | Japonica | NA | | Os12g0506700 | OsR498G1221685500.01.T01 | |
| maker-12-augustus-gene-203.12-mRNA-1 | Japonica | NA | | Os12g0507200 | OsR498G1221687500.01.T01 | Eukaryotic translation initiation factor 5A |
| maker-12-augustus-gene-203.16-mRNA-1 | Japonica | NA | | Os12g0507300 | OsR498G1221688500.01.T01 | WRKY domain class transcription factor |
| maker-12-augustus-gene-203.20-mRNA-1 | Japonica | NA | | Os03g0666200 | OsR498G1119668000.01.T01 | differentially expressed in FDCP 6 homolog isoform X1 |
| maker-12-augustus-gene-203.9-mRNA-1 | Japonica | NA | | Os12g0507000 | OsR498G1221686500.01.T07 | GTP 3',8-cyclase |
| maker-12-augustus-gene-204.34-mRNA-1 | Japonica | NA | | | OsR498G1221689700.01.T08 | Protein TRIGALACTOSYLDIACYLGLYCEROL 5 chloroplastic |
| maker-12-augustus-gene-204.36-mRNA-1 | Japonica | NA | | Os12g0507900 | | DUF223 domain-containing protein |
| maker-12-augustus-gene-204.39-mRNA-1 | Japonica | Japonica | | Os12g0508100 | OsR498G1221690700.01.T01 | Peroxisomal membrane protein MPV17 |
| maker-12-augustus-gene-204.41-mRNA-1 | Japonica | NA | | | OsR498G1221691900.01.T01 | Ribosomal protein S14p/S29e containing protein, expressed |
| maker-12-augustus-gene-204.43-mRNA-1 | Japonica | NA | | | OsR498G1221692700.01.T01 | Transcription factor bHLH85 like |
| maker-12-augustus-gene-204.46-mRNA-1 | Japonica | NA | | | OsR498G1221689600.01.T02 | SWIB domain-containing protein |
| maker-12-augustus-gene-204.49-mRNA-1 | Japonica | NA | | Os12g0507700 | | |
| maker-12-augustus-gene-204.51-mRNA-1 | Japonica | NA | | Os12g0508200 | OsR498G1221691000.01.T01 | |
| maker-12-augustus-gene-204.53-mRNA-1 | Japonica | NA | | Os12g0508266 | OsR498G1221691800.01.T01 | UBP-type domain-containing protein (Fragment) |
| maker-12-augustus-gene-204.55-mRNA-1 | Japonica | NA | | Os12g0508900 | | |
| maker-12-augustus-gene-205.0-mRNA-1 | Japonica | NA | | | | |
| maker-12-augustus-gene-205.13-mRNA-1 | Japonica | Japonica | | Os12g0509300 | OsR498G1221697300.01.T01 | |
| maker-12-augustus-gene-205.16-mRNA-1 | Japonica | NA | | | OsR498G1221699600.01.T05 | |
| maker-12-augustus-gene-205.18-mRNA-1 | Japonica | NA | | Os12g0510200 | | No pollen germination related 1 |
| maker-12-augustus-gene-205.3-mRNA-1 | Japonica | NA | | Os12g0509600 | | |
| maker-12-augustus-gene-205.5-mRNA-1 | Japonica | NA | | Os12g0510000 | | |
| maker-12-augustus-gene-205.9-mRNA-1 | Japonica | NA | | | OsR498G1221702100.01.T01 | Transcription factor LIM |
| maker-12-augustus-gene-206.21-mRNA-1 | Japonica | Japonica | | Os12g0511200 | OsR498G1221702900.01.T01 | Heptahelical transmembrane protein 5 |
| maker-12-augustus-gene-206.23-mRNA-1 | Japonica | NA | | Os12g0511300 | OsR498G1221703100.01.T02 | CRT-like domain-containing protein (Fragment) |
| maker-12-augustus-gene-206.26-mRNA-1 | Japonica | NA | | | OsR498G1221702600.01.T01 | F-box domain containing protein, expressed |
| maker-12-augustus-gene-206.28-mRNA-1 | Japonica | NA | NBS-TM | Os12g0511400 | OsR498G1221703400.01.T01 | NB-ARC domain containing protein, expressed |
| maker-12-augustus-gene-206.31-mRNA-1 | Japonica | NA | CC-NBS-TM | | OsR498G1221703800.01.T01 | NB-ARC domain containing protein, expressed |
| maker-12-augustus-gene-206.34-mRNA-1 | Japonica | Indica | CC-NBS-TM | Os12g0511400 | OsR498G1221704000.01.T02 | NB-ARC domain containing protein, expressed |
| maker-12-augustus-gene-206.36-mRNA-1 | Japonica | NA | NBS-TM | | | NB-ARC domain containing protein, expressed |
| maker-12-augustus-gene-207.32-mRNA-1 | Japonica | Japonica | | Os12g0512000 | OsR498G1221704700.01.T01 | Flavin-containing monooxygenase |
| maker-12-augustus-gene-207.34-mRNA-1 | Japonica | NA | | Os12g0512200 | | Transposon protein, putative, Mariner sub-class |
| maker-12-augustus-gene-207.37-mRNA-1 | Japonica | NA | NBS-TM | | OsR498G1221704200.01.T01 | NB-ARC domain containing protein, expressed |
| maker-12-augustus-gene-207.55-mRNA-1 | Japonica | Indica | | Os03g0850200 | OsR498G1221706400.01.T01 | 4-hydroxyphenylacetaldehyde oxime monooxygenase |
| maker-12-augustus-gene-208.39-mRNA-1 | Japonica | NA | | | | Mitochondrial inner membrane translocase subunit |
| maker-12-augustus-gene-208.41-mRNA-1 | Japonica | NA | | Os12g0514000 | OsR498G1221708200.01.T01 | Polyol transporter 5 |
| maker-12-augustus-gene-208.43-mRNA-1 | Japonica | NA | | | OsR498G1221709000.01.T01 | Stress-associated endoplasmic reticulum protein 2 |
| maker-12-augustus-gene-208.46-mRNA-1 | Japonica | NA | | | OsR498G1221710000.01.T02 | PHD domain-containing protein (Fragment) |
| maker-12-augustus-gene-208.48-mRNA-1 | Japonica | NA | | Os12g0514500 | OsR498G1221710800.01.T01 | Heat shock protein 90-6 mitochondrial |
| maker-12-augustus-gene-208.54-mRNA-1 | Japonica | NA | | Os12g0514600 | OsR498G1221711000.01.T01 | SAM domain-containing protein (Fragment) |

| | | | | | | |
|---|---|---|---|---|---|---|
| maker-12-augustus-gene-209.28-mRNA-1 | Japonica | NA | | Os12g0515300 | OsR498G1221715000.01.T01 | Transcription factor like |
| maker-12-augustus-gene-21.50-mRNA-1 | Japonica | NA | | Os12g0138800 | OsR498G1220908500.01.T01 | Dimethylnonatriene synthase |
| maker-12-augustus-gene-21.52-mRNA-1 | Japonica | Indica | | Os11g0142400 | OsR498G1220908900.01.T01 | Strictosidine synthase |
| maker-12-augustus-gene-21.55-mRNA-1 | Japonica | NA | | Os12g0138900 | OsR498G1220909100.01.T02 | 2-isopropylmalate synthase 1 chloroplastic |
| maker-12-augustus-gene-21.65-mRNA-1 | Japonica | NA | | | | MFS domain-containing protein (Fragment) |
| maker-12-augustus-gene-21.67-mRNA-1 | Japonica | NA | | Os12g0139300 | OsR498G1220910200.01.T01 | CPD brassinosteroid C-23 hydroxylase |
| maker-12-augustus-gene-21.70-mRNA-1 | Japonica | NA | | Os12g0139500 | OsR498G1220911200.01.T01 | RNA polymerase Rpb5, N-terminal domain containing protein, expressed |
| maker-12-augustus-gene-21.73-mRNA-1 | Japonica | NA | | Os11g0143500 | OsR498G1220911800.01.T01 | Mitochondrial L-galactono-1,4-lactone dehydrogenase |
| maker-12-augustus-gene-21.78-mRNA-1 | Japonica | NA | | | | Transcription initiation factor IIE subunit alpha |
| maker-12-augustus-gene-21.84-mRNA-1 | Japonica | Japonica | | Os12g0140300 | OsR498G1220913400.01.T01 | |
| maker-12-augustus-gene-216.57-mRNA-1 | Japonica | Indica | | Os01g0375200 | OsR498G1221758000.01.T05 | Shikimate biosynthesis protein aroDE |
| maker-12-augustus-gene-22.34-mRNA-1 | Japonica | NA | | | OsR498G1119556100.01.T01 | Transposon mutator sub-class |
| maker-12-augustus-gene-22.37-mRNA-1 | Japonica | NA | | Os12g0141100 | OsR498G1220916400.01.T02 | Phosphatidylinositol 4-phosphate 5-kinase |
| maker-12-augustus-gene-22.39-mRNA-1 | Japonica | Indica | | Os01g0776500 | OsR498G1220917600.01.T01 | |
| maker-12-augustus-gene-220.24-mRNA-1 | Japonica | Indica | | Os12g0540700 | OsR498G1221784900.01.T02 | Dynein beta chain, ciliary protein |
| maker-12-augustus-gene-220.27-mRNA-1 | Japonica | NA | | Os12g0540800 | OsR498G1221784900.01.T04 | Acyl-CoA-binding domain-containing protein 4-like isoform X2 |
| maker-12-augustus-gene-220.41-mRNA-1 | Japonica | NA | | Os12g0541500 | OsR498G1221787900.01.T03 | Elongation factor Ts, mitochondrial |
| maker-12-augustus-gene-220.43-mRNA-1 | Japonica | NA | | Os12g0540900 | OsR498G1221785200.01.T01 | Tryptophanyl-tRNA synthetase |
| maker-12-augustus-gene-226.8-mRNA-1 | Japonica | NA | | | | |
| maker-12-augustus-gene-228.36-mRNA-1 | Japonica | NA | | Os12g0553900 | OsR498G1221857500.01.T01 | splicing regulatory glutamine/lysine-rich protein 1 |
| maker-12-augustus-gene-228.50-mRNA-1 | Japonica | NA | | Os12g0516800 | OsR498G1221857800.01.T03 | Trichome birefringence-like 16 |
| maker-12-augustus-gene-229.17-mRNA-1 | Japonica | NA | | Os12g0556300 | OsR498G1221865700.01.T02 | Calmodulin-binding protein, putative, expressed |
| maker-12-augustus-gene-229.19-mRNA-1 | Japonica | Japonica | | Os12g0556400 | OsR498G1221865900.01.T02 | Putative Magnesium transporter |
| maker-12-augustus-gene-229.21-mRNA-1 | Japonica | NA | | Os12g0556500 | OsR498G1221866100.01.T02 | Calmodulin-binding protein, putative, expressed |
| maker-12-augustus-gene-229.25-mRNA-1 | Japonica | Indica | | Os06g0336500 | OsR498G1221863800.01.T01 | Cellulose synthase-like protein D4 |
| maker-12-augustus-gene-229.27-mRNA-1 | Japonica | NA | | Os12g0556600 | OsR498G1221866400.01.T01 | CTP synthase |
| maker-12-augustus-gene-229.4-mRNA-1 | Japonica | Japonica | | Os12g0555400 | OsR498G1221863300.01.T01 | Galactinol-raffinose galactosyltransferase |
| maker-12-augustus-gene-229.6-mRNA-1 | Japonica | NA | | Os12g0555500 | OsR498G1221863500.01.T01 | Major allergen Api g 1, isoallergen 1-like |
| maker-12-augustus-gene-23.0-mRNA-1 | Japonica | NA | | Os12g0143200 | OsR498G1220920500.01.T01 | Set1/Ash2 histone methyltransferase complex subunit ASH2 |
| maker-12-augustus-gene-23.14-mRNA-1 | Japonica | NA | | Os12g0143800 | OsR498G1220924800.01.T03 | DNA meiotic recombinase 1 |
| maker-12-augustus-gene-23.19-mRNA-1 | Japonica | NA | | Os12g0143900 | OsR498G1220925600.01.T03 | Long chain acyl-CoA synthetase 6 peroxisomal |
| maker-12-augustus-gene-23.2-mRNA-1 | Japonica | Indica | | Os11g0147100 | OsR498G1220925900.01.T01 | R3H-associated N-terminal domain-containing protein |
| maker-12-augustus-gene-23.7-mRNA-1 | Japonica | Japonica | | Os12g0143400 | OsR498G1119407400.01.T02 | Suppressor of mec-8 and unc-52 protein 2 |
| maker-12-augustus-gene-230.0-mRNA-1 | Japonica | NA | | Os12g0557900 | OsR498G1221868900.01.T02 | FBD domain-containing protein (Fragment) |
| maker-12-augustus-gene-230.3-mRNA-1 | Japonica | NA | | Os12g0557400 | OsR498G1221867600.01.T01 | Transmembrane protein 205 |
| maker-12-augustus-gene-231.30-mRNA-1 | Japonica | NA | | Os12g0559200 | OsR498G1221874400.01.T02 | Lipoxygenase |
| maker-12-augustus-gene-231.39-mRNA-1 | Japonica | NA | CC-NBS-TM | Os12g0559500 | OsR498G1221875800.01.T01 | Disease resistance protein RPP13 |
| maker-12-augustus-gene-232.31-mRNA-1 | Japonica | Indica | | Os12g0559200 | OsR498G1221876500.01.T01 | Lipoxygenase |
| maker-12-augustus-gene-232.38-mRNA-1 | Japonica | NA | | Os12g0560400 | OsR498G1221877300.01.T01 | DNA-directed RNA polymerase subunit |
| maker-12-augustus-gene-232.41-mRNA-1 | Japonica | NA | | Os12g0560600 | OsR498G1221877900.01.T01 | Ubiquitin-conjugating enzyme E2 37 |
| maker-12-augustus-gene-232.44-mRNA-1 | Japonica | NA | | Os12g0560300 | OsR498G1221877200.01.T01 | GTP-binding protein SAR1A |
| maker-12-augustus-gene-232.47-mRNA-1 | Japonica | NA | | Os12g0560500 | OsR498G1221877800.01.T01 | RNA pseudourine synthase 1 |
| maker-12-augustus-gene-232.49-mRNA-1 | Japonica | NA | | Os12g0560700 | OsR498G1221878200.01.T02 | DNA replication licensing factor MCM7 |
| maker-12-augustus-gene-232.53-mRNA-1 | Japonica | Indica | | Os12g0561000 | OsR498G1221879000.01.T01 | Cytochrome c oxidase polypeptide Vc |
| maker-12-augustus-gene-232.55-mRNA-1 | Japonica | Indica | | Os12g0561200 | OsR498G1221879200.01.T02 | PAPA-1 domain-containing protein (Fragment) |
| maker-12-augustus-gene-233.0-mRNA-1 | Japonica | Indica | | Os12g0562000 | OsR498G1221881500.01.T02 | |
| maker-12-augustus-gene-233.2-mRNA-1 | Japonica | NA | | | OsR498G1221882700.01.T03 | Smr domain-containing protein (Fragment) |
| maker-12-augustus-gene-233.24-mRNA-1 | Japonica | NA | | Os12g0562100 | OsR498G1221882000.01.T04 | Solute carrier family 40 member 2, chloroplastic |
| maker-12-augustus-gene-234.53-mRNA-1 | Japonica | NA | | Os12g0563000 | OsR498G1221886900.01.T01 | PlsC domain-containing protein (Fragment) |
| maker-12-augustus-gene-234.75-mRNA-1 | Japonica | Japonica | CC-NBS-TM | Os12g0565100 | OsR498G1221893300.01.T01 | NB-ARC domain containing protein, expressed |
| maker-12-augustus-gene-234.79-mRNA-1 | Japonica | NA | | Os12g0563700 | OsR498G1221889200.01.T01 | Pectinesterase |
| maker-12-augustus-gene-234.83-mRNA-1 | Japonica | NA | | | OsR498G1221890200.01.T01 | Transcription factor MYB21 |
| maker-12-augustus-gene-234.92-mRNA-1 | Japonica | NA | NBS-TM | Os12g0564800 | OsR498G1221892000.01.T02 | NB-ARC domain containing protein expressed |
| maker-12-augustus-gene-235.0-mRNA-1 | Japonica | NA | CC-LRR-NBS-TM | Os12g0565200 | OsR498G1221893900.01.T01 | NB-ARC domain containing protein, expressed |
| maker-12-augustus-gene-235.10-mRNA-1 | Japonica | NA | | | OsR498G1221895700.01.T01 | Modifier of snc1 |
| maker-12-augustus-gene-235.13-mRNA-1 | Japonica | NA | | Os12g0566300 | OsR498G1221895900.01.T03 | ATP citrate synthase |
| maker-12-augustus-gene-235.17-mRNA-1 | Japonica | Indica | | Os12g0566400 | OsR498G1221896100.01.T02 | Membrane magnesium transporter |
| maker-12-augustus-gene-235.20-mRNA-1 | Japonica | NA | | Os12g0565300 | OsR498G1221894200.01.T01 | Calmodulin-binding protein MPCBP |
| maker-12-augustus-gene-235.25-mRNA-1 | Japonica | Indica | | Os12g0566100 | OsR498G1221895200.01.T01 | LYR motif-containing 5A |
| maker-12-augustus-gene-235.3-mRNA-1 | Japonica | NA | | Os12g0566000 | OsR498G1221894900.01.T01 | Boron transporter like |
| maker-12-augustus-gene-236.44-mRNA-1 | Japonica | NA | | | | |

| | | | | | | |
|---|---|---|---|---|---|---|
| maker-12-augustus-gene-236.51-mRNA-1 | Japonica | NA | | | OsR498G1221897500.01.T04 | Diaminopimelate epimerase |
| maker-12-augustus-gene-236.56-mRNA-1 | Japonica | NA | | Os12g0567700 | OsR498G1221900900.01.T01 | Large subunit ribosomal protein L8e |
| maker-12-augustus-gene-236.58-mRNA-1 | Japonica | Indica | | Os12g0566700 | OsR498G1221896800.01.T01 | GDSL-like Lipase/Acylhydrolase family protein, expressed |
| maker-12-augustus-gene-236.61-mRNA-1 | Japonica | NA | | Os12g0566900 | OsR498G1221897000.01.T03 | |
| maker-12-augustus-gene-236.63-mRNA-1 | Japonica | Indica | | Os12g0566800 | OsR498G1221897000.01.T02 | Putative Ion channel regulatory protein, UNC-93 |
| maker-12-augustus-gene-236.67-mRNA-1 | Japonica | Indica | | Os12g0566900 | OsR498G1221897200.01.T01 | Putative Ion channel regulatory protein, UNC-93 |
| maker-12-augustus-gene-236.68-mRNA-1 | Japonica | NA | | Os12g0566900 | OsR498G1221897200.01.T06 | Ion channel regulatory protein |
| maker-12-augustus-gene-236.77-mRNA-1 | Japonica | NA | | Os12g0567300 | OsR498G1221897800.01.T01 | Myb-related protein MYBAS2 |
| maker-12-augustus-gene-236.80-mRNA-1 | Japonica | Japonica | Kinase-LRR-TM | Os12g0567500 | OsR498G1221899800.01.T01 | Expressed protein kinase |
| maker-12-augustus-gene-236.82-mRNA-1 | Japonica | NA | | Os12g0567800 | OsR498G1221901200.01.T01 | Metallothionein-like protein 1, putative, expressed |
| maker-12-augustus-gene-237.33-mRNA-1 | Japonica | NA | | Os12g0568700 | OsR498G1221903300.01.T01 | |
| maker-12-augustus-gene-237.35-mRNA-1 | Japonica | NA | | Os12g0568800 | OsR498G1221903700.01.T01 | Importin subunit beta-1 like |
| maker-12-augustus-gene-237.37-mRNA-1 | Japonica | Japonica | | Os12g0569700 | OsR498G0306484600.01.T01 | Non-cell-autonomous heat shock cognate protein 70 |
| maker-12-augustus-gene-237.41-mRNA-1 | Japonica | NA | | Os12g0570000 | OsR498G1221907400.01.T01 | RING-type E3 ubiquitin transferase |
| maker-12-augustus-gene-238.0-mRNA-1 | Japonica | NA | | Os12g0570400 | OsR498G1221909300.01.T02 | |
| maker-12-augustus-gene-238.10-mRNA-1 | Japonica | NA | | Os12g0571100 | OsR498G1221910900.01.T01 | Metallothionein-like protein 1, putative, expressed |
| maker-12-augustus-gene-238.13-mRNA-1 | Japonica | NA | | Os12g0571200 | OsR498G1221911300.01.T01 | Mitochondrial import inner membrane translocase subunit Tim9 like |
| maker-12-augustus-gene-238.15-mRNA-1 | Japonica | NA | | Os12g0571900 | OsR498G1221912900.01.T06 | Plant RNA cleavage stimulation factor |
| maker-12-augustus-gene-238.2-mRNA-1 | Japonica | NA | | Os12g0570500 | OsR498G1221909500.01.T03 | SAM domain-containing protein |
| maker-12-augustus-gene-238.22-mRNA-1 | Japonica | NA | | Os12g0572000 | OsR498G1221913200.01.T01 | Protein rough sheath 2 homolog |
| maker-12-augustus-gene-238.7-mRNA-1 | Japonica | Indica | | Os12g0571000 | OsR498G1221910700.01.T01 | Metallothionein-like protein 1, putative, expressed |
| maker-12-augustus-gene-239.25-mRNA-1 | Japonica | NA | | Os12g0572500 | OsR498G1221916500.01.T04 | XH domain containing protein, expressed |
| maker-12-augustus-gene-239.30-mRNA-1 | Japonica | Indica | | Os12g0573000 | OsR498G1221918300.01.T02 | |
| maker-12-augustus-gene-239.37-mRNA-1 | Japonica | NA | | Os12g0573900 | | |
| maker-12-augustus-gene-239.40-mRNA-1 | Japonica | NA | | Os12g0572400 | OsR498G1221916200.01.T01 | 35 kDa SR repressor protein |
| maker-12-augustus-gene-239.45-mRNA-1 | Japonica | Indica | | Os12g0572800 | OsR498G1221917200.01.T01 | HTH OST-type domain-containing protein |
| maker-12-augustus-gene-24.0-mRNA-1 | Japonica | NA | | | OsR498G1220926300.01.T01 | |
| maker-12-augustus-gene-24.12-mRNA-1 | Japonica | Indica | | Os11g0148400 | OsR498G1220932300.01.T01 | Peptidylprolyl isomerase |
| maker-12-augustus-gene-24.15-mRNA-1 | Japonica | NA | | Os12g0145100 | OsR498G1220930000.01.T01 | Stem-specific protein TSJT1 |
| maker-12-augustus-gene-24.19-mRNA-1 | Japonica | NA | | Os12g0145700 | OsR498G1220932600.01.T01 | Pyruvate kinase |
| maker-12-augustus-gene-24.29-mRNA-1 | Japonica | NA | Kinase-LRR-TM | | OsR498G1220933000.01.T02 | Leucine-rich repeat receptor-like protein kinase PXC1 |
| maker-12-augustus-gene-24.3-mRNA-1 | Japonica | NA | | Os12g0144600 | OsR498G1220928100.01.T02 | HMA domain-containing protein (Fragment) |
| maker-12-augustus-gene-24.5-mRNA-1 | Japonica | NA | | Os12g0145400 | OsR498G1220931300.01.T01 | Protein trichome birefringence-like protein 11 |
| maker-12-augustus-gene-24.8-mRNA-1 | Japonica | NA | | Os12g0145500 | OsR498G1220931500.01.T01 | Peptidylprolyl isomerase |
| maker-12-augustus-gene-240.36-mRNA-1 | Japonica | NA | | Os12g0574400 | OsR498G1221921500.01.T05 | PUM-HD domain-containing protein |
| maker-12-augustus-gene-240.39-mRNA-1 | Japonica | NA | | Os12g0574000 | OsR498G1221920800.01.T01 | Lysine histidine transporter 1 |
| maker-12-augustus-gene-240.41-mRNA-1 | Japonica | NA | | Os12g0574900 | OsR498G1221924400.01.T04 | Asparagine synthetase domain-containing protein 1 |
| maker-12-augustus-gene-240.46-mRNA-1 | Japonica | NA | | Os12g0575000 | OsR498G1221924600.01.T01 | Endopolygalacturonase AN8327 (Fragment) |
| maker-12-augustus-gene-241.33-mRNA-1 | Japonica | NA | | | OsR498G1221928900.01.T01 | TPX2 (Targeting protein for Xklp2) protein family |
| maker-12-augustus-gene-241.38-mRNA-1 | Japonica | NA | | Os12g0577100 | OsR498G1221929100.01.T03 | Putative RNA-binding protein 46 isoform X2 |
| maker-12-augustus-gene-241.56-mRNA-1 | Japonica | Indica | | Os12g0576300 | OsR498G1221927000.01.T02 | |
| maker-12-augustus-gene-241.59-mRNA-1 | Japonica | NA | | Os12g0576600 | OsR498G1221927800.01.T02 | Purple acid phosphatase |
| maker-12-augustus-gene-241.64-mRNA-1 | Japonica | NA | | Os12g0576700 | OsR498G1221928000.01.T03 | Purple acid phosphatase |
| maker-12-augustus-gene-241.69-mRNA-1 | Japonica | Indica | | Os12g0576600 | OsR498G1221928600.01.T01 | Purple acid phosphatase |
| maker-12-augustus-gene-241.71-mRNA-1 | Japonica | NA | | Os12g0576900 | OsR498G1221928800.01.T01 | Diacylglycerol kinase |
| maker-12-augustus-gene-243.4-mRNA-1 | Japonica | NA | | Os12g0580500 | OsR498G1221944900.01.T02 | Transcriptional regulator of RNA polII SAGA subunit |
| maker-12-augustus-gene-243.5-mRNA-1 | Japonica | NA | | Os12g0580600 | OsR498G1221945300.01.T04 | Keratin, type II cytoskeletal 2 epidermal-like |
| maker-12-augustus-gene-243.7-mRNA-1 | Japonica | NA | | Os06g0282000 | OsR498G0816353100.01.T01 | Putative E3 ubiquitin-protein ligase RHY1A |
| maker-12-augustus-gene-243.8-mRNA-1 | Japonica | NA | | Os12g0580700 | OsR498G1221945700.01.T01 | UDP-glycosyltransferase 89B1 |
| maker-12-augustus-gene-244.34-mRNA-1 | Japonica | Indica | | Os03g0606100 | OsR498G1221947400.01.T02 | Lipase, putative, expressed |
| maker-12-augustus-gene-245.50-mRNA-1 | Japonica | Indica | | Os12g0584100 | OsR498G1221963400.01.T05 | |
| maker-12-augustus-gene-247.3-mRNA-1 | Japonica | NA | | Os12g0586300 | OsR498G1221989900.01.T01 | Myb family transcription factor EFM |
| maker-12-augustus-gene-247.6-mRNA-1 | Japonica | NA | | Os03g0240900 | OsR498G1221990100.01.T01 | |
| maker-12-augustus-gene-248.31-mRNA-1 | Japonica | NA | | Os12g0586600 | OsR498G1221990700.01.T02 | Calcium-transporting ATPase |
| maker-12-augustus-gene-248.42-mRNA-1 | Japonica | NA | | Os12g0588000 | | |
| maker-12-augustus-gene-248.44-mRNA-1 | Japonica | NA | | | | HTH OST-type domain-containing protein |
| maker-12-augustus-gene-248.45-mRNA-1 | Japonica | NA | | Os12g0572800 | OsR498G1222005100.01.T04 | RNA recognition motif family protein |
| maker-12-augustus-gene-248.48-mRNA-1 | Japonica | NA | | Os12g0588100 | | Retrotransposon protein, putative, Ty1-copia subclass |
| maker-12-augustus-gene-249.33-mRNA-1 | Japonica | Japonica | | Os12g0588400 | OsR498G1221996700.01.T01 | |
| maker-12-augustus-gene-249.35-mRNA-1 | Japonica | Japonica | | Os12g0589400 | OsR498G1222005000.01.T01 | |
| maker-12-augustus-gene-249.36-mRNA-1 | Japonica | NA | | Os12g0588800 | OsR498G1221999600.01.T01 | Cyclin N-terminal domain-containing protein |

| | | | | | | |
|---|---|---|---|---|---|---|
| maker-12-augustus-gene-249.40-mRNA-1 | Japonica | NA | | Os12g0588900 | OsR498G1222000200.01.T02 | Cytoplasmic tRNA 2-thiolation protein 2 |
| maker-12-augustus-gene-249.42-mRNA-1 | Japonica | Indica | | Os03g0617800 | OsR498G1222000400.01.T01 | BHLH domain-containing protein (Fragment) |
| maker-12-augustus-gene-249.44-mRNA-1 | Japonica | NA | | Os12g0589100 | OsR498G1222001000.01.T01 | Adenine phosphoribosyltransferase |
| maker-12-augustus-gene-25.0-mRNA-1 | Japonica | Japonica | | Os12g0147000 | OsR498G1220936100.01.T01 | AT-hook motif nuclear-localized protein |
| maker-12-augustus-gene-25.11-mRNA-1 | Japonica | NA | | | OsR498G1221640400.01.T03 | Formin-like protein 14 |
| maker-12-augustus-gene-25.13-mRNA-1 | Japonica | NA | | | OsR498G1220940200.01.T01 | Retrotransposon protein, putative, unclassified, expressed |
| maker-12-augustus-gene-25.3-mRNA-1 | Japonica | NA | | Os12g0147500 | OsR498G1220937300.01.T01 | DEAD domain-containing protein (Fragment) |
| maker-12-augustus-gene-25.6-mRNA-1 | Japonica | NA | | Os12g0147800 | OsR498G1220938900.01.T01 | Phytosulfokine-alpha |
| maker-12-augustus-gene-25.9-mRNA-1 | Japonica | Indica | | Os11g0148600 | OsR498G1220935000.01.T02 | |
| maker-12-augustus-gene-250.0-mRNA-1 | Japonica | NA | | | OsR498G0511259700.01.T01 | Transposon protein, putative, Mutator sub-class, expressed |
| maker-12-augustus-gene-250.12-mRNA-1 | Japonica | Indica | | Os12g0589400 | OsR498G1222005000.01.T01 | |
| maker-12-augustus-gene-250.15-mRNA-1 | Japonica | NA | | | OsR498G1222005600.01.T02 | Kinesin-like protein KIN-12F |
| maker-12-augustus-gene-250.3-mRNA-1 | Japonica | NA | | | OsR498G1222009900.01.T01 | |
| maker-12-augustus-gene-250.8-mRNA-1 | Japonica | NA | | | OsR498G1222010100.01.T01 | B3 domain-containing protein |
| maker-12-augustus-gene-251.28-mRNA-1 | Japonica | NA | | Os12g0592200 | OsR498G1222012100.01.T01 | |
| maker-12-augustus-gene-251.30-mRNA-1 | Japonica | Indica | | Os12g0589100 | OsR498G1222012900.01.T01 | Adenine phosphoribosyltransferase |
| maker-12-augustus-gene-251.35-mRNA-1 | Japonica | NA | | Os12g0592300 | OsR498G1222012400.01.T02 | B3 domain-containing protein |
| maker-12-augustus-gene-251.37-mRNA-1 | Japonica | Indica | | Os12g0593000 | OsR498G1222022200.01.T02 | Guanine nucleotide-binding protein alpha-2 subunit |
| maker-12-augustus-gene-252.0-mRNA-1 | Japonica | NA | | | | SHR-BD domain-containing protein |
| maker-12-augustus-gene-252.13-mRNA-1 | Japonica | NA | | Os12g0594600 | OsR498G1222027900.01.T01 | Skp1 domain-containing protein |
| maker-12-augustus-gene-252.15-mRNA-1 | Japonica | NA | | Os12g0594700 | | F-box domain containing protein, expressed |
| maker-12-augustus-gene-252.19-mRNA-1 | Japonica | NA | | Os12g0595000 | OsR498G1222029400.01.T01 | SPX and EXS domain-containing protein 1 |
| maker-12-augustus-gene-252.9-mRNA-1 | Japonica | NA | Kinase-TM | Os12g0594300 | OsR498G1222027100.01.T01 | Serine/threonine-protein kinase CTR1 |
| maker-12-augustus-gene-253.0-mRNA-1 | Japonica | NA | Kinase-TM | Os12g0595800 | OsR498G1222032500.01.T02 | Wall-associated receptor kinase-like 14 |
| maker-12-augustus-gene-253.10-mRNA-1 | Japonica | NA | | Os12g0596900 | OsR498G1222034300.01.T02 | Putative glycosylphosphatidylinositol-mannosyltransferase I, PIG-X/PBN1 |
| maker-12-augustus-gene-253.13-mRNA-1 | Japonica | NA | | Os12g0597000 | OsR498G1222034900.01.T05 | Calcineurin b-like protein 3-like |
| maker-12-augustus-gene-253.18-mRNA-1 | Japonica | NA | | | OsR498G1222035300.01.T02 | MATH domain-containing protein (Fragment) |
| maker-12-augustus-gene-253.23-mRNA-1 | Japonica | Japonica | | Os12g0595200 | OsR498G1222028700.01.T01 | F-box domain containing protein, expressed |
| maker-12-augustus-gene-253.25-mRNA-1 | Japonica | NA | | Os12g0595300 | OsR498G1222030800.01.T03 | F-box domain containing protein, expressed |
| maker-12-augustus-gene-253.27-mRNA-1 | Japonica | NA | | Os12g0594700 | OsR498G1222028700.01.T01 | F-box domain containing protein, expressed |
| maker-12-augustus-gene-253.3-mRNA-1 | Japonica | NA | | | OsR498G1222033700.01.T03 | BTB domain-containing protein |
| maker-12-augustus-gene-253.31-mRNA-1 | Japonica | Indica | | Os12g0595600 | OsR498G1222031800.01.T01 | Skp1 domain-containing protein |
| maker-12-augustus-gene-253.34-mRNA-1 | Japonica | NA | | Os12g0596000 | OsR498G1222032800.01.T02 | Lipoyl(octanoyl) transferase |
| maker-12-augustus-gene-253.36-mRNA-1 | Japonica | NA | | Os12g0597400 | OsR498G1222036000.01.T01 | Putative bifunctional amine oxidase |
| maker-12-augustus-gene-253.6-mRNA-1 | Japonica | NA | | Os12g0596800 | OsR498G1222034100.01.T01 | LIM and UIM domain-containing |
| maker-12-augustus-gene-254.35-mRNA-1 | Japonica | NA | | | OsR498G1222038100.01.T01 | Transcription factor PIF4 |
| maker-12-augustus-gene-254.37-mRNA-1 | Japonica | NA | | | | BHLH domain-containing protein |
| maker-12-augustus-gene-254.44-mRNA-1 | Japonica | Indica | | Os12g0597500 | OsR498G1222036200.01.T01 | Ureidoglycolate amidohydrolase |
| maker-12-augustus-gene-254.48-mRNA-1 | Japonica | NA | | Os12g0597700 | OsR498G1222037200.01.T02 | WRKY DNA binding domain containing protein, expressed |
| maker-12-augustus-gene-255.48-mRNA-1 | Japonica | NA | | Os12g0599900 | OsR498G1222046700.01.T05 | Serine/threonine-protein phosphatase 6 regulatory ankyrin repeat subunit B |
| maker-12-augustus-gene-256.11-mRNA-1 | Japonica | NA | | | OsR498G1222052100.01.T01 | Basic leucine zipper 25 |
| maker-12-augustus-gene-256.21-mRNA-1 | Japonica | NA | | | | |
| maker-12-augustus-gene-256.9-mRNA-1 | Japonica | NA | | Os12g0601400 | OsR498G1222051500.01.T01 | Auxin-responsive protein IAA31 |
| maker-12-augustus-gene-259.0-mRNA-1 | Japonica | NA | Kinase | Os12g0605900 | OsR498G1222069500.01.T01 | CXC domain-containing TSO1-like protein 1 |
| maker-12-augustus-gene-26.32-mRNA-1 | Japonica | Japonica | | Os12g0148600 | OsR498G0509623900.01.T03 | |
| maker-12-augustus-gene-26.35-mRNA-1 | Japonica | Japonica | Kinase-TM | Os12g0149700 | OsR498G1119421300.01.T01 | Serine/threonine-protein kinase D6PKL2 |
| maker-12-augustus-gene-26.37-mRNA-1 | Japonica | NA | | Os12g0149800 | OsR498G1220944900.01.T03 | G10 protein/predicted nuclear transcription regulator |
| maker-12-augustus-gene-26.39-mRNA-1 | Japonica | Indica | | Os11g0137300 | OsR498G1220940400.01.T02 | Proteasome-associated protein ECM |
| maker-12-augustus-gene-26.44-mRNA-1 | Japonica | NA | | Os12g0148700 | OsR498G1220941200.01.T01 | Putative 2-carboxy-D-arabinitol-1-phosphatase |
| maker-12-augustus-gene-26.47-mRNA-1 | Japonica | NA | | | | RING-H2 finger protein ATL34 |
| maker-12-augustus-gene-26.49-mRNA-1 | Japonica | NA | | Os12g0149900 | OsR498G1220945200.01.T01 | IQ domain-containing protein IQM3 |
| maker-12-augustus-gene-260.58-mRNA-1 | Japonica | NA | | Os12g0608600 | OsR498G1222079400.01.T01 | Sulfurtransferase |
| maker-12-augustus-gene-260.61-mRNA-1 | Japonica | Japonica | Kinase-TM | Os12g0608700 | OsR498G1222080200.01.T01 | L-type lectin-domain containing receptor kinase IX.1 |
| maker-12-augustus-gene-260.63-mRNA-1 | Japonica | Japonica | Kinase-TM | Os12g0609000 | OsR498G1222081400.01.T02 | Wall-associated receptor kinase-like 21 |
| maker-12-augustus-gene-260.64-mRNA-1 | Japonica | NA | | Os12g0609500 | OsR498G1222082600.01.T01 | (-)-isopiperitenone reductase |
| maker-12-augustus-gene-261.0-mRNA-1 | Japonica | NA | | Os12g0609800 | OsR498G1222082900.01.T01 | WD repeat-containing protein 75 |
| maker-12-augustus-gene-261.11-mRNA-1 | Japonica | NA | | Os12g0610800 | OsR498G1222086200.01.T01 | Casparian strip membrane protein 2 |
| maker-12-augustus-gene-261.13-mRNA-1 | Japonica | NA | | Os12g0611000 | OsR498G1222086600.01.T01 | 18S subunit ribosomal protein (Fragment) |
| maker-12-augustus-gene-261.18-mRNA-1 | Japonica | Indica | Kinase-TM | Os12g0611100 | OsR498G1222086800.01.T03 | Serine/threonine-protein kinase receptor |
| maker-12-augustus-gene-261.22-mRNA-1 | Japonica | NA | | | OsR498G1222087200.01.T02 | ATP-dependent RNA helicase DeaD |
| maker-12-augustus-gene-261.27-mRNA-1 | Japonica | Indica | | Os12g0611300 | OsR498G1222088000.01.T01 | Patatin |

| | | | | | | |
|---|---|---|---|---|---|---|
| maker-12-augustus-gene-261.9-mRNA-1 | Japonica | NA | | Os12g0610500 | OsR498G1222084900.01.T05 | methyltransferase-like protein 25 isoform X2 |
| maker-12-augustus-gene-262.27-mRNA-1 | Japonica | NA | | | OsR498G1222092700.01.T01 | Gamma-thionins family protein |
| maker-12-augustus-gene-262.29-mRNA-1 | Japonica | NA | | Os12g0612100 | OsR498G1222093000.01.T01 | GDP-L-galactose phosphorylase 1 |
| maker-12-augustus-gene-262.32-mRNA-1 | Japonica | NA | | Os12g0612300 | OsR498G1222093200.01.T02 | WPP domain-associated protein-like |
| maker-12-augustus-gene-262.35-mRNA-1 | Japonica | NA | | Os12g0612400 | OsR498G1222093800.01.T02 | Chaperone DnaJ-domain superfamily protein isoform 1 |
| maker-12-augustus-gene-262.39-mRNA-1 | Japonica | NA | | Os12g0612500 | OsR498G1222094400.01.T06 | MTS domain-containing protein |
| maker-12-augustus-gene-262.42-mRNA-1 | Japonica | NA | | Os12g0612700 | OsR498G1222095000.01.T01 | Homeobox-leucine zipper protein ATHB-14 |
| maker-12-augustus-gene-263.0-mRNA-1 | Japonica | NA | | Os12g0613500 | OsR498G1222097900.01.T03 | LisH dimerization motif |
| maker-12-augustus-gene-263.10-mRNA-1 | Japonica | NA | | Os12g0614000 | OsR498G1222099300.01.T05 | Mediator of RNA polymerase II transcription subunit 19 |
| maker-12-augustus-gene-263.13-mRNA-1 | Japonica | NA | | Os12g0613000 | OsR498G1222095800.01.T01 | CCAAT-binding transcription factor B |
| maker-12-augustus-gene-263.17-mRNA-1 | Japonica | Indica | | Os03g0641200 | OsR498G1222096200.01.T01 | High affinity cationic amino acid transporter 1 |
| maker-12-augustus-gene-263.19-mRNA-1 | Japonica | NA | | | | Putative SET-domain containing protein family |
| maker-12-augustus-gene-263.22-mRNA-1 | Japonica | Indica | | Os12g0613250 | OsR498G1222097400.01.T01 | BTB/POZ domain-containing protein At1g30440 isoform X1 |
| maker-12-augustus-gene-263.25-mRNA-1 | Japonica | NA | | Os12g0613300 | OsR498G1222097800.01.T03 | MYB-type transcription factor |
| maker-12-augustus-gene-263.28-mRNA-1 | Japonica | NA | | Os12g0613700 | OsR498G1222099000.01.T02 | Auxin response factor |
| maker-12-augustus-gene-263.5-mRNA-1 | Japonica | NA | | Os12g0613600 | OsR498G1222098700.01.T01 | Remorin, C-terminal region family protein, expressed |
| maker-12-augustus-gene-263.7-mRNA-1 | Japonica | NA | | | OsR498G1222099100.01.T01 | Galectin domain-containing protein (Fragment) |
| maker-12-augustus-gene-264.0-mRNA-1 | Japonica | Japonica | Kinase-TM | Os12g0615000 | OsR498G1222103900.01.T01 | Wall-associated receptor kinase 3 |
| maker-12-augustus-gene-264.11-mRNA-1 | Japonica | NA | | Os12g0615400 | OsR498G1222105000.01.T02 | 2-methyl-6-phytyl-1,4-hydroquinone methyltransferase, chloroplastic |
| maker-12-augustus-gene-264.14-mRNA-1 | Japonica | NA | | | OsR498G1222105200.01.T02 | Metallo-hydrolase/oxidoreductase superfamily protein |
| maker-12-augustus-gene-264.17-mRNA-1 | Japonica | NA | | Os12g0615500 | OsR498G1222105600.01.T01 | Metallo-hydrolase/oxidoreductase superfamily protein |
| maker-12-augustus-gene-264.24-mRNA-1 | Japonica | NA | | | | Kinesin-4 |
| maker-12-augustus-gene-264.29-mRNA-1 | Japonica | NA | | Os12g0616200 | OsR498G1222107000.01.T01 | 50S ribosomal protein HLP, mitochondrial |
| maker-12-augustus-gene-264.3-mRNA-1 | Japonica | NA | Kinase-TM | | | Wall-associated receptor kinase 5 |
| maker-12-augustus-gene-264.5-mRNA-1 | Japonica | NA | | Os12g0615700 | | Protein DETOXIFICATION |
| maker-12-augustus-gene-264.9-mRNA-1 | Japonica | NA | | | OsR498G1222103800.01.T07 | |
| maker-12-augustus-gene-265.21-mRNA-1 | Japonica | NA | | Os12g0616500 | OsR498G1222108900.01.T01 | Cation/H(+) antiporter like |
| maker-12-augustus-gene-265.24-mRNA-1 | Japonica | NA | | | OsR498G1222109300.01.T01 | Serine protease |
| maker-12-augustus-gene-265.30-mRNA-1 | Japonica | NA | | Os12g0616900 | OsR498G1222111900.01.T01 | Pyruvate dehydrogenase (acetyl-transferring) |
| maker-12-augustus-gene-265.35-mRNA-1 | Japonica | NA | | Os12g0617100 | OsR498G1222112800.01.T05 | Translation initiation factor eIF-2B subunit delta |
| maker-12-augustus-gene-268.0-mRNA-1 | Japonica | NA | | Os12g0620900 | | |
| maker-12-augustus-gene-268.14-mRNA-1 | Japonica | NA | Kinase-TM | Os12g0621500 | OsR498G1222131900.01.T03 | Probable serine/threonine protein kinase IREH1 isoform X1 |
| maker-12-augustus-gene-268.17-mRNA-1 | Japonica | NA | | Os12g0621700 | OsR498G1222132700.01.T01 | Protein downstream neighbor of Son |
| maker-12-augustus-gene-268.19-mRNA-1 | Japonica | NA | | | OsR498G1222128200.01.T01 | Cyclin-dependent kinase C-2 |
| maker-12-augustus-gene-268.21-mRNA-1 | Japonica | NA | | Os12g0621600 | OsR498G1222132200.01.T01 | |
| maker-12-augustus-gene-268.6-mRNA-1 | Japonica | NA | | | | |
| maker-12-augustus-gene-268.7-mRNA-1 | Japonica | NA | | Os12g0621100 | OsR498G1222129900.01.T01 | Putative axial regulator YABBY 2 |
| maker-12-augustus-gene-269.0-mRNA-1 | Japonica | NA | | | | COP9 signalosome complex subunit 6 |
| maker-12-augustus-gene-269.10-mRNA-1 | Japonica | NA | | | OsR498G1222136500.01.T01 | |
| maker-12-augustus-gene-269.15-mRNA-1 | Japonica | NA | | | OsR498G1222136900.01.T01 | |
| maker-12-augustus-gene-269.18-mRNA-1 | Japonica | NA | | Os12g0623500 | OsR498G1222137200.01.T02 | Amino acid permease family protein, expressed |
| maker-12-augustus-gene-269.25-mRNA-1 | Japonica | NA | | Os12g0623600 | OsR498G1222137600.01.T01 | Cysteine dioxygenase |
| maker-12-augustus-gene-269.30-mRNA-1 | Japonica | NA | | | | Dihydrofolate synthetase |
| maker-12-augustus-gene-269.5-mRNA-1 | Japonica | NA | | Os12g0622900 | OsR498G1222136100.01.T01 | AP2/ERF domain-containing protein |
| maker-12-augustus-gene-269.9-mRNA-1 | Japonica | NA | | | OsR498G1222136500.01.T01 | PHD domain-containing protein |
| maker-12-augustus-gene-27.37-mRNA-1 | Japonica | NA | | Os12g0151000 | OsR498G1220949100.01.T01 | Purple acid phosphatase |
| maker-12-augustus-gene-27.39-mRNA-1 | Japonica | NA | | Os12g0151900 | OsR498G1220950900.01.T03 | |
| maker-12-augustus-gene-27.41-mRNA-1 | Japonica | NA | | Os12g0152100 | OsR498G1220951300.01.T01 | Putative dihydrolipoyllysine-residue acetyltransferase |
| maker-12-augustus-gene-27.43-mRNA-1 | Japonica | Indica | | Os11g0151700 | OsR498G1220948000.01.T01 | Purple acid phosphatase |
| maker-12-augustus-gene-27.45-mRNA-1 | Japonica | NA | | Os12g0152000 | OsR498G1220951200.01.T01 | Centroradialis |
| maker-12-augustus-gene-270.31-mRNA-1 | Japonica | NA | | Os12g0623900 | OsR498G1222138500.01.T02 | 5-methyltetrahydropteroyltriglutamate--homocysteine S-methyltransferase |
| maker-12-augustus-gene-270.36-mRNA-1 | Japonica | NA | | Os12g0624000 | OsR498G1222139100.01.T03 | 5-methyltetrahydropteroyltriglutamate--homocysteine S-methyltransferase |
| maker-12-augustus-gene-270.42-mRNA-1 | Japonica | NA | | Os12g0624900 | OsR498G1222141300.01.T01 | GATA transcription factor |
| maker-12-augustus-gene-270.45-mRNA-1 | Japonica | NA | | | OsR498G1222140600.01.T01 | BRCT domain-containing protein |
| maker-12-augustus-gene-270.47-mRNA-1 | Japonica | Japonica | | Os12g0624800 | OsR498G1222141000.01.T02 | Copper transport protein ATX1 |
| maker-12-augustus-gene-270.49-mRNA-1 | Japonica | Indica | | Os12g0625000 | OsR498G1222141600.01.T01 | Cytosolic cysteine synthase |
| maker-12-augustus-gene-271.32-mRNA-1 | Japonica | NA | | | OsR498G1222147000.01.T01 | |
| maker-12-augustus-gene-271.34-mRNA-1 | Japonica | Japonica | | Os12g0626000 | OsR498G1222146600.01.T01 | |
| maker-12-augustus-gene-271.36-mRNA-1 | Japonica | NA | | Os12g0626100 | OsR498G1222146100.01.T01 | Deoxyhypusine hydroxylase |
| maker-12-augustus-gene-271.40-mRNA-1 | Japonica | NA | | | OsR498G1222145300.01.T02 | Anaphase-promoting complex subunit 5 |
| maker-12-augustus-gene-271.46-mRNA-1 | Japonica | Indica | | Os12g0626400 | OsR498G1222144900.01.T01 | Phytoene synthase, chloroplastic |

| | | | | | | |
|---|---|---|---|---|---|---|
| maker-12-augustus-gene-272.0-mRNA-1 | Japonica | NA | | | OsR498G1222148300.01.T01 | Putative endoplasmic reticulum insertion protein SEC61 |
| maker-12-augustus-gene-272.10-mRNA-1 | Japonica | NA | | Os12g0628100 | OsR498G1222149200.01.T02 | Actin-depolymerizing factor 11 |
| maker-12-augustus-gene-272.2-mRNA-1 | Japonica | NA | | Os12g0628400 | OsR498G1222149900.01.T04 | Aldehyde oxygenase (deformylating) |
| maker-12-augustus-gene-272.5-mRNA-1 | Japonica | NA | | Os12g0628500 | OsR498G1222150100.01.T01 | Methionine aminopeptidase 2 |
| maker-12-augustus-gene-273.42-mRNA-1 | Japonica | NA | | | OsR498G1222159100.01.T02 | PMD domain-containing protein |
| maker-12-augustus-gene-29.0-mRNA-1 | Japonica | NA | | Os04g0530400 | OsR498G1220960500.01.T01 | SNARE-complex protein Syntaxin-18 N-terminal |
| maker-12-augustus-gene-3.103-mRNA-1 | Japonica | NA | | Os11g0106400 | OsR498G1119292600.01.T02 | E1 ubiquitin-activating enzyme |
| maker-12-augustus-gene-3.107-mRNA-1 | Japonica | NA | | Os12g0106000 | OsR498G1220799800.01.T01 | Ferritin |
| maker-12-augustus-gene-3.35-mRNA-1 | Japonica | NA | | Os12g0104700 | OsR498G1220795300.01.T01 | Protein trichome birefringence-like 28 |
| maker-12-augustus-gene-3.40-mRNA-1 | Japonica | NA | | | OsR498G1119287500.01.T03 | Clathrin heavy chain |
| maker-12-augustus-gene-3.43-mRNA-1 | Japonica | NA | | | OsR498G1220795500.01.T05 | Clathrin heavy chain |
| maker-12-augustus-gene-3.60-mRNA-1 | Japonica | Indica | | Os11g0205500 | OsR498G1220796300.01.T01 | |
| maker-12-augustus-gene-3.62-mRNA-1 | Japonica | Japonica | | Os12g0106100 | OsR498G1220799900.01.T01 | Pentatricopeptide repeat-containing protein At5g12100, mitochondrial (Frag |
| maker-12-augustus-gene-3.69-mRNA-1 | Japonica | NA | | Os12g0106300 | OsR498G1220800900.01.T01 | Protein trichome birefringence-like 34 |
| maker-12-augustus-gene-3.74-mRNA-1 | Japonica | NA | | Os11g0105400 | OsR498G1220797200.01.T01 | Ribosome assembly factor mrt4 |
| maker-12-augustus-gene-3.78-mRNA-1 | Japonica | NA | | Os11g0105750 | OsR498G1220797600.01.T03 | Protein CHUP1 chloroplastic |
| maker-12-augustus-gene-3.85-mRNA-1 | Japonica | NA | | Os12g0105500 | OsR498G1220798000.01.T01 | Phytol kinase 2 |
| maker-12-augustus-gene-3.90-mRNA-1 | Japonica | NA | | Os12g0105600 | OsR498G1220798200.01.T01 | HTH myb-type domain-containing protein (Fragment) |
| maker-12-augustus-gene-3.93-mRNA-1 | Japonica | NA | | Os12g0105700 | OsR498G1119292200.01.T01 | Sorting and assembly machinery component 50 A |
| maker-12-augustus-gene-3.97-mRNA-1 | Japonica | NA | Kinase-TM | | OsR498G1220798800.01.T02 | Putative LRR receptor-like serine/threonine-protein kinase MRH1-like |
| maker-12-augustus-gene-31.0-mRNA-1 | Japonica | Indica | | Os12g0158600 | OsR498G1220981300.01.T01 | |
| maker-12-augustus-gene-32.40-mRNA-1 | Japonica | NA | | Os12g0160400 | OsR498G1220988300.01.T02 | Transcription factor LHW |
| maker-12-augustus-gene-32.45-mRNA-1 | Japonica | NA | | Os12g0160900 | | |
| maker-12-augustus-gene-32.53-mRNA-1 | Japonica | Indica | | Os12g0160500 | OsR498G1220988600.01.T02 | BEL1-like homeodomain protein 1 isoform A |
| maker-12-augustus-gene-33.0-mRNA-1 | Japonica | NA | | Os12g0162500 | OsR498G1221000100.01.T01 | Transcription factor VIP1 |
| maker-12-augustus-gene-33.16-mRNA-1 | Japonica | NA | | Os12g0162400 | OsR498G1220999600.01.T01 | Calcineurin B |
| maker-12-augustus-gene-33.3-mRNA-1 | Japonica | Indica | | Os12g0161500 | OsR498G1220994200.01.T01 | |
| maker-12-augustus-gene-34.12-mRNA-1 | Japonica | NA | | Os12g0163400 | OsR498G1221003000.01.T01 | Tubby-like F-box protein 13 |
| maker-12-augustus-gene-34.15-mRNA-1 | Japonica | NA | | Os12g0163700 | OsR498G1221004400.01.T01 | Actin beta/gamma 1 |
| maker-12-augustus-gene-34.19-mRNA-1 | Japonica | NA | Kinase-TM | | OsR498G1221004600.01.T03 | Serine/threonine-protein kinase STY46 |
| maker-12-augustus-gene-34.2-mRNA-1 | Japonica | NA | | Os12g0163200 | OsR498G1221002500.01.T01 | Nucleolar complex protein 2 |
| maker-12-augustus-gene-34.5-mRNA-1 | Japonica | NA | | | OsR498G1221001800.01.T01 | HTH OST-type domain-containing protein (Fragment) |
| maker-12-augustus-gene-34.7-mRNA-1 | Japonica | NA | | Os12g0163000 | OsR498G1221002000.01.T01 | Cyclic nucleotide-gated ion channel like |
| maker-12-augustus-gene-34.9-mRNA-1 | Japonica | NA | | Os12g0163300 | OsR498G1221002800.01.T02 | Deoxyhypusine hydroxylase-A |
| maker-12-augustus-gene-35.0-mRNA-1 | Japonica | Indica | | Os12g0164300 | OsR498G1221005700.01.T02 | F-box protein interaction domain containing protein, expressed |
| maker-12-augustus-gene-35.11-mRNA-1 | Japonica | NA | | | OsR498G1221010700.01.T04 | nucleolar protein 6 |
| maker-12-augustus-gene-35.16-mRNA-1 | Japonica | NA | | | OsR498G1221007200.01.T05 | DUF676 domain-containing protein |
| maker-12-augustus-gene-35.2-mRNA-1 | Japonica | NA | | Os12g0165400 | | Citrate-binding protein-like |
| maker-12-augustus-gene-35.23-mRNA-1 | Japonica | NA | | Os12g0165000 | OsR498G1221007600.01.T01 | WD repeat-containing protein 48 |
| maker-12-augustus-gene-35.30-mRNA-1 | Japonica | NA | | Os12g0165200 | OsR498G1221007800.01.T01 | SHSP domain-containing protein (Fragment) |
| maker-12-augustus-gene-35.32-mRNA-1 | Japonica | NA | | Os12g0165700 | OsR498G1221008600.01.T01 | Mediator of RNA polymerase II transcription subunit 26c like |
| maker-12-augustus-gene-35.4-mRNA-1 | Japonica | NA | | | OsR498G1221009500.01.T01 | Peptidase S59 domain-containing protein (Fragment) |
| maker-12-augustus-gene-35.8-mRNA-1 | Japonica | NA | | | OsR498G1221009900.01.T01 | Peptidase S59 domain-containing protein |
| maker-12-augustus-gene-36.0-mRNA-1 | Japonica | NA | NBS-TM | | OsR498G1221011500.01.T01 | Disease resistance protein RPM1 |
| maker-12-augustus-gene-36.13-mRNA-1 | Japonica | NA | | | OsR498G1221013000.01.T01 | SAP domain containing protein, expressed |
| maker-12-augustus-gene-36.19-mRNA-1 | Japonica | Japonica | | Os12g0168100 | OsR498G1221014600.01.T01 | Cold-responsive element-binding factor 3 |
| maker-12-augustus-gene-36.2-mRNA-1 | Japonica | NA | | Os12g0167900 | OsR498G1221014100.01.T01 | Ribosomal protein L3 |
| maker-12-augustus-gene-36.21-mRNA-1 | Japonica | NA | | | | Protein-serine/threonine phosphatase |
| maker-12-augustus-gene-36.6-mRNA-1 | Japonica | NA | | Os12g0168000 | OsR498G1221014300.01.T03 | Methenyltetrahydrofolate synthase domain-containing protein (Fragment) |
| maker-12-augustus-gene-36.9-mRNA-1 | Japonica | NA | | Os12g0168400 | OsR498G1221016100.01.T01 | Gametophytic factor 2 |
| maker-12-augustus-gene-37.0-mRNA-1 | Japonica | NA | | Os12g0168700 | OsR498G1221017900.01.T04 | Long chain acyl-CoA synthetase 9 chloroplastic |
| maker-12-augustus-gene-37.11-mRNA-1 | Japonica | Indica | | Os11g0170000 | OsR498G1221019600.01.T03 | Amidase domain-containing protein |
| maker-12-augustus-gene-37.18-mRNA-1 | Japonica | NA | | Os12g0169100 | OsR498G1221020200.01.T02 | Filament-like plant protein 3 |
| maker-12-augustus-gene-37.23-mRNA-1 | Japonica | NA | | Os12g0169400 | OsR498G1221020400.01.T01 | CBS-PB1 domain contining protein |
| maker-12-augustus-gene-37.27-mRNA-1 | Japonica | NA | | Os12g0169800 | OsR498G1221021400.01.T02 | Calcium-dependent protein kinase SK5 |
| maker-12-augustus-gene-37.3-mRNA-1 | Japonica | NA | | Os12g0169700 | OsR498G1221021100.01.T01 | Gamma carbonic anhydrase 2 mitochondrial |
| maker-12-augustus-gene-37.34-mRNA-1 | Japonica | NA | | Os12g0170100 | OsR498G1221022200.01.T02 | Pentatricopeptide repeat-containing protein mitochondrial |
| maker-12-augustus-gene-37.36-mRNA-1 | Japonica | NA | | Os12g0170200 | OsR498G1221022400.01.T01 | Bile acid sodium symporter/ transporter |
| maker-12-augustus-gene-37.8-mRNA-1 | Japonica | NA | | Os12g0168900 | OsR498G1221019400.01.T01 | V-type proton ATPase proteolipid subunit |
| maker-12-augustus-gene-38.30-mRNA-1 | Japonica | NA | | Os12g0170600 | OsR498G1221022900.01.T02 | 4-alpha-L-fucosyltransferase |
| maker-12-augustus-gene-38.33-mRNA-1 | Japonica | NA | | | OsR498G1221023300.01.T03 | RNA cytidine acetyltransferase |

| | | | | | | |
|---|---|---|---|---|---|---|
| maker-12-augustus-gene-38.40-mRNA-1 | Japonica | NA | | Os12g0170800 | OsR498G1221023500.01.T01 | Citrate binding protein, putative, expressed |
| maker-12-augustus-gene-38.42-mRNA-1 | Japonica | NA | | Os12g0171200 | OsR498G1221024300.01.T02 | Polynucleotide adenylyltransferase family protein |
| maker-12-augustus-gene-38.48-mRNA-1 | Japonica | Japonica | | Os12g0171600 | OsR498G1221024900.01.T02 | |
| maker-12-augustus-gene-38.50-mRNA-1 | Japonica | NA | | Os12g0172400 | | DUF1618 domain-containing protein (Fragment) |
| maker-12-augustus-gene-38.52-mRNA-1 | Japonica | NA | | | OsR498G1221024800.01.T03 | |
| maker-12-augustus-gene-39.0-mRNA-1 | Japonica | NA | | Os01g0177400 | OsR498G0917551500.01.T01 | Gibberellin 3-beta-dioxygenase 2-2 |
| maker-12-augustus-gene-39.1-mRNA-1 | Japonica | NA | | Os12g0174000 | OsR498G1221030100.01.T02 | XS domain-containing protein (Fragment) |
| maker-12-augustus-gene-39.3-mRNA-1 | Japonica | NA | | Os12g0174100 | OsR498G1221030300.01.T01 | O-fucosyltransferase family protein (Fragment) |
| maker-12-augustus-gene-39.8-mRNA-1 | Japonica | NA | | Os12g0172500 | OsR498G1221026000.01.T02 | Dystrophia myotonica WD repeat-containing protein-like |
| maker-12-augustus-gene-39.9-mRNA-1 | Japonica | NA | | Os12g0172500 | OsR498G1221026000.01.T05 | Dystrophia myotonica WD repeat-containing protein-like |
| maker-12-augustus-gene-4.46-mRNA-1 | Japonica | NA | | Os12g0106500 | OsR498G1220801500.01.T01 | Nodulin-like domain-containing protein (Fragment) |
| maker-12-augustus-gene-4.50-mRNA-1 | Japonica | NA | | | | Protein DETOXIFICATION |
| maker-12-augustus-gene-4.55-mRNA-1 | Japonica | NA | | Os11g0107500 | OsR498G1119296500.01.T02 | Macrophage migration inhibitory factor |
| maker-12-augustus-gene-4.60-mRNA-1 | Japonica | Indica | Kinase-LRR-TM | Os11g0107700 | OsR498G1220804500.01.T01 | probable leucine-rich repeat receptor-like protein kinase IMK3 |
| maker-12-augustus-gene-4.63-mRNA-1 | Japonica | NA | | Os12g0108000 | OsR498G1220805900.01.T01 | Laccase |
| maker-12-augustus-gene-4.68-mRNA-1 | Japonica | Japonica | | Os12g0106700 | OsR498G1220802400.01.T03 | |
| maker-12-augustus-gene-4.71-mRNA-1 | Japonica | Indica | | Os12g0106900 | OsR498G1220802600.01.T03 | Kinesin-like protein KIN-UA |
| maker-12-augustus-gene-4.76-mRNA-1 | Japonica | NA | | | | E1 ubiquitin-activating enzyme |
| maker-12-augustus-gene-40.0-mRNA-1 | Japonica | NA | | Os12g0175400 | OsR498G1221036900.01.T01 | MYB transcription factor |
| maker-12-augustus-gene-40.16-mRNA-1 | Japonica | NA | | | OsR498G1221034400.01.T01 | Myb domain protein 98 |
| maker-12-augustus-gene-40.18-mRNA-1 | Japonica | NA | | Os12g0175500 | OsR498G1221038000.01.T01 | Bifunctional monothiol glutaredoxin-S16 chloroplastic |
| maker-12-augustus-gene-40.20-mRNA-1 | Japonica | NA | | Os12g0175900 | OsR498G1221038600.01.T03 | Ribonuclease P |
| maker-12-augustus-gene-40.3-mRNA-1 | Japonica | NA | | Os12g0175700 | OsR498G1221038300.01.T03 | Transmembrane 9 superfamily member |
| maker-12-augustus-gene-40.7-mRNA-1 | Japonica | NA | | Os12g0176200 | OsR498G1221038900.01.T01 | NifU-like protein 2 chloroplastic |
| maker-12-augustus-gene-40.9-mRNA-1 | Japonica | NA | | Os12g0174800 | OsR498G1221033800.01.T02 | Protein-tyrosine-phosphatase PTP1 |
| maker-12-augustus-gene-41.34-mRNA-1 | Japonica | NA | | Os12g0176500 | OsR498G1221039500.01.T01 | Replication factor C / DNA polymerase III gamma-tau subunit |
| maker-12-augustus-gene-41.43-mRNA-1 | Japonica | NA | | Os12g0176600 | OsR498G1221039900.01.T04 | SWI/SNF complex subunit SWI3C |
| maker-12-augustus-gene-41.44-mRNA-1 | Japonica | NA | | Os12g0177500 | | Aspartic proteinase Asp1 |
| maker-12-augustus-gene-41.52-mRNA-1 | Japonica | NA | | Os12g0176800 | OsR498G1221040200.01.T01 | Protein-synthesizing GTPase |
| maker-12-augustus-gene-41.56-mRNA-1 | Japonica | NA | | Os12g0178100 | OsR498G1221041600.01.T01 | L-ascorbate peroxidase |
| maker-12-augustus-gene-41.61-mRNA-1 | Japonica | NA | | Os12g0178200 | OsR498G1221042000.01.T01 | L-ascorbate peroxidase |
| maker-12-augustus-gene-42.0-mRNA-1 | Japonica | Indica | | Os09g0254600 | OsR498G1221042700.01.T01 | Bidirectional sugar transporter SWEET |
| maker-12-augustus-gene-42.14-mRNA-1 | Japonica | NA | | Os12g0179800 | OsR498G1221045200.01.T03 | NAD-dependent protein deacylase |
| maker-12-augustus-gene-42.18-mRNA-1 | Japonica | NA | Kinase-TM | | OsR498G1221046200.01.T01 | Protein kinase protein with adenine nucleotide alpha hydrolase-like domain |
| maker-12-augustus-gene-42.2-mRNA-1 | Japonica | Indica | | Os12g0180400 | OsR498G1221045900.01.T01 | DNA-directed RNA polymerase, subunit N/Rpb |
| maker-12-augustus-gene-42.4-mRNA-1 | Japonica | NA | | Os12g0180600 | | |
| maker-12-augustus-gene-42.6-mRNA-1 | Japonica | Indica | | Os12g0178633 | OsR498G1221044000.01.T01 | WD repeat-containing protein 44 |
| maker-12-augustus-gene-42.9-mRNA-1 | Japonica | NA | | Os12g0178700 | OsR498G1221044200.01.T02 | Dynamin-like protein ARC5 |
| maker-12-augustus-gene-43.10-mRNA-1 | Japonica | NA | | | | Protein kinase domain-containing protein |
| maker-12-augustus-gene-43.14-mRNA-1 | Japonica | NA | | Os12g0181300 | OsR498G1221046400.01.T01 | TRAF-type domain-containing protein |
| maker-12-augustus-gene-43.17-mRNA-1 | Japonica | NA | | Os12g0181600 | OsR498G1221047500.01.T01 | Amino acid permease |
| maker-12-augustus-gene-43.2-mRNA-1 | Japonica | NA | | Os12g0181500 | OsR498G1221047500.01.T01 | Amino acid permease |
| maker-12-augustus-gene-43.4-mRNA-1 | Japonica | Japonica | | Os12g0180800 | OsR498G0408793100.01.T02 | BHLH domain-containing protein |
| maker-12-augustus-gene-43.8-mRNA-1 | Japonica | NA | Kinase-TM | | OsR498G1221046200.01.T01 | Protein kinase protein with adenine nucleotide alpha hydrolase-like domain |
| maker-12-augustus-gene-44.0-mRNA-1 | Japonica | NA | | Os12g0182700 | OsR498G1221051500.01.T01 | Histone deacetylase |
| maker-12-augustus-gene-44.10-mRNA-1 | Japonica | NA | | Os12g0183100 | OsR498G1221052100.01.T03 | 2-oxoisovalerate dehydrogenase subunit alpha 2 mitochondrial |
| maker-12-augustus-gene-44.16-mRNA-1 | Japonica | NA | | Os12g0182200 | OsR498G1221050000.01.T01 | Dihydrolipoamide acetyltransferase component of pyruvate dehydrogenase |
| maker-12-augustus-gene-44.21-mRNA-1 | Japonica | NA | Kinase-LRR-TM | Os12g0182300 | OsR498G1221050600.01.T01 | Tyrosine-protein kinase |
| maker-12-augustus-gene-44.24-mRNA-1 | Japonica | NA | | | | cx9C motif-containing protein 4 |
| maker-12-augustus-gene-44.26-mRNA-1 | Japonica | NA | | Os12g0183200 | OsR498G1221052400.01.T03 | 3'(2'),5'-bisphosphate nucleotidase |
| maker-12-augustus-gene-44.30-mRNA-1 | Japonica | NA | | Os12g0183300 | OsR498G1221052800.01.T01 | 3'(2'),5'-bisphosphate nucleotidase |
| maker-12-augustus-gene-44.34-mRNA-1 | Japonica | NA | | | OsR498G1221055000.01.T01 | |
| maker-12-augustus-gene-44.5-mRNA-1 | Japonica | NA | | | OsR498G1221051700.01.T03 | Proteasome assembly chaperone |
| maker-12-augustus-gene-46.35-mRNA-1 | Japonica | NA | | Os12g0188500 | OsR498G1221057100.01.T01 | BTB/POZ domain-containing protein At2g13690 |
| maker-12-augustus-gene-46.38-mRNA-1 | Japonica | NA | | Os12g0187800 | OsR498G1221056000.01.T01 | |
| maker-12-augustus-gene-46.40-mRNA-1 | Japonica | NA | | Os12g0188700 | OsR498G1221058200.01.T02 | Chloroplast thioredoxin M-type |
| maker-12-augustus-gene-47.0-mRNA-1 | Japonica | NA | | | OsR498G1221058300.01.T01 | Small RNA degrading nuclease 5 |
| maker-12-augustus-gene-47.12-mRNA-1 | Japonica | NA | | Os12g0189900 | OsR498G1221061300.01.T01 | Ribosomal biogenesis protein LAS1L |
| maker-12-augustus-gene-47.17-mRNA-1 | Japonica | NA | | Os12g0190100 | OsR498G1221061900.01.T03 | O-fucosyltransferase family protein (Fragment) |
| maker-12-augustus-gene-47.20-mRNA-1 | Japonica | NA | | Os12g0189500 | OsR498G1221059800.01.T01 | Flavin-containing monooxygenase |
| maker-12-augustus-gene-47.23-mRNA-1 | Japonica | NA | | Os12g0190000 | OsR498G1221061600.01.T01 | GDP-L-galactose phosphorylase |

| | | | | | | |
|---|---|---|---|---|---|---|
| maker-12-augustus-gene-47.26-mRNA-1 | Japonica | NA | | Os12g0190200 | OsR498G1221062200.01.T01 | PsbP domain-containing protein 4, chloroplastic |
| maker-12-augustus-gene-47.9-mRNA-1 | Japonica | Japonica | | Os12g0189400 | OsR498G1221059300.01.T01 | Photosystem I reaction center subunit N like |
| maker-12-augustus-gene-48.0-mRNA-1 | Japonica | Indica | | Os01g0729600 | OsR498G1221064700.01.T01 | Putative cysteine desulfurase |
| maker-12-augustus-gene-48.2-mRNA-1 | Japonica | Indica | | Os01g0729600 | OsR498G1221062400.01.T01 | Putative cysteine desulfurase |
| maker-12-augustus-gene-48.4-mRNA-1 | Japonica | Indica | | Os05g0281400 | OsR498G1221064200.01.T06 | |
| maker-12-augustus-gene-48.6-mRNA-1 | Japonica | NA | | Os12g0191500 | OsR498G1221065400.01.T01 | Peroxidase |
| maker-12-augustus-gene-49.0-mRNA-1 | Japonica | NA | | Os12g0192500 | OsR498G1221069500.01.T01 | Thiamine-phosphate pyrophosphorylase |
| maker-12-augustus-gene-49.6-mRNA-1 | Japonica | NA | | | OsR498G1221071100.01.T01 | BRX domain-containing protein |
| maker-12-augustus-gene-49.8-mRNA-1 | Japonica | NA | | | OsR498G1221071400.01.T02 | |
| maker-12-augustus-gene-5.0-mRNA-1 | Japonica | NA | | Os12g0108500 | OsR498G1220808300.01.T01 | F-box/LRR-repeat protein 3 |
| maker-12-augustus-gene-5.11-mRNA-1 | Japonica | NA | | Os12g0108900 | | DNA (Cytosine-5)-methyltransferase DRM2 |
| maker-12-augustus-gene-5.16-mRNA-1 | Japonica | Japonica | Kinase | Os12g0109800 | OsR498G1119298800.01.T04 | Putative serine/threonine-protein kinase |
| maker-12-augustus-gene-5.18-mRNA-1 | Japonica | NA | Kinase-TM | Os12g0108100 | OsR498G1220806200.01.T01 | Putative serine/threonine-protein kinase |
| maker-12-augustus-gene-5.22-mRNA-1 | Japonica | NA | | | OsR498G1220810000.01.T01 | ADP-glucose pyrophosphorylase |
| maker-12-augustus-gene-5.7-mRNA-1 | Japonica | Japonica | | Os12g0108600 | OsR498G1220808500.01.T01 | Catalytic protein serine threonine phosphatase |
| maker-12-augustus-gene-50.40-mRNA-1 | Japonica | Japonica | | Os12g0193800 | OsR498G1221072900.01.T02 | NAC domain-containing protein |
| maker-12-augustus-gene-50.46-mRNA-1 | Japonica | Japonica | | Os12g0194600 | OsR498G1221077900.01.T03 | BZIP type transcription factor |
| maker-12-augustus-gene-50.48-mRNA-1 | Japonica | NA | | | | GTP--RNA guanylyltransferase |
| maker-12-augustus-gene-50.54-mRNA-1 | Japonica | NA | | | | |
| maker-12-augustus-gene-50.58-mRNA-1 | Japonica | NA | | Os12g0194700 | OsR498G1221078200.01.T01 | DNA-directed RNA polymerases I and III subunit like |
| maker-12-augustus-gene-51.37-mRNA-1 | Japonica | NA | | Os12g0194900 | OsR498G1221079100.01.T01 | Amino acid transporter |
| maker-12-augustus-gene-52.0-mRNA-1 | Japonica | NA | | Os12g0197400 | OsR498G1221087100.01.T02 | Chloroplast protein import component |
| maker-12-augustus-gene-52.2-mRNA-1 | Japonica | Japonica | | Os12g0196700 | OsR498G1221085600.01.T01 | Cytochrome P450 family protein, expressed |
| maker-12-augustus-gene-52.4-mRNA-1 | Japonica | NA | | Os12g0197100 | OsR498G1221086600.01.T01 | Glycinamide ribonucleotide synthetase |
| maker-12-augustus-gene-52.6-mRNA-1 | Japonica | NA | | Os12g0197500 | OsR498G1221087400.01.T01 | Suppressor of gene silencing 3 |
| maker-12-augustus-gene-52.8-mRNA-1 | Japonica | Japonica | | Os12g0197700 | OsR498G1221087800.01.T01 | Suppressor of gene silencing 3 |
| maker-12-augustus-gene-53.34-mRNA-1 | Japonica | Indica | | Os11g0240900 | OsR498G1221087900.01.T02 | CCR4-NOT transcription complex subunit 11 |
| maker-12-augustus-gene-53.41-mRNA-1 | Japonica | Indica | CC-NBS-TM | Os09g0517200 | OsR498G1221092300.01.T02 | Disease resistance protein RPM1 |
| maker-12-augustus-gene-53.42-mRNA-1 | Japonica | NA | | Os12g0198200 | OsR498G1221088800.01.T01 | PPM-type phosphatase domain-containing protein |
| maker-12-augustus-gene-53.44-mRNA-1 | Japonica | NA | | Os12g0198400 | | |
| maker-12-augustus-gene-53.45-mRNA-1 | Japonica | NA | | | OsR498G1221090600.01.T01 | |
| maker-12-augustus-gene-54.35-mRNA-1 | Japonica | NA | | | | Caffeate O-methyltransferase-1 (Fragment) |
| maker-12-augustus-gene-56.10-mRNA-1 | Japonica | NA | | | OsR498G1221096000.01.T05 | Methyltransferase |
| maker-12-augustus-gene-56.2-mRNA-1 | Japonica | Japonica | | Os12g0202800 | OsR498G1221094300.01.T02 | Putative inactive methyltransferase |
| maker-12-augustus-gene-56.4-mRNA-1 | Japonica | NA | Kinase-TM | Os11g0242500 | OsR498G1119688900.01.T03 | Serine/threonine-protein kinase |
| maker-12-augustus-gene-56.8-mRNA-1 | Japonica | NA | | | OsR498G1221096900.01.T01 | Aquaporin |
| maker-12-augustus-gene-58.16-mRNA-1 | Japonica | NA | | Os12g0207000 | OsR498G1221103500.01.T01 | MADS domain transporter AGL11 |
| maker-12-augustus-gene-58.18-mRNA-1 | Japonica | NA | | Os12g0207200 | OsR498G1221103700.01.T01 | Transposon protein, putative, CACTA, En/Spm sub-class, expressed |
| maker-12-augustus-gene-58.20-mRNA-1 | Japonica | NA | | Os12g0207300 | OsR498G1221104100.01.T02 | AP complex subunit sigma |
| maker-12-augustus-gene-59.23-mRNA-1 | Japonica | NA | | | OsR498G1221106700.01.T04 | 28S ribosomal protein S23 |
| maker-12-augustus-gene-59.26-mRNA-1 | Japonica | NA | | Os12g0209200 | OsR498G1221107500.01.T01 | B-box zinc finger protein 20 |
| maker-12-augustus-gene-59.28-mRNA-1 | Japonica | NA | | Os12g0209700 | OsR498G1221108500.01.T01 | PHD domain-containing protein |
| maker-12-augustus-gene-59.35-mRNA-1 | Japonica | NA | | Os12g0209000 | OsR498G1221106400.01.T02 | AAA domain-containing protein (Fragment) |
| maker-12-augustus-gene-59.41-mRNA-1 | Japonica | NA | | Os12g0209300 | OsR498G1221107800.01.T02 | ATPase, AAA family protein, expressed |
| maker-12-augustus-gene-6.0-mRNA-1 | Japonica | Indica | | Os12g0110400 | OsR498G1220815500.01.T04 | Plastid transcriptionally active 6 |
| maker-12-augustus-gene-6.10-mRNA-1 | Japonica | Japonica | | Os11g0112150 | OsR498G1220816700.01.T02 | Proteoglycan 4-like |
| maker-12-augustus-gene-6.16-mRNA-1 | Japonica | NA | | Os12g0112000 | OsR498G1220818100.01.T01 | Peroxidase |
| maker-12-augustus-gene-6.25-mRNA-1 | Japonica | NA | | Os12g0112300 | OsR498G1220818700.01.T05 | ADP,ATP carrier protein (Fragment) |
| maker-12-augustus-gene-6.33-mRNA-1 | Japonica | NA | | | OsR498G1220819100.01.T01 | AAI domain-containing protein (Fragment) |
| maker-12-augustus-gene-6.35-mRNA-1 | Japonica | NA | | | OsR498G1220814400.01.T03 | DRBM domain-containing protein |
| maker-12-augustus-gene-6.39-mRNA-1 | Japonica | NA | | Os12g0110000 | OsR498G1220814600.01.T02 | Regulatory-associated protein of TOR 1 |
| maker-12-augustus-gene-6.5-mRNA-1 | Japonica | Indica | | Os11g0111800 | OsR498G1220815900.01.T01 | Transcription factor bHLH61 like |
| maker-12-augustus-gene-6.53-mRNA-1 | Japonica | NA | | Os12g0110100 | OsR498G1220815000.01.T04 | Alpha/beta-Hydrolases superfamily protein |
| maker-12-augustus-gene-6.57-mRNA-1 | Japonica | NA | | Os12g0110300 | OsR498G1220815400.01.T01 | Iwr1 domain-containing protein |
| maker-12-augustus-gene-6.63-mRNA-1 | Japonica | NA | | Os12g0111500 | OsR498G1119309400.01.T01 | BTB domain-containing protein (Fragment) |
| maker-12-augustus-gene-6.65-mRNA-1 | Japonica | NA | | Os11g0112000 | OsR498G1119309800.01.T03 | HIG1 domain-containing protein (Fragment) |
| maker-12-augustus-gene-6.67-mRNA-1 | Japonica | Japonica | | Os12g0111900 | OsR498G1119310800.01.T01 | Biogenesis of lysosome-related organelles complex 1 subunit 1 |
| maker-12-augustus-gene-60.24-mRNA-1 | Japonica | NA | | Os12g0210200 | OsR498G1221109700.01.T07 | Glutathione transferase18 |
| maker-12-augustus-gene-60.27-mRNA-1 | Japonica | Japonica | | Os12g0210300 | OsR498G1221109700.01.T07 | Glutathione transferase18 |
| maker-12-augustus-gene-60.29-mRNA-1 | Japonica | NA | Kinase-LRR-TM | Os12g0210400 | OsR498G1221109900.01.T01 | Leucine-rich repeat receptor-like protein kinase |
| maker-12-augustus-gene-60.38-mRNA-1 | Japonica | NA | | Os07g0652800 | | 3-deoxy-8-phosphooctulonate synthase |

| | | | | | | |
|---|---|---|---|---|---|---|
| maker-12-augustus-gene-60.44-mRNA-1 | Japonica | Japonica | | Os12g0211000 | OsR498G1221112500.01.T01 | RING-type domain-containing protein |
| maker-12-augustus-gene-60.46-mRNA-1 | Japonica | NA | | Os12g0211100 | OsR498G1221113500.01.T01 | Peptidase A1 domain-containing protein |
| maker-12-augustus-gene-60.49-mRNA-1 | Japonica | NA | | | OsR498G1221114100.01.T01 | Retrotransposon protein, putative, unclassified |
| maker-12-augustus-gene-60.52-mRNA-1 | Japonica | NA | | | | |
| maker-12-augustus-gene-60.55-mRNA-1 | Japonica | NA | | | | Retrotransposon protein, putative, unclassified |
| maker-12-augustus-gene-60.57-mRNA-1 | Japonica | NA | | Os12g0211400 | OsR498G1221115300.01.T01 | Adenine DNA glycosylase |
| maker-12-augustus-gene-64.30-mRNA-1 | Japonica | NA | | Os12g0217300 | OsR498G1221121400.01.T01 | |
| maker-12-augustus-gene-65.27-mRNA-1 | Japonica | NA | | | OsR498G1221129600.01.T03 | Reverse transcriptase domain-containing protein |
| maker-12-augustus-gene-66.35-mRNA-1 | Japonica | NA | | Os04g0184800 | OsR498G0204883800.01.T01 | Transposon protein, putative, Mutator sub-class |
| maker-12-augustus-gene-67.20-mRNA-1 | Japonica | NA | | Os03g0154300 | OsR498G1119934800.01.T01 | |
| maker-12-augustus-gene-69.0-mRNA-1 | Japonica | NA | | Os12g0223300 | OsR498G1221135300.01.T01 | Cytochrome b5-like Heme/Steroid binding domain containing protein, expre |
| maker-12-augustus-gene-69.3-mRNA-1 | Japonica | NA | | | OsR498G1221137200.01.T04 | Diacylglycerol kinase |
| maker-12-augustus-gene-69.8-mRNA-1 | Japonica | Indica | | Os12g0224400 | OsR498G1221138400.01.T01 | probable xyloglucan galactosyltransferase GT19 |
| maker-12-augustus-gene-7.0-mRNA-1 | Japonica | NA | | Os12g0112600 | OsR498G1220819500.01.T01 | Stress response protein nst1-like isoform X1 |
| maker-12-augustus-gene-7.13-mRNA-1 | Japonica | NA | | Os11g0114700 | OsR498G1220847100.01.T02 | Nucleotidyltransferase family protein, putative, expressed |
| maker-12-augustus-gene-7.2-mRNA-1 | Japonica | NA | | Os11g0113300 | OsR498G1220819900.01.T01 | Serine/arginine-rich splicing factor like |
| maker-12-augustus-gene-7.4-mRNA-1 | Japonica | Indica | | Os12g0114500 | OsR498G1220846600.01.T01 | Non-specific lipid-transfer protein (Fragment) |
| maker-12-augustus-gene-7.6-mRNA-1 | Japonica | NA | | Os11g0113100 | OsR498G1119313200.01.T01 | Phosphoglucan phosphatase |
| maker-12-augustus-gene-7.9-mRNA-1 | Japonica | NA | Kinase | Os12g0114100 | OsR498G1220847300.01.T01 | probable serine/threonine-protein kinase WNK5 |
| maker-12-augustus-gene-70.0-mRNA-1 | Japonica | Indica | | Os12g0225200 | OsR498G1221142300.01.T01 | Outer envelope pore protein 37 chloroplastic |
| maker-12-augustus-gene-70.11-mRNA-1 | Japonica | Indica | | Os11g0248200 | OsR498G1221142200.01.T01 | Transposon protein, putative, Mutator sub-class, expressed |
| maker-12-augustus-gene-70.6-mRNA-1 | Japonica | NA | | Os12g0224500 | OsR498G1221138800.01.T02 | Putative histone-lysine N-methyltransferase chromatin regulator PHD family |
| maker-12-augustus-gene-71.0-mRNA-1 | Japonica | Indica | | Os12g0226900 | OsR498G1221145100.01.T04 | NADP-dependent oxidoreductase P1, putative, expressed |
| maker-12-augustus-gene-71.10-mRNA-1 | Japonica | Indica | | Os12g0226300 | OsR498G1221146500.01.T01 | |
| maker-12-augustus-gene-71.12-mRNA-1 | Japonica | NA | | Os12g0226700 | OsR498G1221146700.01.T01 | NADP-dependent oxidoreductase P2, putative, expressed |
| maker-12-augustus-gene-71.16-mRNA-1 | Japonica | NA | | Os12g0226900 | OsR498G1221146900.01.T01 | NADP-dependent alkenal double bond reductase P1 |
| maker-12-augustus-gene-71.22-mRNA-1 | Japonica | NA | | Os12g0227500 | OsR498G1221147900.01.T01 | Dirigent protein (Fragment) |
| maker-12-augustus-gene-71.4-mRNA-1 | Japonica | NA | | | | Putative NADP-dependent oxidoreductase P1 |
| maker-12-augustus-gene-72.0-mRNA-1 | Japonica | NA | | | | Jacalin-like lectin domain containing protein, expressed |
| maker-12-augustus-gene-72.11-mRNA-1 | Japonica | NA | | | OsR498G1221152200.01.T02 | Leucine Rich Repeat family protein, expressed |
| maker-12-augustus-gene-72.17-mRNA-1 | Japonica | NA | | | | Transposon protein, putative, CACTA, En/Spm sub-class |
| maker-12-augustus-gene-72.2-mRNA-1 | Japonica | NA | | | OsR498G1221150600.01.T01 | Carboxymethylenebutenolidase homolog |
| maker-12-augustus-gene-72.4-mRNA-1 | Japonica | NA | | | OsR498G1221151000.01.T03 | Putative chaperonin Cpn60/TCP-1 family, groEL-like apical domain, groEL-like |
| maker-12-augustus-gene-72.8-mRNA-1 | Japonica | NA | | | | NAM-associated domain-containing protein |
| maker-12-augustus-gene-73.27-mRNA-1 | Japonica | NA | | | OsR498G1221155300.01.T01 | PGG domain-containing protein |
| maker-12-augustus-gene-73.29-mRNA-1 | Japonica | NA | | Os12g0230200 | OsR498G1221156900.01.T01 | Calcium-dependent protein kinase |
| maker-12-augustus-gene-73.31-mRNA-1 | Japonica | NA | | Os12g0230600 | OsR498G1221157700.01.T01 | |
| maker-12-augustus-gene-73.34-mRNA-1 | Japonica | NA | | Os12g0230100 | OsR498G1221155800.01.T02 | ATP-dependent Clp protease ATP-binding subunit clpA like CD4B, chloroplas |
| maker-12-augustus-gene-74.1-mRNA-1 | Japonica | NA | | Os12g0231000 | OsR498G1221158500.01.T01 | Peptide transporter PTR5 |
| maker-12-augustus-gene-74.11-mRNA-1 | Japonica | NA | | | OsR498G1221158800.01.T01 | KAT8 regulatory NSL complex subunit 2 |
| maker-12-augustus-gene-74.13-mRNA-1 | Japonica | NA | | | | RWP-RK domain containing protein |
| maker-12-augustus-gene-74.4-mRNA-1 | Japonica | NA | | Os01g0650700 | | |
| maker-12-augustus-gene-74.6-mRNA-1 | Japonica | NA | | | OsR498G0714279400.01.T01 | Hydroxyproline-rich glycoprotein-like |
| maker-12-augustus-gene-74.9-mRNA-1 | Japonica | Indica | | Os01g0218500 | OsR498G1221161500.01.T01 | TWIN SISTER of FT protein, putative, expressed |
| maker-12-augustus-gene-75.0-mRNA-1 | Japonica | Indica | | Os12g0233100 | OsR498G1221165700.01.T01 | WW domain containing protein, expressed |
| maker-12-augustus-gene-75.10-mRNA-1 | Japonica | NA | | | OsR498G1221166500.01.T01 | Secretory pathway Sec39 |
| maker-12-augustus-gene-75.14-mRNA-1 | Japonica | NA | | | OsR498G0407513400.01.T01 | Hydroxyproline-rich glycoprotein-like |
| maker-12-augustus-gene-75.16-mRNA-1 | Japonica | NA | | Os12g0233800 | OsR498G1221167000.01.T02 | Transcription factor HBP-1a |
| maker-12-augustus-gene-75.2-mRNA-1 | Japonica | NA | | Os12g0233300 | OsR498G1221165900.01.T03 | HIT domain-containing protein (Fragment) |
| maker-12-augustus-gene-75.7-mRNA-1 | Japonica | NA | | Os12g0233400 | OsR498G1221166100.01.T06 | C2H2-type domain-containing protein (Fragment) |
| maker-12-augustus-gene-76.35-mRNA-1 | Japonica | Indica | Kinase-LRR-TM | Os11g0300600 | OsR498G1221171100.01.T01 | Putative inactive leucine-rich repeat receptor-like protein kinase |
| maker-12-augustus-gene-76.37-mRNA-1 | Japonica | NA | | Os12g0235800 | OsR498G1221171700.01.T01 | Citrulline--aspartate ligase |
| maker-12-augustus-gene-76.42-mRNA-1 | Japonica | NA | | | OsR498G1221169000.01.T02 | |
| maker-12-augustus-gene-76.44-mRNA-1 | Japonica | NA | | | OsR498G1221172000.01.T02 | C2 NT-type domain-containing protein |
| maker-12-augustus-gene-77.0-mRNA-1 | Japonica | NA | | Os12g0236400 | OsR498G1221174100.01.T01 | Adenylate kinase |
| maker-12-augustus-gene-77.11-mRNA-1 | Japonica | NA | | | OsR498G1221172800.01.T01 | Cullin, N-terminal |
| maker-12-augustus-gene-77.13-mRNA-1 | Japonica | NA | | Os12g0236500 | OsR498G1221175000.01.T02 | Zn-dependent exopeptidases superfamily protein (Fragment) |
| maker-12-augustus-gene-77.3-mRNA-1 | Japonica | NA | | Os09g0278300 | | 1-phosphatidylinositol-3-phosphate 5-kinase |
| maker-12-augustus-gene-77.8-mRNA-1 | Japonica | NA | | Os12g0236900 | OsR498G1221179700.01.T01 | Histone-lysine N-methyltransferase |
| maker-12-augustus-gene-78.0-mRNA-1 | Japonica | NA | | | OsR498G1221180400.01.T01 | |
| maker-12-augustus-gene-78.2-mRNA-1 | Japonica | NA | | Os12g0238000 | OsR498G1221183200.01.T01 | Myb-like DNA-binding domain containing protein, expressed |

| | | | | | | |
|---|---|---|---|---|---|---|
| maker-12-augustus-gene-78.9-mRNA-1 | Japonica | NA | | Os12g0238100 | OsR498G1221184600.01.T01 | Exocyst complex component 5 |
| maker-12-augustus-gene-79.16-mRNA-1 | Japonica | Japonica | | Os12g0238900 | OsR498G1221185500.01.T02 | Glycosyltransferase, HGA-like,putative,expressed |
| maker-12-augustus-gene-79.18-mRNA-1 | Japonica | NA | | Os12g0239000 | OsR498G1221186200.01.T01 | Proline-rich family protein |
| maker-12-augustus-gene-79.23-mRNA-1 | Japonica | Indica | | Os12g0239200 | OsR498G1221186800.01.T03 | Proline-rich family protein |
| maker-12-augustus-gene-79.26-mRNA-1 | Japonica | NA | | | | ABC transporter domain-containing protein |
| maker-12-augustus-gene-8.47-mRNA-1 | Japonica | NA | | Os12g0115900 | OsR498G1220842800.01.T03 | EFP domain-containing protein |
| maker-12-augustus-gene-8.54-mRNA-1 | Japonica | NA | | Os01g0874300 | OsR498G0409243500.01.T01 | MYB transcription factor MYB5 |
| maker-12-augustus-gene-8.56-mRNA-1 | Japonica | NA | | Os12g0115700 | OsR498G1220843100.01.T03 | Chalcone-flavonone isomerase family protein (Fragment) |
| maker-12-augustus-gene-8.63-mRNA-1 | Japonica | NA | | Os12g0116000 | OsR498G1220842300.01.T01 | Vacuolar-sorting receptor 1 |
| maker-12-augustus-gene-8.67-mRNA-1 | Japonica | NA | | Os11g0116900 | OsR498G1220841100.01.T01 | WRKY DNA binding domain containing protein, expressed |
| maker-12-augustus-gene-8.70-mRNA-1 | Japonica | NA | | Os11g0117400 | OsR498G1119327000.01.T01 | WRKY DNA binding domain containing protein, expressed |
| maker-12-augustus-gene-8.72-mRNA-1 | Japonica | Indica | | Os11g0117500 | OsR498G1220839300.01.T01 | WRKY transcription factor 64 |
| maker-12-augustus-gene-8.75-mRNA-1 | Japonica | Indica | | Os11g0117600 | OsR498G1220838500.01.T02 | WRKY DNA binding domain containing protein, expressed |
| maker-12-augustus-gene-80.24-mRNA-1 | Japonica | NA | | Os04g0597400 | OsR498G0409209500.01.T01 | Peptide transporter PTR2 |
| maker-12-augustus-gene-80.25-mRNA-1 | Japonica | NA | | Os12g0240900 | OsR498G1221191800.01.T01 | Tricetin 3',4',5'-O-trimethyltransferase |
| maker-12-augustus-gene-81.28-mRNA-1 | Japonica | Indica | | Os12g0242700 | OsR498G1221198900.01.T04 | Beta-ketoacyl-acyl carrier protein reductase |
| maker-12-augustus-gene-81.31-mRNA-1 | Japonica | NA | | Os12g0242900 | OsR498G1221199400.01.T01 | DNA polymerase alpha subunit B |
| maker-12-augustus-gene-82.0-mRNA-1 | Japonica | NA | | | | |
| maker-12-augustus-gene-82.10-mRNA-1 | Japonica | NA | | Os12g0244500 | OsR498G1221201400.01.T01 | Ribosome biogenesis protein slx9-like |
| maker-12-augustus-gene-82.2-mRNA-1 | Japonica | NA | | Os12g0244100 | OsR498G1221200700.01.T01 | Molecular chaperones mortalin/PBP74/GRP75, HSP70 superfamily |
| maker-12-augustus-gene-82.6-mRNA-1 | Japonica | NA | | Os12g0244400 | OsR498G1221201100.01.T01 | Lysine histidine transporter like |
| maker-12-augustus-gene-82.8-mRNA-1 | Japonica | Japonica | | Os12g0245100 | OsR498G1221203300.01.T01 | |
| maker-12-augustus-gene-85.0-mRNA-1 | Japonica | NA | | | OsR498G0612461700.01.T02 | |
| maker-12-augustus-gene-85.2-mRNA-1 | Japonica | Indica | | Os03g0853200 | OsR498G1221215500.01.T01 | FeS cluster assembly protein sufB |
| maker-12-augustus-gene-85.4-mRNA-1 | Japonica | Japonica | Kinase-TM | Os12g0248000 | OsR498G1221217600.01.T01 | Interleukin-1 receptor-associated kinase 4 |
| maker-12-augustus-gene-86.0-mRNA-1 | Japonica | Indica | Kinase-TM | Os12g0249900 | OsR498G1221216600.01.T01 | Protein kinase domain-containing protein |
| maker-12-augustus-gene-86.2-mRNA-1 | Japonica | NA | Kinase-TM | Os12g0249900 | | Proline-rich receptor-like protein kinase PERK2 isoform X2 |
| maker-12-augustus-gene-9.0-mRNA-1 | Japonica | NA | | Os12g0117100 | OsR498G1220836800.01.T01 | Methylesterase 17 |
| maker-12-augustus-gene-9.18-mRNA-1 | Japonica | Indica | | Os12g0118900 | OsR498G1220832200.01.T02 | Secologanin synthase |
| maker-12-augustus-gene-9.21-mRNA-1 | Japonica | NA | | Os12g0119000 | OsR498G1220832200.01.T01 | Secologanin synthase |
| maker-12-augustus-gene-9.23-mRNA-1 | Japonica | Indica | | Os12g0119700 | OsR498G1119333300.01.T02 | Retrotransposon protein, putative, Ty1-copia subclass |
| maker-12-augustus-gene-9.25-mRNA-1 | Japonica | NA | | Os12g0119500 | OsR498G1220831200.01.T01 | Eukaryotic translation initiation factor 3 subunit B |
| maker-12-augustus-gene-9.27-mRNA-1 | Japonica | NA | | Os11g0117700 | OsR498G1220837700.01.T01 | |
| maker-12-augustus-gene-9.29-mRNA-1 | Japonica | NA | | Os12g0117250 | OsR498G1220836100.01.T01 | Mannan endo-1,4-beta-mannosidase |
| maker-12-augustus-gene-9.32-mRNA-1 | Japonica | NA | | Os12g0117600 | OsR498G1220834900.01.T01 | BTB/POZ domain-containing protein At3g08570 |
| maker-12-augustus-gene-9.34-mRNA-1 | Japonica | NA | | Os12g0117800 | OsR498G1119331000.01.T02 | NAM-associated domain-containing protein |
| maker-12-augustus-gene-9.35-mRNA-1 | Japonica | NA | | Os11g0118800 | OsR498G1220834100.01.T01 | Zinc finger CCCH domain protein |
| maker-12-augustus-gene-9.4-mRNA-1 | Japonica | NA | | Os12g0117200 | OsR498G1220836400.01.T03 | AB hydrolase-1 domain-containing protein |
| maker-12-augustus-gene-9.9-mRNA-1 | Japonica | Japonica | | Os12g0118400 | OsR498G1119332100.01.T03 | 2-hydroxy-6-oxononadienedioate/2-hydroxy-6- oxononatrienedioate hydrol |
| maker-12-augustus-gene-90.37-mRNA-1 | Japonica | NA | | Os12g0256000 | OsR498G1221220100.01.T01 | CXE carboxylesterase |
| maker-12-augustus-gene-90.39-mRNA-1 | Japonica | Japonica | | Os12g0256300 | OsR498G1221220300.01.T01 | Putative pre-mRNA-splicing factor cwc-22 |
| maker-12-augustus-gene-90.46-mRNA-1 | Japonica | NA | | | OsR498G1221221500.01.T01 | PMD domain-containing protein |
| maker-12-augustus-gene-90.50-mRNA-1 | Japonica | NA | | Os12g0257000 | OsR498G1221222200.01.T03 | Carboxypeptidase |
| maker-12-augustus-gene-91.0-mRNA-1 | Japonica | Indica | | Os12g0259800 | OsR498G1221223500.01.T01 | Laccase |
| maker-12-augustus-gene-91.3-mRNA-1 | Japonica | NA | Kinase-TM | Os12g0257900 | OsR498G1221224100.01.T01 | Non-specific serine/threonine protein kinase |
| maker-12-augustus-gene-91.5-mRNA-1 | Japonica | NA | | Os12g0258200 | OsR498G1221224400.01.T03 | DnaJ protein ERDJ7 |
| maker-12-augustus-gene-92.32-mRNA-1 | Japonica | NA | | Os12g0258700 | OsR498G1221227900.01.T01 | Laccase |
| maker-12-augustus-gene-93.0-mRNA-1 | Japonica | NA | | Os12g0259800 | OsR498G1221230900.01.T01 | Laccase |
| maker-12-augustus-gene-93.3-mRNA-1 | Japonica | NA | | Os08g0514800 | | Ptta/En/Spm family plant transposase |
| maker-12-augustus-gene-93.6-mRNA-1 | Japonica | NA | | Os12g0260500 | OsR498G1221233200.01.T01 | Putative sex determination protein tasselseed 2 |
| maker-12-augustus-gene-94.20-mRNA-1 | Japonica | Indica | | Os12g0551200 | OsR498G1221234700.01.T01 | Disease resistance protein |
| maker-12-augustus-gene-94.23-mRNA-1 | Japonica | NA | | Os12g0261300 | OsR498G1221234900.01.T01 | Retrotransposon protein, putative, unclassified, expressed |
| maker-12-augustus-gene-94.25-mRNA-1 | Japonica | NA | | | OsR498G1221237400.01.T01 | CCT domain-containing protein (Fragment) |
| maker-12-augustus-gene-95.32-mRNA-1 | Japonica | NA | | Os12g0262700 | OsR498G1221238700.01.T01 | Caffeoylshikimate esterase |
| maker-12-augustus-gene-95.36-mRNA-1 | Japonica | Indica | | Os12g0263000 | OsR498G1221239800.01.T02 | Glutathione synthetase |
| maker-12-augustus-gene-95.46-mRNA-1 | Japonica | NA | | Os12g0263100 | OsR498G1221240800.01.T01 | S-acyltransferase |
| maker-12-augustus-gene-95.48-mRNA-1 | Japonica | Indica | | Os12g0265100 | OsR498G1221241000.01.T01 | S-acyltransferase |
| maker-12-augustus-gene-95.50-mRNA-1 | Japonica | NA | | Os12g0263200 | OsR498G1221241000.01.T03 | NmrA domain-containing protein |
| maker-12-augustus-gene-95.52-mRNA-1 | Japonica | Indica | | Os12g0263200 | OsR498G1221242000.01.T03 | S-acyltransferase |
| maker-12-augustus-gene-95.60-mRNA-1 | Japonica | NA | | | OsR498G1221244600.01.T01 | Cytokinin inducible protein-like |
| maker-12-augustus-gene-96.0-mRNA-1 | Japonica | NA | | | | 3-hydroxyisobutyryl-coenzyme A hydrolase, putative, expressed |

| | | | | | | |
|---|---|---|---|---|---|---|
| maker-12-augustus-gene-96.18-mRNA-1 | Japonica | NA | | Os12g0265100 | OsR498G1221249000.01.T01 | NmrA domain-containing protein (Fragment) |
| maker-12-augustus-gene-97.0-mRNA-1 | Japonica | NA | | | | Hydroxyproline O-galactosyltransferase |
| maker-12-augustus-gene-97.1-mRNA-1 | Japonica | Indica | | Os12g0265600 | OsR498G1221250200.01.T04 | Galectin domain-containing protein (Fragment) |
| maker-12-augustus-gene-97.10-mRNA-1 | Japonica | NA | | Os12g0266200 | OsR498G1221252200.01.T03 | Wall-associated receptor kinase 3 |
| maker-12-augustus-gene-97.8-mRNA-1 | Japonica | Japonica | CC-Kinase-TM | Os12g0265900 | OsR498G1221251800.01.T01 | Wall-associated receptor kinase 2 |
| maker-12-augustus-gene-98.0-mRNA-1 | Japonica | Japonica | | Os12g0267100 | OsR498G0714282700.01.T02 | Cyclopropane fatty acid synthase |
| maker-12-augustus-gene-98.2-mRNA-1 | Japonica | Japonica | | Os12g0267200 | OsR498G1221257500.01.T01 | Mycolic acid cyclopropane synthase |
| maker-12-augustus-gene-98.6-mRNA-1 | Japonica | NA | | Os12g0267900 | OsR498G1221260700.01.T01 | Zinc finger, RING-type |
| maker-12-augustus-gene-98.9-mRNA-1 | Japonica | NA | | | OsR498G1221261800.01.T01 | Tryptamine 5-hydroxylase |
| maker-2-augustus-gene-1.42-mRNA-1 | Japonica | Indica | | Os02g0101900 | OsR498G0202802900.01.T01 | Homeobox protein engrailed-1 |
| maker-2-augustus-gene-1.45-mRNA-1 | Japonica | NA | | Os02g0102200 | OsR498G0202803900.01.T01 | Amino acid transporter, transmembrane |
| maker-2-augustus-gene-1.48-mRNA-1 | Japonica | NA | | Os02g0102300 | OsR498G0202804500.01.T01 | Rhodanese domain-containing protein |
| maker-2-augustus-gene-1.50-mRNA-1 | Japonica | NA | | Os02g0102600 | OsR498G0202805100.01.T01 | ATP-dependent Clp protease proteolytic subunit 4 |
| maker-2-augustus-gene-1.52-mRNA-1 | Japonica | NA | | Os02g0102800 | OsR498G0202805500.01.T01 | Protein ENHANCED DISEASE RESISTANCE 2 |
| maker-2-augustus-gene-1.54-mRNA-1 | Japonica | NA | | Os02g0102900 | OsR498G0202806100.01.T05 | RuBisCO large subunit-binding protein subunit beta |
| maker-2-augustus-gene-1.82-mRNA-1 | Japonica | NA | | | OsR498G0202804800.01.T01 | Ribosomal protein S5 |
| maker-2-augustus-gene-1.87-mRNA-1 | Japonica | NA | | Os02g0102700 | OsR498G0202805400.01.T01 | Small nuclear ribonucleoprotein Sm D3 |
| maker-2-augustus-gene-100.28-mRNA-1 | Japonica | NA | | Os02g0273000 | OsR498G0203396500.01.T01 | Uridine kinase |
| maker-2-augustus-gene-100.35-mRNA-1 | Japonica | Indica | CC-LRR-NBS-TM | Os02g0272900 | OsR498G0203396700.01.T01 | AAA domain-containing protein |
| maker-2-augustus-gene-100.39-mRNA-1 | Japonica | NA | | Os02g0273700 | OsR498G0203398500.01.T02 | VHS and GAT domain protein |
| maker-2-augustus-gene-100.44-mRNA-1 | Japonica | Indica | | Os02g0274000 | OsR498G0203399500.01.T01 | |
| maker-2-augustus-gene-100.48-mRNA-1 | Japonica | NA | | Os02g0274100 | OsR498G0203400100.01.T02 | 3-hydroxyacyl-CoA dehydrogenase |
| maker-2-augustus-gene-100.53-mRNA-1 | Japonica | NA | | Os02g0273800 | OsR498G0203399200.01.T03 | Ribonuclease P |
| maker-2-augustus-gene-100.55-mRNA-1 | Japonica | Japonica | | Os02g0273900 | OsR498G0612559700.01.T02 | Ubiquitin carrier protein E2 21 isoform 1 |
| maker-2-augustus-gene-100.57-mRNA-1 | Japonica | NA | LRR-TM | Os02g0274200 | | Receptor-like protein 12 |
| maker-2-augustus-gene-101.41-mRNA-1 | Japonica | NA | | | | Putative plastidic glucose transporter 2 |
| maker-2-augustus-gene-101.47-mRNA-1 | Japonica | NA | | Os02g0275100 | OsR498G0203402100.01.T02 | Cytochrome c-type biogenesis protein (Fragment) |
| maker-2-augustus-gene-101.49-mRNA-1 | Japonica | NA | | | OsR498G0203400800.01.T03 | CRC domain-containing protein |
| maker-2-augustus-gene-101.53-mRNA-1 | Japonica | NA | | Os02g0275200 | OsR498G0203402400.01.T01 | Fucosyltransferase |
| maker-2-augustus-gene-102.10-mRNA-1 | Japonica | NA | | Os02g0276400 | OsR498G0203404200.01.T01 | Putative inactive nicotinamidase |
| maker-2-augustus-gene-102.14-mRNA-1 | Japonica | NA | | Os02g0276500 | OsR498G0203404400.01.T03 | Tocopherol cyclase, chloroplastic |
| maker-2-augustus-gene-102.18-mRNA-1 | Japonica | NA | | Os02g0276900 | OsR498G0203406200.01.T05 | Transcription factor bHLH68 |
| maker-2-augustus-gene-102.24-mRNA-1 | Japonica | NA | Kinase-LRR-TM | | OsR498G0203410200.01.T02 | Putative leucine-rich repeat receptor-like protein kinase |
| maker-2-augustus-gene-102.7-mRNA-1 | Japonica | NA | | | OsR498G0203403000.01.T01 | Putative inactive nicotinamidase isoform B |
| maker-2-augustus-gene-103.0-mRNA-1 | Japonica | Japonica | | Os02g0278400 | OsR498G0203410500.01.T01 | Ent-isokaurene C2-hydroxylase |
| maker-2-augustus-gene-103.2-mRNA-1 | Japonica | NA | | Os02g0279600 | OsR498G0203413500.01.T01 | Tobamovirus multiplication protein 2B |
| maker-2-augustus-gene-103.4-mRNA-1 | Japonica | NA | | Os02g0278700 | OsR498G0203410800.01.T01 | Ent-copalyl diphosphate synthase |
| maker-2-augustus-gene-104.0-mRNA-1 | Japonica | NA | | | | |
| maker-2-augustus-gene-104.12-mRNA-1 | Japonica | Japonica | | Os02g0280700 | OsR498G0611713700.01.T03 | Fe2OG dioxygenase domain-containing protein (Fragment) |
| maker-2-augustus-gene-104.16-mRNA-1 | Japonica | NA | | Os02g0281000 | OsR498G0203419100.01.T05 | Protein-serine/threonine phosphatase |
| maker-2-augustus-gene-104.2-mRNA-1 | Japonica | NA | | Os02g0280200 | OsR498G0203415300.01.T01 | Xyloglucan endotransglucosylase/hydrolase |
| maker-2-augustus-gene-104.20-mRNA-1 | Japonica | NA | | Os02g0280100 | OsR498G0203415200.01.T02 | MFAP1 domain-containing protein |
| maker-2-augustus-gene-104.22-mRNA-1 | Japonica | Indica | | Os05g0230900 | OsR498G0203417000.01.T02 | Glyoxalase I |
| maker-2-augustus-gene-104.23-mRNA-1 | Japonica | Indica | | Os05g0230900 | OsR498G0203417000.01.T02 | Glyoxalase I |
| maker-2-augustus-gene-104.6-mRNA-1 | Japonica | NA | | Os02g0280400 | OsR498G0203416700.01.T05 | Casein kinase I |
| maker-2-augustus-gene-105.26-mRNA-1 | Japonica | NA | | | OsR498G0203419700.01.T02 | RNI-like superfamily protein isoform 1 |
| maker-2-augustus-gene-105.32-mRNA-1 | Japonica | Japonica | CC-NBS-TM | Os02g0281900 | OsR498G0203420100.01.T02 | Disease resistance protein RGA2 |
| maker-2-augustus-gene-105.34-mRNA-1 | Japonica | NA | | Os02g0282100 | OsR498G0203421200.01.T01 | 39S ribosomal protein L53 |
| maker-2-augustus-gene-106.0-mRNA-1 | Japonica | NA | | Os02g0282900 | OsR498G0203421800.01.T01 | ATP-binding cassette sub-family E member 1 |
| maker-2-augustus-gene-111.39-mRNA-1 | Japonica | NA | | Os02g0292100 | | GDSL-motif lipase/hydrolase-like |
| maker-2-augustus-gene-111.42-mRNA-1 | Japonica | NA | | Os02g0292600 | OsR498G0203459900.01.T02 | GDSL esterase/lipase |
| maker-2-augustus-gene-111.54-mRNA-1 | Japonica | NA | | Os02g0292800 | OsR498G0203461200.01.T01 | DUF599 domain-containing protein |
| maker-2-augustus-gene-112.0-mRNA-1 | Japonica | NA | | | OsR498G0203461500.01.T01 | Factor of DNA methylation 2 |
| maker-2-augustus-gene-112.10-mRNA-1 | Japonica | Indica | | Os01g0886600 | OsR498G0203462400.01.T01 | ATP-dependent Clp protease ATP-binding subunit clpX |
| maker-2-augustus-gene-112.19-mRNA-1 | Japonica | NA | | Os02g0295700 | OsR498G0203464600.01.T03 | Topoisomerase II-associated protein PAT1 |
| maker-2-augustus-gene-112.2-mRNA-1 | Japonica | Indica | | Os02g0293800 | OsR498G0203463300.01.T01 | zf-RVT domain-containing protein |
| maker-2-augustus-gene-112.4-mRNA-1 | Japonica | NA | | | OsR498G0203464300.01.T01 | WD repeat-containing protein 26 |
| maker-2-augustus-gene-112.8-mRNA-1 | Japonica | NA | | Os02g0293400 | OsR498G0203461800.01.T01 | RING-type E3 ubiquitin transferase |
| maker-2-augustus-gene-113.0-mRNA-1 | Japonica | NA | | Os02g0295700 | OsR498G0203474200.01.T03 | Topoisomerase II-associated protein PAT1 |
| maker-2-augustus-gene-113.3-mRNA-1 | Japonica | NA | | Os02g0296300 | OsR498G0203475200.01.T01 | |
| maker-2-augustus-gene-113.6-mRNA-1 | Japonica | Indica | | Os04g0613400 | OsR498G0203475400.01.T01 | CCR4-NOT transcription complex subunit 9-like |

| | | | | | | |
|---|---|---|---|---|---|---|
| maker-2-augustus-gene-114.30-mRNA-1 | Japonica | NA | | | OsR498G0203722500.01.T01 | Cytokinin inducible protein-like |
| maker-2-augustus-gene-114.31-mRNA-1 | Japonica | NA | | Os02g0297600 | OsR498G0203478900.01.T06 | Lysine ketoglutarate reductase trans-splicing related 1 |
| maker-2-augustus-gene-114.38-mRNA-1 | Japonica | NA | | | OsR498G0203476400.01.T02 | Cobalamin (Vitamin B12) biosynthesis CbiX |
| maker-2-augustus-gene-115.0-mRNA-1 | Japonica | NA | | Os02g0299200 | OsR498G0203486100.01.T02 | Calmodulin-binding protein-like |
| maker-2-augustus-gene-115.1-mRNA-1 | Japonica | NA | | Os02g0299200 | OsR498G0203486100.01.T03 | Calmodulin-binding protein-like |
| maker-2-augustus-gene-115.12-mRNA-1 | Japonica | NA | | Os02g0299600 | OsR498G0203488400.01.T01 | |
| maker-2-augustus-gene-115.14-mRNA-1 | Japonica | NA | | | | Phosphate synthase-like |
| maker-2-augustus-gene-115.6-mRNA-1 | Japonica | NA | CC-LRR-NBS-TM | | OsR498G0203491300.01.T01 | Putative stripe rust resistance protein Yr10 |
| maker-2-augustus-gene-115.8-mRNA-1 | Japonica | Indica | | Os02g0299300 | OsR498G0203486400.01.T02 | AB hydrolase-1 domain-containing protein (Fragment) |
| maker-2-augustus-gene-116.30-mRNA-1 | Japonica | NA | | Os02g0301000 | OsR498G0203494100.01.T01 | Zinc finger CCCH domain-containing protein 1 |
| maker-2-augustus-gene-116.32-mRNA-1 | Japonica | NA | | Os02g0301100 | OsR498G0203495200.01.T02 | Bidirectional sugar transporter SWEET |
| maker-2-augustus-gene-117.41-mRNA-1 | Japonica | Indica | | Os06g0343100 | OsR498G0203498500.01.T01 | RNA helicase |
| maker-2-augustus-gene-117.44-mRNA-1 | Japonica | Indica | | Os04g0613400 | OsR498G0203500500.01.T01 | CCR4-NOT transcription complex subunit 9 |
| maker-2-augustus-gene-117.46-mRNA-1 | Japonica | NA | | Os02g0302400 | | probable aminotransferase TAT2 isoform X3 |
| maker-2-augustus-gene-117.48-mRNA-1 | Japonica | NA | | Os02g0301400 | | Zinc finger MYM-type protein 1 |
| maker-2-augustus-gene-117.50-mRNA-1 | Japonica | NA | CC-NBS-TM | | OsR498G0203501200.01.T03 | LZ-NBS-LRR class RGA |
| maker-2-augustus-gene-117.53-mRNA-1 | Japonica | NA | | Os01g0704400 | | zf-RVT domain-containing protein |
| maker-2-augustus-gene-118.37-mRNA-1 | Japonica | NA | | Os02g0302700 | OsR498G0203502900.01.T01 | Tyrosine transaminase family protein isoform 1 |
| maker-2-augustus-gene-118.40-mRNA-1 | Japonica | NA | | Os02g0302900 | OsR498G0203503300.01.T02 | Reticulon domain-containing protein (Fragment) |
| maker-2-augustus-gene-119.11-mRNA-1 | Japonica | NA | | Os02g0305600 | OsR498G0203510000.01.T04 | |
| maker-2-augustus-gene-119.6-mRNA-1 | Japonica | NA | Kinase-TM | | | probable serine/threonine-protein kinase At1g54610 |
| maker-2-augustus-gene-119.8-mRNA-1 | Japonica | NA | | Os02g0304900 | OsR498G0203508800.01.T01 | Drought-induced 19 |
| maker-2-augustus-gene-120.0-mRNA-1 | Japonica | NA | | Os02g0306900 | OsR498G0203521900.01.T01 | Heat-shock protein DnaJ |
| maker-2-augustus-gene-120.11-mRNA-1 | Japonica | NA | | | OsR498G0203521800.01.T03 | |
| maker-2-augustus-gene-120.14-mRNA-1 | Japonica | NA | | Os02g0307000 | OsR498G0203522400.01.T02 | GB1/RHD3-type G domain-containing protein (Fragment) |
| maker-2-augustus-gene-120.2-mRNA-1 | Japonica | NA | | | OsR498G0203524100.01.T01 | DNA excision repair protein like |
| maker-2-augustus-gene-120.21-mRNA-1 | Japonica | Japonica | | Os02g0307300 | OsR498G0102370200.01.T01 | General transcription factor 3C polypeptide 2 |
| maker-2-augustus-gene-120.7-mRNA-1 | Japonica | NA | | | OsR498G0203521400.01.T03 | Nicotianamine aminotransferase A |
| maker-2-augustus-gene-121.33-mRNA-1 | Japonica | NA | | Os02g0307800 | OsR498G0203525300.01.T01 | Bidirectional sugar transporter SWEET4 (Fragment) |
| maker-2-augustus-gene-122.0-mRNA-1 | Japonica | NA | | | OsR498G0203533000.01.T01 | |
| maker-2-augustus-gene-123.0-mRNA-1 | Japonica | NA | | Os02g0312500 | OsR498G0203538300.01.T01 | optic atrophy 3 protein homolog |
| maker-2-augustus-gene-123.10-mRNA-1 | Japonica | NA | | Os02g0312600 | OsR498G0203538600.01.T01 | Rac-like GTP-binding protein 7 |
| maker-2-augustus-gene-123.15-mRNA-1 | Japonica | Indica | | Os02g0312700 | OsR498G0203539200.01.T01 | ATP synthase mitochondrial F1 complex assembly factor |
| maker-2-augustus-gene-123.18-mRNA-1 | Japonica | NA | | Os02g0312800 | OsR498G0203539800.01.T01 | Negative regulator of systemic acquired resistance |
| maker-2-augustus-gene-123.3-mRNA-1 | Japonica | Indica | | Os02g0313400 | OsR498G0203541100.01.T01 | Apoptosis inhibitory |
| maker-2-augustus-gene-124.24-mRNA-1 | Japonica | NA | | Os02g0313500 | | CHCH domain-containing protein (Fragment) |
| maker-2-augustus-gene-124.26-mRNA-1 | Japonica | NA | | | OsR498G0203542100.01.T06 | DDT domain-containing protein |
| maker-2-augustus-gene-124.35-mRNA-1 | Japonica | NA | | Os02g0313700 | OsR498G0203542600.01.T02 | Calcium-dependent lipid-binding (CaLB domain) family protein |
| maker-2-augustus-gene-124.41-mRNA-1 | Japonica | NA | | | | High affinity cationic amino acid transporter 1 |
| maker-2-augustus-gene-124.43-mRNA-1 | Japonica | NA | | | | P-type H(+)-exporting transporter |
| maker-2-augustus-gene-125.33-mRNA-1 | Japonica | Indica | | Os02g0633100 | OsR498G0203550700.01.T03 | B-block binding subunit of TFIIIC |
| maker-2-augustus-gene-125.35-mRNA-1 | Japonica | NA | | Os02g0316200 | OsR498G0203549800.01.T01 | F-boxkelch-repeat protein or23 |
| maker-2-augustus-gene-126.33-mRNA-1 | Japonica | Indica | | Os02g0633100 | OsR498G0203550700.01.T05 | |
| maker-2-augustus-gene-126.36-mRNA-1 | Japonica | NA | | Os02g0317100 | OsR498G0203551300.01.T01 | DUF295 domain-containing protein |
| maker-2-augustus-gene-126.38-mRNA-1 | Japonica | NA | | Os02g0317400 | OsR498G0203553300.01.T04 | Coatomer subunit zeta |
| maker-2-augustus-gene-126.41-mRNA-1 | Japonica | NA | | Os02g0317700 | OsR498G0203553900.01.T02 | |
| maker-2-augustus-gene-126.45-mRNA-1 | Japonica | Indica | | Os02g0318100 | OsR498G0203556100.01.T02 | Ubiquinol oxidase |
| maker-2-augustus-gene-126.49-mRNA-1 | Japonica | NA | | Os02g0317500 | OsR498G0203553600.01.T01 | F-box protein SKIP19 |
| maker-2-augustus-gene-126.51-mRNA-1 | Japonica | Indica | | Os02g0317800 | OsR498G0203555200.01.T01 | Major pollen allergen |
| maker-2-augustus-gene-127.36-mRNA-1 | Japonica | NA | | Os02g0319100 | OsR498G0203560900.01.T01 | RNA recognition motif domain-containing protein |
| maker-2-augustus-gene-127.42-mRNA-1 | Japonica | NA | | | | Cysteine dioxygenase |
| maker-2-augustus-gene-127.44-mRNA-1 | Japonica | Japonica | | Os02g0320300 | OsR498G0203563400.01.T02 | Chalcone--flavonone isomerase |
| maker-2-augustus-gene-127.48-mRNA-1 | Japonica | Indica | NBS-TM | Os02g0318500 | OsR498G0203559000.01.T05 | PDR-type ABC transporter |
| maker-2-augustus-gene-127.54-mRNA-1 | Japonica | NA | | | OsR498G0203561600.01.T01 | GST C-terminal domain-containing protein (Fragment) |
| maker-2-augustus-gene-127.58-mRNA-1 | Japonica | NA | | Os02g0319800 | OsR498G0203562000.01.T03 | WD repeat-containing protein 25 |
| maker-2-augustus-gene-127.62-mRNA-1 | Japonica | Indica | | Os02g0320100 | OsR498G0203562400.01.T02 | 28S rRNA (Cytosine-C(5))-methyltransferase |
| maker-2-augustus-gene-128.35-mRNA-1 | Japonica | Japonica | | Os02g0320800 | OsR498G0203566300.01.T01 | Fe2OG dioxygenase domain-containing protein (Fragment) |
| maker-2-augustus-gene-128.39-mRNA-1 | Japonica | NA | | Os02g0321500 | OsR498G0203567300.01.T02 | CRAL-TRIO domain-containing protein C3H8.02-like isoform X1 |
| maker-2-augustus-gene-128.44-mRNA-1 | Japonica | NA | | Os02g0321800 | OsR498G0203567900.01.T01 | Endoplasmic reticulum transmembrane protein (Fragment) |
| maker-2-augustus-gene-128.46-mRNA-1 | Japonica | NA | | Os02g0321900 | OsR498G0203568300.01.T02 | OVATE domain-containing protein |
| maker-2-augustus-gene-129.0-mRNA-1 | Japonica | NA | Kinase-TM | Os02g0322400 | OsR498G0203569900.01.T01 | Putative MAPKKK family protein kinase |

| | | | | | | |
|---|---|---|---|---|---|---|
| maker-2-augustus-gene-129.1-mRNA-1 | Japonica | NA | | | OsR498G0203571800.01.T01 | Multidrug resistance protein 1, 2, putative |
| maker-2-augustus-gene-130.0-mRNA-1 | Japonica | NA | | Os02g0324300 | OsR498G0203578100.01.T02 | Nuclear protein-like |
| maker-2-augustus-gene-130.4-mRNA-1 | Japonica | NA | | Os02g0324700 | OsR498G0203580700.01.T01 | Senescence-associated protein |
| maker-2-augustus-gene-130.6-mRNA-1 | Japonica | NA | CC-NBS-TM | Os02g0325100 | OsR498G0203583700.01.T01 | 26S proteasome regulatory subunit 6B homolog |
| maker-2-augustus-gene-131.32-mRNA-1 | Japonica | Japonica | | Os02g0325401 | OsR498G1119390000.01.T01 | Phosphoglycerate mutase family protein, expressed |
| maker-2-augustus-gene-131.34-mRNA-1 | Japonica | NA | | Os02g0325600 | OsR498G0203585900.01.T01 | Myb family transcription factor EFM |
| maker-2-augustus-gene-131.38-mRNA-1 | Japonica | NA | | Os02g0326200 | OsR498G0203590900.01.T01 | RNA recognition motif (RRM)-containing protein-like |
| maker-2-augustus-gene-131.40-mRNA-1 | Japonica | Indica | | Os02g0326600 | OsR498G0203591500.01.T01 | ARF guanine-nucleotide exchange factor GNOM |
| maker-2-augustus-gene-131.44-mRNA-1 | Japonica | NA | | Os02g0326700 | OsR498G0203592300.01.T04 | RHOMBOID-like protein 9 chloroplastic |
| maker-2-augustus-gene-131.47-mRNA-1 | Japonica | NA | | Os02g0327000 | OsR498G0203594100.01.T01 | C2 calcium-dependent membrane targeting |
| maker-2-augustus-gene-131.50-mRNA-1 | Japonica | NA | | | OsR498G0203584200.01.T03 | Plasma membrane ATPase |
| maker-2-augustus-gene-131.52-mRNA-1 | Japonica | Indica | | Os10g0561900 | OsR498G0203584200.01.T04 | Dual-specificity protein-like phosphatase 1 |
| maker-2-augustus-gene-131.54-mRNA-1 | Japonica | NA | | Os02g0326000 | OsR498G0203590400.01.T02 | Putative acyl-activating enzyme 19 |
| maker-2-augustus-gene-132.0-mRNA-1 | Japonica | NA | | | OsR498G1120154400.01.T01 | Endonuclease |
| maker-2-augustus-gene-132.12-mRNA-1 | Japonica | NA | | Os02g0327700 | OsR498G0203596400.01.T01 | Protein O-linked-mannose beta-1,4-N-acetylglucosaminyltransferase 2-like |
| maker-2-augustus-gene-132.6-mRNA-1 | Japonica | NA | | | OsR498G1120154400.01.T01 | Retrotransposon protein, putative, Ty3-gypsy sub-class |
| maker-2-augustus-gene-132.8-mRNA-1 | Japonica | NA | | Os02g0327100 | OsR498G0203594600.01.T01 | ADP-ribosylation factor-like protein 2 |
| maker-2-augustus-gene-133.0-mRNA-1 | Japonica | NA | | Os02g0328300 | OsR498G0203601100.01.T03 | Fruit protein like |
| maker-2-augustus-gene-133.11-mRNA-1 | Japonica | NA | | Os04g0590366 | OsR498G0203601400.01.T01 | Actin-7 |
| maker-2-augustus-gene-133.13-mRNA-1 | Japonica | NA | | | OsR498G0407699700.01.T05 | |
| maker-2-augustus-gene-133.4-mRNA-1 | Japonica | Indica | | Os02g0224900 | OsR498G0203602700.01.T04 | AP-5 complex subunit zeta-1 |
| maker-2-augustus-gene-133.6-mRNA-1 | Japonica | NA | | | | AUGMIN subunit 6 |
| maker-2-augustus-gene-133.9-mRNA-1 | Japonica | NA | | Os02g0329800 | OsR498G0203609300.01.T01 | Glycosyltransferase family 61 protein |
| maker-2-augustus-gene-134.38-mRNA-1 | Japonica | NA | | Os02g0330200 | OsR498G0203612300.01.T01 | Glycosyltransferase |
| maker-2-augustus-gene-134.41-mRNA-1 | Japonica | NA | | | | |
| maker-2-augustus-gene-135.0-mRNA-1 | Japonica | NA | | Os02g0331200 | OsR498G0203614100.01.T05 | Glycosyltransferase, putative, expressed |
| maker-2-augustus-gene-135.3-mRNA-1 | Japonica | Indica | | Os08g0387050 | OsR498G0203621100.01.T01 | |
| maker-2-augustus-gene-135.6-mRNA-1 | Japonica | NA | | | OsR498G0203614400.01.T01 | |
| maker-2-augustus-gene-135.8-mRNA-1 | Japonica | NA | | Os02g0332200 | OsR498G0203620800.01.T01 | T-complex protein 1 subunit delta |
| maker-2-augustus-gene-137.8-mRNA-1 | Japonica | NA | | | OsR498G1220780000.01.T03 | Tetratricopeptide repeat (TPR)-like superfamily protein |
| maker-2-augustus-gene-140.19-mRNA-1 | Japonica | NA | | Os02g0433600 | OsR498G0203625700.01.T04 | BHLH transcription factor |
| maker-2-augustus-gene-141.32-mRNA-1 | Japonica | NA | | Os02g0435000 | OsR498G0203632400.01.T01 | Translocation protein SEC62 |
| maker-2-augustus-gene-141.38-mRNA-1 | Japonica | NA | | | OsR498G0203633600.01.T04 | |
| maker-2-augustus-gene-141.42-mRNA-1 | Japonica | NA | | Os02g0436400 | OsR498G0203639000.01.T01 | 4-hydroxy-tetrahydrodipicolinate reductase 2 chloroplastic |
| maker-2-augustus-gene-142.0-mRNA-1 | Japonica | NA | | Os02g0438200 | OsR498G0203646900.01.T02 | CDK9 kinase-activating protein cyclin T |
| maker-2-augustus-gene-142.13-mRNA-1 | Japonica | NA | | | OsR498G0203643800.01.T03 | t-SNARE coiled-coil homology domain-containing protein |
| maker-2-augustus-gene-142.15-mRNA-1 | Japonica | NA | | Os02g0437800 | OsR498G0203645800.01.T07 | Vacuolar protein sorting-associated protein 45 isogeny |
| maker-2-augustus-gene-142.5-mRNA-1 | Japonica | Indica | OsSNAP32 | Os02g0437200 | OsR498G0203643200.01.T06 | SNAPous protein SNAP33 |
| maker-2-augustus-gene-143.0-mRNA-1 | Japonica | NA | | | OsR498G0203648500.01.T01 | Reverse transcriptase Ty1/copia-type domain-containing protein |
| maker-2-augustus-gene-143.2-mRNA-1 | Japonica | NA | | Os02g0439200 | OsR498G0203648900.01.T01 | Putative WEB family protein chloroplastic |
| maker-2-augustus-gene-143.4-mRNA-1 | Japonica | NA | | Os02g0440000 | OsR498G0203653400.01.T02 | Diaminopimelate decarboxylase |
| maker-2-augustus-gene-144.0-mRNA-1 | Japonica | Indica | | Os10g0122600 | OsR498G0203657700.01.T01 | Phosphatidylinositol/phosphatidylcholine transfer protein SFH12-like |
| maker-2-augustus-gene-146.2-mRNA-1 | Japonica | NA | | | | KARI N-terminal Rossmann domain-containing protein |
| maker-2-augustus-gene-146.6-mRNA-1 | Japonica | Indica | | Os02g0701833 | OsR498G0203665800.01.T01 | ATP-dependent DNA helicase |
| maker-2-augustus-gene-147.0-mRNA-1 | Japonica | NA | | | | Transposon protein, putative, CACTA, En/Spm sub-class |
| maker-2-augustus-gene-148.0-mRNA-1 | Japonica | NA | | | OsR498G0203677700.01.T01 | SWIM-type domain-containing protein |
| maker-2-augustus-gene-148.3-mRNA-1 | Japonica | NA | | Os02g0448400 | OsR498G0203676600.01.T01 | Synaptotagmin-2 |
| maker-2-augustus-gene-151.31-mRNA-1 | Japonica | NA | | Os01g0372100 | OsR498G1018392400.01.T01 | |
| maker-2-augustus-gene-152.0-mRNA-1 | Japonica | NA | | Os02g0452800 | | Epimerase domain-containing protein |
| maker-2-augustus-gene-152.1-mRNA-1 | Japonica | NA | | | OsR498G0203707200.01.T05 | SNARE-interacting protein KEULE |
| maker-2-augustus-gene-152.7-mRNA-1 | Japonica | NA | | Os02g0453600 | OsR498G0203710800.01.T01 | Glutelin type-B 5 |
| maker-2-augustus-gene-152.9-mRNA-1 | Japonica | Indica | | Os08g0114100 | OsR498G0203711000.01.T04 | |
| maker-2-augustus-gene-153.32-mRNA-1 | Japonica | NA | | | | |
| maker-2-augustus-gene-154.38-mRNA-1 | Japonica | Indica | | Os07g0165500 | OsR498G0203722500.01.T01 | Cytokinin inducible protein-like |
| maker-2-augustus-gene-154.40-mRNA-1 | Japonica | NA | | | OsR498G0203722700.01.T02 | Glutelin type-A 1 |
| maker-2-augustus-gene-154.43-mRNA-1 | Japonica | Indica | | Os02g0456000 | OsR498G0203722400.01.T02 | Component of oligomeric Golgi complex 4 |
| maker-2-augustus-gene-154.46-mRNA-1 | Japonica | NA | | Os02g0456200 | OsR498G0203723000.01.T05 | Eukaryotic peptide chain release factor GTP-binding subunit ERF3A |
| maker-2-augustus-gene-154.54-mRNA-1 | Japonica | NA | | | | Histone domain-containing protein |
| maker-2-augustus-gene-155.0-mRNA-1 | Japonica | NA | | Os02g0458800 | | GDSL-motif lipase/hydrolase family protein |
| maker-2-augustus-gene-155.2-mRNA-1 | Japonica | NA | | Os02g0457500 | OsR498G0203727200.01.T01 | ankyrin repeat domain-containing protein 17-like |
| maker-2-augustus-gene-155.4-mRNA-1 | Japonica | NA | | | | Sesquiterpene cyclase |

| | | | | | | |
|---|---|---|---|---|---|---|
| maker-2-augustus-gene-155.6-mRNA-1 | Japonica | NA | | | OsR498G0203733000.01.T02 | Terpene synthase TPS3 |
| maker-2-augustus-gene-156.40-mRNA-1 | Japonica | Indica | | Os02g0459800 | OsR498G0203738100.01.T01 | |
| maker-2-augustus-gene-156.42-mRNA-1 | Japonica | Japonica | | Os02g0459300 | OsR498G0203736400.01.T01 | Microtubule-binding protein TANGLED |
| maker-2-augustus-gene-157.2-mRNA-1 | Japonica | Japonica | | Os02g0462000 | OsR498G0102635500.01.T01 | |
| maker-2-augustus-gene-157.4-mRNA-1 | Japonica | NA | | Os02g0462800 | OsR498G0203747100.01.T01 | HKT12 transporter |
| maker-2-augustus-gene-157.6-mRNA-1 | Japonica | NA | | Os02g0461900 | OsR498G0203745800.01.T02 | Actin associated protein |
| maker-2-augustus-gene-157.9-mRNA-1 | Japonica | NA | | | | Putative extracellular ribonuclease |
| maker-2-augustus-gene-158.2-mRNA-1 | Japonica | NA | | | OsR498G0203749800.01.T03 | Carboxypeptidase |
| maker-2-augustus-gene-162.21-mRNA-1 | Japonica | NA | | Os02g0471300 | OsR498G0203781300.01.T02 | 3-hydroxybenzoate 6-hydroxylase 1 |
| maker-2-augustus-gene-162.25-mRNA-1 | Japonica | NA | | | | C2 domain-containing protein |
| maker-2-augustus-gene-162.31-mRNA-1 | Japonica | NA | | Os02g0471500 | OsR498G0203782000.01.T01 | Catalytic/protein phosphatase type 2C |
| maker-2-augustus-gene-163.26-mRNA-1 | Japonica | NA | Kinase-TM | Os02g0472700 | OsR498G0203785700.01.T05 | Putative pathogenesis-related protein 1 |
| maker-2-augustus-gene-163.30-mRNA-1 | Japonica | NA | | | | |
| maker-2-augustus-gene-163.32-mRNA-1 | Japonica | NA | | | OsR498G0917607000.01.T01 | |
| maker-2-augustus-gene-163.34-mRNA-1 | Japonica | NA | | Os02g0473000 | OsR498G0203786400.01.T03 | 5-amino-6-(5-phosphoribosylamino)uracil reductase |
| maker-2-augustus-gene-163.39-mRNA-1 | Japonica | Japonica | | Os02g0473200 | OsR498G0203787200.01.T04 | Peptidase A1 domain-containing protein |
| maker-2-augustus-gene-164.0-mRNA-1 | Japonica | NA | | | OsR498G0203789900.01.T01 | DNA topoisomerase |
| maker-2-augustus-gene-164.9-mRNA-1 | Japonica | NA | | | | Importin N-terminal domain-containing protein (Fragment) |
| maker-2-augustus-gene-165.23-mRNA-1 | Japonica | NA | | Os02g0475300 | OsR498G0203794900.01.T02 | MACPF domain-containing protein isoform X1 |
| maker-2-augustus-gene-165.28-mRNA-1 | Japonica | NA | | Os02g0475400 | OsR498G0203795300.01.T02 | Sodium/metabolite cotransporter like |
| maker-2-augustus-gene-166.0-mRNA-1 | Japonica | NA | | | OsR498G0203801600.01.T02 | Brevis plant1 |
| maker-2-augustus-gene-173.3-mRNA-1 | Japonica | NA | | | OsR498G0203840000.01.T02 | Probable UDP-N-acetylglucosamine--peptide N-acetylglucosaminyltransfera |
| maker-2-augustus-gene-174.35-mRNA-1 | Japonica | NA | | Os02g0489800 | | Kinesin-like protein KIN-12A |
| maker-2-augustus-gene-174.38-mRNA-1 | Japonica | NA | | Os02g0490000 | OsR498G0203841100.01.T01 | RING-type E3 ubiquitin transferase |
| maker-2-augustus-gene-174.40-mRNA-1 | Japonica | NA | | Os02g0490500 | OsR498G0203842300.01.T01 | UDP-glucuronosyl/UDP-glucosyltransferase |
| maker-2-augustus-gene-174.42-mRNA-1 | Japonica | Indica | | Os02g0491700 | OsR498G0203848100.01.T01 | |
| maker-2-augustus-gene-174.45-mRNA-1 | Japonica | NA | | Os02g0491400 | OsR498G0203847400.01.T01 | Peptidylprolyl isomerase |
| maker-2-augustus-gene-174.50-mRNA-1 | Japonica | NA | | Os02g0491600 | OsR498G0203847800.01.T01 | |
| maker-2-augustus-gene-175.31-mRNA-1 | Japonica | NA | | | | Kinase D-interacting substrate of 220 kDa |
| maker-2-augustus-gene-175.33-mRNA-1 | Japonica | Indica | | Os02g0494400 | OsR498G0203851400.01.T01 | Putative ribosomal RNA apurinic site specific lyase |
| maker-2-augustus-gene-175.35-mRNA-1 | Japonica | Indica | | Os02g0493300 | OsR498G0203851600.01.T03 | Ankyrin-2 |
| maker-2-augustus-gene-175.37-mRNA-1 | Japonica | NA | | Os02g0492900 | OsR498G0203852200.01.T01 | Ankyrin-like protein-like protein |
| maker-2-augustus-gene-175.41-mRNA-1 | Japonica | Indica | | Os02g0493050 | OsR498G0203852400.01.T02 | Serine/threonine-protein phosphatase 6 regulatory ankyrin repeat subunit B |
| maker-2-augustus-gene-176.0-mRNA-1 | Japonica | NA | | | | Cortactin-binding protein 2 |
| maker-2-augustus-gene-176.11-mRNA-1 | Japonica | Japonica | | Os02g0493300 | OsR498G0203853000.01.T02 | Serine/threonine-protein phosphatase 6 regulatory ankyrin repeat subunit B |
| maker-2-augustus-gene-176.12-mRNA-1 | Japonica | NA | | Os02g0493300 | OsR498G0203853000.01.T05 | Ankyrin-like protein-like protein |
| maker-2-augustus-gene-176.21-mRNA-1 | Japonica | Indica | | Os02g0494400 | OsR498G0203854200.01.T03 | Putative ribosomal RNA apurinic site specific lyase |
| maker-2-augustus-gene-176.22-mRNA-1 | Japonica | Japonica | | Os02g0494400 | OsR498G0203857500.01.T02 | Putative ribosomal RNA apurinic site specific lyase |
| maker-2-augustus-gene-176.6-mRNA-1 | Japonica | NA | | Os02g0494700 | | Repressor of silencing 3 |
| maker-2-augustus-gene-176.8-mRNA-1 | Japonica | Indica | | Os06g0492900 | OsR498G0203852800.01.T02 | Putative ribosomal RNA apurinic site specific lyase |
| maker-2-augustus-gene-177.0-mRNA-1 | Japonica | NA | | | | Transcription factor bHLH14 |
| maker-2-augustus-gene-177.12-mRNA-1 | Japonica | NA | | | | BED-type domain-containing protein |
| maker-2-augustus-gene-177.2-mRNA-1 | Japonica | NA | | Os02g0496100 | OsR498G0203858900.01.T01 | Heat shock transcription factor |
| maker-2-augustus-gene-177.4-mRNA-1 | Japonica | Japonica | | Os02g0496500 | OsR498G0203859700.01.T06 | Transcriptional activator DEMETER |
| maker-2-augustus-gene-178.0-mRNA-1 | Japonica | Indica | | Os02g0498300 | OsR498G0203864300.01.T02 | Probable magnesium transporter |
| maker-2-augustus-gene-178.13-mRNA-1 | Japonica | Indica | | Os04g0372800 | OsR498G0203864000.01.T01 | Nuclear transport factor 2 (NTF2) family protein with RNA binding (RRM-RBD |
| maker-2-augustus-gene-178.17-mRNA-1 | Japonica | NA | | Os02g0498700 | OsR498G0203866200.01.T01 | Hexosyltransferase |
| maker-2-augustus-gene-178.7-mRNA-1 | Japonica | NA | | Os02g0497500 | OsR498G0203863600.01.T02 | DNA repair protein RAD50 |
| maker-2-augustus-gene-179.30-mRNA-1 | Japonica | NA | | | OsR498G0203867900.01.T01 | Ethylene-responsive transcription factor-like protein At4g13040 |
| maker-2-augustus-gene-181.0-mRNA-1 | Japonica | Indica | | Os09g0441400 | OsR498G0203881300.01.T01 | Flavonoid 3'-monooxygenase |
| maker-2-augustus-gene-182.28-mRNA-1 | Japonica | NA | CC-NBS-TM | | OsR498G0203882300.01.T01 | CC-NBS-LRR type disease resistance protein NLR2-V |
| maker-2-augustus-gene-182.32-mRNA-1 | Japonica | Japonica | | Os02g0503700 | OsR498G0203882700.01.T01 | Isoflavone 2'-hydroxylase |
| maker-2-augustus-gene-183.11-mRNA-1 | Japonica | Indica | | Os02g0504800 | OsR498G0203887400.01.T01 | Acyl-[acyl-carrier-protein] desaturase |
| maker-2-augustus-gene-183.6-mRNA-1 | Japonica | Japonica | | Os02g0504700 | OsR498G0203887100.01.T01 | AvrRpt-cleavage domain-containing protein (Fragment) |
| maker-2-augustus-gene-183.9-mRNA-1 | Japonica | NA | | | OsR498G0203891300.01.T01 | |
| maker-2-augustus-gene-184.0-mRNA-1 | Japonica | NA | | Os02g0506500 | | Ubiquitin-like modifier-activating enzyme 5 |
| maker-2-augustus-gene-184.2-mRNA-1 | Japonica | NA | | Os02g0506400 | OsR498G0203893200.01.T01 | Putative F-box domain, Zinc finger, MYND-type, tetratricopeptide-like helica |
| maker-2-augustus-gene-184.4-mRNA-1 | Japonica | NA | | | OsR498G0815948900.01.T01 | Transposon protein, putative, CACTA, En/Spm sub-class |
| maker-2-augustus-gene-185.0-mRNA-1 | Japonica | NA | | Os02g0507400 | OsR498G0203899300.01.T01 | Fibrous sheath-interacting protein |
| maker-2-augustus-gene-185.15-mRNA-1 | Japonica | NA | | Os02g0510300 | OsR498G0203903800.01.T02 | Proline-rich protein HaeIII subfamily 1 |
| maker-2-augustus-gene-190.3-mRNA-1 | Japonica | NA | LRR-TM | | | |

| | | | | | | |
|---|---|---|---|---|---|---|
| maker-2-augustus-gene-192.41-mRNA-1 | Japonica | NA | | Os05g0471800 | | Far-red impaired response protein-like |
| maker-2-augustus-gene-192.48-mRNA-1 | Japonica | NA | | | | ZF-HD dimerization-type domain-containing protein |
| maker-2-augustus-gene-194.1-mRNA-1 | Japonica | NA | | | | Putative AC transposase |
| maker-2-augustus-gene-194.11-mRNA-1 | Japonica | NA | | Os02g0521700 | OsR498G0203955900.01.T01 | Acyl-CoA thioesterase, putative |
| maker-2-augustus-gene-194.15-mRNA-1 | Japonica | NA | | Os02g0520800 | OsR498G0203954000.01.T01 | Cytochrome b-c1 complex subunit Rieske, mitochondrial |
| maker-2-augustus-gene-194.17-mRNA-1 | Japonica | NA | | | OsR498G0203954400.01.T01 | Ethylene-responsive transcription factor ERF115 |
| maker-2-augustus-gene-194.19-mRNA-1 | Japonica | NA | | Os02g0521300 | OsR498G0203954800.01.T04 | Calcium-dependent phospholipid-binding Copine family protein (Fragment) |
| maker-2-augustus-gene-194.3-mRNA-1 | Japonica | NA | | | OsR498G0203955500.01.T03 | Ubiquitinyl hydrolase 1 |
| maker-2-augustus-gene-194.7-mRNA-1 | Japonica | NA | | | OsR498G0203955700.01.T02 | Ubiquitinyl hydrolase 1 |
| maker-2-augustus-gene-195.35-mRNA-1 | Japonica | NA | | | | Ubiquitinyl hydrolase 1 |
| maker-2-augustus-gene-195.41-mRNA-1 | Japonica | NA | | Os02g0523300 | OsR498G0203961600.01.T02 | Gamma-tubulin complex component |
| maker-2-augustus-gene-195.49-mRNA-1 | Japonica | NA | | Os02g0523500 | OsR498G0203961800.01.T03 | Ribonuclease |
| maker-2-augustus-gene-196.0-mRNA-1 | Japonica | NA | | | OsR498G0203969700.01.T05 | Adenylyltransferase and sulfurtransferase MOCS3 |
| maker-2-augustus-gene-199.12-mRNA-1 | Japonica | NA | | | | Voltage-dependent anion channel |
| maker-2-augustus-gene-2.37-mRNA-1 | Japonica | NA | | Os02g0103600 | OsR498G0202807200.01.T01 | Ferredoxin--NADP(+) reductase |
| maker-2-augustus-gene-2.39-mRNA-1 | Japonica | NA | | Os02g0103700 | OsR498G0202807400.01.T01 | 60S ribosomal protein L9 |
| maker-2-augustus-gene-2.41-mRNA-1 | Japonica | NA | | Os02g0103800 | OsR498G0202807600.01.T01 | Ferredoxin--NADP reductase, chloroplastic |
| maker-2-augustus-gene-2.44-mRNA-1 | Japonica | NA | | Os02g0103900 | OsR498G0202807800.01.T01 | U3 small nucleolar RNA-associated protein 6 isogeny |
| maker-2-augustus-gene-2.47-mRNA-1 | Japonica | Indica | | Os02g0104500 | OsR498G0202810200.01.T01 | Trihelix transcription factor GT-2 |
| maker-2-augustus-gene-20.59-mRNA-1 | Japonica | NA | | Os02g0137800 | OsR498G0202927100.01.T01 | Guanine nucleotide-binding protein subunit gamma 2 |
| maker-2-augustus-gene-21.34-mRNA-1 | Japonica | NA | | | OsR498G0202932700.01.T04 | Vacuolar cation/proton exchanger |
| maker-2-augustus-gene-21.38-mRNA-1 | Japonica | NA | | Os02g0139300 | OsR498G0202934300.01.T01 | Glucan endo-1,3-beta-D-glucosidase |
| maker-2-augustus-gene-21.40-mRNA-1 | Japonica | Indica | | Os06g0703500 | OsR498G0202935500.01.T01 | Squamosa promoter-binding-like protein 3 |
| maker-2-augustus-gene-21.43-mRNA-1 | Japonica | NA | | | OsR498G0202936700.01.T04 | Aspartyl-tRNA synthetase |
| maker-2-augustus-gene-21.48-mRNA-1 | Japonica | NA | | Os02g0139000 | OsR498G0202933000.01.T03 | Protein PHOSPHATE STARVATION RESPONSE 3 |
| maker-2-augustus-gene-21.61-mRNA-1 | Japonica | NA | | | | Cycloartenol synthase |
| maker-2-augustus-gene-21.70-mRNA-1 | Japonica | NA | | Os02g0139700 | OsR498G0202937000.01.T01 | Cycloartenol synthase |
| maker-2-augustus-gene-21.76-mRNA-1 | Japonica | NA | | | | Terpene cyclase/mutase family member |
| maker-2-augustus-gene-210.34-mRNA-1 | Japonica | NA | Kinase-TM | Os02g0549200 | OsR498G0204084500.01.T02 | probable serine/threonine-protein kinase At1g01540 |
| maker-2-augustus-gene-211.0-mRNA-1 | Japonica | NA | | Os02g0549600 | OsR498G0204085100.01.T01 | Ribosomal protein S10 |
| maker-2-augustus-gene-211.11-mRNA-1 | Japonica | NA | | Os02g0550300 | OsR498G0204089100.01.T01 | Thioredoxin |
| maker-2-augustus-gene-211.14-mRNA-1 | Japonica | NA | | Os02g0550100 | OsR498G0204088600.01.T01 | V-type proton ATPase proteolipid subunit |
| maker-2-augustus-gene-211.16-mRNA-1 | Japonica | NA | | Os02g0550600 | OsR498G0204090800.01.T02 | Alkaline/neutral invertase |
| maker-2-augustus-gene-211.18-mRNA-1 | Japonica | Indica | | Os02g0550700 | OsR498G0204091600.01.T02 | Exosome complex component RRP45B like |
| maker-2-augustus-gene-211.4-mRNA-1 | Japonica | NA | CC-NBS | | OsR498G0204086500.01.T01 | |
| maker-2-augustus-gene-211.8-mRNA-1 | Japonica | NA | | Os02g0550000 | OsR498G0204088100.01.T01 | |
| maker-2-augustus-gene-212.0-mRNA-1 | Japonica | NA | | Os02g0550800 | OsR498G0204091900.01.T02 | Ammonium transporter |
| maker-2-augustus-gene-212.12-mRNA-1 | Japonica | NA | Kinase-TM | Os02g0551100 | OsR498G0204092600.01.T01 | Serine/threonine-protein kinase SAPK7 |
| maker-2-augustus-gene-212.18-mRNA-1 | Japonica | NA | | Os02g0551400 | OsR498G0204093800.01.T02 | HTH myb-type domain-containing protein |
| maker-2-augustus-gene-212.22-mRNA-1 | Japonica | NA | | | OsR498G0204095000.01.T01 | Zinc finger protein Pegasus |
| maker-2-augustus-gene-212.3-mRNA-1 | Japonica | NA | | Os02g0551900 | | zinc finger transcription factor YY1 |
| maker-2-augustus-gene-212.5-mRNA-1 | Japonica | NA | | Os02g0552000 | OsR498G0204096700.01.T01 | Transmembrane 9 superfamily member |
| maker-2-augustus-gene-213.32-mRNA-1 | Japonica | NA | | | OsR498G0204101300.01.T01 | Tetratricopeptide repeat protein like |
| maker-2-augustus-gene-213.35-mRNA-1 | Japonica | NA | | Os02g0553600 | OsR498G0204101700.01.T02 | RNA polymerase II transcriptional coactivator KIWI like |
| maker-2-augustus-gene-213.44-mRNA-1 | Japonica | NA | | Os02g0554100 | OsR498G0204103500.01.T01 | Ultraviolet-B receptor like |
| maker-2-augustus-gene-213.50-mRNA-1 | Japonica | NA | | Os02g0553200 | OsR498G0204100600.01.T01 | Thylakoid-bound ascorbate peroxidase |
| maker-2-augustus-gene-214.29-mRNA-1 | Japonica | NA | | Os02g0554400 | | |
| maker-2-augustus-gene-214.33-mRNA-1 | Japonica | NA | | Os02g0555300 | OsR498G0204107300.01.T02 | NAC transcription factor |
| maker-2-augustus-gene-214.36-mRNA-1 | Japonica | NA | | Os02g0555600 | OsR498G0204107700.01.T05 | ACT domain repeat 3 |
| maker-2-augustus-gene-214.41-mRNA-1 | Japonica | Japonica | | Os02g0554900 | OsR498G0204106600.01.T01 | Protein disulfide-isomerase |
| maker-2-augustus-gene-214.43-mRNA-1 | Japonica | NA | | Os02g0555000 | OsR498G0204106800.01.T03 | GPN-loop GTPase 2 |
| maker-2-augustus-gene-214.47-mRNA-1 | Japonica | NA | | | | |
| maker-2-augustus-gene-214.50-mRNA-1 | Japonica | NA | | Os02g0555700 | OsR498G0204108000.01.T02 | DnaJ domain |
| maker-2-augustus-gene-214.54-mRNA-1 | Japonica | NA | Kinase-TM | Os02g0555900 | OsR498G0204108200.01.T03 | Mitogen-activated protein kinase kinase kinase YODA |
| maker-2-augustus-gene-214.60-mRNA-1 | Japonica | NA | | Os02g0556000 | OsR498G0204108600.01.T01 | Hexosyltransferase |
| maker-2-augustus-gene-215.32-mRNA-1 | Japonica | Japonica | | Os02g0556100 | OsR498G0204109300.01.T01 | Catalytic/ coenzyme binding protein |
| maker-2-augustus-gene-215.38-mRNA-1 | Japonica | NA | | Os02g0556700 | OsR498G0204110300.01.T01 | T-complex protein 11 |
| maker-2-augustus-gene-215.41-mRNA-1 | Japonica | NA | | Os02g0557100 | OsR498G0204111700.01.T02 | AIG1-type G domain-containing protein (Fragment) |
| maker-2-augustus-gene-215.43-mRNA-1 | Japonica | NA | | Os02g0557200 | OsR498G0204112700.01.T01 | Auxin response factor |
| maker-2-augustus-gene-215.49-mRNA-1 | Japonica | Indica | | Os02g0557500 | OsR498G0204113100.01.T01 | zinc finger CCCH domain-containing protein 5 |
| maker-2-augustus-gene-215.54-mRNA-1 | Japonica | NA | | Os02g0557700 | OsR498G0204113500.01.T01 | NADPH-dependent pterin aldehyde reductase |

| | | | | | | |
|---|---|---|---|---|---|---|
| maker-2-augustus-gene-215.58-mRNA-1 | Japonica | NA | | | OsR498G0204109800.01.T03 | tRNA guanine10-N2-methyltransferase |
| maker-2-augustus-gene-215.65-mRNA-1 | Japonica | NA | | | OsR498G0204110200.01.T02 | SPla/RYanodine receptor (SPRY) domain-containing protein |
| maker-2-augustus-gene-215.70-mRNA-1 | Japonica | NA | | Os02g0556800 | OsR498G0204110800.01.T01 | protein MAINTENANCE OF PSII UNDER HIGH LIGHT 1 |
| maker-2-augustus-gene-215.72-mRNA-1 | Japonica | NA | | Os02g0556900 | OsR498G0204111200.01.T01 | DDT transcription factor |
| maker-2-augustus-gene-215.75-mRNA-1 | Japonica | NA | | Os02g0557300 | OsR498G0204113000.01.T01 | Ring-h2 finger protein atl58 |
| maker-2-augustus-gene-215.77-mRNA-1 | Japonica | NA | | Os02g0557600 | OsR498G0204113400.01.T02 | Ligatin |
| maker-2-augustus-gene-215.80-mRNA-1 | Japonica | Japonica | | Os02g0557800 | OsR498G0204113800.01.T02 | Two-component response regulator ARR16 |
| maker-2-augustus-gene-216.0-mRNA-1 | Japonica | NA | CC-NBS-TM | | OsR498G0204117300.01.T01 | Disease resistance protein RPM1 |
| maker-2-augustus-gene-216.10-mRNA-1 | Japonica | Indica | | Os02g0558500 | OsR498G0204117800.01.T01 | |
| maker-2-augustus-gene-216.12-mRNA-1 | Japonica | NA | | Os02g0558600 | OsR498G0204118000.01.T02 | TPX2 (Targeting protein for Xklp2) protein family |
| maker-2-augustus-gene-216.17-mRNA-1 | Japonica | NA | | Os02g0559400 | OsR498G0204120400.01.T05 | 12-oxophytodienoate reductase |
| maker-2-augustus-gene-216.2-mRNA-1 | Japonica | NA | CC-Kinase-TM | Os02g0559300 | OsR498G0204120100.01.T02 | Putative serine/threonine-protein kinase |
| maker-2-augustus-gene-216.5-mRNA-1 | Japonica | Indica | | Os02g0558100 | OsR498G0204116600.01.T04 | Chloride channel protein CLC-c |
| maker-2-augustus-gene-217.0-mRNA-1 | Japonica | Indica | | Os02g0471300 | OsR498G0204121100.01.T04 | Monooxygenase 1 |
| maker-2-augustus-gene-217.11-mRNA-1 | Japonica | Indica | | Os02g0562400 | OsR498G0204123800.01.T01 | Ankyrin repeat-containing domain-containing protein |
| maker-2-augustus-gene-217.13-mRNA-1 | Japonica | NA | | | OsR498G0204124000.01.T05 | |
| maker-2-augustus-gene-217.2-mRNA-1 | Japonica | NA | | Os02g0562300 | OsR498G0204123500.01.T03 | Calmodulin binding protein1 |
| maker-2-augustus-gene-217.7-mRNA-1 | Japonica | NA | | Os02g0562700 | OsR498G0204125300.01.T04 | Glyoxylate reductase 1 |
| maker-2-augustus-gene-218.0-mRNA-1 | Japonica | Indica | | Os04g0444900 | OsR498G0204130100.01.T01 | PHD finger protein Alfin1 |
| maker-2-augustus-gene-218.11-mRNA-1 | Japonica | NA | | Os02g0565100 | OsR498G0204131700.01.T04 | Nucleoside diphosphate kinase |
| maker-2-augustus-gene-218.15-mRNA-1 | Japonica | NA | | Os02g0564000 | OsR498G0204130000.01.T03 | Glutathione S-transferase |
| maker-2-augustus-gene-218.21-mRNA-1 | Japonica | NA | | Os02g0564500 | OsR498G0204130800.01.T01 | Vesicle transport protein (Fragment) |
| maker-2-augustus-gene-218.26-mRNA-1 | Japonica | NA | | Os02g0565200 | OsR498G0204132400.01.T02 | Signal peptidase complex subunit 2 |
| maker-2-augustus-gene-218.4-mRNA-1 | Japonica | Indica | | Os01g0886600 | OsR498G0204130500.01.T01 | AAA+ ATPase domain |
| maker-2-augustus-gene-218.9-mRNA-1 | Japonica | NA | | | OsR498G0204130900.01.T01 | protein SENESCENCE-ASSOCIATED GENE 21, mitochondrial-like |
| maker-2-augustus-gene-219.0-mRNA-1 | Japonica | NA | | Os02g0566400 | OsR498G0204135900.01.T01 | Extracellular ligand-gated ion channel |
| maker-2-augustus-gene-219.12-mRNA-1 | Japonica | NA | | Os02g0566900 | OsR498G0204137200.01.T01 | Transmembrane protein 50 |
| maker-2-augustus-gene-219.14-mRNA-1 | Japonica | NA | | Os02g0567000 | OsR498G0204137400.01.T01 | Salt tolerant protein |
| maker-2-augustus-gene-219.16-mRNA-1 | Japonica | NA | | Os02g0567100 | OsR498G0204137600.01.T02 | Thioredoxin-like 4, chloroplastic |
| maker-2-augustus-gene-219.18-mRNA-1 | Japonica | NA | | Os02g0567200 | OsR498G0204138000.01.T04 | PPM-type phosphatase domain-containing protein |
| maker-2-augustus-gene-219.2-mRNA-1 | Japonica | NA | | Os02g0566700 | OsR498G0204136700.01.T01 | |
| maker-2-augustus-gene-219.5-mRNA-1 | Japonica | NA | | Os02g0565600 | OsR498G0204133800.01.T01 | Homeodomain leucine-zipper protein Hox7 |
| maker-2-augustus-gene-219.7-mRNA-1 | Japonica | NA | | Os02g0566500 | OsR498G0204136200.01.T02 | WRKY domain-containing protein |
| maker-2-augustus-gene-219.8-mRNA-1 | Japonica | Indica | | Os09g0452900 | OsR498G0204137000.01.T01 | Hexosyltransferase |
| maker-2-augustus-gene-22.32-mRNA-1 | Japonica | NA | | Os02g0140400 | OsR498G0202938800.01.T03 | Camelliol C synthase |
| maker-2-augustus-gene-22.44-mRNA-1 | Japonica | NA | | Os02g0141300 | OsR498G0202943400.01.T02 | GHMP kinase N-terminal domain |
| maker-2-augustus-gene-22.51-mRNA-1 | Japonica | NA | | Os02g0141500 | OsR498G0202944200.01.T01 | |
| maker-2-augustus-gene-220.40-mRNA-1 | Japonica | Indica | | Os04g0449900 | OsR498G0204139900.01.T02 | Polyadenylate-binding protein RBP47 |
| maker-2-augustus-gene-220.44-mRNA-1 | Japonica | NA | | Os02g0568200 | OsR498G0204140800.01.T01 | Coleoptile phototropism protein 1 |
| maker-2-augustus-gene-220.46-mRNA-1 | Japonica | NA | | | OsR498G0204142600.01.T01 | Terpene synthase family, metal binding domain containing protein, expresse |
| maker-2-augustus-gene-220.48-mRNA-1 | Japonica | NA | | | OsR498G0204142800.01.T01 | Ent-kaurene synthase |
| maker-2-augustus-gene-220.49-mRNA-1 | Japonica | NA | | | OsR498G1119549200.01.T02 | |
| maker-2-augustus-gene-221.0-mRNA-1 | Japonica | NA | | | | Ent-cassa-12,15-diene synthase |
| maker-2-augustus-gene-221.6-mRNA-1 | Japonica | NA | | Os02g0570500 | OsR498G0204148100.01.T01 | Ent-isokaurene C2-hydroxylase |
| maker-2-augustus-gene-222.33-mRNA-1 | Japonica | Indica | | Os02g0571300 | OsR498G0204152600.01.T01 | Ent-isokaurene synthase |
| maker-2-augustus-gene-222.34-mRNA-1 | Japonica | NA | | Os02g0571800 | OsR498G0204152600.01.T02 | Ent-isokaurene synthase |
| maker-2-augustus-gene-223.0-mRNA-1 | Japonica | NA | | Os02g0573200 | OsR498G0204159700.01.T01 | Photolyase/cryptochrome alpha/beta domain-containing protein |
| maker-2-augustus-gene-223.14-mRNA-1 | Japonica | NA | | Os02g0573400 | OsR498G0204160200.01.T01 | Ubiquitin carboxyl-terminal hydrolase |
| maker-2-augustus-gene-223.3-mRNA-1 | Japonica | NA | | Os02g0572400 | OsR498G0204155800.01.T02 | GTP cyclohydrolase II |
| maker-2-augustus-gene-223.6-mRNA-1 | Japonica | NA | | Os02g0572600 | OsR498G0204156800.01.T01 | Myosin heavy chain-related protein |
| maker-2-augustus-gene-223.9-mRNA-1 | Japonica | NA | | | OsR498G0204160000.01.T04 | Nucleotide-sugar transporter/ sugar porter |
| maker-2-augustus-gene-224.36-mRNA-1 | Japonica | NA | | Os02g0573500 | OsR498G0204160900.01.T01 | Sugar transport protein 5 |
| maker-2-augustus-gene-224.38-mRNA-1 | Japonica | NA | | Os02g0574100 | OsR498G0204161900.01.T01 | Sugar transport protein 5 |
| maker-2-augustus-gene-224.41-mRNA-1 | Japonica | NA | | Os02g0574100 | OsR498G0204162100.01.T01 | Sugar transport protein 5 |
| maker-2-augustus-gene-224.43-mRNA-1 | Japonica | NA | | Os02g0575500 | OsR498G0204166100.01.T02 | Protein ACTIVITY OF BC1 COMPLEX KINASE 8, chloroplastic isoform A |
| maker-2-augustus-gene-224.47-mRNA-1 | Japonica | NA | | Os02g0574500 | OsR498G0204163400.01.T02 | Sugar transport protein 5 |
| maker-2-augustus-gene-224.49-mRNA-1 | Japonica | NA | | Os02g0574600 | OsR498G0204164000.01.T04 | Putative hematological and neurological expressed 1-like |
| maker-2-augustus-gene-224.52-mRNA-1 | Japonica | NA | | Os02g0575200 | OsR498G0204165800.01.T01 | Phosphopantothenate--cysteine ligase |
| maker-2-augustus-gene-225.0-mRNA-1 | Japonica | NA | | Os02g0575800 | OsR498G0204167100.01.T01 | Aldose 1-epimerase |
| maker-2-augustus-gene-225.14-mRNA-1 | Japonica | NA | | Os02g0577100 | OsR498G0204171000.01.T04 | Zinc finger, RING-CH-type |
| maker-2-augustus-gene-225.5-mRNA-1 | Japonica | NA | | | | |

| | | | | | | |
|---|---|---|---|---|---|---|
| maker-2-augustus-gene-225.7-mRNA-1 | Japonica | NA | | Os02g0576600 | OsR498G0204170400.01.T01 | Sucrose Transporter |
| maker-2-augustus-gene-225.9-mRNA-1 | Japonica | NA | | Os02g0576700 | OsR498G0204170600.01.T01 | Nucleosome/chromatin assembly factor A |
| maker-2-augustus-gene-226.40-mRNA-1 | Japonica | NA | | Os02g0577300 | OsR498G0204171700.01.T03 | Galectin domain-containing protein |
| maker-2-augustus-gene-226.44-mRNA-1 | Japonica | NA | | Os02g0577700 | OsR498G0204172300.01.T01 | Glycosyltransferase |
| maker-2-augustus-gene-226.46-mRNA-1 | Japonica | Indica | | Os02g0578100 | OsR498G0204172500.01.T01 | Glycosyltransferase |
| maker-2-augustus-gene-226.48-mRNA-1 | Japonica | NA | | Os02g0578300 | OsR498G0204173100.01.T01 | Glycosyltransferase |
| maker-2-augustus-gene-226.50-mRNA-1 | Japonica | Indica | | Os04g0460400 | OsR498G0204173500.01.T01 | Casparian strip membrane protein 7 |
| maker-2-augustus-gene-226.52-mRNA-1 | Japonica | Indica | | Os02g0579000 | OsR498G0204175500.01.T05 | NAC transcription factor |
| maker-2-augustus-gene-226.55-mRNA-1 | Japonica | NA | | Os02g0579300 | OsR498G0204176500.01.T01 | Transcription factor MYB74 |
| maker-2-augustus-gene-226.57-mRNA-1 | Japonica | NA | | Os02g0578400 | OsR498G0204173800.01.T01 | Photosynthetic NDH subunit of lumenal location 2 chloroplastic |
| maker-2-augustus-gene-227.0-mRNA-1 | Japonica | NA | | Os02g0579600 | OsR498G0204179700.01.T03 | MADS-box transcription factor |
| maker-2-augustus-gene-227.11-mRNA-1 | Japonica | NA | | Os02g0580700 | OsR498G0204183100.01.T01 | Flavin-containing monooxygenase |
| maker-2-augustus-gene-227.13-mRNA-1 | Japonica | NA | | Os02g0579800 | OsR498G0204180600.01.T01 | Cell number regulator 2 |
| maker-2-augustus-gene-227.16-mRNA-1 | Japonica | Indica | | Os02g0580000 | OsR498G0204181200.01.T01 | Plant cadmium resistance 2 |
| maker-2-augustus-gene-227.19-mRNA-1 | Japonica | NA | | Os02g0580100 | OsR498G0204181600.01.T04 | Plasma-membrane choline transporter family protein |
| maker-2-augustus-gene-227.24-mRNA-1 | Japonica | NA | | Os02g0580400 | OsR498G0204182200.01.T01 | Dynein light chain, cytoplasmic |
| maker-2-augustus-gene-227.4-mRNA-1 | Japonica | NA | | Os02g0580300 | OsR498G0204181700.01.T06 | General regulatory factor2 |
| maker-2-augustus-gene-227.8-mRNA-1 | Japonica | Indica | | Os02g0580600 | OsR498G0204182700.01.T01 | Flavin-containing monooxygenase |
| maker-2-augustus-gene-228.0-mRNA-1 | Japonica | Indica | | Os02g0581000 | OsR498G0204183900.01.T01 | GPI ethanolamine phosphate transferase 1 |
| maker-2-augustus-gene-228.20-mRNA-1 | Japonica | NA | | Os02g0581800 | OsR498G0204186500.01.T01 | |
| maker-2-augustus-gene-228.22-mRNA-1 | Japonica | NA | | Os02g0580900 | OsR498G0204183600.01.T01 | Nitrate transporter 1.3 |
| maker-2-augustus-gene-228.26-mRNA-1 | Japonica | NA | | Os02g0581300 | OsR498G0204185600.01.T01 | LAG1 longevity assurance homolog 3 |
| maker-2-augustus-gene-228.32-mRNA-1 | Japonica | Indica | | Os02g0582150 | OsR498G0204187200.01.T02 | 18S pre-ribosomal assembly protein gar2-related |
| maker-2-augustus-gene-228.35-mRNA-1 | Japonica | Indica | | Os05g0587400 | OsR498G0204187400.01.T01 | |
| maker-2-augustus-gene-228.38-mRNA-1 | Japonica | NA | | Os02g0582400 | OsR498G0204187800.01.T01 | |
| maker-2-augustus-gene-228.40-mRNA-1 | Japonica | NA | | Os02g0582600 | OsR498G0204188600.01.T01 | |
| maker-2-augustus-gene-228.8-mRNA-1 | Japonica | NA | | | | AB hydrolase-1 domain-containing protein |
| maker-2-augustus-gene-229.10-mRNA-1 | Japonica | NA | | Os02g0584200 | | |
| maker-2-augustus-gene-229.12-mRNA-1 | Japonica | NA | | Os02g0584700 | OsR498G0204192200.01.T01 | HMA domain-containing protein (Fragment) |
| maker-2-augustus-gene-229.13-mRNA-1 | Japonica | NA | | Os02g0584800 | OsR498G0204192400.01.T02 | HMA domain-containing protein (Fragment) |
| maker-2-augustus-gene-229.14-mRNA-1 | Japonica | Japonica | | Os02g0585100 | OsR498G0204193000.01.T01 | HMA domain-containing protein |
| maker-2-augustus-gene-229.16-mRNA-1 | Japonica | NA | | Os02g0585700 | | glycine-rich RNA-binding protein 2-like |
| maker-2-augustus-gene-229.6-mRNA-1 | Japonica | Indica | | Os02g0582900 | OsR498G0204189400.01.T01 | |
| maker-2-augustus-gene-229.8-mRNA-1 | Japonica | NA | | Os02g0583000 | OsR498G0204190000.01.T01 | Fanconi anemia group E protein |
| maker-2-augustus-gene-23.0-mRNA-1 | Japonica | NA | | | | |
| maker-2-augustus-gene-23.2-mRNA-1 | Japonica | NA | | Os05g0269800 | | DDE Tnp4 domain-containing protein |
| maker-2-augustus-gene-23.3-mRNA-1 | Japonica | Indica | | Os05g0269800 | OsR498G0202947900.01.T02 | Transposon protein, putative, CACTA, En/Spm sub-class |
| maker-2-augustus-gene-230.54-mRNA-1 | Japonica | NA | | Os02g0586800 | OsR498G0204197700.01.T01 | Protein FLUORESCENT IN BLUE LIGHT chloroplastic |
| maker-2-augustus-gene-230.58-mRNA-1 | Japonica | NA | | Os02g0586900 | OsR498G0204198300.01.T01 | glycine-rich protein 3-like |
| maker-2-augustus-gene-230.60-mRNA-1 | Japonica | NA | | Os02g0587000 | OsR498G0204198700.01.T01 | Glycine-rich cell wall structural protein-like |
| maker-2-augustus-gene-230.64-mRNA-1 | Japonica | NA | | Os02g0587600 | OsR498G0204199700.01.T01 | Xylan 14-beta-xylosidase |
| maker-2-augustus-gene-230.66-mRNA-1 | Japonica | NA | | Os02g0587800 | OsR498G0204200500.01.T01 | TATA-binding protein-associated factor 2N |
| maker-2-augustus-gene-230.68-mRNA-1 | Japonica | NA | | | OsR498G0204200900.01.T01 | Interleukin enhancer binding factor 3 |
| maker-2-augustus-gene-230.70-mRNA-1 | Japonica | NA | | | OsR498G0204201100.01.T01 | Low complexity |
| maker-2-augustus-gene-230.72-mRNA-1 | Japonica | NA | | | | Glycine-rich cell wall structural protein |
| maker-2-augustus-gene-230.74-mRNA-1 | Japonica | NA | | Os02g0586500 | OsR498G0204196600.01.T01 | Small nuclear ribonucleoprotein Sm D1 |
| maker-2-augustus-gene-230.76-mRNA-1 | Japonica | NA | | Os02g0588300 | OsR498G0204202000.01.T03 | Fimbrin 1 |
| maker-2-augustus-gene-231.33-mRNA-1 | Japonica | NA | | Os02g0588500 | OsR498G0204203300.01.T01 | Glycerophosphodiester phosphodiesterase |
| maker-2-augustus-gene-231.39-mRNA-1 | Japonica | NA | | Os02g0588600 | OsR498G0204203500.01.T01 | PTB domain-containing engulfment adapter protein 1 isoform 1 |
| maker-2-augustus-gene-231.42-mRNA-1 | Japonica | NA | | Os02g0589700 | OsR498G0204209400.01.T01 | Lecithin-cholesterol acyltransferase-like 1 |
| maker-2-augustus-gene-232.0-mRNA-1 | Japonica | NA | | | OsR498G0204210500.01.T06 | |
| maker-2-augustus-gene-232.1-mRNA-1 | Japonica | Indica | | Os02g0591500 | OsR498G0204215900.01.T02 | 60S ribosomal protein L7-3 |
| maker-2-augustus-gene-232.7-mRNA-1 | Japonica | NA | Kinase | | OsR498G0204210800.01.T01 | Serine/threonine-protein kinase Nek6 |
| maker-2-augustus-gene-233.15-mRNA-1 | Japonica | NA | | | | Putative WD40/YVTN repeat-like-containing domain-containing protein |
| maker-2-augustus-gene-233.22-mRNA-1 | Japonica | NA | | Os02g0591700 | OsR498G0204216600.01.T01 | 60S ribosomal protein L6 |
| maker-2-augustus-gene-233.24-mRNA-1 | Japonica | NA | | Os02g0591800 | OsR498G0204216800.01.T01 | Brix domain-containing protein 2 |
| maker-2-augustus-gene-234.12-mRNA-1 | Japonica | Indica | | Os04g0477000 | OsR498G0204228500.01.T02 | NPH3 domain-containing protein (Fragment) |
| maker-2-augustus-gene-234.20-mRNA-1 | Japonica | NA | | Os02g0594800 | OsR498G0204230000.01.T01 | NAC domain-containing protein 8 |
| maker-2-augustus-gene-234.22-mRNA-1 | Japonica | NA | | Os02g0594900 | OsR498G0204230400.01.T01 | N-acetylglucosaminyltransferase III |
| maker-2-augustus-gene-235.39-mRNA-1 | Japonica | NA | | Os02g0595800 | OsR498G0204235100.01.T02 | Eukaryotic translation initiation factor 4B |
| maker-2-augustus-gene-235.41-mRNA-1 | Japonica | NA | | | OsR498G0204238100.01.T01 | BTB/POZ and TAZ domain-containing protein 2 |

| | | | | | | |
|---|---|---|---|---|---|---|
| maker-2-augustus-gene-235.43-mRNA-1 | Japonica | NA | | Os02g0596900 | OsR498G0204238700.01.T01 | Actin-related protein 3 isoform A |
| maker-2-augustus-gene-235.48-mRNA-1 | Japonica | NA | | Os02g0595100 | OsR498G0204231800.01.T01 | N-acetylglucosaminyltransferase III |
| maker-2-augustus-gene-235.50-mRNA-1 | Japonica | Japonica | | Os02g0595300 | OsR498G0204233200.01.T05 | |
| maker-2-augustus-gene-235.55-mRNA-1 | Japonica | NA | | Os02g0595500 | OsR498G0204234400.01.T01 | Isocitrate dehydrogenase |
| maker-2-augustus-gene-235.57-mRNA-1 | Japonica | NA | | Os02g0595900 | OsR498G0204235400.01.T01 | High-affinity nitrate transporter |
| maker-2-augustus-gene-235.59-mRNA-1 | Japonica | NA | | Os02g0596000 | OsR498G0204236200.01.T02 | Rhodanese-like domain-containing protein 11 chloroplastic |
| maker-2-augustus-gene-235.64-mRNA-1 | Japonica | NA | | Os02g0596100 | OsR498G0204236600.01.T05 | Nucleoporin GLE1 |
| maker-2-augustus-gene-235.69-mRNA-1 | Japonica | NA | | Os02g0596500 | OsR498G0204237600.01.T01 | Vacuolar protein sorting-associated protein 20 isogeny 2 |
| maker-2-augustus-gene-236.11-mRNA-1 | Japonica | Indica | | Os02g0599100 | OsR498G0204243700.01.T01 | Embryo defective 2423 |
| maker-2-augustus-gene-236.15-mRNA-1 | Japonica | NA | | Os02g0597000 | OsR498G0204239200.01.T01 | Ultraviolet-B receptor UVR8 |
| maker-2-augustus-gene-236.8-mRNA-1 | Japonica | NA | | | OsR498G0204243500.01.T01 | Intracellular protein transport protein USO1 |
| maker-2-augustus-gene-237.135-mRNA-1 | Japonica | NA | | Os02g0601700 | OsR498G0204252100.01.T04 | E3 ubiquitin-protein ligase mind-bomb-like |
| maker-2-augustus-gene-237.145-mRNA-1 | Japonica | NA | | Os02g0601400 | OsR498G0204251600.01.T01 | Premnaspirodiene oxygenase |
| maker-2-augustus-gene-237.147-mRNA-1 | Japonica | Indica | | Os02g0601500 | OsR498G0204251600.01.T01 | Premnaspirodiene oxygenase |
| maker-2-augustus-gene-237.46-mRNA-1 | Japonica | Indica | | Os02g0598400 | OsR498G0204244900.01.T01 | Telomere length regulation protein TEL |
| maker-2-augustus-gene-237.49-mRNA-1 | Japonica | NA | | | | Protein-serine/threonine phosphatase |
| maker-2-augustus-gene-237.51-mRNA-1 | Japonica | Indica | | Os02g0598500 | OsR498G0204246300.01.T03 | PPM-type phosphatase domain-containing protein |
| maker-2-augustus-gene-238.40-mRNA-1 | Japonica | NA | Kinase | Os02g0602100 | OsR498G0204253300.01.T07 | Cyclin-dependent kinase G-1 |
| maker-2-augustus-gene-238.47-mRNA-1 | Japonica | Japonica | | Os02g0602600 | OsR498G0204253900.01.T01 | RRM domain-containing protein |
| maker-2-augustus-gene-238.52-mRNA-1 | Japonica | NA | | | OsR498G0204258300.01.T05 | Transcription factor bHLH128 |
| maker-2-augustus-gene-238.56-mRNA-1 | Japonica | Indica | | Os02g0601800 | OsR498G0204252400.01.T05 | Transcription factor GTE9 |
| maker-2-augustus-gene-238.61-mRNA-1 | Japonica | Indica | | Os04g0488400 | OsR498G0204254200.01.T01 | Protein TWIN SISTER of FT (Fragment) |
| maker-2-augustus-gene-238.65-mRNA-1 | Japonica | NA | | Os02g0603800 | OsR498G0204258800.01.T02 | Hydroxymethylbutenyl 4-diphosphate synthase |
| maker-2-augustus-gene-239.0-mRNA-1 | Japonica | NA | | Os02g0604300 | OsR498G0204261100.01.T01 | UDP-galactose transporter |
| maker-2-augustus-gene-239.13-mRNA-1 | Japonica | NA | | Os02g0605600 | OsR498G0204265700.01.T01 | RNA-binding protein Luc7-like 2 |
| maker-2-augustus-gene-239.19-mRNA-1 | Japonica | NA | | Os02g0606000 | OsR498G0204267500.01.T03 | Chitinase |
| maker-2-augustus-gene-239.22-mRNA-1 | Japonica | NA | | Os02g0605500 | OsR498G0204265600.01.T03 | TRAF-type domain-containing protein (Fragment) |
| maker-2-augustus-gene-239.24-mRNA-1 | Japonica | NA | | Os02g0605900 | OsR498G0204267400.01.T01 | Class IV chitinase |
| maker-2-augustus-gene-239.26-mRNA-1 | Japonica | NA | | | OsR498G0204267800.01.T01 | Eukaryotic translation initiation factor 2A |
| maker-2-augustus-gene-239.32-mRNA-1 | Japonica | NA | | | OsR498G0204269600.01.T03 | tRNA (guanine(37)-N1)-methyltransferase |
| maker-2-augustus-gene-240.0-mRNA-1 | Japonica | NA | | Os02g0606700 | OsR498G0204270100.01.T03 | Isochorismatase domain-containing protein |
| maker-2-augustus-gene-240.10-mRNA-1 | Japonica | NA | | Os02g0608300 | | SWIM-type domain-containing protein |
| maker-2-augustus-gene-240.17-mRNA-1 | Japonica | Indica | | Os02g0606900 | OsR498G0204272000.01.T01 | PPM-type phosphatase domain-containing protein |
| maker-2-augustus-gene-240.20-mRNA-1 | Japonica | NA | | | | Protein-serine/threonine phosphatase |
| maker-2-augustus-gene-240.23-mRNA-1 | Japonica | NA | | Os02g0607700 | OsR498G0204272400.01.T01 | (+)-neomenthol dehydrogenase |
| maker-2-augustus-gene-240.3-mRNA-1 | Japonica | NA | | Os02g0606800 | OsR498G0204270300.01.T01 | Isochorismatase hydrolase |
| maker-2-augustus-gene-240.8-mRNA-1 | Japonica | NA | | Os02g0608100 | | |
| maker-2-augustus-gene-241.12-mRNA-1 | Japonica | NA | | Os02g0608400 | OsR498G0204275400.01.T02 | Leucine zipper-EF-hand-containing transmembrane protein 1 |
| maker-2-augustus-gene-241.17-mRNA-1 | Japonica | NA | Kinase-TM | Os02g0608500 | OsR498G0204275600.01.T03 | Dual specificity protein kinase shkC |
| maker-2-augustus-gene-241.22-mRNA-1 | Japonica | NA | | Os02g0608900 | OsR498G0204276800.01.T01 | Outer envelope pore protein like |
| maker-2-augustus-gene-241.28-mRNA-1 | Japonica | Indica | | Os02g0212600 | OsR498G0204279800.01.T04 | |
| maker-2-augustus-gene-241.5-mRNA-1 | Japonica | NA | | Os02g0609200 | OsR498G0204277100.01.T01 | Monopolar spindle |
| maker-2-augustus-gene-241.7-mRNA-1 | Japonica | NA | | Os02g0609400 | OsR498G0204277700.01.T01 | |
| maker-2-augustus-gene-242.27-mRNA-1 | Japonica | NA | | Os02g0610500 | OsR498G0204282700.01.T01 | COLD |
| maker-2-augustus-gene-242.29-mRNA-1 | Japonica | NA | | Os02g0610800 | OsR498G0204283700.01.T01 | Protein TAB2 homolog, chloroplastic |
| maker-2-augustus-gene-242.31-mRNA-1 | Japonica | NA | | Os02g0611200 | OsR498G0204285300.01.T01 | S-adenosylmethionine decarboxylase proenzyme |
| maker-2-augustus-gene-242.32-mRNA-1 | Japonica | NA | | Os02g0611400 | OsR498G0204285700.01.T01 | Protein NUCLEAR FUSION DEFECTIVE 5 mitochondrial |
| maker-2-augustus-gene-242.34-mRNA-1 | Japonica | NA | | Os02g0610400 | OsR498G0204282000.01.T02 | La protein 1 |
| maker-2-augustus-gene-242.38-mRNA-1 | Japonica | NA | | Os02g0610600 | OsR498G0204283200.01.T03 | Splicing factor, arginine/serine-rich 7 |
| maker-2-augustus-gene-242.42-mRNA-1 | Japonica | NA | | Os02g0610700 | OsR498G0204283600.01.T01 | Early light-induced, chloroplastic |
| maker-2-augustus-gene-242.45-mRNA-1 | Japonica | Indica | | Os02g0611000 | OsR498G0204285000.01.T01 | Secologanin synthase |
| maker-2-augustus-gene-242.47-mRNA-1 | Japonica | NA | | | OsR498G0204285600.01.T01 | Histone-lysine N-methyltransferase ASHR3 |
| maker-2-augustus-gene-243.0-mRNA-1 | Japonica | NA | | Os02g0611800 | OsR498G0204287700.01.T01 | Hydroxycinnamoyl-Coenzyme A shikimate/quinate hydroxycinnamoyltransfe |
| maker-2-augustus-gene-243.13-mRNA-1 | Japonica | NA | | Os02g0611500 | OsR498G0204286200.01.T01 | MI domain-containing protein |
| maker-2-augustus-gene-243.17-mRNA-1 | Japonica | NA | | Os02g0612300 | OsR498G0204289000.01.T01 | Nuclear cap-binding protein subunit 2 |
| maker-2-augustus-gene-243.25-mRNA-1 | Japonica | NA | | Os02g0612800 | OsR498G0204292000.01.T03 | sister chromatid cohesion protein PDS5 homolog B-B-like |
| maker-2-augustus-gene-243.30-mRNA-1 | Japonica | Indica | | Os04g0109900 | OsR498G0204293200.01.T01 | Exostosin domain-containing protein (Fragment) |
| maker-2-augustus-gene-243.5-mRNA-1 | Japonica | NA | | Os02g0612100 | OsR498G0204288500.01.T01 | Phosphatidyl-N-methylethanolamine N-methyltransferase |
| maker-2-augustus-gene-243.7-mRNA-1 | Japonica | Indica | | Os02g0613000 | OsR498G0204292900.01.T01 | Calcium uptake protein, mitochondrial |
| maker-2-augustus-gene-244.26-mRNA-1 | Japonica | NA | | Os02g0613600 | OsR498G0204293900.01.T04 | Trafficking protein particle complex subunit 2 |
| maker-2-augustus-gene-244.45-mRNA-1 | Japonica | NA | LRR-TM | Os02g0613200 | OsR498G0204293400.01.T01 | Regulatory subunit |

| | | | | | | |
|---|---|---|---|---|---|---|
| maker-2-augustus-gene-244.50-mRNA-1 | Japonica | NA | | | OsR498G0204293600.01.T01 | Fanconi anaemia protein FANCD |
| maker-2-augustus-gene-252.34-mRNA-1 | Japonica | NA | | | OsR498G0204322800.01.T01 | Phosphatidylinositol N-acetylglucosaminlytransferase subunit P-related |
| maker-2-augustus-gene-253.14-mRNA-1 | Japonica | NA | | Os02g0618200 | OsR498G0204327700.01.T01 | Two-component response regulator-like PRR1 |
| maker-2-augustus-gene-254.0-mRNA-1 | Japonica | NA | | Os02g0620100 | OsR498G0204332500.01.T01 | Kinetochore protein-like |
| maker-2-augustus-gene-254.12-mRNA-1 | Japonica | NA | | Os02g0621300 | OsR498G0204336600.01.T01 | Aldehyde oxygenase (deformylating) |
| maker-2-augustus-gene-254.3-mRNA-1 | Japonica | NA | | Os02g0620400 | OsR498G0204333300.01.T01 | Enzyme of the cupin superfamily |
| maker-2-augustus-gene-254.5-mRNA-1 | Japonica | NA | | | | Putative SET-domain containing protein family |
| maker-2-augustus-gene-254.9-mRNA-1 | Japonica | NA | | Os02g0619600 | OsR498G0204331600.01.T01 | Putative RING zinc finger and VWF domain family protein |
| maker-2-augustus-gene-255.33-mRNA-1 | Japonica | NA | | Os02g0621700 | OsR498G0204337500.01.T01 | Succinate--CoA ligase [ADP-forming] subunit beta, mitochondrial |
| maker-2-augustus-gene-255.40-mRNA-1 | Japonica | NA | | Os02g0622100 | OsR498G0204338900.01.T01 | Serine/threonine protein kinase |
| maker-2-augustus-gene-255.46-mRNA-1 | Japonica | NA | | | OsR498G0204339500.01.T05 | GLTP domain-containing protein (Fragment) |
| maker-2-augustus-gene-255.62-mRNA-1 | Japonica | NA | | Os02g0621500 | OsR498G0204337000.01.T01 | E3 ubiquitin-protein ligase PRT1 |
| maker-2-augustus-gene-255.64-mRNA-1 | Japonica | NA | | Os02g0621800 | OsR498G0204338000.01.T01 | Long-chain-alcohol oxidase |
| maker-2-augustus-gene-255.69-mRNA-1 | Japonica | Indica | | Os08g0567200 | OsR498G0204340000.01.T02 | RNA-binding protein 42 |
| maker-2-augustus-gene-256.0-mRNA-1 | Japonica | NA | | Os02g0624400 | OsR498G0204347900.01.T02 | Hexosyltransferase |
| maker-2-augustus-gene-256.11-mRNA-1 | Japonica | NA | | Os02g0623500 | OsR498G0204343600.01.T04 | Lysine--tRNA ligase chloroplastic/mitochondrial |
| maker-2-augustus-gene-256.18-mRNA-1 | Japonica | Indica | Kinase-TM | Os02g0623600 | OsR498G0204344200.01.T02 | Wall-associated receptor kinase-like 8 |
| maker-2-augustus-gene-256.20-mRNA-1 | Japonica | NA | Kinase-TM | | OsR498G0204345600.01.T01 | Putative wall-associated receptor kinase-like 16 |
| maker-2-augustus-gene-256.22-mRNA-1 | Japonica | NA | | Os02g0624300 | OsR498G0204346000.01.T01 | Transcription factor MYB4 |
| maker-2-augustus-gene-256.6-mRNA-1 | Japonica | NA | | | OsR498G0204349900.01.T04 | Photolyase/cryptochrome alpha/beta domain-containing protein (Fragment |
| maker-2-augustus-gene-257.43-mRNA-1 | Japonica | NA | | | OsR498G0204352100.01.T02 | |
| maker-2-augustus-gene-257.46-mRNA-1 | Japonica | NA | | Os02g0625500 | OsR498G0204351800.01.T03 | Adenosine kinase |
| maker-2-augustus-gene-257.50-mRNA-1 | Japonica | NA | | Os02g0626100 | OsR498G0204353600.01.T01 | Phenylalanine ammonia-lyase |
| maker-2-augustus-gene-257.51-mRNA-1 | Japonica | NA | | Os02g0626400 | OsR498G0204354400.01.T02 | Phenylalanine ammonia-lyase |
| maker-2-augustus-gene-257.60-mRNA-1 | Japonica | NA | | Os02g0627100 | OsR498G0204355400.01.T01 | Phenylalanine ammonia-lyase |
| maker-2-augustus-gene-258.0-mRNA-1 | Japonica | NA | | Os02g0627700 | OsR498G0204357100.01.T01 | Cyclic nucleotide-binding domain |
| maker-2-augustus-gene-258.2-mRNA-1 | Japonica | Indica | | Os04g0518800 | OsR498G0204358700.01.T03 | Cytokinin riboside 5'-monophosphate phosphoribohydrolase |
| maker-2-augustus-gene-258.5-mRNA-1 | Japonica | NA | | Os01g0281200 | OsR498G0100675400.01.T01 | CYCLIN domain-containing protein (Fragment) |
| maker-2-augustus-gene-258.9-mRNA-1 | Japonica | NA | | | | |
| maker-2-augustus-gene-259.45-mRNA-1 | Japonica | Indica | | Os02g0628600 | OsR498G0204360900.01.T01 | Auxin response factor |
| maker-2-augustus-gene-259.47-mRNA-1 | Japonica | NA | | | OsR498G0204362700.01.T01 | |
| maker-2-augustus-gene-259.50-mRNA-1 | Japonica | NA | | Os02g0629200 | OsR498G0204364100.01.T01 | PIP aquaporin |
| maker-2-augustus-gene-260.12-mRNA-1 | Japonica | NA | | | OsR498G0204370600.01.T01 | Regulator of nonsense transcripts 2 |
| maker-2-augustus-gene-260.6-mRNA-1 | Japonica | Japonica | Kinase-TM | Os02g0632100 | OsR498G0509684800.01.T02 | Wall-associated receptor kinase 3 |
| maker-2-augustus-gene-260.9-mRNA-1 | Japonica | NA | | Os02g0631200 | OsR498G0204370400.01.T05 | Putative homeodomain-like transcription factor superfamily protein |
| maker-2-augustus-gene-261.32-mRNA-1 | Japonica | NA | | Os02g0632500 | OsR498G0204376100.01.T06 | ADP-ribosylation factor GTPase-activating protein AGD4-like |
| maker-2-augustus-gene-262.11-mRNA-1 | Japonica | NA | | Os02g0634900 | OsR498G0204383100.01.T02 | Proteasome endopeptidase complex |
| maker-2-augustus-gene-262.15-mRNA-1 | Japonica | NA | | Os02g0635000 | OsR498G0204383500.01.T02 | Bifunctional nitrilase/nitrile hydratase NIT4 |
| maker-2-augustus-gene-262.18-mRNA-1 | Japonica | NA | | Os02g0635200 | OsR498G0204383700.01.T01 | Beta-cyano-L-alanine hydratase/nitrilase |
| maker-2-augustus-gene-262.24-mRNA-1 | Japonica | Indica | | Os02g0635333 | OsR498G0204383900.01.T01 | |
| maker-2-augustus-gene-262.27-mRNA-1 | Japonica | Indica | Kinase-TM | Os02g0633066 | OsR498G0204379000.01.T01 | Wall-associated receptor kinase 2-like |
| maker-2-augustus-gene-262.38-mRNA-1 | Japonica | NA | | | | B-block binding subunit of TFIIIC |
| maker-2-augustus-gene-262.4-mRNA-1 | Japonica | NA | | Os02g0634600 | OsR498G0204382300.01.T03 | SET domain-containing protein 9 |
| maker-2-augustus-gene-262.48-mRNA-1 | Japonica | NA | | | OsR498G0204381800.01.T01 | Glycosyltransferase |
| maker-2-augustus-gene-262.7-mRNA-1 | Japonica | NA | | Os02g0634700 | OsR498G0204382700.01.T05 | Carboxypeptidase |
| maker-2-augustus-gene-263.37-mRNA-1 | Japonica | Indica | Kinase-LRR-TM | Os11g0569800 | OsR498G0204384300.01.T02 | Putative LRR receptor-like serine/threonine-protein kinase |
| maker-2-augustus-gene-263.38-mRNA-1 | Japonica | NA | | Os02g0635700 | OsR498G0204384500.01.T01 | C-terminal |
| maker-2-augustus-gene-263.40-mRNA-1 | Japonica | NA | | | OsR498G0204386200.01.T01 | F-box/LRR-repeat protein 2-like |
| maker-2-augustus-gene-263.42-mRNA-1 | Japonica | NA | | Os02g0636600 | OsR498G0204387000.01.T01 | ABA-responsive protein-like |
| maker-2-augustus-gene-263.44-mRNA-1 | Japonica | NA | | Os02g0636700 | OsR498G0204387200.01.T01 | ABA-responsive protein-like |
| maker-2-augustus-gene-263.47-mRNA-1 | Japonica | NA | | Os02g0637700 | OsR498G0204391000.01.T01 | S-nitrosoglutathione reductase |
| maker-2-augustus-gene-263.52-mRNA-1 | Japonica | NA | | Os02g0637800 | OsR498G0204391200.01.T01 | DUF639 domain-containing protein |
| maker-2-augustus-gene-264.35-mRNA-1 | Japonica | NA | | Os02g0637900 | OsR498G0204391300.01.T05 | Ankyrin repeat domain-containing protein 50 isoform 1 |
| maker-2-augustus-gene-264.40-mRNA-1 | Japonica | Indica | | Os02g0638900 | OsR498G0204394700.01.T03 | WD repeat-containing protein 44 |
| maker-2-augustus-gene-264.46-mRNA-1 | Japonica | NA | | Os02g0639000 | OsR498G0204395100.01.T01 | Protein-serine/threonine phosphatase |
| maker-2-augustus-gene-264.52-mRNA-1 | Japonica | Indica | | Os02g0639300 | OsR498G0204395900.01.T02 | CBS domain-containing protein CBSX5 |
| maker-2-augustus-gene-264.54-mRNA-1 | Japonica | Indica | | Os04g0530100 | OsR498G0204396500.01.T01 | Beta-expansin 3 |
| maker-2-augustus-gene-264.57-mRNA-1 | Japonica | NA | | Os02g0639600 | OsR498G0204396700.01.T01 | Sphingolipid 4-desaturase |
| maker-2-augustus-gene-264.60-mRNA-1 | Japonica | Indica | | Os02g0639700 | OsR498G0204396900.01.T02 | Putative nuclear RNA export factor SDE5 |
| maker-2-augustus-gene-264.65-mRNA-1 | Japonica | NA | | Os02g0638000 | OsR498G0204391600.01.T01 | Hydroxyproline-rich glycoprotein family protein |
| maker-2-augustus-gene-264.69-mRNA-1 | Japonica | NA | | | OsR498G0204392000.01.T01 | Serine/arginine repetitive matrix protein 1-like protein |

| | | | | | | |
|---|---|---|---|---|---|---|
| maker-2-augustus-gene-264.71-mRNA-1 | Japonica | NA | | Os02g0638400 | OsR498G0204392600.01.T01 | Vacuolar protein-sorting protein bro1 |
| maker-2-augustus-gene-264.73-mRNA-1 | Japonica | Japonica | Kinase-TM | Os02g0639100 | OsR498G0204395600.01.T01 | cold-responsive protein kinase 1 |
| maker-2-augustus-gene-265.0-mRNA-1 | Japonica | NA | | | | Elicitor-responsive protein 3 |
| maker-2-augustus-gene-265.10-mRNA-1 | Japonica | Indica | | Os02g0641300 | OsR498G0204402500.01.T01 | MYB transcription factor |
| maker-2-augustus-gene-265.14-mRNA-1 | Japonica | Indica | | Os02g0641800 | OsR498G0204402700.01.T02 | DEAD-box ATP-dependent RNA helicase 8 |
| maker-2-augustus-gene-265.22-mRNA-1 | Japonica | Indica | | Os02g0640300 | OsR498G0204398800.01.T02 | Membrane steroid-binding protein 1 |
| maker-2-augustus-gene-265.26-mRNA-1 | Japonica | NA | | Os02g0641000 | OsR498G0204401000.01.T01 | LIM transcription factor |
| maker-2-augustus-gene-265.3-mRNA-1 | Japonica | NA | | Os02g0640200 | OsR498G0204398300.01.T01 | tRNA (Guanine(26)-N(2))-dimethyltransferase |
| maker-2-augustus-gene-265.6-mRNA-1 | Japonica | NA | | | OsR498G0204400500.01.T01 | (+)-neomenthol dehydrogenase-like isoform X1 |
| maker-2-augustus-gene-266.30-mRNA-1 | Japonica | Japonica | | Os02g0641900 | OsR498G0204403500.01.T01 | Transcription factor MYB74 |
| maker-2-augustus-gene-266.32-mRNA-1 | Japonica | Indica | | Os02g0642700 | OsR498G0204405900.01.T02 | CAP10 domain-containing protein |
| maker-2-augustus-gene-266.37-mRNA-1 | Japonica | NA | | Os02g0643500 | OsR498G0204409300.01.T01 | Thylakoid lumenal protein like |
| maker-2-augustus-gene-266.43-mRNA-1 | Japonica | NA | | Os02g0642200 | OsR498G0204404000.01.T01 | Remorin |
| maker-2-augustus-gene-266.46-mRNA-1 | Japonica | Indica | | Os04g0533500 | OsR498G0204404400.01.T01 | Ascorbate-specific transmembrane electron transporter 2 |
| maker-2-augustus-gene-266.49-mRNA-1 | Japonica | NA | | Os02g0642600 | OsR498G0204405600.01.T01 | Shortage in chiasmata 1 |
| maker-2-augustus-gene-266.56-mRNA-1 | Japonica | NA | | Os02g0643200 | OsR498G0204408000.01.T01 | Axial regulator YABBY 1 |
| maker-2-augustus-gene-266.59-mRNA-1 | Japonica | NA | | | OsR498G0204409600.01.T01 | Secondary wall NAC master switch |
| maker-2-augustus-gene-267.0-mRNA-1 | Japonica | Indica | | Os04g0537800 | OsR498G0204410700.01.T01 | Cysteine-rich repeat secretory protein 15 |
| maker-2-augustus-gene-267.14-mRNA-1 | Japonica | NA | | Os02g0644500 | OsR498G0204412600.01.T01 | ATG13 domain-containing protein (Fragment) |
| maker-2-augustus-gene-267.17-mRNA-1 | Japonica | NA | | Os02g0645100 | OsR498G0204416000.01.T02 | Kinesin-related protein1 |
| maker-2-augustus-gene-267.19-mRNA-1 | Japonica | NA | | Os11g0660000 | OsR498G0204415200.01.T01 | Magnesiumproton exchanger |
| maker-2-augustus-gene-267.2-mRNA-1 | Japonica | NA | | Os02g0644100 | OsR498G0204411300.01.T02 | Hsp organizing protein/stress-inducible protein |
| maker-2-augustus-gene-267.27-mRNA-1 | Japonica | NA | | Os02g0645100 | OsR498G0204416000.01.T02 | Kinesin-like protein KIN-7C |
| maker-2-augustus-gene-267.7-mRNA-1 | Japonica | Indica | | Os04g0537900 | OsR498G0204411200.01.T01 | Vacuolar-processing enzyme |
| maker-2-augustus-gene-268.0-mRNA-1 | Japonica | NA | | Os02g0645600 | OsR498G0204416700.01.T01 | GATA transcription factor |
| maker-2-augustus-gene-268.13-mRNA-1 | Japonica | NA | | Os02g0646500 | OsR498G0204418800.01.T01 | Aldehyde dehydrogenase |
| maker-2-augustus-gene-268.14-mRNA-1 | Japonica | Indica | | Os02g0646500 | OsR498G0204418800.01.T03 | Aldehyde dehydrogenase |
| maker-2-augustus-gene-268.2-mRNA-1 | Japonica | NA | | Os02g0646200 | OsR498G0204418300.01.T01 | B-box zinc finger protein 21 |
| maker-2-augustus-gene-268.4-mRNA-1 | Japonica | NA | | Os02g0646400 | OsR498G0204418500.01.T01 | Monothiol glutaredoxin-S6 |
| maker-2-augustus-gene-268.8-mRNA-1 | Japonica | NA | | Os02g0647900 | OsR498G0204420900.01.T02 | Aldehyde dehydrogenase |
| maker-2-augustus-gene-269.31-mRNA-1 | Japonica | NA | | Os02g0649700 | OsR498G0204425300.01.T01 | ATP-dependent zinc metalloprotease FTSH 7 chloroplastic |
| maker-2-augustus-gene-269.36-mRNA-1 | Japonica | NA | Kinase-TM | Os02g0648100 | OsR498G0204421800.01.T01 | Putative LRR receptor-like serine/threonine-protein kinase |
| maker-2-augustus-gene-269.38-mRNA-1 | Japonica | NA | | | OsR498G0204422600.01.T01 | Putative homeodomain-like transcription factor superfamily protein isoform |
| maker-2-augustus-gene-269.41-mRNA-1 | Japonica | NA | | | | RING-type domain-containing protein |
| maker-2-augustus-gene-269.43-mRNA-1 | Japonica | NA | | Os02g0649400 | OsR498G0204425200.01.T02 | Embryogenesis-associated protein EMB8 |
| maker-2-augustus-gene-269.48-mRNA-1 | Japonica | NA | | Os02g0649800 | OsR498G0204425600.01.T01 | Cytochrome b5 heme-binding domain-containing protein |
| maker-2-augustus-gene-269.50-mRNA-1 | Japonica | NA | | Os02g0649900 | OsR498G0204426800.01.T01 | Iron-phytosiderophore transporter yellow stripe 1 |
| maker-2-augustus-gene-27.20-mRNA-1 | Japonica | NA | | Os02g0148000 | OsR498G0202971900.01.T01 | CCT domain-containing protein (Fragment) |
| maker-2-augustus-gene-27.22-mRNA-1 | Japonica | NA | | | OsR498G0202973100.01.T01 | COP1-interactive protein 1-like isoform X2 |
| maker-2-augustus-gene-27.27-mRNA-1 | Japonica | NA | | Os02g0148400 | OsR498G0202973500.01.T01 | FMN-binding split barrel |
| maker-2-augustus-gene-27.30-mRNA-1 | Japonica | Indica | | Os02g0148500 | OsR498G0202973700.01.T03 | GATA transcription factor 17 |
| maker-2-augustus-gene-27.41-mRNA-1 | Japonica | NA | | | | |
| maker-2-augustus-gene-27.43-mRNA-1 | Japonica | NA | Kinase-TM | Os02g0148100 | OsR498G0202972400.01.T02 | Mitogen-activated protein kinase |
| maker-2-augustus-gene-270.0-mRNA-1 | Japonica | NA | | | OsR498G0204429900.01.T01 | Mutant required to maintain repression 1 |
| maker-2-augustus-gene-270.11-mRNA-1 | Japonica | NA | Kinase-TM | Os02g0650500 | OsR498G0204428800.01.T03 | probable serine/threonine-protein kinase PBL19 |
| maker-2-augustus-gene-270.14-mRNA-1 | Japonica | NA | | | OsR498G0204430800.01.T02 | U6 snRNA phosphodiesterase |
| maker-2-augustus-gene-270.18-mRNA-1 | Japonica | NA | | Os02g0651300 | OsR498G0204431000.01.T03 | Protein ORANGE-GREEN, chloroplastic |
| maker-2-augustus-gene-270.2-mRNA-1 | Japonica | NA | | Os02g0650900 | OsR498G0204430300.01.T01 | Glutamate dehydrogenase |
| maker-2-augustus-gene-270.22-mRNA-1 | Japonica | NA | | Os02g0652000 | OsR498G0204432800.01.T01 | Cyclin |
| maker-2-augustus-gene-270.25-mRNA-1 | Japonica | NA | | | OsR498G0204433400.01.T01 | WRKY transcription factor |
| maker-2-augustus-gene-270.4-mRNA-1 | Japonica | Indica | | Os04g0542800 | OsR498G0204428200.01.T01 | Iron-phytosiderophore transporter yellow stripe 1 |
| maker-2-augustus-gene-271.0-mRNA-1 | Japonica | NA | | Os02g0652800 | OsR498G0204435700.01.T01 | Glycerol-3-phosphate transporter |
| maker-2-augustus-gene-271.12-mRNA-1 | Japonica | NA | | Os02g0652300 | OsR498G0204434600.01.T01 | ELMO domain-containing protein A |
| maker-2-augustus-gene-271.15-mRNA-1 | Japonica | Indica | | Os10g0549100 | OsR498G0204435000.01.T01 | Vesicle-associated protein 2-1 |
| maker-2-augustus-gene-271.21-mRNA-1 | Japonica | NA | | Os02g0652600 | OsR498G0204435600.01.T01 | Mitochondrial/chloroplast ribosomal protein L19 |
| maker-2-augustus-gene-271.26-mRNA-1 | Japonica | NA | | Os02g0653200 | OsR498G0204438000.01.T01 | Phytocyanin domain-containing protein |
| maker-2-augustus-gene-271.28-mRNA-1 | Japonica | NA | | Os02g0653400 | OsR498G0204438200.01.T01 | Agmatine coumaroyltransferase-2 |
| maker-2-augustus-gene-271.3-mRNA-1 | Japonica | NA | | Os02g0653800 | OsR498G0204439300.01.T03 | GTP-binding protein YPTM2 |
| maker-2-augustus-gene-271.6-mRNA-1 | Japonica | NA | | | OsR498G0204441100.01.T02 | ATP-dependent caseinolytic protease/crotonase family protein |
| maker-2-augustus-gene-272.32-mRNA-1 | Japonica | Indica | | Os02g0654100 | OsR498G0204441300.01.T02 | Methylglutaconyl-CoA hydratase, mitochondrial-like isoform X2 |
| maker-2-augustus-gene-272.35-mRNA-1 | Japonica | NA | Kinase | Os02g0654300 | OsR498G0204441700.01.T01 | Serine/threonine-protein kinase |

| | | | | | | |
|---|---|---|---|---|---|---|
| maker-2-augustus-gene-272.37-mRNA-1 | Japonica | NA | | Os02g0654500 | OsR498G0204442100.01.T01 | Ubiquitin carboxyl-terminal hydrolase |
| maker-2-augustus-gene-272.41-mRNA-1 | Japonica | NA | | Os02g0655100 | OsR498G0204443300.01.T01 | Brix domain-containing protein |
| maker-2-augustus-gene-272.43-mRNA-1 | Japonica | NA | | Os02g0655500 | OsR498G0204445300.01.T01 | DUF862 domain-containing protein |
| maker-2-augustus-gene-272.45-mRNA-1 | Japonica | NA | | Os02g0655700 | OsR498G0204446300.01.T01 | YfnA |
| maker-2-augustus-gene-272.49-mRNA-1 | Japonica | NA | | | | Transcription initiation factor TFIID subunit 4b |
| maker-2-augustus-gene-272.54-mRNA-1 | Japonica | Japonica | | Os02g0654600 | OsR498G0204442400.01.T01 | DUF632 domain-containing protein |
| maker-2-augustus-gene-272.56-mRNA-1 | Japonica | NA | | | OsR498G0204445600.01.T04 | DUF676 domain-containing protein |
| maker-2-augustus-gene-273.0-mRNA-1 | Japonica | NA | | Os02g0656300 | OsR498G0204447900.01.T01 | Anaphase-promoting complex subunit 8 like |
| maker-2-augustus-gene-273.10-mRNA-1 | Japonica | NA | | | OsR498G0204447800.01.T03 | Ceramide kinase |
| maker-2-augustus-gene-273.16-mRNA-1 | Japonica | NA | | Os02g0657700 | OsR498G0204452600.01.T03 | |
| maker-2-augustus-gene-273.20-mRNA-1 | Japonica | NA | | | OsR498G0204453600.01.T01 | PH domain-containing protein (Fragment) |
| maker-2-augustus-gene-273.4-mRNA-1 | Japonica | NA | | Os02g0656500 | OsR498G0204448500.01.T04 | DnaJ protein like |
| maker-2-augustus-gene-273.8-mRNA-1 | Japonica | NA | Kinase-TM | Os02g0655800 | OsR498G0204447000.01.T01 | Nodulation receptor kinase |
| maker-2-augustus-gene-274.27-mRNA-1 | Japonica | NA | | Os02g0658300 | | protein NUCLEAR FUSION DEFECTIVE 6, chloroplastic/mitochondrial |
| maker-2-augustus-gene-274.29-mRNA-1 | Japonica | NA | | Os02g0658600 | OsR498G0204456300.01.T01 | Expansin-like EG45 domain-containing protein |
| maker-2-augustus-gene-274.31-mRNA-1 | Japonica | NA | | Os02g0658800 | OsR498G0204457300.01.T01 | Beta-expansin 4 |
| maker-2-augustus-gene-274.33-mRNA-1 | Japonica | NA | | Os02g0658100 | OsR498G0204454200.01.T01 | Aquaporin TIP2-1 |
| maker-2-augustus-gene-274.36-mRNA-1 | Japonica | Indica | | Os02g0658200 | OsR498G0204454800.01.T02 | Zinc finger, FYVE/PHD-type |
| maker-2-augustus-gene-274.39-mRNA-1 | Japonica | Indica | | Os02g0658500 | OsR498G0204455800.01.T01 | F-box domain-containing protein |
| maker-2-augustus-gene-274.46-mRNA-1 | Japonica | NA | | Os02g0658900 | OsR498G0204458200.01.T02 | Vesicle transport protein SEC20 |
| maker-2-augustus-gene-275.11-mRNA-1 | Japonica | NA | | Os02g0661100 | OsR498G0204464600.01.T05 | Trehalose 6-phosphate phosphatase |
| maker-2-augustus-gene-275.15-mRNA-1 | Japonica | NA | | | OsR498G0204465800.01.T01 | PLATZ transcription factor family protein |
| maker-2-augustus-gene-275.2-mRNA-1 | Japonica | NA | | | OsR498G0204462000.01.T01 | 4HBT domain-containing protein |
| maker-2-augustus-gene-275.4-mRNA-1 | Japonica | NA | | | OsR498G0204462000.01.T01 | 4HBT domain-containing protein |
| maker-2-augustus-gene-275.6-mRNA-1 | Japonica | NA | | | OsR498G0204463400.01.T01 | 4HBT domain-containing protein |
| maker-2-augustus-gene-275.8-mRNA-1 | Japonica | NA | | Os02g0661000 | OsR498G0204464200.01.T01 | 40 kDa farnesylated protein associated with peroxisome |
| maker-2-augustus-gene-276.0-mRNA-1 | Japonica | NA | | | OsR498G0204467100.01.T02 | Vesicle-associated membraneprotein-like |
| maker-2-augustus-gene-276.11-mRNA-1 | Japonica | Indica | | Os02g0662300 | OsR498G0204469400.01.T01 | |
| maker-2-augustus-gene-276.14-mRNA-1 | Japonica | NA | | | | BEACH-type PH domain-containing protein |
| maker-2-augustus-gene-276.7-mRNA-1 | Japonica | NA | | Os02g0662200 | OsR498G0204468900.01.T01 | Nitrate transporter 1.1-like |
| maker-2-augustus-gene-277.14-mRNA-1 | Japonica | NA | | Os02g0664300 | OsR498G0204475100.01.T01 | Tripeptidyl-peptidase II |
| maker-2-augustus-gene-277.20-mRNA-1 | Japonica | Japonica | | Os02g0663800 | OsR498G0409054800.01.T01 | Actin-dipolymerizing factor |
| maker-2-augustus-gene-277.22-mRNA-1 | Japonica | NA | | Os02g0663900 | OsR498G0204474000.01.T02 | Phosphoribosylanthranilate transferase |
| maker-2-augustus-gene-277.24-mRNA-1 | Japonica | NA | | | OsR498G0204476600.01.T02 | |
| maker-2-augustus-gene-277.28-mRNA-1 | Japonica | NA | | | OsR498G0204477000.01.T06 | Skin secretory protein xP2-like |
| maker-2-augustus-gene-277.32-mRNA-1 | Japonica | NA | | Os02g0665000 | OsR498G0204477200.01.T04 | NADP-dependent malic enzyme, chloroplastic |
| maker-2-augustus-gene-277.4-mRNA-1 | Japonica | NA | | | | Glycoprotein glucosyltransferase-like protein |
| maker-2-augustus-gene-278.0-mRNA-1 | Japonica | NA | | Os02g0665200 | OsR498G0204477500.01.T01 | Putative S-adenosylmethionine carrier 2, chloroplastic isoform X1 |
| maker-2-augustus-gene-278.11-mRNA-1 | Japonica | NA | | Os02g0666200 | OsR498G0204480100.01.T02 | Plasma membrane intrinsic protein 1 |
| maker-2-augustus-gene-278.15-mRNA-1 | Japonica | NA | Kinase-TM | Os02g0666300 | OsR498G0204480400.01.T02 | Putative MAPKKK family protein kinase isoform 1 |
| maker-2-augustus-gene-278.16-mRNA-1 | Japonica | NA | | Os02g0666300 | OsR498G0204480400.01.T05 | Putative MAPKKK family protein kinase isoform 1 |
| maker-2-augustus-gene-278.25-mRNA-1 | Japonica | NA | | | OsR498G0204481800.01.T01 | |
| maker-2-augustus-gene-278.3-mRNA-1 | Japonica | NA | CC-Kinase-TM | | OsR498G0204478300.01.T02 | E3 ubiquitin ligase |
| maker-2-augustus-gene-278.7-mRNA-1 | Japonica | NA | CC-Kinase-TM | | OsR498G0204478700.01.T01 | E3 ubiquitin ligase |
| maker-2-augustus-gene-279.0-mRNA-1 | Japonica | NA | | | OsR498G0204483700.01.T02 | tetracycline resistance protein, class D-like isoform X2 |
| maker-2-augustus-gene-279.11-mRNA-1 | Japonica | NA | | Os02g0667600 | OsR498G0204484000.01.T01 | Late embryogenisis abundant protein 5 |
| maker-2-augustus-gene-279.13-mRNA-1 | Japonica | Indica | | Os02g0668500 | OsR498G0204485800.01.T02 | Cellular retinaldehyde-binding/triple function |
| maker-2-augustus-gene-279.16-mRNA-1 | Japonica | NA | | | OsR498G0204488000.01.T01 | Putative anter-specific proline-rich protein APG |
| maker-2-augustus-gene-279.18-mRNA-1 | Japonica | NA | | Os02g0669000 | OsR498G0204488200.01.T01 | GSDL-motif protein lipase |
| maker-2-augustus-gene-279.20-mRNA-1 | Japonica | NA | | Os02g0669700 | OsR498G0204491200.01.T03 | RRM domain-containing protein (Fragment) |
| maker-2-augustus-gene-279.5-mRNA-1 | Japonica | NA | | Os02g0668000 | OsR498G0204484700.01.T01 | Mechanosensitive ion channel protein |
| maker-2-augustus-gene-279.9-mRNA-1 | Japonica | NA | | Os02g0668400 | OsR498G0204485500.01.T02 | tRNA pseudouridine synthase A |
| maker-2-augustus-gene-28.0-mRNA-1 | Japonica | NA | | Os02g0149600 | OsR498G0202977300.01.T01 | Serrate RNA effector molecule |
| maker-2-augustus-gene-28.10-mRNA-1 | Japonica | NA | | Os02g0150300 | OsR498G0202980900.01.T01 | Apoptosis-inducing factor B like |
| maker-2-augustus-gene-28.22-mRNA-1 | Japonica | Indica | | Os02g0150100 | OsR498G0202980400.01.T02 | DEAD-box ATP-dependent RNA helicase 35A |
| maker-2-augustus-gene-28.3-mRNA-1 | Japonica | NA | | Os02g0149800 | OsR498G0202977900.01.T01 | Protein-serine/threonine phosphatase |
| maker-2-augustus-gene-28.8-mRNA-1 | Japonica | NA | | | OsR498G0202979100.01.T01 | Putative homeobox-leucine zipper protein HOX26 |
| maker-2-augustus-gene-280.0-mRNA-1 | Japonica | NA | | | OsR498G0409073100.01.T01 | DUF632 domain-containing protein |
| maker-2-augustus-gene-280.10-mRNA-1 | Japonica | NA | Kinase | Os02g0670100 | OsR498G0204492800.01.T01 | probable serine/threonine-protein kinase PIX13 |
| maker-2-augustus-gene-280.13-mRNA-1 | Japonica | NA | | Os02g0671300 | OsR498G0204495600.01.T02 | BHLH domain-containing protein (Fragment) |
| maker-2-augustus-gene-280.2-mRNA-1 | Japonica | NA | | Os02g0670400 | OsR498G0204493100.01.T01 | High mobility group box protein 2 |

| | | | | | | |
|---|---|---|---|---|---|---|
| maker-2-augustus-gene-280.6-mRNA-1 | Japonica | NA | | | | Lazarus |
| maker-2-augustus-gene-281.0-mRNA-1 | Japonica | Japonica | | Os02g0672100 | OsR498G0409085900.01.T05 | Naked endosperm2 |
| maker-2-augustus-gene-281.15-mRNA-1 | Japonica | NA | | | OsR498G0204500600.01.T01 | THUMP domain-containing protein |
| maker-2-augustus-gene-281.17-mRNA-1 | Japonica | NA | | Os02g0672400 | OsR498G0204500800.01.T06 | THUMP domain-containing protein |
| maker-2-augustus-gene-281.2-mRNA-1 | Japonica | NA | | | OsR498G0204500300.01.T01 | HTH myb-type domain-containing protein |
| maker-2-augustus-gene-281.24-mRNA-1 | Japonica | NA | Kinase-TM | Os02g0672800 | OsR498G0204501800.01.T01 | Serine/threonine-protein kinase WNK8 |
| maker-2-augustus-gene-281.29-mRNA-1 | Japonica | NA | | Os02g0673100 | OsR498G0204503000.01.T02 | Aluminum-activated malate transporter 10 |
| maker-2-augustus-gene-281.4-mRNA-1 | Japonica | NA | | Os02g0672600 | OsR498G0204501100.01.T01 | N6-adenosine-methyltransferase MT-A70-like |
| maker-2-augustus-gene-281.8-mRNA-1 | Japonica | NA | | Os02g0672200 | OsR498G0204500000.01.T01 | Cytochrome f |
| maker-2-augustus-gene-282.24-mRNA-1 | Japonica | NA | | Os02g0673500 | OsR498G0204505500.01.T02 | Transcription factor bHLH157 |
| maker-2-augustus-gene-282.29-mRNA-1 | Japonica | NA | | Os02g0673600 | OsR498G0204505700.01.T01 | ORMDL |
| maker-2-augustus-gene-282.32-mRNA-1 | Japonica | NA | | Os02g0674700 | OsR498G0204510200.01.T01 | E3 ubiquitin-protein ligase |
| maker-2-augustus-gene-282.36-mRNA-1 | Japonica | NA | | Os02g0674800 | OsR498G0204510600.01.T01 | Homeobox-leucine zipper protein ROC5 |
| maker-2-augustus-gene-283.29-mRNA-1 | Japonica | NA | LRR | Os02g0675800 | OsR498G0204514700.01.T03 | RING/U-box superfamily protein |
| maker-2-augustus-gene-283.32-mRNA-1 | Japonica | NA | | Os02g0676000 | OsR498G0204515700.01.T02 | Lysophospholipid acyltransferase |
| maker-2-augustus-gene-283.37-mRNA-1 | Japonica | Indica | | Os04g0570000 | OsR498G0204512600.01.T01 | Cytochrome P450 family 87 subfamily A polypeptide 2 |
| maker-2-augustus-gene-283.40-mRNA-1 | Japonica | NA | | Os02g0675700 | OsR498G0204514000.01.T01 | Methyltransferase |
| maker-2-augustus-gene-284.29-mRNA-1 | Japonica | Japonica | | Os02g0676500 | OsR498G0204517300.01.T01 | Putative RING-H2 finger protein ATL62 |
| maker-2-augustus-gene-284.32-mRNA-1 | Japonica | NA | | | OsR498G0204522900.01.T01 | Zinc finger CCCH domain-containing protein 17 |
| maker-2-augustus-gene-284.37-mRNA-1 | Japonica | NA | | Os02g0676600 | | Harbinger transposon protein |
| maker-2-augustus-gene-284.39-mRNA-1 | Japonica | NA | | Os02g0677600 | OsR498G0204522800.01.T01 | Auxin transport protein like |
| maker-2-augustus-gene-284.42-mRNA-1 | Japonica | NA | | | OsR498G0204523200.01.T01 | Cytoplasmatic ribosomal protein S13 |
| maker-2-augustus-gene-285.46-mRNA-1 | Japonica | Indica | | Os04g0573000 | OsR498G0204524700.01.T05 | SPX domain-containing membrane protein |
| maker-2-augustus-gene-285.51-mRNA-1 | Japonica | Indica | | Os04g0573100 | OsR498G0204524900.01.T01 | Mandelonitrile lyase |
| maker-2-augustus-gene-285.55-mRNA-1 | Japonica | NA | | Os02g0678400 | OsR498G0204525700.01.T03 | UDP-L-rhamnose synthase |
| maker-2-augustus-gene-285.58-mRNA-1 | Japonica | NA | | Os02g0678800 | OsR498G0204526100.01.T01 | Growth-regulating factor |
| maker-2-augustus-gene-285.60-mRNA-1 | Japonica | Indica | | Os02g0679500 | OsR498G0204527700.01.T01 | Rho GTPase-activating protein gacA |
| maker-2-augustus-gene-285.64-mRNA-1 | Japonica | NA | | Os02g0680400 | OsR498G0204529300.01.T01 | CAAX prenyl protease |
| maker-2-augustus-gene-285.69-mRNA-1 | Japonica | NA | | Os02g0679200 | OsR498G0204527200.01.T03 | Pentatricopeptide repeat |
| maker-2-augustus-gene-285.70-mRNA-1 | Japonica | NA | | Os02g0680600 | OsR498G0204529800.01.T01 | 2-C-methyl-D-erythritol 2,4-cyclodiphosphate synthase |
| maker-2-augustus-gene-285.72-mRNA-1 | Japonica | NA | | | | HTH myb-type domain-containing protein |
| maker-2-augustus-gene-286.0-mRNA-1 | Japonica | NA | | Os02g0681000 | OsR498G0204531300.01.T04 | Mechanosensitive ion channel protein MscS |
| maker-2-augustus-gene-286.14-mRNA-1 | Japonica | NA | | Os02g0681100 | OsR498G0204531600.01.T01 | Putative DUF604-domain containing/glycosyltransferase-related family prot |
| maker-2-augustus-gene-286.7-mRNA-1 | Japonica | Indica | Kinase-TM | Os02g0681700 | OsR498G0204533500.01.T01 | Serine/threonine receptor-like protein kinase NFP |
| maker-2-augustus-gene-286.9-mRNA-1 | Japonica | NA | | Os02g0682200 | OsR498G0204536100.01.T01 | MADS box transcription factor |
| maker-2-augustus-gene-287.33-mRNA-1 | Japonica | Japonica | | Os02g0682700 | OsR498G0204538300.01.T02 | RING-H2 zinc finger protein-like |
| maker-2-augustus-gene-287.37-mRNA-1 | Japonica | NA | | Os02g0683800 | OsR498G0204543900.01.T02 | HMA domain-containing protein (Fragment) |
| maker-2-augustus-gene-287.39-mRNA-1 | Japonica | NA | | Os02g0683900 | OsR498G0204544100.01.T04 | Tobamovirus multiplication protein 3 |
| maker-2-augustus-gene-287.44-mRNA-1 | Japonica | Indica | | Os02g0684300 | OsR498G0204545100.01.T01 | Nucleoporin protein Ndc1-Nup |
| maker-2-augustus-gene-287.47-mRNA-1 | Japonica | NA | | Os02g0682600 | OsR498G0204538000.01.T01 | Mitochondrial import inner membrane translocase subunit |
| maker-2-augustus-gene-287.51-mRNA-1 | Japonica | NA | | Os02g0684200 | | p-loop containing nucleoside triphosphate hydrolase superfamily protein |
| maker-2-augustus-gene-287.53-mRNA-1 | Japonica | NA | | Os02g0684400 | OsR498G0204545400.01.T01 | Oxidative stress 3 |
| maker-2-augustus-gene-288.0-mRNA-1 | Japonica | NA | | | OsR498G0204546300.01.T04 | Transaldolase |
| maker-2-augustus-gene-288.10-mRNA-1 | Japonica | NA | | Os02g0685200 | OsR498G0204548800.01.T01 | HTH myb-type domain-containing protein (Fragment) |
| maker-2-augustus-gene-288.14-mRNA-1 | Japonica | NA | | | OsR498G1120528300.01.T01 | PMD domain-containing protein |
| maker-2-augustus-gene-288.17-mRNA-1 | Japonica | NA | | Os02g0685600 | OsR498G0204550600.01.T05 | Protein-serine/threonine phosphatase |
| maker-2-augustus-gene-288.2-mRNA-1 | Japonica | Indica | | Os04g0583700 | OsR498G0204547300.01.T02 | Regulator of chromosome condensation (RCC1) family with FYVE zinc finger |
| maker-2-augustus-gene-288.6-mRNA-1 | Japonica | NA | Kinase-TM | Os02g0685900 | OsR498G0204551100.01.T01 | Calcium-dependent protein kinase |
| maker-2-augustus-gene-289.0-mRNA-1 | Japonica | NA | | Os02g0686400 | OsR498G0204553900.01.T01 | Aspartyl-tRNA synthetase |
| maker-2-augustus-gene-289.10-mRNA-1 | Japonica | NA | | Os02g0686600 | OsR498G0204554400.01.T01 | Metal-dependent protein hydrolase |
| maker-2-augustus-gene-289.14-mRNA-1 | Japonica | NA | | | | FLZ-type domain-containing protein (Fragment) |
| maker-2-augustus-gene-289.16-mRNA-1 | Japonica | NA | | Os02g0686800 | OsR498G0204555800.01.T01 | FLZ-type domain-containing protein (Fragment) |
| maker-2-augustus-gene-289.18-mRNA-1 | Japonica | Indica | | Os04g0586000 | OsR498G0204556400.01.T01 | FLZ-type domain-containing protein (Fragment) |
| maker-2-augustus-gene-289.20-mRNA-1 | Japonica | NA | | | OsR498G0204557000.01.T01 | RRM domain-containing protein |
| maker-2-augustus-gene-289.22-mRNA-1 | Japonica | NA | | Os02g0687200 | OsR498G0204557600.01.T01 | FLZ-type domain-containing protein (Fragment) |
| maker-2-augustus-gene-289.24-mRNA-1 | Japonica | NA | | | OsR498G0204558400.01.T02 | S-ribonuclease binding protein SBP1 |
| maker-2-augustus-gene-289.4-mRNA-1 | Japonica | NA | | | OsR498G0204554100.01.T01 | F-box/WD repeat-containing protein pof10 |
| maker-2-augustus-gene-289.6-mRNA-1 | Japonica | NA | | Os02g0686300 | | Glycosyltransferase |
| maker-2-augustus-gene-290.0-mRNA-1 | Japonica | NA | | Os02g0687900 | OsR498G0204560700.01.T05 | |
| maker-2-augustus-gene-290.11-mRNA-1 | Japonica | NA | | | OsR498G0204568700.01.T02 | VAN3-binding protein-like |
| maker-2-augustus-gene-290.13-mRNA-1 | Japonica | NA | | Os02g0688900 | OsR498G0204565000.01.T02 | phosphatidylinositol glycan anchor biosynthesis class U protein isoform X1 |

| | | | | | | |
|---|---|---|---|---|---|---|
| maker-2-augustus-gene-290.6-mRNA-1 | Japonica | NA | | Os02g0688500 | OsR498G0204562900.01.T01 | Alpha-1,3-glucosyltransferase |
| maker-2-augustus-gene-290.9-mRNA-1 | Japonica | NA | | Os02g0689200 | OsR498G0204565900.01.T02 | |
| maker-2-augustus-gene-291.13-mRNA-1 | Japonica | Indica | | Os02g0691500 | OsR498G0204574100.01.T01 | Transcription factor bHLH71 |
| maker-2-augustus-gene-291.16-mRNA-1 | Japonica | Indica | | Os02g0689800 | OsR498G0204569400.01.T02 | Protein PHOTOPERIOD-INDEPENDENT EARLY FLOWERING |
| maker-2-augustus-gene-291.21-mRNA-1 | Japonica | NA | | Os02g0689900 | OsR498G0204569800.01.T04 | Nitrate transporter 1.5 |
| maker-2-augustus-gene-291.3-mRNA-1 | Japonica | NA | | Os02g0690700 | OsR498G0204572300.01.T01 | MHD domain-containing protein |
| maker-2-augustus-gene-291.8-mRNA-1 | Japonica | NA | | Os02g0690800 | OsR498G0204572900.01.T01 | Metalloexopeptidase |
| maker-2-augustus-gene-292.41-mRNA-1 | Japonica | NA | | | OsR498G0204580300.01.T04 | USP domain-containing protein (Fragment) |
| maker-2-augustus-gene-292.47-mRNA-1 | Japonica | Japonica | | Os02g0692700 | OsR498G0204577600.01.T01 | PLATZ transcription factor |
| maker-2-augustus-gene-292.50-mRNA-1 | Japonica | NA | | Os02g0693200 | OsR498G0204580000.01.T02 | J domain-containing protein |
| maker-2-augustus-gene-292.53-mRNA-1 | Japonica | Japonica | | Os02g0693500 | OsR498G0204580600.01.T01 | SNARE associated Golgi protein family |
| maker-2-augustus-gene-293.0-mRNA-1 | Japonica | NA | | | OsR498G0204582900.01.T08 | Auxin response factor |
| maker-2-augustus-gene-293.11-mRNA-1 | Japonica | NA | | Os02g0694700 | OsR498G0204583700.01.T01 | E3 ubiquitin-protein ligase RNF14 |
| maker-2-augustus-gene-293.13-mRNA-1 | Japonica | NA | | Os02g0695600 | OsR498G0204586500.01.T01 | |
| maker-2-augustus-gene-293.15-mRNA-1 | Japonica | NA | | Os02g0693700 | OsR498G0204581000.01.T01 | ABC transporter |
| maker-2-augustus-gene-293.23-mRNA-1 | Japonica | NA | | Os02g0694100 | OsR498G0204582200.01.T01 | FBD domain-containing protein (Fragment) |
| maker-2-augustus-gene-293.25-mRNA-1 | Japonica | NA | | | OsR498G0204582800.01.T06 | Histone acetyltransferase MCC1 |
| maker-2-augustus-gene-293.29-mRNA-1 | Japonica | Indica | | Os02g0694800 | OsR498G0204584000.01.T02 | Protein kinase domain-containing protein |
| maker-2-augustus-gene-293.3-mRNA-1 | Japonica | NA | | Os02g0694400 | OsR498G0204583300.01.T05 | RBR-type E3 ubiquitin transferase |
| maker-2-augustus-gene-293.33-mRNA-1 | Japonica | NA | | Os02g0695200 | OsR498G0204585000.01.T01 | Myb domain protein 58 |
| maker-2-augustus-gene-293.35-mRNA-1 | Japonica | Indica | | Os04g0594800 | OsR498G0204586800.01.T01 | Oligopeptide transporter |
| maker-2-augustus-gene-293.5-mRNA-1 | Japonica | NA | | | OsR498G0204583300.01.T01 | RBR-type E3 ubiquitin transferase |
| maker-2-augustus-gene-293.8-mRNA-1 | Japonica | NA | | | OsR498G0204583500.01.T01 | RWD domain-containing protein |
| maker-2-augustus-gene-294.34-mRNA-1 | Japonica | NA | | Os02g0696500 | OsR498G0204588700.01.T01 | Xyloglucan endotransglucosylase/hydrolase |
| maker-2-augustus-gene-294.36-mRNA-1 | Japonica | NA | | Os02g0696700 | OsR498G0204589700.01.T04 | Lysine-specific demethylase lid |
| maker-2-augustus-gene-294.42-mRNA-1 | Japonica | NA | | | | SANT domain-containing protein |
| maker-2-augustus-gene-294.46-mRNA-1 | Japonica | NA | | Os02g0697200 | OsR498G0204592100.01.T02 | Survival motor neuron protein |
| maker-2-augustus-gene-294.50-mRNA-1 | Japonica | NA | | Os02g0697300 | OsR498G0204592300.01.T07 | MYB transcription factor 9 |
| maker-2-augustus-gene-294.53-mRNA-1 | Japonica | NA | | Os02g0697600 | OsR498G0204592900.01.T01 | 26S protease regulatory subunit 6A like |
| maker-2-augustus-gene-294.56-mRNA-1 | Japonica | NA | | Os02g0696600 | | |
| maker-2-augustus-gene-294.58-mRNA-1 | Japonica | NA | | Os02g0697400 | OsR498G0204592600.01.T01 | Coumaroyl-CoA ligase |
| maker-2-augustus-gene-294.61-mRNA-1 | Japonica | NA | | | OsR498G0204592800.01.T01 | Pentatricopeptide repeat |
| maker-2-augustus-gene-295.0-mRNA-1 | Japonica | Indica | | Os04g0595800 | OsR498G0204593300.01.T01 | Secretory carrier-associated membrane protein 5 |
| maker-2-augustus-gene-295.12-mRNA-1 | Japonica | NA | | Os02g0699000 | OsR498G0204597100.01.T02 | |
| maker-2-augustus-gene-295.15-mRNA-1 | Japonica | Indica | | Os10g0580200 | OsR498G0204597500.01.T03 | ADP-ribosylation factor |
| maker-2-augustus-gene-295.20-mRNA-1 | Japonica | NA | | | | GTP diphosphokinase |
| maker-2-augustus-gene-295.23-mRNA-1 | Japonica | NA | | | OsR498G0204598100.01.T01 | Helicase swr-1 |
| maker-2-augustus-gene-295.27-mRNA-1 | Japonica | NA | | Os02g0697700 | OsR498G0204593200.01.T01 | Histone acetyltransferase mst1 |
| maker-2-augustus-gene-295.31-mRNA-1 | Japonica | Indica | | Os02g0699700 | OsR498G0204598600.01.T01 | DNA topoisomerase 2 |
| maker-2-augustus-gene-295.38-mRNA-1 | Japonica | NA | | Os02g0700000 | OsR498G0204599600.01.T03 | PWWP domain |
| maker-2-augustus-gene-295.5-mRNA-1 | Japonica | NA | | Os02g0698000 | OsR498G0204593700.01.T02 | Phosphoribulokinase |
| maker-2-augustus-gene-295.9-mRNA-1 | Japonica | NA | | Os02g0698800 | OsR498G0204594500.01.T01 | DNA-binding WRKY |
| maker-2-augustus-gene-296.0-mRNA-1 | Japonica | NA | | Os02g0700100 | OsR498G0204599700.01.T03 | Cell division cycle protein 20 |
| maker-2-augustus-gene-296.10-mRNA-1 | Japonica | NA | | Os02g0701600 | OsR498G0204604100.01.T01 | Gamma tocopherol methyltransferase |
| maker-2-augustus-gene-296.14-mRNA-1 | Japonica | NA | | Os02g0700400 | OsR498G0204600600.01.T01 | Ubiquinol oxidase |
| maker-2-augustus-gene-296.16-mRNA-1 | Japonica | NA | | Os02g0700700 | OsR498G0204601200.01.T03 | Protein BREVIS RADIX |
| maker-2-augustus-gene-296.18-mRNA-1 | Japonica | NA | | Os02g0701300 | OsR498G0204603200.01.T01 | Growth-regulating factor |
| maker-2-augustus-gene-296.4-mRNA-1 | Japonica | NA | | Os02g0700300 | OsR498G0204600300.01.T01 | HTH myb-type domain-containing protein (Fragment) |
| maker-2-augustus-gene-297.47-mRNA-1 | Japonica | NA | | Os02g0701900 | OsR498G0204605500.01.T01 | 3-dehydrosphinganine reductase TSC10B |
| maker-2-augustus-gene-297.49-mRNA-1 | Japonica | NA | | Os02g0702000 | OsR498G0204605700.01.T01 | Homing endonuclease, LAGLIDADG |
| maker-2-augustus-gene-297.51-mRNA-1 | Japonica | NA | | Os02g0702400 | OsR498G0204607500.01.T01 | Pectin acetylesterase |
| maker-2-augustus-gene-297.58-mRNA-1 | Japonica | Indica | | Os04g0601700 | OsR498G0204604400.01.T05 | V-type proton ATPase subunit G |
| maker-2-augustus-gene-297.66-mRNA-1 | Japonica | NA | | Os02g0702500 | OsR498G0204608000.01.T04 | Protein kinase domain-containing protein |
| maker-2-augustus-gene-297.73-mRNA-1 | Japonica | NA | | | | Plant specific Rop nucleotide exchanger, PRONE |
| maker-2-augustus-gene-297.76-mRNA-1 | Japonica | NA | | | OsR498G0204609400.01.T01 | Allantoate deiminase isoform X1 |
| maker-2-augustus-gene-297.78-mRNA-1 | Japonica | NA | | Os02g0702800 | OsR498G0204609600.01.T01 | t-SNARE coiled-coil homology domain-containing protein (Fragment) |
| maker-2-augustus-gene-298.0-mRNA-1 | Japonica | Indica | | Os02g0703600 | OsR498G0204611500.01.T01 | Abscisic acid 8'-hydroxylase |
| maker-2-augustus-gene-298.10-mRNA-1 | Japonica | NA | | Os02g0704500 | OsR498G0204613900.01.T01 | Lipid phosphate phosphatase epsilon 2 chloroplastic |
| maker-2-augustus-gene-298.14-mRNA-1 | Japonica | NA | | | OsR498G0204614500.01.T01 | Ornithine transcarbamylase |
| maker-2-augustus-gene-298.16-mRNA-1 | Japonica | Japonica | | Os02g0704300 | OsR498G0916748200.01.T04 | DNA-binding protein SMUBP-2 |
| maker-2-augustus-gene-298.18-mRNA-1 | Japonica | NA | | Os02g0704600 | OsR498G0204614200.01.T01 | |

| | | | | | | |
|---|---|---|---|---|---|---|
| maker-2-augustus-gene-298.20-mRNA-1 | Japonica | NA | | Os02g0704900 | OsR498G0204614800.01.T04 | Soluble inorganic pyrophosphatase |
| maker-2-augustus-gene-298.4-mRNA-1 | Japonica | NA | | Os05g0394100 | | NAM-associated domain-containing protein |
| maker-2-augustus-gene-298.6-mRNA-1 | Japonica | Japonica | | Os02g0703900 | OsR498G0204612100.01.T04 | Auxin-induced protein 5NG4 |
| maker-2-augustus-gene-299.0-mRNA-1 | Japonica | NA | | Os02g0705300 | OsR498G0204615900.01.T02 | Tubby-like F-box protein 5 |
| maker-2-augustus-gene-299.11-mRNA-1 | Japonica | NA | | Os02g0705100 | OsR498G0204615400.01.T01 | Pheophytinase, chloroplastic |
| maker-2-augustus-gene-299.14-mRNA-1 | Japonica | NA | | Os02g0705500 | OsR498G0204616600.01.T02 | BHLH transcription factor |
| maker-2-augustus-gene-299.18-mRNA-1 | Japonica | NA | | Os02g0705600 | OsR498G0204618400.01.T03 | RING-type E3 ubiquitin transferase |
| maker-2-augustus-gene-299.3-mRNA-1 | Japonica | NA | | Os02g0705400 | OsR498G0204616300.01.T01 | Usp domain-containing protein (Fragment) |
| maker-2-augustus-gene-299.6-mRNA-1 | Japonica | NA | | | OsR498G0204615200.01.T02 | Replication factor C subunit 3 |
| maker-2-augustus-gene-299.8-mRNA-1 | Japonica | NA | | Os02g0705000 | OsR498G0204615200.01.T06 | NmrA domain-containing protein (Fragment) |
| maker-2-augustus-gene-3.0-mRNA-1 | Japonica | NA | | Os02g0104700 | OsR498G0202812300.01.T03 | Nitric oxide synthase-associated protein I |
| maker-2-augustus-gene-3.14-mRNA-1 | Japonica | NA | | | OsR498G0202813300.01.T07 | Protein transport protein sec16 |
| maker-2-augustus-gene-3.26-mRNA-1 | Japonica | NA | | Os02g0105400 | OsR498G0202813900.01.T01 | L-lactate dehydrogenase A |
| maker-2-augustus-gene-3.28-mRNA-1 | Japonica | NA | | Os02g0105500 | OsR498G0202814300.01.T01 | BAG family molecular chaperone regulator 7 |
| maker-2-augustus-gene-3.30-mRNA-1 | Japonica | NA | | | OsR498G0202814900.01.T02 | 40S ribosomal subunit protein S14 |
| maker-2-augustus-gene-3.31-mRNA-1 | Japonica | NA | | Os02g0106100 | OsR498G0202817500.01.T01 | Acid beta-fructofuranosidase |
| maker-2-augustus-gene-3.4-mRNA-1 | Japonica | Indica | | Os02g0104800 | OsR498G0202812500.01.T01 | RNA-binding protein 48 |
| maker-2-augustus-gene-3.41-mRNA-1 | Japonica | NA | | Os02g0105900 | OsR498G0202815400.01.T01 | S4 RNA-binding domain-containing protein (Fragment) |
| maker-2-augustus-gene-3.9-mRNA-1 | Japonica | NA | | Os02g0105000 | OsR498G0202812900.01.T02 | RNA-binding protein 48 |
| maker-2-augustus-gene-300.36-mRNA-1 | Japonica | NA | | Os02g0706500 | OsR498G0204620100.01.T01 | Putative AAA-type ATPase |
| maker-2-augustus-gene-300.40-mRNA-1 | Japonica | Indica | | Os02g0708100 | OsR498G0204625500.01.T02 | Glutamine amidotransferase type-1 domain-containing protein (Fragment) |
| maker-2-augustus-gene-300.46-mRNA-1 | Japonica | NA | | Os02g0708200 | OsR498G0204625700.01.T01 | Uridine-cytidine kinase C |
| maker-2-augustus-gene-300.51-mRNA-1 | Japonica | Indica | | Os02g0708300 | OsR498G0204625900.01.T03 | RING-box protein 1a isoform B |
| maker-2-augustus-gene-300.55-mRNA-1 | Japonica | Indica | | Os02g0706900 | OsR498G0204621800.01.T01 | AB hydrolase-1 domain-containing protein (Fragment) |
| maker-2-augustus-gene-300.57-mRNA-1 | Japonica | Indica | | Os02g0707000 | OsR498G0204622200.01.T01 | Monodehydroascorbate reductase |
| maker-2-augustus-gene-300.59-mRNA-1 | Japonica | NA | | Os02g0707100 | OsR498G0204622400.01.T01 | Monodehydroascorbate reductase |
| maker-2-augustus-gene-300.63-mRNA-1 | Japonica | NA | | Os02g0707200 | OsR498G0204622600.01.T02 | Dof zinc finger protein 12 |
| maker-2-augustus-gene-300.65-mRNA-1 | Japonica | NA | | Os02g0707900 | OsR498G0204624800.01.T03 | Ethylene response protein |
| maker-2-augustus-gene-301.26-mRNA-1 | Japonica | NA | | | OsR498G0204626700.01.T01 | Putative SET-domain transcriptional regulator |
| maker-2-augustus-gene-301.34-mRNA-1 | Japonica | NA | | Os02g0709000 | OsR498G0204628700.01.T01 | C2H2L domain class transcription factor |
| maker-2-augustus-gene-301.36-mRNA-1 | Japonica | NA | | Os02g0709400 | OsR498G0204630500.01.T02 | Calpain-type cysteine protease DEK1 |
| maker-2-augustus-gene-301.42-mRNA-1 | Japonica | Indica | | Os05g0577100 | OsR498G0204631100.01.T02 | Plant-specific domain TIGR01570 family protein |
| maker-2-augustus-gene-301.44-mRNA-1 | Japonica | NA | | Os02g0708400 | OsR498G0204626200.01.T02 | Kinesin light chain |
| maker-2-augustus-gene-301.47-mRNA-1 | Japonica | NA | | | OsR498G0204629000.01.T01 | |
| maker-2-augustus-gene-301.49-mRNA-1 | Japonica | NA | | Os02g0709200 | OsR498G0204629400.01.T01 | Histidinol-phosphate aminotransferase 2 chloroplastic |
| maker-2-augustus-gene-301.52-mRNA-1 | Japonica | NA | | Os02g0709800 | OsR498G0204632400.01.T06 | RabGAP/TBC domain-containing protein (Fragment) |
| maker-2-augustus-gene-301.58-mRNA-1 | Japonica | NA | | Os02g0709900 | OsR498G0204632600.01.T03 | Protein CROWDED NUCLEI 1 |
| maker-2-augustus-gene-302.30-mRNA-1 | Japonica | NA | | Os02g0710300 | OsR498G0204634900.01.T01 | Transcription factor bHLH83 |
| maker-2-augustus-gene-302.32-mRNA-1 | Japonica | NA | Kinase-TM | Os02g0710500 | OsR498G0204635700.01.T01 | Liguleless narrow |
| maker-2-augustus-gene-302.37-mRNA-1 | Japonica | NA | | Os02g0710700 | OsR498G0204636700.01.T01 | Laccase |
| maker-2-augustus-gene-302.39-mRNA-1 | Japonica | NA | | | OsR498G0204636900.01.T02 | ATP-dependent DNA helicase RecG |
| maker-2-augustus-gene-302.45-mRNA-1 | Japonica | NA | | Os02g0710900 | OsR498G0204637100.01.T01 | Heat shock protein 17 |
| maker-2-augustus-gene-302.50-mRNA-1 | Japonica | NA | | | OsR498G0204637500.01.T03 | p-loop containing nucleoside triphosphate hydrolases superfamily protein is |
| maker-2-augustus-gene-302.53-mRNA-1 | Japonica | NA | | | OsR498G0204633400.01.T01 | |
| maker-2-augustus-gene-302.55-mRNA-1 | Japonica | Indica | | Os02g0711000 | OsR498G0204637400.01.T04 | Pentatricopeptide repeat-containing protein (Fragment) |
| maker-2-augustus-gene-303.0-mRNA-1 | Japonica | NA | | Os02g0713400 | OsR498G0204643100.01.T01 | Thioredoxin reductase NTRB |
| maker-2-augustus-gene-303.13-mRNA-1 | Japonica | NA | | Os02g0712400 | | AP2 domain class transcription factor |
| maker-2-augustus-gene-303.14-mRNA-1 | Japonica | NA | | Os02g0712500 | OsR498G0204640800.01.T01 | Glycosyltransferase family 92 protein (Fragment) |
| maker-2-augustus-gene-303.16-mRNA-1 | Japonica | NA | Kinase-TM | Os02g0712600 | OsR498G0204641600.01.T01 | Lectin receptor kinase |
| maker-2-augustus-gene-303.18-mRNA-1 | Japonica | NA | Kinase-TM | Os02g0712700 | OsR498G0204641800.01.T01 | Lectin receptor kinase 1 |
| maker-2-augustus-gene-303.2-mRNA-1 | Japonica | NA | | Os02g0713600 | | Zinc finger BED domain-containing protein RICESLEEPER 2 |
| maker-2-augustus-gene-303.21-mRNA-1 | Japonica | NA | | Os02g0713500 | | F-box domain-containing protein |
| maker-2-augustus-gene-303.23-mRNA-1 | Japonica | NA | | Os02g0713700 | OsR498G0204645000.01.T02 | PPC domain-containing protein (Fragment) |
| maker-2-augustus-gene-303.4-mRNA-1 | Japonica | NA | | Os02g0711900 | OsR498G0204639600.01.T01 | Trithorax group protein osa-like |
| maker-2-augustus-gene-303.6-mRNA-1 | Japonica | Indica | | Os02g0712000 | OsR498G0204639800.01.T04 | Pro-apoptotic serine protease NMA111 |
| maker-2-augustus-gene-304.31-mRNA-1 | Japonica | NA | | Os02g0714000 | OsR498G0204646900.01.T01 | RRM domain-containing protein |
| maker-2-augustus-gene-304.37-mRNA-1 | Japonica | NA | | Os02g0714600 | OsR498G0204648300.01.T01 | Ribose-phosphate pyrophosphokinase 4 |
| maker-2-augustus-gene-304.41-mRNA-1 | Japonica | NA | | | | Peptidase S1 and S6 chymotrypsin/Hap |
| maker-2-augustus-gene-304.44-mRNA-1 | Japonica | NA | | Os02g0715000 | OsR498G0204650300.01.T01 | Cysteine protease XCP1 |
| maker-2-augustus-gene-304.46-mRNA-1 | Japonica | NA | | Os02g0715200 | OsR498G0204650700.01.T02 | Short-chain dehydrogenase/reductase family 42E member 1 |
| maker-2-augustus-gene-304.50-mRNA-1 | Japonica | Indica | | Os08g0512700 | OsR498G0204646800.01.T02 | 3-hydroxy-3-methylglutaryl coenzyme A reductase |

| | | | | | | |
|---|---|---|---|---|---|---|
| maker-2-augustus-gene-304.54-mRNA-1 | Japonica | NA | | Os02g0714100 | OsR498G0204647200.01.T04 | Acyltransferase |
| maker-2-augustus-gene-304.59-mRNA-1 | Japonica | NA | | Os02g0714200 | OsR498G0204647600.01.T02 | Pyrophosphate--fructose 6-phosphate 1-phosphotransferase subunit alpha |
| maker-2-augustus-gene-304.65-mRNA-1 | Japonica | NA | | Os02g0714300 | OsR498G0204647800.01.T04 | AT-rich interactive domain-containing protein 3 |
| maker-2-augustus-gene-305.0-mRNA-1 | Japonica | NA | | Os02g0717100 | OsR498G0204654300.01.T02 | Transcriptional regulator ATRX-like |
| maker-2-augustus-gene-305.11-mRNA-1 | Japonica | NA | | Os02g0717400 | OsR498G0204656200.01.T02 | Clustered mitochondria protein homolog |
| maker-2-augustus-gene-305.2-mRNA-1 | Japonica | NA | | Os02g0717300 | OsR498G0204655300.01.T02 | Outer envelope pore protein like |
| maker-2-augustus-gene-305.4-mRNA-1 | Japonica | NA | | Os02g0717500 | OsR498G0204656700.01.T01 | Flavonol synthase/flavanone 3-hydroxylase |
| maker-2-augustus-gene-305.8-mRNA-1 | Japonica | NA | | Os02g0716800 | OsR498G0204653800.01.T02 | Nitrate transporter 1.5 |
| maker-2-augustus-gene-306.0-mRNA-1 | Japonica | NA | | Os02g0718900 | OsR498G0204659500.01.T02 | ADP,ATP carrier protein, mitochondrial |
| maker-2-augustus-gene-306.12-mRNA-1 | Japonica | NA | | Os02g0719600 | OsR498G0204661700.01.T01 | Anthranilate O-methyltransferase 2 |
| maker-2-augustus-gene-306.2-mRNA-1 | Japonica | NA | | Os02g0719000 | OsR498G0204659900.01.T01 | Rho protein GDP-dissociation inhibitor |
| maker-2-augustus-gene-306.20-mRNA-1 | Japonica | NA | | | OsR498G0204662700.01.T02 | PHD-type domain-containing protein |
| maker-2-augustus-gene-306.22-mRNA-1 | Japonica | NA | | Os02g0720200 | OsR498G0204663300.01.T01 | 65-kDa microtubule-associated protein 1 |
| maker-2-augustus-gene-306.25-mRNA-1 | Japonica | NA | | Os02g0720300 | OsR498G0204663500.01.T01 | Protein-tyrosine-phosphatase IBR5 |
| maker-2-augustus-gene-306.28-mRNA-1 | Japonica | NA | | | OsR498G0204657000.01.T01 | Cobyric acid synthase |
| maker-2-augustus-gene-306.34-mRNA-1 | Japonica | Indica | | Os02g0717800 | OsR498G0204657600.01.T01 | 60S ribosomal protein L31 |
| maker-2-augustus-gene-306.36-mRNA-1 | Japonica | NA | | Os02g0720100 | OsR498G0204663200.01.T01 | Phytocyanin domain-containing protein (Fragment) |
| maker-2-augustus-gene-306.7-mRNA-1 | Japonica | NA | | Os02g0719100 | OsR498G0204660100.01.T04 | Fimbrin 1 |
| maker-2-augustus-gene-307.0-mRNA-1 | Japonica | Indica | | Os06g0610800 | OsR498G0204664300.01.T02 | Putative chloroplast nucleoid DNA-binding protein cnd41 |
| maker-2-augustus-gene-307.14-mRNA-1 | Japonica | NA | | Os02g0721100 | OsR498G0204666800.01.T01 | Putative ubiquitin-conjugating enzyme 11 |
| maker-2-augustus-gene-307.16-mRNA-1 | Japonica | Indica | | Os02g0721100 | OsR498G0204669000.01.T01 | UBC core domain-containing protein (Fragment) |
| maker-2-augustus-gene-307.18-mRNA-1 | Japonica | NA | | Os02g0721600 | OsR498G0204669200.01.T02 | WD-repeat protein-like |
| maker-2-augustus-gene-307.19-mRNA-1 | Japonica | NA | | Os02g0721600 | OsR498G0204669200.01.T03 | WD-repeat protein-like |
| maker-2-augustus-gene-307.2-mRNA-1 | Japonica | NA | | Os02g0720900 | | Peptidase A1 domain-containing protein (Fragment) |
| maker-2-augustus-gene-307.26-mRNA-1 | Japonica | NA | | Os02g0721700 | OsR498G0204670000.01.T01 | GPI-anchored protein LLG1 |
| maker-2-augustus-gene-307.28-mRNA-1 | Japonica | NA | | Os02g0721800 | OsR498G0204670200.01.T03 | Phosphatidylinositol/phosphatidylcholine transfer protein SFH13 |
| maker-2-augustus-gene-307.33-mRNA-1 | Japonica | Indica | | Os07g0675400 | OsR498G0204671400.01.T03 | Tyrosyl-tRNA synthetase |
| maker-2-augustus-gene-307.36-mRNA-1 | Japonica | Indica | | Os02g0722200 | OsR498G0204671400.01.T04 | Tyrosyl-tRNA synthetase |
| maker-2-augustus-gene-307.4-mRNA-1 | Japonica | Japonica | | Os02g0789700 | OsR498G1120753400.01.T01 | |
| maker-2-augustus-gene-307.7-mRNA-1 | Japonica | Japonica | | Os02g0720600 | OsR498G0611518800.01.T01 | Peptidase A1 domain-containing protein (Fragment) |
| maker-2-augustus-gene-307.9-mRNA-1 | Japonica | NA | | Os02g0720700 | OsR498G0204665200.01.T01 | Chloride channel protein CLC-f |
| maker-2-augustus-gene-308.34-mRNA-1 | Japonica | NA | CC-Kinase | | OsR498G0204671700.01.T01 | Non-specific serine/threonine protein kinase |
| maker-2-augustus-gene-308.37-mRNA-1 | Japonica | NA | | Os02g0722400 | OsR498G0204672700.01.T01 | Amino acid permease 7 |
| maker-2-augustus-gene-308.39-mRNA-1 | Japonica | NA | | | OsR498G0204674100.01.T04 | KH domain-containing protein |
| maker-2-augustus-gene-308.42-mRNA-1 | Japonica | NA | | Os02g0723700 | OsR498G0204678100.01.T01 | Bushy growth protein (Fragment) |
| maker-2-augustus-gene-308.47-mRNA-1 | Japonica | NA | | Os02g0722500 | OsR498G0204673000.01.T02 | ADP-ribosylation factor GTPase-activating protein AGD12 |
| maker-2-augustus-gene-308.52-mRNA-1 | Japonica | NA | | Os02g0722800 | OsR498G0204674600.01.T01 | WD40, G-beta domain containing family protein, family |
| maker-2-augustus-gene-308.56-mRNA-1 | Japonica | NA | | | OsR498G0204677000.01.T05 | RPOL4c domain-containing protein |
| maker-2-augustus-gene-308.62-mRNA-1 | Japonica | NA | | | | DNA-directed RNA polymerases I and III subunit RPAC1 |
| maker-2-augustus-gene-309.0-mRNA-1 | Japonica | Indica | | Os02g0724000 | OsR498G0204681300.01.T06 | Zinc finger protein CONSTANS-LIKE 9 |
| maker-2-augustus-gene-309.15-mRNA-1 | Japonica | NA | Kinase-TM | Os02g0725000 | OsR498G0204682900.01.T03 | serine/threonine-protein kinase D6PK |
| maker-2-augustus-gene-309.23-mRNA-1 | Japonica | NA | | Os02g0725300 | OsR498G0204683900.01.T01 | Putative cellulose synthase (UDP-forming) |
| maker-2-augustus-gene-309.3-mRNA-1 | Japonica | Indica | | Os02g0724500 | OsR498G0204681900.01.T03 | TPT domain-containing protein (Fragment) |
| maker-2-augustus-gene-309.33-mRNA-1 | Japonica | Indica | | Os02g0725600 | OsR498G0204684900.01.T01 | tRNA (guanine(9)-N(1))-methyltransferase |
| maker-2-augustus-gene-309.36-mRNA-1 | Japonica | NA | | Os06g0682750 | | AAI domain-containing protein (Fragment) |
| maker-2-augustus-gene-309.38-mRNA-1 | Japonica | NA | | Os02g0725100 | OsR498G0204683400.01.T01 | Retrotransposon protein Ty1-copia subclass |
| maker-2-augustus-gene-309.42-mRNA-1 | Japonica | NA | | Os02g0725500 | OsR498G0204684600.01.T01 | Phytocyanin domain-containing protein |
| maker-2-augustus-gene-309.8-mRNA-1 | Japonica | Indica | | Os09g0551300 | OsR498G0204682100.01.T01 | tRNA and rRNA cytosine-C5-methylase (Nucleolar protein NOL1/NOP2) |
| maker-2-augustus-gene-31.56-mRNA-1 | Japonica | NA | | Os02g0156100 | OsR498G0202996300.01.T01 | Protein kinase domain-containing protein |
| maker-2-augustus-gene-31.64-mRNA-1 | Japonica | NA | LRR-TM | Os02g0156800 | OsR498G0203001200.01.T02 | Tyrosine-sulfated glycopeptide receptor 1 |
| maker-2-augustus-gene-310.35-mRNA-1 | Japonica | NA | | Os02g0726400 | OsR498G0204688500.01.T01 | BSD domain-containing protein (Fragment) |
| maker-2-augustus-gene-310.40-mRNA-1 | Japonica | Indica | | Os06g0284200 | OsR498G0204688900.01.T01 | O-fucosyltransferase family protein |
| maker-2-augustus-gene-310.43-mRNA-1 | Japonica | NA | | Os02g0727300 | OsR498G0204690300.01.T02 | RING-type E3 ubiquitin transferase |
| maker-2-augustus-gene-310.45-mRNA-1 | Japonica | NA | | Os02g0725900 | OsR498G0204686400.01.T01 | CCAAT-binding transcription factor |
| maker-2-augustus-gene-310.47-mRNA-1 | Japonica | NA | | | | Transcription factor BIM3 |
| maker-2-augustus-gene-310.53-mRNA-1 | Japonica | NA | | Os02g0727100 | OsR498G0204690000.01.T01 | System N amino acid transporter |
| maker-2-augustus-gene-310.57-mRNA-1 | Japonica | NA | | Os02g0728100 | OsR498G0204693400.01.T05 | Signal peptide peptidase isoform 1 |
| maker-2-augustus-gene-311.51-mRNA-1 | Japonica | Indica | | Os02g0581300 | OsR498G0204693700.01.T02 | LAG1 longevity assurance homolog 3 |
| maker-2-augustus-gene-311.54-mRNA-1 | Japonica | NA | | Os02g0728700 | OsR498G0204695100.01.T02 | RRP7 domain-containing protein (Fragment) |
| maker-2-augustus-gene-311.57-mRNA-1 | Japonica | NA | | | OsR498G0204695300.01.T01 | Putative senescence-associated protein 5 |
| maker-2-augustus-gene-311.59-mRNA-1 | Japonica | NA | | Os02g0728900 | | Mediator of RNA polymerase II transcription subunit 11 |

| | | | | | | |
|---|---|---|---|---|---|---|
| maker-2-augustus-gene-311.63-mRNA-1 | Japonica | NA | | Os02g0189000 | OsR498G0203126400.01.T01 | |
| maker-2-augustus-gene-311.65-mRNA-1 | Japonica | NA | | Os02g0729100 | OsR498G0204696500.01.T01 | Leaf senescence protein-like |
| maker-2-augustus-gene-311.68-mRNA-1 | Japonica | NA | | Os02g0729700 | OsR498G0204701500.01.T01 | Homeobox-leucine zipper protein HOX16 |
| maker-2-augustus-gene-311.70-mRNA-1 | Japonica | Indica | Kinase-LRR-TM | Os06g0274500 | OsR498G0204694200.01.T01 | Putative somatic embryogenesis protein kinase 1 |
| maker-2-augustus-gene-311.77-mRNA-1 | Japonica | NA | | Os02g0728600 | OsR498G0204695000.01.T01 | Ribonucloprotein (Fragment) |
| maker-2-augustus-gene-311.78-mRNA-1 | Japonica | Indica | | Os06g0272900 | OsR498G0204697000.01.T01 | Leaf senescence protein-like |
| maker-2-augustus-gene-311.80-mRNA-1 | Japonica | NA | | | OsR498G0204698800.01.T01 | Rhodanese domain-containing protein |
| maker-2-augustus-gene-311.83-mRNA-1 | Japonica | NA | | Os02g0729400 | OsR498G0204698800.01.T04 | Extracellular calcium sensing receptor |
| maker-2-augustus-gene-311.85-mRNA-1 | Japonica | NA | | Os02g0730000 | OsR498G0204703200.01.T02 | Aldehyde dehydrogenase2 |
| maker-2-augustus-gene-311.89-mRNA-1 | Japonica | Japonica | | Os02g0730100 | OsR498G0204703400.01.T02 | Nop domain-containing protein (Fragment) |
| maker-2-augustus-gene-312.41-mRNA-1 | Japonica | NA | | Os02g0730400 | OsR498G0204705500.01.T01 | NHL repeat-containing protein 2 |
| maker-2-augustus-gene-312.46-mRNA-1 | Japonica | Indica | | Os06g0268800 | OsR498G0204706100.01.T01 | Aluminum-activated malate transporter like |
| maker-2-augustus-gene-312.48-mRNA-1 | Japonica | NA | | Os02g0730800 | OsR498G0204706700.01.T04 | Organelle RRM domain-containing protein 1, chloroplastic |
| maker-2-augustus-gene-312.53-mRNA-1 | Japonica | NA | | Os02g0731200 | OsR498G0204707300.01.T06 | MADS-box transcription factor |
| maker-2-augustus-gene-312.58-mRNA-1 | Japonica | NA | | | | CAAD domain-containing protein (Fragment) |
| maker-2-augustus-gene-312.60-mRNA-1 | Japonica | NA | | Os02g0730500 | OsR498G0204705800.01.T01 | rRNA-processing protein fcf2 |
| maker-2-augustus-gene-312.63-mRNA-1 | Japonica | NA | | Os02g0730700 | | Aspartyl protease APCB1 |
| maker-2-augustus-gene-312.65-mRNA-1 | Japonica | Indica | | Os02g0731500 | OsR498G0204708400.01.T02 | ABA induced plasma membrane protein PM 19 |
| maker-2-augustus-gene-312.68-mRNA-1 | Japonica | Indica | | Os02g0731700 | OsR498G0204708800.01.T01 | B-box zinc finger protein 10 |
| maker-2-augustus-gene-313.43-mRNA-1 | Japonica | NA | | Os02g0732200 | OsR498G0204711700.01.T03 | RING-type E3 ubiquitin transferase |
| maker-2-augustus-gene-313.47-mRNA-1 | Japonica | Indica | | Os06g0260000 | OsR498G0204712100.01.T01 | Tify domain-containing protein |
| maker-2-augustus-gene-313.49-mRNA-1 | Japonica | NA | | | OsR498G0204712500.01.T02 | Tify domain-containing protein |
| maker-2-augustus-gene-313.53-mRNA-1 | Japonica | NA | | Os02g0732900 | OsR498G0204714500.01.T01 | APO protein 2 chloroplastic |
| maker-2-augustus-gene-313.55-mRNA-1 | Japonica | NA | | Os02g0733300 | OsR498G0204716300.01.T01 | Endoglucanase |
| maker-2-augustus-gene-313.59-mRNA-1 | Japonica | NA | | Os02g0733800 | OsR498G0204717700.01.T03 | Rubis-subs-bind domain-containing protein (Fragment) |
| maker-2-augustus-gene-313.65-mRNA-1 | Japonica | NA | | Os02g0732600 | OsR498G0204713400.01.T01 | Myb-related transcription factor |
| maker-2-augustus-gene-313.67-mRNA-1 | Japonica | NA | | | | Mediator of RNA polymerase II transcription subunit 23 |
| maker-2-augustus-gene-313.75-mRNA-1 | Japonica | Indica | | Os06g0257600 | OsR498G0204714400.01.T01 | GDSL esterase/lipase |
| maker-2-augustus-gene-313.79-mRNA-1 | Japonica | NA | | | OsR498G0204714800.01.T01 | Dynein light chain |
| maker-2-augustus-gene-313.81-mRNA-1 | Japonica | NA | | Os02g0733400 | OsR498G0204716800.01.T01 | |
| maker-2-augustus-gene-314.37-mRNA-1 | Japonica | NA | | Os02g0734300 | OsR498G0204719900.01.T01 | Nudix hydrolase 17 mitochondrial |
| maker-2-augustus-gene-314.39-mRNA-1 | Japonica | Indica | | Os02g0734400 | OsR498G0204720300.01.T01 | probable peroxygenase 5 |
| maker-2-augustus-gene-314.42-mRNA-1 | Japonica | Indica | | Os02g0734400 | OsR498G0204720700.01.T01 | Caleosin |
| maker-2-augustus-gene-314.45-mRNA-1 | Japonica | NA | | Os02g0734600 | OsR498G0204720900.01.T01 | Peroxygenase |
| maker-2-augustus-gene-314.50-mRNA-1 | Japonica | NA | | | | TPR repeat-containing protein YfgC |
| maker-2-augustus-gene-314.52-mRNA-1 | Japonica | NA | | Os02g0735100 | OsR498G0204723300.01.T01 | Mitochondrial metalloendopeptidase OMA1 |
| maker-2-augustus-gene-314.54-mRNA-1 | Japonica | NA | | Os02g0735700 | OsR498G0204725300.01.T01 | Ankyrin repeat |
| maker-2-augustus-gene-314.57-mRNA-1 | Japonica | NA | | Os02g0735200 | OsR498G0204723800.01.T02 | Glutamine synthetase cytosolic isozyme 2 |
| maker-2-augustus-gene-314.63-mRNA-1 | Japonica | NA | | | OsR498G0204725800.01.T03 | E3 ubiquitin-protein ligase At4g11680 family |
| maker-2-augustus-gene-315.0-mRNA-1 | Japonica | NA | | Os02g0736100 | OsR498G0204726900.01.T01 | Microtubule-associated protein like |
| maker-2-augustus-gene-315.10-mRNA-1 | Japonica | NA | | Os02g0736300 | OsR498G0204727600.01.T01 | MACPF domain-containing protein (Fragment) |
| maker-2-augustus-gene-315.14-mRNA-1 | Japonica | NA | | Os02g0736400 | OsR498G0204727800.01.T02 | Pyrimidine 1 isoform 1 |
| maker-2-augustus-gene-315.18-mRNA-1 | Japonica | NA | | Os02g0736500 | OsR498G0204728000.01.T03 | Nicastrin |
| maker-2-augustus-gene-315.24-mRNA-1 | Japonica | NA | | Os02g0736700 | | |
| maker-2-augustus-gene-315.6-mRNA-1 | Japonica | NA | | Os02g0736600 | OsR498G0204728100.01.T03 | RNA helicase |
| maker-2-augustus-gene-316.0-mRNA-1 | Japonica | NA | | | | |
| maker-2-augustus-gene-316.14-mRNA-1 | Japonica | NA | | Os02g0738900 | OsR498G0204736200.01.T01 | Dynamin GTPase |
| maker-2-augustus-gene-316.2-mRNA-1 | Japonica | NA | | | OsR498G0204733700.01.T01 | Histidine kinase |
| maker-2-augustus-gene-316.21-mRNA-1 | Japonica | Indica | | Os02g0739100 | OsR498G0204737000.01.T01 | Formin-like protein 16 |
| maker-2-augustus-gene-316.5-mRNA-1 | Japonica | NA | | Os02g0738600 | OsR498G0204735300.01.T01 | Endoglucanase |
| maker-2-augustus-gene-316.9-mRNA-1 | Japonica | NA | | Os02g0739000 | OsR498G0204736700.01.T01 | Increased size exclusion limit 2 |
| maker-2-augustus-gene-317.0-mRNA-1 | Japonica | NA | | Os02g0740500 | OsR498G0204743700.01.T01 | |
| maker-2-augustus-gene-317.12-mRNA-1 | Japonica | NA | | Os02g0739700 | OsR498G0204741400.01.T01 | Transcription factor E2F7 |
| maker-2-augustus-gene-317.16-mRNA-1 | Japonica | Indica | | Os06g0244700 | OsR498G0204741800.01.T01 | Microtubule-associated protein TORTIFOLIA1 |
| maker-2-augustus-gene-317.18-mRNA-1 | Japonica | NA | | Os02g0740300 | OsR498G0204743000.01.T01 | meiotic spindle formation protein mei-1 isoform X1 |
| maker-2-augustus-gene-317.2-mRNA-1 | Japonica | NA | | Os02g0741100 | OsR498G0204745100.01.T01 | Chaperone protein dnaJ 16 |
| maker-2-augustus-gene-317.22-mRNA-1 | Japonica | NA | | Os02g0740400 | OsR498G0204743200.01.T01 | GDSL esterase/lipase APG |
| maker-2-augustus-gene-317.8-mRNA-1 | Japonica | NA | | Os02g0739600 | OsR498G0204741000.01.T01 | Pyruvate dehydrogenase E1 component subunit alpha, mitochondrial |
| maker-2-augustus-gene-318.24-mRNA-1 | Japonica | NA | | Os02g0741300 | OsR498G0204745700.01.T02 | alpha-1,2-Mannosidase |
| maker-2-augustus-gene-318.28-mRNA-1 | Japonica | NA | | Os02g0742000 | OsR498G0204748300.01.T01 | BAH domain |
| maker-2-augustus-gene-318.30-mRNA-1 | Japonica | NA | | | OsR498G0204746200.01.T01 | Nuclear-pore anchor |

| | | | | | | |
|---|---|---|---|---|---|---|
| maker-2-augustus-gene-318.39-mRNA-1 | Japonica | NA | | Os02g0741700 | OsR498G0204746400.01.T01 | |
| maker-2-augustus-gene-318.42-mRNA-1 | Japonica | NA | | Os02g0741800 | OsR498G0204747400.01.T02 | Nucleobase-ascorbate transporter 6 |
| maker-2-augustus-gene-318.47-mRNA-1 | Japonica | Japonica | | Os02g0741900 | OsR498G0204747800.01.T01 | AD domain-containing protein (Fragment) |
| maker-2-augustus-gene-318.49-mRNA-1 | Japonica | NA | | Os02g0742200 | OsR498G0204749000.01.T01 | Rac-like GTP-binding protein 3 |
| maker-2-augustus-gene-318.53-mRNA-1 | Japonica | Indica | | Os06g0234100 | OsR498G0204750000.01.T02 | Protease Do-like protein 9 |
| maker-2-augustus-gene-319.34-mRNA-1 | Japonica | Indica | CC-Kinase-TM | Os02g0743500 | OsR498G0204756500.01.T01 | Serine/threonine-protein kinase CTR1 |
| maker-2-augustus-gene-319.39-mRNA-1 | Japonica | NA | | | OsR498G0204757300.01.T01 | Latex abundant protein 1 |
| maker-2-augustus-gene-319.41-mRNA-1 | Japonica | NA | | Os02g0743700 | OsR498G0204757700.01.T01 | NEP1-interacting protein-like 2 |
| maker-2-augustus-gene-319.45-mRNA-1 | Japonica | NA | | Os02g0744000 | OsR498G0204758500.01.T02 | Photosynthetic NDH subunit of subcomplex B 4 chloroplastic |
| maker-2-augustus-gene-319.47-mRNA-1 | Japonica | NA | | | OsR498G0204751200.01.T03 | p-loop containing nucleoside triphosphate hydrolase superfamily protein |
| maker-2-augustus-gene-319.54-mRNA-1 | Japonica | NA | | Os02g0743400 | OsR498G0204755400.01.T01 | Auxin efflux carrier component |
| maker-2-augustus-gene-319.56-mRNA-1 | Japonica | NA | | | | Casparian strip membrane protein 4 |
| maker-2-augustus-gene-320.0-mRNA-1 | Japonica | NA | | Os02g0744100 | OsR498G0204759300.01.T01 | Methyltransferase-like protein 7A |
| maker-2-augustus-gene-320.11-mRNA-1 | Japonica | NA | | Os02g0745100 | OsR498G0204764100.01.T01 | Aquaporin NIP2-1 |
| maker-2-augustus-gene-320.14-mRNA-1 | Japonica | NA | | | OsR498G0204765100.01.T01 | NAC domain class transcription factor |
| maker-2-augustus-gene-320.16-mRNA-1 | Japonica | NA | | Os02g0745400 | OsR498G0204765300.01.T02 | Hexosyltransferase |
| maker-2-augustus-gene-320.18-mRNA-1 | Japonica | NA | | Os02g0745600 | OsR498G0204765700.01.T01 | DCD domain-containing protein (Fragment) |
| maker-2-augustus-gene-320.21-mRNA-1 | Japonica | NA | | Os02g0745700 | OsR498G0204765900.01.T01 | Aldehyde oxygenase (deformylating) |
| maker-2-augustus-gene-320.25-mRNA-1 | Japonica | NA | | | OsR498G0204761200.01.T01 | Putative mannan synthase |
| maker-2-augustus-gene-320.3-mRNA-1 | Japonica | NA | | Os02g0744200 | OsR498G0204760300.01.T01 | Expansin |
| maker-2-augustus-gene-320.32-mRNA-1 | Japonica | NA | | Os02g0744700 | OsR498G0204761600.01.T01 | Starch synthase, chloroplastic/amyloplastic |
| maker-2-augustus-gene-320.37-mRNA-1 | Japonica | NA | | | OsR498G0204762600.01.T01 | RWP-RK domain-containing protein |
| maker-2-augustus-gene-320.39-mRNA-1 | Japonica | Indica | | Os02g0745000 | OsR498G0204763000.01.T01 | ATPase ARSA1 isoform A |
| maker-2-augustus-gene-320.5-mRNA-1 | Japonica | NA | | | OsR498G0509807100.01.T01 | Putative LRR receptor-like serine/threonine-protein kinase IRK |
| maker-2-augustus-gene-320.7-mRNA-1 | Japonica | NA | | Os02g0744501 | | |
| maker-2-augustus-gene-320.9-mRNA-1 | Japonica | NA | | Os02g0744900 | OsR498G0204762300.01.T01 | Geranylgeranyl diphosphate reductase, chloroplastic |
| maker-2-augustus-gene-321.31-mRNA-1 | Japonica | NA | | | OsR498G0204766300.01.T02 | Aldehyde oxygenase (deformylating) |
| maker-2-augustus-gene-321.36-mRNA-1 | Japonica | NA | | Os02g0746000 | OsR498G0204766600.01.T03 | Cullin-3 |
| maker-2-augustus-gene-326.37-mRNA-1 | Japonica | NA | | Os06g0677700 | OsR498G0613174100.01.T01 | Cleavage and polyadenylation specificity factor like |
| maker-2-augustus-gene-331.52-mRNA-1 | Japonica | NA | | Os11g0205500 | OsR498G1119581200.01.T01 | |
| maker-2-augustus-gene-332.28-mRNA-1 | Japonica | NA | | | | Copper-transporting ATPase RAN1 |
| maker-2-augustus-gene-337.42-mRNA-1 | Japonica | NA | | Os02g0747500 | OsR498G0204772500.01.T03 | |
| maker-2-augustus-gene-337.46-mRNA-1 | Japonica | NA | | Os02g0749000 | OsR498G0815725100.01.T05 | AAA domain-containing protein |
| maker-2-augustus-gene-337.52-mRNA-1 | Japonica | NA | | | | AP2-like ethylene-responsive transcription factor AIL6 isoform A |
| maker-2-augustus-gene-337.57-mRNA-1 | Japonica | Indica | | Os06g0226500 | OsR498G0204774000.01.T01 | Transcription factor ILI5 |
| maker-2-augustus-gene-337.59-mRNA-1 | Japonica | NA | | Os02g0748100 | OsR498G0204775600.01.T03 | RAD50-interacting protein 1 (Fragment) |
| maker-2-augustus-gene-338.37-mRNA-1 | Japonica | NA | | | OsR498G0204779500.01.T01 | AAA domain-containing protein (Fragment) |
| maker-2-augustus-gene-338.42-mRNA-1 | Japonica | Indica | | Os02g0749300 | OsR498G0204780700.01.T01 | Shikimate kinase |
| maker-2-augustus-gene-338.47-mRNA-1 | Japonica | NA | | | OsR498G0204783300.01.T01 | Tetratricopeptide repeat (TPR)-like superfamily protein isoform 1 |
| maker-2-augustus-gene-338.49-mRNA-1 | Japonica | NA | | Os02g0751100 | | Aspartic proteinase nepenthesin-1 |
| maker-2-augustus-gene-338.51-mRNA-1 | Japonica | NA | | | OsR498G0204781600.01.T02 | Exocyst complex component Sec6 |
| maker-2-augustus-gene-338.59-mRNA-1 | Japonica | Indica | | Os01g0374600 | OsR498G0204782000.01.T01 | Laccase |
| maker-2-augustus-gene-338.62-mRNA-1 | Japonica | NA | | Os02g0750500 | OsR498G0204783600.01.T01 | tRNA (Carboxymethyluridine(34)-5-O)-methyltransferase isoform X1 |
| maker-2-augustus-gene-338.64-mRNA-1 | Japonica | NA | | | | Membrane-anchored ubiquitin-fold protein |
| maker-2-augustus-gene-339.27-mRNA-1 | Japonica | NA | | Os02g0751300 | OsR498G0204786700.01.T01 | FLZ-type domain-containing protein (Fragment) |
| maker-2-augustus-gene-339.29-mRNA-1 | Japonica | NA | | Os02g0751600 | OsR498G0204787500.01.T01 | Peptidylprolyl isomerase |
| maker-2-augustus-gene-34.48-mRNA-1 | Japonica | Indica | | Os06g0681300 | OsR498G0203020500.01.T01 | CRIB domain-containing protein RIC1 |
| maker-2-augustus-gene-34.50-mRNA-1 | Japonica | NA | | Os02g0162200 | OsR498G0203020900.01.T01 | Phytocyanin domain-containing protein |
| maker-2-augustus-gene-34.52-mRNA-1 | Japonica | Japonica | | Os02g0162400 | OsR498G0203021300.01.T01 | Phytocyanin domain-containing protein |
| maker-2-augustus-gene-34.56-mRNA-1 | Japonica | Indica | | Os02g0161800 | OsR498G0203019200.01.T02 | Peroxidase |
| maker-2-augustus-gene-34.59-mRNA-1 | Japonica | NA | | | OsR498G0203019800.01.T01 | N utilization substance B |
| maker-2-augustus-gene-34.63-mRNA-1 | Japonica | NA | | | | Ribosomal protein S14 |
| maker-2-augustus-gene-34.66-mRNA-1 | Japonica | NA | | | | BEACH domain-containing protein lvsC |
| maker-2-augustus-gene-344.16-mRNA-1 | Japonica | NA | Kinase-TM | Os06g0654500 | OsR498G0613085100.01.T01 | Concanavalin A-like lectin/glucanase domain, Serine/threonine-protein kina |
| maker-2-augustus-gene-344.5-mRNA-1 | Japonica | Indica | | Os02g0178100 | OsR498G0613085400.01.T01 | COLE |
| maker-2-augustus-gene-346.8-mRNA-1 | Japonica | NA | | Os06g0650800 | OsR498G0613073200.01.T01 | Magnesium transporter |
| maker-2-augustus-gene-35.30-mRNA-1 | Japonica | NA | | Os02g0164000 | | |
| maker-2-augustus-gene-35.8-mRNA-1 | Japonica | NA | | | OsR498G0203027300.01.T01 | Ubiquitinyl hydrolase 1 |
| maker-2-augustus-gene-351.42-mRNA-1 | Japonica | NA | | Os11g0635500 | OsR498G0613042200.01.T01 | Premnaspirodiene oxygenase |
| maker-2-augustus-gene-351.44-mRNA-1 | Japonica | NA | | Os11g0635500 | OsR498G0613042000.01.T01 | Cytochrome P450 family protein, expressed |
| maker-2-augustus-gene-355.12-mRNA-1 | Japonica | NA | | Os02g0757900 | OsR498G0204807600.01.T02 | Polyadenylate-binding protein 3 |

| | | | | | | | |
|---|---|---|---|---|---|---|---|
| maker-2-augustus-gene-355.3-mRNA-1 | Japonica | NA | | Os02g0758000 | OsR498G0204807700.01.T01 | Small heat shock protein HSP |
| maker-2-augustus-gene-355.5-mRNA-1 | Japonica | NA | | Os02g0758100 | OsR498G0204807900.01.T04 | LMBR1 domain-containing protein A |
| maker-2-augustus-gene-356.41-mRNA-1 | Japonica | NA | | Os02g0759700 | OsR498G0204813300.01.T02 | F-box protein FBX14 |
| maker-2-augustus-gene-356.44-mRNA-1 | Japonica | NA | | Os02g0759900 | OsR498G0204814300.01.T01 | CAAD domain-containing protein |
| maker-2-augustus-gene-356.47-mRNA-1 | Japonica | NA | | Os02g0760200 | OsR498G0204815300.01.T05 | Rhomboid-like protease 5 |
| maker-2-augustus-gene-356.52-mRNA-1 | Japonica | NA | | Os02g0761000 | OsR498G0204817100.01.T01 | MADS-box transcription factor 30 |
| maker-2-augustus-gene-356.56-mRNA-1 | Japonica | NA | | Os02g0758800 | OsR498G0204809800.01.T01 | Phytocyanin domain-containing protein |
| maker-2-augustus-gene-356.58-mRNA-1 | Japonica | Indica | | Os09g0468700 | OsR498G0204811000.01.T02 | Transcription factor bHLH96 isoform A |
| maker-2-augustus-gene-356.62-mRNA-1 | Japonica | NA | | | OsR498G0204814600.01.T03 | Glucose-6-phosphate isomerase isoform 1 |
| maker-2-augustus-gene-356.67-mRNA-1 | Japonica | NA | | Os02g0760300 | OsR498G0204815600.01.T01 | Peptidyl-prolyl cis-trans isomerase |
| maker-2-augustus-gene-356.70-mRNA-1 | Japonica | NA | | | OsR498G0204815800.01.T03 | Stress response protein nhaX (Fragment) |
| maker-2-augustus-gene-357.37-mRNA-1 | Japonica | NA | | Os02g0761100 | OsR498G0204817700.01.T01 | Peptidylprolyl isomerase |
| maker-2-augustus-gene-357.41-mRNA-1 | Japonica | NA | | | OsR498G0204818700.01.T01 | S-ribosylhomocysteinase (LuxS) |
| maker-2-augustus-gene-357.48-mRNA-1 | Japonica | NA | | Os02g0762400 | OsR498G0204821100.01.T01 | Cyclin-dependent kinase inhibitor |
| maker-2-augustus-gene-357.50-mRNA-1 | Japonica | NA | | Os02g0761200 | OsR498G0204818000.01.T02 | Armadillo repeat-containing protein 6 |
| maker-2-augustus-gene-357.53-mRNA-1 | Japonica | NA | | Os02g0761600 | OsR498G0204819000.01.T01 | Dimethylallyl, adenosine tRNA methylthiotransferase |
| maker-2-augustus-gene-357.58-mRNA-1 | Japonica | NA | | | OsR498G0204819200.01.T06 | Methionine aminopeptidase |
| maker-2-augustus-gene-357.65-mRNA-1 | Japonica | NA | | Os02g0762200 | OsR498G0204820000.01.T01 | TIMELESS-interacting protein isoform X1 |
| maker-2-augustus-gene-357.67-mRNA-1 | Japonica | NA | | Os02g0762300 | OsR498G0204820200.01.T04 | Cytoplasmic tRNA 2-thiolation protein 1 |
| maker-2-augustus-gene-357.72-mRNA-1 | Japonica | NA | | Os02g0762600 | OsR498G0204822200.01.T01 | |
| maker-2-augustus-gene-357.73-mRNA-1 | Japonica | NA | | Os02g0762800 | OsR498G0204822400.01.T03 | Protein CHROMATIN REMODELING like |
| maker-2-augustus-gene-358.0-mRNA-1 | Japonica | NA | | Os02g0763000 | OsR498G0204823100.01.T02 | Cell number regulator 1 |
| maker-2-augustus-gene-358.11-mRNA-1 | Japonica | NA | | Os02g0765600 | OsR498G0204827200.01.T01 | 1,4-alpha-D-glucan glucanohydrolase |
| maker-2-augustus-gene-358.13-mRNA-1 | Japonica | NA | | Os02g0764500 | OsR498G0204826000.01.T02 | Chlorophyll a-b binding protein, chloroplastic |
| maker-2-augustus-gene-358.18-mRNA-1 | Japonica | NA | | Os02g0765600 | OsR498G0204827200.01.T01 | Alpha-amylase type B isozyme |
| maker-2-augustus-gene-358.2-mRNA-1 | Japonica | NA | | Os02g0763200 | OsR498G0204823700.01.T01 | Fucosyltransferase |
| maker-2-augustus-gene-358.4-mRNA-1 | Japonica | NA | | Os02g0763900 | OsR498G0204824700.01.T02 | Fucosyltransferase |
| maker-2-augustus-gene-358.7-mRNA-1 | Japonica | NA | | Os02g0764200 | OsR498G0204825300.01.T01 | Fucosyltransferase |
| maker-2-augustus-gene-358.9-mRNA-1 | Japonica | Japonica | | Os02g0764400 | OsR498G0204825700.01.T01 | Fucosyltransferase |
| maker-2-augustus-gene-359.18-mRNA-1 | Japonica | NA | | Os02g0766700 | OsR498G0204829400.01.T01 | ABA responsive element binding factor 1 |
| maker-2-augustus-gene-359.2-mRNA-1 | Japonica | Indica | | Os02g0767100 | OsR498G0204831100.01.T02 | THO complex subunit 5 B |
| maker-2-augustus-gene-359.22-mRNA-1 | Japonica | NA | | Os02g0766900 | OsR498G0204830000.01.T01 | Mannan endo-1,4-beta-mannosidase |
| maker-2-augustus-gene-359.24-mRNA-1 | Japonica | Indica | | Os02g0767000 | OsR498G0204830200.01.T03 | |
| maker-2-augustus-gene-359.27-mRNA-1 | Japonica | NA | | Os02g0767200 | OsR498G0204831400.01.T01 | Triacylglycerol lipase-like 1 |
| maker-2-augustus-gene-359.29-mRNA-1 | Japonica | NA | | Os02g0767300 | OsR498G0204832000.01.T01 | Flavonol synthase/flavanone 3-hydroxylase |
| maker-2-augustus-gene-359.32-mRNA-1 | Japonica | NA | | Os02g0767500 | OsR498G0204832600.01.T01 | Mitochondrial phosphate transporter |
| maker-2-augustus-gene-359.6-mRNA-1 | Japonica | Indica | | Os02g0765800 | OsR498G0204827400.01.T01 | non-specific lipid-transfer protein-like 1 |
| maker-2-augustus-gene-359.7-mRNA-1 | Japonica | NA | | Os01g0357100 | OsR498G0204827600.01.T01 | Ferredoxin--nitrite reductase chloroplastic |
| maker-2-augustus-gene-360.0-mRNA-1 | Japonica | Indica | | Os02g0768600 | OsR498G0204835900.01.T01 | Inorganic diphosphatase |
| maker-2-augustus-gene-360.12-mRNA-1 | Japonica | NA | | Os02g0768200 | OsR498G0204835200.01.T01 | Transmembrane emp24 domain-containing protein 10 |
| maker-2-augustus-gene-360.14-mRNA-1 | Japonica | NA | | Os02g0768300 | OsR498G0204835400.01.T01 | |
| maker-2-augustus-gene-360.16-mRNA-1 | Japonica | Indica | | Os02g0769200 | OsR498G0204839600.01.T03 | Erwinia induced protein 1 |
| maker-2-augustus-gene-360.3-mRNA-1 | Japonica | NA | | | OsR498G0204836900.01.T01 | Putative PHD-finger domain containing protein family |
| maker-2-augustus-gene-360.9-mRNA-1 | Japonica | Japonica | | Os02g0768000 | OsR498G0204835000.01.T01 | |
| maker-2-augustus-gene-361.35-mRNA-1 | Japonica | NA | Kinase-TM | Os02g0769700 | OsR498G0204841300.01.T04 | Mitogen-activated protein kinase kinase kinase 1 |
| maker-2-augustus-gene-361.36-mRNA-1 | Japonica | NA | | | OsR498G0204841300.01.T01 | Mitogen-activated protein kinase kinase kinase |
| maker-2-augustus-gene-361.43-mRNA-1 | Japonica | NA | Kinase | | OsR498G0204841500.01.T02 | Mitogen-activated protein kinase kinase kinase |
| maker-2-augustus-gene-361.49-mRNA-1 | Japonica | NA | | Os02g0770000 | OsR498G0204842000.01.T01 | Proteasome endopeptidase complex |
| maker-2-augustus-gene-361.55-mRNA-1 | Japonica | NA | | Os02g0770100 | OsR498G0204842400.01.T03 | |
| maker-2-augustus-gene-361.58-mRNA-1 | Japonica | NA | | | | Separase |
| maker-2-augustus-gene-361.63-mRNA-1 | Japonica | NA | | Os02g0770800 | OsR498G0204845000.01.T01 | Nitrate reductase |
| maker-2-augustus-gene-361.65-mRNA-1 | Japonica | NA | | Os02g0771100 | OsR498G0204846000.01.T01 | CopI |
| maker-2-augustus-gene-361.71-mRNA-1 | Japonica | NA | | Os02g0771400 | OsR498G0204846400.01.T02 | probable tyrosine-protein phosphatase DSP4 |
| maker-2-augustus-gene-362.0-mRNA-1 | Japonica | NA | | Os02g0771600 | OsR498G0204848700.01.T01 | 1-aminocyclopropane-1-carboxylate oxidase |
| maker-2-augustus-gene-362.13-mRNA-1 | Japonica | NA | | Os02g0772500 | OsR498G0204850900.01.T01 | SiRNA-mediated silencing protein NRDE-2 |
| maker-2-augustus-gene-362.18-mRNA-1 | Japonica | NA | | Os02g0771700 | OsR498G0204849000.01.T01 | Glucan endo-1,3-beta-D-glucosidase |
| maker-2-augustus-gene-362.22-mRNA-1 | Japonica | NA | | | OsR498G0204849400.01.T01 | (Rice Genome Annotation Project) expressed protein |
| maker-2-augustus-gene-362.27-mRNA-1 | Japonica | NA | | Os02g0772600 | OsR498G0204851200.01.T01 | |
| maker-2-augustus-gene-362.4-mRNA-1 | Japonica | NA | | | OsR498G0611830400.01.T01 | Transcription elongation factor SPT5 |
| maker-2-augustus-gene-362.9-mRNA-1 | Japonica | Indica | | Os02g0772300 | OsR498G0204850500.01.T05 | N-alpha-acetyltransferase 40 |
| maker-2-augustus-gene-363.0-mRNA-1 | Japonica | NA | | Os02g0773200 | OsR498G0204853500.01.T02 | Usp domain-containing protein |

| | | | | | | |
|---|---|---|---|---|---|---|
| maker-2-augustus-gene-363.11-mRNA-1 | Japonica | NA | | Os02g0774700 | OsR498G0204856700.01.T02 | CobW C-terminal domain-containing protein |
| maker-2-augustus-gene-363.14-mRNA-1 | Japonica | Indica | | Os02g0775100 | OsR498G0204857500.01.T01 | Metal tolerance protein 3 |
| maker-2-augustus-gene-363.19-mRNA-1 | Japonica | NA | | Os02g0773300 | OsR498G0204853800.01.T02 | Bifunctional D-cysteine desulfhydrase/1-aminocyclopropane-1-carboxylate d |
| maker-2-augustus-gene-363.28-mRNA-1 | Japonica | Indica | | Os06g0207500 | OsR498G0204854800.01.T01 | Protein trichome birefringence like |
| maker-2-augustus-gene-363.3-mRNA-1 | Japonica | NA | | Os02g0773400 | OsR498G0204853900.01.T01 | Cyclic nucleotide-gated ion channel |
| maker-2-augustus-gene-363.30-mRNA-1 | Japonica | NA | | Os02g0774300 | OsR498G0204856200.01.T01 | Mitochondrial HSO70 2 |
| maker-2-augustus-gene-363.35-mRNA-1 | Japonica | NA | | Os02g0774500 | OsR498G0204856400.01.T01 | 3-methyladenine DNA glycosidase |
| maker-2-augustus-gene-363.38-mRNA-1 | Japonica | NA | | | OsR498G0204856600.01.T01 | 3-methyladenine DNA glycosidase |
| maker-2-augustus-gene-363.40-mRNA-1 | Japonica | Indica | | Os02g0774800 | OsR498G0204857000.01.T01 | |
| maker-2-augustus-gene-363.43-mRNA-1 | Japonica | NA | | Os02g0775200 | OsR498G0204857800.01.T04 | Replication factor C subunit |
| maker-2-augustus-gene-363.47-mRNA-1 | Japonica | NA | | | OsR498G0204858400.01.T02 | Kinesin-like protein KIN-7D, chloroplastic |
| maker-2-augustus-gene-363.8-mRNA-1 | Japonica | NA | | Os02g0774100 | OsR498G0204855900.01.T01 | Thioredoxin-like 3-1 chloroplastic |
| maker-2-augustus-gene-364.0-mRNA-1 | Japonica | NA | | | | Telomere repeat-binding protein 6 |
| maker-2-augustus-gene-364.11-mRNA-1 | Japonica | NA | | Os02g0776600 | | |
| maker-2-augustus-gene-364.12-mRNA-1 | Japonica | NA | | Os02g0776800 | OsR498G0204866800.01.T01 | Replication protein A subunit |
| maker-2-augustus-gene-364.14-mRNA-1 | Japonica | NA | | Os02g0776900 | OsR498G0204867400.01.T01 | Growth-regulating factor |
| maker-2-augustus-gene-364.3-mRNA-1 | Japonica | NA | | Os02g0775900 | OsR498G0204862800.01.T01 | |
| maker-2-augustus-gene-364.5-mRNA-1 | Japonica | NA | | | OsR498G0204863400.01.T01 | |
| maker-2-augustus-gene-365.0-mRNA-1 | Japonica | Indica | Kinase-LRR-TM | Os06g0203800 | OsR498G0204869500.01.T02 | LRR receptor-like serine/threonine-protein kinase ERECTA |
| maker-2-augustus-gene-365.13-mRNA-1 | Japonica | NA | | Os02g0777100 | OsR498G0204868200.01.T08 | UDENN domain-containing protein |
| maker-2-augustus-gene-365.16-mRNA-1 | Japonica | Indica | Kinase-TM | Os06g0202900 | OsR498G0204870400.01.T01 | Mitogen-activated protein kinase kinase kinase |
| maker-2-augustus-gene-365.18-mRNA-1 | Japonica | NA | | | | |
| maker-2-augustus-gene-365.6-mRNA-1 | Japonica | NA | | | OsR498G0204870100.01.T02 | Opaque endosperm1 |
| maker-2-augustus-gene-366.33-mRNA-1 | Japonica | NA | | Os02g0778200 | OsR498G0204872300.01.T05 | Isoleucyl-tRNA synthetase |
| maker-2-augustus-gene-366.37-mRNA-1 | Japonica | NA | | Os02g0778300 | OsR498G0204872500.01.T02 | Putative FRIGIDA interacting protein 1 |
| maker-2-augustus-gene-366.41-mRNA-1 | Japonica | NA | | Os02g0778400 | OsR498G0204872500.01.T06 | Adenylate kinase |
| maker-2-augustus-gene-366.45-mRNA-1 | Japonica | NA | | Os02g0778500 | OsR498G0204873100.01.T01 | Chalcone--flavonone isomerase |
| maker-2-augustus-gene-366.48-mRNA-1 | Japonica | NA | | Os02g0778600 | OsR498G0204873300.01.T01 | Endoglucanase |
| maker-2-augustus-gene-366.50-mRNA-1 | Japonica | NA | | | OsR498G0204873700.01.T01 | Myotubularin-related 2 |
| maker-2-augustus-gene-366.52-mRNA-1 | Japonica | NA | | Os02g0779000 | OsR498G0204874500.01.T01 | Cucumisin isoform B |
| maker-2-augustus-gene-367.0-mRNA-1 | Japonica | NA | | | | ARM repeat superfamily protein |
| maker-2-augustus-gene-367.1-mRNA-1 | Japonica | NA | | Os02g0780500 | OsR498G0204878300.01.T01 | ARM repeat superfamily protein |
| maker-2-augustus-gene-367.13-mRNA-1 | Japonica | Indica | | Os06g0199200 | OsR498G0204879100.01.T06 | Smr domain-containing protein (Fragment) |
| maker-2-augustus-gene-367.17-mRNA-1 | Japonica | NA | | | | mRNA (guanine-N(7)-)-methyltransferase |
| maker-2-augustus-gene-367.21-mRNA-1 | Japonica | NA | | Os02g0780700 | OsR498G0204880100.01.T05 | Putative lipase/calmodulin-binding heat-shock protein |
| maker-2-augustus-gene-367.26-mRNA-1 | Japonica | Indica | | Os02g0781000 | OsR498G0204882100.01.T02 | Polygalacturonase |
| maker-2-augustus-gene-367.53-mRNA-1 | Japonica | NA | | Os02g0781400 | OsR498G0204884600.01.T02 | Chaperonin, chloroplastic |
| maker-2-augustus-gene-367.55-mRNA-1 | Japonica | NA | | Os02g0781700 | OsR498G0204885000.01.T02 | MADS box interactor-like |
| maker-2-augustus-gene-367.60-mRNA-1 | Japonica | Indica | | Os05g0482600 | OsR498G0204885400.01.T01 | |
| maker-2-augustus-gene-368.22-mRNA-1 | Japonica | Japonica | | Os02g0781900 | OsR498G0204886300.01.T01 | |
| maker-2-augustus-gene-368.26-mRNA-1 | Japonica | Indica | | Os02g0782100 | OsR498G0204887100.01.T01 | NUC173 domain-containing protein |
| maker-2-augustus-gene-368.33-mRNA-1 | Japonica | NA | | Os02g0782500 | OsR498G0204889900.01.T01 | 18. class III heat shock protein (Fragment) |
| maker-2-augustus-gene-368.35-mRNA-1 | Japonica | Indica | | Os06g0195600 | OsR498G0204890700.01.T06 | SAC domain-containing protein |
| maker-2-augustus-gene-368.42-mRNA-1 | Japonica | NA | | | OsR498G0204891500.01.T01 | Putative acetyltransferase A, auxiliary subunit, leucine-rich repeat domain, L |
| maker-2-augustus-gene-368.50-mRNA-1 | Japonica | Indica | | Os02g0782900 | OsR498G0204892100.01.T01 | |
| maker-2-augustus-gene-368.52-mRNA-1 | Japonica | NA | | Os02g0782200 | OsR498G0204889400.01.T04 | NOT2/NOT3/NOT5 family protein, expressed |
| maker-2-augustus-gene-368.59-mRNA-1 | Japonica | NA | | | OsR498G0204889600.01.T01 | J domain-containing protein (Fragment) |
| maker-2-augustus-gene-368.64-mRNA-1 | Japonica | NA | | Os02g0782700 | OsR498G0204891000.01.T02 | Ethylene-responsive transcription factor |
| maker-2-augustus-gene-368.66-mRNA-1 | Japonica | NA | | Os02g0783000 | OsR498G0204892400.01.T01 | Pectinesterase |
| maker-2-augustus-gene-369.38-mRNA-1 | Japonica | NA | | | OsR498G0204896300.01.T01 | |
| maker-2-augustus-gene-369.42-mRNA-1 | Japonica | NA | | | | Lysine-ketoglutarate reductase/saccharopine dehydrogenase1 |
| maker-2-augustus-gene-369.50-mRNA-1 | Japonica | NA | | | OsR498G0204891500.01.T01 | Protein TONSOKU like |
| maker-2-augustus-gene-369.58-mRNA-1 | Japonica | Indica | | Os06g0192600 | OsR498G0204898000.01.T01 | 26S protease regulatory subunit 7 homolog A |
| maker-2-augustus-gene-369.64-mRNA-1 | Japonica | NA | | | OsR498G0204898200.01.T01 | |
| maker-2-augustus-gene-370.21-mRNA-1 | Japonica | Indica | Kinase-TM | Os02g0786200 | OsR498G0204902300.01.T02 | Serine/threonine-protein kinase STY8 (Fragment) |
| maker-2-augustus-gene-370.25-mRNA-1 | Japonica | Japonica | | Os02g0786300 | OsR498G0204903100.01.T01 | Transcription factor like |
| maker-2-augustus-gene-370.27-mRNA-1 | Japonica | NA | | | | F-box domain-containing protein |
| maker-2-augustus-gene-370.29-mRNA-1 | Japonica | Japonica | | Os02g0787100 | OsR498G0204905700.01.T03 | DUF676 domain-containing protein |
| maker-2-augustus-gene-370.35-mRNA-1 | Japonica | NA | | | | Transposon protein, putative, Pong sub-class, expressed |
| maker-2-augustus-gene-370.4-mRNA-1 | Japonica | Japonica | | Os02g0785900 | OsR498G0204901100.01.T03 | Anaphase-promoting complex subunit 4 |
| maker-2-augustus-gene-370.9-mRNA-1 | Japonica | NA | | | OsR498G0204901500.01.T07 | Quinoprotein amine dehydrogenase isoform 1 |

| | | | | | | |
|---|---|---|---|---|---|---|
| maker-2-augustus-gene-371.0-mRNA-1 | Japonica | NA | CC-Kinase-TM | Os02g0787200 | OsR498G0204905900.01.T01 | Serine threonine kinase |
| maker-2-augustus-gene-371.10-mRNA-1 | Japonica | NA | | Os02g0788400 | OsR498G0204910500.01.T01 | Eukaryotic translation initiation factor 3 subunit G |
| maker-2-augustus-gene-371.12-mRNA-1 | Japonica | NA | | Os02g0787800 | OsR498G0204909000.01.T06 | Diacylglycerol kinase |
| maker-2-augustus-gene-371.16-mRNA-1 | Japonica | NA | | | OsR498G0204910200.01.T01 | Desumoylating isopeptidase 1 |
| maker-2-augustus-gene-371.18-mRNA-1 | Japonica | NA | | Os02g0788500 | OsR498G0204911000.01.T01 | F-box protein SKIP8 |
| maker-2-augustus-gene-371.2-mRNA-1 | Japonica | Indica | | Os02g0787500 | OsR498G0204907700.01.T03 | E3 ubiquitin-protein ligase |
| maker-2-augustus-gene-371.20-mRNA-1 | Japonica | NA | | Os02g0788600 | OsR498G0204911800.01.T02 | Chaperone protein ClpB-like |
| maker-2-augustus-gene-371.23-mRNA-1 | Japonica | NA | | Os02g0788800 | OsR498G0204912400.01.T06 | Amino acid transporter, transmembrane |
| maker-2-augustus-gene-371.5-mRNA-1 | Japonica | NA | | Os02g0787600 | OsR498G0204908700.01.T01 | Ionotropic glutamate receptor |
| maker-2-augustus-gene-371.8-mRNA-1 | Japonica | Japonica | | Os02g0788300 | OsR498G0204910500.01.T01 | Eukaryotic translation initiation factor 3 subunit G |
| maker-2-augustus-gene-372.46-mRNA-1 | Japonica | NA | | Os02g0789600 | OsR498G0204913700.01.T01 | Vacuolar import and degradation protein VID27 |
| maker-2-augustus-gene-372.50-mRNA-1 | Japonica | Japonica | | Os02g0789700 | OsR498G0917982000.01.T01 | WW domain-containing protein |
| maker-2-augustus-gene-372.51-mRNA-1 | Japonica | Indica | | Os02g0790500 | OsR498G0204915700.01.T01 | Alpha,alpha-trehalose-phosphate synthase 9 |
| maker-2-augustus-gene-372.58-mRNA-1 | Japonica | NA | | Os02g0791300 | OsR498G0204917900.01.T02 | Transcription factor IBH1-like |
| maker-2-augustus-gene-372.59-mRNA-1 | Japonica | NA | | Os02g0789100 | OsR498G0204913200.01.T03 | Cyclic nucleotide-binding domain-containing protein (Fragment) |
| maker-2-augustus-gene-372.63-mRNA-1 | Japonica | NA | | Os02g0789400 | OsR498G0204913400.01.T03 | Serine/arginine-rich splicing factor 7 |
| maker-2-augustus-gene-372.66-mRNA-1 | Japonica | Indica | | Os02g0791400 | OsR498G0204918400.01.T02 | Cytochrome c oxidase subunit |
| maker-2-augustus-gene-373.1-mRNA-1 | Japonica | Indica | CC-Kinase-TM | Os02g0791700 | OsR498G0204919500.01.T02 | Serine/threonine-protein kinase BLUS1 |
| maker-2-augustus-gene-373.11-mRNA-1 | Japonica | NA | | Os02g0793300 | OsR498G0204924300.01.T05 | Nudix hydrolase 3 |
| maker-2-augustus-gene-373.17-mRNA-1 | Japonica | NA | | Os02g0791800 | OsR498G0204919800.01.T01 | Autophagy-related protein 18b |
| maker-2-augustus-gene-373.22-mRNA-1 | Japonica | NA | | Os02g0792900 | OsR498G0204923600.01.T01 | Serine incorporator 3 |
| maker-2-augustus-gene-373.25-mRNA-1 | Japonica | NA | | Os02g0793000 | OsR498G0204923800.01.T01 | Zinc finger CCCH domain-containing protein 18 |
| maker-2-augustus-gene-373.28-mRNA-1 | Japonica | Japonica | | Os02g0793500 | OsR498G0204925200.01.T02 | Methyltransferase-like protein 16 |
| maker-2-augustus-gene-373.7-mRNA-1 | Japonica | NA | | Os02g0793100 | OsR498G0204924100.01.T01 | Protein TPLATE like |
| maker-2-augustus-gene-374.36-mRNA-1 | Japonica | NA | | Os02g0793800 | | Homeobox protein cut-like |
| maker-2-augustus-gene-374.39-mRNA-1 | Japonica | NA | | | OsR498G0204926700.01.T02 | AUGMIN subunit 8 |
| maker-2-augustus-gene-374.44-mRNA-1 | Japonica | NA | | Os02g0794500 | OsR498G0204928300.01.T01 | Carboxypeptidase |
| maker-2-augustus-gene-374.49-mRNA-1 | Japonica | Japonica | | Os02g0795000 | OsR498G0204929900.01.T02 | Ubiquitin carboxyl-terminal hydrolase |
| maker-2-augustus-gene-374.57-mRNA-1 | Japonica | NA | | Os02g0795200 | OsR498G0204930100.01.T01 | Protein POLAR LOCALIZATION DURING ASYMMETRIC DIVISION AND REDISTR |
| maker-2-augustus-gene-374.59-mRNA-1 | Japonica | NA | | Os02g0793900 | OsR498G0204926200.01.T01 | Protein HEADING DATE REPRESSOR like |
| maker-2-augustus-gene-374.61-mRNA-1 | Japonica | NA | | Os02g0794000 | OsR498G0204926400.01.T01 | Putative RING zinc finger domain superfamily protein isoform 1 |
| maker-2-augustus-gene-374.63-mRNA-1 | Japonica | Indica | | Os02g0794400 | OsR498G0204927000.01.T02 | Translation initiation factor IF-3, chloroplastic |
| maker-2-augustus-gene-374.67-mRNA-1 | Japonica | Indica | | Os02g0794700 | OsR498G0204929000.01.T01 | Leucine aminopeptidase 2 chloroplastic |
| maker-2-augustus-gene-374.75-mRNA-1 | Japonica | Indica | | Os02g0794900 | OsR498G0204929400.01.T01 | Formin-like protein 7 |
| maker-2-augustus-gene-374.89-mRNA-1 | Japonica | NA | | Os02g0795300 | OsR498G0204930600.01.T01 | E3 ubiquitin-protein ligase CHFR |
| maker-2-augustus-gene-374.94-mRNA-1 | Japonica | NA | | Os02g0795600 | | Neprosin domain-containing protein |
| maker-2-augustus-gene-375.33-mRNA-1 | Japonica | NA | | | OsR498G0204932100.01.T02 | Transcription factor bHLH69 |
| maker-2-augustus-gene-375.35-mRNA-1 | Japonica | NA | | Os02g0795900 | OsR498G0204932400.01.T04 | DEAD-box ATP-dependent RNA helicase |
| maker-2-augustus-gene-375.39-mRNA-1 | Japonica | Japonica | | Os02g0796400 | OsR498G0204934400.01.T01 | Pentatricopeptide repeat-containing protein |
| maker-2-augustus-gene-375.41-mRNA-1 | Japonica | NA | | Os02g0796500 | OsR498G0204934600.01.T03 | Two-component response regulator |
| maker-2-augustus-gene-375.45-mRNA-1 | Japonica | NA | | Os02g0796600 | OsR498G0204936000.01.T01 | Protein kinase family protein / WD-40 repeat family protein |
| maker-2-augustus-gene-375.49-mRNA-1 | Japonica | NA | | | OsR498G0204936400.01.T02 | Putative serine/threonine-protein kinase CCRP1 |
| maker-2-augustus-gene-375.54-mRNA-1 | Japonica | NA | | | OsR498G0204936800.01.T01 | |
| maker-2-augustus-gene-375.56-mRNA-1 | Japonica | NA | | Os02g0797100 | OsR498G0204937800.01.T01 | Ethylene-responsive transcription factor ERF003 |
| maker-2-augustus-gene-376.34-mRNA-1 | Japonica | NA | | Os02g0797500 | OsR498G0204939300.01.T01 | Aspartate aminotransferase |
| maker-2-augustus-gene-376.38-mRNA-1 | Japonica | NA | | | | |
| maker-2-augustus-gene-376.42-mRNA-1 | Japonica | NA | | Os02g0798200 | OsR498G0204940900.01.T06 | RING-H2 finger protein ATL80 |
| maker-2-augustus-gene-376.44-mRNA-1 | Japonica | NA | | Os02g0798700 | OsR498G0204943700.01.T03 | Neurochondrin |
| maker-2-augustus-gene-376.48-mRNA-1 | Japonica | NA | | Os02g0797400 | OsR498G0204939200.01.T04 | DNA helicase |
| maker-2-augustus-gene-376.53-mRNA-1 | Japonica | Indica | | Os02g0798600 | OsR498G0204943000.01.T01 | RING/U-box superfamily protein isoform 1 |
| maker-2-augustus-gene-377.11-mRNA-1 | Japonica | NA | | | OsR498G0204945000.01.T06 | Shugoshin-1 |
| maker-2-augustus-gene-377.15-mRNA-1 | Japonica | NA | | Os02g0800100 | OsR498G0204949200.01.T01 | Proteasome maturation factor UMP |
| maker-2-augustus-gene-377.4-mRNA-1 | Japonica | NA | | Os02g0799300 | | Plasma membrane fusion protein |
| maker-2-augustus-gene-377.6-mRNA-1 | Japonica | NA | | | OsR498G0204948900.01.T01 | Plastid transcriptionally active 17 |
| maker-2-augustus-gene-378.41-mRNA-1 | Japonica | NA | | Os02g0802200 | OsR498G0204958300.01.T03 | Heparanase-like protein 3 |
| maker-2-augustus-gene-378.44-mRNA-1 | Japonica | Indica | | Os02g0802700 | OsR498G0204960500.01.T01 | Monogalactosyldiacylglycerol synthase |
| maker-2-augustus-gene-378.50-mRNA-1 | Japonica | NA | | Os02g0803200 | OsR498G0204961500.01.T01 | Cell wall protein RBR3 isoform X1 |
| maker-2-augustus-gene-378.53-mRNA-1 | Japonica | NA | | | OsR498G0204961700.01.T01 | N-alpha-acetyltransferase 35, NatC auxiliary subunit like |
| maker-2-augustus-gene-378.60-mRNA-1 | Japonica | NA | | Os02g0803300 | OsR498G0204962300.01.T01 | ENTH domain-containing protein (Fragment) |
| maker-2-augustus-gene-378.66-mRNA-1 | Japonica | Indica | | Os02g0803600 | OsR498G0204962700.01.T02 | Vesicle-associated membrane protein 713 |
| maker-2-augustus-gene-378.68-mRNA-1 | Japonica | Indica | | Os08g0442300 | OsR498G0204959200.01.T02 | Calcineurin subunit B like |

| | | | | | | |
|---|---|---|---|---|---|---|
| maker-2-augustus-gene-378.73-mRNA-1 | Japonica | NA | | Os02g0802500 | OsR498G0204959400.01.T01 | H(+)-exporting diphosphatase |
| maker-2-augustus-gene-378.75-mRNA-1 | Japonica | Indica | | Os02g0803400 | OsR498G0204962600.01.T02 | Vacuolar protein sorting |
| maker-2-augustus-gene-378.77-mRNA-1 | Japonica | NA | | Os02g0803700 | OsR498G0204963000.01.T03 | 26S proteasome regulatory particle triple-A ATPase protein |
| maker-2-augustus-gene-379.40-mRNA-1 | Japonica | NA | | Os02g0804300 | OsR498G0204964700.01.T03 | 18S rRNA (Guanine-N(7))-methyltransferase |
| maker-2-augustus-gene-379.44-mRNA-1 | Japonica | NA | | Os02g0804400 | OsR498G0204964900.01.T01 | Homoserine O-acetyltransferase |
| maker-2-augustus-gene-379.46-mRNA-1 | Japonica | Indica | | Os06g0168600 | OsR498G0204965900.01.T02 | Ribonucleoside-diphosphate reductase |
| maker-2-augustus-gene-379.48-mRNA-1 | Japonica | NA | | Os02g0805100 | OsR498G0204967300.01.T03 | Auxin-responsive protein |
| maker-2-augustus-gene-379.51-mRNA-1 | Japonica | NA | | Os02g0805200 | OsR498G0204967700.01.T01 | Proliferating cell nuclear antigen |
| maker-2-augustus-gene-379.57-mRNA-1 | Japonica | NA | | Os02g0804500 | OsR498G0204965200.01.T01 | DnaJ-like 1, mitochondrial |
| maker-2-augustus-gene-379.63-mRNA-1 | Japonica | NA | | | OsR498G0204965600.01.T02 | Adenylyltransferase and sulfurtransferase MOCS3 1 Molybdopterin-synthase |
| maker-2-augustus-gene-379.68-mRNA-1 | Japonica | Indica | | Os02g0805000 | OsR498G0204966400.01.T04 | AP-1 complex subunit gamma |
| maker-2-augustus-gene-379.76-mRNA-1 | Japonica | NA | | | OsR498G0204968000.01.T04 | Transcription factor SPATULA |
| maker-2-augustus-gene-379.81-mRNA-1 | Japonica | NA | | Os02g0805700 | OsR498G0204969200.01.T01 | Intermediate filament protein ifc-1 |
| maker-2-augustus-gene-379.84-mRNA-1 | Japonica | Japonica | | Os02g0805800 | OsR498G0204969400.01.T03 | Protein kinase domain-containing protein (Fragment) |
| maker-2-augustus-gene-380.14-mRNA-1 | Japonica | NA | | | OsR498G0204972300.01.T03 | GATA transcription factor |
| maker-2-augustus-gene-380.16-mRNA-1 | Japonica | NA | | Os02g0806900 | OsR498G0204976100.01.T01 | Long chain base biosynthesis protein 1 |
| maker-2-augustus-gene-380.23-mRNA-1 | Japonica | NA | | Os02g0805900 | OsR498G0204969600.01.T01 | mRNA-decapping enzyme subunit like |
| maker-2-augustus-gene-380.29-mRNA-1 | Japonica | NA | | Os02g0806500 | OsR498G0204973200.01.T01 | Glycosyltransferase family protein 2 |
| maker-2-augustus-gene-380.35-mRNA-1 | Japonica | NA | | Os02g0806600 | OsR498G0204973800.01.T01 | Clathrin interactor EPSIN like |
| maker-2-augustus-gene-380.38-mRNA-1 | Japonica | NA | | Os02g0806700 | OsR498G0204974200.01.T01 | E3 ubiquitin-protein ligase WAV3 |
| maker-2-augustus-gene-380.40-mRNA-1 | Japonica | NA | Kinase-TM | Os02g0807000 | OsR498G0204976400.01.T01 | Calcium-dependent protein kinase, isoform AK1 |
| maker-2-augustus-gene-381.34-mRNA-1 | Japonica | Indica | | Os02g0807100 | OsR498G0204976700.01.T01 | glycogen synthase isoform X1 |
| maker-2-augustus-gene-381.38-mRNA-1 | Japonica | NA | | | | zf-LSD1 domain-containing protein |
| maker-2-augustus-gene-381.46-mRNA-1 | Japonica | NA | Kinase-TM | Os02g0811200 | OsR498G0204979300.01.T01 | Wall-associated receptor kinase 3 |
| maker-2-augustus-gene-381.48-mRNA-1 | Japonica | NA | Kinase-TM | Os02g0807200 | | Wall-associated receptor kinase 3 |
| maker-2-augustus-gene-381.49-mRNA-1 | Japonica | Indica | Kinase-TM | Os02g0807800 | OsR498G0204977600.01.T01 | Wall-associated receptor kinase 3 |
| maker-2-augustus-gene-381.50-mRNA-1 | Japonica | Indica | Kinase-TM | Os02g0811200 | OsR498G0204978800.01.T01 | Wall-associated receptor kinase 3 |
| maker-2-augustus-gene-382.26-mRNA-1 | Japonica | NA | | Os02g0809500 | OsR498G0204981900.01.T05 | Isocitrate dehydrogenase [NADP], mitochondrial |
| maker-2-augustus-gene-382.29-mRNA-1 | Japonica | NA | | Os02g0809800 | OsR498G0204983100.01.T01 | Phosphate transporter PHO1 |
| maker-2-augustus-gene-382.36-mRNA-1 | Japonica | NA | | | OsR498G0204984700.01.T01 | Kinesin-like protein KIN-10A |
| maker-2-augustus-gene-382.40-mRNA-1 | Japonica | NA | | Os02g0810300 | OsR498G0204985100.01.T02 | ABC transporter domain-containing protein (Fragment) |
| maker-2-augustus-gene-382.45-mRNA-1 | Japonica | NA | Kinase | Os02g0810400 | OsR498G0204985500.01.T01 | Diphthamide synthase |
| maker-2-augustus-gene-382.50-mRNA-1 | Japonica | Indica | | Os02g0810450 | OsR498G0204985700.01.T05 | Diphthamide synthase |
| maker-2-augustus-gene-382.55-mRNA-1 | Japonica | NA | | Os02g0810500 | OsR498G0204985900.01.T03 | Diphthamide synthase |
| maker-2-augustus-gene-382.60-mRNA-1 | Japonica | NA | | Os02g0810900 | OsR498G0204987500.01.T01 | NAC transcription factor |
| maker-2-augustus-gene-382.62-mRNA-1 | Japonica | NA | | | OsR498G0204983600.01.T01 | Transducin/WD40 repeat-like superfamily protein, putative isoform 1 |
| maker-2-augustus-gene-382.65-mRNA-1 | Japonica | Indica | | Os07g0436000 | OsR498G0204983800.01.T01 | Ankyrin repeat domain-containing protein 13C-B |
| maker-2-augustus-gene-383.13-mRNA-1 | Japonica | NA | | | OsR498G0204993700.01.T01 | F-box domain, Leucine-rich repeat domain, L domain-like protein |
| maker-2-augustus-gene-383.15-mRNA-1 | Japonica | NA | | | | Trimeric LpxA-like enzyme |
| maker-2-augustus-gene-383.18-mRNA-1 | Japonica | NA | | Os02g0812500 | OsR498G0204994300.01.T01 | F-box domain-containing protein |
| maker-2-augustus-gene-383.2-mRNA-1 | Japonica | Japonica | | Os02g0811600 | OsR498G0204992300.01.T01 | Epimerase domain-containing protein (Fragment) |
| maker-2-augustus-gene-383.21-mRNA-1 | Japonica | NA | | Os02g0812600 | OsR498G0204994500.01.T01 | Phloem-specific lectin |
| maker-2-augustus-gene-383.22-mRNA-1 | Japonica | Japonica | | Os02g0812600 | OsR498G0204994500.01.T01 | Phloem-specific lectin |
| maker-2-augustus-gene-383.29-mRNA-1 | Japonica | Indica | | Os02g0811101 | OsR498G0204990200.01.T03 | SAP-like protein BP-73 |
| maker-2-augustus-gene-383.5-mRNA-1 | Japonica | NA | | Os02g0811800 | OsR498G0204992300.01.T01 | Epimerase domain-containing protein (Fragment) |
| maker-2-augustus-gene-383.6-mRNA-1 | Japonica | NA | | Os02g0812000 | OsR498G0204992500.01.T01 | Epimerase domain-containing protein (Fragment) |
| maker-2-augustus-gene-384.0-mRNA-1 | Japonica | NA | | Os02g0813133 | | F-box domain-containing protein |
| maker-2-augustus-gene-384.1-mRNA-1 | Japonica | NA | | Os02g0813166 | | Phloem-specific lectin |
| maker-2-augustus-gene-384.10-mRNA-1 | Japonica | NA | | Os06g0595700 | | DDE Tnp4 domain-containing protein (Fragment) |
| maker-2-augustus-gene-384.18-mRNA-1 | Japonica | NA | | Os02g0813300 | OsR498G0204995500.01.T01 | Phloem-specific lectin |
| maker-2-augustus-gene-384.21-mRNA-1 | Japonica | NA | | Os02g0813500 | OsR498G0204995900.01.T07 | Cytosolic glutathione reductase |
| maker-2-augustus-gene-384.27-mRNA-1 | Japonica | Japonica | | Os02g0814300 | OsR498G0205000100.01.T03 | Cytochrome c domain-containing protein |
| maker-2-augustus-gene-384.28-mRNA-1 | Japonica | Indica | | Os02g0814400 | OsR498G0205000100.01.T03 | Cytochrome c domain-containing protein |
| maker-2-augustus-gene-384.35-mRNA-1 | Japonica | NA | | Os02g0814800 | OsR498G0205000900.01.T01 | GST C-terminal domain-containing protein (Fragment) |
| maker-2-augustus-gene-384.37-mRNA-1 | Japonica | Indica | | Os02g0815001 | OsR498G0205001300.01.T01 | Ribosomal protein L37 |
| maker-2-augustus-gene-384.41-mRNA-1 | Japonica | NA | | Os02g0813800 | OsR498G0204997000.01.T03 | Transcriptional coregulator |
| maker-2-augustus-gene-384.45-mRNA-1 | Japonica | NA | | Os02g0814000 | OsR498G0204998600.01.T03 | DUF862 domain-containing protein (Fragment) |
| maker-2-augustus-gene-384.47-mRNA-1 | Japonica | Indica | | Os02g0814100 | OsR498G0204998800.01.T03 | Phospholipid--sterol O-acyltransferase |
| maker-2-augustus-gene-384.54-mRNA-1 | Japonica | NA | | Os02g0814200 | OsR498G0204999000.01.T01 | Aldehyde oxygenase (deformylating) |
| maker-2-augustus-gene-384.57-mRNA-1 | Japonica | NA | | | | HTH myb-type domain-containing protein |
| maker-2-augustus-gene-384.61-mRNA-1 | Japonica | NA | | Os02g0814900 | OsR498G0205001200.01.T04 | Nicotinamide-nucleotide adenylyltransferase |

| | | | | | | |
|---|---|---|---|---|---|---|
| maker-2-augustus-gene-385.0-mRNA-1 | Japonica | NA | | Os02g0815200 | OsR498G0205001700.01.T01 | 28 kDa ribonucleoprotein chloroplastic |
| maker-2-augustus-gene-385.15-mRNA-1 | Japonica | NA | | Os02g0816300 | OsR498G0205005900.01.T07 | CLIP-associated protein isoform X1 |
| maker-2-augustus-gene-385.2-mRNA-1 | Japonica | NA | | Os02g0815400 | OsR498G0205002100.01.T03 | S-(hydroxymethyl)glutathione dehydrogenase |
| maker-2-augustus-gene-385.23-mRNA-1 | Japonica | NA | | Os02g0816400 | OsR498G0205006100.01.T04 | Phosphatidylinositol N-acetyglucosaminlytransferase subunit P-related |
| maker-2-augustus-gene-385.25-mRNA-1 | Japonica | NA | | | | Tubulin-specific chaperone A |
| maker-2-augustus-gene-385.38-mRNA-1 | Japonica | NA | Kinase-TM | Os02g0815900 | OsR498G0205003400.01.T01 | Receptor-like serine/threonine-protein kinase ALE2 isoform A |
| maker-2-augustus-gene-385.42-mRNA-1 | Japonica | NA | | Os02g0816100 | OsR498G0205004200.01.T01 | Catalytic/ hydrolase/ phosphoglycolate phosphatase |
| maker-2-augustus-gene-385.45-mRNA-1 | Japonica | NA | | Os02g0816200 | OsR498G0205004600.01.T01 | Gdsl esteraselipase |
| maker-2-augustus-gene-385.47-mRNA-1 | Japonica | NA | Kinase-TM | | OsR498G0205006800.01.T01 | AarF domain-containing kinase |
| maker-2-augustus-gene-385.54-mRNA-1 | Japonica | NA | | Os02g0816700 | OsR498G0205007000.01.T01 | Transmembrane protein, putative |
| maker-2-augustus-gene-385.6-mRNA-1 | Japonica | NA | | Os02g0815500 | OsR498G0205002300.01.T01 | S-(hydroxymethyl)glutathione dehydrogenase |
| maker-2-augustus-gene-386.0-mRNA-1 | Japonica | NA | | Os02g0816900 | OsR498G0205007700.01.T03 | Dilute domain-containing protein |
| maker-2-augustus-gene-386.10-mRNA-1 | Japonica | NA | | Os02g0817500 | OsR498G0205009300.01.T01 | Voltage-gated potassium channel beta subunit |
| maker-2-augustus-gene-386.13-mRNA-1 | Japonica | Indica | | Os02g0818000 | OsR498G0205010700.01.T05 | CBS domain-containing protein CBSX1, chloroplastic |
| maker-2-augustus-gene-386.18-mRNA-1 | Japonica | NA | | Os02g0817200 | OsR498G0205009000.01.T02 | Actin-related protein 2/3 complex subunit |
| maker-2-augustus-gene-386.23-mRNA-1 | Japonica | NA | | Os02g0817600 | OsR498G0205010000.01.T02 | Auxin-responsive protein IAA10 |
| maker-2-augustus-gene-386.32-mRNA-1 | Japonica | Indica | | Os02g0817800 | OsR498G0205010400.01.T03 | Initiator binding protein1 |
| maker-2-augustus-gene-386.37-mRNA-1 | Japonica | NA | | Os02g0817900 | OsR498G0205010600.01.T01 | protein LUTEIN DEFICIENT 5, chloroplastic |
| maker-2-augustus-gene-386.9-mRNA-1 | Japonica | NA | | | | |
| maker-2-augustus-gene-387.0-mRNA-1 | Japonica | Japonica | | Os02g0818900 | OsR498G0205012900.01.T01 | Flagellar motor switch phosphatase FliY |
| maker-2-augustus-gene-387.11-mRNA-1 | Japonica | NA | | | OsR498G0205016300.01.T01 | Pumilio-like protein 4 |
| maker-2-augustus-gene-387.17-mRNA-1 | Japonica | NA | | | OsR498G0205012400.01.T02 | Protein N-lysine methyltransferase |
| maker-2-augustus-gene-387.2-mRNA-1 | Japonica | Japonica | | Os02g0819000 | OsR498G0205013100.01.T01 | HMA domain-containing protein |
| maker-2-augustus-gene-387.22-mRNA-1 | Japonica | Indica | | Os02g0819400 | OsR498G0205016600.01.T01 | P-loop NTPase domain-containing protein LPA1 |
| maker-2-augustus-gene-387.26-mRNA-1 | Japonica | Indica | | Os02g0819500 | OsR498G0205017400.01.T06 | OTU domain-containing protein (Fragment) |
| maker-2-augustus-gene-387.32-mRNA-1 | Japonica | NA | | Os02g0819700 | OsR498G0205019200.01.T01 | DNL-type domain-containing protein (Fragment) |
| maker-2-augustus-gene-387.34-mRNA-1 | Japonica | NA | | Os02g0820000 | OsR498G0205020200.01.T03 | Serine/threonine-protein phosphatase |
| maker-2-augustus-gene-387.4-mRNA-1 | Japonica | NA | | Os02g0819100 | OsR498G0205015900.01.T06 | S-acyltransferase |
| maker-2-augustus-gene-387.9-mRNA-1 | Japonica | NA | | Os02g0819200 | OsR498G0205016100.01.T01 | Thioredoxin-like 3-2 chloroplastic |
| maker-2-augustus-gene-388.0-mRNA-1 | Japonica | NA | | Os02g0820900 | OsR498G0205023300.01.T02 | Ethylene receptor |
| maker-2-augustus-gene-388.11-mRNA-1 | Japonica | NA | | Os02g0821900 | OsR498G0205025300.01.T02 | ATG8-interacting protein 1-like isoform X1 |
| maker-2-augustus-gene-388.13-mRNA-1 | Japonica | NA | | Os02g0822000 | OsR498G0205025500.01.T03 | Hydroxyproline-rich glycoprotein family protein |
| maker-2-augustus-gene-388.16-mRNA-1 | Japonica | NA | | Os02g0822100 | OsR498G0205025700.01.T01 | Transporter arsB like |
| maker-2-augustus-gene-388.19-mRNA-1 | Japonica | NA | | Os02g0822200 | OsR498G0205026100.01.T05 | Ubiquitin carboxyl-terminal hydrolase |
| maker-2-augustus-gene-388.2-mRNA-1 | Japonica | Indica | | Os02g0821500 | OsR498G0205024900.01.T01 | Protein DETOXIFICATION |
| maker-2-augustus-gene-388.24-mRNA-1 | Japonica | NA | | | OsR498G0205026300.01.T02 | K Homology domain |
| maker-2-augustus-gene-388.29-mRNA-1 | Japonica | NA | | Os02g0822400 | OsR498G0205026500.01.T01 | NAC transcription factor |
| maker-2-augustus-gene-388.32-mRNA-1 | Japonica | NA | | Os02g0820300 | OsR498G0205020800.01.T02 | Kinesin light chain 3 |
| maker-2-augustus-gene-388.35-mRNA-1 | Japonica | NA | | Os02g0820500 | OsR498G0205021200.01.T01 | LOB domain-containing protein 16 |
| maker-2-augustus-gene-388.37-mRNA-1 | Japonica | NA | | Os02g0820600 | OsR498G0205021600.01.T01 | Nucleolar/coiled-body phosphoprotein |
| maker-2-augustus-gene-388.4-mRNA-1 | Japonica | NA | | | OsR498G0205025100.01.T01 | Mediator of RNA polymerase II transcription subunit 36a |
| maker-2-augustus-gene-388.41-mRNA-1 | Japonica | Indica | | Os02g0821200 | OsR498G0205023600.01.T01 | 60S ribosomal protein l28 |
| maker-2-augustus-gene-388.45-mRNA-1 | Japonica | NA | Kinase | Os02g0821400 | OsR498G0205024600.01.T01 | Serine/threonine protein kinase |
| maker-2-augustus-gene-388.47-mRNA-1 | Japonica | NA | | Os02g0822500 | OsR498G0205026800.01.T03 | Phosphatidylinositol 4-phosphate 5-kinase |
| maker-2-augustus-gene-389.0-mRNA-1 | Japonica | NA | CC-Kinase | | OsR498G0205028700.01.T03 | U-box domain-containing protein 33 |
| maker-2-augustus-gene-389.10-mRNA-1 | Japonica | NA | | Os02g0823100 | OsR498G0205029100.01.T01 | Plasma membrane intrinsic protein 1 |
| maker-2-augustus-gene-389.12-mRNA-1 | Japonica | Indica | | Os04g0166600 | OsR498G0205029500.01.T02 | Histone deacetylase complex subunit SAP30/SAP30-like |
| maker-2-augustus-gene-389.17-mRNA-1 | Japonica | NA | | Os02g0823300 | OsR498G0205030100.01.T02 | RING/U-box superfamily protein |
| maker-2-augustus-gene-389.20-mRNA-1 | Japonica | NA | | Os02g0823400 | OsR498G0205030500.01.T01 | Caffeic acid O-methyltransferase |
| maker-2-augustus-gene-389.23-mRNA-1 | Japonica | Japonica | | Os02g0823800 | OsR498G0205031500.01.T03 | |
| maker-2-augustus-gene-389.25-mRNA-1 | Japonica | NA | | Os02g0822600 | OsR498G0205027800.01.T01 | 50S ribosomal protein L9, chloroplastic |
| maker-2-augustus-gene-389.28-mRNA-1 | Japonica | NA | | Os02g0822800 | OsR498G0205028600.01.T02 | Acyl-CoA-binding domain-containing protein 4 |
| maker-2-augustus-gene-389.33-mRNA-1 | Japonica | NA | | Os02g0823600 | | Translation initiation factor IF-2 like (Fragment) |
| maker-2-augustus-gene-389.35-mRNA-1 | Japonica | Indica | | Os10g0545500 | OsR498G0205031400.01.T01 | Xyloglucan endotransglucosylase/hydrolase |
| maker-2-augustus-gene-389.37-mRNA-1 | Japonica | Japonica | | Os02g0824000 | OsR498G0205032200.01.T06 | Pentatricopeptide repeat-containing protein mitochondrial-like |
| maker-2-augustus-gene-389.4-mRNA-1 | Japonica | NA | | Os02g0823000 | OsR498G0205028900.01.T06 | PA domain-containing protein |
| maker-2-augustus-gene-389.40-mRNA-1 | Japonica | NA | | Os02g0824300 | OsR498G0205033400.01.T01 | AT-hook motif nuclear-localized protein (Fragment) |
| maker-2-augustus-gene-39.34-mRNA-1 | Japonica | NA | | Os02g0170100 | OsR498G0203054900.01.T01 | Glycine cleavage system H protein (Fragment) |
| maker-2-augustus-gene-39.37-mRNA-1 | Japonica | NA | | Os02g0171100 | OsR498G0203057100.01.T01 | Glyceraldehyde-3-phosphate dehydrogenase |
| maker-2-augustus-gene-39.43-mRNA-1 | Japonica | NA | | Os02g0170200 | OsR498G0203055200.01.T01 | Style cell-cycle inhibitor 1-A |
| maker-2-augustus-gene-39.46-mRNA-1 | Japonica | NA | | Os02g0170300 | OsR498G0203055400.01.T01 | MADS-box protein ZMM17 |

| | | | | | | |
|---|---|---|---|---|---|---|
| maker-2-augustus-gene-39.48-mRNA-1 | Japonica | NA | | | | Transglycosylase SLT domain containing protein expressed |
| maker-2-augustus-gene-390.104-mRNA-1 | Japonica | Japonica | | Os02g0826000 | OsR498G0205036400.01.T01 | F-box domain-containing protein |
| maker-2-augustus-gene-390.106-mRNA-1 | Japonica | Indica | | Os02g0825100 | OsR498G0205036600.01.T01 | F-box domain-containing protein |
| maker-2-augustus-gene-390.119-mRNA-1 | Japonica | NA | | Os02g0826100 | OsR498G0205036800.01.T02 | ATP-dependent RNA helicase pitchoune |
| maker-2-augustus-gene-390.54-mRNA-1 | Japonica | Japonica | | Os02g0824500 | OsR498G0408962200.01.T01 | Remorin |
| maker-2-augustus-gene-390.56-mRNA-1 | Japonica | NA | | Os02g0824700 | OsR498G0205034100.01.T01 | V-type proton ATPase subunit F |
| maker-2-augustus-gene-390.63-mRNA-1 | Japonica | NA | | Os02g0826200 | OsR498G0205037100.01.T01 | Methionine adenosyltransferase 2 subunit beta like |
| maker-2-augustus-gene-390.72-mRNA-1 | Japonica | Japonica | | Os02g0824400 | OsR498G0205033600.01.T01 | mediator of RNA polymerase II transcription subunit 2-like |
| maker-2-augustus-gene-390.75-mRNA-1 | Japonica | NA | | | OsR498G0205034000.01.T01 | |
| maker-2-augustus-gene-390.79-mRNA-1 | Japonica | Indica | | Os02g0825100 | OsR498G0205034700.01.T02 | F-box domain-containing protein |
| maker-2-augustus-gene-390.95-mRNA-1 | Japonica | NA | | | | Transposon protein, putative, unclassified |
| maker-2-augustus-gene-390.97-mRNA-1 | Japonica | NA | | | | Signal peptide peptidase |
| maker-2-augustus-gene-391.0-mRNA-1 | Japonica | Indica | | Os10g0497800 | OsR498G0205041700.01.T05 | Prolyl 4-hydroxylase alpha subunit |
| maker-2-augustus-gene-391.13-mRNA-1 | Japonica | NA | | Os02g0827300 | OsR498G0205042100.01.T01 | PSP domain-containing protein |
| maker-2-augustus-gene-391.19-mRNA-1 | Japonica | NA | | Os02g0827400 | OsR498G0205042300.01.T01 | DUF789 domain-containing protein |
| maker-2-augustus-gene-391.21-mRNA-1 | Japonica | NA | | | OsR498G0205042500.01.T01 | SMP-LTD domain-containing protein |
| maker-2-augustus-gene-391.24-mRNA-1 | Japonica | NA | | | | HAT family dimerisation domain containing protein |
| maker-2-augustus-gene-391.26-mRNA-1 | Japonica | NA | | | OsR498G0205038400.01.T03 | ABC transporter F family member 3 |
| maker-2-augustus-gene-391.32-mRNA-1 | Japonica | NA | CC-LRR-TM | Os02g0826600 | OsR498G0205039000.01.T01 | Plant intracellular Ras-group-related LRR protein 9 |
| maker-2-augustus-gene-391.39-mRNA-1 | Japonica | NA | | Os02g0827600 | OsR498G0205042800.01.T02 | Alpha-galactosidase-like |
| maker-2-augustus-gene-391.42-mRNA-1 | Japonica | NA | | | OsR498G0205043000.01.T04 | Pentatricopeptide repeat |
| maker-2-augustus-gene-391.43-mRNA-1 | Japonica | NA | | Os02g0827900 | OsR498G0205043000.01.T06 | Signal peptidase I |
| maker-2-augustus-gene-391.5-mRNA-1 | Japonica | NA | | Os02g0827200 | OsR498G0205041900.01.T02 | Sucrose transporter |
| maker-2-augustus-gene-391.54-mRNA-1 | Japonica | Indica | | Os02g0828100 | OsR498G0205043200.01.T01 | Vacuolar protein-sorting protein BRO1 |
| maker-2-augustus-gene-392.39-mRNA-1 | Japonica | NA | | Os02g0828900 | OsR498G0205045700.01.T01 | Transcription factor jumonji (JmjC) domain-containing protein |
| maker-2-augustus-gene-392.45-mRNA-1 | Japonica | NA | | Os02g0829400 | OsR498G0205047300.01.T01 | AP-5 complex subunit beta-1 |
| maker-2-augustus-gene-392.47-mRNA-1 | Japonica | NA | | Os02g0829100 | OsR498G0205046800.01.T01 | Replication protein A subunit A |
| maker-2-augustus-gene-392.52-mRNA-1 | Japonica | Indica | | Os02g0829200 | OsR498G0205047200.01.T02 | Calcium-dependent lipid-binding (CaLB domain) family protein isoform 2 |
| maker-2-augustus-gene-392.54-mRNA-1 | Japonica | NA | | Os02g0829500 | OsR498G0205047600.01.T04 | Zn-dependent hydrolase, including glyoxylase |
| maker-2-augustus-gene-392.59-mRNA-1 | Japonica | Japonica | | Os02g0829600 | OsR498G0205047600.01.T01 | Zn-dependent hydrolases, including glyoxylases |
| maker-2-augustus-gene-392.62-mRNA-1 | Japonica | NA | | | OsR498G0205047800.01.T05 | U-box domain-containing protein 62 |
| maker-2-augustus-gene-392.65-mRNA-1 | Japonica | NA | | Os02g0829900 | OsR498G0205049000.01.T01 | Matrilin, coiled-coil trimerization domain-containing protein |
| maker-2-augustus-gene-392.67-mRNA-1 | Japonica | NA | | Os02g0830100 | OsR498G0205049200.01.T01 | Metallopeptidase, catalytic domain-containing protein |
| maker-2-augustus-gene-392.72-mRNA-1 | Japonica | Japonica | | Os02g0830200 | OsR498G0204371000.01.T01 | Two-component response regulator ORR5 |
| maker-2-augustus-gene-392.74-mRNA-1 | Japonica | NA | | | OsR498G0205049600.01.T01 | Response regulatory domain-containing protein |
| maker-2-augustus-gene-392.78-mRNA-1 | Japonica | NA | | | OsR498G0205050000.01.T01 | |
| maker-2-augustus-gene-392.80-mRNA-1 | Japonica | NA | Kinase-LRR-TM | | OsR498G0205050200.01.T01 | Inactive leucine-rich repeat receptor-like serine/threonine-protein kinase |
| maker-2-augustus-gene-393.0-mRNA-1 | Japonica | NA | | | OsR498G0205050500.01.T02 | ARM repeat superfamily protein |
| maker-2-augustus-gene-393.10-mRNA-1 | Japonica | NA | | Os02g0831500 | OsR498G0205052100.01.T02 | Sucrose synthase |
| maker-2-augustus-gene-393.15-mRNA-1 | Japonica | NA | | Os02g0832000 | OsR498G0205053100.01.T01 | Calcium-dependent/calmodulin-independent protein kinase |
| maker-2-augustus-gene-393.17-mRNA-1 | Japonica | NA | | | OsR498G0205054100.01.T01 | 1,3-beta-glucan synthase |
| maker-2-augustus-gene-393.34-mRNA-1 | Japonica | NA | | | OsR498G0205056100.01.T01 | Protein DETOXIFICATION |
| maker-2-augustus-gene-393.38-mRNA-1 | Japonica | Indica | | Os02g0833900 | OsR498G0205058300.01.T01 | Peroxidase |
| maker-2-augustus-gene-393.41-mRNA-1 | Japonica | NA | | Os02g0834000 | OsR498G0205059100.01.T01 | Rac-like GTP-binding protein 6 |
| maker-2-augustus-gene-393.54-mRNA-1 | Japonica | NA | | Os02g0831200 | OsR498G0205051000.01.T01 | Large ribosomal RNA subunit accumulation protein YCED 1, chloroplastic |
| maker-2-augustus-gene-393.56-mRNA-1 | Japonica | NA | | Os02g0831300 | OsR498G0205051200.01.T02 | Beta-catenin-like protein 1 isoform A |
| maker-2-augustus-gene-393.60-mRNA-1 | Japonica | Indica | | Os02g0831400 | OsR498G0205051800.01.T01 | SKIP interacting protein 16 |
| maker-2-augustus-gene-393.63-mRNA-1 | Japonica | NA | | | | Piwi domain-containing protein (Fragment) |
| maker-2-augustus-gene-393.68-mRNA-1 | Japonica | NA | | Os02g0831700 | | Phospholipase A |
| maker-2-augustus-gene-393.7-mRNA-1 | Japonica | Indica | | Os02g0831100 | OsR498G0205050700.01.T01 | Zinc finger CCCH domain-containing protein 19 |
| maker-2-augustus-gene-393.74-mRNA-1 | Japonica | NA | | | | Alpha-13-mannosyl-glycoprotein 2-beta-N-acetylglucosaminyltransferase |
| maker-2-augustus-gene-393.81-mRNA-1 | Japonica | NA | | Os02g0833400 | OsR498G0205056800.01.T01 | probable serine/threonine-protein kinase roco9 |
| maker-2-augustus-gene-393.85-mRNA-1 | Japonica | NA | | Os02g0833600 | OsR498G0205057600.01.T01 | BZIP transcription factor |
| maker-2-augustus-gene-393.88-mRNA-1 | Japonica | NA | | Os02g0834300 | OsR498G0205060600.01.T01 | Ferredoxin-related |
| maker-2-augustus-gene-4.0-mRNA-1 | Japonica | NA | | Os02g0106700 | OsR498G0202818900.01.T04 | Zinc finger with UFM1-specific peptidase domain protein isoform X1 |
| maker-2-augustus-gene-4.13-mRNA-1 | Japonica | NA | | | OsR498G0202820900.01.T02 | Diphosphomevalonate decarboxylase |
| maker-2-augustus-gene-40.29-mRNA-1 | Japonica | NA | | Os02g0172800 | OsR498G0203062100.01.T01 | B box-type domain-containing protein |
| maker-2-augustus-gene-40.31-mRNA-1 | Japonica | NA | | | OsR498G0203064100.01.T02 | Carotene beta-ring hydroxylase |
| maker-2-augustus-gene-40.39-mRNA-1 | Japonica | Indica | | Os06g0665100 | OsR498G0203065100.01.T04 | Haloacid dehalogenase-like hydrolase domain-containing protein 3 |
| maker-2-augustus-gene-40.41-mRNA-1 | Japonica | Indica | | Os02g0173700 | OsR498G0203065300.01.T01 | PUA domain-containing protein (Fragment) |
| maker-2-augustus-gene-40.53-mRNA-1 | Japonica | NA | | | OsR498G0203064000.01.T03 | Urease accessory protein UreD |

| | | | | | | |
|---|---|---|---|---|---|---|
| maker-2-augustus-gene-40.59-mRNA-1 | Japonica | NA | | Os02g0173800 | OsR498G0203065800.01.T01 | ALA-interacting subunit |
| maker-2-augustus-gene-42.33-mRNA-1 | Japonica | NA | | | OsR498G0203076200.01.T01 | Transmembrane protein 230 |
| maker-2-augustus-gene-42.36-mRNA-1 | Japonica | NA | | | | Calcium-transporting ATPase |
| maker-2-augustus-gene-42.44-mRNA-1 | Japonica | Indica | | Os02g0176900 | OsR498G0203078800.01.T03 | Glucose-6-phosphate 1-epimerase |
| maker-2-augustus-gene-43.30-mRNA-1 | Japonica | NA | | Os02g0177300 | OsR498G0203079900.01.T01 | Squamosa promoter-binding-like protein 5 |
| maker-2-augustus-gene-43.32-mRNA-1 | Japonica | NA | | Os02g0177600 | OsR498G0203081900.01.T03 | 4-coumarate coenzyme A ligase |
| maker-2-augustus-gene-43.37-mRNA-1 | Japonica | NA | | | | FGGY carbohydrate kinase domain-containing protein isoform X3 |
| maker-2-augustus-gene-43.43-mRNA-1 | Japonica | Indica | | Os02g0178100 | OsR498G0203084300.01.T02 | COLE |
| maker-2-augustus-gene-43.45-mRNA-1 | Japonica | NA | | | OsR498G0203084500.01.T01 | FBD domain-containing protein (Fragment) |
| maker-2-augustus-gene-43.47-mRNA-1 | Japonica | Indica | | Os02g0178400 | OsR498G0203085500.01.T02 | Protein transport protein Sec61 subunit gamma |
| maker-2-augustus-gene-43.49-mRNA-1 | Japonica | NA | | Os02g0177400 | OsR498G0203080800.01.T02 | |
| maker-2-augustus-gene-43.53-mRNA-1 | Japonica | NA | | Os02g0177500 | OsR498G0203081200.01.T01 | Sm protein F |
| maker-2-augustus-gene-43.58-mRNA-1 | Japonica | NA | | Os02g0177700 | OsR498G0203082200.01.T01 | |
| maker-2-augustus-gene-43.60-mRNA-1 | Japonica | NA | | Os02g0177800 | OsR498G0203082600.01.T02 | Calmodulin-binding protein 60 B like |
| maker-2-augustus-gene-43.67-mRNA-1 | Japonica | NA | Kinase-TM | Os02g0178000 | OsR498G0203083200.01.T03 | Non-specific serine/threonine protein kinase |
| maker-2-augustus-gene-43.74-mRNA-1 | Japonica | NA | | Os02g0178500 | OsR498G0203085800.01.T01 | Vacuolar import and degradation protein VID27 |
| maker-2-augustus-gene-44.0-mRNA-1 | Japonica | NA | | Os02g0178700 | OsR498G0203086300.01.T01 | BHLH domain-containing protein |
| maker-2-augustus-gene-44.15-mRNA-1 | Japonica | NA | | Os02g0178800 | OsR498G0203086600.01.T01 | Aldehyde oxygenase (deformylating) |
| maker-2-augustus-gene-47.38-mRNA-1 | Japonica | NA | | Os02g0184400 | OsR498G0203107300.01.T02 | UDP-glucose-fructose-phosphate glucosyltransferase |
| maker-2-augustus-gene-49.64-mRNA-1 | Japonica | NA | | | | Cinnamyl alcohol dehydrogenase |
| maker-2-augustus-gene-49.65-mRNA-1 | Japonica | NA | | | OsR498G0203120200.01.T04 | Cinnamoyl alcohol dehydrogenase |
| maker-2-augustus-gene-5.10-mRNA-1 | Japonica | Indica | | Os02g0109400 | OsR498G0202829400.01.T01 | Transcription factor jumonji (JmjC) domain-containing protein |
| maker-2-augustus-gene-5.14-mRNA-1 | Japonica | NA | | Os02g0109800 | OsR498G0202831000.01.T01 | Putative nephrocystin-3 |
| maker-2-augustus-gene-50.55-mRNA-1 | Japonica | Indica | | Os02g0190000 | OsR498G0203130400.01.T02 | ABC transporter family protein isoform 1 |
| maker-2-augustus-gene-51.0-mRNA-1 | Japonica | NA | Kinase-LRR-TM | Os02g0190500 | OsR498G0203132100.01.T01 | Protein STRUBBELIG-RECEPTOR FAMILY 8 isoform X1 |
| maker-2-augustus-gene-51.10-mRNA-1 | Japonica | NA | | Os02g0190800 | OsR498G0203133000.01.T02 | Alpha/beta hydrolase domain-containing protein 17B (Fragment) |
| maker-2-augustus-gene-51.14-mRNA-1 | Japonica | Indica | | Os02g0190900 | OsR498G0203133400.01.T01 | Bioproteinsis of lysosome-related organelles complex 1 subunit 2 |
| maker-2-augustus-gene-51.16-mRNA-1 | Japonica | NA | | Os02g0191300 | OsR498G0203134400.01.T04 | Sodium-coupled neutral amino acid transporter 1 |
| maker-2-augustus-gene-51.19-mRNA-1 | Japonica | Indica | | Os10g0476600 | OsR498G0203135000.01.T02 | Serine/threonine-protein phosphatase 2A regulatory subunit B'' subunit alph |
| maker-2-augustus-gene-51.6-mRNA-1 | Japonica | NA | | Os02g0191500 | OsR498G0203134500.01.T01 | E3 ubiquitin-protein ligase CIP8 |
| maker-2-augustus-gene-51.8-mRNA-1 | Japonica | NA | | Os02g0190700 | OsR498G0203132800.01.T01 | |
| maker-2-augustus-gene-52.31-mRNA-1 | Japonica | NA | | | OsR498G0203138300.01.T01 | Methyl-CpG-binding domain-containing protein 9 |
| maker-2-augustus-gene-52.34-mRNA-1 | Japonica | Indica | | Os02g0192100 | OsR498G0203137600.01.T01 | |
| maker-2-augustus-gene-52.36-mRNA-1 | Japonica | NA | | Os02g0192500 | OsR498G0203138600.01.T01 | Glucomannan 4-beta-mannosyltransferase |
| maker-2-augustus-gene-53.28-mRNA-1 | Japonica | NA | | Os02g0193600 | OsR498G0203142300.01.T01 | Mitotic checkpoint serine/threonine-protein kinase BUB1 |
| maker-2-augustus-gene-53.33-mRNA-1 | Japonica | NA | | | OsR498G0203143700.01.T02 | SWI/SNF complex subunit SMARCC2 |
| maker-2-augustus-gene-53.38-mRNA-1 | Japonica | NA | | Os02g0194200 | OsR498G0203144300.01.T01 | Zinc finger CCCH domain-containing protein 14 |
| maker-2-augustus-gene-53.43-mRNA-1 | Japonica | NA | Kinase-TM | Os02g0194600 | OsR498G0203145700.01.T02 | Putative STRUBBELIG family receptor protein kinase |
| maker-2-augustus-gene-53.44-mRNA-1 | Japonica | NA | | | OsR498G0203143200.01.T01 | SEC7 domain-containing protein |
| maker-2-augustus-gene-53.49-mRNA-1 | Japonica | NA | | Os02g0194100 | OsR498G0203144000.01.T04 | Citrate synthase |
| maker-2-augustus-gene-53.57-mRNA-1 | Japonica | Indica | | Os02g0194300 | OsR498G0203144600.01.T01 | Elongation factor Ts, mitochondrial |
| maker-2-augustus-gene-53.60-mRNA-1 | Japonica | Indica | Kinase-LRR-TM | Os01g0917500 | OsR498G0203145000.01.T01 | Leucine-rich repeat receptor protein kinase EXS |
| maker-2-augustus-gene-53.61-mRNA-1 | Japonica | NA | | Os02g0194700 | OsR498G0203146000.01.T01 | Lipoxygenase |
| maker-2-augustus-gene-53.64-mRNA-1 | Japonica | NA | | Os02g0194800 | OsR498G0203146600.01.T03 | TBCC domain-containing protein 1 |
| maker-2-augustus-gene-54.42-mRNA-1 | Japonica | NA | | Os02g0195500 | OsR498G0203148300.01.T03 | AbfB domain-containing protein |
| maker-2-augustus-gene-54.47-mRNA-1 | Japonica | NA | | Os02g0195800 | OsR498G0203148900.01.T01 | J domain-containing protein (Fragment) |
| maker-2-augustus-gene-54.50-mRNA-1 | Japonica | NA | | Os02g0196000 | OsR498G0203149300.01.T02 | Zinc transporter like |
| maker-2-augustus-gene-54.56-mRNA-1 | Japonica | NA | | Os02g0195000 | OsR498G0203147400.01.T01 | Polygalacturonase inhibitor |
| maker-2-augustus-gene-54.58-mRNA-1 | Japonica | NA | | | OsR498G0203147600.01.T06 | DnaJ protein P58IPK homolog A |
| maker-2-augustus-gene-54.63-mRNA-1 | Japonica | NA | | Os02g0196100 | | Zinc finger protein WIP2 |
| maker-2-augustus-gene-54.65-mRNA-1 | Japonica | Indica | | Os02g0196300 | OsR498G0203150600.01.T02 | Outer envelope protein 80 chloroplastic |
| maker-2-augustus-gene-55.58-mRNA-1 | Japonica | NA | | Os02g0196800 | OsR498G0203153000.01.T01 | Fumarylacetoacetase |
| maker-2-augustus-gene-55.62-mRNA-1 | Japonica | NA | | Os02g0196900 | OsR498G0203153400.01.T01 | 3-oxoacyl-acyl-carrier-protein synthase |
| maker-2-augustus-gene-57.38-mRNA-1 | Japonica | NA | | Os02g0202200 | OsR498G0203173700.01.T01 | SPX domain-containing protein 2 |
| maker-2-augustus-gene-57.46-mRNA-1 | Japonica | NA | | | OsR498G0203169000.01.T01 | SHSP domain-containing protein (Fragment) |
| maker-2-augustus-gene-57.48-mRNA-1 | Japonica | Indica | | Os06g0604500 | OsR498G0203171000.01.T03 | Calcium-binding mitochondrial carrier protein like |
| maker-2-augustus-gene-57.51-mRNA-1 | Japonica | NA | | | OsR498G0203172400.01.T07 | DEAD-box ATP-dependent RNA helicase |
| maker-2-augustus-gene-58.23-mRNA-1 | Japonica | NA | | | OsR498G0203174000.01.T04 | Nuclear pore complex protein NUP205 |
| maker-2-augustus-gene-58.28-mRNA-1 | Japonica | Indica | | Os06g0602600 | OsR498G0203174600.01.T01 | Alba domain-containing protein (Fragment) |
| maker-2-augustus-gene-58.32-mRNA-1 | Japonica | NA | | Os02g0202600 | OsR498G0203175200.01.T01 | Binding |
| maker-2-augustus-gene-58.37-mRNA-1 | Japonica | NA | | Os02g0202800 | | SWIM-type domain-containing protein |

| | | | | | | |
|---|---|---|---|---|---|---|
| maker-2-augustus-gene-58.39-mRNA-1 | Japonica | NA | | Os02g0202900 | OsR498G0203176000.01.T01 | Galactose oxidase/kelch repeat superfamily protein isoform 1 |
| maker-2-augustus-gene-58.40-mRNA-1 | Japonica | NA | | Os02g0203000 | | cAMP response element binding (CREB) protein |
| maker-2-augustus-gene-58.42-mRNA-1 | Japonica | NA | CC-NBS-TM | Os02g0203500 | OsR498G0203179000.01.T01 | NB-ARC domain-containing protein (Fragment) |
| maker-2-augustus-gene-58.45-mRNA-1 | Japonica | NA | | Os02g0203700 | OsR498G0203179800.01.T03 | TATA-binding protein-associated factor 2N |
| maker-2-augustus-gene-59.16-mRNA-1 | Japonica | NA | | | OsR498G0203181300.01.T07 | Peptidylprolyl isomerase |
| maker-2-augustus-gene-59.21-mRNA-1 | Japonica | NA | | Os02g0204400 | OsR498G0203181500.01.T01 | Photolyase/cryptochrome alpha/beta domain-containing protein |
| maker-2-augustus-gene-59.25-mRNA-1 | Japonica | NA | | | OsR498G0203181700.01.T08 | ENT domain-containing protein (Fragment) |
| maker-2-augustus-gene-59.29-mRNA-1 | Japonica | NA | | Os02g0204700 | OsR498G0203183800.01.T01 | |
| maker-2-augustus-gene-6.35-mRNA-1 | Japonica | Indica | | Os01g0580100 | OsR498G0202834900.01.T01 | Mannosyltransferase |
| maker-2-augustus-gene-6.41-mRNA-1 | Japonica | NA | | Os02g0111700 | OsR498G0202835300.01.T01 | Ribosomal protein L37 |
| maker-2-augustus-gene-6.44-mRNA-1 | Japonica | NA | Kinase-TM | | OsR498G0202833400.01.T05 | Receptor-like serine/threonine-protein kinase NCRK |
| maker-2-augustus-gene-6.47-mRNA-1 | Japonica | NA | | | OsR498G0202833800.01.T04 | Helicase protein MOM1 |
| maker-2-augustus-gene-6.52-mRNA-1 | Japonica | Japonica | Kinase-TM | Os02g0111600 | OsR498G0202835200.01.T02 | Wall-associated receptor kinase 5 |
| maker-2-augustus-gene-6.55-mRNA-1 | Japonica | Indica | | Os02g0112000 | OsR498G0202836400.01.T01 | |
| maker-2-augustus-gene-60.0-mRNA-1 | Japonica | NA | | Os02g0205400 | OsR498G0203187500.01.T01 | serine-threonine kinase receptor-associated protein isoform X1 |
| maker-2-augustus-gene-60.15-mRNA-1 | Japonica | NA | | Os02g0205500 | OsR498G0203188000.01.T01 | Very-long-chain 3-oxoacyl-CoA synthase |
| maker-2-augustus-gene-60.2-mRNA-1 | Japonica | NA | | Os02g0205000 | OsR498G0203186200.01.T01 | 50S ribosomal protein like |
| maker-2-augustus-gene-60.6-mRNA-1 | Japonica | NA | | Os02g0205200 | OsR498G0203186600.01.T02 | Allergen Act d 3 |
| maker-2-augustus-gene-60.9-mRNA-1 | Japonica | NA | | Os02g0205300 | OsR498G0203187400.01.T01 | 26S proteasome regulatory particle triple-A ATPase subunit6 |
| maker-2-augustus-gene-61.25-mRNA-1 | Japonica | Indica | | Os01g0647100 | OsR498G0203194900.01.T01 | ADP,ATP carrier protein (Fragment) |
| maker-2-augustus-gene-61.34-mRNA-1 | Japonica | Indica | | Os01g0609300 | OsR498G0203195500.01.T02 | Pleiotropic drug resistance protein 7 |
| maker-2-augustus-gene-61.39-mRNA-1 | Japonica | Indica | | Os02g0208600 | OsR498G0203196500.01.T01 | SPOC domain / Transcription elongation factor S-II protein |
| maker-2-augustus-gene-62.0-mRNA-1 | Japonica | NA | | Os02g0208900 | OsR498G0203199300.01.T05 | NSP (Nuclear shuttle protein)-interacting GTPase |
| maker-2-augustus-gene-62.14-mRNA-1 | Japonica | Indica | | Os06g0143900 | OsR498G0203199800.01.T03 | Coatomer subunit beta' |
| maker-2-augustus-gene-62.15-mRNA-1 | Japonica | Indica | | Os06g0143900 | OsR498G0203200200.01.T03 | Coatomer subunit beta' |
| maker-2-augustus-gene-62.42-mRNA-1 | Japonica | NA | | | | SUN domain-containing protein |
| maker-2-augustus-gene-62.44-mRNA-1 | Japonica | NA | | Os02g0209900 | OsR498G0203201200.01.T01 | Syntaxin-123 |
| maker-2-augustus-gene-62.46-mRNA-1 | Japonica | NA | | | OsR498G0203201800.01.T01 | |
| maker-2-augustus-gene-62.5-mRNA-1 | Japonica | NA | Kinase-LRR-TM | Os02g0210700 | OsR498G0203202700.01.T01 | Putative LRR receptor-like serine/threonine-protein kinase |
| maker-2-augustus-gene-62.7-mRNA-1 | Japonica | NA | | | OsR498G0203203100.01.T06 | UDP-Glycosyltransferase superfamily protein |
| maker-2-augustus-gene-63.11-mRNA-1 | Japonica | Japonica | | Os02g0212600 | OsR498G0203211700.01.T06 | Kinesin-II 85 kDa subunit isoform X2 |
| maker-2-augustus-gene-63.14-mRNA-1 | Japonica | NA | Kinase-LRR-TM | Os02g0211200 | | Putative LRR receptor-like serine/threonine-protein kinase |
| maker-2-augustus-gene-63.16-mRNA-1 | Japonica | NA | Kinase-LRR-TM | Os02g0211200 | | Putative LRR receptor-like serine/threonine-protein kinase |
| maker-2-augustus-gene-63.3-mRNA-1 | Japonica | Japonica | | Os02g0211100 | OsR498G0202823400.01.T01 | |
| maker-2-augustus-gene-63.5-mRNA-1 | Japonica | NA | | Os02g0212400 | | |
| maker-2-augustus-gene-63.7-mRNA-1 | Japonica | NA | | Os02g0212500 | | |
| maker-2-augustus-gene-64.39-mRNA-1 | Japonica | NA | Kinase | | OsR498G1120698300.01.T01 | Receptor-like serine/threonine-protein kinase |
| maker-2-augustus-gene-64.47-mRNA-1 | Japonica | NA | | | OsR498G0203214000.01.T01 | |
| maker-2-augustus-gene-65.36-mRNA-1 | Japonica | NA | | Os02g0214300 | OsR498G0203216700.01.T02 | Nuclear ribonuclease Z |
| maker-2-augustus-gene-65.39-mRNA-1 | Japonica | NA | | Os02g0214500 | OsR498G0203217100.01.T01 | NAC domain class transcription factor |
| maker-2-augustus-gene-65.41-mRNA-1 | Japonica | NA | | Os02g0214900 | OsR498G0203218700.01.T02 | Histone deacetylase |
| maker-2-augustus-gene-65.48-mRNA-1 | Japonica | Japonica | Kinase-LRR-TM | Os02g0215500 | OsR498G0203219100.01.T01 | Putative LRR receptor-like serine/threonine-protein kinase |
| maker-2-augustus-gene-65.50-mRNA-1 | Japonica | NA | Kinase-LRR-TM | | OsR498G0203220300.01.T01 | Putative LRR receptor-like serine/threonine-protein kinase |
| maker-2-augustus-gene-65.52-mRNA-1 | Japonica | NA | Kinase-LRR-TM | Os02g0215700 | | Putative LRR receptor-like serine/threonine-protein kinase |
| maker-2-augustus-gene-65.54-mRNA-1 | Japonica | NA | | Os02g0214900 | OsR498G0203217600.01.T02 | Histone deacetylase |
| maker-2-augustus-gene-65.57-mRNA-1 | Japonica | NA | | Os02g0215000 | | p-loop containing nucleoside triphosphate hydrolase superfamily protein |
| maker-2-augustus-gene-66.45-mRNA-1 | Japonica | NA | | Os07g0658550 | | |
| maker-2-augustus-gene-66.46-mRNA-1 | Japonica | Indica | | Os02g0217400 | OsR498G0203223700.01.T01 | Premnaspirodiene oxygenase |
| maker-2-augustus-gene-66.48-mRNA-1 | Japonica | NA | | Os02g0217400 | OsR498G0203224500.01.T01 | Premnaspirodiene oxygenase |
| maker-2-augustus-gene-66.50-mRNA-1 | Japonica | NA | | Os02g0217500 | OsR498G0203225100.01.T01 | |
| maker-2-augustus-gene-66.54-mRNA-1 | Japonica | NA | | | | 28S rRNA (Cytosine-C(5))-methyltransferase |
| maker-2-augustus-gene-66.58-mRNA-1 | Japonica | Japonica | Kinase-LRR-TM | Os02g0215700 | OsR498G0203219100.01.T01 | Putative LRR receptor-like serine/threonine-protein kinase |
| maker-2-augustus-gene-66.69-mRNA-1 | Japonica | NA | | Os02g0217600 | OsR498G0203225400.01.T01 | Serine/threonine-protein phosphatase |
| maker-2-augustus-gene-67.40-mRNA-1 | Japonica | NA | | Os02g0218200 | OsR498G0203226300.01.T06 | Aminopeptidase |
| maker-2-augustus-gene-67.47-mRNA-1 | Japonica | Indica | CC-Kinase-TM | Os02g0218600 | OsR498G0203227300.01.T01 | E3 ubiquitin ligase |
| maker-2-augustus-gene-67.52-mRNA-1 | Japonica | NA | | Os02g0219700 | OsR498G0203230900.01.T01 | Senescence-associated protein 5 |
| maker-2-augustus-gene-67.54-mRNA-1 | Japonica | NA | | | OsR498G0203231500.01.T01 | Cytokinin dehydrogenase |
| maker-2-augustus-gene-67.56-mRNA-1 | Japonica | NA | | | OsR498G0203232100.01.T01 | Cytokinin dehydrogenase |
| maker-2-augustus-gene-67.58-mRNA-1 | Japonica | NA | CC-Kinase-TM | Os02g0218400 | OsR498G0203227000.01.T01 | U-box domain-containing protein 52 |
| maker-2-augustus-gene-67.61-mRNA-1 | Japonica | Indica | | Os02g0219200 | OsR498G0203229800.01.T01 | Beta-galactosidase 9 |
| maker-2-augustus-gene-67.69-mRNA-1 | Japonica | NA | | Os02g0219400 | OsR498G0203230600.01.T02 | GPI-anchor transamidase |

| | | | | | | |
|---|---|---|---|---|---|---|
| maker-2-augustus-gene-67.73-mRNA-1 | Japonica | NA | | | OsR498G0203231200.01.T01 | |
| maker-2-augustus-gene-68.0-mRNA-1 | Japonica | NA | | Os02g0220500 | OsR498G0203233300.01.T01 | Elongation factor 1-gamma |
| maker-2-augustus-gene-68.1-mRNA-1 | Japonica | NA | | Os02g0220600 | OsR498G0203233300.01.T08 | Elongation factor 1-gamma |
| maker-2-augustus-gene-68.14-mRNA-1 | Japonica | NA | | Os02g0220400 | OsR498G0203233000.01.T01 | GATA transcription factor 20 |
| maker-2-augustus-gene-68.16-mRNA-1 | Japonica | NA | Kinase-TM | | OsR498G0203233800.01.T02 | (Rice Genome Annotation Project) MAP3K delta-1 protein kinase |
| maker-2-augustus-gene-68.23-mRNA-1 | Japonica | Japonica | | Os02g0221100 | OsR498G0203235600.01.T01 | BHLH domain-containing protein (Fragment) |
| maker-2-augustus-gene-69.29-mRNA-1 | Japonica | NA | | | OsR498G0203237300.01.T03 | Glycolipid permease LtaA |
| maker-2-augustus-gene-69.35-mRNA-1 | Japonica | NA | | Os02g0221800 | OsR498G0203237700.01.T02 | calumenin |
| maker-2-augustus-gene-69.46-mRNA-1 | Japonica | NA | | Os02g0221900 | OsR498G0203238000.01.T02 | Thromboxane-A synthase |
| maker-2-augustus-gene-7.0-mRNA-1 | Japonica | NA | | | OsR498G0202837900.01.T01 | Pathogenesis-related protein 5 |
| maker-2-augustus-gene-7.12-mRNA-1 | Japonica | NA | | Os02g0113800 | OsR498G0202840900.01.T02 | DUF1618 domain-containing protein (Fragment) |
| maker-2-augustus-gene-7.16-mRNA-1 | Japonica | NA | | Os02g0112900 | OsR498G0202838200.01.T01 | Gamma-aminobutyric acid transaminase |
| maker-2-augustus-gene-7.19-mRNA-1 | Japonica | Indica | | Os09g0120033 | OsR498G0202840400.01.T01 | |
| maker-2-augustus-gene-7.21-mRNA-1 | Japonica | NA | | | OsR498G0202841400.01.T01 | ATP-dependent helicase BRM |
| maker-2-augustus-gene-7.3-mRNA-1 | Japonica | Japonica | | Os02g0113200 | OsR498G0202839100.01.T01 | Obtusifoliol 14-alpha demethylase |
| maker-2-augustus-gene-7.6-mRNA-1 | Japonica | NA | | Os02g0113300 | OsR498G0202839700.01.T01 | DUF1618 domain-containing protein (Fragment) |
| maker-2-augustus-gene-7.8-mRNA-1 | Japonica | Japonica | | Os02g0113400 | OsR498G0202839900.01.T01 | |
| maker-2-augustus-gene-70.0-mRNA-1 | Japonica | NA | Kinase-LRR-TM | | OsR498G0203239100.01.T01 | receptor-like protein kinase 5 isoform X1 |
| maker-2-augustus-gene-70.2-mRNA-1 | Japonica | NA | Kinase-LRR-TM | | OsR498G0203240500.01.T01 | Serine/threonine protein kinase |
| maker-2-augustus-gene-71.0-mRNA-1 | Japonica | Indica | | Os02g0224300 | OsR498G0203249500.01.T02 | Far upstream element-binding protein 1 |
| maker-2-augustus-gene-71.12-mRNA-1 | Japonica | NA | | Os02g0224200 | OsR498G0203248800.01.T04 | Serine/threonine-protein phosphatase 2A 55 kDa regulatory subunit B |
| maker-2-augustus-gene-71.5-mRNA-1 | Japonica | NA | | Os02g0224400 | | Metallopeptidase M24 family protein isoform 1 |
| maker-2-augustus-gene-71.7-mRNA-1 | Japonica | NA | | Os02g0224800 | OsR498G0203254500.01.T01 | Pollen-specific protein (Fragment) |
| maker-2-augustus-gene-72.0-mRNA-1 | Japonica | NA | | Os09g0508900 | OsR498G0203254900.01.T01 | Peroxisomal nicotinamide adenine dinucleotide carrier |
| maker-2-augustus-gene-72.10-mRNA-1 | Japonica | NA | | Os02g0226200 | OsR498G0203259700.01.T01 | Inorganic pyrophosphatase 2 |
| maker-2-augustus-gene-72.18-mRNA-1 | Japonica | NA | | | | F-box family protein, putative, expressed |
| maker-2-augustus-gene-72.22-mRNA-1 | Japonica | Indica | | Os02g0225300 | OsR498G0203256200.01.T01 | ATPase, vacuolar ER assembly factor, Vma |
| maker-2-augustus-gene-72.25-mRNA-1 | Japonica | NA | | Os02g0226000 | OsR498G0203258800.01.T01 | Peroxisomal membrane protein PMP22 |
| maker-2-augustus-gene-74.39-mRNA-1 | Japonica | NA | LRR-TM | Os02g0230100 | OsR498G0203274700.01.T03 | Protein phosphatase 1 regulatory subunit pprA |
| maker-2-augustus-gene-74.45-mRNA-1 | Japonica | NA | | Os02g0228900 | OsR498G0203270600.01.T01 | Auxin-responsive protein IAA7 |
| maker-2-augustus-gene-74.47-mRNA-1 | Japonica | NA | | Os02g0229000 | OsR498G0203271400.01.T01 | 40S ribosomal protein S24 |
| maker-2-augustus-gene-74.50-mRNA-1 | Japonica | Japonica | | Os02g0229600 | OsR498G0203272800.01.T05 | Kinesin motor domain-containing protein |
| maker-2-augustus-gene-74.59-mRNA-1 | Japonica | Indica | | Os02g0230000 | OsR498G0203274600.01.T03 | |
| maker-2-augustus-gene-75.16-mRNA-1 | Japonica | NA | | Os02g0230300 | OsR498G0203275400.01.T01 | Meiosis 5 |
| maker-2-augustus-gene-75.18-mRNA-1 | Japonica | NA | | | OsR498G0203275800.01.T01 | BHLH domain-containing protein |
| maker-2-augustus-gene-76.0-mRNA-1 | Japonica | NA | | Os02g0232000 | OsR498G0203284300.01.T01 | Heat stress transcription factor |
| maker-2-augustus-gene-76.10-mRNA-1 | Japonica | NA | Kinase-LRR-TM | | | Mitogen-activated protein kinase kinase kinase |
| maker-2-augustus-gene-76.17-mRNA-1 | Japonica | NA | | | OsR498G0203282200.01.T01 | Phytocyanin domain-containing protein |
| maker-2-augustus-gene-76.2-mRNA-1 | Japonica | Indica | | Os06g0157500 | OsR498G0203285700.01.T01 | |
| maker-2-augustus-gene-76.20-mRNA-1 | Japonica | NA | Kinase-LRR-TM | Os02g0231700 | OsR498G0203282600.01.T01 | Leucine-rich repeat protein kinase family protein |
| maker-2-augustus-gene-76.22-mRNA-1 | Japonica | NA | | Os02g0232100 | OsR498G0203284600.01.T02 | E3 ubiquitin-protein like |
| maker-2-augustus-gene-76.25-mRNA-1 | Japonica | NA | | Os02g0232900 | OsR498G0203286800.01.T02 | Aquaporin NIP |
| maker-2-augustus-gene-76.4-mRNA-1 | Japonica | NA | | Os02g0232400 | OsR498G0203286100.01.T01 | Citrate synthase |
| maker-2-augustus-gene-77.25-mRNA-1 | Japonica | NA | | Os02g0233100 | OsR498G0203288500.01.T01 | FYVE-type domain-containing protein (Fragment) |
| maker-2-augustus-gene-77.29-mRNA-1 | Japonica | NA | | Os02g0234200 | OsR498G0203290500.01.T01 | Putative six-bladed beta-propeller, TolB |
| maker-2-augustus-gene-77.32-mRNA-1 | Japonica | Indica | | Os02g0233200 | OsR498G0203288800.01.T01 | BES1/BZR1 like protein 4 |
| maker-2-augustus-gene-77.36-mRNA-1 | Japonica | NA | | | | |
| maker-2-augustus-gene-77.38-mRNA-1 | Japonica | NA | | | | TTF-type domain-containing protein |
| maker-2-augustus-gene-78.18-mRNA-1 | Japonica | Indica | | Os02g0235900 | OsR498G0203297900.01.T01 | Berberine bridge enzyme-like protein 50 |
| maker-2-augustus-gene-78.19-mRNA-1 | Japonica | NA | | Os02g0236000 | OsR498G0203299500.01.T02 | Aspartate aminotransferase |
| maker-2-augustus-gene-78.24-mRNA-1 | Japonica | Japonica | | Os02g0234700 | OsR498G0612730300.01.T02 | |
| maker-2-augustus-gene-78.26-mRNA-1 | Japonica | NA | | Os02g0234800 | OsR498G0203293800.01.T01 | U2 small nuclear ribonucleoprotein A' |
| maker-2-augustus-gene-78.31-mRNA-1 | Japonica | NA | Kinase-LRR-TM | Os02g0236100 | OsR498G0203300000.01.T02 | LRR receptor-like serine/threonine-protein kinase |
| maker-2-augustus-gene-78.5-mRNA-1 | Japonica | Indica | | Os02g0235000 | OsR498G0203294900.01.T05 | Actin-related protein 2/3 complex subunit |
| maker-2-augustus-gene-78.9-mRNA-1 | Japonica | NA | | Os02g0235600 | OsR498G0203297700.01.T04 | 60S ribosomal protein L11 |
| maker-2-augustus-gene-79.0-mRNA-1 | Japonica | Indica | | Os06g0547400 | OsR498G0203302100.01.T01 | Peroxidase |
| maker-2-augustus-gene-79.10-mRNA-1 | Japonica | NA | Kinase-TM | Os02g0236200 | OsR498G0203300400.01.T01 | Non-specific serine/threonine protein kinase |
| maker-2-augustus-gene-79.16-mRNA-1 | Japonica | NA | | Os02g0237100 | OsR498G0203303600.01.T03 | PABS domain-containing protein |
| maker-2-augustus-gene-79.4-mRNA-1 | Japonica | NA | | Os02g0236800 | OsR498G0203302700.01.T02 | Peroxidase |
| maker-2-augustus-gene-79.8-mRNA-1 | Japonica | NA | | Os02g0237700 | | BED-type domain-containing protein (Fragment) |
| maker-2-augustus-gene-8.28-mRNA-1 | Japonica | NA | | Os02g0114700 | OsR498G0202843500.01.T01 | D-site 20S pre-rRNA nuclease Nob |

| | | | | | | |
|---|---|---|---|---|---|---|
| maker-2-augustus-gene-8.31-mRNA-1 | Japonica | NA | | Os02g0115600 | OsR498G0202846700.01.T02 | S1 motif domain-containing protein (Fragment) |
| maker-2-augustus-gene-8.34-mRNA-1 | Japonica | NA | | Os02g0114050 | OsR498G0202842000.01.T01 | |
| maker-2-augustus-gene-8.37-mRNA-1 | Japonica | NA | | Os02g0114200 | OsR498G0202842400.01.T01 | Carboxypeptidase |
| maker-2-augustus-gene-8.41-mRNA-1 | Japonica | NA | | Os02g0114400 | OsR498G0202842600.01.T01 | Glycerol-3-phosphate 2-O-acyltransferase 4 |
| maker-2-augustus-gene-8.43-mRNA-1 | Japonica | NA | | Os02g0114600 | OsR498G0202843000.01.T02 | F-box/kelch-repeat protein SKIP4 |
| maker-2-augustus-gene-8.45-mRNA-1 | Japonica | NA | | Os02g0114800 | OsR498G0202843800.01.T01 | transcription repressor MYB6 |
| maker-2-augustus-gene-8.47-mRNA-1 | Japonica | Japonica | | Os02g0115700 | OsR498G0305175000.01.T01 | Catalase isozyme A |
| maker-2-augustus-gene-80.23-mRNA-1 | Japonica | NA | | Os08g0487300 | OsR498G0816350400.01.T01 | Transposon protein, putative, Mutator sub-class |
| maker-2-augustus-gene-80.26-mRNA-1 | Japonica | NA | | Os04g0599400 | OsR498G0203306400.01.T02 | Phospholipid scramblase |
| maker-2-augustus-gene-81.0-mRNA-1 | Japonica | NA | | Os02g0240100 | OsR498G0203308300.01.T01 | Peroxidase |
| maker-2-augustus-gene-81.15-mRNA-1 | Japonica | NA | Kinase-LRR-TM | Os02g0241100 | OsR498G0203310000.01.T01 | Serine/threonine protein kinase |
| maker-2-augustus-gene-81.2-mRNA-1 | Japonica | NA | | Os02g0240300 | OsR498G0203308700.01.T01 | Peroxidase |
| maker-2-augustus-gene-81.4-mRNA-1 | Japonica | Japonica | | Os02g0240500 | OsR498G0203309700.01.T01 | Peroxidase |
| maker-2-augustus-gene-81.6-mRNA-1 | Japonica | NA | | | OsR498G0203310700.01.T01 | HTH myb-type domain-containing protein |
| maker-2-augustus-gene-81.8-mRNA-1 | Japonica | NA | Kinase-TM | Os02g0241600 | OsR498G0203312300.01.T01 | Serine/threonine-protein kinase EDR1 |
| maker-2-augustus-gene-82.0-mRNA-1 | Japonica | NA | | Os02g0242600 | OsR498G0203314300.01.T01 | Glutelin type-B |
| maker-2-augustus-gene-82.2-mRNA-1 | Japonica | Japonica | | Os02g0243900 | OsR498G0203315300.01.T01 | Potassium channel KAT1 |
| maker-2-augustus-gene-83.14-mRNA-1 | Japonica | NA | | Os02g0244100 | OsR498G0203315700.01.T02 | RING-type E3 ubiquitin ligase |
| maker-2-augustus-gene-83.18-mRNA-1 | Japonica | NA | | Os02g0244300 | OsR498G0203317100.01.T01 | Ubiquitin carboxyl-terminal hydrolase 15 |
| maker-2-augustus-gene-83.24-mRNA-1 | Japonica | NA | | Os02g0245000 | OsR498G0203319700.01.T05 | Splicing factor 3A subunit 1 |
| maker-2-augustus-gene-83.26-mRNA-1 | Japonica | NA | | Os02g0244700 | OsR498G0203318800.01.T02 | Phosphoenolpyruvate carboxylase |
| maker-2-augustus-gene-84.0-mRNA-1 | Japonica | NA | | | OsR498G0203324700.01.T02 | 1,3-beta-glucan synthase |
| maker-2-augustus-gene-84.2-mRNA-1 | Japonica | NA | | | | 1,3-beta-glucan synthase |
| maker-2-augustus-gene-84.6-mRNA-1 | Japonica | Indica | | Os02g0245800 | OsR498G0203322200.01.T03 | Potassium channel KAT1 |
| maker-2-augustus-gene-85.43-mRNA-1 | Japonica | NA | | Os02g0247100 | OsR498G0203325700.01.T01 | Basic-leucine zipper domain |
| maker-2-augustus-gene-85.45-mRNA-1 | Japonica | Indica | | Os01g0212400 | OsR498G0203327500.01.T01 | Sodium/calcium exchanger |
| maker-2-augustus-gene-86.34-mRNA-1 | Japonica | Japonica | | Os02g0249600 | OsR498G0203334100.01.T01 | Glutelin type-B |
| maker-2-augustus-gene-86.37-mRNA-1 | Japonica | NA | | Os02g0249900 | OsR498G0203334100.01.T01 | Glutelin type-B |
| maker-2-augustus-gene-86.40-mRNA-1 | Japonica | Indica | | Os02g0250300 | OsR498G0203336300.01.T03 | Transcription factor GTE4 |
| maker-2-augustus-gene-86.43-mRNA-1 | Japonica | NA | | Os02g0250400 | OsR498G0203336700.01.T01 | GDSL esterase/lipase |
| maker-2-augustus-gene-86.47-mRNA-1 | Japonica | NA | | Os02g0249700 | | F-box domain-containing protein |
| maker-2-augustus-gene-86.49-mRNA-1 | Japonica | NA | | Os02g0249900 | OsR498G0203334100.01.T01 | Glutelin type-B |
| maker-2-augustus-gene-87.34-mRNA-1 | Japonica | NA | | Os02g0252200 | OsR498G0203341300.01.T01 | NAC domain-containing protein 76 |
| maker-2-augustus-gene-87.36-mRNA-1 | Japonica | Indica | | Os02g0252100 | OsR498G0203340800.01.T01 | Pre-mRNA splicing factor |
| maker-2-augustus-gene-87.41-mRNA-1 | Japonica | Indica | | Os02g0252600 | OsR498G0203342400.01.T02 | D-aminoacid aminotransferase-like PLP-dependent enzymes superfamily pro |
| maker-2-augustus-gene-88.13-mRNA-1 | Japonica | NA | | | OsR498G0203346200.01.T01 | |
| maker-2-augustus-gene-88.16-mRNA-1 | Japonica | Indica | | Os06g0528600 | OsR498G0203346800.01.T01 | PABS domain-containing protein (Fragment) |
| maker-2-augustus-gene-88.29-mRNA-1 | Japonica | NA | | Os02g0255200 | OsR498G0203348200.01.T01 | 30S ribosomal protein S8, chloroplastic |
| maker-2-augustus-gene-88.6-mRNA-1 | Japonica | Indica | | Os02g0255500 | OsR498G0203349500.01.T01 | Abscisic acid receptor like |
| maker-2-augustus-gene-88.8-mRNA-1 | Japonica | NA | | Os02g0255500 | OsR498G0203350900.01.T01 | Abscisic acid receptor PYR/PYL family |
| maker-2-augustus-gene-89.0-mRNA-1 | Japonica | NA | | Os02g0256100 | OsR498G0203351900.01.T01 | Pectin lyase-like superfamily protein (Fragment) |
| maker-2-augustus-gene-89.10-mRNA-1 | Japonica | NA | | | OsR498G0203351800.01.T03 | clustered mitochondria protein isoform X4 |
| maker-2-augustus-gene-89.18-mRNA-1 | Japonica | Japonica | | Os02g0256800 | OsR498G0203353400.01.T01 | Phytocyanin domain-containing protein (Fragment) |
| maker-2-augustus-gene-89.20-mRNA-1 | Japonica | NA | | Os02g0257100 | OsR498G0203353400.01.T01 | Phytocyanin domain-containing protein (Fragment) |
| maker-2-augustus-gene-89.22-mRNA-1 | Japonica | Japonica | | Os02g0257200 | OsR498G0203353600.01.T01 | Polypyrimidine tract-binding protein-like protein 2 |
| maker-2-augustus-gene-89.24-mRNA-1 | Japonica | NA | | | OsR498G0203354000.01.T01 | BHLH transcription factor |
| maker-2-augustus-gene-89.3-mRNA-1 | Japonica | NA | | Os02g0257001 | | |
| maker-2-augustus-gene-89.5-mRNA-1 | Japonica | NA | | Os02g0257300 | OsR498G0203353700.01.T01 | Rhodanese-like domain-containing protein 4 chloroplastic |
| maker-2-augustus-gene-90.0-mRNA-1 | Japonica | Indica | | Os02g0258200 | OsR498G0203358000.01.T01 | High mobility group B protein 6 |
| maker-2-augustus-gene-90.10-mRNA-1 | Japonica | NA | | Os02g0259600 | OsR498G0203362800.01.T01 | 50S ribosomal protein L21 chloroplastic |
| maker-2-augustus-gene-90.2-mRNA-1 | Japonica | NA | | Os02g0258300 | OsR498G0203358200.01.T01 | Extra-large G-protein-like |
| maker-2-augustus-gene-90.5-mRNA-1 | Japonica | NA | | Os02g0258900 | OsR498G0203361000.01.T06 | GTP 3',8-cyclase, mitochondrial isoform C |
| maker-2-augustus-gene-91.14-mRNA-1 | Japonica | NA | | | OsR498G0203363300.01.T02 | BAG family molecular chaperone regulator 6 |
| maker-2-augustus-gene-91.18-mRNA-1 | Japonica | Indica | | Os02g0260200 | OsR498G0203364900.01.T02 | F-box/kelch-repeat protein At3g61590 |
| maker-2-augustus-gene-91.20-mRNA-1 | Japonica | NA | | Os02g0260500 | OsR498G0203365500.01.T01 | Intracellular protein transport protein USO1-related |
| maker-2-augustus-gene-91.23-mRNA-1 | Japonica | NA | | Os02g0260700 | OsR498G0203366500.01.T01 | BTB/POZ domain-containing protein POB1 |
| maker-2-augustus-gene-91.27-mRNA-1 | Japonica | NA | | Os02g0261000 | OsR498G0203367900.01.T02 | Late embryogenesis abundant (LEA) hydroxyproline-rich glycoprotein family |
| maker-2-augustus-gene-91.28-mRNA-1 | Japonica | Indica | | Os11g0706100 | OsR498G0203365400.01.T05 | |
| maker-2-augustus-gene-92.25-mRNA-1 | Japonica | NA | | Os02g0261100 | OsR498G0203368500.01.T03 | UBC core domain-containing protein (Fragment) |
| maker-2-augustus-gene-93.0-mRNA-1 | Japonica | NA | | Os02g0262700 | | Putative bacterial blight-resistance protein Xa1 |
| maker-2-augustus-gene-94.40-mRNA-1 | Japonica | NA | | Os02g0264300 | OsR498G0203370000.01.T01 | Plastid division protein PDV |

| | | | | | | | |
|---|---|---|---|---|---|---|---|
| maker-2-augustus-gene-94.42-mRNA-1 | Japonica | NA | | Os02g0264800 | OsR498G0203372600.01.T01 | Arabinogalactan peptide |
| maker-2-augustus-gene-95.29-mRNA-1 | Japonica | NA | | Os02g0265200 | OsR498G0203373900.01.T01 | WRKY transcription factor |
| maker-2-augustus-gene-95.31-mRNA-1 | Japonica | Indica | | Os02g0265700 | OsR498G0203376100.01.T02 | |
| maker-2-augustus-gene-95.35-mRNA-1 | Japonica | NA | | Os02g0265900 | OsR498G0203376500.01.T02 | Reticulon-like protein (Fragment) |
| maker-2-augustus-gene-95.39-mRNA-1 | Japonica | NA | | | OsR498G0203376700.01.T01 | Phosphoribosylanthranilate isomerase |
| maker-2-augustus-gene-95.42-mRNA-1 | Japonica | NA | | Os02g0266200 | OsR498G0203377100.01.T01 | Pentatricopeptide repeat-containing protein, putative |
| maker-2-augustus-gene-95.44-mRNA-1 | Japonica | NA | | Os02g0265400 | OsR498G0203375600.01.T01 | Vacuolar protein sorting-associated protein 29 |
| maker-2-augustus-gene-95.53-mRNA-1 | Japonica | Japonica | | Os02g0266100 | OsR498G0203377000.01.T01 | U1 small nuclear ribonucleoprotein C |
| maker-2-augustus-gene-95.56-mRNA-1 | Japonica | NA | | Os02g0266300 | OsR498G0203377400.01.T02 | Putative ankyrin repeat-containing domain, P-loop containing nucleoside tri |
| maker-2-augustus-gene-96.0-mRNA-1 | Japonica | NA | | | OsR498G0203378100.01.T01 | tRNA(Ile)-2-lysyl-cytidine synthase |
| maker-2-augustus-gene-96.11-mRNA-1 | Japonica | NA | | Os02g0267200 | OsR498G0203379500.01.T01 | Expansin |
| maker-2-augustus-gene-96.13-mRNA-1 | Japonica | NA | | Os02g0267700 | OsR498G0203380900.01.T01 | Expansin |
| maker-2-augustus-gene-96.15-mRNA-1 | Japonica | Indica | | Os02g0268600 | OsR498G0203381700.01.T01 | Expansin |
| maker-2-augustus-gene-96.17-mRNA-1 | Japonica | Indica | | Os02g0267700 | OsR498G0203383000.01.T01 | Expansin |
| maker-2-augustus-gene-96.19-mRNA-1 | Japonica | NA | | Os02g0268300 | OsR498G0203382800.01.T01 | Glutelin type-B |
| maker-2-augustus-gene-96.21-mRNA-1 | Japonica | NA | | Os02g0266800 | OsR498G0203378400.01.T01 | Basic leucine zipper 9 |
| maker-2-augustus-gene-96.25-mRNA-1 | Japonica | NA | Kinase-TM | Os01g0711200 | OsR498G0101870300.01.T01 | Putative leucine-rich repeat receptor-like protein kinase |
| maker-2-augustus-gene-96.29-mRNA-1 | Japonica | Indica | | Os02g0267700 | OsR498G0203383000.01.T01 | Expansin |
| maker-2-augustus-gene-96.31-mRNA-1 | Japonica | NA | | Os02g0268600 | OsR498G0203383600.01.T01 | Expansin |
| maker-2-augustus-gene-96.33-mRNA-1 | Japonica | NA | | Os02g0269000 | OsR498G0203384400.01.T01 | Protein CONTINUOUS VASCULAR RING 1 |
| maker-2-augustus-gene-96.6-mRNA-1 | Japonica | NA | | Os02g0267000 | OsR498G0203379300.01.T01 | Chloroplast processing peptidase |
| maker-2-augustus-gene-97.27-mRNA-1 | Japonica | NA | | Os02g0269600 | OsR498G0203385800.01.T01 | Subtilisin-like protease |
| maker-2-augustus-gene-97.29-mRNA-1 | Japonica | Indica | | Os02g0270933 | OsR498G0203388600.01.T02 | Subtilisin-like protease |
| maker-2-augustus-gene-98.1-mRNA-1 | Japonica | Indica | | Os02g0270933 | OsR498G0203389000.01.T01 | Subtilisin-like protease |
| maker-2-augustus-gene-98.10-mRNA-1 | Japonica | NA | | Os02g0271000 | OsR498G0203389600.01.T01 | Subtilisin-like protease |
| maker-2-augustus-gene-98.14-mRNA-1 | Japonica | NA | | Os02g0271600 | OsR498G0203389800.01.T01 | Subtilisin-like protease |
| maker-2-augustus-gene-99.0-mRNA-1 | Japonica | NA | | Os02g0271900 | OsR498G0203390500.01.T02 | HTH myb-type domain-containing protein |
| maker-2-augustus-gene-99.11-mRNA-1 | Japonica | NA | | Os02g0273100 | OsR498G0203395500.01.T01 | D-amino-acid transaminase, chloroplastic |
| maker-2-augustus-gene-99.13-mRNA-1 | Japonica | NA | | Os02g0272400 | OsR498G0203393000.01.T01 | Eukaryotic translation initiation factor 3 subunit L-like |
| maker-2-augustus-gene-99.3-mRNA-1 | Japonica | NA | | | OsR498G0203391700.01.T01 | Flavin-containing monooxygenase |
| maker-2-augustus-gene-99.5-mRNA-1 | Japonica | NA | | Os02g0272300 | OsR498G0203392500.01.T01 | Rho guanine nucleotide exchange factor like |
| maker-2-augustus-gene-99.7-mRNA-1 | Japonica | NA | | Os02g0272600 | OsR498G0203393900.01.T01 | Gamma-secretase subunit Aph-1 |
| maker-3-augustus-gene-1.35-mRNA-1 | Japonica | Indica | | Os05g0413200 | OsR498G0305083300.01.T01 | Tubulin beta chain |
| maker-3-augustus-gene-1.37-mRNA-1 | Japonica | NA | | Os03g0105500 | | |
| maker-3-augustus-gene-1.38-mRNA-1 | Japonica | NA | | Os03g0105700 | OsR498G0305083600.01.T01 | AT-hook motif nuclear-localized protein (Fragment) |
| maker-3-augustus-gene-1.41-mRNA-1 | Japonica | NA | | Os03g0105800 | OsR498G0305083800.01.T02 | PPC domain-containing protein |
| maker-3-augustus-gene-1.43-mRNA-1 | Japonica | NA | | Os03g0106000 | OsR498G0305084600.01.T02 | UPF0187 protein chloroplastic |
| maker-3-augustus-gene-1.46-mRNA-1 | Japonica | NA | | Os03g0106200 | OsR498G0305085200.01.T03 | Phosphatidylinositol N-acetyglucosaminlytransferase subunit P-related |
| maker-3-augustus-gene-1.49-mRNA-1 | Japonica | NA | | Os03g0106300 | OsR498G0305085400.01.T01 | |
| maker-3-augustus-gene-1.51-mRNA-1 | Japonica | NA | | Os03g0106400 | OsR498G0305085600.01.T02 | Branched-chain-amino-acid aminotransferase |
| maker-3-augustus-gene-1.56-mRNA-1 | Japonica | Japonica | | Os03g0106700 | OsR498G0305087100.01.T01 | Group I pollen allergen |
| maker-3-augustus-gene-10.20-mRNA-1 | Japonica | NA | Kinase-TM | Os03g0122000 | OsR498G0305141900.01.T02 | Serine/threonine-protein kinase ATG1a |
| maker-3-augustus-gene-10.25-mRNA-1 | Japonica | NA | | | OsR498G0305142500.01.T01 | Transcription factor bHLH70 |
| maker-3-augustus-gene-10.28-mRNA-1 | Japonica | NA | | | OsR498G0305141600.01.T02 | Endoribonuclease Dicer like 1 |
| maker-3-augustus-gene-10.40-mRNA-1 | Japonica | NA | | Os03g0122200 | OsR498G0305143000.01.T01 | Plastid ribosomal protein l11 |
| maker-3-augustus-gene-10.43-mRNA-1 | Japonica | NA | | Os03g0122300 | OsR498G0305143200.01.T02 | Flavanone 3-dioxygenase |
| maker-3-augustus-gene-10.46-mRNA-1 | Japonica | NA | | | | Transposon protein, putative, Mutator sub-class, expressed |
| maker-3-augustus-gene-100.0-mRNA-1 | Japonica | NA | | | OsR498G0305746100.01.T02 | Pherophorin like protein |
| maker-3-augustus-gene-100.13-mRNA-1 | Japonica | Indica | | Os03g0293850 | OsR498G0305744600.01.T08 | |
| maker-3-augustus-gene-100.17-mRNA-1 | Japonica | Indica | | Os05g0353400 | OsR498G0305747000.01.T01 | Importin subunit beta-1 like |
| maker-3-augustus-gene-100.19-mRNA-1 | Japonica | NA | Kinase-TM | Os03g0294800 | OsR498G0305747600.01.T01 | Putative LRR receptor-like serine/threonine-protein kinase MRH1 |
| maker-3-augustus-gene-100.2-mRNA-1 | Japonica | Indica | | Os03g0294200 | OsR498G0305746300.01.T01 | 6-phosphofructo-2-kinase/fructose-26-bisphosphatase |
| maker-3-augustus-gene-100.9-mRNA-1 | Japonica | NA | | Os03g0293500 | OsR498G0305742800.01.T01 | Pyruvate decarboxylase |
| maker-3-augustus-gene-101.0-mRNA-1 | Japonica | NA | | Os03g0295500 | OsR498G0305749300.01.T02 | CHCH domain containing protein, expressed |
| maker-3-augustus-gene-101.11-mRNA-1 | Japonica | NA | | Os03g0296400 | OsR498G0305752700.01.T01 | Eukaryotic translation initiation factor 2 subunit alpha |
| maker-3-augustus-gene-101.17-mRNA-1 | Japonica | NA | | Os03g0296800 | OsR498G0305753700.01.T02 | Mitoferrin |
| maker-3-augustus-gene-101.2-mRNA-1 | Japonica | NA | | Os03g0295700 | OsR498G0305750700.01.T04 | Rab3 GTPase-activating protein catalytic subunit |
| maker-3-augustus-gene-101.20-mRNA-1 | Japonica | NA | | Os03g0297100 | OsR498G0305754100.01.T01 | 40S ribosomal protein S7 |
| maker-3-augustus-gene-101.23-mRNA-1 | Japonica | NA | | Os03g0297100 | OsR498G0305754500.01.T01 | 40S ribosomal protein S7 |
| maker-3-augustus-gene-101.25-mRNA-1 | Japonica | NA | | Os03g0295400 | OsR498G0305749200.01.T02 | DNA-directed RNA polymerases I and III subunit RPAC1 |
| maker-3-augustus-gene-101.30-mRNA-1 | Japonica | NA | Kinase-TM | Os03g0295600 | OsR498G0305749600.01.T01 | Serine/threonine-protein kinase STY46 |

| | | | | | | |
|---|---|---|---|---|---|---|
| maker-3-augustus-gene-101.32-mRNA-1 | Japonica | Indica | | Os03g0295800 | OsR498G0305751000.01.T02 | Gamma interferon inducible lysosomal thiol reductase GILT |
| maker-3-augustus-gene-101.37-mRNA-1 | Japonica | NA | | Os03g0296000 | OsR498G0305751800.01.T01 | Myb transcription factor |
| maker-3-augustus-gene-101.40-mRNA-1 | Japonica | NA | | | | Mitochondrial import inner membrane translocase |
| maker-3-augustus-gene-101.43-mRNA-1 | Japonica | NA | | | | [Acyl-carrier-protein] S-malonyltransferase |
| maker-3-augustus-gene-101.9-mRNA-1 | Japonica | NA | | Os03g0296200 | OsR498G0305752300.01.T01 | |
| maker-3-augustus-gene-102.11-mRNA-1 | Japonica | NA | | Os03g0298400 | OsR498G0305759700.01.T01 | 26S protease regulatory subunit 4 isogeny |
| maker-3-augustus-gene-102.15-mRNA-1 | Japonica | NA | | Os03g0299200 | OsR498G0305761900.01.T01 | Tasselseed2-like short-chain dehydrogenase/reductase (Fragment) |
| maker-3-augustus-gene-102.17-mRNA-1 | Japonica | NA | | Os03g0298600 | OsR498G0305760000.01.T02 | Iron regulated 1 protein |
| maker-3-augustus-gene-102.19-mRNA-1 | Japonica | NA | | Os03g0298700 | OsR498G0305760200.01.T01 | |
| maker-3-augustus-gene-102.25-mRNA-1 | Japonica | NA | | Os03g0298800 | OsR498G0305760400.01.T01 | U1 small nuclear ribonucleoprotein A/U2 small nuclear ribonucleoprotein B |
| maker-3-augustus-gene-102.3-mRNA-1 | Japonica | Indica | CC-Kinase-LRR-TM | Os03g0297800 | OsR498G0305757700.01.T01 | Probably inactive leucine-rich repeat receptor-like protein kinase IMK2 |
| maker-3-augustus-gene-102.5-mRNA-1 | Japonica | NA | | Os03g0297900 | | Laccase |
| maker-3-augustus-gene-102.8-mRNA-1 | Japonica | NA | | Os03g0298300 | OsR498G0305759300.01.T01 | AAA domain-containing protein |
| maker-3-augustus-gene-103.34-mRNA-1 | Japonica | NA | | Os03g0299900 | OsR498G0305765300.01.T01 | LL-diaminopimelate aminotransferase chloroplastic |
| maker-3-augustus-gene-103.39-mRNA-1 | Japonica | NA | | Os03g0300300 | OsR498G0305766500.01.T01 | Utp12 domain-containing protein |
| maker-3-augustus-gene-103.43-mRNA-1 | Japonica | NA | | Os03g0300400 | OsR498G0305766700.01.T01 | Pathogenesis related protein 10 |
| maker-3-augustus-gene-103.45-mRNA-1 | Japonica | NA | | Os03g0301400 | OsR498G0305771300.01.T02 | three prime repair exonuclease 2 |
| maker-3-augustus-gene-103.47-mRNA-1 | Japonica | NA | | | OsR498G0305771500.01.T02 | Zinc finger CCCH domain-containing protein 9 |
| maker-3-augustus-gene-103.49-mRNA-1 | Japonica | NA | | Os03g0300200 | OsR498G0305766400.01.T04 | Programmed cell death protein 2 |
| maker-3-augustus-gene-103.54-mRNA-1 | Japonica | NA | | Os03g0300600 | OsR498G0305767800.01.T01 | J domain-containing protein |
| maker-3-augustus-gene-104.32-mRNA-1 | Japonica | NA | | | OsR498G0305771500.01.T04 | Calmodulin-binding protein 60 D |
| maker-3-augustus-gene-104.37-mRNA-1 | Japonica | NA | | Os03g0302200 | OsR498G0305773700.01.T06 | Remodeling and spacing factor 1-like |
| maker-3-augustus-gene-104.39-mRNA-1 | Japonica | NA | | Os03g0302500 | OsR498G0305774300.01.T01 | Cytoplasmic dynein 1 light intermediate chain 2 |
| maker-3-augustus-gene-104.40-mRNA-1 | Japonica | NA | | Os03g0302900 | OsR498G0305775700.01.T02 | DUF4378 domain-containing protein (Fragment) |
| maker-3-augustus-gene-104.43-mRNA-1 | Japonica | NA | | Os03g0301700 | OsR498G0305771800.01.T03 | PPM-type phosphatase domain-containing protein |
| maker-3-augustus-gene-104.48-mRNA-1 | Japonica | NA | | | | p-loop nucleoside triphosphate hydrolase superfamily protein with CH (Calp |
| maker-3-augustus-gene-104.54-mRNA-1 | Japonica | NA | | | OsR498G0305776800.01.T01 | Myb-related protein P |
| maker-3-augustus-gene-105.25-mRNA-1 | Japonica | NA | | Os03g0304400 | OsR498G0305778900.01.T02 | BRCA1 C Terminus domain containing protein, expressed |
| maker-3-augustus-gene-105.29-mRNA-1 | Japonica | NA | | Os03g0304100 | OsR498G0305778600.01.T01 | Glycine-rich protein family |
| maker-3-augustus-gene-105.31-mRNA-1 | Japonica | NA | | | OsR498G0305778800.01.T01 | |
| maker-3-augustus-gene-105.33-mRNA-1 | Japonica | NA | | Os03g0304500 | OsR498G0305780400.01.T01 | J domain-containing protein |
| maker-3-augustus-gene-105.36-mRNA-1 | Japonica | Indica | | Os03g0304800 | OsR498G0305782200.01.T03 | Negatively light-regulated protein, putative, expressed |
| maker-3-augustus-gene-106.0-mRNA-1 | Japonica | NA | | Os03g0305500 | OsR498G0305786100.01.T02 | Fumarate lyase |
| maker-3-augustus-gene-106.10-mRNA-1 | Japonica | NA | | | OsR498G0305787700.01.T01 | |
| maker-3-augustus-gene-106.11-mRNA-1 | Japonica | NA | | Os03g0306700 | OsR498G0305789700.01.T01 | BZIP transcription factor |
| maker-3-augustus-gene-106.12-mRNA-1 | Japonica | NA | | Os03g0307000 | OsR498G0305790900.01.T02 | Nicotianamine synthase6 |
| maker-3-augustus-gene-106.14-mRNA-1 | Japonica | Indica | | Os03g0305100 | OsR498G0305783600.01.T01 | Acetate-CoA ligase |
| maker-3-augustus-gene-106.16-mRNA-1 | Japonica | NA | | Os03g0305100 | OsR498G0305784600.01.T02 | Acetate-CoA ligase |
| maker-3-augustus-gene-106.18-mRNA-1 | Japonica | NA | | Os03g0305400 | OsR498G0305786000.01.T04 | Usp domain-containing protein (Fragment) |
| maker-3-augustus-gene-106.21-mRNA-1 | Japonica | NA | | | OsR498G0305788200.01.T01 | WD40/YVTN repeat-like-containing domain |
| maker-3-augustus-gene-106.29-mRNA-1 | Japonica | NA | CC-NBS-TM | Os03g0306400 | OsR498G0305788600.01.T02 | Lon protease, mitochondrial |
| maker-3-augustus-gene-106.3-mRNA-1 | Japonica | NA | | Os03g0305600 | OsR498G0305786300.01.T04 | Outer envelope pore protein 16-2 chloroplastic |
| maker-3-augustus-gene-106.35-mRNA-1 | Japonica | NA | | Os03g0306900 | OsR498G0305790600.01.T01 | Seed maturation protein PM36 |
| maker-3-augustus-gene-106.6-mRNA-1 | Japonica | NA | | Os03g0305700 | OsR498G0305786500.01.T01 | PCRF domain-containing protein (Fragment) |
| maker-3-augustus-gene-107.40-mRNA-1 | Japonica | NA | | Os03g0307100 | OsR498G0305791100.01.T01 | Probable glutamyl endopeptidase, chloroplastic |
| maker-3-augustus-gene-107.44-mRNA-1 | Japonica | NA | | Os03g0307400 | OsR498G0305792900.01.T01 | Calcium-dependent phosphotriesterase superfamily protein |
| maker-3-augustus-gene-107.46-mRNA-1 | Japonica | NA | | | OsR498G0305792900.01.T02 | Putative ARM repeat-containing protein containing family protein |
| maker-3-augustus-gene-107.48-mRNA-1 | Japonica | Japonica | | Os03g0307700 | OsR498G0305793500.01.T02 | Protein trichome birefringence |
| maker-3-augustus-gene-107.56-mRNA-1 | Japonica | NA | | Os03g0308500 | OsR498G0305794900.01.T01 | DEAD-box ATP-dependent RNA helicase |
| maker-3-augustus-gene-107.59-mRNA-1 | Japonica | Indica | | Os03g0308600 | OsR498G0305795100.01.T02 | |
| maker-3-augustus-gene-107.62-mRNA-1 | Japonica | Indica | | Os03g0308700 | OsR498G0305795300.01.T01 | Energy transducer TonB |
| maker-3-augustus-gene-107.64-mRNA-1 | Japonica | NA | | Os03g0307800 | OsR498G0305793800.01.T01 | Histone-lysine N-methyltransferase EZ3 |
| maker-3-augustus-gene-107.68-mRNA-1 | Japonica | NA | | Os03g0308100 | OsR498G0305794200.01.T01 | ATP-dependent Clp protease proteolytic subunit |
| maker-3-augustus-gene-107.73-mRNA-1 | Japonica | NA | | Os03g0308200 | OsR498G0305794600.01.T06 | ARM repeat superfamily protein |
| maker-3-augustus-gene-107.78-mRNA-1 | Japonica | NA | | Os03g0308800 | OsR498G0305795600.01.T01 | High chlorophyll fluorescent 107 |
| maker-3-augustus-gene-107.82-mRNA-1 | Japonica | NA | | | OsR498G0305796000.01.T01 | Pre-mRNA 3'-end-processing factor FIP1 |
| maker-3-augustus-gene-107.85-mRNA-1 | Japonica | NA | | Os03g0309000 | OsR498G0305796200.01.T02 | Heme binding |
| maker-3-augustus-gene-108.0-mRNA-1 | Japonica | NA | | Os03g0309200 | OsR498G0305797300.01.T04 | Phytochrome B |
| maker-3-augustus-gene-108.11-mRNA-1 | Japonica | NA | | Os03g0310400 | | Crotonase superfamily |
| maker-3-augustus-gene-108.13-mRNA-1 | Japonica | NA | | | OsR498G0305801100.01.T01 | Protein-serine/threonine phosphatase (Fragment) |
| maker-3-augustus-gene-108.20-mRNA-1 | Japonica | NA | | Os03g0309400 | OsR498G0305798400.01.T01 | Pectinesterase |

| | | | | | | |
|---|---|---|---|---|---|---|
| maker-3-augustus-gene-108.22-mRNA-1 | Japonica | NA | | Os03g0310900 | | |
| maker-3-augustus-gene-108.9-mRNA-1 | Japonica | NA | | Os03g0310000 | OsR498G0305800500.01.T01 | GDSL-like Lipase/Acylhydrolase family protein, expressed |
| maker-3-augustus-gene-109.34-mRNA-1 | Japonica | NA | | | | NHL repeat-containing protein 2 |
| maker-3-augustus-gene-109.40-mRNA-1 | Japonica | NA | | Os03g0312600 | OsR498G0305809900.01.T06 | GPN-loop GTPase |
| maker-3-augustus-gene-109.43-mRNA-1 | Japonica | NA | | Os03g0313000 | OsR498G0305810300.01.T01 | NADH-ubiquinone oxidoreductase 13 kDa-B subunit |
| maker-3-augustus-gene-109.45-mRNA-1 | Japonica | NA | | Os03g0312300 | OsR498G0305808600.01.T01 | Cysteine-rich repeat secretory protein 55 |
| maker-3-augustus-gene-109.47-mRNA-1 | Japonica | NA | | Os03g0313100 | OsR498G0305810800.01.T01 | AP2-EREBP transcription factor |
| maker-3-augustus-gene-109.49-mRNA-1 | Japonica | NA | | | | Polynucleotide adenylyltransferase |
| maker-3-augustus-gene-11.0-mRNA-1 | Japonica | NA | | Os03g0123100 | OsR498G0305147500.01.T04 | UBC core domain-containing protein (Fragment) |
| maker-3-augustus-gene-11.12-mRNA-1 | Japonica | Indica | | Os03g0123500 | OsR498G0305149500.01.T01 | Transcription factor HD1 |
| maker-3-augustus-gene-11.17-mRNA-1 | Japonica | NA | | Os03g0123800 | OsR498G0305150500.01.T01 | MENTAL domain-containing protein |
| maker-3-augustus-gene-11.19-mRNA-1 | Japonica | NA | | Os03g0124000 | OsR498G0305151300.01.T01 | Associated with HOX family protein, expressed |
| maker-3-augustus-gene-11.21-mRNA-1 | Japonica | Japonica | | Os03g0122600 | OsR498G1019100900.01.T01 | K-box domain-containing protein (Fragment) |
| maker-3-augustus-gene-11.22-mRNA-1 | Japonica | Japonica | | Os03g0122600 | OsR498G1019100900.01.T03 | MADS box transcription factor 5 |
| maker-3-augustus-gene-11.29-mRNA-1 | Japonica | NA | | Os03g0123600 | OsR498G0305149800.01.T01 | Putative diphthine synthase-like |
| maker-3-augustus-gene-11.4-mRNA-1 | Japonica | NA | | Os03g0123200 | OsR498G0305147700.01.T01 | Phragmoplastin interacting protein 1 |
| maker-3-augustus-gene-11.8-mRNA-1 | Japonica | NA | | Os03g0123300 | OsR498G0305148100.01.T03 | Cell division cycle 20-like protein 1, cofactor of APC complex |
| maker-3-augustus-gene-110.0-mRNA-1 | Japonica | NA | | Os03g0313600 | OsR498G0305813300.01.T03 | Adenylosuccinate lyase |
| maker-3-augustus-gene-110.13-mRNA-1 | Japonica | NA | | Os03g0314700 | OsR498G0305816700.01.T01 | NifU-like protein 1 chloroplastic |
| maker-3-augustus-gene-110.16-mRNA-1 | Japonica | NA | | Os03g0313800 | | |
| maker-3-augustus-gene-110.18-mRNA-1 | Japonica | NA | | Os03g0314200 | OsR498G0305815000.01.T03 | Protein REDUCED WALL ACETYLATION 3 isoform A |
| maker-3-augustus-gene-110.25-mRNA-1 | Japonica | NA | | Os03g0314800 | OsR498G0305817600.01.T02 | Alkaline/neutral invertase |
| maker-3-augustus-gene-110.5-mRNA-1 | Japonica | Indica | | Os03g0314100 | OsR498G0305814700.01.T03 | RNA helicase |
| maker-3-augustus-gene-111.0-mRNA-1 | Japonica | NA | | Os03g0316200 | OsR498G0305823100.01.T01 | Hexosyltransferase |
| maker-3-augustus-gene-111.11-mRNA-1 | Japonica | NA | | Os03g0315800 | OsR498G0305822400.01.T01 | Tex protein-relatedtranscription accessory protein, putative |
| maker-3-augustus-gene-111.3-mRNA-1 | Japonica | NA | | Os03g0316900 | OsR498G0305824900.01.T01 | Non-intrinsic abc protein 9 |
| maker-3-augustus-gene-111.6-mRNA-1 | Japonica | NA | | Os03g0317000 | OsR498G0305825100.01.T04 | YTH domain-containing family protein 2 |
| maker-3-augustus-gene-111.9-mRNA-1 | Japonica | NA | | Os03g0315400 | OsR498G0305819800.01.T01 | Myb domain protein 78 |
| maker-3-augustus-gene-112.30-mRNA-1 | Japonica | NA | | | | Transposon protein, putative, Mutator sub-class, expressed |
| maker-3-augustus-gene-112.32-mRNA-1 | Japonica | NA | | Os03g0318500 | OsR498G0305829400.01.T05 | Glucose-6-phosphate 1-dehydrogenase |
| maker-3-augustus-gene-112.35-mRNA-1 | Japonica | NA | | Os03g0318600 | OsR498G0305829600.01.T04 | BZIP transcription factor |
| maker-3-augustus-gene-113.26-mRNA-1 | Japonica | Indica | | Os07g0687300 | OsR498G0305831100.01.T01 | 5\'-AMP-activated protein kinase beta-2 subunit protein isoform 1 |
| maker-3-augustus-gene-113.30-mRNA-1 | Japonica | NA | | | OsR498G0305831500.01.T01 | ESX-1 secretion-associated protein EspI-like |
| maker-3-augustus-gene-113.32-mRNA-1 | Japonica | NA | | Os03g0319300 | OsR498G0305832300.01.T01 | Calmodulin |
| maker-3-augustus-gene-113.34-mRNA-1 | Japonica | NA | | Os03g0320400 | OsR498G0305835500.01.T01 | |
| maker-3-augustus-gene-113.36-mRNA-1 | Japonica | NA | Kinase-LRR-TM | | OsR498G0305838900.01.T01 | Putative inactive receptor kinase |
| maker-3-augustus-gene-113.42-mRNA-1 | Japonica | NA | | Os03g0321000 | OsR498G0305839700.01.T01 | Membrane steroid-binding protein 2 |
| maker-3-augustus-gene-113.44-mRNA-1 | Japonica | NA | Kinase-TM | Os03g0319400 | OsR498G0305833000.01.T03 | CBL-interacting protein kinase |
| maker-3-augustus-gene-113.54-mRNA-1 | Japonica | NA | | Os03g0320000 | OsR498G0305835000.01.T01 | Aquaporin SIP |
| maker-3-augustus-gene-113.56-mRNA-1 | Japonica | NA | | | OsR498G0305835200.01.T01 | Non-reducing end alpha-L-arabinofuranosidase |
| maker-3-augustus-gene-114.0-mRNA-1 | Japonica | NA | | Os03g0321700 | OsR498G0305844700.01.T01 | WRKY DNA binding domain containing protein, expressed |
| maker-3-augustus-gene-114.14-mRNA-1 | Japonica | NA | | Os03g0322600 | OsR498G0305846700.01.T03 | HIT domain-containing protein |
| maker-3-augustus-gene-114.16-mRNA-1 | Japonica | NA | | Os03g0322800 | OsR498G0305847900.01.T01 | ELMO domain-containing protein A |
| maker-3-augustus-gene-114.2-mRNA-1 | Japonica | Japonica | | Os03g0321900 | OsR498G0305845100.01.T03 | tRNA-specific adenosine deaminase |
| maker-3-augustus-gene-114.20-mRNA-1 | Japonica | Indica | | Os04g0455900 | OsR498G0305842400.01.T03 | Serologically defined breast cancer antigen NY-BR-84 |
| maker-3-augustus-gene-114.26-mRNA-1 | Japonica | NA | | Os03g0321500 | OsR498G0305842800.01.T02 | DUF632 domain-containing protein |
| maker-3-augustus-gene-114.30-mRNA-1 | Japonica | Indica | | Os07g0686400 | OsR498G0305845600.01.T01 | Centromere protein-like |
| maker-3-augustus-gene-114.33-mRNA-1 | Japonica | Indica | | Os07g0686100 | OsR498G0305847200.01.T01 | ABSCISIC ACID-INSENSITIVE 5-like protein 2 isoform D |
| maker-3-augustus-gene-114.7-mRNA-1 | Japonica | NA | | Os03g0322500 | OsR498G0305846500.01.T01 | 14 kDa zinc-binding protein, putative, expressed |
| maker-3-augustus-gene-117.30-mRNA-1 | Japonica | NA | | | OsR498G0305067900.01.T05 | tRNA/rRNA methyltransferase (SpoU) family protein |
| maker-3-augustus-gene-117.37-mRNA-1 | Japonica | NA | | Os03g0100800 | OsR498G0305068300.01.T03 | Plasma membrane ATPase |
| maker-3-augustus-gene-118.36-mRNA-1 | Japonica | Indica | | Os08g0155400 | OsR498G0305075700.01.T01 | Solute carrier family 15 (Peptide/histidine transporter), member 3/4 |
| maker-3-augustus-gene-118.38-mRNA-1 | Japonica | NA | | Os03g0103800 | OsR498G0305081700.01.T01 | |
| maker-3-augustus-gene-118.40-mRNA-1 | Japonica | NA | | | | PPM-type phosphatase domain-containing protein |
| maker-3-augustus-gene-119.0-mRNA-1 | Japonica | NA | | Os10g0557600 | OsR498G1019180300.01.T04 | GATA transcription factor |
| maker-3-augustus-gene-12.0-mRNA-1 | Japonica | NA | | Os03g0124100 | OsR498G0305151500.01.T01 | Beta-1,3-N-acetylglucosaminyltransferase lunatic fringe |
| maker-3-augustus-gene-12.14-mRNA-1 | Japonica | NA | | | | Sphingoid long-chain bases kinase |
| maker-3-augustus-gene-12.18-mRNA-1 | Japonica | Indica | Kinase-TM | Os10g0533150 | OsR498G0305155100.01.T01 | probable serine/threonine-protein kinase At1g01540 |
| maker-3-augustus-gene-12.23-mRNA-1 | Japonica | NA | | Os03g0125800 | OsR498G0305156500.01.T01 | Metal transporter CNNM |
| maker-3-augustus-gene-12.3-mRNA-1 | Japonica | NA | | Os03g0124500 | OsR498G0305152300.01.T01 | PORR domain-containing protein |
| maker-3-augustus-gene-12.31-mRNA-1 | Japonica | NA | | Os03g0125900 | OsR498G0305156900.01.T01 | |

| | | | | | | |
|---|---|---|---|---|---|---|
| maker-3-augustus-gene-12.33-mRNA-1 | Japonica | NA | | Os03g0125000 | OsR498G0305153600.01.T01 | 50S ribosomal protein L5, chloroplastic |
| maker-3-augustus-gene-12.35-mRNA-1 | Japonica | Japonica | | Os03g0125100 | OsR498G0305153800.01.T01 | Beta-carotene 3-hydroxylase |
| maker-3-augustus-gene-12.39-mRNA-1 | Japonica | NA | | Os03g0125700 | OsR498G0305155800.01.T02 | Cleavage and polyadenylation specificity factor subunit 1 |
| maker-3-augustus-gene-12.44-mRNA-1 | Japonica | NA | | Os03g0126000 | OsR498G0305157200.01.T02 | Anthranilate phosphoribosyltransferase |
| maker-3-augustus-gene-12.49-mRNA-1 | Japonica | Indica | | Os03g0126100 | OsR498G0305157400.01.T03 | C-terminal binding protein AN |
| maker-3-augustus-gene-12.5-mRNA-1 | Japonica | NA | | Os03g0124900 | OsR498G0305153300.01.T01 | Polygalacturonase |
| maker-3-augustus-gene-12.9-mRNA-1 | Japonica | NA | | Os03g0125300 | OsR498G0305154500.01.T01 | G patch domain-containing protein 4 isoform X2 |
| maker-3-augustus-gene-120.4-mRNA-1 | Japonica | NA | | Os10g0558200 | OsR498G1019184400.01.T01 | S-norcoclaurine synthase 1-like isoform X2 |
| maker-3-augustus-gene-121.0-mRNA-1 | Japonica | NA | | | OsR498G1019194100.01.T01 | Sphingomyelin phosphodiesterase like |
| maker-3-augustus-gene-121.22-mRNA-1 | Japonica | NA | | Os01g0200000 | OsR498G0100350800.01.T02 | Autophagy-related protein 3 |
| maker-3-augustus-gene-125.11-mRNA-1 | Japonica | NA | | Os10g0569000 | OsR498G1019225700.01.T02 | |
| maker-3-augustus-gene-125.25-mRNA-1 | Japonica | NA | | | OsR498G1019222000.01.T01 | Transposon protein, putative, Pong sub-class |
| maker-3-augustus-gene-125.27-mRNA-1 | Japonica | NA | | Os10g0567800 | OsR498G1019222200.01.T02 | Dynamin-related protein 1E |
| maker-3-augustus-gene-125.36-mRNA-1 | Japonica | NA | | | OsR498G1019223000.01.T05 | DUF295 domain-containing protein |
| maker-3-augustus-gene-125.37-mRNA-1 | Japonica | NA | | Os10g0568300 | OsR498G1019223400.01.T01 | VAN3-binding protein-like |
| maker-3-augustus-gene-125.41-mRNA-1 | Japonica | NA | LRR-NBS-TM | Os10g0568600 | OsR498G1019224400.01.T01 | NB-ARC domain-containing protein (Fragment) |
| maker-3-augustus-gene-125.43-mRNA-1 | Japonica | NA | | Os10g0569100 | OsR498G1019226000.01.T01 | PIG-P domain-containing protein |
| maker-3-augustus-gene-125.46-mRNA-1 | Japonica | NA | | Os10g0569200 | OsR498G1019226200.01.T01 | Eukaryotic translation initiation factor 3 subunit B |
| maker-3-augustus-gene-126.39-mRNA-1 | Japonica | NA | | Os10g0569300 | OsR498G1019226300.01.T02 | Methyltransferase |
| maker-3-augustus-gene-126.42-mRNA-1 | Japonica | NA | | Os10g0569500 | OsR498G1019227700.01.T03 | |
| maker-3-augustus-gene-126.46-mRNA-1 | Japonica | NA | | Os10g0571100 | OsR498G1019231700.01.T02 | NADH dehydrogenase [ubiquinone] 1 alpha subcomplex assembly factor 3 |
| maker-3-augustus-gene-126.53-mRNA-1 | Japonica | NA | | Os10g0571200 | OsR498G1019231900.01.T01 | Pyruvate kinase |
| maker-3-augustus-gene-126.57-mRNA-1 | Japonica | NA | Kinase-TM | Os10g0571300 | OsR498G1019232500.01.T01 | probable serine/threonine-protein kinase At4g35230 |
| maker-3-augustus-gene-126.61-mRNA-1 | Japonica | NA | | | OsR498G1019231600.01.T03 | |
| maker-3-augustus-gene-127.33-mRNA-1 | Japonica | NA | | Os10g0572000 | OsR498G1019234700.01.T01 | G domain-containing protein (Fragment) |
| maker-3-augustus-gene-127.37-mRNA-1 | Japonica | NA | CC-LRR | Os10g0572300 | OsR498G1019235500.01.T03 | Plant intracellular Ras-group-related LRR protein 5 |
| maker-3-augustus-gene-127.39-mRNA-1 | Japonica | NA | | Os10g0573000 | OsR498G1019238100.01.T01 | PfkB-like carbohydrate kinase family protein isoform 1 |
| maker-3-augustus-gene-127.43-mRNA-1 | Japonica | NA | | Os10g0573100 | OsR498G1019238300.01.T05 | AMMECR1 domain-containing protein |
| maker-3-augustus-gene-127.48-mRNA-1 | Japonica | NA | | Os10g0572100 | OsR498G1019235000.01.T01 | Aluminum-activated malate transporter like |
| maker-3-augustus-gene-127.50-mRNA-1 | Japonica | NA | | | OsR498G1019236000.01.T03 | RING/FYVE/PHD zinc finger superfamily protein |
| maker-3-augustus-gene-127.60-mRNA-1 | Japonica | NA | | Os12g0264500 | OsR498G1221246300.01.T05 | Enoyl-CoA hydratase/isomerase, HIBYL-CoA-H type |
| maker-3-augustus-gene-127.72-mRNA-1 | Japonica | NA | | | OsR498G1019237200.01.T02 | AT-hook motif nuclear-localized protein (Fragment) |
| maker-3-augustus-gene-128.0-mRNA-1 | Japonica | NA | | Os10g0573700 | OsR498G1019239600.01.T04 | Mitochondrial substrate/solute carrier |
| maker-3-augustus-gene-128.2-mRNA-1 | Japonica | NA | | Os10g0574500 | OsR498G1019241600.01.T02 | Replication factor C subunit |
| maker-3-augustus-gene-128.6-mRNA-1 | Japonica | NA | | Os10g0574700 | OsR498G1019242000.01.T01 | PLATZ transcription factor |
| maker-3-augustus-gene-128.9-mRNA-1 | Japonica | NA | | Os10g0574800 | OsR498G1019243200.01.T01 | ADP-ribosylation factor GTPase-activating protein AGD8 |
| maker-3-augustus-gene-129.34-mRNA-1 | Japonica | NA | | Os10g0575700 | OsR498G1019246300.01.T03 | plastoglobulin-1, chloroplastic |
| maker-3-augustus-gene-129.37-mRNA-1 | Japonica | NA | | | OsR498G1019246900.01.T01 | |
| maker-3-augustus-gene-129.39-mRNA-1 | Japonica | NA | | | OsR498G1019247300.01.T02 | NADH dehydrogenase ubiquinone 1 beta subcomplex subunit 10-B |
| maker-3-augustus-gene-129.41-mRNA-1 | Japonica | NA | | Os10g0575200 | OsR498G1019244400.01.T01 | DnaJ homolog subfamily C member 13 |
| maker-3-augustus-gene-129.45-mRNA-1 | Japonica | NA | | Os10g0575600 | OsR498G1019245800.01.T02 | Homeobox-leucine zipper protein ROC3 |
| maker-3-augustus-gene-129.47-mRNA-1 | Japonica | NA | | Os10g0575800 | OsR498G1019246600.01.T02 | ARM repeat superfamily protein isoform 2 |
| maker-3-augustus-gene-129.52-mRNA-1 | Japonica | NA | | Os10g0576600 | OsR498G1019249000.01.T01 | Tetratricopeptide repeat-containing domain |
| maker-3-augustus-gene-13.0-mRNA-1 | Japonica | Japonica | | Os03g0126700 | OsR498G0305158100.01.T02 | Heavy metal-associated domain containing protein, expressed |
| maker-3-augustus-gene-13.1-mRNA-1 | Japonica | Japonica | | Os03g0126700 | OsR498G0305158100.01.T02 | Heavy metal-associated domain containing protein, expressed |
| maker-3-augustus-gene-13.11-mRNA-1 | Japonica | NA | | | OsR498G0305161700.01.T02 | Hydroxyproline-rich glycoprotein family protein |
| maker-3-augustus-gene-13.13-mRNA-1 | Japonica | Japonica | | Os03g0126300 | OsR498G0305157800.01.T02 | C-terminal binding protein AN |
| maker-3-augustus-gene-13.17-mRNA-1 | Japonica | Indica | Kinase-TM | Os11g0134300 | OsR498G0305158600.01.T04 | Non-specific serine/threonine protein kinase |
| maker-3-augustus-gene-13.23-mRNA-1 | Japonica | Indica | | Os03g0127000 | OsR498G0305159200.01.T05 | Cell growth defect factor 2 |
| maker-3-augustus-gene-13.26-mRNA-1 | Japonica | Japonica | | Os03g0127200 | OsR498G1019087100.01.T01 | NAC domain class transcription factor |
| maker-3-augustus-gene-13.28-mRNA-1 | Japonica | NA | | Os03g0127600 | OsR498G0305161400.01.T02 | FHA-transcription factor 1 |
| maker-3-augustus-gene-13.34-mRNA-1 | Japonica | NA | | | | Potassium efflux antiporter |
| maker-3-augustus-gene-13.40-mRNA-1 | Japonica | NA | | | OsR498G0305163200.01.T04 | Callose synthase 3 |
| maker-3-augustus-gene-13.6-mRNA-1 | Japonica | Japonica | | Os03g0127500 | OsR498G0305161100.01.T01 | BZIP-type transcription factor (Fragment) |
| maker-3-augustus-gene-13.9-mRNA-1 | Japonica | NA | Kinase-LRR-TM | Os03g0127700 | OsR498G0305161700.01.T01 | Leucine-rich repeat transmembrane protein kinase, putative, expressed |
| maker-3-augustus-gene-130.32-mRNA-1 | Japonica | NA | | Os10g0576900 | OsR498G1019249900.01.T01 | Cinnamoyl-CoA reductase-like protein SNL6 |
| maker-3-augustus-gene-130.34-mRNA-1 | Japonica | NA | | | | Lysine-specific demethylase REF6 |
| maker-3-augustus-gene-130.37-mRNA-1 | Japonica | NA | | Os10g0577700 | OsR498G1019253500.01.T01 | CS domain-containing protein (Fragment) |
| maker-3-augustus-gene-130.40-mRNA-1 | Japonica | NA | | Os10g0577900 | OsR498G1019253900.01.T07 | Glycerol-3-phosphate acyltransferase, chloroplastic |
| maker-3-augustus-gene-130.46-mRNA-1 | Japonica | NA | | Os10g0578000 | OsR498G1019254100.01.T01 | Hepatocyte growth factor-regulated tyrosine kinase substrate-like protein |
| maker-3-augustus-gene-130.48-mRNA-1 | Japonica | NA | | Os10g0577000 | OsR498G1019250200.01.T02 | RAP annotation release 2, NERD domain protein |

| | | | | | | |
|---|---|---|---|---|---|---|
| maker-3-augustus-gene-130.53-mRNA-1 | Japonica | NA | | Os10g0577400 | OsR498G1019251400.01.T01 | Chalcone-flavanone isomerase family protein, putative isoform 1 |
| maker-3-augustus-gene-130.55-mRNA-1 | Japonica | NA | | Os10g0577500 | OsR498G1019251600.01.T01 | Xyloglucan endotransglucosylase/hydrolase |
| maker-3-augustus-gene-130.58-mRNA-1 | Japonica | NA | | Os10g0577800 | OsR498G1019253800.01.T01 | PARP catalytic domain-containing protein |
| maker-3-augustus-gene-131.42-mRNA-1 | Japonica | NA | | Os10g0579400 | OsR498G1019256300.01.T02 | WRKY DNA binding domain containing protein, expressed |
| maker-3-augustus-gene-131.44-mRNA-1 | Japonica | NA | | Os10g0579600 | OsR498G1019256700.01.T01 | POT family protein, expressed |
| maker-3-augustus-gene-131.49-mRNA-1 | Japonica | NA | | Os10g0579800 | OsR498G1019258300.01.T01 | POT family protein, expressed |
| maker-3-augustus-gene-131.54-mRNA-1 | Japonica | NA | | Os10g0580200 | OsR498G1019259300.01.T05 | ADP-ribosylation factor B1B |
| maker-3-augustus-gene-131.60-mRNA-1 | Japonica | NA | | Os10g0580400 | OsR498G1019260300.01.T01 | Urea-proton symporter like |
| maker-3-augustus-gene-131.62-mRNA-1 | Japonica | NA | | Os10g0578300 | OsR498G0102411000.01.T05 | |
| maker-3-augustus-gene-131.64-mRNA-1 | Japonica | NA | | | | LrgB-like family protein, expressed |
| maker-3-augustus-gene-131.70-mRNA-1 | Japonica | NA | | Os10g0578900 | OsR498G1019255400.01.T01 | Phosphorylcholine transferase/cholinephosphate cytidylyltransferase |
| maker-3-augustus-gene-131.72-mRNA-1 | Japonica | NA | | Os10g0578950 | OsR498G1019255600.01.T01 | 4-coumarate--CoA ligase-like protein 6 |
| maker-3-augustus-gene-131.75-mRNA-1 | Japonica | NA | | Os10g0579100 | OsR498G1019255800.01.T01 | Calcium-dependent channel |
| maker-3-augustus-gene-131.80-mRNA-1 | Japonica | NA | | Os10g0579200 | OsR498G1019256000.01.T01 | D-xylose-proton symporter-like 2 |
| maker-3-augustus-gene-131.85-mRNA-1 | Japonica | NA | | Os10g0579300 | OsR498G1019256200.01.T02 | NADH dehydrogenase [ubiquinone] 1 alpha subcomplex subunit 12 |
| maker-3-augustus-gene-131.88-mRNA-1 | Japonica | NA | | Os10g0579500 | OsR498G1019256600.01.T01 | Ribosomal protein L35 containing protein, expressed |
| maker-3-augustus-gene-131.90-mRNA-1 | Japonica | NA | Kinase-TM | | | Cyclin-dependent kinase E |
| maker-3-augustus-gene-132.39-mRNA-1 | Japonica | NA | | Os10g0580700 | OsR498G1019262100.01.T01 | tankyrase |
| maker-3-augustus-gene-132.41-mRNA-1 | Japonica | NA | | Os10g0580800 | OsR498G1019262500.01.T01 | ATP-dependent Clp protease proteolytic subunit |
| maker-3-augustus-gene-132.50-mRNA-1 | Japonica | NA | | Os10g0580500 | OsR498G1019260600.01.T03 | Essential protein Yae1 N-terminal |
| maker-3-augustus-gene-132.52-mRNA-1 | Japonica | NA | | Os07g0633000 | OsR498G1019262000.01.T01 | |
| maker-3-augustus-gene-132.54-mRNA-1 | Japonica | NA | | Os10g0580900 | OsR498G1019262800.01.T01 | Pseudouridine synthase, RsuA/RluB/C/D/E/F |
| maker-3-augustus-gene-132.58-mRNA-1 | Japonica | NA | | Os10g0581000 | OsR498G1019263000.01.T04 | |
| maker-3-augustus-gene-132.60-mRNA-1 | Japonica | NA | | Os04g0218600 | OsR498G1019263000.01.T06 | Expressed protein (Fragment) |
| maker-3-augustus-gene-132.64-mRNA-1 | Japonica | NA | | | OsR498G1019263400.01.T03 | Methyltransferase |
| maker-3-augustus-gene-132.67-mRNA-1 | Japonica | NA | | | OsR498G1019264200.01.T05 | Glycosyltransferase-like KOBITO 1 |
| maker-3-augustus-gene-133.0-mRNA-1 | Japonica | NA | | Os10g0527400 | OsR498G1019075300.01.T01 | Glutathione S-transferase, N-terminal domain containing protein, expressed |
| maker-3-augustus-gene-133.2-mRNA-1 | Japonica | NA | | Os10g0527800 | OsR498G1019075900.01.T01 | Glutathione S-transferase, N-terminal domain containing protein, expressed |
| maker-3-augustus-gene-134.167-mRNA-1 | Japonica | NA | | Os10g0530400 | OsR498G1019083000.01.T01 | Glutathione-S-transferase Cla47 |
| maker-3-augustus-gene-134.68-mRNA-1 | Japonica | NA | | Os10g0528200 | OsR498G1019078900.01.T01 | Glutathione S-transferase, N-terminal domain containing protein, expressed |
| maker-3-augustus-gene-134.69-mRNA-1 | Japonica | NA | | Os10g0528300 | OsR498G1019079100.01.T01 | Glutathione S-transferase GSTU6 |
| maker-3-augustus-gene-134.70-mRNA-1 | Japonica | NA | | Os10g0529300 | OsR498G1019080300.01.T01 | Glutathione S-transferase, N-terminal domain containing protein, expressed |
| maker-3-augustus-gene-134.71-mRNA-1 | Japonica | NA | | Os10g0529700 | OsR498G1019080500.01.T01 | Glutathione S-transferase U16 |
| maker-3-augustus-gene-134.72-mRNA-1 | Japonica | NA | | Os10g0529500 | OsR498G1019080700.01.T01 | Glutathione S-transferase 1 |
| maker-3-augustus-gene-134.73-mRNA-1 | Japonica | NA | | Os10g0529700 | OsR498G1019080900.01.T01 | Glutathione S-transferase U16 |
| maker-3-augustus-gene-134.74-mRNA-1 | Japonica | NA | | Os10g0529800 | OsR498G1019081700.01.T01 | Glutathione S-transferase |
| maker-3-augustus-gene-134.75-mRNA-1 | Japonica | NA | | Os10g0529900 | OsR498G1019081700.01.T01 | Glutathione S-transferase (Fragment) |
| maker-3-augustus-gene-134.76-mRNA-1 | Japonica | NA | | Os10g0530200 | OsR498G1019082500.01.T01 | Glutathione-S-transferase 2 |
| maker-3-augustus-gene-135.28-mRNA-1 | Japonica | NA | | Os10g0532100 | OsR498G1019087300.01.T01 | SWIRM domain-containing protein (Fragment) |
| maker-3-augustus-gene-135.31-mRNA-1 | Japonica | NA | | Os10g0533000 | OsR498G1019091500.01.T04 | C-terminal binding protein AN |
| maker-3-augustus-gene-135.36-mRNA-1 | Japonica | NA | | Os10g0533500 | OsR498G1019092700.01.T01 | Aldehyde oxygenase (deformylating) |
| maker-3-augustus-gene-135.40-mRNA-1 | Japonica | NA | Kinase | Os10g0533600 | OsR498G1019094300.01.T01 | Mitogen-activated protein kinase |
| maker-3-augustus-gene-135.42-mRNA-1 | Japonica | NA | | Os10g0532200 | OsR498G1019087800.01.T01 | Lysophospholipase BODYGUARD 3 like |
| maker-3-augustus-gene-135.46-mRNA-1 | Japonica | Japonica | Kinase-TM | Os10g0533150 | OsR498G0305155100.01.T01 | Protein kinase domain containing protein, expressed |
| maker-3-augustus-gene-136.31-mRNA-1 | Japonica | NA | | Os10g0533900 | OsR498G1019094700.01.T02 | DUF862 domain-containing protein (Fragment) |
| maker-3-augustus-gene-136.34-mRNA-1 | Japonica | NA | | | OsR498G1019097500.01.T01 | |
| maker-3-augustus-gene-136.36-mRNA-1 | Japonica | NA | | Os10g0535800 | OsR498G1019100000.01.T01 | |
| maker-3-augustus-gene-136.41-mRNA-1 | Japonica | NA | | Os01g0274500 | OsR498G1019099800.01.T01 | Expansin |
| maker-3-augustus-gene-136.43-mRNA-1 | Japonica | NA | | Os10g0534100 | OsR498G1019095000.01.T02 | |
| maker-3-augustus-gene-136.46-mRNA-1 | Japonica | NA | | Os10g0534400 | OsR498G1019095400.01.T01 | Putative membrane-associated kinase regulator 6 |
| maker-3-augustus-gene-136.48-mRNA-1 | Japonica | NA | | Os10g0534700 | OsR498G1019096200.01.T01 | Beta-1,3-N-acetylglucosaminyltransferase lunatic fringe |
| maker-3-augustus-gene-136.50-mRNA-1 | Japonica | NA | | Os10g0534900 | OsR498G1019096600.01.T01 | Associated with HOX family protein, expressed |
| maker-3-augustus-gene-136.54-mRNA-1 | Japonica | NA | | Os10g0535600 | OsR498G1019100500.01.T01 | MENTAL domain-containing protein |
| maker-3-augustus-gene-136.56-mRNA-1 | Japonica | NA | | Os10g0536000 | OsR498G1019099500.01.T01 | SUMO-conjugating protein SCE1 |
| maker-3-augustus-gene-137.32-mRNA-1 | Japonica | NA | | Os10g0536100 | OsR498G1019100900.01.T01 | MADS-box transcription factor |
| maker-3-augustus-gene-137.35-mRNA-1 | Japonica | Indica | | Os03g0122300 | OsR498G1019102500.01.T01 | Flavanone 3-dioxygenase 2 |
| maker-3-augustus-gene-137.40-mRNA-1 | Japonica | NA | | Os10g0536500 | OsR498G1019102900.01.T01 | Thylakoid membrane phosphoprotein 14 kDa |
| maker-3-augustus-gene-137.44-mRNA-1 | Japonica | NA | | Os10g0536600 | OsR498G1019103100.01.T01 | Peroxidase |
| maker-3-augustus-gene-137.46-mRNA-1 | Japonica | NA | | Os10g0536700 | OsR498G1019103900.01.T01 | Peroxidase |
| maker-3-augustus-gene-137.48-mRNA-1 | Japonica | Indica | | Os03g0120400 | OsR498G1019105300.01.T01 | Heavy metal-associated isoprenylated plant protein 30 |
| maker-3-augustus-gene-137.50-mRNA-1 | Japonica | NA | | Os10g0537500 | OsR498G1019105700.01.T01 | Tobamovirus multiplication protein like |

| | | | | | | |
|---|---|---|---|---|---|---|
| maker-3-augustus-gene-137.55-mRNA-1 | Japonica | NA | | Os10g0537300 | OsR498G1019105200.01.T01 | |
| maker-3-augustus-gene-137.60-mRNA-1 | Japonica | NA | | Os10g0537600 | OsR498G1019106000.01.T03 | Putative P-loop containing nucleoside triphosphate hydrolase |
| maker-3-augustus-gene-138.0-mRNA-1 | Japonica | NA | Kinase-TM | Os10g0539600 | OsR498G1019112100.01.T04 | Calcium-dependent protein kinase isoform 1 |
| maker-3-augustus-gene-138.4-mRNA-1 | Japonica | NA | | Os10g0539900 | OsR498G1019114700.01.T01 | Monosaccharide-sensing protein 2 |
| maker-3-augustus-gene-139.34-mRNA-1 | Japonica | NA | | | OsR498G1019117300.01.T03 | |
| maker-3-augustus-gene-139.40-mRNA-1 | Japonica | NA | | Os10g0541200 | OsR498G1019118900.01.T01 | PPM-type phosphatase domain-containing protein |
| maker-3-augustus-gene-139.43-mRNA-1 | Japonica | NA | | Os10g0541000 | OsR498G1019118600.01.T03 | |
| maker-3-augustus-gene-139.50-mRNA-1 | Japonica | NA | | | | HTH myb-type domain-containing protein |
| maker-3-augustus-gene-139.52-mRNA-1 | Japonica | NA | | | OsR498G1019119800.01.T01 | Carboxypeptidase |
| maker-3-augustus-gene-14.0-mRNA-1 | Japonica | NA | Kinase-TM | Os03g0128700 | OsR498G0305164700.01.T02 | Calcium-dependent protein kinase |
| maker-3-augustus-gene-14.15-mRNA-1 | Japonica | NA | | Os03g0128300 | OsR498G0305163600.01.T01 | |
| maker-3-augustus-gene-14.17-mRNA-1 | Japonica | NA | | Os03g0128500 | OsR498G0305164000.01.T02 | DNA polymerase delta small subunit like |
| maker-3-augustus-gene-14.22-mRNA-1 | Japonica | NA | | Os03g0128800 | OsR498G0305165000.01.T02 | Putative acetyl-ornithine/Acetyl-lysine deacetylase |
| maker-3-augustus-gene-14.24-mRNA-1 | Japonica | NA | | Os03g0129100 | OsR498G0305166000.01.T01 | |
| maker-3-augustus-gene-14.29-mRNA-1 | Japonica | Indica | | Os03g0129200 | OsR498G0305166600.01.T03 | Exosome complex exonuclease RRP44 A |
| maker-3-augustus-gene-14.34-mRNA-1 | Japonica | NA | | Os03g0129350 | OsR498G0305167400.01.T01 | Glycosyltransferase |
| maker-3-augustus-gene-14.37-mRNA-1 | Japonica | Japonica | | Os03g0129800 | OsR498G0305167800.01.T01 | HTH myb-type domain-containing protein |
| maker-3-augustus-gene-14.6-mRNA-1 | Japonica | NA | | Os03g0129000 | OsR498G0305165700.01.T04 | Phosphatidylinositol-glycan biosynthesis class F protein |
| maker-3-augustus-gene-140.0-mRNA-1 | Japonica | NA | | Os10g0542100 | OsR498G1019122100.01.T01 | EC protein III |
| maker-3-augustus-gene-140.10-mRNA-1 | Japonica | NA | | Os10g0542900 | OsR498G1019125300.01.T01 | Acidic endochitinase Q |
| maker-3-augustus-gene-140.15-mRNA-1 | Japonica | NA | | Os10g0543500 | OsR498G1019125700.01.T01 | Strictosidine synthase |
| maker-3-augustus-gene-140.18-mRNA-1 | Japonica | NA | | Os10g0544500 | OsR498G1019128500.01.T01 | DDB1-and CUL4-associated factor |
| maker-3-augustus-gene-140.2-mRNA-1 | Japonica | NA | | Os10g0542400 | OsR498G1019123300.01.T01 | |
| maker-3-augustus-gene-140.21-mRNA-1 | Japonica | NA | | Os10g0542200 | OsR498G1019122600.01.T02 | Ubiquitin family protein, expressed |
| maker-3-augustus-gene-140.25-mRNA-1 | Japonica | NA | | Os10g0561800 | OsR498G1019126000.01.T01 | Homeobox-leucine zipper protein ATHB-4 |
| maker-3-augustus-gene-140.27-mRNA-1 | Japonica | NA | | Os10g0543800 | OsR498G1019126800.01.T01 | Glutathione transferase11 |
| maker-3-augustus-gene-140.29-mRNA-1 | Japonica | NA | | Os10g0544200 | OsR498G1019128000.01.T01 | Transcription factor bHLH93 like |
| maker-3-augustus-gene-140.5-mRNA-1 | Japonica | Indica | Kinase-TM | Os03g0132800 | OsR498G1019125100.01.T01 | Serine/threonine-protein kinase BSK1-2 |
| maker-3-augustus-gene-141.40-mRNA-1 | Japonica | NA | | Os10g0544600 | OsR498G1019131100.01.T02 | NEP1-interacting protein 1 isoform A |
| maker-3-augustus-gene-141.43-mRNA-1 | Japonica | NA | | Os10g0544900 | OsR498G1019131700.01.T01 | Protein-serine/threonine phosphatase |
| maker-3-augustus-gene-141.46-mRNA-1 | Japonica | NA | | Os10g0545100 | OsR498G1019132300.01.T01 | Vacuolar acid trehalase |
| maker-3-augustus-gene-141.51-mRNA-1 | Japonica | NA | | Os10g0545200 | OsR498G1019133100.01.T01 | Aldehyde oxygenase (deformylating) |
| maker-3-augustus-gene-141.54-mRNA-1 | Japonica | NA | | Os10g0545500 | OsR498G1019133700.01.T01 | Xyloglucan endotransglucosylase/hydrolase |
| maker-3-augustus-gene-141.57-mRNA-1 | Japonica | NA | | Os10g0545700 | OsR498G1019134100.01.T01 | Rhodanese domain-containing protein (Fragment) |
| maker-3-augustus-gene-141.60-mRNA-1 | Japonica | NA | | Os10g0546100 | OsR498G1019134900.01.T01 | Pistil-specific extensin-like protein |
| maker-3-augustus-gene-141.62-mRNA-1 | Japonica | NA | | | | UDP-Glycosyltransferase superfamily protein |
| maker-3-augustus-gene-141.68-mRNA-1 | Japonica | NA | | Os10g0546300 | OsR498G1019135500.01.T03 | Syndetin like |
| maker-3-augustus-gene-141.72-mRNA-1 | Japonica | NA | | Os10g0545000 | OsR498G1019132200.01.T01 | Magnesium transporter |
| maker-3-augustus-gene-141.76-mRNA-1 | Japonica | NA | | Os10g0545300 | OsR498G1019133400.01.T01 | Coiled-coil domain-containing protein 90B |
| maker-3-augustus-gene-141.78-mRNA-1 | Japonica | NA | | | OsR498G1019134000.01.T01 | E3 ubiquitin-protein ligase RFWD3 |
| maker-3-augustus-gene-142.0-mRNA-1 | Japonica | NA | | Os10g0546600 | OsR498G1019136700.01.T06 | Carotene epsilon-ring hydroxylase |
| maker-3-augustus-gene-142.12-mRNA-1 | Japonica | NA | | | | Oxidoreductase, short chain dehydrogenase/reductase family protein, expre |
| maker-3-augustus-gene-142.15-mRNA-1 | Japonica | NA | | Os10g0548000 | OsR498G1019139800.01.T03 | Oxidoreductase, short chain dehydrogenase/reductase family protein, expre |
| maker-3-augustus-gene-142.18-mRNA-1 | Japonica | NA | | Os10g0548000 | OsR498G1019140000.01.T05 | Short-chain dehydrogenase TIC 32 chloroplastic |
| maker-3-augustus-gene-142.23-mRNA-1 | Japonica | NA | | | | Putative UDP-N-acetylglucosamine--peptide N-acetylglucosaminyltransferas |
| maker-3-augustus-gene-142.4-mRNA-1 | Japonica | NA | | Os10g0547500 | OsR498G1019138700.01.T02 | Silicon efflux transporter |
| maker-3-augustus-gene-142.6-mRNA-1 | Japonica | NA | | Os10g0548600 | OsR498G1019140700.01.T01 | Beta-expansin |
| maker-3-augustus-gene-142.8-mRNA-1 | Japonica | NA | | Os10g0547000 | OsR498G1019137800.01.T01 | C2 NT-type domain-containing protein |
| maker-3-augustus-gene-143.39-mRNA-1 | Japonica | NA | | Os05g0558400 | OsR498G1019142700.01.T04 | Branched-chain-amino-acid aminotransferase |
| maker-3-augustus-gene-143.40-mRNA-1 | Japonica | NA | | Os05g0558400 | OsR498G1019142700.01.T02 | Branched-chain-amino-acid aminotransferase |
| maker-3-augustus-gene-143.56-mRNA-1 | Japonica | NA | | Os10g0549850 | OsR498G1019143900.01.T01 | |
| maker-3-augustus-gene-143.57-mRNA-1 | Japonica | NA | | Os10g0549850 | | |
| maker-3-augustus-gene-143.72-mRNA-1 | Japonica | NA | CC-Kinase-TM | Os10g0548700 | OsR498G1019141000.01.T03 | E3 ubiquitin ligase |
| maker-3-augustus-gene-143.78-mRNA-1 | Japonica | NA | | Os10g0548850 | OsR498G1019141800.01.T01 | E3 ubiquitin ligase |
| maker-3-augustus-gene-143.82-mRNA-1 | Japonica | NA | | Os10g0548900 | OsR498G1019142000.01.T03 | UDP-N-acetylmuramoyl-L-alanyl-D-glutamate--2,6-diaminopimelate ligase M |
| maker-3-augustus-gene-143.85-mRNA-1 | Japonica | NA | | Os10g0549100 | OsR498G1019142200.01.T01 | Vesicle-associated protein 2-1 |
| maker-3-augustus-gene-143.86-mRNA-1 | Japonica | NA | | | | Vesicle-associated protein 2-1 |
| maker-3-augustus-gene-144.46-mRNA-1 | Japonica | Indica | | Os03g0105800 | OsR498G1019147700.01.T04 | PPC domain-containing protein |
| maker-3-augustus-gene-144.50-mRNA-1 | Japonica | NA | | Os10g0551600 | OsR498G1019151700.01.T03 | NADH dehydrogenase [ubiquinone] 1 beta subcomplex subunit 8 mitochond |
| maker-3-augustus-gene-144.54-mRNA-1 | Japonica | NA | | Os10g0550300 | OsR498G1019146000.01.T01 | Plant specific Rop nucleotide exchanger, PRONE |
| maker-3-augustus-gene-144.56-mRNA-1 | Japonica | NA | | | OsR498G1019147200.01.T02 | Pectin acetylesterase |

| | | | | | | |
|---|---|---|---|---|---|---|
| maker-3-augustus-gene-144.60-mRNA-1 | Japonica | NA | | | OsR498G1222155600.01.T01 | UDP-glucose-fructose-phosphate glucosyltransferase |
| maker-3-augustus-gene-145.40-mRNA-1 | Japonica | NA | | Os10g0552400 | OsR498G1019155100.01.T01 | E3 ubiquitin ligase |
| maker-3-augustus-gene-145.43-mRNA-1 | Japonica | NA | | Os10g0553900 | OsR498G1019160100.01.T02 | |
| maker-3-augustus-gene-145.45-mRNA-1 | Japonica | NA | | | OsR498G1019161700.01.T03 | Oxysterol-binding protein-related protein 2A |
| maker-3-augustus-gene-145.52-mRNA-1 | Japonica | NA | | Os10g0552900 | OsR498G1019158000.01.T02 | 3-hydroxyisobutyryl-CoA hydrolase, mitochondrial |
| maker-3-augustus-gene-145.57-mRNA-1 | Japonica | NA | | Os10g0553300 | OsR498G1019158200.01.T01 | Trehalose 6-phosphate phosphatase |
| maker-3-augustus-gene-145.60-mRNA-1 | Japonica | NA | | Os10g0553800 | OsR498G1019159800.01.T05 | Flavin-containing monooxygenase |
| maker-3-augustus-gene-145.64-mRNA-1 | Japonica | NA | | Os10g0554200 | OsR498G1019162400.01.T01 | Nitrate/chlorate transporter, putative, expressed |
| maker-3-augustus-gene-146.0-mRNA-1 | Japonica | NA | | Os10g0555100 | OsR498G1019166100.01.T01 | Glucuronosyltransferase |
| maker-3-augustus-gene-146.10-mRNA-1 | Japonica | NA | | Os10g0554900 | OsR498G1019164800.01.T01 | QWRF motif-containing protein 7 |
| maker-3-augustus-gene-146.13-mRNA-1 | Japonica | NA | | Os10g0555200 | OsR498G1019166400.01.T01 | YqgFc domain-containing protein (Fragment) |
| maker-3-augustus-gene-146.7-mRNA-1 | Japonica | NA | | Os10g0554300 | OsR498G1019163600.01.T02 | Flavin-containing monooxygenase |
| maker-3-augustus-gene-148.9-mRNA-1 | Japonica | NA | | Os03g0323800 | OsR498G0305852100.01.T02 | Mitochondrial import receptor subunit TOM40-1, N-terminally processed lik |
| maker-3-augustus-gene-149.33-mRNA-1 | Japonica | NA | | Os03g0325200 | | |
| maker-3-augustus-gene-149.35-mRNA-1 | Japonica | NA | | Os03g0325500 | OsR498G0305857300.01.T01 | HTH myb-type domain-containing protein |
| maker-3-augustus-gene-149.39-mRNA-1 | Japonica | NA | | | OsR498G0305859300.01.T01 | QXW lectin repeat family protein |
| maker-3-augustus-gene-149.44-mRNA-1 | Japonica | NA | | Os03g0324800 | OsR498G0305855200.01.T01 | |
| maker-3-augustus-gene-149.48-mRNA-1 | Japonica | Japonica | | Os03g0325000 | OsR498G0305856200.01.T01 | Pyruvate kinase |
| maker-3-augustus-gene-149.51-mRNA-1 | Japonica | NA | | Os03g0325300 | | Transposon protein, putative, Mutator sub-class |
| maker-3-augustus-gene-149.54-mRNA-1 | Japonica | NA | | | | |
| maker-3-augustus-gene-149.56-mRNA-1 | Japonica | Indica | | Os01g0277600 | OsR498G0305859800.01.T01 | Phospholipid-transporting ATPase |
| maker-3-augustus-gene-149.60-mRNA-1 | Japonica | NA | | Os03g0326200 | OsR498G0305860200.01.T01 | Phospholipid-transporting ATPase |
| maker-3-augustus-gene-149.62-mRNA-1 | Japonica | NA | | Os03g0326300 | OsR498G0305861000.01.T03 | RING/U-box superfamily protein, putative isoform 1 |
| maker-3-augustus-gene-15.0-mRNA-1 | Japonica | NA | | Os03g0130100 | OsR498G0305169100.01.T01 | Benzoate--CoA ligase, peroxisomal |
| maker-3-augustus-gene-15.13-mRNA-1 | Japonica | NA | Kinase-TM | Os03g0130900 | OsR498G0305172900.01.T01 | Putative serine/threonine-protein kinase PBL21 |
| maker-3-augustus-gene-15.2-mRNA-1 | Japonica | NA | | Os03g0130300 | OsR498G0305170100.01.T01 | Gamma-thionin/defensin |
| maker-3-augustus-gene-15.20-mRNA-1 | Japonica | NA | | | OsR498G0305169600.01.T01 | |
| maker-3-augustus-gene-15.21-mRNA-1 | Japonica | NA | | Os03g0130500 | OsR498G0305170800.01.T01 | EF hand family protein, expressed |
| maker-3-augustus-gene-15.27-mRNA-1 | Japonica | NA | | Os03g0130700 | OsR498G0305172000.01.T02 | AAA domain-containing protein |
| maker-3-augustus-gene-15.31-mRNA-1 | Japonica | NA | | Os03g0131100 | OsR498G0305173400.01.T05 | Truncated nodule inception protein |
| maker-3-augustus-gene-15.34-mRNA-1 | Japonica | NA | | Os03g0131200 | OsR498G0305175000.01.T01 | Chloroplast catalase |
| maker-3-augustus-gene-15.37-mRNA-1 | Japonica | NA | | Os03g0131300 | OsR498G0305175200.01.T03 | Ubiquitin family protein, expressed |
| maker-3-augustus-gene-15.4-mRNA-1 | Japonica | NA | | Os03g0130400 | OsR498G0305170500.01.T01 | Adenylate kinase |
| maker-3-augustus-gene-15.7-mRNA-1 | Japonica | NA | | Os03g0130800 | OsR498G0305172300.01.T01 | Bromo domain-containing protein (Fragment) |
| maker-3-augustus-gene-150.32-mRNA-1 | Japonica | NA | | | OsR498G0305870500.01.T02 | Guanine nucleotide exchange factor SPIKE 1 |
| maker-3-augustus-gene-150.38-mRNA-1 | Japonica | Indica | | Os07g0684100 | OsR498G0305862400.01.T01 | Thioredoxin-like 1-1 chloroplastic |
| maker-3-augustus-gene-150.43-mRNA-1 | Japonica | NA | | Os03g0326600 | OsR498G0305862600.01.T03 | U11/U12 small nuclear ribonucleoprotein 65 kDa protein isoform X1 |
| maker-3-augustus-gene-150.47-mRNA-1 | Japonica | NA | | Os03g0327100 | OsR498G0305863600.01.T01 | NAC transcription factor |
| maker-3-augustus-gene-150.49-mRNA-1 | Japonica | NA | | Os03g0327600 | OsR498G0305865600.01.T01 | Hydroxyproline-rich glycoprotein family protein |
| maker-3-augustus-gene-150.52-mRNA-1 | Japonica | NA | | | OsR498G0305867600.01.T01 | NAC domain transcription factor |
| maker-3-augustus-gene-151.0-mRNA-1 | Japonica | NA | | | OsR498G0305871300.01.T01 | GOLD domain-containing protein |
| maker-3-augustus-gene-151.10-mRNA-1 | Japonica | NA | | Os03g0328100 | OsR498G0305870800.01.T01 | O-fucosyltransferase family protein (Fragment) |
| maker-3-augustus-gene-151.16-mRNA-1 | Japonica | Indica | | Os07g0682800 | OsR498G0305871600.01.T01 | Apyrase |
| maker-3-augustus-gene-151.2-mRNA-1 | Japonica | NA | | Os03g0328900 | OsR498G0305873500.01.T02 | Zinc finger CCCH domain-containing protein 22 |
| maker-3-augustus-gene-151.5-mRNA-1 | Japonica | NA | | Os03g0329200 | OsR498G0305874500.01.T04 | Zinc finger CCCH domain-containing protein 53 |
| maker-3-augustus-gene-152.0-mRNA-1 | Japonica | NA | Kinase-LRR-TM | | OsR498G0305877700.01.T01 | probable leucine-rich repeat receptor-like protein kinase At5g49770 |
| maker-3-augustus-gene-152.16-mRNA-1 | Japonica | NA | | Os03g0329500 | OsR498G0305876600.01.T01 | Endoglucanase |
| maker-3-augustus-gene-152.20-mRNA-1 | Japonica | NA | | Os03g0330000 | OsR498G0305878200.01.T01 | Hexosyltransferase |
| maker-3-augustus-gene-152.23-mRNA-1 | Japonica | NA | | Os03g0330300 | OsR498G0305879600.01.T02 | Serine/arginine repetitive matrix protein 1-like |
| maker-3-augustus-gene-152.30-mRNA-1 | Japonica | NA | | Os02g0791200 | OsR498G0408232100.01.T01 | Probable E3 ubiquitin-protein ligase XBOS32 |
| maker-3-augustus-gene-152.5-mRNA-1 | Japonica | NA | | Os03g0329900 | OsR498G0305877900.01.T01 | HTH myb-type domain-containing protein |
| maker-3-augustus-gene-152.9-mRNA-1 | Japonica | NA | | Os03g0330200 | OsR498G0305879300.01.T01 | 2,3-bisphosphoglycerate-independent phosphoglycerate mutase |
| maker-3-augustus-gene-153.0-mRNA-1 | Japonica | Indica | | Os03g0331600 | OsR498G0305882100.01.T01 | Pheophorbide a oxygenase family protein |
| maker-3-augustus-gene-153.13-mRNA-1 | Japonica | NA | | Os03g0332000 | OsR498G0305884200.01.T02 | Secologanin synthase |
| maker-3-augustus-gene-153.16-mRNA-1 | Japonica | NA | | Os03g0332100 | OsR498G0305884210.01.T01 | Secologanin synthase |
| maker-3-augustus-gene-153.18-mRNA-1 | Japonica | NA | | Os03g0332400 | OsR498G0305886000.01.T02 | Metallo-hydrolase/oxidoreductase superfamily protein (Fragment) |
| maker-3-augustus-gene-153.23-mRNA-1 | Japonica | Indica | | Os11g0220800 | OsR498G0305886400.01.T01 | 60S ribosomal protein L10 isoform B |
| maker-3-augustus-gene-153.25-mRNA-1 | Japonica | Indica | | Os07g0681200 | OsR498G0305886600.01.T01 | HAD superfamily subfamily IIIB acid phosphatase |
| maker-3-augustus-gene-153.28-mRNA-1 | Japonica | NA | | Os03g0332700 | OsR498G0305886800.01.T02 | ABC transporter ATPase component |
| maker-3-augustus-gene-153.3-mRNA-1 | Japonica | NA | | | | Bromo domain-containing protein (Fragment) |
| maker-3-augustus-gene-153.33-mRNA-1 | Japonica | NA | | Os03g0333000 | OsR498G0305887800.01.T02 | |

| | | | | | | |
|---|---|---|---|---|---|---|
| maker-3-augustus-gene-153.7-mRNA-1 | Japonica | NA | | Os03g0333100 | OsR498G0305887900.01.T02 | Bms1-type G domain-containing protein |
| maker-3-augustus-gene-154.10-mRNA-1 | Japonica | NA | | Os03g0334200 | OsR498G0305891500.01.T02 | |
| maker-3-augustus-gene-154.12-mRNA-1 | Japonica | NA | | Os08g0389800 | OsR498G0305892300.01.T01 | Protein DETOXIFICATION |
| maker-3-augustus-gene-154.14-mRNA-1 | Japonica | Indica | | Os03g0334700 | OsR498G0305893500.01.T05 | Phospholipid-transporting ATPase |
| maker-3-augustus-gene-154.17-mRNA-1 | Japonica | Japonica | | Os03g0335200 | OsR498G0305894900.01.T01 | WRKY transcription factor 47 |
| maker-3-augustus-gene-154.19-mRNA-1 | Japonica | Japonica | | Os03g0333800 | OsR498G0305890200.01.T01 | DUF1618 domain-containing protein (Fragment) |
| maker-3-augustus-gene-154.23-mRNA-1 | Japonica | NA | Kinase | Os03g0334000 | OsR498G0305890800.01.T02 | Non-specific serine/threonine protein kinase |
| maker-3-augustus-gene-154.26-mRNA-1 | Japonica | NA | | Os03g0334800 | OsR498G0305893800.01.T01 | Lung seven transmembrane receptor family protein, expressed |
| maker-3-augustus-gene-154.5-mRNA-1 | Japonica | Indica | | Os03g0334100 | OsR498G0305891300.01.T02 | PRC domain-containing protein (Fragment) |
| maker-3-augustus-gene-155.32-mRNA-1 | Japonica | NA | | Os03g0335300 | OsR498G0305895300.01.T01 | Vacuolar-sorting receptor 1 |
| maker-3-augustus-gene-155.37-mRNA-1 | Japonica | NA | Kinase-LRR-TM | Os03g0335500 | OsR498G0305895900.01.T01 | Mitogen-activated protein kinase kinase kinase |
| maker-3-augustus-gene-155.39-mRNA-1 | Japonica | NA | | Os03g0336200 | OsR498G0305898100.01.T02 | BZIP-type transcription factor (Fragment) |
| maker-3-augustus-gene-155.43-mRNA-1 | Japonica | NA | | Os03g0336300 | OsR498G0305898300.01.T01 | Nardilysin |
| maker-3-augustus-gene-155.49-mRNA-1 | Japonica | NA | | Os03g0336500 | OsR498G0305899100.01.T01 | Macro domain |
| maker-3-augustus-gene-155.53-mRNA-1 | Japonica | NA | | | | Nucleotidyltransferase domain containing protein, expressed |
| maker-3-augustus-gene-155.57-mRNA-1 | Japonica | Indica | | Os03g0694900 | OsR498G0305896800.01.T05 | Valyl-tRNA synthetase |
| maker-3-augustus-gene-155.63-mRNA-1 | Japonica | NA | | | OsR498G0305897800.01.T01 | Vicilin-like seed storage protein |
| maker-3-augustus-gene-155.65-mRNA-1 | Japonica | NA | | Os03g0336600 | OsR498G0305899400.01.T01 | Reticulon-like protein (Fragment) |
| maker-3-augustus-gene-156.0-mRNA-1 | Japonica | Indica | | Os07g0679000 | OsR498G0305903100.01.T06 | Potassium transporter |
| maker-3-augustus-gene-156.10-mRNA-1 | Japonica | NA | | Os03g0338400 | OsR498G0305906500.01.T01 | BHLH transcription factor |
| maker-3-augustus-gene-156.20-mRNA-1 | Japonica | NA | | Os03g0337700 | OsR498G0305903600.01.T01 | GPN-loop GTPase 3 |
| maker-3-augustus-gene-156.28-mRNA-1 | Japonica | NA | | Os03g0337800 | OsR498G0305903800.01.T01 | Ribosomal protein L19 |
| maker-3-augustus-gene-156.33-mRNA-1 | Japonica | NA | | | OsR498G0305904000.01.T01 | Fumarate hydratase |
| maker-3-augustus-gene-156.40-mRNA-1 | Japonica | NA | | Os03g0338000 | OsR498G0305904400.01.T01 | Aminotransferase class-III |
| maker-3-augustus-gene-156.42-mRNA-1 | Japonica | NA | | Os03g0338600 | OsR498G0305907000.01.T02 | tRNA pseudouridine synthase |
| maker-3-augustus-gene-156.6-mRNA-1 | Japonica | NA | | Os03g0337600 | OsR498G0305903300.01.T01 | Uroporphyrinogen decarboxylase |
| maker-3-augustus-gene-157.35-mRNA-1 | Japonica | NA | | Os03g0339300 | OsR498G0305908500.01.T01 | Peroxidase |
| maker-3-augustus-gene-157.37-mRNA-1 | Japonica | NA | | Os03g0339700 | OsR498G0305910100.01.T01 | DUF827 domain-containing protein (Fragment) |
| maker-3-augustus-gene-157.39-mRNA-1 | Japonica | NA | | Os03g0340600 | | |
| maker-3-augustus-gene-157.41-mRNA-1 | Japonica | NA | | Os03g0340700 | OsR498G0305916700.01.T01 | Sperm-associated antigen 1 |
| maker-3-augustus-gene-157.43-mRNA-1 | Japonica | NA | | Os03g0340900 | OsR498G0305916900.01.T03 | GTP diphosphokinase |
| maker-3-augustus-gene-157.51-mRNA-1 | Japonica | Indica | | Os07g0677600 | OsR498G0305908800.01.T01 | Peroxidase |
| maker-3-augustus-gene-157.57-mRNA-1 | Japonica | NA | | Os03g0340500 | OsR498G0305915600.01.T02 | Sucrose synthase |
| maker-3-augustus-gene-157.64-mRNA-1 | Japonica | NA | | | OsR498G0101949000.01.T02 | |
| maker-3-augustus-gene-158.44-mRNA-1 | Japonica | NA | | Os03g0341000 | OsR498G0305918100.01.T01 | Ethylene-responsive transcription factor RAP2-3 |
| maker-3-augustus-gene-158.46-mRNA-1 | Japonica | NA | | Os03g0341300 | OsR498G0305919100.01.T04 | Bidirectional sugar transporter SWEET |
| maker-3-augustus-gene-158.62-mRNA-1 | Japonica | NA | | Os03g0341100 | OsR498G0305918400.01.T01 | 60S ribosomal protein L18 |
| maker-3-augustus-gene-158.67-mRNA-1 | Japonica | NA | | Os03g0341200 | OsR498G0305918600.01.T01 | Fanconi anemia group I protein homolog |
| maker-3-augustus-gene-158.72-mRNA-1 | Japonica | NA | | Os03g0341600 | OsR498G0305920400.01.T02 | Phylloplanin-like |
| maker-3-augustus-gene-158.74-mRNA-1 | Japonica | NA | | Os03g0342100 | OsR498G0305920600.01.T01 | |
| maker-3-augustus-gene-158.76-mRNA-1 | Japonica | Indica | | Os03g0342900 | OsR498G0305921800.01.T05 | Dormancy/auxin associated protein, expressed |
| maker-3-augustus-gene-16.48-mRNA-1 | Japonica | NA | | Os03g0131500 | OsR498G0305175500.01.T01 | Thiol-disulfide oxidoreductase LTO1 |
| maker-3-augustus-gene-16.51-mRNA-1 | Japonica | Indica | | Os04g0561600 | OsR498G0305175900.01.T01 | Coiled-coil domain-containing protein 124 |
| maker-3-augustus-gene-16.53-mRNA-1 | Japonica | NA | | Os03g0132000 | OsR498G0305176700.01.T01 | 4-coumarate--CoA ligase-like 4 |
| maker-3-augustus-gene-16.75-mRNA-1 | Japonica | NA | | Os03g0131400 | OsR498G0305175400.01.T01 | Polynucleotide adenylyltransferase family protein |
| maker-3-augustus-gene-160.40-mRNA-1 | Japonica | NA | | | OsR498G0305931400.01.T02 | HMA domain-containing protein (Fragment) |
| maker-3-augustus-gene-162.5-mRNA-1 | Japonica | NA | CSD1 | Os03g0351500 | OsR498G0305951300.01.T01 | Superoxide dismutase |
| maker-3-augustus-gene-163.36-mRNA-1 | Japonica | NA | | Os03g0352200 | OsR498G0305954100.01.T05 | SAC3 family protein C |
| maker-3-augustus-gene-163.40-mRNA-1 | Japonica | NA | | Os03g0352400 | OsR498G0305954500.01.T01 | Nuclear/nucleolar GTPase 2 |
| maker-3-augustus-gene-163.45-mRNA-1 | Japonica | NA | | | OsR498G0305955100.01.T01 | Chromatin-remodeling ATPase INO80 |
| maker-3-augustus-gene-163.52-mRNA-1 | Japonica | NA | | Os03g0353000 | OsR498G0305955900.01.T01 | |
| maker-3-augustus-gene-163.54-mRNA-1 | Japonica | Indica | | Os12g0594700 | OsR498G0305956300.01.T01 | F-box domain containing protein, expressed |
| maker-3-augustus-gene-163.56-mRNA-1 | Japonica | Japonica | | Os03g0353400 | OsR498G0305957500.01.T02 | EARLY responsive TO dehydration protein, putative |
| maker-3-augustus-gene-163.59-mRNA-1 | Japonica | NA | | Os03g0353500 | OsR498G0305957900.01.T01 | ribosomal RNA-processing protein 17 |
| maker-3-augustus-gene-163.61-mRNA-1 | Japonica | NA | | Os03g0352300 | OsR498G0305954400.01.T01 | Nop domain-containing protein |
| maker-3-augustus-gene-163.67-mRNA-1 | Japonica | NA | | Os03g0352800 | OsR498G0305955800.01.T01 | Acyl carrier protein 2, mitochondrial |
| maker-3-augustus-gene-164.0-mRNA-1 | Japonica | NA | | | OsR498G0305958300.01.T04 | Aberrant pollen transmission1 |
| maker-3-augustus-gene-164.10-mRNA-1 | Japonica | NA | | Os03g0353900 | OsR498G0305959300.01.T04 | ArgoN domain-containing protein |
| maker-3-augustus-gene-165.28-mRNA-1 | Japonica | NA | | Os05g0267732 | OsR498G0203607000.01.T01 | |
| maker-3-augustus-gene-169.36-mRNA-1 | Japonica | NA | | | OsR498G0305965900.01.T01 | U4/U6 small nuclear ribonucleoprotein Prp4 |
| maker-3-augustus-gene-169.39-mRNA-1 | Japonica | Japonica | | Os03g0355700 | OsR498G0305967300.01.T01 | IQ-domain 11 |

| | | | | | | |
|---|---|---|---|---|---|---|
| maker-3-augustus-gene-169.41-mRNA-1 | Japonica | NA | | Os03g0355600 | OsR498G0305966400.01.T02 | Beta-adaptin-like protein C |
| maker-3-augustus-gene-169.46-mRNA-1 | Japonica | NA | | Os03g0355800 | OsR498G0305967800.01.T01 | Diphthine methyl ester synthase |
| maker-3-augustus-gene-169.49-mRNA-1 | Japonica | NA | | Os03g0355900 | OsR498G0305968000.01.T01 | Sanguinarine reductase |
| maker-3-augustus-gene-169.54-mRNA-1 | Japonica | NA | | Os03g0356300 | OsR498G0305969000.01.T01 | 50S ribosomal protein L6 chloroplastic |
| maker-3-augustus-gene-169.56-mRNA-1 | Japonica | NA | | Os03g0356414 | OsR498G0305969400.01.T01 | RING-type E3 ubiquitin transferase |
| maker-3-augustus-gene-169.58-mRNA-1 | Japonica | NA | | Os03g0356470 | | |
| maker-3-augustus-gene-169.63-mRNA-1 | Japonica | NA | | Os03g0356484 | OsR498G0305971000.01.T02 | Mitochondria fission 1 protein (Fragment) |
| maker-3-augustus-gene-17.43-mRNA-1 | Japonica | NA | | | | IQ-domain 20 |
| maker-3-augustus-gene-17.45-mRNA-1 | Japonica | NA | | Os03g0134900 | OsR498G0305185200.01.T01 | Glutathione S-transferase, N-terminal domain containing protein, expressed |
| maker-3-augustus-gene-17.47-mRNA-1 | Japonica | NA | | Os03g0135100 | OsR498G0305185800.01.T01 | Glutathione transferase |
| maker-3-augustus-gene-17.50-mRNA-1 | Japonica | Indica | | Os03g0135200 | OsR498G0305186000.01.T01 | Glutathione transferase |
| maker-3-augustus-gene-17.53-mRNA-1 | Japonica | NA | | Os03g0135300 | OsR498G0305186200.01.T01 | Glutathione transferase |
| maker-3-augustus-gene-17.55-mRNA-1 | Japonica | NA | | Os03g0135400 | OsR498G0305186800.01.T01 | F-box/LRR-repeat protein 10 |
| maker-3-augustus-gene-17.57-mRNA-1 | Japonica | Indica | | Os03g0135600 | OsR498G0305187200.01.T09 | Ankyrin repeat domain-containing protein chloroplastic |
| maker-3-augustus-gene-17.61-mRNA-1 | Japonica | Japonica | RAI1 | Os03g0135700 | OsR498G0305187800.01.T01 | BHLH transcription factor |
| maker-3-augustus-gene-170.27-mRNA-1 | Japonica | NA | | | | Hydrolase, putative, expressed |
| maker-3-augustus-gene-170.30-mRNA-1 | Japonica | NA | | Os03g0356582 | OsR498G0305973900.01.T01 | Phosphatidylinositol 4-phosphate 5-kinase |
| maker-3-augustus-gene-170.33-mRNA-1 | Japonica | Japonica | | Os03g0356596 | OsR498G0714888800.01.T01 | Phosphatidylinositol 4-phosphate 5-kinase |
| maker-3-augustus-gene-170.35-mRNA-1 | Japonica | NA | | | OsR498G0305976300.01.T01 | F-box domain containing protein, expressed |
| maker-3-augustus-gene-170.37-mRNA-1 | Japonica | NA | | | OsR498G0409243500.01.T01 | HTH myb-type domain-containing protein |
| maker-3-augustus-gene-170.39-mRNA-1 | Japonica | NA | | Os03g0356638 | OsR498G0305974800.01.T02 | Pleckstriny domain-containing protein, putative, expressed |
| maker-3-augustus-gene-170.47-mRNA-1 | Japonica | NA | | | OsR498G0305975400.01.T06 | TRAF-type zinc finger domain-containing protein 1 |
| maker-3-augustus-gene-171.0-mRNA-1 | Japonica | NA | | Os03g0356700 | OsR498G0305980300.01.T06 | HP domain-containing protein |
| maker-3-augustus-gene-171.10-mRNA-1 | Japonica | NA | | | OsR498G0305979000.01.T07 | Alpha-N-acetylglucosaminidase isoform X1 |
| maker-3-augustus-gene-171.11-mRNA-1 | Japonica | NA | | | OsR498G0305979000.01.T04 | Alpha-N-acetylglucosaminidase isoform X1 |
| maker-3-augustus-gene-171.7-mRNA-1 | Japonica | NA | | | | |
| maker-3-augustus-gene-172.0-mRNA-1 | Japonica | NA | | Os03g0358100 | OsR498G0305983500.01.T01 | Glutathione peroxidase |
| maker-3-augustus-gene-172.13-mRNA-1 | Japonica | Indica | | Os07g0655400 | OsR498G0305983800.01.T01 | Rhomboid-like protein 19 |
| maker-3-augustus-gene-172.5-mRNA-1 | Japonica | NA | | | | |
| maker-3-augustus-gene-172.8-mRNA-1 | Japonica | NA | | | OsR498G0305983200.01.T03 | CID domain-containing protein |
| maker-3-augustus-gene-173.48-mRNA-1 | Japonica | NA | | | OsR498G0305988200.01.T02 | branched-chain-amino-acid aminotransferase-like protein 1 |
| maker-3-augustus-gene-174.46-mRNA-1 | Japonica | NA | | Os05g0368300 | OsR498G0510685100.01.T01 | S4 RNA-binding domain-containing protein (Fragment) |
| maker-3-augustus-gene-175.0-mRNA-1 | Japonica | NA | | | | HEAT repeat family protein, expressed |
| maker-3-augustus-gene-175.10-mRNA-1 | Japonica | NA | | | | Endo/exonuclease/phosphatase domain-containing protein (Fragment) |
| maker-3-augustus-gene-175.11-mRNA-1 | Japonica | Indica | | Os07g0645500 | OsR498G0305991300.01.T01 | Endonuclease/exonuclease/phosphatase |
| maker-3-augustus-gene-175.14-mRNA-1 | Japonica | Japonica | | Os03g0361100 | OsR498G0305992500.01.T01 | Terpene synthase family, metal binding domain containing protein, expresse |
| maker-3-augustus-gene-175.22-mRNA-1 | Japonica | Japonica | | Os03g0361500 | OsR498G0305993600.01.T01 | Terpene synthase family, metal binding domain containing protein, expresse |
| maker-3-augustus-gene-175.5-mRNA-1 | Japonica | NA | | | OsR498G0305991100.01.T03 | Methionine sulfoxide reductase |
| maker-3-augustus-gene-176.0-mRNA-1 | Japonica | NA | | Os03g0362200 | OsR498G0305997700.01.T01 | Armadillo |
| maker-3-augustus-gene-176.14-mRNA-1 | Japonica | NA | | Os03g0361700 | OsR498G0305996000.01.T01 | Terpene synthase family, metal binding domain containing protein, expresse |
| maker-3-augustus-gene-176.17-mRNA-1 | Japonica | Indica | | Os03g0361100 | OsR498G0305996700.01.T01 | Terpene synthase family, metal binding domain containing protein, expresse |
| maker-3-augustus-gene-176.19-mRNA-1 | Japonica | Indica | | Os03g0361700 | OsR498G0305996500.01.T01 | Terpene synthase family, metal binding domain containing protein, expresse |
| maker-3-augustus-gene-176.2-mRNA-1 | Japonica | Japonica | | Os03g0363100 | OsR498G0305997700.01.T01 | Armadillo/beta-catenin-like repeat protein |
| maker-3-augustus-gene-176.21-mRNA-1 | Japonica | NA | | Os03g0363500 | OsR498G0305999000.01.T02 | MFS domain-containing protein (Fragment) |
| maker-3-augustus-gene-176.4-mRNA-1 | Japonica | NA | | Os03g0363350 | OsR498G0305998300.01.T01 | Rieske domain-containing protein |
| maker-3-augustus-gene-176.7-mRNA-1 | Japonica | NA | | Os03g0363400 | OsR498G0305998700.01.T03 | Atherin |
| maker-3-augustus-gene-177.10-mRNA-1 | Japonica | NA | Kinase-TM | Os03g0364400 | OsR498G0306001300.01.T01 | probable serine/threonine-protein kinase PBL15 |
| maker-3-augustus-gene-177.14-mRNA-1 | Japonica | NA | | | OsR498G0306002500.01.T02 | TFIIS N-terminal domain-containing protein |
| maker-3-augustus-gene-177.20-mRNA-1 | Japonica | Japonica | | Os03g0363600 | OsR498G0305999200.01.T01 | MFS domain-containing protein |
| maker-3-augustus-gene-177.26-mRNA-1 | Japonica | NA | | Os03g0363950 | OsR498G0306000200.01.T02 | Sulfotransferase |
| maker-3-augustus-gene-177.31-mRNA-1 | Japonica | NA | | | | Polyubiquitin 9 |
| maker-3-augustus-gene-177.35-mRNA-1 | Japonica | NA | | | | Stomatal closure-related actin-binding protein 1 |
| maker-3-augustus-gene-177.4-mRNA-1 | Japonica | NA | | Os03g0363900 | OsR498G0305999900.01.T01 | S-acyltransferase |
| maker-3-augustus-gene-178.0-mRNA-1 | Japonica | NA | | Os03g0365800 | OsR498G0306006500.01.T01 | GDSL esterase/lipase |
| maker-3-augustus-gene-178.1-mRNA-1 | Japonica | Japonica | | Os03g0365800 | OsR498G0306006500.01.T01 | GDSL-lipase |
| maker-3-augustus-gene-178.12-mRNA-1 | Japonica | NA | Kinase-TM | Os03g0366200 | OsR498G0306008700.01.T02 | Calcium/calmodulin-dependent serine/threonine-protein kinase 1 |
| maker-3-augustus-gene-178.18-mRNA-1 | Japonica | Indica | | Os07g0641000 | OsR498G0306009300.01.T03 | O-fucosyltransferase family protein (Fragment) |
| maker-3-augustus-gene-178.22-mRNA-1 | Japonica | NA | | Os03g0366000 | OsR498G0306007000.01.T02 | Chaperonin, mitochondrial |
| maker-3-augustus-gene-178.8-mRNA-1 | Japonica | Indica | | Os03g0365900 | OsR498G0306006700.01.T01 | GDSL-like Lipase/Acylhydrolase family protein, expressed |
| maker-3-augustus-gene-179.0-mRNA-1 | Japonica | NA | | Os03g0367000 | OsR498G0306010100.01.T01 | Peptidylprolyl isomerase |
| maker-3-augustus-gene-179.11-mRNA-1 | Japonica | NA | | Os03g0367900 | OsR498G0306013100.01.T03 | Pyrrolidone-carboxylate peptidase-like |

| | | | | | | |
|---|---|---|---|---|---|---|
| maker-3-augustus-gene-179.15-mRNA-1 | Japonica | NA | | Os03g0368300 | OsR498G0306013500.01.T01 | Peroxidase |
| maker-3-augustus-gene-179.17-mRNA-1 | Japonica | Japonica | | Os03g0368600 | OsR498G0306015100.01.T01 | Peroxidase |
| maker-3-augustus-gene-179.20-mRNA-1 | Japonica | NA | | Os03g0368300 | OsR498G0306013500.01.T01 | Peroxidase |
| maker-3-augustus-gene-179.7-mRNA-1 | Japonica | NA | | | OsR498G0306011500.01.T01 | Flavonoid hydroxylase |
| maker-3-augustus-gene-179.9-mRNA-1 | Japonica | NA | | Os03g0367800 | | SWR1 complex subunit like |
| maker-3-augustus-gene-18.26-mRNA-1 | Japonica | NA | | Os03g0137200 | OsR498G0305193500.01.T03 | Protein-serine/threonine phosphatase |
| maker-3-augustus-gene-18.33-mRNA-1 | Japonica | NA | | Os03g0137600 | OsR498G0305194700.01.T02 | GATA zinc finger domain-containing protein 10 |
| maker-3-augustus-gene-18.35-mRNA-1 | Japonica | NA | | Os03g0137800 | OsR498G0305197100.01.T01 | Cyclin-dependent kinase inhibitor |
| maker-3-augustus-gene-18.38-mRNA-1 | Japonica | NA | | Os03g0138000 | OsR498G0305197900.01.T01 | No pollen germination related 2 |
| maker-3-augustus-gene-18.40-mRNA-1 | Japonica | NA | | Os03g0136800 | OsR498G0305190800.01.T01 | J domain-containing protein |
| maker-3-augustus-gene-18.42-mRNA-1 | Japonica | NA | | Os03g0136900 | OsR498G0305191400.01.T01 | Aconitate hydratase |
| maker-3-augustus-gene-18.48-mRNA-1 | Japonica | Indica | | Os03g0137300 | OsR498G0305193800.01.T01 | protein CHAPERONE-LIKE PROTEIN OF POR1, chloroplastic-like |
| maker-3-augustus-gene-18.51-mRNA-1 | Japonica | NA | | Os03g0137700 | OsR498G0305195000.01.T01 | Magnesium transporter |
| maker-3-augustus-gene-180.0-mRNA-1 | Japonica | NA | | Os03g0368900 | OsR498G0306016300.01.T01 | Peroxidase |
| maker-3-augustus-gene-180.13-mRNA-1 | Japonica | NA | | | OsR498G0306020300.01.T01 | NYN domain-containing protein (Fragment) |
| maker-3-augustus-gene-180.16-mRNA-1 | Japonica | NA | | Os03g0370900 | OsR498G0306024100.01.T01 | Cytochrome P450 family protein, expressed |
| maker-3-augustus-gene-180.18-mRNA-1 | Japonica | NA | | Os03g0369800 | OsR498G0306019600.01.T02 | t-SNARE coiled-coil homology domain-containing protein |
| maker-3-augustus-gene-180.2-mRNA-1 | Japonica | NA | | Os03g0369000 | OsR498G0306017500.01.T01 | Peroxidase |
| maker-3-augustus-gene-180.23-mRNA-1 | Japonica | NA | | | | Stress-induced hydrophobic peptide |
| maker-3-augustus-gene-180.4-mRNA-1 | Japonica | NA | | Os03g0369100 | OsR498G0306018100.01.T01 | AAI domain-containing protein |
| maker-3-augustus-gene-180.6-mRNA-1 | Japonica | NA | | Os03g0369200 | OsR498G0306018300.01.T01 | Peroxidase |
| maker-3-augustus-gene-180.8-mRNA-1 | Japonica | NA | | Os03g0369400 | OsR498G0306018500.01.T01 | Peroxidase |
| maker-3-augustus-gene-181.19-mRNA-1 | Japonica | NA | | | | Secologanin synthase |
| maker-3-augustus-gene-181.21-mRNA-1 | Japonica | NA | | | OsR498G0306027800.01.T01 | Transcription factor MYB32 |
| maker-3-augustus-gene-182.0-mRNA-1 | Japonica | NA | | Os03g0372500 | OsR498G0306030300.01.T02 | PPM-type phosphatase domain-containing protein (Fragment) |
| maker-3-augustus-gene-182.10-mRNA-1 | Japonica | NA | | | OsR498G0306030800.01.T01 | Inositol-pentakisphosphate 2-kinase |
| maker-3-augustus-gene-182.12-mRNA-1 | Japonica | NA | | Os03g0374100 | OsR498G0306031800.01.T01 | Hepatocellular carcinoma-associated antigen 59 |
| maker-3-augustus-gene-182.3-mRNA-1 | Japonica | NA | | Os03g0372700 | OsR498G0306030900.01.T01 | Low temperature viability protein, expressed |
| maker-3-augustus-gene-182.5-mRNA-1 | Japonica | NA | | Os03g0374400 | OsR498G0306032700.01.T01 | TVP38/TMEM64 family membrane protein like |
| maker-3-augustus-gene-183.33-mRNA-1 | Japonica | Indica | | Os03g0374900 | OsR498G0306036800.01.T01 | Polyamine transporter |
| maker-3-augustus-gene-184.0-mRNA-1 | Japonica | NA | | Os03g0376900 | OsR498G0306042300.01.T01 | Polypyrimidine tract-binding protein like 1 |
| maker-3-augustus-gene-184.16-mRNA-1 | Japonica | NA | | | OsR498G0306044100.01.T02 | Nuclear control of ATPase protein 2 |
| maker-3-augustus-gene-184.24-mRNA-1 | Japonica | Japonica | | Os03g0378000 | OsR498G0306046100.01.T03 | probable apyrase 6 |
| maker-3-augustus-gene-184.28-mRNA-1 | Japonica | NA | | Os03g0378200 | OsR498G0306046700.01.T03 | Transferase transferring acyl groups |
| maker-3-augustus-gene-184.33-mRNA-1 | Japonica | NA | | Os03g0376600 | OsR498G0306042000.01.T01 | Ribonucleoprotein B, chloroplastic |
| maker-3-augustus-gene-184.35-mRNA-1 | Japonica | NA | | Os03g0376800 | OsR498G0306042200.01.T02 | RNA-binding KH domain-containing protein RCF3 |
| maker-3-augustus-gene-184.38-mRNA-1 | Japonica | NA | | Os03g0377700 | OsR498G0306044400.01.T02 | Putative mannan synthase 7 |
| maker-3-augustus-gene-184.7-mRNA-1 | Japonica | NA | | Os03g0377100 | OsR498G0306042500.01.T01 | Expansin |
| maker-3-augustus-gene-184.9-mRNA-1 | Japonica | NA | | | OsR498G0306043500.01.T04 | |
| maker-3-augustus-gene-185.0-mRNA-1 | Japonica | NA | | Os03g0379100 | OsR498G0306048300.01.T02 | Methyltransferase |
| maker-3-augustus-gene-185.2-mRNA-1 | Japonica | NA | | Os03g0379300 | OsR498G0306048500.01.T01 | Transcription factor bHLH47 |
| maker-3-augustus-gene-186.11-mRNA-1 | Japonica | NA | | | OsR498G0306053700.01.T01 | |
| maker-3-augustus-gene-186.13-mRNA-1 | Japonica | NA | | Os08g0487300 | OsR498G0816350400.01.T01 | Transposon protein, putative, Mutator sub-class |
| maker-3-augustus-gene-186.16-mRNA-1 | Japonica | NA | | Os03g0381000 | OsR498G0306052600.01.T01 | Aldose 1-epimerase |
| maker-3-augustus-gene-186.18-mRNA-1 | Japonica | Indica | | Os03g0381300 | OsR498G0306053600.01.T01 | CS domain-containing protein (Fragment) |
| maker-3-augustus-gene-186.23-mRNA-1 | Japonica | NA | | Os03g0381500 | OsR498G0306054000.01.T01 | |
| maker-3-augustus-gene-186.6-mRNA-1 | Japonica | NA | | | OsR498G0306052900.01.T05 | tRNA pseudouridine synthase |
| maker-3-augustus-gene-186.8-mRNA-1 | Japonica | NA | | Os03g0381200 | OsR498G0306053300.01.T01 | F-actin-capping protein subunit beta |
| maker-3-augustus-gene-187.38-mRNA-1 | Japonica | NA | | Os03g0383900 | OsR498G0306057500.01.T03 | HMA domain-containing protein |
| maker-3-augustus-gene-187.40-mRNA-1 | Japonica | NA | | | OsR498G0306056800.01.T03 | SAP domain-containing protein (Fragment) |
| maker-3-augustus-gene-188.33-mRNA-1 | Japonica | NA | | Os03g0385400 | OsR498G0306060700.01.T01 | AAI domain-containing protein |
| maker-3-augustus-gene-188.45-mRNA-1 | Japonica | NA | | Os03g0385301 | | Zinc finger CCCH domain-containing protein 15 |
| maker-3-augustus-gene-188.47-mRNA-1 | Japonica | Japonica | | Os03g0385900 | OsR498G0306062000.01.T02 | Small subunit ribosomal protein (Fragment) |
| maker-3-augustus-gene-189.22-mRNA-1 | Japonica | NA | | Os03g0386500 | OsR498G0306065700.01.T03 | Trehalose 6-phosphate phosphatase |
| maker-3-augustus-gene-189.26-mRNA-1 | Japonica | NA | | | OsR498G0306067500.01.T03 | Carboxypeptidase |
| maker-3-augustus-gene-189.28-mRNA-1 | Japonica | NA | | Os03g0386800 | OsR498G0306067900.01.T02 | Carboxypeptidase |
| maker-3-augustus-gene-189.32-mRNA-1 | Japonica | NA | | | OsR498G0306069100.01.T01 | Intron-binding protein aquarius |
| maker-3-augustus-gene-189.39-mRNA-1 | Japonica | NA | | Os03g0387100 | OsR498G0306069500.01.T01 | Proteasome endopeptidase complex |
| maker-3-augustus-gene-189.45-mRNA-1 | Japonica | NA | | Os03g0387800 | OsR498G0306072700.01.T01 | Naringenin,2-oxoglutarate 3-dioxygenase |
| maker-3-augustus-gene-19.40-mRNA-1 | Japonica | NA | | Os03g0138500 | OsR498G0305199500.01.T01 | |
| maker-3-augustus-gene-19.42-mRNA-1 | Japonica | NA | | Os03g0139100 | OsR498G0305201500.01.T01 | Ribosomal protein L17 |

| | | | | | | |
|---|---|---|---|---|---|---|
| maker-3-augustus-gene-19.46-mRNA-1 | Japonica | NA | | | OsR498G0305198400.01.T03 | RNA recognition motif family protein, expressed |
| maker-3-augustus-gene-19.50-mRNA-1 | Japonica | Japonica | | Os03g0138600 | OsR498G0305200000.01.T02 | MHD1 domain-containing protein |
| maker-3-augustus-gene-19.54-mRNA-1 | Japonica | NA | | Os03g0138700 | OsR498G0305200400.01.T01 | Peptide transporter 3 |
| maker-3-augustus-gene-19.56-mRNA-1 | Japonica | NA | | Os03g0139200 | OsR498G0305201800.01.T03 | CCT domain-containing protein |
| maker-3-augustus-gene-19.59-mRNA-1 | Japonica | NA | | Os03g0139500 | OsR498G0305202800.01.T01 | CCT motif family protein, expressed |
| maker-3-augustus-gene-190.0-mRNA-1 | Japonica | NA | | Os03g0387900 | OsR498G0306073500.01.T04 | |
| maker-3-augustus-gene-190.15-mRNA-1 | Japonica | Indica | | Os03g0388000 | OsR498G0306073800.01.T03 | Serine/arginine-rich splicing factor like |
| maker-3-augustus-gene-190.17-mRNA-1 | Japonica | NA | | Os03g0388100 | OsR498G0306074200.01.T02 | ATP-dependent RNA helicase DBP2-A |
| maker-3-augustus-gene-190.20-mRNA-1 | Japonica | NA | | | OsR498G0306077000.01.T01 | MYb transcription factor 18 |
| maker-3-augustus-gene-190.27-mRNA-1 | Japonica | NA | | Os03g0389100 | OsR498G0306081000.01.T01 | zf-LSD1 domain-containing protein |
| maker-3-augustus-gene-190.5-mRNA-1 | Japonica | NA | | | OsR498G0306076100.01.T01 | Calmodulin-binding transcription activator 2 |
| maker-3-augustus-gene-191.0-mRNA-1 | Japonica | NA | | Os03g0389700 | OsR498G0306083900.01.T01 | Phospho-2-dehydro-3-deoxyheptonate aldolase |
| maker-3-augustus-gene-191.10-mRNA-1 | Japonica | NA | | Os03g0390400 | OsR498G0306085500.01.T01 | Cytochrome c oxidase subunit |
| maker-3-augustus-gene-191.13-mRNA-1 | Japonica | NA | | Os03g0390600 | OsR498G0306085700.01.T05 | Histone domain-containing protein (Fragment) |
| maker-3-augustus-gene-191.16-mRNA-1 | Japonica | Indica | | Os03g0390700 | OsR498G0306085900.01.T02 | Hydroxyisourate hydrolase |
| maker-3-augustus-gene-191.20-mRNA-1 | Japonica | Indica | | Os04g0682000 | OsR498G0306086100.01.T02 | Cysteine protease |
| maker-3-augustus-gene-191.27-mRNA-1 | Japonica | NA | | Os03g0390000 | OsR498G0306085000.01.T01 | 40S ribosomal protein S6 |
| maker-3-augustus-gene-191.3-mRNA-1 | Japonica | NA | | | | JmjC domain-containing protein |
| maker-3-augustus-gene-191.31-mRNA-1 | Japonica | NA | Kinase-TM | Os03g0390200 | OsR498G0306085400.01.T01 | Serine/threonine-protein kinase SRK2C |
| maker-3-augustus-gene-191.35-mRNA-1 | Japonica | NA | | Os11g0432800 | OsR498G1120015800.01.T02 | Protein RETICULATA chloroplastic |
| maker-3-augustus-gene-192.0-mRNA-1 | Japonica | Indica | | Os05g0171000 | OsR498G0306086700.01.T01 | Phospholipase D |
| maker-3-augustus-gene-195.10-mRNA-1 | Japonica | NA | | Os03g0395300 | OsR498G0306105600.01.T01 | Protein WEAK CHLOROPLAST MOVEMENT UNDER BLUE LIGHT 1 |
| maker-3-augustus-gene-195.7-mRNA-1 | Japonica | NA | | Os03g0395000 | OsR498G0306105000.01.T01 | Inactive heme oxygenase |
| maker-3-augustus-gene-198.2-mRNA-1 | Japonica | Japonica | | Os03g0399300 | OsR498G0713610100.01.T02 | Inwardly rectifying potassium channel subunit |
| maker-3-augustus-gene-2.0-mRNA-1 | Japonica | Japonica | | Os03g0107100 | OsR498G0305090700.01.T04 | Plasma membrane ATPase |
| maker-3-augustus-gene-2.14-mRNA-1 | Japonica | NA | | Os04g0119300 | OsR498G0305093300.01.T01 | DUF1990 domain-containing protein (Fragment) |
| maker-3-augustus-gene-2.17-mRNA-1 | Japonica | NA | | Os03g0107900 | OsR498G0305094500.01.T01 | Glucuronoxylan glucuronosyltransferase |
| maker-3-augustus-gene-2.20-mRNA-1 | Japonica | NA | | Os10g0545600 | | Zinc finger, C3HC4 type family protein, expressed |
| maker-3-augustus-gene-2.28-mRNA-1 | Japonica | Indica | | Os03g0108400 | OsR498G0305095900.01.T01 | Vacuolar protein sorting-associated protein 1 |
| maker-3-augustus-gene-2.39-mRNA-1 | Japonica | NA | | Os03g0107300 | OsR498G0305091200.01.T02 | Putative transporter arsB |
| maker-3-augustus-gene-2.4-mRNA-1 | Japonica | Indica | | Os03g0107400 | OsR498G0305091900.01.T04 | Myosin-2 heavy chain-like isoform X1 |
| maker-3-augustus-gene-2.43-mRNA-1 | Japonica | NA | | | OsR498G0305094000.01.T02 | Phosphoglucan phosphatase |
| maker-3-augustus-gene-2.49-mRNA-1 | Japonica | NA | | Os03g0108000 | OsR498G0305094800.01.T02 | Rhodanese domain-containing protein (Fragment) |
| maker-3-augustus-gene-2.51-mRNA-1 | Japonica | NA | | Os03g0108300 | OsR498G0305095600.01.T01 | Xyloglucan endotransglucosylase/hydrolase |
| maker-3-augustus-gene-2.53-mRNA-1 | Japonica | NA | | Os03g0108500 | OsR498G0305096200.01.T01 | Aldehyde oxygenase (deformylating) |
| maker-3-augustus-gene-2.9-mRNA-1 | Japonica | NA | | Os03g0107500 | | Ring/U-Box superfamily protein, putative isoform 3 |
| maker-3-augustus-gene-20.0-mRNA-1 | Japonica | NA | | Os03g0140700 | OsR498G0305209100.01.T01 | TPR transcription factor |
| maker-3-augustus-gene-20.14-mRNA-1 | Japonica | NA | | Os01g0838725 | OsR498G1018411500.01.T01 | |
| maker-3-augustus-gene-20.17-mRNA-1 | Japonica | Japonica | | Os03g0141100 | OsR498G0714417100.01.T01 | Transposase (Fragment) |
| maker-3-augustus-gene-20.2-mRNA-1 | Japonica | NA | | Os03g0140900 | OsR498G0305210300.01.T01 | |
| maker-3-augustus-gene-20.4-mRNA-1 | Japonica | NA | | Os03g0141000 | OsR498G0305210500.01.T01 | 60S ribosomal protein L21 isoform 1 |
| maker-3-augustus-gene-20.6-mRNA-1 | Japonica | NA | | Os03g0141200 | OsR498G0305210700.01.T01 | Beta-amylase 2, chloroplastic |
| maker-3-augustus-gene-20.9-mRNA-1 | Japonica | Japonica | | Os03g0141700 | OsR498G0305211900.01.T02 | |
| maker-3-augustus-gene-200.34-mRNA-1 | Japonica | Indica | | Os07g0616600 | OsR498G0306136300.01.T01 | 40S ribosomal protein SA |
| maker-3-augustus-gene-200.39-mRNA-1 | Japonica | Indica | | Os07g0616200 | OsR498G0306136700.01.T04 | Beta-ketoacyl-[acyl-carrier-protein] synthase I |
| maker-3-augustus-gene-200.44-mRNA-1 | Japonica | NA | | Os03g0402000 | OsR498G0306136000.01.T01 | Trafficking protein particle complex subunit |
| maker-3-augustus-gene-200.49-mRNA-1 | Japonica | NA | | | OsR498G0306137800.01.T01 | Transcription factor btd-like |
| maker-3-augustus-gene-200.51-mRNA-1 | Japonica | NA | | | OsR498G0306139200.01.T01 | |
| maker-3-augustus-gene-201.0-mRNA-1 | Japonica | NA | | Os03g0402800 | OsR498G0306144200.01.T01 | Tify domain-containing protein |
| maker-3-augustus-gene-202.32-mRNA-1 | Japonica | NA | | Os03g0403100 | OsR498G0306144900.01.T05 | DNA-directed RNA polymerase subunit beta |
| maker-3-augustus-gene-203.34-mRNA-1 | Japonica | NA | | Os03g0404800 | OsR498G0306150300.01.T02 | 3beta-hydroxysteroid-dehydrogenase/decarboxylase isoform 1 |
| maker-3-augustus-gene-203.38-mRNA-1 | Japonica | NA | | Os03g0405000 | OsR498G0306152100.01.T04 | Sterol-4-alpha-carboxylate 3-dehydrogenase, decarboxylating |
| maker-3-augustus-gene-203.45-mRNA-1 | Japonica | NA | | Os03g0405100 | OsR498G0306152500.01.T02 | Ubiquinone biosynthesis protein (Fragment) |
| maker-3-augustus-gene-203.48-mRNA-1 | Japonica | NA | | Os03g0405500 | OsR498G0306153100.01.T01 | Nucleoredoxin like |
| maker-3-augustus-gene-203.52-mRNA-1 | Japonica | NA | | Os03g0405550 | | |
| maker-3-augustus-gene-203.54-mRNA-1 | Japonica | NA | | | OsR498G0306157700.01.T01 | Cotton nucleoredoxin 1 |
| maker-3-augustus-gene-204.0-mRNA-1 | Japonica | NA | | Os03g0406100 | OsR498G0306158900.01.T01 | SPX domain-containing protein 5 |
| maker-3-augustus-gene-204.2-mRNA-1 | Japonica | Indica | | Os03g0406200 | OsR498G0306159500.01.T02 | Translation elongation factor 1-delta 1 |
| maker-3-augustus-gene-204.8-mRNA-1 | Japonica | NA | | | OsR498G0306160000.01.T01 | Nucleoside-diphosphate kinase |
| maker-3-augustus-gene-21.0-mRNA-1 | Japonica | Japonica | | Os03g0141800 | OsR498G0305212900.01.T01 | |
| maker-3-augustus-gene-21.10-mRNA-1 | Japonica | NA | | Os03g0142900 | OsR498G0305215800.01.T01 | Vacuolar protein sorting-associated protein 62 |

| | | | | | | |
|---|---|---|---|---|---|---|
| maker-3-augustus-gene-21.12-mRNA-1 | Japonica | NA | | | OsR498G0305216000.01.T01 | Methyltransferase-like protein 6 isoform B |
| maker-3-augustus-gene-21.19-mRNA-1 | Japonica | NA | | | | Nuclear transcription factor Y subunit A-8 |
| maker-3-augustus-gene-21.2-mRNA-1 | Japonica | NA | | Os03g0142500 | OsR498G0305214100.01.T01 | Zinc finger (C3HC4-type RING finger) family protein |
| maker-3-augustus-gene-21.4-mRNA-1 | Japonica | NA | | Os03g0142800 | OsR498G0305215500.01.T01 | ABC transporter C family MRP4 |
| maker-3-augustus-gene-21.8-mRNA-1 | Japonica | NA | | Os03g0142600 | OsR498G0305214400.01.T01 | Transcription factor MYB57 |
| maker-3-augustus-gene-210.31-mRNA-1 | Japonica | NA | | | | Mitochondrial import inner membrane translocase subunit Tim17 family pro |
| maker-3-augustus-gene-210.33-mRNA-1 | Japonica | NA | | Os03g0416200 | OsR498G0306209100.01.T02 | COBRA-like protein 5 |
| maker-3-augustus-gene-210.35-mRNA-1 | Japonica | NA | | Os03g0416300 | OsR498G0306209400.01.T01 | COBRA-like protein 1 |
| maker-3-augustus-gene-211.0-mRNA-1 | Japonica | Indica | | Os07g0178700 | OsR498G0306215800.01.T01 | Early light-inducible protein ELIP |
| maker-3-augustus-gene-211.11-mRNA-1 | Japonica | NA | | Os03g0418600 | OsR498G0306222400.01.T04 | |
| maker-3-augustus-gene-211.2-mRNA-1 | Japonica | Japonica | | Os03g0417800 | OsR498G0306216800.01.T02 | Nitrilase-associated protein |
| maker-3-augustus-gene-211.5-mRNA-1 | Japonica | NA | | Os03g0417900 | OsR498G0306219200.01.T01 | Vacuolar protein sorting-associated protein 52 isogeny |
| maker-3-augustus-gene-212.48-mRNA-1 | Japonica | NA | | Os03g0418700 | OsR498G0306223300.01.T02 | |
| maker-3-augustus-gene-212.52-mRNA-1 | Japonica | NA | | Os03g0418800 | OsR498G0306223900.01.T01 | Ras GTPase-activating protein-binding protein 1 |
| maker-3-augustus-gene-212.56-mRNA-1 | Japonica | NA | | Os03g0419100 | OsR498G0306224500.01.T02 | Ninja-family protein 6 |
| maker-3-augustus-gene-213.39-mRNA-1 | Japonica | NA | | | OsR498G0307311800.01.T04 | Acylpyruvase FAHD1, mitochondrial |
| maker-3-augustus-gene-213.40-mRNA-1 | Japonica | NA | | Os03g0421700 | OsR498G0306232900.01.T01 | Acylpyruvase FAHD1, mitochondrial |
| maker-3-augustus-gene-213.49-mRNA-1 | Japonica | Japonica | | Os03g0422600 | OsR498G0306234500.01.T01 | 3-oxo-Delta(45)-steroid 5-beta-reductase |
| maker-3-augustus-gene-213.51-mRNA-1 | Japonica | Indica | | Os03g0421400 | OsR498G0306232800.01.T02 | Erlin-2-B isoform A |
| maker-3-augustus-gene-213.55-mRNA-1 | Japonica | NA | | Os03g0421800 | OsR498G0306233400.01.T01 | Predicted pectin lyase-like family protein with X8 domain |
| maker-3-augustus-gene-213.58-mRNA-1 | Japonica | NA | | | OsR498G0306236600.01.T05 | UDP-N-acetylglucosamine transferase subunit ALG |
| maker-3-augustus-gene-214.34-mRNA-1 | Japonica | Indica | Kinase-TM | Os03g0424200 | OsR498G0306238100.01.T01 | U-box domain-containing protein 57 |
| maker-3-augustus-gene-214.36-mRNA-1 | Japonica | NA | | Os03g0423800 | OsR498G0306238300.01.T06 | Mediator of RNA polymerase II transcription subunit 8 |
| maker-3-augustus-gene-214.39-mRNA-1 | Japonica | NA | | Os03g0424500 | OsR498G0306240900.01.T02 | 40S ribosomal protein S19 |
| maker-3-augustus-gene-214.43-mRNA-1 | Japonica | Japonica | CC-Kinase-TM | Os03g0424000 | OsR498G0306240800.01.T02 | E3 ubiquitin ligase |
| maker-3-augustus-gene-214.46-mRNA-1 | Japonica | NA | | | OsR498G0306238800.01.T06 | Ubiquitinyl hydrolase 1 |
| maker-3-augustus-gene-214.48-mRNA-1 | Japonica | NA | Kinase-TM | Os03g0424200 | OsR498G0306240800.01.T02 | U-box domain-containing protein 32 |
| maker-3-augustus-gene-214.52-mRNA-1 | Japonica | NA | | | OsR498G0306240800.01.T06 | U-box domain-containing protein 57 |
| maker-3-augustus-gene-214.58-mRNA-1 | Japonica | NA | | Os03g0424600 | | Zinc finger BED domain-containing protein RICESLEEPER 2-like |
| maker-3-augustus-gene-215.37-mRNA-1 | Japonica | NA | | Os03g0424500 | OsR498G0306241700.01.T02 | 40S ribosomal protein S19 |
| maker-3-augustus-gene-215.41-mRNA-1 | Japonica | Indica | | Os03g0425000 | OsR498G0306241900.01.T02 | RAP domain-containing protein chloroplastic |
| maker-3-augustus-gene-215.44-mRNA-1 | Japonica | NA | | | OsR498G0306242900.01.T03 | Diacylglycerol kinase |
| maker-3-augustus-gene-215.51-mRNA-1 | Japonica | NA | | Os03g0425800 | OsR498G0306244500.01.T01 | SANT domain-containing protein |
| maker-3-augustus-gene-215.53-mRNA-1 | Japonica | NA | | Os03g0425200 | OsR498G0306242600.01.T02 | probable uridine nucleosidase 2 |
| maker-3-augustus-gene-216.13-mRNA-1 | Japonica | NA | | | OsR498G0306251000.01.T01 | Beta-caryophyllene synthase |
| maker-3-augustus-gene-216.2-mRNA-1 | Japonica | NA | | Os03g0426900 | OsR498G0306246900.01.T01 | Chaperone protein ClpB3 chloroplastic |
| maker-3-augustus-gene-216.6-mRNA-1 | Japonica | NA | | Os03g0427300 | OsR498G0306248900.01.T01 | Glutelin type-A 3 |
| maker-3-augustus-gene-216.8-mRNA-1 | Japonica | NA | | | OsR498G0306250100.01.T02 | Ubiquitin conjugation factor like |
| maker-3-augustus-gene-217.1-mRNA-1 | Japonica | NA | | | OsR498G0306253300.01.T03 | ATP-dependent protease Clp ATPase subunit |
| maker-3-augustus-gene-217.11-mRNA-1 | Japonica | NA | | Os03g0430000 | OsR498G0306256500.01.T06 | nuclear envelope integral membrane protein 1 isoform X3 |
| maker-3-augustus-gene-217.16-mRNA-1 | Japonica | NA | | | OsR498G0306251800.01.T01 | C2 tensin-type domain-containing protein |
| maker-3-augustus-gene-217.4-mRNA-1 | Japonica | NA | | Os03g0429800 | OsR498G0306255700.01.T01 | Xanthine dehydrogenase |
| maker-3-augustus-gene-218.36-mRNA-1 | Japonica | NA | | | OsR498G0306257500.01.T01 | JmjC domain containing protein, expressed |
| maker-3-augustus-gene-218.39-mRNA-1 | Japonica | Indica | | Os02g0828900 | OsR498G0306257900.01.T01 | Transcription factor jumonji (JmjC) domain-containing protein |
| maker-3-augustus-gene-218.50-mRNA-1 | Japonica | Japonica | | Os03g0431200 | OsR498G0714654200.01.T04 | Amino-acid N-acetyltransferase |
| maker-3-augustus-gene-218.56-mRNA-1 | Japonica | NA | | Os03g0431500 | OsR498G0306263800.01.T05 | Ubiquitin-like domain-containing protein |
| maker-3-augustus-gene-218.57-mRNA-1 | Japonica | NA | | Os01g0444100 | | |
| maker-3-augustus-gene-219.34-mRNA-1 | Japonica | NA | | | OsR498G0306265310.01.T02 | TATA element modulatory factor isoform X1 |
| maker-3-augustus-gene-219.37-mRNA-1 | Japonica | NA | | Os03g0431932 | OsR498G0306265310.01.T05 | Myosin heavy chain-related |
| maker-3-augustus-gene-219.40-mRNA-1 | Japonica | NA | | | OsR498G0306269500.01.T04 | Mechanosensitive ion channel protein 3 chloroplastic |
| maker-3-augustus-gene-219.48-mRNA-1 | Japonica | NA | | Os03g0432100 | OsR498G0306265800.01.T02 | Pyruvate, phosphate dikinase |
| maker-3-augustus-gene-22.48-mRNA-1 | Japonica | NA | | Os03g0143700 | OsR498G0305218700.01.T05 | Phosphatidylinositol N-acetyglucosaminlytransferase subunit P-related |
| maker-3-augustus-gene-22.52-mRNA-1 | Japonica | NA | | | | Transcription activators isoform 1 |
| maker-3-augustus-gene-22.59-mRNA-1 | Japonica | NA | | Os03g0143400 | OsR498G0305217600.01.T01 | Heat shock protein 60 isoform 1 |
| maker-3-augustus-gene-22.67-mRNA-1 | Japonica | NA | | | OsR498G0305219200.01.T01 | |
| maker-3-augustus-gene-221.27-mRNA-1 | Japonica | NA | | Os03g0434400 | OsR498G0306276700.01.T01 | Putative PhzC/PhzF-type epimerase |
| maker-3-augustus-gene-221.32-mRNA-1 | Japonica | NA | | Os03g0435200 | | Pentatricopeptide, putative |
| maker-3-augustus-gene-221.34-mRNA-1 | Japonica | NA | | Os03g0435300 | OsR498G0306279500.01.T01 | U-box domain-containing protein 4 |
| maker-3-augustus-gene-221.36-mRNA-1 | Japonica | Japonica | | Os03g0434500 | OsR498G0306278000.01.T01 | Peroxidase |
| maker-3-augustus-gene-221.37-mRNA-1 | Japonica | NA | | | | Peroxidase |
| maker-3-augustus-gene-222.36-mRNA-1 | Japonica | NA | | Os03g0436300 | OsR498G0306282100.01.T01 | Calmodulin-binding protein, putative, expressed |

| | | | | | | |
|---|---|---|---|---|---|---|
| maker-3-augustus-gene-222.41-mRNA-1 | Japonica | NA | | Os03g0437600 | OsR498G0306286600.01.T01 | Alpha/beta hydroxylase D14 |
| maker-3-augustus-gene-223.38-mRNA-1 | Japonica | NA | | Os03g0439500 | OsR498G0306294500.01.T01 | Fe2OG dioxygenase domain-containing protein (Fragment) |
| maker-3-augustus-gene-223.40-mRNA-1 | Japonica | NA | | Os03g0438100 | OsR498G0306289400.01.T01 | Allene-oxide cyclase |
| maker-3-augustus-gene-224.0-mRNA-1 | Japonica | NA | LRR-TM | Os03g0440900 | OsR498G0306297500.01.T01 | Somatic embryogenesis receptor kinase 1 |
| maker-3-augustus-gene-224.17-mRNA-1 | Japonica | NA | | | OsR498G0306297800.01.T01 | RST domain-containing protein |
| maker-3-augustus-gene-224.5-mRNA-1 | Japonica | NA | | Os03g0439700 | OsR498G0306295000.01.T02 | protein CONSERVED IN THE GREEN LINEAGE AND DIATOMS 27, chloroplastic |
| maker-3-augustus-gene-225.30-mRNA-1 | Japonica | NA | | Os03g0442900 | OsR498G0306305300.01.T02 | |
| maker-3-augustus-gene-225.32-mRNA-1 | Japonica | NA | | Os08g0487300 | OsR498G0816350400.01.T01 | Transposon protein, putative, Mutator sub-class |
| maker-3-augustus-gene-226.27-mRNA-1 | Japonica | NA | | | | HGWP repeat containing protein-like |
| maker-3-augustus-gene-227.4-mRNA-1 | Japonica | NA | | Os03g0445700 | OsR498G0306326100.01.T01 | LOB domain-containing protein 37 |
| maker-3-augustus-gene-229.24-mRNA-1 | Japonica | Japonica | | Os03g0448500 | OsR498G0203451400.01.T02 | Calcineurin B-like protein 7 |
| maker-3-augustus-gene-23.30-mRNA-1 | Japonica | NA | | Os03g0145200 | OsR498G0305222100.01.T01 | GATA transcription factor |
| maker-3-augustus-gene-23.32-mRNA-1 | Japonica | NA | | | OsR498G0305223500.01.T04 | |
| maker-3-augustus-gene-23.35-mRNA-1 | Japonica | NA | | Os03g0145700 | OsR498G0305223500.01.T02 | |
| maker-3-augustus-gene-23.37-mRNA-1 | Japonica | NA | | | | E3 ubiquitin-protein ligase miel1 |
| maker-3-augustus-gene-23.45-mRNA-1 | Japonica | NA | | Os03g0146000 | OsR498G0305224700.01.T01 | GTP-binding protein yptV3 |
| maker-3-augustus-gene-23.49-mRNA-1 | Japonica | NA | | | OsR498G0305226300.01.T02 | Cyclin-dependent kinases regulatory subunit |
| maker-3-augustus-gene-23.53-mRNA-1 | Japonica | Indica | | Os03g0146400 | OsR498G0305226500.01.T01 | Pheophorbide a oxygenase chloroplastic |
| maker-3-augustus-gene-23.58-mRNA-1 | Japonica | NA | | Os03g0146500 | OsR498G0305226700.01.T02 | (RAP Annotation release2) Galactose-binding like domain containing protein |
| maker-3-augustus-gene-23.63-mRNA-1 | Japonica | NA | | Os03g0145500 | OsR498G0305222800.01.T01 | UDENN domain-containing protein |
| maker-3-augustus-gene-23.70-mRNA-1 | Japonica | NA | | | | Zinc finger CCCH domain-containing protein 48-like |
| maker-3-augustus-gene-23.72-mRNA-1 | Japonica | Indica | | Os03g0145800 | OsR498G0305224200.01.T01 | Tankyrase-1 |
| maker-3-augustus-gene-23.75-mRNA-1 | Japonica | Indica | | Os03g0146100 | OsR498G0305225000.01.T02 | Tonoplast intrinsic protein 1 |
| maker-3-augustus-gene-230.21-mRNA-1 | Japonica | NA | | | OsR498G0306318000.01.T01 | Exonuclease/helicase-like |
| maker-3-augustus-gene-230.23-mRNA-1 | Japonica | NA | | | | Mitotic checkpoint protein BUB3 |
| maker-3-augustus-gene-230.28-mRNA-1 | Japonica | NA | | Os03g0448700 | OsR498G0306320700.01.T03 | Interferon-related developmental regulator family protein / IFRD protein fa[illegible] |
| maker-3-augustus-gene-232.30-mRNA-1 | Japonica | NA | | Os03g0452300 | OsR498G0306330000.01.T02 | 30S ribosomal protein S5, chloroplastic |
| maker-3-augustus-gene-233.0-mRNA-1 | Japonica | NA | | Os03g0453800 | OsR498G0306337300.01.T02 | |
| maker-3-augustus-gene-233.13-mRNA-1 | Japonica | NA | | Os12g0468900 | OsR498G0510193500.01.T01 | Cell wall protein-like |
| maker-3-augustus-gene-233.15-mRNA-1 | Japonica | NA | | Os03g0454100 | OsR498G0306337600.01.T01 | Serine incorporator 3 |
| maker-3-augustus-gene-233.2-mRNA-1 | Japonica | NA | | | OsR498G0306333700.01.T03 | |
| maker-3-augustus-gene-233.6-mRNA-1 | Japonica | NA | | | OsR498G0306338700.01.T03 | LON peptidase N-terminal domain and RING finger protein 1 |
| maker-3-augustus-gene-234.0-mRNA-1 | Japonica | NA | | | OsR498G0306340300.01.T01 | DUF1336 domain containing protein expressed |
| maker-3-augustus-gene-24.1-mRNA-1 | Japonica | NA | | | | Protein SHOOT GRAVITROPISM 6 isoform D |
| maker-3-augustus-gene-24.12-mRNA-1 | Japonica | NA | | Os03g0147400 | OsR498G0305229600.01.T02 | CitMHS domain-containing protein |
| maker-3-augustus-gene-24.16-mRNA-1 | Japonica | Indica | | Os01g0267100 | OsR498G0305231800.01.T01 | Methyltransferase-like protein 1 isoform A |
| maker-3-augustus-gene-24.21-mRNA-1 | Japonica | NA | | Os03g0148000 | OsR498G0305232200.01.T01 | Homogentisate phytyltransferase 1 |
| maker-3-augustus-gene-24.24-mRNA-1 | Japonica | NA | | | OsR498G0305232600.01.T01 | Mesoderm development candidate |
| maker-3-augustus-gene-24.8-mRNA-1 | Japonica | NA | | Os03g0147900 | OsR498G0305231900.01.T03 | Mitochondrial inner membrane protease subunit 2 |
| maker-3-augustus-gene-240.34-mRNA-1 | Japonica | NA | | | OsR498G0306444400.01.T01 | GDSL esterase/lipase |
| maker-3-augustus-gene-249.0-mRNA-1 | Japonica | NA | | Os03g0595200 | OsR498G0306493500.01.T01 | Cysteine synthase 2-like isoform X1 |
| maker-3-augustus-gene-25.38-mRNA-1 | Japonica | NA | Kinase | Os03g0148700 | OsR498G0305233300.01.T01 | Serine/threonine protein kinase |
| maker-3-augustus-gene-25.43-mRNA-1 | Japonica | Indica | | Os03g0149400 | OsR498G0305237100.01.T05 | TPR Domain containing protein, expressed |
| maker-3-augustus-gene-25.45-mRNA-1 | Japonica | NA | | Os03g0148800 | | Lysine-specific demethylase SE14 |
| maker-3-augustus-gene-25.47-mRNA-1 | Japonica | NA | | Os03g0149200 | OsR498G0305235800.01.T01 | HR-like lesion-inducing protein-related |
| maker-3-augustus-gene-25.51-mRNA-1 | Japonica | NA | | | OsR498G0305236200.01.T02 | |
| maker-3-augustus-gene-26.38-mRNA-1 | Japonica | Indica | | Os03g0150700 | OsR498G0305239700.01.T01 | |
| maker-3-augustus-gene-26.40-mRNA-1 | Japonica | NA | | | OsR498G0305240700.01.T02 | Putative lysine-specific demethylase ELF6 |
| maker-3-augustus-gene-26.44-mRNA-1 | Japonica | NA | | Os03g0151700 | OsR498G0305241500.01.T01 | WD repeat-containing protein 3 |
| maker-3-augustus-gene-26.48-mRNA-1 | Japonica | NA | | Os03g0152100 | OsR498G0305244100.01.T02 | Transcription factor-like protein DPB |
| maker-3-augustus-gene-26.53-mRNA-1 | Japonica | NA | | Os03g0152300 | OsR498G0305244300.01.T01 | Peroxidase |
| maker-3-augustus-gene-26.55-mRNA-1 | Japonica | NA | | | | Appr-1-p processing enzyme family protein, expressed |
| maker-3-augustus-gene-26.62-mRNA-1 | Japonica | NA | | Os03g0151800 | OsR498G0305242000.01.T01 | Cell division cycle protein 48 like |
| maker-3-augustus-gene-26.67-mRNA-1 | Japonica | NA | | Os03g0151900 | OsR498G0305242200.01.T01 | Ras-related protein RABF1 |
| maker-3-augustus-gene-26.70-mRNA-1 | Japonica | NA | | Os03g0152000 | OsR498G0305243800.01.T01 | HMA domain-containing protein |
| maker-3-augustus-gene-26.75-mRNA-1 | Japonica | NA | | | OsR498G0305245000.01.T01 | 4-coumarate--CoA ligase-like protein 7 |
| maker-3-augustus-gene-26.78-mRNA-1 | Japonica | NA | | Os03g0152600 | OsR498G0305245400.01.T03 | D-tagatose-1,6-bisphosphate aldolase subunit |
| maker-3-augustus-gene-265.15-mRNA-1 | Japonica | NA | | Os10g0425500 | OsR498G1018754900.01.T01 | BTB/POZ and MATH domain-containing protein 1 |
| maker-3-augustus-gene-265.2-mRNA-1 | Japonica | NA | | | OsR498G0409123600.01.T01 | |
| maker-3-augustus-gene-265.3-mRNA-1 | Japonica | NA | | Os10g0426800 | OsR498G1018758700.01.T01 | BTB domain-containing protein |
| maker-3-augustus-gene-265.6-mRNA-1 | Japonica | NA | | Os10g0425900 | OsR498G1018755900.01.T01 | BTB/POZ and MATH domain-containing protein 2 |

| | | | | | | |
|---|---|---|---|---|---|---|
| maker-3-augustus-gene-266.0-mRNA-1 | Japonica | NA | | Os10g0423600 | OsR498G1018758400.01.T01 | Speckle-type POZ protein-like protein A |
| maker-3-augustus-gene-267.15-mRNA-1 | Japonica | NA | | Os10g0422200 | OsR498G1018745900.01.T01 | Hcy-binding domain-containing protein |
| maker-3-augustus-gene-267.3-mRNA-1 | Japonica | NA | | Os10g0422566 | OsR498G1018748000.01.T03 | Pentatricopeptide repeat-containing protein mitochondrial |
| maker-3-augustus-gene-267.4-mRNA-1 | Japonica | NA | | | OsR498G1018745200.01.T01 | NAB domain-containing protein (Fragment) |
| maker-3-augustus-gene-267.7-mRNA-1 | Japonica | NA | | Os10g0422500 | OsR498G1018747300.01.T03 | Type IV inositol polyphosphate 5-phosphatase |
| maker-3-augustus-gene-268.41-mRNA-1 | Japonica | NA | | | | |
| maker-3-augustus-gene-268.43-mRNA-1 | Japonica | NA | | | OsR498G1018741700.01.T01 | CAF1 family ribonuclease containing protein |
| maker-3-augustus-gene-269.0-mRNA-1 | Japonica | NA | | Os10g0420000 | OsR498G1018738600.01.T01 | Alkylated DNA repair protein alkB like 6 |
| maker-3-augustus-gene-269.11-mRNA-1 | Japonica | NA | | Os10g0419400 | OsR498G1018736600.01.T01 | 1,2-dihydroxy-3-keto-5-methylthiopentene dioxygenase |
| maker-3-augustus-gene-269.13-mRNA-1 | Japonica | NA | | Os10g0419300 | OsR498G1018736000.01.T03 | Heat shock transcription factor |
| maker-3-augustus-gene-269.15-mRNA-1 | Japonica | NA | | Os10g0419200 | | zinc finger protein JACKDAW |
| maker-3-augustus-gene-269.17-mRNA-1 | Japonica | NA | | Os10g0420400 | OsR498G1018739300.01.T01 | Sulfate anion transporter |
| maker-3-augustus-gene-269.21-mRNA-1 | Japonica | NA | | | OsR498G1018738700.01.T02 | IQ calmodulin-binding motif family protein, expressed |
| maker-3-augustus-gene-269.24-mRNA-1 | Japonica | NA | | Os10g0419100 | OsR498G1018735300.01.T02 | Perakine reductase |
| maker-3-augustus-gene-269.7-mRNA-1 | Japonica | NA | | Os10g0419600 | OsR498G1018737400.01.T01 | Chlorophyllase |
| maker-3-augustus-gene-269.9-mRNA-1 | Japonica | NA | | Os10g0419500 | OsR498G1018737000.01.T01 | 1,2-dihydroxy-3-keto-5-methylthiopentene dioxygenase |
| maker-3-augustus-gene-27.46-mRNA-1 | Japonica | NA | | Os03g0152700 | OsR498G0305245500.01.T03 | S4 RNA-binding domain-containing protein (Fragment) |
| maker-3-augustus-gene-27.52-mRNA-1 | Japonica | NA | | Os03g0152800 | OsR498G0305245700.01.T06 | S4 RNA-binding domain-containing protein |
| maker-3-augustus-gene-27.59-mRNA-1 | Japonica | Japonica | | Os03g0152900 | OsR498G0305245900.01.T02 | Kinesin-like protein KIN-UA |
| maker-3-augustus-gene-27.65-mRNA-1 | Japonica | Indica | | Os03g0153500 | OsR498G0305246500.01.T01 | FAD binding domain containing protein, expressed |
| maker-3-augustus-gene-27.67-mRNA-1 | Japonica | NA | | Os03g0153500 | OsR498G0305247100.01.T02 | FAD binding domain containing protein, expressed |
| maker-3-augustus-gene-27.70-mRNA-1 | Japonica | NA | | Os03g0153900 | OsR498G0305247700.01.T03 | FAD binding domain containing protein, expressed |
| maker-3-augustus-gene-27.74-mRNA-1 | Japonica | NA | | Os03g0154100 | OsR498G0305248100.01.T01 | FAD binding domain containing protein, expressed |
| maker-3-augustus-gene-27.76-mRNA-1 | Japonica | NA | | Os03g0154700 | OsR498G0305248900.01.T01 | 30S ribosomal protein S4, chloroplastic |
| maker-3-augustus-gene-27.79-mRNA-1 | Japonica | NA | | Os03g0154100 | OsR498G0305249300.01.T01 | FAD binding domain containing protein, expressed |
| maker-3-augustus-gene-27.80-mRNA-1 | Japonica | NA | | Os03g0155300 | OsR498G0305249700.01.T01 | Expansin |
| maker-3-augustus-gene-27.82-mRNA-1 | Japonica | NA | | | OsR498G0305246200.01.T02 | Splicing factor isoform 1 |
| maker-3-augustus-gene-27.85-mRNA-1 | Japonica | Indica | | Os10g0439200 | OsR498G0305250200.01.T01 | Expansin |
| maker-3-augustus-gene-270.27-mRNA-1 | Japonica | NA | | Os10g0417800 | OsR498G1018729400.01.T01 | Plant-specific domain TIGR01615 family protein |
| maker-3-augustus-gene-270.29-mRNA-1 | Japonica | NA | | Os10g0418000 | OsR498G1018731500.01.T01 | Probable histone H2A variant 2 |
| maker-3-augustus-gene-270.31-mRNA-1 | Japonica | NA | | Os10g0417600 | OsR498G1018728100.01.T02 | GDP-mannose 3,5-epimerase 1 |
| maker-3-augustus-gene-271.32-mRNA-1 | Japonica | NA | | Os10g0415900 | OsR498G1018721000.01.T03 | Histone acetyltransferase |
| maker-3-augustus-gene-271.36-mRNA-1 | Japonica | NA | | Os10g0415400 | OsR498G1018719200.01.T03 | |
| maker-3-augustus-gene-271.38-mRNA-1 | Japonica | NA | | Os10g0415300 | OsR498G1018718600.01.T01 | Glutathione reductase, chloroplastic |
| maker-3-augustus-gene-271.43-mRNA-1 | Japonica | NA | | Os10g0415800 | | Acylamino-acid-releasing enzyme like |
| maker-3-augustus-gene-271.45-mRNA-1 | Japonica | NA | | Os10g0415600 | OsR498G1018720700.01.T05 | Acylaminoacyl-peptidase |
| maker-3-augustus-gene-272.0-mRNA-1 | Japonica | NA | | Os10g0414500 | OsR498G1018716600.01.T02 | Putative 16S rRNA (Cytidine(1402)-2'-O)-methyltransferase |
| maker-3-augustus-gene-272.11-mRNA-1 | Japonica | NA | | Os10g0414800 | OsR498G1018717500.01.T01 | Myosin-binding protein 1 |
| maker-3-augustus-gene-272.13-mRNA-1 | Japonica | NA | | Os10g0414700 | OsR498G1018717300.01.T01 | KH domain containing protein, expressed |
| maker-3-augustus-gene-272.16-mRNA-1 | Japonica | NA | | Os10g0414000 | OsR498G1018712900.01.T01 | NAC domain-containing protein |
| maker-3-augustus-gene-272.18-mRNA-1 | Japonica | NA | | Os10g0413900 | OsR498G1018712500.01.T02 | NAC domain-containing protein |
| maker-3-augustus-gene-272.21-mRNA-1 | Japonica | NA | | Os10g0413700 | OsR498G1018711900.01.T01 | NAC domain-containing protein |
| maker-3-augustus-gene-272.23-mRNA-1 | Japonica | NA | | Os10g0413400 | OsR498G1018710900.01.T01 | Glycerol-3-phosphate 2-O-acyltransferase |
| maker-3-augustus-gene-272.4-mRNA-1 | Japonica | NA | | Os10g0413500 | OsR498G1018711200.01.T01 | Procollagen-proline 4-dioxygenase |
| maker-3-augustus-gene-272.9-mRNA-1 | Japonica | NA | | Os10g0415100 | OsR498G1018717900.01.T01 | GABA transporter 1 |
| maker-3-augustus-gene-273.24-mRNA-1 | Japonica | NA | | Os10g0412700 | OsR498G1018709400.01.T01 | Thaumatin |
| maker-3-augustus-gene-273.26-mRNA-1 | Japonica | NA | | Os10g0412100 | OsR498G1018708600.01.T03 | Carbon catabolite repressor protein 2 |
| maker-3-augustus-gene-273.31-mRNA-1 | Japonica | NA | | | OsR498G1018708400.01.T02 | Phospholipid-transporting ATPase |
| maker-3-augustus-gene-273.42-mRNA-1 | Japonica | NA | | | | 40S ribosomal protein S28 |
| maker-3-augustus-gene-274.31-mRNA-1 | Japonica | Indica | | Os03g0167400 | OsR498G1018705400.01.T01 | Lipase, putative, expressed |
| maker-3-augustus-gene-274.36-mRNA-1 | Japonica | NA | | Os10g0410700 | OsR498G1018704600.01.T02 | Histone-lysine N-methyltransferase ATXR4 |
| maker-3-augustus-gene-274.37-mRNA-1 | Japonica | NA | | Os10g0410700 | OsR498G1018704600.01.T01 | Histone-lysine N-methyltransferase ATXR4 |
| maker-3-augustus-gene-274.42-mRNA-1 | Japonica | NA | | Os10g0409900 | OsR498G1018704000.01.T01 | Kelch motif family protein, expressed |
| maker-3-augustus-gene-274.43-mRNA-1 | Japonica | NA | | Os10g0411500 | OsR498G1018706700.01.T01 | IQ domain-containing protein IQM6 |
| maker-3-augustus-gene-274.47-mRNA-1 | Japonica | NA | | | OsR498G1018704900.01.T01 | Transposable element protein, putative, MuDR |
| maker-3-augustus-gene-274.48-mRNA-1 | Japonica | NA | | Os01g0204900 | OsR498G0203659800.01.T01 | rRNA N-glycosidase |
| maker-3-augustus-gene-274.61-mRNA-1 | Japonica | NA | | Os10g0410600 | OsR498G1018704300.01.T02 | Serine/threonine-protein phosphatase |
| maker-3-augustus-gene-275.20-mRNA-1 | Japonica | NA | | Os10g0409400 | OsR498G1018700800.01.T01 | Polygalacturonase 2 |
| maker-3-augustus-gene-277.0-mRNA-1 | Japonica | NA | | Os10g0407200 | OsR498G1018685800.01.T01 | Putative endoplasmic reticulum membrane protein C16E8.02 |
| maker-3-augustus-gene-277.11-mRNA-1 | Japonica | NA | | | OsR498G1018686900.01.T02 | Exonuclease |
| maker-3-augustus-gene-277.15-mRNA-1 | Japonica | NA | | Os10g0406800 | OsR498G1018684300.01.T01 | |

| | | | | | | |
|---|---|---|---|---|---|---|
| maker-3-augustus-gene-277.2-mRNA-1 | Japonica | NA | | Os10g0407100 | OsR498G1018685600.01.T02 | 50S ribosomal protein L32, chloroplastic |
| maker-3-augustus-gene-277.4-mRNA-1 | Japonica | NA | | | OsR498G1018685200.01.T03 | Pectinesterase |
| maker-3-augustus-gene-277.7-mRNA-1 | Japonica | NA | | Os10g0407500 | OsR498G1018687100.01.T01 | Ribulose-phosphate 3-epimerase |
| maker-3-augustus-gene-278.0-mRNA-1 | Japonica | NA | | | OsR498G1018681600.01.T01 | Putative mannan synthase 2 |
| maker-3-augustus-gene-278.14-mRNA-1 | Japonica | NA | | Os10g0405600 | OsR498G1018678000.01.T01 | ATP-dependent 6-phosphofructokinase 5, chloroplastic |
| maker-3-augustus-gene-278.18-mRNA-1 | Japonica | NA | | Os10g0405500 | OsR498G1018677700.01.T01 | Meiosis-specific protein PAIR3 |
| maker-3-augustus-gene-278.22-mRNA-1 | Japonica | NA | | Os10g0405300 | OsR498G1018677300.01.T01 | AvrRpt-cleavage domain-containing protein (Fragment) |
| maker-3-augustus-gene-278.24-mRNA-1 | Japonica | NA | Kinase-TM | Os10g0405100 | OsR498G1018676700.01.T05 | probable serine/threonine-protein kinase PBL3 |
| maker-3-augustus-gene-278.8-mRNA-1 | Japonica | NA | | Os10g0406200 | OsR498G1018679400.01.T03 | Putative methyltransferase-like protein 7B-like |
| maker-3-augustus-gene-28.0-mRNA-1 | Japonica | Indica | | Os03g0156000 | OsR498G0305250700.01.T01 | Expansin |
| maker-3-augustus-gene-28.10-mRNA-1 | Japonica | NA | | Os03g0157300 | OsR498G0305254100.01.T03 | Cell number regulator 13 |
| maker-3-augustus-gene-28.17-mRNA-1 | Japonica | NA | | Os03g0157500 | OsR498G0305254900.01.T02 | FH protein interacting protein FIP2 |
| maker-3-augustus-gene-28.20-mRNA-1 | Japonica | Indica | | Os04g0228400 | OsR498G0305250400.01.T01 | Expansin |
| maker-3-augustus-gene-28.22-mRNA-1 | Japonica | NA | | Os03g0156600 | OsR498G0305252000.01.T01 | Heavy metal-associated isoprenylated protein 1 |
| maker-3-augustus-gene-28.24-mRNA-1 | Japonica | NA | | Os03g0156700 | OsR498G0305252200.01.T02 | Nickel/cobalt transporter, high-affinity |
| maker-3-augustus-gene-28.27-mRNA-1 | Japonica | NA | | Os03g0157400 | OsR498G0305254600.01.T01 | ABC transporter domain-containing protein (Fragment) |
| maker-3-augustus-gene-28.3-mRNA-1 | Japonica | NA | | Os03g0156000 | OsR498G0305251100.01.T01 | Expansin |
| maker-3-augustus-gene-28.5-mRNA-1 | Japonica | NA | | Os03g0156500 | OsR498G0305251700.01.T01 | HTH La-type RNA-binding domain-containing protein |
| maker-3-augustus-gene-281.18-mRNA-1 | Japonica | NA | | Os10g0401100 | OsR498G1018661100.01.T01 | Endonuclease/Exonuclease/phosphatase family protein, expressed |
| maker-3-augustus-gene-283.0-mRNA-1 | Japonica | NA | | | OsR498G1018649200.01.T01 | Delta(24)-sterol reductase |
| maker-3-augustus-gene-283.2-mRNA-1 | Japonica | NA | | Os10g0397200 | OsR498G1018648800.01.T04 | Transcription initiation factor TFIIE beta subunit |
| maker-3-augustus-gene-284.0-mRNA-1 | Japonica | NA | | Os10g0396400 | OsR498G1018647400.01.T02 | F-box/LRR-repeat protein 15 |
| maker-3-augustus-gene-284.12-mRNA-1 | Japonica | NA | | Os10g0402100 | OsR498G1018645200.01.T01 | No apical meristem protein, expressed |
| maker-3-augustus-gene-284.15-mRNA-1 | Japonica | NA | | Os10g0395400 | OsR498G1018644800.01.T01 | Bronze-2 protein (Fragment) |
| maker-3-augustus-gene-284.17-mRNA-1 | Japonica | NA | | | | Stress enhanced protein 1 chloroplastic |
| maker-3-augustus-gene-284.21-mRNA-1 | Japonica | NA | | | | Myosin-15 isoform A |
| maker-3-augustus-gene-284.28-mRNA-1 | Japonica | NA | | Os10g0395500 | OsR498G1018644900.01.T05 | Proteasome assembly chaperone 3 |
| maker-3-augustus-gene-284.7-mRNA-1 | Japonica | NA | | Os10g0396300 | OsR498G1018646800.01.T02 | MOB kinase activator family |
| maker-3-augustus-gene-285.37-mRNA-1 | Japonica | NA | Kinase | Os10g0395000 | OsR498G1018643000.01.T01 | Serine/threonine-protein kinase RIPK |
| maker-3-augustus-gene-285.44-mRNA-1 | Japonica | NA | CC-NBS-TM | | OsR498G1018641100.01.T02 | Putative disease resistance protein RGA3 |
| maker-3-augustus-gene-285.47-mRNA-1 | Japonica | Indica | | Os03g0605300 | OsR498G1018640500.01.T01 | Subtilisin-like protease |
| maker-3-augustus-gene-285.52-mRNA-1 | Japonica | NA | | Os10g0393800 | OsR498G1018638500.01.T01 | GDSL-like Lipase/Acylhydrolase family protein, expressed |
| maker-3-augustus-gene-286.38-mRNA-1 | Japonica | NA | | Os10g0393100 | OsR498G1018638000.01.T01 | Presenilin/signal peptide peptidase |
| maker-3-augustus-gene-286.40-mRNA-1 | Japonica | NA | | | OsR498G1018636800.01.T02 | Translation initiation factor eIF-2B beta subunit |
| maker-3-augustus-gene-286.45-mRNA-1 | Japonica | NA | | Os10g0392500 | | |
| maker-3-augustus-gene-286.46-mRNA-1 | Japonica | NA | | Os10g0392400 | OsR498G1018635200.01.T01 | Tify domain-containing protein (Fragment) |
| maker-3-augustus-gene-286.48-mRNA-1 | Japonica | NA | | Os10g0393500 | OsR498G1018638100.01.T01 | GDSL esterase/lipase |
| maker-3-augustus-gene-286.51-mRNA-1 | Japonica | NA | | Os10g0392900 | OsR498G1018637700.01.T01 | GDSL-like Lipase/Acylhydrolase family protein, expressed |
| maker-3-augustus-gene-286.53-mRNA-1 | Japonica | NA | | Os10g0392600 | OsR498G1018636500.01.T01 | SPX domain-containing protein 3 |
| maker-3-augustus-gene-287.17-mRNA-1 | Japonica | NA | | Os10g0390800 | OsR498G1018627100.01.T02 | Ethylene-responsive element binding protein 2 |
| maker-3-augustus-gene-287.19-mRNA-1 | Japonica | NA | | Os10g0390650 | | |
| maker-3-augustus-gene-287.21-mRNA-1 | Japonica | NA | | Os10g0390600 | OsR498G1018626700.01.T03 | Alanine aminotransferase5 |
| maker-3-augustus-gene-287.26-mRNA-1 | Japonica | NA | | Os10g0390500 | OsR498G1018626300.01.T02 | Alanine aminotransferase |
| maker-3-augustus-gene-287.4-mRNA-1 | Japonica | NA | | | OsR498G1018628000.01.T03 | |
| maker-3-augustus-gene-287.7-mRNA-1 | Japonica | NA | | Os10g0390900 | OsR498G1018628000.01.T01 | SAC domain-containing protein |
| maker-3-augustus-gene-287.9-mRNA-1 | Japonica | NA | | Os10g0390100 | OsR498G1018626200.01.T02 | Pumilio homolog 23 |
| maker-3-augustus-gene-288.11-mRNA-1 | Japonica | NA | | Os10g0389200 | OsR498G1018622600.01.T01 | Red chlorophyll catabolite reductase chloroplastic |
| maker-3-augustus-gene-288.12-mRNA-1 | Japonica | NA | | | | Red chlorophyll catabolite reductase |
| maker-3-augustus-gene-288.18-mRNA-1 | Japonica | NA | | Os10g0389000 | OsR498G1018621400.01.T02 | Probable calcium-binding protein CML8 |
| maker-3-augustus-gene-288.2-mRNA-1 | Japonica | NA | Kinase-LRR-TM | Os10g0389800 | OsR498G1018625400.01.T01 | Strubbelig-receptor family 6 |
| maker-3-augustus-gene-288.28-mRNA-1 | Japonica | NA | | Os10g0388900 | OsR498G1018621200.01.T02 | Eukaryotic initiation factor 4a (Fragment) |
| maker-3-augustus-gene-288.31-mRNA-1 | Japonica | NA | | | OsR498G1221458900.01.T01 | Retrotransposon protein, putative, unclassified, expressed |
| maker-3-augustus-gene-288.9-mRNA-1 | Japonica | NA | | | OsR498G1018623600.01.T02 | Red chlorophyll catabolite reductase chloroplastic |
| maker-3-augustus-gene-289.0-mRNA-1 | Japonica | NA | | Os10g0387000 | OsR498G1018615200.01.T01 | Ethanolamine-phosphate cytidylyltransferase-like |
| maker-3-augustus-gene-289.6-mRNA-1 | Japonica | NA | | | | ATP-dependent DNA helicase |
| maker-3-augustus-gene-29.45-mRNA-1 | Japonica | NA | | Os03g0157700 | OsR498G0305255500.01.T01 | |
| maker-3-augustus-gene-29.47-mRNA-1 | Japonica | NA | | | | HRDC domain-containing protein (Fragment) |
| maker-3-augustus-gene-29.53-mRNA-1 | Japonica | NA | | | OsR498G0305256500.01.T01 | DEAD-box ATP-dependent RNA helicase 38 |
| maker-3-augustus-gene-29.61-mRNA-1 | Japonica | NA | | Os03g0159200 | OsR498G0305258100.01.T02 | XS domain containing protein expressed |
| maker-3-augustus-gene-29.63-mRNA-1 | Japonica | NA | | Os03g0159700 | OsR498G0305259300.01.T06 | Pentatricopeptide repeat-containing protein, mitochondrial |
| maker-3-augustus-gene-29.65-mRNA-1 | Japonica | NA | Kinase-TM | Os03g0160100 | OsR498G0305260500.01.T01 | Serine/threonine-protein kinase EDR1 |

| | | | | | | | |
|---|---|---|---|---|---|---|---|
| maker-3-augustus-gene-29.70-mRNA-1 | Japonica | NA | | Os03g0158300 | | Photosynthetic NDH subunit of subcomplex B 2 chloroplastic |
| maker-3-augustus-gene-29.72-mRNA-1 | Japonica | NA | | Os03g0158500 | OsR498G0305257000.01.T02 | Evolutionarily conserved C-terminal region 2 |
| maker-3-augustus-gene-29.78-mRNA-1 | Japonica | NA | | | OsR498G0305257800.01.T03 | Subtilisin-like protease |
| maker-3-augustus-gene-29.79-mRNA-1 | Japonica | NA | | Os03g0159000 | | Inhibitor I9 domain-containing protein |
| maker-3-augustus-gene-29.88-mRNA-1 | Japonica | NA | | Os03g0159600 | OsR498G0305259200.01.T01 | Late embryogenesis abundant |
| maker-3-augustus-gene-292.25-mRNA-1 | Japonica | NA | | Os10g0384600 | OsR498G1018602400.01.T01 | F-box domain containing protein, expressed |
| maker-3-augustus-gene-292.27-mRNA-1 | Japonica | NA | | | OsR498G0101949000.01.T01 | |
| maker-3-augustus-gene-292.31-mRNA-1 | Japonica | NA | | Os03g0340600 | | |
| maker-3-augustus-gene-293.0-mRNA-1 | Japonica | NA | | | | F-box domain containing protein |
| maker-3-augustus-gene-294.0-mRNA-1 | Japonica | NA | | Os10g0381501 | | |
| maker-3-augustus-gene-294.2-mRNA-1 | Japonica | NA | | Os10g0382300 | OsR498G1018596500.01.T01 | CTLH domain-containing protein |
| maker-3-augustus-gene-294.7-mRNA-1 | Japonica | NA | | Os07g0165500 | OsR498G0203722500.01.T01 | Cytokinin inducible protein-like |
| maker-3-augustus-gene-295.37-mRNA-1 | Japonica | NA | | Os10g0379100 | OsR498G1018591600.01.T01 | Tryptamine hydroxycinnamoyl transferase |
| maker-3-augustus-gene-295.38-mRNA-1 | Japonica | NA | | Os10g0379100 | OsR498G1018590600.01.T01 | Tryptamine hydroxycinnamoyl transferase |
| maker-3-augustus-gene-295.45-mRNA-1 | Japonica | NA | | Os10g0379100 | OsR498G1018592300.01.T01 | Tryptamine hydroxycinnamoyl transferase |
| maker-3-augustus-gene-296.23-mRNA-1 | Japonica | NA | | Os10g0378100 | OsR498G1018586200.01.T02 | Ent-kaurenoic acid oxidase like |
| maker-3-augustus-gene-296.30-mRNA-1 | Japonica | NA | | Os10g0378300 | OsR498G1018586300.01.T01 | Protein CutA, chloroplastic isoform C |
| maker-3-augustus-gene-296.35-mRNA-1 | Japonica | NA | | Os10g0378075 | | |
| maker-3-augustus-gene-297.0-mRNA-1 | Japonica | NA | | Os10g0378050 | OsR498G1018586200.01.T02 | Ent-kaurenoic acid oxidase like |
| maker-3-augustus-gene-297.13-mRNA-1 | Japonica | Indica | | Os03g0188900 | OsR498G1018583100.01.T01 | Homeodomain-leucine zipper transcription factor TaHDZipI-1 |
| maker-3-augustus-gene-297.3-mRNA-1 | Japonica | NA | | Os10g0377800 | OsR498G1018585200.01.T01 | NAD(P)H-hydrate epimerase |
| maker-3-augustus-gene-3.0-mRNA-1 | Japonica | NA | | Os03g0108600 | OsR498G0305096500.01.T01 | DEAD-box ATP-dependent RNA helicase |
| maker-3-augustus-gene-3.12-mRNA-1 | Japonica | NA | | Os03g0108901 | | |
| maker-3-augustus-gene-3.13-mRNA-1 | Japonica | NA | | Os03g0109000 | OsR498G0305097500.01.T01 | Vascular related NAC-domain protein 1 |
| maker-3-augustus-gene-3.18-mRNA-1 | Japonica | NA | | Os03g0109300 | OsR498G0305099300.01.T01 | Cytokinin riboside 5'-monophosphate phosphoribohydrolase |
| maker-3-augustus-gene-3.22-mRNA-1 | Japonica | NA | | Os03g0109400 | OsR498G0305101300.01.T03 | Rolled leaf2 |
| maker-3-augustus-gene-3.38-mRNA-1 | Japonica | NA | | Os03g0108700 | OsR498G0305096800.01.T01 | Aldehyde oxygenase (deformylating) |
| maker-3-augustus-gene-3.4-mRNA-1 | Japonica | Indica | Kinase-TM | Os03g0118400 | OsR498G0305096900.01.T04 | Cell division control protein A |
| maker-3-augustus-gene-3.40-mRNA-1 | Japonica | NA | | Os03g0109600 | OsR498G0305101800.01.T01 | Nascent polypeptide-associated complex subunit beta |
| maker-3-augustus-gene-3.43-mRNA-1 | Japonica | NA | | | | Nascent polypeptide-associated complex subunit beta |
| maker-3-augustus-gene-3.45-mRNA-1 | Japonica | NA | | Os03g0109700 | OsR498G0305102600.01.T04 | CemA-like proton extrusion protein-related |
| maker-3-augustus-gene-3.50-mRNA-1 | Japonica | NA | | | | |
| maker-3-augustus-gene-30.0-mRNA-1 | Japonica | NA | | Os03g0161000 | OsR498G0305262700.01.T01 | |
| maker-3-augustus-gene-30.13-mRNA-1 | Japonica | NA | | | | F-box domain-containing protein |
| maker-3-augustus-gene-30.15-mRNA-1 | Japonica | NA | | Os03g0161200 | OsR498G0305263600.01.T01 | STAS domain |
| maker-3-augustus-gene-30.4-mRNA-1 | Japonica | NA | | Os03g0161100 | | NAB domain-containing protein |
| maker-3-augustus-gene-30.7-mRNA-1 | Japonica | NA | | Os03g0160400 | OsR498G0305261200.01.T02 | p-loop containing nucleoside triphosphate hydrolases superfamily protein is |
| maker-3-augustus-gene-30.9-mRNA-1 | Japonica | Indica | | Os10g0422500 | OsR498G0305261600.01.T06 | Type IV inositol polyphosphate 5-phosphatase |
| maker-3-augustus-gene-300.0-mRNA-1 | Japonica | NA | | Os10g0370800 | OsR498G1018561600.01.T01 | Mannan endo-1,4-beta-mannosidase |
| maker-3-augustus-gene-300.4-mRNA-1 | Japonica | NA | | Os10g0370700 | OsR498G1018561400.01.T07 | Putative LeOPT1-oligopeptide transporter |
| maker-3-augustus-gene-300.7-mRNA-1 | Japonica | NA | | Os10g0371000 | OsR498G1018562700.01.T01 | Pollen-specific protein C13 |
| maker-3-augustus-gene-301.22-mRNA-1 | Japonica | NA | | Os10g0370500 | OsR498G1018560400.01.T01 | Mannan endo-1,4-beta-mannosidase |
| maker-3-augustus-gene-302.0-mRNA-1 | Japonica | NA | | Os10g0370000 | OsR498G1018558800.01.T04 | PPM-type phosphatase domain-containing protein |
| maker-3-augustus-gene-302.9-mRNA-1 | Japonica | NA | | Os10g0368800 | OsR498G1018556410.01.T06 | Pentatricopeptide, putative, expressed |
| maker-3-augustus-gene-303.24-mRNA-1 | Japonica | NA | | Os09g0368200 | OsR498G1018555800.01.T01 | Polyamine oxidase |
| maker-3-augustus-gene-304.8-mRNA-1 | Japonica | NA | | Os10g0365800 | OsR498G1018554500.01.T01 | |
| maker-3-augustus-gene-308.11-mRNA-1 | Japonica | NA | | Os10g0358600 | OsR498G1018527900.01.T01 | Histone deacetylase complex subunit SAP30 |
| maker-3-augustus-gene-308.7-mRNA-1 | Japonica | NA | | Os10g0358700 | OsR498G1018528200.01.T01 | Pentatricopeptide repeat-containing protein |
| maker-3-augustus-gene-308.9-mRNA-1 | Japonica | NA | | | OsR498G0204226400.01.T01 | Plant disease resistance polyprotein-like |
| maker-3-augustus-gene-31.11-mRNA-1 | Japonica | NA | | Os03g0161900 | OsR498G0305270500.01.T08 | Heat shock transcription factor |
| maker-3-augustus-gene-31.19-mRNA-1 | Japonica | NA | | Os03g0162200 | OsR498G0305273100.01.T01 | Probable histone H2A variant 1 |
| maker-3-augustus-gene-31.21-mRNA-1 | Japonica | Indica | | Os03g0162800 | OsR498G0305275500.01.T01 | Very-long-chain 3-oxoacyl-CoA synthase |
| maker-3-augustus-gene-310.20-mRNA-1 | Japonica | NA | | Os10g0352000 | OsR498G1018524000.01.T01 | TRIO and F-actin-binding protein |
| maker-3-augustus-gene-310.22-mRNA-1 | Japonica | NA | | Os10g0351700 | OsR498G1018523500.01.T01 | GH10 domain-containing protein (Fragment) |
| maker-3-augustus-gene-315.15-mRNA-1 | Japonica | NA | | Os10g0345900 | | |
| maker-3-augustus-gene-316.21-mRNA-1 | Japonica | NA | | Os10g0345100 | OsR498G1018505600.01.T02 | Protein DETOXIFICATION |
| maker-3-augustus-gene-316.25-mRNA-1 | Japonica | NA | | Os02g0325401 | OsR498G1220897000.01.T02 | |
| maker-3-augustus-gene-316.27-mRNA-1 | Japonica | NA | | Os10g0345600 | OsR498G1018508100.01.T02 | Qwrf motif-containing protein 2 |
| maker-3-augustus-gene-316.32-mRNA-1 | Japonica | NA | | Os10g0344900 | OsR498G1018504700.01.T01 | Protein DETOXIFICATION |
| maker-3-augustus-gene-317.37-mRNA-1 | Japonica | NA | | Os10g0344900 | OsR498G1018502800.01.T01 | Protein DETOXIFICATION |
| maker-3-augustus-gene-317.40-mRNA-1 | Japonica | NA | | Os10g0343900 | OsR498G1018501800.01.T05 | Glutaredoxin domain-containing cysteine-rich protein 1 |

| | | | | | | |
|---|---|---|---|---|---|---|
| maker-3-augustus-gene-317.48-mRNA-1 | Japonica | NA | | Os10g0343400 | OsR498G1018500800.01.T01 | Mixed-linked glucan synthase 2 |
| maker-3-augustus-gene-317.50-mRNA-1 | Japonica | NA | | Os10g0343200 | OsR498G1018500200.01.T01 | |
| maker-3-augustus-gene-317.52-mRNA-1 | Japonica | NA | | Os10g0343100 | OsR498G1018499900.01.T03 | Syntaxin/t-SNARE family protein |
| maker-3-augustus-gene-318.33-mRNA-1 | Japonica | NA | | Os01g0533400 | OsR498G1018495200.01.T02 | Beta-galactosidase 13 |
| maker-3-augustus-gene-318.39-mRNA-1 | Japonica | NA | | Os10g0341700 | OsR498G1018496500.01.T02 | Cellulose synthase family protein, expressed |
| maker-3-augustus-gene-319.0-mRNA-1 | Japonica | NA | | | OsR498G1018487000.01.T01 | Large secreted protein, putative, expressed |
| maker-3-augustus-gene-32.40-mRNA-1 | Japonica | NA | | Os03g0163100 | OsR498G0305276100.01.T02 | CASTOR/POLLUX/SYM8 ion channel |
| maker-3-augustus-gene-32.47-mRNA-1 | Japonica | NA | | Os03g0163200 | OsR498G0305276300.01.T01 | |
| maker-3-augustus-gene-32.49-mRNA-1 | Japonica | NA | | Os03g0163300 | OsR498G0305276500.01.T03 | Glutathione reductase, chloroplastic |
| maker-3-augustus-gene-32.54-mRNA-1 | Japonica | NA | | Os03g0164700 | OsR498G0305280300.01.T01 | Carbohydrate kinase PfkB |
| maker-3-augustus-gene-32.57-mRNA-1 | Japonica | Japonica | | Os03g0163400 | OsR498G0305276800.01.T02 | Altered inheritance of mitochondria protein 24, mitochondrial |
| maker-3-augustus-gene-32.59-mRNA-1 | Japonica | NA | | | | GRF zinc finger family protein, expressed |
| maker-3-augustus-gene-32.61-mRNA-1 | Japonica | Japonica | | Os03g0164400 | OsR498G0204262300.01.T01 | Transposon protein, putative, unclassified, expressed |
| maker-3-augustus-gene-32.63-mRNA-1 | Japonica | NA | | | OsR498G0305280600.01.T01 | Myosin-binding protein 1 isoform X1 |
| maker-3-augustus-gene-32.65-mRNA-1 | Japonica | NA | | Os03g0165000 | OsR498G0305280800.01.T05 | DNA topoisomerase |
| maker-3-augustus-gene-32.70-mRNA-1 | Japonica | Japonica | | Os03g0165100 | OsR498G0305281400.01.T02 | Rf1 protein, mitochondrial, putative |
| maker-3-augustus-gene-320.32-mRNA-1 | Japonica | NA | | | | Large secreted protein, putative, expressed |
| maker-3-augustus-gene-320.35-mRNA-1 | Japonica | NA | | Os02g0777700 | OsR498G0204870100.01.T02 | Opaque endosperm1 |
| maker-3-augustus-gene-321.32-mRNA-1 | Japonica | NA | | | OsR498G1018483400.01.T01 | FAR1 domain-containing protein |
| maker-3-augustus-gene-321.34-mRNA-1 | Japonica | Indica | | Os03g0209400 | OsR498G1018481400.01.T02 | Golgi transport 1 protein B |
| maker-3-augustus-gene-321.37-mRNA-1 | Japonica | NA | | Os10g0338000 | OsR498G1018482100.01.T01 | |
| maker-3-augustus-gene-321.39-mRNA-1 | Japonica | NA | | Os10g0337500 | OsR498G1018481100.01.T02 | S-acyltransferase |
| maker-3-augustus-gene-323.0-mRNA-1 | Japonica | NA | | Os10g0331700 | OsR498G1018472400.01.T01 | UDP-glucoronosyl and UDP-glucosyl transferase family protein, expressed |
| maker-3-augustus-gene-323.2-mRNA-1 | Japonica | NA | | Os02g0156100 | OsR498G0202996300.01.T02 | Protein kinase domain-containing protein |
| maker-3-augustus-gene-324.39-mRNA-1 | Japonica | NA | | | | UDP-glucoronosyl and UDP-glucosyl transferase family protein, expressed |
| maker-3-augustus-gene-324.41-mRNA-1 | Japonica | NA | | | OsR498G0408446100.01.T01 | Far-red impaired response-like |
| maker-3-augustus-gene-325.29-mRNA-1 | Japonica | NA | Kinase-TM | Os10g0329700 | OsR498G1018459800.01.T01 | Receptor-like serine/threonine-protein kinase |
| maker-3-augustus-gene-325.34-mRNA-1 | Japonica | NA | | | OsR498G0509715500.01.T01 | Ulp1 protease family, C-terminal catalytic domain containing protein |
| maker-3-augustus-gene-325.36-mRNA-1 | Japonica | NA | | | | |
| maker-3-augustus-gene-327.30-mRNA-1 | Japonica | NA | Kinase-TM | Os10g0327000 | OsR498G1018449400.01.T02 | Receptor-like serine/threonine-protein kinase |
| maker-3-augustus-gene-33.35-mRNA-1 | Japonica | Japonica | | Os03g0165266 | OsR498G0305281500.01.T01 | SNF2 domain-containing protein / helicase domain-containing protein |
| maker-3-augustus-gene-33.38-mRNA-1 | Japonica | NA | | Os03g0165800 | OsR498G0305284300.01.T01 | Vesicle-associated membrane protein 726 |
| maker-3-augustus-gene-33.41-mRNA-1 | Japonica | Indica | | Os10g0413500 | OsR498G0305285500.01.T01 | Procollagen-proline 4-dioxygenase |
| maker-3-augustus-gene-33.44-mRNA-1 | Japonica | NA | | Os03g0165300 | OsR498G0305282000.01.T01 | Benzothiadiazole-induced homeodomain protein 1 |
| maker-3-augustus-gene-33.46-mRNA-1 | Japonica | NA | | Os03g0165400 | OsR498G0305283000.01.T01 | Beta-galactosidase |
| maker-3-augustus-gene-33.52-mRNA-1 | Japonica | NA | | | OsR498G0305284000.01.T02 | Inactive ubiquitin carboxyl-terminal hydrolase 54 |
| maker-3-augustus-gene-33.58-mRNA-1 | Japonica | NA | | Os03g0165900 | OsR498G0305284600.01.T01 | Protein SULFUR DEFICIENCY-INDUCED like |
| maker-3-augustus-gene-33.60-mRNA-1 | Japonica | Indica | | Os03g0166000 | OsR498G0305285000.01.T01 | Alba domain-containing protein |
| maker-3-augustus-gene-33.65-mRNA-1 | Japonica | Indica | | Os03g0166600 | OsR498G0305287000.01.T01 | PlsC domain-containing protein (Fragment) |
| maker-3-augustus-gene-330.0-mRNA-1 | Japonica | NA | | | | Transposon protein, putative, Mutator sub-class, expressed |
| maker-3-augustus-gene-330.12-mRNA-1 | Japonica | NA | | Os10g0322250 | | Inorganic phosphate transporter 6 |
| maker-3-augustus-gene-335.21-mRNA-1 | Japonica | NA | | | | |
| maker-3-augustus-gene-337.0-mRNA-1 | Japonica | NA | | | | Putative ATP synthase subunit |
| maker-3-augustus-gene-338.0-mRNA-1 | Japonica | NA | | | | |
| maker-3-augustus-gene-339.0-mRNA-1 | Japonica | NA | | Os03g0563100 | OsR498G0306381300.01.T01 | L-gulonolactone oxidase |
| maker-3-augustus-gene-339.2-mRNA-1 | Japonica | NA | | Os03g0563600 | OsR498G0306383900.01.T01 | Peroxidase |
| maker-3-augustus-gene-339.5-mRNA-1 | Japonica | Indica | | Os03g0564200 | OsR498G0306387300.01.T03 | Boron transporter 1-like |
| maker-3-augustus-gene-339.9-mRNA-1 | Japonica | NA | | Os03g0563300 | OsR498G0306382200.01.T01 | Mg-protoporphyrin IX chelatase |
| maker-3-augustus-gene-34.52-mRNA-1 | Japonica | NA | | Os03g0166800 | OsR498G0305287500.01.T02 | Carbon catabolite repressor protein 2 |
| maker-3-augustus-gene-34.57-mRNA-1 | Japonica | Japonica | | Os03g0167000 | OsR498G0305287700.01.T02 | AAI domain-containing protein (Fragment) |
| maker-3-augustus-gene-34.59-mRNA-1 | Japonica | NA | | Os03g0167400 | OsR498G0305288700.01.T01 | Lipase, putative, expressed |
| maker-3-augustus-gene-34.61-mRNA-1 | Japonica | NA | | | OsR498G0305289500.01.T04 | Receptor y region, transmembrane domain-and RING domain-containing pro |
| maker-3-augustus-gene-34.65-mRNA-1 | Japonica | NA | | Os03g0167600 | OsR498G0305289900.01.T01 | Fatty acyl-CoA reductase |
| maker-3-augustus-gene-34.70-mRNA-1 | Japonica | NA | | Os03g0167800 | OsR498G0305290500.01.T01 | Kelch motif family protein, expressed |
| maker-3-augustus-gene-34.72-mRNA-1 | Japonica | NA | | Os03g0168000 | OsR498G0305291100.01.T01 | Major facilitator superfamily domain-containing protein 12 |
| maker-3-augustus-gene-34.75-mRNA-1 | Japonica | NA | | Os03g0168200 | OsR498G0305291500.01.T01 | tRNA pseudouridine synthase A, mitochondrial (Fragment) |
| maker-3-augustus-gene-34.81-mRNA-1 | Japonica | NA | | Os03g0167200 | OsR498G0305288200.01.T05 | IQ domain-containing protein IQM2 |
| maker-3-augustus-gene-34.84-mRNA-1 | Japonica | NA | | Os03g0805300 | OsR498G0307234100.01.T02 | Serine/threonine-protein phosphatase |
| maker-3-augustus-gene-34.89-mRNA-1 | Japonica | NA | | Os03g0168100 | OsR498G0305291400.01.T01 | Embryonic protein DC-8 |
| maker-3-augustus-gene-34.91-mRNA-1 | Japonica | NA | | Os03g0168300 | OsR498G0305291800.01.T02 | Pentatricopeptide repeat-containing protein |
| maker-3-augustus-gene-34.95-mRNA-1 | Japonica | NA | | | OsR498G0305292600.01.T01 | Thioredoxin-like 1-1 chloroplastic |

| | | | | | | |
|---|---|---|---|---|---|---|
| maker-3-augustus-gene-34.98-mRNA-1 | Japonica | NA | | | OsR498G0305293200.01.T01 | Cytochrome P450 family protein, expressed |
| maker-3-augustus-gene-340.0-mRNA-1 | Japonica | NA | | Os03g0565200 | OsR498G0306392300.01.T01 | Haloacid dehalogenase-like hydrolase domain-containing protein isoform X1 |
| maker-3-augustus-gene-340.4-mRNA-1 | Japonica | NA | | Os03g0565300 | OsR498G0306392700.01.T06 | PMD domain-containing protein |
| maker-3-augustus-gene-340.6-mRNA-1 | Japonica | NA | | Os03g0565500 | OsR498G0306393900.01.T01 | Elongation factor G, mitochondrial |
| maker-3-augustus-gene-341.39-mRNA-1 | Japonica | NA | | Os03g0566600 | OsR498G0306396500.01.T02 | Nodulation protein noel |
| maker-3-augustus-gene-341.41-mRNA-1 | Japonica | NA | | | | Tobamovirus multiplication protein 1 |
| maker-3-augustus-gene-341.47-mRNA-1 | Japonica | NA | | Os03g0565900 | OsR498G0306396000.01.T01 | PMD domain-containing protein |
| maker-3-augustus-gene-341.49-mRNA-1 | Japonica | NA | | Os03g0566500 | OsR498G0306396400.01.T01 | La-related protein 7 |
| maker-3-augustus-gene-341.56-mRNA-1 | Japonica | NA | | Os03g0567600 | OsR498G0306399600.01.T01 | Glycosyltransferase |
| maker-3-augustus-gene-342.11-mRNA-1 | Japonica | NA | | | OsR498G0306403600.01.T01 | |
| maker-3-augustus-gene-342.3-mRNA-1 | Japonica | NA | Kinase-TM | Os03g0568800 | OsR498G0306402700.01.T01 | Proline-rich receptor-like protein kinase PERK13 |
| maker-3-augustus-gene-342.5-mRNA-1 | Japonica | Japonica | | Os03g0568500 | OsR498G0306402000.01.T03 | Protein FATTY ACID EXPORT like |
| maker-3-augustus-gene-342.8-mRNA-1 | Japonica | NA | | Os03g0568900 | OsR498G0306403000.01.T01 | Purple acid phosphatase |
| maker-3-augustus-gene-343.0-mRNA-1 | Japonica | NA | | | OsR498G0509784600.01.T01 | SANT domain-containing protein |
| maker-3-augustus-gene-343.5-mRNA-1 | Japonica | NA | | Os03g0570200 | OsR498G0203772900.01.T07 | |
| maker-3-augustus-gene-35.42-mRNA-1 | Japonica | NA | | Os03g0168900 | OsR498G0305294100.01.T01 | Ribulose-phosphate 3-epimerase |
| maker-3-augustus-gene-35.44-mRNA-1 | Japonica | NA | | Os03g0169100 | OsR498G0305294500.01.T01 | Ribulose-phosphate 3-epimerase, chloroplastic |
| maker-3-augustus-gene-35.48-mRNA-1 | Japonica | Indica | | Os03g0169500 | OsR498G0305295700.01.T01 | Glucomannan 4-beta-mannosyltransferase |
| maker-3-augustus-gene-35.53-mRNA-1 | Japonica | NA | | Os03g0169600 | OsR498G0305296300.01.T01 | Cyclic dof factor 2 |
| maker-3-augustus-gene-35.55-mRNA-1 | Japonica | NA | | Os03g0170300 | OsR498G0305297900.01.T02 | 3-dehydroquinate synthase |
| maker-3-augustus-gene-35.60-mRNA-1 | Japonica | NA | | Os03g0169000 | OsR498G0305294400.01.T02 | O-fucosyltransferase family protein (Fragment) |
| maker-3-augustus-gene-35.65-mRNA-1 | Japonica | NA | | Os03g0169400 | OsR498G0305295600.01.T01 | 50S ribosomal protein L18, chloroplastic |
| maker-3-augustus-gene-35.67-mRNA-1 | Japonica | NA | | Os03g0169800 | OsR498G0305296600.01.T05 | HNHc domain-containing protein (Fragment) |
| maker-3-augustus-gene-35.68-mRNA-1 | Japonica | NA | | Os03g0169900 | OsR498G0305297000.01.T01 | AvrRpt-cleavage domain-containing protein (Fragment) |
| maker-3-augustus-gene-35.71-mRNA-1 | Japonica | NA | | Os03g0170200 | | |
| maker-3-augustus-gene-35.82-mRNA-1 | Japonica | NA | | | | Homeobox-leucine zipper protein like |
| maker-3-augustus-gene-35.84-mRNA-1 | Japonica | NA | | Os03g0170900 | OsR498G0305302600.01.T03 | Sucrose transporter 6 |
| maker-3-augustus-gene-350.37-mRNA-1 | Japonica | Indica | | Os03g0581800 | OsR498G0306451900.01.T03 | 1D-myo-inositol 2-amino-2-deoxy-alpha-D-glucopyranoside ligase |
| maker-3-augustus-gene-358.2-mRNA-1 | Japonica | NA | | Os03g0595200 | OsR498G0306493500.01.T01 | Cysteine synthase 2-like isoform X1 |
| maker-3-augustus-gene-359.55-mRNA-1 | Japonica | NA | | Os03g0598200 | OsR498G0306503000.01.T01 | Nucleolar protein 14 |
| maker-3-augustus-gene-36.0-mRNA-1 | Japonica | NA | | Os03g0171700 | OsR498G0305305500.01.T01 | Transcription factor ILI5 |
| maker-3-augustus-gene-36.10-mRNA-1 | Japonica | NA | | Os03g0171300 | OsR498G0305303400.01.T03 | Transcription factor like |
| maker-3-augustus-gene-36.14-mRNA-1 | Japonica | NA | | Os03g0172200 | OsR498G0305306400.01.T01 | Proteinase inhibitor I25, cystatin |
| maker-3-augustus-gene-36.2-mRNA-1 | Japonica | NA | | Os03g0171900 | OsR498G0305305900.01.T03 | Alanine-glyoxylate aminotransferase |
| maker-3-augustus-gene-36.7-mRNA-1 | Japonica | NA | | Os03g0172000 | OsR498G0305306100.01.T01 | Peptidase S59 domain-containing protein (Fragment) |
| maker-3-augustus-gene-361.2-mRNA-1 | Japonica | NA | | | | RRM domain-containing protein |
| maker-3-augustus-gene-361.4-mRNA-1 | Japonica | Japonica | | Os03g0600500 | OsR498G1018781700.01.T01 | Glucan endo-1,3-beta-glucosidase GII |
| maker-3-augustus-gene-364.0-mRNA-1 | Japonica | Japonica | | Os03g0606100 | OsR498G0306528600.01.T04 | Lipase, putative, expressed |
| maker-3-augustus-gene-366.9-mRNA-1 | Japonica | NA | | Os03g0609800 | OsR498G0306545400.01.T01 | BTB/POZ domain-containing protein NPY2 |
| maker-3-augustus-gene-37.0-mRNA-1 | Japonica | NA | | Os03g0173900 | OsR498G0305309500.01.T04 | E3 ubiquitin-protein ligase BIG BROTHER |
| maker-3-augustus-gene-37.10-mRNA-1 | Japonica | NA | | Os03g0174200 | OsR498G0305309900.01.T01 | Polyadenylate-binding protein RBP47B |
| maker-3-augustus-gene-37.13-mRNA-1 | Japonica | Indica | | Os03g0174300 | OsR498G0305310300.01.T04 | Putative glucuronoxylan glucuronosyltransferase F8H |
| maker-3-augustus-gene-37.18-mRNA-1 | Japonica | NA | | Os03g0174600 | OsR498G0305311100.01.T03 | tRNA-5-taurinomethyluridine 2-sulfurtransferase |
| maker-3-augustus-gene-37.24-mRNA-1 | Japonica | NA | | Os03g0173500 | OsR498G0713747300.01.T07 | Protein-serine/threonine phosphatase |
| maker-3-augustus-gene-37.26-mRNA-1 | Japonica | NA | | Os03g0173700 | OsR498G0305308400.01.T02 | RNase H domain-containing protein |
| maker-3-augustus-gene-37.29-mRNA-1 | Japonica | NA | | Os03g0174500 | OsR498G0305311000.01.T01 | Adenylosuccinate synthetase, chloroplastic |
| maker-3-augustus-gene-37.31-mRNA-1 | Japonica | NA | | Os03g0174900 | OsR498G0305311800.01.T01 | CCAAT-binding transcription factor B |
| maker-3-augustus-gene-37.5-mRNA-1 | Japonica | NA | | Os03g0174100 | OsR498G0305309700.01.T01 | Polyadenylate-binding protein RBP47B |
| maker-3-augustus-gene-373.5-mRNA-1 | Japonica | Japonica | | Os03g0623100 | OsR498G0306583300.01.T01 | |
| maker-3-augustus-gene-374.0-mRNA-1 | Japonica | Indica | | Os03g0624000 | OsR498G0306586700.01.T02 | probable transcription factor KAN4 |
| maker-3-augustus-gene-374.2-mRNA-1 | Japonica | NA | | Os03g0624600 | OsR498G0306589500.01.T01 | Protein CUP-SHAPED COTYLEDON 2 |
| maker-3-augustus-gene-374.8-mRNA-1 | Japonica | Japonica | | Os03g0625500 | OsR498G0306592200.01.T02 | FBD domain-containing protein |
| maker-3-augustus-gene-375.17-mRNA-1 | Japonica | NA | | Os03g0625800 | OsR498G0306593100.01.T03 | Putative E3 ubiquitin-protein ligase ARI2 |
| maker-3-augustus-gene-375.19-mRNA-1 | Japonica | Indica | | Os03g0626100 | OsR498G0306594300.01.T01 | Putative E3 ubiquitin-protein ligase ARI2 |
| maker-3-augustus-gene-375.21-mRNA-1 | Japonica | Japonica | | Os03g0626100 | OsR498G0306594700.01.T01 | Putative E3 ubiquitin-protein ligase ARI1 |
| maker-3-augustus-gene-375.23-mRNA-1 | Japonica | NA | | | OsR498G0306595700.01.T05 | Inositol polyphosphate 5-phosphatase |
| maker-3-augustus-gene-375.29-mRNA-1 | Japonica | NA | | Os03g0626600 | OsR498G0306596100.01.T06 | LIM zinc-binding domain-containing protein |
| maker-3-augustus-gene-375.31-mRNA-1 | Japonica | NA | | | OsR498G0306593000.01.T01 | Exocyst complex |
| maker-3-augustus-gene-375.39-mRNA-1 | Japonica | NA | | Os03g0625900 | OsR498G0306593400.01.T01 | U3 snoRNP-associated protein-like YAOH |
| maker-3-augustus-gene-375.41-mRNA-1 | Japonica | NA | | Os03g0626700 | OsR498G0306597000.01.T01 | Protein DETOXIFICATION |
| maker-3-augustus-gene-376.34-mRNA-1 | Japonica | NA | | Os03g0626800 | OsR498G0306597900.01.T01 | Calcineurin B |

| | | | | | | |
|---|---|---|---|---|---|---|
| maker-3-augustus-gene-376.41-mRNA-1 | Japonica | NA | | | OsR498G0306598900.01.T04 | Iron-sulfur protein NUBPL |
| maker-3-augustus-gene-377.38-mRNA-1 | Japonica | NA | | Os03g0628800 | OsR498G0306604300.01.T01 | Toxin PezT |
| maker-3-augustus-gene-377.41-mRNA-1 | Japonica | NA | | | OsR498G0306608300.01.T01 | |
| maker-3-augustus-gene-377.42-mRNA-1 | Japonica | NA | | Os03g0628900 | OsR498G0306604800.01.T01 | Prefoldin subunit 4 |
| maker-3-augustus-gene-377.44-mRNA-1 | Japonica | NA | | | OsR498G0306607400.01.T01 | F-box domain-containing protein (Fragment) |
| maker-3-augustus-gene-378.36-mRNA-1 | Japonica | Indica | | Os12g0600200 | OsR498G0306609600.01.T01 | E3 ubiquitin protein ligase DRIP2 |
| maker-3-augustus-gene-379.26-mRNA-1 | Japonica | NA | | Os03g0633400 | OsR498G0306610500.01.T02 | F-box/LRR-repeat protein 17-like isoform X1 |
| maker-3-augustus-gene-379.31-mRNA-1 | Japonica | NA | | Os03g0633800 | OsR498G0306611700.01.T01 | Auxin-responsive protein IAA12 |
| maker-3-augustus-gene-379.33-mRNA-1 | Japonica | NA | | Os03g0633500 | OsR498G0306610800.01.T01 | Auxin-responsive protein IAA11 |
| maker-3-augustus-gene-379.35-mRNA-1 | Japonica | NA | | Os03g0633900 | OsR498G0306612000.01.T01 | Primosome PriB/single-strand DNA-binding protein |
| maker-3-augustus-gene-379.39-mRNA-1 | Japonica | NA | | Os03g0634000 | OsR498G0306612400.01.T01 | Sec-independent protein translocase protein TatA |
| maker-3-augustus-gene-379.41-mRNA-1 | Japonica | NA | | Os03g0635100 | | GTP binding protein gamma subunit |
| maker-3-augustus-gene-379.43-mRNA-1 | Japonica | Indica | | Os03g0635800 | OsR498G0306615200.01.T01 | VAN3-binding protein isoform X4 |
| maker-3-augustus-gene-38.0-mRNA-1 | Japonica | Indica | | Os03g0175600 | OsR498G0305315100.01.T01 | Nitrilase/cyanide hydratase and apolipoprotein N-acyltransferase family pro |
| maker-3-augustus-gene-38.14-mRNA-1 | Japonica | NA | | Os03g0177300 | OsR498G0305320100.01.T04 | RING-type E3 ubiquitin transferase |
| maker-3-augustus-gene-38.18-mRNA-1 | Japonica | NA | | Os03g0177400 | OsR498G0305320300.01.T01 | Elongation factor 1-alpha |
| maker-3-augustus-gene-38.19-mRNA-1 | Japonica | NA | | Os03g0177500 | OsR498G0305320300.01.T04 | Elongation factor 1-alpha |
| maker-3-augustus-gene-38.24-mRNA-1 | Japonica | NA | | | OsR498G0305317800.01.T02 | AP2-EREBP-type transcription factor |
| maker-3-augustus-gene-38.26-mRNA-1 | Japonica | NA | | Os03g0176600 | OsR498G0305318800.01.T01 | Protein kinase domain-containing protein |
| maker-3-augustus-gene-38.29-mRNA-1 | Japonica | Japonica | | Os03g0176800 | OsR498G0305319000.01.T02 | Cupin type-1 domain-containing protein |
| maker-3-augustus-gene-38.34-mRNA-1 | Japonica | Indica | | Os09g0357250 | OsR498G0305320000.01.T01 | Zinc finger protein ZFPM2 |
| maker-3-augustus-gene-38.5-mRNA-1 | Japonica | Japonica | | Os03g0176100 | OsR498G1018648800.01.T04 | Transcription initiation factor IIE subunit beta |
| maker-3-augustus-gene-38.8-mRNA-1 | Japonica | NA | | Os03g0177000 | OsR498G0305319700.01.T01 | Acyl-CoA N-acyltransferase (NAT) superfamily protein |
| maker-3-augustus-gene-380.20-mRNA-1 | Japonica | NA | | Os09g0401600 | OsR498G0917411600.01.T01 | Plant UBX domain-containing protein 2 |
| maker-3-augustus-gene-380.24-mRNA-1 | Japonica | Japonica | | Os03g0636700 | OsR498G1222064900.01.T01 | IQ motif, EF-hand binding site |
| maker-3-augustus-gene-380.27-mRNA-1 | Japonica | Indica | Kinase-TM | Os12g0604700 | OsR498G0306621200.01.T01 | Serine/threonine-protein kinase Nek2 |
| maker-3-augustus-gene-381.0-mRNA-1 | Japonica | NA | | Os03g0638200 | OsR498G0306626300.01.T08 | Organic cation/carnitine transporter 7 |
| maker-3-augustus-gene-381.11-mRNA-1 | Japonica | NA | | | | CRC domain-containing protein TSO1 isoform X1 |
| maker-3-augustus-gene-381.5-mRNA-1 | Japonica | Indica | Kinase | Os12g0605900 | OsR498G0306628100.01.T01 | Serinethreonine-protein kinase sty17 |
| maker-3-augustus-gene-381.8-mRNA-1 | Japonica | NA | | | OsR498G0306624800.01.T01 | NAB domain-containing protein |
| maker-3-augustus-gene-382.0-mRNA-1 | Japonica | NA | | | OsR498G0306632300.01.T02 | DIRP family protein, expressed |
| maker-3-augustus-gene-382.11-mRNA-1 | Japonica | NA | | Os03g0639600 | OsR498G0306636000.01.T01 | LSD1-type zinc finger protein |
| maker-3-augustus-gene-382.5-mRNA-1 | Japonica | NA | | Os03g0639300 | OsR498G0306633100.01.T01 | Transcription factor PIF1 (Fragment) |
| maker-3-augustus-gene-382.6-mRNA-1 | Japonica | Indica | | Os12g0610200 | OsR498G0306633100.01.T01 | transcription factor PHYTOCHROME INTERACTING FACTOR-LIKE 13-like isofo |
| maker-3-augustus-gene-383.28-mRNA-1 | Japonica | NA | | Os03g0639700 | OsR498G0306637300.01.T02 | DNA repair protein recA, chloroplastic |
| maker-3-augustus-gene-383.32-mRNA-1 | Japonica | NA | | Os03g0639800 | OsR498G0306637600.01.T01 | Vacuolar protein sorting-associated protein 2 like 2 |
| maker-3-augustus-gene-383.38-mRNA-1 | Japonica | Indica | | Os03g0640100 | OsR498G0306639600.01.T02 | Histone-binding protein MSI1 |
| maker-3-augustus-gene-383.39-mRNA-1 | Japonica | NA | | Os03g0640100 | OsR498G0306639600.01.T03 | Histone-binding protein MSI1 |
| maker-3-augustus-gene-383.46-mRNA-1 | Japonica | Japonica | | Os03g0640200 | OsR498G0306640800.01.T01 | KxDL motif-containing protein 1 |
| maker-3-augustus-gene-383.48-mRNA-1 | Japonica | NA | | | OsR498G0306640000.01.T03 | PIG-P domain-containing protein |
| maker-3-augustus-gene-383.50-mRNA-1 | Japonica | NA | | Os03g0640400 | OsR498G0306640800.01.T01 | KxDL motif-containing protein 1 |
| maker-3-augustus-gene-383.52-mRNA-1 | Japonica | NA | | Os03g0640800 | OsR498G0306641800.01.T01 | Homeobox-leucine zipper protein ATHB-14 |
| maker-3-augustus-gene-384.33-mRNA-1 | Japonica | NA | | Os03g0641500 | OsR498G0306646300.01.T01 | |
| maker-3-augustus-gene-384.35-mRNA-1 | Japonica | NA | | Os03g0641700 | OsR498G0306647100.01.T03 | Mediator of RNA polymerase II transcription subunit 19a |
| maker-3-augustus-gene-384.39-mRNA-1 | Japonica | NA | | Os03g0641200 | OsR498G0306643800.01.T01 | High affinity cationic amino acid transporter 1 |
| maker-3-augustus-gene-384.41-mRNA-1 | Japonica | Indica | | Os12g0613250 | OsR498G0306644400.01.T01 | BTB/POZ domain-containing protein At1g30440 |
| maker-3-augustus-gene-384.43-mRNA-1 | Japonica | NA | Kinase-TM | | OsR498G0306653000.01.T01 | Wall-associated receptor kinase 5 |
| maker-3-augustus-gene-385.33-mRNA-1 | Japonica | NA | | Os03g0644000 | OsR498G0306653900.01.T03 | Beclin 1 protein (Fragment) |
| maker-3-augustus-gene-385.37-mRNA-1 | Japonica | NA | | Os03g0642900 | OsR498G0306651200.01.T03 | Phosphonate metabolism protein, putative |
| maker-3-augustus-gene-385.42-mRNA-1 | Japonica | NA | | Os03g0643100 | OsR498G0713408500.01.T01 | WAC domain-containing protein |
| maker-3-augustus-gene-385.45-mRNA-1 | Japonica | NA | Kinase-TM | Os03g0643250 | | Phosphonate metabolism protein, putative |
| maker-3-augustus-gene-385.49-mRNA-1 | Japonica | NA | | Os03g0643300 | OsR498G0306653600.01.T01 | Ornithine--oxo-acid aminotransferase |
| maker-3-augustus-gene-385.55-mRNA-1 | Japonica | NA | | Os03g0643900 | | |
| maker-3-augustus-gene-385.56-mRNA-1 | Japonica | NA | | Os03g0644400 | OsR498G0306655000.01.T01 | Proline transporter |
| maker-3-augustus-gene-386.0-mRNA-1 | Japonica | NA | | Os03g0645000 | OsR498G0306657300.01.T02 | Beta-expansin 4 |
| maker-3-augustus-gene-386.6-mRNA-1 | Japonica | Indica | | Os03g0645100 | OsR498G0306657700.01.T01 | Pyruvate dehydrogenase (acetyl-transferring) |
| maker-3-augustus-gene-386.8-mRNA-1 | Japonica | Indica | | Os12g0617100 | OsR498G0306658000.01.T01 | Translation initiation factor eIF-2B delta subunit |
| maker-3-augustus-gene-387.0-mRNA-1 | Japonica | NA | | Os03g0646100 | OsR498G0306664100.01.T01 | Tubulin GTPase |
| maker-3-augustus-gene-387.14-mRNA-1 | Japonica | NA | | Os03g0646400 | OsR498G0306664800.01.T03 | EXPERA domain-containing protein (Fragment) |
| maker-3-augustus-gene-387.16-mRNA-1 | Japonica | Indica | | Os12g0617900 | OsR498G0306665600.01.T01 | Serine/threonine-protein phosphatase |
| maker-3-augustus-gene-387.24-mRNA-1 | Japonica | NA | | Os03g0647600 | OsR498G0306667600.01.T01 | CCAAT-binding transcription factor B |

| | | | | | | | |
|---|---|---|---|---|---|---|---|
| maker-3-augustus-gene-387.7-mRNA-1 | Japonica | NA | | | OsR498G0306665300.01.T05 | DNA-directed RNA polymerase subunit beta |
| maker-3-augustus-gene-388.14-mRNA-1 | Japonica | NA | | Os03g0648500 | OsR498G0306670600.01.T01 | Phytocyanin domain-containing protein |
| maker-3-augustus-gene-388.16-mRNA-1 | Japonica | Indica | | Os12g0620600 | OsR498G0306672200.01.T01 | C2 NT-type domain-containing protein |
| maker-3-augustus-gene-388.4-mRNA-1 | Japonica | NA | | Os03g0648900 | OsR498G0306671900.01.T01 | 5'-nucleotidase |
| maker-3-augustus-gene-388.6-mRNA-1 | Japonica | NA | | Os03g0647800 | OsR498G0306669200.01.T01 | CSL zinc finger domain-containing protein |
| maker-3-augustus-gene-388.8-mRNA-1 | Japonica | Japonica | | Os03g0648300 | OsR498G0306670200.01.T01 | IQ calmodulin-binding motif family protein, expressed |
| maker-3-augustus-gene-389.13-mRNA-1 | Japonica | NA | | Os03g0651000 | OsR498G0306681100.01.T01 | Glutamyl-tRNA(Gln) amidotransferase subunit C, chloroplastic/mitochondria |
| maker-3-augustus-gene-39.0-mRNA-1 | Japonica | NA | | Os03g0177900 | OsR498G0305321100.01.T01 | Elongation factor 1-alpha |
| maker-3-augustus-gene-39.11-mRNA-1 | Japonica | NA | Kinase-TM | Os03g0179400 | OsR498G0305326300.01.T01 | Serine/threonine-protein kinase RIPK |
| maker-3-augustus-gene-39.15-mRNA-1 | Japonica | NA | | Os03g0179900 | OsR498G0305327300.01.T01 | Lipoxygenase |
| maker-3-augustus-gene-39.19-mRNA-1 | Japonica | NA | | Os03g0180300 | OsR498G0305328700.01.T05 | Smr domain-containing protein |
| maker-3-augustus-gene-39.2-mRNA-1 | Japonica | NA | | Os03g0178000 | OsR498G0305321500.01.T02 | Elongation factor 1-alpha |
| maker-3-augustus-gene-39.23-mRNA-1 | Japonica | NA | | | OsR498G0305321800.01.T02 | Putative copper-transporting ATPase PAA1 |
| maker-3-augustus-gene-39.30-mRNA-1 | Japonica | NA | | Os03g0178300 | OsR498G0305322600.01.T01 | AB hydrolase-1 domain-containing protein (Fragment) |
| maker-3-augustus-gene-39.32-mRNA-1 | Japonica | NA | | Os03g0178400 | OsR498G0305322800.01.T02 | AB hydrolase-1 domain-containing protein (Fragment) |
| maker-3-augustus-gene-39.36-mRNA-1 | Japonica | NA | | Os03g0178500 | OsR498G0305323000.01.T01 | 2-hydroxy-6-oxononadienedioate/2-hydroxy-6-oxononatrienedioate hydrola |
| maker-3-augustus-gene-39.4-mRNA-1 | Japonica | NA | | Os03g0178200 | OsR498G0305322300.01.T01 | ylmG homolog protein 2, chloroplastic |
| maker-3-augustus-gene-39.40-mRNA-1 | Japonica | NA | | Os03g0179700 | OsR498G0305326600.01.T01 | LURP1-related protein domain containing protein |
| maker-3-augustus-gene-39.6-mRNA-1 | Japonica | NA | | | | Tubulin--tyrosine ligase-like protein 12 |
| maker-3-augustus-gene-390.36-mRNA-1 | Japonica | Japonica | | Os03g0651800 | OsR498G0102706900.01.T02 | Germin-like protein 1-3 |
| maker-3-augustus-gene-390.38-mRNA-1 | Japonica | NA | | Os03g0652100 | OsR498G0306685700.01.T01 | General negative regulator of transcription subunit 3 isoform X2 |
| maker-3-augustus-gene-390.42-mRNA-1 | Japonica | Indica | | Os03g0651100 | OsR498G0306681400.01.T01 | UBA domain-containing protein |
| maker-3-augustus-gene-390.49-mRNA-1 | Japonica | NA | | Os03g0651300 | OsR498G0306682200.01.T01 | Plasma-membrane choline transporter family protein |
| maker-3-augustus-gene-390.53-mRNA-1 | Japonica | NA | | | OsR498G0306690300.01.T01 | F-box domain-containing protein (Fragment) |
| maker-3-augustus-gene-390.54-mRNA-1 | Japonica | Indica | | Os03g0652100 | OsR498G0306688600.01.T01 | Transcription regulator NOT2/NOT3/NOT5 family protein |
| maker-3-augustus-gene-390.56-mRNA-1 | Japonica | NA | | | OsR498G0306688800.01.T03 | AAI domain-containing protein |
| maker-3-augustus-gene-391.28-mRNA-1 | Japonica | NA | | Os03g0653900 | OsR498G0306691400.01.T02 | rRNA N-glycosidase |
| maker-3-augustus-gene-392.39-mRNA-1 | Japonica | NA | | Os03g0654500 | OsR498G0306694000.01.T02 | High affinity cationic amino acid transporter 1 |
| maker-3-augustus-gene-392.45-mRNA-1 | Japonica | NA | | | OsR498G0306694200.01.T02 | Protein PHOSPHATE STARVATION RESPONSE 3 |
| maker-3-augustus-gene-392.50-mRNA-1 | Japonica | NA | | | OsR498G0306694400.01.T02 | Chlorophyllide b reductase NOLic isoform X2 |
| maker-3-augustus-gene-399.52-mRNA-1 | Japonica | NA | | Os05g0394100 | | Glutathione S-transferase T3-like |
| maker-3-augustus-gene-4.0-mRNA-1 | Japonica | NA | | | OsR498G0305105100.01.T01 | LysM domain containing protein, expressed |
| maker-3-augustus-gene-4.16-mRNA-1 | Japonica | NA | | Os03g0111500 | OsR498G0305107700.01.T01 | Valyl-tRNA synthetase |
| maker-3-augustus-gene-4.19-mRNA-1 | Japonica | NA | | Os03g0694900 | OsR498G0306856700.01.T01 | Valyl-tRNA synthetase |
| maker-3-augustus-gene-4.28-mRNA-1 | Japonica | NA | | Os03g0111800 | OsR498G0305108300.01.T02 | Transducin family protein / WD-40 repeat family protein |
| maker-3-augustus-gene-4.3-mRNA-1 | Japonica | NA | | Os03g0110800 | OsR498G0305105900.01.T02 | DNA (cytosine-5)-methyltransferase DRM2 |
| maker-3-augustus-gene-4.34-mRNA-1 | Japonica | NA | | | | AP-2 complex subunit alpha |
| maker-3-augustus-gene-4.40-mRNA-1 | Japonica | NA | | Os03g0111100 | OsR498G0305106600.01.T02 | Folylpoly-gamma-glutamate synthetase |
| maker-3-augustus-gene-4.48-mRNA-1 | Japonica | NA | | Os03g0111200 | OsR498G0305107000.01.T01 | Remorin |
| maker-3-augustus-gene-4.51-mRNA-1 | Japonica | Japonica | | Os03g0111400 | OsR498G0305107600.01.T01 | Heavy-metal-associated domain-containing protein, putative, expressed |
| maker-3-augustus-gene-4.54-mRNA-1 | Japonica | NA | | Os03g0111600 | OsR498G0305108000.01.T01 | DUF1618 domain-containing protein (Fragment) |
| maker-3-augustus-gene-4.9-mRNA-1 | Japonica | NA | | | OsR498G0305106100.01.T08 | Stress responsive alpha-beta barrel |
| maker-3-augustus-gene-40.35-mRNA-1 | Japonica | NA | | Os03g0180900 | OsR498G0305330900.01.T01 | Tify domain-containing protein (Fragment) |
| maker-3-augustus-gene-40.37-mRNA-1 | Japonica | NA | | Os03g0181500 | OsR498G0305333900.01.T01 | Very-long-chain 3-oxoacyl-CoA synthase |
| maker-3-augustus-gene-40.40-mRNA-1 | Japonica | NA | | Os03g0181600 | | |
| maker-3-augustus-gene-40.42-mRNA-1 | Japonica | NA | | | OsR498G0305334900.01.T01 | Multidrug/pheromone exporter, MDR family, ABC transporter family |
| maker-3-augustus-gene-40.44-mRNA-1 | Japonica | NA | | | OsR498G0305335100.01.T01 | Multidrug/pheromone exporter, MDR family, ABC transporter family |
| maker-3-augustus-gene-40.46-mRNA-1 | Japonica | NA | | Os03g0181800 | OsR498G0305335900.01.T04 | DUF936 domain-containing protein |
| maker-3-augustus-gene-40.48-mRNA-1 | Japonica | NA | | Os03g0180400 | OsR498G0305329000.01.T01 | Proteasome endopeptidase complex |
| maker-3-augustus-gene-40.53-mRNA-1 | Japonica | NA | | | OsR498G0612787000.01.T03 | UDP-glycosyltransferase TURAN |
| maker-3-augustus-gene-401.6-mRNA-1 | Japonica | Indica | | Os03g0670700 | OsR498G0306764900.01.T01 | Glycine-rich RNA-binding protein GRP1A |
| maker-3-augustus-gene-402.0-mRNA-1 | Japonica | NA | | Os03g0671800 | OsR498G0306767000.01.T01 | Transcription factor bHLH28 |
| maker-3-augustus-gene-402.11-mRNA-1 | Japonica | NA | | | OsR498G0306769800.01.T02 | Myb-like protein O |
| maker-3-augustus-gene-402.2-mRNA-1 | Japonica | NA | | Os03g0672300 | OsR498G0306768400.01.T02 | Pyruvate kinase |
| maker-3-augustus-gene-402.5-mRNA-1 | Japonica | NA | | Os03g0672400 | OsR498G0306768800.01.T01 | Pyruvate kinase |
| maker-3-augustus-gene-402.9-mRNA-1 | Japonica | NA | | | OsR498G0306769200.01.T01 | EPIDERMAL PATTERNING FACTOR-like protein 2 |
| maker-3-augustus-gene-403.0-mRNA-1 | Japonica | NA | | Os03g0672900 | OsR498G0306771100.01.T01 | GH10 domain-containing protein (Fragment) |
| maker-3-augustus-gene-403.12-mRNA-1 | Japonica | NA | | Os03g0847401 | | HAT family dimerisation domain containing protein |
| maker-3-augustus-gene-403.14-mRNA-1 | Japonica | NA | | | OsR498G0306773600.01.T01 | KNOTTED1 homeodomain protein (Fragment) |
| maker-3-augustus-gene-403.16-mRNA-1 | Japonica | Indica | | Os03g0673800 | OsR498G0306774000.01.T03 | Expressed protein (With alternative splicing) |
| maker-3-augustus-gene-403.2-mRNA-1 | Japonica | Indica | | Os03g0125800 | OsR498G0306774900.01.T01 | DUF21 domain-containing protein isoform A |

| | | | | | | | |
|---|---|---|---|---|---|---|---|
| maker-3-augustus-gene-403.8-mRNA-1 | Japonica | NA | | | OsR498G0306771400.01.T01 | Homeotic protein knotted-1 |
| maker-3-augustus-gene-404.43-mRNA-1 | Japonica | NA | | Os03g0674700 | OsR498G0306775700.01.T01 | Growth-regulating factor 9 |
| maker-3-augustus-gene-404.46-mRNA-1 | Japonica | NA | | Os03g0675600 | OsR498G0306779900.01.T01 | Phytosulfokine-alpha |
| maker-3-augustus-gene-404.48-mRNA-1 | Japonica | NA | | | | |
| maker-3-augustus-gene-404.50-mRNA-1 | Japonica | Japonica | | Os03g0675300 | OsR498G0306777400.01.T01 | Proline synthase co-transcribed bacterial homolog protein |
| maker-3-augustus-gene-405.0-mRNA-1 | Japonica | NA | | | OsR498G0306786700.01.T02 | rRNA N-glycosidase |
| maker-3-augustus-gene-405.3-mRNA-1 | Japonica | NA | | Os03g0678400 | OsR498G0306792100.01.T02 | RING finger protein 5 |
| maker-3-augustus-gene-406.0-mRNA-1 | Japonica | NA | | Os01g0144300 | OsR498G0100163200.01.T01 | |
| maker-3-augustus-gene-406.14-mRNA-1 | Japonica | NA | | Os01g0233900 | OsR498G0100477300.01.T01 | UBC core domain-containing protein (Fragment) |
| maker-3-augustus-gene-406.3-mRNA-1 | Japonica | NA | | Os03g0679800 | OsR498G0306798700.01.T01 | Ankyrin repeat family protein |
| maker-3-augustus-gene-406.8-mRNA-1 | Japonica | NA | | Os03g0679000 | OsR498G0306795400.01.T03 | Glucose-induced degradation protein 4 |
| maker-3-augustus-gene-407.26-mRNA-1 | Japonica | Japonica | | Os03g0680100 | OsR498G0306798700.01.T01 | TPR Domain containing protein, expressed |
| maker-3-augustus-gene-407.30-mRNA-1 | Japonica | NA | | Os03g0680200 | OsR498G0306799900.01.T02 | TPR Domain containing protein, expressed |
| maker-3-augustus-gene-407.35-mRNA-1 | Japonica | Japonica | | Os03g0680700 | OsR498G0306801400.01.T01 | Homeodomain transcription factor |
| maker-3-augustus-gene-407.38-mRNA-1 | Japonica | NA | | Os03g0680800 | OsR498G0306802400.01.T06 | Benzothiadiazole-induced homeodomain protein 1 |
| maker-3-augustus-gene-408.28-mRNA-1 | Japonica | NA | | | OsR498G0306805100.01.T01 | Golgi-body localization protein domain |
| maker-3-augustus-gene-408.36-mRNA-1 | Japonica | NA | | Os03g0681700 | OsR498G0306805900.01.T03 | Target of rapamycin complex subunit LST8 |
| maker-3-augustus-gene-408.41-mRNA-1 | Japonica | Indica | | Os04g0485100 | OsR498G0306806200.01.T02 | Serine/threonine protein phosphatase 2A regulatory subunit |
| maker-3-augustus-gene-408.43-mRNA-1 | Japonica | NA | | Os03g0681900 | OsR498G0306806600.01.T01 | Glycine-rich RNA-binding protein RZ1C |
| maker-3-augustus-gene-409.36-mRNA-1 | Japonica | NA | | | | MHD1 domain-containing protein |
| maker-3-augustus-gene-409.42-mRNA-1 | Japonica | NA | | Os03g0683800 | OsR498G0306812100.01.T01 | Gdsl esteraselipase |
| maker-3-augustus-gene-409.44-mRNA-1 | Japonica | NA | | Os03g0682600 | | Argonaute 5 |
| maker-3-augustus-gene-41.36-mRNA-1 | Japonica | NA | | | OsR498G0305336100.01.T01 | Flavin-containing monooxygenase |
| maker-3-augustus-gene-41.39-mRNA-1 | Japonica | NA | | Os03g0182400 | OsR498G0305336500.01.T02 | SAC domain-containing protein |
| maker-3-augustus-gene-41.53-mRNA-1 | Japonica | Indica | | Os07g0616600 | OsR498G0305337000.01.T01 | 40S ribosomal protein SA |
| maker-3-augustus-gene-41.57-mRNA-1 | Japonica | NA | | Os03g0182700 | OsR498G0305337200.01.T01 | Eukaryotic translation initiation factor 3 subunit K |
| maker-3-augustus-gene-41.60-mRNA-1 | Japonica | NA | | Os03g0182800 | OsR498G0305337400.01.T01 | Ethylene-responsive transcription factor |
| maker-3-augustus-gene-41.63-mRNA-1 | Japonica | NA | | Os03g0183000 | OsR498G0305337800.01.T02 | Jasmonic acid and ethylene responsive factor |
| maker-3-augustus-gene-41.65-mRNA-1 | Japonica | NA | | Os03g0183100 | OsR498G0305338600.01.T05 | SAP-like protein BP-73 |
| maker-3-augustus-gene-41.66-mRNA-1 | Japonica | NA | | Os03g0183500 | OsR498G0305340600.01.T01 | FLZ-type domain-containing protein |
| maker-3-augustus-gene-41.68-mRNA-1 | Japonica | Indica | | Os10g0390500 | OsR498G0305341400.01.T04 | Alanine aminotransferase |
| maker-3-augustus-gene-410.0-mRNA-1 | Japonica | NA | | Os03g0684300 | OsR498G0306813500.01.T01 | OB-fold nucleic acid binding domain containing protein, expressed |
| maker-3-augustus-gene-410.13-mRNA-1 | Japonica | NA | | Os03g0685000 | OsR498G0306815500.01.T03 | Ferredoxin |
| maker-3-augustus-gene-410.17-mRNA-1 | Japonica | NA | | Os03g0685100 | OsR498G0306815700.01.T01 | Putative 23S rRNA (Guanosine(2251)-2'-O)-methyltransferase |
| maker-3-augustus-gene-410.19-mRNA-1 | Japonica | NA | | Os03g0685300 | OsR498G0306817500.01.T01 | Gamma-glutamyl peptidase |
| maker-3-augustus-gene-410.29-mRNA-1 | Japonica | NA | | | OsR498G0306812400.01.T02 | HECT domain-containing protein (Fragment) |
| maker-3-augustus-gene-410.3-mRNA-1 | Japonica | NA | | Os03g0684400 | OsR498G0306813700.01.T02 | Magnesium transporter |
| maker-3-augustus-gene-410.34-mRNA-1 | Japonica | NA | | Os03g0683900 | OsR498G0306812600.01.T02 | Ubiquitin-protein ligase E3 B |
| maker-3-augustus-gene-410.37-mRNA-1 | Japonica | NA | | Os03g0684000 | OsR498G0306812800.01.T01 | GATA transcription factor |
| maker-3-augustus-gene-410.41-mRNA-1 | Japonica | NA | | Os03g0684100 | OsR498G0306813400.01.T01 | ARM repeat superfamily protein |
| maker-3-augustus-gene-410.47-mRNA-1 | Japonica | NA | | Os03g0684700 | OsR498G0306815000.01.T01 | HPP family protein, expressed |
| maker-3-augustus-gene-410.49-mRNA-1 | Japonica | NA | | Os03g0685500 | OsR498G0306818600.01.T01 | CHCH domain containing protein, expressed |
| maker-3-augustus-gene-410.53-mRNA-1 | Japonica | NA | | Os03g0685600 | OsR498G0306818800.01.T02 | Angio-associated migratory cell protein isoform X2 |
| maker-3-augustus-gene-410.9-mRNA-1 | Japonica | NA | | Os03g0684500 | OsR498G0306814500.01.T01 | Glycosyltransferase family protein 47 |
| maker-3-augustus-gene-411.38-mRNA-1 | Japonica | NA | | | | |
| maker-3-augustus-gene-411.41-mRNA-1 | Japonica | Indica | | Os03g0686900 | OsR498G0306821500.01.T01 | Protein FLOURY ENDOSPERM 6, chloroplastic |
| maker-3-augustus-gene-411.45-mRNA-1 | Japonica | NA | | Os03g0688200 | OsR498G0306828100.01.T01 | |
| maker-3-augustus-gene-411.52-mRNA-1 | Japonica | NA | | Os03g0687000 | OsR498G0306821800.01.T01 | Solute carrier family 15 (Peptide/histidine transporter), member 3/4 |
| maker-3-augustus-gene-411.57-mRNA-1 | Japonica | Indica | | Os03g0687200 | OsR498G0306824200.01.T03 | Formin-like protein 14 |
| maker-3-augustus-gene-411.62-mRNA-1 | Japonica | Japonica | | Os03g0688000 | OsR498G0306827000.01.T01 | rRNA N-glycosidase |
| maker-3-augustus-gene-412.0-mRNA-1 | Japonica | NA | | Os03g0688300 | OsR498G0306829300.01.T01 | Calmodulin domain protein kinase, putative |
| maker-3-augustus-gene-412.14-mRNA-1 | Japonica | NA | CC-LRR-NBS-TM | Os03g0689800 | OsR498G0306832100.01.T01 | NB-ARC domain containing protein |
| maker-3-augustus-gene-412.16-mRNA-1 | Japonica | Indica | | Os03g0690000 | OsR498G0306833100.01.T01 | Costars domain-containing protein (Fragment) |
| maker-3-augustus-gene-412.19-mRNA-1 | Japonica | NA | CC-LRR-NBS-TM | Os03g0689400 | | Disease resistance protein RGA2 |
| maker-3-augustus-gene-412.2-mRNA-1 | Japonica | NA | | Os03g0689100 | OsR498G0306829900.01.T01 | Inositol hexakisphosphate and diphosphoinositol-pentakisphosphate kinase |
| maker-3-augustus-gene-412.21-mRNA-1 | Japonica | Indica | | Os03g0689900 | OsR498G0306832800.01.T01 | Tetratricopeptide repeat protein 38 |
| maker-3-augustus-gene-413.22-mRNA-1 | Japonica | Japonica | | Os03g0691500 | OsR498G0306844300.01.T01 | BHLH family protein, putative, expressed |
| maker-3-augustus-gene-413.25-mRNA-1 | Japonica | NA | | Os03g0691400 | OsR498G0306843600.01.T01 | 4HBT domain-containing protein |
| maker-3-augustus-gene-414.0-mRNA-1 | Japonica | NA | | Os03g0691800 | OsR498G0306845300.01.T01 | GSDH domain-containing protein (Fragment) |
| maker-3-augustus-gene-414.11-mRNA-1 | Japonica | Japonica | | Os03g0692500 | OsR498G0306849000.01.T03 | Hydroxyproline O-galactosyltransferase GALT4 |
| maker-3-augustus-gene-414.2-mRNA-1 | Japonica | NA | | Os03g0692000 | OsR498G0306845900.01.T01 | Beta-glucuronosyltransferase |

| | | | | | | |
|---|---|---|---|---|---|---|
| maker-3-augustus-gene-414.5-mRNA-1 | Japonica | NA | | | OsR498G0306846100.01.T02 | |
| maker-3-augustus-gene-414.8-mRNA-1 | Japonica | NA | | Os03g0692400 | OsR498G0306848200.01.T05 | Protein UPSTREAM OF FLC like |
| maker-3-augustus-gene-415.42-mRNA-1 | Japonica | NA | | Os03g0693600 | OsR498G0306852900.01.T01 | Indole-3-acetate beta-glucosyltransferase |
| maker-3-augustus-gene-415.44-mRNA-1 | Japonica | NA | | Os03g0694500 | OsR498G0306855500.01.T02 | Nucleobase-ascorbate transporter LPE1 |
| maker-3-augustus-gene-415.53-mRNA-1 | Japonica | NA | | Os03g0694900 | OsR498G0306856700.01.T01 | Valyl-tRNA synthetase |
| maker-3-augustus-gene-415.59-mRNA-1 | Japonica | NA | | | OsR498G0713858700.01.T01 | Kinase D-interacting substrate of |
| maker-3-augustus-gene-415.62-mRNA-1 | Japonica | NA | | Os03g0695500 | OsR498G0306859100.01.T01 | Myelin-associated oligodendrocyte basic protein isoform 1 |
| maker-3-augustus-gene-416.30-mRNA-1 | Japonica | NA | | Os03g0696000 | OsR498G0306860100.01.T02 | WHy domain-containing protein |
| maker-3-augustus-gene-416.32-mRNA-1 | Japonica | NA | | Os03g0696300 | OsR498G0306861700.01.T08 | CCAAT-binding transcription factor |
| maker-3-augustus-gene-416.37-mRNA-1 | Japonica | NA | | Os03g0695600 | OsR498G0306859400.01.T01 | Proteasome endopeptidase complex |
| maker-3-augustus-gene-416.40-mRNA-1 | Japonica | NA | | | OsR498G0306859600.01.T03 | Chloride channel protein CLC-d |
| maker-3-augustus-gene-417.35-mRNA-1 | Japonica | NA | | | | C2H2-type domain-containing protein |
| maker-3-augustus-gene-417.37-mRNA-1 | Japonica | NA | | Os03g0698500 | OsR498G0306867700.01.T06 | Yippee domain-containing protein |
| maker-3-augustus-gene-417.41-mRNA-1 | Japonica | NA | | Os03g0699200 | OsR498G0306870300.01.T01 | Pescadillo homolog |
| maker-3-augustus-gene-417.47-mRNA-1 | Japonica | NA | | Os03g0699300 | OsR498G0306870500.01.T02 | Adenylosuccinate synthetase, chloroplastic |
| maker-3-augustus-gene-417.50-mRNA-1 | Japonica | NA | | Os03g0699600 | OsR498G0306870900.01.T01 | LysM domain containing protein, expressed |
| maker-3-augustus-gene-417.52-mRNA-1 | Japonica | NA | | Os03g0699700 | OsR498G0306871300.01.T01 | Lipoxygenase |
| maker-3-augustus-gene-417.56-mRNA-1 | Japonica | Indica | | Os03g0698350 | OsR498G0306867200.01.T01 | ABC1 domain-containing protein |
| maker-3-augustus-gene-417.62-mRNA-1 | Japonica | NA | | | OsR498G0306870200.01.T01 | denticleless protein homolog A |
| maker-3-augustus-gene-417.65-mRNA-1 | Japonica | NA | | Os03g0699400 | OsR498G0306870800.01.T01 | N-alpha-acetyltransferase 20 |
| maker-3-augustus-gene-418.39-mRNA-1 | Japonica | NA | | Os03g0700400 | OsR498G0306873300.01.T03 | Lipoxygenase |
| maker-3-augustus-gene-418.42-mRNA-1 | Japonica | NA | | Os03g0700700 | OsR498G0306875300.01.T02 | Lipoxygenase |
| maker-3-augustus-gene-418.47-mRNA-1 | Japonica | NA | | Os03g0700800 | OsR498G0306875700.01.T02 | Ethylene insensitive 2 |
| maker-3-augustus-gene-418.52-mRNA-1 | Japonica | Indica | | Os03g0701000 | OsR498G0306876300.01.T02 | TOG domain-containing protein (Fragment) |
| maker-3-augustus-gene-418.57-mRNA-1 | Japonica | NA | | Os03g0701200 | OsR498G0306876900.01.T01 | Suppressor of disruption of TFIIS |
| maker-3-augustus-gene-418.62-mRNA-1 | Japonica | Indica | | Os03g0701300 | OsR498G0306877500.01.T02 | |
| maker-3-augustus-gene-418.65-mRNA-1 | Japonica | NA | | | OsR498G0306877900.01.T03 | Pentatricopeptide repeat-containing protein |
| maker-3-augustus-gene-418.66-mRNA-1 | Japonica | NA | | | OsR498G0306876800.01.T03 | Pre-mRNA-splicing factor 18 |
| maker-3-augustus-gene-419.0-mRNA-1 | Japonica | NA | | Os03g0701800 | OsR498G0306880300.01.T07 | Phosphatidylinositol 4-phosphate 5-kinase |
| maker-3-augustus-gene-419.11-mRNA-1 | Japonica | NA | | Os03g0701600 | OsR498G0306879000.01.T01 | CHZ domain-containing protein |
| maker-3-augustus-gene-419.15-mRNA-1 | Japonica | NA | | Os03g0701700 | OsR498G0306879800.01.T01 | Ethylene responsive factor |
| maker-3-augustus-gene-419.19-mRNA-1 | Japonica | NA | | | OsR498G0306880600.01.T01 | Pinin/SDK/memA/ protein conserved region containing protein |
| maker-3-augustus-gene-419.25-mRNA-1 | Japonica | NA | | Os03g0702000 | OsR498G0306881400.01.T01 | Glycosyltransferase |
| maker-3-augustus-gene-419.27-mRNA-1 | Japonica | NA | | Os03g0703000 | OsR498G0306884400.01.T02 | Beta-glucosidase 7 |
| maker-3-augustus-gene-419.34-mRNA-1 | Japonica | NA | | Os03g0703100 | OsR498G0306884800.01.T02 | Beta-glucosidase 8 |
| maker-3-augustus-gene-419.41-mRNA-1 | Japonica | Indica | Kinase-LRR-TM | Os03g0703200 | OsR498G0306885000.01.T01 | LRR receptor-like serine/threonine-protein kinase |
| maker-3-augustus-gene-419.6-mRNA-1 | Japonica | NA | | | | Organic anion transporter |
| maker-3-augustus-gene-42.40-mRNA-1 | Japonica | NA | | Os03g0184100 | OsR498G0305343300.01.T06 | 15-cis-phytoene desaturase chloroplastic/chromoplastic |
| maker-3-augustus-gene-42.44-mRNA-1 | Japonica | NA | | Os10g0352200 | | |
| maker-3-augustus-gene-42.46-mRNA-1 | Japonica | Indica | | Os03g0184300 | OsR498G0305344900.01.T01 | Hexosyltransferase |
| maker-3-augustus-gene-42.49-mRNA-1 | Japonica | NA | LRR-TM | Os03g0184400 | OsR498G0305345100.01.T01 | Leucine-rich repeat receptor-like serine/threonine-protein kinase |
| maker-3-augustus-gene-42.52-mRNA-1 | Japonica | NA | | | OsR498G0305345700.01.T01 | NAD-dependent epimerase/dehydratase |
| maker-3-augustus-gene-42.59-mRNA-1 | Japonica | NA | | | OsR498G0305348700.01.T02 | GPI inositol-deacylase |
| maker-3-augustus-gene-42.64-mRNA-1 | Japonica | NA | | Os03g0186100 | OsR498G0305349500.01.T01 | Uroporphyrinogen-III synthase |
| maker-3-augustus-gene-42.71-mRNA-1 | Japonica | NA | | Os03g0186500 | OsR498G0305350300.01.T01 | Transmembrane protein 1 (Fragment) |
| maker-3-augustus-gene-42.73-mRNA-1 | Japonica | NA | | Os03g0184500 | OsR498G0305345400.01.T01 | B3 DNA binding domain |
| maker-3-augustus-gene-42.75-mRNA-1 | Japonica | NA | | Os03g0184700 | OsR498G0305346200.01.T01 | Origin of replication complex subunit like |
| maker-3-augustus-gene-420.0-mRNA-1 | Japonica | NA | | Os03g0703900 | OsR498G0306887100.01.T01 | SNARE associated Golgi protein family |
| maker-3-augustus-gene-420.11-mRNA-1 | Japonica | NA | | | OsR498G0306889100.01.T05 | Oxysterol-binding protein-related protein 1C |
| maker-3-augustus-gene-420.15-mRNA-1 | Japonica | NA | | Os03g0703300 | OsR498G0306885200.01.T01 | Opaque endosperm10 |
| maker-3-augustus-gene-420.19-mRNA-1 | Japonica | NA | Kinase-TM | Os03g0703400 | OsR498G0306885800.01.T03 | Mitogen-activated protein kinase kinase kinase 1 |
| maker-3-augustus-gene-420.23-mRNA-1 | Japonica | NA | | | OsR498G0306887800.01.T03 | Histone-lysine N-methyltransferase ASHR1 |
| maker-3-augustus-gene-420.3-mRNA-1 | Japonica | Indica | | Os03g0704000 | OsR498G0306887300.01.T02 | Putative plastid-lipid-associated protein 4, chloroplastic |
| maker-3-augustus-gene-420.5-mRNA-1 | Japonica | NA | | Os03g0704100 | OsR498G0306887500.01.T02 | probable plastid-lipid-associated protein 4, chloroplastic |
| maker-3-augustus-gene-420.9-mRNA-1 | Japonica | Indica | Kinase-TM | Os03g0704400 | OsR498G0306887900.01.T01 | Serine/threonine-protein kinase mph1 |
| maker-3-augustus-gene-421.24-mRNA-1 | Japonica | NA | | Os03g0705300 | OsR498G0306890800.01.T01 | Phosphatidylinositol 4-phosphate 5-kinase |
| maker-3-augustus-gene-421.29-mRNA-1 | Japonica | NA | | Os03g0706900 | OsR498G0306899000.01.T03 | Transmembrane Fragile-X-F-associated protein isoform 1 |
| maker-3-augustus-gene-422.1-mRNA-1 | Japonica | Indica | | Os03g0707200 | OsR498G0306899500.01.T03 | Drug/metabolite transporter |
| maker-3-augustus-gene-422.15-mRNA-1 | Japonica | NA | | Os03g0707900 | OsR498G0306903200.01.T01 | Outer envelope protein 64, chloroplastic |
| maker-3-augustus-gene-422.19-mRNA-1 | Japonica | NA | | | OsR498G0306904000.01.T05 | Phytanoyl-CoA dioxygenase |
| maker-3-augustus-gene-422.6-mRNA-1 | Japonica | Japonica | | Os03g0708000 | OsR498G1120323400.01.T01 | Secretory phospholipase A2 |

| | | | | | | |
|---|---|---|---|---|---|---|
| maker-3-augustus-gene-422.9-mRNA-1 | Japonica | NA | | Os03g0707300 | OsR498G0306899800.01.T01 | |
| maker-3-augustus-gene-423.0-mRNA-1 | Japonica | NA | | Os03g0708400 | OsR498G0306904700.01.T01 | FKBP-type peptidyl-prolyl cis-trans isomerase 5 isoform 2 |
| maker-3-augustus-gene-423.10-mRNA-1 | Japonica | NA | | Os03g0710000 | OsR498G0306910500.01.T02 | Putative vacuolar protein sorting-associated protein Ist1 |
| maker-3-augustus-gene-423.13-mRNA-1 | Japonica | Japonica | | Os03g0710500 | OsR498G0306911100.01.T01 | Luminal-binding protein 5 |
| maker-3-augustus-gene-423.16-mRNA-1 | Japonica | NA | | Os03g0708500 | OsR498G0306905000.01.T02 | Peptidylprolyl isomerase |
| maker-3-augustus-gene-423.18-mRNA-1 | Japonica | Japonica | | Os03g0708700 | OsR498G0306905400.01.T01 | Myosin-binding protein 2 isoform X3 |
| maker-3-augustus-gene-423.20-mRNA-1 | Japonica | NA | | Os03g0709000 | OsR498G0306907200.01.T01 | MAPEG family protein, expressed |
| maker-3-augustus-gene-423.23-mRNA-1 | Japonica | NA | | Os03g0709100 | OsR498G0306907600.01.T01 | Phytocyanin domain-containing protein (Fragment) |
| maker-3-augustus-gene-423.25-mRNA-1 | Japonica | NA | | Os03g0709300 | OsR498G0306908200.01.T01 | Chemocyanin |
| maker-3-augustus-gene-423.27-mRNA-1 | Japonica | NA | | | OsR498G0306910800.01.T01 | Homologous-pairing protein 2 homolog |
| maker-3-augustus-gene-423.29-mRNA-1 | Japonica | NA | CC-Kinase | | OsR498G0306910800.01.T02 | Homologous-pairing protein 2 homolog |
| maker-3-augustus-gene-423.6-mRNA-1 | Japonica | NA | | Os03g0708900 | OsR498G0306906900.01.T01 | Zn-finger in Ran binding protein and others, putative |
| maker-3-augustus-gene-424.0-mRNA-1 | Japonica | NA | | Os03g0710800 | OsR498G0306912100.01.T04 | |
| maker-3-augustus-gene-424.14-mRNA-1 | Japonica | NA | | Os03g0710700 | OsR498G0306911600.01.T02 | GLTP domain-containing protein |
| maker-3-augustus-gene-424.2-mRNA-1 | Japonica | NA | | Os03g0710900 | OsR498G0306912500.01.T01 | |
| maker-3-augustus-gene-424.21-mRNA-1 | Japonica | NA | | | OsR498G0306914000.01.T02 | Phosphatidylinositol 4-kinase alpha 1 |
| maker-3-augustus-gene-424.23-mRNA-1 | Japonica | NA | Kinase-TM | Os03g0711300 | OsR498G0306914200.01.T02 | Serinethreonine-protein kinase grik2 |
| maker-3-augustus-gene-424.29-mRNA-1 | Japonica | NA | | Os03g0711400 | OsR498G0306914600.01.T01 | Coatomer subunit alpha |
| maker-3-augustus-gene-424.30-mRNA-1 | Japonica | NA | | Os03g0711400 | OsR498G0306915200.01.T01 | Coatomer subunit alpha |
| maker-3-augustus-gene-424.35-mRNA-1 | Japonica | Indica | | Os03g0711650 | OsR498G0306916000.01.T02 | AT hook motif family protein, expressed |
| maker-3-augustus-gene-424.8-mRNA-1 | Japonica | NA | | Os03g0711100 | OsR498G0306913700.01.T01 | Zinc finger protein like |
| maker-3-augustus-gene-425.30-mRNA-1 | Japonica | NA | | | OsR498G0306917100.01.T01 | Calmodulin-binding heat-shock protein, putative |
| maker-3-augustus-gene-425.32-mRNA-1 | Japonica | NA | | Os03g0712100 | OsR498G0306917300.01.T01 | Cell division cycle-associated protein 7 |
| maker-3-augustus-gene-425.35-mRNA-1 | Japonica | NA | | Os03g0712200 | OsR498G0306917500.01.T01 | Zinc finger protein VAR3, chloroplastic |
| maker-3-augustus-gene-425.39-mRNA-1 | Japonica | NA | | Os03g0712300 | OsR498G0306917700.01.T01 | UBC core domain-containing protein (Fragment) |
| maker-3-augustus-gene-425.42-mRNA-1 | Japonica | NA | | Os03g0712700 | OsR498G0306919300.01.T01 | Phosphoglucomutase (alpha-D-glucose-1,6-bisphosphate-dependent) |
| maker-3-augustus-gene-425.48-mRNA-1 | Japonica | NA | | | OsR498G0306921700.01.T01 | Threonine dehydratase 1 biosynthetic, chloroplastic |
| maker-3-augustus-gene-425.55-mRNA-1 | Japonica | Indica | | Os10g0567800 | OsR498G0306922100.01.T01 | Dynamin-related protein 1C isoform A |
| maker-3-augustus-gene-425.62-mRNA-1 | Japonica | NA | Kinase-TM | Os03g0711800 | OsR498G0306916400.01.T04 | probable serine/threonine protein kinase IREH1 |
| maker-3-augustus-gene-425.67-mRNA-1 | Japonica | NA | Kinase-LRR-TM | | OsR498G0306918600.01.T01 | Atypical receptor-like kinase MARK |
| maker-3-augustus-gene-425.69-mRNA-1 | Japonica | NA | | Os03g0712600 | OsR498G0306919200.01.T01 | Leucine Rich Repeat, putative |
| maker-3-augustus-gene-425.72-mRNA-1 | Japonica | NA | | Os03g0712800 | OsR498G0306920200.01.T02 | Glutamine synthetase nodule isozyme |
| maker-3-augustus-gene-425.77-mRNA-1 | Japonica | Indica | | Os03g0713200 | OsR498G0306922400.01.T01 | NADH-ubiquinone oxidoreductase 75 kDa subunit, mitochondrial |
| maker-3-augustus-gene-426.35-mRNA-1 | Japonica | NA | | Os03g0713700 | OsR498G0306924300.01.T04 | WAS protein family 1 (Fragment) |
| maker-3-augustus-gene-426.40-mRNA-1 | Japonica | NA | | Os03g0337700 | OsR498G0306926100.01.T01 | GPN-loop GTPase 3 |
| maker-3-augustus-gene-426.48-mRNA-1 | Japonica | NA | | Os03g0713400 | OsR498G0306922600.01.T01 | NADH-ubiquinone oxidoreductase 75 kDa subunit, mitochondrial |
| maker-3-augustus-gene-426.50-mRNA-1 | Japonica | NA | | Os03g0713600 | OsR498G0306924000.01.T02 | Binding partner of ACD11 1 |
| maker-3-augustus-gene-427.32-mRNA-1 | Japonica | NA | | Os03g0715400 | OsR498G0306930300.01.T05 | Protein POLLEN DEFECTIVE IN GUIDANCE 1 isoform C |
| maker-3-augustus-gene-427.38-mRNA-1 | Japonica | NA | | | OsR498G0306930700.01.T01 | Calmodulin |
| maker-3-augustus-gene-427.41-mRNA-1 | Japonica | NA | | | OsR498G0306932700.01.T02 | |
| maker-3-augustus-gene-427.43-mRNA-1 | Japonica | NA | | Os03g0717200 | OsR498G0306935300.01.T01 | Transmembrane ascorbate ferrireductase 1 |
| maker-3-augustus-gene-427.45-mRNA-1 | Japonica | NA | | Os09g0434600 | OsR498G0204973200.01.T01 | Glycosyltransferase family protein 2 |
| maker-3-augustus-gene-427.47-mRNA-1 | Japonica | NA | | | | CNH domain-containing protein |
| maker-3-augustus-gene-427.51-mRNA-1 | Japonica | NA | | Os03g0716200 | OsR498G0306931400.01.T01 | PHD finger protein MALE MEIOCYTE DEATH 1 |
| maker-3-augustus-gene-427.53-mRNA-1 | Japonica | Indica | Kinase-LRR-TM | Os03g0717000 | OsR498G0306934600.01.T01 | Receptor-like kinase TMK2 |
| maker-3-augustus-gene-427.55-mRNA-1 | Japonica | NA | | Os03g0717600 | OsR498G0306936200.01.T01 | Matrin-type domain-containing protein |
| maker-3-augustus-gene-428.0-mRNA-1 | Japonica | NA | | | OsR498G0306936500.01.T02 | Histidine kinase |
| maker-3-augustus-gene-428.14-mRNA-1 | Japonica | NA | | Os03g0718000 | OsR498G0306937800.01.T02 | Anthranilate synthase/para-aminobenzoate synthase, glutamine amidotrans |
| maker-3-augustus-gene-428.24-mRNA-1 | Japonica | Indica | | Os03g0719000 | OsR498G0306942400.01.T01 | 65-kDa microtubule-associated protein 6 |
| maker-3-augustus-gene-428.29-mRNA-1 | Japonica | NA | | Os03g0719300 | OsR498G0306943600.01.T02 | 3,4-dihydroxy-2-butanone kinase |
| maker-3-augustus-gene-428.4-mRNA-1 | Japonica | NA | | Os03g0717900 | OsR498G0306937500.01.T01 | UV-B-induced protein chloroplastic |
| maker-3-augustus-gene-429.30-mRNA-1 | Japonica | NA | | Os03g0720300 | OsR498G0306946900.01.T01 | Glutamate decarboxylase |
| maker-3-augustus-gene-429.34-mRNA-1 | Japonica | NA | | Os03g0720800 | OsR498G0306953100.01.T01 | Transcription factor MYB52 |
| maker-3-augustus-gene-429.36-mRNA-1 | Japonica | NA | | Os03g0719400 | OsR498G0306943800.01.T01 | Monoglyceride lipase |
| maker-3-augustus-gene-429.41-mRNA-1 | Japonica | NA | CC-Kinase | | OsR498G0306944200.01.T01 | Putative serine/threonine-protein kinase dyrk2 |
| maker-3-augustus-gene-429.44-mRNA-1 | Japonica | NA | | Os03g0719800 | OsR498G0306945000.01.T01 | Phytochrome A |
| maker-3-augustus-gene-429.46-mRNA-1 | Japonica | NA | Kinase-TM | Os03g0719850 | OsR498G0306945400.01.T01 | Protein kinase domain containing protein |
| maker-3-augustus-gene-429.48-mRNA-1 | Japonica | NA | | Os03g0719900 | OsR498G0306945600.01.T01 | Proton-dependent oligopeptide transporter |
| maker-3-augustus-gene-429.53-mRNA-1 | Japonica | NA | Kinase | Os11g0620500 | OsR498G0510972100.01.T01 | Pollen receptor-like kinase 6 |
| maker-3-augustus-gene-429.55-mRNA-1 | Japonica | NA | | | OsR498G0407709200.01.T01 | |
| maker-3-augustus-gene-43.0-mRNA-1 | Japonica | NA | | Os03g0186800 | OsR498G0305351100.01.T01 | VPS37 C-terminal domain-containing protein |

| | | | | | | |
|---|---|---|---|---|---|---|
| maker-3-augustus-gene-43.10-mRNA-1 | Japonica | Indica | | Os03g0187300 | OsR498G0305353100.01.T01 | WD and tetratricopeptide repeats protein 1 |
| maker-3-augustus-gene-43.15-mRNA-1 | Japonica | NA | | Os03g0187400 | OsR498G0305354100.01.T01 | Myb-like protein H isoform X1 |
| maker-3-augustus-gene-43.17-mRNA-1 | Japonica | NA | | Os03g0187600 | OsR498G0305356500.01.T01 | GRAM domain-containing protein (Fragment) |
| maker-3-augustus-gene-43.21-mRNA-1 | Japonica | Indica | | Os08g0480000 | OsR498G0305358500.01.T01 | Protein DETOXIFICATION |
| maker-3-augustus-gene-43.24-mRNA-1 | Japonica | NA | | Os03g0186600 | OsR498G0305350600.01.T02 | JOINTLESS like protein MdJOINTLESSb |
| maker-3-augustus-gene-43.3-mRNA-1 | Japonica | NA | | Os03g0187000 | OsR498G0305352100.01.T01 | Protein DCL, chloroplastic |
| maker-3-augustus-gene-43.31-mRNA-1 | Japonica | NA | | Os03g0186950 | OsR498G0305352000.01.T02 | Protein CutA chloroplastic |
| maker-3-augustus-gene-43.33-mRNA-1 | Japonica | NA | | | OsR498G0305355400.01.T01 | F-box protein FBX14 |
| maker-3-augustus-gene-430.34-mRNA-1 | Japonica | NA | | | | ILITYHIA |
| maker-3-augustus-gene-430.39-mRNA-1 | Japonica | NA | | Os03g0721900 | OsR498G0306955700.01.T01 | |
| maker-3-augustus-gene-430.41-mRNA-1 | Japonica | NA | | | OsR498G0306956300.01.T01 | SWI/SNF complex subunit SWI3D |
| maker-3-augustus-gene-430.47-mRNA-1 | Japonica | NA | | | OsR498G0306956900.01.T03 | Chromatin complex subunit A 106 |
| maker-3-augustus-gene-430.51-mRNA-1 | Japonica | NA | | Os03g0721300 | OsR498G0306954600.01.T01 | Pre-mRNA processing protein PRP39, putative |
| maker-3-augustus-gene-430.53-mRNA-1 | Japonica | NA | | | | |
| maker-3-augustus-gene-430.55-mRNA-1 | Japonica | Indica | | Os03g0722600 | OsR498G0306957400.01.T01 | Adenylyl cyclase-associated protein (Fragment) |
| maker-3-augustus-gene-430.60-mRNA-1 | Japonica | NA | | Os03g0722800 | OsR498G0306958200.01.T03 | FBD domain-containing protein |
| maker-3-augustus-gene-431.32-mRNA-1 | Japonica | NA | | Os03g0723400 | OsR498G0306960100.01.T01 | Late embryogenesis abundant protein (Fragment) |
| maker-3-augustus-gene-431.35-mRNA-1 | Japonica | NA | | Os03g0723600 | OsR498G0306960300.01.T01 | BTB/POZ domain-containing protein isoform A |
| maker-3-augustus-gene-431.37-mRNA-1 | Japonica | NA | | Os03g0724100 | OsR498G0306963100.01.T02 | CRAL-TRIO domain-containing protein |
| maker-3-augustus-gene-431.40-mRNA-1 | Japonica | NA | Kinase-LRR-TM | | OsR498G0306963400.01.T02 | Lrr receptor protein kinase |
| maker-3-augustus-gene-432.45-mRNA-1 | Japonica | Japonica | | Os03g0724900 | OsR498G0306965800.01.T01 | |
| maker-3-augustus-gene-432.47-mRNA-1 | Japonica | Indica | | Os03g0725100 | OsR498G0306966300.01.T01 | Fip1 domain-containing protein |
| maker-3-augustus-gene-432.54-mRNA-1 | Japonica | NA | | | OsR498G0306966900.01.T03 | DBR1 domain-containing protein (Fragment) |
| maker-3-augustus-gene-432.60-mRNA-1 | Japonica | NA | | Os03g0725400 | OsR498G0306967100.01.T04 | COMPASS-like protein H3K4 histone methylase component WDR5A |
| maker-3-augustus-gene-432.62-mRNA-1 | Japonica | NA | | Os03g0725800 | OsR498G0306969900.01.T01 | Symbiotic ammonium transporter |
| maker-3-augustus-gene-432.65-mRNA-1 | Japonica | Indica | | Os03g0726100 | OsR498G0306970700.01.T02 | Tubulin alpha chain |
| maker-3-augustus-gene-432.69-mRNA-1 | Japonica | NA | | Os03g0726200 | OsR498G0306971100.01.T01 | Inositol-tetrakisphosphate 1-kinase |
| maker-3-augustus-gene-432.74-mRNA-1 | Japonica | NA | | Os03g0724500 | OsR498G0306965200.01.T01 | SANT domain-containing protein (Fragment) |
| maker-3-augustus-gene-432.76-mRNA-1 | Japonica | NA | | Os03g0724600 | OsR498G0306965400.01.T01 | |
| maker-3-augustus-gene-432.78-mRNA-1 | Japonica | NA | | Os03g0724800 | OsR498G0306965800.01.T01 | |
| maker-3-augustus-gene-433.26-mRNA-1 | Japonica | NA | | Os03g0727100 | OsR498G0306976100.01.T01 | E3 ubiquitin-protein ligase BRE1-like |
| maker-3-augustus-gene-433.29-mRNA-1 | Japonica | NA | | Os03g0726300 | OsR498G0306972000.01.T01 | Expressed protein (With alternative splicing) |
| maker-3-augustus-gene-433.31-mRNA-1 | Japonica | NA | | Os03g0726500 | OsR498G0306973000.01.T01 | Transmembrane protein TauE like |
| maker-3-augustus-gene-433.36-mRNA-1 | Japonica | NA | | | OsR498G0306974600.01.T01 | EPIDERMAL PATTERNING FACTOR-like protein 2 |
| maker-3-augustus-gene-433.38-mRNA-1 | Japonica | NA | | Os03g0726800 | OsR498G0306974800.01.T02 | DUF676 domain-containing protein (Fragment) |
| maker-3-augustus-gene-433.42-mRNA-1 | Japonica | Japonica | | Os03g0726900 | OsR498G0306975200.01.T03 | WD-3 domain-containing protein |
| maker-3-augustus-gene-433.49-mRNA-1 | Japonica | Indica | | Os03g0727000 | OsR498G0306975400.01.T02 | Homeotic protein like |
| maker-3-augustus-gene-434.0-mRNA-1 | Japonica | NA | | Os03g0728100 | | J domain-containing protein |
| maker-3-augustus-gene-434.17-mRNA-1 | Japonica | Indica | | Os03g0728600 | OsR498G0306979600.01.T02 | Putative DNA-binding protein-like |
| maker-3-augustus-gene-434.3-mRNA-1 | Japonica | NA | | Os03g0727600 | OsR498G0306977200.01.T01 | Pollen-specific 1-aminocyclopropane-1-carboxylate synthase |
| maker-3-augustus-gene-434.5-mRNA-1 | Japonica | NA | | Os03g0727900 | OsR498G0306979000.01.T01 | Hflx-type G domain-containing protein (Fragment) |
| maker-3-augustus-gene-434.6-mRNA-1 | Japonica | NA | | Os03g0727900 | OsR498G0306979000.01.T02 | Hflx-type G domain-containing protein (Fragment) |
| maker-3-augustus-gene-435.0-mRNA-1 | Japonica | NA | | Os03g0728800 | OsR498G0306980100.01.T02 | DEAD-box ATP-dependent RNA helicase |
| maker-3-augustus-gene-435.13-mRNA-1 | Japonica | Japonica | | Os03g0729800 | OsR498G0306986100.01.T02 | ACT domain containing protein, expressed |
| maker-3-augustus-gene-435.16-mRNA-1 | Japonica | NA | | Os03g0728900 | OsR498G0306980400.01.T03 | Transcription factor bHLH74 |
| maker-3-augustus-gene-435.20-mRNA-1 | Japonica | NA | | Os03g0729000 | OsR498G0306981600.01.T01 | Ethylene-dependent gravitropism-deficient and yellow-green-like 3 |
| maker-3-augustus-gene-435.25-mRNA-1 | Japonica | NA | | | OsR498G0306982000.01.T03 | Transmembrane protein |
| maker-3-augustus-gene-435.26-mRNA-1 | Japonica | NA | | Os03g0729200 | OsR498G0306982400.01.T04 | Thioredoxin-like protein AAED1, chloroplastic |
| maker-3-augustus-gene-435.30-mRNA-1 | Japonica | NA | | Os03g0729300 | OsR498G0306982600.01.T01 | Thioredoxin-like protein AAED1 chloroplastic |
| maker-3-augustus-gene-435.34-mRNA-1 | Japonica | NA | | | OsR498G0306986400.01.T01 | BSD domain containing protein, expressed |
| maker-3-augustus-gene-435.37-mRNA-1 | Japonica | NA | | Os03g0730000 | OsR498G0306986800.01.T01 | Lecithin-cholesterol acyltransferase-like protein 1 |
| maker-3-augustus-gene-435.6-mRNA-1 | Japonica | Indica | | Os03g0729500 | OsR498G0306985100.01.T01 | Growth-regulating factor |
| maker-3-augustus-gene-435.9-mRNA-1 | Japonica | NA | | Os03g0729700 | OsR498G0306985700.01.T01 | 16S rRNA (uracil(1498)-N(3))-methyltransferase |
| maker-3-augustus-gene-436.0-mRNA-1 | Japonica | NA | | | OsR498G0306987700.01.T04 | Serine carboxypeptidase family protein, expressed |
| maker-3-augustus-gene-436.11-mRNA-1 | Japonica | Indica | | Os03g0730500 | OsR498G0306989100.01.T01 | Serine carboxypeptidase family protein, expressed |
| maker-3-augustus-gene-436.13-mRNA-1 | Japonica | Indica | | Os03g0731050 | OsR498G0306989900.01.T01 | Core-2/I-branching beta-16-N-acetylglucosaminyltransferase family protein |
| maker-3-augustus-gene-436.15-mRNA-1 | Japonica | NA | | Os03g0731100 | OsR498G0306990100.01.T02 | Photosystem I reaction center subunit N |
| maker-3-augustus-gene-436.17-mRNA-1 | Japonica | NA | | Os03g0731500 | OsR498G0306990900.01.T01 | Metalloendopeptidase/zinc ion-binding protein |
| maker-3-augustus-gene-436.23-mRNA-1 | Japonica | NA | | | OsR498G0713488800.01.T01 | Pathogenesis-related protein 5 |
| maker-3-augustus-gene-436.26-mRNA-1 | Japonica | Indica | | Os03g0730800 | OsR498G0306989600.01.T02 | Calcium-transporting ATPase |
| maker-3-augustus-gene-436.4-mRNA-1 | Japonica | NA | | Os03g0730500 | OsR498G0306988300.01.T02 | Serine carboxypeptidase-like 18 |

| | | | | | | |
|---|---|---|---|---|---|---|
| maker-3-augustus-gene-437.0-mRNA-1 | Japonica | Indica | | Os03g0731800 | OsR498G0306991100.01.T01 | Nucleotide-diphospho-sugar transferase |
| maker-3-augustus-gene-437.10-mRNA-1 | Japonica | Japonica | | Os03g0732000 | OsR498G0306991800.01.T01 | Hydroxymethylbutenyl diphosphate reductase |
| maker-3-augustus-gene-437.23-mRNA-1 | Japonica | NA | | | OsR498G0306992200.01.T04 | Dynamin GTPase |
| maker-3-augustus-gene-437.25-mRNA-1 | Japonica | Japonica | | Os03g0733000 | OsR498G0101678500.01.T03 | |
| maker-3-augustus-gene-437.5-mRNA-1 | Japonica | NA | | Os03g0731900 | OsR498G0306991400.01.T01 | Putative chloroplast 4-hydroxy-3-methylbut-2-en-1-yl diphosphate reductas |
| maker-3-augustus-gene-438.0-mRNA-1 | Japonica | NA | | Os03g0734000 | | |
| maker-3-augustus-gene-438.11-mRNA-1 | Japonica | NA | | Os03g0733600 | OsR498G0306998000.01.T01 | GRF-interacting factor 1 |
| maker-3-augustus-gene-438.13-mRNA-1 | Japonica | NA | | | OsR498G0306999400.01.T02 | Endoplasmic reticulum oxidoreductin |
| maker-3-augustus-gene-438.19-mRNA-1 | Japonica | NA | | | | Mitochondrial folate transporter/carrier |
| maker-3-augustus-gene-438.23-mRNA-1 | Japonica | NA | | Os03g0734900 | OsR498G0307002400.01.T02 | GATA transcription factor |
| maker-3-augustus-gene-438.33-mRNA-1 | Japonica | NA | | Os03g0735300 | OsR498G0307003400.01.T01 | Alba DNA/RNA-binding protein isoform 3 (Fragment) |
| maker-3-augustus-gene-438.4-mRNA-1 | Japonica | Japonica | | Os03g0734200 | OsR498G0307000500.01.T02 | |
| maker-3-augustus-gene-438.6-mRNA-1 | Japonica | NA | | | OsR498G0307002900.01.T01 | Cirhin |
| maker-3-augustus-gene-439.40-mRNA-1 | Japonica | Indica | | Os04g0413200 | OsR498G0307004900.01.T01 | Beta-fructofuranosidase, insoluble isoenzyme 1 like |
| maker-3-augustus-gene-439.42-mRNA-1 | Japonica | NA | | Os03g0737600 | OsR498G0307009500.01.T05 | Cyclin-H |
| maker-3-augustus-gene-439.46-mRNA-1 | Japonica | NA | | Os03g0735900 | OsR498G0307005200.01.T01 | Glycerol-3-phosphate 2-O-acyltransferase |
| maker-3-augustus-gene-439.48-mRNA-1 | Japonica | NA | | Os03g0736000 | OsR498G0307005800.01.T04 | CCR4-NOT transcription complex subunit 2 |
| maker-3-augustus-gene-439.49-mRNA-1 | Japonica | Indica | | Os02g0782200 | OsR498G0307005800.01.T06 | NOT2/NOT3/NOT5 family protein, expressed |
| maker-3-augustus-gene-439.62-mRNA-1 | Japonica | NA | | Os03g0736300 | OsR498G0307006400.01.T02 | Endoglucanase |
| maker-3-augustus-gene-439.67-mRNA-1 | Japonica | NA | | Os03g0736400 | OsR498G0307007000.01.T03 | Putative rRNA methyltransferase YlbH |
| maker-3-augustus-gene-439.71-mRNA-1 | Japonica | NA | | Os03g0737000 | OsR498G0307008400.01.T01 | Cystathionine beta-synthase family protein |
| maker-3-augustus-gene-439.75-mRNA-1 | Japonica | NA | | | | ELYS domain-containing protein (Fragment) |
| maker-3-augustus-gene-439.85-mRNA-1 | Japonica | NA | | Os03g0737300 | OsR498G0307008800.01.T01 | Magnesium-dependent phosphatase-1 |
| maker-3-augustus-gene-439.87-mRNA-1 | Japonica | NA | | Os03g0737400 | OsR498G0307009400.01.T01 | Magnesium-dependent phosphatase |
| maker-3-augustus-gene-44.36-mRNA-1 | Japonica | NA | | Os03g0188400 | OsR498G0305360300.01.T01 | Transcription factor MYC2 |
| maker-3-augustus-gene-44.38-mRNA-1 | Japonica | NA | | | | Homeodomain-leucine zipper transcription factor TaHDZipI-1 |
| maker-3-augustus-gene-44.40-mRNA-1 | Japonica | NA | | Os03g0189400 | OsR498G0305364400.01.T01 | GroES-like zinc-binding dehydrogenase family protein isoform 3 |
| maker-3-augustus-gene-44.44-mRNA-1 | Japonica | NA | | Os03g0189600 | OsR498G0305364800.01.T03 | Alcohol dehydrogenase 1 |
| maker-3-augustus-gene-44.48-mRNA-1 | Japonica | NA | | Os03g0190100 | OsR498G0305366400.01.T02 | 1,4-dihydroxy-2-naphthoate polyprenyltransferase, chloroplastic |
| maker-3-augustus-gene-44.53-mRNA-1 | Japonica | Indica | LRR-TM | Os03g0190300 | OsR498G0305366800.01.T02 | Dynein assembly factor 1, axonemal |
| maker-3-augustus-gene-440.0-mRNA-1 | Japonica | NA | | Os03g0737800 | OsR498G0307010100.01.T02 | Vps51/Vps67 family (Components of vesicular transport) protein |
| maker-3-augustus-gene-440.13-mRNA-1 | Japonica | NA | | | OsR498G0307011300.01.T06 | Non-specific serine/threonine protein kinase |
| maker-3-augustus-gene-440.24-mRNA-1 | Japonica | NA | | Os03g0738400 | OsR498G0307011500.01.T02 | Glycine hydroxymethyltransferase |
| maker-3-augustus-gene-440.27-mRNA-1 | Japonica | NA | | Os03g0738600 | OsR498G0307013100.01.T01 | Lipoxygenase |
| maker-3-augustus-gene-440.29-mRNA-1 | Japonica | NA | | Os03g0738900 | OsR498G0307014300.01.T01 | Phototropic-responsive NPH3 family protein isoform 2 |
| maker-3-augustus-gene-440.31-mRNA-1 | Japonica | NA | | | OsR498G0307010400.01.T01 | BAT2 domain-containing protein 1 |
| maker-3-augustus-gene-440.7-mRNA-1 | Japonica | NA | | Os03g0738000 | OsR498G0307011100.01.T01 | Telomerase Cajal body protein 1 |
| maker-3-augustus-gene-441.0-mRNA-1 | Japonica | NA | | Os03g0739700 | OsR498G0307016500.01.T01 | Pre-mRNA-splicing factor ATP-dependent RNA helicase |
| maker-3-augustus-gene-441.12-mRNA-1 | Japonica | NA | | Os03g0741100 | OsR498G0307020100.01.T01 | BHLH transcription factor (Fragment) |
| maker-3-augustus-gene-441.14-mRNA-1 | Japonica | NA | | | OsR498G0307022300.01.T03 | |
| maker-3-augustus-gene-441.15-mRNA-1 | Japonica | NA | | Os03g0741400 | OsR498G0307022300.01.T01 | WRKY transcription factor 21 |
| maker-3-augustus-gene-441.3-mRNA-1 | Japonica | NA | | Os03g0740600 | OsR498G0307018900.01.T04 | Dehydration responsive element binding protein 1 |
| maker-3-augustus-gene-441.6-mRNA-1 | Japonica | NA | | Os03g0740700 | OsR498G0307019100.01.T01 | CwfJ C-terminus 1 containing protein, expressed |
| maker-3-augustus-gene-441.9-mRNA-1 | Japonica | NA | | Os03g0740800 | OsR498G0307019700.01.T01 | X-ray repair cross-complementing 4 |
| maker-3-augustus-gene-442.0-mRNA-1 | Japonica | NA | | Os03g0742300 | OsR498G0307024500.01.T01 | Two-component response regulator ORR41 |
| maker-3-augustus-gene-442.15-mRNA-1 | Japonica | NA | | | OsR498G0307023400.01.T01 | Zinc finger, C3HC4 type family protein, expressed |
| maker-3-augustus-gene-442.19-mRNA-1 | Japonica | Indica | | Os03g0742400 | OsR498G0307024800.01.T03 | Magnesium transporter |
| maker-3-augustus-gene-442.2-mRNA-1 | Japonica | NA | | Os03g0742800 | OsR498G0307026300.01.T02 | Protein transport protein SEC23 |
| maker-3-augustus-gene-442.27-mRNA-1 | Japonica | NA | | Os03g0742900 | OsR498G0307026600.01.T01 | Auxin-responsive protein IAA13 |
| maker-3-augustus-gene-442.29-mRNA-1 | Japonica | NA | | Os03g0743900 | OsR498G0307030400.01.T01 | Sulfate adenylyltransferase |
| maker-3-augustus-gene-442.4-mRNA-1 | Japonica | NA | | | OsR498G0306510800.01.T03 | |
| maker-3-augustus-gene-442.6-mRNA-1 | Japonica | NA | | Os03g0743400 | OsR498G0307029300.01.T04 | Probable histone H2A variant 3 |
| maker-3-augustus-gene-442.8-mRNA-1 | Japonica | NA | | Os03g0743500 | OsR498G0307029700.01.T02 | Calmodulin |
| maker-3-augustus-gene-443.0-mRNA-1 | Japonica | NA | | Os03g0744700 | OsR498G0307033700.01.T05 | |
| maker-3-augustus-gene-443.11-mRNA-1 | Japonica | NA | | Os03g0744600 | OsR498G0307032000.01.T01 | Stem-specific protein TSJT1 |
| maker-3-augustus-gene-443.15-mRNA-1 | Japonica | Japonica | | Os03g0744650 | OsR498G0307032200.01.T02 | BEACH domain-containing protein lvsA |
| maker-3-augustus-gene-443.20-mRNA-1 | Japonica | NA | | Os03g0744800 | OsR498G0307034000.01.T01 | Transmembrane emp24 domain-containing protein A |
| maker-3-augustus-gene-443.23-mRNA-1 | Japonica | NA | | Os03g0745000 | OsR498G0307034400.01.T01 | Heat shock transcription factor |
| maker-3-augustus-gene-443.25-mRNA-1 | Japonica | NA | | Os03g0745200 | OsR498G0307035000.01.T01 | Transferase family protein, expressed |
| maker-3-augustus-gene-443.8-mRNA-1 | Japonica | NA | Kinase-LRR-TM | | OsR498G0307031400.01.T02 | Serine/threonine protein kinase |
| maker-3-augustus-gene-444.43-mRNA-1 | Japonica | NA | | Os03g0746600 | OsR498G0307041100.01.T01 | Autophagy-related protein 16 isoform A |

| | | | | | | |
|---|---|---|---|---|---|---|
| maker-3-augustus-gene-444.46-mRNA-1 | Japonica | NA | | | OsR498G0307042900.01.T01 | sigma non-opioid intracellular receptor 1 |
| maker-3-augustus-gene-444.48-mRNA-1 | Japonica | NA | | Os03g0747300 | OsR498G0307043300.01.T01 | Altered inheritance of mitochondria protein 24, mitochondrial |
| maker-3-augustus-gene-444.52-mRNA-1 | Japonica | NA | | Os03g0746500 | OsR498G0307041000.01.T04 | DExH-box ATP-dependent RNA helicase DExH16 mitochondrial |
| maker-3-augustus-gene-444.59-mRNA-1 | Japonica | NA | | Os03g0746800 | OsR498G0307041800.01.T01 | tRNA (guanine-N(7)-)-methyltransferase non-catalytic subunit |
| maker-3-augustus-gene-444.64-mRNA-1 | Japonica | NA | | | OsR498G0307043200.01.T04 | |
| maker-3-augustus-gene-445.100-mRNA-1 | Japonica | NA | | | | RNA helicase |
| maker-3-augustus-gene-445.42-mRNA-1 | Japonica | NA | | | OsR498G0307044300.01.T02 | Acyl-CoA N-acyltransferase with RING/FYVE/PHD-type zinc finger protein |
| maker-3-augustus-gene-445.47-mRNA-1 | Japonica | NA | | Os03g0747800 | OsR498G0307045300.01.T03 | Cytosolic cysteine synthase |
| maker-3-augustus-gene-445.53-mRNA-1 | Japonica | NA | | Os03g0747900 | OsR498G0307045500.01.T01 | Myosin-J heavy chain |
| maker-3-augustus-gene-445.65-mRNA-1 | Japonica | NA | | Os03g0748200 | OsR498G0307046300.01.T04 | PHD finger protein ING |
| maker-3-augustus-gene-445.69-mRNA-1 | Japonica | NA | Kinase | Os03g0748400 | OsR498G0307046900.01.T02 | Serine/threonine-protein kinase AFC3 |
| maker-3-augustus-gene-445.73-mRNA-1 | Japonica | NA | | Os03g0748600 | OsR498G0307047300.01.T01 | MFAP1 domain-containing protein |
| maker-3-augustus-gene-445.76-mRNA-1 | Japonica | NA | | Os03g0748900 | OsR498G0307048300.01.T02 | Binding partner of ACD11 1 isoform A |
| maker-3-augustus-gene-445.79-mRNA-1 | Japonica | NA | | | OsR498G0307048700.01.T01 | Ammonium transporter |
| maker-3-augustus-gene-445.81-mRNA-1 | Japonica | NA | | Os03g0749100 | OsR498G0307049100.01.T01 | Glycosyl hydrolase family 3 N terminal domain containing protein, expressed |
| maker-3-augustus-gene-445.83-mRNA-1 | Japonica | Japonica | | Os03g0747400 | OsR498G0307043600.01.T01 | Late embryogenesis abundant protein D-34 |
| maker-3-augustus-gene-445.86-mRNA-1 | Japonica | NA | | Os03g0747500 | OsR498G0307044000.01.T01 | late embryogenesis abundant protein 3 |
| maker-3-augustus-gene-445.88-mRNA-1 | Japonica | NA | | Os03g0747700 | OsR498G0307044800.01.T01 | photosystem II D1 precursor processing protein PSB27-H2, chloroplastic |
| maker-3-augustus-gene-445.90-mRNA-1 | Japonica | NA | | Os03g0748300 | OsR498G0307046600.01.T01 | NDH-dependent cyclic electron flow 5 |
| maker-3-augustus-gene-445.92-mRNA-1 | Japonica | NA | | Os03g0748500 | OsR498G0307047200.01.T01 | NAD(P)H dehydrogenase (quinone) |
| maker-3-augustus-gene-445.94-mRNA-1 | Japonica | NA | | Os03g0748700 | OsR498G0307047600.01.T01 | Cytosolic Fe-S cluster assembly factor narfl |
| maker-3-augustus-gene-446.0-mRNA-1 | Japonica | NA | | Os03g0749300 | OsR498G0307050300.01.T09 | Lysosomal beta glucosidase |
| maker-3-augustus-gene-446.13-mRNA-1 | Japonica | NA | | Os03g0749900 | OsR498G0307052300.01.T01 | Protein-lysine N-methyltransferase |
| maker-3-augustus-gene-446.15-mRNA-1 | Japonica | NA | | Os03g0750200 | OsR498G0307053100.01.T01 | Kinesin-like protein KIN-12E |
| maker-3-augustus-gene-446.22-mRNA-1 | Japonica | NA | CC-Kinase-TM | Os03g0749800 | OsR498G0307052200.01.T02 | Serine/threonine-protein kinase TOUSLED |
| maker-3-augustus-gene-446.27-mRNA-1 | Japonica | Indica | | Os03g0750100 | OsR498G0307052800.01.T02 | TPR Domain containing protein, expressed |
| maker-3-augustus-gene-446.31-mRNA-1 | Japonica | NA | | Os03g0750700 | | Strictosidine synthase |
| maker-3-augustus-gene-446.6-mRNA-1 | Japonica | NA | | Os03g0749500 | OsR498G0307051700.01.T07 | Beta-glucosidase BoGH3B |
| maker-3-augustus-gene-447.42-mRNA-1 | Japonica | Indica | | Os03g0751000 | OsR498G0307054700.01.T03 | Transmembrane protein 256 |
| maker-3-augustus-gene-447.45-mRNA-1 | Japonica | NA | | | | COR domain-containing protein |
| maker-3-augustus-gene-447.48-mRNA-1 | Japonica | NA | | Os03g0751400 | OsR498G0307056700.01.T01 | 50S ribosomal protein L6 chloroplastic |
| maker-3-augustus-gene-447.52-mRNA-1 | Japonica | NA | | Os03g0751600 | OsR498G0307057300.01.T03 | Protein PYRICULARIA ORYZAE RESISTANCE 21 |
| maker-3-augustus-gene-447.54-mRNA-1 | Japonica | NA | | Os03g0752100 | OsR498G0307058300.01.T01 | Phytochrome C |
| maker-3-augustus-gene-447.56-mRNA-1 | Japonica | NA | | Os03g0752200 | OsR498G0307058500.01.T02 | Initiator of meiotic double stranded breaks SPO11-1 |
| maker-3-augustus-gene-447.60-mRNA-1 | Japonica | Indica | | Os04g0208200 | OsR498G0307059100.01.T01 | Cysteine proteinase |
| maker-3-augustus-gene-447.62-mRNA-1 | Japonica | NA | | Os03g0752700 | OsR498G0307059500.01.T03 | J domain-containing protein |
| maker-3-augustus-gene-447.65-mRNA-1 | Japonica | NA | | Os03g0750800 | OsR498G0307054200.01.T01 | Transcriptional adapter |
| maker-3-augustus-gene-447.66-mRNA-1 | Japonica | NA | | Os03g0750800 | OsR498G0307054200.01.T04 | Transcriptional adapter |
| maker-3-augustus-gene-447.77-mRNA-1 | Japonica | NA | | | OsR498G0307054400.01.T01 | Transcriptional adapter ADA2b |
| maker-3-augustus-gene-447.78-mRNA-1 | Japonica | NA | | Os03g0750900 | OsR498G0307054400.01.T01 | Transcriptional adapter ADA2b |
| maker-3-augustus-gene-447.83-mRNA-1 | Japonica | NA | | Os03g0751100 | OsR498G0307055400.01.T01 | Oligopeptide transporter |
| maker-3-augustus-gene-447.85-mRNA-1 | Japonica | NA | | Os03g0752300 | OsR498G0307058800.01.T02 | Outward-rectifying potassium channel |
| maker-3-augustus-gene-447.90-mRNA-1 | Japonica | NA | | Os03g0752800 | OsR498G0307059800.01.T04 | MADS-box protein BM5A |
| maker-3-augustus-gene-448.26-mRNA-1 | Japonica | NA | | Os03g0753500 | OsR498G0307062200.01.T01 | |
| maker-3-augustus-gene-448.28-mRNA-1 | Japonica | NA | | Os03g0753100 | OsR498G0307061000.01.T01 | MADS-box transcription factor |
| maker-3-augustus-gene-448.31-mRNA-1 | Japonica | Indica | | Os03g0753500 | OsR498G0307061200.01.T01 | |
| maker-3-augustus-gene-449.0-mRNA-1 | Japonica | NA | | | OsR498G0307063500.01.T02 | Cytochrome b5 heme-binding domain-containing protein (Fragment) |
| maker-3-augustus-gene-449.11-mRNA-1 | Japonica | NA | Kinase-TM | Os03g0755000 | OsR498G0307065100.01.T02 | Serine/threonine-protein kinase dst1 |
| maker-3-augustus-gene-449.17-mRNA-1 | Japonica | Indica | | Os05g0386800 | OsR498G0307064000.01.T01 | COBRA-like protein 3 |
| maker-3-augustus-gene-449.2-mRNA-1 | Japonica | NA | | Os03g0754800 | OsR498G0307064700.01.T01 | Solute carrier family 25 member like |
| maker-3-augustus-gene-449.7-mRNA-1 | Japonica | NA | | Os03g0754900 | OsR498G0307064900.01.T01 | Cleavage stimulation factor subunit like |
| maker-3-augustus-gene-45.30-mRNA-1 | Japonica | Japonica | | Os03g0191400 | OsR498G0305369900.01.T02 | RAB GTPase homolog H1E |
| maker-3-augustus-gene-45.35-mRNA-1 | Japonica | Japonica | | Os03g0192100 | OsR498G0305371900.01.T01 | Carboxypeptidase |
| maker-3-augustus-gene-45.38-mRNA-1 | Japonica | NA | | Os03g0190800 | OsR498G0305367400.01.T01 | USP domain-containing protein |
| maker-3-augustus-gene-45.42-mRNA-1 | Japonica | NA | | | | Transmembrane protein like |
| maker-3-augustus-gene-45.47-mRNA-1 | Japonica | NA | | Os03g0191000 | OsR498G0305367800.01.T02 | Calmodulin-binding transcription activator 2 |
| maker-3-augustus-gene-45.51-mRNA-1 | Japonica | NA | | Os03g0191100 | OsR498G0305368200.01.T01 | Mitochondrial adenine nucleotide transporter like |
| maker-3-augustus-gene-45.53-mRNA-1 | Japonica | NA | | Os03g0191200 | OsR498G0305369000.01.T01 | WW domain containing protein expressed |
| maker-3-augustus-gene-45.55-mRNA-1 | Japonica | NA | | Os03g0192000 | OsR498G0305371800.01.T01 | Polymerase/histidinol phosphatase-like |
| maker-3-augustus-gene-45.59-mRNA-1 | Japonica | NA | | Os03g0192300 | OsR498G0305372200.01.T01 | Upstream of FLC-like protein isoform X2 |
| maker-3-augustus-gene-45.63-mRNA-1 | Japonica | NA | | Os03g0192400 | OsR498G0305373600.01.T01 | NADH-ubiquinone oxidoreductase B16.6 subunit |

| | | | | | | |
|---|---|---|---|---|---|---|
| maker-3-augustus-gene-450.0-mRNA-1 | Japonica | NA | NBS-TM | Os03g0755100 | OsR498G0307065300.01.T01 | Transporter associated with antigen processing protein 2 |
| maker-3-augustus-gene-450.12-mRNA-1 | Japonica | NA | | Os03g0756000 | OsR498G0307069700.01.T01 | Putative ribosomal protein L13 |
| maker-3-augustus-gene-450.16-mRNA-1 | Japonica | Japonica | | Os03g0755700 | OsR498G0307068000.01.T02 | Large subunit ribosomal protein L13Ae |
| maker-3-augustus-gene-450.21-mRNA-1 | Japonica | Indica | | Os03g0755600 | OsR498G0307068600.01.T02 | FCP1 homology domain-containing protein (Fragment) |
| maker-3-augustus-gene-450.27-mRNA-1 | Japonica | NA | | Os03g0756300 | OsR498G0307070600.01.T01 | X8 domain-containing protein (Fragment) |
| maker-3-augustus-gene-450.29-mRNA-1 | Japonica | NA | | Os03g0756400 | OsR498G0307070800.01.T01 | Transmembrane protein adipocyte-associated |
| maker-3-augustus-gene-450.6-mRNA-1 | Japonica | NA | | Os03g0755600 | OsR498G0307066700.01.T01 | FCP1 homology domain-containing protein (Fragment) |
| maker-3-augustus-gene-451.40-mRNA-1 | Japonica | NA | | Os03g0756800 | OsR498G0307071300.01.T01 | Glycosyltransferase |
| maker-3-augustus-gene-451.42-mRNA-1 | Japonica | NA | | Os03g0757500 | OsR498G0307073700.01.T01 | UDP-glycosyltransferase 83A1 |
| maker-3-augustus-gene-451.46-mRNA-1 | Japonica | NA | | Os03g0758300 | OsR498G0307078700.01.T02 | Cyclic nucleotide gated channel, other eukaryote |
| maker-3-augustus-gene-451.56-mRNA-1 | Japonica | NA | | Os03g0757200 | OsR498G0307073400.01.T02 | UDP-glucoronosyl and UDP-glucosyl transferase family protein, expressed |
| maker-3-augustus-gene-451.58-mRNA-1 | Japonica | NA | | Os03g0758000 | OsR498G0307077000.01.T01 | WRKY DNA binding domain containing protein, expressed |
| maker-3-augustus-gene-451.60-mRNA-1 | Japonica | NA | | | OsR498G0307078200.01.T03 | Alpha-glucan phosphorylase 1 |
| maker-3-augustus-gene-452.0-mRNA-1 | Japonica | NA | | | OsR498G0307078900.01.T01 | 26S proteasome non-ATPase regulatory subunit like |
| maker-3-augustus-gene-452.10-mRNA-1 | Japonica | NA | | Os03g0758800 | OsR498G0307081100.01.T01 | Eukaryotic translation initiation factor 5A |
| maker-3-augustus-gene-452.16-mRNA-1 | Japonica | NA | | Os03g0758900 | | WRKY DNA binding domain containing protein, expressed |
| maker-3-augustus-gene-452.25-mRNA-1 | Japonica | NA | | Os03g0759500 | OsR498G0307082500.01.T01 | Origin of replication complex subunit like |
| maker-3-augustus-gene-452.3-mRNA-1 | Japonica | Indica | | Os03g0758600 | OsR498G0307080500.01.T05 | |
| maker-3-augustus-gene-452.35-mRNA-1 | Japonica | NA | | Os03g0760200 | OsR498G0307084500.01.T01 | Isoflavone 2'-hydroxylase |
| maker-3-augustus-gene-452.37-mRNA-1 | Japonica | Indica | | Os03g0760500 | OsR498G0307084700.01.T03 | Isoflavone 2'-hydroxylase |
| maker-3-augustus-gene-452.39-mRNA-1 | Japonica | NA | | Os03g0758500 | OsR498G0307079200.01.T01 | Phytocyanin domain-containing protein (Fragment) |
| maker-3-augustus-gene-452.41-mRNA-1 | Japonica | NA | | | OsR498G0307081600.01.T02 | DUF1336 domain-containing protein (Fragment) |
| maker-3-augustus-gene-452.5-mRNA-1 | Japonica | NA | | | OsR498G0307080900.01.T03 | Mitotic checkpoint protein BUB3.3 |
| maker-3-augustus-gene-453.0-mRNA-1 | Japonica | Japonica | | Os03g0760500 | OsR498G0307084700.01.T03 | Isoflavone 2'-hydroxylase |
| maker-3-augustus-gene-453.10-mRNA-1 | Japonica | NA | | Os03g0761900 | OsR498G0307088300.01.T02 | Prolyl 4-hydroxylase alpha subunit |
| maker-3-augustus-gene-453.15-mRNA-1 | Japonica | NA | | Os03g0762300 | OsR498G0307090300.01.T01 | Peroxidase |
| maker-3-augustus-gene-453.18-mRNA-1 | Japonica | NA | | Os03g0762400 | OsR498G0307090900.01.T02 | Peroxidase |
| maker-3-augustus-gene-453.2-mRNA-1 | Japonica | NA | | Os03g0760700 | OsR498G0307085100.01.T01 | Aspartate-semialdehyde dehydrogenase |
| maker-3-augustus-gene-453.21-mRNA-1 | Japonica | NA | | Os03g0762500 | OsR498G0307091500.01.T02 | |
| maker-3-augustus-gene-453.23-mRNA-1 | Japonica | NA | | Os03g0760600 | OsR498G0307085000.01.T01 | Tonoplast intrinsic protein 41 |
| maker-3-augustus-gene-453.27-mRNA-1 | Japonica | NA | | Os03g0760800 | OsR498G0307085600.01.T01 | Peamaclein |
| maker-3-augustus-gene-453.29-mRNA-1 | Japonica | Indica | | Os03g0761000 | OsR498G0307086400.01.T01 | SWIB domain-containing protein (Fragment) |
| maker-3-augustus-gene-453.31-mRNA-1 | Japonica | Indica | | Os07g0114000 | OsR498G0307086600.01.T01 | Protein-serine/threonine phosphatase |
| maker-3-augustus-gene-453.35-mRNA-1 | Japonica | NA | | Os03g0761200 | OsR498G0307086800.01.T01 | Protein arginine N-methyltransferase 2 |
| maker-3-augustus-gene-453.37-mRNA-1 | Japonica | Indica | Kinase-TM | Os07g0114400 | OsR498G0307088600.01.T01 | Casein kinase II alpha subunit |
| maker-3-augustus-gene-453.42-mRNA-1 | Japonica | NA | | Os03g0762100 | OsR498G0307089000.01.T01 | Glycosyltransferase-like protein |
| maker-3-augustus-gene-453.7-mRNA-1 | Japonica | NA | | Os03g0761700 | OsR498G0307088100.01.T04 | J domain-containing protein |
| maker-3-augustus-gene-454.0-mRNA-1 | Japonica | NA | | Os03g0762900 | OsR498G0307093700.01.T01 | Protein SNOWY COTYLEDON 3 |
| maker-3-augustus-gene-454.10-mRNA-1 | Japonica | NA | | | OsR498G0307097300.01.T01 | BHLH domain-containing protein (Fragment) |
| maker-3-augustus-gene-454.12-mRNA-1 | Japonica | NA | | | OsR498G0307097900.01.T02 | Upstream activation factor subunit spp27 like |
| maker-3-augustus-gene-454.18-mRNA-1 | Japonica | NA | | Os03g0764600 | OsR498G0307099100.01.T01 | HTH myb-type domain-containing protein |
| maker-3-augustus-gene-454.22-mRNA-1 | Japonica | NA | Kinase-TM | Os03g0764300 | OsR498G0307097600.01.T01 | Mitogen-activated protein kinase kinase kinase 5 |
| maker-3-augustus-gene-454.4-mRNA-1 | Japonica | NA | Kinase-TM | Os03g0763000 | OsR498G0307094500.01.T01 | Casein kinase II subunit alpha |
| maker-3-augustus-gene-454.7-mRNA-1 | Japonica | NA | | Os03g0764000 | OsR498G0307096300.01.T01 | probable N-acetyltransferase HLS1 |
| maker-3-augustus-gene-455.0-mRNA-1 | Japonica | NA | Kinase-TM | Os03g0764800 | OsR498G0307099900.01.T01 | Serine/threonine-protein kinase SAPK8 |
| maker-3-augustus-gene-455.13-mRNA-1 | Japonica | NA | | | OsR498G0307103200.01.T02 | Nuclear pore complex protein isoform 1 |
| maker-3-augustus-gene-455.15-mRNA-1 | Japonica | NA | | Os03g0765400 | OsR498G0307103200.01.T04 | CCG-binding protein 1 |
| maker-3-augustus-gene-455.18-mRNA-1 | Japonica | NA | | | OsR498G0307103400.01.T01 | CCG-binding protein 1 |
| maker-3-augustus-gene-455.22-mRNA-1 | Japonica | Japonica | | Os03g0766000 | OsR498G0307105200.01.T02 | Transmembrane protein 147 |
| maker-3-augustus-gene-455.4-mRNA-1 | Japonica | NA | | | OsR498G0307101900.01.T03 | Non-specific serine/threonine protein kinase |
| maker-3-augustus-gene-455.7-mRNA-1 | Japonica | NA | | | OsR498G0307102300.01.T01 | Non-specific serine/threonine protein kinase |
| maker-3-augustus-gene-455.9-mRNA-1 | Japonica | NA | | Os03g0765200 | OsR498G0307102600.01.T02 | Thylakoidal processing peptidase 1 chloroplastic |
| maker-3-augustus-gene-456.0-mRNA-1 | Japonica | NA | | Os03g0766500 | OsR498G0307106700.01.T01 | probable transcription factor KAN4 |
| maker-3-augustus-gene-456.12-mRNA-1 | Japonica | NA | | Os03g0767500 | OsR498G0307108800.01.T01 | Thioredoxin-like protein HCF164, chloroplastic |
| maker-3-augustus-gene-456.15-mRNA-1 | Japonica | NA | | Os03g0769000 | OsR498G0307111200.01.T01 | Ternary complex factor MIP1 |
| maker-3-augustus-gene-456.19-mRNA-1 | Japonica | NA | | | OsR498G0307111800.01.T04 | Forkhead box protein like |
| maker-3-augustus-gene-456.5-mRNA-1 | Japonica | NA | | Os03g0767800 | OsR498G0307109500.01.T01 | Cold-regulated plasma membrane protein 2 |
| maker-3-augustus-gene-456.9-mRNA-1 | Japonica | NA | | Os03g0767900 | OsR498G0307109900.01.T01 | BTB domain-containing protein |
| maker-3-augustus-gene-457.0-mRNA-1 | Japonica | NA | | Os03g0769400 | OsR498G0307113100.01.T04 | BTB domain-containing protein |
| maker-3-augustus-gene-457.14-mRNA-1 | Japonica | Indica | | Os03g0770800 | OsR498G0307119200.01.T01 | Xyloglucan 6-xylosyltransferase |
| maker-3-augustus-gene-457.17-mRNA-1 | Japonica | NA | | Os03g0770900 | OsR498G0307119800.01.T01 | Neurofilament heavy polypeptide isoform X1 |

| | | | | | | | |
|---|---|---|---|---|---|---|---|
| maker-3-augustus-gene-457.2-mRNA-1 | Japonica | Indica | | Os03g0769800 | OsR498G0307114700.01.T05 | Homeobox-leucine zipper protein REVOLUTA |
| maker-3-augustus-gene-457.5-mRNA-1 | Japonica | NA | | | OsR498G0307118700.01.T01 | AP2-like ethylene-responsive transcription factor ANT |
| maker-3-augustus-gene-457.9-mRNA-1 | Japonica | NA | TM | Os03g0769600 | OsR498G0307113800.01.T02 | C-type cytochrome synthesis protein1 |
| maker-3-augustus-gene-458.0-mRNA-1 | Japonica | NA | | Os03g0771500 | OsR498G0307121900.01.T01 | Homeobox protein knotted-1-like 8 |
| maker-3-augustus-gene-458.1-mRNA-1 | Japonica | Indica | | Os03g0771500 | OsR498G0307123100.01.T01 | Homeobox protein rough sheath 1 |
| maker-3-augustus-gene-458.10-mRNA-1 | Japonica | NA | | Os03g0772800 | OsR498G0307126300.01.T01 | Cytochrome c oxidase subunit VIa |
| maker-3-augustus-gene-458.15-mRNA-1 | Japonica | NA | | Os03g0771100 | OsR498G0307120600.01.T01 | Transcription factor RAX3 |
| maker-3-augustus-gene-458.17-mRNA-1 | Japonica | Indica | | Os03g0241100 | OsR498G0307128400.01.T02 | TSL-kinase interacting protein 1 |
| maker-3-augustus-gene-458.26-mRNA-1 | Japonica | Indica | | Os03g0773150 | OsR498G0307128600.01.T01 | Ribosomal protein S14p/S29e containing protein, expressed |
| maker-3-augustus-gene-458.28-mRNA-1 | Japonica | NA | Kinase-LRR-TM | Os03g0773300 | OsR498G0307128800.01.T01 | Putative LRR receptor-like serine/threonine-protein kinase |
| maker-3-augustus-gene-459.39-mRNA-1 | Japonica | Indica | Kinase-LRR-TM | Os03g0773700 | OsR498G0307131300.01.T03 | Leucine-rich repeat receptor-like serine/threonine-protein kinase BAM1 |
| maker-3-augustus-gene-459.41-mRNA-1 | Japonica | NA | | | | |
| maker-3-augustus-gene-459.44-mRNA-1 | Japonica | NA | | Os03g0774600 | OsR498G0307133500.01.T02 | |
| maker-3-augustus-gene-459.47-mRNA-1 | Japonica | NA | | Os03g0775200 | OsR498G0307136100.01.T01 | tRNA 4-demethylwyosine synthase (AdoMet-dependent) |
| maker-3-augustus-gene-459.53-mRNA-1 | Japonica | NA | | Os03g0773800 | OsR498G0307131800.01.T01 | Malate dehydrogenase |
| maker-3-augustus-gene-459.57-mRNA-1 | Japonica | NA | | Os03g0774200 | OsR498G0307132600.01.T01 | NADH-ubiquinone oxidoreductase 23 kDa subunit |
| maker-3-augustus-gene-459.61-mRNA-1 | Japonica | Indica | | Os03g0774300 | OsR498G0307133000.01.T01 | 5'-nucleotidase domain-containing protein 4 isoform X1 |
| maker-3-augustus-gene-459.69-mRNA-1 | Japonica | NA | | Os03g0774700 | | |
| maker-3-augustus-gene-459.71-mRNA-1 | Japonica | Japonica | | Os03g0775000 | OsR498G0307135200.01.T01 | S-adenosyl-L-methionine-dependent methyltransferase |
| maker-3-augustus-gene-46.0-mRNA-1 | Japonica | NA | | Os03g0192500 | OsR498G0305373900.01.T02 | PPM-type phosphatase domain-containing protein |
| maker-3-augustus-gene-46.19-mRNA-1 | Japonica | NA | | Os03g0193075 | OsR498G0305375500.01.T01 | Yippee domain-containing protein |
| maker-3-augustus-gene-46.21-mRNA-1 | Japonica | NA | | | | Pumilio domain-containing protein |
| maker-3-augustus-gene-46.24-mRNA-1 | Japonica | Japonica | | Os03g0193400 | OsR498G0917271000.01.T02 | Flavin containing polyamine oxidase |
| maker-3-augustus-gene-46.29-mRNA-1 | Japonica | NA | | Os03g0192600 | OsR498G0305374200.01.T01 | AAI domain-containing protein (Fragment) |
| maker-3-augustus-gene-46.32-mRNA-1 | Japonica | NA | | Os03g0192700 | OsR498G0305374400.01.T04 | Myo-inositol 1-phosphate synthase |
| maker-3-augustus-gene-46.40-mRNA-1 | Japonica | NA | | Os03g0193225 | OsR498G0305376000.01.T01 | |
| maker-3-augustus-gene-46.42-mRNA-1 | Japonica | NA | | | OsR498G0305376800.01.T01 | Mutator-like transposase |
| maker-3-augustus-gene-46.6-mRNA-1 | Japonica | NA | | | OsR498G0305374900.01.T06 | Ubiquitinyl hydrolase 1 |
| maker-3-augustus-gene-460.49-mRNA-1 | Japonica | Indica | | Os03g0775300 | OsR498G0307136300.01.T01 | S-adenosyl-L-methionine-dependent tRNA 4-demethylwyosine synthase |
| maker-3-augustus-gene-460.55-mRNA-1 | Japonica | NA | | Os03g0775500 | OsR498G0307136700.01.T01 | DUF59 domain-containing protein |
| maker-3-augustus-gene-460.59-mRNA-1 | Japonica | NA | | Os03g0775600 | OsR498G0307137300.01.T01 | 2-hydroxyacid dehydrogenase |
| maker-3-augustus-gene-460.61-mRNA-1 | Japonica | NA | | Os03g0775700 | OsR498G0307137500.01.T01 | VARLMGL domain-containing protein (Fragment) |
| maker-3-augustus-gene-460.64-mRNA-1 | Japonica | NA | | Os03g0776000 | OsR498G0307138100.01.T06 | Glucose-6-phosphate isomerase |
| maker-3-augustus-gene-460.74-mRNA-1 | Japonica | Indica | | Os03g0776900 | OsR498G0307140500.01.T01 | Mitochondrial import inner membrane translocase subunit TIM14 |
| maker-3-augustus-gene-460.78-mRNA-1 | Japonica | NA | ONAC066 | Os03g0777000 | OsR498G0307142500.01.T01 | Transcription factor JUNGBRUNNEN 1 |
| maker-3-augustus-gene-460.80-mRNA-1 | Japonica | NA | Kinase-TM | Os03g0776100 | OsR498G0307138400.01.T01 | proline-rich receptor-like protein kinase PERK4 |
| maker-3-augustus-gene-460.82-mRNA-1 | Japonica | Indica | | Os03g0777100 | OsR498G0307142800.01.T01 | Dentin sialophosphoprotein-like protein |
| maker-3-augustus-gene-460.87-mRNA-1 | Japonica | NA | | Os03g0777500 | OsR498G0307143600.01.T01 | Hydroperoxide lyase, putative isoform 1 |
| maker-3-augustus-gene-461.36-mRNA-1 | Japonica | Indica | | Os03g0777600 | OsR498G0307143900.01.T01 | Hexosyltransferase |
| maker-3-augustus-gene-461.40-mRNA-1 | Japonica | NA | | Os03g0778000 | OsR498G0307145300.01.T04 | Protein kinase domain-containing protein |
| maker-3-augustus-gene-461.46-mRNA-1 | Japonica | NA | | | | protein FAR-RED IMPAIRED RESPONSE 1-like |
| maker-3-augustus-gene-463.0-mRNA-1 | Japonica | NA | | | OsR498G0307158500.01.T05 | Actin-related protein 7 isoform 1 |
| maker-3-augustus-gene-463.10-mRNA-1 | Japonica | NA | | Os03g0783700 | OsR498G0307165000.01.T01 | AP complex subunit sigma |
| maker-3-augustus-gene-463.13-mRNA-1 | Japonica | NA | | Os03g0784100 | OsR498G0307166200.01.T01 | Patatin-like protein 3 |
| maker-3-augustus-gene-464.34-mRNA-1 | Japonica | NA | | Os03g0784400 | OsR498G0307166900.01.T01 | Endoplasmic reticulum vesicle transporter, C-terminal |
| maker-3-augustus-gene-464.40-mRNA-1 | Japonica | NA | | Os03g0785500 | OsR498G0307168700.01.T01 | ALA-interacting subunit |
| maker-3-augustus-gene-464.44-mRNA-1 | Japonica | NA | | Os03g0786000 | OsR498G0307170100.01.T01 | (S)-2-hydroxy-acid oxidase |
| maker-3-augustus-gene-464.46-mRNA-1 | Japonica | NA | | Os03g0786100 | OsR498G0307170300.01.T03 | (S)-2-hydroxy-acid oxidase |
| maker-3-augustus-gene-464.52-mRNA-1 | Japonica | NA | | Os03g0784300 | | |
| maker-3-augustus-gene-464.57-mRNA-1 | Japonica | Indica | | Os07g0147800 | OsR498G0307167200.01.T05 | WPP domain-associated protein-like |
| maker-3-augustus-gene-464.60-mRNA-1 | Japonica | NA | | Os03g0784700 | OsR498G0307167400.01.T01 | Ferredoxin--NADP reductase, chloroplastic |
| maker-3-augustus-gene-464.64-mRNA-1 | Japonica | Indica | | Os04g0613400 | OsR498G0307167600.01.T01 | CCR4-NOT transcription complex subunit 9 |
| maker-3-augustus-gene-464.69-mRNA-1 | Japonica | Indica | | Os07g0150500 | OsR498G0307168600.01.T03 | C3HC-type domain-containing protein (Fragment) |
| maker-3-augustus-gene-465.0-mRNA-1 | Japonica | NA | | Os03g0786700 | OsR498G0307172700.01.T01 | tRNA (guanine(26)-N(2))-dimethyltransferase |
| maker-3-augustus-gene-465.13-mRNA-1 | Japonica | NA | | Os03g0786900 | OsR498G0307173100.01.T07 | Protein with predicted involvement in meiosis (GSG1) |
| maker-3-augustus-gene-465.24-mRNA-1 | Japonica | NA | | Os03g0788200 | OsR498G0307176300.01.T01 | MD-2-related lipid recognition domain-containing protein / ML domain-cont |
| maker-3-augustus-gene-465.30-mRNA-1 | Japonica | Indica | | Os03g0647800 | OsR498G0307173600.01.T01 | Putative glycosyl transferase Gly (Fragment) |
| maker-3-augustus-gene-465.33-mRNA-1 | Japonica | NA | | Os03g0787200 | OsR498G0307173800.01.T01 | IQ-domain 5 |
| maker-3-augustus-gene-465.37-mRNA-1 | Japonica | NA | | Os03g0813700 | OsR498G0307175200.01.T01 | |
| maker-3-augustus-gene-465.6-mRNA-1 | Japonica | NA | | | OsR498G0307172900.01.T03 | Cullin-4 isoform B |
| maker-3-augustus-gene-466.43-mRNA-1 | Japonica | NA | | Os03g0788500 | OsR498G0307176900.01.T01 | Calcium-dependent protein kinase |

| | | | | | | |
|---|---|---|---|---|---|---|
| maker-3-augustus-gene-466.46-mRNA-1 | Japonica | NA | | | OsR498G0307180500.01.T03 | Putative post-transcriptional gene silencing PAZ-Argonaute family |
| maker-3-augustus-gene-466.53-mRNA-1 | Japonica | NA | | | OsR498G1221698500.01.T04 | Phosphate synthase-like |
| maker-3-augustus-gene-466.57-mRNA-1 | Japonica | NA | | Os03g0788400 | | |
| maker-3-augustus-gene-466.59-mRNA-1 | Japonica | NA | | Os03g0788700 | OsR498G0307177800.01.T02 | Fasciclin-like arabinogalactan protein 16 |
| maker-3-augustus-gene-466.62-mRNA-1 | Japonica | NA | | Os03g0788800 | OsR498G0307178000.01.T02 | E3 ubiquitin-protein ligase RHF2A isoform B |
| maker-3-augustus-gene-466.67-mRNA-1 | Japonica | NA | | Os03g0789000 | OsR498G0307178600.01.T01 | Calcium-dependent protein kinase |
| maker-3-augustus-gene-466.69-mRNA-1 | Japonica | NA | | Os03g0789200 | OsR498G0307179400.01.T01 | GTD-binding domain-containing protein (Fragment) |
| maker-3-augustus-gene-466.72-mRNA-1 | Japonica | NA | | Os06g0507101 | OsR498G1120660600.01.T01 | |
| maker-3-augustus-gene-466.74-mRNA-1 | Japonica | NA | | Os03g0789900 | OsR498G0307181400.01.T05 | |
| maker-3-augustus-gene-467.1-mRNA-1 | Japonica | NA | | | OsR498G0307183500.01.T05 | Glutamate carboxypeptidase II |
| maker-3-augustus-gene-467.11-mRNA-1 | Japonica | NA | Kinase-LRR-TM | Os03g0791700 | OsR498G0307187900.01.T02 | Leucine-rich repeat transmembrane protein kinase, putative, expressed |
| maker-3-augustus-gene-467.15-mRNA-1 | Japonica | NA | | Os03g0790900 | OsR498G0307183800.01.T01 | Indole-3-acetaldehyde oxidase |
| maker-3-augustus-gene-467.16-mRNA-1 | Japonica | NA | | Os03g0790900 | OsR498G0307184200.01.T01 | Indole-3-acetaldehyde oxidase |
| maker-3-augustus-gene-467.2-mRNA-1 | Japonica | NA | | | | Lob domain-containing protein 4 |
| maker-3-augustus-gene-467.31-mRNA-1 | Japonica | NA | | Os03g0790700 | OsR498G0307184200.01.T02 | Aldehyde oxidase2 |
| maker-3-augustus-gene-467.33-mRNA-1 | Japonica | NA | | | OsR498G0307185600.01.T01 | Phytocyanin domain-containing protein (Fragment) |
| maker-3-augustus-gene-467.35-mRNA-1 | Japonica | NA | | | OsR498G0307186400.01.T02 | 25.3 kDa vesicle transport protein, putative, expressed |
| maker-3-augustus-gene-467.41-mRNA-1 | Japonica | NA | | | OsR498G0307188200.01.T05 | UBC core domain-containing protein (Fragment) |
| maker-3-augustus-gene-468.48-mRNA-1 | Japonica | NA | | Os03g0792400 | OsR498G0307189300.01.T05 | Sterol regulatory element-binding protein site 2 protease |
| maker-3-augustus-gene-468.54-mRNA-1 | Japonica | NA | | Os03g0792500 | OsR498G0307189500.01.T03 | BTB/POZ and MATH domain-containing protein 4 |
| maker-3-augustus-gene-468.59-mRNA-1 | Japonica | Indica | | Os03g0792800 | OsR498G0307190300.01.T02 | Glucan endo-1,3-beta-D-glucosidase |
| maker-3-augustus-gene-468.61-mRNA-1 | Japonica | NA | | Os03g0793500 | OsR498G0307192700.01.T08 | Casein kinase (Serine/threonine/tyrosine protein kinase) |
| maker-3-augustus-gene-468.69-mRNA-1 | Japonica | Indica | | Os03g0793600 | OsR498G0307193300.01.T03 | Type IV inositol polyphosphate 5-phosphatase 6 |
| maker-3-augustus-gene-468.78-mRNA-1 | Japonica | NA | | Os03g0793700 | OsR498G0307193900.01.T01 | Globulin-2 |
| maker-3-augustus-gene-468.80-mRNA-1 | Japonica | NA | | Os03g0792600 | OsR498G0307189800.01.T01 | Replication factor C subunit like |
| maker-3-augustus-gene-468.85-mRNA-1 | Japonica | NA | | Os03g0792900 | OsR498G0307190600.01.T03 | Zinc finger A20 and AN1 domain-containing stress-associated protein 6 |
| maker-3-augustus-gene-468.86-mRNA-1 | Japonica | NA | | Os03g0793100 | OsR498G1119421100.01.T02 | Glycosyltransferases |
| maker-3-augustus-gene-468.89-mRNA-1 | Japonica | NA | | Os03g0793800 | OsR498G0307194200.01.T02 | AAI domain-containing protein |
| maker-3-augustus-gene-468.91-mRNA-1 | Japonica | Indica | | Os03g0793900 | OsR498G0307194800.01.T02 | AAI domain-containing protein (Fragment) |
| maker-3-augustus-gene-468.94-mRNA-1 | Japonica | NA | | Os03g0794500 | OsR498G0307196200.01.T02 | GdhA protein (Fragment) |
| maker-3-augustus-gene-469.0-mRNA-1 | Japonica | NA | | Os03g0794800 | OsR498G0307196700.01.T01 | 5'-3' exoribonuclease |
| maker-3-augustus-gene-469.14-mRNA-1 | Japonica | NA | | Os03g0795900 | OsR498G0307199500.01.T02 | Heat shock transcription factor |
| maker-3-augustus-gene-469.16-mRNA-1 | Japonica | Indica | | Os03g0796000 | OsR498G0307199900.01.T03 | Stem-specific protein TSJT1 |
| maker-3-augustus-gene-469.20-mRNA-1 | Japonica | NA | | Os03g0794700 | OsR498G0307196600.01.T01 | 40S ribosomal protein S18 |
| maker-3-augustus-gene-469.22-mRNA-1 | Japonica | Japonica | | Os03g0795000 | OsR498G0307197200.01.T03 | Folate-biopterin transporter 1 chloroplastic |
| maker-3-augustus-gene-469.25-mRNA-1 | Japonica | NA | LRR-TM | Os03g0795300 | OsR498G0307198000.01.T01 | Leucine-rich repeat extensin-like protein 6 |
| maker-3-augustus-gene-469.27-mRNA-1 | Japonica | NA | | Os03g0795600 | OsR498G0307199000.01.T01 | Transmembrane BAX inhibitor motif-containing protein 4 |
| maker-3-augustus-gene-469.29-mRNA-1 | Japonica | NA | | Os03g0795800 | OsR498G0307199400.01.T01 | Transmembrane BAX inhibitor motif-containing protein 4 |
| maker-3-augustus-gene-469.32-mRNA-1 | Japonica | NA | | Os03g0796400 | OsR498G0307202000.01.T02 | ENT domain-containing protein (Fragment) |
| maker-3-augustus-gene-469.34-mRNA-1 | Japonica | NA | | Os03g0796501 | OsR498G0307202600.01.T02 | Ribosomal protein L4/L1 family |
| maker-3-augustus-gene-469.5-mRNA-1 | Japonica | NA | | Os03g0794900 | OsR498G0307196900.01.T01 | 5'-3' exoribonuclease 4 |
| maker-3-augustus-gene-469.9-mRNA-1 | Japonica | NA | | Os03g0795500 | OsR498G0307198700.01.T01 | PITH domain-containing protein isoform A |
| maker-3-augustus-gene-47.41-mRNA-1 | Japonica | Japonica | | Os03g0193900 | OsR498G0101213400.01.T05 | |
| maker-3-augustus-gene-47.43-mRNA-1 | Japonica | NA | | Os03g0194400 | OsR498G0305379300.01.T04 | N-acetyltransferase 9-like protein isoform X2 |
| maker-3-augustus-gene-47.46-mRNA-1 | Japonica | NA | | | OsR498G0305379500.01.T01 | Mitochondrial import inner membrane translocase subunit TIM22 |
| maker-3-augustus-gene-47.49-mRNA-1 | Japonica | NA | | Os03g0194900 | OsR498G0305380500.01.T01 | Cytochrome b561 and DOMON domain-containing protein |
| maker-3-augustus-gene-47.51-mRNA-1 | Japonica | NA | | Os03g0195100 | OsR498G0305381100.01.T01 | LL-diaminopimelate aminotransferase, chloroplastic |
| maker-3-augustus-gene-47.53-mRNA-1 | Japonica | NA | | Os03g0195200 | OsR498G0305381400.01.T02 | 18S rRNA aminocarboxypropyltransferase |
| maker-3-augustus-gene-47.58-mRNA-1 | Japonica | NA | | Os03g0195300 | OsR498G0305381800.01.T01 | Low affinity sulfate transporter 3-like |
| maker-3-augustus-gene-47.62-mRNA-1 | Japonica | NA | | | OsR498G0305382000.01.T01 | Low affinity sulfate transporter like |
| maker-3-augustus-gene-470.0-mRNA-1 | Japonica | NA | | Os03g0796600 | OsR498G0307206300.01.T02 | Plant-specific domain TIGR01615 family protein |
| maker-3-augustus-gene-470.14-mRNA-1 | Japonica | NA | | Os03g0797700 | OsR498G0307212300.01.T01 | rRNA-processing protein EFG1 |
| maker-3-augustus-gene-470.19-mRNA-1 | Japonica | NA | | Os03g0796900 | OsR498G0307208200.01.T01 | Opaque-2 heterodimerizing protein 1b |
| maker-3-augustus-gene-470.2-mRNA-1 | Japonica | Indica | | Os07g0181700 | OsR498G0307207900.01.T01 | GB1/RHD3-type G domain-containing protein |
| maker-3-augustus-gene-470.26-mRNA-1 | Japonica | Indica | | Os03g0797550 | OsR498G0307210400.01.T01 | Tryptophan synthase |
| maker-3-augustus-gene-470.30-mRNA-1 | Japonica | Japonica | | Os03g0797550 | OsR498G0307211000.01.T02 | Tryptophan synthase |
| maker-3-augustus-gene-471.42-mRNA-1 | Japonica | NA | | Os03g0798200 | OsR498G0307214900.01.T02 | E3 ubiquitin protein ligase DRIP2 |
| maker-3-augustus-gene-471.47-mRNA-1 | Japonica | NA | | | OsR498G0307215300.01.T03 | DNA (cytosine-5)-methyltransferase |
| maker-3-augustus-gene-471.50-mRNA-1 | Japonica | Japonica | | Os03g0798500 | OsR498G0307216100.01.T01 | WRKY DNA binding domain containing protein, expressed |
| maker-3-augustus-gene-471.53-mRNA-1 | Japonica | NA | | Os03g0799000 | OsR498G0307217700.01.T01 | H15 domain-containing protein (Fragment) |
| maker-3-augustus-gene-471.55-mRNA-1 | Japonica | NA | | Os03g0799100 | OsR498G0307217900.01.T01 | TPX2 (Targeting protein for Xklp2) proteinfamily |

| | | | | | | |
|---|---|---|---|---|---|---|
| maker-3-augustus-gene-471.59-mRNA-1 | Japonica | NA | | Os03g0799200 | OsR498G0307218100.01.T04 | GTD-binding domain-containing protein (Fragment) |
| maker-3-augustus-gene-471.61-mRNA-1 | Japonica | NA | | Os03g0799500 | OsR498G0307218900.01.T01 | C-repeat binding transcription factor |
| maker-3-augustus-gene-471.63-mRNA-1 | Japonica | NA | | Os03g0799600 | OsR498G0307219100.01.T02 | Chromatin remodeling protein EBS |
| maker-3-augustus-gene-471.66-mRNA-1 | Japonica | NA | | Os03g0799700 | OsR498G0307219300.01.T01 | Probable GTP-binding protein OBGC2 |
| maker-3-augustus-gene-471.70-mRNA-1 | Japonica | NA | | Os03g0797800 | OsR498G0307212800.01.T01 | Auxin-responsive protein IAA14 |
| maker-3-augustus-gene-471.72-mRNA-1 | Japonica | NA | | Os03g0797901 | OsR498G0307183800.01.T02 | Aldehyde oxidase2 |
| maker-3-augustus-gene-471.74-mRNA-1 | Japonica | Japonica | | Os03g0798000 | OsR498G0307214400.01.T01 | Aldehyde oxidase1 |
| maker-3-augustus-gene-471.77-mRNA-1 | Japonica | NA | | Os03g0798600 | OsR498G0307216400.01.T01 | Ribosomal protein S19/S15 |
| maker-3-augustus-gene-472.0-mRNA-1 | Japonica | Indica | | Os07g0187700 | OsR498G0307220100.01.T01 | |
| maker-3-augustus-gene-472.17-mRNA-1 | Japonica | Indica | | Os03g0801500 | OsR498G0307223100.01.T02 | |
| maker-3-augustus-gene-472.21-mRNA-1 | Japonica | NA | | Os03g0801600 | OsR498G0307223300.01.T01 | Vacuolar protein sorting-associated protein 35 |
| maker-3-augustus-gene-472.27-mRNA-1 | Japonica | NA | | Os03g0801800 | OsR498G0307223700.01.T02 | RNA-binding protein 28 |
| maker-3-augustus-gene-472.3-mRNA-1 | Japonica | NA | | | OsR498G0307220900.01.T02 | Cactin C-terminal domain-containing protein |
| maker-3-augustus-gene-472.37-mRNA-1 | Japonica | NA | | | OsR498G0307221200.01.T02 | Argonaute/Dicer protein |
| maker-3-augustus-gene-472.43-mRNA-1 | Japonica | NA | | Os03g0800500 | OsR498G0307221800.01.T01 | Xaa-Pro aminopeptidase 2 |
| maker-3-augustus-gene-472.45-mRNA-1 | Japonica | NA | | Os03g0800700 | OsR498G0307222000.01.T01 | Thioredoxin like |
| maker-3-augustus-gene-472.47-mRNA-1 | Japonica | NA | | Os03g0800800 | OsR498G0307222200.01.T03 | FHA domain containing protein, expressed |
| maker-3-augustus-gene-472.51-mRNA-1 | Japonica | NA | | Os03g0801700 | OsR498G0307223600.01.T02 | FAD synthetase, chloroplastic |
| maker-3-augustus-gene-473.44-mRNA-1 | Japonica | NA | | Os03g0801900 | OsR498G0307224100.01.T01 | DUF569 domain-containing protein |
| maker-3-augustus-gene-473.48-mRNA-1 | Japonica | NA | | Os03g0803000 | OsR498G0307227300.01.T01 | Vesicle-associated membrane protein 721 |
| maker-3-augustus-gene-473.52-mRNA-1 | Japonica | NA | | Os03g0802300 | OsR498G0307225800.01.T03 | BTP domain-containing protein |
| maker-3-augustus-gene-473.57-mRNA-1 | Japonica | NA | | | OsR498G0307226800.01.T01 | DEAD-box ATP-dependent RNA helicase 27 |
| maker-3-augustus-gene-473.65-mRNA-1 | Japonica | Indica | | Os07g0193600 | OsR498G0307227000.01.T01 | DDT domain-containing protein DDR4 |
| maker-3-augustus-gene-473.72-mRNA-1 | Japonica | NA | | Os03g0802900 | OsR498G0307227200.01.T01 | Transcription factor BHLH089 |
| maker-3-augustus-gene-476.53-mRNA-1 | Japonica | NA | | Os03g0808600 | OsR498G0307247900.01.T01 | Calcium-dependent protein kinase |
| maker-3-augustus-gene-476.58-mRNA-1 | Japonica | Indica | | Os07g0209100 | OsR498G0307248900.01.T01 | Galactinol--sucrose galactosyltransferase |
| maker-3-augustus-gene-476.62-mRNA-1 | Japonica | NA | | | OsR498G0307249700.01.T01 | BZIP transcription factor |
| maker-3-augustus-gene-476.77-mRNA-1 | Japonica | NA | | Os03g0809100 | OsR498G0307249400.01.T01 | MFS domain-containing protein (Fragment) |
| maker-3-augustus-gene-477.0-mRNA-1 | Japonica | NA | | Os03g0809700 | OsR498G0307250900.01.T01 | Protein-serine/threonine phosphatase |
| maker-3-augustus-gene-477.12-mRNA-1 | Japonica | NA | | | OsR498G0307250200.01.T01 | Protein-serine/threonine phosphatase |
| maker-3-augustus-gene-477.16-mRNA-1 | Japonica | NA | | | OsR498G0307251200.01.T01 | Zinc finger, RING-type |
| maker-3-augustus-gene-477.20-mRNA-1 | Japonica | Indica | | Os07g0212300 | OsR498G0307253200.01.T01 | NUDIX hydrolase domain |
| maker-3-augustus-gene-477.23-mRNA-1 | Japonica | NA | | Os03g0810600 | OsR498G0307253800.01.T01 | Mitochondrial Rho GTPase |
| maker-3-augustus-gene-477.29-mRNA-1 | Japonica | Indica | | Os03g0810600 | OsR498G0307254000.01.T01 | EF hand family protein, expressed |
| maker-3-augustus-gene-477.31-mRNA-1 | Japonica | NA | | Os03g0810900 | OsR498G0307254600.01.T01 | PNPLA domain-containing protein (Fragment) |
| maker-3-augustus-gene-477.5-mRNA-1 | Japonica | NA | | Os03g0809900 | OsR498G0307251300.01.T01 | RNA-binding protein 8A |
| maker-3-augustus-gene-477.8-mRNA-1 | Japonica | NA | | Os03g0810800 | OsR498G0307254300.01.T01 | Xanthoxin dehydrogenase |
| maker-3-augustus-gene-477.9-mRNA-1 | Japonica | NA | | Os03g0809300 | OsR498G0307250000.01.T01 | Protein-serine/threonine phosphatase |
| maker-3-augustus-gene-478.30-mRNA-1 | Japonica | NA | | Os03g0811100 | OsR498G0307254700.01.T01 | Mg-protoporphyrin IX chelatase |
| maker-3-augustus-gene-478.35-mRNA-1 | Japonica | NA | | Os03g0811200 | OsR498G0307254900.01.T02 | BRCA1 C Terminus domain containing protein expressed |
| maker-3-augustus-gene-478.39-mRNA-1 | Japonica | NA | | Os03g0811300 | OsR498G0307255100.01.T01 | AP complex subunit sigma |
| maker-3-augustus-gene-478.42-mRNA-1 | Japonica | NA | | Os03g0811400 | OsR498G0307255700.01.T02 | Transcription factor bHLH106 |
| maker-3-augustus-gene-478.44-mRNA-1 | Japonica | NA | | Os03g0811900 | OsR498G0307258500.01.T05 | ADP-ribosylation factor A1F |
| maker-3-augustus-gene-478.48-mRNA-1 | Japonica | NA | | Os03g0811500 | OsR498G0307257200.01.T01 | PAPA-1 domain-containing protein (Fragment) |
| maker-3-augustus-gene-478.51-mRNA-1 | Japonica | NA | | | OsR498G0307257600.01.T04 | Peptidyl-prolyl cis-trans isomerase |
| maker-3-augustus-gene-478.56-mRNA-1 | Japonica | Indica | | Os03g0812000 | OsR498G0307258800.01.T02 | DNA gyrase subunit A chloroplastic/mitochondrial |
| maker-3-augustus-gene-479.35-mRNA-1 | Japonica | NA | | | | NAC domain-containing protein |
| maker-3-augustus-gene-479.37-mRNA-1 | Japonica | NA | | Os03g0813700 | OsR498G0307262100.01.T01 | |
| maker-3-augustus-gene-479.39-mRNA-1 | Japonica | NA | | Os03g0813200 | | Prohibitin |
| maker-3-augustus-gene-479.42-mRNA-1 | Japonica | NA | | | OsR498G0307266000.01.T02 | Prohibitin |
| maker-3-augustus-gene-48.24-mRNA-1 | Japonica | NA | | Os03g0195800 | OsR498G0305383900.01.T02 | High affinity sulfate transporter 2 |
| maker-3-augustus-gene-48.27-mRNA-1 | Japonica | NA | | Os03g0196000 | OsR498G0305385100.01.T01 | Sulfate anion transporter |
| maker-3-augustus-gene-48.32-mRNA-1 | Japonica | NA | | | OsR498G0305394200.01.T01 | Small subunit ribosomal protein S10 |
| maker-3-augustus-gene-480.0-mRNA-1 | Japonica | NA | | Os03g0815100 | OsR498G0307266700.01.T01 | Stress-induced transcription factor NAC1 |
| maker-3-augustus-gene-480.13-mRNA-1 | Japonica | NA | | Os03g0815200 | OsR498G0307267000.01.T01 | Methylenetetrahydrofolate reductase |
| maker-3-augustus-gene-480.19-mRNA-1 | Japonica | NA | | Os03g0815900 | OsR498G0307269000.01.T02 | Transcription elongation factor TFIIS |
| maker-3-augustus-gene-480.2-mRNA-1 | Japonica | NA | | Os03g0815400 | OsR498G0307267300.01.T02 | Large subunit ribosomal protein L17 |
| maker-3-augustus-gene-480.21-mRNA-1 | Japonica | Japonica | | Os03g0816000 | OsR498G0307269200.01.T01 | U-box domain-containing protein 62 |
| maker-3-augustus-gene-480.25-mRNA-1 | Japonica | Indica | Kinase-TM | Os03g0816100 | OsR498G0307269400.01.T01 | Non-specific lipid-transfer protein-like isoform 1 |
| maker-3-augustus-gene-480.31-mRNA-1 | Japonica | NA | | | OsR498G0307271600.01.T01 | Plant mobile domain protein family |
| maker-3-augustus-gene-480.33-mRNA-1 | Japonica | NA | | Os03g0816400 | OsR498G0307272000.01.T02 | EngB-type G domain-containing protein |

| | | | | | | | |
|---|---|---|---|---|---|---|---|
| maker-3-augustus-gene-480.37-mRNA-1 | Japonica | NA | | Os03g0816700 | OsR498G0307273800.01.T01 | |
| maker-3-augustus-gene-480.4-mRNA-1 | Japonica | NA | | Os03g0815700 | OsR498G0307268300.01.T01 | KH domain, STAR protein, homodimerization region |
| maker-3-augustus-gene-480.8-mRNA-1 | Japonica | NA | | Os03g0816600 | OsR498G0307273500.01.T01 | Pentatricopeptide repeat-containing protein (Fragment) |
| maker-3-augustus-gene-481.32-mRNA-1 | Japonica | Indica | | Os03g0816900 | OsR498G0307274900.01.T01 | WH2 domain-containing protein |
| maker-3-augustus-gene-481.40-mRNA-1 | Japonica | NA | | Os03g0818000 | | Protein FLC EXPRESSOR |
| maker-3-augustus-gene-481.43-mRNA-1 | Japonica | NA | | Os03g0818100 | OsR498G0307279900.01.T02 | Multiple inositol polyphosphate phosphatase PhyIIc |
| maker-3-augustus-gene-481.49-mRNA-1 | Japonica | NA | | Os03g0818200 | | Epimerase domain-containing protein |
| maker-3-augustus-gene-481.51-mRNA-1 | Japonica | NA | | Os03g0817100 | OsR498G0307275400.01.T01 | |
| maker-3-augustus-gene-481.53-mRNA-1 | Japonica | NA | | Os03g0817500 | OsR498G0307277400.01.T01 | Trichome birefringence-like 34 |
| maker-3-augustus-gene-481.55-mRNA-1 | Japonica | NA | | | | |
| maker-3-augustus-gene-481.58-mRNA-1 | Japonica | NA | | | OsR498G0307278600.01.T02 | Protein trichome birefringence-like 33 |
| maker-3-augustus-gene-481.62-mRNA-1 | Japonica | NA | | Os03g0817900 | OsR498G0307279400.01.T01 | Protein trichome birefringence-like protein 33 |
| maker-3-augustus-gene-481.66-mRNA-1 | Japonica | NA | | Os03g0818300 | OsR498G0307280200.01.T01 | ALFIN-like transcription factor |
| maker-3-augustus-gene-481.68-mRNA-1 | Japonica | NA | | Os03g0818400 | OsR498G0307280400.01.T01 | 40S ribosomal protein S23 |
| maker-3-augustus-gene-482.42-mRNA-1 | Japonica | Japonica | | Os03g0818800 | OsR498G0307281900.01.T01 | Floral homeotic protein APETALA 2 |
| maker-3-augustus-gene-482.48-mRNA-1 | Japonica | NA | | Os03g0819100 | OsR498G0307282900.01.T01 | Leukotriene A-4 hydrolase |
| maker-3-augustus-gene-482.51-mRNA-1 | Japonica | NA | | Os03g0819300 | OsR498G0307283100.01.T01 | Glycine-rich protein A3 |
| maker-3-augustus-gene-482.53-mRNA-1 | Japonica | NA | | | | RAB GTPase homolog 8A |
| maker-3-augustus-gene-482.61-mRNA-1 | Japonica | NA | | Os03g0820500 | OsR498G0307286300.01.T01 | Actin-depolymerizing factor |
| maker-3-augustus-gene-482.64-mRNA-1 | Japonica | NA | | Os03g0821100 | OsR498G0307287500.01.T01 | Non-cell-autonomous heat shock cognate protein 70 |
| maker-3-augustus-gene-482.66-mRNA-1 | Japonica | NA | | | OsR498G0307281400.01.T01 | |
| maker-3-augustus-gene-482.67-mRNA-1 | Japonica | NA | | Os03g0819400 | OsR498G0307283400.01.T01 | Protein SODIUM POTASSIUM ROOT DEFECTIVE 2 like |
| maker-3-augustus-gene-482.70-mRNA-1 | Japonica | NA | | | | Peptidase A1 domain-containing protein |
| maker-3-augustus-gene-482.71-mRNA-1 | Japonica | NA | | Os03g0819600 | OsR498G0307284200.01.T01 | Chalcone--flavonone isomerase |
| maker-3-augustus-gene-482.75-mRNA-1 | Japonica | NA | | Os03g0819700 | OsR498G0307284400.01.T01 | DUF4378 domain-containing protein (Fragment) |
| maker-3-augustus-gene-482.78-mRNA-1 | Japonica | NA | | Os03g0820700 | OsR498G0307286800.01.T01 | Serine/arginine-rich splicing factor 7 (Fragment) |
| maker-3-augustus-gene-482.82-mRNA-1 | Japonica | NA | | Os03g0820900 | OsR498G0307287200.01.T02 | SEC14 cytosolic factor family protein / phosphoglyceride transfer family prot |
| maker-3-augustus-gene-483.0-mRNA-1 | Japonica | NA | | Os03g0821200 | OsR498G0307287900.01.T01 | Dof-type domain-containing protein |
| maker-3-augustus-gene-483.10-mRNA-1 | Japonica | NA | | Os03g0822300 | OsR498G0307294300.01.T03 | Ribosomal RNA methyltransferase FtsJ domain |
| maker-3-augustus-gene-483.14-mRNA-1 | Japonica | NA | | Os03g0822700 | OsR498G0307294700.01.T01 | TOG domain-containing protein (Fragment) |
| maker-3-augustus-gene-483.18-mRNA-1 | Japonica | Indica | | Os03g0822800 | OsR498G0307294900.01.T01 | DnaJ subfamily A member 3 |
| maker-3-augustus-gene-483.23-mRNA-1 | Japonica | NA | | Os03g0821300 | OsR498G0307290000.01.T01 | PPM-type phosphatase domain-containing protein |
| maker-3-augustus-gene-483.27-mRNA-1 | Japonica | Indica | | Os03g0821800 | OsR498G0307292200.01.T02 | Dolichyl-phosphate beta-glucosyltransferase |
| maker-3-augustus-gene-483.33-mRNA-1 | Japonica | NA | | Os03g0822000 | OsR498G0307292800.01.T01 | Expansin |
| maker-3-augustus-gene-483.36-mRNA-1 | Japonica | NA | | Os03g0822200 | OsR498G0307294200.01.T01 | Sanguinarine reductase |
| maker-3-augustus-gene-483.7-mRNA-1 | Japonica | NA | | Os07g0624100 | | BED zinc finger family protein, expressed |
| maker-3-augustus-gene-484.43-mRNA-1 | Japonica | NA | | Os03g0824200 | OsR498G0307298700.01.T01 | HemK methyltransferase family member 2 |
| maker-3-augustus-gene-484.45-mRNA-1 | Japonica | Indica | | Os03g0824300 | OsR498G0307299100.01.T03 | RNA-binding protein 28 |
| maker-3-augustus-gene-484.52-mRNA-1 | Japonica | NA | | Os03g0824400 | OsR498G0307299300.01.T02 | Dolichol-phosphate mannosyltransferase subunit 1 |
| maker-3-augustus-gene-484.56-mRNA-1 | Japonica | Indica | | Os03g0823200 | OsR498G0307295800.01.T03 | D-xylose-proton symporter-like 3 chloroplastic |
| maker-3-augustus-gene-484.62-mRNA-1 | Japonica | NA | | Os03g0823500 | OsR498G0307296800.01.T01 | Nitrate transporter 1/peptide transporter family protein |
| maker-3-augustus-gene-484.68-mRNA-1 | Japonica | NA | | | | nucleobase-ascorbate transporter 7 |
| maker-3-augustus-gene-484.75-mRNA-1 | Japonica | Japonica | | Os03g0823900 | OsR498G0306282100.01.T02 | Calmodulin-binding protein, putative, expressed |
| maker-3-augustus-gene-484.79-mRNA-1 | Japonica | Indica | | Os03g0824100 | OsR498G0307298600.01.T01 | Smr domain-containing protein (Fragment) |
| maker-3-augustus-gene-484.83-mRNA-1 | Japonica | NA | | Os03g0824350 | OsR498G0100977700.01.T05 | NnrU domain-containing protein |
| maker-3-augustus-gene-484.85-mRNA-1 | Japonica | NA | | | OsR498G0307300000.01.T01 | |
| maker-3-augustus-gene-485.0-mRNA-1 | Japonica | Indica | Kinase-TM | Os03g0825300 | OsR498G0307301100.01.T01 | Putative serine/threonine-protein kinase BSK3 |
| maker-3-augustus-gene-485.11-mRNA-1 | Japonica | NA | | Os03g0826400 | OsR498G0307305100.01.T01 | Zinc finger CCCH domain-containing protein 25 |
| maker-3-augustus-gene-485.15-mRNA-1 | Japonica | Indica | | Os03g0826600 | OsR498G0307305700.01.T01 | Phospholipase C-type enzymes |
| maker-3-augustus-gene-485.17-mRNA-1 | Japonica | NA | | Os03g0825400 | OsR498G0307301400.01.T01 | Mitochondrial import inner membrane translocase |
| maker-3-augustus-gene-485.21-mRNA-1 | Japonica | NA | Kinase | Os03g0825800 | OsR498G0307302800.01.T02 | Receptor-like protein kinase ANXUR2 |
| maker-3-augustus-gene-485.23-mRNA-1 | Japonica | NA | | Os03g0826000 | OsR498G0307304400.01.T03 | Phosphatidylinositol N-acetylglucosaminlytransferase subunit P-related |
| maker-3-augustus-gene-485.25-mRNA-1 | Japonica | NA | | | OsR498G0307305000.01.T01 | GONST2 Golgi Nucleotide sugar transporter |
| maker-3-augustus-gene-485.31-mRNA-1 | Japonica | Indica | | Os03g0826700 | OsR498G0307306000.01.T01 | S-adenosyl-L-methionine-dependent methyltransferases superfamily protei |
| maker-3-augustus-gene-485.35-mRNA-1 | Japonica | NA | | Os03g0826800 | OsR498G0307306200.01.T01 | Isoflavone reductase-like protein-like |
| maker-3-augustus-gene-485.38-mRNA-1 | Japonica | Indica | | Os03g0826900 | OsR498G0307306400.01.T01 | |
| maker-3-augustus-gene-485.4-mRNA-1 | Japonica | NA | | Os03g0825500 | OsR498G0307301500.01.T02 | KOW motif family protein, expressed |
| maker-3-augustus-gene-485.6-mRNA-1 | Japonica | NA | | Os03g0825700 | OsR498G0307302500.01.T02 | Defective in exine formation |
| maker-3-augustus-gene-486.44-mRNA-1 | Japonica | Indica | | Os03g0827500 | OsR498G0307307900.01.T02 | SPX domain-containing protein 4 |
| maker-3-augustus-gene-486.48-mRNA-1 | Japonica | NA | | Os03g0827700 | OsR498G0307308700.01.T02 | DEAD-box ATP-dependent RNA helicase 3, chloroplastic |
| maker-3-augustus-gene-486.52-mRNA-1 | Japonica | NA | | Os03g0828100 | OsR498G0307311600.01.T01 | Large subunit ribosomal protein L18 |

| | | | | | | |
|---|---|---|---|---|---|---|
| maker-3-augustus-gene-486.54-mRNA-1 | Japonica | Indica | | Os03g0828500 | OsR498G0307311400.01.T01 | Mannan endo-1,4-beta-mannosidase |
| maker-3-augustus-gene-486.56-mRNA-1 | Japonica | Japonica | | Os03g0828500 | OsR498G0307311000.01.T01 | Mannan endo-1,4-beta-mannosidase |
| maker-3-augustus-gene-486.58-mRNA-1 | Japonica | NA | | Os03g0828600 | OsR498G0307310800.01.T01 | Cation/H(+) antiporter 19 |
| maker-3-augustus-gene-486.60-mRNA-1 | Japonica | NA | | | OsR498G0307308200.01.T01 | Transmembrane protein like |
| maker-3-augustus-gene-486.65-mRNA-1 | Japonica | NA | | | OsR498G0307311800.01.T04 | Acylpyruvase FAHD1, mitochondrial |
| maker-3-augustus-gene-486.70-mRNA-1 | Japonica | NA | | Os03g0829100 | OsR498G0307312000.01.T01 | AB hydrolase-1 domain-containing protein (Fragment) |
| maker-3-augustus-gene-486.72-mRNA-1 | Japonica | NA | | Os03g0829200 | OsR498G0307312200.01.T01 | AB hydrolase-1 domain-containing protein (Fragment) |
| maker-3-augustus-gene-487.0-mRNA-1 | Japonica | NA | | | OsR498G0307315300.01.T01 | J domain-containing protein (Fragment) |
| maker-3-augustus-gene-487.14-mRNA-1 | Japonica | Indica | | Os03g0829800 | OsR498G0307313000.01.T01 | Cell number regulator 2 |
| maker-3-augustus-gene-487.16-mRNA-1 | Japonica | NA | | Os03g0830200 | OsR498G0307313600.01.T01 | Cell number regulator 7 |
| maker-3-augustus-gene-487.18-mRNA-1 | Japonica | Indica | | Os03g0830401 | OsR498G0307313800.01.T01 | Cell number regulator 7 |
| maker-3-augustus-gene-487.2-mRNA-1 | Japonica | NA | | Os03g0831200 | OsR498G0307315700.01.T01 | GATA-type domain-containing protein |
| maker-3-augustus-gene-487.20-mRNA-1 | Japonica | NA | | Os03g0830500 | OsR498G0307314400.01.T01 | Cell number regulator 2 |
| maker-3-augustus-gene-487.22-mRNA-1 | Japonica | NA | | | | PMEI domain-containing protein |
| maker-3-augustus-gene-487.23-mRNA-1 | Japonica | NA | | Os03g0830900 | OsR498G0307315200.01.T01 | Mono-/di-acylglycerol lipase N-terminal |
| maker-3-augustus-gene-487.28-mRNA-1 | Japonica | NA | | Os03g0831100 | OsR498G0307315600.01.T02 | Pumilio homology domain family member 6 |
| maker-3-augustus-gene-489.65-mRNA-1 | Japonica | NA | | Os03g0128700 | OsR498G0305164700.01.T04 | |
| maker-3-augustus-gene-49.38-mRNA-1 | Japonica | NA | | Os03g0197100 | OsR498G0305395700.01.T01 | Polyol transporter protein 4 |
| maker-3-augustus-gene-49.40-mRNA-1 | Japonica | NA | | Os03g0197300 | OsR498G0305397500.01.T01 | Vicilin-like seed storage protein |
| maker-3-augustus-gene-49.42-mRNA-1 | Japonica | NA | | Os03g0198300 | OsR498G0305402100.01.T04 | J domain-containing protein required for chloroplast accumulation response |
| maker-3-augustus-gene-49.47-mRNA-1 | Japonica | NA | | Os03g0198400 | OsR498G0305402300.01.T03 | Seryl-tRNA synthetase |
| maker-3-augustus-gene-49.54-mRNA-1 | Japonica | NA | | Os03g0198500 | OsR498G0305402700.01.T01 | DUF862 domain-containing protein |
| maker-3-augustus-gene-49.57-mRNA-1 | Japonica | Indica | | Os03g0196900 | OsR498G0305394400.01.T01 | Plant-specific TFIIB-related protein 1 |
| maker-3-augustus-gene-49.61-mRNA-1 | Japonica | NA | | Os03g0197000 | OsR498G0305394600.01.T05 | Ditrans,polycis-polyprenyl diphosphate synthase ((2E,6E)-farnesyldiphospha |
| maker-3-augustus-gene-49.66-mRNA-1 | Japonica | NA | | Os03g0197400 | OsR498G0305398000.01.T01 | COP9 signalosome complex subunit 4 |
| maker-3-augustus-gene-49.71-mRNA-1 | Japonica | NA | | Os03g0197900 | OsR498G0305401000.01.T01 | Transcription repressor |
| maker-3-augustus-gene-49.72-mRNA-1 | Japonica | NA | | Os03g0198600 | OsR498G0305403200.01.T01 | Homeodomain leucine zipper protein class 1 |
| maker-3-augustus-gene-490.43-mRNA-1 | Japonica | Indica | | Os03g0836800 | OsR498G0307335100.01.T02 | IAA-amino acid hydrolase ILR1-like protein 8 |
| maker-3-augustus-gene-490.46-mRNA-1 | Japonica | NA | | Os03g0836900 | OsR498G0307335500.01.T01 | IAA-amino acid hydrolase ILR1 |
| maker-3-augustus-gene-490.48-mRNA-1 | Japonica | NA | | Os03g0837100 | OsR498G0307336500.01.T03 | Cellulose synthase |
| maker-3-augustus-gene-490.56-mRNA-1 | Japonica | NA | | Os03g0837300 | OsR498G0307337900.01.T01 | Nicotinate phosphoribosyltransferase |
| maker-3-augustus-gene-490.62-mRNA-1 | Japonica | NA | | | | Coenzyme Q-binding protein COQ10, START domain containing protein (Frag |
| maker-3-augustus-gene-490.65-mRNA-1 | Japonica | Indica | | Os03g0838400 | OsR498G0307339700.01.T01 | Ammonium transporter |
| maker-3-augustus-gene-490.67-mRNA-1 | Japonica | NA | | Os03g0838500 | OsR498G0307339900.01.T01 | Inhibitor I9 domain-containing protein |
| maker-3-augustus-gene-490.69-mRNA-1 | Japonica | NA | | Os03g0837000 | OsR498G0307335800.01.T02 | |
| maker-3-augustus-gene-490.75-mRNA-1 | Japonica | NA | | | OsR498G0307337800.01.T01 | Myb-like DNA-binding domain containing protein |
| maker-3-augustus-gene-490.77-mRNA-1 | Japonica | Indica | | Os03g0837800 | OsR498G0307338600.01.T02 | Transmembrane and coiled-coil domain-containing protein 5B |
| maker-3-augustus-gene-491.0-mRNA-1 | Japonica | NA | | | OsR498G0307341100.01.T01 | Protein DETOXIFICATION |
| maker-3-augustus-gene-491.12-mRNA-1 | Japonica | NA | | Os03g0839100 | OsR498G0307341000.01.T01 | Ubiquinone biosynthesis monooxygenase COQ6, mitochondrial |
| maker-3-augustus-gene-491.2-mRNA-1 | Japonica | NA | | | OsR498G0307342100.01.T01 | |
| maker-3-augustus-gene-491.22-mRNA-1 | Japonica | NA | Kinase-TM | Os03g0839900 | OsR498G0307344800.01.T01 | Protein kinase protein with adenine nucleotide alpha hydrolase-like domain |
| maker-3-augustus-gene-491.5-mRNA-1 | Japonica | NA | | Os03g0838900 | OsR498G0307340800.01.T02 | Transcription termination factor like |
| maker-3-augustus-gene-491.7-mRNA-1 | Japonica | NA | | Os03g0839000 | OsR498G0307340800.01.T04 | E3 ubiquitin-protein ligase RNF170 |
| maker-3-augustus-gene-492.0-mRNA-1 | Japonica | NA | | | OsR498G0307349700.01.T01 | |
| maker-3-augustus-gene-492.16-mRNA-1 | Japonica | NA | | | OsR498G0307352700.01.T05 | RNA-binding protein containing a PIN domain |
| maker-3-augustus-gene-492.2-mRNA-1 | Japonica | NA | | | OsR498G0307350100.01.T01 | Calcium-dependent protein kinase 6, putative |
| maker-3-augustus-gene-492.21-mRNA-1 | Japonica | NA | | Os03g0843400 | OsR498G0307353300.01.T01 | 30S ribosomal protein S6 alpha chloroplastic |
| maker-3-augustus-gene-492.24-mRNA-1 | Japonica | NA | | Os03g0840400 | OsR498G0307346800.01.T01 | SANT domain-containing protein |
| maker-3-augustus-gene-492.37-mRNA-1 | Japonica | Indica | | Os03g0840800 | OsR498G0307347800.01.T01 | Phospholipase D |
| maker-3-augustus-gene-492.41-mRNA-1 | Japonica | NA | CC-Kinase-TM | Os03g0841100 | OsR498G0307348200.01.T01 | Wall-associated receptor kinase 3 |
| maker-3-augustus-gene-492.45-mRNA-1 | Japonica | NA | | Os03g0841700 | OsR498G0307348600.01.T01 | Prohibitin |
| maker-3-augustus-gene-492.48-mRNA-1 | Japonica | NA | Kinase | Os03g0841800 | OsR498G0307348800.01.T01 | Non-specific serine/threonine protein kinase |
| maker-3-augustus-gene-492.50-mRNA-1 | Japonica | Japonica | | Os03g0841900 | OsR498G0307349200.01.T04 | Prolycopene isomerase chloroplastic |
| maker-3-augustus-gene-493.1-mRNA-1 | Japonica | NA | | Os03g0843600 | OsR498G0307353700.01.T04 | Ion channel CASTOR |
| maker-3-augustus-gene-493.15-mRNA-1 | Japonica | Indica | | Os08g0116800 | OsR498G0307356100.01.T02 | Exosome complex component |
| maker-3-augustus-gene-493.19-mRNA-1 | Japonica | NA | | Os03g0844500 | OsR498G0307356300.01.T02 | Serine/threonine protein phosphatase 2A regulatory subunit |
| maker-3-augustus-gene-493.21-mRNA-1 | Japonica | NA | | | OsR498G0307356900.01.T01 | ALBINO3-like protein 1, chloroplastic isoform A |
| maker-3-augustus-gene-493.31-mRNA-1 | Japonica | NA | | Os03g0843500 | OsR498G0307353600.01.T09 | RNA-binding protein Luc7-like 2 |
| maker-3-augustus-gene-493.45-mRNA-1 | Japonica | NA | | Os03g0844600 | OsR498G0307356600.01.T01 | Gdsl esteraselipase |
| maker-3-augustus-gene-493.53-mRNA-1 | Japonica | Indica | | Os03g0845000 | OsR498G0307357800.01.T03 | 30S ribosomal protein S1 isoform X1 |
| maker-3-augustus-gene-493.7-mRNA-1 | Japonica | NA | | | OsR498G0307354300.01.T02 | Thiol methyltransferase |

| | | | | | | |
|---|---|---|---|---|---|---|
| maker-3-augustus-gene-494.24-mRNA-1 | Japonica | NA | | Os03g0846400 | OsR498G0307362100.01.T03 | Putative polyprotein from transposon TNT |
| maker-3-augustus-gene-494.29-mRNA-1 | Japonica | NA | | Os04g0668900 | OsR498G0409459400.01.T06 | Ubiquinol oxidase |
| maker-3-augustus-gene-494.32-mRNA-1 | Japonica | NA | Kinase-TM | Os03g0847600 | OsR498G0307365500.01.T05 | Serine/threonine protein kinase |
| maker-3-augustus-gene-494.37-mRNA-1 | Japonica | NA | | Os03g0845500 | OsR498G0307359200.01.T04 | Acyl-activating enzyme 16 |
| maker-3-augustus-gene-494.44-mRNA-1 | Japonica | Indica | | Os03g0845700 | OsR498G0307360000.01.T03 | Nucleic acid-binding, OB-fold (Fragment) |
| maker-3-augustus-gene-494.47-mRNA-1 | Japonica | NA | | Os03g0846900 | | |
| maker-3-augustus-gene-495.0-mRNA-1 | Japonica | NA | | | OsR498G0307366100.01.T02 | Ankyrin repeat domain-containing protein 11-like |
| maker-3-augustus-gene-495.13-mRNA-1 | Japonica | NA | | Os03g0848100 | OsR498G0307367000.01.T04 | Ser/Thr protein phosphatase family protein, expressed |
| maker-3-augustus-gene-495.15-mRNA-1 | Japonica | NA | | Os03g0848200 | OsR498G0307367200.01.T01 | Purple acid phosphatase |
| maker-3-augustus-gene-495.17-mRNA-1 | Japonica | NA | | Os03g0848400 | OsR498G0307367600.01.T03 | Endoplasmic reticulum transmembrane protein (Fragment) |
| maker-3-augustus-gene-495.19-mRNA-1 | Japonica | Japonica | CC-NBS-TM | Os03g0848700 | OsR498G0307368000.01.T01 | Powdery mildew resistance protein PM3 variant |
| maker-3-augustus-gene-495.28-mRNA-1 | Japonica | Japonica | | Os03g0849600 | OsR498G0307368400.01.T01 | Component of oligomeric Golgi complex 2 |
| maker-3-augustus-gene-495.3-mRNA-1 | Japonica | NA | | | OsR498G0307366700.01.T01 | ROP-interactive CRIB motif-containing protein 4 |
| maker-3-augustus-gene-495.5-mRNA-1 | Japonica | NA | | Os03g0848300 | OsR498G0307367300.01.T01 | Actin-related protein 2/3 complex subunit 4 |
| maker-3-augustus-gene-495.9-mRNA-1 | Japonica | NA | | Os03g0848600 | OsR498G0307367700.01.T04 | AvrRpt-cleavage domain-containing protein (Fragment) |
| maker-3-augustus-gene-496.42-mRNA-1 | Japonica | Japonica | | Os03g0849700 | OsR498G0307368500.01.T03 | Sec16 domain-containing protein |
| maker-3-augustus-gene-496.44-mRNA-1 | Japonica | Indica | | Os03g0849800 | OsR498G0307369100.01.T03 | Nucleotide-diphospho-sugar transferase family protein |
| maker-3-augustus-gene-496.48-mRNA-1 | Japonica | NA | | Os03g0850200 | OsR498G0307370500.01.T01 | 4-hydroxyphenylacetaldehyde oxime monooxygenase |
| maker-3-augustus-gene-496.50-mRNA-1 | Japonica | Indica | | Os03g0850400 | OsR498G0307371900.01.T01 | Aspartate kinase |
| maker-3-augustus-gene-496.60-mRNA-1 | Japonica | Indica | | Os03g0851400 | OsR498G0307375500.01.T02 | |
| maker-3-augustus-gene-496.63-mRNA-1 | Japonica | NA | | Os03g0850000 | OsR498G0307370200.01.T01 | Carbonyl reductase (NADPH) 2 |
| maker-3-augustus-gene-496.65-mRNA-1 | Japonica | NA | | Os03g0850100 | OsR498G0307370400.01.T02 | FCP1 homology domain-containing protein (Fragment) |
| maker-3-augustus-gene-496.68-mRNA-1 | Japonica | NA | | Os03g0850600 | OsR498G0307373000.01.T01 | Perilipin-3 |
| maker-3-augustus-gene-496.73-mRNA-1 | Japonica | NA | | Os03g0850800 | OsR498G0307373800.01.T01 | Transferring glycosyl group transferase |
| maker-3-augustus-gene-497.38-mRNA-1 | Japonica | NA | | Os03g0851500 | OsR498G0307375500.01.T05 | DNA repair RAD52-like protein 2, chloroplastic |
| maker-3-augustus-gene-497.40-mRNA-1 | Japonica | Indica | | Os03g0851600 | OsR498G0307375700.01.T02 | U5 small nuclear ribonucleoprotein 40 kDa protein isoform X1 |
| maker-3-augustus-gene-497.45-mRNA-1 | Japonica | NA | | Os03g0851700 | OsR498G0307375900.01.T01 | Ankyrin repeat domain-containing protein 2 |
| maker-3-augustus-gene-497.47-mRNA-1 | Japonica | NA | | Os03g0851800 | OsR498G0307376100.01.T01 | Pantoate--beta-alanine ligase |
| maker-3-augustus-gene-497.49-mRNA-1 | Japonica | NA | | Os03g0852300 | OsR498G0307377100.01.T04 | Histone-like transcription factor and archaeal histone family protein, express |
| maker-3-augustus-gene-497.51-mRNA-1 | Japonica | NA | | Os03g0852600 | OsR498G0307377700.01.T01 | |
| maker-3-augustus-gene-497.53-mRNA-1 | Japonica | NA | | | OsR498G0307379100.01.T01 | Tetraspanin-3 |
| maker-3-augustus-gene-497.55-mRNA-1 | Japonica | NA | | Os03g0853200 | OsR498G0307379500.01.T01 | Tetraspanin/Peripherin |
| maker-3-augustus-gene-497.57-mRNA-1 | Japonica | NA | | Os03g0851900 | OsR498G0307376600.01.T05 | lactation elevated protein 1 isoform X3 |
| maker-3-augustus-gene-497.62-mRNA-1 | Japonica | NA | | Os03g0852000 | OsR498G0307376600.01.T07 | Transcription factor bHLH25-like |
| maker-3-augustus-gene-497.64-mRNA-1 | Japonica | NA | | Os03g0852200 | OsR498G0307377000.01.T01 | Derlin |
| maker-3-augustus-gene-497.68-mRNA-1 | Japonica | NA | | Os03g0852500 | OsR498G0307377600.01.T01 | |
| maker-3-augustus-gene-497.70-mRNA-1 | Japonica | NA | | Os03g0852900 | OsR498G0307378800.01.T01 | Cleavage and polyadenylation specificity factor |
| maker-3-augustus-gene-498.31-mRNA-1 | Japonica | Indica | | Os03g0853500 | OsR498G0307381100.01.T01 | Brevis radix (BRX) domain |
| maker-3-augustus-gene-498.36-mRNA-1 | Japonica | NA | | Os03g0854300 | OsR498G0307383300.01.T02 | RNA-binding protein 34 |
| maker-3-augustus-gene-498.39-mRNA-1 | Japonica | NA | | Os03g0854500 | OsR498G0307383900.01.T04 | Heat shock transcription factor |
| maker-3-augustus-gene-498.41-mRNA-1 | Japonica | Indica | | Os03g0853600 | OsR498G0307381600.01.T02 | Ribonuclease P protein subunit P38-related |
| maker-3-augustus-gene-498.44-mRNA-1 | Japonica | NA | | Os03g0853900 | OsR498G0307382600.01.T02 | Sinigrinase |
| maker-3-augustus-gene-498.48-mRNA-1 | Japonica | NA | | Os03g0854100 | OsR498G0307383000.01.T04 | probable ADP-ribosylation factor GTPase-activating protein AGD8 |
| maker-3-augustus-gene-498.53-mRNA-1 | Japonica | NA | | Os03g0854200 | OsR498G0307383200.01.T02 | Exosome complex exonuclease RRP46 like |
| maker-3-augustus-gene-498.57-mRNA-1 | Japonica | NA | | Os03g0854600 | OsR498G0307384200.01.T01 | Xyloglucan endotransglucosylase/hydrolase |
| maker-3-augustus-gene-498.59-mRNA-1 | Japonica | Indica | | Os03g0854800 | OsR498G0307385000.01.T05 | PARP catalytic domain-containing protein |
| maker-3-augustus-gene-499.0-mRNA-1 | Japonica | NA | | Os03g0855100 | OsR498G0307385900.01.T01 | WRKY transcription factor 14 |
| maker-3-augustus-gene-499.10-mRNA-1 | Japonica | NA | | | OsR498G0307391700.01.T05 | Coatomer subunit zeta |
| maker-3-augustus-gene-499.13-mRNA-1 | Japonica | NA | | | OsR498G0307391700.01.T02 | ATP-dependent DNA helicase 2 subunit KU80 |
| maker-3-augustus-gene-5.0-mRNA-1 | Japonica | NA | | | OsR498G0305108900.01.T01 | RDR2-independent DNA methylation protein |
| maker-3-augustus-gene-5.11-mRNA-1 | Japonica | NA | | Os03g0113800 | OsR498G0305113100.01.T01 | Hop-interacting protein THI002 |
| maker-3-augustus-gene-5.17-mRNA-1 | Japonica | NA | | | OsR498G0305113900.01.T01 | Kinesin-like protein KIN-14E |
| maker-3-augustus-gene-5.29-mRNA-1 | Japonica | NA | | Os03g0112800 | OsR498G0305109200.01.T01 | Putative DUF26 domain receptor-like protein kinase |
| maker-3-augustus-gene-5.35-mRNA-1 | Japonica | NA | | Os03g0112900 | OsR498G0305109400.01.T01 | Ferulic acid 5-hydroxylase |
| maker-3-augustus-gene-5.37-mRNA-1 | Japonica | NA | Kinase-TM | Os03g0113000 | OsR498G0305110000.01.T01 | Receptor protein kinase CRINKLY4 |
| maker-3-augustus-gene-5.39-mRNA-1 | Japonica | NA | | | OsR498G0305111400.01.T01 | Trihelix transcription factor GT-2 |
| maker-3-augustus-gene-5.41-mRNA-1 | Japonica | NA | | | OsR498G0305112400.01.T01 | |
| maker-3-augustus-gene-5.43-mRNA-1 | Japonica | NA | | Os03g0113700 | OsR498G0305113000.01.T01 | Heat shock 70 kDa protein, mitochondrial |
| maker-3-augustus-gene-5.7-mRNA-1 | Japonica | NA | | Os03g0113100 | OsR498G0305110300.01.T01 | Thymidine kinase |
| maker-3-augustus-gene-5.9-mRNA-1 | Japonica | NA | | Os03g0113200 | OsR498G0305110500.01.T02 | F-box domain containing protein-like |
| maker-3-augustus-gene-50.33-mRNA-1 | Japonica | Indica | | Os12g0511300 | OsR498G0305405300.01.T02 | CRT-like domain-containing protein (Fragment) |

| | | | | | | |
|---|---|---|---|---|---|---|
| maker-3-augustus-gene-50.38-mRNA-1 | Japonica | Indica | | Os12g0511200 | OsR498G0305408900.01.T01 | Heptahelical transmembrane protein 5 |
| maker-3-augustus-gene-50.40-mRNA-1 | Japonica | NA | | | | |
| maker-3-augustus-gene-50.42-mRNA-1 | Japonica | NA | | Os03g0200200 | OsR498G0305409500.01.T02 | TRIO and F-actin-binding protein |
| maker-3-augustus-gene-50.44-mRNA-1 | Japonica | Indica | | Os03g0200400 | OsR498G0305410100.01.T01 | CYSTM domain-containing protein |
| maker-3-augustus-gene-50.47-mRNA-1 | Japonica | NA | | Os03g0200500 | OsR498G0305410500.01.T01 | |
| maker-3-augustus-gene-50.51-mRNA-1 | Japonica | NA | | Os03g0198900 | OsR498G0305405200.01.T02 | 26S proteasome non-ATPase regulatory subunit 9-like |
| maker-3-augustus-gene-50.53-mRNA-1 | Japonica | NA | | | OsR498G0305405200.01.T07 | Methyltransferase-like protein 2 isoform A |
| maker-3-augustus-gene-50.56-mRNA-1 | Japonica | Indica | | Os03g0199500 | OsR498G0305407200.01.T01 | zf-RVT domain-containing protein |
| maker-3-augustus-gene-50.59-mRNA-1 | Japonica | NA | | | OsR498G0305410800.01.T03 | No pollen germination related 1 |
| maker-3-augustus-gene-500.37-mRNA-1 | Japonica | NA | | Os03g0858400 | OsR498G0307398100.01.T01 | Transducin beta-like protein 2-like |
| maker-3-augustus-gene-500.41-mRNA-1 | Japonica | Indica | | Os03g0857400 | OsR498G0307396400.01.T02 | PGR5-like protein 1B chloroplastic |
| maker-3-augustus-gene-500.45-mRNA-1 | Japonica | NA | | Os03g0857500 | OsR498G0307396600.01.T02 | SASA domain-containing protein (Fragment) |
| maker-3-augustus-gene-500.49-mRNA-1 | Japonica | NA | | Os03g0858100 | OsR498G0307397600.01.T03 | Importin N-terminal domain-containing protein (Fragment) |
| maker-3-augustus-gene-501.46-mRNA-1 | Japonica | NA | | Os03g0859900 | OsR498G0307400900.01.T01 | DUF547 domain-containing protein |
| maker-3-augustus-gene-501.52-mRNA-1 | Japonica | NA | | | OsR498G0307401100.01.T04 | ASCH domain-containing protein |
| maker-3-augustus-gene-501.56-mRNA-1 | Japonica | NA | | Os03g0858700 | | ATP-dependent DNA helicase 2 subunit KU80-like |
| maker-3-augustus-gene-501.58-mRNA-1 | Japonica | NA | | Os03g0859100 | OsR498G0307399600.01.T01 | GDSL esterase/lipase EXL3 |
| maker-3-augustus-gene-501.60-mRNA-1 | Japonica | NA | | Os03g0859300 | OsR498G0307400000.01.T01 | La domain containing protein, expressed |
| maker-3-augustus-gene-501.63-mRNA-1 | Japonica | NA | | Os03g0859500 | OsR498G0307400200.01.T01 | ABC-2 type transporter family protein isoform 1 |
| maker-3-augustus-gene-501.67-mRNA-1 | Japonica | Indica | | Os12g0277500 | OsR498G0307400200.01.T05 | RuBisCO large subunit-binding protein subunit alpha |
| maker-3-augustus-gene-501.72-mRNA-1 | Japonica | Indica | | Os03g0859800 | OsR498G0307400600.01.T02 | OTU-like cysteine protease family protein, putative, expressed |
| maker-3-augustus-gene-502.58-mRNA-1 | Japonica | NA | | | OsR498G0307404300.01.T04 | Transcriptional corepressor LEUNIG |
| maker-3-augustus-gene-502.65-mRNA-1 | Japonica | Japonica | | Os03g0861100 | OsR498G0307404700.01.T03 | |
| maker-3-augustus-gene-502.73-mRNA-1 | Japonica | NA | | Os03g0862100 | OsR498G0307406700.01.T03 | SLH domain-containing protein |
| maker-3-augustus-gene-502.81-mRNA-1 | Japonica | NA | | Os03g0861400 | OsR498G0307405400.01.T02 | Heavy metal-associated domain containing protein, expressed |
| maker-3-augustus-gene-502.91-mRNA-1 | Japonica | NA | | Os03g0862200 | OsR498G0713369200.01.T01 | Kinesin motor domain-containing protein |
| maker-3-augustus-gene-51.0-mRNA-1 | Japonica | NA | | Os03g0201000 | OsR498G0305411500.01.T01 | |
| maker-3-augustus-gene-51.12-mRNA-1 | Japonica | NA | | Os03g0202500 | OsR498G0305415900.01.T02 | FBD domain-containing protein |
| maker-3-augustus-gene-51.16-mRNA-1 | Japonica | NA | | Os03g0200700 | OsR498G0305411000.01.T01 | |
| maker-3-augustus-gene-51.2-mRNA-1 | Japonica | NA | | Os03g0201200 | OsR498G0305411900.01.T01 | Anti-sigma-I factor RsgI6 |
| maker-3-augustus-gene-51.22-mRNA-1 | Japonica | Indica | | Os03g0200800 | OsR498G0305411200.01.T01 | ADP-ribosylation factor-like protein 8a |
| maker-3-augustus-gene-51.25-mRNA-1 | Japonica | Indica | | Os03g0201100 | OsR498G0305411800.01.T04 | Peptidylprolyl isomerase |
| maker-3-augustus-gene-51.33-mRNA-1 | Japonica | Indica | | Os03g0201600 | OsR498G0305413000.01.T03 | YrdC domain-containing protein, mitochondrial |
| maker-3-augustus-gene-51.36-mRNA-1 | Japonica | NA | | Os03g0201800 | OsR498G0305413400.01.T01 | GH10 domain-containing protein (Fragment) |
| maker-3-augustus-gene-51.38-mRNA-1 | Japonica | Indica | | Os03g0201901 | OsR498G0305413600.01.T01 | GH10 domain-containing protein (Fragment) |
| maker-3-augustus-gene-51.40-mRNA-1 | Japonica | Indica | NBS-TM | Os07g0573100 | OsR498G0305414000.01.T04 | Adenylyl-sulfate kinase |
| maker-3-augustus-gene-51.44-mRNA-1 | Japonica | NA | | Os03g0202200 | OsR498G0305414200.01.T01 | Outer mitochondrial membrane protein porin |
| maker-3-augustus-gene-51.47-mRNA-1 | Japonica | NA | | Os03g0202300 | OsR498G0305414400.01.T01 | Ribosomal RNA large subunit methyltransferase N |
| maker-3-augustus-gene-51.5-mRNA-1 | Japonica | Indica | | Os03g0201400 | OsR498G0305412700.01.T01 | GH10 domain-containing protein (Fragment) |
| maker-3-augustus-gene-51.8-mRNA-1 | Japonica | NA | | Os03g0201700 | OsR498G0305413100.01.T01 | Elongator complex protein 5 |
| maker-3-augustus-gene-52.36-mRNA-1 | Japonica | NA | | Os09g0506800 | OsR498G0917770500.01.T06 | HORMA domain-containing protein 1 |
| maker-3-augustus-gene-52.41-mRNA-1 | Japonica | NA | | | | Urb2 domain-containing protein |
| maker-3-augustus-gene-52.44-mRNA-1 | Japonica | NA | | Os03g0203200 | OsR498G0305418300.01.T06 | Strigolactone esterase |
| maker-3-augustus-gene-52.46-mRNA-1 | Japonica | NA | | Os03g0203700 | OsR498G0305418900.01.T01 | Calcium-transporting ATPase |
| maker-3-augustus-gene-52.48-mRNA-1 | Japonica | NA | | Os03g0204100 | OsR498G0305420500.01.T04 | Formin-like protein 8 |
| maker-3-augustus-gene-52.50-mRNA-1 | Japonica | NA | | | OsR498G0305422100.01.T01 | PGG domain-containing protein |
| maker-3-augustus-gene-52.52-mRNA-1 | Japonica | NA | | | OsR498G0305423500.01.T01 | F-box domain-containing protein |
| maker-3-augustus-gene-52.54-mRNA-1 | Japonica | NA | | Os03g0203000 | OsR498G0305416600.01.T01 | Somatic embryogenesis related protein |
| maker-3-augustus-gene-52.57-mRNA-1 | Japonica | NA | | Os03g0203800 | OsR498G0305419200.01.T01 | D-type cyclin |
| maker-3-augustus-gene-52.61-mRNA-1 | Japonica | NA | | Os03g0204500 | OsR498G0305422800.01.T01 | |
| maker-3-augustus-gene-53.26-mRNA-1 | Japonica | NA | | Os03g0205400 | OsR498G0305425700.01.T01 | DNA repair protein UVH3 |
| maker-3-augustus-gene-53.31-mRNA-1 | Japonica | Japonica | | Os03g0205500 | OsR498G0305425900.01.T01 | Membrane-associated progesterone-binding protein 4 |
| maker-3-augustus-gene-53.36-mRNA-1 | Japonica | NA | | Os03g0205700 | OsR498G0305426900.01.T02 | BTB/POZ domain-containing protein NPY2 |
| maker-3-augustus-gene-53.38-mRNA-1 | Japonica | NA | | | | |
| maker-3-augustus-gene-53.40-mRNA-1 | Japonica | NA | | Os03g0205000 | OsR498G0305424000.01.T01 | CUE domain containing protein, expressed |
| maker-3-augustus-gene-53.43-mRNA-1 | Japonica | NA | | | OsR498G0305424200.01.T02 | Retrotransposon protein, putative, Ty1-copia subclass |
| maker-3-augustus-gene-53.46-mRNA-1 | Japonica | NA | | | OsR498G0305424600.01.T01 | Transcription factor bHLH85 like |
| maker-3-augustus-gene-53.48-mRNA-1 | Japonica | NA | | Os03g0205800 | OsR498G0305427400.01.T01 | probable N-acetyltransferase HLS1 |
| maker-3-augustus-gene-53.52-mRNA-1 | Japonica | NA | | | | ATP-dependent RNA helicase DDX18 |
| maker-3-augustus-gene-53.54-mRNA-1 | Japonica | Indica | | Os03g0206300 | OsR498G0305429200.01.T05 | Sulfhydryl oxidase |
| maker-3-augustus-gene-53.60-mRNA-1 | Japonica | NA | | Os03g0206400 | | |

| | | | | | | |
|---|---|---|---|---|---|---|
| maker-3-augustus-gene-54.40-mRNA-1 | Japonica | NA | | Os03g0207400 | OsR498G0305432300.01.T01 | Protein-serine/threonine phosphatase |
| maker-3-augustus-gene-54.42-mRNA-1 | Japonica | NA | | Os03g0207700 | | |
| maker-3-augustus-gene-54.44-mRNA-1 | Japonica | NA | | | OsR498G0305433300.01.T01 | Tetratricopeptide repeat protein 1 |
| maker-3-augustus-gene-54.46-mRNA-1 | Japonica | NA | | | OsR498G0305435100.01.T01 | Cyclin N-terminal domain-containing protein |
| maker-3-augustus-gene-54.47-mRNA-1 | Japonica | NA | | | OsR498G0305435100.01.T01 | Cyclin N-terminal domain-containing protein |
| maker-3-augustus-gene-54.63-mRNA-1 | Japonica | NA | | Os03g0207200 | OsR498G0305431600.01.T04 | Syntaxin/t-SNARE family protein |
| maker-3-augustus-gene-54.66-mRNA-1 | Japonica | NA | | Os03g0207250 | OsR498G0305431800.01.T01 | 54S ribosomal protein L51, mitochondrial |
| maker-3-augustus-gene-54.69-mRNA-1 | Japonica | NA | Kinase-TM | Os03g0207300 | OsR498G0305432000.01.T01 | Protein kinase CK2 alpha chain |
| maker-3-augustus-gene-54.74-mRNA-1 | Japonica | NA | | Os03g0207900 | OsR498G0305433600.01.T01 | Retinoblastoma-binding protein-like |
| maker-3-augustus-gene-54.77-mRNA-1 | Japonica | NA | | Os03g0208500 | OsR498G0305434000.01.T01 | Divalent metal transporter 1 |
| maker-3-augustus-gene-55.36-mRNA-1 | Japonica | NA | | | | Vesicle transport protein GOT1 |
| maker-3-augustus-gene-55.39-mRNA-1 | Japonica | NA | | Os03g0209600 | OsR498G0305438500.01.T01 | Methionyl-tRNA synthetase |
| maker-3-augustus-gene-55.49-mRNA-1 | Japonica | Indica | | Os03g0256800 | OsR498G0305604600.01.T02 | Rho GTPase activation protein (RhoGAP) with PH domain |
| maker-3-augustus-gene-55.59-mRNA-1 | Japonica | NA | | | OsR498G0305440700.01.T04 | Pentatricopeptide repeat-containing family protein |
| maker-3-augustus-gene-55.63-mRNA-1 | Japonica | NA | | | OsR498G0305441300.01.T02 | Acetyl-CoA decarbonylase/synthase complex subunit alpha 1 |
| maker-3-augustus-gene-55.66-mRNA-1 | Japonica | NA | | Os03g0208900 | OsR498G0305435600.01.T02 | ADP-glucose pyrophosphorylase |
| maker-3-augustus-gene-55.72-mRNA-1 | Japonica | NA | | | | S-acyltransferase |
| maker-3-augustus-gene-55.77-mRNA-1 | Japonica | NA | | Os03g0210400 | OsR498G0305440600.01.T03 | Pre-mRNA-processing factor like |
| maker-3-augustus-gene-56.40-mRNA-1 | Japonica | NA | | | OsR498G0305442500.01.T01 | ABC transporter permease protein YtcP |
| maker-3-augustus-gene-56.42-mRNA-1 | Japonica | Indica | | Os03g0211600 | OsR498G0305443900.01.T01 | Pentatricopeptide repeat |
| maker-3-augustus-gene-56.44-mRNA-1 | Japonica | NA | | Os03g0211700 | OsR498G0305444100.01.T03 | Glucuronidase 3 |
| maker-3-augustus-gene-56.49-mRNA-1 | Japonica | NA | LRR | | OsR498G0305445900.01.T02 | Plant intracellular Ras-group-related LRR protein 7 |
| maker-3-augustus-gene-56.54-mRNA-1 | Japonica | NA | | | | SNAP25 isogenyous protein SNAP33 |
| maker-3-augustus-gene-56.57-mRNA-1 | Japonica | NA | | | | TPX2 (Targeting protein for Xklp2) protein family |
| maker-3-augustus-gene-56.60-mRNA-1 | Japonica | NA | | Os03g0212700 | OsR498G0305446900.01.T01 | Mitochondrial-processing peptidase subunit beta like |
| maker-3-augustus-gene-56.64-mRNA-1 | Japonica | Indica | | Os03g0210800 | OsR498G0305441600.01.T04 | Nucleotidyltransferase domain containing protein, expressed |
| maker-3-augustus-gene-56.72-mRNA-1 | Japonica | Indica | | Os12g0578500 | OsR498G0305444400.01.T01 | Hexosyltransferase |
| maker-3-augustus-gene-56.75-mRNA-1 | Japonica | NA | LRR-TM | Os03g0211900 | OsR498G0305445400.01.T01 | probable leucine-rich repeat receptor-like protein kinase At1g35710 |
| maker-3-augustus-gene-56.79-mRNA-1 | Japonica | NA | | Os03g0212800 | OsR498G0305447400.01.T02 | Sinigrinase |
| maker-3-augustus-gene-57.0-mRNA-1 | Japonica | NA | | Os03g0213100 | OsR498G0305448700.01.T01 | Transport protein Sec61 subunit alpha-like |
| maker-3-augustus-gene-57.10-mRNA-1 | Japonica | NA | | | OsR498G0305449700.01.T03 | |
| maker-3-augustus-gene-57.12-mRNA-1 | Japonica | Japonica | | Os03g0213600 | OsR498G0305449900.01.T02 | |
| maker-3-augustus-gene-57.18-mRNA-1 | Japonica | NA | | | OsR498G0305450900.01.T06 | Hydroxyproline-rich glycoprotein family protein, putative, expressed |
| maker-3-augustus-gene-57.25-mRNA-1 | Japonica | NA | | Os03g0214000 | OsR498G0305451100.01.T02 | Purple acid phosphatase |
| maker-3-augustus-gene-57.30-mRNA-1 | Japonica | NA | | Os03g0214100 | OsR498G0305451300.01.T01 | Replication protein A subunit |
| maker-3-augustus-gene-57.32-mRNA-1 | Japonica | NA | | | OsR498G0305449400.01.T03 | DExH-box ATP-dependent RNA helicase DExH14 |
| maker-3-augustus-gene-57.41-mRNA-1 | Japonica | NA | | | OsR498G0305450200.01.T04 | Protein root UVB sensitive 3 |
| maker-3-augustus-gene-57.6-mRNA-1 | Japonica | NA | | Os03g0213300 | OsR498G0305449100.01.T01 | |
| maker-3-augustus-gene-58.0-mRNA-1 | Japonica | NA | | Os03g0214600 | OsR498G0305452300.01.T01 | Proteasome component (PCI) domain |
| maker-3-augustus-gene-58.12-mRNA-1 | Japonica | NA | | Os03g0214200 | OsR498G0305451600.01.T01 | Ninja-family protein 1 |
| maker-3-augustus-gene-58.15-mRNA-1 | Japonica | NA | | Os03g0214400 | OsR498G0305452200.01.T02 | Digalactosyldiacylglycerol synthase |
| maker-3-augustus-gene-58.20-mRNA-1 | Japonica | NA | | Os03g0214900 | OsR498G0305452600.01.T01 | Zinc finger CCCH domain-containing protein 55 |
| maker-3-augustus-gene-58.24-mRNA-1 | Japonica | NA | | Os03g0215400 | OsR498G0305455800.01.T01 | Leafy hull sterile 1 (Fragment) |
| maker-3-augustus-gene-58.5-mRNA-1 | Japonica | NA | | Os03g0215200 | OsR498G0305454900.01.T02 | DROOPING LEAF |
| maker-3-augustus-gene-58.8-mRNA-1 | Japonica | NA | | Os03g0215700 | OsR498G0305456700.01.T02 | microtubule-associated protein 70-5-like isoform X1 |
| maker-3-augustus-gene-59.0-mRNA-1 | Japonica | NA | | Os03g0215800 | OsR498G0305457100.01.T01 | PALP domain-containing protein (Fragment) |
| maker-3-augustus-gene-59.11-mRNA-1 | Japonica | NA | | Os03g0216800 | OsR498G0305461700.01.T06 | Polygalacturonase |
| maker-3-augustus-gene-59.14-mRNA-1 | Japonica | Indica | | Os03g0216900 | OsR498G0305462100.01.T02 | |
| maker-3-augustus-gene-59.17-mRNA-1 | Japonica | NA | | Os03g0217200 | OsR498G0305462500.01.T04 | FBD domain-containing protein |
| maker-3-augustus-gene-59.22-mRNA-1 | Japonica | NA | | Os03g0216700 | OsR498G0305460600.01.T03 | Protein DETOXIFICATION |
| maker-3-augustus-gene-59.6-mRNA-1 | Japonica | NA | | Os03g0216600 | OsR498G0305460300.01.T01 | Glucan 1,3-alpha-glucosidase |
| maker-3-augustus-gene-6.6-mRNA-1 | Japonica | NA | | Os03g0115400 | OsR498G0305118700.01.T02 | WD repeat-containing protein 44 |
| maker-3-augustus-gene-60.37-mRNA-1 | Japonica | NA | | | OsR498G0305463500.01.T02 | |
| maker-3-augustus-gene-60.41-mRNA-1 | Japonica | NA | | Os03g0219100 | OsR498G0305466300.01.T01 | CRAL/TRIO domain containing protein, expressed |
| maker-3-augustus-gene-60.44-mRNA-1 | Japonica | Indica | | Os03g0217200 | OsR498G0305463200.01.T03 | FBD domain-containing protein |
| maker-3-augustus-gene-60.47-mRNA-1 | Japonica | Indica | | Os03g0218200 | OsR498G0305463200.01.T04 | FBD domain-containing protein |
| maker-3-augustus-gene-60.50-mRNA-1 | Japonica | NA | | Os03g0219200 | OsR498G0305466600.01.T01 | Superoxide dismutase |
| maker-3-augustus-gene-60.55-mRNA-1 | Japonica | NA | | Os03g0219300 | OsR498G0305466800.01.T01 | Tubulin alpha chain |
| maker-3-augustus-gene-60.58-mRNA-1 | Japonica | NA | | Os03g0219400 | OsR498G0305467000.01.T01 | Beta-N-acetylhexosaminidase |
| maker-3-augustus-gene-61.24-mRNA-1 | Japonica | NA | | | OsR498G0305467700.01.T02 | SprT-like domain-containing protein Spartan (projected from Caenorhabditis |
| maker-3-augustus-gene-61.29-mRNA-1 | Japonica | NA | | Os03g0219900 | OsR498G0305467900.01.T05 | Large subunit ribosomal protein L15 |

| | | | | | | |
|---|---|---|---|---|---|---|
| maker-3-augustus-gene-61.31-mRNA-1 | Japonica | NA | | | | |
| maker-3-augustus-gene-61.34-mRNA-1 | Japonica | Indica | | Os03g0221200 | OsR498G0305472100.01.T01 | Hcy-binding domain-containing protein (Fragment) |
| maker-3-augustus-gene-61.37-mRNA-1 | Japonica | NA | | Os03g0221500 | OsR498G0305472900.01.T01 | Glucan endo-1,3-beta-D-glucosidase |
| maker-3-augustus-gene-61.40-mRNA-1 | Japonica | NA | | Os03g0220100 | OsR498G0305468600.01.T01 | Very-long-chain 3-oxoacyl-CoA synthase |
| maker-3-augustus-gene-61.42-mRNA-1 | Japonica | Indica | | Os03g0221300 | OsR498G0305472400.01.T01 | NAC domain-containing protein 8 |
| maker-3-augustus-gene-62.49-mRNA-1 | Japonica | NA | | | OsR498G0305476700.01.T07 | Chaperone DnaJ |
| maker-3-augustus-gene-62.54-mRNA-1 | Japonica | NA | | | OsR498G0305477100.01.T03 | Chaperone protein DnaJ |
| maker-3-augustus-gene-62.58-mRNA-1 | Japonica | NA | Kinase-LRR-TM | Os03g0223000 | OsR498G0305477500.01.T01 | Atypical receptor-like kinase MARK |
| maker-3-augustus-gene-62.61-mRNA-1 | Japonica | Indica | | Os03g0223200 | OsR498G0305478700.01.T01 | Cinnamyl-alcohol dehydrogenase |
| maker-3-augustus-gene-62.63-mRNA-1 | Japonica | Indica | | Os01g0862800 | OsR498G0305479700.01.T01 | NAC domain-containing |
| maker-3-augustus-gene-62.65-mRNA-1 | Japonica | NA | | | OsR498G0305473400.01.T03 | Dynein light chain 1, axonemal |
| maker-3-augustus-gene-62.72-mRNA-1 | Japonica | NA | | Os03g0222100 | OsR498G0305474200.01.T01 | Neoplastic transformation suppressor Pdcd4/MA-3, contains MA3 domain |
| maker-3-augustus-gene-62.74-mRNA-1 | Japonica | NA | | Os03g0223400 | OsR498G0305479200.01.T01 | Glutamine synthetase cytosolic isozyme |
| maker-3-augustus-gene-62.80-mRNA-1 | Japonica | NA | | | | |
| maker-3-augustus-gene-63.0-mRNA-1 | Japonica | NA | | Os03g0223900 | OsR498G0305481700.01.T01 | UDP-3-O-acyl-N-acetylglucosamine deacetylase |
| maker-3-augustus-gene-63.11-mRNA-1 | Japonica | Indica | | Os03g0224501 | OsR498G0305484500.01.T01 | |
| maker-3-augustus-gene-63.14-mRNA-1 | Japonica | NA | | Os03g0224700 | OsR498G0305484900.01.T03 | Heat stress transcription factor A-9 |
| maker-3-augustus-gene-63.16-mRNA-1 | Japonica | NA | | | | PLATZ transcription factor family protein |
| maker-3-augustus-gene-63.18-mRNA-1 | Japonica | NA | | Os03g0225500 | OsR498G0305487700.01.T01 | Nuclear pore complex protein NUP133 |
| maker-3-augustus-gene-63.21-mRNA-1 | Japonica | Indica | | Os03g0224300 | OsR498G0305483400.01.T02 | Trehalose phosphate synthase |
| maker-3-augustus-gene-63.23-mRNA-1 | Japonica | NA | | Os03g0225200 | OsR498G0305487000.01.T05 | Cyclin N-terminal domain-containing protein (Fragment) |
| maker-3-augustus-gene-63.6-mRNA-1 | Japonica | NA | | Os03g0224200 | OsR498G0305483100.01.T03 | Two-component response regulator ORR21 |
| maker-3-augustus-gene-64.0-mRNA-1 | Japonica | NA | | | OsR498G0305487900.01.T03 | Conserved oligomeric Golgi complex subunit 6 |
| maker-3-augustus-gene-64.10-mRNA-1 | Japonica | Japonica | | Os03g0226400 | OsR498G0305490500.01.T01 | Metal tolerance protein 4 |
| maker-3-augustus-gene-64.15-mRNA-1 | Japonica | NA | | Os03g0226600 | OsR498G0305491100.01.T01 | |
| maker-3-augustus-gene-64.25-mRNA-1 | Japonica | NA | Kinase-TM | | OsR498G0305491900.01.T01 | Proline-rich receptor-like protein kinase PERK4 isoform A |
| maker-3-augustus-gene-64.27-mRNA-1 | Japonica | NA | | Os05g0471800 | | Far-red impaired response protein-like |
| maker-3-augustus-gene-64.30-mRNA-1 | Japonica | NA | | Os03g0226200 | OsR498G0305489600.01.T01 | Non-symbiotic hemoglobin |
| maker-3-augustus-gene-64.32-mRNA-1 | Japonica | NA | | Os03g0226700 | OsR498G0305491400.01.T01 | |
| maker-3-augustus-gene-64.4-mRNA-1 | Japonica | NA | Kinase-TM | Os03g0225700 | OsR498G0305488100.01.T01 | Wall-associated receptor kinase |
| maker-3-augustus-gene-64.6-mRNA-1 | Japonica | NA | | | | |
| maker-3-augustus-gene-64.8-mRNA-1 | Japonica | NA | Kinase-TM | Os03g0226300 | OsR498G0305489900.01.T01 | Pto kinase interactor |
| maker-3-augustus-gene-65.0-mRNA-1 | Japonica | NA | | Os03g0227000 | OsR498G0305492100.01.T03 | Coatomer subunit gamma |
| maker-3-augustus-gene-65.14-mRNA-1 | Japonica | NA | | Os03g0227500 | OsR498G0305494100.01.T01 | |
| maker-3-augustus-gene-65.16-mRNA-1 | Japonica | NA | | Os03g0227700 | OsR498G0305496100.01.T01 | Brassinosteroid synthesis1 |
| maker-3-augustus-gene-65.20-mRNA-1 | Japonica | NA | Kinase-TM | Os03g0227900 | OsR498G0305497400.01.T02 | probable serine/threonine-protein kinase At1g01540 |
| maker-3-augustus-gene-65.7-mRNA-1 | Japonica | NA | | Os03g0227300 | OsR498G0305493300.01.T02 | DNA-directed DNA polymerase |
| maker-3-augustus-gene-66.0-mRNA-1 | Japonica | NA | | Os03g0228400 | OsR498G0305498900.01.T01 | AP complex subunit sigma |
| maker-3-augustus-gene-66.10-mRNA-1 | Japonica | NA | | Os03g0227966 | OsR498G0305505200.01.T01 | Protein DETOXIFICATION |
| maker-3-augustus-gene-66.4-mRNA-1 | Japonica | NA | | Os03g0228500 | OsR498G0305499100.01.T01 | |
| maker-3-augustus-gene-66.6-mRNA-1 | Japonica | NA | | Os03g0228200 | OsR498G0305498600.01.T03 | Erythronate-4-phosphate dehydrogenase family protein isoform 1 |
| maker-3-augustus-gene-66.8-mRNA-1 | Japonica | NA | Kinase-LRR-TM | Os03g0228800 | OsR498G0305501000.01.T01 | Leucine-rich repeat receptor-like serine/threonine-protein kinase BAM1 |
| maker-3-augustus-gene-67.14-mRNA-1 | Japonica | NA | | | OsR498G0305506000.01.T01 | Maf family protein, putative, expressed |
| maker-3-augustus-gene-67.20-mRNA-1 | Japonica | NA | | Os03g0231700 | OsR498G0305514600.01.T05 | Squalene monooxygenase |
| maker-3-augustus-gene-67.23-mRNA-1 | Japonica | Indica | | Os03g0231800 | OsR498G0305514800.01.T01 | Squalene monooxygenase |
| maker-3-augustus-gene-68.0-mRNA-1 | Japonica | NA | | Os03g0232400 | OsR498G0305519700.01.T01 | Phytosulfokine-alpha |
| maker-3-augustus-gene-68.10-mRNA-1 | Japonica | NA | | Os03g0232200 | OsR498G0305517400.01.T02 | AP2-like ethylene-responsive transcription factor ANT |
| maker-3-augustus-gene-68.14-mRNA-1 | Japonica | NA | | Os03g0232800 | OsR498G0305522200.01.T01 | 1-O-acylceramide synthase |
| maker-3-augustus-gene-68.16-mRNA-1 | Japonica | NA | | Os03g0233200 | OsR498G0305524800.01.T01 | Pathogenesis-related protein 5 |
| maker-3-augustus-gene-68.2-mRNA-1 | Japonica | NA | | Os03g0232500 | OsR498G0305520300.01.T04 | EngB-type G domain-containing protein (Fragment) |
| maker-3-augustus-gene-68.5-mRNA-1 | Japonica | NA | | Os03g0233000 | OsR498G0305524100.01.T01 | Calcium uniporter protein 2, mitochondrial |
| maker-3-augustus-gene-68.7-mRNA-1 | Japonica | NA | | | OsR498G0305516600.01.T02 | Transcription factor bHLH62 |
| maker-3-augustus-gene-69.43-mRNA-1 | Japonica | NA | | Os03g0233500 | OsR498G0305525500.01.T01 | probable E3 ubiquitin-protein ligase ARI2 |
| maker-3-augustus-gene-69.46-mRNA-1 | Japonica | NA | | Os03g0233800 | OsR498G0305526500.01.T05 | Ternary complex factor MIP1 |
| maker-3-augustus-gene-69.52-mRNA-1 | Japonica | NA | | Os03g0233900 | OsR498G0305526700.01.T01 | Non-symbiotic hemoglobin |
| maker-3-augustus-gene-69.54-mRNA-1 | Japonica | NA | | Os03g0234000 | OsR498G0305526900.01.T01 | GLOBIN domain-containing protein |
| maker-3-augustus-gene-69.56-mRNA-1 | Japonica | NA | | Os03g0234100 | OsR498G0305527100.01.T01 | GLOBIN domain-containing protein |
| maker-3-augustus-gene-69.58-mRNA-1 | Japonica | Indica | | Os10g0109300 | OsR498G0305527500.01.T01 | Peroxidase |
| maker-3-augustus-gene-69.60-mRNA-1 | Japonica | NA | | Os03g0235100 | OsR498G0305528700.01.T03 | LIS motif containing protein |
| maker-3-augustus-gene-69.63-mRNA-1 | Japonica | NA | | | OsR498G0305527800.01.T02 | Transposon protein, putative, unclassified, expressed |
| maker-3-augustus-gene-69.67-mRNA-1 | Japonica | NA | | Os03g0234900 | OsR498G0305528200.01.T01 | Peroxidase |

| | | | | | | |
|---|---|---|---|---|---|---|
| maker-3-augustus-gene-69.68-mRNA-1 | Japonica | NA | | Os03g0235000 | OsR498G0305528200.01.T04 | Peroxidase |
| maker-3-augustus-gene-69.78-mRNA-1 | Japonica | NA | | | OsR498G0305529200.01.T05 | Peptide transporter PTR2 |
| maker-3-augustus-gene-69.82-mRNA-1 | Japonica | NA | | Os03g0235700 | OsR498G0305529600.01.T01 | Proton-dependent oligopeptide transporter |
| maker-3-augustus-gene-69.87-mRNA-1 | Japonica | NA | | Os03g0235900 | OsR498G0305529800.01.T02 | Low affinity nitrate transporter (Fragment) |
| maker-3-augustus-gene-7.0-mRNA-1 | Japonica | Indica | | Os03g0116000 | OsR498G0305121100.01.T01 | Mitochondrial inner membrane protein OXA1 |
| maker-3-augustus-gene-7.11-mRNA-1 | Japonica | NA | | Os03g0116400 | OsR498G0305122300.01.T01 | RHOMBOID-like protein (Fragment) |
| maker-3-augustus-gene-7.14-mRNA-1 | Japonica | NA | | Os03g0116700 | OsR498G0305123300.01.T01 | F-box family protein, putative, expressed |
| maker-3-augustus-gene-7.17-mRNA-1 | Japonica | NA | | Os03g0116800 | OsR498G0305123500.01.T01 | F-box family protein, putative, expressed |
| maker-3-augustus-gene-7.22-mRNA-1 | Japonica | NA | | Os03g0117200 | OsR498G0305124300.01.T01 | HTP reductase |
| maker-3-augustus-gene-7.25-mRNA-1 | Japonica | NA | | Os03g0117800 | OsR498G0305125100.01.T02 | Protein-ribulosamine 3-kinase, chloroplastic |
| maker-3-augustus-gene-7.28-mRNA-1 | Japonica | NA | | Os03g0118100 | OsR498G0305125700.01.T01 | |
| maker-3-augustus-gene-7.30-mRNA-1 | Japonica | NA | | Os03g0118200 | OsR498G0305126100.01.T03 | UDP-galactose/UDP-glucose transporter like |
| maker-3-augustus-gene-7.34-mRNA-1 | Japonica | NA | | Os03g0116500 | OsR498G0305122600.01.T01 | COP9 signalosome complex subunit |
| maker-3-augustus-gene-7.37-mRNA-1 | Japonica | Indica | | Os03g0118600 | OsR498G0305124000.01.T02 | Dihydrodipicolinate reductase |
| maker-3-augustus-gene-7.44-mRNA-1 | Japonica | NA | | Os03g0117100 | OsR498G0305124200.01.T02 | Peroxisomal biogenesis factor |
| maker-3-augustus-gene-70.25-mRNA-1 | Japonica | NA | | Os03g0236200 | OsR498G0305530900.01.T01 | Glutamate decarboxylase |
| maker-3-augustus-gene-70.29-mRNA-1 | Japonica | NA | | | OsR498G0305533900.01.T01 | Anaphase promoting complex/cyclosome subunit |
| maker-3-augustus-gene-70.34-mRNA-1 | Japonica | NA | | Os03g0237000 | OsR498G0305534100.01.T01 | Transmembrane 9 superfamily member |
| maker-3-augustus-gene-70.38-mRNA-1 | Japonica | NA | | Os03g0237250 | | Protein SHOOT GRAVITROPISM 5 |
| maker-3-augustus-gene-71.0-mRNA-1 | Japonica | NA | | Os03g0238600 | OsR498G0305541500.01.T01 | Purple acid phosphatase |
| maker-3-augustus-gene-71.10-mRNA-1 | Japonica | NA | | Os03g0237900 | OsR498G0305538400.01.T01 | Cyclin N-terminal domain-containing protein (Fragment) |
| maker-3-augustus-gene-71.14-mRNA-1 | Japonica | NA | | Os03g0238300 | OsR498G0305539600.01.T01 | Type I inositol polyphosphate 5-phosphatase |
| maker-3-augustus-gene-71.16-mRNA-1 | Japonica | Indica | | Os03g0238700 | OsR498G0305541800.01.T01 | Procollagen-proline 3-dioxygenase |
| maker-3-augustus-gene-71.2-mRNA-1 | Japonica | NA | | Os03g0239200 | OsR498G0305542900.01.T01 | CONSTANS interacting protein 6 |
| maker-3-augustus-gene-71.20-mRNA-1 | Japonica | Indica | | Os03g0238800 | OsR498G0305542000.01.T01 | Hydroxyproline-rich glycoprotein family protein, putative, expressed |
| maker-3-augustus-gene-71.25-mRNA-1 | Japonica | Indica | | Os03g0239000 | OsR498G0305542400.01.T01 | Xyloglucan endotransglucosylase/hydrolase |
| maker-3-augustus-gene-71.28-mRNA-1 | Japonica | NA | | Os03g0239400 | OsR498G0305543400.01.T02 | MFMR domain-containing protein (Fragment) |
| maker-3-augustus-gene-71.4-mRNA-1 | Japonica | NA | | Os03g0237600 | OsR498G0305537200.01.T03 | 65-kDa microtubule-associated protein 7 |
| maker-3-augustus-gene-72.14-mRNA-1 | Japonica | NA | | Os03g0240900 | OsR498G0305547000.01.T01 | Misato Segment II tubulin-like domain |
| maker-3-augustus-gene-72.15-mRNA-1 | Japonica | NA | | Os03g0240900 | OsR498G0305547000.01.T02 | HTH myb-type domain-containing protein |
| maker-3-augustus-gene-72.3-mRNA-1 | Japonica | NA | | Os03g0240500 | OsR498G0305546200.01.T03 | Translocase of chloroplast |
| maker-3-augustus-gene-72.9-mRNA-1 | Japonica | NA | | Os03g0240700 | OsR498G0305546600.01.T02 | Endoplasmic reticulum transmembrane protein |
| maker-3-augustus-gene-73.0-mRNA-1 | Japonica | NA | | Os03g0241100 | OsR498G0305547500.01.T02 | SANT domain-containing protein |
| maker-3-augustus-gene-73.11-mRNA-1 | Japonica | Indica | | Os03g0241400 | OsR498G0305547900.01.T01 | Stress inducible protein coi6.1 |
| maker-3-augustus-gene-73.14-mRNA-1 | Japonica | NA | | | | Cell cycle checkpoint protein RAD17 |
| maker-3-augustus-gene-73.20-mRNA-1 | Japonica | Indica | | Os03g0242200 | OsR498G0305550300.01.T05 | GTP-binding protein, putative, expressed |
| maker-3-augustus-gene-73.26-mRNA-1 | Japonica | NA | Kinase-TM | | OsR498G0305548400.01.T03 | serine/threonine-protein kinase RIPK-like isoform X1 |
| maker-3-augustus-gene-73.32-mRNA-1 | Japonica | Japonica | | Os03g0241800 | OsR498G0305549600.01.T04 | Pentatricopeptide repeat-containing protein, putative |
| maker-3-augustus-gene-73.34-mRNA-1 | Japonica | NA | | Os03g0241900 | OsR498G0305549800.01.T01 | Senescence domain-containing protein (Fragment) |
| maker-3-augustus-gene-73.37-mRNA-1 | Japonica | Indica | | Os03g0243100 | OsR498G0305553600.01.T04 | Actin-depolymerizing factor 5 like |
| maker-3-augustus-gene-73.7-mRNA-1 | Japonica | NA | | Os03g0241200 | OsR498G0305547700.01.T01 | Ribonucloprotein (Fragment) |
| maker-3-augustus-gene-74.0-mRNA-1 | Japonica | NA | | Os03g0243600 | OsR498G0305555700.01.T02 | ACB domain-containing protein |
| maker-3-augustus-gene-74.20-mRNA-1 | Japonica | NA | | Os03g0244000 | OsR498G0305556500.01.T08 | Protein disulfide-isomerase LQY1, chloroplastic isoform A |
| maker-3-augustus-gene-74.27-mRNA-1 | Japonica | NA | | Os03g0244600 | OsR498G0305557700.01.T01 | Auxin import carrier1 |
| maker-3-augustus-gene-74.29-mRNA-1 | Japonica | NA | | Os01g0874300 | OsR498G0409243500.01.T01 | Transcription factor myb-like protein (Fragment) |
| maker-3-augustus-gene-74.31-mRNA-1 | Japonica | NA | | | | Beta-Casp domain-containing protein |
| maker-3-augustus-gene-74.38-mRNA-1 | Japonica | NA | | Os03g0243300 | OsR498G0305555000.01.T02 | Proteasome subunit Rpn |
| maker-3-augustus-gene-74.4-mRNA-1 | Japonica | Indica | | Os03g0243700 | OsR498G0305556100.01.T07 | GH10 domain-containing protein (Fragment) |
| maker-3-augustus-gene-74.42-mRNA-1 | Japonica | NA | | Os03g0243900 | OsR498G0305556400.01.T01 | Thaumatin-like protein 1 |
| maker-3-augustus-gene-74.5-mRNA-1 | Japonica | NA | | | | Calmodulin-lysine N-methyltransferase |
| maker-3-augustus-gene-75.0-mRNA-1 | Japonica | NA | | Os03g0245100 | OsR498G0305559500.01.T03 | 4-hydroxy-tetrahydrodipicolinate reductase |
| maker-3-augustus-gene-75.10-mRNA-1 | Japonica | NA | | Os03g0245200 | OsR498G0305559800.01.T01 | Proline-rich protein 4 |
| maker-3-augustus-gene-75.12-mRNA-1 | Japonica | NA | | Os03g0245300 | OsR498G0305560000.01.T01 | Proline-rich protein, putative |
| maker-3-augustus-gene-75.14-mRNA-1 | Japonica | NA | | | OsR498G0305562200.01.T01 | RRM domain-containing protein |
| maker-3-augustus-gene-75.16-mRNA-1 | Japonica | NA | | Os03g0246800 | OsR498G0305566600.01.T04 | Guanine-nucleotide-exchange protein |
| maker-3-augustus-gene-75.19-mRNA-1 | Japonica | NA | | Os03g0246900 | OsR498G0305567000.01.T01 | LOB domain-containing protein 18 |
| maker-3-augustus-gene-75.5-mRNA-1 | Japonica | NA | | Os03g0246500 | OsR498G0305565900.01.T03 | Filament-like plant protein 4 |
| maker-3-augustus-gene-76.0-mRNA-1 | Japonica | NA | | Os03g0247300 | OsR498G0305572100.01.T01 | |
| maker-3-augustus-gene-76.14-mRNA-1 | Japonica | NA | | Os03g0248000 | OsR498G0305574600.01.T02 | Autophagy-related protein 9 |
| maker-3-augustus-gene-76.18-mRNA-1 | Japonica | Indica | | Os03g0248200 | OsR498G0305575000.01.T02 | Geraniol 8-hydroxylase |
| maker-3-augustus-gene-76.2-mRNA-1 | Japonica | NA | | Os03g0247400 | | |

| | | | | | | |
|---|---|---|---|---|---|---|
| maker-3-augustus-gene-76.21-mRNA-1 | Japonica | Indica | | Os03g0248200 | OsR498G0305575800.01.T01 | Cytochrome P450 family 76 subfamily C polypeptide 7 |
| maker-3-augustus-gene-76.23-mRNA-1 | Japonica | NA | | Os03g0248600 | OsR498G0305576600.01.T01 | Phosphopyruvate hydratase |
| maker-3-augustus-gene-76.4-mRNA-1 | Japonica | NA | | | OsR498G0305573700.01.T01 | Amino acid binding protein, putative |
| maker-3-augustus-gene-76.8-mRNA-1 | Japonica | NA | | | | |
| maker-3-augustus-gene-76.9-mRNA-1 | Japonica | NA | | Os03g0247000 | OsR498G0305567600.01.T01 | Clp amino terminal domain containing protein, expressed |
| maker-3-augustus-gene-77.34-mRNA-1 | Japonica | NA | | Os03g0249100 | OsR498G0305577700.01.T01 | IRK-interacting protein |
| maker-3-augustus-gene-77.36-mRNA-1 | Japonica | NA | | Os03g0249200 | OsR498G0305577900.01.T03 | Immature colon carcinoma transcript 1 protein, putative, expressed |
| maker-3-augustus-gene-77.38-mRNA-1 | Japonica | NA | | Os03g0249300 | OsR498G0305578100.01.T01 | Chloride conductance regulatory protein ICln |
| maker-3-augustus-gene-77.41-mRNA-1 | Japonica | NA | | Os03g0249400 | OsR498G0305578300.01.T01 | 40S ribosomal protein S20 |
| maker-3-augustus-gene-77.44-mRNA-1 | Japonica | Indica | | Os10g0115550 | OsR498G0305579100.01.T01 | Gibberellin-regulated protein 12 |
| maker-3-augustus-gene-77.46-mRNA-1 | Japonica | NA | | Os03g0249900 | OsR498G0305580700.01.T01 | Transcriptional regulator ATRX isoform X1 |
| maker-3-augustus-gene-77.49-mRNA-1 | Japonica | NA | | Os03g0250900 | | |
| maker-3-augustus-gene-77.51-mRNA-1 | Japonica | NA | | | OsR498G0305579400.01.T01 | Geraniol 8-hydroxylase |
| maker-3-augustus-gene-77.53-mRNA-1 | Japonica | Indica | | Os10g0177200 | OsR498G0305581000.01.T02 | Glycerol-3-phosphate dehydrogenase-2C mitochondrial |
| maker-3-augustus-gene-77.57-mRNA-1 | Japonica | NA | | | OsR498G0305581600.01.T03 | |
| maker-3-augustus-gene-78.40-mRNA-1 | Japonica | NA | | | OsR498G0305591300.01.T05 | Piwi domain containing protein, expressed |
| maker-3-augustus-gene-78.43-mRNA-1 | Japonica | Indica | | Os07g0409100 | OsR498G0305587400.01.T01 | Synaptotagmin-5 |
| maker-3-augustus-gene-78.45-mRNA-1 | Japonica | NA | | | | Transcription initiation factor IIF subunit alpha |
| maker-3-augustus-gene-78.48-mRNA-1 | Japonica | NA | | Os03g0252800 | OsR498G0305591600.01.T04 | Long chain base biosynthesis protein 1 |
| maker-3-augustus-gene-78.54-mRNA-1 | Japonica | Indica | | Os05g0579600 | OsR498G0305592600.01.T01 | Myb family transcription factor, putative |
| maker-3-augustus-gene-79.34-mRNA-1 | Japonica | NA | | Os03g0254200 | OsR498G0305596500.01.T01 | Tryptophan/tyrosine permease |
| maker-3-augustus-gene-79.40-mRNA-1 | Japonica | NA | | Os03g0253100 | OsR498G0305593200.01.T02 | Phosphomevalonate kinase |
| maker-3-augustus-gene-79.44-mRNA-1 | Japonica | NA | | Os03g0253400 | | MADS-box transcription factor 31 |
| maker-3-augustus-gene-79.46-mRNA-1 | Japonica | NA | | Os03g0253800 | OsR498G0305595600.01.T01 | Mitochondrial import inner membrane translocase subunit |
| maker-3-augustus-gene-79.50-mRNA-1 | Japonica | Indica | CC-LRR-NBS-TM | Os03g0254000 | OsR498G0305596400.01.T01 | Disease resistance protein RPS2 |
| maker-3-augustus-gene-8.0-mRNA-1 | Japonica | NA | Kinase-TM | Os03g0118400 | OsR498G0305126900.01.T01 | Cell division control protein A |
| maker-3-augustus-gene-8.12-mRNA-1 | Japonica | NA | | Os03g0118700 | OsR498G0305127700.01.T01 | Putative mandelonitrile lyase |
| maker-3-augustus-gene-8.14-mRNA-1 | Japonica | NA | | Os03g0118800 | OsR498G0305128500.01.T01 | Hydroxymethylglutaryl-CoA synthase |
| maker-3-augustus-gene-8.20-mRNA-1 | Japonica | NA | | Os03g0118900 | OsR498G0305128700.01.T01 | Ataxin-10 |
| maker-3-augustus-gene-8.22-mRNA-1 | Japonica | NA | | | OsR498G0305132300.01.T04 | 1,3-beta-glucan synthase |
| maker-3-augustus-gene-8.25-mRNA-1 | Japonica | Indica | | Os03g0119500 | OsR498G0305132300.01.T03 | 1,3-beta-glucan synthase |
| maker-3-augustus-gene-8.28-mRNA-1 | Japonica | Japonica | | Os03g0119966 | OsR498G0305133300.01.T01 | NAC domain class transcription factor |
| maker-3-augustus-gene-8.30-mRNA-1 | Japonica | NA | | Os03g0119000 | OsR498G0305129000.01.T01 | Oxoglutarate/iron-dependent dioxygenase |
| maker-3-augustus-gene-8.32-mRNA-1 | Japonica | NA | | Os03g0119100 | OsR498G0305129400.01.T01 | Phospholipase D |
| maker-3-augustus-gene-8.6-mRNA-1 | Japonica | NA | | Os03g0118600 | OsR498G0305127300.01.T02 | Dihydrodipicolinate reductase |
| maker-3-augustus-gene-80.11-mRNA-1 | Japonica | NA | | Os03g0254800 | OsR498G0305598600.01.T01 | Chorismate synthase |
| maker-3-augustus-gene-80.17-mRNA-1 | Japonica | NA | | Os03g0255100 | OsR498G0305600000.01.T02 | Beta-galactosidase |
| maker-3-augustus-gene-80.34-mRNA-1 | Japonica | NA | | Os03g0256400 | OsR498G0305603400.01.T03 | Imidazole glycerol phosphate synthase hisHF |
| maker-3-augustus-gene-80.4-mRNA-1 | Japonica | NA | | Os03g0254700 | OsR498G0305598000.01.T01 | TOPLESS |
| maker-3-augustus-gene-81.0-mRNA-1 | Japonica | NA | | Os03g0257900 | | COX assembly mitochondrial protein (Fragment) |
| maker-3-augustus-gene-81.14-mRNA-1 | Japonica | NA | | Os03g0257500 | OsR498G0305606000.01.T01 | Regulator of Vps4 activity in the MVB pathway protein |
| maker-3-augustus-gene-81.16-mRNA-1 | Japonica | Japonica | | Os03g0257600 | OsR498G0305606200.01.T02 | Regulator of Vps4 activity in the MVB pathway protein |
| maker-3-augustus-gene-82.13-mRNA-1 | Japonica | NA | | Os03g0261500 | OsR498G0305616500.01.T03 | DnaJ subfamily C member like |
| maker-3-augustus-gene-82.5-mRNA-1 | Japonica | NA | | Os03g0259900 | OsR498G0305613300.01.T01 | GTD-binding domain-containing protein (Fragment) |
| maker-3-augustus-gene-82.7-mRNA-1 | Japonica | NA | | Os03g0260600 | OsR498G0305615300.01.T01 | Transcription factor AIG1 |
| maker-3-augustus-gene-82.9-mRNA-1 | Japonica | Indica | | Os03g0261100 | OsR498G0305615900.01.T01 | Secretory phospholipase A2 |
| maker-3-augustus-gene-83.0-mRNA-1 | Japonica | NA | | | OsR498G0305619900.01.T04 | 1-phosphatidylinositol-4-phosphate 5-kinase |
| maker-3-augustus-gene-83.13-mRNA-1 | Japonica | NA | Kinase-TM | | OsR498G0305621000.01.T02 | MAP kinase kinase kinase10 |
| maker-3-augustus-gene-83.17-mRNA-1 | Japonica | NA | | | OsR498G0305621600.01.T04 | p-loop containing nucleoside triphosphate hydrolase superfamily protein |
| maker-3-augustus-gene-83.23-mRNA-1 | Japonica | NA | | Os03g0262400 | OsR498G0305621800.01.T02 | mRNA cap-binding protein (Fragment) |
| maker-3-augustus-gene-83.26-mRNA-1 | Japonica | NA | | | OsR498G0305622000.01.T05 | J domain-containing protein |
| maker-3-augustus-gene-83.5-mRNA-1 | Japonica | NA | | Os03g0262100 | OsR498G0305620700.01.T01 | Mago-bind domain-containing protein |
| maker-3-augustus-gene-83.7-mRNA-1 | Japonica | NA | | | OsR498G0305619800.01.T01 | HEAT repeat family protein, expressed |
| maker-3-augustus-gene-84.48-mRNA-1 | Japonica | Japonica | | Os03g0262700 | OsR498G0305622900.01.T01 | Harpin-induced protein |
| maker-3-augustus-gene-84.54-mRNA-1 | Japonica | Indica | | Os03g0263400 | OsR498G0305625900.01.T02 | Mitochondrial phosphate carrier protein 3 mitochondrial |
| maker-3-augustus-gene-84.58-mRNA-1 | Japonica | NA | | Os03g0263700 | OsR498G0305626500.01.T04 | |
| maker-3-augustus-gene-84.61-mRNA-1 | Japonica | NA | | Os03g0263800 | OsR498G0305626700.01.T01 | CONSTANS interacting protein 4 |
| maker-3-augustus-gene-84.64-mRNA-1 | Japonica | NA | Kinase | | OsR498G0305629300.01.T03 | Putative LRR receptor-like serine/threonine-protein kinase |
| maker-3-augustus-gene-84.68-mRNA-1 | Japonica | NA | | Os03g0263500 | OsR498G0305626200.01.T01 | Splicing factor 3A subunit like |
| maker-3-augustus-gene-84.72-mRNA-1 | Japonica | NA | | Os03g0263600 | OsR498G0305626400.01.T01 | Strictosidine synthase |
| maker-3-augustus-gene-84.74-mRNA-1 | Japonica | NA | | | OsR498G0305627400.01.T04 | LAG1 longevity assurance homolog 2 |

| | | | | | | |
|---|---|---|---|---|---|---|
| maker-3-augustus-gene-84.78-mRNA-1 | Japonica | NA | | Os03g0264400 | OsR498G0305629600.01.T01 | Anthranilate synthase alpha subunit 2, chloroplastic |
| maker-3-augustus-gene-85.33-mRNA-1 | Japonica | NA | | Os03g0265100 | OsR498G0305632700.01.T01 | Sulfoquinovosyl transferase SQD2 |
| maker-3-augustus-gene-85.39-mRNA-1 | Japonica | NA | | Os03g0265300 | OsR498G0305633700.01.T01 | Peroxisomal nicotinamide adenine dinucleotide carrier |
| maker-3-augustus-gene-85.43-mRNA-1 | Japonica | NA | | Os03g0265400 | OsR498G0305633900.01.T02 | 50S ribosomal protein uL |
| maker-3-augustus-gene-85.45-mRNA-1 | Japonica | NA | | Os03g0265500 | OsR498G0305634700.01.T01 | Coronatine-insensitive protein 1 |
| maker-3-augustus-gene-85.48-mRNA-1 | Japonica | NA | | Os03g0265700 | OsR498G0305635300.01.T01 | SH3 domain containing protein, expressed |
| maker-3-augustus-gene-85.60-mRNA-1 | Japonica | NA | | Os03g0266100 | OsR498G0305636300.01.T01 | GATA type zinc finger transcription factor family protein |
| maker-3-augustus-gene-85.63-mRNA-1 | Japonica | Indica | CC-NBS-TM | Os03g0264800 | OsR498G0305631400.01.T02 | AAA domain-containing protein (Fragment) |
| maker-3-augustus-gene-85.66-mRNA-1 | Japonica | NA | | Os03g0265200 | OsR498G0305633000.01.T01 | Ubiquitinyl hydrolase 1 |
| maker-3-augustus-gene-85.68-mRNA-1 | Japonica | NA | | | OsR498G0305635000.01.T03 | Serine/arginine-rich splicing factor SR45a isoform B |
| maker-3-augustus-gene-85.72-mRNA-1 | Japonica | NA | | Os03g0265900 | OsR498G0305635800.01.T01 | Maternal effect embryo arrest 59 |
| maker-3-augustus-gene-85.75-mRNA-1 | Japonica | Indica | | Os03g0266200 | OsR498G0305636600.01.T01 | Phosphopyruvate hydratase |
| maker-3-augustus-gene-86.1-mRNA-1 | Japonica | NA | Kinase-LRR-TM | Os03g0266800 | OsR498G0305638100.01.T01 | LRR receptor-like serine/threonine-protein kinase FEI 1 |
| maker-3-augustus-gene-86.20-mRNA-1 | Japonica | NA | | Os03g0267500 | OsR498G0305641300.01.T01 | Lipase, putative, expressed |
| maker-3-augustus-gene-86.22-mRNA-1 | Japonica | NA | | Os03g0267700 | OsR498G0305642700.01.T01 | Mitochondrial carrier protein, expressed |
| maker-3-augustus-gene-86.27-mRNA-1 | Japonica | NA | | Os03g0268000 | OsR498G0305643500.01.T06 | Phosphoprotein phosphatase |
| maker-3-augustus-gene-86.30-mRNA-1 | Japonica | NA | | Os03g0267300 | OsR498G0305639600.01.T01 | D-fructose-1,6-bisphosphate 1-phosphohydrolase |
| maker-3-augustus-gene-86.32-mRNA-1 | Japonica | NA | | Os03g0267600 | OsR498G0305641600.01.T03 | |
| maker-3-augustus-gene-86.34-mRNA-1 | Japonica | NA | | Os03g0267800 | OsR498G0305643000.01.T03 | LIM zinc-binding domain-containing protein (Fragment) |
| maker-3-augustus-gene-86.38-mRNA-1 | Japonica | NA | | Os03g0268100 | OsR498G0305643800.01.T03 | tropomyosin isoform X2 |
| maker-3-augustus-gene-86.45-mRNA-1 | Japonica | NA | | | OsR498G0305644000.01.T01 | Serine/threonine-protein kinase ATG1c |
| maker-3-augustus-gene-86.49-mRNA-1 | Japonica | NA | Kinase-TM | | OsR498G0305644000.01.T04 | Serine/threonine-protein kinase ATG1c isoform C |
| maker-3-augustus-gene-86.51-mRNA-1 | Japonica | NA | | Os03g0268300 | OsR498G0305644400.01.T02 | Digalactosyldiacylglycerol synthase |
| maker-3-augustus-gene-86.54-mRNA-1 | Japonica | NA | | Os03g0268400 | OsR498G0305644600.01.T03 | Mannose-1-phosphate guanylyltransferase |
| maker-3-augustus-gene-86.6-mRNA-1 | Japonica | NA | | Os03g0266700 | OsR498G0305637700.01.T01 | SH3 domain-containing protein 1 |
| maker-3-augustus-gene-87.0-mRNA-1 | Japonica | NA | | Os03g0269900 | OsR498G0305650300.01.T01 | Ribulose bisphosphate carboxylase/oxygenase activase 1 |
| maker-3-augustus-gene-87.10-mRNA-1 | Japonica | NA | | Os03g0269000 | OsR498G0305647200.01.T05 | Tropinone reductase |
| maker-3-augustus-gene-87.14-mRNA-1 | Japonica | Indica | | Os03g0269100 | OsR498G0305647600.01.T01 | Tropinone reductase |
| maker-3-augustus-gene-87.18-mRNA-1 | Japonica | NA | Kinase-TM | Os03g0269300 | OsR498G0305649000.01.T01 | Serine/threonine protein kinase |
| maker-3-augustus-gene-87.2-mRNA-1 | Japonica | NA | | Os03g0268600 | OsR498G0305645600.01.T01 | Protein-serine/threonine phosphatase |
| maker-3-augustus-gene-87.5-mRNA-1 | Japonica | NA | | Os03g0268900 | OsR498G0305646600.01.T01 | Tropinone reductase |
| maker-3-augustus-gene-88.44-mRNA-1 | Japonica | NA | | Os03g0270700 | OsR498G0305652300.01.T04 | Hydroxyproline-rich glycoprotein family protein |
| maker-3-augustus-gene-88.47-mRNA-1 | Japonica | NA | | Os03g0271100 | OsR498G0305653500.01.T01 | RNA polymerase sigma factor |
| maker-3-augustus-gene-88.50-mRNA-1 | Japonica | NA | | Os03g0271200 | OsR498G0305653700.01.T01 | Chloroplast envelope protein translocase, IAP |
| maker-3-augustus-gene-88.54-mRNA-1 | Japonica | NA | | | OsR498G0305651800.01.T03 | Splicing factor PWI domain-containing protein |
| maker-3-augustus-gene-88.55-mRNA-1 | Japonica | Indica | | Os03g0270200 | OsR498G0305651800.01.T03 | Splicing factor PWI domain-containing protein |
| maker-3-augustus-gene-88.71-mRNA-1 | Japonica | NA | | Os03g0271400 | OsR498G0305654200.01.T03 | TOG domain-containing protein (Fragment) |
| maker-3-augustus-gene-89.0-mRNA-1 | Japonica | Indica | | Os03g0272300 | OsR498G0305659100.01.T02 | E3 ubiquitin-protein ligase |
| maker-3-augustus-gene-89.14-mRNA-1 | Japonica | NA | | Os03g0273200 | OsR498G0305661600.01.T01 | Laccase |
| maker-3-augustus-gene-89.6-mRNA-1 | Japonica | NA | | Os03g0272400 | OsR498G0305659300.01.T01 | Putative tRNA (Guanine(26)-N(2))-dimethyltransferase 1 |
| maker-3-augustus-gene-89.8-mRNA-1 | Japonica | NA | | Os03g0273800 | OsR498G0305664100.01.T01 | Suppressor of disruption of TFIIS-like protein |
| maker-3-augustus-gene-9.0-mRNA-1 | Japonica | Japonica | | Os03g0120000 | OsR498G0305134500.01.T04 | Putative reverse transcriptase |
| maker-3-augustus-gene-9.12-mRNA-1 | Japonica | NA | | Os03g0120200 | OsR498G0305135000.01.T01 | |
| maker-3-augustus-gene-9.14-mRNA-1 | Japonica | NA | | Os03g0120300 | OsR498G0305135800.01.T03 | Tobamovirus multiplication protein 3 |
| maker-3-augustus-gene-9.19-mRNA-1 | Japonica | NA | | Os03g0120400 | OsR498G0305136000.01.T01 | Putative heavy metal-associated domain, HMA |
| maker-3-augustus-gene-9.2-mRNA-1 | Japonica | NA | | Os03g0120800 | OsR498G0305137100.01.T01 | |
| maker-3-augustus-gene-9.21-mRNA-1 | Japonica | NA | | Os03g0121200 | OsR498G0305140000.01.T01 | Peroxidase |
| maker-3-augustus-gene-9.23-mRNA-1 | Japonica | NA | | Os03g0121300 | OsR498G0305140600.01.T01 | Peroxidase |
| maker-3-augustus-gene-9.25-mRNA-1 | Japonica | NA | | | | Syntaxin of plants SYP5 |
| maker-3-augustus-gene-9.27-mRNA-1 | Japonica | Indica | | Os03g0121200 | OsR498G0305141200.01.T01 | L-ascorbate peroxidase |
| maker-3-augustus-gene-9.4-mRNA-1 | Japonica | NA | | Os03g0121700 | OsR498G0305141300.01.T01 | NAC-A/B domain-containing protein |
| maker-3-augustus-gene-9.7-mRNA-1 | Japonica | NA | | Os03g0120100 | OsR498G0305134800.01.T02 | ALA-interacting subunit |
| maker-3-augustus-gene-90.26-mRNA-1 | Japonica | NA | | Os03g0274000 | OsR498G0305665500.01.T01 | oxysterol-binding protein-related protein 3C-like isoform X2 |
| maker-3-augustus-gene-90.40-mRNA-1 | Japonica | NA | | Os03g0275100 | OsR498G0305670300.01.T01 | Pyruvate dehydrogenase phosphatase |
| maker-3-augustus-gene-90.42-mRNA-1 | Japonica | NA | | Os03g0275400 | OsR498G0305672500.01.T03 | S-acyltransferase |
| maker-3-augustus-gene-90.50-mRNA-1 | Japonica | NA | Kinase | Os03g0274800 | OsR498G0305669400.01.T01 | receptor-like cytoplasmic kinase 176 |
| maker-3-augustus-gene-90.54-mRNA-1 | Japonica | Indica | | Os01g0974400 | OsR498G0305671600.01.T01 | E3 ubiquitin-protein ligase XB3 |
| maker-3-augustus-gene-91.0-mRNA-1 | Japonica | NA | | Os03g0275900 | OsR498G0305674500.01.T01 | RING-type E3 ubiquitin transferase |
| maker-3-augustus-gene-91.14-mRNA-1 | Japonica | Indica | | Os03g0276900 | OsR498G0305679600.01.T01 | Beta-glucuronosyltransferase |
| maker-3-augustus-gene-92.59-mRNA-1 | Japonica | Indica | | Os03g0278300 | OsR498G0305684800.01.T05 | THO complex subunit 4 |
| maker-3-augustus-gene-95.47-mRNA-1 | Japonica | NA | | Os03g0284600 | OsR498G0305711700.01.T01 | Thioredoxin-like protein AAED1, chloroplastic |

| | | | | | | |
|---|---|---|---|---|---|---|
| maker-3-augustus-gene-96.0-mRNA-1 | Japonica | NA | | Os03g0285200 | | |
| maker-3-augustus-gene-96.12-mRNA-1 | Japonica | Indica | | Os03g0286800 | OsR498G0305720700.01.T02 | DDB1-and CUL4-associated factor 8 |
| maker-3-augustus-gene-96.15-mRNA-1 | Japonica | NA | | Os03g0284900 | OsR498G0305713010.01.T01 | Pentatricopeptide repeat-containing protein chloroplastic |
| maker-3-augustus-gene-96.19-mRNA-1 | Japonica | NA | Kinase-TM | Os03g0285800 | OsR498G0305716200.01.T01 | Mitogen-activated protein kinase |
| maker-3-augustus-gene-96.2-mRNA-1 | Japonica | NA | | Os03g0285700 | OsR498G0305715700.01.T02 | Cytosolic ascorbate peroxidase |
| maker-3-augustus-gene-96.24-mRNA-1 | Japonica | NA | | | | Alternative splicing regulator |
| maker-3-augustus-gene-96.28-mRNA-1 | Japonica | NA | | Os03g0286500 | OsR498G0305718800.01.T01 | probable RNA-binding protein ARP1 isoform X2 |
| maker-3-augustus-gene-96.30-mRNA-1 | Japonica | Indica | | Os03g0287100 | OsR498G0305721600.01.T04 | Phosphatidylinositol transfer protein expressed |
| maker-3-augustus-gene-96.36-mRNA-1 | Japonica | NA | | Os03g0287400 | OsR498G0305722400.01.T01 | LOB domain-containing protein 1 |
| maker-3-augustus-gene-96.7-mRNA-1 | Japonica | NA | | Os03g0286200 | OsR498G0305717700.01.T02 | Arogenate dehydratase/prephenate dehydratase 2 chloroplastic |
| maker-3-augustus-gene-97.30-mRNA-1 | Japonica | Indica | | Os03g0826000 | OsR498G0305723500.01.T03 | Phosphatidylinositol N-acetyglucosaminlytransferase subunit P-related |
| maker-3-augustus-gene-97.34-mRNA-1 | Japonica | NA | | Os03g0287800 | OsR498G0305724100.01.T03 | Glycosyltransferases |
| maker-3-augustus-gene-97.40-mRNA-1 | Japonica | NA | | Os03g0288300 | OsR498G0305726100.01.T01 | AB hydrolase-1 domain-containing protein |
| maker-3-augustus-gene-97.44-mRNA-1 | Japonica | NA | | | | Cytochrome c oxidase assembly protein COX |
| maker-3-augustus-gene-97.48-mRNA-1 | Japonica | NA | | | OsR498G0305726700.01.T01 | Pseudouridine synthase |
| maker-3-augustus-gene-97.54-mRNA-1 | Japonica | NA | | Os03g0288600 | OsR498G0305726900.01.T04 | Taxilin |
| maker-3-augustus-gene-97.59-mRNA-1 | Japonica | NA | | Os03g0288800 | OsR498G0305727500.01.T01 | Transmembrane protein 209 |
| maker-3-augustus-gene-97.62-mRNA-1 | Japonica | NA | | Os03g0288900 | OsR498G0305727700.01.T02 | Nascent polypeptide-associated complex subunit alpha, muscle-specific forn |
| maker-3-augustus-gene-97.64-mRNA-1 | Japonica | NA | CC-Kinase-TM | Os03g0289100 | OsR498G0305728500.01.T03 | Serine/threonine protein kinase OSK4 |
| maker-3-augustus-gene-97.71-mRNA-1 | Japonica | NA | | Os03g0287900 | OsR498G0305724400.01.T02 | Thioredoxin domain-containing protein 5 |
| maker-3-augustus-gene-97.74-mRNA-1 | Japonica | NA | | Os03g0288700 | OsR498G0305727200.01.T01 | Lipid phosphate phosphatase gammaic |
| maker-3-augustus-gene-98.25-mRNA-1 | Japonica | NA | | Os03g0289300 | OsR498G0305729500.01.T02 | Phosphoinositide phospholipase C |
| maker-3-augustus-gene-98.29-mRNA-1 | Japonica | Indica | | Os03g0289400 | OsR498G0305729900.01.T01 | Rhodanese-like domain-containing protein 10 |
| maker-3-augustus-gene-98.31-mRNA-1 | Japonica | NA | | Os03g0290900 | OsR498G0305735700.01.T01 | Putative PMR5 domain, PC-Esterase, trichome birefringence-like family |
| maker-3-augustus-gene-98.33-mRNA-1 | Japonica | NA | | Os03g0291200 | OsR498G0305736100.01.T01 | Protein trichome birefringence-like |
| maker-3-augustus-gene-98.36-mRNA-1 | Japonica | NA | | Os03g0289800 | OsR498G0305731000.01.T01 | Jasmonate-regulated gene 21 |
| maker-3-augustus-gene-98.40-mRNA-1 | Japonica | Indica | | Os03g0290300 | OsR498G0305733600.01.T01 | Temperature-sensitive omega-3 fatty acid desaturase, chloroplastic |
| maker-3-augustus-gene-98.45-mRNA-1 | Japonica | Indica | | Os07g0693700 | OsR498G0305734600.01.T01 | V-SNARE coiled-coil homology domain-containing protein |
| maker-3-augustus-gene-98.47-mRNA-1 | Japonica | NA | | Os03g0290500 | OsR498G0305734800.01.T01 | Phosphoinositide phosphatase SAC8 |
| maker-3-augustus-gene-99.25-mRNA-1 | Japonica | NA | | Os03g0291800 | OsR498G0305738500.01.T01 | Protein trichome birefringence-like 28 |
| maker-3-augustus-gene-99.29-mRNA-1 | Japonica | NA | | Os03g0292100 | OsR498G0305739300.01.T01 | PPM-type phosphatase domain-containing protein |
| maker-3-augustus-gene-99.32-mRNA-1 | Japonica | NA | | Os03g0293000 | OsR498G0305742100.01.T01 | J domain-containing protein (Fragment) |
| maker-3-augustus-gene-99.34-mRNA-1 | Japonica | NA | | Os03g0293100 | OsR498G0305742300.01.T02 | UDP-Glycosyltransferase superfamily protein isoform 1 |
| maker-3-augustus-gene-99.37-mRNA-1 | Japonica | NA | NBS-TM | | OsR498G0305742500.01.T01 | Macro domain-containing protein |
| maker-3-augustus-gene-99.40-mRNA-1 | Japonica | NA | | | OsR498G0305736400.01.T01 | Asparagine synthetase [glutamine-hydrolyzing] |
| maker-3-augustus-gene-99.46-mRNA-1 | Japonica | NA | | Os03g0292800 | OsR498G0305741800.01.T01 | Ran-binding protein c |
| maker-3-augustus-gene-99.49-mRNA-1 | Japonica | NA | | | OsR498G0305742000.01.T01 | Importin-beta |
| maker-4-augustus-gene-0.3-mRNA-1 | Japonica | NA | | | OsR498G0816152000.01.T01 | |
| maker-4-augustus-gene-1.0-mRNA-1 | Japonica | NA | | | OsR498G1221334400.01.T01 | |
| maker-4-augustus-gene-10.0-mRNA-1 | Japonica | NA | | Os04g0184100 | OsR498G0407660100.01.T01 | Amidase domain-containing protein (Fragment) |
| maker-4-augustus-gene-10.1-mRNA-1 | Japonica | Indica | | Os04g0183500 | OsR498G0407660700.01.T01 | Amidase domain-containing protein (Fragment) |
| maker-4-augustus-gene-10.12-mRNA-1 | Japonica | Japonica | | Os04g0185100 | OsR498G0407661200.01.T01 | CDT1 domain-containing protein |
| maker-4-augustus-gene-10.6-mRNA-1 | Japonica | Indica | | Os04g0185000 | OsR498G0407660700.01.T01 | Amidase |
| maker-4-augustus-gene-109.8-mRNA-1 | Japonica | NA | | | OsR498G0408213900.01.T02 | 40S ribosomal protein S27 |
| maker-4-augustus-gene-11.0-mRNA-1 | Japonica | NA | | Os04g0186800 | OsR498G0407668300.01.T01 | H(+)/Pi cotransporter |
| maker-4-augustus-gene-110.34-mRNA-1 | Japonica | NA | | | | 40S ribosomal protein S27 |
| maker-4-augustus-gene-110.35-mRNA-1 | Japonica | NA | | | OsR498G0408219300.01.T01 | 40S ribosomal protein S27 |
| maker-4-augustus-gene-110.38-mRNA-1 | Japonica | NA | | | OsR498G0408219900.01.T01 | MAU2 chromatid cohesion factor like |
| maker-4-augustus-gene-118.35-mRNA-1 | Japonica | Indica | | Os08g0114300 | OsR498G0408279200.01.T02 | L-gulonolactone oxidase |
| maker-4-augustus-gene-12.0-mRNA-1 | Japonica | Indica | | Os04g0187800 | OsR498G0407673800.01.T01 | |
| maker-4-augustus-gene-121.10-mRNA-1 | Japonica | NA | | | | B3 domain-containing protein |
| maker-4-augustus-gene-121.4-mRNA-1 | Japonica | NA | | Os02g0530900 | OsR498G0203991600.01.T01 | |
| maker-4-augustus-gene-123.1-mRNA-1 | Japonica | Indica | Kinase-TM | Os04g0366000 | OsR498G0408332800.01.T02 | Calcium binding EGF domain containing protein, expressed |
| maker-4-augustus-gene-124.35-mRNA-1 | Japonica | NA | Kinase-TM | Os04g0368000 | OsR498G0408337400.01.T01 | Wall-associated receptor kinase 2 |
| maker-4-augustus-gene-125.39-mRNA-1 | Japonica | NA | CC-Kinase-TM | | OsR498G0408339000.01.T01 | Jacalin-like lectin domain containing protein |
| maker-4-augustus-gene-13.33-mRNA-1 | Japonica | NA | | Os04g0189400 | OsR498G0407680000.01.T01 | Knot1 domain-containing protein (Fragment) |
| maker-4-augustus-gene-132.0-mRNA-1 | Japonica | NA | | Os04g0380500 | | DUF1618 domain-containing protein (Fragment) |
| maker-4-augustus-gene-138.20-mRNA-1 | Japonica | NA | | Os04g0391900 | OsR498G0408442500.01.T01 | 4-sulfomuconolactone hydrolase |
| maker-4-augustus-gene-138.25-mRNA-1 | Japonica | NA | | | | |
| maker-4-augustus-gene-138.26-mRNA-1 | Japonica | NA | | Os04g0392500 | | GLTSCR1 domain-containing protein |
| maker-4-augustus-gene-138.30-mRNA-1 | Japonica | NA | | | OsR498G0408443600.01.T01 | |

| | | | | | | |
|---|---|---|---|---|---|---|
| maker-4-augustus-gene-139.33-mRNA-1 | Japonica | NA | Kinase-TM | Os04g0393900 | OsR498G0408448500.01.T01 | Serine/threonine-protein kinase NAK |
| maker-4-augustus-gene-139.36-mRNA-1 | Japonica | NA | | Os04g0394100 | OsR498G0408448900.01.T01 | Glycerophosphodiester phosphodiesterase |
| maker-4-augustus-gene-139.37-mRNA-1 | Japonica | NA | | Os04g0394100 | OsR498G0408448900.01.T01 | Glycerophosphodiester phosphodiesterase |
| maker-4-augustus-gene-139.49-mRNA-1 | Japonica | NA | | Os04g0393300 | OsR498G0408447200.01.T01 | Membrane-anchored ubiquitin-fold protein 4-like |
| maker-4-augustus-gene-139.53-mRNA-1 | Japonica | NA | | Os04g0394300 | OsR498G0408449400.01.T01 | Zinc finger CCCH domain-containing protein 27 |
| maker-4-augustus-gene-140.0-mRNA-1 | Japonica | NA | | Os04g0394700 | OsR498G0408451300.01.T01 | BAR domain-containing protein |
| maker-4-augustus-gene-140.25-mRNA-1 | Japonica | Japonica | | Os04g0395466 | OsR498G0306852400.01.T01 | Transposon protein, putative, CACTA, En/Spm sub-class |
| maker-4-augustus-gene-140.27-mRNA-1 | Japonica | NA | | | OsR498G0408453600.01.T01 | |
| maker-4-augustus-gene-140.29-mRNA-1 | Japonica | NA | | Os04g0395600 | OsR498G0408453800.01.T02 | Auxin signaling F-box 2 |
| maker-4-augustus-gene-141.10-mRNA-1 | Japonica | NA | | Os04g0395800 | OsR498G0408455300.01.T01 | Tify domain-containing protein |
| maker-4-augustus-gene-141.13-mRNA-1 | Japonica | NA | | | OsR498G0408459500.01.T01 | E3 ubiquitin protein ligase DRIP2 |
| maker-4-augustus-gene-141.15-mRNA-1 | Japonica | NA | | Os04g0395900 | OsR498G0408455600.01.T01 | Polynucleotide adenylyltransferase family protein |
| maker-4-augustus-gene-142.20-mRNA-1 | Japonica | NA | | Os04g0396800 | OsR498G0408461300.01.T01 | Carboxypeptidase |
| maker-4-augustus-gene-142.25-mRNA-1 | Japonica | NA | | Os04g0397000 | OsR498G0408461900.01.T01 | Dolichyl-diphosphooligosaccharide--protein glycosyltransferase subunit DAD |
| maker-4-augustus-gene-142.28-mRNA-1 | Japonica | NA | | | OsR498G0408462300.01.T01 | |
| maker-4-augustus-gene-142.29-mRNA-1 | Japonica | NA | | Os04g0397700 | OsR498G0408463500.01.T01 | Centromere protein S |
| maker-4-augustus-gene-142.34-mRNA-1 | Japonica | Indica | | Os08g0538000 | OsR498G0408467900.01.T02 | Diadenosine tetraphosphate synthetase |
| maker-4-augustus-gene-142.38-mRNA-1 | Japonica | Indica | | Os12g0230100 | OsR498G0408462200.01.T03 | ATP-dependent Clp protease ATP-binding subunit clpA like CD4B, chloroplas |
| maker-4-augustus-gene-142.47-mRNA-1 | Japonica | NA | | Os04g0397500 | OsR498G0408462600.01.T01 | Trihelix transcription factor ASR3 |
| maker-4-augustus-gene-142.49-mRNA-1 | Japonica | Indica | | Os04g0397901 | OsR498G0408464800.01.T01 | |
| maker-4-augustus-gene-143.0-mRNA-1 | Japonica | NA | | Os04g0398500 | OsR498G0408468300.01.T01 | Cytochrome b-c1 complex subunit Rieske, mitochondrial |
| maker-4-augustus-gene-143.11-mRNA-1 | Japonica | NA | | Os04g0398700 | OsR498G0408469200.01.T01 | Pollen allergen Phl p 11 |
| maker-4-augustus-gene-143.13-mRNA-1 | Japonica | NA | | | OsR498G0408469400.01.T01 | WASH complex subunit strumpellin-like protein |
| maker-4-augustus-gene-143.16-mRNA-1 | Japonica | NA | | Os04g0398800 | OsR498G0408469600.01.T05 | WASH complex subunit strumpellin-like protein |
| maker-4-augustus-gene-143.2-mRNA-1 | Japonica | NA | | Os04g0398600 | OsR498G0408468900.01.T02 | Exostosin domain-containing protein |
| maker-4-augustus-gene-143.4-mRNA-1 | Japonica | Indica | | Os04g0346100 | OsR498G0408469900.01.T01 | 40S ribosomal protein S27 |
| maker-4-augustus-gene-143.7-mRNA-1 | Japonica | NA | | Os04g0399300 | OsR498G0408470900.01.T01 | NUDIX hydrolase domain |
| maker-4-augustus-gene-144.35-mRNA-1 | Japonica | NA | | Os04g0400000 | OsR498G0408473800.01.T01 | Protein PYRICULARIA ORYZAE RESISTANCE 21 |
| maker-4-augustus-gene-144.37-mRNA-1 | Japonica | Japonica | | Os04g0400600 | OsR498G0408475400.01.T01 | |
| maker-4-augustus-gene-144.38-mRNA-1 | Japonica | Indica | | Os04g0400600 | OsR498G0408475800.01.T01 | HMA domain-containing protein |
| maker-4-augustus-gene-144.43-mRNA-1 | Japonica | Japonica | | Os04g0400800 | OsR498G0408476600.01.T02 | |
| maker-4-augustus-gene-144.45-mRNA-1 | Japonica | Japonica | | Os04g0401100 | OsR498G0408478200.01.T01 | Protein PYRICULARIA ORYZAE RESISTANCE 21 |
| maker-4-augustus-gene-144.47-mRNA-1 | Japonica | NA | | Os04g0401300 | OsR498G0408479400.01.T01 | Sucrose nonfermenting 4-like protein |
| maker-4-augustus-gene-144.54-mRNA-1 | Japonica | NA | | | | DDE Tnp4 domain-containing protein |
| maker-4-augustus-gene-145.11-mRNA-1 | Japonica | NA | | Os04g0402100 | OsR498G0408483600.01.T01 | Calnexin |
| maker-4-augustus-gene-145.16-mRNA-1 | Japonica | Japonica | | Os04g0402500 | OsR498G0408485400.01.T02 | Inhibitor of apoptosis-like protein |
| maker-4-augustus-gene-145.4-mRNA-1 | Japonica | NA | | | OsR498G0408485100.01.T04 | OTU domain-containing protein isoform A |
| maker-4-augustus-gene-145.8-mRNA-1 | Japonica | NA | | Os04g0401800 | OsR498G0408482200.01.T03 | DRMBL domain-containing protein |
| maker-4-augustus-gene-15.24-mRNA-1 | Japonica | Indica | | Os03g0558600 | OsR498G0407689300.01.T02 | |
| maker-4-augustus-gene-155.43-mRNA-1 | Japonica | NA | | Os04g0421900 | OsR498G0408565700.01.T01 | Exocyst complex component 2 |
| maker-4-augustus-gene-155.54-mRNA-1 | Japonica | NA | | Os04g0422000 | OsR498G0408565900.01.T01 | probable plastid-lipid-associated protein 7, chloroplastic |
| maker-4-augustus-gene-155.56-mRNA-1 | Japonica | Indica | | Os04g0422600 | OsR498G0408567100.01.T01 | |
| maker-4-augustus-gene-155.62-mRNA-1 | Japonica | Indica | | Os04g0423100 | OsR498G0408568100.01.T01 | 3-hydroxybenzoate 6-hydroxylase (Fragment) |
| maker-4-augustus-gene-155.65-mRNA-1 | Japonica | NA | | Os04g0422300 | OsR498G0408566200.01.T01 | Nascent polypeptide-associated complex subunit beta |
| maker-4-augustus-gene-155.68-mRNA-1 | Japonica | Japonica | | Os04g0422700 | OsR498G0408567400.01.T01 | Leucine-rich repeat extensin-like protein 5 |
| maker-4-augustus-gene-155.71-mRNA-1 | Japonica | NA | | Os04g0423400 | OsR498G0408569000.01.T02 | Abscisic stress ripening |
| maker-4-augustus-gene-155.73-mRNA-1 | Japonica | NA | | | | SET domain-containing protein (Fragment) |
| maker-4-augustus-gene-156.11-mRNA-1 | Japonica | NA | | Os04g0425100 | OsR498G0408572300.01.T05 | CHD3-type chromatin-remodeling factor PICKLE-like isoform X1 |
| maker-4-augustus-gene-156.14-mRNA-1 | Japonica | Japonica | | Os04g0423700 | OsR498G0408569400.01.T01 | Sphingomyelin synthetase family protein isoform 1 |
| maker-4-augustus-gene-158.0-mRNA-1 | Japonica | NA | | Os04g0428900 | OsR498G0408582200.01.T01 | COP9 signalosome complex subunit 8 |
| maker-4-augustus-gene-159.36-mRNA-1 | Japonica | Japonica | | Os04g0429100 | OsR498G0408583100.01.T01 | Histone-lysine N-methyltransferase ASHH1 |
| maker-4-augustus-gene-159.42-mRNA-1 | Japonica | NA | | | OsR498G0408583500.01.T05 | USP domain-containing protein |
| maker-4-augustus-gene-159.50-mRNA-1 | Japonica | Indica | | Os04g0429300 | OsR498G0408583700.01.T01 | BHLH domain-containing protein |
| maker-4-augustus-gene-159.52-mRNA-1 | Japonica | NA | | Os04g0429400 | OsR498G0408583900.01.T01 | Transcription factor bHLH95 like |
| maker-4-augustus-gene-159.60-mRNA-1 | Japonica | Indica | Kinase-TM | Os04g0430000 | OsR498G0408585300.01.T02 | probable serine/threonine-protein kinase At1g01540 |
| maker-4-augustus-gene-159.64-mRNA-1 | Japonica | NA | | Os04g0430100 | OsR498G0408585700.01.T01 | Ribosomal protein S10 |
| maker-4-augustus-gene-159.68-mRNA-1 | Japonica | Indica | Kinase-TM | Os04g0430400 | OsR498G0408586700.01.T01 | Protein kinase domain-containing protein |
| maker-4-augustus-gene-159.72-mRNA-1 | Japonica | NA | | Os04g0429500 | OsR498G0408584200.01.T01 | Cellulose synthase A |
| maker-4-augustus-gene-159.73-mRNA-1 | Japonica | NA | | Os04g0429600 | | Cellulose synthase family protein, expressed |
| maker-4-augustus-gene-159.90-mRNA-1 | Japonica | NA | | Os04g0430200 | OsR498G0408586000.01.T01 | PI-PLC X domain-containing protein |
| maker-4-augustus-gene-16.0-mRNA-1 | Japonica | NA | | | OsR498G0407689900.01.T01 | Methyl-CpG-binding domain-containing protein 13 |

| | | | | | | |
|---|---|---|---|---|---|---|
| maker-4-augustus-gene-16.4-mRNA-1 | Japonica | NA | | | OsR498G0407689900.01.T01 | MBD domain-containing protein |
| maker-4-augustus-gene-160.0-mRNA-1 | Japonica | Japonica | | Os04g0430600 | OsR498G0408587500.01.T01 | Late embryogenesis abundant protein, LEA-14 |
| maker-4-augustus-gene-160.11-mRNA-1 | Japonica | NA | Kinase-TM | Os04g0432000 | OsR498G0408595100.01.T04 | Serine/threonine-protein kinase SAPK7 |
| maker-4-augustus-gene-160.15-mRNA-1 | Japonica | NA | | | | 4-alpha-L-fucosyltransferase |
| maker-4-augustus-gene-160.17-mRNA-1 | Japonica | NA | | Os04g0430800 | | Thioredoxin M3, chloroplastic |
| maker-4-augustus-gene-160.2-mRNA-1 | Japonica | NA | | | OsR498G0408589300.01.T01 | Magnesium transporter |
| maker-4-augustus-gene-160.20-mRNA-1 | Japonica | NA | | Os04g0431100 | OsR498G0408590000.01.T01 | GrpE protein homolog |
| maker-4-augustus-gene-160.22-mRNA-1 | Japonica | NA | | Os04g0431300 | OsR498G0408592800.01.T01 | Acetyltransferase |
| maker-4-augustus-gene-160.23-mRNA-1 | Japonica | NA | | Os04g0431800 | OsR498G0408593400.01.T01 | Brain acid soluble protein 1-like protein |
| maker-4-augustus-gene-160.27-mRNA-1 | Japonica | Japonica | | Os04g0432300 | OsR498G0408597400.01.T03 | DExH-box ATP-dependent RNA helicase DExH7 chloroplastic |
| maker-4-augustus-gene-160.4-mRNA-1 | Japonica | NA | | | OsR498G0408589700.01.T01 | |
| maker-4-augustus-gene-160.7-mRNA-1 | Japonica | NA | | Os04g0431200 | OsR498G0408590100.01.T02 | F-box protein 7 |
| maker-4-augustus-gene-161.0-mRNA-1 | Japonica | NA | | | OsR498G0408600900.01.T06 | Etoposide-induced 2.4 |
| maker-4-augustus-gene-161.13-mRNA-1 | Japonica | NA | | | OsR498G0408602600.01.T01 | |
| maker-4-augustus-gene-161.4-mRNA-1 | Japonica | NA | | Os04g0432400 | OsR498G0408598000.01.T01 | Alkaline/neutral invertase |
| maker-4-augustus-gene-161.7-mRNA-1 | Japonica | NA | | Os04g0432500 | OsR498G0408599000.01.T01 | Protein disulfide isomerase11 |
| maker-4-augustus-gene-161.9-mRNA-1 | Japonica | NA | | Os04g0432900 | OsR498G0408599600.01.T01 | BTB/POZ and MATH domain-containing protein 1 |
| maker-4-augustus-gene-162.41-mRNA-1 | Japonica | NA | | | OsR498G0408603200.01.T01 | ATP-dependent DNA helicase |
| maker-4-augustus-gene-162.50-mRNA-1 | Japonica | NA | | | OsR498G0408603800.01.T01 | Increased DNA methylation 1 |
| maker-4-augustus-gene-162.54-mRNA-1 | Japonica | NA | | Os04g0434300 | OsR498G0408604400.01.T01 | Threonylcarbamoyladenosine tRNA methylthiotransferase |
| maker-4-augustus-gene-162.58-mRNA-1 | Japonica | NA | | | OsR498G0408605800.01.T02 | Kinesin-like protein KIN-7C mitochondrial |
| maker-4-augustus-gene-165.0-mRNA-1 | Japonica | Indica | | Os04g0439100 | OsR498G0408621300.01.T01 | T-complex protein 11 |
| maker-4-augustus-gene-165.11-mRNA-1 | Japonica | NA | | Os04g0439900 | OsR498G0408623000.01.T01 | Protein TIC 40 chloroplastic |
| maker-4-augustus-gene-165.4-mRNA-1 | Japonica | Japonica | | Os04g0439200 | OsR498G0408621500.01.T01 | T-complex protein 11 |
| maker-4-augustus-gene-165.6-mRNA-1 | Japonica | Indica | | Os02g0556900 | OsR498G0408621800.01.T02 | DDT transcription factor |
| maker-4-augustus-gene-166.34-mRNA-1 | Japonica | NA | | Os04g0442000 | OsR498G0408632900.01.T03 | Auxin response factor |
| maker-4-augustus-gene-166.39-mRNA-1 | Japonica | NA | | Os04g0442100 | OsR498G0408633300.01.T02 | Smr protein/MutS2 C-terminal |
| maker-4-augustus-gene-166.41-mRNA-1 | Japonica | NA | | Os04g0442200 | OsR498G0408633500.01.T04 | Monocarboxylate transporter 12 like |
| maker-4-augustus-gene-168.39-mRNA-1 | Japonica | NA | | Os04g0444200 | OsR498G0408647100.01.T05 | Calmodulin-binding protein 60 C |
| maker-4-augustus-gene-168.77-mRNA-1 | Japonica | NA | | | OsR498G0408644200.01.T03 | COX assembly mitochondrial protein (Fragment) |
| maker-4-augustus-gene-168.80-mRNA-1 | Japonica | NA | | | OsR498G0408644600.01.T01 | NAC domain-containing protein 12 |
| maker-4-augustus-gene-168.82-mRNA-1 | Japonica | NA | | | OsR498G0408645400.01.T01 | RuBisCO large subunit-binding protein subunit beta |
| maker-4-augustus-gene-169.0-mRNA-1 | Japonica | NA | | Os04g0444800 | OsR498G0408648900.01.T01 | Ferric reduction oxidase 7 chloroplastic |
| maker-4-augustus-gene-169.10-mRNA-1 | Japonica | NA | | Os04g0445800 | OsR498G0408651700.01.T01 | U6 snRNA-associated Sm-like protein LSm1 |
| maker-4-augustus-gene-169.13-mRNA-1 | Japonica | NA | | Os04g0446000 | OsR498G0408652100.01.T02 | LURP1-related protein domain containing protein |
| maker-4-augustus-gene-169.19-mRNA-1 | Japonica | Japonica | | Os04g0444400 | OsR498G0408647800.01.T03 | Pentatricopeptide repeat-containing protein |
| maker-4-augustus-gene-169.2-mRNA-1 | Japonica | NA | | Os04g0444900 | OsR498G0408649100.01.T01 | PHD finger protein ALFIN-LIKE 5, N-terminally processed like |
| maker-4-augustus-gene-169.24-mRNA-1 | Japonica | Indica | | Os04g0445000 | OsR498G0408649400.01.T01 | Potassium channel SKOR |
| maker-4-augustus-gene-169.27-mRNA-1 | Japonica | NA | | Os04g0445200 | OsR498G0408650000.01.T01 | Enzyme of the cupin superfamily |
| maker-4-augustus-gene-169.29-mRNA-1 | Japonica | NA | | Os04g0445700 | OsR498G0408651600.01.T01 | Beta-ketoacyl-[acyl-carrier-protein] synthase I |
| maker-4-augustus-gene-169.33-mRNA-1 | Japonica | NA | | Os04g0446300 | OsR498G0408653400.01.T01 | Signal peptidase complex, subunit SPC25 |
| maker-4-augustus-gene-169.34-mRNA-1 | Japonica | NA | | Os04g0446300 | OsR498G0408653400.01.T04 | Signal peptidase complex subunit 2 |
| maker-4-augustus-gene-17.0-mRNA-1 | Japonica | NA | | | OsR498G0407689900.01.T01 | MBD domain-containing protein |
| maker-4-augustus-gene-17.15-mRNA-1 | Japonica | NA | | Os04g0194500 | OsR498G0407698800.01.T01 | White-brown-complex ABC transporter family |
| maker-4-augustus-gene-17.5-mRNA-1 | Japonica | NA | | | OsR498G0407699900.01.T01 | |
| maker-4-augustus-gene-17.7-mRNA-1 | Japonica | NA | | Os04g0195000 | OsR498G0407700300.01.T02 | Vacuolar protein sorting-associated protein 41 homolog |
| maker-4-augustus-gene-170.0-mRNA-1 | Japonica | NA | | Os04g0446500 | OsR498G0408653700.01.T02 | Peptidylprolyl isomerase |
| maker-4-augustus-gene-170.15-mRNA-1 | Japonica | NA | | Os04g0447600 | OsR498G0408659900.01.T01 | Non-functional NADPH-dependent codeinone reductase 2 |
| maker-4-augustus-gene-170.19-mRNA-1 | Japonica | Japonica | | Os04g0447700 | OsR498G0408659900.01.T01 | NAD(P)H-dependent 6'-deoxychalcone synthase |
| maker-4-augustus-gene-170.22-mRNA-1 | Japonica | Indica | | Os04g0448000 | OsR498G0408661900.01.T01 | |
| maker-4-augustus-gene-170.24-mRNA-1 | Japonica | NA | | Os04g0448200 | OsR498G0408662500.01.T02 | |
| maker-4-augustus-gene-170.5-mRNA-1 | Japonica | NA | | Os04g0446600 | | |
| maker-4-augustus-gene-171.0-mRNA-1 | Japonica | NA | | Os04g0448800 | OsR498G0408663900.01.T01 | Mitochondrial phosphate carrier protein like |
| maker-4-augustus-gene-171.12-mRNA-1 | Japonica | NA | | Os04g0449000 | OsR498G0408664500.01.T02 | Mitochondrial uncoupling protein 3 |
| maker-4-augustus-gene-171.18-mRNA-1 | Japonica | NA | | Os04g0450000 | OsR498G0408668500.01.T01 | Hepatoma-derived growth factor-related protein 2-like |
| maker-4-augustus-gene-171.20-mRNA-1 | Japonica | NA | | Os04g0450100 | OsR498G0408669700.01.T01 | Phospholipase/carboxyhydrolase |
| maker-4-augustus-gene-171.24-mRNA-1 | Japonica | Indica | | Os04g0450200 | OsR498G0408669900.01.T03 | Pentatricopeptide repeat-containing protein mitochondrial |
| maker-4-augustus-gene-171.26-mRNA-1 | Japonica | NA | | Os04g0450500 | OsR498G0408670900.01.T01 | Nucleic acid-binding |
| maker-4-augustus-gene-171.30-mRNA-1 | Japonica | NA | | Os04g0450600 | OsR498G0408671100.01.T01 | Pterin-binding domain-containing protein |
| maker-4-augustus-gene-171.32-mRNA-1 | Japonica | NA | | Os04g0448600 | OsR498G0408663600.01.T01 | Gamma-glutamylcyclotransferase |
| maker-4-augustus-gene-171.36-mRNA-1 | Japonica | NA | | | OsR498G0408663800.01.T01 | F-box protein FBW2 |

| | | | | | | |
|---|---|---|---|---|---|---|
| maker-4-augustus-gene-171.38-mRNA-1 | Japonica | NA | | Os04g0449100 | OsR498G0408664800.01.T01 | Fucosyltransferase |
| maker-4-augustus-gene-171.40-mRNA-1 | Japonica | NA | | Os04g0449200 | OsR498G0408665000.01.T01 | Fucosyltransferase |
| maker-4-augustus-gene-171.42-mRNA-1 | Japonica | NA | | | OsR498G0408665200.01.T04 | PPM-type phosphatase domain-containing protein |
| maker-4-augustus-gene-171.47-mRNA-1 | Japonica | NA | | Os04g0449500 | OsR498G0408665600.01.T01 | Steroid nuclear receptor ligand-binding |
| maker-4-augustus-gene-171.5-mRNA-1 | Japonica | NA | | Os04g0448900 | OsR498G0408664300.01.T03 | Zeaxanthin epoxidase, chloroplastic |
| maker-4-augustus-gene-172.46-mRNA-1 | Japonica | NA | | Os04g0452100 | OsR498G0408676900.01.T02 | Cryptochrome |
| maker-4-augustus-gene-172.51-mRNA-1 | Japonica | NA | | | OsR498G0408675800.01.T04 | PPM-type phosphatase domain-containing protein |
| maker-4-augustus-gene-172.55-mRNA-1 | Japonica | NA | | | OsR498G0408512800.01.T01 | IQ calmodulin-binding motif family protein, expressed |
| maker-4-augustus-gene-172.59-mRNA-1 | Japonica | NA | | Os04g0452400 | OsR498G0408677600.01.T01 | USP domain-containing protein |
| maker-4-augustus-gene-173.0-mRNA-1 | Japonica | Japonica | | Os04g0452500 | OsR498G0408677700.01.T02 | Synaptonemal complex protein 1 |
| maker-4-augustus-gene-173.10-mRNA-1 | Japonica | NA | | Os04g0453350 | OsR498G0408679700.01.T01 | Sugar transport protein 5 |
| maker-4-augustus-gene-173.13-mRNA-1 | Japonica | NA | | Os04g0453400 | OsR498G0408680000.01.T01 | Sugar transport protein 5 |
| maker-4-augustus-gene-173.4-mRNA-1 | Japonica | NA | | Os04g0452600 | OsR498G0408677900.01.T01 | Sugar transport protein 5 |
| maker-4-augustus-gene-173.6-mRNA-1 | Japonica | NA | | Os04g0452700 | OsR498G0408678300.01.T01 | Sugar transport protein 5 |
| maker-4-augustus-gene-173.8-mRNA-1 | Japonica | NA | | Os04g0453200 | OsR498G0408679300.01.T01 | Sugar transport protein 5 |
| maker-4-augustus-gene-174.0-mRNA-1 | Japonica | NA | | Os04g0454200 | OsR498G0408683900.01.T01 | Sugar transport protein 5 |
| maker-4-augustus-gene-174.2-mRNA-1 | Japonica | NA | | | OsR498G0408684100.01.T01 | |
| maker-4-augustus-gene-175.48-mRNA-1 | Japonica | Indica | | Os04g0456200 | OsR498G0408687300.01.T01 | PLAT domain-containing protein 1 |
| maker-4-augustus-gene-175.50-mRNA-1 | Japonica | Japonica | | Os04g0456700 | OsR498G0408687500.01.T01 | PLAT domain-containing protein 3 |
| maker-4-augustus-gene-175.56-mRNA-1 | Japonica | Indica | | Os04g0457700 | OsR498G0408689900.01.T01 | Epidermal patterning factor |
| maker-4-augustus-gene-175.58-mRNA-1 | Japonica | NA | | Os04g0458300 | OsR498G0408691700.01.T02 | Aldose 1-epimerase |
| maker-4-augustus-gene-175.61-mRNA-1 | Japonica | NA | | Os04g0455800 | OsR498G0408686000.01.T01 | Lipoyl synthase, mitochondrial |
| maker-4-augustus-gene-175.63-mRNA-1 | Japonica | NA | | | OsR498G0408686200.01.T01 | Endoplasmic reticulum-Golgi intermediate compartment protein 3 |
| maker-4-augustus-gene-175.70-mRNA-1 | Japonica | NA | | Os04g0457000 | OsR498G0408688400.01.T01 | Chlorophyll a-b binding protein, chloroplastic |
| maker-4-augustus-gene-175.72-mRNA-1 | Japonica | Indica | | Os04g0457200 | OsR498G0408688600.01.T01 | 11-beta-hydroxysteroid dehydrogenase-like 4B |
| maker-4-augustus-gene-175.74-mRNA-1 | Japonica | NA | | | OsR498G0408689800.01.T01 | Gamma-glutamyltranspeptidase 1 |
| maker-4-augustus-gene-175.77-mRNA-1 | Japonica | NA | Kinase-LRR-TM | Os04g0457800 | OsR498G0408690200.01.T01 | Non-specific serine/threonine protein kinase |
| maker-4-augustus-gene-176.0-mRNA-1 | Japonica | Indica | | Os04g0458600 | OsR498G0408692700.01.T01 | Aldose 1-epimerase |
| maker-4-augustus-gene-176.24-mRNA-1 | Japonica | NA | | Os04g0458700 | | |
| maker-4-augustus-gene-18.0-mRNA-1 | Japonica | NA | | Os04g0196200 | OsR498G0407701900.01.T01 | Acetylserotonin O-methyltransferase 1 |
| maker-4-augustus-gene-18.2-mRNA-1 | Japonica | Indica | | Os04g0196600 | OsR498G0407703600.01.T01 | Putative glycosyltransferase AGO61 |
| maker-4-augustus-gene-181.32-mRNA-1 | Japonica | NA | | Os04g0470100 | OsR498G0408739900.01.T01 | Ras-related protein Rab-2-B |
| maker-4-augustus-gene-181.46-mRNA-1 | Japonica | NA | | | | Small nuclear ribonucleoprotein Sm D1 |
| maker-4-augustus-gene-182.43-mRNA-1 | Japonica | NA | | | OsR498G0408741600.01.T01 | Transcription factor MYB80 |
| maker-4-augustus-gene-182.46-mRNA-1 | Japonica | Indica | | Os04g0470700 | OsR498G0408741800.01.T02 | Amino acid transporter, transmembrane |
| maker-4-augustus-gene-182.47-mRNA-1 | Japonica | Indica | | Os04g0470700 | OsR498G0408741800.01.T06 | Amino acid permease 2 |
| maker-4-augustus-gene-182.62-mRNA-1 | Japonica | NA | | Os06g0190900 | | CAI-1 autoinducer sensor kinase/phosphatase cqsS isoform 1 |
| maker-4-augustus-gene-182.65-mRNA-1 | Japonica | NA | | Os04g0471300 | OsR498G0408743600.01.T01 | WPP domain-interacting protein 2 |
| maker-4-augustus-gene-182.67-mRNA-1 | Japonica | NA | | Os04g0471400 | OsR498G0408744600.01.T03 | Hapless 8 |
| maker-4-augustus-gene-182.69-mRNA-1 | Japonica | NA | | Os04g0471700 | OsR498G0408746400.01.T03 | WRKY transcription factor |
| maker-4-augustus-gene-182.73-mRNA-1 | Japonica | NA | | | OsR498G0408747600.01.T01 | FAS1 domain-containing protein |
| maker-4-augustus-gene-183.0-mRNA-1 | Japonica | NA | | Os04g0472300 | OsR498G0408748500.01.T02 | Glycerophosphodiester phosphodiesterase |
| maker-4-augustus-gene-183.11-mRNA-1 | Japonica | Japonica | | Os04g0472700 | OsR498G0408749700.01.T01 | |
| maker-4-augustus-gene-183.14-mRNA-1 | Japonica | NA | | Os04g0473400 | OsR498G0408753000.01.T01 | 60S ribosomal protein L6 |
| maker-4-augustus-gene-183.16-mRNA-1 | Japonica | NA | | | OsR498G0408753200.01.T01 | |
| maker-4-augustus-gene-183.3-mRNA-1 | Japonica | NA | | | OsR498G0408749100.01.T02 | RNA binding (RRM/RBD/RNP motifs) family protein |
| maker-4-augustus-gene-183.9-mRNA-1 | Japonica | NA | Kinase-LRR-TM | Os04g0472500 | OsR498G0408749300.01.T01 | Inactive leucine-rich repeat receptor-like protein kinase |
| maker-4-augustus-gene-184.36-mRNA-1 | Japonica | NA | | | OsR498G0100584200.01.T01 | IF rod domain-containing protein |
| maker-4-augustus-gene-184.39-mRNA-1 | Japonica | NA | | | OsR498G0408758900.01.T01 | BRX domain-containing protein |
| maker-4-augustus-gene-184.42-mRNA-1 | Japonica | Japonica | | Os04g0473900 | OsR498G0408754200.01.T01 | 4-coumarate--CoA ligase |
| maker-4-augustus-gene-184.45-mRNA-1 | Japonica | Indica | | Os04g0474800 | OsR498G0408754800.01.T01 | Non-cyanogenic beta-glucosidase |
| maker-4-augustus-gene-184.47-mRNA-1 | Japonica | Indica | | Os04g0474500 | OsR498G0408754800.01.T03 | 4-hydroxy-7-methoxy-3-oxo-3,4-dihydro-2H-1,4-benzoxazin-2-yl glucosidebe |
| maker-4-augustus-gene-184.49-mRNA-1 | Japonica | Japonica | | Os04g0474500 | OsR498G0408756000.01.T03 | Beta-glucosidase 12 |
| maker-4-augustus-gene-184.50-mRNA-1 | Japonica | Japonica | | Os04g0474500 | OsR498G0408756000.01.T03 | Beta-glucosidase 12 |
| maker-4-augustus-gene-184.53-mRNA-1 | Japonica | Indica | | Os04g0474500 | OsR498G0408756000.01.T03 | Beta-glucosidase 12 |
| maker-4-augustus-gene-184.61-mRNA-1 | Japonica | NA | | Os04g0474900 | OsR498G0408757800.01.T03 | 4-hydroxy-7-methoxy-3-oxo-3,4-dihydro-2H-1,4-benzoxazin-2-yl glucosidebe |
| maker-4-augustus-gene-184.63-mRNA-1 | Japonica | NA | | Os04g0475500 | OsR498G0408759800.01.T01 | protein THYLAKOID ASSEMBLY 8, chloroplastic |
| maker-4-augustus-gene-185.0-mRNA-1 | Japonica | NA | | Os04g0476000 | OsR498G0408761700.01.T01 | F-box protein isoform A |
| maker-4-augustus-gene-185.10-mRNA-1 | Japonica | NA | | | OsR498G0408763700.01.T01 | C2 calcium-dependent membrane targeting |
| maker-4-augustus-gene-185.12-mRNA-1 | Japonica | Indica | CC-LRR | Os04g0476700 | OsR498G0408763900.01.T01 | Plant intracellular Ras-group-related LRR protein 3 |
| maker-4-augustus-gene-185.15-mRNA-1 | Japonica | Japonica | | Os04g0477000 | OsR498G0408764700.01.T04 | NPH3 domain-containing protein (Fragment) |

| | | | | | | |
|---|---|---|---|---|---|---|
| maker-4-augustus-gene-185.19-mRNA-1 | Japonica | NA | | Os04g0475600 | OsR498G0408760000.01.T01 | 2-oxoglutarate-dependent dioxygenase DAO |
| maker-4-augustus-gene-185.24-mRNA-1 | Japonica | Indica | | Os04g0476100 | OsR498G0408762000.01.T01 | Amine oxidase |
| maker-4-augustus-gene-185.35-mRNA-1 | Japonica | Indica | | Os02g0594800 | OsR498G0408765200.01.T02 | NAC domain-containing protein 8 |
| maker-4-augustus-gene-185.4-mRNA-1 | Japonica | NA | | | OsR498G0408763100.01.T01 | Ribonuclease H2 subunit B |
| maker-4-augustus-gene-186.37-mRNA-1 | Japonica | Indica | | Os04g0479000 | OsR498G0408766900.01.T02 | Pachytene checkpoint protein 2 like |
| maker-4-augustus-gene-186.41-mRNA-1 | Japonica | NA | | Os04g0477500 | OsR498G0408765800.01.T01 | beta-1,4-mannosyl-glycoprotein 4-beta-N-acetylglucosaminyltransferase-like |
| maker-4-augustus-gene-186.44-mRNA-1 | Japonica | NA | | Os04g0479100 | OsR498G0408767200.01.T01 | Transglutaminase |
| maker-4-augustus-gene-186.47-mRNA-1 | Japonica | NA | | Os04g0479200 | OsR498G0408767400.01.T01 | Isocitrate dehydrogenase |
| maker-4-augustus-gene-186.49-mRNA-1 | Japonica | NA | | Os04g0479300 | OsR498G0408767400.01.T03 | |
| maker-4-augustus-gene-186.52-mRNA-1 | Japonica | NA | | Os04g0479500 | OsR498G0408768000.01.T03 | FBD domain-containing protein |
| maker-4-augustus-gene-187.0-mRNA-1 | Japonica | NA | | Os04g0479800 | OsR498G0408768100.01.T01 | FBD domain-containing protein |
| maker-4-augustus-gene-187.12-mRNA-1 | Japonica | NA | LRR-TM | Os04g0480500 | OsR498G0408770900.01.T01 | Serine/threonine-protein kinase BRI1-like protein 2 |
| maker-4-augustus-gene-187.14-mRNA-1 | Japonica | NA | | Os04g0480900 | OsR498G0408772500.01.T02 | Mannan endo-1,4-beta-mannosidase |
| maker-4-augustus-gene-187.18-mRNA-1 | Japonica | Indica | | Os04g0480900 | OsR498G0408772900.01.T01 | Mannan endo-1,4-beta-mannosidase |
| maker-4-augustus-gene-187.20-mRNA-1 | Japonica | NA | | Os04g0481300 | OsR498G0408773700.01.T01 | 31-kDa RNA binding protein |
| maker-4-augustus-gene-187.22-mRNA-1 | Japonica | Indica | | Os04g0481700 | OsR498G0408776100.01.T02 | p-glycoprotein 11 |
| maker-4-augustus-gene-187.25-mRNA-1 | Japonica | NA | | Os04g0480200 | OsR498G0408770000.01.T01 | High-affinity nitrate transporter |
| maker-4-augustus-gene-187.27-mRNA-1 | Japonica | NA | | Os04g0480700 | OsR498G0408771400.01.T01 | Cytochrome P450, E-class, group I |
| maker-4-augustus-gene-187.29-mRNA-1 | Japonica | NA | | Os04g0480700 | OsR498G0408771800.01.T01 | Cytochrome P450, family 71, subfamily B, polypeptide 37 |
| maker-4-augustus-gene-187.3-mRNA-1 | Japonica | Indica | | Os04g0480100 | OsR498G0408769300.01.T01 | Eukaryotic translation initiation factor 4B |
| maker-4-augustus-gene-187.31-mRNA-1 | Japonica | NA | | Os04g0481100 | OsR498G0408773400.01.T01 | Galactinol--sucrose galactosyltransferase |
| maker-4-augustus-gene-187.37-mRNA-1 | Japonica | Indica | | Os04g0481400 | OsR498G0408774000.01.T01 | Protein-L-isoaspartate(D-aspartate) O-methyltransferase |
| maker-4-augustus-gene-187.39-mRNA-1 | Japonica | Indica | | Os04g0481500 | OsR498G0408774200.01.T01 | Centromere protein like |
| maker-4-augustus-gene-187.5-mRNA-1 | Japonica | NA | | Os04g0480300 | OsR498G0408770300.01.T01 | SWI/SNF complex subunit SWI3A |
| maker-4-augustus-gene-187.9-mRNA-1 | Japonica | NA | | Os04g0480400 | OsR498G0408770500.01.T02 | SWI/SNF complex subunit SWI3A |
| maker-4-augustus-gene-188.16-mRNA-1 | Japonica | NA | | Os04g0482000 | OsR498G0408777000.01.T01 | Peptide methionine sulfoxide reductase A2-1 |
| maker-4-augustus-gene-19.43-mRNA-1 | Japonica | NA | Kinase-TM | Os04g0197200 | OsR498G0407706500.01.T02 | Cysteine-rich receptor-like protein kinase 6 |
| maker-4-augustus-gene-19.50-mRNA-1 | Japonica | Indica | Kinase-TM | Os04g0197200 | OsR498G0407709800.01.T01 | Cysteine-rich receptor-like protein kinase 6 |
| maker-4-augustus-gene-191.27-mRNA-1 | Japonica | NA | | Os04g0489600 | OsR498G0408806900.01.T01 | BHLH domain-containing protein (Fragment) |
| maker-4-augustus-gene-191.30-mRNA-1 | Japonica | NA | | Os04g0489800 | OsR498G0408807500.01.T01 | RING-type E3 ubiquitin transferase |
| maker-4-augustus-gene-191.32-mRNA-1 | Japonica | NA | | | OsR498G0408807700.01.T03 | Mitochondrial protoporphyrinogen IX oxidase |
| maker-4-augustus-gene-191.39-mRNA-1 | Japonica | NA | Kinase-TM | Os04g0488700 | OsR498G0408802200.01.T01 | Serine/threonine-protein kinase OXI1 |
| maker-4-augustus-gene-191.41-mRNA-1 | Japonica | NA | | Os04g0489100 | OsR498G0408804800.01.T04 | Serine/threonine-protein kinase OXI1 |
| maker-4-augustus-gene-192.41-mRNA-1 | Japonica | NA | | | OsR498G0408808900.01.T01 | ankyrin-3 isoform X1 |
| maker-4-augustus-gene-192.44-mRNA-1 | Japonica | Indica | Kinase | Os04g0490500 | OsR498G0408809700.01.T02 | Tyrosine-protein kinase |
| maker-4-augustus-gene-192.48-mRNA-1 | Japonica | Japonica | | Os04g0490900 | OsR498G0408810900.01.T01 | Amino acid transporter, transmembrane |
| maker-4-augustus-gene-192.56-mRNA-1 | Japonica | NA | | Os04g0490400 | OsR498G0408809200.01.T01 | Ribosome-recycling factor, chloroplastic |
| maker-4-augustus-gene-192.59-mRNA-1 | Japonica | Indica | | Os04g0490600 | OsR498G0408810000.01.T02 | CMP-sialic acid transporter 4 |
| maker-4-augustus-gene-192.66-mRNA-1 | Japonica | NA | | Os04g0490800 | OsR498G0408810600.01.T02 | 2-phosphoglycolate phosphatase, eukaryotic |
| maker-4-augustus-gene-192.69-mRNA-1 | Japonica | Japonica | | Os04g0491200 | OsR498G0408812200.01.T02 | Nitrate transporter 1.2 |
| maker-4-augustus-gene-192.74-mRNA-1 | Japonica | NA | | Os04g0491500 | OsR498G0408813800.01.T01 | Nitrate transporter 1.2 |
| maker-4-augustus-gene-192.78-mRNA-1 | Japonica | NA | | Os04g0491700 | OsR498G0408814000.01.T01 | Inositol transporter like |
| maker-4-augustus-gene-193.0-mRNA-1 | Japonica | NA | | | | DNA-directed RNA polymerase subunit |
| maker-4-augustus-gene-193.16-mRNA-1 | Japonica | NA | | Os04g0492900 | OsR498G0408818300.01.T01 | Putative CRM domain-containing protein, chloroplastic |
| maker-4-augustus-gene-193.20-mRNA-1 | Japonica | NA | | Os04g0493100 | OsR498G0408820400.01.T04 | Transcription factor bHLH153 |
| maker-4-augustus-gene-193.22-mRNA-1 | Japonica | NA | | Os04g0493400 | OsR498G0408821800.01.T01 | Chitinase |
| maker-4-augustus-gene-193.8-mRNA-1 | Japonica | NA | | Os04g0492400 | OsR498G0408817100.01.T04 | S-adenosyl-L-methionine-dependent methyltransferase |
| maker-4-augustus-gene-194.0-mRNA-1 | Japonica | NA | | | OsR498G0815948900.01.T01 | Hydroxyproline-rich glycoprotein-like |
| maker-4-augustus-gene-194.10-mRNA-1 | Japonica | Indica | | Os01g0611000 | OsR498G0408824300.01.T01 | DUF642 domain-containing protein (Fragment) |
| maker-4-augustus-gene-194.19-mRNA-1 | Japonica | NA | | | OsR498G0408826500.01.T01 | Cytochrome b561 and DOMON domain-containing protein |
| maker-4-augustus-gene-194.21-mRNA-1 | Japonica | NA | | Os04g0494100 | OsR498G0408822600.01.T01 | Chitinase |
| maker-4-augustus-gene-194.23-mRNA-1 | Japonica | NA | | | | Transcription factor RF2a |
| maker-4-augustus-gene-194.26-mRNA-1 | Japonica | Indica | | Os05g0305700 | OsR498G0408827000.01.T01 | Homeobox protein SIX3-like isoform X2 |
| maker-4-augustus-gene-194.29-mRNA-1 | Japonica | NA | | | | Transposon protein, putative, Mutator sub-class, expressed |
| maker-4-augustus-gene-194.4-mRNA-1 | Japonica | NA | | Os04g0494600 | OsR498G0408823500.01.T01 | Allergen Act d 3 |
| maker-4-augustus-gene-194.8-mRNA-1 | Japonica | NA | | Os04g0494900 | OsR498G0408824100.01.T01 | |
| maker-4-augustus-gene-195.0-mRNA-1 | Japonica | NA | | Os04g0495800 | OsR498G0408827100.01.T03 | NLP transcription factor (Fragment) |
| maker-4-augustus-gene-195.10-mRNA-1 | Japonica | Indica | | Os02g0607700 | OsR498G0408828200.01.T01 | (+)-neomenthol dehydrogenase |
| maker-4-augustus-gene-195.13-mRNA-1 | Japonica | NA | | Os04g0496600 | OsR498G0408829600.01.T02 | CBF1 interacting corepressor |
| maker-4-augustus-gene-195.17-mRNA-1 | Japonica | NA | | Os04g0496800 | OsR498G0408830200.01.T02 | Leucine zipper-EF-hand-containing transmembrane protein 1 |
| maker-4-augustus-gene-195.2-mRNA-1 | Japonica | NA | | Os04g0495900 | OsR498G0408827700.01.T01 | Glycerol-3-phosphatase 1 |

| | | | | | | |
|---|---|---|---|---|---|---|
| maker-4-augustus-gene-195.22-mRNA-1 | Japonica | NA | | Os04g0497000 | OsR498G0408830600.01.T01 | NADP-dependent alkenal double bond reductase P1 |
| maker-4-augustus-gene-195.25-mRNA-1 | Japonica | NA | | | OsR498G0408830800.01.T01 | Endoglucanase |
| maker-4-augustus-gene-195.27-mRNA-1 | Japonica | Indica | | Os04g0497350 | OsR498G0408831600.01.T01 | Ubiquitin-like-conjugating enzyme ATG10 |
| maker-4-augustus-gene-195.32-mRNA-1 | Japonica | NA | | Os04g0497400 | OsR498G0408831800.01.T01 | 6,7-dimethyl-8-ribityllumazine synthase |
| maker-4-augustus-gene-195.35-mRNA-1 | Japonica | NA | | Os04g0497600 | OsR498G0408832200.01.T02 | La protein 1 |
| maker-4-augustus-gene-195.4-mRNA-1 | Japonica | NA | | Os04g0496400 | OsR498G0408828900.01.T02 | HIV Tat-specific factor like |
| maker-4-augustus-gene-195.8-mRNA-1 | Japonica | Japonica | | Os04g0497300 | OsR498G0408831100.01.T01 | Stress-inducible protein |
| maker-4-augustus-gene-196.42-mRNA-1 | Japonica | NA | | | OsR498G0408832900.01.T03 | VAN3-binding protein isoform X1 |
| maker-4-augustus-gene-196.46-mRNA-1 | Japonica | NA | | Os04g0498800 | OsR498G0408834700.01.T05 | Cell division control protein B |
| maker-4-augustus-gene-196.54-mRNA-1 | Japonica | Japonica | | Os04g0499100 | OsR498G0408835700.01.T02 | probable folate-biopterin transporter 2 |
| maker-4-augustus-gene-196.58-mRNA-1 | Japonica | Japonica | | Os04g0499800 | OsR498G0408837100.01.T04 | |
| maker-4-augustus-gene-196.60-mRNA-1 | Japonica | NA | | Os04g0500300 | OsR498G0408838100.01.T01 | GPI-anchored protein LLG2 |
| maker-4-augustus-gene-196.62-mRNA-1 | Japonica | NA | | Os04g0500400 | OsR498G0408838500.01.T01 | |
| maker-4-augustus-gene-196.64-mRNA-1 | Japonica | Indica | | Os04g0498200 | OsR498G0408833600.01.T01 | Cytochrome c oxidase subunit |
| maker-4-augustus-gene-196.67-mRNA-1 | Japonica | Japonica | | Os04g0498700 | OsR498G0408834600.01.T01 | Peroxidase |
| maker-4-augustus-gene-196.73-mRNA-1 | Japonica | Japonica | | Os04g0499200 | OsR498G0204286000.01.T01 | Enhancer of rudimentary homolog |
| maker-4-augustus-gene-196.75-mRNA-1 | Japonica | Indica | | Os04g0499300 | OsR498G0408836000.01.T02 | eukaryotic translation initiation factor |
| maker-4-augustus-gene-197.39-mRNA-1 | Japonica | NA | | Os04g0500700 | OsR498G0408840900.01.T05 | Hydroxycinnamoyl-Coenzyme A shikimate/quinate hydroxycinnamoyltransfe |
| maker-4-augustus-gene-197.41-mRNA-1 | Japonica | Indica | | Os04g0500900 | OsR498G0408841300.01.T01 | Serine/threonine protein phosphatase |
| maker-4-augustus-gene-197.45-mRNA-1 | Japonica | NA | | Os04g0501100 | OsR498G0408841700.01.T03 | Magnesium transporter |
| maker-4-augustus-gene-197.49-mRNA-1 | Japonica | NA | | | OsR498G0408841900.01.T01 | GuaA |
| maker-4-augustus-gene-197.52-mRNA-1 | Japonica | NA | | Os04g0501700 | OsR498G0408843900.01.T06 | Gamma-tubulin complex component |
| maker-4-augustus-gene-197.58-mRNA-1 | Japonica | NA | | Os04g0501800 | OsR498G0408844300.01.T01 | Calmodulin-binding protein isoform 1 |
| maker-4-augustus-gene-197.61-mRNA-1 | Japonica | Japonica | | Os04g0502100 | OsR498G0408845100.01.T01 | |
| maker-4-augustus-gene-197.63-mRNA-1 | Japonica | NA | | Os04g0502200 | OsR498G0408845300.01.T02 | Trafficking protein particle complex subunit 9 |
| maker-4-augustus-gene-197.69-mRNA-1 | Japonica | NA | | Os04g0501000 | OsR498G0408841600.01.T04 | 50S ribosomal protein L23, chloroplastic |
| maker-4-augustus-gene-197.71-mRNA-1 | Japonica | NA | | Os04g0501600 | OsR498G0408843400.01.T01 | |
| maker-4-augustus-gene-197.76-mRNA-1 | Japonica | NA | | | OsR498G0408844800.01.T01 | HMA domain-containing protein |
| maker-4-augustus-gene-198.0-mRNA-1 | Japonica | NA | | Os04g0502800 | OsR498G0408846900.01.T01 | Nodulin-like domain-containing protein (Fragment) |
| maker-4-augustus-gene-198.14-mRNA-1 | Japonica | Indica | | Os04g0503800 | OsR498G0408848700.01.T01 | TRAPP 20 K subunit |
| maker-4-augustus-gene-198.17-mRNA-1 | Japonica | NA | | | OsR498G0408850300.01.T03 | Adenine phosphoribosyltransferase1 |
| maker-4-augustus-gene-198.20-mRNA-1 | Japonica | NA | | | OsR498G0408851500.01.T01 | AP2-like ethylene-responsive transcription factor BBM2 |
| maker-4-augustus-gene-198.23-mRNA-1 | Japonica | Indica | | Os04g0386600 | OsR498G0408852300.01.T02 | Phosphoenolpyruvate carboxylase family protein isoform 2 |
| maker-4-augustus-gene-198.28-mRNA-1 | Japonica | NA | | | OsR498G0408847800.01.T04 | Calcium-dependent protein kinase 5 |
| maker-4-augustus-gene-198.3-mRNA-1 | Japonica | NA | | Os04g0502900 | OsR498G0408847100.01.T02 | Calcium uptake protein, mitochondrial |
| maker-4-augustus-gene-198.30-mRNA-1 | Japonica | NA | LRR-TM | Os04g0503500 | OsR498G0408848000.01.T07 | F-box/LRR-repeat protein 14-like isoform X2 |
| maker-4-augustus-gene-198.36-mRNA-1 | Japonica | NA | | Os04g0503700 | OsR498G0408848600.01.T02 | |
| maker-4-augustus-gene-198.39-mRNA-1 | Japonica | NA | | | OsR498G0408852600.01.T01 | Putative non-LTR retroelement reverse transcriptase |
| maker-4-augustus-gene-198.9-mRNA-1 | Japonica | Indica | Kinase | Os09g0123300 | OsR498G0408848300.01.T03 | Mitogen-activated protein kinase kinase kinase |
| maker-4-augustus-gene-199.0-mRNA-1 | Japonica | NA | | Os04g0505000 | OsR498G0408853300.01.T02 | Filament-like plant protein 7 |
| maker-4-augustus-gene-199.2-mRNA-1 | Japonica | NA | | Os04g0504800 | OsR498G0408852800.01.T03 | Polyadenylate-binding protein (Fragment) |
| maker-4-augustus-gene-199.9-mRNA-1 | Japonica | NA | | Os04g0506300 | OsR498G0408859600.01.T02 | Serine incorporator |
| maker-4-augustus-gene-2.0-mRNA-1 | Japonica | NA | | Os04g0171800 | OsR498G0407629800.01.T01 | Isoleucine N-monooxygenase 2-like (Fragment) |
| maker-4-augustus-gene-20.0-mRNA-1 | Japonica | NA | | | | TTF-type domain-containing protein (Fragment) |
| maker-4-augustus-gene-200.36-mRNA-1 | Japonica | NA | | Os04g0507700 | OsR498G0408864500.01.T01 | Anther-specific proline-rich protein APG |
| maker-4-augustus-gene-200.38-mRNA-1 | Japonica | NA | | Os04g0507900 | OsR498G0408864900.01.T06 | Diphthamide biosynthesis protein 7-like protein (Fragment) |
| maker-4-augustus-gene-200.40-mRNA-1 | Japonica | NA | | Os04g0508100 | | Isocitrate dehydrogenase [NADP] |
| maker-4-augustus-gene-200.42-mRNA-1 | Japonica | NA | | | OsR498G0408865700.01.T01 | Isocitrate dehydrogenase |
| maker-4-augustus-gene-200.47-mRNA-1 | Japonica | NA | | Os04g0506900 | OsR498G0408861800.01.T02 | Phosphatidylinositol N-acetylglucosaminlytransferase subunit P-related |
| maker-4-augustus-gene-200.50-mRNA-1 | Japonica | NA | | Os04g0507000 | OsR498G0408862600.01.T04 | GIY-YIG nuclease superfamily |
| maker-4-augustus-gene-200.54-mRNA-1 | Japonica | NA | | | | GIY-YIG nuclease superfamily |
| maker-4-augustus-gene-200.60-mRNA-1 | Japonica | NA | | | OsR498G0408863400.01.T06 | CLIP-associated protein-like |
| maker-4-augustus-gene-200.73-mRNA-1 | Japonica | NA | | Os04g0508000 | OsR498G0408865400.01.T01 | Protein trichome birefringence |
| maker-4-augustus-gene-201.35-mRNA-1 | Japonica | Indica | | Os04g0508300 | OsR498G0408866100.01.T01 | Glutaredoxin GRX |
| maker-4-augustus-gene-201.39-mRNA-1 | Japonica | NA | Kinase-TM | | OsR498G0408871500.01.T01 | Receptor-like serinethreonine-protein kinase ale2 |
| maker-4-augustus-gene-202.59-mRNA-1 | Japonica | Indica | | Os04g0510500 | OsR498G0408877100.01.T02 | Nuclear transport factor 2 (NTF2) family protein with RNA binding (RRM-RBD |
| maker-4-augustus-gene-202.71-mRNA-1 | Japonica | NA | | Os04g0510600 | OsR498G0408877400.01.T01 | Anaphase-promoting complex subunit like |
| maker-4-augustus-gene-203.13-mRNA-1 | Japonica | NA | | Os04g0512400 | OsR498G0408881200.01.T01 | protein BREAST CANCER SUSCEPTIBILITY 1 homolog |
| maker-4-augustus-gene-208.54-mRNA-1 | Japonica | NA | | Os04g0521800 | OsR498G0408918300.01.T02 | IAA-amino acid hydrolase |
| maker-4-augustus-gene-208.57-mRNA-1 | Japonica | Indica | | Os02g0629800 | OsR498G0408918500.01.T01 | Knot1 domain-containing protein (Fragment) |
| maker-4-augustus-gene-209.24-mRNA-1 | Japonica | Indica | | Os04g0525100 | OsR498G0408929200.01.T01 | Glycosyltransferase |

| | | | | | | |
|---|---|---|---|---|---|---|
| maker-4-augustus-gene-209.27-mRNA-1 | Japonica | NA | | Os04g0525700 | OsR498G0408931600.01.T01 | Carboxypeptidase |
| maker-4-augustus-gene-21.0-mRNA-1 | Japonica | NA | | | OsR498G0407714700.01.T02 | Retrotransposon protein, putative, LINE sub-class |
| maker-4-augustus-gene-21.3-mRNA-1 | Japonica | Indica | | Os04g0330200 | OsR498G0407712200.01.T01 | Jacalin-type lectin domain-containing protein |
| maker-4-augustus-gene-21.5-mRNA-1 | Japonica | NA | | | | NB-ARC domain-containing protein |
| maker-4-augustus-gene-21.7-mRNA-1 | Japonica | NA | | Os04g0201200 | OsR498G0407716800.01.T01 | Pumilio |
| maker-4-augustus-gene-210.41-mRNA-1 | Japonica | NA | | | OsR498G0408932500.01.T01 | Sphingolipid transporter spinster like |
| maker-4-augustus-gene-211.19-mRNA-1 | Japonica | Indica | | Os04g0528600 | OsR498G0408943800.01.T01 | Serine/arginine repetitive matrix protein 2-like |
| maker-4-augustus-gene-212.49-mRNA-1 | Japonica | NA | | Os04g0531900 | OsR498G0408957400.01.T01 | Salutaridine reductase |
| maker-4-augustus-gene-218.10-mRNA-1 | Japonica | NA | | Os04g0542900 | OsR498G0409008100.01.T01 | XendoU domain-containing protein |
| maker-4-augustus-gene-218.15-mRNA-1 | Japonica | NA | | | OsR498G0409008500.01.T01 | |
| maker-4-augustus-gene-218.17-mRNA-1 | Japonica | NA | | Os04g0543200 | OsR498G0409008700.01.T03 | RNA recognition motif protein 1 |
| maker-4-augustus-gene-218.19-mRNA-1 | Japonica | NA | | Os04g0542200 | OsR498G0409006200.01.T01 | Probable metal-nicotianamine transporter YSL9 |
| maker-4-augustus-gene-218.22-mRNA-1 | Japonica | NA | | | OsR498G0409006400.01.T01 | |
| maker-4-augustus-gene-218.23-mRNA-1 | Japonica | NA | | Os04g0542800 | OsR498G0409008000.01.T03 | Iron-phytosiderophore transporter yellow stripe 1 |
| maker-4-augustus-gene-218.4-mRNA-1 | Japonica | NA | | Os04g0541700 | OsR498G0409002900.01.T01 | Homeodomain-containing transcription factor S1 |
| maker-4-augustus-gene-218.6-mRNA-1 | Japonica | Indica | | Os04g0541900 | OsR498G0409004300.01.T01 | Cysteine-type endopeptidase/ ubiquitin thiolesterase |
| maker-4-augustus-gene-219.36-mRNA-1 | Japonica | NA | | Os04g0543700 | OsR498G0409009900.01.T05 | Subtilisin-like protease |
| maker-4-augustus-gene-219.40-mRNA-1 | Japonica | Indica | | Os02g0650900 | OsR498G0409010500.01.T01 | Glutamate dehydrogenase |
| maker-4-augustus-gene-219.45-mRNA-1 | Japonica | Indica | | Os04g0543600 | OsR498G0409009600.01.T02 | Cationic amino acid transporter like |
| maker-4-augustus-gene-219.49-mRNA-1 | Japonica | NA | | Os04g0544200 | OsR498G0409012200.01.T01 | Cyclin |
| maker-4-augustus-gene-219.51-mRNA-1 | Japonica | NA | | Os04g0544400 | OsR498G0409012400.01.T01 | Pentatricopeptide repeat-containing protein (Fragment) |
| maker-4-augustus-gene-219.54-mRNA-1 | Japonica | NA | | Os04g0544500 | OsR498G0409012600.01.T01 | GATA-type domain-containing protein |
| maker-4-augustus-gene-219.57-mRNA-1 | Japonica | NA | | | | Mannosyltransferase |
| maker-4-augustus-gene-219.62-mRNA-1 | Japonica | NA | | Os04g0545000 | OsR498G0409013800.01.T01 | WRKY transcription factor 4 |
| maker-4-augustus-gene-22.41-mRNA-1 | Japonica | NA | | Os04g0203500 | OsR498G0407720100.01.T01 | Senescence-specific cysteine protease SAG39 |
[truncated: 1,938,041 more chars]
