# Supplementary material for: Development and Genome-Wide Analysis of a Blast-Resistant japonica Rice Variety
Source: Plants (Basel). 2023 Oct 11;12(20):3536. doi: 10.3390/plants12203536 (PMC10667994; doi:10.3390/plants12203536)
Supplement: Supplementary file 1 [file plants-12-03536-s001.zip › Supplementary Material Escol_Suppl Table S3 Homologues in jap and ind.pdf]

Supplemental Table S3. Genes identified in the genome of COPSEMAR9. Only COPSEMAR9 genes for which homologues are identified in *japonica* (IRGSP-1.0) and *indica* (R498) are listed.

Maker_ID	Chromosome_Query	Indica_ID	vs Indica_Id	vs Indica_evalue	vs Indica_Bitscore	Japonica_ID	vs Japonica_Id	vs Japonica_evalue	vs Japonica_Bitscore	Japonica_vs_Indica_Bitscore	Assignment_Gene	Assignment_Genome	Resistance Gene Class
augustus_masked-1-processed-gene-10.16-mRNA-1	1	OsR498G0100063000.01.T01	99,19	0,00E+00	1962	Os01g0119500	82,50	0,00E+00	1121	-841	Indica	Japonica	
augustus_masked-1-processed-gene-108.23-mRNA-1	1	OsR498G0100722000.01.T02	99,92	0,00E+00	2144	Os01g0293000	100,00	0,00E+00	2149	5	NA	Japonica	
augustus_masked-1-processed-gene-11.28-mRNA-1	1	OsR498G0100066200.01.T03	100,00	0,00E+00	1548	Os01g0120700	100,00	0,00E+00	1548	0	NA	NA	
augustus_masked-1-processed-gene-110.37-mRNA-1	1	OsR498G0100732200.01.T01	99,20	0,00E+00	2201	Os05g0539300	75,11	0,00E+00	777	-1424	Indica	Japonica	
augustus_masked-1-processed-gene-111.61-mRNA-1	1	OsR498G0100738800.01.T01	78,93	6,45E-137	488	Os01g0299700	100,00	0,00E+00	1173	685	Japonica	Japonica	
augustus_masked-1-processed-gene-112.19-mRNA-1	1	OsR498G0100745200.01.T01	99,34	1,69E-152	538	Os01g0301500	100,00	1,13E-155	547	9	NA	Japonica	
augustus_masked-1-processed-gene-118.52-mRNA-1	1	OsR498G0100779400.01.T01	99,71	0,00E+00	1865	Os01g0311600	100,00	0,00E+00	1878	13	NA	Japonica	
augustus_masked-1-processed-gene-12.2-mRNA-1	1	OsR498G0100070800.01.T01	100,00	0,00E+00	861	Os01g0121500	100,00	4,54E-144	509	-352	Indica	NA	
augustus_masked-1-processed-gene-125.33-mRNA-1	1	OsR498G0100820800.01.T01	99,75	0,00E+00	2124	Os01g0293000	83,80	0,00E+00	1258	-866	Indica	Japonica	
augustus_masked-1-processed-gene-125.39-mRNA-1	1	OsR498G0100826200.01.T01	99,08	0,00E+00	957	Os08g0545400	83,19	1,82E-143	508	-449	Indica	Japonica	
augustus_masked-1-processed-gene-13.29-mRNA-1	1	OsR498G0100081000.01.T01	100,00	0,00E+00	1407	Os01g0124100	100,00	0,00E+00	1407	0	NA	NA	
augustus_masked-1-processed-gene-13.30-mRNA-1	1	OsR498G0100081600.01.T01	100,00	0,00E+00	1364	Os01g0124200	99,74	0,00E+00	1355	-9	NA	NA	
augustus_masked-1-processed-gene-13.31-mRNA-1	1	OsR498G0100081800.01.T01	100,00	0,00E+00	1380	Os01g0124400	100,00	0,00E+00	1380	0	NA	NA	
augustus_masked-1-processed-gene-13.36-mRNA-1	1	OsR498G0100084600.01.T04	99,72	1,72E-179	629	Os01g0125500	100,00	1,95E-173	607	-22	NA	Japonica	
augustus_masked-1-processed-gene-13.9-mRNA-1	1	OsR498G0100080200.01.T02	100,00	0,00E+00	1153	Os01g0123900	100,00	0,00E+00	1050	-103	NA	NA	
augustus_masked-1-processed-gene-135.45-mRNA-1	1	OsR498G0100874800.01.T01	75,60	3,77E-109	396	Os01g0340600	100,00	0,00E+00	1526	1130	Japonica	Japonica	
augustus_masked-1-processed-gene-136.12-mRNA-1	1	OsR498G0100874800.01.T01	94,69	0,00E+00	1865	Os01g0341200	100,00	0,00E+00	2560	695	Japonica	Japonica	
augustus_masked-1-processed-gene-136.22-mRNA-1	1	OsR498G0100876900.01.T01	99,38	0,00E+00	1439	Os06g0489700	90,55	2,11E-157	554	-885	Indica	Japonica	
augustus_masked-1-processed-gene-137.26-mRNA-1	1	OsR498G0509881000.01.T01	89,91	0,00E+00	1738	Os01g0343900	97,18	0,00E+00	3067	1329	Japonica	NA	
augustus_masked-1-processed-gene-137.5-mRNA-1	1	OsR498G0100882700.01.T01	98,09	0,00E+00	2511	Os01g0343100	99,95	0,00E+00	3956	1445	Japonica	Japonica	
augustus_masked-1-processed-gene-139.38-mRNA-1	1	OsR498G0100895200.01.T01	98,31	0,00E+00	918	Os01g0346700	100,00	0,00E+00	958	40	NA	Japonica	
augustus_masked-1-processed-gene-139.41-mRNA-1	1	OsR498G0100897800.01.T01	93,75	0,00E+00	2270	Os01g0347200	100,00	0,00E+00	2679	409	Japonica	Japonica	
augustus_masked-1-processed-gene-146.27-mRNA-1	1	OsR498G0204493300.01.T02	92,01	1,32E-135	483	Os01g0357900	100,00	0,00E+00	639	156	Japonica	Japonica	
augustus_masked-1-processed-gene-147.20-mRNA-1	1	OsR498G0100940200.01.T05	98,33	0,00E+00	2501	Os01g0359400	100,00	0,00E+00	2560	59	NA	Japonica	NBS-TM
augustus_masked-1-processed-gene-147.22-mRNA-1	1	OsR498G0100941400.01.T02	99,77	0,00E+00	5379	Os01g0359800	100,00	0,00E+00	6729	1350	Japonica	Japonica	LRR-NBS-TM
augustus_masked-1-processed-gene-149.26-mRNA-1	1	OsR498G0100949800.01.T01	99,09	0,00E+00	1945	Os01g0362100	100,00	0,00E+00	1970	25	NA	Japonica	
augustus_masked-1-processed-gene-15.20-mRNA-1	1	OsR498G0100094800.01.T02	99,85	0,00E+00	1176	Os01g0128300	99,85	0,00E+00	1176	0	NA	NA	
augustus_masked-1-processed-gene-150.19-mRNA-1	1	OsR498G0100955800.01.T01	98,70	0,00E+00	1611	Os01g0364400	100,00	0,00E+00	1665	54	NA	Japonica	
augustus_masked-1-processed-gene-155.37-mRNA-1	1	OsR498G0714828400.01.T01	99,14	0,00E+00	2457	Os01g0373400	100,00	0,00E+00	2040	-417	Indica	Japonica	
augustus_masked-1-processed-gene-156.37-mRNA-1	1	OsR498G0100985200.01.T03	99,60	0,00E+00	5405	Os01g0375100	100,00	0,00E+00	5460	55	NA	Japonica	
augustus_masked-1-processed-gene-16.17-mRNA-1	1	OsR498G0100106000.01.T01	100,00	0,00E+00	948	Os01g0130900	98,86	0,00E+00	921	-27	NA	Indica	
augustus_masked-1-processed-gene-161.25-mRNA-1	1	OsR498G0100996100.01.T01	97,42	0,00E+00	851	Os01g0382400	100,00	0,00E+00	910	59	NA	Japonica	
augustus_masked-1-processed-gene-17.43-mRNA-1	1	OsR498G0100112800.01.T01	100,00	0,00E+00	1310	Os01g0132200	99,86	0,00E+00	1306	-4	NA	Indica	
augustus_masked-1-processed-gene-178.20-mRNA-1	1	OsR498G0101052000.01.T01	99,81	0,00E+00	2892	Os01g0498300	100,00	0,00E+00	2906	14	NA	Japonica	
augustus_masked-1-processed-gene-182.10-mRNA-1	1	OsR498G0101070200.01.T01	99,91	0,00E+00	2052	Os01g0504100	100,00	0,00E+00	2057	5	NA	Japonica	
augustus_masked-1-processed-gene-183.15-mRNA-1	1	OsR498G0101075200.01.T01	99,82	0,00E+00	2914	Os01g0505500	100,00	0,00E+00	2928	14	NA	Japonica	
augustus_masked-1-processed-gene-186.29-mRNA-1	1	OsR498G0101096000.01.T01	100,00	5,82E-178	623	Os01g0510200	100,00	2,02E-178	623	0	NA	Japonica	
augustus_masked-1-processed-gene-187.21-mRNA-1	1	OsR498G0101102600.01.T01	99,63	0,00E+00	1432	Os01g0511200	100,00	0,00E+00	1445	13	NA	Japonica	
augustus_masked-1-processed-gene-187.3-mRNA-1	1	OsR498G0101099600.01.T01	99,30	0,00E+00	1764	Os01g0511000	92,27	0,00E+00	923	-841	Indica	Japonica	
augustus_masked-1-processed-gene-19.51-mRNA-1	1	OsR498G0100124800.01.T01	100,00	0,00E+00	850	Os01g0136300	99,36	0,00E+00	837	-13	NA	Indica	
augustus_masked-1-processed-gene-191.39-mRNA-1	1	OsR498G0101126800.01.T04	100,00	0,00E+00	2019	Os01g0519100	100,00	0,00E+00	1824	-195	NA	NA	
augustus_masked-1-processed-gene-198.24-mRNA-1	1	OsR498G0101164000.01.T01	99,68	0,00E+00	2206	Os01g0530500	100,00	0,00E+00	1440	-766	Indica	Japonica	
augustus_masked-1-processed-gene-199.34-mRNA-1	1	OsR498G0101169200.01.T01	100,00	0,00E+00	1359	Os01g0532300	100,00	0,00E+00	1359	0	NA	Japonica	
augustus_masked-1-processed-gene-200.17-mRNA-1	1	OsR498G0101174600.01.T01	99,75	0,00E+00	721	Os02g0822300	87,13	8,91E-146	516	-205	Indica	Japonica	
augustus_masked-1-processed-gene-21.64-mRNA-1	1	OsR498G0100139600.01.T02	99,61	3,05E-129	462	Os01g0139900	99,61	1,07E-129	462	0	NA	NA	
augustus_masked-1-processed-gene-210.48-mRNA-1	1	OsR498G0102290100.01.T01	75,75	2,71E-150	534	Os01g0546800	100,00	0,00E+00	1975	1441	Japonica	NA	
augustus_masked-1-processed-gene-212.31-mRNA-1	1	OsR498G0101272200.01.T01	100,00	0,00E+00	807	Os09g0292400	83,29	8,14E-111	399	-408	Indica	NA	
augustus_masked-1-processed-gene-213.4-mRNA-1	1	OsR498G0101278700.01.T04	100,00	0,00E+00	2324	Os01g0550000	100,00	0,00E+00	2324	0	NA	NA	
augustus_masked-1-processed-gene-220.40-mRNA-1	1	OsR498G0101341800.01.T01	99,74	0,00E+00	2787	Os01g0561600	100,00	0,00E+00	2841	54	NA	Japonica	
augustus_masked-1-processed-gene-224.11-mRNA-1	1	OsR498G0101357600.01.T01	97,87	0,00E+00	2416	Os01g0566800	100,00	0,00E+00	2572	156	NA	Japonica	
augustus_masked-1-processed-gene-229.0-mRNA-1	1	OsR498G0101383200.01.T01	96,46	0,00E+00	4391	Os01g0575500	95,92	0,00E+00	5992	1601	Japonica	Japonica	
augustus_masked-1-processed-gene-231.69-mRNA-1	1	OsR498G0307172900.01.T05	92,70	1,93E-69	263	Os01g0580300	100,00	6,27E-159	558	295	Japonica	Japonica	
augustus_masked-1-processed-gene-245.13-mRNA-1	1	OsR498G0101494400.01.T03	98,13	0,00E+00	1297	Os01g0604500	100,00	0,00E+00	1353	56	NA	NA	
augustus_masked-1-processed-gene-245.40-mRNA-1	1	OsR498G0101500000.01.T01	100,00	0,00E+00	1801	Os01g0606100	100,00	0,00E+00	2646	845	Japonica	NA	
augustus_masked-1-processed-gene-250.40-mRNA-1	1	OsR498G0101534400.01.T01	98,40	0,00E+00	644	Os05g0468700	91,84	4,00E-156	550	-94	Indica	NA	
augustus_masked-1-processed-gene-250.64-mRNA-1	1	OsR498G0101538800.01.T03	99,78	0,00E+00	2411	Os01g0614500	100,00	0,00E+00	2425	14	NA	NA	
augustus_masked-1-processed-gene-251.5-mRNA-1	1	OsR498G0101542000.01.T01	99,95	0,00E+00	3318	Os01g0615500	100,00	0,00E+00	3236	-82	NA	NA	NBS-TM
augustus_masked-1-processed-gene-252.18-mRNA-1	1	OsR498G0101550000.01.T03	99,86	0,00E+00	2480	Os01g0617600	100,00	0,00E+00	2527	47	NA	NA	
augustus_masked-1-processed-gene-254.25-mRNA-1	1	OsR498G0101559000.01.T01	95,14	0,00E+00	1594	Os01g0620300	100,00	0,00E+00	2603	1009	Japonica	NA	
augustus_masked-1-processed-gene-256.41-mRNA-1	1	OsR498G0511414500.01.T01	73,79	1,72E-89	332	Os01g0625900	100,00	0,00E+00	1986	1654	Japonica	NA	
augustus_masked-1-processed-gene-257.24-mRNA-1	1	OsR498G0101580000.01.T02	99,45	0,00E+00	1303	Os01g0626900	100,00	0,00E+00	1321	18	NA	NA	
augustus_masked-1-processed-gene-259.49-mRNA-1	1	OsR498G0101589400.01.T01	98,88	0,00E+00	935	Os01g0630300	97,75	0,00E+00	913	-22	NA	NA	
augustus_masked-1-processed-gene-26.22-mRNA-1	1	OsR498G0100175200.01.T01	99,75	0,00E+00	2151	Os01g0148500	99,58	0,00E+00	2142	-9	NA	Indica	
augustus_masked-1-processed-gene-261.33-mRNA-1	1	OsR498G0101603800.01.T02	99,86	0,00E+00	1257	Os01g0635550	100,00	0,00E+00	1261	4	NA	Japonica	
augustus_masked-1-processed-gene-266.42-mRNA-1	1	OsR498G0101637800.01.T01	100,00	5,05E-113	407	Os01g0644200	100,00	1,77E-113	407	0	NA	Japonica	
augustus_masked-1-processed-gene-267.44-mRNA-1	1	OsR498G0101644400.01.T01	99,57	0,00E+00	2484	Os01g0645900	100,00	0,00E+00	2511	27	NA	Japonica	
augustus_masked-1-processed-gene-268.38-mRNA-1	1	OsR498G0101650800.01.T02	100,00	0,00E+00	639	Os01g0647800	100,00	0,00E+00	639	0	NA	NA	
augustus_masked-1-processed-gene-268.45-mRNA-1	1	OsR498G0101654400.01.T01	99,86	0,00E+00	1275	Os01g0648700	100,00	0,00E+00	1279	4	NA	Japonica	
augustus_masked-1-processed-gene-275.26-mRNA-1	1	OsR498G0101694200.01.T01	97,52	0,00E+00	750	Os01g0660000	99,80	0,00E+00	878	128	Japonica	Japonica	
augustus_masked-1-processed-gene-276.12-mRNA-1	1	OsR498G0101696800.01.T01	99,92	0,00E+00	2214	Os01g0660700	100,00	0,00E+00	2219	5	NA	Japonica	
augustus_masked-1-processed-gene-276.15-mRNA-1	1	OsR498G0101697800.01.T01	99,27	0,00E+00	1451	Os01g0660800	100,00	0,00E+00	1083	-368	Indica	NA	
augustus_masked-1-processed-gene-277.15-mRNA-1	1	OsR498G0101702400.01.T01	99,64	5,56E-139	494	Os07g0443500	90,79	3,09E-86	317	-177	Indica	Japonica	
augustus_masked-1-processed-gene-28.60-mRNA-1	1	OsR498G0100191200.01.T01	97,19	0,00E+00	775	Os01g0152300	99,78	0,00E+00	829	54	NA	Japonica	
augustus_masked-1-processed-gene-28.63-mRNA-1	1	OsR498G0100190400.01.T01	98,61	0,00E+00	2257	Os01g0152950	99,77	0,00E+00	2324	67	NA	NA	
augustus_masked-1-processed-gene-280.18-mRNA-1	1	OsR498G0101726500.01.T01	73,94	0,00E+00	862	Os01g0668600	100,00	0,00E+00	4318	3456	Japonica	Japonica	Kinase-TM
augustus_masked-1-processed-gene-280.20-mRNA-1	1	OsR498G0101727800.01.T01	98,50	0,00E+00	4274	Os01g0669100	100,00	0,00E+00	4437	163	NA	Japonica	Kinase-TM
augustus_masked-1-processed-gene-281.23-mRNA-1	1	OsR498G0101730000.01.T01	99,92	0,00E+00	4401	Os01g0670100	100,00	0,00E+00	4410	9	NA	Japonica	Kinase-TM
augustus_masked-1-processed-gene-283.22-mRNA-1	1	OsR498G0101745200.01.T01	99,70	0,00E+00	1789	Os01g0674000	99,80	0,00E+00	1793	4	NA	Japonica	
augustus_masked-1-processed-gene-283.24-mRNA-1	1	OsR498G0101747200.01.T01	99,81	0,00E+00	1923	Os05g0560400	79,09	0,00E+00	876	-1047	Indica	Japonica	
augustus_masked-1-processed-gene-284.23-mRNA-1	1	OsR498G0101751200.01.T01	98,87	0,00E+00	1550	Os01g0675800	100,00	0,00E+00	1575	25	NA	Japonica	
augustus_masked-1-processed-gene-285.32-mRNA-1	1	OsR498G0101756200.01.T02	99,82	0,00E+00	976	Os01g0677100	100,00	0,00E+00	931	-45	NA	Japonica	
augustus_masked-1-processed-gene-29.15-mRNA-1	1	OsR498G0100191600.01.T05	99,52	0,00E+00	3737	Os01g0153200	99,58	0,00E+00	4685	948	Japonica	NA	
augustus_masked-1-processed-gene-29.44-mRNA-1	1	OsR498G0100193100.01.T01	81,56	0,00E+00	1028	Os01g0154900	94,91	0,00E+00	1518	490	Japonica	Indica	
augustus_masked-1-processed-gene-29.45-mRNA-1	1	OsR498G0100195800.01.T01	100,00	0,00E+00	1829	Os01g0155000	99,61	0,00E+00	1811	-18	NA	Indica	
augustus_masked-1-processed-gene-290.62-mRNA-1	1	OsR498G0101789800.01.T01	99,89	0,00E+00	1636	Os01g0687400	100,00	0,00E+00	1640	4	NA	Japonica	
augustus_masked-1-processed-gene-291.60-mRNA-1	1	OsR498G0101799400.01.T02	90,65	0,00E+00	1184	Os01g0689900	100,00	0,00E+00	1557	373	Japonica	Japonica	
augustus_masked-1-processed-gene-291.61-mRNA-1	1	OsR498G0101800200.01.T01	97,23	0,00E+00	1333	Os01g0690200	100,00	0,00E+00	1422	89	NA	Japonica	
augustus_masked-1-processed-gene-292.73-mRNA-1	1	OsR498G0101801400.01.T01	99,69	1,10E-161	570	Os01g0691300	100,00	3,59E-175	612	42	NA	Japonica	
augustus_masked-1-processed-gene-293.29-mRNA-1	1	OsR498G0101809000.01.T03	99,84	0,00E+00	1118	Os01g0693400	100,00	0,00E+00	2132	1014	Japonica	Japonica	
augustus_masked-1-processed-gene-293.42-mRNA-1	1	OsR498G0101813200.01.T01	99,94	0,00E+00	2842	Os01g0693800	100,00	0,00E+00	2971	129	NA	Japonica	
augustus_masked-1-processed-gene-298.71-mRNA-1	1	OsR498G0101846200.01.T01	99,61	0,00E+00	2288	Os01g0704300	100,00	0,00E+00	1175	-1113	Indica	Japonica	
augustus_masked-1-processed-gene-30.85-mRNA-1	1	OsR498G0100204400.01.T01	100,00	0,00E+00	1397	Os01g0158100	99,75	0,00E+00	1443	46	NA	Indica	
augustus_masked-1-processed-gene-300.49-mRNA-1	1	OsR498G0101859800.01.T02	99,66	0,00E+00	2118	Os01g0707500	100,00	0,00E+00	2132	14	NA	Japonica	
augustus_masked-1-processed-gene-302.56-mRNA-1	1	OsR498G0101867800.01.T01	99,08	0,00E+00	2684	Os01g0710200	100,00	0,00E+00	2776	92	NA	Japonica	
augustus_masked-1-processed-gene-302.57-mRNA-1	1	OsR498G0101869400.01.T04	99,20	0,00E+00	2424	Os01g0710700	100,00	0,00E+00	2468	44	NA	Japonica	
augustus_masked-1-processed-gene-303.6-mRNA-1	1	OsR498G0101875000.01.T01	99,57	0,00E+00	1676	Os01g0712700	100,00	0,00E+00	1694	18	NA	Japonica	
augustus_masked-1-processed-gene-304.65-mRNA-1	1	OsR498G0101886400.01.T01	99,95	0,00E+00	3291	Os01g0715900	100,00	0,00E+00	3296	5	NA	Japonica	
augustus_masked-1-processed-gene-306.22-mRNA-1	1	OsR498G0101902000.01.T01	99,79	0,00E+00	6039	Os01g0718300	100,00	0,00E+00	6071	32	NA	Japonica	Kinase-LRR-TM
augustus_masked-1-processed-gene-306.23-mRNA-1	1	OsR498G0101903800.01.T02	99,69	0,00E+00	1735	Os01g0718900	100,00	0,00E+00	1905	170	NA	Japonica	
augustus_masked-1-processed-gene-307.62-mRNA-1	1	OsR498G0101912800.01.T01	98,69	0,00E+00	1062	Os10g0160600	95,81	0,00E+00	809	-253	Indica	Japonica	
augustus_masked-1-processed-gene-31.28-mRNA-1	1	OsR498G0100209000.01.T03	100,00	0,00E+00	1462	Os01g0159200	99,65	0,00E+00	1016	-446	Indica	Indica	
augustus_masked-1-processed-gene-31.6-mRNA-1	1	OsR498G0100207000.01.T01	100,00	0,00E+00	1900	Os02g0144200	72,33	4,40E-85	315	-1585	Indica	NA	
augustus_masked-1-processed-gene-311.29-mRNA-1	1	OsR498G0101936400.01.T01	99,81	0,00E+00	1876	Os01g0730200	100,00	0,00E+00	1003	-873	Indica	Japonica	
augustus_masked-1-processed-gene-311.31-mRNA-1	1	OsR498G0101938000.01.T01	98,43	0,00E+00	1102	Os01g0730600	100,00	0,00E+00	823	-279	Indica	Japonica	
augustus_masked-1-processed-gene-314.35-mRNA-1	1	OsR498G0101953400.01.T01	98,89	0,00E+00	2520	Os01g0735900	100,00	0,00E+00	2592	72	NA	Japonica	
augustus_masked-1-processed-gene-314.37-mRNA-1	1	OsR498G0101953800.01.T01	98,74	0,00E+00	2489	Os01g0736100	100,00	0,00E+00	2571	82	NA	Japonica	
augustus_masked-1-processed-gene-314.38-mRNA-1	1	OsR498G0101954200.01.T01	97,43	0,00E+00	1641	Os01g0736300	100,00	0,00E+00	2554	913	Japonica	Japonica	
augustus_masked-1-processed-gene-314.69-mRNA-1	1	OsR498G0101955400.01.T01	99,66	0,00E+00	1063	Os01g0736600	100,00	0,00E+00	1072	9	NA	Japonica	
augustus_masked-1-processed-gene-314.70-mRNA-1	1	OsR498G0101956800.01.T01	95,97	0,00E+00	1524	Os01g0737100	100,00	0,00E+00	1002	-522	Indica	Japonica	
augustus_masked-1-processed-gene-316.55-mRNA-1	1	OsR498G0204773100.01.T01	91,80	0,00E+00	724	Os01g0740700	100,00	0,00E+00	1272	548	Japonica	Japonica	
augustus_masked-1-processed-gene-320.69-mRNA-1	1	OsR498G0101990600.01.T01	99,81	0,00E+00	1923	Os01g0748200	100,00	0,00E+00	1932	9	NA	Japonica	
augustus_masked-1-processed-gene-320.70-mRNA-1	1	OsR498G0101991000.01.T01	99,77	0,00E+00	770	Os01g0748300	100,00	0,00E+00	774	4	NA	Japonica	
augustus_masked-1-processed-gene-323.30-mRNA-1	1	OsR498G0102012800.01.T01	99,95	0,00E+00	3544	Os01g0754701	99,95	0,00E+00	3544	0	NA	Japonica	
augustus_masked-1-processed-gene-324.53-mRNA-1	1	OsR498G0102018800.01.T01	100,00	0,00E+00	1050	Os04g0208500	82,91	2,55E-91	334	-716	Indica	Japonica	
augustus_masked-1-processed-gene-325.60-mRNA-1	1	OsR498G0102023400.01.T01	99,74	0,00E+00	2043	Os01g0757600	100,00	0,00E+00	1817	-226	Indica	Japonica	
augustus_masked-1-processed-gene-326.20-mRNA-1	1	OsR498G0102036200.01.T01	99,37	0,00E+00	2544	Os01g0759200	100,00	0,00E+00	2581	37	NA	Japonica	Kinase-TM
augustus_masked-1-processed-gene-326.21-mRNA-1	1	OsR498G0102036600.01.T01	99,74	0,00E+00	2798	Os01g0759400	100,00	0,00E+00	2816	18	NA	Japonica	Kinase-TM
augustus_masked-1-processed-gene-327.6-mRNA-1	1	OsR498G0102039800.01.T01	99,73	0,00E+00	5387	Os01g0760400	100,00	0,00E+00	5427	40	NA	Japonica	TIR-TM
augustus_masked-1-processed-gene-328.45-mRNA-1	1	OsR498G0102046000.01.T04	99,89	0,00E+00	1663	Os01g0763200	100,00	0,00E+00	1667	4	NA	Japonica	
augustus_masked-1-processed-gene-329.58-mRNA-1	1	OsR498G0102052200.01.T05	99,93	0,00E+00	2609	Os01g0764950	100,00	0,00E+00	2614	5	NA	Japonica	
augustus_masked-1-processed-gene-329.67-mRNA-1	1	OsR498G0102054200.01.T02	100,00	0,00E+00	1218	Os01g0766200	100,00	0,00E+00	1218	0	NA	Japonica	
augustus_masked-1-processed-gene-330.37-mRNA-1	1	OsR498G0102055400.01.T01	99,27	0,00E+00	973	Os01g0766300	99,82	0,00E+00	986	13	NA	Japonica	
augustus_masked-1-processed-gene-332.20-mRNA-1	1	OsR498G0102067800.01.T01	100,00	0,00E+00	1234	Os01g0771000	100,00	0,00E+00	1234	0	NA	Japonica	
augustus_masked-1-processed-gene-332.4-mRNA-1	1	OsR498G0102066200.01.T01	99,38	0,00E+00	2845	644750	100,00	0,00E+00	2890	45	NA	Japonica	
augustus_masked-1-processed-gene-332.5-mRNA-1	1	OsR498G0102066400.01.T02	99,66	0,00E+00	2131	Os01g0770400	100,00	0,00E+00	2149	18	NA	Japonica	
augustus_masked-1-processed-gene-336.52-mRNA-1	1	OsR498G0102092400.01.T04	97,00	0,00E+00	1290	Os01g0777500	94,73	0,00E+00	1365	75	NA	NA	
augustus_masked-1-processed-gene-336.77-mRNA-1	1	OsR498G0102096000.01.T01	100,00	3,91E-121	434	Os01g0778900	100,00	1,37E-121	434	0	NA	Japonica	
augustus_masked-1-processed-gene-337.19-mRNA-1	1	OsR498G0102098200.01.T01	99,41	0,00E+00	1491	Os01g0779800	100,00	0,00E+00	1510	19	NA	Japonica	
augustus_masked-1-processed-gene-338.56-mRNA-1	1	OsR498G0102108600.01.T01	99,41	2,25E-170	598	Os01g0783700	100,00	1,51E-173	607	9	NA	Japonica	
augustus_masked-1-processed-gene-339.36-mRNA-1	1	OsR498G0511115700.01.T02	71,63	2,01E-86	321	Os01g0785600	100,00	0,00E+00	1294	973	Japonica	Japonica	
augustus_masked-1-processed-gene-341.24-mRNA-1	1	OsR498G0102125800.01.T03	99,85	0,00E+00	4906	Os01g0788500	100,00	0,00E+00	4924	18	NA	Japonica	CC-NBS-TM
augustus_masked-1-processed-gene-341.25-mRNA-1	1	OsR498G0102126800.01.T01	98,37	0,00E+00	3489	Os01g0788900	100,00	0,00E+00	3631	142	NA	Japonica	
augustus_masked-1-processed-gene-343.4-mRNA-1	1	OsR498G0102129800.01.T01	99,71	4,39E-173	608	Os01g0793800	100,00	3,59E-175	612	4	NA	Japonica	
augustus_masked-1-processed-gene-343.41-mRNA-1	1	OsR498G0102133400.01.T01	99,12	5,40E-172	604	Os10g0402400	80,16	2,99E-62	237	-367	Indica	Japonica	
augustus_masked-1-processed-gene-343.6-mRNA-1	1	OsR498G0102130200.01.T01	99,69	2,45E-163	575	Os01g0793900	100,00	2,00E-165	580	5	NA	Japonica	
augustus_masked-1-processed-gene-343.7-mRNA-1	1	OsR498G0102130600.01.T01	99,71	2,48E-176	618	Os01g0794100	100,00	2,02E-178	623	5	NA	Japonica	
augustus_masked-1-processed-gene-345.30-mRNA-1	1	OsR498G0102148800.01.T04	100,00	0,00E+00	1786	Os01g0800300	100,00	0,00E+00	1786	0	NA	Japonica	
augustus_masked-1-processed-gene-347.7-mRNA-1	1	OsR498G0511097100.01.T04	88,01	0,00E+00	1338	Os01g0802850	100,00	0,00E+00	1959	621	Japonica	Japonica	
augustus_masked-1-processed-gene-35.34-mRNA-1	1	OsR498G0100225000.01.T04	100,00	0,00E+00	3214	Os01g0168100	99,72	0,00E+00	3192	-22	NA	Indica	
augustus_masked-1-processed-gene-35.53-mRNA-1	1	OsR498G0100232000.01.T02	99,74	0,00E+00	1361	Os01g0169400	99,08	0,00E+00	1336	-25	NA	Indica	
augustus_masked-1-processed-gene-353.78-mRNA-1	1	OsR498G0102199000.01.T01	100,00	0,00E+00	1884	Os01g0812900	99,52	0,00E+00	1865	-19	NA	Indica	
augustus_masked-1-processed-gene-354.66-mRNA-1	1	OsR498G0102207400.01.T02	100,00	0,00E+00	2940	Os01g0815900	99,76	0,00E+00	2922	-18	NA	Indica	
augustus_masked-1-processed-gene-359.33-mRNA-1	1	OsR498G0102239000.01.T04	100,00	0,00E+00	2370	Os01g0823700	99,77	0,00E+00	2357	-13	NA	Indica	
augustus_masked-1-processed-gene-359.34-mRNA-1	1	OsR498G0102239800.01.T01	100,00	0,00E+00	3685	Os01g0823900	99,80	0,00E+00	3667	-18	NA	Indica	
augustus_masked-1-processed-gene-359.9-mRNA-1	1	OsR498G0102234200.01.T02	100,00	0,00E+00	655	Os01g0822900	99,45	0,00E+00	646	-9	NA	Indica	
augustus_masked-1-processed-gene-36.28-mRNA-1	1	OsR498G0100238200.01.T01	99,12	1,89E-171	602	Os01g0170900	99,12	6,55E-172	602	0	NA	NA	
augustus_masked-1-processed-gene-362.25-mRNA-1	1	OsR498G0102261800.01.T03	100,00	0,00E+00	3041	Os01g0830200	99,76	0,00E+00	3023	-18	NA	Indica	
augustus_masked-1-processed-gene-365.44-mRNA-1	1	OsR498G0102284000.01.T03	100,00	0,00E+00	1537	Os01g0835600	99,88	0,00E+00	1533	-4	NA	NA	
augustus_masked-1-processed-gene-366.27-mRNA-1	1	OsR498G0102289000.01.T01	100,00	0,00E+00	2435	Os01g0836800	99,93	0,00E+00	2431	-4	NA	Indica	
augustus_masked-1-processed-gene-366.31-mRNA-1	1	OsR498G0102292400.01.T02	100,00	0,00E+00	2251	Os01g0837800	100,00	0,00E+00	2251	0	NA	Indica	
augustus_masked-1-processed-gene-367.40-mRNA-1	1	OsR498G0102294600.01.T01	100,00	1,31E-140	499	Os01g0838200	99,28	2,37E-138	489	-10	NA	Indica	
augustus_masked-1-processed-gene-367.41-mRNA-1	1	OsR498G0102296000.01.T01	100,00	0,00E+00	1180	Os01g0838600	99,69	0,00E+00	1171	-9	NA	Indica	
augustus_masked-1-processed-gene-367.60-mRNA-1	1	OsR498G0305687300.01.T01	84,41	3,99E-143	508	Os01g0839500	99,52	0,00E+00	744	236	Japonica	Indica	
augustus_masked-1-processed-gene-368.24-mRNA-1	1	OsR498G0102302600.01.T01	100,00	0,00E+00	2825	Os01g0840200	99,94	0,00E+00	2820	-5	NA	Indica	
augustus_masked-1-processed-gene-368.38-mRNA-1	1	OsR498G0102308200.01.T01	100,00	0,00E+00	2316	Os01g0841100	99,92	0,00E+00	2312	-4	NA	NA	
augustus_masked-1-processed-gene-370.13-mRNA-1	1	OsR498G0102316200.01.T01	100,00	0,00E+00	1186	Os10g0447400	76,97	2,42E-170	598	-588	Indica	NA	
augustus_masked-1-processed-gene-372.35-mRNA-1	1	OsR498G0102331600.01.T02	100,00	0,00E+00	3577	Os01g0848300	99,95	0,00E+00	3572	-5	NA	NA	
augustus_masked-1-processed-gene-372.51-mRNA-1	1	OsR498G0102333400.01.T01	70,33	2,38E-40	168	Os01g0848550	70,33	8,26E-41	168	0	NA	NA	
augustus_masked-1-processed-gene-373.43-mRNA-1	1	OsR498G0102339200.01.T01	100,00	0,00E+00	964	Os01g0850200	97,75	0,00E+00	916	-48	NA	NA	
augustus_masked-1-processed-gene-374.22-mRNA-1	1	OsR498G0102347000.01.T01	100,00	0,00E+00	3512	Os01g0852400	99,44	0,00E+00	3482	-30	NA	NA	
augustus_masked-1-processed-gene-376.15-mRNA-1	1	OsR498G0102361800.01.T01	100,00	0,00E+00	2571	Os01g0855600	99,86	0,00E+00	2562	-9	NA	NA	
augustus_masked-1-processed-gene-377.24-mRNA-1	1	OsR498G0102370800.01.T01	100,00	0,00E+00	2841	Os01g0858350	99,87	0,00E+00	2832	-9	NA	NA	
augustus_masked-1-processed-gene-378.18-mRNA-1	1	OsR498G0102377800.01.T05	100,00	0,00E+00	2917	Os01g0859500	99,75	0,00E+00	2902	-15	NA	NA	
augustus_masked-1-processed-gene-38.21-mRNA-1	1	OsR498G0100247800.01.T01	99,70	1,85E-171	602	Os01g0174100	99,70	6,43E-172	602	0	NA	Japonica	
augustus_masked-1-processed-gene-381.70-mRNA-1	1	OsR498G0102395800.01.T01	100,00	0,00E+00	2565	Os01g0865400	99,65	0,00E+00	2543	-22	NA	NA	
augustus_masked-1-processed-gene-381.71-mRNA-1	1	OsR498G0102396600.01.T01	100,00	0,00E+00	2084	Os01g0865700	99,67	0,00E+00	2174	90	NA	NA	
augustus_masked-1-processed-gene-381.72-mRNA-1	1	OsR498G0102397600.01.T01	100,00	0,00E+00	1786	Os01g0865800	99,79	0,00E+00	1728	-58	NA	NA	
augustus_masked-1-processed-gene-385.68-mRNA-1	1	OsR498G0102423600.01.T01	100,00	0,00E+00	2982	Os05g0430800	83,60	0,00E+00	1715	-1267	Indica	NA	
augustus_masked-1-processed-gene-388.7-mRNA-1	1	OsR498G0102444400.01.T01	100,00	0,00E+00	785	Os01g0877900	99,54	0,00E+00	776	-9	NA	NA	
augustus_masked-1-processed-gene-389.45-mRNA-1	1	OsR498G0102455600.01.T01	100,00	0,00E+00	2825	Os01g0881100	99,62	0,00E+00	2798	-27	NA	NA	
augustus_masked-1-processed-gene-391.49-mRNA-1	1	OsR498G0102471600.01.T01	100,00	0,00E+00	1671	Os01g0885200	99,89	0,00E+00	1666	-5	NA	NA	
augustus_masked-1-processed-gene-393.24-mRNA-1	1	OsR498G0102484600.01.T02	100,00	0,00E+00	7683	Os01g0888500	100,00	0,00E+00	4296	-3387	Indica	NA	
augustus_masked-1-processed-gene-4.64-mRNA-1	1	OsR498G0100023600.01.T01	99,08	0,00E+00	1149	Os01g0106500	100,00	0,00E+00	1164	15	NA	Japonica	
augustus_masked-1-processed-gene-4.65-mRNA-1	1	OsR498G0100023800.01.T01	95,13	0,00E+00	1417	Os01g0106600	100,00	0,00E+00	1586	169	Japonica	Japonica	
augustus_masked-1-processed-gene-40.8-mRNA-1	1	OsR498G0100258600.01.T01	99,82	0,00E+00	2015	Os01g0177400	98,93	0,00E+00	1974	-41	NA	Japonica	
augustus_masked-1-processed-gene-402.71-mRNA-1	1	OsR498G0101572500.01.T01	99,11	0,00E+00	1392	Os01g0623900	100,00	0,00E+00	1478	86	NA	NA	
augustus_masked-1-processed-gene-403.43-mRNA-1	1	OsR498G0101564900.01.T01	99,74	0,00E+00	3501	Os01g0621900	99,95	0,00E+00	3497	-4	NA	NA	
augustus_masked-1-processed-gene-404.20-mRNA-1	1	OsR498G0101560500.01.T01	99,67	0,00E+00	2727	Os01g0620800	99,93	0,00E+00	2745	18	NA	NA	
augustus_masked-1-processed-gene-406.62-mRNA-1	1	OsR498G0101547500.01.T01	99,30	0,00E+00	1266	Os01g0616800	99,58	0,00E+00	1275	9	NA	NA	
augustus_masked-1-processed-gene-407.5-mRNA-1	1	OsR498G0101546700.01.T01	99,56	0,00E+00	2027	Os01g0616600	99,91	0,00E+00	2045	18	NA	NA	
augustus_masked-1-processed-gene-412.43-mRNA-1	1	OsR498G0101506700.01.T01	99,93	0,00E+00	2514	Os01g0607800	100,00	0,00E+00	1829	-685	Indica	NA	
augustus_masked-1-processed-gene-412.57-mRNA-1	1	OsR498G0101503900.01.T02	96,21	0,00E+00	1225	Os01g0607300	96,34	0,00E+00	1230	5	NA	NA	
augustus_masked-1-processed-gene-412.63-mRNA-1	1	OsR498G0101502500.01.T01	99,38	0,00E+00	858	Os01g0607000	100,00	0,00E+00	910	52	NA	NA	
augustus_masked-1-processed-gene-414.8-mRNA-1	1	OsR498G0101493300.01.T01	99,14	0,00E+00	1226	Os01g0604300	100,00	0,00E+00	672	-554	Indica	NA	
augustus_masked-1-processed-gene-415.18-mRNA-1	1	OsR498G0101476300.01.T01	99,65	0,00E+00	1544	Os01g0600900	100,00	0,00E+00	1418	-126	NA	NA	
augustus_masked-1-processed-gene-416.62-mRNA-1	1	OsR498G0101471900.01.T01	99,87	0,00E+00	2702	Os01g0598600	100,00	0,00E+00	2711	9	NA	NA	
augustus_masked-1-processed-gene-417.5-mRNA-1	1	OsR498G0101469300.01.T01	97,72	0,00E+00	2540	Os01g0597800	100,00	0,00E+00	2695	155	NA	NA	
augustus_masked-1-processed-gene-418.57-mRNA-1	1	OsR498G0101457300.01.T01	99,59	0,00E+00	3042	Os01g0594300	94,86	0,00E+00	2736	-306	Indica	NA	LRR-TM
augustus_masked-1-processed-gene-42.29-mRNA-1	1	OsR498G0100270600.01.T01	99,74	0,00E+00	2801	Os01g0180000	100,00	0,00E+00	2819	18	NA	Japonica	LRR-TM
augustus_masked-1-processed-gene-420.6-mRNA-1	1	OsR498G0102308200.01.T01	100,00	0,00E+00	2316	Os01g0841100	99,92	0,00E+00	2312	-4	NA	NA	
augustus_masked-1-processed-gene-421.26-mRNA-1	1	OsR498G0102316200.01.T01	100,00	0,00E+00	1186	Os10g0447400	76,97	2,42E-170	598	-588	Indica	NA	
augustus_masked-1-processed-gene-423.54-mRNA-1	1	OsR498G0102331600.01.T02	100,00	0,00E+00	3577	Os01g0848300	99,95	0,00E+00	3572	-5	NA	NA	
augustus_masked-1-processed-gene-423.56-mRNA-1	1	OsR498G0102333400.01.T01	70,33	2,38E-40	168	Os01g0848550	70,33	8,26E-41	168	0	NA	NA	
augustus_masked-1-processed-gene-424.56-mRNA-1	1	OsR498G0102339200.01.T01	100,00	0,00E+00	964	Os01g0850200	97,75	0,00E+00	916	-48	NA	NA	
augustus_masked-1-processed-gene-425.51-mRNA-1	1	OsR498G0102347000.01.T01	100,00	0,00E+00	3512	Os01g0852400	99,44	0,00E+00	3482	-30	NA	NA	
augustus_masked-1-processed-gene-427.18-mRNA-1	1	OsR498G0102361800.01.T01	100,00	0,00E+00	2571	Os01g0855600	99,86	0,00E+00	2562	-9	NA	NA	
augustus_masked-1-processed-gene-429.44-mRNA-1	1	OsR498G0102370800.01.T01	100,00	0,00E+00	2841	Os01g0858350	99,87	0,00E+00	2832	-9	NA	NA	
augustus_masked-1-processed-gene-430.2-mRNA-1	1	OsR498G0102377800.01.T05	100,00	0,00E+00	2917	Os01g0859500	99,75	0,00E+00	2902	-15	NA	NA	
augustus_masked-1-processed-gene-432.44-mRNA-1	1	OsR498G0102395800.01.T01	100,00	0,00E+00	2565	Os01g0865400	99,65	0,00E+00	2543	-22	NA	NA	
augustus_masked-1-processed-gene-432.45-mRNA-1	1	OsR498G0102396600.01.T01	100,00	0,00E+00	2084	Os01g0865700	99,67	0,00E+00	2174	90	NA	NA	
augustus_masked-1-processed-gene-432.46-mRNA-1	1	OsR498G0102397600.01.T01	100,00	0,00E+00	1786	Os01g0865800	99,79	0,00E+00	1728	-58	NA	NA	
augustus_masked-1-processed-gene-436.34-mRNA-1	1	OsR498G0102423600.01.T01	100,00	0,00E+00	2982	Os05g0430800	83,60	0,00E+00	1715	-1267	Indica	NA	
augustus_masked-1-processed-gene-439.52-mRNA-1	1	OsR498G0102444400.01.T01	100,00	0,00E+00	785	Os01g0877900	99,54	0,00E+00	776	-9	NA	NA	
augustus_masked-1-processed-gene-44.32-mRNA-1	1	OsR498G0100278600.01.T01	99,64	0,00E+00	2510	Os01g0182400	99,93	0,00E+00	2528	18	NA	Japonica	
augustus_masked-1-processed-gene-440.25-mRNA-1	1	OsR498G0102455600.01.T01	100,00	0,00E+00	2825	Os01g0881100	99,62	0,00E+00	2798	-27	NA	NA	
augustus_masked-1-processed-gene-444.18-mRNA-1	1	OsR498G0102484600.01.T02	100,00	0,00E+00	7683	Os01g0888500	100,00	0,00E+00	4296	-3387	Indica	NA	
augustus_masked-1-processed-gene-454.33-mRNA-1	1	OsR498G0101572500.01.T01	99,11	0,00E+00	1392	Os01g0623900	100,00	0,00E+00	1478	86	NA	NA	

augustus_masked-1-processed-gene-455.19-mRNA-1	1	OsR498G0101560500.01.T01	99,67	0,00E+00	2727	Os01g0620800	99,93	0,00E+00	2745	18	NA	NA	
augustus_masked-1-processed-gene-455.4-mRNA-1	1	OsR498G0101564900.01.T01	99,74	0,00E+00	3501	Os01g0621900	99,95	0,00E+00	3497	-4	NA	NA	
augustus_masked-1-processed-gene-458.33-mRNA-1	1	OsR498G0101547500.01.T01	99,30	0,00E+00	1266	Os01g0616800	99,58	0,00E+00	1275	9	NA	NA	
augustus_masked-1-processed-gene-458.41-mRNA-1	1	OsR498G0101546700.01.T01	99,56	0,00E+00	2027	Os01g0616600	99,91	0,00E+00	2045	18	NA	NA	
augustus_masked-1-processed-gene-463.50-mRNA-1	1	OsR498G0101506700.01.T01	99,93	0,00E+00	2514	Os01g0607800	100,00	0,00E+00	1829	-685	Indica	NA	
augustus_masked-1-processed-gene-463.53-mRNA-1	1	OsR498G0101503900.01.T02	96,21	0,00E+00	1225	Os01g0607300	96,34	0,00E+00	1230	5	NA	NA	
augustus_masked-1-processed-gene-464.34-mRNA-1	1	OsR498G0101502500.01.T01	99,38	0,00E+00	858	Os01g0607000	100,00	0,00E+00	910	52	NA	NA	
augustus_masked-1-processed-gene-465.31-mRNA-1	1	OsR498G0101493300.01.T01	99,14	0,00E+00	1226	Os01g0604300	100,00	0,00E+00	672	-554	Indica	NA	
augustus_masked-1-processed-gene-467.38-mRNA-1	1	OsR498G0101476300.01.T01	99,65	0,00E+00	1544	Os01g0600900	100,00	0,00E+00	1418	-126	NA	NA	
augustus_masked-1-processed-gene-467.65-mRNA-1	1	OsR498G0101471900.01.T01	99,87	0,00E+00	2702	Os01g0598600	100,00	0,00E+00	2711	9	NA	NA	
augustus_masked-1-processed-gene-468.25-mRNA-1	1	OsR498G0101469300.01.T01	97,72	0,00E+00	2540	Os01g0597800	100,00	0,00E+00	2695	155	NA	NA	
augustus_masked-1-processed-gene-470.25-mRNA-1	1	OsR498G0102538200.01.T01	99,91	0,00E+00	1939	Os01g0905800	99,63	0,00E+00	1925	-14	NA	Indica	
augustus_masked-1-processed-gene-470.30-mRNA-1	1	OsR498G0102541200.01.T01	99,19	0,00E+00	870	Os01g0906425	98,17	0,00E+00	847	-23	NA	Indica	
augustus_masked-1-processed-gene-470.9-mRNA-1	1	OsR498G0101457300.01.T01	99,59	0,00E+00	3042	Os01g0594300	94,86	0,00E+00	2736	-306	Indica	NA	LRR-TM
augustus_masked-1-processed-gene-472.64-mRNA-1	1	OsR498G0102552000.01.T01	100,00	0,00E+00	1775	Os01g0909400	99,29	0,00E+00	1745	-30	NA	Indica	
augustus_masked-1-processed-gene-475.29-mRNA-1	1	OsR498G0102562800.01.T01	99,44	0,00E+00	3816	Os01g0913400	100,00	0,00E+00	3858	42	NA	Japonica	
augustus_masked-1-processed-gene-475.35-mRNA-1	1	OsR498G0102565000.01.T05	99,72	0,00E+00	3170	Os01g0914600	100,00	0,00E+00	3193	23	NA	Japonica	
augustus_masked-1-processed-gene-475.7-mRNA-1	1	OsR498G0102561600.01.T02	99,88	0,00E+00	2881	Os01g0912900	100,00	0,00E+00	2890	9	NA	Japonica	
augustus_masked-1-processed-gene-475.8-mRNA-1	1	OsR498G0102562000.01.T02	100,00	0,00E+00	861	Os01g0913100	100,00	0,00E+00	848	-13	NA	Japonica	
augustus_masked-1-processed-gene-476.60-mRNA-1	1	OsR498G0102571400.01.T03	83,00	0,00E+00	987	Os01g0915900	99,18	0,00E+00	1507	520	Japonica	Japonica	
augustus_masked-1-processed-gene-476.61-mRNA-1	1	OsR498G0102571600.01.T02	98,75	0,00E+00	1404	Os01g0916000	100,00	0,00E+00	1445	41	NA	Japonica	
augustus_masked-1-processed-gene-476.62-mRNA-1	1	OsR498G0102571800.01.T01	98,64	0,00E+00	1157	Os01g0916100	99,61	0,00E+00	919	-238	Indica	Japonica	
augustus_masked-1-processed-gene-477.46-mRNA-1	1	OsR498G0102578800.01.T01	97,82	0,00E+00	1653	Os01g0917900	100,00	0,00E+00	1700	47	NA	Japonica	
augustus_masked-1-processed-gene-48.30-mRNA-1	1	OsR498G0100305000.01.T02	100,00	0,00E+00	2700	Os01g0189800	100,00	0,00E+00	2700	0	NA	NA	
augustus_masked-1-processed-gene-48.6-mRNA-1	1	OsR498G0100302800.01.T01	99,93	0,00E+00	2597	Os01g0188800	99,93	0,00E+00	2597	0	NA	NA	
augustus_masked-1-processed-gene-480.13-mRNA-1	1	OsR498G0102598800.01.T01	99,68	0,00E+00	2250	Os01g0922100	99,92	0,00E+00	2263	13	NA	Japonica	
augustus_masked-1-processed-gene-482.15-mRNA-1	1	OsR498G0102616400.01.T01	99,44	0,00E+00	945	Os01g0924966	99,81	0,00E+00	934	-11	NA	Japonica	
augustus_masked-1-processed-gene-487.45-mRNA-1	1	OsR498G0102649000.01.T02	99,81	0,00E+00	1934	Os01g0934100	100,00	0,00E+00	1943	9	NA	Japonica	
augustus_masked-1-processed-gene-487.62-mRNA-1	1	OsR498G0102652200.01.T01	99,32	0,00E+00	1036	Os01g0935200	75,97	3,55E-64	245	-791	Indica	Japonica	
augustus_masked-1-processed-gene-488.16-mRNA-1	1	OsR498G0102660600.01.T01	72,23	6,52E-178	625	Os01g0936900	78,98	0,00E+00	956	331	Japonica	Japonica	
augustus_masked-1-processed-gene-488.17-mRNA-1	1	OsR498G0102660200.01.T01	99,79	0,00E+00	1745	Os01g0936900	73,92	0,00E+00	645	-1100	Indica	Japonica	
augustus_masked-1-processed-gene-488.18-mRNA-1	1	OsR498G0102660600.01.T01	99,77	0,00E+00	2330	Os01g0936900	92,90	0,00E+00	1681	-649	Indica	Japonica	
augustus_masked-1-processed-gene-488.20-mRNA-1	1	OsR498G0102660800.01.T01	99,79	0,00E+00	1739	Os01g0937000	100,00	0,00E+00	2143	404	Japonica	Japonica	
augustus_masked-1-processed-gene-488.22-mRNA-1	1	OsR498G0102661800.01.T01	99,68	0,00E+00	2255	Os01g0937100	100,00	0,00E+00	2273	18	NA	Japonica	
augustus_masked-1-processed-gene-488.23-mRNA-1	1	OsR498G0102662000.01.T03	91,75	0,00E+00	2310	Os01g0937300	99,94	0,00E+00	2909	599	Japonica	Japonica	CC-NBS-TM
augustus_masked-1-processed-gene-489.31-mRNA-1	1	OsR498G0102666600.01.T01	100,00	0,00E+00	921	Os01g0938400	100,00	0,00E+00	921	0	NA	Japonica	
augustus_masked-1-processed-gene-490.8-mRNA-1	1	OsR498G0102672200.01.T02	96,01	0,00E+00	1574	Os01g0940100	96,22	0,00E+00	1583	9	NA	Japonica	
augustus_masked-1-processed-gene-491.62-mRNA-1	1	OsR498G0102675100.01.T01	99,79	0,00E+00	1676	Os01g0941200	100,00	0,00E+00	1685	9	NA	Indica	
augustus_masked-1-processed-gene-491.74-mRNA-1	1	OsR498G0102678200.01.T01	100,00	0,00E+00	2387	Os01g0942200	99,85	0,00E+00	2378	-9	NA	NA	
augustus_masked-1-processed-gene-494.27-mRNA-1	1	OsR498G0102694000.01.T01	99,79	0,00E+00	2618	Os01g0948500	100,00	0,00E+00	2632	14	NA	Japonica	
augustus_masked-1-processed-gene-496.89-mRNA-1	1	OsR498G0510739700.01.T01	76,77	0,00E+00	1149	Os01g0951500	99,70	0,00E+00	2938	1789	Japonica	NA	
augustus_masked-1-processed-gene-498.14-mRNA-1	1	OsR498G0102720400.01.T01	99,93	0,00E+00	2409	Os01g0954900	99,93	0,00E+00	2409	0	NA	Japonica	
augustus_masked-1-processed-gene-498.16-mRNA-1	1	OsR498G0102721000.01.T01	99,56	0,00E+00	814	Os01g0955100	100,00	0,00E+00	823	9	NA	Japonica	
augustus_masked-1-processed-gene-498.17-mRNA-1	1	OsR498G0102721800.01.T01	99,56	0,00E+00	814	Os01g0955100	77,94	3,56E-88	324	-490	Indica	Japonica	
augustus_masked-1-processed-gene-50.13-mRNA-1	1	OsR498G0100316400.01.T03	99,83	0,00E+00	2085	Os01g0192000	99,83	0,00E+00	2085	0	NA	NA	
augustus_masked-1-processed-gene-505.62-mRNA-1	1	OsR498G0102772400.01.T02	100,00	0,00E+00	3106	Os01g0970900	100,00	0,00E+00	3106	0	NA	NA	
augustus_masked-1-processed-gene-506.35-mRNA-1	1	OsR498G0102775000.01.T01	99,44	0,00E+00	1276	Os01g0971800	100,00	0,00E+00	1294	18	NA	Japonica	
augustus_masked-1-processed-gene-508.23-mRNA-1	1	OsR498G0102788800.01.T01	98,85	0,00E+00	1987	Os01g0976200	100,00	0,00E+00	2046	59	NA	Japonica	
augustus_masked-1-processed-gene-52.19-mRNA-1	1	OsR498G0100339800.01.T01	98,35	0,00E+00	1049	Os01g0196500	100,00	0,00E+00	1094	45	NA	Japonica	
augustus_masked-1-processed-gene-53.47-mRNA-1	1	OsR498G0100343800.01.T01	99,77	0,00E+00	3067	Os01g0197500	100,00	0,00E+00	3378	311	NA	Japonica	
augustus_masked-1-processed-gene-57.34-mRNA-1	1	OsR498G0100367600.01.T05	98,85	9,05E-173	607	Os01g0203300	100,00	1,43E-177	621	14	NA	Japonica	
augustus_masked-1-processed-gene-57.38-mRNA-1	1	OsR498G0100369400.01.T02	99,74	0,00E+00	2801	Os01g0203800	99,94	0,00E+00	2815	14	NA	Japonica	
augustus_masked-1-processed-gene-58.86-mRNA-1	1	OsR498G0100377600.01.T01	100,00	0,00E+00	2500	Os01g0206700	100,00	0,00E+00	2500	0	NA	NA	Kinase-TM
augustus_masked-1-processed-gene-59.25-mRNA-1	1	OsR498G0100379200.01.T01	100,00	0,00E+00	3377	Os01g0207200	100,00	0,00E+00	3377	0	NA	NA	
augustus_masked-1-processed-gene-59.28-mRNA-1	1	OsR498G0100380800.01.T01	100,00	0,00E+00	3317	Os01g0207600	100,00	0,00E+00	3317	0	NA	NA	
augustus_masked-1-processed-gene-59.53-mRNA-1	1	OsR498G0100383000.01.T01	100,00	0,00E+00	3339	Os01g0208400	100,00	0,00E+00	3339	0	NA	NA	
augustus_masked-1-processed-gene-60.23-mRNA-1	1	OsR498G0100387600.01.T03	99,88	0,00E+00	3070	Os01g0209500	100,00	0,00E+00	3079	9	NA	Japonica	
augustus_masked-1-processed-gene-61.14-mRNA-1	1	OsR498G0100397000.01.T01	100,00	0,00E+00	2733	Os01g0211200	100,00	0,00E+00	2733	0	NA	NA	
augustus_masked-1-processed-gene-61.3-mRNA-1	1	OsR498G0100395200.01.T01	90,01	0,00E+00	2044	Os01g0210900	100,00	0,00E+00	2771	727	Japonica	NA	
augustus_masked-1-processed-gene-61.4-mRNA-1	1	OsR498G0100395200.01.T01	100,00	0,00E+00	2776	Os01g0211000	100,00	0,00E+00	2776	0	NA	NA	
augustus_masked-1-processed-gene-68.24-mRNA-1	1	OsR498G0100448000.01.T02	99,89	0,00E+00	1654	Os01g0223700	94,56	0,00E+00	1433	-221	Indica	Japonica	Kinase-TM
augustus_masked-1-processed-gene-68.25-mRNA-1	1	OsR498G0100448100.01.T01	84,21	0,00E+00	2599	Os01g0224000	100,00	0,00E+00	4361	1762	Japonica	Japonica	Kinase-TM
augustus_masked-1-processed-gene-7.32-mRNA-1	1	OsR498G0100037600.01.T01	98,69	0,00E+00	929	Os01g0111700	100,00	0,00E+00	953	24	NA	Japonica	
augustus_masked-1-processed-gene-7.51-mRNA-1	1	OsR498G0100039200.01.T02	100,00	0,00E+00	2021	Os01g0112100	99,20	0,00E+00	2003	-18	NA	Indica	
augustus_masked-1-processed-gene-7.53-mRNA-1	1	OsR498G0100040400.01.T02	100,00	0,00E+00	1243	Os01g0112400	99,86	0,00E+00	1239	-4	NA	Indica	
augustus_masked-1-processed-gene-70.37-mRNA-1	1	OsR498G0100462600.01.T01	99,75	0,00E+00	4334	Os01g0226400	99,88	0,00E+00	4346	12	NA	Japonica	
augustus_masked-1-processed-gene-72.63-mRNA-1	1	OsR498G0100476800.01.T01	99,81	0,00E+00	1909	Os01g0229800	100,00	0,00E+00	1645	-264	Indica	Japonica	
augustus_masked-1-processed-gene-75.31-mRNA-1	1	OsR498G0100494600.01.T01	99,97	0,00E+00	5320	Os01g0235400	100,00	0,00E+00	2938	-2382	Indica	Japonica	
augustus_masked-1-processed-gene-8.12-mRNA-1	1	OsR498G0100045000.01.T04	100,00	0,00E+00	2727	Os01g0113400	98,48	0,00E+00	2624	-103	NA	Indica	Kinase-TM
augustus_masked-1-processed-gene-80.27-mRNA-1	1	OsR498G0100533600.01.T02	99,91	0,00E+00	2090	Os01g0244400	99,91	0,00E+00	2086	-4	NA	NA	
augustus_masked-1-processed-gene-81.23-mRNA-1	1	OsR498G0100537400.01.T01	99,71	0,00E+00	2434	Os01g0244400	75,83	0,00E+00	680	-1754	Indica	NA	
augustus_masked-1-processed-gene-81.28-mRNA-1	1	OsR498G0100539200.01.T05	99,91	0,00E+00	4019	Os01g0246100	99,91	0,00E+00	4019	0	NA	Indica	
augustus_masked-1-processed-gene-85.2-mRNA-1	1	OsR498G0100568000.01.T01	100,00	0,00E+00	1689	Os01g0254300	100,00	0,00E+00	1229	-460	Indica	Japonica	
augustus_masked-1-processed-gene-85.22-mRNA-1	1	OsR498G0100570800.01.T01	100,00	0,00E+00	1478	Os01g0254900	100,00	0,00E+00	1478	0	NA	NA	
augustus_masked-1-processed-gene-86.10-mRNA-1	1	OsR498G0100576000.01.T03	99,63	1,45E-134	480	Os01g0256500	100,00	2,00E-165	580	100	Japonica	NA	
augustus_masked-1-processed-gene-86.24-mRNA-1	1	OsR498G0100578800.01.T01	100,00	0,00E+00	715	Os01g0257100	100,00	0,00E+00	715	0	NA	NA	
augustus_masked-1-processed-gene-86.26-mRNA-1	1	OsR498G0100579400.01.T03	100,00	7,71E-170	596	Os01g0257300	100,00	2,68E-170	596	0	NA	NA	
augustus_masked-1-processed-gene-9.31-mRNA-1	1	OsR498G0100056000.01.T03	100,00	0,00E+00	2040	Os01g0117400	90,36	0,00E+00	1549	-491	Indica	Indica	Kinase-TM
augustus_masked-1-processed-gene-9.4-mRNA-1	1	OsR498G0100051200.01.T01	100,00	0,00E+00	2140	Os01g0115700	98,45	0,00E+00	2019	-121	NA	Indica	Kinase-TM
augustus_masked-1-processed-gene-90.25-mRNA-1	1	OsR498G0100607600.01.T01	100,00	0,00E+00	1424	Os04g0423200	88,96	5,88E-177	619	-805	Indica	NA	
augustus_masked-1-processed-gene-91.58-mRNA-1	1	OsR498G0100614400.01.T03	99,59	0,00E+00	2631	Os01g0265800	100,00	0,00E+00	2841	210	NA	NA	
augustus_masked-1-processed-gene-97.17-mRNA-1	1	OsR498G0100655000.01.T01	99,65	0,00E+00	2570	Os01g0276400	100,00	0,00E+00	1905	-665	Indica	Japonica	
augustus_masked-1-processed-gene-98.24-mRNA-1	1	OsR498G0100660800.01.T01	97,77	0,00E+00	1231	Os01g0278000	98,75	0,00E+00	1257	26	NA	Japonica	
augustus-1-processed-gene-1.72-mRNA-1	1	OsR498G0100006300.01.T06	99,90	0,00E+00	5284	Os01g0101600	100,00	0,00E+00	5297	13	NA	Japonica	
augustus-1-processed-gene-100.51-mRNA-1	1	OsR498G0509898500.01.T01	89,99	0,00E+00	897	Os01g0862500	94,31	0,00E+00	1150	253	Japonica	Japonica	
augustus-1-processed-gene-102.35-mRNA-1	1	OsR498G0100680700.01.T01	100,00	1,83E-171	601	Os01g0283400	100,00	6,37E-172	601	0	NA	Japonica	
augustus-1-processed-gene-106.19-mRNA-1	1	OsR498G0100711500.01.T01	100,00	0,00E+00	1864	Os01g0290600	100,00	0,00E+00	2567	703	Japonica	Japonica	
augustus-1-processed-gene-107.17-mRNA-1	1	OsR498G0100721100.01.T03	99,26	0,00E+00	1427	Os01g0292700	100,00	0,00E+00	1456	29	NA	Japonica	
augustus-1-processed-gene-108.20-mRNA-1	1	OsR498G0100723300.01.T03	99,68	0,00E+00	2239	Os01g0293200	100,00	0,00E+00	2250	11	NA	Japonica	
augustus-1-processed-gene-110.23-mRNA-1	1	OsR498G0100734500.01.T01	99,77	0,00E+00	1582	Os01g0298400	100,00	0,00E+00	1591	9	NA	Japonica	
augustus-1-processed-gene-113.11-mRNA-1	1	OsR498G0100517100.01.T01	80,55	5,54E-70	264	Os01g0302900	100,00	6,27E-159	558	294	Japonica	Japonica	
augustus-1-processed-gene-113.13-mRNA-1	1	OsR498G0100750300.01.T01	99,90	0,00E+00	1814	Os01g0303100	100,00	0,00E+00	1819	5	NA	Japonica	
augustus-1-processed-gene-115.16-mRNA-1	1	OsR498G0100760300.01.T01	99,85	0,00E+00	1199	Os01g0306400	99,86	0,00E+00	1327	128	NA	Japonica	
augustus-1-processed-gene-117.14-mRNA-1	1	OsR498G0100773300.01.T01	97,24	0,00E+00	979	Os01g0309100	100,00	0,00E+00	1045	66	NA	Japonica	
augustus-1-processed-gene-118.39-mRNA-1	1	OsR498G0100778900.01.T01	100,00	0,00E+00	1077	Os01g0311400	100,00	0,00E+00	1077	0	NA	Japonica	
augustus-1-processed-gene-12.15-mRNA-1	1	OsR498G0100074300.01.T01	100,00	0,00E+00	1283	Os01g0122300	100,00	0,00E+00	1656	373	Japonica	NA	
augustus-1-processed-gene-120.1-mRNA-1	1	OsR498G0100787900.01.T01	98,08	2,75E-98	358	Os03g0341300	71,18	5,70E-13	74,3	-283,7	Indica	Japonica	
augustus-1-processed-gene-122.10-mRNA-1	1	OsR498G0100798100.01.T01	99,39	0,00E+00	1759	Os01g0317800	100,00	0,00E+00	1786	27	NA	Japonica	
augustus-1-processed-gene-127.18-mRNA-1	1	OsR498G0100832700.01.T01	97,18	0,00E+00	713	Os01g0327000	100,00	0,00E+00	823	110	Japonica	Japonica	
augustus-1-processed-gene-129.4-mRNA-1	1	OsR498G0100844100.01.T01	98,54	0,00E+00	2629	Os01g0330500	100,00	0,00E+00	2915	286	NA	Japonica	
augustus-1-processed-gene-13.21-mRNA-1	1	OsR498G0100084700.01.T01	99,76	0,00E+00	2265	Os01g0125600	100,00	0,00E+00	2278	13	NA	Japonica	
augustus-1-processed-gene-134.35-mRNA-1	1	OsR498G0100861300.01.T01	99,76	0,00E+00	3668	Os01g0337500	100,00	0,00E+00	3690	22	NA	Japonica	
augustus-1-processed-gene-134.47-mRNA-1	1	OsR498G0100863300.01.T01	99,77	0,00E+00	2379	Os01g0338200	100,00	0,00E+00	2392	13	NA	Japonica	
augustus-1-processed-gene-137.0-mRNA-1	1	OsR498G0100880900.01.T01	98,81	0,00E+00	7256	Os01g0342700	98,91	0,00E+00	7274	18	NA	Japonica	
augustus-1-processed-gene-137.12-mRNA-1	1	OsR498G0100883700.01.T06	94,19	0,00E+00	2757	Os01g0343200	95,90	0,00E+00	2892	135	NA	Japonica	
augustus-1-processed-gene-139.30-mRNA-1	1	OsR498G0100897900.01.T02	87,41	0,00E+00	1390	Os01g0347300	100,00	0,00E+00	2035	645	Japonica	Japonica	
augustus-1-processed-gene-140.15-mRNA-1	1	OsR498G0100901500.01.T01	98,26	0,00E+00	792	Os01g0348700	100,00	0,00E+00	829	37	NA	Japonica	
augustus-1-processed-gene-141.8-mRNA-1	1	OsR498G0100905900.01.T01	99,64	0,00E+00	2445	Os01g0350000	100,00	0,00E+00	2468	23	NA	Japonica	
augustus-1-processed-gene-142.10-mRNA-1	1	OsR498G0100909500.01.T03	99,79	0,00E+00	4312	Os01g0351300	100,00	0,00E+00	4334	22	NA	Japonica	
augustus-1-processed-gene-142.12-mRNA-1	1	OsR498G0100911700.01.T01	100,00	0,00E+00	1867	Os01g0351800	100,00	0,00E+00	1867	0	NA	Japonica	
augustus-1-processed-gene-144.31-mRNA-1	1	OsR498G0100917100.01.T04	98,75	0,00E+00	4602	Os01g0355000	100,00	0,00E+00	4751	149	NA	Japonica	
augustus-1-processed-gene-144.32-mRNA-1	1	OsR498G0100918100.01.T01	94,94	0,00E+00	686	Os01g0355100	100,00	0,00E+00	785	99	Japonica	NA	
augustus-1-processed-gene-146.10-mRNA-1	1	OsR498G0100931900.01.T01	97,78	0,00E+00	840	Os01g0357100	93,27	2,44E-179	627	-213	Indica	Japonica	
augustus-1-processed-gene-146.13-mRNA-1	1	OsR498G0100933100.01.T05	99,83	0,00E+00	4338	Os01g0357800	99,96	0,00E+00	4351	13	NA	Japonica	
augustus-1-processed-gene-15.2-mRNA-1	1	OsR498G0100091300.01.T01	100,00	0,00E+00	755	Os01g0127600	99,44	0,00E+00	957	202	Japonica	Indica	
augustus-1-processed-gene-151.21-mRNA-1	1	OsR498G0100957400.01.T01	97,40	0,00E+00	1361	Os01g0364400	78,40	3,62E-149	527	-834	Indica	Japonica	CC-Kinase-TM
augustus-1-processed-gene-152.17-mRNA-1	1	OsR498G0100962700.01.T05	99,88	0,00E+00	3016	Os01g0367700	100,00	0,00E+00	3025	9	NA	Japonica	CC-Kinase
augustus-1-processed-gene-153.3-mRNA-1	1	OsR498G0100966100.01.T01	99,38	0,00E+00	1151	Os01g0368700	100,00	0,00E+00	1169	18	NA	Japonica	
augustus-1-processed-gene-154.7-mRNA-1	1	OsR498G0100971500.01.T01	100,00	1,03E-174	612	Os01g0370600	100,00	3,59E-175	612	0	NA	Japonica	
augustus-1-processed-gene-160.8-mRNA-1	1	OsR498G0100999600.01.T01	98,30	0,00E+00	2031	Os01g0380800	100,00	0,00E+00	2122	91	NA	Japonica	
augustus-1-processed-gene-162.24-mRNA-1	1	OsR498G0101004300.01.T01	99,08	0,00E+00	3268	Os01g0383900	100,00	0,00E+00	3344	76	NA	Japonica	
augustus-1-processed-gene-162.25-mRNA-1	1	OsR498G0101005100.01.T01	99,69	0,00E+00	3440	Os01g0384300	100,00	0,00E+00	2516	-924	Indica	Japonica	Kinase-TM
augustus-1-processed-gene-166.18-mRNA-1	1	OsR498G0101026700.01.T01	99,72	0,00E+00	1296	Os01g0389700	100,00	0,00E+00	1305	9	NA	Japonica	
augustus-1-processed-gene-166.23-mRNA-1	1	OsR498G0101029900.01.T05	99,80	0,00E+00	3543	Os01g0390600	100,00	0,00E+00	3561	18	NA	Japonica	
augustus-1-processed-gene-166.3-mRNA-1	1	OsR498G0101025500.01.T01	99,37	0,00E+00	1412	Os01g0389200	100,00	0,00E+00	1434	22	NA	Japonica	
augustus-1-processed-gene-17.20-mRNA-1	1	OsR498G0100108700.01.T01	100,00	0,00E+00	859	Os01g0131300	99,24	0,00E+00	933	74	NA	Indica	
augustus-1-processed-gene-17.23-mRNA-1	1	OsR498G0100109900.01.T06	100,00	0,00E+00	1840	Os01g0131600	99,61	0,00E+00	1820	-20	NA	Indica	
augustus-1-processed-gene-180.6-mRNA-1	1	OsR498G0101059100.01.T01	99,93	0,00E+00	2691	Os01g0500900	100,00	0,00E+00	2695	4	NA	Japonica	
augustus-1-processed-gene-184.10-mRNA-1	1	OsR498G0101082100.01.T01	100,00	0,00E+00	1732	Os01g0507500	100,00	0,00E+00	1732	0	NA	Japonica	
augustus-1-processed-gene-185.0-mRNA-1	1	OsR498G0101085900.01.T01	99,80	0,00E+00	878	Os01g0508100	100,00	0,00E+00	883	5	NA	Japonica	
augustus-1-processed-gene-188.1-mRNA-1	1	OsR498G0101106700.01.T01	99,74	0,00E+00	678	Os01g0512700	100,00	0,00E+00	1315	637	Japonica	Japonica	
augustus-1-processed-gene-19.26-mRNA-1	1	OsR498G0100121300.01.T01	100,00	0,00E+00	818	Os01g0135800	100,00	0,00E+00	818	0	NA	Indica	
augustus-1-processed-gene-19.37-mRNA-1	1	OsR498G0100119800.01.T01	100,00	0,00E+00	3050	Os01g0134800	99,91	0,00E+00	1993	-1057	Indica	Indica	
augustus-1-processed-gene-19.38-mRNA-1	1	OsR498G0100120000.01.T01	100,00	0,00E+00	3020	Os01g0134900	99,91	0,00E+00	2002	-1018	Indica	Indica	
augustus-1-processed-gene-19.42-mRNA-1	1	OsR498G0100124300.01.T01	100,00	0,00E+00	794	Os01g0136200	99,78	0,00E+00	808	14	NA	Indica	
augustus-1-processed-gene-191.22-mRNA-1	1	OsR498G0102362500.01.T01	79,93	3,53E-72	271	Os01g0518800	100,00	8,26E-151	531	260	Japonica	NA	
augustus-1-processed-gene-191.30-mRNA-1	1	OsR498G0101973200.01.T01	89,22	8,37E-72	269	Os01g0742300	88,73	1,24E-70	265	-4	NA	NA	
augustus-1-processed-gene-198.5-mRNA-1	1	OsR498G0101163500.01.T07	90,93	0,00E+00	820	Os01g0530400	91,10	0,00E+00	825	5	NA	Japonica	
augustus-1-processed-gene-199.19-mRNA-1	1	OsR498G0101168500.01.T01	99,46	0,00E+00	2643	Os01g0532100	100,00	0,00E+00	2679	36	NA	Japonica	
augustus-1-processed-gene-2.43-mRNA-1	1	OsR498G0100011300.01.T01	98,93	0,00E+00	2626	Os01g0102400	99,92	0,00E+00	2276	-350	Indica	Japonica	
augustus-1-processed-gene-2.48-mRNA-1	1	OsR498G0100013300.01.T01	99,78	0,00E+00	3267	Os01g0103100	100,00	0,00E+00	3285	18	NA	Japonica	
augustus-1-processed-gene-20.29-mRNA-1	1	OsR498G0100127300.01.T02	95,68	0,00E+00	1080	Os01g0137100	97,62	0,00E+00	1139	59	NA	NA	
augustus-1-processed-gene-204.0-mRNA-1	1	OsR498G0101198100.01.T05	100,00	0,00E+00	1272	Os01g0539900	83,05	6,40E-119	426	-846	Indica	NA	
augustus-1-processed-gene-208.18-mRNA-1	1	OsR498G0101243900.01.T04	90,99	7,63E-178	623	Os01g0543600	100,00	0,00E+00	801	178	Japonica	NA	
augustus-1-processed-gene-21.32-mRNA-1	1	OsR498G0100133900.01.T01	100,00	0,00E+00	1389	Os01g0138300	99,74	0,00E+00	1380	-9	NA	Indica	
augustus-1-processed-gene-21.48-mRNA-1	1	OsR498G0100138300.01.T01	99,68	0,00E+00	1104	Os01g0139600	99,51	0,00E+00	1100	-4	NA	Indica	
augustus-1-processed-gene-21.50-mRNA-1	1	OsR498G0101471900.01.T01	70,38	1,32E-130	469	Os01g0140100	99,67	0,00E+00	2714	2245	Japonica	NA	
augustus-1-processed-gene-210.27-mRNA-1	1	OsR498G0101254300.01.T01	100,00	0,00E+00	2527	Os01g0545900	100,00	0,00E+00	3085	558	Japonica	NA	
augustus-1-processed-gene-213.2-mRNA-1	1	OsR498G0101280300.01.T01	100,00	0,00E+00	1591	Os01g0550200	100,00	0,00E+00	1591	0	NA	NA	
augustus-1-processed-gene-215.21-mRNA-1	1	OsR498G0101299100.01.T04	100,00	0,00E+00	827	Os01g0553400	99,78	0,00E+00	810	-17	NA	NA	
augustus-1-processed-gene-215.22-mRNA-1	1	OsR498G0101301100.01.T01	100,00	2,87E-103	374	Os05g0471800	91,71	2,68E-79	294	-80	Indica	NA	
augustus-1-processed-gene-216.22-mRNA-1	1	OsR498G0101311500.01.T03	100,00	0,00E+00	758	Os01g0555100	100,00	0,00E+00	758	0	NA	NA	
augustus-1-processed-gene-217.18-mRNA-1	1	OsR498G0101321300.01.T01	100,00	0,00E+00	3168	Os01g0556700	100,00	0,00E+00	3068	-100	NA	NA	
augustus-1-processed-gene-217.21-mRNA-1	1	OsR498G0101324300.01.T01	100,00	0,00E+00	2370	Os01g0557400	100,00	0,00E+00	2370	0	NA	NA	
augustus-1-processed-gene-217.9-mRNA-1	1	OsR498G0101315500.01.T03	100,00	0,00E+00	2062	Os01g0556400	100,00	0,00E+00	2062	0	NA	NA	
augustus-1-processed-gene-218.10-mRNA-1	1	OsR498G0101332700.01.T02	100,00	0,00E+00	2127	Os01g0558500	99,92	0,00E+00	2122	-5	NA	NA	
augustus-1-processed-gene-218.9-mRNA-1	1	OsR498G0101330900.01.T01	100,00	0,00E+00	3335	Os01g0558200	100,00	0,00E+00	3335	0	NA	NA	
augustus-1-processed-gene-22.10-mRNA-1	1	OsR498G0100148300.01.T03	97,48	0,00E+00	1874	Os01g0141000	97,48	0,00E+00	1874	0	NA	NA	
augustus-1-processed-gene-22.9-mRNA-1	1	OsR498G0100143300.01.T01	98,75	0,00E+00	1676	Os01g0141000	85,85	0,00E+00	839	-837	Indica	NA	
augustus-1-processed-gene-221.13-mRNA-1	1	OsR498G0101346000.01.T07	97,41	1,21E-108	392	Os01g0563000	100,00	3,06E-117	419	27	NA	Japonica	
augustus-1-processed-gene-222.16-mRNA-1	1	OsR498G0101347500.01.T01	86,43	4,61E-90	331	Os01g0564300	100,00	1,50E-141	500	169	Japonica	Indica	
augustus-1-processed-gene-23.56-mRNA-1	1	OsR498G0100150400.01.T02	98,59	0,00E+00	2354	Os01g0141700	98,76	0,00E+00	3103	749	Japonica	Indica	
augustus-1-processed-gene-23.61-mRNA-1	1	OsR498G0101158000.01.T01	98,44	0,00E+00	4004	Os02g0547100	85,26	0,00E+00	3484	-520	Indica	Indica	
augustus-1-processed-gene-231.54-mRNA-1	1	OsR498G0101399700.01.T01	97,47	0,00E+00	736	Os01g0579800	100,00	0,00E+00	785	49	NA	Japonica	
augustus-1-processed-gene-234.11-mRNA-1	1	OsR498G0101417500.01.T01	100,00	0,00E+00	682	Os01g0585200	99,74	0,00E+00	678	-4	NA	Indica	
augustus-1-processed-gene-235.12-mRNA-1	1	OsR498G0101422900.01.T01	98,63	0,00E+00	2153	Os01g0597400	88,03	0,00E+00	1587	-566	Indica	Japonica	
augustus-1-processed-gene-237.19-mRNA-1	1	OsR498G0101436500.01.T01	93,86	0,00E+00	1365	Os01g0589300	94,83	0,00E+00	1385	20	NA	Japonica	
augustus-1-processed-gene-239.8-mRNA-1	1	OsR498G0101455300.01.T01	100,00	5,28E-114	410	Os01g0593600	100,00	1,85E-114	410	0	NA	Japonica	
augustus-1-processed-gene-24.23-mRNA-1	1	OsR498G0100167700.01.T01	80,53	4,77E-84	311	Os01g0145000	91,37	2,80E-132	471	160	Japonica	NA	
augustus-1-processed-gene-240.28-mRNA-1	1	OsR498G0101457300.01.T01	99,59	0,00E+00	3042	Os01g0594300	94,86	0,00E+00	2736	-306	Indica	NA	LRR-TM
augustus-1-processed-gene-241.17-mRNA-1	1	OsR498G0101469300.01.T01	97,72	0,00E+00	2540	Os01g0597800	100,00	0,00E+00	2695	155	NA	NA	
augustus-1-processed-gene-242.30-mRNA-1	1	OsR498G0101471900.01.T01	99,87	0,00E+00	2702	Os01g0598600	100,00	0,00E+00	2711	9	NA	NA	
augustus-1-processed-gene-242.56-mRNA-1	1	OsR498G0101471400.01.T01	99,68	0,00E+00	2786	Os01g0598400	100,00	0,00E+00	2809	23	NA	NA	
augustus-1-processed-gene-243.13-mRNA-1	1	OsR498G0101477000.01.T01	99,85	0,00E+00	2351	Os01g0601000	99,08	0,00E+00	3052	701	Japonica	NA	
augustus-1-processed-gene-243.2-mRNA-1	1	OsR498G0101476300.01.T01	99,65	0,00E+00	1544	Os01g0600900	100,00	0,00E+00	1418	-126	NA	NA	
augustus-1-processed-gene-245.1-mRNA-1	1	OsR498G0101493300.01.T01	99,14	0,00E+00	1226	Os01g0604300	100,00	0,00E+00	672	-554	Indica	NA	
augustus-1-processed-gene-246.1-mRNA-1	1	OsR498G0101502500.01.T01	99,38	0,00E+00	858	Os01g0607000	100,00	0,00E+00	910	52	NA	NA	
augustus-1-processed-gene-246.15-mRNA-1	1	OsR498G0101503900.01.T02	96,21	0,00E+00	1225	Os01g0607300	96,34	0,00E+00	1230	5	NA	NA	
augustus-1-processed-gene-248.70-mRNA-1	1	OsR498G0101521400.01.T01	92,18	0,00E+00	832	Os01g0610800	99,74	0,00E+00	682	-150	Indica	NA	
augustus-1-processed-gene-252.0-mRNA-1	1	OsR498G0101547500.01.T01	99,72	0,00E+00	1285	Os01g0616800	100,00	0,00E+00	1294	9	NA	NA	
augustus-1-processed-gene-254.30-mRNA-1	1	OsR498G0101560500.01.T01	99,67	0,00E+00	2727	Os01g0620800	99,93	0,00E+00	2745	18	NA	NA	
augustus-1-processed-gene-255.1-mRNA-1	1	OsR498G0101564900.01.T01	99,74	0,00E+00	3501	Os01g0621900	99,95	0,00E+00	3497	-4	NA	NA	
augustus-1-processed-gene-256.45-mRNA-1	1	OsR498G0101574800.01.T01	99,71	0,00E+00	1875	Os01g0624700	99,90	0,00E+00	1884	9	NA	NA	
augustus-1-processed-gene-258.26-mRNA-1	1	OsR498G0511411500.01.T01	72,34	0,00E+00	640	Os01g0629600	100,00	0,00E+00	2452	1812	Japonica	Japonica	
augustus-1-processed-gene-258.30-mRNA-1	1	OsR498G0101589100.01.T01	99,91	0,00E+00	4082	Os04g0594800	73,86	0,00E+00	1285	-2797	Indica	NA	
augustus-1-processed-gene-260.34-mRNA-1	1	OsR498G0101598900.01.T01	99,89	0,00E+00	3400	Os01g0633200	99,74	0,00E+00	3387	-13	NA	Indica	
augustus-1-processed-gene-263.18-mRNA-1	1	OsR498G0101614100.01.T01	99,80	0,00E+00	2649	Os01g0638000	100,00	0,00E+00	2663	14	NA	Japonica	
augustus-1-processed-gene-263.38-mRNA-1	1	OsR498G0101615700.01.T01	99,73	0,00E+00	2672	Os01g0638600	100,00	0,00E+00	2690	18	NA	Japonica	
augustus-1-processed-gene-265.0-mRNA-1	1	OsR498G0101626900.01.T01	100,00	0,00E+00	1763	Os01g0641800	100,00	0,00E+00	1763	0	NA	NA	
augustus-1-processed-gene-265.1-mRNA-1	1	OsR498G0101626900.01.T01	100,00	0,00E+00	1027	Os01g0641800	100,00	0,00E+00	1027	0	NA	NA	
augustus-1-processed-gene-266.23-mRNA-1	1	OsR498G0511392000.01.T01	70,37	5,94E-47	188	Os01g0643900	100,00	0,00E+00	915	727	Japonica	Japonica	
augustus-1-processed-gene-266.30-mRNA-1	1	OsR498G0101640300.01.T01	100,00	0,00E+00	958	Os01g0644900	100,00	0,00E+00	958	0	NA	Japonica	
augustus-1-processed-gene-267.50-mRNA-1	1	OsR498G0101645600.01.T01	99,93	0,00E+00	2680	Os01g0646300	100,00	0,00E+00	2673	-7	NA	Japonica	
augustus-1-processed-gene-269.39-mRNA-1	1	OsR498G0101656100.01.T02	84,43	7,25E-88	324	Os01g0649300	100,00	2,68E-170	596	272	Japonica	Japonica	
augustus-1-processed-gene-269.58-mRNA-1	1	OsR498G0101658300.01.T01	94,23	0,00E+00	909	Os01g0650200	94,22	0,00E+00	907	-2	NA	Japonica	
augustus-1-processed-gene-270.28-mRNA-1	1	OsR498G0101660900.01.T05	99,82	0,00E+00	3064	Os01g0651100	100,00	0,00E+00	2563	-501	Indica	Japonica	
augustus-1-processed-gene-270.42-mRNA-1	1	OsR498G0101661700.01.T01	99,91	0,00E+00	1928	Os01g0651500	100,00	0,00E+00	1889	-39	NA	Japonica	
augustus-1-processed-gene-271.11-mRNA-1	1	OsR498G0101668900.01.T01	100,00	0,00E+00	2727	Os01g0653200	100,00	0,00E+00	2727	0	NA	Japonica	
augustus-1-processed-gene-271.2-mRNA-1	1	OsR498G0101665900.01.T01	99,76	0,00E+00	737	Os01g0652700	100,00	0,00E+00	742	5	NA	Japonica	
augustus-1-processed-gene-275.15-mRNA-1	1	OsR498G0101693700.01.T01	100,00	0,00E+00	1856	Os01g0659800	100,00	0,00E+00	1608	-248	Indica	Japonica	
augustus-1-processed-gene-275.17-mRNA-1	1	OsR498G0101694700.01.T03	100,00	0,00E+00	1635	Os01g0660200	100,00	0,00E+00	1635	0	NA	Japonica	
augustus-1-processed-gene-277.26-mRNA-1	1	OsR498G0101705900.01.T02	99,88	0,00E+00	3016	Os01g0664100	100,00	0,00E+00	3025	9	NA	Japonica	
augustus-1-processed-gene-28.37-mRNA-1	1	OsR498G0100183100.01.T06	100,00	0,00E+00	2875	Os01g0151100	99,81	0,00E+00	2862	-13	NA	Indica	
augustus-1-processed-gene-28.49-mRNA-1	1	OsR498G0100184700.01.T01	99,94	0,00E+00	2896	Os01g0151900	99,69	0,00E+00	2876	-20	NA	Japonica	
augustus-1-processed-gene-285.22-mRNA-1	1	OsR498G0101757700.01.T01	99,89	0,00E+00	4910	Os01g0677900	99,96	0,00E+00	4139	-771	Indica	Japonica	
augustus-1-processed-gene-285.23-mRNA-1	1	OsR498G0101758100.01.T01	99,72	0,00E+00	3241	Os01g0678000	99,96	0,00E+00	4871	1630	Japonica	Japonica	
augustus-1-processed-gene-286.15-mRNA-1	1	OsR498G0101765100.01.T01	99,66	0,00E+00	2082	Os01g0680200	100,00	0,00E+00	2100	18	NA	Japonica	
augustus-1-processed-gene-287.1-mRNA-1	1	OsR498G0101767700.01.T01	99,78	0,00E+00	1626	Os01g0680600	100,00	0,00E+00	1635	9	NA	Japonica	
augustus-1-processed-gene-289.49-mRNA-1	1	OsR498G0101785300.01.T01	100,00	0,00E+00	1023	Os01g0686000	100,00	0,00E+00	1223	200	Japonica	Japonica	
augustus-1-processed-gene-289.51-mRNA-1	1	OsR498G0101785900.01.T01	97,55	0,00E+00	2369	Os01g0686200	100,00	0,00E+00	2311	-58	NA	Japonica	
augustus-1-processed-gene-29.2-mRNA-1	1	OsR498G0100190500.01.T02	93,35	0,00E+00	1052	Os01g0153000	99,26	0,00E+00	1199	147	Japonica	NA	LRR-TM
augustus-1-processed-gene-29.20-mRNA-1	1	OsR498G0100193100.01.T01	99,71	0,00E+00	1839	Os01g0153800	99,07	0,00E+00	1893	54	NA	Indica	

augustus-1-processed-gene-29.21-mRNA-1	1	OsR498G0100194100.01.T01	100,00	0,00E+00	1283	Os01g0154300	99,56	0,00E+00	1221	-62	NA	Indica	
augustus-1-processed-gene-29.4-mRNA-1	1	OsR498G0100191300.01.T01	97,47	0,00E+00	801	Os04g0625700	86,90	3,70E-164	577	-224	Indica	NA	
augustus-1-processed-gene-290.33-mRNA-1	1	OsR498G0101786500.01.T01	99,79	0,00E+00	2579	Os01g0686300	100,00	0,00E+00	2592	13	NA	Japonica	
augustus-1-processed-gene-290.35-mRNA-1	1	OsR498G0101787300.01.T01	98,98	0,00E+00	2236	Os01g0686500	100,00	0,00E+00	1431	-805	Indica	Japonica	
augustus-1-processed-gene-290.55-mRNA-1	1	OsR498G0101792300.01.T01	99,91	0,00E+00	2047	Os05g0551700	82,30	0,00E+00	837	-1210	Indica	Japonica	
augustus-1-processed-gene-292.59-mRNA-1	1	OsR498G0101801900.01.T03	98,71	0,00E+00	4084	Os01g0691600	98,47	0,00E+00	3080	-1004	Indica	Japonica	
augustus-1-processed-gene-295.15-mRNA-1	1	OsR498G0101823100.01.T01	99,93	0,00E+00	2452	Os01g0696800	99,59	0,00E+00	1736	-716	Indica	Japonica	
augustus-1-processed-gene-296.25-mRNA-1	1	OsR498G0101831600.01.T01	80,44	0,00E+00	872	Os01g0699400	100,00	0,00E+00	2233	1361	Japonica	Japonica	Kinase-TM
augustus-1-processed-gene-298.58-mRNA-1	1	OsR498G0101845100.01.T01	99,81	0,00E+00	2784	Os01g0704100	100,00	0,00E+00	2296	-488	Indica	Japonica	
augustus-1-processed-gene-299.40-mRNA-1	1	OsR498G0101853300.01.T01	99,65	0,00E+00	2543	Os01g0705700	97,26	0,00E+00	2420	-123	NA	Japonica	
augustus-1-processed-gene-3.52-mRNA-1	1	OsR498G0100016300.01.T01	95,78	0,00E+00	1324	Os01g0104400	100,00	0,00E+00	1452	128	NA	Japonica	
augustus-1-processed-gene-30.34-mRNA-1	1	OsR498G0100197900.01.T01	99,96	0,00E+00	4849	Os01g0155500	97,74	0,00E+00	4581	-268	NA	Indica	Kinase-TM
augustus-1-processed-gene-300.27-mRNA-1	1	OsR498G0101856700.01.T01	99,61	1,58E-126	453	Os01g0706600	100,00	2,51E-131	466	13	NA	Japonica	
augustus-1-processed-gene-301.49-mRNA-1	1	OsR498G0101861900.01.T01	100,00	0,00E+00	2370	Os01g0708100	100,00	0,00E+00	2370	0	NA	Japonica	
augustus-1-processed-gene-304.43-mRNA-1	1	OsR498G0101882900.01.T01	93,22	0,00E+00	708	Os01g0715200	100,00	3,01E-178	623	-85	Indica	Japonica	
augustus-1-processed-gene-304.48-mRNA-1	1	OsR498G0101885900.01.T01	99,93	0,00E+00	2496	Os01g0715700	100,00	0,00E+00	3788	1292	Japonica	Japonica	
augustus-1-processed-gene-307.41-mRNA-1	1	OsR498G0101476300.01.T01	92,36	0,00E+00	1158	Os01g0720500	100,00	0,00E+00	1440	282	Japonica	Japonica	
augustus-1-processed-gene-307.44-mRNA-1	1	OsR498G0101910700.01.T05	99,19	0,00E+00	2629	Os01g0721000	100,00	0,00E+00	2646	17	NA	Japonica	
augustus-1-processed-gene-307.47-mRNA-1	1	OsR498G0101911900.01.T01	99,71	0,00E+00	2477	Os01g0721500	80,94	0,00E+00	1412	-1065	Indica	Japonica	
augustus-1-processed-gene-308.60-mRNA-1	1	OsR498G0101916100.01.T01	99,80	0,00E+00	911	Os01g0722800	100,00	0,00E+00	915	4	NA	Japonica	
augustus-1-processed-gene-308.66-mRNA-1	1	OsR498G0101918500.01.T03	99,38	0,00E+00	2588	Os01g0723400	99,91	0,00E+00	2099	-489	Indica	Japonica	
augustus-1-processed-gene-309.9-mRNA-1	1	OsR498G0101922900.01.T01	99,59	0,00E+00	870	Os01g0724700	100,00	0,00E+00	1207	337	Japonica	Japonica	
augustus-1-processed-gene-31.35-mRNA-1	1	OsR498G0100208400.01.T01	100,00	1,46E-156	553	Os05g0163100	78,06	1,13E-95	350	-203	Indica	Indica	
augustus-1-processed-gene-311.1-mRNA-1	1	OsR498G0101933900.01.T01	99,71	0,00E+00	2444	Os01g0729400	100,00	0,00E+00	2462	18	NA	Japonica	LRR-TM
augustus-1-processed-gene-312.15-mRNA-1	1	OsR498G0101942300.01.T01	99,49	0,00E+00	1729	Os01g0732300	100,00	0,00E+00	1754	25	NA	Japonica	
augustus-1-processed-gene-313.24-mRNA-1	1	OsR498G0101946900.01.T03	99,54	0,00E+00	2300	Os01g0733500	100,00	0,00E+00	2327	27	NA	Japonica	
augustus-1-processed-gene-313.39-mRNA-1	1	OsR498G0101949900.01.T03	99,79	0,00E+00	2584	Os01g0734600	100,00	0,00E+00	2598	14	NA	Japonica	
augustus-1-processed-gene-313.43-mRNA-1	1	OsR498G0101951500.01.T01	99,39	0,00E+00	2622	Os01g0735300	100,00	0,00E+00	2663	41	NA	Japonica	
augustus-1-processed-gene-314.54-mRNA-1	1	OsR498G0101957500.01.T01	98,40	0,00E+00	1081	Os01g0737600	100,00	0,00E+00	1126	45	NA	Japonica	
augustus-1-processed-gene-314.55-mRNA-1	1	OsR498G0101957700.01.T01	99,04	0,00E+00	1103	Os01g0737700	99,85	0,00E+00	1197	94	NA	Japonica	
augustus-1-processed-gene-315.5-mRNA-1	1	OsR498G0101960500.01.T01	99,56	0,00E+00	1210	Os01g0738400	100,00	0,00E+00	1223	13	NA	Japonica	
augustus-1-processed-gene-322.48-mRNA-1	1	OsR498G0511182600.01.T01	75,20	2,92E-31	136	Os01g0751400	100,00	0,00E+00	818	682	Japonica	Japonica	
augustus-1-processed-gene-323.15-mRNA-1	1	OsR498G0102010900.01.T02	99,42	0,00E+00	1851	Os01g0753800	100,00	0,00E+00	1878	27	NA	Japonica	
augustus-1-processed-gene-324.26-mRNA-1	1	OsR498G0102015300.01.T01	99,86	0,00E+00	1289	Os01g0755700	100,00	0,00E+00	1294	5	NA	Japonica	
augustus-1-processed-gene-327.0-mRNA-1	1	OsR498G0102038900.01.T02	100,00	7,41E-144	509	Os01g0760000	100,00	1,75E-70	264	-245	Indica	Japonica	
augustus-1-processed-gene-327.1-mRNA-1	1	OsR498G0102039300.01.T02	99,74	0,00E+00	3403	Os01g0760300	100,00	0,00E+00	2825	-578	Indica	Japonica	
augustus-1-processed-gene-327.10-mRNA-1	1	OsR498G0102040200.01.T01	99,87	0,00E+00	1392	Os01g0760600	100,00	0,00E+00	1397	5	NA	Japonica	
augustus-1-processed-gene-327.12-mRNA-1	1	OsR498G0102040900.01.T03	99,75	0,00E+00	719	Os01g0761000	100,00	0,00E+00	724	5	NA	Japonica	
augustus-1-processed-gene-328.52-mRNA-1	1	OsR498G0102047500.01.T02	98,97	3,61E-144	512	Os01g0763650	100,00	0,00E+00	823	311	Japonica	Japonica	
augustus-1-processed-gene-328.53-mRNA-1	1	OsR498G0102047500.01.T03	99,67	0,00E+00	3236	Os01g0763700	100,00	0,00E+00	3263	27	NA	Japonica	
augustus-1-processed-gene-33.59-mRNA-1	1	OsR498G0100216100.01.T03	99,75	0,00E+00	1404	Os01g0165000	99,75	0,00E+00	1404	0	NA	Indica	
augustus-1-processed-gene-331.48-mRNA-1	1	OsR498G0102062300.01.T06	99,79	0,00E+00	4360	Os01g0769000	100,00	0,00E+00	4383	23	NA	Japonica	
augustus-1-processed-gene-331.52-mRNA-1	1	OsR498G0102064500.01.T01	99,89	0,00E+00	4840	Os01g0769700	100,00	0,00E+00	4854	14	NA	Japonica	Kinase-TM
augustus-1-processed-gene-336.57-mRNA-1	1	OsR498G0102093700.01.T01	99,51	0,00E+00	2177	Os01g0778100	100,00	0,00E+00	1240	-937	Indica	Japonica	
augustus-1-processed-gene-337.1-mRNA-1	1	OsR498G0102097100.01.T01	99,71	0,00E+00	3674	Os01g0779300	100,00	0,00E+00	3772	98	NA	Japonica	Kinase-TM
augustus-1-processed-gene-338.31-mRNA-1	1	OsR498G0102101700.01.T01	100,00	0,00E+00	1159	Os01g0781000	98,60	0,00E+00	1118	-41	NA	NA	
augustus-1-processed-gene-338.41-mRNA-1	1	OsR498G0102106700.01.T04	99,95	0,00E+00	3702	Os01g0783100	100,00	0,00E+00	3707	5	NA	Japonica	
augustus-1-processed-gene-339.2-mRNA-1	1	OsR498G0102109300.01.T01	94,99	0,00E+00	2104	Os01g0784200	99,67	0,00E+00	4267	2163	Japonica	Japonica	Kinase-TM
augustus-1-processed-gene-339.20-mRNA-1	1	OsR498G0102111100.01.T02	100,00	0,00E+00	1063	Os01g0784800	100,00	0,00E+00	1063	0	NA	Japonica	
augustus-1-processed-gene-339.26-mRNA-1	1	OsR498G0102115700.01.T05	99,92	0,00E+00	2350	Os01g0785700	100,00	0,00E+00	2354	4	NA	Japonica	
augustus-1-processed-gene-34.49-mRNA-1	1	OsR498G0100220900.01.T02	100,00	0,00E+00	1294	Os01g0166700	94,70	0,00E+00	1150	-144	Indica	Indica	
augustus-1-processed-gene-34.53-mRNA-1	1	OsR498G0100222500.01.T01	100,00	0,00E+00	1498	Os01g0167400	99,51	0,00E+00	1449	-49	NA	Indica	
augustus-1-processed-gene-342.1-mRNA-1	1	OsR498G0102128300.01.T02	98,81	0,00E+00	2053	Os01g0793200	100,00	0,00E+00	1278	-775	Indica	Japonica	
augustus-1-processed-gene-343.21-mRNA-1	1	OsR498G0102133700.01.T02	92,50	0,00E+00	3243	Os01g0794800	100,00	0,00E+00	3993	750	Japonica	NA	
augustus-1-processed-gene-343.25-mRNA-1	1	OsR498G0102135900.01.T01	100,00	2,22E-124	444	Os01g0795600	100,00	7,78E-125	444	0	NA	Japonica	
augustus-1-processed-gene-343.28-mRNA-1	1	OsR498G0102135900.01.T01	72,37	1,47E-25	116	Os01g0796001	100,00	1,05E-129	461	345	Japonica	Japonica	
augustus-1-processed-gene-344.34-mRNA-1	1	OsR498G0102141700.01.T01	99,86	0,00E+00	1273	Os01g0797600	100,00	0,00E+00	1278	5	NA	Japonica	
augustus-1-processed-gene-345.19-mRNA-1	1	OsR498G0102184800.01.T02	99,92	0,00E+00	2382	Os01g0800400	100,00	0,00E+00	2387	5	NA	Japonica	
augustus-1-processed-gene-345.2-mRNA-1	1	OsR498G0102144900.01.T02	99,90	0,00E+00	5327	Os01g0799100	100,00	0,00E+00	5341	14	NA	Japonica	NBS-TM
augustus-1-processed-gene-346.33-mRNA-1	1	OsR498G0102154100.01.T04	99,89	0,00E+00	1560	Os05g0495200	72,63	2,27E-81	302	-1258	Indica	Japonica	
augustus-1-processed-gene-346.34-mRNA-1	1	OsR498G0102154700.01.T01	99,00	0,00E+00	1753	Os01g0802000	100,00	0,00E+00	1786	33	NA	Japonica	
augustus-1-processed-gene-348.38-mRNA-1	1	OsR498G0102165700.01.T01	99,93	0,00E+00	2458	Os01g0805100	100,00	0,00E+00	1407	-1051	Indica	Japonica	
augustus-1-processed-gene-352.13-mRNA-1	1	OsR498G0102193100.01.T01	100,00	0,00E+00	720	Os01g0811200	96,74	0,00E+00	668	-52	NA	Indica	
augustus-1-processed-gene-352.14-mRNA-1	1	OsR498G0102193500.01.T01	100,00	0,00E+00	3988	Os01g0811300	99,91	0,00E+00	3979	-9	NA	Indica	
augustus-1-processed-gene-353.52-mRNA-1	1	OsR498G0102197300.01.T01	100,00	0,00E+00	1916	Os01g0812200	99,81	0,00E+00	1907	-9	NA	Indica	
augustus-1-processed-gene-353.68-mRNA-1	1	OsR498G0102200300.01.T05	100,00	0,00E+00	985	Os01g0813400	99,82	0,00E+00	981	-4	NA	Indica	
augustus-1-processed-gene-353.71-mRNA-1	1	OsR498G0102202300.01.T01	100,00	0,00E+00	1018	Os01g0814100	99,78	0,00E+00	813	-205	Indica	Indica	
augustus-1-processed-gene-354.59-mRNA-1	1	OsR498G0102207500.01.T01	100,00	0,00E+00	2863	Os01g0816000	99,18	0,00E+00	2816	-47	NA	Indica	
augustus-1-processed-gene-355.34-mRNA-1	1	OsR498G0102211100.01.T01	100,00	0,00E+00	2998	Os01g0816700	99,64	0,00E+00	2971	-27	NA	Indica	
augustus-1-processed-gene-361.11-mRNA-1	1	OsR498G0102253700.01.T05	100,00	0,00E+00	3534	Os01g0827500	99,80	0,00E+00	3516	-18	NA	Indica	
augustus-1-processed-gene-362.10-mRNA-1	1	OsR498G0102260500.01.T01	100,00	0,00E+00	2695	Os01g0829800	99,66	0,00E+00	2131	-564	Indica	Indica	
augustus-1-processed-gene-362.11-mRNA-1	1	OsR498G0102261300.01.T03	100,00	0,00E+00	2473	Os01g0830100	100,00	0,00E+00	2473	0	NA	Indica	
augustus-1-processed-gene-364.40-mRNA-1	1	OsR498G0102273300.01.T03	100,00	0,00E+00	2874	Os01g0832900	99,87	0,00E+00	2865	-9	NA	Indica	
augustus-1-processed-gene-365.39-mRNA-1	1	OsR498G0102286500.01.T01	100,00	1,80E-158	558	Os01g0835900	99,68	2,67E-157	554	-4	NA	Indica	
augustus-1-processed-gene-366.1-mRNA-1	1	OsR498G0102288300.01.T01	100,00	0,00E+00	4058	Os01g0836600	99,91	0,00E+00	4049	-9	NA	Indica	
augustus-1-processed-gene-367.49-mRNA-1	1	OsR498G0102299500.01.T01	100,00	0,00E+00	2473	Os01g0839400	99,85	0,00E+00	2464	-9	NA	Indica	
augustus-1-processed-gene-369.38-mRNA-1	1	OsR498G0102309900.01.T01	100,00	0,00E+00	850	Os01g0841700	98,13	0,00E+00	829	-21	NA	NA	
augustus-1-processed-gene-369.50-mRNA-1	1	OsR498G0102311900.01.T03	100,00	0,00E+00	4442	Os01g0842200	99,84	0,00E+00	4424	-18	NA	NA	
augustus-1-processed-gene-37.62-mRNA-1	1	OsR498G0100239400.01.T02	99,24	0,00E+00	1380	Os01g0171200	99,58	0,00E+00	2119	739	Japonica	NA	
augustus-1-processed-gene-370.21-mRNA-1	1	OsR498G0102321300.01.T01	100,00	0,00E+00	1375	Os01g0844900	100,00	0,00E+00	1191	-184	Indica	NA	
augustus-1-processed-gene-371.68-mRNA-1	1	OsR498G0916643600.01.T01	90,36	0,00E+00	881	Os01g0630700	86,63	0,00E+00	774	-107	Indica	NA	
augustus-1-processed-gene-373.34-mRNA-1	1	OsR498G0102000100.01.T01	98,73	0,00E+00	1923	Os03g0110200	86,58	0,00E+00	1515	-408	Indica	NA	
augustus-1-processed-gene-374.1-mRNA-1	1	OsR498G0102344100.01.T01	100,00	0,00E+00	1388	Os01g0851600	99,63	0,00E+00	1443	55	NA	NA	
augustus-1-processed-gene-374.14-mRNA-1	1	OsR498G0102348900.01.T02	100,00	0,00E+00	3810	Os01g0852900	99,91	0,00E+00	3800	-10	NA	NA	
augustus-1-processed-gene-375.16-mRNA-1	1	OsR498G0102354500.01.T01	100,00	0,00E+00	2619	Os05g0449200	81,36	0,00E+00	1323	-1296	Indica	NA	
augustus-1-processed-gene-375.19-mRNA-1	1	OsR498G0102357300.01.T01	100,00	0,00E+00	2955	Os01g0854800	99,94	0,00E+00	2950	-5	NA	NA	
augustus-1-processed-gene-375.7-mRNA-1	1	OsR498G0102351500.01.T06	100,00	0,00E+00	710	Os01g0853600	100,00	0,00E+00	682	-28	NA	NA	
augustus-1-processed-gene-375.8-mRNA-1	1	OsR498G0102352900.01.T01	100,00	0,00E+00	1775	Os01g0853800	99,90	0,00E+00	1771	-4	NA	NA	
augustus-1-processed-gene-377.1-mRNA-1	1	OsR498G0102367500.01.T02	100,00	0,00E+00	861	Os01g0856800	89,52	0,00E+00	674	-187	Indica	NA	
augustus-1-processed-gene-378.2-mRNA-1	1	OsR498G0102373300.01.T01	100,00	0,00E+00	2132	Os01g0858900	99,75	0,00E+00	2119	-13	NA	NA	
augustus-1-processed-gene-378.7-mRNA-1	1	OsR498G0102372800.01.T02	100,00	0,00E+00	1927	Os01g0858500	96,88	0,00E+00	1563	-364	Indica	NA	
augustus-1-processed-gene-379.1-mRNA-1	1	OsR498G0102379100.01.T01	97,70	0,00E+00	1116	Os02g0321900	89,56	0,00E+00	877	-239	Indica	NA	
augustus-1-processed-gene-379.21-mRNA-1	1	OsR498G0102384500.01.T02	100,00	0,00E+00	1191	Os01g0862600	100,00	0,00E+00	1191	0	NA	NA	
augustus-1-processed-gene-379.3-mRNA-1	1	OsR498G0102379300.01.T01	75,46	5,69E-158	559	Os01g0860500	99,24	0,00E+00	1625	1066	Japonica	NA	
augustus-1-processed-gene-379.4-mRNA-1	1	OsR498G0408525900.01.T04	79,72	4,78E-67	255	Os01g0860601	99,60	0,00E+00	890	635	Japonica	NA	
augustus-1-processed-gene-379.6-mRNA-1	1	OsR498G0102380900.01.T06	100,00	0,00E+00	2159	Os01g0860800	99,83	0,00E+00	2150	-9	NA	NA	
augustus-1-processed-gene-380.21-mRNA-1	1	OsR498G0102390500.01.T01	100,00	0,00E+00	823	Os01g0864200	99,56	0,00E+00	814	-9	NA	NA	
augustus-1-processed-gene-381.58-mRNA-1	1	OsR498G0611661000.01.T01	93,78	1,54E-173	609	Os01g0866200	100,00	0,00E+00	737	128	Japonica	NA	
augustus-1-processed-gene-383.60-mRNA-1	1	OsR498G0102409900.01.T01	100,00	0,00E+00	4112	Os01g0868900	99,61	0,00E+00	4075	-37	NA	NA	
augustus-1-processed-gene-383.64-mRNA-1	1	OsR498G0102413500.01.T01	100,00	0,00E+00	1168	Os01g0869900	100,00	0,00E+00	1168	0	NA	NA	Kinase-TM
augustus-1-processed-gene-384.53-mRNA-1	1	OsR498G0102416100.01.T01	100,00	0,00E+00	4078	Os01g0889900	72,30	0,00E+00	1183	-2895	Indica	NA	Kinase-TM
augustus-1-processed-gene-385.63-mRNA-1	1	OsR498G0102425300.01.T01	99,73	0,00E+00	1960	Os01g0873700	99,54	0,00E+00	1951	-9	NA	NA	
augustus-1-processed-gene-388.16-mRNA-1	1	OsR498G0102448100.01.T01	100,00	0,00E+00	1061	Os01g0879200	99,66	0,00E+00	1052	-9	NA	NA	
augustus-1-processed-gene-388.17-mRNA-1	1	OsR498G0102448300.01.T01	100,00	0,00E+00	1240	Os01g0879200	75,20	6,23E-100	364	-876	Indica	NA	
augustus-1-processed-gene-388.18-mRNA-1	1	OsR498G0102448300.01.T03	100,00	0,00E+00	2574	Os01g0879400	99,93	0,00E+00	2555	-19	NA	NA	
augustus-1-processed-gene-388.2-mRNA-1	1	OsR498G0102446100.01.T01	100,00	0,00E+00	5222	Os01g0878300	99,79	0,00E+00	5194	-28	NA	NA	Kinase-LRR-TM
augustus-1-processed-gene-389.30-mRNA-1	1	OsR498G0102458500.01.T01	100,00	0,00E+00	3416	Os01g0881400	99,78	0,00E+00	2454	-962	Indica	NA	
augustus-1-processed-gene-389.33-mRNA-1	1	OsR498G0611598700.01.T01	82,69	6,82E-88	324	Os01g0881800	98,08	7,23E-152	535	211	Japonica	NA	
augustus-1-processed-gene-39.50-mRNA-1	1	OsR498G0100251500.01.T01	96,22	0,00E+00	2345	Os01g0175500	100,00	0,00E+00	2565	220	NA	Japonica	
augustus-1-processed-gene-39.73-mRNA-1	1	OsR498G0100256900.01.T01	99,76	0,00E+00	5883	Os01g0176800	99,88	0,00E+00	5901	18	NA	Japonica	
augustus-1-processed-gene-390.54-mRNA-1	1	OsR498G0102463700.01.T01	100,00	0,00E+00	1191	Os10g0492400	88,09	8,89E-162	569	-622	Indica	NA	Kinase-TM
augustus-1-processed-gene-392.0-mRNA-1	1	OsR498G0102473900.01.T01	100,00	0,00E+00	4475	Os01g0885700	99,68	0,00E+00	4439	-36	NA	NA	Kinase-TM
augustus-1-processed-gene-393.14-mRNA-1	1	OsR498G0102485500.01.T01	100,00	0,00E+00	801	Os01g0888800	100,00	0,00E+00	801	0	NA	NA	
augustus-1-processed-gene-393.17-mRNA-1	1	OsR498G0102487900.01.T03	100,00	0,00E+00	1462	Os01g0889400	100,00	0,00E+00	1462	0	NA	NA	
augustus-1-processed-gene-394.52-mRNA-1	1	OsR498G0102493700.01.T01	100,00	0,00E+00	1662	Os01g0891700	100,00	0,00E+00	1662	0	NA	NA	LRR-TM
augustus-1-processed-gene-395.38-mRNA-1	1	OsR498G0102495900.01.T01	90,77	8,57E-138	490	Os01g0892200	99,70	6,55E-172	602	112	Japonica	NA	
augustus-1-processed-gene-395.39-mRNA-1	1	OsR498G0102496500.01.T01	100,00	0,00E+00	1516	Os01g0892300	99,64	0,00E+00	1502	-14	NA	NA	LRR-TM
augustus-1-processed-gene-395.54-mRNA-1	1	OsR498G0102498100.01.T02	100,00	0,00E+00	2587	Os01g0892800	100,00	0,00E+00	2587	0	NA	NA	Kinase-TM
augustus-1-processed-gene-396.14-mRNA-1	1	OsR498G0102505500.01.T01	100,00	0,00E+00	1483	Os01g0895500	99,88	0,00E+00	1479	-4	NA	NA	
augustus-1-processed-gene-396.16-mRNA-1	1	OsR498G0102505900.01.T01	100,00	0,00E+00	3030	Os01g0896200	98,94	0,00E+00	1650	-1380	Indica	NA	
augustus-1-processed-gene-4.45-mRNA-1	1	OsR498G0100022900.01.T01	99,27	0,00E+00	964	Os01g0106200	100,00	0,00E+00	982	18	NA	Japonica	
augustus-1-processed-gene-40.0-mRNA-1	1	OsR498G0100258100.01.T04	98,64	0,00E+00	2055	Os01g0177100	98,21	0,00E+00	2033	-22	NA	Japonica	
augustus-1-processed-gene-400.99-mRNA-1	1	OsR498G0102533400.01.T01	99,47	0,00E+00	6077	Os01g0904400	99,47	0,00E+00	6077	0	NA	NA	
augustus-1-processed-gene-401.33-mRNA-1	1	OsR498G0101582200.01.T01	99,82	0,00E+00	1943	Os01g0627933	82,51	0,00E+00	1086	-857	Indica	NA	
augustus-1-processed-gene-401.35-mRNA-1	1	OsR498G0101580000.01.T02	99,45	0,00E+00	1303	Os01g0626900	100,00	0,00E+00	1321	18	NA	NA	
augustus-1-processed-gene-402.34-mRNA-1	1	OsR498G0511414500.01.T01	73,79	1,72E-89	332	Os01g0625900	100,00	0,00E+00	1986	1654	Japonica	NA	
augustus-1-processed-gene-402.51-mRNA-1	1	OsR498G0101574800.01.T01	99,71	0,00E+00	1875	Os01g0624700	99,90	0,00E+00	1884	9	NA	NA	
augustus-1-processed-gene-402.54-mRNA-1	1	OsR498G0101573400.01.T02	96,55	0,00E+00	4076	Os01g0624000	96,76	0,00E+00	4099	23	NA	NA	
augustus-1-processed-gene-404.12-mRNA-1	1	OsR498G0101559000.01.T01	95,14	0,00E+00	1594	Os01g0620300	100,00	0,00E+00	2603	1009	Japonica	NA	
augustus-1-processed-gene-407.14-mRNA-1	1	OsR498G0101542000.01.T01	99,95	0,00E+00	3318	Os01g0615500	100,00	0,00E+00	3236	-82	NA	NA	NBS-TM
augustus-1-processed-gene-408.30-mRNA-1	1	OsR498G0101538800.01.T03	99,78	0,00E+00	2411	Os01g0614500	100,00	0,00E+00	2425	14	NA	NA	
augustus-1-processed-gene-408.46-mRNA-1	1	OsR498G0101534400.01.T01	98,40	0,00E+00	644	Os05g0468700	91,84	4,00E-156	550	-94	Indica	NA	
augustus-1-processed-gene-409.0-mRNA-1	1	OsR498G0101530400.01.T06	99,90	0,00E+00	1715	Os01g0612600	100,00	0,00E+00	1719	4	NA	NA	
augustus-1-processed-gene-409.8-mRNA-1	1	OsR498G0101526600.01.T05	99,88	0,00E+00	4321	Os01g0611900	100,00	0,00E+00	4334	13	NA	NA	
augustus-1-processed-gene-41.38-mRNA-1	1	OsR498G0100265100.01.T01	99,63	0,00E+00	2440	Os01g0178600	99,93	0,00E+00	2458	18	NA	Japonica	
augustus-1-processed-gene-41.46-mRNA-1	1	OsR498G0100266100.01.T05	99,84	0,00E+00	2188	Os01g0178700	100,00	0,00E+00	2197	9	NA	Japonica	
augustus-1-processed-gene-410.48-mRNA-1	1	OsR498G0101517800.01.T03	99,26	4,17E-134	477	Os01g0342700	75,09	8,66E-49	193	-284	Indica	NA	
augustus-1-processed-gene-412.37-mRNA-1	1	OsR498G0101507200.01.T01	99,85	0,00E+00	3693	Os05g0588250	74,72	0,00E+00	865	-2828	Indica	NA	Kinase-LRR-TM
augustus-1-processed-gene-413.2-mRNA-1	1	OsR498G0101500000.01.T01	100,00	0,00E+00	1801	Os01g0606100	100,00	0,00E+00	2646	845	Japonica	NA	
augustus-1-processed-gene-413.21-mRNA-1	1	OsR498G0101494400.01.T03	98,13	0,00E+00	1297	Os01g0604500	100,00	0,00E+00	1353	56	NA	NA	
augustus-1-processed-gene-415.8-mRNA-1	1	OsR498G0101477000.01.T01	99,85	0,00E+00	2351	Os01g0601000	99,08	0,00E+00	3052	701	Japonica	NA	
augustus-1-processed-gene-416.48-mRNA-1	1	OsR498G0101471400.01.T01	99,68	0,00E+00	2786	Os01g0598400	100,00	0,00E+00	2809	23	NA	NA	
augustus-1-processed-gene-418.44-mRNA-1	1	OsR498G0101459200.01.T01	99,61	0,00E+00	1356	Os01g0594900	100,00	0,00E+00	1370	14	NA	NA	
augustus-1-processed-gene-420.13-mRNA-1	1	OsR498G0102309900.01.T01	100,00	0,00E+00	850	Os01g0841700	98,13	0,00E+00	829	-21	NA	NA	
augustus-1-processed-gene-420.15-mRNA-1	1	OsR498G0102311900.01.T03	100,00	0,00E+00	4442	Os01g0842200	99,84	0,00E+00	4424	-18	NA	NA	
augustus-1-processed-gene-422.0-mRNA-1	1	OsR498G0102321300.01.T01	100,00	0,00E+00	1375	Os01g0844900	100,00	0,00E+00	1191	-184	Indica	NA	
augustus-1-processed-gene-425.30-mRNA-1	1	OsR498G0102000100.01.T01	98,73	0,00E+00	1923	Os08g0487300	90,49	0,00E+00	1562	-361	Indica	NA	
augustus-1-processed-gene-425.42-mRNA-1	1	OsR498G0102344100.01.T01	100,00	0,00E+00	1388	Os01g0851600	99,63	0,00E+00	1443	55	NA	NA	
augustus-1-processed-gene-426.0-mRNA-1	1	OsR498G0102348900.01.T02	100,00	0,00E+00	3810	Os01g0852900	99,91	0,00E+00	3800	-10	NA	NA	
augustus-1-processed-gene-426.14-mRNA-1	1	OsR498G0102352900.01.T01	100,00	0,00E+00	1775	Os01g0853800	99,90	0,00E+00	1771	-4	NA	NA	
augustus-1-processed-gene-426.16-mRNA-1	1	OsR498G0102354500.01.T01	100,00	0,00E+00	2619	Os05g0449200	81,36	0,00E+00	1323	-1296	Indica	NA	
augustus-1-processed-gene-426.3-mRNA-1	1	OsR498G0102351500.01.T06	100,00	0,00E+00	710	Os01g0853600	100,00	0,00E+00	682	-28	NA	NA	
augustus-1-processed-gene-427.0-mRNA-1	1	OsR498G0102357300.01.T01	100,00	0,00E+00	2955	Os01g0854800	99,94	0,00E+00	2950	-5	NA	NA	
augustus-1-processed-gene-428.9-mRNA-1	1	OsR498G0102367500.01.T02	100,00	0,00E+00	861	Os01g0856800	89,52	0,00E+00	674	-187	Indica	NA	
augustus-1-processed-gene-429.51-mRNA-1	1	OsR498G0102373300.01.T01	100,00	0,00E+00	2132	Os01g0858900	99,75	0,00E+00	2119	-13	NA	NA	
augustus-1-processed-gene-429.63-mRNA-1	1	OsR498G0102372800.01.T02	100,00	0,00E+00	1927	Os01g0858500	96,88	0,00E+00	1563	-364	Indica	NA	
augustus-1-processed-gene-430.13-mRNA-1	1	OsR498G0102379100.01.T01	97,70	0,00E+00	1116	Os02g0321900	89,56	0,00E+00	877	-239	Indica	NA	
augustus-1-processed-gene-430.15-mRNA-1	1	OsR498G0102379300.01.T01	75,46	5,69E-158	559	Os01g0860500	99,24	0,00E+00	1625	1066	Japonica	NA	
augustus-1-processed-gene-430.16-mRNA-1	1	OsR498G0408525900.01.T04	79,72	4,78E-67	255	Os01g0860601	99,60	0,00E+00	890	635	Japonica	NA	
augustus-1-processed-gene-430.18-mRNA-1	1	OsR498G0102380900.01.T06	100,00	0,00E+00	2159	Os01g0860800	99,83	0,00E+00	2150	-9	NA	NA	
augustus-1-processed-gene-431.15-mRNA-1	1	OsR498G0102384500.01.T02	100,00	0,00E+00	1191	Os01g0862600	100,00	0,00E+00	1191	0	NA	NA	
augustus-1-processed-gene-432.18-mRNA-1	1	OsR498G0102390500.01.T01	100,00	0,00E+00	823	Os01g0864200	99,56	0,00E+00	814	-9	NA	NA	
augustus-1-processed-gene-433.60-mRNA-1	1	OsR498G0611661000.01.T01	93,78	1,54E-173	609	Os01g0866200	100,00	0,00E+00	737	128	Japonica	NA	
augustus-1-processed-gene-434.9-mRNA-1	1	OsR498G0102409900.01.T01	100,00	0,00E+00	4112	Os01g0868900	99,61	0,00E+00	4075	-37	NA	NA	
augustus-1-processed-gene-435.41-mRNA-1	1	OsR498G0102413500.01.T01	100,00	0,00E+00	1168	Os01g0869900	100,00	0,00E+00	1168	0	NA	NA	Kinase-TM
augustus-1-processed-gene-435.54-mRNA-1	1	OsR498G0102416100.01.T01	100,00	0,00E+00	4432	Os01g0889900	72,53	0,00E+00	1266	-3166	Indica	NA	Kinase-TM
augustus-1-processed-gene-437.29-mRNA-1	1	OsR498G0102425300.01.T01	99,73	0,00E+00	1960	Os01g0873700	99,54	0,00E+00	1951	-9	NA	NA	
augustus-1-processed-gene-439.40-mRNA-1	1	OsR498G0102446100.01.T01	100,00	0,00E+00	5222	Os01g0878300	99,79	0,00E+00	5194	-28	NA	NA	Kinase-LRR-TM
augustus-1-processed-gene-439.43-mRNA-1	1	OsR498G0102448100.01.T01	100,00	0,00E+00	1061	Os01g0879200	99,66	0,00E+00	1052	-9	NA	NA	
augustus-1-processed-gene-439.44-mRNA-1	1	OsR498G0102448300.01.T01	100,00	0,00E+00	1240	Os01g0879200	75,20	6,23E-100	364	-876	Indica	NA	
augustus-1-processed-gene-439.45-mRNA-1	1	OsR498G0102448300.01.T03	100,00	0,00E+00	2574	Os01g0879400	99,93	0,00E+00	2555	-19	NA	NA	
augustus-1-processed-gene-441.21-mRNA-1	1	OsR498G0102463700.01.T01	100,00	0,00E+00	1191	Os10g0492400	88,09	8,89E-162	569	-622	Indica	NA	Kinase-TM
augustus-1-processed-gene-441.4-mRNA-1	1	OsR498G0611598700.01.T01	82,69	6,82E-88	324	Os01g0881800	98,08	7,23E-152	535	211	Japonica	NA	
augustus-1-processed-gene-443.42-mRNA-1	1	OsR498G0102473900.01.T01	100,00	0,00E+00	4475	Os01g0885700	99,68	0,00E+00	4439	-36	NA	NA	Kinase-TM
augustus-1-processed-gene-444.12-mRNA-1	1	OsR498G0102485500.01.T01	100,00	0,00E+00	801	Os01g0888800	100,00	0,00E+00	801	0	NA	NA	
augustus-1-processed-gene-445.37-mRNA-1	1	OsR498G0102487900.01.T03	100,00	0,00E+00	1462	Os01g0889400	100,00	0,00E+00	1462	0	NA	NA	
augustus-1-processed-gene-446.16-mRNA-1	1	OsR498G0102496500.01.T01	100,00	0,00E+00	1516	Os01g0892300	99,64	0,00E+00	1502	-14	NA	NA	LRR-TM
augustus-1-processed-gene-446.17-mRNA-1	1	OsR498G0102498100.01.T02	100,00	0,00E+00	2587	Os01g0892800	100,00	0,00E+00	2587	0	NA	NA	Kinase-TM
augustus-1-processed-gene-446.3-mRNA-1	1	OsR498G0102493700.01.T01	100,00	0,00E+00	1662	Os01g0891700	100,00	0,00E+00	1662	0	NA	NA	LRR-TM
augustus-1-processed-gene-447.15-mRNA-1	1	OsR498G0102505500.01.T01	100,00	0,00E+00	1483	Os01g0895500	99,88	0,00E+00	1479	-4	NA	NA	
augustus-1-processed-gene-447.17-mRNA-1	1	OsR498G0102505900.01.T01	100,00	0,00E+00	3030	Os01g0896200	98,94	0,00E+00	1650	-1380	Indica	NA	
augustus-1-processed-gene-45.13-mRNA-1	1	OsR498G0100284500.01.T01	99,63	0,00E+00	1930	Os01g0183800	99,91	0,00E+00	1944	14	NA	Japonica	
augustus-1-processed-gene-45.20-mRNA-1	1	OsR498G0100286300.01.T01	97,59	0,00E+00	916	Os01g0184700	99,84	0,00E+00	1136	220	Japonica	Japonica	
augustus-1-processed-gene-452.46-mRNA-1	1	OsR498G0102533400.01.T01	99,47	0,00E+00	6077	Os01g0904400	99,47	0,00E+00	6077	0	NA	NA	
augustus-1-processed-gene-452.52-mRNA-1	1	OsR498G0101582200.01.T01	99,82	0,00E+00	1943	Os01g0627933	82,51	0,00E+00	1086	-857	Indica	NA	
augustus-1-processed-gene-452.54-mRNA-1	1	OsR498G0101580000.01.T02	99,45	0,00E+00	1303	Os01g0626900	100,00	0,00E+00	1321	18	NA	NA	
augustus-1-processed-gene-453.34-mRNA-1	1	OsR498G0511414500.01.T01	73,79	1,72E-89	332	Os01g0625900	100,00	0,00E+00	1986	1654	Japonica	NA	
augustus-1-processed-gene-453.39-mRNA-1	1	OsR498G0101574800.01.T01	99,71	0,00E+00	1875	Os01g0624700	99,90	0,00E+00	1884	9	NA	NA	
augustus-1-processed-gene-454.25-mRNA-1	1	OsR498G0101573400.01.T02	96,55	0,00E+00	4076	Os01g0624000	96,76	0,00E+00	4099	23	NA	NA	
augustus-1-processed-gene-456.0-mRNA-1	1	OsR498G0101559000.01.T01	95,14	0,00E+00	1594	Os01g0620300	100,00	0,00E+00	2603	1009	Japonica	NA	
augustus-1-processed-gene-459.1-mRNA-1	1	OsR498G0101542000.01.T01	99,95	0,00E+00	3318	Os01g0615500	100,00	0,00E+00	3236	-82	NA	NA	NBS-TM
augustus-1-processed-gene-459.14-mRNA-1	1	OsR498G0101538800.01.T03	99,78	0,00E+00	2411	Os01g0614500	100,00	0,00E+00	2425	14	NA	NA	
augustus-1-processed-gene-459.20-mRNA-1	1	OsR498G0101534400.01.T01	98,40	0,00E+00	644	Os05g0468700	91,84	4,00E-156	550	-94	Indica	NA	
augustus-1-processed-gene-46.17-mRNA-1	1	OsR498G0100291700.01.T01	100,00	0,00E+00	1131	Os01g0186600	100,00	0,00E+00	1131	0	NA	Indica	
augustus-1-processed-gene-460.36-mRNA-1	1	OsR498G0101530400.01.T06	99,90	0,00E+00	1715	Os01g0612600	100,00	0,00E+00	1719	4	NA	NA	
augustus-1-processed-gene-460.39-mRNA-1	1	OsR498G0101526600.01.T05	99,88	0,00E+00	4321	Os01g0611900	100,00	0,00E+00	4334	13	NA	NA	
augustus-1-processed-gene-462.20-mRNA-1	1	OsR498G0101517800.01.T03	99,26	4,17E-134	477	Os01g0342700	75,09	8,66E-49	193	-284	Indica	NA	
augustus-1-processed-gene-463.44-mRNA-1	1	OsR498G0101507200.01.T01	99,85	0,00E+00	3693	Os05g0588250	74,72	0,00E+00	865	-2828	Indica	NA	Kinase-LRR-TM
augustus-1-processed-gene-464.42-mRNA-1	1	OsR498G0101500000.01.T01	100,00	0,00E+00	1801	Os01g0606100	100,00	0,00E+00	2646	845	Japonica	NA	
augustus-1-processed-gene-465.21-mRNA-1	1	OsR498G0101494400.01.T03	98,13	0,00E+00	1297	Os01g0604500	100,00	0,00E+00	1353	56	NA	NA	
augustus-1-processed-gene-467.32-mRNA-1	1	OsR498G0101477000.01.T01	99,85	0,00E+00	2351	Os01g0601000	99,08	0,00E+00	3052	701	Japonica	NA	
augustus-1-processed-gene-469.13-mRNA-1	1	OsR498G0101459200.01.T01	99,61	0,00E+00	1356	Os01g0594900	100,00	0,00E+00	1370	14	NA	NA	
augustus-1-processed-gene-470.16-mRNA-1	1	OsR498G0408694600.01.T01	74,80	7,74E-39	162	Os01g0905550	90,36	0,00E+00	679	517	Japonica	NA	
augustus-1-processed-gene-470.17-mRNA-1	1	OsR498G0102537700.01.T01	100,00	0,00E+00	1370	Os01g0905700	100,00	0,00E+00	1370	0	NA	Indica	
augustus-1-processed-gene-472.32-mRNA-1	1	OsR498G0102549700.01.T02	100,00	0,00E+00	2744	Os01g0908800	99,74	0,00E+00	2726	-18	NA	Indica	
augustus-1-processed-gene-475.12-mRNA-1	1	OsR498G0102563100.01.T01	99,53	0,00E+00	2647	Os01g0913800	100,00	0,00E+00	2679	32	NA	Japonica	
augustus-1-processed-gene-478.3-mRNA-1	1	OsR498G0102583100.01.T01	73,47	5,77E-102	372	Os01g0919300	77,38	3,39E-131	469	97	Japonica	Japonica	
augustus-1-processed-gene-479.17-mRNA-1	1	OsR498G0102592100.01.T01	95,64	0,00E+00	1886	Os01g0921100	100,00	0,00E+00	2111	225	Japonica	Japonica	
augustus-1-processed-gene-479.20-mRNA-1	1	OsR498G0102593100.01.T01	99,28	0,00E+00	3458	Os01g0921400	97,21	0,00E+00	2920	-538	Indica	Japonica	
augustus-1-processed-gene-481.12-mRNA-1	1	OsR498G0102610500.01.T01	99,87	0,00E+00	4018	Os01g0923800	100,00	0,00E+00	4031	13	NA	Japonica	
augustus-1-processed-gene-481.14-mRNA-1	1	OsR498G0102612100.01.T01	99,47	0,00E+00	1010	Os01g0924200	100,00	0,00E+00	1023	13	NA	Japonica	
augustus-1-processed-gene-482.0-mRNA-1	1	OsR498G0102613700.01.T03	99,48	0,00E+00	1702	Os01g0924400	99,79	0,00E+00	1712	10	NA	Japonica	

augustus-1-processed-gene-482.2-mRNA-1	1	OsR498G0102614900.01.T01	99,91	0,00E+00	2034	Os01g0924900	100,00	0,00E+00	2039	5	NA	Japonica	
augustus-1-processed-gene-484.34-mRNA-1	1	OsR498G0102625300.01.T03	99,36	0,00E+00	1660	Os01g0927500	100,00	0,00E+00	1689	29	NA	Japonica	Kinase-TM
augustus-1-processed-gene-488.1-mRNA-1	1	OsR498G0102656100.01.T01	100,00	1,24E-161	569	Os01g0936000	99,74	0,00E+00	678	109	Japonica	Japonica	
augustus-1-processed-gene-489.1-mRNA-1	1	OsR498G0102663900.01.T01	99,92	0,00E+00	2339	Os01g0937600	99,92	0,00E+00	2339	0	NA	Japonica	
augustus-1-processed-gene-489.2-mRNA-1	1	OsR498G0102664300.01.T01	99,85	0,00E+00	2345	Os01g0937800	99,92	0,00E+00	2321	-24	NA	Japonica	
augustus-1-processed-gene-489.22-mRNA-1	1	OsR498G0102668500.01.T01	99,77	0,00E+00	2351	Os01g0939500	100,00	0,00E+00	2365	14	NA	Japonica	
augustus-1-processed-gene-489.3-mRNA-1	1	OsR498G0102664700.01.T01	99,17	0,00E+00	847	Os01g0937900	100,00	0,00E+00	861	14	NA	Japonica	
augustus-1-processed-gene-491.57-mRNA-1	1	OsR498G0102675900.01.T01	100,00	0,00E+00	1489	Os01g0941500	100,00	0,00E+00	1727	238	Japonica	NA	
augustus-1-processed-gene-492.26-mRNA-1	1	OsR498G0102681300.01.T01	100,00	7,24E-173	607	Os01g0943000	100,00	2,52E-173	607	0	NA	NA	
augustus-1-processed-gene-493.2-mRNA-1	1	OsR498G0102675700.01.T01	99,71	0,00E+00	1216	Os01g0941400	99,85	0,00E+00	1221	5	NA	Japonica	
augustus-1-processed-gene-495.27-mRNA-1	1	OsR498G0102700100.01.T01	99,84	0,00E+00	1105	Os01g0949300	100,00	0,00E+00	1110	5	NA	NA	
augustus-1-processed-gene-496.59-mRNA-1	1	OsR498G0102702900.01.T01	99,02	0,00E+00	1260	Os01g0950300	99,86	0,00E+00	1289	29	NA	Japonica	
augustus-1-processed-gene-497.0-mRNA-1	1	OsR498G0102709300.01.T01	99,86	0,00E+00	1257	Os01g0952500	100,00	0,00E+00	1261	4	NA	NA	
augustus-1-processed-gene-497.6-mRNA-1	1	OsR498G0510749100.01.T01	75,85	1,32E-113	410	Os01g0952900	99,70	0,00E+00	1177	767	Japonica	NA	
augustus-1-processed-gene-497.8-mRNA-1	1	OsR498G0102710700.01.T01	99,77	0,00E+00	3839	Os01g0953100	99,81	0,00E+00	3844	5	NA	Japonica	
augustus-1-processed-gene-498.4-mRNA-1	1	OsR498G0102722100.01.T01	99,56	0,00E+00	801	Os01g0955100	76,05	3,28E-82	304	-497	Indica	Japonica	
augustus-1-processed-gene-5.14-mRNA-1	1	OsR498G0101358900.01.T01	93,75	5,81E-100	364	Os01g0108500	99,58	5,82E-120	429	65	Japonica	Japonica	
augustus-1-processed-gene-500.77-mRNA-1	1	OsR498G0102735100.01.T01	99,86	0,00E+00	2502	Os01g0958200	99,86	0,00E+00	2502	0	NA	NA	
augustus-1-processed-gene-500.93-mRNA-1	1	OsR498G0102739900.01.T01	99,58	0,00E+00	841	Os01g0960000	99,79	0,00E+00	846	5	NA	Japonica	
augustus-1-processed-gene-503.48-mRNA-1	1	OsR498G0102752500.01.T01	99,89	0,00E+00	4818	Os01g0964800	100,00	0,00E+00	2684	-2134	Indica	Japonica	
augustus-1-processed-gene-503.49-mRNA-1	1	OsR498G0102752700.01.T01	99,64	0,00E+00	2006	Os01g0964900	100,00	0,00E+00	1954	-52	NA	Japonica	
augustus-1-processed-gene-503.63-mRNA-1	1	OsR498G0102757100.01.T05	99,59	0,00E+00	1311	Os01g0966300	99,86	0,00E+00	1320	9	NA	Japonica	
augustus-1-processed-gene-504.2-mRNA-1	1	OsR498G0102759300.01.T01	99,33	0,00E+00	1059	Os07g0533600	96,18	0,00E+00	642	-417	Indica	NA	
augustus-1-processed-gene-504.23-mRNA-1	1	OsR498G0102764300.01.T01	99,70	0,00E+00	1182	Os01g0968800	100,00	0,00E+00	1191	9	NA	Japonica	
augustus-1-processed-gene-506.28-mRNA-1	1	OsR498G0102775500.01.T01	99,01	0,00E+00	1769	Os01g0972000	99,90	0,00E+00	1809	40	NA	Japonica	
augustus-1-processed-gene-507.75-mRNA-1	1	OsR498G0204247700.01.T03	90,87	9,10E-92	336	Os01g0974900	100,00	3,39E-136	482	146	Japonica	Japonica	
augustus-1-processed-gene-52.11-mRNA-1	1	OsR498G0100340100.01.T01	99,86	0,00E+00	2589	Os01g0196600	99,93	0,00E+00	2593	4	NA	Japonica	
augustus-1-processed-gene-53.40-mRNA-1	1	OsR498G0100343500.01.T03	99,25	0,00E+00	705	Os01g0197350	100,00	0,00E+00	717	12	NA	Japonica	
augustus-1-processed-gene-54.42-mRNA-1	1	OsR498G0100351500.01.T01	97,74	0,00E+00	1059	Os01g0200200	99,77	0,00E+00	1539	480	Japonica	NA	
augustus-1-processed-gene-55.21-mRNA-1	1	OsR498G0100352500.01.T01	99,80	0,00E+00	1766	Os01g0200500	99,80	0,00E+00	1766	0	NA	NA	
augustus-1-processed-gene-57.25-mRNA-1	1	OsR498G0100368300.01.T01	97,67	2,50E-144	512	Os01g0203400	100,00	1,13E-168	590	78	Japonica	Japonica	
augustus-1-processed-gene-58.64-mRNA-1	1	OsR498G0100374100.01.T03	100,00	0,00E+00	926	Os01g0205300	99,03	0,00E+00	723	-203	Indica	NA	
augustus-1-processed-gene-58.66-mRNA-1	1	OsR498G0511225400.01.T02	71,12	4,91E-50	200	Os01g0205600	100,00	0,00E+00	1342	1142	Japonica	NA	
augustus-1-processed-gene-58.69-mRNA-1	1	OsR498G0100377100.01.T01	99,94	0,00E+00	2766	Os01g0206300	100,00	0,00E+00	1838	-928	Indica	NA	Kinase-TM
augustus-1-processed-gene-59.22-mRNA-1	1	OsR498G0100380900.01.T01	100,00	0,00E+00	2019	Os01g0207700	100,00	0,00E+00	2019	0	NA	NA	
augustus-1-processed-gene-6.45-mRNA-1	1	OsR498G0100031500.01.T03	99,39	0,00E+00	1168	Os01g0109500	100,00	0,00E+00	1283	115	NA	Japonica	
augustus-1-processed-gene-66.29-mRNA-1	1	OsR498G0100429300.01.T01	98,95	0,00E+00	1007	Os01g0219300	100,00	0,00E+00	1034	27	NA	Japonica	
augustus-1-processed-gene-67.44-mRNA-1	1	OsR498G0100443500.01.T01	100,00	0,00E+00	3220	Os01g0222800	100,00	0,00E+00	2724	-496	Indica	NA	Kinase-TM
augustus-1-processed-gene-68.14-mRNA-1	1	OsR498G0100448100.01.T01	99,80	0,00E+00	4382	Os01g0223900	100,00	0,00E+00	4405	23	NA	Japonica	Kinase-TM
augustus-1-processed-gene-68.16-mRNA-1	1	OsR498G0100451500.01.T01	99,48	0,00E+00	2044	Os01g0224100	99,65	0,00E+00	2048	4	NA	Japonica	
augustus-1-processed-gene-68.2-mRNA-1	1	OsR498G0100445300.01.T01	100,00	0,00E+00	1894	Os01g0223300	100,00	0,00E+00	1894	0	NA	NA	
augustus-1-processed-gene-72.51-mRNA-1	1	OsR498G0100474900.01.T01	99,72	0,00E+00	5684	Os01g0229300	100,00	0,00E+00	5725	41	NA	Japonica	
augustus-1-processed-gene-73.17-mRNA-1	1	OsR498G0100481100.01.T06	99,61	0,00E+00	2310	Os01g0231500	100,00	0,00E+00	2333	23	NA	Japonica	Kinase
augustus-1-processed-gene-73.19-mRNA-1	1	OsR498G0100481700.01.T05	99,56	4,36E-111	402	Os01g0231800	100,00	3,56E-113	407	5	NA	Japonica	
augustus-1-processed-gene-75.1-mRNA-1	1	OsR498G0100490700.01.T01	98,17	0,00E+00	1511	Os01g0234300	100,00	0,00E+00	2080	569	Japonica	Japonica	
augustus-1-processed-gene-75.18-mRNA-1	1	OsR498G0100494900.01.T01	99,13	2,19E-172	606	Os01g0235500	100,00	0,00E+00	856	250	Japonica	Japonica	
augustus-1-processed-gene-75.2-mRNA-1	1	OsR498G0100490700.01.T01	99,72	0,00E+00	1281	Os01g0234300	100,00	0,00E+00	1290	9	NA	Japonica	
augustus-1-processed-gene-75.20-mRNA-1	1	OsR498G0100497300.01.T01	99,24	0,00E+00	2088	Os01g0235900	100,00	0,00E+00	2122	34	NA	Japonica	
augustus-1-processed-gene-75.21-mRNA-1	1	OsR498G0100497500.01.T02	99,67	0,00E+00	1090	Os01g0236000	100,00	0,00E+00	1099	9	NA	Japonica	
augustus-1-processed-gene-77.55-mRNA-1	1	OsR498G0100510100.01.T01	99,81	0,00E+00	1885	Os01g0239100	100,00	0,00E+00	1894	9	NA	Japonica	
augustus-1-processed-gene-78.1-mRNA-1	1	OsR498G0100512300.01.T01	99,80	0,00E+00	5400	Os01g0239700	100,00	0,00E+00	5427	27	NA	Japonica	Kinase-LRR-TM
augustus-1-processed-gene-8.0-mRNA-1	1	OsR498G0100044500.01.T01	100,00	0,00E+00	3534	Os01g0113350	98,32	0,00E+00	2571	-963	Indica	Indica	CC-Kinase-TM
augustus-1-processed-gene-8.3-mRNA-1	1	OsR498G0100044500.01.T01	82,62	0,00E+00	2013	Os01g0113650	99,90	0,00E+00	3539	1526	Japonica	Indica	Kinase-TM
augustus-1-processed-gene-83.16-mRNA-1	1	OsR498G0100557300.01.T01	100,00	0,00E+00	1732	Os01g0251100	100,00	0,00E+00	1732	0	NA	NA	
augustus-1-processed-gene-84.15-mRNA-1	1	OsR498G0100563100.01.T01	100,00	0,00E+00	771	Os01g0253200	100,00	0,00E+00	897	126	Japonica	NA	
augustus-1-processed-gene-84.17-mRNA-1	1	OsR498G0100564100.01.T01	99,07	0,00E+00	1706	Os01g0253500	100,00	0,00E+00	1727	21	NA	NA	
augustus-1-processed-gene-89.32-mRNA-1	1	OsR498G0100598700.01.T01	91,20	6,48E-173	607	Os01g0261600	91,20	2,26E-173	607	0	NA	NA	
augustus-1-processed-gene-92.47-mRNA-1	1	OsR498G0100622500.01.T01	100,00	0,00E+00	2295	Os01g0267300	100,00	0,00E+00	2836	541	Japonica	NA	
augustus-1-processed-gene-93.35-mRNA-1	1	OsR498G0100624700.01.T01	100,00	0,00E+00	2149	Os01g0267800	100,00	0,00E+00	2149	0	NA	NA	Kinase-TM
augustus-1-processed-gene-93.46-mRNA-1	1	OsR498G0100628700.01.T01	98,98	0,00E+00	1549	Os01g0268800	98,98	0,00E+00	1549	0	NA	NA	
augustus-1-processed-gene-94.11-mRNA-1	1	OsR498G0100633700.01.T01	99,66	0,00E+00	2645	Os01g0270700	99,94	0,00E+00	2820	175	NA	Japonica	
augustus-1-processed-gene-95.16-mRNA-1	1	OsR498G0100640100.01.T01	99,63	0,00E+00	2884	Os01g0273300	100,00	0,00E+00	2911	27	NA	Japonica	
augustus-1-processed-gene-96.22-mRNA-1	1	OsR498G0100644500.01.T01	99,73	0,00E+00	1339	Os01g0274500	99,87	0,00E+00	1343	4	NA	Japonica	
augustus-1-processed-gene-98.12-mRNA-1	1	OsR498G0100658300.01.T01	99,66	0,00E+00	2638	Os01g0277500	100,00	0,00E+00	2657	19	NA	Japonica	
maker-1-augustus-gene-0.26-mRNA-1	1	OsR498G0100127300.01.T01	100,00	0,00E+00	1499	Os01g0137100	93,32	0,00E+00	1195	-304	Indica	NA	
maker-1-augustus-gene-0.28-mRNA-1	1	OsR498G0100126800.01.T01	99,01	0,00E+00	3562	Os01g0136800	98,27	0,00E+00	3495	-67	NA	NA	Kinase-TM
maker-1-augustus-gene-1.12-mRNA-1	1	OsR498G0100001700.01.T05	99,94	0,00E+00	2837	Os01g0100500	100,00	0,00E+00	2841	4	NA	Japonica	
maker-1-augustus-gene-1.16-mRNA-1	1	OsR498G0100002100.01.T01	99,49	0,00E+00	1742	Os01g0100600	99,90	0,00E+00	1760	18	NA	Japonica	
maker-1-augustus-gene-1.20-mRNA-1	1	OsR498G0100002300.01.T01	100,00	0,00E+00	1088	Os01g0100700	100,00	0,00E+00	1088	0	NA	Japonica	
maker-1-augustus-gene-1.29-mRNA-1	1	OsR498G0100003100.01.T01	98,84	0,00E+00	1816	Os01g0100900	98,84	0,00E+00	1816	0	NA	Japonica	
maker-1-augustus-gene-1.3-mRNA-1	1	OsR498G0100000700.01.T01	98,81	0,00E+00	3553	Os01g0100100	100,00	0,00E+00	2129	-1424	Indica	Japonica	
maker-1-augustus-gene-1.38-mRNA-1	1	OsR498G0100248000.01.T01	99,89	0,00E+00	1684	Os01g0174300	100,00	0,00E+00	1689	5	NA	NA	
maker-1-augustus-gene-1.42-mRNA-1	1	OsR498G0100248600.01.T03	95,22	0,00E+00	1155	Os01g0174500	95,36	0,00E+00	1159	4	NA	NA	
maker-1-augustus-gene-1.47-mRNA-1	1	OsR498G0100004600.01.T01	99,76	0,00E+00	2195	Os01g0101300	100,00	0,00E+00	2208	13	NA	Japonica	
maker-1-augustus-gene-1.8-mRNA-1	1	OsR498G0100001500.01.T01	99,74	0,00E+00	3392	Os01g0100400	100,00	0,00E+00	3047	-345	Indica	Japonica	
maker-1-augustus-gene-10.28-mRNA-1	1	OsR498G0100057100.01.T04	100,00	0,00E+00	2035	Os01g0117900	98,67	0,00E+00	1967	-68	NA	Indica	
maker-1-augustus-gene-10.31-mRNA-1	1	OsR498G1119486400.01.T02	74,47	0,00E+00	658	Os01g0118000	99,83	0,00E+00	2096	1438	Japonica	Indica	
maker-1-augustus-gene-10.45-mRNA-1	1	OsR498G0100063300.01.T02	99,71	0,00E+00	2509	Os01g0119500	100,00	0,00E+00	2527	18	NA	Japonica	
maker-1-augustus-gene-10.47-mRNA-1	1	OsR498G0100059400.01.T01	99,77	0,00E+00	3158	Os01g0118400	99,83	0,00E+00	2111	-1047	Indica	Japonica	
maker-1-augustus-gene-10.50-mRNA-1	1	OsR498G0100060400.01.T01	99,56	0,00E+00	2425	Os01g0118600	99,93	0,00E+00	2449	24	NA	Japonica	
maker-1-augustus-gene-10.53-mRNA-1	1	OsR498G0100060600.01.T01	99,49	0,00E+00	2773	Os01g0118700	100,00	0,00E+00	2809	36	NA	Japonica	
maker-1-augustus-gene-10.57-mRNA-1	1	OsR498G0100061000.01.T01	99,93	0,00E+00	2609	Os01g0119000	100,00	0,00E+00	2614	5	NA	Japonica	
maker-1-augustus-gene-10.59-mRNA-1	1	OsR498G0100062400.01.T02	99,06	0,00E+00	3573	Os01g0119100	100,00	0,00E+00	3021	-552	Indica	Japonica	
maker-1-augustus-gene-100.0-mRNA-1	1	OsR498G0100668900.01.T01	92,75	0,00E+00	739	Os01g0279300	93,17	0,00E+00	748	9	NA	Japonica	
maker-1-augustus-gene-100.10-mRNA-1	1	OsR498G0100672300.01.T01	99,67	0,00E+00	3223	Os12g0170100	97,28	0,00E+00	3030	-193	NA	Japonica	
maker-1-augustus-gene-100.13-mRNA-1	1	OsR498G0100669200.01.T01	99,84	0,00E+00	2286	Os01g0279400	100,00	0,00E+00	2295	9	NA	Japonica	
maker-1-augustus-gene-100.2-mRNA-1	1	OsR498G0100670100.01.T03	99,78	0,00E+00	1602	Os01g0279700	100,00	0,00E+00	1609	7	NA	Japonica	
maker-1-augustus-gene-100.20-mRNA-1	1	OsR498G0100670400.01.T01	100,00	0,00E+00	1164	Os01g0279800	100,00	0,00E+00	1164	0	NA	Japonica	LRR-TM
maker-1-augustus-gene-100.23-mRNA-1	1	OsR498G0100671400.01.T02	99,79	0,00E+00	864	Os01g0280000	100,00	0,00E+00	868	4	NA	Japonica	
maker-1-augustus-gene-100.27-mRNA-1	1	OsR498G0100672600.01.T01	93,39	0,00E+00	1155	Os01g0280500	93,52	0,00E+00	1159	4	NA	Japonica	
maker-1-augustus-gene-100.6-mRNA-1	1	OsR498G0100671100.01.T04	100,00	0,00E+00	1786	Os01g0279900	100,00	0,00E+00	1786	0	NA	Japonica	
maker-1-augustus-gene-101.0-mRNA-1	1	OsR498G0100674900.01.T01	99,76	0,00E+00	2238	Os01g0281000	100,00	0,00E+00	2251	13	NA	Japonica	
maker-1-augustus-gene-101.13-mRNA-1	1	OsR498G0100676600.01.T01	100,00	0,00E+00	1364	Os01g0281600	100,00	0,00E+00	1364	0	NA	Japonica	
maker-1-augustus-gene-101.3-mRNA-1	1	OsR498G0100675200.01.T01	98,29	0,00E+00	1629	Os01g0281100	100,00	0,00E+00	1438	-191	Indica	Japonica	
maker-1-augustus-gene-101.7-mRNA-1	1	OsR498G0100675400.01.T01	96,71	0,00E+00	2450	Os01g0281200	96,39	0,00E+00	2083	-367	Indica	Japonica	
maker-1-augustus-gene-101.9-mRNA-1	1	OsR498G0100676000.01.T02	99,72	0,00E+00	5110	Os11g0174000	87,39	0,00E+00	3523	-1587	Indica	Japonica	
maker-1-augustus-gene-102.0-mRNA-1	1	OsR498G0100679900.01.T01	99,75	0,00E+00	1420	Os01g0283100	100,00	0,00E+00	1429	9	NA	Japonica	
maker-1-augustus-gene-102.10-mRNA-1	1	OsR498G0100678600.01.T01	99,85	0,00E+00	2415	Os01g0282800	100,00	0,00E+00	2425	10	NA	Japonica	
maker-1-augustus-gene-102.12-mRNA-1	1	OsR498G0100679800.01.T02	99,67	0,00E+00	3274	Os01g0283000	100,00	0,00E+00	3301	27	NA	Japonica	
maker-1-augustus-gene-102.14-mRNA-1	1	OsR498G0100681000.01.T01	99,15	0,00E+00	1860	Os01g0283500	100,00	0,00E+00	1900	40	NA	Japonica	
maker-1-augustus-gene-102.16-mRNA-1	1	OsR498G0100684800.01.T01	99,85	0,00E+00	1170	Os01g0284500	100,00	0,00E+00	1175	5	NA	Japonica	
maker-1-augustus-gene-102.5-mRNA-1	1	OsR498G0100681500.01.T01	99,69	0,00E+00	1756	Os01g0283600	100,00	0,00E+00	1763	7	NA	Japonica	
maker-1-augustus-gene-102.8-mRNA-1	1	OsR498G0100683300.01.T01	99,26	0,00E+00	1443	Os01g0283700	100,00	0,00E+00	1471	28	NA	Japonica	
maker-1-augustus-gene-103.44-mRNA-1	1	OsR498G0100687900.01.T04	95,06	0,00E+00	1174	Os05g0103200	71,47	4,56E-51	202	-972	Indica	Japonica	
maker-1-augustus-gene-103.51-mRNA-1	1	OsR498G0100688400.01.T01	97,92	0,00E+00	1572	Os01g0284900	100,00	0,00E+00	1613	41	NA	Japonica	
maker-1-augustus-gene-103.55-mRNA-1	1	OsR498G0100690200.01.T01	99,77	0,00E+00	2308	Os01g0285300	100,00	0,00E+00	2322	14	NA	Japonica	
maker-1-augustus-gene-103.58-mRNA-1	1	OsR498G0100694000.01.T01	99,84	0,00E+00	3435	Os01g0286100	100,00	0,00E+00	3449	14	NA	Japonica	
maker-1-augustus-gene-104.0-mRNA-1	1	OsR498G0100695700.01.T01	99,71	0,00E+00	1881	Os01g0286600	100,00	0,00E+00	1894	13	NA	Japonica	
maker-1-augustus-gene-104.10-mRNA-1	1	OsR498G0100702400.01.T01	99,66	0,00E+00	1565	Os01g0287600	100,00	0,00E+00	1575	10	NA	Japonica	
maker-1-augustus-gene-104.6-mRNA-1	1	OsR498G0100698500.01.T01	99,19	0,00E+00	1088	Os01g0286900	98,74	5,55E-157	554	-534	Indica	Japonica	
maker-1-augustus-gene-104.8-mRNA-1	1	OsR498G0100695400.01.T01	99,09	0,00E+00	778	Os06g0620000	74,83	7,46E-59	226	-552	Indica	Japonica	
maker-1-augustus-gene-107.29-mRNA-1	1	OsR498G0100720500.01.T01	99,86	0,00E+00	2491	Os01g0292200	100,00	0,00E+00	2500	9	NA	Japonica	Kinase-TM
maker-1-augustus-gene-107.39-mRNA-1	1	OsR498G0100715000.01.T01	99,85	0,00E+00	2372	Os01g0291500	100,00	0,00E+00	2381	9	NA	Japonica	
maker-1-augustus-gene-108.0-mRNA-1	1	OsR498G0100721700.01.T06	96,23	0,00E+00	2569	Os01g0292900	96,48	0,00E+00	2587	18	NA	Japonica	
maker-1-augustus-gene-108.11-mRNA-1	1	OsR498G0100724000.01.T01	93,96	0,00E+00	1643	Os01g0293900	100,00	0,00E+00	1851	208	Japonica	Japonica	
maker-1-augustus-gene-108.13-mRNA-1	1	OsR498G0100725400.01.T01	86,67	0,00E+00	1288	Os01g0294700	70,66	4,99E-65	248	-1040	Indica	Japonica	
maker-1-augustus-gene-108.15-mRNA-1	1	OsR498G0100725400.01.T01	99,90	0,00E+00	1868	Os05g0134800	70,63	3,56E-105	381	-1487	Indica	Japonica	
maker-1-augustus-gene-108.17-mRNA-1	1	OsR498G0100726000.01.T01	99,71	0,00E+00	1870	Os01g0294700	100,00	0,00E+00	1884	14	NA	Japonica	
maker-1-augustus-gene-108.5-mRNA-1	1	OsR498G0100722600.01.T01	99,04	0,00E+00	2005	Os01g0293100	100,00	0,00E+00	2057	52	NA	Japonica	
maker-1-augustus-gene-108.7-mRNA-1	1	OsR498G0100723800.01.T01	96,39	0,00E+00	1611	Os07g0157000	95,10	0,00E+00	1471	-140	NA	Japonica	
maker-1-augustus-gene-109.10-mRNA-1	1	OsR498G0100728900.01.T01	99,51	0,00E+00	2197	Os01g0296200	100,00	0,00E+00	2727	530	Japonica	Japonica	
maker-1-augustus-gene-109.17-mRNA-1	1	OsR498G0100727600.01.T01	99,91	0,00E+00	2058	Os01g0295700	100,00	0,00E+00	2062	4	NA	Japonica	
maker-1-augustus-gene-109.26-mRNA-1	1	OsR498G0100728600.01.T01	99,61	0,00E+00	1822	Os01g0296000	100,00	0,00E+00	1689	-133	NA	Japonica	Kinase-TM
maker-1-augustus-gene-109.5-mRNA-1	1	OsR498G0100728700.01.T04	99,67	0,00E+00	2195	Os01g0296100	100,00	0,00E+00	2214	19	NA	Japonica	Kinase-TM
maker-1-augustus-gene-11.49-mRNA-1	1	OsR498G0100066900.01.T01	97,91	0,00E+00	1810	Os01g0121100	97,26	0,00E+00	1364	-446	Indica	NA	
maker-1-augustus-gene-11.64-mRNA-1	1	OsR498G0100065000.01.T01	100,00	0,00E+00	1434	Os01g0120400	100,00	0,00E+00	1434	0	NA	NA	
maker-1-augustus-gene-11.70-mRNA-1	1	OsR498G0100065400.01.T01	100,00	0,00E+00	1380	Os01g0120500	100,00	0,00E+00	1380	0	NA	NA	
maker-1-augustus-gene-11.73-mRNA-1	1	OsR498G0100066400.01.T02	99,76	0,00E+00	5310	Os01g0120800	99,73	0,00E+00	5305	-5	NA	NA	
maker-1-augustus-gene-11.79-mRNA-1	1	OsR498G0100067400.01.T03	96,43	0,00E+00	653	Os01g0121200	96,43	0,00E+00	653	0	NA	NA	
maker-1-augustus-gene-110.50-mRNA-1	1	OsR498G0100730500.01.T01	99,84	0,00E+00	4414	Os01g0296700	100,00	0,00E+00	2830	-1584	Indica	Japonica	
maker-1-augustus-gene-110.52-mRNA-1	1	OsR498G0100730900.01.T01	99,43	0,00E+00	1249	Os01g0296900	100,00	0,00E+00	1943	694	Japonica	Japonica	
maker-1-augustus-gene-110.55-mRNA-1	1	OsR498G0100731500.01.T01	99,81	0,00E+00	2844	Os01g0297200	100,00	0,00E+00	2857	13	NA	Japonica	
maker-1-augustus-gene-110.57-mRNA-1	1	OsR498G0100733500.01.T02	98,86	0,00E+00	1996	Os01g0297700	100,00	0,00E+00	2051	55	NA	Japonica	
maker-1-augustus-gene-111.13-mRNA-1	1	OsR498G0202947900.01.T01	89,45	6,69E-142	504	Os01g0299100	100,00	0,00E+00	1029	525	Japonica	Japonica	
maker-1-augustus-gene-111.16-mRNA-1	1	OsR498G0100737000.01.T01	99,79	0,00E+00	2568	Os01g0299300	100,00	0,00E+00	3108	540	Japonica	Japonica	
maker-1-augustus-gene-111.18-mRNA-1	1	OsR498G0100737600.01.T03	99,71	0,00E+00	1214	Os01g0299500	100,00	0,00E+00	935	-279	Indica	Japonica	
maker-1-augustus-gene-111.2-mRNA-1	1	OsR498G0100737300.01.T01	100,00	2,28E-118	425	Os01g0299400	100,00	7,98E-119	425	0	NA	Japonica	
maker-1-augustus-gene-111.21-mRNA-1	1	OsR498G0100740400.01.T01	99,89	0,00E+00	3287	Os01g0300200	100,00	0,00E+00	3296	9	NA	Japonica	
maker-1-augustus-gene-112.30-mRNA-1	1	OsR498G0100743700.01.T01	99,36	0,00E+00	3328	Os01g0300900	100,00	0,00E+00	3382	54	NA	Japonica	
maker-1-augustus-gene-112.33-mRNA-1	1	OsR498G0100744000.01.T01	99,75	0,00E+00	2812	Os01g0301000	100,00	0,00E+00	2830	18	NA	Japonica	
maker-1-augustus-gene-112.35-mRNA-1	1	OsR498G0100745200.01.T01	99,10	0,00E+00	1773	Os01g0301500	100,00	0,00E+00	2078	305	Japonica	Japonica	
maker-1-augustus-gene-112.37-mRNA-1	1	OsR498G0100745600.01.T02	99,80	0,00E+00	2719	Os01g0301700	100,00	0,00E+00	2733	14	NA	Japonica	
maker-1-augustus-gene-113.39-mRNA-1	1	OsR498G0100750500.01.T05	99,83	0,00E+00	5215	Os01g0303200	100,00	0,00E+00	5238	23	NA	Japonica	
maker-1-augustus-gene-113.45-mRNA-1	1	OsR498G0100752500.01.T04	99,82	0,00E+00	986	Os01g0303800	100,00	0,00E+00	991	5	NA	Japonica	
maker-1-augustus-gene-113.48-mRNA-1	1	OsR498G0100750800.01.T02	99,62	0,00E+00	928	Os03g0305600	71,43	3,14E-64	245	-683	Indica	Japonica	
maker-1-augustus-gene-113.53-mRNA-1	1	OsR498G0100751600.01.T01	99,70	0,00E+00	1187	Os01g0303600	100,00	0,00E+00	1196	9	NA	Japonica	
maker-1-augustus-gene-114.0-mRNA-1	1	OsR498G0100753700.01.T01	99,78	0,00E+00	3261	Os05g0531500	72,71	0,00E+00	634	-2627	Indica	Japonica	
maker-1-augustus-gene-115.0-mRNA-1	1	OsR498G0100758100.01.T01	98,95	0,00E+00	1674	Os01g0305900	98,70	0,00E+00	1205	-469	Indica	NA	
maker-1-augustus-gene-115.3-mRNA-1	1	OsR498G0100761500.01.T03	99,87	0,00E+00	1425	Os01g0306800	100,00	0,00E+00	1429	4	NA	Japonica	
maker-1-augustus-gene-115.7-mRNA-1	1	OsR498G0100759200.01.T01	99,32	0,00E+00	1302	Os01g0306100	100,00	0,00E+00	1321	19	NA	NA	
maker-1-augustus-gene-115.9-mRNA-1	1	OsR498G0100762200.01.T01	99,35	0,00E+00	1910	Os01g0306900	100,00	0,00E+00	1938	28	NA	Japonica	
maker-1-augustus-gene-116.31-mRNA-1	1	OsR498G0100770300.01.T01	99,80	0,00E+00	5315	Os01g0308300	100,00	0,00E+00	5575	260	NA	Japonica	LRR-NBS-TM
maker-1-augustus-gene-116.33-mRNA-1	1	OsR498G0100765000.01.T01	99,64	0,00E+00	2976	Os01g0307500	99,94	0,00E+00	2999	23	NA	Japonica	
maker-1-augustus-gene-116.35-mRNA-1	1	OsR498G0100767800.01.T04	97,94	0,00E+00	1508	Os05g0357800	75,58	2,85E-169	595	-913	Indica	Japonica	
maker-1-augustus-gene-117.0-mRNA-1	1	OsR498G0100775700.01.T02	99,73	0,00E+00	1339	Os01g0309800	100,00	0,00E+00	1348	9	NA	Japonica	
maker-1-augustus-gene-117.2-mRNA-1	1	OsR498G0100776100.01.T01	99,91	0,00E+00	2072	Os01g0309900	100,00	0,00E+00	2076	4	NA	Japonica	
maker-1-augustus-gene-118.0-mRNA-1	1	OsR498G0100776900.01.T03	99,67	0,00E+00	3272	Os01g0310400	100,00	0,00E+00	4729	1457	Japonica	Japonica	Kinase-TM
maker-1-augustus-gene-118.12-mRNA-1	1	OsR498G0100779100.01.T02	99,71	0,00E+00	3636	Os01g0311500	99,96	0,00E+00	4045	409	Japonica	Japonica	
maker-1-augustus-gene-118.18-mRNA-1	1	OsR498G0100779700.01.T01	99,49	0,00E+00	1053	Os01g0311700	100,00	0,00E+00	1067	14	NA	Japonica	
maker-1-augustus-gene-118.27-mRNA-1	1	OsR498G0100780000.01.T01	99,82	0,00E+00	3006	Os01g0311800	100,00	0,00E+00	3020	14	NA	Japonica	
maker-1-augustus-gene-118.4-mRNA-1	1	OsR498G0409269800.01.T05	71,84	2,90E-69	262	Os01g0310500	100,00	8,79E-172	601	339	Japonica	Japonica	
maker-1-augustus-gene-118.6-mRNA-1	1	OsR498G0100778100.01.T02	99,77	0,00E+00	5527	Os01g0310400	86,38	0,00E+00	2169	-3358	Indica	Japonica	Kinase-TM
maker-1-augustus-gene-119.0-mRNA-1	1	OsR498G0100781700.01.T01	99,94	0,00E+00	3161	Os01g0312500	100,00	0,00E+00	2311	-850	Indica	Japonica	
maker-1-augustus-gene-119.14-mRNA-1	1	OsR498G0100786800.01.T01	93,22	0,00E+00	847	Os01g0314300	93,41	0,00E+00	851	4	NA	Japonica	
maker-1-augustus-gene-119.4-mRNA-1	1	OsR498G0100782500.01.T01	98,29	0,00E+00	2649	Os01g0312800	98,43	0,00E+00	2658	9	NA	Japonica	
maker-1-augustus-gene-119.6-mRNA-1	1	OsR498G0100785100.01.T01	99,36	0,00E+00	1108	Os01g0313300	100,00	0,00E+00	1126	18	NA	Japonica	
maker-1-augustus-gene-12.36-mRNA-1	1	OsR498G0100071500.01.T03	100,00	0,00E+00	1272	Os01g0121800	100,00	0,00E+00	1254	-18	NA	NA	
maker-1-augustus-gene-12.39-mRNA-1	1	OsR498G0100071900.01.T01	99,91	0,00E+00	1931	Os01g0121900	100,00	0,00E+00	3528	1597	Japonica	NA	
maker-1-augustus-gene-12.46-mRNA-1	1	OsR498G0100072700.01.T01	100,00	0,00E+00	1802	Os01g0122000	100,00	0,00E+00	969	-833	Indica	NA	
maker-1-augustus-gene-12.48-mRNA-1	1	OsR498G0100073300.01.T01	100,00	0,00E+00	1829	Os01g0122200	100,00	0,00E+00	1829	0	NA	NA	
maker-1-augustus-gene-12.50-mRNA-1	1	OsR498G0100071400.01.T01	98,09	0,00E+00	3545	Os01g0121700	98,09	0,00E+00	3545	0	NA	NA	
maker-1-augustus-gene-120.24-mRNA-1	1	OsR498G0100791300.01.T02	99,52	0,00E+00	1838	Os05g0363100	84,16	0,00E+00	929	-909	Indica	Japonica	
maker-1-augustus-gene-120.31-mRNA-1	1	OsR498G0100789400.01.T01	100,00	0,00E+00	937	Os01g0314800	100,00	4,97E-144	509	-428	Indica	Japonica	
maker-1-augustus-gene-120.33-mRNA-1	1	OsR498G0100791600.01.T01	99,92	0,00E+00	2301	Os01g0315800	100,00	0,00E+00	2305	4	NA	Japonica	
maker-1-augustus-gene-121.0-mRNA-1	1	OsR498G0100794100.01.T01	94,67	0,00E+00	989	Os01g0316600	99,81	0,00E+00	934	-55	NA	Japonica	
maker-1-augustus-gene-121.16-mRNA-1	1	OsR498G0100793800.01.T02	99,87	0,00E+00	1333	Os01g0316500	100,00	0,00E+00	1337	4	NA	Japonica	
maker-1-augustus-gene-121.9-mRNA-1	1	OsR498G0100795100.01.T03	99,63	0,00E+00	1911	Os01g0316900	100,00	0,00E+00	1647	-264	Indica	Japonica	
maker-1-augustus-gene-122.40-mRNA-1	1	OsR498G0100800900.01.T01	99,92	0,00E+00	2263	Os01g0319000	100,00	0,00E+00	2268	5	NA	Japonica	
maker-1-augustus-gene-122.47-mRNA-1	1	OsR498G0100803600.01.T01	99,71	0,00E+00	2473	Os01g0319200	100,00	0,00E+00	2491	18	NA	Japonica	
maker-1-augustus-gene-123.24-mRNA-1	1	OsR498G0100809600.01.T01	99,37	1,25E-160	565	Os01g0320400	100,00	8,42E-164	574	9	NA	Japonica	
maker-1-augustus-gene-124.0-mRNA-1	1	OsR498G0100814500.01.T01	100,00	0,00E+00	1142	Os01g0321800	100,00	0,00E+00	1142	0	NA	Japonica	
maker-1-augustus-gene-124.14-mRNA-1	1	OsR498G0100814400.01.T01	99,42	0,00E+00	4622	Os01g0321700	100,00	0,00E+00	4686	64	NA	Japonica	
maker-1-augustus-gene-124.17-mRNA-1	1	OsR498G0100817200.01.T01	99,91	0,00E+00	1984	Os01g0322800	100,00	0,00E+00	1988	4	NA	Japonica	
maker-1-augustus-gene-124.2-mRNA-1	1	OsR498G0100818500.01.T02	99,81	0,00E+00	2784	Os01g0323000	100,00	0,00E+00	2798	14	NA	Japonica	Kinase-TM
maker-1-augustus-gene-125.7-mRNA-1	1	OsR498G0100818800.01.T01	99,82	0,00E+00	2004	Os01g0323100	100,00	0,00E+00	2013	9	NA	Japonica	Kinase-TM
maker-1-augustus-gene-126.0-mRNA-1	1	OsR498G0100830700.01.T01	99,63	0,00E+00	1920	Os01g0326000	100,00	0,00E+00	1938	18	NA	Japonica	
maker-1-augustus-gene-126.2-mRNA-1	1	OsR498G0100832100.01.T01	100,00	4,19E-103	374	Os01g0326100	100,00	1,46E-103	374	0	NA	Japonica	
maker-1-augustus-gene-126.3-mRNA-1	1	OsR498G0100832100.01.T01	99,71	0,00E+00	1214	Os01g0326000	85,46	0,00E+00	897	-317	Indica	Japonica	
maker-1-augustus-gene-127.0-mRNA-1	1	OsR498G0100832700.01.T01	98,77	0,00E+00	1137	Os01g0327000	100,00	0,00E+00	1301	164	Japonica	Japonica	
maker-1-augustus-gene-127.14-mRNA-1	1	OsR498G0100834400.01.T01	83,92	0,00E+00	1259	Os01g0327900	100,00	0,00E+00	2273	1014	Japonica	Japonica	
maker-1-augustus-gene-127.3-mRNA-1	1	OsR498G0100833100.01.T01	97,74	0,00E+00	1810	Os01g0327100	100,00	0,00E+00	1916	106	NA	Japonica	
maker-1-augustus-gene-127.5-mRNA-1	1	OsR498G0100833700.01.T03	96,97	0,00E+00	1763	Os01g0327400	100,00	0,00E+00	1905	142	NA	Japonica	
maker-1-augustus-gene-128.33-mRNA-1	1	OsR498G0100834900.01.T03	99,75	0,00E+00	1425	Os01g0328500	100,00	0,00E+00	1434	9	NA	Japonica	
maker-1-augustus-gene-128.35-mRNA-1	1	OsR498G0100835100.01.T06	99,23	6,28E-129	461	Os01g0328600	100,00	4,22E-132	470	9	NA	Japonica	
maker-1-augustus-gene-128.41-mRNA-1	1	OsR498G0100840300.01.T01	99,78	0,00E+00	4090	Os01g0329300	100,00	0,00E+00	4112	22	NA	Japonica	
maker-1-augustus-gene-128.43-mRNA-1	1	OsR498G0100841100.01.T01	97,11	0,00E+00	3686	Os01g0329800	97,66	0,00E+00	3740	54	NA	Japonica	
maker-1-augustus-gene-128.45-mRNA-1	1	OsR498G0100835400.01.T01	99,80	0,00E+00	2714	Os01g0328700	100,00	0,00E+00	2727	13	NA	Japonica	
maker-1-augustus-gene-128.47-mRNA-1	1	OsR498G0100836400.01.T01	99,12	0,00E+00	3195	Os01g0328900	99,50	0,00E+00	3226	31	NA	Japonica	
maker-1-augustus-gene-128.52-mRNA-1	1	OsR498G0100836600.01.T01	99,82	0,00E+00	3012	Os01g0329000	100,00	0,00E+00	2944	-68	NA	Japonica	
maker-1-augustus-gene-128.54-mRNA-1	1	OsR498G0100840600.01.T01	99,60	0,00E+00	1768	Os01g0329400	100,00	0,00E+00	1786	18	NA	Japonica	
maker-1-augustus-gene-129.40-mRNA-1	1	OsR498G0100843300.01.T01	98,73	0,00E+00	1935	Os01g0330300	100,00	0,00E+00	1992	57	NA	Japonica	
maker-1-augustus-gene-129.42-mRNA-1	1	OsR498G0714577900.01.T01	86,39	7,59E-147	520	Os01g0330500	88,79	7,09E-161	566	46	NA	Japonica	
maker-1-augustus-gene-129.44-mRNA-1	1	OsR498G0100845700.01.T01	99,83	0,00E+00	2075	Os01g0329900	71,09	9,33E-126	451	-1624	Indica	Japonica	
maker-1-augustus-gene-129.46-mRNA-1	1	OsR498G0100843200.01.T01	99,63	0,00E+00	1920	Os01g0330200	100,00	0,00E+00	1829	-91	NA	Japonica	
maker-1-augustus-gene-13.48-mRNA-1	1	OsR498G0100078900.01.T02	100,00	0,00E+00	1225	Os01g0123500	100,00	0,00E+00	1532	307	Japonica	NA	
maker-1-augustus-gene-13.54-mRNA-1	1	OsR498G0100078800.01.T01	100,00	0,00E+00	2376	Os01g0119500	92,99	0,00E+00	1978	-398	Indica	NA	
maker-1-augustus-gene-13.56-mRNA-1	1	OsR498G0100080000.01.T01	100,00	0,00E+00	1640	Os01g0123500	77,51	1,41E-166	585	-1055	Indica	NA	
maker-1-augustus-gene-13.58-mRNA-1	1	OsR498G0100082200.01.T01	97,63	0,00E+00	3612	Os05g0524500	77,26	0,00E+00	1644	-1968	Indica	NA	Kinase-LRR-TM
maker-1-augustus-gene-13.60-mRNA-1	1	OsR498G0100082800.01.T01	100,00	0,00E+00	1007	Os01g0124601	100,00	0,00E+00	1041	34	NA	Japonica	
maker-1-augustus-gene-130.26-mRNA-1	1	OsR498G0100847500.01.T01	99,63	0,00E+00	960	Os01g0331900	100,00	0,00E+00	969	9	NA	Japonica	
maker-1-augustus-gene-130.30-mRNA-1	1	OsR498G0100848700.01.T01	99,79	0,00E+00	1667	Os01g0332100	100,00	0,00E+00	1676	9	NA	Japonica	
maker-1-augustus-gene-130.36-mRNA-1	1	OsR498G0100849700.01.T01	99,83	0,00E+00	2073	Os01g0332500	100,00	0,00E+00	2082	9	NA	Japonica	

maker-1-augustus-gene-130.42-mRNA-1	1	OsR498G0100850400.01.T01	99,79	0,00E+00	1685	Os01g0332800	100,00	0,00E+00	1694	9	NA	Japonica	
maker-1-augustus-gene-130.45-mRNA-1	1	OsR498G0100851000.01.T01	99,70	0,00E+00	1198	Os01g0332900	100,00	0,00E+00	1007	-191	Indica	Japonica	
maker-1-augustus-gene-133.42-mRNA-1	1	OsR498G0100857600.01.T01	99,84	0,00E+00	4452	Os01g0335700	100,00	0,00E+00	4470	18	NA	Japonica	CC-LRR-NBS-TM
maker-1-augustus-gene-134.0-mRNA-1	1	OsR498G0100861300.01.T01	99,64	1,88E-139	496	Os01g0337500	100,00	1,54E-141	500	4	NA	Japonica	
maker-1-augustus-gene-134.10-mRNA-1	1	OsR498G0100862700.01.T01	99,83	0,00E+00	1046	Os01g0338000	100,00	0,00E+00	1050	4	NA	Japonica	
maker-1-augustus-gene-134.21-mRNA-1	1	OsR498G0100861600.01.T01	99,04	0,00E+00	2378	Os01g0337600	96,77	0,00E+00	2335	-43	NA	Japonica	
maker-1-augustus-gene-134.26-mRNA-1	1	OsR498G0100864800.01.T03	99,18	0,00E+00	1517	Os01g0338600	100,00	0,00E+00	1537	20	NA	Japonica	
maker-1-augustus-gene-134.4-mRNA-1	1	OsR498G0100862300.01.T01	100,00	0,00E+00	3041	Os01g0337900	100,00	0,00E+00	3041	0	NA	Japonica	
maker-1-augustus-gene-135.1-mRNA-1	1	OsR498G0100866300.01.T03	98,38	0,00E+00	643	Os01g0339600	98,01	3,02E-120	431	-212	Indica	Japonica	
maker-1-augustus-gene-135.11-mRNA-1	1	OsR498G0100866200.01.T01	99,71	0,00E+00	3073	Os01g0339500	100,00	0,00E+00	2100	-973	Indica	Japonica	
maker-1-augustus-gene-135.4-mRNA-1	1	OsR498G0100866500.01.T01	99,82	0,00E+00	2004	Os01g0339900	100,00	0,00E+00	2013	9	NA	Japonica	
maker-1-augustus-gene-137.38-mRNA-1	1	OsR498G0100882300.01.T01	96,67	0,00E+00	2978	Os01g0342900	100,00	0,00E+00	3236	258	NA	Japonica	
maker-1-augustus-gene-137.54-mRNA-1	1	OsR498G0100881200.01.T01	94,57	1,07E-122	440	Os01g0342800	94,57	3,76E-123	440	0	NA	Japonica	
maker-1-augustus-gene-139.11-mRNA-1	1	OsR498G0100897400.01.T02	97,88	0,00E+00	3900	Os01g0347100	96,75	0,00E+00	2585	-1315	Indica	Japonica	
maker-1-augustus-gene-139.4-mRNA-1	1	OsR498G0100894900.01.T01	91,88	0,00E+00	1012	Os01g0346600	100,00	0,00E+00	1267	255	Japonica	Japonica	
maker-1-augustus-gene-139.5-mRNA-1	1	OsR498G0100897100.01.T01	99,69	0,00E+00	4682	Os01g0347000	100,00	0,00E+00	4718	36	NA	Japonica	
maker-1-augustus-gene-14.41-mRNA-1	1	OsR498G0100085300.01.T01	99,84	0,00E+00	3293	Os01g0125800	100,00	0,00E+00	3306	13	NA	Japonica	
maker-1-augustus-gene-14.45-mRNA-1	1	OsR498G0100085500.01.T03	99,82	0,00E+00	5855	Os01g0125900	100,00	0,00E+00	3963	-1892	Indica	Japonica	
maker-1-augustus-gene-14.49-mRNA-1	1	OsR498G0100086500.01.T01	99,83	0,00E+00	3141	Os01g0126100	100,00	0,00E+00	3155	14	NA	Japonica	
maker-1-augustus-gene-14.51-mRNA-1	1	OsR498G0100086900.01.T05	99,83	0,00E+00	3228	Os01g0126200	100,00	0,00E+00	3097	-131	NA	Japonica	
maker-1-augustus-gene-14.55-mRNA-1	1	OsR498G0100087500.01.T01	99,66	0,00E+00	3160	Os01g0126900	100,00	0,00E+00	3090	-70	NA	Japonica	
maker-1-augustus-gene-14.58-mRNA-1	1	OsR498G0100085200.01.T02	100,00	0,00E+00	1710	Os01g0125700	100,00	0,00E+00	1710	0	NA	Japonica	
maker-1-augustus-gene-14.60-mRNA-1	1	OsR498G0100088600.01.T01	98,83	0,00E+00	3435	Os01g0127000	100,00	0,00E+00	2030	-1405	Indica	Indica	
maker-1-augustus-gene-14.63-mRNA-1	1	OsR498G0100089400.01.T01	100,00	0,00E+00	3447	Os01g0127200	97,54	0,00E+00	2539	-908	Indica	Indica	
maker-1-augustus-gene-14.66-mRNA-1	1	OsR498G0100089600.01.T01	100,00	0,00E+00	2614	Os01g0127300	98,62	0,00E+00	2524	-90	NA	Indica	
maker-1-augustus-gene-140.41-mRNA-1	1	OsR498G0100899700.01.T01	98,91	0,00E+00	1774	Os01g0330200	86,64	0,00E+00	1234	-540	Indica	Japonica	
maker-1-augustus-gene-140.43-mRNA-1	1	OsR498G0100899600.01.T01	98,80	0,00E+00	1901	Os01g0348000	99,91	0,00E+00	1955	54	NA	Japonica	
maker-1-augustus-gene-140.45-mRNA-1	1	OsR498G0100901300.01.T01	99,13	0,00E+00	3854	Os01g0348600	100,00	0,00E+00	3939	85	NA	Japonica	
maker-1-augustus-gene-140.53-mRNA-1	1	OsR498G0100899600.01.T01	98,90	0,00E+00	1905	Os01g0347500	99,45	0,00E+00	1932	27	NA	Japonica	
maker-1-augustus-gene-140.55-mRNA-1	1	OsR498G0100900600.01.T01	100,00	0,00E+00	1856	Os01g0330200	86,22	0,00E+00	1218	-638	Indica	Japonica	
maker-1-augustus-gene-140.57-mRNA-1	1	OsR498G0100902200.01.T02	99,77	0,00E+00	786	Os01g0348900	100,00	0,00E+00	791	5	NA	Japonica	
maker-1-augustus-gene-141.26-mRNA-1	1	OsR498G0100904900.01.T01	98,93	0,00E+00	2779	Os01g0349800	100,00	0,00E+00	2836	57	NA	Japonica	
maker-1-augustus-gene-141.29-mRNA-1	1	OsR498G0100907900.01.T03	98,80	0,00E+00	2770	Os05g0154600	73,40	0,00E+00	795	-1975	Indica	Japonica	
maker-1-augustus-gene-141.34-mRNA-1	1	OsR498G0100904600.01.T01	99,80	0,00E+00	1799	Os01g0349400	100,00	0,00E+00	1721	-78	NA	Japonica	
maker-1-augustus-gene-141.39-mRNA-1	1	OsR498G0100907200.01.T01	100,00	6,82E-147	520	Os01g0350500	100,00	2,38E-147	520	0	NA	Japonica	
maker-1-augustus-gene-142.27-mRNA-1	1	OsR498G0100908700.01.T01	99,09	0,00E+00	1752	Os01g0351100	97,01	0,00E+00	1691	-61	NA	Japonica	
maker-1-augustus-gene-142.40-mRNA-1	1	OsR498G0100913700.01.T01	99,24	0,00E+00	1864	Os01g0352100	100,00	0,00E+00	1900	36	NA	Japonica	
maker-1-augustus-gene-143.37-mRNA-1	1	OsR498G0100914900.01.T01	96,78	0,00E+00	1085	Os01g0353400	100,00	0,00E+00	1180	95	NA	Japonica	
maker-1-augustus-gene-144.14-mRNA-1	1	OsR498G0100918400.01.T02	96,90	0,00E+00	4871	Os01g0355500	99,97	0,00E+00	5130	259	NA	Japonica	
maker-1-augustus-gene-145.14-mRNA-1	1	OsR498G0100923200.01.T02	99,87	0,00E+00	1414	Os01g0356500	100,00	0,00E+00	1234	-180	Indica	Japonica	
maker-1-augustus-gene-145.5-mRNA-1	1	OsR498G0100920300.01.T01	99,62	0,00E+00	1410	Os01g0355800	100,00	0,00E+00	1418	8	NA	Japonica	
maker-1-augustus-gene-145.7-mRNA-1	1	OsR498G0100920600.01.T01	98,33	0,00E+00	2592	Os01g0355900	100,00	0,00E+00	2695	103	NA	Japonica	
maker-1-augustus-gene-146.38-mRNA-1	1	OsR498G0100930500.01.T02	98,89	0,00E+00	3307	Os01g0357100	99,43	0,00E+00	1875	-1432	Indica	Japonica	
maker-1-augustus-gene-146.39-mRNA-1	1	OsR498G0100930700.01.T02	98,66	0,00E+00	783	Os02g0765800	95,21	1,62E-169	595	-188	Indica	Japonica	
maker-1-augustus-gene-146.53-mRNA-1	1	OsR498G0100931500.01.T01	98,87	0,00E+00	1238	Os02g0765600	95,27	0,00E+00	879	-359	Indica	Japonica	
maker-1-augustus-gene-146.54-mRNA-1	1	OsR498G0100931500.01.T01	98,36	0,00E+00	739	Os01g0357400	98,83	0,00E+00	748	9	NA	Japonica	
maker-1-augustus-gene-146.60-mRNA-1	1	OsR498G0100935300.01.T01	99,75	0,00E+00	1431	Os01g0358400	100,00	0,00E+00	1440	9	NA	Japonica	
maker-1-augustus-gene-146.63-mRNA-1	1	OsR498G0100935200.01.T02	99,85	0,00E+00	2459	Os01g0358300	100,00	0,00E+00	2468	9	NA	Japonica	
maker-1-augustus-gene-148.26-mRNA-1	1	OsR498G0100942600.01.T01	99,77	0,00E+00	2351	Os01g0359800	89,63	0,00E+00	1752	-599	Indica	Japonica	NBS-TM
maker-1-augustus-gene-148.29-mRNA-1	1	OsR498G0100943000.01.T04	99,89	0,00E+00	4889	Os01g0360200	100,00	0,00E+00	4902	13	NA	Japonica	
maker-1-augustus-gene-148.34-mRNA-1	1	OsR498G0100944800.01.T01	99,49	0,00E+00	1048	Os01g0360600	99,83	0,00E+00	1057	9	NA	Japonica	
maker-1-augustus-gene-149.44-mRNA-1	1	OsR498G0100952300.01.T01	99,38	0,00E+00	1151	Os01g0363300	100,00	0,00E+00	1169	18	NA	Japonica	
maker-1-augustus-gene-149.47-mRNA-1	1	OsR498G0100948400.01.T04	100,00	0,00E+00	2067	Os01g0361500	99,91	0,00E+00	2063	-4	NA	Japonica	
maker-1-augustus-gene-15.29-mRNA-1	1	OsR498G0100089700.01.T04	100,00	0,00E+00	2316	Os01g0127400	98,95	0,00E+00	2008	-308	Indica	Indica	
maker-1-augustus-gene-15.31-mRNA-1	1	OsR498G0100089900.01.T01	99,90	0,00E+00	1825	Os01g0127500	99,80	0,00E+00	1820	-5	NA	Indica	
maker-1-augustus-gene-15.35-mRNA-1	1	OsR498G0100091900.01.T02	100,00	0,00E+00	2322	Os01g0127900	99,85	0,00E+00	2313	-9	NA	Indica	
maker-1-augustus-gene-15.38-mRNA-1	1	OsR498G0100094500.01.T01	99,89	0,00E+00	1668	Os01g0128200	99,89	0,00E+00	1668	0	NA	NA	
maker-1-augustus-gene-15.44-mRNA-1	1	OsR498G0100095900.01.T02	99,86	0,00E+00	3773	Os01g0128800	99,89	0,00E+00	3324	-449	Indica	NA	
maker-1-augustus-gene-15.51-mRNA-1	1	OsR498G0100098300.01.T01	96,40	0,00E+00	1297	Os01g0129000	99,78	0,00E+00	824	-473	Indica	NA	
maker-1-augustus-gene-15.54-mRNA-1	1	OsR498G0100093200.01.T01	99,91	0,00E+00	1895	Os01g0128000	99,91	0,00E+00	1895	0	NA	NA	
maker-1-augustus-gene-15.56-mRNA-1	1	OsR498G0100094400.01.T01	97,37	0,00E+00	1489	Os01g0128100	97,37	0,00E+00	1489	0	NA	NA	
maker-1-augustus-gene-15.64-mRNA-1	1	OsR498G0100095000.01.T01	99,83	0,00E+00	3163	Os01g0128400	99,83	0,00E+00	3163	0	NA	NA	
maker-1-augustus-gene-150.31-mRNA-1	1	OsR498G0100952700.01.T02	98,34	0,00E+00	6353	Os01g0363900	100,00	0,00E+00	5155	-1198	Indica	Japonica	
maker-1-augustus-gene-150.40-mRNA-1	1	OsR498G0100956900.01.T01	99,59	0,00E+00	2196	Os01g0364800	100,00	0,00E+00	2219	23	NA	Japonica	Kinase-TM
maker-1-augustus-gene-151.6-mRNA-1	1	OsR498G0100959800.01.T07	100,00	3,84E-151	535	Os01g0367100	100,00	1,34E-151	535	0	NA	Japonica	
maker-1-augustus-gene-152.40-mRNA-1	1	OsR498G0100964300.01.T01	99,41	0,00E+00	1211	Os01g0368000	98,26	0,00E+00	2393	1182	Japonica	Japonica	
maker-1-augustus-gene-152.44-mRNA-1	1	OsR498G0100960200.01.T07	100,00	0,00E+00	1526	Os01g0367300	100,00	0,00E+00	1526	0	NA	Japonica	
maker-1-augustus-gene-152.46-mRNA-1	1	OsR498G0100961200.01.T04	99,86	0,00E+00	1269	Os01g0367400	99,05	0,00E+00	1106	-163	Indica	Japonica	
maker-1-augustus-gene-152.52-mRNA-1	1	OsR498G0100964000.01.T02	99,88	0,00E+00	5977	Os01g0367900	100,00	0,00E+00	5995	18	NA	Japonica	
maker-1-augustus-gene-153.34-mRNA-1	1	OsR498G0100968500.01.T01	99,91	0,00E+00	4033	Os01g0369200	100,00	0,00E+00	4042	9	NA	Japonica	
maker-1-augustus-gene-153.36-mRNA-1	1	OsR498G0100968000.01.T03	99,91	0,00E+00	4022	Os01g0369000	100,00	0,00E+00	4031	9	NA	Japonica	
maker-1-augustus-gene-153.43-mRNA-1	1	OsR498G0100969800.01.T01	99,31	0,00E+00	1286	Os01g0369700	100,00	0,00E+00	1305	19	NA	Japonica	
maker-1-augustus-gene-153.45-mRNA-1	1	OsR498G0100970600.01.T01	96,31	0,00E+00	1876	Os01g0369900	100,00	0,00E+00	1445	-431	Indica	Japonica	
maker-1-augustus-gene-154.30-mRNA-1	1	OsR498G0100975500.01.T01	100,00	0,00E+00	1175	Os01g0371500	100,00	0,00E+00	1175	0	NA	Japonica	
maker-1-augustus-gene-154.32-mRNA-1	1	OsR498G0100971400.01.T01	97,36	0,00E+00	701	Os01g0370200	100,00	0,00E+00	1186	485	Japonica	Japonica	
maker-1-augustus-gene-154.35-mRNA-1	1	OsR498G0100972400.01.T01	99,40	0,00E+00	1178	Os01g0371200	82,28	0,00E+00	653	-525	Indica	Japonica	
maker-1-augustus-gene-154.38-mRNA-1	1	OsR498G0100974400.01.T01	100,00	0,00E+00	1191	Os01g0371200	100,00	0,00E+00	1191	0	NA	Japonica	
maker-1-augustus-gene-155.2-mRNA-1	1	OsR498G0100978100.01.T01	100,00	0,00E+00	1202	Os01g0372400	100,00	0,00E+00	1202	0	NA	Japonica	
maker-1-augustus-gene-155.4-mRNA-1	1	OsR498G0100979000.01.T01	99,58	0,00E+00	2999	Os01g0372700	100,00	0,00E+00	3030	31	NA	Japonica	
maker-1-augustus-gene-156.0-mRNA-1	1	OsR498G0100980700.01.T01	99,69	0,00E+00	1155	Os01g0374000	100,00	0,00E+00	1164	9	NA	Japonica	
maker-1-augustus-gene-156.12-mRNA-1	1	OsR498G0100984800.01.T01	99,10	0,00E+00	3131	Os01g0375000	100,00	0,00E+00	3204	73	NA	Japonica	
maker-1-augustus-gene-156.2-mRNA-1	1	OsR498G0100983700.01.T01	95,09	0,00E+00	2654	Os01g0374600	100,00	0,00E+00	2232	-422	Indica	Japonica	
maker-1-augustus-gene-156.5-mRNA-1	1	OsR498G0100984500.01.T01	98,63	0,00E+00	1651	Os01g0374900	100,00	0,00E+00	1700	49	NA	Japonica	
maker-1-augustus-gene-156.7-mRNA-1	1	OsR498G0100985300.01.T01	99,35	0,00E+00	2715	Os01g0375200	100,00	0,00E+00	3101	386	Japonica	Japonica	
maker-1-augustus-gene-157.0-mRNA-1	1	OsR498G0100985700.01.T01	99,40	0,00E+00	2352	Os01g0375200	93,19	0,00E+00	1979	-373	Indica	Japonica	
maker-1-augustus-gene-157.4-mRNA-1	1	OsR498G0100989200.01.T02	96,65	0,00E+00	2205	Os01g0376700	96,80	0,00E+00	2214	9	NA	Japonica	
maker-1-augustus-gene-158.0-mRNA-1	1	OsR498G0100990900.01.T01	99,44	0,00E+00	2220	Os01g0377700	100,00	0,00E+00	2251	31	NA	Japonica	
maker-1-augustus-gene-158.2-mRNA-1	1	OsR498G0100990800.01.T01	98,46	0,00E+00	1577	Os01g0377500	100,00	0,00E+00	1640	63	NA	Japonica	
maker-1-augustus-gene-158.5-mRNA-1	1	OsR498G0100992000.01.T01	99,52	0,00E+00	1832	Os12g0530100	71,69	1,01E-124	446	-1386	Indica	Japonica	
maker-1-augustus-gene-16.28-mRNA-1	1	OsR498G0100101300.01.T01	98,81	0,00E+00	1618	Os01g0129600	98,81	0,00E+00	1618	0	NA	NA	
maker-1-augustus-gene-16.32-mRNA-1	1	OsR498G0100104900.01.T01	99,96	0,00E+00	5044	Os01g0130400	99,89	0,00E+00	5035	-9	NA	Indica	
maker-1-augustus-gene-16.34-mRNA-1	1	OsR498G0100099600.01.T01	98,95	0,00E+00	3350	Os01g0129500	98,95	0,00E+00	3350	0	NA	NA	
maker-1-augustus-gene-16.36-mRNA-1	1	OsR498G0100102600.01.T02	100,00	0,00E+00	1040	Os01g0130000	100,00	0,00E+00	1040	0	NA	Indica	
maker-1-augustus-gene-161.34-mRNA-1	1	OsR498G0100996400.01.T01	99,06	0,00E+00	2431	Os01g0382200	100,00	0,00E+00	2387	-44	NA	Japonica	
maker-1-augustus-gene-161.36-mRNA-1	1	OsR498G0101000100.01.T02	93,58	0,00E+00	1645	Os01g0382700	95,75	0,00E+00	1739	94	NA	Japonica	
maker-1-augustus-gene-162.0-mRNA-1	1	OsR498G0101004100.01.T01	99,60	0,00E+00	1772	Os05g0144100	79,37	1,55E-128	459	-1313	Indica	Japonica	
maker-1-augustus-gene-163.26-mRNA-1	1	OsR498G0101009400.01.T01	98,87	2,28E-177	621	Os01g0385000	100,00	0,00E+00	639	18	NA	Japonica	
maker-1-augustus-gene-163.28-mRNA-1	1	OsR498G0101010400.01.T02	98,53	0,00E+00	3330	Os01g0385400	98,58	0,00E+00	3334	4	NA	Japonica	
maker-1-augustus-gene-163.31-mRNA-1	1	OsR498G0101011200.01.T01	94,97	0,00E+00	1374	Os01g0385600	94,95	0,00E+00	1055	-319	Indica	Japonica	
maker-1-augustus-gene-165.11-mRNA-1	1	OsR498G0101021000.01.T02	99,82	0,00E+00	2925	Os02g0204700	75,48	0,00E+00	1117	-1808	Indica	Japonica	
maker-1-augustus-gene-167.0-mRNA-1	1	OsR498G0101031000.01.T01	100,00	3,96E-149	527	Os01g0390900	100,00	1,38E-149	527	0	NA	Japonica	
maker-1-augustus-gene-168.40-mRNA-1	1	OsR498G0101039100.01.T02	99,54	7,51E-106	384	Os01g0392100	100,00	6,15E-108	389	5	NA	Japonica	
maker-1-augustus-gene-169.11-mRNA-1	1	OsR498G0101043400.01.T01	100,00	0,00E+00	1808	Os01g0393100	100,00	0,00E+00	1669	-139	NA	Japonica	
maker-1-augustus-gene-169.2-mRNA-1	1	OsR498G0101041900.01.T05	98,29	6,66E-52	203	Os01g0104600	97,56	2,33E-33	140	-63	Indica	Japonica	
maker-1-augustus-gene-169.9-mRNA-1	1	OsR498G0101043700.01.T03	100,00	1,59E-174	612	Os01g0393400	100,00	0,00E+00	782	170	Japonica	Japonica	
maker-1-augustus-gene-17.11-mRNA-1	1	OsR498G0100111200.01.T01	87,26	4,16E-103	375	Os01g0132000	100,00	1,11E-155	547	172	Japonica	Indica	
maker-1-augustus-gene-17.13-mRNA-1	1	OsR498G0100113200.01.T01	100,00	0,00E+00	2149	Os01g0132400	99,25	0,00E+00	2120	-29	NA	Indica	
maker-1-augustus-gene-17.15-mRNA-1	1	OsR498G0100113600.01.T05	94,27	0,00E+00	860	Os01g0132700	99,78	0,00E+00	802	-58	NA	Indica	
maker-1-augustus-gene-17.2-mRNA-1	1	OsR498G0100110900.01.T01	100,00	0,00E+00	915	Os01g0131800	100,00	0,00E+00	915	0	NA	Indica	
maker-1-augustus-gene-17.7-mRNA-1	1	OsR498G0100113300.01.T01	100,00	0,00E+00	1153	Os01g0132500	99,65	0,00E+00	1025	-128	Indica	Indica	
maker-1-augustus-gene-17.9-mRNA-1	1	OsR498G0100111200.01.T01	100,00	7,59E-157	553	Os01g0131900	100,00	2,64E-157	553	0	NA	Indica	
maker-1-augustus-gene-174.0-mRNA-1	1	OsR498G0101112100.01.T01	97,99	0,00E+00	4260	Os01g0514300	98,07	0,00E+00	4269	9	NA	NA	
maker-1-augustus-gene-174.5-mRNA-1	1	OsR498G0101112000.01.T02	90,29	0,00E+00	788	Os01g0514000	90,66	0,00E+00	797	9	NA	NA	
maker-1-augustus-gene-176.8-mRNA-1	1	OsR498G1018408000.01.T01	99,02	0,00E+00	1262	Os01g0495200	100,00	0,00E+00	1299	37	NA	NA	
maker-1-augustus-gene-177.36-mRNA-1	1	OsR498G0101046700.01.T01	99,76	0,00E+00	3776	Os01g0495900	100,00	0,00E+00	3799	23	NA	Japonica	
maker-1-augustus-gene-177.42-mRNA-1	1	OsR498G0101049400.01.T01	99,80	0,00E+00	2660	Os01g0496900	100,00	0,00E+00	2673	13	NA	Japonica	
maker-1-augustus-gene-179.25-mRNA-1	1	OsR498G0101054600.01.T01	99,63	0,00E+00	1947	Os01g0499300	100,00	0,00E+00	1965	18	NA	Japonica	
maker-1-augustus-gene-179.31-mRNA-1	1	OsR498G1119310800.01.T01	83,17	8,67E-21	100	Os01g0499600	100,00	1,66E-100	363	263	Japonica	Japonica	
maker-1-augustus-gene-18.0-mRNA-1	1	OsR498G0100115900.01.T01	100,00	0,00E+00	4794	Os01g0133100	99,95	0,00E+00	3533	-1261	Indica	Indica	
maker-1-augustus-gene-18.13-mRNA-1	1	OsR498G0100117100.01.T01	100,00	0,00E+00	1905	Os01g0133700	83,09	0,00E+00	1317	-588	Indica	Indica	
maker-1-augustus-gene-18.15-mRNA-1	1	OsR498G0100117500.01.T01	100,00	0,00E+00	1948	Os01g0133700	99,59	0,00E+00	2169	221	Japonica	Indica	
maker-1-augustus-gene-18.17-mRNA-1	1	OsR498G0100117900.01.T02	100,00	0,00E+00	3777	Os01g0133900	99,90	0,00E+00	3761	-16	NA	Indica	Kinase-LRR-TM
maker-1-augustus-gene-18.22-mRNA-1	1	OsR498G0100119100.01.T01	100,00	0,00E+00	1489	Os01g0134500	100,00	0,00E+00	1489	0	NA	Indica	
maker-1-augustus-gene-18.3-mRNA-1	1	OsR498G0100116700.01.T02	100,00	0,00E+00	3030	Os01g0133400	99,88	0,00E+00	2929	-101	NA	Indica	
maker-1-augustus-gene-18.9-mRNA-1	1	OsR498G0100116900.01.T07	95,43	0,00E+00	1502	Os01g0133500	95,00	0,00E+00	1484	-18	NA	Indica	
maker-1-augustus-gene-180.31-mRNA-1	1	OsR498G0101060700.01.T01	100,00	0,00E+00	1808	Os01g0501800	100,00	0,00E+00	1808	0	NA	Japonica	
maker-1-augustus-gene-180.38-mRNA-1	1	OsR498G0101060600.01.T01	99,82	0,00E+00	1939	Os01g0501700	100,00	0,00E+00	1948	9	NA	Japonica	
maker-1-augustus-gene-181.10-mRNA-1	1	OsR498G0101068400.01.T03	100,00	0,00E+00	2051	Os01g0503400	100,00	0,00E+00	2051	0	NA	Japonica	NRAMP6
maker-1-augustus-gene-181.2-mRNA-1	1	OsR498G0101064300.01.T02	100,00	0,00E+00	1141	Os01g0502400	100,00	0,00E+00	1141	0	NA	Japonica	
maker-1-augustus-gene-181.8-mRNA-1	1	OsR498G0101065300.01.T01	100,00	0,00E+00	866	Os01g0502700	100,00	0,00E+00	866	0	NA	Japonica	
maker-1-augustus-gene-182.34-mRNA-1	1	OsR498G0101071600.01.T03	95,97	0,00E+00	1971	Os01g0504500	95,97	0,00E+00	1971	0	NA	Japonica	
maker-1-augustus-gene-183.3-mRNA-1	1	OsR498G0101078100.01.T03	99,61	0,00E+00	2774	Os01g0506100	100,00	0,00E+00	2801	27	NA	Japonica	
maker-1-augustus-gene-183.4-mRNA-1	1	OsR498G0101079200.01.T04	100,00	0,00E+00	1613	Os01g0506200	100,00	0,00E+00	1561	-52	NA	Japonica	
maker-1-augustus-gene-184.27-mRNA-1	1	OsR498G0101084100.01.T03	100,00	0,00E+00	823	Os01g0507700	100,00	0,00E+00	823	0	NA	Japonica	
maker-1-augustus-gene-184.29-mRNA-1	1	OsR498G0101084700.01.T02	99,89	0,00E+00	1700	Os01g0507900	100,00	0,00E+00	1705	5	NA	Japonica	
maker-1-augustus-gene-184.34-mRNA-1	1	OsR498G0101085300.01.T01	95,43	0,00E+00	2472	Os01g0508000	95,69	0,00E+00	2490	18	NA	Japonica	
maker-1-augustus-gene-186.36-mRNA-1	1	OsR498G0101094500.01.T01	99,91	0,00E+00	2003	Os01g0509900	100,00	0,00E+00	2960	957	Japonica	Japonica	
maker-1-augustus-gene-186.51-mRNA-1	1	OsR498G0101097800.01.T01	99,59	0,00E+00	1741	Os01g0510600	100,00	0,00E+00	1759	18	NA	Japonica	
maker-1-augustus-gene-187.33-mRNA-1	1	OsR498G0101105500.01.T01	100,00	0,00E+00	2186	Os01g0512100	100,00	0,00E+00	2186	0	NA	Japonica	
maker-1-augustus-gene-187.45-mRNA-1	1	OsR498G0101102400.01.T01	100,00	0,00E+00	910	Os01g0511100	100,00	0,00E+00	910	0	NA	Japonica	
maker-1-augustus-gene-187.48-mRNA-1	1	OsR498G0101102800.01.T01	99,57	0,00E+00	2067	Os01g0511300	100,00	0,00E+00	2089	22	NA	Japonica	
maker-1-augustus-gene-187.53-mRNA-1	1	OsR498G0101103600.01.T02	98,46	0,00E+00	1927	Os01g0511600	100,00	0,00E+00	1581	-346	Indica	Japonica	
maker-1-augustus-gene-188.26-mRNA-1	1	OsR498G0101110500.01.T01	99,11	0,00E+00	1973	Os01g0513400	99,20	0,00E+00	1977	4	NA	Japonica	
maker-1-augustus-gene-188.29-mRNA-1	1	OsR498G0101111700.01.T03	99,97	0,00E+00	5163	Os01g0513900	100,00	0,00E+00	5167	4	NA	Japonica	
maker-1-augustus-gene-188.46-mRNA-1	1	OsR498G0101111200.01.T01	100,00	0,00E+00	975	Os01g0513700	99,45	0,00E+00	965	-10	NA	Japonica	
maker-1-augustus-gene-188.49-mRNA-1	1	OsR498G0101111400.01.T01	100,00	0,00E+00	1683	Os01g0513800	100,00	0,00E+00	1683	0	NA	Japonica	
maker-1-augustus-gene-188.55-mRNA-1	1	OsR498G0101112000.01.T02	90,29	0,00E+00	788	Os01g0514000	90,66	0,00E+00	797	9	NA	NA	
maker-1-augustus-gene-189.0-mRNA-1	1	OsR498G0101112100.01.T01	98,33	0,00E+00	5078	Os01g0514300	98,40	0,00E+00	5087	9	NA	NA	
maker-1-augustus-gene-189.11-mRNA-1	1	OsR498G0101114500.01.T01	99,39	0,00E+00	3762	Os01g0514700	100,00	0,00E+00	3820	58	NA	Japonica	Kinase-LRR-TM
maker-1-augustus-gene-189.5-mRNA-1	1	OsR498G0101113500.01.T01	99,95	0,00E+00	3762	Os01g0514600	100,00	0,00E+00	3766	4	NA	Japonica	
maker-1-augustus-gene-19.0-mRNA-1	1	OsR498G0100119500.01.T01	100,00	0,00E+00	2376	Os01g0134700	99,85	0,00E+00	2367	-9	NA	Indica	
maker-1-augustus-gene-19.10-mRNA-1	1	OsR498G0100125200.01.T03	100,00	0,00E+00	1377	Os01g0136400	95,01	0,00E+00	1241	-136	NA	Indica	
maker-1-augustus-gene-19.21-mRNA-1	1	OsR498G0100126800.01.T01	100,00	0,00E+00	3609	Os01g0136800	99,25	0,00E+00	3542	-67	NA	Indica	Kinase-TM
maker-1-augustus-gene-19.3-mRNA-1	1	OsR498G0100127300.01.T01	100,00	0,00E+00	1499	Os01g0137100	93,32	0,00E+00	1195	-304	Indica	Indica	
maker-1-augustus-gene-190.0-mRNA-1	1	OsR498G0101116900.01.T01	100,00	2,00E-95	349	Os01g0516400	100,00	6,95E-96	349	0	NA	Japonica	
maker-1-augustus-gene-190.20-mRNA-1	1	OsR498G0101120600.01.T01	99,18	0,00E+00	2163	Os01g0517500	100,00	0,00E+00	2208	45	NA	Japonica	
maker-1-augustus-gene-190.4-mRNA-1	1	OsR498G0101117500.01.T01	100,00	2,45E-176	618	Os01g0516600	100,00	8,53E-177	618	0	NA	Japonica	
maker-1-augustus-gene-190.6-mRNA-1	1	OsR498G0101118000.01.T03	99,08	0,00E+00	2296	Os01g0516700	100,00	0,00E+00	1696	-600	Indica	Japonica	
maker-1-augustus-gene-191.2-mRNA-1	1	OsR498G0101120800.01.T01	99,54	0,00E+00	776	Os01g0517800	100,00	0,00E+00	1283	507	Japonica	Japonica	
maker-1-augustus-gene-191.5-mRNA-1	1	OsR498G0101123600.01.T01	100,00	0,00E+00	2024	Os01g0518500	100,00	0,00E+00	2024	0	NA	NA	
maker-1-augustus-gene-193.0-mRNA-1	1	OsR498G0101133900.01.T01	99,83	0,00E+00	3087	Os01g0521400	100,00	0,00E+00	2192	-895	Indica	Japonica	
maker-1-augustus-gene-193.10-mRNA-1	1	OsR498G0101136300.01.T01	99,66	0,00E+00	2662	Os01g0522433	100,00	0,00E+00	3355	693	Japonica	Japonica	
maker-1-augustus-gene-193.3-mRNA-1	1	OsR498G0101134300.01.T01	99,78	0,00E+00	3250	Os01g0521500	100,00	0,00E+00	3268	18	NA	Japonica	
maker-1-augustus-gene-193.9-mRNA-1	1	OsR498G0101136300.01.T01	100,00	2,51E-137	488	Os01g0522100	100,00	2,50E-138	489	1	NA	Japonica	
maker-1-augustus-gene-194.6-mRNA-1	1	OsR498G0101142900.01.T01	98,48	0,00E+00	1605	Os01g0524500	100,00	0,00E+00	1393	-212	Indica	Japonica	
maker-1-augustus-gene-195.0-mRNA-1	1	OsR498G0101143700.01.T02	99,57	0,00E+00	4548	Os01g0524700	100,00	0,00E+00	4594	46	NA	Japonica	
maker-1-augustus-gene-195.6-mRNA-1	1	OsR498G0101149100.01.T01	100,00	0,00E+00	2273	Os01g0526200	100,00	0,00E+00	2273	0	NA	Japonica	
maker-1-augustus-gene-196.2-mRNA-1	1	OsR498G0101154900.01.T04	99,76	0,00E+00	2953	Os01g0527400	99,38	0,00E+00	3464	511	Japonica	Japonica	
maker-1-augustus-gene-197.14-mRNA-1	1	OsR498G0101155400.01.T01	99,54	0,00E+00	2336	Os01g0527700	100,00	0,00E+00	2360	24	NA	Japonica	
maker-1-augustus-gene-197.18-mRNA-1	1	OsR498G0101155600.01.T01	98,44	0,00E+00	2215	Os06g0521000	98,74	0,00E+00	1531	-684	Indica	Japonica	
maker-1-augustus-gene-197.7-mRNA-1	1	OsR498G0101158700.01.T01	99,41	0,00E+00	1797	Os01g0528800	100,00	0,00E+00	1824	27	NA	Japonica	
maker-1-augustus-gene-198.37-mRNA-1	1	OsR498G0101163100.01.T01	99,63	0,00E+00	1916	Os01g0530300	100,00	0,00E+00	1108	-808	Indica	Japonica	
maker-1-augustus-gene-198.48-mRNA-1	1	OsR498G0101166100.01.T01	99,88	0,00E+00	1522	Os01g0531500	100,00	0,00E+00	1526	4	NA	Japonica	
maker-1-augustus-gene-198.52-mRNA-1	1	OsR498G0101165600.01.T01	99,92	0,00E+00	2128	Os01g0541600	96,11	0,00E+00	1925	-203	NA	Japonica	
maker-1-augustus-gene-198.55-mRNA-1	1	OsR498G0101960700.01.T03	99,13	0,00E+00	4044	Os01g0531200	100,00	0,00E+00	3707	-337	NA	Japonica	
maker-1-augustus-gene-198.60-mRNA-1	1	OsR498G0102017700.01.T01	93,67	0,00E+00	1847	Os01g0756600	93,75	0,00E+00	1851	4	NA	Japonica	
maker-1-augustus-gene-199.0-mRNA-1	1	OsR498G0613256400.01.T02	95,69	0,00E+00	2462	Os01g0532600	100,00	0,00E+00	2760	298	Japonica	Japonica	
maker-1-augustus-gene-2.0-mRNA-1	1	OsR498G0100006700.01.T02	99,65	0,00E+00	1025	Os01g0101700	100,00	0,00E+00	771	-254	Indica	Japonica	
maker-1-augustus-gene-2.14-mRNA-1	1	OsR498G0100011900.01.T01	99,35	0,00E+00	1096	Os01g0102700	99,35	0,00E+00	1096	0	NA	Japonica	
maker-1-augustus-gene-2.17-mRNA-1	1	OsR498G0100012100.01.T01	99,83	0,00E+00	6482	Os01g0102800	100,00	0,00E+00	6428	-54	NA	Japonica	
maker-1-augustus-gene-2.19-mRNA-1	1	OsR498G0100012800.01.T01	99,23	0,00E+00	685	Os01g0102900	100,00	0,00E+00	699	14	NA	Japonica	
maker-1-augustus-gene-2.2-mRNA-1	1	OsR498G0100006900.01.T01	99,66	0,00E+00	3632	Os01g0101800	100,00	0,00E+00	3663	31	NA	Japonica	
maker-1-augustus-gene-2.22-mRNA-1	1	OsR498G0100013000.01.T02	100,00	0,00E+00	1261	Os01g0102950	100,00	0,00E+00	1261	0	NA	Japonica	
maker-1-augustus-gene-2.26-mRNA-1	1	OsR498G0100014200.01.T01	99,61	4,01E-128	458	Os01g0103600	100,00	3,29E-130	462	4	NA	Japonica	
maker-1-augustus-gene-2.27-mRNA-1	1	OsR498G0100014200.01.T03	100,00	0,00E+00	731	Os01g0103600	100,00	0,00E+00	729	-2	NA	Japonica	
maker-1-augustus-gene-2.4-mRNA-1	1	OsR498G0100010900.01.T01	99,10	0,00E+00	1377	Os01g0102300	100,00	0,00E+00	1397	20	NA	Japonica	
maker-1-augustus-gene-2.6-mRNA-1	1	OsR498G0100011500.01.T01	99,80	0,00E+00	1793	Os01g0102500	100,00	0,00E+00	1802	9	NA	Japonica	
maker-1-augustus-gene-2.9-mRNA-1	1	OsR498G0100011700.01.T01	99,59	0,00E+00	872	Os01g0102600	100,00	0,00E+00	881	9	NA	Japonica	
maker-1-augustus-gene-20.20-mRNA-1	1	OsR498G0100130200.01.T01	100,00	0,00E+00	1543	Os06g0586600	98,86	0,00E+00	921	-622	Indica	Indica	
maker-1-augustus-gene-200.26-mRNA-1	1	OsR498G0101176700.01.T02	99,60	0,00E+00	6653	Os01g0533900	100,00	0,00E+00	4484	-2169	Indica	Japonica	
maker-1-augustus-gene-200.39-mRNA-1	1	OsR498G0101174400.01.T01	99,84	0,00E+00	4462	Os01g0533400	100,00	0,00E+00	4480	18	NA	Japonica	
maker-1-augustus-gene-200.41-mRNA-1	1	OsR498G0101174800.01.T01	99,64	0,00E+00	3009	Os01g0533500	100,00	0,00E+00	3036	27	NA	Japonica	
maker-1-augustus-gene-201.0-mRNA-1	1	OsR498G0101178700.01.T01	99,92	0,00E+00	6396	Os01g0534700	100,00	0,00E+00	6311	-85	NA	NA	
maker-1-augustus-gene-201.2-mRNA-1	1	OsR498G0101179100.01.T01	100,00	0,00E+00	3085	Os01g0534800	99,94	0,00E+00	3080	-5	NA	NA	
maker-1-augustus-gene-201.4-mRNA-1	1	OsR498G0101179400.01.T03	100,00	0,00E+00	2239	Os01g0534900	100,00	0,00E+00	2239	0	NA	NA	
maker-1-augustus-gene-202.45-mRNA-1	1	OsR498G0101187200.01.T01	100,00	0,00E+00	2149	Os01g0536400	100,00	0,00E+00	2149	0	NA	NA	
maker-1-augustus-gene-202.47-mRNA-1	1	OsR498G0101190400.01.T01	100,00	0,00E+00	1023	Os01g0537250	100,00	0,00E+00	708	-315	Indica	NA	
maker-1-augustus-gene-204.24-mRNA-1	1	OsR498G0101198500.01.T01	100,00	0,00E+00	646	Os01g0538200	99,51	0,00E+00	729	83	Japonica	NA	
maker-1-augustus-gene-206.11-mRNA-1	1	OsR498G0101219000.01.T01	99,92	0,00E+00	2128	Os01g0541600	100,00	0,00E+00	2132	4	NA	NA	
maker-1-augustus-gene-206.3-mRNA-1	1	OsR498G0101216300.01.T08	99,67	2,76E-151	535	Os01g0541000	96,75	8,98E-146	515	-20	NA	NA	
maker-1-augustus-gene-206.6-mRNA-1	1	OsR498G0101225100.01.T01	100,00	0,00E+00	1321	Os01g0541800	100,00	0,00E+00	1321	0	NA	NA	
maker-1-augustus-gene-206.8-mRNA-1	1	OsR498G0101218400.01.T01	100,00	6,26E-174	610	Os11g0471200	92,00	1,79E-156	551	-59	NA	NA	
maker-1-augustus-gene-207.34-mRNA-1	1	OsR498G0101225400.01.T04	100,00	0,00E+00	3534	Os01g0541900	99,24	0,00E+00	3502	-32	NA	NA	Kinase
maker-1-augustus-gene-208.0-mRNA-1	1	OsR498G0101234300.01.T01	100,00	0,00E+00	1846	Os01g0543100	100,00	0,00E+00	1846	0	NA	NA	
maker-1-augustus-gene-209.31-mRNA-1	1	OsR498G0101251800.01.T02	100,00	0,00E+00	1397	Os01g0545500	100,00	0,00E+00	1397	0	NA	NA	
maker-1-augustus-gene-21.10-mRNA-1	1	OsR498G0100136300.01.T01	100,00	0,00E+00	1159	Os01g0139000	99,84	0,00E+00	1154	-5	NA	Indica	
maker-1-augustus-gene-21.16-mRNA-1	1	OsR498G0100135400.01.T03	99,80	0,00E+00	909	Os01g0138600	99,80	0,00E+00	909	0	NA	Indica	
maker-1-augustus-gene-21.2-mRNA-1	1	OsR498G0100134300.01.T01	99,95	0,00E+00	3659	Os01g0138400	99,41	0,00E+00	3613	-46	NA	Indica	Kinase-TM
maker-1-augustus-gene-21.23-mRNA-1	1	OsR498G0100136000.01.T03	100,00	0,00E+00	2408	Os01g0138900	99,78	0,00E+00	2395	-13	NA	Indica	
maker-1-augustus-gene-21.27-mRNA-1	1	OsR498G0100136800.01.T05	100,00	0,00E+00	3631	Os01g0139200	99,75	0,00E+00	3612	-19	NA	Indica	
maker-1-augustus-gene-21.4-mRNA-1	1	OsR498G0100135100.01.T03	100,00	5,03E-176	618	Os01g0138500	99,70	4,69E-171	600	-18	NA	Indica	
maker-1-augustus-gene-21.7-mRNA-1	1	OsR498G0100135700.01.T01	100,00	0,00E+00	1862	Os01g0138800	98,66	0,00E+00	1822	-40	NA	Indica	

maker-1-augustus-gene-210.10-mRNA-1	1	OsR498G0101254800.01.T01	100,00	0,00E+00	1867	Os01g0546100	100,00	0,00E+00	1090	-777	Indica	NA	
maker-1-augustus-gene-210.14-mRNA-1	1	OsR498G0101257000.01.T02	100,00	0,00E+00	1867	Os01g0546400	100,00	0,00E+00	1867	0	NA	NA	
maker-1-augustus-gene-210.16-mRNA-1	1	OsR498G0101257000.01.T05	99,67	3,78E-151	535	Os01g0546400	75,37	2,24E-47	189	-346	Indica	NA	
maker-1-augustus-gene-210.19-mRNA-1	1	OsR498G0101259400.01.T01	100,00	0,00E+00	2300	Os01g0547133	100,00	0,00E+00	2300	0	NA	NA	
maker-1-augustus-gene-212.2-mRNA-1	1	OsR498G0101275700.01.T01	100,00	0,00E+00	4154	Os01g0549400	100,00	0,00E+00	4154	0	NA	NA	
maker-1-augustus-gene-212.20-mRNA-1	1	OsR498G0101275600.01.T01	100,00	0,00E+00	2111	Os01g0549300	100,00	0,00E+00	2111	0	NA	NA	
maker-1-augustus-gene-212.23-mRNA-1	1	OsR498G0101276600.01.T03	100,00	0,00E+00	1420	Os01g0549500	100,00	0,00E+00	1420	0	NA	NA	
maker-1-augustus-gene-212.7-mRNA-1	1	OsR498G0101276700.01.T01	100,00	0,00E+00	1930	Os01g0549700	100,00	0,00E+00	1930	0	NA	NA	
maker-1-augustus-gene-213.43-mRNA-1	1	OsR498G0101282300.01.T01	100,00	0,00E+00	3155	Os01g0550600	99,96	0,00E+00	4725	1570	Japonica	NA	
maker-1-augustus-gene-213.45-mRNA-1	1	OsR498G0101284300.01.T01	99,92	0,00E+00	2198	Os01g0550800	100,00	0,00E+00	2203	5	NA	NA	
maker-1-augustus-gene-213.46-mRNA-1	1	OsR498G0102779300.01.T01	88,72	0,00E+00	758	Os01g0550900	99,84	0,00E+00	1095	337	Japonica	NA	
maker-1-augustus-gene-213.61-mRNA-1	1	OsR498G0101286900.01.T01	100,00	0,00E+00	1492	Os01g0551000	100,00	0,00E+00	1492	0	NA	NA	
maker-1-augustus-gene-213.63-mRNA-1	1	OsR498G0101279600.01.T01	93,80	0,00E+00	2272	Os01g0550100	93,80	0,00E+00	2272	0	NA	NA	
maker-1-augustus-gene-213.68-mRNA-1	1	OsR498G0101280600.01.T01	100,00	0,00E+00	1911	Os01g0550300	99,15	0,00E+00	1453	-458	Indica	NA	
maker-1-augustus-gene-213.72-mRNA-1	1	OsR498G0101287200.01.T02	100,00	0,00E+00	1391	Os01g0551100	100,00	0,00E+00	1391	0	NA	NA	
maker-1-augustus-gene-214.30-mRNA-1	1	OsR498G0101292100.01.T05	98,61	0,00E+00	1132	Os01g0552300	98,01	0,00E+00	1120	-12	NA	NA	
maker-1-augustus-gene-215.4-mRNA-1	1	OsR498G0101301300.01.T01	100,00	0,00E+00	1391	Os05g0471800	98,62	0,00E+00	1258	-133	NA	NA	
maker-1-augustus-gene-215.5-mRNA-1	1	OsR498G0101296800.01.T02	99,90	0,00E+00	1776	Os01g0553301	100,00	0,00E+00	1775	-1	NA	NA	
maker-1-augustus-gene-216.2-mRNA-1	1	OsR498G0101311800.01.T01	100,00	0,00E+00	1498	Os01g0555200	100,00	0,00E+00	1498	0	NA	NA	
maker-1-augustus-gene-216.4-mRNA-1	1	OsR498G0101312000.01.T01	100,00	0,00E+00	955	Os01g0555300	100,00	0,00E+00	955	0	NA	NA	
maker-1-augustus-gene-216.6-mRNA-1	1	OsR498G0101312200.01.T01	100,00	0,00E+00	1159	Os01g0555600	100,00	0,00E+00	1159	0	NA	NA	
maker-1-augustus-gene-217.5-mRNA-1	1	OsR498G0101322400.01.T01	100,00	0,00E+00	1440	Os01g0557100	100,00	0,00E+00	1440	0	NA	NA	
maker-1-augustus-gene-218.51-mRNA-1	1	OsR498G0101334100.01.T01	100,00	0,00E+00	1110	Os01g0559000	100,00	0,00E+00	1110	0	NA	Japonica	
maker-1-augustus-gene-218.56-mRNA-1	1	OsR498G0101324800.01.T02	100,00	0,00E+00	2516	Os01g0557500	100,00	0,00E+00	2324	-192	NA	NA	
maker-1-augustus-gene-218.60-mRNA-1	1	OsR498G0101331200.01.T01	100,00	0,00E+00	1332	Os01g0558300	100,00	0,00E+00	1332	0	NA	NA	
maker-1-augustus-gene-218.63-mRNA-1	1	OsR498G0101333000.01.T01	100,00	0,00E+00	1074	Os01g0558600	100,00	0,00E+00	1074	0	NA	NA	
maker-1-augustus-gene-218.67-mRNA-1	1	OsR498G0101333800.01.T02	100,00	0,00E+00	1526	Os01g0558800	100,00	0,00E+00	1526	0	NA	NA	
maker-1-augustus-gene-219.0-mRNA-1	1	OsR498G0101334700.01.T01	95,22	0,00E+00	2131	Os03g0198400	92,46	0,00E+00	1950	-181	NA	NA	
maker-1-augustus-gene-219.11-mRNA-1	1	OsR498G0101335100.01.T01	100,00	0,00E+00	1061	Os01g0559300	100,00	0,00E+00	1061	0	NA	Japonica	
maker-1-augustus-gene-219.14-mRNA-1	1	OsR498G0101336300.01.T05	99,39	0,00E+00	3219	Os01g0559500	100,00	0,00E+00	3714	495	Japonica	Japonica	
maker-1-augustus-gene-219.18-mRNA-1	1	OsR498G0101337500.01.T01	100,00	0,00E+00	2275	Os01g0559600	100,00	0,00E+00	2275	0	NA	Japonica	
maker-1-augustus-gene-219.22-mRNA-1	1	OsR498G0101339800.01.T01	99,85	0,00E+00	2388	Os01g0560000	100,00	0,00E+00	2397	9	NA	Japonica	
maker-1-augustus-gene-219.7-mRNA-1	1	OsR498G0101334900.01.T01	100,00	0,00E+00	1499	Os01g0559200	100,00	0,00E+00	1499	0	NA	Japonica	
maker-1-augustus-gene-22.25-mRNA-1	1	OsR498G0100141500.01.T06	99,67	0,00E+00	3222	Os01g0140400	99,72	0,00E+00	3226	4	NA	NA	LRR-TM
maker-1-augustus-gene-22.32-mRNA-1	1	OsR498G0100149000.01.T02	99,77	0,00E+00	2335	Os01g0141100	96,24	0,00E+00	1636	-699	Indica	NA	
maker-1-augustus-gene-22.37-mRNA-1	1	OsR498G0100149600.01.T02	100,00	0,00E+00	2576	Os01g0141100	79,16	0,00E+00	873	-1703	Indica	NA	
maker-1-augustus-gene-220.0-mRNA-1	1	OsR498G0101340800.01.T01	99,85	0,00E+00	1197	Os01g0560200	100,00	0,00E+00	1202	5	NA	NA	
maker-1-augustus-gene-221.30-mRNA-1	1	OsR498G0101342100.01.T02	90,51	0,00E+00	1841	Os01g0562400	100,00	0,00E+00	2246	405	Japonica	Japonica	
maker-1-augustus-gene-221.35-mRNA-1	1	OsR498G0101346000.01.T04	95,11	0,00E+00	1555	Os01g0563000	100,00	0,00E+00	1773	218	Japonica	Japonica	
maker-1-augustus-gene-221.42-mRNA-1	1	OsR498G0612516300.01.T06	96,22	2,51E-146	518	Os01g0563500	99,77	0,00E+00	770	252	Japonica	NA	
maker-1-augustus-gene-223.34-mRNA-1	1	OsR498G0101352200.01.T02	97,36	0,00E+00	895	Os01g0565600	99,81	0,00E+00	954	59	NA	Japonica	
maker-1-augustus-gene-223.37-mRNA-1	1	OsR498G0101352600.01.T01	99,03	0,00E+00	903	Os01g0565800	100,00	0,00E+00	926	23	NA	Japonica	
maker-1-augustus-gene-223.39-mRNA-1	1	OsR498G0101353600.01.T04	98,88	0,00E+00	3929	Os01g0566100	100,00	0,00E+00	2141	-1788	Indica	Japonica	
maker-1-augustus-gene-223.42-mRNA-1	1	OsR498G0101357200.01.T02	95,41	0,00E+00	2746	Os01g0566500	99,94	0,00E+00	2988	242	NA	Japonica	
maker-1-augustus-gene-224.0-mRNA-1	1	OsR498G0101359700.01.T01	99,74	0,00E+00	2764	Os01g0567500	100,00	0,00E+00	2782	18	NA	Japonica	
maker-1-augustus-gene-224.2-mRNA-1	1	OsR498G0101358200.01.T04	99,78	0,00E+00	1624	Os01g0566900	94,48	0,00E+00	1561	-63	NA	Japonica	
maker-1-augustus-gene-224.8-mRNA-1	1	OsR498G0101360200.01.T01	99,68	0,00E+00	2754	Os01g0567600	100,00	0,00E+00	2776	22	NA	Japonica	
maker-1-augustus-gene-226.0-mRNA-1	1	OsR498G0101367300.01.T01	99,79	0,00E+00	2546	Os01g0570500	99,93	0,00E+00	2555	9	NA	Japonica	
maker-1-augustus-gene-226.13-mRNA-1	1	OsR498G0101370700.01.T01	97,96	0,00E+00	759	Os01g0571200	99,77	0,00E+00	792	33	NA	Japonica	
maker-1-augustus-gene-226.17-mRNA-1	1	OsR498G0101370900.01.T01	100,00	0,00E+00	1348	Os01g0571300	100,00	0,00E+00	1348	0	NA	Japonica	
maker-1-augustus-gene-226.23-mRNA-1	1	OsR498G0101369200.01.T01	99,42	0,00E+00	3039	Os01g0570800	100,00	0,00E+00	3085	46	NA	Japonica	
maker-1-augustus-gene-226.28-mRNA-1	1	OsR498G0101369800.01.T03	99,12	0,00E+00	1408	Os01g0571100	100,00	0,00E+00	861	-547	Indica	Japonica	
maker-1-augustus-gene-226.7-mRNA-1	1	OsR498G0101369500.01.T01	99,90	0,00E+00	1818	Os01g0571000	100,00	0,00E+00	1586	-232	Indica	Japonica	
maker-1-augustus-gene-228.33-mRNA-1	1	OsR498G0101378500.01.T03	99,86	0,00E+00	3828	Os01g0574400	100,00	0,00E+00	3842	14	NA	Japonica	NBS-TM
maker-1-augustus-gene-228.36-mRNA-1	1	OsR498G0101378700.01.T01	99,72	0,00E+00	3847	Os01g0574500	100,00	0,00E+00	3874	27	NA	Japonica	NBS-TM
maker-1-augustus-gene-228.39-mRNA-1	1	OsR498G0101381500.01.T01	99,86	0,00E+00	3789	Os01g0575000	100,00	0,00E+00	3802	13	NA	Japonica	
maker-1-augustus-gene-228.46-mRNA-1	1	OsR498G0101382500.01.T01	99,77	0,00E+00	1582	Os01g0575200	100,00	0,00E+00	1231	-351	Indica	Japonica	
maker-1-augustus-gene-228.47-mRNA-1	1	OsR498G0101382500.01.T01	99,59	0,00E+00	866	Os01g0575200	100,00	0,00E+00	895	29	NA	Japonica	
maker-1-augustus-gene-228.52-mRNA-1	1	OsR498G0101379000.01.T01	99,90	0,00E+00	1847	Os01g0574600	100,00	0,00E+00	1851	4	NA	Japonica	
maker-1-augustus-gene-229.29-mRNA-1	1	OsR498G0101393500.01.T01	91,47	0,00E+00	711	Os04g0557200	81,17	8,97E-131	467	-244	Indica	Japonica	
maker-1-augustus-gene-229.30-mRNA-1	1	OsR498G0101387300.01.T01	98,46	0,00E+00	1688	Os01g0576100	100,00	0,00E+00	1622	-66	NA	Japonica	
maker-1-augustus-gene-23.0-mRNA-1	1	OsR498G0100150500.01.T02	95,38	0,00E+00	2414	Os01g0141600	95,61	0,00E+00	1593	-821	Indica	Indica	
maker-1-augustus-gene-23.10-mRNA-1	1	OsR498G0100151100.01.T03	100,00	0,00E+00	655	Os01g0141900	95,66	4,61E-168	589	-66	Indica	Indica	
maker-1-augustus-gene-23.14-mRNA-1	1	OsR498G0100151500.01.T05	100,00	0,00E+00	937	Os01g0142200	99,81	0,00E+00	932	-5	NA	Indica	
maker-1-augustus-gene-23.18-mRNA-1	1	OsR498G0100151700.01.T01	100,00	0,00E+00	2365	Os01g0142300	99,77	0,00E+00	2351	-14	NA	Indica	
maker-1-augustus-gene-23.23-mRNA-1	1	OsR498G0100152900.01.T04	100,00	0,00E+00	3144	Os01g0142800	99,83	0,00E+00	3131	-13	NA	Indica	
maker-1-augustus-gene-23.33-mRNA-1	1	OsR498G0100151400.01.T01	100,00	0,00E+00	2955	Os01g0142100	99,57	0,00E+00	2927	-28	NA	Indica	
maker-1-augustus-gene-23.41-mRNA-1	1	OsR498G0100152200.01.T02	100,00	0,00E+00	1597	Os01g0142500	99,66	0,00E+00	1583	-14	NA	Indica	
maker-1-augustus-gene-230.10-mRNA-1	1	OsR498G0101394600.01.T01	98,79	0,00E+00	3619	Os01g0577600	100,00	0,00E+00	3734	115	NA	Japonica	Kinase-LRR-TM
maker-1-augustus-gene-230.13-mRNA-1	1	OsR498G0101395400.01.T01	99,43	0,00E+00	1867	Os01g0578000	100,00	0,00E+00	1894	27	NA	Japonica	
maker-1-augustus-gene-230.18-mRNA-1	1	OsR498G0101395800.01.T03	98,81	0,00E+00	2205	Os03g0300200	70,78	1,16E-137	489	-1716	Indica	Japonica	
maker-1-augustus-gene-231.15-mRNA-1	1	OsR498G0101400300.01.T03	98,30	0,00E+00	2239	Os01g0580100	97,99	0,00E+00	2929	690	Japonica	Japonica	
maker-1-augustus-gene-231.21-mRNA-1	1	OsR498G0101400900.01.T02	98,15	0,00E+00	4283	Os01g0580200	99,52	0,00E+00	4433	150	NA	Japonica	
maker-1-augustus-gene-231.28-mRNA-1	1	OsR498G0101400000.01.T01	99,40	0,00E+00	1471	Os01g0579900	100,00	0,00E+00	1494	23	NA	Japonica	
maker-1-augustus-gene-231.33-mRNA-1	1	OsR498G0101400200.01.T02	99,69	0,00E+00	1746	Os01g0580000	100,00	0,00E+00	1759	13	NA	Japonica	
maker-1-augustus-gene-231.6-mRNA-1	1	OsR498G0101399300.01.T01	99,10	0,00E+00	977	Os01g0711500	75,31	2,17E-92	339	-638	Indica	Japonica	
maker-1-augustus-gene-232.13-mRNA-1	1	OsR498G0101402200.01.T01	99,55	0,00E+00	2352	Os01g0580800	95,57	0,00E+00	2024	-328	Indica	Japonica	
maker-1-augustus-gene-232.18-mRNA-1	1	OsR498G0101404800.01.T01	99,69	0,00E+00	1728	Os01g0581800	100,00	0,00E+00	2381	653	Japonica	Japonica	
maker-1-augustus-gene-232.2-mRNA-1	1	OsR498G0101403500.01.T04	96,93	0,00E+00	1416	Os01g0581300	100,00	0,00E+00	1523	107	NA	Japonica	
maker-1-augustus-gene-232.20-mRNA-1	1	OsR498G0101405000.01.T02	100,00	0,00E+00	3020	Os01g0581900	100,00	0,00E+00	1739	-1281	Indica	NA	
maker-1-augustus-gene-232.7-mRNA-1	1	OsR498G0101403700.01.T01	98,65	0,00E+00	4011	Os01g0581400	100,00	0,00E+00	4145	134	NA	Japonica	CC-Kinase-TM
maker-1-augustus-gene-232.9-mRNA-1	1	OsR498G0101401800.01.T01	97,79	0,00E+00	2462	Os01g0580400	100,00	0,00E+00	2397	-65	NA	Japonica	
maker-1-augustus-gene-233.29-mRNA-1	1	OsR498G0101409500.01.T01	99,64	0,00E+00	2510	Os05g0592800	74,26	0,00E+00	742	-1768	Indica	Japonica	
maker-1-augustus-gene-233.37-mRNA-1	1	OsR498G0101407000.01.T02	96,27	0,00E+00	2577	Os01g0582400	96,33	0,00E+00	2581	4	NA	Japonica	
maker-1-augustus-gene-234.41-mRNA-1	1	OsR498G0101416500.01.T01	100,00	0,00E+00	1337	Os01g0584900	100,00	0,00E+00	1337	0	NA	Indica	
maker-1-augustus-gene-234.43-mRNA-1	1	OsR498G0101417300.01.T03	100,00	0,00E+00	2013	Os01g0585100	100,00	0,00E+00	2013	0	NA	Indica	
maker-1-augustus-gene-234.49-mRNA-1	1	OsR498G0101420100.01.T02	100,00	0,00E+00	1934	Os01g0585600	100,00	0,00E+00	1934	0	NA	Japonica	
maker-1-augustus-gene-234.60-mRNA-1	1	OsR498G0101418800.01.T01	99,82	0,00E+00	1013	Os01g0585300	100,00	0,00E+00	1018	5	NA	Indica	
maker-1-augustus-gene-235.26-mRNA-1	1	OsR498G0101420300.01.T04	100,00	0,00E+00	2296	Os01g0585700	100,00	0,00E+00	2296	0	NA	NA	
maker-1-augustus-gene-235.35-mRNA-1	1	OsR498G0101423200.01.T04	99,78	0,00E+00	2427	Os01g0586600	100,00	0,00E+00	2441	14	NA	Japonica	
maker-1-augustus-gene-235.40-mRNA-1	1	OsR498G0101425200.01.T03	100,00	0,00E+00	1232	Os01g0587000	100,00	0,00E+00	1227	-5	NA	Japonica	
maker-1-augustus-gene-236.0-mRNA-1	1	OsR498G0101430700.01.T01	100,00	0,00E+00	1777	Os01g0588700	100,00	0,00E+00	1777	0	NA	Japonica	
maker-1-augustus-gene-236.11-mRNA-1	1	OsR498G0101427400.01.T01	99,75	0,00E+00	1452	Os01g0588000	99,63	0,00E+00	1448	-4	NA	NA	
maker-1-augustus-gene-236.13-mRNA-1	1	OsR498G0101428400.01.T02	100,00	0,00E+00	1494	Os01g0588200	100,00	0,00E+00	1494	0	NA	Japonica	
maker-1-augustus-gene-236.17-mRNA-1	1	OsR498G0101428600.01.T01	99,35	0,00E+00	1626	Os01g0588400	100,00	0,00E+00	1689	63	NA	Japonica	
maker-1-augustus-gene-236.19-mRNA-1	1	OsR498G0101429400.01.T01	99,73	0,00E+00	2661	Os01g0588500	99,80	0,00E+00	2665	4	NA	Japonica	Kinase
maker-1-augustus-gene-236.22-mRNA-1	1	OsR498G0101433200.01.T01	100,00	0,00E+00	1315	Os01g0588900	100,00	0,00E+00	1315	0	NA	Japonica	
maker-1-augustus-gene-236.4-mRNA-1	1	OsR498G0101432900.01.T06	99,51	0,00E+00	2199	Os01g0588800	99,84	0,00E+00	2214	15	NA	Japonica	
maker-1-augustus-gene-236.9-mRNA-1	1	OsR498G0101426800.01.T01	99,69	0,00E+00	4601	Os01g0587400	100,00	0,00E+00	4156	-445	NA	Japonica	Kinase-TM
maker-1-augustus-gene-237.0-mRNA-1	1	OsR498G0511248800.01.T01	71,32	7,37E-10	65,3	Os01g0589100	100,00	2,41E-118	423	357,7	Japonica	Japonica	
maker-1-augustus-gene-237.11-mRNA-1	1	OsR498G0101435800.01.T03	96,96	0,00E+00	1204	Os01g0589000	100,00	0,00E+00	1104	-100	NA	Japonica	
maker-1-augustus-gene-237.12-mRNA-1	1	OsR498G0101437000.01.T01	100,00	0,00E+00	1088	Os01g0589500	100,00	0,00E+00	1326	238	Japonica	Japonica	
maker-1-augustus-gene-237.14-mRNA-1	1	OsR498G0101439000.01.T01	96,03	0,00E+00	1333	Os01g0590200	96,28	0,00E+00	1342	9	NA	Japonica	
maker-1-augustus-gene-237.9-mRNA-1	1	OsR498G0101437300.01.T01	97,91	0,00E+00	984	Os01g0589800	100,00	0,00E+00	1061	77	NA	Japonica	
maker-1-augustus-gene-238.42-mRNA-1	1	OsR498G0101443500.01.T01	95,11	0,00E+00	1522	Os01g0590900	97,17	0,00E+00	1674	152	NA	Japonica	
maker-1-augustus-gene-238.48-mRNA-1	1	OsR498G0101443900.01.T01	99,27	0,00E+00	2672	Os01g0591000	100,00	0,00E+00	2722	50	NA	Japonica	
maker-1-augustus-gene-238.55-mRNA-1	1	OsR498G0101445100.01.T02	98,06	0,00E+00	2666	Os01g0591300	100,00	0,00E+00	2749	83	NA	Japonica	
maker-1-augustus-gene-238.60-mRNA-1	1	OsR498G0101443000.01.T01	99,21	0,00E+00	669	Os01g0590800	100,00	4,40E-171	600	-69	Indica	Japonica	
maker-1-augustus-gene-238.63-mRNA-1	1	OsR498G0101443400.01.T01	98,36	1,82E-146	519	Os01g0590800	100,00	1,22E-149	527	8	NA	Japonica	
maker-1-augustus-gene-239.28-mRNA-1	1	OsR498G0101451500.01.T05	99,26	0,00E+00	6433	Os01g0593000	100,00	0,00E+00	6543	110	NA	Japonica	
maker-1-augustus-gene-239.36-mRNA-1	1	OsR498G0101453300.01.T05	99,87	0,00E+00	1408	Os01g0593200	100,00	0,00E+00	1413	5	NA	Japonica	
maker-1-augustus-gene-239.40-mRNA-1	1	OsR498G0101455700.01.T01	99,39	0,00E+00	3517	Os01g0593700	100,00	0,00E+00	3571	54	NA	Japonica	
maker-1-augustus-gene-239.42-mRNA-1	1	OsR498G0101450600.01.T01	99,36	0,00E+00	5258	Os01g0592900	100,00	0,00E+00	4745	-513	NA	Japonica	
maker-1-augustus-gene-239.47-mRNA-1	1	OsR498G0714656200.01.T02	93,37	0,00E+00	786	Os01g0593100	100,00	0,00E+00	1061	275	Japonica	Japonica	
maker-1-augustus-gene-239.49-mRNA-1	1	OsR498G0101455200.01.T01	99,63	0,00E+00	1459	Os01g0593500	100,00	0,00E+00	1472	13	NA	Japonica	
maker-1-augustus-gene-24.50-mRNA-1	1	OsR498G0306795400.01.T03	97,72	0,00E+00	1118	Os01g0144200	99,39	0,00E+00	1168	50	NA	Japonica	
maker-1-augustus-gene-24.56-mRNA-1	1	OsR498G0100164300.01.T04	95,67	2,87E-113	409	Os01g0144600	95,67	9,98E-114	409	0	NA	NA	
maker-1-augustus-gene-24.60-mRNA-1	1	OsR498G0100159200.01.T05	100,00	0,00E+00	868	Os01g0143100	100,00	0,00E+00	866	-2	NA	Indica	
maker-1-augustus-gene-24.64-mRNA-1	1	OsR498G0100159600.01.T01	99,87	0,00E+00	1430	Os01g0143300	99,87	0,00E+00	1430	0	NA	Japonica	
maker-1-augustus-gene-24.66-mRNA-1	1	OsR498G0100161600.01.T01	99,77	0,00E+00	786	Os01g0143700	99,74	0,00E+00	1398	612	Japonica	Indica	
maker-1-augustus-gene-24.69-mRNA-1	1	OsR498G0100162200.01.T01	99,76	0,00E+00	1507	Os01g0143800	99,51	0,00E+00	1452	-55	NA	Indica	
maker-1-augustus-gene-24.73-mRNA-1	1	OsR498G0100162600.01.T01	100,00	0,00E+00	1137	Os01g0144000	100,00	0,00E+00	1137	0	NA	NA	
maker-1-augustus-gene-24.78-mRNA-1	1	OsR498G0100162800.01.T01	99,38	0,00E+00	860	Os01g0144100	100,00	0,00E+00	874	14	NA	Japonica	
maker-1-augustus-gene-24.81-mRNA-1	1	OsR498G0100163200.01.T01	99,41	0,00E+00	1802	Os01g0144300	99,41	0,00E+00	1802	0	NA	NA	
maker-1-augustus-gene-240.10-mRNA-1	1	OsR498G0101462300.01.T01	96,94	0,00E+00	2354	Os01g0595700	97,76	0,00E+00	2835	481	Japonica	NA	
maker-1-augustus-gene-240.13-mRNA-1	1	OsR498G0101462500.01.T01	98,42	0,00E+00	2626	Os01g0595800	100,00	0,00E+00	2722	96	NA	NA	
maker-1-augustus-gene-240.15-mRNA-1	1	OsR498G0101462700.01.T01	98,53	0,00E+00	2836	Os01g0595900	100,00	0,00E+00	2944	108	NA	NA	
maker-1-augustus-gene-240.17-mRNA-1	1	OsR498G0101462900.01.T01	100,00	0,00E+00	1559	Os01g0596000	100,00	0,00E+00	2727	1168	Japonica	NA	
maker-1-augustus-gene-240.19-mRNA-1	1	OsR498G0101463300.01.T01	99,94	0,00E+00	2815	Os01g0596100	100,00	0,00E+00	2819	4	NA	NA	
maker-1-augustus-gene-240.21-mRNA-1	1	OsR498G0101457800.01.T01	99,14	0,00E+00	1022	Os01g0594500	100,00	0,00E+00	1045	23	NA	NA	
maker-1-augustus-gene-240.25-mRNA-1	1	OsR498G0101459200.01.T01	99,61	0,00E+00	1356	Os01g0594900	100,00	0,00E+00	1370	14	NA	NA	
maker-1-augustus-gene-240.4-mRNA-1	1	OsR498G0101460100.01.T01	99,75	0,00E+00	710	Os01g0719600	73,08	8,25E-26	116	-594	Indica	NA	
maker-1-augustus-gene-240.7-mRNA-1	1	OsR498G0101462100.01.T01	95,30	0,00E+00	1452	Os01g0595600	100,00	0,00E+00	1640	188	Japonica	NA	
maker-1-augustus-gene-241.34-mRNA-1	1	OsR498G0101463900.01.T01	99,93	0,00E+00	2566	Os01g0596300	100,00	0,00E+00	2571	5	NA	NA	
maker-1-augustus-gene-241.38-mRNA-1	1	OsR498G0101466500.01.T01	99,37	0,00E+00	2828	Os01g0596700	100,00	0,00E+00	2874	46	NA	NA	
maker-1-augustus-gene-241.40-mRNA-1	1	OsR498G0101468700.01.T01	96,35	0,00E+00	1884	Os01g0597400	100,00	0,00E+00	2403	519	Japonica	NA	
maker-1-augustus-gene-241.42-mRNA-1	1	OsR498G0101469100.01.T01	97,99	0,00E+00	2124	Os01g0597600	100,00	0,00E+00	2233	109	NA	NA	
maker-1-augustus-gene-241.43-mRNA-1	1	OsR498G0101469500.01.T01	97,51	0,00E+00	1359	Os01g0597800	86,89	0,00E+00	973	-386	Indica	NA	
maker-1-augustus-gene-242.0-mRNA-1	1	OsR498G0101471100.01.T02	99,85	0,00E+00	1181	Os01g0598200	100,00	0,00E+00	1186	5	NA	NA	
maker-1-augustus-gene-242.13-mRNA-1	1	OsR498G0101473200.01.T03	92,24	0,00E+00	1697	Os01g0598900	100,00	0,00E+00	1745	48	NA	NA	
maker-1-augustus-gene-242.17-mRNA-1	1	OsR498G0101474200.01.T03	100,00	0,00E+00	666	Os01g0600000	100,00	0,00E+00	666	0	NA	NA	
maker-1-augustus-gene-242.3-mRNA-1	1	OsR498G0101473900.01.T01	93,22	0,00E+00	2581	Os01g0599900	99,94	0,00E+00	2907	326	Japonica	NA	
maker-1-augustus-gene-243.37-mRNA-1	1	OsR498G0101474700.01.T01	99,75	0,00E+00	2823	Os01g0600300	99,78	0,00E+00	1608	-1215	Indica	NA	
maker-1-augustus-gene-243.42-mRNA-1	1	OsR498G0101477700.01.T01	99,72	0,00E+00	2558	Os01g0601200	99,68	0,00E+00	1681	-877	Indica	NA	Kinase
maker-1-augustus-gene-243.44-mRNA-1	1	OsR498G0101475400.01.T01	100,00	0,00E+00	1543	Os02g0226200	71,87	7,83E-62	238	-1305	Indica	NA	
maker-1-augustus-gene-243.49-mRNA-1	1	OsR498G0101482200.01.T02	93,26	0,00E+00	2371	Os01g0602400	94,71	0,00E+00	2517	146	NA	NA	
maker-1-augustus-gene-243.51-mRNA-1	1	OsR498G0101483000.01.T01	98,48	0,00E+00	2736	Os01g0602400	93,30	0,00E+00	2407	-329	Indica	NA	
maker-1-augustus-gene-243.79-mRNA-1	1	OsR498G0101483200.01.T01	99,86	0,00E+00	1250	Os11g0566800	72,72	4,89E-155	548	-702	Indica	NA	
maker-1-augustus-gene-244.5-mRNA-1	1	OsR498G0101487900.01.T01	99,56	0,00E+00	1617	Os01g0603300	100,00	0,00E+00	1635	18	NA	NA	
maker-1-augustus-gene-244.7-mRNA-1	1	OsR498G0100035900.01.T05	89,21	7,96E-102	370	Os01g0604100	100,00	0,00E+00	729	359	Japonica	NA	
maker-1-augustus-gene-244.9-mRNA-1	1	OsR498G0101486800.01.T01	100,00	0,00E+00	1014	Os01g0603000	100,00	0,00E+00	1014	0	NA	NA	
maker-1-augustus-gene-245.52-mRNA-1	1	OsR498G0101497700.01.T02	100,00	0,00E+00	2636	Os01g0605100	99,63	0,00E+00	1947	-689	Indica	NA	
maker-1-augustus-gene-245.54-mRNA-1	1	OsR498G0101497900.01.T01	99,94	0,00E+00	3010	Os01g0605300	100,00	0,00E+00	3014	4	NA	NA	
maker-1-augustus-gene-245.56-mRNA-1	1	OsR498G0101497900.01.T02	99,78	0,00E+00	1617	Os01g0605400	98,23	0,00E+00	1548	-69	NA	NA	
maker-1-augustus-gene-245.61-mRNA-1	1	OsR498G0101498500.01.T01	99,48	0,00E+00	1362	Os01g0605700	100,00	0,00E+00	1380	18	NA	NA	
maker-1-augustus-gene-245.63-mRNA-1	1	OsR498G0101499300.01.T01	99,23	0,00E+00	1380	Os01g0606000	100,00	0,00E+00	1407	27	NA	NA	
maker-1-augustus-gene-245.65-mRNA-1	1	OsR498G0101495000.01.T01	99,35	0,00E+00	1896	Os01g0604700	99,63	0,00E+00	1910	14	NA	NA	
maker-1-augustus-gene-245.69-mRNA-1	1	OsR498G0101497400.01.T04	89,91	9,17E-177	619	Os01g0605200	89,91	3,18E-177	619	0	NA	NA	
maker-1-augustus-gene-245.72-mRNA-1	1	OsR498G0101498200.01.T01	99,90	0,00E+00	3579	Os01g0605500	100,00	0,00E+00	4145	566	Japonica	NA	
maker-1-augustus-gene-246.30-mRNA-1	1	OsR498G0101502300.01.T01	98,48	0,00E+00	1029	Os01g0606900	100,00	0,00E+00	1072	43	NA	NA	
maker-1-augustus-gene-246.35-mRNA-1	1	OsR498G0101503300.01.T01	98,49	0,00E+00	803	Os01g0607200	100,00	0,00E+00	744	-59	NA	NA	
maker-1-augustus-gene-246.36-mRNA-1	1	OsR498G0101503700.01.T01	99,86	0,00E+00	2607	Os01g0607200	96,63	0,00E+00	2389	-218	NA	NA	
maker-1-augustus-gene-246.53-mRNA-1	1	OsR498G0101504200.01.T03	99,87	0,00E+00	4094	Os01g0607400	100,00	0,00E+00	4107	13	NA	NA	
maker-1-augustus-gene-246.65-mRNA-1	1	OsR498G0101507200.01.T01	99,85	0,00E+00	3693	Os05g0588250	74,72	0,00E+00	865	-2828	Indica	NA	Kinase-LRR-TM
maker-1-augustus-gene-247.21-mRNA-1	1	OsR498G0611518800.01.T01	70,11	2,78E-113	410	Os01g0608366	100,00	0,00E+00	2452	2042	Japonica	NA	
maker-1-augustus-gene-247.28-mRNA-1	1	OsR498G0101514000.01.T02	99,48	0,00E+00	7845	Os01g0609066	100,00	0,00E+00	7946	101	NA	Japonica	
maker-1-augustus-gene-247.31-mRNA-1	1	OsR498G0101515200.01.T01	99,79	0,00E+00	7852	Os01g0609300	100,00	0,00E+00	7889	37	NA	NA	
maker-1-augustus-gene-248.0-mRNA-1	1	OsR498G0101518900.01.T01	100,00	0,00E+00	958	Os01g0610100	100,00	0,00E+00	958	0	NA	NA	
maker-1-augustus-gene-248.11-mRNA-1	1	OsR498G0101522300.01.T01	99,82	0,00E+00	2015	Os01g0611000	100,00	0,00E+00	2165	150	NA	NA	
maker-1-augustus-gene-248.13-mRNA-1	1	OsR498G0101522700.01.T01	99,85	0,00E+00	1194	Os01g0611100	100,00	0,00E+00	1198	4	NA	NA	
maker-1-augustus-gene-248.16-mRNA-1	1	OsR498G0101517800.01.T03	99,66	0,00E+00	5755	Os01g0609900	99,80	0,00E+00	5442	-313	NA	NA	
maker-1-augustus-gene-248.20-mRNA-1	1	OsR498G0101520600.01.T01	99,88	0,00E+00	1475	Os01g0610500	100,00	0,00E+00	1480	5	NA	NA	
maker-1-augustus-gene-248.23-mRNA-1	1	OsR498G0101521000.01.T03	98,41	0,00E+00	4726	Os01g0610600	98,67	0,00E+00	4757	31	NA	NA	
maker-1-augustus-gene-248.29-mRNA-1	1	OsR498G0101521200.01.T01	98,17	0,00E+00	1610	Os01g0610700	100,00	0,00E+00	1678	68	NA	NA	
maker-1-augustus-gene-248.3-mRNA-1	1	OsR498G0101519900.01.T03	100,00	0,00E+00	2630	Os01g0610300	100,00	0,00E+00	2630	0	NA	NA	
maker-1-augustus-gene-248.9-mRNA-1	1	OsR498G0101520100.01.T02	99,63	0,00E+00	966	Os01g0610400	99,68	1,39E-157	555	-411	Indica	NA	
maker-1-augustus-gene-249.1-mRNA-1	1	OsR498G0101526300.01.T01	99,42	0,00E+00	2746	Os07g0218200	82,67	0,00E+00	1002	-1744	Indica	NA	
maker-1-augustus-gene-249.19-mRNA-1	1	OsR498G0101530400.01.T06	99,90	0,00E+00	1715	Os01g0612600	100,00	0,00E+00	1719	4	NA	NA	
maker-1-augustus-gene-249.3-mRNA-1	1	OsR498G0306062000.01.T02	86,57	0,00E+00	793	Os01g0612100	100,00	0,00E+00	1397	604	Japonica	NA	
maker-1-augustus-gene-249.5-mRNA-1	1	OsR498G0101528100.01.T01	100,00	0,00E+00	921	Os01g0612200	100,00	0,00E+00	921	0	NA	NA	
maker-1-augustus-gene-249.9-mRNA-1	1	OsR498G0101530100.01.T01	99,73	0,00E+00	1328	Os01g0612500	100,00	0,00E+00	1178	-150	Indica	NA	
maker-1-augustus-gene-25.6-mRNA-1	1	OsR498G0100171500.01.T01	99,21	0,00E+00	1567	Os11g0704800	71,45	3,92E-105	382	-1185	Indica	Japonica	
maker-1-augustus-gene-250.0-mRNA-1	1	OsR498G0101532700.01.T03	99,72	0,00E+00	5708	Os01g0612700	100,00	0,00E+00	4103	-1605	Indica	NA	
maker-1-augustus-gene-250.12-mRNA-1	1	OsR498G0101538300.01.T01	98,21	0,00E+00	2501	Os01g0614300	100,00	0,00E+00	2598	97	NA	NA	
maker-1-augustus-gene-250.14-mRNA-1	1	OsR498G0101539500.01.T01	99,89	0,00E+00	1679	Os01g0614700	100,00	0,00E+00	969	-710	Indica	NA	
maker-1-augustus-gene-250.19-mRNA-1	1	OsR498G0101536000.01.T01	99,91	0,00E+00	1949	Os01g0613500	100,00	0,00E+00	1954	5	NA	NA	
maker-1-augustus-gene-250.21-mRNA-1	1	OsR498G0101536800.01.T01	99,35	0,00E+00	1917	Os01g0613800	100,00	0,00E+00	1948	31	NA	NA	
maker-1-augustus-gene-250.23-mRNA-1	1	OsR498G0101537000.01.T02	99,88	0,00E+00	1497	Os01g0613900	100,00	0,00E+00	1501	4	NA	NA	
maker-1-augustus-gene-250.30-mRNA-1	1	OsR498G0101539800.01.T06	100,00	0,00E+00	2663	Os01g0614900	99,93	0,00E+00	2642	-21	NA	NA	
maker-1-augustus-gene-250.4-mRNA-1	1	OsR498G0101532900.01.T03	100,00	1,38E-179	628	Os01g0612800	100,00	4,80E-180	628	0	NA	NA	
maker-1-augustus-gene-250.9-mRNA-1	1	OsR498G0101535700.01.T01	100,00	0,00E+00	1137	Os01g0613300	100,00	0,00E+00	1131	-6	NA	NA	
maker-1-augustus-gene-251.27-mRNA-1	1	OsR498G0101542500.01.T07	99,69	0,00E+00	3481	Os01g0616100	100,00	0,00E+00	3508	27	NA	NA	
maker-1-augustus-gene-251.34-mRNA-1	1	OsR498G0101545900.01.T02	99,86	0,00E+00	3736	Os05g0587300	75,85	0,00E+00	1160	-2576	Indica	NA	
maker-1-augustus-gene-251.38-mRNA-1	1	OsR498G0101546500.01.T01	99,88	0,00E+00	1506	Os01g0616500	100,00	0,00E+00	1510	4	NA	NA	
maker-1-augustus-gene-251.43-mRNA-1	1	OsR498G0101541000.01.T02	99,70	0,00E+00	2379	Os01g0615200	100,00	0,00E+00	2419	40	NA	NA	
maker-1-augustus-gene-251.45-mRNA-1	1	OsR498G0101541200.01.T01	99,92	0,00E+00	2296	Os01g0615300	100,00	0,00E+00	2300	4	NA	NA	
maker-1-augustus-gene-251.47-mRNA-1	1	OsR498G0101546400.01.T02	99,71	0,00E+00	2488	Os01g0616400	99,78	0,00E+00	2490	2	NA	NA	
maker-1-augustus-gene-252.30-mRNA-1	1	OsR498G0101550500.01.T04	99,63	0,00E+00	3876	Os01g0617700	99,95	0,00E+00	3908	32	NA	NA	
maker-1-augustus-gene-252.36-mRNA-1	1	OsR498G0101550900.01.T01	99,79	0,00E+00	1701	Os01g0617900	100,00	0,00E+00	1775	74	NA	NA	
maker-1-augustus-gene-252.39-mRNA-1	1	OsR498G0101551700.01.T02	98,01	0,00E+00	1994	Os01g0618200	96,35	0,00E+00	959	-1035	Indica	NA	
maker-1-augustus-gene-252.41-mRNA-1	1	OsR498G0101547800.01.T01	99,89	0,00E+00	3270	Os05g0573300	77,91	0,00E+00	1345	-1925	Indica	NA	
maker-1-augustus-gene-252.47-mRNA-1	1	OsR498G0101549200.01.T01	99,03	0,00E+00	4732	Os01g0617500	99,07	0,00E+00	4736	4	NA	NA	
maker-1-augustus-gene-252.54-mRNA-1	1	OsR498G0101550800.01.T01	98,74	0,00E+00	968	Os01g0617800	99,10	0,00E+00	977	9	NA	NA	
maker-1-augustus-gene-252.58-mRNA-1	1	OsR498G0101551600.01.T02	100,00	0,00E+00	1867	Os01g0618100	100,00	0,00E+00	1867	0	NA	NA	
maker-1-augustus-gene-253.27-mRNA-1	1	OsR498G0101552900.01.T01	96,76	0,00E+00	1713	Os01g0618400	96,76	0,00E+00	1713	0	NA	NA	
maker-1-augustus-gene-253.31-mRNA-1	1	OsR498G0101553100.01.T01	99,69	0,00E+00	2883	Os01g0618500	99,94	0,00E+00	2902	19	NA	NA	
maker-1-augustus-gene-253.38-mRNA-1	1	OsR498G0101554700.01.T01	99,63	0,00E+00	2391	Os01g0618900	100,00	0,00E+00	1673	-718	Indica	NA	
maker-1-augustus-gene-253.42-mRNA-1	1	OsR498G0101552600.01.T01	99,46	0,00E+00	4630	Os01g0618300	99,92	0,00E+00	4543	-87	NA	NA	
maker-1-augustus-gene-253.51-mRNA-1	1	OsR498G0101555000.01.T01	99,94	0,00E+00	2999	Os01g0619000	100,00	0,00E+00	2300	-699	Indica	NA	
maker-1-augustus-gene-254.0-mRNA-1	1	OsR498G0101557500.01.T02	99,77	0,00E+00	2346	Os01g0619800	99,58	0,00E+00	1263	-1083	Indica	NA	
maker-1-augustus-gene-254.15-mRNA-1	1	OsR498G0101562800.01.T02	99,47	0,00E+00	3715	Os01g0621600	100,00	0,00E+00	3766	51	NA	NA	Kinase-TM
maker-1-augustus-gene-254.3-mRNA-1	1	OsR498G0101558200.01.T01	99,49	0,00E+00	1737	Os01g0620100	100,00	0,00E+00	1759	22	NA	NA	

maker-1-augustus-gene-254.9-mRNA-1	1	OsR498G0101561600.01.T01	99,92	0,00E+00	2297	Os01g0621200	100,00	0,00E+00	1882	-415	Indica	NA	
maker-1-augustus-gene-255.24-mRNA-1	1	OsR498G0101563100.01.T01	99,70	0,00E+00	1198	Os01g0621700	100,00	0,00E+00	1207	9	NA	NA	
maker-1-augustus-gene-255.29-mRNA-1	1	OsR498G0101565900.01.T01	99,68	0,00E+00	2790	Os05g0585500	84,73	0,00E+00	1696	-1094	Indica	NA	Kinase-TM
maker-1-augustus-gene-255.40-mRNA-1	1	OsR498G0101565600.01.T03	99,56	0,00E+00	2007	Os01g0622300	99,71	0,00E+00	1881	-126	NA	NA	
maker-1-augustus-gene-255.42-mRNA-1	1	OsR498G0101566200.01.T01	100,00	0,00E+00	758	Os01g0622700	100,00	0,00E+00	758	0	NA	NA	
maker-1-augustus-gene-255.46-mRNA-1	1	OsR498G0101568400.01.T03	99,35	0,00E+00	3544	Os01g0623200	99,94	0,00E+00	2790	-754	Indica	NA	
maker-1-augustus-gene-256.52-mRNA-1	1	OsR498G0101571300.01.T01	99,24	0,00E+00	3931	Os01g0623500	100,00	0,00E+00	3990	59	NA	NA	
maker-1-augustus-gene-256.57-mRNA-1	1	OsR498G0101572500.01.T01	99,06	0,00E+00	1494	Os01g0623900	100,00	0,00E+00	1420	-74	NA	NA	
maker-1-augustus-gene-256.58-mRNA-1	1	OsR498G0101574300.01.T02	100,00	0,00E+00	980	Os01g0624400	100,00	0,00E+00	980	0	NA	NA	
maker-1-augustus-gene-256.60-mRNA-1	1	OsR498G0101574500.01.T01	97,35	0,00E+00	1925	Os01g0624500	97,61	0,00E+00	1939	14	NA	NA	
maker-1-augustus-gene-256.71-mRNA-1	1	OsR498G0101571600.01.T01	99,76	0,00E+00	2254	Os01g0891100	70,16	4,04E-137	489	-1765	Indica	NA	
maker-1-augustus-gene-256.73-mRNA-1	1	OsR498G0101573400.01.T02	96,55	0,00E+00	4076	Os01g0624000	96,76	0,00E+00	4099	23	NA	NA	
maker-1-augustus-gene-257.44-mRNA-1	1	OsR498G0101577900.01.T01	99,48	0,00E+00	2033	Os01g0626400	99,91	0,00E+00	2052	19	NA	NA	
maker-1-augustus-gene-257.46-mRNA-1	1	OsR498G0101580700.01.T01	99,75	0,00E+00	2915	Os01g0627400	99,94	0,00E+00	2929	14	NA	NA	
maker-1-augustus-gene-257.48-mRNA-1	1	OsR498G0101582300.01.T01	99,75	0,00E+00	2110	Os01g0627800	100,00	0,00E+00	2123	13	NA	NA	
maker-1-augustus-gene-257.50-mRNA-1	1	OsR498G0101582500.01.T01	99,12	0,00E+00	2800	Os01g0627900	99,56	0,00E+00	2831	31	NA	NA	
maker-1-augustus-gene-257.52-mRNA-1	1	OsR498G0101582500.01.T01	90,39	0,00E+00	1597	Os01g0627933	100,00	0,00E+00	2154	557	Japonica	Japonica	
maker-1-augustus-gene-257.53-mRNA-1	1	OsR498G0101582500.01.T01	81,72	5,61E-100	365	Os01g0627966	99,47	0,00E+00	1330	965	Japonica	Japonica	
maker-1-augustus-gene-257.60-mRNA-1	1	OsR498G0101583500.01.T01	99,94	0,00E+00	2874	Os01g0627900	77,74	0,00E+00	1260	-1614	Indica	Japonica	
maker-1-augustus-gene-257.64-mRNA-1	1	OsR498G0101581000.01.T01	100,00	0,00E+00	926	Os01g0627500	100,00	0,00E+00	926	0	NA	NA	
maker-1-augustus-gene-257.67-mRNA-1	1	OsR498G0101582200.01.T01	99,82	0,00E+00	1943	Os01g0627933	82,51	0,00E+00	1086	-857	Indica	NA	
maker-1-augustus-gene-258.0-mRNA-1	1	OsR498G0101585300.01.T01	99,94	0,00E+00	2858	Os01g0628900	99,81	0,00E+00	2849	-9	NA	NA	
maker-1-augustus-gene-258.10-mRNA-1	1	OsR498G0101587800.01.T02	97,98	0,00E+00	3326	Os01g0629900	99,89	0,00E+00	3261	-65	NA	Japonica	Kinase-TM
maker-1-augustus-gene-259.2-mRNA-1	1	OsR498G0101590100.01.T01	98,69	0,00E+00	2656	Os01g0630700	99,90	0,00E+00	1760	-896	Indica	Japonica	
maker-1-augustus-gene-259.20-mRNA-1	1	OsR498G0101592900.01.T01	99,94	0,00E+00	2858	Os01g0631100	100,00	0,00E+00	2863	5	NA	Japonica	
maker-1-augustus-gene-259.26-mRNA-1	1	OsR498G0101593100.01.T03	99,74	0,00E+00	2070	Os01g0631200	100,00	0,00E+00	1168	-902	Indica	Japonica	
maker-1-augustus-gene-259.29-mRNA-1	1	OsR498G0101593500.01.T01	99,69	0,00E+00	2927	Os01g0631400	100,00	0,00E+00	1786	-1141	Indica	Japonica	
maker-1-augustus-gene-259.3-mRNA-1	1	OsR498G0102324800.01.T02	90,71	1,07E-66	254	Os01g0630700	100,00	3,25E-93	340	86	Japonica	Japonica	
maker-1-augustus-gene-259.31-mRNA-1	1	OsR498G0101593900.01.T01	100,00	0,00E+00	1526	Os01g0631500	99,29	0,00E+00	1500	-26	NA	Japonica	
maker-1-augustus-gene-259.33-mRNA-1	1	OsR498G0101594500.01.T03	99,93	0,00E+00	2517	Os01g0631700	100,00	0,00E+00	2522	5	NA	Japonica	Kinase-TM
maker-1-augustus-gene-259.42-mRNA-1	1	OsR498G0101591800.01.T01	98,69	0,00E+00	1063	Os12g0539800	83,26	5,14E-132	471	-592	Indica	Japonica	NBS
maker-1-augustus-gene-26.30-mRNA-1	1	OsR498G0100174700.01.T01	99,89	0,00E+00	1583	Os01g0148400	99,66	0,00E+00	1578	-5	NA	Indica	
maker-1-augustus-gene-26.33-mRNA-1	1	OsR498G0100172400.01.T01	93,19	0,00E+00	1237	Os01g0147600	99,62	0,00E+00	931	-306	Indica	Japonica	
maker-1-augustus-gene-26.40-mRNA-1	1	OsR498G0100172800.01.T01	99,95	0,00E+00	3789	Os01g0147700	98,57	0,00E+00	3658	-131	NA	Indica	
maker-1-augustus-gene-26.48-mRNA-1	1	OsR498G0100173200.01.T01	99,72	0,00E+00	1296	Os01g0147900	99,72	0,00E+00	1296	0	NA	Indica	
maker-1-augustus-gene-26.55-mRNA-1	1	OsR498G0100174000.01.T04	96,04	0,00E+00	1419	Os01g0148100	96,52	0,00E+00	1680	261	Japonica	NA	
maker-1-augustus-gene-260.13-mRNA-1	1	OsR498G0101599100.01.T01	99,54	0,00E+00	1919	Os01g0633400	99,72	0,00E+00	1928	9	NA	NA	
maker-1-augustus-gene-260.17-mRNA-1	1	OsR498G0101599900.01.T01	99,55	0,00E+00	1996	Os01g0633500	99,59	0,00E+00	1736	-260	Indica	NA	
maker-1-augustus-gene-260.2-mRNA-1	1	OsR498G0101597500.01.T03	92,96	0,00E+00	897	Os01g0632700	92,34	0,00E+00	850	-47	NA	Indica	
maker-1-augustus-gene-260.20-mRNA-1	1	OsR498G0101598600.01.T01	100,00	0,00E+00	737	Os01g0633000	100,00	0,00E+00	737	0	NA	NA	
maker-1-augustus-gene-260.7-mRNA-1	1	OsR498G0101598700.01.T09	98,30	0,00E+00	2755	Os01g0633100	98,23	0,00E+00	2751	-4	NA	Indica	
maker-1-augustus-gene-261.42-mRNA-1	1	OsR498G0101602500.01.T02	100,00	0,00E+00	1131	Os01g0634900	99,87	0,00E+00	1397	266	Japonica	NA	
maker-1-augustus-gene-261.45-mRNA-1	1	OsR498G0101603500.01.T02	99,88	0,00E+00	1526	Os01g0635400	99,88	0,00E+00	1526	0	NA	Japonica	
maker-1-augustus-gene-261.51-mRNA-1	1	OsR498G0101600400.01.T03	99,66	0,00E+00	7931	Os01g0634300	99,68	0,00E+00	7855	-76	NA	Japonica	
maker-1-augustus-gene-261.55-mRNA-1	1	OsR498G0101601600.01.T01	99,76	0,00E+00	3029	Os01g0842500	71,12	0,00E+00	760	-2269	Indica	Japonica	
maker-1-augustus-gene-261.57-mRNA-1	1	OsR498G0101601800.01.T01	98,48	0,00E+00	1722	Os01g0634600	98,48	0,00E+00	1722	0	NA	Japonica	
maker-1-augustus-gene-262.0-mRNA-1	1	OsR498G0101607300.01.T03	100,00	0,00E+00	2917	Os01g0636500	100,00	0,00E+00	2917	0	NA	NA	
maker-1-augustus-gene-262.17-mRNA-1	1	OsR498G0101609600.01.T02	100,00	0,00E+00	1912	Os01g0637100	100,00	0,00E+00	2206	294	Japonica	NA	
maker-1-augustus-gene-262.4-mRNA-1	1	OsR498G0101606000.01.T01	100,00	0,00E+00	1829	Os02g0706900	71,25	3,71E-92	338	-1491	Indica	Japonica	
maker-1-augustus-gene-262.7-mRNA-1	1	OsR498G0101612800.01.T01	99,18	0,00E+00	1075	Os01g0637600	99,18	0,00E+00	1075	0	NA	Japonica	
maker-1-augustus-gene-263.0-mRNA-1	1	OsR498G0101613100.01.T01	99,85	0,00E+00	1235	Os01g0637800	100,00	0,00E+00	1240	5	NA	Japonica	
maker-1-augustus-gene-263.10-mRNA-1	1	OsR498G0101612800.01.T01	100,00	0,00E+00	1462	Os01g0637600	100,00	0,00E+00	1462	0	NA	Japonica	
maker-1-augustus-gene-263.14-mRNA-1	1	OsR498G0101616800.01.T01	98,42	0,00E+00	1982	Os01g0639200	100,00	0,00E+00	2057	75	NA	Japonica	
maker-1-augustus-gene-263.2-mRNA-1	1	OsR498G0101616500.01.T03	100,00	0,00E+00	1530	Os01g0639100	100,00	0,00E+00	1530	0	NA	Japonica	
maker-1-augustus-gene-264.35-mRNA-1	1	OsR498G0101621700.01.T03	99,64	0,00E+00	1468	Os01g0639900	100,00	0,00E+00	1526	58	NA	Japonica	
maker-1-augustus-gene-264.42-mRNA-1	1	OsR498G0101621900.01.T01	100,00	9,69E-136	482	Os01g0640000	100,00	6,13E-133	471	-11	NA	NA	
maker-1-augustus-gene-264.45-mRNA-1	1	OsR498G0101621700.01.T08	73,63	9,07E-58	224	Os01g0640132	100,00	0,00E+00	996	772	Japonica	NA	
maker-1-augustus-gene-264.47-mRNA-1	1	OsR498G0101624100.01.T02	99,93	0,00E+00	2701	Os01g0640600	99,60	0,00E+00	2691	-10	NA	NA	
maker-1-augustus-gene-264.49-mRNA-1	1	OsR498G0101624900.01.T01	100,00	0,00E+00	2105	Os01g0641000	100,00	0,00E+00	2105	0	NA	Japonica	Kinase-TM
maker-1-augustus-gene-264.56-mRNA-1	1	OsR498G0101623200.01.T01	100,00	0,00E+00	3061	Os01g0640200	100,00	0,00E+00	3061	0	NA	NA	
maker-1-augustus-gene-264.57-mRNA-1	1	OsR498G0101624600.01.T04	99,92	0,00E+00	2193	Os01g0640800	100,00	0,00E+00	2197	4	NA	Japonica	
maker-1-augustus-gene-265.34-mRNA-1	1	OsR498G0101627300.01.T07	99,89	0,00E+00	1581	Os01g0642000	100,00	0,00E+00	1395	-186	Indica	NA	
maker-1-augustus-gene-265.39-mRNA-1	1	OsR498G0101632900.01.T01	100,00	0,00E+00	3350	Os01g0643300	99,75	0,00E+00	2130	-1220	Indica	Japonica	
maker-1-augustus-gene-265.51-mRNA-1	1	OsR498G0101632000.01.T03	100,00	0,00E+00	1218	Os01g0642900	100,00	0,00E+00	1218	0	NA	NA	
maker-1-augustus-gene-266.0-mRNA-1	1	OsR498G0101637100.01.T01	100,00	0,00E+00	710	Os01g0644000	100,00	0,00E+00	710	0	NA	Japonica	
maker-1-augustus-gene-266.12-mRNA-1	1	OsR498G0101640600.01.T01	99,90	0,00E+00	1803	Os01g0645000	100,00	0,00E+00	1801	-2	NA	Japonica	
maker-1-augustus-gene-266.2-mRNA-1	1	OsR498G0101634400.01.T01	100,00	0,00E+00	1202	Os01g0643600	100,00	0,00E+00	1202	0	NA	Japonica	
maker-1-augustus-gene-267.0-mRNA-1	1	OsR498G0101641700.01.T02	96,67	0,00E+00	2117	Os01g0645200	96,67	0,00E+00	2117	0	NA	Japonica	
maker-1-augustus-gene-267.11-mRNA-1	1	OsR498G0101647000.01.T05	99,69	0,00E+00	2336	Os08g0112000	91,80	0,00E+00	2604	268	Japonica	NA	
maker-1-augustus-gene-267.9-mRNA-1	1	OsR498G0101647300.01.T01	100,00	0,00E+00	2268	Os01g0647000	100,00	0,00E+00	2268	0	NA	Japonica	
maker-1-augustus-gene-268.0-mRNA-1	1	OsR498G0101650500.01.T04	100,00	0,00E+00	2623	Os01g0647700	100,00	0,00E+00	2623	0	NA	Japonica	
maker-1-augustus-gene-268.12-mRNA-1	1	OsR498G0101651000.01.T01	97,78	0,00E+00	2862	Os01g0648000	98,02	0,00E+00	2880	18	NA	Japonica	
maker-1-augustus-gene-268.4-mRNA-1	1	OsR498G0101653700.01.T03	100,00	0,00E+00	796	Os05g0347000	91,08	2,82E-127	454	-342	Indica	NA	
maker-1-augustus-gene-268.7-mRNA-1	1	OsR498G0101647600.01.T01	99,74	0,00E+00	3461	Os01g0647100	100,00	0,00E+00	3479	18	NA	Japonica	
maker-1-augustus-gene-269.10-mRNA-1	1	OsR498G0101655500.01.T01	98,76	0,00E+00	2272	Os01g0649900	85,65	0,00E+00	1350	-922	Indica	Japonica	
maker-1-augustus-gene-269.16-mRNA-1	1	OsR498G0101655700.01.T01	99,45	0,00E+00	1607	Os01g0649400	100,00	0,00E+00	1662	55	NA	Japonica	
maker-1-augustus-gene-269.19-mRNA-1	1	OsR498G0101656100.01.T01	100,00	0,00E+00	2170	Os01g0649900	100,00	0,00E+00	2170	0	NA	Japonica	
maker-1-augustus-gene-269.22-mRNA-1	1	OsR498G0101656300.01.T02	100,00	0,00E+00	796	Os01g0650000	100,00	0,00E+00	958	162	Japonica	Japonica	
maker-1-augustus-gene-269.26-mRNA-1	1	OsR498G0101655500.01.T02	88,65	2,34E-100	365	Os01g0649200	89,34	3,47E-99	361	-4	NA	NA	
maker-1-augustus-gene-269.6-mRNA-1	1	OsR498G0101655100.01.T01	99,84	0,00E+00	1100	Os01g0649100	100,00	0,00E+00	1104	4	NA	Japonica	
maker-1-augustus-gene-27.39-mRNA-1	1	OsR498G0100175700.01.T02	99,65	0,00E+00	2058	Os01g0148700	99,48	0,00E+00	2063	5	NA	NA	
maker-1-augustus-gene-27.40-mRNA-1	1	OsR498G0100180100.01.T02	96,85	0,00E+00	1740	Os01g0150100	99,82	0,00E+00	992	-748	Indica	Indica	
maker-1-augustus-gene-27.45-mRNA-1	1	OsR498G0100180300.01.T03	96,24	0,00E+00	1150	Os01g0150200	95,81	0,00E+00	1137	-13	NA	Indica	
maker-1-augustus-gene-27.51-mRNA-1	1	OsR498G0100180500.01.T03	100,00	0,00E+00	682	Os01g0150500	86,24	1,71E-125	448	-234	Indica	NA	
maker-1-augustus-gene-27.57-mRNA-1	1	OsR498G0100180900.01.T01	85,39	0,00E+00	869	Os01g0150500	92,34	0,00E+00	1068	199	Japonica	NA	
maker-1-augustus-gene-27.62-mRNA-1	1	OsR498G0100178400.01.T01	99,94	0,00E+00	5689	Os01g0149700	99,39	0,00E+00	3800	-1889	Indica	Indica	Kinase-LRR-TM
maker-1-augustus-gene-27.65-mRNA-1	1	OsR498G0100176800.01.T01	98,50	3,16E-129	461	Os01g0149800	100,00	2,00E-126	450	-11	NA	Indica	
maker-1-augustus-gene-27.67-mRNA-1	1	OsR498G0100179000.01.T01	99,87	0,00E+00	4007	Os01g0149900	99,96	0,00E+00	4016	9	NA	Indica	
maker-1-augustus-gene-27.71-mRNA-1	1	OsR498G0100179800.01.T01	100,00	0,00E+00	1425	Os01g0150000	99,75	0,00E+00	1416	-9	NA	Indica	
maker-1-augustus-gene-270.0-mRNA-1	1	OsR498G0101660700.01.T01	74,74	0,00E+00	692	Os01g0650900	100,00	0,00E+00	2062	1370	Japonica	Japonica	
maker-1-augustus-gene-270.11-mRNA-1	1	OsR498G0101662900.01.T01	99,76	0,00E+00	2265	Os01g0651800	100,00	0,00E+00	2278	13	NA	Japonica	
maker-1-augustus-gene-270.21-mRNA-1	1	OsR498G0101664600.01.T01	100,00	0,00E+00	2127	Os01g0652100	100,00	0,00E+00	2127	0	NA	Japonica	
maker-1-augustus-gene-270.5-mRNA-1	1	OsR498G0101661100.01.T03	99,84	0,00E+00	2177	Os01g0651200	100,00	0,00E+00	2214	37	NA	Japonica	
maker-1-augustus-gene-270.8-mRNA-1	1	OsR498G0101661500.01.T02	100,00	1,63E-103	376	Os01g0651400	99,53	5,68E-104	377	1	NA	Japonica	
maker-1-augustus-gene-271.26-mRNA-1	1	OsR498G0101665700.01.T03	99,89	0,00E+00	3140	Os01g0652600	100,00	0,00E+00	2857	-283	NA	Japonica	
maker-1-augustus-gene-271.32-mRNA-1	1	OsR498G0101665200.01.T02	100,00	0,00E+00	2149	Os05g0574100	71,33	5,31E-167	588	-1561	Indica	Japonica	
maker-1-augustus-gene-271.34-mRNA-1	1	OsR498G0101666400.01.T01	99,92	0,00E+00	2187	Os01g0652800	100,00	0,00E+00	2192	5	NA	Japonica	
maker-1-augustus-gene-271.36-mRNA-1	1	OsR498G0101668200.01.T01	100,00	0,00E+00	2132	Os01g0653100	100,00	0,00E+00	2132	0	NA	Japonica	
maker-1-augustus-gene-271.40-mRNA-1	1	OsR498G0101671400.01.T03	100,00	0,00E+00	3741	Os01g0653800	100,00	0,00E+00	3741	0	NA	Japonica	
maker-1-augustus-gene-272.0-mRNA-1	1	OsR498G0101676300.01.T01	94,67	0,00E+00	2984	Os01g0654100	94,67	0,00E+00	2984	0	NA	NA	
maker-1-augustus-gene-272.15-mRNA-1	1	OsR498G0101676600.01.T04	100,00	0,00E+00	1838	Os01g0654200	100,00	0,00E+00	1797	-41	NA	NA	
maker-1-augustus-gene-272.20-mRNA-1	1	OsR498G0101677000.01.T01	100,00	0,00E+00	2235	Os01g0654500	100,00	0,00E+00	2235	0	NA	Japonica	
maker-1-augustus-gene-273.19-mRNA-1	1	OsR498G0101681300.01.T01	100,00	0,00E+00	3523	Os01g0655500	100,00	0,00E+00	3523	0	NA	Japonica	
maker-1-augustus-gene-273.25-mRNA-1	1	OsR498G0101682500.01.T08	98,06	0,00E+00	2950	Os01g0655800	98,06	0,00E+00	2950	0	NA	Japonica	
maker-1-augustus-gene-273.32-mRNA-1	1	OsR498G0101683700.01.T01	100,00	0,00E+00	2186	Os01g0656200	100,00	0,00E+00	2186	0	NA	NA	
maker-1-augustus-gene-274.0-mRNA-1	1	OsR498G0101687300.01.T02	99,93	0,00E+00	2420	Os01g0656900	100,00	0,00E+00	4210	1790	Japonica	Japonica	
maker-1-augustus-gene-274.3-mRNA-1	1	OsR498G0203372600.01.T01	74,82	1,35E-18	94,2	Os01g0657000	100,00	2,28E-105	379	284,8	Japonica	Japonica	
maker-1-augustus-gene-274.5-mRNA-1	1	OsR498G0101685000.01.T01	99,88	0,00E+00	1484	Os01g0656400	100,00	0,00E+00	1489	5	NA	NA	
maker-1-augustus-gene-274.9-mRNA-1	1	OsR498G0101687800.01.T01	99,94	0,00E+00	3004	Os01g0657100	100,00	0,00E+00	3009	5	NA	Japonica	
maker-1-augustus-gene-275.29-mRNA-1	1	OsR498G0101691100.01.T01	94,95	0,00E+00	1374	Os06g0330400	76,52	6,83E-88	324	-1050	Indica	Japonica	
maker-1-augustus-gene-275.31-mRNA-1	1	OsR498G0101691900.01.T02	99,71	0,00E+00	1242	Os01g0659200	100,00	0,00E+00	1251	9	NA	Japonica	
maker-1-augustus-gene-275.36-mRNA-1	1	OsR498G0101693900.01.T01	99,92	0,00E+00	2225	Os01g0659900	99,92	0,00E+00	2225	0	NA	Japonica	
maker-1-augustus-gene-275.39-mRNA-1	1	OsR498G0101690600.01.T03	99,78	0,00E+00	797	Os01g0658400	100,00	0,00E+00	801	4	NA	Japonica	
maker-1-augustus-gene-275.45-mRNA-1	1	OsR498G0101691400.01.T01	99,89	0,00E+00	1700	Os01g0658700	99,89	0,00E+00	1700	0	NA	Japonica	
maker-1-augustus-gene-275.49-mRNA-1	1	OsR498G0101691600.01.T01	99,72	0,00E+00	1940	Os01g0658900	99,91	0,00E+00	1949	9	NA	Japonica	
maker-1-augustus-gene-276.26-mRNA-1	1	OsR498G0101697900.01.T02	99,89	0,00E+00	1581	Os01g0660900	100,00	0,00E+00	1535	-46	NA	Japonica	
maker-1-augustus-gene-276.30-mRNA-1	1	OsR498G0101698100.01.T03	99,63	0,00E+00	1936	Os01g0661000	99,81	0,00E+00	1842	-94	NA	Japonica	
maker-1-augustus-gene-276.32-mRNA-1	1	OsR498G0101698300.01.T04	99,85	0,00E+00	1210	Os01g0661400	99,85	0,00E+00	1210	0	NA	Japonica	
maker-1-augustus-gene-276.37-mRNA-1	1	OsR498G0101698300.01.T05	97,63	0,00E+00	1011	Os01g0661500	100,00	0,00E+00	1018	7	NA	Japonica	
maker-1-augustus-gene-276.39-mRNA-1	1	OsR498G0101695000.01.T01	97,01	0,00E+00	3050	Os10g0571200	73,55	0,00E+00	797	-2253	Indica	Japonica	
maker-1-augustus-gene-277.46-mRNA-1	1	OsR498G0101703900.01.T01	99,73	0,00E+00	2633	Os01g0663300	99,90	0,00E+00	1713	-920	Indica	Japonica	
maker-1-augustus-gene-277.50-mRNA-1	1	OsR498G0101704500.01.T04	100,00	0,00E+00	681	Os01g0663500	98,22	0,00E+00	680	-1	NA	Japonica	
maker-1-augustus-gene-277.56-mRNA-1	1	OsR498G0101705300.01.T02	99,74	0,00E+00	1370	Os01g0664000	99,61	0,00E+00	1365	-5	NA	Japonica	
maker-1-augustus-gene-277.65-mRNA-1	1	OsR498G0101701400.01.T01	99,61	0,00E+00	922	Os01g0662600	100,00	0,00E+00	931	9	NA	Japonica	
maker-1-augustus-gene-277.67-mRNA-1	1	OsR498G0101701600.01.T01	99,70	0,00E+00	1783	Os01g0662700	99,90	0,00E+00	1792	9	NA	Japonica	
maker-1-augustus-gene-277.71-mRNA-1	1	OsR498G0101701800.01.T01	99,77	0,00E+00	781	Os01g0662800	99,73	0,00E+00	1306	525	Japonica	Japonica	
maker-1-augustus-gene-277.75-mRNA-1	1	OsR498G0101704800.01.T03	99,79	0,00E+00	2499	Os01g0663800	99,79	0,00E+00	2499	0	NA	Japonica	
maker-1-augustus-gene-277.80-mRNA-1	1	OsR498G0101706400.01.T01	99,78	0,00E+00	2490	Os01g0664200	99,86	0,00E+00	2495	5	NA	Japonica	Kinase-TM
maker-1-augustus-gene-278.36-mRNA-1	1	OsR498G0101707900.01.T05	99,77	0,00E+00	770	Os01g0664500	99,77	0,00E+00	770	0	NA	NA	
maker-1-augustus-gene-278.40-mRNA-1	1	OsR498G0101709700.01.T01	99,94	0,00E+00	3194	Os01g0665200	99,89	0,00E+00	3189	-5	NA	Japonica	Kinase-TM
maker-1-augustus-gene-278.45-mRNA-1	1	OsR498G0101710100.01.T02	99,70	0,00E+00	1807	Os01g0665400	99,80	0,00E+00	1811	4	NA	Japonica	
maker-1-augustus-gene-278.50-mRNA-1	1	OsR498G0101710000.01.T02	99,55	0,00E+00	2354	Os01g0665300	99,85	0,00E+00	2372	18	NA	Japonica	
maker-1-augustus-gene-278.55-mRNA-1	1	OsR498G0101711000.01.T01	99,74	0,00E+00	2076	Os01g0665500	99,71	0,00E+00	3131	1055	Japonica	Japonica	
maker-1-augustus-gene-278.58-mRNA-1	1	OsR498G0101714600.01.T02	99,78	0,00E+00	1593	Os01g0666000	100,00	0,00E+00	1602	9	NA	Japonica	
maker-1-augustus-gene-279.30-mRNA-1	1	OsR498G0101715500.01.T01	99,47	0,00E+00	1004	Os01g0666600	100,00	0,00E+00	1018	14	NA	Japonica	
maker-1-augustus-gene-279.37-mRNA-1	1	OsR498G0101720100.01.T01	99,85	0,00E+00	1214	Os01g0667600	100,00	0,00E+00	1218	4	NA	Japonica	
maker-1-augustus-gene-279.39-mRNA-1	1	OsR498G0101715200.01.T01	95,44	0,00E+00	2003	Os01g0666400	95,84	0,00E+00	2025	22	NA	Japonica	
maker-1-augustus-gene-279.41-mRNA-1	1	OsR498G0101715400.01.T02	99,65	0,00E+00	1545	Os01g0666500	100,00	0,00E+00	1559	14	NA	Japonica	
maker-1-augustus-gene-279.43-mRNA-1	1	OsR498G0101717400.01.T01	99,81	0,00E+00	965	Os01g0666900	100,00	0,00E+00	969	4	NA	Japonica	
maker-1-augustus-gene-279.46-mRNA-1	1	OsR498G0101718000.01.T01	99,53	0,00E+00	1145	Os01g0667100	100,00	0,00E+00	1007	-138	Indica	Japonica	
maker-1-augustus-gene-279.48-mRNA-1	1	OsR498G0101718200.01.T01	94,25	0,00E+00	1463	Os01g0667200	94,25	0,00E+00	1463	0	NA	Japonica	
maker-1-augustus-gene-279.55-mRNA-1	1	OsR498G0101720400.01.T03	99,76	0,00E+00	1471	Os01g0667700	100,00	0,00E+00	1480	9	NA	Japonica	
maker-1-augustus-gene-28.11-mRNA-1	1	OsR498G0100183300.01.T03	99,93	0,00E+00	2490	Os01g0151200	99,71	0,00E+00	2475	-15	NA	Indica	
maker-1-augustus-gene-28.15-mRNA-1	1	OsR498G0100183500.01.T01	99,53	0,00E+00	2667	Os01g0151400	97,86	0,00E+00	2246	-421	Indica	Japonica	
maker-1-augustus-gene-28.17-mRNA-1	1	OsR498G0100183500.01.T01	92,18	0,00E+00	2019	Os01g0151400	90,74	0,00E+00	1826	-193	NA	NA	
maker-1-augustus-gene-28.19-mRNA-1	1	OsR498G0100183900.01.T01	99,68	0,00E+00	1657	Os01g0151600	99,89	0,00E+00	1666	9	NA	NA	
maker-1-augustus-gene-28.25-mRNA-1	1	OsR498G0100184500.01.T02	95,45	0,00E+00	1653	Os01g0151700	95,35	0,00E+00	1648	-5	NA	Indica	
maker-1-augustus-gene-28.3-mRNA-1	1	OsR498G0100182100.01.T03	99,48	0,00E+00	679	Os01g0150800	91,32	3,48E-113	407	-272	Indica	NA	
maker-1-augustus-gene-28.30-mRNA-1	1	OsR498G0100185300.01.T01	96,87	0,00E+00	5315	Os01g0152000	99,97	0,00E+00	5763	448	NA	Japonica	Kinase-LRR-TM
maker-1-augustus-gene-280.33-mRNA-1	1	OsR498G0101723500.01.T01	99,79	0,00E+00	856	Os01g0668000	100,00	0,00E+00	861	5	NA	Japonica	
maker-1-augustus-gene-281.1-mRNA-1	1	OsR498G0101731800.01.T01	98,07	0,00E+00	3736	Os01g0670800	99,45	0,00E+00	3865	129	NA	Japonica	
maker-1-augustus-gene-282.23-mRNA-1	1	OsR498G0101736100.01.T02	99,46	0,00E+00	9927	Os05g0562400	75,11	0,00E+00	2819	-7108	Indica	Japonica	
maker-1-augustus-gene-282.35-mRNA-1	1	OsR498G0101736500.01.T01	99,73	0,00E+00	1328	Os01g0672400	99,87	0,00E+00	1333	5	NA	Japonica	
maker-1-augustus-gene-283.34-mRNA-1	1	OsR498G0101737900.01.T01	98,10	0,00E+00	3275	Os01g0672700	98,27	0,00E+00	3021	-254	NA	Japonica	
maker-1-augustus-gene-283.40-mRNA-1	1	OsR498G0101738400.01.T01	99,75	0,00E+00	2910	Os01g0672800	100,00	0,00E+00	2327	-583	Indica	Japonica	
maker-1-augustus-gene-283.48-mRNA-1	1	OsR498G0101740200.01.T01	99,78	0,00E+00	829	Os01g0673600	100,00	0,00E+00	834	5	NA	Japonica	
maker-1-augustus-gene-283.52-mRNA-1	1	OsR498G0101741200.01.T06	99,73	0,00E+00	1962	Os01g0673800	98,73	0,00E+00	1660	-302	Indica	Japonica	
maker-1-augustus-gene-284.46-mRNA-1	1	OsR498G0101748500.01.T01	99,90	0,00E+00	1728	Os01g0675000	100,00	0,00E+00	1732	4	NA	Japonica	
maker-1-augustus-gene-284.48-mRNA-1	1	OsR498G0101750900.01.T03	99,58	0,00E+00	1282	Os01g0675700	100,00	0,00E+00	1292	10	NA	Japonica	
maker-1-augustus-gene-284.51-mRNA-1	1	OsR498G0101748800.01.T01	100,00	0,00E+00	883	Os01g0675100	100,00	0,00E+00	883	0	NA	Japonica	
maker-1-augustus-gene-284.54-mRNA-1	1	OsR498G0101749600.01.T01	99,85	0,00E+00	2410	Os01g0675500	100,00	0,00E+00	2419	9	NA	Japonica	
maker-1-augustus-gene-285.0-mRNA-1	1	OsR498G0101755300.01.T02	99,59	2,00E-122	440	Os01g0676900	100,00	0,00E+00	700	260	Japonica	Japonica	
maker-1-augustus-gene-285.4-mRNA-1	1	OsR498G0101755700.01.T01	99,68	0,00E+00	3355	Os01g0677000	100,00	0,00E+00	3382	27	NA	Japonica	
maker-1-augustus-gene-286.30-mRNA-1	1	OsR498G0101760500.01.T01	99,83	0,00E+00	1062	Os01g0678600	100,00	0,00E+00	1067	5	NA	Japonica	
maker-1-augustus-gene-286.32-mRNA-1	1	OsR498G0101761900.01.T03	98,42	0,00E+00	2527	Os01g0679000	98,75	0,00E+00	2530	3	NA	Japonica	
maker-1-augustus-gene-286.36-mRNA-1	1	OsR498G0101763500.01.T01	99,70	4,84E-169	595	Os01g0679600	100,00	3,96E-171	600	5	NA	Japonica	
maker-1-augustus-gene-286.38-mRNA-1	1	OsR498G0101764500.01.T01	99,67	0,00E+00	3274	Os01g0679900	100,00	0,00E+00	3301	27	NA	Japonica	
maker-1-augustus-gene-286.46-mRNA-1	1	OsR498G0101760800.01.T01	98,77	0,00E+00	1572	Os01g0678700	99,66	0,00E+00	1587	15	NA	Japonica	
maker-1-augustus-gene-286.50-mRNA-1	1	OsR498G0101761000.01.T01	98,40	0,00E+00	760	Os01g0678800	100,00	0,00E+00	791	31	NA	Japonica	
maker-1-augustus-gene-286.52-mRNA-1	1	OsR498G0101763800.01.T01	100,00	3,93E-141	500	Os01g0679700	100,00	1,37E-141	500	0	NA	Japonica	
maker-1-augustus-gene-287.31-mRNA-1	1	OsR498G0101767300.01.T01	96,54	0,00E+00	1058	Os01g0680400	97,17	0,00E+00	1078	20	NA	Japonica	
maker-1-augustus-gene-287.34-mRNA-1	1	OsR498G0101770300.01.T01	99,31	0,00E+00	2820	Os05g0556100	83,88	0,00E+00	1971	-849	Indica	Japonica	
maker-1-augustus-gene-287.38-mRNA-1	1	OsR498G0101771700.01.T01	99,85	0,00E+00	2399	Os01g0681600	100,00	0,00E+00	2408	9	NA	Japonica	
maker-1-augustus-gene-287.40-mRNA-1	1	OsR498G0101768200.01.T01	99,74	0,00E+00	2070	Os01g0680700	100,00	0,00E+00	2084	14	NA	Japonica	
maker-1-augustus-gene-287.45-mRNA-1	1	OsR498G0101769600.01.T01	99,89	0,00E+00	1702	Os01g0680900	99,51	0,00E+00	1093	-609	Indica	Japonica	
maker-1-augustus-gene-287.48-mRNA-1	1	OsR498G0101770000.01.T01	99,81	0,00E+00	2865	Os01g0681000	100,00	0,00E+00	2879	14	NA	Japonica	
maker-1-augustus-gene-287.51-mRNA-1	1	OsR498G0101770600.01.T03	99,92	0,00E+00	2140	Os01g0681200	100,00	0,00E+00	2145	5	NA	Japonica	
maker-1-augustus-gene-287.57-mRNA-1	1	OsR498G0101771000.01.T03	99,80	0,00E+00	2730	Os01g0681400	98,82	0,00E+00	2674	-56	NA	Japonica	
maker-1-augustus-gene-287.60-mRNA-1	1	OsR498G0101772400.01.T03	99,21	0,00E+00	8731	Os01g0681900	99,37	0,00E+00	8767	36	NA	Japonica	
maker-1-augustus-gene-288.28-mRNA-1	1	OsR498G0101775100.01.T01	98,25	0,00E+00	2183	Os01g0682500	98,33	0,00E+00	2187	4	NA	Japonica	
maker-1-augustus-gene-288.33-mRNA-1	1	OsR498G0101775900.01.T01	99,87	0,00E+00	2805	Os01g0683100	100,00	0,00E+00	2814	9	NA	Japonica	
maker-1-augustus-gene-288.35-mRNA-1	1	OsR498G0101778300.01.T06	99,93	0,00E+00	2541	Os01g0683400	100,00	0,00E+00	2549	8	NA	Japonica	
maker-1-augustus-gene-288.47-mRNA-1	1	OsR498G0101778900.01.T02	98,44	0,00E+00	1330	Os01g0683700	99,47	0,00E+00	1351	21	NA	Japonica	
maker-1-augustus-gene-288.49-mRNA-1	1	OsR498G0101779900.01.T08	95,76	0,00E+00	2016	Os01g0684200	96,25	0,00E+00	2043	27	NA	Japonica	
maker-1-augustus-gene-288.59-mRNA-1	1	OsR498G0101778600.01.T03	99,69	0,00E+00	1155	Os05g0554400	82,61	0,00E+00	634	-521	Indica	Japonica	
maker-1-augustus-gene-289.0-mRNA-1	1	OsR498G0101781300.01.T02	99,09	0,00E+00	1360	Os01g0684200	96,63	0,00E+00	1129	-231	Indica	Japonica	
maker-1-augustus-gene-289.10-mRNA-1	1	OsR498G0101784700.01.T01	99,88	0,00E+00	3011	Os01g0685800	100,00	0,00E+00	3020	9	NA	Japonica	
maker-1-augustus-gene-289.16-mRNA-1	1	OsR498G0101784900.01.T01	96,76	0,00E+00	3461	Os01g0685900	96,81	0,00E+00	3465	4	NA	Japonica	
maker-1-augustus-gene-289.20-mRNA-1	1	OsR498G0101781600.01.T03	99,56	0,00E+00	1624	Os01g0684900	99,67	0,00E+00	1630	6	NA	Japonica	
maker-1-augustus-gene-289.8-mRNA-1	1	OsR498G0101782700.01.T01	99,79	0,00E+00	1718	Os01g0685400	100,00	0,00E+00	1254	-464	Indica	Japonica	
maker-1-augustus-gene-29.59-mRNA-1	1	OsR498G0100187100.01.T01	97,69	0,00E+00	5079	Os01g0152600	99,59	0,00E+00	6521	1442	Japonica	Japonica	Kinase-LRR-TM
maker-1-augustus-gene-29.60-mRNA-1	1	OsR498G0100189500.01.T01	95,05	0,00E+00	4026	Os01g0152800	99,94	0,00E+00	5683	1657	Japonica	Japonica	Kinase-LRR-TM
maker-1-augustus-gene-29.61-mRNA-1	1	OsR498G0100190500.01.T02	99,66	0,00E+00	4228	Os01g0153000	99,70	0,00E+00	4232	4	NA	NA	Kinase-LRR-TM
maker-1-augustus-gene-29.83-mRNA-1	1	OsR498G0100192100.01.T01	98,81	0,00E+00	1318	Os01g0153500	89,22	0,00E+00	971	-347	Indica	Japonica	
maker-1-augustus-gene-29.88-mRNA-1	1	OsR498G0100194300.01.T01	100,00	0,00E+00	1294	Os01g0154500	99,58	0,00E+00	1280	-14	NA	Indica	
maker-1-augustus-gene-29.90-mRNA-1	1	OsR498G0100194700.01.T02	100,00	0,00E+00	1310	Os01g0154600	99,45	0,00E+00	1292	-18	NA	Indica	
maker-1-augustus-gene-290.11-mRNA-1	1	OsR498G0101790100.01.T05	99,88	0,00E+00	1439	Os01g0687500	100,00	0,00E+00	1443	4	NA	Japonica	
maker-1-augustus-gene-290.16-mRNA-1	1	OsR498G0101788000.01.T01	93,59	0,00E+00	1168	Os01g0686700	100,00	0,00E+00	1002	-166	Indica	Japonica	
maker-1-augustus-gene-290.19-mRNA-1	1	OsR498G0101790400.01.T02	95,94	0,00E+00	1780	Os01g0687600	97,49	0,00E+00	1841	61	NA	Japonica	
maker-1-augustus-gene-290.21-mRNA-1	1	OsR498G0101790400.01.T04	99,76	0,00E+00	1501	Os01g0687700	97,86	0,00E+00	1690	189	Japonica	Japonica	
maker-1-augustus-gene-290.26-mRNA-1	1	OsR498G0101791000.01.T01	99,72	0,00E+00	3186	Os01g0687800	100,00	0,00E+00	3209	23	NA	Japonica	
maker-1-augustus-gene-290.28-mRNA-1	1	OsR498G0101791800.01.T02	100,00	0,00E+00	1597	Os01g0688200	100,00	0,00E+00	1597	0	NA	Japonica	
maker-1-augustus-gene-290.5-mRNA-1	1	OsR498G0101788300.01.T02	99,70	0,00E+00	1800	Os01g0686800	100,00	0,00E+00	1813	13	NA	Japonica	
maker-1-augustus-gene-290.7-mRNA-1	1	OsR498G0101789300.01.T05	99,74	0,00E+00	4112	Os01g0687300	100,00	0,00E+00	4140	28	NA	Japonica	
maker-1-augustus-gene-291.11-mRNA-1	1	OsR498G0101794400.01.T01	98,04	0,00E+00	1407	Os01g0688600	100,00	0,00E+00	1126	-281	Indica	Japonica	
maker-1-augustus-gene-291.13-mRNA-1	1	OsR498G0101795400.01.T02	99,70	0,00E+00	2365	Os05g0460000	74,57	0,00E+00	797	-1568	Indica	Japonica	
maker-1-augustus-gene-291.15-mRNA-1	1	OsR498G0101796800.01.T03	99,94	0,00E+00	5532	Os05g0551000	76,21	0,00E+00	1361	-4171	Indica	Japonica	
maker-1-augustus-gene-291.20-mRNA-1	1	OsR498G0101798600.01.T05	99,54	0,00E+00	782	Os01g0689600	99,75	0,00E+00	718	-64	NA	Japonica	
maker-1-augustus-gene-291.26-mRNA-1	1	OsR498G0101799400.01.T01	99,62	0,00E+00	3741	Os01g0689900	100,00	0,00E+00	2201	-1540	Indica	Japonica	Kinase-TM
maker-1-augustus-gene-291.4-mRNA-1	1	OsR498G0101795900.01.T01	99,73	0,00E+00	1323	Os01g0689000	100,00	0,00E+00	1332	9	NA	Japonica	
maker-1-augustus-gene-291.6-mRNA-1	1	OsR498G0101799100.01.T01	100,00	0,00E+00	1975	Os01g0689800	100,00	0,00E+00	1975	0	NA	Japonica	
maker-1-augustus-gene-292.1-mRNA-1	1	OsR498G0101800500.01.T01	98,71	0,00E+00	1484	Os01g0690800	100,00	0,00E+00	1534	50	NA	Japonica	Kinase-TM

maker-1-augustus-gene-292.17-mRNA-1	1	OsR498G0101802300.01.T01	99,88	0,00E+00	1495	Os01g0691700	100,00	0,00E+00	1499	4	NA	Japonica	
maker-1-augustus-gene-292.22-mRNA-1	1	OsR498G0101803900.01.T01	99,74	0,00E+00	3403	Os01g0692300	93,95	0,00E+00	2892	-511	Indica	Japonica	
maker-1-augustus-gene-292.25-mRNA-1	1	OsR498G0101804100.01.T01	99,73	0,00E+00	3305	Os01g0692600	100,00	0,00E+00	3328	23	NA	Japonica	
maker-1-augustus-gene-292.29-mRNA-1	1	OsR498G0101801800.01.T01	95,75	0,00E+00	1974	Os01g0691500	100,00	0,00E+00	1469	-505	Indica	Japonica	
maker-1-augustus-gene-292.40-mRNA-1	1	OsR498G0101803400.01.T01	99,58	0,00E+00	1275	Os01g0692100	100,00	0,00E+00	1288	13	NA	Japonica	
maker-1-augustus-gene-292.42-mRNA-1	1	OsR498G0101804400.01.T01	99,80	0,00E+00	1828	Os01g0692700	100,00	0,00E+00	1025	-803	Indica	Japonica	
maker-1-augustus-gene-292.7-mRNA-1	1	OsR498G0101801500.01.T01	94,83	0,00E+00	1122	Os01g0691400	100,00	0,00E+00	922	-200	Indica	Japonica	
maker-1-augustus-gene-293.0-mRNA-1	1	OsR498G0101806500.01.T03	100,00	0,00E+00	1025	Os01g0693300	100,00	0,00E+00	1025	0	NA	Japonica	
maker-1-augustus-gene-293.15-mRNA-1	1	OsR498G0101815200.01.T03	99,64	0,00E+00	1471	Os01g0694200	100,00	0,00E+00	1485	14	NA	Japonica	
maker-1-augustus-gene-293.8-mRNA-1	1	OsR498G0101813400.01.T03	99,87	0,00E+00	1354	Os01g0693900	100,00	0,00E+00	1359	5	NA	Japonica	
maker-1-augustus-gene-294.0-mRNA-1	1	OsR498G0101817300.01.T05	99,63	0,00E+00	1444	Os01g0694900	100,00	0,00E+00	1447	3	NA	Japonica	
maker-1-augustus-gene-294.10-mRNA-1	1	OsR498G0101818100.01.T01	100,00	0,00E+00	2127	Os01g0695200	100,00	0,00E+00	1902	-225	Indica	Japonica	
maker-1-augustus-gene-294.16-mRNA-1	1	OsR498G0101819100.01.T06	99,89	0,00E+00	3166	Os01g0695700	100,00	0,00E+00	3175	9	NA	Japonica	
maker-1-augustus-gene-294.22-mRNA-1	1	OsR498G0101820100.01.T01	99,88	0,00E+00	1489	Os01g0695900	100,00	0,00E+00	1494	5	NA	Japonica	
maker-1-augustus-gene-294.27-mRNA-1	1	OsR498G0101818600.01.T03	98,04	0,00E+00	1839	Os01g0695300	100,00	0,00E+00	1622	-217	Indica	Japonica	
maker-1-augustus-gene-294.48-mRNA-1	1	OsR498G0101820800.01.T02	99,67	0,00E+00	2179	Os01g0696100	100,00	0,00E+00	2084	-95	NA	Japonica	
maker-1-augustus-gene-294.5-mRNA-1	1	OsR498G0101817900.01.T01	99,93	0,00E+00	2649	Os01g0695100	100,00	0,00E+00	2654	5	NA	Japonica	
maker-1-augustus-gene-295.0-mRNA-1	1	OsR498G0101822500.01.T01	99,77	0,00E+00	6858	Os01g0695700	71,99	0,00E+00	2010	-4848	Indica	Japonica	
maker-1-augustus-gene-295.5-mRNA-1	1	OsR498G0101826700.01.T03	99,85	0,00E+00	4733	Os01g0698100	100,00	0,00E+00	4751	18	NA	Japonica	
maker-1-augustus-gene-296.0-mRNA-1	1	OsR498G0101828700.01.T01	99,27	0,00E+00	1445	Os01g0698900	100,00	0,00E+00	1319	-126	NA	Japonica	
maker-1-augustus-gene-296.11-mRNA-1	1	OsR498G0101835300.01.T04	99,95	0,00E+00	3349	Os01g0700200	100,00	0,00E+00	3353	4	NA	Japonica	
maker-1-augustus-gene-296.16-mRNA-1	1	OsR498G0101835700.01.T01	88,86	0,00E+00	1916	Os01g0700500	100,00	0,00E+00	2219	303	Japonica	Japonica	
maker-1-augustus-gene-296.18-mRNA-1	1	OsR498G0101828000.01.T01	98,90	0,00E+00	958	Os01g0698800	100,00	0,00E+00	829	-129	Indica	Japonica	
maker-1-augustus-gene-296.20-mRNA-1	1	OsR498G0101831600.01.T01	99,74	0,00E+00	2099	Os01g0699500	100,00	0,00E+00	2113	14	NA	Japonica	Kinase
maker-1-augustus-gene-296.21-mRNA-1	1	OsR498G0101835600.01.T01	99,56	0,00E+00	2029	Os01g0701700	83,25	0,00E+00	1179	-850	Indica	Japonica	
maker-1-augustus-gene-296.4-mRNA-1	1	OsR498G0101833700.01.T01	100,00	0,00E+00	850	Os01g0699900	100,00	0,00E+00	850	0	NA	Japonica	
maker-1-augustus-gene-296.6-mRNA-1	1	OsR498G0101834700.01.T02	99,62	0,00E+00	1399	Os01g0700000	100,00	0,00E+00	1413	14	NA	Japonica	
maker-1-augustus-gene-296.8-mRNA-1	1	OsR498G0101835100.01.T01	99,86	0,00E+00	1246	Os01g0700100	100,00	0,00E+00	1251	5	NA	Japonica	
maker-1-augustus-gene-297.0-mRNA-1	1	OsR498G0101836900.01.T01	86,17	0,00E+00	1401	Os01g0701300	100,00	0,00E+00	2221	820	Japonica	NA	
maker-1-augustus-gene-297.1-mRNA-1	1	OsR498G0101836700.01.T01	89,93	5,87E-160	563	Os01g0701300	100,00	0,00E+00	699	136	Japonica	Japonica	
maker-1-augustus-gene-297.13-mRNA-1	1	OsR498G0101838600.01.T03	99,80	0,00E+00	1788	Os01g0702000	100,00	0,00E+00	1797	9	NA	Japonica	
maker-1-augustus-gene-297.4-mRNA-1	1	OsR498G0101836900.01.T01	98,83	0,00E+00	2831	Os01g0701500	100,00	0,00E+00	2067	-764	Indica	Japonica	
maker-1-augustus-gene-297.8-mRNA-1	1	OsR498G0101838400.01.T03	99,58	0,00E+00	3360	Os01g0701900	100,00	0,00E+00	2457	-903	Indica	Japonica	
maker-1-augustus-gene-298.0-mRNA-1	1	OsR498G0101839700.01.T01	97,67	0,00E+00	3787	Os01g0702300	100,00	0,00E+00	3754	-33	NA	Japonica	
maker-1-augustus-gene-298.14-mRNA-1	1	OsR498G0101840500.01.T01	98,85	0,00E+00	1215	Os01g0702500	100,00	0,00E+00	1240	25	NA	Japonica	
maker-1-augustus-gene-298.18-mRNA-1	1	OsR498G0101844100.01.T01	99,81	0,00E+00	1907	Os01g0703400	100,00	0,00E+00	1916	9	NA	Japonica	
maker-1-augustus-gene-298.23-mRNA-1	1	OsR498G0101844300.01.T01	99,77	0,00E+00	2314	Os01g0703600	100,00	0,00E+00	2327	13	NA	Japonica	
maker-1-augustus-gene-298.3-mRNA-1	1	OsR498G0101839900.01.T01	99,61	0,00E+00	2756	Os01g0702400	99,87	0,00E+00	2774	18	NA	Japonica	
maker-1-augustus-gene-298.35-mRNA-1	1	OsR498G0101841800.01.T01	99,56	0,00E+00	1620	Os01g0702700	99,67	0,00E+00	1625	5	NA	Japonica	
maker-1-augustus-gene-298.38-mRNA-1	1	OsR498G0101844800.01.T01	99,67	0,00E+00	1095	Os01g0704000	100,00	0,00E+00	1104	9	NA	Japonica	
maker-1-augustus-gene-298.9-mRNA-1	1	OsR498G0101840300.01.T01	99,80	0,00E+00	1815	Os01g0702450	99,84	0,00E+00	1143	-672	Indica	Japonica	
maker-1-augustus-gene-299.16-mRNA-1	1	OsR498G0101849200.01.T01	99,85	0,00E+00	1165	Os01g0705200	100,00	0,00E+00	1169	4	NA	Japonica	
maker-1-augustus-gene-299.18-mRNA-1	1	OsR498G0101850000.01.T01	99,10	1,63E-167	590	Os01g0705300	100,00	2,57E-172	603	13	NA	Japonica	
maker-1-augustus-gene-299.20-mRNA-1	1	OsR498G0101854200.01.T01	99,64	0,00E+00	993	Os01g0706000	100,00	0,00E+00	1002	9	NA	Japonica	
maker-1-augustus-gene-299.6-mRNA-1	1	OsR498G0101854900.01.T01	99,74	0,00E+00	2092	Os01g0706100	100,00	0,00E+00	2309	217	NA	Japonica	
maker-1-augustus-gene-3.15-mRNA-1	1	OsR498G0100015500.01.T03	96,93	0,00E+00	1982	Os01g0104100	96,81	0,00E+00	1901	-81	NA	Japonica	
maker-1-augustus-gene-3.17-mRNA-1	1	OsR498G0100015900.01.T01	97,46	0,00E+00	1741	Os01g0104200	100,00	0,00E+00	1012	-729	Indica	Japonica	
maker-1-augustus-gene-3.21-mRNA-1	1	OsR498G0100017900.01.T04	99,89	0,00E+00	1663	Os01g0104800	100,00	0,00E+00	1667	4	NA	Japonica	
maker-1-augustus-gene-3.26-mRNA-1	1	OsR498G0100015400.01.T01	99,70	0,00E+00	2984	Os01g0104000	100,00	0,00E+00	3009	25	NA	Japonica	Kinase-TM
maker-1-augustus-gene-3.29-mRNA-1	1	OsR498G0100017400.01.T04	100,00	0,00E+00	1326	Os01g0104600	100,00	0,00E+00	1326	0	NA	Japonica	
maker-1-augustus-gene-3.33-mRNA-1	1	OsR498G0100018400.01.T01	94,13	0,00E+00	2193	Os01g0104900	100,00	0,00E+00	2538	345	Japonica	Japonica	
maker-1-augustus-gene-3.36-mRNA-1	1	OsR498G0100018800.01.T01	98,95	0,00E+00	833	Os01g0104900	98,14	0,00E+00	738	-95	Indica	Japonica	
maker-1-augustus-gene-3.4-mRNA-1	1	OsR498G0100014700.01.T02	99,94	0,00E+00	2755	Os01g0103800	100,00	0,00E+00	2760	5	NA	Japonica	
maker-1-augustus-gene-3.8-mRNA-1	1	OsR498G0100015100.01.T01	96,69	0,00E+00	2684	Os01g0103900	95,16	0,00E+00	1707	-977	Indica	Japonica	
maker-1-augustus-gene-30.0-mRNA-1	1	OsR498G0100198300.01.T01	100,00	0,00E+00	742	Os01g0155800	95,54	2,29E-173	608	-134	Indica	Indica	
maker-1-augustus-gene-30.13-mRNA-1	1	OsR498G0100197800.01.T04	100,00	0,00E+00	778	Os01g0155400	100,00	0,00E+00	778	0	NA	Indica	
maker-1-augustus-gene-30.27-mRNA-1	1	OsR498G0509618800.01.T01	78,16	2,61E-118	426	Os01g0155600	99,80	0,00E+00	1750	1324	Japonica	Indica	
maker-1-augustus-gene-30.29-mRNA-1	1	OsR498G0100198600.01.T01	96,10	0,00E+00	1657	Os01g0156000	92,92	0,00E+00	1457	-200	Indica	Indica	
maker-1-augustus-gene-30.31-mRNA-1	1	OsR498G0100205600.01.T02	100,00	0,00E+00	1326	Os01g0158400	98,64	0,00E+00	1281	-45	NA	Indica	
maker-1-augustus-gene-30.4-mRNA-1	1	OsR498G0100204100.01.T01	100,00	0,00E+00	2863	Os01g0158000	99,69	0,00E+00	2840	-23	NA	Indica	
maker-1-augustus-gene-30.8-mRNA-1	1	OsR498G0100204500.01.T01	99,91	0,00E+00	2077	Os01g0158200	97,75	0,00E+00	1968	-109	NA	Indica	
maker-1-augustus-gene-300.0-mRNA-1	1	OsR498G0101856100.01.T01	99,77	0,00E+00	4691	Os01g0706400	100,00	0,00E+00	4715	24	NA	Japonica	
maker-1-augustus-gene-300.11-mRNA-1	1	OsR498G0101856600.01.T04	99,07	0,00E+00	2079	Os01g0706700	99,24	0,00E+00	2088	9	NA	Japonica	
maker-1-augustus-gene-300.17-mRNA-1	1	OsR498G0101857000.01.T01	99,84	0,00E+00	1150	Os01g0706800	100,00	0,00E+00	1104	-46	NA	Japonica	
maker-1-augustus-gene-300.19-mRNA-1	1	OsR498G0101857400.01.T01	99,71	0,00E+00	2455	Os01g0706900	100,00	0,00E+00	2473	18	NA	Japonica	
maker-1-augustus-gene-300.4-mRNA-1	1	OsR498G0101856300.01.T02	99,32	0,00E+00	777	Os01g0706500	100,00	0,00E+00	791	14	NA	Japonica	
maker-1-augustus-gene-300.7-mRNA-1	1	OsR498G0101858100.01.T01	100,00	0,00E+00	1202	Os01g0707300	100,00	0,00E+00	1202	0	NA	Japonica	
maker-1-augustus-gene-301.0-mRNA-1	1	OsR498G0101861700.01.T02	99,89	0,00E+00	1657	Os01g0708000	99,35	0,00E+00	1636	-21	NA	Japonica	
maker-1-augustus-gene-301.12-mRNA-1	1	OsR498G0101862300.01.T01	98,27	0,00E+00	2507	Os05g0593800	75,00	0,00E+00	881	-1626	Indica	Japonica	Kinase
maker-1-augustus-gene-301.21-mRNA-1	1	OsR498G0101864100.01.T01	99,80	0,00E+00	1820	Os01g0708900	100,00	0,00E+00	1829	9	NA	Japonica	
maker-1-augustus-gene-301.26-mRNA-1	1	OsR498G0101864900.01.T01	99,35	0,00E+00	1640	Os01g0709000	100,00	0,00E+00	1667	27	NA	Japonica	
maker-1-augustus-gene-301.29-mRNA-1	1	OsR498G0101865300.01.T01	99,60	0,00E+00	2249	Os01g0709400	99,76	0,00E+00	2258	9	NA	Japonica	
maker-1-augustus-gene-301.32-mRNA-1	1	OsR498G0101862600.01.T04	99,77	0,00E+00	786	Os01g0708500	100,00	0,00E+00	791	5	NA	Japonica	
maker-1-augustus-gene-301.38-mRNA-1	1	OsR498G0101863200.01.T02	99,55	0,00E+00	2363	Os01g0708700	100,00	0,00E+00	2392	29	NA	Japonica	
maker-1-augustus-gene-301.40-mRNA-1	1	OsR498G0101865600.01.T04	98,59	0,00E+00	3090	Os01g0709500	100,00	0,00E+00	3191	101	NA	Japonica	Kinase-TM
maker-1-augustus-gene-302.11-mRNA-1	1	OsR498G0101869900.01.T01	99,61	0,00E+00	2711	Os01g0711100	100,00	0,00E+00	1754	-957	Indica	Japonica	
maker-1-augustus-gene-302.17-mRNA-1	1	OsR498G0101870300.01.T01	97,68	0,00E+00	3367	Os01g0711200	100,00	0,00E+00	3555	188	NA	Japonica	Kinase-LRR-TM
maker-1-augustus-gene-302.19-mRNA-1	1	OsR498G0101870900.01.T01	99,45	0,00E+00	5518	Os01g0711400	100,00	0,00E+00	5595	77	NA	Japonica	
maker-1-augustus-gene-302.29-mRNA-1	1	OsR498G0101871100.01.T02	98,63	0,00E+00	1396	Os01g0711500	100,00	0,00E+00	1348	-48	NA	Japonica	
maker-1-augustus-gene-302.35-mRNA-1	1	OsR498G0101871300.01.T01	99,48	0,00E+00	1019	Os01g0711600	99,83	0,00E+00	1028	9	NA	Japonica	
maker-1-augustus-gene-302.37-mRNA-1	1	OsR498G0101868400.01.T02	100,00	0,00E+00	677	Os01g0924700	94,15	1,82E-141	501	-176	Indica	Japonica	
maker-1-augustus-gene-302.39-mRNA-1	1	OsR498G0101872000.01.T01	98,12	0,00E+00	1556	Os01g0711800	100,00	0,00E+00	1613	57	NA	Japonica	
maker-1-augustus-gene-302.41-mRNA-1	1	OsR498G0101874800.01.T02	98,10	0,00E+00	1533	Os01g0712500	100,00	0,00E+00	2776	1243	Japonica	Japonica	
maker-1-augustus-gene-302.5-mRNA-1	1	OsR498G0101869700.01.T02	97,93	0,00E+00	2575	Os01g0711000	98,19	0,00E+00	2593	18	NA	Japonica	
maker-1-augustus-gene-303.32-mRNA-1	1	OsR498G0101877100.01.T01	99,81	0,00E+00	1880	Os01g0713200	100,00	0,00E+00	1835	-45	NA	Japonica	
maker-1-augustus-gene-303.34-mRNA-1	1	OsR498G0101878300.01.T01	96,25	0,00E+00	2082	Os01g0713600	95,40	0,00E+00	1489	-593	Indica	Japonica	
maker-1-augustus-gene-303.36-mRNA-1	1	OsR498G0101878900.01.T01	99,80	0,00E+00	1754	Os01g0713700	100,00	0,00E+00	2129	375	Japonica	Japonica	
maker-1-augustus-gene-304.11-mRNA-1	1	OsR498G0101881600.01.T01	91,61	0,00E+00	926	Os01g0714900	91,77	0,00E+00	930	4	NA	Japonica	
maker-1-augustus-gene-304.15-mRNA-1	1	OsR498G0101881800.01.T03	99,76	0,00E+00	1480	Os01g0715000	100,00	0,00E+00	1061	-419	Indica	Japonica	
maker-1-augustus-gene-304.20-mRNA-1	1	OsR498G0101884600.01.T01	97,48	0,00E+00	1630	Os01g0715500	97,18	0,00E+00	1625	-5	NA	Japonica	
maker-1-augustus-gene-304.26-mRNA-1	1	OsR498G0101886200.01.T02	100,00	0,00E+00	1115	Os01g0715800	100,00	0,00E+00	1115	0	NA	Japonica	
maker-1-augustus-gene-304.3-mRNA-1	1	OsR498G0101884300.01.T03	99,80	0,00E+00	2734	Os01g0715400	100,00	0,00E+00	2452	-282	Indica	Japonica	
maker-1-augustus-gene-304.9-mRNA-1	1	OsR498G0101881000.01.T01	97,16	0,00E+00	708	Os01g0714800	100,00	0,00E+00	1332	624	Japonica	Japonica	
maker-1-augustus-gene-305.27-mRNA-1	1	OsR498G0101888500.01.T04	99,94	0,00E+00	2874	Os01g0716400	100,00	0,00E+00	2879	5	NA	Japonica	
maker-1-augustus-gene-305.30-mRNA-1	1	OsR498G0101888500.01.T01	99,75	0,00E+00	1420	Os01g0716500	100,00	0,00E+00	1429	9	NA	Japonica	
maker-1-augustus-gene-305.35-mRNA-1	1	OsR498G0101896900.01.T01	99,68	0,00E+00	1688	Os01g0717400	100,00	0,00E+00	1701	13	NA	Japonica	
maker-1-augustus-gene-305.38-mRNA-1	1	OsR498G0101888000.01.T05	100,00	0,00E+00	3155	Os01g0716200	100,00	0,00E+00	3048	-107	NA	Japonica	
maker-1-augustus-gene-305.42-mRNA-1	1	OsR498G0101888400.01.T01	94,32	0,00E+00	1171	Os01g0716300	99,19	0,00E+00	1306	135	Japonica	Japonica	
maker-1-augustus-gene-305.45-mRNA-1	1	OsR498G0101891000.01.T01	99,91	0,00E+00	1993	Os01g0717000	100,00	0,00E+00	1997	4	NA	Japonica	
maker-1-augustus-gene-305.49-mRNA-1	1	OsR498G0101897400.01.T01	99,22	0,00E+00	902	Os01g0718000	84,85	1,30E-145	515	-387	Indica	Japonica	
maker-1-augustus-gene-306.30-mRNA-1	1	OsR498G0101898100.01.T01	99,93	0,00E+00	2620	Os01g0717700	100,00	0,00E+00	2625	5	NA	Japonica	
maker-1-augustus-gene-306.35-mRNA-1	1	OsR498G0101904300.01.T01	99,87	0,00E+00	1419	Os01g0719000	100,00	0,00E+00	1424	5	NA	Japonica	
maker-1-augustus-gene-306.37-mRNA-1	1	OsR498G0101904900.01.T02	98,53	1,18E-167	590	Os01g0719250	100,00	7,92E-133	471	-119	Indica	Japonica	
maker-1-augustus-gene-306.39-mRNA-1	1	OsR498G0101906500.01.T02	99,72	0,00E+00	1935	Os01g0719300	99,81	0,00E+00	1930	-5	NA	Japonica	
maker-1-augustus-gene-306.40-mRNA-1	1	OsR498G0101906500.01.T01	99,68	0,00E+00	1670	Os01g0719300	100,00	0,00E+00	1683	13	NA	Japonica	
maker-1-augustus-gene-306.49-mRNA-1	1	OsR498G0101904800.01.T06	92,90	0,00E+00	737	Os01g0719100	92,90	0,00E+00	737	0	NA	Japonica	
maker-1-augustus-gene-307.0-mRNA-1	1	OsR498G0101906700.01.T02	96,12	0,00E+00	3432	Os01g0719400	94,32	0,00E+00	1915	-1517	Indica	Japonica	
maker-1-augustus-gene-307.11-mRNA-1	1	OsR498G0101907300.01.T01	96,77	0,00E+00	2669	Os01g0719700	98,12	0,00E+00	1737	-932	Indica	Japonica	
maker-1-augustus-gene-307.16-mRNA-1	1	OsR498G0101910100.01.T02	99,73	0,00E+00	2612	Os01g0720800	100,00	0,00E+00	2623	11	NA	Japonica	
maker-1-augustus-gene-307.18-mRNA-1	1	OsR498G0101908000.01.T01	99,58	0,00E+00	841	Os05g0533800	79,78	2,41E-84	312	-529	Indica	Japonica	
maker-1-augustus-gene-307.20-mRNA-1	1	OsR498G0101908200.01.T01	99,67	0,00E+00	1074	Os05g0533700	91,83	0,00E+00	668	-406	Indica	Japonica	
maker-1-augustus-gene-307.22-mRNA-1	1	OsR498G0101909600.01.T03	94,18	0,00E+00	2678	Os01g0720600	95,03	0,00E+00	2727	49	NA	Japonica	
maker-1-augustus-gene-307.8-mRNA-1	1	OsR498G0101907100.01.T01	99,77	0,00E+00	770	Os01g0719600	100,00	0,00E+00	774	4	NA	Japonica	
maker-1-augustus-gene-308.0-mRNA-1	1	OsR498G0101914500.01.T07	97,13	0,00E+00	885	Os01g0722100	97,13	0,00E+00	885	0	NA	Japonica	
maker-1-augustus-gene-308.12-mRNA-1	1	OsR498G0101915900.01.T01	99,54	0,00E+00	2691	Os01g0722700	100,00	0,00E+00	1525	-1166	Indica	Japonica	
maker-1-augustus-gene-308.21-mRNA-1	1	OsR498G0101917500.01.T02	98,06	0,00E+00	1063	Os01g0723100	100,00	0,00E+00	1113	50	NA	Japonica	
maker-1-augustus-gene-308.29-mRNA-1	1	OsR498G0101918700.01.T01	99,67	0,00E+00	2161	Os01g0723500	100,00	0,00E+00	2181	20	NA	Japonica	
maker-1-augustus-gene-308.32-mRNA-1	1	OsR498G0101918900.01.T01	99,27	0,00E+00	2179	Os01g0723600	100,00	0,00E+00	2219	40	NA	Japonica	
maker-1-augustus-gene-308.35-mRNA-1	1	OsR498G0101919300.01.T03	100,00	0,00E+00	1779	Os01g0723700	99,60	0,00E+00	1780	1	NA	Japonica	
maker-1-augustus-gene-308.37-mRNA-1	1	OsR498G0101914200.01.T01	99,82	0,00E+00	2046	Os01g0721900	100,00	0,00E+00	2055	9	NA	Japonica	
maker-1-augustus-gene-308.41-mRNA-1	1	OsR498G0101917000.01.T03	99,96	0,00E+00	4617	Os01g0723000	100,00	0,00E+00	4621	4	NA	Japonica	
maker-1-augustus-gene-308.43-mRNA-1	1	OsR498G0101917800.01.T01	99,76	0,00E+00	748	Os01g0723200	100,00	0,00E+00	753	5	NA	Japonica	
maker-1-augustus-gene-308.6-mRNA-1	1	OsR498G0101914900.01.T01	99,89	0,00E+00	1614	Os01g0722300	100,00	0,00E+00	1618	4	NA	Japonica	
maker-1-augustus-gene-309.44-mRNA-1	1	OsR498G0101925700.01.T03	100,00	0,00E+00	1110	Os01g0725400	100,00	0,00E+00	1110	0	NA	Japonica	
maker-1-augustus-gene-309.45-mRNA-1	1	OsR498G0101926100.01.T03	98,93	0,00E+00	2966	Os01g0725600	99,05	0,00E+00	2975	9	NA	Japonica	
maker-1-augustus-gene-309.56-mRNA-1	1	OsR498G0101926500.01.T05	98,87	0,00E+00	2782	Os01g0725800	100,00	0,00E+00	2482	-300	Indica	Japonica	CC-Kinase-TM
maker-1-augustus-gene-309.60-mRNA-1	1	OsR498G0101919800.01.T01	99,89	0,00E+00	6664	Os01g0723800	100,00	0,00E+00	6682	18	NA	Japonica	
maker-1-augustus-gene-309.62-mRNA-1	1	OsR498G0101926800.01.T01	100,00	0,00E+00	921	Os01g0725900	100,00	0,00E+00	921	0	NA	Japonica	
maker-1-augustus-gene-309.64-mRNA-1	1	OsR498G0101925400.01.T02	100,00	0,00E+00	1180	Os01g0726100	100,00	0,00E+00	1180	0	NA	Japonica	
maker-1-augustus-gene-309.66-mRNA-1	1	OsR498G0101927400.01.T02	100,00	0,00E+00	1049	Os01g0726400	100,00	0,00E+00	1049	0	NA	Japonica	
maker-1-augustus-gene-31.44-mRNA-1	1	OsR498G0100210300.01.T02	100,00	0,00E+00	1402	Os01g0159800	99,87	0,00E+00	1397	-5	NA	Indica	
maker-1-augustus-gene-31.46-mRNA-1	1	OsR498G0100208600.01.T06	100,00	0,00E+00	2168	Os01g0158900	98,18	0,00E+00	2089	-79	NA	Indica	
maker-1-augustus-gene-31.51-mRNA-1	1	OsR498G0100208800.01.T01	100,00	0,00E+00	1462	Os01g0159000	98,52	0,00E+00	1407	-55	NA	Indica	
maker-1-augustus-gene-31.53-mRNA-1	1	OsR498G0100209200.01.T02	100,00	0,00E+00	2717	Os01g0159300	100,00	0,00E+00	2717	0	NA	Indica	
maker-1-augustus-gene-31.59-mRNA-1	1	OsR498G0100209800.01.T01	100,00	0,00E+00	2349	Os01g0159400	100,00	0,00E+00	2349	0	NA	Indica	
maker-1-augustus-gene-31.63-mRNA-1	1	OsR498G0100211200.01.T01	100,00	0,00E+00	3236	Os01g0160100	98,72	0,00E+00	3145	-91	NA	Indica	
maker-1-augustus-gene-310.44-mRNA-1	1	OsR498G0101927700.01.T01	77,28	0,00E+00	793	Os01g0726700	77,18	0,00E+00	789	-4	NA	Japonica	
maker-1-augustus-gene-310.47-mRNA-1	1	OsR498G0101929300.01.T01	96,42	0,00E+00	2747	Os01g0727100	96,54	0,00E+00	2756	9	NA	Japonica	
maker-1-augustus-gene-310.50-mRNA-1	1	OsR498G0101929500.01.T05	100,00	0,00E+00	1245	Os01g0727400	100,00	0,00E+00	1245	0	NA	Japonica	
maker-1-augustus-gene-310.56-mRNA-1	1	OsR498G0101931300.01.T03	100,00	0,00E+00	1196	Os01g0727900	100,00	0,00E+00	1196	0	NA	Japonica	
maker-1-augustus-gene-310.58-mRNA-1	1	OsR498G0101931500.01.T02	99,91	0,00E+00	1966	Os05g0518300	81,51	0,00E+00	957	-1009	Indica	Japonica	
maker-1-augustus-gene-310.62-mRNA-1	1	OsR498G0101931000.01.T01	99,92	0,00E+00	4228	Os01g0727800	100,00	0,00E+00	2100	-2128	Indica	Japonica	
maker-1-augustus-gene-310.69-mRNA-1	1	OsR498G0101932200.01.T01	100,00	0,00E+00	921	Os01g0728300	100,00	0,00E+00	921	0	NA	Japonica	
maker-1-augustus-gene-311.42-mRNA-1	1	OsR498G0101933500.01.T02	99,89	0,00E+00	1560	Os01g0729100	99,65	0,00E+00	1549	-11	NA	Japonica	
maker-1-augustus-gene-311.45-mRNA-1	1	OsR498G0101935700.01.T05	99,88	0,00E+00	1522	Os01g0729900	100,00	0,00E+00	1526	4	NA	Japonica	
maker-1-augustus-gene-311.50-mRNA-1	1	OsR498G0101936700.01.T01	99,89	0,00E+00	4743	Os01g0730300	100,00	0,00E+00	4756	13	NA	Japonica	
maker-1-augustus-gene-311.53-mRNA-1	1	OsR498G0101937700.01.T02	91,94	0,00E+00	728	Os01g0730500	91,94	0,00E+00	728	0	NA	Japonica	
maker-1-augustus-gene-311.57-mRNA-1	1	OsR498G0101932800.01.T01	99,87	0,00E+00	1354	Os01g0728700	100,00	0,00E+00	1359	5	NA	Japonica	
maker-1-augustus-gene-311.59-mRNA-1	1	OsR498G0101934800.01.T01	99,89	0,00E+00	3330	Os01g0729600	100,00	0,00E+00	3339	9	NA	Japonica	
maker-1-augustus-gene-311.61-mRNA-1	1	OsR498G0101936200.01.T01	99,55	0,00E+00	2381	Os01g0730100	100,00	0,00E+00	2408	27	NA	Japonica	
maker-1-augustus-gene-311.63-mRNA-1	1	OsR498G0101938600.01.T01	99,79	0,00E+00	1723	Os01g0730700	100,00	0,00E+00	1732	9	NA	Japonica	
maker-1-augustus-gene-312.38-mRNA-1	1	OsR498G0101939300.01.T04	99,72	0,00E+00	1290	Os01g0730800	100,00	0,00E+00	1299	9	NA	Japonica	
maker-1-augustus-gene-312.42-mRNA-1	1	OsR498G0101942100.01.T02	99,79	0,00E+00	1707	Os01g0732200	100,00	0,00E+00	1716	9	NA	Japonica	
maker-1-augustus-gene-312.44-mRNA-1	1	OsR498G0101944100.01.T01	99,85	0,00E+00	2388	Os01g0732600	100,00	0,00E+00	1377	-1011	Indica	Japonica	
maker-1-augustus-gene-312.46-mRNA-1	1	OsR498G0101946100.01.T01	99,73	0,00E+00	1350	Os01g0733200	100,00	0,00E+00	1359	9	NA	Japonica	
maker-1-augustus-gene-312.48-mRNA-1	1	OsR498G0101939600.01.T03	99,53	0,00E+00	3754	Os01g0730900	100,00	0,00E+00	3164	-590	Indica	Japonica	
maker-1-augustus-gene-312.52-mRNA-1	1	OsR498G0101940000.01.T01	100,00	1,75E-122	439	Os01g0731000	100,00	6,07E-123	439	0	NA	Japonica	
maker-1-augustus-gene-312.56-mRNA-1	1	OsR498G0101940200.01.T01	99,38	0,00E+00	1423	Os01g0731100	100,00	0,00E+00	1445	22	NA	Japonica	
maker-1-augustus-gene-312.58-mRNA-1	1	OsR498G0101941200.01.T01	100,00	0,00E+00	1434	Os01g0731100	94,84	0,00E+00	1248	-186	Indica	Japonica	
maker-1-augustus-gene-312.60-mRNA-1	1	OsR498G0101941400.01.T01	99,50	0,00E+00	717	Os01g0731800	100,00	0,00E+00	785	68	NA	Japonica	
maker-1-augustus-gene-313.13-mRNA-1	1	OsR498G0101951500.01.T01	91,90	0,00E+00	1492	Os01g0735500	100,00	0,00E+00	1885	393	Japonica	Japonica	
maker-1-augustus-gene-313.14-mRNA-1	1	OsR498G0101947200.01.T02	99,78	0,00E+00	1597	Os01g0733600	100,00	0,00E+00	1606	9	NA	Japonica	
maker-1-augustus-gene-313.2-mRNA-1	1	OsR498G0101947500.01.T01	100,00	0,00E+00	1380	Os01g0734000	100,00	0,00E+00	1380	0	NA	Japonica	
maker-1-augustus-gene-313.21-mRNA-1	1	OsR498G0101949000.01.T01	99,53	0,00E+00	760	Os06g0279950	97,72	7,59E-103	373	-387	Indica	Japonica	
maker-1-augustus-gene-313.4-mRNA-1	1	OsR498G0101948700.01.T02	99,79	0,00E+00	3344	Os01g0734200	100,00	0,00E+00	3362	18	NA	Japonica	
maker-1-augustus-gene-314.0-mRNA-1	1	OsR498G0101954300.01.T01	99,14	0,00E+00	1232	Os10g0560900	87,97	0,00E+00	881	-351	Indica	Japonica	
maker-1-augustus-gene-314.18-mRNA-1	1	OsR498G0101957300.01.T01	95,89	0,00E+00	2479	Os01g0737500	99,77	0,00E+00	1541	-938	Indica	Japonica	
maker-1-augustus-gene-314.21-mRNA-1	1	OsR498G0101958100.01.T01	98,95	0,00E+00	4196	Os01g0737900	100,00	0,00E+00	4313	117	NA	Japonica	
maker-1-augustus-gene-314.23-mRNA-1	1	OsR498G0101958000.01.T02	97,19	0,00E+00	1498	Os01g0737800	100,00	0,00E+00	1606	108	NA	Japonica	
maker-1-augustus-gene-314.8-mRNA-1	1	OsR498G0101956100.01.T03	98,69	0,00E+00	1330	Os01g0736900	100,00	0,00E+00	1375	45	NA	Japonica	
maker-1-augustus-gene-315.34-mRNA-1	1	OsR498G0101958300.01.T01	97,92	9,05E-139	493	Os01g0738000	100,00	2,26E-147	520	27	NA	Japonica	
maker-1-augustus-gene-315.36-mRNA-1	1	OsR498G0101960100.01.T01	99,51	0,00E+00	3603	Os01g0738300	100,00	0,00E+00	3636	33	NA	Japonica	Kinase-TM
maker-1-augustus-gene-315.40-mRNA-1	1	OsR498G0101960700.01.T03	99,82	0,00E+00	3062	Os01g0531200	99,06	0,00E+00	3003	-59	NA	Japonica	
maker-1-augustus-gene-315.42-mRNA-1	1	OsR498G0101960900.01.T03	99,42	0,00E+00	3374	Os01g0738600	100,00	0,00E+00	3404	30	NA	Japonica	
maker-1-augustus-gene-315.48-mRNA-1	1	OsR498G0101963300.01.T01	95,79	0,00E+00	1410	Os01g0739200	95,79	0,00E+00	1410	0	NA	Japonica	
maker-1-augustus-gene-315.55-mRNA-1	1	OsR498G0101961400.01.T03	96,62	0,00E+00	2278	Os05g0524400	82,29	0,00E+00	1316	-962	Indica	Japonica	
maker-1-augustus-gene-315.63-mRNA-1	1	OsR498G0101962600.01.T01	99,53	0,00E+00	2674	Os01g0739000	100,00	0,00E+00	2706	32	NA	Japonica	
maker-1-augustus-gene-316.13-mRNA-1	1	OsR498G0101964800.01.T01	99,24	0,00E+00	935	Os01g0739500	100,00	0,00E+00	953	18	NA	Japonica	
maker-1-augustus-gene-316.19-mRNA-1	1	OsR498G0101968800.01.T01	97,17	0,00E+00	3126	Os01g0743300	73,61	0,00E+00	884	-2242	Indica	Japonica	
maker-1-augustus-gene-316.24-mRNA-1	1	OsR498G0101969600.01.T01	99,07	0,00E+00	2475	Os01g0741200	100,00	0,00E+00	2278	-197	NA	Japonica	Kinase-TM
maker-1-augustus-gene-316.5-mRNA-1	1	OsR498G0101966500.01.T01	99,80	0,00E+00	2660	Os01g0739700	100,00	0,00E+00	2673	13	NA	Japonica	
maker-1-augustus-gene-316.7-mRNA-1	1	OsR498G0101967900.01.T01	100,00	0,00E+00	2419	Os01g0740400	100,00	0,00E+00	1243	-1176	Indica	Japonica	
maker-1-augustus-gene-316.9-mRNA-1	1	OsR498G0101971900.01.T04	99,80	0,00E+00	1810	Os01g0741900	100,00	0,00E+00	1819	9	NA	Japonica	
maker-1-augustus-gene-317.33-mRNA-1	1	OsR498G0101972900.01.T02	99,68	0,00E+00	4439	Os01g0742200	100,00	0,00E+00	4475	36	NA	Japonica	
maker-1-augustus-gene-317.35-mRNA-1	1	OsR498G0101973900.01.T01	97,35	0,00E+00	5402	Os01g0742400	100,00	0,00E+00	5773	371	NA	Japonica	Kinase-LRR-TM
maker-1-augustus-gene-317.37-mRNA-1	1	OsR498G0101975300.01.T01	99,70	0,00E+00	2405	Os01g0743100	100,00	0,00E+00	2051	-354	Indica	Japonica	
maker-1-augustus-gene-317.39-mRNA-1	1	OsR498G0101976900.01.T03	99,68	0,00E+00	3913	Os01g0743300	100,00	0,00E+00	3945	32	NA	Japonica	
maker-1-augustus-gene-317.44-mRNA-1	1	OsR498G0101977300.01.T01	99,77	0,00E+00	3153	Os01g0743500	100,00	0,00E+00	3171	18	NA	Japonica	
maker-1-augustus-gene-317.48-mRNA-1	1	OsR498G0101974200.01.T01	99,89	0,00E+00	1605	Os01g0742500	100,00	0,00E+00	1609	4	NA	Japonica	
maker-1-augustus-gene-317.53-mRNA-1	1	OsR498G0101976000.01.T01	99,83	0,00E+00	2069	Os01g0743200	99,91	0,00E+00	2074	5	NA	Japonica	
maker-1-augustus-gene-317.57-mRNA-1	1	OsR498G0101977200.01.T03	99,83	0,00E+00	2156	Os01g0743400	98,50	0,00E+00	2095	-61	NA	Japonica	
maker-1-augustus-gene-318.0-mRNA-1	1	OsR498G0101978300.01.T01	100,00	0,00E+00	715	Os01g0743800	100,00	0,00E+00	1018	303	Japonica	Japonica	
maker-1-augustus-gene-318.10-mRNA-1	1	OsR498G0101979100.01.T01	99,66	0,00E+00	2131	Os01g0744200	99,92	0,00E+00	2166	35	NA	Japonica	
maker-1-augustus-gene-318.14-mRNA-1	1	OsR498G0101977600.01.T03	99,81	0,00E+00	2887	Os01g0743600	100,00	0,00E+00	2901	14	NA	Japonica	
maker-1-augustus-gene-318.19-mRNA-1	1	OsR498G0101979400.01.T04	99,86	0,00E+00	2612	Os01g0744300	100,00	0,00E+00	2619	7	NA	Japonica	
maker-1-augustus-gene-318.26-mRNA-1	1	OsR498G0101979800.01.T03	99,70	0,00E+00	2966	Os01g0744400	100,00	0,00E+00	2989	23	NA	Japonica	
maker-1-augustus-gene-318.3-mRNA-1	1	OsR498G0101978700.01.T04	99,89	0,00E+00	3238	Os01g0744000	100,00	0,00E+00	3247	9	NA	Japonica	
maker-1-augustus-gene-318.33-mRNA-1	1	OsR498G0101980400.01.T01	99,78	0,00E+00	819	Os01g0200200	73,80	5,66E-35	148	-671	Indica	Japonica	
maker-1-augustus-gene-318.35-mRNA-1	1	OsR498G0101982800.01.T02	99,74	0,00E+00	2086	Os01g0745700	100,00	0,00E+00	2100	14	NA	Japonica	
maker-1-augustus-gene-319.37-mRNA-1	1	OsR498G0101986700.01.T01	99,75	0,00E+00	1452	Os01g0747300	100,00	0,00E+00	735	-717	Indica	Japonica	
maker-1-augustus-gene-319.40-mRNA-1	1	OsR498G0101984600.01.T01	99,83	0,00E+00	3071	Os01g0746400	100,00	0,00E+00	3085	14	NA	Japonica	
maker-1-augustus-gene-319.42-mRNA-1	1	OsR498G0101985200.01.T01	99,78	0,00E+00	2400	Os01g0746700	100,00	0,00E+00	2414	14	NA	Japonica	
maker-1-augustus-gene-319.46-mRNA-1	1	OsR498G0101987200.01.T01	99,58	0,00E+00	3416	Os01g0747400	100,00	0,00E+00	3452	36	NA	Japonica	Kinase-TM
maker-1-augustus-gene-32.51-mRNA-1	1	OsR498G0100211800.01.T01	100,00	0,00E+00	1537	Os01g0160800	99,30	0,00E+00	1508	-29	NA	Indica	
maker-1-augustus-gene-32.52-mRNA-1	1	OsR498G0100212800.01.T01	100,00	0,00E+00	1169	Os01g0160800	79,15	1,51E-159	562	-607	Indica	Indica	
maker-1-augustus-gene-320.0-mRNA-1	1	OsR498G0101987900.01.T06	95,33	0,00E+00	1985	Os01g0747500	95,50	0,00E+00	1994	9	NA	Japonica	
maker-1-augustus-gene-320.10-mRNA-1	1	OsR498G0101993100.01.T02	99,88	0,00E+00	2989	Os01g0748900	100,00	0,00E+00	2998	9	NA	Japonica	
maker-1-augustus-gene-320.16-mRNA-1	1	OsR498G0101993500.01.T01	99,89	0,00E+00	1587	Os01g0749000	100,00	0,00E+00	1370	-217	Indica	Japonica	
maker-1-augustus-gene-320.22-mRNA-1	1	OsR498G0101994100.01.T01	100,00	0,00E+00	1267	Os01g0749200	100,00	0,00E+00	1267	0	NA	Japonica	
maker-1-augustus-gene-320.26-mRNA-1	1	OsR498G0101994300.01.T01	99,77	0,00E+00	2373	Os01g0749300	100,00	0,00E+00	2387	14	NA	Japonica	
maker-1-augustus-gene-320.32-mRNA-1	1	OsR498G0101990200.01.T01	99,44	0,00E+00	1270	Os01g0748150	100,00	0,00E+00	1288	18	NA	Japonica	
maker-1-augustus-gene-320.40-mRNA-1	1	OsR498G0101994600.01.T02	99,78	0,00E+00	4922	Os01g0749400	100,00	0,00E+00	4946	24	NA	Japonica	

maker-1-augustus-gene-320.5-mRNA-1	1	OsR498G0101991700.01.T01	99,76	0,00E+00	2216	Os05g0518300	82,61	0,00E+00	1049	-1167	Indica	Japonica	
maker-1-augustus-gene-320.8-mRNA-1	1	OsR498G0101992300.01.T01	97,56	4,72E-95	348	Os01g0748800	100,00	1,45E-102	370	22	NA	Japonica	
maker-1-augustus-gene-321.0-mRNA-1	1	OsR498G0101996500.01.T01	99,75	0,00E+00	1454	Os01g0749900	100,00	0,00E+00	1458	4	NA	Japonica	
maker-1-augustus-gene-321.10-mRNA-1	1	OsR498G0101999100.01.T02	99,66	0,00E+00	5312	Os01g0750300	100,00	0,00E+00	5357	45	NA	Japonica	
maker-1-augustus-gene-321.16-mRNA-1	1	OsR498G0102001400.01.T01	99,85	0,00E+00	1165	Os01g0750500	100,00	0,00E+00	1169	4	NA	Japonica	
maker-1-augustus-gene-321.19-mRNA-1	1	OsR498G0102002200.01.T01	99,18	0,00E+00	3655	Os01g0750600	100,00	0,00E+00	3696	41	NA	Japonica	Kinase-TM
maker-1-augustus-gene-321.8-mRNA-1	1	OsR498G0101997100.01.T01	100,00	0,00E+00	1202	Os01g0750000	100,00	0,00E+00	1202	0	NA	Japonica	
maker-1-augustus-gene-322.0-mRNA-1	1	OsR498G0102004500.01.T02	99,61	0,00E+00	1383	Os01g0751300	100,00	0,00E+00	1319	-64	NA	Japonica	
maker-1-augustus-gene-322.11-mRNA-1	1	OsR498G0102008100.01.T01	99,74	0,00E+00	3475	Os01g0752700	100,00	0,00E+00	3498	23	NA	Japonica	
maker-1-augustus-gene-322.17-mRNA-1	1	OsR498G0102005200.01.T01	99,51	0,00E+00	2174	Os01g0751600	100,00	0,00E+00	2230	56	NA	Japonica	
maker-1-augustus-gene-322.19-mRNA-1	1	OsR498G0102006800.01.T01	94,72	0,00E+00	908	Os01g0752200	95,25	0,00E+00	923	15	NA	Japonica	
maker-1-augustus-gene-322.23-mRNA-1	1	OsR498G0102007200.01.T01	99,63	0,00E+00	960	Os01g0752300	100,00	0,00E+00	969	9	NA	Japonica	
maker-1-augustus-gene-322.26-mRNA-1	1	OsR498G0102007400.01.T02	99,55	0,00E+00	1179	Os01g0752400	100,00	0,00E+00	1196	17	NA	Japonica	
maker-1-augustus-gene-322.30-mRNA-1	1	OsR498G0102008000.01.T04	99,70	0,00E+00	1195	Os01g0752600	100,00	0,00E+00	1204	9	NA	Japonica	
maker-1-augustus-gene-322.8-mRNA-1	1	OsR498G0102006500.01.T01	98,59	0,00E+00	2359	Os01g0752100	100,00	0,00E+00	2408	49	NA	Japonica	
maker-1-augustus-gene-323.40-mRNA-1	1	OsR498G0102009100.01.T01	99,91	0,00E+00	1976	Os01g0753100	100,00	0,00E+00	1981	5	NA	Japonica	
maker-1-augustus-gene-323.48-mRNA-1	1	OsR498G0102011100.01.T06	99,28	2,57E-138	491	Os01g0754000	100,00	4,05E-143	506	15	NA	Japonica	
maker-1-augustus-gene-323.50-mRNA-1	1	OsR498G0102008400.01.T02	100,00	0,00E+00	841	Os01g0752800	100,00	0,00E+00	841	0	NA	Japonica	
maker-1-augustus-gene-323.54-mRNA-1	1	OsR498G0102009000.01.T03	97,93	0,00E+00	2993	Os05g0515700	78,95	0,00E+00	893	-2100	Indica	Japonica	
maker-1-augustus-gene-323.56-mRNA-1	1	OsR498G0102009600.01.T01	99,66	4,29E-149	528	Os01g0753150	100,00	1,22E-150	531	3	NA	Japonica	
maker-1-augustus-gene-323.58-mRNA-1	1	OsR498G0102009800.01.T01	99,86	0,00E+00	1311	Os02g0788500	75,98	2,17E-93	343	-968	Indica	Japonica	
maker-1-augustus-gene-323.60-mRNA-1	1	OsR498G0102010200.01.T02	99,69	0,00E+00	4075	Os01g0753500	100,00	0,00E+00	3101	-974	Indica	Japonica	
maker-1-augustus-gene-323.66-mRNA-1	1	OsR498G0102011400.01.T01	98,58	0,00E+00	734	Os01g0754100	100,00	0,00E+00	764	30	NA	Japonica	
maker-1-augustus-gene-323.68-mRNA-1	1	OsR498G0102012200.01.T02	99,66	0,00E+00	3198	Os01g0754500	99,83	0,00E+00	3210	12	NA	Japonica	
maker-1-augustus-gene-324.0-mRNA-1	1	OsR498G0102014300.01.T01	99,69	0,00E+00	2302	Os01g0755500	100,00	0,00E+00	2320	18	NA	Japonica	
maker-1-augustus-gene-324.10-mRNA-1	1	OsR498G0102018500.01.T01	99,87	0,00E+00	2713	Os01g0756700	100,00	0,00E+00	2722	9	NA	Japonica	
maker-1-augustus-gene-324.12-mRNA-1	1	OsR498G0102013800.01.T03	99,71	0,00E+00	2502	Os01g0755100	100,00	0,00E+00	2520	18	NA	Japonica	
maker-1-augustus-gene-324.2-mRNA-1	1	OsR498G0102016300.01.T01	99,75	0,00E+00	1431	Os01g0756200	100,00	0,00E+00	1440	9	NA	Japonica	
maker-1-augustus-gene-324.6-mRNA-1	1	OsR498G0102017700.01.T01	99,82	0,00E+00	1979	Os01g0531400	94,85	0,00E+00	2435	456	Japonica	Japonica	
maker-1-augustus-gene-325.0-mRNA-1	1	OsR498G0102021500.01.T01	99,90	0,00E+00	1771	Os01g0757200	100,00	0,00E+00	1775	4	NA	Japonica	
maker-1-augustus-gene-325.15-mRNA-1	1	OsR498G0102022800.01.T01	99,85	0,00E+00	1224	Os01g0757500	100,00	0,00E+00	1229	5	NA	Japonica	
maker-1-augustus-gene-325.2-mRNA-1	1	OsR498G0102022500.01.T02	99,90	0,00E+00	1830	Os01g0757400	100,00	0,00E+00	1835	5	NA	Japonica	NBS
maker-1-augustus-gene-325.32-mRNA-1	1	OsR498G0102025600.01.T01	97,88	0,00E+00	810	Os01g0758000	100,00	0,00E+00	852	42	NA	Japonica	
maker-1-augustus-gene-325.34-mRNA-1	1	OsR498G0102028800.01.T01	99,86	0,00E+00	5214	Os01g0758300	100,00	0,00E+00	4630	-584	Indica	Japonica	
maker-1-augustus-gene-325.38-mRNA-1	1	OsR498G0102029400.01.T05	97,62	0,00E+00	2372	Os01g0758400	97,54	0,00E+00	2085	-287	Indica	Japonica	
maker-1-augustus-gene-325.9-mRNA-1	1	OsR498G0102025100.01.T01	99,09	0,00E+00	2906	Os01g0757800	99,08	0,00E+00	1922	-984	Indica	Japonica	
maker-1-augustus-gene-326.32-mRNA-1	1	OsR498G0102034300.01.T01	98,94	0,00E+00	3646	Os01g0758900	98,99	0,00E+00	3654	8	NA	Japonica	
maker-1-augustus-gene-326.34-mRNA-1	1	OsR498G0102034900.01.T01	98,89	0,00E+00	1416	Os01g0759000	100,00	0,00E+00	1456	40	NA	Japonica	
maker-1-augustus-gene-326.36-mRNA-1	1	OsR498G0102038300.01.T02	99,85	0,00E+00	2394	Os01g0759700	100,00	0,00E+00	1364	-1030	Indica	Japonica	
maker-1-augustus-gene-326.40-mRNA-1	1	OsR498G0102038700.01.T02	99,75	0,00E+00	2823	Os01g0759900	100,00	0,00E+00	2430	-393	Indica	Japonica	
maker-1-augustus-gene-326.48-mRNA-1	1	OsR498G0102035200.01.T02	99,46	0,00E+00	1321	Os01g0759100	100,00	0,00E+00	1997	676	Japonica	Japonica	
maker-1-augustus-gene-327.52-mRNA-1	1	OsR498G0102043900.01.T01	99,64	0,00E+00	1502	Os01g0761900	100,00	0,00E+00	1516	14	NA	Japonica	
maker-1-augustus-gene-327.54-mRNA-1	1	OsR498G0102044500.01.T01	99,40	0,00E+00	2665	Os01g0762500	100,00	0,00E+00	2706	41	NA	Japonica	
maker-1-augustus-gene-327.65-mRNA-1	1	OsR498G0102040800.01.T01	94,53	0,00E+00	1283	Os01g0760900	95,15	0,00E+00	1306	23	NA	Japonica	
maker-1-augustus-gene-327.69-mRNA-1	1	OsR498G0102041200.01.T01	98,72	0,00E+00	3279	Os01g0761100	99,36	0,00E+00	3329	50	NA	Japonica	
maker-1-augustus-gene-327.72-mRNA-1	1	OsR498G0102042000.01.T02	99,59	0,00E+00	3080	Os01g0761400	100,00	0,00E+00	3112	32	NA	Japonica	
maker-1-augustus-gene-327.75-mRNA-1	1	OsR498G0102042600.01.T01	99,83	0,00E+00	3185	Os01g0761500	100,00	0,00E+00	3198	13	NA	Japonica	
maker-1-augustus-gene-327.77-mRNA-1	1	OsR498G0102044200.01.T01	99,87	0,00E+00	5636	Os01g0762000	100,00	0,00E+00	5402	-234	NA	Japonica	
maker-1-augustus-gene-327.79-mRNA-1	1	OsR498G0102044400.01.T01	95,98	0,00E+00	655	Os01g0762300	100,00	0,00E+00	719	64	NA	Japonica	
maker-1-augustus-gene-328.0-mRNA-1	1	OsR498G0102045300.01.T04	92,43	0,00E+00	1047	Os01g0763100	89,11	0,00E+00	1314	267	Japonica	Japonica	
maker-1-augustus-gene-328.13-mRNA-1	1	OsR498G0102049700.01.T01	99,83	0,00E+00	2064	Os07g0694800	74,60	1,91E-115	416	-1648	Indica	Japonica	
maker-1-augustus-gene-328.18-mRNA-1	1	OsR498G0102044400.01.T06	99,51	0,00E+00	724	Os01g0762600	99,83	0,00E+00	1040	316	Japonica	Japonica	
maker-1-augustus-gene-328.21-mRNA-1	1	OsR498G0102045200.01.T01	100,00	0,00E+00	1040	Os01g0763000	100,00	0,00E+00	1040	0	NA	Japonica	
maker-1-augustus-gene-328.27-mRNA-1	1	OsR498G0102048000.01.T02	98,46	0,00E+00	902	Os01g0763900	98,46	0,00E+00	902	0	NA	Japonica	
maker-1-augustus-gene-328.30-mRNA-1	1	OsR498G0102048200.01.T01	96,43	0,00E+00	1122	Os01g0764000	96,43	0,00E+00	1122	0	NA	Japonica	
maker-1-augustus-gene-328.32-mRNA-1	1	OsR498G0102050000.01.T01	99,68	0,00E+00	1686	Os01g0764400	100,00	0,00E+00	1700	14	NA	Japonica	
maker-1-augustus-gene-328.6-mRNA-1	1	OsR498G0102047300.01.T01	99,80	0,00E+00	1763	Os01g0763600	100,00	0,00E+00	1772	9	NA	Japonica	
maker-1-augustus-gene-329.0-mRNA-1	1	OsR498G0102050900.01.T05	98,27	0,00E+00	897	Os01g0764700	98,65	0,00E+00	906	9	NA	Japonica	
maker-1-augustus-gene-329.16-mRNA-1	1	OsR498G0102050800.01.T02	99,90	0,00E+00	1814	Os01g0764600	100,00	0,00E+00	1819	5	NA	Japonica	
maker-1-augustus-gene-329.25-mRNA-1	1	OsR498G0102052400.01.T01	99,54	0,00E+00	769	Os01g0765000	99,77	0,00E+00	774	5	NA	Japonica	
maker-1-augustus-gene-329.3-mRNA-1	1	OsR498G0102051900.01.T01	99,78	0,00E+00	3310	Os01g0764800	100,00	0,00E+00	3328	18	NA	Japonica	
maker-1-augustus-gene-329.32-mRNA-1	1	OsR498G0102053200.01.T01	99,60	0,00E+00	2207	Os01g0765400	100,00	0,00E+00	2230	23	NA	Japonica	
maker-1-augustus-gene-329.34-mRNA-1	1	OsR498G0102053400.01.T01	99,81	0,00E+00	3819	Os01g0765500	100,00	0,00E+00	3837	18	NA	Japonica	
maker-1-augustus-gene-329.36-mRNA-1	1	OsR498G0102054000.01.T01	99,83	0,00E+00	2156	Os01g0766000	100,00	0,00E+00	2587	431	Japonica	Japonica	
maker-1-augustus-gene-33.11-mRNA-1	1	OsR498G0100215400.01.T01	100,00	0,00E+00	3512	Os01g0164500	98,87	0,00E+00	3404	-108	NA	Indica	
maker-1-augustus-gene-33.16-mRNA-1	1	OsR498G0100215600.01.T01	100,00	0,00E+00	1588	Os01g0164600	99,32	0,00E+00	1561	-27	NA	Indica	
maker-1-augustus-gene-33.29-mRNA-1	1	OsR498G0100216000.01.T05	99,82	0,00E+00	2945	Os01g0164900	97,81	0,00E+00	2502	-443	Indica	Indica	
maker-1-augustus-gene-33.31-mRNA-1	1	OsR498G0100216400.01.T01	100,00	0,00E+00	2722	Os01g0165100	100,00	0,00E+00	2722	0	NA	Indica	
maker-1-augustus-gene-33.33-mRNA-1	1	OsR498G0100216600.01.T03	100,00	0,00E+00	3057	Os01g0165200	99,94	0,00E+00	2966	-91	NA	Indica	
maker-1-augustus-gene-330.0-mRNA-1	1	OsR498G0102055700.01.T01	100,00	0,00E+00	829	Os01g0766400	100,00	0,00E+00	829	0	NA	Japonica	
maker-1-augustus-gene-330.12-mRNA-1	1	OsR498G0102060100.01.T03	99,63	0,00E+00	3410	Os01g0767900	100,00	0,00E+00	3442	32	NA	Japonica	
maker-1-augustus-gene-330.16-mRNA-1	1	OsR498G0102057400.01.T01	99,81	0,00E+00	3721	Os03g0770800	75,64	0,00E+00	1244	-2477	Indica	Japonica	
maker-1-augustus-gene-330.19-mRNA-1	1	OsR498G0102058400.01.T01	99,87	0,00E+00	5328	Os01g0767000	99,93	0,00E+00	5337	9	NA	Japonica	
maker-1-augustus-gene-330.23-mRNA-1	1	OsR498G0102058600.01.T02	99,74	0,00E+00	2101	Os01g0767100	99,83	0,00E+00	2105	4	NA	Japonica	
maker-1-augustus-gene-330.3-mRNA-1	1	OsR498G0102055900.01.T01	98,59	0,00E+00	2094	Os01g0766600	100,00	0,00E+00	2154	60	NA	Japonica	
maker-1-augustus-gene-330.5-mRNA-1	1	OsR498G0102058900.01.T01	99,69	0,00E+00	2271	Os01g0767600	99,84	0,00E+00	2280	9	NA	Japonica	
maker-1-augustus-gene-330.7-mRNA-1	1	OsR498G0102059100.01.T04	99,80	0,00E+00	4431	Os01g0767700	99,76	0,00E+00	4426	-5	NA	Japonica	
maker-1-augustus-gene-331.15-mRNA-1	1	OsR498G0102062700.01.T02	99,91	0,00E+00	2003	Os01g0769100	100,00	0,00E+00	2008	5	NA	Japonica	
maker-1-augustus-gene-331.20-mRNA-1	1	OsR498G0102064100.01.T01	99,65	0,00E+00	4559	Os01g0769200	100,00	0,00E+00	4599	40	NA	Japonica	
maker-1-augustus-gene-331.22-mRNA-1	1	OsR498G0102064700.01.T04	99,76	0,00E+00	2969	Os01g0769900	100,00	0,00E+00	2987	18	NA	Japonica	
maker-1-augustus-gene-331.26-mRNA-1	1	OsR498G0102060800.01.T03	100,00	0,00E+00	1386	Os01g0768200	100,00	0,00E+00	1157	-229	Indica	Japonica	
maker-1-augustus-gene-332.32-mRNA-1	1	OsR498G0102068700.01.T01	99,67	0,00E+00	1068	Os01g0771200	100,00	0,00E+00	1077	9	NA	Japonica	
maker-1-augustus-gene-332.34-mRNA-1	1	OsR498G0102065000.01.T01	99,62	0,00E+00	2782	Os01g0770000	100,00	0,00E+00	2809	27	NA	Japonica	
maker-1-augustus-gene-332.48-mRNA-1	1	OsR498G0102066600.01.T01	96,20	0,00E+00	774	Os01g0770500	96,41	0,00E+00	779	5	NA	Japonica	
maker-1-augustus-gene-332.50-mRNA-1	1	OsR498G0102068600.01.T01	99,86	0,00E+00	1316	Os01g0771100	100,00	0,00E+00	1321	5	NA	Japonica	
maker-1-augustus-gene-333.28-mRNA-1	1	OsR498G0102072700.01.T02	99,81	0,00E+00	932	Os01g0772400	100,00	0,00E+00	1397	465	Japonica	NA	
maker-1-augustus-gene-333.32-mRNA-1	1	OsR498G0102073700.01.T02	99,71	0,00E+00	3755	Os01g0772600	99,95	0,00E+00	3778	23	NA	Japonica	
maker-1-augustus-gene-333.46-mRNA-1	1	OsR498G0102074300.01.T01	95,51	0,00E+00	2395	Os01g0772800	95,57	0,00E+00	2399	4	NA	Japonica	
maker-1-augustus-gene-333.51-mRNA-1	1	OsR498G0102075300.01.T02	99,91	0,00E+00	3860	Os01g0773100	99,95	0,00E+00	3865	5	NA	Japonica	
maker-1-augustus-gene-333.56-mRNA-1	1	OsR498G0102070800.01.T06	99,35	0,00E+00	2196	Os01g0771900	99,50	0,00E+00	2140	-56	NA	Japonica	
maker-1-augustus-gene-333.64-mRNA-1	1	OsR498G0102071600.01.T01	99,19	0,00E+00	1305	Os01g0772100	100,00	0,00E+00	756	-549	Indica	Japonica	
maker-1-augustus-gene-333.67-mRNA-1	1	OsR498G0102071800.01.T01	99,90	0,00E+00	1830	Os01g0772150	100,00	0,00E+00	1252	-578	Indica	Japonica	
maker-1-augustus-gene-333.69-mRNA-1	1	OsR498G0102072200.01.T01	100,00	0,00E+00	1499	Os05g0509900	79,20	0,00E+00	730	-769	Indica	NA	
maker-1-augustus-gene-333.71-mRNA-1	1	OsR498G0102073000.01.T01	99,75	0,00E+00	2178	Os01g0772500	99,92	0,00E+00	2187	9	NA	Japonica	
maker-1-augustus-gene-333.73-mRNA-1	1	OsR498G0102074800.01.T02	100,00	0,00E+00	1781	Os01g0772900	99,90	0,00E+00	1776	-5	NA	Japonica	
maker-1-augustus-gene-334.12-mRNA-1	1	OsR498G0102082700.01.T01	99,93	0,00E+00	4845	Os01g0775200	100,00	0,00E+00	4854	9	NA	Japonica	
maker-1-augustus-gene-334.15-mRNA-1	1	OsR498G0102077400.01.T01	100,00	0,00E+00	677	Os01g0773700	100,00	0,00E+00	677	0	NA	Japonica	
maker-1-augustus-gene-334.17-mRNA-1	1	OsR498G0102081600.01.T01	99,72	0,00E+00	1935	Os01g0774500	100,00	0,00E+00	2760	825	Japonica	Japonica	
maker-1-augustus-gene-334.2-mRNA-1	1	OsR498G0102077100.01.T02	98,10	0,00E+00	3537	Os01g0773600	98,20	0,00E+00	3546	9	NA	Japonica	
maker-1-augustus-gene-334.20-mRNA-1	1	OsR498G0102081800.01.T02	99,85	0,00E+00	4830	Os01g0775100	100,00	0,00E+00	4242	-588	Indica	Japonica	
maker-1-augustus-gene-334.8-mRNA-1	1	OsR498G0102078100.01.T01	95,31	0,00E+00	1318	Os01g0773800	100,00	0,00E+00	1458	140	NA	Japonica	
maker-1-augustus-gene-335.31-mRNA-1	1	OsR498G0102088500.01.T02	99,21	0,00E+00	3140	Os01g0776500	100,00	0,00E+00	2089	-1051	Indica	Japonica	
maker-1-augustus-gene-335.37-mRNA-1	1	OsR498G0102089900.01.T01	98,59	0,00E+00	2470	Os01g0776800	100,00	0,00E+00	2554	84	NA	Japonica	
maker-1-augustus-gene-335.40-mRNA-1	1	OsR498G0102090300.01.T03	99,73	0,00E+00	3947	Os01g0777000	99,96	0,00E+00	3969	22	NA	Japonica	
maker-1-augustus-gene-335.45-mRNA-1	1	OsR498G0102083400.01.T02	98,52	0,00E+00	5311	Os01g0775300	98,72	0,00E+00	5338	27	NA	Japonica	
maker-1-augustus-gene-335.50-mRNA-1	1	OsR498G0102083800.01.T01	99,88	0,00E+00	2886	Os01g0775400	100,00	0,00E+00	2895	9	NA	Japonica	
maker-1-augustus-gene-335.53-mRNA-1	1	OsR498G0102085600.01.T01	99,61	0,00E+00	3183	Os01g0775500	100,00	0,00E+00	1891	-1292	Indica	Japonica	
maker-1-augustus-gene-335.56-mRNA-1	1	OsR498G0102085800.01.T03	99,84	0,00E+00	1109	Os01g0775600	100,00	0,00E+00	1110	1	NA	Japonica	
maker-1-augustus-gene-335.65-mRNA-1	1	OsR498G0102089000.01.T01	99,86	0,00E+00	2513	Os01g0776600	100,00	0,00E+00	2522	9	NA	Japonica	
maker-1-augustus-gene-335.68-mRNA-1	1	OsR498G0102089400.01.T01	99,59	0,00E+00	874	Os01g0776700	100,00	4,98E-131	466	-408	Indica	Japonica	
maker-1-augustus-gene-336.0-mRNA-1	1	OsR498G0102091500.01.T01	99,80	0,00E+00	2719	Os01g0776900	100,00	0,00E+00	2012	-707	Indica	Japonica	
maker-1-augustus-gene-336.13-mRNA-1	1	OsR498G0102093100.01.T01	100,00	1,14E-101	369	Os01g0777900	100,00	3,98E-102	369	0	NA	Japonica	
maker-1-augustus-gene-336.18-mRNA-1	1	OsR498G0102095500.01.T01	99,89	0,00E+00	1700	Os01g0778700	100,00	0,00E+00	1705	5	NA	Japonica	
maker-1-augustus-gene-336.31-mRNA-1	1	OsR498G0102092400.01.T02	98,77	0,00E+00	5559	Os03g0689100	75,83	0,00E+00	1672	-3887	Indica	Japonica	
maker-1-augustus-gene-336.42-mRNA-1	1	OsR498G0102095000.01.T01	100,00	0,00E+00	645	Os01g0778500	100,00	2,74E-88	324	-321	Indica	Japonica	
maker-1-augustus-gene-336.6-mRNA-1	1	OsR498G0102091700.01.T01	95,92	0,00E+00	2225	Os01g0777300	96,22	0,00E+00	2243	18	NA	Japonica	
maker-1-augustus-gene-337.44-mRNA-1	1	OsR498G0102099700.01.T01	95,56	0,00E+00	1474	Os01g0780500	95,67	0,00E+00	1479	5	NA	Japonica	
maker-1-augustus-gene-337.50-mRNA-1	1	OsR498G0102097400.01.T02	99,73	0,00E+00	6673	Os01g0779400	100,00	0,00E+00	5793	-880	Indica	Japonica	
maker-1-augustus-gene-337.55-mRNA-1	1	OsR498G0102100000.01.T01	98,26	0,00E+00	3478	Os01g0780600	100,00	0,00E+00	2305	-1173	Indica	Japonica	
maker-1-augustus-gene-337.58-mRNA-1	1	OsR498G0102100200.01.T01	99,86	0,00E+00	1273	Os01g0780800	100,00	0,00E+00	1278	5	NA	Japonica	
maker-1-augustus-gene-338.11-mRNA-1	1	OsR498G0102105000.01.T01	99,76	0,00E+00	2281	Os01g0782700	99,84	0,00E+00	2286	5	NA	Japonica	
maker-1-augustus-gene-338.12-mRNA-1	1	OsR498G0511120900.01.T03	74,51	2,87E-72	274	Os01g0782700	100,00	0,00E+00	939	665	Japonica	Japonica	
maker-1-augustus-gene-338.21-mRNA-1	1	OsR498G0102107000.01.T01	99,45	0,00E+00	2603	Os01g0783200	99,86	0,00E+00	2632	29	NA	Japonica	
maker-1-augustus-gene-338.30-mRNA-1	1	OsR498G0102107800.01.T01	99,87	0,00E+00	1419	Os01g0783500	100,00	0,00E+00	1424	5	NA	Japonica	
maker-1-augustus-gene-338.4-mRNA-1	1	OsR498G0102103600.01.T02	99,74	0,00E+00	2731	Os01g0782200	99,93	0,00E+00	2745	14	NA	Japonica	
maker-1-augustus-gene-338.9-mRNA-1	1	OsR498G0102103800.01.T03	99,83	0,00E+00	2156	Os01g0782500	100,00	0,00E+00	2165	9	NA	Japonica	
maker-1-augustus-gene-339.39-mRNA-1	1	OsR498G0102108700.01.T06	84,91	0,00E+00	1458	Os01g0783800	100,00	0,00E+00	3059	1601	Japonica	Japonica	Kinase-TM
maker-1-augustus-gene-339.51-mRNA-1	1	OsR498G0102109900.01.T01	99,81	0,00E+00	1934	Os01g0784600	100,00	0,00E+00	1651	-283	Indica	Japonica	
maker-1-augustus-gene-339.58-mRNA-1	1	OsR498G0102114500.01.T01	99,78	0,00E+00	3288	Os01g0785400	100,00	0,00E+00	3306	18	NA	Japonica	
maker-1-augustus-gene-339.61-mRNA-1	1	OsR498G0102110200.01.T02	99,96	0,00E+00	4579	Os01g0784700	98,70	0,00E+00	4460	-119	NA	Japonica	Kinase-TM
maker-1-augustus-gene-339.64-mRNA-1	1	OsR498G0102111800.01.T01	99,75	0,00E+00	2113	Os01g0784900	99,92	0,00E+00	2122	9	NA	Japonica	
maker-1-augustus-gene-34.0-mRNA-1	1	OsR498G0100219100.01.T01	100,00	0,00E+00	1808	Os01g0166100	98,40	0,00E+00	1737	-71	NA	Indica	
maker-1-augustus-gene-34.13-mRNA-1	1	OsR498G0100221500.01.T06	100,00	3,15E-144	511	Os01g0167100	100,00	2,12E-109	394	-117	Indica	Indica	
maker-1-augustus-gene-34.20-mRNA-1	1	OsR498G0100221200.01.T01	100,00	0,00E+00	3236	Os01g0166800	99,94	0,00E+00	3232	-4	NA	Indica	
maker-1-augustus-gene-34.24-mRNA-1	1	OsR498G0100222800.01.T01	100,00	0,00E+00	1932	Os01g0167500	99,72	0,00E+00	1919	-13	NA	Indica	
maker-1-augustus-gene-340.0-mRNA-1	1	OsR498G0102121700.01.T01	99,32	0,00E+00	777	Os01g0787400	100,00	0,00E+00	791	14	NA	Japonica	
maker-1-augustus-gene-340.10-mRNA-1	1	OsR498G0102119200.01.T01	99,67	0,00E+00	2136	Os01g0786700	100,00	0,00E+00	1072	-1064	Indica	Japonica	
maker-1-augustus-gene-340.13-mRNA-1	1	OsR498G0102119400.01.T01	99,78	0,00E+00	2487	Os01g0786800	100,00	0,00E+00	2354	-133	NA	Japonica	
maker-1-augustus-gene-340.16-mRNA-1	1	OsR498G0102119600.01.T03	100,00	0,00E+00	4713	Os01g0786900	100,00	0,00E+00	4713	0	NA	Japonica	
maker-1-augustus-gene-340.21-mRNA-1	1	OsR498G0102120400.01.T01	99,51	0,00E+00	1801	Os01g0787000	100,00	0,00E+00	1824	23	NA	Japonica	
maker-1-augustus-gene-340.4-mRNA-1	1	OsR498G0102122700.01.T01	99,75	0,00E+00	1420	Os01g0787600	100,00	0,00E+00	1429	9	NA	Japonica	
maker-1-augustus-gene-340.6-mRNA-1	1	OsR498G0102116800.01.T01	98,98	0,00E+00	2590	Os01g0785900	100,00	0,00E+00	2630	40	NA	Japonica	
maker-1-augustus-gene-340.8-mRNA-1	1	OsR498G0102118600.01.T01	99,87	0,00E+00	1338	Os01g0786500	100,00	0,00E+00	1054	-284	Indica	Japonica	
maker-1-augustus-gene-341.36-mRNA-1	1	OsR498G0102124700.01.T01	97,68	0,00E+00	737	Os01g0788200	97,96	0,00E+00	759	22	NA	Japonica	
maker-1-augustus-gene-341.38-mRNA-1	1	OsR498G0102125500.01.T01	99,70	0,00E+00	2938	Os01g0788400	100,00	0,00E+00	2960	22	NA	Japonica	
maker-1-augustus-gene-341.40-mRNA-1	1	OsR498G0102126500.01.T01	99,20	0,00E+00	2862	Os01g0788800	100,00	0,00E+00	2917	55	NA	Japonica	
maker-1-augustus-gene-341.46-mRNA-1	1	OsR498G0102127000.01.T01	99,25	0,00E+00	1174	Os03g0115200	88,73	0,00E+00	895	-279	Indica	Japonica	
maker-1-augustus-gene-341.48-mRNA-1	1	OsR498G0102127200.01.T01	98,96	0,00E+00	842	Os01g0789000	100,00	0,00E+00	821	-21	NA	Japonica	
maker-1-augustus-gene-341.51-mRNA-1	1	OsR498G0102127400.01.T01	98,79	0,00E+00	1730	Os01g0789100	100,00	0,00E+00	1786	56	NA	Japonica	
maker-1-augustus-gene-343.104-mRNA-1	1	OsR498G0102132600.01.T02	100,00	1,38E-179	628	Os01g0794500	100,00	4,80E-180	628	0	NA	Japonica	
maker-1-augustus-gene-343.59-mRNA-1	1	OsR498G0102132300.01.T02	99,75	0,00E+00	2124	Os01g0794400	100,00	0,00E+00	2138	14	NA	Japonica	
maker-1-augustus-gene-343.64-mRNA-1	1	OsR498G0102133700.01.T02	98,43	0,00E+00	3521	Os01g0795000	100,00	0,00E+00	3665	144	NA	Japonica	
maker-1-augustus-gene-343.78-mRNA-1	1	OsR498G0102134700.01.T03	99,96	0,00E+00	4000	Os01g0795200	97,70	0,00E+00	3816	-184	NA	NA	
maker-1-augustus-gene-343.84-mRNA-1	1	OsR498G0102134900.01.T06	99,87	0,00E+00	3985	Os01g0795200	79,79	0,00E+00	1999	-1986	Indica	Japonica	
maker-1-augustus-gene-344.0-mRNA-1	1	OsR498G0102139500.01.T01	99,31	0,00E+00	770	Os01g0796400	99,31	0,00E+00	770	0	NA	Japonica	
maker-1-augustus-gene-344.10-mRNA-1	1	OsR498G0102140600.01.T05	99,56	0,00E+00	1629	Os01g0796700	100,00	0,00E+00	1647	18	NA	Japonica	
maker-1-augustus-gene-344.11-mRNA-1	1	OsR498G0102140600.01.T06	100,00	2,81E-136	484	Os01g0796900	100,00	3,43E-136	482	-2	NA	Japonica	
maker-1-augustus-gene-344.3-mRNA-1	1	OsR498G0102143900.01.T01	99,66	0,00E+00	1572	Os01g0798500	100,00	0,00E+00	1586	14	NA	Japonica	
maker-1-augustus-gene-345.35-mRNA-1	1	OsR498G0102146300.01.T01	99,83	0,00E+00	2113	Os01g0799500	100,00	0,00E+00	2122	9	NA	Japonica	
maker-1-augustus-gene-345.39-mRNA-1	1	OsR498G0102147300.01.T01	99,68	0,00E+00	2244	Os01g0799900	100,00	0,00E+00	2262	18	NA	Japonica	
maker-1-augustus-gene-345.41-mRNA-1	1	OsR498G0511102000.01.T04	78,99	8,59E-164	579	Os01g0800266	100,00	0,00E+00	2089	1510	Japonica	Japonica	
maker-1-augustus-gene-345.43-mRNA-1	1	OsR498G0102149100.01.T01	99,84	0,00E+00	3287	Os01g0800500	100,00	0,00E+00	3301	14	NA	Japonica	
maker-1-augustus-gene-345.47-mRNA-1	1	OsR498G0102144600.01.T01	99,90	0,00E+00	3719	Os01g0798800	100,00	0,00E+00	3728	9	NA	Japonica	
maker-1-augustus-gene-346.0-mRNA-1	1	OsR498G0102151700.01.T05	98,92	0,00E+00	1300	Os01g0801000	99,19	0,00E+00	1309	9	NA	Japonica	
maker-1-augustus-gene-346.12-mRNA-1	1	OsR498G0102152400.01.T01	99,81	0,00E+00	1842	Os01g0801200	100,00	0,00E+00	1851	9	NA	Japonica	
maker-1-augustus-gene-346.14-mRNA-1	1	OsR498G0102153200.01.T01	100,00	0,00E+00	1829	Os01g0801500	100,00	0,00E+00	1829	0	NA	Japonica	
maker-1-augustus-gene-346.16-mRNA-1	1	OsR498G0102153400.01.T03	99,86	0,00E+00	2524	Os01g0801600	100,00	0,00E+00	2533	9	NA	Japonica	
maker-1-augustus-gene-346.19-mRNA-1	1	OsR498G0102153800.01.T02	100,00	0,00E+00	1873	Os01g0801700	100,00	0,00E+00	1873	0	NA	Japonica	
maker-1-augustus-gene-346.21-mRNA-1	1	OsR498G0102156000.01.T01	99,92	0,00E+00	2306	Os01g0802700	100,00	0,00E+00	2311	5	NA	Japonica	
maker-1-augustus-gene-346.5-mRNA-1	1	OsR498G0102152100.01.T03	100,00	0,00E+00	1683	Os01g0801100	100,00	0,00E+00	1683	0	NA	Japonica	
maker-1-augustus-gene-346.7-mRNA-1	1	OsR498G0102154900.01.T05	99,75	0,00E+00	2162	Os01g0802100	100,00	0,00E+00	2176	14	NA	Japonica	
maker-1-augustus-gene-347.50-mRNA-1	1	OsR498G0102157700.01.T05	99,53	0,00E+00	755	Os01g0803200	100,00	0,00E+00	764	9	NA	Japonica	
maker-1-augustus-gene-347.52-mRNA-1	1	OsR498G0102159600.01.T01	98,44	0,00E+00	2118	Os01g0803300	100,00	0,00E+00	1797	-321	Indica	Japonica	
maker-1-augustus-gene-348.0-mRNA-1	1	OsR498G0102166300.01.T01	99,27	0,00E+00	1462	Os01g0805300	99,27	0,00E+00	1462	0	NA	Japonica	
maker-1-augustus-gene-348.10-mRNA-1	1	OsR498G0102165600.01.T01	99,80	0,00E+00	905	Os01g0805000	99,65	1,38E-144	512	-393	Indica	Japonica	
maker-1-augustus-gene-348.12-mRNA-1	1	OsR498G0102166000.01.T01	92,30	0,00E+00	997	Os01g0805200	92,44	0,00E+00	1002	5	NA	Japonica	
maker-1-augustus-gene-348.14-mRNA-1	1	OsR498G0102167000.01.T01	99,85	0,00E+00	2426	Os01g0805600	100,00	0,00E+00	1781	-645	Indica	Japonica	
maker-1-augustus-gene-348.17-mRNA-1	1	OsR498G0102167200.01.T01	99,93	0,00E+00	2669	Os01g0805700	100,00	0,00E+00	2673	4	NA	Japonica	
maker-1-augustus-gene-348.2-mRNA-1	1	OsR498G0102166500.01.T01	99,01	0,00E+00	2489	Os01g0805400	100,00	0,00E+00	2549	60	NA	Japonica	
maker-1-augustus-gene-348.24-mRNA-1	1	OsR498G0102167600.01.T01	100,00	0,00E+00	1142	Os01g0805800	100,00	0,00E+00	1142	0	NA	Indica	
maker-1-augustus-gene-348.28-mRNA-1	1	OsR498G0102168400.01.T03	100,00	0,00E+00	839	Os01g0268800	70,69	2,75E-48	193	-646	Indica	Indica	
maker-1-augustus-gene-348.31-mRNA-1	1	OsR498G0102168600.01.T01	100,00	0,00E+00	2300	Os01g0806200	99,14	0,00E+00	2270	-30	NA	Indica	
maker-1-augustus-gene-348.4-mRNA-1	1	OsR498G0102166700.01.T02	96,09	0,00E+00	1992	Os01g0805500	100,00	0,00E+00	2614	622	Japonica	Japonica	
maker-1-augustus-gene-348.6-mRNA-1	1	OsR498G0102167900.01.T01	100,00	0,00E+00	2690	Os01g0805900	99,78	0,00E+00	2411	-279	Indica	Indica	
maker-1-augustus-gene-349.44-mRNA-1	1	OsR498G0102176900.01.T03	100,00	0,00E+00	2922	Os01g0807900	99,69	0,00E+00	2900	-22	NA	Indica	
maker-1-augustus-gene-349.48-mRNA-1	1	OsR498G0102177400.01.T02	100,00	0,00E+00	1445	Os01g0279900	76,28	9,03E-165	580	-865	Indica	Indica	
maker-1-augustus-gene-35.0-mRNA-1	1	OsR498G0100226300.01.T01	100,00	0,00E+00	2305	Os01g0168400	99,46	0,00E+00	2286	-19	NA	Indica	
maker-1-augustus-gene-35.18-mRNA-1	1	OsR498G0100224600.01.T02	100,00	0,00E+00	2343	Os01g0167900	99,77	0,00E+00	2330	-13	NA	Indica	
maker-1-augustus-gene-35.20-mRNA-1	1	OsR498G0100225200.01.T02	100,00	0,00E+00	715	Os01g0168200	99,50	0,00E+00	706	-9	NA	Indica	
maker-1-augustus-gene-35.23-mRNA-1	1	OsR498G0100225600.01.T01	100,00	0,00E+00	2062	Os01g0168300	99,56	0,00E+00	2039	-23	NA	Indica	
maker-1-augustus-gene-35.25-mRNA-1	1	OsR498G0100227000.01.T02	100,00	0,00E+00	1824	Os01g0168800	99,60	0,00E+00	1806	-18	NA	Indica	
maker-1-augustus-gene-35.6-mRNA-1	1	OsR498G0100226500.01.T03	100,00	0,00E+00	2262	Os01g0168500	99,92	0,00E+00	2258	-4	NA	Indica	
maker-1-augustus-gene-35.8-mRNA-1	1	OsR498G0100226700.01.T02	99,89	0,00E+00	1627	Os01g0168600	99,88	0,00E+00	1461	-166	Indica	Indica	
maker-1-augustus-gene-351.12-mRNA-1	1	OsR498G0102186800.01.T02	100,00	0,00E+00	1245	Os01g0810000	99,85	0,00E+00	1233	-12	NA	Indica	
maker-1-augustus-gene-351.17-mRNA-1	1	OsR498G0102187200.01.T01	99,94	0,00E+00	2856	Os01g0810100	99,87	0,00E+00	2852	-4	NA	Indica	
maker-1-augustus-gene-351.2-mRNA-1	1	OsR498G0102186500.01.T01	100,00	0,00E+00	2554	Os01g0809900	99,52	0,00E+00	2230	-324	Indica	Indica	
maker-1-augustus-gene-351.20-mRNA-1	1	OsR498G0102188400.01.T04	99,93	0,00E+00	2721	Os01g0810533	96,87	0,00E+00	2340	-381	Indica	Indica	Kinase-LRR-TM
maker-1-augustus-gene-351.6-mRNA-1	1	OsR498G0102187300.01.T02	100,00	0,00E+00	1229	Os01g0810200	99,85	0,00E+00	1224	-5	NA	Indica	
maker-1-augustus-gene-351.8-mRNA-1	1	OsR498G0102187500.01.T02	100,00	0,00E+00	1018	Os01g0810300	98,40	0,00E+00	977	-41	NA	Indica	
maker-1-augustus-gene-352.35-mRNA-1	1	OsR498G0102192900.01.T01	100,00	0,00E+00	1353	Os01g0811100	99,73	0,00E+00	1344	-9	NA	Indica	
maker-1-augustus-gene-352.41-mRNA-1	1	OsR498G0102195500.01.T02	100,00	0,00E+00	2998	Os01g0812000	99,94	0,00E+00	2993	-5	NA	Indica	
maker-1-augustus-gene-352.45-mRNA-1	1	OsR498G0102195900.01.T01	100,00	0,00E+00	7359	Os01g0812050	98,41	0,00E+00	7104	-255	NA	Indica	
maker-1-augustus-gene-352.57-mRNA-1	1	OsR498G0102192000.01.T04	99,85	0,00E+00	2432	Os01g0810900	99,48	0,00E+00	2409	-23	NA	Indica	Kinase-LRR-TM
maker-1-augustus-gene-352.63-mRNA-1	1	OsR498G0102193800.01.T02	100,00	0,00E+00	2711	Os01g0811400	99,66	0,00E+00	2600	-111	NA	Indica	
maker-1-augustus-gene-353.15-mRNA-1	1	OsR498G0102201900.01.T01	100,00	0,00E+00	2890	Os01g0814000	99,13	0,00E+00	2839	-51	NA	Indica	
maker-1-augustus-gene-353.22-mRNA-1	1	OsR498G0102198200.01.T01	100,00	0,00E+00	1148	Os01g0812600	99,84	0,00E+00	1143	-5	NA	Indica	
maker-1-augustus-gene-353.26-mRNA-1	1	OsR498G0102199200.01.T02	92,18	0,00E+00	813	Os03g0322500	73,26	8,75E-40	164	-649	Indica	Indica	
maker-1-augustus-gene-353.31-mRNA-1	1	OsR498G0102199400.01.T02	99,80	0,00E+00	1745	Os01g0813100	97,31	0,00E+00	1638	-107	NA	Indica	
maker-1-augustus-gene-353.35-mRNA-1	1	OsR498G0102200600.01.T01	100,00	0,00E+00	2320	Os01g0813500	99,46	0,00E+00	2288	-32	NA	Indica	
maker-1-augustus-gene-353.40-mRNA-1	1	OsR498G0102200800.01.T01	100,00	0,00E+00	2358	Os01g0930800	74,09	0,00E+00	776	-1582	Indica	Indica	
maker-1-augustus-gene-353.41-mRNA-1	1	OsR498G0102200800.01.T01	100,00	2,52E-97	354	Os01g0930800	78,30	2,07E-23	107	-247	Indica	Indica	
maker-1-augustus-gene-353.44-mRNA-1	1	OsR498G0102201400.01.T03	100,00	0,00E+00	1355	Os01g0930800	77,71	1,49E-154	545	-810	Indica	Indica	

maker-1-augustus-gene-353.8-mRNA-1	1	OsR498G0102201700.01.T06	100,00	0,00E+00	2834	Os01g0813900	99,81	0,00E+00	2820	-14	NA	Indica	
maker-1-augustus-gene-354.10-mRNA-1	1	OsR498G0102202600.01.T02	99,76	0,00E+00	2935	Os05g0489000	74,88	0,00E+00	1078	-1857	Indica	Indica	
maker-1-augustus-gene-354.14-mRNA-1	1	OsR498G0102203000.01.T01	100,00	0,00E+00	2030	Os01g0814400	100,00	0,00E+00	2030	0	NA	Indica	
maker-1-augustus-gene-354.16-mRNA-1	1	OsR498G0102203400.01.T05	100,00	0,00E+00	1310	Os01g0814700	98,77	0,00E+00	994	-316	Indica	Indica	
maker-1-augustus-gene-354.2-mRNA-1	1	OsR498G0102202700.01.T01	99,52	0,00E+00	1868	Os01g0814300	98,30	0,00E+00	1829	-39	NA	Indica	
maker-1-augustus-gene-354.20-mRNA-1	1	OsR498G0102203600.01.T01	100,00	0,00E+00	2127	Os01g0814800	99,75	0,00E+00	2113	-14	NA	Indica	
maker-1-augustus-gene-354.23-mRNA-1	1	OsR498G0102204200.01.T01	100,00	0,00E+00	1516	Os01g0814900	100,00	0,00E+00	1516	0	NA	Indica	
maker-1-augustus-gene-354.27-mRNA-1	1	OsR498G0511077900.01.T01	70,04	6,84E-18	91,5	Os01g0815400	99,66	3,51E-149	526	434,5	Japonica	Indica	
maker-1-augustus-gene-354.30-mRNA-1	1	OsR498G0102207800.01.T02	100,00	0,00E+00	1727	Os01g0816100	99,90	0,00E+00	1722	-5	NA	Indica	
maker-1-augustus-gene-354.32-mRNA-1	1	OsR498G0102209000.01.T04	99,80	0,00E+00	10532	Os01g0816400	99,56	0,00E+00	10468	-64	NA	Indica	
maker-1-augustus-gene-354.4-mRNA-1	1	OsR498G0102206700.01.T01	100,00	0,00E+00	2318	Os01g0815700	99,77	0,00E+00	2305	-13	NA	Indica	
maker-1-augustus-gene-354.8-mRNA-1	1	OsR498G0102206900.01.T03	100,00	0,00E+00	883	Os01g0815800	100,00	0,00E+00	883	0	NA	Indica	
maker-1-augustus-gene-355.13-mRNA-1	1	OsR498G0102213900.01.T04	100,00	0,00E+00	1698	Os01g0817700	99,79	0,00E+00	1689	-9	NA	Indica	
maker-1-augustus-gene-355.18-mRNA-1	1	OsR498G0102210200.01.T01	100,00	0,00E+00	1635	Os01g0816400	88,87	0,00E+00	935	-700	Indica	Indica	
maker-1-augustus-gene-355.21-mRNA-1	1	OsR498G0102211400.01.T01	100,00	0,00E+00	1656	Os01g0817000	99,78	0,00E+00	1647	-9	NA	Indica	
maker-1-augustus-gene-355.24-mRNA-1	1	OsR498G0102211800.01.T05	100,00	0,00E+00	1413	Os01g0817100	99,75	0,00E+00	1404	-9	NA	Indica	
maker-1-augustus-gene-355.26-mRNA-1	1	OsR498G0102214200.01.T01	100,00	0,00E+00	2462	Os01g0817800	99,78	0,00E+00	2449	-13	NA	Indica	
maker-1-augustus-gene-355.9-mRNA-1	1	OsR498G0102212100.01.T01	100,00	0,00E+00	4740	Os01g0817400	99,82	0,00E+00	3026	-1714	Indica	Indica	
maker-1-augustus-gene-356.0-mRNA-1	1	OsR498G0102215500.01.T06	100,00	0,00E+00	2343	Os01g0818000	99,85	0,00E+00	2334	-9	NA	Indica	
maker-1-augustus-gene-356.11-mRNA-1	1	OsR498G0102216700.01.T02	100,00	0,00E+00	1153	Os01g0818400	99,84	0,00E+00	1149	-4	NA	Indica	
maker-1-augustus-gene-356.13-mRNA-1	1	OsR498G0102218300.01.T01	100,00	0,00E+00	3139	Os01g0819000	99,66	0,00E+00	3112	-27	NA	Indica	
maker-1-augustus-gene-356.16-mRNA-1	1	OsR498G0102218900.01.T03	100,00	0,00E+00	3452	Os05g0480400	79,55	0,00E+00	1624	-1828	Indica	Indica	Kinase-LRR-TM
maker-1-augustus-gene-356.25-mRNA-1	1	OsR498G0102219200.01.T01	100,00	0,00E+00	2819	Os09g0538800	80,72	0,00E+00	1532	-1287	Indica	Indica	
maker-1-augustus-gene-356.37-mRNA-1	1	OsR498G0102219400.01.T03	99,08	0,00E+00	2885	Os09g0538800	86,57	0,00E+00	1944	-941	Indica	Indica	
maker-1-augustus-gene-356.7-mRNA-1	1	OsR498G0102216100.01.T01	97,26	0,00E+00	1489	Os01g0818300	97,26	0,00E+00	1489	0	NA	Indica	
maker-1-augustus-gene-357.0-mRNA-1	1	OsR498G0102220900.01.T02	97,25	1,31E-82	306	Os01g0819400	98,43	1,15E-90	332	26	NA	Indica	
maker-1-augustus-gene-357.1-mRNA-1	1	OsR498G0102221700.01.T02	96,79	4,49E-115	414	Os01g0819400	100,00	2,17E-126	450	36	NA	Indica	
maker-1-augustus-gene-357.16-mRNA-1	1	OsR498G0102222600.01.T01	100,00	0,00E+00	4470	Os01g0819900	99,84	0,00E+00	4452	-18	NA	Indica	
maker-1-augustus-gene-357.20-mRNA-1	1	OsR498G0510486800.01.T01	79,26	0,00E+00	1310	Os01g0820000	99,36	0,00E+00	1948	638	Japonica	Indica	
maker-1-augustus-gene-357.6-mRNA-1	1	OsR498G0102220900.01.T02	100,00	0,00E+00	807	Os01g0819400	96,87	0,00E+00	744	-63	NA	Indica	
maker-1-augustus-gene-357.8-mRNA-1	1	OsR498G0102222100.01.T02	100,00	0,00E+00	2516	Os01g0819700	99,00	0,00E+00	2473	-43	NA	NA	
maker-1-augustus-gene-358.0-mRNA-1	1	OsR498G0102228300.01.T01	100,00	0,00E+00	1505	Os01g0821600	98,58	0,00E+00	1471	-34	NA	Indica	
maker-1-augustus-gene-358.13-mRNA-1	1	OsR498G0102228800.01.T01	100,00	0,00E+00	2219	Os01g0821800	99,84	0,00E+00	2210	-9	NA	Indica	
maker-1-augustus-gene-358.18-mRNA-1	1	OsR498G0102230200.01.T01	100,00	0,00E+00	5130	Os01g0821900	99,70	0,00E+00	4167	-963	Indica	Indica	Kinase-LRR-TM
maker-1-augustus-gene-358.20-mRNA-1	1	OsR498G0102231000.01.T04	99,75	0,00E+00	2169	Os01g0822200	99,67	0,00E+00	2165	-4	NA	Indica	Kinase-TM
maker-1-augustus-gene-358.4-mRNA-1	1	OsR498G0102227200.01.T01	100,00	0,00E+00	1932	Os01g0821300	97,97	0,00E+00	1862	-70	NA	Indica	
maker-1-augustus-gene-359.42-mRNA-1	1	OsR498G0102236100.01.T01	100,00	0,00E+00	1364	Os01g0823100	99,74	0,00E+00	1355	-9	NA	Indica	
maker-1-augustus-gene-359.49-mRNA-1	1	OsR498G0102236500.01.T01	100,00	0,00E+00	722	Os01g0823300	99,50	0,00E+00	713	-9	NA	Indica	
maker-1-augustus-gene-359.51-mRNA-1	1	OsR498G0102236900.01.T02	100,00	0,00E+00	1970	Os01g0823400	97,75	0,00E+00	1910	-60	NA	Indica	
maker-1-augustus-gene-359.54-mRNA-1	1	OsR498G0102238700.01.T01	100,00	0,00E+00	926	Os01g0823600	100,00	0,00E+00	926	0	NA	Indica	
maker-1-augustus-gene-36.36-mRNA-1	1	OsR498G0100236300.01.T02	99,94	0,00E+00	6040	Os01g0170300	99,69	0,00E+00	5163	-877	Indica	Indica	Kinase-LRR-TM
maker-1-augustus-gene-36.42-mRNA-1	1	OsR498G0100237500.01.T01	99,40	0,00E+00	2378	Os01g0170700	99,59	0,00E+00	2166	-212	NA	Japonica	
maker-1-augustus-gene-36.46-mRNA-1	1	OsR498G0100232800.01.T02	99,80	0,00E+00	5247	Os01g0169500	99,86	0,00E+00	5256	9	NA	Indica	
maker-1-augustus-gene-36.50-mRNA-1	1	OsR498G0100233200.01.T01	99,80	0,00E+00	2736	Os05g0169300	77,08	0,00E+00	896	-1840	Indica	NA	
maker-1-augustus-gene-36.53-mRNA-1	1	OsR498G0100234400.01.T01	99,94	0,00E+00	3140	Os01g0169900	99,94	0,00E+00	3140	0	NA	NA	
maker-1-augustus-gene-36.58-mRNA-1	1	OsR498G0100236600.01.T01	99,60	0,00E+00	1329	Os01g0170500	99,73	0,00E+00	1333	4	NA	NA	
maker-1-augustus-gene-360.12-mRNA-1	1	OsR498G0102245400.01.T03	100,00	0,00E+00	2928	Os01g0825500	99,94	0,00E+00	2923	-5	NA	Indica	
maker-1-augustus-gene-360.14-mRNA-1	1	OsR498G0102246600.01.T01	100,00	0,00E+00	3231	Os01g0825700	99,67	0,00E+00	3207	-24	NA	Indica	
maker-1-augustus-gene-360.20-mRNA-1	1	OsR498G0102247400.01.T01	97,83	0,00E+00	2377	Os01g0825800	88,41	0,00E+00	1651	-726	Indica	Indica	
maker-1-augustus-gene-360.21-mRNA-1	1	OsR498G0102247400.01.T02	92,44	0,00E+00	907	Os01g0825800	92,10	0,00E+00	898	-9	NA	Indica	
maker-1-augustus-gene-360.3-mRNA-1	1	OsR498G0102242100.01.T06	100,00	0,00E+00	2583	Os01g0824600	100,00	0,00E+00	2405	-178	NA	Indica	Kinase-TM
maker-1-augustus-gene-360.4-mRNA-1	1	OsR498G0102242700.01.T02	100,00	0,00E+00	2170	Os01g0824800	99,92	0,00E+00	2166	-4	NA	Indica	
maker-1-augustus-gene-360.6-mRNA-1	1	OsR498G0102243500.01.T01	100,00	0,00E+00	1364	Os01g0825000	99,87	0,00E+00	1360	-4	NA	Indica	
maker-1-augustus-gene-360.8-mRNA-1	1	OsR498G0102247900.01.T01	100,00	1,14E-101	369	Os01g0826000	99,51	1,69E-100	364	-5	NA	Indica	
maker-1-augustus-gene-361.38-mRNA-1	1	OsR498G0102249300.01.T01	100,00	0,00E+00	3009	Os01g0826400	99,58	0,00E+00	2977	-32	NA	Indica	
maker-1-augustus-gene-361.43-mRNA-1	1	OsR498G0102253300.01.T01	100,00	0,00E+00	1956	Os01g0827300	99,91	0,00E+00	1951	-5	NA	Indica	
maker-1-augustus-gene-361.51-mRNA-1	1	OsR498G0102255700.01.T04	100,00	0,00E+00	1930	Os01g0828300	99,81	0,00E+00	1921	-9	NA	Indica	
maker-1-augustus-gene-361.60-mRNA-1	1	OsR498G0102252200.01.T01	100,00	0,00E+00	3918	Os01g0826900	99,77	0,00E+00	3895	-23	NA	Indica	
maker-1-augustus-gene-361.64-mRNA-1	1	OsR498G0102252800.01.T01	100,00	0,00E+00	2733	Os01g0827200	99,54	0,00E+00	2701	-32	NA	Indica	
maker-1-augustus-gene-361.68-mRNA-1	1	OsR498G0102254000.01.T01	100,00	0,00E+00	1900	Os01g0827700	99,42	0,00E+00	1535	-365	Indica	Indica	
maker-1-augustus-gene-362.39-mRNA-1	1	OsR498G0102257700.01.T02	100,00	0,00E+00	1721	Os01g0829000	99,79	0,00E+00	1712	-9	NA	Indica	
maker-1-augustus-gene-362.48-mRNA-1	1	OsR498G0102265100.01.T04	100,00	0,00E+00	2830	Os01g0830700	99,94	0,00E+00	2826	-4	NA	Indica	
maker-1-augustus-gene-362.53-mRNA-1	1	OsR498G0102258000.01.T01	100,00	0,00E+00	2414	Os01g0829100	99,61	0,00E+00	2288	-126	NA	Indica	
maker-1-augustus-gene-362.57-mRNA-1	1	OsR498G0102261000.01.T01	99,83	0,00E+00	3069	Os01g0829900	99,77	0,00E+00	3065	-4	NA	Indica	
maker-1-augustus-gene-362.59-mRNA-1	1	OsR498G0102261200.01.T01	100,00	0,00E+00	2949	Os01g0830000	99,94	0,00E+00	2945	-4	NA	Indica	
maker-1-augustus-gene-362.61-mRNA-1	1	OsR498G0102262400.01.T01	100,00	0,00E+00	2114	Os01g0830500	99,74	0,00E+00	2101	-13	NA	Indica	
maker-1-augustus-gene-363.0-mRNA-1	1	OsR498G0102268500.01.T02	100,00	0,00E+00	1099	Os01g0831200	99,67	0,00E+00	1090	-9	NA	NA	
maker-1-augustus-gene-363.14-mRNA-1	1	OsR498G0102271500.01.T02	94,82	0,00E+00	1847	Os01g0832600	94,73	0,00E+00	1843	-4	NA	Indica	
maker-1-augustus-gene-363.16-mRNA-1	1	OsR498G0102268800.01.T01	100,00	0,00E+00	2695	Os01g0831900	87,56	0,00E+00	1851	-844	Indica	Indica	
maker-1-augustus-gene-363.18-mRNA-1	1	OsR498G0102269200.01.T01	100,00	0,00E+00	2717	Os01g0831900	100,00	0,00E+00	2717	0	NA	Indica	
maker-1-augustus-gene-363.25-mRNA-1	1	OsR498G0102270000.01.T01	100,00	0,00E+00	1240	Os05g0467300	82,37	9,68E-137	487	-753	Indica	Indica	
maker-1-augustus-gene-363.6-mRNA-1	1	OsR498G0102269700.01.T01	100,00	0,00E+00	1975	Os01g0832100	99,54	0,00E+00	1953	-22	NA	Indica	
maker-1-augustus-gene-363.8-mRNA-1	1	OsR498G0102270900.01.T05	100,00	0,00E+00	2987	Os05g0467000	86,70	0,00E+00	2010	-977	Indica	Indica	
maker-1-augustus-gene-364.11-mRNA-1	1	OsR498G0102276700.01.T05	98,90	0,00E+00	2880	Os01g0834200	98,84	0,00E+00	2875	-5	NA	Indica	
maker-1-augustus-gene-364.18-mRNA-1	1	OsR498G0102273800.01.T01	100,00	0,00E+00	1694	Os01g0833100	99,47	0,00E+00	1672	-22	NA	Indica	
maker-1-augustus-gene-364.22-mRNA-1	1	OsR498G0102274800.01.T01	100,00	0,00E+00	2462	Os01g0833500	99,93	0,00E+00	2458	-4	NA	Indica	
maker-1-augustus-gene-364.25-mRNA-1	1	OsR498G0102275000.01.T01	100,00	0,00E+00	975	Os01g0833600	99,62	0,00E+00	928	-47	NA	Indica	
maker-1-augustus-gene-364.27-mRNA-1	1	OsR498G0102275200.01.T03	100,00	0,00E+00	2809	Os01g0833700	99,74	0,00E+00	2791	-18	NA	Indica	
maker-1-augustus-gene-364.32-mRNA-1	1	OsR498G0102275400.01.T05	97,85	0,00E+00	2647	Os01g0833800	97,66	0,00E+00	2634	-13	NA	Indica	
maker-1-augustus-gene-364.35-mRNA-1	1	OsR498G0102275800.01.T02	100,00	0,00E+00	2857	Os01g0834100	98,98	0,00E+00	1892	-965	Indica	Indica	
maker-1-augustus-gene-364.6-mRNA-1	1	OsR498G0102274100.01.T02	100,00	0,00E+00	1018	Os01g0833200	99,82	0,00E+00	1013	-5	NA	Indica	
maker-1-augustus-gene-364.9-mRNA-1	1	OsR498G0102274300.01.T02	100,00	0,00E+00	856	Os01g0833400	99,79	0,00E+00	851	-5	NA	Indica	
maker-1-augustus-gene-365.0-mRNA-1	1	OsR498G0102279700.01.T01	96,46	0,00E+00	1841	Os01g0834700	96,37	0,00E+00	1837	-4	NA	Indica	
maker-1-augustus-gene-365.11-mRNA-1	1	OsR498G0102279400.01.T04	99,10	0,00E+00	783	Os01g0834500	99,77	0,00E+00	763	-20	NA	Indica	
maker-1-augustus-gene-365.13-mRNA-1	1	OsR498G0102279400.01.T04	100,00	0,00E+00	801	Os01g0834601	99,53	0,00E+00	758	-43	NA	Indica	
maker-1-augustus-gene-365.15-mRNA-1	1	OsR498G0102281200.01.T01	100,00	0,00E+00	4464	Os05g0465100	76,17	0,00E+00	1681	-2783	Indica	Indica	
maker-1-augustus-gene-365.20-mRNA-1	1	OsR498G0102285600.01.T01	100,00	0,00E+00	1813	Os01g0835700	99,41	0,00E+00	1790	-23	NA	Indica	
maker-1-augustus-gene-365.22-mRNA-1	1	OsR498G0102286000.01.T02	100,00	0,00E+00	4551	Os01g0835800	99,79	0,00E+00	4362	-189	NA	Indica	
maker-1-augustus-gene-365.3-mRNA-1	1	OsR498G0102283700.01.T01	100,00	0,00E+00	4567	Os01g0835500	99,96	0,00E+00	4562	-5	NA	Indica	
maker-1-augustus-gene-365.9-mRNA-1	1	OsR498G0102279000.01.T01	98,00	0,00E+00	856	Os05g0463800	79,64	1,28E-94	346	-510	Indica	Indica	
maker-1-augustus-gene-366.40-mRNA-1	1	OsR498G0102287100.01.T04	100,00	0,00E+00	3204	Os01g0836400	99,94	0,00E+00	2752	-452	Indica	Indica	
maker-1-augustus-gene-366.49-mRNA-1	1	OsR498G0102291100.01.T01	100,00	0,00E+00	2224	Os01g0837300	99,92	0,00E+00	2220	-4	NA	Indica	
maker-1-augustus-gene-366.53-mRNA-1	1	OsR498G0102291300.01.T04	100,00	0,00E+00	2468	Os01g0837500	99,64	0,00E+00	2445	-23	NA	Indica	
maker-1-augustus-gene-366.59-mRNA-1	1	OsR498G0102291500.01.T01	100,00	0,00E+00	684	Os01g0837600	100,00	1,10E-158	558	-126	Indica	Indica	
maker-1-augustus-gene-366.63-mRNA-1	1	OsR498G0102290000.01.T02	100,00	0,00E+00	3014	Os01g0837000	99,84	0,00E+00	2242	-772	Indica	Indica	
maker-1-augustus-gene-366.73-mRNA-1	1	OsR498G0102292600.01.T02	100,00	0,00E+00	2354	Os01g0837900	99,92	0,00E+00	2350	-4	NA	Indica	
maker-1-augustus-gene-367.1-mRNA-1	1	OsR498G0102294100.01.T02	99,56	0,00E+00	816	Os01g0838100	98,25	0,00E+00	789	-27	NA	Indica	
maker-1-augustus-gene-367.16-mRNA-1	1	OsR498G0102297100.01.T01	100,00	0,00E+00	964	Os01g0838800	100,00	0,00E+00	964	0	NA	Indica	
maker-1-augustus-gene-367.28-mRNA-1	1	OsR498G0102299000.01.T01	100,00	0,00E+00	2246	Os01g0839200	98,96	0,00E+00	2191	-55	NA	Indica	
maker-1-augustus-gene-367.30-mRNA-1	1	OsR498G0102300200.01.T01	96,08	0,00E+00	823	Os01g0839700	96,08	0,00E+00	823	0	NA	Indica	
maker-1-augustus-gene-368.0-mRNA-1	1	OsR498G0102302100.01.T02	100,00	0,00E+00	3512	Os01g0840100	99,64	0,00E+00	3480	-32	NA	Indica	
maker-1-augustus-gene-368.15-mRNA-1	1	OsR498G0102308400.01.T02	99,71	0,00E+00	1249	Os01g0841200	99,57	0,00E+00	1244	-5	NA	NA	
maker-1-augustus-gene-368.7-mRNA-1	1	OsR498G0102304200.01.T01	100,00	0,00E+00	1700	Os01g0840300	99,89	0,00E+00	1695	-5	NA	Indica	
maker-1-augustus-gene-368.9-mRNA-1	1	OsR498G0102306200.01.T01	100,00	1,83E-171	601	Os01g0840700	99,40	3,30E-169	592	-9	NA	NA	
maker-1-augustus-gene-369.0-mRNA-1	1	OsR498G0102309500.01.T01	98,82	0,00E+00	3140	Os01g0841500	98,66	0,00E+00	3126	-14	NA	NA	
maker-1-augustus-gene-369.12-mRNA-1	1	OsR498G0102310200.01.T01	97,79	0,00E+00	3507	Os01g0841800	97,14	0,00E+00	2786	-721	Indica	NA	
maker-1-augustus-gene-369.19-mRNA-1	1	OsR498G0102312600.01.T01	100,00	0,00E+00	3139	Os01g0842400	99,89	0,00E+00	3130	-9	NA	NA	
maker-1-augustus-gene-369.21-mRNA-1	1	OsR498G0102313000.01.T01	100,00	0,00E+00	3128	Os01g0842500	99,71	0,00E+00	3105	-23	NA	NA	
maker-1-augustus-gene-369.26-mRNA-1	1	OsR498G0102313400.01.T01	100,00	0,00E+00	4345	Os01g0842600	99,88	0,00E+00	4332	-13	NA	NA	
maker-1-augustus-gene-369.4-mRNA-1	1	OsR498G0102309700.01.T01	100,00	0,00E+00	1386	Os01g0841600	99,61	0,00E+00	1372	-14	NA	NA	
maker-1-augustus-gene-37.2-mRNA-1	1	OsR498G0100242100.01.T02	100,00	0,00E+00	1516	Os01g0172100	99,88	0,00E+00	1511	-5	NA	Japonica	
maker-1-augustus-gene-37.22-mRNA-1	1	OsR498G0100240600.01.T03	99,83	0,00E+00	1051	Os01g0171800	99,83	0,00E+00	1051	0	NA	NA	
maker-1-augustus-gene-37.33-mRNA-1	1	OsR498G0100242400.01.T02	97,59	0,00E+00	2201	Os01g0172200	97,44	0,00E+00	2195	-6	NA	Indica	
maker-1-augustus-gene-37.36-mRNA-1	1	OsR498G0100242600.01.T02	98,42	0,00E+00	992	Os01g0172300	98,42	0,00E+00	992	0	NA	Indica	
maker-1-augustus-gene-37.39-mRNA-1	1	OsR498G0100242800.01.T02	99,96	0,00E+00	4395	Os01g0172400	99,92	0,00E+00	4390	-5	NA	Indica	
maker-1-augustus-gene-37.42-mRNA-1	1	OsR498G0100243400.01.T05	99,83	0,00E+00	1051	Os01g0173000	99,83	0,00E+00	1051	0	NA	Japonica	
maker-1-augustus-gene-37.46-mRNA-1	1	OsR498G0100243800.01.T01	100,00	0,00E+00	829	Os01g0173100	100,00	0,00E+00	829	0	NA	Japonica	
maker-1-augustus-gene-370.38-mRNA-1	1	OsR498G0102315900.01.T01	100,00	0,00E+00	2284	Os03g0810600	85,20	0,00E+00	1429	-855	Indica	NA	
maker-1-augustus-gene-370.43-mRNA-1	1	OsR498G0102317300.01.T01	100,00	0,00E+00	3149	Os01g0844050	99,61	0,00E+00	2267	-882	Indica	NA	
maker-1-augustus-gene-370.46-mRNA-1	1	OsR498G0102318300.01.T05	100,00	0,00E+00	1007	Os01g0844300	99,82	0,00E+00	1003	-4	NA	NA	
maker-1-augustus-gene-370.49-mRNA-1	1	OsR498G0102321100.01.T02	100,00	0,00E+00	5519	Os01g0844800	99,54	0,00E+00	5468	-51	NA	NA	
maker-1-augustus-gene-370.56-mRNA-1	1	OsR498G0102318600.01.T04	100,00	0,00E+00	695	Os01g0844400	100,00	0,00E+00	695	0	NA	NA	
maker-1-augustus-gene-371.12-mRNA-1	1	OsR498G0102326300.01.T04	100,00	0,00E+00	1270	Os01g0846500	99,85	0,00E+00	1188	-82	NA	NA	
maker-1-augustus-gene-371.22-mRNA-1	1	OsR498G0102328500.01.T02	100,00	0,00E+00	1959	Os01g0847200	100,00	0,00E+00	1959	0	NA	NA	
maker-1-augustus-gene-371.25-mRNA-1	1	OsR498G0102329100.01.T03	100,00	0,00E+00	1689	Os01g0847700	89,96	0,00E+00	1265	-424	Indica	NA	
maker-1-augustus-gene-371.28-mRNA-1	1	OsR498G0102329500.01.T01	100,00	0,00E+00	1689	Os01g0847700	99,90	0,00E+00	1755	66	NA	NA	
maker-1-augustus-gene-371.3-mRNA-1	1	OsR498G0102325500.01.T01	100,00	0,00E+00	2246	Os01g0846300	99,92	0,00E+00	2241	-5	NA	NA	
maker-1-augustus-gene-371.34-mRNA-1	1	OsR498G0102324800.01.T02	94,83	0,00E+00	930	Os03g0855700	86,86	1,58E-176	618	-312	Indica	NA	
maker-1-augustus-gene-371.37-mRNA-1	1	OsR498G1221780100.01.T02	96,14	4,86E-130	465	Os01g0630700	84,03	6,76E-85	313	-152	Indica	NA	
maker-1-augustus-gene-371.43-mRNA-1	1	OsR498G0102327000.01.T02	99,90	0,00E+00	1843	Os01g0846600	99,41	0,00E+00	1820	-23	NA	NA	
maker-1-augustus-gene-371.46-mRNA-1	1	OsR498G0102328000.01.T03	100,00	0,00E+00	2870	Os01g0846900	99,14	0,00E+00	2458	-412	Indica	NA	
maker-1-augustus-gene-371.50-mRNA-1	1	OsR498G0102328800.01.T01	100,00	1,07E-170	600	Os01g0847300	100,00	3,72E-171	600	0	NA	NA	
maker-1-augustus-gene-372.0-mRNA-1	1	OsR498G0102329700.01.T02	100,00	0,00E+00	1501	Os05g0456300	70,16	1,33E-103	376	-1125	Indica	NA	
maker-1-augustus-gene-372.11-mRNA-1	1	OsR498G0102334700.01.T01	100,00	0,00E+00	850	Os01g0848900	95,57	3,02E-165	580	-270	Indica	NA	
maker-1-augustus-gene-372.16-mRNA-1	1	OsR498G0102330000.01.T02	100,00	0,00E+00	659	Os01g0847900	100,00	0,00E+00	659	0	NA	NA	
maker-1-augustus-gene-372.18-mRNA-1	1	OsR498G0102332000.01.T01	100,00	0,00E+00	3317	Os01g0848400	99,89	0,00E+00	3308	-9	NA	NA	
maker-1-augustus-gene-372.22-mRNA-1	1	OsR498G0102333600.01.T01	100,00	0,00E+00	1175	Os01g0848700	100,00	0,00E+00	1175	0	NA	NA	
maker-1-augustus-gene-372.24-mRNA-1	1	OsR498G0102335200.01.T01	100,00	0,00E+00	2960	Os01g0849100	99,82	0,00E+00	2947	-13	NA	NA	
maker-1-augustus-gene-372.26-mRNA-1	1	OsR498G0102335800.01.T01	100,00	0,00E+00	883	Os01g0849600	100,00	0,00E+00	883	0	NA	NA	
maker-1-augustus-gene-372.4-mRNA-1	1	OsR498G0102331100.01.T02	100,00	0,00E+00	3472	Os01g0848200	99,95	0,00E+00	3468	-4	NA	NA	
maker-1-augustus-gene-373.0-mRNA-1	1	OsR498G0102338100.01.T02	100,00	0,00E+00	1970	Os01g0850000	99,73	0,00E+00	1957	-13	NA	NA	
maker-1-augustus-gene-373.10-mRNA-1	1	OsR498G0102339900.01.T01	100,00	0,00E+00	3144	Os12g0259800	73,93	0,00E+00	984	-2160	Indica	NA	
maker-1-augustus-gene-373.13-mRNA-1	1	OsR498G0102340300.01.T01	100,00	0,00E+00	3030	Os01g0850700	99,82	0,00E+00	3017	-13	NA	NA	
maker-1-augustus-gene-373.15-mRNA-1	1	OsR498G0102340700.01.T01	100,00	0,00E+00	3003	Os01g0850800	99,88	0,00E+00	2994	-9	NA	Japonica	
maker-1-augustus-gene-373.17-mRNA-1	1	OsR498G0102341500.01.T03	100,00	0,00E+00	2879	Os01g0851000	99,81	0,00E+00	2865	-14	NA	NA	
maker-1-augustus-gene-373.19-mRNA-1	1	OsR498G0102339600.01.T01	100,00	0,00E+00	2241	Os01g0850400	99,68	0,00E+00	2223	-18	NA	NA	
maker-1-augustus-gene-373.3-mRNA-1	1	OsR498G0102338900.01.T06	99,86	0,00E+00	1266	Os01g0850100	99,71	0,00E+00	1245	-21	NA	NA	
maker-1-augustus-gene-374.33-mRNA-1	1	OsR498G0102350700.01.T02	100,00	0,00E+00	3415	Os01g0853400	99,95	0,00E+00	3410	-5	NA	NA	
maker-1-augustus-gene-374.37-mRNA-1	1	OsR498G0102343200.01.T02	100,00	0,00E+00	1104	Os01g0851300	99,51	0,00E+00	1091	-13	NA	NA	
maker-1-augustus-gene-374.40-mRNA-1	1	OsR498G0102343400.01.T01	100,00	0,00E+00	1824	Os01g0851400	99,60	0,00E+00	1806	-18	NA	NA	
maker-1-augustus-gene-374.42-mRNA-1	1	OsR498G0102345000.01.T01	100,00	0,00E+00	4556	Os01g0851700	99,84	0,00E+00	4538	-18	NA	NA	
maker-1-augustus-gene-374.48-mRNA-1	1	OsR498G0102345600.01.T02	100,00	0,00E+00	2457	Os03g0825800	71,23	1,44E-92	341	-2116	Indica	NA	Kinase-TM
maker-1-augustus-gene-374.52-mRNA-1	1	OsR498G0102346000.01.T01	100,00	0,00E+00	2814	Os01g0852200	99,81	0,00E+00	1907	-907	Indica	NA	
maker-1-augustus-gene-374.57-mRNA-1	1	OsR498G0102348400.01.T03	99,91	0,00E+00	2023	Os01g0852500	99,79	0,00E+00	1716	-307	Indica	NA	
maker-1-augustus-gene-374.60-mRNA-1	1	OsR498G0102349200.01.T01	100,00	0,00E+00	1407	Os01g0853000	99,36	0,00E+00	1388	-19	NA	NA	
maker-1-augustus-gene-375.0-mRNA-1	1	OsR498G0102355900.01.T01	100,00	0,00E+00	693	Os01g0854400	100,00	0,00E+00	693	0	NA	NA	
maker-1-augustus-gene-375.1-mRNA-1	1	OsR498G0102352200.01.T01	100,00	0,00E+00	1624	Os01g0853700	99,89	0,00E+00	1619	-5	NA	NA	
maker-1-augustus-gene-375.3-mRNA-1	1	OsR498G0102356600.01.T01	100,00	0,00E+00	1088	Os01g0854500	99,67	0,00E+00	1079	-9	NA	NA	
maker-1-augustus-gene-375.5-mRNA-1	1	OsR498G0102357800.01.T01	100,00	0,00E+00	2695	Os01g0855000	99,93	0,00E+00	2691	-4	NA	NA	
maker-1-augustus-gene-376.22-mRNA-1	1	OsR498G0102360100.01.T01	100,00	0,00E+00	2760	Os01g0855200	99,16	0,00E+00	2721	-39	NA	NA	
maker-1-augustus-gene-376.24-mRNA-1	1	OsR498G0102361100.01.T01	100,00	0,00E+00	2430	Os01g0855400	99,77	0,00E+00	2292	-138	NA	NA	
maker-1-augustus-gene-376.26-mRNA-1	1	OsR498G0102362900.01.T03	100,00	0,00E+00	1622	Os01g0856000	99,89	0,00E+00	1618	-4	NA	NA	
maker-1-augustus-gene-376.29-mRNA-1	1	OsR498G0102366700.01.T01	100,00	0,00E+00	2668	Os01g0856500	99,80	0,00E+00	2654	-14	NA	NA	
maker-1-augustus-gene-377.33-mRNA-1	1	OsR498G0102367900.01.T01	100,00	0,00E+00	2127	Os01g0856900	99,91	0,00E+00	2052	-75	NA	NA	
maker-1-augustus-gene-377.56-mRNA-1	1	OsR498G0102368200.01.T03	100,00	0,00E+00	3961	Os01g0857000	99,82	0,00E+00	3943	-18	NA	NA	
maker-1-augustus-gene-377.61-mRNA-1	1	OsR498G0102368400.01.T02	100,00	0,00E+00	1602	Os01g0857200	99,78	0,00E+00	1593	-9	NA	NA	
maker-1-augustus-gene-377.69-mRNA-1	1	OsR498G0102368800.01.T01	100,00	0,00E+00	2479	Os01g0857400	99,85	0,00E+00	2470	-9	NA	NA	
maker-1-augustus-gene-377.72-mRNA-1	1	OsR498G0102369600.01.T01	99,72	0,00E+00	3854	Os01g0857600	99,64	0,00E+00	2950	-904	Indica	NA	
maker-1-augustus-gene-377.75-mRNA-1	1	OsR498G0102370200.01.T01	98,28	0,00E+00	5496	Os01g0858000	99,72	0,00E+00	5715	219	NA	NA	
maker-1-augustus-gene-378.32-mRNA-1	1	OsR498G0102372900.01.T01	100,00	0,00E+00	915	Os01g0858700	98,23	0,00E+00	875	-40	NA	NA	
maker-1-augustus-gene-378.34-mRNA-1	1	OsR498G0102374900.01.T05	100,00	0,00E+00	1916	Os01g0859200	99,90	0,00E+00	1879	-37	NA	NA	
maker-1-augustus-gene-378.37-mRNA-1	1	OsR498G0102377300.01.T01	100,00	0,00E+00	2105	Os01g0859300	99,49	0,00E+00	1731	-374	Indica	NA	
maker-1-augustus-gene-378.39-mRNA-1	1	OsR498G0102377500.01.T01	100,00	0,00E+00	1407	Os01g0859400	99,87	0,00E+00	1403	-4	NA	NA	
maker-1-augustus-gene-379.50-mRNA-1	1	OsR498G0102379300.01.T01	100,00	0,00E+00	1613	Os01g0860400	99,78	0,00E+00	1604	-9	NA	NA	
maker-1-augustus-gene-379.55-mRNA-1	1	OsR498G0100480900.01.T01	97,34	0,00E+00	950	Os01g0861400	92,15	0,00E+00	954	4	NA	NA	
maker-1-augustus-gene-379.57-mRNA-1	1	OsR498G0102383100.01.T01	100,00	0,00E+00	1952	Os01g0861700	99,82	0,00E+00	1943	-9	NA	NA	
maker-1-augustus-gene-379.60-mRNA-1	1	OsR498G0102383900.01.T01	100,00	0,00E+00	1012	Os01g0862200	99,64	0,00E+00	1003	-9	NA	NA	
maker-1-augustus-gene-379.62-mRNA-1	1	OsR498G0102385500.01.T01	100,00	0,00E+00	1097	Os01g0862800	99,67	0,00E+00	1088	-9	NA	NA	
maker-1-augustus-gene-379.65-mRNA-1	1	OsR498G0102383600.01.T01	100,00	0,00E+00	2657	Os01g0861900	99,46	0,00E+00	2621	-36	NA	NA	
maker-1-augustus-gene-379.67-mRNA-1	1	OsR498G0102383800.01.T04	97,73	0,00E+00	1819	Os01g0862000	96,72	0,00E+00	974	-845	Indica	NA	
maker-1-augustus-gene-379.72-mRNA-1	1	OsR498G0102384200.01.T01	99,25	0,00E+00	2128	Os01g0862300	99,17	0,00E+00	2123	-5	NA	NA	
maker-1-augustus-gene-38.31-mRNA-1	1	OsR498G0100245300.01.T01	99,69	0,00E+00	1166	Os01g0173600	99,54	0,00E+00	1159	-7	NA	Japonica	
maker-1-augustus-gene-38.35-mRNA-1	1	OsR498G0100246500.01.T02	99,90	0,00E+00	5632	Os01g0173900	99,97	0,00E+00	5641	9	NA	Japonica	
maker-1-augustus-gene-38.38-mRNA-1	1	OsR498G0100249700.01.T02	93,94	0,00E+00	938	Os01g0174900	93,94	0,00E+00	938	0	NA	NA	
maker-1-augustus-gene-38.42-mRNA-1	1	OsR498G0100246800.01.T01	100,00	0,00E+00	1104	Os01g0174000	100,00	0,00E+00	1104	0	NA	Japonica	
maker-1-augustus-gene-38.44-mRNA-1	1	OsR498G0100248000.01.T01	99,89	0,00E+00	1684	Os01g0174300	100,00	0,00E+00	1689	5	NA	Japonica	
maker-1-augustus-gene-38.48-mRNA-1	1	OsR498G0100248600.01.T01	96,55	0,00E+00	1697	Os01g0174500	96,51	0,00E+00	1581	-116	NA	NA	
maker-1-augustus-gene-38.52-mRNA-1	1	OsR498G0100248800.01.T01	100,00	0,00E+00	1516	Os01g0174600	100,00	0,00E+00	1516	0	NA	Japonica	
maker-1-augustus-gene-38.54-mRNA-1	1	OsR498G0100250000.01.T02	100,00	0,00E+00	1397	Os01g0175000	100,00	0,00E+00	1400	3	NA	Japonica	
maker-1-augustus-gene-380.0-mRNA-1	1	OsR498G0102387900.01.T01	100,00	0,00E+00	942	Os05g0442400	78,46	6,51E-111	399	-543	Indica	NA	
maker-1-augustus-gene-380.3-mRNA-1	1	OsR498G0102388200.01.T01	100,00	0,00E+00	1618	Os01g0863400	99,67	0,00E+00	1605	-13	NA	NA	
maker-1-augustus-gene-380.5-mRNA-1	1	OsR498G0102390000.01.T01	100,00	0,00E+00	1240	Os01g0864000	99,45	0,00E+00	974	-266	Indica	NA	
maker-1-augustus-gene-381.0-mRNA-1	1	OsR498G0102396300.01.T01	100,00	0,00E+00	2435	Os01g0865600	97,82	0,00E+00	2369	-66	NA	NA	
maker-1-augustus-gene-381.12-mRNA-1	1	OsR498G0102400900.01.T01	99,42	0,00E+00	1825	Os01g0866400	99,32	0,00E+00	1820	-5	NA	NA	
maker-1-augustus-gene-381.23-mRNA-1	1	OsR498G0102395000.01.T01	100,00	0,00E+00	1667	Os01g0865100	99,89	0,00E+00	1663	-4	NA	NA	
maker-1-augustus-gene-381.29-mRNA-1	1	OsR498G0102401200.01.T02	100,00	0,00E+00	1153	Os01g0866500	99,84	0,00E+00	1149	-4	NA	NA	
maker-1-augustus-gene-381.32-mRNA-1	1	OsR498G0102401600.01.T01	100,00	1,76E-145	515	Os01g0866600	100,00	6,13E-146	515	0	NA	NA	
maker-1-augustus-gene-381.34-mRNA-1	1	OsR498G0102401800.01.T01	100,00	1,02E-161	569	Os01g0866700	100,00	3,54E-162	569	0	NA	NA	
maker-1-augustus-gene-381.5-mRNA-1	1	OsR498G0102400100.01.T02	100,00	0,00E+00	2040	Os05g0438800	86,27	0,00E+00	1338	-702	Indica	NA	
maker-1-augustus-gene-381.7-mRNA-1	1	OsR498G0102400700.01.T04	100,00	0,00E+00	755	Os01g0866300	100,00	0,00E+00	755	0	NA	NA	
maker-1-augustus-gene-382.0-mRNA-1	1	OsR498G0102403100.01.T05	100,00	0,00E+00	2430	Os01g0866800	99,93	0,00E+00	2425	-5	NA	NA	
maker-1-augustus-gene-382.14-mRNA-1	1	OsR498G0102404900.01.T01	96,09	0,00E+00	2030	Os01g0867600	96,09	0,00E+00	2030	0	NA	NA	
maker-1-augustus-gene-382.21-mRNA-1	1	OsR498G0102405100.01.T01	100,00	0,00E+00	1478	Os01g0867700	100,00	0,00E+00	1478	0	NA	NA	
maker-1-augustus-gene-382.23-mRNA-1	1	OsR498G0102405300.01.T10	100,00	0,00E+00	1164	Os01g0867800	99,69	0,00E+00	1155	-9	NA	NA	
maker-1-augustus-gene-382.28-mRNA-1	1	OsR498G0102404000.01.T03	100,00	0,00E+00	1445	Os01g0867300	99,63	0,00E+00	1432	-13	NA	NA	
maker-1-augustus-gene-382.32-mRNA-1	1	OsR498G0102405600.01.T01	100,00	0,00E+00	1220	Os01g0867900	99,85	0,00E+00	1215	-5	NA	NA	
maker-1-augustus-gene-382.36-mRNA-1	1	OsR498G0102406000.01.T01	100,00	0,00E+00	1510	Os01g0868000	100,00	0,00E+00	1905	395	Japonica	NA	
maker-1-augustus-gene-382.6-mRNA-1	1	OsR498G0102403500.01.T04	100,00	0,00E+00	1040	Os07g0675400	75,79	1,89E-159	562	-478	Indica	NA	
maker-1-augustus-gene-382.9-mRNA-1	1	OsR498G0102403700.01.T03	100,00	0,00E+00	1959	Os01g0867200	99,63	0,00E+00	1941	-18	NA	NA	
maker-1-augustus-gene-383.1-mRNA-1	1	OsR498G0102410700.01.T01	100,00	0,00E+00	2268	Os01g0869200	99,92	0,00E+00	2263	-5	NA	Indica	
maker-1-augustus-gene-383.27-mRNA-1	1	OsR498G0102410200.01.T01	100,00	0,00E+00	3232	Os01g0869000	99,94	0,00E+00	3228	-4	NA	NA	
maker-1-augustus-gene-383.3-mRNA-1	1	OsR498G0102412100.01.T01	100,00	0,00E+00	1532	Os01g0869600	98,35	0,00E+00	1475	-57	NA	NA	

maker-1-augustus-gene-383.32-mRNA-1	1	OsR498G0102410400.01.T01	100,00	0,00E+00	1153	Os07g0232900	90,80	0,00E+00	830	-323	Indica	Indica	
maker-1-augustus-gene-383.39-mRNA-1	1	OsR498G0102412000.01.T01	100,00	0,00E+00	1797	Os01g0869500	99,90	0,00E+00	1792	-5	NA	NA	
maker-1-augustus-gene-383.43-mRNA-1	1	OsR498G0102414000.01.T01	100,00	0,00E+00	5227	Os01g0870100	99,86	0,00E+00	5209	-18	NA	NA	
maker-1-augustus-gene-383.6-mRNA-1	1	OsR498G0102412300.01.T01	100,00	0,00E+00	1456	Os01g0869800	100,00	0,00E+00	1456	0	NA	NA	
maker-1-augustus-gene-384.0-mRNA-1	1	OsR498G0102414300.01.T01	100,00	0,00E+00	1981	Os02g0688900	92,11	0,00E+00	1579	-402	Indica	NA	
maker-1-augustus-gene-384.17-mRNA-1	1	OsR498G0102417100.01.T01	100,00	0,00E+00	2673	Os01g0871300	100,00	0,00E+00	2484	-189	NA	NA	
maker-1-augustus-gene-384.21-mRNA-1	1	OsR498G0102418100.01.T03	100,00	0,00E+00	2955	Os01g0871500	99,94	0,00E+00	2950	-5	NA	NA	
maker-1-augustus-gene-384.25-mRNA-1	1	OsR498G0102418700.01.T01	100,00	0,00E+00	2971	Os01g0871600	99,88	0,00E+00	2875	-96	NA	NA	
maker-1-augustus-gene-384.28-mRNA-1	1	OsR498G0102418900.01.T01	100,00	0,00E+00	1315	Os01g0872100	76,61	1,18E-141	503	-812	Indica	NA	
maker-1-augustus-gene-384.30-mRNA-1	1	OsR498G0102419100.01.T01	100,00	1,03E-174	612	Os01g0871800	99,71	1,53E-173	608	-4	NA	NA	
maker-1-augustus-gene-384.32-mRNA-1	1	OsR498G0102420300.01.T02	85,43	0,00E+00	1504	Os01g0871900	96,63	0,00E+00	2233	729	Japonica	NA	
maker-1-augustus-gene-384.34-mRNA-1	1	OsR498G0102414800.01.T01	100,00	0,00E+00	4374	Os01g0889900	71,62	0,00E+00	1196	-3178	Indica	NA	Kinase-TM
maker-1-augustus-gene-384.8-mRNA-1	1	OsR498G0102414500.01.T06	100,00	0,00E+00	2700	Os01g0870300	100,00	0,00E+00	2700	0	NA	NA	
maker-1-augustus-gene-385.1-mRNA-1	1	OsR498G0102420300.01.T02	100,00	0,00E+00	2944	Os01g0872100	99,33	0,00E+00	2895	-49	NA	NA	
maker-1-augustus-gene-385.21-mRNA-1	1	OsR498G0102422400.01.T01	100,00	0,00E+00	5784	Os01g0872700	99,81	0,00E+00	5757	-27	NA	NA	
maker-1-augustus-gene-385.27-mRNA-1	1	OsR498G0102422600.01.T01	100,00	0,00E+00	2700	Os01g0872800	99,80	0,00E+00	2687	-13	NA	NA	Kinase
maker-1-augustus-gene-385.34-mRNA-1	1	OsR498G0102425600.01.T01	100,00	0,00E+00	7580	Os01g0873800	99,03	0,00E+00	7423	-157	NA	NA	
maker-1-augustus-gene-385.38-mRNA-1	1	OsR498G0102426200.01.T01	98,80	0,00E+00	3502	Os05g0430300	78,72	0,00E+00	1352	-2150	Indica	NA	
maker-1-augustus-gene-385.4-mRNA-1	1	OsR498G0102420700.01.T01	100,00	0,00E+00	726	Os01g0871800	84,96	1,32E-105	382	-344	Indica	NA	
maker-1-augustus-gene-385.6-mRNA-1	1	OsR498G0102420900.01.T01	100,00	0,00E+00	942	Os01g0872000	80,94	1,40E-158	559	-383	Indica	NA	
maker-1-augustus-gene-386.0-mRNA-1	1	OsR498G0102429100.01.T01	100,00	0,00E+00	1559	Os01g0874300	100,00	0,00E+00	1559	0	NA	NA	
maker-1-augustus-gene-386.17-mRNA-1	1	OsR498G0102428000.01.T01	97,89	0,00E+00	2696	Os01g0874100	97,34	0,00E+00	2302	-394	Indica	NA	
maker-1-augustus-gene-386.2-mRNA-1	1	OsR498G0102431700.01.T01	100,00	0,00E+00	3283	Os01g0874700	99,02	0,00E+00	3222	-61	NA	NA	
maker-1-augustus-gene-386.20-mRNA-1	1	OsR498G0102432600.01.T02	100,00	0,00E+00	1925	Os01g0874800	99,53	0,00E+00	1902	-23	NA	NA	
maker-1-augustus-gene-386.22-mRNA-1	1	OsR498G0102433600.01.T01	100,00	0,00E+00	1727	Os01g0875000	99,48	0,00E+00	1704	-23	NA	NA	
maker-1-augustus-gene-386.24-mRNA-1	1	OsR498G0102436000.01.T02	100,00	0,00E+00	1900	Os01g0875400	100,00	0,00E+00	1900	0	NA	NA	
maker-1-augustus-gene-386.5-mRNA-1	1	OsR498G0102433100.01.T01	100,00	0,00E+00	2055	Os01g0874900	99,91	0,00E+00	2050	-5	NA	NA	
maker-1-augustus-gene-386.8-mRNA-1	1	OsR498G0102435700.01.T01	100,00	0,00E+00	1299	Os01g0875300	99,04	0,00E+00	1279	-20	NA	NA	
maker-1-augustus-gene-387.0-mRNA-1	1	OsR498G0102437500.01.T01	100,00	0,00E+00	1775	Os01g0875700	99,80	0,00E+00	1766	-9	NA	NA	
maker-1-augustus-gene-387.13-mRNA-1	1	OsR498G0102441900.01.T01	100,00	0,00E+00	2205	Os01g0877400	99,76	0,00E+00	2191	-14	NA	NA	
maker-1-augustus-gene-387.16-mRNA-1	1	OsR498G0102442700.01.T01	100,00	0,00E+00	3880	Os01g0877500	99,91	0,00E+00	3871	-9	NA	NA	
maker-1-augustus-gene-387.19-mRNA-1	1	OsR498G0102437800.01.T01	100,00	0,00E+00	2084	Os01g0875800	99,83	0,00E+00	2075	-9	NA	NA	
maker-1-augustus-gene-387.2-mRNA-1	1	OsR498G0102438100.01.T01	100,00	0,00E+00	4296	Os08g0300300	72,66	0,00E+00	682	-3614	Indica	NA	
maker-1-augustus-gene-387.21-mRNA-1	1	OsR498G0102439000.01.T01	100,00	0,00E+00	2964	Os01g0876400	97,90	0,00E+00	2782	-182	NA	NA	
maker-1-augustus-gene-387.25-mRNA-1	1	OsR498G0102441200.01.T02	100,00	0,00E+00	1023	Os01g0876900	99,82	0,00E+00	1019	-4	NA	NA	
maker-1-augustus-gene-387.4-mRNA-1	1	OsR498G0102439100.01.T04	98,64	6,64E-106	384	Os01g0876500	99,59	3,44E-123	440	56	Japonica	NA	
maker-1-augustus-gene-387.9-mRNA-1	1	OsR498G0102441700.01.T01	100,00	0,00E+00	3901	Os01g0877300	99,58	0,00E+00	3861	-40	NA	NA	
maker-1-augustus-gene-388.48-mRNA-1	1	OsR498G0102444800.01.T02	100,00	0,00E+00	861	Os01g0878000	99,16	0,00E+00	843	-18	NA	NA	
maker-1-augustus-gene-388.50-mRNA-1	1	OsR498G0102446400.01.T01	100,00	0,00E+00	2522	Os01g0878400	99,79	0,00E+00	2508	-14	NA	NA	
maker-1-augustus-gene-388.52-mRNA-1	1	OsR498G0102447200.01.T02	100,00	0,00E+00	2527	Os01g0878700	99,86	0,00E+00	2518	-9	NA	NA	
maker-1-augustus-gene-388.54-mRNA-1	1	OsR498G0102447400.01.T01	100,00	0,00E+00	1748	Os01g0878800	99,17	0,00E+00	1712	-36	NA	NA	
maker-1-augustus-gene-388.56-mRNA-1	1	OsR498G0102447600.01.T02	100,00	0,00E+00	1521	Os01g0878900	99,76	0,00E+00	1512	-9	NA	NA	
maker-1-augustus-gene-389.0-mRNA-1	1	OsR498G0102453500.01.T01	100,00	0,00E+00	3442	Os01g0880200	99,69	0,00E+00	3415	-27	NA	NA	
maker-1-augustus-gene-389.13-mRNA-1	1	OsR498G0102454200.01.T01	100,00	0,00E+00	2928	Os01g0880300	99,69	0,00E+00	2905	-23	NA	NA	
maker-1-augustus-gene-389.15-mRNA-1	1	OsR498G0102454400.01.T03	100,00	0,00E+00	2354	Os01g0880400	99,76	0,00E+00	2260	-94	NA	NA	
maker-1-augustus-gene-389.18-mRNA-1	1	OsR498G0102455200.01.T01	100,00	0,00E+00	3227	Os01g0881000	99,16	0,00E+00	3163	-64	NA	NA	
maker-1-augustus-gene-389.20-mRNA-1	1	OsR498G0102459000.01.T01	100,00	0,00E+00	2998	Os01g0881500	99,88	0,00E+00	2989	-9	NA	NA	
maker-1-augustus-gene-389.3-mRNA-1	1	OsR498G0102454900.01.T01	100,00	0,00E+00	2067	Os01g0880800	99,74	0,00E+00	2054	-13	NA	NA	
maker-1-augustus-gene-389.5-mRNA-1	1	OsR498G0102458300.01.T01	100,00	0,00E+00	1483	Os01g0881300	99,88	0,00E+00	1479	-4	NA	NA	
maker-1-augustus-gene-39.0-mRNA-1	1	OsR498G0510826100.01.T01	99,74	0,00E+00	4116	Os01g0175300	100,00	0,00E+00	2836	-1280	Indica	Japonica	
maker-1-augustus-gene-39.12-mRNA-1	1	OsR498G0100256100.01.T02	99,79	0,00E+00	1678	Os01g0176700	99,89	0,00E+00	1682	4	NA	Japonica	
maker-1-augustus-gene-39.16-mRNA-1	1	OsR498G0100251400.01.T05	99,60	0,00E+00	888	Os01g0175400	100,00	0,00E+00	805	-83	NA	Japonica	
maker-1-augustus-gene-39.3-mRNA-1	1	OsR498G0100253300.01.T01	98,67	0,00E+00	2614	Os01g0176000	100,00	0,00E+00	2700	86	NA	Japonica	
maker-1-augustus-gene-39.41-mRNA-1	1	OsR498G0100252000.01.T02	98,49	0,00E+00	1948	Os01g0175600	100,00	0,00E+00	2024	76	NA	Japonica	
maker-1-augustus-gene-39.43-mRNA-1	1	OsR498G0100253000.01.T01	99,82	0,00E+00	2015	Os01g0175600	95,28	0,00E+00	1785	-230	Indica	Japonica	
maker-1-augustus-gene-39.47-mRNA-1	1	OsR498G0100254600.01.T01	99,80	0,00E+00	4485	Os01g0176500	99,92	0,00E+00	4498	13	NA	Indica	
maker-1-augustus-gene-39.5-mRNA-1	1	OsR498G0100253700.01.T01	99,47	0,00E+00	2681	Os01g0176100	100,00	0,00E+00	2717	36	NA	Japonica	
maker-1-augustus-gene-39.7-mRNA-1	1	OsR498G0100254100.01.T01	99,87	0,00E+00	2686	Os01g0176200	100,00	0,00E+00	2695	9	NA	Japonica	
maker-1-augustus-gene-39.9-mRNA-1	1	OsR498G0100254300.01.T01	99,86	0,00E+00	2632	Os05g0178000	73,11	0,00E+00	836	-1796	Indica	Indica	
maker-1-augustus-gene-390.0-mRNA-1	1	OsR498G0102460500.01.T01	100,00	0,00E+00	834	Os01g0882100	100,00	3,38E-139	493	-341	Indica	NA	
maker-1-augustus-gene-390.15-mRNA-1	1	OsR498G0102466500.01.T01	100,00	0,00E+00	1056	Os01g0883800	99,66	0,00E+00	1047	-9	NA	NA	
maker-1-augustus-gene-390.17-mRNA-1	1	OsR498G0102460200.01.T04	100,00	0,00E+00	2057	Os01g0881900	100,00	0,00E+00	2057	0	NA	NA	
maker-1-augustus-gene-390.26-mRNA-1	1	OsR498G0102462400.01.T01	100,00	0,00E+00	1148	Os01g0882500	100,00	0,00E+00	1148	0	NA	NA	
maker-1-augustus-gene-390.28-mRNA-1	1	OsR498G0102462800.01.T01	100,00	0,00E+00	2646	Os01g0882800	99,93	0,00E+00	2642	-4	NA	NA	
maker-1-augustus-gene-390.3-mRNA-1	1	OsR498G0102462100.01.T03	100,00	0,00E+00	1954	Os01g0882300	99,82	0,00E+00	1945	-9	NA	NA	
maker-1-augustus-gene-390.30-mRNA-1	1	OsR498G0102463400.01.T04	100,00	0,00E+00	888	Os01g0883100	99,84	0,00E+00	1132	244	Japonica	NA	
maker-1-augustus-gene-391.0-mRNA-1	1	OsR498G0102469100.01.T01	100,00	0,00E+00	2697	Os01g0884400	99,80	0,00E+00	2683	-14	NA	NA	
maker-1-augustus-gene-391.11-mRNA-1	1	OsR498G0102467600.01.T01	100,00	0,00E+00	1645	Os01g0884300	99,78	0,00E+00	1636	-9	NA	NA	
maker-1-augustus-gene-391.14-mRNA-1	1	OsR498G0102469400.01.T04	100,00	0,00E+00	2756	Os01g0884500	99,94	0,00E+00	2752	-4	NA	NA	
maker-1-augustus-gene-391.20-mRNA-1	1	OsR498G0102470200.01.T01	100,00	0,00E+00	1364	Os01g0884700	99,73	0,00E+00	1324	-40	NA	NA	
maker-1-augustus-gene-391.26-mRNA-1	1	OsR498G0102472000.01.T04	99,75	0,00E+00	2169	Os01g0885300	99,67	0,00E+00	2154	-15	NA	NA	
maker-1-augustus-gene-391.3-mRNA-1	1	OsR498G0102470900.01.T02	100,00	0,00E+00	785	Os01g0885000	100,00	2,39E-167	587	-198	Indica	NA	
maker-1-augustus-gene-391.6-mRNA-1	1	OsR498G0102466800.01.T02	100,00	0,00E+00	4367	Os01g0883900	99,84	0,00E+00	4349	-18	NA	NA	
maker-1-augustus-gene-392.41-mRNA-1	1	OsR498G0102475700.01.T01	100,00	0,00E+00	1440	Os01g0886200	100,00	0,00E+00	1613	173	Japonica	NA	
maker-1-augustus-gene-392.45-mRNA-1	1	OsR498G0102477100.01.T07	100,00	0,00E+00	1575	Os01g0886300	100,00	0,00E+00	1575	0	NA	NA	
maker-1-augustus-gene-392.49-mRNA-1	1	OsR498G0102478500.01.T01	100,00	0,00E+00	2755	Os05g0419200	84,17	0,00E+00	1329	-1426	Indica	NA	
maker-1-augustus-gene-392.60-mRNA-1	1	OsR498G0102475000.01.T01	96,47	0,00E+00	1847	Os01g0886000	96,38	0,00E+00	1842	-5	NA	NA	
maker-1-augustus-gene-392.64-mRNA-1	1	OsR498G0102477400.01.T04	100,00	0,00E+00	737	Os01g0886500	100,00	0,00E+00	737	0	NA	NA	
maker-1-augustus-gene-392.66-mRNA-1	1	OsR498G0102477600.01.T01	97,91	0,00E+00	3221	Os01g0886600	96,65	0,00E+00	2498	-723	Indica	NA	
maker-1-augustus-gene-392.75-mRNA-1	1	OsR498G0102480200.01.T01	100,00	0,00E+00	1478	Os01g0887700	99,88	0,00E+00	1473	-5	NA	NA	
maker-1-augustus-gene-393.41-mRNA-1	1	OsR498G0102483900.01.T01	100,00	0,00E+00	2452	Os01g0888300	100,00	0,00E+00	2452	0	NA	NA	
maker-1-augustus-gene-393.43-mRNA-1	1	OsR498G0102485100.01.T01	100,00	0,00E+00	1364	Os01g0888600	100,00	0,00E+00	1364	0	NA	NA	
maker-1-augustus-gene-393.46-mRNA-1	1	OsR498G0102486500.01.T01	100,00	0,00E+00	3631	Os01g0889000	99,85	0,00E+00	3608	-23	NA	NA	
maker-1-augustus-gene-393.52-mRNA-1	1	OsR498G0102485400.01.T01	100,00	0,00E+00	2663	Os01g0888700	99,93	0,00E+00	2658	-5	NA	NA	
maker-1-augustus-gene-393.58-mRNA-1	1	OsR498G0102487400.01.T06	100,00	0,00E+00	666	Os01g0889200	99,73	0,00E+00	662	-4	NA	NA	
maker-1-augustus-gene-394.0-mRNA-1	1	OsR498G0102489700.01.T01	100,00	0,00E+00	1620	Os01g0890600	79,22	0,00E+00	756	-864	Indica	NA	Kinase-TM
maker-1-augustus-gene-394.10-mRNA-1	1	OsR498G0102492500.01.T01	100,00	0,00E+00	1489	Os01g0891300	99,88	0,00E+00	1484	-5	NA	NA	
maker-1-augustus-gene-394.17-mRNA-1	1	OsR498G0102488600.01.T02	100,00	0,00E+00	1862	Os01g0889800	99,71	0,00E+00	1848	-14	NA	NA	
maker-1-augustus-gene-394.19-mRNA-1	1	OsR498G0102488800.01.T01	100,00	0,00E+00	4453	Os01g0889900	99,19	0,00E+00	4391	-62	NA	NA	Kinase-TM
maker-1-augustus-gene-394.25-mRNA-1	1	OsR498G0102491600.01.T02	100,00	0,00E+00	2852	Os01g0891000	100,00	0,00E+00	2852	0	NA	NA	
maker-1-augustus-gene-394.3-mRNA-1	1	OsR498G0102491100.01.T02	100,00	0,00E+00	3535	Os01g0890600	99,82	0,00E+00	2939	-596	Indica	NA	Kinase-TM
maker-1-augustus-gene-394.30-mRNA-1	1	OsR498G0102492800.01.T03	100,00	0,00E+00	1299	Os01g0661400	98,75	0,00E+00	1259	-40	NA	NA	
maker-1-augustus-gene-394.9-mRNA-1	1	OsR498G0102492100.01.T01	100,00	0,00E+00	2192	Os01g0891100	100,00	0,00E+00	2214	22	NA	NA	
maker-1-augustus-gene-395.13-mRNA-1	1	OsR498G0102500100.01.T01	100,00	0,00E+00	2168	Os01g0893400	96,34	0,00E+00	1968	-200	NA	NA	
maker-1-augustus-gene-395.17-mRNA-1	1	OsR498G0102496800.01.T01	100,00	0,00E+00	2219	Os01g0892600	90,72	0,00E+00	1628	-591	Indica	NA	
maker-1-augustus-gene-395.22-mRNA-1	1	OsR498G0102497400.01.T01	100,00	0,00E+00	2149	Os01g0892600	85,71	0,00E+00	1324	-825	Indica	NA	
maker-1-augustus-gene-395.27-mRNA-1	1	OsR498G0102497600.01.T02	100,00	0,00E+00	2085	Os01g0892600	99,48	0,00E+00	2058	-27	NA	NA	
maker-1-augustus-gene-395.34-mRNA-1	1	OsR498G0102500600.01.T01	100,00	0,00E+00	1645	Os01g0893700	96,46	0,00E+00	1489	-156	NA	NA	
maker-1-augustus-gene-396.46-mRNA-1	1	OsR498G0102501300.01.T02	100,00	0,00E+00	3972	Os01g0894000	98,59	0,00E+00	3832	-140	NA	NA	
maker-1-augustus-gene-396.49-mRNA-1	1	OsR498G0102502500.01.T03	100,00	0,00E+00	883	Os01g0894500	99,59	0,00E+00	874	-9	NA	NA	
maker-1-augustus-gene-396.53-mRNA-1	1	OsR498G0102504300.01.T01	100,00	0,00E+00	2370	Os01g0895200	99,70	0,00E+00	2352	-18	NA	NA	
maker-1-augustus-gene-396.55-mRNA-1	1	OsR498G0102505100.01.T01	100,00	0,00E+00	2067	Os01g0895300	99,52	0,00E+00	1492	-575	Indica	NA	
maker-1-augustus-gene-396.63-mRNA-1	1	OsR498G0102506100.01.T02	100,00	0,00E+00	5092	Os01g0896300	99,39	0,00E+00	3472	-1620	Indica	NA	
maker-1-augustus-gene-396.66-mRNA-1	1	OsR498G0102506700.01.T01	100,00	0,00E+00	1647	Os01g0896800	97,59	0,00E+00	1548	-99	NA	NA	
maker-1-augustus-gene-396.70-mRNA-1	1	OsR498G0102506900.01.T01	100,00	0,00E+00	1645	Os01g0896800	99,89	0,00E+00	1641	-4	NA	NA	
maker-1-augustus-gene-396.72-mRNA-1	1	OsR498G0102501800.01.T01	100,00	0,00E+00	1754	Os01g0894300	99,90	0,00E+00	1749	-5	NA	NA	
maker-1-augustus-gene-396.77-mRNA-1	1	OsR498G0102502800.01.T04	100,00	0,00E+00	1278	Os01g0894600	99,85	0,00E+00	1185	-93	NA	NA	
maker-1-augustus-gene-396.84-mRNA-1	1	OsR498G0102504200.01.T01	100,00	0,00E+00	1721	Os01g0895100	99,48	0,00E+00	1699	-22	NA	NA	
maker-1-augustus-gene-396.87-mRNA-1	1	OsR498G0102505800.01.T01	100,00	0,00E+00	2394	Os05g0507300	84,37	0,00E+00	1164	-1230	Indica	NA	
maker-1-augustus-gene-396.93-mRNA-1	1	OsR498G0102506400.01.T03	97,65	0,00E+00	3291	Os01g0896500	97,39	0,00E+00	3268	-23	NA	NA	
maker-1-augustus-gene-397.32-mRNA-1	1	OsR498G0102507700.01.T02	100,00	0,00E+00	2637	Os01g0897100	99,86	0,00E+00	2628	-9	NA	NA	
maker-1-augustus-gene-397.39-mRNA-1	1	OsR498G0102513500.01.T02	100,00	0,00E+00	1131	Os01g0898400	97,93	0,00E+00	1079	-52	NA	NA	
maker-1-augustus-gene-397.43-mRNA-1	1	OsR498G0102513700.01.T01	100,00	0,00E+00	2230	Os01g0898500	99,84	0,00E+00	2221	-9	NA	NA	
maker-1-augustus-gene-397.48-mRNA-1	1	OsR498G0102507600.01.T01	100,00	0,00E+00	1645	Os08g0512600	70,37	9,53E-112	403	-1242	Indica	NA	Kinase
maker-1-augustus-gene-397.52-mRNA-1	1	OsR498G0102508000.01.T03	100,00	0,00E+00	776	Os01g0897200	99,77	0,00E+00	770	-6	NA	NA	
maker-1-augustus-gene-397.56-mRNA-1	1	OsR498G0102508400.01.T02	98,99	0,00E+00	1216	Os01g0897300	91,87	0,00E+00	897	-319	Indica	NA	
maker-1-augustus-gene-397.61-mRNA-1	1	OsR498G0102511200.01.T04	100,00	0,00E+00	3272	Os01g0897800	99,95	0,00E+00	3268	-4	NA	NA	
maker-1-augustus-gene-397.69-mRNA-1	1	OsR498G0102513000.01.T01	98,54	0,00E+00	4781	Os01g0898300	98,32	0,00E+00	4754	-27	NA	NA	
maker-1-augustus-gene-397.75-mRNA-1	1	OsR498G0102514000.01.T01	100,00	0,00E+00	639	Os01g0898600	99,15	5,96E-179	626	-13	NA	NA	
maker-1-augustus-gene-398.0-mRNA-1	1	OsR498G0102514700.01.T02	94,35	0,00E+00	846	Os01g0898800	100,00	0,00E+00	818	-28	NA	NA	
maker-1-augustus-gene-398.16-mRNA-1	1	OsR498G0102515200.01.T03	100,00	0,00E+00	2003	Os01g0899000	99,55	0,00E+00	1980	-23	NA	NA	Kinase-TM
maker-1-augustus-gene-398.2-mRNA-1	1	OsR498G0102514900.01.T04	100,00	0,00E+00	2181	Os01g0898900	99,84	0,00E+00	2172	-9	NA	NA	
maker-1-augustus-gene-398.24-mRNA-1	1	OsR498G0102518200.01.T04	100,00	0,00E+00	2430	Os01g0899500	99,78	0,00E+00	2416	-14	NA	NA	
maker-1-augustus-gene-398.29-mRNA-1	1	OsR498G0102518400.01.T01	100,00	0,00E+00	2235	Os01g0899700	99,92	0,00E+00	2231	-4	NA	NA	
maker-1-augustus-gene-398.31-mRNA-1	1	OsR498G0102518800.01.T01	99,57	0,00E+00	3724	Os01g0899800	99,81	0,00E+00	1853	-1871	Indica	NA	
maker-1-augustus-gene-398.7-mRNA-1	1	OsR498G0102515500.01.T01	100,00	6,97E-114	410	Os01g0899100	99,56	1,03E-112	406	-4	NA	NA	
maker-1-augustus-gene-399.0-mRNA-1	1	OsR498G0102525300.01.T01	100,00	0,00E+00	1287	Os01g0900800	99,72	0,00E+00	1278	-9	NA	NA	
maker-1-augustus-gene-399.20-mRNA-1	1	OsR498G0102525600.01.T02	99,63	4,40E-135	481	Os01g0900900	99,63	1,53E-135	481	0	NA	NA	
maker-1-augustus-gene-399.24-mRNA-1	1	OsR498G0102526000.01.T03	100,00	0,00E+00	4323	Os01g0901000	99,58	0,00E+00	4290	-33	NA	NA	
maker-1-augustus-gene-399.28-mRNA-1	1	OsR498G0102527000.01.T01	100,00	0,00E+00	2186	Os01g0901200	99,67	0,00E+00	2168	-18	NA	NA	
maker-1-augustus-gene-4.0-mRNA-1	1	OsR498G0100018900.01.T04	99,10	4,43E-167	588	Os01g0513900	93,04	3,63E-93	340	-248	Indica	Japonica	
maker-1-augustus-gene-4.19-mRNA-1	1	OsR498G0100024900.01.T01	100,00	0,00E+00	688	Os01g0106800	100,00	0,00E+00	688	0	NA	Japonica	
maker-1-augustus-gene-4.21-mRNA-1	1	OsR498G0100021200.01.T03	99,28	0,00E+00	739	Os01g0105800	100,00	0,00E+00	753	14	NA	Japonica	
maker-1-augustus-gene-4.23-mRNA-1	1	OsR498G0100021400.01.T01	99,91	0,00E+00	2052	Os01g0105900	100,00	0,00E+00	2057	5	NA	Japonica	
maker-1-augustus-gene-4.28-mRNA-1	1	OsR498G0100023200.01.T01	99,79	0,00E+00	1718	Os01g0106300	100,00	0,00E+00	1727	9	NA	Japonica	
maker-1-augustus-gene-4.30-mRNA-1	1	OsR498G0100023400.01.T01	99,89	0,00E+00	1700	Os01g0106400	100,00	0,00E+00	1705	5	NA	Japonica	
maker-1-augustus-gene-4.32-mRNA-1	1	OsR498G0100024800.01.T01	99,86	0,00E+00	2556	Os01g0106900	100,00	0,00E+00	2565	9	NA	Japonica	
maker-1-augustus-gene-4.37-mRNA-1	1	OsR498G0100025600.01.T02	98,82	0,00E+00	2380	Os01g0107000	100,00	0,00E+00	2446	66	NA	Japonica	
maker-1-augustus-gene-4.4-mRNA-1	1	OsR498G0100020700.01.T01	99,74	0,00E+00	2092	Os01g0105700	99,91	0,00E+00	2101	9	NA	Japonica	
maker-1-augustus-gene-40.26-mRNA-1	1	OsR498G0100258300.01.T01	98,87	0,00E+00	4192	Os01g0177200	99,20	0,00E+00	4228	36	NA	Japonica	
maker-1-augustus-gene-40.42-mRNA-1	1	OsR498G0100262600.01.T06	97,61	0,00E+00	2076	Os01g0178000	97,77	0,00E+00	2085	9	NA	Japonica	
maker-1-augustus-gene-400.0-mRNA-1	1	OsR498G0102529300.01.T01	99,77	0,00E+00	2324	Os01g0902200	99,61	0,00E+00	2315	-9	NA	NA	
maker-1-augustus-gene-400.1-mRNA-1	1	OsR498G0102529300.01.T04	99,45	0,00E+00	1301	Os01g0902200	99,59	0,00E+00	1306	5	NA	NA	
maker-1-augustus-gene-400.12-mRNA-1	1	OsR498G0102530100.01.T01	98,69	0,00E+00	2935	Os01g0902800	84,54	0,00E+00	1811	-1124	Indica	NA	
maker-1-augustus-gene-400.14-mRNA-1	1	OsR498G0102530300.01.T01	99,88	0,00E+00	2892	Os01g0902800	99,07	0,00E+00	2845	-47	NA	NA	
maker-1-augustus-gene-400.18-mRNA-1	1	OsR498G0102533100.01.T01	99,28	1,63E-139	495	Os01g0904300	100,00	3,16E-86	316	-179	Indica	NA	
maker-1-augustus-gene-400.23-mRNA-1	1	OsR498G0102528400.01.T02	99,81	0,00E+00	927	Os01g0901800	100,00	0,00E+00	931	4	NA	NA	
maker-1-augustus-gene-400.27-mRNA-1	1	OsR498G0102528600.01.T01	99,80	0,00E+00	3510	Os01g0901900	99,15	0,00E+00	2498	-1012	Indica	NA	
maker-1-augustus-gene-400.39-mRNA-1	1	OsR498G0102529600.01.T01	100,00	0,00E+00	2224	Os01g0902300	99,76	0,00E+00	2211	-13	NA	NA	
maker-1-augustus-gene-400.43-mRNA-1	1	OsR498G0102533000.01.T06	93,97	0,00E+00	1047	Os01g0904200	93,67	0,00E+00	1038	-9	NA	NA	
maker-1-augustus-gene-400.52-mRNA-1	1	OsR498G0102533800.01.T02	99,81	0,00E+00	1894	Os01g0904500	99,81	0,00E+00	1894	0	NA	NA	
maker-1-augustus-gene-401.11-mRNA-1	1	OsR498G0101582300.01.T01	99,66	0,00E+00	2102	Os01g0627800	99,92	0,00E+00	2115	13	NA	NA	
maker-1-augustus-gene-401.13-mRNA-1	1	OsR498G0101580700.01.T01	99,75	0,00E+00	2915	Os01g0627400	99,94	0,00E+00	2929	14	NA	NA	
maker-1-augustus-gene-401.15-mRNA-1	1	OsR498G0101577900.01.T01	99,48	0,00E+00	2033	Os01g0626400	99,91	0,00E+00	2052	19	NA	NA	
maker-1-augustus-gene-401.4-mRNA-1	1	OsR498G0101581000.01.T01	100,00	0,00E+00	926	Os01g0627500	100,00	0,00E+00	926	0	NA	NA	
maker-1-augustus-gene-401.7-mRNA-1	1	OsR498G0102534400.01.T01	99,54	0,00E+00	3119	Os01g0904700	99,72	0,00E+00	1935	-1184	Indica	NA	
maker-1-augustus-gene-402.11-mRNA-1	1	OsR498G0101575700.01.T01	98,73	0,00E+00	1788	Os01g0625300	100,00	0,00E+00	1840	52	NA	NA	
maker-1-augustus-gene-402.20-mRNA-1	1	OsR498G0101574500.01.T01	97,35	0,00E+00	1925	Os01g0624500	97,61	0,00E+00	1939	14	NA	NA	
maker-1-augustus-gene-402.24-mRNA-1	1	OsR498G0101574300.01.T02	99,82	0,00E+00	972	Os01g0624400	99,82	0,00E+00	972	0	NA	NA	
maker-1-augustus-gene-402.27-mRNA-1	1	OsR498G0101571300.01.T01	99,24	0,00E+00	3931	Os01g0623500	100,00	0,00E+00	3990	59	NA	NA	
maker-1-augustus-gene-402.9-mRNA-1	1	OsR498G0101571600.01.T01	99,76	0,00E+00	2254	Os01g0891100	70,16	4,04E-137	489	-1765	Indica	NA	
maker-1-augustus-gene-403.0-mRNA-1	1	OsR498G0101568400.01.T03	99,36	0,00E+00	3609	Os01g0623200	99,94	0,00E+00	2790	-819	Indica	NA	
maker-1-augustus-gene-403.15-mRNA-1	1	OsR498G0101565900.01.T01	98,23	0,00E+00	2737	Os05g0585500	83,45	0,00E+00	1644	-1093	Indica	NA	
maker-1-augustus-gene-403.23-mRNA-1	1	OsR498G0101563100.01.T01	99,70	0,00E+00	1198	Os01g0621700	100,00	0,00E+00	1207	9	NA	NA	
maker-1-augustus-gene-403.3-mRNA-1	1	OsR498G0101566200.01.T01	100,00	0,00E+00	758	Os01g0622700	100,00	0,00E+00	758	0	NA	NA	
maker-1-augustus-gene-403.8-mRNA-1	1	OsR498G0101565600.01.T03	99,56	0,00E+00	2007	Os01g0622300	99,71	0,00E+00	1881	-126	NA	NA	
maker-1-augustus-gene-404.31-mRNA-1	1	OsR498G0101562800.01.T02	99,47	0,00E+00	3715	Os01g0621600	100,00	0,00E+00	3766	51	NA	NA	Kinase-TM
maker-1-augustus-gene-404.36-mRNA-1	1	OsR498G0101561600.01.T01	99,92	0,00E+00	2297	Os01g0621200	100,00	0,00E+00	1882	-415	Indica	NA	
maker-1-augustus-gene-404.39-mRNA-1	1	OsR498G0101558200.01.T01	99,49	0,00E+00	1737	Os01g0620100	100,00	0,00E+00	1759	22	NA	NA	
maker-1-augustus-gene-404.45-mRNA-1	1	OsR498G0101557500.01.T02	99,77	0,00E+00	2346	Os01g0619800	99,58	0,00E+00	1263	-1083	Indica	NA	
maker-1-augustus-gene-405.31-mRNA-1	1	OsR498G0101555000.01.T01	99,94	0,00E+00	2999	Os01g0619000	100,00	0,00E+00	2300	-699	Indica	NA	
maker-1-augustus-gene-405.42-mRNA-1	1	OsR498G0101554700.01.T01	99,63	0,00E+00	2391	Os01g0618900	100,00	0,00E+00	1673	-718	Indica	NA	
maker-1-augustus-gene-405.50-mRNA-1	1	OsR498G0101553100.01.T01	99,69	0,00E+00	2883	Os01g0618500	99,94	0,00E+00	2902	19	NA	NA	
maker-1-augustus-gene-405.53-mRNA-1	1	OsR498G0101552900.01.T01	96,76	0,00E+00	1713	Os01g0618400	96,76	0,00E+00	1713	0	NA	NA	
maker-1-augustus-gene-406.0-mRNA-1	1	OsR498G0101552600.01.T01	98,36	0,00E+00	5046	Os01g0618300	98,73	0,00E+00	4959	-87	NA	NA	
maker-1-augustus-gene-406.11-mRNA-1	1	OsR498G0101550000.01.T03	99,34	0,00E+00	2409	Os01g0617600	99,49	0,00E+00	2418	9	NA	NA	
maker-1-augustus-gene-406.12-mRNA-1	1	OsR498G0101549200.01.T01	99,93	0,00E+00	4823	Os01g0617500	99,96	0,00E+00	4828	5	NA	NA	
maker-1-augustus-gene-406.19-mRNA-1	1	OsR498G0101547800.01.T01	99,89	0,00E+00	3270	Os05g0573300	77,91	0,00E+00	1345	-1925	Indica	NA	
maker-1-augustus-gene-406.2-mRNA-1	1	OsR498G0101551600.01.T02	100,00	0,00E+00	1867	Os01g0618100	100,00	0,00E+00	1867	0	NA	NA	
maker-1-augustus-gene-406.25-mRNA-1	1	OsR498G0101551700.01.T02	98,01	0,00E+00	1994	Os01g0618200	96,35	0,00E+00	959	-1035	Indica	NA	
maker-1-augustus-gene-406.27-mRNA-1	1	OsR498G0101550900.01.T01	99,79	0,00E+00	1701	Os01g0617900	100,00	0,00E+00	1775	74	NA	NA	
maker-1-augustus-gene-406.30-mRNA-1	1	OsR498G0101550500.01.T04	99,63	0,00E+00	3876	Os01g0617700	99,95	0,00E+00	3908	32	NA	NA	
maker-1-augustus-gene-406.7-mRNA-1	1	OsR498G0101550800.01.T01	98,74	0,00E+00	968	Os01g0617800	99,10	0,00E+00	977	9	NA	NA	
maker-1-augustus-gene-407.29-mRNA-1	1	OsR498G0101546400.01.T02	99,71	0,00E+00	2488	Os01g0616400	99,78	0,00E+00	2490	2	NA	NA	
maker-1-augustus-gene-407.33-mRNA-1	1	OsR498G0101541200.01.T01	99,92	0,00E+00	2296	Os01g0615300	100,00	0,00E+00	2300	4	NA	NA	
maker-1-augustus-gene-407.35-mRNA-1	1	OsR498G0101541000.01.T02	99,70	0,00E+00	2379	Os01g0615200	100,00	0,00E+00	2419	40	NA	NA	
maker-1-augustus-gene-407.39-mRNA-1	1	OsR498G0101546500.01.T01	99,88	0,00E+00	1506	Os01g0616500	100,00	0,00E+00	1510	4	NA	NA	
maker-1-augustus-gene-407.42-mRNA-1	1	OsR498G0101545900.01.T02	99,13	0,00E+00	3673	Os05g0587300	75,12	0,00E+00	1111	-2562	Indica	NA	
maker-1-augustus-gene-407.46-mRNA-1	1	OsR498G0101542500.01.T07	99,69	0,00E+00	4007	Os01g0616100	100,00	0,00E+00	3512	-495	Indica	NA	
maker-1-augustus-gene-408.0-mRNA-1	1	OsR498G0101539800.01.T03	99,73	0,00E+00	2628	Os01g0614900	99,93	0,00E+00	2642	14	NA	NA	
maker-1-augustus-gene-408.10-mRNA-1	1	OsR498G0101536800.01.T01	99,35	0,00E+00	1917	Os01g0613800	100,00	0,00E+00	1948	31	NA	NA	
maker-1-augustus-gene-408.12-mRNA-1	1	OsR498G0101536000.01.T01	99,91	0,00E+00	1949	Os01g0613500	100,00	0,00E+00	1954	5	NA	NA	
maker-1-augustus-gene-408.14-mRNA-1	1	OsR498G0101539500.01.T01	99,89	0,00E+00	1679	Os01g0614700	100,00	0,00E+00	969	-710	Indica	NA	
maker-1-augustus-gene-408.19-mRNA-1	1	OsR498G0101538300.01.T01	98,00	0,00E+00	2496	Os01g0614300	99,79	0,00E+00	2588	92	NA	NA	
maker-1-augustus-gene-408.21-mRNA-1	1	OsR498G0101535700.01.T01	100,00	0,00E+00	1137	Os01g0613300	100,00	0,00E+00	1131	-6	NA	NA	
maker-1-augustus-gene-408.27-mRNA-1	1	OsR498G0101532900.01.T03	100,00	1,38E-179	628	Os01g0612800	100,00	4,80E-180	628	0	NA	NA	
maker-1-augustus-gene-408.3-mRNA-1	1	OsR498G0101537000.01.T02	99,88	0,00E+00	1497	Os01g0613900	100,00	0,00E+00	1501	4	NA	NA	
maker-1-augustus-gene-409.31-mRNA-1	1	OsR498G0101532700.01.T03	99,72	0,00E+00	5708	Os01g0612700	100,00	0,00E+00	4103	-1605	Indica	NA	
maker-1-augustus-gene-409.35-mRNA-1	1	OsR498G0101530100.01.T01	99,73	0,00E+00	1328	Os01g0612500	100,00	0,00E+00	1178	-150	Indica	NA	
maker-1-augustus-gene-409.39-mRNA-1	1	OsR498G0101528100.01.T01	100,00	0,00E+00	921	Os01g0612200	100,00	0,00E+00	921	0	NA	NA	
maker-1-augustus-gene-409.43-mRNA-1	1	OsR498G0306062000.01.T02	86,57	0,00E+00	793	Os01g0612100	100,00	0,00E+00	1397	604	Japonica	NA	
maker-1-augustus-gene-409.45-mRNA-1	1	OsR498G0101526300.01.T01	99,42	0,00E+00	2746	Os07g0218200	82,67	0,00E+00	1002	-1744	Indica	NA	
maker-1-augustus-gene-41.16-mRNA-1	1	OsR498G0100268100.01.T01	99,81	0,00E+00	1858	Os01g0179200	100,00	0,00E+00	1867	9	NA	Japonica	
maker-1-augustus-gene-41.20-mRNA-1	1	OsR498G0100268300.01.T01	98,91	0,00E+00	1767	Os01g0179300	100,00	0,00E+00	1797	30	NA	Japonica	
maker-1-augustus-gene-41.29-mRNA-1	1	OsR498G0100268900.01.T02	99,91	0,00E+00	4008	Os01g0179500	100,00	0,00E+00	4017	9	NA	Japonica	
maker-1-augustus-gene-41.31-mRNA-1	1	OsR498G0100264200.01.T01	96,81	0,00E+00	2515	Os05g0178400	78,78	0,00E+00	1177	-1338	Indica	Japonica	
maker-1-augustus-gene-41.33-mRNA-1	1	OsR498G0100264600.01.T01	100,00	0,00E+00	1083	Os01g0178500	100,00	0,00E+00	1083	0	NA	Japonica	
maker-1-augustus-gene-41.6-mRNA-1	1	OsR498G0100263500.01.T05	100,00	0,00E+00	1808	Os01g0178200	100,00	0,00E+00	1808	0	NA	Japonica	
maker-1-augustus-gene-410.0-mRNA-1	1	OsR498G0101521400.01.T01	100,00	0,00E+00	681	Os01g0610800	100,00	0,00E+00	681	0	NA	NA	
maker-1-augustus-gene-410.12-mRNA-1	1	OsR498G0101520600.01.T01	99,88	0,00E+00	1475	Os01g0610500	100,00	0,00E+00	1480	5	NA	NA	
maker-1-augustus-gene-410.15-mRNA-1	1	OsR498G0101517800.01.T02	99,88	0,00E+00	7222	Os01g0609900	100,00	0,00E+00	7245	23	NA	NA	
maker-1-augustus-gene-410.22-mRNA-1	1	OsR498G0101522300.01.T01	99,82	0,00E+00	2015	Os01g0611000	100,00	0,00E+00	2165	150	NA	NA	

maker-1-augustus-gene-410.24-mRNA-1	1	OsR498G0101520100.01.T02	99,63	0,00E+00	966	Os01g0610400	99,68	1,39E-157	555	-411	Indica	NA	
maker-1-augustus-gene-410.26-mRNA-1	1	OsR498G0101519900.01.T03	100,00	0,00E+00	2630	Os01g0610300	100,00	0,00E+00	2630	0	NA	NA	
maker-1-augustus-gene-410.3-mRNA-1	1	OsR498G0101521200.01.T01	98,17	0,00E+00	1610	Os01g0610700	100,00	0,00E+00	1678	68	NA	NA	
maker-1-augustus-gene-410.32-mRNA-1	1	OsR498G0101518900.01.T01	100,00	0,00E+00	958	Os01g0610100	100,00	0,00E+00	958	0	NA	NA	
maker-1-augustus-gene-410.6-mRNA-1	1	OsR498G0101521000.01.T03	98,41	0,00E+00	4726	Os01g0610600	98,67	0,00E+00	4757	31	NA	NA	
maker-1-augustus-gene-411.19-mRNA-1	1	OsR498G0101515200.01.T01	99,79	0,00E+00	7852	Os01g0609300	100,00	0,00E+00	7889	37	NA	NA	
maker-1-augustus-gene-411.23-mRNA-1	1	OsR498G0101514400.01.T01	98,49	0,00E+00	4938	Os01g0609066	91,91	0,00E+00	4094	-844	Indica	NA	
maker-1-augustus-gene-411.24-mRNA-1	1	OsR498G0101514400.01.T01	98,45	0,00E+00	1897	Os01g0609200	100,00	0,00E+00	1974	77	NA	NA	
maker-1-augustus-gene-411.28-mRNA-1	1	OsR498G0101514000.01.T02	99,48	0,00E+00	7845	Os01g0609066	100,00	0,00E+00	7946	101	NA	NA	
maker-1-augustus-gene-412.22-mRNA-1	1	OsR498G0101503700.01.T01	99,86	0,00E+00	2607	Os01g0607200	96,63	0,00E+00	2389	-218	NA	NA	
maker-1-augustus-gene-412.23-mRNA-1	1	OsR498G0101503300.01.T01	98,49	0,00E+00	803	Os01g0607200	100,00	0,00E+00	744	-59	NA	NA	
maker-1-augustus-gene-412.34-mRNA-1	1	OsR498G0101502300.01.T01	98,48	0,00E+00	1027	Os01g0606900	99,83	0,00E+00	1064	37	NA	NA	
maker-1-augustus-gene-412.6-mRNA-1	1	OsR498G0101504200.01.T03	99,87	0,00E+00	4094	Os01g0607400	100,00	0,00E+00	4107	13	NA	NA	
maker-1-augustus-gene-413.50-mRNA-1	1	OsR498G0101498200.01.T01	99,90	0,00E+00	3579	Os01g0605500	100,00	0,00E+00	4145	566	Japonica	NA	
maker-1-augustus-gene-413.53-mRNA-1	1	OsR498G0101497400.01.T04	89,91	9,17E-177	619	Os01g0605200	89,91	3,18E-177	619	0	NA	NA	
maker-1-augustus-gene-413.56-mRNA-1	1	OsR498G0101495000.01.T01	99,35	0,00E+00	1896	Os01g0604700	99,63	0,00E+00	1910	14	NA	NA	
maker-1-augustus-gene-413.60-mRNA-1	1	OsR498G0101499300.01.T01	99,23	0,00E+00	1380	Os01g0606000	100,00	0,00E+00	1407	27	NA	NA	
maker-1-augustus-gene-413.62-mRNA-1	1	OsR498G0101498500.01.T01	98,83	0,00E+00	1342	Os01g0605700	99,35	0,00E+00	1361	19	NA	NA	
maker-1-augustus-gene-413.67-mRNA-1	1	OsR498G0101497900.01.T02	99,78	0,00E+00	1617	Os01g0605400	98,23	0,00E+00	1548	-69	NA	NA	
maker-1-augustus-gene-413.69-mRNA-1	1	OsR498G0101497900.01.T01	99,94	0,00E+00	3010	Os01g0605300	100,00	0,00E+00	3014	4	NA	NA	
maker-1-augustus-gene-413.71-mRNA-1	1	OsR498G0101497700.01.T02	100,00	0,00E+00	2636	Os01g0605100	99,63	0,00E+00	1947	-689	Indica	NA	
maker-1-augustus-gene-414.36-mRNA-1	1	OsR498G0101486800.01.T01	100,00	0,00E+00	1014	Os01g0603000	100,00	0,00E+00	1014	0	NA	NA	
maker-1-augustus-gene-414.41-mRNA-1	1	OsR498G0100035900.01.T05	80,94	4,00E-74	279	Os01g0604100	94,48	0,00E+00	690	411	Japonica	NA	
maker-1-augustus-gene-414.43-mRNA-1	1	OsR498G0101487900.01.T01	99,56	0,00E+00	1617	Os01g0603300	100,00	0,00E+00	1635	18	NA	NA	
maker-1-augustus-gene-415.23-mRNA-1	1	OsR498G0101483200.01.T01	99,86	0,00E+00	1250	Os11g0566800	72,72	4,89E-155	548	-702	Indica	NA	
maker-1-augustus-gene-415.40-mRNA-1	1	OsR498G0101582500.01.T01	82,08	0,00E+00	1391	Os01g0602400	99,93	0,00E+00	2546	1155	Japonica	Japonica	
maker-1-augustus-gene-415.44-mRNA-1	1	OsR498G0101482200.01.T03	94,13	0,00E+00	1251	Os01g0602200	99,77	0,00E+00	1526	275	Japonica	NA	
maker-1-augustus-gene-415.56-mRNA-1	1	OsR498G0101477700.01.T01	99,54	0,00E+00	2725	Os01g0601200	99,33	0,00E+00	1839	-886	Indica	NA	Kinase-TM
maker-1-augustus-gene-416.0-mRNA-1	1	OsR498G0101475400.01.T01	100,00	0,00E+00	1543	Os02g0226200	71,87	7,83E-62	238	-1305	Indica	NA	
maker-1-augustus-gene-416.11-mRNA-1	1	OsR498G0101474700.01.T01	99,75	0,00E+00	2823	Os01g0600300	99,78	0,00E+00	1608	-1215	Indica	NA	
maker-1-augustus-gene-416.15-mRNA-1	1	OsR498G0101474500.01.T01	99,87	0,00E+00	2740	Os02g0224200	74,70	0,00E+00	995	-1745	Indica	NA	
maker-1-augustus-gene-416.2-mRNA-1	1	OsR498G0101474200.01.T03	100,00	0,00E+00	666	Os01g0600000	100,00	0,00E+00	666	0	NA	NA	
maker-1-augustus-gene-416.20-mRNA-1	1	OsR498G0101473900.01.T01	93,15	0,00E+00	2574	Os01g0599900	99,88	0,00E+00	2899	325	Japonica	NA	
maker-1-augustus-gene-416.26-mRNA-1	1	OsR498G0101471100.01.T02	99,85	0,00E+00	1181	Os01g0598200	100,00	0,00E+00	1186	5	NA	NA	
maker-1-augustus-gene-416.6-mRNA-1	1	OsR498G0101473200.01.T03	93,52	0,00E+00	1562	Os01g0598900	96,02	0,00E+00	1662	100	NA	NA	
maker-1-augustus-gene-417.38-mRNA-1	1	OsR498G0101469500.01.T01	97,51	0,00E+00	1359	Os01g0597800	86,89	0,00E+00	973	-386	Indica	NA	
maker-1-augustus-gene-417.40-mRNA-1	1	OsR498G0101469100.01.T01	97,99	0,00E+00	2124	Os01g0597600	100,00	0,00E+00	2233	109	NA	NA	
maker-1-augustus-gene-417.41-mRNA-1	1	OsR498G0101468700.01.T01	96,35	0,00E+00	1884	Os01g0597400	100,00	0,00E+00	2403	519	Japonica	NA	
maker-1-augustus-gene-417.43-mRNA-1	1	OsR498G0101466500.01.T01	99,37	0,00E+00	2828	Os01g0596700	100,00	0,00E+00	2874	46	NA	NA	
maker-1-augustus-gene-418.10-mRNA-1	1	OsR498G0101462900.01.T01	100,00	0,00E+00	1559	Os01g0596000	100,00	0,00E+00	2727	1168	Japonica	NA	
maker-1-augustus-gene-418.12-mRNA-1	1	OsR498G0101462700.01.T01	94,67	0,00E+00	2573	Os01g0595900	96,14	0,00E+00	2681	108	NA	NA	
maker-1-augustus-gene-418.14-mRNA-1	1	OsR498G0101462500.01.T01	98,42	0,00E+00	2626	Os01g0595800	100,00	0,00E+00	2722	96	NA	NA	
maker-1-augustus-gene-418.16-mRNA-1	1	OsR498G0101462300.01.T01	96,94	0,00E+00	2354	Os01g0595700	97,76	0,00E+00	2835	481	Japonica	NA	
maker-1-augustus-gene-418.19-mRNA-1	1	OsR498G0101462100.01.T01	95,30	0,00E+00	1452	Os01g0595600	100,00	0,00E+00	1640	188	Japonica	NA	
maker-1-augustus-gene-418.22-mRNA-1	1	OsR498G0101460100.01.T01	99,75	0,00E+00	710	Os01g0719600	73,08	8,25E-26	116	-594	Indica	NA	
maker-1-augustus-gene-418.4-mRNA-1	1	OsR498G0101457800.01.T01	98,96	0,00E+00	1014	Os01g0594500	99,83	0,00E+00	1037	23	NA	NA	
maker-1-augustus-gene-418.6-mRNA-1	1	OsR498G0101463900.01.T01	100,00	0,00E+00	1413	Os01g0596300	100,00	0,00E+00	1413	0	NA	NA	
maker-1-augustus-gene-418.8-mRNA-1	1	OsR498G0101463300.01.T01	99,94	0,00E+00	2815	Os01g0596100	100,00	0,00E+00	2819	4	NA	NA	
maker-1-augustus-gene-419.30-mRNA-1	1	OsR498G0102306200.01.T01	100,00	1,83E-171	601	Os01g0840700	99,40	3,30E-169	592	-9	NA	NA	
maker-1-augustus-gene-42.0-mRNA-1	1	OsR498G0100269800.01.T05	100,00	0,00E+00	1213	Os01g0179700	100,00	0,00E+00	1079	-134	Indica	Japonica	
maker-1-augustus-gene-42.11-mRNA-1	1	OsR498G0100270400.01.T02	97,14	0,00E+00	650	Os01g0179901	99,59	2,70E-120	431	-219	Indica	Japonica	
maker-1-augustus-gene-42.13-mRNA-1	1	OsR498G0100271400.01.T01	99,51	0,00E+00	4362	Os01g0180300	99,70	0,00E+00	3604	-758	Indica	NA	
maker-1-augustus-gene-42.5-mRNA-1	1	OsR498G0100270200.01.T01	95,21	0,00E+00	2023	Os01g0179800	95,37	0,00E+00	2032	9	NA	Japonica	
maker-1-augustus-gene-420.40-mRNA-1	1	OsR498G0102309500.01.T01	96,55	0,00E+00	3059	Os01g0841500	96,39	0,00E+00	3046	-13	NA	NA	
maker-1-augustus-gene-420.43-mRNA-1	1	OsR498G0102309700.01.T01	100,00	0,00E+00	1386	Os01g0841600	99,61	0,00E+00	1372	-14	NA	NA	
maker-1-augustus-gene-420.51-mRNA-1	1	OsR498G0102308400.01.T02	99,71	0,00E+00	1249	Os01g0841200	99,57	0,00E+00	1244	-5	NA	NA	
maker-1-augustus-gene-420.56-mRNA-1	1	OsR498G0102310200.01.T01	97,79	0,00E+00	3507	Os01g0841800	97,14	0,00E+00	2786	-721	Indica	NA	
maker-1-augustus-gene-420.63-mRNA-1	1	OsR498G0102312600.01.T01	100,00	0,00E+00	3139	Os01g0842400	99,89	0,00E+00	3130	-9	NA	NA	
maker-1-augustus-gene-420.65-mRNA-1	1	OsR498G0102313000.01.T01	100,00	0,00E+00	3128	Os01g0842500	99,71	0,00E+00	3105	-23	NA	NA	
maker-1-augustus-gene-420.70-mRNA-1	1	OsR498G0102313400.01.T01	100,00	0,00E+00	4345	Os01g0842600	99,88	0,00E+00	4332	-13	NA	NA	
maker-1-augustus-gene-421.38-mRNA-1	1	OsR498G0102315900.01.T01	100,00	0,00E+00	2284	Os03g0810600	85,20	0,00E+00	1429	-855	Indica	NA	
maker-1-augustus-gene-421.43-mRNA-1	1	OsR498G0102317300.01.T01	100,00	0,00E+00	3149	Os01g0844050	99,61	0,00E+00	2267	-882	Indica	NA	
maker-1-augustus-gene-421.46-mRNA-1	1	OsR498G0102318300.01.T05	100,00	0,00E+00	1007	Os01g0844300	99,82	0,00E+00	1003	-4	NA	NA	
maker-1-augustus-gene-421.49-mRNA-1	1	OsR498G0102321100.01.T02	100,00	0,00E+00	5519	Os01g0844800	99,54	0,00E+00	5468	-51	NA	NA	
maker-1-augustus-gene-421.59-mRNA-1	1	OsR498G0102318600.01.T04	100,00	0,00E+00	695	Os01g0844400	100,00	0,00E+00	695	0	NA	NA	
maker-1-augustus-gene-422.43-mRNA-1	1	OsR498G0102325500.01.T01	100,00	0,00E+00	2246	Os01g0846300	99,92	0,00E+00	2241	-5	NA	NA	
maker-1-augustus-gene-422.52-mRNA-1	1	OsR498G0102326300.01.T04	100,00	0,00E+00	1270	Os01g0846500	99,85	0,00E+00	1188	-82	NA	NA	
maker-1-augustus-gene-422.62-mRNA-1	1	OsR498G0102324800.01.T02	100,00	0,00E+00	1867	Os03g0855700	86,44	0,00E+00	1223	-644	Indica	Indica	
maker-1-augustus-gene-422.66-mRNA-1	1	OsR498G1221780100.01.T02	96,14	4,86E-130	465	Os01g0630700	84,03	6,76E-85	313	-152	Indica	Indica	
maker-1-augustus-gene-422.72-mRNA-1	1	OsR498G0102327000.01.T02	99,90	0,00E+00	1843	Os01g0846600	99,41	0,00E+00	1820	-23	NA	NA	
maker-1-augustus-gene-422.75-mRNA-1	1	OsR498G0102328000.01.T03	100,00	0,00E+00	2360	Os01g0846900	99,09	0,00E+00	2315	-45	NA	NA	
maker-1-augustus-gene-423.0-mRNA-1	1	OsR498G0102328500.01.T02	100,00	0,00E+00	1959	Os01g0847200	100,00	0,00E+00	1959	0	NA	NA	
maker-1-augustus-gene-423.10-mRNA-1	1	OsR498G0102329700.01.T02	100,00	0,00E+00	1501	Os05g0456300	70,16	1,33E-103	376	-1125	Indica	NA	
maker-1-augustus-gene-423.14-mRNA-1	1	OsR498G0102331100.01.T02	100,00	0,00E+00	3472	Os01g0848200	99,95	0,00E+00	3468	-4	NA	NA	
maker-1-augustus-gene-423.21-mRNA-1	1	OsR498G0102328800.01.T01	100,00	1,07E-170	600	Os01g0847300	100,00	3,72E-171	600	0	NA	NA	
maker-1-augustus-gene-423.23-mRNA-1	1	OsR498G0102330000.01.T02	100,00	0,00E+00	659	Os01g0847900	100,00	0,00E+00	659	0	NA	NA	
maker-1-augustus-gene-423.25-mRNA-1	1	OsR498G0102332000.01.T01	100,00	0,00E+00	3317	Os01g0848400	99,89	0,00E+00	3308	-9	NA	NA	
maker-1-augustus-gene-423.29-mRNA-1	1	OsR498G0102333600.01.T01	100,00	0,00E+00	1175	Os01g0848700	100,00	0,00E+00	1175	0	NA	NA	
maker-1-augustus-gene-423.3-mRNA-1	1	OsR498G0102329100.01.T03	100,00	0,00E+00	1689	Os01g0847700	89,96	0,00E+00	1265	-424	Indica	NA	
maker-1-augustus-gene-423.6-mRNA-1	1	OsR498G0102329500.01.T01	100,00	0,00E+00	1689	Os01g0847700	99,90	0,00E+00	1755	66	NA	NA	
maker-1-augustus-gene-424.0-mRNA-1	1	OsR498G0102334700.01.T01	100,00	0,00E+00	850	Os01g0848900	95,57	3,02E-165	580	-270	Indica	NA	
maker-1-augustus-gene-424.15-mRNA-1	1	OsR498G0102339900.01.T01	100,00	0,00E+00	3144	Os12g0259800	73,93	0,00E+00	984	-2160	Indica	NA	
maker-1-augustus-gene-424.18-mRNA-1	1	OsR498G0102340300.01.T01	100,00	0,00E+00	3030	Os01g0850700	99,82	0,00E+00	3017	-13	NA	NA	
maker-1-augustus-gene-424.20-mRNA-1	1	OsR498G0102335200.01.T01	100,00	0,00E+00	2960	Os01g0849100	99,82	0,00E+00	2947	-13	NA	NA	
maker-1-augustus-gene-424.22-mRNA-1	1	OsR498G0102335800.01.T01	100,00	0,00E+00	883	Os01g0849600	100,00	0,00E+00	883	0	NA	NA	
maker-1-augustus-gene-424.24-mRNA-1	1	OsR498G0102339600.01.T01	100,00	0,00E+00	2241	Os01g0850400	99,68	0,00E+00	2223	-18	NA	NA	
maker-1-augustus-gene-424.5-mRNA-1	1	OsR498G0102338100.01.T02	100,00	0,00E+00	1970	Os01g0850000	99,73	0,00E+00	1957	-13	NA	NA	
maker-1-augustus-gene-424.8-mRNA-1	1	OsR498G0102338900.01.T06	99,86	0,00E+00	1266	Os01g0850100	99,71	0,00E+00	1245	-21	NA	NA	
maker-1-augustus-gene-425.0-mRNA-1	1	OsR498G0102340700.01.T01	100,00	0,00E+00	3003	Os01g0850800	99,88	0,00E+00	2994	-9	NA	Indica	
maker-1-augustus-gene-425.11-mRNA-1	1	OsR498G0102343400.01.T01	100,00	0,00E+00	1824	Os01g0851400	99,60	0,00E+00	1806	-18	NA	NA	
maker-1-augustus-gene-425.13-mRNA-1	1	OsR498G0102345000.01.T01	100,00	0,00E+00	4556	Os01g0851700	99,84	0,00E+00	4538	-18	NA	NA	
maker-1-augustus-gene-425.19-mRNA-1	1	OsR498G0102345600.01.T02	100,00	0,00E+00	2457	Os03g0825800	71,23	1,44E-92	341	-2116	Indica	NA	Kinase-TM
maker-1-augustus-gene-425.2-mRNA-1	1	OsR498G0102341500.01.T03	100,00	0,00E+00	2879	Os01g0851000	99,81	0,00E+00	2865	-14	NA	NA	
maker-1-augustus-gene-425.23-mRNA-1	1	OsR498G0102346000.01.T01	100,00	0,00E+00	2814	Os01g0852200	99,81	0,00E+00	1907	-907	Indica	NA	
maker-1-augustus-gene-425.8-mRNA-1	1	OsR498G0102343200.01.T02	100,00	0,00E+00	1104	Os01g0851300	99,51	0,00E+00	1091	-13	NA	NA	
maker-1-augustus-gene-426.38-mRNA-1	1	OsR498G0102350700.01.T02	100,00	0,00E+00	3415	Os01g0853400	99,95	0,00E+00	3410	-5	NA	NA	
maker-1-augustus-gene-426.42-mRNA-1	1	OsR498G0102355900.01.T01	100,00	0,00E+00	693	Os01g0854400	100,00	0,00E+00	693	0	NA	NA	
maker-1-augustus-gene-426.43-mRNA-1	1	OsR498G0102348400.01.T03	99,91	0,00E+00	2023	Os01g0852500	99,79	0,00E+00	1716	-307	Indica	NA	
maker-1-augustus-gene-426.46-mRNA-1	1	OsR498G0102349200.01.T01	100,00	0,00E+00	1407	Os01g0853000	99,36	0,00E+00	1388	-19	NA	NA	
maker-1-augustus-gene-426.48-mRNA-1	1	OsR498G0102352200.01.T01	100,00	0,00E+00	1624	Os01g0853700	99,89	0,00E+00	1619	-5	NA	NA	
maker-1-augustus-gene-426.50-mRNA-1	1	OsR498G0102356600.01.T01	100,00	0,00E+00	1088	Os01g0854500	99,67	0,00E+00	1079	-9	NA	NA	
maker-1-augustus-gene-427.24-mRNA-1	1	OsR498G0102360100.01.T01	100,00	0,00E+00	2760	Os01g0855200	99,16	0,00E+00	2721	-39	NA	NA	
maker-1-augustus-gene-427.26-mRNA-1	1	OsR498G0102361100.01.T01	100,00	0,00E+00	2430	Os01g0855400	99,77	0,00E+00	2292	-138	NA	NA	
maker-1-augustus-gene-427.28-mRNA-1	1	OsR498G0102362900.01.T03	100,00	0,00E+00	1622	Os01g0856000	99,89	0,00E+00	1618	-4	NA	NA	
maker-1-augustus-gene-427.31-mRNA-1	1	OsR498G0102357800.01.T01	100,00	0,00E+00	2695	Os01g0855000	99,93	0,00E+00	2691	-4	NA	NA	
maker-1-augustus-gene-428.27-mRNA-1	1	OsR498G0102366700.01.T01	100,00	0,00E+00	2668	Os01g0856500	99,80	0,00E+00	2654	-14	NA	NA	
maker-1-augustus-gene-428.32-mRNA-1	1	OsR498G0102367900.01.T01	100,00	0,00E+00	2127	Os01g0856900	99,91	0,00E+00	2052	-75	NA	NA	
maker-1-augustus-gene-428.46-mRNA-1	1	OsR498G0102368200.01.T03	100,00	0,00E+00	3961	Os01g0857000	99,82	0,00E+00	3943	-18	NA	NA	
maker-1-augustus-gene-428.51-mRNA-1	1	OsR498G0102368400.01.T02	100,00	0,00E+00	1602	Os01g0857200	99,78	0,00E+00	1593	-9	NA	NA	
maker-1-augustus-gene-428.59-mRNA-1	1	OsR498G0102368800.01.T01	100,00	0,00E+00	2479	Os01g0857400	99,85	0,00E+00	2470	-9	NA	NA	
maker-1-augustus-gene-428.62-mRNA-1	1	OsR498G0102369600.01.T01	99,72	0,00E+00	3854	Os01g0857600	99,64	0,00E+00	2950	-904	Indica	NA	
maker-1-augustus-gene-429.11-mRNA-1	1	OsR498G0102374900.01.T05	100,00	0,00E+00	1916	Os01g0859200	99,90	0,00E+00	1879	-37	NA	NA	
maker-1-augustus-gene-429.14-mRNA-1	1	OsR498G0102377300.01.T01	100,00	0,00E+00	2105	Os01g0859300	99,49	0,00E+00	1731	-374	Indica	NA	
maker-1-augustus-gene-429.16-mRNA-1	1	OsR498G0102377500.01.T01	100,00	0,00E+00	1407	Os01g0859400	99,87	0,00E+00	1403	-4	NA	NA	
maker-1-augustus-gene-429.19-mRNA-1	1	OsR498G0102370200.01.T01	98,28	0,00E+00	5496	Os01g0858000	99,72	0,00E+00	5715	219	NA	NA	
maker-1-augustus-gene-429.9-mRNA-1	1	OsR498G0102372900.01.T01	100,00	0,00E+00	915	Os01g0858700	98,23	0,00E+00	875	-40	NA	NA	
maker-1-augustus-gene-43.0-mRNA-1	1	OsR498G0100274100.01.T04	99,80	0,00E+00	4555	Os01g0180800	100,00	0,00E+00	4578	23	NA	Japonica	
maker-1-augustus-gene-43.10-mRNA-1	1	OsR498G0100275400.01.T01	99,89	0,00E+00	3216	Os01g0181000	100,00	0,00E+00	3173	-43	NA	Japonica	
maker-1-augustus-gene-43.4-mRNA-1	1	OsR498G0100274900.01.T01	95,12	0,00E+00	1094	Os01g0180900	97,08	0,00E+00	1795	701	Japonica	Japonica	
maker-1-augustus-gene-430.52-mRNA-1	1	OsR498G0102379300.01.T01	100,00	0,00E+00	1613	Os01g0860400	99,78	0,00E+00	1604	-9	NA	NA	
maker-1-augustus-gene-430.57-mRNA-1	1	OsR498G0100480900.01.T01	97,34	0,00E+00	950	Os01g0861400	92,15	0,00E+00	954	4	NA	NA	
maker-1-augustus-gene-430.59-mRNA-1	1	OsR498G0102383100.01.T01	100,00	0,00E+00	1952	Os01g0861700	99,82	0,00E+00	1943	-9	NA	NA	
maker-1-augustus-gene-430.62-mRNA-1	1	OsR498G0102383900.01.T01	100,00	0,00E+00	1012	Os01g0862200	99,64	0,00E+00	1003	-9	NA	NA	
maker-1-augustus-gene-430.75-mRNA-1	1	OsR498G0102383600.01.T01	100,00	0,00E+00	2657	Os01g0861900	99,46	0,00E+00	2621	-36	NA	NA	
maker-1-augustus-gene-430.77-mRNA-1	1	OsR498G0102383800.01.T04	97,73	0,00E+00	1819	Os01g0862000	96,72	0,00E+00	974	-845	Indica	NA	
maker-1-augustus-gene-431.0-mRNA-1	1	OsR498G0102385500.01.T01	100,00	0,00E+00	1097	Os01g0862800	99,67	0,00E+00	1088	-9	NA	NA	
maker-1-augustus-gene-431.12-mRNA-1	1	OsR498G0102388200.01.T01	100,00	0,00E+00	1618	Os01g0863400	99,67	0,00E+00	1605	-13	NA	NA	
maker-1-augustus-gene-431.3-mRNA-1	1	OsR498G0102387900.01.T01	100,00	0,00E+00	942	Os05g0442400	78,46	6,51E-111	399	-543	Indica	NA	
maker-1-augustus-gene-431.6-mRNA-1	1	OsR498G0102384200.01.T01	99,25	0,00E+00	2128	Os01g0862300	99,17	0,00E+00	2123	-5	NA	NA	
maker-1-augustus-gene-432.0-mRNA-1	1	OsR498G0102396300.01.T01	100,00	0,00E+00	2435	Os01g0865600	97,82	0,00E+00	2369	-66	NA	NA	
maker-1-augustus-gene-432.12-mRNA-1	1	OsR498G0102395000.01.T01	100,00	0,00E+00	1667	Os01g0865100	99,89	0,00E+00	1663	-4	NA	NA	
maker-1-augustus-gene-432.5-mRNA-1	1	OsR498G0102390000.01.T01	100,00	0,00E+00	1240	Os01g0864000	99,45	0,00E+00	974	-266	Indica	NA	
maker-1-augustus-gene-433.0-mRNA-1	1	OsR498G0102400100.01.T02	100,00	0,00E+00	2040	Os05g0438800	86,27	0,00E+00	1338	-702	Indica	NA	
maker-1-augustus-gene-433.12-mRNA-1	1	OsR498G0102403100.01.T05	100,00	0,00E+00	2430	Os01g0866800	99,93	0,00E+00	2425	-5	NA	NA	
maker-1-augustus-gene-433.18-mRNA-1	1	OsR498G0102403500.01.T04	100,00	0,00E+00	1040	Os07g0675400	75,79	1,89E-159	562	-478	Indica	NA	
maker-1-augustus-gene-433.2-mRNA-1	1	OsR498G0102400700.01.T04	100,00	0,00E+00	755	Os01g0866300	100,00	0,00E+00	755	0	NA	NA	
maker-1-augustus-gene-433.21-mRNA-1	1	OsR498G0102403700.01.T03	100,00	0,00E+00	1959	Os01g0867200	99,63	0,00E+00	1941	-18	NA	NA	
maker-1-augustus-gene-433.26-mRNA-1	1	OsR498G0102404900.01.T01	96,09	0,00E+00	2030	Os01g0867600	96,09	0,00E+00	2030	0	NA	NA	
maker-1-augustus-gene-433.33-mRNA-1	1	OsR498G0102405100.01.T01	100,00	0,00E+00	1478	Os01g0867700	100,00	0,00E+00	1478	0	NA	NA	
maker-1-augustus-gene-433.35-mRNA-1	1	OsR498G0102405300.01.T10	100,00	0,00E+00	1164	Os01g0867800	99,69	0,00E+00	1155	-9	NA	NA	
maker-1-augustus-gene-433.40-mRNA-1	1	OsR498G0102401200.01.T02	100,00	0,00E+00	1153	Os01g0866500	99,84	0,00E+00	1149	-4	NA	NA	
maker-1-augustus-gene-433.43-mRNA-1	1	OsR498G0102401600.01.T01	100,00	1,76E-145	515	Os01g0866600	100,00	6,13E-146	515	0	NA	NA	
maker-1-augustus-gene-433.45-mRNA-1	1	OsR498G0102401800.01.T01	100,00	1,02E-161	569	Os01g0866700	100,00	3,54E-162	569	0	NA	NA	
maker-1-augustus-gene-433.48-mRNA-1	1	OsR498G0102404000.01.T03	100,00	0,00E+00	1445	Os01g0867300	99,63	0,00E+00	1432	-13	NA	NA	
maker-1-augustus-gene-433.52-mRNA-1	1	OsR498G0102405600.01.T01	100,00	0,00E+00	1220	Os01g0867900	99,85	0,00E+00	1215	-5	NA	NA	
maker-1-augustus-gene-433.56-mRNA-1	1	OsR498G0102406000.01.T01	100,00	0,00E+00	1510	Os01g0868000	100,00	0,00E+00	1905	395	Japonica	NA	
maker-1-augustus-gene-433.7-mRNA-1	1	OsR498G0102400900.01.T01	99,42	0,00E+00	1825	Os01g0866400	99,32	0,00E+00	1820	-5	NA	NA	
maker-1-augustus-gene-434.37-mRNA-1	1	OsR498G0102410700.01.T01	100,00	0,00E+00	2268	Os01g0869200	99,92	0,00E+00	2263	-5	NA	NA	
maker-1-augustus-gene-434.39-mRNA-1	1	OsR498G0102412100.01.T01	100,00	0,00E+00	1532	Os01g0869600	98,35	0,00E+00	1475	-57	NA	NA	
maker-1-augustus-gene-434.42-mRNA-1	1	OsR498G0102412300.01.T01	100,00	0,00E+00	1456	Os01g0869800	100,00	0,00E+00	1456	0	NA	NA	
maker-1-augustus-gene-434.56-mRNA-1	1	OsR498G0102410200.01.T01	100,00	0,00E+00	3232	Os01g0869000	99,94	0,00E+00	3228	-4	NA	NA	
maker-1-augustus-gene-434.61-mRNA-1	1	OsR498G0102410400.01.T01	100,00	0,00E+00	1153	Os07g0232900	90,80	0,00E+00	830	-323	Indica	NA	
maker-1-augustus-gene-434.68-mRNA-1	1	OsR498G0102412000.01.T01	100,00	0,00E+00	1797	Os01g0869500	99,90	0,00E+00	1792	-5	NA	NA	
maker-1-augustus-gene-435.15-mRNA-1	1	OsR498G0102414500.01.T06	100,00	0,00E+00	2700	Os01g0870300	100,00	0,00E+00	2700	0	NA	NA	
maker-1-augustus-gene-435.24-mRNA-1	1	OsR498G0102417100.01.T01	100,00	0,00E+00	2673	Os01g0871300	100,00	0,00E+00	2484	-189	NA	NA	
maker-1-augustus-gene-435.28-mRNA-1	1	OsR498G0102418100.01.T03	100,00	0,00E+00	2955	Os01g0871500	99,94	0,00E+00	2950	-5	NA	NA	
maker-1-augustus-gene-435.32-mRNA-1	1	OsR498G0102414000.01.T01	100,00	0,00E+00	5227	Os01g0870100	99,86	0,00E+00	5209	-18	NA	NA	
maker-1-augustus-gene-435.37-mRNA-1	1	OsR498G0102414800.01.T01	100,00	0,00E+00	4464	Os01g0889900	71,62	0,00E+00	1196	-3268	Indica	NA	Kinase-TM
maker-1-augustus-gene-435.7-mRNA-1	1	OsR498G0102414300.01.T01	100,00	0,00E+00	1981	Os02g0688900	92,11	0,00E+00	1579	-402	Indica	NA	
maker-1-augustus-gene-436.47-mRNA-1	1	OsR498G0102418700.01.T01	100,00	0,00E+00	2971	Os01g0871600	99,88	0,00E+00	2875	-96	NA	NA	
maker-1-augustus-gene-436.50-mRNA-1	1	OsR498G0102418900.01.T01	100,00	0,00E+00	1315	Os01g0872100	76,61	1,18E-141	503	-812	Indica	NA	
maker-1-augustus-gene-436.52-mRNA-1	1	OsR498G0102419100.01.T01	100,00	1,03E-174	612	Os01g0871800	99,71	1,53E-173	608	-4	NA	NA	
maker-1-augustus-gene-436.54-mRNA-1	1	OsR498G0102420300.01.T02	85,43	0,00E+00	1504	Os01g0871900	96,63	0,00E+00	2233	729	Japonica	NA	
maker-1-augustus-gene-436.57-mRNA-1	1	OsR498G0102420300.01.T02	100,00	0,00E+00	2944	Os01g0872100	99,33	0,00E+00	2895	-49	NA	NA	
maker-1-augustus-gene-436.60-mRNA-1	1	OsR498G0102420700.01.T01	100,00	0,00E+00	726	Os01g0871800	84,96	1,32E-105	382	-344	Indica	NA	
maker-1-augustus-gene-436.62-mRNA-1	1	OsR498G0102420900.01.T01	100,00	0,00E+00	942	Os01g0872000	80,94	1,40E-158	559	-383	Indica	NA	
maker-1-augustus-gene-436.72-mRNA-1	1	OsR498G0102422400.01.T01	100,00	0,00E+00	5784	Os01g0872700	99,81	0,00E+00	5757	-27	NA	NA	
maker-1-augustus-gene-436.78-mRNA-1	1	OsR498G0102422600.01.T01	100,00	0,00E+00	2700	Os01g0872800	99,80	0,00E+00	2687	-13	NA	NA	Kinase
maker-1-augustus-gene-437.10-mRNA-1	1	OsR498G0102433100.01.T01	100,00	0,00E+00	2055	Os01g0874900	99,91	0,00E+00	2050	-5	NA	NA	
maker-1-augustus-gene-437.13-mRNA-1	1	OsR498G0102425600.01.T01	100,00	0,00E+00	7580	Os01g0873800	99,03	0,00E+00	7423	-157	NA	NA	
maker-1-augustus-gene-437.17-mRNA-1	1	OsR498G0102426200.01.T01	98,80	0,00E+00	3502	Os05g0430300	78,72	0,00E+00	1352	-2150	Indica	NA	
maker-1-augustus-gene-437.22-mRNA-1	1	OsR498G0102428000.01.T01	97,89	0,00E+00	2696	Os01g0874100	97,34	0,00E+00	2302	-394	Indica	NA	
maker-1-augustus-gene-437.25-mRNA-1	1	OsR498G0102432600.01.T02	100,00	0,00E+00	1925	Os01g0874800	99,53	0,00E+00	1902	-23	NA	NA	
maker-1-augustus-gene-437.27-mRNA-1	1	OsR498G0102433600.01.T01	100,00	0,00E+00	1727	Os01g0875000	99,48	0,00E+00	1704	-23	NA	NA	
maker-1-augustus-gene-437.5-mRNA-1	1	OsR498G0102429100.01.T01	100,00	0,00E+00	1559	Os01g0874300	100,00	0,00E+00	1559	0	NA	NA	
maker-1-augustus-gene-437.7-mRNA-1	1	OsR498G0102431700.01.T01	100,00	0,00E+00	3283	Os01g0874700	99,02	0,00E+00	3222	-61	NA	NA	
maker-1-augustus-gene-438.0-mRNA-1	1	OsR498G0102435700.01.T01	100,00	0,00E+00	1299	Os01g0875300	99,04	0,00E+00	1279	-20	NA	NA	
maker-1-augustus-gene-438.11-mRNA-1	1	OsR498G0102438100.01.T01	100,00	0,00E+00	4296	Os08g0300300	72,66	0,00E+00	682	-3614	Indica	NA	
maker-1-augustus-gene-438.13-mRNA-1	1	OsR498G0102439100.01.T04	98,64	6,64E-106	384	Os01g0876500	99,59	3,44E-123	440	56	Japonica	NA	
maker-1-augustus-gene-438.18-mRNA-1	1	OsR498G0102436000.01.T02	100,00	0,00E+00	1900	Os01g0875400	100,00	0,00E+00	1900	0	NA	NA	
maker-1-augustus-gene-438.22-mRNA-1	1	OsR498G0102437800.01.T01	100,00	0,00E+00	2084	Os01g0875800	99,83	0,00E+00	2075	-9	NA	NA	
maker-1-augustus-gene-438.24-mRNA-1	1	OsR498G0102439000.01.T01	100,00	0,00E+00	2964	Os01g0876400	97,90	0,00E+00	2782	-182	NA	NA	
maker-1-augustus-gene-438.28-mRNA-1	1	OsR498G0102441200.01.T02	100,00	0,00E+00	1023	Os01g0876900	99,82	0,00E+00	1019	-4	NA	NA	
maker-1-augustus-gene-438.9-mRNA-1	1	OsR498G0102437500.01.T01	100,00	0,00E+00	1775	Os01g0875700	99,80	0,00E+00	1766	-9	NA	NA	
maker-1-augustus-gene-439.0-mRNA-1	1	OsR498G0102441700.01.T01	100,00	0,00E+00	3901	Os01g0877300	99,58	0,00E+00	3861	-40	NA	NA	
maker-1-augustus-gene-439.15-mRNA-1	1	OsR498G0102444800.01.T02	100,00	0,00E+00	861	Os01g0878000	99,16	0,00E+00	843	-18	NA	NA	
maker-1-augustus-gene-439.17-mRNA-1	1	OsR498G0102446400.01.T01	100,00	0,00E+00	2522	Os01g0878400	99,79	0,00E+00	2508	-14	NA	NA	
maker-1-augustus-gene-439.19-mRNA-1	1	OsR498G0102447200.01.T02	100,00	0,00E+00	2527	Os01g0878700	99,86	0,00E+00	2518	-9	NA	NA	
maker-1-augustus-gene-439.21-mRNA-1	1	OsR498G0102447400.01.T01	100,00	0,00E+00	1748	Os01g0878800	99,17	0,00E+00	1712	-36	NA	NA	
maker-1-augustus-gene-439.23-mRNA-1	1	OsR498G0102447600.01.T02	100,00	0,00E+00	1521	Os01g0878900	99,76	0,00E+00	1512	-9	NA	NA	
maker-1-augustus-gene-439.4-mRNA-1	1	OsR498G0102441900.01.T01	100,00	0,00E+00	2205	Os01g0877400	99,76	0,00E+00	2191	-14	NA	NA	
maker-1-augustus-gene-439.7-mRNA-1	1	OsR498G0102442700.01.T01	100,00	0,00E+00	3880	Os01g0877500	99,91	0,00E+00	3871	-9	NA	NA	
maker-1-augustus-gene-44.0-mRNA-1	1	OsR498G0100277300.01.T01	99,85	0,00E+00	2336	Os01g0182100	99,92	0,00E+00	2341	5	NA	NA	
maker-1-augustus-gene-44.12-mRNA-1	1	OsR498G0100277600.01.T02	99,87	0,00E+00	1333	Os01g0182200	100,00	0,00E+00	1337	4	NA	Japonica	
maker-1-augustus-gene-44.18-mRNA-1	1	OsR498G0100279000.01.T05	99,78	0,00E+00	3209	Os01g0182600	99,89	0,00E+00	3218	9	NA	Japonica	
maker-1-augustus-gene-440.36-mRNA-1	1	OsR498G0102453500.01.T01	100,00	0,00E+00	3442	Os01g0880200	99,69	0,00E+00	3415	-27	NA	NA	
maker-1-augustus-gene-440.39-mRNA-1	1	OsR498G0102454900.01.T01	100,00	0,00E+00	2067	Os01g0880800	99,74	0,00E+00	2054	-13	NA	NA	
maker-1-augustus-gene-440.41-mRNA-1	1	OsR498G0102454200.01.T01	100,00	0,00E+00	2928	Os01g0880300	99,69	0,00E+00	2905	-23	NA	NA	
maker-1-augustus-gene-440.43-mRNA-1	1	OsR498G0102454400.01.T03	100,00	0,00E+00	2354	Os01g0880400	99,76	0,00E+00	2260	-94	NA	NA	
maker-1-augustus-gene-440.46-mRNA-1	1	OsR498G0102455200.01.T01	100,00	0,00E+00	3227	Os01g0881000	99,16	0,00E+00	3163	-64	NA	NA	
maker-1-augustus-gene-441.46-mRNA-1	1	OsR498G0102458300.01.T01	100,00	0,00E+00	1483	Os01g0881300	99,88	0,00E+00	1479	-4	NA	NA	
maker-1-augustus-gene-441.51-mRNA-1	1	OsR498G0102458500.01.T01	100,00	0,00E+00	3766	Os01g0881400	99,78	0,00E+00	2454	-1312	Indica	NA	
maker-1-augustus-gene-441.54-mRNA-1	1	OsR498G0102460500.01.T01	100,00	0,00E+00	834	Os01g0882100	100,00	3,38E-139	493	-341	Indica	NA	
maker-1-augustus-gene-441.57-mRNA-1	1	OsR498G0102462100.01.T03	100,00	0,00E+00	1954	Os01g0882300	99,82	0,00E+00	1945	-9	NA	NA	
maker-1-augustus-gene-441.69-mRNA-1	1	OsR498G0102459000.01.T01	100,00	0,00E+00	2998	Os01g0881500	99,88	0,00E+00	2989	-9	NA	NA	
maker-1-augustus-gene-441.71-mRNA-1	1	OsR498G0102460200.01.T04	100,00	0,00E+00	2057	Os01g0881900	100,00	0,00E+00	2057	0	NA	NA	
maker-1-augustus-gene-441.80-mRNA-1	1	OsR498G0102462400.01.T01	100,00	0,00E+00	1148	Os01g0882500	100,00	0,00E+00	1148	0	NA	NA	
maker-1-augustus-gene-441.82-mRNA-1	1	OsR498G0102462800.01.T01	100,00	0,00E+00	2646	Os01g0882800	99,93	0,00E+00	2642	-4	NA	NA	
maker-1-augustus-gene-441.84-mRNA-1	1	OsR498G0102463400.01.T04	100,00	0,00E+00	888	Os01g0883100	99,84	0,00E+00	1132	244	Japonica	NA	
maker-1-augustus-gene-442.27-mRNA-1	1	OsR498G0102466500.01.T01	100,00	0,00E+00	1056	Os01g0883800	99,66	0,00E+00	1047	-9	NA	NA	
maker-1-augustus-gene-442.29-mRNA-1	1	OsR498G0102469100.01.T01	100,00	0,00E+00	2697	Os01g0884400	99,80	0,00E+00	2683	-14	NA	NA	
maker-1-augustus-gene-442.32-mRNA-1	1	OsR498G0102470900.01.T02	100,00	0,00E+00	785	Os01g0885000	100,00	2,39E-167	587	-198	Indica	NA	

maker-1-augustus-gene-442.35-mRNA-1	1	OsR498G0102466800.01.T02	100,00	0,00E+00	4367	Os01g0883900	99,84	0,00E+00	4349	-18	NA	NA	
maker-1-augustus-gene-442.40-mRNA-1	1	OsR498G0102467600.01.T01	100,00	0,00E+00	1645	Os01g0884300	99,78	0,00E+00	1636	-9	NA	NA	
maker-1-augustus-gene-442.43-mRNA-1	1	OsR498G0102469400.01.T04	100,00	0,00E+00	2756	Os01g0884500	99,94	0,00E+00	2752	-4	NA	NA	
maker-1-augustus-gene-442.50-mRNA-1	1	OsR498G0102470200.01.T01	100,00	0,00E+00	1364	Os01g0884700	99,73	0,00E+00	1324	-40	NA	NA	
maker-1-augustus-gene-443.13-mRNA-1	1	OsR498G0102478500.01.T01	100,00	0,00E+00	2755	Os05g0419200	84,17	0,00E+00	1329	-1426	Indica	NA	
maker-1-augustus-gene-443.24-mRNA-1	1	OsR498G0102472000.01.T04	99,75	0,00E+00	2169	Os01g0885300	99,67	0,00E+00	2154	-15	NA	NA	
maker-1-augustus-gene-443.29-mRNA-1	1	OsR498G0102475000.01.T01	96,47	0,00E+00	1847	Os01g0886000	96,38	0,00E+00	1842	-5	NA	NA	
maker-1-augustus-gene-443.33-mRNA-1	1	OsR498G0102477400.01.T04	100,00	0,00E+00	737	Os01g0886500	100,00	0,00E+00	737	0	NA	NA	
maker-1-augustus-gene-443.35-mRNA-1	1	OsR498G0102477600.01.T01	97,91	0,00E+00	3221	Os01g0886600	96,65	0,00E+00	2498	-723	Indica	NA	
maker-1-augustus-gene-443.5-mRNA-1	1	OsR498G0102475700.01.T01	100,00	0,00E+00	1440	Os01g0886200	100,00	0,00E+00	1613	173	Japonica	NA	
maker-1-augustus-gene-443.9-mRNA-1	1	OsR498G0102477100.01.T07	100,00	0,00E+00	1575	Os01g0886300	100,00	0,00E+00	1575	0	NA	NA	
maker-1-augustus-gene-444.35-mRNA-1	1	OsR498G0102483900.01.T01	100,00	0,00E+00	2452	Os01g0888300	100,00	0,00E+00	2452	0	NA	NA	
maker-1-augustus-gene-444.37-mRNA-1	1	OsR498G0102485100.01.T01	100,00	0,00E+00	1364	Os01g0888600	100,00	0,00E+00	1364	0	NA	NA	
maker-1-augustus-gene-444.43-mRNA-1	1	OsR498G0102480200.01.T01	100,00	0,00E+00	1478	Os01g0887700	99,88	0,00E+00	1473	-5	NA	NA	
maker-1-augustus-gene-444.47-mRNA-1	1	OsR498G0102485400.01.T01	100,00	0,00E+00	2663	Os01g0888700	99,93	0,00E+00	2658	-5	NA	NA	
maker-1-augustus-gene-445.0-mRNA-1	1	OsR498G0102486500.01.T01	100,00	0,00E+00	3631	Os01g0889000	99,85	0,00E+00	3608	-23	NA	NA	
maker-1-augustus-gene-445.15-mRNA-1	1	OsR498G0102487400.01.T06	100,00	0,00E+00	666	Os01g0889200	99,73	0,00E+00	662	-4	NA	NA	
maker-1-augustus-gene-445.22-mRNA-1	1	OsR498G0102488600.01.T02	100,00	0,00E+00	1862	Os01g0889800	99,71	0,00E+00	1848	-14	NA	NA	
maker-1-augustus-gene-445.24-mRNA-1	1	OsR498G0102488800.01.T01	100,00	0,00E+00	4453	Os01g0889900	99,19	0,00E+00	4391	-62	NA	NA	Kinase-TM
maker-1-augustus-gene-445.30-mRNA-1	1	OsR498G0102491600.01.T02	100,00	0,00E+00	2852	Os01g0891000	100,00	0,00E+00	2852	0	NA	NA	
maker-1-augustus-gene-445.6-mRNA-1	1	OsR498G0102489700.01.T01	100,00	0,00E+00	1620	Os01g0890600	79,22	0,00E+00	756	-864	Indica	NA	Kinase-TM
maker-1-augustus-gene-445.9-mRNA-1	1	OsR498G0102491100.01.T02	100,00	0,00E+00	3535	Os01g0890600	99,82	0,00E+00	2939	-596	Indica	Indica	Kinase-TM
maker-1-augustus-gene-446.47-mRNA-1	1	OsR498G0102492100.01.T01	100,00	0,00E+00	2192	Os01g0891100	100,00	0,00E+00	2214	22	NA	NA	
maker-1-augustus-gene-446.48-mRNA-1	1	OsR498G0102492500.01.T01	100,00	0,00E+00	1489	Os01g0891300	99,88	0,00E+00	1484	-5	NA	NA	
maker-1-augustus-gene-446.55-mRNA-1	1	OsR498G0102495900.01.T01	95,37	0,00E+00	1091	Os01g0892200	99,73	0,00E+00	667	-424	Indica	NA	
maker-1-augustus-gene-446.70-mRNA-1	1	OsR498G0102492800.01.T03	100,00	0,00E+00	1299	Os01g0661400	98,75	0,00E+00	1259	-40	NA	NA	
maker-1-augustus-gene-446.74-mRNA-1	1	OsR498G0102496800.01.T01	100,00	0,00E+00	2219	Os01g0892600	90,72	0,00E+00	1628	-591	Indica	NA	
maker-1-augustus-gene-446.79-mRNA-1	1	OsR498G0102497400.01.T01	100,00	0,00E+00	2149	Os01g0892600	85,71	0,00E+00	1324	-825	Indica	NA	
maker-1-augustus-gene-446.84-mRNA-1	1	OsR498G0102497600.01.T02	100,00	0,00E+00	2085	Os01g0892600	99,48	0,00E+00	2058	-27	NA	NA	
maker-1-augustus-gene-447.44-mRNA-1	1	OsR498G0102500100.01.T01	100,00	0,00E+00	2168	Os01g0893400	96,34	0,00E+00	1968	-200	NA	NA	
maker-1-augustus-gene-447.48-mRNA-1	1	OsR498G0102501300.01.T02	100,00	0,00E+00	3972	Os01g0894000	98,59	0,00E+00	3832	-140	NA	NA	
maker-1-augustus-gene-447.51-mRNA-1	1	OsR498G0102502500.01.T03	100,00	0,00E+00	883	Os01g0894500	99,59	0,00E+00	874	-9	NA	NA	
maker-1-augustus-gene-447.55-mRNA-1	1	OsR498G0102504300.01.T01	100,00	0,00E+00	2370	Os01g0895200	99,70	0,00E+00	2352	-18	NA	NA	
maker-1-augustus-gene-447.57-mRNA-1	1	OsR498G0102505100.01.T01	100,00	0,00E+00	2067	Os01g0895300	99,52	0,00E+00	1492	-575	Indica	NA	
maker-1-augustus-gene-447.67-mRNA-1	1	OsR498G0102500600.01.T01	100,00	0,00E+00	1645	Os01g0893700	96,46	0,00E+00	1489	-156	NA	NA	
maker-1-augustus-gene-447.69-mRNA-1	1	OsR498G0102501800.01.T01	100,00	0,00E+00	1754	Os01g0894300	99,90	0,00E+00	1749	-5	NA	NA	
maker-1-augustus-gene-447.74-mRNA-1	1	OsR498G0102502800.01.T04	100,00	0,00E+00	1278	Os01g0894600	99,85	0,00E+00	1185	-93	NA	NA	
maker-1-augustus-gene-447.81-mRNA-1	1	OsR498G0102504200.01.T01	100,00	0,00E+00	1721	Os01g0895100	99,48	0,00E+00	1699	-22	NA	NA	
maker-1-augustus-gene-447.84-mRNA-1	1	OsR498G0102505800.01.T01	100,00	0,00E+00	2394	Os05g0507300	84,37	0,00E+00	1164	-1230	Indica	NA	
maker-1-augustus-gene-448.0-mRNA-1	1	OsR498G0102506100.01.T02	100,00	0,00E+00	5092	Os01g0896300	99,39	0,00E+00	3472	-1620	Indica	NA	
maker-1-augustus-gene-448.11-mRNA-1	1	OsR498G0102507700.01.T02	100,00	0,00E+00	2637	Os01g0897100	99,86	0,00E+00	2628	-9	NA	NA	
maker-1-augustus-gene-448.18-mRNA-1	1	OsR498G0102506400.01.T03	97,65	0,00E+00	3291	Os01g0896500	97,39	0,00E+00	3268	-23	NA	NA	
maker-1-augustus-gene-448.27-mRNA-1	1	OsR498G0102508000.01.T03	100,00	0,00E+00	776	Os01g0897200	99,77	0,00E+00	770	-6	NA	NA	
maker-1-augustus-gene-448.3-mRNA-1	1	OsR498G0102506700.01.T01	100,00	0,00E+00	1647	Os01g0896800	97,59	0,00E+00	1548	-99	NA	NA	
maker-1-augustus-gene-448.31-mRNA-1	1	OsR498G0102508400.01.T02	98,99	0,00E+00	1216	Os01g0897300	91,87	0,00E+00	897	-319	Indica	NA	
maker-1-augustus-gene-448.36-mRNA-1	1	OsR498G0102511200.01.T04	100,00	0,00E+00	3272	Os01g0897800	99,95	0,00E+00	3268	-4	NA	NA	
maker-1-augustus-gene-448.7-mRNA-1	1	OsR498G0102506900.01.T01	100,00	0,00E+00	1645	Os01g0896800	99,89	0,00E+00	1641	-4	NA	NA	
maker-1-augustus-gene-449.24-mRNA-1	1	OsR498G0102513500.01.T02	100,00	0,00E+00	1131	Os01g0898400	97,93	0,00E+00	1079	-52	NA	NA	
maker-1-augustus-gene-449.28-mRNA-1	1	OsR498G0102513700.01.T01	100,00	0,00E+00	2230	Os01g0898500	99,84	0,00E+00	2221	-9	NA	NA	
maker-1-augustus-gene-449.31-mRNA-1	1	OsR498G0102514700.01.T02	94,35	0,00E+00	846	Os01g0898800	100,00	0,00E+00	818	-28	NA	NA	
maker-1-augustus-gene-449.33-mRNA-1	1	OsR498G0102514900.01.T04	100,00	0,00E+00	2181	Os01g0898900	99,84	0,00E+00	2172	-9	NA	NA	
maker-1-augustus-gene-449.38-mRNA-1	1	OsR498G0102515500.01.T01	100,00	6,97E-114	410	Os01g0899100	99,56	1,03E-112	406	-4	NA	NA	
maker-1-augustus-gene-449.40-mRNA-1	1	OsR498G0102513000.01.T01	98,54	0,00E+00	4781	Os01g0898300	98,32	0,00E+00	4754	-27	NA	NA	
maker-1-augustus-gene-449.46-mRNA-1	1	OsR498G0102514000.01.T01	100,00	0,00E+00	639	Os01g0898600	99,15	5,96E-179	626	-13	NA	NA	
maker-1-augustus-gene-449.48-mRNA-1	1	OsR498G0102515200.01.T03	100,00	0,00E+00	2003	Os01g0899000	99,55	0,00E+00	1980	-23	NA	NA	Kinase-TM
maker-1-augustus-gene-449.56-mRNA-1	1	OsR498G0102518200.01.T04	100,00	0,00E+00	2430	Os01g0899500	99,78	0,00E+00	2416	-14	NA	NA	
maker-1-augustus-gene-449.61-mRNA-1	1	OsR498G0102518400.01.T01	100,00	0,00E+00	2235	Os01g0899700	99,92	0,00E+00	2231	-4	NA	NA	
maker-1-augustus-gene-449.63-mRNA-1	1	OsR498G0102518800.01.T01	99,57	0,00E+00	3724	Os01g0899800	99,81	0,00E+00	1853	-1871	Indica	NA	
maker-1-augustus-gene-45.45-mRNA-1	1	OsR498G0511247700.01.T02	70,84	4,40E-123	443	Os01g0183000	99,46	0,00E+00	1966	1523	Japonica	NA	
maker-1-augustus-gene-45.51-mRNA-1	1	OsR498G0100282100.01.T01	99,94	0,00E+00	2891	Os01g0183100	99,81	0,00E+00	2882	-9	NA	Japonica	
maker-1-augustus-gene-45.56-mRNA-1	1	OsR498G0100285700.01.T01	99,74	0,00E+00	2043	Os01g0184000	99,91	0,00E+00	2052	9	NA	Japonica	
maker-1-augustus-gene-45.64-mRNA-1	1	OsR498G0100286700.01.T01	99,73	0,00E+00	3361	Os01g0184900	99,95	0,00E+00	3379	18	NA	Japonica	
maker-1-augustus-gene-45.70-mRNA-1	1	OsR498G0100282600.01.T01	99,71	0,00E+00	3060	Os01g0183300	99,82	0,00E+00	3069	9	NA	Japonica	
maker-1-augustus-gene-45.76-mRNA-1	1	OsR498G0100286200.01.T02	96,56	0,00E+00	3125	Os01g0184500	99,84	0,00E+00	3374	249	NA	Japonica	
maker-1-augustus-gene-45.81-mRNA-1	1	OsR498G0100286600.01.T01	94,40	0,00E+00	1242	Os01g0184800	96,18	0,00E+00	1306	64	NA	Japonica	
maker-1-augustus-gene-450.11-mRNA-1	1	OsR498G0102525600.01.T02	99,63	4,40E-135	481	Os01g0900900	99,63	1,53E-135	481	0	NA	NA	
maker-1-augustus-gene-450.15-mRNA-1	1	OsR498G0102526000.01.T03	100,00	0,00E+00	4323	Os01g0901000	99,58	0,00E+00	4290	-33	NA	NA	
maker-1-augustus-gene-450.19-mRNA-1	1	OsR498G0102527000.01.T01	100,00	0,00E+00	2186	Os01g0901200	99,67	0,00E+00	2168	-18	NA	NA	
maker-1-augustus-gene-450.7-mRNA-1	1	OsR498G0102525300.01.T01	100,00	0,00E+00	1287	Os01g0900800	99,72	0,00E+00	1278	-9	NA	NA	
maker-1-augustus-gene-451.16-mRNA-1	1	OsR498G0102529300.01.T01	99,77	0,00E+00	2324	Os01g0902200	99,61	0,00E+00	2315	-9	NA	NA	
maker-1-augustus-gene-451.17-mRNA-1	1	OsR498G0102529300.01.T04	99,45	0,00E+00	1301	Os01g0902200	99,59	0,00E+00	1306	5	NA	NA	
maker-1-augustus-gene-451.28-mRNA-1	1	OsR498G0102530100.01.T01	98,69	0,00E+00	2935	Os01g0902800	84,54	0,00E+00	1811	-1124	Indica	NA	
maker-1-augustus-gene-451.30-mRNA-1	1	OsR498G0102530300.01.T01	99,88	0,00E+00	2892	Os01g0902800	99,07	0,00E+00	2845	-47	NA	NA	
maker-1-augustus-gene-451.34-mRNA-1	1	OsR498G0102528400.01.T02	99,81	0,00E+00	927	Os01g0901800	100,00	0,00E+00	931	4	NA	NA	
maker-1-augustus-gene-451.38-mRNA-1	1	OsR498G0102528600.01.T01	99,80	0,00E+00	3510	Os01g0901900	99,15	0,00E+00	2498	-1012	Indica	NA	
maker-1-augustus-gene-451.50-mRNA-1	1	OsR498G0102529600.01.T01	100,00	0,00E+00	2224	Os01g0902300	99,76	0,00E+00	2211	-13	NA	NA	
maker-1-augustus-gene-452.0-mRNA-1	1	OsR498G0102533100.01.T01	99,28	1,63E-139	495	Os01g0904300	100,00	3,16E-86	316	-179	Indica	NA	
maker-1-augustus-gene-452.14-mRNA-1	1	OsR498G0102533000.01.T06	93,97	0,00E+00	1047	Os01g0904200	93,67	0,00E+00	1038	-9	NA	NA	
maker-1-augustus-gene-452.23-mRNA-1	1	OsR498G0102533800.01.T02	99,81	0,00E+00	1894	Os01g0904500	99,81	0,00E+00	1894	0	NA	NA	
maker-1-augustus-gene-452.28-mRNA-1	1	OsR498G0102534400.01.T01	99,54	0,00E+00	3119	Os01g0904700	99,72	0,00E+00	1935	-1184	Indica	NA	
maker-1-augustus-gene-452.32-mRNA-1	1	OsR498G0101582300.01.T01	99,66	0,00E+00	2102	Os01g0627800	99,92	0,00E+00	2115	13	NA	NA	
maker-1-augustus-gene-452.34-mRNA-1	1	OsR498G0101580700.01.T01	99,75	0,00E+00	2915	Os01g0627400	99,94	0,00E+00	2929	14	NA	NA	
maker-1-augustus-gene-452.9-mRNA-1	1	OsR498G0101581000.01.T01	100,00	0,00E+00	926	Os01g0627500	100,00	0,00E+00	926	0	NA	NA	
maker-1-augustus-gene-453.13-mRNA-1	1	OsR498G0101575700.01.T01	98,73	0,00E+00	1788	Os01g0625300	100,00	0,00E+00	1840	52	NA	NA	
maker-1-augustus-gene-453.19-mRNA-1	1	OsR498G0101574500.01.T01	97,35	0,00E+00	1925	Os01g0624500	97,61	0,00E+00	1939	14	NA	NA	
maker-1-augustus-gene-453.23-mRNA-1	1	OsR498G0101574300.01.T02	99,82	0,00E+00	972	Os01g0624400	99,82	0,00E+00	972	0	NA	NA	
maker-1-augustus-gene-453.4-mRNA-1	1	OsR498G0101577900.01.T01	99,48	0,00E+00	2033	Os01g0626400	99,91	0,00E+00	2052	19	NA	NA	
maker-1-augustus-gene-454.10-mRNA-1	1	OsR498G0101566200.01.T01	100,00	0,00E+00	758	Os01g0622700	100,00	0,00E+00	758	0	NA	NA	
maker-1-augustus-gene-454.15-mRNA-1	1	OsR498G0101571300.01.T01	99,24	0,00E+00	3931	Os01g0623500	100,00	0,00E+00	3990	59	NA	NA	
maker-1-augustus-gene-454.20-mRNA-1	1	OsR498G0101565900.01.T01	98,23	0,00E+00	2737	Os05g0585500	83,45	0,00E+00	1644	-1093	Indica	NA	
maker-1-augustus-gene-454.5-mRNA-1	1	OsR498G0101571600.01.T01	99,76	0,00E+00	2254	Os01g0891100	70,16	4,04E-137	489	-1765	Indica	NA	
maker-1-augustus-gene-454.7-mRNA-1	1	OsR498G0101568400.01.T03	99,36	0,00E+00	3609	Os01g0623200	99,94	0,00E+00	2790	-819	Indica	NA	
maker-1-augustus-gene-455.30-mRNA-1	1	OsR498G0101565600.01.T03	99,56	0,00E+00	2007	Os01g0622300	99,71	0,00E+00	1881	-126	NA	NA	
maker-1-augustus-gene-455.37-mRNA-1	1	OsR498G0101562800.01.T02	99,47	0,00E+00	3715	Os01g0621600	100,00	0,00E+00	3766	51	NA	NA	Kinase-TM
maker-1-augustus-gene-455.42-mRNA-1	1	OsR498G0101561600.01.T01	99,92	0,00E+00	2297	Os01g0621200	100,00	0,00E+00	1882	-415	Indica	NA	
maker-1-augustus-gene-455.48-mRNA-1	1	OsR498G0101563100.01.T01	99,70	0,00E+00	1198	Os01g0621700	100,00	0,00E+00	1207	9	NA	NA	
maker-1-augustus-gene-456.29-mRNA-1	1	OsR498G0101558200.01.T01	99,49	0,00E+00	1737	Os01g0620100	100,00	0,00E+00	1759	22	NA	NA	
maker-1-augustus-gene-456.37-mRNA-1	1	OsR498G0101555000.01.T01	99,94	0,00E+00	2999	Os01g0619000	100,00	0,00E+00	2300	-699	Indica	NA	
maker-1-augustus-gene-456.41-mRNA-1	1	OsR498G0101557500.01.T02	99,77	0,00E+00	2346	Os01g0619800	99,58	0,00E+00	1263	-1083	Indica	NA	
maker-1-augustus-gene-456.44-mRNA-1	1	OsR498G0101554700.01.T01	99,63	0,00E+00	2391	Os01g0618900	100,00	0,00E+00	1673	-718	Indica	NA	
maker-1-augustus-gene-457.14-mRNA-1	1	OsR498G0101550800.01.T01	98,74	0,00E+00	968	Os01g0617800	99,10	0,00E+00	977	9	NA	NA	
maker-1-augustus-gene-457.18-mRNA-1	1	OsR498G0101550000.01.T03	99,34	0,00E+00	2409	Os01g0617600	99,49	0,00E+00	2418	9	NA	NA	
maker-1-augustus-gene-457.19-mRNA-1	1	OsR498G0101549200.01.T01	99,93	0,00E+00	4823	Os01g0617500	99,96	0,00E+00	4828	5	NA	NA	
maker-1-augustus-gene-457.30-mRNA-1	1	OsR498G0101553100.01.T01	99,69	0,00E+00	2883	Os01g0618500	99,94	0,00E+00	2902	19	NA	NA	
maker-1-augustus-gene-457.33-mRNA-1	1	OsR498G0101552900.01.T01	96,76	0,00E+00	1713	Os01g0618400	96,76	0,00E+00	1713	0	NA	NA	
maker-1-augustus-gene-457.37-mRNA-1	1	OsR498G0101551700.01.T02	98,01	0,00E+00	1994	Os01g0618200	96,35	0,00E+00	959	-1035	Indica	NA	
maker-1-augustus-gene-457.39-mRNA-1	1	OsR498G0101550900.01.T01	99,79	0,00E+00	1701	Os01g0617900	100,00	0,00E+00	1775	74	NA	NA	
maker-1-augustus-gene-457.42-mRNA-1	1	OsR498G0101550500.01.T04	99,63	0,00E+00	3876	Os01g0617700	99,95	0,00E+00	3908	32	NA	NA	
maker-1-augustus-gene-457.7-mRNA-1	1	OsR498G0101552600.01.T01	98,36	0,00E+00	5046	Os01g0618300	98,73	0,00E+00	4959	-87	NA	NA	
maker-1-augustus-gene-457.9-mRNA-1	1	OsR498G0101551600.01.T02	100,00	0,00E+00	1867	Os01g0618100	100,00	0,00E+00	1867	0	NA	NA	
maker-1-augustus-gene-458.0-mRNA-1	1	OsR498G0101547800.01.T01	99,89	0,00E+00	3270	Os05g0573300	77,91	0,00E+00	1345	-1925	Indica	NA	
maker-1-augustus-gene-458.12-mRNA-1	1	OsR498G0101546500.01.T01	99,88	0,00E+00	1506	Os01g0616500	100,00	0,00E+00	1510	4	NA	NA	
maker-1-augustus-gene-458.15-mRNA-1	1	OsR498G0101545900.01.T02	99,13	0,00E+00	3673	Os05g0587300	75,12	0,00E+00	1111	-2562	Indica	NA	
maker-1-augustus-gene-458.19-mRNA-1	1	OsR498G0101542500.01.T07	99,69	0,00E+00	4007	Os01g0616100	100,00	0,00E+00	3512	-495	Indica	NA	
maker-1-augustus-gene-458.6-mRNA-1	1	OsR498G0101546400.01.T02	99,71	0,00E+00	2488	Os01g0616400	99,78	0,00E+00	2490	2	NA	NA	
maker-1-augustus-gene-459.43-mRNA-1	1	OsR498G0101541200.01.T01	99,92	0,00E+00	2296	Os01g0615300	100,00	0,00E+00	2300	4	NA	NA	
maker-1-augustus-gene-459.45-mRNA-1	1	OsR498G0101541000.01.T02	99,70	0,00E+00	2379	Os01g0615200	100,00	0,00E+00	2419	40	NA	NA	
maker-1-augustus-gene-459.47-mRNA-1	1	OsR498G0101539800.01.T03	99,73	0,00E+00	2628	Os01g0614900	99,93	0,00E+00	2642	14	NA	NA	
maker-1-augustus-gene-459.50-mRNA-1	1	OsR498G0101537000.01.T02	99,88	0,00E+00	1497	Os01g0613900	100,00	0,00E+00	1501	4	NA	NA	
maker-1-augustus-gene-459.57-mRNA-1	1	OsR498G0101536800.01.T01	99,35	0,00E+00	1917	Os01g0613800	100,00	0,00E+00	1948	31	NA	NA	
maker-1-augustus-gene-459.59-mRNA-1	1	OsR498G0101536000.01.T01	99,91	0,00E+00	1949	Os01g0613500	100,00	0,00E+00	1954	5	NA	NA	
maker-1-augustus-gene-459.61-mRNA-1	1	OsR498G0101539500.01.T01	99,89	0,00E+00	1679	Os01g0614700	100,00	0,00E+00	969	-710	Indica	NA	
maker-1-augustus-gene-459.66-mRNA-1	1	OsR498G0101538300.01.T01	98,00	0,00E+00	2496	Os01g0614300	99,79	0,00E+00	2588	92	NA	NA	
maker-1-augustus-gene-459.68-mRNA-1	1	OsR498G0101535700.01.T01	100,00	0,00E+00	1137	Os01g0613300	100,00	0,00E+00	1131	-6	NA	NA	
maker-1-augustus-gene-46.32-mRNA-1	1	OsR498G0100287700.01.T02	99,92	0,00E+00	2269	Os01g0185300	99,76	0,00E+00	2260	-9	NA	Japonica	
maker-1-augustus-gene-46.34-mRNA-1	1	OsR498G0100287900.01.T03	99,74	0,00E+00	2758	Os01g0185400	100,00	0,00E+00	2174	-584	Indica	Japonica	
maker-1-augustus-gene-46.42-mRNA-1	1	OsR498G0100288900.01.T01	99,74	0,00E+00	2731	Os01g0185900	99,93	0,00E+00	2745	14	NA	Japonica	
maker-1-augustus-gene-46.44-mRNA-1	1	OsR498G0100287200.01.T04	99,70	0,00E+00	2394	Os01g0185200	99,76	0,00E+00	2263	-131	NA	NA	
maker-1-augustus-gene-46.50-mRNA-1	1	OsR498G0100290000.01.T02	99,58	0,00E+00	2121	Os01g0186200	100,00	0,00E+00	2143	22	NA	Japonica	
maker-1-augustus-gene-460.10-mRNA-1	1	OsR498G0101532900.01.T03	100,00	1,38E-179	628	Os01g0612800	100,00	4,80E-180	628	0	NA	NA	
maker-1-augustus-gene-460.12-mRNA-1	1	OsR498G0101532700.01.T03	99,72	0,00E+00	5708	Os01g0612700	100,00	0,00E+00	4103	-1605	Indica	NA	
maker-1-augustus-gene-460.16-mRNA-1	1	OsR498G0101530100.01.T01	99,73	0,00E+00	1328	Os01g0612500	100,00	0,00E+00	1178	-150	Indica	NA	
maker-1-augustus-gene-460.20-mRNA-1	1	OsR498G0101528100.01.T01	100,00	0,00E+00	921	Os01g0612200	100,00	0,00E+00	921	0	NA	NA	
maker-1-augustus-gene-460.24-mRNA-1	1	OsR498G0306062000.01.T02	86,57	0,00E+00	793	Os01g0612100	100,00	0,00E+00	1397	604	Japonica	NA	
maker-1-augustus-gene-460.26-mRNA-1	1	OsR498G0101526300.01.T01	99,42	0,00E+00	2746	Os07g0218200	82,67	0,00E+00	1002	-1744	Indica	NA	
maker-1-augustus-gene-461.0-mRNA-1	1	OsR498G0101521400.01.T01	100,00	0,00E+00	681	Os01g0610800	100,00	0,00E+00	681	0	NA	NA	
maker-1-augustus-gene-461.12-mRNA-1	1	OsR498G0101520600.01.T01	99,88	0,00E+00	1475	Os01g0610500	100,00	0,00E+00	1480	5	NA	NA	
maker-1-augustus-gene-461.18-mRNA-1	1	OsR498G0101522300.01.T01	99,82	0,00E+00	2015	Os01g0611000	100,00	0,00E+00	2165	150	NA	NA	
maker-1-augustus-gene-461.20-mRNA-1	1	OsR498G0101520100.01.T02	99,63	0,00E+00	966	Os01g0610400	99,68	1,39E-157	555	-411	Indica	NA	
maker-1-augustus-gene-461.22-mRNA-1	1	OsR498G0101519900.01.T03	100,00	0,00E+00	2630	Os01g0610300	100,00	0,00E+00	2630	0	NA	NA	
maker-1-augustus-gene-461.28-mRNA-1	1	OsR498G0101518900.01.T01	100,00	0,00E+00	958	Os01g0610100	100,00	0,00E+00	958	0	NA	NA	
maker-1-augustus-gene-461.3-mRNA-1	1	OsR498G0101521200.01.T01	98,17	0,00E+00	1610	Os01g0610700	100,00	0,00E+00	1678	68	NA	NA	
maker-1-augustus-gene-461.6-mRNA-1	1	OsR498G0101521000.01.T03	98,41	0,00E+00	4726	Os01g0610600	98,67	0,00E+00	4757	31	NA	NA	
maker-1-augustus-gene-462.0-mRNA-1	1	OsR498G0101517800.01.T02	99,88	0,00E+00	7222	Os01g0609900	100,00	0,00E+00	7245	23	NA	NA	
maker-1-augustus-gene-462.13-mRNA-1	1	OsR498G0101514000.01.T02	99,48	0,00E+00	7845	Os01g0609066	100,00	0,00E+00	7946	101	NA	NA	
maker-1-augustus-gene-462.4-mRNA-1	1	OsR498G0101515200.01.T01	99,79	0,00E+00	7852	Os01g0609300	100,00	0,00E+00	7889	37	NA	NA	
maker-1-augustus-gene-462.8-mRNA-1	1	OsR498G0101514400.01.T01	98,49	0,00E+00	4938	Os01g0609066	91,91	0,00E+00	4094	-844	Indica	NA	
maker-1-augustus-gene-462.9-mRNA-1	1	OsR498G0101514400.01.T01	98,45	0,00E+00	1897	Os01g0609200	100,00	0,00E+00	1974	77	NA	NA	
maker-1-augustus-gene-463.22-mRNA-1	1	OsR498G0101503700.01.T01	99,86	0,00E+00	2607	Os01g0607200	96,63	0,00E+00	2389	-218	NA	NA	
maker-1-augustus-gene-463.23-mRNA-1	1	OsR498G0101503300.01.T01	98,49	0,00E+00	803	Os01g0607200	100,00	0,00E+00	744	-59	NA	NA	
maker-1-augustus-gene-463.6-mRNA-1	1	OsR498G0101504200.01.T03	99,87	0,00E+00	4094	Os01g0607400	100,00	0,00E+00	4107	13	NA	NA	
maker-1-augustus-gene-464.10-mRNA-1	1	OsR498G0101502300.01.T01	98,48	0,00E+00	1027	Os01g0606900	99,83	0,00E+00	1064	37	NA	NA	
maker-1-augustus-gene-464.13-mRNA-1	1	OsR498G0101499300.01.T01	99,23	0,00E+00	1380	Os01g0606000	100,00	0,00E+00	1407	27	NA	NA	
maker-1-augustus-gene-464.15-mRNA-1	1	OsR498G0101498500.01.T01	98,83	0,00E+00	1342	Os01g0605700	99,35	0,00E+00	1361	19	NA	NA	
maker-1-augustus-gene-464.20-mRNA-1	1	OsR498G0101497900.01.T02	99,78	0,00E+00	1617	Os01g0605400	98,23	0,00E+00	1548	-69	NA	NA	
maker-1-augustus-gene-464.22-mRNA-1	1	OsR498G0101497900.01.T01	99,94	0,00E+00	3010	Os01g0605300	100,00	0,00E+00	3014	4	NA	NA	
maker-1-augustus-gene-464.24-mRNA-1	1	OsR498G0101497700.01.T02	100,00	0,00E+00	2636	Os01g0605100	99,63	0,00E+00	1947	-689	Indica	NA	
maker-1-augustus-gene-464.4-mRNA-1	1	OsR498G0101498200.01.T01	99,90	0,00E+00	3579	Os01g0605500	100,00	0,00E+00	4145	566	Japonica	NA	
maker-1-augustus-gene-464.7-mRNA-1	1	OsR498G0101497400.01.T04	89,91	9,17E-177	619	Os01g0605200	89,91	3,18E-177	619	0	NA	NA	
maker-1-augustus-gene-465.0-mRNA-1	1	OsR498G0101495000.01.T01	99,35	0,00E+00	1896	Os01g0604700	99,63	0,00E+00	1910	14	NA	NA	
maker-1-augustus-gene-465.11-mRNA-1	1	OsR498G0100035900.01.T05	80,94	4,00E-74	279	Os01g0604100	94,48	0,00E+00	690	411	Japonica	NA	
maker-1-augustus-gene-465.13-mRNA-1	1	OsR498G0101487900.01.T01	99,56	0,00E+00	1617	Os01g0603300	100,00	0,00E+00	1635	18	NA	NA	
maker-1-augustus-gene-465.6-mRNA-1	1	OsR498G0101486800.01.T01	100,00	0,00E+00	1014	Os01g0603000	100,00	0,00E+00	1014	0	NA	NA	
maker-1-augustus-gene-466.17-mRNA-1	1	OsR498G0101483200.01.T01	99,86	0,00E+00	1250	Os11g0566800	72,72	4,89E-155	548	-702	Indica	NA	
maker-1-augustus-gene-466.34-mRNA-1	1	OsR498G0101582500.01.T01	82,08	0,00E+00	1391	Os01g0602400	99,93	0,00E+00	2546	1155	Japonica	NA	
maker-1-augustus-gene-466.38-mRNA-1	1	OsR498G0101482200.01.T03	94,13	0,00E+00	1251	Os01g0602200	99,77	0,00E+00	1526	275	Japonica	NA	
maker-1-augustus-gene-466.47-mRNA-1	1	OsR498G0101477700.01.T01	99,54	0,00E+00	2725	Os01g0601200	99,33	0,00E+00	1839	-886	Indica	NA	Kinase-TM
maker-1-augustus-gene-467.12-mRNA-1	1	OsR498G0101471400.01.T01	99,68	0,00E+00	2786	Os01g0598400	100,00	0,00E+00	2809	23	NA	NA	
maker-1-augustus-gene-467.14-mRNA-1	1	OsR498G0101474700.01.T01	99,75	0,00E+00	2823	Os01g0600300	99,78	0,00E+00	1608	-1215	Indica	NA	
maker-1-augustus-gene-467.18-mRNA-1	1	OsR498G0101474500.01.T01	99,87	0,00E+00	2740	Os02g0224200	74,70	0,00E+00	995	-1745	Indica	NA	
maker-1-augustus-gene-467.23-mRNA-1	1	OsR498G0101473900.01.T01	93,15	0,00E+00	2574	Os01g0599900	99,88	0,00E+00	2899	325	Japonica	NA	
maker-1-augustus-gene-467.29-mRNA-1	1	OsR498G0101471100.01.T02	99,85	0,00E+00	1181	Os01g0598200	100,00	0,00E+00	1186	5	NA	NA	
maker-1-augustus-gene-467.3-mRNA-1	1	OsR498G0101475400.01.T01	100,00	0,00E+00	1543	Os02g0226200	71,87	7,83E-62	238	-1305	Indica	NA	
maker-1-augustus-gene-467.5-mRNA-1	1	OsR498G0101474200.01.T03	100,00	0,00E+00	666	Os01g0600000	100,00	0,00E+00	666	0	NA	NA	
maker-1-augustus-gene-467.9-mRNA-1	1	OsR498G0101473200.01.T03	93,52	0,00E+00	1562	Os01g0598900	96,02	0,00E+00	1662	100	NA	NA	
maker-1-augustus-gene-468.0-mRNA-1	1	OsR498G0101469500.01.T01	97,51	0,00E+00	1359	Os01g0597800	86,89	0,00E+00	973	-386	Indica	NA	
maker-1-augustus-gene-468.2-mRNA-1	1	OsR498G0101469100.01.T01	97,99	0,00E+00	2124	Os01g0597600	100,00	0,00E+00	2233	109	NA	NA	
maker-1-augustus-gene-468.3-mRNA-1	1	OsR498G0101468700.01.T01	96,35	0,00E+00	1884	Os01g0597400	100,00	0,00E+00	2403	519	Japonica	NA	
maker-1-augustus-gene-468.5-mRNA-1	1	OsR498G0101466500.01.T01	99,37	0,00E+00	2828	Os01g0596700	100,00	0,00E+00	2874	46	NA	NA	
maker-1-augustus-gene-469.39-mRNA-1	1	OsR498G0101463900.01.T01	100,00	0,00E+00	1413	Os01g0596300	100,00	0,00E+00	1413	0	NA	NA	
maker-1-augustus-gene-469.41-mRNA-1	1	OsR498G0101463300.01.T01	99,94	0,00E+00	2815	Os01g0596100	100,00	0,00E+00	2819	4	NA	NA	
maker-1-augustus-gene-469.43-mRNA-1	1	OsR498G0101462900.01.T01	100,00	0,00E+00	1559	Os01g0596000	100,00	0,00E+00	2727	1168	Japonica	NA	
maker-1-augustus-gene-469.45-mRNA-1	1	OsR498G0101462700.01.T01	94,67	0,00E+00	2573	Os01g0595900	96,14	0,00E+00	2681	108	NA	NA	
maker-1-augustus-gene-469.47-mRNA-1	1	OsR498G0101462500.01.T01	98,42	0,00E+00	2626	Os01g0595800	100,00	0,00E+00	2722	96	NA	NA	
maker-1-augustus-gene-469.49-mRNA-1	1	OsR498G0101462300.01.T01	96,94	0,00E+00	2354	Os01g0595700	97,76	0,00E+00	2835	481	Japonica	NA	
maker-1-augustus-gene-469.52-mRNA-1	1	OsR498G0101462100.01.T01	95,30	0,00E+00	1452	Os01g0595600	100,00	0,00E+00	1640	188	Japonica	NA	
maker-1-augustus-gene-469.55-mRNA-1	1	OsR498G0101460100.01.T01	99,75	0,00E+00	710	Os01g0719600	73,08	8,25E-26	116	-594	Indica	NA	
maker-1-augustus-gene-47.22-mRNA-1	1	OsR498G0100294100.01.T01	100,00	0,00E+00	1510	Os01g0187000	99,17	0,00E+00	1489	-21	NA	Indica	
maker-1-augustus-gene-47.42-mRNA-1	1	OsR498G0100292200.01.T03	100,00	0,00E+00	2857	Os01g0186700	96,88	0,00E+00	2761	-96	NA	Indica	Kinase-TM
maker-1-augustus-gene-47.52-mRNA-1	1	OsR498G0100298600.01.T02	100,00	0,00E+00	1986	Os01g0187900	100,00	0,00E+00	1986	0	NA	NA	
maker-1-augustus-gene-470.37-mRNA-1	1	OsR498G0101457800.01.T01	98,96	0,00E+00	1014	Os01g0594500	99,83	0,00E+00	1037	23	NA	NA	
maker-1-augustus-gene-470.39-mRNA-1	1	OsR498G0102535900.01.T01	99,88	0,00E+00	3021	Os01g0905200	99,41	0,00E+00	2986	-35	NA	Indica	
maker-1-augustus-gene-470.41-mRNA-1	1	OsR498G0102536100.01.T01	99,95	0,00E+00	3296	Os01g0905300	99,74	0,00E+00	2054	-1242	Indica	Indica	
maker-1-augustus-gene-470.47-mRNA-1	1	OsR498G0102536400.01.T01	99,36	0,00E+00	3617	Os01g0905400	99,02	0,00E+00	3585	-32	NA	Indica	
maker-1-augustus-gene-471.0-mRNA-1	1	OsR498G0102542100.01.T01	100,00	0,00E+00	937	Os01g0907200	100,00	0,00E+00	937	0	NA	Japonica	
maker-1-augustus-gene-471.10-mRNA-1	1	OsR498G0102547300.01.T01	100,00	0,00E+00	1884	Os01g0908200	99,81	0,00E+00	1874	-10	NA	Indica	
maker-1-augustus-gene-471.2-mRNA-1	1	OsR498G0102542500.01.T02	98,98	0,00E+00	3289	Os01g0907300	99,09	0,00E+00	3298	9	NA	Indica	
maker-1-augustus-gene-471.21-mRNA-1	1	OsR498G0102541400.01.T01	100,00	0,00E+00	3198	Os01g0906600	99,94	0,00E+00	3194	-4	NA	NA	
maker-1-augustus-gene-471.32-mRNA-1	1	OsR498G0102547800.01.T01	99,93	0,00E+00	2404	Os01g0908500	99,89	0,00E+00	1700	-704	Indica	Indica	
maker-1-augustus-gene-471.37-mRNA-1	1	OsR498G0102548800.01.T01	100,00	0,00E+00	2576	Os01g0908700	99,75	0,00E+00	2106	-470	Indica	Indica	
maker-1-augustus-gene-471.8-mRNA-1	1	OsR498G0102544500.01.T01	99,85	0,00E+00	3672	Os01g0907900	99,17	0,00E+00	3628	-44	NA	Indica	
maker-1-augustus-gene-472.11-mRNA-1	1	OsR498G0102553900.01.T09	99,56	0,00E+00	1223	Os01g0910400	93,60	0,00E+00	1123	-100	NA	Indica	
maker-1-augustus-gene-472.17-mRNA-1	1	OsR498G0102551400.01.T02	100,00	0,00E+00	7815	Os01g0909200	99,79	0,00E+00	7774	-41	NA	Indica	
maker-1-augustus-gene-473.0-mRNA-1	1	OsR498G0102555700.01.T01	99,91	0,00E+00	2081	Os01g0911200	99,91	0,00E+00	2081	0	NA	Indica	
maker-1-augustus-gene-473.15-mRNA-1	1	OsR498G0102556000.01.T01	95,05	0,00E+00	1564	Os01g0911300	95,05	0,00E+00	1564	0	NA	Indica	NBS-TM
maker-1-augustus-gene-474.33-mRNA-1	1	OsR498G0102558100.01.T01	100,00	0,00E+00	4118	Os01g0911800	99,83	0,00E+00	4100	-18	NA	Indica	
maker-1-augustus-gene-474.44-mRNA-1	1	OsR498G0102560100.01.T03	100,00	0,00E+00	1694	Os01g0912700	100,00	0,00E+00	1694	0	NA	Indica	
maker-1-augustus-gene-474.48-mRNA-1	1	OsR498G0102558000.01.T02	100,00	0,00E+00	3939	Os01g0911700	99,57	0,00E+00	3736	-203	NA	Indica	
maker-1-augustus-gene-475.48-mRNA-1	1	OsR498G0102561700.01.T01	99,82	0,00E+00	1013	Os01g0913000	100,00	0,00E+00	1018	5	NA	Japonica	
maker-1-augustus-gene-475.53-mRNA-1	1	OsR498G0102565100.01.T01	100,00	0,00E+00	672	Os01g0914400	100,00	0,00E+00	672	0	NA	Japonica	
maker-1-augustus-gene-475.56-mRNA-1	1	OsR498G0102565500.01.T01	99,81	0,00E+00	2822	Os01g0914700	100,00	0,00E+00	2836	14	NA	Japonica	
maker-1-augustus-gene-475.58-mRNA-1	1	OsR498G0102565700.01.T01	99,80	0,00E+00	2709	Os01g0914800	100,00	0,00E+00	2722	13	NA	Japonica	

maker-1-augustus-gene-475.69-mRNA-1	1	OsR498G0102563000.01.T01	99,69	0,00E+00	1171	Os01g0913600	100,00	0,00E+00	1180	9	NA	Japonica	
maker-1-augustus-gene-475.71-mRNA-1	1	OsR498G0102563800.01.T02	100,00	0,00E+00	699	Os01g0914000	100,00	0,00E+00	661	-38	NA	Japonica	
maker-1-augustus-gene-476.0-mRNA-1	1	OsR498G0102572700.01.T01	99,93	0,00E+00	2609	Os01g0916400	100,00	0,00E+00	2614	5	NA	Japonica	
maker-1-augustus-gene-476.17-mRNA-1	1	OsR498G0102568600.01.T04	99,93	0,00E+00	2604	Os01g0915600	100,00	0,00E+00	2608	4	NA	Japonica	
maker-1-augustus-gene-476.20-mRNA-1	1	OsR498G0102571000.01.T01	99,79	0,00E+00	856	Os01g0915800	100,00	0,00E+00	861	5	NA	Japonica	
maker-1-augustus-gene-476.25-mRNA-1	1	OsR498G0102572000.01.T01	99,86	0,00E+00	5128	Os01g0916200	99,93	0,00E+00	5137	9	NA	Japonica	
maker-1-augustus-gene-476.31-mRNA-1	1	OsR498G0102572200.01.T01	99,03	0,00E+00	2535	Os01g0916300	99,29	0,00E+00	2495	-40	NA	Japonica	
maker-1-augustus-gene-476.37-mRNA-1	1	OsR498G0102573800.01.T02	99,75	0,00E+00	7059	Os01g0916800	99,27	0,00E+00	8987	1928	Japonica	Japonica	
maker-1-augustus-gene-476.6-mRNA-1	1	OsR498G0102573300.01.T05	99,78	0,00E+00	813	Os01g0916600	99,56	0,00E+00	809	-4	NA	Japonica	
maker-1-augustus-gene-476.9-mRNA-1	1	OsR498G0102573500.01.T01	99,76	0,00E+00	759	Os01g0916700	100,00	0,00E+00	764	5	NA	Japonica	
maker-1-augustus-gene-477.11-mRNA-1	1	OsR498G0102576200.01.T01	100,00	0,00E+00	921	Os01g0917400	100,00	0,00E+00	921	0	NA	Japonica	
maker-1-augustus-gene-477.15-mRNA-1	1	OsR498G0102577800.01.T02	99,47	0,00E+00	997	Os01g0917700	100,00	0,00E+00	1012	15	NA	Japonica	
maker-1-augustus-gene-477.17-mRNA-1	1	OsR498G0102580200.01.T01	99,53	0,00E+00	1530	Os01g0918100	96,30	0,00E+00	1484	-46	NA	Japonica	
maker-1-augustus-gene-477.2-mRNA-1	1	OsR498G0102575500.01.T01	99,82	0,00E+00	2968	Os01g0917200	100,00	0,00E+00	2513	-455	Indica	Japonica	
maker-1-augustus-gene-477.20-mRNA-1	1	OsR498G0102580600.01.T01	100,00	7,59E-157	553	Os01g0918200	100,00	2,64E-157	553	0	NA	Japonica	
maker-1-augustus-gene-477.22-mRNA-1	1	OsR498G0102580600.01.T02	100,00	3,19E-155	547	Os01g0918300	100,00	1,11E-155	547	0	NA	Japonica	
maker-1-augustus-gene-477.25-mRNA-1	1	OsR498G0102581400.01.T01	98,91	0,00E+00	641	Os01g0918500	100,00	2,57E-171	600	-41	NA	Japonica	
maker-1-augustus-gene-477.5-mRNA-1	1	OsR498G0102576500.01.T05	99,89	0,00E+00	6691	Os01g0917500	100,00	0,00E+00	6709	18	NA	Japonica	Kinase-LRR-TM
maker-1-augustus-gene-478.33-mRNA-1	1	OsR498G0102585300.01.T01	99,59	0,00E+00	1297	Os01g0919500	100,00	0,00E+00	1310	13	NA	Japonica	
maker-1-augustus-gene-478.35-mRNA-1	1	OsR498G0102586700.01.T02	99,77	0,00E+00	2346	Os01g0919600	99,92	0,00E+00	2355	9	NA	Japonica	
maker-1-augustus-gene-478.38-mRNA-1	1	OsR498G0102586900.01.T02	99,65	0,00E+00	2026	Os01g0919700	100,00	0,00E+00	2044	18	NA	Japonica	
maker-1-augustus-gene-478.45-mRNA-1	1	OsR498G0102589300.01.T01	99,86	0,00E+00	1251	Os01g0920100	100,00	0,00E+00	1256	5	NA	Japonica	
maker-1-augustus-gene-478.50-mRNA-1	1	OsR498G0102587200.01.T02	98,54	0,00E+00	1907	Os01g0919800	98,63	0,00E+00	1911	4	NA	Japonica	
maker-1-augustus-gene-478.52-mRNA-1	1	OsR498G0102588000.01.T01	99,92	0,00E+00	2166	Os01g0919900	100,00	0,00E+00	2170	4	NA	Japonica	
maker-1-augustus-gene-478.54-mRNA-1	1	OsR498G0102588400.01.T01	99,84	0,00E+00	2183	Os01g0920000	100,00	0,00E+00	1934	-249	Indica	Japonica	
maker-1-augustus-gene-478.56-mRNA-1	1	OsR498G0102589600.01.T01	94,68	0,00E+00	783	Os01g0920200	100,00	2,58E-128	457	-326	Indica	Japonica	
maker-1-augustus-gene-479.43-mRNA-1	1	OsR498G0102590500.01.T01	99,64	0,00E+00	1979	Os03g0849900	71,49	2,10E-89	330	-1649	Indica	Japonica	
maker-1-augustus-gene-479.45-mRNA-1	1	OsR498G0102591100.01.T01	99,23	0,00E+00	1141	Os01g0920700	100,00	0,00E+00	2003	862	Japonica	Japonica	
maker-1-augustus-gene-479.48-mRNA-1	1	OsR498G0102591900.01.T01	99,82	0,00E+00	2010	Os01g0921000	100,00	0,00E+00	2019	9	NA	Japonica	
maker-1-augustus-gene-479.52-mRNA-1	1	OsR498G0102592700.01.T01	99,37	0,00E+00	2516	Os01g0921200	96,33	0,00E+00	2452	-64	NA	Japonica	
maker-1-augustus-gene-479.53-mRNA-1	1	OsR498G0102592900.01.T01	99,92	0,00E+00	2366	Os01g0921300	100,00	0,00E+00	2370	4	NA	Japonica	
maker-1-augustus-gene-479.70-mRNA-1	1	OsR498G0102593500.01.T01	99,34	0,00E+00	1079	Os01g0921600	100,00	0,00E+00	1099	20	NA	Japonica	
maker-1-augustus-gene-479.76-mRNA-1	1	OsR498G0102590200.01.T01	98,55	0,00E+00	4350	Os01g0920400	98,91	0,00E+00	4378	28	NA	Japonica	
maker-1-augustus-gene-479.81-mRNA-1	1	OsR498G0102593400.01.T01	99,92	0,00E+00	2355	Os01g0921500	100,00	0,00E+00	2360	5	NA	Japonica	
maker-1-augustus-gene-479.83-mRNA-1	1	OsR498G0102595400.01.T01	99,57	0,00E+00	2086	Os01g0921800	100,00	0,00E+00	2003	-83	NA	Japonica	
maker-1-augustus-gene-48.38-mRNA-1	1	OsR498G0100304700.01.T02	88,29	2,35E-134	480	Os01g0175200	79,02	3,27E-89	327	-153	Indica	NA	
maker-1-augustus-gene-48.42-mRNA-1	1	OsR498G0100305100.01.T02	100,00	0,00E+00	1151	Os01g0190000	100,00	0,00E+00	1151	0	NA	NA	
maker-1-augustus-gene-48.46-mRNA-1	1	OsR498G0100306900.01.T01	100,00	0,00E+00	1294	Os01g0190300	100,00	0,00E+00	1294	0	NA	NA	
maker-1-augustus-gene-48.48-mRNA-1	1	OsR498G0100307700.01.T01	100,00	0,00E+00	2522	Os01g0190500	100,00	0,00E+00	2522	0	NA	NA	
maker-1-augustus-gene-48.56-mRNA-1	1	OsR498G0100300400.01.T01	100,00	0,00E+00	3463	Os01g0188400	100,00	0,00E+00	3463	0	NA	NA	
maker-1-augustus-gene-48.63-mRNA-1	1	OsR498G0100251400.01.T01	95,32	0,00E+00	1003	Os01g0189100	88,73	0,00E+00	820	-183	Indica	NA	
maker-1-augustus-gene-48.73-mRNA-1	1	OsR498G0100303800.01.T02	97,73	0,00E+00	2269	Os01g0189100	97,73	0,00E+00	2269	0	NA	NA	
maker-1-augustus-gene-48.77-mRNA-1	1	OsR498G0100307200.01.T02	100,00	0,00E+00	1521	Os01g0190400	100,00	0,00E+00	1700	179	Japonica	NA	
maker-1-augustus-gene-480.0-mRNA-1	1	OsR498G0102601900.01.T01	99,92	0,00E+00	2362	Os01g0922600	100,00	0,00E+00	1689	-673	Indica	Japonica	
maker-1-augustus-gene-481.28-mRNA-1	1	OsR498G0102607100.01.T06	99,03	0,00E+00	2183	Os01g0923600	99,85	0,00E+00	2381	198	NA	Japonica	
maker-1-augustus-gene-481.33-mRNA-1	1	OsR498G0102611700.01.T01	94,98	0,00E+00	998	Os01g0924000	95,15	0,00E+00	1003	5	NA	Japonica	
maker-1-augustus-gene-481.41-mRNA-1	1	OsR498G0102612600.01.T02	96,55	0,00E+00	1600	Os01g0924300	96,87	0,00E+00	1614	14	NA	Japonica	
maker-1-augustus-gene-482.27-mRNA-1	1	OsR498G0102614300.01.T01	99,44	1,17E-180	632	Os01g0924700	100,00	0,00E+00	645	13	NA	Japonica	
maker-1-augustus-gene-482.35-mRNA-1	1	OsR498G0102617500.01.T01	97,93	0,00E+00	1489	Os01g0925200	100,00	0,00E+00	1581	92	NA	Japonica	
maker-1-augustus-gene-482.42-mRNA-1	1	OsR498G0102614600.01.T01	99,78	0,00E+00	802	Os01g0924800	95,90	0,00E+00	1208	406	Japonica	Japonica	
maker-1-augustus-gene-482.45-mRNA-1	1	OsR498G0102617200.01.T02	98,70	0,00E+00	2019	Os01g0925000	100,00	0,00E+00	2075	56	NA	Japonica	
maker-1-augustus-gene-482.53-mRNA-1	1	OsR498G0102617800.01.T01	97,75	0,00E+00	1966	Os01g0925300	100,00	0,00E+00	2084	118	NA	Japonica	
maker-1-augustus-gene-483.34-mRNA-1	1	OsR498G0102618900.01.T01	95,06	0,00E+00	877	Os01g0925600	96,10	5,87E-156	550	-327	Indica	Japonica	
maker-1-augustus-gene-483.39-mRNA-1	1	OsR498G0102620500.01.T03	99,70	0,00E+00	1177	Os01g0926200	94,06	0,00E+00	1093	-84	NA	Japonica	
maker-1-augustus-gene-483.41-mRNA-1	1	OsR498G0102622700.01.T01	99,73	0,00E+00	1333	Os01g0926800	99,87	0,00E+00	1338	5	NA	Japonica	
maker-1-augustus-gene-483.44-mRNA-1	1	OsR498G0102623300.01.T01	99,80	0,00E+00	3575	Os01g0927000	99,95	0,00E+00	3589	14	NA	Japonica	
maker-1-augustus-gene-483.49-mRNA-1	1	OsR498G0102623900.01.T01	89,33	0,00E+00	710	Os01g0927200	94,32	0,00E+00	1512	802	Japonica	Japonica	
maker-1-augustus-gene-483.51-mRNA-1	1	OsR498G0102619200.01.T01	99,70	0,00E+00	3008	Os03g0349200	70,13	2,16E-110	399	-2609	Indica	Japonica	Kinase-TM
maker-1-augustus-gene-483.55-mRNA-1	1	OsR498G0102620000.01.T01	99,83	0,00E+00	1057	Os01g0925800	100,00	0,00E+00	1061	4	NA	Japonica	
maker-1-augustus-gene-483.58-mRNA-1	1	OsR498G0102620800.01.T01	99,77	0,00E+00	2330	Os01g0926300	100,00	0,00E+00	2343	13	NA	Japonica	
maker-1-augustus-gene-483.60-mRNA-1	1	OsR498G0102621000.01.T01	99,92	0,00E+00	2285	Os01g0926400	100,00	0,00E+00	2289	4	NA	Japonica	
maker-1-augustus-gene-483.62-mRNA-1	1	OsR498G0102622000.01.T02	99,76	0,00E+00	2238	Os01g0926600	100,00	0,00E+00	2251	13	NA	Japonica	
maker-1-augustus-gene-483.65-mRNA-1	1	OsR498G0102622400.01.T02	99,84	0,00E+00	2253	Os01g0926600	80,80	0,00E+00	1093	-1160	Indica	Japonica	
maker-1-augustus-gene-484.0-mRNA-1	1	OsR498G0102626700.01.T03	99,96	0,00E+00	4234	Os01g0927600	100,00	0,00E+00	4378	144	NA	Japonica	
maker-1-augustus-gene-484.11-mRNA-1	1	OsR498G0102624400.01.T03	99,30	0,00E+00	755	Os01g0927300	100,00	0,00E+00	769	14	NA	Japonica	
maker-1-augustus-gene-484.14-mRNA-1	1	OsR498G0102627600.01.T02	99,77	0,00E+00	2321	Os01g0927900	100,00	0,00E+00	2334	13	NA	Japonica	
maker-1-augustus-gene-484.19-mRNA-1	1	OsR498G0102627800.01.T02	99,13	0,00E+00	2023	Os01g0928000	100,00	0,00E+00	2067	44	NA	Japonica	
maker-1-augustus-gene-484.22-mRNA-1	1	OsR498G0102629600.01.T01	99,91	0,00E+00	1966	Os01g0928100	100,00	0,00E+00	1963	-3	NA	Japonica	
maker-1-augustus-gene-484.28-mRNA-1	1	OsR498G0102630800.01.T01	95,63	0,00E+00	1050	Os01g0928400	98,04	0,00E+00	1502	452	Japonica	Japonica	
maker-1-augustus-gene-484.30-mRNA-1	1	OsR498G0102631800.01.T01	99,73	0,00E+00	2688	Os01g0928700	83,88	0,00E+00	1596	-1092	Indica	Japonica	
maker-1-augustus-gene-484.32-mRNA-1	1	OsR498G0102632000.01.T01	99,92	0,00E+00	2305	Os01g0928700	100,00	0,00E+00	2309	4	NA	Japonica	
maker-1-augustus-gene-485.0-mRNA-1	1	OsR498G0102632900.01.T04	99,86	0,00E+00	2637	Os01g0929000	100,00	0,00E+00	2646	9	NA	Japonica	
maker-1-augustus-gene-485.11-mRNA-1	1	OsR498G0102636900.01.T01	99,80	0,00E+00	1750	Os01g0930400	100,00	0,00E+00	1759	9	NA	Japonica	
maker-1-augustus-gene-485.13-mRNA-1	1	OsR498G0102638700.01.T06	99,74	0,00E+00	2034	Os01g0930800	100,00	0,00E+00	1961	-73	NA	Japonica	
maker-1-augustus-gene-485.16-mRNA-1	1	OsR498G0102638900.01.T01	99,65	0,00E+00	1513	Os01g0930900	100,00	0,00E+00	1526	13	NA	Japonica	
maker-1-augustus-gene-485.18-mRNA-1	1	OsR498G0102639100.01.T01	99,64	0,00E+00	1491	Os01g0931000	100,00	0,00E+00	1505	14	NA	Japonica	
maker-1-augustus-gene-485.20-mRNA-1	1	OsR498G0102632600.01.T01	99,80	0,00E+00	2638	Os01g0928800	100,00	0,00E+00	2652	14	NA	Japonica	
maker-1-augustus-gene-485.29-mRNA-1	1	OsR498G0102639400.01.T05	96,38	0,00E+00	2532	Os01g0931100	96,64	0,00E+00	2550	18	NA	Japonica	
maker-1-augustus-gene-485.35-mRNA-1	1	OsR498G0102639800.01.T01	99,37	0,00E+00	1698	Os01g0931200	99,69	0,00E+00	1711	13	NA	Japonica	
maker-1-augustus-gene-485.37-mRNA-1	1	OsR498G0102640000.01.T01	99,34	0,00E+00	2704	Os01g0931300	100,00	0,00E+00	2749	45	NA	Japonica	
maker-1-augustus-gene-485.41-mRNA-1	1	OsR498G0102640200.01.T04	99,37	0,00E+00	1416	Os01g0931400	99,62	0,00E+00	1425	9	NA	Japonica	
maker-1-augustus-gene-485.6-mRNA-1	1	OsR498G0102635300.01.T03	99,32	0,00E+00	777	Os01g0930000	100,00	0,00E+00	791	14	NA	Japonica	
maker-1-augustus-gene-485.9-mRNA-1	1	OsR498G0102635500.01.T01	98,30	0,00E+00	911	Os01g0930200	100,00	0,00E+00	937	26	NA	Japonica	
maker-1-augustus-gene-486.11-mRNA-1	1	OsR498G0102645300.01.T01	98,55	0,00E+00	717	Os01g0932825	100,00	1,28E-176	618	-99	Indica	Japonica	
maker-1-augustus-gene-486.15-mRNA-1	1	OsR498G0102644600.01.T01	95,58	0,00E+00	3695	Os01g0932500	96,48	0,00E+00	3758	63	NA	Japonica	
maker-1-augustus-gene-486.17-mRNA-1	1	OsR498G0102646200.01.T02	99,60	0,00E+00	1806	Os01g0933200	100,00	0,00E+00	1824	18	NA	Japonica	
maker-1-augustus-gene-486.20-mRNA-1	1	OsR498G0102647600.01.T01	100,00	0,00E+00	962	Os01g0933600	100,00	0,00E+00	962	0	NA	Japonica	
maker-1-augustus-gene-486.23-mRNA-1	1	OsR498G0102648200.01.T01	99,85	0,00E+00	1197	Os01g0933900	100,00	0,00E+00	1202	5	NA	Japonica	
maker-1-augustus-gene-486.25-mRNA-1	1	OsR498G0102648800.01.T01	99,42	0,00E+00	2443	Os01g0934000	100,00	0,00E+00	2479	36	NA	Japonica	
maker-1-augustus-gene-486.6-mRNA-1	1	OsR498G0102645100.01.T02	99,96	0,00E+00	4433	Os01g0932600	98,42	0,00E+00	4292	-141	NA	Japonica	
maker-1-augustus-gene-487.0-mRNA-1	1	OsR498G0102649300.01.T07	100,00	0,00E+00	1813	Os01g0934200	99,90	0,00E+00	1778	-35	NA	Japonica	
maker-1-augustus-gene-487.12-mRNA-1	1	OsR498G0102649700.01.T01	99,88	0,00E+00	1533	Os01g0934400	100,00	0,00E+00	980	-553	Indica	Japonica	
maker-1-augustus-gene-487.15-mRNA-1	1	OsR498G0102653900.01.T06	99,80	0,00E+00	889	Os01g0935300	100,00	0,00E+00	893	4	NA	Japonica	
maker-1-augustus-gene-487.19-mRNA-1	1	OsR498G0102654700.01.T01	99,84	0,00E+00	2271	Os01g0935500	100,00	0,00E+00	2280	9	NA	Japonica	
maker-1-augustus-gene-487.22-mRNA-1	1	OsR498G0102650600.01.T01	78,02	0,00E+00	680	Os01g0934600	100,00	0,00E+00	1299	619	Japonica	Japonica	
maker-1-augustus-gene-487.25-mRNA-1	1	OsR498G0102650800.01.T01	99,63	0,00E+00	1443	Os01g0934800	100,00	0,00E+00	1424	-19	NA	Japonica	
maker-1-augustus-gene-487.26-mRNA-1	1	OsR498G0102650800.01.T03	98,65	0,00E+00	773	Os01g0934900	100,00	0,00E+00	737	-36	NA	Japonica	
maker-1-augustus-gene-487.37-mRNA-1	1	OsR498G0102654200.01.T01	99,64	0,00E+00	1968	Os01g0935400	100,00	0,00E+00	1986	18	NA	Japonica	
maker-1-augustus-gene-487.8-mRNA-1	1	OsR498G0102649500.01.T04	99,49	0,00E+00	3129	Os01g0934300	99,94	0,00E+00	3165	36	NA	Japonica	
maker-1-augustus-gene-488.36-mRNA-1	1	OsR498G0102655100.01.T05	100,00	0,00E+00	1667	Os01g0935700	99,03	0,00E+00	1630	-37	NA	Japonica	
maker-1-augustus-gene-488.40-mRNA-1	1	OsR498G0102657500.01.T01	99,73	0,00E+00	2645	Os01g0936100	99,93	0,00E+00	2658	13	NA	Japonica	Kinase
maker-1-augustus-gene-488.53-mRNA-1	1	OsR498G0102655400.01.T01	99,74	0,00E+00	2070	Os01g0935800	100,00	0,00E+00	1272	-798	Indica	Japonica	Kinase-TM
maker-1-augustus-gene-488.56-mRNA-1	1	OsR498G0102655800.01.T01	99,84	0,00E+00	1143	Os01g0935900	100,00	0,00E+00	1148	5	NA	Japonica	
maker-1-augustus-gene-488.58-mRNA-1	1	OsR498G0102657800.01.T02	96,02	0,00E+00	1785	Os01g0936200	93,61	0,00E+00	956	-829	Indica	Japonica	
maker-1-augustus-gene-488.63-mRNA-1	1	OsR498G0102658400.01.T01	98,80	0,00E+00	2333	Os01g0936700	100,00	0,00E+00	2549	216	NA	Japonica	
maker-1-augustus-gene-488.65-mRNA-1	1	OsR498G0102658800.01.T02	99,74	0,00E+00	2049	Os01g0936800	100,00	0,00E+00	2062	13	NA	Japonica	
maker-1-augustus-gene-488.70-mRNA-1	1	OsR498G0102663200.01.T06	99,74	0,00E+00	2746	Os01g0937400	100,00	0,00E+00	2553	-193	NA	Japonica	CC-NBS-TM
maker-1-augustus-gene-489.37-mRNA-1	1	OsR498G0102664300.01.T01	78,62	0,00E+00	930	Os01g0937500	100,00	0,00E+00	2403	1473	Japonica	Japonica	
maker-1-augustus-gene-489.39-mRNA-1	1	OsR498G0102666300.01.T01	99,82	0,00E+00	1994	Os01g0938200	100,00	0,00E+00	2003	9	NA	Japonica	
maker-1-augustus-gene-489.44-mRNA-1	1	OsR498G0102667300.01.T02	99,84	0,00E+00	1095	Os01g0938900	100,00	0,00E+00	1099	4	NA	Japonica	
maker-1-augustus-gene-489.48-mRNA-1	1	OsR498G0102667700.01.T01	99,90	0,00E+00	5635	Os01g0939100	100,00	0,00E+00	5649	14	NA	Japonica	
maker-1-augustus-gene-489.53-mRNA-1	1	OsR498G0102668700.01.T03	99,72	0,00E+00	2526	Os01g0939600	100,00	0,00E+00	2477	-49	NA	Japonica	
maker-1-augustus-gene-489.56-mRNA-1	1	OsR498G0102666800.01.T01	100,00	0,00E+00	1240	Os01g0938600	100,00	0,00E+00	1240	0	NA	Japonica	
maker-1-augustus-gene-489.58-mRNA-1	1	OsR498G0102668000.01.T02	99,75	0,00E+00	2173	Os01g0939200	100,00	0,00E+00	1847	-326	Indica	Japonica	
maker-1-augustus-gene-489.60-mRNA-1	1	OsR498G0102668200.01.T01	99,73	0,00E+00	2628	Os01g0939300	100,00	0,00E+00	2646	18	NA	Japonica	
maker-1-augustus-gene-489.62-mRNA-1	1	OsR498G0102668400.01.T01	99,60	0,00E+00	884	Os01g0939400	100,00	0,00E+00	893	9	NA	Japonica	
maker-1-augustus-gene-49.0-mRNA-1	1	OsR498G0100309500.01.T03	100,00	0,00E+00	682	Os01g0191100	100,00	2,23E-178	623	-59	NA	NA	
maker-1-augustus-gene-49.10-mRNA-1	1	OsR498G0100312900.01.T08	99,88	0,00E+00	2957	Os01g0191700	97,02	0,00E+00	2783	-174	NA	NA	
maker-1-augustus-gene-49.16-mRNA-1	1	OsR498G0100309400.01.T03	99,44	0,00E+00	945	Os01g0191000	99,59	0,00E+00	863	-82	NA	NA	
maker-1-augustus-gene-49.18-mRNA-1	1	OsR498G0100310200.01.T01	99,34	0,00E+00	1618	Os01g0191200	99,34	0,00E+00	1618	0	NA	NA	
maker-1-augustus-gene-49.20-mRNA-1	1	OsR498G0100311400.01.T05	99,46	0,00E+00	2607	Os01g0191300	99,54	0,00E+00	2329	-278	Indica	NA	
maker-1-augustus-gene-49.26-mRNA-1	1	OsR498G0100313200.01.T04	100,00	0,00E+00	710	Os01g0191800	100,00	0,00E+00	704	-6	NA	NA	
maker-1-augustus-gene-49.32-mRNA-1	1	OsR498G0100313800.01.T01	99,50	0,00E+00	1415	Os01g0191900	99,50	0,00E+00	1415	0	NA	NA	
maker-1-augustus-gene-49.4-mRNA-1	1	OsR498G0100311700.01.T01	99,93	0,00E+00	2599	Os01g0191500	99,93	0,00E+00	2599	0	NA	NA	
maker-1-augustus-gene-490.13-mRNA-1	1	OsR498G0102668910.01.T07	99,80	0,00E+00	889	Os01g0939700	100,00	0,00E+00	1586	697	Japonica	Japonica	
maker-1-augustus-gene-490.20-mRNA-1	1	OsR498G0102671700.01.T03	100,00	0,00E+00	2868	Os01g0940000	100,00	0,00E+00	2868	0	NA	Japonica	
maker-1-augustus-gene-491.0-mRNA-1	1	OsR498G0102673700.01.T01	100,00	0,00E+00	791	Os01g0940600	100,00	0,00E+00	791	0	NA	Japonica	
maker-1-augustus-gene-491.1-mRNA-1	1	OsR498G0102674100.01.T01	99,90	0,00E+00	1787	Os01g0941400	88,76	0,00E+00	1312	-475	Indica	Japonica	
maker-1-augustus-gene-491.12-mRNA-1	1	OsR498G0102675700.01.T01	99,21	0,00E+00	1793	Os01g0941400	99,31	0,00E+00	1798	5	NA	Japonica	
maker-1-augustus-gene-491.18-mRNA-1	1	OsR498G0102677000.01.T01	100,00	0,00E+00	2073	Os01g0941800	100,00	0,00E+00	2073	0	NA	NA	
maker-1-augustus-gene-491.2-mRNA-1	1	OsR498G0102674300.01.T01	99,80	0,00E+00	1793	Os01g0940800	100,00	0,00E+00	1802	9	NA	NA	
maker-1-augustus-gene-492.6-mRNA-1	1	OsR498G0102679000.01.T01	99,81	0,00E+00	1880	Os05g0375400	74,57	1,52E-179	628	-1252	Indica	Japonica	
maker-1-augustus-gene-492.8-mRNA-1	1	OsR498G0102681800.01.T01	99,53	1,43E-103	377	Os01g0176400	88,43	6,90E-77	287	-90	Indica	NA	
maker-1-augustus-gene-493.101-mRNA-1	1	OsR498G0102687600.01.T01	99,25	0,00E+00	2135	Os01g0945766	92,29	0,00E+00	1779	-356	Indica	Japonica	
maker-1-augustus-gene-493.102-mRNA-1	1	OsR498G0102688000.01.T03	97,77	0,00E+00	2667	Os01g0945766	98,02	0,00E+00	2708	41	NA	Japonica	
maker-1-augustus-gene-493.115-mRNA-1	1	OsR498G0102688200.01.T02	99,90	0,00E+00	1854	Os01g0945800	99,90	0,00E+00	1854	0	NA	Japonica	
maker-1-augustus-gene-493.54-mRNA-1	1	OsR498G0102683900.01.T04	99,43	0,00E+00	1554	Os01g0944200	99,00	0,00E+00	2287	733	Japonica	Japonica	
maker-1-augustus-gene-493.58-mRNA-1	1	OsR498G0102688700.01.T01	99,64	0,00E+00	1984	Os01g0946300	99,91	0,00E+00	1966	-18	NA	Japonica	
maker-1-augustus-gene-493.60-mRNA-1	1	OsR498G0102685400.01.T01	99,90	0,00E+00	1809	Os01g0944700	100,00	0,00E+00	1813	4	NA	Japonica	
maker-1-augustus-gene-493.70-mRNA-1	1	OsR498G0102686800.01.T01	99,87	0,00E+00	2816	Os01g0945200	90,38	0,00E+00	2094	-722	Indica	Japonica	
maker-1-augustus-gene-493.74-mRNA-1	1	OsR498G0102687000.01.T04	95,30	0,00E+00	2504	Os01g0945200	100,00	0,00E+00	2798	294	Japonica	Japonica	
maker-1-augustus-gene-493.75-mRNA-1	1	OsR498G0102687000.01.T06	100,00	0,00E+00	2098	Os01g0945300	100,00	0,00E+00	2098	0	NA	Japonica	
maker-1-augustus-gene-493.76-mRNA-1	1	OsR498G0102687000.01.T08	100,00	0,00E+00	690	Os01g0945300	100,00	0,00E+00	690	0	NA	Japonica	
maker-1-augustus-gene-494.38-mRNA-1	1	OsR498G0102690700.01.T01	97,78	0,00E+00	3227	Os01g0947000	100,00	0,00E+00	3416	189	NA	Japonica	
maker-1-augustus-gene-494.40-mRNA-1	1	OsR498G0102690900.01.T02	99,24	0,00E+00	3509	Os01g0948100	100,00	0,00E+00	3577	68	NA	Japonica	
maker-1-augustus-gene-494.46-mRNA-1	1	OsR498G0102693300.01.T01	99,87	0,00E+00	1370	Os01g0948300	100,00	0,00E+00	1375	5	NA	Japonica	
maker-1-augustus-gene-494.50-mRNA-1	1	OsR498G0102693800.01.T02	99,88	0,00E+00	1538	Os01g0948400	100,00	0,00E+00	1434	-104	NA	Japonica	
maker-1-augustus-gene-495.0-mRNA-1	1	OsR498G0102701900.01.T03	99,44	0,00E+00	1272	Os01g0949700	99,58	0,00E+00	1277	5	NA	Indica	
maker-1-augustus-gene-495.2-mRNA-1	1	OsR498G0102702700.01.T01	75,56	4,20E-120	432	Os01g0949850	100,00	0,00E+00	1234	802	Japonica	NA	
maker-1-augustus-gene-495.4-mRNA-1	1	OsR498G0102696200.01.T01	99,48	0,00E+00	2719	Os01g0948900	99,70	0,00E+00	1778	-941	Indica	Japonica	
maker-1-augustus-gene-496.0-mRNA-1	1	OsR498G0102702700.01.T01	99,85	0,00E+00	1235	Os01g0950000	100,00	0,00E+00	1240	5	NA	Japonica	
maker-1-augustus-gene-496.11-mRNA-1	1	OsR498G0102704700.01.T01	99,91	0,00E+00	2063	Os01g0951100	99,91	0,00E+00	2063	0	NA	Japonica	
maker-1-augustus-gene-496.17-mRNA-1	1	OsR498G0102704900.01.T02	100,00	0,00E+00	2168	Os01g0951200	99,92	0,00E+00	2164	-4	NA	NA	
maker-1-augustus-gene-496.2-mRNA-1	1	OsR498G0102703700.01.T01	98,70	0,00E+00	2284	Os01g0950700	99,54	0,00E+00	2327	43	NA	NA	
maker-1-augustus-gene-496.21-mRNA-1	1	OsR498G0102705300.01.T01	97,07	0,00E+00	2483	Os01g0951400	96,55	0,00E+00	1892	-591	Indica	NA	
maker-1-augustus-gene-496.25-mRNA-1	1	OsR498G0102706900.01.T02	99,70	0,00E+00	1204	Os01g0952000	99,55	0,00E+00	1199	-5	NA	Japonica	
maker-1-augustus-gene-496.27-mRNA-1	1	OsR498G0102707100.01.T01	99,44	0,00E+00	1260	Os01g0952100	100,00	0,00E+00	1278	18	NA	Japonica	
maker-1-augustus-gene-496.29-mRNA-1	1	OsR498G0102707300.01.T01	99,90	0,00E+00	1805	Os01g0952200	99,80	0,00E+00	1801	-4	NA	Japonica	
maker-1-augustus-gene-496.32-mRNA-1	1	OsR498G0102704000.01.T04	99,68	0,00E+00	1133	Os01g0950800	99,68	0,00E+00	1133	0	NA	NA	
maker-1-augustus-gene-496.36-mRNA-1	1	OsR498G0102704200.01.T01	99,86	0,00E+00	1322	Os05g0150000	77,66	3,03E-142	505	-817	Indica	NA	
maker-1-augustus-gene-496.4-mRNA-1	1	OsR498G0102704300.01.T03	96,94	0,00E+00	2320	Os01g0950900	97,02	0,00E+00	2324	4	NA	NA	
maker-1-augustus-gene-496.47-mRNA-1	1	OsR498G0102706400.01.T01	100,00	0,00E+00	888	Os01g0951800	100,00	0,00E+00	888	0	NA	Japonica	
maker-1-augustus-gene-496.49-mRNA-1	1	OsR498G0102707600.01.T01	99,93	0,00E+00	4834	Os01g0952300	98,37	0,00E+00	5020	186	NA	Japonica	
maker-1-augustus-gene-497.52-mRNA-1	1	OsR498G0102709900.01.T01	99,77	0,00E+00	3072	Os01g0952700	99,94	0,00E+00	3085	13	NA	NA	
maker-1-augustus-gene-497.55-mRNA-1	1	OsR498G0102712500.01.T01	99,31	0,00E+00	1785	Os01g0953400	99,89	0,00E+00	3205	1420	Japonica	Japonica	LRR-NBS-TM
maker-1-augustus-gene-497.56-mRNA-1	1	OsR498G0102714500.01.T01	99,84	0,00E+00	1100	Os01g0954000	100,00	0,00E+00	1104	4	NA	Japonica	
maker-1-augustus-gene-497.62-mRNA-1	1	OsR498G0102710200.01.T03	100,00	0,00E+00	1348	Os01g0952800	99,60	0,00E+00	1333	-15	NA	Japonica	
maker-1-augustus-gene-497.64-mRNA-1	1	OsR498G0102713200.01.T01	99,83	0,00E+00	1067	Os01g0953600	100,00	0,00E+00	1072	5	NA	Indica	
maker-1-augustus-gene-498.36-mRNA-1	1	OsR498G0102717800.01.T04	99,61	0,00E+00	1828	Os01g0954500	100,00	0,00E+00	1846	18	NA	Japonica	
maker-1-augustus-gene-498.39-mRNA-1	1	OsR498G0102720600.01.T01	99,81	0,00E+00	2806	Os01g0955000	100,00	0,00E+00	2819	13	NA	Japonica	
maker-1-augustus-gene-499.0-mRNA-1	1	OsR498G0102727900.01.T02	99,57	0,00E+00	3339	Os01g0956500	99,73	0,00E+00	1949	-1390	Indica	Japonica	
maker-1-augustus-gene-499.14-mRNA-1	1	OsR498G0102731100.01.T01	98,82	0,00E+00	5336	Os01g0957100	99,96	0,00E+00	4995	-341	NA	Japonica	Kinase-LRR-TM
maker-1-augustus-gene-499.16-mRNA-1	1	OsR498G0102731500.01.T01	99,92	0,00E+00	2106	Os01g0957200	100,00	0,00E+00	2111	5	NA	Japonica	
maker-1-augustus-gene-499.21-mRNA-1	1	OsR498G0102729800.01.T01	100,00	0,00E+00	1716	Os01g0956700	99,90	0,00E+00	1711	-5	NA	Indica	
maker-1-augustus-gene-499.6-mRNA-1	1	OsR498G0102728900.01.T01	99,89	0,00E+00	3222	Os01g0956600	99,95	0,00E+00	3277	55	NA	Japonica	
maker-1-augustus-gene-5.29-mRNA-1	1	OsR498G0100027300.01.T01	96,43	0,00E+00	1296	Os01g0107700	100,00	0,00E+00	1388	92	NA	Japonica	
maker-1-augustus-gene-5.32-mRNA-1	1	OsR498G0100027900.01.T01	96,76	0,00E+00	2743	Os01g0108000	98,32	0,00E+00	4032	1289	Japonica	Japonica	
maker-1-augustus-gene-5.36-mRNA-1	1	OsR498G0100028100.01.T01	99,50	0,00E+00	3906	Os01g0108200	100,00	0,00E+00	3956	50	NA	Japonica	
maker-1-augustus-gene-5.40-mRNA-1	1	OsR498G0100028700.01.T02	93,20	0,00E+00	1049	Os01g0108400	99,86	0,00E+00	1268	219	Japonica	Japonica	
maker-1-augustus-gene-5.43-mRNA-1	1	OsR498G0100029700.01.T01	99,56	0,00E+00	1205	Os01g0108600	91,11	0,00E+00	976	-229	Indica	Japonica	
maker-1-augustus-gene-50.0-mRNA-1	1	OsR498G0100319400.01.T01	99,79	0,00E+00	1674	Os01g0192300	99,79	0,00E+00	1674	0	NA	NA	
maker-1-augustus-gene-50.2-mRNA-1	1	OsR498G0100320400.01.T02	99,33	0,00E+00	1852	Os01g0192600	99,33	0,00E+00	1852	0	NA	NA	
maker-1-augustus-gene-50.6-mRNA-1	1	OsR498G0100322800.01.T01	100,00	0,00E+00	2500	Os01g0192900	100,00	0,00E+00	2500	0	NA	NA	
maker-1-augustus-gene-50.8-mRNA-1	1	OsR498G0100325000.01.T01	100,00	0,00E+00	1418	Os01g0193500	100,00	0,00E+00	2457	1039	Japonica	NA	
maker-1-augustus-gene-500.12-mRNA-1	1	OsR498G0102735300.01.T04	100,00	0,00E+00	2262	Os01g0958400	100,00	0,00E+00	2116	-146	NA	NA	
maker-1-augustus-gene-500.16-mRNA-1	1	OsR498G0102735500.01.T01	99,67	0,00E+00	1618	Os01g0958500	99,67	0,00E+00	1618	0	NA	Indica	
maker-1-augustus-gene-500.20-mRNA-1	1	OsR498G0102736100.01.T01	99,78	0,00E+00	3278	Os01g0958700	100,00	0,00E+00	3296	18	NA	Japonica	
maker-1-augustus-gene-500.26-mRNA-1	1	OsR498G0102736500.01.T03	99,82	0,00E+00	3905	Os01g0958900	100,00	0,00E+00	3923	18	NA	Japonica	
maker-1-augustus-gene-500.39-mRNA-1	1	OsR498G0102736700.01.T03	99,80	0,00E+00	891	Os01g0959000	99,80	0,00E+00	891	0	NA	Japonica	
maker-1-augustus-gene-500.42-mRNA-1	1	OsR498G0102737100.01.T01	99,68	4,81E-160	564	Os01g0959200	99,71	2,48E-177	620	56	NA	Japonica	
maker-1-augustus-gene-500.44-mRNA-1	1	OsR498G0102738500.01.T01	99,84	0,00E+00	3417	Os01g0959600	99,88	0,00E+00	2924	-493	Indica	NA	
maker-1-augustus-gene-500.46-mRNA-1	1	OsR498G0102739700.01.T01	100,00	0,00E+00	893	Os01g0959900	99,80	0,00E+00	889	-4	NA	Japonica	
maker-1-augustus-gene-500.50-mRNA-1	1	OsR498G0102740300.01.T02	99,85	0,00E+00	2405	Os01g0960200	99,72	0,00E+00	4487	2082	Japonica	NA	
maker-1-augustus-gene-500.53-mRNA-1	1	OsR498G0102734800.01.T04	100,00	0,00E+00	2170	Os01g0958100	100,00	0,00E+00	1438	-732	Indica	NA	
maker-1-augustus-gene-500.6-mRNA-1	1	OsR498G0102734300.01.T01	97,66	0,00E+00	2708	Os01g0958000	97,59	0,00E+00	2703	-5	NA	NA	CC-Kinase-TM
maker-1-augustus-gene-500.60-mRNA-1	1	OsR498G0102736400.01.T02	99,58	0,00E+00	1714	Os01g0958800	99,84	0,00E+00	1089	-625	Indica	Japonica	
maker-1-augustus-gene-500.66-mRNA-1	1	OsR498G0102737000.01.T01	99,66	4,24E-147	521	Os01g0959100	100,00	3,47E-149	526	5	NA	Japonica	
maker-1-augustus-gene-501.0-mRNA-1	1	OsR498G0102743100.01.T01	97,38	0,00E+00	2801	Os01g0961200	97,32	0,00E+00	2796	-5	NA	Japonica	
maker-1-augustus-gene-501.16-mRNA-1	1	OsR498G0102745100.01.T01	99,80	0,00E+00	894	Os01g0962300	99,80	0,00E+00	894	0	NA	Japonica	
maker-1-augustus-gene-501.18-mRNA-1	1	OsR498G0102740600.01.T01	99,77	0,00E+00	3863	Os01g0960300	99,86	0,00E+00	3872	9	NA	Japonica	
maker-1-augustus-gene-501.23-mRNA-1	1	OsR498G0102740800.01.T02	99,79	0,00E+00	5139	Os01g0960400	99,83	0,00E+00	5138	-1	NA	Japonica	Kinase-LRR-TM
maker-1-augustus-gene-501.38-mRNA-1	1	OsR498G0102742000.01.T01	98,63	0,00E+00	2028	Os01g0960500	99,57	0,00E+00	2077	49	NA	Japonica	
maker-1-augustus-gene-501.43-mRNA-1	1	OsR498G0102742200.01.T02	99,02	0,00E+00	1081	Os01g0956500	83,30	1,32E-160	566	-515	Indica	Japonica	
maker-1-augustus-gene-501.46-mRNA-1	1	OsR498G0102742400.01.T01	98,83	6,58E-171	600	Os01g0960800	100,00	8,53E-177	618	18	NA	Japonica	
maker-1-augustus-gene-501.50-mRNA-1	1	OsR498G0102743800.01.T01	99,84	0,00E+00	2226	Os01g0961600	99,68	0,00E+00	2217	-9	NA	Indica	
maker-1-augustus-gene-501.55-mRNA-1	1	OsR498G0102744800.01.T01	98,81	0,00E+00	1773	Os01g0962200	98,98	0,00E+00	1560	-213	Indica	Indica	
maker-1-augustus-gene-501.58-mRNA-1	1	OsR498G0102745400.01.T01	100,00	1,80E-158	558	Os01g0962400	100,00	6,27E-159	558	0	NA	Japonica	
maker-1-augustus-gene-501.6-mRNA-1	1	OsR498G0102743300.01.T03	100,00	0,00E+00	1014	Os01g0961300	100,00	0,00E+00	1014	0	NA	Japonica	
maker-1-augustus-gene-501.61-mRNA-1	1	OsR498G0102745600.01.T01	99,88	0,00E+00	2978	Os01g0962500	100,00	0,00E+00	2987	9	NA	Japonica	
maker-1-augustus-gene-501.64-mRNA-1	1	OsR498G0102745800.01.T01	99,44	0,00E+00	961	Os01g0962600	99,82	0,00E+00	970	9	NA	Japonica	
maker-1-augustus-gene-501.8-mRNA-1	1	OsR498G0102743500.01.T03	99,88	0,00E+00	3011	Os07g0651500	73,00	2,48E-179	629	-2382	Indica	Indica	
maker-1-augustus-gene-502.0-mRNA-1	1	OsR498G0102746100.01.T01	97,88	0,00E+00	1944	Os01g0962700	97,80	0,00E+00	1939	-5	NA	Japonica	

maker-1-augustus-gene-502.12-mRNA-1	1	OsR498G0102749300.01.T01	100,00	0,00E+00	991	Os01g0963600	99,82	0,00E+00	986	-5	NA	Japonica	
maker-1-augustus-gene-502.16-mRNA-1	1	OsR498G0102748600.01.T01	93,55	0,00E+00	897	Os01g0963300	93,73	0,00E+00	902	5	NA	Japonica	
maker-1-augustus-gene-502.2-mRNA-1	1	OsR498G0102746700.01.T01	99,32	0,00E+00	1820	Os01g0962900	95,10	0,00E+00	1654	-166	NA	Japonica	
maker-1-augustus-gene-502.21-mRNA-1	1	OsR498G0102750400.01.T01	97,52	0,00E+00	1101	Os01g0964000	97,67	0,00E+00	1105	4	NA	Japonica	
maker-1-augustus-gene-502.24-mRNA-1	1	OsR498G0102750600.01.T01	99,91	0,00E+00	2041	Os05g0106600	88,18	0,00E+00	1442	-599	Indica	Japonica	
maker-1-augustus-gene-502.4-mRNA-1	1	OsR498G0102748300.01.T01	99,54	0,00E+00	1920	Os01g0963200	99,82	0,00E+00	1004	-916	Indica	Japonica	
maker-1-augustus-gene-502.6-mRNA-1	1	OsR498G0102748700.01.T02	100,00	0,00E+00	915	Os01g0963400	100,00	0,00E+00	666	-249	Indica	Japonica	
maker-1-augustus-gene-503.25-mRNA-1	1	OsR498G0102757500.01.T02	100,00	0,00E+00	682	Os01g0966500	100,00	9,50E-177	618	-64	NA	NA	
maker-1-augustus-gene-503.29-mRNA-1	1	OsR498G0102757700.01.T06	99,77	0,00E+00	1534	Os01g0966700	99,77	0,00E+00	1534	0	NA	NA	
maker-1-augustus-gene-503.34-mRNA-1	1	OsR498G0102755000.01.T01	99,62	0,00E+00	1416	Os01g0965600	100,00	0,00E+00	1429	13	NA	Japonica	
maker-1-augustus-gene-503.37-mRNA-1	1	OsR498G0102755800.01.T01	99,43	3,26E-177	621	Os01g0965900	100,00	2,19E-180	630	9	NA	Japonica	
maker-1-augustus-gene-503.43-mRNA-1	1	OsR498G0102757000.01.T01	99,33	0,00E+00	1054	Os01g0966200	100,00	0,00E+00	1072	18	NA	NA	
maker-1-augustus-gene-503.45-mRNA-1	1	OsR498G0102757400.01.T01	99,29	0,00E+00	2486	Os01g0966400	99,29	0,00E+00	2486	0	NA	Japonica	LRR-TM
maker-1-augustus-gene-503.5-mRNA-1	1	OsR498G0102754100.01.T01	93,28	0,00E+00	1756	Os01g0965400	93,37	0,00E+00	1761	5	NA	Japonica	
maker-1-augustus-gene-504.45-mRNA-1	1	OsR498G0102759700.01.T01	99,81	0,00E+00	6710	Os01g0967100	99,89	0,00E+00	6724	14	NA	NA	
maker-1-augustus-gene-504.51-mRNA-1	1	OsR498G0102761300.01.T01	100,00	0,00E+00	1402	Os01g0967200	99,87	0,00E+00	1397	-5	NA	NA	
maker-1-augustus-gene-504.53-mRNA-1	1	OsR498G0102761500.01.T01	99,22	0,00E+00	2027	Os01g0967800	98,41	0,00E+00	1959	-68	NA	Japonica	
maker-1-augustus-gene-504.55-mRNA-1	1	OsR498G0102761700.01.T01	97,76	0,00E+00	2510	Os01g0967900	99,80	0,00E+00	2645	135	NA	Japonica	
maker-1-augustus-gene-504.60-mRNA-1	1	OsR498G0102762700.01.T01	100,00	0,00E+00	1792	Os01g0968300	97,38	0,00E+00	1674	-118	NA	Indica	
maker-1-augustus-gene-504.62-mRNA-1	1	OsR498G0102762900.01.T01	98,64	0,00E+00	1788	Os01g0968400	98,76	0,00E+00	1273	-515	Indica	NA	
maker-1-augustus-gene-504.69-mRNA-1	1	OsR498G0102758400.01.T01	99,30	0,00E+00	2785	Os01g0966900	99,42	0,00E+00	2465	-320	Indica	NA	
maker-1-augustus-gene-504.74-mRNA-1	1	OsR498G0102762000.01.T01	96,62	0,00E+00	1915	Os01g0968000	99,81	0,00E+00	1880	-35	NA	Japonica	
maker-1-augustus-gene-504.76-mRNA-1	1	OsR498G0102762200.01.T01	97,85	0,00E+00	1663	Os01g0968100	99,69	0,00E+00	1744	81	NA	Japonica	
maker-1-augustus-gene-504.78-mRNA-1	1	OsR498G0102762600.01.T01	98,47	0,00E+00	1697	Os01g0968300	93,31	0,00E+00	1431	-266	Indica	Indica	
maker-1-augustus-gene-504.80-mRNA-1	1	OsR498G0102764600.01.T01	99,38	2,98E-162	571	Os01g0969000	100,00	2,00E-165	580	9	NA	Japonica	
maker-1-augustus-gene-504.82-mRNA-1	1	OsR498G0102764800.01.T01	99,09	0,00E+00	2115	Os01g0969100	99,92	0,00E+00	2155	40	NA	Japonica	
maker-1-augustus-gene-505.2-mRNA-1	1	OsR498G0102770700.01.T01	100,00	0,00E+00	1234	Os01g0970400	100,00	0,00E+00	1234	0	NA	NA	
maker-1-augustus-gene-505.29-mRNA-1	1	OsR498G0102771600.01.T04	100,00	0,00E+00	1775	Os01g0970700	100,00	0,00E+00	1775	0	NA	NA	
maker-1-augustus-gene-505.35-mRNA-1	1	OsR498G0102773200.01.T01	100,00	0,00E+00	2181	Os01g0971200	100,00	0,00E+00	2181	0	NA	NA	
maker-1-augustus-gene-505.6-mRNA-1	1	OsR498G0102771100.01.T01	100,00	8,40E-170	596	Os01g0970500	100,00	2,92E-170	596	0	NA	NA	
maker-1-augustus-gene-505.8-mRNA-1	1	OsR498G0102772900.01.T03	100,00	0,00E+00	1099	Os01g0971100	100,00	0,00E+00	1099	0	NA	NA	
maker-1-augustus-gene-506.0-mRNA-1	1	OsR498G0102773700.01.T01	100,00	0,00E+00	1981	Os01g0971400	100,00	0,00E+00	1981	0	NA	NA	
maker-1-augustus-gene-506.10-mRNA-1	1	OsR498G0102774400.01.T03	100,00	0,00E+00	866	Os01g0971600	100,00	0,00E+00	866	0	NA	NA	
maker-1-augustus-gene-506.16-mRNA-1	1	OsR498G0102774800.01.T03	100,00	0,00E+00	1618	Os01g0971700	100,00	0,00E+00	1429	-189	Indica	Japonica	
maker-1-augustus-gene-506.18-mRNA-1	1	OsR498G0102775200.01.T02	99,91	0,00E+00	3871	Os01g0971900	99,95	0,00E+00	3875	4	NA	Japonica	
maker-1-augustus-gene-506.2-mRNA-1	1	OsR498G0102774100.01.T02	100,00	0,00E+00	852	Os01g0971500	100,00	0,00E+00	737	-115	Indica	NA	
maker-1-augustus-gene-506.5-mRNA-1	1	OsR498G0102778700.01.T01	98,06	0,00E+00	2137	Os01g0972800	98,78	0,00E+00	2166	29	NA	Japonica	
maker-1-augustus-gene-506.7-mRNA-1	1	OsR498G0102778900.01.T01	96,94	0,00E+00	2427	Os01g0972900	97,08	0,00E+00	2436	9	NA	Japonica	
maker-1-augustus-gene-506.9-mRNA-1	1	OsR498G0102774400.01.T02	100,00	0,00E+00	1119	Os01g0971600	100,00	0,00E+00	1025	-94	NA	NA	
maker-1-augustus-gene-507.0-mRNA-1	1	OsR498G0102779100.01.T03	99,88	0,00E+00	1522	Os01g0973000	99,65	0,00E+00	1513	-9	NA	Japonica	
maker-1-augustus-gene-507.12-mRNA-1	1	OsR498G0102779700.01.T01	99,75	0,00E+00	4311	Os01g0973300	99,84	0,00E+00	3443	-868	Indica	Japonica	
maker-1-augustus-gene-507.18-mRNA-1	1	OsR498G0102780500.01.T01	99,90	0,00E+00	1825	Os01g0973600	100,00	0,00E+00	1829	4	NA	Japonica	
maker-1-augustus-gene-507.20-mRNA-1	1	OsR498G0102780900.01.T01	99,82	0,00E+00	1999	Os01g0974000	100,00	0,00E+00	2008	9	NA	Japonica	
maker-1-augustus-gene-507.23-mRNA-1	1	OsR498G0102781100.01.T02	99,32	0,00E+00	777	Os05g0111200	74,02	7,53E-65	246	-531	Indica	Japonica	
maker-1-augustus-gene-507.28-mRNA-1	1	OsR498G0102782900.01.T04	99,72	1,39E-179	629	Os01g0974600	100,00	0,00E+00	634	5	NA	Japonica	
maker-1-augustus-gene-507.31-mRNA-1	1	OsR498G0102779600.01.T01	96,07	0,00E+00	1645	Os01g0973200	100,00	0,00E+00	1755	110	NA	Japonica	
maker-1-augustus-gene-507.36-mRNA-1	1	OsR498G0102780000.01.T01	99,75	0,00E+00	2140	Os01g0973400	99,92	0,00E+00	2150	10	NA	Japonica	
maker-1-augustus-gene-507.39-mRNA-1	1	OsR498G0102780200.01.T01	99,84	0,00E+00	2205	Os01g0973500	99,92	0,00E+00	2209	4	NA	Japonica	Kinase-TM
maker-1-augustus-gene-507.4-mRNA-1	1	OsR498G0102779300.01.T02	98,61	0,00E+00	2132	Os01g0550800	87,37	0,00E+00	1526	-606	Indica	Japonica	
maker-1-augustus-gene-507.48-mRNA-1	1	OsR498G0102782800.01.T03	99,77	0,00E+00	2319	Os01g0974500	100,00	0,00E+00	2333	14	NA	Japonica	
maker-1-augustus-gene-507.51-mRNA-1	1	OsR498G0102783200.01.T04	99,69	0,00E+00	1171	Os05g0114000	86,74	0,00E+00	710	-461	Indica	Japonica	
maker-1-augustus-gene-507.55-mRNA-1	1	OsR498G0102784200.01.T01	99,71	0,00E+00	1843	Os01g0975000	100,00	0,00E+00	1856	13	NA	Japonica	
maker-1-augustus-gene-508.34-mRNA-1	1	OsR498G0102785500.01.T01	99,88	0,00E+00	1495	Os01g0975300	100,00	0,00E+00	1202	-293	Indica	Japonica	
maker-1-augustus-gene-508.42-mRNA-1	1	OsR498G0102789100.01.T03	97,67	0,00E+00	4820	Os01g0976500	99,61	0,00E+00	4988	168	NA	Japonica	
maker-1-augustus-gene-508.44-mRNA-1	1	OsR498G0102787800.01.T01	99,61	0,00E+00	1356	Os01g0975900	100,00	0,00E+00	1370	14	NA	Japonica	
maker-1-augustus-gene-508.46-mRNA-1	1	OsR498G0102788400.01.T03	99,94	0,00E+00	3069	Os01g0976000	100,00	0,00E+00	3074	5	NA	Japonica	
maker-1-augustus-gene-508.49-mRNA-1	1	OsR498G0102788600.01.T03	99,83	0,00E+00	7533	Os03g0280000	76,01	0,00E+00	3032	-4501	Indica	Japonica	
maker-1-augustus-gene-508.54-mRNA-1	1	OsR498G0102789400.01.T01	99,74	0,00E+00	1388	Os01g0976600	100,00	0,00E+00	1397	9	NA	Japonica	
maker-1-augustus-gene-508.57-mRNA-1	1	OsR498G0102790200.01.T01	95,41	0,00E+00	693	Os01g0976800	97,93	0,00E+00	746	53	NA	Japonica	
maker-1-augustus-gene-509.0-mRNA-1	1	OsR498G0102790900.01.T01	99,88	0,00E+00	1554	Os01g0977100	100,00	0,00E+00	1512	-42	NA	NA	
maker-1-augustus-gene-509.16-mRNA-1	1	OsR498G0102790800.01.T02	97,73	0,00E+00	5027	Os01g0976900	99,87	0,00E+00	2805	-2222	Indica	Japonica	Kinase-LRR-TM
maker-1-augustus-gene-509.21-mRNA-1	1	OsR498G0102792600.01.T01	99,89	0,00E+00	4728	Os01g0977600	100,00	0,00E+00	4742	14	NA	Japonica	
maker-1-augustus-gene-509.26-mRNA-1	1	OsR498G0102794600.01.T01	99,19	0,00E+00	2194	Os01g0978100	100,00	0,00E+00	1389	-805	Indica	Japonica	
maker-1-augustus-gene-51.0-mRNA-1	1	OsR498G0100325700.01.T06	99,40	0,00E+00	2957	Os01g0193900	99,40	0,00E+00	2957	0	NA	NA	
maker-1-augustus-gene-51.11-mRNA-1	1	OsR498G0100326000.01.T02	100,00	0,00E+00	3095	Os01g0194000	100,00	0,00E+00	2527	-568	Indica	NA	
maker-1-augustus-gene-51.14-mRNA-1	1	OsR498G0100328000.01.T02	97,15	0,00E+00	2323	Os01g0194200	97,15	0,00E+00	2323	0	NA	NA	
maker-1-augustus-gene-51.16-mRNA-1	1	OsR498G0100328800.01.T01	100,00	0,00E+00	3155	Os01g0194300	100,00	0,00E+00	3155	0	NA	NA	
maker-1-augustus-gene-51.7-mRNA-1	1	OsR498G0100325200.01.T03	100,00	0,00E+00	3020	Os01g0193600	100,00	0,00E+00	3020	0	NA	NA	
maker-1-augustus-gene-510.0-mRNA-1	1	OsR498G0102222100.01.T02	100,00	0,00E+00	2516	Os01g0819700	99,00	0,00E+00	2473	-43	NA	NA	
maker-1-augustus-gene-510.11-mRNA-1	1	OsR498G0102703700.01.T01	98,70	0,00E+00	2284	Os01g0950700	99,54	0,00E+00	2327	43	NA	NA	
maker-1-augustus-gene-510.13-mRNA-1	1	OsR498G0102704300.01.T03	96,94	0,00E+00	2320	Os01g0950900	97,02	0,00E+00	2324	4	NA	NA	
maker-1-augustus-gene-510.2-mRNA-1	1	OsR498G0102268500.01.T02	100,00	0,00E+00	1099	Os01g0831200	99,67	0,00E+00	1090	-9	NA	NA	
maker-1-augustus-gene-510.20-mRNA-1	1	OsR498G0102268500.01.T02	100,00	0,00E+00	1099	Os01g0831200	99,67	0,00E+00	1090	-9	NA	NA	
maker-1-augustus-gene-510.26-mRNA-1	1	OsR498G0102304200.01.T01	100,00	0,00E+00	1700	Os01g0840300	99,89	0,00E+00	1695	-5	NA	NA	
maker-1-augustus-gene-510.28-mRNA-1	1	OsR498G0102704000.01.T04	99,68	0,00E+00	1133	Os01g0950800	99,68	0,00E+00	1133	0	NA	NA	
maker-1-augustus-gene-510.32-mRNA-1	1	OsR498G0102704200.01.T01	99,86	0,00E+00	1322	Os05g0150000	77,66	3,03E-142	505	-817	Indica	NA	
maker-1-augustus-gene-510.37-mRNA-1	1	OsR498G0102704600.01.T03	99,66	0,00E+00	1571	Os01g0951000	100,00	0,00E+00	1584	13	NA	NA	
maker-1-augustus-gene-52.26-mRNA-1	1	OsR498G0100335900.01.T01	92,84	0,00E+00	1500	Os01g0195700	96,07	0,00E+00	1608	108	NA	Japonica	
maker-1-augustus-gene-52.29-mRNA-1	1	OsR498G0100335800.01.T01	100,00	0,00E+00	1083	Os01g0195500	99,33	0,00E+00	1065	-18	NA	Indica	
maker-1-augustus-gene-53.0-mRNA-1	1	OsR498G0100341300.01.T01	99,53	0,00E+00	2626	Os01g0197100	99,93	0,00E+00	2653	27	NA	Japonica	
maker-1-augustus-gene-53.14-mRNA-1	1	OsR498G0100344000.01.T02	100,00	0,00E+00	1559	Os01g0197500	100,00	0,00E+00	1535	-24	NA	Japonica	
maker-1-augustus-gene-53.19-mRNA-1	1	OsR498G0100345200.01.T02	84,11	0,00E+00	1380	Os01g0197900	100,00	0,00E+00	2100	720	Japonica	Japonica	
maker-1-augustus-gene-53.21-mRNA-1	1	OsR498G0100345000.01.T05	99,48	0,00E+00	1720	Os01g0198100	79,09	0,00E+00	709	-1011	Indica	Japonica	
maker-1-augustus-gene-53.22-mRNA-1	1	OsR498G0100345000.01.T05	99,33	0,00E+00	1056	Os01g0198100	75,42	1,34E-120	432	-624	Indica	Japonica	
maker-1-augustus-gene-53.32-mRNA-1	1	OsR498G0100345600.01.T01	99,63	0,00E+00	3415	Os01g0198200	100,00	0,00E+00	3447	32	NA	Japonica	
maker-1-augustus-gene-53.4-mRNA-1	1	OsR498G0100343500.01.T04	99,86	0,00E+00	1306	Os01g0197400	100,00	0,00E+00	1310	4	NA	Japonica	
maker-1-augustus-gene-54.0-mRNA-1	1	OsR498G0100350300.01.T03	98,47	0,00E+00	3309	Os01g0199900	98,47	0,00E+00	3309	0	NA	NA	
maker-1-augustus-gene-54.10-mRNA-1	1	OsR498G0100346600.01.T01	100,00	0,00E+00	2152	Os01g0198500	100,00	0,00E+00	1288	-864	Indica	NA	
maker-1-augustus-gene-54.11-mRNA-1	1	OsR498G0100348800.01.T01	99,92	0,00E+00	2139	Os01g0199400	99,92	0,00E+00	2139	0	NA	NA	
maker-1-augustus-gene-54.16-mRNA-1	1	OsR498G0100350800.01.T02	99,77	0,00E+00	1537	Os01g0200000	99,77	0,00E+00	1537	0	NA	NA	
maker-1-augustus-gene-55.11-mRNA-1	1	OsR498G0100352400.01.T02	99,72	0,00E+00	636	Os01g0200400	99,72	0,00E+00	636	0	NA	NA	
maker-1-augustus-gene-55.18-mRNA-1	1	OsR498G0100357200.01.T04	99,72	0,00E+00	2598	Os01g0201200	99,86	0,00E+00	2607	9	NA	Japonica	Kinase-TM
maker-1-augustus-gene-55.3-mRNA-1	1	OsR498G0100356100.01.T01	100,00	0,00E+00	3442	Os08g0112500	74,12	0,00E+00	663	-2779	Indica	NA	Kinase-TM
maker-1-augustus-gene-55.7-mRNA-1	1	OsR498G0100356900.01.T01	99,79	0,00E+00	2590	Os03g0692000	72,34	1,62E-136	486	-2104	Indica	Japonica	
maker-1-augustus-gene-56.0-mRNA-1	1	OsR498G0100361000.01.T01	98,79	0,00E+00	1597	Os01g0201600	100,00	0,00E+00	1635	38	NA	Japonica	
maker-1-augustus-gene-56.2-mRNA-1	1	OsR498G0100361800.01.T03	99,64	0,00E+00	1486	Os01g0201700	100,00	0,00E+00	807	-679	Indica	Japonica	
maker-1-augustus-gene-56.6-mRNA-1	1	OsR498G0100362800.01.T07	99,01	0,00E+00	4439	Os01g0201900	99,94	0,00E+00	3185	-1254	Indica	Japonica	
maker-1-augustus-gene-57.0-mRNA-1	1	OsR498G0100364900.01.T01	98,66	0,00E+00	1816	Os01g0202500	100,00	0,00E+00	1864	48	NA	Japonica	
maker-1-augustus-gene-57.16-mRNA-1	1	OsR498G0100370600.01.T01	99,87	0,00E+00	2767	Os01g0204000	100,00	0,00E+00	2776	9	NA	Japonica	
maker-1-augustus-gene-57.2-mRNA-1	1	OsR498G0100365100.01.T01	100,00	0,00E+00	980	Os06g0157500	75,00	1,60E-55	216	-764	Indica	Japonica	
maker-1-augustus-gene-57.4-mRNA-1	1	OsR498G0100366800.01.T02	100,00	1,45E-140	499	Os01g0202800	100,00	5,04E-141	499	0	NA	Japonica	
maker-1-augustus-gene-57.6-mRNA-1	1	OsR498G0100367000.01.T01	99,73	0,00E+00	1967	Os01g0203000	99,82	0,00E+00	1966	-1	NA	Japonica	
maker-1-augustus-gene-58.0-mRNA-1	1	OsR498G0100372900.01.T01	94,83	0,00E+00	1667	Os01g0205100	93,48	0,00E+00	1578	-89	NA	Japonica	
maker-1-augustus-gene-58.20-mRNA-1	1	OsR498G0100374300.01.T01	100,00	0,00E+00	991	Os01g0205500	100,00	0,00E+00	991	0	NA	NA	
maker-1-augustus-gene-58.24-mRNA-1	1	OsR498G0100376100.01.T01	100,00	0,00E+00	1036	Os01g0205900	100,00	0,00E+00	1036	0	NA	NA	
maker-1-augustus-gene-58.29-mRNA-1	1	OsR498G0203659800.01.T01	96,10	0,00E+00	1291	Os01g0204900	99,82	0,00E+00	1019	-272	Indica	NA	
maker-1-augustus-gene-58.31-mRNA-1	1	OsR498G0100374600.01.T04	100,00	0,00E+00	2208	Os05g0207500	83,20	0,00E+00	1270	-938	Indica	NA	Kinase-TM
maker-1-augustus-gene-58.38-mRNA-1	1	OsR498G0100376800.01.T01	94,27	0,00E+00	1084	Os01g0206200	93,84	0,00E+00	997	-87	NA	NA	
maker-1-augustus-gene-58.40-mRNA-1	1	OsR498G0100378000.01.T01	100,00	0,00E+00	3669	Os01g0206800	99,12	0,00E+00	3632	-37	NA	NA	Kinase-LRR-TM
maker-1-augustus-gene-59.12-mRNA-1	1	OsR498G0100384500.01.T01	100,00	0,00E+00	3332	Os01g0208700	100,00	0,00E+00	3332	0	NA	Japonica	
maker-1-augustus-gene-59.16-mRNA-1	1	OsR498G0100385100.01.T03	99,75	0,00E+00	2144	Os01g0209000	100,00	0,00E+00	2158	14	NA	Japonica	
maker-1-augustus-gene-59.2-mRNA-1	1	OsR498G0100381300.01.T01	97,66	0,00E+00	1982	Os01g0207900	100,00	0,00E+00	2084	102	NA	NA	
maker-1-augustus-gene-59.4-mRNA-1	1	OsR498G0100381700.01.T01	99,91	0,00E+00	2036	Os01g0207900	80,58	0,00E+00	1067	-969	Indica	NA	
maker-1-augustus-gene-59.6-mRNA-1	1	OsR498G0100384300.01.T02	100,00	0,00E+00	7223	Os01g0208600	100,00	0,00E+00	7223	0	NA	NA	
maker-1-augustus-gene-6.10-mRNA-1	1	OsR498G0100033900.01.T03	99,67	0,00E+00	2177	Os01g0110400	99,92	0,00E+00	2191	14	NA	Japonica	
maker-1-augustus-gene-6.15-mRNA-1	1	OsR498G0100035700.01.T01	97,80	0,00E+00	3204	Os01g0111200	98,01	0,00E+00	3223	19	NA	Japonica	
maker-1-augustus-gene-6.19-mRNA-1	1	OsR498G0100031800.01.T01	99,06	0,00E+00	7520	Os01g0109700	99,25	0,00E+00	7067	-453	NA	Japonica	
maker-1-augustus-gene-6.26-mRNA-1	1	OsR498G0100032000.01.T04	99,52	0,00E+00	738	Os01g0109750	100,00	0,00E+00	747	9	NA	Japonica	
maker-1-augustus-gene-6.29-mRNA-1	1	OsR498G0100033600.01.T01	99,74	0,00E+00	1377	Os01g0110200	100,00	0,00E+00	1305	-72	NA	Japonica	
maker-1-augustus-gene-6.31-mRNA-1	1	OsR498G0100034200.01.T01	99,81	0,00E+00	3764	Os01g0110500	100,00	0,00E+00	3782	18	NA	Japonica	Kinase-TM
maker-1-augustus-gene-6.35-mRNA-1	1	OsR498G0100034600.01.T01	99,76	0,00E+00	5298	Os01g0110700	100,00	0,00E+00	5330	32	NA	Japonica	
maker-1-augustus-gene-6.37-mRNA-1	1	OsR498G0100035200.01.T01	99,86	0,00E+00	2578	Os01g0110800	97,38	0,00E+00	1223	-1355	Indica	Japonica	LRR-TM
maker-1-augustus-gene-6.40-mRNA-1	1	OsR498G0100035600.01.T01	99,78	0,00E+00	1642	Os01g0111100	100,00	0,00E+00	1597	-45	NA	Japonica	
maker-1-augustus-gene-6.6-mRNA-1	1	OsR498G0100033300.01.T01	99,88	0,00E+00	4315	Os06g0493600	71,95	0,00E+00	812	-3503	Indica	Japonica	
maker-1-augustus-gene-60.12-mRNA-1	1	OsR498G0100389000.01.T01	99,21	0,00E+00	1786	Os01g0209700	100,00	0,00E+00	1819	33	NA	Japonica	
maker-1-augustus-gene-60.14-mRNA-1	1	OsR498G0100389600.01.T01	100,00	0,00E+00	3339	Os01g0209800	100,00	0,00E+00	3339	0	NA	Japonica	
maker-1-augustus-gene-60.16-mRNA-1	1	OsR498G0100391800.01.T01	93,93	0,00E+00	2351	Os01g0210400	100,00	0,00E+00	2706	355	Japonica	Japonica	
maker-1-augustus-gene-60.18-mRNA-1	1	OsR498G0100392000.01.T01	100,00	0,00E+00	1186	Os01g0210500	100,00	0,00E+00	1186	0	NA	Japonica	
maker-1-augustus-gene-60.3-mRNA-1	1	OsR498G0100391500.01.T01	99,79	0,00E+00	835	Os01g0210300	100,00	0,00E+00	1570	735	Japonica	Japonica	
maker-1-augustus-gene-60.6-mRNA-1	1	OsR498G0100386600.01.T01	99,63	0,00E+00	1444	Os01g0209400	100,00	0,00E+00	1458	14	NA	Japonica	
maker-1-augustus-gene-61.24-mRNA-1	1	OsR498G0100394100.01.T01	97,22	0,00E+00	2385	Os01g0210800	100,00	0,00E+00	1640	-745	Indica	Japonica	
maker-1-augustus-gene-61.29-mRNA-1	1	OsR498G0100393600.01.T03	99,80	0,00E+00	1815	Os01g0210700	99,90	0,00E+00	1819	4	NA	Japonica	
maker-1-augustus-gene-61.32-mRNA-1	1	OsR498G0100400400.01.T04	100,00	0,00E+00	1759	Os01g0211800	99,18	0,00E+00	1726	-33	NA	NA	
maker-1-augustus-gene-62.0-mRNA-1	1	OsR498G0100402100.01.T04	100,00	0,00E+00	2610	Os01g0212100	100,00	0,00E+00	2610	0	NA	NA	
maker-1-augustus-gene-62.5-mRNA-1	1	OsR498G0100402300.01.T01	100,00	0,00E+00	3166	Os01g0212400	100,00	0,00E+00	3166	0	NA	Japonica	
maker-1-augustus-gene-63.11-mRNA-1	1	OsR498G0100411100.01.T01	99,65	0,00E+00	1526	Os01g0214600	94,02	0,00E+00	1351	-175	Indica	Japonica	
maker-1-augustus-gene-63.13-mRNA-1	1	OsR498G0100411900.01.T01	95,26	0,00E+00	1821	Os01g0214800	95,44	0,00E+00	1830	9	NA	Japonica	
maker-1-augustus-gene-63.15-mRNA-1	1	OsR498G0100444200.01.T01	72,77	6,77E-95	350	Os01g0215000	100,00	0,00E+00	2089	1739	Japonica	Japonica	
maker-1-augustus-gene-63.20-mRNA-1	1	OsR498G0100410000.01.T01	99,58	0,00E+00	1280	Os01g0214200	100,00	0,00E+00	1294	14	NA	Japonica	
maker-1-augustus-gene-63.7-mRNA-1	1	OsR498G0100410700.01.T01	100,00	6,87E-118	423	Os01g0214500	100,00	2,41E-118	423	0	NA	Japonica	
maker-1-augustus-gene-64.0-mRNA-1	1	OsR498G0100415100.01.T01	100,00	0,00E+00	2051	Os01g0215700	100,00	0,00E+00	2051	0	NA	Japonica	
maker-1-augustus-gene-64.16-mRNA-1	1	OsR498G0100417300.01.T01	99,69	0,00E+00	1733	Os01g0216900	99,74	0,00E+00	2068	335	Japonica	NA	
maker-1-augustus-gene-64.18-mRNA-1	1	OsR498G0100419100.01.T01	100,00	0,00E+00	834	Os01g0217100	100,00	0,00E+00	771	-63	NA	NA	
maker-1-augustus-gene-64.2-mRNA-1	1	OsR498G0100416300.01.T01	99,83	0,00E+00	2145	Os01g0216000	99,92	0,00E+00	2150	5	NA	Japonica	
maker-1-augustus-gene-64.21-mRNA-1	1	OsR498G0100419700.01.T01	100,00	0,00E+00	1986	Os01g0217300	100,00	0,00E+00	1986	0	NA	NA	
maker-1-augustus-gene-64.24-mRNA-1	1	OsR498G0100420900.01.T01	100,00	0,00E+00	2143	Os01g0217500	100,00	0,00E+00	2143	0	NA	NA	
maker-1-augustus-gene-64.29-mRNA-1	1	OsR498G0100418200.01.T01	100,00	0,00E+00	2143	Os01g0217000	100,00	0,00E+00	2143	0	NA	NA	
maker-1-augustus-gene-64.32-mRNA-1	1	OsR498G0100419400.01.T01	100,00	0,00E+00	2143	Os01g0330200	85,99	0,00E+00	1204	-939	Indica	NA	
maker-1-augustus-gene-64.4-mRNA-1	1	OsR498G0100416500.01.T01	97,31	0,00E+00	2022	Os01g0216300	97,48	0,00E+00	2031	9	NA	Japonica	
maker-1-augustus-gene-64.6-mRNA-1	1	OsR498G0100416700.01.T02	100,00	0,00E+00	2073	Os01g0216400	100,00	0,00E+00	2073	0	NA	NA	
maker-1-augustus-gene-64.7-mRNA-1	1	OsR498G0100416700.01.T03	100,00	0,00E+00	2051	Os01g0216900	89,81	0,00E+00	1523	-528	Indica	NA	
maker-1-augustus-gene-65.0-mRNA-1	1	OsR498G0100422900.01.T01	100,00	0,00E+00	2322	Os01g0217800	100,00	0,00E+00	2322	0	NA	NA	
maker-1-augustus-gene-65.12-mRNA-1	1	OsR498G0100424900.01.T01	99,88	0,00E+00	1446	Os01g0218100	100,00	0,00E+00	1451	5	NA	Japonica	
maker-1-augustus-gene-65.14-mRNA-1	1	OsR498G0100426100.01.T01	99,95	0,00E+00	3637	Os01g0218200	100,00	0,00E+00	3642	5	NA	Japonica	
maker-1-augustus-gene-65.19-mRNA-1	1	OsR498G0100427300.01.T01	99,64	0,00E+00	1486	Os01g0218500	100,00	0,00E+00	1499	13	NA	Japonica	
maker-1-augustus-gene-65.23-mRNA-1	1	OsR498G0100427600.01.T02	99,87	0,00E+00	4164	Os01g0218700	100,00	0,00E+00	4177	13	NA	Japonica	
maker-1-augustus-gene-65.34-mRNA-1	1	OsR498G0100428600.01.T03	99,84	0,00E+00	2188	Os01g0218900	100,00	0,00E+00	2197	9	NA	Japonica	
maker-1-augustus-gene-66.0-mRNA-1	1	OsR498G0100428900.01.T03	99,80	0,00E+00	900	Os01g0219100	100,00	0,00E+00	904	4	NA	Japonica	
maker-1-augustus-gene-66.10-mRNA-1	1	OsR498G0100430000.01.T01	99,27	0,00E+00	3409	Os01g0219600	100,00	0,00E+00	3469	60	NA	Japonica	
maker-1-augustus-gene-66.12-mRNA-1	1	OsR498G0100430400.01.T02	99,53	0,00E+00	3428	Os01g0220100	100,00	0,00E+00	3469	41	NA	Japonica	
maker-1-augustus-gene-66.14-mRNA-1	1	OsR498G0100431000.01.T02	99,82	0,00E+00	1983	Os05g0212400	76,46	0,00E+00	642	-1341	Indica	Japonica	
maker-1-augustus-gene-66.19-mRNA-1	1	OsR498G0100433200.01.T01	90,74	2,17E-132	472	Os01g0220500	88,89	3,00E-125	446	-26	NA	Japonica	
maker-1-augustus-gene-66.21-mRNA-1	1	OsR498G0100435800.01.T03	99,67	7,85E-153	541	Os01g0220700	88,55	3,80E-164	577	36	NA	Japonica	
maker-1-augustus-gene-66.6-mRNA-1	1	OsR498G0100429700.01.T01	99,74	0,00E+00	689	Os01g0219500	100,00	0,00E+00	693	4	NA	Japonica	
maker-1-augustus-gene-67.0-mRNA-1	1	OsR498G0100439900.01.T01	100,00	0,00E+00	1074	Os05g0215600	78,01	1,20E-141	503	-571	Indica	Indica	
maker-1-augustus-gene-67.2-mRNA-1	1	OsR498G0100440500.01.T08	100,00	0,00E+00	2841	Os01g0221600	99,94	0,00E+00	2837	-4	NA	Indica	
maker-1-augustus-gene-67.23-mRNA-1	1	OsR498G0100443200.01.T02	100,00	0,00E+00	1011	Os01g0222500	100,00	0,00E+00	1011	0	NA	NA	
maker-1-augustus-gene-67.27-mRNA-1	1	OsR498G0100444200.01.T01	98,41	0,00E+00	1090	Os01g0223000	99,07	0,00E+00	1898	808	Japonica	NA	
maker-1-augustus-gene-67.9-mRNA-1	1	OsR498G0100439000.01.T01	100,00	0,00E+00	2679	Os01g0221300	99,87	0,00E+00	2670	-9	NA	Indica	
maker-1-augustus-gene-68.34-mRNA-1	1	OsR498G0100444800.01.T01	99,47	0,00E+00	2030	Os01g0223200	99,47	0,00E+00	2030	0	NA	NA	
maker-1-augustus-gene-68.38-mRNA-1	1	OsR498G0100446400.01.T02	100,00	0,00E+00	948	Os01g0223200	77,13	5,74E-99	360	-588	Indica	NA	
maker-1-augustus-gene-68.41-mRNA-1	1	OsR498G0100446600.01.T01	100,00	0,00E+00	3474	Os01g0223600	100,00	0,00E+00	2668	-806	Indica	NA	Kinase-LRR-TM
maker-1-augustus-gene-69.0-mRNA-1	1	OsR498G0100453300.01.T01	100,00	1,56E-106	385	Os01g0224300	100,00	5,44E-107	385	0	NA	Japonica	
maker-1-augustus-gene-69.26-mRNA-1	1	OsR498G0100453200.01.T01	99,74	0,00E+00	1377	Os01g0224200	100,00	0,00E+00	1350	-27	NA	Japonica	
maker-1-augustus-gene-69.28-mRNA-1	1	OsR498G0100454000.01.T01	99,54	0,00E+00	1172	Os01g0224500	100,00	0,00E+00	1186	14	NA	Japonica	
maker-1-augustus-gene-69.31-mRNA-1	1	OsR498G0100456800.01.T01	100,00	0,00E+00	1748	Os01g0225000	100,00	0,00E+00	1748	0	NA	Japonica	
maker-1-augustus-gene-69.7-mRNA-1	1	OsR498G0100455300.01.T01	99,92	0,00E+00	2377	Os01g0224700	100,00	0,00E+00	2381	4	NA	Japonica	
maker-1-augustus-gene-7.0-mRNA-1	1	OsR498G0100035900.01.T03	99,46	0,00E+00	983	Os01g0111250	100,00	0,00E+00	899	-84	NA	Japonica	
maker-1-augustus-gene-7.11-mRNA-1	1	OsR498G0100036600.01.T01	97,76	0,00E+00	1535	Os01g0111500	99,33	0,00E+00	1587	52	NA	Japonica	
maker-1-augustus-gene-7.14-mRNA-1	1	OsR498G0100040000.01.T01	93,63	0,00E+00	1677	Os05g0103700	98,44	0,00E+00	1110	-567	Indica	Indica	
maker-1-augustus-gene-7.25-mRNA-1	1	OsR498G0100043400.01.T02	100,00	0,00E+00	3344	Os01g0113200	99,62	0,00E+00	3313	-31	NA	Indica	Kinase-TM
maker-1-augustus-gene-7.7-mRNA-1	1	OsR498G0100037100.01.T01	99,43	0,00E+00	934	Os01g0111600	100,00	0,00E+00	948	14	NA	Japonica	
maker-1-augustus-gene-7.9-mRNA-1	1	OsR498G0100043700.01.T01	100,00	0,00E+00	1956	Os01g0113300	99,08	0,00E+00	1911	-45	NA	Indica	Kinase-TM
maker-1-augustus-gene-70.0-mRNA-1	1	OsR498G0100462300.01.T01	99,64	0,00E+00	1502	Os01g0226300	100,00	0,00E+00	969	-533	Indica	Japonica	
maker-1-augustus-gene-70.4-mRNA-1	1	OsR498G0100462700.01.T05	96,06	0,00E+00	2140	Os01g0226500	96,51	0,00E+00	2164	24	NA	Japonica	
maker-1-augustus-gene-70.7-mRNA-1	1	OsR498G0100463300.01.T03	99,84	0,00E+00	2233	Os01g0226600	100,00	0,00E+00	2242	9	NA	Japonica	
maker-1-augustus-gene-70.9-mRNA-1	1	OsR498G0100461600.01.T01	98,72	0,00E+00	2186	Os01g0226200	99,76	0,00E+00	2214	28	NA	Japonica	
maker-1-augustus-gene-71.28-mRNA-1	1	OsR498G0100466700.01.T02	99,67	0,00E+00	2710	Os01g0227100	100,00	0,00E+00	2733	23	NA	Japonica	
maker-1-augustus-gene-71.33-mRNA-1	1	OsR498G0100467900.01.T01	98,99	0,00E+00	1913	Os01g0227200	100,00	0,00E+00	1959	46	NA	Japonica	Kinase-TM
maker-1-augustus-gene-71.37-mRNA-1	1	OsR498G0100468100.01.T01	100,00	0,00E+00	1126	Os01g0227300	100,00	0,00E+00	1126	0	NA	Japonica	
maker-1-augustus-gene-71.41-mRNA-1	1	OsR498G0100468300.01.T01	98,57	0,00E+00	2570	Os01g0227500	84,54	0,00E+00	1886	-684	Indica	Japonica	
maker-1-augustus-gene-71.43-mRNA-1	1	OsR498G0100468500.01.T01	99,54	0,00E+00	3120	Os01g0227500	100,00	0,00E+00	3041	-79	NA	Japonica	
maker-1-augustus-gene-71.45-mRNA-1	1	OsR498G0100468900.01.T01	96,74	0,00E+00	2666	Os01g0227800	82,07	0,00E+00	1498	-1168	Indica	Japonica	
maker-1-augustus-gene-71.47-mRNA-1	1	OsR498G0100469700.01.T01	98,09	0,00E+00	2782	Os01g0227800	99,94	0,00E+00	2903	121	NA	Japonica	
maker-1-augustus-gene-71.50-mRNA-1	1	OsR498G0100470100.01.T01	99,40	0,00E+00	5029	Os01g0228200	100,00	0,00E+00	5984	955	Japonica	Japonica	Kinase-LRR-TM
maker-1-augustus-gene-71.53-mRNA-1	1	OsR498G0100471100.01.T02	99,81	0,00E+00	1900	Os01g0228400	100,00	0,00E+00	1909	9	NA	Japonica	
maker-1-augustus-gene-71.55-mRNA-1	1	OsR498G0100470400.01.T01	99,80	0,00E+00	889	Os01g0228300	99,80	0,00E+00	889	0	NA	Japonica	
maker-1-augustus-gene-71.57-mRNA-1	1	OsR498G0100471400.01.T04	99,60	0,00E+00	1772	Os01g0228500	100,00	0,00E+00	1792	20	NA	Japonica	
maker-1-augustus-gene-71.59-mRNA-1	1	OsR498G0100471600.01.T02	100,00	0,00E+00	1494	Os01g0228600	86,73	0,00E+00	829	-665	Indica	Japonica	
maker-1-augustus-gene-71.62-mRNA-1	1	OsR498G0100472000.01.T02	100,00	0,00E+00	2551	Os01g0228800	100,00	0,00E+00	2551	0	NA	Japonica	
maker-1-augustus-gene-72.0-mRNA-1	1	OsR498G0100472700.01.T01	96,38	0,00E+00	4956	Os12g0238000	86,83	0,00E+00	3682	-1274	Indica	Japonica	
maker-1-augustus-gene-72.14-mRNA-1	1	OsR498G0100476300.01.T01	100,00	0,00E+00	910	Os01g0229500	100,00	0,00E+00	910	0	NA	Japonica	
maker-1-augustus-gene-72.18-mRNA-1	1	OsR498G0100477300.01.T01	99,85	0,00E+00	1190	Os01g0233900	96,05	0,00E+00	858	-332	Indica	Japonica	
maker-1-augustus-gene-72.27-mRNA-1	1	OsR498G0100473000.01.T01	99,81	0,00E+00	2811	Os01g0229100	100,00	0,00E+00	2825	14	NA	Japonica	
maker-1-augustus-gene-72.31-mRNA-1	1	OsR498G0100475200.01.T01	94,57	0,00E+00	1090	Os01g0229400	94,71	0,00E+00	1095	5	NA	Japonica	
maker-1-augustus-gene-72.34-mRNA-1	1	OsR498G0100476600.01.T01	99,52	0,00E+00	7445	Os01g0229700	99,98	0,00E+00	7533	88	NA	Japonica	
maker-1-augustus-gene-72.7-mRNA-1	1	OsR498G0100473100.01.T04	99,53	0,00E+00	3828	Os01g0229200	100,00	0,00E+00	3853	25	NA	Japonica	
maker-1-augustus-gene-73.38-mRNA-1	1	OsR498G0100479700.01.T01	95,19	0,00E+00	1350	Os01g0231000	95,31	0,00E+00	1354	4	NA	Japonica	
maker-1-augustus-gene-73.55-mRNA-1	1	OsR498G0100482300.01.T01	99,60	0,00E+00	1351	Os01g0232000	100,00	0,00E+00	1364	13	NA	Japonica	
maker-1-augustus-gene-73.57-mRNA-1	1	OsR498G0100482700.01.T03	100,00	0,00E+00	1391	Os01g0232100	100,00	0,00E+00	1391	0	NA	Japonica	
maker-1-augustus-gene-73.60-mRNA-1	1	OsR498G0100477800.01.T05	99,64	0,00E+00	1493	Os01g0230200	100,00	0,00E+00	1507	14	NA	Japonica	
maker-1-augustus-gene-73.71-mRNA-1	1	OsR498G0100481600.01.T02	100,00	0,00E+00	650	Os01g0231700	100,00	0,00E+00	650	0	NA	Japonica	

maker-1-augustus-gene-74.28-mRNA-1	1	OsR498G0100484700.01.T02	99,87	0,00E+00	2807	Os01g0232300	100,00	0,00E+00	2816	9	NA	Japonica	
maker-1-augustus-gene-74.34-mRNA-1	1	OsR498G0100484900.01.T01	99,76	0,00E+00	3733	Os01g0232400	100,00	0,00E+00	3755	22	NA	Japonica	
maker-1-augustus-gene-74.36-mRNA-1	1	OsR498G0100485700.01.T02	99,78	0,00E+00	1654	Os01g0232500	100,00	0,00E+00	1663	9	NA	Japonica	
maker-1-augustus-gene-74.39-mRNA-1	1	OsR498G0100486100.01.T02	98,34	0,00E+00	2509	Os01g0232700	98,55	0,00E+00	2523	14	NA	Japonica	
maker-1-augustus-gene-74.44-mRNA-1	1	OsR498G0100488100.01.T04	98,62	0,00E+00	884	Os01g0233400	100,00	0,00E+00	915	31	NA	NA	
maker-1-augustus-gene-74.47-mRNA-1	1	OsR498G0100488900.01.T01	99,94	0,00E+00	2880	Os01g0233800	100,00	0,00E+00	2884	4	NA	Japonica	Kinase-TM
maker-1-augustus-gene-74.55-mRNA-1	1	OsR498G0100487200.01.T02	100,00	0,00E+00	1110	Os01g0233000	99,84	0,00E+00	1105	-5	NA	Japonica	
maker-1-augustus-gene-74.58-mRNA-1	1	OsR498G0100488600.01.T01	96,84	0,00E+00	2573	Os01g0233500	99,74	0,00E+00	2740	167	NA	Japonica	
maker-1-augustus-gene-74.73-mRNA-1	1	OsR498G0100489200.01.T05	100,00	0,00E+00	2208	Os01g0233900	100,00	0,00E+00	2030	-178	NA	Japonica	
maker-1-augustus-gene-75.42-mRNA-1	1	OsR498G0100489900.01.T01	94,44	0,00E+00	2385	Os01g0234100	94,58	0,00E+00	2394	9	NA	Japonica	
maker-1-augustus-gene-75.46-mRNA-1	1	OsR498G0100493500.01.T01	96,52	0,00E+00	900	Os01g0235100	94,57	0,00E+00	914	14	NA	Japonica	
maker-1-augustus-gene-75.49-mRNA-1	1	OsR498G0100494100.01.T01	100,00	0,00E+00	1164	Os01g0235300	100,00	0,00E+00	1164	0	NA	Japonica	
maker-1-augustus-gene-75.63-mRNA-1	1	OsR498G0100492600.01.T01	98,94	0,00E+00	2993	Os01g0234850	100,00	0,00E+00	3052	59	NA	Japonica	
maker-1-augustus-gene-75.66-mRNA-1	1	OsR498G0100493200.01.T02	99,62	0,00E+00	1865	Os01g0234900	100,00	0,00E+00	1884	19	NA	Japonica	
maker-1-augustus-gene-75.68-mRNA-1	1	OsR498G0100493800.01.T02	99,84	0,00E+00	2206	Os01g0235200	95,77	0,00E+00	2139	-67	NA	Japonica	
maker-1-augustus-gene-75.76-mRNA-1	1	OsR498G0100495800.01.T01	99,78	0,00E+00	3326	Os01g0235700	100,00	0,00E+00	3344	18	NA	Japonica	
maker-1-augustus-gene-76.32-mRNA-1	1	OsR498G0100499700.01.T01	99,95	0,00E+00	3764	Os01g0236300	100,00	0,00E+00	3768	4	NA	Japonica	
maker-1-augustus-gene-76.38-mRNA-1	1	OsR498G0100500300.01.T01	100,00	0,00E+00	666	Os01g0236400	100,00	0,00E+00	666	0	NA	Japonica	
maker-1-augustus-gene-76.42-mRNA-1	1	OsR498G0100502100.01.T04	99,62	0,00E+00	3325	Os01g0236700	100,00	0,00E+00	3357	32	NA	Japonica	
maker-1-augustus-gene-76.47-mRNA-1	1	OsR498G0100503300.01.T01	93,10	0,00E+00	805	Os01g0237000	93,68	0,00E+00	819	14	NA	Japonica	
maker-1-augustus-gene-76.49-mRNA-1	1	OsR498G0100502400.01.T01	99,18	0,00E+00	3009	Os01g0236800	100,00	0,00E+00	3002	-7	NA	Japonica	
maker-1-augustus-gene-76.54-mRNA-1	1	OsR498G0100503600.01.T01	96,70	0,00E+00	1218	Os01g0237100	97,11	0,00E+00	1232	14	NA	Japonica	
maker-1-augustus-gene-77.0-mRNA-1	1	OsR498G0100507700.01.T01	100,00	0,00E+00	1829	Os01g0238500	100,00	0,00E+00	1802	-27	NA	Japonica	
maker-1-augustus-gene-77.18-mRNA-1	1	OsR498G0100505800.01.T02	99,74	0,00E+00	678	Os01g0237500	100,00	1,14E-150	531	-147	Indica	Japonica	
maker-1-augustus-gene-77.19-mRNA-1	1	OsR498G0100505800.01.T02	99,50	0,00E+00	1076	Os01g0237366	70,64	6,19E-64	244	-832	Indica	Japonica	
maker-1-augustus-gene-77.26-mRNA-1	1	OsR498G0100509200.01.T02	98,28	0,00E+00	2815	Os01g0239000	99,75	0,00E+00	2908	93	NA	Japonica	
maker-1-augustus-gene-77.5-mRNA-1	1	OsR498G0100507900.01.T01	99,77	0,00E+00	765	Os01g0238600	100,00	0,00E+00	769	4	NA	Japonica	
maker-1-augustus-gene-77.7-mRNA-1	1	OsR498G0100508300.01.T01	99,58	0,00E+00	3796	Os01g0238700	100,00	0,00E+00	3837	41	NA	Japonica	
maker-1-augustus-gene-78.41-mRNA-1	1	OsR498G0100514900.01.T01	98,89	0,00E+00	1419	Os01g0240700	89,88	0,00E+00	1082	-337	Indica	Japonica	
maker-1-augustus-gene-78.43-mRNA-1	1	OsR498G0100515300.01.T01	99,36	0,00E+00	1385	Os01g0240700	100,00	0,00E+00	2064	679	Japonica	Japonica	
maker-1-augustus-gene-78.46-mRNA-1	1	OsR498G0100519100.01.T01	99,93	0,00E+00	2523	Os01g0241000	100,00	0,00E+00	2078	-445	Indica	Japonica	
maker-1-augustus-gene-78.48-mRNA-1	1	OsR498G0100510600.01.T02	99,84	0,00E+00	2284	Os01g0239200	100,00	0,00E+00	2237	-47	NA	Japonica	
maker-1-augustus-gene-79.12-mRNA-1	1	OsR498G0100522400.01.T02	99,84	0,00E+00	1136	Os01g0242500	100,00	0,00E+00	964	-172	Indica	Japonica	
maker-1-augustus-gene-79.16-mRNA-1	1	OsR498G0100522600.01.T02	99,73	0,00E+00	2608	Os01g0242600	100,00	0,00E+00	2626	18	NA	Japonica	
maker-1-augustus-gene-79.2-mRNA-1	1	OsR498G0100521900.01.T03	98,42	5,33E-122	437	Os01g0242300	98,81	1,52E-123	442	5	NA	Japonica	
maker-1-augustus-gene-79.22-mRNA-1	1	OsR498G0100524200.01.T01	99,82	0,00E+00	1939	Os01g0243000	100,00	0,00E+00	2278	339	Japonica	Japonica	
maker-1-augustus-gene-79.24-mRNA-1	1	OsR498G0100525200.01.T01	99,74	0,00E+00	2704	Os01g0243200	100,00	0,00E+00	2298	-406	Indica	Japonica	
maker-1-augustus-gene-79.5-mRNA-1	1	OsR498G0100523900.01.T01	99,04	0,00E+00	733	Os01g0242900	100,00	0,00E+00	753	20	NA	Japonica	
maker-1-augustus-gene-8.130-mRNA-1	1	OsR498G1119343800.01.T03	85,40	6,20E-137	488	Os01g0115200	100,00	0,00E+00	1068	580	Japonica	Indica	
maker-1-augustus-gene-8.133-mRNA-1	1	OsR498G0100045600.01.T01	100,00	0,00E+00	2771	Os01g0113400	85,03	0,00E+00	1523	-1248	Indica	Indica	Kinase-TM
maker-1-augustus-gene-8.134-mRNA-1	1	OsR498G0100046400.01.T01	100,00	0,00E+00	2782	Os01g0114100	97,95	0,00E+00	3333	551	Japonica	Indica	Kinase-TM
maker-1-augustus-gene-8.139-mRNA-1	1	OsR498G0100046400.01.T07	100,00	0,00E+00	3874	Os01g0116200	94,74	0,00E+00	3407	-467	Indica	Indica	Kinase-TM
maker-1-augustus-gene-8.141-mRNA-1	1	OsR498G0100047600.01.T01	100,00	0,00E+00	2013	Os01g0114600	99,63	0,00E+00	3381	1368	Japonica	Indica	Kinase-TM
maker-1-augustus-gene-8.144-mRNA-1	1	OsR498G0100050600.01.T01	100,00	0,00E+00	1727	Os01g0115750	72,48	4,26E-110	398	-1329	Indica	Indica	
maker-1-augustus-gene-8.47-mRNA-1	1	OsR498G0100045100.01.T01	100,00	0,00E+00	3442	Os01g0113800	84,60	0,00E+00	2084	-1358	Indica	Indica	Kinase-TM
maker-1-augustus-gene-8.48-mRNA-1	1	OsR498G0100045700.01.T01	99,95	0,00E+00	3529	Os01g0113800	99,95	0,00E+00	3410	-119	NA	Indica	Kinase-TM
maker-1-augustus-gene-8.49-mRNA-1	1	OsR498G0100046700.01.T01	100,00	0,00E+00	1981	Os01g0115900	98,23	0,00E+00	2050	69	NA	Indica	Kinase-TM
maker-1-augustus-gene-8.50-mRNA-1	1	OsR498G0100047300.01.T01	97,80	0,00E+00	2028	Os01g0114500	99,89	0,00E+00	3135	1107	Japonica	Indica	Kinase-TM
maker-1-augustus-gene-8.51-mRNA-1	1	OsR498G0100047900.01.T01	99,93	0,00E+00	2487	Os01g0114700	99,71	0,00E+00	2457	-30	NA	Indica	Kinase-TM
maker-1-augustus-gene-80.44-mRNA-1	1	OsR498G0101278400.01.T01	97,98	1,23E-168	593	Os02g0140500	97,40	7,71E-166	582	-11	NA	Indica	
maker-1-augustus-gene-80.46-mRNA-1	1	OsR498G0100529000.01.T01	100,00	0,00E+00	661	Os01g0243700	100,00	0,00E+00	661	0	NA	NA	
maker-1-augustus-gene-80.50-mRNA-1	1	OsR498G0100529200.01.T01	100,00	0,00E+00	1824	Os01g0243900	100,00	0,00E+00	2286	462	Japonica	Japonica	
maker-1-augustus-gene-80.53-mRNA-1	1	OsR498G0100529800.01.T02	100,00	0,00E+00	2397	Os01g0244000	100,00	0,00E+00	2372	-25	NA	NA	
maker-1-augustus-gene-80.55-mRNA-1	1	OsR498G0100533600.01.T01	79,32	0,00E+00	988	Os01g0244400	79,32	0,00E+00	988	0	NA	Indica	
maker-1-augustus-gene-81.0-mRNA-1	1	OsR498G0510259700.01.T02	72,90	2,89E-85	316	Os01g0245600	99,51	0,00E+00	1449	1133	Japonica	NA	
maker-1-augustus-gene-81.15-mRNA-1	1	OsR498G0100540000.01.T01	100,00	0,00E+00	987	Os01g0246500	100,00	0,00E+00	987	0	NA	NA	
maker-1-augustus-gene-81.2-mRNA-1	1	OsR498G0100539500.01.T01	100,00	0,00E+00	1099	Os01g0246400	100,00	0,00E+00	1099	0	NA	NA	
maker-1-augustus-gene-81.4-mRNA-1	1	OsR498G0100541900.01.T01	100,00	0,00E+00	3214	Os01g0246700	99,39	0,00E+00	2615	-599	Indica	Indica	
maker-1-augustus-gene-82.0-mRNA-1	1	OsR498G0100548500.01.T01	100,00	0,00E+00	1305	Os01g0248300	100,00	0,00E+00	1305	0	NA	NA	
maker-1-augustus-gene-82.15-mRNA-1	1	OsR498G0100547000.01.T01	99,81	0,00E+00	927	Os01g0247900	100,00	0,00E+00	931	4	NA	NA	
maker-1-augustus-gene-82.17-mRNA-1	1	OsR498G0100550200.01.T01	99,86	0,00E+00	1300	Os01g0248500	100,00	0,00E+00	1305	5	NA	NA	
maker-1-augustus-gene-82.2-mRNA-1	1	OsR498G0100549900.01.T07	100,00	0,00E+00	1662	Os01g0248400	100,00	0,00E+00	1662	0	NA	NA	
maker-1-augustus-gene-82.21-mRNA-1	1	OsR498G0100550400.01.T04	93,78	0,00E+00	1050	Os01g0248600	93,78	0,00E+00	1050	0	NA	NA	
maker-1-augustus-gene-82.23-mRNA-1	1	OsR498G0100551800.01.T01	100,00	0,00E+00	1364	Os01g0248900	100,00	0,00E+00	1364	0	NA	NA	
maker-1-augustus-gene-82.25-mRNA-1	1	OsR498G0100552600.01.T01	100,00	0,00E+00	1380	Os01g0249100	100,00	0,00E+00	1380	0	NA	NA	
maker-1-augustus-gene-82.27-mRNA-1	1	OsR498G0100552800.01.T01	100,00	0,00E+00	1234	Os01g0249200	100,00	0,00E+00	1234	0	NA	NA	
maker-1-augustus-gene-82.29-mRNA-1	1	OsR498G0100553200.01.T05	100,00	0,00E+00	688	Os01g0249300	100,00	0,00E+00	688	0	NA	NA	
maker-1-augustus-gene-82.9-mRNA-1	1	OsR498G0100545400.01.T01	100,00	0,00E+00	1900	Os01g0247500	100,00	0,00E+00	1900	0	NA	NA	Kinase-TM
maker-1-augustus-gene-83.38-mRNA-1	1	OsR498G0100556300.01.T01	100,00	3,19E-155	547	Os01g0250600	100,00	1,11E-155	547	0	NA	NA	
maker-1-augustus-gene-83.49-mRNA-1	1	OsR498G0100557500.01.T02	100,00	0,00E+00	2152	Os01g0251200	100,00	0,00E+00	2152	0	NA	NA	
maker-1-augustus-gene-84.39-mRNA-1	1	OsR498G0100559700.01.T07	100,00	0,00E+00	2214	Os01g0252100	100,00	0,00E+00	2214	0	NA	NA	Kinase-TM
maker-1-augustus-gene-84.56-mRNA-1	1	OsR498G1221886900.01.T01	71,33	8,28E-115	416	Os01g0252700	100,00	0,00E+00	3090	2674	Japonica	NA	
maker-1-augustus-gene-84.58-mRNA-1	1	OsR498G0100563300.01.T01	100,00	0,00E+00	2852	Os01g0253300	100,00	0,00E+00	2852	0	NA	NA	
maker-1-augustus-gene-84.65-mRNA-1	1	OsR498G0100564900.01.T01	100,00	0,00E+00	3831	Os01g0253900	100,00	0,00E+00	3227	-604	Indica	NA	
maker-1-augustus-gene-84.68-mRNA-1	1	OsR498G0100562400.01.T04	99,82	0,00E+00	994	Os01g0252900	99,82	0,00E+00	994	0	NA	NA	
maker-1-augustus-gene-84.72-mRNA-1	1	OsR498G0100562800.01.T03	100,00	0,00E+00	2432	Os01g0253100	100,00	0,00E+00	2432	0	NA	NA	Kinase-TM
maker-1-augustus-gene-84.79-mRNA-1	1	OsR498G0100563600.01.T01	100,00	0,00E+00	1613	Os01g0253400	100,00	0,00E+00	1613	0	NA	NA	
maker-1-augustus-gene-84.81-mRNA-1	1	OsR498G0100564400.01.T03	100,00	4,35E-173	607	Os01g0253600	100,00	2,10E-165	580	-27	NA	NA	
maker-1-augustus-gene-84.83-mRNA-1	1	OsR498G0100564600.01.T01	100,00	0,00E+00	893	Os01g0253800	100,00	0,00E+00	893	0	NA	NA	
maker-1-augustus-gene-84.85-mRNA-1	1	OsR498G0100565800.01.T01	100,00	0,00E+00	1050	Os01g0254000	100,00	0,00E+00	1050	0	NA	NA	
maker-1-augustus-gene-85.32-mRNA-1	1	OsR498G0100571700.01.T01	100,00	0,00E+00	1748	Os01g0255000	100,00	0,00E+00	1748	0	NA	NA	
maker-1-augustus-gene-85.33-mRNA-1	1	OsR498G0100571700.01.T04	100,00	0,00E+00	1007	Os01g0255100	97,60	0,00E+00	1711	704	Japonica	NA	
maker-1-augustus-gene-85.45-mRNA-1	1	OsR498G0100566200.01.T04	100,00	0,00E+00	3873	Os01g0254100	100,00	0,00E+00	3873	0	NA	NA	
maker-1-augustus-gene-85.51-mRNA-1	1	OsR498G0100567600.01.T02	100,00	3,95E-121	434	Os01g0254200	100,00	6,71E-94	342	-92	Indica	NA	
maker-1-augustus-gene-86.37-mRNA-1	1	OsR498G0100576100.01.T02	100,00	5,66E-159	560	Os01g0256400	100,00	1,97E-159	560	0	NA	NA	
maker-1-augustus-gene-86.39-mRNA-1	1	OsR498G0100575600.01.T01	100,00	0,00E+00	2132	Os01g0255700	100,00	0,00E+00	2114	-18	NA	NA	
maker-1-augustus-gene-86.51-mRNA-1	1	OsR498G0100578600.01.T01	100,00	0,00E+00	760	Os01g0256900	100,00	0,00E+00	760	0	NA	NA	
maker-1-augustus-gene-86.54-mRNA-1	1	OsR498G0100580800.01.T02	94,90	0,00E+00	790	Os01g0257400	94,90	0,00E+00	790	0	NA	NA	
maker-1-augustus-gene-87.31-mRNA-1	1	OsR498G0100585300.01.T03	100,00	3,33E-37	154	Os01g0258600	100,00	1,16E-37	154	0	NA	NA	
maker-1-augustus-gene-87.33-mRNA-1	1	OsR498G0100587300.01.T01	97,02	0,00E+00	2388	Os01g0259200	97,02	0,00E+00	2388	0	NA	NA	Kinase-TM
maker-1-augustus-gene-87.40-mRNA-1	1	OsR498G0100589500.01.T01	100,00	0,00E+00	1202	Os01g0259900	100,00	0,00E+00	1180	-22	NA	NA	
maker-1-augustus-gene-87.47-mRNA-1	1	OsR498G0100585200.01.T01	99,93	0,00E+00	2577	Os01g0258700	100,00	0,00E+00	2581	4	NA	NA	
maker-1-augustus-gene-87.52-mRNA-1	1	OsR498G0100587800.01.T01	100,00	0,00E+00	6444	Os01g0259400	100,00	0,00E+00	6444	0	NA	NA	Kinase-TM
maker-1-augustus-gene-87.58-mRNA-1	1	OsR498G0100588000.01.T01	99,73	0,00E+00	1337	Os01g0259600	99,71	0,00E+00	1247	-90	NA	NA	
maker-1-augustus-gene-88.18-mRNA-1	1	OsR498G0100594800.01.T02	100,00	0,00E+00	1496	Os01g0261200	100,00	0,00E+00	1440	-56	NA	NA	
maker-1-augustus-gene-88.21-mRNA-1	1	OsR498G0100598700.01.T01	99,86	0,00E+00	1250	Os01g0261600	100,00	0,00E+00	2462	1212	Japonica	NA	
maker-1-augustus-gene-88.7-mRNA-1	1	OsR498G0100594500.01.T01	100,00	0,00E+00	2338	Os01g0261100	100,00	0,00E+00	2338	0	NA	NA	
maker-1-augustus-gene-89.0-mRNA-1	1	OsR498G0101011200.01.T01	78,49	2,68E-128	459	Os09g0241700	90,23	0,00E+00	643	184	Japonica	NA	
maker-1-augustus-gene-89.15-mRNA-1	1	OsR498G0100605100.01.T02	100,00	0,00E+00	1819	Os01g0263300	100,00	0,00E+00	1819	0	NA	NA	
maker-1-augustus-gene-89.19-mRNA-1	1	OsR498G0100603200.01.T01	100,00	0,00E+00	2614	Os01g0262600	100,00	0,00E+00	2614	0	NA	NA	
maker-1-augustus-gene-89.2-mRNA-1	1	OsR498G0100598700.01.T01	99,86	0,00E+00	1250	Os01g0261600	100,00	0,00E+00	2462	1212	Japonica	NA	
maker-1-augustus-gene-89.24-mRNA-1	1	OsR498G0100603800.01.T01	100,00	0,00E+00	1884	Os01g0263000	100,00	0,00E+00	1884	0	NA	NA	
maker-1-augustus-gene-89.4-mRNA-1	1	OsR498G0100602700.01.T01	97,53	0,00E+00	829	Os01g0262500	97,52	0,00E+00	824	-5	NA	NA	
maker-1-augustus-gene-89.7-mRNA-1	1	OsR498G0100603300.01.T05	100,00	0,00E+00	1716	Os01g0262700	99,89	0,00E+00	1681	-35	NA	NA	
maker-1-augustus-gene-9.101-mRNA-1	1	OsR498G0100053600.01.T01	100,00	0,00E+00	2030	Os01g0116400	99,29	0,00E+00	1992	-38	NA	Indica	Kinase-TM
maker-1-augustus-gene-9.102-mRNA-1	1	OsR498G0100054400.01.T01	100,00	0,00E+00	3669	Os01g0116600	99,53	0,00E+00	2667	-1002	Indica	Indica	
maker-1-augustus-gene-9.103-mRNA-1	1	OsR498G0100053600.01.T01	93,73	0,00E+00	901	Os01g0116400	93,90	0,00E+00	905	4	NA	Indica	
maker-1-augustus-gene-9.105-mRNA-1	1	OsR498G0100056600.01.T01	98,20	0,00E+00	3667	Os01g0117400	89,25	0,00E+00	2805	-862	Indica	Indica	Kinase-TM
maker-1-augustus-gene-9.181-mRNA-1	1	OsR498G0100057000.01.T04	98,86	0,00E+00	2305	Os01g0117850	96,85	0,00E+00	2129	-176	NA	Indica	
maker-1-augustus-gene-9.48-mRNA-1	1	OsR498G0100050900.01.T02	100,00	0,00E+00	827	Os01g0115600	99,56	0,00E+00	818	-9	NA	Indica	
maker-1-augustus-gene-9.51-mRNA-1	1	OsR498G0100052100.01.T01	100,00	0,00E+00	1979	Os01g0115900	99,66	0,00E+00	2127	148	NA	Indica	Kinase-TM
maker-1-augustus-gene-9.52-mRNA-1	1	OsR498G0100045700.01.T01	79,30	0,00E+00	1620	Os01g0114500	83,14	0,00E+00	1848	228	Japonica	Indica	Kinase-TM
maker-1-augustus-gene-9.53-mRNA-1	1	OsR498G0100054700.01.T03	97,18	0,00E+00	655	Os01g0116500	99,37	0,00E+00	847	192	Japonica	Indica	
maker-1-augustus-gene-9.75-mRNA-1	1	OsR498G0100056100.01.T02	98,94	0,00E+00	2330	Os01g0117500	84,23	0,00E+00	2031	-299	Indica	Indica	Kinase-TM
maker-1-augustus-gene-9.81-mRNA-1	1	OsR498G0100056700.01.T02	100,00	0,00E+00	2554	Os01g0117100	95,07	0,00E+00	3139	585	Japonica	Indica	Kinase-TM
maker-1-augustus-gene-9.86-mRNA-1	1	OsR498G0100051800.01.T01	99,92	0,00E+00	2281	Os01g0115750	99,13	0,00E+00	2231	-50	NA	Indica	Kinase-TM
maker-1-augustus-gene-9.99-mRNA-1	1	OsR498G0100052000.01.T01	100,00	0,00E+00	2852	Os01g0116000	81,16	0,00E+00	1897	-955	Indica	Indica	Kinase-TM
maker-1-augustus-gene-90.41-mRNA-1	1	OsR498G0100605500.01.T02	95,16	0,00E+00	2021	Os01g0263500	99,89	0,00E+00	1576	-445	Indica	NA	
maker-1-augustus-gene-90.55-mRNA-1	1	OsR498G0100606400.01.T01	100,00	0,00E+00	2987	Os01g0263900	97,89	0,00E+00	2530	-457	Indica	NA	
maker-1-augustus-gene-91.0-mRNA-1	1	OsR498G0100609500.01.T01	87,77	5,10E-160	564	Os01g0264500	87,77	1,77E-160	564	0	NA	NA	
maker-1-augustus-gene-91.12-mRNA-1	1	OsR498G0100611400.01.T05	100,00	0,00E+00	1077	Os01g0264700	100,00	0,00E+00	1077	0	NA	NA	
maker-1-augustus-gene-91.17-mRNA-1	1	OsR498G0100612200.01.T06	100,00	0,00E+00	1402	Os01g0265100	100,00	0,00E+00	975	-427	Indica	NA	
maker-1-augustus-gene-91.21-mRNA-1	1	OsR498G0100612600.01.T02	100,00	0,00E+00	2067	Os01g0265200	100,00	0,00E+00	2067	0	NA	NA	
maker-1-augustus-gene-91.29-mRNA-1	1	OsR498G0100614000.01.T02	100,00	0,00E+00	1310	Os01g0265700	100,00	0,00E+00	1310	0	NA	NA	
maker-1-augustus-gene-91.32-mRNA-1	1	OsR498G0100614600.01.T03	99,79	0,00E+00	846	Os01g0265900	100,00	0,00E+00	825	-21	NA	NA	
maker-1-augustus-gene-91.7-mRNA-1	1	OsR498G0100614900.01.T01	99,95	0,00E+00	3859	Os01g0266000	99,95	0,00E+00	3859	0	NA	NA	
maker-1-augustus-gene-92.13-mRNA-1	1	OsR498G0100618000.01.T02	99,85	0,00E+00	1217	Os01g0266500	100,00	0,00E+00	1222	5	NA	NA	
maker-1-augustus-gene-92.17-mRNA-1	1	OsR498G0100618400.01.T01	100,00	0,00E+00	1077	Os01g0266600	100,00	0,00E+00	1077	0	NA	NA	
maker-1-augustus-gene-92.21-mRNA-1	1	OsR498G0100620200.01.T09	100,00	0,00E+00	852	Os01g0266800	100,00	0,00E+00	852	0	NA	NA	
maker-1-augustus-gene-92.28-mRNA-1	1	OsR498G0100622800.01.T01	100,00	0,00E+00	681	Os01g0267400	100,00	0,00E+00	681	0	NA	NA	
maker-1-augustus-gene-92.3-mRNA-1	1	OsR498G0100621700.01.T04	100,00	0,00E+00	4140	Os01g0267100	100,00	0,00E+00	4140	0	NA	NA	
maker-1-augustus-gene-92.31-mRNA-1	1	OsR498G0100623200.01.T01	100,00	0,00E+00	2468	Os01g0267600	100,00	0,00E+00	2468	0	NA	NA	
maker-1-augustus-gene-92.8-mRNA-1	1	OsR498G0100622100.01.T01	100,00	0,00E+00	1667	Os01g0267200	100,00	0,00E+00	1667	0	NA	NA	
maker-1-augustus-gene-93.14-mRNA-1	1	OsR498G0100628900.01.T03	100,00	0,00E+00	1559	Os01g0268900	100,00	0,00E+00	1559	0	NA	NA	
maker-1-augustus-gene-93.17-mRNA-1	1	OsR498G0100625000.01.T01	100,00	0,00E+00	677	Os01g0267900	100,00	0,00E+00	677	0	NA	NA	
maker-1-augustus-gene-93.19-mRNA-1	1	OsR498G0100625600.01.T02	100,00	0,00E+00	1371	Os01g0268100	100,00	0,00E+00	1371	0	NA	NA	
maker-1-augustus-gene-93.26-mRNA-1	1	OsR498G0100629200.01.T01	100,00	0,00E+00	2489	Os01g0269000	100,00	0,00E+00	2489	0	NA	NA	
maker-1-augustus-gene-93.29-mRNA-1	1	OsR498G0100629400.01.T07	99,87	0,00E+00	1423	Os01g0269100	99,87	0,00E+00	1423	0	NA	NA	
maker-1-augustus-gene-93.6-mRNA-1	1	OsR498G0100627900.01.T01	100,00	0,00E+00	5048	Os01g0268500	100,00	0,00E+00	5048	0	NA	NA	
maker-1-augustus-gene-94.39-mRNA-1	1	OsR498G0100629900.01.T03	100,00	0,00E+00	4814	Os01g0269500	99,10	0,00E+00	4723	-91	NA	Indica	CC-NBS-TM
maker-1-augustus-gene-94.41-mRNA-1	1	OsR498G0100631100.01.T01	99,93	0,00E+00	5467	Os01g0269800	99,93	0,00E+00	5467	0	NA	NA	CC-NBS-TM
maker-1-augustus-gene-94.43-mRNA-1	1	OsR498G0100632700.01.T01	99,59	0,00E+00	1741	Os01g0270300	100,00	0,00E+00	1759	18	NA	Japonica	
maker-1-augustus-gene-94.45-mRNA-1	1	OsR498G0100633900.01.T01	99,15	0,00E+00	1249	Os01g0271000	98,96	0,00E+00	1174	-75	NA	Japonica	
maker-1-augustus-gene-94.49-mRNA-1	1	OsR498G0100630400.01.T01	100,00	0,00E+00	4675	Os01g0269800	84,25	0,00E+00	3306	-1369	Indica	NA	CC-NBS-TM
maker-1-augustus-gene-94.52-mRNA-1	1	OsR498G0100631600.01.T05	100,00	0,00E+00	2327	Os01g0269900	100,00	0,00E+00	2327	0	NA	NA	
maker-1-augustus-gene-94.55-mRNA-1	1	OsR498G0100632000.01.T01	100,00	0,00E+00	1359	Os01g0270100	100,00	0,00E+00	1359	0	NA	Japonica	
maker-1-augustus-gene-94.57-mRNA-1	1	OsR498G0100634200.01.T01	99,81	0,00E+00	3856	Os01g0271200	100,00	0,00E+00	3874	18	NA	Japonica	
maker-1-augustus-gene-94.59-mRNA-1	1	OsR498G0100634400.01.T03	99,44	0,00E+00	1596	Os01g0271400	100,00	0,00E+00	1618	22	NA	Japonica	
maker-1-augustus-gene-95.33-mRNA-1	1	OsR498G0100634700.01.T03	99,86	0,00E+00	1286	Os01g0271500	100,00	0,00E+00	1290	4	NA	Japonica	
maker-1-augustus-gene-95.37-mRNA-1	1	OsR498G0100638100.01.T01	99,82	0,00E+00	2010	Os01g0272600	100,00	0,00E+00	2019	9	NA	Japonica	
maker-1-augustus-gene-95.58-mRNA-1	1	OsR498G0100639400.01.T01	99,57	1,22E-114	413	Os01g0273000	100,00	1,01E-116	417	4	NA	Japonica	
maker-1-augustus-gene-96.0-mRNA-1	1	OsR498G0100642500.01.T01	99,75	0,00E+00	2146	Os01g0273800	100,00	0,00E+00	2159	13	NA	Japonica	
maker-1-augustus-gene-96.6-mRNA-1	1	OsR498G0100643300.01.T01	99,35	0,00E+00	2194	Os01g0274100	99,92	0,00E+00	2225	31	NA	Japonica	
maker-1-augustus-gene-97.24-mRNA-1	1	OsR498G0100653900.01.T05	99,91	0,00E+00	1960	Os01g0276100	100,00	0,00E+00	1965	5	NA	Japonica	
maker-1-augustus-gene-97.29-mRNA-1	1	OsR498G0100654700.01.T01	100,00	0,00E+00	1310	Os01g0276300	100,00	0,00E+00	1310	0	NA	Japonica	
maker-1-augustus-gene-97.41-mRNA-1	1	OsR498G0100651200.01.T03	99,71	0,00E+00	4861	Os01g0275600	100,00	0,00E+00	4897	36	NA	Japonica	
maker-1-augustus-gene-97.55-mRNA-1	1	OsR498G0100653000.01.T01	99,70	2,56E-171	602	Os01g0276000	100,00	2,10E-173	607	5	NA	Japonica	
maker-1-augustus-gene-97.60-mRNA-1	1	OsR498G0100654200.01.T01	98,71	0,00E+00	1772	Os01g0276200	98,81	0,00E+00	1776	4	NA	Japonica	
maker-1-augustus-gene-98.28-mRNA-1	1	OsR498G0100658900.01.T01	99,86	0,00E+00	1297	Os01g0277700	100,00	0,00E+00	1310	13	NA	Japonica	
maker-1-augustus-gene-98.31-mRNA-1	1	OsR498G0100660500.01.T01	98,66	0,00E+00	1042	Os01g0277900	100,00	0,00E+00	1074	32	NA	Japonica	
maker-1-augustus-gene-98.34-mRNA-1	1	OsR498G0100655200.01.T01	99,89	0,00E+00	1657	Os01g0276500	100,00	0,00E+00	1662	5	NA	Japonica	
maker-1-augustus-gene-98.38-mRNA-1	1	OsR498G0100655600.01.T01	99,67	0,00E+00	2743	Os01g0276700	100,00	0,00E+00	2765	22	NA	Japonica	
maker-1-augustus-gene-98.41-mRNA-1	1	OsR498G0100656000.01.T01	97,77	0,00E+00	3240	Os01g0276800	97,93	0,00E+00	3253	13	NA	Japonica	
maker-1-augustus-gene-98.47-mRNA-1	1	OsR498G0100658600.01.T01	99,76	0,00E+00	5114	Os01g0277600	100,00	0,00E+00	5146	32	NA	Japonica	
maker-1-augustus-gene-99.33-mRNA-1	1	OsR498G0100667700.01.T02	100,00	0,00E+00	780	Os01g0278900	100,00	0,00E+00	780	0	NA	Japonica	
maker-1-augustus-gene-99.37-mRNA-1	1	OsR498G0100668100.01.T01	99,92	0,00E+00	2241	Os01g0279000	100,00	0,00E+00	2154	-87	NA	Japonica	
maker-1-augustus-gene-99.40-mRNA-1	1	OsR498G0100668500.01.T01	99,92	0,00E+00	2209	Os01g0279100	100,00	0,00E+00	2214	5	NA	Japonica	
maker-1-augustus-gene-99.44-mRNA-1	1	OsR498G0100668700.01.T02	99,85	0,00E+00	2356	Os01g0279200	100,00	0,00E+00	2365	9	NA	Japonica	
augustus_masked-2-processed-gene-120.57-mRNA-1	2	OsR498G0203525000.01.T01	99,50	0,00E+00	1764	Os08g0543050	74,06	2,43E-170	598	-1166	Indica	Japonica	
augustus_masked-2-processed-gene-128.31-mRNA-1	2	OsR498G0203567200.01.T02	99,02	0,00E+00	2866	Os02g0321000	100,00	0,00E+00	2938	72	NA	Japonica	
augustus_masked-2-processed-gene-195.15-mRNA-1	2	OsR498G0203962500.01.T02	100,00	0,00E+00	1602	Os02g0523800	99,89	0,00E+00	1598	-4	NA	Japonica	
augustus_masked-2-processed-gene-258.41-mRNA-1	2	OsR498G0204358400.01.T01	99,81	0,00E+00	1849	Os01g0721800	79,43	0,00E+00	915	-934	Indica	Japonica	
augustus_masked-2-processed-gene-367.74-mRNA-1	2	OsR498G0204877800.01.T01	99,38	0,00E+00	4001	Os02g0780200	100,00	0,00E+00	4064	63	NA	Japonica	
augustus_masked-2-processed-gene-376.26-mRNA-1	2	OsR498G0204942600.01.T01	98,97	0,00E+00	686	Os02g0798450	100,00	0,00E+00	704	18	NA	Japonica	
augustus_masked-2-processed-gene-393.133-mRNA-1	2	OsR498G0205054700.01.T03	99,71	0,00E+00	1870	Os02g0832700	99,39	0,00E+00	1172	-698	Indica	Japonica	
augustus_masked-2-processed-gene-54.4-mRNA-1	2	OsR498G0203147100.01.T01	99,14	0,00E+00	2676	Os02g0194900	99,72	0,00E+00	1890	-786	Indica	Japonica	
augustus_masked-2-processed-gene-78.49-mRNA-1	2	OsR498G0203297700.01.T02	99,72	4,01E-180	631	Os02g0235600	100,00	0,00E+00	634	3	NA	Japonica	
augustus_masked-2-processed-gene-88.59-mRNA-1	2	OsR498G0203347800.01.T01	99,40	0,00E+00	1471	Os02g0255000	99,52	0,00E+00	1475	4	NA	Japonica	
augustus-2-processed-gene-0.29-mRNA-1	2	OsR498G0202798700.01.T01	100,00	0,00E+00	1164	Os02g0100600	100,00	0,00E+00	1164	0	NA	NA	
augustus-2-processed-gene-10.15-mRNA-1	2	OsR498G0202857500.01.T01	99,85	0,00E+00	3637	Os02g0119000	99,95	0,00E+00	3387	-250	NA	NA	
augustus-2-processed-gene-10.28-mRNA-1	2	OsR498G0202860400.01.T05	100,00	0,00E+00	1992	Os02g0119700	100,00	0,00E+00	1992	0	NA	NA	
augustus-2-processed-gene-101.2-mRNA-1	2	OsR498G0203400900.01.T01	98,12	0,00E+00	2010	Os02g0274700	100,00	0,00E+00	1267	-743	Indica	Japonica	
augustus-2-processed-gene-104.36-mRNA-1	2	OsR498G0203416300.01.T01	99,66	0,00E+00	1567	Os02g0280300	100,00	0,00E+00	1581	14	NA	Japonica	
augustus-2-processed-gene-108.24-mRNA-1	2	OsR498G0203432300.01.T01	99,89	0,00E+00	1679	Os02g0286200	99,89	0,00E+00	1679	0	NA	NA	
augustus-2-processed-gene-109.13-mRNA-1	2	OsR498G0203439500.01.T01	100,00	0,00E+00	2771	Os02g0288200	99,66	0,00E+00	3108	337	Japonica	NA	
augustus-2-processed-gene-109.8-mRNA-1	2	OsR498G0203438600.01.T01	100,00	0,00E+00	2289	Os02g0287900	100,00	0,00E+00	2289	0	NA	NA	
augustus-2-processed-gene-109.9-mRNA-1	2	OsR498G0203438800.01.T02	100,00	0,00E+00	2235	Os02g0288000	100,00	0,00E+00	2235	0	NA	NA	
augustus-2-processed-gene-11.65-mRNA-1	2	OsR498G0202868300.01.T04	100,00	0,00E+00	1526	Os03g0132300	77,92	0,00E+00	684	-842	Indica	NA	
augustus-2-processed-gene-11.67-mRNA-1	2	OsR498G0202869700.01.T01	100,00	0,00E+00	3382	Os02g0121900	100,00	0,00E+00	3382	0	NA	NA	
augustus-2-processed-gene-112.52-mRNA-1	2	OsR498G0203464000.01.T02	99,45	0,00E+00	2263	Os02g0294100	100,00	0,00E+00	2295	32	NA	Japonica	
augustus-2-processed-gene-114.14-mRNA-1	2	OsR498G0203485500.01.T01	95,09	0,00E+00	2996	Os02g0298200	99,93	0,00E+00	2490	-506	Indica	Japonica	Kinase-TM
augustus-2-processed-gene-114.7-mRNA-1	2	OsR498G0203489200.01.T01	72,82	0,00E+00	960	Os02g0297200	99,96	0,00E+00	4130	3170	Japonica	NA	
augustus-2-processed-gene-115.18-mRNA-1	2	OsR498G0203485500.01.T01	99,63	0,00E+00	3383	Os02g0298200	95,32	0,00E+00	2214	-1169	Indica	Japonica	Kinase-TM
augustus-2-processed-gene-115.36-mRNA-1	2	OsR498G0203489200.01.T01	99,76	0,00E+00	2942	Os02g0297200	72,70	0,00E+00	951	-1991	Indica	Japonica	
augustus-2-processed-gene-117.13-mRNA-1	2	OsR498G0203521400.01.T03	70,74	1,47E-20	101	Os02g0302400	100,00	7,54E-152	535	434	Japonica	Japonica	
augustus-2-processed-gene-118.30-mRNA-1	2	OsR498G0203504400.01.T01	97,26	0,00E+00	1104	Os02g0303500	100,00	0,00E+00	1186	82	NA	Japonica	
augustus-2-processed-gene-119.16-mRNA-1	2	OsR498G0203508300.01.T04	99,75	0,00E+00	2913	Os02g0304800	100,00	0,00E+00	2809	-104	NA	Japonica	
augustus-2-processed-gene-121.20-mRNA-1	2	OsR498G0203527800.01.T01	99,75	0,00E+00	1447	Os02g0308400	70,77	3,14E-60	233	-1214	Indica	Japonica	
augustus-2-processed-gene-121.9-mRNA-1	2	OsR498G0203527200.01.T01	99,88	0,00E+00	1446	Os02g0308400	100,00	0,00E+00	1451	5	NA	Japonica	
augustus-2-processed-gene-124.9-mRNA-1	2	OsR498G0203543900.01.T09	99,50	0,00E+00	1069	Os02g0314100	99,83	0,00E+00	1078	9	NA	Japonica	
augustus-2-processed-gene-125.1-mRNA-1	2	OsR498G0203547100.01.T01	99,81	0,00E+00	959	Os02g0315400	100,00	0,00E+00	964	5	NA	Japonica	
augustus-2-processed-gene-126.2-mRNA-1	2	OsR498G0203551700.01.T01	99,92	0,00E+00	2263	Os02g0317200	100,00	0,00E+00	2210	-53	NA	Japonica	
augustus-2-processed-gene-126.23-mRNA-1	2	OsR498G0203556600.01.T01	98,76	0,00E+00	2260	Os02g0318200	99,92	0,00E+00	2301	41	NA	Japonica	
augustus-2-processed-gene-126.25-mRNA-1	2	OsR498G0203558200.01.T01	100,00	0,00E+00	901	Os02g0318400	100,00	0,00E+00	948	47	NA	Japonica	
augustus-2-processed-gene-126.8-mRNA-1	2	OsR498G0203552200.01.T01	99,90	0,00E+00	1776	Os02g0317300	100,00	0,00E+00	1781	5	NA	Japonica	
augustus-2-processed-gene-128.15-mRNA-1	2	OsR498G0203567500.01.T01	99,73	0,00E+00	674	Os02g0321600	100,00	0,00E+00	679	5	NA	Japonica	
augustus-2-processed-gene-130.30-mRNA-1	2	OsR498G0203578500.01.T01	99,50	0,00E+00	706	Os02g0324400	100,00	0,00E+00	715	9	NA	Japonica	
augustus-2-processed-gene-130.43-mRNA-1	2	OsR498G0203579600.01.T01	99,65	3,42E-144	512	Os02g0324500	100,00	0,00E+00	791	279	Japonica	Japonica	
augustus-2-processed-gene-132.17-mRNA-1	2	OsR498G0203595100.01.T01	99,92	0,00E+00	2377	Os02g0327500	100,00	0,00E+00	2381	4	NA	Japonica	
augustus-2-processed-gene-136.12-mRNA-1	2	OsR498G0714227900.01.T02	92,96	4,77E-108	391	Os02g0332700	100,00	3,92E-91	333	-58	Indica	Japonica	
augustus-2-processed-gene-14.10-mRNA-1	2	OsR498G0202882900.01.T04	100,00	0,00E+00	2316	Os02g0126500	100,00	0,00E+00	2316	0	NA	NA	
augustus-2-processed-gene-143.19-mRNA-1	2	OsR498G0203652100.01.T01	99,62	0,00E+00	2359	Os02g0439700	100,00	0,00E+00	2392	33	NA	Japonica	

augustus-2-processed-gene-146.33-mRNA-1	2	OsR498G1018968000.01.T01	92,56	1,83E-97	356	Os02g0701833	96,69	1,71E-111	401	45	Japonica	Japonica	
augustus-2-processed-gene-147.12-mRNA-1	2	OsR498G0203675100.01.T01	99,79	0,00E+00	1707	Os02g0448000	100,00	0,00E+00	1716	9	NA	Japonica	
augustus-2-processed-gene-149.12-mRNA-1	2	OsR498G0203686900.01.T01	99,88	0,00E+00	4375	Os02g0450000	100,00	0,00E+00	4388	13	NA	Japonica	
augustus-2-processed-gene-15.53-mRNA-1	2	OsR498G0202895300.01.T05	97,93	0,00E+00	2252	Os02g0129600	97,63	0,00E+00	1957	-295	Indica	NA	
augustus-2-processed-gene-153.1-mRNA-1	2	OsR498G0203714300.01.T01	100,00	0,00E+00	1911	Os02g0454500	100,00	0,00E+00	1911	0	NA	Japonica	
augustus-2-processed-gene-153.20-mRNA-1	2	OsR498G0203719700.01.T01	99,55	0,00E+00	1181	Os02g0455400	100,00	0,00E+00	1191	10	NA	Japonica	
augustus-2-processed-gene-154.0-mRNA-1	2	OsR498G0203720900.01.T01	99,38	0,00E+00	1428	Os02g0455800	100,00	0,00E+00	1451	23	NA	Japonica	
augustus-2-processed-gene-154.19-mRNA-1	2	OsR498G0203724300.01.T01	99,82	0,00E+00	5871	Os02g0456800	100,00	0,00E+00	5898	27	NA	Japonica	CC-LRR-NBS-TM
augustus-2-processed-gene-155.18-mRNA-1	2	OsR498G0613026600.01.T01	72,53	9,47E-51	202	Os02g0458900	100,00	0,00E+00	915	713	Japonica	Japonica	
augustus-2-processed-gene-156.10-mRNA-1	2	OsR498G0203737300.01.T03	99,33	0,00E+00	3713	Os02g0459600	100,00	0,00E+00	3804	91	NA	Japonica	Kinase-TM
augustus-2-processed-gene-156.32-mRNA-1	2	OsR498G0203738600.01.T01	99,46	0,00E+00	657	Os02g0460200	100,00	0,00E+00	666	9	NA	Japonica	
augustus-2-processed-gene-157.18-mRNA-1	2	OsR498G0203744600.01.T01	99,89	0,00E+00	1646	Os02g0461500	100,00	0,00E+00	1651	5	NA	Japonica	
augustus-2-processed-gene-157.35-mRNA-1	2	OsR498G0203746200.01.T01	99,77	0,00E+00	2351	Os02g0462200	100,00	0,00E+00	2365	14	NA	Japonica	
augustus-2-processed-gene-158.28-mRNA-1	2	OsR498G0203753500.01.T01	100,00	0,00E+00	645	Os12g0506100	72,80	5,31E-34	143	-502	Indica	NA	
augustus-2-processed-gene-158.36-mRNA-1	2	OsR498G0203750400.01.T01	99,86	0,00E+00	3960	Os02g0463700	99,29	0,00E+00	5494	1534	Japonica	NA	
augustus-2-processed-gene-159.16-mRNA-1	2	OsR498G0203760300.01.T01	100,00	0,00E+00	1927	Os02g0466400	100,00	0,00E+00	2116	189	NA	NA	
augustus-2-processed-gene-159.27-mRNA-1	2	OsR498G0203757400.01.T01	100,00	0,00E+00	2176	Os02g0465600	100,00	0,00E+00	2176	0	NA	NA	
augustus-2-processed-gene-16.0-mRNA-1	2	OsR498G0202897300.01.T01	97,78	0,00E+00	1625	Os02g0129900	95,56	0,00E+00	1549	-76	NA	NA	
augustus-2-processed-gene-16.14-mRNA-1	2	OsR498G0202903900.01.T01	100,00	7,17E-131	466	Os02g0131600	100,00	2,51E-131	466	0	NA	NA	
augustus-2-processed-gene-16.23-mRNA-1	2	OsR498G0202901600.01.T01	100,00	0,00E+00	764	Os02g0131000	100,00	0,00E+00	764	0	NA	NA	
augustus-2-processed-gene-160.16-mRNA-1	2	OsR498G0203768900.01.T01	100,00	0,00E+00	1570	Os02g0468500	100,00	0,00E+00	2744	1174	Japonica	NA	
augustus-2-processed-gene-161.34-mRNA-1	2	OsR498G0203772700.01.T01	100,00	0,00E+00	1364	Os02g0469000	100,00	0,00E+00	1333	-31	NA	NA	
augustus-2-processed-gene-161.38-mRNA-1	2	OsR498G0203772600.01.T01	100,00	0,00E+00	3268	Os02g0468800	100,00	0,00E+00	3268	0	NA	NA	
augustus-2-processed-gene-161.48-mRNA-1	2	OsR498G0203776900.01.T02	100,00	0,00E+00	1180	Os02g0469900	100,00	0,00E+00	1180	0	NA	Japonica	
augustus-2-processed-gene-161.55-mRNA-1	2	OsR498G0203777200.01.T01	99,69	0,00E+00	3490	Os02g0470000	70,03	2,06E-130	467	-3023	Indica	Japonica	
augustus-2-processed-gene-166.8-mRNA-1	2	OsR498G0816166700.01.T02	90,43	0,00E+00	854	Os02g0477401	100,00	0,00E+00	1359	505	Japonica	Japonica	
augustus-2-processed-gene-167.54-mRNA-1	2	OsR498G0202966300.01.T03	72,57	9,18E-65	248	Os02g0478900	96,89	0,00E+00	1076	828	Japonica	Indica	
augustus-2-processed-gene-167.55-mRNA-1	2	OsR498G0203805900.01.T02	100,00	0,00E+00	5449	Os02g0478900	99,75	0,00E+00	4996	-453	NA	Indica	
augustus-2-processed-gene-169.3-mRNA-1	2	OsR498G0917892000.01.T01	70,35	3,65E-82	306	Os02g0482400	99,16	0,00E+00	1055	749	Japonica	Indica	
augustus-2-processed-gene-17.56-mRNA-1	2	OsR498G0202907000.01.T03	100,00	0,00E+00	888	Os02g0132500	100,00	0,00E+00	888	0	NA	NA	
augustus-2-processed-gene-170.0-mRNA-1	2	OsR498G0203823300.01.T01	99,78	0,00E+00	2460	Os02g0484600	99,20	0,00E+00	2649	189	NA	Indica	
augustus-2-processed-gene-170.23-mRNA-1	2	OsR498G0203826500.01.T01	99,78	0,00E+00	2492	Os02g0485800	96,72	0,00E+00	2349	-143	NA	Indica	
augustus-2-processed-gene-172.16-mRNA-1	2	OsR498G0203832900.01.T01	99,87	0,00E+00	1430	Os02g0225200	89,08	0,00E+00	922	-508	Indica	Indica	
augustus-2-processed-gene-173.18-mRNA-1	2	OsR498G0203838700.01.T01	99,79	0,00E+00	1745	Os02g0489500	99,28	0,00E+00	1722	-23	NA	NA	
augustus-2-processed-gene-174.15-mRNA-1	2	OsR498G0203846900.01.T01	99,72	0,00E+00	3138	Os02g0491300	100,00	0,00E+00	3160	22	NA	Japonica	
augustus-2-processed-gene-174.17-mRNA-1	2	OsR498G0203848300.01.T01	98,51	0,00E+00	1168	Os02g0491800	100,00	0,00E+00	1213	45	NA	Japonica	
augustus-2-processed-gene-18.61-mRNA-1	2	OsR498G0202913200.01.T01	100,00	0,00E+00	1386	Os02g0134200	100,00	0,00E+00	1386	0	NA	NA	
augustus-2-processed-gene-180.12-mRNA-1	2	OsR498G0203880700.01.T01	98,35	0,00E+00	3664	Os02g0502500	100,00	0,00E+00	3822	158	NA	Japonica	
augustus-2-processed-gene-181.13-mRNA-1	2	OsR498G0203881700.01.T01	100,00	2,83E-150	531	Os02g0503300	100,00	1,28E-174	610	79	Japonica	Indica	
augustus-2-processed-gene-184.15-mRNA-1	2	OsR498G0203894500.01.T02	84,30	2,15E-120	432	Os02g0506600	100,00	0,00E+00	655	223	Japonica	Japonica	
augustus-2-processed-gene-185.18-mRNA-1	2	OsR498G0203900100.01.T01	98,64	0,00E+00	1407	Os02g0507600	100,00	0,00E+00	1456	49	NA	Japonica	
augustus-2-processed-gene-185.27-mRNA-1	2	OsR498G1018954900.01.T01	70,21	7,92E-35	150	Os02g0507900	99,60	0,00E+00	1349	1199	Japonica	NA	
augustus-2-processed-gene-185.36-mRNA-1	2	OsR498G0203902900.01.T01	99,91	0,00E+00	3968	Os02g0510000	98,28	0,00E+00	3806	-162	NA	Indica	
augustus-2-processed-gene-185.37-mRNA-1	2	OsR498G0203903500.01.T02	99,72	0,00E+00	2596	Os02g0510200	98,40	0,00E+00	3351	755	Japonica	Indica	
augustus-2-processed-gene-185.38-mRNA-1	2	OsR498G0203903900.01.T01	100,00	0,00E+00	1775	Os02g0510600	97,37	0,00E+00	1666	-109	NA	Indica	
augustus-2-processed-gene-185.55-mRNA-1	2	OsR498G1019116500.01.T01	86,55	8,78E-139	494	Os02g0212975	84,64	4,84E-124	443	-51	Indica	Japonica	
augustus-2-processed-gene-187.10-mRNA-1	2	OsR498G0203905300.01.T04	99,85	0,00E+00	1214	Os02g0511400	99,40	0,00E+00	1788	574	Japonica	Indica	
augustus-2-processed-gene-187.9-mRNA-1	2	OsR498G0203905300.01.T03	100,00	2,03E-92	338	Os02g0511400	100,00	7,08E-93	338	0	NA	Indica	
augustus-2-processed-gene-19.45-mRNA-1	2	OsR498G0202919300.01.T04	100,00	0,00E+00	1656	Os02g0135800	100,00	0,00E+00	1656	0	NA	NA	
augustus-2-processed-gene-19.46-mRNA-1	2	OsR498G0202919500.01.T01	100,00	0,00E+00	839	Os09g0125100	92,17	6,55E-180	628	-211	Indica	NA	
augustus-2-processed-gene-19.68-mRNA-1	2	OsR498G0202924400.01.T04	100,00	0,00E+00	1905	Os02g0136800	100,00	0,00E+00	1905	0	NA	NA	
augustus-2-processed-gene-190.44-mRNA-1	2	OsR498G0203926000.01.T02	100,00	0,00E+00	1462	Os02g0516400	98,18	0,00E+00	1425	-37	NA	Indica	
augustus-2-processed-gene-192.13-mRNA-1	2	OsR498G0203943500.01.T02	100,00	2,37E-150	531	Os02g0302500	78,62	1,01E-73	275	-256	Indica	Indica	
augustus-2-processed-gene-192.20-mRNA-1	2	OsR498G0203938000.01.T01	100,00	0,00E+00	1018	Os02g0517700	99,29	0,00E+00	1000	-18	NA	Indica	
augustus-2-processed-gene-192.23-mRNA-1	2	OsR498G0203939200.01.T01	99,71	0,00E+00	1848	Os02g0518000	99,90	0,00E+00	1857	9	NA	Indica	
augustus-2-processed-gene-196.28-mRNA-1	2	OsR498G0203968000.01.T02	99,11	0,00E+00	2184	Os02g0524600	98,84	0,00E+00	2269	85	NA	NA	
augustus-2-processed-gene-196.31-mRNA-1	2	OsR498G0203968800.01.T01	100,00	3,25E-168	590	Os02g0525100	100,00	1,13E-168	590	0	NA	NA	
augustus-2-processed-gene-197.24-mRNA-1	2	OsR498G0203974100.01.T01	99,50	0,00E+00	2127	Os02g0526600	99,57	0,00E+00	2072	-55	NA	NA	
augustus-2-processed-gene-197.27-mRNA-1	2	OsR498G0203975000.01.T01	99,40	0,00E+00	2381	Os02g0526700	99,40	0,00E+00	2381	0	NA	NA	
augustus-2-processed-gene-198.0-mRNA-1	2	OsR498G0203980700.01.T01	99,36	0,00E+00	2789	Os02g0527900	99,36	0,00E+00	2782	-7	NA	NA	
augustus-2-processed-gene-199.33-mRNA-1	2	OsR498G0203986200.01.T04	99,52	0,00E+00	2575	Os02g0529600	99,52	0,00E+00	2575	0	NA	NA	
augustus-2-processed-gene-199.39-mRNA-1	2	OsR498G0408521900.01.T01	79,91	3,05E-47	188	Os02g0530500	100,00	6,28E-133	471	283	Japonica	NA	
augustus-2-processed-gene-2.18-mRNA-1	2	OsR498G0202808500.01.T03	99,03	1,40E-153	543	Os10g0536100	85,71	8,29E-50	196	-347	Indica	Japonica	
augustus-2-processed-gene-2.4-mRNA-1	2	OsR498G0202807000.01.T01	99,03	0,00E+00	2000	Os02g0103500	100,00	0,00E+00	1608	-392	Indica	Japonica	
augustus-2-processed-gene-20.13-mRNA-1	2	OsR498G0202926700.01.T01	100,00	0,00E+00	2365	Os02g0137600	100,00	0,00E+00	2365	0	NA	NA	
augustus-2-processed-gene-20.27-mRNA-1	2	OsR498G0202928200.01.T01	100,00	0,00E+00	3761	Os02g0138000	100,00	0,00E+00	3761	0	NA	Japonica	LRR-TM
augustus-2-processed-gene-201.47-mRNA-1	2	OsR498G0204000400.01.T01	100,00	0,00E+00	948	Os02g0534000	100,00	0,00E+00	1029	81	NA	NA	
augustus-2-processed-gene-202.13-mRNA-1	2	OsR498G0204010100.01.T06	100,00	0,00E+00	3555	Os02g0536400	99,95	0,00E+00	3551	-4	NA	NA	
augustus-2-processed-gene-204.31-mRNA-1	2	OsR498G0204021400.01.T01	99,67	1,15E-154	546	Os05g0578100	88,42	6,36E-102	369	-177	Indica	NA	
augustus-2-processed-gene-204.42-mRNA-1	2	OsR498G0204025500.01.T01	100,00	0,00E+00	1471	Os02g0539500	100,00	0,00E+00	1494	23	NA	NA	
augustus-2-processed-gene-204.58-mRNA-1	2	OsR498G0204023600.01.T01	100,00	0,00E+00	2397	Os02g0539200	100,00	0,00E+00	2397	0	NA	NA	
augustus-2-processed-gene-205.10-mRNA-1	2	OsR498G0408554900.01.T01	79,06	0,00E+00	1155	Os02g0540700	99,93	0,00E+00	2734	1579	Japonica	NA	
augustus-2-processed-gene-206.18-mRNA-1	2	OsR498G0204039900.01.T01	99,92	0,00E+00	2220	Os02g0542400	99,92	0,00E+00	2220	0	NA	NA	
augustus-2-processed-gene-206.41-mRNA-1	2	OsR498G0204043600.01.T01	100,00	0,00E+00	3377	Os02g0543300	100,00	0,00E+00	5443	2066	Japonica	NA	
augustus-2-processed-gene-21.0-mRNA-1	2	OsR498G0202932100.01.T01	98,14	0,00E+00	1933	Os02g0138600	100,00	0,00E+00	2035	102	NA	Japonica	
augustus-2-processed-gene-21.8-mRNA-1	2	OsR498G0202933600.01.T01	99,84	0,00E+00	1095	Os02g0139100	100,00	0,00E+00	1099	4	NA	Japonica	
augustus-2-processed-gene-21.9-mRNA-1	2	OsR498G0202933800.01.T02	99,89	0,00E+00	3249	Os02g0139200	100,00	0,00E+00	3258	9	NA	Japonica	
augustus-2-processed-gene-210.25-mRNA-1	2	OsR498G0204080800.01.T01	99,84	0,00E+00	2253	Os02g0548700	100,00	0,00E+00	2262	9	NA	Japonica	
augustus-2-processed-gene-212.41-mRNA-1	2	OsR498G0204094500.01.T01	99,69	0,00E+00	1166	Os02g0551600	100,00	0,00E+00	1175	9	NA	Japonica	
augustus-2-processed-gene-212.53-mRNA-1	2	OsR498G0204097400.01.T01	99,90	0,00E+00	1836	Os02g0552200	89,90	0,00E+00	1402	-434	Indica	NA	
augustus-2-processed-gene-213.25-mRNA-1	2	OsR498G0204104000.01.T02	99,48	0,00E+00	3405	Os02g0554300	99,76	0,00E+00	2988	-417	Indica	Japonica	
augustus-2-processed-gene-213.6-mRNA-1	2	OsR498G0204100400.01.T01	99,85	0,00E+00	1192	Os01g0296000	74,25	1,19E-96	353	-839	Indica	Japonica	Kinase-LRR-TM
augustus-2-processed-gene-214.3-mRNA-1	2	OsR498G0204106300.01.T01	95,78	0,00E+00	1159	Os02g0554800	100,00	0,00E+00	1267	108	NA	Japonica	
augustus-2-processed-gene-215.16-mRNA-1	2	OsR498G0204111500.01.T01	99,06	0,00E+00	1877	Os02g0557000	100,00	0,00E+00	1023	-854	Indica	Japonica	
augustus-2-processed-gene-216.37-mRNA-1	2	OsR498G0204118400.01.T01	100,00	0,00E+00	926	Os04g0549700	72,17	2,60E-40	165	-761	Indica	Japonica	
augustus-2-processed-gene-217.25-mRNA-1	2	OsR498G0204121600.01.T01	97,87	0,00E+00	1689	Os02g0559800	99,90	0,00E+00	1760	71	NA	Japonica	
augustus-2-processed-gene-217.31-mRNA-1	2	OsR498G0204123300.01.T02	99,64	0,00E+00	1489	Os02g0562100	100,00	0,00E+00	1575	86	NA	Japonica	
augustus-2-processed-gene-217.51-mRNA-1	2	OsR498G0204127000.01.T01	88,85	0,00E+00	1656	Os02g0563301	89,13	0,00E+00	1658	2	NA	Japonica	
augustus-2-processed-gene-218.55-mRNA-1	2	OsR498G0204131600.01.T01	99,68	0,00E+00	2253	Os02g0565000	99,92	0,00E+00	2269	16	NA	Japonica	
augustus-2-processed-gene-218.61-mRNA-1	2	OsR498G0204133200.01.T01	99,82	0,00E+00	1961	Os02g0565500	100,00	0,00E+00	1970	9	NA	Japonica	Kinase-TM
augustus-2-processed-gene-22.0-mRNA-1	2	OsR498G0202938500.01.T01	99,49	0,00E+00	1037	Os02g0140300	100,00	0,00E+00	1050	13	NA	Japonica	
augustus-2-processed-gene-22.18-mRNA-1	2	OsR498G0202944000.01.T01	99,70	0,00E+00	1792	Os02g0141400	99,92	0,00E+00	2306	514	Japonica	Japonica	
augustus-2-processed-gene-22.6-mRNA-1	2	OsR498G0202940300.01.T01	98,95	0,00E+00	1838	Os02g0140800	96,48	0,00E+00	1805	-33	NA	Japonica	
augustus-2-processed-gene-220.14-mRNA-1	2	OsR498G0204143300.01.T01	99,07	0,00E+00	2654	Os02g0569000	100,00	0,00E+00	2717	63	NA	Japonica	
augustus-2-processed-gene-221.21-mRNA-1	2	OsR498G0204146300.01.T01	99,73	0,00E+00	2641	Os02g0569900	99,93	0,00E+00	2701	60	NA	Japonica	
augustus-2-processed-gene-221.8-mRNA-1	2	OsR498G0204145300.01.T01	98,34	0,00E+00	2599	Os02g0569400	100,00	0,00E+00	2711	112	NA	Japonica	
augustus-2-processed-gene-222.18-mRNA-1	2	OsR498G0204151800.01.T05	95,56	0,00E+00	2309	Os02g0571300	95,77	0,00E+00	2323	14	NA	NA	
augustus-2-processed-gene-223.24-mRNA-1	2	OsR498G0204154400.01.T01	98,83	0,00E+00	2840	Os02g0571900	97,98	0,00E+00	2628	-212	NA	Japonica	
augustus-2-processed-gene-223.38-mRNA-1	2	OsR498G0204155600.01.T01	100,00	0,00E+00	2003	Os02g0572350	97,71	0,00E+00	1561	-442	Indica	Japonica	
augustus-2-processed-gene-224.15-mRNA-1	2	OsR498G0204164900.01.T01	99,86	0,00E+00	2486	Os02g0574900	100,00	0,00E+00	2495	9	NA	Japonica	
augustus-2-processed-gene-225.20-mRNA-1	2	OsR498G0204166900.01.T01	98,99	0,00E+00	3496	Os02g0575700	100,00	0,00E+00	3550	54	NA	Japonica	
augustus-2-processed-gene-225.22-mRNA-1	2	OsR498G0204167700.01.T01	99,49	0,00E+00	1388	Os02g0575900	99,87	0,00E+00	1401	13	NA	Japonica	
augustus-2-processed-gene-227.49-mRNA-1	2	OsR498G0408710600.01.T03	85,44	0,00E+00	947	Os02g0580500	99,76	0,00E+00	1517	570	Japonica	Japonica	
augustus-2-processed-gene-229.23-mRNA-1	2	OsR498G0204189200.01.T01	99,81	0,00E+00	945	Os02g0582800	100,00	0,00E+00	949	4	NA	Japonica	
augustus-2-processed-gene-229.46-mRNA-1	2	OsR498G0204171500.01.T01	79,78	1,17E-66	253	Os03g0721001	96,92	1,02E-169	595	342	Japonica	NA	
augustus-2-processed-gene-229.53-mRNA-1	2	OsR498G0204193200.01.T01	97,49	0,00E+00	677	Os02g0585200	100,00	0,00E+00	719	42	NA	Japonica	
augustus-2-processed-gene-23.24-mRNA-1	2	OsR498G0202945800.01.T01	99,61	0,00E+00	2283	Os02g0144800	70,36	2,27E-121	435	-1848	Indica	Japonica	
augustus-2-processed-gene-23.31-mRNA-1	2	OsR498G0815259700.01.T01	84,53	0,00E+00	699	Os02g0142800	99,81	0,00E+00	1874	1175	Japonica	NA	
augustus-2-processed-gene-230.0-mRNA-1	2	OsR498G0204196300.01.T01	99,84	0,00E+00	1138	Os02g0586400	100,00	0,00E+00	1142	4	NA	Japonica	
augustus-2-processed-gene-231.22-mRNA-1	2	OsR498G0204208400.01.T01	100,00	0,00E+00	2338	Os02g0589400	99,75	0,00E+00	2122	-216	NA	NA	
augustus-2-processed-gene-232.18-mRNA-1	2	OsR498G0204209400.01.T01	83,94	0,00E+00	919	Os02g0590250	100,00	0,00E+00	1543	624	Japonica	NA	
augustus-2-processed-gene-233.64-mRNA-1	2	OsR498G0204219300.01.T08	100,00	0,00E+00	4150	Os02g0592600	100,00	0,00E+00	4150	0	NA	NA	
augustus-2-processed-gene-235.10-mRNA-1	2	OsR498G0204234900.01.T01	99,64	0,00E+00	2510	Os02g0595700	100,00	0,00E+00	2533	23	NA	Japonica	
augustus-2-processed-gene-235.12-mRNA-1	2	OsR498G0204236900.01.T01	99,92	0,00E+00	2137	Os02g0596200	99,91	0,00E+00	1928	-209	NA	Japonica	
augustus-2-processed-gene-235.13-mRNA-1	2	OsR498G0204236900.01.T01	99,12	0,00E+00	997	Os02g0596200	99,66	1,06E-146	519	-478	Indica	Japonica	
augustus-2-processed-gene-235.26-mRNA-1	2	OsR498G0204237200.01.T02	99,39	0,00E+00	2927	Os02g0596300	99,58	0,00E+00	2104	-823	Indica	NA	
augustus-2-processed-gene-235.4-mRNA-1	2	OsR498G0204234200.01.T01	99,67	0,00E+00	1090	Os02g0595400	100,00	0,00E+00	1099	9	NA	Japonica	
augustus-2-processed-gene-236.44-mRNA-1	2	OsR498G0204244400.01.T01	99,74	0,00E+00	2032	Os02g0598600	99,91	0,00E+00	2041	9	NA	NA	
augustus-2-processed-gene-237.21-mRNA-1	2	OsR498G0204251700.01.T01	99,73	0,00E+00	2693	Os02g0601600	99,85	0,00E+00	2415	-278	Indica	NA	
augustus-2-processed-gene-238.18-mRNA-1	2	OsR498G0204259500.01.T01	100,00	0,00E+00	704	Os02g0604100	100,00	0,00E+00	704	0	NA	Japonica	
augustus-2-processed-gene-238.26-mRNA-1	2	OsR498G0204255800.01.T01	99,63	0,00E+00	2402	Os02g0603000	99,78	0,00E+00	2411	9	NA	Japonica	Kinase-TM
augustus-2-processed-gene-238.27-mRNA-1	2	OsR498G0204256200.01.T01	99,79	0,00E+00	3337	Os02g0603100	100,00	0,00E+00	3158	-179	NA	Japonica	LRR-TM
augustus-2-processed-gene-238.31-mRNA-1	2	OsR498G0204259400.01.T01	98,03	0,00E+00	1316	Os02g0604000	100,00	0,00E+00	2203	887	Japonica	Japonica	
augustus-2-processed-gene-238.9-mRNA-1	2	OsR498G0204253800.01.T01	99,89	0,00E+00	1684	Os02g0602500	100,00	0,00E+00	1104	-580	Indica	Japonica	
augustus-2-processed-gene-239.59-mRNA-1	2	OsR498G0204264300.01.T01	98,87	0,00E+00	1397	Os02g0605000	100,00	0,00E+00	2419	1022	Japonica	Japonica	
augustus-2-processed-gene-239.61-mRNA-1	2	OsR498G0204265100.01.T04	99,78	0,00E+00	1658	Os02g0605400	94,89	0,00E+00	1891	233	Japonica	Japonica	
augustus-2-processed-gene-24.18-mRNA-1	2	OsR498G0202957700.01.T01	99,81	0,00E+00	1934	Os02g0144200	99,72	0,00E+00	1929	-5	NA	NA	
augustus-2-processed-gene-24.2-mRNA-1	2	OsR498G0202945800.01.T01	90,99	0,00E+00	1789	Os02g0144800	70,49	2,15E-134	479	-1310	Indica	NA	
augustus-2-processed-gene-24.8-mRNA-1	2	OsR498G0202954000.01.T01	99,75	0,00E+00	705	Os02g0143400	99,49	0,00E+00	700	-5	NA	Indica	
augustus-2-processed-gene-240.35-mRNA-1	2	OsR498G0204270600.01.T03	99,76	0,00E+00	1508	Os02g0606900	100,00	0,00E+00	1063	-445	Indica	Japonica	
augustus-2-processed-gene-240.36-mRNA-1	2	OsR498G0204270800.01.T01	99,75	0,00E+00	1431	Os02g0605000	95,67	0,00E+00	1448	17	NA	Japonica	
augustus-2-processed-gene-241.31-mRNA-1	2	OsR498G0204275900.01.T01	99,52	0,00E+00	2577	Os02g0608600	99,80	0,00E+00	1831	-746	Indica	Japonica	
augustus-2-processed-gene-241.38-mRNA-1	2	OsR498G0204276600.01.T01	99,92	0,00E+00	2182	Os02g0608801	100,00	0,00E+00	2186	4	NA	Japonica	
augustus-2-processed-gene-244.8-mRNA-1	2	OsR498G0204296100.01.T03	99,84	0,00E+00	1105	Os02g0613900	100,00	0,00E+00	919	-186	Indica	Japonica	
augustus-2-processed-gene-245.29-mRNA-1	2	OsR498G0204300200.01.T01	100,00	0,00E+00	3773	Os06g0272601	97,80	0,00E+00	3865	92	NA	Indica	
augustus-2-processed-gene-247.15-mRNA-1	2	OsR498G0204309700.01.T01	100,00	0,00E+00	2571	Os02g0615300	85,43	0,00E+00	1525	-1046	Indica	Indica	LRR-TM
augustus-2-processed-gene-25.11-mRNA-1	2	OsR498G0202960700.01.T01	99,91	0,00E+00	1944	Os02g0144800	99,82	0,00E+00	1939	-5	NA	NA	
augustus-2-processed-gene-252.14-mRNA-1	2	OsR498G0204322200.01.T01	99,93	0,00E+00	2660	Os02g0616800	99,30	0,00E+00	2031	-629	Indica	NA	
augustus-2-processed-gene-254.39-mRNA-1	2	OsR498G0204334300.01.T01	99,93	0,00E+00	2696	Os02g0620500	100,00	0,00E+00	2700	4	NA	Japonica	
augustus-2-processed-gene-254.41-mRNA-1	2	OsR498G0204334900.01.T01	99,80	0,00E+00	2676	Os02g0620600	100,00	0,00E+00	2690	14	NA	Japonica	
augustus-2-processed-gene-254.53-mRNA-1	2	OsR498G0204335200.01.T04	99,69	0,00E+00	2851	Os02g0620800	99,94	0,00E+00	2869	18	NA	Japonica	
augustus-2-processed-gene-255.22-mRNA-1	2	OsR498G0204339200.01.T03	99,89	0,00E+00	1603	Os02g0622200	100,00	0,00E+00	1608	5	NA	NA	
augustus-2-processed-gene-255.5-mRNA-1	2	OsR498G0204338000.01.T02	98,63	0,00E+00	636	Os02g0621800	100,00	5,04E-142	502	-134	Indica	Japonica	
augustus-2-processed-gene-256.29-mRNA-1	2	OsR498G0204348500.01.T01	92,14	1,12E-155	549	Os10g0460000	88,89	1,77E-122	438	-111	Indica	Japonica	
augustus-2-processed-gene-256.35-mRNA-1	2	OsR498G0204350100.01.T01	99,81	0,00E+00	1885	Os02g0625100	100,00	0,00E+00	2673	788	Japonica	Japonica	
augustus-2-processed-gene-257.1-mRNA-1	2	OsR498G0204350900.01.T01	100,00	0,00E+00	1559	Os02g0625300	100,00	0,00E+00	1867	308	Japonica	Japonica	CC-Kinase-TM
augustus-2-processed-gene-257.31-mRNA-1	2	OsR498G0204355400.01.T01	89,77	0,00E+00	2873	Os02g0626600	100,00	0,00E+00	3864	991	Japonica	Japonica	
augustus-2-processed-gene-259.14-mRNA-1	2	OsR498G0204366500.01.T01	99,75	0,00E+00	2168	Os02g0630000	70,75	5,07E-104	378	-1790	Indica	NA	
augustus-2-processed-gene-259.22-mRNA-1	2	OsR498G0204363000.01.T01	99,81	0,00E+00	921	Os02g0629000	100,00	0,00E+00	926	5	NA	Japonica	
augustus-2-processed-gene-259.25-mRNA-1	2	OsR498G0204364800.01.T01	99,97	0,00E+00	5693	Os02g0629400	99,94	0,00E+00	5689	-4	NA	Japonica	Kinase-LRR-TM
augustus-2-processed-gene-259.26-mRNA-1	2	OsR498G0204365600.01.T01	97,48	0,00E+00	664	Os08g0114000	96,52	0,00E+00	709	45	NA	Japonica	
augustus-2-processed-gene-259.5-mRNA-1	2	OsR498G0204362400.01.T02	99,77	0,00E+00	786	Os02g0628800	100,00	1,55E-111	401	-385	Indica	NA	
augustus-2-processed-gene-26.11-mRNA-1	2	OsR498G0202966300.01.T03	100,00	0,00E+00	6947	Os02g0147300	98,32	0,00E+00	4105	-2842	Indica	Indica	
augustus-2-processed-gene-26.25-mRNA-1	2	OsR498G0202967600.01.T01	99,73	0,00E+00	1987	Os02g0147600	99,01	0,00E+00	1963	-24	NA	Indica	
augustus-2-processed-gene-260.21-mRNA-1	2	OsR498G0204370100.01.T03	99,72	0,00E+00	645	Os02g0631100	100,00	0,00E+00	650	5	NA	Japonica	
augustus-2-processed-gene-261.6-mRNA-1	2	OsR498G0204378000.01.T02	92,16	0,00E+00	1813	Os02g0632800	92,81	0,00E+00	1749	-64	NA	NA	Kinase-TM WAK14
augustus-2-processed-gene-262.64-mRNA-1	2	OsR498G0204382100.01.T04	99,78	0,00E+00	1609	Os02g0634500	100,00	0,00E+00	1618	9	NA	Japonica	
augustus-2-processed-gene-263.17-mRNA-1	2	OsR498G0204386000.01.T01	99,71	0,00E+00	3084	Os02g0636300	100,00	0,00E+00	3106	22	NA	Japonica	
augustus-2-processed-gene-264.29-mRNA-1	2	OsR498G0204397200.01.T01	99,35	0,00E+00	1629	Os02g0639800	99,88	0,00E+00	1549	-80	NA	Japonica	
augustus-2-processed-gene-264.4-mRNA-1	2	OsR498G0204392200.01.T03	100,00	0,00E+00	812	Os02g0638300	100,00	0,00E+00	812	0	NA	Japonica	
augustus-2-processed-gene-264.6-mRNA-1	2	OsR498G0204392800.01.T01	99,16	0,00E+00	2512	Os02g0638650	100,00	0,00E+00	2565	53	NA	Japonica	
augustus-2-processed-gene-265.32-mRNA-1	2	OsR498G0204397600.01.T01	99,81	0,00E+00	938	Os02g0639900	100,00	0,00E+00	942	4	NA	Japonica	
augustus-2-processed-gene-265.40-mRNA-1	2	OsR498G0204400100.01.T01	99,55	0,00E+00	2799	Os02g0640700	100,00	0,00E+00	2830	31	NA	Japonica	
augustus-2-processed-gene-265.58-mRNA-1	2	OsR498G0204399600.01.T01	99,80	0,00E+00	3635	Os02g0640500	100,00	0,00E+00	3653	18	NA	Japonica	Kinase-TM
augustus-2-processed-gene-266.17-mRNA-1	2	OsR498G0204410500.01.T01	99,61	0,00E+00	919	Os02g0643800	100,00	0,00E+00	1034	115	Japonica	Japonica	
augustus-2-processed-gene-267.45-mRNA-1	2	OsR498G0204412400.01.T01	99,43	0,00E+00	1242	Os02g0644200	100,00	0,00E+00	699	-543	Indica	Japonica	
augustus-2-processed-gene-268.30-mRNA-1	2	OsR498G0204419300.01.T01	100,00	0,00E+00	1186	Os02g0646900	100,00	0,00E+00	1065	-121	Indica	Japonica	
augustus-2-processed-gene-268.32-mRNA-1	2	OsR498G0204419500.01.T01	99,40	0,00E+00	880	Os01g0346700	89,68	0,00E+00	659	-221	Indica	Japonica	
augustus-2-processed-gene-27.49-mRNA-1	2	OsR498G0202969600.01.T01	99,73	0,00E+00	1987	Os02g0147600	99,10	0,00E+00	1967	-20	NA	NA	
augustus-2-processed-gene-27.57-mRNA-1	2	OsR498G0202973900.01.T01	99,87	0,00E+00	1399	Os02g0148600	100,00	0,00E+00	1391	-8	NA	Japonica	
augustus-2-processed-gene-272.16-mRNA-1	2	OsR498G0204444300.01.T01	99,62	0,00E+00	1853	Os02g0655200	100,00	0,00E+00	1867	14	NA	Japonica	
augustus-2-processed-gene-272.26-mRNA-1	2	OsR498G0204445200.01.T02	99,74	0,00E+00	1384	Os02g0655300	100,00	0,00E+00	1397	13	NA	Japonica	
augustus-2-processed-gene-272.8-mRNA-1	2	OsR498G0204443200.01.T01	99,89	0,00E+00	1641	Os02g0654700	100,00	0,00E+00	1645	4	NA	Japonica	
augustus-2-processed-gene-273.34-mRNA-1	2	OsR498G0204448900.01.T01	97,73	0,00E+00	1513	Os02g0656600	100,00	0,00E+00	1559	46	NA	Japonica	
augustus-2-processed-gene-273.35-mRNA-1	2	OsR498G0204450900.01.T01	100,00	0,00E+00	1223	Os02g0657000	100,00	0,00E+00	1223	0	NA	Japonica	
augustus-2-processed-gene-274.15-mRNA-1	2	OsR498G0204458500.01.T01	99,79	0,00E+00	3337	Os02g0659100	100,00	0,00E+00	3355	18	NA	Japonica	
augustus-2-processed-gene-274.18-mRNA-1	2	OsR498G0204460300.01.T01	99,81	0,00E+00	1842	Os02g0659500	100,00	0,00E+00	1851	9	NA	Japonica	
augustus-2-processed-gene-275.17-mRNA-1	2	OsR498G0204461300.01.T01	99,62	0,00E+00	1392	Os02g0659600	100,00	0,00E+00	1406	14	NA	Japonica	
augustus-2-processed-gene-275.45-mRNA-1	2	OsR498G0204465000.01.T03	99,43	0,00E+00	1243	Os02g0661300	100,00	0,00E+00	1261	18	NA	Japonica	
augustus-2-processed-gene-276.22-mRNA-1	2	OsR498G0204467700.01.T01	99,75	0,00E+00	721	Os02g0662000	100,00	0,00E+00	726	5	NA	Japonica	
augustus-2-processed-gene-276.23-mRNA-1	2	OsR498G0204468300.01.T01	99,74	0,00E+00	694	Os02g0662100	100,00	0,00E+00	699	5	NA	Japonica	
augustus-2-processed-gene-276.30-mRNA-1	2	OsR498G0204470900.01.T01	99,11	0,00E+00	3768	Os02g0662700	100,00	0,00E+00	2879	-889	Indica	Japonica	
augustus-2-processed-gene-276.32-mRNA-1	2	OsR498G0204471300.01.T01	99,67	0,00E+00	3843	Os02g0663100	100,00	0,00E+00	3874	31	NA	Japonica	
augustus-2-processed-gene-277.54-mRNA-1	2	OsR498G0204474300.01.T01	99,58	0,00E+00	1280	Os02g0664000	100,00	0,00E+00	1294	14	NA	Japonica	
augustus-2-processed-gene-278.30-mRNA-1	2	OsR498G0204477900.01.T01	99,79	0,00E+00	4263	Os02g0665300	100,00	0,00E+00	4286	23	NA	Japonica	
augustus-2-processed-gene-278.52-mRNA-1	2	OsR498G0204480600.01.T02	99,88	0,00E+00	2875	Os02g0666500	100,00	0,00E+00	2884	9	NA	Japonica	
augustus-2-processed-gene-279.23-mRNA-1	2	OsR498G0204484100.01.T01	99,07	0,00E+00	756	Os02g0667701	100,00	0,00E+00	1159	403	Japonica	Japonica	
augustus-2-processed-gene-279.35-mRNA-1	2	OsR498G0204485000.01.T02	99,42	0,00E+00	1841	Os02g0668100	100,00	0,00E+00	1894	53	NA	Japonica	
augustus-2-processed-gene-279.44-mRNA-1	2	OsR498G0204490700.01.T01	99,62	0,00E+00	1903	Os04g0562500	74,90	0,00E+00	667	-1236	Indica	Japonica	
augustus-2-processed-gene-279.54-mRNA-1	2	OsR498G0204487800.01.T01	99,16	0,00E+00	1048	Os02g0111900	87,83	1,07E-121	435	-613	Indica	Japonica	
augustus-2-processed-gene-28.25-mRNA-1	2	OsR498G0202976900.01.T01	99,49	0,00E+00	2419	Os02g0149500	100,00	0,00E+00	1256	-1163	Indica	Japonica	
augustus-2-processed-gene-28.33-mRNA-1	2	OsR498G0202977600.01.T03	99,95	0,00E+00	3594	Os02g0149700	100,00	0,00E+00	2679	-915	Indica	Japonica	
augustus-2-processed-gene-28.53-mRNA-1	2	OsR498G0202979600.01.T01	98,86	0,00E+00	919	Os02g0150000	99,37	0,00E+00	847	-72	NA	Japonica	
augustus-2-processed-gene-280.27-mRNA-1	2	OsR498G0204494100.01.T03	99,69	0,00E+00	2271	Os02g0670900	100,00	0,00E+00	2289	18	NA	Japonica	
augustus-2-processed-gene-281.52-mRNA-1	2	OsR498G0204501000.01.T01	99,83	0,00E+00	1078	Os02g0672500	100,00	0,00E+00	1083	5	NA	Japonica	
augustus-2-processed-gene-282.9-mRNA-1	2	OsR498G0204506800.01.T01	99,46	0,00E+00	2301	Os02g0673700	100,00	0,00E+00	2024	-277	Indica	Japonica	
augustus-2-processed-gene-283.14-mRNA-1	2	OsR498G0204516700.01.T01	99,79	0,00E+00	2590	Os02g0676400	100,00	0,00E+00	2976	386	Japonica	Japonica	
augustus-2-processed-gene-284.1-mRNA-1	2	OsR498G0204519700.01.T01	99,49	0,00E+00	1400	Os02g0676800	99,87	0,00E+00	1414	14	NA	Japonica	
augustus-2-processed-gene-284.11-mRNA-1	2	OsR498G0204522100.01.T01	99,26	0,00E+00	1196	Os02g0677300	100,00	0,00E+00	1218	22	NA	Japonica	
augustus-2-processed-gene-285.12-mRNA-1	2	OsR498G0204528100.01.T01	98,71	0,00E+00	1497	Os02g0679700	100,00	0,00E+00	1526	29	NA	Japonica	
augustus-2-processed-gene-285.30-mRNA-1	2	OsR498G0204527400.01.T01	98,70	0,00E+00	799	Os02g0559800	89,34	6,02E-136	482	-317	Indica	Japonica	
augustus-2-processed-gene-286.35-mRNA-1	2	OsR498G0204533500.01.T01	99,37	0,00E+00	1399	Os02g0681632	100,00	0,00E+00	1424	25	NA	Japonica	
augustus-2-processed-gene-286.46-mRNA-1	2	OsR498G0204535200.01.T01	99,79	0,00E+00	3332	Os02g0681900	100,00	0,00E+00	3350	18	NA	Japonica	
augustus-2-processed-gene-287.15-mRNA-1	2	OsR498G0204544300.01.T02	100,00	0,00E+00	1278	Os02g0684000	100,00	0,00E+00	1278	0	NA	Japonica	
augustus-2-processed-gene-287.23-mRNA-1	2	OsR498G0204540600.01.T02	99,35	0,00E+00	2197	Os02g0683500	100,00	0,00E+00	2235	38	NA	Japonica	
augustus-2-processed-gene-287.4-mRNA-1	2	OsR498G0204537800.01.T02	99,53	0,00E+00	1904	Os02g0682500	100,00	0,00E+00	1927	23	NA	Japonica	
augustus-2-processed-gene-288.25-mRNA-1	2	OsR498G0204546200.01.T01	100,00	1,80E-158	558	Os02g0684500	100,00	6,27E-159	558	0	NA	Japonica	
augustus-2-processed-gene-288.49-mRNA-1	2	OsR498G0204552000.01.T01	100,00	0,00E+00	1029	Os02g0686100	100,00	0,00E+00	1029	0	NA	Japonica	
augustus-2-processed-gene-29.29-mRNA-1	2	OsR498G0202983100.01.T04	99,92	0,00E+00	4666	Os02g0151000	99,89	0,00E+00	4981	315	NA	NA	
augustus-2-processed-gene-29.41-mRNA-1	2	OsR498G0202986100.01.T03	100,00	0,00E+00	1884	Os02g0152200	100,00	0,00E+00	1884	0	NA	NA	
augustus-2-processed-gene-290.41-mRNA-1	2	OsR498G0204564700.01.T01	99,82	0,00E+00	2015	Os02g0688800	100,00	0,00E+00	2024	9	NA	Japonica	
augustus-2-processed-gene-290.58-mRNA-1	2	OsR498G0204567600.01.T02	100,00	0,00E+00	1575	Os02g0689500	100,00	0,00E+00	1575	0	NA	Japonica	

augustus-2-processed-gene-291.32-mRNA-1	2	OsR498G0204571100.01.T02	99,61	1,28E-127	456	Os02g0690200	100,00	1,05E-129	461	5	NA	Japonica	
augustus-2-processed-gene-291.33-mRNA-1	2	OsR498G0204571900.01.T01	99,88	0,00E+00	3002	Os02g0690500	100,00	0,00E+00	3011	9	NA	Japonica	
augustus-2-processed-gene-291.34-mRNA-1	2	OsR498G0204572100.01.T01	99,92	0,00E+00	2301	Os02g0690600	100,00	0,00E+00	2305	4	NA	Japonica	
augustus-2-processed-gene-292.9-mRNA-1	2	OsR498G0204575600.01.T01	99,83	0,00E+00	2102	Os02g0692000	100,00	0,00E+00	2111	9	NA	Japonica	
augustus-2-processed-gene-293.65-mRNA-1	2	OsR498G0409312500.01.T01	95,74	1,20E-115	416	Os02g0695700	100,00	8,07E-138	488	72	Japonica	Japonica	
augustus-2-processed-gene-295.63-mRNA-1	2	OsR498G0204598300.01.T02	100,00	0,00E+00	904	Os02g0699600	100,00	0,00E+00	904	0	NA	Japonica	
augustus-2-processed-gene-296.27-mRNA-1	2	OsR498G0204600800.01.T01	99,93	0,00E+00	2656	Os02g0700500	100,00	0,00E+00	2661	5	NA	Japonica	
augustus-2-processed-gene-297.26-mRNA-1	2	OsR498G0204609700.01.T01	99,84	0,00E+00	1095	Os02g0703300	100,00	0,00E+00	1099	4	NA	Japonica	
augustus-2-processed-gene-298.33-mRNA-1	2	OsR498G0204612000.01.T01	94,95	0,00E+00	1325	Os02g0703800	100,00	0,00E+00	1462	137	NA	Japonica	
augustus-2-processed-gene-298.47-mRNA-1	2	OsR498G1221463200.01.T01	70,14	0,00E+00	701	Os02g0704000	100,00	0,00E+00	3458	2757	Japonica	Japonica	
augustus-2-processed-gene-3.48-mRNA-1	2	OsR498G0202813500.01.T05	98,84	0,00E+00	2272	Os02g0105200	99,31	0,00E+00	2299	27	NA	Japonica	
augustus-2-processed-gene-3.67-mRNA-1	2	OsR498G0202815200.01.T04	96,35	0,00E+00	2150	Os02g0105800	96,43	0,00E+00	2155	5	NA	Japonica	
augustus-2-processed-gene-3.71-mRNA-1	2	OsR498G0202817800.01.T05	99,91	0,00E+00	4060	Os02g0106300	100,00	0,00E+00	4069	9	NA	Japonica	
augustus-2-processed-gene-30.34-mRNA-1	2	OsR498G0202991900.01.T04	99,91	0,00E+00	1998	Os02g0154600	97,48	0,00E+00	1876	-122	NA	Indica	
augustus-2-processed-gene-30.39-mRNA-1	2	OsR498G0202989000.01.T01	100,00	0,00E+00	5692	Os02g0153100	98,45	0,00E+00	5471	-221	NA	Indica	Kinase-LRR-TM
augustus-2-processed-gene-30.40-mRNA-1	2	OsR498G0202989400.01.T01	99,97	0,00E+00	5688	Os02g0153200	97,81	0,00E+00	5379	-309	NA	Indica	Kinase-LRR-TM
augustus-2-processed-gene-30.42-mRNA-1	2	OsR498G0202991600.01.T01	78,36	0,00E+00	2551	Os02g0153700	98,79	0,00E+00	5499	2948	Japonica	Indica	Kinase-LRR-TM
augustus-2-processed-gene-30.44-mRNA-1	2	OsR498G0202990800.01.T01	100,00	0,00E+00	5676	Os02g0153900	99,81	0,00E+00	5665	-11	NA	Indica	Kinase-LRR-TM
augustus-2-processed-gene-300.0-mRNA-1	2	OsR498G0204619500.01.T03	98,04	1,05E-148	526	Os02g0706400	100,00	2,64E-157	553	27	NA	Japonica	
augustus-2-processed-gene-300.6-mRNA-1	2	OsR498G0204620600.01.T04	99,38	0,00E+00	2292	Os02g0706600	100,00	0,00E+00	2473	181	NA	Japonica	
augustus-2-processed-gene-301.16-mRNA-1	2	OsR498G0204629800.01.T01	98,20	0,00E+00	957	Os02g0709300	100,00	0,00E+00	1002	45	NA	Japonica	
augustus-2-processed-gene-305.27-mRNA-1	2	OsR498G0204654900.01.T03	100,00	0,00E+00	774	Os02g0717200	100,00	0,00E+00	774	0	NA	Japonica	
augustus-2-processed-gene-306.38-mRNA-1	2	OsR498G0204657300.01.T02	97,66	0,00E+00	878	Os02g0717700	100,00	0,00E+00	856	-22	NA	Japonica	
augustus-2-processed-gene-306.62-mRNA-1	2	OsR498G0204662500.01.T03	99,93	0,00E+00	2624	Os02g0719800	100,00	0,00E+00	2628	4	NA	Japonica	
augustus-2-processed-gene-307.43-mRNA-1	2	OsR498G0204663700.01.T01	99,88	0,00E+00	1554	Os02g0720400	100,00	0,00E+00	1559	5	NA	Japonica	
augustus-2-processed-gene-307.53-mRNA-1	2	OsR498G1120753400.01.T01	94,92	2,09E-158	558	Os02g0789700	97,18	1,01E-169	594	36	NA	Japonica	
augustus-2-processed-gene-308.12-mRNA-1	2	OsR498G1120322800.01.T01	73,71	0,00E+00	668	Os02g0723200	100,00	0,00E+00	2425	1757	Japonica	Japonica	
augustus-2-processed-gene-309.50-mRNA-1	2	OsR498G0204681800.01.T01	71,46	7,62E-54	212	Os02g0724100	100,00	0,00E+00	1315	1103	Japonica	Japonica	
augustus-2-processed-gene-309.58-mRNA-1	2	OsR498G0204683700.01.T02	99,87	0,00E+00	1329	Os02g0725200	100,00	0,00E+00	1333	4	NA	Japonica	
augustus-2-processed-gene-31.36-mRNA-1	2	OsR498G0202998000.01.T01	99,91	0,00E+00	3882	Os02g0156400	100,00	0,00E+00	3891	9	NA	Japonica	LRR-TM
augustus-2-processed-gene-31.8-mRNA-1	2	OsR498G0202992400.01.T01	100,00	2,37E-150	531	Os05g0523100	80,36	1,01E-92	339	-192	Indica	Indica	
augustus-2-processed-gene-310.13-mRNA-1	2	OsR498G0204691300.01.T01	99,15	0,00E+00	1664	Os02g0727700	99,15	0,00E+00	1664	0	NA	NA	
augustus-2-processed-gene-310.3-mRNA-1	2	OsR498G0204687000.01.T01	99,88	0,00E+00	1441	Os02g0726000	100,00	0,00E+00	1445	4	NA	Japonica	
augustus-2-processed-gene-310.4-mRNA-1	2	OsR498G0204687800.01.T01	99,53	0,00E+00	1517	Os02g0726300	100,00	0,00E+00	1532	15	NA	Japonica	
augustus-2-processed-gene-311.20-mRNA-1	2	OsR498G0204702700.01.T01	100,00	0,00E+00	816	Os06g0271600	70,64	1,94E-47	188	-628	Indica	Japonica	
augustus-2-processed-gene-311.21-mRNA-1	2	OsR498G0204702900.01.T01	100,00	0,00E+00	791	Os02g0729900	100,00	0,00E+00	791	0	NA	Japonica	
augustus-2-processed-gene-311.8-mRNA-1	2	OsR498G0204693600.01.T01	98,55	0,00E+00	957	Os03g0351300	80,26	8,40E-148	523	-434	Indica	Japonica	
augustus-2-processed-gene-312.0-mRNA-1	2	OsR498G0204704700.01.T04	99,44	0,00E+00	4137	Os02g0730300	99,87	0,00E+00	4162	25	NA	Japonica	
augustus-2-processed-gene-312.18-mRNA-1	2	OsR498G0204710500.01.T01	99,73	0,00E+00	2612	Os02g0731900	100,00	0,00E+00	2630	18	NA	Japonica	
augustus-2-processed-gene-313.10-mRNA-1	2	OsR498G0204713000.01.T03	99,80	0,00E+00	902	Os02g0732500	100,00	0,00E+00	906	4	NA	Japonica	
augustus-2-processed-gene-313.26-mRNA-1	2	OsR498G0204717000.01.T02	100,00	0,00E+00	948	Os02g0733500	100,00	0,00E+00	948	0	NA	Japonica	
augustus-2-processed-gene-314.13-mRNA-1	2	OsR498G0204722100.01.T01	97,87	0,00E+00	1369	Os02g0734900	100,00	0,00E+00	720	-649	Indica	Japonica	
augustus-2-processed-gene-314.16-mRNA-1	2	OsR498G0204724500.01.T01	99,65	0,00E+00	1524	Os03g0589100	70,06	6,83E-107	387	-1137	Indica	Japonica	
augustus-2-processed-gene-316.47-mRNA-1	2	OsR498G0204738600.01.T01	99,82	0,00E+00	2015	Os02g0739400	100,00	0,00E+00	2024	9	NA	Japonica	
augustus-2-processed-gene-317.49-mRNA-1	2	OsR498G0204744400.01.T01	99,73	0,00E+00	2005	Os02g0740700	100,00	0,00E+00	2019	14	NA	Japonica	
augustus-2-processed-gene-318.5-mRNA-1	2	OsR498G0204748700.01.T01	99,82	0,00E+00	8730	Os02g0742000	85,64	0,00E+00	3123	-5607	Indica	Japonica	
augustus-2-processed-gene-319.0-mRNA-1	2	OsR498G0204750500.01.T01	100,00	0,00E+00	1824	Os02g0742700	100,00	0,00E+00	1824	0	NA	Japonica	
augustus-2-processed-gene-319.8-mRNA-1	2	OsR498G0204753500.01.T01	99,89	0,00E+00	1695	Os02g0743100	100,00	0,00E+00	2149	454	Japonica	Japonica	
augustus-2-processed-gene-32.1-mRNA-1	2	OsR498G0409507600.01.T01	73,28	3,62E-56	220	Os02g0452800	92,57	1,34E-157	554	334	Japonica	NA	
augustus-2-processed-gene-32.19-mRNA-1	2	OsR498G0203005900.01.T01	100,00	0,00E+00	2078	Os02g0158800	100,00	0,00E+00	2078	0	NA	Indica	
augustus-2-processed-gene-32.4-mRNA-1	2	OsR498G0203003900.01.T01	100,00	0,00E+00	931	Os08g0114000	89,24	0,00E+00	714	-217	Indica	Indica	
augustus-2-processed-gene-33.34-mRNA-1	2	OsR498G0203015000.01.T02	100,00	0,00E+00	2673	Os02g0161000	99,93	0,00E+00	2669	-4	NA	Indica	Kinase-TM
augustus-2-processed-gene-337.13-mRNA-1	2	OsR498G0204773100.01.T01	99,84	0,00E+00	1154	Os01g0740700	91,80	0,00E+00	724	-430	Indica	Japonica	
augustus-2-processed-gene-337.2-mRNA-1	2	OsR498G0204771300.01.T01	99,84	0,00E+00	2215	Os02g0747400	100,00	0,00E+00	2224	9	NA	Japonica	
augustus-2-processed-gene-338.10-mRNA-1	2	OsR498G0204782500.01.T01	99,83	0,00E+00	2064	Os02g0749900	100,00	0,00E+00	2073	9	NA	Japonica	
augustus-2-processed-gene-34.13-mRNA-1	2	OsR498G0203023100.01.T01	96,37	0,00E+00	725	Os02g0162600	100,00	0,00E+00	796	71	NA	Japonica	
augustus-2-processed-gene-34.2-mRNA-1	2	OsR498G0203018700.01.T01	99,94	0,00E+00	2929	Os02g0161700	99,96	0,00E+00	3973	1044	Japonica	Indica	LRR-TM
augustus-2-processed-gene-341.11-mRNA-1	2	OsR498G0204800300.01.T03	99,75	0,00E+00	718	Os02g0755400	99,75	0,00E+00	718	0	NA	NA	
augustus-2-processed-gene-341.13-mRNA-1	2	OsR498G0204800000.01.T01	99,80	0,00E+00	4573	Os02g0755200	99,96	0,00E+00	4591	18	NA	NA	
augustus-2-processed-gene-341.38-mRNA-1	2	OsR498G0204802800.01.T01	100,00	0,00E+00	1673	Os02g0756200	100,00	0,00E+00	1673	0	NA	NA	
augustus-2-processed-gene-341.39-mRNA-1	2	OsR498G0204803000.01.T01	99,89	0,00E+00	1695	Os02g0756600	100,00	0,00E+00	1700	5	NA	NA	
augustus-2-processed-gene-341.40-mRNA-1	2	OsR498G0204803600.01.T01	99,80	0,00E+00	1772	Os02g0756800	100,00	0,00E+00	1781	9	NA	NA	
augustus-2-processed-gene-35.50-mRNA-1	2	OsR498G0203023900.01.T03	98,50	0,00E+00	2089	Os06g0679500	79,66	0,00E+00	1023	-1066	Indica	Japonica	
augustus-2-processed-gene-35.56-mRNA-1	2	OsR498G0203026500.01.T06	100,00	0,00E+00	2581	Os02g0164600	100,00	0,00E+00	2581	0	NA	Japonica	
augustus-2-processed-gene-35.58-mRNA-1	2	OsR498G0203029300.01.T01	100,00	0,00E+00	4919	Os02g0164900	100,00	0,00E+00	4919	0	NA	NA	
augustus-2-processed-gene-35.72-mRNA-1	2	OsR498G0203029600.01.T03	100,00	0,00E+00	2058	Os02g0165000	100,00	0,00E+00	2058	0	NA	NA	
augustus-2-processed-gene-353.11-mRNA-1	2	OsR498G0204795900.01.T01	99,80	0,00E+00	894	Os02g0754300	100,00	0,00E+00	899	5	NA	NA	
augustus-2-processed-gene-353.7-mRNA-1	2	OsR498G0204793700.01.T01	99,89	0,00E+00	1690	Os02g0753400	100,00	0,00E+00	1694	4	NA	NA	
augustus-2-processed-gene-354.28-mRNA-1	2	OsR498G0204800000.01.T01	99,84	0,00E+00	4581	Os02g0755200	100,00	0,00E+00	4599	18	NA	NA	
augustus-2-processed-gene-354.46-mRNA-1	2	OsR498G0204802800.01.T01	100,00	0,00E+00	1673	Os02g0756200	100,00	0,00E+00	1673	0	NA	NA	
augustus-2-processed-gene-354.47-mRNA-1	2	OsR498G0204803000.01.T01	99,89	0,00E+00	1695	Os02g0756600	100,00	0,00E+00	1700	5	NA	NA	
augustus-2-processed-gene-354.48-mRNA-1	2	OsR498G0204803600.01.T01	99,80	0,00E+00	1772	Os02g0756800	100,00	0,00E+00	1781	9	NA	NA	
augustus-2-processed-gene-355.36-mRNA-1	2	OsR498G0204807100.01.T06	99,90	0,00E+00	1755	Os02g0757700	100,00	0,00E+00	1759	4	NA	Japonica	
augustus-2-processed-gene-356.1-mRNA-1	2	OsR498G0204812100.01.T01	99,70	0,00E+00	2352	Os02g0759400	100,00	0,00E+00	2370	18	NA	Japonica	
augustus-2-processed-gene-356.2-mRNA-1	2	OsR498G0204812500.01.T01	99,64	0,00E+00	2982	Os02g0759500	100,00	0,00E+00	3009	27	NA	Japonica	
augustus-2-processed-gene-356.28-mRNA-1	2	OsR498G0204814200.01.T01	99,78	0,00E+00	3272	Os02g0759800	100,00	0,00E+00	3290	18	NA	Japonica	
augustus-2-processed-gene-357.24-mRNA-1	2	OsR498G0204819800.01.T01	98,82	0,00E+00	888	Os02g0762100	100,00	0,00E+00	915	27	NA	Japonica	
augustus-2-processed-gene-358.55-mRNA-1	2	OsR498G0204826400.01.T01	99,73	0,00E+00	1306	Os02g0764700	100,00	0,00E+00	1315	9	NA	Japonica	
augustus-2-processed-gene-359.43-mRNA-1	2	OsR498G0204829100.01.T01	99,64	0,00E+00	998	Os02g0766600	100,00	0,00E+00	1007	9	NA	Japonica	
augustus-2-processed-gene-359.68-mRNA-1	2	OsR498G0204832400.01.T01	99,45	0,00E+00	4838	Os02g0767400	99,96	0,00E+00	4898	60	NA	Japonica	Kinase-TM
augustus-2-processed-gene-359.70-mRNA-1	2	OsR498G0204833000.01.T03	99,90	0,00E+00	1749	Os02g0767700	99,38	0,00E+00	1723	-26	NA	Japonica	
augustus-2-processed-gene-36.8-mRNA-1	2	OsR498G0203033100.01.T01	99,92	0,00E+00	2198	Os02g0165500	100,00	0,00E+00	2203	5	NA	NA	
augustus-2-processed-gene-360.31-mRNA-1	2	OsR498G0204836200.01.T01	98,53	0,00E+00	1544	Os02g0768700	100,00	0,00E+00	1543	-1	NA	Japonica	
augustus-2-processed-gene-361.17-mRNA-1	2	OsR498G0204844300.01.T02	99,52	0,00E+00	1850	Os02g0770600	99,90	0,00E+00	1868	18	NA	Japonica	
augustus-2-processed-gene-361.28-mRNA-1	2	OsR498G0204846200.01.T01	98,75	0,00E+00	1682	Os02g0771200	100,00	0,00E+00	1716	34	NA	Japonica	
augustus-2-processed-gene-361.8-mRNA-1	2	OsR498G0204841800.01.T01	99,68	0,00E+00	2835	Os02g0769900	99,92	0,00E+00	2371	-464	Indica	Japonica	
augustus-2-processed-gene-363.60-mRNA-1	2	OsR498G0204854400.01.T01	96,24	0,00E+00	1236	Os02g0773600	96,37	0,00E+00	1241	5	NA	Japonica	
augustus-2-processed-gene-363.83-mRNA-1	2	OsR498G0204858200.01.T02	100,00	0,00E+00	845	Os02g0775300	100,00	0,00E+00	845	0	NA	Japonica	
augustus-2-processed-gene-364.17-mRNA-1	2	OsR498G0204860400.01.T01	97,64	0,00E+00	1449	Os02g0775600	100,00	0,00E+00	1516	67	NA	Japonica	
augustus-2-processed-gene-366.26-mRNA-1	2	OsR498G0204874800.01.T01	99,32	0,00E+00	4195	Os02g0779200	99,32	0,00E+00	4201	6	NA	Japonica	
augustus-2-processed-gene-366.8-mRNA-1	2	OsR498G0204874500.01.T01	93,12	0,00E+00	844	Os02g0778900	100,00	0,00E+00	1332	488	Japonica	Japonica	
augustus-2-processed-gene-367.78-mRNA-1	2	OsR498G0204884700.01.T01	97,83	0,00E+00	2458	Os02g0781500	97,63	0,00E+00	1957	-501	Indica	Japonica	
augustus-2-processed-gene-368.18-mRNA-1	2	OsR498G0204892600.01.T01	99,86	0,00E+00	2616	Os02g0783100	100,00	0,00E+00	2625	9	NA	Japonica	
augustus-2-processed-gene-37.57-mRNA-1	2	OsR498G0203043100.01.T03	100,00	0,00E+00	663	Os06g0671150	77,12	1,24E-86	319	-344	Indica	NA	
augustus-2-processed-gene-37.60-mRNA-1	2	OsR498G0203044100.01.T04	100,00	0,00E+00	1418	Os02g0168000	100,00	0,00E+00	1586	168	Japonica	NA	
augustus-2-processed-gene-37.63-mRNA-1	2	OsR498G0203045700.01.T03	100,00	0,00E+00	2392	Os02g0168500	99,85	0,00E+00	2383	-9	NA	Indica	
augustus-2-processed-gene-370.38-mRNA-1	2	OsR498G0204900900.01.T02	99,71	4,39E-173	608	Os02g0785800	99,71	1,53E-173	608	0	NA	Japonica	
augustus-2-processed-gene-370.47-mRNA-1	2	OsR498G0204900600.01.T01	78,19	0,00E+00	704	Os02g0785000	70,43	1,54E-77	289	-415	Indica	Japonica	
augustus-2-processed-gene-371.37-mRNA-1	2	OsR498G0204906600.01.T01	99,91	0,00E+00	1998	Os02g0787300	100,00	0,00E+00	2192	194	NA	Japonica	Kinase
augustus-2-processed-gene-372.27-mRNA-1	2	OsR498G0204917500.01.T02	96,69	0,00E+00	2681	Os02g0791200	96,87	0,00E+00	2695	14	NA	Japonica	
augustus-2-processed-gene-372.29-mRNA-1	2	OsR498G0204919300.01.T01	99,92	0,00E+00	2366	Os02g0791500	100,00	0,00E+00	2370	4	NA	Japonica	
augustus-2-processed-gene-372.39-mRNA-1	2	OsR498G0204916600.01.T01	99,69	0,00E+00	2304	Os02g0790600	100,00	0,00E+00	2322	18	NA	Japonica	
augustus-2-processed-gene-373.46-mRNA-1	2	OsR498G0204924100.01.T02	99,57	0,00E+00	2934	Os02g0793200	100,00	0,00E+00	2565	-369	Indica	Japonica	
augustus-2-processed-gene-376.0-mRNA-1	2	OsR498G0204938900.01.T01	99,30	0,00E+00	5067	Os02g0797300	99,36	0,00E+00	5038	-29	NA	Japonica	
augustus-2-processed-gene-376.19-mRNA-1	2	OsR498G0204942600.01.T01	98,97	0,00E+00	686	Os02g0798450	100,00	0,00E+00	704	18	NA	Japonica	
augustus-2-processed-gene-377.26-mRNA-1	2	OsR498G0204944700.01.T02	99,81	0,00E+00	1912	Os02g0799000	100,00	0,00E+00	1921	9	NA	Japonica	
augustus-2-processed-gene-377.29-mRNA-1	2	OsR498G0204947300.01.T01	99,78	0,00E+00	824	Os02g0799600	100,00	2,11E-173	607	-217	Indica	Japonica	
augustus-2-processed-gene-377.42-mRNA-1	2	OsR498G0204954900.01.T01	99,48	0,00E+00	684	Os02g0800300	99,48	0,00E+00	684	0	NA	Japonica	
augustus-2-processed-gene-379.16-mRNA-1	2	OsR498G0204968900.01.T01	99,82	0,00E+00	997	Os02g0805600	100,00	0,00E+00	1002	5	NA	Japonica	
augustus-2-processed-gene-379.30-mRNA-1	2	OsR498G0204968800.01.T01	99,65	0,00E+00	2017	Os02g0805500	99,04	0,00E+00	2206	189	NA	Japonica	
augustus-2-processed-gene-381.26-mRNA-1	2	OsR498G0204980200.01.T01	99,87	0,00E+00	2740	Os02g0809100	100,00	0,00E+00	2749	9	NA	Japonica	
augustus-2-processed-gene-382.2-mRNA-1	2	OsR498G0204983100.01.T04	98,40	0,00E+00	1084	Os02g0809800	99,68	0,00E+00	1108	24	NA	Japonica	
augustus-2-processed-gene-382.3-mRNA-1	2	OsR498G0204983100.01.T04	99,50	0,00E+00	719	Os02g0809800	100,00	0,00E+00	728	9	NA	Japonica	
augustus-2-processed-gene-383.42-mRNA-1	2	OsR498G0204994700.01.T01	98,33	0,00E+00	723	Os02g0812700	100,00	0,00E+00	1223	500	Japonica	Japonica	
augustus-2-processed-gene-383.44-mRNA-1	2	OsR498G0204994900.01.T01	95,63	0,00E+00	786	Os02g0813300	82,40	1,22E-160	566	-220	Indica	Japonica	
augustus-2-processed-gene-384.80-mRNA-1	2	OsR498G0205000500.01.T01	100,00	0,00E+00	764	Os02g0814700	100,00	0,00E+00	764	0	NA	Japonica	
augustus-2-processed-gene-385.69-mRNA-1	2	OsR498G0205002600.01.T01	99,35	0,00E+00	2461	Os02g0815600	100,00	0,00E+00	2504	43	NA	NA	
augustus-2-processed-gene-385.76-mRNA-1	2	OsR498G0205003700.01.T01	99,87	0,00E+00	5393	Os02g0816000	100,00	0,00E+00	5411	18	NA	Japonica	
augustus-2-processed-gene-385.80-mRNA-1	2	OsR498G0205007100.01.T01	99,76	0,00E+00	2200	Os02g0816800	100,00	0,00E+00	2214	14	NA	Japonica	
augustus-2-processed-gene-386.43-mRNA-1	2	OsR498G0205007900.01.T02	99,70	0,00E+00	4688	Os02g0817000	100,00	0,00E+00	4724	36	NA	Japonica	
augustus-2-processed-gene-386.56-mRNA-1	2	OsR498G0205010200.01.T01	98,66	0,00E+00	2355	Os02g0817700	99,11	0,00E+00	2382	27	NA	Japonica	
augustus-2-processed-gene-387.52-mRNA-1	2	OsR498G0205020500.01.T01	99,59	0,00E+00	1719	Os02g0820200	100,00	0,00E+00	1737	18	NA	Japonica	
augustus-2-processed-gene-388.51-mRNA-1	2	OsR498G0205022500.01.T01	99,38	0,00E+00	1423	Os02g0820800	100,00	0,00E+00	1445	22	NA	Japonica	
augustus-2-processed-gene-388.56-mRNA-1	2	OsR498G0205021000.01.T01	99,71	0,00E+00	2439	Os02g0820400	100,00	0,00E+00	2457	18	NA	Japonica	
augustus-2-processed-gene-389.59-mRNA-1	2	OsR498G0100001000.01.T01	79,28	0,00E+00	825	Os02g0824100	100,00	0,00E+00	2879	2054	Japonica	Japonica	
augustus-2-processed-gene-39.9-mRNA-1	2	OsR498G0203056200.01.T03	100,00	0,00E+00	1407	Os02g0170500	100,00	0,00E+00	1407	0	NA	Japonica	
augustus-2-processed-gene-390.14-mRNA-1	2	OsR498G0205034900.01.T01	99,76	0,00E+00	746	Os02g0825100	87,28	3,03E-136	484	-262	Indica	Japonica	
augustus-2-processed-gene-390.4-mRNA-1	2	OsR498G0205034900.01.T01	95,08	0,00E+00	997	Os02g0825500	100,00	0,00E+00	1137	140	Japonica	Japonica	
augustus-2-processed-gene-390.40-mRNA-1	2	OsR498G0205034700.01.T03	91,67	1,59E-160	566	Os02g0825700	100,00	0,00E+00	715	149	Japonica	NA	
augustus-2-processed-gene-390.41-mRNA-1	2	OsR498G0205034700.01.T02	83,64	1,26E-144	514	Os02g0825100	79,65	6,55E-67	253	-261	Indica	NA	
augustus-2-processed-gene-390.45-mRNA-1	2	OsR498G0205038000.01.T03	100,00	5,30E-126	450	Os02g0826400	100,00	1,86E-126	450	0	NA	Japonica	
augustus-2-processed-gene-391.79-mRNA-1	2	OsR498G0205043500.01.T03	99,76	0,00E+00	2260	Os02g0828500	72,81	0,00E+00	650	-1610	Indica	Japonica	
augustus-2-processed-gene-391.80-mRNA-1	2	OsR498G0205044100.01.T01	99,68	0,00E+00	2215	Os02g0828500	99,92	0,00E+00	2241	26	NA	Japonica	
augustus-2-processed-gene-392.20-mRNA-1	2	OsR498G0205048000.01.T01	99,92	0,00E+00	2290	Os02g0829800	100,00	0,00E+00	2295	5	NA	Japonica	
augustus-2-processed-gene-393.111-mRNA-1	2	OsR498G0205054500.01.T02	100,00	0,00E+00	1905	Os02g0832600	100,00	0,00E+00	1905	0	NA	Japonica	
augustus-2-processed-gene-393.118-mRNA-1	2	OsR498G0205056900.01.T01	99,83	0,00E+00	4177	Os02g0833500	100,00	0,00E+00	4195	18	NA	Japonica	
augustus-2-processed-gene-393.152-mRNA-1	2	OsR498G0205056400.01.T05	99,84	0,00E+00	2172	Os02g0833300	100,00	0,00E+00	2181	9	NA	Japonica	
augustus-2-processed-gene-393.161-mRNA-1	2	OsR498G0205002600.01.T01	99,35	0,00E+00	2461	Os02g0815600	100,00	0,00E+00	2504	43	NA	NA	
augustus-2-processed-gene-4.49-mRNA-1	2	OsR498G0202821800.01.T04	99,83	0,00E+00	2064	Os02g0107600	100,00	0,00E+00	2073	9	NA	Japonica	
augustus-2-processed-gene-43.21-mRNA-1	2	OsR498G0203084800.01.T01	99,56	0,00E+00	814	Os02g0178300	100,00	0,00E+00	1005	191	Japonica	Japonica	
augustus-2-processed-gene-44.48-mRNA-1	2	OsR498G0613076300.01.T01	79,91	0,00E+00	1174	Os02g0179600	99,70	0,00E+00	3528	2354	Japonica	NA	
augustus-2-processed-gene-45.1-mRNA-1	2	OsR498G0203091300.01.T03	100,00	0,00E+00	1386	Os02g0180200	99,79	0,00E+00	871	-515	Indica	Indica	
augustus-2-processed-gene-48.1-mRNA-1	2	OsR498G0203109500.01.T01	99,81	0,00E+00	2779	Os02g0184900	84,18	0,00E+00	1688	-1091	Indica	Japonica	
augustus-2-processed-gene-49.23-mRNA-1	2	OsR498G0203122000.01.T02	99,44	0,00E+00	2551	Os02g0188000	100,00	0,00E+00	2232	-319	Indica	Japonica	
augustus-2-processed-gene-49.4-mRNA-1	2	OsR498G0203117500.01.T02	92,24	0,00E+00	952	Os02g0187100	98,80	0,00E+00	730	-222	Indica	Indica	
augustus-2-processed-gene-5.21-mRNA-1	2	OsR498G0202827500.01.T01	99,91	0,00E+00	2095	Os02g0108400	100,00	0,00E+00	2100	5	NA	Japonica	
augustus-2-processed-gene-5.24-mRNA-1	2	OsR498G0202827500.01.T01	89,20	0,00E+00	1575	Os02g0108900	100,00	0,00E+00	2062	487	Japonica	NA	
augustus-2-processed-gene-50.9-mRNA-1	2	OsR498G0203126600.01.T04	100,00	3,62E-84	311	Os02g0189100	100,00	1,27E-84	311	0	NA	Indica	
augustus-2-processed-gene-51.25-mRNA-1	2	OsR498G0203132400.01.T02	99,80	0,00E+00	2638	Os02g0190600	100,00	0,00E+00	1451	-1187	Indica	Japonica	
augustus-2-processed-gene-51.31-mRNA-1	2	OsR498G0613274200.01.T01	90,67	0,00E+00	3036	Os02g0191200	100,00	0,00E+00	3936	900	Japonica	Japonica	
augustus-2-processed-gene-51.36-mRNA-1	2	OsR498G0203022300.01.T01	87,36	0,00E+00	3375	Os02g0191000	100,00	0,00E+00	4946	1571	Japonica	Japonica	CC-LRR-NBS-TM
augustus-2-processed-gene-52.19-mRNA-1	2	OsR498G0203140400.01.T01	98,44	0,00E+00	1112	Os02g0193100	100,00	0,00E+00	1137	25	NA	Japonica	
augustus-2-processed-gene-52.20-mRNA-1	2	OsR498G0203141200.01.T01	99,67	0,00E+00	1079	Os02g0193200	100,00	0,00E+00	1088	9	NA	Japonica	
augustus-2-processed-gene-53.2-mRNA-1	2	OsR498G0203143300.01.T01	99,36	0,00E+00	1113	Os02g0193900	100,00	0,00E+00	1131	18	NA	Japonica	
augustus-2-processed-gene-55.14-mRNA-1	2	OsR498G0203157500.01.T04	99,46	0,00E+00	1639	Os06g0609700	72,30	7,80E-146	517	-1122	Indica	Indica	
augustus-2-processed-gene-56.64-mRNA-1	2	OsR498G0203161400.01.T02	100,00	0,00E+00	1050	Os02g0198900	100,00	0,00E+00	1050	0	NA	NA	
augustus-2-processed-gene-57.16-mRNA-1	2	OsR498G0203172500.01.T01	99,84	0,00E+00	1111	Os02g0202000	100,00	0,00E+00	1115	4	NA	Japonica	
augustus-2-processed-gene-58.4-mRNA-1	2	OsR498G0203177300.01.T01	99,73	0,00E+00	2699	Os02g0203300	100,00	0,00E+00	2717	18	NA	Japonica	
augustus-2-processed-gene-6.0-mRNA-1	2	OsR498G0202833200.01.T02	99,73	0,00E+00	4020	Os02g0110400	100,00	0,00E+00	4048	28	NA	Japonica	
augustus-2-processed-gene-6.11-mRNA-1	2	OsR498G0202836100.01.T01	99,96	0,00E+00	5017	Os02g0111900	100,00	0,00E+00	5021	4	NA	Japonica	
augustus-2-processed-gene-6.12-mRNA-1	2	OsR498G0202837400.01.T01	99,81	0,00E+00	2876	Os02g0112600	100,00	0,00E+00	2890	14	NA	Japonica	
augustus-2-processed-gene-6.27-mRNA-1	2	OsR498G0202835600.01.T01	99,84	0,00E+00	5610	Os02g0111800	100,00	0,00E+00	5633	23	NA	Japonica	Kinase-LRR-TM
augustus-2-processed-gene-61.21-mRNA-1	2	OsR498G0203194400.01.T01	99,90	0,00E+00	1857	Os02g0207900	100,00	0,00E+00	2003	146	NA	Japonica	
augustus-2-processed-gene-61.22-mRNA-1	2	OsR498G0203197400.01.T01	99,65	0,00E+00	2049	Os02g0208700	100,00	0,00E+00	1714	-335	Indica	Japonica	
augustus-2-processed-gene-62.56-mRNA-1	2	OsR498G1019116500.01.T01	91,93	6,14E-141	500	Os02g0212975	84,83	1,54E-130	464	-36	NA	Japonica	
augustus-2-processed-gene-62.71-mRNA-1	2	OsR498G0203200800.01.T02	95,29	0,00E+00	877	Os02g0209400	100,00	0,00E+00	980	103	Japonica	Japonica	
augustus-2-processed-gene-63.19-mRNA-1	2	OsR498G0203203700.01.T01	96,52	0,00E+00	4803	Os02g0211000	100,00	0,00E+00	5222	419	NA	Japonica	
augustus-2-processed-gene-65.14-mRNA-1	2	OsR498G0203218300.01.T01	92,37	2,08E-159	562	Os02g0215100	100,00	0,00E+00	764	202	Japonica	Japonica	
augustus-2-processed-gene-67.10-mRNA-1	2	OsR498G0203227900.01.T01	97,21	0,00E+00	2527	Os02g0218700	100,00	0,00E+00	2711	184	NA	Japonica	
augustus-2-processed-gene-67.11-mRNA-1	2	OsR498G0203228700.01.T01	97,41	0,00E+00	2613	Os02g0218800	100,00	0,00E+00	2765	152	NA	Japonica	
augustus-2-processed-gene-67.28-mRNA-1	2	OsR498G0203229000.01.T01	99,36	0,00E+00	2492	Os02g0219000	100,00	0,00E+00	2533	41	NA	Japonica	
augustus-2-processed-gene-67.29-mRNA-1	2	OsR498G0816363900.01.T01	95,81	0,00E+00	2080	Os02g0219050	100,00	0,00E+00	2468	388	Japonica	Japonica	
augustus-2-processed-gene-69.10-mRNA-1	2	OsR498G0203238300.01.T03	99,74	0,00E+00	1402	Os06g0564500	73,37	3,38E-130	464	-938	Indica	Japonica	
augustus-2-processed-gene-69.18-mRNA-1	2	OsR498G0203237200.01.T01	96,41	0,00E+00	1992	Os02g0221500	96,90	0,00E+00	2015	23	NA	Japonica	
augustus-2-processed-gene-72.60-mRNA-1	2	OsR498G0612770700.01.T01	83,39	3,80E-177	622	Os02g0226801	100,00	0,00E+00	1126	504	Japonica	NA	
augustus-2-processed-gene-74.10-mRNA-1	2	OsR498G0203272500.01.T04	99,78	0,00E+00	4020	Os02g0229400	100,00	0,00E+00	4042	22	NA	Japonica	
augustus-2-processed-gene-75.0-mRNA-1	2	OsR498G0203275100.01.T05	99,81	0,00E+00	1902	Os02g0230200	100,00	0,00E+00	1911	9	NA	Japonica	
augustus-2-processed-gene-77.9-mRNA-1	2	OsR498G0203292500.01.T01	99,82	0,00E+00	3927	Os02g0234300	100,00	0,00E+00	3149	-778	Indica	Japonica	
augustus-2-processed-gene-78.37-mRNA-1	2	OsR498G0203293100.01.T04	99,83	0,00E+00	3168	Os02g0234500	100,00	0,00E+00	3182	14	NA	Japonica	
augustus-2-processed-gene-79.29-mRNA-1	2	OsR498G0203303300.01.T01	99,53	0,00E+00	1514	Os02g0237000	100,00	0,00E+00	1835	321	Japonica	Japonica	
augustus-2-processed-gene-81.50-mRNA-1	2	OsR498G0203313200.01.T01	93,15	0,00E+00	1773	Os02g0241700	100,00	0,00E+00	2100	327	Japonica	Japonica	
augustus-2-processed-gene-82.10-mRNA-1	2	OsR498G0203313200.01.T01	97,87	0,00E+00	2480	Os02g0242100	100,00	0,00E+00	2625	145	NA	Japonica	
augustus-2-processed-gene-82.27-mRNA-1	2	OsR498G0203314800.01.T01	96,34	0,00E+00	2465	Os02g0242900	100,00	0,00E+00	2700	235	NA	Japonica	
augustus-2-processed-gene-82.31-mRNA-1	2	OsR498G0203315000.01.T02	97,35	0,00E+00	2480	Os02g0243300	100,00	0,00E+00	2652	172	NA	Japonica	
augustus-2-processed-gene-83.9-mRNA-1	2	OsR498G0203320200.01.T01	99,79	0,00E+00	1739	Os02g0245100	99,90	0,00E+00	1744	5	NA	Japonica	
augustus-2-processed-gene-85.20-mRNA-1	2	OsR498G0203329000.01.T01	99,54	0,00E+00	1156	Os02g0248320	73,54	3,25E-84	312	-844	Indica	Japonica	
augustus-2-processed-gene-85.4-mRNA-1	2	OsR498G0203326000.01.T01	98,54	0,00E+00	2389	Os02g0247200	98,93	0,00E+00	2961	572	Japonica	Japonica	
augustus-2-processed-gene-86.0-mRNA-1	2	OsR498G0203331500.01.T01	99,50	0,00E+00	1069	Os02g0249300	100,00	0,00E+00	1083	14	NA	Japonica	
augustus-2-processed-gene-87.0-mRNA-1	2	OsR498G0203339500.01.T02	100,00	0,00E+00	1050	Os02g0251700	100,00	0,00E+00	1050	0	NA	Japonica	
augustus-2-processed-gene-87.20-mRNA-1	2	OsR498G0203342200.01.T01	99,55	0,00E+00	2002	Os02g0252400	99,91	0,00E+00	2020	18	NA	Japonica	
augustus-2-processed-gene-88.44-mRNA-1	2	OsR498G0203347900.01.T01	97,25	0,00E+00	984	Os02g0255100	97,28	0,00E+00	936	-48	NA	Japonica	
augustus-2-processed-gene-9.69-mRNA-1	2	OsR498G0202850700.01.T01	99,97	0,00E+00	5736	Os02g0116700	100,00	0,00E+00	5741	5	NA	NA	Kinase-LRR-TM
augustus-2-processed-gene-9.97-mRNA-1	2	OsR498G0202853400.01.T01	100,00	0,00E+00	2636	Os02g0118000	100,00	0,00E+00	2636	0	NA	NA	
augustus-2-processed-gene-90.22-mRNA-1	2	OsR498G0203360700.01.T03	97,22	2,95E-136	485	Os02g0258800	100,00	2,59E-144	509	24	NA	Japonica	
augustus-2-processed-gene-90.30-mRNA-1	2	OsR498G0203361400.01.T03	99,24	0,00E+00	696	Os06g0523100	75,57	9,33E-38	156	-540	Indica	Japonica	
augustus-2-processed-gene-93.14-mRNA-1	2	OsR498G0203369300.01.T01	97,95	0,00E+00	3920	Os02g0262800	100,00	0,00E+00	7548	3628	Japonica	Japonica	NBS-TM
augustus-2-processed-gene-95.1-mRNA-1	2	OsR498G0203374300.01.T01	91,78	0,00E+00	658	Os02g0265300	99,77	0,00E+00	770	112	Japonica	Japonica	
augustus-2-processed-gene-98.19-mRNA-1	2	OsR498G0203388600.01.T02	92,48	0,00E+00	2423	Os02g0270933	81,09	0,00E+00	1047	-1376	Indica	Indica	
augustus-2-processed-gene-99.35-mRNA-1	2	OsR498G0203391400.01.T01	98,67	0,00E+00	2094	Os10g0130200	77,86	5,01E-180	630	-1464	Indica	Japonica	
maker-2-augustus-gene-0.0-mRNA-1	2	OsR498G0202797100.01.T02	100,00	0,00E+00	2219	Os02g0100300	99,76	0,00E+00	2204	-15	NA	NA	
maker-2-augustus-gene-0.11-mRNA-1	2	OsR498G0202796800.01.T01	98,60	0,00E+00	3004	Os02g0100100	98,60	0,00E+00	3004	0	NA	NA	
maker-2-augustus-gene-0.15-mRNA-1	2	OsR498G0202797000.01.T03	99,96	0,00E+00	4595	Os02g0100200	100,00	0,00E+00	4599	4	NA	NA	
maker-2-augustus-gene-0.18-mRNA-1	2	OsR498G0202799600.01.T06	97,61	0,00E+00	2371	Os02g0101000	97,61	0,00E+00	2371	0	NA	NA	
maker-2-augustus-gene-0.5-mRNA-1	2	OsR498G0202798900.01.T05	100,00	0,00E+00	2040	Os02g0100700	100,00	0,00E+00	2040	0	NA	NA	
maker-2-augustus-gene-1.37-mRNA-1	2	OsR498G0202800100.01.T01	100,00	0,00E+00	2814	Os02g0101100	100,00	0,00E+00	2814	0	NA	NA	
maker-2-augustus-gene-1.40-mRNA-1	2	OsR498G0202801700.01.T02	100,00	0,00E+00	1948	Os02g0101400	100,00	0,00E+00	1532	-416	Indica	NA	
maker-2-augustus-gene-1.42-mRNA-1	2	OsR498G0202802900.01.T01	100,00	0,00E+00	1927	Os02g0101900	100,00	0,00E+00	1429	-498	Indica	Japonica	
maker-2-augustus-gene-1.45-mRNA-1	2	OsR498G0202803900.01.T01	99,87	0,00E+00	2704	Os02g0102200	100,00	0,00E+00	2713	9	NA	Japonica	
maker-2-augustus-gene-1.48-mRNA-1	2	OsR498G0202804500.01.T01	84,85	3,05E-134	479	Os02g0102300	85,35	8,69E-136	483	4	NA	Japonica	
maker-2-augustus-gene-1.50-mRNA-1	2	OsR498G0202805100.01.T01	99,80	0,00E+00	900	Os02g0102600	100,00	0,00E+00	904	4	NA	Japonica	
maker-2-augustus-gene-1.52-mRNA-1	2	OsR498G0202805500.01.T01	100,00	0,00E+00	1947	Os02g0102800	100,00	0,00E+00	1947	0	NA	Japonica	
maker-2-augustus-gene-1.54-mRNA-1	2	OsR498G0202806100.01.T05	99,14	0,00E+00	3303	Os02g0102900	99,17	0,00E+00	3210	-93	NA	Japonica	

maker-2-augustus-gene-1.87-mRNA-1	2	OsR498G0202805400.01.T01	100,00	0,00E+00	720	Os02g0102700	100,00	0,00E+00	720	0	NA	Japonica	
maker-2-augustus-gene-10.36-mRNA-1	2	OsR498G0202854500.01.T01	100,00	0,00E+00	2381	Os02g0118400	100,00	0,00E+00	2381	0	NA	NA	
maker-2-augustus-gene-10.55-mRNA-1	2	OsR498G0202858300.01.T01	99,94	0,00E+00	3091	Os02g0119200	100,00	0,00E+00	3095	4	NA	NA	
maker-2-augustus-gene-10.57-mRNA-1	2	OsR498G0202858500.01.T01	100,00	0,00E+00	2360	Os02g0119300	100,00	0,00E+00	2360	0	NA	NA	
maker-2-augustus-gene-10.65-mRNA-1	2	OsR498G0202860500.01.T03	100,00	0,00E+00	1081	Os02g0119800	100,00	0,00E+00	1081	0	NA	NA	
maker-2-augustus-gene-10.77-mRNA-1	2	OsR498G0202859000.01.T03	100,00	0,00E+00	1261	Os02g0119400	100,00	0,00E+00	1261	0	NA	NA	
maker-2-augustus-gene-10.84-mRNA-1	2	OsR498G0202860800.01.T06	100,00	0,00E+00	2690	Os02g0120000	100,00	0,00E+00	2690	0	NA	NA	
maker-2-augustus-gene-10.86-mRNA-1	2	OsR498G0202861000.01.T04	99,91	0,00E+00	1917	Os02g0120100	100,00	0,00E+00	1921	4	NA	NA	Kinase-TM
maker-2-augustus-gene-100.28-mRNA-1	2	OsR498G0203396500.01.T01	99,59	0,00E+00	2608	Os02g0273000	100,00	0,00E+00	2636	28	NA	Japonica	
maker-2-augustus-gene-100.35-mRNA-1	2	OsR498G0203396700.01.T01	97,82	0,00E+00	4690	Os02g0272900	99,91	0,00E+00	3963	-727	Indica	Japonica	CC-LRR-NBS-TM
maker-2-augustus-gene-100.39-mRNA-1	2	OsR498G0203398500.01.T02	99,53	0,00E+00	3396	Os02g0273700	100,00	0,00E+00	3436	40	NA	Japonica	
maker-2-augustus-gene-100.44-mRNA-1	2	OsR498G0203399500.01.T01	99,79	0,00E+00	856	Os02g0274000	99,68	2,35E-160	564	-292	Indica	Japonica	
maker-2-augustus-gene-100.48-mRNA-1	2	OsR498G0203400100.01.T02	99,86	0,00E+00	3920	Os02g0274100	100,00	0,00E+00	3934	14	NA	Japonica	
maker-2-augustus-gene-100.53-mRNA-1	2	OsR498G0203399200.01.T03	96,57	0,00E+00	3575	Os02g0273800	100,00	0,00E+00	3896	321	NA	Japonica	
maker-2-augustus-gene-100.55-mRNA-1	2	OsR498G0612559700.01.T02	74,69	3,31E-40	166	Os02g0273900	100,00	9,48E-118	421	255	Japonica	Japonica	
maker-2-augustus-gene-101.47-mRNA-1	2	OsR498G0203402100.01.T02	100,00	0,00E+00	829	Os02g0275100	100,00	0,00E+00	829	0	NA	Japonica	
maker-2-augustus-gene-101.53-mRNA-1	2	OsR498G0203402400.01.T01	96,69	0,00E+00	3002	Os02g0275200	100,00	0,00E+00	3258	256	NA	Japonica	
maker-2-augustus-gene-102.0-mRNA-1	2	OsR498G0203408300.01.T02	100,00	0,00E+00	2706	Os02g0277600	100,00	0,00E+00	2706	0	NA	NA	
maker-2-augustus-gene-102.10-mRNA-1	2	OsR498G0203404200.01.T01	99,85	0,00E+00	1215	Os02g0276400	100,00	0,00E+00	1166	-49	NA	Japonica	
maker-2-augustus-gene-102.14-mRNA-1	2	OsR498G0203404400.01.T03	99,67	0,00E+00	1097	Os02g0276500	99,84	0,00E+00	1104	7	NA	Japonica	
maker-2-augustus-gene-102.18-mRNA-1	2	OsR498G0203406200.01.T05	100,00	0,00E+00	1485	Os02g0276900	99,88	0,00E+00	1484	-1	NA	Japonica	
maker-2-augustus-gene-103.0-mRNA-1	2	OsR498G0203410500.01.T01	96,21	0,00E+00	2017	Os02g0278400	99,81	0,00E+00	2838	821	Japonica	Japonica	
maker-2-augustus-gene-103.2-mRNA-1	2	OsR498G0203413500.01.T01	100,00	0,00E+00	710	Os02g0279600	100,00	0,00E+00	710	0	NA	Japonica	
maker-2-augustus-gene-103.4-mRNA-1	2	OsR498G0203410800.01.T01	98,29	0,00E+00	3054	Os02g0278700	100,00	0,00E+00	3169	115	NA	Japonica	
maker-2-augustus-gene-104.12-mRNA-1	2	OsR498G0611713700.01.T03	92,61	0,00E+00	1552	Os02g0280700	100,00	0,00E+00	1905	353	Japonica	Japonica	
maker-2-augustus-gene-104.16-mRNA-1	2	OsR498G0203419100.01.T05	98,28	0,00E+00	4041	Os02g0281000	98,41	0,00E+00	4055	14	NA	Japonica	
maker-2-augustus-gene-104.2-mRNA-1	2	OsR498G0203415300.01.T01	98,31	0,00E+00	1527	Os02g0280200	100,00	0,00E+00	1608	81	NA	Japonica	
maker-2-augustus-gene-104.20-mRNA-1	2	OsR498G0203415200.01.T02	98,70	0,00E+00	2287	Os02g0280100	100,00	0,00E+00	2360	73	NA	Japonica	
maker-2-augustus-gene-104.22-mRNA-1	2	OsR498G0203417000.01.T02	99,82	0,00E+00	992	Os05g0230900	71,82	3,38E-77	288	-704	Indica	Japonica	
maker-2-augustus-gene-104.23-mRNA-1	2	OsR498G0203417000.01.T02	97,72	0,00E+00	899	Os05g0230900	76,15	3,17E-45	182	-717	Indica	Japonica	
maker-2-augustus-gene-104.6-mRNA-1	2	OsR498G0203416700.01.T05	100,00	0,00E+00	2122	Os02g0280400	100,00	0,00E+00	2122	0	NA	Japonica	
maker-2-augustus-gene-105.32-mRNA-1	2	OsR498G0203420100.01.T02	80,51	0,00E+00	1400	Os02g0281900	99,94	0,00E+00	2835	1435	Japonica	Japonica	CC-NBS-TM
maker-2-augustus-gene-105.34-mRNA-1	2	OsR498G0203421200.01.T01	96,78	0,00E+00	723	Os02g0282100	100,00	0,00E+00	785	62	NA	Japonica	
maker-2-augustus-gene-106.0-mRNA-1	2	OsR498G0203421800.01.T01	99,01	0,00E+00	3205	Os02g0282900	100,00	0,00E+00	3296	91	NA	Japonica	
maker-2-augustus-gene-107.34-mRNA-1	2	OsR498G0203424500.01.T01	100,00	0,00E+00	3290	Os02g0283800	100,00	0,00E+00	3290	0	NA	NA	Kinase-LRR-TM
maker-2-augustus-gene-107.42-mRNA-1	2	OsR498G0203429800.01.T01	100,00	0,00E+00	1256	Os02g0285300	99,57	0,00E+00	1246	-10	NA	NA	
maker-2-augustus-gene-108.0-mRNA-1	2	OsR498G0203431100.01.T03	100,00	0,00E+00	3626	Os02g0285800	100,00	0,00E+00	3626	0	NA	NA	
maker-2-augustus-gene-108.17-mRNA-1	2	OsR498G0203436600.01.T01	100,00	0,00E+00	1418	Os02g0287000	100,00	0,00E+00	1418	0	NA	NA	
maker-2-augustus-gene-108.7-mRNA-1	2	OsR498G0203433300.01.T01	100,00	0,00E+00	1326	Os03g0711650	96,64	0,00E+00	636	-690	Indica	NA	
maker-2-augustus-gene-109.44-mRNA-1	2	OsR498G0203439000.01.T01	99,11	0,00E+00	2972	Os02g0288100	99,11	0,00E+00	2972	0	NA	NA	
maker-2-augustus-gene-109.48-mRNA-1	2	OsR498G0203440600.01.T01	97,37	0,00E+00	4160	Os02g0288733	100,00	0,00E+00	3382	-778	Indica	NA	
maker-2-augustus-gene-11.0-mRNA-1	2	OsR498G0202862500.01.T01	100,00	0,00E+00	1375	Os02g0120300	100,00	0,00E+00	1375	0	NA	NA	
maker-2-augustus-gene-11.11-mRNA-1	2	OsR498G0202869300.01.T01	100,00	0,00E+00	3225	Os02g0121700	100,00	0,00E+00	3225	0	NA	NA	
maker-2-augustus-gene-11.14-mRNA-1	2	OsR498G0202869500.01.T01	88,24	2,05E-172	606	Os02g0121800	88,24	7,12E-173	606	0	NA	NA	
maker-2-augustus-gene-11.15-mRNA-1	2	OsR498G0202862800.01.T02	100,00	0,00E+00	1094	Os02g0120400	100,00	0,00E+00	769	-325	Indica	NA	
maker-2-augustus-gene-11.19-mRNA-1	2	OsR498G0202863200.01.T01	99,82	0,00E+00	2983	Os02g0120500	99,82	0,00E+00	2983	0	NA	NA	
maker-2-augustus-gene-11.22-mRNA-1	2	OsR498G0202863800.01.T02	100,00	1,19E-151	536	Os02g0120800	100,00	4,13E-152	536	0	NA	NA	
maker-2-augustus-gene-11.25-mRNA-1	2	OsR498G0202864000.01.T01	100,00	0,00E+00	2013	Os02g0120900	100,00	0,00E+00	2013	0	NA	NA	
maker-2-augustus-gene-11.27-mRNA-1	2	OsR498G0202864400.01.T01	100,00	0,00E+00	3074	Os02g0121000	100,00	0,00E+00	3074	0	NA	NA	
maker-2-augustus-gene-11.34-mRNA-1	2	OsR498G0202865800.01.T02	100,00	0,00E+00	2399	Os02g0121200	100,00	0,00E+00	2399	0	NA	NA	
maker-2-augustus-gene-11.41-mRNA-1	2	OsR498G0202867800.01.T01	99,80	0,00E+00	2636	Os02g0121500	99,85	0,00E+00	3513	877	Japonica	NA	
maker-2-augustus-gene-11.5-mRNA-1	2	OsR498G0202863500.01.T01	100,00	0,00E+00	807	Os02g0120600	100,00	0,00E+00	807	0	NA	NA	
maker-2-augustus-gene-110.0-mRNA-1	2	OsR498G0203449900.01.T01	100,00	0,00E+00	1543	Os02g0290400	100,00	0,00E+00	1543	0	NA	NA	
maker-2-augustus-gene-110.12-mRNA-1	2	OsR498G0203450200.01.T01	100,00	0,00E+00	3063	Os02g0290500	100,00	0,00E+00	3063	0	NA	NA	
maker-2-augustus-gene-110.14-mRNA-1	2	OsR498G0203450400.01.T01	98,77	0,00E+00	4281	Os02g0290600	98,33	0,00E+00	3118	-1163	Indica	NA	
maker-2-augustus-gene-110.4-mRNA-1	2	OsR498G0203451100.01.T01	100,00	0,00E+00	1478	Os02g0290900	97,60	0,00E+00	2218	740	Japonica	NA	
maker-2-augustus-gene-110.9-mRNA-1	2	OsR498G0203449400.01.T03	98,48	0,00E+00	4140	Os02g0290300	98,48	0,00E+00	4140	0	NA	NA	
maker-2-augustus-gene-111.42-mRNA-1	2	OsR498G0203459900.01.T02	97,32	0,00E+00	2093	Os02g0292600	97,56	0,00E+00	2106	13	NA	Japonica	
maker-2-augustus-gene-111.54-mRNA-1	2	OsR498G0203461200.01.T01	99,87	0,00E+00	1354	Os02g0292800	100,00	0,00E+00	1359	5	NA	Japonica	
maker-2-augustus-gene-112.10-mRNA-1	2	OsR498G0203462400.01.T01	99,46	0,00E+00	2621	Os01g0886600	71,77	3,21E-139	496	-2125	Indica	Japonica	
maker-2-augustus-gene-112.19-mRNA-1	2	OsR498G0203464600.01.T03	99,52	0,00E+00	4442	Os02g0295700	97,95	0,00E+00	4263	-179	NA	Japonica	
maker-2-augustus-gene-112.2-mRNA-1	2	OsR498G0203463300.01.T01	99,40	0,00E+00	3854	Os02g0293800	100,00	0,00E+00	3398	-456	Indica	Japonica	
maker-2-augustus-gene-112.8-mRNA-1	2	OsR498G0203461800.01.T01	99,81	0,00E+00	1885	Os02g0293400	100,00	0,00E+00	1894	9	NA	Japonica	
maker-2-augustus-gene-113.0-mRNA-1	2	OsR498G0203474200.01.T03	99,61	0,00E+00	4110	Os02g0295700	99,96	0,00E+00	4146	36	NA	Japonica	
maker-2-augustus-gene-113.3-mRNA-1	2	OsR498G0203475200.01.T01	99,75	0,00E+00	2137	Os02g0296300	96,35	0,00E+00	2288	151	NA	Japonica	
maker-2-augustus-gene-113.6-mRNA-1	2	OsR498G0203475400.01.T01	88,11	0,00E+00	1276	Os04g0613400	74,73	8,16E-100	363	-913	Indica	Japonica	
maker-2-augustus-gene-114.31-mRNA-1	2	OsR498G0203478900.01.T06	99,75	0,00E+00	2108	Os02g0297600	100,00	0,00E+00	2122	14	NA	Japonica	
maker-2-augustus-gene-115.0-mRNA-1	2	OsR498G0203486100.01.T02	97,25	0,00E+00	739	Os02g0299200	100,00	0,00E+00	787	48	NA	Japonica	
maker-2-augustus-gene-115.1-mRNA-1	2	OsR498G0203486100.01.T03	99,71	0,00E+00	1859	Os02g0299200	100,00	0,00E+00	1847	-12	NA	Japonica	
maker-2-augustus-gene-115.12-mRNA-1	2	OsR498G0203488400.01.T01	99,53	8,22E-104	377	Os02g0299600	100,00	6,73E-106	381	4	NA	Japonica	
maker-2-augustus-gene-115.8-mRNA-1	2	OsR498G0203486400.01.T02	97,26	0,00E+00	2027	Os02g0299300	96,27	0,00E+00	1448	-579	Indica	Japonica	
maker-2-augustus-gene-116.30-mRNA-1	2	OsR498G0203494100.01.T01	99,77	0,00E+00	1566	Os02g0301000	99,89	0,00E+00	1571	5	NA	Japonica	
maker-2-augustus-gene-116.32-mRNA-1	2	OsR498G0203495200.01.T02	99,49	0,00E+00	1389	Os02g0301100	100,00	0,00E+00	1407	18	NA	Japonica	
maker-2-augustus-gene-117.41-mRNA-1	2	OsR498G0203498500.01.T01	99,71	0,00E+00	6665	Os06g0343100	75,04	0,00E+00	1954	-4711	Indica	Japonica	
maker-2-augustus-gene-117.44-mRNA-1	2	OsR498G0203500500.01.T01	99,83	0,00E+00	1078	Os04g0613400	76,44	1,61E-133	476	-602	Indica	Japonica	
maker-2-augustus-gene-118.37-mRNA-1	2	OsR498G0203502900.01.T01	99,58	0,00E+00	2140	Os02g0302700	100,00	0,00E+00	2163	23	NA	Japonica	
maker-2-augustus-gene-118.40-mRNA-1	2	OsR498G0203503300.01.T02	96,52	0,00E+00	2232	Os02g0302900	100,00	0,00E+00	2441	209	NA	Japonica	
maker-2-augustus-gene-119.1-mRNA-1	2	OsR498G0203517300.01.T04	100,00	7,15E-160	563	Os02g0305801	100,00	2,49E-160	563	0	NA	NA	
maker-2-augustus-gene-119.11-mRNA-1	2	OsR498G0203510000.01.T04	100,00	0,00E+00	1267	Os02g0305600	100,00	0,00E+00	1267	0	NA	Japonica	
maker-2-augustus-gene-119.8-mRNA-1	2	OsR498G0203508800.01.T01	99,74	0,00E+00	696	Os02g0304900	100,00	0,00E+00	700	4	NA	Japonica	
maker-2-augustus-gene-12.101-mRNA-1	2	OsR498G0202874400.01.T02	99,44	2,29E-179	628	Os02g0123600	99,44	7,98E-180	628	0	NA	NA	
maker-2-augustus-gene-12.47-mRNA-1	2	OsR498G0202873300.01.T01	100,00	0,00E+00	2040	Os02g0123100	100,00	0,00E+00	1582	-458	Indica	NA	Kinase
maker-2-augustus-gene-12.54-mRNA-1	2	OsR498G0202873700.01.T01	100,00	0,00E+00	5054	Os07g0124500	93,65	0,00E+00	4266	-788	Indica	NA	
maker-2-augustus-gene-12.58-mRNA-1	2	OsR498G0202874500.01.T01	100,00	0,00E+00	2727	Os02g0123700	100,00	0,00E+00	2727	0	NA	NA	
maker-2-augustus-gene-12.63-mRNA-1	2	OsR498G0202870200.01.T02	100,00	0,00E+00	829	Os02g0122000	100,00	0,00E+00	829	0	NA	NA	
maker-2-augustus-gene-12.75-mRNA-1	2	OsR498G0202870800.01.T02	100,00	0,00E+00	1012	Os02g0122300	100,00	0,00E+00	1012	0	NA	NA	
maker-2-augustus-gene-12.85-mRNA-1	2	OsR498G0202872600.01.T02	99,88	0,00E+00	1511	Os02g0122800	100,00	0,00E+00	1516	5	NA	NA	
maker-2-augustus-gene-12.93-mRNA-1	2	OsR498G0202874000.01.T05	99,96	0,00E+00	4314	Os02g0123400	99,94	0,00E+00	2892	-1422	Indica	NA	
maker-2-augustus-gene-12.96-mRNA-1	2	OsR498G0202874200.01.T01	98,17	0,00E+00	1993	Os02g0123500	98,17	0,00E+00	1993	0	NA	NA	
maker-2-augustus-gene-120.0-mRNA-1	2	OsR498G0203521900.01.T01	99,15	0,00E+00	1874	Os02g0306900	100,00	0,00E+00	1894	20	NA	Japonica	
maker-2-augustus-gene-120.14-mRNA-1	2	OsR498G0203522400.01.T02	99,89	0,00E+00	3285	Os02g0307000	100,00	0,00E+00	3294	9	NA	Japonica	
maker-2-augustus-gene-120.21-mRNA-1	2	OsR498G0102370200.01.T01	95,91	0,00E+00	1517	Os02g0307300	95,86	0,00E+00	2256	739	Japonica	Japonica	
maker-2-augustus-gene-121.33-mRNA-1	2	OsR498G0203525300.01.T01	99,76	0,00E+00	1474	Os02g0307800	100,00	0,00E+00	1483	9	NA	Japonica	
maker-2-augustus-gene-123.0-mRNA-1	2	OsR498G0203538300.01.T01	99,80	0,00E+00	900	Os02g0312500	100,00	0,00E+00	904	4	NA	Japonica	
maker-2-augustus-gene-123.10-mRNA-1	2	OsR498G0203538600.01.T01	99,83	0,00E+00	1067	Os02g0312600	99,83	0,00E+00	1067	0	NA	Japonica	
maker-2-augustus-gene-123.15-mRNA-1	2	OsR498G0203539200.01.T01	99,86	0,00E+00	1278	Os02g0312700	100,00	0,00E+00	733	-545	Indica	Japonica	
maker-2-augustus-gene-123.18-mRNA-1	2	OsR498G0203539800.01.T01	97,10	0,00E+00	2395	Os02g0312800	97,24	0,00E+00	2404	9	NA	Japonica	
maker-2-augustus-gene-123.3-mRNA-1	2	OsR498G0203541100.01.T01	99,88	0,00E+00	2902	Os02g0313400	100,00	0,00E+00	1472	-1430	Indica	Japonica	
maker-2-augustus-gene-124.35-mRNA-1	2	OsR498G0203542600.01.T02	99,54	0,00E+00	3489	Os02g0313700	100,00	0,00E+00	3530	41	NA	Japonica	
maker-2-augustus-gene-125.33-mRNA-1	2	OsR498G0203550700.01.T03	99,78	0,00E+00	1631	Os02g0633100	74,02	4,91E-160	563	-1068	Indica	Japonica	
maker-2-augustus-gene-125.35-mRNA-1	2	OsR498G0203549800.01.T01	99,69	0,00E+00	2282	Os02g0316200	100,00	0,00E+00	2300	18	NA	Japonica	
maker-2-augustus-gene-126.33-mRNA-1	2	OsR498G0203550700.01.T05	99,54	0,00E+00	1566	Os02g0633100	79,44	0,00E+00	784	-782	Indica	Japonica	
maker-2-augustus-gene-126.36-mRNA-1	2	OsR498G0203551300.01.T01	99,92	0,00E+00	2272	Os02g0317100	100,00	0,00E+00	2435	163	NA	Japonica	
maker-2-augustus-gene-126.38-mRNA-1	2	OsR498G0203553300.01.T04	100,00	0,00E+00	991	Os02g0317400	100,00	0,00E+00	991	0	NA	Japonica	
maker-2-augustus-gene-126.41-mRNA-1	2	OsR498G0203553900.01.T02	99,73	0,00E+00	1319	Os02g0317700	100,00	0,00E+00	1328	9	NA	Japonica	
maker-2-augustus-gene-126.45-mRNA-1	2	OsR498G0203556100.01.T02	100,00	0,00E+00	1581	Os02g0318100	100,00	0,00E+00	996	-585	Indica	Japonica	
maker-2-augustus-gene-126.49-mRNA-1	2	OsR498G0203553600.01.T01	99,71	0,00E+00	1227	Os02g0317500	100,00	0,00E+00	1236	9	NA	Japonica	
maker-2-augustus-gene-126.51-mRNA-1	2	OsR498G0203555200.01.T01	99,82	0,00E+00	992	Os02g0317800	100,00	0,00E+00	845	-147	Indica	Japonica	
maker-2-augustus-gene-127.36-mRNA-1	2	OsR498G0203560900.01.T01	99,76	0,00E+00	2935	Os02g0319100	100,00	0,00E+00	2953	18	NA	Japonica	
maker-2-augustus-gene-127.44-mRNA-1	2	OsR498G0203563400.01.T02	94,44	0,00E+00	1315	Os02g0320300	100,00	0,00E+00	1521	206	Japonica	Japonica	
maker-2-augustus-gene-127.48-mRNA-1	2	OsR498G0203559000.01.T05	99,03	0,00E+00	4013	Os02g0318500	96,57	0,00E+00	3220	-793	Indica	Japonica	NBS-TM
maker-2-augustus-gene-127.58-mRNA-1	2	OsR498G0203562000.01.T03	99,78	0,00E+00	2433	Os02g0319800	99,93	0,00E+00	2442	9	NA	Japonica	
maker-2-augustus-gene-127.62-mRNA-1	2	OsR498G0203562400.01.T02	99,83	0,00E+00	2129	Os02g0320100	100,00	0,00E+00	1784	-345	Indica	Japonica	
maker-2-augustus-gene-128.35-mRNA-1	2	OsR498G0203566300.01.T01	95,29	0,00E+00	1450	Os02g0320800	100,00	0,00E+00	1640	190	Japonica	Japonica	
maker-2-augustus-gene-128.39-mRNA-1	2	OsR498G0203567300.01.T02	98,73	0,00E+00	1373	Os02g0321500	100,00	0,00E+00	1418	45	NA	Japonica	
maker-2-augustus-gene-128.44-mRNA-1	2	OsR498G0203567900.01.T01	99,42	0,00E+00	1214	Os02g0321800	100,00	0,00E+00	1229	15	NA	Japonica	
maker-2-augustus-gene-128.46-mRNA-1	2	OsR498G0203568300.01.T02	99,54	0,00E+00	780	Os02g0321900	100,00	0,00E+00	708	-72	NA	Japonica	
maker-2-augustus-gene-129.0-mRNA-1	2	OsR498G0203569900.01.T01	99,92	0,00E+00	2241	Os02g0322400	98,38	0,00E+00	2263	22	NA	Japonica	Kinase-TM
maker-2-augustus-gene-13.10-mRNA-1	2	OsR498G0202878100.01.T02	100,00	0,00E+00	2105	Os02g0125200	100,00	0,00E+00	2105	0	NA	NA	
maker-2-augustus-gene-13.13-mRNA-1	2	OsR498G0202878700.01.T01	100,00	0,00E+00	1353	Os02g0125300	100,00	0,00E+00	1353	0	NA	NA	
maker-2-augustus-gene-13.17-mRNA-1	2	OsR498G0202878900.01.T01	100,00	0,00E+00	1375	Os02g0125400	100,00	0,00E+00	767	-608	Indica	NA	
maker-2-augustus-gene-13.21-mRNA-1	2	OsR498G0202879300.01.T03	100,00	0,00E+00	2607	Os04g0432400	71,30	1,82E-180	633	-1974	Indica	NA	
maker-2-augustus-gene-13.28-mRNA-1	2	OsR498G0202876000.01.T01	100,00	0,00E+00	3436	Os02g0124600	88,66	0,00E+00	2229	-1207	Indica	NA	
maker-2-augustus-gene-13.30-mRNA-1	2	OsR498G0202876200.01.T01	100,00	0,00E+00	2771	Os02g0124600	100,00	0,00E+00	3247	476	Japonica	NA	
maker-2-augustus-gene-13.32-mRNA-1	2	OsR498G0202876600.01.T01	98,26	0,00E+00	2194	Os04g0119500	71,22	7,24E-96	351	-1843	Indica	NA	
maker-2-augustus-gene-13.36-mRNA-1	2	OsR498G0202878000.01.T01	100,00	0,00E+00	2787	Os02g0125100	100,00	0,00E+00	2787	0	NA	NA	
maker-2-augustus-gene-13.8-mRNA-1	2	OsR498G0202877500.01.T01	99,80	0,00E+00	887	Os02g0125000	99,80	0,00E+00	887	0	NA	NA	
maker-2-augustus-gene-130.0-mRNA-1	2	OsR498G0203578100.01.T02	99,89	0,00E+00	1673	Os02g0324300	100,00	0,00E+00	1678	5	NA	Japonica	
maker-2-augustus-gene-130.4-mRNA-1	2	OsR498G0203580700.01.T01	99,78	0,00E+00	824	Os02g0324700	100,00	0,00E+00	829	5	NA	Japonica	
maker-2-augustus-gene-130.6-mRNA-1	2	OsR498G0203583700.01.T01	100,00	0,00E+00	2273	Os02g0325100	100,00	0,00E+00	2273	0	NA	Japonica	CC-NBS-TM
maker-2-augustus-gene-131.32-mRNA-1	2	OsR498G1119390000.01.T01	92,00	1,65E-34	145	Os02g0325401	100,00	2,98E-51	199	54	Japonica	Japonica	
maker-2-augustus-gene-131.34-mRNA-1	2	OsR498G0203585900.01.T01	99,60	0,00E+00	2216	Os02g0325600	100,00	0,00E+00	2235	19	NA	Japonica	
maker-2-augustus-gene-131.38-mRNA-1	2	OsR498G0203590900.01.T01	99,29	0,00E+00	1495	Os02g0326200	100,00	0,00E+00	1521	26	NA	Japonica	
maker-2-augustus-gene-131.40-mRNA-1	2	OsR498G0203591500.01.T01	99,93	0,00E+00	7697	Os02g0326600	100,00	0,00E+00	5395	-2302	Indica	Japonica	
maker-2-augustus-gene-131.44-mRNA-1	2	OsR498G0203592300.01.T04	97,71	0,00E+00	1724	Os02g0326700	100,00	0,00E+00	1739	15	NA	Japonica	
maker-2-augustus-gene-131.47-mRNA-1	2	OsR498G0203594100.01.T01	100,00	0,00E+00	899	Os02g0327000	100,00	0,00E+00	899	0	NA	Japonica	
maker-2-augustus-gene-131.52-mRNA-1	2	OsR498G0203584200.01.T04	90,85	3,80E-57	221	Os10g0561900	80,35	1,33E-38	158	-63	Indica	Japonica	
maker-2-augustus-gene-131.54-mRNA-1	2	OsR498G0203590400.01.T02	99,79	0,00E+00	835	Os02g0326000	100,00	0,00E+00	839	4	NA	Japonica	
maker-2-augustus-gene-132.12-mRNA-1	2	OsR498G0203596400.01.T01	99,86	0,00E+00	2599	Os02g0327700	100,00	0,00E+00	2608	9	NA	Japonica	
maker-2-augustus-gene-132.8-mRNA-1	2	OsR498G0203594600.01.T01	99,82	0,00E+00	1003	Os02g0327100	100,00	0,00E+00	1007	4	NA	Japonica	
maker-2-augustus-gene-133.0-mRNA-1	2	OsR498G0203601100.01.T03	100,00	0,00E+00	1458	Os02g0328300	100,00	0,00E+00	1447	-11	NA	Japonica	
maker-2-augustus-gene-133.11-mRNA-1	2	OsR498G0203601400.01.T01	99,51	0,00E+00	1819	Os04g0590366	96,47	0,00E+00	1680	-139	NA	Japonica	
maker-2-augustus-gene-133.4-mRNA-1	2	OsR498G0203602700.01.T04	99,65	7,50E-144	510	Os02g0224900	87,10	3,89E-47	187	-323	Indica	Japonica	
maker-2-augustus-gene-133.9-mRNA-1	2	OsR498G0203609300.01.T01	99,94	0,00E+00	3064	Os02g0329800	100,00	0,00E+00	3068	4	NA	Japonica	
maker-2-augustus-gene-134.38-mRNA-1	2	OsR498G0203612300.01.T01	99,94	0,00E+00	2894	Os02g0330200	100,00	0,00E+00	2899	5	NA	Japonica	
maker-2-augustus-gene-135.0-mRNA-1	2	OsR498G0203614100.01.T05	99,74	0,00E+00	2715	Os02g0331200	100,00	0,00E+00	2733	18	NA	Japonica	
maker-2-augustus-gene-135.3-mRNA-1	2	OsR498G0203621100.01.T01	97,91	0,00E+00	737	Os08g0387050	98,32	5,35E-175	612	-125	Indica	Japonica	
maker-2-augustus-gene-135.8-mRNA-1	2	OsR498G0203620800.01.T01	96,98	0,00E+00	2795	Os02g0332200	97,10	0,00E+00	2804	9	NA	Japonica	
maker-2-augustus-gene-14.43-mRNA-1	2	OsR498G0202882700.01.T02	99,73	0,00E+00	2002	Os02g0126400	99,20	0,00E+00	1986	-16	NA	NA	
maker-2-augustus-gene-14.52-mRNA-1	2	OsR498G0202883300.01.T01	100,00	0,00E+00	1342	Os02g0126800	100,00	0,00E+00	1342	0	NA	NA	
maker-2-augustus-gene-14.55-mRNA-1	2	OsR498G0202883700.01.T01	100,00	0,00E+00	1683	Os02g0127000	100,00	0,00E+00	1229	-454	Indica	NA	
maker-2-augustus-gene-14.58-mRNA-1	2	OsR498G0202885900.01.T01	100,00	0,00E+00	1846	Os02g0127800	100,00	0,00E+00	1846	0	NA	NA	
maker-2-augustus-gene-14.62-mRNA-1	2	OsR498G0202886900.01.T01	100,00	0,00E+00	921	Os02g0128100	100,00	0,00E+00	832	-89	NA	NA	
maker-2-augustus-gene-14.65-mRNA-1	2	OsR498G0202882200.01.T01	100,00	0,00E+00	3030	Os02g0126300	100,00	0,00E+00	3030	0	NA	NA	
maker-2-augustus-gene-14.67-mRNA-1	2	OsR498G0202883200.01.T01	100,00	9,69E-136	482	Os02g0126700	100,00	3,39E-136	482	0	NA	NA	
maker-2-augustus-gene-14.71-mRNA-1	2	OsR498G0202883600.01.T01	97,50	0,00E+00	1024	Os02g0126900	100,00	0,00E+00	1965	941	Japonica	NA	
maker-2-augustus-gene-140.19-mRNA-1	2	OsR498G0203625700.01.T04	95,11	0,00E+00	1756	Os02g0433600	95,29	0,00E+00	1765	9	NA	Japonica	
maker-2-augustus-gene-141.32-mRNA-1	2	OsR498G0203632400.01.T01	99,64	0,00E+00	2001	Os02g0435000	100,00	0,00E+00	2019	18	NA	Japonica	
maker-2-augustus-gene-141.42-mRNA-1	2	OsR498G0203639000.01.T01	99,90	0,00E+00	1857	Os02g0436400	100,00	0,00E+00	1862	5	NA	Japonica	
maker-2-augustus-gene-142.0-mRNA-1	2	OsR498G0203646900.01.T02	99,81	0,00E+00	1838	Os02g0438200	100,00	0,00E+00	1847	9	NA	Japonica	
maker-2-augustus-gene-142.15-mRNA-1	2	OsR498G0203645800.01.T07	100,00	0,00E+00	1712	Os02g0437800	100,00	0,00E+00	1712	0	NA	Japonica	
maker-2-augustus-gene-142.5-mRNA-1	2	OsR498G0203643200.01.T06	100,00	0,00E+00	1573	Os02g0437200	100,00	0,00E+00	1357	-216	Indica	Japonica	
maker-2-augustus-gene-143.2-mRNA-1	2	OsR498G0203648900.01.T01	99,59	0,00E+00	3872	Os02g0439200	100,00	0,00E+00	3912	40	NA	Japonica	
maker-2-augustus-gene-143.4-mRNA-1	2	OsR498G0203653400.01.T02	99,86	0,00E+00	2648	Os02g0440000	99,93	0,00E+00	2653	5	NA	Japonica	
maker-2-augustus-gene-144.0-mRNA-1	2	OsR498G0203657700.01.T01	98,19	0,00E+00	3145	Os10g0122600	94,06	0,00E+00	2807	-338	Indica	Japonica	
maker-2-augustus-gene-145.0-mRNA-1	2	OsR498G0407986600.01.T01	98,91	0,00E+00	802	Os02g0443000	100,00	0,00E+00	1045	243	Japonica	NA	
maker-2-augustus-gene-146.6-mRNA-1	2	OsR498G0203665800.01.T01	98,92	0,00E+00	2919	Os02g0701833	93,85	0,00E+00	2551	-368	Indica	Japonica	
maker-2-augustus-gene-148.3-mRNA-1	2	OsR498G0203676600.01.T01	99,88	0,00E+00	2908	Os02g0448400	99,94	0,00E+00	2912	4	NA	Japonica	
maker-2-augustus-gene-15.0-mRNA-1	2	OsR498G0202887300.01.T05	100,00	0,00E+00	1884	Os02g0128200	100,00	0,00E+00	1884	0	NA	NA	
maker-2-augustus-gene-15.22-mRNA-1	2	OsR498G0202887600.01.T05	99,96	0,00E+00	4270	Os02g0128300	99,92	0,00E+00	2382	-1888	Indica	NA	
maker-2-augustus-gene-15.27-mRNA-1	2	OsR498G0202888400.01.T03	100,00	0,00E+00	1673	Os02g0128800	100,00	0,00E+00	1673	0	NA	NA	
maker-2-augustus-gene-15.32-mRNA-1	2	OsR498G0202895000.01.T04	100,00	0,00E+00	1317	Os02g0129300	100,00	0,00E+00	847	-470	Indica	NA	
maker-2-augustus-gene-152.7-mRNA-1	2	OsR498G0203710800.01.T01	99,22	0,00E+00	2711	Os02g0453600	100,00	0,00E+00	2765	54	NA	Japonica	
maker-2-augustus-gene-152.9-mRNA-1	2	OsR498G0203711000.01.T04	98,57	9,45E-137	487	Os08g0114100	77,78	1,60E-33	141	-346	Indica	Japonica	
maker-2-augustus-gene-154.38-mRNA-1	2	OsR498G0203722500.01.T01	98,70	0,00E+00	1205	Os07g0165500	88,55	0,00E+00	785	-420	Indica	Japonica	
maker-2-augustus-gene-154.43-mRNA-1	2	OsR498G0203722400.01.T02	99,83	0,00E+00	4105	Os02g0456000	100,00	0,00E+00	2989	-1116	Indica	Japonica	
maker-2-augustus-gene-154.46-mRNA-1	2	OsR498G0203723000.01.T05	99,82	0,00E+00	1939	Os02g0456200	99,90	0,00E+00	1807	-132	NA	Japonica	
maker-2-augustus-gene-155.2-mRNA-1	2	OsR498G0203727200.01.T01	99,74	0,00E+00	4176	Os02g0457500	100,00	0,00E+00	4199	23	NA	Japonica	
maker-2-augustus-gene-156.40-mRNA-1	2	OsR498G0203738100.01.T01	98,02	0,00E+00	1298	Os02g0459800	100,00	0,00E+00	1137	-161	Indica	Japonica	
maker-2-augustus-gene-156.42-mRNA-1	2	OsR498G0203736400.01.T01	95,73	0,00E+00	1682	Os02g0459300	100,00	0,00E+00	2289	607	Japonica	Japonica	
maker-2-augustus-gene-157.2-mRNA-1	2	OsR498G0102635500.01.T01	90,91	3,54E-46	185	Os02g0462000	100,00	6,41E-63	239	54	Japonica	Japonica	
maker-2-augustus-gene-157.4-mRNA-1	2	OsR498G0203747100.01.T01	99,74	0,00E+00	1366	Os02g0462800	100,00	0,00E+00	1375	9	NA	Japonica	
maker-2-augustus-gene-157.6-mRNA-1	2	OsR498G0203745800.01.T02	100,00	0,00E+00	899	Os02g0461900	99,80	0,00E+00	894	-5	NA	Japonica	
maker-2-augustus-gene-158.0-mRNA-1	2	OsR498G0203752300.01.T01	100,00	0,00E+00	4962	Os02g0464500	100,00	0,00E+00	4962	0	NA	NA	
maker-2-augustus-gene-158.10-mRNA-1	2	OsR498G0203754200.01.T01	100,00	0,00E+00	4210	Os02g0464900	100,00	0,00E+00	2982	-1228	Indica	NA	
maker-2-augustus-gene-159.41-mRNA-1	2	OsR498G0816133900.01.T03	94,84	1,16E-109	397	Os02g0465112	100,00	1,09E-142	504	107	Japonica	NA	
maker-2-augustus-gene-159.43-mRNA-1	2	OsR498G0203756600.01.T02	99,90	0,00E+00	1787	Os02g0465400	99,90	0,00E+00	1787	0	NA	NA	
maker-2-augustus-gene-159.48-mRNA-1	2	OsR498G0203757000.01.T01	100,00	0,00E+00	1342	Os02g0465500	100,00	0,00E+00	1342	0	NA	NA	
maker-2-augustus-gene-159.53-mRNA-1	2	OsR498G0203758400.01.T03	100,00	0,00E+00	1175	Os02g0465900	100,00	0,00E+00	1175	0	NA	NA	
maker-2-augustus-gene-159.61-mRNA-1	2	OsR498G0203763000.01.T01	100,00	0,00E+00	2890	Os02g0467000	100,00	0,00E+00	2890	0	NA	NA	
maker-2-augustus-gene-16.39-mRNA-1	2	OsR498G0202900900.01.T03	100,00	0,00E+00	1965	Os02g0130600	100,00	0,00E+00	1965	0	NA	NA	
maker-2-augustus-gene-16.44-mRNA-1	2	OsR498G0202901100.01.T01	100,00	0,00E+00	650	Os02g0130700	97,04	6,56E-179	626	-24	NA	NA	
maker-2-augustus-gene-16.58-mRNA-1	2	OsR498G0202902900.01.T01	100,00	0,00E+00	1305	Os02g0131300	100,00	0,00E+00	1305	0	NA	NA	
maker-2-augustus-gene-16.63-mRNA-1	2	OsR498G0202897600.01.T01	100,00	0,00E+00	715	Os02g0130000	100,00	0,00E+00	715	0	NA	NA	
maker-2-augustus-gene-16.65-mRNA-1	2	OsR498G0202897800.01.T01	100,00	0,00E+00	1386	Os02g0130100	100,00	0,00E+00	1386	0	NA	NA	
maker-2-augustus-gene-16.70-mRNA-1	2	OsR498G0202898000.01.T01	98,63	0,00E+00	2674	Os02g0130200	98,63	0,00E+00	2674	0	NA	NA	
maker-2-augustus-gene-16.72-mRNA-1	2	OsR498G0202898200.01.T03	100,00	0,00E+00	1862	Os02g0130300	100,00	0,00E+00	1862	0	NA	NA	
maker-2-augustus-gene-16.80-mRNA-1	2	OsR498G0202903600.01.T01	100,00	0,00E+00	3360	Os02g0131400	100,00	0,00E+00	3360	0	NA	NA	
maker-2-augustus-gene-160.35-mRNA-1	2	OsR498G0203764700.01.T01	100,00	0,00E+00	2890	Os02g0467600	100,00	0,00E+00	2890	0	NA	NA	
maker-2-augustus-gene-160.37-mRNA-1	2	OsR498G0203764900.01.T06	100,00	0,00E+00	641	Os02g0467700	100,00	0,00E+00	641	0	NA	NA	
maker-2-augustus-gene-160.40-mRNA-1	2	OsR498G0203767300.01.T01	99,92	0,00E+00	2341	Os02g0468400	100,00	0,00E+00	2345	4	NA	NA	
maker-2-augustus-gene-160.43-mRNA-1	2	OsR498G0203767000.01.T02	100,00	0,00E+00	1514	Os02g0468200	100,00	0,00E+00	1337	-177	Indica	NA	
maker-2-augustus-gene-161.0-mRNA-1	2	OsR498G0203772900.01.T01	98,13	0,00E+00	4164	Os02g0469200	99,68	0,00E+00	2819	-1345	Indica	NA	
maker-2-augustus-gene-161.10-mRNA-1	2	OsR498G0203773700.01.T10	100,00	1,55E-95	349	Os02g0469300	100,00	5,39E-96	349	0	NA	NA	
maker-2-augustus-gene-161.25-mRNA-1	2	OsR498G0203775300.01.T01	100,00	0,00E+00	2024	Os02g0469600	100,00	0,00E+00	2024	0	NA	NA	
maker-2-augustus-gene-161.9-mRNA-1	2	OsR498G0203773700.01.T09	97,08	0,00E+00	2613	Os02g0469300	96,89	0,00E+00	2442	-171	NA	NA	
maker-2-augustus-gene-162.21-mRNA-1	2	OsR498G0203781300.01.T02	99,60	0,00E+00	2254	Os02g0471300	100,00	0,00E+00	2273	19	NA	Japonica	
maker-2-augustus-gene-162.31-mRNA-1	2	OsR498G0203782000.01.T01	98,98	0,00E+00	2756	Os02g0471500	100,00	0,00E+00	2809	53	NA	Japonica	
maker-2-augustus-gene-163.26-mRNA-1	2	OsR498G0203785700.01.T05	99,05	0,00E+00	2037	Os02g0472700	97,14	0,00E+00	2198	161	NA	Japonica	Kinase-TM
maker-2-augustus-gene-163.34-mRNA-1	2	OsR498G0203786400.01.T03	97,06	0,00E+00	2762	Os02g0473000	97,23	0,00E+00	2577	-185	NA	Japonica	
maker-2-augustus-gene-163.39-mRNA-1	2	OsR498G0203787200.01.T04	99,83	0,00E+00	2140	Os02g0473200	100,00	0,00E+00	2679	539	Japonica	Japonica	
maker-2-augustus-gene-165.23-mRNA-1	2	OsR498G0203794900.01.T02	99,70	0,00E+00	2965	Os02g0475300	100,00	0,00E+00	2987	22	NA	Japonica	

maker-2-augustus-gene-165.28-mRNA-1	2	OsR498G0203795300.01.T02	99,69	0,00E+00	2277	Os02g0475400	100,00	0,00E+00	2295	18	NA	Japonica	
maker-2-augustus-gene-167.0-mRNA-1	2	OsR498G0203804900.01.T01	100,00	0,00E+00	1678	Os02g0478550	99,74	0,00E+00	1386	-292	Indica	Indica	
maker-2-augustus-gene-167.18-mRNA-1	2	OsR498G0203807200.01.T01	100,00	0,00E+00	937	Os02g0479300	99,62	0,00E+00	928	-9	NA	Indica	
maker-2-augustus-gene-167.3-mRNA-1	2	OsR498G0203805100.01.T02	100,00	0,00E+00	710	Os02g0478600	99,49	0,00E+00	700	-10	NA	Indica	
maker-2-augustus-gene-167.35-mRNA-1	2	OsR498G0203808800.01.T04	100,00	0,00E+00	1398	Os02g0480100	88,37	0,00E+00	1253	-145	Indica	Indica	
maker-2-augustus-gene-167.5-mRNA-1	2	OsR498G0203805300.01.T02	100,00	3,12E-131	468	Os02g0478700	99,61	4,62E-130	463	-5	NA	Indica	
maker-2-augustus-gene-168.16-mRNA-1	2	OsR498G0203811600.01.T02	100,00	0,00E+00	2686	Os02g0480900	99,33	0,00E+00	2642	-44	NA	Indica	
maker-2-augustus-gene-168.21-mRNA-1	2	OsR498G0203812000.01.T02	100,00	5,75E-165	580	Os02g0481000	95,76	6,12E-153	539	-41	NA	Indica	
maker-2-augustus-gene-168.3-mRNA-1	2	OsR498G0203816900.01.T01	99,82	0,00E+00	4851	Os02g0481900	99,32	0,00E+00	3897	-954	Indica	Indica	
maker-2-augustus-gene-169.37-mRNA-1	2	OsR498G0203817800.01.T01	96,17	0,00E+00	952	Os02g0482400	95,47	0,00E+00	934	-18	NA	Indica	
maker-2-augustus-gene-17.0-mRNA-1	2	OsR498G0202905900.01.T02	100,00	0,00E+00	1539	Os02g0132300	100,00	0,00E+00	1431	-108	NA	NA	
maker-2-augustus-gene-17.11-mRNA-1	2	OsR498G0202904800.01.T01	100,00	0,00E+00	2955	Os02g0131800	100,00	0,00E+00	2955	0	NA	NA	
maker-2-augustus-gene-17.16-mRNA-1	2	OsR498G0202905400.01.T04	100,00	0,00E+00	3826	Os02g0132100	100,00	0,00E+00	4718	892	Japonica	NA	
maker-2-augustus-gene-17.18-mRNA-1	2	OsR498G0202905600.01.T01	100,00	0,00E+00	3009	Os02g0132200	100,00	0,00E+00	3009	0	NA	NA	
maker-2-augustus-gene-17.34-mRNA-1	2	OsR498G0202911000.01.T01	100,00	0,00E+00	2114	Os02g0133200	100,00	0,00E+00	2114	0	NA	NA	
maker-2-augustus-gene-17.5-mRNA-1	2	OsR498G0202910700.01.T02	98,58	0,00E+00	3329	Os02g0133100	97,92	0,00E+00	2241	-1088	Indica	NA	
maker-2-augustus-gene-172.39-mRNA-1	2	OsR498G0203832700.01.T01	99,58	0,00E+00	2124	Os02g0487300	99,16	0,00E+00	2100	-24	NA	NA	
maker-2-augustus-gene-172.43-mRNA-1	2	OsR498G0203835200.01.T03	99,93	0,00E+00	2528	Os02g0488701	99,84	0,00E+00	2286	-242	NA	Indica	
maker-2-augustus-gene-173.0-mRNA-1	2	OsR498G0203838500.01.T01	100,00	0,00E+00	1191	Os02g0489400	99,70	0,00E+00	1182	-9	NA	NA	
maker-2-augustus-gene-174.38-mRNA-1	2	OsR498G0203841100.01.T01	99,72	0,00E+00	3154	Os02g0490000	100,00	0,00E+00	3177	23	NA	Japonica	
maker-2-augustus-gene-174.40-mRNA-1	2	OsR498G0203842300.01.T01	99,66	0,00E+00	2656	Os02g0490500	100,00	0,00E+00	2679	23	NA	Japonica	
maker-2-augustus-gene-174.42-mRNA-1	2	OsR498G0203848100.01.T01	98,66	0,00E+00	1172	Os02g0491700	100,00	0,00E+00	1003	-169	Indica	Japonica	
maker-2-augustus-gene-174.45-mRNA-1	2	OsR498G0203847400.01.T01	98,88	0,00E+00	3604	Os02g0491400	100,00	0,00E+00	3696	92	NA	Japonica	
maker-2-augustus-gene-174.50-mRNA-1	2	OsR498G0203847800.01.T01	99,69	0,00E+00	1166	Os02g0491600	100,00	0,00E+00	1175	9	NA	Japonica	
maker-2-augustus-gene-175.33-mRNA-1	2	OsR498G0203851400.01.T01	98,72	3,05E-113	409	Os02g0494400	73,59	2,34E-33	142	-267	Indica	Japonica	
maker-2-augustus-gene-175.35-mRNA-1	2	OsR498G0203851600.01.T03	99,68	7,75E-160	564	Os02g0493300	72,51	2,38E-53	208	-356	Indica	Japonica	
maker-2-augustus-gene-175.37-mRNA-1	2	OsR498G0203852200.01.T01	99,70	0,00E+00	1187	Os02g0492900	99,85	0,00E+00	1192	5	NA	Japonica	
maker-2-augustus-gene-175.41-mRNA-1	2	OsR498G0203852400.01.T02	95,74	0,00E+00	1542	Os02g0493050	95,49	0,00E+00	1306	-236	Indica	Japonica	
maker-2-augustus-gene-176.11-mRNA-1	2	OsR498G0203853000.01.T02	99,29	2,11E-140	499	Os02g0493300	100,00	0,00E+00	740	241	Japonica	Japonica	
maker-2-augustus-gene-176.12-mRNA-1	2	OsR498G0203853000.01.T05	99,62	0,00E+00	1426	Os02g0493300	100,00	0,00E+00	1440	14	NA	Japonica	
maker-2-augustus-gene-176.21-mRNA-1	2	OsR498G0203854200.01.T03	99,87	0,00E+00	1334	Os02g0494400	90,07	0,00E+00	1007	-327	Indica	Japonica	
maker-2-augustus-gene-176.22-mRNA-1	2	OsR498G0203857500.01.T02	99,37	0,00E+00	1119	Os02g0494400	100,00	0,00E+00	1339	220	Japonica	Japonica	
maker-2-augustus-gene-176.8-mRNA-1	2	OsR498G0203852800.01.T02	99,48	0,00E+00	3451	Os06g0492900	70,70	2,90E-179	628	-2823	Indica	Japonica	
maker-2-augustus-gene-177.2-mRNA-1	2	OsR498G0203858900.01.T01	99,72	0,00E+00	2558	Os02g0496100	100,00	0,00E+00	2576	18	NA	Japonica	
maker-2-augustus-gene-177.4-mRNA-1	2	OsR498G0203859700.01.T06	97,73	0,00E+00	5687	Os02g0496500	99,97	0,00E+00	6532	845	Japonica	Japonica	
maker-2-augustus-gene-178.0-mRNA-1	2	OsR498G0203864300.01.T02	99,69	0,00E+00	1756	Os02g0498300	100,00	0,00E+00	1456	-300	Indica	Japonica	
maker-2-augustus-gene-178.13-mRNA-1	2	OsR498G0203864000.01.T01	98,69	0,00E+00	2529	Os04g0372800	73,81	1,25E-131	470	-2059	Indica	Japonica	
maker-2-augustus-gene-178.17-mRNA-1	2	OsR498G0203866200.01.T01	99,75	0,00E+00	2872	Os02g0498700	100,00	0,00E+00	3052	180	NA	Japonica	
maker-2-augustus-gene-178.7-mRNA-1	2	OsR498G0203863600.01.T02	98,39	0,00E+00	6914	Os02g0497500	99,25	0,00E+00	7067	153	NA	Japonica	
maker-2-augustus-gene-18.0-mRNA-1	2	OsR498G0202912300.01.T01	100,00	0,00E+00	1467	Os02g0133800	100,00	0,00E+00	1467	0	NA	NA	
maker-2-augustus-gene-18.2-mRNA-1	2	OsR498G0202912500.01.T01	100,00	0,00E+00	2062	Os02g0133900	100,00	0,00E+00	2062	0	NA	NA	
maker-2-augustus-gene-18.27-mRNA-1	2	OsR498G0202914200.01.T01	100,00	2,42E-163	574	Os02g0134300	100,00	8,42E-164	574	0	NA	NA	
maker-2-augustus-gene-18.32-mRNA-1	2	OsR498G0202915600.01.T03	100,00	0,00E+00	704	Os02g0134700	100,00	0,00E+00	747	43	NA	NA	
maker-2-augustus-gene-18.33-mRNA-1	2	OsR498G0202916400.01.T05	100,00	0,00E+00	2744	Os02g0135200	100,00	0,00E+00	2744	0	NA	NA	Kinase
maker-2-augustus-gene-18.5-mRNA-1	2	OsR498G0202912700.01.T01	92,63	4,24E-176	618	Os02g0134000	100,00	0,00E+00	733	115	Japonica	NA	
maker-2-augustus-gene-18.8-mRNA-1	2	OsR498G0202914700.01.T02	100,00	0,00E+00	1945	Os02g0134400	100,00	0,00E+00	1945	0	NA	NA	
maker-2-augustus-gene-181.0-mRNA-1	2	OsR498G0203881300.01.T01	99,63	0,00E+00	2884	Os09g0441400	76,67	0,00E+00	1127	-1757	Indica	Japonica	
maker-2-augustus-gene-181.2-mRNA-1	2	OsR498G0203881700.01.T01	100,00	3,21E-111	401	Os02g0503300	99,00	1,77E-96	351	-50	Indica	Indica	
maker-2-augustus-gene-181.4-mRNA-1	2	OsR498G0203881900.01.T01	99,19	0,00E+00	658	Os02g0503400	99,73	0,00E+00	667	9	NA	NA	
maker-2-augustus-gene-182.32-mRNA-1	2	OsR498G0203882700.01.T01	99,19	0,00E+00	2177	Os02g0503700	99,11	0,00E+00	2784	607	Japonica	Japonica	
maker-2-augustus-gene-182.35-mRNA-1	2	OsR498G0203883100.01.T01	96,63	0,00E+00	2597	Os02g0503800	96,64	0,00E+00	2646	49	NA	NA	
maker-2-augustus-gene-182.36-mRNA-1	2	OsR498G0203883100.01.T02	99,75	0,00E+00	2823	Os02g0503900	99,68	0,00E+00	2819	-4	NA	Indica	
maker-2-augustus-gene-182.43-mRNA-1	2	OsR498G0203883500.01.T01	99,93	0,00E+00	2530	Os02g0503900	88,17	0,00E+00	1699	-831	Indica	Indica	
maker-2-augustus-gene-183.0-mRNA-1	2	OsR498G0203886100.01.T01	99,76	0,00E+00	2232	Os02g0504500	99,68	0,00E+00	2228	-4	NA	Indica	
maker-2-augustus-gene-183.11-mRNA-1	2	OsR498G0203887400.01.T01	99,73	0,00E+00	1969	Os02g0504800	100,00	0,00E+00	1213	-756	Indica	Japonica	
maker-2-augustus-gene-183.13-mRNA-1	2	OsR498G0203887600.01.T02	99,91	0,00E+00	2085	Os02g0504900	99,74	0,00E+00	2076	-9	NA	Indica	
maker-2-augustus-gene-183.6-mRNA-1	2	OsR498G0203887100.01.T01	99,86	0,00E+00	1266	Os02g0504700	99,51	0,00E+00	1463	197	Japonica	Japonica	
maker-2-augustus-gene-184.2-mRNA-1	2	OsR498G0203893200.01.T01	99,74	0,00E+00	2043	Os02g0506400	99,47	0,00E+00	2030	-13	NA	Japonica	
maker-2-augustus-gene-185.0-mRNA-1	2	OsR498G0203899300.01.T01	100,00	0,00E+00	648	Os02g0507400	100,00	0,00E+00	648	0	NA	Japonica	
maker-2-augustus-gene-185.12-mRNA-1	2	OsR498G0203903200.01.T01	100,00	0,00E+00	645	Os02g0510100	94,50	5,29E-129	460	-185	Indica	Indica	
maker-2-augustus-gene-185.15-mRNA-1	2	OsR498G0203903800.01.T02	98,15	0,00E+00	1684	Os02g0510300	97,74	0,00E+00	1666	-18	NA	Indica	
maker-2-augustus-gene-185.5-mRNA-1	2	OsR498G0203902500.01.T01	99,85	0,00E+00	1159	Os02g0509600	96,12	0,00E+00	1055	-104	NA	Indica	
maker-2-augustus-gene-187.27-mRNA-1	2	OsR498G0203904700.01.T01	99,91	0,00E+00	1966	Os02g0511400	82,11	0,00E+00	1058	-908	Indica	Indica	
maker-2-augustus-gene-187.35-mRNA-1	2	OsR498G0203906100.01.T01	99,75	0,00E+00	716	Os02g0511600	99,50	0,00E+00	711	-5	NA	Indica	
maker-2-augustus-gene-187.42-mRNA-1	2	OsR498G0203906400.01.T02	97,17	0,00E+00	1974	Os02g0511800	98,63	0,00E+00	1525	-449	Indica	Indica	
maker-2-augustus-gene-189.0-mRNA-1	2	OsR498G0203912300.01.T01	100,00	0,00E+00	2852	Os02g0513000	99,81	0,00E+00	2838	-14	NA	Indica	Kinase-TM
maker-2-augustus-gene-189.10-mRNA-1	2	OsR498G0203915600.01.T06	99,62	0,00E+00	1394	Os02g0513800	99,66	0,00E+00	1578	184	Japonica	NA	
maker-2-augustus-gene-189.8-mRNA-1	2	OsR498G0203912800.01.T01	100,00	0,00E+00	1732	Os02g0513100	99,48	0,00E+00	1709	-23	NA	Indica	
maker-2-augustus-gene-19.0-mRNA-1	2	OsR498G0202917300.01.T01	100,00	0,00E+00	2630	Os02g0135500	100,00	0,00E+00	2630	0	NA	NA	
maker-2-augustus-gene-19.12-mRNA-1	2	OsR498G0202924100.01.T01	100,00	0,00E+00	4112	Os02g0136900	100,00	0,00E+00	3647	-465	Indica	NA	Kinase-LRR-TM
maker-2-augustus-gene-19.2-mRNA-1	2	OsR498G0202918500.01.T01	100,00	0,00E+00	4085	Os02g0135700	100,00	0,00E+00	4085	0	NA	NA	
maker-2-augustus-gene-19.20-mRNA-1	2	OsR498G0202918200.01.T01	100,00	0,00E+00	1829	Os02g0135600	100,00	0,00E+00	1829	0	NA	NA	
maker-2-augustus-gene-19.7-mRNA-1	2	OsR498G0202919700.01.T02	97,15	0,00E+00	1608	Os02g0135900	97,15	0,00E+00	1608	0	NA	NA	
maker-2-augustus-gene-190.0-mRNA-1	2	OsR498G0203918700.01.T01	99,66	0,00E+00	1040	Os02g0514766	98,99	0,00E+00	1043	3	NA	NA	
maker-2-augustus-gene-190.10-mRNA-1	2	OsR498G0203921600.01.T01	99,73	0,00E+00	3334	Os02g0515200	99,57	0,00E+00	3321	-13	NA	NA	
maker-2-augustus-gene-190.16-mRNA-1	2	OsR498G0203923000.01.T01	100,00	0,00E+00	839	Os02g0515600	99,79	0,00E+00	835	-4	NA	Indica	
maker-2-augustus-gene-190.8-mRNA-1	2	OsR498G0203919000.01.T01	100,00	1,14E-101	369	Os02g0514800	100,00	2,69E-66	250	-119	Indica	NA	
maker-2-augustus-gene-191.0-mRNA-1	2	OsR498G0203929500.01.T02	100,00	0,00E+00	4415	Os02g0517300	99,84	0,00E+00	4397	-18	NA	Indica	
maker-2-augustus-gene-191.13-mRNA-1	2	OsR498G0203927400.01.T01	100,00	0,00E+00	2030	Os02g0516800	99,47	0,00E+00	2003	-27	NA	Indica	
maker-2-augustus-gene-191.9-mRNA-1	2	OsR498G0203926800.01.T01	99,18	0,00E+00	3668	Os02g0516600	99,08	0,00E+00	3659	-9	NA	Indica	
maker-2-augustus-gene-192.35-mRNA-1	2	OsR498G0203934500.01.T01	100,00	0,00E+00	5422	Os02g0517531	99,97	0,00E+00	5417	-5	NA	Indica	
maker-2-augustus-gene-192.50-mRNA-1	2	OsR498G0203939400.01.T01	100,00	0,00E+00	2278	Os02g0518100	99,05	0,00E+00	2231	-47	NA	Indica	
maker-2-augustus-gene-192.56-mRNA-1	2	OsR498G0203941800.01.T02	100,00	0,00E+00	4020	Os02g0518600	100,00	0,00E+00	4020	0	NA	Indica	
maker-2-augustus-gene-193.0-mRNA-1	2	OsR498G0203948900.01.T06	100,00	0,00E+00	4567	Os02g0519900	100,00	0,00E+00	4567	0	NA	Indica	
maker-2-augustus-gene-193.1-mRNA-1	2	OsR498G0203945200.01.T01	100,00	0,00E+00	4048	Os02g0519100	100,00	0,00E+00	4048	0	NA	Indica	
maker-2-augustus-gene-193.10-mRNA-1	2	OsR498G0203951000.01.T02	100,00	0,00E+00	1110	Os02g0520100	99,84	0,00E+00	1105	-5	NA	Indica	
maker-2-augustus-gene-193.3-mRNA-1	2	OsR498G0203946800.01.T01	96,16	0,00E+00	1728	Os04g0661300	86,63	0,00E+00	1244	-484	Indica	Indica	
maker-2-augustus-gene-194.11-mRNA-1	2	OsR498G0203955900.01.T01	100,00	0,00E+00	991	Os02g0521700	100,00	0,00E+00	991	0	NA	Japonica	
maker-2-augustus-gene-194.13-mRNA-1	2	OsR498G0203953400.01.T01	100,00	0,00E+00	2354	Os04g0398600	82,59	0,00E+00	1238	-1116	Indica	NA	
maker-2-augustus-gene-194.15-mRNA-1	2	OsR498G0203954000.01.T01	100,00	0,00E+00	1478	Os02g0520800	100,00	0,00E+00	1478	0	NA	Japonica	
maker-2-augustus-gene-194.19-mRNA-1	2	OsR498G0203954800.01.T04	99,90	0,00E+00	1744	Os02g0521300	100,00	0,00E+00	1748	4	NA	Japonica	
maker-2-augustus-gene-195.33-mRNA-1	2	OsR498G0203959100.01.T05	99,82	0,00E+00	970	Os02g0522700	100,00	4,90E-170	596	-374	Indica	NA	
maker-2-augustus-gene-195.41-mRNA-1	2	OsR498G0203961600.01.T02	99,94	0,00E+00	5564	Os02g0523300	100,00	0,00E+00	5573	9	NA	Japonica	
maker-2-augustus-gene-195.49-mRNA-1	2	OsR498G0203961800.01.T03	99,97	0,00E+00	5336	Os02g0523500	100,00	0,00E+00	5341	5	NA	Japonica	
maker-2-augustus-gene-196.11-mRNA-1	2	OsR498G0203973700.01.T01	99,86	0,00E+00	5063	Os02g0526400	99,79	0,00E+00	5054	-9	NA	NA	
maker-2-augustus-gene-196.18-mRNA-1	2	OsR498G0203972200.01.T01	99,93	0,00E+00	2393	Os02g0526000	100,00	0,00E+00	2040	-353	Indica	Indica	
maker-2-augustus-gene-196.5-mRNA-1	2	OsR498G0203971900.01.T01	99,95	0,00E+00	3816	Os02g0525900	99,86	0,00E+00	3807	-9	NA	Indica	
maker-2-augustus-gene-197.10-mRNA-1	2	OsR498G0203976400.01.T02	99,50	0,00E+00	1075	Os02g0527200	99,50	0,00E+00	1075	0	NA	NA	
maker-2-augustus-gene-197.13-mRNA-1	2	OsR498G0203977800.01.T02	99,73	0,00E+00	2682	Os02g0527300	99,73	0,00E+00	2682	0	NA	NA	
maker-2-augustus-gene-197.7-mRNA-1	2	OsR498G0203974000.01.T03	100,00	0,00E+00	1238	Os04g0405100	77,92	6,58E-157	553	-685	Indica	NA	
maker-2-augustus-gene-198.42-mRNA-1	2	OsR498G0102641700.01.T01	75,57	6,58E-99	361	Os02g0528500	99,44	0,00E+00	943	582	Japonica	NA	
maker-2-augustus-gene-198.44-mRNA-1	2	OsR498G0203981000.01.T01	99,69	4,81E-161	568	Os02g0528000	99,76	0,00E+00	748	180	Japonica	NA	
maker-2-augustus-gene-198.46-mRNA-1	2	OsR498G0203981400.01.T01	95,43	0,00E+00	856	Os02g0528100	95,43	0,00E+00	856	0	NA	NA	
maker-2-augustus-gene-198.48-mRNA-1	2	OsR498G0203981600.01.T02	99,80	0,00E+00	4447	Os02g0528200	99,80	0,00E+00	4447	0	NA	NA	
maker-2-augustus-gene-199.0-mRNA-1	2	OsR498G0203985100.01.T01	99,84	0,00E+00	3271	Os02g0529400	99,78	0,00E+00	3267	-4	NA	NA	
maker-2-augustus-gene-199.11-mRNA-1	2	OsR498G0203987400.01.T02	100,00	7,06E-160	563	Os02g0530100	100,00	2,46E-160	563	0	NA	NA	
maker-2-augustus-gene-199.19-mRNA-1	2	OsR498G0203990200.01.T01	100,00	0,00E+00	4498	Os02g0530600	100,00	0,00E+00	4498	0	NA	NA	
maker-2-augustus-gene-199.5-mRNA-1	2	OsR498G0203985500.01.T01	99,74	0,00E+00	1398	Os02g0529500	99,74	0,00E+00	1398	0	NA	NA	
maker-2-augustus-gene-199.7-mRNA-1	2	OsR498G0100309500.01.T03	81,63	1,45E-90	334	Os02g0529700	100,00	8,53E-177	618	284	Japonica	NA	
maker-2-augustus-gene-199.9-mRNA-1	2	OsR498G0203986400.01.T03	100,00	0,00E+00	2782	Os02g0529800	100,00	0,00E+00	2782	0	NA	NA	
maker-2-augustus-gene-2.37-mRNA-1	2	OsR498G0202807200.01.T01	99,91	0,00E+00	1911	Os02g0103600	100,00	0,00E+00	1916	5	NA	Japonica	
maker-2-augustus-gene-2.39-mRNA-1	2	OsR498G0202807400.01.T01	100,00	0,00E+00	1045	Os02g0103700	100,00	0,00E+00	1045	0	NA	Japonica	
maker-2-augustus-gene-2.41-mRNA-1	2	OsR498G0202807600.01.T01	99,64	0,00E+00	1968	Os02g0103800	95,64	0,00E+00	1809	-159	NA	Japonica	
maker-2-augustus-gene-2.44-mRNA-1	2	OsR498G0202807800.01.T01	99,75	0,00E+00	2845	Os02g0103900	100,00	0,00E+00	2863	18	NA	Japonica	
maker-2-augustus-gene-2.47-mRNA-1	2	OsR498G0202810200.01.T01	99,72	0,00E+00	3826	Os02g0104500	100,00	0,00E+00	2008	-1818	Indica	Japonica	
maker-2-augustus-gene-20.31-mRNA-1	2	OsR498G0202924500.01.T03	100,00	0,00E+00	6502	Os02g0137000	99,96	0,00E+00	4018	-2484	Indica	NA	
maker-2-augustus-gene-20.38-mRNA-1	2	OsR498G0202925100.01.T02	97,08	0,00E+00	1565	Os02g0137100	97,08	0,00E+00	1565	0	NA	NA	
maker-2-augustus-gene-20.42-mRNA-1	2	OsR498G0202925700.01.T01	100,00	0,00E+00	6682	Os02g0137400	100,00	0,00E+00	4578	-2104	Indica	NA	
maker-2-augustus-gene-20.53-mRNA-1	2	OsR498G0202926900.01.T02	100,00	0,00E+00	3241	Os06g0704700	83,01	0,00E+00	1569	-1672	Indica	NA	
maker-2-augustus-gene-20.59-mRNA-1	2	OsR498G0202927100.01.T01	100,00	0,00E+00	818	Os02g0137800	100,00	0,00E+00	818	0	NA	Japonica	
maker-2-augustus-gene-20.62-mRNA-1	2	OsR498G0202929500.01.T01	100,00	0,00E+00	2993	Os02g0138200	100,00	0,00E+00	2993	0	NA	NA	
maker-2-augustus-gene-20.67-mRNA-1	2	OsR498G0202925400.01.T03	100,00	0,00E+00	1483	Os02g0137200	100,00	0,00E+00	1285	-198	Indica	NA	
maker-2-augustus-gene-200.37-mRNA-1	2	OsR498G0203991700.01.T01	100,00	0,00E+00	2295	Os02g0531200	100,00	0,00E+00	2295	0	NA	NA	
maker-2-augustus-gene-200.42-mRNA-1	2	OsR498G0203995700.01.T01	99,91	0,00E+00	2014	Os02g0532300	99,91	0,00E+00	2014	0	NA	NA	
maker-2-augustus-gene-200.44-mRNA-1	2	OsR498G0203998100.01.T01	100,00	0,00E+00	2122	Os02g0532900	100,00	0,00E+00	2122	0	NA	NA	
maker-2-augustus-gene-200.46-mRNA-1	2	OsR498G0203991600.01.T01	100,00	0,00E+00	1353	Os02g0530900	99,74	0,00E+00	687	-666	Indica	NA	
maker-2-augustus-gene-200.49-mRNA-1	2	OsR498G0203993200.01.T01	100,00	0,00E+00	942	Os02g0531600	100,00	0,00E+00	942	0	NA	NA	
maker-2-augustus-gene-201.0-mRNA-1	2	OsR498G0203998700.01.T02	100,00	0,00E+00	1104	Os02g0533200	100,00	0,00E+00	1559	455	Japonica	NA	
maker-2-augustus-gene-201.12-mRNA-1	2	OsR498G0204003500.01.T05	100,00	0,00E+00	3112	Os02g0534400	99,48	0,00E+00	3091	-21	NA	NA	
maker-2-augustus-gene-201.30-mRNA-1	2	OsR498G0204005600.01.T01	93,25	0,00E+00	759	Os02g0534800	93,25	0,00E+00	759	0	NA	NA	
maker-2-augustus-gene-201.33-mRNA-1	2	OsR498G0204005800.01.T01	100,00	0,00E+00	1119	Os02g0535000	100,00	0,00E+00	1119	0	NA	NA	
maker-2-augustus-gene-201.4-mRNA-1	2	OsR498G0203998900.01.T01	100,00	0,00E+00	1451	Os02g0533300	99,63	0,00E+00	1435	-16	NA	NA	
maker-2-augustus-gene-201.7-mRNA-1	2	OsR498G0204000100.01.T01	98,69	0,00E+00	1608	Os02g0533900	98,69	0,00E+00	1608	0	NA	NA	
maker-2-augustus-gene-202.35-mRNA-1	2	OsR498G0204009300.01.T02	100,00	5,75E-165	580	Os02g0536100	100,00	2,00E-165	580	0	NA	NA	
maker-2-augustus-gene-202.37-mRNA-1	2	OsR498G0204009900.01.T04	100,00	0,00E+00	1894	Os02g0537200	81,27	0,00E+00	1271	-623	Indica	NA	
maker-2-augustus-gene-202.43-mRNA-1	2	OsR498G0204013200.01.T01	100,00	0,00E+00	1218	Os02g0537200	84,11	0,00E+00	729	-489	Indica	NA	
maker-2-augustus-gene-203.11-mRNA-1	2	OsR498G0204016000.01.T01	99,82	0,00E+00	3064	Os02g0537200	99,82	0,00E+00	3064	0	NA	NA	
maker-2-augustus-gene-203.13-mRNA-1	2	OsR498G0204017200.01.T01	100,00	0,00E+00	2576	Os02g0537500	100,00	0,00E+00	2576	0	NA	NA	
maker-2-augustus-gene-203.4-mRNA-1	2	OsR498G0204017500.01.T01	100,00	0,00E+00	1418	Os02g0537700	100,00	0,00E+00	1418	0	NA	Indica	
maker-2-augustus-gene-203.8-mRNA-1	2	OsR498G0204018500.01.T01	100,00	0,00E+00	2690	Os02g0537800	100,00	0,00E+00	3106	416	Japonica	Indica	
maker-2-augustus-gene-204.12-mRNA-1	2	OsR498G0204019400.01.T01	100,00	0,00E+00	2152	Os02g0537900	100,00	0,00E+00	2152	0	NA	NA	
maker-2-augustus-gene-204.22-mRNA-1	2	OsR498G0204022400.01.T01	99,90	0,00E+00	1765	Os02g0538900	99,90	0,00E+00	1765	0	NA	Indica	
maker-2-augustus-gene-204.4-mRNA-1	2	OsR498G0204022900.01.T01	100,00	0,00E+00	4345	Os02g0539100	100,00	0,00E+00	4345	0	NA	NA	
maker-2-augustus-gene-205.28-mRNA-1	2	OsR498G0204036700.01.T01	100,00	0,00E+00	2040	Os02g0541300	100,00	0,00E+00	1126	-914	Indica	NA	
maker-2-augustus-gene-206.0-mRNA-1	2	OsR498G0204038300.01.T01	100,00	0,00E+00	715	Os02g0541900	100,00	0,00E+00	801	86	Japonica	NA	
maker-2-augustus-gene-206.11-mRNA-1	2	OsR498G0204042000.01.T01	100,00	2,42E-163	574	Os02g0543000	95,58	2,76E-100	363	-211	Indica	NA	
maker-2-augustus-gene-206.13-mRNA-1	2	OsR498G0204042600.01.T01	100,00	0,00E+00	1927	Os02g0543100	100,00	0,00E+00	2282	355	Japonica	NA	
maker-2-augustus-gene-206.2-mRNA-1	2	OsR498G0204040300.01.T01	100,00	0,00E+00	1710	Os02g0542500	99,69	0,00E+00	1700	-10	NA	NA	
maker-2-augustus-gene-206.4-mRNA-1	2	OsR498G0204043700.01.T06	100,00	0,00E+00	1492	Os02g0543400	100,00	0,00E+00	1492	0	NA	NA	
maker-2-augustus-gene-206.9-mRNA-1	2	OsR498G0204040800.01.T01	100,00	0,00E+00	1159	Os02g0542700	100,00	0,00E+00	1159	0	NA	NA	
maker-2-augustus-gene-208.0-mRNA-1	2	OsR498G0204065700.01.T01	99,20	0,00E+00	6212	Os02g0816900	71,17	0,00E+00	1625	-4587	Indica	Indica	
maker-2-augustus-gene-209.0-mRNA-1	2	OsR498G0204070700.01.T01	100,00	4,70E-82	304	Os02g0545700	100,00	5,74E-120	428	124	Japonica	NA	
maker-2-augustus-gene-209.3-mRNA-1	2	OsR498G0611818300.01.T01	78,38	1,41E-101	371	Os02g0547300	99,03	0,00E+00	907	536	Japonica	NA	
maker-2-augustus-gene-209.5-mRNA-1	2	OsR498G0204073600.01.T01	97,40	0,00E+00	1113	Os02g0546600	97,40	0,00E+00	1113	0	NA	NA	
maker-2-augustus-gene-209.7-mRNA-1	2	OsR498G0204073600.01.T01	89,97	0,00E+00	928	Os02g0546800	99,69	0,00E+00	1166	238	Japonica	NA	
maker-2-augustus-gene-21.38-mRNA-1	2	OsR498G0202934300.01.T01	99,19	0,00E+00	2617	Os02g0139300	100,00	0,00E+00	2652	35	NA	Japonica	
maker-2-augustus-gene-21.40-mRNA-1	2	OsR498G0202935500.01.T01	100,00	0,00E+00	2544	Os06g0703500	71,53	3,74E-171	602	-1942	Indica	Japonica	
maker-2-augustus-gene-21.48-mRNA-1	2	OsR498G0202933000.01.T03	99,93	0,00E+00	2528	Os02g0139000	100,00	0,00E+00	2533	5	NA	Japonica	
maker-2-augustus-gene-21.70-mRNA-1	2	OsR498G0202937000.01.T01	99,94	0,00E+00	2898	Os02g0139700	100,00	0,00E+00	2902	4	NA	Japonica	
maker-2-augustus-gene-210.34-mRNA-1	2	OsR498G0204084500.01.T02	98,85	0,00E+00	2587	Os02g0549200	100,00	0,00E+00	2643	56	NA	Japonica	Kinase-TM
maker-2-augustus-gene-211.0-mRNA-1	2	OsR498G0204085100.01.T01	97,65	0,00E+00	945	Os02g0549600	97,65	0,00E+00	945	0	NA	Japonica	
maker-2-augustus-gene-211.11-mRNA-1	2	OsR498G0204089100.01.T01	99,77	0,00E+00	2292	Os02g0550300	100,00	0,00E+00	2305	13	NA	Japonica	
maker-2-augustus-gene-211.14-mRNA-1	2	OsR498G0204088600.01.T01	99,80	0,00E+00	905	Os02g0550100	100,00	0,00E+00	910	5	NA	Japonica	
maker-2-augustus-gene-211.16-mRNA-1	2	OsR498G0204090800.01.T02	99,76	0,00E+00	3029	Os02g0550600	100,00	0,00E+00	3047	18	NA	Japonica	
maker-2-augustus-gene-211.18-mRNA-1	2	OsR498G0204091600.01.T02	99,78	0,00E+00	2420	Os02g0550700	100,00	0,00E+00	1783	-637	Indica	Japonica	
maker-2-augustus-gene-211.8-mRNA-1	2	OsR498G0204088100.01.T01	99,67	0,00E+00	4341	Os02g0550000	99,96	0,00E+00	4373	32	NA	Japonica	
maker-2-augustus-gene-212.0-mRNA-1	2	OsR498G0204091900.01.T02	99,79	0,00E+00	2590	Os02g0550800	100,00	0,00E+00	2603	13	NA	Japonica	
maker-2-augustus-gene-212.10-mRNA-1	2	OsR498G0204096900.01.T03	99,71	0,00E+00	3035	Os02g0552100	99,82	0,00E+00	3044	9	NA	NA	
maker-2-augustus-gene-212.12-mRNA-1	2	OsR498G0204092600.01.T02	91,35	0,00E+00	955	Os02g0551100	91,50	0,00E+00	959	4	NA	Japonica	Kinase-TM
maker-2-augustus-gene-212.18-mRNA-1	2	OsR498G0204093800.01.T02	99,77	0,00E+00	3874	Os02g0551400	100,00	0,00E+00	3896	22	NA	Japonica	
maker-2-augustus-gene-212.5-mRNA-1	2	OsR498G0204096700.01.T01	99,77	0,00E+00	3180	Os02g0552000	100,00	0,00E+00	3198	18	NA	Japonica	
maker-2-augustus-gene-213.35-mRNA-1	2	OsR498G0204101700.01.T02	100,00	7,59E-157	553	Os02g0553600	100,00	2,64E-157	553	0	NA	Japonica	
maker-2-augustus-gene-213.44-mRNA-1	2	OsR498G0204103500.01.T01	99,85	0,00E+00	2448	Os02g0554100	100,00	0,00E+00	2457	9	NA	Japonica	
maker-2-augustus-gene-213.50-mRNA-1	2	OsR498G0204100600.01.T01	94,56	0,00E+00	2373	Os02g0553200	94,69	0,00E+00	2382	9	NA	Japonica	
maker-2-augustus-gene-214.33-mRNA-1	2	OsR498G0204107300.01.T02	99,51	0,00E+00	1096	Os02g0555300	100,00	0,00E+00	1110	14	NA	Japonica	
maker-2-augustus-gene-214.36-mRNA-1	2	OsR498G0204107700.01.T05	99,35	0,00E+00	2466	Os02g0555600	100,00	0,00E+00	2506	40	NA	Japonica	
maker-2-augustus-gene-214.41-mRNA-1	2	OsR498G0204106600.01.T01	99,73	0,00E+00	2650	Os02g0554900	100,00	0,00E+00	2955	305	Japonica	Japonica	
maker-2-augustus-gene-214.43-mRNA-1	2	OsR498G0204106800.01.T03	99,86	0,00E+00	1286	Os02g0555000	99,86	0,00E+00	1286	0	NA	Japonica	
maker-2-augustus-gene-214.50-mRNA-1	2	OsR498G0204108000.01.T02	99,25	0,00E+00	1885	Os02g0555700	99,91	0,00E+00	1917	32	NA	Japonica	
maker-2-augustus-gene-214.54-mRNA-1	2	OsR498G0204108200.01.T03	96,32	0,00E+00	2239	Os02g0555900	100,00	0,00E+00	2434	195	NA	Japonica	Kinase-TM
maker-2-augustus-gene-214.60-mRNA-1	2	OsR498G0204108600.01.T01	97,91	0,00E+00	3427	Os02g0556000	99,95	0,00E+00	3500	73	NA	Japonica	
maker-2-augustus-gene-215.32-mRNA-1	2	OsR498G0204109300.01.T01	99,05	0,00E+00	1106	Os02g0556100	100,00	0,00E+00	1586	480	Japonica	Japonica	
maker-2-augustus-gene-215.38-mRNA-1	2	OsR498G0204110300.01.T01	99,72	0,00E+00	6334	Os02g0556700	98,95	0,00E+00	6240	-94	NA	Japonica	
maker-2-augustus-gene-215.41-mRNA-1	2	OsR498G0204111700.01.T02	99,80	0,00E+00	1783	Os02g0557100	100,00	0,00E+00	1792	9	NA	Japonica	
maker-2-augustus-gene-215.43-mRNA-1	2	OsR498G0204112700.01.T01	98,16	0,00E+00	3571	Os02g0557200	98,69	0,00E+00	3621	50	NA	Japonica	
maker-2-augustus-gene-215.49-mRNA-1	2	OsR498G0204113100.01.T01	98,73	0,00E+00	3852	Os02g0557500	100,00	0,00E+00	2176	-1676	Indica	Japonica	
maker-2-augustus-gene-215.54-mRNA-1	2	OsR498G0204113500.01.T01	99,73	0,00E+00	1333	Os02g0557700	100,00	0,00E+00	1218	-115	NA	Japonica	
maker-2-augustus-gene-215.70-mRNA-1	2	OsR498G0204110800.01.T01	99,86	0,00E+00	1306	Os02g0556800	100,00	0,00E+00	1310	4	NA	Japonica	
maker-2-augustus-gene-215.72-mRNA-1	2	OsR498G0204111200.01.T01	99,73	0,00E+00	3322	Os02g0556900	100,00	0,00E+00	3344	22	NA	Japonica	
maker-2-augustus-gene-215.75-mRNA-1	2	OsR498G0204113000.01.T01	99,86	0,00E+00	1262	Os02g0557300	100,00	0,00E+00	1267	5	NA	Japonica	
maker-2-augustus-gene-215.77-mRNA-1	2	OsR498G0204113400.01.T02	97,08	0,00E+00	3071	Os02g0557600	97,19	0,00E+00	3080	9	NA	Japonica	
maker-2-augustus-gene-215.80-mRNA-1	2	OsR498G0204113800.01.T02	92,46	0,00E+00	1007	Os02g0557800	94,51	0,00E+00	1313	306	Japonica	Japonica	
maker-2-augustus-gene-216.10-mRNA-1	2	OsR498G0204117800.01.T01	99,81	0,00E+00	1885	Os02g0558500	100,00	0,00E+00	1175	-710	Indica	Japonica	
maker-2-augustus-gene-216.12-mRNA-1	2	OsR498G0204118000.01.T02	99,60	0,00E+00	3134	Os02g0558600	100,00	0,00E+00	3166	32	NA	Japonica	
maker-2-augustus-gene-216.17-mRNA-1	2	OsR498G0204120400.01.T05	97,59	0,00E+00	2131	Os02g0559400	97,99	0,00E+00	2153	22	NA	Japonica	
maker-2-augustus-gene-216.2-mRNA-1	2	OsR498G0204120100.01.T02	99,27	0,00E+00	3863	Os02g0559300	100,00	0,00E+00	3905	42	NA	Japonica	CC-Kinase-TM
maker-2-augustus-gene-216.5-mRNA-1	2	OsR498G0204116600.01.T04	99,88	0,00E+00	4342	Os02g0558100	99,75	0,00E+00	3549	-793	Indica	Japonica	
maker-2-augustus-gene-217.0-mRNA-1	2	OsR498G0204121100.01.T04	99,80	0,00E+00	894	Os02g0471300	75,87	3,91E-88	324	-570	Indica	Japonica	
maker-2-augustus-gene-217.11-mRNA-1	2	OsR498G0204123800.01.T01	99,83	0,00E+00	3212	Os02g0562400	100,00	0,00E+00	2603	-609	Indica	Japonica	
maker-2-augustus-gene-217.2-mRNA-1	2	OsR498G0204123500.01.T03	99,90	0,00E+00	3530	Os02g0562300	99,85	0,00E+00	3524	-6	NA	Japonica	
maker-2-augustus-gene-217.7-mRNA-1	2	OsR498G0204125300.01.T04	99,77	0,00E+00	1582	Os02g0562700	100,00	0,00E+00	1591	9	NA	Japonica	
maker-2-augustus-gene-218.0-mRNA-1	2	OsR498G0204130100.01.T01	100,00	0,00E+00	1451	Os04g0444900	79,84	0,00E+00	655	-796	Indica	Japonica	
maker-2-augustus-gene-218.11-mRNA-1	2	OsR498G0204131700.01.T04	100,00	1,66E-154	545	Os02g0565100	100,00	5,76E-155	545	0	NA	Japonica	
maker-2-augustus-gene-218.15-mRNA-1	2	OsR498G0204130000.01.T03	100,00	0,00E+00	1288	Os02g0564000	100,00	0,00E+00	1288	0	NA	Japonica	
maker-2-augustus-gene-218.21-mRNA-1	2	OsR498G0204130800.01.T01	100,00	0,00E+00	695	Os02g0564500	100,00	0,00E+00	695	0	NA	Japonica	
maker-2-augustus-gene-218.26-mRNA-1	2	OsR498G0204132400.01.T02	99,70	5,18E-167	588	Os02g0565200	100,00	4,23E-169	592	4	NA	Japonica	
maker-2-augustus-gene-218.4-mRNA-1	2	OsR498G0204130500.01.T01	99,95	0,00E+00	3491	Os01g0886600	73,07	0,00E+00	685	-2806	Indica	Japonica	
maker-2-augustus-gene-219.0-mRNA-1	2	OsR498G0204135900.01.T01	96,21	0,00E+00	2320	Os02g0566400	96,57	0,00E+00	2344	24	NA	Japonica	
maker-2-augustus-gene-219.12-mRNA-1	2	OsR498G0204137200.01.T01	100,00	0,00E+00	774	Os02g0566900	100,00	0,00E+00	774	0	NA	Japonica	
maker-2-augustus-gene-219.14-mRNA-1	2	OsR498G0204137400.01.T01	99,77	0,00E+00	792	Os02g0567000	100,00	0,00E+00	796	4	NA	Japonica	
maker-2-augustus-gene-219.16-mRNA-1	2	OsR498G0204137600.01.T02	99,52	0,00E+00	1107	Os02g0567100	100,00	0,00E+00	1113	6	NA	Japonica	
maker-2-augustus-gene-219.18-mRNA-1	2	OsR498G0204138000.01.T04	99,52	0,00E+00	1104	Os02g0567200	99,84	0,00E+00	1116	12	NA	Japonica	

maker-2-augustus-gene-219.2-mRNA-1	2	OsR498G0204136700.01.T01	99,65	0,00E+00	3529	Os02g0566700	99,95	0,00E+00	3556	27	NA	Japonica	
maker-2-augustus-gene-219.5-mRNA-1	2	OsR498G0204133800.01.T01	99,52	0,00E+00	1872	Os02g0565600	100,00	0,00E+00	1894	22	NA	Japonica	
maker-2-augustus-gene-219.7-mRNA-1	2	OsR498G0204136200.01.T02	99,63	0,00E+00	1914	Os02g0566500	100,00	0,00E+00	1932	18	NA	Japonica	
maker-2-augustus-gene-219.8-mRNA-1	2	OsR498G0204137000.01.T01	100,00	0,00E+00	2170	Os09g0452900	74,09	0,00E+00	729	-1441	Indica	Japonica	
maker-2-augustus-gene-22.30-mRNA-1	2	OsR498G0202940900.01.T03	100,00	0,00E+00	834	Os02g0141000	100,00	0,00E+00	834	0	NA	NA	
maker-2-augustus-gene-22.32-mRNA-1	2	OsR498G0202938800.01.T03	99,55	0,00E+00	1982	Os02g0140400	96,93	0,00E+00	2037	55	NA	Japonica	
maker-2-augustus-gene-22.44-mRNA-1	2	OsR498G0202943400.01.T02	99,67	0,00E+00	5348	Os02g0141300	99,87	0,00E+00	5375	27	NA	Japonica	
maker-2-augustus-gene-22.51-mRNA-1	2	OsR498G0202944200.01.T01	92,95	0,00E+00	1139	Os02g0141500	92,95	0,00E+00	1139	0	NA	Japonica	
maker-2-augustus-gene-220.40-mRNA-1	2	OsR498G0204139900.01.T02	99,73	0,00E+00	1323	Os04g0449900	84,82	0,00E+00	827	-496	Indica	Japonica	
maker-2-augustus-gene-220.44-mRNA-1	2	OsR498G0204140800.01.T01	99,74	0,00E+00	4102	Os02g0568200	99,96	0,00E+00	4124	22	NA	Japonica	
maker-2-augustus-gene-221.6-mRNA-1	2	OsR498G0204148100.01.T01	97,45	0,00E+00	2652	Os02g0570500	99,59	0,00E+00	2625	-27	NA	Japonica	
maker-2-augustus-gene-222.28-mRNA-1	2	OsR498G0204150700.01.T01	99,00	0,00E+00	4226	Os02g0571100	99,00	0,00E+00	4226	0	NA	NA	
maker-2-augustus-gene-222.30-mRNA-1	2	OsR498G0204149200.01.T01	99,62	0,00E+00	2782	Os02g0570700	99,74	0,00E+00	2791	9	NA	NA	
maker-2-augustus-gene-222.33-mRNA-1	2	OsR498G0204152600.01.T01	95,20	0,00E+00	2954	Os02g0571300	91,06	0,00E+00	2607	-347	Indica	Japonica	
maker-2-augustus-gene-222.34-mRNA-1	2	OsR498G0204152600.01.T02	98,30	0,00E+00	1114	Os02g0571800	99,85	0,00E+00	1159	45	NA	Japonica	
maker-2-augustus-gene-223.0-mRNA-1	2	OsR498G0204159700.01.T01	98,61	0,00E+00	3775	Os02g0573200	98,75	0,00E+00	3789	14	NA	Japonica	
maker-2-augustus-gene-223.14-mRNA-1	2	OsR498G0204160200.01.T01	99,76	0,00E+00	2238	Os02g0573400	100,00	0,00E+00	2251	13	NA	Japonica	
maker-2-augustus-gene-223.3-mRNA-1	2	OsR498G0204155800.01.T02	99,76	0,00E+00	2980	Os02g0572400	100,00	0,00E+00	2998	18	NA	Japonica	
maker-2-augustus-gene-223.6-mRNA-1	2	OsR498G0204156800.01.T01	99,80	0,00E+00	2671	Os02g0572600	100,00	0,00E+00	2684	13	NA	Japonica	
maker-2-augustus-gene-224.36-mRNA-1	2	OsR498G0204160900.01.T01	99,87	0,00E+00	2848	Os02g0573500	100,00	0,00E+00	2857	9	NA	Japonica	
maker-2-augustus-gene-224.38-mRNA-1	2	OsR498G0204161900.01.T01	98,19	0,00E+00	2666	Os02g0574100	94,30	0,00E+00	2417	-249	NA	Japonica	
maker-2-augustus-gene-224.40-mRNA-1	2	OsR498G0204161900.01.T01	97,35	0,00E+00	2606	Os02g0574100	95,25	0,00E+00	2481	-125	NA	NA	
maker-2-augustus-gene-224.41-mRNA-1	2	OsR498G0204162100.01.T01	99,36	0,00E+00	2764	Os02g0574100	100,00	0,00E+00	2809	45	NA	Japonica	
maker-2-augustus-gene-224.43-mRNA-1	2	OsR498G0204166100.01.T02	98,03	0,00E+00	4044	Os02g0575500	98,07	0,00E+00	4048	4	NA	Japonica	
maker-2-augustus-gene-224.47-mRNA-1	2	OsR498G0204163400.01.T02	97,29	0,00E+00	1988	Os02g0574500	100,00	0,00E+00	2132	144	NA	Japonica	
maker-2-augustus-gene-224.49-mRNA-1	2	OsR498G0204164000.01.T04	99,79	0,00E+00	1674	Os02g0574600	99,89	0,00E+00	1679	5	NA	Japonica	
maker-2-augustus-gene-224.52-mRNA-1	2	OsR498G0204165800.01.T01	99,23	0,00E+00	1827	Os02g0575200	99,90	0,00E+00	1738	-89	NA	Japonica	
maker-2-augustus-gene-225.0-mRNA-1	2	OsR498G0204167100.01.T01	99,47	0,00E+00	2024	Os02g0575800	99,82	0,00E+00	2042	18	NA	Japonica	
maker-2-augustus-gene-225.14-mRNA-1	2	OsR498G0204171000.01.T04	99,87	0,00E+00	1354	Os02g0577100	100,00	0,00E+00	1359	5	NA	Japonica	
maker-2-augustus-gene-225.7-mRNA-1	2	OsR498G0204170400.01.T01	100,00	0,00E+00	2351	Os02g0576600	99,92	0,00E+00	2346	-5	NA	Japonica	
maker-2-augustus-gene-225.9-mRNA-1	2	OsR498G0204170600.01.T01	99,34	0,00E+00	1347	Os02g0576700	100,00	0,00E+00	1370	23	NA	Japonica	
maker-2-augustus-gene-226.40-mRNA-1	2	OsR498G0204171700.01.T03	98,83	0,00E+00	3299	Os02g0577300	99,95	0,00E+00	3363	64	NA	Japonica	
maker-2-augustus-gene-226.44-mRNA-1	2	OsR498G0204172300.01.T01	97,40	0,00E+00	2587	Os02g0577700	100,00	0,00E+00	2755	168	NA	Japonica	
maker-2-augustus-gene-226.46-mRNA-1	2	OsR498G0204172500.01.T01	99,52	0,00E+00	2609	Os02g0578100	100,00	0,00E+00	1719	-890	Indica	Japonica	
maker-2-augustus-gene-226.48-mRNA-1	2	OsR498G0204173100.01.T01	99,53	0,00E+00	2626	Os02g0578300	100,00	0,00E+00	2657	31	NA	Japonica	
maker-2-augustus-gene-226.50-mRNA-1	2	OsR498G0204173500.01.T01	100,00	0,00E+00	1094	Os04g0460400	81,40	3,23E-128	457	-637	Indica	Japonica	
maker-2-augustus-gene-226.52-mRNA-1	2	OsR498G0204175500.01.T05	99,29	0,00E+00	2004	Os02g0579000	98,56	0,00E+00	1208	-796	Indica	Japonica	
maker-2-augustus-gene-226.55-mRNA-1	2	OsR498G0204176500.01.T01	100,00	0,00E+00	1856	Os02g0579300	100,00	0,00E+00	1856	0	NA	Japonica	
maker-2-augustus-gene-226.57-mRNA-1	2	OsR498G0204173800.01.T01	99,56	0,00E+00	1210	Os02g0578400	99,85	0,00E+00	1219	9	NA	Japonica	
maker-2-augustus-gene-227.0-mRNA-1	2	OsR498G0204179700.01.T03	99,30	5,25E-143	508	Os02g0579600	99,65	1,50E-144	512	4	NA	Japonica	
maker-2-augustus-gene-227.11-mRNA-1	2	OsR498G0204183100.01.T01	99,77	0,00E+00	3117	Os02g0580700	99,94	0,00E+00	3131	14	NA	Japonica	
maker-2-augustus-gene-227.13-mRNA-1	2	OsR498G0204180600.01.T01	99,80	0,00E+00	878	Os02g0579800	100,00	0,00E+00	883	5	NA	Japonica	
maker-2-augustus-gene-227.16-mRNA-1	2	OsR498G0204181200.01.T01	99,12	0,00E+00	805	Os02g0580000	100,00	0,00E+00	675	-130	Indica	Japonica	
maker-2-augustus-gene-227.19-mRNA-1	2	OsR498G0204181600.01.T04	99,61	0,00E+00	1813	Os02g0580100	100,00	0,00E+00	1828	15	NA	Japonica	
maker-2-augustus-gene-227.24-mRNA-1	2	OsR498G0204182200.01.T01	98,85	0,00E+00	1063	Os02g0580400	99,83	0,00E+00	1078	15	NA	Japonica	
maker-2-augustus-gene-227.4-mRNA-1	2	OsR498G0204181700.01.T06	99,87	0,00E+00	1419	Os02g0580300	100,00	0,00E+00	1424	5	NA	Japonica	
maker-2-augustus-gene-227.8-mRNA-1	2	OsR498G0204182700.01.T01	99,30	0,00E+00	2771	Os02g0580600	100,00	0,00E+00	2426	-345	Indica	Japonica	
maker-2-augustus-gene-228.0-mRNA-1	2	OsR498G0204183900.01.T01	99,66	0,00E+00	5247	Os02g0581000	100,00	0,00E+00	3083	-2164	Indica	Japonica	
maker-2-augustus-gene-228.20-mRNA-1	2	OsR498G0204186500.01.T01	100,00	2,99E-163	574	Os02g0581800	100,00	1,04E-163	574	0	NA	Japonica	
maker-2-augustus-gene-228.22-mRNA-1	2	OsR498G0204183600.01.T01	99,73	0,00E+00	2672	Os02g0580900	99,93	0,00E+00	2685	13	NA	Japonica	
maker-2-augustus-gene-228.26-mRNA-1	2	OsR498G0204185600.01.T01	96,27	0,00E+00	1068	Os02g0581300	96,42	0,00E+00	1073	5	NA	Japonica	
maker-2-augustus-gene-228.32-mRNA-1	2	OsR498G0204187200.01.T02	99,86	0,00E+00	2529	Os02g0582150	95,83	0,00E+00	1384	-1145	Indica	Japonica	
maker-2-augustus-gene-228.35-mRNA-1	2	OsR498G0204187400.01.T01	99,81	0,00E+00	1842	Os05g0587400	82,56	0,00E+00	1037	-805	Indica	Japonica	
maker-2-augustus-gene-228.38-mRNA-1	2	OsR498G0204187800.01.T01	97,75	0,00E+00	1211	Os02g0582400	100,00	0,00E+00	1283	72	NA	Japonica	
maker-2-augustus-gene-228.40-mRNA-1	2	OsR498G0204188600.01.T01	99,87	0,00E+00	1333	Os02g0582600	100,00	0,00E+00	1337	4	NA	Japonica	
maker-2-augustus-gene-229.12-mRNA-1	2	OsR498G0204192200.01.T01	97,76	4,60E-173	607	Os02g0584700	100,00	0,00E+00	639	32	NA	Japonica	
maker-2-augustus-gene-229.13-mRNA-1	2	OsR498G0204192400.01.T02	96,75	1,37E-173	609	Os02g0584800	100,00	0,00E+00	661	52	NA	Japonica	
maker-2-augustus-gene-229.14-mRNA-1	2	OsR498G0204193000.01.T01	98,79	3,31E-164	578	Os02g0585100	100,00	0,00E+00	926	348	Japonica	Japonica	
maker-2-augustus-gene-229.6-mRNA-1	2	OsR498G0204189400.01.T01	99,81	0,00E+00	932	Os02g0582900	100,00	1,57E-176	618	-314	Indica	Japonica	
maker-2-augustus-gene-229.8-mRNA-1	2	OsR498G0204190000.01.T01	89,74	1,19E-169	596	Os02g0583000	100,00	9,75E-172	601	5	NA	Japonica	
maker-2-augustus-gene-23.3-mRNA-1	2	OsR498G0202947900.01.T02	99,36	0,00E+00	1108	Os05g0269800	89,59	0,00E+00	845	-263	Indica	Japonica	
maker-2-augustus-gene-230.54-mRNA-1	2	OsR498G0204197700.01.T01	99,79	0,00E+00	1723	Os02g0586800	100,00	0,00E+00	1732	9	NA	Japonica	
maker-2-augustus-gene-230.58-mRNA-1	2	OsR498G0204198300.01.T01	99,68	4,33E-160	564	Os02g0586900	99,68	1,51E-160	564	0	NA	Japonica	
maker-2-augustus-gene-230.60-mRNA-1	2	OsR498G0204198700.01.T01	99,45	0,00E+00	646	Os02g0587000	100,00	0,00E+00	655	9	NA	Japonica	
maker-2-augustus-gene-230.64-mRNA-1	2	OsR498G0204199700.01.T01	99,58	0,00E+00	841	Os02g0587600	99,36	0,00E+00	837	-4	NA	Indica	
maker-2-augustus-gene-230.66-mRNA-1	2	OsR498G0204200500.01.T01	100,00	0,00E+00	758	Os02g0587800	100,00	0,00E+00	758	0	NA	Japonica	
maker-2-augustus-gene-230.74-mRNA-1	2	OsR498G0204196600.01.T01	99,42	3,02E-175	614	Os02g0586500	100,00	2,02E-178	623	9	NA	Japonica	
maker-2-augustus-gene-230.76-mRNA-1	2	OsR498G0204202000.01.T03	99,93	0,00E+00	2508	Os02g0588300	99,93	0,00E+00	2508	0	NA	Japonica	
maker-2-augustus-gene-231.33-mRNA-1	2	OsR498G0204203300.01.T01	99,91	0,00E+00	4049	Os02g0588500	100,00	0,00E+00	4058	9	NA	Japonica	
maker-2-augustus-gene-231.39-mRNA-1	2	OsR498G0204203500.01.T01	99,79	0,00E+00	851	Os02g0588600	100,00	0,00E+00	856	5	NA	Japonica	
maker-2-augustus-gene-231.42-mRNA-1	2	OsR498G0204209400.01.T01	99,62	0,00E+00	2337	Os02g0589700	99,92	0,00E+00	2355	18	NA	Japonica	
maker-2-augustus-gene-232.1-mRNA-1	2	OsR498G0204215900.01.T02	99,72	0,00E+00	1263	Os02g0591500	100,00	0,00E+00	805	-458	Indica	Japonica	
maker-2-augustus-gene-232.3-mRNA-1	2	OsR498G0204209400.01.T01	73,76	3,02E-79	297	Os02g0590000	96,18	0,00E+00	1217	920	Japonica	NA	
maker-2-augustus-gene-232.5-mRNA-1	2	OsR498G0204207000.01.T01	77,79	0,00E+00	1061	Os02g0590400	100,00	0,00E+00	2387	1326	Japonica	NA	
maker-2-augustus-gene-233.0-mRNA-1	2	OsR498G0204217300.01.T05	96,76	0,00E+00	3343	Os02g0591900	100,00	0,00E+00	1885	-1458	Indica	NA	CC-Kinase-TM
maker-2-augustus-gene-233.12-mRNA-1	2	OsR498G0204223300.01.T01	100,00	0,00E+00	3101	Os02g0593500	100,00	0,00E+00	3101	0	NA	NA	
maker-2-augustus-gene-233.22-mRNA-1	2	OsR498G0204216600.01.T01	99,85	0,00E+00	1187	Os02g0591700	100,00	0,00E+00	1191	4	NA	Japonica	
maker-2-augustus-gene-233.24-mRNA-1	2	OsR498G0204216800.01.T01	100,00	0,00E+00	1797	Os02g0591800	100,00	0,00E+00	1797	0	NA	Japonica	
maker-2-augustus-gene-233.28-mRNA-1	2	OsR498G0204218000.01.T01	100,00	0,00E+00	1618	Os02g0592000	100,00	0,00E+00	1009	-609	Indica	NA	
maker-2-augustus-gene-233.31-mRNA-1	2	OsR498G0204218200.01.T01	100,00	0,00E+00	2462	Os02g0592200	99,93	0,00E+00	2458	-4	NA	NA	
maker-2-augustus-gene-233.35-mRNA-1	2	OsR498G0204218400.01.T01	96,68	0,00E+00	4704	Os02g0592300	96,68	0,00E+00	4704	0	NA	NA	
maker-2-augustus-gene-233.39-mRNA-1	2	OsR498G0204219200.01.T02	100,00	0,00E+00	1613	Os02g0592500	100,00	0,00E+00	1613	0	NA	NA	
maker-2-augustus-gene-233.41-mRNA-1	2	OsR498G0204219600.01.T01	100,00	0,00E+00	688	Os02g0592800	99,74	0,00E+00	683	-5	NA	NA	
maker-2-augustus-gene-233.49-mRNA-1	2	OsR498G0204223600.01.T01	100,00	9,98E-149	526	Os02g0593400	100,00	3,47E-149	526	0	NA	NA	
maker-2-augustus-gene-233.6-mRNA-1	2	OsR498G0204218900.01.T01	96,43	1,98E-158	559	Os02g0592400	96,43	6,87E-159	559	0	NA	NA	
maker-2-augustus-gene-234.0-mRNA-1	2	OsR498G0204224100.01.T01	100,00	0,00E+00	2717	Os02g0593600	100,00	0,00E+00	2717	0	NA	NA	LRR-TM
maker-2-augustus-gene-234.12-mRNA-1	2	OsR498G0204228500.01.T02	99,64	0,00E+00	3507	Os04g0477000	74,66	0,00E+00	1249	-2258	Indica	Japonica	
maker-2-augustus-gene-234.15-mRNA-1	2	OsR498G0204224400.01.T07	100,00	0,00E+00	2232	Os02g0593700	100,00	0,00E+00	2232	0	NA	NA	
maker-2-augustus-gene-234.20-mRNA-1	2	OsR498G0204230000.01.T01	99,71	0,00E+00	1222	Os02g0594800	100,00	0,00E+00	1231	9	NA	Japonica	
maker-2-augustus-gene-234.22-mRNA-1	2	OsR498G0204230400.01.T01	99,83	0,00E+00	2113	Os02g0594900	100,00	0,00E+00	2122	9	NA	Japonica	
maker-2-augustus-gene-234.5-mRNA-1	2	OsR498G0204224700.01.T03	88,78	0,00E+00	840	Os02g0593900	89,00	0,00E+00	838	-2	NA	NA	
maker-2-augustus-gene-234.7-mRNA-1	2	OsR498G0204225300.01.T02	100,00	0,00E+00	1911	Os02g0594100	100,00	0,00E+00	1911	0	NA	NA	Kinase-TM
maker-2-augustus-gene-235.39-mRNA-1	2	OsR498G0204235100.01.T02	99,68	0,00E+00	2791	Os02g0595800	100,00	0,00E+00	2814	23	NA	Japonica	
maker-2-augustus-gene-235.43-mRNA-1	2	OsR498G0204238700.01.T01	99,92	0,00E+00	2317	Os02g0596900	100,00	0,00E+00	2322	5	NA	Japonica	
maker-2-augustus-gene-235.48-mRNA-1	2	OsR498G0204231800.01.T01	99,83	0,00E+00	2102	Os02g0595100	100,00	0,00E+00	1943	-159	NA	Japonica	
maker-2-augustus-gene-235.50-mRNA-1	2	OsR498G0204233200.01.T05	99,51	0,00E+00	1087	Os02g0595300	100,00	0,00E+00	1846	759	Japonica	Japonica	
maker-2-augustus-gene-235.55-mRNA-1	2	OsR498G0204234400.01.T01	99,82	0,00E+00	2042	Os02g0595500	100,00	0,00E+00	2051	9	NA	Japonica	
maker-2-augustus-gene-235.57-mRNA-1	2	OsR498G0204235400.01.T01	99,36	0,00E+00	1106	Os02g0595900	99,52	0,00E+00	1111	5	NA	Japonica	
maker-2-augustus-gene-235.59-mRNA-1	2	OsR498G0204236200.01.T02	96,47	0,00E+00	1457	Os02g0596000	96,47	0,00E+00	1457	0	NA	Japonica	
maker-2-augustus-gene-235.64-mRNA-1	2	OsR498G0204236600.01.T01	99,62	0,00E+00	3284	Os02g0596100	99,95	0,00E+00	3311	27	NA	Japonica	
maker-2-augustus-gene-235.69-mRNA-1	2	OsR498G0204237600.01.T01	99,85	0,00E+00	1235	Os02g0596500	100,00	0,00E+00	1240	5	NA	Japonica	
maker-2-augustus-gene-236.0-mRNA-1	2	OsR498G0204241500.01.T01	92,11	0,00E+00	765	Os02g0597700	99,61	0,00E+00	906	141	Japonica	NA	
maker-2-augustus-gene-236.11-mRNA-1	2	OsR498G0204243700.01.T01	99,13	2,64E-111	401	Os02g0599100	92,83	4,19E-97	353	-48	Indica	Japonica	
maker-2-augustus-gene-236.13-mRNA-1	2	OsR498G0204245100.01.T01	89,31	0,00E+00	2132	Os02g0598500	89,25	0,00E+00	2128	-4	NA	NA	
maker-2-augustus-gene-236.15-mRNA-1	2	OsR498G0204239200.01.T01	99,57	0,00E+00	2461	Os02g0597000	99,86	0,00E+00	2479	18	NA	Japonica	
maker-2-augustus-gene-236.17-mRNA-1	2	OsR498G0204242200.01.T01	99,69	0,00E+00	1751	Os04g0483500	82,42	0,00E+00	948	-803	Indica	NA	
maker-2-augustus-gene-236.19-mRNA-1	2	OsR498G0204243000.01.T02	99,10	0,00E+00	5451	Os02g0598200	98,90	0,00E+00	5422	-29	NA	NA	
maker-2-augustus-gene-236.2-mRNA-1	2	OsR498G0204241900.01.T05	99,94	0,00E+00	3167	Os02g0597800	99,89	0,00E+00	3162	-5	NA	NA	
maker-2-augustus-gene-237.122-mRNA-1	2	OsR498G0204247700.01.T01	99,91	0,00E+00	2101	Os02g0600100	99,83	0,00E+00	2096	-5	NA	NA	
maker-2-augustus-gene-237.126-mRNA-1	2	OsR498G0204248100.01.T05	99,74	0,00E+00	2050	Os02g0600400	99,04	0,00E+00	2014	-36	NA	Indica	
maker-2-augustus-gene-237.129-mRNA-1	2	OsR498G0204250900.01.T01	100,00	0,00E+00	1829	Os02g0601300	100,00	0,00E+00	1829	0	NA	NA	
maker-2-augustus-gene-237.135-mRNA-1	2	OsR498G0204252100.01.T04	99,77	0,00E+00	3863	Os02g0601700	99,91	0,00E+00	3876	13	NA	Japonica	
maker-2-augustus-gene-237.139-mRNA-1	2	OsR498G0204248000.01.T03	100,00	0,00E+00	1422	Os02g0600200	99,87	0,00E+00	1416	-6	NA	Indica	
maker-2-augustus-gene-237.145-mRNA-1	2	OsR498G0204251600.01.T01	84,35	0,00E+00	1733	Os02g0601400	99,79	0,00E+00	1716	-17	NA	Japonica	
maker-2-augustus-gene-237.147-mRNA-1	2	OsR498G0204251600.01.T01	99,37	0,00E+00	2807	Os02g0601500	100,00	0,00E+00	1712	-1095	Indica	Japonica	
maker-2-augustus-gene-237.46-mRNA-1	2	OsR498G0204244900.01.T01	99,78	0,00E+00	4895	Os02g0598400	99,72	0,00E+00	3779	-1116	Indica	Japonica	
maker-2-augustus-gene-237.51-mRNA-1	2	OsR498G0204246300.01.T03	99,93	0,00E+00	2391	Os02g0598500	99,91	0,00E+00	1982	-409	Indica	Japonica	
maker-2-augustus-gene-238.40-mRNA-1	2	OsR498G0204253300.01.T07	99,66	0,00E+00	3724	Os02g0602100	99,95	0,00E+00	3751	27	NA	Japonica	Kinase
maker-2-augustus-gene-238.47-mRNA-1	2	OsR498G0204253900.01.T01	99,51	0,00E+00	1807	Os02g0602600	99,92	0,00E+00	2227	420	Japonica	Japonica	
maker-2-augustus-gene-238.49-mRNA-1	2	OsR498G0204254300.01.T05	99,74	0,00E+00	682	Os02g0602800	99,74	0,00E+00	682	0	NA	NA	
maker-2-augustus-gene-238.56-mRNA-1	2	OsR498G0204252400.01.T05	99,82	0,00E+00	3997	Os02g0601800	99,89	0,00E+00	3384	-613	Indica	Japonica	
maker-2-augustus-gene-238.61-mRNA-1	2	OsR498G0204254200.01.T01	100,00	2,23E-151	535	Os04g0488400	85,81	7,78E-95	345	-190	Indica	Japonica	
maker-2-augustus-gene-238.65-mRNA-1	2	OsR498G0204258800.01.T02	99,91	0,00E+00	3970	Os02g0603800	99,96	0,00E+00	3975	5	NA	Japonica	
maker-2-augustus-gene-239.0-mRNA-1	2	OsR498G0204261100.01.T01	100,00	0,00E+00	1586	Os02g0604300	100,00	0,00E+00	1586	0	NA	Japonica	
maker-2-augustus-gene-239.13-mRNA-1	2	OsR498G0204265700.01.T01	99,27	0,00E+00	2190	Os02g0605600	99,52	0,00E+00	2204	14	NA	Japonica	
maker-2-augustus-gene-239.19-mRNA-1	2	OsR498G0204267500.01.T03	99,87	0,00E+00	1354	Os02g0606000	100,00	0,00E+00	1359	5	NA	Japonica	
maker-2-augustus-gene-239.22-mRNA-1	2	OsR498G0204265600.01.T03	99,91	0,00E+00	1922	Os02g0605500	99,91	0,00E+00	1922	0	NA	Japonica	
maker-2-augustus-gene-239.24-mRNA-1	2	OsR498G0204267400.01.T01	99,76	0,00E+00	1463	Os02g0605900	100,00	0,00E+00	1472	9	NA	Japonica	
maker-2-augustus-gene-24.32-mRNA-1	2	OsR498G0202953100.01.T05	99,76	0,00E+00	2281	Os02g0143100	99,76	0,00E+00	2281	0	NA	NA	
maker-2-augustus-gene-24.37-mRNA-1	2	OsR498G0202953500.01.T02	99,11	0,00E+00	5548	Os02g0143200	99,11	0,00E+00	5548	0	NA	Indica	
maker-2-augustus-gene-240.0-mRNA-1	2	OsR498G0204270100.01.T03	100,00	0,00E+00	1321	Os02g0606700	100,00	0,00E+00	1321	0	NA	Japonica	
maker-2-augustus-gene-240.17-mRNA-1	2	OsR498G0204272000.01.T01	97,24	0,00E+00	2023	Os02g0606900	79,66	4,06E-162	571	-1452	Indica	Japonica	
maker-2-augustus-gene-240.23-mRNA-1	2	OsR498G0204272400.01.T01	99,88	0,00E+00	1540	Os02g0607700	100,00	0,00E+00	1544	4	NA	Japonica	
maker-2-augustus-gene-240.3-mRNA-1	2	OsR498G0204270300.01.T01	98,70	0,00E+00	1344	Os02g0606800	100,00	0,00E+00	1370	26	NA	Japonica	
maker-2-augustus-gene-241.12-mRNA-1	2	OsR498G0204275400.01.T02	99,96	0,00E+00	4151	Os02g0608400	100,00	0,00E+00	4156	5	NA	Japonica	
maker-2-augustus-gene-241.17-mRNA-1	2	OsR498G0204275600.01.T03	97,34	0,00E+00	2366	Os02g0608500	97,83	0,00E+00	2386	20	NA	Japonica	Kinase-TM
maker-2-augustus-gene-241.22-mRNA-1	2	OsR498G0204276800.01.T01	99,37	0,00E+00	1973	Os02g0608900	100,00	0,00E+00	2008	35	NA	Japonica	
maker-2-augustus-gene-241.28-mRNA-1	2	OsR498G0204279800.01.T04	96,61	0,00E+00	686	Os02g0212600	96,33	3,22E-152	536	-150	Indica	Japonica	
maker-2-augustus-gene-241.5-mRNA-1	2	OsR498G0204277100.01.T01	100,00	1,64E-119	428	Os02g0609200	100,00	5,74E-120	428	0	NA	Japonica	
maker-2-augustus-gene-241.7-mRNA-1	2	OsR498G0204277700.01.T01	99,79	0,00E+00	856	Os02g0609400	100,00	0,00E+00	861	5	NA	Japonica	
maker-2-augustus-gene-242.27-mRNA-1	2	OsR498G0204282700.01.T01	99,60	0,00E+00	1784	Os02g0610500	100,00	0,00E+00	1802	18	NA	Japonica	
maker-2-augustus-gene-242.29-mRNA-1	2	OsR498G0204283700.01.T01	99,83	0,00E+00	2118	Os02g0610800	100,00	0,00E+00	2127	9	NA	Japonica	
maker-2-augustus-gene-242.31-mRNA-1	2	OsR498G0204285300.01.T01	99,66	0,00E+00	2084	Os02g0611200	100,00	0,00E+00	2102	18	NA	Japonica	
maker-2-augustus-gene-242.32-mRNA-1	2	OsR498G0204285700.01.T01	100,00	0,00E+00	3701	Os02g0611400	100,00	0,00E+00	3701	0	NA	Japonica	
maker-2-augustus-gene-242.34-mRNA-1	2	OsR498G0204282000.01.T02	99,79	0,00E+00	2519	Os02g0610400	100,00	0,00E+00	2533	14	NA	Japonica	
maker-2-augustus-gene-242.38-mRNA-1	2	OsR498G0204283200.01.T03	99,84	0,00E+00	1100	Os02g0610600	98,53	0,00E+00	1067	-33	NA	Japonica	
maker-2-augustus-gene-242.42-mRNA-1	2	OsR498G0204283600.01.T01	84,77	1,81E-161	569	Os02g0610700	84,77	6,30E-162	569	0	NA	Japonica	
maker-2-augustus-gene-242.45-mRNA-1	2	OsR498G0204285000.01.T01	96,04	0,00E+00	942	Os02g0611000	99,78	0,00E+00	824	-118	Indica	Japonica	
maker-2-augustus-gene-243.0-mRNA-1	2	OsR498G0204287700.01.T01	99,93	0,00E+00	2393	Os02g0611800	100,00	0,00E+00	2397	4	NA	Japonica	
maker-2-augustus-gene-243.13-mRNA-1	2	OsR498G0204286200.01.T01	99,66	0,00E+00	4190	Os02g0611500	99,96	0,00E+00	4222	32	NA	Japonica	
maker-2-augustus-gene-243.17-mRNA-1	2	OsR498G0204289000.01.T01	100,00	0,00E+00	1321	Os02g0612300	100,00	0,00E+00	1321	0	NA	Japonica	
maker-2-augustus-gene-243.25-mRNA-1	2	OsR498G0204292000.01.T03	99,30	0,00E+00	4047	Os02g0612800	99,39	0,00E+00	4056	9	NA	Japonica	
maker-2-augustus-gene-243.30-mRNA-1	2	OsR498G0204293200.01.T01	99,90	0,00E+00	1868	Os04g0109900	71,09	1,17E-144	514	-1354	Indica	Japonica	
maker-2-augustus-gene-243.5-mRNA-1	2	OsR498G0204288500.01.T01	99,80	0,00E+00	911	Os02g0612100	100,00	0,00E+00	915	4	NA	Japonica	
maker-2-augustus-gene-243.7-mRNA-1	2	OsR498G0204292900.01.T01	99,86	0,00E+00	2491	Os02g0613000	100,00	0,00E+00	2176	-315	Indica	Japonica	
maker-2-augustus-gene-244.26-mRNA-1	2	OsR498G0204293900.01.T04	99,57	5,31E-115	415	Os02g0613600	100,00	4,34E-117	419	4	NA	Japonica	
maker-2-augustus-gene-244.34-mRNA-1	2	OsR498G0204296700.01.T01	100,00	0,00E+00	1878	Os02g0614100	99,90	0,00E+00	1874	-4	NA	Indica	
maker-2-augustus-gene-244.43-mRNA-1	2	OsR498G0204298500.01.T02	100,00	0,00E+00	1397	Os02g0614400	99,87	0,00E+00	1392	-5	NA	Indica	
maker-2-augustus-gene-244.45-mRNA-1	2	OsR498G0204293400.01.T01	99,77	0,00E+00	3137	Os02g0613200	100,00	0,00E+00	3155	18	NA	Japonica	LRR-TM
maker-2-augustus-gene-244.54-mRNA-1	2	OsR498G0204297200.01.T01	100,00	0,00E+00	785	Os02g0614200	99,77	0,00E+00	781	-4	NA	Indica	
maker-2-augustus-gene-245.0-mRNA-1	2	OsR498G0204299100.01.T02	100,00	0,00E+00	4259	Os02g0614600	97,51	0,00E+00	4016	-243	NA	Indica	
maker-2-augustus-gene-245.16-mRNA-1	2	OsR498G0204298800.01.T01	100,00	0,00E+00	1797	Os02g0614500	99,70	0,00E+00	1783	-14	NA	Indica	
maker-2-augustus-gene-245.21-mRNA-1	2	OsR498G0204299400.01.T01	100,00	0,00E+00	1905	Os02g0614700	97,47	0,00E+00	2613	708	Japonica	Indica	
maker-2-augustus-gene-245.8-mRNA-1	2	OsR498G0204301900.01.T02	100,00	0,00E+00	2150	Os02g0615500	74,94	0,00E+00	830	-1320	Indica	NA	Kinase-LRR-TM
maker-2-augustus-gene-247.5-mRNA-1	2	OsR498G0204310500.01.T04	100,00	0,00E+00	668	Os02g0615800	75,51	1,89E-40	165	-503	Indica	NA	LRR-TM
maker-2-augustus-gene-247.8-mRNA-1	2	OsR498G0204301900.01.T02	83,59	0,00E+00	2911	Os02g0615500	70,63	0,00E+00	1206	-1705	Indica	NA	Kinase-TM
maker-2-augustus-gene-248.0-mRNA-1	2	OsR498G0204314300.01.T03	100,00	0,00E+00	1131	Os02g0615300	73,11	5,74E-88	324	-807	Indica	NA	LRR-TM
maker-2-augustus-gene-249.0-mRNA-1	2	OsR498G0204315700.01.T02	99,93	0,00E+00	5384	Os02g0615300	70,34	0,00E+00	1323	-4061	Indica	NA	Kinase-LRR-TM
maker-2-augustus-gene-252.36-mRNA-1	2	OsR498G0204323600.01.T04	99,87	0,00E+00	2684	Os02g0617200	99,80	0,00E+00	2676	-8	NA	NA	
maker-2-augustus-gene-253.0-mRNA-1	2	OsR498G0204325500.01.T01	99,91	0,00E+00	1987	Os02g0617400	99,73	0,00E+00	1978	-9	NA	NA	
maker-2-augustus-gene-253.12-mRNA-1	2	OsR498G0204327500.01.T01	100,00	0,00E+00	726	Os02g0618100	99,49	0,00E+00	692	-34	NA	NA	
maker-2-augustus-gene-253.14-mRNA-1	2	OsR498G0204327700.01.T01	99,52	0,00E+00	1857	Os02g0618200	99,71	0,00E+00	1866	9	NA	Japonica	
maker-2-augustus-gene-253.18-mRNA-1	2	OsR498G0204329700.01.T01	99,50	0,00E+00	2485	Os02g0618700	99,93	0,00E+00	2512	27	NA	NA	
maker-2-augustus-gene-253.28-mRNA-1	2	OsR498G0204328800.01.T01	99,77	0,00E+00	1561	Os02g0618400	99,77	0,00E+00	1561	0	NA	NA	
maker-2-augustus-gene-253.8-mRNA-1	2	OsR498G0204327100.01.T01	100,00	4,86E-155	547	Os02g0617900	98,30	8,73E-172	602	55	NA	Indica	
maker-2-augustus-gene-254.0-mRNA-1	2	OsR498G0204332500.01.T01	99,58	0,00E+00	1280	Os02g0620100	100,00	0,00E+00	1294	14	NA	Japonica	
maker-2-augustus-gene-254.12-mRNA-1	2	OsR498G0204336600.01.T01	99,79	0,00E+00	3337	Os02g0621300	99,68	0,00E+00	3328	-9	NA	Japonica	
maker-2-augustus-gene-254.3-mRNA-1	2	OsR498G0204333300.01.T01	100,00	2,45E-176	618	Os02g0620400	100,00	8,53E-177	618	0	NA	Japonica	
maker-2-augustus-gene-254.9-mRNA-1	2	OsR498G0204331600.01.T01	99,81	0,00E+00	3824	Os02g0619600	100,00	0,00E+00	3842	18	NA	Japonica	
maker-2-augustus-gene-255.33-mRNA-1	2	OsR498G0204337500.01.T01	99,76	0,00E+00	2276	Os02g0621700	100,00	0,00E+00	2289	13	NA	Japonica	
maker-2-augustus-gene-255.40-mRNA-1	2	OsR498G0204338900.01.T01	99,93	0,00E+00	2507	Os02g0622100	100,00	0,00E+00	2511	4	NA	Japonica	
maker-2-augustus-gene-255.57-mRNA-1	2	OsR498G0204341900.01.T01	100,00	0,00E+00	1597	Os04g0515900	84,85	7,56E-170	596	-1001	Indica	Indica	
maker-2-augustus-gene-255.62-mRNA-1	2	OsR498G0204337000.01.T01	99,66	0,00E+00	1565	Os02g0621500	100,00	0,00E+00	1579	14	NA	Japonica	
maker-2-augustus-gene-255.64-mRNA-1	2	OsR498G0204338000.01.T01	99,66	0,00E+00	3124	Os02g0621800	99,83	0,00E+00	3138	14	NA	Japonica	
maker-2-augustus-gene-255.66-mRNA-1	2	OsR498G0204339400.01.T02	99,71	0,00E+00	1214	Os04g0515300	91,24	2,61E-155	547	-667	Indica	NA	
maker-2-augustus-gene-255.69-mRNA-1	2	OsR498G0204340000.01.T02	99,86	0,00E+00	1268	Os08g0567200	71,47	5,62E-50	198	-1070	Indica	Japonica	
maker-2-augustus-gene-256.0-mRNA-1	2	OsR498G0204347900.01.T02	99,38	0,00E+00	1727	Os02g0624400	99,59	0,00E+00	1736	9	NA	Japonica	
maker-2-augustus-gene-256.11-mRNA-1	2	OsR498G0204343600.01.T04	96,04	0,00E+00	1461	Os02g0623500	96,26	0,00E+00	1470	9	NA	Japonica	
maker-2-augustus-gene-256.18-mRNA-1	2	OsR498G0204344200.01.T02	99,96	0,00E+00	4103	Os02g0623600	100,00	0,00E+00	2089	-2014	Indica	Japonica	Kinase-TM
maker-2-augustus-gene-256.22-mRNA-1	2	OsR498G0204346000.01.T01	99,87	0,00E+00	1397	Os02g0624300	100,00	0,00E+00	1402	5	NA	Japonica	
maker-2-augustus-gene-256.9-mRNA-1	2	OsR498G0204342200.01.T05	100,00	0,00E+00	1130	Os02g0623400	100,00	0,00E+00	1130	0	NA	NA	
maker-2-augustus-gene-257.46-mRNA-1	2	OsR498G0204351800.01.T03	96,52	0,00E+00	1777	Os02g0625500	96,92	0,00E+00	1971	194	NA	Japonica	
maker-2-augustus-gene-257.50-mRNA-1	2	OsR498G0204353600.01.T01	99,91	0,00E+00	3790	Os02g0626100	99,86	0,00E+00	3785	-5	NA	Japonica	
maker-2-augustus-gene-257.51-mRNA-1	2	OsR498G0204354400.01.T02	99,77	0,00E+00	3868	Os02g0626400	99,86	0,00E+00	3877	9	NA	Japonica	
maker-2-augustus-gene-257.60-mRNA-1	2	OsR498G0204355400.01.T01	99,77	0,00E+00	3841	Os02g0627100	99,91	0,00E+00	3855	14	NA	Japonica	
maker-2-augustus-gene-258.0-mRNA-1	2	OsR498G0204357100.01.T01	99,72	0,00E+00	3777	Os02g0627700	99,37	0,00E+00	3687	-90	NA	Japonica	
maker-2-augustus-gene-258.2-mRNA-1	2	OsR498G0204358700.01.T03	100,00	0,00E+00	1326	Os04g0518800	83,63	0,00E+00	720	-606	Indica	Japonica	
maker-2-augustus-gene-259.45-mRNA-1	2	OsR498G0204360900.01.T01	99,65	0,00E+00	3533	Os02g0628600	99,81	0,00E+00	1911	-1622	Indica	Japonica	
maker-2-augustus-gene-259.50-mRNA-1	2	OsR498G0204364100.01.T01	99,89	0,00E+00	1560	Os02g0629200	99,89	0,00E+00	1560	0	NA	Japonica	
maker-2-augustus-gene-259.54-mRNA-1	2	OsR498G0204365900.01.T01	100,00	9,32E-123	439	Os02g0629800	100,00	3,26E-123	439	0	NA	Indica	
maker-2-augustus-gene-259.56-mRNA-1	2	OsR498G0204367200.01.T01	96,16	0,00E+00	1856	Os04g0522500	77,40	0,00E+00	674	-1182	Indica	NA	
maker-2-augustus-gene-26.37-mRNA-1	2	OsR498G0202963300.01.T06	100,00	0,00E+00	1099	Os02g0145600	99,84	0,00E+00	1095	-4	NA	Indica	
maker-2-augustus-gene-26.47-mRNA-1	2	OsR498G0202966700.01.T02	100,00	0,00E+00	2246	Os02g0146600	100,00	0,00E+00	2246	0	NA	Indica	
maker-2-augustus-gene-26.49-mRNA-1	2	OsR498G0202967300.01.T01	99,56	0,00E+00	4814	Os02g0146700	99,44	0,00E+00	4800	-14	NA	Indica	
maker-2-augustus-gene-26.56-mRNA-1	2	OsR498G0202969700.01.T02	99,57	0,00E+00	1663	Os02g0147700	99,57	0,00E+00	1663	0	NA	Indica	
maker-2-augustus-gene-26.74-mRNA-1	2	OsR498G0202968200.01.T02	99,79	0,00E+00	4213	Os02g0147300	98,82	0,00E+00	6114	1901	Japonica	Indica	
maker-2-augustus-gene-260.0-mRNA-1	2	OsR498G0204369900.01.T01	99,74	0,00E+00	2097	Os02g0631000	99,74	0,00E+00	2097	0	NA	NA	
maker-2-augustus-gene-260.16-mRNA-1	2	OsR498G0204371000.01.T01	99,78	0,00E+00	797	Os02g0631700	99,78	0,00E+00	797	0	NA	NA	
maker-2-augustus-gene-260.6-mRNA-1	2	OsR498G0509684800.01.T02	81,99	0,00E+00	1233	Os02g0632100	99,42	0,00E+00	2457	1224	Japonica	Indica	Kinase-TM
maker-2-augustus-gene-260.9-mRNA-1	2	OsR498G0204370400.01.T05	99,77	0,00E+00	1566	Os02g0631200	99,54	0,00E+00	1557	-9	NA	Japonica	
maker-2-augustus-gene-261.32-mRNA-1	2	OsR498G0204376100.01.T06	98,51	0,00E+00	2920	Os02g0632500	98,51	0,00E+00	2920	0	NA	Japonica	
maker-2-augustus-gene-262.0-mRNA-1	2	OsR498G0204380300.01.T01	99,91	0,00E+00	3941	Os04g0524600	80,82	0,00E+00	1602	-2339	Indica	Indica	

maker-2-augustus-gene-262.11-mRNA-1	2	OsR498G0204383100.01.T02	99,60	0,00E+00	1324	Os02g0634900	100,00	0,00E+00	1278	-46	NA	Japonica	
maker-2-augustus-gene-262.15-mRNA-1	2	OsR498G0204383500.01.T02	99,72	0,00E+00	1924	Os02g0635000	100,00	0,00E+00	1938	14	NA	Japonica	
maker-2-augustus-gene-262.18-mRNA-1	2	OsR498G0204383700.01.T01	99,73	0,00E+00	1342	Os02g0635200	100,00	0,00E+00	1352	10	NA	Japonica	
maker-2-augustus-gene-262.24-mRNA-1	2	OsR498G0204383900.01.T01	99,42	1,13E-175	616	Os02g0635333	100,00	1,29E-131	468	-148	Indica	Japonica	
maker-2-augustus-gene-262.27-mRNA-1	2	OsR498G0204379000.01.T01	99,29	0,00E+00	2996	Os02g0633066	99,33	0,00E+00	2646	-350	Indica	Japonica	Kinase-TM
maker-2-augustus-gene-262.4-mRNA-1	2	OsR498G0204382300.01.T03	99,92	0,00E+00	2193	Os02g0634600	100,00	0,00E+00	2197	4	NA	Japonica	
maker-2-augustus-gene-262.44-mRNA-1	2	OsR498G0204380600.01.T01	99,80	0,00E+00	885	Os02g0633400	100,00	0,00E+00	890	5	NA	NA	
maker-2-augustus-gene-262.7-mRNA-1	2	OsR498G0204382700.01.T05	99,69	0,00E+00	1744	Os02g0634700	99,80	0,00E+00	1748	4	NA	Japonica	
maker-2-augustus-gene-263.37-mRNA-1	2	OsR498G0204384300.01.T02	99,76	0,00E+00	2202	Os11g0569800	71,09	0,00E+00	659	-1543	Indica	Japonica	Kinase-LRR-TM
maker-2-augustus-gene-263.38-mRNA-1	2	OsR498G0204384500.01.T01	99,61	0,00E+00	906	Os02g0635700	100,00	0,00E+00	915	9	NA	Japonica	
maker-2-augustus-gene-263.42-mRNA-1	2	OsR498G0204387000.01.T01	99,71	0,00E+00	1236	Os02g0636600	100,00	0,00E+00	1245	9	NA	Japonica	
maker-2-augustus-gene-263.44-mRNA-1	2	OsR498G0204387200.01.T01	100,00	0,00E+00	1261	Os02g0636700	100,00	0,00E+00	1261	0	NA	Japonica	
maker-2-augustus-gene-263.47-mRNA-1	2	OsR498G0204391000.01.T01	99,73	0,00E+00	1980	Os02g0637700	100,00	0,00E+00	1994	14	NA	Japonica	
maker-2-augustus-gene-263.52-mRNA-1	2	OsR498G0204391200.01.T01	98,17	0,00E+00	1798	Os02g0637800	98,56	0,00E+00	1816	18	NA	Japonica	
maker-2-augustus-gene-264.35-mRNA-1	2	OsR498G0204391300.01.T05	99,19	0,00E+00	655	Os02g0637900	99,73	0,00E+00	664	9	NA	Japonica	
maker-2-augustus-gene-264.40-mRNA-1	2	OsR498G0204394700.01.T03	99,81	0,00E+00	2896	Os02g0638900	99,31	0,00E+00	2557	-339	Indica	Japonica	
maker-2-augustus-gene-264.46-mRNA-1	2	OsR498G0204395100.01.T01	99,47	0,00E+00	5028	Os02g0639000	100,00	0,00E+00	5092	64	NA	Japonica	
maker-2-augustus-gene-264.52-mRNA-1	2	OsR498G0204395900.01.T02	99,20	0,00E+00	2217	Os02g0639300	100,00	0,00E+00	1602	-615	Indica	Japonica	
maker-2-augustus-gene-264.54-mRNA-1	2	OsR498G0204396500.01.T01	100,00	0,00E+00	1478	Os04g0530100	81,49	1,54E-146	518	-960	Indica	Japonica	
maker-2-augustus-gene-264.57-mRNA-1	2	OsR498G0204396700.01.T01	99,70	0,00E+00	1767	Os02g0639600	100,00	0,00E+00	1781	14	NA	Japonica	
maker-2-augustus-gene-264.60-mRNA-1	2	OsR498G0204396900.01.T02	99,89	0,00E+00	1607	Os02g0639700	100,00	0,00E+00	1333	-274	Indica	Japonica	
maker-2-augustus-gene-264.65-mRNA-1	2	OsR498G0204391600.01.T01	99,68	0,00E+00	1686	Os02g0638000	100,00	0,00E+00	1700	14	NA	Japonica	
maker-2-augustus-gene-264.71-mRNA-1	2	OsR498G0204392600.01.T01	99,72	0,00E+00	1902	Os02g0638400	100,00	0,00E+00	1916	14	NA	Japonica	
maker-2-augustus-gene-264.73-mRNA-1	2	OsR498G0204395600.01.T01	98,88	0,00E+00	1873	Os02g0639100	100,00	0,00E+00	2603	730	Japonica	Japonica	Kinase-TM
maker-2-augustus-gene-265.10-mRNA-1	2	OsR498G0204402500.01.T01	99,27	0,00E+00	1701	Os02g0641300	99,86	0,00E+00	1244	-457	Indica	Japonica	
maker-2-augustus-gene-265.14-mRNA-1	2	OsR498G0204402700.01.T02	99,87	0,00E+00	2746	Os02g0641800	99,86	0,00E+00	2471	-275	Indica	Japonica	
maker-2-augustus-gene-265.22-mRNA-1	2	OsR498G0204398800.01.T02	99,57	0,00E+00	2867	Os02g0640300	100,00	0,00E+00	1808	-1059	Indica	Japonica	
maker-2-augustus-gene-265.26-mRNA-1	2	OsR498G0204401000.01.T01	99,84	0,00E+00	1116	Os02g0641000	100,00	0,00E+00	1121	5	NA	Japonica	
maker-2-augustus-gene-265.3-mRNA-1	2	OsR498G0204398300.01.T01	99,38	0,00E+00	1439	Os02g0640200	100,00	0,00E+00	1462	23	NA	Japonica	
maker-2-augustus-gene-266.30-mRNA-1	2	OsR498G0204403500.01.T01	100,00	0,00E+00	1629	Os02g0641900	99,91	0,00E+00	2030	401	Japonica	Japonica	
maker-2-augustus-gene-266.32-mRNA-1	2	OsR498G0204405900.01.T02	96,31	0,00E+00	2116	Os02g0642700	96,01	0,00E+00	1783	-333	Indica	Japonica	
maker-2-augustus-gene-266.37-mRNA-1	2	OsR498G0204409300.01.T01	99,28	0,00E+00	1484	Os02g0643500	99,64	0,00E+00	1495	11	NA	Japonica	
maker-2-augustus-gene-266.43-mRNA-1	2	OsR498G0204404000.01.T01	100,00	0,00E+00	1104	Os02g0642200	100,00	0,00E+00	1104	0	NA	Japonica	
maker-2-augustus-gene-266.46-mRNA-1	2	OsR498G0204404400.01.T01	99,86	0,00E+00	1268	Os04g0533500	78,76	1,08E-166	586	-682	Indica	Japonica	
maker-2-augustus-gene-266.49-mRNA-1	2	OsR498G0204405600.01.T01	99,76	0,00E+00	5965	Os02g0642600	99,25	0,00E+00	5948	-17	NA	Japonica	
maker-2-augustus-gene-266.52-mRNA-1	2	OsR498G0204407200.01.T01	99,87	0,00E+00	1403	Os02g0643000	99,74	0,00E+00	1398	-5	NA	NA	
maker-2-augustus-gene-266.56-mRNA-1	2	OsR498G0204408000.01.T01	99,74	0,00E+00	1382	Os02g0643200	100,00	0,00E+00	1391	9	NA	Japonica	
maker-2-augustus-gene-267.0-mRNA-1	2	OsR498G0204410700.01.T01	96,62	0,00E+00	1420	Os04g0537800	78,60	0,00E+00	641	-779	Indica	Japonica	
maker-2-augustus-gene-267.14-mRNA-1	2	OsR498G0204412600.01.T01	99,63	0,00E+00	2922	Os02g0644500	100,00	0,00E+00	2949	27	NA	Japonica	
maker-2-augustus-gene-267.17-mRNA-1	2	OsR498G0204416000.01.T02	99,81	0,00E+00	2831	Os02g0645100	100,00	0,00E+00	2845	14	NA	Japonica	
maker-2-augustus-gene-267.19-mRNA-1	2	OsR498G0204415200.01.T01	99,36	0,00E+00	2791	Os11g0660000	98,47	0,00E+00	2727	-64	NA	Japonica	
maker-2-augustus-gene-267.2-mRNA-1	2	OsR498G0204411300.01.T02	99,83	0,00E+00	3120	Os02g0644100	99,94	0,00E+00	3129	9	NA	Japonica	
maker-2-augustus-gene-267.27-mRNA-1	2	OsR498G0204416000.01.T02	99,69	0,00E+00	3498	Os02g0645100	99,95	0,00E+00	3520	22	NA	Japonica	
maker-2-augustus-gene-267.7-mRNA-1	2	OsR498G0204411200.01.T01	99,69	0,00E+00	1162	Os04g0537900	77,97	1,49E-142	506	-656	Indica	Japonica	
maker-2-augustus-gene-268.0-mRNA-1	2	OsR498G0204416700.01.T01	99,00	0,00E+00	2288	Os02g0645600	99,66	0,00E+00	2085	-203	NA	Japonica	
maker-2-augustus-gene-268.13-mRNA-1	2	OsR498G0204418800.01.T01	99,39	0,00E+00	1164	Os02g0646500	100,00	0,00E+00	1182	18	NA	Japonica	
maker-2-augustus-gene-268.14-mRNA-1	2	OsR498G0204418800.01.T03	99,42	0,00E+00	923	Os02g0646500	100,00	2,57E-141	500	-423	Indica	Japonica	
maker-2-augustus-gene-268.2-mRNA-1	2	OsR498G0204418300.01.T01	99,14	0,00E+00	1434	Os02g0646200	100,00	0,00E+00	1462	28	NA	Japonica	
maker-2-augustus-gene-268.4-mRNA-1	2	OsR498G0204418500.01.T01	100,00	0,00E+00	715	Os02g0646400	100,00	0,00E+00	715	0	NA	Japonica	
maker-2-augustus-gene-268.8-mRNA-1	2	OsR498G0204420900.01.T02	99,59	0,00E+00	1315	Os02g0647900	100,00	0,00E+00	1328	13	NA	Japonica	
maker-2-augustus-gene-269.31-mRNA-1	2	OsR498G0204425300.01.T01	99,88	0,00E+00	4440	Os02g0649700	100,00	0,00E+00	4453	13	NA	Japonica	
maker-2-augustus-gene-269.36-mRNA-1	2	OsR498G0204421800.01.T01	99,09	0,00E+00	1939	Os02g0648100	100,00	0,00E+00	1981	42	NA	Japonica	Kinase-TM
maker-2-augustus-gene-269.43-mRNA-1	2	OsR498G0204425200.01.T02	99,58	0,00E+00	2101	Os02g0649400	99,92	0,00E+00	2119	18	NA	Japonica	
maker-2-augustus-gene-269.48-mRNA-1	2	OsR498G0204425600.01.T01	100,00	0,00E+00	753	Os02g0649800	100,00	0,00E+00	753	0	NA	Japonica	
maker-2-augustus-gene-269.50-mRNA-1	2	OsR498G0204426800.01.T01	99,80	0,00E+00	1764	Os02g0649900	99,90	0,00E+00	1765	1	NA	Japonica	
maker-2-augustus-gene-27.15-mRNA-1	2	OsR498G0202970100.01.T01	95,85	0,00E+00	2027	Os02g0147800	95,85	0,00E+00	2027	0	NA	NA	
maker-2-augustus-gene-27.20-mRNA-1	2	OsR498G0202971900.01.T01	99,36	0,00E+00	2491	Os02g0148000	100,00	0,00E+00	2554	63	NA	Japonica	
maker-2-augustus-gene-27.27-mRNA-1	2	OsR498G0202973500.01.T01	92,64	0,00E+00	1160	Os02g0148400	92,77	0,00E+00	1165	5	NA	Japonica	
maker-2-augustus-gene-27.30-mRNA-1	2	OsR498G0202973700.01.T03	99,89	0,00E+00	1646	Os02g0148500	100,00	0,00E+00	1462	-184	Indica	Japonica	
maker-2-augustus-gene-27.4-mRNA-1	2	OsR498G0202969700.01.T02	99,90	0,00E+00	1852	Os02g0147700	99,90	0,00E+00	1852	0	NA	NA	
maker-2-augustus-gene-27.43-mRNA-1	2	OsR498G0202972400.01.T02	99,82	0,00E+00	1999	Os02g0148100	100,00	0,00E+00	2008	9	NA	Japonica	Kinase-TM
maker-2-augustus-gene-270.11-mRNA-1	2	OsR498G0204428800.01.T03	99,64	0,00E+00	2499	Os02g0650500	100,00	0,00E+00	2522	23	NA	Japonica	Kinase-TM
maker-2-augustus-gene-270.18-mRNA-1	2	OsR498G0204431000.01.T03	99,62	0,00E+00	1401	Os02g0651300	100,00	0,00E+00	1415	14	NA	Japonica	
maker-2-augustus-gene-270.2-mRNA-1	2	OsR498G0204430300.01.T01	100,00	0,00E+00	2230	Os02g0650900	100,00	0,00E+00	2230	0	NA	Japonica	
maker-2-augustus-gene-270.22-mRNA-1	2	OsR498G0204432800.01.T01	99,38	0,00E+00	1139	Os02g0652000	100,00	0,00E+00	1153	14	NA	Japonica	
maker-2-augustus-gene-270.4-mRNA-1	2	OsR498G0204428200.01.T01	99,60	0,00E+00	3606	Os04g0542800	76,26	0,00E+00	1440	-2166	Indica	Japonica	
maker-2-augustus-gene-271.0-mRNA-1	2	OsR498G0204435700.01.T01	99,73	0,00E+00	2661	Os02g0652800	100,00	0,00E+00	2679	18	NA	Japonica	
maker-2-augustus-gene-271.12-mRNA-1	2	OsR498G0204434600.01.T01	99,45	0,00E+00	1295	Os02g0652300	99,45	0,00E+00	1295	0	NA	Japonica	
maker-2-augustus-gene-271.15-mRNA-1	2	OsR498G0204435000.01.T01	99,48	0,00E+00	1033	Os10g0549100	91,71	0,00E+00	829	-204	Indica	Japonica	
maker-2-augustus-gene-271.21-mRNA-1	2	OsR498G0204435600.01.T01	99,86	0,00E+00	1257	Os02g0652600	100,00	0,00E+00	1272	15	NA	Japonica	
maker-2-augustus-gene-271.26-mRNA-1	2	OsR498G0204438000.01.T01	99,84	0,00E+00	1095	Os02g0653200	100,00	0,00E+00	1099	4	NA	Japonica	
maker-2-augustus-gene-271.28-mRNA-1	2	OsR498G0204438200.01.T01	99,83	0,00E+00	2080	Os02g0653400	100,00	0,00E+00	2089	9	NA	Japonica	
maker-2-augustus-gene-271.3-mRNA-1	2	OsR498G0204439300.01.T03	99,36	0,00E+00	1113	Os02g0653800	100,00	0,00E+00	1104	-9	NA	Japonica	
maker-2-augustus-gene-272.32-mRNA-1	2	OsR498G0204441300.01.T02	96,49	0,00E+00	1664	Os02g0654100	95,17	0,00E+00	1109	-555	Indica	Japonica	
maker-2-augustus-gene-272.35-mRNA-1	2	OsR498G0204441700.01.T01	99,36	0,00E+00	1942	Os02g0654300	100,00	0,00E+00	1974	32	NA	Japonica	Kinase
maker-2-augustus-gene-272.37-mRNA-1	2	OsR498G0204442100.01.T01	94,96	0,00E+00	1090	Os02g0654500	95,11	0,00E+00	1095	5	NA	Japonica	
maker-2-augustus-gene-272.41-mRNA-1	2	OsR498G0204443300.01.T01	99,54	0,00E+00	1926	Os02g0655100	100,00	0,00E+00	1943	17	NA	Japonica	
maker-2-augustus-gene-272.43-mRNA-1	2	OsR498G0204445300.01.T01	100,00	0,00E+00	1002	Os02g0655500	100,00	0,00E+00	1070	68	NA	Japonica	
maker-2-augustus-gene-272.45-mRNA-1	2	OsR498G0204446300.01.T01	99,73	0,00E+00	3257	Os02g0655700	100,00	0,00E+00	3279	22	NA	Japonica	
maker-2-augustus-gene-272.54-mRNA-1	2	OsR498G0204442400.01.T01	99,92	0,00E+00	2142	Os02g0654600	99,95	0,00E+00	3296	1154	Japonica	Japonica	
maker-2-augustus-gene-273.0-mRNA-1	2	OsR498G0204447900.01.T01	99,77	0,00E+00	3054	Os02g0656300	100,00	0,00E+00	3076	22	NA	Japonica	
maker-2-augustus-gene-273.16-mRNA-1	2	OsR498G0204452600.01.T03	99,90	0,00E+00	1771	Os02g0657700	99,90	0,00E+00	1771	0	NA	Japonica	
maker-2-augustus-gene-273.4-mRNA-1	2	OsR498G0204448500.01.T04	99,62	0,00E+00	1421	Os02g0656500	100,00	0,00E+00	1321	-100	NA	Japonica	
maker-2-augustus-gene-273.8-mRNA-1	2	OsR498G0204447000.01.T01	99,92	0,00E+00	2225	Os02g0655800	100,00	0,00E+00	2230	5	NA	Japonica	Kinase-TM
maker-2-augustus-gene-274.29-mRNA-1	2	OsR498G0204456300.01.T01	99,76	0,00E+00	1474	Os02g0658600	100,00	0,00E+00	1483	9	NA	Japonica	
maker-2-augustus-gene-274.31-mRNA-1	2	OsR498G0204457300.01.T01	99,89	0,00E+00	1581	Os02g0658800	100,00	0,00E+00	1586	5	NA	Japonica	
maker-2-augustus-gene-274.33-mRNA-1	2	OsR498G0204454200.01.T01	99,87	0,00E+00	1343	Os02g0658100	100,00	0,00E+00	1348	5	NA	Japonica	
maker-2-augustus-gene-274.36-mRNA-1	2	OsR498G0204454800.01.T02	100,00	0,00E+00	774	Os02g0658200	88,11	6,43E-167	586	-188	Indica	Japonica	
maker-2-augustus-gene-274.39-mRNA-1	2	OsR498G0204455800.01.T01	99,73	0,00E+00	2699	Os02g0658500	99,92	0,00E+00	2117	-582	Indica	Japonica	
maker-2-augustus-gene-274.46-mRNA-1	2	OsR498G0204458200.01.T02	99,77	0,00E+00	1582	Os02g0658900	100,00	0,00E+00	1537	-45	NA	Japonica	
maker-2-augustus-gene-275.11-mRNA-1	2	OsR498G0204464600.01.T05	100,00	0,00E+00	1072	Os02g0661100	100,00	0,00E+00	1072	0	NA	Japonica	
maker-2-augustus-gene-275.8-mRNA-1	2	OsR498G0204464200.01.T01	93,39	0,00E+00	1130	Os02g0661000	93,40	0,00E+00	1064	-66	NA	Japonica	
maker-2-augustus-gene-276.11-mRNA-1	2	OsR498G0204469400.01.T01	100,00	0,00E+00	872	Os02g0662300	99,32	1,08E-145	516	-356	Indica	Japonica	
maker-2-augustus-gene-276.7-mRNA-1	2	OsR498G0204468900.01.T01	99,86	0,00E+00	1246	Os02g0662200	99,86	0,00E+00	1246	0	NA	Japonica	
maker-2-augustus-gene-277.14-mRNA-1	2	OsR498G0204475100.01.T01	99,68	0,00E+00	7177	Os02g0664300	100,00	0,00E+00	7236	59	NA	Japonica	
maker-2-augustus-gene-277.20-mRNA-1	2	OsR498G0409054800.01.T01	84,14	7,70E-127	453	Os02g0663800	100,00	0,00E+00	755	302	Japonica	Japonica	
maker-2-augustus-gene-277.22-mRNA-1	2	OsR498G0204474000.01.T02	99,49	0,00E+00	4167	Os02g0663900	100,00	0,00E+00	4221	54	NA	Japonica	
maker-2-augustus-gene-277.32-mRNA-1	2	OsR498G0204477200.01.T04	99,29	0,00E+00	994	Os02g0665000	99,82	0,00E+00	1008	14	NA	Japonica	
maker-2-augustus-gene-278.0-mRNA-1	2	OsR498G0204477500.01.T01	99,89	0,00E+00	3128	Os02g0665200	100,00	0,00E+00	3137	9	NA	Japonica	
maker-2-augustus-gene-278.11-mRNA-1	2	OsR498G0204480100.01.T02	99,89	0,00E+00	1565	Os02g0666200	99,89	0,00E+00	1565	0	NA	Japonica	
maker-2-augustus-gene-278.15-mRNA-1	2	OsR498G0204480400.01.T02	99,88	0,00E+00	1518	Os02g0666300	100,00	0,00E+00	1523	5	NA	Japonica	Kinase-TM
maker-2-augustus-gene-278.16-mRNA-1	2	OsR498G0204480400.01.T05	99,93	0,00E+00	2591	Os02g0666300	100,00	0,00E+00	2596	5	NA	Japonica	
maker-2-augustus-gene-279.11-mRNA-1	2	OsR498G0204484000.01.T01	99,90	0,00E+00	1825	Os02g0667600	100,00	0,00E+00	1829	4	NA	Japonica	
maker-2-augustus-gene-279.13-mRNA-1	2	OsR498G0204485800.01.T02	99,31	0,00E+00	1287	Os02g0668500	100,00	0,00E+00	899	-388	Indica	Japonica	
maker-2-augustus-gene-279.18-mRNA-1	2	OsR498G0204488200.01.T01	99,76	0,00E+00	1483	Os02g0669000	100,00	0,00E+00	1492	9	NA	Japonica	
maker-2-augustus-gene-279.20-mRNA-1	2	OsR498G0204491200.01.T03	99,51	0,00E+00	1080	Os02g0669700	100,00	0,00E+00	1094	14	NA	Japonica	
maker-2-augustus-gene-279.5-mRNA-1	2	OsR498G0204484700.01.T01	99,59	0,00E+00	5222	Os02g0668000	100,00	0,00E+00	5276	54	NA	Japonica	
maker-2-augustus-gene-279.9-mRNA-1	2	OsR498G0204485500.01.T02	99,71	0,00E+00	3123	Os02g0668400	100,00	0,00E+00	3149	26	NA	Japonica	
maker-2-augustus-gene-28.0-mRNA-1	2	OsR498G0202977300.01.T01	99,65	0,00E+00	3576	Os02g0149600	100,00	0,00E+00	3609	33	NA	Japonica	
maker-2-augustus-gene-28.10-mRNA-1	2	OsR498G0202980900.01.T01	99,91	0,00E+00	1933	Os02g0150300	100,00	0,00E+00	1745	-188	NA	Japonica	
maker-2-augustus-gene-28.15-mRNA-1	2	OsR498G0202981900.01.T02	100,00	0,00E+00	3144	Os02g0150700	100,00	0,00E+00	3144	0	NA	NA	
maker-2-augustus-gene-28.19-mRNA-1	2	OsR498G0202982300.01.T05	99,79	0,00E+00	3416	Os02g0150800	99,93	0,00E+00	2582	-834	Indica	NA	
maker-2-augustus-gene-28.22-mRNA-1	2	OsR498G0202980400.01.T02	99,84	0,00E+00	3385	Os02g0150100	100,00	0,00E+00	2495	-890	Indica	Japonica	
maker-2-augustus-gene-28.3-mRNA-1	2	OsR498G0202977900.01.T01	99,64	0,00E+00	1468	Os02g0149800	100,00	0,00E+00	1481	13	NA	Japonica	
maker-2-augustus-gene-280.10-mRNA-1	2	OsR498G0204492800.01.T01	99,78	0,00E+00	2470	Os02g0670100	100,00	0,00E+00	2484	14	NA	Japonica	Kinase
maker-2-augustus-gene-280.13-mRNA-1	2	OsR498G0204495600.01.T02	99,92	0,00E+00	2171	Os02g0671300	99,92	0,00E+00	2171	0	NA	Japonica	
maker-2-augustus-gene-280.2-mRNA-1	2	OsR498G0204493100.01.T01	99,77	0,00E+00	786	Os02g0670400	100,00	0,00E+00	791	5	NA	Japonica	
maker-2-augustus-gene-281.0-mRNA-1	2	OsR498G0409085900.01.T05	76,89	0,00E+00	1423	Os02g0672100	100,00	0,00E+00	3333	1910	Japonica	Japonica	
maker-2-augustus-gene-281.17-mRNA-1	2	OsR498G0204500800.01.T06	99,78	0,00E+00	799	Os02g0672400	100,00	0,00E+00	803	4	NA	Japonica	
maker-2-augustus-gene-281.24-mRNA-1	2	OsR498G0204501800.01.T01	99,89	0,00E+00	3308	Os02g0672800	99,95	0,00E+00	3313	5	NA	Japonica	Kinase-TM
maker-2-augustus-gene-281.29-mRNA-1	2	OsR498G0204503000.01.T02	99,73	0,00E+00	2628	Os02g0673100	100,00	0,00E+00	2646	18	NA	Japonica	
maker-2-augustus-gene-281.4-mRNA-1	2	OsR498G0204501100.01.T01	99,76	0,00E+00	3803	Os02g0672600	100,00	0,00E+00	3826	23	NA	Japonica	
maker-2-augustus-gene-281.8-mRNA-1	2	OsR498G0204500000.01.T01	99,88	0,00E+00	5842	Os02g0672200	100,00	0,00E+00	5860	18	NA	Japonica	
maker-2-augustus-gene-282.24-mRNA-1	2	OsR498G0204505500.01.T02	99,75	0,00E+00	4264	Os02g0673500	100,00	0,00E+00	4291	27	NA	Japonica	
maker-2-augustus-gene-282.29-mRNA-1	2	OsR498G0204505700.01.T01	99,58	0,00E+00	847	Os02g0673600	100,00	0,00E+00	856	9	NA	Japonica	
maker-2-augustus-gene-282.32-mRNA-1	2	OsR498G0204510200.01.T01	99,74	0,00E+00	2043	Os02g0674700	100,00	0,00E+00	2057	14	NA	Japonica	
maker-2-augustus-gene-282.36-mRNA-1	2	OsR498G0204510600.01.T01	98,16	0,00E+00	4199	Os02g0674800	98,32	0,00E+00	4217	18	NA	Japonica	
maker-2-augustus-gene-283.29-mRNA-1	2	OsR498G0204514700.01.T03	100,00	0,00E+00	838	Os02g0675800	100,00	0,00E+00	838	0	NA	Japonica	LRR
maker-2-augustus-gene-283.32-mRNA-1	2	OsR498G0204515700.01.T02	99,83	0,00E+00	1040	Os02g0676000	100,00	0,00E+00	1045	5	NA	Japonica	
maker-2-augustus-gene-283.37-mRNA-1	2	OsR498G0204512600.01.T01	99,72	0,00E+00	2585	Os04g0570000	84,86	0,00E+00	1542	-1043	Indica	Japonica	
maker-2-augustus-gene-283.40-mRNA-1	2	OsR498G0204514000.01.T01	99,54	0,00E+00	3461	Os02g0675700	100,00	0,00E+00	3501	40	NA	Japonica	
maker-2-augustus-gene-284.29-mRNA-1	2	OsR498G0204517300.01.T01	99,82	0,00E+00	970	Os02g0676500	100,00	0,00E+00	1142	172	Japonica	Japonica	
maker-2-augustus-gene-284.39-mRNA-1	2	OsR498G0204522800.01.T01	99,87	0,00E+00	1387	Os02g0677600	100,00	0,00E+00	1391	4	NA	Japonica	
maker-2-augustus-gene-285.46-mRNA-1	2	OsR498G0204524700.01.T05	99,92	0,00E+00	2119	Os04g0573000	81,83	0,00E+00	1148	-971	Indica	Japonica	
maker-2-augustus-gene-285.51-mRNA-1	2	OsR498G0204524900.01.T01	99,94	0,00E+00	3156	Os04g0573100	81,24	0,00E+00	1583	-1573	Indica	Japonica	
maker-2-augustus-gene-285.55-mRNA-1	2	OsR498G0204525700.01.T03	99,68	0,00E+00	1675	Os02g0678400	100,00	0,00E+00	1689	14	NA	Japonica	
maker-2-augustus-gene-285.58-mRNA-1	2	OsR498G0204526100.01.T01	99,68	0,00E+00	1128	Os02g0678800	99,06	0,00E+00	1122	-6	NA	Japonica	
maker-2-augustus-gene-285.60-mRNA-1	2	OsR498G0204527700.01.T01	99,85	0,00E+00	2434	Os02g0679500	100,00	0,00E+00	1804	-630	Indica	Japonica	
maker-2-augustus-gene-285.64-mRNA-1	2	OsR498G0204529300.01.T01	99,60	0,00E+00	2227	Os02g0680400	100,00	0,00E+00	2250	23	NA	Japonica	
maker-2-augustus-gene-285.69-mRNA-1	2	OsR498G0204527200.01.T03	99,63	0,00E+00	2400	Os02g0679200	100,00	0,00E+00	2423	23	NA	Japonica	
maker-2-augustus-gene-285.70-mRNA-1	2	OsR498G0204529800.01.T01	99,14	0,00E+00	820	Os02g0680600	100,00	0,00E+00	838	18	NA	Japonica	
maker-2-augustus-gene-286.0-mRNA-1	2	OsR498G0204531300.01.T04	99,48	0,00E+00	2729	Os02g0681000	100,00	0,00E+00	2765	36	NA	Japonica	
maker-2-augustus-gene-286.14-mRNA-1	2	OsR498G0204531600.01.T01	99,74	0,00E+00	2780	Os02g0681100	100,00	0,00E+00	2798	18	NA	Japonica	
maker-2-augustus-gene-286.7-mRNA-1	2	OsR498G0204533500.01.T01	99,82	0,00E+00	1956	Os02g0681700	100,00	0,00E+00	1362	-594	Indica	Japonica	Kinase-TM
maker-2-augustus-gene-286.9-mRNA-1	2	OsR498G0204536100.01.T01	99,73	0,00E+00	1350	Os02g0682200	100,00	0,00E+00	1359	9	NA	Japonica	
maker-2-augustus-gene-287.33-mRNA-1	2	OsR498G0204538300.01.T02	98,89	0,00E+00	940	Os02g0682700	100,00	0,00E+00	1083	143	Japonica	Japonica	
maker-2-augustus-gene-287.37-mRNA-1	2	OsR498G0204543900.01.T02	99,78	0,00E+00	813	Os02g0683800	97,24	0,00E+00	799	-14	NA	Japonica	
maker-2-augustus-gene-287.39-mRNA-1	2	OsR498G0204544100.01.T04	100,00	6,41E-168	590	Os02g0683900	100,00	2,23E-168	590	0	NA	Japonica	
maker-2-augustus-gene-287.44-mRNA-1	2	OsR498G0204545100.01.T01	100,00	0,00E+00	1496	Os02g0684300	99,86	0,00E+00	1284	-212	Indica	Japonica	
maker-2-augustus-gene-287.47-mRNA-1	2	OsR498G0204538000.01.T01	99,61	1,28E-127	456	Os02g0682600	100,00	1,05E-129	461	5	NA	Japonica	
maker-2-augustus-gene-287.53-mRNA-1	2	OsR498G0204545400.01.T01	99,43	0,00E+00	939	Os02g0684400	100,00	0,00E+00	953	14	NA	Japonica	
maker-2-augustus-gene-288.10-mRNA-1	2	OsR498G0204548800.01.T01	97,10	0,00E+00	2559	Os02g0685200	97,62	0,00E+00	2593	34	NA	Japonica	
maker-2-augustus-gene-288.17-mRNA-1	2	OsR498G0204550600.01.T05	100,00	0,00E+00	2100	Os02g0685600	100,00	0,00E+00	2100	0	NA	Japonica	
maker-2-augustus-gene-288.2-mRNA-1	2	OsR498G0204547300.01.T02	99,74	0,00E+00	4844	Os04g0583700	72,35	0,00E+00	1007	-3837	Indica	Japonica	
maker-2-augustus-gene-288.6-mRNA-1	2	OsR498G0204551100.01.T01	99,76	0,00E+00	2957	Os02g0685900	100,00	0,00E+00	2976	19	NA	Japonica	Kinase-TM
maker-2-augustus-gene-289.0-mRNA-1	2	OsR498G0204553900.01.T01	99,88	0,00E+00	2967	Os02g0686400	100,00	0,00E+00	2976	9	NA	Japonica	
maker-2-augustus-gene-289.10-mRNA-1	2	OsR498G0204554400.01.T01	99,13	0,00E+00	2031	Os02g0686600	100,00	0,00E+00	1911	-120	NA	Japonica	
maker-2-augustus-gene-289.16-mRNA-1	2	OsR498G0204555800.01.T01	100,00	0,00E+00	796	Os02g0686800	100,00	0,00E+00	796	0	NA	Japonica	
maker-2-augustus-gene-289.18-mRNA-1	2	OsR498G0204556400.01.T01	99,47	0,00E+00	673	Os04g0586000	72,10	6,88E-33	140	-533	Indica	Japonica	
maker-2-augustus-gene-289.22-mRNA-1	2	OsR498G0204557600.01.T01	100,00	5,75E-165	580	Os02g0687200	100,00	2,00E-165	580	0	NA	Japonica	
maker-2-augustus-gene-29.0-mRNA-1	2	OsR498G0202985100.01.T06	99,63	0,00E+00	1440	Os02g0151400	99,63	0,00E+00	1440	0	NA	NA	
maker-2-augustus-gene-29.11-mRNA-1	2	OsR498G0202986700.01.T01	100,00	0,00E+00	996	Os02g0152400	100,00	0,00E+00	996	0	NA	NA	
maker-2-augustus-gene-29.13-mRNA-1	2	OsR498G0202982600.01.T02	99,81	0,00E+00	1891	Os02g0150900	99,79	0,00E+00	1728	-163	NA	NA	
maker-2-augustus-gene-29.22-mRNA-1	2	OsR498G0202983600.01.T01	95,27	0,00E+00	2252	Os02g0151300	95,27	0,00E+00	2252	0	NA	NA	
maker-2-augustus-gene-29.27-mRNA-1	2	OsR498G0202986600.01.T02	99,96	0,00E+00	4023	Os02g0152500	99,91	0,00E+00	4019	-4	NA	NA	
maker-2-augustus-gene-29.8-mRNA-1	2	OsR498G0202986300.01.T01	100,00	0,00E+00	1757	Os02g0152300	100,00	0,00E+00	1757	0	NA	NA	CC-Kinase-TM
maker-2-augustus-gene-290.0-mRNA-1	2	OsR498G0204560700.01.T05	98,33	0,00E+00	1350	Os02g0687900	100,00	0,00E+00	1407	57	NA	Japonica	
maker-2-augustus-gene-290.13-mRNA-1	2	OsR498G0204565000.01.T02	98,00	0,00E+00	2152	Os02g0688900	98,00	0,00E+00	2152	0	NA	Japonica	
maker-2-augustus-gene-290.6-mRNA-1	2	OsR498G0204562900.01.T01	99,81	0,00E+00	2795	Os02g0688500	99,94	0,00E+00	2788	-7	NA	Japonica	
maker-2-augustus-gene-290.9-mRNA-1	2	OsR498G0204565900.01.T02	94,88	0,00E+00	1944	Os02g0689200	100,00	0,00E+00	1965	21	NA	Japonica	
maker-2-augustus-gene-291.13-mRNA-1	2	OsR498G0204574100.01.T01	99,82	0,00E+00	2015	Os02g0691500	100,00	0,00E+00	1337	-678	Indica	Japonica	
maker-2-augustus-gene-291.16-mRNA-1	2	OsR498G0204569400.01.T02	99,45	0,00E+00	10963	Os02g0689800	99,97	0,00E+00	5731	-5232	Indica	Japonica	
maker-2-augustus-gene-291.21-mRNA-1	2	OsR498G0204569800.01.T04	99,89	0,00E+00	3292	Os02g0689900	100,00	0,00E+00	3301	9	NA	Japonica	
maker-2-augustus-gene-291.3-mRNA-1	2	OsR498G0204572300.01.T01	99,84	0,00E+00	2205	Os02g0690700	100,00	0,00E+00	2214	9	NA	Japonica	
maker-2-augustus-gene-291.8-mRNA-1	2	OsR498G0204572900.01.T01	99,93	0,00E+00	2398	Os02g0690800	100,00	0,00E+00	2403	5	NA	Japonica	
maker-2-augustus-gene-292.47-mRNA-1	2	OsR498G0204577600.01.T01	99,35	0,00E+00	1372	Os02g0692700	100,00	0,00E+00	1559	187	Japonica	Japonica	
maker-2-augustus-gene-292.50-mRNA-1	2	OsR498G0204580000.01.T02	99,73	0,00E+00	656	Os02g0693200	100,00	0,00E+00	661	5	NA	Japonica	
maker-2-augustus-gene-292.53-mRNA-1	2	OsR498G0204580600.01.T01	99,61	0,00E+00	1372	Os02g0693500	100,00	0,00E+00	1526	154	Japonica	Japonica	
maker-2-augustus-gene-293.11-mRNA-1	2	OsR498G0204583700.01.T01	99,37	0,00E+00	1691	Os02g0694700	100,00	0,00E+00	1718	27	NA	Japonica	
maker-2-augustus-gene-293.13-mRNA-1	2	OsR498G0204586500.01.T01	100,00	4,19E-147	520	Os02g0695600	100,00	1,46E-147	520	0	NA	Japonica	
maker-2-augustus-gene-293.15-mRNA-1	2	OsR498G0204581000.01.T01	99,90	0,00E+00	6827	Os02g0693700	100,00	0,00E+00	6845	18	NA	Japonica	
maker-2-augustus-gene-293.23-mRNA-1	2	OsR498G0204582200.01.T01	99,86	0,00E+00	2567	Os02g0694100	99,93	0,00E+00	2571	4	NA	Japonica	
maker-2-augustus-gene-293.29-mRNA-1	2	OsR498G0204584000.01.T02	99,74	0,00E+00	1353	Os02g0694800	100,00	0,00E+00	1178	-175	Indica	Japonica	
maker-2-augustus-gene-293.3-mRNA-1	2	OsR498G0204583300.01.T05	99,65	1,32E-143	510	Os02g0694400	100,00	4,04E-132	470	-40	NA	Japonica	
maker-2-augustus-gene-293.33-mRNA-1	2	OsR498G0204585000.01.T01	98,65	0,00E+00	1810	Os02g0695200	99,90	0,00E+00	1841	31	NA	Japonica	
maker-2-augustus-gene-293.35-mRNA-1	2	OsR498G0204586800.01.T01	99,25	0,00E+00	3996	Os04g0594800	71,66	0,00E+00	1086	-2910	Indica	Japonica	
maker-2-augustus-gene-294.34-mRNA-1	2	OsR498G0204588700.01.T01	98,56	0,00E+00	1334	Os02g0696500	100,00	0,00E+00	1364	30	NA	Japonica	
maker-2-augustus-gene-294.36-mRNA-1	2	OsR498G0204589700.01.T04	99,81	0,00E+00	1869	Os02g0696700	100,00	0,00E+00	1878	9	NA	Japonica	
maker-2-augustus-gene-294.46-mRNA-1	2	OsR498G0204592100.01.T02	100,00	0,00E+00	737	Os02g0697200	100,00	0,00E+00	733	-4	NA	Japonica	
maker-2-augustus-gene-294.50-mRNA-1	2	OsR498G0204592300.01.T07	99,83	0,00E+00	2118	Os02g0697300	100,00	0,00E+00	2127	9	NA	Japonica	
maker-2-augustus-gene-294.53-mRNA-1	2	OsR498G0204592900.01.T01	99,79	0,00E+00	3451	Os02g0697600	99,90	0,00E+00	3460	9	NA	Japonica	
maker-2-augustus-gene-294.58-mRNA-1	2	OsR498G0204592600.01.T01	99,83	0,00E+00	3071	Os02g0697400	100,00	0,00E+00	3085	14	NA	Japonica	
maker-2-augustus-gene-295.0-mRNA-1	2	OsR498G0204593300.01.T01	99,40	0,00E+00	1470	Os04g0595800	77,04	0,00E+00	637	-833	Indica	Japonica	
maker-2-augustus-gene-295.12-mRNA-1	2	OsR498G0204597100.01.T02	99,59	0,00E+00	3080	Os02g0699000	100,00	0,00E+00	3112	32	NA	Japonica	
maker-2-augustus-gene-295.15-mRNA-1	2	OsR498G0204597500.01.T03	100,00	0,00E+00	993	Os10g0580200	81,17	2,23E-143	508	-485	Indica	Japonica	
maker-2-augustus-gene-295.27-mRNA-1	2	OsR498G0204593200.01.T01	100,00	0,00E+00	850	Os02g0697700	100,00	0,00E+00	850	0	NA	Japonica	
maker-2-augustus-gene-295.31-mRNA-1	2	OsR498G0204598600.01.T01	99,80	0,00E+00	8214	Os02g0699700	100,00	0,00E+00	5175	-3039	Indica	Japonica	
maker-2-augustus-gene-295.38-mRNA-1	2	OsR498G0204599600.01.T03	99,92	0,00E+00	2368	Os02g0700000	100,00	0,00E+00	2372	4	NA	Japonica	
maker-2-augustus-gene-295.5-mRNA-1	2	OsR498G0204593700.01.T02	99,75	0,00E+00	2173	Os02g0698000	99,92	0,00E+00	2182	9	NA	Japonica	
maker-2-augustus-gene-295.9-mRNA-1	2	OsR498G0204594500.01.T01	98,83	0,00E+00	2695	Os02g0698800	99,54	0,00E+00	2719	24	NA	Japonica	
maker-2-augustus-gene-296.0-mRNA-1	2	OsR498G0204599700.01.T03	99,79	0,00E+00	2530	Os02g0700100	100,00	0,00E+00	2544	14	NA	Japonica	
maker-2-augustus-gene-296.10-mRNA-1	2	OsR498G0204604100.01.T01	99,82	0,00E+00	1956	Os02g0701600	100,00	0,00E+00	1965	9	NA	Japonica	
maker-2-augustus-gene-296.14-mRNA-1	2	OsR498G0204600600.01.T01	99,81	0,00E+00	1864	Os02g0700400	100,00	0,00E+00	1873	9	NA	Japonica	
maker-2-augustus-gene-296.16-mRNA-1	2	OsR498G0204601200.01.T03	98,24	0,00E+00	2167	Os02g0700700	98,80	0,00E+00	2198	31	NA	Japonica	
maker-2-augustus-gene-296.18-mRNA-1	2	OsR498G0204603200.01.T01	99,66	0,00E+00	2109	Os02g0701300	100,00	0,00E+00	2127	18	NA	Japonica	
maker-2-augustus-gene-296.4-mRNA-1	2	OsR498G0204600300.01.T01	99,77	0,00E+00	1555	Os02g0700300	100,00	0,00E+00	1564	9	NA	Japonica	
maker-2-augustus-gene-296.6-mRNA-1	2	OsR498G0204600900.01.T03	95,84	0,00E+00	1703	Os02g0700600	95,93	0,00E+00	1708	5	NA	NA	Kinase-TM
maker-2-augustus-gene-297.47-mRNA-1	2	OsR498G0204605500.01.T01	99,90	0,00E+00	1771	Os02g0701900	100,00	0,00E+00	1775	4	NA	Japonica	
maker-2-augustus-gene-297.49-mRNA-1	2	OsR498G0204605700.01.T01	99,45	0,00E+00	4220	Os02g0702000	100,00	0,00E+00	4280	60	NA	Japonica	
maker-2-augustus-gene-297.51-mRNA-1	2	OsR498G0204607500.01.T01	95,39	0,00E+00	1946	Os02g0702400	95,48	0,00E+00	1950	4	NA	Japonica	
maker-2-augustus-gene-297.58-mRNA-1	2	OsR498G0204604400.01.T05	100,00	7,59E-157	553	Os04g0601700	80,62	1,20E-85	315	-238	Indica	Japonica	
maker-2-augustus-gene-297.66-mRNA-1	2	OsR498G0204608000.01.T04	98,02	0,00E+00	2701	Os02g0702500	98,21	0,00E+00	2713	12	NA	Japonica	

maker-2-augustus-gene-297.78-mRNA-1	2	OsR498G0204609600.01.T01	89,47	0,00E+00	1190	Os02g0702800	89,47	0,00E+00	1190	0	NA	Japonica	
maker-2-augustus-gene-298.0-mRNA-1	2	OsR498G0204611500.01.T01	99,93	0,00E+00	2550	Os02g0703600	100,00	0,00E+00	1710	-840	Indica	Japonica	
maker-2-augustus-gene-298.10-mRNA-1	2	OsR498G0204613900.01.T01	99,64	0,00E+00	1479	Os02g0704500	99,88	0,00E+00	1489	10	NA	Japonica	
maker-2-augustus-gene-298.16-mRNA-1	2	OsR498G0916748200.01.T04	76,88	0,00E+00	910	Os02g0704300	100,00	0,00E+00	1797	887	Japonica	Japonica	
maker-2-augustus-gene-298.18-mRNA-1	2	OsR498G0204614200.01.T01	99,71	0,00E+00	2455	Os02g0704600	100,00	0,00E+00	2473	18	NA	Japonica	
maker-2-augustus-gene-298.20-mRNA-1	2	OsR498G0204614800.01.T04	99,38	0,00E+00	1146	Os02g0704900	100,00	0,00E+00	1164	18	NA	Japonica	
maker-2-augustus-gene-298.6-mRNA-1	2	OsR498G0204612100.01.T04	92,09	0,00E+00	1010	Os02g0703900	100,00	0,00E+00	1225	215	Japonica	Japonica	
maker-2-augustus-gene-299.0-mRNA-1	2	OsR498G0204615900.01.T02	99,84	0,00E+00	1089	Os02g0705300	100,00	0,00E+00	1094	5	NA	Japonica	
maker-2-augustus-gene-299.11-mRNA-1	2	OsR498G0204615400.01.T01	99,65	0,00E+00	2042	Os02g0705100	100,00	0,00E+00	1912	-130	NA	Japonica	
maker-2-augustus-gene-299.14-mRNA-1	2	OsR498G0204616600.01.T02	99,91	0,00E+00	1955	Os02g0705500	100,00	0,00E+00	1959	4	NA	Japonica	
maker-2-augustus-gene-299.18-mRNA-1	2	OsR498G0204618400.01.T03	99,83	0,00E+00	4154	Os02g0705600	100,00	0,00E+00	4172	18	NA	Japonica	
maker-2-augustus-gene-299.3-mRNA-1	2	OsR498G0204616300.01.T01	98,02	0,00E+00	957	Os02g0705400	98,20	0,00E+00	961	4	NA	Japonica	
maker-2-augustus-gene-299.8-mRNA-1	2	OsR498G0204615200.01.T06	99,77	0,00E+00	1553	Os02g0705000	100,00	0,00E+00	1563	10	NA	Japonica	
maker-2-augustus-gene-3.0-mRNA-1	2	OsR498G0202812300.01.T03	99,94	0,00E+00	2961	Os02g0104700	100,00	0,00E+00	2966	5	NA	Japonica	
maker-2-augustus-gene-3.26-mRNA-1	2	OsR498G0202813900.01.T01	99,92	0,00E+00	2122	Os02g0105400	100,00	0,00E+00	2127	5	NA	Japonica	
maker-2-augustus-gene-3.28-mRNA-1	2	OsR498G0202814300.01.T01	99,67	0,00E+00	2141	Os02g0105500	100,00	0,00E+00	2159	18	NA	Japonica	
maker-2-augustus-gene-3.31-mRNA-1	2	OsR498G0202817500.01.T01	100,00	0,00E+00	3588	Os02g0106100	100,00	0,00E+00	3588	0	NA	Japonica	
maker-2-augustus-gene-3.4-mRNA-1	2	OsR498G0202812500.01.T01	97,12	0,00E+00	1343	Os02g0104800	97,52	0,00E+00	1103	-240	Indica	Japonica	
maker-2-augustus-gene-3.41-mRNA-1	2	OsR498G0202815400.01.T01	99,88	0,00E+00	1435	Os02g0105900	100,00	0,00E+00	1440	5	NA	Japonica	
maker-2-augustus-gene-3.9-mRNA-1	2	OsR498G0202812900.01.T02	99,70	0,00E+00	1187	Os02g0105000	100,00	0,00E+00	1196	9	NA	Japonica	
maker-2-augustus-gene-30.16-mRNA-1	2	OsR498G0202988400.01.T01	100,00	0,00E+00	899	Os02g0152900	100,00	0,00E+00	899	0	NA	Indica	
maker-2-augustus-gene-30.20-mRNA-1	2	OsR498G0202991600.01.T01	100,00	0,00E+00	5600	Os02g0154000	92,05	0,00E+00	4487	-1113	Indica	Indica	Kinase-LRR-TM
maker-2-augustus-gene-300.36-mRNA-1	2	OsR498G0204620100.01.T01	99,84	0,00E+00	3325	Os02g0706500	100,00	0,00E+00	3339	14	NA	Japonica	
maker-2-augustus-gene-300.40-mRNA-1	2	OsR498G0204625500.01.T02	99,58	0,00E+00	2538	Os02g0708100	100,00	0,00E+00	2186	-352	Indica	Japonica	
maker-2-augustus-gene-300.46-mRNA-1	2	OsR498G0204625700.01.T01	99,80	0,00E+00	3516	Os02g0708200	100,00	0,00E+00	3534	18	NA	Japonica	
maker-2-augustus-gene-300.51-mRNA-1	2	OsR498G0204625900.01.T03	98,59	3,10E-175	615	Os02g0708300	100,00	3,77E-118	423	-192	Indica	Japonica	
maker-2-augustus-gene-300.55-mRNA-1	2	OsR498G0204621800.01.T01	99,80	0,00E+00	1831	Os02g0706900	100,00	0,00E+00	1279	-552	Indica	Japonica	
maker-2-augustus-gene-300.57-mRNA-1	2	OsR498G0204622200.01.T01	99,66	0,00E+00	2122	Os02g0707000	100,00	0,00E+00	1261	-861	Indica	Japonica	
maker-2-augustus-gene-300.59-mRNA-1	2	OsR498G0204622400.01.T01	100,00	0,00E+00	2581	Os02g0707100	100,00	0,00E+00	2581	0	NA	Japonica	
maker-2-augustus-gene-300.63-mRNA-1	2	OsR498G0204622600.01.T02	98,51	0,00E+00	1640	Os02g0707200	98,97	0,00E+00	1544	-96	NA	Japonica	
maker-2-augustus-gene-300.65-mRNA-1	2	OsR498G0204624800.01.T03	100,00	0,00E+00	964	Os02g0707900	100,00	0,00E+00	964	0	NA	Japonica	
maker-2-augustus-gene-301.34-mRNA-1	2	OsR498G0204628700.01.T01	100,00	0,00E+00	1829	Os02g0709000	97,92	0,00E+00	1744	-85	NA	Japonica	
maker-2-augustus-gene-301.36-mRNA-1	2	OsR498G0204630500.01.T02	99,34	0,00E+00	11594	Os02g0709400	99,49	0,00E+00	11639	45	NA	Japonica	
maker-2-augustus-gene-301.42-mRNA-1	2	OsR498G0204631100.01.T02	98,63	0,00E+00	1527	Os05g0577100	71,89	5,04E-77	288	-1239	Indica	Japonica	
maker-2-augustus-gene-301.44-mRNA-1	2	OsR498G0204626200.01.T02	99,91	0,00E+00	3844	Os02g0708400	100,00	0,00E+00	3853	9	NA	Japonica	
maker-2-augustus-gene-301.49-mRNA-1	2	OsR498G0204629400.01.T01	99,61	0,00E+00	2283	Os02g0709200	100,00	0,00E+00	2305	22	NA	Japonica	
maker-2-augustus-gene-301.52-mRNA-1	2	OsR498G0204632400.01.T06	99,11	0,00E+00	1775	Os02g0709800	100,00	0,00E+00	1815	40	NA	Japonica	
maker-2-augustus-gene-301.58-mRNA-1	2	OsR498G0204632600.01.T03	99,63	0,00E+00	6196	Os02g0709900	99,97	0,00E+00	6250	54	NA	Japonica	
maker-2-augustus-gene-302.30-mRNA-1	2	OsR498G0204634900.01.T01	99,12	0,00E+00	1597	Os02g0710300	100,00	0,00E+00	1629	32	NA	Japonica	
maker-2-augustus-gene-302.32-mRNA-1	2	OsR498G0204635700.01.T01	99,84	0,00E+00	2302	Os02g0710500	100,00	0,00E+00	2311	9	NA	Japonica	Kinase-TM
maker-2-augustus-gene-302.37-mRNA-1	2	OsR498G0204636700.01.T01	99,72	0,00E+00	1892	Os02g0710700	99,91	0,00E+00	1901	9	NA	Japonica	
maker-2-augustus-gene-302.45-mRNA-1	2	OsR498G0204637100.01.T01	99,74	0,00E+00	4855	Os02g0710900	100,00	0,00E+00	4886	31	NA	Japonica	
maker-2-augustus-gene-302.55-mRNA-1	2	OsR498G0204637400.01.T04	95,04	0,00E+00	2050	Os02g0711000	97,21	0,00E+00	1825	-225	Indica	Japonica	
maker-2-augustus-gene-303.0-mRNA-1	2	OsR498G0204643100.01.T01	100,00	0,00E+00	1797	Os02g0713400	100,00	0,00E+00	1797	0	NA	Japonica	
maker-2-augustus-gene-303.14-mRNA-1	2	OsR498G0204640800.01.T01	99,94	0,00E+00	2804	Os02g0712500	100,00	0,00E+00	2809	5	NA	Japonica	
maker-2-augustus-gene-303.16-mRNA-1	2	OsR498G0204641600.01.T01	99,59	0,00E+00	3937	Os02g0712600	100,00	0,00E+00	3977	40	NA	Japonica	Kinase-TM
maker-2-augustus-gene-303.18-mRNA-1	2	OsR498G0204641800.01.T01	99,69	0,00E+00	4014	Os02g0712700	100,00	0,00E+00	4048	34	NA	Japonica	Kinase-TM
maker-2-augustus-gene-303.23-mRNA-1	2	OsR498G0204645000.01.T02	99,70	0,00E+00	1810	Os02g0713700	100,00	0,00E+00	1824	14	NA	Japonica	
maker-2-augustus-gene-303.4-mRNA-1	2	OsR498G0204639600.01.T01	99,87	0,00E+00	1349	Os02g0711900	100,00	0,00E+00	1353	4	NA	Japonica	
maker-2-augustus-gene-303.6-mRNA-1	2	OsR498G0204639800.01.T04	99,87	0,00E+00	4294	Os02g0712000	100,00	0,00E+00	3344	-950	Indica	Japonica	
maker-2-augustus-gene-304.31-mRNA-1	2	OsR498G0204646900.01.T01	99,63	0,00E+00	955	Os02g0714000	100,00	0,00E+00	964	9	NA	Japonica	
maker-2-augustus-gene-304.37-mRNA-1	2	OsR498G0204648300.01.T01	96,45	0,00E+00	1695	Os02g0714600	96,35	0,00E+00	1691	-4	NA	Japonica	
maker-2-augustus-gene-304.44-mRNA-1	2	OsR498G0204650300.01.T01	99,81	0,00E+00	1929	Os02g0715000	100,00	0,00E+00	1986	57	NA	Japonica	
maker-2-augustus-gene-304.46-mRNA-1	2	OsR498G0204650700.01.T02	98,40	0,00E+00	1079	Os02g0715200	99,52	0,00E+00	1111	32	NA	Japonica	
maker-2-augustus-gene-304.50-mRNA-1	2	OsR498G0204646800.01.T02	99,75	0,00E+00	2904	Os08g0512700	74,44	0,00E+00	782	-2122	Indica	Japonica	
maker-2-augustus-gene-304.54-mRNA-1	2	OsR498G0204647200.01.T04	99,80	0,00E+00	884	Os02g0714100	100,00	0,00E+00	883	-1	NA	Japonica	
maker-2-augustus-gene-304.59-mRNA-1	2	OsR498G0204647600.01.T02	99,84	0,00E+00	2215	Os02g0714200	100,00	0,00E+00	2224	9	NA	Japonica	
maker-2-augustus-gene-304.65-mRNA-1	2	OsR498G0204647800.01.T04	100,00	0,00E+00	1656	Os02g0714300	100,00	0,00E+00	1656	0	NA	Japonica	
maker-2-augustus-gene-305.0-mRNA-1	2	OsR498G0204654300.01.T02	100,00	0,00E+00	1213	Os02g0717100	100,00	0,00E+00	1213	0	NA	Japonica	
maker-2-augustus-gene-305.11-mRNA-1	2	OsR498G0204656200.01.T02	98,83	0,00E+00	7353	Os02g0717400	99,00	0,00E+00	7385	32	NA	Japonica	
maker-2-augustus-gene-305.2-mRNA-1	2	OsR498G0204655300.01.T02	99,56	0,00E+00	809	Os02g0717300	100,00	0,00E+00	818	9	NA	Japonica	
maker-2-augustus-gene-305.4-mRNA-1	2	OsR498G0204656700.01.T01	99,72	0,00E+00	1929	Os02g0717500	100,00	0,00E+00	1943	14	NA	Japonica	
maker-2-augustus-gene-305.8-mRNA-1	2	OsR498G0204653800.01.T02	99,88	0,00E+00	2931	Os02g0716800	100,00	0,00E+00	2940	9	NA	Japonica	
maker-2-augustus-gene-306.0-mRNA-1	2	OsR498G0204659500.01.T02	99,57	0,00E+00	2050	Os02g0718900	100,00	0,00E+00	2073	23	NA	Japonica	
maker-2-augustus-gene-306.12-mRNA-1	2	OsR498G0204661700.01.T01	99,91	0,00E+00	2058	Os02g0719600	100,00	0,00E+00	2062	4	NA	Japonica	
maker-2-augustus-gene-306.2-mRNA-1	2	OsR498G0204659900.01.T01	99,82	0,00E+00	2037	Os02g0719000	100,00	0,00E+00	2046	9	NA	Japonica	
maker-2-augustus-gene-306.22-mRNA-1	2	OsR498G0204663300.01.T01	99,94	0,00E+00	3188	Os02g0720200	100,00	0,00E+00	3193	5	NA	Japonica	
maker-2-augustus-gene-306.25-mRNA-1	2	OsR498G0204663500.01.T01	99,57	0,00E+00	834	Os02g0720300	100,00	0,00E+00	843	9	NA	Japonica	
maker-2-augustus-gene-306.34-mRNA-1	2	OsR498G0204657600.01.T01	99,19	0,00E+00	658	Os02g0717800	100,00	1,22E-143	508	-150	Indica	Japonica	
maker-2-augustus-gene-306.36-mRNA-1	2	OsR498G0204663200.01.T01	99,85	0,00E+00	1181	Os02g0720100	100,00	0,00E+00	1186	5	NA	Japonica	
maker-2-augustus-gene-306.7-mRNA-1	2	OsR498G0204660100.01.T04	99,76	0,00E+00	3803	Os02g0719100	100,00	0,00E+00	3826	23	NA	Japonica	
maker-2-augustus-gene-307.0-mRNA-1	2	OsR498G0204664300.01.T02	99,72	0,00E+00	2518	Os06g0610800	70,83	6,29E-110	398	-2120	Indica	Japonica	
maker-2-augustus-gene-307.14-mRNA-1	2	OsR498G0204666800.01.T01	99,57	0,00E+00	1259	Os02g0721100	100,00	0,00E+00	1272	13	NA	Japonica	
maker-2-augustus-gene-307.16-mRNA-1	2	OsR498G0204669000.01.T01	99,70	0,00E+00	1209	Os02g0721100	78,30	4,13E-116	418	-791	Indica	Japonica	
maker-2-augustus-gene-307.18-mRNA-1	2	OsR498G0204669200.01.T02	100,00	0,00E+00	1263	Os02g0721600	100,00	0,00E+00	1263	0	NA	Japonica	
maker-2-augustus-gene-307.19-mRNA-1	2	OsR498G0204669200.01.T03	99,22	0,00E+00	1136	Os02g0721600	100,00	0,00E+00	1159	23	NA	Japonica	
maker-2-augustus-gene-307.26-mRNA-1	2	OsR498G0204670000.01.T01	100,00	0,00E+00	910	Os02g0721700	100,00	0,00E+00	910	0	NA	Japonica	
maker-2-augustus-gene-307.28-mRNA-1	2	OsR498G0204670200.01.T03	99,09	0,00E+00	3307	Os02g0721800	99,18	0,00E+00	3241	-66	NA	Japonica	
maker-2-augustus-gene-307.33-mRNA-1	2	OsR498G0204671400.01.T03	99,35	5,05E-73	274	Os07g0675400	78,57	1,12E-12	71,6	-202,4	Indica	Japonica	
maker-2-augustus-gene-307.36-mRNA-1	2	OsR498G0204671400.01.T04	99,82	0,00E+00	994	Os02g0722200	93,76	0,00E+00	865	-129	Indica	Japonica	
maker-2-augustus-gene-307.4-mRNA-1	2	OsR498G1120753400.01.T01	96,69	0,00E+00	901	Os02g0789700	98,55	0,00E+00	1080	179	Japonica	Japonica	
maker-2-augustus-gene-307.7-mRNA-1	2	OsR498G0611518800.01.T01	70,00	8,16E-114	413	Os02g0720600	100,00	0,00E+00	2511	2098	Japonica	Japonica	
maker-2-augustus-gene-307.9-mRNA-1	2	OsR498G0204665200.01.T01	96,20	0,00E+00	1971	Os02g0720700	96,20	0,00E+00	1971	0	NA	Japonica	
maker-2-augustus-gene-308.37-mRNA-1	2	OsR498G0204672700.01.T01	97,44	0,00E+00	1729	Os02g0722400	97,63	0,00E+00	1738	9	NA	Japonica	
maker-2-augustus-gene-308.42-mRNA-1	2	OsR498G0204678100.01.T01	99,59	0,00E+00	1297	Os02g0723700	99,85	0,00E+00	1221	-76	NA	Japonica	
maker-2-augustus-gene-308.47-mRNA-1	2	OsR498G0204673000.01.T02	99,90	0,00E+00	1798	Os02g0722500	100,00	0,00E+00	1802	4	NA	Japonica	
maker-2-augustus-gene-308.52-mRNA-1	2	OsR498G0204674600.01.T01	99,65	0,00E+00	7187	Os02g0722800	100,00	0,00E+00	7250	63	NA	Japonica	
maker-2-augustus-gene-309.0-mRNA-1	2	OsR498G0204681300.01.T06	99,72	0,00E+00	2544	Os02g0724000	100,00	0,00E+00	2208	-336	Indica	Japonica	
maker-2-augustus-gene-309.15-mRNA-1	2	OsR498G0204682900.01.T03	99,89	0,00E+00	3178	Os02g0725000	100,00	0,00E+00	3187	9	NA	Japonica	Kinase-TM
maker-2-augustus-gene-309.23-mRNA-1	2	OsR498G0204683900.01.T01	100,00	0,00E+00	3492	Os02g0725300	100,00	0,00E+00	3492	0	NA	Japonica	
maker-2-augustus-gene-309.3-mRNA-1	2	OsR498G0204681900.01.T03	99,91	0,00E+00	1902	Os02g0724500	98,37	0,00E+00	1481	-421	Indica	Japonica	
maker-2-augustus-gene-309.33-mRNA-1	2	OsR498G0204684900.01.T01	100,00	0,00E+00	2186	Os02g0725600	100,00	0,00E+00	1202	-984	Indica	Japonica	
maker-2-augustus-gene-309.38-mRNA-1	2	OsR498G0204683400.01.T01	100,00	0,00E+00	2343	Os02g0725100	100,00	0,00E+00	2343	0	NA	Japonica	
maker-2-augustus-gene-309.42-mRNA-1	2	OsR498G0204684600.01.T01	100,00	0,00E+00	1186	Os02g0725500	100,00	0,00E+00	1186	0	NA	Japonica	
maker-2-augustus-gene-309.8-mRNA-1	2	OsR498G0204682100.01.T01	99,33	0,00E+00	3699	Os09g0551300	90,69	0,00E+00	2938	-761	Indica	Japonica	
maker-2-augustus-gene-31.56-mRNA-1	2	OsR498G0202996300.01.T01	99,77	0,00E+00	788	Os02g0156100	100,00	0,00E+00	792	4	NA	Japonica	
maker-2-augustus-gene-31.61-mRNA-1	2	OsR498G0203001700.01.T01	99,80	0,00E+00	916	Os02g0157600	100,00	0,00E+00	758	-158	Indica	NA	
maker-2-augustus-gene-31.64-mRNA-1	2	OsR498G0203001200.01.T02	100,00	0,00E+00	1595	Os02g0156800	100,00	0,00E+00	1595	0	NA	Japonica	LRR-TM
maker-2-augustus-gene-31.67-mRNA-1	2	OsR498G0202990800.01.T01	71,22	0,00E+00	746	Os02g0157200	83,65	0,00E+00	1596	850	Japonica	NA	LRR-TM
maker-2-augustus-gene-310.35-mRNA-1	2	OsR498G0204688500.01.T01	99,67	0,00E+00	1632	Os02g0726400	99,89	0,00E+00	1641	9	NA	Japonica	
maker-2-augustus-gene-310.40-mRNA-1	2	OsR498G0204688900.01.T01	99,61	0,00E+00	2738	Os06g0284200	82,10	0,00E+00	1541	-1197	Indica	Japonica	
maker-2-augustus-gene-310.43-mRNA-1	2	OsR498G0204690300.01.T02	99,73	0,00E+00	5232	Os02g0727300	99,90	0,00E+00	5257	25	NA	Japonica	
maker-2-augustus-gene-310.45-mRNA-1	2	OsR498G0204686400.01.T01	99,11	0,00E+00	990	Os02g0725900	100,00	0,00E+00	1012	22	NA	Japonica	
maker-2-augustus-gene-310.53-mRNA-1	2	OsR498G0204690000.01.T01	99,70	0,00E+00	2412	Os02g0727100	99,93	0,00E+00	2425	13	NA	Japonica	
maker-2-augustus-gene-310.55-mRNA-1	2	OsR498G0204691000.01.T01	99,51	0,00E+00	728	Os02g0727500	99,76	0,00E+00	732	4	NA	NA	
maker-2-augustus-gene-310.57-mRNA-1	2	OsR498G0204693400.01.T05	99,80	0,00E+00	3635	Os02g0728100	100,00	0,00E+00	3653	18	NA	Japonica	
maker-2-augustus-gene-311.51-mRNA-1	2	OsR498G0204693700.01.T02	99,67	0,00E+00	1632	Os02g0581300	75,44	1,96E-177	621	-1011	Indica	Japonica	
maker-2-augustus-gene-311.54-mRNA-1	2	OsR498G0204695100.01.T02	100,00	0,00E+00	1961	Os02g0728700	100,00	0,00E+00	1961	0	NA	Japonica	
maker-2-augustus-gene-311.63-mRNA-1	2	OsR498G0203126400.01.T01	72,73	7,74E-45	182	Os02g0189000	72,73	2,69E-45	182	0	NA	Japonica	
maker-2-augustus-gene-311.65-mRNA-1	2	OsR498G0204696500.01.T01	99,92	0,00E+00	2252	Os02g0729100	100,00	0,00E+00	2257	5	NA	Japonica	
maker-2-augustus-gene-311.68-mRNA-1	2	OsR498G0204701500.01.T01	99,33	0,00E+00	1842	Os02g0729700	100,00	0,00E+00	1862	20	NA	Japonica	
maker-2-augustus-gene-311.70-mRNA-1	2	OsR498G0204694200.01.T01	99,89	0,00E+00	3389	Os06g0274500	75,09	0,00E+00	1137	-2252	Indica	Japonica	Kinase-LRR-TM
maker-2-augustus-gene-311.77-mRNA-1	2	OsR498G0204695000.01.T01	90,56	0,00E+00	723	Os02g0728600	90,96	0,00E+00	732	9	NA	Japonica	
maker-2-augustus-gene-311.78-mRNA-1	2	OsR498G0204697000.01.T01	99,90	0,00E+00	1722	Os06g0272900	70,77	8,29E-127	454	-1268	Indica	Japonica	
maker-2-augustus-gene-311.83-mRNA-1	2	OsR498G0204698800.01.T04	100,00	0,00E+00	1626	Os02g0729400	100,00	0,00E+00	1626	0	NA	Japonica	
maker-2-augustus-gene-311.85-mRNA-1	2	OsR498G0204703200.01.T02	99,76	0,00E+00	2980	Os02g0730000	100,00	0,00E+00	2998	18	NA	Japonica	
maker-2-augustus-gene-311.89-mRNA-1	2	OsR498G0204703400.01.T02	99,87	0,00E+00	1352	Os02g0730100	100,00	0,00E+00	1712	360	Japonica	Japonica	
maker-2-augustus-gene-312.41-mRNA-1	2	OsR498G0204705500.01.T01	99,80	0,00E+00	2741	Os02g0730400	100,00	0,00E+00	2755	14	NA	Japonica	
maker-2-augustus-gene-312.46-mRNA-1	2	OsR498G0204706100.01.T01	100,00	0,00E+00	2803	Os06g0268800	78,88	0,00E+00	805	-1998	Indica	Japonica	
maker-2-augustus-gene-312.48-mRNA-1	2	OsR498G0204706700.01.T04	99,87	0,00E+00	1383	Os02g0730800	99,87	0,00E+00	1383	0	NA	Japonica	
maker-2-augustus-gene-312.53-mRNA-1	2	OsR498G0204707300.01.T06	100,00	0,00E+00	1288	Os02g0731200	100,00	0,00E+00	1281	-7	NA	Japonica	
maker-2-augustus-gene-312.60-mRNA-1	2	OsR498G0204705800.01.T01	99,49	0,00E+00	1048	Os02g0730500	100,00	0,00E+00	1061	13	NA	Japonica	
maker-2-augustus-gene-312.65-mRNA-1	2	OsR498G0204708400.01.T02	99,46	0,00E+00	994	Os02g0731500	100,00	4,12E-158	556	-438	Indica	Japonica	
maker-2-augustus-gene-312.68-mRNA-1	2	OsR498G0204708800.01.T01	99,63	0,00E+00	2429	Os02g0731700	100,00	0,00E+00	1754	-675	Indica	Japonica	
maker-2-augustus-gene-313.43-mRNA-1	2	OsR498G0204711700.01.T03	98,81	0,00E+00	3401	Os02g0732200	98,92	0,00E+00	3410	9	NA	Japonica	
maker-2-augustus-gene-313.47-mRNA-1	2	OsR498G0204712100.01.T01	99,87	0,00E+00	1333	Os06g0260000	77,22	3,49E-135	480	-853	Indica	Japonica	
maker-2-augustus-gene-313.53-mRNA-1	2	OsR498G0204714500.01.T01	99,85	0,00E+00	2426	Os02g0732900	100,00	0,00E+00	2435	9	NA	Japonica	
maker-2-augustus-gene-313.55-mRNA-1	2	OsR498G0204716300.01.T01	99,74	0,00E+00	2736	Os02g0733300	100,00	0,00E+00	2755	19	NA	Japonica	
maker-2-augustus-gene-313.59-mRNA-1	2	OsR498G0204717700.01.T03	99,84	0,00E+00	1141	Os02g0733800	100,00	0,00E+00	1146	5	NA	Japonica	
maker-2-augustus-gene-313.65-mRNA-1	2	OsR498G0204713400.01.T01	99,48	0,00E+00	1366	Os02g0732600	100,00	0,00E+00	1386	20	NA	Japonica	
maker-2-augustus-gene-313.75-mRNA-1	2	OsR498G0204714400.01.T01	97,90	0,00E+00	1958	Os06g0257600	80,45	0,00E+00	940	-1018	Indica	Japonica	
maker-2-augustus-gene-313.81-mRNA-1	2	OsR498G0204716800.01.T01	99,48	0,00E+00	1024	Os02g0733400	100,00	0,00E+00	1034	10	NA	Japonica	
maker-2-augustus-gene-314.37-mRNA-1	2	OsR498G0204719900.01.T01	99,38	0,00E+00	858	Os02g0734300	100,00	0,00E+00	872	14	NA	Japonica	
maker-2-augustus-gene-314.39-mRNA-1	2	OsR498G0204720300.01.T01	99,09	0,00E+00	1157	Os02g0734400	100,00	0,00E+00	677	-480	Indica	Japonica	
maker-2-augustus-gene-314.42-mRNA-1	2	OsR498G0204720700.01.T01	89,74	0,00E+00	801	Os02g0734400	80,35	6,29E-86	317	-484	Indica	Japonica	
maker-2-augustus-gene-314.45-mRNA-1	2	OsR498G0204720900.01.T01	99,55	0,00E+00	2002	Os02g0734600	100,00	0,00E+00	2024	22	NA	Japonica	
maker-2-augustus-gene-314.52-mRNA-1	2	OsR498G0204723300.01.T01	97,11	0,00E+00	2342	Os02g0735100	97,40	0,00E+00	2360	18	NA	Japonica	
maker-2-augustus-gene-314.54-mRNA-1	2	OsR498G0204725300.01.T01	99,87	0,00E+00	2843	Os02g0735700	99,94	0,00E+00	2847	4	NA	Japonica	
maker-2-augustus-gene-314.57-mRNA-1	2	OsR498G0204723800.01.T02	99,91	0,00E+00	1928	Os02g0735200	100,00	0,00E+00	1932	4	NA	Japonica	
maker-2-augustus-gene-315.0-mRNA-1	2	OsR498G0204726900.01.T01	99,89	0,00E+00	3243	Os02g0736100	99,83	0,00E+00	3237	-6	NA	Japonica	
maker-2-augustus-gene-315.10-mRNA-1	2	OsR498G0204727600.01.T01	99,79	0,00E+00	3342	Os02g0736300	100,00	0,00E+00	3360	18	NA	Japonica	
maker-2-augustus-gene-315.14-mRNA-1	2	OsR498G0204727800.01.T02	99,84	0,00E+00	2237	Os02g0736400	100,00	0,00E+00	2246	9	NA	Japonica	
maker-2-augustus-gene-315.18-mRNA-1	2	OsR498G0204728000.01.T03	100,00	0,00E+00	2403	Os02g0736500	100,00	0,00E+00	2397	-6	NA	Japonica	
maker-2-augustus-gene-315.6-mRNA-1	2	OsR498G0204728100.01.T03	99,49	0,00E+00	3501	Os02g0736600	100,00	0,00E+00	3543	42	NA	Japonica	
maker-2-augustus-gene-316.14-mRNA-1	2	OsR498G0204736200.01.T01	98,37	0,00E+00	4910	Os02g0738900	98,40	0,00E+00	4914	4	NA	Japonica	
maker-2-augustus-gene-316.21-mRNA-1	2	OsR498G0204737000.01.T01	99,82	0,00E+00	4885	Os02g0739100	100,00	0,00E+00	2625	-2260	Indica	Japonica	
maker-2-augustus-gene-316.5-mRNA-1	2	OsR498G0204735300.01.T01	99,88	0,00E+00	2989	Os02g0738600	100,00	0,00E+00	2998	9	NA	Japonica	
maker-2-augustus-gene-316.9-mRNA-1	2	OsR498G0204736700.01.T01	99,13	0,00E+00	6283	Os02g0739000	99,63	0,00E+00	6354	71	NA	Japonica	
maker-2-augustus-gene-317.0-mRNA-1	2	OsR498G0204743700.01.T01	99,70	3,51E-168	591	Os02g0740500	100,00	2,87E-170	596	5	NA	Japonica	
maker-2-augustus-gene-317.12-mRNA-1	2	OsR498G0204741400.01.T01	99,93	0,00E+00	2388	Os02g0739700	100,00	0,00E+00	2392	4	NA	Japonica	
maker-2-augustus-gene-317.16-mRNA-1	2	OsR498G0204741800.01.T01	99,84	0,00E+00	3286	Os06g0244700	73,94	0,00E+00	1121	-2165	Indica	Japonica	
maker-2-augustus-gene-317.18-mRNA-1	2	OsR498G0204743000.01.T01	99,62	0,00E+00	3280	Os02g0740300	99,95	0,00E+00	3307	27	NA	Japonica	
maker-2-augustus-gene-317.2-mRNA-1	2	OsR498G0204745100.01.T01	100,00	0,00E+00	2397	Os02g0741100	100,00	0,00E+00	2397	0	NA	Japonica	
maker-2-augustus-gene-317.22-mRNA-1	2	OsR498G0204743200.01.T01	99,91	0,00E+00	2074	Os02g0740400	100,00	0,00E+00	2078	4	NA	Japonica	
maker-2-augustus-gene-317.8-mRNA-1	2	OsR498G0204741000.01.T01	99,83	0,00E+00	2107	Os02g0739600	100,00	0,00E+00	2116	9	NA	Japonica	
maker-2-augustus-gene-318.24-mRNA-1	2	OsR498G0204745700.01.T02	99,77	0,00E+00	3153	Os02g0741300	100,00	0,00E+00	3171	18	NA	Japonica	
maker-2-augustus-gene-318.28-mRNA-1	2	OsR498G0204748300.01.T01	99,76	0,00E+00	7485	Os02g0742000	99,98	0,00E+00	7527	42	NA	Japonica	
maker-2-augustus-gene-318.39-mRNA-1	2	OsR498G0204746400.01.T01	96,65	0,00E+00	949	Os02g0741700	99,79	0,00E+00	858	-91	NA	Japonica	
maker-2-augustus-gene-318.42-mRNA-1	2	OsR498G0204747400.01.T02	100,00	0,00E+00	2917	Os02g0741800	100,00	0,00E+00	2917	0	NA	Japonica	
maker-2-augustus-gene-318.47-mRNA-1	2	OsR498G0204747800.01.T01	94,13	0,00E+00	917	Os02g0741900	94,93	0,00E+00	1051	134	Japonica	Japonica	
maker-2-augustus-gene-318.49-mRNA-1	2	OsR498G0204749000.01.T01	100,00	0,00E+00	1164	Os02g0742200	100,00	0,00E+00	1164	0	NA	Japonica	
maker-2-augustus-gene-318.53-mRNA-1	2	OsR498G0204750000.01.T02	99,71	0,00E+00	3051	Os06g0234100	82,41	0,00E+00	1590	-1461	Indica	Japonica	
maker-2-augustus-gene-319.34-mRNA-1	2	OsR498G0204756500.01.T01	99,23	0,00E+00	5049	Os02g0743500	100,00	0,00E+00	4455	-594	Indica	Japonica	CC-Kinase-TM
maker-2-augustus-gene-319.41-mRNA-1	2	OsR498G0204757700.01.T01	99,63	0,00E+00	975	Os02g0743700	100,00	0,00E+00	976	1	NA	Japonica	
maker-2-augustus-gene-319.45-mRNA-1	2	OsR498G0204758500.01.T02	93,77	0,00E+00	857	Os02g0744000	93,96	0,00E+00	862	5	NA	Japonica	
maker-2-augustus-gene-319.54-mRNA-1	2	OsR498G0204755400.01.T01	99,73	0,00E+00	2018	Os02g0743400	100,00	0,00E+00	2031	13	NA	Japonica	
maker-2-augustus-gene-32.53-mRNA-1	2	OsR498G0203004900.01.T01	100,00	0,00E+00	1867	Os02g0158300	100,00	0,00E+00	1867	0	NA	Indica	
maker-2-augustus-gene-32.58-mRNA-1	2	OsR498G0203006100.01.T01	100,00	0,00E+00	2284	Os02g0158900	100,00	0,00E+00	2284	0	NA	Indica	
maker-2-augustus-gene-32.60-mRNA-1	2	OsR498G0203007700.01.T01	100,00	0,00E+00	3658	Os02g0159200	99,90	0,00E+00	3649	-9	NA	Indica	
maker-2-augustus-gene-32.66-mRNA-1	2	OsR498G0203008900.01.T01	99,60	0,00E+00	1342	Os01g0141100	73,91	0,00E+00	649	-693	Indica	Indica	
maker-2-augustus-gene-32.73-mRNA-1	2	OsR498G0203003800.01.T01	100,00	0,00E+00	2810	Os02g0158100	99,94	0,00E+00	2806	-4	NA	Indica	
maker-2-augustus-gene-32.82-mRNA-1	2	OsR498G0203005400.01.T01	100,00	0,00E+00	2127	Os02g0158500	99,74	0,00E+00	1389	-738	Indica	Indica	
maker-2-augustus-gene-32.84-mRNA-1	2	OsR498G0203005600.01.T01	100,00	0,00E+00	969	Os02g0158600	100,00	0,00E+00	969	0	NA	NA	
maker-2-augustus-gene-32.91-mRNA-1	2	OsR498G0203008600.01.T04	97,70	0,00E+00	1792	Os02g0159700	97,64	0,00E+00	1016	-776	Indica	Indica	
maker-2-augustus-gene-320.0-mRNA-1	2	OsR498G0204759300.01.T01	99,68	0,00E+00	1126	Os02g0744100	99,84	0,00E+00	1131	5	NA	Japonica	
maker-2-augustus-gene-320.11-mRNA-1	2	OsR498G0204764100.01.T01	100,00	0,00E+00	1618	Os02g0745100	99,89	0,00E+00	1614	-4	NA	Japonica	
maker-2-augustus-gene-320.16-mRNA-1	2	OsR498G0204765300.01.T02	99,80	0,00E+00	2660	Os02g0745400	100,00	0,00E+00	2673	13	NA	Japonica	
maker-2-augustus-gene-320.18-mRNA-1	2	OsR498G0204765700.01.T01	99,59	0,00E+00	2619	Os02g0745600	100,00	0,00E+00	2646	27	NA	Japonica	
maker-2-augustus-gene-320.21-mRNA-1	2	OsR498G0204765900.01.T01	99,88	0,00E+00	1435	Os02g0745700	100,00	0,00E+00	1440	5	NA	Japonica	
maker-2-augustus-gene-320.3-mRNA-1	2	OsR498G0204760300.01.T01	99,20	0,00E+00	1550	Os02g0744200	100,00	0,00E+00	1581	31	NA	Japonica	
maker-2-augustus-gene-320.32-mRNA-1	2	OsR498G0204761600.01.T01	99,66	0,00E+00	3729	Os02g0744700	100,00	0,00E+00	3662	-67	NA	Japonica	
maker-2-augustus-gene-320.39-mRNA-1	2	OsR498G0204763000.01.T01	94,63	0,00E+00	2000	Os02g0745000	96,72	0,00E+00	1381	-619	Indica	Japonica	
maker-2-augustus-gene-320.9-mRNA-1	2	OsR498G0204762300.01.T01	99,78	0,00E+00	2498	Os02g0744900	100,00	0,00E+00	2652	154	NA	Japonica	
maker-2-augustus-gene-321.36-mRNA-1	2	OsR498G0204766600.01.T03	99,65	0,00E+00	3070	Os02g0746000	99,83	0,00E+00	3084	14	NA	Japonica	
maker-2-augustus-gene-323.16-mRNA-1	2	OsR498G0204682400.01.T01	82,59	3,06E-59	229	Os06g0682750	100,00	1,30E-115	414	185	Japonica	NA	
maker-2-augustus-gene-325.21-mRNA-1	2	OsR498G0204447900.01.T01	96,55	0,00E+00	2810	Os02g0656300	96,67	0,00E+00	2817	7	NA	NA	
maker-2-augustus-gene-33.0-mRNA-1	2	OsR498G0203010500.01.T01	99,75	0,00E+00	1431	Os02g0160100	98,92	0,00E+00	1783	352	Japonica	Indica	
maker-2-augustus-gene-33.3-mRNA-1	2	OsR498G0203010900.01.T01	100,00	0,00E+00	2684	Os02g0160200	99,53	0,00E+00	2653	-31	NA	Indica	
maker-2-augustus-gene-33.5-mRNA-1	2	OsR498G0203015300.01.T01	100,00	0,00E+00	4773	Os02g0161100	99,92	0,00E+00	4763	-10	NA	Indica	
maker-2-augustus-gene-337.42-mRNA-1	2	OsR498G0204772500.01.T03	99,83	0,00E+00	1040	Os02g0747500	100,00	0,00E+00	1045	5	NA	Japonica	
maker-2-augustus-gene-337.46-mRNA-1	2	OsR498G0815725100.01.T05	98,77	3,67E-161	567	Os02g0749000	100,00	4,75E-167	585	18	NA	Japonica	
maker-2-augustus-gene-337.57-mRNA-1	2	OsR498G0204774000.01.T01	100,00	9,69E-136	482	Os06g0226500	90,46	2,79E-99	361	-121	Indica	Japonica	
maker-2-augustus-gene-337.59-mRNA-1	2	OsR498G0204775600.01.T03	100,00	0,00E+00	4237	Os02g0748100	100,00	0,00E+00	4237	0	NA	Japonica	
maker-2-augustus-gene-338.42-mRNA-1	2	OsR498G0204780700.01.T01	99,89	0,00E+00	1581	Os02g0749300	100,00	0,00E+00	1032	-549	Indica	Japonica	
maker-2-augustus-gene-338.59-mRNA-1	2	OsR498G0204782000.01.T01	99,20	0,00E+00	3076	Os01g0374600	88,85	0,00E+00	2374	-702	Indica	Japonica	
maker-2-augustus-gene-338.62-mRNA-1	2	OsR498G0204783600.01.T01	99,82	0,00E+00	1983	Os02g0750500	99,91	0,00E+00	1987	4	NA	Japonica	
maker-2-augustus-gene-339.27-mRNA-1	2	OsR498G0204786700.01.T01	99,55	0,00E+00	792	Os02g0751300	100,00	0,00E+00	801	9	NA	Japonica	
maker-2-augustus-gene-339.29-mRNA-1	2	OsR498G0204787500.01.T01	99,86	0,00E+00	1246	Os02g0751600	100,00	0,00E+00	1251	5	NA	Japonica	
maker-2-augustus-gene-339.31-mRNA-1	2	OsR498G0204787900.01.T02	100,00	0,00E+00	1384	Os02g0751800	100,00	0,00E+00	1384	0	NA	NA	
maker-2-augustus-gene-339.40-mRNA-1	2	OsR498G0204791500.01.T01	99,64	0,00E+00	1974	Os02g0753000	100,00	0,00E+00	1992	18	NA	NA	
maker-2-augustus-gene-339.43-mRNA-1	2	OsR498G0204788400.01.T02	100,00	0,00E+00	2051	Os02g0752000	100,00	0,00E+00	2051	0	NA	NA	
maker-2-augustus-gene-339.46-mRNA-1	2	OsR498G0204788600.01.T01	99,70	0,00E+00	4198	Os02g0752200	99,87	0,00E+00	4216	18	NA	NA	
maker-2-augustus-gene-339.49-mRNA-1	2	OsR498G0204789600.01.T01	99,70	0,00E+00	1189	Os02g0752300	100,00	0,00E+00	1198	9	NA	NA	
maker-2-augustus-gene-34.48-mRNA-1	2	OsR498G0203020500.01.T01	99,37	0,00E+00	846	Os06g0681300	76,50	1,39E-74	279	-567	Indica	Japonica	
maker-2-augustus-gene-34.50-mRNA-1	2	OsR498G0203020900.01.T01	99,75	0,00E+00	1409	Os02g0162200	100,00	0,00E+00	1418	9	NA	Japonica	
maker-2-augustus-gene-34.52-mRNA-1	2	OsR498G0203021300.01.T01	93,86	0,00E+00	1000	Os02g0162400	100,00	0,00E+00	1164	164	Japonica	Japonica	
maker-2-augustus-gene-34.56-mRNA-1	2	OsR498G0203019200.01.T02	99,73	0,00E+00	2021	Os02g0161800	99,83	0,00E+00	1078	-943	Indica	Japonica	
maker-2-augustus-gene-340.28-mRNA-1	2	OsR498G0204792500.01.T01	100,00	0,00E+00	1397	Os02g0753200	100,00	0,00E+00	1353	-44	NA	NA	
maker-2-augustus-gene-340.30-mRNA-1	2	OsR498G0204792700.01.T01	99,83	0,00E+00	1046	Os02g0753300	100,00	0,00E+00	1050	4	NA	NA	
maker-2-augustus-gene-340.32-mRNA-1	2	OsR498G0204795300.01.T03	99,54	0,00E+00	2349	Os02g0753900	100,00	0,00E+00	2320	-29	NA	NA	
maker-2-augustus-gene-340.37-mRNA-1	2	OsR498G0204795500.01.T01	97,65	0,00E+00	1892	Os02g0754000	97,74	0,00E+00	1896	4	NA	NA	
maker-2-augustus-gene-340.41-mRNA-1	2	OsR498G0204795900.01.T01	99,60	0,00E+00	886	Os02g0754300	99,80	0,00E+00	891	5	NA	NA	

maker-2-augustus-gene-340.43-mRNA-1	2	OsR498G0204797900.01.T01	99,36	0,00E+00	1940	Os02g0754500	100,00	0,00E+00	1972	32	NA	NA	
maker-2-augustus-gene-340.50-mRNA-1	2	OsR498G0204798100.01.T01	99,56	0,00E+00	1633	Os02g0754600	100,00	0,00E+00	2062	429	Japonica	NA	
maker-2-augustus-gene-340.55-mRNA-1	2	OsR498G0204795200.01.T02	99,79	0,00E+00	1696	Os02g0753800	100,00	0,00E+00	1705	9	NA	NA	
maker-2-augustus-gene-340.58-mRNA-1	2	OsR498G0204795800.01.T02	98,00	0,00E+00	5521	Os02g0754100	98,25	0,00E+00	5557	36	NA	NA	
maker-2-augustus-gene-340.66-mRNA-1	2	OsR498G0204798400.01.T03	98,92	0,00E+00	2605	Os02g0754700	98,99	0,00E+00	2609	4	NA	NA	
maker-2-augustus-gene-340.71-mRNA-1	2	OsR498G0204798800.01.T01	99,68	0,00E+00	1675	Os02g0754900	100,00	0,00E+00	1689	14	NA	NA	
maker-2-augustus-gene-341.3-mRNA-1	2	OsR498G0204801700.01.T01	99,79	0,00E+00	2617	Os02g0755900	100,00	0,00E+00	2630	13	NA	NA	
maker-2-augustus-gene-341.5-mRNA-1	2	OsR498G0204800600.01.T01	99,52	0,00E+00	2604	Os02g0755500	100,00	0,00E+00	2636	32	NA	NA	
maker-2-augustus-gene-341.7-mRNA-1	2	OsR498G0204801000.01.T01	100,00	0,00E+00	2614	Os02g0755600	100,00	0,00E+00	2614	0	NA	NA	
maker-2-augustus-gene-341.9-mRNA-1	2	OsR498G0204802400.01.T01	100,00	1,83E-171	601	Os02g0756100	100,00	6,37E-172	601	0	NA	NA	
maker-2-augustus-gene-344.5-mRNA-1	2	OsR498G0613085400.01.T01	90,13	0,00E+00	1581	Os02g0178100	72,25	3,41E-80	298	-1283	Indica	Japonica	
maker-2-augustus-gene-349.14-mRNA-1	2	OsR498G0613053500.01.T02	99,43	0,00E+00	6284	Os02g0183700	79,62	0,00E+00	1726	-4558	Indica	NA	
maker-2-augustus-gene-35.20-mRNA-1	2	OsR498G0203030300.01.T07	97,10	0,00E+00	1576	Os02g0165100	93,23	0,00E+00	1442	-134	NA	NA	Kinase-TM
maker-2-augustus-gene-350.32-mRNA-1	2	OsR498G0613048800.01.T01	99,83	0,00E+00	5269	Os02g0184400	82,73	0,00E+00	2993	-2276	Indica	NA	
maker-2-augustus-gene-350.70-mRNA-1	2	OsR498G0613044600.01.T01	98,56	0,00E+00	2303	Os02g0186800	71,32	0,00E+00	649	-1654	Indica	NA	
maker-2-augustus-gene-352.27-mRNA-1	2	OsR498G0204787900.01.T02	100,00	0,00E+00	1384	Os02g0751800	100,00	0,00E+00	1384	0	NA	NA	
maker-2-augustus-gene-352.36-mRNA-1	2	OsR498G0204788400.01.T02	100,00	0,00E+00	2051	Os02g0752000	100,00	0,00E+00	2051	0	NA	NA	
maker-2-augustus-gene-352.39-mRNA-1	2	OsR498G0204788600.01.T01	99,70	0,00E+00	4198	Os02g0752200	99,87	0,00E+00	4216	18	NA	NA	
maker-2-augustus-gene-352.42-mRNA-1	2	OsR498G0204789600.01.T01	99,70	0,00E+00	1189	Os02g0752300	100,00	0,00E+00	1198	9	NA	NA	
maker-2-augustus-gene-353.27-mRNA-1	2	OsR498G0204791500.01.T01	99,64	0,00E+00	1974	Os02g0753000	100,00	0,00E+00	1992	18	NA	NA	
maker-2-augustus-gene-353.30-mRNA-1	2	OsR498G0204792500.01.T01	100,00	0,00E+00	1397	Os02g0753200	100,00	0,00E+00	1353	-44	NA	NA	
maker-2-augustus-gene-353.32-mRNA-1	2	OsR498G0204792700.01.T01	99,83	0,00E+00	1046	Os02g0753300	100,00	0,00E+00	1050	4	NA	NA	
maker-2-augustus-gene-353.34-mRNA-1	2	OsR498G0204795300.01.T03	99,54	0,00E+00	2349	Os02g0753900	100,00	0,00E+00	2320	-29	NA	NA	
maker-2-augustus-gene-353.39-mRNA-1	2	OsR498G0204795500.01.T01	97,65	0,00E+00	1892	Os02g0754000	97,74	0,00E+00	1896	4	NA	NA	
maker-2-augustus-gene-353.45-mRNA-1	2	OsR498G0204797900.01.T01	99,36	0,00E+00	1940	Os02g0754500	100,00	0,00E+00	1972	32	NA	NA	
maker-2-augustus-gene-353.52-mRNA-1	2	OsR498G0204798100.01.T01	99,56	0,00E+00	1633	Os02g0754600	100,00	0,00E+00	2062	429	Japonica	NA	
maker-2-augustus-gene-353.57-mRNA-1	2	OsR498G0204795200.01.T02	99,79	0,00E+00	1696	Os02g0753800	100,00	0,00E+00	1705	9	NA	NA	
maker-2-augustus-gene-353.60-mRNA-1	2	OsR498G0204795800.01.T02	98,00	0,00E+00	5521	Os02g0754100	98,25	0,00E+00	5557	36	NA	NA	
maker-2-augustus-gene-354.0-mRNA-1	2	OsR498G0204800300.01.T01	96,57	0,00E+00	1655	Os02g0755400	100,00	0,00E+00	955	-700	Indica	NA	
maker-2-augustus-gene-354.10-mRNA-1	2	OsR498G0204798800.01.T01	99,68	0,00E+00	1675	Os02g0754900	100,00	0,00E+00	1689	14	NA	NA	
maker-2-augustus-gene-354.18-mRNA-1	2	OsR498G0204800600.01.T01	99,52	0,00E+00	2604	Os02g0755500	100,00	0,00E+00	2636	32	NA	NA	
maker-2-augustus-gene-354.20-mRNA-1	2	OsR498G0204801000.01.T01	100,00	0,00E+00	2614	Os02g0755600	100,00	0,00E+00	2614	0	NA	NA	
maker-2-augustus-gene-354.22-mRNA-1	2	OsR498G0204802400.01.T01	100,00	1,83E-171	601	Os02g0756100	100,00	6,37E-172	601	0	NA	NA	
maker-2-augustus-gene-354.3-mRNA-1	2	OsR498G0204801700.01.T01	99,79	0,00E+00	2617	Os02g0755900	100,00	0,00E+00	2630	13	NA	NA	
maker-2-augustus-gene-354.5-mRNA-1	2	OsR498G0204798400.01.T03	98,92	0,00E+00	2605	Os02g0754700	98,99	0,00E+00	2609	4	NA	NA	
maker-2-augustus-gene-355.12-mRNA-1	2	OsR498G0204807600.01.T02	100,00	0,00E+00	1175	Os02g0757900	98,16	0,00E+00	1127	-48	NA	Japonica	
maker-2-augustus-gene-355.3-mRNA-1	2	OsR498G0204807700.01.T01	100,00	0,00E+00	1196	Os02g0758000	100,00	0,00E+00	1196	0	NA	Japonica	
maker-2-augustus-gene-355.5-mRNA-1	2	OsR498G0204807900.01.T04	96,32	0,00E+00	1913	Os02g0758100	96,49	0,00E+00	1922	9	NA	Japonica	
maker-2-augustus-gene-356.41-mRNA-1	2	OsR498G0204813300.01.T02	99,10	0,00E+00	3540	Os02g0759700	100,00	0,00E+00	3452	-88	NA	Japonica	
maker-2-augustus-gene-356.44-mRNA-1	2	OsR498G0204814300.01.T01	99,35	0,00E+00	1097	Os02g0759900	100,00	0,00E+00	1115	18	NA	Japonica	
maker-2-augustus-gene-356.47-mRNA-1	2	OsR498G0204815300.01.T05	99,91	0,00E+00	4167	Os02g0760200	100,00	0,00E+00	4176	9	NA	Japonica	
maker-2-augustus-gene-356.52-mRNA-1	2	OsR498G0204817100.01.T01	99,40	0,00E+00	1182	Os02g0761000	100,00	0,00E+00	1196	14	NA	Japonica	
maker-2-augustus-gene-356.56-mRNA-1	2	OsR498G0204809800.01.T01	99,60	0,00E+00	1324	Os02g0758800	100,00	0,00E+00	1337	13	NA	Japonica	
maker-2-augustus-gene-356.58-mRNA-1	2	OsR498G0204811000.01.T02	99,70	0,00E+00	1800	Os09g0468700	74,21	2,47E-132	471	-1329	Indica	Japonica	
maker-2-augustus-gene-356.67-mRNA-1	2	OsR498G0204815600.01.T01	100,00	1,03E-174	612	Os02g0760300	100,00	3,59E-175	612	0	NA	Japonica	
maker-2-augustus-gene-357.37-mRNA-1	2	OsR498G0204817700.01.T01	99,75	0,00E+00	2153	Os02g0761100	100,00	0,00E+00	2167	14	NA	Japonica	
maker-2-augustus-gene-357.48-mRNA-1	2	OsR498G0204821100.01.T01	99,75	0,00E+00	1415	Os02g0762400	100,00	0,00E+00	1424	9	NA	Japonica	
maker-2-augustus-gene-357.50-mRNA-1	2	OsR498G0204818000.01.T02	99,86	0,00E+00	2502	Os02g0761200	100,00	0,00E+00	2511	9	NA	Japonica	
maker-2-augustus-gene-357.53-mRNA-1	2	OsR498G0204819000.01.T01	98,31	0,00E+00	1751	Os02g0761600	98,51	0,00E+00	1760	9	NA	Japonica	
maker-2-augustus-gene-357.65-mRNA-1	2	OsR498G0204820000.01.T01	98,97	0,00E+00	1714	Os02g0762200	100,00	0,00E+00	1759	45	NA	Japonica	
maker-2-augustus-gene-357.67-mRNA-1	2	OsR498G0204820200.01.T04	92,34	0,00E+00	992	Os02g0762300	92,65	0,00E+00	1001	9	NA	Japonica	
maker-2-augustus-gene-357.72-mRNA-1	2	OsR498G0204822200.01.T01	100,00	0,00E+00	834	Os02g0762600	100,00	0,00E+00	834	0	NA	Japonica	
maker-2-augustus-gene-357.73-mRNA-1	2	OsR498G0204822400.01.T03	99,59	0,00E+00	3071	Os02g0762800	100,00	0,00E+00	3099	28	NA	Japonica	
maker-2-augustus-gene-358.0-mRNA-1	2	OsR498G0204823100.01.T02	99,82	0,00E+00	981	Os02g0763000	100,00	0,00E+00	985	4	NA	Japonica	
maker-2-augustus-gene-358.11-mRNA-1	2	OsR498G0204827200.01.T01	95,03	0,00E+00	2033	Os02g0765600	95,26	0,00E+00	2047	14	NA	Japonica	
maker-2-augustus-gene-358.13-mRNA-1	2	OsR498G0204826000.01.T02	99,87	0,00E+00	1414	Os02g0764500	100,00	0,00E+00	1418	4	NA	Japonica	
maker-2-augustus-gene-358.18-mRNA-1	2	OsR498G0204827200.01.T01	99,62	0,00E+00	2332	Os02g0765600	100,00	0,00E+00	2354	22	NA	Japonica	
maker-2-augustus-gene-358.2-mRNA-1	2	OsR498G0204823700.01.T01	99,83	0,00E+00	3103	Os02g0763200	100,00	0,00E+00	3117	14	NA	Japonica	
maker-2-augustus-gene-358.4-mRNA-1	2	OsR498G0204824700.01.T02	96,52	0,00E+00	2689	Os02g0763900	98,76	0,00E+00	2681	-8	NA	Japonica	
maker-2-augustus-gene-358.7-mRNA-1	2	OsR498G0204825300.01.T01	99,73	0,00E+00	3273	Os02g0764200	100,00	0,00E+00	3296	23	NA	Japonica	
maker-2-augustus-gene-358.9-mRNA-1	2	OsR498G0204825700.01.T01	99,75	0,00E+00	2108	Os02g0764400	100,00	0,00E+00	3068	960	Japonica	Japonica	
maker-2-augustus-gene-359.18-mRNA-1	2	OsR498G0204829400.01.T01	98,98	0,00E+00	1908	Os02g0766700	100,00	0,00E+00	1938	30	NA	Japonica	
maker-2-augustus-gene-359.2-mRNA-1	2	OsR498G0204831100.01.T02	99,87	0,00E+00	4074	Os02g0767100	100,00	0,00E+00	2628	-1446	Indica	Japonica	
maker-2-augustus-gene-359.22-mRNA-1	2	OsR498G0204830000.01.T01	100,00	0,00E+00	2208	Os02g0766900	100,00	0,00E+00	2208	0	NA	Japonica	
maker-2-augustus-gene-359.24-mRNA-1	2	OsR498G0204830200.01.T03	99,76	0,00E+00	2985	Os02g0767000	100,00	0,00E+00	2113	-872	Indica	Japonica	
maker-2-augustus-gene-359.27-mRNA-1	2	OsR498G0204831400.01.T01	99,17	0,00E+00	2561	Os02g0767200	100,00	0,00E+00	2614	53	NA	Japonica	
maker-2-augustus-gene-359.29-mRNA-1	2	OsR498G0204832000.01.T01	99,90	0,00E+00	1792	Os02g0767300	99,90	0,00E+00	1792	0	NA	Japonica	
maker-2-augustus-gene-359.32-mRNA-1	2	OsR498G0204832600.01.T01	99,82	0,00E+00	1988	Os02g0767500	100,00	0,00E+00	1997	9	NA	Japonica	
maker-2-augustus-gene-359.6-mRNA-1	2	OsR498G0204827400.01.T01	100,00	0,00E+00	861	Os02g0765800	100,00	0,00E+00	666	-195	Indica	Japonica	
maker-2-augustus-gene-359.7-mRNA-1	2	OsR498G0204827600.01.T01	99,73	0,00E+00	2699	Os01g0357100	97,35	0,00E+00	2541	-158	NA	Japonica	
maker-2-augustus-gene-36.2-mRNA-1	2	OsR498G0203032000.01.T02	100,00	0,00E+00	1759	Os06g0675600	76,26	2,74E-106	385	-1374	Indica	NA	
maker-2-augustus-gene-360.0-mRNA-1	2	OsR498G0204835900.01.T01	98,14	0,00E+00	1490	Os02g0768600	98,06	0,00E+00	1338	-152	Indica	Japonica	
maker-2-augustus-gene-360.12-mRNA-1	2	OsR498G0204835200.01.T01	99,54	0,00E+00	1172	Os02g0768200	100,00	0,00E+00	1186	14	NA	Japonica	
maker-2-augustus-gene-360.14-mRNA-1	2	OsR498G0204835400.01.T01	99,83	0,00E+00	2075	Os02g0768300	100,00	0,00E+00	2084	9	NA	Japonica	
maker-2-augustus-gene-360.16-mRNA-1	2	OsR498G0204839600.01.T03	99,50	0,00E+00	1771	Os02g0769200	99,43	0,00E+00	1236	-535	Indica	Japonica	
maker-2-augustus-gene-360.9-mRNA-1	2	OsR498G0204835000.01.T01	99,50	0,00E+00	708	Os02g0768000	100,00	0,00E+00	1274	566	Japonica	Japonica	
maker-2-augustus-gene-361.35-mRNA-1	2	OsR498G0204841300.01.T04	99,46	0,00E+00	995	Os02g0769700	100,00	0,00E+00	1009	14	NA	Japonica	Kinase-TM
maker-2-augustus-gene-361.47-mRNA-1	2	OsR498G0204843700.01.T01	99,71	0,00E+00	3094	Os02g0770500	99,84	0,00E+00	3439	345	Japonica	NA	
maker-2-augustus-gene-361.49-mRNA-1	2	OsR498G0204842000.01.T01	100,00	0,00E+00	710	Os02g0770000	100,00	0,00E+00	710	0	NA	Japonica	
maker-2-augustus-gene-361.55-mRNA-1	2	OsR498G0204842400.01.T03	99,64	0,00E+00	1970	Os02g0770100	100,00	0,00E+00	1983	13	NA	Japonica	
maker-2-augustus-gene-361.63-mRNA-1	2	OsR498G0204845000.01.T01	99,25	0,00E+00	4754	Os02g0770800	100,00	0,00E+00	4816	62	NA	Japonica	
maker-2-augustus-gene-361.65-mRNA-1	2	OsR498G0204846000.01.T01	99,85	0,00E+00	3699	Os02g0771100	100,00	0,00E+00	3712	13	NA	Japonica	
maker-2-augustus-gene-361.71-mRNA-1	2	OsR498G0204846400.01.T02	99,84	0,00E+00	1105	Os02g0771400	100,00	0,00E+00	1110	5	NA	Japonica	
maker-2-augustus-gene-362.0-mRNA-1	2	OsR498G0204848700.01.T01	98,27	0,00E+00	1702	Os02g0771600	100,00	0,00E+00	1743	41	NA	Japonica	
maker-2-augustus-gene-362.13-mRNA-1	2	OsR498G0204850900.01.T01	99,71	0,00E+00	4360	Os02g0772500	100,00	0,00E+00	4392	32	NA	Japonica	
maker-2-augustus-gene-362.18-mRNA-1	2	OsR498G0204849000.01.T01	99,73	0,00E+00	2628	Os02g0771700	99,86	0,00E+00	2637	9	NA	Japonica	
maker-2-augustus-gene-362.27-mRNA-1	2	OsR498G0204851200.01.T01	99,64	0,00E+00	1961	Os02g0772600	99,73	0,00E+00	1966	5	NA	Japonica	
maker-2-augustus-gene-362.9-mRNA-1	2	OsR498G0204850500.01.T05	94,33	0,00E+00	1372	Os02g0772300	94,53	0,00E+00	1141	-231	Indica	Japonica	
maker-2-augustus-gene-363.0-mRNA-1	2	OsR498G0204853500.01.T02	99,60	0,00E+00	890	Os02g0773200	100,00	0,00E+00	899	9	NA	Japonica	
maker-2-augustus-gene-363.11-mRNA-1	2	OsR498G0204856700.01.T02	89,18	9,35E-166	583	Os02g0774700	100,00	3,04E-160	563	-20	NA	Japonica	
maker-2-augustus-gene-363.14-mRNA-1	2	OsR498G0204857500.01.T01	98,96	0,00E+00	2194	Os02g0775100	100,00	0,00E+00	1110	-1084	Indica	Japonica	
maker-2-augustus-gene-363.19-mRNA-1	2	OsR498G0204853800.01.T02	99,77	0,00E+00	2297	Os02g0773300	100,00	0,00E+00	2089	-208	NA	Japonica	
maker-2-augustus-gene-363.28-mRNA-1	2	OsR498G0204854800.01.T01	99,67	0,00E+00	3762	Os06g0207500	80,15	0,00E+00	959	-2803	Indica	Japonica	
maker-2-augustus-gene-363.3-mRNA-1	2	OsR498G0204853900.01.T01	99,87	0,00E+00	2749	Os02g0773400	100,00	0,00E+00	2758	9	NA	Japonica	
maker-2-augustus-gene-363.30-mRNA-1	2	OsR498G0204856200.01.T01	99,66	0,00E+00	3648	Os02g0774300	100,00	0,00E+00	3680	32	NA	Japonica	
maker-2-augustus-gene-363.35-mRNA-1	2	OsR498G0204856400.01.T01	99,77	0,00E+00	1544	Os02g0774500	100,00	0,00E+00	1553	9	NA	Japonica	
maker-2-augustus-gene-363.40-mRNA-1	2	OsR498G0204857000.01.T01	99,60	0,00E+00	3093	Os02g0774800	100,00	0,00E+00	2194	-899	Indica	Japonica	
maker-2-augustus-gene-363.43-mRNA-1	2	OsR498G0204857800.01.T04	94,88	0,00E+00	976	Os02g0775200	94,88	0,00E+00	976	0	NA	Japonica	
maker-2-augustus-gene-363.8-mRNA-1	2	OsR498G0204855900.01.T01	100,00	0,00E+00	1050	Os02g0774100	96,91	0,00E+00	981	-69	NA	Japonica	
maker-2-augustus-gene-364.12-mRNA-1	2	OsR498G0204866800.01.T01	99,75	0,00E+00	3542	Os02g0776800	100,00	0,00E+00	3555	13	NA	Japonica	
maker-2-augustus-gene-364.14-mRNA-1	2	OsR498G0204867400.01.T01	99,41	0,00E+00	2112	Os02g0776900	99,92	0,00E+00	2140	28	NA	Japonica	
maker-2-augustus-gene-364.3-mRNA-1	2	OsR498G0204862800.01.T01	99,53	0,00E+00	753	Os02g0775900	100,00	0,00E+00	762	9	NA	Japonica	
maker-2-augustus-gene-365.0-mRNA-1	2	OsR498G0204869500.01.T02	99,61	0,00E+00	2722	Os06g0203800	76,95	0,00E+00	1337	-1385	Indica	Japonica	Kinase-LRR-TM
maker-2-augustus-gene-365.13-mRNA-1	2	OsR498G0204868200.01.T08	99,76	0,00E+00	4480	Os02g0777100	100,00	0,00E+00	4507	27	NA	Japonica	
maker-2-augustus-gene-365.16-mRNA-1	2	OsR498G0204870400.01.T01	99,26	0,00E+00	2402	Os06g0202900	79,97	0,00E+00	1064	-1338	Indica	Japonica	Kinase-TM
maker-2-augustus-gene-366.33-mRNA-1	2	OsR498G0204872300.01.T05	99,68	0,00E+00	3377	Os02g0778200	99,95	0,00E+00	3399	22	NA	Japonica	
maker-2-augustus-gene-366.37-mRNA-1	2	OsR498G0204872500.01.T02	99,72	0,00E+00	1919	Os02g0778300	100,00	0,00E+00	1932	13	NA	Japonica	
maker-2-augustus-gene-366.41-mRNA-1	2	OsR498G0204872500.01.T06	99,70	0,00E+00	1198	Os02g0778400	100,00	0,00E+00	1151	-47	NA	Japonica	
maker-2-augustus-gene-366.45-mRNA-1	2	OsR498G0204873100.01.T01	99,92	0,00E+00	2312	Os02g0778500	100,00	0,00E+00	2316	4	NA	Japonica	
maker-2-augustus-gene-366.48-mRNA-1	2	OsR498G0204873300.01.T01	99,93	0,00E+00	2712	Os02g0778600	100,00	0,00E+00	2717	5	NA	Japonica	
maker-2-augustus-gene-366.52-mRNA-1	2	OsR498G0204874500.01.T01	99,72	0,00E+00	1884	Os02g0779000	100,00	0,00E+00	1898	14	NA	Japonica	
maker-2-augustus-gene-367.1-mRNA-1	2	OsR498G0204878300.01.T01	99,53	0,00E+00	3045	Os02g0780500	99,88	0,00E+00	3072	27	NA	Japonica	
maker-2-augustus-gene-367.13-mRNA-1	2	OsR498G0204879100.01.T06	99,86	0,00E+00	2621	Os06g0199200	71,10	2,15E-122	439	-2182	Indica	Japonica	
maker-2-augustus-gene-367.21-mRNA-1	2	OsR498G0204880100.01.T05	99,49	0,00E+00	3516	Os02g0780700	100,00	0,00E+00	3561	45	NA	Japonica	
maker-2-augustus-gene-367.26-mRNA-1	2	OsR498G0204882100.01.T02	96,69	0,00E+00	2617	Os02g0781000	94,13	0,00E+00	1251	-1366	Indica	Japonica	
maker-2-augustus-gene-367.53-mRNA-1	2	OsR498G0204884600.01.T02	95,63	0,00E+00	1313	Os02g0781400	95,88	0,00E+00	1322	9	NA	Japonica	
maker-2-augustus-gene-367.55-mRNA-1	2	OsR498G0204885000.01.T02	99,75	0,00E+00	1431	Os02g0781700	100,00	0,00E+00	1440	9	NA	Japonica	
maker-2-augustus-gene-367.60-mRNA-1	2	OsR498G0204885400.01.T01	100,00	0,00E+00	2652	Os05g0482600	71,21	1,27E-131	471	-2181	Indica	Japonica	
maker-2-augustus-gene-368.22-mRNA-1	2	OsR498G0204886300.01.T01	98,98	0,00E+00	3105	Os02g0781900	100,00	0,00E+00	3463	358	Japonica	Japonica	
maker-2-augustus-gene-368.26-mRNA-1	2	OsR498G0204887100.01.T01	99,69	0,00E+00	6834	Os02g0782100	100,00	0,00E+00	5514	-1320	Indica	Japonica	
maker-2-augustus-gene-368.33-mRNA-1	2	OsR498G0204889900.01.T01	99,62	0,00E+00	928	Os02g0782500	100,00	0,00E+00	937	9	NA	Japonica	
maker-2-augustus-gene-368.35-mRNA-1	2	OsR498G0204890700.01.T06	99,73	0,00E+00	3984	Os06g0195600	75,32	0,00E+00	1519	-2465	Indica	Japonica	
maker-2-augustus-gene-368.50-mRNA-1	2	OsR498G0204892100.01.T01	99,37	0,00E+00	842	Os02g0782900	100,00	0,00E+00	720	-122	Indica	Japonica	
maker-2-augustus-gene-368.52-mRNA-1	2	OsR498G0204889400.01.T04	99,68	0,00E+00	3312	Os02g0782200	100,00	0,00E+00	3339	27	NA	Japonica	
maker-2-augustus-gene-368.64-mRNA-1	2	OsR498G0204891000.01.T02	99,91	0,00E+00	1976	Os02g0782700	100,00	0,00E+00	1981	5	NA	Japonica	
maker-2-augustus-gene-368.66-mRNA-1	2	OsR498G0204892400.01.T01	99,48	0,00E+00	3094	Os02g0783000	99,94	0,00E+00	3131	37	NA	Japonica	
maker-2-augustus-gene-369.58-mRNA-1	2	OsR498G0204898000.01.T01	99,92	0,00E+00	2306	Os06g0192600	84,06	0,00E+00	1389	-917	Indica	Japonica	
maker-2-augustus-gene-37.10-mRNA-1	2	OsR498G0203041500.01.T01	100,00	0,00E+00	2425	Os02g0167300	100,00	0,00E+00	2425	0	NA	NA	
maker-2-augustus-gene-37.13-mRNA-1	2	OsR498G0203044700.01.T01	100,00	0,00E+00	1604	Os02g0168300	99,89	0,00E+00	1599	-5	NA	Indica	
maker-2-augustus-gene-37.20-mRNA-1	2	OsR498G0203041800.01.T04	100,00	0,00E+00	3267	Os02g0167500	100,00	0,00E+00	2938	-329	Indica	NA	
maker-2-augustus-gene-37.30-mRNA-1	2	OsR498G0203043400.01.T01	98,01	0,00E+00	6339	Os02g0167700	98,01	0,00E+00	6339	0	NA	NA	
maker-2-augustus-gene-37.39-mRNA-1	2	OsR498G0203044400.01.T01	100,00	0,00E+00	2419	Os02g0168100	100,00	0,00E+00	1299	-1120	Indica	NA	
maker-2-augustus-gene-37.41-mRNA-1	2	OsR498G0203044600.01.T01	100,00	0,00E+00	1278	Os02g0168200	99,86	0,00E+00	1273	-5	NA	Indica	
maker-2-augustus-gene-37.5-mRNA-1	2	OsR498G0203040500.01.T02	100,00	0,00E+00	1667	Os02g0167100	100,00	0,00E+00	1824	157	NA	NA	
maker-2-augustus-gene-370.21-mRNA-1	2	OsR498G0204902300.01.T02	99,85	0,00E+00	5843	Os02g0786200	100,00	0,00E+00	3422	-2421	Indica	Japonica	Kinase-TM
maker-2-augustus-gene-370.25-mRNA-1	2	OsR498G0204903100.01.T01	95,14	0,00E+00	1770	Os02g0786300	100,00	0,00E+00	3052	1282	Japonica	Japonica	
maker-2-augustus-gene-370.29-mRNA-1	2	OsR498G0204905700.01.T03	99,81	0,00E+00	1864	Os02g0787100	100,00	0,00E+00	2343	479	Japonica	Japonica	
maker-2-augustus-gene-370.4-mRNA-1	2	OsR498G0204901100.01.T03	99,78	0,00E+00	2433	Os02g0785900	97,30	0,00E+00	3033	600	Japonica	Japonica	
maker-2-augustus-gene-371.0-mRNA-1	2	OsR498G0204905900.01.T01	96,68	0,00E+00	3878	Os02g0787200	97,26	0,00E+00	3921	43	NA	Japonica	CC-Kinase-TM
maker-2-augustus-gene-371.10-mRNA-1	2	OsR498G0204910500.01.T01	99,77	0,00E+00	1561	Os02g0788400	100,00	0,00E+00	1570	9	NA	Japonica	
maker-2-augustus-gene-371.12-mRNA-1	2	OsR498G0204909000.01.T06	99,12	0,00E+00	1804	Os02g0787800	100,00	0,00E+00	1844	40	NA	Japonica	
maker-2-augustus-gene-371.18-mRNA-1	2	OsR498G0204911000.01.T01	99,86	0,00E+00	1300	Os02g0788500	100,00	0,00E+00	1305	5	NA	Japonica	
maker-2-augustus-gene-371.2-mRNA-1	2	OsR498G0204907700.01.T03	99,84	0,00E+00	2177	Os02g0787500	96,26	0,00E+00	1780	-397	Indica	Japonica	
maker-2-augustus-gene-371.20-mRNA-1	2	OsR498G0204911800.01.T02	99,86	0,00E+00	5209	Os02g0788600	100,00	0,00E+00	5227	18	NA	Japonica	
maker-2-augustus-gene-371.23-mRNA-1	2	OsR498G0204912400.01.T06	99,60	0,00E+00	1329	Os02g0788800	100,00	0,00E+00	1342	13	NA	Japonica	
maker-2-augustus-gene-371.5-mRNA-1	2	OsR498G0204908700.01.T01	99,63	0,00E+00	4793	Os02g0787600	99,96	0,00E+00	4833	40	NA	Japonica	
maker-2-augustus-gene-371.8-mRNA-1	2	OsR498G0204910500.01.T01	70,22	2,79E-98	361	Os02g0788300	100,00	0,00E+00	1570	1209	Japonica	Japonica	
maker-2-augustus-gene-372.46-mRNA-1	2	OsR498G0204913700.01.T01	99,95	0,00E+00	3340	Os02g0789600	100,00	0,00E+00	3344	4	NA	Japonica	
maker-2-augustus-gene-372.50-mRNA-1	2	OsR498G0917982000.01.T01	75,26	0,00E+00	713	Os02g0789700	100,00	0,00E+00	2305	1592	Japonica	Japonica	
maker-2-augustus-gene-372.51-mRNA-1	2	OsR498G0204915700.01.T01	99,84	0,00E+00	4498	Os02g0790500	100,00	0,00E+00	2383	-2115	Indica	Japonica	
maker-2-augustus-gene-372.58-mRNA-1	2	OsR498G0204917900.01.T02	99,51	0,00E+00	1089	Os02g0791300	99,84	0,00E+00	1100	11	NA	Japonica	
maker-2-augustus-gene-372.59-mRNA-1	2	OsR498G0204913200.01.T03	99,70	0,00E+00	1207	Os02g0789100	100,00	0,00E+00	1213	6	NA	Japonica	
maker-2-augustus-gene-372.63-mRNA-1	2	OsR498G0204913400.01.T03	99,82	0,00E+00	1003	Os02g0789400	100,00	0,00E+00	1007	4	NA	Japonica	
maker-2-augustus-gene-372.66-mRNA-1	2	OsR498G0204918400.01.T02	100,00	7,82E-119	426	Os02g0791400	89,86	4,04E-79	294	-132	Indica	Japonica	
maker-2-augustus-gene-373.1-mRNA-1	2	OsR498G0204919500.01.T02	99,67	0,00E+00	3767	Os02g0791700	100,00	0,00E+00	2224	-1543	Indica	Japonica	CC-Kinase-TM
maker-2-augustus-gene-373.11-mRNA-1	2	OsR498G0204924300.01.T05	99,92	0,00E+00	2364	Os02g0793300	100,00	0,00E+00	2369	5	NA	Japonica	
maker-2-augustus-gene-373.17-mRNA-1	2	OsR498G0204919800.01.T01	99,47	0,00E+00	2003	Os02g0791800	100,00	0,00E+00	2030	27	NA	Japonica	
maker-2-augustus-gene-373.22-mRNA-1	2	OsR498G0204923600.01.T01	99,76	0,00E+00	2232	Os02g0792900	100,00	0,00E+00	2246	14	NA	Japonica	
maker-2-augustus-gene-373.25-mRNA-1	2	OsR498G0204923800.01.T01	99,74	0,00E+00	2715	Os02g0793000	100,00	0,00E+00	2733	18	NA	Japonica	
maker-2-augustus-gene-373.28-mRNA-1	2	OsR498G0204925200.01.T02	99,51	0,00E+00	1805	Os02g0793500	100,00	0,00E+00	2571	766	Japonica	Japonica	
maker-2-augustus-gene-373.7-mRNA-1	2	OsR498G0204924100.01.T01	99,89	0,00E+00	6231	Os02g0793100	100,00	0,00E+00	6249	18	NA	Japonica	
maker-2-augustus-gene-374.44-mRNA-1	2	OsR498G0204928300.01.T01	99,44	0,00E+00	2526	Os02g0794500	100,00	0,00E+00	2565	39	NA	Japonica	
maker-2-augustus-gene-374.49-mRNA-1	2	OsR498G0204929900.01.T02	98,31	0,00E+00	3894	Os02g0795000	100,00	0,00E+00	6417	2523	Japonica	Japonica	
maker-2-augustus-gene-374.57-mRNA-1	2	OsR498G0204930100.01.T01	98,95	0,00E+00	1838	Os02g0795200	100,00	0,00E+00	1889	51	NA	Japonica	
maker-2-augustus-gene-374.59-mRNA-1	2	OsR498G0204926200.01.T01	99,68	0,00E+00	1133	Os02g0793900	100,00	0,00E+00	1142	9	NA	Japonica	
maker-2-augustus-gene-374.61-mRNA-1	2	OsR498G0204926400.01.T01	99,49	0,00E+00	2782	Os02g0794000	100,00	0,00E+00	2819	37	NA	Japonica	
maker-2-augustus-gene-374.63-mRNA-1	2	OsR498G0204927000.01.T02	99,78	0,00E+00	1647	Os02g0794400	100,00	0,00E+00	1088	-559	Indica	Japonica	
maker-2-augustus-gene-374.67-mRNA-1	2	OsR498G0204929000.01.T01	100,00	0,00E+00	2740	Os02g0794700	100,00	0,00E+00	1716	-1024	Indica	Japonica	
maker-2-augustus-gene-374.75-mRNA-1	2	OsR498G0204929400.01.T01	99,88	0,00E+00	7693	Os02g0794900	99,45	0,00E+00	5814	-1879	Indica	Japonica	
maker-2-augustus-gene-374.89-mRNA-1	2	OsR498G0204930600.01.T01	99,82	0,00E+00	1972	Os02g0795300	100,00	0,00E+00	1981	9	NA	Japonica	
maker-2-augustus-gene-375.35-mRNA-1	2	OsR498G0204932400.01.T04	96,36	0,00E+00	2603	Os02g0795900	96,42	0,00E+00	2608	5	NA	Japonica	
maker-2-augustus-gene-375.39-mRNA-1	2	OsR498G0204934400.01.T01	99,84	0,00E+00	2221	Os02g0796400	100,00	0,00E+00	2803	582	Japonica	Japonica	
maker-2-augustus-gene-375.41-mRNA-1	2	OsR498G0204934600.01.T03	99,64	0,00E+00	1966	Os02g0796500	99,91	0,00E+00	1980	14	NA	Japonica	
maker-2-augustus-gene-375.45-mRNA-1	2	OsR498G0204936000.01.T01	99,60	0,00E+00	901	Os02g0796600	99,80	0,00E+00	905	4	NA	Japonica	
maker-2-augustus-gene-375.56-mRNA-1	2	OsR498G0204937800.01.T01	100,00	0,00E+00	937	Os02g0797100	100,00	0,00E+00	937	0	NA	Japonica	
maker-2-augustus-gene-376.34-mRNA-1	2	OsR498G0204939300.01.T01	99,93	0,00E+00	2480	Os02g0797500	100,00	0,00E+00	2484	4	NA	Japonica	
maker-2-augustus-gene-376.42-mRNA-1	2	OsR498G0204940900.01.T06	99,16	0,00E+00	839	Os02g0798200	100,00	0,00E+00	859	20	NA	Japonica	
maker-2-augustus-gene-376.44-mRNA-1	2	OsR498G0204943700.01.T03	99,71	0,00E+00	1852	Os02g0798700	100,00	0,00E+00	1865	13	NA	Japonica	
maker-2-augustus-gene-376.48-mRNA-1	2	OsR498G0204939200.01.T04	99,63	0,00E+00	1920	Os02g0797400	100,00	0,00E+00	1938	18	NA	Japonica	
maker-2-augustus-gene-376.53-mRNA-1	2	OsR498G0204943000.01.T01	99,86	0,00E+00	2484	Os02g0798600	97,95	0,00E+00	2020	-464	Indica	Japonica	
maker-2-augustus-gene-377.15-mRNA-1	2	OsR498G0204949200.01.T01	100,00	0,00E+00	769	Os02g0800100	100,00	0,00E+00	769	0	NA	Japonica	
maker-2-augustus-gene-378.41-mRNA-1	2	OsR498G0204958300.01.T03	99,88	0,00E+00	2924	Os02g0802200	100,00	0,00E+00	2933	9	NA	Japonica	
maker-2-augustus-gene-378.44-mRNA-1	2	OsR498G0204960500.01.T01	99,67	0,00E+00	2732	Os02g0802700	100,00	0,00E+00	1773	-959	Indica	Japonica	
maker-2-augustus-gene-378.50-mRNA-1	2	OsR498G0204961500.01.T01	99,63	0,00E+00	2406	Os02g0803200	99,78	0,00E+00	2415	9	NA	Japonica	
maker-2-augustus-gene-378.60-mRNA-1	2	OsR498G0204962300.01.T01	99,71	0,00E+00	3066	Os02g0803300	99,83	0,00E+00	3075	9	NA	Japonica	
maker-2-augustus-gene-378.66-mRNA-1	2	OsR498G0204962700.01.T02	99,59	0,00E+00	1313	Os02g0803600	100,00	0,00E+00	1070	-243	Indica	Japonica	
maker-2-augustus-gene-378.68-mRNA-1	2	OsR498G0204959200.01.T02	100,00	0,00E+00	726	Os08g0442300	77,11	5,87E-68	257	-469	Indica	Japonica	
maker-2-augustus-gene-378.73-mRNA-1	2	OsR498G0204959400.01.T01	99,96	0,00E+00	4124	Os02g0802500	100,00	0,00E+00	4129	5	NA	Japonica	
maker-2-augustus-gene-378.75-mRNA-1	2	OsR498G0204962600.01.T02	99,78	0,00E+00	833	Os02g0803400	100,00	0,00E+00	742	-91	Indica	Japonica	
maker-2-augustus-gene-378.77-mRNA-1	2	OsR498G0204963000.01.T01	99,92	0,00E+00	2323	Os02g0803700	100,00	0,00E+00	2327	4	NA	Japonica	
maker-2-augustus-gene-379.40-mRNA-1	2	OsR498G0204964700.01.T03	99,78	0,00E+00	1593	Os02g0804300	100,00	0,00E+00	1602	9	NA	Japonica	
maker-2-augustus-gene-379.44-mRNA-1	2	OsR498G0204964900.01.T01	99,73	0,00E+00	662	Os02g0804400	100,00	0,00E+00	666	4	NA	Japonica	
maker-2-augustus-gene-379.46-mRNA-1	2	OsR498G0204965900.01.T02	99,96	0,00E+00	4384	Os06g0168600	85,52	0,00E+00	2811	-1573	Indica	Japonica	
maker-2-augustus-gene-379.48-mRNA-1	2	OsR498G0204967300.01.T03	98,91	0,00E+00	965	Os02g0805100	100,00	0,00E+00	991	26	NA	Japonica	
maker-2-augustus-gene-379.51-mRNA-1	2	OsR498G0204967700.01.T01	100,00	0,00E+00	1429	Os02g0805200	100,00	0,00E+00	1429	0	NA	Japonica	
maker-2-augustus-gene-379.57-mRNA-1	2	OsR498G0204965200.01.T01	99,64	0,00E+00	1975	Os02g0804500	100,00	0,00E+00	1994	19	NA	Japonica	
maker-2-augustus-gene-379.68-mRNA-1	2	OsR498G0204966400.01.T04	98,12	0,00E+00	3876	Os02g0805000	97,41	0,00E+00	2571	-1305	Indica	Japonica	
maker-2-augustus-gene-379.81-mRNA-1	2	OsR498G0204969200.01.T01	99,30	0,00E+00	761	Os02g0805700	100,00	0,00E+00	785	24	NA	Japonica	
maker-2-augustus-gene-379.84-mRNA-1	2	OsR498G0204969400.01.T03	99,39	0,00E+00	4370	Os02g0805800	100,00	0,00E+00	5205	835	Japonica	Japonica	
maker-2-augustus-gene-38.0-mRNA-1	2	OsR498G0203046700.01.T01	96,68	0,00E+00	1869	Os02g0168800	96,68	0,00E+00	1869	0	NA	Indica	
maker-2-augustus-gene-38.14-mRNA-1	2	OsR498G0203043400.01.T03	93,98	0,00E+00	3029	Os02g0167700	93,98	0,00E+00	3029	0	NA	Indica	
maker-2-augustus-gene-38.23-mRNA-1	2	OsR498G0203053700.01.T02	99,67	0,00E+00	3248	Os02g0170000	99,75	0,00E+00	2140	-1108	Indica	Indica	
maker-2-augustus-gene-38.28-mRNA-1	2	OsR498G0203046000.01.T03	100,00	0,00E+00	661	Os02g0168600	99,45	0,00E+00	652	-9	NA	Indica	
maker-2-augustus-gene-38.31-mRNA-1	2	OsR498G0203046400.01.T01	100,00	0,00E+00	1159	Os02g0168700	100,00	0,00E+00	1159	0	NA	Indica	
maker-2-augustus-gene-38.33-mRNA-1	2	OsR498G0203047200.01.T01	100,00	1,67E-153	542	Os02g0168900	100,00	5,79E-154	542	0	NA	Indica	
maker-2-augustus-gene-38.35-mRNA-1	2	OsR498G0203047400.01.T01	100,00	0,00E+00	2030	Os02g0169000	100,00	0,00E+00	2100	70	NA	NA	
maker-2-augustus-gene-38.4-mRNA-1	2	OsR498G0203048100.01.T02	100,00	0,00E+00	2181	Os02g0169300	100,00	0,00E+00	2181	0	NA	Indica	
maker-2-augustus-gene-38.45-mRNA-1	2	OsR498G0203053600.01.T04	100,00	0,00E+00	933	Os02g0169900	97,14	0,00E+00	888	-45	NA	Indica	
maker-2-augustus-gene-380.16-mRNA-1	2	OsR498G0204976100.01.T01	98,76	0,00E+00	2527	Os02g0806900	100,00	0,00E+00	2608	81	NA	Japonica	
maker-2-augustus-gene-380.23-mRNA-1	2	OsR498G0204969600.01.T01	99,59	0,00E+00	1736	Os02g0805900	100,00	0,00E+00	1754	18	NA	Japonica	
maker-2-augustus-gene-380.29-mRNA-1	2	OsR498G0204973200.01.T01	99,96	0,00E+00	5022	Os02g0806500	99,96	0,00E+00	5022	0	NA	Japonica	
maker-2-augustus-gene-380.35-mRNA-1	2	OsR498G0204973800.01.T01	99,01	0,00E+00	5012	Os02g0806600	100,00	0,00E+00	5119	107	NA	Japonica	

maker-2-augustus-gene-380.38-mRNA-1	2	OsR498G0204974200.01.T01	99,72	0,00E+00	3891	Os02g0806700	100,00	0,00E+00	3918	27	NA	Japonica	
maker-2-augustus-gene-380.40-mRNA-1	2	OsR498G0204976400.01.T01	98,51	0,00E+00	1511	Os02g0807000	99,89	0,00E+00	1565	54	NA	Japonica	Kinase-TM
maker-2-augustus-gene-381.34-mRNA-1	2	OsR498G0204976700.01.T01	98,86	0,00E+00	3688	Os02g0807100	100,00	0,00E+00	2179	-1509	Indica	Japonica	
maker-2-augustus-gene-381.46-mRNA-1	2	OsR498G0204979300.01.T01	87,56	0,00E+00	1992	Os02g0811200	78,51	0,00E+00	1887	-105	NA	Japonica	Kinase-TM
maker-2-augustus-gene-381.49-mRNA-1	2	OsR498G0204977600.01.T01	96,96	0,00E+00	3789	Os02g0807800	99,67	0,00E+00	2176	-1613	Indica	Japonica	Kinase-TM
maker-2-augustus-gene-381.50-mRNA-1	2	OsR498G0204978800.01.T01	99,15	0,00E+00	2071	Os02g0811200	82,39	0,00E+00	1659	-412	Indica	Japonica	Kinase-TM
maker-2-augustus-gene-382.26-mRNA-1	2	OsR498G0204981900.01.T05	99,77	0,00E+00	1588	Os02g0809500	100,00	0,00E+00	1597	9	NA	Japonica	
maker-2-augustus-gene-382.29-mRNA-1	2	OsR498G0204983100.01.T01	99,72	0,00E+00	1893	Os02g0809800	99,91	0,00E+00	1902	9	NA	Japonica	
maker-2-augustus-gene-382.40-mRNA-1	2	OsR498G0204985100.01.T02	99,66	0,00E+00	1572	Os02g0810300	100,00	0,00E+00	1586	14	NA	Japonica	
maker-2-augustus-gene-382.45-mRNA-1	2	OsR498G0204985500.01.T01	97,71	0,00E+00	2173	Os02g0810400	97,71	0,00E+00	2173	0	NA	Japonica	Kinase
maker-2-augustus-gene-382.50-mRNA-1	2	OsR498G0204985700.01.T05	99,81	0,00E+00	2795	Os02g0810450	99,75	0,00E+00	2164	-631	Indica	Japonica	
maker-2-augustus-gene-382.55-mRNA-1	2	OsR498G0204985900.01.T03	99,73	0,00E+00	1324	Os02g0810500	100,00	0,00E+00	1333	9	NA	Japonica	
maker-2-augustus-gene-382.60-mRNA-1	2	OsR498G0204987500.01.T01	98,52	0,00E+00	1765	Os02g0810900	99,70	0,00E+00	1783	18	NA	Japonica	
maker-2-augustus-gene-382.65-mRNA-1	2	OsR498G0204983800.01.T01	99,84	0,00E+00	3423	Os07g0436000	72,99	9,15E-154	544	-2879	Indica	Japonica	
maker-2-augustus-gene-383.0-mRNA-1	2	OsR498G0204979300.01.T01	88,80	0,00E+00	1535	Os02g0811200	99,58	0,00E+00	2115	580	Japonica	NA	Kinase-TM
maker-2-augustus-gene-383.18-mRNA-1	2	OsR498G0204994300.01.T01	99,80	0,00E+00	1793	Os02g0812500	99,90	0,00E+00	1798	5	NA	Japonica	
maker-2-augustus-gene-383.2-mRNA-1	2	OsR498G0204992300.01.T01	86,40	0,00E+00	1166	Os02g0811600	99,91	0,00E+00	1917	751	Japonica	Japonica	
maker-2-augustus-gene-383.21-mRNA-1	2	OsR498G0204994500.01.T01	98,78	0,00E+00	1425	Os02g0812600	94,25	0,00E+00	1299	-126	NA	Japonica	
maker-2-augustus-gene-383.22-mRNA-1	2	OsR498G0204994500.01.T01	70,93	1,33E-37	158	Os02g0812600	99,40	0,00E+00	1169	1011	Japonica	Japonica	
maker-2-augustus-gene-383.29-mRNA-1	2	OsR498G0204990200.01.T03	99,15	7,88E-178	623	Os02g0811101	100,00	4,36E-144	509	-114	Indica	Japonica	
maker-2-augustus-gene-383.5-mRNA-1	2	OsR498G0204992300.01.T01	97,09	0,00E+00	1733	Os02g0811800	100,00	0,00E+00	1840	107	NA	Japonica	
maker-2-augustus-gene-383.6-mRNA-1	2	OsR498G0204992500.01.T01	97,44	0,00E+00	1719	Os02g0812000	100,00	0,00E+00	1813	94	NA	Japonica	
maker-2-augustus-gene-384.18-mRNA-1	2	OsR498G0204995500.01.T01	99,55	0,00E+00	1595	Os02g0813300	100,00	0,00E+00	1613	18	NA	Japonica	
maker-2-augustus-gene-384.21-mRNA-1	2	OsR498G0204995900.01.T07	99,80	0,00E+00	2676	Os02g0813500	100,00	0,00E+00	2690	14	NA	Japonica	
maker-2-augustus-gene-384.27-mRNA-1	2	OsR498G0205000100.01.T03	74,70	4,27E-73	275	Os02g0814300	100,00	0,00E+00	758	483	Japonica	Japonica	
maker-2-augustus-gene-384.28-mRNA-1	2	OsR498G0205000100.01.T03	96,66	0,00E+00	1199	Os02g0814400	100,00	0,00E+00	715	-484	Indica	Japonica	
maker-2-augustus-gene-384.35-mRNA-1	2	OsR498G0205000900.01.T01	99,75	0,00E+00	2178	Os02g0814800	100,00	0,00E+00	2192	14	NA	Japonica	
maker-2-augustus-gene-384.37-mRNA-1	2	OsR498G0205001300.01.T01	99,57	0,00E+00	825	Os02g0815001	100,00	2,43E-147	520	-305	Indica	Japonica	
maker-2-augustus-gene-384.41-mRNA-1	2	OsR498G0204997000.01.T03	97,09	0,00E+00	2151	Os02g0813800	97,40	0,00E+00	2169	18	NA	Japonica	
maker-2-augustus-gene-384.45-mRNA-1	2	OsR498G0204998600.01.T03	100,00	0,00E+00	1375	Os02g0814000	99,87	0,00E+00	1352	-23	NA	Japonica	
maker-2-augustus-gene-384.47-mRNA-1	2	OsR498G0204998800.01.T03	97,92	0,00E+00	3398	Os02g0814100	100,00	0,00E+00	2282	-1116	Indica	Japonica	
maker-2-augustus-gene-384.54-mRNA-1	2	OsR498G0204999000.01.T01	99,74	0,00E+00	3419	Os02g0814200	100,00	0,00E+00	3442	23	NA	Japonica	
maker-2-augustus-gene-384.61-mRNA-1	2	OsR498G0205001200.01.T04	99,27	0,00E+00	1682	Os02g0814900	99,90	0,00E+00	1706	24	NA	Japonica	
maker-2-augustus-gene-385.0-mRNA-1	2	OsR498G0205001700.01.T01	99,06	0,00E+00	1687	Os02g0815200	100,00	0,00E+00	1716	29	NA	Japonica	
maker-2-augustus-gene-385.15-mRNA-1	2	OsR498G0205005900.01.T07	99,89	0,00E+00	4759	Os02g0816300	99,96	0,00E+00	4768	9	NA	Japonica	
maker-2-augustus-gene-385.2-mRNA-1	2	OsR498G0205002100.01.T03	99,74	0,00E+00	676	Os02g0815400	100,00	0,00E+00	681	5	NA	Japonica	
maker-2-augustus-gene-385.23-mRNA-1	2	OsR498G0205006100.01.T04	99,79	0,00E+00	5032	Os02g0816400	100,00	0,00E+00	5059	27	NA	Japonica	
maker-2-augustus-gene-385.34-mRNA-1	2	OsR498G0205003000.01.T01	100,00	0,00E+00	1072	Os02g0815800	100,00	0,00E+00	1072	0	NA	NA	
maker-2-augustus-gene-385.38-mRNA-1	2	OsR498G0205003400.01.T01	99,91	0,00E+00	4001	Os02g0815900	100,00	0,00E+00	3999	-2	NA	Japonica	Kinase-TM
maker-2-augustus-gene-385.42-mRNA-1	2	OsR498G0205004200.01.T01	99,61	0,00E+00	1388	Os02g0816100	100,00	0,00E+00	1402	14	NA	Japonica	
maker-2-augustus-gene-385.45-mRNA-1	2	OsR498G0205004600.01.T01	100,00	0,00E+00	2003	Os02g0816200	99,91	0,00E+00	1998	-5	NA	Japonica	
maker-2-augustus-gene-385.54-mRNA-1	2	OsR498G0205007000.01.T01	99,57	0,00E+00	836	Os02g0816700	100,00	0,00E+00	845	9	NA	Japonica	
maker-2-augustus-gene-385.6-mRNA-1	2	OsR498G0205002300.01.T01	99,91	0,00E+00	1994	Os02g0815500	100,00	0,00E+00	1999	5	NA	Japonica	
maker-2-augustus-gene-386.0-mRNA-1	2	OsR498G0205007700.01.T03	98,39	0,00E+00	5311	Os02g0816900	98,52	0,00E+00	5329	18	NA	Japonica	
maker-2-augustus-gene-386.10-mRNA-1	2	OsR498G0205009300.01.T01	99,70	0,00E+00	1767	Os02g0817500	100,00	0,00E+00	1781	14	NA	Japonica	
maker-2-augustus-gene-386.13-mRNA-1	2	OsR498G0205010700.01.T05	100,00	0,00E+00	1153	Os02g0818000	100,00	0,00E+00	987	-166	Indica	Japonica	
maker-2-augustus-gene-386.18-mRNA-1	2	OsR498G0205009000.01.T02	100,00	0,00E+00	2035	Os02g0817200	100,00	0,00E+00	2035	0	NA	Japonica	
maker-2-augustus-gene-386.23-mRNA-1	2	OsR498G0205010000.01.T02	99,12	0,00E+00	1401	Os02g0817600	99,50	0,00E+00	1415	14	NA	Japonica	
maker-2-augustus-gene-386.32-mRNA-1	2	OsR498G0205010400.01.T03	99,65	0,00E+00	2568	Os02g0817800	100,00	0,00E+00	1896	-672	Indica	Japonica	
maker-2-augustus-gene-386.37-mRNA-1	2	OsR498G0205010600.01.T01	99,85	0,00E+00	3471	Os02g0817900	100,00	0,00E+00	3485	14	NA	Japonica	
maker-2-augustus-gene-387.0-mRNA-1	2	OsR498G0205012900.01.T01	90,37	0,00E+00	906	Os02g0818900	100,00	0,00E+00	1094	188	Japonica	Japonica	
maker-2-augustus-gene-387.2-mRNA-1	2	OsR498G0205013100.01.T01	99,74	0,00E+00	1370	Os02g0819000	100,00	0,00E+00	1988	618	Japonica	Japonica	
maker-2-augustus-gene-387.22-mRNA-1	2	OsR498G0205016600.01.T01	99,30	0,00E+00	3819	Os02g0819400	100,00	0,00E+00	2778	-1041	Indica	Japonica	
maker-2-augustus-gene-387.26-mRNA-1	2	OsR498G0205017400.01.T06	94,84	0,00E+00	1524	Os02g0819500	93,80	0,00E+00	1066	-458	Indica	Japonica	
maker-2-augustus-gene-387.32-mRNA-1	2	OsR498G0205019200.01.T01	99,30	0,00E+00	1005	Os02g0819700	99,65	0,00E+00	1014	9	NA	Japonica	
maker-2-augustus-gene-387.34-mRNA-1	2	OsR498G0205020200.01.T03	99,90	0,00E+00	1738	Os02g0820000	100,00	0,00E+00	1829	91	NA	Japonica	
maker-2-augustus-gene-387.4-mRNA-1	2	OsR498G0205015900.01.T06	99,81	0,00E+00	959	Os02g0819100	100,00	0,00E+00	964	5	NA	Japonica	
maker-2-augustus-gene-387.9-mRNA-1	2	OsR498G0205016100.01.T01	99,65	0,00E+00	1014	Os02g0819200	100,00	0,00E+00	1023	9	NA	Japonica	
maker-2-augustus-gene-388.0-mRNA-1	2	OsR498G0205023300.01.T02	99,87	0,00E+00	4157	Os02g0820900	100,00	0,00E+00	4170	13	NA	Japonica	
maker-2-augustus-gene-388.11-mRNA-1	2	OsR498G0205025300.01.T02	99,89	0,00E+00	1576	Os02g0821900	100,00	0,00E+00	1581	5	NA	Japonica	
maker-2-augustus-gene-388.13-mRNA-1	2	OsR498G0205025500.01.T03	99,83	0,00E+00	2116	Os02g0822000	100,00	0,00E+00	2125	9	NA	Japonica	
maker-2-augustus-gene-388.16-mRNA-1	2	OsR498G0205025700.01.T01	99,69	0,00E+00	2851	Os02g0822100	100,00	0,00E+00	2874	23	NA	Japonica	
maker-2-augustus-gene-388.19-mRNA-1	2	OsR498G0205026100.01.T05	100,00	0,00E+00	1797	Os02g0822200	100,00	0,00E+00	1797	0	NA	Japonica	
maker-2-augustus-gene-388.2-mRNA-1	2	OsR498G0205024900.01.T01	96,70	0,00E+00	2703	Os02g0821500	100,00	0,00E+00	1801	-902	Indica	Japonica	
maker-2-augustus-gene-388.29-mRNA-1	2	OsR498G0205026500.01.T01	99,32	0,00E+00	3370	Os02g0822400	100,00	0,00E+00	3425	55	NA	Japonica	
maker-2-augustus-gene-388.32-mRNA-1	2	OsR498G0205020800.01.T02	99,52	0,00E+00	3325	Os02g0820300	100,00	0,00E+00	3366	41	NA	Japonica	
maker-2-augustus-gene-388.35-mRNA-1	2	OsR498G0205021200.01.T01	100,00	0,00E+00	1175	Os02g0820500	100,00	0,00E+00	1175	0	NA	Japonica	
maker-2-augustus-gene-388.37-mRNA-1	2	OsR498G0205021600.01.T01	92,58	0,00E+00	1914	Os02g0820600	95,89	0,00E+00	2052	138	NA	Japonica	
maker-2-augustus-gene-388.41-mRNA-1	2	OsR498G0205023600.01.T01	99,32	0,00E+00	788	Os02g0821200	99,64	7,61E-141	499	-289	Indica	Japonica	
maker-2-augustus-gene-388.45-mRNA-1	2	OsR498G0205024600.01.T01	99,79	0,00E+00	2546	Os02g0821400	100,00	0,00E+00	2560	14	NA	Japonica	Kinase
maker-2-augustus-gene-388.47-mRNA-1	2	OsR498G0205026800.01.T03	99,76	0,00E+00	4437	Os02g0822500	100,00	0,00E+00	4464	27	NA	Japonica	
maker-2-augustus-gene-389.10-mRNA-1	2	OsR498G0205029100.01.T01	99,77	0,00E+00	1555	Os02g0823100	100,00	0,00E+00	1564	9	NA	Japonica	
maker-2-augustus-gene-389.12-mRNA-1	2	OsR498G0205029500.01.T02	99,41	0,00E+00	1193	Os04g0166600	94,20	0,00E+00	1033	-160	Indica	Japonica	
maker-2-augustus-gene-389.17-mRNA-1	2	OsR498G0205030100.01.T02	99,82	0,00E+00	2957	Os02g0823300	100,00	0,00E+00	2971	14	NA	Japonica	
maker-2-augustus-gene-389.20-mRNA-1	2	OsR498G0205030500.01.T01	99,55	0,00E+00	1958	Os02g0823400	100,00	0,00E+00	1981	23	NA	Japonica	
maker-2-augustus-gene-389.23-mRNA-1	2	OsR498G0205031500.01.T03	99,02	0,00E+00	5557	Os02g0823800	99,31	0,00E+00	6202	645	Japonica	Japonica	
maker-2-augustus-gene-389.25-mRNA-1	2	OsR498G0205027800.01.T01	96,89	0,00E+00	973	Os02g0822600	97,41	0,00E+00	986	13	NA	Japonica	
maker-2-augustus-gene-389.28-mRNA-1	2	OsR498G0205028600.01.T02	99,74	0,00E+00	2796	Os02g0822800	100,00	0,00E+00	2814	18	NA	Japonica	
maker-2-augustus-gene-389.35-mRNA-1	2	OsR498G0205031400.01.T01	99,49	0,00E+00	1753	Os10g0545500	73,60	2,42E-113	409	-1344	Indica	Japonica	
maker-2-augustus-gene-389.37-mRNA-1	2	OsR498G0205032200.01.T06	99,57	0,00E+00	3709	Os02g0824000	100,00	0,00E+00	4758	1049	Japonica	Japonica	
maker-2-augustus-gene-389.4-mRNA-1	2	OsR498G0205028900.01.T06	99,88	0,00E+00	2946	Os02g0823000	100,00	0,00E+00	2955	9	NA	Japonica	
maker-2-augustus-gene-389.40-mRNA-1	2	OsR498G0205033400.01.T01	95,63	0,00E+00	2028	Os02g0824300	95,87	0,00E+00	2041	13	NA	Japonica	
maker-2-augustus-gene-39.34-mRNA-1	2	OsR498G0203054900.01.T01	100,00	0,00E+00	861	Os02g0170100	99,79	0,00E+00	856	-5	NA	Japonica	
maker-2-augustus-gene-39.37-mRNA-1	2	OsR498G0203057100.01.T01	96,62	0,00E+00	1894	Os02g0171100	96,70	0,00E+00	1899	5	NA	Japonica	
maker-2-augustus-gene-39.43-mRNA-1	2	OsR498G0203055200.01.T01	99,39	0,00E+00	869	Os02g0170200	100,00	0,00E+00	883	14	NA	Japonica	
maker-2-augustus-gene-39.46-mRNA-1	2	OsR498G0203055400.01.T01	99,75	0,00E+00	1404	Os02g0170300	99,87	0,00E+00	1408	4	NA	Japonica	
maker-2-augustus-gene-390.104-mRNA-1	2	OsR498G0205036400.01.T01	98,12	0,00E+00	2189	Os02g0826000	100,00	0,00E+00	3842	1653	Japonica	Japonica	
maker-2-augustus-gene-390.106-mRNA-1	2	OsR498G0205036600.01.T01	98,83	0,00E+00	899	Os02g0825100	91,09	0,00E+00	725	-174	Indica	Japonica	
maker-2-augustus-gene-390.119-mRNA-1	2	OsR498G0205036800.01.T02	100,00	0,00E+00	4394	Os02g0826100	100,00	0,00E+00	4394	0	NA	Japonica	
maker-2-augustus-gene-390.54-mRNA-1	2	OsR498G0408962200.01.T01	74,91	2,75E-45	184	Os02g0824500	98,36	0,00E+00	954	770	Japonica	Japonica	
maker-2-augustus-gene-390.56-mRNA-1	2	OsR498G0205034100.01.T01	100,00	0,00E+00	710	Os02g0824700	100,00	0,00E+00	710	0	NA	Japonica	
maker-2-augustus-gene-390.63-mRNA-1	2	OsR498G0205037100.01.T01	96,94	0,00E+00	1654	Os02g0826200	97,25	0,00E+00	1668	14	NA	Japonica	
maker-2-augustus-gene-390.72-mRNA-1	2	OsR498G0205033600.01.T01	100,00	1,20E-89	329	Os02g0824400	100,00	1,37E-121	434	105	Japonica	Japonica	
maker-2-augustus-gene-390.78-mRNA-1	2	OsR498G0205036400.01.T01	89,25	0,00E+00	1767	Os02g0825100	100,00	0,00E+00	2663	896	Japonica	NA	
maker-2-augustus-gene-390.79-mRNA-1	2	OsR498G0205034700.01.T02	96,20	0,00E+00	2366	Os02g0825100	85,11	0,00E+00	1624	-742	Indica	Japonica	
maker-2-augustus-gene-391.0-mRNA-1	2	OsR498G0205041700.01.T05	99,79	0,00E+00	1674	Os10g0497800	77,52	2,01E-139	496	-1178	Indica	Japonica	
maker-2-augustus-gene-391.13-mRNA-1	2	OsR498G0205042100.01.T01	99,71	0,00E+00	3131	Os02g0827300	100,00	0,00E+00	3155	24	NA	Japonica	
maker-2-augustus-gene-391.19-mRNA-1	2	OsR498G0205042300.01.T01	99,89	0,00E+00	1619	Os02g0827400	100,00	0,00E+00	1624	5	NA	Japonica	
maker-2-augustus-gene-391.32-mRNA-1	2	OsR498G0205039000.01.T01	100,00	0,00E+00	2542	Os02g0826600	100,00	0,00E+00	2542	0	NA	Japonica	CC-LRR-TM
maker-2-augustus-gene-391.39-mRNA-1	2	OsR498G0205042800.01.T02	100,00	0,00E+00	645	Os02g0827600	100,00	0,00E+00	645	0	NA	Japonica	
maker-2-augustus-gene-391.43-mRNA-1	2	OsR498G0205043000.01.T06	99,78	0,00E+00	810	Os02g0827900	100,00	0,00E+00	812	2	NA	Japonica	
maker-2-augustus-gene-391.5-mRNA-1	2	OsR498G0205041900.01.T02	99,94	0,00E+00	3221	Os02g0827200	100,00	0,00E+00	3225	4	NA	Japonica	
maker-2-augustus-gene-391.54-mRNA-1	2	OsR498G0205043200.01.T01	97,65	0,00E+00	2186	Os02g0828100	97,71	0,00E+00	1873	-313	Indica	Japonica	
maker-2-augustus-gene-392.39-mRNA-1	2	OsR498G0205045700.01.T01	99,63	0,00E+00	4285	Os02g0828900	99,88	0,00E+00	4310	25	NA	Japonica	
maker-2-augustus-gene-392.45-mRNA-1	2	OsR498G0205047300.01.T01	99,91	0,00E+00	5917	Os02g0829400	100,00	0,00E+00	5930	13	NA	Japonica	
maker-2-augustus-gene-392.47-mRNA-1	2	OsR498G0205046800.01.T01	99,88	0,00E+00	1511	Os02g0829100	100,00	0,00E+00	1516	5	NA	Japonica	
maker-2-augustus-gene-392.52-mRNA-1	2	OsR498G0205047200.01.T02	97,77	0,00E+00	3472	Os02g0829200	97,24	0,00E+00	2588	-884	Indica	Japonica	
maker-2-augustus-gene-392.54-mRNA-1	2	OsR498G0205047600.01.T04	99,79	0,00E+00	849	Os02g0829500	100,00	0,00E+00	854	5	NA	Japonica	
maker-2-augustus-gene-392.59-mRNA-1	2	OsR498G0205047600.01.T01	93,50	0,00E+00	1363	Os02g0829600	100,00	0,00E+00	1932	569	Japonica	Japonica	
maker-2-augustus-gene-392.65-mRNA-1	2	OsR498G0205049000.01.T01	100,00	7,17E-131	466	Os02g0829900	100,00	2,51E-131	466	0	NA	Japonica	
maker-2-augustus-gene-392.67-mRNA-1	2	OsR498G0205049200.01.T01	99,94	0,00E+00	2923	Os02g0830100	100,00	0,00E+00	2928	5	NA	Japonica	
maker-2-augustus-gene-392.72-mRNA-1	2	OsR498G0204371000.01.T01	75,99	3,76E-67	256	Os02g0830200	100,00	0,00E+00	715	459	Japonica	Japonica	
maker-2-augustus-gene-393.10-mRNA-1	2	OsR498G0205052100.01.T02	98,85	0,00E+00	4574	Os02g0831500	99,05	0,00E+00	4443	-131	NA	Japonica	
maker-2-augustus-gene-393.15-mRNA-1	2	OsR498G0205053100.01.T01	99,76	0,00E+00	2940	Os02g0832000	100,00	0,00E+00	2955	15	NA	Japonica	
maker-2-augustus-gene-393.38-mRNA-1	2	OsR498G0205058300.01.T01	99,80	0,00E+00	1772	Os02g0833900	98,91	0,00E+00	1122	-650	Indica	Japonica	
maker-2-augustus-gene-393.41-mRNA-1	2	OsR498G0205059100.01.T01	100,00	1,30E-117	423	Os02g0834000	100,00	4,53E-118	423	0	NA	Japonica	
maker-2-augustus-gene-393.45-mRNA-1	2	OsR498G0205002300.01.T01	99,91	0,00E+00	1994	Os02g0815500	100,00	0,00E+00	1999	5	NA	NA	
maker-2-augustus-gene-393.54-mRNA-1	2	OsR498G0205051000.01.T01	99,47	0,00E+00	1677	Os02g0831200	100,00	0,00E+00	1678	1	NA	Japonica	
maker-2-augustus-gene-393.56-mRNA-1	2	OsR498G0205051200.01.T02	99,55	0,00E+00	2778	Os02g0831300	99,62	0,00E+00	2782	4	NA	Japonica	
maker-2-augustus-gene-393.60-mRNA-1	2	OsR498G0205051800.01.T01	99,23	0,00E+00	1386	Os02g0831400	100,00	0,00E+00	996	-390	Indica	Japonica	
maker-2-augustus-gene-393.7-mRNA-1	2	OsR498G0205050700.01.T01	97,05	0,00E+00	3369	Os02g0831100	100,00	0,00E+00	1965	-1404	Indica	Japonica	
maker-2-augustus-gene-393.81-mRNA-1	2	OsR498G0205056800.01.T01	99,70	0,00E+00	1810	Os02g0833400	100,00	0,00E+00	1824	14	NA	Japonica	
maker-2-augustus-gene-393.85-mRNA-1	2	OsR498G0205057600.01.T01	99,77	0,00E+00	770	Os02g0833600	100,00	0,00E+00	774	4	NA	Japonica	
maker-2-augustus-gene-393.88-mRNA-1	2	OsR498G0205060600.01.T01	99,57	0,00E+00	1235	Os02g0834300	100,00	0,00E+00	1251	16	NA	Japonica	
maker-2-augustus-gene-393.90-mRNA-1	2	OsR498G0205003000.01.T01	100,00	0,00E+00	1072	Os02g0815800	100,00	0,00E+00	1072	0	NA	NA	
maker-2-augustus-gene-4.0-mRNA-1	2	OsR498G0202818900.01.T04	100,00	0,00E+00	1972	Os02g0106700	100,00	0,00E+00	1972	0	NA	Japonica	
maker-2-augustus-gene-4.19-mRNA-1	2	OsR498G0202819600.01.T01	100,00	0,00E+00	2203	Os02g0106900	100,00	0,00E+00	2203	0	NA	NA	Kinase-TM
maker-2-augustus-gene-4.2-mRNA-1	2	OsR498G0202819300.01.T03	100,00	0,00E+00	1732	Os02g0106800	100,00	0,00E+00	1658	-74	NA	NA	
maker-2-augustus-gene-40.29-mRNA-1	2	OsR498G0203062100.01.T01	99,17	0,00E+00	1283	Os02g0172800	99,86	0,00E+00	1306	23	NA	Japonica	
maker-2-augustus-gene-40.36-mRNA-1	2	OsR498G0203065100.01.T06	98,21	0,00E+00	1643	Os02g0173500	98,21	0,00E+00	1643	0	NA	NA	
maker-2-augustus-gene-40.39-mRNA-1	2	OsR498G0203065100.01.T04	99,63	0,00E+00	1464	Os06g0665100	77,13	2,78E-162	572	-892	Indica	Japonica	
maker-2-augustus-gene-40.41-mRNA-1	2	OsR498G0203065300.01.T01	99,50	0,00E+00	2489	Os02g0173700	99,90	0,00E+00	1874	-615	Indica	Japonica	
maker-2-augustus-gene-40.48-mRNA-1	2	OsR498G0203061200.01.T02	99,57	0,00E+00	5363	Os02g0172600	98,13	0,00E+00	5277	-86	NA	NA	
maker-2-augustus-gene-40.59-mRNA-1	2	OsR498G0203065800.01.T01	99,91	0,00E+00	1895	Os02g0173800	99,72	0,00E+00	1886	-9	NA	Japonica	
maker-2-augustus-gene-41.11-mRNA-1	2	OsR498G0203068700.01.T01	99,91	0,00E+00	2014	Os02g0174300	99,03	0,00E+00	2186	172	NA	Indica	
maker-2-augustus-gene-41.16-mRNA-1	2	OsR498G0203069700.01.T02	100,00	0,00E+00	1186	Os02g0174800	99,70	0,00E+00	1177	-9	NA	NA	
maker-2-augustus-gene-41.18-mRNA-1	2	OsR498G0203070500.01.T04	100,00	0,00E+00	1505	Os02g0175100	99,69	0,00E+00	1706	201	Japonica	NA	
maker-2-augustus-gene-41.23-mRNA-1	2	OsR498G0203071300.01.T01	100,00	0,00E+00	3366	Os02g0175400	100,00	0,00E+00	3366	0	NA	NA	
maker-2-augustus-gene-41.29-mRNA-1	2	OsR498G0203071500.01.T01	100,00	0,00E+00	2116	Os02g0175500	100,00	0,00E+00	2116	0	NA	NA	
maker-2-augustus-gene-41.34-mRNA-1	2	OsR498G0203067000.01.T01	99,91	0,00E+00	2074	Os02g0174000	100,00	0,00E+00	1927	-147	NA	NA	
maker-2-augustus-gene-41.37-mRNA-1	2	OsR498G0203068200.01.T01	100,00	0,00E+00	2284	Os02g0174200	99,84	0,00E+00	2275	-9	NA	Indica	Kinase-TM
maker-2-augustus-gene-41.41-mRNA-1	2	OsR498G0203072200.01.T01	99,94	0,00E+00	3156	Os02g0175700	100,00	0,00E+00	3160	4	NA	NA	
maker-2-augustus-gene-41.43-mRNA-1	2	OsR498G0203073000.01.T01	100,00	0,00E+00	2305	Os02g0175900	99,92	0,00E+00	2301	-4	NA	NA	
maker-2-augustus-gene-41.7-mRNA-1	2	OsR498G0203067900.01.T02	100,00	0,00E+00	847	Os02g0174100	97,51	0,00E+00	820	-27	NA	Indica	
maker-2-augustus-gene-42.26-mRNA-1	2	OsR498G0203075000.01.T09	100,00	0,00E+00	2957	Os02g0176100	100,00	0,00E+00	2957	0	NA	NA	Kinase-LRR-TM
maker-2-augustus-gene-42.44-mRNA-1	2	OsR498G0203078800.01.T03	100,00	0,00E+00	1020	Os02g0176900	100,00	0,00E+00	838	-182	Indica	Japonica	
maker-2-augustus-gene-43.30-mRNA-1	2	OsR498G0203079900.01.T01	97,16	0,00E+00	2446	Os02g0177300	97,45	0,00E+00	2415	-31	NA	Japonica	
maker-2-augustus-gene-43.32-mRNA-1	2	OsR498G0203081900.01.T03	99,82	0,00E+00	2990	Os02g0177600	100,00	0,00E+00	3003	13	NA	Japonica	
maker-2-augustus-gene-43.43-mRNA-1	2	OsR498G0203084300.01.T02	99,62	0,00E+00	1876	Os02g0178100	100,00	0,00E+00	1094	-782	Indica	Japonica	
maker-2-augustus-gene-43.47-mRNA-1	2	OsR498G0203085500.01.T02	100,00	2,42E-163	574	Os02g0178400	100,00	3,59E-105	379	-195	Indica	Japonica	
maker-2-augustus-gene-43.49-mRNA-1	2	OsR498G0203080800.01.T02	99,93	0,00E+00	2609	Os02g0177400	100,00	0,00E+00	2572	-37	NA	Japonica	
maker-2-augustus-gene-43.53-mRNA-1	2	OsR498G0203081200.01.T01	100,00	1,71E-132	471	Os02g0177500	100,00	3,10E-130	462	-9	NA	Japonica	
maker-2-augustus-gene-43.58-mRNA-1	2	OsR498G0203082200.01.T01	99,32	0,00E+00	1043	Os02g0177700	100,00	0,00E+00	1061	18	NA	Japonica	
maker-2-augustus-gene-43.60-mRNA-1	2	OsR498G0203082600.01.T02	99,75	0,00E+00	3603	Os02g0177800	100,00	0,00E+00	3382	-221	NA	Japonica	
maker-2-augustus-gene-43.67-mRNA-1	2	OsR498G0203083200.01.T03	99,70	0,00E+00	2347	Os02g0178000	100,00	0,00E+00	2365	18	NA	Japonica	Kinase-TM
maker-2-augustus-gene-43.74-mRNA-1	2	OsR498G0203085800.01.T01	99,74	0,00E+00	3495	Os02g0178500	100,00	0,00E+00	3517	22	NA	Japonica	
maker-2-augustus-gene-44.0-mRNA-1	2	OsR498G0203086300.01.T01	98,91	0,00E+00	1122	Os02g0178700	100,00	0,00E+00	1142	20	NA	Japonica	
maker-2-augustus-gene-44.13-mRNA-1	2	OsR498G0203089700.01.T03	100,00	0,00E+00	1236	Os02g0179500	99,85	0,00E+00	1232	-4	NA	Indica	
maker-2-augustus-gene-44.15-mRNA-1	2	OsR498G0203086600.01.T01	97,31	0,00E+00	3295	Os02g0178800	97,52	0,00E+00	3313	18	NA	Japonica	
maker-2-augustus-gene-44.18-mRNA-1	2	OsR498G0203088200.01.T01	100,00	0,00E+00	1629	Os02g0179200	98,67	0,00E+00	1573	-56	NA	Indica	
maker-2-augustus-gene-44.24-mRNA-1	2	OsR498G0203090200.01.T01	99,05	0,00E+00	6110	Os02g0179800	99,48	0,00E+00	4078	-2032	Indica	Indica	
maker-2-augustus-gene-44.5-mRNA-1	2	OsR498G0203087700.01.T04	99,85	0,00E+00	1235	Os02g0179100	99,85	0,00E+00	1235	0	NA	Indica	
maker-2-augustus-gene-44.8-mRNA-1	2	OsR498G0203089100.01.T01	99,91	0,00E+00	1998	Os02g0179300	99,82	0,00E+00	1994	-4	NA	NA	
maker-2-augustus-gene-45.28-mRNA-1	2	OsR498G0203090900.01.T02	99,82	0,00E+00	1956	Os02g0180000	99,45	0,00E+00	1938	-18	NA	Indica	
maker-2-augustus-gene-45.39-mRNA-1	2	OsR498G0203093300.01.T01	99,87	0,00E+00	1383	Os02g0180700	99,87	0,00E+00	1383	0	NA	Indica	
maker-2-augustus-gene-45.42-mRNA-1	2	OsR498G0203096300.01.T06	99,90	0,00E+00	1884	Os02g0181300	99,90	0,00E+00	1884	0	NA	NA	
maker-2-augustus-gene-45.44-mRNA-1	2	OsR498G0203091200.01.T03	99,90	0,00E+00	1845	Os02g0180100	99,80	0,00E+00	1777	-68	NA	Indica	
maker-2-augustus-gene-45.48-mRNA-1	2	OsR498G0203092800.01.T01	100,00	0,00E+00	1386	Os02g0180500	100,00	0,00E+00	1386	0	NA	Indica	
maker-2-augustus-gene-46.29-mRNA-1	2	OsR498G0203098300.01.T01	99,68	0,00E+00	1691	Os02g0181800	99,58	0,00E+00	1687	-4	NA	NA	
maker-2-augustus-gene-46.34-mRNA-1	2	OsR498G0203102700.01.T01	99,87	0,00E+00	1349	Os02g0182700	99,73	0,00E+00	1344	-5	NA	Indica	
maker-2-augustus-gene-46.38-mRNA-1	2	OsR498G0203103100.01.T02	100,00	0,00E+00	1700	Os02g0182800	99,84	0,00E+00	1149	-551	Indica	Indica	
maker-2-augustus-gene-46.44-mRNA-1	2	OsR498G0203099000.01.T01	99,79	0,00E+00	3375	Os02g0182100	99,84	0,00E+00	3379	4	NA	Indica	
maker-2-augustus-gene-46.47-mRNA-1	2	OsR498G0203101400.01.T01	100,00	0,00E+00	1101	Os06g0643100	87,83	0,00E+00	764	-337	Indica	Indica	
maker-2-augustus-gene-46.59-mRNA-1	2	OsR498G0203103600.01.T05	100,00	0,00E+00	796	Os02g0182900	99,74	0,00E+00	701	-95	Indica	NA	
maker-2-augustus-gene-47.36-mRNA-1	2	OsR498G0203106300.01.T01	99,23	0,00E+00	913	Os02g0184100	99,23	0,00E+00	913	0	NA	Indica	
maker-2-augustus-gene-47.38-mRNA-1	2	OsR498G0203107300.01.T02	99,91	0,00E+00	4120	Os02g0184400	99,51	0,00E+00	4346	226	NA	Indica	
maker-2-augustus-gene-47.44-mRNA-1	2	OsR498G0203107900.01.T01	99,88	0,00E+00	1511	Os02g0184500	99,69	0,00E+00	1144	-367	Indica	Indica	
maker-2-augustus-gene-47.47-mRNA-1	2	OsR498G0203104200.01.T01	100,00	0,00E+00	1274	Os02g0183000	100,00	0,00E+00	1261	-13	NA	Indica	
maker-2-augustus-gene-47.58-mRNA-1	2	OsR498G0203106000.01.T01	99,84	0,00E+00	2242	Os02g0183900	99,26	0,00E+00	1200	-1042	Indica	Indica	
maker-2-augustus-gene-47.61-mRNA-1	2	OsR498G0203106200.01.T03	96,70	0,00E+00	2034	Os02g0184000	97,34	0,00E+00	2050	16	NA	Indica	
maker-2-augustus-gene-47.66-mRNA-1	2	OsR498G0203106600.01.T02	100,00	0,00E+00	4172	Os06g0644200	86,81	0,00E+00	2791	-1381	Indica	Indica	
maker-2-augustus-gene-47.72-mRNA-1	2	OsR498G0203112700.01.T01	74,32	0,00E+00	966	Os02g0184600	97,36	0,00E+00	2632	1666	Japonica	Indica	
maker-2-augustus-gene-47.74-mRNA-1	2	OsR498G0203108600.01.T01	99,81	0,00E+00	2801	Os02g0184700	99,74	0,00E+00	2796	-5	NA	Indica	
maker-2-augustus-gene-48.49-mRNA-1	2	OsR498G0203111900.01.T01	99,81	0,00E+00	2817	Os02g0185200	99,61	0,00E+00	2760	-57	NA	Indica	
maker-2-augustus-gene-48.52-mRNA-1	2	OsR498G0203112700.01.T01	99,68	0,00E+00	2802	Os02g0185300	99,55	0,00E+00	2793	-9	NA	NA	
maker-2-augustus-gene-48.54-mRNA-1	2	OsR498G0203112900.01.T02	99,68	0,00E+00	1666	Os02g0184900	91,07	0,00E+00	1157	-509	Indica	Indica	
maker-2-augustus-gene-48.57-mRNA-1	2	OsR498G0203113300.01.T01	99,87	0,00E+00	2783	Os02g0185500	98,27	0,00E+00	1997	-786	Indica	Indica	
maker-2-augustus-gene-48.59-mRNA-1	2	OsR498G0203113700.01.T01	99,48	0,00E+00	2763	Os02g0186100	94,36	0,00E+00	1266	-1497	Indica	Indica	
maker-2-augustus-gene-48.61-mRNA-1	2	OsR498G0203113700.01.T01	92,52	0,00E+00	2276	Os02g0186100	98,53	0,00E+00	1419	-857	Indica	NA	
maker-2-augustus-gene-48.62-mRNA-1	2	OsR498G0203113900.01.T01	84,20	0,00E+00	1435	Os02g0184700	75,74	0,00E+00	1011	-424	Indica	Indica	
maker-2-augustus-gene-48.67-mRNA-1	2	OsR498G0203115400.01.T01	99,87	0,00E+00	4121	Os02g0186400	99,78	0,00E+00	4112	-9	NA	NA	
maker-2-augustus-gene-48.69-mRNA-1	2	OsR498G0203116200.01.T02	99,82	0,00E+00	1990	Os02g0186500	99,82	0,00E+00	1990	0	NA	NA	Kinase
maker-2-augustus-gene-49.32-mRNA-1	2	OsR498G0203116700.01.T02	99,87	0,00E+00	1425	Os02g0186700	99,83	0,00E+00	1080	-345	Indica	Indica	
maker-2-augustus-gene-49.35-mRNA-1	2	OsR498G0203116900.01.T01	99,94	0,00E+00	2755	Os02g0186800	99,74	0,00E+00	2742	-13	NA	Indica	
maker-2-augustus-gene-49.37-mRNA-1	2	OsR498G0203117100.01.T01	99,94	0,00E+00	2831	Os02g0186900	99,47	0,00E+00	1663	-1168	Indica	Indica	
maker-2-augustus-gene-49.39-mRNA-1	2	OsR498G0203117300.01.T01	99,94	0,00E+00	2831	Os02g0187000	99,87	0,00E+00	2827	-4	NA	Indica	
maker-2-augustus-gene-49.45-mRNA-1	2	OsR498G0203119500.01.T01	99,88	0,00E+00	1473	Os02g0187600	100,00	0,00E+00	1478	5	NA	Indica	
maker-2-augustus-gene-49.48-mRNA-1	2	OsR498G0203118200.01.T01	95,27	0,00E+00	2064	Os02g0187200	95,11	0,00E+00	2055	-9	NA	Indica	
maker-2-augustus-gene-49.57-mRNA-1	2	OsR498G0203118600.01.T01	99,96	0,00E+00	4151	Os02g0187400	100,00	0,00E+00	3672	-479	Indica	NA	
maker-2-augustus-gene-49.60-mRNA-1	2	OsR498G0203119400.01.T01	100,00	0,00E+00	785	Os02g0187500	99,54	0,00E+00	776	-9	NA	Indica	
maker-2-augustus-gene-49.74-mRNA-1	2	OsR498G0203124200.01.T01	100,00	0,00E+00	1498	Os02g0188500	100,00	0,00E+00	1498	0	NA	Indica	
maker-2-augustus-gene-5.0-mRNA-1	2	OsR498G0202827500.01.T04	100,00	1,99E-154	545	Os02g0108700	95,03	0,00E+00	868	323	Japonica	NA	
maker-2-augustus-gene-5.10-mRNA-1	2	OsR498G0202829400.01.T01	100,00	0,00E+00	5243	Os02g0109400	100,00	0,00E+00	2621	-2622	Indica	Japonica	
maker-2-augustus-gene-5.14-mRNA-1	2	OsR498G0202831000.01.T01	99,94	0,00E+00	3242	Os02g0109800	100,00	0,00E+00	3247	5	NA	Japonica	
maker-2-augustus-gene-5.16-mRNA-1	2	OsR498G0202831400.01.T01	100,00	0,00E+00	2457	Os02g0110000	100,00	0,00E+00	2457	0	NA	NA	
maker-2-augustus-gene-50.38-mRNA-1	2	OsR498G0203127100.01.T02	99,46	0,00E+00	1990	Os02g0189300	99,91	0,00E+00	1971	-19	NA	Indica	
maker-2-augustus-gene-50.43-mRNA-1	2	OsR498G0203126400.01.T01	100,00	0,00E+00	796	Os02g0189000	99,77	0,00E+00	792	-4	NA	Indica	
maker-2-augustus-gene-50.45-mRNA-1	2	OsR498G0203130400.01.T02	90,48	0,00E+00	3088	Os02g0189800	100,00	0,00E+00	4053	965	Japonica	NA	
maker-2-augustus-gene-50.47-mRNA-1	2	OsR498G0203129800.01.T01	99,97	0,00E+00	6726	Os02g0190000	100,00	0,00E+00	4053	-2673	Indica	NA	
maker-2-augustus-gene-50.55-mRNA-1	2	OsR498G0203130400.01.T02	99,37	0,00E+00	7038	Os02g0190000	96,75	0,00E+00	3724	-3314	Indica	Japonica	
maker-2-augustus-gene-51.0-mRNA-1	2	OsR498G0203132100.01.T01	99,57	0,00E+00	3328	Os02g0190500	100,00	0,00E+00	3364	36	NA	Japonica	Kinase-LRR-TM
maker-2-augustus-gene-51.10-mRNA-1	2	OsR498G0203133000.01.T02	99,88	0,00E+00	1470	Os02g0190800	100,00	0,00E+00	1474	4	NA	Japonica	

maker-2-augustus-gene-51.14-mRNA-1	2	OsR498G0203133400.01.T01	99,04	0,00E+00	912	Os02g0190900	100,00	0,00E+00	661	-251	Indica	Japonica	
maker-2-augustus-gene-51.16-mRNA-1	2	OsR498G0203134400.01.T04	99,93	0,00E+00	2485	Os02g0191300	100,00	0,00E+00	2489	4	NA	Japonica	
maker-2-augustus-gene-51.19-mRNA-1	2	OsR498G0203135000.01.T02	99,04	0,00E+00	2927	Os10g0476600	74,74	0,00E+00	822	-2105	Indica	Japonica	
maker-2-augustus-gene-51.6-mRNA-1	2	OsR498G0203134500.01.T01	99,65	0,00E+00	2017	Os02g0191500	100,00	0,00E+00	2035	18	NA	Japonica	
maker-2-augustus-gene-51.8-mRNA-1	2	OsR498G0203132800.01.T01	100,00	0,00E+00	666	Os02g0190700	100,00	0,00E+00	666	0	NA	Japonica	
maker-2-augustus-gene-52.34-mRNA-1	2	OsR498G0203137600.01.T01	97,81	0,00E+00	1490	Os02g0192100	100,00	0,00E+00	1330	-160	Indica	Japonica	
maker-2-augustus-gene-52.36-mRNA-1	2	OsR498G0203138600.01.T01	99,62	0,00E+00	2798	Os02g0192500	100,00	0,00E+00	2825	27	NA	Japonica	
maker-2-augustus-gene-53.28-mRNA-1	2	OsR498G0203142300.01.T01	99,09	0,00E+00	2130	Os02g0193600	100,00	0,00E+00	2159	29	NA	Japonica	
maker-2-augustus-gene-53.38-mRNA-1	2	OsR498G0203144300.01.T01	100,00	0,00E+00	1629	Os02g0194200	100,00	0,00E+00	1507	-122	NA	Japonica	
maker-2-augustus-gene-53.43-mRNA-1	2	OsR498G0203145700.01.T02	97,64	0,00E+00	3916	Os02g0194600	99,83	0,00E+00	4094	178	NA	Japonica	Kinase-TM
maker-2-augustus-gene-53.49-mRNA-1	2	OsR498G0203144000.01.T04	99,79	0,00E+00	1682	Os02g0194100	100,00	0,00E+00	1691	9	NA	Japonica	
maker-2-augustus-gene-53.57-mRNA-1	2	OsR498G0203144600.01.T01	99,72	0,00E+00	1940	Os02g0194300	100,00	0,00E+00	1003	-937	Indica	Japonica	
maker-2-augustus-gene-53.60-mRNA-1	2	OsR498G0203145000.01.T01	99,77	0,00E+00	7065	Os01g0917500	71,54	0,00E+00	1989	-5076	Indica	Japonica	Kinase-LRR-TM
maker-2-augustus-gene-53.61-mRNA-1	2	OsR498G0203146000.01.T01	99,57	0,00E+00	4962	Os02g0194700	100,00	0,00E+00	5016	54	NA	Japonica	
maker-2-augustus-gene-53.64-mRNA-1	2	OsR498G0203146600.01.T03	99,54	0,00E+00	3103	Os02g0194800	100,00	0,00E+00	3139	36	NA	Japonica	
maker-2-augustus-gene-54.42-mRNA-1	2	OsR498G0203148300.01.T03	99,66	0,00E+00	4786	Os02g0195500	99,96	0,00E+00	4822	36	NA	Japonica	
maker-2-augustus-gene-54.47-mRNA-1	2	OsR498G0203148900.01.T01	99,88	0,00E+00	1527	Os02g0195800	100,00	0,00E+00	1532	5	NA	Japonica	
maker-2-augustus-gene-54.50-mRNA-1	2	OsR498G0203149300.01.T02	99,60	0,00E+00	897	Os02g0196000	100,00	0,00E+00	906	9	NA	Japonica	
maker-2-augustus-gene-54.56-mRNA-1	2	OsR498G0203147400.01.T01	99,73	0,00E+00	1973	Os02g0195000	100,00	0,00E+00	1986	13	NA	Japonica	
maker-2-augustus-gene-54.65-mRNA-1	2	OsR498G0203150600.01.T02	99,85	0,00E+00	3590	Os02g0196300	100,00	0,00E+00	2782	-808	Indica	Japonica	
maker-2-augustus-gene-55.31-mRNA-1	2	OsR498G0203152500.01.T04	100,00	0,00E+00	3584	Os02g0196600	100,00	0,00E+00	3584	0	NA	NA	
maker-2-augustus-gene-55.35-mRNA-1	2	OsR498G0203152700.01.T01	99,92	0,00E+00	2285	Os02g0196700	100,00	0,00E+00	2435	150	NA	NA	
maker-2-augustus-gene-55.37-mRNA-1	2	OsR498G0203154300.01.T02	100,00	0,00E+00	2516	Os02g0197200	99,90	0,00E+00	1821	-695	Indica	Indica	
maker-2-augustus-gene-55.50-mRNA-1	2	OsR498G0203158500.01.T02	100,00	0,00E+00	1472	Os02g0198200	99,71	0,00E+00	1231	-241	Indica	Indica	
maker-2-augustus-gene-55.54-mRNA-1	2	OsR498G0203158700.01.T04	100,00	0,00E+00	1737	Os02g0198300	99,79	0,00E+00	1728	-9	NA	Indica	
maker-2-augustus-gene-55.58-mRNA-1	2	OsR498G0203153000.01.T01	99,85	0,00E+00	2318	Os02g0196800	100,00	0,00E+00	2327	9	NA	Japonica	
maker-2-augustus-gene-55.62-mRNA-1	2	OsR498G0203153400.01.T01	100,00	0,00E+00	1364	Os02g0196900	100,00	0,00E+00	1299	-65	NA	Indica	
maker-2-augustus-gene-55.77-mRNA-1	2	OsR498G0203156800.01.T01	100,00	0,00E+00	1297	Os02g0197700	100,00	0,00E+00	1297	0	NA	Indica	
maker-2-augustus-gene-55.81-mRNA-1	2	OsR498G0203157400.01.T02	99,03	0,00E+00	907	Os02g0197800	100,00	0,00E+00	769	-138	Indica	Indica	
maker-2-augustus-gene-56.0-mRNA-1	2	OsR498G0203160100.01.T05	100,00	0,00E+00	2251	Os02g0198600	100,00	0,00E+00	2251	0	NA	Indica	
maker-2-augustus-gene-56.19-mRNA-1	2	OsR498G0203160400.01.T01	100,00	0,00E+00	4329	Os02g0198700	99,96	0,00E+00	4324	-5	NA	Indica	
maker-2-augustus-gene-56.23-mRNA-1	2	OsR498G0203163200.01.T01	99,84	0,00E+00	1143	Os02g0199300	100,00	0,00E+00	1148	5	NA	NA	
maker-2-augustus-gene-56.29-mRNA-1	2	OsR498G0203165000.01.T01	100,00	0,00E+00	6001	Os02g0199800	100,00	0,00E+00	6001	0	NA	NA	
maker-2-augustus-gene-56.33-mRNA-1	2	OsR498G0203165400.01.T01	100,00	0,00E+00	2170	Os02g0199900	100,00	0,00E+00	2170	0	NA	NA	
maker-2-augustus-gene-56.6-mRNA-1	2	OsR498G0203163700.01.T01	100,00	0,00E+00	2365	Os02g0199500	100,00	0,00E+00	2365	0	NA	NA	
maker-2-augustus-gene-57.32-mRNA-1	2	OsR498G0203167900.01.T02	99,92	0,00E+00	2139	Os02g0200800	100,00	0,00E+00	1813	-326	Indica	NA	
maker-2-augustus-gene-57.38-mRNA-1	2	OsR498G0203173700.01.T01	99,17	0,00E+00	1490	Os02g0202200	100,00	0,00E+00	1521	31	NA	Japonica	
maker-2-augustus-gene-57.40-mRNA-1	2	OsR498G0203166800.01.T01	100,00	0,00E+00	2300	Os02g0200300	99,06	0,00E+00	2252	-48	NA	NA	
maker-2-augustus-gene-57.44-mRNA-1	2	OsR498G0203168200.01.T04	100,00	0,00E+00	3577	Os02g0200900	100,00	0,00E+00	2497	-1080	Indica	NA	
maker-2-augustus-gene-57.48-mRNA-1	2	OsR498G0203171000.01.T03	99,04	0,00E+00	2758	Os06g0604500	82,90	0,00E+00	1595	-1163	Indica	Japonica	
maker-2-augustus-gene-58.28-mRNA-1	2	OsR498G0203174600.01.T01	99,77	0,00E+00	786	Os06g0602600	87,81	9,77E-127	452	-334	Indica	Japonica	
maker-2-augustus-gene-58.32-mRNA-1	2	OsR498G0203175200.01.T01	99,65	0,00E+00	1535	Os02g0202600	100,00	0,00E+00	1548	13	NA	Japonica	
maker-2-augustus-gene-58.39-mRNA-1	2	OsR498G0203176000.01.T01	99,70	0,00E+00	2347	Os02g0202900	100,00	0,00E+00	2365	18	NA	Japonica	
maker-2-augustus-gene-58.42-mRNA-1	2	OsR498G0203179000.01.T01	99,52	0,00E+00	5569	Os02g0203500	100,00	0,00E+00	5838	269	NA	Japonica	CC-NBS-TM
maker-2-augustus-gene-58.45-mRNA-1	2	OsR498G0203179800.01.T03	99,60	0,00E+00	895	Os02g0203700	99,80	0,00E+00	900	5	NA	Japonica	
maker-2-augustus-gene-59.21-mRNA-1	2	OsR498G0203181500.01.T01	97,74	0,00E+00	2884	Os02g0204400	98,40	0,00E+00	2934	50	NA	Japonica	
maker-2-augustus-gene-59.29-mRNA-1	2	OsR498G0203183800.01.T01	99,06	0,00E+00	2430	Os02g0204700	100,00	0,00E+00	2439	9	NA	Japonica	
maker-2-augustus-gene-6.35-mRNA-1	2	OsR498G0202834900.01.T01	99,27	0,00E+00	1447	Os01g0580100	90,99	0,00E+00	1149	-298	Indica	Japonica	
maker-2-augustus-gene-6.41-mRNA-1	2	OsR498G0202835300.01.T01	99,65	9,30E-144	510	Os02g0111700	100,00	7,60E-146	515	5	NA	Japonica	
maker-2-augustus-gene-6.52-mRNA-1	2	OsR498G0202835200.01.T02	99,05	0,00E+00	3316	Os02g0111600	100,00	0,00E+00	3993	677	Japonica	Japonica	Kinase-TM
maker-2-augustus-gene-6.55-mRNA-1	2	OsR498G0202836400.01.T01	99,68	0,00E+00	1112	Os02g0112000	100,00	0,00E+00	682	-430	Indica	Japonica	
maker-2-augustus-gene-60.0-mRNA-1	2	OsR498G0203187500.01.T01	99,90	0,00E+00	1852	Os02g0205400	100,00	0,00E+00	1856	4	NA	Japonica	
maker-2-augustus-gene-60.15-mRNA-1	2	OsR498G0203188000.01.T01	100,00	0,00E+00	2372	Os02g0205500	100,00	0,00E+00	2369	-3	NA	Japonica	
maker-2-augustus-gene-60.2-mRNA-1	2	OsR498G0203186200.01.T01	99,70	0,00E+00	1198	Os02g0205000	100,00	0,00E+00	1207	9	NA	Japonica	
maker-2-augustus-gene-60.6-mRNA-1	2	OsR498G0203186600.01.T02	99,83	0,00E+00	2048	Os02g0205200	100,00	0,00E+00	1900	-148	NA	Japonica	
maker-2-augustus-gene-60.9-mRNA-1	2	OsR498G0203187400.01.T01	99,84	0,00E+00	2291	Os02g0205300	99,92	0,00E+00	2296	5	NA	Japonica	
maker-2-augustus-gene-61.25-mRNA-1	2	OsR498G0203194900.01.T01	99,68	0,00E+00	3388	Os01g0647100	78,79	0,00E+00	1270	-2118	Indica	Japonica	
maker-2-augustus-gene-61.34-mRNA-1	2	OsR498G0203195500.01.T02	99,75	0,00E+00	7769	Os01g0609300	70,42	0,00E+00	1893	-5876	Indica	Japonica	
maker-2-augustus-gene-61.39-mRNA-1	2	OsR498G0203196500.01.T01	99,58	0,00E+00	5946	Os02g0208600	100,00	0,00E+00	4940	-1006	Indica	Japonica	
maker-2-augustus-gene-62.0-mRNA-1	2	OsR498G0203199300.01.T05	98,86	0,00E+00	3844	Os02g0208900	100,00	0,00E+00	3945	101	NA	Japonica	
maker-2-augustus-gene-62.14-mRNA-1	2	OsR498G0203199800.01.T03	99,63	0,00E+00	2917	Os06g0143900	79,84	0,00E+00	1461	-1456	Indica	Japonica	
maker-2-augustus-gene-62.15-mRNA-1	2	OsR498G0203200200.01.T03	99,82	0,00E+00	2929	Os06g0143900	79,71	0,00E+00	1450	-1479	Indica	Japonica	
maker-2-augustus-gene-62.44-mRNA-1	2	OsR498G0203201200.01.T01	97,65	0,00E+00	1593	Os02g0209900	100,00	0,00E+00	1689	96	NA	Japonica	
maker-2-augustus-gene-62.5-mRNA-1	2	OsR498G0203202700.01.T01	99,85	0,00E+00	6173	Os02g0210700	100,00	0,00E+00	6195	22	NA	Japonica	Kinase-LRR-TM
maker-2-augustus-gene-63.11-mRNA-1	2	OsR498G0203211700.01.T06	100,00	0,00E+00	1018	Os02g0212600	100,00	0,00E+00	1256	238	Japonica	Japonica	
maker-2-augustus-gene-63.3-mRNA-1	2	OsR498G0202823400.01.T01	90,11	1,66E-104	380	Os02g0211100	99,25	4,15E-132	470	90	Japonica	Japonica	
maker-2-augustus-gene-64.42-mRNA-1	2	OsR498G0203211700.01.T06	97,87	1,93E-86	318	Os02g0213400	100,00	8,77E-92	334	16	NA	NA	
maker-2-augustus-gene-64.50-mRNA-1	2	OsR498G0202840400.01.T01	91,06	0,00E+00	680	Os09g0120033	87,87	5,41E-175	612	-68	Indica	NA	
maker-2-augustus-gene-65.36-mRNA-1	2	OsR498G0203216700.01.T02	100,00	0,00E+00	1640	Os02g0214300	100,00	0,00E+00	1640	0	NA	Japonica	
maker-2-augustus-gene-65.39-mRNA-1	2	OsR498G0203217100.01.T01	98,95	0,00E+00	1333	Os02g0214500	100,00	0,00E+00	1370	37	NA	Japonica	
maker-2-augustus-gene-65.41-mRNA-1	2	OsR498G0203218700.01.T02	97,64	0,00E+00	2589	Os02g0214900	97,38	0,00E+00	2571	-18	NA	Japonica	
maker-2-augustus-gene-65.48-mRNA-1	2	OsR498G0203219100.01.T01	93,48	0,00E+00	2408	Os02g0215500	99,94	0,00E+00	2873	465	Japonica	Japonica	Kinase-LRR-TM
maker-2-augustus-gene-65.54-mRNA-1	2	OsR498G0203217600.01.T02	98,29	0,00E+00	2517	Os02g0214900	100,00	0,00E+00	2630	113	NA	Japonica	
maker-2-augustus-gene-66.46-mRNA-1	2	OsR498G0203223700.01.T01	99,35	0,00E+00	2449	Os02g0217400	82,33	0,00E+00	1521	-928	Indica	Japonica	
maker-2-augustus-gene-66.48-mRNA-1	2	OsR498G0203224500.01.T01	99,41	0,00E+00	2714	Os02g0217400	99,94	0,00E+00	2750	36	NA	Japonica	
maker-2-augustus-gene-66.50-mRNA-1	2	OsR498G0203225100.01.T01	98,77	0,00E+00	2278	Os02g0217500	99,08	0,00E+00	2296	18	NA	Japonica	
maker-2-augustus-gene-66.58-mRNA-1	2	OsR498G0203219100.01.T01	71,85	0,00E+00	1715	Os02g0215700	100,00	0,00E+00	5211	3496	Japonica	Japonica	Kinase-LRR-TM
maker-2-augustus-gene-66.67-mRNA-1	2	OsR498G0202837900.01.T01	92,74	0,00E+00	719	Os03g0233200	93,00	6,78E-175	613	-106	Indica	Indica	
maker-2-augustus-gene-66.69-mRNA-1	2	OsR498G0203225400.01.T01	99,89	0,00E+00	1663	Os02g0217600	100,00	0,00E+00	1667	4	NA	Japonica	
maker-2-augustus-gene-67.40-mRNA-1	2	OsR498G0203226300.01.T06	99,77	0,00E+00	3863	Os02g0218200	99,95	0,00E+00	3881	18	NA	Japonica	
maker-2-augustus-gene-67.47-mRNA-1	2	OsR498G0203227300.01.T01	99,95	0,00E+00	3852	Os02g0218600	100,00	0,00E+00	2486	-1366	Indica	Japonica	CC-Kinase-TM
maker-2-augustus-gene-67.52-mRNA-1	2	OsR498G0203230900.01.T01	100,00	0,00E+00	1467	Os02g0219700	100,00	0,00E+00	1467	0	NA	Japonica	
maker-2-augustus-gene-67.58-mRNA-1	2	OsR498G0203227000.01.T01	99,75	0,00E+00	4259	Os02g0218400	100,00	0,00E+00	3956	-303	NA	Japonica	CC-Kinase-TM
maker-2-augustus-gene-67.61-mRNA-1	2	OsR498G0203229800.01.T01	99,86	0,00E+00	3937	Os02g0219200	100,00	0,00E+00	2711	-1226	Indica	Japonica	
maker-2-augustus-gene-67.69-mRNA-1	2	OsR498G0203230600.01.T02	100,00	0,00E+00	2123	Os02g0219400	100,00	0,00E+00	2123	0	NA	Japonica	
maker-2-augustus-gene-68.0-mRNA-1	2	OsR498G0203233300.01.T01	99,84	0,00E+00	2221	Os02g0220500	99,92	0,00E+00	2225	4	NA	Japonica	
maker-2-augustus-gene-68.1-mRNA-1	2	OsR498G0203233300.01.T08	99,60	0,00E+00	2232	Os02g0220600	100,00	0,00E+00	2255	23	NA	Japonica	
maker-2-augustus-gene-68.14-mRNA-1	2	OsR498G0203233000.01.T01	97,36	0,00E+00	1805	Os02g0220400	100,00	0,00E+00	1916	111	NA	Japonica	
maker-2-augustus-gene-68.23-mRNA-1	2	OsR498G0203235600.01.T01	99,05	0,00E+00	2399	Os02g0221100	98,97	0,00E+00	3415	1016	Japonica	Japonica	
maker-2-augustus-gene-69.24-mRNA-1	2	OsR498G0203236700.01.T02	99,02	0,00E+00	3238	Os02g0221300	98,04	0,00E+00	2644	-594	Indica	NA	
maker-2-augustus-gene-69.27-mRNA-1	2	OsR498G0203236700.01.T02	98,96	0,00E+00	4709	Os02g0221300	98,23	0,00E+00	2947	-1762	Indica	NA	
maker-2-augustus-gene-69.35-mRNA-1	2	OsR498G0203237700.01.T02	99,39	0,00E+00	2045	Os02g0221800	100,00	0,00E+00	2078	33	NA	Japonica	
maker-2-augustus-gene-69.46-mRNA-1	2	OsR498G0203238000.01.T02	98,67	0,00E+00	2879	Os02g0221900	100,00	0,00E+00	2971	92	NA	Japonica	
maker-2-augustus-gene-7.12-mRNA-1	2	OsR498G0202840900.01.T02	99,93	0,00E+00	2636	Os02g0113800	100,00	0,00E+00	2641	5	NA	Japonica	
maker-2-augustus-gene-7.14-mRNA-1	2	OsR498G0202839700.01.T01	84,86	0,00E+00	1643	Os02g0113900	100,00	0,00E+00	2738	1095	Japonica	NA	
maker-2-augustus-gene-7.16-mRNA-1	2	OsR498G0202838200.01.T01	99,91	0,00E+00	2000	Os02g0112900	100,00	0,00E+00	2004	4	NA	Japonica	
maker-2-augustus-gene-7.19-mRNA-1	2	OsR498G0202840400.01.T01	99,54	0,00E+00	774	Os09g0120033	94,24	0,00E+00	671	-103	Indica	Japonica	
maker-2-augustus-gene-7.3-mRNA-1	2	OsR498G0202839100.01.T01	96,45	0,00E+00	2442	Os02g0113200	100,00	0,00E+00	2852	410	Japonica	Japonica	
maker-2-augustus-gene-7.6-mRNA-1	2	OsR498G0202839700.01.T01	97,68	0,00E+00	2493	Os02g0113300	100,00	0,00E+00	2625	132	NA	Japonica	
maker-2-augustus-gene-7.8-mRNA-1	2	OsR498G0202839900.01.T01	99,83	0,00E+00	2058	Os02g0113400	100,00	0,00E+00	2803	745	Japonica	Japonica	
maker-2-augustus-gene-71.0-mRNA-1	2	OsR498G0203249500.01.T02	99,95	0,00E+00	3783	Os02g0224300	100,00	0,00E+00	2114	-1669	Indica	Japonica	
maker-2-augustus-gene-71.12-mRNA-1	2	OsR498G0203248800.01.T04	99,89	0,00E+00	1627	Os02g0224200	100,00	0,00E+00	1629	2	NA	Japonica	
maker-2-augustus-gene-71.7-mRNA-1	2	OsR498G0203254500.01.T01	99,81	0,00E+00	1880	Os02g0224800	100,00	0,00E+00	1889	9	NA	Japonica	
maker-2-augustus-gene-72.0-mRNA-1	2	OsR498G0203254900.01.T01	98,99	0,00E+00	1920	Os09g0508900	98,55	0,00E+00	1920	0	NA	Japonica	
maker-2-augustus-gene-72.10-mRNA-1	2	OsR498G0203259700.01.T01	100,00	0,00E+00	1608	Os02g0226200	100,00	0,00E+00	1608	0	NA	Japonica	
maker-2-augustus-gene-72.16-mRNA-1	2	OsR498G0203260900.01.T03	100,00	0,00E+00	2758	Os02g0226600	99,80	0,00E+00	1799	-959	Indica	NA	
maker-2-augustus-gene-72.22-mRNA-1	2	OsR498G0203256200.01.T01	99,83	0,00E+00	1078	Os02g0225300	93,97	0,00E+00	811	-267	Indica	Japonica	
maker-2-augustus-gene-72.25-mRNA-1	2	OsR498G0203258800.01.T01	99,36	0,00E+00	1101	Os02g0226000	100,00	0,00E+00	1115	14	NA	Japonica	
maker-2-augustus-gene-73.0-mRNA-1	2	OsR498G0203262700.01.T05	100,00	0,00E+00	1278	Os02g0226900	100,00	0,00E+00	1278	0	NA	NA	
maker-2-augustus-gene-73.12-mRNA-1	2	OsR498G0203263200.01.T03	100,00	0,00E+00	1462	Os02g0227100	100,00	0,00E+00	1083	-379	Indica	NA	
maker-2-augustus-gene-73.14-mRNA-1	2	OsR498G0203263400.01.T01	98,71	0,00E+00	813	Os02g0227200	100,00	5,75E-149	526	-287	Indica	NA	
maker-2-augustus-gene-73.2-mRNA-1	2	OsR498G0203262900.01.T04	98,29	0,00E+00	1830	Os02g0227000	99,32	0,00E+00	1554	-276	Indica	NA	
maker-2-augustus-gene-73.8-mRNA-1	2	OsR498G0203263700.01.T01	99,51	0,00E+00	3656	Os02g0227400	98,58	0,00E+00	3189	-467	Indica	NA	
maker-2-augustus-gene-74.39-mRNA-1	2	OsR498G0203274700.01.T03	99,61	0,00E+00	924	Os02g0230100	100,00	0,00E+00	933	9	NA	Japonica	LRR-TM
maker-2-augustus-gene-74.43-mRNA-1	2	OsR498G0203268600.01.T02	90,23	0,00E+00	4182	Os02g0228300	90,09	0,00E+00	4164	-18	NA	NA	Kinase-LRR-TM
maker-2-augustus-gene-74.45-mRNA-1	2	OsR498G0203270600.01.T01	99,56	0,00E+00	1611	Os02g0228900	100,00	0,00E+00	1629	18	NA	Japonica	
maker-2-augustus-gene-74.47-mRNA-1	2	OsR498G0203271400.01.T01	100,00	0,00E+00	753	Os02g0229000	100,00	0,00E+00	753	0	NA	Japonica	
maker-2-augustus-gene-74.50-mRNA-1	2	OsR498G0203272800.01.T05	99,81	0,00E+00	3712	Os02g0229600	98,78	0,00E+00	4876	1164	Japonica	Japonica	
maker-2-augustus-gene-74.59-mRNA-1	2	OsR498G0203274600.01.T03	100,00	0,00E+00	829	Os02g0230000	100,00	2,58E-134	477	-352	Indica	Japonica	
maker-2-augustus-gene-75.16-mRNA-1	2	OsR498G0203275400.01.T01	99,85	0,00E+00	1187	Os02g0230300	100,00	0,00E+00	1191	4	NA	Japonica	
maker-2-augustus-gene-76.0-mRNA-1	2	OsR498G0203284300.01.T01	98,78	0,00E+00	1577	Os02g0232000	100,00	0,00E+00	1618	41	NA	Japonica	
maker-2-augustus-gene-76.2-mRNA-1	2	OsR498G0203285700.01.T01	99,64	0,00E+00	998	Os06g0157500	79,28	9,10E-78	290	-708	Indica	Japonica	
maker-2-augustus-gene-76.20-mRNA-1	2	OsR498G0203282600.01.T01	99,87	0,00E+00	5420	Os02g0231700	100,00	0,00E+00	5597	177	NA	Japonica	Kinase-LRR-TM
maker-2-augustus-gene-76.22-mRNA-1	2	OsR498G0203284600.01.T02	100,00	0,00E+00	1645	Os02g0232100	100,00	0,00E+00	1645	0	NA	Japonica	
maker-2-augustus-gene-76.25-mRNA-1	2	OsR498G0203286800.01.T02	100,00	0,00E+00	1543	Os02g0232900	99,88	0,00E+00	1538	-5	NA	Japonica	
maker-2-augustus-gene-76.4-mRNA-1	2	OsR498G0203286100.01.T01	99,87	0,00E+00	2702	Os02g0232400	99,75	0,00E+00	2904	202	NA	Japonica	
maker-2-augustus-gene-77.25-mRNA-1	2	OsR498G0203288500.01.T01	99,57	0,00E+00	2869	Os02g0233100	99,94	0,00E+00	2896	27	NA	Japonica	
maker-2-augustus-gene-77.29-mRNA-1	2	OsR498G0203290500.01.T01	99,93	0,00E+00	2669	Os02g0234200	100,00	0,00E+00	2673	4	NA	Japonica	
maker-2-augustus-gene-77.32-mRNA-1	2	OsR498G0203288800.01.T01	99,72	0,00E+00	1895	Os02g0233200	99,88	0,00E+00	1500	-395	Indica	Japonica	
maker-2-augustus-gene-78.18-mRNA-1	2	OsR498G0203297900.01.T01	99,59	0,00E+00	3040	Os02g0235900	100,00	0,00E+00	2203	-837	Indica	Japonica	
maker-2-augustus-gene-78.19-mRNA-1	2	OsR498G0203299500.01.T02	99,77	0,00E+00	2330	Os02g0236000	100,00	0,00E+00	2343	13	NA	Japonica	
maker-2-augustus-gene-78.24-mRNA-1	2	OsR498G0612730300.01.T02	80,65	9,19E-42	170	Os02g0234700	100,00	8,53E-151	531	361	Japonica	Japonica	
maker-2-augustus-gene-78.26-mRNA-1	2	OsR498G0203293800.01.T01	100,00	0,00E+00	1534	Os02g0234800	100,00	0,00E+00	1534	0	NA	Japonica	
maker-2-augustus-gene-78.31-mRNA-1	2	OsR498G0203300000.01.T02	99,72	0,00E+00	1926	Os02g0236100	100,00	0,00E+00	1939	13	NA	Japonica	Kinase-LRR-TM
maker-2-augustus-gene-78.5-mRNA-1	2	OsR498G0203294900.01.T05	93,06	0,00E+00	869	Os02g0235000	90,69	2,42E-174	611	-258	Indica	Japonica	
maker-2-augustus-gene-78.9-mRNA-1	2	OsR498G0203297700.01.T04	100,00	5,92E-99	360	Os02g0235600	100,00	2,06E-99	360	0	NA	Japonica	
maker-2-augustus-gene-79.0-mRNA-1	2	OsR498G0203302100.01.T01	99,90	0,00E+00	1738	Os06g0547400	77,30	0,00E+00	651	-1087	Indica	Japonica	
maker-2-augustus-gene-79.10-mRNA-1	2	OsR498G0203300400.01.T01	99,60	0,00E+00	2223	Os02g0236200	100,00	0,00E+00	2176	-47	NA	Japonica	Kinase-TM
maker-2-augustus-gene-79.16-mRNA-1	2	OsR498G0203303600.01.T03	100,00	0,00E+00	953	Os02g0237100	100,00	0,00E+00	953	0	NA	Japonica	
maker-2-augustus-gene-79.4-mRNA-1	2	OsR498G0203302700.01.T02	99,90	0,00E+00	1765	Os02g0236800	100,00	0,00E+00	1770	5	NA	Japonica	
maker-2-augustus-gene-8.28-mRNA-1	2	OsR498G0202843500.01.T01	99,60	0,00E+00	3091	Os02g0114700	100,00	0,00E+00	3122	31	NA	Japonica	
maker-2-augustus-gene-8.31-mRNA-1	2	OsR498G0202846700.01.T02	98,65	0,00E+00	3764	Os02g0115600	98,74	0,00E+00	3773	9	NA	Japonica	
maker-2-augustus-gene-8.34-mRNA-1	2	OsR498G0202842000.01.T01	100,00	0,00E+00	2094	Os02g0114050	100,00	0,00E+00	2094	0	NA	Japonica	
maker-2-augustus-gene-8.37-mRNA-1	2	OsR498G0202842400.01.T01	99,93	0,00E+00	2707	Os02g0114200	100,00	0,00E+00	2711	4	NA	Japonica	
maker-2-augustus-gene-8.41-mRNA-1	2	OsR498G0202842600.01.T01	99,87	0,00E+00	2735	Os02g0114400	100,00	0,00E+00	2744	9	NA	Japonica	
maker-2-augustus-gene-8.43-mRNA-1	2	OsR498G0202843000.01.T02	99,85	0,00E+00	1230	Os02g0114600	100,00	0,00E+00	1234	4	NA	Japonica	
maker-2-augustus-gene-8.45-mRNA-1	2	OsR498G0202843800.01.T01	100,00	0,00E+00	1613	Os02g0114800	100,00	0,00E+00	1613	0	NA	Japonica	
maker-2-augustus-gene-8.47-mRNA-1	2	OsR498G0305175000.01.T01	82,50	0,00E+00	1332	Os02g0115700	100,00	0,00E+00	2645	1313	Japonica	Japonica	
maker-2-augustus-gene-8.50-mRNA-1	2	OsR498G0202848800.01.T01	100,00	0,00E+00	3604	Os02g0115900	100,00	0,00E+00	3604	0	NA	NA	
maker-2-augustus-gene-80.26-mRNA-1	2	OsR498G0203306400.01.T02	99,71	0,00E+00	1249	Os04g0599400	97,85	0,00E+00	1190	-59	NA	Japonica	
maker-2-augustus-gene-81.0-mRNA-1	2	OsR498G0203308300.01.T01	95,94	0,00E+00	1601	Os02g0240100	100,00	0,00E+00	1775	174	NA	Japonica	
maker-2-augustus-gene-81.15-mRNA-1	2	OsR498G0203310000.01.T01	99,70	0,00E+00	3588	Os02g0241100	100,00	0,00E+00	3615	27	NA	Japonica	Kinase-LRR-TM
maker-2-augustus-gene-81.2-mRNA-1	2	OsR498G0203308700.01.T01	97,10	0,00E+00	1617	Os02g0240300	100,00	0,00E+00	1743	126	NA	Japonica	
maker-2-augustus-gene-81.4-mRNA-1	2	OsR498G0203309700.01.T01	97,50	0,00E+00	1422	Os02g0240500	100,00	0,00E+00	1813	391	Japonica	Japonica	
maker-2-augustus-gene-81.8-mRNA-1	2	OsR498G0203312300.01.T01	97,89	0,00E+00	2864	Os02g0241600	98,01	0,00E+00	2873	9	NA	Japonica	Kinase-TM
maker-2-augustus-gene-82.0-mRNA-1	2	OsR498G0203314300.01.T01	98,79	0,00E+00	2610	Os02g0242600	100,00	0,00E+00	2684	74	NA	Japonica	
maker-2-augustus-gene-82.2-mRNA-1	2	OsR498G0203315300.01.T01	82,51	0,00E+00	699	Os02g0243900	99,10	0,00E+00	1174	475	Japonica	Japonica	
maker-2-augustus-gene-83.14-mRNA-1	2	OsR498G0203315700.01.T02	99,89	0,00E+00	1614	Os02g0244100	99,33	0,00E+00	1592	-22	NA	Japonica	
maker-2-augustus-gene-83.18-mRNA-1	2	OsR498G0203317100.01.T01	99,76	0,00E+00	5248	Os02g0244300	100,00	0,00E+00	5281	33	NA	Japonica	
maker-2-augustus-gene-83.24-mRNA-1	2	OsR498G0203319700.01.T05	99,66	0,00E+00	4255	Os02g0245000	100,00	0,00E+00	4291	36	NA	Japonica	
maker-2-augustus-gene-83.26-mRNA-1	2	OsR498G0203318800.01.T02	99,93	0,00E+00	5234	Os02g0244700	100,00	0,00E+00	5243	9	NA	Japonica	
maker-2-augustus-gene-84.6-mRNA-1	2	OsR498G0203322200.01.T03	99,72	0,00E+00	3864	Os02g0245800	100,00	0,00E+00	2919	-945	Indica	Japonica	
maker-2-augustus-gene-85.43-mRNA-1	2	OsR498G0203325700.01.T01	96,68	0,00E+00	1410	Os02g0247100	98,90	0,00E+00	1441	31	NA	Japonica	
maker-2-augustus-gene-85.45-mRNA-1	2	OsR498G0203327500.01.T01	99,83	0,00E+00	3103	Os01g0212400	72,28	0,00E+00	830	-2273	Indica	Japonica	
maker-2-augustus-gene-86.34-mRNA-1	2	OsR498G0203334100.01.T01	92,73	0,00E+00	2216	Os02g0249600	100,00	0,00E+00	2684	468	Japonica	Japonica	
maker-2-augustus-gene-86.37-mRNA-1	2	OsR498G0203334100.01.T01	99,07	0,00E+00	2643	Os02g0249900	100,00	0,00E+00	2706	63	NA	Japonica	
maker-2-augustus-gene-86.40-mRNA-1	2	OsR498G0203336300.01.T03	99,85	0,00E+00	3542	Os02g0250300	100,00	0,00E+00	3160	-382	Indica	Japonica	
maker-2-augustus-gene-86.43-mRNA-1	2	OsR498G0203336700.01.T01	99,50	0,00E+00	1787	Os02g0250400	98,72	0,00E+00	1779	-8	NA	Japonica	
maker-2-augustus-gene-86.49-mRNA-1	2	OsR498G0203334100.01.T01	99,07	0,00E+00	2643	Os02g0249900	100,00	0,00E+00	2706	63	NA	Japonica	
maker-2-augustus-gene-87.34-mRNA-1	2	OsR498G0203341300.01.T01	99,54	0,00E+00	1926	Os02g0252200	100,00	0,00E+00	1948	22	NA	Japonica	
maker-2-augustus-gene-87.36-mRNA-1	2	OsR498G0203340800.01.T01	99,69	0,00E+00	1702	Os02g0252100	100,00	0,00E+00	1440	-262	Indica	Japonica	
maker-2-augustus-gene-87.41-mRNA-1	2	OsR498G0203342400.01.T02	97,75	0,00E+00	1737	Os02g0252600	97,87	0,00E+00	1042	-695	Indica	Japonica	
maker-2-augustus-gene-88.16-mRNA-1	2	OsR498G0203346800.01.T01	99,90	0,00E+00	1874	Os06g0528600	84,37	0,00E+00	1027	-847	Indica	Japonica	
maker-2-augustus-gene-88.29-mRNA-1	2	OsR498G0203348200.01.T01	99,74	0,00E+00	700	Os02g0255200	100,00	0,00E+00	704	4	NA	Japonica	
maker-2-augustus-gene-88.6-mRNA-1	2	OsR498G0203349500.01.T01	99,63	0,00E+00	971	Os02g0255500	72,85	2,89E-71	269	-702	Indica	Japonica	
maker-2-augustus-gene-88.8-mRNA-1	2	OsR498G0203350900.01.T01	99,84	0,00E+00	1105	Os02g0255500	100,00	0,00E+00	1110	5	NA	Japonica	
maker-2-augustus-gene-89.0-mRNA-1	2	OsR498G0203351900.01.T01	97,71	0,00E+00	1191	Os02g0256100	100,00	0,00E+00	1263	72	NA	Japonica	
maker-2-augustus-gene-89.18-mRNA-1	2	OsR498G0203353400.01.T01	75,48	1,43E-59	231	Os02g0256800	100,00	0,00E+00	688	457	Japonica	Japonica	
maker-2-augustus-gene-89.20-mRNA-1	2	OsR498G0203353400.01.T01	96,51	1,39E-173	609	Os02g0257100	99,19	0,00E+00	656	47	NA	Japonica	
maker-2-augustus-gene-89.22-mRNA-1	2	OsR498G0203353600.01.T01	97,91	0,00E+00	1308	Os02g0257200	100,00	0,00E+00	1651	343	Japonica	Japonica	
maker-2-augustus-gene-89.5-mRNA-1	2	OsR498G0203353700.01.T01	98,88	0,00E+00	2521	Os02g0257300	100,00	0,00E+00	2592	71	NA	Japonica	
maker-2-augustus-gene-9.0-mRNA-1	2	OsR498G0202849700.01.T06	100,00	0,00E+00	3701	Os02g0116300	100,00	0,00E+00	3701	0	NA	NA	
maker-2-augustus-gene-9.12-mRNA-1	2	OsR498G0202851300.01.T01	100,00	0,00E+00	2046	Os02g0117100	100,00	0,00E+00	2046	0	NA	NA	
maker-2-augustus-gene-9.18-mRNA-1	2	OsR498G0202851500.01.T06	100,00	0,00E+00	1012	Os02g0117200	100,00	0,00E+00	1012	0	NA	NA	
maker-2-augustus-gene-9.24-mRNA-1	2	OsR498G0202851700.01.T03	100,00	0,00E+00	1862	Os02g0117400	100,00	0,00E+00	1862	0	NA	NA	
maker-2-augustus-gene-9.32-mRNA-1	2	OsR498G0202852100.01.T02	99,95	0,00E+00	3415	Os02g0117500	100,00	0,00E+00	3413	-2	NA	NA	
maker-2-augustus-gene-9.36-mRNA-1	2	OsR498G0202852500.01.T03	100,00	0,00E+00	1315	Os02g0117600	100,00	0,00E+00	1272	-43	NA	NA	
maker-2-augustus-gene-9.4-mRNA-1	2	OsR498G0202850300.01.T02	99,27	0,00E+00	966	Os02g0116500	99,27	0,00E+00	966	0	NA	NA	
maker-2-augustus-gene-9.40-mRNA-1	2	OsR498G0202854300.01.T01	100,00	0,00E+00	2316	Os02g0118200	100,00	0,00E+00	2316	0	NA	NA	CC-Kinase
maker-2-augustus-gene-9.44-mRNA-1	2	OsR498G0202850000.01.T01	100,00	0,00E+00	3761	Os02g0116400	100,00	0,00E+00	3761	0	NA	NA	
maker-2-augustus-gene-9.46-mRNA-1	2	OsR498G0202850600.01.T01	100,00	0,00E+00	1283	Os02g0116600	100,00	0,00E+00	1283	0	NA	NA	
maker-2-augustus-gene-9.51-mRNA-1	2	OsR498G0202851200.01.T01	100,00	0,00E+00	774	Os02g0116900	100,00	0,00E+00	774	0	NA	NA	
maker-2-augustus-gene-9.53-mRNA-1	2	OsR498G0202852400.01.T02	100,00	0,00E+00	1673	Os02g0117700	100,00	0,00E+00	1673	0	NA	NA	
maker-2-augustus-gene-9.54-mRNA-1	2	OsR498G0202852400.01.T03	100,00	0,00E+00	774	Os02g0117700	100,00	0,00E+00	774	0	NA	NA	
maker-2-augustus-gene-9.64-mRNA-1	2	OsR498G0202853000.01.T01	100,00	0,00E+00	2186	Os02g0117900	100,00	0,00E+00	2186	0	NA	NA	
maker-2-augustus-gene-9.8-mRNA-1	2	OsR498G0202850900.01.T01	96,41	0,00E+00	2537	Os02g0116800	96,35	0,00E+00	2533	-4	NA	NA	
maker-2-augustus-gene-90.0-mRNA-1	2	OsR498G0203358000.01.T01	99,67	0,00E+00	2710	Os02g0258200	100,00	0,00E+00	1462	-1248	Indica	Japonica	
maker-2-augustus-gene-90.10-mRNA-1	2	OsR498G0203362800.01.T01	99,54	0,00E+00	1150	Os02g0259600	100,00	0,00E+00	1164	14	NA	Japonica	
maker-2-augustus-gene-90.2-mRNA-1	2	OsR498G0203358200.01.T01	98,16	0,00E+00	4434	Os02g0258300	98,17	0,00E+00	4467	33	NA	Japonica	
maker-2-augustus-gene-90.5-mRNA-1	2	OsR498G0203361000.01.T06	99,65	0,00E+00	1513	Os02g0258900	100,00	0,00E+00	1516	3	NA	Japonica	
maker-2-augustus-gene-91.18-mRNA-1	2	OsR498G0203364900.01.T02	99,81	0,00E+00	2756	Os02g0260200	100,00	0,00E+00	2214	-542	Indica	Japonica	
maker-2-augustus-gene-91.20-mRNA-1	2	OsR498G0203365500.01.T01	99,86	0,00E+00	3758	Os02g0260500	97,89	0,00E+00	3686	-72	NA	Japonica	
maker-2-augustus-gene-91.23-mRNA-1	2	OsR498G0203366500.01.T01	99,59	0,00E+00	1728	Os02g0260700	100,00	0,00E+00	1746	18	NA	Japonica	
maker-2-augustus-gene-91.27-mRNA-1	2	OsR498G0203367900.01.T02	98,37	0,00E+00	1481	Os02g0261000	100,00	0,00E+00	1548	67	NA	Japonica	
maker-2-augustus-gene-91.28-mRNA-1	2	OsR498G0203365400.01.T05	99,34	3,38E-151	535	Os11g0706100	77,61	1,35E-49	196	-339	Indica	Japonica	
maker-2-augustus-gene-92.25-mRNA-1	2	OsR498G0203368500.01.T03	99,33	0,00E+00	793	Os02g0261100	99,78	0,00E+00	802	9	NA	Japonica	
maker-2-augustus-gene-94.40-mRNA-1	2	OsR498G0203370000.01.T01	99,53	0,00E+00	1519	Os02g0264300	100,00	0,00E+00	1537	18	NA	Japonica	
maker-2-augustus-gene-94.42-mRNA-1	2	OsR498G0203372600.01.T01	100,00	2,12E-111	401	Os02g0264800	100,00	7,41E-112	401	0	NA	Japonica	
maker-2-augustus-gene-95.29-mRNA-1	2	OsR498G0203373900.01.T01	99,54	0,00E+00	1937	Os02g0265200	99,91	0,00E+00	1955	18	NA	Japonica	
maker-2-augustus-gene-95.31-mRNA-1	2	OsR498G0203376100.01.T02	99,30	0,00E+00	3286	Os02g0265700	99,92	0,00E+00	2155	-1131	Indica	Japonica	
maker-2-augustus-gene-95.35-mRNA-1	2	OsR498G0203376500.01.T02	98,27	0,00E+00	1196	Os02g0265900	99,57	0,00E+00	1237	41	NA	Japonica	
maker-2-augustus-gene-95.42-mRNA-1	2	OsR498G0203377100.01.T01	98,21	0,00E+00	3272	Os02g0266200	99,95	0,00E+00	3421	149	NA	Japonica	

maker-2-augustus-gene-95.44-mRNA-1	2	OsR498G0203375600.01.T01	99,65	0,00E+00	1014	Os02g0265400	99,82	0,00E+00	1019	5	NA	Japonica	
maker-2-augustus-gene-95.48-mRNA-1	2	OsR498G0407890300.01.T01	96,35	0,00E+00	1206	Os02g0265550	100,00	0,00E+00	740	-466	Indica	NA	
maker-2-augustus-gene-95.53-mRNA-1	2	OsR498G0203377000.01.T01	94,30	0,00E+00	1099	Os02g0266100	100,00	0,00E+00	1240	141	Japonica	Japonica	
maker-2-augustus-gene-95.56-mRNA-1	2	OsR498G0203377400.01.T02	99,93	0,00E+00	2626	Os02g0266300	100,00	0,00E+00	2630	4	NA	Japonica	
maker-2-augustus-gene-96.11-mRNA-1	2	OsR498G0203379500.01.T01	99,75	0,00E+00	1415	Os02g0267200	100,00	0,00E+00	1424	9	NA	Japonica	
maker-2-augustus-gene-96.13-mRNA-1	2	OsR498G0203380900.01.T01	99,62	0,00E+00	1410	Os02g0267700	100,00	0,00E+00	1424	14	NA	Japonica	
maker-2-augustus-gene-96.15-mRNA-1	2	OsR498G0203381700.01.T01	99,64	0,00E+00	1497	Os02g0268600	88,11	0,00E+00	1062	-435	Indica	Japonica	
maker-2-augustus-gene-96.17-mRNA-1	2	OsR498G0203383000.01.T01	100,00	0,00E+00	1472	Os02g0267700	85,78	0,00E+00	818	-654	Indica	Japonica	
maker-2-augustus-gene-96.19-mRNA-1	2	OsR498G0203382800.01.T01	99,47	0,00E+00	2675	Os02g0268300	99,93	0,00E+00	2707	32	NA	Japonica	
maker-2-augustus-gene-96.21-mRNA-1	2	OsR498G0203378400.01.T01	99,89	0,00E+00	1608	Os02g0266800	99,67	0,00E+00	1603	-5	NA	Japonica	
maker-2-augustus-gene-96.27-mRNA-1	2	OsR498G0203382800.01.T01	99,27	0,00E+00	1205	Os02g0268100	100,00	0,00E+00	1227	22	NA	NA	
maker-2-augustus-gene-96.29-mRNA-1	2	OsR498G0203383000.01.T01	100,00	0,00E+00	1472	Os02g0267700	85,78	0,00E+00	818	-654	Indica	Japonica	
maker-2-augustus-gene-96.31-mRNA-1	2	OsR498G0203383600.01.T01	99,53	0,00E+00	1503	Os02g0268600	100,00	0,00E+00	1521	18	NA	Japonica	
maker-2-augustus-gene-96.33-mRNA-1	2	OsR498G0203384400.01.T01	99,89	0,00E+00	1576	Os02g0269000	100,00	0,00E+00	1581	5	NA	Japonica	
maker-2-augustus-gene-96.6-mRNA-1	2	OsR498G0203379300.01.T01	99,03	2,99E-152	539	Os02g0267000	99,03	1,04E-152	539	0	NA	Japonica	
maker-2-augustus-gene-97.27-mRNA-1	2	OsR498G0203385800.01.T01	99,09	0,00E+00	3855	Os02g0269600	99,05	0,00E+00	3902	47	NA	Japonica	
maker-2-augustus-gene-97.29-mRNA-1	2	OsR498G0203388600.01.T02	94,38	0,00E+00	3429	Os02g0270933	82,81	0,00E+00	1754	-1675	Indica	Japonica	
maker-2-augustus-gene-98.1-mRNA-1	2	OsR498G0203389000.01.T01	97,35	0,00E+00	2668	Os02g0270933	81,15	0,00E+00	1032	-1636	Indica	Japonica	
maker-2-augustus-gene-98.10-mRNA-1	2	OsR498G0203389600.01.T01	98,91	0,00E+00	3392	Os02g0271000	100,00	0,00E+00	3487	95	NA	Japonica	
maker-2-augustus-gene-98.14-mRNA-1	2	OsR498G0203389800.01.T01	98,52	0,00E+00	3892	Os02g0271600	99,06	0,00E+00	3574	-318	NA	Japonica	
maker-2-augustus-gene-99.0-mRNA-1	2	OsR498G0203390500.01.T02	95,99	0,00E+00	1792	Os02g0271900	100,00	0,00E+00	1966	174	NA	Japonica	
maker-2-augustus-gene-99.11-mRNA-1	2	OsR498G0203395500.01.T01	99,66	0,00E+00	2089	Os02g0273100	100,00	0,00E+00	2111	22	NA	Japonica	
maker-2-augustus-gene-99.13-mRNA-1	2	OsR498G0203393000.01.T01	100,00	0,00E+00	1645	Os02g0272400	96,38	0,00E+00	1522	-123	NA	Japonica	
maker-2-augustus-gene-99.5-mRNA-1	2	OsR498G0203392500.01.T01	99,68	0,00E+00	2759	Os02g0272300	99,87	0,00E+00	2773	14	NA	Japonica	
maker-2-augustus-gene-99.7-mRNA-1	2	OsR498G0203393900.01.T01	99,87	0,00E+00	1349	Os02g0272600	99,87	0,00E+00	1349	0	NA	Japonica	
augustus_masked-3-processed-gene-1.1-mRNA-1	3	OsR498G0305082800.01.T01	81,52	0,00E+00	1024	Os03g0105300	100,00	0,00E+00	2219	1195	Japonica	NA	
augustus_masked-3-processed-gene-1.13-mRNA-1	3	OsR498G0305087100.01.T01	99,07	0,00E+00	1333	Os03g0106900	100,00	0,00E+00	1364	31	NA	Japonica	
augustus_masked-3-processed-gene-1.25-mRNA-1	3	OsR498G0305087800.01.T01	98,26	0,00E+00	1388	Os03g0106800	100,00	0,00E+00	1451	63	NA	Japonica	
augustus_masked-3-processed-gene-100.58-mRNA-1	3	OsR498G0305748800.01.T01	99,79	0,00E+00	862	Os03g0295300	100,00	0,00E+00	720	-142	Indica	Japonica	
augustus_masked-3-processed-gene-101.65-mRNA-1	3	OsR498G0305753300.01.T01	98,90	0,00E+00	801	Os03g0296600	100,00	0,00E+00	823	22	NA	Japonica	
augustus_masked-3-processed-gene-101.81-mRNA-1	3	OsR498G0305751000.01.T04	99,29	3,93E-141	500	Os03g0295800	100,00	2,64E-144	509	9	NA	Japonica	
augustus_masked-3-processed-gene-102.36-mRNA-1	3	OsR498G0305756400.01.T01	99,57	0,00E+00	1232	Os03g0297600	100,00	0,00E+00	1245	13	NA	Japonica	
augustus_masked-3-processed-gene-103.14-mRNA-1	3	OsR498G0305770700.01.T01	99,60	0,00E+00	3600	Os07g0690900	71,49	0,00E+00	918	-2682	Indica	Japonica	
augustus_masked-3-processed-gene-103.23-mRNA-1	3	OsR498G0305767200.01.T01	100,00	0,00E+00	3063	Os03g0300500	100,00	0,00E+00	3063	0	NA	Japonica	
augustus_masked-3-processed-gene-103.6-mRNA-1	3	OsR498G0305764800.01.T01	99,84	0,00E+00	3260	Os03g0299800	100,00	0,00E+00	3274	14	NA	Japonica	
augustus_masked-3-processed-gene-103.8-mRNA-1	3	OsR498G0305766000.01.T01	99,78	0,00E+00	2416	Os03g0300000	99,90	0,00E+00	1884	-532	Indica	Japonica	
augustus_masked-3-processed-gene-104.16-mRNA-1	3	OsR498G0305774900.01.T01	99,54	0,00E+00	1150	Os03g0302800	100,00	0,00E+00	1164	14	NA	Japonica	
augustus_masked-3-processed-gene-104.18-mRNA-1	3	OsR498G0305776300.01.T01	99,72	0,00E+00	1911	Os03g0303100	100,00	0,00E+00	1925	14	NA	Japonica	
augustus_masked-3-processed-gene-104.2-mRNA-1	3	OsR498G0305773500.01.T01	99,48	0,00E+00	1362	Os03g0301950	100,00	0,00E+00	1380	18	NA	Japonica	
augustus_masked-3-processed-gene-104.3-mRNA-1	3	OsR498G0305773700.01.T02	100,00	0,00E+00	1315	Os03g0302000	100,00	0,00E+00	1315	0	NA	Japonica	
augustus_masked-3-processed-gene-106.55-mRNA-1	3	OsR498G0305787900.01.T01	99,82	0,00E+00	1977	Os03g0306100	100,00	0,00E+00	2435	458	Japonica	Japonica	
augustus_masked-3-processed-gene-106.57-mRNA-1	3	OsR498G0100750200.01.T01	83,59	7,25E-70	264	Os03g0306800	100,00	0,00E+00	715	451	Japonica	Japonica	
augustus_masked-3-processed-gene-106.65-mRNA-1	3	OsR498G0305787900.01.T01	81,24	0,00E+00	1018	Os03g0305800	100,00	0,00E+00	2619	1601	Japonica	Japonica	
augustus_masked-3-processed-gene-107.1-mRNA-1	3	OsR498G0305791800.01.T01	99,39	0,00E+00	1743	Os03g0307200	99,59	0,00E+00	1752	9	NA	Japonica	
augustus_masked-3-processed-gene-107.12-mRNA-1	3	OsR498G0305793900.01.T03	99,79	0,00E+00	1691	Os03g0308000	99,89	0,00E+00	1695	4	NA	Japonica	
augustus_masked-3-processed-gene-108.42-mRNA-1	3	OsR498G0305799900.01.T04	99,87	0,00E+00	2708	Os03g0309800	99,93	0,00E+00	2712	4	NA	Japonica	
augustus_masked-3-processed-gene-109.2-mRNA-1	3	OsR498G0305805500.01.T01	99,68	0,00E+00	1106	Os03g0311600	100,00	0,00E+00	1115	9	NA	Japonica	
augustus_masked-3-processed-gene-110.41-mRNA-1	3	OsR498G0305815500.01.T05	99,85	0,00E+00	3498	Os03g0314400	100,00	0,00E+00	3512	14	NA	Japonica	
augustus_masked-3-processed-gene-112.0-mRNA-1	3	OsR498G0305825700.01.T04	99,76	0,00E+00	3803	Os03g0317100	100,00	0,00E+00	3826	23	NA	Japonica	
augustus_masked-3-processed-gene-112.1-mRNA-1	3	OsR498G0305826500.01.T01	99,78	0,00E+00	2416	Os03g0317300	100,00	0,00E+00	2430	14	NA	Japonica	
augustus_masked-3-processed-gene-112.15-mRNA-1	3	OsR498G0305828500.01.T01	84,94	0,00E+00	1311	Os03g0318400	100,00	0,00E+00	2354	1043	Japonica	Japonica	
augustus_masked-3-processed-gene-113.14-mRNA-1	3	OsR498G0305839500.01.T03	100,00	0,00E+00	940	Os03g0320900	100,00	0,00E+00	937	-3	NA	Japonica	
augustus_masked-3-processed-gene-113.22-mRNA-1	3	OsR498G0305836200.01.T01	99,85	0,00E+00	1165	Os03g0320500	100,00	0,00E+00	1169	4	NA	Japonica	
augustus_masked-3-processed-gene-114.35-mRNA-1	3	OsR498G0305840900.01.T01	99,74	0,00E+00	2070	Os03g0321300	99,91	0,00E+00	2079	9	NA	Japonica	
augustus_masked-3-processed-gene-114.60-mRNA-1	3	OsR498G0305845000.01.T04	99,59	0,00E+00	1297	Os03g0321800	100,00	0,00E+00	1310	13	NA	Japonica	
augustus_masked-3-processed-gene-115.14-mRNA-1	3	OsR498G0305853900.01.T02	99,74	0,00E+00	3452	Os03g0324300	100,00	0,00E+00	2571	-881	Indica	NA	
augustus_masked-3-processed-gene-118.0-mRNA-1	3	OsR498G0305073300.01.T03	100,00	1,17E-129	462	Os06g0289300	92,26	4,33E-136	482	20	NA	NA	
augustus_masked-3-processed-gene-118.10-mRNA-1	3	OsR498G0305072400.01.T01	100,00	0,00E+00	1499	Os03g0102500	100,00	0,00E+00	1499	0	NA	NA	
augustus_masked-3-processed-gene-118.15-mRNA-1	3	OsR498G0305077300.01.T01	96,04	0,00E+00	914	Os03g0103300	100,00	0,00E+00	1002	88	NA	Japonica	
augustus_masked-3-processed-gene-119.9-mRNA-1	3	OsR498G0305082800.01.T01	91,38	0,00E+00	1688	Os03g0104400	100,00	0,00E+00	2154	466	Japonica	Japonica	
augustus_masked-3-processed-gene-12.55-mRNA-1	3	OsR498G0305151700.01.T01	98,93	0,00E+00	5093	Os03g0124200	100,00	0,00E+00	4594	-499	NA	Japonica	Kinase-TM
augustus_masked-3-processed-gene-12.56-mRNA-1	3	OsR498G0305151900.01.T04	99,64	0,00E+00	2505	Os03g0124300	100,00	0,00E+00	1966	-539	Indica	Japonica	
augustus_masked-3-processed-gene-132.22-mRNA-1	3	OsR498G0305066500.01.T01	100,00	0,00E+00	1104	Os03g0100150	100,00	0,00E+00	1104	0	NA	NA	
augustus_masked-3-processed-gene-14.50-mRNA-1	3	OsR498G0305166900.01.T02	99,93	0,00E+00	2404	Os03g0129300	100,00	0,00E+00	2408	4	NA	Japonica	
augustus_masked-3-processed-gene-148.34-mRNA-1	3	OsR498G0305853900.01.T02	99,74	0,00E+00	3452	Os03g0324300	100,00	0,00E+00	2571	-881	Indica	NA	
augustus_masked-3-processed-gene-148.37-mRNA-1	3	OsR498G0305854900.01.T01	99,54	0,00E+00	2325	Os03g0324700	100,00	0,00E+00	2370	45	NA	Japonica	
augustus_masked-3-processed-gene-148.50-mRNA-1	3	OsR498G0305854800.01.T01	99,88	0,00E+00	5793	Os03g0324600	100,00	0,00E+00	5811	18	NA	Japonica	CC-LRR-NBS-TM
augustus_masked-3-processed-gene-149.0-mRNA-1	3	OsR498G0305855900.01.T02	99,69	0,00E+00	1724	Os03g0324900	100,00	0,00E+00	1737	13	NA	Japonica	
augustus_masked-3-processed-gene-149.24-mRNA-1	3	OsR498G0305857800.01.T02	99,87	0,00E+00	1426	Os03g0325600	100,00	0,00E+00	1431	5	NA	Japonica	
augustus_masked-3-processed-gene-153.48-mRNA-1	3	OsR498G0305887500.01.T03	99,69	0,00E+00	3409	Os03g0332900	100,00	0,00E+00	3436	27	NA	Japonica	Kinase-LRR-TM
augustus_masked-3-processed-gene-154.30-mRNA-1	3	OsR498G0305888100.01.T01	99,66	0,00E+00	3654	Os03g0333200	100,00	0,00E+00	4832	1178	Japonica	Japonica	Kinase-TM
augustus_masked-3-processed-gene-154.31-mRNA-1	3	OsR498G0305888500.01.T01	99,88	0,00E+00	1462	Os03g0333300	100,00	0,00E+00	1467	5	NA	Japonica	
augustus_masked-3-processed-gene-155.25-mRNA-1	3	OsR498G0305898600.01.T01	99,69	0,00E+00	1155	Os03g0336400	100,00	0,00E+00	1164	9	NA	Japonica	
augustus_masked-3-processed-gene-156.55-mRNA-1	3	OsR498G0305902200.01.T01	99,67	0,00E+00	1625	Os03g0336900	100,00	0,00E+00	1635	10	NA	Japonica	
augustus_masked-3-processed-gene-156.60-mRNA-1	3	OsR498G0305907700.01.T06	100,00	0,00E+00	2560	Os03g0339100	100,00	0,00E+00	2560	0	NA	Japonica	
augustus_masked-3-processed-gene-158.20-mRNA-1	3	OsR498G0305924100.01.T01	100,00	0,00E+00	2360	Os03g0343700	100,00	0,00E+00	1954	-406	Indica	NA	
augustus_masked-3-processed-gene-159.36-mRNA-1	3	OsR498G0305924900.01.T01	100,00	0,00E+00	699	Os03g0343900	100,00	0,00E+00	699	0	NA	NA	
augustus_masked-3-processed-gene-159.57-mRNA-1	3	OsR498G0305928800.01.T01	99,91	0,00E+00	1911	Os03g0345300	100,00	0,00E+00	1916	5	NA	NA	
augustus_masked-3-processed-gene-16.22-mRNA-1	3	OsR498G0305178700.01.T01	100,00	0,00E+00	1786	Os03g0132600	100,00	0,00E+00	1786	0	NA	NA	
augustus_masked-3-processed-gene-16.27-mRNA-1	3	OsR498G0305181500.01.T01	100,00	0,00E+00	1204	Os03g0133400	100,00	0,00E+00	1204	0	NA	NA	LRR-TM
augustus_masked-3-processed-gene-160.5-mRNA-1	3	OsR498G0305934000.01.T01	99,70	4,83E-166	584	Os03g0346200	100,00	1,58E-103	374	-210	Indica	Japonica	
augustus_masked-3-processed-gene-160.8-mRNA-1	3	OsR498G0305935700.01.T01	99,62	0,00E+00	934	Os05g0375400	75,19	2,61E-65	248	-686	Indica	NA	
augustus_masked-3-processed-gene-162.62-mRNA-1	3	OsR498G0305948400.01.T01	99,67	0,00E+00	2179	Os03g0351200	99,67	0,00E+00	2179	0	NA	NA	
augustus_masked-3-processed-gene-162.64-mRNA-1	3	OsR498G0305953600.01.T03	100,00	0,00E+00	1537	Os03g0351800	100,00	0,00E+00	1537	0	NA	Japonica	
augustus_masked-3-processed-gene-166.23-mRNA-1	3	OsR498G0305990700.01.T01	100,00	0,00E+00	1635	Os03g0360600	100,00	0,00E+00	1635	0	NA	NA	
augustus_masked-3-processed-gene-169.13-mRNA-1	3	OsR498G0305969100.01.T01	100,00	0,00E+00	1104	Os03g0356400	100,00	0,00E+00	1104	0	NA	Japonica	
augustus_masked-3-processed-gene-169.6-mRNA-1	3	OsR498G0305965400.01.T02	98,95	0,00E+00	2512	Os03g0355100	100,00	0,00E+00	2560	48	NA	Japonica	
augustus_masked-3-processed-gene-169.7-mRNA-1	3	OsR498G0305965400.01.T02	99,72	0,00E+00	2558	Os03g0355300	100,00	0,00E+00	2576	18	NA	NA	
augustus_masked-3-processed-gene-17.6-mRNA-1	3	OsR498G0305184000.01.T01	100,00	0,00E+00	2966	Os03g0134632	100,00	0,00E+00	1853	-1113	Indica	NA	
augustus_masked-3-processed-gene-171.26-mRNA-1	3	OsR498G0305981500.01.T01	99,69	0,00E+00	1751	Os03g0356900	100,00	0,00E+00	1764	13	NA	Japonica	
augustus_masked-3-processed-gene-172.42-mRNA-1	3	OsR498G0305987200.01.T01	99,80	0,00E+00	1772	Os03g0356900	88,30	0,00E+00	1078	-694	Indica	Japonica	
augustus_masked-3-processed-gene-175.39-mRNA-1	3	OsR498G0305990700.01.T01	100,00	0,00E+00	1635	Os03g0360600	100,00	0,00E+00	1635	0	NA	Japonica	
augustus_masked-3-processed-gene-177.41-mRNA-1	3	OsR498G0305999700.01.T01	94,40	0,00E+00	1168	Os03g0363800	94,54	0,00E+00	1172	4	NA	Japonica	
augustus_masked-3-processed-gene-177.48-mRNA-1	3	OsR498G0305999600.01.T05	99,74	0,00E+00	2794	Os03g0363700	100,00	0,00E+00	2812	18	NA	Japonica	
augustus_masked-3-processed-gene-178.42-mRNA-1	3	OsR498G0306007200.01.T01	98,80	1,72E-165	581	Os03g0366100	100,00	6,37E-172	601	20	NA	Japonica	
augustus_masked-3-processed-gene-178.43-mRNA-1	3	OsR498G0306009000.01.T01	97,75	0,00E+00	1969	Os03g0366800	100,00	0,00E+00	2706	737	Japonica	Japonica	
augustus_masked-3-processed-gene-18.0-mRNA-1	3	OsR498G0305190100.01.T01	99,76	0,00E+00	748	Os03g0136500	100,00	0,00E+00	632	-116	Indica	Japonica	
augustus_masked-3-processed-gene-18.13-mRNA-1	3	OsR498G0305193900.01.T01	99,82	0,00E+00	3972	Os03g0137400	100,00	0,00E+00	3990	18	NA	Japonica	
augustus_masked-3-processed-gene-18.4-mRNA-1	3	OsR498G0305190000.01.T01	99,94	0,00E+00	2847	Os03g0136400	100,00	0,00E+00	2852	5	NA	Japonica	
augustus_masked-3-processed-gene-180.38-mRNA-1	3	OsR498G0306019700.01.T02	100,00	0,00E+00	1981	Os03g0370000	99,91	0,00E+00	1976	-5	NA	Japonica	
augustus_masked-3-processed-gene-180.41-mRNA-1	3	OsR498G0306021700.01.T01	97,48	0,00E+00	3012	Os03g0370500	100,00	0,00E+00	3204	192	NA	Japonica	
augustus_masked-3-processed-gene-180.42-mRNA-1	3	OsR498G0408541500.01.T01	74,39	1,10E-75	284	Os03g0370800	100,00	0,00E+00	996	712	Japonica	Japonica	
augustus_masked-3-processed-gene-180.60-mRNA-1	3	OsR498G0306020800.01.T01	99,30	0,00E+00	2021	Os03g0370250	99,76	0,00E+00	2227	206	NA	Indica	
augustus_masked-3-processed-gene-182.28-mRNA-1	3	OsR498G0306031300.01.T01	98,83	0,00E+00	2243	Os03g0372900	100,00	0,00E+00	2311	68	NA	Japonica	
augustus_masked-3-processed-gene-183.5-mRNA-1	3	OsR498G0306036800.01.T01	100,00	0,00E+00	1719	Os03g0375100	100,00	0,00E+00	1792	73	NA	NA	
augustus_masked-3-processed-gene-185.22-mRNA-1	3	OsR498G0305248900.01.T01	75,15	5,59E-104	379	Os03g0379500	93,18	7,26E-110	396	17	NA	Indica	
augustus_masked-3-processed-gene-188.16-mRNA-1	3	OsR498G0306062300.01.T03	97,18	0,00E+00	1607	Os03g0386000	100,00	0,00E+00	1728	121	NA	Japonica	
augustus_masked-3-processed-gene-189.10-mRNA-1	3	OsR498G0306070300.01.T01	99,89	0,00E+00	1560	Os03g0387300	100,00	0,00E+00	1207	-353	Indica	Japonica	
augustus_masked-3-processed-gene-19.0-mRNA-1	3	OsR498G0305198900.01.T01	99,68	0,00E+00	2808	Os03g0138200	99,94	0,00E+00	2826	18	NA	Japonica	
augustus_masked-3-processed-gene-19.1-mRNA-1	3	OsR498G0305199100.01.T02	99,63	9,82E-136	483	Os03g0138400	100,00	2,82E-137	486	3	NA	Japonica	
augustus_masked-3-processed-gene-19.14-mRNA-1	3	OsR498G0305201100.01.T01	99,47	0,00E+00	1666	Os03g0139000	100,00	0,00E+00	1689	23	NA	Japonica	
augustus_masked-3-processed-gene-19.16-mRNA-1	3	OsR498G0305202300.01.T01	99,87	0,00E+00	1376	Os03g0139300	100,00	0,00E+00	1380	4	NA	Japonica	
augustus_masked-3-processed-gene-19.17-mRNA-1	3	OsR498G0305203100.01.T01	99,41	0,00E+00	2692	Os03g0140100	78,82	0,00E+00	1265	-1427	Indica	Japonica	
augustus_masked-3-processed-gene-19.19-mRNA-1	3	OsR498G0305203500.01.T01	99,74	0,00E+00	2731	Os03g0140100	81,13	0,00E+00	1431	-1300	Indica	Japonica	
augustus_masked-3-processed-gene-19.22-mRNA-1	3	OsR498G0305205100.01.T01	99,54	0,00E+00	2723	Os03g0140100	100,00	0,00E+00	2755	32	NA	Japonica	
augustus_masked-3-processed-gene-192.16-mRNA-1	3	OsR498G0306087800.01.T02	100,00	0,00E+00	1710	Os03g0391700	100,00	0,00E+00	1710	0	NA	Japonica	
augustus_masked-3-processed-gene-192.19-mRNA-1	3	OsR498G0306092300.01.T01	100,00	0,00E+00	1104	Os03g0392300	99,84	0,00E+00	1100	-4	NA	Indica	
augustus_masked-3-processed-gene-192.29-mRNA-1	3	OsR498G0306091600.01.T01	100,00	0,00E+00	720	Os04g0465100	79,10	1,01E-62	239	-481	Indica	Indica	
augustus_masked-3-processed-gene-192.30-mRNA-1	3	OsR498G0306092800.01.T02	100,00	0,00E+00	2197	Os03g0392400	99,75	0,00E+00	2184	-13	NA	Indica	
augustus_masked-3-processed-gene-197.1-mRNA-1	3	OsR498G0306113900.01.T02	100,00	0,00E+00	3095	Os03g0397500	99,83	0,00E+00	3082	-13	NA	Indica	
augustus_masked-3-processed-gene-197.14-mRNA-1	3	OsR498G0306118900.01.T01	99,64	0,00E+00	1508	Os03g0398600	100,00	0,00E+00	1451	-57	NA	Japonica	
augustus_masked-3-processed-gene-197.15-mRNA-1	3	OsR498G0306119900.01.T01	99,72	0,00E+00	1931	Os03g0399000	100,00	0,00E+00	1945	14	NA	Japonica	
augustus_masked-3-processed-gene-197.16-mRNA-1	3	OsR498G0306119900.01.T01	100,00	0,00E+00	1326	Os03g0399000	100,00	0,00E+00	1326	0	NA	Japonica	
augustus_masked-3-processed-gene-198.17-mRNA-1	3	OsR498G0306125100.01.T01	90,28	0,00E+00	822	Os03g0399800	100,00	0,00E+00	955	133	Japonica	Indica	
augustus_masked-3-processed-gene-198.18-mRNA-1	3	OsR498G0306125700.01.T01	100,00	0,00E+00	818	Os03g0399900	96,89	0,00E+00	636	-182	Indica	NA	
augustus_masked-3-processed-gene-198.9-mRNA-1	3	OsR498G0306121800.01.T01	99,43	0,00E+00	2182	Os03g0399400	100,00	0,00E+00	2214	32	NA	Japonica	
augustus_masked-3-processed-gene-199.47-mRNA-1	3	OsR498G0306131600.01.T01	99,91	0,00E+00	4044	Os03g0401200	99,60	0,00E+00	4012	-32	NA	Indica	
augustus_masked-3-processed-gene-20.20-mRNA-1	3	OsR498G0305205900.01.T01	99,69	0,00E+00	2862	Os03g0140100	74,38	0,00E+00	819	-2043	Indica	Japonica	
augustus_masked-3-processed-gene-20.21-mRNA-1	3	OsR498G0305207300.01.T01	99,75	0,00E+00	2899	Os03g0140100	72,72	0,00E+00	745	-2154	Indica	Japonica	
augustus_masked-3-processed-gene-20.24-mRNA-1	3	OsR498G0305207800.01.T01	99,65	0,00E+00	1540	Os03g0140500	100,00	0,00E+00	1553	13	NA	Japonica	
augustus_masked-3-processed-gene-202.2-mRNA-1	3	OsR498G0306146500.01.T01	99,51	0,00E+00	2892	Os03g0403600	100,00	0,00E+00	2257	-635	Indica	Japonica	
augustus_masked-3-processed-gene-208.15-mRNA-1	3	OsR498G0306193300.01.T02	100,00	0,00E+00	1191	Os03g0413000	100,00	0,00E+00	1191	0	NA	NA	
augustus_masked-3-processed-gene-208.2-mRNA-1	3	OsR498G0306190700.01.T02	100,00	0,00E+00	706	Os03g0412200	93,18	7,29E-175	613	-93	Indica	NA	
augustus_masked-3-processed-gene-209.14-mRNA-1	3	OsR498G0306203100.01.T01	100,00	0,00E+00	1429	Os03g0414900	100,00	0,00E+00	1429	0	NA	NA	
augustus_masked-3-processed-gene-209.7-mRNA-1	3	OsR498G0306198600.01.T01	100,00	0,00E+00	1959	Os03g0414100	100,00	0,00E+00	1959	0	NA	NA	
augustus_masked-3-processed-gene-210.0-mRNA-1	3	OsR498G0306207300.01.T02	100,00	0,00E+00	1115	Os03g0415500	100,00	0,00E+00	1115	0	NA	Japonica	
augustus_masked-3-processed-gene-211.19-mRNA-1	3	OsR498G0306216600.01.T01	100,00	0,00E+00	1712	Os03g0417700	100,00	0,00E+00	2971	1259	Japonica	Japonica	
augustus_masked-3-processed-gene-211.25-mRNA-1	3	OsR498G0306219500.01.T01	99,90	0,00E+00	1760	Os03g0418000	100,00	0,00E+00	1764	4	NA	Japonica	
augustus_masked-3-processed-gene-213.12-mRNA-1	3	OsR498G0306236100.01.T02	99,60	0,00E+00	1345	Os03g0423000	100,00	0,00E+00	1359	14	NA	Japonica	
augustus_masked-3-processed-gene-213.29-mRNA-1	3	OsR498G0306236800.01.T02	99,78	0,00E+00	2400	Os03g0423300	100,00	0,00E+00	2008	-392	Indica	Japonica	
augustus_masked-3-processed-gene-213.6-mRNA-1	3	OsR498G1120568600.01.T01	75,73	7,12E-38	159	Os03g0422300	100,00	0,00E+00	661	502	Japonica	Japonica	
augustus_masked-3-processed-gene-215.25-mRNA-1	3	OsR498G0306245800.01.T01	99,56	0,00E+00	2023	Os03g0426300	100,00	0,00E+00	1564	-459	Indica	Japonica	Kinase-TM
augustus_masked-3-processed-gene-216.18-mRNA-1	3	OsR498G0306246300.01.T03	99,44	0,00E+00	641	Os03g0426800	100,00	0,00E+00	650	9	NA	Japonica	
augustus_masked-3-processed-gene-216.22-mRNA-1	3	OsR498G0306247200.01.T03	99,73	0,00E+00	1333	Os03g0427000	100,00	0,00E+00	1342	9	NA	Japonica	
augustus_masked-3-processed-gene-216.23-mRNA-1	3	OsR498G0306247800.01.T02	99,75	0,00E+00	1442	Os03g0427100	100,00	0,00E+00	1451	9	NA	Japonica	
augustus_masked-3-processed-gene-217.19-mRNA-1	3	OsR498G0306252300.01.T01	99,74	0,00E+00	1382	Os03g0428700	100,00	0,00E+00	1391	9	NA	Japonica	
augustus_masked-3-processed-gene-217.21-mRNA-1	3	OsR498G0306253900.01.T01	100,00	0,00E+00	672	Os03g0429000	100,00	0,00E+00	672	0	NA	Japonica	
augustus_masked-3-processed-gene-218.21-mRNA-1	3	OsR498G0305421900.01.T01	88,36	0,00E+00	752	Os02g0212975	83,70	0,00E+00	735	-17	NA	Japonica	
augustus_masked-3-processed-gene-218.4-mRNA-1	3	OsR498G0306258700.01.T01	99,37	0,00E+00	4205	Os03g0430500	100,00	0,00E+00	4269	64	NA	Japonica	
augustus_masked-3-processed-gene-22.21-mRNA-1	3	OsR498G0305219500.01.T02	99,87	0,00E+00	2724	Os03g0144300	100,00	0,00E+00	2733	9	NA	Japonica	
augustus_masked-3-processed-gene-22.22-mRNA-1	3	OsR498G0305220100.01.T01	99,74	0,00E+00	2756	Os03g0144500	99,93	0,00E+00	2386	-370	Indica	Japonica	
augustus_masked-3-processed-gene-22.25-mRNA-1	3	OsR498G0305220900.01.T03	99,73	0,00E+00	3251	Os03g0144800	100,00	0,00E+00	3274	23	NA	Japonica	
augustus_masked-3-processed-gene-22.39-mRNA-1	3	OsR498G0305221600.01.T01	99,81	0,00E+00	5552	Os03g0145000	100,00	0,00E+00	5579	27	NA	Japonica	Kinase-LRR-TM
augustus_masked-3-processed-gene-220.10-mRNA-1	3	OsR498G0306272800.01.T01	99,78	0,00E+00	3250	Os03g0433200	100,00	0,00E+00	3268	18	NA	Japonica	
augustus_masked-3-processed-gene-222.22-mRNA-1	3	OsR498G0306284800.01.T01	98,01	0,00E+00	1033	Os03g0437100	100,00	0,00E+00	1088	55	NA	Japonica	
augustus_masked-3-processed-gene-222.23-mRNA-1	3	OsR498G0306284800.01.T01	72,22	2,11E-60	234	Os03g0437200	100,00	0,00E+00	1196	962	Japonica	Japonica	
augustus_masked-3-processed-gene-222.8-mRNA-1	3	OsR498G0306281600.01.T01	99,67	5,71E-152	537	Os03g0435900	100,00	4,67E-154	542	5	NA	Japonica	
augustus_masked-3-processed-gene-222.9-mRNA-1	3	OsR498G0714710500.01.T01	76,46	0,00E+00	898	Os03g0436400	100,00	0,00E+00	2149	1251	Japonica	Japonica	
augustus_masked-3-processed-gene-223.16-mRNA-1	3	OsR498G0306291900.01.T01	99,59	0,00E+00	1719	Os03g0439000	100,00	0,00E+00	1710	-9	NA	Japonica	
augustus_masked-3-processed-gene-225.5-mRNA-1	3	OsR498G0306299200.01.T05	99,80	0,00E+00	4512	Os03g0441400	100,00	0,00E+00	4534	22	NA	Japonica	
augustus_masked-3-processed-gene-225.6-mRNA-1	3	OsR498G0306299600.01.T03	99,77	0,00E+00	3825	Os03g0441500	100,00	0,00E+00	3847	22	NA	Japonica	
augustus_masked-3-processed-gene-227.7-mRNA-1	3	OsR498G0306310300.01.T02	96,93	0,00E+00	2419	Os03g0444900	100,00	0,00E+00	1447	-972	Indica	Japonica	
augustus_masked-3-processed-gene-234.15-mRNA-1	3	OsR498G0306410800.01.T06	99,85	0,00E+00	2324	Os03g0570300	100,00	0,00E+00	2333	9	NA	NA	
augustus_masked-3-processed-gene-238.31-mRNA-1	3	OsR498G0306431100.01.T01	97,83	0,00E+00	707	Os06g0127800	87,08	1,46E-130	465	-242	Indica	NA	
augustus_masked-3-processed-gene-238.41-mRNA-1	3	OsR498G0306432300.01.T04	99,67	1,13E-154	546	Os04g0444600	98,37	3,67E-149	526	-20	NA	NA	
augustus_masked-3-processed-gene-238.42-mRNA-1	3	OsR498G0306432300.01.T04	99,57	0,00E+00	1665	Os03g0576400	100,00	0,00E+00	1683	18	NA	NA	
augustus_masked-3-processed-gene-238.43-mRNA-1	3	OsR498G0306432500.01.T01	98,68	0,00E+00	657	Os03g0576500	100,00	2,10E-134	477	-180	Indica	NA	
augustus_masked-3-processed-gene-239.21-mRNA-1	3	OsR498G0306440800.01.T01	99,84	0,00E+00	2248	Os03g0579000	100,00	0,00E+00	2257	9	NA	NA	
augustus_masked-3-processed-gene-239.6-mRNA-1	3	OsR498G0306438100.01.T07	94,39	6,16E-126	450	Os10g0391500	98,95	1,35E-141	501	51	Japonica	NA	
augustus_masked-3-processed-gene-239.8-mRNA-1	3	OsR498G0306438900.01.T01	99,77	0,00E+00	765	Os03g0578500	100,00	0,00E+00	646	-119	Indica	NA	
augustus_masked-3-processed-gene-24.47-mRNA-1	3	OsR498G0305233000.01.T02	99,58	0,00E+00	2110	Os03g0148400	100,00	0,00E+00	2397	287	Japonica	Japonica	
augustus_masked-3-processed-gene-242.7-mRNA-1	3	OsR498G0305649000.01.T01	93,38	0,00E+00	1772	Os03g0269300	93,63	0,00E+00	1783	11	NA	NA	Kinase-TM
augustus_masked-3-processed-gene-244.0-mRNA-1	3	OsR498G0306468100.01.T01	100,00	0,00E+00	3528	Os03g0586900	100,00	0,00E+00	3528	0	NA	NA	
augustus_masked-3-processed-gene-244.9-mRNA-1	3	OsR498G0306469500.01.T01	99,47	0,00E+00	4032	Os03g0587600	100,00	0,00E+00	4075	43	NA	NA	
augustus_masked-3-processed-gene-245.6-mRNA-1	3	OsR498G0306476800.01.T01	96,44	0,00E+00	1392	Os03g0589100	96,32	0,00E+00	1388	-4	NA	NA	
augustus_masked-3-processed-gene-246.12-mRNA-1	3	OsR498G0306481700.01.T01	99,87	0,00E+00	1425	Os02g0315600	74,64	5,92E-139	493	-932	Indica	NA	
augustus_masked-3-processed-gene-246.15-mRNA-1	3	OsR498G0306476800.01.T01	93,31	0,00E+00	1290	Os03g0589100	93,19	0,00E+00	1286	-4	NA	NA	
augustus_masked-3-processed-gene-247.12-mRNA-1	3	OsR498G0306486700.01.T01	100,00	0,00E+00	742	Os03g0593100	100,00	0,00E+00	742	0	NA	NA	
augustus_masked-3-processed-gene-249.33-mRNA-1	3	OsR498G0306497900.01.T01	99,48	0,00E+00	1379	Os03g0596700	100,00	0,00E+00	1359	-20	NA	NA	
augustus_masked-3-processed-gene-25.1-mRNA-1	3	OsR498G0305234100.01.T01	100,00	6,02E-152	536	Os03g0149000	100,00	2,09E-152	536	0	NA	Japonica	
augustus_masked-3-processed-gene-25.19-mRNA-1	3	OsR498G0305239100.01.T01	99,80	0,00E+00	1802	Os03g0150500	100,00	0,00E+00	2933	1131	Japonica	Japonica	
augustus_masked-3-processed-gene-25.2-mRNA-1	3	OsR498G0305234100.01.T01	98,52	6,19E-132	470	Os03g0149000	99,63	4,18E-135	480	10	NA	Japonica	
augustus_masked-3-processed-gene-25.30-mRNA-1	3	OsR498G0305237600.01.T01	98,00	0,00E+00	1631	Os03g0149700	100,00	0,00E+00	1689	58	NA	Japonica	
augustus_masked-3-processed-gene-25.31-mRNA-1	3	OsR498G0305237800.01.T01	99,56	0,00E+00	1611	Os03g0149800	100,00	0,00E+00	1629	18	NA	Japonica	
augustus_masked-3-processed-gene-250.4-mRNA-1	3	OsR498G0306499400.01.T02	99,73	0,00E+00	3993	Os03g0597200	100,00	0,00E+00	4020	27	NA	NA	
augustus_masked-3-processed-gene-253.1-mRNA-1	3	OsR498G0306516100.01.T01	99,15	0,00E+00	2493	Os03g0602300	99,79	0,00E+00	2530	37	NA	NA	
augustus_masked-3-processed-gene-254.33-mRNA-1	3	OsR498G0306527200.01.T02	98,25	0,00E+00	685	Os08g0130300	89,46	7,28E-166	582	-103	Indica	NA	
augustus_masked-3-processed-gene-254.6-mRNA-1	3	OsR498G0306523600.01.T01	99,66	0,00E+00	2591	Os03g0604200	100,00	0,00E+00	2614	23	NA	NA	
augustus_masked-3-processed-gene-256.16-mRNA-1	3	OsR498G0306537600.01.T01	98,38	0,00E+00	1282	Os03g0607700	100,00	0,00E+00	1332	50	NA	NA	
augustus_masked-3-processed-gene-257.19-mRNA-1	3	OsR498G0306547800.01.T01	99,72	0,00E+00	1285	Os03g0610400	100,00	0,00E+00	1294	9	NA	NA	
augustus_masked-3-processed-gene-258.31-mRNA-1	3	OsR498G0306552900.01.T01	99,03	0,00E+00	1627	Os03g0612400	100,00	0,00E+00	1846	219	Japonica	NA	
augustus_masked-3-processed-gene-26.8-mRNA-1	3	OsR498G0305239400.01.T01	99,87	0,00E+00	2848	Os03g0150600	100,00	0,00E+00	2397	-451	Indica	Japonica	
augustus_masked-3-processed-gene-26.9-mRNA-1	3	OsR498G0305240200.01.T01	99,87	0,00E+00	2854	Os03g0150800	100,00	0,00E+00	2863	9	NA	Japonica	
augustus_masked-3-processed-gene-261.51-mRNA-1	3	OsR498G0306563600.01.T01	99,58	0,00E+00	5027	Os03g0616600	99,94	0,00E+00	6377	1350	Japonica	NA	
augustus_masked-3-processed-gene-262.2-mRNA-1	3	OsR498G0100564100.01.T01	85,16	5,49E-132	471	Os03g0617700	100,00	0,00E+00	1407	936	Japonica	NA	
augustus_masked-3-processed-gene-264.8-mRNA-1	3	OsR498G0306581700.01.T01	97,56	0,00E+00	1317	Os11g0139400	92,00	0,00E+00	865	-452	Indica	NA	
augustus_masked-3-processed-gene-267.27-mRNA-1	3	OsR498G0307042100.01.T01	77,43	1,26E-46	187	Os10g0422800	100,00	8,42E-164	574	387	Japonica	Japonica	
augustus_masked-3-processed-gene-27.13-mRNA-1	3	OsR498G0305247900.01.T01	99,68	0,00E+00	2239	Os03g0154000	100,00	0,00E+00	2257	18	NA	Japonica	
augustus_masked-3-processed-gene-27.15-mRNA-1	3	OsR498G0305248300.01.T01	99,38	0,00E+00	858	Os11g0602400	97,12	0,00E+00	697	-161	Indica	Japonica	
augustus_masked-3-processed-gene-27.35-mRNA-1	3	OsR498G0916883200.01.T02	92,93	8,84E-167	587	Os03g0153400	100,00	0,00E+00	1261	674	Japonica	Japonica	
augustus_masked-3-processed-gene-272.58-mRNA-1	3	OsR498G0305421900.01.T01	87,00	2,70E-106	385	Os10g0460000	85,40	4,56E-98	357	-28	NA	Japonica	
augustus_masked-3-processed-gene-287.31-mRNA-1	3	OsR498G0306438100.01.T03	96,18	0,00E+00	734	Os10g0391500	100,00	9,92E-146	515	-219	Indica	NA	
augustus_masked-3-processed-gene-29.12-mRNA-1	3	OsR498G1221887800.01.T01	97,44	3,25E-129	462	Os03g0158000	100,00	1,92E-139	493	31	NA	Japonica	
augustus_masked-3-processed-gene-29.18-mRNA-1	3	OsR498G0307003200.01.T01	93,73	0,00E+00	1868	Os03g0158900	100,00	0,00E+00	2657	789	Japonica	Japonica	
augustus_masked-3-processed-gene-29.19-mRNA-1	3	OsR498G0305257900.01.T01	95,73	0,00E+00	1733	Os03g0159100	95,73	0,00E+00	1733	0	NA	Japonica	Kinase-TM
augustus_masked-3-processed-gene-29.23-mRNA-1	3	OsR498G0305260700.01.T02	99,82	0,00E+00	1008	Os03g0160200	100,00	0,00E+00	1012	4	NA	Japonica	
augustus_masked-3-processed-gene-29.33-mRNA-1	3	OsR498G0305258600.01.T01	99,85	0,00E+00	1159	Os03g0159400	100,00	0,00E+00	1164	5	NA	Japonica	
augustus_masked-3-processed-gene-29.36-mRNA-1	3	OsR498G0305260000.01.T02	99,80	0,00E+00	878	Os03g0159900	100,00	0,00E+00	738	-140	Indica	Japonica	
augustus_masked-3-processed-gene-30.19-mRNA-1	3	OsR498G0307042100.01.T01	73,02	1,45E-32	141	Os03g0160300	100,00	6,13E-146	515	374	Japonica	Japonica	
augustus_masked-3-processed-gene-30.22-mRNA-1	3	OsR498G0305261800.01.T04	99,75	0,00E+00	1431	Os03g0160900	100,00	0,00E+00	1180	-251	Indica	Japonica	
augustus_masked-3-processed-gene-302.15-mRNA-1	3	OsR498G1018558000.01.T02	99,94	0,00E+00	2929	Os03g0193000	76,03	0,00E+00	1159	-1770	Indica	Indica	
augustus_masked-3-processed-gene-308.31-mRNA-1	3	OsR498G1018531100.01.T01	99,74	0,00E+00	2065	Os03g0199100	77,84	0,00E+00	920	-1145	Indica	Indica	

augustus_masked-3-processed-gene-31.40-mRNA-1	3	OsR498G0305274100.01.T01	100,00	1,71E-172	605	Os03g0162500	100,00	1,49E-180	630	25	NA	Japonica	
augustus_masked-3-processed-gene-32.2-mRNA-1	3	OsR498G0305276900.01.T02	99,75	0,00E+00	4242	Os03g0163500	100,00	0,00E+00	4269	27	NA	Japonica	
augustus_masked-3-processed-gene-32.8-mRNA-1	3	OsR498G0305279700.01.T02	99,61	1,02E-125	451	Os03g0164300	99,61	3,56E-126	451	0	NA	Japonica	
augustus_masked-3-processed-gene-33.21-mRNA-1	3	OsR498G0305285000.01.T04	86,52	3,06E-130	464	Os03g0166000	86,80	8,73E-132	469	5	NA	Japonica	
augustus_masked-3-processed-gene-334.0-mRNA-1	3	OsR498G0306350500.01.T01	99,68	0,00E+00	4982	Os03g0556600	100,00	0,00E+00	4702	-280	NA	Japonica	Kinase-TM
augustus_masked-3-processed-gene-336.16-mRNA-1	3	OsR498G0306366400.01.T01	99,32	0,00E+00	1304	Os03g0559700	100,00	0,00E+00	1326	22	NA	Japonica	
augustus_masked-3-processed-gene-338.27-mRNA-1	3	OsR498G0306377600.01.T01	99,56	0,00E+00	1638	Os03g0562200	100,00	0,00E+00	1656	18	NA	Japonica	
augustus_masked-3-processed-gene-34.24-mRNA-1	3	OsR498G0305292300.01.T06	99,95	0,00E+00	3287	Os03g0168400	100,00	0,00E+00	3292	5	NA	Japonica	
augustus_masked-3-processed-gene-34.41-mRNA-1	3	OsR498G0305293400.01.T01	99,81	0,00E+00	2892	Os03g0168700	100,00	0,00E+00	2906	14	NA	Japonica	
augustus_masked-3-processed-gene-340.16-mRNA-1	3	OsR498G0306388200.01.T01	99,74	0,00E+00	689	Os03g0564600	100,00	0,00E+00	1337	648	Japonica	Japonica	
augustus_masked-3-processed-gene-341.24-mRNA-1	3	OsR498G0306397400.01.T01	95,89	0,00E+00	2005	Os03g0566800	96,05	0,00E+00	2014	9	NA	Japonica	
augustus_masked-3-processed-gene-341.26-mRNA-1	3	OsR498G0306398000.01.T01	99,71	5,88E-178	624	Os03g0567100	100,00	4,80E-180	628	4	NA	Japonica	
augustus_masked-3-processed-gene-342.29-mRNA-1	3	OsR498G0306401700.01.T01	99,20	2,74E-124	444	Os03g0568466	100,00	1,96E-152	536	92	Japonica	Japonica	
augustus_masked-3-processed-gene-342.30-mRNA-1	3	OsR498G0306402100.01.T01	99,53	0,00E+00	2680	Os03g0568600	100,00	0,00E+00	2711	31	NA	Japonica	
augustus_masked-3-processed-gene-343.29-mRNA-1	3	OsR498G0306409400.01.T02	99,59	0,00E+00	2184	Os03g0569900	99,78	0,00E+00	2404	220	NA	Japonica	
augustus_masked-3-processed-gene-343.30-mRNA-1	3	OsR498G0306410000.01.T01	99,48	0,00E+00	3087	Os03g0570100	100,00	0,00E+00	1781	-1306	Indica	Japonica	
augustus_masked-3-processed-gene-343.34-mRNA-1	3	OsR498G0306410800.01.T06	99,85	0,00E+00	2324	Os03g0570300	100,00	0,00E+00	2333	9	NA	NA	
augustus_masked-3-processed-gene-343.8-mRNA-1	3	OsR498G0306408900.01.T01	99,74	0,00E+00	3468	Os03g0569800	100,00	0,00E+00	3479	11	NA	Japonica	
augustus_masked-3-processed-gene-345.38-mRNA-1	3	OsR498G0306422200.01.T01	99,63	0,00E+00	6286	Os03g0573500	99,97	0,00E+00	6851	565	NA	NA	LRR-NBS-TM
augustus_masked-3-processed-gene-347.1-mRNA-1	3	OsR498G0306431100.01.T01	97,83	0,00E+00	707	Os06g0127800	87,08	1,46E-130	465	-242	Indica	NA	
augustus_masked-3-processed-gene-347.13-mRNA-1	3	OsR498G0306432300.01.T04	99,67	1,13E-154	546	Os04g0444600	98,37	3,67E-149	526	-20	NA	NA	
augustus_masked-3-processed-gene-347.14-mRNA-1	3	OsR498G0306432300.01.T04	99,57	0,00E+00	1665	Os03g0576400	100,00	0,00E+00	1683	18	NA	NA	
augustus_masked-3-processed-gene-347.15-mRNA-1	3	OsR498G0306432500.01.T01	98,68	0,00E+00	657	Os03g0576500	100,00	2,10E-134	477	-180	Indica	NA	
augustus_masked-3-processed-gene-348.14-mRNA-1	3	OsR498G0306438100.01.T07	94,39	6,16E-126	450	Os10g0391500	98,95	1,35E-141	501	51	Japonica	NA	
augustus_masked-3-processed-gene-348.16-mRNA-1	3	OsR498G0306438900.01.T01	99,77	0,00E+00	765	Os03g0578500	100,00	0,00E+00	646	-119	Indica	Japonica	
augustus_masked-3-processed-gene-349.28-mRNA-1	3	OsR498G0306440800.01.T01	99,84	0,00E+00	2248	Os03g0579000	100,00	0,00E+00	2257	9	NA	NA	
augustus_masked-3-processed-gene-35.11-mRNA-1	3	OsR498G0305295200.01.T01	99,23	0,00E+00	1150	Os04g0286500	74,50	1,31E-76	286	-864	Indica	Japonica	
augustus_masked-3-processed-gene-35.2-mRNA-1	3	OsR498G0305295300.01.T01	99,61	0,00E+00	2738	Os03g0169300	100,00	0,00E+00	2765	27	NA	Japonica	
augustus_masked-3-processed-gene-35.26-mRNA-1	3	OsR498G0305298200.01.T01	99,69	0,00E+00	2277	Os10g0405100	79,94	0,00E+00	1107	-1170	Indica	Japonica	Kinase-TM
augustus_masked-3-processed-gene-35.27-mRNA-1	3	OsR498G0305299000.01.T01	100,00	0,00E+00	1007	Os03g0170500	100,00	0,00E+00	1003	-4	NA	Japonica	
augustus_masked-3-processed-gene-353.49-mRNA-1	3	OsR498G0306468100.01.T01	100,00	0,00E+00	3528	Os03g0586900	100,00	0,00E+00	3528	0	NA	NA	
augustus_masked-3-processed-gene-353.52-mRNA-1	3	OsR498G0306469500.01.T01	99,47	0,00E+00	4032	Os03g0587600	100,00	0,00E+00	4075	43	NA	NA	
augustus_masked-3-processed-gene-354.1-mRNA-1	3	OsR498G0306473300.01.T01	99,71	0,00E+00	2495	Os03g0588200	100,00	0,00E+00	2513	18	NA	NA	
augustus_masked-3-processed-gene-354.27-mRNA-1	3	OsR498G0306476800.01.T01	99,73	0,00E+00	2021	Os03g0589100	100,00	0,00E+00	1499	-522	Indica	NA	
augustus_masked-3-processed-gene-355.16-mRNA-1	3	OsR498G0306481700.01.T01	99,87	0,00E+00	1425	Os02g0315600	74,64	5,92E-139	493	-932	Indica	NA	
augustus_masked-3-processed-gene-355.23-mRNA-1	3	OsR498G0306476800.01.T01	93,31	0,00E+00	1290	Os03g0589100	93,19	0,00E+00	1286	-4	NA	NA	
augustus_masked-3-processed-gene-356.20-mRNA-1	3	OsR498G0306486700.01.T01	100,00	0,00E+00	742	Os03g0593100	100,00	0,00E+00	742	0	NA	NA	
augustus_masked-3-processed-gene-358.27-mRNA-1	3	OsR498G0306497900.01.T01	99,48	0,00E+00	1379	Os03g0596700	100,00	0,00E+00	1359	-20	NA	NA	
augustus_masked-3-processed-gene-359.24-mRNA-1	3	OsR498G0306500800.01.T01	99,79	0,00E+00	847	Os03g0597600	100,00	0,00E+00	852	5	NA	NA	
augustus_masked-3-processed-gene-359.6-mRNA-1	3	OsR498G0306499400.01.T02	99,73	0,00E+00	3993	Os03g0597200	100,00	0,00E+00	4020	27	NA	NA	
augustus_masked-3-processed-gene-359.7-mRNA-1	3	OsR498G0306500200.01.T01	99,70	0,00E+00	2412	Os03g0597400	99,93	0,00E+00	2452	40	NA	NA	
augustus_masked-3-processed-gene-36.18-mRNA-1	3	OsR498G0305304300.01.T05	99,61	0,00E+00	2315	Os03g0171600	100,00	0,00E+00	2338	23	NA	Japonica	
augustus_masked-3-processed-gene-36.42-mRNA-1	3	OsR498G0305306800.01.T01	98,28	0,00E+00	705	Os03g0172400	78,74	1,04E-62	239	-466	Indica	Japonica	
augustus_masked-3-processed-gene-36.43-mRNA-1	3	OsR498G0305307000.01.T01	94,99	1,59E-179	628	Os03g0172400	100,00	0,00E+00	715	87	Japonica	Japonica	
augustus_masked-3-processed-gene-362.17-mRNA-1	3	OsR498G0306516100.01.T01	99,36	0,00E+00	2507	Os03g0602300	100,00	0,00E+00	2544	37	NA	NA	
augustus_masked-3-processed-gene-362.31-mRNA-1	3	OsR498G0306521200.01.T02	99,60	0,00E+00	1795	Os03g0603300	100,00	0,00E+00	1516	-279	Indica	NA	
augustus_masked-3-processed-gene-363.13-mRNA-1	3	OsR498G0100655000.01.T01	70,60	2,81E-54	214	Os03g0603700	99,89	0,00E+00	1672	1458	Japonica	NA	
augustus_masked-3-processed-gene-363.14-mRNA-1	3	OsR498G0306522900.01.T02	99,54	9,75E-107	388	Os03g0603700	100,00	0,00E+00	785	397	Japonica	NA	
augustus_masked-3-processed-gene-363.34-mRNA-1	3	OsR498G0306523600.01.T01	99,66	0,00E+00	2591	Os03g0604200	100,00	0,00E+00	2614	23	NA	NA	
augustus_masked-3-processed-gene-364.12-mRNA-1	3	OsR498G0306527200.01.T02	98,25	0,00E+00	685	Os08g0130300	89,46	7,28E-166	582	-103	Indica	NA	
augustus_masked-3-processed-gene-365.23-mRNA-1	3	OsR498G0306537600.01.T01	98,38	0,00E+00	1282	Os03g0607700	100,00	0,00E+00	1332	50	NA	NA	
augustus_masked-3-processed-gene-367.19-mRNA-1	3	OsR498G0306547800.01.T01	99,72	0,00E+00	1285	Os03g0610400	100,00	0,00E+00	1294	9	NA	NA	
augustus_masked-3-processed-gene-368.1-mRNA-1	3	OsR498G0306552900.01.T01	99,03	0,00E+00	1627	Os03g0612400	100,00	0,00E+00	1846	219	Japonica	NA	
augustus_masked-3-processed-gene-369.17-mRNA-1	3	OsR498G0306500200.01.T01	76,98	0,00E+00	758	Os03g0615300	100,00	0,00E+00	1736	978	Japonica	NA	
augustus_masked-3-processed-gene-371.5-mRNA-1	3	OsR498G0100564100.01.T01	85,16	5,49E-132	471	Os03g0617700	100,00	0,00E+00	1407	936	Japonica	NA	
augustus_masked-3-processed-gene-374.10-mRNA-1	3	OsR498G0306587700.01.T01	97,48	1,74E-152	539	Os03g0624300	100,00	8,42E-164	574	35	NA	Japonica	
augustus_masked-3-processed-gene-374.21-mRNA-1	3	OsR498G0306589900.01.T06	99,70	0,00E+00	2417	Os03g0624800	100,00	0,00E+00	2435	18	NA	Japonica	
augustus_masked-3-processed-gene-376.15-mRNA-1	3	OsR498G0306600500.01.T02	99,91	0,00E+00	1928	Os03g0627500	99,91	0,00E+00	1928	0	NA	Japonica	
augustus_masked-3-processed-gene-38.66-mRNA-1	3	OsR498G0305319400.01.T01	99,02	5,83E-152	537	Os03g0176900	100,00	9,21E-157	551	14	NA	Japonica	
augustus_masked-3-processed-gene-381.22-mRNA-1	3	OsR498G0306623400.01.T01	99,58	0,00E+00	2115	Os03g0637600	100,00	0,00E+00	2138	23	NA	Japonica	LRR-TM
augustus_masked-3-processed-gene-381.26-mRNA-1	3	OsR498G0306624300.01.T01	100,00	0,00E+00	735	Os03g0637800	100,00	0,00E+00	735	0	NA	Japonica	
augustus_masked-3-processed-gene-381.27-mRNA-1	3	OsR498G0306624300.01.T01	99,82	0,00E+00	4057	Os03g0637800	100,00	0,00E+00	4075	18	NA	Japonica	Kinase-TM
augustus_masked-3-processed-gene-382.18-mRNA-1	3	OsR498G0306629400.01.T01	99,10	0,00E+00	1564	Os03g0638900	100,00	0,00E+00	1597	33	NA	Japonica	
augustus_masked-3-processed-gene-382.26-mRNA-1	3	OsR498G0306635200.01.T01	96,76	0,00E+00	985	Os03g0639400	100,00	0,00E+00	1310	325	Japonica	Japonica	
augustus_masked-3-processed-gene-386.29-mRNA-1	3	OsR498G1222114300.01.T01	87,66	0,00E+00	1960	Os03g0645900	100,00	0,00E+00	3296	1336	Japonica	Japonica	
augustus_masked-3-processed-gene-387.30-mRNA-1	3	OsR498G0306664500.01.T01	99,81	0,00E+00	3835	Os03g0646300	97,89	0,00E+00	3686	-149	NA	Japonica	
augustus_masked-3-processed-gene-388.27-mRNA-1	3	OsR498G0306669800.01.T03	99,63	0,00E+00	1947	Os03g0648100	100,00	0,00E+00	1965	18	NA	Japonica	
augustus_masked-3-processed-gene-388.48-mRNA-1	3	OsR498G0306671600.01.T01	100,00	0,00E+00	1005	Os03g0648800	100,00	0,00E+00	1759	754	Japonica	Japonica	
augustus_masked-3-processed-gene-389.34-mRNA-1	3	OsR498G0306678300.01.T05	99,92	0,00E+00	4249	Os03g0650400	99,92	0,00E+00	4249	0	NA	NA	
augustus_masked-3-processed-gene-389.35-mRNA-1	3	OsR498G0306679300.01.T02	100,00	0,00E+00	5573	Os03g0650700	100,00	0,00E+00	5573	0	NA	NA	
augustus_masked-3-processed-gene-389.43-mRNA-1	3	OsR498G0306676600.01.T01	100,00	0,00E+00	2911	Os03g0650200	100,00	0,00E+00	2966	55	NA	NA	
augustus_masked-3-processed-gene-39.57-mRNA-1	3	OsR498G0305324900.01.T01	99,69	0,00E+00	1708	Os03g0179200	100,00	0,00E+00	1860	152	NA	Japonica	
augustus_masked-3-processed-gene-39.75-mRNA-1	3	OsR498G0305327800.01.T01	99,95	0,00E+00	3459	Os03g0180000	100,00	0,00E+00	3463	4	NA	Japonica	
augustus_masked-3-processed-gene-392.2-mRNA-1	3	OsR498G0306693800.01.T01	99,94	0,00E+00	3039	Os03g0654400	100,00	0,00E+00	3043	4	NA	Japonica	
augustus_masked-3-processed-gene-392.9-mRNA-1	3	OsR498G0306697700.01.T01	100,00	0,00E+00	3014	Os03g0655500	100,00	0,00E+00	3014	0	NA	NA	
augustus_masked-3-processed-gene-393.42-mRNA-1	3	OsR498G0306704300.01.T02	99,73	0,00E+00	662	Os03g0657000	99,73	0,00E+00	664	2	NA	NA	
augustus_masked-3-processed-gene-395.32-mRNA-1	3	OsR498G0306717700.01.T01	100,00	0,00E+00	1164	Os03g0659700	100,00	0,00E+00	666	-498	Indica	NA	
augustus_masked-3-processed-gene-395.34-mRNA-1	3	OsR498G0306718700.01.T01	100,00	2,85E-110	398	Os06g0145950	74,46	8,80E-23	106	-292	Indica	NA	
augustus_masked-3-processed-gene-396.21-mRNA-1	3	OsR498G0306727100.01.T01	100,00	0,00E+00	2316	Os03g0661500	99,92	0,00E+00	2312	-4	NA	NA	
augustus_masked-3-processed-gene-397.1-mRNA-1	3	OsR498G0306729500.01.T01	100,00	0,00E+00	1559	Os03g0661900	100,00	0,00E+00	1559	0	NA	NA	
augustus_masked-3-processed-gene-397.21-mRNA-1	3	OsR498G0306730800.01.T01	98,99	0,00E+00	1390	Os08g0546800	85,56	0,00E+00	887	-503	Indica	NA	
augustus_masked-3-processed-gene-397.23-mRNA-1	3	OsR498G0306732000.01.T01	100,00	0,00E+00	1180	Os03g0663400	100,00	0,00E+00	1180	0	NA	NA	
augustus_masked-3-processed-gene-398.1-mRNA-1	3	OsR498G0306737500.01.T01	100,00	0,00E+00	1321	Os03g0664400	100,00	0,00E+00	706	-615	Indica	NA	
augustus_masked-3-processed-gene-398.13-mRNA-1	3	OsR498G0306740100.01.T02	100,00	0,00E+00	691	Os03g0665000	100,00	0,00E+00	691	0	NA	NA	
augustus_masked-3-processed-gene-398.14-mRNA-1	3	OsR498G0306740100.01.T02	100,00	1,20E-134	479	Os03g0665000	100,00	4,18E-135	479	0	NA	NA	
augustus_masked-3-processed-gene-399.0-mRNA-1	3	OsR498G0307042100.01.T01	70,20	2,59E-24	113	Os03g0665700	100,00	4,94E-180	628	515	Japonica	NA	
augustus_masked-3-processed-gene-399.25-mRNA-1	3	OsR498G0306747200.01.T01	100,00	0,00E+00	2641	Os03g0666600	100,00	0,00E+00	2641	0	NA	NA	
augustus_masked-3-processed-gene-40.12-mRNA-1	3	OsR498G0305331700.01.T01	100,00	0,00E+00	1018	Os03g0181100	100,00	0,00E+00	1018	0	NA	Japonica	
augustus_masked-3-processed-gene-40.14-mRNA-1	3	OsR498G0305333100.01.T01	99,37	0,00E+00	2248	Os03g0181400	100,00	0,00E+00	2284	36	NA	Japonica	
augustus_masked-3-processed-gene-400.22-mRNA-1	3	OsR498G0306751800.01.T01	100,00	0,00E+00	829	Os03g0667500	100,00	0,00E+00	829	0	NA	NA	
augustus_masked-3-processed-gene-400.23-mRNA-1	3	OsR498G0306751800.01.T01	100,00	0,00E+00	1211	Os03g0667500	100,00	0,00E+00	1211	0	NA	NA	
augustus_masked-3-processed-gene-400.36-mRNA-1	3	OsR498G0306756900.01.T03	100,00	0,00E+00	1662	Os03g0668400	100,00	0,00E+00	1662	0	NA	NA	
augustus_masked-3-processed-gene-401.47-mRNA-1	3	OsR498G0306763700.01.T03	100,00	0,00E+00	682	Os03g0670000	100,00	0,00E+00	720	38	NA	NA	
augustus_masked-3-processed-gene-403.24-mRNA-1	3	OsR498G0306772900.01.T01	100,00	0,00E+00	661	Os03g0673100	100,00	5,09E-123	439	-222	Indica	Japonica	
augustus_masked-3-processed-gene-404.32-mRNA-1	3	OsR498G0306781800.01.T01	99,77	0,00E+00	775	Os03g0676400	100,00	0,00E+00	780	5	NA	Japonica	
augustus_masked-3-processed-gene-405.19-mRNA-1	3	OsR498G0306787500.01.T01	99,20	0,00E+00	2856	Os03g0678100	100,00	0,00E+00	2895	39	NA	Japonica	CC-Kinase-TM
augustus_masked-3-processed-gene-406.25-mRNA-1	3	OsR498G0305988900.01.T01	74,35	1,22E-154	548	Os03g0678800	100,00	0,00E+00	1954	1406	Japonica	Japonica	
augustus_masked-3-processed-gene-409.25-mRNA-1	3	OsR498G0306825400.01.T01	78,36	0,00E+00	645	Os03g0683200	100,00	0,00E+00	796	151	Japonica	NA	
augustus_masked-3-processed-gene-409.26-mRNA-1	3	OsR498G1019231300.01.T01	78,86	0,00E+00	673	Os03g0683300	100,00	0,00E+00	1581	908	Japonica	Japonica	
augustus_masked-3-processed-gene-41.17-mRNA-1	3	OsR498G0305341900.01.T01	99,15	0,00E+00	832	Os03g0183700	100,00	0,00E+00	1618	786	Japonica	NA	
augustus_masked-3-processed-gene-41.18-mRNA-1	3	OsR498G0305342500.01.T01	97,73	0,00E+00	3708	Os03g0183800	97,82	0,00E+00	3717	9	NA	Japonica	Kinase-LRR-TM
augustus_masked-3-processed-gene-41.19-mRNA-1	3	OsR498G0305342900.01.T03	99,94	0,00E+00	3129	Os03g0184000	100,00	0,00E+00	3020	-109	NA	Japonica	
augustus_masked-3-processed-gene-411.5-mRNA-1	3	OsR498G0306819800.01.T02	99,82	0,00E+00	8103	Os06g0488200	70,20	0,00E+00	2008	-6095	Indica	Japonica	
augustus_masked-3-processed-gene-412.41-mRNA-1	3	OsR498G0306832100.01.T01	99,81	0,00E+00	954	Os03g0689800	100,00	0,00E+00	731	-223	Indica	Japonica	
augustus_masked-3-processed-gene-413.0-mRNA-1	3	OsR498G0306837400.01.T01	98,44	0,00E+00	2012	Os03g0690500	100,00	0,00E+00	2024	12	NA	Japonica	
augustus_masked-3-processed-gene-413.2-mRNA-1	3	OsR498G0306837600.01.T09	99,95	0,00E+00	3956	Os03g0690600	99,95	0,00E+00	3956	0	NA	NA	
augustus_masked-3-processed-gene-415.15-mRNA-1	3	OsR498G0306856500.01.T03	100,00	5,82E-178	623	Os03g0694800	100,00	2,02E-178	623	0	NA	Japonica	
augustus_masked-3-processed-gene-415.2-mRNA-1	3	OsR498G0306851600.01.T02	99,81	0,00E+00	921	Os03g0693400	100,00	0,00E+00	926	5	NA	Japonica	
augustus_masked-3-processed-gene-415.3-mRNA-1	3	OsR498G0306853200.01.T01	99,85	0,00E+00	1230	Os03g0693700	100,00	0,00E+00	1234	4	NA	Japonica	
augustus_masked-3-processed-gene-415.5-mRNA-1	3	OsR498G0306854600.01.T01	95,65	0,00E+00	1111	Os03g0693900	100,00	0,00E+00	1234	123	NA	Japonica	
augustus_masked-3-processed-gene-417.12-mRNA-1	3	OsR498G0306869300.01.T05	99,78	0,00E+00	4079	Os03g0698800	100,00	0,00E+00	4102	23	NA	Japonica	
augustus_masked-3-processed-gene-418.28-mRNA-1	3	OsR498G0306874200.01.T01	99,58	6,97E-118	424	Os07g0196700	72,05	3,19E-28	123	-301	Indica	Japonica	
augustus_masked-3-processed-gene-419.57-mRNA-1	3	OsR498G0306882500.01.T01	99,79	0,00E+00	2546	Os03g0702500	100,00	0,00E+00	2560	14	NA	Japonica	
augustus_masked-3-processed-gene-419.59-mRNA-1	3	OsR498G0306884100.01.T02	99,92	0,00E+00	2366	Os03g0702800	100,00	0,00E+00	2370	4	NA	Japonica	
augustus_masked-3-processed-gene-42.16-mRNA-1	3	OsR498G0305393200.01.T01	93,25	0,00E+00	1442	Os03g0185000	100,00	0,00E+00	1635	193	Japonica	Japonica	
augustus_masked-3-processed-gene-42.32-mRNA-1	3	OsR498G0305348400.01.T05	99,72	0,00E+00	2540	Os03g0185200	100,00	0,00E+00	2558	18	NA	Japonica	
augustus_masked-3-processed-gene-42.34-mRNA-1	3	OsR498G0305349200.01.T01	99,64	0,00E+00	2451	Os03g0185700	100,00	0,00E+00	2473	22	NA	Japonica	
augustus_masked-3-processed-gene-421.0-mRNA-1	3	OsR498G0306891900.01.T01	100,00	1,89E-114	412	Os03g0705433	100,00	8,53E-177	618	206	Japonica	Japonica	
augustus_masked-3-processed-gene-421.12-mRNA-1	3	OsR498G0306897300.01.T01	99,66	0,00E+00	2091	Os03g0706500	100,00	0,00E+00	2105	14	NA	Japonica	
augustus_masked-3-processed-gene-422.40-mRNA-1	3	OsR498G0306900900.01.T01	99,73	0,00E+00	3365	Os03g0707600	100,00	0,00E+00	3388	23	NA	Japonica	
augustus_masked-3-processed-gene-423.36-mRNA-1	3	OsR498G0306904300.01.T01	99,42	0,00E+00	2128	Os03g0708200	100,00	0,00E+00	2159	31	NA	Japonica	
augustus_masked-3-processed-gene-423.41-mRNA-1	3	OsR498G0306905200.01.T03	98,17	0,00E+00	3878	Os03g0708600	100,00	0,00E+00	3988	110	NA	Japonica	
augustus_masked-3-processed-gene-423.53-mRNA-1	3	OsR498G0306910900.01.T01	99,59	9,45E-123	440	Os09g0552200	96,22	5,25E-108	389	-51	Indica	Japonica	
augustus_masked-3-processed-gene-426.1-mRNA-1	3	OsR498G0306923500.01.T01	99,61	0,00E+00	1844	Os03g0225100	71,15	9,51E-62	238	-1606	Indica	Japonica	Kinase-TM
augustus_masked-3-processed-gene-428.46-mRNA-1	3	OsR498G0306943300.01.T02	99,50	0,00E+00	4271	Os03g0719100	100,00	0,00E+00	4327	56	NA	Japonica	
augustus_masked-3-processed-gene-429.9-mRNA-1	3	OsR498G0306947300.01.T01	99,57	0,00E+00	2511	Os03g0720400	100,00	0,00E+00	2538	27	NA	Japonica	
augustus_masked-3-processed-gene-43.37-mRNA-1	3	OsR498G0305351700.01.T01	99,09	0,00E+00	2333	Os03g0186900	100,00	0,00E+00	2376	43	NA	Japonica	
augustus_masked-3-processed-gene-43.61-mRNA-1	3	OsR498G0305357000.01.T01	99,25	0,00E+00	2122	Os03g0187800	100,00	0,00E+00	2165	43	NA	Japonica	
augustus_masked-3-processed-gene-430.12-mRNA-1	3	OsR498G0306957100.01.T02	100,00	0,00E+00	2298	Os03g0722500	100,00	0,00E+00	2298	0	NA	Japonica	
augustus_masked-3-processed-gene-431.1-mRNA-1	3	OsR498G0306958900.01.T01	99,89	0,00E+00	3124	Os03g0723000	100,00	0,00E+00	3133	9	NA	Japonica	
augustus_masked-3-processed-gene-432.0-mRNA-1	3	OsR498G0306965500.01.T05	99,72	6,38E-180	631	Os03g0724700	100,00	0,00E+00	636	5	NA	Japonica	
augustus_masked-3-processed-gene-433.0-mRNA-1	3	OsR498G0306972700.01.T01	98,16	0,00E+00	2165	Os03g0726400	100,00	0,00E+00	2224	59	NA	Japonica	
augustus_masked-3-processed-gene-434.24-mRNA-1	3	OsR498G0306978500.01.T01	99,55	0,00E+00	2376	Os03g0727800	100,00	0,00E+00	2403	27	NA	Japonica	
augustus_masked-3-processed-gene-434.27-mRNA-1	3	OsR498G0306979100.01.T02	99,84	0,00E+00	3396	Os03g0728200	100,00	0,00E+00	3258	-138	NA	Japonica	
augustus_masked-3-processed-gene-434.28-mRNA-1	3	OsR498G0306979300.01.T01	99,93	0,00E+00	2555	Os03g0728500	100,00	0,00E+00	2560	5	NA	Japonica	
augustus_masked-3-processed-gene-437.47-mRNA-1	3	OsR498G0306994100.01.T01	96,92	0,00E+00	4079	Os03g0732100	100,00	0,00E+00	3382	-697	Indica	Japonica	
augustus_masked-3-processed-gene-438.50-mRNA-1	3	OsR498G0307000500.01.T02	99,12	2,27E-170	599	Os03g0734100	100,00	2,59E-126	450	-149	Indica	Japonica	
augustus_masked-3-processed-gene-438.62-mRNA-1	3	OsR498G0307001400.01.T01	99,05	0,00E+00	929	Os03g0734400	100,00	0,00E+00	953	24	NA	Japonica	
augustus_masked-3-processed-gene-439.13-mRNA-1	3	OsR498G0307006100.01.T01	99,66	0,00E+00	2109	Os03g0736200	100,00	0,00E+00	2127	18	NA	Japonica	
augustus_masked-3-processed-gene-439.14-mRNA-1	3	OsR498G0307007300.01.T01	99,68	0,00E+00	1675	Os03g0787000	70,55	2,16E-88	325	-1350	Indica	Japonica	SYP121
augustus_masked-3-processed-gene-439.15-mRNA-1	3	OsR498G0307007700.01.T01	99,84	0,00E+00	1132	Os03g0736600	100,00	0,00E+00	1137	5	NA	Japonica	
augustus_masked-3-processed-gene-44.14-mRNA-1	3	OsR498G0305365900.01.T01	97,44	0,00E+00	1526	Os03g0189900	100,00	0,00E+00	1348	-178	Indica	Japonica	
augustus_masked-3-processed-gene-44.24-mRNA-1	3	OsR498G0305364200.01.T01	99,51	0,00E+00	2555	Os03g0189300	100,00	0,00E+00	2587	32	NA	Japonica	
augustus_masked-3-processed-gene-441.40-mRNA-1	3	OsR498G0307023100.01.T02	99,88	0,00E+00	1435	Os03g0741600	100,00	0,00E+00	1440	5	NA	Japonica	
augustus_masked-3-processed-gene-442.52-mRNA-1	3	OsR498G0307030000.01.T01	97,70	9,95E-169	592	Os03g0743650	100,00	4,80E-180	628	36	NA	Japonica	
augustus_masked-3-processed-gene-444.32-mRNA-1	3	OsR498G0307041400.01.T01	99,29	0,00E+00	1489	Os03g0746700	98,93	0,00E+00	1484	-5	NA	Japonica	
augustus_masked-3-processed-gene-445.12-mRNA-1	3	OsR498G0307045700.01.T01	99,47	0,00E+00	2992	Os03g0748000	99,82	0,00E+00	3017	25	NA	Japonica	
augustus_masked-3-processed-gene-446.39-mRNA-1	3	OsR498G0307051300.01.T01	99,68	4,60E-160	564	Os03g0749400	100,00	9,45E-170	594	30	NA	Japonica	
augustus_masked-3-processed-gene-446.45-mRNA-1	3	OsR498G0307053300.01.T02	96,65	0,00E+00	843	Os03g0750500	100,00	0,00E+00	910	67	NA	Japonica	
augustus_masked-3-processed-gene-448.17-mRNA-1	3	OsR498G0307062200.01.T01	99,79	0,00E+00	2608	Os03g0753500	100,00	0,00E+00	2621	13	NA	Japonica	
augustus_masked-3-processed-gene-448.18-mRNA-1	3	OsR498G0305603400.01.T05	95,00	1,59E-123	443	Os03g0753901	100,00	0,00E+00	861	418	Japonica	NA	
augustus_masked-3-processed-gene-45.19-mRNA-1	3	OsR498G0305370400.01.T03	99,44	0,00E+00	2556	Os03g0191700	100,00	0,00E+00	2592	36	NA	Japonica	
augustus_masked-3-processed-gene-450.56-mRNA-1	3	OsR498G0307070000.01.T03	99,78	0,00E+00	5650	Os03g0756200	100,00	0,00E+00	5681	31	NA	Japonica	Kinase-LRR-TM
augustus_masked-3-processed-gene-451.17-mRNA-1	3	OsR498G0307074100.01.T01	99,48	0,00E+00	2398	Os03g0757600	100,00	0,00E+00	2430	32	NA	Japonica	
augustus_masked-3-processed-gene-451.28-mRNA-1	3	OsR498G0307073200.01.T01	99,79	0,00E+00	2535	Os03g0757100	100,00	0,00E+00	2549	14	NA	Japonica	
augustus_masked-3-processed-gene-452.60-mRNA-1	3	OsR498G0307082900.01.T01	99,04	0,00E+00	2581	Os03g0759700	100,00	0,00E+00	1721	-860	Indica	Japonica	
augustus_masked-3-processed-gene-452.62-mRNA-1	3	OsR498G0307083700.01.T02	99,68	0,00E+00	1699	Os03g0760000	100,00	0,00E+00	1873	174	NA	Japonica	
augustus_masked-3-processed-gene-452.65-mRNA-1	3	OsR498G0307084100.01.T01	98,94	3,09E-139	496	Os03g0760100	100,00	8,85E-141	499	3	NA	Japonica	
augustus_masked-3-processed-gene-453.48-mRNA-1	3	OsR498G0307086900.01.T01	99,93	0,00E+00	2620	Os03g0761300	100,00	0,00E+00	2625	5	NA	Japonica	
augustus_masked-3-processed-gene-453.78-mRNA-1	3	OsR498G0307087600.01.T01	99,56	0,00E+00	4094	Os03g0761500	100,00	0,00E+00	4140	46	NA	Japonica	
augustus_masked-3-processed-gene-453.81-mRNA-1	3	OsR498G0307092400.01.T01	99,34	0,00E+00	1618	Os03g0762700	100,00	0,00E+00	1645	27	NA	Japonica	
augustus_masked-3-processed-gene-455.35-mRNA-1	3	OsR498G0307100600.01.T01	99,91	0,00E+00	1944	Os03g0764900	100,00	0,00E+00	1948	4	NA	Japonica	
augustus_masked-3-processed-gene-455.39-mRNA-1	3	OsR498G0307104500.01.T01	99,72	0,00E+00	5029	Os03g0765800	100,00	0,00E+00	5065	36	NA	Japonica	
augustus_masked-3-processed-gene-456.26-mRNA-1	3	OsR498G0307107500.01.T04	99,84	0,00E+00	1118	Os03g0766900	100,00	0,00E+00	1122	4	NA	Japonica	
augustus_masked-3-processed-gene-456.33-mRNA-1	3	OsR498G0307107800.01.T01	99,87	0,00E+00	2767	Os03g0767000	100,00	0,00E+00	2776	9	NA	Japonica	
augustus_masked-3-processed-gene-457.23-mRNA-1	3	OsR498G0307115300.01.T01	99,09	0,00E+00	1947	Os03g0769900	100,00	0,00E+00	1992	45	NA	Japonica	
augustus_masked-3-processed-gene-458.45-mRNA-1	3	OsR498G0307124500.01.T01	99,51	0,00E+00	2550	Os03g0772600	100,00	0,00E+00	1505	-1045	Indica	Japonica	Kinase-TM
augustus_masked-3-processed-gene-458.51-mRNA-1	3	OsR498G0307125400.01.T01	98,71	0,00E+00	2305	Os04g0531500	72,40	3,98E-131	469	-1836	Indica	Japonica	Kinase
augustus_masked-3-processed-gene-458.53-mRNA-1	3	OsR498G0307126600.01.T01	99,86	0,00E+00	1300	Os03g0772900	100,00	0,00E+00	1305	5	NA	Japonica	
augustus_masked-3-processed-gene-46.58-mRNA-1	3	OsR498G0305375300.01.T05	99,94	0,00E+00	2896	Os03g0193000	100,00	0,00E+00	2901	5	NA	Japonica	
augustus_masked-3-processed-gene-460.35-mRNA-1	3	OsR498G0307139600.01.T01	99,77	0,00E+00	2292	Os03g0776400	100,00	0,00E+00	2305	13	NA	Japonica	
augustus_masked-3-processed-gene-461.14-mRNA-1	3	OsR498G0307149500.01.T01	100,00	0,00E+00	1121	Os03g0778400	100,00	0,00E+00	1086	-35	NA	Japonica	
augustus_masked-3-processed-gene-462.2-mRNA-1	3	OsR498G0307152300.01.T02	100,00	0,00E+00	2408	Os03g0780600	100,00	0,00E+00	2408	0	NA	NA	
augustus_masked-3-processed-gene-462.21-mRNA-1	3	OsR498G0307156300.01.T03	100,00	0,00E+00	1283	Os03g0782200	100,00	0,00E+00	1283	0	NA	NA	
augustus_masked-3-processed-gene-463.19-mRNA-1	3	OsR498G0307161100.01.T01	99,07	0,00E+00	756	Os03g0783100	85,27	2,57E-102	370	-386	Indica	Japonica	
augustus_masked-3-processed-gene-463.29-mRNA-1	3	OsR498G0307165900.01.T08	94,03	0,00E+00	1415	Os03g0784000	94,03	0,00E+00	1415	0	NA	Japonica	
augustus_masked-3-processed-gene-465.44-mRNA-1	3	OsR498G0307172600.01.T01	99,51	0,00E+00	2168	Os03g0786600	100,00	0,00E+00	2197	29	NA	Japonica	
augustus_masked-3-processed-gene-465.50-mRNA-1	3	OsR498G0307176100.01.T01	99,36	0,00E+00	1113	Os03g0788100	100,00	0,00E+00	1131	18	NA	Japonica	
augustus_masked-3-processed-gene-466.1-mRNA-1	3	OsR498G0307176700.01.T02	99,75	0,00E+00	6371	Os03g0788300	100,00	0,00E+00	6412	41	NA	Japonica	
augustus_masked-3-processed-gene-466.8-mRNA-1	3	OsR498G0307177200.01.T01	99,93	0,00E+00	2434	Os03g0788600	100,00	0,00E+00	2439	5	NA	Japonica	
augustus_masked-3-processed-gene-467.46-mRNA-1	3	OsR498G0307182700.01.T01	99,82	0,00E+00	1945	Os03g0790500	100,00	0,00E+00	1954	9	NA	Japonica	
augustus_masked-3-processed-gene-468.20-mRNA-1	3	OsR498G0307194900.01.T01	98,95	0,00E+00	1000	Os03g0794000	99,47	0,00E+00	1013	13	NA	Japonica	
augustus_masked-3-processed-gene-468.30-mRNA-1	3	OsR498G0307191600.01.T01	99,80	0,00E+00	916	Os03g0793000	100,00	0,00E+00	921	5	NA	Japonica	
augustus_masked-3-processed-gene-469.46-mRNA-1	3	OsR498G0307197700.01.T01	99,89	0,00E+00	3303	Os03g0795200	100,00	0,00E+00	3312	9	NA	Japonica	
augustus_masked-3-processed-gene-469.63-mRNA-1	3	OsR498G0511251800.01.T01	82,59	4,26E-52	205	Os03g0796100	100,00	3,47E-149	526	321	Japonica	Japonica	
augustus_masked-3-processed-gene-47.16-mRNA-1	3	OsR498G0305379000.01.T04	100,00	0,00E+00	2181	Os03g0194100	100,00	0,00E+00	2181	0	NA	Japonica	
augustus_masked-3-processed-gene-47.17-mRNA-1	3	OsR498G0305379200.01.T01	99,92	0,00E+00	2258	Os03g0194300	100,00	0,00E+00	2262	4	NA	Japonica	
augustus_masked-3-processed-gene-47.6-mRNA-1	3	OsR498G0305379700.01.T02	99,49	0,00E+00	1037	Os03g0194600	100,00	0,00E+00	1050	13	NA	Japonica	
augustus_masked-3-processed-gene-470.39-mRNA-1	3	OsR498G0307212100.01.T04	99,89	0,00E+00	1592	Os03g0797600	100,00	0,00E+00	1597	5	NA	Japonica	
augustus_masked-3-processed-gene-470.48-mRNA-1	3	OsR498G0307209000.01.T03	99,73	0,00E+00	1346	Os03g0797000	100,00	0,00E+00	1355	9	NA	Japonica	
augustus_masked-3-processed-gene-471.32-mRNA-1	3	OsR498G0307023100.01.T02	71,67	5,56E-49	196	Os03g0798400	100,00	0,00E+00	1251	1055	Japonica	Japonica	
augustus_masked-3-processed-gene-472.55-mRNA-1	3	OsR498G0307220700.01.T01	99,52	0,00E+00	3363	Os03g0800000	100,00	0,00E+00	2107	-1256	Indica	Japonica	
augustus_masked-3-processed-gene-472.78-mRNA-1	3	OsR498G0307223000.01.T01	97,41	0,00E+00	844	Os03g0801350	100,00	0,00E+00	877	33	NA	Japonica	
augustus_masked-3-processed-gene-473.14-mRNA-1	3	OsR498G0713673300.01.T01	80,04	0,00E+00	1312	Os03g0802500	100,00	0,00E+00	2819	1507	Japonica	Japonica	
augustus_masked-3-processed-gene-473.17-mRNA-1	3	OsR498G0307226500.01.T01	99,41	0,00E+00	2734	Os03g0802600	100,00	0,00E+00	1581	-1153	Indica	Japonica	
augustus_masked-3-processed-gene-473.3-mRNA-1	3	OsR498G0307225100.01.T01	99,78	0,00E+00	2498	Os03g0802150	100,00	0,00E+00	2511	13	NA	Japonica	
augustus_masked-3-processed-gene-473.4-mRNA-1	3	OsR498G0102093100.01.T01	83,33	9,10E-40	163	Os03g0802200	100,00	2,28E-105	379	216	Japonica	Japonica	
augustus_masked-3-processed-gene-474.10-mRNA-1	3	OsR498G0307232300.01.T01	99,86	0,00E+00	1251	Os03g0804700	99,86	0,00E+00	1251	0	NA	NA	
augustus_masked-3-processed-gene-474.4-mRNA-1	3	OsR498G0307229600.01.T07	99,95	0,00E+00	3545	Os03g0803900	99,95	0,00E+00	3545	0	NA	NA	
augustus_masked-3-processed-gene-474.9-mRNA-1	3	OsR498G0307231500.01.T01	100,00	0,00E+00	1267	Os03g0804500	100,00	0,00E+00	1267	0	NA	NA	
augustus_masked-3-processed-gene-475.58-mRNA-1	3	OsR498G0307240900.01.T01	99,88	0,00E+00	1453	Os03g0806700	100,00	0,00E+00	1458	5	NA	NA	
augustus_masked-3-processed-gene-476.15-mRNA-1	3	OsR498G0307245900.01.T01	100,00	0,00E+00	2673	Os03g0808200	100,00	0,00E+00	2673	0	NA	NA	
augustus_masked-3-processed-gene-476.34-mRNA-1	3	OsR498G0307249200.01.T01	100,00	0,00E+00	1012	Os03g0809000	100,00	0,00E+00	1012	0	NA	Japonica	
augustus_masked-3-processed-gene-477.48-mRNA-1	3	OsR498G0307251700.01.T01	99,63	0,00E+00	1920	Os03g0810100	100,00	0,00E+00	1938	18	NA	Japonica	
augustus_masked-3-processed-gene-478.19-mRNA-1	3	OsR498G0307258000.01.T01	100,00	6,64E-105	379	Os03g0811800	100,00	2,31E-105	379	0	NA	Japonica	
augustus_masked-3-processed-gene-478.8-mRNA-1	3	OsR498G0307257700.01.T01	100,00	0,00E+00	702	Os03g0811700	100,00	0,00E+00	710	8	NA	Japonica	
augustus_masked-3-processed-gene-48.14-mRNA-1	3	OsR498G0305387400.01.T01	100,00	0,00E+00	753	Os03g0196250	100,00	0,00E+00	753	0	NA	Japonica	
augustus_masked-3-processed-gene-48.15-mRNA-1	3	OsR498G0305390400.01.T01	99,47	0,00E+00	1341	Os02g0316200	86,09	0,00E+00	696	-645	Indica	Japonica	
augustus_masked-3-processed-gene-48.16-mRNA-1	3	OsR498G0305391600.01.T01	100,00	0,00E+00	969	Os03g0196500	100,00	0,00E+00	969	0	NA	Japonica	
augustus_masked-3-processed-gene-48.17-mRNA-1	3	OsR498G0305393200.01.T01	99,90	0,00E+00	1706	Os03g0196600	100,00	0,00E+00	1710	4	NA	Japonica	
augustus_masked-3-processed-gene-480.66-mRNA-1	3	OsR498G0307268600.01.T01	99,45	0,00E+00	1292	Os03g0815800	100,00	0,00E+00	1310	18	NA	Japonica	
augustus_masked-3-processed-gene-481.1-mRNA-1	3	OsR498G0307275500.01.T01	99,52	0,00E+00	2244	Os03g0817200	100,00	0,00E+00	2268	24	NA	Japonica	

augustus_masked-3-processed-gene-482.11-mRNA-1	3	OsR498G0307285100.01.T04	99,81	0,00E+00	2865	Os03g0820100	100,00	0,00E+00	2879	14	NA	Japonica	
augustus_masked-3-processed-gene-482.15-mRNA-1	3	OsR498G0307286500.01.T01	100,00	0,00E+00	756	Os03g0820600	100,00	0,00E+00	756	0	NA	Japonica	
augustus_masked-3-processed-gene-483.47-mRNA-1	3	OsR498G0307292500.01.T01	100,00	0,00E+00	2156	Os03g0821900	100,00	0,00E+00	2156	0	NA	Japonica	Kinase-TM
augustus_masked-3-processed-gene-483.63-mRNA-1	3	OsR498G0307291800.01.T01	99,72	0,00E+00	3241	Os03g0821700	100,00	0,00E+00	3263	22	NA	Japonica	
augustus_masked-3-processed-gene-484.15-mRNA-1	3	OsR498G0307299700.01.T01	96,89	0,00E+00	2190	Os03g0824600	100,00	0,00E+00	2549	359	Japonica	Japonica	
augustus_masked-3-processed-gene-484.2-mRNA-1	3	OsR498G0307296200.01.T01	99,39	0,00E+00	873	Os03g0823400	100,00	3,36E-133	473	-400	Indica	Japonica	
augustus_masked-3-processed-gene-484.21-mRNA-1	3	OsR498G0307297400.01.T01	99,85	0,00E+00	1165	Os03g0823700	100,00	0,00E+00	1169	4	NA	Japonica	
augustus_masked-3-processed-gene-484.25-mRNA-1	3	OsR498G0307298400.01.T02	98,81	0,00E+00	872	Os03g0824000	100,00	2,65E-147	520	-352	Indica	Japonica	
augustus_masked-3-processed-gene-485.64-mRNA-1	3	OsR498G0307305400.01.T01	97,03	0,00E+00	3022	Os03g0826500	97,14	0,00E+00	3031	9	NA	Japonica	
augustus_masked-3-processed-gene-486.14-mRNA-1	3	OsR498G0307310600.01.T02	99,83	0,00E+00	4300	Os03g0828800	100,00	0,00E+00	4318	18	NA	Japonica	Kinase-TM
augustus_masked-3-processed-gene-486.29-mRNA-1	3	OsR498G0307312600.01.T01	98,76	0,00E+00	847	Os03g0829600	100,00	0,00E+00	888	41	NA	Japonica	
augustus_masked-3-processed-gene-489.15-mRNA-1	3	OsR498G0307332500.01.T02	100,00	0,00E+00	834	Os03g0835900	100,00	0,00E+00	834	0	NA	NA	
augustus_masked-3-processed-gene-490.33-mRNA-1	3	OsR498G0307339000.01.T02	99,88	0,00E+00	4635	Os03g0838100	100,00	0,00E+00	4648	13	NA	Japonica	Kinase-TM
augustus_masked-3-processed-gene-491.40-mRNA-1	3	OsR498G0307345500.01.T01	96,95	0,00E+00	2567	Os03g0840200	100,00	0,00E+00	3728	1161	Japonica	NA	
augustus_masked-3-processed-gene-492.101-mRNA-1	3	OsR498G0307353000.01.T03	100,00	0,00E+00	1707	Os03g0843300	100,00	0,00E+00	1707	0	NA	Japonica	
augustus_masked-3-processed-gene-494.5-mRNA-1	3	OsR498G0307359400.01.T01	99,78	0,00E+00	2487	Os03g0845600	100,00	0,00E+00	2500	13	NA	Japonica	
augustus_masked-3-processed-gene-496.13-mRNA-1	3	OsR498G0306908200.01.T01	84,55	3,57E-105	382	Os03g0850900	100,00	0,00E+00	682	300	Japonica	NA	
augustus_masked-3-processed-gene-496.15-mRNA-1	3	OsR498G0307374700.01.T01	100,00	0,00E+00	814	Os03g0851200	100,00	0,00E+00	814	0	NA	NA	
augustus_masked-3-processed-gene-496.24-mRNA-1	3	OsR498G0307370800.01.T02	100,00	0,00E+00	1391	Os03g0850300	100,00	0,00E+00	1391	0	NA	Japonica	
augustus_masked-3-processed-gene-496.26-mRNA-1	3	OsR498G0307373200.01.T01	100,00	0,00E+00	1418	Os03g0850700	100,00	0,00E+00	1418	0	NA	Japonica	
augustus_masked-3-processed-gene-496.32-mRNA-1	3	OsR498G0307375200.01.T01	99,85	0,00E+00	2394	Os03g0851300	100,00	0,00E+00	2403	9	NA	Japonica	
augustus_masked-3-processed-gene-497.11-mRNA-1	3	OsR498G0307378500.01.T01	99,69	0,00E+00	2838	Os03g0852800	100,00	0,00E+00	2857	19	NA	Japonica	
augustus_masked-3-processed-gene-497.24-mRNA-1	3	OsR498G0307377400.01.T01	100,00	0,00E+00	1191	Os03g0852400	100,00	0,00E+00	1191	0	NA	Japonica	
augustus_masked-3-processed-gene-497.26-mRNA-1	3	OsR498G0307378000.01.T01	99,48	0,00E+00	2756	Os03g0852700	100,00	0,00E+00	2744	-12	NA	Japonica	
augustus_masked-3-processed-gene-498.5-mRNA-1	3	OsR498G0307382200.01.T02	99,86	0,00E+00	5155	Os03g0853700	100,00	0,00E+00	5173	18	NA	Japonica	
augustus_masked-3-processed-gene-499.41-mRNA-1	3	OsR498G0307391300.01.T01	99,54	0,00E+00	1939	Os03g0856000	100,00	0,00E+00	1965	26	NA	Japonica	
augustus_masked-3-processed-gene-499.42-mRNA-1	3	OsR498G0307391500.01.T01	99,43	0,00E+00	3763	Os03g0856100	100,00	0,00E+00	3225	-538	Indica	Japonica	
augustus_masked-3-processed-gene-50.6-mRNA-1	3	OsR498G0305405700.01.T01	99,83	0,00E+00	2096	Os03g0199100	100,00	0,00E+00	2105	9	NA	Japonica	
augustus_masked-3-processed-gene-500.0-mRNA-1	3	OsR498G0307394300.01.T01	99,82	0,00E+00	2010	Os03g0856700	100,00	0,00E+00	2019	9	NA	Japonica	
augustus_masked-3-processed-gene-500.11-mRNA-1	3	OsR498G0307396700.01.T01	99,00	0,00E+00	1410	Os03g0857600	100,00	0,00E+00	1445	35	NA	Japonica	
augustus_masked-3-processed-gene-500.21-mRNA-1	3	OsR498G0307397000.01.T01	100,00	1,80E-158	558	Os03g0857750	100,00	1,21E-142	504	-54	NA	Japonica	
augustus_masked-3-processed-gene-500.26-mRNA-1	3	OsR498G0307399000.01.T01	99,16	0,00E+00	2526	Os03g0858600	100,00	0,00E+00	2576	50	NA	Japonica	
augustus_masked-3-processed-gene-500.9-mRNA-1	3	OsR498G0307395900.01.T01	98,06	3,28E-150	531	Os03g0857100	100,00	0,00E+00	715	184	Japonica	Japonica	
augustus_masked-3-processed-gene-501.21-mRNA-1	3	OsR498G0307403900.01.T01	99,64	0,00E+00	1984	Os03g0860600	100,00	0,00E+00	1959	-25	NA	Japonica	
augustus_masked-3-processed-gene-501.3-mRNA-1	3	OsR498G0307399200.01.T05	99,79	0,00E+00	2584	Os03g0858800	100,00	0,00E+00	2598	14	NA	Japonica	
augustus_masked-3-processed-gene-501.32-mRNA-1	3	OsR498G0307401600.01.T01	100,00	0,00E+00	1223	Os03g0860100	100,00	0,00E+00	1223	0	NA	Japonica	
augustus_masked-3-processed-gene-502.17-mRNA-1	3	OsR498G0307406100.01.T05	99,85	0,00E+00	3515	Os03g0861900	100,00	0,00E+00	3528	13	NA	NA	
augustus_masked-3-processed-gene-502.9-mRNA-1	3	OsR498G0307406100.01.T05	99,85	0,00E+00	3515	Os03g0861900	100,00	0,00E+00	3528	13	NA	NA	
augustus_masked-3-processed-gene-52.21-mRNA-1	3	OsR498G0408675500.01.T01	90,55	1,27E-116	420	Os03g0204201	100,00	8,53E-177	618	198	Japonica	Japonica	
augustus_masked-3-processed-gene-53.17-mRNA-1	3	OsR498G0305428000.01.T03	97,99	0,00E+00	2912	Os03g0206100	99,03	0,00E+00	3094	182	NA	Japonica	
augustus_masked-3-processed-gene-54.18-mRNA-1	3	OsR498G0305434900.01.T01	98,81	0,00E+00	2805	Os03g0208600	100,00	0,00E+00	2879	74	NA	Japonica	
augustus_masked-3-processed-gene-55.10-mRNA-1	3	OsR498G0305438500.01.T05	99,65	3,30E-142	505	Os03g0209600	100,00	2,70E-144	509	4	NA	Japonica	
augustus_masked-3-processed-gene-55.12-mRNA-1	3	OsR498G0305438700.01.T04	99,83	0,00E+00	1076	Os03g0209700	100,00	0,00E+00	836	-240	Indica	Japonica	
augustus_masked-3-processed-gene-55.24-mRNA-1	3	OsR498G0305439800.01.T01	100,00	0,00E+00	639	Os03g0210200	78,69	6,41E-52	203	-436	Indica	Japonica	
augustus_masked-3-processed-gene-55.28-mRNA-1	3	OsR498G0305441000.01.T01	99,66	0,00E+00	2107	Os03g0210600	100,00	0,00E+00	1153	-954	Indica	Japonica	
augustus_masked-3-processed-gene-56.17-mRNA-1	3	OsR498G0305445700.01.T01	98,29	0,00E+00	1525	Os03g0212000	99,93	0,00E+00	2669	1144	Japonica	Japonica	
augustus_masked-3-processed-gene-56.2-mRNA-1	3	OsR498G0305443500.01.T02	99,74	0,00E+00	4150	Os03g0211400	99,91	0,00E+00	4168	18	NA	Japonica	
augustus_masked-3-processed-gene-57.51-mRNA-1	3	OsR498G0305448900.01.T03	93,92	0,00E+00	1451	Os03g0213200	97,02	0,00E+00	1134	-317	Indica	Japonica	
augustus_masked-3-processed-gene-57.73-mRNA-1	3	OsR498G0305450400.01.T01	99,79	0,00E+00	846	Os03g0213800	100,00	0,00E+00	850	4	NA	Japonica	
augustus_masked-3-processed-gene-58.35-mRNA-1	3	OsR498G0305452800.01.T01	100,00	0,00E+00	2006	Os03g0215000	100,00	0,00E+00	2003	-3	NA	Japonica	
augustus_masked-3-processed-gene-59.34-mRNA-1	3	OsR498G0305457400.01.T01	99,71	0,00E+00	3636	Os03g0215900	100,00	0,00E+00	3663	27	NA	Japonica	
augustus_masked-3-processed-gene-59.35-mRNA-1	3	OsR498G0305457800.01.T01	99,89	0,00E+00	1608	Os03g0216000	100,00	0,00E+00	1613	5	NA	Japonica	
augustus_masked-3-processed-gene-59.55-mRNA-1	3	OsR498G0305459600.01.T01	99,81	0,00E+00	1891	Os03g0216500	100,00	0,00E+00	1900	9	NA	Japonica	
augustus_masked-3-processed-gene-60.14-mRNA-1	3	OsR498G0204385500.01.T01	81,25	4,61E-66	251	Os03g0219500	100,00	0,00E+00	720	469	Japonica	Japonica	
augustus_masked-3-processed-gene-60.21-mRNA-1	3	OsR498G0305464200.01.T01	99,89	0,00E+00	3124	Os03g0218500	100,00	0,00E+00	3133	9	NA	Japonica	
augustus_masked-3-processed-gene-61.4-mRNA-1	3	OsR498G0305467400.01.T01	99,71	0,00E+00	3084	Os03g0219700	100,00	0,00E+00	3106	22	NA	Japonica	
augustus_masked-3-processed-gene-62.32-mRNA-1	3	OsR498G0305478400.01.T01	99,88	0,00E+00	2919	Os03g0223100	100,00	0,00E+00	2928	9	NA	Japonica	
augustus_masked-3-processed-gene-63.45-mRNA-1	3	OsR498G0305485800.01.T01	99,90	0,00E+00	1836	Os03g0225100	100,00	0,00E+00	1840	4	NA	NA	Kinase-TM
augustus_masked-3-processed-gene-63.50-mRNA-1	3	OsR498G0305487200.01.T01	99,58	0,00E+00	2522	Os03g0225300	99,93	0,00E+00	2544	22	NA	Japonica	
augustus_masked-3-processed-gene-64.37-mRNA-1	3	OsR498G0305488900.01.T01	99,79	0,00E+00	2579	Os03g0225900	100,00	0,00E+00	2592	13	NA	Japonica	
augustus_masked-3-processed-gene-67.30-mRNA-1	3	OsR498G0305507900.01.T01	100,00	0,00E+00	2511	Os03g0230300	100,00	0,00E+00	2511	0	NA	NA	
augustus_masked-3-processed-gene-68.20-mRNA-1	3	OsR498G0305516600.01.T08	99,24	0,00E+00	2568	Os03g0232000	100,00	0,00E+00	2576	8	NA	Japonica	
augustus_masked-3-processed-gene-68.43-mRNA-1	3	OsR498G0305520600.01.T01	99,78	0,00E+00	2476	Os03g0232600	100,00	0,00E+00	2489	13	NA	Japonica	
augustus_masked-3-processed-gene-69.20-mRNA-1	3	OsR498G0305528900.01.T01	99,81	0,00E+00	2811	Os03g0235200	100,00	0,00E+00	3474	663	Japonica	Japonica	
augustus_masked-3-processed-gene-7.58-mRNA-1	3	OsR498G0305123700.01.T05	99,82	0,00E+00	3039	Os03g0116900	100,00	0,00E+00	3052	13	NA	Japonica	
augustus_masked-3-processed-gene-71.40-mRNA-1	3	OsR498G0305538800.01.T01	100,00	0,00E+00	1288	Os03g0237950	100,00	0,00E+00	845	-443	Indica	Japonica	
augustus_masked-3-processed-gene-71.61-mRNA-1	3	OsR498G0305543200.01.T02	99,86	0,00E+00	1300	Os03g0239300	100,00	0,00E+00	1305	5	NA	Japonica	
augustus_masked-3-processed-gene-72.40-mRNA-1	3	OsR498G0305545900.01.T01	99,81	0,00E+00	4723	Os03g0240400	100,00	0,00E+00	5027	304	NA	Japonica	
augustus_masked-3-processed-gene-72.41-mRNA-1	3	OsR498G0305546700.01.T01	100,00	0,00E+00	720	Os03g0240800	100,00	0,00E+00	720	0	NA	Japonica	
augustus_masked-3-processed-gene-72.47-mRNA-1	3	OsR498G0305546400.01.T01	99,85	0,00E+00	2405	Os03g0240600	100,00	0,00E+00	2414	9	NA	Japonica	
augustus_masked-3-processed-gene-73.67-mRNA-1	3	OsR498G0305550600.01.T01	99,85	0,00E+00	1235	Os03g0242300	100,00	0,00E+00	1240	5	NA	Japonica	
augustus_masked-3-processed-gene-73.69-mRNA-1	3	OsR498G0305553400.01.T01	99,92	0,00E+00	4217	Os03g0242900	100,00	0,00E+00	4226	9	NA	Japonica	
augustus_masked-3-processed-gene-74.55-mRNA-1	3	OsR498G0305555400.01.T01	97,72	0,00E+00	1127	Os03g0243400	100,00	0,00E+00	1856	729	Japonica	Japonica	
augustus_masked-3-processed-gene-74.62-mRNA-1	3	OsR498G0305557100.01.T01	99,80	0,00E+00	1793	Os03g0244200	100,00	0,00E+00	1802	9	NA	Japonica	
augustus_masked-3-processed-gene-74.75-mRNA-1	3	OsR498G0305559400.01.T01	96,69	0,00E+00	1413	Os03g0244950	100,00	0,00E+00	899	-514	Indica	Japonica	
augustus_masked-3-processed-gene-75.42-mRNA-1	3	OsR498G0305563100.01.T01	97,80	1,43E-153	543	Os06g0236300	86,97	2,12E-95	347	-196	Indica	Japonica	
augustus_masked-3-processed-gene-75.51-mRNA-1	3	OsR498G0305562600.01.T01	99,84	0,00E+00	2206	Os03g0246100	100,00	0,00E+00	2215	9	NA	Japonica	
augustus_masked-3-processed-gene-76.30-mRNA-1	3	OsR498G0305567700.01.T01	99,74	0,00E+00	3493	Os03g0247100	100,00	0,00E+00	3512	19	NA	Japonica	
augustus_masked-3-processed-gene-77.26-mRNA-1	3	OsR498G0305584400.01.T01	100,00	0,00E+00	699	Os03g0250400	100,00	0,00E+00	699	0	NA	Japonica	
augustus_masked-3-processed-gene-77.4-mRNA-1	3	OsR498G0305578700.01.T01	99,66	0,00E+00	2602	Os03g0249500	100,00	0,00E+00	2625	23	NA	Japonica	
augustus_masked-3-processed-gene-78.2-mRNA-1	3	OsR498G0305588100.01.T01	99,75	0,00E+00	3641	Os03g0251700	99,95	0,00E+00	3650	9	NA	Japonica	Kinase-TM
augustus_masked-3-processed-gene-78.6-mRNA-1	3	OsR498G0305586800.01.T04	99,87	0,00E+00	1333	Os03g0251350	100,00	0,00E+00	1337	4	NA	Japonica	
augustus_masked-3-processed-gene-79.1-mRNA-1	3	OsR498G0305593900.01.T01	99,80	0,00E+00	2741	Os03g0253200	100,00	0,00E+00	2700	-41	NA	Japonica	Kinase-TM
augustus_masked-3-processed-gene-79.14-mRNA-1	3	OsR498G0305597100.01.T01	99,72	0,00E+00	2526	Os03g0254400	100,00	0,00E+00	2544	18	NA	Japonica	
augustus_masked-3-processed-gene-79.8-mRNA-1	3	OsR498G0305594600.01.T10	99,69	0,00E+00	2304	Os03g0253500	100,00	0,00E+00	2322	18	NA	Japonica	
augustus_masked-3-processed-gene-8.48-mRNA-1	3	OsR498G0305132500.01.T01	99,77	0,00E+00	2287	Os03g0119700	100,00	0,00E+00	2365	78	NA	Japonica	
augustus_masked-3-processed-gene-8.49-mRNA-1	3	OsR498G0305133100.01.T01	100,00	1,80E-158	558	Os03g0119900	100,00	6,27E-159	558	0	NA	Japonica	
augustus_masked-3-processed-gene-8.57-mRNA-1	3	OsR498G0305130200.01.T01	99,91	0,00E+00	4076	Os03g0119300	100,00	0,00E+00	4085	9	NA	Japonica	
augustus_masked-3-processed-gene-82.29-mRNA-1	3	OsR498G0305610500.01.T01	99,71	0,00E+00	3033	Os03g0258900	99,88	0,00E+00	3048	15	NA	Japonica	
augustus_masked-3-processed-gene-83.29-mRNA-1	3	OsR498G0305617800.01.T01	99,16	0,00E+00	2096	Os03g0261800	99,92	0,00E+00	2133	37	NA	Japonica	
augustus_masked-3-processed-gene-84.12-mRNA-1	3	OsR498G0305623600.01.T01	99,36	0,00E+00	1379	Os03g0263000	100,00	0,00E+00	1402	23	NA	Japonica	
augustus_masked-3-processed-gene-84.2-mRNA-1	3	OsR498G0305623100.01.T01	99,86	0,00E+00	2594	Os03g0262900	96,37	0,00E+00	1933	-661	Indica	Japonica	
augustus_masked-3-processed-gene-84.22-mRNA-1	3	OsR498G0305627100.01.T01	99,80	0,00E+00	4571	Os03g0263900	100,00	0,00E+00	4594	23	NA	Japonica	
augustus_masked-3-processed-gene-84.37-mRNA-1	3	OsR498G0305631000.01.T01	99,93	0,00E+00	2458	Os03g0264600	100,00	0,00E+00	2462	4	NA	Japonica	
augustus_masked-3-processed-gene-84.38-mRNA-1	3	OsR498G0305631200.01.T01	99,72	0,00E+00	2590	Os03g0264700	100,00	0,00E+00	2608	18	NA	Japonica	
augustus_masked-3-processed-gene-85.15-mRNA-1	3	OsR498G0305635900.01.T06	100,00	0,00E+00	910	Os03g0266000	100,00	0,00E+00	724	-186	Indica	Japonica	
augustus_masked-3-processed-gene-86.70-mRNA-1	3	OsR498G0305639000.01.T01	99,58	0,00E+00	857	Os03g0267200	100,00	0,00E+00	866	9	NA	Japonica	
augustus_masked-3-processed-gene-88.22-mRNA-1	3	OsR498G0305655100.01.T01	99,76	0,00E+00	1507	Os03g0271600	100,00	0,00E+00	1516	9	NA	Japonica	
augustus_masked-3-processed-gene-9.46-mRNA-1	3	OsR498G0305138300.01.T02	98,72	0,00E+00	1638	Os03g0120900	100,00	0,00E+00	1689	51	NA	Japonica	
augustus_masked-3-processed-gene-90.20-mRNA-1	3	OsR498G0305672800.01.T02	99,69	0,00E+00	3501	Os03g0275500	100,00	0,00E+00	3528	27	NA	Japonica	
augustus_masked-3-processed-gene-90.9-mRNA-1	3	OsR498G0305669100.01.T01	99,89	0,00E+00	1625	Os03g0274700	100,00	0,00E+00	1629	4	NA	Japonica	
augustus_masked-3-processed-gene-91.38-mRNA-1	3	OsR498G0305680500.01.T03	100,00	0,00E+00	2922	Os01g0151100	70,62	1,23E-138	493	-2429	Indica	NA	
augustus_masked-3-processed-gene-92.28-mRNA-1	3	OsR498G0305687200.01.T04	100,00	0,00E+00	1148	Os03g0278900	100,00	0,00E+00	1148	0	NA	NA	
augustus_masked-3-processed-gene-93.20-mRNA-1	3	OsR498G0305698000.01.T01	100,00	0,00E+00	2214	Os03g0281201	100,00	0,00E+00	1694	-520	Indica	NA	
augustus_masked-3-processed-gene-94.36-mRNA-1	3	OsR498G0305699500.01.T01	100,00	0,00E+00	3360	Os03g0281500	100,00	0,00E+00	4545	1185	Japonica	NA	Kinase-TM
augustus_masked-3-processed-gene-94.48-mRNA-1	3	OsR498G0305701100.01.T01	100,00	0,00E+00	4264	Os03g0281900	100,00	0,00E+00	4264	0	NA	NA	
augustus_masked-3-processed-gene-94.53-mRNA-1	3	OsR498G0305706100.01.T01	100,00	0,00E+00	2192	Os03g0282700	100,00	0,00E+00	2192	0	NA	NA	
augustus_masked-3-processed-gene-95.14-mRNA-1	3	OsR498G0305709700.01.T02	100,00	0,00E+00	865	Os03g0284000	100,00	0,00E+00	996	131	Japonica	NA	
augustus_masked-3-processed-gene-95.7-mRNA-1	3	OsR498G0305708200.01.T05	100,00	0,00E+00	3241	Os03g0283500	100,00	0,00E+00	3241	0	NA	NA	
augustus_masked-3-processed-gene-97.8-mRNA-1	3	OsR498G0305724500.01.T01	100,00	8,91E-110	396	Os03g0288000	100,00	3,10E-110	396	0	NA	Japonica	
augustus_masked-3-processed-gene-99.9-mRNA-1	3	OsR498G0305739900.01.T03	99,81	0,00E+00	948	Os03g0292200	100,00	0,00E+00	760	-188	Indica	Japonica	
augustus-3-processed-gene-102.65-mRNA-1	3	OsR498G0305763200.01.T01	99,73	0,00E+00	673	Os03g0299600	100,00	0,00E+00	1229	556	Japonica	Japonica	
augustus-3-processed-gene-110.47-mRNA-1	3	OsR498G0305816300.01.T01	99,59	0,00E+00	3953	Os03g0314500	100,00	0,00E+00	3956	3	NA	Japonica	
augustus-3-processed-gene-118.23-mRNA-1	3	OsR498G0305078700.01.T01	99,55	0,00E+00	1975	Os03g0103400	100,00	0,00E+00	2547	572	Japonica	Japonica	
augustus-3-processed-gene-149.22-mRNA-1	3	OsR498G0305859100.01.T01	87,01	1,76E-162	572	Os03g0325700	100,00	3,86E-178	623	51	NA	Japonica	
augustus-3-processed-gene-151.34-mRNA-1	3	OsR498G0510886900.01.T01	74,53	0,00E+00	655	Os03g0328200	100,00	0,00E+00	2556	1901	Japonica	Japonica	
augustus-3-processed-gene-178.51-mRNA-1	3	OsR498G0306009600.01.T01	97,60	0,00E+00	1566	Os07g0640900	81,81	0,00E+00	659	-907	Indica	Japonica	
augustus-3-processed-gene-208.12-mRNA-1	3	OsR498G0306191200.01.T01	100,00	0,00E+00	1553	Os03g0412400	100,00	0,00E+00	1553	0	NA	NA	
augustus-3-processed-gene-215.19-mRNA-1	3	OsR498G0306243700.01.T01	99,59	0,00E+00	2581	Os03g0425600	100,00	0,00E+00	2522	-59	NA	Japonica	
augustus-3-processed-gene-22.3-mRNA-1	3	OsR498G0305218500.01.T01	98,92	0,00E+00	1300	Os03g0143600	100,00	0,00E+00	1324	24	NA	Japonica	
augustus-3-processed-gene-255.50-mRNA-1	3	OsR498G0306531800.01.T01	99,65	7,50E-144	510	Os03g0607200	100,00	6,13E-146	515	5	NA	NA	
augustus-3-processed-gene-29.11-mRNA-1	3	OsR498G0305257100.01.T02	98,38	0,00E+00	1074	Os03g0158600	100,00	0,00E+00	1142	68	NA	Japonica	
augustus-3-processed-gene-3.69-mRNA-1	3	OsR498G0305103900.01.T02	99,62	0,00E+00	2346	Os03g0110400	100,00	0,00E+00	2370	24	NA	Japonica	
augustus-3-processed-gene-365.14-mRNA-1	3	OsR498G0306531800.01.T01	99,65	7,50E-144	510	Os03g0607200	100,00	6,13E-146	515	5	NA	NA	
augustus-3-processed-gene-373.13-mRNA-1	3	OsR498G0306579100.01.T01	98,95	0,00E+00	2009	Os03g0622200	100,00	0,00E+00	2051	42	NA	NA	
augustus-3-processed-gene-388.39-mRNA-1	3	OsR498G0306670300.01.T02	99,76	0,00E+00	2249	Os03g0648400	100,00	0,00E+00	2262	13	NA	Japonica	
augustus-3-processed-gene-401.41-mRNA-1	3	OsR498G0306761200.01.T01	100,00	0,00E+00	1223	Os03g0669100	100,00	0,00E+00	866	-357	Indica	NA	
augustus-3-processed-gene-412.43-mRNA-1	3	OsR498G0306830100.01.T03	98,22	0,00E+00	3023	Os03g0689300	98,28	0,00E+00	3028	5	NA	Japonica	
augustus-3-processed-gene-416.21-mRNA-1	3	OsR498G0306865100.01.T01	99,86	0,00E+00	1246	Os03g0697200	100,00	0,00E+00	1251	5	NA	Japonica	
augustus-3-processed-gene-417.2-mRNA-1	3	OsR498G0101158000.01.T01	96,52	0,00E+00	3893	Os03g0110200	85,04	0,00E+00	4561	668	Japonica	Japonica	
augustus-3-processed-gene-430.30-mRNA-1	3	OsR498G0306955400.01.T01	99,80	0,00E+00	1815	Os03g0721700	100,00	0,00E+00	1824	9	NA	Japonica	
augustus-3-processed-gene-438.73-mRNA-1	3	OsR498G0307002800.01.T02	96,56	0,00E+00	1799	Os03g0735000	96,56	0,00E+00	1799	0	NA	Japonica	
augustus-3-processed-gene-440.40-mRNA-1	3	OsR498G0307011300.01.T09	99,85	0,00E+00	14664	Os03g0738100	100,00	0,00E+00	15870	1206	NA	Japonica	
augustus-3-processed-gene-459.2-mRNA-1	3	OsR498G0307130100.01.T04	99,90	0,00E+00	3633	Os03g0773600	100,00	0,00E+00	3642	9	NA	Japonica	
augustus-3-processed-gene-46.67-mRNA-1	3	OsR498G0305377500.01.T02	99,50	0,00E+00	715	Os09g0368500	85,15	1,54E-92	338	-377	Indica	Japonica	
augustus-3-processed-gene-465.45-mRNA-1	3	OsR498G0307172400.01.T03	99,57	0,00E+00	2061	Os03g0786500	100,00	0,00E+00	2084	23	NA	Japonica	
augustus-3-processed-gene-465.54-mRNA-1	3	OsR498G0307174300.01.T01	99,84	0,00E+00	2253	Os03g0787300	100,00	0,00E+00	2262	9	NA	Japonica	
augustus-3-processed-gene-469.45-mRNA-1	3	OsR498G0307197400.01.T01	98,80	0,00E+00	730	Os03g0795100	100,00	0,00E+00	755	25	NA	Japonica	
augustus-3-processed-gene-478.14-mRNA-1	3	OsR498G0307259300.01.T02	99,46	0,00E+00	1975	Os03g0812200	100,00	0,00E+00	2003	28	NA	Japonica	
augustus-3-processed-gene-489.36-mRNA-1	3	OsR498G0307331000.01.T01	100,00	0,00E+00	3555	Os03g0835600	99,85	0,00E+00	3540	-15	NA	NA	
augustus-3-processed-gene-493.101-mRNA-1	3	OsR498G0307357600.01.T02	99,78	0,00E+00	1665	Os03g0844900	100,00	0,00E+00	1674	9	NA	Japonica	
augustus-3-processed-gene-493.104-mRNA-1	3	OsR498G0307358600.01.T01	99,95	0,00E+00	3475	Os03g0845300	100,00	0,00E+00	2003	-1472	Indica	Japonica	
augustus-3-processed-gene-493.97-mRNA-1	3	OsR498G0307355200.01.T01	99,91	0,00E+00	1993	Os03g0844100	100,00	0,00E+00	1997	4	NA	Japonica	Kinase
augustus-3-processed-gene-496.5-mRNA-1	3	OsR498G0307369900.01.T01	99,67	0,00E+00	2195	Os03g0849900	100,00	0,00E+00	2214	19	NA	Japonica	
augustus-3-processed-gene-77.33-mRNA-1	3	OsR498G0305585800.01.T01	97,33	0,00E+00	822	Os03g0251000	100,00	0,00E+00	861	39	NA	Japonica	
augustus-3-processed-gene-80.65-mRNA-1	3	OsR498G0305600800.01.T07	99,60	0,00E+00	3561	Os03g0255500	99,60	0,00E+00	3561	0	NA	Japonica	
maker-3-augustus-gene-0.17-mRNA-1	3	OsR498G0305859800.01.T01	98,88	0,00E+00	5634	Os01g0277600	70,21	0,00E+00	1385	-4249	Indica	NA	
maker-3-augustus-gene-0.23-mRNA-1	3	OsR498G0305430600.01.T01	99,89	0,00E+00	3384	Os03g0206700	100,00	0,00E+00	2378	-1006	Indica	NA	
maker-3-augustus-gene-0.3-mRNA-1	3	OsR498G0305430300.01.T01	97,99	0,00E+00	939	Os03g0206600	100,00	0,00E+00	969	30	NA	NA	
maker-3-augustus-gene-0.5-mRNA-1	3	OsR498G0305430700.01.T01	99,15	0,00E+00	1446	Os03g0206900	100,00	0,00E+00	1478	32	NA	NA	
maker-3-augustus-gene-0.7-mRNA-1	3	OsR498G0305067700.01.T03	100,00	0,00E+00	3382	Os03g0100400	100,00	0,00E+00	3382	0	NA	NA	
maker-3-augustus-gene-0.8-mRNA-1	3	OsR498G0305067700.01.T03	99,91	0,00E+00	2016	Os03g0100400	100,00	0,00E+00	2010	-6	NA	NA	
maker-3-augustus-gene-1.35-mRNA-1	3	OsR498G0305083300.01.T01	99,93	0,00E+00	2420	Os05g0413200	90,47	0,00E+00	1815	-605	Indica	Japonica	
maker-3-augustus-gene-1.38-mRNA-1	3	OsR498G0305083600.01.T01	98,46	0,00E+00	1583	Os03g0105700	98,68	0,00E+00	1592	9	NA	Japonica	
maker-3-augustus-gene-1.41-mRNA-1	3	OsR498G0305083800.01.T02	99,86	0,00E+00	3980	Os03g0105800	98,40	0,00E+00	3924	-56	NA	Japonica	
maker-3-augustus-gene-1.43-mRNA-1	3	OsR498G0305084600.01.T02	99,83	0,00E+00	1073	Os03g0106000	100,00	0,00E+00	1077	4	NA	Japonica	
maker-3-augustus-gene-1.46-mRNA-1	3	OsR498G0305085200.01.T03	99,33	0,00E+00	1848	Os03g0106200	100,00	0,00E+00	1880	32	NA	Japonica	
maker-3-augustus-gene-1.49-mRNA-1	3	OsR498G0305085400.01.T01	98,24	0,00E+00	2542	Os03g0106300	98,17	0,00E+00	2541	-1	NA	Japonica	
maker-3-augustus-gene-1.51-mRNA-1	3	OsR498G0305085600.01.T02	100,00	0,00E+00	2197	Os03g0106400	100,00	0,00E+00	2197	0	NA	Japonica	
maker-3-augustus-gene-1.56-mRNA-1	3	OsR498G0305087100.01.T01	95,93	0,00E+00	717	Os03g0106700	100,00	0,00E+00	1240	523	Japonica	Japonica	
maker-3-augustus-gene-10.20-mRNA-1	3	OsR498G0305141900.01.T02	99,90	0,00E+00	3514	Os03g0122000	100,00	0,00E+00	3534	20	NA	Japonica	Kinase-TM
maker-3-augustus-gene-10.40-mRNA-1	3	OsR498G0305143000.01.T01	100,00	0,00E+00	1142	Os03g0122200	100,00	0,00E+00	1142	0	NA	Japonica	
maker-3-augustus-gene-10.43-mRNA-1	3	OsR498G0305143200.01.T02	99,81	0,00E+00	1847	Os03g0122300	100,00	0,00E+00	1856	9	NA	Japonica	
maker-3-augustus-gene-100.13-mRNA-1	3	OsR498G0305744600.01.T08	99,63	4,76E-134	478	Os03g0293850	100,00	5,07E-103	372	-106	Indica	Japonica	
maker-3-augustus-gene-100.17-mRNA-1	3	OsR498G0305747000.01.T01	99,66	0,00E+00	4672	Os05g0353400	71,75	0,00E+00	1269	-3403	Indica	Japonica	
maker-3-augustus-gene-100.19-mRNA-1	3	OsR498G0305747600.01.T01	99,90	0,00E+00	1866	Os03g0294800	100,00	0,00E+00	1871	5	NA	Japonica	Kinase-TM
maker-3-augustus-gene-100.2-mRNA-1	3	OsR498G0305746300.01.T01	99,77	0,00E+00	3910	Os03g0294200	100,00	0,00E+00	2626	-1284	Indica	Japonica	
maker-3-augustus-gene-100.22-mRNA-1	3	OsR498G0305748000.01.T01	99,72	0,00E+00	5722	Os03g0294900	100,00	0,00E+00	5763	41	NA	NA	
maker-3-augustus-gene-100.9-mRNA-1	3	OsR498G0305742800.01.T01	99,45	0,00E+00	3238	Os03g0293500	99,95	0,00E+00	3275	37	NA	Japonica	
maker-3-augustus-gene-101.0-mRNA-1	3	OsR498G0305749300.01.T02	100,00	1,83E-171	601	Os03g0295500	100,00	6,37E-172	601	0	NA	Japonica	
maker-3-augustus-gene-101.11-mRNA-1	3	OsR498G0305752700.01.T01	99,51	0,00E+00	1818	Os03g0296400	100,00	0,00E+00	1840	22	NA	Japonica	
maker-3-augustus-gene-101.17-mRNA-1	3	OsR498G0305753700.01.T02	99,90	0,00E+00	1776	Os03g0296800	100,00	0,00E+00	1781	5	NA	Japonica	
maker-3-augustus-gene-101.2-mRNA-1	3	OsR498G0305750700.01.T04	99,46	0,00E+00	4274	Os03g0295700	100,00	0,00E+00	4334	60	NA	Japonica	
maker-3-augustus-gene-101.20-mRNA-1	3	OsR498G0305754100.01.T01	100,00	0,00E+00	1045	Os03g0297100	100,00	0,00E+00	1045	0	NA	Japonica	
maker-3-augustus-gene-101.23-mRNA-1	3	OsR498G0305754500.01.T01	99,83	0,00E+00	1040	Os03g0297100	98,79	0,00E+00	1013	-27	NA	Japonica	
maker-3-augustus-gene-101.25-mRNA-1	3	OsR498G0305749200.01.T02	99,67	0,00E+00	1099	Os03g0295400	100,00	0,00E+00	1108	9	NA	Japonica	
maker-3-augustus-gene-101.30-mRNA-1	3	OsR498G0305749600.01.T01	99,83	0,00E+00	3195	Os03g0295600	100,00	0,00E+00	3209	14	NA	Japonica	Kinase-TM
maker-3-augustus-gene-101.32-mRNA-1	3	OsR498G0305751000.01.T02	99,48	0,00E+00	1373	Os03g0295800	93,08	0,00E+00	1037	-336	Indica	Japonica	
maker-3-augustus-gene-101.37-mRNA-1	3	OsR498G0305751800.01.T01	99,68	0,00E+00	1652	Os03g0296000	99,68	0,00E+00	1652	0	NA	Japonica	
maker-3-augustus-gene-101.9-mRNA-1	3	OsR498G0305752300.01.T01	99,75	0,00E+00	1409	Os03g0296200	100,00	0,00E+00	1418	9	NA	Japonica	
maker-3-augustus-gene-102.11-mRNA-1	3	OsR498G0305759700.01.T01	99,85	0,00E+00	2432	Os03g0298400	100,00	0,00E+00	2441	9	NA	Japonica	
maker-3-augustus-gene-102.15-mRNA-1	3	OsR498G0305761900.01.T01	99,80	0,00E+00	1799	Os03g0299200	100,00	0,00E+00	1808	9	NA	Japonica	
maker-3-augustus-gene-102.17-mRNA-1	3	OsR498G0305760000.01.T02	99,07	0,00E+00	952	Os03g0298600	100,00	0,00E+00	921	-31	NA	Japonica	
maker-3-augustus-gene-102.19-mRNA-1	3	OsR498G0305760200.01.T01	99,74	0,00E+00	7010	Os03g0298700	98,75	0,00E+00	6873	-137	NA	Japonica	
maker-3-augustus-gene-102.25-mRNA-1	3	OsR498G0305760400.01.T01	99,43	0,00E+00	1242	Os03g0298800	99,57	0,00E+00	1246	4	NA	Japonica	
maker-3-augustus-gene-102.3-mRNA-1	3	OsR498G0305757700.01.T01	99,71	0,00E+00	4308	Os03g0297800	99,86	0,00E+00	2507	-1801	Indica	Japonica	CC-Kinase-LRR-TM
maker-3-augustus-gene-102.8-mRNA-1	3	OsR498G0305759300.01.T01	99,84	0,00E+00	1116	Os03g0298300	100,00	0,00E+00	1121	5	NA	Japonica	
maker-3-augustus-gene-103.34-mRNA-1	3	OsR498G0305765300.01.T01	99,71	0,00E+00	2498	Os03g0299900	100,00	0,00E+00	2516	18	NA	Japonica	
maker-3-augustus-gene-103.39-mRNA-1	3	OsR498G0305766500.01.T01	99,84	0,00E+00	3439	Os03g0300300	100,00	0,00E+00	3452	13	NA	Japonica	
maker-3-augustus-gene-103.43-mRNA-1	3	OsR498G0305766700.01.T01	100,00	0,00E+00	872	Os03g0300400	100,00	0,00E+00	872	0	NA	Japonica	
maker-3-augustus-gene-103.45-mRNA-1	3	OsR498G0305771300.01.T02	99,33	0,00E+00	1595	Os03g0301400	99,89	0,00E+00	1618	23	NA	Japonica	
maker-3-augustus-gene-103.49-mRNA-1	3	OsR498G0305766400.01.T04	99,52	0,00E+00	2246	Os03g0300200	100,00	0,00E+00	2273	27	NA	Japonica	
maker-3-augustus-gene-103.54-mRNA-1	3	OsR498G0305767800.01.T01	100,00	0,00E+00	904	Os03g0300600	100,00	0,00E+00	904	0	NA	Japonica	
maker-3-augustus-gene-104.37-mRNA-1	3	OsR498G0305773700.01.T06	98,58	0,00E+00	4190	Os03g0302200	99,96	0,00E+00	4324	134	NA	Japonica	
maker-3-augustus-gene-104.39-mRNA-1	3	OsR498G0305774300.01.T01	98,91	0,00E+00	641	Os03g0302500	100,00	0,00E+00	655	14	NA	Japonica	
maker-3-augustus-gene-104.40-mRNA-1	3	OsR498G0305775700.01.T02	99,12	0,00E+00	5019	Os03g0302900	100,00	0,00E+00	5113	94	NA	Japonica	
maker-3-augustus-gene-104.43-mRNA-1	3	OsR498G0305771800.01.T03	99,65	0,00E+00	1509	Os03g0301700	99,88	0,00E+00	1518	9	NA	Japonica	
maker-3-augustus-gene-105.25-mRNA-1	3	OsR498G0305778900.01.T02	99,81	0,00E+00	5716	Os03g0304400	100,00	0,00E+00	5743	27	NA	Japonica	
maker-3-augustus-gene-105.29-mRNA-1	3	OsR498G0305778600.01.T01	100,00	0,00E+00	980	Os03g0304100	100,00	0,00E+00	980	0	NA	Japonica	
maker-3-augustus-gene-105.33-mRNA-1	3	OsR498G0305780400.01.T01	99,75	0,00E+00	3598	Os03g0304500	99,95	0,00E+00	3616	18	NA	Japonica	
maker-3-augustus-gene-105.36-mRNA-1	3	OsR498G0305782200.01.T03	97,99	2,84E-169	595	Os03g0304800	99,25	2,84E-132	471	-124	Indica	Japonica	
maker-3-augustus-gene-106.0-mRNA-1	3	OsR498G0305786100.01.T02	99,94	0,00E+00	2810	Os03g0305500	100,00	0,00E+00	2814	4	NA	Japonica	
maker-3-augustus-gene-106.11-mRNA-1	3	OsR498G0305789700.01.T01	99,62	0,00E+00	933	Os03g0306700	100,00	0,00E+00	942	9	NA	Japonica	
maker-3-augustus-gene-106.12-mRNA-1	3	OsR498G0305790900.01.T02	99,74	0,00E+00	1371	Os03g0307000	100,00	0,00E+00	1380	9	NA	Japonica	
maker-3-augustus-gene-106.14-mRNA-1	3	OsR498G0305783600.01.T01	99,82	0,00E+00	3060	Os03g0305100	81,09	0,00E+00	1615	-1445	Indica	Japonica	
maker-3-augustus-gene-106.16-mRNA-1	3	OsR498G0305784600.01.T02	99,88	0,00E+00	3103	Os03g0305100	99,88	0,00E+00	3103	0	NA	Japonica	
maker-3-augustus-gene-106.18-mRNA-1	3	OsR498G0305786000.01.T04	99,71	2,01E-174	613	Os03g0305400	100,00	1,26E-171	601	-12	NA	Japonica	
maker-3-augustus-gene-106.29-mRNA-1	3	OsR498G0305788600.01.T02	99,60	0,00E+00	3158	Os03g0306400	99,64	0,00E+00	3005	-153	NA	Japonica	CC-NBS-TM
maker-3-augustus-gene-106.3-mRNA-1	3	OsR498G0305786300.01.T04	99,02	0,00E+00	714	Os03g0305600	98,77	0,00E+00	710	-4	NA	Japonica	
maker-3-augustus-gene-106.35-mRNA-1	3	OsR498G0305790600.01.T01	100,00	0,00E+00	1218	Os03g0306900	100,00	0,00E+00	1267	49	NA	Japonica	
maker-3-augustus-gene-106.6-mRNA-1	3	OsR498G0305786500.01.T01	99,64	0,00E+00	1471	Os03g0305700	100,00	0,00E+00	1485	14	NA	Japonica	
maker-3-augustus-gene-107.40-mRNA-1	3	OsR498G0305791100.01.T01	99,86	0,00E+00	5061	Os03g0307100	100,00	0,00E+00	5079	18	NA	Japonica	

maker-3-augustus-gene-107.44-mRNA-1	3	OsR498G0305792900.01.T01	88,22	0,00E+00	1266	Os03g0307400	88,42	0,00E+00	1275	9	NA	Japonica	
maker-3-augustus-gene-107.48-mRNA-1	3	OsR498G0305793500.01.T02	99,71	0,00E+00	2461	Os03g0307700	100,00	0,00E+00	3415	954	Japonica	Japonica	
maker-3-augustus-gene-107.56-mRNA-1	3	OsR498G0305794900.01.T01	99,05	0,00E+00	4098	Os03g0308500	99,87	0,00E+00	4162	64	NA	Japonica	
maker-3-augustus-gene-107.59-mRNA-1	3	OsR498G0305795100.01.T02	99,37	0,00E+00	1405	Os03g0308600	100,00	0,00E+00	893	-512	Indica	Japonica	
maker-3-augustus-gene-107.62-mRNA-1	3	OsR498G0305795300.01.T01	96,19	0,00E+00	2389	Os03g0308700	96,20	0,00E+00	2051	-338	Indica	Japonica	
maker-3-augustus-gene-107.64-mRNA-1	3	OsR498G0305793800.01.T01	97,06	0,00E+00	3222	Os03g0307800	97,11	0,00E+00	3226	4	NA	Japonica	
maker-3-augustus-gene-107.68-mRNA-1	3	OsR498G0305794200.01.T01	99,53	0,00E+00	1517	Os03g0308100	100,00	0,00E+00	1532	15	NA	Japonica	
maker-3-augustus-gene-107.73-mRNA-1	3	OsR498G0305794600.01.T06	99,04	0,00E+00	3120	Os03g0308200	99,83	0,00E+00	3177	57	NA	Japonica	
maker-3-augustus-gene-107.78-mRNA-1	3	OsR498G0305795600.01.T01	99,90	0,00E+00	1857	Os03g0308800	100,00	0,00E+00	1862	5	NA	Japonica	
maker-3-augustus-gene-107.85-mRNA-1	3	OsR498G0305796200.01.T02	99,88	0,00E+00	1488	Os03g0309000	100,00	0,00E+00	1379	-109	NA	Japonica	
maker-3-augustus-gene-108.0-mRNA-1	3	OsR498G0305797300.01.T04	99,75	0,00E+00	5797	Os03g0309200	99,97	0,00E+00	5491	-306	NA	Japonica	
maker-3-augustus-gene-108.20-mRNA-1	3	OsR498G0305798400.01.T01	99,71	0,00E+00	1859	Os03g0309400	100,00	0,00E+00	1873	14	NA	Japonica	
maker-3-augustus-gene-108.9-mRNA-1	3	OsR498G0305800500.01.T01	99,55	0,00E+00	1969	Os03g0310000	100,00	0,00E+00	1992	23	NA	Japonica	
maker-3-augustus-gene-109.40-mRNA-1	3	OsR498G0305809900.01.T06	97,54	0,00E+00	2090	Os03g0312600	97,54	0,00E+00	2090	0	NA	Japonica	
maker-3-augustus-gene-109.43-mRNA-1	3	OsR498G0305810300.01.T01	100,00	0,00E+00	890	Os03g0313000	100,00	0,00E+00	890	0	NA	Japonica	
maker-3-augustus-gene-109.45-mRNA-1	3	OsR498G0305808600.01.T01	99,38	0,00E+00	864	Os03g0312300	100,00	0,00E+00	877	13	NA	Japonica	
maker-3-augustus-gene-109.47-mRNA-1	3	OsR498G0305810800.01.T01	98,46	0,00E+00	2260	Os03g0313100	99,08	0,00E+00	2291	31	NA	Japonica	
maker-3-augustus-gene-11.0-mRNA-1	3	OsR498G0305147500.01.T04	100,00	8,83E-87	320	Os03g0123100	100,00	3,07E-87	320	0	NA	Japonica	
maker-3-augustus-gene-11.12-mRNA-1	3	OsR498G0305149500.01.T01	99,89	0,00E+00	1700	Os03g0123500	99,52	0,00E+00	1472	-228	Indica	Japonica	
maker-3-augustus-gene-11.17-mRNA-1	3	OsR498G0305150500.01.T01	99,53	0,00E+00	1140	Os03g0123800	100,00	0,00E+00	1153	13	NA	Japonica	
maker-3-augustus-gene-11.19-mRNA-1	3	OsR498G0305151300.01.T01	99,72	0,00E+00	3181	Os03g0124000	100,00	0,00E+00	3204	23	NA	Japonica	
maker-3-augustus-gene-11.21-mRNA-1	3	OsR498G1019100900.01.T01	73,21	5,73E-48	192	Os03g0122600	100,00	0,00E+00	924	732	Japonica	Japonica	
maker-3-augustus-gene-11.22-mRNA-1	3	OsR498G1019100900.01.T03	90,56	4,49E-65	249	Os03g0122600	100,00	5,81E-90	329	80	Japonica	Japonica	
maker-3-augustus-gene-11.29-mRNA-1	3	OsR498G0305149800.01.T01	92,85	0,00E+00	1256	Os03g0123600	93,09	0,00E+00	1265	9	NA	Japonica	
maker-3-augustus-gene-11.4-mRNA-1	3	OsR498G0305147700.01.T01	99,74	0,00E+00	1361	Os03g0123200	100,00	0,00E+00	1370	9	NA	Japonica	
maker-3-augustus-gene-11.8-mRNA-1	3	OsR498G0305148100.01.T03	99,94	0,00E+00	2831	Os03g0123300	100,00	0,00E+00	2836	5	NA	Japonica	
maker-3-augustus-gene-110.0-mRNA-1	3	OsR498G0305813300.01.T03	99,76	0,00E+00	2204	Os03g0313600	99,92	0,00E+00	2214	10	NA	Japonica	
maker-3-augustus-gene-110.13-mRNA-1	3	OsR498G0305816700.01.T01	99,70	0,00E+00	1209	Os03g0314700	100,00	0,00E+00	1218	9	NA	Japonica	
maker-3-augustus-gene-110.18-mRNA-1	3	OsR498G0305815000.01.T03	99,92	0,00E+00	2351	Os03g0314200	100,00	0,00E+00	2356	5	NA	Japonica	
maker-3-augustus-gene-110.25-mRNA-1	3	OsR498G0305817600.01.T02	99,81	0,00E+00	2801	Os03g0314800	100,00	0,00E+00	2814	13	NA	Japonica	
maker-3-augustus-gene-110.5-mRNA-1	3	OsR498G0305814700.01.T03	99,86	0,00E+00	3899	Os03g0314100	100,00	0,00E+00	2821	-1078	Indica	Japonica	
maker-3-augustus-gene-111.0-mRNA-1	3	OsR498G0305823100.01.T01	99,71	0,00E+00	1838	Os03g0316200	100,00	0,00E+00	1851	13	NA	Japonica	
maker-3-augustus-gene-111.11-mRNA-1	3	OsR498G0305822400.01.T01	99,92	0,00E+00	2177	Os03g0315800	98,87	0,00E+00	2010	-167	NA	Japonica	
maker-3-augustus-gene-111.3-mRNA-1	3	OsR498G0305824900.01.T01	91,29	0,00E+00	1151	Os03g0316900	91,53	0,00E+00	1160	9	NA	Japonica	
maker-3-augustus-gene-111.6-mRNA-1	3	OsR498G0305825100.01.T04	99,67	0,00E+00	3805	Os03g0317000	100,00	0,00E+00	3837	32	NA	Japonica	
maker-3-augustus-gene-111.9-mRNA-1	3	OsR498G0305819800.01.T01	99,70	0,00E+00	1773	Os03g0315400	100,00	0,00E+00	1786	13	NA	Japonica	
maker-3-augustus-gene-112.32-mRNA-1	3	OsR498G0305829400.01.T05	99,89	0,00E+00	3250	Os03g0318500	100,00	0,00E+00	3259	9	NA	Japonica	
maker-3-augustus-gene-112.35-mRNA-1	3	OsR498G0305829600.01.T04	100,00	0,00E+00	1294	Os03g0318600	100,00	0,00E+00	1294	0	NA	Japonica	
maker-3-augustus-gene-113.26-mRNA-1	3	OsR498G0305831100.01.T01	99,89	0,00E+00	1598	Os07g0687300	73,08	9,54E-100	364	-1234	Indica	Japonica	
maker-3-augustus-gene-113.32-mRNA-1	3	OsR498G0305832300.01.T01	99,78	0,00E+00	808	Os03g0319300	100,00	0,00E+00	812	4	NA	Japonica	
maker-3-augustus-gene-113.34-mRNA-1	3	OsR498G0305835500.01.T01	98,89	0,00E+00	634	Os03g0320400	99,72	0,00E+00	647	13	NA	Japonica	
maker-3-augustus-gene-113.42-mRNA-1	3	OsR498G0305839700.01.T01	99,13	0,00E+00	1015	Os03g0321000	100,00	0,00E+00	1040	25	NA	Japonica	
maker-3-augustus-gene-113.44-mRNA-1	3	OsR498G0305833000.01.T03	99,85	0,00E+00	2426	Os03g0319400	100,00	0,00E+00	2435	9	NA	Japonica	Kinase-TM
maker-3-augustus-gene-113.54-mRNA-1	3	OsR498G0305835000.01.T01	99,87	0,00E+00	1354	Os03g0320000	100,00	0,00E+00	1359	5	NA	Japonica	
maker-3-augustus-gene-114.0-mRNA-1	3	OsR498G0305844700.01.T01	99,84	0,00E+00	1138	Os03g0321700	100,00	0,00E+00	1142	4	NA	Japonica	
maker-3-augustus-gene-114.14-mRNA-1	3	OsR498G0305846700.01.T03	93,98	0,00E+00	786	Os03g0322600	93,98	0,00E+00	786	0	NA	Japonica	
maker-3-augustus-gene-114.16-mRNA-1	3	OsR498G0305847900.01.T01	99,69	0,00E+00	1708	Os03g0322800	100,00	0,00E+00	1721	13	NA	Japonica	
maker-3-augustus-gene-114.2-mRNA-1	3	OsR498G0305845100.01.T03	92,08	9,16E-145	513	Os03g0321900	100,00	0,00E+00	737	224	Japonica	Japonica	
maker-3-augustus-gene-114.20-mRNA-1	3	OsR498G0305842400.01.T03	100,00	0,00E+00	829	Os04g0455900	75,68	3,71E-85	315	-514	Indica	Japonica	
maker-3-augustus-gene-114.26-mRNA-1	3	OsR498G0305842800.01.T02	99,92	0,00E+00	4558	Os03g0321500	100,00	0,00E+00	4567	9	NA	Japonica	
maker-3-augustus-gene-114.30-mRNA-1	3	OsR498G0305845600.01.T01	99,87	0,00E+00	4193	Os07g0686400	72,73	0,00E+00	939	-3254	Indica	Japonica	
maker-3-augustus-gene-114.33-mRNA-1	3	OsR498G0305847200.01.T01	99,72	0,00E+00	1279	Os07g0686100	70,31	4,10E-77	288	-991	Indica	Japonica	
maker-3-augustus-gene-114.7-mRNA-1	3	OsR498G0305846500.01.T01	99,74	0,00E+00	700	Os03g0322500	100,00	0,00E+00	704	4	NA	Japonica	
maker-3-augustus-gene-115.26-mRNA-1	3	OsR498G0305849500.01.T01	98,69	0,00E+00	7387	Os03g0323200	98,55	0,00E+00	5796	-1591	Indica	NA	
maker-3-augustus-gene-115.29-mRNA-1	3	OsR498G0305850500.01.T03	99,58	0,00E+00	2992	Os03g0323500	99,81	0,00E+00	2871	-121	NA	NA	
maker-3-augustus-gene-115.35-mRNA-1	3	OsR498G0305852100.01.T02	99,80	0,00E+00	1820	Os03g0323800	100,00	0,00E+00	1829	9	NA	NA	
maker-3-augustus-gene-116.30-mRNA-1	3	OsR498G0305067500.01.T01	100,00	0,00E+00	1797	Os03g0100300	100,00	0,00E+00	1797	0	NA	NA	
maker-3-augustus-gene-116.35-mRNA-1	3	OsR498G0305067700.01.T02	99,97	0,00E+00	5385	Os03g0100400	100,00	0,00E+00	5389	4	NA	NA	
maker-3-augustus-gene-117.37-mRNA-1	3	OsR498G0305068300.01.T03	98,13	0,00E+00	4737	Os03g0100800	98,13	0,00E+00	4737	0	NA	Japonica	
maker-3-augustus-gene-117.44-mRNA-1	3	OsR498G0305068500.01.T03	100,00	1,49E-116	419	Os03g0100900	100,00	5,18E-117	419	0	NA	NA	
maker-3-augustus-gene-117.47-mRNA-1	3	OsR498G0305068900.01.T07	96,69	0,00E+00	1602	Os03g0101100	96,63	0,00E+00	1558	-44	NA	NA	
maker-3-augustus-gene-117.52-mRNA-1	3	OsR498G0305070900.01.T01	100,00	0,00E+00	1992	Os03g0101900	100,00	0,00E+00	1992	0	NA	NA	
maker-3-augustus-gene-117.55-mRNA-1	3	OsR498G0305070900.01.T08	100,00	0,00E+00	1645	Os03g0102100	100,00	0,00E+00	1645	0	NA	NA	
maker-3-augustus-gene-117.69-mRNA-1	3	OsR498G0202858300.01.T01	89,51	5,35E-116	418	Os03g0101000	95,76	0,00E+00	696	278	Japonica	NA	
maker-3-augustus-gene-117.71-mRNA-1	3	OsR498G0305069200.01.T01	98,62	0,00E+00	4178	Os03g0101200	100,00	0,00E+00	4399	221	NA	NA	CC-Kinase-TM
maker-3-augustus-gene-117.76-mRNA-1	3	OsR498G0305069400.01.T01	100,00	0,00E+00	2814	Os03g0101300	100,00	0,00E+00	2814	0	NA	NA	
maker-3-augustus-gene-117.78-mRNA-1	3	OsR498G0305069800.01.T01	100,00	0,00E+00	1407	Os03g0101500	100,00	0,00E+00	1407	0	NA	NA	
maker-3-augustus-gene-117.81-mRNA-1	3	OsR498G0305070000.01.T01	100,00	0,00E+00	2019	Os03g0101600	100,00	0,00E+00	1781	-238	Indica	NA	
maker-3-augustus-gene-117.84-mRNA-1	3	OsR498G0305070200.01.T03	100,00	0,00E+00	2073	Os03g0101700	100,00	0,00E+00	2073	0	NA	NA	
maker-3-augustus-gene-117.91-mRNA-1	3	OsR498G0714254900.01.T02	96,59	0,00E+00	2371	Os03g0102400	100,00	0,00E+00	1849	-522	Indica	NA	
maker-3-augustus-gene-118.34-mRNA-1	3	OsR498G0305073700.01.T01	100,00	0,00E+00	1775	Os03g0102700	100,00	0,00E+00	1775	0	NA	NA	
maker-3-augustus-gene-118.36-mRNA-1	3	OsR498G0305075700.01.T01	99,94	0,00E+00	3188	Os08g0155400	75,32	0,00E+00	1020	-2168	Indica	Japonica	
maker-3-augustus-gene-118.38-mRNA-1	3	OsR498G0305081700.01.T01	97,98	0,00E+00	1780	Os03g0103800	100,00	0,00E+00	1874	94	NA	Japonica	
maker-3-augustus-gene-12.0-mRNA-1	3	OsR498G0305151500.01.T01	99,67	0,00E+00	2721	Os03g0124100	100,00	0,00E+00	2729	8	NA	Japonica	
maker-3-augustus-gene-12.18-mRNA-1	3	OsR498G0305155100.01.T01	99,81	0,00E+00	2860	Os10g0533150	75,03	0,00E+00	641	-2219	Indica	Japonica	Kinase-TM
maker-3-augustus-gene-12.23-mRNA-1	3	OsR498G0305156500.01.T01	96,83	0,00E+00	2233	Os03g0125800	97,06	0,00E+00	2247	14	NA	Japonica	
maker-3-augustus-gene-12.3-mRNA-1	3	OsR498G0305152300.01.T01	99,59	0,00E+00	1302	Os03g0124500	100,00	0,00E+00	1315	13	NA	Japonica	
maker-3-augustus-gene-12.31-mRNA-1	3	OsR498G0305156900.01.T01	99,29	0,00E+00	1239	Os03g0125900	100,00	0,00E+00	1261	22	NA	Japonica	
maker-3-augustus-gene-12.33-mRNA-1	3	OsR498G0305153600.01.T01	99,88	0,00E+00	1511	Os03g0125000	100,00	0,00E+00	1516	5	NA	Japonica	
maker-3-augustus-gene-12.35-mRNA-1	3	OsR498G0305153800.01.T01	99,82	0,00E+00	979	Os03g0125100	100,00	0,00E+00	1732	753	Japonica	Japonica	
maker-3-augustus-gene-12.39-mRNA-1	3	OsR498G0305155800.01.T02	99,69	1,04E-164	581	Os03g0125700	100,00	8,53E-167	585	4	NA	Japonica	
maker-3-augustus-gene-12.44-mRNA-1	3	OsR498G0305157200.01.T02	97,88	0,00E+00	2014	Os03g0126000	100,00	0,00E+00	2127	113	NA	Japonica	
maker-3-augustus-gene-12.49-mRNA-1	3	OsR498G0305157400.01.T03	99,19	0,00E+00	3050	Os03g0126100	100,00	0,00E+00	2035	-1015	Indica	Japonica	
maker-3-augustus-gene-12.5-mRNA-1	3	OsR498G0305153300.01.T01	99,85	0,00E+00	2434	Os03g0124900	100,00	0,00E+00	2443	9	NA	Japonica	
maker-3-augustus-gene-12.9-mRNA-1	3	OsR498G0305154500.01.T01	99,48	0,00E+00	2060	Os03g0125300	99,74	0,00E+00	2074	14	NA	Japonica	
maker-3-augustus-gene-13.0-mRNA-1	3	OsR498G0305158100.01.T02	92,67	0,00E+00	920	Os03g0126700	100,00	0,00E+00	1049	129	Japonica	Japonica	
maker-3-augustus-gene-13.1-mRNA-1	3	OsR498G0305158100.01.T02	92,65	0,00E+00	916	Os03g0126700	100,00	0,00E+00	1045	129	Japonica	Japonica	
maker-3-augustus-gene-13.13-mRNA-1	3	OsR498G0305157800.01.T02	96,96	0,00E+00	1425	Os03g0126300	100,00	0,00E+00	1878	453	Japonica	Japonica	
maker-3-augustus-gene-13.17-mRNA-1	3	OsR498G0305158600.01.T04	99,56	0,00E+00	2437	Os11g0134300	70,41	1,74E-123	444	-1993	Indica	Japonica	Kinase-TM
maker-3-augustus-gene-13.23-mRNA-1	3	OsR498G0305159200.01.T05	100,00	5,82E-178	623	Os03g0127000	100,00	8,07E-133	471	-152	Indica	Japonica	
maker-3-augustus-gene-13.26-mRNA-1	3	OsR498G1019087100.01.T01	76,58	0,00E+00	677	Os03g0127200	100,00	0,00E+00	1986	1309	Japonica	Japonica	
maker-3-augustus-gene-13.28-mRNA-1	3	OsR498G0305161400.01.T02	99,77	0,00E+00	4767	Os03g0127600	99,89	0,00E+00	4779	12	NA	Japonica	
maker-3-augustus-gene-13.6-mRNA-1	3	OsR498G0305161100.01.T01	94,47	0,00E+00	1068	Os03g0127500	97,27	0,00E+00	2241	1173	Japonica	Japonica	
maker-3-augustus-gene-13.9-mRNA-1	3	OsR498G0305161700.01.T01	99,70	0,00E+00	4791	Os03g0127700	100,00	0,00E+00	4827	36	NA	Japonica	Kinase-LRR-TM
maker-3-augustus-gene-135.46-mRNA-1	3	OsR498G0305155100.01.T01	75,03	0,00E+00	641	Os10g0533150	100,00	0,00E+00	2798	2157	Japonica	Japonica	Kinase-TM
maker-3-augustus-gene-137.35-mRNA-1	3	OsR498G1019102500.01.T01	99,61	0,00E+00	1838	Os03g0122300	81,82	0,00E+00	978	-860	Indica	Japonica	
maker-3-augustus-gene-137.48-mRNA-1	3	OsR498G1019105300.01.T01	98,94	0,00E+00	664	Os03g0120400	77,03	2,28E-85	315	-349	Indica	Japonica	
maker-3-augustus-gene-14.0-mRNA-1	3	OsR498G0305164700.01.T02	99,88	0,00E+00	2929	Os03g0128700	100,00	0,00E+00	2938	9	NA	Japonica	Kinase-TM
maker-3-augustus-gene-14.15-mRNA-1	3	OsR498G0305163600.01.T01	99,59	0,00E+00	874	Os03g0128300	100,00	0,00E+00	883	9	NA	Japonica	
maker-3-augustus-gene-14.17-mRNA-1	3	OsR498G0305164000.01.T02	99,78	0,00E+00	2389	Os03g0128500	99,84	0,00E+00	2221	-168	NA	Japonica	
maker-3-augustus-gene-14.22-mRNA-1	3	OsR498G0305165000.01.T02	100,00	0,00E+00	1840	Os03g0128800	100,00	0,00E+00	1840	0	NA	Japonica	
maker-3-augustus-gene-14.24-mRNA-1	3	OsR498G0305166000.01.T01	99,94	0,00E+00	3004	Os03g0129100	100,00	0,00E+00	3009	5	NA	Japonica	
maker-3-augustus-gene-14.29-mRNA-1	3	OsR498G0305166600.01.T03	98,25	0,00E+00	4874	Os03g0129200	100,00	0,00E+00	2545	-2329	Indica	Japonica	
maker-3-augustus-gene-14.34-mRNA-1	3	OsR498G0305167400.01.T01	99,53	0,00E+00	1516	Os03g0129350	100,00	0,00E+00	1647	131	NA	Japonica	
maker-3-augustus-gene-14.37-mRNA-1	3	OsR498G0305167800.01.T01	93,95	0,00E+00	1880	Os03g0129800	99,00	0,00E+00	2122	242	Japonica	Japonica	
maker-3-augustus-gene-14.6-mRNA-1	3	OsR498G0305165700.01.T04	100,00	0,00E+00	982	Os03g0129000	100,00	0,00E+00	982	0	NA	Japonica	
maker-3-augustus-gene-140.5-mRNA-1	3	OsR498G1019125100.01.T01	99,94	0,00E+00	2984	Os03g0132800	82,27	0,00E+00	1417	-1567	Indica	Japonica	Kinase-TM
maker-3-augustus-gene-144.46-mRNA-1	3	OsR498G1019147700.01.T04	99,95	0,00E+00	3951	Os03g0105800	70,30	0,00E+00	979	-2972	Indica	Japonica	
maker-3-augustus-gene-147.29-mRNA-1	3	OsR498G0305748000.01.T01	99,75	0,00E+00	2832	Os03g0294900	99,94	0,00E+00	2846	14	NA	NA	
maker-3-augustus-gene-147.38-mRNA-1	3	OsR498G0305748000.01.T01	99,61	0,00E+00	2767	Os03g0294900	99,94	0,00E+00	2790	23	NA	NA	
maker-3-augustus-gene-148.0-mRNA-1	3	OsR498G0305849500.01.T01	98,69	0,00E+00	7387	Os03g0323200	98,55	0,00E+00	5796	-1591	Indica	NA	
maker-3-augustus-gene-148.17-mRNA-1	3	OsR498G0305850000.01.T01	99,46	1,28E-89	330	Os07g0656200	92,31	4,49E-71	266	-64	Indica	NA	
maker-3-augustus-gene-148.3-mRNA-1	3	OsR498G0305850500.01.T03	99,58	0,00E+00	2992	Os03g0323500	99,81	0,00E+00	2871	-121	NA	NA	
maker-3-augustus-gene-148.9-mRNA-1	3	OsR498G0305852100.01.T02	99,80	0,00E+00	1820	Os03g0323800	100,00	0,00E+00	1829	9	NA	Japonica	
maker-3-augustus-gene-149.35-mRNA-1	3	OsR498G0305857300.01.T01	99,72	0,00E+00	1908	Os03g0325500	100,00	0,00E+00	1921	13	NA	Japonica	
maker-3-augustus-gene-149.44-mRNA-1	3	OsR498G0305855200.01.T01	99,69	0,00E+00	1155	Os03g0324800	100,00	0,00E+00	1164	9	NA	Japonica	
maker-3-augustus-gene-149.48-mRNA-1	3	OsR498G0305856200.01.T01	99,40	0,00E+00	2060	Os03g0325000	100,00	0,00E+00	2343	283	Japonica	Japonica	
maker-3-augustus-gene-149.56-mRNA-1	3	OsR498G0305859800.01.T01	99,73	0,00E+00	6025	Os01g0277600	70,58	0,00E+00	1418	-4607	Indica	Japonica	
maker-3-augustus-gene-149.60-mRNA-1	3	OsR498G0305860200.01.T01	99,81	0,00E+00	3856	Os03g0326200	100,00	0,00E+00	3874	18	NA	Japonica	
maker-3-augustus-gene-149.62-mRNA-1	3	OsR498G0305861000.01.T03	100,00	0,00E+00	2419	Os03g0326300	99,78	0,00E+00	2404	-15	NA	Japonica	
maker-3-augustus-gene-15.0-mRNA-1	3	OsR498G0305169100.01.T01	99,83	0,00E+00	3120	Os03g0130100	100,00	0,00E+00	3133	13	NA	Japonica	
maker-3-augustus-gene-15.13-mRNA-1	3	OsR498G0305172900.01.T01	100,00	0,00E+00	2067	Os03g0130900	100,00	0,00E+00	2067	0	NA	Japonica	Kinase-TM
maker-3-augustus-gene-15.2-mRNA-1	3	OsR498G0305170100.01.T01	100,00	2,22E-124	444	Os03g0130300	100,00	7,78E-125	444	0	NA	Japonica	
maker-3-augustus-gene-15.21-mRNA-1	3	OsR498G0305170800.01.T01	99,84	0,00E+00	5762	Os03g0130500	100,00	0,00E+00	5784	22	NA	Japonica	
maker-3-augustus-gene-15.27-mRNA-1	3	OsR498G0305172000.01.T02	99,67	0,00E+00	4878	Os03g0130700	100,00	0,00E+00	5198	320	NA	Japonica	
maker-3-augustus-gene-15.31-mRNA-1	3	OsR498G0305173400.01.T05	99,85	0,00E+00	3567	Os03g0131100	99,90	0,00E+00	3571	4	NA	Japonica	
maker-3-augustus-gene-15.34-mRNA-1	3	OsR498G0305175000.01.T01	99,66	0,00E+00	2645	Os03g0131200	100,00	0,00E+00	2668	23	NA	Japonica	
maker-3-augustus-gene-15.37-mRNA-1	3	OsR498G0305175200.01.T03	99,13	0,00E+00	2441	Os03g0131300	100,00	0,00E+00	2495	54	NA	Japonica	
maker-3-augustus-gene-15.4-mRNA-1	3	OsR498G0305170500.01.T01	99,32	0,00E+00	1565	Os03g0130400	100,00	0,00E+00	1591	26	NA	Japonica	
maker-3-augustus-gene-15.7-mRNA-1	3	OsR498G0305172300.01.T01	96,43	0,00E+00	3381	Os03g0130800	100,00	0,00E+00	3079	-302	NA	Japonica	
maker-3-augustus-gene-150.38-mRNA-1	3	OsR498G0305862400.01.T01	99,88	0,00E+00	1511	Os07g0684100	79,09	9,33E-156	549	-962	Indica	Japonica	
maker-3-augustus-gene-150.43-mRNA-1	3	OsR498G0305862600.01.T03	100,00	0,00E+00	2533	Os03g0326600	99,93	0,00E+00	2528	-5	NA	Japonica	
maker-3-augustus-gene-150.47-mRNA-1	3	OsR498G0305863600.01.T01	99,72	0,00E+00	1929	Os03g0327100	100,00	0,00E+00	1943	14	NA	Japonica	
maker-3-augustus-gene-150.49-mRNA-1	3	OsR498G0305865600.01.T01	99,82	0,00E+00	2015	Os03g0327600	100,00	0,00E+00	1885	-130	NA	Japonica	
maker-3-augustus-gene-151.10-mRNA-1	3	OsR498G0305870800.01.T01	99,07	0,00E+00	2665	Os03g0328100	100,00	0,00E+00	2700	35	NA	Japonica	
maker-3-augustus-gene-151.16-mRNA-1	3	OsR498G0305871600.01.T01	96,94	0,00E+00	2485	Os07g0682800	74,52	0,00E+00	973	-1512	Indica	Japonica	
maker-3-augustus-gene-151.2-mRNA-1	3	OsR498G0305873500.01.T02	99,56	0,00E+00	3622	Os03g0328900	99,70	0,00E+00	3635	13	NA	Japonica	
maker-3-augustus-gene-151.5-mRNA-1	3	OsR498G0305874500.01.T04	99,74	0,00E+00	2047	Os03g0329200	100,00	0,00E+00	2060	13	NA	Japonica	
maker-3-augustus-gene-152.16-mRNA-1	3	OsR498G0305876600.01.T01	100,00	0,00E+00	3355	Os03g0329500	100,00	0,00E+00	3355	0	NA	Japonica	
maker-3-augustus-gene-152.20-mRNA-1	3	OsR498G0305878200.01.T01	99,72	0,00E+00	3194	Os03g0330000	99,94	0,00E+00	3212	18	NA	Japonica	
maker-3-augustus-gene-152.23-mRNA-1	3	OsR498G0305879600.01.T02	99,72	0,00E+00	5771	Os03g0330300	100,00	0,00E+00	5811	40	NA	Japonica	
maker-3-augustus-gene-152.5-mRNA-1	3	OsR498G0305877900.01.T01	99,85	0,00E+00	2313	Os03g0329900	100,00	0,00E+00	2322	9	NA	Japonica	
maker-3-augustus-gene-152.9-mRNA-1	3	OsR498G0305879300.01.T01	99,79	0,00E+00	1698	Os03g0330200	97,78	0,00E+00	1627	-71	NA	Japonica	
maker-3-augustus-gene-153.0-mRNA-1	3	OsR498G0305882100.01.T01	99,52	0,00E+00	1107	Os03g0331600	100,00	0,00E+00	773	-334	Indica	Japonica	
maker-3-augustus-gene-153.13-mRNA-1	3	OsR498G0305884200.01.T02	95,86	0,00E+00	2036	Os03g0332000	94,94	0,00E+00	2010	-26	NA	Japonica	
maker-3-augustus-gene-153.16-mRNA-1	3	OsR498G0305884210.01.T01	100,00	1,62E-180	632	Os03g0332100	100,00	2,40E-179	627	-5	NA	Japonica	
maker-3-augustus-gene-153.18-mRNA-1	3	OsR498G0305886000.01.T02	90,24	0,00E+00	1242	Os03g0332400	90,24	0,00E+00	1242	0	NA	Japonica	
maker-3-augustus-gene-153.23-mRNA-1	3	OsR498G0305886400.01.T01	98,90	0,00E+00	795	Os11g0220800	74,48	7,85E-105	379	-416	Indica	Japonica	
maker-3-augustus-gene-153.25-mRNA-1	3	OsR498G0305886600.01.T01	99,79	0,00E+00	1745	Os07g0681200	75,50	6,85E-133	473	-1272	Indica	Japonica	
maker-3-augustus-gene-153.28-mRNA-1	3	OsR498G0305886800.01.T02	100,00	0,00E+00	1689	Os03g0332700	100,00	0,00E+00	1689	0	NA	Japonica	
maker-3-augustus-gene-153.33-mRNA-1	3	OsR498G0305887800.01.T02	99,77	0,00E+00	2287	Os03g0333000	100,00	0,00E+00	2300	13	NA	Japonica	
maker-3-augustus-gene-153.7-mRNA-1	3	OsR498G0305887900.01.T02	99,85	0,00E+00	3655	Os03g0333100	100,00	0,00E+00	3669	14	NA	Japonica	
maker-3-augustus-gene-154.10-mRNA-1	3	OsR498G0305891500.01.T02	99,69	0,00E+00	3469	Os03g0334200	99,95	0,00E+00	3491	22	NA	Japonica	
maker-3-augustus-gene-154.12-mRNA-1	3	OsR498G0305892300.01.T01	97,30	0,00E+00	1131	Os08g0389800	99,23	0,00E+00	1149	18	NA	Japonica	
maker-3-augustus-gene-154.14-mRNA-1	3	OsR498G0305893500.01.T05	99,87	0,00E+00	6728	Os03g0334700	100,00	0,00E+00	4246	-2482	Indica	Japonica	
maker-3-augustus-gene-154.17-mRNA-1	3	OsR498G0305894900.01.T01	99,68	0,00E+00	1101	Os03g0335200	93,58	0,00E+00	1447	346	Japonica	Japonica	
maker-3-augustus-gene-154.19-mRNA-1	3	OsR498G0305890200.01.T01	99,86	0,00E+00	1284	Os03g0333800	100,00	0,00E+00	2284	1000	Japonica	Japonica	
maker-3-augustus-gene-154.23-mRNA-1	3	OsR498G0305890800.01.T02	99,72	0,00E+00	2585	Os03g0334000	100,00	0,00E+00	2603	18	NA	Japonica	Kinase
maker-3-augustus-gene-154.26-mRNA-1	3	OsR498G0305893800.01.T01	99,87	0,00E+00	2670	Os03g0334800	100,00	0,00E+00	2679	9	NA	Japonica	
maker-3-augustus-gene-154.5-mRNA-1	3	OsR498G0305891300.01.T02	99,71	0,00E+00	1870	Os03g0334100	99,85	0,00E+00	1214	-656	Indica	Japonica	
maker-3-augustus-gene-155.32-mRNA-1	3	OsR498G0305895300.01.T01	97,67	0,00E+00	3239	Os03g0335300	97,77	0,00E+00	3248	9	NA	Japonica	
maker-3-augustus-gene-155.37-mRNA-1	3	OsR498G0305895900.01.T01	99,76	0,00E+00	5228	Os03g0335500	100,00	0,00E+00	5259	31	NA	Japonica	Kinase-LRR-TM
maker-3-augustus-gene-155.39-mRNA-1	3	OsR498G0305898100.01.T02	100,00	0,00E+00	1786	Os03g0336200	100,00	0,00E+00	1786	0	NA	Japonica	
maker-3-augustus-gene-155.43-mRNA-1	3	OsR498G0305898300.01.T01	100,00	0,00E+00	5613	Os03g0336300	100,00	0,00E+00	5633	20	NA	Japonica	
maker-3-augustus-gene-155.49-mRNA-1	3	OsR498G0305899100.01.T01	100,00	0,00E+00	861	Os03g0336500	100,00	0,00E+00	861	0	NA	Japonica	
maker-3-augustus-gene-155.57-mRNA-1	3	OsR498G0305896800.01.T05	99,66	0,00E+00	3108	Os03g0694900	82,02	0,00E+00	1719	-1389	Indica	Japonica	
maker-3-augustus-gene-155.65-mRNA-1	3	OsR498G0305899400.01.T01	100,00	0,00E+00	1256	Os03g0336600	100,00	0,00E+00	1256	0	NA	Japonica	
maker-3-augustus-gene-156.0-mRNA-1	3	OsR498G0305903100.01.T06	99,73	0,00E+00	2000	Os07g0679000	83,07	0,00E+00	1123	-877	Indica	Japonica	
maker-3-augustus-gene-156.10-mRNA-1	3	OsR498G0305906500.01.T01	98,39	0,00E+00	864	Os03g0338400	100,00	0,00E+00	888	24	NA	Japonica	
maker-3-augustus-gene-156.20-mRNA-1	3	OsR498G0305903600.01.T01	99,37	0,00E+00	1417	Os03g0337700	100,00	0,00E+00	1440	23	NA	Japonica	
maker-3-augustus-gene-156.28-mRNA-1	3	OsR498G0305903800.01.T01	99,68	0,00E+00	1122	Os03g0337800	100,00	0,00E+00	1131	9	NA	Japonica	
maker-3-augustus-gene-156.40-mRNA-1	3	OsR498G0305904400.01.T01	99,71	0,00E+00	2504	Os03g0338000	100,00	0,00E+00	2522	18	NA	Japonica	
maker-3-augustus-gene-156.42-mRNA-1	3	OsR498G0305907000.01.T02	99,87	0,00E+00	2789	Os03g0338600	100,00	0,00E+00	2798	9	NA	Japonica	
maker-3-augustus-gene-156.6-mRNA-1	3	OsR498G0305903300.01.T01	96,86	0,00E+00	2038	Os03g0337600	97,02	0,00E+00	2047	9	NA	Japonica	
maker-3-augustus-gene-157.35-mRNA-1	3	OsR498G0305908500.01.T01	98,96	0,00E+00	1693	Os03g0339300	99,38	0,00E+00	1711	18	NA	Japonica	
maker-3-augustus-gene-157.37-mRNA-1	3	OsR498G0305910100.01.T01	100,00	0,00E+00	2949	Os03g0339700	100,00	0,00E+00	2949	0	NA	Japonica	
maker-3-augustus-gene-157.41-mRNA-1	3	OsR498G0305916700.01.T01	99,59	0,00E+00	2641	Os03g0340700	100,00	0,00E+00	2668	27	NA	Japonica	
maker-3-augustus-gene-157.43-mRNA-1	3	OsR498G0305916900.01.T03	99,80	0,00E+00	3498	Os03g0340900	100,00	0,00E+00	3516	18	NA	Japonica	
maker-3-augustus-gene-157.51-mRNA-1	3	OsR498G0305908800.01.T01	99,39	0,00E+00	1743	Os07g0677600	70,58	1,66E-117	423	-1320	Indica	Japonica	
maker-3-augustus-gene-157.53-mRNA-1	3	OsR498G0305910600.01.T02	100,00	0,00E+00	2484	Os03g0339900	99,85	0,00E+00	2374	-110	NA	NA	Kinase-TM
maker-3-augustus-gene-157.57-mRNA-1	3	OsR498G0305915600.01.T02	99,84	0,00E+00	2253	Os03g0340500	100,00	0,00E+00	2262	9	NA	Japonica	
maker-3-augustus-gene-158.44-mRNA-1	3	OsR498G0305918100.01.T01	99,73	0,00E+00	1312	Os03g0341000	100,00	0,00E+00	1321	9	NA	Japonica	
maker-3-augustus-gene-158.46-mRNA-1	3	OsR498G0305919100.01.T04	99,00	0,00E+00	1755	Os03g0341300	100,00	0,00E+00	1781	26	NA	Japonica	
maker-3-augustus-gene-158.54-mRNA-1	3	OsR498G0305923500.01.T01	100,00	0,00E+00	2489	Os03g0343400	100,00	0,00E+00	2489	0	NA	NA	
maker-3-augustus-gene-158.62-mRNA-1	3	OsR498G0305918400.01.T01	99,82	0,00E+00	1013	Os03g0341100	100,00	0,00E+00	1018	5	NA	Japonica	
maker-3-augustus-gene-158.67-mRNA-1	3	OsR498G0305918600.01.T02	99,20	0,00E+00	3975	Os03g0341200	100,00	0,00E+00	3889	-86	NA	Japonica	
maker-3-augustus-gene-158.72-mRNA-1	3	OsR498G0305920400.01.T02	99,82	0,00E+00	986	Os03g0341600	100,00	0,00E+00	991	5	NA	Japonica	
maker-3-augustus-gene-158.74-mRNA-1	3	OsR498G0305920600.01.T01	99,81	0,00E+00	932	Os03g0342100	100,00	0,00E+00	937	5	NA	Japonica	
maker-3-augustus-gene-158.76-mRNA-1	3	OsR498G0305921800.01.T05	100,00	0,00E+00	659	Os03g0342900	89,56	7,47E-124	443	-216	Indica	Japonica	
maker-3-augustus-gene-158.80-mRNA-1	3	OsR498G0305923800.01.T01	100,00	0,00E+00	715	Os03g0343500	100,00	0,00E+00	715	0	NA	NA	
maker-3-augustus-gene-159.0-mRNA-1	3	OsR498G0305925100.01.T01	100,00	0,00E+00	1553	Os03g0344100	100,00	0,00E+00	1332	-221	Indica	NA	
maker-3-augustus-gene-159.18-mRNA-1	3	OsR498G0305926800.01.T01	100,00	0,00E+00	6011	Os03g0344700	100,00	0,00E+00	3609	-2402	Indica	NA	
maker-3-augustus-gene-159.25-mRNA-1	3	OsR498G0305927400.01.T01	100,00	0,00E+00	1851	Os03g0344900	97,71	0,00E+00	1799	-52	NA	NA	
maker-3-augustus-gene-159.31-mRNA-1	3	OsR498G0305928400.01.T02	98,48	0,00E+00	1829	Os03g0345100	93,90	0,00E+00	1627	-202	Indica	NA	
maker-3-augustus-gene-159.5-mRNA-1	3	OsR498G0305928500.01.T02	93,51	7,48E-171	600	Os03g0345200	100,00	2,97E-126	450	-150	Indica	NA	
maker-3-augustus-gene-16.48-mRNA-1	3	OsR498G0305175500.01.T01	99,82	0,00E+00	2010	Os03g0131500	100,00	0,00E+00	2019	9	NA	Japonica	
maker-3-augustus-gene-16.51-mRNA-1	3	OsR498G0305175900.01.T01	99,42	0,00E+00	1223	Os04g0561600	80,66	0,00E+00	636	-587	Indica	Japonica	
maker-3-augustus-gene-16.53-mRNA-1	3	OsR498G0305176700.01.T01	99,82	0,00E+00	2979	Os03g0132000	100,00	0,00E+00	2993	14	NA	Japonica	
maker-3-augustus-gene-16.57-mRNA-1	3	OsR498G0305177700.01.T03	100,00	0,00E+00	1516	Os03g0132200	100,00	0,00E+00	1516	0	NA	NA	
maker-3-augustus-gene-16.62-mRNA-1	3	OsR498G0305179900.01.T01	100,00	0,00E+00	2879	Os03g0132800	100,00	0,00E+00	2879	0	NA	NA	CC-Kinase-TM
maker-3-augustus-gene-16.66-mRNA-1	3	OsR498G0305180300.01.T01	100,00	0,00E+00	1391	Os03g0132900	100,00	0,00E+00	1391	0	NA	NA	
maker-3-augustus-gene-16.68-mRNA-1	3	OsR498G0305181300.01.T01	97,38	0,00E+00	1544	Os03g0133300	97,38	0,00E+00	1544	0	NA	NA	
maker-3-augustus-gene-16.75-mRNA-1	3	OsR498G0305175400.01.T01	99,75	0,00E+00	2146	Os03g0131400	100,00	0,00E+00	2159	13	NA	Japonica	
maker-3-augustus-gene-16.81-mRNA-1	3	OsR498G0305178200.01.T01	100,00	0,00E+00	1564	Os03g0132300	100,00	0,00E+00	1564	0	NA	NA	
maker-3-augustus-gene-16.84-mRNA-1	3	OsR498G0305180600.01.T01	100,00	0,00E+00	1716	Os03g0133000	100,00	0,00E+00	1716	0	NA	NA	
maker-3-augustus-gene-16.86-mRNA-1	3	OsR498G0305182000.01.T01	100,00	0,00E+00	2982	Os03g0133500	100,00	0,00E+00	2982	0	NA	NA	
maker-3-augustus-gene-16.88-mRNA-1	3	OsR498G0305182200.01.T01	98,68	0,00E+00	2908	Os03g0133600	100,00	0,00E+00	2632	-276	NA	NA	
maker-3-augustus-gene-16.90-mRNA-1	3	OsR498G0305183000.01.T04	100,00	0,00E+00	1649	Os03g0133900	100,00	0,00E+00	1649	0	NA	NA	
maker-3-augustus-gene-160.27-mRNA-1	3	OsR498G0305936900.01.T01	100,00	0,00E+00	2019	Os03g0346800	100,00	0,00E+00	1981	-38	NA	NA	
maker-3-augustus-gene-160.34-mRNA-1	3	OsR498G0305938700.01.T03	100,00	0,00E+00	4114	Os03g0347200	100,00	0,00E+00	4114	0	NA	NA	
maker-3-augustus-gene-160.45-mRNA-1	3	OsR498G0305939000.01.T05	99,56	0,00E+00	7314	Os03g0347300	99,18	0,00E+00	3890	-3424	Indica	NA	
maker-3-augustus-gene-161.0-mRNA-1	3	OsR498G0305940700.01.T02	99,86	0,00E+00	1284	Os03g0347800	100,00	0,00E+00	931	-353	Indica	NA	

maker-3-augustus-gene-161.15-mRNA-1	3	OsR498G0305942000.01.T02	97,59	0,00E+00	2633	Os03g0348200	99,94	0,00E+00	2945	312	Japonica	NA	
maker-3-augustus-gene-161.17-mRNA-1	3	OsR498G0305944000.01.T01	98,93	0,00E+00	1966	Os07g0668700	77,21	0,00E+00	636	-1330	Indica	NA	
maker-3-augustus-gene-161.23-mRNA-1	3	OsR498G0305944200.01.T03	100,00	0,00E+00	1095	Os03g0348900	100,00	0,00E+00	1095	0	NA	NA	
maker-3-augustus-gene-161.5-mRNA-1	3	OsR498G0305939400.01.T01	100,00	0,00E+00	1629	Os03g0347500	100,00	0,00E+00	1629	0	NA	NA	
maker-3-augustus-gene-161.7-mRNA-1	3	OsR498G0305941000.01.T05	99,85	0,00E+00	1226	Os03g0347900	99,85	0,00E+00	1226	0	NA	NA	
maker-3-augustus-gene-162.0-mRNA-1	3	OsR498G0305948100.01.T04	99,92	0,00E+00	2182	Os03g0351100	99,92	0,00E+00	2182	0	NA	NA	
maker-3-augustus-gene-162.14-mRNA-1	3	OsR498G0305945000.01.T02	99,76	0,00E+00	3002	Os03g0349200	99,70	0,00E+00	2378	-624	Indica	NA	CC-Kinase
maker-3-augustus-gene-162.19-mRNA-1	3	OsR498G0305945800.01.T02	100,00	0,00E+00	3079	Os03g0350100	99,94	0,00E+00	3006	-73	NA	NA	
maker-3-augustus-gene-162.23-mRNA-1	3	OsR498G0305946800.01.T01	99,94	0,00E+00	3075	Os03g0350300	99,94	0,00E+00	3075	0	NA	NA	
maker-3-augustus-gene-162.27-mRNA-1	3	OsR498G0305949200.01.T05	99,82	0,00E+00	1012	Os07g0667000	79,93	1,23E-142	506	-506	Indica	NA	
maker-3-augustus-gene-162.3-mRNA-1	3	OsR498G0305948900.01.T01	100,00	0,00E+00	2841	Os03g0351300	100,00	0,00E+00	2841	0	NA	NA	
maker-3-augustus-gene-162.5-mRNA-1	3	OsR498G0305951300.01.T01	100,00	0,00E+00	829	Os03g0351500	100,00	0,00E+00	829	0	NA	Japonica	CSD1
maker-3-augustus-gene-162.8-mRNA-1	3	OsR498G0305944600.01.T01	99,33	0,00E+00	4268	Os03g0349000	99,33	0,00E+00	4268	0	NA	NA	
maker-3-augustus-gene-163.36-mRNA-1	3	OsR498G0305954100.01.T05	99,85	0,00E+00	1177	Os03g0352200	100,00	0,00E+00	1182	5	NA	Japonica	
maker-3-augustus-gene-163.40-mRNA-1	3	OsR498G0305954500.01.T01	99,44	0,00E+00	2860	Os03g0352400	100,00	0,00E+00	2901	41	NA	Japonica	
maker-3-augustus-gene-163.52-mRNA-1	3	OsR498G0305955900.01.T01	97,86	0,00E+00	716	Os03g0353000	100,00	0,00E+00	753	37	NA	Japonica	
maker-3-augustus-gene-163.54-mRNA-1	3	OsR498G0305956300.01.T01	99,67	0,00E+00	2149	Os12g0594700	75,33	0,00E+00	674	-1475	Indica	Japonica	
maker-3-augustus-gene-163.56-mRNA-1	3	OsR498G0305957500.01.T02	99,38	0,00E+00	862	Os03g0353400	100,00	0,00E+00	1104	242	Japonica	Japonica	
maker-3-augustus-gene-163.59-mRNA-1	3	OsR498G0305957900.01.T01	99,42	0,00E+00	1214	Os03g0353500	100,00	0,00E+00	1229	15	NA	Japonica	
maker-3-augustus-gene-163.61-mRNA-1	3	OsR498G0305954400.01.T01	99,66	0,00E+00	2604	Os03g0352300	100,00	0,00E+00	2626	22	NA	Japonica	
maker-3-augustus-gene-163.67-mRNA-1	3	OsR498G0305955800.01.T01	99,76	0,00E+00	732	Os03g0352800	100,00	0,00E+00	737	5	NA	Japonica	
maker-3-augustus-gene-164.10-mRNA-1	3	OsR498G0305959300.01.T04	99,81	0,00E+00	3707	Os03g0353900	99,95	0,00E+00	3720	13	NA	Japonica	
maker-3-augustus-gene-166.0-mRNA-1	3	OsR498G0305990400.01.T01	100,00	0,00E+00	969	Os03g0360500	100,00	0,00E+00	969	0	NA	NA	
maker-3-augustus-gene-169.39-mRNA-1	3	OsR498G0305967300.01.T01	99,90	0,00E+00	1830	Os03g0355700	100,00	0,00E+00	2717	887	Japonica	Japonica	
maker-3-augustus-gene-169.41-mRNA-1	3	OsR498G0305966400.01.T02	99,96	0,00E+00	4849	Os03g0355600	100,00	0,00E+00	4837	-12	NA	Japonica	
maker-3-augustus-gene-169.46-mRNA-1	3	OsR498G0305967800.01.T01	99,88	0,00E+00	1484	Os03g0355800	100,00	0,00E+00	1489	5	NA	Japonica	
maker-3-augustus-gene-169.49-mRNA-1	3	OsR498G0305968000.01.T01	100,00	0,00E+00	1808	Os03g0355900	100,00	0,00E+00	1808	0	NA	Japonica	
maker-3-augustus-gene-169.54-mRNA-1	3	OsR498G0305969000.01.T01	95,89	0,00E+00	1123	Os03g0356300	95,89	0,00E+00	1123	0	NA	Japonica	
maker-3-augustus-gene-169.56-mRNA-1	3	OsR498G0305969400.01.T01	100,00	0,00E+00	1635	Os03g0356414	100,00	0,00E+00	1635	0	NA	Japonica	
maker-3-augustus-gene-169.63-mRNA-1	3	OsR498G0305971000.01.T02	99,81	0,00E+00	938	Os03g0356484	100,00	0,00E+00	942	4	NA	Japonica	
maker-3-augustus-gene-17.38-mRNA-1	3	OsR498G0305183700.01.T01	100,00	0,00E+00	2192	Os03g0134300	100,00	0,00E+00	2192	0	NA	NA	
maker-3-augustus-gene-17.45-mRNA-1	3	OsR498G0305185200.01.T01	99,17	0,00E+00	1708	Os03g0134900	100,00	0,00E+00	1732	24	NA	Japonica	
maker-3-augustus-gene-17.47-mRNA-1	3	OsR498G0305185800.01.T01	99,71	0,00E+00	1214	Os03g0135100	100,00	0,00E+00	1223	9	NA	Japonica	
maker-3-augustus-gene-17.50-mRNA-1	3	OsR498G0305186000.01.T01	99,85	0,00E+00	1203	Os03g0135200	100,00	0,00E+00	704	-499	Indica	Japonica	
maker-3-augustus-gene-17.53-mRNA-1	3	OsR498G0305186200.01.T01	99,56	0,00E+00	1205	Os03g0135300	100,00	0,00E+00	1218	13	NA	Japonica	
maker-3-augustus-gene-17.55-mRNA-1	3	OsR498G0305186800.01.T01	100,00	0,00E+00	3458	Os03g0135400	100,00	0,00E+00	3458	0	NA	Japonica	
maker-3-augustus-gene-17.57-mRNA-1	3	OsR498G0305187200.01.T09	99,49	0,00E+00	2440	Os03g0135600	98,84	0,00E+00	1802	-638	Indica	Japonica	
maker-3-augustus-gene-17.61-mRNA-1	3	OsR498G0305187800.01.T01	98,77	0,00E+00	1850	Os03g0135700	100,00	0,00E+00	2262	412	Japonica	Japonica	
maker-3-augustus-gene-170.30-mRNA-1	3	OsR498G0305973900.01.T01	99,16	0,00E+00	4010	Os03g0356582	100,00	0,00E+00	4091	81	NA	Japonica	
maker-3-augustus-gene-170.33-mRNA-1	3	OsR498G0714888800.01.T01	79,70	0,00E+00	1126	Os03g0356596	100,00	0,00E+00	2560	1434	Japonica	Japonica	
maker-3-augustus-gene-170.39-mRNA-1	3	OsR498G0305974800.01.T02	98,11	0,00E+00	4214	Os03g0356638	99,03	0,00E+00	4371	157	NA	Japonica	
maker-3-augustus-gene-171.0-mRNA-1	3	OsR498G0305980300.01.T06	99,88	0,00E+00	3057	Os03g0356700	100,00	0,00E+00	2942	-115	NA	Japonica	
maker-3-augustus-gene-172.0-mRNA-1	3	OsR498G0305983500.01.T01	100,00	0,00E+00	921	Os03g0358100	100,00	0,00E+00	921	0	NA	Japonica	
maker-3-augustus-gene-172.13-mRNA-1	3	OsR498G0305983800.01.T01	94,00	0,00E+00	1519	Os07g0655400	73,86	1,32E-160	566	-953	Indica	Japonica	
maker-3-augustus-gene-173.41-mRNA-1	3	OsR498G0305987400.01.T01	99,94	0,00E+00	3112	Os03g0359000	99,56	0,00E+00	2439	-673	Indica	Indica	
maker-3-augustus-gene-175.11-mRNA-1	3	OsR498G0305991300.01.T01	99,49	0,00E+00	699	Os07g0645500	77,74	5,26E-54	211	-488	Indica	Japonica	
maker-3-augustus-gene-175.14-mRNA-1	3	OsR498G0305992500.01.T01	99,80	0,00E+00	1754	Os03g0361100	100,00	0,00E+00	3128	1374	Japonica	Japonica	
maker-3-augustus-gene-175.17-mRNA-1	3	OsR498G0305990400.01.T01	100,00	0,00E+00	969	Os03g0360500	100,00	0,00E+00	969	0	NA	NA	
maker-3-augustus-gene-175.22-mRNA-1	3	OsR498G0305993600.01.T01	98,66	0,00E+00	1174	Os03g0361500	100,00	0,00E+00	1754	580	Japonica	Japonica	
maker-3-augustus-gene-176.0-mRNA-1	3	OsR498G0305997700.01.T01	96,55	0,00E+00	4253	Os03g0362200	100,00	0,00E+00	4616	363	NA	Japonica	
maker-3-augustus-gene-176.14-mRNA-1	3	OsR498G0305996000.01.T01	99,24	0,00E+00	3042	Os03g0361700	100,00	0,00E+00	2738	-304	NA	Japonica	
maker-3-augustus-gene-176.17-mRNA-1	3	OsR498G0305996700.01.T01	94,55	0,00E+00	1400	Os03g0361100	80,04	0,00E+00	809	-591	Indica	Japonica	
maker-3-augustus-gene-176.19-mRNA-1	3	OsR498G0305996500.01.T01	88,24	0,00E+00	842	Os03g0361700	76,12	2,60E-117	421	-421	Indica	Japonica	
maker-3-augustus-gene-176.2-mRNA-1	3	OsR498G0305997700.01.T01	86,28	0,00E+00	3078	Os03g0363100	100,00	0,00E+00	4605	1527	Japonica	Japonica	
maker-3-augustus-gene-176.21-mRNA-1	3	OsR498G0305999000.01.T02	99,88	0,00E+00	1497	Os03g0363500	100,00	0,00E+00	1501	4	NA	Japonica	
maker-3-augustus-gene-176.4-mRNA-1	3	OsR498G0305998300.01.T01	98,69	0,00E+00	1867	Os03g0363350	100,00	0,00E+00	1930	63	NA	Japonica	
maker-3-augustus-gene-176.7-mRNA-1	3	OsR498G0305998700.01.T03	95,18	0,00E+00	1196	Os03g0363400	100,00	0,00E+00	1326	130	NA	Japonica	
maker-3-augustus-gene-177.10-mRNA-1	3	OsR498G0306001300.01.T01	99,73	0,00E+00	1321	Os03g0364400	100,00	0,00E+00	1330	9	NA	Japonica	Kinase-TM
maker-3-augustus-gene-177.17-mRNA-1	3	OsR498G0306004300.01.T01	99,45	0,00E+00	652	Os03g0365200	100,00	0,00E+00	688	36	NA	NA	
maker-3-augustus-gene-177.20-mRNA-1	3	OsR498G0305999200.01.T01	100,00	0,00E+00	2381	Os03g0363600	100,00	0,00E+00	2792	411	Japonica	Japonica	
maker-3-augustus-gene-177.26-mRNA-1	3	OsR498G0306000200.01.T02	100,00	0,00E+00	902	Os03g0363950	100,00	0,00E+00	902	0	NA	Japonica	
maker-3-augustus-gene-177.4-mRNA-1	3	OsR498G0305999900.01.T01	95,74	0,00E+00	1885	Os03g0363900	95,99	0,00E+00	1899	14	NA	Japonica	
maker-3-augustus-gene-178.0-mRNA-1	3	OsR498G0306006500.01.T01	80,89	0,00E+00	902	Os03g0365800	82,63	0,00E+00	870	-32	NA	Japonica	
maker-3-augustus-gene-178.1-mRNA-1	3	OsR498G0306006500.01.T01	92,45	0,00E+00	1461	Os03g0365800	100,00	0,00E+00	1698	237	Japonica	Japonica	
maker-3-augustus-gene-178.12-mRNA-1	3	OsR498G0306008700.01.T02	99,91	0,00E+00	2029	Os03g0366200	100,00	0,00E+00	2008	-21	NA	Japonica	Kinase-TM
maker-3-augustus-gene-178.18-mRNA-1	3	OsR498G0306009300.01.T03	99,81	0,00E+00	956	Os07g0641000	81,58	1,63E-146	518	-438	Indica	Japonica	
maker-3-augustus-gene-178.22-mRNA-1	3	OsR498G0306007000.01.T02	100,00	3,38E-135	480	Os03g0366000	98,82	2,97E-124	444	-36	NA	Japonica	
maker-3-augustus-gene-178.8-mRNA-1	3	OsR498G0306006700.01.T01	93,00	0,00E+00	1798	Os03g0365900	100,00	0,00E+00	1101	-697	Indica	Japonica	
maker-3-augustus-gene-179.0-mRNA-1	3	OsR498G0306010100.01.T01	99,83	0,00E+00	2109	Os03g0367000	100,00	0,00E+00	2118	9	NA	Japonica	
maker-3-augustus-gene-179.11-mRNA-1	3	OsR498G0306013100.01.T03	98,95	0,00E+00	1176	Os03g0367900	100,00	0,00E+00	1207	31	NA	Japonica	
maker-3-augustus-gene-179.15-mRNA-1	3	OsR498G0306013500.01.T01	98,15	0,00E+00	1673	Os03g0368300	99,79	0,00E+00	1745	72	NA	Japonica	
maker-3-augustus-gene-179.17-mRNA-1	3	OsR498G0306015100.01.T01	100,00	0,00E+00	1754	Os03g0368600	100,00	0,00E+00	2159	405	Japonica	Japonica	
maker-3-augustus-gene-179.20-mRNA-1	3	OsR498G0306013500.01.T01	98,15	0,00E+00	1673	Os03g0368300	100,00	0,00E+00	1754	81	NA	Japonica	
maker-3-augustus-gene-18.26-mRNA-1	3	OsR498G0305193500.01.T03	99,75	0,00E+00	2151	Os03g0137200	100,00	0,00E+00	2165	14	NA	Japonica	
maker-3-augustus-gene-18.33-mRNA-1	3	OsR498G0305194700.01.T02	100,00	0,00E+00	1364	Os03g0137600	100,00	0,00E+00	1364	0	NA	Japonica	
maker-3-augustus-gene-18.35-mRNA-1	3	OsR498G0305197100.01.T01	98,50	0,00E+00	1160	Os03g0137800	100,00	0,00E+00	1202	42	NA	Japonica	
maker-3-augustus-gene-18.38-mRNA-1	3	OsR498G0305197900.01.T01	99,76	0,00E+00	3776	Os03g0138000	99,75	0,00E+00	3623	-153	NA	Japonica	
maker-3-augustus-gene-18.40-mRNA-1	3	OsR498G0305190800.01.T01	99,78	0,00E+00	1599	Os03g0136800	100,00	0,00E+00	1608	9	NA	Japonica	
maker-3-augustus-gene-18.42-mRNA-1	3	OsR498G0305191400.01.T01	99,87	0,00E+00	5323	Os03g0136900	100,00	0,00E+00	5341	18	NA	Japonica	
maker-3-augustus-gene-18.48-mRNA-1	3	OsR498G0305193800.01.T01	99,76	0,00E+00	1517	Os03g0137300	100,00	0,00E+00	1126	-391	Indica	Japonica	
maker-3-augustus-gene-18.51-mRNA-1	3	OsR498G0305195000.01.T01	99,74	0,00E+00	1370	Os03g0137700	100,00	0,00E+00	1379	9	NA	Japonica	
maker-3-augustus-gene-180.0-mRNA-1	3	OsR498G0306016300.01.T01	100,00	0,00E+00	1802	Os03g0368900	100,00	0,00E+00	1802	0	NA	Japonica	
maker-3-augustus-gene-180.16-mRNA-1	3	OsR498G0306024100.01.T01	99,68	0,00E+00	2786	Os03g0370900	100,00	0,00E+00	2809	23	NA	Japonica	
maker-3-augustus-gene-180.18-mRNA-1	3	OsR498G0306019600.01.T02	99,88	0,00E+00	1486	Os03g0369800	99,88	0,00E+00	1489	3	NA	Japonica	
maker-3-augustus-gene-180.2-mRNA-1	3	OsR498G0306017500.01.T01	99,80	0,00E+00	1837	Os03g0369000	100,00	0,00E+00	1846	9	NA	Japonica	
maker-3-augustus-gene-180.4-mRNA-1	3	OsR498G0306018100.01.T01	100,00	5,82E-178	623	Os03g0369100	100,00	2,02E-178	623	0	NA	Japonica	
maker-3-augustus-gene-180.6-mRNA-1	3	OsR498G0306018300.01.T01	96,70	0,00E+00	1654	Os03g0369200	100,00	0,00E+00	1802	148	NA	Japonica	
maker-3-augustus-gene-180.8-mRNA-1	3	OsR498G0306018500.01.T01	99,71	0,00E+00	1827	Os03g0369400	99,90	0,00E+00	1836	9	NA	Japonica	
maker-3-augustus-gene-182.0-mRNA-1	3	OsR498G0306030300.01.T02	98,86	0,00E+00	5073	Os03g0372500	100,00	0,00E+00	5218	145	NA	Japonica	
maker-3-augustus-gene-182.12-mRNA-1	3	OsR498G0306031800.01.T01	97,05	0,00E+00	959	Os03g0374100	100,00	0,00E+00	1031	72	NA	Japonica	
maker-3-augustus-gene-182.17-mRNA-1	3	OsR498G0306033000.01.T01	100,00	0,00E+00	3128	Os07g0633400	77,70	0,00E+00	948	-2180	Indica	NA	
maker-3-augustus-gene-182.20-mRNA-1	3	OsR498G0306035000.01.T01	99,85	0,00E+00	1174	Os03g0374575	100,00	0,00E+00	1339	165	Japonica	NA	
maker-3-augustus-gene-182.3-mRNA-1	3	OsR498G0306030900.01.T01	99,06	0,00E+00	2632	Os03g0372700	100,00	0,00E+00	2704	72	NA	Japonica	
maker-3-augustus-gene-182.5-mRNA-1	3	OsR498G0306032700.01.T01	100,00	0,00E+00	1743	Os03g0374400	100,00	0,00E+00	1878	135	NA	Japonica	
maker-3-augustus-gene-183.29-mRNA-1	3	OsR498G0306035500.01.T01	97,62	0,00E+00	2525	Os03g0374600	97,62	0,00E+00	2525	0	NA	NA	
maker-3-augustus-gene-183.33-mRNA-1	3	OsR498G0306036800.01.T01	99,34	0,00E+00	1083	Os03g0374900	100,00	0,00E+00	710	-373	Indica	Japonica	
maker-3-augustus-gene-183.36-mRNA-1	3	OsR498G0306036800.01.T01	92,61	0,00E+00	2287	Os03g0375100	91,99	0,00E+00	1410	-877	Indica	NA	
maker-3-augustus-gene-183.41-mRNA-1	3	OsR498G0306040400.01.T02	100,00	0,00E+00	1261	Os03g0376000	100,00	0,00E+00	1261	0	NA	NA	
maker-3-augustus-gene-183.43-mRNA-1	3	OsR498G0306040800.01.T01	100,00	0,00E+00	2765	Os03g0376100	100,00	0,00E+00	1954	-811	Indica	NA	
maker-3-augustus-gene-184.0-mRNA-1	3	OsR498G0306042300.01.T01	99,86	0,00E+00	2507	Os03g0376900	100,00	0,00E+00	2516	9	NA	Japonica	
maker-3-augustus-gene-184.24-mRNA-1	3	OsR498G0306046100.01.T03	99,83	0,00E+00	1030	Os03g0378000	99,88	0,00E+00	1538	508	Japonica	Japonica	
maker-3-augustus-gene-184.28-mRNA-1	3	OsR498G0306046700.01.T03	99,82	0,00E+00	2037	Os03g0378200	99,91	0,00E+00	2041	4	NA	Japonica	
maker-3-augustus-gene-184.33-mRNA-1	3	OsR498G0306042000.01.T01	99,75	0,00E+00	1431	Os03g0376600	100,00	0,00E+00	1440	9	NA	Japonica	
maker-3-augustus-gene-184.35-mRNA-1	3	OsR498G0306042200.01.T02	99,82	0,00E+00	2925	Os03g0376800	99,94	0,00E+00	2934	9	NA	Japonica	
maker-3-augustus-gene-184.38-mRNA-1	3	OsR498G0306044400.01.T02	99,79	0,00E+00	1678	Os03g0377700	100,00	0,00E+00	1687	9	NA	Japonica	
maker-3-augustus-gene-184.7-mRNA-1	3	OsR498G0306042500.01.T01	97,90	0,00E+00	1394	Os03g0377100	100,00	0,00E+00	1434	40	NA	Japonica	
maker-3-augustus-gene-185.0-mRNA-1	3	OsR498G0306048300.01.T02	100,00	0,00E+00	3312	Os03g0379100	100,00	0,00E+00	3312	0	NA	Japonica	
maker-3-augustus-gene-185.10-mRNA-1	3	OsR498G0306048800.01.T01	98,17	0,00E+00	1602	Os03g0379400	98,11	0,00E+00	1550	-52	NA	NA	
maker-3-augustus-gene-185.2-mRNA-1	3	OsR498G0306048500.01.T01	99,88	0,00E+00	1484	Os03g0379300	100,00	0,00E+00	1370	-114	NA	Japonica	
maker-3-augustus-gene-185.7-mRNA-1	3	OsR498G0306049500.01.T01	99,48	0,00E+00	2713	Os07g0501500	72,81	0,00E+00	798	-1915	Indica	NA	NBS-TM
maker-3-augustus-gene-186.16-mRNA-1	3	OsR498G0306052600.01.T01	99,74	0,00E+00	2027	Os03g0381000	99,56	0,00E+00	2018	-9	NA	Japonica	
maker-3-augustus-gene-186.18-mRNA-1	3	OsR498G0306053600.01.T01	96,27	0,00E+00	1331	Os03g0381300	96,13	0,00E+00	900	-431	Indica	Japonica	
maker-3-augustus-gene-186.23-mRNA-1	3	OsR498G0306054000.01.T01	100,00	0,00E+00	1083	Os03g0381500	100,00	0,00E+00	1083	0	NA	Japonica	
maker-3-augustus-gene-186.8-mRNA-1	3	OsR498G0306053300.01.T01	99,87	0,00E+00	1365	Os03g0381200	100,00	0,00E+00	1370	5	NA	Japonica	
maker-3-augustus-gene-187.38-mRNA-1	3	OsR498G0306057500.01.T03	96,69	0,00E+00	803	Os03g0383900	100,00	0,00E+00	856	53	NA	Japonica	
maker-3-augustus-gene-188.33-mRNA-1	3	OsR498G0306060700.01.T01	96,32	0,00E+00	889	Os03g0385400	100,00	0,00E+00	969	80	NA	Japonica	
maker-3-augustus-gene-188.43-mRNA-1	3	OsR498G0306060200.01.T01	99,19	4,87E-56	218	Os03g0384900	100,00	1,30E-108	390	172	Japonica	NA	
maker-3-augustus-gene-188.47-mRNA-1	3	OsR498G0306062000.01.T02	97,91	0,00E+00	1306	Os03g0385900	100,00	0,00E+00	1802	496	Japonica	Japonica	
maker-3-augustus-gene-189.22-mRNA-1	3	OsR498G0306065700.01.T03	99,54	0,00E+00	1167	Os03g0386500	99,85	0,00E+00	1176	9	NA	Japonica	
maker-3-augustus-gene-189.28-mRNA-1	3	OsR498G0306067900.01.T02	99,59	0,00E+00	1750	Os03g0386800	99,89	0,00E+00	1666	-84	NA	Japonica	
maker-3-augustus-gene-189.39-mRNA-1	3	OsR498G0306069500.01.T01	99,70	0,00E+00	1205	Os03g0387100	99,85	0,00E+00	1210	5	NA	Japonica	
maker-3-augustus-gene-189.45-mRNA-1	3	OsR498G0306072700.01.T01	96,46	0,00E+00	893	Os03g0387800	99,81	0,00E+00	963	70	NA	Japonica	
maker-3-augustus-gene-19.40-mRNA-1	3	OsR498G0305199500.01.T01	99,59	0,00E+00	2195	Os03g0138500	100,00	0,00E+00	2176	-19	NA	Japonica	
maker-3-augustus-gene-19.42-mRNA-1	3	OsR498G0305201500.01.T01	99,53	0,00E+00	755	Os03g0139100	100,00	0,00E+00	764	9	NA	Japonica	
maker-3-augustus-gene-19.50-mRNA-1	3	OsR498G0305200000.01.T02	99,70	0,00E+00	2957	Os03g0138600	100,00	0,00E+00	5806	2849	Japonica	Japonica	
maker-3-augustus-gene-19.54-mRNA-1	3	OsR498G0305200400.01.T01	99,89	0,00E+00	3243	Os03g0138700	100,00	0,00E+00	3252	9	NA	Japonica	
maker-3-augustus-gene-19.56-mRNA-1	3	OsR498G0305201800.01.T03	98,73	0,00E+00	3158	Os03g0139200	100,00	0,00E+00	3155	-3	NA	Japonica	
maker-3-augustus-gene-19.59-mRNA-1	3	OsR498G0305202800.01.T01	96,28	0,00E+00	2059	Os03g0139500	95,98	0,00E+00	1893	-166	NA	Japonica	
maker-3-augustus-gene-190.0-mRNA-1	3	OsR498G0306073500.01.T04	98,53	0,00E+00	2956	Os03g0387900	98,95	0,00E+00	3020	64	NA	Japonica	
maker-3-augustus-gene-190.11-mRNA-1	3	OsR498G0306078100.01.T01	100,00	0,00E+00	1835	Os03g0388800	99,90	0,00E+00	1803	-32	NA	NA	
maker-3-augustus-gene-190.15-mRNA-1	3	OsR498G0306073800.01.T03	100,00	0,00E+00	1159	Os03g0388000	100,00	0,00E+00	1034	-125	Indica	Japonica	
maker-3-augustus-gene-190.17-mRNA-1	3	OsR498G0306074200.01.T02	99,68	6,21E-159	561	Os03g0388100	100,00	5,08E-161	565	4	NA	Japonica	
maker-3-augustus-gene-190.22-mRNA-1	3	OsR498G0306078400.01.T01	100,00	0,00E+00	1997	Os03g0388900	100,00	0,00E+00	1186	-811	Indica	NA	
maker-3-augustus-gene-190.25-mRNA-1	3	OsR498G0306080600.01.T01	100,00	0,00E+00	1856	Os03g0389000	100,00	0,00E+00	2170	314	Japonica	NA	
maker-3-augustus-gene-190.27-mRNA-1	3	OsR498G0306081000.01.T01	100,00	0,00E+00	1743	Os03g0389100	100,00	0,00E+00	1640	-103	NA	Japonica	
maker-3-augustus-gene-191.0-mRNA-1	3	OsR498G0306083900.01.T01	99,64	0,00E+00	2982	Os03g0389700	99,82	0,00E+00	2993	11	NA	Japonica	
maker-3-augustus-gene-191.10-mRNA-1	3	OsR498G0306085500.01.T01	98,53	0,00E+00	828	Os03g0390400	100,00	0,00E+00	859	31	NA	Japonica	
maker-3-augustus-gene-191.13-mRNA-1	3	OsR498G0306085700.01.T05	99,51	0,00E+00	733	Os03g0390600	100,00	0,00E+00	742	9	NA	Japonica	
maker-3-augustus-gene-191.16-mRNA-1	3	OsR498G0306085900.01.T02	98,28	0,00E+00	1606	Os03g0390700	100,00	0,00E+00	976	-630	Indica	Japonica	
maker-3-augustus-gene-191.20-mRNA-1	3	OsR498G0306086100.01.T02	98,11	0,00E+00	2447	Os04g0682000	89,42	0,00E+00	1928	-519	Indica	Japonica	
maker-3-augustus-gene-191.25-mRNA-1	3	OsR498G0306083400.01.T02	100,00	0,00E+00	2003	Os03g0389400	100,00	0,00E+00	2003	0	NA	NA	
maker-3-augustus-gene-191.27-mRNA-1	3	OsR498G0306085000.01.T01	100,00	0,00E+00	1317	Os03g0390000	100,00	0,00E+00	1332	15	NA	Japonica	
maker-3-augustus-gene-191.31-mRNA-1	3	OsR498G0306085400.01.T01	99,81	0,00E+00	1847	Os03g0390200	100,00	0,00E+00	1856	9	NA	Japonica	Kinase-TM
maker-3-augustus-gene-192.0-mRNA-1	3	OsR498G0306086700.01.T01	95,83	0,00E+00	4272	Os05g0171000	74,32	0,00E+00	1214	-3058	Indica	Japonica	
maker-3-augustus-gene-192.7-mRNA-1	3	OsR498G0306091200.01.T02	99,91	0,00E+00	1949	Os07g0620800	70,46	2,68E-75	283	-1666	Indica	Indica	
maker-3-augustus-gene-192.9-mRNA-1	3	OsR498G0306093000.01.T01	98,69	0,00E+00	1994	Os03g0392600	98,69	0,00E+00	1994	0	NA	NA	
maker-3-augustus-gene-194.32-mRNA-1	3	OsR498G0306099500.01.T01	99,16	0,00E+00	2314	Os03g0393900	99,16	0,00E+00	2314	0	NA	NA	
maker-3-augustus-gene-194.36-mRNA-1	3	OsR498G0306098600.01.T02	98,97	0,00E+00	2042	Os03g0392600	79,12	0,00E+00	968	-1074	Indica	NA	
maker-3-augustus-gene-194.37-mRNA-1	3	OsR498G0306098600.01.T06	99,29	3,84E-141	500	Os03g0392600	91,28	1,18E-53	209	-291	Indica	NA	
maker-3-augustus-gene-195.0-mRNA-1	3	OsR498G0306105100.01.T01	99,70	0,00E+00	2390	Os02g0567200	73,62	1,41E-111	403	-1987	Indica	NA	
maker-3-augustus-gene-195.10-mRNA-1	3	OsR498G0306105600.01.T01	99,83	0,00E+00	4186	Os03g0395300	99,87	0,00E+00	4191	5	NA	Indica	
maker-3-augustus-gene-195.2-mRNA-1	3	OsR498G0306104200.01.T01	99,00	0,00E+00	1935	Os03g0394900	99,00	0,00E+00	1935	0	NA	NA	
maker-3-augustus-gene-195.7-mRNA-1	3	OsR498G0306105000.01.T01	98,79	0,00E+00	1738	Os03g0395000	98,49	0,00E+00	1728	-10	NA	Japonica	
maker-3-augustus-gene-196.44-mRNA-1	3	OsR498G0306112500.01.T01	100,00	0,00E+00	2915	Os03g0396900	98,60	0,00E+00	3623	708	Japonica	Indica	
maker-3-augustus-gene-196.49-mRNA-1	3	OsR498G0306113300.01.T02	98,23	0,00E+00	2647	Os03g0397300	97,61	0,00E+00	2149	-498	Indica	Indica	
maker-3-augustus-gene-196.54-mRNA-1	3	OsR498G0306111400.01.T01	99,84	0,00E+00	1095	Os03g0396500	99,84	0,00E+00	1095	0	NA	Indica	
maker-3-augustus-gene-197.30-mRNA-1	3	OsR498G0306113700.01.T04	100,00	0,00E+00	2345	Os03g0397400	99,54	0,00E+00	2320	-25	NA	Indica	
maker-3-augustus-gene-197.33-mRNA-1	3	OsR498G0306114400.01.T02	100,00	0,00E+00	4080	Os07g0618400	73,70	0,00E+00	1281	-2799	Indica	Indica	Kinase-LRR-TM
maker-3-augustus-gene-198.2-mRNA-1	3	OsR498G0713610100.01.T02	95,79	5,96E-83	307	Os03g0399300	99,72	4,85E-180	629	322	Japonica	Japonica	
maker-3-augustus-gene-199.10-mRNA-1	3	OsR498G0306129200.01.T01	99,78	0,00E+00	4057	Os03g0400850	99,65	0,00E+00	4044	-13	NA	Indica	LRR-TM
maker-3-augustus-gene-199.12-mRNA-1	3	OsR498G0306132000.01.T03	99,96	0,00E+00	4416	Os03g0401300	99,88	0,00E+00	4407	-9	NA	Indica	
maker-3-augustus-gene-2.0-mRNA-1	3	OsR498G0305090700.01.T04	94,01	0,00E+00	1354	Os03g0107100	100,00	0,00E+00	2690	1336	Japonica	Japonica	
maker-3-augustus-gene-2.14-mRNA-1	3	OsR498G0305093300.01.T01	99,35	0,00E+00	1358	Os04g0119300	100,00	0,00E+00	1223	-135	NA	Japonica	
maker-3-augustus-gene-2.17-mRNA-1	3	OsR498G0305094500.01.T01	100,00	0,00E+00	1288	Os03g0107900	100,00	0,00E+00	1288	0	NA	Japonica	
maker-3-augustus-gene-2.28-mRNA-1	3	OsR498G0305095900.01.T01	98,00	0,00E+00	946	Os03g0108400	97,88	0,00E+00	728	-218	Indica	Japonica	
maker-3-augustus-gene-2.39-mRNA-1	3	OsR498G0305091200.01.T02	99,86	0,00E+00	2551	Os03g0107300	100,00	0,00E+00	2560	9	NA	Japonica	
maker-3-augustus-gene-2.4-mRNA-1	3	OsR498G0305091900.01.T04	99,83	0,00E+00	7349	Os03g0107400	100,00	0,00E+00	5142	-2207	Indica	Japonica	
maker-3-augustus-gene-2.49-mRNA-1	3	OsR498G0305094800.01.T02	100,00	0,00E+00	710	Os03g0108000	100,00	0,00E+00	710	0	NA	Japonica	
maker-3-augustus-gene-2.51-mRNA-1	3	OsR498G0305095600.01.T01	99,79	0,00E+00	1674	Os03g0108300	100,00	0,00E+00	1683	9	NA	Japonica	
maker-3-augustus-gene-2.53-mRNA-1	3	OsR498G0305096200.01.T01	99,34	0,00E+00	1600	Os03g0108500	100,00	0,00E+00	1629	29	NA	Japonica	
maker-3-augustus-gene-20.0-mRNA-1	3	OsR498G0305209100.01.T01	99,31	0,00E+00	1807	Os03g0140700	100,00	0,00E+00	1840	33	NA	Japonica	
maker-3-augustus-gene-20.17-mRNA-1	3	OsR498G0714417100.01.T01	71,21	3,24E-126	454	Os03g0141100	100,00	0,00E+00	1743	1289	Japonica	Japonica	
maker-3-augustus-gene-20.2-mRNA-1	3	OsR498G0305210300.01.T01	99,48	0,00E+00	2037	Os03g0140900	100,00	0,00E+00	2064	27	NA	Japonica	
maker-3-augustus-gene-20.4-mRNA-1	3	OsR498G0305210500.01.T01	100,00	0,00E+00	893	Os03g0141000	100,00	0,00E+00	893	0	NA	Japonica	
maker-3-augustus-gene-20.6-mRNA-1	3	OsR498G0305210700.01.T01	99,64	0,00E+00	2993	Os03g0141200	100,00	0,00E+00	3020	27	NA	Japonica	
maker-3-augustus-gene-20.9-mRNA-1	3	OsR498G0305211900.01.T02	99,53	0,00E+00	1888	Os03g0141700	100,00	0,00E+00	2479	591	Japonica	Japonica	
maker-3-augustus-gene-200.34-mRNA-1	3	OsR498G0306136300.01.T01	98,94	0,00E+00	1658	Os07g0616600	84,41	0,00E+00	931	-727	Indica	Japonica	
maker-3-augustus-gene-200.39-mRNA-1	3	OsR498G0306136700.01.T04	99,86	0,00E+00	1275	Os07g0616200	78,42	1,58E-173	609	-666	Indica	Japonica	
maker-3-augustus-gene-200.44-mRNA-1	3	OsR498G0306136000.01.T01	93,53	0,00E+00	943	Os03g0402000	93,53	0,00E+00	943	0	NA	Japonica	
maker-3-augustus-gene-201.0-mRNA-1	3	OsR498G0306144200.01.T01	100,00	0,00E+00	1240	Os03g0402800	100,00	0,00E+00	1240	0	NA	Japonica	
maker-3-augustus-gene-202.32-mRNA-1	3	OsR498G0306144900.01.T05	97,09	0,00E+00	3395	Os03g0403100	97,24	0,00E+00	3404	9	NA	Japonica	
maker-3-augustus-gene-203.34-mRNA-1	3	OsR498G0306150300.01.T02	99,12	0,00E+00	1397	Os03g0404800	100,00	0,00E+00	1445	48	NA	Japonica	
maker-3-augustus-gene-203.38-mRNA-1	3	OsR498G0306152100.01.T04	99,86	0,00E+00	1278	Os03g0405000	100,00	0,00E+00	1279	1	NA	Japonica	
maker-3-augustus-gene-203.45-mRNA-1	3	OsR498G0306152500.01.T02	99,82	0,00E+00	992	Os03g0405100	100,00	0,00E+00	996	4	NA	Japonica	
maker-3-augustus-gene-203.48-mRNA-1	3	OsR498G0306153100.01.T01	99,83	0,00E+00	3071	Os03g0405500	100,00	0,00E+00	3085	14	NA	Japonica	
maker-3-augustus-gene-204.0-mRNA-1	3	OsR498G0306158900.01.T01	99,60	0,00E+00	1333	Os03g0406100	100,00	0,00E+00	1342	9	NA	Japonica	
maker-3-augustus-gene-204.2-mRNA-1	3	OsR498G0306159500.01.T02	99,61	0,00E+00	1367	Os03g0406200	100,00	0,00E+00	1225	-142	Indica	Japonica	
maker-3-augustus-gene-205.34-mRNA-1	3	OsR498G0306167000.01.T01	100,00	0,00E+00	2205	Os03g0407900	100,00	0,00E+00	2486	281	Japonica	NA	Kinase-TM
maker-3-augustus-gene-206.9-mRNA-1	3	OsR498G0306175000.01.T01	100,00	0,00E+00	1180	Os03g0409400	100,00	0,00E+00	1040	-140	Indica	NA	
maker-3-augustus-gene-207.0-mRNA-1	3	OsR498G0306181500.01.T01	100,00	9,32E-123	439	Os03g0410900	100,00	3,26E-123	439	0	NA	NA	
maker-3-augustus-gene-207.11-mRNA-1	3	OsR498G0306186500.01.T01	92,82	0,00E+00	1128	Os03g0411500	92,82	0,00E+00	1128	0	NA	NA	
maker-3-augustus-gene-207.15-mRNA-1	3	OsR498G0306186700.01.T01	100,00	0,00E+00	969	Os03g0411600	100,00	0,00E+00	969	0	NA	NA	
maker-3-augustus-gene-207.2-mRNA-1	3	OsR498G0306181900.01.T01	100,00	0,00E+00	4177	Os03g0411000	100,00	0,00E+00	4177	0	NA	NA	
maker-3-augustus-gene-207.23-mRNA-1	3	OsR498G0306188000.01.T01	100,00	0,00E+00	1943	Os03g0411800	100,00	0,00E+00	1943	0	NA	NA	
maker-3-augustus-gene-207.26-mRNA-1	3	OsR498G0306190000.01.T02	99,85	0,00E+00	1170	Os03g0411900	100,00	0,00E+00	1370	200	Japonica	NA	
maker-3-augustus-gene-207.6-mRNA-1	3	OsR498G0306182900.01.T09	100,00	0,00E+00	1721	Os03g0411100	100,00	0,00E+00	1721	0	NA	NA	
maker-3-augustus-gene-208.29-mRNA-1	3	OsR498G0306190900.01.T03	100,00	0,00E+00	1440	Os03g0412300	99,11	0,00E+00	1191	-249	Indica	NA	
maker-3-augustus-gene-208.31-mRNA-1	3	OsR498G0306192500.01.T06	97,64	0,00E+00	3048	Os03g0412800	97,64	0,00E+00	3048	0	NA	NA	
maker-3-augustus-gene-208.42-mRNA-1	3	OsR498G0306193900.01.T04	100,00	0,00E+00	2452	Os03g0413100	100,00	0,00E+00	2452	0	NA	NA	
maker-3-augustus-gene-208.52-mRNA-1	3	OsR498G0306195400.01.T01	100,00	0,00E+00	2847	Os03g0413400	100,00	0,00E+00	2847	0	NA	NA	
maker-3-augustus-gene-209.37-mRNA-1	3	OsR498G0306200600.01.T01	100,00	4,35E-173	607	Os03g0414400	100,00	1,51E-173	607	0	NA	NA	
maker-3-augustus-gene-209.39-mRNA-1	3	OsR498G0306205000.01.T03	100,00	0,00E+00	1954	Os03g0415200	100,00	0,00E+00	1954	0	NA	NA	
maker-3-augustus-gene-21.0-mRNA-1	3	OsR498G0305212900.01.T01	99,92	0,00E+00	2198	Os03g0141800	100,00	0,00E+00	3983	1785	Japonica	Japonica	
maker-3-augustus-gene-21.10-mRNA-1	3	OsR498G0305215800.01.T01	99,76	0,00E+00	2953	Os03g0142900	100,00	0,00E+00	2971	18	NA	Japonica	
maker-3-augustus-gene-21.2-mRNA-1	3	OsR498G0305214100.01.T01	99,19	0,00E+00	3704	Os03g0142500	100,00	0,00E+00	3761	57	NA	Japonica	
maker-3-augustus-gene-21.4-mRNA-1	3	OsR498G0305215500.01.T01	99,83	0,00E+00	4248	Os03g0142800	100,00	0,00E+00	4266	18	NA	Japonica	
maker-3-augustus-gene-21.8-mRNA-1	3	OsR498G0305214400.01.T01	99,47	0,00E+00	1328	Os03g0142600	100,00	0,00E+00	1342	14	NA	Japonica	
maker-3-augustus-gene-210.33-mRNA-1	3	OsR498G0306209100.01.T02	100,00	0,00E+00	2538	Os03g0416200	100,00	0,00E+00	2538	0	NA	Japonica	
maker-3-augustus-gene-210.35-mRNA-1	3	OsR498G0306209400.01.T01	99,84	0,00E+00	1123	Os03g0416300	99,84	0,00E+00	1123	0	NA	Japonica	
maker-3-augustus-gene-210.37-mRNA-1	3	OsR498G0306211200.01.T02	100,00	0,00E+00	1608	Os03g0416500	100,00	0,00E+00	1608	0	NA	NA	
maker-3-augustus-gene-211.0-mRNA-1	3	OsR498G0306215800.01.T01	99,33	0,00E+00	1307	Os07g0178700	90,02	7,63E-169	593	-714	Indica	Japonica	
maker-3-augustus-gene-211.11-mRNA-1	3	OsR498G0306222400.01.T04	100,00	0,00E+00	814	Os03g0418600	100,00	0,00E+00	814	0	NA	Japonica	

maker-3-augustus-gene-211.2-mRNA-1	3	OsR498G0306216800.01.T02	100,00	0,00E+00	634	Os03g0417800	99,78	0,00E+00	799	165	Japonica	Japonica	
maker-3-augustus-gene-211.5-mRNA-1	3	OsR498G0306219200.01.T01	97,53	0,00E+00	3611	Os03g0417900	97,58	0,00E+00	3616	5	NA	Japonica	
maker-3-augustus-gene-212.48-mRNA-1	3	OsR498G0306223300.01.T02	99,50	0,00E+00	4947	Os03g0418700	100,00	0,00E+00	5011	64	NA	Japonica	
maker-3-augustus-gene-212.52-mRNA-1	3	OsR498G0306223900.01.T01	99,86	0,00E+00	2637	Os03g0418800	100,00	0,00E+00	2646	9	NA	Japonica	
maker-3-augustus-gene-212.56-mRNA-1	3	OsR498G0306224500.01.T02	99,87	0,00E+00	1410	Os03g0419100	100,00	0,00E+00	1415	5	NA	Japonica	
maker-3-augustus-gene-213.40-mRNA-1	3	OsR498G0306232900.01.T01	99,30	3,04E-143	508	Os03g0421700	98,76	1,57E-160	563	55	NA	Japonica	
maker-3-augustus-gene-213.49-mRNA-1	3	OsR498G0306234500.01.T01	99,60	0,00E+00	1351	Os03g0422600	95,54	0,00E+00	1544	193	Japonica	Japonica	
maker-3-augustus-gene-213.51-mRNA-1	3	OsR498G0306232800.01.T02	94,04	0,00E+00	1778	Os03g0421400	93,93	0,00E+00	1217	-561	Indica	Japonica	
maker-3-augustus-gene-213.55-mRNA-1	3	OsR498G0306233400.01.T01	99,65	0,00E+00	1014	Os03g0421800	100,00	0,00E+00	1023	9	NA	Japonica	
maker-3-augustus-gene-214.34-mRNA-1	3	OsR498G0306238100.01.T01	99,67	0,00E+00	2158	Os03g0424200	78,93	0,00E+00	1003	-1155	Indica	Japonica	Kinase-TM
maker-3-augustus-gene-214.36-mRNA-1	3	OsR498G0306238300.01.T06	99,44	0,00E+00	1572	Os03g0423800	100,00	0,00E+00	1474	-98	NA	Japonica	
maker-3-augustus-gene-214.39-mRNA-1	3	OsR498G0306240900.01.T02	100,00	0,00E+00	796	Os03g0424500	100,00	0,00E+00	796	0	NA	Japonica	
maker-3-augustus-gene-214.43-mRNA-1	3	OsR498G0306240800.01.T02	92,96	0,00E+00	1096	Os03g0424000	100,00	0,00E+00	1333	237	Japonica	Japonica	CC-Kinase-TM
maker-3-augustus-gene-214.48-mRNA-1	3	OsR498G0306240800.01.T02	99,59	0,00E+00	1728	Os03g0424200	100,00	0,00E+00	1746	18	NA	Japonica	Kinase-TM
maker-3-augustus-gene-215.37-mRNA-1	3	OsR498G0306241700.01.T02	99,77	0,00E+00	792	Os03g0424500	98,87	0,00E+00	774	-18	NA	Japonica	
maker-3-augustus-gene-215.41-mRNA-1	3	OsR498G0306241900.01.T02	99,74	0,00E+00	3446	Os03g0425000	100,00	0,00E+00	3041	-405	Indica	Japonica	
maker-3-augustus-gene-215.51-mRNA-1	3	OsR498G0306244500.01.T01	99,81	0,00E+00	2817	Os03g0425800	100,00	0,00E+00	3052	235	NA	Japonica	
maker-3-augustus-gene-215.53-mRNA-1	3	OsR498G0306242600.01.T02	99,90	0,00E+00	1744	Os03g0425200	100,00	0,00E+00	1748	4	NA	Japonica	
maker-3-augustus-gene-216.2-mRNA-1	3	OsR498G0306246900.01.T01	99,97	0,00E+00	5293	Os03g0426900	100,00	0,00E+00	5297	4	NA	Japonica	
maker-3-augustus-gene-216.6-mRNA-1	3	OsR498G0306248900.01.T01	99,67	0,00E+00	2667	Os03g0427300	100,00	0,00E+00	2690	23	NA	Japonica	
maker-3-augustus-gene-217.11-mRNA-1	3	OsR498G0306256500.01.T06	99,80	0,00E+00	2622	Os03g0430000	99,59	0,00E+00	2609	-13	NA	Japonica	
maker-3-augustus-gene-217.4-mRNA-1	3	OsR498G0306255700.01.T01	99,88	0,00E+00	7390	Os03g0429800	100,00	0,00E+00	7413	23	NA	Japonica	
maker-3-augustus-gene-218.39-mRNA-1	3	OsR498G0306257900.01.T01	97,55	0,00E+00	3352	Os02g0828900	78,42	0,00E+00	1313	-2039	Indica	Japonica	
maker-3-augustus-gene-218.50-mRNA-1	3	OsR498G0714654200.01.T04	80,95	0,00E+00	983	Os03g0431200	100,00	0,00E+00	2257	1274	Japonica	Japonica	
maker-3-augustus-gene-218.56-mRNA-1	3	OsR498G0306263800.01.T05	100,00	6,54E-167	587	Os03g0431500	100,00	2,28E-167	587	0	NA	Japonica	
maker-3-augustus-gene-219.37-mRNA-1	3	OsR498G0306265310.01.T05	99,88	0,00E+00	1506	Os03g0431932	100,00	0,00E+00	1510	4	NA	Japonica	
maker-3-augustus-gene-219.48-mRNA-1	3	OsR498G0306265800.01.T02	99,29	0,00E+00	4755	Os03g0432100	99,38	0,00E+00	4281	-474	NA	Japonica	
maker-3-augustus-gene-22.48-mRNA-1	3	OsR498G0305218700.01.T05	99,85	0,00E+00	4863	Os03g0143700	100,00	0,00E+00	4881	18	NA	Japonica	
maker-3-augustus-gene-22.59-mRNA-1	3	OsR498G0305217600.01.T01	99,83	0,00E+00	3114	Os03g0143400	100,00	0,00E+00	3128	14	NA	Japonica	
maker-3-augustus-gene-221.27-mRNA-1	3	OsR498G0306276700.01.T01	99,89	0,00E+00	1636	Os03g0434400	100,00	0,00E+00	1604	-32	NA	Japonica	
maker-3-augustus-gene-221.34-mRNA-1	3	OsR498G0306279500.01.T01	99,72	0,00E+00	1915	Os03g0435300	100,00	0,00E+00	1929	14	NA	Japonica	
maker-3-augustus-gene-221.36-mRNA-1	3	OsR498G0306278000.01.T01	99,49	0,00E+00	700	Os03g0434500	100,00	0,00E+00	958	258	Japonica	Japonica	
maker-3-augustus-gene-222.36-mRNA-1	3	OsR498G0306282100.01.T01	99,88	0,00E+00	3092	Os03g0436300	100,00	0,00E+00	3101	9	NA	Japonica	
maker-3-augustus-gene-222.41-mRNA-1	3	OsR498G0306286600.01.T01	100,00	0,00E+00	1472	Os03g0437600	100,00	0,00E+00	1472	0	NA	Japonica	
maker-3-augustus-gene-223.38-mRNA-1	3	OsR498G0306294500.01.T01	99,46	0,00E+00	1992	Os03g0439500	100,00	0,00E+00	2019	27	NA	Japonica	
maker-3-augustus-gene-223.40-mRNA-1	3	OsR498G0306289400.01.T01	99,86	0,00E+00	1300	Os03g0438100	100,00	0,00E+00	1305	5	NA	Japonica	
maker-3-augustus-gene-224.0-mRNA-1	3	OsR498G0306297500.01.T01	99,85	0,00E+00	1181	Os03g0440900	100,00	0,00E+00	1186	5	NA	Japonica	LRR-TM
maker-3-augustus-gene-224.5-mRNA-1	3	OsR498G0306295000.01.T02	100,00	0,00E+00	1694	Os03g0439700	100,00	0,00E+00	1694	0	NA	Japonica	
maker-3-augustus-gene-225.30-mRNA-1	3	OsR498G0306305300.01.T02	92,59	0,00E+00	949	Os03g0442900	92,75	0,00E+00	954	5	NA	Japonica	
maker-3-augustus-gene-227.4-mRNA-1	3	OsR498G0306326100.01.T01	99,84	0,00E+00	1105	Os03g0445700	100,00	0,00E+00	1110	5	NA	Japonica	
maker-3-augustus-gene-229.24-mRNA-1	3	OsR498G0203451400.01.T02	94,08	6,03E-69	260	Os03g0448500	100,00	4,64E-84	309	49	Japonica	Japonica	
maker-3-augustus-gene-23.30-mRNA-1	3	OsR498G0305222100.01.T01	100,00	0,00E+00	1191	Os03g0145200	100,00	0,00E+00	1191	0	NA	Japonica	
maker-3-augustus-gene-23.35-mRNA-1	3	OsR498G0305223500.01.T02	99,84	0,00E+00	1127	Os03g0145700	100,00	0,00E+00	1113	-14	NA	Japonica	
maker-3-augustus-gene-23.45-mRNA-1	3	OsR498G0305224700.01.T01	99,68	0,00E+00	1133	Os03g0146000	100,00	0,00E+00	1142	9	NA	Japonica	
maker-3-augustus-gene-23.53-mRNA-1	3	OsR498G0305226500.01.T01	99,90	0,00E+00	3535	Os03g0146400	99,94	0,00E+00	2864	-671	Indica	Japonica	
maker-3-augustus-gene-23.58-mRNA-1	3	OsR498G0305226700.01.T02	99,71	0,00E+00	1242	Os03g0146500	100,00	0,00E+00	1251	9	NA	Japonica	
maker-3-augustus-gene-23.63-mRNA-1	3	OsR498G0305222800.01.T01	98,63	0,00E+00	4935	Os03g0145500	98,91	0,00E+00	4971	36	NA	Japonica	
maker-3-augustus-gene-23.72-mRNA-1	3	OsR498G0305224200.01.T01	97,47	0,00E+00	1079	Os03g0145800	100,00	0,00E+00	823	-256	Indica	Japonica	
maker-3-augustus-gene-23.75-mRNA-1	3	OsR498G0305225000.01.T02	100,00	0,00E+00	1359	Os03g0146100	99,81	0,00E+00	921	-438	Indica	Japonica	
maker-3-augustus-gene-230.28-mRNA-1	3	OsR498G0306320700.01.T03	100,00	0,00E+00	2185	Os03g0448700	100,00	0,00E+00	2185	0	NA	Japonica	
maker-3-augustus-gene-232.30-mRNA-1	3	OsR498G0306330000.01.T02	99,90	0,00E+00	1765	Os03g0452300	100,00	0,00E+00	1770	5	NA	Japonica	
maker-3-augustus-gene-233.0-mRNA-1	3	OsR498G0306337300.01.T02	99,07	3,01E-162	572	Os03g0453800	100,00	4,75E-167	585	13	NA	Japonica	
maker-3-augustus-gene-233.15-mRNA-1	3	OsR498G0306337600.01.T01	99,84	0,00E+00	2253	Os03g0454100	100,00	0,00E+00	2262	9	NA	Japonica	
maker-3-augustus-gene-235.36-mRNA-1	3	OsR498G0306412800.01.T03	99,67	0,00E+00	2158	Os03g0570800	100,00	0,00E+00	2188	30	NA	NA	
maker-3-augustus-gene-235.43-mRNA-1	3	OsR498G0306415400.01.T01	99,93	0,00E+00	2388	Os03g0571700	100,00	0,00E+00	1880	-508	Indica	NA	
maker-3-augustus-gene-235.46-mRNA-1	3	OsR498G0306415800.01.T02	99,30	0,00E+00	2508	Os03g0570800	81,02	0,00E+00	1222	-1286	Indica	NA	
maker-3-augustus-gene-235.50-mRNA-1	3	OsR498G1120379000.01.T02	76,41	7,01E-79	296	Os03g0572250	100,00	0,00E+00	1142	846	Japonica	NA	
maker-3-augustus-gene-235.52-mRNA-1	3	OsR498G0306417600.01.T05	99,56	0,00E+00	1203	Os03g0572300	100,00	0,00E+00	1216	13	NA	NA	
maker-3-augustus-gene-236.39-mRNA-1	3	OsR498G0306418600.01.T01	99,80	0,00E+00	2698	Os03g0572900	100,00	0,00E+00	2711	13	NA	NA	
maker-3-augustus-gene-236.44-mRNA-1	3	OsR498G0306422200.01.T01	99,60	0,00E+00	5382	Os03g0573500	99,97	0,00E+00	5942	560	NA	NA	LRR-NBS-TM
maker-3-augustus-gene-237.2-mRNA-1	3	OsR498G0306427000.01.T02	99,67	0,00E+00	4358	Os03g0574900	100,00	0,00E+00	4394	36	NA	NA	
maker-3-augustus-gene-237.7-mRNA-1	3	OsR498G0306428400.01.T01	99,77	0,00E+00	3911	Os03g0575200	100,00	0,00E+00	3934	23	NA	NA	
maker-3-augustus-gene-238.0-mRNA-1	3	OsR498G0306433700.01.T02	99,87	0,00E+00	2655	Os03g0576900	100,00	0,00E+00	2664	9	NA	NA	
maker-3-augustus-gene-238.10-mRNA-1	3	OsR498G0815254000.01.T01	96,21	2,06E-120	432	Os03g0576600	100,00	4,81E-142	502	70	Japonica	NA	
maker-3-augustus-gene-238.14-mRNA-1	3	OsR498G0306433400.01.T01	99,68	0,00E+00	1122	Os03g0576700	100,00	0,00E+00	1131	9	NA	NA	
maker-3-augustus-gene-238.18-mRNA-1	3	OsR498G0306434000.01.T01	99,71	0,00E+00	1231	Os03g0577000	100,00	0,00E+00	1240	9	NA	NA	
maker-3-augustus-gene-238.2-mRNA-1	3	OsR498G0306434900.01.T01	95,13	0,00E+00	1097	Os03g0577200	95,27	0,00E+00	1102	5	NA	NA	
maker-3-augustus-gene-238.21-mRNA-1	3	OsR498G0306434400.01.T02	99,79	0,00E+00	2553	Os03g0577100	100,00	0,00E+00	2567	14	NA	NA	
maker-3-augustus-gene-238.26-mRNA-1	3	OsR498G0306435600.01.T01	99,84	0,00E+00	2188	Os03g0577500	99,92	0,00E+00	2193	5	NA	NA	
maker-3-augustus-gene-238.8-mRNA-1	3	OsR498G0306432000.01.T01	99,75	0,00E+00	4307	Os03g0576200	100,00	0,00E+00	4329	22	NA	NA	
maker-3-augustus-gene-239.27-mRNA-1	3	OsR498G0306440100.01.T01	99,80	0,00E+00	1770	Os03g0578900	100,00	0,00E+00	2646	876	Japonica	NA	
maker-3-augustus-gene-239.32-mRNA-1	3	OsR498G0306437600.01.T02	99,60	5,37E-126	451	Os03g0578200	100,00	4,42E-128	455	4	NA	NA	
maker-3-augustus-gene-239.34-mRNA-1	3	OsR498G0306442600.01.T02	99,52	0,00E+00	1107	Os03g0337800	95,69	0,00E+00	1011	-96	NA	NA	
maker-3-augustus-gene-24.12-mRNA-1	3	OsR498G0305229600.01.T02	99,82	0,00E+00	3006	Os03g0147400	100,00	0,00E+00	3020	14	NA	Japonica	
maker-3-augustus-gene-24.16-mRNA-1	3	OsR498G0305231800.01.T01	99,87	0,00E+00	4066	Os01g0267100	90,46	0,00E+00	3159	-907	Indica	Japonica	
maker-3-augustus-gene-24.21-mRNA-1	3	OsR498G0305232200.01.T01	99,74	0,00E+00	2038	Os03g0148000	100,00	0,00E+00	2051	13	NA	Japonica	
maker-3-augustus-gene-24.8-mRNA-1	3	OsR498G0305231900.01.T03	99,59	5,17E-120	431	Os03g0147900	100,00	1,48E-121	434	3	NA	Japonica	
maker-3-augustus-gene-240.46-mRNA-1	3	OsR498G0306446200.01.T01	99,65	0,00E+00	2039	Os03g0580800	100,00	0,00E+00	2057	18	NA	NA	
maker-3-augustus-gene-241.30-mRNA-1	3	OsR498G0306450100.01.T01	99,78	0,00E+00	1647	Os03g0581400	100,00	0,00E+00	1992	345	Japonica	NA	
maker-3-augustus-gene-241.34-mRNA-1	3	OsR498G0306451100.01.T01	99,85	0,00E+00	1235	Os03g0581600	100,00	0,00E+00	643	-592	Indica	NA	
maker-3-augustus-gene-241.40-mRNA-1	3	OsR498G0306451900.01.T03	100,00	2,22E-124	444	Os03g0581800	100,00	7,30E-81	298	-146	Indica	NA	
maker-3-augustus-gene-241.43-mRNA-1	3	OsR498G0306452500.01.T04	99,89	0,00E+00	1679	Os03g0582000	100,00	0,00E+00	1683	4	NA	NA	
maker-3-augustus-gene-241.45-mRNA-1	3	OsR498G0306453400.01.T03	95,14	0,00E+00	740	Os03g0582200	95,14	0,00E+00	740	0	NA	NA	
maker-3-augustus-gene-241.51-mRNA-1	3	OsR498G0306453600.01.T02	99,82	0,00E+00	976	Os03g0582200	71,81	8,19E-61	233	-743	Indica	NA	
maker-3-augustus-gene-242.41-mRNA-1	3	OsR498G0306460900.01.T06	99,63	0,00E+00	1925	Os03g0583800	100,00	0,00E+00	1943	18	NA	NA	
maker-3-augustus-gene-242.47-mRNA-1	3	OsR498G0306462300.01.T02	99,73	0,00E+00	667	Os03g0584300	100,00	0,00E+00	672	5	NA	NA	
maker-3-augustus-gene-243.0-mRNA-1	3	OsR498G0306466900.01.T01	99,83	0,00E+00	1060	Os03g0586500	100,00	0,00E+00	1065	5	NA	NA	
maker-3-augustus-gene-243.3-mRNA-1	3	OsR498G0306466600.01.T04	96,95	0,00E+00	3152	Os03g0586300	94,59	0,00E+00	1714	-1438	Indica	NA	
maker-3-augustus-gene-243.8-mRNA-1	3	OsR498G0306467200.01.T01	98,74	0,00E+00	692	Os03g0586600	100,00	0,00E+00	711	19	NA	NA	
maker-3-augustus-gene-244.42-mRNA-1	3	OsR498G0306468300.01.T01	100,00	0,00E+00	1451	Os03g0587000	100,00	0,00E+00	1451	0	NA	NA	
maker-3-augustus-gene-244.47-mRNA-1	3	OsR498G0306468500.01.T01	94,74	0,00E+00	1621	Os03g0587100	100,00	0,00E+00	948	-673	Indica	NA	
maker-3-augustus-gene-244.49-mRNA-1	3	OsR498G0306473300.01.T01	99,57	0,00E+00	2497	Os03g0588200	100,00	0,00E+00	2520	23	NA	NA	
maker-3-augustus-gene-244.51-mRNA-1	3	OsR498G0306474500.01.T01	98,72	0,00E+00	3557	Os03g0588600	100,00	0,00E+00	3631	74	NA	NA	
maker-3-augustus-gene-244.56-mRNA-1	3	OsR498G0306467600.01.T01	99,83	0,00E+00	3159	Os03g0586700	99,94	0,00E+00	3168	9	NA	NA	
maker-3-augustus-gene-244.59-mRNA-1	3	OsR498G0306468000.01.T02	99,78	0,00E+00	3245	Os03g0586800	100,00	0,00E+00	3263	18	NA	NA	
maker-3-augustus-gene-244.63-mRNA-1	3	OsR498G0306468800.01.T01	99,94	0,00E+00	6105	Os03g0587200	100,00	0,00E+00	6114	9	NA	NA	
maker-3-augustus-gene-245.31-mRNA-1	3	OsR498G0306474900.01.T01	99,46	0,00E+00	4280	Os03g0588700	100,00	0,00E+00	3422	-858	Indica	NA	
maker-3-augustus-gene-245.35-mRNA-1	3	OsR498G0306477000.01.T02	96,26	0,00E+00	2447	Os03g0589300	100,00	0,00E+00	2562	115	NA	NA	
maker-3-augustus-gene-247.36-mRNA-1	3	OsR498G0306485300.01.T02	99,75	0,00E+00	1420	Os03g0592500	100,00	0,00E+00	1429	9	NA	NA	
maker-3-augustus-gene-247.40-mRNA-1	3	OsR498G0306486600.01.T01	99,80	0,00E+00	3662	Os03g0593200	99,27	0,00E+00	3151	-511	Indica	NA	
maker-3-augustus-gene-248.0-mRNA-1	3	OsR498G0306487100.01.T01	99,38	0,00E+00	2584	Os03g0593600	100,00	0,00E+00	2625	41	NA	NA	
maker-3-augustus-gene-248.10-mRNA-1	3	OsR498G0306492800.01.T01	98,77	0,00E+00	4545	Os03g0594700	100,00	0,00E+00	4675	130	NA	NA	
maker-3-augustus-gene-248.2-mRNA-1	3	OsR498G0306490700.01.T01	99,29	0,00E+00	2727	Os03g0594100	100,00	0,00E+00	2776	49	NA	NA	
maker-3-augustus-gene-248.6-mRNA-1	3	OsR498G0306492900.01.T01	99,87	0,00E+00	2783	Os03g0594900	100,00	0,00E+00	2792	9	NA	NA	
maker-3-augustus-gene-248.8-mRNA-1	3	OsR498G0306491600.01.T01	99,88	0,00E+00	1446	Os03g0594400	100,00	0,00E+00	1451	5	NA	NA	
maker-3-augustus-gene-249.0-mRNA-1	3	OsR498G0306493500.01.T01	97,47	0,00E+00	808	Os03g0595200	100,00	0,00E+00	856	48	NA	Japonica	
maker-3-augustus-gene-249.11-mRNA-1	3	OsR498G0306498400.01.T01	99,70	1,10E-168	593	Os03g0596800	99,70	3,83E-169	593	0	NA	NA	
maker-3-augustus-gene-249.13-mRNA-1	3	OsR498G0306498800.01.T05	99,92	0,00E+00	2303	Os03g0596900	99,92	0,00E+00	2303	0	NA	NA	
maker-3-augustus-gene-249.2-mRNA-1	3	OsR498G0306494800.01.T01	95,34	0,00E+00	1187	Os03g0595600	100,00	0,00E+00	1315	128	NA	NA	
maker-3-augustus-gene-25.38-mRNA-1	3	OsR498G0305233300.01.T01	99,79	0,00E+00	1683	Os03g0148700	100,00	0,00E+00	1692	9	NA	Japonica	Kinase
maker-3-augustus-gene-25.43-mRNA-1	3	OsR498G0305237100.01.T05	99,63	0,00E+00	2877	Os03g0149400	100,00	0,00E+00	1992	-885	Indica	Japonica	
maker-3-augustus-gene-25.47-mRNA-1	3	OsR498G0305235800.01.T01	99,37	0,00E+00	842	Os03g0149200	100,00	0,00E+00	856	14	NA	Japonica	
maker-3-augustus-gene-250.32-mRNA-1	3	OsR498G0306502700.01.T05	99,79	0,00E+00	2568	Os03g0598100	100,00	0,00E+00	2441	-127	NA	NA	
maker-3-augustus-gene-250.36-mRNA-1	3	OsR498G0306004300.01.T01	98,91	0,00E+00	806	Os03g0365200	99,45	0,00E+00	652	-154	Indica	NA	
maker-3-augustus-gene-250.39-mRNA-1	3	OsR498G0306505500.01.T02	95,67	0,00E+00	1096	Os03g0598700	100,00	0,00E+00	1159	63	NA	NA	
maker-3-augustus-gene-250.41-mRNA-1	3	OsR498G0306500800.01.T01	99,79	0,00E+00	847	Os03g0597600	100,00	0,00E+00	852	5	NA	NA	
maker-3-augustus-gene-250.42-mRNA-1	3	OsR498G0306500800.01.T02	97,86	0,00E+00	880	Os03g0597600	97,78	0,00E+00	696	-184	Indica	NA	
maker-3-augustus-gene-250.47-mRNA-1	3	OsR498G0306503000.01.T01	99,57	0,00E+00	4131	Os03g0598200	100,00	0,00E+00	3925	-206	NA	NA	
maker-3-augustus-gene-251.13-mRNA-1	3	OsR498G0306506000.01.T01	100,00	0,00E+00	1110	Os03g0598800	100,00	0,00E+00	1110	0	NA	NA	
maker-3-augustus-gene-251.24-mRNA-1	3	OsR498G0306507000.01.T01	99,39	0,00E+00	2045	Os03g0599000	99,94	0,00E+00	2927	882	Japonica	NA	NBS-TM
maker-3-augustus-gene-251.26-mRNA-1	3	OsR498G0306508200.01.T02	95,68	0,00E+00	1251	Os03g0599800	96,99	0,00E+00	1847	596	Japonica	NA	
maker-3-augustus-gene-251.31-mRNA-1	3	OsR498G1018781700.01.T01	89,18	1,68E-156	554	Os03g0600500	100,00	0,00E+00	722	168	Japonica	NA	
maker-3-augustus-gene-252.24-mRNA-1	3	OsR498G0306512300.01.T01	100,00	1,75E-135	482	Os03g0601500	100,00	6,10E-136	482	0	NA	NA	
maker-3-augustus-gene-253.37-mRNA-1	3	OsR498G0306522300.01.T01	99,91	0,00E+00	2094	Os03g0603600	100,00	0,00E+00	2098	4	NA	NA	
maker-3-augustus-gene-253.43-mRNA-1	3	OsR498G0306519600.01.T01	99,85	0,00E+00	2361	Os03g0603100	100,00	0,00E+00	1700	-661	Indica	NA	
maker-3-augustus-gene-254.43-mRNA-1	3	OsR498G0306524700.01.T01	99,67	0,00E+00	1068	Os03g0604500	100,00	0,00E+00	1191	123	Japonica	NA	
maker-3-augustus-gene-255.0-mRNA-1	3	OsR498G0306532100.01.T01	97,55	0,00E+00	2075	Os12g0581700	72,69	2,82E-164	579	-1496	Indica	NA	
maker-3-augustus-gene-255.4-mRNA-1	3	OsR498G0306528600.01.T04	99,66	0,00E+00	2615	Os03g0606100	100,00	0,00E+00	3220	605	Japonica	NA	
maker-3-augustus-gene-255.9-mRNA-1	3	OsR498G0306532000.01.T01	99,28	0,00E+00	1478	Os12g0581600	79,80	0,00E+00	728	-750	Indica	NA	
maker-3-augustus-gene-256.0-mRNA-1	3	OsR498G0306537300.01.T01	98,57	0,00E+00	1952	Os03g0607600	100,00	0,00E+00	2017	65	NA	NA	
maker-3-augustus-gene-256.4-mRNA-1	3	OsR498G0306542700.01.T04	99,08	0,00E+00	1151	Os03g0608800	100,00	0,00E+00	1178	27	NA	NA	
maker-3-augustus-gene-256.9-mRNA-1	3	OsR498G0306538000.01.T01	99,72	0,00E+00	636	Os03g0607800	99,72	0,00E+00	645	9	NA	NA	
maker-3-augustus-gene-257.28-mRNA-1	3	OsR498G0306543500.01.T01	87,44	1,53E-147	522	Os07g0477000	96,77	6,95E-115	412	-110	Indica	NA	
maker-3-augustus-gene-257.30-mRNA-1	3	OsR498G0306544400.01.T01	98,02	0,00E+00	1216	Os03g0609500	99,86	0,00E+00	1257	41	NA	NA	
maker-3-augustus-gene-257.32-mRNA-1	3	OsR498G0306545400.01.T01	99,63	0,00E+00	3405	Os03g0609800	100,00	0,00E+00	3436	31	NA	NA	
maker-3-augustus-gene-258.0-mRNA-1	3	OsR498G0306548700.01.T01	99,41	0,00E+00	2117	Os03g0610800	87,27	0,00E+00	1482	-635	Indica	NA	
maker-3-augustus-gene-258.11-mRNA-1	3	OsR498G0306549800.01.T02	99,34	0,00E+00	2406	Os03g0610900	99,91	0,00E+00	1960	-446	Indica	NA	Kinase-TM
maker-3-augustus-gene-258.2-mRNA-1	3	OsR498G0306549300.01.T01	99,78	0,00E+00	801	Os03g0610800	99,78	0,00E+00	806	5	NA	NA	
maker-3-augustus-gene-258.3-mRNA-1	3	OsR498G0306548700.01.T01	90,16	0,00E+00	1050	Os03g0610800	100,00	0,00E+00	1400	350	Japonica	NA	
maker-3-augustus-gene-258.6-mRNA-1	3	OsR498G0306550700.01.T05	99,29	0,00E+00	1490	Os03g0611200	99,76	0,00E+00	1508	18	NA	NA	
maker-3-augustus-gene-259.44-mRNA-1	3	OsR498G0102477000.01.T01	71,06	5,41E-35	149	Os03g0615700	93,36	0,00E+00	658	509	Japonica	NA	
maker-3-augustus-gene-26.38-mRNA-1	3	OsR498G0305239700.01.T01	99,59	0,00E+00	875	Os03g0150700	90,88	0,00E+00	783	-92	Indica	Japonica	
maker-3-augustus-gene-26.44-mRNA-1	3	OsR498G0305241500.01.T01	99,86	0,00E+00	5074	Os03g0151700	100,00	0,00E+00	5092	18	NA	Japonica	
maker-3-augustus-gene-26.48-mRNA-1	3	OsR498G0305244100.01.T02	100,00	0,00E+00	2057	Os03g0152100	100,00	0,00E+00	2057	0	NA	Japonica	
maker-3-augustus-gene-26.53-mRNA-1	3	OsR498G0305244300.01.T01	100,00	0,00E+00	2636	Os03g0152300	100,00	0,00E+00	2636	0	NA	Japonica	
maker-3-augustus-gene-26.62-mRNA-1	3	OsR498G0305242000.01.T01	99,96	0,00E+00	4378	Os03g0151800	100,00	0,00E+00	4383	5	NA	Japonica	
maker-3-augustus-gene-26.67-mRNA-1	3	OsR498G0305242200.01.T01	100,00	0,00E+00	1038	Os03g0151900	100,00	0,00E+00	1038	0	NA	Japonica	
maker-3-augustus-gene-26.70-mRNA-1	3	OsR498G0305243800.01.T01	99,62	0,00E+00	1871	Os03g0152000	99,90	0,00E+00	1884	13	NA	Japonica	
maker-3-augustus-gene-26.78-mRNA-1	3	OsR498G0305245400.01.T03	100,00	4,30E-170	598	Os03g0152600	100,00	5,21E-170	596	-2	NA	Japonica	
maker-3-augustus-gene-260.27-mRNA-1	3	OsR498G0102477000.01.T01	72,03	8,88E-39	162	Os03g0615700	99,82	0,00E+00	976	814	Japonica	NA	
maker-3-augustus-gene-261.22-mRNA-1	3	OsR498G0306566200.01.T01	99,47	0,00E+00	1339	Os03g0617500	100,00	0,00E+00	1357	18	NA	NA	
maker-3-augustus-gene-262.49-mRNA-1	3	OsR498G0306571500.01.T02	99,75	0,00E+00	2828	Os05g0556700	84,79	0,00E+00	1103	-1725	Indica	NA	
maker-3-augustus-gene-262.54-mRNA-1	3	OsR498G0306571700.01.T01	99,44	0,00E+00	2865	Os03g0619600	100,00	0,00E+00	2906	41	NA	NA	
maker-3-augustus-gene-262.59-mRNA-1	3	OsR498G0306568000.01.T01	100,00	0,00E+00	1056	Os03g0617800	100,00	0,00E+00	1683	627	Japonica	NA	
maker-3-augustus-gene-262.61-mRNA-1	3	OsR498G1018971300.01.T01	84,65	0,00E+00	693	Os03g0617900	92,92	0,00E+00	914	221	Japonica	NA	
maker-3-augustus-gene-262.66-mRNA-1	3	OsR498G0306569000.01.T01	99,62	0,00E+00	1889	Os03g0618300	99,91	0,00E+00	1902	13	NA	NA	
maker-3-augustus-gene-263.0-mRNA-1	3	OsR498G0306573300.01.T02	99,89	0,00E+00	1583	Os03g0620400	100,00	0,00E+00	1588	5	NA	NA	TIR
maker-3-augustus-gene-263.16-mRNA-1	3	OsR498G0306578700.01.T01	97,74	0,00E+00	1973	Os03g0622100	100,00	0,00E+00	2073	100	NA	NA	
maker-3-augustus-gene-263.19-mRNA-1	3	OsR498G0306579100.01.T01	98,95	0,00E+00	2009	Os03g0622200	100,00	0,00E+00	2051	42	NA	NA	
maker-3-augustus-gene-263.21-mRNA-1	3	OsR498G0306574600.01.T02	99,57	0,00E+00	3341	Os03g0620800	100,00	0,00E+00	3377	36	NA	NA	
maker-3-augustus-gene-263.31-mRNA-1	3	OsR498G1222012100.01.T02	73,00	6,76E-37	154	Os03g0622300	100,00	9,96E-131	464	310	Japonica	NA	
maker-3-augustus-gene-263.5-mRNA-1	3	OsR498G0306575100.01.T02	99,67	0,00E+00	2136	Os02g0494400	71,26	1,63E-154	545	-1591	Indica	NA	
maker-3-augustus-gene-263.9-mRNA-1	3	OsR498G0306575900.01.T01	97,32	0,00E+00	3174	Os03g0621600	97,47	0,00E+00	3163	-11	NA	NA	
maker-3-augustus-gene-27.46-mRNA-1	3	OsR498G0305245500.01.T03	99,84	0,00E+00	2275	Os03g0152700	100,00	0,00E+00	2080	-195	NA	Japonica	
maker-3-augustus-gene-27.52-mRNA-1	3	OsR498G0305245700.01.T06	99,94	0,00E+00	2793	Os03g0152800	100,00	0,00E+00	2798	5	NA	Japonica	
maker-3-augustus-gene-27.59-mRNA-1	3	OsR498G0305245900.01.T02	98,63	0,00E+00	4706	Os03g0152900	98,23	0,00E+00	5284	578	Japonica	Japonica	
maker-3-augustus-gene-27.65-mRNA-1	3	OsR498G0305246500.01.T01	94,50	0,00E+00	2042	Os03g0153500	90,21	0,00E+00	1756	-286	Indica	Japonica	
maker-3-augustus-gene-27.67-mRNA-1	3	OsR498G0305247100.01.T02	99,35	0,00E+00	2195	Os03g0153500	98,19	0,00E+00	2098	-97	NA	Japonica	
maker-3-augustus-gene-27.70-mRNA-1	3	OsR498G0305247700.01.T03	99,92	0,00E+00	2225	Os03g0153900	99,03	0,00E+00	2182	-43	NA	Japonica	
maker-3-augustus-gene-27.74-mRNA-1	3	OsR498G0305248100.01.T01	99,92	0,00E+00	2198	Os03g0154100	100,00	0,00E+00	2203	5	NA	Japonica	
maker-3-augustus-gene-27.76-mRNA-1	3	OsR498G0305248900.01.T01	99,46	0,00E+00	997	Os03g0154700	100,00	0,00E+00	1011	14	NA	Japonica	
maker-3-augustus-gene-27.79-mRNA-1	3	OsR498G0305249300.01.T01	99,32	0,00E+00	788	Os03g0154100	88,70	0,00E+00	730	-58	NA	Japonica	
maker-3-augustus-gene-27.80-mRNA-1	3	OsR498G0305249700.01.T01	99,73	0,00E+00	1350	Os03g0155300	100,00	0,00E+00	1359	9	NA	Japonica	
maker-3-augustus-gene-27.85-mRNA-1	3	OsR498G0305250200.01.T01	100,00	0,00E+00	1386	Os10g0439200	82,50	0,00E+00	711	-675	Indica	Japonica	
maker-3-augustus-gene-274.31-mRNA-1	3	OsR498G1018705400.01.T01	97,22	0,00E+00	1386	Os03g0167400	84,03	0,00E+00	910	-476	Indica	Japonica	
maker-3-augustus-gene-279.7-mRNA-1	3	OsR498G1018672900.01.T01	100,00	1,31E-140	499	Os03g0171300	80,29	3,77E-66	250	-249	Indica	Indica	
maker-3-augustus-gene-28.0-mRNA-1	3	OsR498G0305250700.01.T01	99,60	0,00E+00	1340	Os03g0156000	91,47	0,00E+00	1065	-275	Indica	Japonica	
maker-3-augustus-gene-28.10-mRNA-1	3	OsR498G0305254100.01.T03	99,90	0,00E+00	1749	Os03g0157300	99,89	0,00E+00	1690	-59	NA	Japonica	
maker-3-augustus-gene-28.17-mRNA-1	3	OsR498G0305254900.01.T02	100,00	0,00E+00	897	Os03g0157500	100,00	0,00E+00	897	0	NA	Japonica	
maker-3-augustus-gene-28.20-mRNA-1	3	OsR498G0305250400.01.T01	99,87	0,00E+00	1408	Os04g0228400	80,73	3,02E-174	610	-798	Indica	Japonica	
maker-3-augustus-gene-28.22-mRNA-1	3	OsR498G0305252000.01.T01	99,79	0,00E+00	840	Os03g0156600	100,00	0,00E+00	845	5	NA	Japonica	
maker-3-augustus-gene-28.24-mRNA-1	3	OsR498G0305252200.01.T02	99,81	0,00E+00	1923	Os03g0156700	100,00	0,00E+00	1932	9	NA	Japonica	
maker-3-augustus-gene-28.26-mRNA-1	3	OsR498G0305254600.01.T03	99,70	7,86E-170	597	Os03g0157400	100,00	6,43E-172	601	4	NA	NA	
maker-3-augustus-gene-28.27-mRNA-1	3	OsR498G0305254600.01.T01	99,95	0,00E+00	3580	Os03g0157400	100,00	0,00E+00	3584	4	NA	Japonica	
maker-3-augustus-gene-28.3-mRNA-1	3	OsR498G0305251100.01.T01	99,73	0,00E+00	1344	Os03g0156000	100,00	0,00E+00	1353	9	NA	Japonica	
maker-3-augustus-gene-28.5-mRNA-1	3	OsR498G0305251700.01.T01	97,96	0,00E+00	2542	Os03g0156500	98,17	0,00E+00	2555	13	NA	Japonica	
maker-3-augustus-gene-280.23-mRNA-1	3	OsR498G1018669400.01.T01	100,00	1,01E-135	482	Os03g0171700	89,67	7,27E-88	323	-159	Indica	Indica	
maker-3-augustus-gene-285.47-mRNA-1	3	OsR498G1018640500.01.T01	98,62	0,00E+00	4061	Os03g0605300	70,70	4,45E-165	582	-3479	Indica	Japonica	
maker-3-augustus-gene-29.45-mRNA-1	3	OsR498G0305255500.01.T01	99,71	0,00E+00	3680	Os03g0157700	98,42	0,00E+00	3643	-37	NA	Japonica	
maker-3-augustus-gene-29.61-mRNA-1	3	OsR498G0305258100.01.T02	99,73	0,00E+00	1335	Os03g0159200	100,00	0,00E+00	1344	9	NA	Japonica	
maker-3-augustus-gene-29.63-mRNA-1	3	OsR498G0305259300.01.T06	99,76	0,00E+00	2205	Os03g0159700	100,00	0,00E+00	2219	14	NA	Japonica	
maker-3-augustus-gene-29.65-mRNA-1	3	OsR498G0305260500.01.T01	98,10	0,00E+00	5379	Os03g0160100	98,23	0,00E+00	5397	18	NA	Japonica	Kinase-TM
maker-3-augustus-gene-29.72-mRNA-1	3	OsR498G0305257000.01.T02	99,53	0,00E+00	3791	Os03g0158500	99,81	0,00E+00	3817	26	NA	Japonica	
maker-3-augustus-gene-29.88-mRNA-1	3	OsR498G0305259200.01.T01	99,88	0,00E+00	1495	Os03g0159600	100,00	0,00E+00	1499	4	NA	Japonica	
maker-3-augustus-gene-297.13-mRNA-1	3	OsR498G1018583100.01.T01	98,37	0,00E+00	1595	Os03g0188900	79,22	1,85E-133	475	-1120	Indica	Japonica	
maker-3-augustus-gene-298.38-mRNA-1	3	OsR498G1018576700.01.T01	100,00	0,00E+00	1824	Os03g0388800	70,86	7,44E-121	434	-1390	Indica	Indica	
maker-3-augustus-gene-3.0-mRNA-1	3	OsR498G0305096500.01.T01	99,85	0,00E+00	2374	Os03g0108600	100,00	0,00E+00	2383	9	NA	Japonica	
maker-3-augustus-gene-3.13-mRNA-1	3	OsR498G0305097500.01.T01	98,43	0,00E+00	1551	Os03g0109000	100,00	0,00E+00	1613	62	NA	Japonica	
maker-3-augustus-gene-3.18-mRNA-1	3	OsR498G0305099300.01.T01	99,67	0,00E+00	1086	Os03g0109300	100,00	0,00E+00	1148	62	NA	Japonica	
maker-3-augustus-gene-3.22-mRNA-1	3	OsR498G0305101300.01.T03	95,14	0,00E+00	1922	Os03g0109400	95,31	0,00E+00	1931	9	NA	Japonica	
maker-3-augustus-gene-3.38-mRNA-1	3	OsR498G0305096800.01.T01	97,66	0,00E+00	874	Os03g0108700	99,42	0,00E+00	911	37	NA	Japonica	
maker-3-augustus-gene-3.4-mRNA-1	3	OsR498G0305096900.01.T04	99,52	0,00E+00	2201	Os03g0118400	97,85	0,00E+00	1509	-692	Indica	Japonica	Kinase-TM
maker-3-augustus-gene-3.40-mRNA-1	3	OsR498G0305101800.01.T01	98,31	0,00E+00	916	Os03g0109600	100,00	0,00E+00	953	37	NA	Japonica	
maker-3-augustus-gene-3.45-mRNA-1	3	OsR498G0305102600.01.T04	100,00	0,00E+00	993	Os03g0109700	100,00	0,00E+00	993	0	NA	Japonica	
maker-3-augustus-gene-30.0-mRNA-1	3	OsR498G0305262700.01.T01	96,64	0,00E+00	693	Os03g0161000	100,00	0,00E+00	650	-43	NA	Japonica	
maker-3-augustus-gene-30.15-mRNA-1	3	OsR498G0305263600.01.T01	98,84	0,00E+00	3458	Os03g0161200	100,00	0,00E+00	3561	103	NA	Japonica	
maker-3-augustus-gene-30.7-mRNA-1	3	OsR498G0305261200.01.T02	99,79	0,00E+00	5167	Os03g0160400	100,00	0,00E+00	5194	27	NA	Japonica	
maker-3-augustus-gene-30.9-mRNA-1	3	OsR498G0305261600.01.T06	97,55	0,00E+00	2968	Os10g0422500	77,99	0,00E+00	1452	-1516	Indica	Japonica	
maker-3-augustus-gene-31.0-mRNA-1	3	OsR498G0305266900.01.T01	100,00	0,00E+00	2224	Os03g0161400	100,00	0,00E+00	2192	-32	NA	NA	
maker-3-augustus-gene-31.11-mRNA-1	3	OsR498G0305270500.01.T08	100,00	0,00E+00	2057	Os03g0161900	100,00	0,00E+00	2057	0	NA	Japonica	
maker-3-augustus-gene-31.14-mRNA-1	3	OsR498G0305271700.01.T05	96,20	0,00E+00	1181	Os03g0162000	97,91	0,00E+00	2231	1050	Japonica	NA	
maker-3-augustus-gene-31.19-mRNA-1	3	OsR498G0305273100.01.T01	99,52	0,00E+00	744	Os03g0162200	100,00	0,00E+00	753	9	NA	Japonica	

maker-3-augustus-gene-31.21-mRNA-1	3	OsR498G0305275500.01.T01	99,56	0,00E+00	2810	Os03g0162800	100,00	0,00E+00	1564	-1246	Indica	Japonica	
maker-3-augustus-gene-31.3-mRNA-1	3	OsR498G0305267300.01.T04	100,00	3,23E-150	531	Os03g0161500	100,00	1,12E-150	531	0	NA	NA	
maker-3-augustus-gene-31.7-mRNA-1	3	OsR498G0305270300.01.T04	94,78	0,00E+00	985	Os03g0161800	94,78	0,00E+00	985	0	NA	NA	
maker-3-augustus-gene-32.40-mRNA-1	3	OsR498G0305276100.01.T02	99,77	0,00E+00	2382	Os03g0163100	100,00	0,00E+00	2396	14	NA	Japonica	
maker-3-augustus-gene-32.47-mRNA-1	3	OsR498G0305276300.01.T01	99,22	0,00E+00	2494	Os03g0163200	100,00	0,00E+00	2544	50	NA	Japonica	
maker-3-augustus-gene-32.49-mRNA-1	3	OsR498G0305276500.01.T03	99,94	0,00E+00	3004	Os03g0163300	100,00	0,00E+00	3009	5	NA	Japonica	
maker-3-augustus-gene-32.54-mRNA-1	3	OsR498G0305280300.01.T01	99,72	0,00E+00	2531	Os03g0164700	100,00	0,00E+00	2549	18	NA	Japonica	
maker-3-augustus-gene-32.57-mRNA-1	3	OsR498G0305276800.01.T02	94,22	0,00E+00	1428	Os03g0163400	100,00	0,00E+00	1938	510	Japonica	Japonica	
maker-3-augustus-gene-32.61-mRNA-1	3	OsR498G0204262300.01.T01	74,98	0,00E+00	867	Os03g0164400	100,00	0,00E+00	3393	2526	Japonica	Japonica	
maker-3-augustus-gene-32.65-mRNA-1	3	OsR498G0305280800.01.T05	100,00	0,00E+00	2636	Os03g0165000	100,00	0,00E+00	2636	0	NA	Japonica	
maker-3-augustus-gene-32.70-mRNA-1	3	OsR498G0305281400.01.T02	99,83	0,00E+00	3125	Os03g0165100	99,15	0,00E+00	3729	604	Japonica	Japonica	
maker-3-augustus-gene-321.34-mRNA-1	3	OsR498G1018481400.01.T02	99,10	7,56E-167	588	Os03g0209400	88,00	8,59E-142	502	-86	Indica	Japonica	
maker-3-augustus-gene-321.46-mRNA-1	3	OsR498G0306712000.01.T02	87,92	2,77E-81	303	Os03g0658800	87,92	9,62E-82	303	0	NA	NA	
maker-3-augustus-gene-33.35-mRNA-1	3	OsR498G0305281500.01.T01	99,82	0,00E+00	5042	Os03g0165266	99,54	0,00E+00	5778	736	Japonica	Japonica	
maker-3-augustus-gene-33.38-mRNA-1	3	OsR498G0305284300.01.T01	100,00	0,00E+00	1213	Os03g0165800	100,00	0,00E+00	1213	0	NA	Japonica	
maker-3-augustus-gene-33.41-mRNA-1	3	OsR498G0305285500.01.T01	93,63	0,00E+00	1494	Os10g0413500	70,77	2,67E-125	449	-1045	Indica	Japonica	
maker-3-augustus-gene-33.44-mRNA-1	3	OsR498G0305282000.01.T01	99,83	0,00E+00	3239	Os03g0165300	100,00	0,00E+00	3252	13	NA	Japonica	
maker-3-augustus-gene-33.46-mRNA-1	3	OsR498G0305283000.01.T01	96,40	0,00E+00	4216	Os03g0165400	96,48	0,00E+00	4225	9	NA	Japonica	
maker-3-augustus-gene-33.58-mRNA-1	3	OsR498G0305284600.01.T01	99,79	0,00E+00	1701	Os03g0165900	100,00	0,00E+00	1710	9	NA	Japonica	
maker-3-augustus-gene-33.60-mRNA-1	3	OsR498G0305285000.01.T01	98,42	0,00E+00	1102	Os03g0166000	100,00	0,00E+00	978	-124	Indica	Japonica	
maker-3-augustus-gene-33.65-mRNA-1	3	OsR498G0305287000.01.T01	99,74	0,00E+00	1355	Os03g0166600	100,00	0,00E+00	1198	-157	Indica	Japonica	
maker-3-augustus-gene-331.28-mRNA-1	3	OsR498G0306680500.01.T01	99,75	0,00E+00	2140	Os03g0650800	100,00	0,00E+00	2711	571	Japonica	NA	
maker-3-augustus-gene-331.32-mRNA-1	3	OsR498G0306667400.01.T01	99,73	0,00E+00	3343	Os12g0618300	78,18	0,00E+00	1434	-1909	Indica	NA	
maker-3-augustus-gene-331.34-mRNA-1	3	OsR498G0306680800.01.T02	99,80	0,00E+00	1831	Os03g0650900	100,00	0,00E+00	1840	9	NA	NA	
maker-3-augustus-gene-339.0-mRNA-1	3	OsR498G0306381300.01.T01	99,84	0,00E+00	3260	Os03g0563100	100,00	0,00E+00	3274	14	NA	Japonica	
maker-3-augustus-gene-339.2-mRNA-1	3	OsR498G0306383900.01.T01	99,71	0,00E+00	1875	Os03g0563600	100,00	0,00E+00	1889	14	NA	Japonica	
maker-3-augustus-gene-339.5-mRNA-1	3	OsR498G0306387300.01.T03	99,17	0,00E+00	635	Os03g0564200	100,00	1,53E-91	334	-301	Indica	Japonica	
maker-3-augustus-gene-339.9-mRNA-1	3	OsR498G0306382200.01.T01	99,92	0,00E+00	2247	Os03g0563300	100,00	0,00E+00	2251	4	NA	Japonica	
maker-3-augustus-gene-34.52-mRNA-1	3	OsR498G0305287500.01.T02	99,78	0,00E+00	3272	Os03g0166800	100,00	0,00E+00	3290	18	NA	Japonica	
maker-3-augustus-gene-34.57-mRNA-1	3	OsR498G0305287700.01.T02	99,82	0,00E+00	1013	Os03g0167000	100,00	0,00E+00	1380	367	Japonica	Japonica	
maker-3-augustus-gene-34.59-mRNA-1	3	OsR498G0305288700.01.T01	99,73	0,00E+00	1973	Os03g0167400	100,00	0,00E+00	1986	13	NA	Japonica	
maker-3-augustus-gene-34.65-mRNA-1	3	OsR498G0305289900.01.T01	99,32	0,00E+00	2858	Os03g0167600	100,00	0,00E+00	2904	46	NA	Japonica	
maker-3-augustus-gene-34.70-mRNA-1	3	OsR498G0305290500.01.T01	99,76	0,00E+00	2249	Os03g0167800	100,00	0,00E+00	2259	10	NA	Japonica	
maker-3-augustus-gene-34.72-mRNA-1	3	OsR498G0305291100.01.T01	96,43	0,00E+00	2296	Os03g0168000	96,51	0,00E+00	2301	5	NA	Japonica	
maker-3-augustus-gene-34.75-mRNA-1	3	OsR498G0305291500.01.T01	100,00	0,00E+00	1440	Os03g0168200	100,00	0,00E+00	1440	0	NA	Japonica	
maker-3-augustus-gene-34.81-mRNA-1	3	OsR498G0305288200.01.T05	100,00	0,00E+00	3306	Os03g0167200	100,00	0,00E+00	3306	0	NA	Japonica	
maker-3-augustus-gene-34.84-mRNA-1	3	OsR498G0307234100.01.T02	75,79	5,62E-159	562	Os03g0805300	75,79	1,95E-159	562	0	NA	Japonica	
maker-3-augustus-gene-34.89-mRNA-1	3	OsR498G0305291400.01.T01	99,92	0,00E+00	2166	Os03g0168100	99,92	0,00E+00	2166	0	NA	Japonica	
maker-3-augustus-gene-34.91-mRNA-1	3	OsR498G0305291800.01.T02	99,88	0,00E+00	1517	Os03g0168300	100,00	0,00E+00	1521	4	NA	Japonica	
maker-3-augustus-gene-340.0-mRNA-1	3	OsR498G0306392300.01.T01	100,00	0,00E+00	1737	Os03g0565200	100,00	0,00E+00	1737	0	NA	Japonica	
maker-3-augustus-gene-340.4-mRNA-1	3	OsR498G0306392700.01.T06	99,68	0,00E+00	5534	Os03g0565300	100,00	0,00E+00	5579	45	NA	Japonica	
maker-3-augustus-gene-340.6-mRNA-1	3	OsR498G0306393900.01.T01	97,45	0,00E+00	3875	Os03g0565500	97,54	0,00E+00	3884	9	NA	Japonica	
maker-3-augustus-gene-341.39-mRNA-1	3	OsR498G0306396500.01.T02	99,69	0,00E+00	1751	Os03g0566600	100,00	0,00E+00	1764	13	NA	Japonica	
maker-3-augustus-gene-341.47-mRNA-1	3	OsR498G0306396000.01.T01	99,46	0,00E+00	2319	Os03g0565900	100,00	0,00E+00	2352	33	NA	Japonica	
maker-3-augustus-gene-341.49-mRNA-1	3	OsR498G0306396400.01.T01	99,69	0,00E+00	2309	Os03g0566500	100,00	0,00E+00	2327	18	NA	Japonica	
maker-3-augustus-gene-341.56-mRNA-1	3	OsR498G0306399600.01.T01	99,77	0,00E+00	3104	Os03g0567600	100,00	0,00E+00	3122	18	NA	Japonica	
maker-3-augustus-gene-342.3-mRNA-1	3	OsR498G0306402700.01.T01	99,13	0,00E+00	3666	Os03g0568800	100,00	0,00E+00	3658	-8	NA	Japonica	Kinase-TM
maker-3-augustus-gene-342.5-mRNA-1	3	OsR498G0306402000.01.T03	99,53	1,44E-103	377	Os03g0568500	100,00	0,00E+00	650	273	Japonica	Japonica	
maker-3-augustus-gene-342.8-mRNA-1	3	OsR498G0306403000.01.T01	99,86	0,00E+00	2540	Os03g0568900	100,00	0,00E+00	2549	9	NA	Japonica	
maker-3-augustus-gene-343.5-mRNA-1	3	OsR498G0203772900.01.T07	96,68	0,00E+00	1245	Os03g0570200	98,27	0,00E+00	1306	61	NA	Japonica	
maker-3-augustus-gene-344.38-mRNA-1	3	OsR498G0306412800.01.T03	99,67	0,00E+00	2158	Os03g0570800	100,00	0,00E+00	2188	30	NA	NA	
maker-3-augustus-gene-344.45-mRNA-1	3	OsR498G0306415400.01.T01	99,93	0,00E+00	2388	Os03g0571700	100,00	0,00E+00	1880	-508	Indica	NA	
maker-3-augustus-gene-344.48-mRNA-1	3	OsR498G0306415800.01.T02	99,52	0,00E+00	1870	Os03g0570800	82,78	0,00E+00	1067	-803	Indica	NA	
maker-3-augustus-gene-345.0-mRNA-1	3	OsR498G1120379000.01.T02	76,41	7,01E-79	296	Os03g0572250	100,00	0,00E+00	1142	846	Japonica	NA	
maker-3-augustus-gene-345.2-mRNA-1	3	OsR498G0306417600.01.T05	99,56	0,00E+00	1203	Os03g0572300	100,00	0,00E+00	1216	13	NA	NA	
maker-3-augustus-gene-345.5-mRNA-1	3	OsR498G0306418600.01.T01	99,80	0,00E+00	2698	Os03g0572900	100,00	0,00E+00	2711	13	NA	NA	
maker-3-augustus-gene-346.29-mRNA-1	3	OsR498G0306427000.01.T02	99,67	0,00E+00	4358	Os03g0574900	100,00	0,00E+00	4394	36	NA	NA	
maker-3-augustus-gene-346.34-mRNA-1	3	OsR498G0306428400.01.T01	99,77	0,00E+00	3911	Os03g0575200	100,00	0,00E+00	3934	23	NA	NA	
maker-3-augustus-gene-347.41-mRNA-1	3	OsR498G0306433700.01.T02	99,87	0,00E+00	2655	Os03g0576900	100,00	0,00E+00	2664	9	NA	NA	
maker-3-augustus-gene-347.43-mRNA-1	3	OsR498G0306434900.01.T01	95,13	0,00E+00	1097	Os03g0577200	95,27	0,00E+00	1102	5	NA	NA	
maker-3-augustus-gene-347.49-mRNA-1	3	OsR498G0306432000.01.T01	99,75	0,00E+00	4307	Os03g0576200	100,00	0,00E+00	4329	22	NA	NA	
maker-3-augustus-gene-347.51-mRNA-1	3	OsR498G0815254000.01.T01	96,21	2,06E-120	432	Os03g0576600	100,00	4,81E-142	502	70	Japonica	NA	
maker-3-augustus-gene-347.55-mRNA-1	3	OsR498G0306433400.01.T01	99,68	0,00E+00	1122	Os03g0576700	100,00	0,00E+00	1131	9	NA	NA	
maker-3-augustus-gene-347.59-mRNA-1	3	OsR498G0306434000.01.T01	99,71	0,00E+00	1231	Os03g0577000	100,00	0,00E+00	1240	9	NA	NA	
maker-3-augustus-gene-347.62-mRNA-1	3	OsR498G0306434400.01.T02	99,79	0,00E+00	2553	Os03g0577100	100,00	0,00E+00	2567	14	NA	NA	
maker-3-augustus-gene-347.67-mRNA-1	3	OsR498G0306435600.01.T01	99,84	0,00E+00	2188	Os03g0577500	99,92	0,00E+00	2193	5	NA	NA	
maker-3-augustus-gene-348.0-mRNA-1	3	OsR498G0306440100.01.T01	99,80	0,00E+00	1770	Os03g0578900	100,00	0,00E+00	2646	876	Japonica	NA	
maker-3-augustus-gene-348.2-mRNA-1	3	OsR498G0306437600.01.T02	99,60	5,37E-126	451	Os03g0578200	100,00	4,42E-128	455	4	NA	NA	
maker-3-augustus-gene-349.23-mRNA-1	3	OsR498G0306446200.01.T01	99,65	0,00E+00	2039	Os03g0580800	100,00	0,00E+00	2057	18	NA	NA	
maker-3-augustus-gene-349.6-mRNA-1	3	OsR498G0306442600.01.T02	99,52	0,00E+00	1107	Os03g0337800	95,69	0,00E+00	1011	-96	NA	NA	
maker-3-augustus-gene-35.42-mRNA-1	3	OsR498G0305294100.01.T01	99,71	1,04E-174	613	Os03g0168900	100,00	8,53E-177	618	5	NA	Japonica	
maker-3-augustus-gene-35.44-mRNA-1	3	OsR498G0305294500.01.T01	99,74	0,00E+00	1389	Os03g0169100	100,00	0,00E+00	1398	9	NA	Japonica	
maker-3-augustus-gene-35.48-mRNA-1	3	OsR498G0305295700.01.T01	94,19	0,00E+00	1799	Os03g0169500	94,92	0,00E+00	1432	-367	Indica	Japonica	
maker-3-augustus-gene-35.53-mRNA-1	3	OsR498G0305296300.01.T01	99,85	0,00E+00	2378	Os03g0169600	100,00	0,00E+00	2387	9	NA	Japonica	
maker-3-augustus-gene-35.55-mRNA-1	3	OsR498G0305297900.01.T02	99,92	0,00E+00	2112	Os03g0170300	100,00	0,00E+00	2116	4	NA	Japonica	
maker-3-augustus-gene-35.60-mRNA-1	3	OsR498G0305294400.01.T02	99,79	0,00E+00	2514	Os03g0169000	100,00	0,00E+00	2549	35	NA	Japonica	
maker-3-augustus-gene-35.65-mRNA-1	3	OsR498G0305295600.01.T01	99,81	0,00E+00	948	Os03g0169400	100,00	0,00E+00	953	5	NA	Japonica	
maker-3-augustus-gene-35.67-mRNA-1	3	OsR498G0305296600.01.T05	100,00	0,00E+00	1018	Os03g0169800	100,00	0,00E+00	1018	0	NA	Japonica	
maker-3-augustus-gene-35.68-mRNA-1	3	OsR498G0305297000.01.T01	99,73	0,00E+00	662	Os03g0169900	100,00	0,00E+00	634	-28	NA	Japonica	
maker-3-augustus-gene-35.84-mRNA-1	3	OsR498G0305302600.01.T03	99,67	0,00E+00	2188	Os03g0170900	99,76	0,00E+00	2193	5	NA	Japonica	
maker-3-augustus-gene-350.27-mRNA-1	3	OsR498G0306450100.01.T01	99,78	0,00E+00	1647	Os03g0581400	100,00	0,00E+00	1992	345	Japonica	NA	
maker-3-augustus-gene-350.31-mRNA-1	3	OsR498G0306451100.01.T01	99,85	0,00E+00	1235	Os03g0581600	100,00	0,00E+00	643	-592	Indica	NA	
maker-3-augustus-gene-350.37-mRNA-1	3	OsR498G0306451900.01.T03	100,00	2,22E-124	444	Os03g0581800	100,00	7,30E-81	298	-146	Indica	Japonica	
maker-3-augustus-gene-350.40-mRNA-1	3	OsR498G0306452500.01.T04	99,89	0,00E+00	1679	Os03g0582000	100,00	0,00E+00	1683	4	NA	NA	
maker-3-augustus-gene-350.42-mRNA-1	3	OsR498G0306453400.01.T03	95,14	0,00E+00	740	Os03g0582200	95,14	0,00E+00	740	0	NA	NA	
maker-3-augustus-gene-351.14-mRNA-1	3	OsR498G0306453600.01.T02	99,82	0,00E+00	976	Os03g0582200	71,81	8,19E-61	233	-743	Indica	NA	
maker-3-augustus-gene-351.20-mRNA-1	3	OsR498G0305649000.01.T01	85,95	0,00E+00	1992	Os03g0269300	86,12	0,00E+00	2005	13	NA	NA	Kinase-TM
maker-3-augustus-gene-351.4-mRNA-1	3	OsR498G0917087500.01.T01	99,30	0,00E+00	755	Os03g0583700	99,74	0,00E+00	701	-54	NA	NA	
maker-3-augustus-gene-351.8-mRNA-1	3	OsR498G0306460900.01.T06	99,63	0,00E+00	1925	Os03g0583800	100,00	0,00E+00	1943	18	NA	NA	
maker-3-augustus-gene-352.0-mRNA-1	3	OsR498G0306462300.01.T02	99,73	0,00E+00	667	Os03g0584300	100,00	0,00E+00	672	5	NA	NA	
maker-3-augustus-gene-353.0-mRNA-1	3	OsR498G0306466900.01.T01	99,83	0,00E+00	1060	Os03g0586500	100,00	0,00E+00	1065	5	NA	NA	
maker-3-augustus-gene-353.14-mRNA-1	3	OsR498G0306468500.01.T01	94,74	0,00E+00	1621	Os03g0587100	100,00	0,00E+00	948	-673	Indica	NA	
maker-3-augustus-gene-353.16-mRNA-1	3	OsR498G0306466600.01.T05	99,59	0,00E+00	1295	Os03g0586300	100,00	0,00E+00	1306	11	NA	NA	
maker-3-augustus-gene-353.21-mRNA-1	3	OsR498G0306467200.01.T01	98,74	0,00E+00	692	Os03g0586600	100,00	0,00E+00	711	19	NA	NA	
maker-3-augustus-gene-353.24-mRNA-1	3	OsR498G0306467600.01.T01	99,87	0,00E+00	6779	Os03g0586700	100,00	0,00E+00	6801	22	NA	NA	
maker-3-augustus-gene-353.30-mRNA-1	3	OsR498G0306468000.01.T02	99,78	0,00E+00	3245	Os03g0586800	100,00	0,00E+00	3263	18	NA	NA	
maker-3-augustus-gene-353.34-mRNA-1	3	OsR498G0306468800.01.T01	99,94	0,00E+00	6105	Os03g0587200	100,00	0,00E+00	6114	9	NA	NA	
maker-3-augustus-gene-353.9-mRNA-1	3	OsR498G0306468300.01.T01	100,00	0,00E+00	1451	Os03g0587000	100,00	0,00E+00	1451	0	NA	NA	
maker-3-augustus-gene-354.39-mRNA-1	3	OsR498G0306474500.01.T01	98,72	0,00E+00	3557	Os03g0588600	100,00	0,00E+00	3631	74	NA	NA	
maker-3-augustus-gene-354.41-mRNA-1	3	OsR498G0306474900.01.T01	99,46	0,00E+00	4280	Os03g0588700	100,00	0,00E+00	3422	-858	Indica	NA	
maker-3-augustus-gene-354.45-mRNA-1	3	OsR498G0306477000.01.T02	96,26	0,00E+00	2447	Os03g0589300	100,00	0,00E+00	2562	115	NA	NA	
maker-3-augustus-gene-356.3-mRNA-1	3	OsR498G0306485300.01.T02	99,75	0,00E+00	1420	Os03g0592500	100,00	0,00E+00	1429	9	NA	NA	
maker-3-augustus-gene-357.0-mRNA-1	3	OsR498G0306487100.01.T01	99,38	0,00E+00	2584	Os03g0593600	100,00	0,00E+00	2625	41	NA	NA	
maker-3-augustus-gene-357.12-mRNA-1	3	OsR498G0306491600.01.T01	99,94	0,00E+00	2826	Os03g0594400	100,00	0,00E+00	2830	4	NA	NA	
maker-3-augustus-gene-357.14-mRNA-1	3	OsR498G0306492800.01.T01	98,77	0,00E+00	4545	Os03g0594700	100,00	0,00E+00	4675	130	NA	NA	
maker-3-augustus-gene-357.2-mRNA-1	3	OsR498G0306490700.01.T01	99,29	0,00E+00	2727	Os03g0594100	100,00	0,00E+00	2776	49	NA	NA	
maker-3-augustus-gene-357.6-mRNA-1	3	OsR498G0306486600.01.T01	99,80	0,00E+00	3662	Os03g0593200	99,27	0,00E+00	3151	-511	Indica	NA	
maker-3-augustus-gene-358.0-mRNA-1	3	OsR498G0306492900.01.T01	99,87	0,00E+00	2783	Os03g0594900	100,00	0,00E+00	2792	9	NA	NA	
maker-3-augustus-gene-358.2-mRNA-1	3	OsR498G0306493500.01.T01	97,47	0,00E+00	808	Os03g0595200	100,00	0,00E+00	856	48	NA	Japonica	
maker-3-augustus-gene-358.4-mRNA-1	3	OsR498G0306494800.01.T01	95,34	0,00E+00	1187	Os03g0595600	100,00	0,00E+00	1315	128	NA	NA	
maker-3-augustus-gene-359.37-mRNA-1	3	OsR498G0306502700.01.T05	99,79	0,00E+00	2568	Os03g0598100	100,00	0,00E+00	2441	-127	NA	NA	
maker-3-augustus-gene-359.41-mRNA-1	3	OsR498G0306498400.01.T01	100,00	2,91E-171	601	Os03g0596800	93,79	0,00E+00	810	209	Japonica	NA	
maker-3-augustus-gene-359.43-mRNA-1	3	OsR498G0306498800.01.T05	100,00	0,00E+00	2307	Os03g0596900	100,00	0,00E+00	2307	0	NA	NA	
maker-3-augustus-gene-359.50-mRNA-1	3	OsR498G0306500800.01.T02	97,86	0,00E+00	880	Os03g0597600	97,78	0,00E+00	696	-184	Indica	NA	
maker-3-augustus-gene-359.55-mRNA-1	3	OsR498G0306503000.01.T01	99,57	0,00E+00	4131	Os03g0598200	100,00	0,00E+00	3925	-206	NA	Japonica	
maker-3-augustus-gene-36.0-mRNA-1	3	OsR498G0305305500.01.T01	100,00	1,31E-140	499	Os03g0171700	100,00	4,58E-141	499	0	NA	Japonica	
maker-3-augustus-gene-36.10-mRNA-1	3	OsR498G0305303400.01.T03	99,64	1,32E-140	499	Os03g0171300	100,00	1,09E-142	504	5	NA	Japonica	
maker-3-augustus-gene-36.14-mRNA-1	3	OsR498G0305306400.01.T01	99,72	0,00E+00	1929	Os03g0172200	100,00	0,00E+00	1943	14	NA	Japonica	
maker-3-augustus-gene-36.2-mRNA-1	3	OsR498G0305305900.01.T03	99,59	0,00E+00	2608	Os03g0171900	100,00	0,00E+00	2636	28	NA	Japonica	
maker-3-augustus-gene-36.7-mRNA-1	3	OsR498G0305306100.01.T01	99,83	0,00E+00	5421	Os03g0172000	100,00	0,00E+00	5443	22	NA	Japonica	
maker-3-augustus-gene-360.23-mRNA-1	3	OsR498G0306004300.01.T01	98,91	0,00E+00	806	Os03g0365200	99,45	0,00E+00	652	-154	Indica	NA	
maker-3-augustus-gene-360.26-mRNA-1	3	OsR498G0306505500.01.T02	95,67	0,00E+00	1096	Os03g0598700	100,00	0,00E+00	1159	63	NA	NA	
maker-3-augustus-gene-360.38-mRNA-1	3	OsR498G0306506000.01.T01	100,00	0,00E+00	1110	Os03g0598800	100,00	0,00E+00	1110	0	NA	NA	
maker-3-augustus-gene-360.49-mRNA-1	3	OsR498G0306507000.01.T01	99,61	0,00E+00	3665	Os03g0599000	100,00	0,00E+00	4556	891	Japonica	NA	LRR-NBS-TM
maker-3-augustus-gene-360.51-mRNA-1	3	OsR498G0306508200.01.T02	100,00	0,00E+00	1379	Os03g0599800	100,00	0,00E+00	1975	596	Japonica	NA	
maker-3-augustus-gene-361.0-mRNA-1	3	OsR498G0306512300.01.T01	100,00	1,75E-135	482	Os03g0601500	100,00	6,10E-136	482	0	NA	NA	
maker-3-augustus-gene-361.4-mRNA-1	3	OsR498G1018781700.01.T01	89,18	1,68E-156	554	Os03g0600500	100,00	0,00E+00	722	168	Japonica	Japonica	
maker-3-augustus-gene-362.6-mRNA-1	3	OsR498G0306519600.01.T01	99,85	0,00E+00	2361	Os03g0603100	100,00	0,00E+00	1700	-661	Indica	NA	
maker-3-augustus-gene-363.0-mRNA-1	3	OsR498G0306522300.01.T01	99,91	0,00E+00	2094	Os03g0603600	100,00	0,00E+00	2098	4	NA	NA	
maker-3-augustus-gene-363.6-mRNA-1	3	OsR498G0306524700.01.T01	99,67	0,00E+00	1068	Os03g0604500	100,00	0,00E+00	1191	123	Japonica	NA	
maker-3-augustus-gene-364.0-mRNA-1	3	OsR498G0306528600.01.T04	99,73	0,00E+00	2623	Os03g0606100	100,00	0,00E+00	3220	597	Japonica	Japonica	
maker-3-augustus-gene-365.36-mRNA-1	3	OsR498G0306532100.01.T01	97,55	0,00E+00	2075	Os12g0581700	72,69	2,82E-164	579	-1496	Indica	NA	
maker-3-augustus-gene-365.40-mRNA-1	3	OsR498G0306537300.01.T01	98,57	0,00E+00	1952	Os03g0607600	100,00	0,00E+00	2017	65	NA	NA	
maker-3-augustus-gene-365.46-mRNA-1	3	OsR498G0306532000.01.T01	99,28	0,00E+00	1478	Os12g0581600	79,80	0,00E+00	728	-750	Indica	NA	
maker-3-augustus-gene-365.59-mRNA-1	3	OsR498G0306538000.01.T01	99,72	0,00E+00	636	Os03g0607800	99,72	0,00E+00	645	9	NA	NA	
maker-3-augustus-gene-366.0-mRNA-1	3	OsR498G0306542700.01.T04	99,08	0,00E+00	1151	Os03g0608800	100,00	0,00E+00	1178	27	NA	NA	
maker-3-augustus-gene-366.5-mRNA-1	3	OsR498G0306543500.01.T01	87,44	1,53E-147	522	Os07g0477000	96,77	6,95E-115	412	-110	Indica	NA	
maker-3-augustus-gene-366.7-mRNA-1	3	OsR498G0306544400.01.T01	98,16	0,00E+00	1221	Os03g0609500	100,00	0,00E+00	1261	40	NA	NA	
maker-3-augustus-gene-366.9-mRNA-1	3	OsR498G0306545400.01.T01	99,63	0,00E+00	3405	Os03g0609800	100,00	0,00E+00	3436	31	NA	Japonica	
maker-3-augustus-gene-367.0-mRNA-1	3	OsR498G0306548700.01.T01	99,41	0,00E+00	2117	Os03g0610800	87,27	0,00E+00	1482	-635	Indica	NA	
maker-3-augustus-gene-367.4-mRNA-1	3	OsR498G0306550700.01.T05	99,29	0,00E+00	1490	Os03g0611200	99,76	0,00E+00	1508	18	NA	NA	
maker-3-augustus-gene-367.9-mRNA-1	3	OsR498G0306549800.01.T02	99,41	0,00E+00	2411	Os03g0610900	100,00	0,00E+00	1965	-446	Indica	NA	Kinase-TM
maker-3-augustus-gene-368.44-mRNA-1	3	OsR498G0102477000.01.T01	71,06	5,41E-35	149	Os03g0615700	93,36	0,00E+00	658	509	Japonica	NA	
maker-3-augustus-gene-369.27-mRNA-1	3	OsR498G0102477000.01.T01	72,03	8,88E-39	162	Os03g0615700	99,82	0,00E+00	976	814	Japonica	NA	
maker-3-augustus-gene-37.0-mRNA-1	3	OsR498G0305309500.01.T04	99,76	0,00E+00	1496	Os03g0173900	100,00	0,00E+00	1359	-137	NA	Japonica	
maker-3-augustus-gene-37.10-mRNA-1	3	OsR498G0305309900.01.T01	99,58	0,00E+00	2137	Os03g0174200	100,00	0,00E+00	2159	22	NA	Japonica	
maker-3-augustus-gene-37.13-mRNA-1	3	OsR498G0305310300.01.T04	99,77	0,00E+00	3139	Os03g0174300	100,00	0,00E+00	2645	-494	Indica	Japonica	
maker-3-augustus-gene-37.18-mRNA-1	3	OsR498G0305311100.01.T03	99,83	0,00E+00	2066	Os03g0174600	100,00	0,00E+00	2075	9	NA	Japonica	
maker-3-augustus-gene-37.24-mRNA-1	3	OsR498G0713747300.01.T07	97,79	1,96E-60	233	Os03g0173500	100,00	3,11E-65	246	13	NA	Japonica	
maker-3-augustus-gene-37.26-mRNA-1	3	OsR498G0305308400.01.T02	99,10	0,00E+00	3711	Os03g0173700	100,00	0,00E+00	3799	88	NA	Japonica	
maker-3-augustus-gene-37.29-mRNA-1	3	OsR498G0305311000.01.T01	99,16	0,00E+00	2306	Os03g0174500	99,77	0,00E+00	2344	38	NA	Japonica	
maker-3-augustus-gene-37.31-mRNA-1	3	OsR498G0305311800.01.T01	100,00	0,00E+00	1299	Os03g0174900	100,00	0,00E+00	1299	0	NA	Japonica	
maker-3-augustus-gene-37.5-mRNA-1	3	OsR498G0305309700.01.T01	99,81	0,00E+00	1903	Os03g0174100	100,00	0,00E+00	2031	128	NA	Japonica	
maker-3-augustus-gene-370.10-mRNA-1	3	OsR498G0306563600.01.T01	99,65	0,00E+00	5041	Os03g0616600	100,00	0,00E+00	5748	707	Japonica	NA	
maker-3-augustus-gene-370.6-mRNA-1	3	OsR498G0306563100.01.T01	99,77	0,00E+00	5563	Os03g0616400	100,00	0,00E+00	5595	32	NA	NA	
maker-3-augustus-gene-371.47-mRNA-1	3	OsR498G0306571500.01.T02	99,75	0,00E+00	2828	Os05g0556700	84,79	0,00E+00	1103	-1725	Indica	NA	
maker-3-augustus-gene-371.52-mRNA-1	3	OsR498G0306571700.01.T01	99,44	0,00E+00	2865	Os03g0619600	100,00	0,00E+00	2906	41	NA	NA	
maker-3-augustus-gene-371.54-mRNA-1	3	OsR498G0306566200.01.T01	99,47	0,00E+00	1339	Os03g0617500	100,00	0,00E+00	1357	18	NA	NA	
maker-3-augustus-gene-371.57-mRNA-1	3	OsR498G0306568000.01.T01	100,00	0,00E+00	1056	Os03g0617800	100,00	0,00E+00	1683	627	Japonica	NA	
maker-3-augustus-gene-371.59-mRNA-1	3	OsR498G1018971300.01.T01	84,65	0,00E+00	693	Os03g0617900	92,92	0,00E+00	914	221	Japonica	NA	
maker-3-augustus-gene-371.64-mRNA-1	3	OsR498G0306569000.01.T01	99,72	0,00E+00	1897	Os03g0618300	100,00	0,00E+00	1911	14	NA	NA	
maker-3-augustus-gene-372.12-mRNA-1	3	OsR498G0306575900.01.T01	97,32	0,00E+00	3174	Os03g0621600	97,47	0,00E+00	3163	-11	NA	NA	
maker-3-augustus-gene-372.19-mRNA-1	3	OsR498G0306574600.01.T02	99,57	0,00E+00	3341	Os03g0620800	100,00	0,00E+00	3377	36	NA	NA	
maker-3-augustus-gene-372.3-mRNA-1	3	OsR498G0306573300.01.T02	99,62	0,00E+00	2785	Os03g0620400	100,00	0,00E+00	2814	29	NA	NA	TIR
maker-3-augustus-gene-372.8-mRNA-1	3	OsR498G0306575100.01.T02	99,67	0,00E+00	2136	Os02g0494400	71,26	1,63E-154	545	-1591	Indica	NA	
maker-3-augustus-gene-373.0-mRNA-1	3	OsR498G0306578700.01.T01	97,74	0,00E+00	1973	Os03g0622100	100,00	0,00E+00	2073	100	NA	NA	
maker-3-augustus-gene-373.5-mRNA-1	3	OsR498G0306583300.01.T01	99,58	0,00E+00	841	Os03g0623100	100,00	0,00E+00	1012	171	Japonica	Japonica	
maker-3-augustus-gene-373.7-mRNA-1	3	OsR498G1222012100.01.T02	73,00	6,76E-37	154	Os03g0622300	100,00	9,96E-131	464	310	Japonica	NA	
maker-3-augustus-gene-374.0-mRNA-1	3	OsR498G0306586700.01.T02	94,91	0,00E+00	2178	Os03g0624000	100,00	0,00E+00	1463	-715	Indica	Japonica	
maker-3-augustus-gene-374.2-mRNA-1	3	OsR498G0306589500.01.T01	99,49	0,00E+00	1731	Os03g0624600	100,00	0,00E+00	1754	23	NA	Japonica	
maker-3-augustus-gene-374.8-mRNA-1	3	OsR498G0306592200.01.T02	99,76	0,00E+00	752	Os03g0625500	100,00	0,00E+00	1124	372	Japonica	Japonica	
maker-3-augustus-gene-375.17-mRNA-1	3	OsR498G0306593100.01.T03	99,75	0,00E+00	2910	Os03g0625800	100,00	0,00E+00	2928	18	NA	Japonica	
maker-3-augustus-gene-375.19-mRNA-1	3	OsR498G0306594300.01.T01	99,40	0,00E+00	2671	Os03g0626100	72,53	0,00E+00	884	-1787	Indica	Japonica	
maker-3-augustus-gene-375.21-mRNA-1	3	OsR498G0306594700.01.T01	99,07	0,00E+00	2654	Os03g0626100	99,30	0,00E+00	3038	384	Japonica	Japonica	
maker-3-augustus-gene-375.29-mRNA-1	3	OsR498G0306596100.01.T06	100,00	0,00E+00	1274	Os03g0626600	100,00	0,00E+00	1274	0	NA	Japonica	
maker-3-augustus-gene-375.39-mRNA-1	3	OsR498G0306593400.01.T01	98,70	0,00E+00	2690	Os03g0625900	100,00	0,00E+00	2771	81	NA	Japonica	
maker-3-augustus-gene-375.41-mRNA-1	3	OsR498G0306597000.01.T01	99,79	0,00E+00	2573	Os03g0626700	100,00	0,00E+00	2587	14	NA	Japonica	
maker-3-augustus-gene-376.34-mRNA-1	3	OsR498G0306597900.01.T01	99,85	0,00E+00	1219	Os03g0626800	100,00	0,00E+00	1223	4	NA	Japonica	
maker-3-augustus-gene-377.38-mRNA-1	3	OsR498G0306604300.01.T01	99,70	0,00E+00	2948	Os03g0628800	100,00	0,00E+00	2971	23	NA	Japonica	
maker-3-augustus-gene-377.42-mRNA-1	3	OsR498G0306604800.01.T01	100,00	0,00E+00	688	Os03g0628900	100,00	0,00E+00	688	0	NA	Japonica	
maker-3-augustus-gene-378.36-mRNA-1	3	OsR498G0306609600.01.T01	99,74	0,00E+00	2747	Os12g0600200	73,24	0,00E+00	671	-2076	Indica	Japonica	
maker-3-augustus-gene-379.26-mRNA-1	3	OsR498G0306610500.01.T02	99,67	0,00E+00	3225	Os03g0633400	99,95	0,00E+00	3248	23	NA	Japonica	
maker-3-augustus-gene-379.31-mRNA-1	3	OsR498G0306611700.01.T01	99,85	0,00E+00	1224	Os03g0633800	100,00	0,00E+00	1229	5	NA	Japonica	
maker-3-augustus-gene-379.33-mRNA-1	3	OsR498G0306610800.01.T01	99,84	0,00E+00	1095	Os03g0633500	100,00	0,00E+00	1099	4	NA	Japonica	
maker-3-augustus-gene-379.35-mRNA-1	3	OsR498G0306612000.01.T01	99,79	0,00E+00	1680	Os03g0633900	100,00	0,00E+00	1689	9	NA	Japonica	
maker-3-augustus-gene-379.39-mRNA-1	3	OsR498G0306612400.01.T01	99,23	0,00E+00	911	Os03g0634000	99,81	0,00E+00	921	10	NA	Japonica	
maker-3-augustus-gene-379.43-mRNA-1	3	OsR498G0306615200.01.T01	99,09	0,00E+00	2514	Os03g0635800	100,00	0,00E+00	2008	-506	Indica	Japonica	
maker-3-augustus-gene-38.0-mRNA-1	3	OsR498G0305315100.01.T01	99,78	0,00E+00	1604	Os03g0175600	99,87	0,00E+00	1387	-217	Indica	Japonica	
maker-3-augustus-gene-38.14-mRNA-1	3	OsR498G0305320100.01.T04	100,00	0,00E+00	1456	Os03g0177300	99,88	0,00E+00	1448	-8	NA	Japonica	
maker-3-augustus-gene-38.18-mRNA-1	3	OsR498G0305320300.01.T01	99,70	0,00E+00	2406	Os03g0177400	100,00	0,00E+00	2425	19	NA	Japonica	
maker-3-augustus-gene-38.19-mRNA-1	3	OsR498G0305320300.01.T04	99,93	0,00E+00	2420	Os03g0177500	100,00	0,00E+00	2425	5	NA	Japonica	
maker-3-augustus-gene-38.26-mRNA-1	3	OsR498G0305318800.01.T01	99,77	0,00E+00	795	Os03g0176600	100,00	0,00E+00	800	5	NA	Japonica	
maker-3-augustus-gene-38.29-mRNA-1	3	OsR498G0305319000.01.T02	99,88	0,00E+00	1489	Os03g0176800	100,00	0,00E+00	2560	1071	Japonica	Japonica	
maker-3-augustus-gene-38.34-mRNA-1	3	OsR498G0305320000.01.T01	99,83	0,00E+00	2058	Os09g0357250	98,39	0,00E+00	1390	-668	Indica	Japonica	
maker-3-augustus-gene-38.5-mRNA-1	3	OsR498G1018648800.01.T04	72,83	1,08E-129	465	Os03g0176100	100,00	0,00E+00	1856	1391	Japonica	Japonica	
maker-3-augustus-gene-38.8-mRNA-1	3	OsR498G0305319700.01.T01	100,00	0,00E+00	1662	Os03g0177000	100,00	0,00E+00	1662	0	NA	Japonica	
maker-3-augustus-gene-380.24-mRNA-1	3	OsR498G1222064900.01.T01	71,67	6,05E-109	396	Os03g0636700	100,00	0,00E+00	2425	2029	Japonica	Japonica	
maker-3-augustus-gene-380.27-mRNA-1	3	OsR498G0306621200.01.T01	100,00	0,00E+00	1543	Os12g0604700	86,30	0,00E+00	1013	-530	Indica	Japonica	Kinase-TM
maker-3-augustus-gene-381.0-mRNA-1	3	OsR498G0306626300.01.T08	99,45	0,00E+00	2567	Os03g0638200	100,00	0,00E+00	2603	36	NA	Japonica	
maker-3-augustus-gene-381.5-mRNA-1	3	OsR498G0306628100.01.T01	99,74	0,00E+00	2043	Os12g0605900	74,57	6,61E-172	603	-1440	Indica	Japonica	Kinase
maker-3-augustus-gene-382.11-mRNA-1	3	OsR498G0306636000.01.T01	92,50	4,86E-155	548	Os03g0639600	100,00	8,73E-172	601	53	NA	Japonica	
maker-3-augustus-gene-382.5-mRNA-1	3	OsR498G0306633100.01.T01	99,10	0,00E+00	1561	Os03g0639300	100,00	0,00E+00	1667	106	NA	Japonica	
maker-3-augustus-gene-382.6-mRNA-1	3	OsR498G0306633100.01.T01	100,00	0,00E+00	728	Os12g0610200	79,85	6,27E-93	341	-387	Indica	Japonica	
maker-3-augustus-gene-383.28-mRNA-1	3	OsR498G0306637300.01.T02	100,00	0,00E+00	1889	Os03g0639700	100,00	0,00E+00	1889	0	NA	Japonica	
maker-3-augustus-gene-383.32-mRNA-1	3	OsR498G0306637600.01.T01	99,76	0,00E+00	757	Os03g0639800	100,00	0,00E+00	762	5	NA	Japonica	
maker-3-augustus-gene-383.38-mRNA-1	3	OsR498G0306639600.01.T02	99,83	0,00E+00	2060	Os03g0640100	99,89	0,00E+00	1690	-370	Indica	Japonica	
maker-3-augustus-gene-383.39-mRNA-1	3	OsR498G0306639600.01.T03	99,72	0,00E+00	642	Os03g0640100	100,00	0,00E+00	646	4	NA	Japonica	
maker-3-augustus-gene-383.46-mRNA-1	3	OsR498G0306640800.01.T01	89,54	5,78E-121	434	Os03g0640200	100,00	8,53E-177	618	184	Japonica	Japonica	
maker-3-augustus-gene-383.50-mRNA-1	3	OsR498G0306640800.01.T01	99,73	0,00E+00	667	Os03g0640400	100,00	0,00E+00	672	5	NA	Japonica	
maker-3-augustus-gene-383.52-mRNA-1	3	OsR498G0306641800.01.T01	99,77	0,00E+00	4626	Os03g0640800	100,00	0,00E+00	4653	27	NA	Japonica	
maker-3-augustus-gene-384.33-mRNA-1	3	OsR498G0306646300.01.T01	96,98	0,00E+00	669	Os03g0641500	100,00	0,00E+00	695	26	NA	Japonica	
maker-3-augustus-gene-384.35-mRNA-1	3	OsR498G0306647100.01.T03	100,00	0,00E+00	1196	Os03g0641700	100,00	0,00E+00	1196	0	NA	Japonica	

maker-3-augustus-gene-384.39-mRNA-1	3	OsR498G0306643800.01.T01	99,94	0,00E+00	3210	Os03g0641200	99,94	0,00E+00	3210	0	NA	Japonica	
maker-3-augustus-gene-384.41-mRNA-1	3	OsR498G0306644400.01.T01	99,95	0,00E+00	3378	Os12g0613250	77,37	0,00E+00	1034	-2344	Indica	Japonica	
maker-3-augustus-gene-385.33-mRNA-1	3	OsR498G0306653900.01.T03	98,94	0,00E+00	2648	Os03g0644000	99,80	0,00E+00	2707	59	NA	Japonica	
maker-3-augustus-gene-385.37-mRNA-1	3	OsR498G0306651200.01.T03	98,50	0,00E+00	1157	Os03g0642900	100,00	0,00E+00	1272	115	NA	Japonica	
maker-3-augustus-gene-385.42-mRNA-1	3	OsR498G0713408500.01.T01	100,00	0,00E+00	1750	Os03g0643100	100,00	0,00E+00	1750	0	NA	Japonica	
maker-3-augustus-gene-385.49-mRNA-1	3	OsR498G0306653600.01.T01	99,21	0,00E+00	2235	Os03g0643300	100,00	0,00E+00	2280	45	NA	Japonica	
maker-3-augustus-gene-385.56-mRNA-1	3	OsR498G0306655000.01.T01	99,44	0,00E+00	2516	Os03g0644400	100,00	0,00E+00	2554	38	NA	Japonica	
maker-3-augustus-gene-386.0-mRNA-1	3	OsR498G0306657300.01.T02	99,70	0,00E+00	1792	Os03g0645000	100,00	0,00E+00	1660	-132	NA	Japonica	
maker-3-augustus-gene-386.6-mRNA-1	3	OsR498G0306657700.01.T01	95,46	0,00E+00	1971	Os03g0645100	100,00	0,00E+00	1667	-304	Indica	Japonica	
maker-3-augustus-gene-386.8-mRNA-1	3	OsR498G0306658000.01.T01	99,84	0,00E+00	3304	Os12g0617100	79,87	0,00E+00	1357	-1947	Indica	Japonica	
maker-3-augustus-gene-387.0-mRNA-1	3	OsR498G0306664100.01.T01	99,71	0,00E+00	2434	Os03g0646100	100,00	0,00E+00	2452	18	NA	Japonica	
maker-3-augustus-gene-387.14-mRNA-1	3	OsR498G0306664800.01.T03	99,80	0,00E+00	900	Os03g0646400	100,00	0,00E+00	904	4	NA	Japonica	
maker-3-augustus-gene-387.16-mRNA-1	3	OsR498G0306665600.01.T01	99,87	0,00E+00	5414	Os12g0617900	84,12	0,00E+00	3305	-2109	Indica	Japonica	
maker-3-augustus-gene-387.22-mRNA-1	3	OsR498G0306667400.01.T01	99,62	0,00E+00	1874	Os03g0647500	99,88	0,00E+00	1556	-318	Indica	NA	
maker-3-augustus-gene-387.24-mRNA-1	3	OsR498G0306667600.01.T01	95,65	0,00E+00	1800	Os03g0647600	97,26	0,00E+00	1863	63	NA	Japonica	
maker-3-augustus-gene-388.14-mRNA-1	3	OsR498G0306670600.01.T01	100,00	0,00E+00	774	Os03g0648500	100,00	0,00E+00	774	0	NA	Japonica	
maker-3-augustus-gene-388.16-mRNA-1	3	OsR498G0306672200.01.T01	99,79	0,00E+00	4225	Os12g0620600	75,77	0,00E+00	1436	-2789	Indica	Japonica	
maker-3-augustus-gene-388.4-mRNA-1	3	OsR498G0306671900.01.T01	99,86	0,00E+00	1320	Os03g0648900	100,00	0,00E+00	1324	4	NA	Japonica	
maker-3-augustus-gene-388.6-mRNA-1	3	OsR498G0306669200.01.T01	99,86	0,00E+00	1320	Os03g0647800	100,00	0,00E+00	1429	109	NA	Japonica	
maker-3-augustus-gene-388.8-mRNA-1	3	OsR498G0306670200.01.T01	99,55	0,00E+00	2360	Os03g0648300	100,00	0,00E+00	2722	362	Japonica	Japonica	
maker-3-augustus-gene-389.0-mRNA-1	3	OsR498G0306675300.01.T04	100,00	0,00E+00	657	Os03g0650000	100,00	0,00E+00	657	0	NA	NA	
maker-3-augustus-gene-389.11-mRNA-1	3	OsR498G0306680500.01.T01	99,75	0,00E+00	2140	Os03g0650800	100,00	0,00E+00	2711	571	Japonica	NA	
maker-3-augustus-gene-389.13-mRNA-1	3	OsR498G0306681100.01.T01	100,00	0,00E+00	834	Os03g0651000	100,00	0,00E+00	834	0	NA	Japonica	
maker-3-augustus-gene-389.17-mRNA-1	3	OsR498G0306680800.01.T02	99,80	0,00E+00	1831	Os03g0650900	100,00	0,00E+00	1840	9	NA	NA	
maker-3-augustus-gene-39.0-mRNA-1	3	OsR498G0305321100.01.T01	99,55	0,00E+00	2397	Os03g0177900	100,00	0,00E+00	2425	28	NA	Japonica	
maker-3-augustus-gene-39.11-mRNA-1	3	OsR498G0305326300.01.T01	99,30	0,00E+00	2277	Os03g0179400	100,00	0,00E+00	2305	28	NA	Japonica	Kinase-TM
maker-3-augustus-gene-39.15-mRNA-1	3	OsR498G0305327300.01.T01	98,44	0,00E+00	4809	Os03g0179900	98,59	0,00E+00	4828	19	NA	Japonica	
maker-3-augustus-gene-39.19-mRNA-1	3	OsR498G0305328700.01.T05	100,00	0,00E+00	2782	Os03g0180300	100,00	0,00E+00	2857	75	NA	Japonica	
maker-3-augustus-gene-39.2-mRNA-1	3	OsR498G0305321500.01.T02	99,78	0,00E+00	2411	Os03g0178000	100,00	0,00E+00	2425	14	NA	Japonica	
maker-3-augustus-gene-39.30-mRNA-1	3	OsR498G0305322600.01.T01	99,79	0,00E+00	1712	Os03g0178300	100,00	0,00E+00	1721	9	NA	Japonica	
maker-3-augustus-gene-39.32-mRNA-1	3	OsR498G0305322800.01.T02	100,00	0,00E+00	1781	Os03g0178400	100,00	0,00E+00	1781	0	NA	Japonica	
maker-3-augustus-gene-39.36-mRNA-1	3	OsR498G0305323000.01.T01	100,00	0,00E+00	1792	Os03g0178500	100,00	0,00E+00	1792	0	NA	Japonica	
maker-3-augustus-gene-39.4-mRNA-1	3	OsR498G0305322300.01.T01	99,86	0,00E+00	1289	Os03g0178200	100,00	0,00E+00	1294	5	NA	Japonica	
maker-3-augustus-gene-39.40-mRNA-1	3	OsR498G0305326600.01.T01	99,67	0,00E+00	1090	Os03g0179700	100,00	0,00E+00	1153	63	NA	Japonica	
maker-3-augustus-gene-390.36-mRNA-1	3	OsR498G0102706900.01.T02	93,90	0,00E+00	1026	Os03g0651800	100,00	0,00E+00	1207	181	Japonica	Japonica	
maker-3-augustus-gene-390.38-mRNA-1	3	OsR498G0306685700.01.T01	99,87	0,00E+00	4007	Os03g0652100	100,00	0,00E+00	4020	13	NA	Japonica	
maker-3-augustus-gene-390.42-mRNA-1	3	OsR498G0306681400.01.T01	99,92	0,00E+00	2157	Os03g0651100	98,82	0,00E+00	1188	-969	Indica	Japonica	
maker-3-augustus-gene-390.49-mRNA-1	3	OsR498G0306682200.01.T01	99,79	0,00E+00	2606	Os03g0651300	100,00	0,00E+00	2619	13	NA	Japonica	
maker-3-augustus-gene-390.54-mRNA-1	3	OsR498G0306688600.01.T01	99,48	0,00E+00	1032	Os03g0652100	95,06	0,00E+00	697	-335	Indica	Japonica	
maker-3-augustus-gene-391.28-mRNA-1	3	OsR498G0306691400.01.T02	99,25	0,00E+00	1880	Os03g0653900	100,00	0,00E+00	1916	36	NA	Japonica	
maker-3-augustus-gene-392.39-mRNA-1	3	OsR498G0306694000.01.T02	99,82	0,00E+00	2925	Os03g0654500	99,45	0,00E+00	2902	-23	NA	Japonica	
maker-3-augustus-gene-392.53-mRNA-1	3	OsR498G0306694600.01.T01	96,12	0,00E+00	1798	Os03g0654700	96,12	0,00E+00	1798	0	NA	NA	
maker-3-augustus-gene-392.55-mRNA-1	3	OsR498G0306696800.01.T02	100,00	0,00E+00	1326	Os03g0655200	100,00	0,00E+00	1326	0	NA	NA	
maker-3-augustus-gene-393.18-mRNA-1	3	OsR498G0306699600.01.T01	100,00	0,00E+00	1213	Os03g0656000	100,00	0,00E+00	1209	-4	NA	NA	
maker-3-augustus-gene-393.21-mRNA-1	3	OsR498G0306701000.01.T01	95,75	0,00E+00	734	Os03g0656201	95,75	0,00E+00	734	0	NA	NA	
maker-3-augustus-gene-393.23-mRNA-1	3	OsR498G0306705400.01.T01	100,00	0,00E+00	755	Os03g0657400	100,00	0,00E+00	774	19	NA	NA	
maker-3-augustus-gene-393.7-mRNA-1	3	OsR498G0306703500.01.T01	99,93	0,00E+00	2663	Os03g0656800	99,93	0,00E+00	2663	0	NA	Indica	
maker-3-augustus-gene-393.9-mRNA-1	3	OsR498G0306703700.01.T01	100,00	0,00E+00	1559	Os03g0656900	100,00	0,00E+00	1559	0	NA	NA	
maker-3-augustus-gene-394.3-mRNA-1	3	OsR498G0306712000.01.T03	93,27	0,00E+00	1179	Os03g0658800	93,27	0,00E+00	1179	0	NA	NA	
maker-3-augustus-gene-395.15-mRNA-1	3	OsR498G0306718200.01.T01	100,00	0,00E+00	3305	Os03g0659900	100,00	0,00E+00	3305	0	NA	NA	
maker-3-augustus-gene-395.4-mRNA-1	3	OsR498G0306714600.01.T03	100,00	0,00E+00	697	Os03g0659300	100,00	0,00E+00	691	-6	NA	NA	
maker-3-augustus-gene-395.7-mRNA-1	3	OsR498G0306714800.01.T01	100,00	0,00E+00	3777	Os03g0659400	100,00	0,00E+00	3777	0	NA	NA	
maker-3-augustus-gene-396.0-mRNA-1	3	OsR498G0306725900.01.T01	100,00	0,00E+00	2419	Os03g0661300	100,00	0,00E+00	2419	0	NA	NA	
maker-3-augustus-gene-396.4-mRNA-1	3	OsR498G0306722400.01.T01	100,00	0,00E+00	1267	Os03g0660700	100,00	0,00E+00	838	-429	Indica	NA	
maker-3-augustus-gene-397.37-mRNA-1	3	OsR498G0306730500.01.T03	100,00	0,00E+00	1673	Os03g0663100	100,00	0,00E+00	1673	0	NA	NA	
maker-3-augustus-gene-397.44-mRNA-1	3	OsR498G0306729000.01.T04	100,00	0,00E+00	1544	Os03g0661800	100,00	0,00E+00	1544	0	NA	NA	
maker-3-augustus-gene-397.50-mRNA-1	3	OsR498G0306736000.01.T01	100,00	0,00E+00	1240	Os03g0663900	100,00	0,00E+00	1240	0	NA	NA	
maker-3-augustus-gene-398.40-mRNA-1	3	OsR498G0306738200.01.T02	99,90	0,00E+00	1883	Os03g0664700	99,90	0,00E+00	1883	0	NA	NA	
maker-3-augustus-gene-398.42-mRNA-1	3	OsR498G0306740400.01.T02	100,00	0,00E+00	1640	Os03g0665200	100,00	0,00E+00	1640	0	NA	NA	
maker-3-augustus-gene-399.38-mRNA-1	3	OsR498G0306744300.01.T02	94,72	0,00E+00	1187	Os03g0665800	94,72	0,00E+00	1187	0	NA	NA	
maker-3-augustus-gene-399.42-mRNA-1	3	OsR498G0306745100.01.T01	100,00	0,00E+00	7634	Os03g0666100	100,00	0,00E+00	6340	-1294	Indica	NA	
maker-3-augustus-gene-399.47-mRNA-1	3	OsR498G0306746900.01.T04	100,00	1,65E-101	369	Os03g0666500	100,00	5,76E-102	369	0	NA	NA	
maker-3-augustus-gene-399.48-mRNA-1	3	OsR498G0306746900.01.T03	100,00	0,00E+00	899	Os03g0666500	100,00	0,00E+00	744	-155	Indica	NA	
maker-3-augustus-gene-399.54-mRNA-1	3	OsR498G0306745400.01.T02	100,00	0,00E+00	2158	Os03g0666200	100,00	0,00E+00	2158	0	NA	NA	
maker-3-augustus-gene-399.58-mRNA-1	3	OsR498G0306747400.01.T01	97,33	0,00E+00	1401	Os03g0666700	97,33	0,00E+00	1401	0	NA	NA	
maker-3-augustus-gene-399.63-mRNA-1	3	OsR498G0306747800.01.T06	99,04	1,21E-154	546	Os03g0666850	100,00	1,68E-109	394	-152	Indica	NA	
maker-3-augustus-gene-399.64-mRNA-1	3	OsR498G0306749000.01.T07	99,94	0,00E+00	3188	Os03g0667100	99,94	0,00E+00	3094	-94	NA	NA	
maker-3-augustus-gene-399.69-mRNA-1	3	OsR498G0306751200.01.T01	100,00	0,00E+00	2008	Os03g0667300	100,00	0,00E+00	2008	0	NA	NA	
maker-3-augustus-gene-4.16-mRNA-1	3	OsR498G0305107700.01.T01	99,73	0,00E+00	1346	Os03g0111500	100,00	0,00E+00	1297	-49	NA	Japonica	
maker-3-augustus-gene-4.19-mRNA-1	3	OsR498G0306856700.01.T01	93,44	0,00E+00	4677	Os03g0694900	93,34	0,00E+00	4663	-14	NA	Japonica	
maker-3-augustus-gene-4.28-mRNA-1	3	OsR498G0305108300.01.T02	99,63	0,00E+00	2904	Os03g0111800	100,00	0,00E+00	2931	27	NA	Japonica	
maker-3-augustus-gene-4.3-mRNA-1	3	OsR498G0305105900.01.T02	99,89	0,00E+00	3232	Os03g0110800	99,83	0,00E+00	3226	-6	NA	Japonica	
maker-3-augustus-gene-4.40-mRNA-1	3	OsR498G0305106600.01.T02	99,74	0,00E+00	2083	Os03g0111100	100,00	0,00E+00	2093	10	NA	Japonica	
maker-3-augustus-gene-4.48-mRNA-1	3	OsR498G0305107000.01.T01	99,81	0,00E+00	948	Os03g0111200	100,00	0,00E+00	953	5	NA	Japonica	
maker-3-augustus-gene-4.51-mRNA-1	3	OsR498G0305107600.01.T01	98,74	7,57E-158	556	Os03g0111400	99,18	0,00E+00	645	89	Japonica	Japonica	
maker-3-augustus-gene-4.54-mRNA-1	3	OsR498G0305108000.01.T01	98,49	0,00E+00	2877	Os03g0111600	100,00	0,00E+00	3117	240	NA	Japonica	
maker-3-augustus-gene-40.32-mRNA-1	3	OsR498G0305329500.01.T01	100,00	0,00E+00	1133	Os03g0180600	100,00	0,00E+00	1496	363	Japonica	NA	
maker-3-augustus-gene-40.35-mRNA-1	3	OsR498G0305330900.01.T01	100,00	0,00E+00	1137	Os03g0180900	100,00	0,00E+00	1137	0	NA	Japonica	
maker-3-augustus-gene-40.37-mRNA-1	3	OsR498G0305333900.01.T01	99,88	0,00E+00	2929	Os03g0181500	100,00	0,00E+00	2938	9	NA	Japonica	
maker-3-augustus-gene-40.46-mRNA-1	3	OsR498G0305335900.01.T04	99,83	0,00E+00	3093	Os03g0181800	100,00	0,00E+00	3106	13	NA	Japonica	
maker-3-augustus-gene-40.48-mRNA-1	3	OsR498G0305329000.01.T01	100,00	0,00E+00	1337	Os03g0180400	100,00	0,00E+00	1337	0	NA	Japonica	
maker-3-augustus-gene-400.0-mRNA-1	3	OsR498G0306754100.01.T02	100,00	0,00E+00	1745	Os03g0668000	100,00	0,00E+00	1745	0	NA	NA	
maker-3-augustus-gene-400.16-mRNA-1	3	OsR498G0306753800.01.T01	100,00	6,67E-152	536	Os03g0667800	100,00	2,65E-126	450	-86	Indica	NA	
maker-3-augustus-gene-400.7-mRNA-1	3	OsR498G0306760300.01.T01	100,00	0,00E+00	1478	Os03g0668900	100,00	0,00E+00	1478	0	NA	NA	
maker-3-augustus-gene-401.0-mRNA-1	3	OsR498G0306763100.01.T01	100,00	0,00E+00	5925	Os03g0669800	100,00	0,00E+00	4947	-978	Indica	NA	
maker-3-augustus-gene-401.23-mRNA-1	3	OsR498G0306761600.01.T01	100,00	0,00E+00	2809	Os03g0722500	75,58	0,00E+00	641	-2168	Indica	NA	
maker-3-augustus-gene-401.26-mRNA-1	3	OsR498G0306762800.01.T01	100,00	0,00E+00	1386	Os03g0669700	100,00	0,00E+00	2697	1311	Japonica	NA	
maker-3-augustus-gene-401.28-mRNA-1	3	OsR498G0306764000.01.T02	100,00	0,00E+00	993	Os03g0670100	100,00	0,00E+00	993	0	NA	NA	NBS
maker-3-augustus-gene-401.3-mRNA-1	3	OsR498G0306764100.01.T03	100,00	0,00E+00	675	Os03g0670200	100,00	0,00E+00	675	0	NA	NA	
maker-3-augustus-gene-401.6-mRNA-1	3	OsR498G0306764900.01.T01	97,98	0,00E+00	847	Os03g0670700	99,02	0,00E+00	711	-136	Indica	Japonica	
maker-3-augustus-gene-401.8-mRNA-1	3	OsR498G0306760600.01.T02	100,00	0,00E+00	2560	Os03g0669000	100,00	0,00E+00	2560	0	NA	NA	
maker-3-augustus-gene-402.0-mRNA-1	3	OsR498G0306767000.01.T01	98,10	0,00E+00	1999	Os03g0671800	100,00	0,00E+00	2089	90	NA	Japonica	
maker-3-augustus-gene-402.2-mRNA-1	3	OsR498G0306768400.01.T02	100,00	0,00E+00	2971	Os03g0672300	100,00	0,00E+00	2971	0	NA	Japonica	
maker-3-augustus-gene-402.5-mRNA-1	3	OsR498G0306768800.01.T01	99,78	0,00E+00	4018	Os03g0672400	99,96	0,00E+00	4173	155	NA	Japonica	
maker-3-augustus-gene-403.0-mRNA-1	3	OsR498G0306771100.01.T01	99,82	0,00E+00	4078	Os03g0672900	100,00	0,00E+00	4096	18	NA	Japonica	
maker-3-augustus-gene-403.16-mRNA-1	3	OsR498G0306774000.01.T03	99,27	0,00E+00	4131	Os03g0673800	100,00	0,00E+00	2679	-1452	Indica	Japonica	
maker-3-augustus-gene-403.2-mRNA-1	3	OsR498G0306774900.01.T01	99,76	0,00E+00	2265	Os03g0125800	70,04	2,40E-108	392	-1873	Indica	Japonica	
maker-3-augustus-gene-404.43-mRNA-1	3	OsR498G0306775700.01.T01	99,77	0,00E+00	2297	Os03g0674700	100,00	0,00E+00	2311	14	NA	Japonica	
maker-3-augustus-gene-404.46-mRNA-1	3	OsR498G0306779900.01.T01	100,00	1,21E-114	412	Os03g0675600	100,00	4,23E-115	412	0	NA	Japonica	
maker-3-augustus-gene-404.50-mRNA-1	3	OsR498G0306777400.01.T01	94,96	0,00E+00	1526	Os03g0675300	100,00	0,00E+00	1748	222	Japonica	Japonica	
maker-3-augustus-gene-405.3-mRNA-1	3	OsR498G0306792100.01.T02	99,86	0,00E+00	1262	Os03g0678400	100,00	0,00E+00	1267	5	NA	Japonica	
maker-3-augustus-gene-406.3-mRNA-1	3	OsR498G0306798700.01.T01	99,69	0,00E+00	1166	Os03g0679800	99,85	0,00E+00	1170	4	NA	Japonica	
maker-3-augustus-gene-406.8-mRNA-1	3	OsR498G0306795400.01.T03	100,00	0,00E+00	1186	Os03g0679000	100,00	0,00E+00	1186	0	NA	Japonica	
maker-3-augustus-gene-407.26-mRNA-1	3	OsR498G0306798700.01.T01	95,13	0,00E+00	1462	Os03g0680100	100,00	0,00E+00	1856	394	Japonica	Japonica	
maker-3-augustus-gene-407.30-mRNA-1	3	OsR498G0306799900.01.T02	99,28	0,00E+00	1485	Os03g0680200	100,00	0,00E+00	1512	27	NA	Japonica	
maker-3-augustus-gene-407.35-mRNA-1	3	OsR498G0306801400.01.T01	99,66	0,00E+00	1061	Os03g0680700	100,00	0,00E+00	1878	817	Japonica	Japonica	
maker-3-augustus-gene-407.38-mRNA-1	3	OsR498G0306802400.01.T06	99,66	0,00E+00	3144	Os03g0680800	100,00	0,00E+00	3171	27	NA	Japonica	
maker-3-augustus-gene-408.36-mRNA-1	3	OsR498G0306805900.01.T03	100,00	0,00E+00	1523	Os03g0681700	100,00	0,00E+00	1523	0	NA	Japonica	
maker-3-augustus-gene-408.41-mRNA-1	3	OsR498G0306806200.01.T02	100,00	0,00E+00	2901	Os04g0485100	71,08	2,69E-115	416	-2485	Indica	Japonica	
maker-3-augustus-gene-408.43-mRNA-1	3	OsR498G0306806600.01.T01	95,17	0,00E+00	1578	Os03g0681900	95,37	0,00E+00	1587	9	NA	Japonica	
maker-3-augustus-gene-409.42-mRNA-1	3	OsR498G0306812100.01.T01	99,81	0,00E+00	1929	Os03g0683800	100,00	0,00E+00	1938	9	NA	Japonica	
maker-3-augustus-gene-41.39-mRNA-1	3	OsR498G0305336500.01.T02	99,90	0,00E+00	3458	Os03g0182400	100,00	0,00E+00	3467	9	NA	Japonica	
maker-3-augustus-gene-41.53-mRNA-1	3	OsR498G0305337000.01.T01	99,67	0,00E+00	1643	Os07g0616600	85,17	0,00E+00	751	-892	Indica	Japonica	
maker-3-augustus-gene-41.57-mRNA-1	3	OsR498G0305337200.01.T01	100,00	0,00E+00	1229	Os03g0182700	100,00	0,00E+00	1229	0	NA	Japonica	
maker-3-augustus-gene-41.60-mRNA-1	3	OsR498G0305337400.01.T01	99,49	0,00E+00	1747	Os03g0182800	100,00	0,00E+00	1770	23	NA	Japonica	
maker-3-augustus-gene-41.63-mRNA-1	3	OsR498G0305337800.01.T02	99,70	0,00E+00	1800	Os03g0183000	100,00	0,00E+00	1813	13	NA	Japonica	
maker-3-augustus-gene-41.65-mRNA-1	3	OsR498G0305338600.01.T05	99,91	0,00E+00	1942	Os03g0183100	100,00	0,00E+00	1947	5	NA	Japonica	
maker-3-augustus-gene-41.66-mRNA-1	3	OsR498G0305340600.01.T01	95,53	0,00E+00	803	Os03g0183500	100,00	0,00E+00	888	85	NA	Japonica	
maker-3-augustus-gene-41.68-mRNA-1	3	OsR498G0305341400.01.T04	99,82	0,00E+00	3006	Os10g0390500	81,47	0,00E+00	1362	-1644	Indica	Japonica	
maker-3-augustus-gene-410.0-mRNA-1	3	OsR498G0306813500.01.T01	100,00	0,00E+00	953	Os03g0684300	100,00	0,00E+00	953	0	NA	Japonica	
maker-3-augustus-gene-410.13-mRNA-1	3	OsR498G0306815500.01.T03	99,78	0,00E+00	831	Os03g0685000	99,77	0,00E+00	790	-41	NA	Japonica	
maker-3-augustus-gene-410.17-mRNA-1	3	OsR498G0306815700.01.T01	99,68	0,00E+00	1673	Os03g0685100	100,00	0,00E+00	1687	14	NA	Japonica	
maker-3-augustus-gene-410.19-mRNA-1	3	OsR498G0306817500.01.T01	99,39	0,00E+00	876	Os03g0685300	100,00	0,00E+00	890	14	NA	Japonica	
maker-3-augustus-gene-410.3-mRNA-1	3	OsR498G0306813700.01.T02	99,60	0,00E+00	1347	Os03g0684400	100,00	0,00E+00	1350	3	NA	Japonica	
maker-3-augustus-gene-410.34-mRNA-1	3	OsR498G0306812600.01.T02	100,00	8,31E-74	277	Os03g0683900	100,00	2,89E-74	277	0	NA	Japonica	
maker-3-augustus-gene-410.37-mRNA-1	3	OsR498G0306812800.01.T01	99,58	0,00E+00	1714	Os03g0684000	100,00	0,00E+00	1732	18	NA	Japonica	
maker-3-augustus-gene-410.41-mRNA-1	3	OsR498G0306813400.01.T01	99,57	0,00E+00	3323	Os03g0684100	100,00	0,00E+00	3360	37	NA	Japonica	
maker-3-augustus-gene-410.47-mRNA-1	3	OsR498G0306815000.01.T01	99,56	0,00E+00	814	Os03g0684700	100,00	0,00E+00	823	9	NA	Japonica	
maker-3-augustus-gene-410.49-mRNA-1	3	OsR498G0306818600.01.T01	99,76	0,00E+00	748	Os03g0685500	100,00	0,00E+00	753	5	NA	Japonica	
maker-3-augustus-gene-410.53-mRNA-1	3	OsR498G0306818800.01.T02	95,78	0,00E+00	1951	Os03g0685600	95,95	0,00E+00	1960	9	NA	Japonica	
maker-3-augustus-gene-410.9-mRNA-1	3	OsR498G0306814500.01.T01	99,85	0,00E+00	3515	Os03g0684500	100,00	0,00E+00	3534	19	NA	Japonica	
maker-3-augustus-gene-411.41-mRNA-1	3	OsR498G0306821500.01.T01	99,68	0,00E+00	2766	Os03g0686900	99,82	0,00E+00	2033	-733	Indica	Japonica	
maker-3-augustus-gene-411.45-mRNA-1	3	OsR498G0306828100.01.T01	96,27	0,00E+00	2354	Os03g0688200	97,22	0,00E+00	2380	26	NA	Japonica	
maker-3-augustus-gene-411.52-mRNA-1	3	OsR498G0306821800.01.T01	99,89	0,00E+00	3144	Os03g0687000	99,35	0,00E+00	3302	158	NA	Japonica	
maker-3-augustus-gene-411.57-mRNA-1	3	OsR498G0306824200.01.T03	99,54	0,00E+00	1546	Os03g0687200	99,84	0,00E+00	1134	-412	Indica	Japonica	
maker-3-augustus-gene-411.62-mRNA-1	3	OsR498G0306827000.01.T01	99,60	0,00E+00	895	Os03g0688000	100,00	0,00E+00	1159	264	Japonica	Japonica	
maker-3-augustus-gene-412.0-mRNA-1	3	OsR498G0306829300.01.T01	97,80	0,00E+00	2030	Os03g0688300	97,97	0,00E+00	2039	9	NA	Japonica	
maker-3-augustus-gene-412.14-mRNA-1	3	OsR498G0306832100.01.T01	99,85	0,00E+00	3641	Os03g0689800	99,90	0,00E+00	3644	3	NA	Japonica	CC-LRR-NBS-TM
maker-3-augustus-gene-412.16-mRNA-1	3	OsR498G0306833100.01.T01	99,62	0,00E+00	933	Os03g0690000	100,00	2,95E-134	477	-456	Indica	Japonica	
maker-3-augustus-gene-412.2-mRNA-1	3	OsR498G0306829900.01.T01	99,90	0,00E+00	5641	Os03g0689100	99,97	0,00E+00	5650	9	NA	Japonica	
maker-3-augustus-gene-412.21-mRNA-1	3	OsR498G0306832800.01.T01	99,86	0,00E+00	2578	Os03g0689900	100,00	0,00E+00	2154	-424	Indica	Japonica	
maker-3-augustus-gene-413.22-mRNA-1	3	OsR498G0306844300.01.T01	99,57	0,00E+00	4947	Os03g0691500	100,00	0,00E+00	5937	990	Japonica	Japonica	
maker-3-augustus-gene-413.25-mRNA-1	3	OsR498G0306843600.01.T01	100,00	0,00E+00	893	Os03g0691400	100,00	0,00E+00	893	0	NA	Japonica	
maker-3-augustus-gene-414.0-mRNA-1	3	OsR498G0306845300.01.T01	99,67	0,00E+00	3751	Os03g0691800	100,00	0,00E+00	3782	31	NA	Japonica	
maker-3-augustus-gene-414.11-mRNA-1	3	OsR498G0306849000.01.T03	99,81	0,00E+00	1923	Os03g0692500	100,00	0,00E+00	2232	309	Japonica	Japonica	
maker-3-augustus-gene-414.2-mRNA-1	3	OsR498G0306845900.01.T01	99,85	0,00E+00	2426	Os03g0692000	100,00	0,00E+00	2435	9	NA	Japonica	
maker-3-augustus-gene-414.8-mRNA-1	3	OsR498G0306848200.01.T05	99,81	0,00E+00	2811	Os03g0692400	100,00	0,00E+00	2825	14	NA	Japonica	
maker-3-augustus-gene-415.42-mRNA-1	3	OsR498G0306852900.01.T01	99,53	0,00E+00	2626	Os03g0693600	99,93	0,00E+00	2728	102	NA	Japonica	
maker-3-augustus-gene-415.44-mRNA-1	3	OsR498G0306855500.01.T02	94,51	0,00E+00	1394	Os03g0694500	94,51	0,00E+00	1394	0	NA	Japonica	
maker-3-augustus-gene-415.53-mRNA-1	3	OsR498G0306856700.01.T01	99,59	0,00E+00	5662	Os03g0694900	99,81	0,00E+00	5693	31	NA	Japonica	
maker-3-augustus-gene-415.62-mRNA-1	3	OsR498G0306859100.01.T01	99,92	0,00E+00	2155	Os03g0695500	100,00	0,00E+00	2159	4	NA	Japonica	
maker-3-augustus-gene-416.30-mRNA-1	3	OsR498G0306860100.01.T02	92,80	0,00E+00	1151	Os03g0696000	93,00	0,00E+00	1127	-24	NA	Japonica	
maker-3-augustus-gene-416.32-mRNA-1	3	OsR498G0306861700.01.T08	99,87	0,00E+00	1397	Os03g0696300	100,00	0,00E+00	1402	5	NA	Japonica	
maker-3-augustus-gene-416.37-mRNA-1	3	OsR498G0306859400.01.T01	100,00	0,00E+00	1153	Os03g0695600	100,00	0,00E+00	1153	0	NA	Japonica	
maker-3-augustus-gene-417.37-mRNA-1	3	OsR498G0306867700.01.T06	100,00	0,00E+00	704	Os03g0698500	100,00	0,00E+00	704	0	NA	Japonica	
maker-3-augustus-gene-417.41-mRNA-1	3	OsR498G0306870300.01.T01	99,65	0,00E+00	2039	Os03g0699200	99,83	0,00E+00	2048	9	NA	Japonica	
maker-3-augustus-gene-417.47-mRNA-1	3	OsR498G0306870500.01.T02	99,93	0,00E+00	2647	Os03g0699300	100,00	0,00E+00	2652	5	NA	Japonica	
maker-3-augustus-gene-417.50-mRNA-1	3	OsR498G0306870900.01.T01	99,70	0,00E+00	1198	Os03g0699600	99,85	0,00E+00	1203	5	NA	Japonica	
maker-3-augustus-gene-417.52-mRNA-1	3	OsR498G0306871300.01.T01	99,88	0,00E+00	4662	Os03g0699700	100,00	0,00E+00	4675	13	NA	Japonica	
maker-3-augustus-gene-417.56-mRNA-1	3	OsR498G0306867200.01.T01	99,94	0,00E+00	2869	Os03g0698350	100,00	0,00E+00	2307	-562	Indica	Japonica	
maker-3-augustus-gene-417.65-mRNA-1	3	OsR498G0306870800.01.T01	100,00	0,00E+00	948	Os03g0699400	100,00	0,00E+00	948	0	NA	Japonica	
maker-3-augustus-gene-418.39-mRNA-1	3	OsR498G0306873300.01.T03	99,89	0,00E+00	4678	Os03g0700400	100,00	0,00E+00	4691	13	NA	Japonica	
maker-3-augustus-gene-418.42-mRNA-1	3	OsR498G0306875300.01.T02	99,77	0,00E+00	4724	Os03g0700700	100,00	0,00E+00	4264	-460	NA	Japonica	
maker-3-augustus-gene-418.47-mRNA-1	3	OsR498G0306875700.01.T02	98,51	0,00E+00	6219	Os03g0700800	98,79	0,00E+00	6264	45	NA	Japonica	
maker-3-augustus-gene-418.52-mRNA-1	3	OsR498G0306876300.01.T02	98,62	0,00E+00	5582	Os03g0701000	98,38	0,00E+00	3108	-2474	Indica	Japonica	
maker-3-augustus-gene-418.57-mRNA-1	3	OsR498G0306876900.01.T01	99,52	0,00E+00	1481	Os03g0701200	100,00	0,00E+00	1499	18	NA	Japonica	
maker-3-augustus-gene-418.62-mRNA-1	3	OsR498G0306877500.01.T02	98,55	1,10E-169	597	Os03g0701300	86,92	2,96E-127	454	-143	Indica	Japonica	
maker-3-augustus-gene-419.0-mRNA-1	3	OsR498G0306880300.01.T07	99,74	0,00E+00	2054	Os03g0701800	99,91	0,00E+00	2063	9	NA	Japonica	
maker-3-augustus-gene-419.11-mRNA-1	3	OsR498G0306879000.01.T01	99,62	0,00E+00	1416	Os03g0701600	98,11	0,00E+00	1370	-46	NA	Japonica	
maker-3-augustus-gene-419.15-mRNA-1	3	OsR498G0306879800.01.T01	99,90	0,00E+00	3438	Os03g0701700	100,00	0,00E+00	3447	9	NA	Japonica	
maker-3-augustus-gene-419.25-mRNA-1	3	OsR498G0306881400.01.T01	99,86	0,00E+00	2497	Os03g0702000	100,00	0,00E+00	2506	9	NA	Japonica	
maker-3-augustus-gene-419.27-mRNA-1	3	OsR498G0306884400.01.T02	96,10	0,00E+00	1701	Os03g0703000	96,49	0,00E+00	1859	158	NA	Japonica	
maker-3-augustus-gene-419.34-mRNA-1	3	OsR498G0306884800.01.T02	97,75	0,00E+00	2902	Os03g0703100	97,87	0,00E+00	2911	9	NA	Japonica	
maker-3-augustus-gene-419.41-mRNA-1	3	OsR498G0306885000.01.T01	97,61	0,00E+00	2831	Os03g0703200	100,00	0,00E+00	2401	-430	Indica	Japonica	Kinase-LRR-TM
maker-3-augustus-gene-42.40-mRNA-1	3	OsR498G0305343300.01.T06	99,69	0,00E+00	1144	Os03g0184100	100,00	0,00E+00	1153	9	NA	Japonica	
maker-3-augustus-gene-42.46-mRNA-1	3	OsR498G0305344900.01.T01	99,51	0,00E+00	3286	Os03g0184300	100,00	0,00E+00	2711	-575	Indica	Japonica	
maker-3-augustus-gene-42.49-mRNA-1	3	OsR498G0305345100.01.T01	98,76	0,00E+00	2685	Os03g0184400	100,00	0,00E+00	2769	84	NA	Japonica	LRR-TM
maker-3-augustus-gene-42.64-mRNA-1	3	OsR498G0305349500.01.T01	98,69	0,00E+00	1608	Os03g0186100	98,91	0,00E+00	1618	10	NA	Japonica	
maker-3-augustus-gene-42.71-mRNA-1	3	OsR498G0305350300.01.T01	99,67	0,00E+00	7028	Os03g0186500	100,00	0,00E+00	7083	55	NA	Japonica	
maker-3-augustus-gene-42.73-mRNA-1	3	OsR498G0305345400.01.T01	100,00	0,00E+00	1288	Os03g0184500	100,00	0,00E+00	1288	0	NA	Japonica	
maker-3-augustus-gene-42.75-mRNA-1	3	OsR498G0305346200.01.T01	99,63	0,00E+00	1432	Os03g0184700	99,88	0,00E+00	1441	9	NA	Japonica	
maker-3-augustus-gene-420.0-mRNA-1	3	OsR498G0306887100.01.T01	99,76	0,00E+00	1496	Os03g0703900	100,00	0,00E+00	1505	9	NA	Japonica	
maker-3-augustus-gene-420.15-mRNA-1	3	OsR498G0306885200.01.T01	99,88	0,00E+00	2924	Os03g0703300	100,00	0,00E+00	2933	9	NA	Japonica	
maker-3-augustus-gene-420.19-mRNA-1	3	OsR498G0306885800.01.T03	99,80	0,00E+00	3525	Os03g0703400	100,00	0,00E+00	3544	19	NA	Japonica	Kinase-TM
maker-3-augustus-gene-420.3-mRNA-1	3	OsR498G0306887300.01.T02	99,48	0,00E+00	690	Os03g0704000	88,01	1,88E-160	563	-127	Indica	Japonica	
maker-3-augustus-gene-420.5-mRNA-1	3	OsR498G0306887500.01.T02	99,65	0,00E+00	1031	Os03g0704100	99,83	0,00E+00	1035	4	NA	Japonica	
maker-3-augustus-gene-420.9-mRNA-1	3	OsR498G0306887900.01.T01	99,66	0,00E+00	3654	Os03g0704400	99,93	0,00E+00	2402	-1252	Indica	Japonica	Kinase-TM
maker-3-augustus-gene-421.24-mRNA-1	3	OsR498G0306890800.01.T01	99,95	0,00E+00	3399	Os03g0705300	100,00	0,00E+00	3404	5	NA	Japonica	
maker-3-augustus-gene-421.29-mRNA-1	3	OsR498G0306899000.01.T03	99,83	0,00E+00	1062	Os03g0706900	100,00	0,00E+00	1067	5	NA	Japonica	
maker-3-augustus-gene-422.1-mRNA-1	3	OsR498G0306899500.01.T03	99,76	0,00E+00	2238	Os03g0707200	100,00	0,00E+00	1335	-903	Indica	Japonica	
maker-3-augustus-gene-422.15-mRNA-1	3	OsR498G0306903200.01.T01	99,94	0,00E+00	3172	Os03g0707900	100,00	0,00E+00	3177	5	NA	Japonica	
maker-3-augustus-gene-422.6-mRNA-1	3	OsR498G1120323400.01.T01	73,45	5,04E-54	212	Os03g0708000	100,00	0,00E+00	888	676	Japonica	Japonica	
maker-3-augustus-gene-422.9-mRNA-1	3	OsR498G0306899800.01.T01	96,94	0,00E+00	2818	Os03g0707300	97,12	0,00E+00	2831	13	NA	Japonica	
maker-3-augustus-gene-423.0-mRNA-1	3	OsR498G0306904700.01.T01	99,89	0,00E+00	1576	Os03g0708400	100,00	0,00E+00	1581	5	NA	Japonica	
maker-3-augustus-gene-423.10-mRNA-1	3	OsR498G0306910500.01.T02	99,72	0,00E+00	1913	Os03g0710000	100,00	0,00E+00	1927	14	NA	Japonica	
maker-3-augustus-gene-423.13-mRNA-1	3	OsR498G0306911100.01.T01	98,19	0,00E+00	2664	Os03g0710500	100,00	0,00E+00	3626	962	Japonica	Japonica	
maker-3-augustus-gene-423.16-mRNA-1	3	OsR498G0306905000.01.T02	98,68	0,00E+00	1328	Os03g0708500	100,00	0,00E+00	1370	42	NA	Japonica	
maker-3-augustus-gene-423.18-mRNA-1	3	OsR498G0306905400.01.T01	99,66	0,00E+00	4168	Os03g0708700	100,00	0,00E+00	5184	1016	Japonica	Japonica	
maker-3-augustus-gene-423.20-mRNA-1	3	OsR498G0306907200.01.T01	99,55	0,00E+00	798	Os03g0709000	100,00	0,00E+00	807	9	NA	Japonica	
maker-3-augustus-gene-423.23-mRNA-1	3	OsR498G0306907600.01.T01	99,24	0,00E+00	701	Os03g0709100	100,00	0,00E+00	715	14	NA	Japonica	
maker-3-augustus-gene-423.25-mRNA-1	3	OsR498G0306908200.01.T01	100,00	0,00E+00	704	Os03g0709300	100,00	0,00E+00	704	0	NA	Japonica	
maker-3-augustus-gene-423.6-mRNA-1	3	OsR498G0306906900.01.T01	99,44	0,00E+00	1587	Os03g0708900	100,00	0,00E+00	1611	24	NA	Japonica	
maker-3-augustus-gene-424.0-mRNA-1	3	OsR498G0306912100.01.T04	99,62	0,00E+00	1399	Os03g0710800	100,00	0,00E+00	1413	14	NA	Japonica	
maker-3-augustus-gene-424.14-mRNA-1	3	OsR498G0306911600.01.T02	100,00	0,00E+00	1261	Os03g0710700	100,00	0,00E+00	1261	0	NA	Japonica	
maker-3-augustus-gene-424.2-mRNA-1	3	OsR498G0306912500.01.T01	99,95	0,00E+00	3789	Os03g0710900	100,00	0,00E+00	3507	-282	NA	Japonica	
maker-3-augustus-gene-424.23-mRNA-1	3	OsR498G0306914200.01.T02	99,84	0,00E+00	2302	Os03g0711300	100,00	0,00E+00	2311	9	NA	Japonica	Kinase-TM
maker-3-augustus-gene-424.29-mRNA-1	3	OsR498G0306914600.01.T01	99,73	0,00E+00	6551	Os03g0711400	99,97	0,00E+00	6591	40	NA	Japonica	
maker-3-augustus-gene-424.30-mRNA-1	3	OsR498G0306915200.01.T01	99,92	0,00E+00	6582	Os03g0711400	97,57	0,00E+00	6194	-388	NA	Japonica	
maker-3-augustus-gene-424.35-mRNA-1	3	OsR498G0306916000.01.T02	99,55	0,00E+00	1201	Os03g0711650	100,00	0,00E+00	962	-239	Indica	Japonica	
maker-3-augustus-gene-424.8-mRNA-1	3	OsR498G0306913700.01.T01	99,92	0,00E+00	2279	Os03g0711100	100,00	0,00E+00	2284	5	NA	Japonica	
maker-3-augustus-gene-425.32-mRNA-1	3	OsR498G0306917300.01.T01	99,55	0,00E+00	1593	Os03g0712100	100,00	0,00E+00	1613	20	NA	Japonica	
maker-3-augustus-gene-425.35-mRNA-1	3	OsR498G0306917500.01.T01	99,62	0,00E+00	2807	Os03g0712200	100,00	0,00E+00	2836	29	NA	Japonica	

maker-3-augustus-gene-425.39-mRNA-1	3	OsR498G0306917700.01.T01	100,00	0,00E+00	809	Os03g0712300	100,00	0,00E+00	809	0	NA	Japonica	
maker-3-augustus-gene-425.42-mRNA-1	3	OsR498G0306919300.01.T01	99,94	0,00E+00	3150	Os03g0712700	100,00	0,00E+00	3155	5	NA	Japonica	
maker-3-augustus-gene-425.55-mRNA-1	3	OsR498G0306922100.01.T01	99,95	0,00E+00	3307	Os10g0567800	73,44	0,00E+00	1085	-2222	Indica	Japonica	
maker-3-augustus-gene-425.62-mRNA-1	3	OsR498G0306916400.01.T04	99,47	0,00E+00	3988	Os03g0711800	100,00	0,00E+00	4040	52	NA	Japonica	Kinase-TM
maker-3-augustus-gene-425.69-mRNA-1	3	OsR498G0306919200.01.T01	99,77	0,00E+00	788	Os03g0712600	100,00	0,00E+00	792	4	NA	Japonica	
maker-3-augustus-gene-425.72-mRNA-1	3	OsR498G0306920200.01.T02	99,91	0,00E+00	2003	Os03g0712800	100,00	0,00E+00	2008	5	NA	Japonica	
maker-3-augustus-gene-425.77-mRNA-1	3	OsR498G0306922400.01.T01	99,49	0,00E+00	1042	Os03g0713200	100,00	0,00E+00	926	-116	Indica	Japonica	
maker-3-augustus-gene-426.35-mRNA-1	3	OsR498G0306924300.01.T04	97,42	0,00E+00	1045	Os03g0713700	100,00	0,00E+00	1122	77	NA	Japonica	
maker-3-augustus-gene-426.40-mRNA-1	3	OsR498G0306926100.01.T01	99,88	0,00E+00	1441	Os03g0337700	98,11	0,00E+00	1365	-76	NA	Japonica	
maker-3-augustus-gene-426.48-mRNA-1	3	OsR498G0306922600.01.T01	99,96	0,00E+00	4043	Os03g0713400	99,96	0,00E+00	4043	0	NA	Japonica	
maker-3-augustus-gene-426.50-mRNA-1	3	OsR498G0306924000.01.T02	100,00	0,00E+00	1543	Os03g0713600	100,00	0,00E+00	1543	0	NA	Japonica	PIBP1
maker-3-augustus-gene-427.32-mRNA-1	3	OsR498G0306930300.01.T05	99,67	0,00E+00	1619	Os03g0715400	99,89	0,00E+00	1628	9	NA	Japonica	
maker-3-augustus-gene-427.43-mRNA-1	3	OsR498G0306935300.01.T01	99,85	0,00E+00	1235	Os03g0717200	100,00	0,00E+00	1240	5	NA	Japonica	
maker-3-augustus-gene-427.51-mRNA-1	3	OsR498G0306931400.01.T01	99,33	0,00E+00	3718	Os03g0716200	100,00	0,00E+00	3519	-199	NA	Japonica	
maker-3-augustus-gene-427.53-mRNA-1	3	OsR498G0306934600.01.T01	99,65	0,00E+00	5166	Os03g0717000	100,00	0,00E+00	4363	-803	Indica	Japonica	Kinase-LRR-TM
maker-3-augustus-gene-427.55-mRNA-1	3	OsR498G0306936200.01.T01	99,74	0,00E+00	2731	Os03g0717600	100,00	0,00E+00	2749	18	NA	Japonica	
maker-3-augustus-gene-428.14-mRNA-1	3	OsR498G0306937800.01.T02	100,00	0,00E+00	1483	Os03g0718000	100,00	0,00E+00	1483	0	NA	Japonica	
maker-3-augustus-gene-428.24-mRNA-1	3	OsR498G0306942400.01.T01	99,26	0,00E+00	3358	Os03g0719000	100,00	0,00E+00	2560	-798	Indica	Japonica	
maker-3-augustus-gene-428.29-mRNA-1	3	OsR498G0306943600.01.T02	99,78	0,00E+00	3202	Os03g0719300	100,00	0,00E+00	3220	18	NA	Japonica	
maker-3-augustus-gene-428.4-mRNA-1	3	OsR498G0306937500.01.T01	99,85	0,00E+00	2459	Os03g0717900	100,00	0,00E+00	2468	9	NA	Japonica	
maker-3-augustus-gene-428.6-mRNA-1	3	OsR498G0306938500.01.T02	100,00	0,00E+00	2046	Os03g0718100	100,00	0,00E+00	2046	0	NA	NA	
maker-3-augustus-gene-429.30-mRNA-1	3	OsR498G0306946900.01.T01	98,60	0,00E+00	2617	Os03g0720300	98,80	0,00E+00	2631	14	NA	Japonica	
maker-3-augustus-gene-429.34-mRNA-1	3	OsR498G0306953100.01.T01	99,86	0,00E+00	1327	Os03g0720800	100,00	0,00E+00	1332	5	NA	Japonica	
maker-3-augustus-gene-429.36-mRNA-1	3	OsR498G0306943800.01.T01	99,49	0,00E+00	2101	Os03g0719400	100,00	0,00E+00	2080	-21	NA	Japonica	
maker-3-augustus-gene-429.44-mRNA-1	3	OsR498G0306945000.01.T01	99,94	0,00E+00	6100	Os03g0719800	100,00	0,00E+00	6109	9	NA	Japonica	
maker-3-augustus-gene-429.46-mRNA-1	3	OsR498G0306945400.01.T01	99,81	0,00E+00	1918	Os03g0719850	100,00	0,00E+00	1927	9	NA	Japonica	Kinase-TM
maker-3-augustus-gene-429.48-mRNA-1	3	OsR498G0306945600.01.T01	99,94	0,00E+00	3210	Os03g0719900	100,00	0,00E+00	3214	4	NA	Japonica	
maker-3-augustus-gene-43.0-mRNA-1	3	OsR498G0305351100.01.T01	99,56	0,00E+00	1215	Os03g0186800	100,00	0,00E+00	1222	7	NA	Japonica	
maker-3-augustus-gene-43.10-mRNA-1	3	OsR498G0305353100.01.T01	99,82	0,00E+00	4084	Os03g0187300	100,00	0,00E+00	2084	-2000	Indica	Japonica	
maker-3-augustus-gene-43.15-mRNA-1	3	OsR498G0305354100.01.T01	99,83	0,00E+00	1084	Os03g0187400	100,00	0,00E+00	1088	4	NA	Japonica	
maker-3-augustus-gene-43.17-mRNA-1	3	OsR498G0305356500.01.T01	99,75	0,00E+00	1425	Os03g0187600	99,87	0,00E+00	1430	5	NA	Japonica	
maker-3-augustus-gene-43.21-mRNA-1	3	OsR498G0305358500.01.T01	97,70	0,00E+00	2496	Os08g0480000	73,00	0,00E+00	755	-1741	Indica	Japonica	
maker-3-augustus-gene-43.24-mRNA-1	3	OsR498G0305350600.01.T02	99,73	0,00E+00	1350	Os03g0186600	99,20	0,00E+00	1333	-17	NA	Japonica	
maker-3-augustus-gene-43.3-mRNA-1	3	OsR498G0305352100.01.T01	100,00	0,00E+00	1261	Os03g0187000	100,00	0,00E+00	1261	0	NA	Japonica	
maker-3-augustus-gene-43.31-mRNA-1	3	OsR498G0305352000.01.T02	96,14	0,00E+00	684	Os03g0186950	100,00	0,00E+00	639	-45	NA	Japonica	
maker-3-augustus-gene-430.39-mRNA-1	3	OsR498G0306955700.01.T01	100,00	0,00E+00	747	Os03g0721900	100,00	0,00E+00	747	0	NA	Japonica	
maker-3-augustus-gene-430.51-mRNA-1	3	OsR498G0306954600.01.T01	99,48	0,00E+00	3082	Os03g0721300	100,00	0,00E+00	3122	40	NA	Japonica	
maker-3-augustus-gene-430.55-mRNA-1	3	OsR498G0306957400.01.T01	99,93	0,00E+00	2663	Os03g0722600	99,90	0,00E+00	1850	-813	Indica	Japonica	
maker-3-augustus-gene-430.60-mRNA-1	3	OsR498G0306958200.01.T03	99,93	0,00E+00	2501	Os03g0722800	100,00	0,00E+00	2533	32	NA	Japonica	
maker-3-augustus-gene-431.32-mRNA-1	3	OsR498G0306960100.01.T01	99,23	0,00E+00	924	Os03g0723400	100,00	0,00E+00	942	18	NA	Japonica	
maker-3-augustus-gene-431.35-mRNA-1	3	OsR498G0306960300.01.T01	99,86	0,00E+00	2583	Os03g0723600	100,00	0,00E+00	2592	9	NA	Japonica	
maker-3-augustus-gene-431.37-mRNA-1	3	OsR498G0306963100.01.T02	99,89	0,00E+00	1619	Os03g0724100	100,00	0,00E+00	1624	5	NA	Japonica	
maker-3-augustus-gene-432.45-mRNA-1	3	OsR498G0306965800.01.T01	81,37	0,00E+00	890	Os03g0724900	99,88	0,00E+00	1527	637	Japonica	Japonica	
maker-3-augustus-gene-432.47-mRNA-1	3	OsR498G0306966300.01.T01	99,74	0,00E+00	6994	Os03g0725100	100,00	0,00E+00	3582	-3412	Indica	Japonica	
maker-3-augustus-gene-432.60-mRNA-1	3	OsR498G0306967100.01.T04	99,08	0,00E+00	1722	Os03g0725400	100,00	0,00E+00	1759	37	NA	Japonica	
maker-3-augustus-gene-432.62-mRNA-1	3	OsR498G0306969900.01.T01	99,45	0,00E+00	1925	Os03g0725800	100,00	0,00E+00	1948	23	NA	Japonica	
maker-3-augustus-gene-432.65-mRNA-1	3	OsR498G0306970700.01.T02	98,96	0,00E+00	2538	Os03g0726100	100,00	0,00E+00	2280	-258	Indica	Japonica	
maker-3-augustus-gene-432.69-mRNA-1	3	OsR498G0306971100.01.T01	99,39	0,00E+00	1737	Os03g0726200	99,59	0,00E+00	1746	9	NA	Japonica	
maker-3-augustus-gene-432.74-mRNA-1	3	OsR498G0306965200.01.T01	100,00	4,19E-147	520	Os03g0724500	100,00	1,46E-147	520	0	NA	Japonica	
maker-3-augustus-gene-432.76-mRNA-1	3	OsR498G0306965400.01.T01	100,00	1,03E-174	612	Os03g0724600	100,00	6,49E-172	601	-11	NA	Japonica	
maker-3-augustus-gene-432.78-mRNA-1	3	OsR498G0306965800.01.T01	99,44	0,00E+00	2200	Os03g0724800	99,92	0,00E+00	2231	31	NA	Japonica	
maker-3-augustus-gene-433.26-mRNA-1	3	OsR498G0306976100.01.T01	100,00	0,00E+00	1153	Os03g0727100	100,00	0,00E+00	1153	0	NA	Japonica	
maker-3-augustus-gene-433.29-mRNA-1	3	OsR498G0306972000.01.T01	99,78	0,00E+00	2395	Os03g0726300	100,00	0,00E+00	2408	13	NA	Japonica	
maker-3-augustus-gene-433.31-mRNA-1	3	OsR498G0306973000.01.T01	99,86	0,00E+00	2567	Os03g0726500	100,00	0,00E+00	2576	9	NA	Japonica	
maker-3-augustus-gene-433.38-mRNA-1	3	OsR498G0306974800.01.T02	100,00	0,00E+00	1463	Os03g0726800	100,00	0,00E+00	1463	0	NA	Japonica	
maker-3-augustus-gene-433.42-mRNA-1	3	OsR498G0306975200.01.T03	90,39	0,00E+00	662	Os03g0726900	90,29	0,00E+00	759	97	Japonica	Japonica	
maker-3-augustus-gene-433.49-mRNA-1	3	OsR498G0306975400.01.T02	100,00	0,00E+00	1281	Os03g0727000	100,00	0,00E+00	1061	-220	Indica	Japonica	
maker-3-augustus-gene-434.17-mRNA-1	3	OsR498G0306979600.01.T02	99,85	0,00E+00	1187	Os03g0728600	100,00	0,00E+00	758	-429	Indica	Japonica	
maker-3-augustus-gene-434.3-mRNA-1	3	OsR498G0306977200.01.T01	99,93	0,00E+00	2636	Os03g0727600	100,00	0,00E+00	2641	5	NA	Japonica	
maker-3-augustus-gene-434.5-mRNA-1	3	OsR498G0306979000.01.T01	99,84	0,00E+00	1140	Os03g0727900	100,00	0,00E+00	1144	4	NA	Japonica	
maker-3-augustus-gene-434.6-mRNA-1	3	OsR498G0306979000.01.T02	99,69	0,00E+00	1148	Os03g0727900	100,00	0,00E+00	1153	5	NA	Japonica	
maker-3-augustus-gene-435.0-mRNA-1	3	OsR498G0306980100.01.T02	95,79	0,00E+00	1838	Os03g0728800	95,97	0,00E+00	1847	9	NA	Japonica	
maker-3-augustus-gene-435.13-mRNA-1	3	OsR498G0306986100.01.T02	97,55	0,00E+00	1952	Os03g0729800	100,00	0,00E+00	2987	1035	Japonica	Japonica	
maker-3-augustus-gene-435.16-mRNA-1	3	OsR498G0306980400.01.T03	99,80	0,00E+00	1766	Os03g0728900	100,00	0,00E+00	1775	9	NA	Japonica	
maker-3-augustus-gene-435.20-mRNA-1	3	OsR498G0306981600.01.T01	99,83	0,00E+00	3163	Os03g0729000	100,00	0,00E+00	3177	14	NA	Japonica	
maker-3-augustus-gene-435.26-mRNA-1	3	OsR498G0306982400.01.T04	99,74	0,00E+00	1393	Os03g0729200	100,00	0,00E+00	1402	9	NA	Japonica	
maker-3-augustus-gene-435.30-mRNA-1	3	OsR498G0306982600.01.T01	99,62	0,00E+00	1394	Os03g0729300	100,00	0,00E+00	1407	13	NA	Japonica	
maker-3-augustus-gene-435.37-mRNA-1	3	OsR498G0306986800.01.T01	99,86	0,00E+00	2513	Os03g0730000	100,00	0,00E+00	2522	9	NA	Japonica	
maker-3-augustus-gene-435.6-mRNA-1	3	OsR498G0306985100.01.T01	99,67	0,00E+00	3241	Os03g0729500	100,00	0,00E+00	2473	-768	Indica	Japonica	
maker-3-augustus-gene-435.9-mRNA-1	3	OsR498G0306985700.01.T01	95,94	0,00E+00	1507	Os03g0729700	95,57	0,00E+00	1403	-104	NA	Japonica	
maker-3-augustus-gene-436.11-mRNA-1	3	OsR498G0306989100.01.T01	99,11	0,00E+00	2589	Os03g0730500	76,08	0,00E+00	820	-1769	Indica	Japonica	
maker-3-augustus-gene-436.13-mRNA-1	3	OsR498G0306989900.01.T01	99,92	0,00E+00	2155	Os03g0731050	100,00	0,00E+00	1305	-850	Indica	Japonica	
maker-3-augustus-gene-436.15-mRNA-1	3	OsR498G0306990100.01.T02	99,49	0,00E+00	695	Os03g0731100	100,00	0,00E+00	677	-18	NA	Japonica	
maker-3-augustus-gene-436.17-mRNA-1	3	OsR498G0306990900.01.T01	99,84	0,00E+00	4560	Os03g0731500	100,00	0,00E+00	4578	18	NA	Japonica	
maker-3-augustus-gene-436.26-mRNA-1	3	OsR498G0306989600.01.T02	99,91	0,00E+00	3793	Os03g0730800	99,94	0,00E+00	2950	-843	Indica	Japonica	
maker-3-augustus-gene-436.4-mRNA-1	3	OsR498G0306988300.01.T02	96,93	0,00E+00	1845	Os03g0730500	98,19	0,00E+00	1902	57	NA	Japonica	
maker-3-augustus-gene-437.0-mRNA-1	3	OsR498G0306991100.01.T01	99,77	0,00E+00	2284	Os03g0731800	100,00	0,00E+00	1905	-379	Indica	Japonica	
maker-3-augustus-gene-437.10-mRNA-1	3	OsR498G0306991800.01.T01	99,67	0,00E+00	2179	Os03g0732000	100,00	0,00E+00	2614	435	Japonica	Japonica	
maker-3-augustus-gene-437.25-mRNA-1	3	OsR498G0101678500.01.T03	86,50	1,71E-49	196	Os03g0733000	100,00	1,01E-116	417	221	Japonica	Japonica	
maker-3-augustus-gene-437.5-mRNA-1	3	OsR498G0306991400.01.T01	99,86	0,00E+00	2480	Os03g0731900	100,00	0,00E+00	2489	9	NA	Japonica	
maker-3-augustus-gene-438.11-mRNA-1	3	OsR498G0306998000.01.T01	97,98	0,00E+00	1189	Os03g0733600	100,00	0,00E+00	1234	45	NA	Japonica	
maker-3-augustus-gene-438.23-mRNA-1	3	OsR498G0307002400.01.T02	100,00	0,00E+00	1472	Os03g0734900	100,00	0,00E+00	1472	0	NA	Japonica	
maker-3-augustus-gene-438.33-mRNA-1	3	OsR498G0307003400.01.T01	94,88	0,00E+00	1514	Os03g0735300	94,99	0,00E+00	1518	4	NA	Japonica	
maker-3-augustus-gene-438.4-mRNA-1	3	OsR498G0307000500.01.T02	73,77	9,58E-35	148	Os03g0734200	100,00	1,09E-142	504	356	Japonica	Japonica	
maker-3-augustus-gene-439.40-mRNA-1	3	OsR498G0307004900.01.T01	99,85	0,00E+00	1230	Os04g0413200	76,89	0,00E+00	737	-493	Indica	Japonica	
maker-3-augustus-gene-439.42-mRNA-1	3	OsR498G0307009500.01.T05	99,90	0,00E+00	1787	Os03g0737600	100,00	0,00E+00	1792	5	NA	Japonica	
maker-3-augustus-gene-439.46-mRNA-1	3	OsR498G0307005200.01.T01	99,03	0,00E+00	2726	Os03g0735900	100,00	0,00E+00	2787	61	NA	Japonica	
maker-3-augustus-gene-439.48-mRNA-1	3	OsR498G0307005800.01.T04	100,00	4,80E-115	414	Os03g0736000	100,00	1,67E-115	414	0	NA	Japonica	
maker-3-augustus-gene-439.49-mRNA-1	3	OsR498G0307005800.01.T06	99,94	0,00E+00	2939	Os02g0782200	93,23	0,00E+00	2453	-486	Indica	Japonica	
maker-3-augustus-gene-439.62-mRNA-1	3	OsR498G0307006400.01.T02	99,92	0,00E+00	2314	Os03g0736300	100,00	0,00E+00	2318	4	NA	Japonica	
maker-3-augustus-gene-439.67-mRNA-1	3	OsR498G0307007000.01.T03	100,00	0,00E+00	942	Os03g0736400	95,40	0,00E+00	851	-91	NA	Japonica	
maker-3-augustus-gene-439.71-mRNA-1	3	OsR498G0307008400.01.T01	100,00	0,00E+00	1209	Os03g0737000	100,00	0,00E+00	1115	-94	NA	Japonica	
maker-3-augustus-gene-439.85-mRNA-1	3	OsR498G0307008800.01.T01	99,45	0,00E+00	972	Os03g0737300	100,00	0,00E+00	985	13	NA	Japonica	
maker-3-augustus-gene-439.87-mRNA-1	3	OsR498G0307009400.01.T01	100,00	0,00E+00	746	Os03g0737400	100,00	0,00E+00	746	0	NA	Japonica	
maker-3-augustus-gene-44.36-mRNA-1	3	OsR498G0305360300.01.T01	99,80	0,00E+00	1777	Os03g0188400	100,00	0,00E+00	1786	9	NA	Japonica	
maker-3-augustus-gene-44.40-mRNA-1	3	OsR498G0305364400.01.T01	94,49	0,00E+00	1482	Os03g0189400	94,49	0,00E+00	1482	0	NA	Japonica	
maker-3-augustus-gene-44.44-mRNA-1	3	OsR498G0305364800.01.T03	93,84	0,00E+00	1044	Os03g0189600	94,41	0,00E+00	1057	13	NA	Japonica	
maker-3-augustus-gene-44.48-mRNA-1	3	OsR498G0305366400.01.T02	99,82	0,00E+00	1994	Os03g0190100	100,00	0,00E+00	2003	9	NA	Japonica	
maker-3-augustus-gene-44.53-mRNA-1	3	OsR498G0305366800.01.T02	99,79	0,00E+00	3364	Os03g0190300	99,73	0,00E+00	1967	-1397	Indica	Japonica	LRR-TM
maker-3-augustus-gene-440.0-mRNA-1	3	OsR498G0307010100.01.T02	99,82	0,00E+00	2997	Os03g0737800	99,42	0,00E+00	2752	-245	NA	Japonica	
maker-3-augustus-gene-440.24-mRNA-1	3	OsR498G0307011500.01.T02	99,94	0,00E+00	2777	Os03g0738400	100,00	0,00E+00	2782	5	NA	Japonica	
maker-3-augustus-gene-440.27-mRNA-1	3	OsR498G0307013100.01.T01	99,89	0,00E+00	4699	Os03g0738600	100,00	0,00E+00	4713	14	NA	Japonica	
maker-3-augustus-gene-440.29-mRNA-1	3	OsR498G0307014300.01.T01	99,60	0,00E+00	3577	Os03g0738900	100,00	0,00E+00	3615	38	NA	Japonica	
maker-3-augustus-gene-440.7-mRNA-1	3	OsR498G0307011100.01.T01	99,90	0,00E+00	1729	Os03g0738000	100,00	0,00E+00	1734	5	NA	Japonica	
maker-3-augustus-gene-441.0-mRNA-1	3	OsR498G0307016500.01.T01	99,81	0,00E+00	1858	Os03g0739700	100,00	0,00E+00	1867	9	NA	Japonica	
maker-3-augustus-gene-441.12-mRNA-1	3	OsR498G0307020100.01.T01	98,44	0,00E+00	1567	Os03g0741100	100,00	0,00E+00	1624	57	NA	Japonica	
maker-3-augustus-gene-441.15-mRNA-1	3	OsR498G0307022300.01.T01	99,57	0,00E+00	2077	Os03g0741400	100,00	0,00E+00	2100	23	NA	Japonica	
maker-3-augustus-gene-441.3-mRNA-1	3	OsR498G0307018900.01.T04	99,57	0,00E+00	1250	Os03g0740600	100,00	0,00E+00	1263	13	NA	Japonica	
maker-3-augustus-gene-441.6-mRNA-1	3	OsR498G0307019100.01.T01	98,64	0,00E+00	3987	Os03g0740700	99,04	0,00E+00	4028	41	NA	Japonica	
maker-3-augustus-gene-441.9-mRNA-1	3	OsR498G0307019700.01.T01	100,00	0,00E+00	1489	Os03g0740800	100,00	0,00E+00	1489	0	NA	Japonica	
maker-3-augustus-gene-442.0-mRNA-1	3	OsR498G0307024500.01.T01	99,53	2,47E-105	382	Os03g0742300	99,53	8,64E-106	382	0	NA	Japonica	
maker-3-augustus-gene-442.19-mRNA-1	3	OsR498G0307024800.01.T03	96,54	0,00E+00	1929	Os03g0742400	100,00	0,00E+00	1353	-576	Indica	Japonica	
maker-3-augustus-gene-442.2-mRNA-1	3	OsR498G0307026300.01.T02	99,74	0,00E+00	4075	Os03g0742800	100,00	0,00E+00	4102	27	NA	Japonica	
maker-3-augustus-gene-442.27-mRNA-1	3	OsR498G0307026600.01.T01	100,00	0,00E+00	1283	Os03g0742900	100,00	0,00E+00	1283	0	NA	Japonica	
maker-3-augustus-gene-442.29-mRNA-1	3	OsR498G0307030400.01.T01	99,86	0,00E+00	2578	Os03g0743900	100,00	0,00E+00	2587	9	NA	Japonica	
maker-3-augustus-gene-442.6-mRNA-1	3	OsR498G0307029300.01.T04	99,76	0,00E+00	743	Os03g0743400	100,00	0,00E+00	747	4	NA	Japonica	
maker-3-augustus-gene-442.8-mRNA-1	3	OsR498G0307029700.01.T02	99,80	0,00E+00	889	Os03g0743500	100,00	0,00E+00	839	-50	NA	Japonica	
maker-3-augustus-gene-443.0-mRNA-1	3	OsR498G0307033700.01.T05	99,48	0,00E+00	1017	Os03g0744700	100,00	0,00E+00	942	-75	NA	Japonica	
maker-3-augustus-gene-443.11-mRNA-1	3	OsR498G0307032000.01.T01	99,47	0,00E+00	1346	Os03g0744600	100,00	0,00E+00	1364	18	NA	Japonica	
maker-3-augustus-gene-443.15-mRNA-1	3	OsR498G0307032200.01.T02	99,89	0,00E+00	14133	Os03g0744650	99,53	0,00E+00	15823	1690	Japonica	Japonica	
maker-3-augustus-gene-443.20-mRNA-1	3	OsR498G0307034000.01.T01	99,68	0,00E+00	1133	Os03g0744800	100,00	0,00E+00	1142	9	NA	Japonica	
maker-3-augustus-gene-443.23-mRNA-1	3	OsR498G0307034400.01.T01	98,32	0,00E+00	1962	Os03g0745000	100,00	0,00E+00	2040	78	NA	Japonica	
maker-3-augustus-gene-443.25-mRNA-1	3	OsR498G0307035000.01.T01	99,86	0,00E+00	2551	Os03g0745200	100,00	0,00E+00	2527	-24	NA	Japonica	
maker-3-augustus-gene-443.6-mRNA-1	3	OsR498G0307037100.01.T01	100,00	0,00E+00	1353	Os03g0745600	100,00	0,00E+00	1353	0	NA	NA	
maker-3-augustus-gene-444.40-mRNA-1	3	OsR498G0307037500.01.T01	100,00	0,00E+00	2208	Os03g0745700	100,00	0,00E+00	1193	-1015	Indica	NA	Kinase-TM
maker-3-augustus-gene-444.43-mRNA-1	3	OsR498G0307041100.01.T01	99,79	0,00E+00	2566	Os03g0746600	100,00	0,00E+00	2580	14	NA	Japonica	
maker-3-augustus-gene-444.48-mRNA-1	3	OsR498G0307043300.01.T01	100,00	0,00E+00	1532	Os03g0747300	100,00	0,00E+00	1532	0	NA	Japonica	
maker-3-augustus-gene-444.52-mRNA-1	3	OsR498G0307041000.01.T04	99,85	0,00E+00	1159	Os03g0746500	100,00	0,00E+00	1164	5	NA	Japonica	
maker-3-augustus-gene-444.59-mRNA-1	3	OsR498G0307041800.01.T01	99,77	0,00E+00	2346	Os03g0746800	100,00	0,00E+00	2360	14	NA	Japonica	
maker-3-augustus-gene-445.47-mRNA-1	3	OsR498G0307045300.01.T03	100,00	0,00E+00	1764	Os03g0747800	100,00	0,00E+00	1764	0	NA	Japonica	
maker-3-augustus-gene-445.53-mRNA-1	3	OsR498G0307045500.01.T01	99,86	0,00E+00	6624	Os03g0747900	100,00	0,00E+00	6646	22	NA	Japonica	
maker-3-augustus-gene-445.65-mRNA-1	3	OsR498G0307046300.01.T04	100,00	0,00E+00	1196	Os03g0748200	100,00	0,00E+00	1196	0	NA	Japonica	
maker-3-augustus-gene-445.69-mRNA-1	3	OsR498G0307046900.01.T02	99,76	0,00E+00	3000	Os03g0748400	100,00	0,00E+00	3020	20	NA	Japonica	Kinase
maker-3-augustus-gene-445.73-mRNA-1	3	OsR498G0307047300.01.T01	99,81	0,00E+00	927	Os03g0748600	100,00	0,00E+00	931	4	NA	Japonica	
maker-3-augustus-gene-445.76-mRNA-1	3	OsR498G0307048300.01.T02	100,00	0,00E+00	1225	Os03g0748900	100,00	0,00E+00	1108	-117	NA	Japonica	
maker-3-augustus-gene-445.81-mRNA-1	3	OsR498G0307049100.01.T01	99,74	0,00E+00	3468	Os03g0749100	100,00	0,00E+00	3490	22	NA	Japonica	
maker-3-augustus-gene-445.83-mRNA-1	3	OsR498G0307043600.01.T01	99,76	0,00E+00	739	Os03g0747400	93,41	0,00E+00	1208	469	Japonica	Japonica	
maker-3-augustus-gene-445.86-mRNA-1	3	OsR498G0307044000.01.T01	99,54	0,00E+00	1150	Os03g0747500	100,00	0,00E+00	1164	14	NA	Japonica	
maker-3-augustus-gene-445.88-mRNA-1	3	OsR498G0307044800.01.T01	99,84	0,00E+00	1149	Os03g0747700	100,00	0,00E+00	1153	4	NA	Japonica	
maker-3-augustus-gene-445.90-mRNA-1	3	OsR498G0307046600.01.T01	99,03	0,00E+00	2177	Os03g0748300	100,00	0,00E+00	2230	53	NA	Japonica	
maker-3-augustus-gene-445.92-mRNA-1	3	OsR498G0307047200.01.T01	96,71	0,00E+00	1271	Os03g0748500	100,00	0,00E+00	1370	99	NA	Japonica	
maker-3-augustus-gene-445.94-mRNA-1	3	OsR498G0307047600.01.T01	99,77	0,00E+00	2292	Os03g0748700	100,00	0,00E+00	2305	13	NA	Japonica	
maker-3-augustus-gene-446.0-mRNA-1	3	OsR498G0307050300.01.T09	99,95	0,00E+00	3383	Os03g0749300	100,00	0,00E+00	3388	5	NA	Japonica	
maker-3-augustus-gene-446.13-mRNA-1	3	OsR498G0307052300.01.T01	100,00	0,00E+00	1310	Os03g0749900	100,00	0,00E+00	1310	0	NA	Japonica	
maker-3-augustus-gene-446.15-mRNA-1	3	OsR498G0307053100.01.T01	99,78	0,00E+00	5791	Os03g0750200	100,00	0,00E+00	5822	31	NA	Japonica	
maker-3-augustus-gene-446.22-mRNA-1	3	OsR498G0307052200.01.T02	99,95	0,00E+00	3670	Os03g0749800	100,00	0,00E+00	3674	4	NA	Japonica	CC-Kinase-TM
maker-3-augustus-gene-446.27-mRNA-1	3	OsR498G0307052800.01.T02	99,86	0,00E+00	1286	Os03g0750100	100,00	0,00E+00	960	-326	Indica	Japonica	
maker-3-augustus-gene-446.6-mRNA-1	3	OsR498G0307051700.01.T07	99,79	0,00E+00	2617	Os03g0749500	100,00	0,00E+00	2630	13	NA	Japonica	
maker-3-augustus-gene-447.42-mRNA-1	3	OsR498G0307054700.01.T03	99,33	0,00E+00	799	Os03g0751000	100,00	0,00E+00	655	-144	Indica	Japonica	
maker-3-augustus-gene-447.48-mRNA-1	3	OsR498G0307056700.01.T01	99,71	0,00E+00	1220	Os03g0751400	100,00	0,00E+00	1229	9	NA	Japonica	
maker-3-augustus-gene-447.52-mRNA-1	3	OsR498G0307057300.01.T03	98,33	0,00E+00	1251	Os03g0751600	100,00	0,00E+00	1279	28	NA	Japonica	
maker-3-augustus-gene-447.54-mRNA-1	3	OsR498G0307058300.01.T01	99,88	0,00E+00	6139	Os03g0752100	100,00	0,00E+00	6157	18	NA	Japonica	
maker-3-augustus-gene-447.56-mRNA-1	3	OsR498G0307058500.01.T03	99,25	0,00E+00	1181	Os03g0752200	99,85	0,00E+00	1199	18	NA	Japonica	
maker-3-augustus-gene-447.60-mRNA-1	3	OsR498G0307059100.01.T01	99,81	0,00E+00	1891	Os04g0208200	78,97	3,37E-137	489	-1402	Indica	Japonica	
maker-3-augustus-gene-447.62-mRNA-1	3	OsR498G0307059500.01.T03	98,87	0,00E+00	3259	Os03g0752700	100,00	0,00E+00	3317	58	NA	Japonica	
maker-3-augustus-gene-447.65-mRNA-1	3	OsR498G0307054200.01.T01	100,00	0,00E+00	1703	Os03g0750800	100,00	0,00E+00	1689	-14	NA	Japonica	
maker-3-augustus-gene-447.66-mRNA-1	3	OsR498G0307054200.01.T04	99,82	0,00E+00	1015	Os03g0750800	100,00	0,00E+00	1020	5	NA	Japonica	
maker-3-augustus-gene-447.78-mRNA-1	3	OsR498G0307054400.01.T01	96,27	0,00E+00	1109	Os03g0750900	99,55	0,00E+00	1176	67	NA	Japonica	
maker-3-augustus-gene-447.83-mRNA-1	3	OsR498G0307055400.01.T01	99,66	0,00E+00	2644	Os03g0751100	100,00	0,00E+00	2663	19	NA	Japonica	
maker-3-augustus-gene-447.85-mRNA-1	3	OsR498G0307058800.01.T02	99,90	0,00E+00	1879	Os03g0752300	100,00	0,00E+00	1884	5	NA	Japonica	
maker-3-augustus-gene-447.90-mRNA-1	3	OsR498G0307059800.01.T04	91,58	0,00E+00	745	Os03g0752800	94,14	0,00E+00	742	-3	NA	Japonica	
maker-3-augustus-gene-448.26-mRNA-1	3	OsR498G0307062200.01.T01	98,49	0,00E+00	2414	Os03g0753500	98,42	0,00E+00	2409	-5	NA	Japonica	
maker-3-augustus-gene-448.28-mRNA-1	3	OsR498G0307061000.01.T01	100,00	0,00E+00	1299	Os03g0753100	100,00	0,00E+00	1299	0	NA	Japonica	
maker-3-augustus-gene-448.31-mRNA-1	3	OsR498G0307061200.01.T01	98,99	0,00E+00	2432	Os03g0753500	71,96	1,82E-161	569	-1863	Indica	Japonica	
maker-3-augustus-gene-449.11-mRNA-1	3	OsR498G0307065100.01.T02	99,92	0,00E+00	4552	Os03g0755000	99,64	0,00E+00	4525	-27	NA	Japonica	Kinase-TM
maker-3-augustus-gene-449.17-mRNA-1	3	OsR498G0307064000.01.T01	100,00	0,00E+00	2479	Os05g0386800	91,04	0,00E+00	1921	-558	Indica	Japonica	
maker-3-augustus-gene-449.2-mRNA-1	3	OsR498G0307064700.01.T01	99,90	0,00E+00	1738	Os03g0754800	100,00	0,00E+00	1743	5	NA	Japonica	
maker-3-augustus-gene-449.7-mRNA-1	3	OsR498G0307064900.01.T01	99,62	0,00E+00	2335	Os03g0754900	99,79	0,00E+00	2539	204	NA	Japonica	
maker-3-augustus-gene-45.30-mRNA-1	3	OsR498G0305369900.01.T02	98,48	0,00E+00	801	Os03g0191400	100,00	0,00E+00	1029	228	Japonica	Japonica	
maker-3-augustus-gene-45.35-mRNA-1	3	OsR498G0305371900.01.T01	99,73	0,00E+00	1989	Os03g0192100	100,00	0,00E+00	2493	504	Japonica	Japonica	
maker-3-augustus-gene-45.38-mRNA-1	3	OsR498G0305367400.01.T01	99,40	0,00E+00	889	Os03g0190800	99,40	0,00E+00	889	0	NA	Japonica	
maker-3-augustus-gene-45.47-mRNA-1	3	OsR498G0305367800.01.T02	99,96	0,00E+00	4337	Os03g0191000	100,00	0,00E+00	4343	6	NA	Japonica	
maker-3-augustus-gene-45.51-mRNA-1	3	OsR498G0305368200.01.T01	99,92	0,00E+00	2279	Os03g0191100	100,00	0,00E+00	2284	5	NA	Japonica	
maker-3-augustus-gene-45.53-mRNA-1	3	OsR498G0305369000.01.T01	100,00	0,00E+00	1326	Os03g0191200	100,00	0,00E+00	1326	0	NA	Japonica	
maker-3-augustus-gene-45.55-mRNA-1	3	OsR498G0305371800.01.T01	99,69	0,00E+00	2309	Os03g0192000	100,00	0,00E+00	2327	18	NA	Japonica	
maker-3-augustus-gene-45.59-mRNA-1	3	OsR498G0305372200.01.T01	99,65	0,00E+00	2055	Os03g0192300	100,00	0,00E+00	1867	-188	NA	Japonica	
maker-3-augustus-gene-45.63-mRNA-1	3	OsR498G0305373600.01.T01	99,53	0,00E+00	765	Os03g0192400	100,00	0,00E+00	774	9	NA	Japonica	
maker-3-augustus-gene-450.0-mRNA-1	3	OsR498G0307065300.01.T01	98,44	0,00E+00	3354	Os03g0755100	98,77	0,00E+00	3426	72	NA	Japonica	NBS-TM
maker-3-augustus-gene-450.12-mRNA-1	3	OsR498G0307069700.01.T01	99,84	0,00E+00	1116	Os03g0756000	100,00	0,00E+00	1121	5	NA	Japonica	
maker-3-augustus-gene-450.16-mRNA-1	3	OsR498G0307068000.01.T02	100,00	1,09E-156	553	Os03g0755700	100,00	0,00E+00	728	175	Japonica	Japonica	
maker-3-augustus-gene-450.21-mRNA-1	3	OsR498G0307068600.01.T02	99,40	0,00E+00	2947	Os03g0755600	100,00	0,00E+00	1988	-959	Indica	Japonica	
maker-3-augustus-gene-450.27-mRNA-1	3	OsR498G0307070600.01.T01	100,00	0,00E+00	953	Os03g0756300	100,00	0,00E+00	953	0	NA	Japonica	
maker-3-augustus-gene-450.29-mRNA-1	3	OsR498G0307070800.01.T01	99,79	0,00E+00	1669	Os03g0756400	100,00	0,00E+00	1678	9	NA	Japonica	
maker-3-augustus-gene-450.6-mRNA-1	3	OsR498G0307066700.01.T01	97,74	0,00E+00	1975	Os03g0755600	100,00	0,00E+00	1986	11	NA	Japonica	
maker-3-augustus-gene-451.40-mRNA-1	3	OsR498G0307071300.01.T01	99,64	3,84E-141	501	Os03g0756800	100,00	3,16E-143	506	5	NA	Japonica	
maker-3-augustus-gene-451.42-mRNA-1	3	OsR498G0307073700.01.T01	99,78	0,00E+00	2465	Os03g0757500	100,00	0,00E+00	2479	14	NA	Japonica	
maker-3-augustus-gene-451.46-mRNA-1	3	OsR498G0307078700.01.T02	99,64	0,00E+00	2465	Os03g0758300	99,86	0,00E+00	2479	14	NA	Japonica	
maker-3-augustus-gene-451.56-mRNA-1	3	OsR498G0307073400.01.T02	99,57	0,00E+00	2468	Os03g0757200	100,00	0,00E+00	2495	27	NA	Japonica	
maker-3-augustus-gene-451.58-mRNA-1	3	OsR498G0307077000.01.T01	99,15	0,00E+00	1670	Os03g0758000	100,00	0,00E+00	1705	35	NA	Japonica	
maker-3-augustus-gene-452.10-mRNA-1	3	OsR498G0307081100.01.T01	99,59	0,00E+00	868	Os03g0758800	100,00	0,00E+00	877	9	NA	Japonica	
maker-3-augustus-gene-452.25-mRNA-1	3	OsR498G0307082500.01.T01	99,74	0,00E+00	2735	Os03g0759500	100,00	0,00E+00	2753	18	NA	Japonica	
maker-3-augustus-gene-452.3-mRNA-1	3	OsR498G0307080500.01.T05	99,56	0,00E+00	5304	Os03g0758600	100,00	0,00E+00	3570	-1734	Indica	Japonica	
maker-3-augustus-gene-452.35-mRNA-1	3	OsR498G0307084500.01.T01	99,74	0,00E+00	2764	Os03g0760200	100,00	0,00E+00	2782	18	NA	Japonica	
maker-3-augustus-gene-452.37-mRNA-1	3	OsR498G0307084700.01.T03	99,81	0,00E+00	2844	Os03g0760500	85,38	0,00E+00	1824	-1020	Indica	Japonica	
maker-3-augustus-gene-452.39-mRNA-1	3	OsR498G0307079200.01.T01	99,80	0,00E+00	894	Os03g0758500	100,00	0,00E+00	899	5	NA	Japonica	
maker-3-augustus-gene-453.0-mRNA-1	3	OsR498G0307084700.01.T03	85,44	0,00E+00	1824	Os03g0760500	100,00	0,00E+00	2809	985	Japonica	Japonica	
maker-3-augustus-gene-453.10-mRNA-1	3	OsR498G0307088300.01.T02	99,15	0,00E+00	1659	Os03g0761900	100,00	0,00E+00	1683	24	NA	Japonica	
maker-3-augustus-gene-453.15-mRNA-1	3	OsR498G0307090300.01.T01	99,90	0,00E+00	1809	Os03g0762300	100,00	0,00E+00	1813	4	NA	Japonica	
maker-3-augustus-gene-453.18-mRNA-1	3	OsR498G0307090900.01.T02	99,80	0,00E+00	1750	Os03g0762400	100,00	0,00E+00	1759	9	NA	Japonica	
maker-3-augustus-gene-453.2-mRNA-1	3	OsR498G0307085100.01.T01	99,82	0,00E+00	2026	Os03g0760700	100,00	0,00E+00	2035	9	NA	Japonica	
maker-3-augustus-gene-453.21-mRNA-1	3	OsR498G0307091500.01.T02	100,00	1,76E-145	515	Os03g0762500	100,00	6,13E-146	515	0	NA	Japonica	
maker-3-augustus-gene-453.23-mRNA-1	3	OsR498G0307085000.01.T01	99,77	0,00E+00	1566	Os03g0760600	100,00	0,00E+00	1575	9	NA	Japonica	
maker-3-augustus-gene-453.27-mRNA-1	3	OsR498G0307085600.01.T01	100,00	7,41E-144	509	Os03g0760800	100,00	2,59E-144	509	0	NA	Japonica	
maker-3-augustus-gene-453.29-mRNA-1	3	OsR498G0307086400.01.T01	100,00	0,00E+00	785	Os03g0761000	100,00	1,44E-162	571	-214	Indica	Japonica	
maker-3-augustus-gene-453.31-mRNA-1	3	OsR498G0307086600.01.T01	99,56	0,00E+00	2039	Os07g0114000	71,61	9,21E-164	577	-1462	Indica	Japonica	
maker-3-augustus-gene-453.35-mRNA-1	3	OsR498G0307086800.01.T01	99,80	0,00E+00	1810	Os03g0761200	100,00	0,00E+00	1819	9	NA	Japonica	
maker-3-augustus-gene-453.37-mRNA-1	3	OsR498G0307088600.01.T01	100,00	0,00E+00	2105	Os07g0114400	87,91	0,00E+00	1253	-852	Indica	Japonica	Kinase-TM
maker-3-augustus-gene-453.42-mRNA-1	3	OsR498G0307089000.01.T01	99,86	0,00E+00	1246	Os03g0762100	100,00	0,00E+00	1251	5	NA	Japonica	
maker-3-augustus-gene-453.7-mRNA-1	3	OsR498G0307088100.01.T04	96,23	0,00E+00	2199	Os03g0761700	96,38	0,00E+00	2208	9	NA	Japonica	
maker-3-augustus-gene-454.0-mRNA-1	3	OsR498G0307093700.01.T01	99,74	0,00E+00	2049	Os03g0762900	100,00	0,00E+00	2062	13	NA	Japonica	
maker-3-augustus-gene-454.18-mRNA-1	3	OsR498G0307099100.01.T01	99,62	0,00E+00	1869	Os03g0764600	100,00	0,00E+00	1889	20	NA	Japonica	
maker-3-augustus-gene-454.22-mRNA-1	3	OsR498G0307097600.01.T02	99,70	0,00E+00	4178	Os03g0764300	100,00	0,00E+00	4210	32	NA	Japonica	Kinase-TM

maker-3-augustus-gene-454.4-mRNA-1	3	OsR498G0307094500.01.T01	99,67	0,00E+00	2195	Os03g0763000	100,00	0,00E+00	2214	19	NA	Japonica	Kinase-TM
maker-3-augustus-gene-454.7-mRNA-1	3	OsR498G0307096300.01.T01	99,84	0,00E+00	2232	Os03g0764000	100,00	0,00E+00	2165	-67	NA	Japonica	
maker-3-augustus-gene-455.0-mRNA-1	3	OsR498G0307099900.01.T01	97,38	0,00E+00	1955	Os03g0764800	97,38	0,00E+00	1955	0	NA	Japonica	Kinase-TM
maker-3-augustus-gene-455.15-mRNA-1	3	OsR498G0307103200.01.T04	99,85	0,00E+00	1170	Os03g0765400	100,00	0,00E+00	1175	5	NA	Japonica	
maker-3-augustus-gene-455.22-mRNA-1	3	OsR498G0307105200.01.T02	99,59	0,00E+00	877	Os03g0766000	100,00	0,00E+00	1097	220	Japonica	Japonica	
maker-3-augustus-gene-455.9-mRNA-1	3	OsR498G0307102600.01.T02	99,45	0,00E+00	2581	Os03g0765200	100,00	0,00E+00	2549	-32	NA	Japonica	
maker-3-augustus-gene-456.0-mRNA-1	3	OsR498G0307106700.01.T01	99,41	0,00E+00	2094	Os03g0766500	100,00	0,00E+00	2127	33	NA	Japonica	
maker-3-augustus-gene-456.12-mRNA-1	3	OsR498G0307108800.01.T01	99,87	0,00E+00	1419	Os03g0767500	100,00	0,00E+00	1424	5	NA	Japonica	
maker-3-augustus-gene-456.15-mRNA-1	3	OsR498G0307111200.01.T01	99,80	0,00E+00	3499	Os03g0769000	100,00	0,00E+00	3577	78	NA	Japonica	
maker-3-augustus-gene-456.5-mRNA-1	3	OsR498G0307109500.01.T01	99,84	0,00E+00	1138	Os03g0767800	100,00	0,00E+00	1142	4	NA	Japonica	
maker-3-augustus-gene-456.9-mRNA-1	3	OsR498G0307109900.01.T01	100,00	1,60E-83	309	Os03g0767900	100,00	5,55E-84	309	0	NA	Japonica	
maker-3-augustus-gene-457.0-mRNA-1	3	OsR498G0307113100.01.T04	99,94	0,00E+00	2826	Os03g0769400	100,00	0,00E+00	2830	4	NA	Japonica	
maker-3-augustus-gene-457.14-mRNA-1	3	OsR498G0307119200.01.T01	99,67	0,00E+00	3773	Os03g0770800	100,00	0,00E+00	3166	-607	Indica	Japonica	
maker-3-augustus-gene-457.17-mRNA-1	3	OsR498G0307119800.01.T01	99,51	0,00E+00	2919	Os03g0770900	100,00	0,00E+00	2955	36	NA	Japonica	
maker-3-augustus-gene-457.2-mRNA-1	3	OsR498G0307114700.01.T05	99,77	0,00E+00	775	Os03g0769800	100,00	0,00E+00	648	-127	Indica	Japonica	
maker-3-augustus-gene-457.9-mRNA-1	3	OsR498G0307113800.01.T02	99,06	0,00E+00	2990	Os03g0769600	100,00	0,00E+00	3057	67	NA	Japonica	TM
maker-3-augustus-gene-458.0-mRNA-1	3	OsR498G0307121900.01.T01	96,14	0,00E+00	1759	Os03g0771500	100,00	0,00E+00	1851	92	NA	Japonica	
maker-3-augustus-gene-458.1-mRNA-1	3	OsR498G0307123100.01.T01	97,14	0,00E+00	1813	Os03g0771500	89,01	0,00E+00	1438	-375	Indica	Japonica	
maker-3-augustus-gene-458.10-mRNA-1	3	OsR498G0307126300.01.T01	100,00	5,64E-152	536	Os03g0772800	100,00	1,96E-152	536	0	NA	Japonica	
maker-3-augustus-gene-458.15-mRNA-1	3	OsR498G0307120600.01.T01	99,90	0,00E+00	1738	Os03g0771100	100,00	0,00E+00	1743	5	NA	Japonica	
maker-3-augustus-gene-458.17-mRNA-1	3	OsR498G0307128400.01.T02	99,71	0,00E+00	3093	Os03g0241100	87,80	0,00E+00	2168	-925	Indica	Japonica	
maker-3-augustus-gene-458.26-mRNA-1	3	OsR498G0307128600.01.T01	100,00	3,66E-90	331	Os03g0773150	100,00	3,22E-79	293	-38	Indica	Japonica	
maker-3-augustus-gene-458.28-mRNA-1	3	OsR498G0307128800.01.T01	99,52	0,00E+00	4068	Os03g0773300	100,00	0,00E+00	4118	50	NA	Japonica	Kinase-LRR-TM
maker-3-augustus-gene-459.39-mRNA-1	3	OsR498G0307131300.01.T03	99,61	0,00E+00	5519	Os03g0773700	100,00	0,00E+00	4794	-725	Indica	Japonica	Kinase-LRR-TM
maker-3-augustus-gene-459.44-mRNA-1	3	OsR498G0307133500.01.T02	99,37	2,01E-75	283	Os03g0774600	100,00	1,64E-77	288	5	NA	Japonica	
maker-3-augustus-gene-459.47-mRNA-1	3	OsR498G0307136100.01.T01	99,82	0,00E+00	3932	Os03g0775200	100,00	0,00E+00	3950	18	NA	Japonica	
maker-3-augustus-gene-459.53-mRNA-1	3	OsR498G0307131800.01.T01	99,81	0,00E+00	1912	Os03g0773800	100,00	0,00E+00	1921	9	NA	Japonica	
maker-3-augustus-gene-459.57-mRNA-1	3	OsR498G0307132600.01.T01	99,56	0,00E+00	1203	Os03g0774200	99,56	0,00E+00	1203	0	NA	Japonica	
maker-3-augustus-gene-459.61-mRNA-1	3	OsR498G0307133000.01.T01	98,84	0,00E+00	3039	Os03g0774300	98,31	0,00E+00	1852	-1187	Indica	Japonica	
maker-3-augustus-gene-459.71-mRNA-1	3	OsR498G0307135200.01.T01	99,78	0,00E+00	1599	Os03g0775000	100,00	0,00E+00	1873	274	Japonica	Japonica	
maker-3-augustus-gene-46.0-mRNA-1	3	OsR498G0305373900.01.T02	99,63	0,00E+00	2866	Os03g0192500	100,00	0,00E+00	2890	24	NA	Japonica	
maker-3-augustus-gene-46.19-mRNA-1	3	OsR498G0305375500.01.T01	100,00	1,83E-171	601	Os03g0193075	100,00	6,37E-172	601	0	NA	Japonica	
maker-3-augustus-gene-46.24-mRNA-1	3	OsR498G0917271000.01.T02	88,54	0,00E+00	730	Os03g0193400	100,00	0,00E+00	1905	1175	Japonica	Japonica	
maker-3-augustus-gene-46.29-mRNA-1	3	OsR498G0305374200.01.T01	100,00	0,00E+00	1148	Os03g0192600	100,00	0,00E+00	1148	0	NA	Japonica	
maker-3-augustus-gene-46.32-mRNA-1	3	OsR498G0305374400.01.T04	99,74	0,00E+00	2747	Os03g0192700	100,00	0,00E+00	2765	18	NA	Japonica	
maker-3-augustus-gene-46.40-mRNA-1	3	OsR498G0305376000.01.T01	99,42	0,00E+00	2746	Os03g0193225	100,00	0,00E+00	2787	41	NA	Japonica	
maker-3-augustus-gene-460.49-mRNA-1	3	OsR498G0307136300.01.T01	99,95	0,00E+00	3534	Os03g0775300	100,00	0,00E+00	2666	-868	Indica	Japonica	
maker-3-augustus-gene-460.55-mRNA-1	3	OsR498G0307136700.01.T01	100,00	0,00E+00	839	Os03g0775500	100,00	0,00E+00	839	0	NA	Japonica	
maker-3-augustus-gene-460.59-mRNA-1	3	OsR498G0307137300.01.T01	99,89	0,00E+00	1641	Os03g0775600	100,00	0,00E+00	1645	4	NA	Japonica	
maker-3-augustus-gene-460.61-mRNA-1	3	OsR498G0307137500.01.T01	99,82	0,00E+00	5085	Os03g0775700	100,00	0,00E+00	5108	23	NA	Japonica	
maker-3-augustus-gene-460.64-mRNA-1	3	OsR498G0307138100.01.T06	94,67	0,00E+00	2614	Os03g0776000	94,85	0,00E+00	2627	13	NA	Japonica	
maker-3-augustus-gene-460.74-mRNA-1	3	OsR498G0307140500.01.T01	99,74	0,00E+00	691	Os03g0776900	100,00	2,15E-172	603	-88	Indica	Japonica	
maker-3-augustus-gene-460.78-mRNA-1	3	OsR498G0307142500.01.T01	98,91	0,00E+00	1939	Os03g0777000	100,00	0,00E+00	1965	26	NA	Japonica	NAC66
maker-3-augustus-gene-460.80-mRNA-1	3	OsR498G0307138400.01.T01	99,88	0,00E+00	3000	Os03g0776100	100,00	0,00E+00	3009	9	NA	Japonica	Kinase-TM
maker-3-augustus-gene-460.82-mRNA-1	3	OsR498G0307142800.01.T01	99,73	0,00E+00	2666	Os03g0777100	99,80	0,00E+00	1825	-841	Indica	Japonica	
maker-3-augustus-gene-460.87-mRNA-1	3	OsR498G0307143600.01.T01	100,00	0,00E+00	996	Os03g0777500	100,00	0,00E+00	996	0	NA	Japonica	
maker-3-augustus-gene-461.36-mRNA-1	3	OsR498G0307143900.01.T01	99,47	0,00E+00	1997	Os03g0777600	100,00	0,00E+00	1544	-453	Indica	Japonica	
maker-3-augustus-gene-461.40-mRNA-1	3	OsR498G0307145300.01.T04	99,54	0,00E+00	1926	Os03g0778000	100,00	0,00E+00	1755	-171	NA	Japonica	
maker-3-augustus-gene-462.56-mRNA-1	3	OsR498G0307152900.01.T04	100,00	0,00E+00	1359	Os03g0780800	100,00	0,00E+00	1288	-71	NA	NA	
maker-3-augustus-gene-462.67-mRNA-1	3	OsR498G0307151600.01.T03	100,00	0,00E+00	755	Os03g0780400	100,00	0,00E+00	753	-2	NA	NA	
maker-3-augustus-gene-462.69-mRNA-1	3	OsR498G0307153200.01.T01	100,00	0,00E+00	1808	Os03g0780900	100,00	0,00E+00	1808	0	NA	NA	
maker-3-augustus-gene-462.82-mRNA-1	3	OsR498G0307154000.01.T01	100,00	1,83E-171	601	Os03g0781300	100,00	6,37E-172	601	0	NA	NA	
maker-3-augustus-gene-462.84-mRNA-1	3	OsR498G0307154200.01.T04	98,00	0,00E+00	1554	Os03g0781400	100,00	0,00E+00	1624	70	NA	NA	
maker-3-augustus-gene-462.87-mRNA-1	3	OsR498G0307154800.01.T03	100,00	0,00E+00	1342	Os03g0781700	100,00	0,00E+00	1342	0	NA	NA	
maker-3-augustus-gene-463.10-mRNA-1	3	OsR498G0307165000.01.T01	99,79	0,00E+00	873	Os03g0783700	100,00	0,00E+00	874	1	NA	Japonica	
maker-3-augustus-gene-463.13-mRNA-1	3	OsR498G0307166200.01.T01	99,78	0,00E+00	2496	Os03g0784100	100,00	0,00E+00	2506	10	NA	Japonica	
maker-3-augustus-gene-464.34-mRNA-1	3	OsR498G0307166900.01.T01	100,00	0,00E+00	1900	Os03g0784400	100,00	0,00E+00	1900	0	NA	Japonica	
maker-3-augustus-gene-464.40-mRNA-1	3	OsR498G0307168700.01.T01	99,60	0,00E+00	1790	Os03g0785500	100,00	0,00E+00	1808	18	NA	Japonica	
maker-3-augustus-gene-464.44-mRNA-1	3	OsR498G0307170100.01.T01	100,00	0,00E+00	1462	Os03g0786000	100,00	0,00E+00	1462	0	NA	Japonica	
maker-3-augustus-gene-464.46-mRNA-1	3	OsR498G0307170300.01.T03	99,91	0,00E+00	1998	Os03g0786100	100,00	0,00E+00	2003	5	NA	Japonica	
maker-3-augustus-gene-464.57-mRNA-1	3	OsR498G0307167200.01.T05	99,69	0,00E+00	4034	Os07g0147800	70,15	0,00E+00	866	-3168	Indica	Japonica	
maker-3-augustus-gene-464.60-mRNA-1	3	OsR498G0307167400.01.T01	99,65	0,00E+00	2033	Os03g0784700	100,00	0,00E+00	2051	18	NA	Japonica	
maker-3-augustus-gene-464.64-mRNA-1	3	OsR498G0307167600.01.T01	99,50	0,00E+00	1767	Os04g0613400	80,83	0,00E+00	914	-853	Indica	Japonica	
maker-3-augustus-gene-464.69-mRNA-1	3	OsR498G0307168600.01.T03	99,88	0,00E+00	4534	Os07g0150500	75,67	0,00E+00	1730	-2804	Indica	Japonica	
maker-3-augustus-gene-465.0-mRNA-1	3	OsR498G0307172700.01.T01	99,95	0,00E+00	3291	Os03g0786700	100,00	0,00E+00	3296	5	NA	Japonica	
maker-3-augustus-gene-465.13-mRNA-1	3	OsR498G0307173100.01.T07	97,24	0,00E+00	4862	Os03g0786900	97,48	0,00E+00	4893	31	NA	Japonica	
maker-3-augustus-gene-465.24-mRNA-1	3	OsR498G0307176300.01.T01	100,00	0,00E+00	850	Os03g0788200	100,00	0,00E+00	850	0	NA	Japonica	
maker-3-augustus-gene-465.30-mRNA-1	3	OsR498G0307173600.01.T01	99,87	0,00E+00	1370	Os03g0647800	72,41	1,34E-102	372	-998	Indica	Japonica	
maker-3-augustus-gene-465.33-mRNA-1	3	OsR498G0307173800.01.T01	99,37	0,00E+00	2251	Os03g0787200	100,00	0,00E+00	2289	38	NA	Japonica	
maker-3-augustus-gene-465.37-mRNA-1	3	OsR498G0307175200.01.T01	99,48	0,00E+00	686	Os03g0813700	96,17	0,00E+00	640	-46	NA	Japonica	
maker-3-augustus-gene-466.43-mRNA-1	3	OsR498G0307176900.01.T01	99,94	0,00E+00	3242	Os03g0788500	100,00	0,00E+00	3247	5	NA	Japonica	
maker-3-augustus-gene-466.59-mRNA-1	3	OsR498G0307177800.01.T02	99,78	0,00E+00	2451	Os03g0788700	100,00	0,00E+00	2464	13	NA	Japonica	
maker-3-augustus-gene-466.62-mRNA-1	3	OsR498G0307178000.01.T02	99,55	0,00E+00	1996	Os03g0788800	100,00	0,00E+00	2019	23	NA	Japonica	
maker-3-augustus-gene-466.67-mRNA-1	3	OsR498G0307178600.01.T01	99,94	0,00E+00	3118	Os03g0789000	100,00	0,00E+00	3122	4	NA	Japonica	
maker-3-augustus-gene-466.69-mRNA-1	3	OsR498G0307179400.01.T01	95,52	0,00E+00	1754	Os03g0789200	95,61	0,00E+00	1758	4	NA	Japonica	
maker-3-augustus-gene-466.74-mRNA-1	3	OsR498G0307181400.01.T05	99,73	0,00E+00	1961	Os03g0789900	100,00	0,00E+00	1974	13	NA	Japonica	
maker-3-augustus-gene-467.11-mRNA-1	3	OsR498G0307187900.01.T02	99,80	0,00E+00	4523	Os03g0791700	100,00	0,00E+00	4545	22	NA	Japonica	Kinase-LRR-TM
maker-3-augustus-gene-467.15-mRNA-1	3	OsR498G0307183800.01.T01	99,85	0,00E+00	7315	Os03g0790900	96,37	0,00E+00	6673	-642	NA	Japonica	
maker-3-augustus-gene-467.16-mRNA-1	3	OsR498G0307184200.01.T01	99,44	0,00E+00	7233	Os03g0790900	100,00	0,00E+00	7445	212	NA	Japonica	
maker-3-augustus-gene-467.31-mRNA-1	3	OsR498G0307184200.01.T02	93,25	8,10E-178	624	Os03g0790700	92,77	1,46E-175	615	-9	NA	Japonica	
maker-3-augustus-gene-468.48-mRNA-1	3	OsR498G0307189300.01.T05	98,58	0,00E+00	1966	Os03g0792400	98,70	0,00E+00	2020	54	NA	Japonica	
maker-3-augustus-gene-468.54-mRNA-1	3	OsR498G0307189500.01.T03	100,00	0,00E+00	1021	Os03g0792500	100,00	0,00E+00	1021	0	NA	Japonica	
maker-3-augustus-gene-468.59-mRNA-1	3	OsR498G0307190300.01.T02	100,00	0,00E+00	2663	Os03g0792800	99,91	0,00E+00	2011	-652	Indica	Japonica	
maker-3-augustus-gene-468.61-mRNA-1	3	OsR498G0307192700.01.T08	99,81	0,00E+00	3813	Os03g0793500	100,00	0,00E+00	3831	18	NA	Japonica	
maker-3-augustus-gene-468.69-mRNA-1	3	OsR498G0307193300.01.T03	99,82	0,00E+00	3031	Os03g0793600	99,32	0,00E+00	2626	-405	Indica	Japonica	
maker-3-augustus-gene-468.78-mRNA-1	3	OsR498G0307193900.01.T01	99,79	0,00E+00	2535	Os03g0793700	99,93	0,00E+00	2544	9	NA	Japonica	
maker-3-augustus-gene-468.80-mRNA-1	3	OsR498G0307189800.01.T01	99,91	0,00E+00	1917	Os03g0792600	100,00	0,00E+00	1921	4	NA	Japonica	
maker-3-augustus-gene-468.85-mRNA-1	3	OsR498G0307190600.01.T03	99,59	0,00E+00	863	Os03g0792900	100,00	0,00E+00	872	9	NA	Japonica	
maker-3-augustus-gene-468.86-mRNA-1	3	OsR498G1119421100.01.T02	98,26	0,00E+00	1480	Os03g0793100	100,00	0,00E+00	1456	-24	NA	Japonica	
maker-3-augustus-gene-468.89-mRNA-1	3	OsR498G0307194200.01.T02	100,00	0,00E+00	964	Os03g0793800	100,00	0,00E+00	964	0	NA	Japonica	
maker-3-augustus-gene-468.91-mRNA-1	3	OsR498G0307194800.01.T01	100,00	0,00E+00	1083	Os03g0793900	93,85	0,00E+00	921	-162	Indica	Japonica	
maker-3-augustus-gene-468.94-mRNA-1	3	OsR498G0307196200.01.T02	99,90	0,00E+00	1733	Os03g0794500	100,00	0,00E+00	1737	4	NA	Japonica	
maker-3-augustus-gene-469.0-mRNA-1	3	OsR498G0307196700.01.T01	99,83	0,00E+00	5329	Os03g0794800	100,00	0,00E+00	5351	22	NA	Japonica	
maker-3-augustus-gene-469.14-mRNA-1	3	OsR498G0307199500.01.T02	99,72	0,00E+00	1924	Os03g0795900	100,00	0,00E+00	1938	14	NA	Japonica	
maker-3-augustus-gene-469.16-mRNA-1	3	OsR498G0307199900.01.T03	100,00	0,00E+00	1353	Os03g0796000	100,00	0,00E+00	1002	-351	Indica	Japonica	
maker-3-augustus-gene-469.20-mRNA-1	3	OsR498G0307196600.01.T01	88,64	0,00E+00	665	Os03g0794700	88,64	0,00E+00	665	0	NA	Japonica	
maker-3-augustus-gene-469.22-mRNA-1	3	OsR498G0307197200.01.T03	100,00	0,00E+00	1851	Os03g0795000	100,00	0,00E+00	2342	491	Japonica	Japonica	
maker-3-augustus-gene-469.25-mRNA-1	3	OsR498G0307198000.01.T01	98,56	0,00E+00	2065	Os03g0795300	100,00	0,00E+00	2132	67	NA	Japonica	LRR-TM
maker-3-augustus-gene-469.27-mRNA-1	3	OsR498G0307199000.01.T01	99,73	0,00E+00	1333	Os03g0795600	100,00	0,00E+00	1342	9	NA	Japonica	
maker-3-augustus-gene-469.29-mRNA-1	3	OsR498G0307199400.01.T01	99,86	0,00E+00	1306	Os03g0795800	100,00	0,00E+00	1310	4	NA	Japonica	
maker-3-augustus-gene-469.32-mRNA-1	3	OsR498G0307202000.01.T02	99,78	0,00E+00	1609	Os03g0796400	100,00	0,00E+00	1618	9	NA	Japonica	
maker-3-augustus-gene-469.34-mRNA-1	3	OsR498G0307202600.01.T02	99,92	0,00E+00	2187	Os03g0796501	99,92	0,00E+00	2187	0	NA	Japonica	
maker-3-augustus-gene-469.5-mRNA-1	3	OsR498G0307196900.01.T01	99,70	0,00E+00	2959	Os03g0794900	100,00	0,00E+00	2982	23	NA	Japonica	
maker-3-augustus-gene-469.9-mRNA-1	3	OsR498G0307198700.01.T01	100,00	0,00E+00	1040	Os03g0795500	100,00	0,00E+00	1040	0	NA	Japonica	
maker-3-augustus-gene-47.41-mRNA-1	3	OsR498G0101213400.01.T05	77,39	4,56E-60	232	Os03g0193900	100,00	0,00E+00	758	526	Japonica	Japonica	
maker-3-augustus-gene-47.43-mRNA-1	3	OsR498G0305379300.01.T04	99,49	0,00E+00	704	Os03g0194400	100,00	0,00E+00	645	-59	NA	Japonica	
maker-3-augustus-gene-47.49-mRNA-1	3	OsR498G0305380500.01.T01	99,13	0,00E+00	2039	Os03g0194900	100,00	0,00E+00	2084	45	NA	Japonica	
maker-3-augustus-gene-47.51-mRNA-1	3	OsR498G0305381100.01.T01	99,40	0,00E+00	2351	Os03g0195100	100,00	0,00E+00	2387	36	NA	Japonica	
maker-3-augustus-gene-47.53-mRNA-1	3	OsR498G0305381400.01.T02	99,70	0,00E+00	1178	Os03g0195200	100,00	0,00E+00	1187	9	NA	Japonica	
maker-3-augustus-gene-47.58-mRNA-1	3	OsR498G0305381800.01.T01	99,87	0,00E+00	2848	Os03g0195300	100,00	0,00E+00	2857	9	NA	Japonica	
maker-3-augustus-gene-470.0-mRNA-1	3	OsR498G0307206300.01.T02	99,67	0,00E+00	2179	Os03g0796600	100,00	0,00E+00	2197	18	NA	Japonica	
maker-3-augustus-gene-470.14-mRNA-1	3	OsR498G0307212300.01.T01	99,58	0,00E+00	1705	Os03g0797700	99,90	0,00E+00	1718	13	NA	Japonica	
maker-3-augustus-gene-470.19-mRNA-1	3	OsR498G0307208200.01.T01	99,45	0,00E+00	2264	Os03g0796900	99,53	0,00E+00	2279	15	NA	Japonica	
maker-3-augustus-gene-470.2-mRNA-1	3	OsR498G0307207900.01.T01	96,78	0,00E+00	3162	Os07g0181700	80,17	0,00E+00	1089	-2073	Indica	Japonica	
maker-3-augustus-gene-470.26-mRNA-1	3	OsR498G0307210400.01.T01	98,68	0,00E+00	1715	Os03g0797550	86,18	0,00E+00	1071	-644	Indica	Japonica	
maker-3-augustus-gene-470.28-mRNA-1	3	OsR498G0307211000.01.T02	99,69	0,00E+00	1746	Os03g0797400	100,00	0,00E+00	1759	13	NA	NA	
maker-3-augustus-gene-470.30-mRNA-1	3	OsR498G0307211000.01.T02	90,15	0,00E+00	1324	Os03g0797550	100,00	0,00E+00	1754	430	Japonica	Japonica	
maker-3-augustus-gene-471.42-mRNA-1	3	OsR498G0307214900.01.T02	99,63	0,00E+00	953	Os03g0798200	100,00	0,00E+00	962	9	NA	Japonica	
maker-3-augustus-gene-471.50-mRNA-1	3	OsR498G0307216100.01.T01	99,88	0,00E+00	1527	Os03g0798500	100,00	0,00E+00	2062	535	Japonica	Japonica	
maker-3-augustus-gene-471.53-mRNA-1	3	OsR498G0307217700.01.T01	99,77	0,00E+00	1582	Os03g0799000	100,00	0,00E+00	1591	9	NA	Japonica	
maker-3-augustus-gene-471.55-mRNA-1	3	OsR498G0307217900.01.T01	99,82	0,00E+00	1939	Os03g0799100	100,00	0,00E+00	1948	9	NA	Japonica	
maker-3-augustus-gene-471.59-mRNA-1	3	OsR498G0307218100.01.T04	99,36	0,00E+00	3876	Os03g0799200	100,00	0,00E+00	3939	63	NA	Japonica	
maker-3-augustus-gene-471.61-mRNA-1	3	OsR498G0307218900.01.T01	99,68	0,00E+00	1101	Os03g0799500	100,00	0,00E+00	1110	9	NA	Japonica	
maker-3-augustus-gene-471.63-mRNA-1	3	OsR498G0307219100.01.T02	99,80	0,00E+00	891	Os03g0799600	100,00	0,00E+00	895	4	NA	Japonica	
maker-3-augustus-gene-471.66-mRNA-1	3	OsR498G0307219300.01.T01	99,67	0,00E+00	2710	Os03g0799700	100,00	0,00E+00	2733	23	NA	Japonica	
maker-3-augustus-gene-471.70-mRNA-1	3	OsR498G0307212800.01.T01	99,83	0,00E+00	1067	Os03g0797800	100,00	0,00E+00	1072	5	NA	Japonica	
maker-3-augustus-gene-471.72-mRNA-1	3	OsR498G0307183800.01.T02	90,32	1,87E-121	435	Os03g0797901	99,62	3,84E-131	467	32	NA	Japonica	
maker-3-augustus-gene-471.74-mRNA-1	3	OsR498G0307214400.01.T01	99,51	2,97E-99	362	Os03g0798000	100,00	3,59E-175	612	250	Japonica	Japonica	
maker-3-augustus-gene-471.77-mRNA-1	3	OsR498G0307216400.01.T01	99,78	0,00E+00	831	Os03g0798600	100,00	0,00E+00	836	5	NA	Japonica	
maker-3-augustus-gene-472.0-mRNA-1	3	OsR498G0307220100.01.T01	99,14	0,00E+00	1432	Os07g0187700	81,38	0,00E+00	749	-683	Indica	Japonica	
maker-3-augustus-gene-472.17-mRNA-1	3	OsR498G0307223100.01.T02	98,99	0,00E+00	1214	Os03g0801500	100,00	0,00E+00	668	-546	Indica	Japonica	
maker-3-augustus-gene-472.21-mRNA-1	3	OsR498G0307223300.01.T01	99,74	0,00E+00	2727	Os03g0801600	100,00	0,00E+00	2746	19	NA	Japonica	
maker-3-augustus-gene-472.27-mRNA-1	3	OsR498G0307223700.01.T02	97,92	0,00E+00	5048	Os03g0801800	98,36	0,00E+00	5103	55	NA	Japonica	
maker-3-augustus-gene-472.43-mRNA-1	3	OsR498G0307221800.01.T01	99,18	0,00E+00	1724	Os03g0800500	99,90	0,00E+00	1744	20	NA	Japonica	
maker-3-augustus-gene-472.45-mRNA-1	3	OsR498G0307222000.01.T01	100,00	0,00E+00	731	Os03g0800700	100,00	0,00E+00	731	0	NA	Japonica	
maker-3-augustus-gene-472.47-mRNA-1	3	OsR498G0307222200.01.T03	99,83	0,00E+00	4235	Os03g0800800	100,00	0,00E+00	4253	18	NA	Japonica	
maker-3-augustus-gene-472.51-mRNA-1	3	OsR498G0307223600.01.T02	99,66	0,00E+00	2104	Os03g0801700	100,00	0,00E+00	2122	18	NA	Japonica	
maker-3-augustus-gene-473.44-mRNA-1	3	OsR498G0307224100.01.T01	96,64	0,00E+00	1797	Os03g0801900	96,92	0,00E+00	1810	13	NA	Japonica	
maker-3-augustus-gene-473.48-mRNA-1	3	OsR498G0307227300.01.T01	99,85	0,00E+00	1192	Os03g0803000	100,00	0,00E+00	1196	4	NA	Japonica	
maker-3-augustus-gene-473.52-mRNA-1	3	OsR498G0307225800.01.T03	99,74	0,00E+00	2097	Os03g0802300	100,00	0,00E+00	2109	12	NA	Japonica	
maker-3-augustus-gene-473.65-mRNA-1	3	OsR498G0307227000.01.T01	99,36	0,00E+00	3606	Os07g0193600	74,15	0,00E+00	928	-2678	Indica	Japonica	
maker-3-augustus-gene-473.72-mRNA-1	3	OsR498G0307227200.01.T01	99,25	0,00E+00	1414	Os03g0802900	100,00	0,00E+00	1440	26	NA	Japonica	
maker-3-augustus-gene-473.77-mRNA-1	3	OsR498G0307229000.01.T01	100,00	0,00E+00	1683	Os07g0194500	75,61	3,62E-163	575	-1108	Indica	NA	
maker-3-augustus-gene-473.80-mRNA-1	3	OsR498G0307229200.01.T02	100,00	0,00E+00	1624	Os03g0803500	100,00	0,00E+00	1624	0	NA	NA	
maker-3-augustus-gene-473.82-mRNA-1	3	OsR498G0307229400.01.T04	100,00	0,00E+00	3528	Os03g0803900	72,35	0,00E+00	799	-2729	Indica	NA	
maker-3-augustus-gene-474.36-mRNA-1	3	OsR498G0307230100.01.T03	100,00	0,00E+00	1126	Os03g0804100	100,00	0,00E+00	1126	0	NA	NA	
maker-3-augustus-gene-474.41-mRNA-1	3	OsR498G0307230500.01.T01	100,00	0,00E+00	1061	Os03g0804200	100,00	4,33E-177	619	-442	Indica	NA	
maker-3-augustus-gene-474.43-mRNA-1	3	OsR498G0307231300.01.T01	100,00	0,00E+00	4177	Os03g0804400	100,00	0,00E+00	3303	-874	Indica	NA	
maker-3-augustus-gene-474.49-mRNA-1	3	OsR498G0307232500.01.T01	99,94	0,00E+00	2956	Os03g0804800	100,00	0,00E+00	2960	4	NA	NA	
maker-3-augustus-gene-474.54-mRNA-1	3	OsR498G0307233300.01.T06	97,05	0,00E+00	1722	Os03g0805100	97,05	0,00E+00	1722	0	NA	NA	
maker-3-augustus-gene-474.60-mRNA-1	3	OsR498G0307234100.01.T02	100,00	0,00E+00	1667	Os03g0805300	100,00	0,00E+00	1667	0	NA	NA	
maker-3-augustus-gene-474.68-mRNA-1	3	OsR498G0307235100.01.T01	100,00	0,00E+00	2917	Os03g0805700	77,45	0,00E+00	1341	-1576	Indica	NA	
maker-3-augustus-gene-474.79-mRNA-1	3	OsR498G0307231000.01.T04	100,00	2,33E-179	628	Os03g0804300	100,00	8,12E-180	628	0	NA	NA	
maker-3-augustus-gene-474.84-mRNA-1	3	OsR498G0307232000.01.T01	98,99	2,02E-94	346	Os03g0804650	100,00	1,11E-155	547	201	Japonica	NA	
maker-3-augustus-gene-474.87-mRNA-1	3	OsR498G0307233000.01.T01	100,00	0,00E+00	2560	Os03g0804900	100,00	0,00E+00	2673	113	NA	NA	
maker-3-augustus-gene-474.89-mRNA-1	3	OsR498G0307233600.01.T01	100,00	0,00E+00	3452	Os03g0805200	100,00	0,00E+00	3452	0	NA	NA	
maker-3-augustus-gene-474.93-mRNA-1	3	OsR498G0307234400.01.T01	100,00	0,00E+00	2163	Os03g0805400	100,00	0,00E+00	2163	0	NA	NA	
maker-3-augustus-gene-474.97-mRNA-1	3	OsR498G0307234600.01.T01	100,00	0,00E+00	3388	Os03g0805500	100,00	0,00E+00	3214	-174	NA	NA	
maker-3-augustus-gene-475.0-mRNA-1	3	OsR498G0307236100.01.T01	97,07	0,00E+00	2253	Os03g0805700	96,67	0,00E+00	2715	462	Japonica	NA	
maker-3-augustus-gene-475.12-mRNA-1	3	OsR498G0307236100.01.T01	88,02	2,59E-178	626	Os03g0805900	88,45	7,92E-148	522	-104	Indica	NA	
maker-3-augustus-gene-475.14-mRNA-1	3	OsR498G0307238900.01.T01	100,00	0,00E+00	1635	Os03g0806500	100,00	0,00E+00	1635	0	NA	NA	
maker-3-augustus-gene-475.20-mRNA-1	3	OsR498G0307241300.01.T01	100,00	0,00E+00	688	Os03g0806900	100,00	0,00E+00	688	0	NA	NA	
maker-3-augustus-gene-475.23-mRNA-1	3	OsR498G0307242100.01.T01	100,00	0,00E+00	2224	Os07g0205500	81,94	0,00E+00	1204	-1020	Indica	NA	
maker-3-augustus-gene-475.3-mRNA-1	3	OsR498G0307236300.01.T01	100,00	0,00E+00	1213	Os03g0805900	84,91	0,00E+00	814	-399	Indica	NA	
maker-3-augustus-gene-475.35-mRNA-1	3	OsR498G0307242400.01.T01	100,00	0,00E+00	1142	Os03g0807200	100,00	0,00E+00	1142	0	NA	NA	
maker-3-augustus-gene-475.38-mRNA-1	3	OsR498G0307318800.01.T02	87,13	0,00E+00	778	Os03g0807400	97,81	0,00E+00	1098	320	Japonica	NA	
maker-3-augustus-gene-475.4-mRNA-1	3	OsR498G0307236300.01.T01	100,00	0,00E+00	1187	Os03g0805700	85,29	0,00E+00	723	-464	Indica	NA	
maker-3-augustus-gene-475.9-mRNA-1	3	OsR498G0307237100.01.T01	98,66	0,00E+00	2617	Os03g0805900	99,27	0,00E+00	1455	-1162	Indica	NA	
maker-3-augustus-gene-476.43-mRNA-1	3	OsR498G0307244100.01.T01	100,00	0,00E+00	2132	Os03g0807700	100,00	0,00E+00	2132	0	NA	NA	
maker-3-augustus-gene-476.45-mRNA-1	3	OsR498G0307244300.01.T02	100,00	0,00E+00	1489	Os03g0807800	100,00	0,00E+00	1489	0	NA	NA	
maker-3-augustus-gene-476.47-mRNA-1	3	OsR498G0307244500.01.T01	100,00	0,00E+00	1061	Os03g0807900	100,00	0,00E+00	1061	0	NA	NA	
maker-3-augustus-gene-476.50-mRNA-1	3	OsR498G0307245500.01.T01	97,79	0,00E+00	1630	Os03g0808000	98,43	0,00E+00	2328	698	Japonica	NA	
maker-3-augustus-gene-476.53-mRNA-1	3	OsR498G0307247900.01.T01	100,00	0,00E+00	2807	Os03g0808600	100,00	0,00E+00	2807	0	NA	Japonica	
maker-3-augustus-gene-476.58-mRNA-1	3	OsR498G0307248900.01.T01	98,88	0,00E+00	4082	Os07g0209100	82,42	0,00E+00	2418	-1664	Indica	Japonica	
maker-3-augustus-gene-476.65-mRNA-1	3	OsR498G0307243400.01.T01	100,00	0,00E+00	1620	Os03g0807600	100,00	0,00E+00	1620	0	NA	NA	
maker-3-augustus-gene-476.68-mRNA-1	3	OsR498G0307245800.01.T01	100,00	0,00E+00	4370	Os03g0808100	100,00	0,00E+00	4370	0	NA	NA	
maker-3-augustus-gene-476.73-mRNA-1	3	OsR498G0307246200.01.T01	96,61	0,00E+00	1484	Os03g0808300	96,61	0,00E+00	1484	0	NA	NA	
maker-3-augustus-gene-476.77-mRNA-1	3	OsR498G0307249400.01.T01	99,86	0,00E+00	2535	Os03g0809100	100,00	0,00E+00	2544	9	NA	Japonica	
maker-3-augustus-gene-477.0-mRNA-1	3	OsR498G0307250900.01.T01	99,76	0,00E+00	2231	Os03g0809700	100,00	0,00E+00	2244	13	NA	Japonica	
maker-3-augustus-gene-477.20-mRNA-1	3	OsR498G0307253200.01.T01	99,59	0,00E+00	871	Os07g0212300	81,17	4,37E-119	426	-445	Indica	Japonica	
maker-3-augustus-gene-477.23-mRNA-1	3	OsR498G0307253800.01.T01	99,84	0,00E+00	3466	Os03g0810600	100,00	0,00E+00	3479	13	NA	Japonica	
maker-3-augustus-gene-477.29-mRNA-1	3	OsR498G0307254000.01.T01	99,20	0,00E+00	886	Os03g0810600	77,50	3,87E-129	462	-424	Indica	Japonica	
maker-3-augustus-gene-477.31-mRNA-1	3	OsR498G0307254600.01.T01	99,92	0,00E+00	4433	Os03g0810900	100,00	0,00E+00	4442	9	NA	Japonica	
maker-3-augustus-gene-477.5-mRNA-1	3	OsR498G0307251300.01.T01	99,81	0,00E+00	965	Os03g0809900	100,00	0,00E+00	969	4	NA	Japonica	
maker-3-augustus-gene-477.8-mRNA-1	3	OsR498G0307254300.01.T01	100,00	0,00E+00	1503	Os03g0810800	100,00	0,00E+00	1526	23	NA	Japonica	
maker-3-augustus-gene-477.9-mRNA-1	3	OsR498G0307250000.01.T01	99,52	0,00E+00	2578	Os03g0809300	100,00	0,00E+00	2598	20	NA	Japonica	
maker-3-augustus-gene-478.30-mRNA-1	3	OsR498G0307254700.01.T01	98,72	0,00E+00	3975	Os03g0811100	98,81	0,00E+00	3984	9	NA	Japonica	
maker-3-augustus-gene-478.35-mRNA-1	3	OsR498G0307254900.01.T02	99,81	0,00E+00	2840	Os03g0811200	100,00	0,00E+00	2854	14	NA	Japonica	
maker-3-augustus-gene-478.39-mRNA-1	3	OsR498G0307255100.01.T01	100,00	9,54E-157	553	Os03g0811300	100,00	3,32E-157	553	0	NA	Japonica	
maker-3-augustus-gene-478.42-mRNA-1	3	OsR498G0307255700.01.T02	96,78	0,00E+00	1352	Os03g0811400	100,00	0,00E+00	1418	66	NA	Japonica	
maker-3-augustus-gene-478.44-mRNA-1	3	OsR498G0307258500.01.T05	99,82	0,00E+00	972	Os03g0811900	100,00	0,00E+00	976	4	NA	Japonica	
maker-3-augustus-gene-478.48-mRNA-1	3	OsR498G0307257200.01.T01	99,87	0,00E+00	2827	Os03g0811500	100,00	0,00E+00	2836	9	NA	Japonica	
maker-3-augustus-gene-478.56-mRNA-1	3	OsR498G0307258800.01.T02	99,78	0,00E+00	3223	Os03g0812000	100,00	0,00E+00	2376	-847	Indica	Japonica	
maker-3-augustus-gene-479.37-mRNA-1	3	OsR498G0307262100.01.T01	99,84	0,00E+00	1116	Os03g0813700	100,00	0,00E+00	1121	5	NA	Japonica	
maker-3-augustus-gene-48.24-mRNA-1	3	OsR498G0305383900.01.T02	99,80	0,00E+00	3570	Os03g0195800	100,00	0,00E+00	3588	18	NA	Japonica	
maker-3-augustus-gene-48.27-mRNA-1	3	OsR498G0305385100.01.T01	99,24	0,00E+00	3479	Os03g0196000	100,00	0,00E+00	3534	55	NA	Japonica	
maker-3-augustus-gene-480.0-mRNA-1	3	OsR498G0307266700.01.T01	99,79	0,00E+00	1696	Os03g0815100	100,00	0,00E+00	1716	20	NA	Japonica	
maker-3-augustus-gene-480.13-mRNA-1	3	OsR498G0307267000.01.T01	99,89	0,00E+00	3211	Os03g0815200	100,00	0,00E+00	3220	9	NA	Japonica	
maker-3-augustus-gene-480.19-mRNA-1	3	OsR498G0307269000.01.T02	99,82	0,00E+00	1983	Os03g0815900	100,00	0,00E+00	1992	9	NA	Japonica	
maker-3-augustus-gene-480.2-mRNA-1	3	OsR498G0307267300.01.T02	99,68	0,00E+00	1133	Os03g0815400	100,00	0,00E+00	1142	9	NA	Japonica	
maker-3-augustus-gene-480.21-mRNA-1	3	OsR498G0307269200.01.T01	93,06	0,00E+00	1076	Os03g0816000	96,68	0,00E+00	1819	743	Japonica	Japonica	
maker-3-augustus-gene-480.25-mRNA-1	3	OsR498G0307269400.01.T01	99,74	0,00E+00	2065	Os03g0816100	100,00	0,00E+00	1521	-544	Indica	Japonica	Kinase-TM
maker-3-augustus-gene-480.33-mRNA-1	3	OsR498G0307272000.01.T02	99,71	0,00E+00	3073	Os03g0816400	100,00	0,00E+00	3095	22	NA	Japonica	
maker-3-augustus-gene-480.37-mRNA-1	3	OsR498G0307273800.01.T01	99,17	0,00E+00	1276	Os03g0816700	100,00	0,00E+00	1283	7	NA	Japonica	
maker-3-augustus-gene-480.4-mRNA-1	3	OsR498G0307268300.01.T01	100,00	0,00E+00	690	Os03g0815700	100,00	0,00E+00	690	0	NA	Japonica	
maker-3-augustus-gene-480.8-mRNA-1	3	OsR498G0307273500.01.T01	99,73	0,00E+00	3323	Os03g0816600	100,00	0,00E+00	3681	358	NA	Japonica	
maker-3-augustus-gene-481.32-mRNA-1	3	OsR498G0307274900.01.T01	99,87	0,00E+00	11046	Os03g0816900	100,00	0,00E+00	6150	-4896	Indica	Japonica	
maker-3-augustus-gene-481.43-mRNA-1	3	OsR498G0307279900.01.T02	99,81	0,00E+00	2801	Os03g0818100	100,00	0,00E+00	2814	13	NA	Japonica	
maker-3-augustus-gene-481.51-mRNA-1	3	OsR498G0307275400.01.T01	100,00	0,00E+00	850	Os03g0817100	100,00	0,00E+00	850	0	NA	Japonica	
maker-3-augustus-gene-481.53-mRNA-1	3	OsR498G0307277400.01.T01	99,58	0,00E+00	2538	Os03g0817500	100,00	0,00E+00	2565	27	NA	Japonica	
maker-3-augustus-gene-481.62-mRNA-1	3	OsR498G0307279400.01.T01	99,85	0,00E+00	2383	Os03g0817900	100,00	0,00E+00	2392	9	NA	Japonica	
maker-3-augustus-gene-481.66-mRNA-1	3	OsR498G0307280200.01.T01	99,87	0,00E+00	1338	Os03g0818300	100,00	0,00E+00	1342	4	NA	Japonica	

maker-3-augustus-gene-481.68-mRNA-1	3	OsR498G0307280400.01.T01	100,00	0,00E+00	767	Os03g0818400	100,00	0,00E+00	767	0	NA	Japonica	
maker-3-augustus-gene-482.42-mRNA-1	3	OsR498G0307281900.01.T01	99,87	0,00E+00	1338	Os03g0818800	100,00	0,00E+00	1687	349	Japonica	Japonica	
maker-3-augustus-gene-482.48-mRNA-1	3	OsR498G0307282900.01.T01	99,78	0,00E+00	3294	Os03g0819100	100,00	0,00E+00	3312	18	NA	Japonica	
maker-3-augustus-gene-482.51-mRNA-1	3	OsR498G0307283100.01.T01	96,24	0,00E+00	921	Os03g0819300	97,31	0,00E+00	948	27	NA	Japonica	
maker-3-augustus-gene-482.61-mRNA-1	3	OsR498G0307286300.01.T01	99,56	0,00E+00	805	Os03g0820500	100,00	0,00E+00	814	9	NA	Japonica	
maker-3-augustus-gene-482.64-mRNA-1	3	OsR498G0307287500.01.T01	99,90	0,00E+00	3508	Os03g0821100	100,00	0,00E+00	3517	9	NA	Japonica	
maker-3-augustus-gene-482.67-mRNA-1	3	OsR498G0307283400.01.T01	100,00	0,00E+00	1104	Os03g0819400	100,00	0,00E+00	1104	0	NA	Japonica	
maker-3-augustus-gene-482.71-mRNA-1	3	OsR498G0307284200.01.T01	100,00	0,00E+00	1267	Os03g0819600	100,00	0,00E+00	1267	0	NA	Japonica	
maker-3-augustus-gene-482.75-mRNA-1	3	OsR498G0307284400.01.T01	98,09	0,00E+00	1719	Os03g0819700	100,00	0,00E+00	1793	74	NA	Japonica	
maker-3-augustus-gene-482.78-mRNA-1	3	OsR498G0307286800.01.T01	99,61	0,00E+00	908	Os03g0820700	86,97	0,00E+00	927	19	NA	Japonica	
maker-3-augustus-gene-482.82-mRNA-1	3	OsR498G0307287200.01.T02	100,00	0,00E+00	899	Os03g0820900	100,00	0,00E+00	899	0	NA	Japonica	
maker-3-augustus-gene-483.0-mRNA-1	3	OsR498G0307287900.01.T01	99,12	0,00E+00	2200	Os03g0821200	100,00	0,00E+00	2246	46	NA	Japonica	
maker-3-augustus-gene-483.10-mRNA-1	3	OsR498G0307294300.01.T03	100,00	0,00E+00	1124	Os03g0822300	100,00	0,00E+00	1124	0	NA	Japonica	
maker-3-augustus-gene-483.14-mRNA-1	3	OsR498G0307294700.01.T01	99,83	0,00E+00	2048	Os03g0822700	100,00	0,00E+00	2057	9	NA	Japonica	
maker-3-augustus-gene-483.18-mRNA-1	3	OsR498G0307294900.01.T01	99,63	0,00E+00	1443	Os03g0822800	100,00	0,00E+00	908	-535	Indica	Japonica	
maker-3-augustus-gene-483.23-mRNA-1	3	OsR498G0307290000.01.T01	99,84	0,00E+00	3450	Os03g0821300	100,00	0,00E+00	3463	13	NA	Japonica	
maker-3-augustus-gene-483.27-mRNA-1	3	OsR498G0307292200.01.T02	99,74	0,00E+00	2049	Os03g0821800	97,14	0,00E+00	1758	-291	Indica	Japonica	
maker-3-augustus-gene-483.33-mRNA-1	3	OsR498G0307292800.01.T01	99,75	0,00E+00	1425	Os03g0822000	100,00	0,00E+00	1434	9	NA	Japonica	
maker-3-augustus-gene-483.36-mRNA-1	3	OsR498G0307294200.01.T01	99,74	0,00E+00	1388	Os03g0822200	100,00	0,00E+00	1397	9	NA	Japonica	
maker-3-augustus-gene-484.43-mRNA-1	3	OsR498G0307298700.01.T01	99,82	0,00E+00	979	Os03g0824200	100,00	0,00E+00	984	5	NA	Japonica	
maker-3-augustus-gene-484.45-mRNA-1	3	OsR498G0307299100.01.T03	96,31	0,00E+00	3516	Os03g0824300	100,00	0,00E+00	3057	-459	Indica	Japonica	
maker-3-augustus-gene-484.52-mRNA-1	3	OsR498G0307299300.01.T02	99,03	0,00E+00	903	Os03g0824400	100,00	0,00E+00	926	23	NA	Japonica	
maker-3-augustus-gene-484.56-mRNA-1	3	OsR498G0307295800.01.T03	99,93	0,00E+00	2653	Os03g0823200	100,00	0,00E+00	1645	-1008	Indica	Japonica	
maker-3-augustus-gene-484.62-mRNA-1	3	OsR498G0307296800.01.T01	96,35	0,00E+00	3002	Os03g0823500	97,07	0,00E+00	3061	59	NA	Japonica	
maker-3-augustus-gene-484.75-mRNA-1	3	OsR498G0306282100.01.T02	92,18	0,00E+00	721	Os03g0823900	98,10	0,00E+00	1551	830	Japonica	Japonica	
maker-3-augustus-gene-484.79-mRNA-1	3	OsR498G0307298600.01.T01	99,73	0,00E+00	4671	Os03g0824100	97,88	0,00E+00	3659	-1012	Indica	Japonica	
maker-3-augustus-gene-484.83-mRNA-1	3	OsR498G0100977700.01.T05	97,43	4,32E-168	591	Os03g0824350	100,00	1,08E-176	618	27	NA	Japonica	
maker-3-augustus-gene-485.0-mRNA-1	3	OsR498G0307301100.01.T01	99,93	0,00E+00	2523	Os03g0825300	100,00	0,00E+00	1388	-1135	Indica	Japonica	Kinase-TM
maker-3-augustus-gene-485.11-mRNA-1	3	OsR498G0307305100.01.T01	99,89	0,00E+00	1690	Os03g0826400	100,00	0,00E+00	1694	4	NA	Japonica	
maker-3-augustus-gene-485.15-mRNA-1	3	OsR498G0307305700.01.T01	99,82	0,00E+00	2941	Os03g0826600	100,00	0,00E+00	2219	-722	Indica	Japonica	
maker-3-augustus-gene-485.17-mRNA-1	3	OsR498G0307301400.01.T01	99,52	3,44E-102	371	Os03g0825400	99,52	1,20E-102	371	0	NA	Japonica	
maker-3-augustus-gene-485.21-mRNA-1	3	OsR498G0307302800.01.T02	99,70	0,00E+00	2378	Os03g0825800	99,77	0,00E+00	2380	2	NA	Japonica	Kinase
maker-3-augustus-gene-485.23-mRNA-1	3	OsR498G0307304400.01.T03	100,00	0,00E+00	2663	Os03g0826000	100,00	0,00E+00	2663	0	NA	Japonica	
maker-3-augustus-gene-485.31-mRNA-1	3	OsR498G0307306000.01.T01	94,41	0,00E+00	1480	Os03g0826700	94,36	0,00E+00	1273	-207	Indica	Japonica	
maker-3-augustus-gene-485.35-mRNA-1	3	OsR498G0307306200.01.T01	99,31	0,00E+00	766	Os03g0826800	100,00	0,00E+00	780	14	NA	Japonica	
maker-3-augustus-gene-485.38-mRNA-1	3	OsR498G0307306400.01.T01	99,54	0,00E+00	1167	Os03g0826900	100,00	0,00E+00	895	-272	Indica	Japonica	
maker-3-augustus-gene-485.4-mRNA-1	3	OsR498G0307301500.01.T02	99,80	0,00E+00	882	Os03g0825500	100,00	0,00E+00	886	4	NA	Japonica	
maker-3-augustus-gene-485.6-mRNA-1	3	OsR498G0307302500.01.T02	98,83	0,00E+00	4495	Os03g0825700	98,94	0,00E+00	4508	13	NA	Japonica	
maker-3-augustus-gene-486.44-mRNA-1	3	OsR498G0307307900.01.T02	99,90	0,00E+00	1733	Os03g0827500	99,87	0,00E+00	1331	-402	Indica	Japonica	
maker-3-augustus-gene-486.48-mRNA-1	3	OsR498G0307308700.01.T02	98,87	0,00E+00	4042	Os03g0827700	100,00	0,00E+00	3867	-175	NA	Japonica	
maker-3-augustus-gene-486.52-mRNA-1	3	OsR498G0307311600.01.T01	99,42	0,00E+00	912	Os03g0828100	100,00	0,00E+00	926	14	NA	Japonica	
maker-3-augustus-gene-486.54-mRNA-1	3	OsR498G0307311400.01.T01	96,71	0,00E+00	2378	Os03g0828500	87,58	0,00E+00	1696	-682	Indica	Japonica	
maker-3-augustus-gene-486.56-mRNA-1	3	OsR498G0307311000.01.T01	97,51	0,00E+00	2236	Os03g0828500	100,00	0,00E+00	2500	264	Japonica	Japonica	
maker-3-augustus-gene-486.58-mRNA-1	3	OsR498G0307310800.01.T01	99,66	0,00E+00	4190	Os03g0828600	100,00	0,00E+00	4226	36	NA	Japonica	
maker-3-augustus-gene-486.70-mRNA-1	3	OsR498G0307312000.01.T01	98,30	0,00E+00	1722	Os03g0829100	100,00	0,00E+00	1808	86	NA	Japonica	
maker-3-augustus-gene-486.72-mRNA-1	3	OsR498G0307312200.01.T01	96,48	0,00E+00	1689	Os03g0829200	100,00	0,00E+00	1835	146	NA	Japonica	
maker-3-augustus-gene-487.11-mRNA-1	3	OsR498G0307317900.01.T01	100,00	0,00E+00	1077	Os03g0831900	100,00	0,00E+00	1077	0	NA	NA	
maker-3-augustus-gene-487.14-mRNA-1	3	OsR498G0307313000.01.T01	98,65	2,55E-106	386	Os03g0829800	88,94	4,60E-85	314	-72	Indica	Japonica	
maker-3-augustus-gene-487.16-mRNA-1	3	OsR498G0307313600.01.T01	98,68	0,00E+00	791	Os03g0830200	100,00	0,00E+00	818	27	NA	Japonica	
maker-3-augustus-gene-487.18-mRNA-1	3	OsR498G0307313800.01.T01	96,30	0,00E+00	1012	Os03g0830401	100,00	0,00E+00	807	-205	Indica	Japonica	
maker-3-augustus-gene-487.2-mRNA-1	3	OsR498G0307315700.01.T01	100,00	0,00E+00	742	Os03g0831200	100,00	0,00E+00	742	0	NA	Japonica	
maker-3-augustus-gene-487.20-mRNA-1	3	OsR498G0307314400.01.T01	99,06	0,00E+00	751	Os03g0830500	100,00	0,00E+00	769	18	NA	Japonica	
maker-3-augustus-gene-487.23-mRNA-1	3	OsR498G0307315200.01.T01	98,13	0,00E+00	2487	Os03g0830900	100,00	0,00E+00	2608	121	NA	Japonica	
maker-3-augustus-gene-487.28-mRNA-1	3	OsR498G0307315600.01.T02	99,92	0,00E+00	2281	Os03g0831100	99,92	0,00E+00	2281	0	NA	Japonica	
maker-3-augustus-gene-487.33-mRNA-1	3	OsR498G0307316600.01.T01	100,00	0,00E+00	2159	Os03g0831500	100,00	0,00E+00	2159	0	NA	NA	
maker-3-augustus-gene-487.4-mRNA-1	3	OsR498G0307316100.01.T04	100,00	0,00E+00	2291	Os03g0831300	100,00	0,00E+00	1692	-599	Indica	NA	
maker-3-augustus-gene-487.8-mRNA-1	3	OsR498G0307317500.01.T02	100,00	0,00E+00	4800	Os03g0831700	100,00	0,00E+00	4800	0	NA	NA	
maker-3-augustus-gene-488.0-mRNA-1	3	OsR498G0307319500.01.T01	100,00	0,00E+00	1099	Os03g0832400	100,00	0,00E+00	1099	0	NA	NA	
maker-3-augustus-gene-488.12-mRNA-1	3	OsR498G0307320700.01.T01	100,00	0,00E+00	2933	Os03g0832800	100,00	0,00E+00	2933	0	NA	NA	
maker-3-augustus-gene-488.14-mRNA-1	3	OsR498G0307321500.01.T01	100,00	0,00E+00	1510	Os03g0833100	100,00	0,00E+00	1440	-70	NA	NA	
maker-3-augustus-gene-488.16-mRNA-1	3	OsR498G0307321700.01.T01	100,00	0,00E+00	2383	Os03g0833200	100,00	0,00E+00	1337	-1046	Indica	NA	
maker-3-augustus-gene-488.23-mRNA-1	3	OsR498G0307324500.01.T02	100,00	0,00E+00	2549	Os03g0833800	100,00	0,00E+00	2549	0	NA	NA	
maker-3-augustus-gene-488.28-mRNA-1	3	OsR498G0307318800.01.T01	100,00	0,00E+00	2397	Os03g0832200	100,00	0,00E+00	2397	0	NA	NA	
maker-3-augustus-gene-488.33-mRNA-1	3	OsR498G0307319400.01.T01	100,00	0,00E+00	693	Os03g0832300	100,00	0,00E+00	693	0	NA	NA	
maker-3-augustus-gene-488.36-mRNA-1	3	OsR498G0307321200.01.T01	100,00	0,00E+00	2392	Os03g0832900	100,00	0,00E+00	1440	-952	Indica	NA	
maker-3-augustus-gene-488.41-mRNA-1	3	OsR498G0307322000.01.T01	100,00	0,00E+00	5249	Os03g0833300	100,00	0,00E+00	5249	0	NA	NA	
maker-3-augustus-gene-488.47-mRNA-1	3	OsR498G0307323800.01.T01	96,92	0,00E+00	1976	Os03g0833700	96,92	0,00E+00	1976	0	NA	NA	
maker-3-augustus-gene-488.49-mRNA-1	3	OsR498G0307324800.01.T02	100,00	0,00E+00	984	Os03g0833900	100,00	0,00E+00	984	0	NA	NA	
maker-3-augustus-gene-488.6-mRNA-1	3	OsR498G0307320100.01.T01	100,00	0,00E+00	2744	Os03g0832600	100,00	0,00E+00	2744	0	NA	NA	
maker-3-augustus-gene-489.41-mRNA-1	3	OsR498G0307325300.01.T01	100,00	0,00E+00	1198	Os03g0834000	100,00	0,00E+00	1198	0	NA	NA	
maker-3-augustus-gene-489.49-mRNA-1	3	OsR498G0307329500.01.T01	100,00	0,00E+00	6451	Os03g0835100	100,00	0,00E+00	3957	-2494	Indica	NA	
maker-3-augustus-gene-489.50-mRNA-1	3	OsR498G0307330500.01.T01	100,00	0,00E+00	1943	Os03g0835400	100,00	0,00E+00	1943	0	NA	NA	
maker-3-augustus-gene-489.56-mRNA-1	3	OsR498G0307331900.01.T06	94,90	0,00E+00	1140	Os03g0835800	94,90	0,00E+00	1140	0	NA	NA	
maker-3-augustus-gene-489.63-mRNA-1	3	OsR498G0307333100.01.T01	98,19	0,00E+00	1716	Os03g0836100	100,00	0,00E+00	2336	620	Japonica	NA	
maker-3-augustus-gene-489.65-mRNA-1	3	OsR498G0305164700.01.T04	84,56	1,90E-36	152	Os03g0128700	84,56	6,67E-37	152	0	NA	Japonica	
maker-3-augustus-gene-489.67-mRNA-1	3	OsR498G0307325600.01.T01	100,00	0,00E+00	1012	Os03g0295300	85,27	6,15E-118	424	-588	Indica	NA	
maker-3-augustus-gene-489.76-mRNA-1	3	OsR498G0307333400.01.T07	100,00	0,00E+00	1115	Os03g0836200	100,00	0,00E+00	1115	0	NA	NA	
maker-3-augustus-gene-49.38-mRNA-1	3	OsR498G0305395700.01.T01	99,94	0,00E+00	2799	Os03g0197100	100,00	0,00E+00	2803	4	NA	Japonica	
maker-3-augustus-gene-49.40-mRNA-1	3	OsR498G0305397500.01.T01	98,99	0,00E+00	3461	Os03g0197300	100,00	0,00E+00	3258	-203	NA	Japonica	
maker-3-augustus-gene-49.42-mRNA-1	3	OsR498G0305402100.01.T04	99,84	0,00E+00	2296	Os03g0198300	100,00	0,00E+00	2300	4	NA	Japonica	
maker-3-augustus-gene-49.47-mRNA-1	3	OsR498G0305402300.01.T03	99,56	0,00E+00	1224	Os03g0198400	100,00	0,00E+00	1202	-22	NA	Japonica	
maker-3-augustus-gene-49.54-mRNA-1	3	OsR498G0305402700.01.T01	94,22	0,00E+00	1182	Os03g0198500	94,36	0,00E+00	1187	5	NA	Japonica	
maker-3-augustus-gene-49.57-mRNA-1	3	OsR498G0305394400.01.T01	99,62	0,00E+00	2845	Os03g0196900	100,00	0,00E+00	1602	-1243	Indica	Japonica	
maker-3-augustus-gene-49.61-mRNA-1	3	OsR498G0305394600.01.T05	95,65	0,00E+00	1469	Os03g0197000	100,00	0,00E+00	1353	-116	NA	Japonica	
maker-3-augustus-gene-49.66-mRNA-1	3	OsR498G0305398000.01.T01	99,91	0,00E+00	1915	Os03g0197400	100,00	0,00E+00	1920	5	NA	Japonica	
maker-3-augustus-gene-49.71-mRNA-1	3	OsR498G0305401000.01.T01	99,15	0,00E+00	1455	Os03g0197900	100,00	0,00E+00	1487	32	NA	Japonica	
maker-3-augustus-gene-49.72-mRNA-1	3	OsR498G0305403200.01.T01	99,58	0,00E+00	1286	Os03g0198600	100,00	0,00E+00	1299	13	NA	Japonica	
maker-3-augustus-gene-490.43-mRNA-1	3	OsR498G0307335100.01.T02	99,88	0,00E+00	1511	Os03g0836800	100,00	0,00E+00	1332	-179	Indica	Japonica	
maker-3-augustus-gene-490.46-mRNA-1	3	OsR498G0307335500.01.T01	99,44	0,00E+00	2216	Os03g0836900	100,00	0,00E+00	2246	30	NA	Japonica	
maker-3-augustus-gene-490.48-mRNA-1	3	OsR498G0307336500.01.T03	99,88	0,00E+00	5896	Os03g0837100	100,00	0,00E+00	5914	18	NA	Japonica	
maker-3-augustus-gene-490.56-mRNA-1	3	OsR498G0307337900.01.T01	99,72	0,00E+00	2596	Os03g0837300	100,00	0,00E+00	2614	18	NA	Japonica	
maker-3-augustus-gene-490.65-mRNA-1	3	OsR498G0307339700.01.T01	99,93	0,00E+00	2593	Os03g0838400	100,00	0,00E+00	1984	-609	Indica	Japonica	
maker-3-augustus-gene-490.67-mRNA-1	3	OsR498G0307339900.01.T01	99,72	1,39E-179	629	Os03g0838500	100,00	0,00E+00	634	5	NA	Japonica	
maker-3-augustus-gene-490.69-mRNA-1	3	OsR498G0307335800.01.T02	95,38	0,00E+00	2167	Os03g0837000	97,31	0,00E+00	2263	96	NA	Japonica	
maker-3-augustus-gene-490.77-mRNA-1	3	OsR498G0307338600.01.T02	100,00	0,00E+00	1386	Os03g0837800	100,00	0,00E+00	1175	-211	Indica	Japonica	
maker-3-augustus-gene-491.12-mRNA-1	3	OsR498G0307341000.01.T01	97,48	0,00E+00	2718	Os03g0839100	97,04	0,00E+00	2562	-156	NA	Japonica	
maker-3-augustus-gene-491.22-mRNA-1	3	OsR498G0307344800.01.T01	99,79	0,00E+00	5054	Os03g0839900	100,00	0,00E+00	5081	27	NA	Japonica	Kinase-TM
maker-3-augustus-gene-491.5-mRNA-1	3	OsR498G0307340800.01.T02	99,85	0,00E+00	1181	Os03g0838900	100,00	0,00E+00	1186	5	NA	Japonica	
maker-3-augustus-gene-491.7-mRNA-1	3	OsR498G0307340800.01.T04	99,83	0,00E+00	1035	Os03g0839000	100,00	0,00E+00	1040	5	NA	Japonica	
maker-3-augustus-gene-492.21-mRNA-1	3	OsR498G0307353300.01.T01	99,85	0,00E+00	1187	Os03g0843400	100,00	0,00E+00	1191	4	NA	Japonica	
maker-3-augustus-gene-492.24-mRNA-1	3	OsR498G0307346800.01.T01	98,28	0,00E+00	3924	Os03g0840400	100,00	0,00E+00	3956	32	NA	Japonica	
maker-3-augustus-gene-492.37-mRNA-1	3	OsR498G0307347800.01.T01	98,82	0,00E+00	4454	Os03g0840800	100,00	0,00E+00	2902	-1552	Indica	Japonica	
maker-3-augustus-gene-492.41-mRNA-1	3	OsR498G0307348200.01.T01	99,29	0,00E+00	2506	Os03g0841100	100,00	0,00E+00	2735	229	NA	Japonica	CC-Kinase-TM
maker-3-augustus-gene-492.45-mRNA-1	3	OsR498G0307348600.01.T01	99,43	0,00E+00	1553	Os03g0841700	100,00	0,00E+00	1575	22	NA	Japonica	
maker-3-augustus-gene-492.48-mRNA-1	3	OsR498G0307348800.01.T01	99,77	0,00E+00	2287	Os03g0841800	100,00	0,00E+00	2300	13	NA	Japonica	Kinase
maker-3-augustus-gene-492.50-mRNA-1	3	OsR498G0307349200.01.T04	99,74	0,00E+00	2081	Os03g0841900	98,19	0,00E+00	3172	1091	Japonica	Japonica	
maker-3-augustus-gene-492.58-mRNA-1	3	OsR498G0307352200.01.T01	100,00	0,00E+00	1927	Os03g0842900	100,00	0,00E+00	1927	0	NA	NA	
maker-3-augustus-gene-492.60-mRNA-1	3	OsR498G0307352600.01.T01	100,00	0,00E+00	1213	Os03g0843100	100,00	0,00E+00	1213	0	NA	NA	
maker-3-augustus-gene-493.1-mRNA-1	3	OsR498G0307353700.01.T04	99,95	0,00E+00	3441	Os03g0843600	98,28	0,00E+00	3284	-157	NA	Japonica	
maker-3-augustus-gene-493.12-mRNA-1	3	OsR498G0307354900.01.T06	99,95	0,00E+00	3567	Os03g0844000	100,00	0,00E+00	3571	4	NA	NA	
maker-3-augustus-gene-493.15-mRNA-1	3	OsR498G0307356100.01.T02	99,49	0,00E+00	1395	Os08g0116800	94,35	0,00E+00	1207	-188	Indica	Japonica	
maker-3-augustus-gene-493.19-mRNA-1	3	OsR498G0307356300.01.T02	100,00	0,00E+00	2803	Os03g0844500	100,00	0,00E+00	2803	0	NA	Japonica	
maker-3-augustus-gene-493.31-mRNA-1	3	OsR498G0307353600.01.T09	100,00	0,00E+00	1683	Os03g0843500	100,00	0,00E+00	1683	0	NA	Japonica	
maker-3-augustus-gene-493.37-mRNA-1	3	OsR498G0307354600.01.T02	100,00	0,00E+00	2760	Os03g0843900	100,00	0,00E+00	1451	-1309	Indica	NA	
maker-3-augustus-gene-493.45-mRNA-1	3	OsR498G0307356600.01.T01	99,91	0,00E+00	1967	Os03g0844600	100,00	0,00E+00	1992	25	NA	Japonica	
maker-3-augustus-gene-493.53-mRNA-1	3	OsR498G0307357800.01.T03	99,75	0,00E+00	2117	Os03g0845000	100,00	0,00E+00	1692	-425	Indica	Japonica	
maker-3-augustus-gene-494.24-mRNA-1	3	OsR498G0307362100.01.T03	100,00	0,00E+00	1851	Os03g0846400	99,42	0,00E+00	1825	-26	NA	Japonica	
maker-3-augustus-gene-494.32-mRNA-1	3	OsR498G0307365500.01.T05	100,00	0,00E+00	1397	Os03g0847600	100,00	0,00E+00	1397	0	NA	Japonica	Kinase-TM
maker-3-augustus-gene-494.37-mRNA-1	3	OsR498G0307359200.01.T04	99,96	0,00E+00	4059	Os03g0845500	100,00	0,00E+00	4064	5	NA	Japonica	
maker-3-augustus-gene-494.44-mRNA-1	3	OsR498G0307360000.01.T03	100,00	1,06E-130	466	Os03g0845700	100,00	4,81E-117	419	-47	Indica	Japonica	
maker-3-augustus-gene-495.13-mRNA-1	3	OsR498G0307367000.01.T04	99,86	0,00E+00	2598	Os03g0848100	100,00	0,00E+00	2607	9	NA	Japonica	
maker-3-augustus-gene-495.15-mRNA-1	3	OsR498G0307367200.01.T01	99,92	0,00E+00	2124	Os03g0848200	100,00	0,00E+00	2129	5	NA	Japonica	
maker-3-augustus-gene-495.17-mRNA-1	3	OsR498G0307367600.01.T03	94,49	0,00E+00	1102	Os03g0848400	94,78	0,00E+00	1111	9	NA	Japonica	
maker-3-augustus-gene-495.19-mRNA-1	3	OsR498G0307368000.01.T01	93,00	0,00E+00	2874	Os03g0848700	100,00	0,00E+00	3483	609	Japonica	Japonica	CC-NBS-TM
maker-3-augustus-gene-495.20-mRNA-1	3	OsR498G0307368000.01.T01	86,15	0,00E+00	4669	Os03g0849100	100,00	0,00E+00	7041	2372	Japonica	NA	LRR-NBS-TM
maker-3-augustus-gene-495.21-mRNA-1	3	OsR498G0307368000.01.T01	91,30	0,00E+00	665	Os03g0848700	89,17	6,46E-177	620	-45	NA	NA	
maker-3-augustus-gene-495.28-mRNA-1	3	OsR498G0307368400.01.T01	95,26	0,00E+00	3626	Os03g0849600	100,00	0,00E+00	4085	459	Japonica	Japonica	
maker-3-augustus-gene-495.5-mRNA-1	3	OsR498G0307367300.01.T01	100,00	0,00E+00	921	Os03g0848300	100,00	0,00E+00	921	0	NA	Japonica	
maker-3-augustus-gene-495.9-mRNA-1	3	OsR498G0307367700.01.T04	99,86	0,00E+00	1300	Os03g0848600	100,00	0,00E+00	1305	5	NA	Japonica	
maker-3-augustus-gene-496.42-mRNA-1	3	OsR498G0307368500.01.T03	99,01	0,00E+00	3385	Os03g0849700	100,00	0,00E+00	3912	527	Japonica	Japonica	
maker-3-augustus-gene-496.44-mRNA-1	3	OsR498G0307369100.01.T03	96,95	0,00E+00	1417	Os03g0849800	100,00	0,00E+00	1247	-170	Indica	Japonica	
maker-3-augustus-gene-496.48-mRNA-1	3	OsR498G0307370500.01.T01	99,62	0,00E+00	2830	Os03g0850200	100,00	0,00E+00	2857	27	NA	Japonica	
maker-3-augustus-gene-496.50-mRNA-1	3	OsR498G0307371900.01.T01	99,94	0,00E+00	2921	Os03g0850400	100,00	0,00E+00	2628	-293	Indica	Japonica	
maker-3-augustus-gene-496.57-mRNA-1	3	OsR498G0307374300.01.T03	91,82	0,00E+00	716	Os03g0851000	91,82	0,00E+00	716	0	NA	NA	
maker-3-augustus-gene-496.60-mRNA-1	3	OsR498G0307375500.01.T02	99,92	0,00E+00	2371	Os03g0851400	99,89	0,00E+00	1583	-788	Indica	Japonica	
maker-3-augustus-gene-496.63-mRNA-1	3	OsR498G0307370200.01.T01	100,00	0,00E+00	1505	Os03g0850000	100,00	0,00E+00	1505	0	NA	Japonica	
maker-3-augustus-gene-496.65-mRNA-1	3	OsR498G0307370400.01.T02	99,85	0,00E+00	2399	Os03g0850100	100,00	0,00E+00	2408	9	NA	Japonica	
maker-3-augustus-gene-496.68-mRNA-1	3	OsR498G0307373000.01.T01	100,00	0,00E+00	1553	Os03g0850600	100,00	0,00E+00	1553	0	NA	Japonica	
maker-3-augustus-gene-496.73-mRNA-1	3	OsR498G0307373800.01.T01	99,69	0,00E+00	1155	Os03g0850800	100,00	0,00E+00	1164	9	NA	Japonica	
maker-3-augustus-gene-496.75-mRNA-1	3	OsR498G0307374600.01.T01	100,00	0,00E+00	2457	Os03g0851100	100,00	0,00E+00	2457	0	NA	NA	
maker-3-augustus-gene-497.38-mRNA-1	3	OsR498G0307375500.01.T05	99,48	0,00E+00	1031	Os03g0851500	100,00	0,00E+00	1045	14	NA	Japonica	
maker-3-augustus-gene-497.40-mRNA-1	3	OsR498G0307375700.01.T02	99,82	0,00E+00	2920	Os03g0851600	100,00	0,00E+00	2581	-339	Indica	Japonica	
maker-3-augustus-gene-497.45-mRNA-1	3	OsR498G0307375900.01.T01	99,90	0,00E+00	1874	Os03g0851700	100,00	0,00E+00	1878	4	NA	Japonica	
maker-3-augustus-gene-497.47-mRNA-1	3	OsR498G0307376100.01.T01	99,90	0,00E+00	1801	Os03g0851800	100,00	0,00E+00	1700	-101	NA	Japonica	
maker-3-augustus-gene-497.49-mRNA-1	3	OsR498G0307377100.01.T04	97,64	0,00E+00	1599	Os03g0852300	97,75	0,00E+00	1603	4	NA	Japonica	
maker-3-augustus-gene-497.51-mRNA-1	3	OsR498G0307377700.01.T01	99,84	0,00E+00	1116	Os03g0852600	100,00	0,00E+00	1119	3	NA	Japonica	
maker-3-augustus-gene-497.55-mRNA-1	3	OsR498G0307379500.01.T01	96,39	0,00E+00	1420	Os03g0853200	100,00	0,00E+00	1548	128	NA	Japonica	
maker-3-augustus-gene-497.57-mRNA-1	3	OsR498G0307376600.01.T05	99,57	0,00E+00	1644	Os03g0851900	100,00	0,00E+00	1662	18	NA	Japonica	
maker-3-augustus-gene-497.62-mRNA-1	3	OsR498G0307376600.01.T07	99,78	0,00E+00	808	Os03g0852000	100,00	0,00E+00	812	4	NA	Japonica	
maker-3-augustus-gene-497.64-mRNA-1	3	OsR498G0307377000.01.T01	100,00	0,00E+00	1353	Os03g0852200	100,00	0,00E+00	1353	0	NA	Japonica	
maker-3-augustus-gene-497.68-mRNA-1	3	OsR498G0307377600.01.T01	100,00	0,00E+00	861	Os03g0852500	100,00	0,00E+00	948	87	NA	Japonica	
maker-3-augustus-gene-497.70-mRNA-1	3	OsR498G0307378800.01.T01	99,86	0,00E+00	3780	Os03g0852900	100,00	0,00E+00	3793	13	NA	Japonica	
maker-3-augustus-gene-498.31-mRNA-1	3	OsR498G0307381100.01.T01	100,00	0,00E+00	2003	Os03g0853500	100,00	0,00E+00	1777	-226	Indica	Japonica	
maker-3-augustus-gene-498.34-mRNA-1	3	OsR498G0307382300.01.T01	100,00	0,00E+00	1413	Os03g0853800	100,00	0,00E+00	904	-509	Indica	NA	
maker-3-augustus-gene-498.36-mRNA-1	3	OsR498G0307383300.01.T02	99,85	0,00E+00	2383	Os03g0854300	100,00	0,00E+00	2392	9	NA	Japonica	
maker-3-augustus-gene-498.39-mRNA-1	3	OsR498G0307383900.01.T04	100,00	0,00E+00	2744	Os03g0854500	100,00	0,00E+00	2744	0	NA	Japonica	
maker-3-augustus-gene-498.41-mRNA-1	3	OsR498G0307381600.01.T02	99,93	0,00E+00	2490	Os03g0853600	100,00	0,00E+00	1404	-1086	Indica	Japonica	
maker-3-augustus-gene-498.44-mRNA-1	3	OsR498G0307382600.01.T02	100,00	0,00E+00	2111	Os03g0853900	100,00	0,00E+00	1943	-168	NA	Japonica	
maker-3-augustus-gene-498.48-mRNA-1	3	OsR498G0307383000.01.T04	100,00	0,00E+00	1635	Os03g0854100	100,00	0,00E+00	1635	0	NA	Japonica	
maker-3-augustus-gene-498.53-mRNA-1	3	OsR498G0307383200.01.T02	96,23	0,00E+00	1182	Os03g0854200	96,37	0,00E+00	1187	5	NA	Japonica	
maker-3-augustus-gene-498.57-mRNA-1	3	OsR498G0307384200.01.T01	99,63	0,00E+00	1941	Os03g0854600	100,00	0,00E+00	1959	18	NA	Japonica	
maker-3-augustus-gene-498.59-mRNA-1	3	OsR498G0307385000.01.T05	100,00	0,00E+00	3166	Os03g0854800	100,00	0,00E+00	1954	-1212	Indica	Japonica	
maker-3-augustus-gene-499.0-mRNA-1	3	OsR498G0307385900.01.T01	100,00	0,00E+00	2035	Os03g0855100	100,00	0,00E+00	2035	0	NA	Japonica	
maker-3-augustus-gene-499.17-mRNA-1	3	OsR498G0307392300.01.T02	100,00	0,00E+00	2507	Os03g0856400	100,00	0,00E+00	2507	0	NA	NA	
maker-3-augustus-gene-499.23-mRNA-1	3	OsR498G0307392900.01.T03	100,00	0,00E+00	1678	Os03g0856500	100,00	0,00E+00	1678	0	NA	NA	
maker-3-augustus-gene-499.25-mRNA-1	3	OsR498G0307392200.01.T01	99,76	0,00E+00	759	Os03g0856300	100,00	0,00E+00	764	5	NA	NA	
maker-3-augustus-gene-5.11-mRNA-1	3	OsR498G0305113100.01.T01	99,74	0,00E+00	2095	Os03g0113800	100,00	0,00E+00	2109	14	NA	Japonica	
maker-3-augustus-gene-5.25-mRNA-1	3	OsR498G0305114700.01.T01	95,53	0,00E+00	1638	Os03g0114300	95,63	0,00E+00	1643	5	NA	NA	Kinase-TM
maker-3-augustus-gene-5.29-mRNA-1	3	OsR498G0305109200.01.T01	99,65	0,00E+00	3551	Os03g0112800	100,00	0,00E+00	3582	31	NA	Japonica	
maker-3-augustus-gene-5.35-mRNA-1	3	OsR498G0305109400.01.T01	99,74	0,00E+00	2796	Os03g0112900	100,00	0,00E+00	2814	18	NA	Japonica	
maker-3-augustus-gene-5.37-mRNA-1	3	OsR498G0305110000.01.T01	99,90	0,00E+00	1857	Os03g0113000	100,00	0,00E+00	1862	5	NA	Japonica	Kinase-TM
maker-3-augustus-gene-5.43-mRNA-1	3	OsR498G0305113000.01.T01	99,75	0,00E+00	3641	Os03g0113700	100,00	0,00E+00	3663	22	NA	Japonica	
maker-3-augustus-gene-5.47-mRNA-1	3	OsR498G0305114400.01.T01	100,00	0,00E+00	910	Os03g0114200	100,00	0,00E+00	910	0	NA	NA	
maker-3-augustus-gene-5.7-mRNA-1	3	OsR498G0305110300.01.T01	99,16	0,00E+00	1475	Os03g0113100	100,00	0,00E+00	1499	24	NA	Japonica	
maker-3-augustus-gene-5.9-mRNA-1	3	OsR498G0305110500.01.T02	99,62	0,00E+00	2371	Os03g0113200	100,00	0,00E+00	2378	7	NA	Japonica	
maker-3-augustus-gene-50.33-mRNA-1	3	OsR498G0305405300.01.T02	100,00	0,00E+00	1759	Os12g0511300	71,51	4,98E-136	485	-1274	Indica	Japonica	
maker-3-augustus-gene-50.38-mRNA-1	3	OsR498G0305408900.01.T01	100,00	0,00E+00	2057	Os12g0511200	72,91	7,57E-89	328	-1729	Indica	Japonica	
maker-3-augustus-gene-50.42-mRNA-1	3	OsR498G0305409500.01.T02	99,23	0,00E+00	1147	Os03g0200200	100,00	0,00E+00	1169	22	NA	Japonica	
maker-3-augustus-gene-50.44-mRNA-1	3	OsR498G0305410100.01.T01	95,10	0,00E+00	884	Os03g0200400	95,55	0,00E+00	734	-150	Indica	Japonica	
maker-3-augustus-gene-50.47-mRNA-1	3	OsR498G0305410500.01.T01	99,75	0,00E+00	1415	Os03g0200500	100,00	0,00E+00	1424	9	NA	Japonica	
maker-3-augustus-gene-50.51-mRNA-1	3	OsR498G0305405200.01.T02	99,92	0,00E+00	2258	Os03g0198900	100,00	0,00E+00	2262	4	NA	Japonica	
maker-3-augustus-gene-50.56-mRNA-1	3	OsR498G0305407200.01.T01	99,84	0,00E+00	1143	Os03g0199500	100,00	0,00E+00	733	-410	Indica	Japonica	
maker-3-augustus-gene-500.37-mRNA-1	3	OsR498G0307398100.01.T01	99,85	0,00E+00	2396	Os03g0858400	100,00	0,00E+00	2408	12	NA	Japonica	
maker-3-augustus-gene-500.41-mRNA-1	3	OsR498G0307396400.01.T02	93,75	0,00E+00	1747	Os03g0857400	93,85	0,00E+00	1526	-221	Indica	Japonica	
maker-3-augustus-gene-500.45-mRNA-1	3	OsR498G0307396600.01.T02	99,87	0,00E+00	1403	Os03g0857500	100,00	0,00E+00	1370	-33	NA	Japonica	
maker-3-augustus-gene-500.49-mRNA-1	3	OsR498G0307397600.01.T03	99,76	0,00E+00	3023	Os03g0858100	99,82	0,00E+00	3028	5	NA	Japonica	
maker-3-augustus-gene-501.46-mRNA-1	3	OsR498G0307400900.01.T01	99,71	0,00E+00	3122	Os03g0859900	99,94	0,00E+00	2939	-183	NA	Japonica	
maker-3-augustus-gene-501.58-mRNA-1	3	OsR498G0307399600.01.T01	100,00	0,00E+00	1981	Os03g0859100	100,00	0,00E+00	1981	0	NA	Japonica	
maker-3-augustus-gene-501.60-mRNA-1	3	OsR498G0307400000.01.T01	99,74	0,00E+00	2065	Os03g0859300	100,00	0,00E+00	2078	13	NA	Japonica	
maker-3-augustus-gene-501.63-mRNA-1	3	OsR498G0307400200.01.T01	99,92	0,00E+00	2216	Os03g0859500	99,92	0,00E+00	2216	0	NA	Japonica	
maker-3-augustus-gene-501.67-mRNA-1	3	OsR498G0307400200.01.T05	99,83	0,00E+00	3152	Os12g0277500	78,22	0,00E+00	1314	-1838	Indica	Japonica	
maker-3-augustus-gene-501.72-mRNA-1	3	OsR498G0307400600.01.T02	99,58	0,00E+00	1691	Os03g0859800	99,88	0,00E+00	1453	-238	Indica	Japonica	
maker-3-augustus-gene-502.101-mRNA-1	3	OsR498G0307405800.01.T01	99,88	0,00E+00	1479	Os03g0861700	100,00	0,00E+00	1406	-73	NA	NA	
maker-3-augustus-gene-502.65-mRNA-1	3	OsR498G0307404700.01.T03	98,67	0,00E+00	5792	Os03g0861100	98,90	0,00E+00	6721	929	Japonica	Japonica	
maker-3-augustus-gene-502.70-mRNA-1	3	OsR498G0307405900.01.T01	99,65	0,00E+00	2028	Os03g0861800	97,94	0,00E+00	1675	-353	Indica	NA	
maker-3-augustus-gene-502.73-mRNA-1	3	OsR498G0307406700.01.T03	99,88	0,00E+00	2902	Os03g0862100	100,00	0,00E+00	2911	9	NA	Japonica	
maker-3-augustus-gene-502.78-mRNA-1	3	OsR498G0307405900.01.T01	99,65	0,00E+00	2028	Os03g0861800	97,94	0,00E+00	1675	-353	Indica	NA	
maker-3-augustus-gene-502.81-mRNA-1	3	OsR498G0307405400.01.T02	99,25	0,00E+00	2121	Os03g0861400	100,00	0,00E+00	2154	33	NA	Japonica	
maker-3-augustus-gene-502.83-mRNA-1	3	OsR498G0307405800.01.T01	99,88	0,00E+00	1479	Os03g0861700	100,00	0,00E+00	1406	-73	NA	NA	
maker-3-augustus-gene-502.91-mRNA-1	3	OsR498G0713369200.01.T01	98,04	0,00E+00	1136	Os03g0862200	100,00	0,00E+00	1195	59	NA	Japonica	
maker-3-augustus-gene-502.94-mRNA-1	3	OsR498G0307211000.01.T02	99,69	0,00E+00	1746	Os03g0797400	100,00	0,00E+00	1759	13	NA	NA	
maker-3-augustus-gene-51.0-mRNA-1	3	OsR498G0305411500.01.T01	99,62	0,00E+00	928	Os03g0201000	100,00	0,00E+00	937	9	NA	Japonica	
maker-3-augustus-gene-51.12-mRNA-1	3	OsR498G0305415900.01.T02	99,68	0,00E+00	1663	Os03g0202500	98,92	0,00E+00	1635	-28	NA	Japonica	
maker-3-augustus-gene-51.16-mRNA-1	3	OsR498G0305411000.01.T01	99,61	0,00E+00	1837	Os03g0200700	99,81	0,00E+00	1846	9	NA	Japonica	
maker-3-augustus-gene-51.2-mRNA-1	3	OsR498G0305411900.01.T01	99,72	0,00E+00	1258	Os03g0201200	100,00	0,00E+00	1267	9	NA	Japonica	
maker-3-augustus-gene-51.22-mRNA-1	3	OsR498G0305411200.01.T01	99,29	0,00E+00	1499	Os03g0200800	100,00	0,00E+00	1002	-497	Indica	Japonica	
maker-3-augustus-gene-51.25-mRNA-1	3	OsR498G0305411800.01.T04	98,60	0,00E+00	3124	Os03g0201100	100,00	0,00E+00	2479	-645	Indica	Japonica	
maker-3-augustus-gene-51.33-mRNA-1	3	OsR498G0305413000.01.T03	99,88	0,00E+00	1484	Os03g0201600	100,00	0,00E+00	951	-533	Indica	Japonica	
maker-3-augustus-gene-51.36-mRNA-1	3	OsR498G0305413400.01.T01	99,94	0,00E+00	3150	Os03g0201800	100,00	0,00E+00	3155	5	NA	Japonica	
maker-3-augustus-gene-51.38-mRNA-1	3	OsR498G0305413600.01.T01	99,60	0,00E+00	3107	Os03g0201901	100,00	0,00E+00	2217	-890	Indica	Japonica	
maker-3-augustus-gene-51.40-mRNA-1	3	OsR498G0305414000.01.T04	99,84	0,00E+00	1154	Os07g0573100	74,31	1,99E-95	350	-804	Indica	Japonica	NBS-TM
maker-3-augustus-gene-51.44-mRNA-1	3	OsR498G0305414200.01.T01	100,00	0,00E+00	1505	Os03g0202200	100,00	0,00E+00	1505	0	NA	Japonica	

maker-3-augustus-gene-51.47-mRNA-1	3	OsR498G0305414400.01.T01	99,61	0,00E+00	1354	Os03g0202300	99,87	0,00E+00	1363	9	NA	Japonica	
maker-3-augustus-gene-51.5-mRNA-1	3	OsR498G0305412700.01.T01	98,09	0,00E+00	2959	Os03g0201400	100,00	0,00E+00	1840	-1119	Indica	Japonica	
maker-3-augustus-gene-51.8-mRNA-1	3	OsR498G0305413100.01.T01	99,83	0,00E+00	2075	Os03g0201700	99,91	0,00E+00	2079	4	NA	Japonica	
maker-3-augustus-gene-52.44-mRNA-1	3	OsR498G0305418300.01.T06	99,79	0,00E+00	1718	Os03g0203200	100,00	0,00E+00	1727	9	NA	Japonica	
maker-3-augustus-gene-52.46-mRNA-1	3	OsR498G0305418900.01.T01	100,00	0,00E+00	5519	Os03g0203700	100,00	0,00E+00	5519	0	NA	Japonica	
maker-3-augustus-gene-52.48-mRNA-1	3	OsR498G0305420500.01.T04	99,70	0,00E+00	4796	Os03g0204100	100,00	0,00E+00	4832	36	NA	Japonica	
maker-3-augustus-gene-52.54-mRNA-1	3	OsR498G0305416600.01.T01	99,82	0,00E+00	1961	Os03g0203000	100,00	0,00E+00	1970	9	NA	Japonica	
maker-3-augustus-gene-52.57-mRNA-1	3	OsR498G0305419200.01.T01	99,84	0,00E+00	1122	Os03g0203800	100,00	0,00E+00	1126	4	NA	Japonica	
maker-3-augustus-gene-52.61-mRNA-1	3	OsR498G0305422800.01.T01	100,00	5,75E-165	580	Os03g0204500	100,00	2,00E-165	580	0	NA	Japonica	
maker-3-augustus-gene-53.26-mRNA-1	3	OsR498G0305425700.01.T01	99,80	0,00E+00	7918	Os03g0205400	100,00	0,00E+00	7959	41	NA	Japonica	
maker-3-augustus-gene-53.31-mRNA-1	3	OsR498G0305425900.01.T01	87,05	0,00E+00	1031	Os03g0205500	100,00	0,00E+00	1379	348	Japonica	Japonica	
maker-3-augustus-gene-53.36-mRNA-1	3	OsR498G0305426900.01.T02	99,62	0,00E+00	3248	Os03g0205700	100,00	0,00E+00	3279	31	NA	Japonica	
maker-3-augustus-gene-53.40-mRNA-1	3	OsR498G0305424000.01.T01	99,74	0,00E+00	3396	Os03g0205000	100,00	0,00E+00	3418	22	NA	Japonica	
maker-3-augustus-gene-53.48-mRNA-1	3	OsR498G0305427400.01.T01	99,51	0,00E+00	2168	Os03g0205800	100,00	0,00E+00	2188	20	NA	Japonica	
maker-3-augustus-gene-53.54-mRNA-1	3	OsR498G0305429200.01.T05	100,00	7,69E-179	627	Os03g0206300	94,24	5,90E-156	550	-77	Indica	Japonica	
maker-3-augustus-gene-54.36-mRNA-1	3	OsR498G0305430300.01.T01	97,99	0,00E+00	939	Os03g0206600	100,00	0,00E+00	969	30	NA	NA	
maker-3-augustus-gene-54.38-mRNA-1	3	OsR498G0305430700.01.T01	99,15	0,00E+00	1446	Os03g0206900	100,00	0,00E+00	1478	32	NA	NA	
maker-3-augustus-gene-54.40-mRNA-1	3	OsR498G0305432300.01.T01	99,66	0,00E+00	2107	Os03g0207400	100,00	0,00E+00	2127	20	NA	Japonica	
maker-3-augustus-gene-54.58-mRNA-1	3	OsR498G0305430600.01.T01	99,89	0,00E+00	3384	Os03g0206700	100,00	0,00E+00	2378	-1006	Indica	NA	
maker-3-augustus-gene-54.63-mRNA-1	3	OsR498G0305431600.01.T04	100,00	0,00E+00	2051	Os03g0207200	100,00	0,00E+00	1923	-128	NA	Japonica	
maker-3-augustus-gene-54.66-mRNA-1	3	OsR498G0305431800.01.T01	99,44	0,00E+00	641	Os03g0207250	100,00	0,00E+00	650	9	NA	Japonica	
maker-3-augustus-gene-54.69-mRNA-1	3	OsR498G0305432000.01.T01	90,53	0,00E+00	1480	Os03g0207300	90,63	0,00E+00	1485	5	NA	Japonica	Kinase-TM
maker-3-augustus-gene-54.74-mRNA-1	3	OsR498G0305433600.01.T01	99,88	0,00E+00	3027	Os03g0207900	100,00	0,00E+00	3036	9	NA	Japonica	
maker-3-augustus-gene-54.77-mRNA-1	3	OsR498G0305434000.01.T01	99,43	0,00E+00	2801	Os03g0208500	100,00	0,00E+00	2841	40	NA	Japonica	
maker-3-augustus-gene-55.39-mRNA-1	3	OsR498G0305438500.01.T01	99,94	0,00E+00	2755	Os03g0209600	100,00	0,00E+00	2760	5	NA	Japonica	
maker-3-augustus-gene-55.49-mRNA-1	3	OsR498G0305604600.01.T02	89,64	0,00E+00	3117	Os03g0256800	89,16	0,00E+00	2704	-413	Indica	Japonica	
maker-3-augustus-gene-55.66-mRNA-1	3	OsR498G0305435600.01.T02	98,17	0,00E+00	1795	Os03g0208900	98,26	0,00E+00	1800	5	NA	Japonica	
maker-3-augustus-gene-55.77-mRNA-1	3	OsR498G0305440600.01.T03	99,66	0,00E+00	4239	Os03g0210400	100,00	0,00E+00	4275	36	NA	Japonica	
maker-3-augustus-gene-56.42-mRNA-1	3	OsR498G0305443900.01.T01	99,71	0,00E+00	3132	Os03g0211600	100,00	0,00E+00	2320	-812	Indica	Japonica	
maker-3-augustus-gene-56.44-mRNA-1	3	OsR498G0305444100.01.T03	96,65	0,00E+00	1746	Os03g0211700	97,12	0,00E+00	1765	19	NA	Japonica	
maker-3-augustus-gene-56.60-mRNA-1	3	OsR498G0305446900.01.T01	99,69	0,00E+00	2867	Os03g0212700	100,00	0,00E+00	2890	23	NA	Japonica	
maker-3-augustus-gene-56.64-mRNA-1	3	OsR498G0305441600.01.T04	99,90	0,00E+00	7117	Os03g0210800	100,00	0,00E+00	4697	-2420	Indica	Japonica	
maker-3-augustus-gene-56.72-mRNA-1	3	OsR498G0305444400.01.T01	99,77	0,00E+00	3110	Os12g0578500	72,00	0,00E+00	790	-2320	Indica	Japonica	
maker-3-augustus-gene-56.75-mRNA-1	3	OsR498G0305445400.01.T01	99,90	0,00E+00	1814	Os03g0211900	100,00	0,00E+00	1819	5	NA	Japonica	LRR-TM
maker-3-augustus-gene-56.79-mRNA-1	3	OsR498G0305447400.01.T02	99,81	0,00E+00	2811	Os03g0212800	100,00	0,00E+00	2825	14	NA	Japonica	
maker-3-augustus-gene-57.0-mRNA-1	3	OsR498G0305448700.01.T01	99,86	0,00E+00	2567	Os03g0213100	100,00	0,00E+00	2576	9	NA	Japonica	
maker-3-augustus-gene-57.12-mRNA-1	3	OsR498G0305449900.01.T02	99,59	0,00E+00	3908	Os03g0213600	99,78	0,00E+00	5635	1727	Japonica	Japonica	
maker-3-augustus-gene-57.25-mRNA-1	3	OsR498G0305451100.01.T02	99,95	0,00E+00	3320	Os03g0214000	100,00	0,00E+00	3333	13	NA	Japonica	
maker-3-augustus-gene-57.30-mRNA-1	3	OsR498G0305451300.01.T01	99,89	0,00E+00	3406	Os03g0214100	100,00	0,00E+00	3415	9	NA	Japonica	
maker-3-augustus-gene-57.6-mRNA-1	3	OsR498G0305449100.01.T01	99,83	0,00E+00	8242	Os03g0213300	100,00	0,00E+00	8278	36	NA	Japonica	
maker-3-augustus-gene-58.0-mRNA-1	3	OsR498G0305452300.01.T01	99,80	0,00E+00	1815	Os03g0214600	100,00	0,00E+00	1824	9	NA	Japonica	
maker-3-augustus-gene-58.12-mRNA-1	3	OsR498G0305451600.01.T01	99,67	0,00E+00	2158	Os03g0214200	100,00	0,00E+00	2176	18	NA	Japonica	
maker-3-augustus-gene-58.15-mRNA-1	3	OsR498G0305452200.01.T02	99,89	0,00E+00	1574	Os03g0214400	100,00	0,00E+00	1579	5	NA	Japonica	
maker-3-augustus-gene-58.20-mRNA-1	3	OsR498G0305452600.01.T01	99,79	0,00E+00	5222	Os03g0214900	100,00	0,00E+00	5249	27	NA	Japonica	
maker-3-augustus-gene-58.24-mRNA-1	3	OsR498G0305455800.01.T01	100,00	0,00E+00	1397	Os03g0215400	100,00	0,00E+00	1397	0	NA	Japonica	
maker-3-augustus-gene-58.5-mRNA-1	3	OsR498G0305454900.01.T02	99,83	0,00E+00	1062	Os03g0215200	98,99	0,00E+00	1040	-22	NA	Japonica	
maker-3-augustus-gene-58.8-mRNA-1	3	OsR498G0305456700.01.T02	99,91	0,00E+00	2002	Os03g0215700	99,91	0,00E+00	2002	0	NA	Japonica	
maker-3-augustus-gene-59.0-mRNA-1	3	OsR498G0305457100.01.T01	98,97	0,00E+00	2401	Os03g0215800	99,71	0,00E+00	2426	25	NA	Japonica	
maker-3-augustus-gene-59.11-mRNA-1	3	OsR498G0305461700.01.T06	97,51	0,00E+00	2261	Os03g0216800	97,74	0,00E+00	2275	14	NA	Japonica	
maker-3-augustus-gene-59.14-mRNA-1	3	OsR498G0305462100.01.T02	100,00	0,00E+00	2199	Os03g0216900	100,00	0,00E+00	1824	-375	Indica	Japonica	
maker-3-augustus-gene-59.17-mRNA-1	3	OsR498G0305462500.01.T04	99,85	0,00E+00	1190	Os03g0217200	100,00	0,00E+00	1195	5	NA	Japonica	
maker-3-augustus-gene-59.22-mRNA-1	3	OsR498G0305460600.01.T03	99,71	0,00E+00	3073	Os03g0216700	100,00	0,00E+00	3095	22	NA	Japonica	
maker-3-augustus-gene-59.6-mRNA-1	3	OsR498G0305460300.01.T01	99,85	0,00E+00	3553	Os03g0216600	100,00	0,00E+00	3258	-295	NA	Japonica	
maker-3-augustus-gene-6.0-mRNA-1	3	OsR498G0305116100.01.T01	99,78	0,00E+00	2465	Os03g0114800	99,93	0,00E+00	2474	9	NA	NA	
maker-3-augustus-gene-6.15-mRNA-1	3	OsR498G0305116800.01.T02	99,75	0,00E+00	5770	Os03g0115100	99,66	0,00E+00	5756	-14	NA	NA	
maker-3-augustus-gene-6.19-mRNA-1	3	OsR498G0305117000.01.T01	100,00	0,00E+00	1234	Os03g0115200	99,56	0,00E+00	1221	-13	NA	NA	
maker-3-augustus-gene-6.21-mRNA-1	3	OsR498G0305117000.01.T04	99,81	0,00E+00	1860	Os03g0115300	99,17	0,00E+00	2337	477	Japonica	Indica	
maker-3-augustus-gene-6.23-mRNA-1	3	OsR498G0305119600.01.T01	100,00	0,00E+00	1802	Os03g0115700	99,90	0,00E+00	1798	-4	NA	NA	
maker-3-augustus-gene-6.4-mRNA-1	3	OsR498G0305116500.01.T01	99,83	0,00E+00	1073	Os03g0115000	99,66	0,00E+00	1058	-15	NA	NA	
maker-3-augustus-gene-6.6-mRNA-1	3	OsR498G0305118700.01.T02	99,78	0,00E+00	4020	Os03g0115400	99,96	0,00E+00	4038	18	NA	Japonica	
maker-3-augustus-gene-60.41-mRNA-1	3	OsR498G0305466300.01.T01	100,00	0,00E+00	2376	Os03g0219100	100,00	0,00E+00	2376	0	NA	Japonica	
maker-3-augustus-gene-60.44-mRNA-1	3	OsR498G0305463200.01.T03	98,71	0,00E+00	1084	Os03g0217200	81,62	0,00E+00	685	-399	Indica	Japonica	
maker-3-augustus-gene-60.47-mRNA-1	3	OsR498G0305463200.01.T04	88,80	1,48E-154	546	Os03g0218200	87,22	1,13E-131	468	-78	Indica	Japonica	
maker-3-augustus-gene-60.50-mRNA-1	3	OsR498G0305466600.01.T01	100,00	0,00E+00	893	Os03g0219200	100,00	0,00E+00	883	-10	NA	Japonica	
maker-3-augustus-gene-60.55-mRNA-1	3	OsR498G0305466800.01.T01	99,93	0,00E+00	2431	Os03g0219300	100,00	0,00E+00	2435	4	NA	Japonica	
maker-3-augustus-gene-60.58-mRNA-1	3	OsR498G0305467000.01.T01	97,90	0,00E+00	3196	Os03g0219400	98,06	0,00E+00	3210	14	NA	Japonica	
maker-3-augustus-gene-61.29-mRNA-1	3	OsR498G0305467900.01.T05	99,62	0,00E+00	1416	Os03g0219900	100,00	0,00E+00	1429	13	NA	Japonica	
maker-3-augustus-gene-61.34-mRNA-1	3	OsR498G0305472100.01.T01	99,80	0,00E+00	1777	Os03g0221200	100,00	0,00E+00	1489	-288	Indica	Japonica	
maker-3-augustus-gene-61.37-mRNA-1	3	OsR498G0305472900.01.T01	99,94	0,00E+00	2801	Os03g0221500	100,00	0,00E+00	2733	-68	NA	Japonica	
maker-3-augustus-gene-61.40-mRNA-1	3	OsR498G0305468600.01.T01	99,87	0,00E+00	2670	Os03g0220100	100,00	0,00E+00	2679	9	NA	Japonica	
maker-3-augustus-gene-61.42-mRNA-1	3	OsR498G0305472400.01.T01	99,85	0,00E+00	4785	Os03g0221300	100,00	0,00E+00	3658	-1127	Indica	Japonica	
maker-3-augustus-gene-62.58-mRNA-1	3	OsR498G0305477500.01.T01	99,80	0,00E+00	3640	Os03g0223000	100,00	0,00E+00	3864	224	NA	Japonica	Kinase-LRR-TM
maker-3-augustus-gene-62.61-mRNA-1	3	OsR498G0305478700.01.T01	99,82	0,00E+00	1956	Os03g0223200	100,00	0,00E+00	1593	-363	Indica	Japonica	
maker-3-augustus-gene-62.63-mRNA-1	3	OsR498G0305479700.01.T01	98,71	8,25E-153	540	Os01g0862800	93,63	3,50E-133	473	-67	Indica	Japonica	
maker-3-augustus-gene-62.72-mRNA-1	3	OsR498G0305474200.01.T01	99,79	0,00E+00	3440	Os03g0222100	100,00	0,00E+00	3458	18	NA	Japonica	
maker-3-augustus-gene-62.74-mRNA-1	3	OsR498G0305479200.01.T01	95,53	0,00E+00	1760	Os03g0223400	95,53	0,00E+00	1760	0	NA	Japonica	
maker-3-augustus-gene-63.0-mRNA-1	3	OsR498G0305481700.01.T01	95,45	0,00E+00	1653	Os03g0223900	95,55	0,00E+00	1657	4	NA	Japonica	
maker-3-augustus-gene-63.11-mRNA-1	3	OsR498G0305484500.01.T01	99,68	1,10E-156	554	Os03g0224501	90,84	2,12E-103	374	-180	Indica	Japonica	
maker-3-augustus-gene-63.14-mRNA-1	3	OsR498G0305484900.01.T03	99,92	0,00E+00	2198	Os03g0224700	99,84	0,00E+00	2185	-13	NA	Japonica	
maker-3-augustus-gene-63.18-mRNA-1	3	OsR498G0305487700.01.T01	99,72	0,00E+00	6936	Os03g0225500	100,00	0,00E+00	6985	49	NA	Japonica	
maker-3-augustus-gene-63.21-mRNA-1	3	OsR498G0305483400.01.T02	99,88	0,00E+00	4645	Os03g0224300	100,00	0,00E+00	4096	-549	Indica	Japonica	
maker-3-augustus-gene-63.23-mRNA-1	3	OsR498G0305487000.01.T05	99,63	0,00E+00	2386	Os03g0225200	100,00	0,00E+00	2405	19	NA	Japonica	
maker-3-augustus-gene-63.6-mRNA-1	3	OsR498G0305483100.01.T03	99,95	0,00E+00	3740	Os03g0224200	100,00	0,00E+00	3745	5	NA	Japonica	
maker-3-augustus-gene-64.10-mRNA-1	3	OsR498G0305490500.01.T01	99,67	0,00E+00	2136	Os03g0226400	99,85	0,00E+00	2384	248	Japonica	Japonica	
maker-3-augustus-gene-64.15-mRNA-1	3	OsR498G0305491100.01.T01	99,80	0,00E+00	6160	Os03g0226600	100,00	0,00E+00	6192	32	NA	Japonica	
maker-3-augustus-gene-64.30-mRNA-1	3	OsR498G0305489600.01.T01	99,80	0,00E+00	916	Os03g0226200	100,00	0,00E+00	921	5	NA	Japonica	
maker-3-augustus-gene-64.32-mRNA-1	3	OsR498G0305491400.01.T01	99,62	0,00E+00	1410	Os03g0226700	100,00	0,00E+00	1424	14	NA	Japonica	
maker-3-augustus-gene-64.4-mRNA-1	3	OsR498G0305488100.01.T01	99,76	0,00E+00	3792	Os03g0225700	100,00	0,00E+00	3815	23	NA	Japonica	Kinase-TM
maker-3-augustus-gene-64.8-mRNA-1	3	OsR498G0305489900.01.T01	99,27	0,00E+00	1939	Os03g0226300	99,91	0,00E+00	1971	32	NA	Japonica	Kinase-TM
maker-3-augustus-gene-65.0-mRNA-1	3	OsR498G0305492100.01.T03	99,93	0,00E+00	4850	Os03g0227000	100,00	0,00E+00	4859	9	NA	Japonica	
maker-3-augustus-gene-65.14-mRNA-1	3	OsR498G0305494100.01.T01	100,00	5,75E-165	580	Os03g0227500	100,00	2,00E-165	580	0	NA	Japonica	
maker-3-augustus-gene-65.16-mRNA-1	3	OsR498G0305496100.01.T01	99,93	0,00E+00	2739	Os03g0227700	100,00	0,00E+00	2744	5	NA	Japonica	
maker-3-augustus-gene-65.20-mRNA-1	3	OsR498G0305497400.01.T02	99,64	0,00E+00	2008	Os03g0227900	100,00	0,00E+00	2026	18	NA	Japonica	Kinase-TM
maker-3-augustus-gene-65.7-mRNA-1	3	OsR498G0305493300.01.T02	99,92	0,00E+00	2279	Os03g0227300	100,00	0,00E+00	2284	5	NA	Japonica	
maker-3-augustus-gene-66.0-mRNA-1	3	OsR498G0305498900.01.T01	99,80	0,00E+00	900	Os03g0228400	100,00	0,00E+00	904	4	NA	Japonica	
maker-3-augustus-gene-66.10-mRNA-1	3	OsR498G0305505200.01.T01	99,33	0,00E+00	3435	Os03g0227966	100,00	0,00E+00	3490	55	NA	Japonica	
maker-3-augustus-gene-66.4-mRNA-1	3	OsR498G0305499100.01.T01	100,00	0,00E+00	1175	Os03g0228500	100,00	0,00E+00	1175	0	NA	Japonica	
maker-3-augustus-gene-66.6-mRNA-1	3	OsR498G0305498600.01.T03	99,89	0,00E+00	1619	Os03g0228200	100,00	0,00E+00	1624	5	NA	Japonica	
maker-3-augustus-gene-66.8-mRNA-1	3	OsR498G0305501000.01.T01	99,18	0,00E+00	5374	Os03g0228800	100,00	0,00E+00	5454	80	NA	Japonica	Kinase-LRR-TM
maker-3-augustus-gene-67.10-mRNA-1	3	OsR498G0305514300.01.T01	100,00	0,00E+00	2251	Os03g0231600	100,00	0,00E+00	2251	0	NA	NA	
maker-3-augustus-gene-67.2-mRNA-1	3	OsR498G0305508500.01.T01	100,00	0,00E+00	1894	Os03g0230500	100,00	0,00E+00	1894	0	NA	NA	
maker-3-augustus-gene-67.20-mRNA-1	3	OsR498G0305514600.01.T05	100,00	0,00E+00	2830	Os03g0231700	100,00	0,00E+00	2823	-7	NA	Japonica	
maker-3-augustus-gene-67.23-mRNA-1	3	OsR498G0305514800.01.T01	99,87	0,00E+00	2828	Os03g0231800	100,00	0,00E+00	2388	-440	Indica	Japonica	
maker-3-augustus-gene-68.0-mRNA-1	3	OsR498G0305519700.01.T01	100,00	7,59E-157	553	Os03g0232400	100,00	2,64E-157	553	0	NA	Japonica	
maker-3-augustus-gene-68.10-mRNA-1	3	OsR498G0305517400.01.T02	99,53	0,00E+00	3443	Os03g0232200	99,53	0,00E+00	3443	0	NA	Japonica	
maker-3-augustus-gene-68.14-mRNA-1	3	OsR498G0305522200.01.T01	99,59	0,00E+00	2200	Os03g0232800	100,00	0,00E+00	2251	51	NA	Japonica	
maker-3-augustus-gene-68.16-mRNA-1	3	OsR498G0305524800.01.T01	99,62	0,00E+00	1426	Os03g0233200	100,00	0,00E+00	1440	14	NA	Japonica	
maker-3-augustus-gene-68.2-mRNA-1	3	OsR498G0305520300.01.T04	98,95	0,00E+00	1002	Os03g0232500	100,00	0,00E+00	1023	21	NA	Japonica	
maker-3-augustus-gene-68.5-mRNA-1	3	OsR498G0305524100.01.T01	99,72	0,00E+00	1886	Os03g0233000	100,00	0,00E+00	1900	14	NA	Japonica	
maker-3-augustus-gene-69.43-mRNA-1	3	OsR498G0305525500.01.T01	99,89	0,00E+00	3308	Os03g0233500	100,00	0,00E+00	3317	9	NA	Japonica	
maker-3-augustus-gene-69.46-mRNA-1	3	OsR498G0305526500.01.T05	99,51	0,00E+00	3280	Os03g0233800	100,00	0,00E+00	3317	37	NA	Japonica	
maker-3-augustus-gene-69.52-mRNA-1	3	OsR498G0305526700.01.T01	100,00	0,00E+00	904	Os03g0233900	100,00	0,00E+00	904	0	NA	Japonica	
maker-3-augustus-gene-69.54-mRNA-1	3	OsR498G0305526900.01.T01	100,00	0,00E+00	921	Os03g0234000	100,00	0,00E+00	921	0	NA	Japonica	
maker-3-augustus-gene-69.56-mRNA-1	3	OsR498G0305527100.01.T01	100,00	0,00E+00	910	Os03g0234100	100,00	0,00E+00	910	0	NA	Japonica	
maker-3-augustus-gene-69.58-mRNA-1	3	OsR498G0305527500.01.T01	98,43	0,00E+00	1764	Os10g0109300	70,51	8,18E-126	451	-1313	Indica	Japonica	
maker-3-augustus-gene-69.60-mRNA-1	3	OsR498G0305528700.01.T03	100,00	1,20E-169	596	Os03g0235100	100,00	4,19E-170	596	0	NA	Japonica	
maker-3-augustus-gene-69.67-mRNA-1	3	OsR498G0305528200.01.T01	99,80	0,00E+00	1799	Os03g0234900	100,00	0,00E+00	1808	9	NA	Japonica	
maker-3-augustus-gene-69.68-mRNA-1	3	OsR498G0305528200.01.T04	99,80	0,00E+00	1810	Os03g0235000	100,00	0,00E+00	1819	9	NA	Japonica	
maker-3-augustus-gene-69.82-mRNA-1	3	OsR498G0305529600.01.T01	99,93	0,00E+00	2487	Os03g0235700	100,00	0,00E+00	2491	4	NA	Japonica	
maker-3-augustus-gene-69.87-mRNA-1	3	OsR498G0305529800.01.T02	100,00	0,00E+00	1485	Os03g0235900	100,00	0,00E+00	1485	0	NA	Japonica	
maker-3-augustus-gene-7.0-mRNA-1	3	OsR498G0305121100.01.T01	98,97	0,00E+00	2215	Os03g0116000	99,85	0,00E+00	1170	-1045	Indica	Japonica	
maker-3-augustus-gene-7.11-mRNA-1	3	OsR498G0305122300.01.T01	100,00	0,00E+00	1764	Os03g0116400	100,00	0,00E+00	1764	0	NA	Japonica	
maker-3-augustus-gene-7.14-mRNA-1	3	OsR498G0305123300.01.T01	100,00	0,00E+00	1613	Os03g0116700	100,00	0,00E+00	1613	0	NA	Japonica	
maker-3-augustus-gene-7.17-mRNA-1	3	OsR498G0305123500.01.T01	99,75	0,00E+00	2135	Os03g0116800	100,00	0,00E+00	2149	14	NA	Japonica	
maker-3-augustus-gene-7.22-mRNA-1	3	OsR498G0305124300.01.T01	99,76	0,00E+00	2216	Os03g0117200	100,00	0,00E+00	2230	14	NA	Japonica	
maker-3-augustus-gene-7.25-mRNA-1	3	OsR498G0305125100.01.T02	99,61	0,00E+00	1838	Os03g0117800	100,00	0,00E+00	1856	18	NA	Japonica	
maker-3-augustus-gene-7.28-mRNA-1	3	OsR498G0305125700.01.T01	98,69	0,00E+00	2397	Os03g0118100	100,00	0,00E+00	2462	65	NA	Japonica	
maker-3-augustus-gene-7.30-mRNA-1	3	OsR498G0305126100.01.T03	94,66	0,00E+00	1765	Os03g0118200	94,75	0,00E+00	1770	5	NA	Japonica	
maker-3-augustus-gene-7.34-mRNA-1	3	OsR498G0305122600.01.T01	96,42	0,00E+00	2276	Os03g0116500	96,56	0,00E+00	2285	9	NA	Japonica	
maker-3-augustus-gene-7.37-mRNA-1	3	OsR498G0305124000.01.T02	99,79	0,00E+00	858	Os03g0118600	94,77	0,00E+00	748	-110	Indica	Japonica	
maker-3-augustus-gene-7.44-mRNA-1	3	OsR498G0305124200.01.T02	99,86	0,00E+00	1284	Os03g0117100	100,00	0,00E+00	1288	4	NA	Japonica	
maker-3-augustus-gene-7.5-mRNA-1	3	OsR498G0305121500.01.T02	99,37	1,64E-160	565	Os10g0521400	78,76	1,35E-48	192	-373	Indica	NA	
maker-3-augustus-gene-7.6-mRNA-1	3	OsR498G0305121500.01.T01	92,92	0,00E+00	1453	Os10g0521400	78,19	0,00E+00	783	-670	Indica	NA	
maker-3-augustus-gene-70.25-mRNA-1	3	OsR498G0305530900.01.T01	99,73	0,00E+00	2650	Os03g0236200	100,00	0,00E+00	2668	18	NA	Japonica	
maker-3-augustus-gene-70.34-mRNA-1	3	OsR498G0305534100.01.T01	99,89	0,00E+00	3216	Os03g0237000	100,00	0,00E+00	3225	9	NA	Japonica	
maker-3-augustus-gene-71.0-mRNA-1	3	OsR498G0305541500.01.T01	100,00	0,00E+00	1840	Os03g0238600	100,00	0,00E+00	1840	0	NA	Japonica	
maker-3-augustus-gene-71.10-mRNA-1	3	OsR498G0305538400.01.T01	99,62	0,00E+00	1405	Os03g0237900	100,00	0,00E+00	1418	13	NA	Japonica	
maker-3-augustus-gene-71.14-mRNA-1	3	OsR498G0305539600.01.T01	96,91	0,00E+00	2510	Os03g0238300	97,18	0,00E+00	2528	18	NA	Japonica	
maker-3-augustus-gene-71.16-mRNA-1	3	OsR498G0305541800.01.T01	99,67	0,00E+00	2163	Os03g0238700	100,00	0,00E+00	1611	-552	Indica	Japonica	
maker-3-augustus-gene-71.2-mRNA-1	3	OsR498G0305542900.01.T01	99,77	0,00E+00	3920	Os03g0239200	100,00	0,00E+00	3939	19	NA	Japonica	
maker-3-augustus-gene-71.20-mRNA-1	3	OsR498G0305542000.01.T01	99,95	0,00E+00	3562	Os03g0238800	100,00	0,00E+00	3050	-512	Indica	Japonica	
maker-3-augustus-gene-71.25-mRNA-1	3	OsR498G0305542400.01.T01	99,75	0,00E+00	2173	Os03g0239000	100,00	0,00E+00	1835	-338	Indica	Japonica	
maker-3-augustus-gene-71.28-mRNA-1	3	OsR498G0305543400.01.T02	99,83	0,00E+00	2069	Os03g0239400	100,00	0,00E+00	2078	9	NA	Japonica	
maker-3-augustus-gene-71.4-mRNA-1	3	OsR498G0305537200.01.T03	99,95	0,00E+00	3302	Os03g0237600	100,00	0,00E+00	3306	4	NA	Japonica	
maker-3-augustus-gene-72.14-mRNA-1	3	OsR498G0305547000.01.T01	99,51	0,00E+00	2528	Os03g0240900	100,00	0,00E+00	2560	32	NA	Japonica	
maker-3-augustus-gene-72.15-mRNA-1	3	OsR498G0305547000.01.T02	99,57	1,98E-115	416	Os03g0240900	100,00	1,62E-117	421	5	NA	Japonica	
maker-3-augustus-gene-72.3-mRNA-1	3	OsR498G0305546200.01.T03	100,00	0,00E+00	1775	Os03g0240500	100,00	0,00E+00	1775	0	NA	Japonica	
maker-3-augustus-gene-72.9-mRNA-1	3	OsR498G0305546600.01.T02	100,00	0,00E+00	704	Os03g0240700	100,00	0,00E+00	704	0	NA	Japonica	
maker-3-augustus-gene-73.0-mRNA-1	3	OsR498G0305547500.01.T02	99,82	0,00E+00	3878	Os03g0241100	100,00	0,00E+00	3896	18	NA	Japonica	
maker-3-augustus-gene-73.11-mRNA-1	3	OsR498G0305547900.01.T01	99,11	0,00E+00	2369	Os03g0241400	100,00	0,00E+00	1965	-404	Indica	Japonica	
maker-3-augustus-gene-73.20-mRNA-1	3	OsR498G0305550300.01.T05	99,90	0,00E+00	1865	Os03g0242200	100,00	0,00E+00	1642	-223	Indica	Japonica	
maker-3-augustus-gene-73.32-mRNA-1	3	OsR498G0305549600.01.T04	99,79	0,00E+00	2588	Os03g0241800	100,00	0,00E+00	3196	608	Japonica	Japonica	
maker-3-augustus-gene-73.34-mRNA-1	3	OsR498G0305549800.01.T01	99,86	0,00E+00	2605	Os03g0241900	100,00	0,00E+00	2614	9	NA	Japonica	
maker-3-augustus-gene-73.37-mRNA-1	3	OsR498G0305553600.01.T04	100,00	0,00E+00	928	Os03g0243100	100,00	0,00E+00	780	-148	Indica	Japonica	
maker-3-augustus-gene-73.7-mRNA-1	3	OsR498G0305547700.01.T01	100,00	0,00E+00	699	Os03g0241200	100,00	0,00E+00	699	0	NA	Japonica	
maker-3-augustus-gene-74.0-mRNA-1	3	OsR498G0305555700.01.T02	99,94	0,00E+00	3080	Os03g0243600	100,00	0,00E+00	3085	5	NA	Japonica	
maker-3-augustus-gene-74.20-mRNA-1	3	OsR498G0305556500.01.T08	100,00	0,00E+00	800	Os03g0244000	100,00	0,00E+00	800	0	NA	Japonica	
maker-3-augustus-gene-74.27-mRNA-1	3	OsR498G0305557700.01.T01	99,78	0,00E+00	2496	Os03g0244600	100,00	0,00E+00	2509	13	NA	Japonica	
maker-3-augustus-gene-74.38-mRNA-1	3	OsR498G0305555000.01.T02	99,75	0,00E+00	2150	Os03g0243300	100,00	0,00E+00	2163	13	NA	Japonica	
maker-3-augustus-gene-74.4-mRNA-1	3	OsR498G0305556100.01.T07	100,00	0,00E+00	3025	Os03g0243700	100,00	0,00E+00	2176	-849	Indica	Japonica	
maker-3-augustus-gene-74.42-mRNA-1	3	OsR498G0305556400.01.T01	100,00	0,00E+00	1824	Os03g0243900	100,00	0,00E+00	1824	0	NA	Japonica	
maker-3-augustus-gene-75.0-mRNA-1	3	OsR498G0305559500.01.T03	99,59	0,00E+00	1746	Os03g0245100	100,00	0,00E+00	1759	13	NA	Japonica	
maker-3-augustus-gene-75.10-mRNA-1	3	OsR498G0305559800.01.T01	99,86	0,00E+00	1284	Os03g0245200	100,00	0,00E+00	1288	4	NA	Japonica	
maker-3-augustus-gene-75.12-mRNA-1	3	OsR498G0305560000.01.T01	99,60	0,00E+00	1351	Os03g0245300	100,00	0,00E+00	1364	13	NA	Japonica	
maker-3-augustus-gene-75.16-mRNA-1	3	OsR498G0305566600.01.T04	99,89	0,00E+00	4912	Os03g0246800	100,00	0,00E+00	4926	14	NA	Japonica	
maker-3-augustus-gene-75.19-mRNA-1	3	OsR498G0305567000.01.T01	99,64	0,00E+00	1484	Os03g0246900	100,00	0,00E+00	1494	10	NA	Japonica	
maker-3-augustus-gene-75.5-mRNA-1	3	OsR498G0305565900.01.T03	99,97	0,00E+00	5607	Os03g0246500	100,00	0,00E+00	5611	4	NA	Japonica	
maker-3-augustus-gene-76.0-mRNA-1	3	OsR498G0305572100.01.T01	97,74	0,00E+00	2180	Os03g0247300	100,00	0,00E+00	2316	136	NA	Japonica	
maker-3-augustus-gene-76.14-mRNA-1	3	OsR498G0305574600.01.T02	99,75	0,00E+00	2852	Os03g0248000	100,00	0,00E+00	2870	18	NA	Japonica	
maker-3-augustus-gene-76.18-mRNA-1	3	OsR498G0305575000.01.T02	99,87	0,00E+00	2708	Os03g0248200	100,00	0,00E+00	2365	-343	Indica	Japonica	
maker-3-augustus-gene-76.21-mRNA-1	3	OsR498G0305575800.01.T01	99,73	0,00E+00	2693	Os03g0248200	83,82	0,00E+00	1398	-1295	Indica	Japonica	
maker-3-augustus-gene-76.23-mRNA-1	3	OsR498G0305576600.01.T01	92,77	0,00E+00	1353	Os03g0248600	93,11	0,00E+00	1367	14	NA	Japonica	
maker-3-augustus-gene-76.9-mRNA-1	3	OsR498G0305567600.01.T01	100,00	0,00E+00	1261	Os03g0247000	100,00	0,00E+00	1261	0	NA	Japonica	
maker-3-augustus-gene-77.34-mRNA-1	3	OsR498G0305577700.01.T01	99,85	0,00E+00	2405	Os03g0249100	100,00	0,00E+00	2414	9	NA	Japonica	
maker-3-augustus-gene-77.36-mRNA-1	3	OsR498G0305577900.01.T03	99,71	2,15E-173	609	Os03g0249200	100,00	1,76E-175	614	5	NA	Japonica	
maker-3-augustus-gene-77.38-mRNA-1	3	OsR498G0305578100.01.T01	99,43	0,00E+00	1243	Os03g0249300	100,00	0,00E+00	1261	18	NA	Japonica	
maker-3-augustus-gene-77.41-mRNA-1	3	OsR498G0305578300.01.T01	99,74	0,00E+00	689	Os03g0249400	100,00	0,00E+00	693	4	NA	Japonica	
maker-3-augustus-gene-77.44-mRNA-1	3	OsR498G0305579100.01.T01	99,67	3,23E-155	548	Os10g0115550	71,37	2,33E-31	135	-413	Indica	Japonica	
maker-3-augustus-gene-77.46-mRNA-1	3	OsR498G0305580700.01.T01	95,51	0,00E+00	1562	Os03g0249900	95,93	0,00E+00	1581	19	NA	Japonica	
maker-3-augustus-gene-77.53-mRNA-1	3	OsR498G0305581000.01.T02	99,58	0,00E+00	2104	Os10g0177200	72,17	1,84E-166	586	-1518	Indica	Japonica	
maker-3-augustus-gene-78.43-mRNA-1	3	OsR498G0305587400.01.T01	99,91	0,00E+00	1901	Os07g0409100	71,35	4,23E-132	472	-1429	Indica	Japonica	
maker-3-augustus-gene-78.48-mRNA-1	3	OsR498G0305591600.01.T04	99,93	0,00E+00	2626	Os03g0252800	100,00	0,00E+00	2630	4	NA	Japonica	
maker-3-augustus-gene-78.54-mRNA-1	3	OsR498G0305592600.01.T01	98,10	1,39E-153	543	Os05g0579600	70,97	1,59E-14	79,7	-463,3	Indica	Japonica	
maker-3-augustus-gene-79.34-mRNA-1	3	OsR498G0305596500.01.T01	99,76	0,00E+00	2931	Os03g0254200	100,00	0,00E+00	2949	18	NA	Japonica	
maker-3-augustus-gene-79.40-mRNA-1	3	OsR498G0305593200.01.T02	97,89	0,00E+00	2696	Os03g0253100	98,28	0,00E+00	2723	27	NA	Japonica	
maker-3-augustus-gene-79.46-mRNA-1	3	OsR498G0305595600.01.T01	99,50	0,00E+00	1422	Os03g0253800	100,00	0,00E+00	1440	18	NA	Japonica	
maker-3-augustus-gene-79.50-mRNA-1	3	OsR498G0305596400.01.T01	99,78	0,00E+00	4935	Os03g0254000	100,00	0,00E+00	3025	-1910	Indica	Japonica	CC-LRR-NBS-TM
maker-3-augustus-gene-8.0-mRNA-1	3	OsR498G0305126900.01.T01	99,75	0,00E+00	1422	Os03g0118400	99,87	0,00E+00	1426	4	NA	Japonica	Kinase-TM
maker-3-augustus-gene-8.12-mRNA-1	3	OsR498G0305127700.01.T01	98,17	0,00E+00	3126	Os03g0118700	100,00	0,00E+00	3198	72	NA	Japonica	
maker-3-augustus-gene-8.14-mRNA-1	3	OsR498G0305128500.01.T01	99,86	0,00E+00	2502	Os03g0118800	99,93	0,00E+00	2507	5	NA	Japonica	
maker-3-augustus-gene-8.20-mRNA-1	3	OsR498G0305128700.01.T01	99,86	0,00E+00	2502	Os03g0118900	100,00	0,00E+00	2511	9	NA	Japonica	
maker-3-augustus-gene-8.25-mRNA-1	3	OsR498G0305132300.01.T03	99,86	0,00E+00	6211	Os03g0119500	100,00	0,00E+00	3301	-2910	Indica	Japonica	
maker-3-augustus-gene-8.28-mRNA-1	3	OsR498G0305133300.01.T01	99,32	0,00E+00	2359	Os03g0119966	100,00	0,00E+00	3523	1164	Japonica	Japonica	
maker-3-augustus-gene-8.30-mRNA-1	3	OsR498G0305129000.01.T01	99,87	0,00E+00	1387	Os03g0119000	100,00	0,00E+00	1391	4	NA	Japonica	
maker-3-augustus-gene-8.32-mRNA-1	3	OsR498G0305129400.01.T01	99,80	0,00E+00	2645	Os03g0119100	100,00	0,00E+00	2659	14	NA	Japonica	
maker-3-augustus-gene-8.6-mRNA-1	3	OsR498G0305127300.01.T02	96,89	0,00E+00	1570	Os03g0118600	97,21	0,00E+00	1583	13	NA	Japonica	
maker-3-augustus-gene-80.0-mRNA-1	3	OsR498G0305600500.01.T06	99,81	0,00E+00	3764	Os03g0255400	99,86	0,00E+00	3769	5	NA	NA	
maker-3-augustus-gene-80.11-mRNA-1	3	OsR498G0305598600.01.T01	99,70	0,00E+00	2358	Os03g0254800	99,70	0,00E+00	2358	0	NA	Japonica	
maker-3-augustus-gene-80.15-mRNA-1	3	OsR498G0305598800.01.T01	99,77	0,00E+00	2319	Os03g0254900	99,66	0,00E+00	1562	-757	Indica	NA	
maker-3-augustus-gene-80.17-mRNA-1	3	OsR498G0305600000.01.T02	97,27	0,00E+00	4985	Os03g0255100	97,41	0,00E+00	4995	10	NA	Japonica	
maker-3-augustus-gene-80.25-mRNA-1	3	OsR498G0305600400.01.T01	99,76	0,00E+00	1523	Os03g0255200	99,76	0,00E+00	1523	0	NA	NA	
maker-3-augustus-gene-80.32-mRNA-1	3	OsR498G0305602400.01.T01	99,66	0,00E+00	1047	Os03g0255900	100,00	2,10E-149	527	-520	Indica	NA	
maker-3-augustus-gene-80.34-mRNA-1	3	OsR498G0305603400.01.T03	99,88	0,00E+00	3070	Os03g0256400	99,77	0,00E+00	3061	-9	NA	Japonica	
maker-3-augustus-gene-80.4-mRNA-1	3	OsR498G0305598000.01.T01	99,94	0,00E+00	6127	Os03g0254700	99,97	0,00E+00	6131	4	NA	Japonica	
maker-3-augustus-gene-81.12-mRNA-1	3	OsR498G0305604800.01.T01	100,00	0,00E+00	1050	Os03g0257000	100,00	0,00E+00	948	-102	NA	NA	
maker-3-augustus-gene-81.14-mRNA-1	3	OsR498G0305606000.01.T01	99,66	0,00E+00	1041	Os03g0257500	100,00	0,00E+00	1050	9	NA	Japonica	
maker-3-augustus-gene-81.16-mRNA-1	3	OsR498G0305606200.01.T02	99,79	0,00E+00	2584	Os03g0257600	99,94	0,00E+00	3075	491	Japonica	Japonica	
maker-3-augustus-gene-81.2-mRNA-1	3	OsR498G0305604600.01.T02	99,80	0,00E+00	4566	Os03g0256800	99,78	0,00E+00	4068	-498	Indica	NA	
maker-3-augustus-gene-82.0-mRNA-1	3	OsR498G0305609300.01.T01	99,70	0,00E+00	2419	Os03g0258500	99,61	0,00E+00	2740	321	Japonica	NA	
maker-3-augustus-gene-82.1-mRNA-1	3	OsR498G0305611500.01.T01	99,73	0,00E+00	651	Os03g0259100	99,73	0,00E+00	651	0	NA	NA	
maker-3-augustus-gene-82.13-mRNA-1	3	OsR498G0305616500.01.T03	99,64	0,00E+00	987	Os03g0261500	100,00	0,00E+00	991	4	NA	Japonica	
maker-3-augustus-gene-82.17-mRNA-1	3	OsR498G0305612000.01.T01	99,48	0,00E+00	1371	Os03g0259400	100,00	0,00E+00	1943	572	Japonica	NA	

maker-3-augustus-gene-82.20-mRNA-1	3	OsR498G0305612600.01.T01	99,79	0,00E+00	3429	Os03g0259700	100,00	0,00E+00	2516	-913	Indica	NA	
maker-3-augustus-gene-82.24-mRNA-1	3	OsR498G0305614000.01.T02	99,81	0,00E+00	929	Os03g0260100	100,00	0,00E+00	935	6	NA	NA	
maker-3-augustus-gene-82.3-mRNA-1	3	OsR498G0305611700.01.T03	100,00	0,00E+00	3759	Os03g0259300	99,90	0,00E+00	3750	-9	NA	Indica	
maker-3-augustus-gene-82.5-mRNA-1	3	OsR498G0305613300.01.T01	99,64	0,00E+00	4974	Os03g0259900	96,55	0,00E+00	4626	-348	NA	Japonica	
maker-3-augustus-gene-82.7-mRNA-1	3	OsR498G0305615300.01.T01	99,43	0,00E+00	2191	Os03g0260600	99,59	0,00E+00	2200	9	NA	Japonica	
maker-3-augustus-gene-82.9-mRNA-1	3	OsR498G0305615900.01.T01	99,80	0,00E+00	894	Os03g0261100	93,90	0,00E+00	775	-119	Indica	Japonica	
maker-3-augustus-gene-83.23-mRNA-1	3	OsR498G0305621800.01.T02	99,81	0,00E+00	950	Os03g0262400	100,00	0,00E+00	955	5	NA	Japonica	
maker-3-augustus-gene-83.5-mRNA-1	3	OsR498G0305620700.01.T01	99,25	0,00E+00	1177	Os03g0262100	100,00	0,00E+00	1202	25	NA	Japonica	
maker-3-augustus-gene-84.48-mRNA-1	3	OsR498G0305622900.01.T01	99,86	0,00E+00	1289	Os03g0262700	100,00	0,00E+00	1553	264	Japonica	Japonica	
maker-3-augustus-gene-84.54-mRNA-1	3	OsR498G0305625900.01.T02	99,91	0,00E+00	2025	Os03g0263400	94,56	0,00E+00	1325	-700	Indica	Japonica	
maker-3-augustus-gene-84.58-mRNA-1	3	OsR498G0305626500.01.T04	89,47	1,46E-135	482	Os03g0263700	89,77	4,16E-137	487	5	NA	Japonica	
maker-3-augustus-gene-84.61-mRNA-1	3	OsR498G0305626700.01.T01	99,81	0,00E+00	1847	Os03g0263800	100,00	0,00E+00	1856	9	NA	Japonica	
maker-3-augustus-gene-84.68-mRNA-1	3	OsR498G0305626200.01.T01	99,63	0,00E+00	1464	Os03g0263500	100,00	0,00E+00	1474	10	NA	Japonica	
maker-3-augustus-gene-84.72-mRNA-1	3	OsR498G0305626400.01.T01	99,15	0,00E+00	2287	Os03g0263600	100,00	0,00E+00	2316	29	NA	Japonica	
maker-3-augustus-gene-84.78-mRNA-1	3	OsR498G0305629600.01.T01	99,84	0,00E+00	3271	Os03g0264400	100,00	0,00E+00	3285	14	NA	Japonica	
maker-3-augustus-gene-85.33-mRNA-1	3	OsR498G0305632700.01.T01	99,84	0,00E+00	2242	Os03g0265100	100,00	0,00E+00	2251	9	NA	Japonica	
maker-3-augustus-gene-85.39-mRNA-1	3	OsR498G0305633700.01.T01	99,90	0,00E+00	1863	Os03g0265300	100,00	0,00E+00	1867	4	NA	Japonica	
maker-3-augustus-gene-85.43-mRNA-1	3	OsR498G0305633900.01.T02	99,28	0,00E+00	1726	Os03g0265400	100,00	0,00E+00	1754	28	NA	Japonica	
maker-3-augustus-gene-85.45-mRNA-1	3	OsR498G0305634700.01.T01	99,89	0,00E+00	3184	Os03g0265500	100,00	0,00E+00	3193	9	NA	Japonica	
maker-3-augustus-gene-85.48-mRNA-1	3	OsR498G0305635300.01.T01	96,99	0,00E+00	6053	Os03g0265700	98,29	0,00E+00	5488	-565	NA	Japonica	
maker-3-augustus-gene-85.60-mRNA-1	3	OsR498G0305636300.01.T01	100,00	4,49E-137	488	Os03g0266100	100,00	1,56E-137	488	0	NA	Japonica	
maker-3-augustus-gene-85.63-mRNA-1	3	OsR498G0305631400.01.T02	98,29	0,00E+00	4321	Os03g0264800	99,06	0,00E+00	2824	-1497	Indica	Japonica	CC-NBS-TM
maker-3-augustus-gene-85.66-mRNA-1	3	OsR498G0305633000.01.T01	99,51	0,00E+00	1086	Os03g0265200	100,00	0,00E+00	1099	13	NA	Japonica	
maker-3-augustus-gene-85.72-mRNA-1	3	OsR498G0305635800.01.T01	99,59	0,00E+00	874	Os03g0265900	100,00	0,00E+00	883	9	NA	Japonica	
maker-3-augustus-gene-85.75-mRNA-1	3	OsR498G0305636600.01.T01	98,63	0,00E+00	2546	Os03g0266200	97,72	0,00E+00	1354	-1192	Indica	Japonica	
maker-3-augustus-gene-86.1-mRNA-1	3	OsR498G0305638100.01.T01	99,94	0,00E+00	3048	Os03g0266800	100,00	0,00E+00	3052	4	NA	Japonica	Kinase-LRR-TM
maker-3-augustus-gene-86.20-mRNA-1	3	OsR498G0305641300.01.T01	99,55	0,00E+00	2369	Os03g0267500	100,00	0,00E+00	2392	23	NA	Japonica	
maker-3-augustus-gene-86.22-mRNA-1	3	OsR498G0305642700.01.T01	100,00	0,00E+00	1878	Os03g0267700	100,00	0,00E+00	1878	0	NA	Japonica	
maker-3-augustus-gene-86.27-mRNA-1	3	OsR498G0305643500.01.T06	100,00	5,97E-162	571	Os03g0268000	100,00	2,07E-162	571	0	NA	Japonica	
maker-3-augustus-gene-86.30-mRNA-1	3	OsR498G0305639600.01.T01	99,84	0,00E+00	2194	Os03g0267300	100,00	0,00E+00	2203	9	NA	Japonica	
maker-3-augustus-gene-86.32-mRNA-1	3	OsR498G0305641600.01.T03	99,83	0,00E+00	4334	Os03g0267600	100,00	0,00E+00	4352	18	NA	Japonica	
maker-3-augustus-gene-86.34-mRNA-1	3	OsR498G0305643000.01.T03	99,58	0,00E+00	1280	Os03g0267800	100,00	0,00E+00	1294	14	NA	Japonica	
maker-3-augustus-gene-86.38-mRNA-1	3	OsR498G0305643800.01.T03	99,87	0,00E+00	1343	Os03g0268100	100,00	0,00E+00	1348	5	NA	Japonica	
maker-3-augustus-gene-86.51-mRNA-1	3	OsR498G0305644400.01.T02	99,93	0,00E+00	2507	Os03g0268300	100,00	0,00E+00	2511	4	NA	Japonica	
maker-3-augustus-gene-86.54-mRNA-1	3	OsR498G0305644600.01.T03	99,82	0,00E+00	1950	Os03g0268400	100,00	0,00E+00	1959	9	NA	Japonica	
maker-3-augustus-gene-86.6-mRNA-1	3	OsR498G0305637700.01.T01	100,00	0,00E+00	2138	Os03g0266700	100,00	0,00E+00	2138	0	NA	Japonica	
maker-3-augustus-gene-87.0-mRNA-1	3	OsR498G0305650300.01.T01	99,75	0,00E+00	2839	Os03g0269900	100,00	0,00E+00	2857	18	NA	Japonica	
maker-3-augustus-gene-87.10-mRNA-1	3	OsR498G0305647200.01.T05	99,68	1,26E-156	554	Os03g0269000	100,00	1,03E-158	558	4	NA	Japonica	
maker-3-augustus-gene-87.14-mRNA-1	3	OsR498G0305647600.01.T01	99,78	0,00E+00	1663	Os03g0269100	100,00	0,00E+00	1303	-360	Indica	Japonica	
maker-3-augustus-gene-87.18-mRNA-1	3	OsR498G0305649000.01.T01	99,71	0,00E+00	3057	Os03g0269300	100,00	0,00E+00	3079	22	NA	Japonica	Kinase-TM
maker-3-augustus-gene-87.2-mRNA-1	3	OsR498G0305645600.01.T01	98,78	0,00E+00	2142	Os03g0268600	100,00	0,00E+00	2192	50	NA	Japonica	
maker-3-augustus-gene-87.5-mRNA-1	3	OsR498G0305646600.01.T01	99,75	0,00E+00	1458	Os03g0268900	100,00	0,00E+00	1467	9	NA	Japonica	
maker-3-augustus-gene-88.44-mRNA-1	3	OsR498G0305652300.01.T04	99,86	0,00E+00	2488	Os03g0270700	96,81	0,00E+00	2696	208	NA	Japonica	
maker-3-augustus-gene-88.47-mRNA-1	3	OsR498G0305653500.01.T01	99,94	0,00E+00	2923	Os03g0271100	100,00	0,00E+00	2928	5	NA	Japonica	
maker-3-augustus-gene-88.50-mRNA-1	3	OsR498G0305653700.01.T01	99,84	0,00E+00	4408	Os03g0271200	100,00	0,00E+00	4426	18	NA	Japonica	
maker-3-augustus-gene-88.55-mRNA-1	3	OsR498G0305651800.01.T03	100,00	0,00E+00	1061	Os03g0270200	100,00	0,00E+00	883	-178	Indica	Japonica	
maker-3-augustus-gene-88.71-mRNA-1	3	OsR498G0305654200.01.T03	99,72	0,00E+00	1920	Os03g0271400	100,00	0,00E+00	1934	14	NA	Japonica	
maker-3-augustus-gene-89.0-mRNA-1	3	OsR498G0305659100.01.T02	99,88	0,00E+00	1517	Os03g0272300	100,00	0,00E+00	1267	-250	Indica	Japonica	
maker-3-augustus-gene-89.14-mRNA-1	3	OsR498G0305661600.01.T01	99,19	0,00E+00	2845	Os03g0273200	100,00	0,00E+00	2897	52	NA	Japonica	
maker-3-augustus-gene-89.6-mRNA-1	3	OsR498G0305659300.01.T01	99,88	0,00E+00	1435	Os03g0272400	100,00	0,00E+00	1440	5	NA	Japonica	
maker-3-augustus-gene-89.8-mRNA-1	3	OsR498G0305664100.01.T01	99,88	0,00E+00	1533	Os03g0273800	100,00	0,00E+00	1537	4	NA	Japonica	
maker-3-augustus-gene-9.0-mRNA-1	3	OsR498G0305134500.01.T04	99,73	0,00E+00	662	Os03g0120000	100,00	0,00E+00	774	112	Japonica	Japonica	
maker-3-augustus-gene-9.12-mRNA-1	3	OsR498G0305135000.01.T01	99,84	0,00E+00	2286	Os03g0120200	100,00	0,00E+00	2295	9	NA	Japonica	
maker-3-augustus-gene-9.14-mRNA-1	3	OsR498G0305135800.01.T03	99,80	0,00E+00	918	Os03g0120300	100,00	0,00E+00	922	4	NA	Japonica	
maker-3-augustus-gene-9.19-mRNA-1	3	OsR498G0305136000.01.T01	100,00	0,00E+00	1050	Os03g0120400	100,00	0,00E+00	1050	0	NA	Japonica	
maker-3-augustus-gene-9.2-mRNA-1	3	OsR498G0305137100.01.T01	99,90	0,00E+00	3499	Os03g0120800	100,00	0,00E+00	3508	9	NA	Japonica	
maker-3-augustus-gene-9.21-mRNA-1	3	OsR498G0305140000.01.T01	100,00	0,00E+00	1797	Os03g0121200	100,00	0,00E+00	1797	0	NA	Japonica	
maker-3-augustus-gene-9.23-mRNA-1	3	OsR498G0305140600.01.T01	100,00	0,00E+00	1748	Os03g0121300	100,00	0,00E+00	1748	0	NA	Japonica	
maker-3-augustus-gene-9.27-mRNA-1	3	OsR498G0305141200.01.T01	100,00	0,00E+00	1840	Os03g0121200	72,24	8,21E-126	451	-1389	Indica	Japonica	
maker-3-augustus-gene-9.4-mRNA-1	3	OsR498G0305141300.01.T01	100,00	0,00E+00	1202	Os03g0121700	100,00	0,00E+00	1202	0	NA	Japonica	
maker-3-augustus-gene-9.7-mRNA-1	3	OsR498G0305134800.01.T02	99,67	0,00E+00	1097	Os03g0120100	100,00	0,00E+00	1106	9	NA	Japonica	
maker-3-augustus-gene-90.26-mRNA-1	3	OsR498G0305665500.01.T01	100,00	0,00E+00	2511	Os03g0274000	100,00	0,00E+00	2511	0	NA	Japonica	
maker-3-augustus-gene-90.40-mRNA-1	3	OsR498G0305670300.01.T01	99,63	0,00E+00	3388	Os03g0275100	99,90	0,00E+00	3411	23	NA	Japonica	
maker-3-augustus-gene-90.42-mRNA-1	3	OsR498G0305672500.01.T03	97,54	0,00E+00	1528	Os03g0275400	99,88	0,00E+00	1480	-48	NA	Japonica	
maker-3-augustus-gene-90.50-mRNA-1	3	OsR498G0305669400.01.T01	100,00	0,00E+00	2030	Os03g0274800	100,00	0,00E+00	2030	0	NA	Japonica	Kinase
maker-3-augustus-gene-90.54-mRNA-1	3	OsR498G0305671600.01.T01	98,06	0,00E+00	2750	Os01g0974400	73,49	0,00E+00	719	-2031	Indica	Japonica	
maker-3-augustus-gene-91.0-mRNA-1	3	OsR498G0305674500.01.T01	100,00	0,00E+00	3934	Os03g0275900	100,00	0,00E+00	3934	0	NA	Japonica	
maker-3-augustus-gene-91.12-mRNA-1	3	OsR498G0305679400.01.T01	100,00	0,00E+00	3036	Os03g0276800	100,00	0,00E+00	3036	0	NA	NA	
maker-3-augustus-gene-91.14-mRNA-1	3	OsR498G0305679600.01.T01	100,00	0,00E+00	2181	Os03g0276900	100,00	0,00E+00	1139	-1042	Indica	Japonica	
maker-3-augustus-gene-91.18-mRNA-1	3	OsR498G0305680000.01.T01	100,00	0,00E+00	2425	Os03g0277000	100,00	0,00E+00	2425	0	NA	NA	
maker-3-augustus-gene-91.20-mRNA-1	3	OsR498G0305681400.01.T01	100,00	0,00E+00	3539	Os03g0276500	89,57	0,00E+00	2591	-948	Indica	NA	
maker-3-augustus-gene-91.22-mRNA-1	3	OsR498G0305682000.01.T01	100,00	0,00E+00	758	Os03g0277500	100,00	0,00E+00	758	0	NA	NA	
maker-3-augustus-gene-91.3-mRNA-1	3	OsR498G0305678500.01.T01	100,00	0,00E+00	3523	Os03g0276500	100,00	0,00E+00	3523	0	NA	NA	
maker-3-augustus-gene-91.8-mRNA-1	3	OsR498G0305679000.01.T01	95,65	0,00E+00	1498	Os03g0276600	95,65	0,00E+00	1498	0	NA	NA	
maker-3-augustus-gene-92.39-mRNA-1	3	OsR498G0305683700.01.T01	100,00	0,00E+00	1900	Os03g0278000	100,00	0,00E+00	1900	0	NA	NA	
maker-3-augustus-gene-92.44-mRNA-1	3	OsR498G0305684300.01.T01	100,00	0,00E+00	3676	Os03g0278200	100,00	0,00E+00	3658	-18	NA	NA	
maker-3-augustus-gene-92.46-mRNA-1	3	OsR498G0305685300.01.T02	100,00	0,00E+00	2030	Os03g0278500	100,00	0,00E+00	2030	0	NA	NA	
maker-3-augustus-gene-92.55-mRNA-1	3	OsR498G0305682600.01.T01	99,88	0,00E+00	1435	Os03g0277600	100,00	0,00E+00	1440	5	NA	NA	
maker-3-augustus-gene-92.57-mRNA-1	3	OsR498G0305682800.01.T01	100,00	0,00E+00	1440	Os03g0277700	100,00	0,00E+00	1440	0	NA	NA	
maker-3-augustus-gene-92.59-mRNA-1	3	OsR498G0305684800.01.T05	99,88	0,00E+00	1479	Os03g0278300	99,84	0,00E+00	1143	-336	Indica	Japonica	
maker-3-augustus-gene-92.63-mRNA-1	3	OsR498G0305685200.01.T02	100,00	0,00E+00	2473	Os03g0278400	99,34	0,00E+00	2436	-37	NA	NA	
maker-3-augustus-gene-92.66-mRNA-1	3	OsR498G0305687200.01.T01	100,00	0,00E+00	942	Os03g0278800	100,00	0,00E+00	942	0	NA	NA	
maker-3-augustus-gene-92.69-mRNA-1	3	OsR498G0305688000.01.T01	100,00	0,00E+00	866	Os03g0279200	100,00	0,00E+00	866	0	NA	NA	
maker-3-augustus-gene-92.71-mRNA-1	3	OsR498G0305689200.01.T01	97,07	0,00E+00	2431	Os03g0279400	97,07	0,00E+00	2431	0	NA	NA	
maker-3-augustus-gene-93.29-mRNA-1	3	OsR498G0305693100.01.T01	99,89	0,00E+00	1652	Os03g0279900	100,00	0,00E+00	1656	4	NA	NA	
maker-3-augustus-gene-93.31-mRNA-1	3	OsR498G0305693300.01.T06	95,28	0,00E+00	1138	Os03g0279950	95,28	0,00E+00	1138	0	NA	NA	
maker-3-augustus-gene-93.36-mRNA-1	3	OsR498G0305694300.01.T01	100,00	1,70E-119	428	Os03g0280400	100,00	5,96E-120	428	0	NA	NA	
maker-3-augustus-gene-93.39-mRNA-1	3	OsR498G0305695500.01.T01	98,96	0,00E+00	5601	Os03g0280700	100,00	0,00E+00	4071	-1530	Indica	NA	
maker-3-augustus-gene-93.43-mRNA-1	3	OsR498G0305696300.01.T01	100,00	0,00E+00	3696	Os03g0281000	100,00	0,00E+00	3696	0	NA	NA	
maker-3-augustus-gene-93.46-mRNA-1	3	OsR498G0305696900.01.T01	100,00	0,00E+00	3063	Os03g0281100	100,00	0,00E+00	3063	0	NA	NA	
maker-3-augustus-gene-93.52-mRNA-1	3	OsR498G0305693600.01.T04	100,00	0,00E+00	7640	Os03g0280000	100,00	0,00E+00	7640	0	NA	NA	
maker-3-augustus-gene-93.55-mRNA-1	3	OsR498G0305696000.01.T03	97,00	0,00E+00	2198	Os03g0280800	89,92	0,00E+00	1814	-384	Indica	NA	
maker-3-augustus-gene-94.0-mRNA-1	3	OsR498G0305706500.01.T01	100,00	0,00E+00	1294	Os03g0282900	100,00	0,00E+00	1294	0	NA	NA	
maker-3-augustus-gene-94.20-mRNA-1	3	OsR498G0305706400.01.T01	100,00	0,00E+00	1218	Os03g0282800	100,00	0,00E+00	1218	0	NA	NA	
maker-3-augustus-gene-94.23-mRNA-1	3	OsR498G0305706800.01.T01	95,25	0,00E+00	1441	Os03g0283000	95,25	0,00E+00	1441	0	NA	NA	
maker-3-augustus-gene-94.26-mRNA-1	3	OsR498G0305707200.01.T01	100,00	0,00E+00	1635	Os03g0283100	100,00	0,00E+00	1310	-325	Indica	NA	
maker-3-augustus-gene-94.32-mRNA-1	3	OsR498G0305707600.01.T01	100,00	0,00E+00	1306	Os03g0283200	100,00	0,00E+00	1306	0	NA	NA	
maker-3-augustus-gene-94.4-mRNA-1	3	OsR498G0305698200.01.T01	99,87	0,00E+00	1387	Os03g0281300	99,87	0,00E+00	1387	0	NA	NA	
maker-3-augustus-gene-94.6-mRNA-1	3	OsR498G0305699400.01.T03	100,00	0,00E+00	5752	Os03g0281600	100,00	0,00E+00	4574	-1178	Indica	NA	
maker-3-augustus-gene-95.34-mRNA-1	3	OsR498G0305707900.01.T01	100,00	0,00E+00	901	Os03g0283300	100,00	0,00E+00	901	0	NA	NA	
maker-3-augustus-gene-95.47-mRNA-1	3	OsR498G0305711700.01.T01	100,00	0,00E+00	1061	Os03g0284600	100,00	0,00E+00	1061	0	NA	Japonica	
maker-3-augustus-gene-95.53-mRNA-1	3	OsR498G0305708400.01.T01	100,00	0,00E+00	1786	Os03g0283600	100,00	0,00E+00	1786	0	NA	NA	
maker-3-augustus-gene-95.56-mRNA-1	3	OsR498G0305709600.01.T01	100,00	0,00E+00	2100	Os03g0283900	100,00	0,00E+00	2100	0	NA	NA	Kinase-TM
maker-3-augustus-gene-95.62-mRNA-1	3	OsR498G0305710000.01.T07	99,91	0,00E+00	2012	Os03g0284100	99,91	0,00E+00	2012	0	NA	NA	
maker-3-augustus-gene-95.64-mRNA-1	3	OsR498G0305711600.01.T01	100,00	0,00E+00	2111	Os03g0284500	100,00	0,00E+00	2111	0	NA	NA	
maker-3-augustus-gene-96.12-mRNA-1	3	OsR498G0305720700.01.T02	99,54	0,00E+00	1926	Os03g0286800	99,89	0,00E+00	1583	-343	Indica	Japonica	
maker-3-augustus-gene-96.15-mRNA-1	3	OsR498G0305713010.01.T01	97,09	0,00E+00	3059	Os03g0284900	97,21	0,00E+00	3012	-47	NA	Japonica	
maker-3-augustus-gene-96.19-mRNA-1	3	OsR498G0305716200.01.T01	99,91	0,00E+00	1998	Os03g0285800	100,00	0,00E+00	2003	5	NA	Japonica	Kinase-TM
maker-3-augustus-gene-96.2-mRNA-1	3	OsR498G0305715700.01.T02	99,60	0,00E+00	1345	Os03g0285700	100,00	0,00E+00	1359	14	NA	Japonica	
maker-3-augustus-gene-96.28-mRNA-1	3	OsR498G0305718800.01.T01	99,26	0,00E+00	1196	Os03g0286500	99,56	0,00E+00	1205	9	NA	Japonica	
maker-3-augustus-gene-96.30-mRNA-1	3	OsR498G0305721600.01.T04	99,76	0,00E+00	1499	Os03g0287100	97,35	0,00E+00	1028	-471	Indica	Japonica	
maker-3-augustus-gene-96.36-mRNA-1	3	OsR498G0305722400.01.T01	99,86	0,00E+00	1295	Os03g0287400	100,00	0,00E+00	1299	4	NA	Japonica	
maker-3-augustus-gene-96.7-mRNA-1	3	OsR498G0305717700.01.T02	99,75	0,00E+00	2151	Os03g0286200	100,00	0,00E+00	2165	14	NA	Japonica	
maker-3-augustus-gene-97.30-mRNA-1	3	OsR498G0305723500.01.T03	99,64	0,00E+00	3000	Os03g0826000	82,25	0,00E+00	2363	-637	Indica	Japonica	
maker-3-augustus-gene-97.34-mRNA-1	3	OsR498G0305724100.01.T03	99,91	0,00E+00	1933	Os03g0287800	100,00	0,00E+00	1824	-109	NA	Japonica	
maker-3-augustus-gene-97.40-mRNA-1	3	OsR498G0305726100.01.T01	100,00	0,00E+00	2906	Os03g0288300	100,00	0,00E+00	2906	0	NA	Japonica	
maker-3-augustus-gene-97.54-mRNA-1	3	OsR498G0305726900.01.T04	98,70	0,00E+00	2292	Os03g0288600	97,51	0,00E+00	2264	-28	NA	Japonica	
maker-3-augustus-gene-97.59-mRNA-1	3	OsR498G0305727500.01.T01	99,85	0,00E+00	3585	Os03g0288800	100,00	0,00E+00	3599	14	NA	Japonica	
maker-3-augustus-gene-97.62-mRNA-1	3	OsR498G0305727700.01.T02	99,82	0,00E+00	2995	Os03g0288900	100,00	0,00E+00	2917	-78	NA	Japonica	
maker-3-augustus-gene-97.64-mRNA-1	3	OsR498G0305728500.01.T03	99,90	0,00E+00	1729	Os03g0289100	100,00	0,00E+00	1734	5	NA	Japonica	CC-Kinase-TM
maker-3-augustus-gene-97.71-mRNA-1	3	OsR498G0305724400.01.T02	100,00	0,00E+00	801	Os03g0287900	100,00	0,00E+00	801	0	NA	Japonica	
maker-3-augustus-gene-97.74-mRNA-1	3	OsR498G0305727200.01.T01	99,70	0,00E+00	1209	Os03g0288700	100,00	0,00E+00	1218	9	NA	Japonica	
maker-3-augustus-gene-98.25-mRNA-1	3	OsR498G0305729500.01.T02	99,94	0,00E+00	3210	Os03g0289300	99,83	0,00E+00	3204	-6	NA	Japonica	
maker-3-augustus-gene-98.29-mRNA-1	3	OsR498G0305729900.01.T01	99,36	0,00E+00	1103	Os03g0289400	100,00	0,00E+00	803	-300	Indica	Japonica	
maker-3-augustus-gene-98.31-mRNA-1	3	OsR498G0305735700.01.T01	98,41	0,00E+00	2078	Os03g0290900	99,24	0,00E+00	2112	34	NA	Japonica	
maker-3-augustus-gene-98.33-mRNA-1	3	OsR498G0305736100.01.T01	97,89	0,00E+00	2288	Os03g0291200	98,19	0,00E+00	2306	18	NA	Japonica	
maker-3-augustus-gene-98.36-mRNA-1	3	OsR498G0305731000.01.T01	99,57	0,00E+00	2077	Os03g0289800	100,00	0,00E+00	2100	23	NA	Japonica	
maker-3-augustus-gene-98.40-mRNA-1	3	OsR498G0305733600.01.T01	99,64	0,00E+00	2461	Os03g0290300	100,00	0,00E+00	1499	-962	Indica	Japonica	
maker-3-augustus-gene-98.45-mRNA-1	3	OsR498G0305734600.01.T01	100,00	0,00E+00	704	Os07g0693700	75,20	5,46E-66	250	-454	Indica	Japonica	
maker-3-augustus-gene-98.47-mRNA-1	3	OsR498G0305734800.01.T02	100,00	0,00E+00	1631	Os03g0290500	99,67	0,00E+00	1616	-15	NA	Japonica	
maker-3-augustus-gene-99.25-mRNA-1	3	OsR498G0305738500.01.T01	99,88	0,00E+00	3016	Os03g0291800	100,00	0,00E+00	3112	96	NA	Japonica	
maker-3-augustus-gene-99.29-mRNA-1	3	OsR498G0305739300.01.T01	99,92	0,00E+00	2117	Os03g0292100	100,00	0,00E+00	2062	-55	NA	Japonica	
maker-3-augustus-gene-99.32-mRNA-1	3	OsR498G0305742100.01.T01	99,93	0,00E+00	2431	Os03g0293000	100,00	0,00E+00	2435	4	NA	Japonica	
maker-3-augustus-gene-99.34-mRNA-1	3	OsR498G0305742300.01.T02	99,27	0,00E+00	1443	Os03g0293100	100,00	0,00E+00	1326	-117	NA	Japonica	
maker-3-augustus-gene-99.46-mRNA-1	3	OsR498G0305741800.01.T01	99,84	0,00E+00	1132	Os03g0292800	100,00	0,00E+00	1137	5	NA	Japonica	
augustus_masked-4-processed-gene-0.19-mRNA-1	4	OsR498G0407624000.01.T02	99,88	0,00E+00	3032	Os04g0169500	99,68	0,00E+00	2761	-271	NA	Indica	
augustus_masked-4-processed-gene-1.39-mRNA-1	4	OsR498G0407698300.01.T01	71,30	3,47E-137	490	Os04g0172400	95,48	0,00E+00	2094	1604	Japonica	Indica	
augustus_masked-4-processed-gene-1.41-mRNA-1	4	OsR498G0407627000.01.T01	74,06	2,63E-59	231	Os04g0173800	73,53	4,73E-57	222	-9	NA	NA	
augustus_masked-4-processed-gene-101.5-mRNA-1	4	OsR498G0408149200.01.T01	100,00	0,00E+00	1359	Os04g0332700	100,00	0,00E+00	1359	0	NA	NA	
augustus_masked-4-processed-gene-101.6-mRNA-1	4	OsR498G0408149800.01.T01	100,00	9,98E-149	526	Os09g0317600	76,68	2,86E-36	150	-376	Indica	NA	
augustus_masked-4-processed-gene-106.11-mRNA-1	4	OsR498G0408185800.01.T01	99,08	7,15E-164	577	Os08g0364700	91,91	2,85E-81	301	-276	Indica	NA	
augustus_masked-4-processed-gene-112.15-mRNA-1	4	OsR498G0408228000.01.T01	100,00	0,00E+00	823	Os04g0350100	89,60	6,42E-180	629	-194	Indica	Indica	
augustus_masked-4-processed-gene-117.10-mRNA-1	4	OsR498G0408267000.01.T01	100,00	0,00E+00	1965	Os04g0359300	100,00	0,00E+00	1943	-22	NA	NA	
augustus_masked-4-processed-gene-127.3-mRNA-1	4	OsR498G0408350000.01.T01	92,28	0,00E+00	2881	Os04g0371700	78,72	0,00E+00	1060	-1821	Indica	NA	CC-Kinase-TM
augustus_masked-4-processed-gene-129.60-mRNA-1	4	OsR498G0408366000.01.T01	96,90	0,00E+00	2356	Os08g0111800	93,80	0,00E+00	2158	-198	NA	NA	
augustus_masked-4-processed-gene-133.17-mRNA-1	4	OsR498G0408404100.01.T01	100,00	0,00E+00	731	Os04g0382900	100,00	0,00E+00	731	0	NA	Indica	
augustus_masked-4-processed-gene-136.25-mRNA-1	4	OsR498G0408425200.01.T01	100,00	6,53E-105	379	Os04g0387600	100,00	2,28E-105	379	0	NA	Indica	
augustus_masked-4-processed-gene-140.52-mRNA-1	4	OsR498G0408454800.01.T02	96,22	0,00E+00	917	Os04g0395700	99,85	0,00E+00	1156	239	Japonica	Japonica	
augustus_masked-4-processed-gene-15.18-mRNA-1	4	OsR498G0407683800.01.T05	99,48	0,00E+00	2071	Os04g0191800	70,64	3,84E-172	605	-1466	Indica	Japonica	
augustus_masked-4-processed-gene-15.6-mRNA-1	4	OsR498G0407682200.01.T02	99,44	0,00E+00	636	Os04g0191400	100,00	0,00E+00	645	9	NA	Japonica	
augustus_masked-4-processed-gene-154.33-mRNA-1	4	OsR498G0408563300.01.T01	70,56	0,00E+00	1017	Os04g0420900	97,44	0,00E+00	4089	3072	Japonica	Indica	Kinase-TM
augustus_masked-4-processed-gene-161.40-mRNA-1	4	OsR498G0408600400.01.T01	99,30	0,00E+00	2008	Os04g0432900	78,95	0,00E+00	963	-1045	Indica	NA	
augustus_masked-4-processed-gene-161.41-mRNA-1	4	OsR498G0408600600.01.T01	97,15	0,00E+00	1877	Os04g0433100	99,91	0,00E+00	2009	132	NA	NA	
augustus_masked-4-processed-gene-163.19-mRNA-1	4	OsR498G0408611000.01.T03	99,95	0,00E+00	3929	Os04g0436350	100,00	0,00E+00	3712	-217	NA	NA	
augustus_masked-4-processed-gene-163.20-mRNA-1	4	OsR498G0408612600.01.T01	100,00	0,00E+00	3885	Os04g0436800	100,00	0,00E+00	3885	0	NA	NA	
augustus_masked-4-processed-gene-165.41-mRNA-1	4	OsR498G0408622600.01.T01	99,88	0,00E+00	4440	Os04g0439600	99,96	0,00E+00	4449	9	NA	Japonica	Kinase-TM
augustus_masked-4-processed-gene-166.28-mRNA-1	4	OsR498G0408633800.01.T01	98,42	0,00E+00	1210	Os04g0442300	98,71	0,00E+00	1219	9	NA	Japonica	
augustus_masked-4-processed-gene-167.30-mRNA-1	4	OsR498G0408639600.01.T02	100,00	0,00E+00	1727	Os04g0443700	100,00	0,00E+00	1727	0	NA	NA	
augustus_masked-4-processed-gene-169.50-mRNA-1	4	OsR498G0408648400.01.T01	99,69	0,00E+00	2309	Os04g0444700	100,00	0,00E+00	2430	121	NA	Japonica	
augustus_masked-4-processed-gene-169.76-mRNA-1	4	OsR498G0408652800.01.T02	99,09	0,00E+00	3089	Os04g0446100	100,00	0,00E+00	3160	71	NA	Japonica	
augustus_masked-4-processed-gene-17.37-mRNA-1	4	OsR498G0407697400.01.T01	99,64	0,00E+00	1990	Os04g0194000	100,00	0,00E+00	2008	18	NA	Japonica	
augustus_masked-4-processed-gene-17.39-mRNA-1	4	OsR498G0407699600.01.T01	99,64	0,00E+00	993	Os04g0194600	100,00	0,00E+00	958	-35	NA	Japonica	
augustus_masked-4-processed-gene-175.36-mRNA-1	4	OsR498G0204165200.01.T01	73,60	5,04E-12	72,5	Os04g0457400	100,00	8,52E-118	421	348,5	Japonica	Japonica	
augustus_masked-4-processed-gene-178.40-mRNA-1	4	OsR498G0408710600.01.T03	100,00	0,00E+00	1543	Os04g0462900	98,01	0,00E+00	1442	-101	NA	NA	
augustus_masked-4-processed-gene-179.18-mRNA-1	4	OsR498G0408720800.01.T01	100,00	0,00E+00	1819	Os04g0465100	100,00	0,00E+00	1819	0	NA	NA	
augustus_masked-4-processed-gene-179.23-mRNA-1	4	OsR498G0408724400.01.T03	100,00	0,00E+00	1223	Os04g0465500	100,00	0,00E+00	1223	0	NA	NA	
augustus_masked-4-processed-gene-18.10-mRNA-1	4	OsR498G0407700600.01.T01	99,83	0,00E+00	2064	Os04g0195100	100,00	0,00E+00	2073	9	NA	Japonica	
augustus_masked-4-processed-gene-182.30-mRNA-1	4	OsR498G0408747800.01.T01	99,49	0,00E+00	1393	Os04g0472200	100,00	0,00E+00	1478	85	NA	Japonica	
augustus_masked-4-processed-gene-184.25-mRNA-1	4	OsR498G0408756800.01.T01	99,76	0,00E+00	2232	Os04g0474800	99,92	0,00E+00	2110	-122	NA	Japonica	
augustus_masked-4-processed-gene-184.27-mRNA-1	4	OsR498G0408759400.01.T01	99,62	0,00E+00	949	Os04g0475300	100,00	0,00E+00	958	9	NA	Japonica	
augustus_masked-4-processed-gene-185.74-mRNA-1	4	OsR498G0408764200.01.T01	97,43	0,00E+00	4466	Os04g0476800	100,00	0,00E+00	4773	307	NA	Japonica	
augustus_masked-4-processed-gene-188.46-mRNA-1	4	OsR498G0408776400.01.T01	100,00	0,00E+00	1894	Os04g0481800	100,00	0,00E+00	1894	0	NA	Japonica	
augustus_masked-4-processed-gene-188.47-mRNA-1	4	OsR498G0408776400.01.T01	85,62	0,00E+00	1199	Os04g0481900	100,00	0,00E+00	1873	674	Japonica	Japonica	
augustus_masked-4-processed-gene-19.21-mRNA-1	4	OsR498G0407707200.01.T01	93,47	0,00E+00	1155	Os04g0197500	99,88	0,00E+00	1553	398	Japonica	Japonica	
augustus_masked-4-processed-gene-193.51-mRNA-1	4	OsR498G0408821800.01.T03	100,00	1,31E-140	499	Os04g0493600	100,00	4,58E-141	499	0	NA	Japonica	
augustus_masked-4-processed-gene-199.35-mRNA-1	4	OsR498G0408855200.01.T01	99,94	0,00E+00	3026	Os04g0505400	100,00	0,00E+00	3030	4	NA	Japonica	
augustus_masked-4-processed-gene-199.37-mRNA-1	4	OsR498G0408856000.01.T01	99,70	0,00E+00	3001	Os04g0505700	100,00	0,00E+00	2251	-750	Indica	Japonica	
augustus_masked-4-processed-gene-2.9-mRNA-1	4	OsR498G0407629800.01.T01	99,69	0,00E+00	1153	Os04g0174100	99,69	0,00E+00	1153	0	NA	Japonica	
augustus_masked-4-processed-gene-201.11-mRNA-1	4	OsR498G0408869400.01.T01	100,00	0,00E+00	3837	Os04g0508800	100,00	0,00E+00	3837	0	NA	NA	
augustus_masked-4-processed-gene-202.26-mRNA-1	4	OsR498G0408876800.01.T01	97,86	0,00E+00	4341	Os04g0510400	97,86	0,00E+00	4341	0	NA	Japonica	
augustus_masked-4-processed-gene-202.6-mRNA-1	4	OsR498G0408875200.01.T02	100,00	0,00E+00	746	Os02g0582100	93,81	0,00E+00	765	19	NA	NA	
augustus_masked-4-processed-gene-203.42-mRNA-1	4	OsR498G0408882000.01.T01	100,00	0,00E+00	715	Os04g0512500	100,00	0,00E+00	715	0	NA	Japonica	
augustus_masked-4-processed-gene-204.35-mRNA-1	4	OsR498G1120733700.01.T01	84,83	4,46E-100	366	Os04g0514101	98,76	2,98E-160	563	197	Japonica	NA	
augustus_masked-4-processed-gene-204.64-mRNA-1	4	OsR498G0408895000.01.T03	100,00	0,00E+00	1099	Os04g0516200	100,00	0,00E+00	1099	0	NA	Indica	
augustus_masked-4-processed-gene-207.21-mRNA-1	4	OsR498G0408916400.01.T01	100,00	0,00E+00	948	Os04g0520700	100,00	0,00E+00	948	0	NA	NA	
augustus_masked-4-processed-gene-208.18-mRNA-1	4	OsR498G0713495500.01.T01	71,00	1,35E-29	131	Os04g0521900	100,00	0,00E+00	883	752	Japonica	Japonica	
augustus_masked-4-processed-gene-209.41-mRNA-1	4	OsR498G0408924200.01.T01	100,00	0,00E+00	2722	Os04g0523600	99,93	0,00E+00	2718	-4	NA	Indica	
augustus_masked-4-processed-gene-209.42-mRNA-1	4	OsR498G0408924600.01.T01	100,00	0,00E+00	2673	Os04g0523700	99,23	0,00E+00	2534	-139	NA	Indica	
augustus_masked-4-processed-gene-210.14-mRNA-1	4	OsR498G0408934200.01.T01	100,00	0,00E+00	1088	Os04g0526600	100,00	0,00E+00	1088	0	NA	Japonica	
augustus_masked-4-processed-gene-211.40-mRNA-1	4	OsR498G0408944800.01.T01	100,00	0,00E+00	893	Os04g0528800	100,00	0,00E+00	893	0	NA	NA	
augustus_masked-4-processed-gene-216.9-mRNA-1	4	OsR498G0408984200.01.T01	100,00	0,00E+00	1434	Os04g0538400	99,87	0,00E+00	1336	-98	NA	NA	
augustus_masked-4-processed-gene-219.18-mRNA-1	4	OsR498G0409011400.01.T01	99,76	0,00E+00	4529	Os04g0544100	100,00	0,00E+00	4556	27	NA	Japonica	
augustus_masked-4-processed-gene-22.27-mRNA-1	4	OsR498G0407718200.01.T01	99,51	0,00E+00	4341	Os04g0202350	99,61	0,00E+00	3649	-692	Indica	Indica	Kinase-TM
augustus_masked-4-processed-gene-220.56-mRNA-1	4	OsR498G0409019800.01.T01	99,27	0,00E+00	1707	Os04g0546800	100,00	0,00E+00	1727	20	NA	Japonica	
augustus_masked-4-processed-gene-221.11-mRNA-1	4	OsR498G0409024200.01.T01	99,83	0,00E+00	5129	Os04g0548100	100,00	0,00E+00	4361	-768	Indica	Japonica	LRR-NBS-TM
augustus_masked-4-processed-gene-225.47-mRNA-1	4	OsR498G0409056600.01.T01	99,82	0,00E+00	2021	Os04g0556400	100,00	0,00E+00	2057	36	NA	Japonica	
augustus_masked-4-processed-gene-225.48-mRNA-1	4	OsR498G0409058200.01.T01	99,86	0,00E+00	2502	Os04g0556500	100,00	0,00E+00	2511	9	NA	Japonica	
augustus_masked-4-processed-gene-225.49-mRNA-1	4	OsR498G0409058600.01.T01	99,57	0,00E+00	2484	Os04g0556600	100,00	0,00E+00	2511	27	NA	Japonica	
augustus_masked-4-processed-gene-226.51-mRNA-1	4	OsR498G0409063400.01.T01	99,91	0,00E+00	4109	Os04g0559000	100,00	0,00E+00	4118	9	NA	NA	
augustus_masked-4-processed-gene-227.65-mRNA-1	4	OsR498G0409067000.01.T01	99,88	0,00E+00	2946	Os04g0560100	100,00	0,00E+00	2955	9	NA	NA	
augustus_masked-4-processed-gene-230.23-mRNA-1	4	OsR498G0409082600.01.T01	99,92	0,00E+00	2301	Os02g0670900	84,60	0,00E+00	1310	-991	Indica	NA	
augustus_masked-4-processed-gene-230.27-mRNA-1	4	OsR498G0409083800.01.T03	99,73	0,00E+00	656	Os04g0566000	100,00	2,31E-165	580	-76	Indica	NA	
augustus_masked-4-processed-gene-232.22-mRNA-1	4	OsR498G0409093200.01.T04	99,64	0,00E+00	2001	Os02g0673700	72,99	0,00E+00	657	-1344	Indica	NA	
augustus_masked-4-processed-gene-239.16-mRNA-1	4	OsR498G0409138800.01.T01	99,69	0,00E+00	1144	Os04g0579200	100,00	0,00E+00	1153	9	NA	Japonica	
augustus_masked-4-processed-gene-239.31-mRNA-1	4	OsR498G0409142800.01.T01	99,89	0,00E+00	3346	Os04g0580300	100,00	0,00E+00	3355	9	NA	Japonica	
augustus_masked-4-processed-gene-239.34-mRNA-1	4	OsR498G0409144800.01.T01	99,81	0,00E+00	1847	Os04g0580500	99,51	0,00E+00	1792	-55	NA	Japonica	
augustus_masked-4-processed-gene-240.33-mRNA-1	4	OsR498G0409149800.01.T02	99,69	0,00E+00	2277	Os04g0581400	99,90	0,00E+00	1709	-568	Indica	Japonica	
augustus_masked-4-processed-gene-241.31-mRNA-1	4	OsR498G0409163000.01.T01	100,00	1,80E-158	558	Os04g0583600	100,00	6,27E-159	558	0	NA	Japonica	
augustus_masked-4-processed-gene-245.15-mRNA-1	4	OsR498G0409187800.01.T01	99,89	0,00E+00	1652	Os04g0590200	100,00	0,00E+00	1656	4	NA	Japonica	
augustus_masked-4-processed-gene-245.30-mRNA-1	4	OsR498G0409190600.01.T01	99,83	0,00E+00	2069	Os04g0590900	100,00	0,00E+00	2078	9	NA	Japonica	
augustus_masked-4-processed-gene-246.37-mRNA-1	4	OsR498G0409198000.01.T01	96,67	8,07E-111	400	Os01g0235900	83,82	8,12E-36	150	-250	Indica	Japonica	
augustus_masked-4-processed-gene-252.41-mRNA-1	4	OsR498G0409239000.01.T01	100,00	0,00E+00	2527	Os04g0606000	100,00	0,00E+00	2527	0	NA	Japonica	

augustus_masked-4-processed-gene-258.8-mRNA-1	4	OsR498G0409271000.01.T03	99,93	0,00E+00	2658	Os04g0613900	99,86	0,00E+00	2654	-4	NA	Japonica	
augustus_masked-4-processed-gene-260.62-mRNA-1	4	OsR498G0409288800.01.T03	99,89	0,00E+00	1614	Os04g0618500	100,00	0,00E+00	1618	4	NA	Japonica	
augustus_masked-4-processed-gene-261.39-mRNA-1	4	OsR498G0409294000.01.T01	99,96	0,00E+00	4568	Os04g0619600	100,00	0,00E+00	4572	4	NA	Japonica	Kinase-TM
augustus_masked-4-processed-gene-261.40-mRNA-1	4	OsR498G0409294200.01.T01	99,94	0,00E+00	3042	Os04g0619700	100,00	0,00E+00	3047	5	NA	Japonica	
augustus_masked-4-processed-gene-265.58-mRNA-1	4	OsR498G0409317600.01.T01	99,20	0,00E+00	1104	Os04g0626100	100,00	0,00E+00	1126	22	NA	Japonica	
augustus_masked-4-processed-gene-266.79-mRNA-1	4	OsR498G0409323200.01.T01	99,73	0,00E+00	1312	Os04g0627900	99,73	0,00E+00	1315	3	NA	Japonica	
augustus_masked-4-processed-gene-269.30-mRNA-1	4	OsR498G0409343400.01.T01	99,17	0,00E+00	1058	Os04g0635800	99,50	0,00E+00	1064	6	NA	Japonica	
augustus_masked-4-processed-gene-269.6-mRNA-1	4	OsR498G0409340800.01.T01	99,66	1,01E-148	526	Os04g0635000	100,00	8,26E-151	531	5	NA	Japonica	
augustus_masked-4-processed-gene-270.6-mRNA-1	4	OsR498G0409346400.01.T03	99,74	0,00E+00	2773	Os04g0636900	99,81	0,00E+00	2777	4	NA	Japonica	
augustus_masked-4-processed-gene-273.88-mRNA-1	4	OsR498G0409366400.01.T01	100,00	0,00E+00	1736	Os04g0644200	100,00	0,00E+00	2360	624	Japonica	Japonica	
augustus_masked-4-processed-gene-275.25-mRNA-1	4	OsR498G0409376000.01.T01	99,82	0,00E+00	3033	Os04g0647300	98,71	0,00E+00	3056	23	NA	Japonica	
augustus_masked-4-processed-gene-276.20-mRNA-1	4	OsR498G0409388800.01.T01	99,66	1,01E-148	526	Os04g0649600	100,00	8,26E-151	531	5	NA	Japonica	
augustus_masked-4-processed-gene-277.81-mRNA-1	4	OsR498G0409398000.01.T07	100,00	4,11E-180	630	Os04g0652400	100,00	1,43E-180	630	0	NA	NA	
augustus_masked-4-processed-gene-279.52-mRNA-1	4	OsR498G0409406000.01.T02	100,00	8,38E-150	529	Os04g0654400	100,00	2,91E-150	529	0	NA	Japonica	
augustus_masked-4-processed-gene-279.53-mRNA-1	4	OsR498G0409406200.01.T01	99,01	0,00E+00	713	Os04g0654500	100,00	0,00E+00	731	18	NA	Japonica	
augustus_masked-4-processed-gene-28.4-mRNA-1	4	OsR498G0407751100.01.T01	99,77	0,00E+00	4762	Os04g0218100	100,00	0,00E+00	4821	59	NA	Japonica	
augustus_masked-4-processed-gene-281.28-mRNA-1	4	OsR498G0409421400.01.T01	99,34	0,00E+00	2968	Os04g0658600	100,00	0,00E+00	2982	14	NA	Japonica	
augustus_masked-4-processed-gene-283.84-mRNA-1	4	OsR498G0409433600.01.T01	100,00	0,00E+00	765	Os04g0662200	100,00	0,00E+00	780	15	NA	Japonica	
augustus_masked-4-processed-gene-284.50-mRNA-1	4	OsR498G0409438200.01.T03	100,00	1,83E-171	601	Os04g0663300	100,00	6,37E-172	601	0	NA	NA	
augustus_masked-4-processed-gene-285.35-mRNA-1	4	OsR498G0409443400.01.T01	100,00	0,00E+00	2435	Os04g0664600	99,70	0,00E+00	2417	-18	NA	Indica	
augustus_masked-4-processed-gene-286.79-mRNA-1	4	OsR498G0409456800.01.T01	100,00	0,00E+00	834	Os04g0668000	100,00	3,53E-165	580	-254	Indica	NA	
augustus_masked-4-processed-gene-287.8-mRNA-1	4	OsR498G0409458800.01.T02	100,00	0,00E+00	3279	Os04g0668700	100,00	0,00E+00	3279	0	NA	NA	
augustus_masked-4-processed-gene-288.13-mRNA-1	4	OsR498G0409467000.01.T01	98,36	0,00E+00	3690	Os04g0671800	98,60	0,00E+00	3712	22	NA	Japonica	
augustus_masked-4-processed-gene-288.15-mRNA-1	4	OsR498G0409468600.01.T01	99,67	0,00E+00	5436	Os04g0672100	100,00	0,00E+00	5481	45	NA	Japonica	Kinase-LRR-TM
augustus_masked-4-processed-gene-289.5-mRNA-1	4	OsR498G0409468600.01.T01	95,14	0,00E+00	2930	Os04g0672600	100,00	0,00E+00	3901	971	Japonica	Japonica	LRR-TM
augustus_masked-4-processed-gene-289.6-mRNA-1	4	OsR498G0409471000.01.T04	99,68	0,00E+00	3865	Os04g0672700	100,00	0,00E+00	3896	31	NA	Japonica	
augustus_masked-4-processed-gene-29.12-mRNA-1	4	OsR498G1119645400.01.T01	74,98	0,00E+00	1304	Os04g0219600	99,62	0,00E+00	3763	2459	Japonica	Indica	CC-NBS-TM
augustus_masked-4-processed-gene-290.76-mRNA-1	4	OsR498G0409484400.01.T05	99,69	0,00E+00	2909	Os04g0675600	100,00	0,00E+00	2928	19	NA	Japonica	
augustus_masked-4-processed-gene-291.21-mRNA-1	4	OsR498G0409489200.01.T01	99,15	2,57E-178	626	Os04g0677033	100,00	3,57E-152	536	-90	Indica	Japonica	
augustus_masked-4-processed-gene-296.29-mRNA-1	4	OsR498G0409520600.01.T01	99,65	0,00E+00	1513	Os04g0685700	100,00	0,00E+00	879	-634	Indica	Japonica	
augustus_masked-4-processed-gene-296.39-mRNA-1	4	OsR498G0409522400.01.T01	99,53	0,00E+00	2645	Os04g0686000	100,00	0,00E+00	2241	-404	Indica	Japonica	
augustus_masked-4-processed-gene-296.42-mRNA-1	4	OsR498G0409524600.01.T01	99,71	0,00E+00	3712	Os04g0686500	100,00	0,00E+00	3739	27	NA	Japonica	
augustus_masked-4-processed-gene-296.45-mRNA-1	4	OsR498G0409525000.01.T02	98,33	0,00E+00	2175	Os04g0686700	99,53	0,00E+00	2264	89	NA	Japonica	
augustus_masked-4-processed-gene-299.27-mRNA-1	4	OsR498G0409539000.01.T01	94,85	0,00E+00	1393	Os08g0103900	75,40	1,73E-95	349	-1044	Indica	Japonica	
augustus_masked-4-processed-gene-3.22-mRNA-1	4	OsR498G0407632800.01.T01	99,47	0,00E+00	2697	Os04g0174800	100,00	0,00E+00	1624	-1073	Indica	Japonica	
augustus_masked-4-processed-gene-30.25-mRNA-1	4	OsR498G0407755600.01.T06	99,84	0,00E+00	2205	Os04g0221300	99,22	0,00E+00	1353	-852	Indica	Indica	
augustus_masked-4-processed-gene-34.26-mRNA-1	4	OsR498G0407765200.01.T01	99,53	0,00E+00	1132	Os04g0225100	98,74	0,00E+00	1110	-22	NA	Indica	
augustus_masked-4-processed-gene-4.8-mRNA-1	4	OsR498G0407635400.01.T01	99,54	0,00E+00	2311	Os04g0175500	100,00	0,00E+00	2338	27	NA	Japonica	
augustus_masked-4-processed-gene-40.25-mRNA-1	4	OsR498G0407811800.01.T01	100,00	1,38E-179	628	Os04g0243700	99,71	2,04E-178	624	-4	NA	NA	
augustus_masked-4-processed-gene-49.24-mRNA-1	4	OsR498G0407853000.01.T01	97,87	5,64E-160	563	Os04g0257500	99,48	0,00E+00	690	127	Japonica	NA	
augustus_masked-4-processed-gene-51.9-mRNA-1	4	OsR498G0407867000.01.T01	99,85	0,00E+00	1201	Os04g0258900	100,00	0,00E+00	2214	1013	Japonica	Indica	
augustus_masked-4-processed-gene-55.20-mRNA-1	4	OsR498G0407887800.01.T01	99,45	0,00E+00	2594	Os04g0267000	99,59	0,00E+00	2603	9	NA	NA	
augustus_masked-4-processed-gene-55.22-mRNA-1	4	OsR498G0407891700.01.T01	99,87	0,00E+00	2767	Os04g0267600	99,59	0,00E+00	2184	-583	Indica	NA	
augustus_masked-4-processed-gene-57.27-mRNA-1	4	OsR498G0407902000.01.T01	99,80	0,00E+00	2676	Os04g0270900	100,00	0,00E+00	2809	133	NA	Japonica	
augustus_masked-4-processed-gene-58.26-mRNA-1	4	OsR498G0407905000.01.T01	96,35	0,00E+00	2204	Os04g0271700	99,93	0,00E+00	2663	459	Japonica	Japonica	
augustus_masked-4-processed-gene-58.30-mRNA-1	4	OsR498G0407905600.01.T01	99,60	0,00E+00	2213	Os04g0272400	99,92	0,00E+00	2231	18	NA	Japonica	
augustus_masked-4-processed-gene-6.13-mRNA-1	4	OsR498G0407645000.01.T01	99,49	0,00E+00	1402	Os04g0179100	100,00	0,00E+00	1420	18	NA	Japonica	
augustus_masked-4-processed-gene-60.12-mRNA-1	4	OsR498G0407914000.01.T01	99,79	0,00E+00	4166	Os04g0275100	99,96	0,00E+00	4184	18	NA	Japonica	Kinase-TM
augustus_masked-4-processed-gene-78.23-mRNA-1	4	OsR498G0407999200.01.T01	85,14	0,00E+00	2498	Os04g0303300	99,96	0,00E+00	3967	1469	Japonica	Japonica	Kinase-TM
augustus_masked-4-processed-gene-79.42-mRNA-1	4	OsR498G0408006600.01.T01	99,80	0,00E+00	1755	Os11g0566800	89,43	0,00E+00	1285	-470	Indica	Japonica	
augustus_masked-4-processed-gene-81.46-mRNA-1	4	OsR498G0408014500.01.T02	99,84	0,00E+00	2250	Os04g0307500	100,00	0,00E+00	2259	9	NA	Japonica	Kinase-TM
augustus_masked-4-processed-gene-84.30-mRNA-1	4	OsR498G0408030600.01.T03	99,67	0,00E+00	2694	Os04g0312100	99,67	0,00E+00	2698	4	NA	NA	
augustus_masked-4-processed-gene-87.12-mRNA-1	4	OsR498G0408099900.01.T01	99,93	0,00E+00	2745	Os04g0324100	99,93	0,00E+00	2745	0	NA	Indica	
augustus_masked-4-processed-gene-89.15-mRNA-1	4	OsR498G0408139100.01.T01	98,87	2,54E-177	621	Os04g0329300	98,87	8,84E-178	621	0	NA	NA	
augustus_masked-4-processed-gene-89.16-mRNA-1	4	OsR498G0408137500.01.T01	98,02	4,60E-173	608	Os04g0317500	99,44	1,40E-180	630	22	NA	Indica	
augustus_masked-4-processed-gene-89.32-mRNA-1	4	OsR498G0408135500.01.T01	97,65	0,00E+00	720	Os04g0317800	99,72	0,00E+00	635	-85	Indica	Japonica	
augustus_masked-4-processed-gene-95.3-mRNA-1	4	OsR498G0408115800.01.T01	99,93	0,00E+00	2685	Os04g0319800	99,67	0,00E+00	2667	-18	NA	NA	
augustus_masked-4-processed-gene-97.11-mRNA-1	4	OsR498G0408115800.01.T01	100,00	0,00E+00	2690	Os04g0319800	99,60	0,00E+00	2663	-27	NA	NA	
augustus-4-processed-gene-1.21-mRNA-1	4	OsR498G0407629800.01.T01	88,00	0,00E+00	1993	Os04g0171600	95,10	0,00E+00	2260	267	Japonica	Indica	
augustus-4-processed-gene-104.14-mRNA-1	4	OsR498G0408172900.01.T01	100,00	0,00E+00	2765	Os04g0341500	72,35	1,73E-136	487	-2278	Indica	Indica	
augustus-4-processed-gene-109.27-mRNA-1	4	OsR498G0408213500.01.T05	99,71	1,41E-175	617	Os04g0346000	99,71	4,92E-176	617	0	NA	Japonica	
augustus-4-processed-gene-11.2-mRNA-1	4	OsR498G0407666500.01.T01	99,88	0,00E+00	2908	Os04g0186400	100,00	0,00E+00	2917	9	NA	Japonica	
augustus-4-processed-gene-111.34-mRNA-1	4	OsR498G0408222700.01.T01	100,00	0,00E+00	733	Os04g0348600	90,78	0,00E+00	693	-40	NA	Indica	
augustus-4-processed-gene-112.0-mRNA-1	4	OsR498G0408226900.01.T02	100,00	0,00E+00	4048	Os04g0351333	93,19	0,00E+00	2482	-1566	Indica	Indica	
augustus-4-processed-gene-112.22-mRNA-1	4	OsR498G0408232300.01.T02	100,00	0,00E+00	3635	Os04g0351333	100,00	0,00E+00	4010	375	NA	NA	
augustus-4-processed-gene-116.13-mRNA-1	4	OsR498G0408255600.01.T02	100,00	0,00E+00	6942	Os10g0192300	70,25	0,00E+00	1707	-5235	Indica	NA	
augustus-4-processed-gene-117.2-mRNA-1	4	OsR498G0408266700.01.T01	100,00	0,00E+00	1242	Os04g0359100	100,00	0,00E+00	1242	0	NA	NA	
augustus-4-processed-gene-118.13-mRNA-1	4	OsR498G0408287300.01.T01	100,00	0,00E+00	2603	Os04g0361500	100,00	0,00E+00	1943	-660	Indica	Indica	
augustus-4-processed-gene-12.15-mRNA-1	4	OsR498G0407674500.01.T02	99,69	2,45E-163	575	Os04g0188500	100,00	9,71E-138	488	-87	Indica	Japonica	
augustus-4-processed-gene-120.22-mRNA-1	4	OsR498G0204388300.01.T01	86,60	0,00E+00	719	Os04g0364800	100,00	0,00E+00	996	277	Japonica	Japonica	
augustus-4-processed-gene-125.16-mRNA-1	4	OsR498G0408346500.01.T01	99,86	0,00E+00	2605	Os04g0370500	98,52	0,00E+00	2461	-144	NA	Indica	
augustus-4-processed-gene-125.17-mRNA-1	4	OsR498G0408346900.01.T02	99,86	0,00E+00	2589	Os04g0370600	99,86	0,00E+00	2589	0	NA	NA	
augustus-4-processed-gene-128.22-mRNA-1	4	OsR498G0408359900.01.T01	100,00	0,00E+00	1721	Os04g0373000	100,00	0,00E+00	1721	0	NA	NA	
augustus-4-processed-gene-129.53-mRNA-1	4	OsR498G0408366900.01.T01	91,53	0,00E+00	2434	Os04g0375300	89,94	0,00E+00	2304	-130	NA	NA	NBS-TM
augustus-4-processed-gene-131.46-mRNA-1	4	OsR498G0408386100.01.T01	100,00	0,00E+00	1166	Os04g0379600	100,00	0,00E+00	1166	0	NA	Japonica	
augustus-4-processed-gene-133.3-mRNA-1	4	OsR498G0408400900.01.T01	100,00	0,00E+00	3728	Os04g0382200	100,00	0,00E+00	3728	0	NA	NA	
augustus-4-processed-gene-134.19-mRNA-1	4	OsR498G0408408700.01.T01	100,00	0,00E+00	715	Os04g0384850	100,00	0,00E+00	715	0	NA	NA	
augustus-4-processed-gene-134.2-mRNA-1	4	OsR498G0408405300.01.T01	100,00	1,03E-174	612	Os04g0383700	100,00	3,59E-175	612	0	NA	Indica	
augustus-4-processed-gene-135.3-mRNA-1	4	OsR498G0203898100.01.T01	81,56	4,67E-74	279	Os04g0386300	96,74	4,96E-157	553	274	Japonica	Indica	
augustus-4-processed-gene-136.6-mRNA-1	4	OsR498G0408424300.01.T04	100,00	1,56E-106	385	Os04g0387400	94,42	1,46E-82	305	-80	Indica	Indica	
augustus-4-processed-gene-137.0-mRNA-1	4	OsR498G0408428100.01.T01	97,98	0,00E+00	4614	Os04g0388500	97,98	0,00E+00	4614	0	NA	NA	
augustus-4-processed-gene-137.11-mRNA-1	4	OsR498G0408432100.01.T01	100,00	0,00E+00	3593	Os04g0389800	100,00	0,00E+00	3463	-130	NA	NA	
augustus-4-processed-gene-137.2-mRNA-1	4	OsR498G0408429700.01.T01	100,00	0,00E+00	3983	Os04g0388800	100,00	0,00E+00	3241	-742	Indica	Indica	
augustus-4-processed-gene-139.19-mRNA-1	4	OsR498G0408449100.01.T04	96,52	0,00E+00	2210	Os04g0394200	96,37	0,00E+00	2209	-1	NA	Japonica	
augustus-4-processed-gene-145.29-mRNA-1	4	OsR498G0407604900.01.T01	71,43	4,80E-05	49,1	Os04g0402700	100,00	1,66E-100	363	313,9	Japonica	Indica	
augustus-4-processed-gene-147.53-mRNA-1	4	OsR498G0408505100.01.T01	100,00	0,00E+00	2176	Os04g0405700	99,92	0,00E+00	2196	20	NA	NA	
augustus-4-processed-gene-148.27-mRNA-1	4	OsR498G0408513300.01.T01	100,00	0,00E+00	1894	Os04g0407500	100,00	0,00E+00	1007	-887	Indica	Indica	
augustus-4-processed-gene-148.59-mRNA-1	4	OsR498G0408518400.01.T04	100,00	0,00E+00	1076	Os04g0409500	100,00	0,00E+00	1076	0	NA	Indica	
augustus-4-processed-gene-149.46-mRNA-1	4	OsR498G0408521900.01.T01	100,00	1,34E-153	542	Os04g0411200	100,00	4,67E-154	542	0	NA	Indica	
augustus-4-processed-gene-149.48-mRNA-1	4	OsR498G0408675500.01.T01	91,53	4,61E-122	437	Os08g0561800	89,49	3,77E-124	444	7	NA	NA	
augustus-4-processed-gene-149.49-mRNA-1	4	OsR498G0408524100.01.T01	99,73	0,00E+00	1957	Os04g0411800	99,81	0,00E+00	1864	-93	NA	Indica	
augustus-4-processed-gene-149.70-mRNA-1	4	OsR498G0408522400.01.T01	100,00	0,00E+00	942	Os04g0411400	99,81	0,00E+00	938	-4	NA	Indica	
augustus-4-processed-gene-150.1-mRNA-1	4	OsR498G0408525300.01.T01	100,00	0,00E+00	1180	Os04g0412100	100,00	0,00E+00	1180	0	NA	Indica	
augustus-4-processed-gene-150.15-mRNA-1	4	OsR498G0408527900.01.T01	99,62	0,00E+00	1405	Os04g0412700	99,75	0,00E+00	1409	4	NA	NA	
augustus-4-processed-gene-150.16-mRNA-1	4	OsR498G0408528100.01.T01	100,00	0,00E+00	2430	Os04g0412800	100,00	0,00E+00	2430	0	NA	NA	
augustus-4-processed-gene-150.3-mRNA-1	4	OsR498G0408525900.01.T04	100,00	0,00E+00	829	Os04g0412200	100,00	0,00E+00	829	0	NA	NA	
augustus-4-processed-gene-151.11-mRNA-1	4	OsR498G0408537500.01.T03	100,00	0,00E+00	2955	Os04g0414800	99,88	0,00E+00	2946	-9	NA	Indica	
augustus-4-processed-gene-151.14-mRNA-1	4	OsR498G0408540100.01.T01	93,82	0,00E+00	966	Os04g0415200	94,15	0,00E+00	976	10	NA	NA	
augustus-4-processed-gene-151.15-mRNA-1	4	OsR498G0408540300.01.T01	100,00	5,82E-178	623	Os01g0660000	70,36	2,04E-26	119	-504	Indica	NA	
augustus-4-processed-gene-151.16-mRNA-1	4	OsR498G0408541500.01.T01	99,10	0,00E+00	979	Os04g0415600	99,02	0,00E+00	900	-79	NA	NA	
augustus-4-processed-gene-152.18-mRNA-1	4	OsR498G0408545900.01.T05	100,00	0,00E+00	4049	Os02g0539100	76,10	0,00E+00	1654	-2395	Indica	NA	
augustus-4-processed-gene-154.22-mRNA-1	4	OsR498G0408563300.01.T01	100,00	0,00E+00	4318	Os04g0420600	99,00	0,00E+00	4210	-108	NA	Indica	Kinase-TM
augustus-4-processed-gene-155.21-mRNA-1	4	OsR498G0408568300.01.T01	95,75	0,00E+00	1799	Os04g0423200	99,45	0,00E+00	1920	121	NA	NA	
augustus-4-processed-gene-159.17-mRNA-1	4	OsR498G0408584900.01.T04	99,90	0,00E+00	1778	Os04g0429800	98,94	0,00E+00	1649	-129	NA	Indica	
augustus-4-processed-gene-160.37-mRNA-1	4	OsR498G0408588300.01.T01	99,83	0,00E+00	4192	Os04g0430700	99,96	0,00E+00	4205	13	NA	Japonica	
augustus-4-processed-gene-160.53-mRNA-1	4	OsR498G0408597500.01.T01	97,81	0,00E+00	1017	Os04g0432366	100,00	0,00E+00	1067	50	NA	Japonica	
augustus-4-processed-gene-161.32-mRNA-1	4	OsR498G0408601300.01.T01	96,27	0,00E+00	1359	Os04g0433300	100,00	0,00E+00	1213	-146	Indica	Japonica	
augustus-4-processed-gene-164.26-mRNA-1	4	OsR498G0408617100.01.T01	100,00	0,00E+00	2603	Os04g0438400	99,91	0,00E+00	2041	-562	Indica	NA	
augustus-4-processed-gene-168.12-mRNA-1	4	OsR498G0408646700.01.T01	99,81	0,00E+00	948	Os04g0444100	100,00	0,00E+00	953	5	NA	Japonica	
augustus-4-processed-gene-169.39-mRNA-1	4	OsR498G0408647300.01.T03	97,77	0,00E+00	917	Os04g0444300	100,00	0,00E+00	969	52	NA	Japonica	
augustus-4-processed-gene-169.41-mRNA-1	4	OsR498G0408648100.01.T02	99,92	0,00E+00	2301	Os04g0444600	100,00	0,00E+00	2305	4	NA	Japonica	
augustus-4-processed-gene-169.57-mRNA-1	4	OsR498G0408650500.01.T01	100,00	2,72E-139	495	Os04g0445400	100,00	9,47E-140	495	0	NA	Japonica	
augustus-4-processed-gene-169.58-mRNA-1	4	OsR498G0408651100.01.T01	99,52	0,00E+00	2235	Os04g0445600	100,00	0,00E+00	2262	27	NA	Japonica	
augustus-4-processed-gene-169.61-mRNA-1	4	OsR498G0408653100.01.T01	99,02	0,00E+00	1073	Os04g0446200	100,00	0,00E+00	783	-290	Indica	Japonica	
augustus-4-processed-gene-17.30-mRNA-1	4	OsR498G0407698300.01.T01	99,55	0,00E+00	2365	Os04g0194400	100,00	0,00E+00	2392	27	NA	NA	
augustus-4-processed-gene-171.69-mRNA-1	4	OsR498G0101579700.01.T01	91,08	0,00E+00	761	Os04g0449800	100,00	0,00E+00	1721	960	Japonica	Japonica	
augustus-4-processed-gene-171.70-mRNA-1	4	OsR498G0408667100.01.T02	96,34	0,00E+00	2309	Os04g0449900	96,13	0,00E+00	1921	-388	Indica	Japonica	
augustus-4-processed-gene-175.2-mRNA-1	4	OsR498G0408687100.01.T01	100,00	0,00E+00	1002	Os04g0456200	100,00	0,00E+00	1002	0	NA	Japonica	
augustus-4-processed-gene-175.21-mRNA-1	4	OsR498G0408689100.01.T02	99,91	0,00E+00	2025	Os04g0457300	100,00	0,00E+00	1451	-574	Indica	Japonica	
augustus-4-processed-gene-175.25-mRNA-1	4	OsR498G0408691500.01.T02	99,79	0,00E+00	3402	Os04g0458200	100,00	0,00E+00	3420	18	NA	Japonica	
augustus-4-processed-gene-178.49-mRNA-1	4	OsR498G0408714300.01.T02	100,00	0,00E+00	645	Os04g0464100	100,00	0,00E+00	645	0	NA	NA	
augustus-4-processed-gene-180.38-mRNA-1	4	OsR498G0408727300.01.T01	100,00	0,00E+00	780	Os04g0467000	100,00	0,00E+00	780	0	NA	NA	
augustus-4-processed-gene-182.0-mRNA-1	4	OsR498G0408741100.01.T01	98,50	0,00E+00	2549	Os04g0470500	100,00	0,00E+00	5027	2478	Japonica	Japonica	CC-NBS-TM
augustus-4-processed-gene-183.27-mRNA-1	4	OsR498G0408750100.01.T01	99,77	0,00E+00	4605	Os04g0472900	100,00	0,00E+00	4632	27	NA	Japonica	
augustus-4-processed-gene-185.46-mRNA-1	4	OsR498G0408760900.01.T01	99,76	0,00E+00	761	Os04g0475900	100,00	0,00E+00	969	208	Japonica	Japonica	
augustus-4-processed-gene-186.0-mRNA-1	4	OsR498G0511078900.01.T01	73,63	6,18E-19	95,1	Os04g0477900	100,00	1,87E-159	560	464,9	Japonica	Japonica	
augustus-4-processed-gene-189.13-mRNA-1	4	OsR498G0408792300.01.T01	100,00	0,00E+00	2300	Os04g0486300	100,00	0,00E+00	2300	0	NA	NA	
augustus-4-processed-gene-190.60-mRNA-1	4	OsR498G0408795300.01.T01	100,00	0,00E+00	3371	Os04g0487200	100,00	0,00E+00	3371	0	NA	NA	Kinase-LRR-TM
augustus-4-processed-gene-190.66-mRNA-1	4	OsR498G0408799700.01.T02	99,92	0,00E+00	2274	Os04g0488200	100,00	0,00E+00	3241	967	Japonica	NA	
augustus-4-processed-gene-192.20-mRNA-1	4	OsR498G0408811900.01.T02	99,96	0,00E+00	4968	Os04g0491100	100,00	0,00E+00	4973	5	NA	Japonica	CC-LRR-NBS-TM
augustus-4-processed-gene-192.23-mRNA-1	4	OsR498G0408815100.01.T01	99,76	0,00E+00	2254	Os04g0492100	99,92	0,00E+00	2263	9	NA	Japonica	
augustus-4-processed-gene-192.3-mRNA-1	4	OsR498G0408810300.01.T01	99,90	0,00E+00	3568	Os04g0490700	100,00	0,00E+00	3577	9	NA	Japonica	
augustus-4-processed-gene-193.26-mRNA-1	4	OsR498G0408817300.01.T05	99,91	0,00E+00	2063	Os04g0492500	100,00	0,00E+00	1873	-190	NA	Japonica	
augustus-4-processed-gene-193.34-mRNA-1	4	OsR498G0408817300.01.T06	100,00	0,00E+00	1092	Os04g0492600	100,00	0,00E+00	1092	0	NA	Japonica	
augustus-4-processed-gene-193.35-mRNA-1	4	OsR498G0408817700.01.T01	99,34	0,00E+00	1340	Os04g0492800	100,00	0,00E+00	1359	19	NA	Japonica	
augustus-4-processed-gene-194.48-mRNA-1	4	OsR498G0408823900.01.T01	99,74	0,00E+00	2043	Os04g0494800	100,00	0,00E+00	2057	14	NA	Japonica	
augustus-4-processed-gene-195.60-mRNA-1	4	OsR498G0408832900.01.T01	99,71	0,00E+00	2509	Os04g0497900	99,95	0,00E+00	3399	890	Japonica	Japonica	
augustus-4-processed-gene-196.2-mRNA-1	4	OsR498G0408834300.01.T04	99,17	0,00E+00	2114	Os04g0498600	100,00	0,00E+00	2159	45	NA	Japonica	
augustus-4-processed-gene-197.34-mRNA-1	4	OsR498G0408845600.01.T01	97,93	0,00E+00	830	Os04g0502300	98,14	0,00E+00	835	5	NA	Japonica	
augustus-4-processed-gene-201.1-mRNA-1	4	OsR498G0408868100.01.T01	100,00	0,00E+00	2441	Os04g0508600	100,00	0,00E+00	2441	0	NA	Indica	
augustus-4-processed-gene-202.0-mRNA-1	4	OsR498G0204334900.01.T01	86,91	0,00E+00	1760	Os04g0509600	100,00	0,00E+00	2700	940	Japonica	NA	
augustus-4-processed-gene-206.27-mRNA-1	4	OsR498G0408903500.01.T01	100,00	0,00E+00	704	Os04g0517900	100,00	0,00E+00	704	0	NA	NA	
augustus-4-processed-gene-207.17-mRNA-1	4	OsR498G0408917300.01.T06	100,00	0,00E+00	2516	Os04g0520900	100,00	0,00E+00	2516	0	NA	NA	
augustus-4-processed-gene-209.57-mRNA-1	4	OsR498G0408931100.01.T02	100,00	0,00E+00	1678	Os04g0525600	100,00	0,00E+00	1678	0	NA	Japonica	
augustus-4-processed-gene-210.1-mRNA-1	4	OsR498G0408933100.01.T01	99,68	0,00E+00	1112	Os04g0526000	100,00	0,00E+00	1121	9	NA	Japonica	
augustus-4-processed-gene-210.23-mRNA-1	4	OsR498G0408940300.01.T01	100,00	0,00E+00	839	Os04g0528200	100,00	0,00E+00	839	0	NA	NA	
augustus-4-processed-gene-210.3-mRNA-1	4	OsR498G0408933900.01.T01	100,00	0,00E+00	1878	Os04g0526300	100,00	0,00E+00	1878	0	NA	Japonica	
augustus-4-processed-gene-211.32-mRNA-1	4	OsR498G0408948900.01.T01	98,44	0,00E+00	4083	Os02g0638900	75,79	0,00E+00	957	-3126	Indica	NA	
augustus-4-processed-gene-212.51-mRNA-1	4	OsR498G0408950500.01.T01	100,00	0,00E+00	2516	Os04g0529700	100,00	0,00E+00	2516	0	NA	NA	
augustus-4-processed-gene-212.65-mRNA-1	4	OsR498G0408956500.01.T01	100,00	0,00E+00	3988	Os04g0531500	100,00	0,00E+00	3988	0	NA	NA	Kinase-TM
augustus-4-processed-gene-212.67-mRNA-1	4	OsR498G0408957100.01.T05	100,00	1,80E-158	558	Os04g0531800	100,00	6,27E-159	558	0	NA	NA	
augustus-4-processed-gene-213.17-mRNA-1	4	OsR498G0408961700.01.T01	96,14	0,00E+00	1549	Os02g0641900	74,20	4,42E-173	607	-942	Indica	NA	
augustus-4-processed-gene-214.46-mRNA-1	4	OsR498G0408968100.01.T02	100,00	0,00E+00	728	Os04g0535600	100,00	0,00E+00	728	0	NA	NA	
augustus-4-processed-gene-215.16-mRNA-1	4	OsR498G0408974700.01.T01	100,00	0,00E+00	803	Os04g0537100	100,00	0,00E+00	910	107	Japonica	NA	
augustus-4-processed-gene-215.22-mRNA-1	4	OsR498G0408981700.01.T01	100,00	0,00E+00	1499	Os04g0537800	100,00	0,00E+00	1499	0	NA	NA	
augustus-4-processed-gene-218.28-mRNA-1	4	OsR498G0409002300.01.T01	100,00	0,00E+00	2408	Os04g0541500	100,00	0,00E+00	2408	0	NA	Japonica	
augustus-4-processed-gene-219.1-mRNA-1	4	OsR498G0409009300.01.T01	97,94	0,00E+00	1085	Os04g0543500	100,00	0,00E+00	1115	30	NA	Japonica	
augustus-4-processed-gene-221.0-mRNA-1	4	OsR498G0409022300.01.T01	99,70	0,00E+00	1767	Os04g0547600	100,00	0,00E+00	1781	14	NA	Japonica	
augustus-4-processed-gene-222.20-mRNA-1	4	OsR498G0409030100.01.T02	99,77	0,00E+00	1534	Os04g0549700	100,00	0,00E+00	1543	9	NA	Japonica	
augustus-4-processed-gene-222.27-mRNA-1	4	OsR498G0409032100.01.T01	99,85	0,00E+00	1176	Os04g0550200	100,00	0,00E+00	1180	4	NA	Japonica	
augustus-4-processed-gene-222.28-mRNA-1	4	OsR498G0409033700.01.T01	99,81	0,00E+00	1853	Os04g0550500	100,00	0,00E+00	1862	9	NA	Japonica	
augustus-4-processed-gene-223.11-mRNA-1	4	OsR498G0409041500.01.T02	100,00	0,00E+00	1180	Os04g0552300	100,00	0,00E+00	1180	0	NA	Japonica	
augustus-4-processed-gene-223.12-mRNA-1	4	OsR498G0409042500.01.T01	99,53	0,00E+00	3054	Os04g0552400	99,94	0,00E+00	3085	31	NA	Japonica	
augustus-4-processed-gene-223.13-mRNA-1	4	OsR498G0409043500.01.T01	99,68	0,00E+00	1675	Os04g0552700	100,00	0,00E+00	1689	14	NA	Japonica	
augustus-4-processed-gene-223.17-mRNA-1	4	OsR498G0409045300.01.T02	99,90	0,00E+00	1868	Os04g0553000	99,62	0,00E+00	1844	-24	NA	Japonica	
augustus-4-processed-gene-223.8-mRNA-1	4	OsR498G0409039700.01.T01	97,55	0,00E+00	2863	Os04g0551800	97,79	0,00E+00	2881	18	NA	Japonica	
augustus-4-processed-gene-224.13-mRNA-1	4	OsR498G0409049300.01.T01	95,40	1,94E-178	625	Os04g0554600	100,00	0,00E+00	715	90	Japonica	Japonica	
augustus-4-processed-gene-224.15-mRNA-1	4	OsR498G0409052300.01.T01	99,86	0,00E+00	3839	Os04g0555000	100,00	0,00E+00	3853	14	NA	Japonica	
augustus-4-processed-gene-225.42-mRNA-1	4	OsR498G0409059700.01.T03	99,80	0,00E+00	1790	Os04g0557000	100,00	0,00E+00	1799	9	NA	Japonica	
augustus-4-processed-gene-226.38-mRNA-1	4	OsR498G0409063100.01.T01	99,87	0,00E+00	4283	Os04g0558900	100,00	0,00E+00	4296	13	NA	NA	
augustus-4-processed-gene-228.10-mRNA-1	4	OsR498G0204490700.01.T01	74,70	0,00E+00	658	Os04g0562500	100,00	0,00E+00	1905	1247	Japonica	NA	
augustus-4-processed-gene-230.3-mRNA-1	4	OsR498G0409082100.01.T01	88,98	0,00E+00	1820	Os04g0565200	100,00	0,00E+00	2516	696	Japonica	NA	
augustus-4-processed-gene-234.18-mRNA-1	4	OsR498G0409109900.01.T01	99,94	0,00E+00	3031	Os04g0571600	100,00	0,00E+00	3036	5	NA	Japonica	
augustus-4-processed-gene-235.41-mRNA-1	4	OsR498G0409113500.01.T01	99,68	0,00E+00	1112	Os02g0676800	74,44	7,29E-105	379	-733	Indica	Japonica	
augustus-4-processed-gene-235.42-mRNA-1	4	OsR498G0204522100.01.T01	78,77	3,10E-153	543	Os04g0572400	100,00	0,00E+00	1191	648	Japonica	Japonica	
augustus-4-processed-gene-236.10-mRNA-1	4	OsR498G0612527500.01.T01	73,41	0,00E+00	675	Os04g0574100	100,00	0,00E+00	2571	1896	Japonica	Japonica	
augustus-4-processed-gene-236.15-mRNA-1	4	OsR498G0409122500.01.T01	99,83	0,00E+00	3120	Os04g0574700	100,00	0,00E+00	3133	13	NA	Japonica	
augustus-4-processed-gene-237.18-mRNA-1	4	OsR498G0409129100.01.T01	99,71	0,00E+00	6221	Os04g0576900	100,00	0,00E+00	3371	-2850	Indica	Japonica	Kinase-LRR-TM
augustus-4-processed-gene-237.19-mRNA-1	4	OsR498G0409129500.01.T02	99,79	0,00E+00	1728	Os04g0577000	99,69	0,00E+00	1724	-4	NA	Japonica	
augustus-4-processed-gene-237.4-mRNA-1	4	OsR498G0408675500.01.T01	93,58	1,92E-139	496	Os10g0531750	92,21	1,06E-124	444	-52	Indica	Japonica	
augustus-4-processed-gene-238.39-mRNA-1	4	OsR498G0409132300.01.T04	100,00	0,00E+00	1992	Os04g0577700	100,00	0,00E+00	1992	0	NA	Japonica	
augustus-4-processed-gene-238.56-mRNA-1	4	OsR498G0409134900.01.T01	99,79	0,00E+00	1669	Os04g0578400	100,00	0,00E+00	1678	9	NA	Japonica	
augustus-4-processed-gene-241.16-mRNA-1	4	OsR498G0409160700.01.T06	99,45	0,00E+00	3251	Os04g0583150	100,00	0,00E+00	3285	34	NA	Japonica	
augustus-4-processed-gene-243.14-mRNA-1	4	OsR498G0409175700.01.T01	99,68	0,00E+00	2759	Os04g0586500	100,00	0,00E+00	2782	23	NA	Japonica	
augustus-4-processed-gene-243.15-mRNA-1	4	OsR498G0409176100.01.T01	99,91	0,00E+00	1944	Os04g0586700	100,00	0,00E+00	1948	4	NA	Japonica	
augustus-4-processed-gene-243.19-mRNA-1	4	OsR498G0409177300.01.T01	100,00	0,00E+00	1023	Os04g0587100	100,00	0,00E+00	1023	0	NA	Japonica	
augustus-4-processed-gene-243.20-mRNA-1	4	OsR498G0409177500.01.T01	99,47	0,00E+00	1015	Os04g0587200	100,00	0,00E+00	1012	-3	NA	Japonica	
augustus-4-processed-gene-243.24-mRNA-1	4	OsR498G0409178500.01.T02	99,82	0,00E+00	2028	Os04g0587400	70,41	6,71E-115	414	-1614	Indica	Japonica	
augustus-4-processed-gene-244.46-mRNA-1	4	OsR498G0409185300.01.T01	99,76	0,00E+00	759	Os04g0589500	100,00	7,73E-128	455	-304	Indica	Japonica	
augustus-4-processed-gene-244.48-mRNA-1	4	OsR498G0409185900.01.T01	99,53	0,00E+00	2257	Os04g0589700	100,00	0,00E+00	2284	27	NA	Japonica	
augustus-4-processed-gene-245.11-mRNA-1	4	OsR498G0203601400.01.T01	95,52	0,00E+00	1074	Os04g0590366	99,70	0,00E+00	1200	126	Japonica	NA	
augustus-4-processed-gene-245.12-mRNA-1	4	OsR498G0409189100.01.T01	99,74	0,00E+00	3424	Os04g0590400	100,00	0,00E+00	3447	23	NA	Japonica	
augustus-4-processed-gene-247.8-mRNA-1	4	OsR498G0409204100.01.T01	99,68	0,00E+00	3962	Os04g0594800	100,00	0,00E+00	4129	167	NA	Japonica	
augustus-4-processed-gene-247.9-mRNA-1	4	OsR498G0409204700.01.T01	99,01	0,00E+00	2480	Os04g0595000	100,00	0,00E+00	2554	74	NA	Japonica	
augustus-4-processed-gene-248.10-mRNA-1	4	OsR498G0409211100.01.T01	99,41	0,00E+00	3929	Os04g0597900	99,85	0,00E+00	2352	-1577	Indica	Japonica	
augustus-4-processed-gene-248.3-mRNA-1	4	OsR498G0409209900.01.T01	99,88	0,00E+00	3108	Os04g0597600	100,00	0,00E+00	3034	-74	NA	Japonica	
augustus-4-processed-gene-248.9-mRNA-1	4	OsR498G0409210900.01.T01	99,94	0,00E+00	3210	Os04g0597800	100,00	0,00E+00	3214	4	NA	Japonica	
augustus-4-processed-gene-254.28-mRNA-1	4	OsR498G0409250600.01.T01	98,91	0,00E+00	5323	Os08g0224700	79,70	0,00E+00	2700	-2623	Indica	Indica	
augustus-4-processed-gene-256.1-mRNA-1	4	OsR498G0409258900.01.T01	99,70	0,00E+00	1198	Os04g0610400	100,00	0,00E+00	1207	9	NA	Japonica	
augustus-4-processed-gene-256.16-mRNA-1	4	OsR498G0409264300.01.T01	98,20	0,00E+00	2312	Os04g0611700	98,12	0,00E+00	2307	-5	NA	Japonica	
augustus-4-processed-gene-257.0-mRNA-1	4	OsR498G0409264900.01.T05	99,65	0,00E+00	2049	Os04g0611800	100,00	0,00E+00	2067	18	NA	Japonica	
augustus-4-processed-gene-257.10-mRNA-1	4	OsR498G0409266300.01.T01	96,46	0,00E+00	799	Os04g0612300	88,73	1,04E-126	452	-347	Indica	Japonica	
augustus-4-processed-gene-260.31-mRNA-1	4	OsR498G0409282900.01.T01	99,40	0,00E+00	884	Os04g0616800	99,60	0,00E+00	888	4	NA	Japonica	
augustus-4-processed-gene-260.32-mRNA-1	4	OsR498G0409283300.01.T01	99,30	0,00E+00	1521	Os04g0616900	100,00	0,00E+00	1544	23	NA	Japonica	
augustus-4-processed-gene-261.28-mRNA-1	4	OsR498G0409292300.01.T01	99,85	0,00E+00	2459	Os04g0619300	100,00	0,00E+00	2468	9	NA	Japonica	
augustus-4-processed-gene-261.33-mRNA-1	4	OsR498G0409294900.01.T01	99,76	0,00E+00	1469	Os04g0619900	99,51	0,00E+00	1460	-9	NA	Japonica	
augustus-4-processed-gene-262.14-mRNA-1	4	OsR498G0409301900.01.T01	76,37	0,00E+00	1026	Os04g0621750	99,89	0,00E+00	3326	2300	Japonica	Japonica	CC-NBS-TM
augustus-4-processed-gene-262.16-mRNA-1	4	OsR498G0409303900.01.T01	79,34	0,00E+00	1684	Os04g0621900	100,00	0,00E+00	3507	1823	Japonica	Japonica	
augustus-4-processed-gene-262.8-mRNA-1	4	OsR498G0409297100.01.T04	89,15	0,00E+00	1308	Os04g0621300	100,00	0,00E+00	2035	727	Japonica	Japonica	Disease Resisatnce RGA protein fragment (Pi63)
augustus-4-processed-gene-264.34-mRNA-1	4	OsR498G0409310700.01.T01	99,36	0,00E+00	1111	Os04g0624400	100,00	0,00E+00	1131	20	NA	Japonica	
augustus-4-processed-gene-265.33-mRNA-1	4	OsR498G0409315900.01.T01	99,74	0,00E+00	2103	Os04g0625600	100,00	0,00E+00	2116	13	NA	Japonica	
augustus-4-processed-gene-265.36-mRNA-1	4	OsR498G0409316900.01.T03	97,67	0,00E+00	2278	Os04g0625800	100,00	0,00E+00	2300	22	NA	Japonica	

augustus-4-processed-gene-266.66-mRNA-1	4	OsR498G0409325300.01.T03	100,00	0,00E+00	742	Os04g0628700	100,00	0,00E+00	742	0	NA	Japonica	
augustus-4-processed-gene-266.67-mRNA-1	4	OsR498G0409325300.01.T01	100,00	0,00E+00	700	Os04g0628800	100,00	0,00E+00	1072	372	Japonica	Japonica	
augustus-4-processed-gene-268.79-mRNA-1	4	OsR498G0409334600.01.T02	83,08	0,00E+00	2564	Os04g0633800	99,92	0,00E+00	4444	1880	Japonica	Japonica	Kinase-TM
augustus-4-processed-gene-270.13-mRNA-1	4	OsR498G0409350100.01.T01	99,89	0,00E+00	1630	Os04g0638800	100,00	0,00E+00	1635	5	NA	NA	
augustus-4-processed-gene-271.17-mRNA-1	4	OsR498G0409355300.01.T01	99,63	0,00E+00	3421	Os04g0640900	99,95	0,00E+00	3448	27	NA	Japonica	
augustus-4-processed-gene-273.58-mRNA-1	4	OsR498G0409364700.01.T04	99,77	0,00E+00	2384	Os04g0643700	100,00	0,00E+00	2397	13	NA	Japonica	
augustus-4-processed-gene-275.39-mRNA-1	4	OsR498G0409380300.01.T01	99,88	0,00E+00	1446	Os04g0648600	100,00	0,00E+00	1451	5	NA	Japonica	
augustus-4-processed-gene-276.11-mRNA-1	4	OsR498G0409389300.01.T01	99,58	0,00E+00	1707	Os04g0649900	100,00	0,00E+00	1727	20	NA	Japonica	
augustus-4-processed-gene-277.63-mRNA-1	4	OsR498G0409393500.01.T01	99,30	0,00E+00	1776	Os04g0650700	100,00	0,00E+00	1007	-769	Indica	Japonica	
augustus-4-processed-gene-277.68-mRNA-1	4	OsR498G0409397100.01.T01	100,00	0,00E+00	1050	Os09g0566000	95,83	3,77E-165	580	-470	Indica	NA	
augustus-4-processed-gene-279.43-mRNA-1	4	OsR498G0409408100.01.T01	99,84	0,00E+00	3342	Os04g0655000	100,00	0,00E+00	4410	1068	Japonica	Japonica	Kinase-TM
augustus-4-processed-gene-280.30-mRNA-1	4	OsR498G0409413100.01.T01	100,00	0,00E+00	5151	Os04g0656100	100,00	0,00E+00	5151	0	NA	NA	
augustus-4-processed-gene-281.13-mRNA-1	4	OsR498G0409419300.01.T06	99,46	0,00E+00	2290	Os04g0658100	100,00	0,00E+00	2322	32	NA	Japonica	
augustus-4-processed-gene-284.33-mRNA-1	4	OsR498G0409436100.01.T01	95,70	0,00E+00	2695	Os04g0662800	95,76	0,00E+00	2700	5	NA	Japonica	
augustus-4-processed-gene-284.34-mRNA-1	4	OsR498G0409437500.01.T05	99,88	0,00E+00	3021	Os04g0663100	99,82	0,00E+00	3017	-4	NA	Indica	
augustus-4-processed-gene-285.29-mRNA-1	4	OsR498G0409443100.01.T01	100,00	0,00E+00	2419	Os04g0664500	95,26	0,00E+00	1924	-495	Indica	Indica	
augustus-4-processed-gene-286.65-mRNA-1	4	OsR498G0409458500.01.T01	100,00	0,00E+00	2278	Os04g0668600	99,67	0,00E+00	2188	-90	NA	NA	
augustus-4-processed-gene-289.0-mRNA-1	4	OsR498G0409471300.01.T02	99,60	0,00E+00	888	Os04g0672800	100,00	0,00E+00	895	7	NA	Japonica	
augustus-4-processed-gene-290.57-mRNA-1	4	OsR498G0409505400.01.T01	98,12	0,00E+00	1370	Os04g0674400	100,00	0,00E+00	1440	70	NA	Japonica	
augustus-4-processed-gene-295.1-mRNA-1	4	OsR498G0409514300.01.T06	99,87	0,00E+00	2670	Os04g0684200	99,84	0,00E+00	2185	-485	Indica	Japonica	Kinase-TM
augustus-4-processed-gene-295.13-mRNA-1	4	OsR498G0409516900.01.T01	99,70	0,00E+00	1773	Os04g0684900	97,63	0,00E+00	1513	-260	Indica	Japonica	
augustus-4-processed-gene-295.9-mRNA-1	4	OsR498G0409515700.01.T01	98,74	0,00E+00	692	Os04g0684400	100,00	2,51E-146	517	-175	Indica	Japonica	
augustus-4-processed-gene-296.36-mRNA-1	4	OsR498G0409525900.01.T01	100,00	0,00E+00	1131	Os04g0686800	99,83	0,00E+00	1076	-55	NA	Japonica	
augustus-4-processed-gene-297.16-mRNA-1	4	OsR498G0409528100.01.T01	99,92	0,00E+00	2231	Os04g0687800	100,00	0,00E+00	2235	4	NA	Japonica	
augustus-4-processed-gene-297.18-mRNA-1	4	OsR498G0409529300.01.T01	99,84	0,00E+00	2296	Os04g0688000	99,49	0,00E+00	2440	144	NA	Japonica	
augustus-4-processed-gene-299.19-mRNA-1	4	OsR498G0409540700.01.T01	99,71	0,00E+00	5489	Os04g0691800	100,00	0,00E+00	5530	41	NA	Japonica	
augustus-4-processed-gene-3.15-mRNA-1	4	OsR498G0407633900.01.T01	99,54	0,00E+00	782	Os04g0175400	100,00	0,00E+00	791	9	NA	Japonica	
augustus-4-processed-gene-301.62-mRNA-1	4	OsR498G0409049300.01.T01	95,40	1,94E-178	625	Os04g0554600	100,00	0,00E+00	715	90	Japonica	NA	
augustus-4-processed-gene-34.22-mRNA-1	4	OsR498G0407767500.01.T01	99,78	0,00E+00	1613	Os04g0225700	96,24	0,00E+00	1474	-139	NA	Indica	
augustus-4-processed-gene-43.16-mRNA-1	4	OsR498G0407698300.01.T01	99,55	0,00E+00	2365	Os04g0194400	100,00	0,00E+00	2392	27	NA	NA	
augustus-4-processed-gene-5.20-mRNA-1	4	OsR498G0407643700.01.T01	99,52	0,00E+00	4106	Os04g0178300	99,96	0,00E+00	4151	45	NA	Japonica	
augustus-4-processed-gene-52.15-mRNA-1	4	OsR498G0203686900.01.T01	75,97	0,00E+00	1372	Os04g0261400	99,78	0,00E+00	4097	2725	Japonica	NA	
augustus-4-processed-gene-56.9-mRNA-1	4	OsR498G0407894900.01.T01	99,30	0,00E+00	1266	Os04g0268700	99,58	0,00E+00	1275	9	NA	NA	
augustus-4-processed-gene-59.5-mRNA-1	4	OsR498G0407907100.01.T01	99,59	0,00E+00	2612	Os04g0272700	99,80	0,00E+00	2626	14	NA	Japonica	
augustus-4-processed-gene-65.0-mRNA-1	4	OsR498G0407938700.01.T01	99,76	0,00E+00	1501	Os02g0461500	70,21	7,18E-56	217	-1284	Indica	Japonica	
augustus-4-processed-gene-66.18-mRNA-1	4	OsR498G0916692600.01.T01	99,76	0,00E+00	2249	Os04g0286500	100,00	0,00E+00	2262	13	NA	NA	
augustus-4-processed-gene-72.16-mRNA-1	4	OsR498G0407959000.01.T02	97,00	3,81E-174	610	Os04g0295000	100,00	0,00E+00	645	35	NA	Japonica	
augustus-4-processed-gene-74.12-mRNA-1	4	OsR498G0407971100.01.T01	97,16	0,00E+00	885	Os04g0297800	100,00	0,00E+00	908	23	NA	Japonica	
augustus-4-processed-gene-80.14-mRNA-1	4	OsR498G0408009100.01.T01	99,66	0,00E+00	2656	Os04g0305700	100,00	0,00E+00	2782	126	NA	Japonica	
augustus-4-processed-gene-81.17-mRNA-1	4	OsR498G0408010700.01.T01	99,88	0,00E+00	1484	Os04g0306400	100,00	0,00E+00	1489	5	NA	Japonica	
augustus-4-processed-gene-82.4-mRNA-1	4	OsR498G0408016900.01.T01	93,53	0,00E+00	1888	Os04g0307900	100,00	0,00E+00	2257	369	Japonica	Japonica	Kinase-TM
augustus-4-processed-gene-84.19-mRNA-1	4	OsR498G0612066600.01.T01	80,14	0,00E+00	1153	Os04g0312000	98,86	0,00E+00	3992	2839	Japonica	NA	CC-NBS-TM
augustus-4-processed-gene-86.0-mRNA-1	4	OsR498G0408075100.01.T01	99,66	1,48E-146	519	Os04g0319800	99,66	5,15E-147	519	0	NA	NA	
augustus-4-processed-gene-86.1-mRNA-1	4	OsR498G0408075100.01.T01	99,91	0,00E+00	1975	Os04g0319800	99,91	0,00E+00	1975	0	NA	NA	
augustus-4-processed-gene-88.17-mRNA-1	4	OsR498G0408050900.01.T01	99,40	0,00E+00	1786	Os04g0316800	99,56	0,00E+00	1622	-164	NA	NA	
augustus-4-processed-gene-91.4-mRNA-1	4	OsR498G0408073100.01.T01	99,86	0,00E+00	2572	Os04g0319700	99,86	0,00E+00	2572	0	NA	NA	
augustus-4-processed-gene-91.9-mRNA-1	4	OsR498G0408075100.01.T01	100,00	0,00E+00	2690	Os04g0319800	100,00	0,00E+00	2690	0	NA	NA	
augustus-4-processed-gene-94.0-mRNA-1	4	OsR498G0408099900.01.T01	100,00	0,00E+00	2749	Os04g0324100	100,00	0,00E+00	2749	0	NA	NA	
augustus-4-processed-gene-99.1-mRNA-1	4	OsR498G0408137500.01.T01	100,00	0,00E+00	639	Os04g0329200	100,00	0,00E+00	639	0	NA	NA	
augustus-4-processed-gene-99.2-mRNA-1	4	OsR498G0408139100.01.T01	100,00	0,00E+00	639	Os04g0329300	100,00	0,00E+00	639	0	NA	NA	
maker-4-augustus-gene-0.0-mRNA-1	4	OsR498G0407621300.01.T01	100,00	0,00E+00	4134	Os04g0169100	99,55	0,00E+00	4360	226	NA	NA	
maker-4-augustus-gene-10.0-mRNA-1	4	OsR498G0407660100.01.T01	99,41	0,00E+00	2704	Os04g0184100	100,00	0,00E+00	2841	137	NA	Japonica	
maker-4-augustus-gene-10.1-mRNA-1	4	OsR498G0407660700.01.T01	100,00	4,47E-76	284	Os04g0183500	89,24	9,88E-54	209	-75	Indica	Japonica	
maker-4-augustus-gene-10.12-mRNA-1	4	OsR498G0407661200.01.T01	98,65	0,00E+00	2834	Os04g0185100	100,00	0,00E+00	3187	353	Japonica	Japonica	
maker-4-augustus-gene-10.6-mRNA-1	4	OsR498G0407660700.01.T01	95,88	0,00E+00	1296	Os04g0185000	100,00	0,00E+00	1009	-287	Indica	Japonica	
maker-4-augustus-gene-10.8-mRNA-1	4	OsR498G0407663700.01.T03	100,00	0,00E+00	3036	Os04g0185500	100,00	0,00E+00	3036	0	NA	NA	
maker-4-augustus-gene-100.0-mRNA-1	4	OsR498G0408168600.01.T04	76,57	3,42E-41	168	Os04g0334700	95,73	1,27E-104	378	210	Japonica	NA	
maker-4-augustus-gene-103.29-mRNA-1	4	OsR498G0408169100.01.T06	100,00	0,00E+00	5573	Os02g0474700	82,69	0,00E+00	2077	-3496	Indica	Indica	
maker-4-augustus-gene-103.36-mRNA-1	4	OsR498G0408169700.01.T01	94,78	0,00E+00	1574	Os04g0337500	94,68	0,00E+00	1570	-4	NA	Indica	
maker-4-augustus-gene-103.39-mRNA-1	4	OsR498G0408170900.01.T04	90,16	1,65E-168	593	Os04g0337700	95,72	0,00E+00	921	328	Japonica	Indica	
maker-4-augustus-gene-103.41-mRNA-1	4	OsR498G0408170900.01.T04	100,00	0,00E+00	1905	Os04g0338000	99,91	0,00E+00	1901	-4	NA	Indica	
maker-4-augustus-gene-103.45-mRNA-1	4	OsR498G0408171100.01.T04	100,00	0,00E+00	1056	Os04g0338000	93,54	0,00E+00	914	-142	Indica	Indica	
maker-4-augustus-gene-103.48-mRNA-1	4	OsR498G0408168600.01.T03	99,81	0,00E+00	2788	Os04g0337000	98,43	0,00E+00	1536	-1252	Indica	NA	
maker-4-augustus-gene-104.11-mRNA-1	4	OsR498G0408176200.01.T01	100,00	0,00E+00	2354	Os04g0340300	72,26	1,38E-150	533	-1821	Indica	NA	
maker-4-augustus-gene-104.7-mRNA-1	4	OsR498G0408175300.01.T01	100,00	0,00E+00	1961	Os04g0339000	100,00	0,00E+00	1961	0	NA	NA	
maker-4-augustus-gene-104.9-mRNA-1	4	OsR498G0408175900.01.T01	100,00	0,00E+00	1927	Os04g0339400	100,00	0,00E+00	1927	0	NA	NA	
maker-4-augustus-gene-105.9-mRNA-1	4	OsR498G0408179000.01.T02	99,94	0,00E+00	3242	Os04g0340300	99,94	0,00E+00	3242	0	NA	Indica	
maker-4-augustus-gene-106.0-mRNA-1	4	OsR498G0408186000.01.T01	98,56	0,00E+00	3130	Os04g0340300	97,72	0,00E+00	3062	-68	NA	NA	
maker-4-augustus-gene-106.5-mRNA-1	4	OsR498G0408187800.01.T01	95,13	0,00E+00	2861	Os04g0341800	94,55	0,00E+00	2373	-488	Indica	NA	
maker-4-augustus-gene-108.32-mRNA-1	4	OsR498G0408202600.01.T03	94,81	0,00E+00	1358	Os04g0341800	74,56	2,63E-152	539	-819	Indica	Indica	
maker-4-augustus-gene-108.34-mRNA-1	4	OsR498G0408206800.01.T01	99,29	0,00E+00	1503	Os04g0345400	75,28	3,30E-155	547	-956	Indica	NA	
maker-4-augustus-gene-109.11-mRNA-1	4	OsR498G0408210400.01.T01	99,76	0,00E+00	2980	Os04g0345400	87,73	0,00E+00	1889	-1091	Indica	NA	
maker-4-augustus-gene-11.0-mRNA-1	4	OsR498G0407668300.01.T01	99,74	0,00E+00	2736	Os04g0186800	100,00	0,00E+00	2755	19	NA	Japonica	
maker-4-augustus-gene-110.32-mRNA-1	4	OsR498G0408216300.01.T02	97,54	0,00E+00	893	Os04g0346800	99,62	1,85E-130	465	-428	Indica	NA	
maker-4-augustus-gene-110.50-mRNA-1	4	OsR498G0408220900.01.T01	100,00	0,00E+00	3431	Os04g0347900	99,95	0,00E+00	3426	-5	NA	NA	
maker-4-augustus-gene-110.54-mRNA-1	4	OsR498G0408221300.01.T05	99,91	0,00E+00	1944	Os04g0348000	95,45	0,00E+00	1795	-149	NA	NA	
maker-4-augustus-gene-110.61-mRNA-1	4	OsR498G0408218400.01.T01	100,00	0,00E+00	1570	Os04g0347200	100,00	0,00E+00	897	-673	Indica	Indica	
maker-4-augustus-gene-111.0-mRNA-1	4	OsR498G0408222300.01.T01	100,00	0,00E+00	5265	Os04g0348300	99,91	0,00E+00	4150	-1115	Indica	Indica	
maker-4-augustus-gene-111.12-mRNA-1	4	OsR498G0408221600.01.T01	99,88	0,00E+00	1533	Os04g0348100	99,88	0,00E+00	1533	0	NA	NA	
maker-4-augustus-gene-111.15-mRNA-1	4	OsR498G0408222600.01.T01	100,00	0,00E+00	2489	Os04g0348400	98,91	0,00E+00	2422	-67	NA	Indica	
maker-4-augustus-gene-111.22-mRNA-1	4	OsR498G0408225600.01.T01	100,00	0,00E+00	1196	Os04g0349500	99,40	0,00E+00	1178	-18	NA	Indica	
maker-4-augustus-gene-112.37-mRNA-1	4	OsR498G0408228100.01.T01	100,00	0,00E+00	4286	Os04g0350300	99,20	0,00E+00	4200	-86	NA	Indica	
maker-4-augustus-gene-112.40-mRNA-1	4	OsR498G0408232100.01.T01	100,00	0,00E+00	747	Os02g0791200	91,23	3,80E-146	517	-230	Indica	NA	
maker-4-augustus-gene-112.45-mRNA-1	4	OsR498G0408227200.01.T02	100,00	0,00E+00	5735	Os04g0349700	97,87	0,00E+00	4637	-1098	Indica	Indica	LRR-TM
maker-4-augustus-gene-112.47-mRNA-1	4	OsR498G0408230800.01.T01	99,77	0,00E+00	1566	Os04g0350700	99,87	0,00E+00	1425	-141	NA	Indica	
maker-4-augustus-gene-113.36-mRNA-1	4	OsR498G0408236200.01.T01	100,00	0,00E+00	3193	Os04g0352400	99,32	0,00E+00	3145	-48	NA	Indica	
maker-4-augustus-gene-114.32-mRNA-1	4	OsR498G0408241100.01.T02	100,00	0,00E+00	2695	Os04g0354600	94,00	0,00E+00	2301	-394	Indica	NA	
maker-4-augustus-gene-114.50-mRNA-1	4	OsR498G0408245500.01.T02	100,00	0,00E+00	2365	Os04g0354600	99,93	0,00E+00	2701	336	Japonica	NA	
maker-4-augustus-gene-117.32-mRNA-1	4	OsR498G0408268900.01.T01	98,95	0,00E+00	1847	Os04g0359700	96,22	0,00E+00	1982	135	NA	NA	
maker-4-augustus-gene-118.35-mRNA-1	4	OsR498G0408279200.01.T02	99,87	0,00E+00	2664	Os08g0114300	78,31	0,00E+00	1436	-1228	Indica	Japonica	
maker-4-augustus-gene-12.0-mRNA-1	4	OsR498G0407673800.01.T01	98,55	1,54E-134	479	Os04g0187800	100,00	1,75E-90	331	-148	Indica	Japonica	
maker-4-augustus-gene-122.31-mRNA-1	4	OsR498G0408325600.01.T01	100,00	0,00E+00	2246	Os04g0366000	99,92	0,00E+00	2241	-5	NA	NA	Kinase-TM
maker-4-augustus-gene-123.1-mRNA-1	4	OsR498G0408332800.01.T02	97,74	0,00E+00	1802	Os04g0366000	82,17	0,00E+00	1056	-746	Indica	Japonica	Kinase-TM
maker-4-augustus-gene-124.31-mRNA-1	4	OsR498G0408336300.01.T01	99,83	0,00E+00	1030	Os04g0367400	99,83	0,00E+00	1030	0	NA	Indica	
maker-4-augustus-gene-124.35-mRNA-1	4	OsR498G0408337400.01.T01	95,95	0,00E+00	3599	Os04g0368000	97,07	0,00E+00	3711	112	NA	Japonica	Kinase-TM
maker-4-augustus-gene-128.0-mRNA-1	4	OsR498G0408358300.01.T01	99,80	0,00E+00	1815	Os09g0502500	70,51	1,47E-122	439	-1376	Indica	NA	
maker-4-augustus-gene-128.8-mRNA-1	4	OsR498G0408358600.01.T01	99,93	0,00E+00	2631	Os04g0372800	99,93	0,00E+00	2631	0	NA	NA	
maker-4-augustus-gene-129.0-mRNA-1	4	OsR498G0408361700.01.T01	98,24	0,00E+00	2258	Os04g0373400	98,51	0,00E+00	1400	-858	Indica	NA	
maker-4-augustus-gene-129.11-mRNA-1	4	OsR498G0408364900.01.T02	97,18	0,00E+00	2321	Os04g0374175	98,07	0,00E+00	1956	-365	Indica	NA	NBS-TM
maker-4-augustus-gene-129.15-mRNA-1	4	OsR498G0408372800.01.T01	96,48	0,00E+00	3588	Os04g0375900	96,48	0,00E+00	3588	0	NA	NA	
maker-4-augustus-gene-129.22-mRNA-1	4	OsR498G0408373000.01.T01	99,46	0,00E+00	663	Os04g0376000	99,46	0,00E+00	663	0	NA	NA	
maker-4-augustus-gene-129.25-mRNA-1	4	OsR498G0408373400.01.T01	100,00	0,00E+00	1072	Os04g0376100	100,00	0,00E+00	1072	0	NA	NA	
maker-4-augustus-gene-129.27-mRNA-1	4	OsR498G0408374600.01.T02	99,79	0,00E+00	1718	Os04g0376300	100,00	0,00E+00	915	-803	Indica	NA	
maker-4-augustus-gene-129.32-mRNA-1	4	OsR498G0408375400.01.T01	100,00	0,00E+00	2706	Os04g0376400	99,86	0,00E+00	2589	-117	NA	NA	
maker-4-augustus-gene-13.33-mRNA-1	4	OsR498G0407680000.01.T01	100,00	4,35E-173	607	Os04g0189400	100,00	1,51E-173	607	0	NA	Japonica	
maker-4-augustus-gene-130.13-mRNA-1	4	OsR498G0408376600.01.T01	100,00	0,00E+00	1884	Os04g0376500	100,00	0,00E+00	1884	0	NA	NA	
maker-4-augustus-gene-130.20-mRNA-1	4	OsR498G0408376800.01.T03	99,72	0,00E+00	1279	Os09g0401900	97,63	0,00E+00	644	-635	Indica	NA	
maker-4-augustus-gene-130.29-mRNA-1	4	OsR498G0408377200.01.T01	99,56	3,34E-112	406	Os04g0376700	99,56	1,16E-112	406	0	NA	NA	
maker-4-augustus-gene-130.33-mRNA-1	4	OsR498G0408382200.01.T03	99,54	7,42E-106	384	Os03g0857200	76,61	4,71E-27	121	-263	Indica	NA	
maker-4-augustus-gene-130.4-mRNA-1	4	OsR498G0408380900.01.T01	99,71	0,00E+00	2426	Os04g0377600	99,88	0,00E+00	1484	-942	Indica	Indica	
maker-4-augustus-gene-130.9-mRNA-1	4	OsR498G0408381500.01.T01	92,70	0,00E+00	1073	Os04g0378200	92,70	0,00E+00	1073	0	NA	NA	
maker-4-augustus-gene-131.0-mRNA-1	4	OsR498G0408384700.01.T01	99,56	0,00E+00	4022	Os04g0379300	99,56	0,00E+00	4022	0	NA	NA	
maker-4-augustus-gene-131.11-mRNA-1	4	OsR498G0408388100.01.T01	100,00	0,00E+00	1559	Os04g0380200	100,00	0,00E+00	1559	0	NA	NA	
maker-4-augustus-gene-131.14-mRNA-1	4	OsR498G0408385000.01.T05	100,00	0,00E+00	726	Os04g0379400	99,74	0,00E+00	692	-34	NA	NA	
maker-4-augustus-gene-131.18-mRNA-1	4	OsR498G0408386400.01.T02	99,86	0,00E+00	2529	Os04g0379700	100,00	0,00E+00	2538	9	NA	NA	
maker-4-augustus-gene-131.22-mRNA-1	4	OsR498G0408386800.01.T01	100,00	0,00E+00	1918	Os04g0379900	100,00	0,00E+00	1918	0	NA	Indica	
maker-4-augustus-gene-131.31-mRNA-1	4	OsR498G0408388600.01.T06	100,00	0,00E+00	2035	Os04g0380300	100,00	0,00E+00	2035	0	NA	NA	
maker-4-augustus-gene-131.8-mRNA-1	4	OsR498G0408386500.01.T01	97,15	0,00E+00	2144	Os04g0379800	97,15	0,00E+00	2144	0	NA	NA	
maker-4-augustus-gene-132.11-mRNA-1	4	OsR498G0408397700.01.T02	99,87	0,00E+00	1334	Os04g0381600	100,00	0,00E+00	1339	5	NA	NA	
maker-4-augustus-gene-132.14-mRNA-1	4	OsR498G0408395400.01.T05	100,00	0,00E+00	2444	Os04g0381000	100,00	0,00E+00	2444	0	NA	Indica	
maker-4-augustus-gene-132.2-mRNA-1	4	OsR498G0408396100.01.T06	100,00	0,00E+00	2203	Os04g0381100	100,00	0,00E+00	2203	0	NA	NA	
maker-4-augustus-gene-133.29-mRNA-1	4	OsR498G0408400500.01.T01	100,00	0,00E+00	2971	Os04g0382100	100,00	0,00E+00	1878	-1093	Indica	NA	
maker-4-augustus-gene-133.31-mRNA-1	4	OsR498G0408403100.01.T01	99,94	0,00E+00	3242	Os04g0382700	99,94	0,00E+00	3242	0	NA	Indica	
maker-4-augustus-gene-133.33-mRNA-1	4	OsR498G0408403700.01.T02	99,04	0,00E+00	915	Os04g0382800	100,00	0,00E+00	939	24	NA	Indica	
maker-4-augustus-gene-133.35-mRNA-1	4	OsR498G0408401400.01.T01	100,00	0,00E+00	2305	Os04g0382300	100,00	0,00E+00	2305	0	NA	NA	
maker-4-augustus-gene-134.45-mRNA-1	4	OsR498G0408412400.01.T01	100,00	0,00E+00	2143	Os04g0385600	100,00	0,00E+00	2143	0	NA	NA	
maker-4-augustus-gene-135.28-mRNA-1	4	OsR498G0408420100.01.T03	100,00	0,00E+00	1196	Os04g0386500	97,04	0,00E+00	910	-286	Indica	NA	
maker-4-augustus-gene-135.31-mRNA-1	4	OsR498G0408420400.01.T01	100,00	0,00E+00	2111	Os04g0386600	100,00	0,00E+00	2111	0	NA	NA	
maker-4-augustus-gene-135.36-mRNA-1	4	OsR498G0408421400.01.T01	100,00	0,00E+00	1153	Os04g0386900	100,00	0,00E+00	1029	-124	Indica	NA	
maker-4-augustus-gene-136.0-mRNA-1	4	OsR498G0408424910.01.T01	82,61	3,43E-61	236	Os04g0387300	100,00	0,00E+00	699	463	Japonica	NA	
maker-4-augustus-gene-136.2-mRNA-1	4	OsR498G0408424910.01.T01	100,00	0,00E+00	742	Os04g0387500	100,00	1,19E-171	601	-141	Indica	NA	
maker-4-augustus-gene-137.34-mRNA-1	4	OsR498G0408429300.01.T01	100,00	0,00E+00	3177	Os08g0532400	70,44	0,00E+00	810	-2367	Indica	Indica	
maker-4-augustus-gene-137.37-mRNA-1	4	OsR498G0408431700.01.T02	99,82	0,00E+00	1013	Os07g0484700	95,95	0,00E+00	788	-225	Indica	NA	
maker-4-augustus-gene-137.45-mRNA-1	4	OsR498G0408432700.01.T01	100,00	0,00E+00	5503	Os04g0390000	100,00	0,00E+00	5418	-85	NA	NA	
maker-4-augustus-gene-137.51-mRNA-1	4	OsR498G0408433300.01.T01	100,00	0,00E+00	2343	Os02g0510600	76,58	0,00E+00	669	-1674	Indica	NA	
maker-4-augustus-gene-137.56-mRNA-1	4	OsR498G0408428400.01.T02	100,00	0,00E+00	1137	Os04g0388601	100,00	0,00E+00	893	-244	Indica	NA	
maker-4-augustus-gene-137.58-mRNA-1	4	OsR498G0203903200.01.T01	86,05	2,19E-94	346	Os04g0388900	100,00	9,27E-151	531	185	Japonica	NA	
maker-4-augustus-gene-138.12-mRNA-1	4	OsR498G0408437700.01.T01	100,00	0,00E+00	1997	Os04g0390700	100,00	0,00E+00	1997	0	NA	Indica	
maker-4-augustus-gene-138.14-mRNA-1	4	OsR498G0408438300.01.T01	100,00	0,00E+00	1095	Os04g0390800	99,36	0,00E+00	1103	8	NA	Indica	
maker-4-augustus-gene-138.15-mRNA-1	4	OsR498G0408438300.01.T01	100,00	0,00E+00	834	Os04g0390800	100,00	0,00E+00	834	0	NA	Indica	
maker-4-augustus-gene-138.18-mRNA-1	4	OsR498G0408441700.01.T02	99,93	0,00E+00	2544	Os04g0391500	100,00	0,00E+00	2549	5	NA	NA	
maker-4-augustus-gene-138.20-mRNA-1	4	OsR498G0408442500.01.T01	99,83	0,00E+00	1024	Os04g0391900	99,83	0,00E+00	1024	0	NA	Japonica	
maker-4-augustus-gene-138.27-mRNA-1	4	OsR498G0408438600.01.T03	100,00	0,00E+00	2590	Os04g0391000	99,72	0,00E+00	2571	-19	NA	Indica	
maker-4-augustus-gene-139.33-mRNA-1	4	OsR498G0408448500.01.T01	99,86	0,00E+00	2562	Os04g0393900	99,78	0,00E+00	2391	-171	NA	Japonica	Kinase-TM
maker-4-augustus-gene-139.36-mRNA-1	4	OsR498G0408448900.01.T01	97,61	0,00E+00	927	Os04g0394100	93,26	0,00E+00	968	41	NA	Japonica	
maker-4-augustus-gene-139.37-mRNA-1	4	OsR498G0408448900.01.T01	99,48	0,00E+00	1021	Os04g0394100	100,00	0,00E+00	1034	13	NA	Japonica	
maker-4-augustus-gene-139.49-mRNA-1	4	OsR498G0408447200.01.T01	99,76	0,00E+00	732	Os04g0393300	100,00	0,00E+00	737	5	NA	Japonica	
maker-4-augustus-gene-139.53-mRNA-1	4	OsR498G0408449400.01.T01	99,74	0,00E+00	4864	Os04g0394300	100,00	0,00E+00	4967	103	NA	Japonica	
maker-4-augustus-gene-140.0-mRNA-1	4	OsR498G0408451300.01.T01	98,67	0,00E+00	3295	Os04g0394700	98,88	0,00E+00	3313	18	NA	Japonica	
maker-4-augustus-gene-140.11-mRNA-1	4	OsR498G0408450800.01.T01	99,87	0,00E+00	1342	Os04g0394500	99,87	0,00E+00	1342	0	NA	NA	
maker-4-augustus-gene-140.18-mRNA-1	4	OsR498G0407856000.01.T01	99,88	0,00E+00	1464	Os04g0394500	100,00	0,00E+00	1499	35	NA	NA	
maker-4-augustus-gene-140.25-mRNA-1	4	OsR498G0306852400.01.T01	86,42	1,16E-98	361	Os04g0395466	100,00	1,15E-175	614	253	Japonica	Japonica	
maker-4-augustus-gene-140.29-mRNA-1	4	OsR498G0408453800.01.T02	99,77	0,00E+00	3099	Os04g0395600	100,00	0,00E+00	3117	18	NA	Japonica	
maker-4-augustus-gene-140.4-mRNA-1	4	OsR498G0407856000.01.T01	99,88	0,00E+00	1464	Os04g0394500	100,00	0,00E+00	1499	35	NA	NA	
maker-4-augustus-gene-141.10-mRNA-1	4	OsR498G0408455300.01.T01	99,65	0,00E+00	1014	Os04g0395800	100,00	0,00E+00	1023	9	NA	Japonica	
maker-4-augustus-gene-141.15-mRNA-1	4	OsR498G0408455600.01.T01	99,80	0,00E+00	1786	Os04g0395900	100,00	0,00E+00	1795	9	NA	Japonica	
maker-4-augustus-gene-142.20-mRNA-1	4	OsR498G0408461300.01.T01	99,86	0,00E+00	2540	Os04g0396800	100,00	0,00E+00	2549	9	NA	Japonica	
maker-4-augustus-gene-142.25-mRNA-1	4	OsR498G0408461900.01.T01	100,00	5,82E-178	623	Os04g0397000	100,00	2,02E-178	623	0	NA	Japonica	
maker-4-augustus-gene-142.29-mRNA-1	4	OsR498G0408463500.01.T01	99,76	0,00E+00	743	Os04g0397700	100,00	0,00E+00	722	-21	NA	Japonica	
maker-4-augustus-gene-142.34-mRNA-1	4	OsR498G0408467900.01.T02	99,82	0,00E+00	1947	Os08g0538000	74,46	0,00E+00	659	-1288	Indica	Japonica	
maker-4-augustus-gene-142.38-mRNA-1	4	OsR498G0408462200.01.T03	99,96	0,00E+00	4968	Os12g0230100	80,47	0,00E+00	2265	-2703	Indica	Japonica	
maker-4-augustus-gene-142.47-mRNA-1	4	OsR498G0408462600.01.T01	99,19	0,00E+00	1754	Os04g0397500	99,70	0,00E+00	1776	22	NA	Japonica	
maker-4-augustus-gene-142.49-mRNA-1	4	OsR498G0408464800.01.T01	97,78	0,00E+00	691	Os04g0397901	100,00	1,33E-124	444	-247	Indica	Japonica	
maker-4-augustus-gene-143.0-mRNA-1	4	OsR498G0408468300.01.T01	100,00	0,00E+00	1510	Os04g0398500	100,00	0,00E+00	1510	0	NA	Japonica	
maker-4-augustus-gene-143.11-mRNA-1	4	OsR498G0408469200.01.T01	99,60	0,00E+00	890	Os04g0398700	100,00	0,00E+00	899	9	NA	Japonica	
maker-4-augustus-gene-143.16-mRNA-1	4	OsR498G0408469600.01.T05	98,63	0,00E+00	2034	Os04g0398800	100,00	0,00E+00	2100	66	NA	Japonica	
maker-4-augustus-gene-143.2-mRNA-1	4	OsR498G0408468900.01.T02	99,68	0,00E+00	2260	Os04g0398600	100,00	0,00E+00	2278	18	NA	Japonica	
maker-4-augustus-gene-143.4-mRNA-1	4	OsR498G0408469900.01.T01	99,62	1,13E-130	467	Os04g0346100	93,28	4,47E-105	380	-87	Indica	Japonica	
maker-4-augustus-gene-143.7-mRNA-1	4	OsR498G0408470900.01.T01	98,80	0,00E+00	1172	Os04g0399300	100,00	0,00E+00	1207	35	NA	Japonica	
maker-4-augustus-gene-144.35-mRNA-1	4	OsR498G0408473800.01.T01	99,85	0,00E+00	1165	Os04g0400000	100,00	0,00E+00	1169	4	NA	Japonica	
maker-4-augustus-gene-144.37-mRNA-1	4	OsR498G0408475400.01.T01	91,86	0,00E+00	1031	Os04g0400600	99,85	0,00E+00	1219	188	Japonica	Japonica	
maker-4-augustus-gene-144.38-mRNA-1	4	OsR498G0408475800.01.T01	96,21	0,00E+00	1225	Os04g0400600	84,83	6,45E-138	489	-736	Indica	Japonica	
maker-4-augustus-gene-144.43-mRNA-1	4	OsR498G0408476600.01.T02	95,47	0,00E+00	1319	Os04g0400800	99,53	0,00E+00	1517	198	Japonica	Japonica	
maker-4-augustus-gene-144.45-mRNA-1	4	OsR498G0408478200.01.T01	99,82	0,00E+00	981	Os04g0401100	99,85	0,00E+00	1168	187	Japonica	Japonica	
maker-4-augustus-gene-144.47-mRNA-1	4	OsR498G0408479400.01.T01	99,71	0,00E+00	1870	Os04g0401300	99,90	0,00E+00	1879	9	NA	Japonica	
maker-4-augustus-gene-145.0-mRNA-1	4	OsR498G0408481900.01.T05	99,83	0,00E+00	4322	Os04g0401700	98,75	0,00E+00	4224	-98	NA	NA	
maker-4-augustus-gene-145.11-mRNA-1	4	OsR498G0408483600.01.T01	99,69	0,00E+00	2889	Os04g0402100	99,88	0,00E+00	2902	13	NA	Japonica	
maker-4-augustus-gene-145.14-mRNA-1	4	OsR498G0408484600.01.T01	99,94	0,00E+00	5710	Os02g0523500	71,49	0,00E+00	1116	-4594	Indica	NA	
maker-4-augustus-gene-145.16-mRNA-1	4	OsR498G0408485400.01.T02	99,43	0,00E+00	1252	Os04g0402500	99,90	0,00E+00	1711	459	Japonica	Japonica	
maker-4-augustus-gene-145.8-mRNA-1	4	OsR498G0408482200.01.T03	98,68	0,00E+00	2527	Os04g0401800	98,75	0,00E+00	2532	5	NA	Japonica	
maker-4-augustus-gene-146.28-mRNA-1	4	OsR498G0408492100.01.T01	100,00	0,00E+00	1884	Os04g0403200	99,81	0,00E+00	1874	-10	NA	NA	
maker-4-augustus-gene-146.30-mRNA-1	4	OsR498G0408493900.01.T01	100,00	0,00E+00	2230	Os04g0403300	100,00	0,00E+00	2367	137	NA	NA	
maker-4-augustus-gene-146.31-mRNA-1	4	OsR498G0408494100.01.T01	100,00	0,00E+00	4069	Os04g0403400	100,00	0,00E+00	4069	0	NA	NA	
maker-4-augustus-gene-146.54-mRNA-1	4	OsR498G0408494500.01.T01	98,30	0,00E+00	1631	Os04g0403500	98,40	0,00E+00	1636	5	NA	NA	
maker-4-augustus-gene-146.57-mRNA-1	4	OsR498G0408494800.01.T07	100,00	0,00E+00	2819	Os04g0403701	100,00	0,00E+00	2111	-708	Indica	NA	
maker-4-augustus-gene-146.60-mRNA-1	4	OsR498G0408497600.01.T01	100,00	0,00E+00	773	Os04g0403901	100,00	1,95E-168	590	-183	Indica	NA	
maker-4-augustus-gene-146.62-mRNA-1	4	OsR498G0408499600.01.T02	100,00	0,00E+00	1754	Os04g0404400	100,00	0,00E+00	1602	-152	NA	NA	
maker-4-augustus-gene-147.0-mRNA-1	4	OsR498G0408501100.01.T02	99,95	0,00E+00	3443	Os02g0525900	82,30	0,00E+00	1921	-1522	Indica	NA	
maker-4-augustus-gene-147.16-mRNA-1	4	OsR498G0408506500.01.T02	100,00	0,00E+00	2874	Os04g0405800	100,00	0,00E+00	1635	-1239	Indica	NA	
maker-4-augustus-gene-147.21-mRNA-1	4	OsR498G0408501400.01.T01	99,77	0,00E+00	2377	Os04g0404900	99,77	0,00E+00	2377	0	NA	NA	
maker-4-augustus-gene-147.25-mRNA-1	4	OsR498G0408502400.01.T02	98,16	0,00E+00	1410	Os04g0405100	100,00	0,00E+00	1667	257	Japonica	NA	
maker-4-augustus-gene-147.30-mRNA-1	4	OsR498G0408503600.01.T01	100,00	0,00E+00	1494	Os04g0405300	100,00	0,00E+00	1494	0	NA	NA	
maker-4-augustus-gene-147.32-mRNA-1	4	OsR498G0408505000.01.T03	100,00	0,00E+00	2275	Os04g0405600	100,00	0,00E+00	2255	-20	NA	NA	
maker-4-augustus-gene-147.38-mRNA-1	4	OsR498G0408507000.01.T03	100,00	0,00E+00	1305	Os02g0527200	75,72	1,11E-90	333	-972	Indica	Indica	
maker-4-augustus-gene-147.7-mRNA-1	4	OsR498G0408502100.01.T04	100,00	0,00E+00	2978	Os02g0526400	81,37	0,00E+00	1578	-1400	Indica	NA	
maker-4-augustus-gene-148.0-mRNA-1	4	OsR498G0408516600.01.T01	100,00	0,00E+00	921	Os04g0408600	100,00	0,00E+00	921	0	NA	Indica	
maker-4-augustus-gene-148.6-mRNA-1	4	OsR498G0408518200.01.T01	100,00	0,00E+00	4556	Os02g0528200	83,22	0,00E+00	2214	-2342	Indica	Indica	
maker-4-augustus-gene-149.0-mRNA-1	4	OsR498G0408518500.01.T07	100,00	0,00E+00	1714	Os04g0409600	99,76	0,00E+00	1467	-247	Indica	NA	
maker-4-augustus-gene-149.11-mRNA-1	4	OsR498G0408520300.01.T03	100,00	0,00E+00	962	Os04g0410300	99,81	0,00E+00	957	-5	NA	Indica	
maker-4-augustus-gene-149.15-mRNA-1	4	OsR498G0408520500.01.T01	100,00	0,00E+00	1927	Os04g0410400	99,53	0,00E+00	1904	-23	NA	Indica	
maker-4-augustus-gene-149.20-mRNA-1	4	OsR498G0408519000.01.T02	100,00	0,00E+00	2706	Os04g0410200	100,00	0,00E+00	2706	0	NA	Indica	
maker-4-augustus-gene-149.28-mRNA-1	4	OsR498G0408520800.01.T01	100,00	0,00E+00	2581	Os04g0410600	99,93	0,00E+00	2445	-136	NA	Indica	
maker-4-augustus-gene-149.6-mRNA-1	4	OsR498G0408518700.01.T01	100,00	0,00E+00	3112	Os02g0529400	82,05	0,00E+00	1386	-1726	Indica	NA	
maker-4-augustus-gene-15.24-mRNA-1	4	OsR498G0407689300.01.T02	99,55	0,00E+00	798	Os03g0558600	95,10	8,70E-166	581	-217	Indica	Japonica	
maker-4-augustus-gene-150.36-mRNA-1	4	OsR498G0408524500.01.T01	99,81	0,00E+00	1856	Os04g0411800	95,26	0,00E+00	1643	-213	Indica	NA	
maker-4-augustus-gene-150.38-mRNA-1	4	OsR498G0408526500.01.T02	99,83	0,00E+00	2123	Os04g0412300	99,83	0,00E+00	2123	0	NA	Indica	
maker-4-augustus-gene-150.44-mRNA-1	4	OsR498G0408528700.01.T01	99,82	0,00E+00	3986	Os04g0412900	99,82	0,00E+00	3986	0	NA	NA	
maker-4-augustus-gene-150.48-mRNA-1	4	OsR498G0408529500.01.T01	99,38	0,00E+00	3131	Os04g0413200	99,38	0,00E+00	3131	0	NA	NA	
maker-4-augustus-gene-150.50-mRNA-1	4	OsR498G0408530100.01.T01	99,39	0,00E+00	3195	Os04g0413500	99,39	0,00E+00	3195	0	NA	NA	
maker-4-augustus-gene-150.56-mRNA-1	4	OsR498G0408530800.01.T02	100,00	0,00E+00	1023	Os04g0413600	100,00	0,00E+00	820	-203	Indica	NA	
maker-4-augustus-gene-151.43-mRNA-1	4	OsR498G0408533300.01.T03	100,00	0,00E+00	2982	Os04g0414100	100,00	0,00E+00	2982	0	NA	NA	
maker-4-augustus-gene-151.54-mRNA-1	4	OsR498G0408539300.01.T01	99,52	0,00E+00	1845	Os04g0415100	99,86	0,00E+00	1318	-527	Indica	NA	

maker-4-augustus-gene-152.35-mRNA-1	4	OsR498G0408542500.01.T01	99,70	1,53E-172	606	Os04g0415800	99,70	5,33E-173	606	0	NA	NA	
maker-4-augustus-gene-152.37-mRNA-1	4	OsR498G0408543100.01.T01	100,00	0,00E+00	672	Os04g0415900	100,00	0,00E+00	672	0	NA	Indica	
maker-4-augustus-gene-152.39-mRNA-1	4	OsR498G0408543300.01.T01	100,00	0,00E+00	1018	Os04g0416000	100,00	0,00E+00	1018	0	NA	Indica	
maker-4-augustus-gene-152.43-mRNA-1	4	OsR498G0204017500.01.T01	86,16	7,49E-70	265	Os04g0416400	100,00	0,00E+00	737	472	Japonica	NA	
maker-4-augustus-gene-152.45-mRNA-1	4	OsR498G0408544300.01.T01	99,81	0,00E+00	4727	Os04g0416500	99,84	0,00E+00	3282	-1445	Indica	NA	
maker-4-augustus-gene-152.50-mRNA-1	4	OsR498G0408543600.01.T04	95,66	0,00E+00	1582	Os02g0537500	70,72	7,96E-127	454	-1128	Indica	Indica	
maker-4-augustus-gene-152.57-mRNA-1	4	OsR498G0408545200.01.T01	100,00	0,00E+00	1196	Os04g0416700	100,00	0,00E+00	1196	0	NA	NA	
maker-4-augustus-gene-152.59-mRNA-1	4	OsR498G0408546200.01.T02	99,85	0,00E+00	2459	Os04g0417000	100,00	0,00E+00	2468	9	NA	NA	
maker-4-augustus-gene-153.37-mRNA-1	4	OsR498G0408552100.01.T01	100,00	0,00E+00	1045	Os04g0417900	99,86	0,00E+00	1306	261	Japonica	Indica	
maker-4-augustus-gene-153.39-mRNA-1	4	OsR498G0408552500.01.T01	100,00	0,00E+00	1256	Os04g0418000	99,86	0,00E+00	1251	-5	NA	Indica	
maker-4-augustus-gene-153.41-mRNA-1	4	OsR498G0408551400.01.T02	100,00	0,00E+00	3685	Os04g0417800	100,00	0,00E+00	3685	0	NA	Indica	
maker-4-augustus-gene-155.43-mRNA-1	4	OsR498G0408565700.01.T01	98,65	0,00E+00	5847	Os04g0421900	98,65	0,00E+00	5851	4	NA	Japonica	
maker-4-augustus-gene-155.54-mRNA-1	4	OsR498G0408565900.01.T01	92,00	0,00E+00	1230	Os04g0422000	92,00	0,00E+00	1230	0	NA	Japonica	
maker-4-augustus-gene-155.56-mRNA-1	4	OsR498G0408567100.01.T01	99,44	0,00E+00	1911	Os04g0422600	100,00	0,00E+00	1290	-621	Indica	Japonica	
maker-4-augustus-gene-155.62-mRNA-1	4	OsR498G0408568100.01.T01	96,31	0,00E+00	2351	Os04g0423100	100,00	0,00E+00	1480	-871	Indica	Japonica	
maker-4-augustus-gene-155.65-mRNA-1	4	OsR498G0408566200.01.T01	99,55	0,00E+00	1572	Os04g0422300	100,00	0,00E+00	1590	18	NA	Japonica	
maker-4-augustus-gene-155.68-mRNA-1	4	OsR498G0408567400.01.T01	92,57	0,00E+00	664	Os04g0422700	99,60	0,00E+00	892	228	Japonica	Japonica	
maker-4-augustus-gene-155.71-mRNA-1	4	OsR498G0408569000.01.T02	89,95	0,00E+00	1041	Os04g0423400	92,27	0,00E+00	966	-75	NA	Japonica	
maker-4-augustus-gene-156.11-mRNA-1	4	OsR498G0408572300.01.T05	99,90	0,00E+00	1819	Os04g0425100	100,00	0,00E+00	1819	0	NA	Japonica	
maker-4-augustus-gene-156.14-mRNA-1	4	OsR498G0408569400.01.T01	99,49	0,00E+00	1755	Os04g0423700	100,00	0,00E+00	2565	810	Japonica	Japonica	
maker-4-augustus-gene-156.3-mRNA-1	4	OsR498G0408570300.01.T02	99,63	0,00E+00	3833	Os06g0310200	97,38	0,00E+00	4341	508	Japonica	NA	
maker-4-augustus-gene-158.0-mRNA-1	4	OsR498G0408582200.01.T01	93,12	0,00E+00	854	Os04g0428900	93,30	0,00E+00	858	4	NA	Japonica	
maker-4-augustus-gene-159.36-mRNA-1	4	OsR498G0408583100.01.T01	99,49	0,00E+00	2776	Os04g0429100	100,00	0,00E+00	3317	541	Japonica	Japonica	
maker-4-augustus-gene-159.50-mRNA-1	4	OsR498G0408583700.01.T01	98,58	0,00E+00	1957	Os04g0429300	99,85	0,00E+00	1206	-751	Indica	Japonica	
maker-4-augustus-gene-159.52-mRNA-1	4	OsR498G0408583900.01.T01	99,80	0,00E+00	1799	Os04g0429400	100,00	0,00E+00	1808	9	NA	Japonica	
maker-4-augustus-gene-159.60-mRNA-1	4	OsR498G0408585300.01.T02	100,00	0,00E+00	2852	Os04g0430000	100,00	0,00E+00	1689	-1163	Indica	Japonica	Kinase-TM
maker-4-augustus-gene-159.64-mRNA-1	4	OsR498G0408585700.01.T01	99,82	0,00E+00	986	Os04g0430100	100,00	0,00E+00	991	5	NA	Japonica	
maker-4-augustus-gene-159.68-mRNA-1	4	OsR498G0408586700.01.T01	99,90	0,00E+00	3730	Os04g0430400	99,42	0,00E+00	2433	-1297	Indica	Japonica	Kinase-TM
maker-4-augustus-gene-159.72-mRNA-1	4	OsR498G0408584200.01.T01	99,60	0,00E+00	4063	Os04g0429500	99,96	0,00E+00	4124	61	NA	Japonica	
maker-4-augustus-gene-159.90-mRNA-1	4	OsR498G0408586000.01.T01	97,18	0,00E+00	2108	Os04g0430200	97,34	0,00E+00	2117	9	NA	Japonica	
maker-4-augustus-gene-160.0-mRNA-1	4	OsR498G0408587500.01.T01	99,61	0,00E+00	1811	Os04g0430600	99,91	0,00E+00	2025	214	Japonica	Japonica	
maker-4-augustus-gene-160.11-mRNA-1	4	OsR498G0408595100.01.T04	94,40	0,00E+00	1793	Os04g0432000	94,40	0,00E+00	1793	0	NA	Japonica	Kinase-TM
maker-4-augustus-gene-160.20-mRNA-1	4	OsR498G0408590000.01.T01	99,31	0,00E+00	1549	Os04g0431100	100,00	0,00E+00	1575	26	NA	Japonica	
maker-4-augustus-gene-160.22-mRNA-1	4	OsR498G0408592800.01.T01	92,41	3,47E-131	468	Os04g0431300	97,67	8,68E-121	433	-35	NA	Japonica	
maker-4-augustus-gene-160.23-mRNA-1	4	OsR498G0408593400.01.T01	99,43	0,00E+00	939	Os04g0431800	100,00	0,00E+00	856	-83	NA	Japonica	
maker-4-augustus-gene-160.27-mRNA-1	4	OsR498G0408597400.01.T03	99,65	0,00E+00	4053	Os04g0432300	99,23	0,00E+00	5071	1018	Japonica	Japonica	
maker-4-augustus-gene-160.7-mRNA-1	4	OsR498G0408590100.01.T02	99,65	0,00E+00	1007	Os04g0431200	100,00	0,00E+00	1020	13	NA	Japonica	
maker-4-augustus-gene-161.4-mRNA-1	4	OsR498G0408598000.01.T01	98,84	0,00E+00	3021	Os04g0432400	100,00	0,00E+00	3112	91	NA	Japonica	
maker-4-augustus-gene-161.7-mRNA-1	4	OsR498G0408599000.01.T01	99,51	0,00E+00	1827	Os04g0432500	99,90	0,00E+00	1841	14	NA	Japonica	
maker-4-augustus-gene-161.9-mRNA-1	4	OsR498G0408599600.01.T01	99,64	0,00E+00	1979	Os04g0432900	99,91	0,00E+00	1993	14	NA	Japonica	
maker-4-augustus-gene-162.35-mRNA-1	4	OsR498G0408606300.01.T04	100,00	0,00E+00	1375	Os04g0434900	100,00	0,00E+00	931	-444	Indica	NA	
maker-4-augustus-gene-162.54-mRNA-1	4	OsR498G0408604400.01.T01	97,82	0,00E+00	3239	Os04g0434300	97,91	0,00E+00	3221	-18	NA	Japonica	
maker-4-augustus-gene-162.65-mRNA-1	4	OsR498G0408606000.01.T03	100,00	0,00E+00	1948	Os04g0434800	100,00	0,00E+00	1948	0	NA	NA	
maker-4-augustus-gene-163.28-mRNA-1	4	OsR498G0408606900.01.T01	100,00	0,00E+00	2874	Os04g0435100	100,00	0,00E+00	2874	0	NA	NA	
maker-4-augustus-gene-163.30-mRNA-1	4	OsR498G0408609900.01.T01	100,00	0,00E+00	2457	Os04g0435700	100,00	0,00E+00	2457	0	NA	NA	
maker-4-augustus-gene-163.37-mRNA-1	4	OsR498G0408612900.01.T01	100,00	0,00E+00	872	Os04g0437000	99,83	0,00E+00	1084	212	Japonica	NA	
maker-4-augustus-gene-163.39-mRNA-1	4	OsR498G0408608400.01.T03	99,88	0,00E+00	1435	Os04g0435500	99,88	0,00E+00	1435	0	NA	NA	
maker-4-augustus-gene-163.43-mRNA-1	4	OsR498G0408610800.01.T06	100,00	0,00E+00	1719	Os04g0436300	100,00	0,00E+00	1719	0	NA	NA	
maker-4-augustus-gene-164.0-mRNA-1	4	OsR498G0408615000.01.T01	99,74	0,00E+00	2079	Os04g0437600	99,74	0,00E+00	2079	0	NA	NA	Kinase
maker-4-augustus-gene-164.11-mRNA-1	4	OsR498G0408618200.01.T02	100,00	0,00E+00	1537	Os04g0438600	95,02	0,00E+00	1006	-531	Indica	NA	
maker-4-augustus-gene-164.6-mRNA-1	4	OsR498G0408617000.01.T02	99,90	0,00E+00	1771	Os04g0438300	100,00	0,00E+00	1775	4	NA	NA	
maker-4-augustus-gene-164.9-mRNA-1	4	OsR498G0408617600.01.T01	100,00	0,00E+00	1851	Os04g0438500	100,00	0,00E+00	1851	0	NA	NA	
maker-4-augustus-gene-165.0-mRNA-1	4	OsR498G0408621300.01.T01	99,61	0,00E+00	6012	Os04g0439100	100,00	0,00E+00	5286	-726	Indica	Japonica	
maker-4-augustus-gene-165.11-mRNA-1	4	OsR498G0408623000.01.T01	100,00	0,00E+00	1772	Os04g0439900	100,00	0,00E+00	1772	0	NA	Japonica	
maker-4-augustus-gene-165.16-mRNA-1	4	OsR498G0408623400.01.T01	100,00	0,00E+00	1786	Os04g0440000	100,00	0,00E+00	1786	0	NA	NA	
maker-4-augustus-gene-165.21-mRNA-1	4	OsR498G0408623600.01.T04	99,65	0,00E+00	1545	Os04g0440100	97,56	0,00E+00	1470	-75	NA	NA	
maker-4-augustus-gene-165.27-mRNA-1	4	OsR498G0408625400.01.T02	100,00	0,00E+00	2149	Os04g0440300	99,93	0,00E+00	2388	239	Japonica	NA	
maker-4-augustus-gene-165.4-mRNA-1	4	OsR498G0408621500.01.T01	99,48	0,00E+00	3754	Os04g0439200	100,00	0,00E+00	4356	602	Japonica	Japonica	
maker-4-augustus-gene-165.6-mRNA-1	4	OsR498G0408621800.01.T02	99,79	0,00E+00	2568	Os02g0556900	72,33	0,00E+00	1058	-1510	Indica	Japonica	
maker-4-augustus-gene-166.34-mRNA-1	4	OsR498G0408632900.01.T03	100,00	0,00E+00	2129	Os04g0442000	100,00	0,00E+00	2129	0	NA	Japonica	
maker-4-augustus-gene-166.39-mRNA-1	4	OsR498G0408633300.01.T02	100,00	0,00E+00	2100	Os04g0442100	100,00	0,00E+00	2100	0	NA	Japonica	
maker-4-augustus-gene-166.41-mRNA-1	4	OsR498G0408633500.01.T04	99,91	0,00E+00	2027	Os04g0442200	100,00	0,00E+00	2031	4	NA	Japonica	
maker-4-augustus-gene-166.48-mRNA-1	4	OsR498G0408629800.01.T01	99,95	0,00E+00	3307	Os04g0441800	100,00	0,00E+00	3312	5	NA	NA	
maker-4-augustus-gene-166.50-mRNA-1	4	OsR498G0408631200.01.T01	98,36	0,00E+00	3177	Os04g0441900	98,25	0,00E+00	3070	-107	NA	NA	
maker-4-augustus-gene-167.12-mRNA-1	4	OsR498G0408639000.01.T01	100,00	0,00E+00	2765	Os04g0443300	100,00	0,00E+00	2711	-54	NA	NA	
maker-4-augustus-gene-167.2-mRNA-1	4	OsR498G0408639300.01.T04	100,00	0,00E+00	1862	Os04g0443500	100,00	0,00E+00	1862	0	NA	NA	
maker-4-augustus-gene-167.7-mRNA-1	4	OsR498G0408636000.01.T04	100,00	0,00E+00	2205	Os02g0558100	80,78	0,00E+00	1131	-1074	Indica	NA	
maker-4-augustus-gene-168.36-mRNA-1	4	OsR498G0408641900.01.T05	100,00	0,00E+00	715	Os01g0558600	75,40	1,24E-69	263	-452	Indica	NA	
maker-4-augustus-gene-168.39-mRNA-1	4	OsR498G0408647100.01.T05	97,36	0,00E+00	3420	Os04g0444200	97,61	0,00E+00	3443	23	NA	Japonica	
maker-4-augustus-gene-168.42-mRNA-1	4	OsR498G0408642400.01.T01	99,81	0,00E+00	927	Os08g0112700	70,23	1,96E-54	213	-714	Indica	NA	
maker-4-augustus-gene-168.45-mRNA-1	4	OsR498G0408643400.01.T01	99,84	0,00E+00	1105	Os08g0112700	70,07	8,25E-60	231	-874	Indica	NA	
maker-4-augustus-gene-168.73-mRNA-1	4	OsR498G0408644000.01.T01	100,00	0,00E+00	2203	Os01g0369900	72,60	3,69E-93	342	-1861	Indica	NA	
maker-4-augustus-gene-168.75-mRNA-1	4	OsR498G0408644200.01.T02	100,00	0,00E+00	2040	Os01g0369900	70,27	5,40E-78	292	-1748	Indica	NA	
maker-4-augustus-gene-169.0-mRNA-1	4	OsR498G0408648900.01.T01	99,74	0,00E+00	4080	Os04g0444800	100,00	0,00E+00	4107	27	NA	Japonica	
maker-4-augustus-gene-169.10-mRNA-1	4	OsR498G0408651700.01.T01	93,66	2,62E-153	542	Os04g0445800	93,95	7,48E-155	546	4	NA	Japonica	
maker-4-augustus-gene-169.13-mRNA-1	4	OsR498G0408652100.01.T02	100,00	0,00E+00	1083	Os04g0446000	100,00	0,00E+00	1083	0	NA	Japonica	
maker-4-augustus-gene-169.19-mRNA-1	4	OsR498G0408647800.01.T03	91,71	0,00E+00	1878	Os04g0444400	100,00	0,00E+00	2495	617	Japonica	Japonica	
maker-4-augustus-gene-169.2-mRNA-1	4	OsR498G0408649100.01.T01	99,87	0,00E+00	1387	Os04g0444900	100,00	0,00E+00	1391	4	NA	Japonica	
maker-4-augustus-gene-169.24-mRNA-1	4	OsR498G0408649400.01.T01	99,82	0,00E+00	3878	Os04g0445000	100,00	0,00E+00	2446	-1432	Indica	Japonica	
maker-4-augustus-gene-169.27-mRNA-1	4	OsR498G0408650000.01.T01	99,79	0,00E+00	835	Os04g0445200	100,00	0,00E+00	839	4	NA	Japonica	
maker-4-augustus-gene-169.29-mRNA-1	4	OsR498G0408651600.01.T01	99,64	0,00E+00	2483	Os04g0445700	99,54	0,00E+00	2302	-181	NA	Japonica	
maker-4-augustus-gene-169.33-mRNA-1	4	OsR498G0408653400.01.T01	100,00	4,76E-120	430	Os04g0446300	100,00	1,67E-120	430	0	NA	Japonica	
maker-4-augustus-gene-169.34-mRNA-1	4	OsR498G0408653400.01.T04	100,00	2,31E-137	488	Os04g0446300	100,00	1,46E-134	477	-11	NA	Japonica	
maker-4-augustus-gene-17.15-mRNA-1	4	OsR498G0407698800.01.T01	98,85	0,00E+00	5645	Os04g0194500	99,44	0,00E+00	5731	86	NA	Japonica	
maker-4-augustus-gene-17.7-mRNA-1	4	OsR498G0407700300.01.T02	99,72	0,00E+00	4411	Os04g0195000	100,00	0,00E+00	4439	28	NA	Japonica	
maker-4-augustus-gene-170.0-mRNA-1	4	OsR498G0408653700.01.T02	98,80	0,00E+00	2770	Os04g0446500	100,00	0,00E+00	2819	49	NA	Japonica	
maker-4-augustus-gene-170.12-mRNA-1	4	OsR498G0408659700.01.T02	100,00	0,00E+00	1808	Os04g0447500	100,00	0,00E+00	1808	0	NA	NA	
maker-4-augustus-gene-170.15-mRNA-1	4	OsR498G0408659900.01.T01	100,00	0,00E+00	1754	Os04g0447600	100,00	0,00E+00	1754	0	NA	Japonica	
maker-4-augustus-gene-170.19-mRNA-1	4	OsR498G0408659900.01.T01	86,70	0,00E+00	1167	Os04g0447700	100,00	0,00E+00	1938	771	Japonica	Japonica	
maker-4-augustus-gene-170.22-mRNA-1	4	OsR498G0408661900.01.T01	99,85	0,00E+00	2388	Os04g0448000	100,00	0,00E+00	1537	-851	Indica	Japonica	
maker-4-augustus-gene-170.24-mRNA-1	4	OsR498G0408662500.01.T02	99,84	0,00E+00	3385	Os04g0448200	99,89	0,00E+00	3308	-77	NA	Japonica	
maker-4-augustus-gene-170.31-mRNA-1	4	OsR498G0408659400.01.T01	100,00	0,00E+00	2625	Os04g0447400	100,00	0,00E+00	2625	0	NA	NA	
maker-4-augustus-gene-170.7-mRNA-1	4	OsR498G0408658500.01.T02	99,95	0,00E+00	3334	Os04g0447100	100,00	0,00E+00	1966	-1368	Indica	NA	
maker-4-augustus-gene-171.0-mRNA-1	4	OsR498G0408663900.01.T01	97,90	0,00E+00	1779	Os04g0448800	100,00	0,00E+00	1779	0	NA	Japonica	
maker-4-augustus-gene-171.12-mRNA-1	4	OsR498G0408664500.01.T02	99,02	0,00E+00	1610	Os04g0449000	99,89	0,00E+00	1619	9	NA	Japonica	
maker-4-augustus-gene-171.18-mRNA-1	4	OsR498G0408668500.01.T01	99,84	0,00E+00	1143	Os04g0450000	100,00	0,00E+00	1148	5	NA	Japonica	
maker-4-augustus-gene-171.20-mRNA-1	4	OsR498G0408669700.01.T01	99,59	0,00E+00	1318	Os04g0450100	100,00	0,00E+00	1332	14	NA	Japonica	
maker-4-augustus-gene-171.24-mRNA-1	4	OsR498G0408669900.01.T03	99,92	0,00E+00	2328	Os04g0450200	100,00	0,00E+00	1586	-742	Indica	Japonica	
maker-4-augustus-gene-171.26-mRNA-1	4	OsR498G0408670900.01.T01	99,86	0,00E+00	1286	Os04g0450500	100,00	0,00E+00	1290	4	NA	Japonica	
maker-4-augustus-gene-171.30-mRNA-1	4	OsR498G0408671100.01.T01	99,66	0,00E+00	1058	Os04g0450600	100,00	0,00E+00	1029	-29	NA	Japonica	
maker-4-augustus-gene-171.32-mRNA-1	4	OsR498G0408663600.01.T01	99,65	0,00E+00	1009	Os04g0448600	100,00	0,00E+00	1018	9	NA	Japonica	
maker-4-augustus-gene-171.38-mRNA-1	4	OsR498G0408664800.01.T01	99,66	0,00E+00	3149	Os04g0449100	99,94	0,00E+00	3172	23	NA	Japonica	
maker-4-augustus-gene-171.40-mRNA-1	4	OsR498G0408665000.01.T01	99,77	0,00E+00	3137	Os04g0449200	100,00	0,00E+00	3155	18	NA	Japonica	
maker-4-augustus-gene-171.47-mRNA-1	4	OsR498G0408665600.01.T01	99,77	0,00E+00	3142	Os04g0449500	100,00	0,00E+00	3160	18	NA	Japonica	
maker-4-augustus-gene-171.5-mRNA-1	4	OsR498G0408664300.01.T03	99,91	0,00E+00	2067	Os04g0448900	95,52	0,00E+00	2033	-34	NA	Japonica	
maker-4-augustus-gene-172.36-mRNA-1	4	OsR498G0408671900.01.T03	100,00	0,00E+00	742	Os03g0390600	90,49	2,01E-160	564	-178	Indica	NA	
maker-4-augustus-gene-172.44-mRNA-1	4	OsR498G0408673700.01.T01	99,39	0,00E+00	2626	Os04g0451200	99,66	0,00E+00	2644	18	NA	Indica	
maker-4-augustus-gene-172.46-mRNA-1	4	OsR498G0408676900.01.T02	99,86	0,00E+00	3866	Os04g0452100	100,00	0,00E+00	3788	-78	NA	Japonica	
maker-4-augustus-gene-172.49-mRNA-1	4	OsR498G0408672600.01.T01	99,63	0,00E+00	2861	Os04g0450900	99,72	0,00E+00	2535	-326	Indica	NA	
maker-4-augustus-gene-172.59-mRNA-1	4	OsR498G0408677600.01.T01	100,00	0,00E+00	1992	Os04g0452400	99,91	0,00E+00	1987	-5	NA	Japonica	
maker-4-augustus-gene-173.0-mRNA-1	4	OsR498G0408677700.01.T02	99,75	0,00E+00	3542	Os04g0452500	97,14	0,00E+00	4197	655	Japonica	Japonica	
maker-4-augustus-gene-173.10-mRNA-1	4	OsR498G0408679700.01.T01	99,81	0,00E+00	2790	Os04g0453350	99,93	0,00E+00	2523	-267	NA	Japonica	
maker-4-augustus-gene-173.13-mRNA-1	4	OsR498G0408680000.01.T01	99,94	0,00E+00	2772	Os04g0453400	99,94	0,00E+00	2772	0	NA	Japonica	
maker-4-augustus-gene-173.4-mRNA-1	4	OsR498G0408677900.01.T01	99,94	0,00E+00	2772	Os04g0452600	100,00	0,00E+00	2776	4	NA	Japonica	
maker-4-augustus-gene-173.6-mRNA-1	4	OsR498G0408678300.01.T01	99,94	0,00E+00	2799	Os04g0452700	99,94	0,00E+00	2799	0	NA	Japonica	
maker-4-augustus-gene-173.8-mRNA-1	4	OsR498G0408679300.01.T01	99,67	0,00E+00	2727	Os04g0453200	99,93	0,00E+00	2745	18	NA	Japonica	
maker-4-augustus-gene-174.0-mRNA-1	4	OsR498G0408683900.01.T01	99,81	0,00E+00	2790	Os04g0454200	100,00	0,00E+00	2803	13	NA	Japonica	
maker-4-augustus-gene-174.10-mRNA-1	4	OsR498G0408685600.01.T01	100,00	0,00E+00	1656	Os04g0455600	100,00	0,00E+00	1656	0	NA	NA	
maker-4-augustus-gene-174.5-mRNA-1	4	OsR498G0408685000.01.T02	99,54	0,00E+00	1163	Os04g0455350	99,85	0,00E+00	1172	9	NA	NA	
maker-4-augustus-gene-175.48-mRNA-1	4	OsR498G0408687300.01.T01	99,30	0,00E+00	1016	Os04g0456200	83,75	6,26E-131	467	-549	Indica	Japonica	
maker-4-augustus-gene-175.50-mRNA-1	4	OsR498G0408687500.01.T01	94,61	0,00E+00	933	Os04g0456700	100,00	0,00E+00	1050	117	Japonica	Japonica	
maker-4-augustus-gene-175.56-mRNA-1	4	OsR498G0408689900.01.T01	96,13	0,00E+00	717	Os04g0457700	100,00	0,00E+00	643	-74	Indica	Japonica	
maker-4-augustus-gene-175.58-mRNA-1	4	OsR498G0408691700.01.T02	99,48	0,00E+00	2035	Os04g0458300	99,83	0,00E+00	2053	18	NA	Japonica	
maker-4-augustus-gene-175.61-mRNA-1	4	OsR498G0408686000.01.T01	99,74	0,00E+00	2059	Os04g0455800	99,91	0,00E+00	2068	9	NA	Japonica	
maker-4-augustus-gene-175.70-mRNA-1	4	OsR498G0408688400.01.T01	99,21	0,00E+00	1342	Os04g0457000	100,00	0,00E+00	1370	28	NA	Japonica	
maker-4-augustus-gene-175.72-mRNA-1	4	OsR498G0408688600.01.T01	99,66	0,00E+00	1589	Os04g0457200	100,00	0,00E+00	1272	-317	Indica	Japonica	
maker-4-augustus-gene-175.77-mRNA-1	4	OsR498G0408690200.01.T01	96,65	0,00E+00	3244	Os04g0457800	97,27	0,00E+00	3298	54	NA	Japonica	Kinase-LRR-TM
maker-4-augustus-gene-176.0-mRNA-1	4	OsR498G0408692700.01.T01	99,62	0,00E+00	2353	Os04g0458600	99,45	0,00E+00	1941	-412	Indica	Japonica	
maker-4-augustus-gene-176.14-mRNA-1	4	OsR498G0408699100.01.T01	100,00	0,00E+00	2192	Os04g0460300	71,89	2,46E-171	602	-1590	Indica	NA	
maker-4-augustus-gene-176.16-mRNA-1	4	OsR498G0408700100.01.T01	91,94	0,00E+00	954	Os04g0460400	91,67	0,00E+00	916	-38	NA	NA	
maker-4-augustus-gene-176.18-mRNA-1	4	OsR498G0408700300.01.T04	100,00	0,00E+00	1218	Os04g0460500	100,00	0,00E+00	1157	-61	NA	NA	
maker-4-augustus-gene-176.21-mRNA-1	4	OsR498G0408701300.01.T02	100,00	0,00E+00	1862	Os04g0460600	100,00	0,00E+00	1862	0	NA	NA	
maker-4-augustus-gene-176.27-mRNA-1	4	OsR498G0408693200.01.T01	99,68	4,33E-160	564	Os04g0458800	100,00	3,54E-162	569	5	NA	NA	
maker-4-augustus-gene-176.29-mRNA-1	4	OsR498G0408693600.01.T01	98,59	0,00E+00	6702	Os04g0459000	98,57	0,00E+00	6698	-4	NA	NA	
maker-4-augustus-gene-176.32-mRNA-1	4	OsR498G0408696400.01.T01	100,00	0,00E+00	1034	Os04g0459600	94,09	0,00E+00	965	-69	NA	NA	
maker-4-augustus-gene-176.38-mRNA-1	4	OsR498G0408696800.01.T01	100,00	0,00E+00	1407	Os04g0459700	100,00	0,00E+00	1407	0	NA	NA	
maker-4-augustus-gene-176.4-mRNA-1	4	OsR498G0408696100.01.T04	100,00	0,00E+00	2181	Os04g0459500	100,00	0,00E+00	2181	0	NA	NA	
maker-4-augustus-gene-176.41-mRNA-1	4	OsR498G0408697000.01.T03	100,00	0,00E+00	3977	Os04g0459800	99,89	0,00E+00	3166	-811	Indica	NA	
maker-4-augustus-gene-176.7-mRNA-1	4	OsR498G0408697500.01.T01	100,00	0,00E+00	2414	Os04g0459900	100,00	0,00E+00	2414	0	NA	NA	
maker-4-augustus-gene-177.0-mRNA-1	4	OsR498G0408703700.01.T01	100,00	0,00E+00	1873	Os04g0461000	100,00	0,00E+00	1873	0	NA	NA	
maker-4-augustus-gene-177.13-mRNA-1	4	OsR498G0408706800.01.T01	100,00	0,00E+00	1131	Os04g0461600	100,00	0,00E+00	975	-156	Indica	NA	
maker-4-augustus-gene-177.17-mRNA-1	4	OsR498G0408707600.01.T01	100,00	0,00E+00	4513	Os04g0461800	100,00	0,00E+00	4513	0	NA	NA	
maker-4-augustus-gene-177.2-mRNA-1	4	OsR498G0408703900.01.T01	100,00	0,00E+00	996	Os04g0461100	100,00	0,00E+00	996	0	NA	NA	
maker-4-augustus-gene-177.21-mRNA-1	4	OsR498G0408708600.01.T03	97,33	0,00E+00	828	Os04g0462300	97,33	0,00E+00	828	0	NA	NA	
maker-4-augustus-gene-177.23-mRNA-1	4	OsR498G0408709000.01.T02	100,00	0,00E+00	2408	Os04g0462400	100,00	0,00E+00	1939	-469	Indica	NA	
maker-4-augustus-gene-177.4-mRNA-1	4	OsR498G0408705300.01.T01	100,00	0,00E+00	1305	Os02g0579600	83,43	0,00E+00	758	-547	Indica	NA	
maker-4-augustus-gene-178.0-mRNA-1	4	OsR498G0408709700.01.T06	100,00	0,00E+00	1424	Os04g0462500	100,00	0,00E+00	1424	0	NA	NA	
maker-4-augustus-gene-178.10-mRNA-1	4	OsR498G0408711000.01.T01	100,00	0,00E+00	3626	Os04g0463000	100,00	0,00E+00	3626	0	NA	NA	Kinase-LRR-TM
maker-4-augustus-gene-178.12-mRNA-1	4	OsR498G0408712400.01.T01	100,00	0,00E+00	1370	Os04g0463400	100,00	0,00E+00	1370	0	NA	NA	
maker-4-augustus-gene-178.14-mRNA-1	4	OsR498G0408712800.01.T02	93,54	0,00E+00	1337	Os04g0463500	93,54	0,00E+00	1337	0	NA	NA	
maker-4-augustus-gene-178.19-mRNA-1	4	OsR498G0408713600.01.T02	100,00	0,00E+00	1775	Os04g0463700	100,00	0,00E+00	1775	0	NA	NA	
maker-4-augustus-gene-178.21-mRNA-1	4	OsR498G0408713600.01.T07	100,00	0,00E+00	4152	Os04g0463800	100,00	0,00E+00	4152	0	NA	NA	
maker-4-augustus-gene-178.22-mRNA-1	4	OsR498G0408715400.01.T03	100,00	0,00E+00	1651	Os04g0464200	100,00	0,00E+00	1651	0	NA	NA	
maker-4-augustus-gene-178.27-mRNA-1	4	OsR498G0408715800.01.T01	100,00	0,00E+00	3209	Os04g0464400	100,00	0,00E+00	3209	0	NA	NA	
maker-4-augustus-gene-178.3-mRNA-1	4	OsR498G0408713300.01.T01	100,00	0,00E+00	1317	Os04g0463600	100,00	0,00E+00	1317	0	NA	NA	
maker-4-augustus-gene-178.5-mRNA-1	4	OsR498G0408716900.01.T01	100,00	0,00E+00	1364	Os04g0464500	100,00	0,00E+00	1364	0	NA	NA	
maker-4-augustus-gene-178.8-mRNA-1	4	OsR498G0408710000.01.T01	100,00	0,00E+00	1034	Os04g0462600	100,00	0,00E+00	1034	0	NA	NA	
maker-4-augustus-gene-179.34-mRNA-1	4	OsR498G0408720500.01.T01	100,00	0,00E+00	1766	Os04g0465000	100,00	0,00E+00	1766	0	NA	NA	
maker-4-augustus-gene-179.38-mRNA-1	4	OsR498G0408724700.01.T01	100,00	0,00E+00	856	Os04g0465600	100,00	0,00E+00	856	0	NA	NA	
maker-4-augustus-gene-179.44-mRNA-1	4	OsR498G0408725500.01.T01	100,00	0,00E+00	681	Os04g0465800	100,00	0,00E+00	681	0	NA	NA	
maker-4-augustus-gene-179.47-mRNA-1	4	OsR498G0408719400.01.T03	99,00	0,00E+00	3185	Os04g0464800	100,00	0,00E+00	3292	107	NA	NA	
maker-4-augustus-gene-179.52-mRNA-1	4	OsR498G0408720200.01.T01	100,00	0,00E+00	2576	Os04g0464966	100,00	0,00E+00	2576	0	NA	NA	
maker-4-augustus-gene-179.54-mRNA-1	4	OsR498G0408721800.01.T01	100,00	2,42E-163	574	Os04g0465300	100,00	8,42E-164	574	0	NA	NA	
maker-4-augustus-gene-179.56-mRNA-1	4	OsR498G0408725800.01.T01	100,00	0,00E+00	1934	Os04g0465900	100,00	0,00E+00	2305	371	Japonica	NA	Kinase-TM
maker-4-augustus-gene-18.0-mRNA-1	4	OsR498G0407701900.01.T01	99,61	0,00E+00	1378	Os04g0196200	100,00	0,00E+00	1391	13	NA	Japonica	
maker-4-augustus-gene-18.2-mRNA-1	4	OsR498G0407703600.01.T01	95,93	0,00E+00	2690	Os04g0196600	100,00	0,00E+00	2296	-394	Indica	Japonica	
maker-4-augustus-gene-180.15-mRNA-1	4	OsR498G0408727600.01.T01	100,00	0,00E+00	3561	Os04g0467100	100,00	0,00E+00	3561	0	NA	NA	
maker-4-augustus-gene-180.22-mRNA-1	4	OsR498G0408731200.01.T01	99,29	0,00E+00	2236	Os04g0467700	100,00	0,00E+00	1337	-899	Indica	NA	
maker-4-augustus-gene-180.9-mRNA-1	4	OsR498G0408727000.01.T03	97,05	0,00E+00	2930	Os04g0466700	97,05	0,00E+00	2930	0	NA	NA	
maker-4-augustus-gene-181.30-mRNA-1	4	OsR498G0408737900.01.T01	100,00	0,00E+00	2587	Os04g0469500	99,86	0,00E+00	2628	41	NA	NA	
maker-4-augustus-gene-181.32-mRNA-1	4	OsR498G0408739900.01.T01	100,00	0,00E+00	1070	Os04g0470100	100,00	0,00E+00	1070	0	NA	Japonica	
maker-4-augustus-gene-181.36-mRNA-1	4	OsR498G0408734000.01.T04	100,00	0,00E+00	1094	Os04g0468600	100,00	0,00E+00	1031	-63	NA	NA	
maker-4-augustus-gene-181.39-mRNA-1	4	OsR498G0408735400.01.T01	100,00	0,00E+00	659	Os04g0469000	100,00	0,00E+00	659	0	NA	NA	
maker-4-augustus-gene-181.41-mRNA-1	4	OsR498G0408737000.01.T01	100,00	0,00E+00	720	Os04g0469300	100,00	0,00E+00	720	0	NA	NA	
maker-4-augustus-gene-181.42-mRNA-1	4	OsR498G0408738400.01.T01	100,00	0,00E+00	1330	Os04g0469800	100,00	0,00E+00	1330	0	NA	NA	
maker-4-augustus-gene-182.39-mRNA-1	4	OsR498G0408740400.01.T03	99,69	2,67E-165	582	Os04g0470300	100,00	2,18E-167	587	5	NA	NA	
maker-4-augustus-gene-182.46-mRNA-1	4	OsR498G0408741800.01.T02	99,79	0,00E+00	2514	Os04g0470700	92,78	0,00E+00	1920	-594	Indica	Japonica	
maker-4-augustus-gene-182.47-mRNA-1	4	OsR498G0408741800.01.T06	99,44	0,00E+00	956	Os04g0470700	100,00	0,00E+00	764	-192	Indica	Japonica	
maker-4-augustus-gene-182.65-mRNA-1	4	OsR498G0408743600.01.T01	99,78	0,00E+00	1631	Os04g0471300	100,00	0,00E+00	1640	9	NA	Japonica	
maker-4-augustus-gene-182.67-mRNA-1	4	OsR498G0408744600.01.T03	99,73	0,00E+00	6664	Os04g0471400	100,00	0,00E+00	6709	45	NA	Japonica	
maker-4-augustus-gene-182.69-mRNA-1	4	OsR498G0408746400.01.T03	98,67	0,00E+00	5246	Os04g0471700	99,70	0,00E+00	5380	134	NA	Japonica	
maker-4-augustus-gene-183.0-mRNA-1	4	OsR498G0408748500.01.T02	99,67	0,00E+00	2174	Os04g0472300	100,00	0,00E+00	2192	18	NA	Japonica	
maker-4-augustus-gene-183.11-mRNA-1	4	OsR498G0408749700.01.T01	100,00	0,00E+00	1153	Os04g0472700	100,00	0,00E+00	1884	731	Japonica	Japonica	
maker-4-augustus-gene-183.14-mRNA-1	4	OsR498G0408753000.01.T01	99,85	0,00E+00	1203	Os04g0473400	100,00	0,00E+00	1207	4	NA	Japonica	
maker-4-augustus-gene-183.9-mRNA-1	4	OsR498G0408749300.01.T01	99,72	0,00E+00	3874	Os04g0472500	99,69	0,00E+00	4063	189	NA	Japonica	Kinase-LRR-TM
maker-4-augustus-gene-184.42-mRNA-1	4	OsR498G0408754200.01.T01	99,74	0,00E+00	8322	Os04g0473900	99,80	0,00E+00	12537	4215	Japonica	Japonica	
maker-4-augustus-gene-184.45-mRNA-1	4	OsR498G0408754800.01.T01	99,61	0,00E+00	1367	Os04g0474800	82,16	0,00E+00	747	-620	Indica	Japonica	
maker-4-augustus-gene-184.47-mRNA-1	4	OsR498G0408754800.01.T03	100,00	0,00E+00	953	Os04g0474500	91,81	4,43E-170	596	-357	Indica	Japonica	
maker-4-augustus-gene-184.49-mRNA-1	4	OsR498G0408756000.01.T03	92,96	0,00E+00	739	Os04g0474500	99,60	0,00E+00	888	149	Japonica	Japonica	
maker-4-augustus-gene-184.50-mRNA-1	4	OsR498G0408756000.01.T03	89,36	1,25E-83	310	Os04g0474500	99,57	1,43E-115	415	105	Japonica	Japonica	
maker-4-augustus-gene-184.53-mRNA-1	4	OsR498G0408756000.01.T03	91,94	0,00E+00	1021	Os04g0474500	85,17	0,00E+00	801	-220	Indica	Japonica	
maker-4-augustus-gene-184.61-mRNA-1	4	OsR498G0408757800.01.T03	96,39	0,00E+00	2510	Os04g0474900	96,66	0,00E+00	2528	18	NA	Japonica	
maker-4-augustus-gene-184.63-mRNA-1	4	OsR498G0408759800.01.T01	98,85	0,00E+00	1368	Os04g0475500	100,00	0,00E+00	1240	-128	NA	Japonica	
maker-4-augustus-gene-185.0-mRNA-1	4	OsR498G0408761700.01.T01	99,31	0,00E+00	2043	Os04g0476000	100,00	0,00E+00	2067	24	NA	Japonica	
maker-4-augustus-gene-185.12-mRNA-1	4	OsR498G0408763900.01.T01	97,84	0,00E+00	2617	Os04g0476700	100,00	0,00E+00	1472	-1145	Indica	Japonica	CC-LRR
maker-4-augustus-gene-185.15-mRNA-1	4	OsR498G0408764700.01.T04	95,22	0,00E+00	3037	Os04g0477000	100,00	0,00E+00	3398	361	Japonica	Japonica	
maker-4-augustus-gene-185.19-mRNA-1	4	OsR498G0408760000.01.T01	99,67	0,00E+00	1616	Os04g0475600	100,00	0,00E+00	1629	13	NA	Japonica	
maker-4-augustus-gene-185.24-mRNA-1	4	OsR498G0408762000.01.T01	99,96	0,00E+00	4286	Os04g0476100	100,00	0,00E+00	2809	-1477	Indica	Japonica	
maker-4-augustus-gene-185.35-mRNA-1	4	OsR498G0408765200.01.T02	99,82	0,00E+00	1941	Os02g0594800	73,19	1,62E-103	376	-1565	Indica	Japonica	
maker-4-augustus-gene-186.37-mRNA-1	4	OsR498G0408766900.01.T02	98,88	0,00E+00	2507	Os04g0479000	98,21	0,00E+00	2038	-469	Indica	Japonica	
maker-4-augustus-gene-186.41-mRNA-1	4	OsR498G0408765800.01.T01	99,83	0,00E+00	2053	Os04g0477500	100,00	0,00E+00	2062	9	NA	Japonica	
maker-4-augustus-gene-186.44-mRNA-1	4	OsR498G0408767200.01.T01	99,43	0,00E+00	2171	Os04g0479100	100,00	0,00E+00	2203	32	NA	Japonica	
maker-4-augustus-gene-186.47-mRNA-1	4	OsR498G0408767400.01.T01	96,89	0,00E+00	2255	Os04g0479200	99,84	0,00E+00	2241	-14	NA	Japonica	
maker-4-augustus-gene-186.49-mRNA-1	4	OsR498G0408767400.01.T03	98,70	0,00E+00	1482	Os04g0479300	100,00	0,00E+00	1483	1	NA	Japonica	
maker-4-augustus-gene-186.52-mRNA-1	4	OsR498G0408768000.01.T03	99,27	0,00E+00	723	Os04g0479500	100,00	0,00E+00	737	14	NA	Japonica	
maker-4-augustus-gene-187.0-mRNA-1	4	OsR498G0408768100.01.T01	98,76	0,00E+00	1981	Os04g0479800	100,00	0,00E+00	2044	63	NA	Japonica	
maker-4-augustus-gene-187.12-mRNA-1	4	OsR498G0408770900.01.T01	99,38	0,00E+00	5753	Os04g0480500	100,00	0,00E+00	5838	85	NA	Japonica	LRR-TM
maker-4-augustus-gene-187.14-mRNA-1	4	OsR498G0408772500.01.T02	99,82	0,00E+00	2990	Os04g0480900	100,00	0,00E+00	3003	13	NA	Japonica	
maker-4-augustus-gene-187.18-mRNA-1	4	OsR498G0408772900.01.T01	99,28	0,00E+00	2958	Os04g0480900	89,43	0,00E+00	2211	-747	Indica	Japonica	
maker-4-augustus-gene-187.20-mRNA-1	4	OsR498G0408773700.01.T01	99,88	0,00E+00	1452	Os04g0481300	100,00	0,00E+00	1456	4	NA	Japonica	
maker-4-augustus-gene-187.22-mRNA-1	4	OsR498G0408776100.01.T02	99,89	0,00E+00	6740	Os04g0481700	100,00	0,00E+00	3631	-3109	Indica	Japonica	
maker-4-augustus-gene-187.25-mRNA-1	4	OsR498G0408770000.01.T01	99,68	0,00E+00	1133	Os04g0480200	100,00	0,00E+00	1142	9	NA	Japonica	

maker-4-augustus-gene-187.27-mRNA-1	4	OsR498G0408771400.01.T01	99,24	0,00E+00	1624	Os04g0480700	96,49	0,00E+00	1499	-125	NA	Japonica	
maker-4-augustus-gene-187.29-mRNA-1	4	OsR498G0408771800.01.T01	99,74	0,00E+00	2753	Os04g0480700	100,00	0,00E+00	2771	18	NA	Japonica	
maker-4-augustus-gene-187.3-mRNA-1	4	OsR498G0408769300.01.T01	99,81	0,00E+00	2828	Os04g0480100	100,00	0,00E+00	2116	-712	Indica	Japonica	
maker-4-augustus-gene-187.31-mRNA-1	4	OsR498G0408773400.01.T01	95,47	0,00E+00	2415	Os04g0481100	95,74	0,00E+00	2434	19	NA	Japonica	
maker-4-augustus-gene-187.37-mRNA-1	4	OsR498G0408774000.01.T01	99,65	0,00E+00	1540	Os04g0481400	100,00	0,00E+00	789	-751	Indica	Japonica	
maker-4-augustus-gene-187.39-mRNA-1	4	OsR498G0408774200.01.T01	100,00	4,35E-173	607	Os04g0481500	97,64	6,88E-121	432	-175	Indica	Japonica	
maker-4-augustus-gene-187.5-mRNA-1	4	OsR498G0408770300.01.T01	99,87	0,00E+00	2865	Os04g0480300	100,00	0,00E+00	2874	9	NA	Japonica	
maker-4-augustus-gene-187.9-mRNA-1	4	OsR498G0408770500.01.T02	99,64	0,00E+00	1480	Os04g0480400	100,00	0,00E+00	1624	144	NA	Japonica	
maker-4-augustus-gene-188.0-mRNA-1	4	OsR498G0408778500.01.T01	100,00	0,00E+00	1927	Os04g0482300	100,00	0,00E+00	1927	0	NA	NA	
maker-4-augustus-gene-188.11-mRNA-1	4	OsR498G0612992800.01.T01	99,48	0,00E+00	1362	Os04g0483700	100,00	0,00E+00	883	-479	Indica	NA	
maker-4-augustus-gene-188.16-mRNA-1	4	OsR498G0408777000.01.T01	100,00	0,00E+00	1018	Os04g0482000	100,00	0,00E+00	1018	0	NA	Japonica	
maker-4-augustus-gene-188.18-mRNA-1	4	OsR498G0408777400.01.T01	100,00	0,00E+00	1034	Os04g0482000	92,29	0,00E+00	813	-221	Indica	NA	
maker-4-augustus-gene-188.20-mRNA-1	4	OsR498G0408780600.01.T02	96,23	0,00E+00	836	Os04g0482700	96,23	0,00E+00	836	0	NA	NA	
maker-4-augustus-gene-188.24-mRNA-1	4	OsR498G0408780800.01.T01	99,95	0,00E+00	3583	Os04g0482800	99,85	0,00E+00	3572	-11	NA	NA	
maker-4-augustus-gene-188.26-mRNA-1	4	OsR498G0408781000.01.T02	98,26	0,00E+00	997	Os04g0482900	100,00	0,00E+00	682	-315	Indica	NA	
maker-4-augustus-gene-188.29-mRNA-1	4	OsR498G0408782800.01.T01	100,00	0,00E+00	4504	Os04g0483400	100,00	0,00E+00	4340	-164	NA	NA	
maker-4-augustus-gene-188.3-mRNA-1	4	OsR498G0408781500.01.T01	100,00	0,00E+00	910	Os02g0597700	82,85	8,70E-141	499	-411	Indica	NA	
maker-4-augustus-gene-188.32-mRNA-1	4	OsR498G0408783200.01.T01	100,00	0,00E+00	1727	Os04g0483500	100,00	0,00E+00	1727	0	NA	NA	
maker-4-augustus-gene-188.34-mRNA-1	4	OsR498G0408783400.01.T02	100,00	0,00E+00	2958	Os04g0483600	100,00	0,00E+00	2958	0	NA	NA	
maker-4-augustus-gene-188.5-mRNA-1	4	OsR498G0408782300.01.T02	100,00	0,00E+00	3604	Os04g0483200	100,00	0,00E+00	3187	-417	Indica	NA	
maker-4-augustus-gene-189.28-mRNA-1	4	OsR498G0408787100.01.T02	100,00	0,00E+00	4336	Os04g0484800	100,00	0,00E+00	4336	0	NA	NA	
maker-4-augustus-gene-189.33-mRNA-1	4	OsR498G0408787500.01.T04	100,00	0,00E+00	1849	Os04g0485000	100,00	0,00E+00	1849	0	NA	NA	
maker-4-augustus-gene-189.38-mRNA-1	4	OsR498G0408788500.01.T02	100,00	0,00E+00	1618	Os04g0485200	100,00	0,00E+00	1615	-3	NA	NA	
maker-4-augustus-gene-189.43-mRNA-1	4	OsR498G0408789100.01.T01	100,00	0,00E+00	1308	Os04g0485300	100,00	0,00E+00	1308	0	NA	NA	
maker-4-augustus-gene-189.49-mRNA-1	4	OsR498G0408790300.01.T01	100,00	0,00E+00	1544	Os08g0236700	97,48	0,00E+00	1410	-134	NA	NA	
maker-4-augustus-gene-189.51-mRNA-1	4	OsR498G0408787400.01.T01	99,94	0,00E+00	3102	Os04g0484900	100,00	0,00E+00	3106	4	NA	NA	
maker-4-augustus-gene-189.57-mRNA-1	4	OsR498G0408787800.01.T01	100,00	0,00E+00	2760	Os04g0485100	100,00	0,00E+00	2760	0	NA	NA	
maker-4-augustus-gene-189.63-mRNA-1	4	OsR498G0408790600.01.T02	100,00	0,00E+00	2105	Os04g0485800	100,00	0,00E+00	1911	-194	NA	NA	
maker-4-augustus-gene-19.43-mRNA-1	4	OsR498G0407706500.01.T02	99,56	0,00E+00	2011	Os04g0197200	100,00	0,00E+00	2033	22	NA	Japonica	Kinase-TM
maker-4-augustus-gene-19.50-mRNA-1	4	OsR498G0407709800.01.T01	99,31	0,00E+00	1548	Os04g0197200	82,09	0,00E+00	787	-761	Indica	Japonica	Kinase-TM
maker-4-augustus-gene-190.0-mRNA-1	4	OsR498G0408792500.01.T01	100,00	0,00E+00	2089	Os04g0486400	100,00	0,00E+00	2089	0	NA	NA	
maker-4-augustus-gene-190.11-mRNA-1	4	OsR498G0408793100.01.T01	99,88	0,00E+00	3097	Os04g0486800	99,94	0,00E+00	3102	5	NA	NA	
maker-4-augustus-gene-190.15-mRNA-1	4	OsR498G0408793300.01.T01	100,00	0,00E+00	834	Os04g0486900	100,00	0,00E+00	834	0	NA	NA	
maker-4-augustus-gene-190.18-mRNA-1	4	OsR498G0408794500.01.T01	100,00	0,00E+00	1586	Os04g0487100	100,00	0,00E+00	1586	0	NA	NA	
maker-4-augustus-gene-190.22-mRNA-1	4	OsR498G0408798300.01.T05	100,00	0,00E+00	3847	Os04g0488000	100,00	0,00E+00	3847	0	NA	NA	Kinase-TM
maker-4-augustus-gene-190.25-mRNA-1	4	OsR498G0408799300.01.T02	100,00	0,00E+00	3699	Os04g0488100	100,00	0,00E+00	3699	0	NA	NA	
maker-4-augustus-gene-190.31-mRNA-1	4	OsR498G0408792800.01.T02	97,78	0,00E+00	1070	Os04g0486500	97,78	0,00E+00	1070	0	NA	NA	
maker-4-augustus-gene-190.38-mRNA-1	4	OsR498G0408794000.01.T02	100,00	2,98E-92	338	Os04g0487000	100,00	1,03E-92	338	0	NA	NA	
maker-4-augustus-gene-190.4-mRNA-1	4	OsR498G0408792900.01.T01	100,00	0,00E+00	1829	Os04g0486600	100,00	0,00E+00	1829	0	NA	NA	
maker-4-augustus-gene-191.27-mRNA-1	4	OsR498G0408806900.01.T01	97,71	0,00E+00	1937	Os04g0489600	100,00	0,00E+00	2003	66	NA	Japonica	
maker-4-augustus-gene-191.30-mRNA-1	4	OsR498G0408807500.01.T01	99,83	0,00E+00	4316	Os04g0489800	100,00	0,00E+00	4334	18	NA	Japonica	
maker-4-augustus-gene-191.39-mRNA-1	4	OsR498G0408802200.01.T01	99,78	0,00E+00	2492	Os04g0488700	100,00	0,00E+00	2506	14	NA	Japonica	Kinase-TM
maker-4-augustus-gene-191.41-mRNA-1	4	OsR498G0408804800.01.T04	99,63	0,00E+00	2397	Os04g0489100	99,93	0,00E+00	2415	18	NA	Japonica	
maker-4-augustus-gene-192.44-mRNA-1	4	OsR498G0408809700.01.T02	99,91	0,00E+00	1982	Os04g0490500	100,00	0,00E+00	1350	-632	Indica	Japonica	Kinase
maker-4-augustus-gene-192.48-mRNA-1	4	OsR498G0408810900.01.T01	100,00	0,00E+00	1207	Os04g0490900	96,64	0,00E+00	1498	291	Japonica	Japonica	
maker-4-augustus-gene-192.56-mRNA-1	4	OsR498G0408809200.01.T01	100,00	0,00E+00	1027	Os04g0490400	100,00	0,00E+00	1027	0	NA	Japonica	
maker-4-augustus-gene-192.59-mRNA-1	4	OsR498G0408810000.01.T02	99,84	0,00E+00	2188	Os04g0490600	95,97	0,00E+00	1520	-668	Indica	Japonica	
maker-4-augustus-gene-192.66-mRNA-1	4	OsR498G0408810600.01.T02	99,73	0,00E+00	1967	Os04g0490800	100,00	0,00E+00	1981	14	NA	Japonica	
maker-4-augustus-gene-192.69-mRNA-1	4	OsR498G0408812200.01.T02	99,51	0,00E+00	2523	Os04g0491200	100,00	0,00E+00	4632	2109	Japonica	Japonica	
maker-4-augustus-gene-192.74-mRNA-1	4	OsR498G0408813800.01.T01	99,88	0,00E+00	3011	Os04g0491500	100,00	0,00E+00	3020	9	NA	Japonica	
maker-4-augustus-gene-192.78-mRNA-1	4	OsR498G0408814000.01.T01	99,74	0,00E+00	2726	Os04g0491700	100,00	0,00E+00	2744	18	NA	Japonica	
maker-4-augustus-gene-193.16-mRNA-1	4	OsR498G0408818300.01.T01	99,11	0,00E+00	2567	Os04g0492900	99,17	0,00E+00	2571	4	NA	Japonica	
maker-4-augustus-gene-193.20-mRNA-1	4	OsR498G0408820400.01.T04	99,79	0,00E+00	846	Os04g0493100	100,00	0,00E+00	850	4	NA	Japonica	
maker-4-augustus-gene-193.22-mRNA-1	4	OsR498G0408821800.01.T01	99,71	0,00E+00	1236	Os04g0493400	100,00	0,00E+00	1245	9	NA	Japonica	
maker-4-augustus-gene-193.8-mRNA-1	4	OsR498G0408817100.01.T04	99,52	0,00E+00	1870	Os04g0492400	100,00	0,00E+00	1878	8	NA	Japonica	
maker-4-augustus-gene-194.10-mRNA-1	4	OsR498G0408824300.01.T01	91,94	0,00E+00	1608	Os01g0611000	77,26	0,00E+00	838	-770	Indica	Japonica	
maker-4-augustus-gene-194.21-mRNA-1	4	OsR498G0408822600.01.T01	99,89	0,00E+00	1560	Os04g0494100	100,00	0,00E+00	1564	4	NA	Japonica	
maker-4-augustus-gene-194.26-mRNA-1	4	OsR498G0408827000.01.T01	99,87	0,00E+00	1333	Os05g0305700	70,70	1,00E-78	293	-1040	Indica	Japonica	
maker-4-augustus-gene-194.4-mRNA-1	4	OsR498G0408823500.01.T01	99,82	0,00E+00	2037	Os04g0494600	100,00	0,00E+00	2046	9	NA	Japonica	
maker-4-augustus-gene-194.8-mRNA-1	4	OsR498G0408824100.01.T01	99,65	0,00E+00	2028	Os04g0494900	100,00	0,00E+00	2046	18	NA	Japonica	
maker-4-augustus-gene-195.0-mRNA-1	4	OsR498G0408827100.01.T03	99,22	0,00E+00	4983	Os04g0495800	99,32	0,00E+00	4996	13	NA	Japonica	
maker-4-augustus-gene-195.10-mRNA-1	4	OsR498G0408828200.01.T01	99,89	0,00E+00	1592	Os02g0607700	81,76	0,00E+00	856	-736	Indica	Japonica	
maker-4-augustus-gene-195.13-mRNA-1	4	OsR498G0408829600.01.T02	97,42	0,00E+00	1058	Os04g0496600	93,56	0,00E+00	972	-86	NA	Japonica	
maker-4-augustus-gene-195.17-mRNA-1	4	OsR498G0408830200.01.T02	99,82	0,00E+00	4062	Os04g0496800	100,00	0,00E+00	4080	18	NA	Japonica	
maker-4-augustus-gene-195.2-mRNA-1	4	OsR498G0408827700.01.T01	95,60	0,00E+00	1338	Os04g0495900	100,00	0,00E+00	1424	86	NA	Japonica	
maker-4-augustus-gene-195.22-mRNA-1	4	OsR498G0408830600.01.T01	99,62	0,00E+00	1855	Os04g0497000	100,00	0,00E+00	1873	18	NA	Japonica	
maker-4-augustus-gene-195.27-mRNA-1	4	OsR498G0408831600.01.T03	98,17	0,00E+00	1138	Os04g0497350	86,78	3,68E-134	478	-660	Indica	Japonica	
maker-4-augustus-gene-195.32-mRNA-1	4	OsR498G0408831800.01.T01	99,81	0,00E+00	936	Os04g0497400	100,00	0,00E+00	940	4	NA	Japonica	
maker-4-augustus-gene-195.35-mRNA-1	4	OsR498G0408832200.01.T02	99,39	0,00E+00	2324	Os04g0497600	100,00	0,00E+00	2360	36	NA	Japonica	
maker-4-augustus-gene-195.4-mRNA-1	4	OsR498G0408828900.01.T02	99,79	0,00E+00	2568	Os04g0496400	100,00	0,00E+00	2581	13	NA	Japonica	
maker-4-augustus-gene-195.8-mRNA-1	4	OsR498G0408831100.01.T01	99,63	1,13E-135	483	Os04g0497300	100,00	2,64E-157	553	70	Japonica	Japonica	
maker-4-augustus-gene-196.46-mRNA-1	4	OsR498G0408834700.01.T05	99,07	0,00E+00	2264	Os04g0498800	100,00	0,00E+00	2318	54	NA	Japonica	
maker-4-augustus-gene-196.54-mRNA-1	4	OsR498G0408835700.01.T02	98,36	0,00E+00	2113	Os04g0499100	100,00	0,00E+00	2857	744	Japonica	Japonica	
maker-4-augustus-gene-196.58-mRNA-1	4	OsR498G0408837100.01.T04	99,42	3,50E-175	614	Os04g0499800	100,00	0,00E+00	715	101	Japonica	Japonica	
maker-4-augustus-gene-196.60-mRNA-1	4	OsR498G0408838100.01.T01	99,71	3,10E-175	615	Os04g0500300	100,00	2,54E-177	619	4	NA	Japonica	
maker-4-augustus-gene-196.62-mRNA-1	4	OsR498G0408838500.01.T01	99,12	0,00E+00	803	Os04g0500400	100,00	0,00E+00	818	15	NA	Japonica	
maker-4-augustus-gene-196.64-mRNA-1	4	OsR498G0408833600.01.T01	97,62	0,00E+00	645	Os04g0498200	100,00	3,95E-118	423	-222	Indica	Japonica	
maker-4-augustus-gene-196.67-mRNA-1	4	OsR498G0408834600.01.T01	95,31	0,00E+00	1782	Os04g0498700	100,00	0,00E+00	1990	208	Japonica	Japonica	
maker-4-augustus-gene-196.73-mRNA-1	4	OsR498G0204286000.01.T01	77,42	4,33E-40	166	Os04g0499200	100,00	6,37E-172	601	435	Japonica	Japonica	
maker-4-augustus-gene-196.75-mRNA-1	4	OsR498G0408836000.01.T02	99,41	0,00E+00	4233	Os04g0499300	98,75	0,00E+00	2534	-1699	Indica	Japonica	
maker-4-augustus-gene-197.39-mRNA-1	4	OsR498G0408840900.01.T05	96,79	0,00E+00	2308	Os04g0500700	96,94	0,00E+00	2317	9	NA	Japonica	
maker-4-augustus-gene-197.41-mRNA-1	4	OsR498G0408841300.01.T01	99,81	0,00E+00	1902	Os04g0500900	96,94	0,00E+00	1322	-580	Indica	Japonica	
maker-4-augustus-gene-197.45-mRNA-1	4	OsR498G0408841700.01.T03	96,27	0,00E+00	2144	Os04g0501100	100,00	0,00E+00	2235	91	NA	Japonica	
maker-4-augustus-gene-197.52-mRNA-1	4	OsR498G0408843900.01.T06	99,58	0,00E+00	2576	Os04g0501700	100,00	0,00E+00	2605	29	NA	Japonica	
maker-4-augustus-gene-197.58-mRNA-1	4	OsR498G0408844300.01.T01	99,50	0,00E+00	1057	Os04g0501800	100,00	0,00E+00	1067	10	NA	Japonica	
maker-4-augustus-gene-197.61-mRNA-1	4	OsR498G0408845100.01.T01	99,64	0,00E+00	1497	Os04g0502100	100,00	0,00E+00	1819	322	Japonica	Japonica	
maker-4-augustus-gene-197.63-mRNA-1	4	OsR498G0408845300.01.T02	99,90	0,00E+00	3699	Os04g0502200	100,00	0,00E+00	3709	10	NA	Japonica	
maker-4-augustus-gene-197.69-mRNA-1	4	OsR498G0408841600.01.T04	100,00	0,00E+00	829	Os04g0501000	100,00	0,00E+00	829	0	NA	Japonica	
maker-4-augustus-gene-197.71-mRNA-1	4	OsR498G0408843400.01.T01	99,69	0,00E+00	4547	Os04g0501600	100,00	0,00E+00	4583	36	NA	Japonica	
maker-4-augustus-gene-198.0-mRNA-1	4	OsR498G0408846900.01.T01	97,78	0,00E+00	2399	Os04g0502800	97,99	0,00E+00	2413	14	NA	Japonica	
maker-4-augustus-gene-198.14-mRNA-1	4	OsR498G0408848700.01.T01	100,00	3,01E-116	417	Os04g0503800	100,00	9,88E-73	271	-146	Indica	Japonica	
maker-4-augustus-gene-198.23-mRNA-1	4	OsR498G0408852300.01.T02	99,54	0,00E+00	2708	Os04g0386600	82,79	0,00E+00	891	-1817	Indica	Japonica	
maker-4-augustus-gene-198.3-mRNA-1	4	OsR498G0408847100.01.T02	99,58	0,00E+00	2531	Os04g0502900	100,00	0,00E+00	2560	29	NA	Japonica	
maker-4-augustus-gene-198.30-mRNA-1	4	OsR498G0408848000.01.T07	99,67	0,00E+00	1630	Os04g0503500	100,00	0,00E+00	1644	14	NA	Japonica	LRR-TM
maker-4-augustus-gene-198.36-mRNA-1	4	OsR498G0408848600.01.T02	99,73	0,00E+00	1342	Os04g0503700	99,87	0,00E+00	1347	5	NA	Japonica	
maker-4-augustus-gene-198.9-mRNA-1	4	OsR498G0408848300.01.T03	99,91	0,00E+00	2030	Os09g0123300	70,30	1,65E-103	377	-1653	Indica	Japonica	Kinase
maker-4-augustus-gene-199.0-mRNA-1	4	OsR498G0408853300.01.T02	99,84	0,00E+00	2291	Os04g0505000	100,00	0,00E+00	2300	9	NA	Japonica	
maker-4-augustus-gene-199.2-mRNA-1	4	OsR498G0408852800.01.T03	99,70	0,00E+00	3544	Os04g0504800	100,00	0,00E+00	3571	27	NA	Japonica	
maker-4-augustus-gene-199.9-mRNA-1	4	OsR498G0408859600.01.T02	99,91	0,00E+00	1890	Os04g0506300	100,00	0,00E+00	1891	1	NA	Japonica	
maker-4-augustus-gene-2.0-mRNA-1	4	OsR498G0407629800.01.T01	99,68	0,00E+00	1677	Os04g0171800	99,80	0,00E+00	1822	145	NA	Japonica	
maker-4-augustus-gene-200.36-mRNA-1	4	OsR498G0408864500.01.T01	100,00	0,00E+00	1997	Os04g0507700	100,00	0,00E+00	1997	0	NA	Japonica	
maker-4-augustus-gene-200.38-mRNA-1	4	OsR498G0408864900.01.T06	100,00	0,00E+00	902	Os04g0507900	100,00	0,00E+00	902	0	NA	Japonica	
maker-4-augustus-gene-200.47-mRNA-1	4	OsR498G0408861800.01.T02	99,97	0,00E+00	5082	Os04g0506900	100,00	0,00E+00	4773	-309	NA	Japonica	
maker-4-augustus-gene-200.50-mRNA-1	4	OsR498G0408862600.01.T04	99,86	0,00E+00	3816	Os04g0507000	99,86	0,00E+00	3816	0	NA	Japonica	
maker-4-augustus-gene-200.73-mRNA-1	4	OsR498G0408865400.01.T01	99,95	0,00E+00	3848	Os04g0508000	100,00	0,00E+00	3853	5	NA	Japonica	
maker-4-augustus-gene-201.35-mRNA-1	4	OsR498G0408866100.01.T01	100,00	0,00E+00	693	Os04g0508300	100,00	4,11E-175	612	-81	Indica	Japonica	
maker-4-augustus-gene-201.44-mRNA-1	4	OsR498G0408871700.01.T01	97,62	0,00E+00	4364	Os04g0509100	100,00	0,00E+00	4810	446	NA	NA	
maker-4-augustus-gene-201.49-mRNA-1	4	OsR498G0408872700.01.T03	91,67	6,96E-152	537	Os04g0509400	91,67	2,42E-152	537	0	NA	NA	
maker-4-augustus-gene-201.51-mRNA-1	4	OsR498G0408867400.01.T02	100,00	0,00E+00	1526	Os04g0508500	99,31	0,00E+00	1275	-251	Indica	NA	
maker-4-augustus-gene-201.58-mRNA-1	4	OsR498G0408872000.01.T04	100,00	4,33E-169	594	Os04g0509200	100,00	1,51E-169	594	0	NA	NA	
maker-4-augustus-gene-202.49-mRNA-1	4	OsR498G0408876500.01.T01	99,87	0,00E+00	4286	Os02g0813800	87,51	0,00E+00	2986	-1300	Indica	NA	
maker-4-augustus-gene-202.59-mRNA-1	4	OsR498G0408877100.01.T02	99,93	0,00E+00	2485	Os04g0510500	100,00	0,00E+00	1371	-1114	Indica	Japonica	
maker-4-augustus-gene-202.71-mRNA-1	4	OsR498G0408877400.01.T01	99,64	0,00E+00	1491	Os04g0510600	100,00	0,00E+00	1505	14	NA	Japonica	
maker-4-augustus-gene-202.76-mRNA-1	4	OsR498G0408877800.01.T01	100,00	0,00E+00	1548	Os04g0510900	94,52	0,00E+00	1374	-174	Indica	NA	
maker-4-augustus-gene-202.78-mRNA-1	4	OsR498G0408878400.01.T01	100,00	0,00E+00	1326	Os04g0511200	100,00	0,00E+00	1326	0	NA	NA	
maker-4-augustus-gene-202.81-mRNA-1	4	OsR498G0408878800.01.T04	100,00	0,00E+00	3149	Os04g0511400	100,00	0,00E+00	3149	0	NA	NA	
maker-4-augustus-gene-202.83-mRNA-1	4	OsR498G0408879200.01.T02	100,00	0,00E+00	3956	Os04g0511600	100,00	0,00E+00	3956	0	NA	NA	
maker-4-augustus-gene-202.89-mRNA-1	4	OsR498G0408879400.01.T01	98,69	0,00E+00	2540	Os04g0511700	98,69	0,00E+00	2406	-134	NA	NA	
maker-4-augustus-gene-203.0-mRNA-1	4	OsR498G0408883500.01.T01	100,00	0,00E+00	910	Os04g0513000	100,00	0,00E+00	765	-145	Indica	NA	
maker-4-augustus-gene-203.13-mRNA-1	4	OsR498G0408881200.01.T01	100,00	0,00E+00	1822	Os04g0512400	100,00	0,00E+00	1822	0	NA	Japonica	
maker-4-augustus-gene-203.16-mRNA-1	4	OsR498G0408883400.01.T01	100,00	0,00E+00	4060	Os04g0512900	100,00	0,00E+00	4066	6	NA	NA	CC-LRR-NBS-TM
maker-4-augustus-gene-203.20-mRNA-1	4	OsR498G0408883800.01.T02	90,98	0,00E+00	1040	Os04g0513100	90,98	0,00E+00	1040	0	NA	NA	
maker-4-augustus-gene-203.23-mRNA-1	4	OsR498G0408884200.01.T01	99,26	0,00E+00	1188	Os03g0399000	94,61	0,00E+00	753	-435	Indica	NA	
maker-4-augustus-gene-203.28-mRNA-1	4	OsR498G0408884400.01.T01	92,30	0,00E+00	1006	Os04g0513700	100,00	0,00E+00	1252	246	Japonica	NA	
maker-4-augustus-gene-203.3-mRNA-1	4	OsR498G0408880600.01.T03	100,00	0,00E+00	1186	Os02g0621300	76,42	1,01E-130	467	-719	Indica	NA	
maker-4-augustus-gene-203.6-mRNA-1	4	OsR498G0408881000.01.T02	100,00	0,00E+00	2046	Os04g0512300	100,00	0,00E+00	1997	-49	NA	NA	
maker-4-augustus-gene-204.0-mRNA-1	4	OsR498G0408886300.01.T01	100,00	0,00E+00	1992	Os04g0514400	99,91	0,00E+00	1987	-5	NA	NA	
maker-4-augustus-gene-204.16-mRNA-1	4	OsR498G0408886200.01.T06	100,00	0,00E+00	5169	Os04g0514500	100,00	0,00E+00	5169	0	NA	NA	
maker-4-augustus-gene-204.17-mRNA-1	4	OsR498G0408888200.01.T02	96,94	0,00E+00	2268	Os04g0514800	96,94	0,00E+00	2268	0	NA	NA	
maker-4-augustus-gene-204.3-mRNA-1	4	OsR498G0408890900.01.T01	100,00	0,00E+00	747	Os04g0515400	100,00	0,00E+00	747	0	NA	NA	
maker-4-augustus-gene-204.6-mRNA-1	4	OsR498G0408893100.01.T01	100,00	0,00E+00	1510	Os04g0515900	100,00	0,00E+00	1510	0	NA	Indica	
maker-4-augustus-gene-205.0-mRNA-1	4	OsR498G0408898700.01.T01	100,00	0,00E+00	975	Os04g0517000	99,79	0,00E+00	842	-133	Indica	NA	
maker-4-augustus-gene-205.11-mRNA-1	4	OsR498G0408897800.01.T02	100,00	0,00E+00	1921	Os04g0516600	100,00	0,00E+00	1921	0	NA	NA	
maker-4-augustus-gene-205.15-mRNA-1	4	OsR498G0408899800.01.T01	100,00	0,00E+00	1397	Os04g0517100	100,00	0,00E+00	1397	0	NA	Indica	
maker-4-augustus-gene-205.17-mRNA-1	4	OsR498G0408901000.01.T01	94,76	0,00E+00	1982	Os04g0517300	94,76	0,00E+00	1982	0	NA	Indica	
maker-4-augustus-gene-205.22-mRNA-1	4	OsR498G0408902600.01.T01	100,00	0,00E+00	4004	Os01g0364800	72,28	0,00E+00	695	-3309	Indica	NA	Kinase-TM
maker-4-augustus-gene-205.3-mRNA-1	4	OsR498G0408901100.01.T01	100,00	0,00E+00	2949	Os04g0517400	98,93	0,00E+00	1470	-1479	Indica	Indica	
maker-4-augustus-gene-205.9-mRNA-1	4	OsR498G0408901300.01.T01	100,00	0,00E+00	1548	Os04g0517500	99,07	0,00E+00	1507	-41	NA	Indica	Kinase-TM
maker-4-augustus-gene-206.0-mRNA-1	4	OsR498G0408908300.01.T02	100,00	0,00E+00	975	Os04g0518800	100,00	0,00E+00	1359	384	Japonica	NA	
maker-4-augustus-gene-206.11-mRNA-1	4	OsR498G0408904400.01.T01	100,00	0,00E+00	3831	Os04g0518100	100,00	0,00E+00	3831	0	NA	NA	
maker-4-augustus-gene-206.16-mRNA-1	4	OsR498G0408906400.01.T01	100,00	0,00E+00	3869	Os04g0518400	100,00	0,00E+00	3869	0	NA	NA	
maker-4-augustus-gene-206.25-mRNA-1	4	OsR498G0407782400.01.T01	70,70	5,61E-50	199	Os11g0519100	99,53	0,00E+00	1499	1300	Japonica	NA	
maker-4-augustus-gene-206.5-mRNA-1	4	OsR498G0408903800.01.T04	99,42	0,00E+00	1841	Os04g0518000	99,42	0,00E+00	1841	0	NA	NA	
maker-4-augustus-gene-207.26-mRNA-1	4	OsR498G0408911500.01.T01	100,00	0,00E+00	3788	Os04g0519700	100,00	0,00E+00	1968	-1820	Indica	NA	
maker-4-augustus-gene-208.46-mRNA-1	4	OsR498G0408917700.01.T01	100,00	0,00E+00	1575	Os04g0521100	100,00	0,00E+00	1575	0	NA	NA	
maker-4-augustus-gene-208.49-mRNA-1	4	OsR498G0408917900.01.T01	100,00	1,34E-153	542	Os04g0521200	100,00	1,99E-133	473	-69	Indica	Indica	
maker-4-augustus-gene-208.54-mRNA-1	4	OsR498G0408918300.01.T02	100,00	0,00E+00	2311	Os04g0521800	100,00	0,00E+00	2311	0	NA	Japonica	
maker-4-augustus-gene-208.57-mRNA-1	4	OsR498G0408918500.01.T01	100,00	2,22E-124	444	Os02g0629800	79,76	8,34E-55	212	-232	Indica	Japonica	
maker-4-augustus-gene-208.61-mRNA-1	4	OsR498G0408921300.01.T02	100,00	0,00E+00	645	Os04g0522800	100,00	0,00E+00	645	0	NA	NA	
maker-4-augustus-gene-208.63-mRNA-1	4	OsR498G0408921900.01.T01	100,00	0,00E+00	1007	Os04g0523000	100,00	0,00E+00	1007	0	NA	NA	
maker-4-augustus-gene-208.65-mRNA-1	4	OsR498G0408919400.01.T01	100,00	0,00E+00	1943	Os04g0522500	100,00	0,00E+00	1943	0	NA	NA	
maker-4-augustus-gene-208.67-mRNA-1	4	OsR498G0408922200.01.T01	100,00	0,00E+00	1591	Os04g0523100	99,66	0,00E+00	1578	-13	NA	NA	
maker-4-augustus-gene-209.0-mRNA-1	4	OsR498G0408923500.01.T01	100,00	0,00E+00	2890	Os10g0483500	71,42	8,74E-166	584	-2306	Indica	NA	
maker-4-augustus-gene-209.11-mRNA-1	4	OsR498G0408928500.01.T01	99,95	0,00E+00	3854	Os04g0524600	73,72	0,00E+00	1052	-2802	Indica	NA	
maker-4-augustus-gene-209.14-mRNA-1	4	OsR498G0408927000.01.T03	99,95	0,00E+00	3919	Os04g0524500	100,00	0,00E+00	3312	-607	Indica	NA	
maker-4-augustus-gene-209.19-mRNA-1	4	OsR498G0408929000.01.T01	100,00	0,00E+00	1424	Os04g0525000	100,00	0,00E+00	1424	0	NA	NA	
maker-4-augustus-gene-209.2-mRNA-1	4	OsR498G0408926300.01.T01	100,00	0,00E+00	731	Os04g0524300	100,00	0,00E+00	731	0	NA	Indica	
maker-4-augustus-gene-209.24-mRNA-1	4	OsR498G0408929200.01.T01	99,94	0,00E+00	2782	Os04g0525100	100,00	0,00E+00	1808	-974	Indica	Japonica	
maker-4-augustus-gene-209.27-mRNA-1	4	OsR498G0408931600.01.T01	99,74	0,00E+00	2065	Os04g0525700	99,48	0,00E+00	2059	-6	NA	Japonica	
maker-4-augustus-gene-209.4-mRNA-1	4	OsR498G0408926700.01.T02	100,00	0,00E+00	1434	Os04g0524400	99,83	0,00E+00	2156	722	Japonica	NA	
maker-4-augustus-gene-209.7-mRNA-1	4	OsR498G0408928100.01.T03	99,92	0,00E+00	2330	Os04g0524600	100,00	0,00E+00	2334	4	NA	NA	
maker-4-augustus-gene-21.3-mRNA-1	4	OsR498G0407712200.01.T01	98,79	0,00E+00	1440	Os04g0330200	97,00	0,00E+00	947	-493	Indica	Japonica	
maker-4-augustus-gene-21.7-mRNA-1	4	OsR498G0407716800.01.T01	99,16	0,00E+00	4217	Os04g0201200	99,79	0,00E+00	4283	66	NA	Japonica	
maker-4-augustus-gene-210.47-mRNA-1	4	OsR498G0408938700.01.T07	100,00	0,00E+00	1856	Os04g0527400	100,00	0,00E+00	1856	0	NA	NA	
maker-4-augustus-gene-210.49-mRNA-1	4	OsR498G0408939100.01.T02	100,00	0,00E+00	823	Os04g0527700	100,00	0,00E+00	823	0	NA	NA	
maker-4-augustus-gene-210.54-mRNA-1	4	OsR498G0408939300.01.T01	99,71	0,00E+00	1825	Os04g0527800	99,71	0,00E+00	1825	0	NA	NA	
maker-4-augustus-gene-210.58-mRNA-1	4	OsR498G0408939500.01.T01	100,00	0,00E+00	1440	Os04g0527900	100,00	0,00E+00	1440	0	NA	NA	
maker-4-augustus-gene-210.60-mRNA-1	4	OsR498G0408940100.01.T07	100,00	0,00E+00	693	Os04g0528100	100,00	0,00E+00	693	0	NA	NA	
maker-4-augustus-gene-210.64-mRNA-1	4	OsR498G0408935400.01.T01	100,00	0,00E+00	1267	Os04g0526800	100,00	0,00E+00	1267	0	NA	NA	
maker-4-augustus-gene-210.66-mRNA-1	4	OsR498G0408935800.01.T01	100,00	0,00E+00	1169	Os04g0527000	100,00	0,00E+00	1169	0	NA	NA	
maker-4-augustus-gene-210.72-mRNA-1	4	OsR498G0408939800.01.T01	100,00	0,00E+00	935	Os04g0528000	100,00	0,00E+00	1700	765	Japonica	NA	
maker-4-augustus-gene-211.0-mRNA-1	4	OsR498G0408940900.01.T01	100,00	0,00E+00	3853	Os04g0528300	100,00	0,00E+00	3853	0	NA	NA	
maker-4-augustus-gene-211.15-mRNA-1	4	OsR498G0408941200.01.T02	100,00	0,00E+00	1770	Os04g0528400	100,00	0,00E+00	1770	0	NA	NA	
maker-4-augustus-gene-211.19-mRNA-1	4	OsR498G0408943800.01.T01	100,00	0,00E+00	3544	Os04g0528600	100,00	0,00E+00	1851	-1693	Indica	Japonica	
maker-4-augustus-gene-211.9-mRNA-1	4	OsR498G0408949500.01.T06	100,00	0,00E+00	3741	Os04g0529500	100,00	0,00E+00	3741	0	NA	NA	
maker-4-augustus-gene-212.13-mRNA-1	4	OsR498G0408955300.01.T02	100,00	0,00E+00	800	Os04g0531100	100,00	0,00E+00	785	-15	NA	NA	
maker-4-augustus-gene-212.17-mRNA-1	4	OsR498G0408955900.01.T03	100,00	0,00E+00	1732	Os04g0531300	100,00	0,00E+00	1732	0	NA	NA	
maker-4-augustus-gene-212.2-mRNA-1	4	OsR498G0408951300.01.T01	98,45	0,00E+00	1800	Os04g0530100	97,04	0,00E+00	912	-888	Indica	NA	
maker-4-augustus-gene-212.26-mRNA-1	4	OsR498G0408956900.01.T01	100,00	0,00E+00	1678	Os04g0531900	94,09	0,00E+00	1426	-252	Indica	NA	
maker-4-augustus-gene-212.30-mRNA-1	4	OsR498G0408950400.01.T02	100,00	0,00E+00	2836	Os04g0529800	100,00	0,00E+00	2836	0	NA	NA	
maker-4-augustus-gene-212.39-mRNA-1	4	OsR498G0408952000.01.T01	96,09	0,00E+00	1782	Os04g0530300	96,09	0,00E+00	1782	0	NA	NA	
maker-4-augustus-gene-212.44-mRNA-1	4	OsR498G0408953600.01.T01	100,00	0,00E+00	980	Os04g0530600	100,00	0,00E+00	980	0	NA	NA	
maker-4-augustus-gene-212.46-mRNA-1	4	OsR498G0408953800.01.T02	100,00	0,00E+00	2120	Os04g0530700	100,00	0,00E+00	2120	0	NA	NA	
maker-4-augustus-gene-212.49-mRNA-1	4	OsR498G0408957400.01.T01	94,16	0,00E+00	1459	Os04g0531900	94,16	0,00E+00	1459	0	NA	Japonica	
maker-4-augustus-gene-212.6-mRNA-1	4	OsR498G0408952100.01.T01	100,00	0,00E+00	1716	Os04g0530400	100,00	0,00E+00	1716	0	NA	NA	
maker-4-augustus-gene-213.48-mRNA-1	4	OsR498G0408960500.01.T01	100,00	0,00E+00	1483	Os04g0532800	100,00	0,00E+00	1483	0	NA	NA	
maker-4-augustus-gene-213.50-mRNA-1	4	OsR498G0408961100.01.T06	100,00	0,00E+00	2700	Os04g0533000	100,00	0,00E+00	2700	0	NA	NA	
maker-4-augustus-gene-213.61-mRNA-1	4	OsR498G0408964300.01.T02	100,00	0,00E+00	2755	Os04g0534000	100,00	0,00E+00	2755	0	NA	Indica	
maker-4-augustus-gene-213.65-mRNA-1	4	OsR498G0408958000.01.T02	98,14	0,00E+00	1673	Os04g0532100	96,27	0,00E+00	1604	-69	NA	NA	
maker-4-augustus-gene-213.69-mRNA-1	4	OsR498G0408958800.01.T03	100,00	0,00E+00	1094	Os04g0532500	100,00	0,00E+00	1094	0	NA	NA	
maker-4-augustus-gene-213.72-mRNA-1	4	OsR498G0408962200.01.T01	100,00	0,00E+00	1121	Os04g0533300	100,00	0,00E+00	1121	0	NA	NA	
maker-4-augustus-gene-213.75-mRNA-1	4	OsR498G0408963000.01.T03	100,00	0,00E+00	1283	Os04g0533500	100,00	0,00E+00	1283	0	NA	NA	
maker-4-augustus-gene-213.78-mRNA-1	4	OsR498G1019009000.01.T01	75,58	6,40E-43	175	Os04g0533900	100,00	4,60E-128	455	280	Japonica	NA	
maker-4-augustus-gene-214.0-mRNA-1	4	OsR498G0408964500.01.T05	100,00	0,00E+00	1966	Os02g0642700	77,16	0,00E+00	665	-1301	Indica	Indica	
maker-4-augustus-gene-214.11-mRNA-1	4	OsR498G0408965100.01.T01	100,00	0,00E+00	1564	Os04g0534300	100,00	0,00E+00	1564	0	NA	NA	
maker-4-augustus-gene-214.15-mRNA-1	4	OsR498G0408968100.01.T01	99,75	0,00E+00	2816	Os04g0535600	99,75	0,00E+00	2816	0	NA	NA	
maker-4-augustus-gene-214.17-mRNA-1	4	OsR498G0408965400.01.T02	100,00	0,00E+00	1370	Os04g0534400	99,21	0,00E+00	1343	-27	NA	NA	
maker-4-augustus-gene-214.21-mRNA-1	4	OsR498G0408965600.01.T02	100,00	0,00E+00	2230	Os04g0534500	100,00	0,00E+00	1981	-249	Indica	NA	
maker-4-augustus-gene-214.25-mRNA-1	4	OsR498G0408967400.01.T06	100,00	0,00E+00	877	Os04g0535400	100,00	0,00E+00	877	0	NA	NA	
maker-4-augustus-gene-215.4-mRNA-1	4	OsR498G0408970400.01.T01	100,00	0,00E+00	893	Os04g0536300	100,00	0,00E+00	893	0	NA	NA	
maker-4-augustus-gene-216.39-mRNA-1	4	OsR498G0408982500.01.T02	100,00	0,00E+00	3112	Os04g0538000	100,00	0,00E+00	3112	0	NA	Indica	
maker-4-augustus-gene-216.43-mRNA-1	4	OsR498G0408987300.01.T01	100,00	0,00E+00	953	Os04g0538900	100,00	0,00E+00	953	0	NA	NA	
maker-4-augustus-gene-216.45-mRNA-1	4	OsR498G0408987700.01.T01	100,00	0,00E+00	1196	Os04g0539000	100,00	0,00E+00	1196	0	NA	NA	
maker-4-augustus-gene-216.47-mRNA-1	4	OsR498G0408990100.01.T03	99,93	0,00E+00	2570	Os02g0645600	71,56	1,71E-180	632	-1938	Indica	NA	
maker-4-augustus-gene-216.50-mRNA-1	4	OsR498G0408982000.01.T02	100,00	0,00E+00	2695	Os04g0537900	100,00	0,00E+00	2695	0	NA	NA	
maker-4-augustus-gene-216.57-mRNA-1	4	OsR498G0408982800.01.T03	100,00	0,00E+00	3777	Os04g0538100	100,00	0,00E+00	2094	-1683	Indica	Indica	
maker-4-augustus-gene-216.59-mRNA-1	4	OsR498G0408985800.01.T01	100,00	0,00E+00	2819	Os04g0538700	100,00	0,00E+00	2819	0	NA	NA	
maker-4-augustus-gene-216.61-mRNA-1	4	OsR498G0408986600.01.T04	100,00	0,00E+00	3847	Os04g0538800	100,00	0,00E+00	3847	0	NA	NA	
maker-4-augustus-gene-216.65-mRNA-1	4	OsR498G0408988000.01.T01	100,00	0,00E+00	1797	Os04g0539100	100,00	0,00E+00	1797	0	NA	NA	
maker-4-augustus-gene-217.25-mRNA-1	4	OsR498G0408992300.01.T02	100,00	0,00E+00	1359	Os04g0540200	100,00	0,00E+00	1359	0	NA	NA	
maker-4-augustus-gene-217.28-mRNA-1	4	OsR498G0408992900.01.T03	100,00	0,00E+00	1021	Os04g0540300	100,00	0,00E+00	2019	998	Japonica	NA	
maker-4-augustus-gene-217.31-mRNA-1	4	OsR498G0408993300.01.T04	87,03	5,02E-150	532	Os04g0540400	98,99	0,00E+00	1044	512	Japonica	NA	

maker-4-augustus-gene-217.34-mRNA-1	4	OsR498G0408991600.01.T01	100,00	0,00E+00	1902	Os10g0512700	71,25	1,74E-147	522	-1380	Indica	NA	
maker-4-augustus-gene-217.41-mRNA-1	4	OsR498G0408993800.01.T01	98,20	0,00E+00	2604	Os04g0540600	98,20	0,00E+00	2604	0	NA	NA	
maker-4-augustus-gene-217.46-mRNA-1	4	OsR498G0408995400.01.T01	100,00	0,00E+00	1959	Os04g0540900	100,00	0,00E+00	1959	0	NA	NA	Kinase-TM
maker-4-augustus-gene-217.48-mRNA-1	4	OsR498G0408998400.01.T01	98,80	0,00E+00	3951	Os04g0541100	98,54	0,00E+00	3226	-725	Indica	NA	
maker-4-augustus-gene-218.10-mRNA-1	4	OsR498G0409008100.01.T01	97,21	0,00E+00	1707	Os04g0542900	97,31	0,00E+00	1711	4	NA	Japonica	
maker-4-augustus-gene-218.17-mRNA-1	4	OsR498G0409008700.01.T03	99,66	0,00E+00	4158	Os04g0543200	99,96	0,00E+00	4189	31	NA	Japonica	
maker-4-augustus-gene-218.19-mRNA-1	4	OsR498G0409006200.01.T01	99,45	0,00E+00	2916	Os04g0542200	99,94	0,00E+00	2952	36	NA	Japonica	
maker-4-augustus-gene-218.23-mRNA-1	4	OsR498G0409008000.01.T03	99,91	0,00E+00	3865	Os04g0542800	100,00	0,00E+00	3658	-207	NA	Japonica	
maker-4-augustus-gene-218.4-mRNA-1	4	OsR498G0409002900.01.T01	100,00	0,00E+00	1499	Os04g0541700	100,00	0,00E+00	1424	-75	NA	Japonica	
maker-4-augustus-gene-218.6-mRNA-1	4	OsR498G0409004300.01.T01	99,90	0,00E+00	1722	Os04g0541900	100,00	0,00E+00	1368	-354	Indica	Japonica	
maker-4-augustus-gene-219.36-mRNA-1	4	OsR498G0409009900.01.T05	99,84	0,00E+00	4511	Os04g0543700	100,00	0,00E+00	4415	-96	NA	Japonica	
maker-4-augustus-gene-219.40-mRNA-1	4	OsR498G0409010500.01.T01	100,00	0,00E+00	2230	Os02g0650900	83,02	0,00E+00	1278	-952	Indica	Japonica	
maker-4-augustus-gene-219.45-mRNA-1	4	OsR498G0409009600.01.T02	99,76	0,00E+00	3023	Os04g0543600	100,00	0,00E+00	2392	-631	Indica	Japonica	
maker-4-augustus-gene-219.49-mRNA-1	4	OsR498G0409012200.01.T01	100,00	0,00E+00	1153	Os04g0544200	100,00	0,00E+00	1153	0	NA	Japonica	
maker-4-augustus-gene-219.51-mRNA-1	4	OsR498G0409012400.01.T01	99,81	0,00E+00	2757	Os04g0544400	100,00	0,00E+00	2693	-64	NA	Japonica	
maker-4-augustus-gene-219.54-mRNA-1	4	OsR498G0409012600.01.T01	99,62	0,00E+00	2346	Os04g0544500	100,00	0,00E+00	2369	23	NA	Japonica	
maker-4-augustus-gene-219.62-mRNA-1	4	OsR498G0409013800.01.T01	92,96	0,00E+00	1384	Os04g0545000	100,00	0,00E+00	1359	-25	NA	Japonica	
maker-4-augustus-gene-22.41-mRNA-1	4	OsR498G0407720100.01.T01	98,33	0,00E+00	1764	Os04g0203500	96,38	0,00E+00	1680	-84	NA	Japonica	
maker-4-augustus-gene-22.46-mRNA-1	4	OsR498G0407717000.01.T02	96,73	0,00E+00	2511	Os02g0101000	71,68	0,00E+00	867	-1644	Indica	Indica	
maker-4-augustus-gene-22.52-mRNA-1	4	OsR498G0407717600.01.T01	99,92	0,00E+00	4331	Os04g0201900	99,02	0,00E+00	4864	533	Japonica	NA	Kinase-TM
maker-4-augustus-gene-22.56-mRNA-1	4	OsR498G0407721000.01.T01	98,63	0,00E+00	2289	Os04g0206450	98,73	0,00E+00	1927	-362	Indica	Japonica	
maker-4-augustus-gene-22.58-mRNA-1	4	OsR498G0407721400.01.T01	99,71	0,00E+00	2488	Os04g0206500	99,71	0,00E+00	2488	0	NA	Japonica	
maker-4-augustus-gene-22.60-mRNA-1	4	OsR498G0407721600.01.T01	99,50	0,00E+00	2472	Os04g0206600	99,71	0,00E+00	2486	14	NA	Japonica	
maker-4-augustus-gene-220.0-mRNA-1	4	OsR498G0409018300.01.T01	99,71	0,00E+00	3721	Os04g0546300	100,00	0,00E+00	3745	24	NA	Japonica	Kinase-TM
maker-4-augustus-gene-220.13-mRNA-1	4	OsR498G0409015800.01.T01	99,72	0,00E+00	2553	Os04g0545200	100,00	0,00E+00	1934	-619	Indica	Japonica	
maker-4-augustus-gene-220.17-mRNA-1	4	OsR498G0409016800.01.T01	99,82	0,00E+00	1003	Os04g0545400	100,00	0,00E+00	1007	4	NA	Japonica	
maker-4-augustus-gene-220.19-mRNA-1	4	OsR498G0409017200.01.T01	99,65	0,00E+00	1031	Os04g0545600	100,00	0,00E+00	1040	9	NA	Japonica	
maker-4-augustus-gene-220.2-mRNA-1	4	OsR498G0409018500.01.T03	100,00	0,00E+00	1213	Os04g0546400	100,00	4,09E-178	623	-590	Indica	Japonica	
maker-4-augustus-gene-220.21-mRNA-1	4	OsR498G0409017400.01.T01	99,74	0,00E+00	2065	Os04g0545700	100,00	0,00E+00	2078	13	NA	Japonica	
maker-4-augustus-gene-220.9-mRNA-1	4	OsR498G0409015600.01.T01	100,00	0,00E+00	1090	Os04g0545100	100,00	0,00E+00	1090	0	NA	Japonica	
maker-4-augustus-gene-221.40-mRNA-1	4	OsR498G0409023700.01.T02	96,30	0,00E+00	1843	Os04g0547900	96,48	0,00E+00	1852	9	NA	Japonica	
maker-4-augustus-gene-221.45-mRNA-1	4	OsR498G0409023900.01.T01	99,68	0,00E+00	1117	Os04g0548000	100,00	0,00E+00	1126	9	NA	Japonica	
maker-4-augustus-gene-221.58-mRNA-1	4	OsR498G0409027900.01.T01	100,00	7,59E-157	553	Os04g0549400	100,00	2,64E-157	553	0	NA	Japonica	
maker-4-augustus-gene-221.65-mRNA-1	4	OsR498G0409024600.01.T01	100,00	0,00E+00	1148	Os02g0655800	79,67	0,00E+00	803	-345	Indica	Japonica	Kinase-TM
maker-4-augustus-gene-221.67-mRNA-1	4	OsR498G0409026000.01.T01	99,73	0,00E+00	1333	Os04g0548700	100,00	0,00E+00	1342	9	NA	Japonica	
maker-4-augustus-gene-221.69-mRNA-1	4	OsR498G0409027800.01.T01	99,61	0,00E+00	2284	Os04g0549300	100,00	0,00E+00	2100	-184	NA	Japonica	
maker-4-augustus-gene-222.0-mRNA-1	4	OsR498G0409029500.01.T01	99,84	0,00E+00	2248	Os04g0549600	100,00	0,00E+00	2257	9	NA	Japonica	
maker-4-augustus-gene-222.14-mRNA-1	4	OsR498G0409034600.01.T01	99,73	0,00E+00	2005	Os04g0550700	100,00	0,00E+00	2019	14	NA	Japonica	
maker-4-augustus-gene-222.17-mRNA-1	4	OsR498G0409035200.01.T01	97,36	0,00E+00	1081	Os04g0550950	100,00	0,00E+00	1353	272	Japonica	Japonica	
maker-4-augustus-gene-222.6-mRNA-1	4	OsR498G0409034300.01.T01	99,90	0,00E+00	1711	Os04g0550600	100,00	0,00E+00	1716	5	NA	Japonica	
maker-4-augustus-gene-223.38-mRNA-1	4	OsR498G0409036300.01.T01	99,62	0,00E+00	1893	Os04g0551200	100,00	0,00E+00	1911	18	NA	Japonica	
maker-4-augustus-gene-223.40-mRNA-1	4	OsR498G0409036900.01.T01	99,63	0,00E+00	1936	Os04g0551500	100,00	0,00E+00	1954	18	NA	Japonica	
maker-4-augustus-gene-223.48-mRNA-1	4	OsR498G0409040100.01.T01	99,87	0,00E+00	1430	Os04g0552000	99,87	0,00E+00	1430	0	NA	Japonica	
maker-4-augustus-gene-223.50-mRNA-1	4	OsR498G0409041300.01.T01	99,88	0,00E+00	1489	Os04g0552200	100,00	0,00E+00	1494	5	NA	Japonica	
maker-4-augustus-gene-223.54-mRNA-1	4	OsR498G0409036600.01.T04	94,93	0,00E+00	2384	Os04g0551300	92,00	0,00E+00	1356	-1028	Indica	Japonica	
maker-4-augustus-gene-223.59-mRNA-1	4	OsR498G0409038800.01.T01	99,73	0,00E+00	673	Os04g0551600	100,00	0,00E+00	677	4	NA	Japonica	
maker-4-augustus-gene-224.34-mRNA-1	4	OsR498G0409048900.01.T01	100,00	0,00E+00	1326	Os04g0554200	100,00	0,00E+00	1326	0	NA	Japonica	
maker-4-augustus-gene-224.37-mRNA-1	4	OsR498G0409053900.01.T07	99,90	0,00E+00	1791	Os04g0555400	100,00	0,00E+00	1732	-59	NA	Japonica	
maker-4-augustus-gene-224.42-mRNA-1	4	OsR498G0409047200.01.T02	99,91	0,00E+00	1911	Os04g0553500	100,00	0,00E+00	1086	-825	Indica	Japonica	
maker-4-augustus-gene-224.44-mRNA-1	4	OsR498G0409048200.01.T01	99,44	0,00E+00	956	Os04g0553800	99,50	0,00E+00	711	-245	Indica	Japonica	
maker-4-augustus-gene-224.46-mRNA-1	4	OsR498G0409048600.01.T01	99,64	0,00E+00	1990	Os02g0661100	76,57	5,29E-173	608	-1382	Indica	Japonica	
maker-4-augustus-gene-224.50-mRNA-1	4	OsR498G0409053200.01.T03	99,42	0,00E+00	2752	Os04g0555300	100,00	0,00E+00	2792	40	NA	Japonica	
maker-4-augustus-gene-225.0-mRNA-1	4	OsR498G0409055700.01.T01	99,87	0,00E+00	5407	Os04g0556000	99,92	0,00E+00	4590	-817	Indica	Japonica	
maker-4-augustus-gene-225.18-mRNA-1	4	OsR498G0409054800.01.T01	100,00	0,00E+00	755	Os04g0555700	100,00	0,00E+00	755	0	NA	Japonica	
maker-4-augustus-gene-225.20-mRNA-1	4	OsR498G0409055000.01.T01	99,76	0,00E+00	1496	Os04g0555800	100,00	0,00E+00	1505	9	NA	Japonica	
maker-4-augustus-gene-225.23-mRNA-1	4	OsR498G0409055200.01.T02	99,80	0,00E+00	1831	Os04g0555900	100,00	0,00E+00	1840	9	NA	Japonica	
maker-4-augustus-gene-225.28-mRNA-1	4	OsR498G0409060800.01.T02	97,54	0,00E+00	2635	Os04g0557200	98,96	0,00E+00	2722	87	NA	Japonica	
maker-4-augustus-gene-225.3-mRNA-1	4	OsR498G0409056100.01.T01	99,78	0,00E+00	824	Os04g0556300	100,00	0,00E+00	829	5	NA	Japonica	
maker-4-augustus-gene-226.16-mRNA-1	4	OsR498G0409062200.01.T01	97,81	0,00E+00	2731	Os04g0557800	98,25	0,00E+00	2763	32	NA	NA	
maker-4-augustus-gene-226.19-mRNA-1	4	OsR498G0409062800.01.T01	99,79	0,00E+00	835	Os04g0558500	100,00	1,04E-126	452	-383	Indica	NA	
maker-4-augustus-gene-226.7-mRNA-1	4	OsR498G0409061000.01.T01	98,20	0,00E+00	1435	Os04g0557500	100,00	0,00E+00	1499	64	NA	Japonica	
maker-4-augustus-gene-227.0-mRNA-1	4	OsR498G0409063900.01.T01	99,86	0,00E+00	2648	Os04g0559100	100,00	0,00E+00	2657	9	NA	NA	
maker-4-augustus-gene-227.18-mRNA-1	4	OsR498G0409068300.01.T01	100,00	2,15E-144	511	Os04g0560700	100,00	7,47E-145	511	0	NA	NA	
maker-4-augustus-gene-227.20-mRNA-1	4	OsR498G0409068700.01.T01	99,82	0,00E+00	5037	Os04g0561000	100,00	0,00E+00	4186	-851	Indica	NA	
maker-4-augustus-gene-227.23-mRNA-1	4	OsR498G0409064200.01.T01	99,77	0,00E+00	4653	Os04g0559200	100,00	0,00E+00	4681	28	NA	NA	
maker-4-augustus-gene-227.25-mRNA-1	4	OsR498G0409065000.01.T01	97,97	0,00E+00	2127	Os04g0559400	98,22	0,00E+00	2140	13	NA	NA	
maker-4-augustus-gene-227.29-mRNA-1	4	OsR498G0409066400.01.T05	99,78	0,00E+00	2458	Os04g0559800	100,00	0,00E+00	2471	13	NA	NA	Kinase
maker-4-augustus-gene-227.34-mRNA-1	4	OsR498G0409067400.01.T01	99,88	0,00E+00	4348	Os04g0560300	99,92	0,00E+00	4352	4	NA	NA	
maker-4-augustus-gene-227.38-mRNA-1	4	OsR498G0409068000.01.T01	100,00	0,00E+00	2890	Os04g0560600	100,00	0,00E+00	2890	0	NA	NA	Kinase-TM
maker-4-augustus-gene-227.42-mRNA-1	4	OsR498G0409069000.01.T01	99,68	0,00E+00	1106	Os04g0561200	100,00	0,00E+00	1229	123	Japonica	NA	
maker-4-augustus-gene-227.5-mRNA-1	4	OsR498G0409066100.01.T01	99,89	0,00E+00	1560	Os04g0559700	99,22	0,00E+00	1355	-205	Indica	NA	
maker-4-augustus-gene-227.8-mRNA-1	4	OsR498G0409067100.01.T01	99,80	0,00E+00	916	Os04g0560200	100,00	0,00E+00	704	-212	Indica	NA	
maker-4-augustus-gene-228.38-mRNA-1	4	OsR498G0409069500.01.T04	99,91	0,00E+00	3995	Os04g0561500	100,00	0,00E+00	4004	9	NA	NA	
maker-4-augustus-gene-228.43-mRNA-1	4	OsR498G0409069700.01.T04	100,00	0,00E+00	1294	Os04g0561600	99,86	0,00E+00	1248	-46	NA	NA	
maker-4-augustus-gene-228.45-mRNA-1	4	OsR498G0409070900.01.T05	99,90	0,00E+00	1879	Os04g0562000	100,00	0,00E+00	1884	5	NA	NA	
maker-4-augustus-gene-228.51-mRNA-1	4	OsR498G0409509300.01.T01	99,49	0,00E+00	1406	Os05g0269800	92,13	0,00E+00	2299	893	Japonica	NA	
maker-4-augustus-gene-228.62-mRNA-1	4	OsR498G0409070000.01.T03	99,59	0,00E+00	1730	Os04g0561700	100,00	0,00E+00	1748	18	NA	NA	
maker-4-augustus-gene-228.71-mRNA-1	4	OsR498G0409071200.01.T02	98,36	0,00E+00	1591	Os04g0562100	99,89	0,00E+00	1648	57	NA	NA	
maker-4-augustus-gene-228.77-mRNA-1	4	OsR498G0409074600.01.T02	100,00	0,00E+00	1011	Os02g0670000	73,48	1,12E-91	337	-674	Indica	NA	
maker-4-augustus-gene-229.25-mRNA-1	4	OsR498G1222106000.01.T03	97,13	2,32E-113	409	Os04g0564300	90,46	1,36E-104	379	-30	NA	NA	
maker-4-augustus-gene-229.26-mRNA-1	4	OsR498G0409079100.01.T03	99,59	2,06E-120	433	Os04g0564200	100,00	6,70E-172	601	168	Japonica	NA	
maker-4-augustus-gene-229.39-mRNA-1	4	OsR498G0409079700.01.T04	100,00	0,00E+00	801	Os04g0564600	100,00	0,00E+00	706	-95	Indica	NA	
maker-4-augustus-gene-229.58-mRNA-1	4	OsR498G0409078000.01.T01	97,76	0,00E+00	2455	Os04g0563900	100,00	0,00E+00	2486	31	NA	NA	Kinase-TM
maker-4-augustus-gene-229.60-mRNA-1	4	OsR498G0409078800.01.T01	99,90	0,00E+00	1836	Os04g0564000	100,00	0,00E+00	1840	4	NA	NA	
maker-4-augustus-gene-23.32-mRNA-1	4	OsR498G0407728500.01.T01	99,91	0,00E+00	1890	Os04g0208200	100,00	0,00E+00	1083	-807	Indica	NA	
maker-4-augustus-gene-23.34-mRNA-1	4	OsR498G0407727000.01.T01	99,71	0,00E+00	1827	Os04g0203500	86,68	0,00E+00	1232	-595	Indica	NA	
maker-4-augustus-gene-23.36-mRNA-1	4	OsR498G0407728800.01.T01	99,87	0,00E+00	2827	Os04g0208400	99,87	0,00E+00	2827	0	NA	NA	
maker-4-augustus-gene-23.38-mRNA-1	4	OsR498G0407729800.01.T01	99,94	0,00E+00	2810	Os04g0208500	99,87	0,00E+00	2805	-5	NA	Japonica	
maker-4-augustus-gene-23.40-mRNA-1	4	OsR498G0407730400.01.T01	95,21	0,00E+00	2507	Os04g0208600	94,73	0,00E+00	2499	-8	NA	NA	
maker-4-augustus-gene-230.35-mRNA-1	4	OsR498G0409082100.01.T02	99,71	0,00E+00	2491	Os04g0565400	99,93	0,00E+00	2505	14	NA	NA	
maker-4-augustus-gene-230.38-mRNA-1	4	OsR498G0409083900.01.T05	99,81	0,00E+00	1876	Os04g0566100	96,67	0,00E+00	1816	-60	NA	NA	
maker-4-augustus-gene-230.43-mRNA-1	4	OsR498G0409080000.01.T02	98,18	0,00E+00	5424	Os04g0564700	97,20	0,00E+00	3266	-2158	Indica	NA	
maker-4-augustus-gene-230.51-mRNA-1	4	OsR498G0409083600.01.T03	99,44	0,00E+00	2234	Os04g0565900	100,00	0,00E+00	2268	34	NA	NA	
maker-4-augustus-gene-231.18-mRNA-1	4	OsR498G0409089300.01.T01	99,57	0,00E+00	1647	Os04g0566600	100,00	0,00E+00	1662	15	NA	NA	
maker-4-augustus-gene-231.37-mRNA-1	4	OsR498G0409090800.01.T01	99,16	0,00E+00	2738	Os04g0567200	100,00	0,00E+00	2782	44	NA	NA	
maker-4-augustus-gene-232.28-mRNA-1	4	OsR498G0409092500.01.T01	99,79	0,00E+00	851	Os04g0567700	100,00	0,00E+00	856	5	NA	NA	
maker-4-augustus-gene-232.31-mRNA-1	4	OsR498G0409097500.01.T01	99,01	0,00E+00	3719	Os04g0568400	100,00	0,00E+00	3782	63	NA	NA	
maker-4-augustus-gene-232.35-mRNA-1	4	OsR498G0409098300.01.T01	99,35	0,00E+00	1631	Os04g0568700	100,00	0,00E+00	1656	25	NA	NA	
maker-4-augustus-gene-232.37-mRNA-1	4	OsR498G0409099500.01.T01	100,00	0,00E+00	1819	Os04g0569000	99,60	0,00E+00	1801	-18	NA	NA	
maker-4-augustus-gene-232.42-mRNA-1	4	OsR498G0409099000.01.T01	99,91	0,00E+00	2058	Os04g0568900	100,00	0,00E+00	1451	-607	Indica	NA	
maker-4-augustus-gene-233.25-mRNA-1	4	OsR498G0409100900.01.T01	99,81	0,00E+00	1847	Os04g0569300	100,00	0,00E+00	1856	9	NA	Japonica	
maker-4-augustus-gene-233.27-mRNA-1	4	OsR498G0409099800.01.T02	98,42	0,00E+00	2756	Os04g0569100	98,83	0,00E+00	2701	-55	NA	NA	
maker-4-augustus-gene-233.29-mRNA-1	4	OsR498G0409101200.01.T02	99,88	0,00E+00	1553	Os04g0569400	100,00	0,00E+00	1828	275	Japonica	Japonica	
maker-4-augustus-gene-233.33-mRNA-1	4	OsR498G0409102000.01.T01	99,86	0,00E+00	2507	Os04g0569900	100,00	0,00E+00	2516	9	NA	Japonica	
maker-4-augustus-gene-233.36-mRNA-1	4	OsR498G0409102800.01.T01	99,61	0,00E+00	2760	Os04g0570000	100,00	0,00E+00	2787	27	NA	Japonica	
maker-4-augustus-gene-234.32-mRNA-1	4	OsR498G0409107500.01.T06	99,67	0,00E+00	1094	Os04g0571200	100,00	0,00E+00	1297	203	Japonica	Japonica	
maker-4-augustus-gene-234.37-mRNA-1	4	OsR498G0409108100.01.T03	99,59	0,00E+00	874	Os04g0571300	100,00	0,00E+00	881	7	NA	Japonica	
maker-4-augustus-gene-234.38-mRNA-1	4	OsR498G0409111700.01.T01	99,31	2,67E-144	511	Os04g0571800	99,65	7,63E-146	516	5	NA	Japonica	
maker-4-augustus-gene-234.42-mRNA-1	4	OsR498G0409104200.01.T01	99,72	0,00E+00	2596	Os04g0570500	100,00	0,00E+00	2614	18	NA	Japonica	
maker-4-augustus-gene-234.44-mRNA-1	4	OsR498G0409105000.01.T02	100,00	0,00E+00	2257	Os04g0570600	100,00	0,00E+00	2257	0	NA	Japonica	
maker-4-augustus-gene-234.50-mRNA-1	4	OsR498G0409105200.01.T02	99,95	0,00E+00	3497	Os04g0570800	100,00	0,00E+00	3501	4	NA	Japonica	
maker-4-augustus-gene-235.24-mRNA-1	4	OsR498G0204522900.01.T02	75,27	2,10E-29	129	Os04g0572700	100,00	8,94E-86	315	186	Japonica	Japonica	
maker-4-augustus-gene-235.26-mRNA-1	4	OsR498G0409117500.01.T03	99,90	0,00E+00	3763	Os04g0573000	100,00	0,00E+00	3772	9	NA	Japonica	
maker-4-augustus-gene-235.31-mRNA-1	4	OsR498G0409117700.01.T01	99,88	0,00E+00	3074	Os04g0573100	100,00	0,00E+00	3083	9	NA	Japonica	
maker-4-augustus-gene-235.33-mRNA-1	4	OsR498G0409117900.01.T03	99,58	0,00E+00	1696	Os04g0573200	99,05	0,00E+00	1673	-23	NA	Japonica	CCSD
maker-4-augustus-gene-235.38-mRNA-1	4	OsR498G0409119100.01.T01	99,87	0,00E+00	2735	Os04g0573900	99,93	0,00E+00	2739	4	NA	Japonica	
maker-4-augustus-gene-236.0-mRNA-1	4	OsR498G0409122100.01.T01	99,16	0,00E+00	1260	Os04g0574500	100,00	0,00E+00	785	-475	Indica	Japonica	
maker-4-augustus-gene-236.8-mRNA-1	4	OsR498G0409122800.01.T01	93,84	0,00E+00	1786	Os04g0574800	100,00	0,00E+00	1483	-303	Indica	Japonica	
maker-4-augustus-gene-237.31-mRNA-1	4	OsR498G0409125200.01.T02	99,44	0,00E+00	1265	Os04g0576500	100,00	0,00E+00	1283	18	NA	Japonica	
maker-4-augustus-gene-237.34-mRNA-1	4	OsR498G0409127700.01.T03	99,45	0,00E+00	1614	Os04g0576700	100,00	0,00E+00	1636	22	NA	Japonica	
maker-4-augustus-gene-237.40-mRNA-1	4	OsR498G0409128100.01.T02	99,81	0,00E+00	1912	Os04g0576800	100,00	0,00E+00	1921	9	NA	NA	
maker-4-augustus-gene-238.0-mRNA-1	4	OsR498G0409130700.01.T01	99,65	0,00E+00	2575	Os04g0577200	100,00	0,00E+00	2598	23	NA	Japonica	
maker-4-augustus-gene-238.16-mRNA-1	4	OsR498G0409136500.01.T03	99,75	0,00E+00	2823	Os04g0578700	99,87	0,00E+00	2832	9	NA	Japonica	
maker-4-augustus-gene-238.22-mRNA-1	4	OsR498G0409131600.01.T03	99,79	0,00E+00	1680	Os04g0577500	100,00	0,00E+00	1689	9	NA	Japonica	
maker-4-augustus-gene-238.25-mRNA-1	4	OsR498G0409132600.01.T01	99,59	0,00E+00	863	Os04g0577800	100,00	0,00E+00	872	9	NA	Japonica	
maker-4-augustus-gene-238.28-mRNA-1	4	OsR498G0409132800.01.T01	99,79	0,00E+00	2606	Os04g0578000	100,00	0,00E+00	2619	13	NA	Japonica	
maker-4-augustus-gene-238.3-mRNA-1	4	OsR498G0409131100.01.T01	99,29	0,00E+00	1492	Os04g0577300	100,00	0,00E+00	2333	841	Japonica	Japonica	
maker-4-augustus-gene-238.30-mRNA-1	4	OsR498G0409136200.01.T03	97,47	0,00E+00	3506	Os04g0578600	99,17	0,00E+00	2746	-760	Indica	Japonica	
maker-4-augustus-gene-238.34-mRNA-1	4	OsR498G0409136800.01.T01	99,87	0,00E+00	2832	Os04g0578800	100,00	0,00E+00	2841	9	NA	Japonica	
maker-4-augustus-gene-238.6-mRNA-1	4	OsR498G0409133900.01.T04	99,71	0,00E+00	1214	Os04g0578200	99,78	0,00E+00	799	-415	Indica	Japonica	
maker-4-augustus-gene-239.0-mRNA-1	4	OsR498G0409141300.01.T01	99,94	0,00E+00	3237	Os04g0579700	100,00	0,00E+00	2890	-347	Indica	Japonica	
maker-4-augustus-gene-239.6-mRNA-1	4	OsR498G0409144200.01.T01	100,00	0,00E+00	872	Os04g0580400	98,94	0,00E+00	664	-208	Indica	Japonica	
maker-4-augustus-gene-24.30-mRNA-1	4	OsR498G0407734600.01.T02	99,91	0,00E+00	5841	Os04g0209300	99,94	0,00E+00	5846	5	NA	Japonica	
maker-4-augustus-gene-240.0-mRNA-1	4	OsR498G0409145300.01.T05	99,87	0,00E+00	1376	Os04g0580700	100,00	0,00E+00	1380	4	NA	Japonica	
maker-4-augustus-gene-240.11-mRNA-1	4	OsR498G0204538000.01.T01	84,06	8,88E-73	274	Os04g0581300	100,00	5,98E-133	471	197	Japonica	Japonica	
maker-4-augustus-gene-240.5-mRNA-1	4	OsR498G0409147310.01.T01	99,47	0,00E+00	1010	Os04g0581000	99,90	0,00E+00	1841	831	Japonica	Japonica	
maker-4-augustus-gene-240.9-mRNA-1	4	OsR498G0409147600.01.T03	99,53	0,00E+00	1888	Os04g0581100	100,00	0,00E+00	1911	23	NA	Japonica	
maker-4-augustus-gene-241.41-mRNA-1	4	OsR498G0409162700.01.T01	100,00	0,00E+00	1397	Os04g0583500	100,00	0,00E+00	1397	0	NA	Japonica	
maker-4-augustus-gene-241.44-mRNA-1	4	OsR498G0409163700.01.T06	99,82	0,00E+00	4862	Os04g0583700	99,96	0,00E+00	4829	-33	NA	Japonica	
maker-4-augustus-gene-241.58-mRNA-1	4	OsR498G0409155200.01.T01	100,00	0,00E+00	666	Os04g0581800	100,00	0,00E+00	666	0	NA	Japonica	
maker-4-augustus-gene-241.63-mRNA-1	4	OsR498G0409156200.01.T02	99,10	0,00E+00	3131	Os04g0582600	99,03	0,00E+00	3831	700	Japonica	Japonica	
maker-4-augustus-gene-241.72-mRNA-1	4	OsR498G0409160000.01.T01	99,75	0,00E+00	727	Os04g0582900	100,00	0,00E+00	720	-7	NA	Japonica	
maker-4-augustus-gene-241.74-mRNA-1	4	OsR498G0409161000.01.T02	99,82	0,00E+00	981	Os04g0583200	99,19	0,00E+00	1089	108	NA	Japonica	
maker-4-augustus-gene-242.11-mRNA-1	4	OsR498G0409167300.01.T01	99,84	0,00E+00	2259	Os04g0584800	100,00	0,00E+00	2268	9	NA	Japonica	
maker-4-augustus-gene-242.14-mRNA-1	4	OsR498G0409164400.01.T02	99,64	0,00E+00	2489	Os04g0583900	100,00	0,00E+00	2511	22	NA	Japonica	
maker-4-augustus-gene-242.17-mRNA-1	4	OsR498G0409164800.01.T01	99,85	0,00E+00	3531	Os04g0584050	100,00	0,00E+00	1824	-1707	Indica	Japonica	Kinase-TM
maker-4-augustus-gene-242.19-mRNA-1	4	OsR498G0409165000.01.T02	99,91	0,00E+00	2101	Os02g0685600	78,84	0,00E+00	985	-1116	Indica	Japonica	
maker-4-augustus-gene-242.21-mRNA-1	4	OsR498G0409167600.01.T01	99,79	0,00E+00	1723	Os04g0584900	100,00	0,00E+00	1732	9	NA	Japonica	
maker-4-augustus-gene-242.24-mRNA-1	4	OsR498G0409169200.01.T02	99,73	0,00E+00	2616	Os04g0585100	100,00	0,00E+00	2634	18	NA	Japonica	
maker-4-augustus-gene-242.4-mRNA-1	4	OsR498G0409166900.01.T02	99,88	0,00E+00	2978	Os04g0584600	100,00	0,00E+00	2527	-451	Indica	Japonica	Kinase-TM
maker-4-augustus-gene-242.41-mRNA-1	4	OsR498G0409169800.01.T01	99,57	0,00E+00	1248	Os04g0585400	100,00	0,00E+00	1056	-192	Indica	Japonica	
maker-4-augustus-gene-242.45-mRNA-1	4	OsR498G0409170000.01.T01	95,50	0,00E+00	1236	Os04g0585600	95,64	0,00E+00	1241	5	NA	Japonica	
maker-4-augustus-gene-242.47-mRNA-1	4	OsR498G0409170200.01.T02	98,90	0,00E+00	793	Os04g0585700	99,78	0,00E+00	813	20	NA	Japonica	
maker-4-augustus-gene-243.46-mRNA-1	4	OsR498G0409177900.01.T01	99,82	0,00E+00	2031	Os04g0587300	100,00	0,00E+00	2040	9	NA	Japonica	
maker-4-augustus-gene-243.48-mRNA-1	4	OsR498G0409178100.01.T01	99,40	0,00E+00	2349	Os04g0587400	100,00	0,00E+00	2214	-135	NA	Japonica	
maker-4-augustus-gene-243.50-mRNA-1	4	OsR498G0409171800.01.T01	96,39	2,43E-170	598	Os04g0585900	100,00	6,08E-141	499	-99	Indica	Japonica	
maker-4-augustus-gene-243.52-mRNA-1	4	OsR498G0409172200.01.T01	100,00	0,00E+00	693	Os04g0586000	100,00	0,00E+00	693	0	NA	Japonica	
maker-4-augustus-gene-244.11-mRNA-1	4	OsR498G0409182300.01.T01	99,77	0,00E+00	7096	Os04g0588700	99,78	0,00E+00	7177	81	NA	Japonica	
maker-4-augustus-gene-244.15-mRNA-1	4	OsR498G0409183700.01.T02	99,28	4,58E-64	245	Os04g0589200	100,00	3,77E-66	250	5	NA	Japonica	
maker-4-augustus-gene-244.18-mRNA-1	4	OsR498G0409185700.01.T01	99,76	0,00E+00	2254	Os04g0589600	99,92	0,00E+00	2263	9	NA	Japonica	
maker-4-augustus-gene-244.21-mRNA-1	4	OsR498G0409187300.01.T01	99,87	0,00E+00	1343	Os04g0590100	100,00	0,00E+00	1348	5	NA	Japonica	
maker-4-augustus-gene-244.27-mRNA-1	4	OsR498G0409186400.01.T04	99,27	0,00E+00	964	Os04g0589900	100,00	0,00E+00	982	18	NA	Japonica	
maker-4-augustus-gene-245.5-mRNA-1	4	OsR498G0409190600.01.T02	99,20	0,00E+00	1547	Os04g0591000	100,00	0,00E+00	1581	34	NA	Japonica	
maker-4-augustus-gene-245.8-mRNA-1	4	OsR498G0409191200.01.T01	100,00	0,00E+00	1252	Os04g0591100	100,00	0,00E+00	1252	0	NA	Japonica	
maker-4-augustus-gene-246.51-mRNA-1	4	OsR498G0409196100.01.T01	99,13	0,00E+00	4048	Os04g0592400	100,00	0,00E+00	4129	81	NA	Japonica	
maker-4-augustus-gene-246.53-mRNA-1	4	OsR498G0409198500.01.T01	99,38	0,00E+00	864	Os04g0593400	99,79	0,00E+00	873	9	NA	Japonica	
maker-4-augustus-gene-246.55-mRNA-1	4	OsR498G0409198700.01.T01	99,23	0,00E+00	913	Os04g0593500	99,81	0,00E+00	927	14	NA	Japonica	
maker-4-augustus-gene-246.61-mRNA-1	4	OsR498G0409199100.01.T01	98,38	0,00E+00	2462	Os04g0593700	98,23	0,00E+00	2451	-11	NA	Japonica	
maker-4-augustus-gene-246.65-mRNA-1	4	OsR498G0409199100.01.T01	86,75	0,00E+00	1409	Os04g0593800	98,66	0,00E+00	1572	163	Japonica	Japonica	
maker-4-augustus-gene-246.67-mRNA-1	4	OsR498G0409196400.01.T02	99,31	0,00E+00	2055	Os04g0592500	99,59	0,00E+00	1309	-746	Indica	Japonica	
maker-4-augustus-gene-246.72-mRNA-1	4	OsR498G0409196800.01.T01	99,74	0,00E+00	1371	Os04g0592600	100,00	0,00E+00	1662	291	Japonica	Japonica	
maker-4-augustus-gene-246.76-mRNA-1	4	OsR498G0409197600.01.T02	99,81	0,00E+00	4804	Os04g0592700	100,00	0,00E+00	4827	23	NA	Japonica	
maker-4-augustus-gene-246.81-mRNA-1	4	OsR498G0409198400.01.T01	100,00	0,00E+00	1846	Os04g0593200	100,00	0,00E+00	1355	-491	Indica	Japonica	
maker-4-augustus-gene-246.85-mRNA-1	4	OsR498G0409200000.01.T02	92,70	0,00E+00	2847	Os04g0594000	97,57	0,00E+00	2515	-332	Indica	Japonica	
maker-4-augustus-gene-246.90-mRNA-1	4	OsR498G0409200600.01.T01	99,64	0,00E+00	1994	Os04g0594100	100,00	0,00E+00	2013	19	NA	Japonica	
maker-4-augustus-gene-247.36-mRNA-1	4	OsR498G0409203000.01.T01	100,00	0,00E+00	1721	Os04g0594400	100,00	0,00E+00	1696	-25	NA	Japonica	
maker-4-augustus-gene-247.40-mRNA-1	4	OsR498G0409203400.01.T01	99,40	0,00E+00	1781	Os04g0594500	100,00	0,00E+00	1663	-118	NA	Japonica	
maker-4-augustus-gene-247.44-mRNA-1	4	OsR498G0409205000.01.T01	100,00	0,00E+00	1943	Os04g0595100	100,00	0,00E+00	1943	0	NA	Japonica	
maker-4-augustus-gene-247.48-mRNA-1	4	OsR498G0409205200.01.T01	91,86	0,00E+00	764	Os04g0595200	92,05	0,00E+00	768	4	NA	Japonica	
maker-4-augustus-gene-247.53-mRNA-1	4	OsR498G0100054400.01.T01	97,26	0,00E+00	3436	Os04g0595300	100,00	0,00E+00	2699	-737	Indica	Japonica	
maker-4-augustus-gene-247.60-mRNA-1	4	OsR498G0409206600.01.T01	98,95	0,00E+00	2003	Os04g0595700	100,00	0,00E+00	2181	178	NA	Japonica	
maker-4-augustus-gene-247.62-mRNA-1	4	OsR498G0409206800.01.T01	99,62	0,00E+00	1412	Os04g0595800	100,00	0,00E+00	1425	13	NA	Japonica	
maker-4-augustus-gene-248.29-mRNA-1	4	OsR498G0409209500.01.T01	99,72	0,00E+00	3186	Os04g0597400	100,00	0,00E+00	3086	-100	NA	Japonica	
maker-4-augustus-gene-248.42-mRNA-1	4	OsR498G0409215700.01.T01	94,54	0,00E+00	3703	Os04g0598800	99,95	0,00E+00	3949	246	NA	Japonica	Kinase-TM
maker-4-augustus-gene-248.45-mRNA-1	4	OsR498G0409211600.01.T01	99,17	0,00E+00	636	Os04g0598000	100,00	0,00E+00	650	14	NA	Japonica	
maker-4-augustus-gene-248.48-mRNA-1	4	OsR498G0409213000.01.T01	99,06	0,00E+00	2061	Os04g0598300	99,74	0,00E+00	2097	36	NA	Japonica	
maker-4-augustus-gene-248.50-mRNA-1	4	OsR498G0409216600.01.T02	99,91	0,00E+00	2038	Os04g0599000	100,00	0,00E+00	2042	4	NA	Japonica	Kinase-TM
maker-4-augustus-gene-249.0-mRNA-1	4	OsR498G0409217700.01.T01	99,41	0,00E+00	1797	Os04g0599400	100,00	0,00E+00	1824	27	NA	Japonica	
maker-4-augustus-gene-249.10-mRNA-1	4	OsR498G0409218700.01.T01	99,92	0,00E+00	2225	Os04g0599700	100,00	0,00E+00	2230	5	NA	NA	
maker-4-augustus-gene-249.14-mRNA-1	4	OsR498G0409218900.01.T01	100,00	0,00E+00	2571	Os04g0599800	100,00	0,00E+00	2300	-271	Indica	Japonica	
maker-4-augustus-gene-249.18-mRNA-1	4	OsR498G0409219300.01.T01	100,00	0,00E+00	3220	Os04g0599900	100,00	0,00E+00	3220	0	NA	Japonica	
maker-4-augustus-gene-249.23-mRNA-1	4	OsR498G0409220100.01.T02	100,00	0,00E+00	756	Os02g0700300	71,66	1,93E-55	216	-540	Indica	Japonica	
maker-4-augustus-gene-249.28-mRNA-1	4	OsR498G0409217400.01.T02	99,79	0,00E+00	2503	Os04g0599300	100,00	0,00E+00	2516	13	NA	Japonica	
maker-4-augustus-gene-249.32-mRNA-1	4	OsR498G0409220400.01.T05	99,11	0,00E+00	1782	Os04g0600200	100,00	0,00E+00	1802	20	NA	Japonica	
maker-4-augustus-gene-249.36-mRNA-1	4	OsR498G0409220600.01.T01	100,00	0,00E+00	1819	Os04g0600300	100,00	0,00E+00	1819	0	NA	Japonica	
maker-4-augustus-gene-249.38-mRNA-1	4	OsR498G0409221000.01.T01	100,00	0,00E+00	1678	Os04g0600400	99,89	0,00E+00	1673	-5	NA	Indica	
maker-4-augustus-gene-249.41-mRNA-1	4	OsR498G0409221200.01.T03	99,76	0,00E+00	2232	Os02g0700700	72,13	0,00E+00	727	-1505	Indica	Indica	
maker-4-augustus-gene-249.50-mRNA-1	4	OsR498G0409222200.01.T03	98,64	0,00E+00	2046	Os02g0701300	75,46	0,00E+00	840	-1206	Indica	Japonica	
maker-4-augustus-gene-25.9-mRNA-1	4	OsR498G0407746300.01.T01	99,50	0,00E+00	711	Os04g0212200	90,61	0,00E+00	759	48	NA	Japonica	
maker-4-augustus-gene-250.0-mRNA-1	4	OsR498G0409223900.01.T01	99,92	0,00E+00	4634	Os04g0602000	91,15	0,00E+00	2019	-2615	Indica	Japonica	
maker-4-augustus-gene-250.13-mRNA-1	4	OsR498G0409226700.01.T01	100,00	0,00E+00	2527	Os04g0602500	100,00	0,00E+00	2527	0	NA	NA	
maker-4-augustus-gene-250.19-mRNA-1	4	OsR498G0409223600.01.T03	100,00	0,00E+00	937	Os04g0601400	100,00	0,00E+00	937	0	NA	Japonica	
maker-4-augustus-gene-250.22-mRNA-1	4	OsR498G0409223800.01.T02	100,00	0,00E+00	904	Os04g0601500	100,00	0,00E+00	1494	590	Japonica	Japonica	
maker-4-augustus-gene-250.26-mRNA-1	4	OsR498G0409224400.01.T03	99,40	9,49E-169	592	Os04g0601700	100,00	6,37E-172	601	9	NA	Japonica	
maker-4-augustus-gene-250.28-mRNA-1	4	OsR498G0409226200.01.T01	100,00	0,00E+00	2911	Os04g0602200	100,00	0,00E+00	1992	-919	Indica	Japonica	
maker-4-augustus-gene-250.3-mRNA-1	4	OsR498G0409224500.01.T01	99,70	0,00E+00	1204	Os06g0116600	84,79	3,36E-141	500	-704	Indica	Japonica	
maker-4-augustus-gene-250.34-mRNA-1	4	OsR498G0409226600.01.T03	99,73	0,00E+00	2020	Os04g0602400	99,73	0,00E+00	2020	0	NA	NA	
maker-4-augustus-gene-250.36-mRNA-1	4	OsR498G0409227200.01.T04	100,00	0,00E+00	921	Os04g0602700	100,00	0,00E+00	1155	234	Japonica	NA	
maker-4-augustus-gene-250.43-mRNA-1	4	OsR498G0409227400.01.T04	98,43	0,00E+00	4339	Os04g0602800	98,43	0,00E+00	4339	0	NA	NA	Kinase-TM
maker-4-augustus-gene-250.7-mRNA-1	4	OsR498G0409223900.01.T01	90,70	0,00E+00	2287	Os04g0602000	100,00	0,00E+00	3669	1382	Japonica	Japonica	
maker-4-augustus-gene-250.9-mRNA-1	4	OsR498G0409225900.01.T01	98,59	0,00E+00	1857	Os04g0602100	98,59	0,00E+00	1857	0	NA	Japonica	
maker-4-augustus-gene-251.44-mRNA-1	4	OsR498G0409228300.01.T05	100,00	0,00E+00	1382	Os04g0658000	74,11	4,66E-122	438	-944	Indica	NA	

maker-4-augustus-gene-251.46-mRNA-1	4	OsR498G0409232700.01.T01	100,00	0,00E+00	1597	Os04g0604800	96,50	0,00E+00	1466	-131	NA	NA	
maker-4-augustus-gene-251.49-mRNA-1	4	OsR498G0409228800.01.T05	100,00	0,00E+00	1781	Os04g0603200	100,00	0,00E+00	1757	-24	NA	NA	
maker-4-augustus-gene-251.54-mRNA-1	4	OsR498G0409230400.01.T02	97,99	0,00E+00	3088	Os04g0604000	98,15	0,00E+00	3102	14	NA	Japonica	
maker-4-augustus-gene-251.58-mRNA-1	4	OsR498G0409230600.01.T01	99,77	0,00E+00	1582	Os04g0604200	100,00	0,00E+00	1591	9	NA	Japonica	
maker-4-augustus-gene-251.61-mRNA-1	4	OsR498G0409231200.01.T01	98,25	0,00E+00	1481	Os04g0604300	100,00	0,00E+00	1521	40	NA	Japonica	
maker-4-augustus-gene-251.66-mRNA-1	4	OsR498G0409232200.01.T02	98,45	2,71E-158	558	Os04g0604600	100,00	0,00E+00	812	254	Japonica	Japonica	
maker-4-augustus-gene-251.68-mRNA-1	4	OsR498G0409233000.01.T01	99,90	0,00E+00	1738	Os04g0604900	100,00	0,00E+00	1743	5	NA	NA	
maker-4-augustus-gene-252.0-mRNA-1	4	OsR498G0409233900.01.T01	100,00	0,00E+00	1678	Os04g0605100	99,89	0,00E+00	1673	-5	NA	NA	
maker-4-augustus-gene-252.2-mRNA-1	4	OsR498G0409238500.01.T05	100,00	0,00E+00	1326	Os04g0605900	100,00	0,00E+00	1326	0	NA	NA	
maker-4-augustus-gene-252.6-mRNA-1	4	OsR498G0409234800.01.T02	100,00	0,00E+00	1911	Os04g0605300	100,00	0,00E+00	1911	0	NA	NA	LRR
maker-4-augustus-gene-252.9-mRNA-1	4	OsR498G0409237000.01.T02	100,00	0,00E+00	2742	Os04g0605500	100,00	0,00E+00	2742	0	NA	Japonica	
maker-4-augustus-gene-253.0-mRNA-1	4	OsR498G0409242100.01.T01	100,00	0,00E+00	1510	Os04g0606800	100,00	0,00E+00	1510	0	NA	Japonica	
maker-4-augustus-gene-253.18-mRNA-1	4	OsR498G0409241000.01.T01	99,93	0,00E+00	2609	Os04g0606500	100,00	0,00E+00	2614	5	NA	Japonica	
maker-4-augustus-gene-253.23-mRNA-1	4	OsR498G0409242000.01.T01	99,78	0,00E+00	799	Os04g0606700	100,00	0,00E+00	803	4	NA	Japonica	
maker-4-augustus-gene-253.27-mRNA-1	4	OsR498G0409242400.01.T03	99,65	0,00E+00	1545	Os04g0606900	100,00	0,00E+00	845	-700	Indica	Japonica	
maker-4-augustus-gene-253.29-mRNA-1	4	OsR498G0409242600.01.T01	100,00	0,00E+00	1191	Os04g0607000	100,00	0,00E+00	1191	0	NA	Japonica	
maker-4-augustus-gene-253.33-mRNA-1	4	OsR498G0409242800.01.T02	99,83	0,00E+00	2064	Os08g0225400	78,14	0,00E+00	801	-1263	Indica	Japonica	
maker-4-augustus-gene-254.30-mRNA-1	4	OsR498G0409250300.01.T01	100,00	0,00E+00	4648	Os04g0608400	99,51	0,00E+00	4707	59	NA	Indica	
maker-4-augustus-gene-254.34-mRNA-1	4	OsR498G0409252300.01.T01	100,00	0,00E+00	3112	Os04g0608800	98,96	0,00E+00	3042	-70	NA	Indica	
maker-4-augustus-gene-254.37-mRNA-1	4	OsR498G0409246400.01.T03	99,92	0,00E+00	2360	Os04g0607600	100,00	0,00E+00	2352	-8	NA	Indica	
maker-4-augustus-gene-254.39-mRNA-1	4	OsR498G0409248000.01.T01	100,00	0,00E+00	2333	Os04g0608100	100,00	0,00E+00	2333	0	NA	NA	
maker-4-augustus-gene-255.0-mRNA-1	4	OsR498G0409254300.01.T02	100,00	0,00E+00	4226	Os04g0609100	99,85	0,00E+00	2466	-1760	Indica	NA	
maker-4-augustus-gene-255.14-mRNA-1	4	OsR498G0409256200.01.T02	100,00	0,00E+00	1743	Os04g0609600	100,00	0,00E+00	1743	0	NA	Japonica	
maker-4-augustus-gene-255.17-mRNA-1	4	OsR498G0409256800.01.T04	97,72	0,00E+00	3390	Os04g0609700	97,69	0,00E+00	3342	-48	NA	Indica	
maker-4-augustus-gene-255.3-mRNA-1	4	OsR498G0409255300.01.T01	99,79	0,00E+00	2514	Os04g0609300	100,00	0,00E+00	2527	13	NA	Japonica	
maker-4-augustus-gene-255.5-mRNA-1	4	OsR498G0409255700.01.T01	99,78	0,00E+00	2465	Os04g0609500	100,00	0,00E+00	2479	14	NA	Japonica	
maker-4-augustus-gene-255.7-mRNA-1	4	OsR498G0409252600.01.T01	100,00	0,00E+00	2289	Os04g0608900	100,00	0,00E+00	2289	0	NA	Indica	Kinase-TM
maker-4-augustus-gene-256.32-mRNA-1	4	OsR498G0409260100.01.T03	100,00	0,00E+00	1489	Os04g0610600	100,00	0,00E+00	1489	0	NA	NA	
maker-4-augustus-gene-256.39-mRNA-1	4	OsR498G0409261700.01.T03	99,96	0,00E+00	4063	Os04g0610900	100,00	0,00E+00	4067	4	NA	Japonica	Kinase-TM
maker-4-augustus-gene-256.43-mRNA-1	4	OsR498G0409263100.01.T01	100,00	0,00E+00	2370	Os04g0611200	100,00	0,00E+00	2370	0	NA	Japonica	
maker-4-augustus-gene-256.48-mRNA-1	4	OsR498G0409263700.01.T01	98,24	0,00E+00	3357	Os04g0611400	98,29	0,00E+00	3361	4	NA	NA	
maker-4-augustus-gene-256.61-mRNA-1	4	OsR498G0409260400.01.T05	100,00	0,00E+00	1878	Os04g0610700	100,00	0,00E+00	1878	0	NA	Japonica	
maker-4-augustus-gene-256.69-mRNA-1	4	OsR498G0409264000.01.T01	100,00	0,00E+00	3999	Os04g0611500	100,00	0,00E+00	2906	-1093	Indica	Japonica	
maker-4-augustus-gene-257.45-mRNA-1	4	OsR498G0409265100.01.T01	99,92	0,00E+00	2245	Os04g0612000	98,39	0,00E+00	3006	761	Japonica	Japonica	
maker-4-augustus-gene-257.48-mRNA-1	4	OsR498G0409267100.01.T01	96,34	0,00E+00	1891	Os04g0612700	100,00	0,00E+00	2057	166	NA	Japonica	
maker-4-augustus-gene-257.51-mRNA-1	4	OsR498G0409267500.01.T01	99,69	6,10E-165	581	Os04g0612900	100,00	4,99E-167	585	4	NA	Japonica	
maker-4-augustus-gene-257.53-mRNA-1	4	OsR498G0409268700.01.T01	97,34	0,00E+00	2545	Os04g0613200	100,00	0,00E+00	2711	166	NA	Japonica	
maker-4-augustus-gene-257.58-mRNA-1	4	OsR498G0409269500.01.T01	98,97	2,97E-93	342	Os04g0613600	98,97	1,03E-93	342	0	NA	Japonica	
maker-4-augustus-gene-257.60-mRNA-1	4	OsR498G0409265400.01.T01	100,00	0,00E+00	1954	Os04g0612100	98,40	0,00E+00	1712	-242	Indica	Japonica	
maker-4-augustus-gene-257.63-mRNA-1	4	OsR498G0409266800.01.T01	100,00	0,00E+00	1559	Os04g0612600	100,00	0,00E+00	1559	0	NA	Japonica	
maker-4-augustus-gene-257.67-mRNA-1	4	OsR498G0409267800.01.T01	98,45	0,00E+00	1899	Os04g0613000	100,00	0,00E+00	1975	76	NA	Japonica	
maker-4-augustus-gene-257.70-mRNA-1	4	OsR498G0409269000.01.T01	99,17	0,00E+00	1495	Os04g0613300	99,88	0,00E+00	1522	27	NA	Japonica	
maker-4-augustus-gene-257.72-mRNA-1	4	OsR498G0409269200.01.T03	100,00	0,00E+00	1276	Os04g0613400	100,00	0,00E+00	1276	0	NA	Japonica	
maker-4-augustus-gene-257.85-mRNA-1	4	OsR498G0409270400.01.T03	99,92	0,00E+00	2133	Os04g0613800	100,00	0,00E+00	2138	5	NA	Japonica	
maker-4-augustus-gene-258.29-mRNA-1	4	OsR498G0409271500.01.T01	99,89	0,00E+00	1619	Os04g0614000	100,00	0,00E+00	1624	5	NA	Japonica	
maker-4-augustus-gene-258.41-mRNA-1	4	OsR498G0202838200.01.T01	83,62	0,00E+00	1364	Os04g0614500	100,00	0,00E+00	2307	943	Japonica	Japonica	
maker-4-augustus-gene-258.61-mRNA-1	4	OsR498G0409276900.01.T04	100,00	0,00E+00	1790	Os04g0615100	100,00	0,00E+00	1656	-134	NA	Japonica	
maker-4-augustus-gene-258.71-mRNA-1	4	OsR498G0409277400.01.T01	98,68	9,27E-110	397	Os04g0615200	100,00	4,23E-115	412	15	NA	Japonica	
maker-4-augustus-gene-259.0-mRNA-1	4	OsR498G0409277900.01.T02	99,69	0,00E+00	2282	Os04g0615500	99,88	0,00E+00	1513	-769	Indica	Japonica	
maker-4-augustus-gene-259.13-mRNA-1	4	OsR498G0409280200.01.T02	99,89	0,00E+00	1690	Os04g0616000	99,89	0,00E+00	1690	0	NA	Japonica	
maker-4-augustus-gene-259.18-mRNA-1	4	OsR498G0409280600.01.T01	99,87	0,00E+00	1432	Os04g0616100	98,46	0,00E+00	1700	268	Japonica	Japonica	
maker-4-augustus-gene-259.2-mRNA-1	4	OsR498G0409278300.01.T01	98,71	0,00E+00	1760	Os04g0615600	100,00	0,00E+00	953	-807	Indica	Japonica	
maker-4-augustus-gene-259.21-mRNA-1	4	OsR498G0409280800.01.T01	99,47	0,00E+00	4386	Os08g0203700	71,62	0,00E+00	1150	-3236	Indica	Japonica	Kinase-LRR-TM
maker-4-augustus-gene-259.22-mRNA-1	4	OsR498G0409280800.01.T06	98,73	0,00E+00	681	Os08g0202300	74,23	1,21E-42	172	-509	Indica	Japonica	
maker-4-augustus-gene-259.29-mRNA-1	4	OsR498G0409282000.01.T05	99,84	0,00E+00	1154	Os04g0616700	75,36	1,06E-77	289	-865	Indica	Japonica	LRR-TM
maker-4-augustus-gene-259.31-mRNA-1	4	OsR498G0409282400.01.T01	89,73	0,00E+00	1416	Os04g0616400	99,44	0,00E+00	1922	506	Japonica	Japonica	Kinase
maker-4-augustus-gene-259.33-mRNA-1	4	OsR498G0409282200.01.T05	95,73	0,00E+00	994	Os04g0616700	77,38	4,64E-69	261	-733	Indica	Japonica	LRR-TM
maker-4-augustus-gene-259.35-mRNA-1	4	OsR498G0409282400.01.T01	99,75	0,00E+00	1438	Os04g0616600	100,00	0,00E+00	1451	13	NA	Japonica	Kinase-TM
maker-4-augustus-gene-259.37-mRNA-1	4	OsR498G0409282400.01.T05	100,00	0,00E+00	921	Os04g0616700	79,27	3,52E-76	285	-636	Indica	Japonica	LRR-TM
maker-4-augustus-gene-259.4-mRNA-1	4	OsR498G0409279300.01.T01	98,56	0,00E+00	5437	Os04g0615700	100,00	0,00E+00	5600	163	NA	Japonica	
maker-4-augustus-gene-26.27-mRNA-1	4	OsR498G0407758000.01.T01	96,73	0,00E+00	1719	Os11g0692100	73,33	7,82E-158	557	-1162	Indica	Indica	Kinase-TM
maker-4-augustus-gene-260.11-mRNA-1	4	OsR498G0409288100.01.T02	99,15	0,00E+00	2076	Os04g0618200	100,00	0,00E+00	2322	246	Japonica	Japonica	
maker-4-augustus-gene-260.17-mRNA-1	4	OsR498G0409288900.01.T04	99,39	0,00E+00	866	Os04g0618600	99,39	0,00E+00	866	0	NA	Japonica	
maker-4-augustus-gene-260.19-mRNA-1	4	OsR498G0409282800.01.T02	84,64	0,00E+00	2158	Os04g0616700	95,30	0,00E+00	2970	812	Japonica	Japonica	Kinase-LRR-TM
maker-4-augustus-gene-260.21-mRNA-1	4	OsR498G0409286200.01.T03	99,75	0,00E+00	5060	Os04g0617600	100,00	0,00E+00	5092	32	NA	Japonica	
maker-4-augustus-gene-260.26-mRNA-1	4	OsR498G0409287800.01.T01	99,09	0,00E+00	1349	Os04g0617900	100,00	0,00E+00	919	-430	Indica	Japonica	
maker-4-augustus-gene-260.7-mRNA-1	4	OsR498G0409287500.01.T01	99,75	0,00E+00	1452	Os04g0617800	100,00	0,00E+00	1462	10	NA	Japonica	
maker-4-augustus-gene-261.0-mRNA-1	4	OsR498G0409293700.01.T01	100,00	0,00E+00	1754	Os04g0619500	99,90	0,00E+00	1749	-5	NA	NA	
maker-4-augustus-gene-261.12-mRNA-1	4	OsR498G0409292800.01.T02	98,50	0,00E+00	1973	Os04g0619400	98,85	0,00E+00	1991	18	NA	Japonica	Kinase-TM
maker-4-augustus-gene-261.7-mRNA-1	4	OsR498G0409289200.01.T01	99,61	0,00E+00	6343	Os04g0618700	99,97	0,00E+00	6402	59	NA	Japonica	Kinase-LRR-TM
maker-4-augustus-gene-261.9-mRNA-1	4	OsR498G0409291200.01.T02	94,11	0,00E+00	1294	Os04g0619000	93,23	0,00E+00	1529	235	Japonica	NA	
maker-4-augustus-gene-262.34-mRNA-1	4	OsR498G0409295700.01.T01	99,62	0,00E+00	2801	Os04g0620200	99,65	0,00E+00	1529	-1272	Indica	Japonica	
maker-4-augustus-gene-262.38-mRNA-1	4	OsR498G0409296300.01.T01	99,79	0,00E+00	4182	Os04g0620400	100,00	0,00E+00	3579	-603	Indica	Japonica	
maker-4-augustus-gene-262.43-mRNA-1	4	OsR498G0409296700.01.T01	98,19	0,00E+00	3727	Os04g0620700	98,61	0,00E+00	3768	41	NA	Japonica	
maker-4-augustus-gene-262.52-mRNA-1	4	OsR498G0409298500.01.T01	98,44	0,00E+00	4472	Os04g0621500	99,85	0,00E+00	8438	3966	Japonica	Japonica	NBS-TM
maker-4-augustus-gene-262.66-mRNA-1	4	OsR498G0409296600.01.T01	100,00	3,11E-142	504	Os04g0620600	100,00	1,09E-142	504	0	NA	Japonica	
maker-4-augustus-gene-262.70-mRNA-1	4	OsR498G0409305600.01.T01	88,43	0,00E+00	744	Os04g0622000	93,90	0,00E+00	1589	845	Japonica	Japonica	
maker-4-augustus-gene-263.42-mRNA-1	4	OsR498G0409301900.01.T01	99,84	0,00E+00	5540	Os04g0622600	100,00	0,00E+00	5912	372	NA	Japonica	CC-LRR-NBS-TM
maker-4-augustus-gene-263.44-mRNA-1	4	OsR498G0409307700.01.T01	100,00	0,00E+00	1992	Os04g0623500	100,00	0,00E+00	1992	0	NA	Japonica	
maker-4-augustus-gene-263.57-mRNA-1	4	OsR498G0409308100.01.T01	100,00	0,00E+00	715	Os04g0623700	100,00	0,00E+00	715	0	NA	Japonica	
maker-4-augustus-gene-263.59-mRNA-1	4	OsR498G0409308500.01.T01	99,76	0,00E+00	2200	Os04g0623800	100,00	0,00E+00	2214	14	NA	Japonica	
maker-4-augustus-gene-263.62-mRNA-1	4	OsR498G0409302400.01.T01	94,66	0,00E+00	1700	Os04g0622700	100,00	0,00E+00	1927	227	Japonica	Japonica	
maker-4-augustus-gene-263.65-mRNA-1	4	OsR498G0409305600.01.T01	99,85	0,00E+00	2464	Os04g0623100	100,00	0,00E+00	2473	9	NA	Japonica	
maker-4-augustus-gene-263.90-mRNA-1	4	OsR498G0409306200.01.T05	99,79	0,00E+00	2588	Os04g0623300	100,00	0,00E+00	2601	13	NA	Japonica	
maker-4-augustus-gene-263.95-mRNA-1	4	OsR498G0409307000.01.T05	99,87	0,00E+00	1408	Os04g0623400	100,00	0,00E+00	1364	-44	NA	Japonica	
maker-4-augustus-gene-264.0-mRNA-1	4	OsR498G0409310700.01.T01	99,84	0,00E+00	1102	Os04g0624300	100,00	0,00E+00	1056	-46	NA	Japonica	
maker-4-augustus-gene-264.1-mRNA-1	4	OsR498G0409313500.01.T01	99,77	0,00E+00	3061	Os04g0625100	100,00	0,00E+00	3081	20	NA	Japonica	
maker-4-augustus-gene-264.10-mRNA-1	4	OsR498G0409312000.01.T01	98,77	0,00E+00	6413	Os04g0624600	98,93	0,00E+00	6440	27	NA	Japonica	
maker-4-augustus-gene-264.16-mRNA-1	4	OsR498G0409312400.01.T01	99,82	0,00E+00	2031	Os04g0624800	100,00	0,00E+00	2040	9	NA	Japonica	
maker-4-augustus-gene-264.18-mRNA-1	4	OsR498G0409312800.01.T01	98,53	0,00E+00	3692	Os08g0190700	73,57	0,00E+00	1096	-2596	Indica	Japonica	
maker-4-augustus-gene-264.3-mRNA-1	4	OsR498G0409314500.01.T02	99,66	0,00E+00	1572	Os04g0625200	100,00	0,00E+00	829	-743	Indica	Japonica	
maker-4-augustus-gene-264.6-mRNA-1	4	OsR498G0409310000.01.T03	100,00	0,00E+00	650	Os04g0624000	100,00	0,00E+00	650	0	NA	Japonica	
maker-4-augustus-gene-265.14-mRNA-1	4	OsR498G0409317400.01.T02	99,84	0,00E+00	2237	Os04g0626000	99,92	0,00E+00	2241	4	NA	Japonica	
maker-4-augustus-gene-265.17-mRNA-1	4	OsR498G0409317800.01.T02	100,00	0,00E+00	1478	Os04g0626400	99,76	0,00E+00	1469	-9	NA	Japonica	
maker-4-augustus-gene-265.20-mRNA-1	4	OsR498G0409319200.01.T02	99,87	0,00E+00	1396	Os04g0626600	99,45	0,00E+00	1303	-93	NA	Japonica	
maker-4-augustus-gene-265.21-mRNA-1	4	OsR498G0409319400.01.T01	96,16	0,00E+00	1813	Os04g0626700	100,00	0,00E+00	1975	162	NA	Japonica	
maker-4-augustus-gene-265.4-mRNA-1	4	OsR498G0409317100.01.T02	98,90	0,00E+00	2382	Os04g0625900	99,78	0,00E+00	2438	56	NA	Japonica	
maker-4-augustus-gene-265.8-mRNA-1	4	OsR498G0409319500.01.T01	96,39	0,00E+00	2866	Os04g0626900	96,28	0,00E+00	2857	-9	NA	Japonica	
maker-4-augustus-gene-266.0-mRNA-1	4	OsR498G0409321700.01.T01	99,82	0,00E+00	992	Os04g0627300	96,16	0,00E+00	949	-43	NA	Japonica	
maker-4-augustus-gene-266.12-mRNA-1	4	OsR498G0409325500.01.T04	99,89	0,00E+00	1571	Os04g0629000	100,00	0,00E+00	1377	-194	Indica	Japonica	
maker-4-augustus-gene-266.17-mRNA-1	4	OsR498G0409325900.01.T01	99,80	0,00E+00	894	Os04g0629200	100,00	0,00E+00	899	5	NA	Japonica	
maker-4-augustus-gene-266.19-mRNA-1	4	OsR498G0409319800.01.T05	99,70	0,00E+00	4216	Os04g0627000	99,96	0,00E+00	4243	27	NA	Japonica	
maker-4-augustus-gene-266.25-mRNA-1	4	OsR498G0409321000.01.T03	99,85	0,00E+00	1199	Os04g0627200	99,67	0,00E+00	1646	447	Japonica	NA	
maker-4-augustus-gene-266.31-mRNA-1	4	OsR498G0409323600.01.T02	99,34	0,00E+00	1074	Os04g0628000	99,67	0,00E+00	1083	9	NA	Japonica	
maker-4-augustus-gene-266.36-mRNA-1	4	OsR498G0409324800.01.T01	99,96	0,00E+00	5071	Os04g0628600	99,93	0,00E+00	5066	-5	NA	Japonica	
maker-4-augustus-gene-266.4-mRNA-1	4	OsR498G0409323900.01.T05	99,90	0,00E+00	1792	Os04g0628200	100,00	0,00E+00	1393	-399	Indica	Japonica	
maker-4-augustus-gene-266.44-mRNA-1	4	OsR498G0409325800.01.T01	99,81	0,00E+00	2790	Os04g0629100	100,00	0,00E+00	2803	13	NA	Japonica	
maker-4-augustus-gene-266.49-mRNA-1	4	OsR498G0409326200.01.T02	99,93	0,00E+00	5387	Os04g0629300	100,00	0,00E+00	5396	9	NA	Japonica	
maker-4-augustus-gene-267.106-mRNA-1	4	OsR498G0409329700.01.T02	97,76	0,00E+00	835	Os04g0631000	97,36	0,00E+00	829	-6	NA	NA	
maker-4-augustus-gene-267.107-mRNA-1	4	OsR498G0409329700.01.T01	96,75	0,00E+00	819	Os04g0631000	96,34	0,00E+00	810	-9	NA	NA	
maker-4-augustus-gene-267.112-mRNA-1	4	OsR498G0409330100.01.T01	99,94	0,00E+00	2820	Os04g0631100	99,72	0,00E+00	1940	-880	Indica	NA	
maker-4-augustus-gene-267.115-mRNA-1	4	OsR498G0409331100.01.T04	99,49	0,00E+00	1757	Os04g0631200	97,14	0,00E+00	1172	-585	Indica	NA	
maker-4-augustus-gene-267.117-mRNA-1	4	OsR498G0409332100.01.T01	99,47	0,00E+00	2361	Os04g0631600	99,02	0,00E+00	2335	-26	NA	NA	
maker-4-augustus-gene-267.122-mRNA-1	4	OsR498G0409327400.01.T01	99,92	0,00E+00	4260	Os04g0629700	99,92	0,00E+00	4260	0	NA	Japonica	
maker-4-augustus-gene-267.42-mRNA-1	4	OsR498G0409327700.01.T05	99,62	0,00E+00	2339	Os04g0630000	100,00	0,00E+00	2361	22	NA	Japonica	
maker-4-augustus-gene-267.48-mRNA-1	4	OsR498G0409328500.01.T03	83,01	0,00E+00	1088	Os04g0630100	96,12	0,00E+00	1661	573	Japonica	Japonica	
maker-4-augustus-gene-267.51-mRNA-1	4	OsR498G0409328500.01.T03	97,93	0,00E+00	1735	Os04g0630300	100,00	0,00E+00	1878	143	NA	Japonica	
maker-4-augustus-gene-267.52-mRNA-1	4	OsR498G0409328900.01.T01	98,94	0,00E+00	1829	Os04g0630400	99,81	0,00E+00	1869	40	NA	Japonica	
maker-4-augustus-gene-267.54-mRNA-1	4	OsR498G0409329100.01.T02	99,73	0,00E+00	1973	Os04g0630800	96,62	0,00E+00	1716	-257	Indica	Indica	
maker-4-augustus-gene-267.55-mRNA-1	4	OsR498G0409329500.01.T01	97,13	0,00E+00	876	Os04g0631000	87,76	0,00E+00	655	-221	Indica	Indica	
maker-4-augustus-gene-268.0-mRNA-1	4	OsR498G0409337500.01.T02	99,88	0,00E+00	4494	Os04g0633800	75,32	0,00E+00	1683	-2811	Indica	Japonica	Kinase-TM
maker-4-augustus-gene-268.13-mRNA-1	4	OsR498G0409334400.01.T03	99,45	0,00E+00	2241	Os04g0632500	98,89	0,00E+00	2209	-32	NA	NA	Kinase-TM
maker-4-augustus-gene-268.16-mRNA-1	4	OsR498G0409334600.01.T02	99,13	0,00E+00	2442	Os04g0632600	99,62	0,00E+00	1898	-544	Indica	NA	Kinase-TM
maker-4-augustus-gene-268.22-mRNA-1	4	OsR498G0409334800.01.T01	98,18	0,00E+00	4084	Os04g0632700	99,83	0,00E+00	3105	-979	Indica	Japonica	Kinase-TM
maker-4-augustus-gene-268.25-mRNA-1	4	OsR498G0409335600.01.T01	99,62	0,00E+00	2366	Os04g0633200	99,89	0,00E+00	4795	2429	Japonica	Japonica	Kinase-TM
maker-4-augustus-gene-268.3-mRNA-1	4	OsR498G0409332600.01.T02	99,76	0,00E+00	3030	Os04g0631800	99,59	0,00E+00	3017	-13	NA	Indica	Kinase-TM
maker-4-augustus-gene-268.31-mRNA-1	4	OsR498G0409336200.01.T01	99,94	0,00E+00	2882	Os04g0633300	99,88	0,00E+00	3103	221	NA	Japonica	Kinase-TM
maker-4-augustus-gene-268.36-mRNA-1	4	OsR498G0409338200.01.T01	97,51	0,00E+00	3708	Os04g0633900	97,37	0,00E+00	3491	-217	NA	NA	Kinase-TM
maker-4-augustus-gene-268.39-mRNA-1	4	OsR498G0409338400.01.T01	99,58	0,00E+00	3398	Os04g0634000	99,68	0,00E+00	4423	1025	Japonica	Indica	Kinase-TM
maker-4-augustus-gene-268.42-mRNA-1	4	OsR498G0409339000.01.T02	99,79	0,00E+00	4209	Os04g0634400	99,92	0,00E+00	4222	13	NA	NA	Kinase-TM
maker-4-augustus-gene-268.7-mRNA-1	4	OsR498G0409332800.01.T04	99,92	0,00E+00	2260	Os04g0632100	99,92	0,00E+00	2260	0	NA	Indica	Kinase-TM
maker-4-augustus-gene-269.51-mRNA-1	4	OsR498G0409339700.01.T05	99,87	0,00E+00	2699	Os04g0634700	100,00	0,00E+00	2706	7	NA	Japonica	
maker-4-augustus-gene-269.56-mRNA-1	4	OsR498G0409343100.01.T01	100,00	0,00E+00	888	Os04g0635700	100,00	0,00E+00	888	0	NA	NA	
maker-4-augustus-gene-269.58-mRNA-1	4	OsR498G0409343500.01.T04	99,87	0,00E+00	2695	Os04g0635900	99,93	0,00E+00	2700	5	NA	Japonica	
maker-4-augustus-gene-269.63-mRNA-1	4	OsR498G0409344100.01.T01	99,76	0,00E+00	2996	Os04g0636100	99,88	0,00E+00	3005	9	NA	NA	
maker-4-augustus-gene-269.65-mRNA-1	4	OsR498G0409345100.01.T02	100,00	0,00E+00	856	Os04g0636400	99,77	0,00E+00	763	-93	Indica	Japonica	
maker-4-augustus-gene-269.69-mRNA-1	4	OsR498G0409345300.01.T01	97,97	0,00E+00	3403	Os04g0636500	98,12	0,00E+00	3416	13	NA	Japonica	
maker-4-augustus-gene-269.71-mRNA-1	4	OsR498G0409339200.01.T03	99,56	0,00E+00	2034	Os01g0783800	70,07	2,03E-64	246	-1788	Indica	Japonica	Kinase-TM
maker-4-augustus-gene-269.82-mRNA-1	4	OsR498G0409343800.01.T01	98,51	0,00E+00	2452	Os04g0636000	98,72	0,00E+00	2466	14	NA	NA	
maker-4-augustus-gene-269.85-mRNA-1	4	OsR498G0409344400.01.T01	99,71	0,00E+00	2466	Os04g0636200	99,85	0,00E+00	2410	-56	NA	NA	
maker-4-augustus-gene-269.90-mRNA-1	4	OsR498G0409345600.01.T01	99,95	0,00E+00	3365	Os04g0636600	99,68	0,00E+00	3332	-33	NA	Japonica	
maker-4-augustus-gene-269.95-mRNA-1	4	OsR498G0409345800.01.T01	99,40	0,00E+00	2680	Os04g0636700	99,60	0,00E+00	2693	13	NA	Japonica	
maker-4-augustus-gene-269.98-mRNA-1	4	OsR498G0409346000.01.T01	100,00	0,00E+00	969	Os04g0636800	99,55	0,00E+00	1199	230	Japonica	Japonica	
maker-4-augustus-gene-270.58-mRNA-1	4	OsR498G0409347700.01.T03	99,90	0,00E+00	3638	Os08g0175300	76,83	0,00E+00	1506	-2132	Indica	Japonica	
maker-4-augustus-gene-270.64-mRNA-1	4	OsR498G0409351100.01.T01	98,19	0,00E+00	1053	Os04g0639200	99,15	0,00E+00	1033	-20	NA	Japonica	
maker-4-augustus-gene-270.67-mRNA-1	4	OsR498G0409348400.01.T02	99,55	0,00E+00	2381	Os04g0638100	99,93	0,00E+00	2404	23	NA	Japonica	
maker-4-augustus-gene-270.68-mRNA-1	4	OsR498G0409349000.01.T01	97,79	0,00E+00	2329	Os04g0638100	83,06	0,00E+00	1394	-935	Indica	Japonica	
maker-4-augustus-gene-270.69-mRNA-1	4	OsR498G0409349400.01.T01	99,36	0,00E+00	826	Os04g0638700	96,67	0,00E+00	803	-23	NA	Japonica	
maker-4-augustus-gene-270.82-mRNA-1	4	OsR498G0409351400.01.T02	99,69	0,00E+00	1751	Os04g0639300	100,00	0,00E+00	942	-809	Indica	Japonica	
maker-4-augustus-gene-271.33-mRNA-1	4	OsR498G0409353100.01.T02	99,58	6,97E-118	424	Os04g0640200	98,62	2,61E-67	253	-171	Indica	Japonica	
maker-4-augustus-gene-271.35-mRNA-1	4	OsR498G0409353700.01.T04	99,77	0,00E+00	2314	Os04g0640500	97,87	0,00E+00	3730	1416	Japonica	Japonica	
maker-4-augustus-gene-271.38-mRNA-1	4	OsR498G0409355100.01.T01	94,06	0,00E+00	1438	Os04g0640800	94,39	0,00E+00	1452	14	NA	Japonica	
maker-4-augustus-gene-271.41-mRNA-1	4	OsR498G0409355500.01.T02	99,75	0,00E+00	4932	Os04g0641000	99,96	0,00E+00	4959	27	NA	Japonica	
maker-4-augustus-gene-271.45-mRNA-1	4	OsR498G0409356300.01.T01	99,15	0,00E+00	5207	Os04g0641200	99,18	0,00E+00	5212	5	NA	Japonica	
maker-4-augustus-gene-271.47-mRNA-1	4	OsR498G0409353400.01.T01	99,75	0,00E+00	1415	Os04g0640300	100,00	0,00E+00	1449	34	NA	NA	
maker-4-augustus-gene-271.50-mRNA-1	4	OsR498G0409354200.01.T01	100,00	0,00E+00	1559	Os04g0640600	100,00	0,00E+00	1559	0	NA	Japonica	NBS
maker-4-augustus-gene-271.56-mRNA-1	4	OsR498G0409354400.01.T01	99,87	0,00E+00	4131	Os04g0640700	99,96	0,00E+00	4140	9	NA	Japonica	
maker-4-augustus-gene-271.60-mRNA-1	4	OsR498G0409356800.01.T05	100,00	0,00E+00	1391	Os04g0641400	100,00	0,00E+00	1364	-27	NA	Japonica	
maker-4-augustus-gene-272.15-mRNA-1	4	OsR498G0409357800.01.T01	100,00	1,02E-161	569	Os03g0171300	72,92	6,88E-32	137	-432	Indica	Japonica	
maker-4-augustus-gene-272.17-mRNA-1	4	OsR498G0409358600.01.T01	99,71	0,00E+00	6820	Os04g0459000	78,34	0,00E+00	3066	-3754	Indica	Japonica	
maker-4-augustus-gene-272.8-mRNA-1	4	OsR498G0409362700.01.T01	99,77	0,00E+00	3938	Os04g0643000	100,00	0,00E+00	3961	23	NA	Japonica	
maker-4-augustus-gene-273.0-mRNA-1	4	OsR498G0409364100.01.T01	99,76	0,00E+00	2200	Os04g0643300	99,84	0,00E+00	2205	5	NA	Japonica	
maker-4-augustus-gene-273.13-mRNA-1	4	OsR498G0409364900.01.T01	94,74	0,00E+00	763	Os04g0643800	95,91	0,00E+00	1008	245	Japonica	Japonica	
maker-4-augustus-gene-273.25-mRNA-1	4	OsR498G0409368700.01.T03	99,78	0,00E+00	1604	Os04g0644700	100,00	0,00E+00	1613	9	NA	Japonica	
maker-4-augustus-gene-273.35-mRNA-1	4	OsR498G0409369300.01.T02	99,94	0,00E+00	3112	Os08g0300200	71,37	0,00E+00	748	-2364	Indica	Japonica	
maker-4-augustus-gene-273.46-mRNA-1	4	OsR498G0409364400.01.T01	99,69	0,00E+00	1756	Os04g0643500	100,00	0,00E+00	1770	14	NA	Japonica	
maker-4-augustus-gene-273.48-mRNA-1	4	OsR498G0409368200.01.T02	98,64	0,00E+00	2061	Os04g0644600	98,98	0,00E+00	2079	18	NA	Japonica	
maker-4-augustus-gene-274.37-mRNA-1	4	OsR498G0409372000.01.T01	98,91	0,00E+00	2746	Os01g0857600	97,03	0,00E+00	1911	-835	Indica	Japonica	
maker-4-augustus-gene-275.12-mRNA-1	4	OsR498G0409380900.01.T01	98,88	0,00E+00	3450	Os04g0648700	99,54	0,00E+00	3502	52	NA	Japonica	
maker-4-augustus-gene-275.17-mRNA-1	4	OsR498G0409377600.01.T01	99,81	0,00E+00	2855	Os04g0647800	99,94	0,00E+00	2864	9	NA	Japonica	
maker-4-augustus-gene-275.19-mRNA-1	4	OsR498G0409379800.01.T02	99,46	0,00E+00	2977	Os04g0648500	100,00	0,00E+00	2875	-102	NA	Japonica	
maker-4-augustus-gene-276.41-mRNA-1	4	OsR498G0409386900.01.T01	99,81	0,00E+00	4784	Os04g0649200	99,96	0,00E+00	4802	18	NA	Japonica	
maker-4-augustus-gene-276.45-mRNA-1	4	OsR498G0409387800.01.T01	99,72	0,00E+00	1274	Os04g0649400	100,00	0,00E+00	1256	-18	NA	Japonica	
maker-4-augustus-gene-276.47-mRNA-1	4	OsR498G0409389000.01.T01	99,44	0,00E+00	3797	Os04g0649700	100,00	0,00E+00	3847	50	NA	Japonica	Kinase-LRR-TM
maker-4-augustus-gene-277.0-mRNA-1	4	OsR498G0409391900.01.T01	99,66	0,00E+00	1578	Os04g0650500	100,00	0,00E+00	1591	13	NA	Japonica	
maker-4-augustus-gene-277.10-mRNA-1	4	OsR498G0409395900.01.T01	99,74	0,00E+00	2796	Os04g0651500	100,00	0,00E+00	4057	1261	Japonica	Japonica	CC-Kinase-TM
maker-4-augustus-gene-277.14-mRNA-1	4	OsR498G0409389600.01.T01	99,86	0,00E+00	2475	Os04g0670200	71,28	4,73E-162	572	-1903	Indica	Japonica	
maker-4-augustus-gene-277.20-mRNA-1	4	OsR498G0409390800.01.T01	99,91	0,00E+00	1922	Os04g0650200	100,00	0,00E+00	1927	5	NA	Japonica	
maker-4-augustus-gene-277.24-mRNA-1	4	OsR498G0409392200.01.T02	99,77	0,00E+00	1590	Os04g0650600	100,00	0,00E+00	1599	9	NA	Japonica	
maker-4-augustus-gene-277.41-mRNA-1	4	OsR498G0409398000.01.T01	100,00	0,00E+00	3252	Os04g0652400	100,00	0,00E+00	2064	-1188	Indica	NA	
maker-4-augustus-gene-277.7-mRNA-1	4	OsR498G0409394100.01.T01	99,73	0,00E+00	3300	Os04g0650800	100,00	0,00E+00	3323	23	NA	Japonica	
maker-4-augustus-gene-278.12-mRNA-1	4	OsR498G0409402300.01.T02	100,00	0,00E+00	1175	Os04g0653000	100,00	0,00E+00	1056	-119	Indica	NA	
maker-4-augustus-gene-278.22-mRNA-1	4	OsR498G0409398600.01.T02	93,30	0,00E+00	2473	Os04g0652500	97,58	0,00E+00	2731	258	NA	NA	
maker-4-augustus-gene-278.31-mRNA-1	4	OsR498G0409400600.01.T04	100,00	0,00E+00	827	Os04g0652900	100,00	0,00E+00	827	0	NA	Indica	
maker-4-augustus-gene-278.35-mRNA-1	4	OsR498G0409402600.01.T02	100,00	0,00E+00	1126	Os04g0653100	99,84	0,00E+00	1122	-4	NA	NA	
maker-4-augustus-gene-278.6-mRNA-1	4	OsR498G0409400300.01.T02	100,00	9,12E-150	529	Os04g0652700	99,66	1,35E-148	525	-4	NA	NA	
maker-4-augustus-gene-279.0-mRNA-1	4	OsR498G0409403100.01.T01	99,41	0,00E+00	907	Os04g0653300	100,00	0,00E+00	921	14	NA	Japonica	
maker-4-augustus-gene-279.14-mRNA-1	4	OsR498G0409406500.01.T01	98,73	0,00E+00	2072	Os04g0654600	100,00	0,00E+00	2122	50	NA	Japonica	Kinase-TM
maker-4-augustus-gene-279.16-mRNA-1	4	OsR498G0409407100.01.T02	100,00	0,00E+00	1247	Os04g0654700	100,00	0,00E+00	1247	0	NA	Japonica	
maker-4-augustus-gene-279.2-mRNA-1	4	OsR498G0409404300.01.T02	98,15	0,00E+00	2724	Os09g0558900	74,32	0,00E+00	886	-1838	Indica	Japonica	
maker-4-augustus-gene-279.21-mRNA-1	4	OsR498G0409409300.01.T01	99,77	0,00E+00	2319	Os04g0655300	100,00	0,00E+00	2230	-89	NA	Japonica	Kinase-TM
maker-4-augustus-gene-279.23-mRNA-1	4	OsR498G0409409600.01.T01	99,85	0,00E+00	2329	Os04g0655400	100,00	0,00E+00	1198	-1131	Indica	Japonica	Kinase-TM
maker-4-augustus-gene-279.9-mRNA-1	4	OsR498G0409405100.01.T01	97,61	0,00E+00	2567	Os04g0653600	98,74	0,00E+00	2635	68	NA	Japonica	
maker-4-augustus-gene-28.42-mRNA-1	4	OsR498G0407752500.01.T03	99,80	0,00E+00	1804	Os04g0218900	99,80	0,00E+00	1804	0	NA	Indica	
maker-4-augustus-gene-28.45-mRNA-1	4	OsR498G0407752500.01.T03	99,70	0,00E+00	1800	Os04g0218900	99,70	0,00E+00	1800	0	NA	NA	
maker-4-augustus-gene-280.0-mRNA-1	4	OsR498G0409410100.01.T01	100,00	0,00E+00	2273	Os04g0655300	74,71	6,40E-179	627	-1646	Indica	Japonica	Kinase-TM
maker-4-augustus-gene-280.15-mRNA-1	4	OsR498G0409415100.01.T01	100,00	0,00E+00	2257	Os04g0656500	99,84	0,00E+00	2248	-9	NA	Indica	
maker-4-augustus-gene-280.19-mRNA-1	4	OsR498G0409416200.01.T01	100,00	0,00E+00	1829	Os04g0656800	98,52	0,00E+00	1771	-58	NA	Japonica	
maker-4-augustus-gene-280.2-mRNA-1	4	OsR498G0409410300.01.T03	99,39	0,00E+00	2320	Os04g0655600	100,00	0,00E+00	2358	38	NA	Japonica	
maker-4-augustus-gene-281.39-mRNA-1	4	OsR498G0409417900.01.T01	99,86	0,00E+00	2497	Os04g0657500	100,00	0,00E+00	2506	9	NA	Japonica	
maker-4-augustus-gene-281.44-mRNA-1	4	OsR498G0409418900.01.T03	99,71	0,00E+00	1854	Os04g0658000	100,00	0,00E+00	1867	13	NA	Japonica	
maker-4-augustus-gene-281.48-mRNA-1	4	OsR498G0409421500.01.T01	99,76	0,00E+00	2238	Os04g0658700	98,74	0,00E+00	2640	402	Japonica	Japonica	Kinase-TM
maker-4-augustus-gene-281.51-mRNA-1	4	OsR498G0409417400.01.T01	99,57	0,00E+00	2054	Os04g0657100	100,00	0,00E+00	2127	73	NA	Japonica	
maker-4-augustus-gene-281.55-mRNA-1	4	OsR498G0409417800.01.T01	100,00	0,00E+00	2208	Os04g0657300	100,00	0,00E+00	2208	0	NA	Japonica	
maker-4-augustus-gene-281.60-mRNA-1	4	OsR498G0613102200.01.T03	71,88	2,25E-17	89,7	Os04g0658200	100,00	1,09E-142	504	414,3	Japonica	Japonica	
maker-4-augustus-gene-282.24-mRNA-1	4	OsR498G0409423900.01.T02	99,74	0,00E+00	683	Os04g0659000	100,00	0,00E+00	688	5	NA	Japonica	
maker-4-augustus-gene-282.26-mRNA-1	4	OsR498G0409425700.01.T06	99,75	0,00E+00	2157	Os04g0659400	100,00	0,00E+00	2170	13	NA	Japonica	
maker-4-augustus-gene-282.33-mRNA-1	4	OsR498G0409426500.01.T01	99,65	0,00E+00	2525	Os04g0470700	74,17	0,00E+00	837	-1688	Indica	Japonica	
maker-4-augustus-gene-282.44-mRNA-1	4	OsR498G0409428700.01.T02	99,90	0,00E+00	1850	Os04g0660400	100,00	0,00E+00	1855	5	NA	Japonica	
maker-4-augustus-gene-282.45-mRNA-1	4	OsR498G0409423800.01.T04	96,81	0,00E+00	2111	Os04g0659100	96,71	0,00E+00	1996	-115	NA	Japonica	
maker-4-augustus-gene-282.51-mRNA-1	4	OsR498G0409424400.01.T01	99,74	0,00E+00	4140	Os04g0659150	100,00	0,00E+00	2206	-1934	Indica	Japonica	
maker-4-augustus-gene-282.54-mRNA-1	4	OsR498G0409426000.01.T02	99,75	0,00E+00	1411	Os04g0659500	100,00	0,00E+00	1195	-216	Indica	Japonica	

maker-4-augustus-gene-282.59-mRNA-1	4	OsR498G0409428400.01.T01	99,63	0,00E+00	3367	Os04g0660200	99,95	0,00E+00	3394	27	NA	Japonica	
maker-4-augustus-gene-283.0-mRNA-1	4	OsR498G0409430500.01.T01	100,00	0,00E+00	3420	Os04g0660900	100,00	0,00E+00	3420	0	NA	Japonica	
maker-4-augustus-gene-283.11-mRNA-1	4	OsR498G0409432700.01.T01	99,89	0,00E+00	1679	Os04g0661900	99,58	0,00E+00	850	-829	Indica	Japonica	
maker-4-augustus-gene-283.17-mRNA-1	4	OsR498G0409432900.01.T03	98,66	0,00E+00	651	Os04g0662000	94,67	1,28E-170	598	-53	NA	Japonica	
maker-4-augustus-gene-283.19-mRNA-1	4	OsR498G0409429000.01.T02	99,82	0,00E+00	3916	Os04g0660500	100,00	0,00E+00	3934	18	NA	Japonica	Kinase-TM OsMAPKKKε
maker-4-augustus-gene-283.25-mRNA-1	4	OsR498G0409429200.01.T01	99,75	0,00E+00	716	Os04g0660600	100,00	4,58E-105	379	-337	Indica	Japonica	
maker-4-augustus-gene-283.30-mRNA-1	4	OsR498G0409431000.01.T01	99,89	0,00E+00	1583	Os04g0661200	100,00	0,00E+00	1588	5	NA	Japonica	
maker-4-augustus-gene-283.34-mRNA-1	4	OsR498G0409431200.01.T02	99,81	0,00E+00	1862	Os04g0661300	100,00	0,00E+00	1871	9	NA	Japonica	
maker-4-augustus-gene-283.39-mRNA-1	4	OsR498G0409432400.01.T02	99,71	0,00E+00	4897	Os04g0661700	99,85	0,00E+00	4915	18	NA	Japonica	
maker-4-augustus-gene-283.46-mRNA-1	4	OsR498G0409432600.01.T02	99,81	0,00E+00	954	Os04g0661800	100,00	0,00E+00	958	4	NA	NA	
maker-4-augustus-gene-284.10-mRNA-1	4	OsR498G0409438300.01.T01	100,00	0,00E+00	666	Os04g0663500	98,40	0,00E+00	653	-13	NA	NA	
maker-4-augustus-gene-284.11-mRNA-1	4	OsR498G0409435600.01.T02	99,73	0,00E+00	2666	Os04g0662700	99,42	0,00E+00	2128	-538	Indica	Japonica	
maker-4-augustus-gene-284.14-mRNA-1	4	OsR498G0409436400.01.T03	100,00	0,00E+00	1218	Os04g0662900	100,00	0,00E+00	1218	0	NA	Japonica	
maker-4-augustus-gene-284.17-mRNA-1	4	OsR498G0409438000.01.T01	100,00	0,00E+00	2544	Os04g0663200	100,00	0,00E+00	2132	-412	Indica	NA	
maker-4-augustus-gene-284.23-mRNA-1	4	OsR498G0409439800.01.T03	100,00	0,00E+00	1413	Os04g0663700	99,87	0,00E+00	1408	-5	NA	Indica	
maker-4-augustus-gene-284.29-mRNA-1	4	OsR498G0409441200.01.T01	100,00	0,00E+00	5173	Os04g0664400	100,00	0,00E+00	5173	0	NA	NA	
maker-4-augustus-gene-285.0-mRNA-1	4	OsR498G0409445100.01.T01	100,00	9,60E-131	466	Os04g0665000	97,64	8,38E-139	491	25	NA	Indica	
maker-4-augustus-gene-285.11-mRNA-1	4	OsR498G0409449300.01.T04	90,48	0,00E+00	1033	Os04g0665800	90,48	0,00E+00	1033	0	NA	NA	
maker-4-augustus-gene-285.14-mRNA-1	4	OsR498G0409452300.01.T01	74,79	0,00E+00	761	Os06g0323100	74,88	0,00E+00	706	-55	NA	Japonica	
maker-4-augustus-gene-285.21-mRNA-1	4	OsR498G0409445400.01.T02	100,00	0,00E+00	2192	Os04g0665200	99,92	0,00E+00	2187	-5	NA	Indica	
maker-4-augustus-gene-285.23-mRNA-1	4	OsR498G0409447000.01.T02	100,00	0,00E+00	1849	Os04g0665400	100,00	0,00E+00	1849	0	NA	Japonica	
maker-4-augustus-gene-285.25-mRNA-1	4	OsR498G0409448200.01.T02	99,92	0,00E+00	2296	Os04g0665600	100,00	0,00E+00	2300	4	NA	Japonica	
maker-4-augustus-gene-285.3-mRNA-1	4	OsR498G0409447900.01.T02	100,00	0,00E+00	2105	Os04g0665500	100,00	0,00E+00	1824	-281	Indica	Japonica	
maker-4-augustus-gene-285.7-mRNA-1	4	OsR498G0409449300.01.T02	100,00	0,00E+00	1530	Os04g0665700	100,00	0,00E+00	1530	0	NA	Indica	
maker-4-augustus-gene-286.0-mRNA-1	4	OsR498G0409451900.01.T01	99,76	0,00E+00	745	Os06g0323100	79,81	1,60E-98	359	-386	Indica	Japonica	
maker-4-augustus-gene-286.2-mRNA-1	4	OsR498G0409452300.01.T01	100,00	0,00E+00	2105	Os06g0323100	89,32	0,00E+00	1401	-704	Indica	Japonica	
maker-4-augustus-gene-286.20-mRNA-1	4	OsR498G0409453400.01.T01	99,80	0,00E+00	916	Os04g0666800	99,82	0,00E+00	968	52	NA	Japonica	
maker-4-augustus-gene-286.22-mRNA-1	4	OsR498G0409453600.01.T03	99,93	0,00E+00	4899	Os04g0666900	100,00	0,00E+00	4908	9	NA	Indica	
maker-4-augustus-gene-286.29-mRNA-1	4	OsR498G0409454200.01.T02	99,78	0,00E+00	1599	Os04g0667000	100,00	0,00E+00	839	-760	Indica	Japonica	
maker-4-augustus-gene-286.33-mRNA-1	4	OsR498G0409455200.01.T01	100,00	0,00E+00	1808	Os04g0667400	100,00	0,00E+00	1808	0	NA	Japonica	
maker-4-augustus-gene-286.41-mRNA-1	4	OsR498G0409456400.01.T05	100,00	0,00E+00	807	Os04g0667800	100,00	0,00E+00	807	0	NA	Japonica	
maker-4-augustus-gene-286.6-mRNA-1	4	OsR498G0409454700.01.T03	99,65	0,00E+00	1021	Os04g0667200	100,00	0,00E+00	1031	10	NA	Japonica	
maker-4-augustus-gene-286.7-mRNA-1	4	OsR498G0409455900.01.T01	99,76	0,00E+00	743	Os04g0667600	100,00	1,46E-168	590	-153	Indica	Japonica	
maker-4-augustus-gene-287.102-mRNA-1	4	OsR498G0409462600.01.T04	99,75	0,00E+00	2177	Os04g0670400	99,25	0,00E+00	1659	-518	Indica	Japonica	
maker-4-augustus-gene-287.108-mRNA-1	4	OsR498G0409463200.01.T06	100,00	0,00E+00	2516	Os04g0670600	100,00	0,00E+00	1467	-1049	Indica	Japonica	
maker-4-augustus-gene-287.49-mRNA-1	4	OsR498G0409459100.01.T01	100,00	0,00E+00	1137	Os04g0668800	100,00	0,00E+00	1137	0	NA	NA	
maker-4-augustus-gene-287.58-mRNA-1	4	OsR498G0409461700.01.T04	100,00	0,00E+00	1240	Os04g0670000	100,00	0,00E+00	998	-242	Indica	NA	
maker-4-augustus-gene-287.61-mRNA-1	4	OsR498G0409462700.01.T01	99,06	0,00E+00	2622	Os04g0670500	100,00	0,00E+00	2657	35	NA	Japonica	
maker-4-augustus-gene-287.68-mRNA-1	4	OsR498G0409459400.01.T07	99,78	0,00E+00	820	Os04g0668900	90,39	0,00E+00	738	-82	Indica	Japonica	
maker-4-augustus-gene-287.73-mRNA-1	4	OsR498G0409459600.01.T01	100,00	0,00E+00	690	Os04g0669100	100,00	0,00E+00	690	0	NA	Japonica	
maker-4-augustus-gene-287.76-mRNA-1	4	OsR498G0409460800.01.T01	97,45	0,00E+00	1403	Os04g0669500	97,45	0,00E+00	1403	0	NA	NA	
maker-4-augustus-gene-287.82-mRNA-1	4	OsR498G0409461000.01.T01	99,18	0,00E+00	644	Os04g0669600	99,18	0,00E+00	644	0	NA	NA	
maker-4-augustus-gene-287.85-mRNA-1	4	OsR498G0409461000.01.T06	99,92	0,00E+00	2328	Os04g0669800	100,00	0,00E+00	2333	5	NA	NA	
maker-4-augustus-gene-287.96-mRNA-1	4	OsR498G0409461600.01.T02	100,00	0,00E+00	2316	Os04g0669800	96,37	0,00E+00	2124	-192	NA	NA	
maker-4-augustus-gene-287.99-mRNA-1	4	OsR498G0409462200.01.T01	100,00	0,00E+00	2527	Os04g0670200	100,00	0,00E+00	2527	0	NA	Japonica	
maker-4-augustus-gene-288.25-mRNA-1	4	OsR498G0409463700.01.T01	100,00	0,00E+00	2489	Os04g0670800	100,00	0,00E+00	1797	-692	Indica	NA	
maker-4-augustus-gene-288.32-mRNA-1	4	OsR498G0409464600.01.T01	100,00	0,00E+00	2116	Os04g0670900	100,00	0,00E+00	2116	0	NA	NA	
maker-4-augustus-gene-288.34-mRNA-1	4	OsR498G0409465400.01.T01	100,00	0,00E+00	1548	Os03g0130400	72,27	2,92E-105	382	-1166	Indica	NA	
maker-4-augustus-gene-288.37-mRNA-1	4	OsR498G0409465600.01.T01	99,93	0,00E+00	2636	Os04g0671200	100,00	0,00E+00	2641	5	NA	NA	
maker-4-augustus-gene-288.39-mRNA-1	4	OsR498G0409465800.01.T01	100,00	0,00E+00	2668	Os04g0671300	100,00	0,00E+00	2668	0	NA	NA	
maker-4-augustus-gene-288.42-mRNA-1	4	OsR498G0409466600.01.T01	99,83	0,00E+00	2091	Os04g0671700	100,00	0,00E+00	2100	9	NA	Japonica	
maker-4-augustus-gene-288.48-mRNA-1	4	OsR498G0409467400.01.T03	97,90	0,00E+00	4189	Os04g0671900	98,27	0,00E+00	4229	40	NA	Japonica	
maker-4-augustus-gene-289.39-mRNA-1	4	OsR498G0409471500.01.T01	99,85	0,00E+00	2421	Os04g0672900	100,00	0,00E+00	2430	9	NA	Japonica	
maker-4-augustus-gene-289.41-mRNA-1	4	OsR498G0409475700.01.T01	99,61	0,00E+00	917	Os04g0673300	100,00	0,00E+00	926	9	NA	Japonica	
maker-4-augustus-gene-289.45-mRNA-1	4	OsR498G0409478300.01.T01	99,85	0,00E+00	1230	Os04g0674200	100,00	0,00E+00	1234	4	NA	Japonica	
maker-4-augustus-gene-289.47-mRNA-1	4	OsR498G0409469600.01.T02	99,70	0,00E+00	5435	Os04g0672200	100,00	0,00E+00	5182	-253	NA	Japonica	
maker-4-augustus-gene-289.53-mRNA-1	4	OsR498G0409470000.01.T02	98,70	0,00E+00	809	Os04g0672300	100,00	0,00E+00	801	-8	NA	Japonica	
maker-4-augustus-gene-289.62-mRNA-1	4	OsR498G0409476600.01.T02	97,48	0,00E+00	1557	Os04g0673400	99,77	0,00E+00	1526	-31	NA	Japonica	
maker-4-augustus-gene-289.66-mRNA-1	4	OsR498G0409477000.01.T01	99,45	0,00E+00	966	Os04g0673800	100,00	0,00E+00	980	14	NA	Japonica	
maker-4-augustus-gene-289.68-mRNA-1	4	OsR498G0409478000.01.T02	99,73	0,00E+00	1350	Os04g0674100	100,00	0,00E+00	792	-558	Indica	Japonica	
maker-4-augustus-gene-290.25-mRNA-1	4	OsR498G0409483700.01.T02	99,59	0,00E+00	2607	Os04g0675400	100,00	0,00E+00	2636	29	NA	Japonica	
maker-4-augustus-gene-290.27-mRNA-1	4	OsR498G0409485100.01.T01	100,00	0,00E+00	980	Os04g0676100	100,00	0,00E+00	980	0	NA	Japonica	
maker-4-augustus-gene-290.29-mRNA-1	4	OsR498G0409478600.01.T02	99,88	0,00E+00	3059	Os04g0674300	100,00	0,00E+00	3068	9	NA	Japonica	
maker-4-augustus-gene-290.32-mRNA-1	4	OsR498G0409480000.01.T02	99,13	0,00E+00	3668	Os04g0674600	100,00	0,00E+00	3837	169	NA	Japonica	
maker-4-augustus-gene-290.33-mRNA-1	4	OsR498G0409480000.01.T03	99,82	0,00E+00	3012	Os04g0674700	100,00	0,00E+00	3025	13	NA	Japonica	
maker-4-augustus-gene-290.40-mRNA-1	4	OsR498G0409480600.01.T01	99,89	0,00E+00	3378	Os04g0674800	100,00	0,00E+00	3388	10	NA	Japonica	
maker-4-augustus-gene-290.42-mRNA-1	4	OsR498G0409481600.01.T02	99,60	0,00E+00	5373	Os04g0675000	99,89	0,00E+00	3296	-2077	Indica	Japonica	
maker-4-augustus-gene-290.47-mRNA-1	4	OsR498G0409484000.01.T01	99,95	0,00E+00	3902	Os04g0675500	100,00	0,00E+00	3907	5	NA	Japonica	
maker-4-augustus-gene-291.34-mRNA-1	4	OsR498G0409485700.01.T04	99,84	0,00E+00	2215	Os04g0676300	100,00	0,00E+00	2089	-126	NA	Japonica	
maker-4-augustus-gene-291.38-mRNA-1	4	OsR498G0409488500.01.T01	98,50	0,00E+00	1856	Os04g0677000	100,00	0,00E+00	1929	73	NA	Japonica	
maker-4-augustus-gene-291.39-mRNA-1	4	OsR498G0409489300.01.T05	99,62	0,00E+00	1865	Os10g0489100	72,20	2,91E-113	408	-1457	Indica	Japonica	
maker-4-augustus-gene-291.45-mRNA-1	4	OsR498G0409486800.01.T02	99,46	0,00E+00	2317	Os04g0676600	99,92	0,00E+00	2344	27	NA	Japonica	
maker-4-augustus-gene-291.47-mRNA-1	4	OsR498G0409487200.01.T01	99,77	0,00E+00	2362	Os04g0676600	85,23	0,00E+00	1471	-891	Indica	Japonica	
maker-4-augustus-gene-291.49-mRNA-1	4	OsR498G0409487400.01.T03	99,11	0,00E+00	2382	Os04g0676700	99,90	0,00E+00	1722	-660	Indica	Japonica	
maker-4-augustus-gene-291.54-mRNA-1	4	OsR498G0409491400.01.T01	99,72	0,00E+00	3855	Os04g0677400	99,91	0,00E+00	3874	19	NA	Japonica	
maker-4-augustus-gene-291.57-mRNA-1	4	OsR498G0409491600.01.T01	97,09	0,00E+00	2672	Os04g0677500	97,34	0,00E+00	2691	19	NA	Japonica	
maker-4-augustus-gene-292.30-mRNA-1	4	OsR498G0409493100.01.T03	100,00	0,00E+00	996	Os04g0678200	100,00	0,00E+00	996	0	NA	Japonica	
maker-4-augustus-gene-292.34-mRNA-1	4	OsR498G0409493700.01.T02	99,00	0,00E+00	2462	Os04g0678300	100,00	0,00E+00	2522	60	NA	Japonica	
maker-4-augustus-gene-292.37-mRNA-1	4	OsR498G0409495300.01.T01	99,91	0,00E+00	2095	Os04g0678700	100,00	0,00E+00	2100	5	NA	Japonica	
maker-4-augustus-gene-292.39-mRNA-1	4	OsR498G0409495500.01.T01	97,05	0,00E+00	1261	Os04g0678800	97,19	0,00E+00	1266	5	NA	Japonica	
maker-4-augustus-gene-292.42-mRNA-1	4	OsR498G0409495700.01.T01	99,81	0,00E+00	2903	Os04g0678900	100,00	0,00E+00	2917	14	NA	Japonica	
maker-4-augustus-gene-292.44-mRNA-1	4	OsR498G0409495900.01.T01	99,88	0,00E+00	2892	Os04g0679000	100,00	0,00E+00	2901	9	NA	Japonica	
maker-4-augustus-gene-292.46-mRNA-1	4	OsR498G0409496700.01.T01	99,78	0,00E+00	1626	Os04g0679100	100,00	0,00E+00	1635	9	NA	Japonica	
maker-4-augustus-gene-292.56-mRNA-1	4	OsR498G0409492800.01.T06	99,81	0,00E+00	3674	Os04g0677800	100,00	0,00E+00	3692	18	NA	Japonica	
maker-4-augustus-gene-292.61-mRNA-1	4	OsR498G0409497000.01.T02	99,73	0,00E+00	2016	Os04g0679200	100,00	0,00E+00	2030	14	NA	Japonica	Kinase-TM
maker-4-augustus-gene-292.65-mRNA-1	4	OsR498G0409497600.01.T02	100,00	0,00E+00	1402	Os04g0679400	99,63	0,00E+00	969	-433	Indica	Japonica	
maker-4-augustus-gene-292.68-mRNA-1	4	OsR498G0409498400.01.T01	98,81	0,00E+00	2065	Os04g0679600	99,50	0,00E+00	2114	49	NA	Japonica	
maker-4-augustus-gene-293.1-mRNA-1	4	OsR498G0409499500.01.T01	100,00	0,00E+00	1348	Os04g0679800	99,79	0,00E+00	849	-499	Indica	Japonica	
maker-4-augustus-gene-293.15-mRNA-1	4	OsR498G0409501900.01.T02	99,38	0,00E+00	4900	Os04g0680700	100,00	0,00E+00	2663	-2237	Indica	Japonica	
maker-4-augustus-gene-293.19-mRNA-1	4	OsR498G0409502100.01.T01	98,49	0,00E+00	3225	Os04g0680800	97,90	0,00E+00	1728	-1497	Indica	Japonica	
maker-4-augustus-gene-293.22-mRNA-1	4	OsR498G0409503100.01.T01	99,37	0,00E+00	1128	Os04g0681500	100,00	0,00E+00	1146	18	NA	Japonica	
maker-4-augustus-gene-293.24-mRNA-1	4	OsR498G0409499800.01.T01	100,00	0,00E+00	1160	Os04g0679900	100,00	0,00E+00	1160	0	NA	Japonica	
maker-4-augustus-gene-293.28-mRNA-1	4	OsR498G0409501400.01.T01	100,00	0,00E+00	753	Os04g0680300	100,00	0,00E+00	753	0	NA	Japonica	
maker-4-augustus-gene-293.34-mRNA-1	4	OsR498G0409503000.01.T01	99,90	0,00E+00	1884	Os04g0681400	100,00	0,00E+00	1889	5	NA	Japonica	
maker-4-augustus-gene-293.38-mRNA-1	4	OsR498G0409503600.01.T01	99,58	0,00E+00	3369	Os04g0681600	99,95	0,00E+00	3401	32	NA	Japonica	
maker-4-augustus-gene-293.42-mRNA-1	4	OsR498G0409504800.01.T01	99,31	0,00E+00	1793	Os04g0681900	99,70	0,00E+00	1810	17	NA	Japonica	
maker-4-augustus-gene-294.0-mRNA-1	4	OsR498G0409505500.01.T01	99,77	0,00E+00	786	Os04g0682100	100,00	0,00E+00	791	5	NA	Japonica	
maker-4-augustus-gene-294.17-mRNA-1	4	OsR498G0409511300.01.T03	96,84	0,00E+00	2048	Os04g0683500	100,00	0,00E+00	1552	-496	Indica	Japonica	
maker-4-augustus-gene-294.31-mRNA-1	4	OsR498G0409505200.01.T02	99,72	0,00E+00	2574	Os04g0682000	100,00	0,00E+00	2592	18	NA	Japonica	
maker-4-augustus-gene-294.37-mRNA-1	4	OsR498G0409505400.01.T02	99,88	0,00E+00	1441	Os04g0674400	98,42	0,00E+00	1315	-126	NA	Japonica	
maker-4-augustus-gene-294.4-mRNA-1	4	OsR498G0409505900.01.T01	93,84	0,00E+00	1252	Os04g0682300	93,96	0,00E+00	1257	5	NA	Japonica	
maker-4-augustus-gene-294.45-mRNA-1	4	OsR498G0409506200.01.T01	98,59	0,00E+00	4837	Os04g0682400	99,89	0,00E+00	4974	137	NA	Japonica	
maker-4-augustus-gene-294.47-mRNA-1	4	OsR498G0409509800.01.T05	99,81	0,00E+00	3775	Os04g0682800	100,00	0,00E+00	3793	18	NA	Japonica	
maker-4-augustus-gene-294.55-mRNA-1	4	OsR498G0409510000.01.T05	99,73	0,00E+00	3349	Os04g0682900	99,79	0,00E+00	3353	4	NA	Japonica	
maker-4-augustus-gene-294.60-mRNA-1	4	OsR498G0409510200.01.T01	100,00	0,00E+00	1099	Os04g0683100	99,87	0,00E+00	1403	304	Japonica	Japonica	
maker-4-augustus-gene-294.64-mRNA-1	4	OsR498G0409512600.01.T01	99,87	0,00E+00	2800	Os04g0683700	100,00	0,00E+00	2809	9	NA	Japonica	
maker-4-augustus-gene-294.9-mRNA-1	4	OsR498G0409506500.01.T02	99,42	0,00E+00	1533	Os04g0682500	99,88	0,00E+00	1553	20	NA	Japonica	
maker-4-augustus-gene-295.30-mRNA-1	4	OsR498G0409512900.01.T01	99,68	0,00E+00	4453	Os04g0683800	99,92	0,00E+00	4480	27	NA	Japonica	
maker-4-augustus-gene-295.31-mRNA-1	4	OsR498G0409513100.01.T01	99,39	7,25E-166	583	Os02g0664000	74,32	1,60E-48	192	-391	Indica	Japonica	
maker-4-augustus-gene-295.42-mRNA-1	4	OsR498G0409515900.01.T03	99,67	0,00E+00	4838	Os04g0684500	100,00	0,00E+00	4881	43	NA	Japonica	
maker-4-augustus-gene-295.49-mRNA-1	4	OsR498G0409516700.01.T01	99,78	0,00E+00	802	Os04g0684800	100,00	0,00E+00	682	-120	Indica	Japonica	
maker-4-augustus-gene-295.51-mRNA-1	4	OsR498G0409513400.01.T01	99,84	0,00E+00	2264	Os04g0683900	100,00	0,00E+00	2273	9	NA	Japonica	
maker-4-augustus-gene-295.54-mRNA-1	4	OsR498G0409518400.01.T01	99,77	0,00E+00	1579	Os04g0685100	100,00	0,00E+00	1588	9	NA	Japonica	
maker-4-augustus-gene-296.0-mRNA-1	4	OsR498G0409520700.01.T01	99,85	0,00E+00	1224	Os04g0685800	100,00	0,00E+00	1207	-17	NA	Japonica	
maker-4-augustus-gene-296.10-mRNA-1	4	OsR498G0409524000.01.T02	99,77	0,00E+00	5480	Os04g0686200	99,97	0,00E+00	5507	27	NA	Japonica	
maker-4-augustus-gene-296.14-mRNA-1	4	OsR498G0409524400.01.T01	96,67	0,00E+00	652	Os04g0686300	100,00	0,00E+00	704	52	NA	Japonica	
maker-4-augustus-gene-296.2-mRNA-1	4	OsR498G0409520200.01.T06	100,00	0,00E+00	1296	Os04g0685500	100,00	0,00E+00	1301	5	NA	Japonica	
maker-4-augustus-gene-296.8-mRNA-1	4	OsR498G0409521200.01.T01	99,75	0,00E+00	5053	Os04g0685900	100,00	0,00E+00	5081	28	NA	Japonica	Kinase-LRR-TM
maker-4-augustus-gene-297.42-mRNA-1	4	OsR498G0409527100.01.T01	98,70	0,00E+00	1342	Os04g0687200	95,38	0,00E+00	965	-377	Indica	Japonica	
maker-4-augustus-gene-297.47-mRNA-1	4	OsR498G0409528900.01.T06	99,35	0,00E+00	2465	Os04g0687900	100,00	0,00E+00	2506	41	NA	Japonica	
maker-4-augustus-gene-297.51-mRNA-1	4	OsR498G0409526800.01.T02	100,00	0,00E+00	1159	Os04g0687100	100,00	0,00E+00	1159	0	NA	Japonica	
maker-4-augustus-gene-297.55-mRNA-1	4	OsR498G0409527400.01.T01	99,27	0,00E+00	1462	Os04g0687300	100,00	0,00E+00	1489	27	NA	Japonica	
maker-4-augustus-gene-297.59-mRNA-1	4	OsR498G0409529600.01.T01	100,00	0,00E+00	1452	Os04g0688100	100,00	0,00E+00	1452	0	NA	Japonica	
maker-4-augustus-gene-297.60-mRNA-1	4	OsR498G0409529600.01.T03	99,52	0,00E+00	1866	Os04g0688200	100,00	0,00E+00	1889	23	NA	Japonica	
maker-4-augustus-gene-298.34-mRNA-1	4	OsR498G0409533700.01.T01	99,75	0,00E+00	3625	Os04g0689400	100,00	0,00E+00	3647	22	NA	Japonica	Kinase-TM
maker-4-augustus-gene-298.39-mRNA-1	4	OsR498G0409533900.01.T01	99,90	0,00E+00	1706	Os04g0689500	100,00	0,00E+00	1889	183	NA	Japonica	
maker-4-augustus-gene-298.41-mRNA-1	4	OsR498G0409534300.01.T03	99,84	0,00E+00	1105	Os04g0689700	100,00	0,00E+00	1110	5	NA	Japonica	
maker-4-augustus-gene-298.47-mRNA-1	4	OsR498G0409535100.01.T01	99,76	0,00E+00	1501	Os04g0689900	100,00	0,00E+00	1510	9	NA	Japonica	
maker-4-augustus-gene-298.49-mRNA-1	4	OsR498G0409535900.01.T01	98,48	0,00E+00	2516	Os04g0690100	99,80	0,00E+00	2691	175	NA	Japonica	
maker-4-augustus-gene-298.51-mRNA-1	4	OsR498G0409530800.01.T02	86,42	0,00E+00	1160	Os04g0688500	100,00	0,00E+00	1911	751	Japonica	NA	
maker-4-augustus-gene-298.55-mRNA-1	4	OsR498G0409533400.01.T01	99,72	0,00E+00	2520	Os04g0689300	100,00	0,00E+00	2538	18	NA	Japonica	
maker-4-augustus-gene-299.37-mRNA-1	4	OsR498G0409536500.01.T01	99,72	0,00E+00	3808	Os04g0690400	99,95	0,00E+00	3879	71	NA	Japonica	
maker-4-augustus-gene-299.40-mRNA-1	4	OsR498G0409536900.01.T02	99,42	0,00E+00	1214	Os04g0690500	100,00	0,00E+00	1234	20	NA	Japonica	
maker-4-augustus-gene-299.42-mRNA-1	4	OsR498G0409538500.01.T02	98,92	0,00E+00	1957	Os04g0691100	100,00	0,00E+00	2008	51	NA	Japonica	Kinase-TM
maker-4-augustus-gene-299.45-mRNA-1	4	OsR498G0409539100.01.T01	99,15	0,00E+00	2713	Os04g0691400	100,00	0,00E+00	2765	52	NA	Japonica	
maker-4-augustus-gene-3.27-mRNA-1	4	OsR498G0407633300.01.T01	99,61	0,00E+00	1367	Os01g0612500	80,41	1,40E-178	625	-742	Indica	Japonica	
maker-4-augustus-gene-30.0-mRNA-1	4	OsR498G0407755400.01.T01	98,59	0,00E+00	2346	Os04g0221000	96,54	0,00E+00	2251	-95	NA	Indica	
maker-4-augustus-gene-30.4-mRNA-1	4	OsR498G0407756000.01.T02	99,81	0,00E+00	4604	Os04g0221600	98,56	0,00E+00	4488	-116	NA	Indica	
maker-4-augustus-gene-300.0-mRNA-1	4	OsR498G0409541500.01.T03	99,85	0,00E+00	3509	Os04g0691900	100,00	0,00E+00	3523	14	NA	Japonica	
maker-4-augustus-gene-300.28-mRNA-1	4	OsR498G0409541800.01.T04	99,83	0,00E+00	1046	Os04g0692000	100,00	0,00E+00	1050	4	NA	Japonica	
maker-4-augustus-gene-300.3-mRNA-1	4	OsR498G0409542100.01.T02	99,48	0,00E+00	1709	Os04g0692200	100,00	0,00E+00	1732	23	NA	Japonica	
maker-4-augustus-gene-300.36-mRNA-1	4	OsR498G0409542400.01.T06	99,30	0,00E+00	1765	Os04g0692300	99,50	0,00E+00	1774	9	NA	Japonica	
maker-4-augustus-gene-300.7-mRNA-1	4	OsR498G0409542700.01.T04	98,97	0,00E+00	3579	Os04g0692400	99,90	0,00E+00	3660	81	NA	Japonica	
maker-4-augustus-gene-301.0-mRNA-1	4	OsR498G0409548500.01.T02	100,00	0,00E+00	2771	Os08g0112500	82,36	0,00E+00	966	-1805	Indica	Japonica	Kinase-TM
maker-4-augustus-gene-301.25-mRNA-1	4	OsR498G0409410300.01.T04	99,41	0,00E+00	2110	Os04g0655600	100,00	0,00E+00	2143	33	NA	NA	
maker-4-augustus-gene-301.31-mRNA-1	4	OsR498G0409533700.01.T02	99,85	0,00E+00	2399	Os04g0689400	100,00	0,00E+00	2378	-21	NA	NA	Kinase-TM
maker-4-augustus-gene-301.45-mRNA-1	4	OsR498G0409533400.01.T01	99,72	0,00E+00	2520	Os04g0689300	100,00	0,00E+00	2538	18	NA	NA	
maker-4-augustus-gene-301.8-mRNA-1	4	OsR498G0409549500.01.T01	99,12	2,27E-170	599	Os05g0420600	78,52	4,70E-66	250	-349	Indica	Japonica	
maker-4-augustus-gene-31.39-mRNA-1	4	OsR498G0407758000.01.T01	99,59	0,00E+00	1304	Os11g0692100	74,39	1,54E-115	416	-888	Indica	Indica	Kinase-TM
maker-4-augustus-gene-33.11-mRNA-1	4	OsR498G0407762400.01.T01	99,87	0,00E+00	2816	Os04g0223901	98,91	0,00E+00	2748	-68	NA	Indica	
maker-4-augustus-gene-33.6-mRNA-1	4	OsR498G0407761800.01.T01	99,77	0,00E+00	1572	Os04g0223300	99,54	0,00E+00	1563	-9	NA	Indica	
maker-4-augustus-gene-33.9-mRNA-1	4	OsR498G0407762000.01.T01	98,85	0,00E+00	2744	Os04g0223500	99,23	0,00E+00	2771	27	NA	NA	
maker-4-augustus-gene-34.0-mRNA-1	4	OsR498G0407764000.01.T02	99,86	0,00E+00	1278	Os04g0224600	98,45	0,00E+00	1233	-45	NA	Indica	
maker-4-augustus-gene-34.4-mRNA-1	4	OsR498G0407764800.01.T05	99,79	0,00E+00	3391	Os04g0224900	99,74	0,00E+00	2038	-1353	Indica	Indica	
maker-4-augustus-gene-35.20-mRNA-1	4	OsR498G0407773100.01.T02	96,89	0,00E+00	3060	Os04g0226600	98,53	0,00E+00	3191	131	NA	Japonica	Kinase-TM
maker-4-augustus-gene-36.31-mRNA-1	4	OsR498G0407777300.01.T02	99,49	0,00E+00	691	Os04g0227500	99,49	0,00E+00	691	0	NA	Japonica	
maker-4-augustus-gene-36.35-mRNA-1	4	OsR498G0407781500.01.T01	100,00	0,00E+00	1418	Os04g0228400	99,87	0,00E+00	1414	-4	NA	NA	
maker-4-augustus-gene-36.37-mRNA-1	4	OsR498G0407780400.01.T01	99,69	0,00E+00	1713	Os04g0228100	99,79	0,00E+00	1718	5	NA	NA	
maker-4-augustus-gene-37.0-mRNA-1	4	OsR498G0407783600.01.T01	100,00	0,00E+00	1954	Os04g0229100	100,00	0,00E+00	1954	0	NA	NA	
maker-4-augustus-gene-39.25-mRNA-1	4	OsR498G0407801400.01.T01	99,76	0,00E+00	1523	Os04g0233400	99,88	0,00E+00	1527	4	NA	NA	
maker-4-augustus-gene-39.28-mRNA-1	4	OsR498G0407805800.01.T01	95,22	0,00E+00	1998	Os04g0234600	95,05	0,00E+00	1989	-9	NA	NA	
maker-4-augustus-gene-4.27-mRNA-1	4	OsR498G0407636100.01.T01	99,65	0,00E+00	2033	Os04g0175600	100,00	0,00E+00	2051	18	NA	Japonica	
maker-4-augustus-gene-4.30-mRNA-1	4	OsR498G0407637100.01.T01	99,60	0,00E+00	1792	Os04g0175900	100,00	0,00E+00	2013	221	Japonica	Japonica	
maker-4-augustus-gene-4.33-mRNA-1	4	OsR498G0407637400.01.T01	99,73	0,00E+00	2011	Os04g0176200	100,00	0,00E+00	1570	-441	Indica	Japonica	
maker-4-augustus-gene-4.36-mRNA-1	4	OsR498G0407639200.01.T02	99,23	0,00E+00	1844	Os04g0176400	100,00	0,00E+00	1880	36	NA	Japonica	
maker-4-augustus-gene-4.41-mRNA-1	4	OsR498G0407640600.01.T01	99,63	0,00E+00	957	Os04g0177100	100,00	0,00E+00	1510	553	Japonica	Japonica	
maker-4-augustus-gene-41.0-mRNA-1	4	OsR498G0407814900.01.T01	99,58	0,00E+00	1714	Os04g0244400	99,79	0,00E+00	1723	9	NA	NA	
maker-4-augustus-gene-41.10-mRNA-1	4	OsR498G0407816200.01.T01	99,79	0,00E+00	840	Os04g0244800	100,00	0,00E+00	845	5	NA	NA	
maker-4-augustus-gene-41.4-mRNA-1	4	OsR498G0407815300.01.T06	99,90	0,00E+00	1838	Os04g0244500	99,80	0,00E+00	1833	-5	NA	Japonica	
maker-4-augustus-gene-43.43-mRNA-1	4	OsR498G0407829000.01.T02	99,50	0,00E+00	4585	Os04g0309600	98,14	0,00E+00	4452	-133	NA	NA	
maker-4-augustus-gene-43.48-mRNA-1	4	OsR498G0407829400.01.T03	99,59	9,84E-123	440	Os04g0249600	99,01	1,13E-97	354	-86	Indica	NA	
maker-4-augustus-gene-43.50-mRNA-1	4	OsR498G0407698800.01.T01	98,89	0,00E+00	5057	Os04g0194500	99,34	0,00E+00	5115	58	NA	NA	
maker-4-augustus-gene-47.23-mRNA-1	4	OsR498G0407841500.01.T01	98,44	0,00E+00	1000	Os04g0253000	98,94	0,00E+00	1155	155	Japonica	Japonica	
maker-4-augustus-gene-48.0-mRNA-1	4	OsR498G0407846900.01.T01	99,91	0,00E+00	2058	Os04g0254000	99,83	0,00E+00	2053	-5	NA	NA	
maker-4-augustus-gene-5.2-mRNA-1	4	OsR498G0407640800.01.T01	98,86	0,00E+00	3375	Os04g0177300	100,00	0,00E+00	3589	214	NA	Japonica	
maker-4-augustus-gene-55.36-mRNA-1	4	OsR498G0407887500.01.T01	99,63	0,00E+00	3833	Os04g0266900	99,91	0,00E+00	3860	27	NA	Japonica	
maker-4-augustus-gene-55.47-mRNA-1	4	OsR498G0407890300.01.T01	97,56	0,00E+00	1249	Os02g0265550	96,83	0,00E+00	682	-567	Indica	NA	
maker-4-augustus-gene-56.0-mRNA-1	4	OsR498G0407896300.01.T01	99,25	0,00E+00	940	Os04g0269100	99,43	0,00E+00	939	-1	NA	Japonica	
maker-4-augustus-gene-57.36-mRNA-1	4	OsR498G0407897300.01.T01	99,60	0,00E+00	4023	Os04g0269600	99,92	0,00E+00	2139	-1884	Indica	Japonica	
maker-4-augustus-gene-57.45-mRNA-1	4	OsR498G0407899500.01.T05	99,75	0,00E+00	2832	Os04g0270100	100,00	0,00E+00	2385	-447	Indica	Japonica	
maker-4-augustus-gene-57.51-mRNA-1	4	OsR498G0407900000.01.T01	99,68	0,00E+00	1128	Os04g0270200	100,00	0,00E+00	1137	9	NA	Japonica	
maker-4-augustus-gene-58.0-mRNA-1	4	OsR498G0407902900.01.T02	99,70	0,00E+00	1193	Os04g0271200	100,00	0,00E+00	1202	9	NA	Japonica	
maker-4-augustus-gene-58.6-mRNA-1	4	OsR498G0407902400.01.T03	98,34	0,00E+00	1359	Os04g0271000	98,08	0,00E+00	1348	-11	NA	Japonica	
maker-4-augustus-gene-6.33-mRNA-1	4	OsR498G0407644200.01.T01	99,21	0,00E+00	2668	Os04g0178400	100,00	0,00E+00	2722	54	NA	NA	
maker-4-augustus-gene-6.34-mRNA-1	4	OsR498G0407644200.01.T01	99,21	0,00E+00	2668	Os04g0178400	100,00	0,00E+00	2722	54	NA	NA	
maker-4-augustus-gene-6.39-mRNA-1	4	OsR498G0407645200.01.T01	97,70	0,00E+00	1403	Os04g0179200	100,00	0,00E+00	1489	86	NA	Japonica	
maker-4-augustus-gene-60.25-mRNA-1	4	OsR498G0407911400.01.T01	98,61	0,00E+00	3530	Os11g0131900	79,77	0,00E+00	1760	-1770	Indica	Japonica	
maker-4-augustus-gene-60.27-mRNA-1	4	OsR498G0407911800.01.T01	95,90	0,00E+00	641	Os04g0274400	95,13	1,90E-179	627	-14	NA	Japonica	
maker-4-augustus-gene-61.30-mRNA-1	4	OsR498G0407919400.01.T03	99,44	0,00E+00	1270	Os04g0276600	99,86	0,00E+00	1284	14	NA	Japonica	
maker-4-augustus-gene-62.38-mRNA-1	4	OsR498G0407921500.01.T01	99,24	0,00E+00	2803	Os04g0278000	99,87	0,00E+00	2848	45	NA	Japonica	
maker-4-augustus-gene-62.41-mRNA-1	4	OsR498G0407922700.01.T01	90,12	0,00E+00	1176	Os04g0278200	89,14	0,00E+00	1005	-171	Indica	Japonica	
maker-4-augustus-gene-62.48-mRNA-1	4	OsR498G0307345500.01.T01	90,73	1,07E-159	563	Os04g0278101	96,14	0,00E+00	1112	549	Japonica	Japonica	
maker-4-augustus-gene-63.10-mRNA-1	4	OsR498G0407929700.01.T01	100,00	0,00E+00	1137	Os04g0281900	100,00	0,00E+00	1137	0	NA	Japonica	
maker-4-augustus-gene-63.13-mRNA-1	4	OsR498G0407927400.01.T01	99,11	0,00E+00	792	Os08g0529800	70,03	1,63E-36	153	-639	Indica	NA	
maker-4-augustus-gene-63.7-mRNA-1	4	OsR498G0407926900.01.T01	93,47	0,00E+00	1123	Os04g0280500	99,07	0,00E+00	1711	588	Japonica	Indica	
maker-4-augustus-gene-64.0-mRNA-1	4	OsR498G0407930900.01.T03	99,44	0,00E+00	1270	Os04g0282200	99,58	0,00E+00	1275	5	NA	Indica	
maker-4-augustus-gene-64.2-mRNA-1	4	OsR498G0407932400.01.T02	99,72	2,15E-179	629	Os04g0282400	99,15	3,88E-177	620	-9	NA	Indica	
maker-4-augustus-gene-65.42-mRNA-1	4	OsR498G0407941200.01.T01	98,51	0,00E+00	1874	Os04g0284600	98,51	0,00E+00	1874	0	NA	Japonica	
maker-4-augustus-gene-65.46-mRNA-1	4	OsR498G0407941400.01.T01	99,58	0,00E+00	2103	Os04g0284900	99,27	0,00E+00	2175	72	NA	NA	
maker-4-augustus-gene-67.0-mRNA-1	4	OsR498G0407949900.01.T01	97,76	0,00E+00	1674	Os04g0287400	100,00	0,00E+00	1770	96	NA	Japonica	
maker-4-augustus-gene-68.40-mRNA-1	4	OsR498G0816248400.01.T01	72,64	2,90E-91	337	Os04g0288100	98,82	0,00E+00	1315	978	Japonica	Indica	
maker-4-augustus-gene-68.42-mRNA-1	4	OsR498G0407951200.01.T01	98,18	0,00E+00	3871	Os04g0288500	100,00	0,00E+00	4042	171	NA	Japonica	Kinase-TM
maker-4-augustus-gene-69.31-mRNA-1	4	OsR498G0407952500.01.T01	98,18	0,00E+00	3865	Os05g0102600	74,71	0,00E+00	922	-2943	Indica	Japonica	
maker-4-augustus-gene-69.43-mRNA-1	4	OsR498G0407953700.01.T06	98,09	0,00E+00	901	Os04g0291000	94,23	0,00E+00	1335	434	Japonica	Japonica	
maker-4-augustus-gene-69.49-mRNA-1	4	OsR498G0407953900.01.T04	98,93	0,00E+00	655	Os04g0291100	99,73	0,00E+00	669	14	NA	Japonica	
maker-4-augustus-gene-7.28-mRNA-1	4	OsR498G0407648100.01.T01	99,31	0,00E+00	2835	Os04g0180400	100,00	0,00E+00	2749	-86	NA	Japonica	
maker-4-augustus-gene-7.30-mRNA-1	4	OsR498G0407647400.01.T01	99,41	0,00E+00	4498	Os04g0179700	100,00	0,00E+00	4562	64	NA	Japonica	
maker-4-augustus-gene-70.0-mRNA-1	4	OsR498G0510192800.01.T08	86,95	0,00E+00	1627	Os04g0291900	100,00	0,00E+00	2365	738	Japonica	Japonica	CC-Kinase-LRR-TM
maker-4-augustus-gene-71.0-mRNA-1	4	OsR498G0407896300.01.T03	89,09	1,24E-76	287	Os04g0293300	100,00	2,41E-118	423	136	Japonica	NA	
maker-4-augustus-gene-72.31-mRNA-1	4	OsR498G0407958700.01.T02	93,25	0,00E+00	754	Os04g0295100	94,27	0,00E+00	748	-6	NA	Japonica	
maker-4-augustus-gene-73.0-mRNA-1	4	OsR498G0407964300.01.T02	98,73	3,68E-115	415	Os04g0295400	100,00	5,85E-101	365	-50	Indica	Japonica	
maker-4-augustus-gene-75.19-mRNA-1	4	OsR498G0407974700.01.T01	96,93	0,00E+00	4396	Os04g0298600	94,62	0,00E+00	3233	-1163	Indica	Japonica	

maker-4-augustus-gene-77.0-mRNA-1	4	OsR498G0407987600.01.T01	98,88	0,00E+00	1716	Os04g0300600	100,00	0,00E+00	1126	-590	Indica	Japonica	
maker-4-augustus-gene-77.2-mRNA-1	4	OsR498G0407993000.01.T01	99,46	0,00E+00	1636	Os04g0301500	99,89	0,00E+00	1679	43	NA	Japonica	
maker-4-augustus-gene-78.0-mRNA-1	4	OsR498G0407995000.01.T02	99,67	0,00E+00	2161	Os04g0301700	99,59	0,00E+00	2152	-9	NA	Japonica	
maker-4-augustus-gene-78.3-mRNA-1	4	OsR498G0408000400.01.T01	99,59	0,00E+00	4387	Os04g0303500	99,96	0,00E+00	4427	40	NA	Japonica	Kinase-TM
maker-4-augustus-gene-79.0-mRNA-1	4	OsR498G0408002900.01.T01	99,87	0,00E+00	1403	Os04g0303900	100,00	0,00E+00	1407	4	NA	Japonica	
maker-4-augustus-gene-79.12-mRNA-1	4	OsR498G0408003400.01.T01	100,00	3,89E-125	448	Os04g0304000	100,00	1,35E-125	448	0	NA	Japonica	
maker-4-augustus-gene-79.2-mRNA-1	4	OsR498G0408005300.01.T01	93,71	0,00E+00	1074	Os04g0304400	93,86	0,00E+00	1078	4	NA	Japonica	
maker-4-augustus-gene-8.0-mRNA-1	4	OsR498G0407654100.01.T01	99,73	0,00E+00	2021	Os04g0182200	100,00	0,00E+00	2035	14	NA	Japonica	
maker-4-augustus-gene-8.2-mRNA-1	4	OsR498G0407651000.01.T01	99,16	0,00E+00	838	Os04g0181100	100,00	0,00E+00	856	18	NA	Japonica	
maker-4-augustus-gene-81.0-mRNA-1	4	OsR498G0408012900.01.T02	99,73	0,00E+00	1994	Os04g0307200	100,00	0,00E+00	2008	14	NA	Japonica	
maker-4-augustus-gene-82.41-mRNA-1	4	OsR498G0408024600.01.T01	98,89	0,00E+00	1111	Os04g0308500	99,53	0,00E+00	1129	18	NA	NA	
maker-4-augustus-gene-82.47-mRNA-1	4	OsR498G0408024700.01.T01	99,54	0,00E+00	1172	Os04g0308401	99,51	0,00E+00	1091	-81	NA	NA	
maker-4-augustus-gene-82.49-mRNA-1	4	OsR498G0408020900.01.T01	99,36	0,00E+00	1933	Os04g0309900	99,91	0,00E+00	1960	27	NA	Japonica	
maker-4-augustus-gene-82.51-mRNA-1	4	OsR498G0407829000.01.T02	99,38	0,00E+00	4571	Os04g0309600	98,49	0,00E+00	4493	-78	NA	NA	
maker-4-augustus-gene-83.34-mRNA-1	4	OsR498G0408020900.01.T01	99,54	0,00E+00	1942	Os04g0308600	100,00	0,00E+00	1965	23	NA	Japonica	
maker-4-augustus-gene-83.36-mRNA-1	4	OsR498G0408024700.01.T01	99,24	0,00E+00	1163	Os04g0308401	99,84	0,00E+00	1100	-63	NA	Japonica	
maker-4-augustus-gene-83.43-mRNA-1	4	OsR498G0408024600.01.T01	98,89	0,00E+00	1111	Os04g0308500	99,53	0,00E+00	1129	18	NA	NA	
maker-4-augustus-gene-83.49-mRNA-1	4	OsR498G0408025600.01.T01	99,44	0,00E+00	947	Os04g0310500	100,00	8,72E-154	542	-405	Indica	Japonica	
maker-4-augustus-gene-83.51-mRNA-1	4	OsR498G0408026400.01.T01	100,00	0,00E+00	3009	Os04g0310700	99,90	0,00E+00	1830	-1179	Indica	Japonica	
maker-4-augustus-gene-84.0-mRNA-1	4	OsR498G0408027100.01.T02	99,59	0,00E+00	4356	Os02g0469300	78,72	0,00E+00	1092	-3264	Indica	Japonica	
maker-4-augustus-gene-84.6-mRNA-1	4	OsR498G0408028500.01.T01	99,65	0,00E+00	2048	Os04g0311400	96,25	0,00E+00	1908	-140	NA	NA	
maker-4-augustus-gene-84.9-mRNA-1	4	OsR498G0408028800.01.T02	99,93	0,00E+00	2696	Os04g0311500	99,80	0,00E+00	1799	-897	Indica	Japonica	
maker-4-augustus-gene-85.0-mRNA-1	4	OsR498G0408032700.01.T01	96,80	0,00E+00	728	Os09g0120033	91,55	2,61E-178	624	-104	Indica	NA	
maker-4-augustus-gene-89.0-mRNA-1	4	OsR498G0408052500.01.T02	99,50	0,00E+00	2508	Os04g0329500	99,41	0,00E+00	2425	-83	NA	Japonica	
maker-4-augustus-gene-9.29-mRNA-1	4	OsR498G0407657100.01.T01	99,48	0,00E+00	2717	Os04g0182900	100,00	0,00E+00	2749	32	NA	Japonica	
maker-4-augustus-gene-9.32-mRNA-1	4	OsR498G0407659900.01.T01	97,90	0,00E+00	2704	Os04g0183500	100,00	0,00E+00	2771	67	NA	Japonica	
maker-4-augustus-gene-9.37-mRNA-1	4	OsR498G0407656600.01.T02	99,61	0,00E+00	1361	Os04g0182800	100,00	0,00E+00	1375	14	NA	Japonica	
maker-4-augustus-gene-9.40-mRNA-1	4	OsR498G0407658000.01.T02	99,76	0,00E+00	748	Os04g0183100	99,75	0,00E+00	707	-41	NA	Japonica	
maker-4-augustus-gene-91.23-mRNA-1	4	OsR498G0408072900.01.T02	100,00	0,00E+00	1775	Os04g0326300	99,17	0,00E+00	1709	-66	NA	NA	
maker-4-augustus-gene-91.40-mRNA-1	4	OsR498G0408115100.01.T01	99,63	0,00E+00	1466	Os04g0326000	99,76	0,00E+00	1469	3	NA	NA	
maker-4-augustus-gene-91.45-mRNA-1	4	OsR498G0408076800.01.T01	100,00	0,00E+00	2841	Os04g0320200	100,00	0,00E+00	2841	0	NA	NA	
maker-4-augustus-gene-92.0-mRNA-1	4	OsR498G0816044700.01.T01	92,25	0,00E+00	2163	Os04g0321100	100,00	0,00E+00	2576	413	Japonica	NA	
maker-4-augustus-gene-92.11-mRNA-1	4	OsR498G0408083200.01.T03	100,00	0,00E+00	2293	Os04g0321700	100,00	0,00E+00	2293	0	NA	NA	
maker-4-augustus-gene-92.18-mRNA-1	4	OsR498G0408083400.01.T01	100,00	0,00E+00	2522	Os04g0321800	98,36	0,00E+00	2387	-135	NA	Indica	
maker-4-augustus-gene-92.6-mRNA-1	4	OsR498G0408082700.01.T01	100,00	0,00E+00	5669	Os04g0321600	100,00	0,00E+00	5669	0	NA	NA	
maker-4-augustus-gene-95.17-mRNA-1	4	OsR498G0408115100.01.T01	97,06	0,00E+00	1957	Os04g0326000	97,41	0,00E+00	1976	19	NA	NA	
maker-4-augustus-gene-96.56-mRNA-1	4	OsR498G0408741100.01.T01	98,50	0,00E+00	2549	Os04g0470500	100,00	0,00E+00	4603	2054	Japonica	NA	CC-NBS-TM
maker-4-augustus-gene-96.59-mRNA-1	4	OsR498G0408740400.01.T03	99,69	2,67E-165	582	Os04g0470300	100,00	2,18E-167	587	5	NA	NA	
maker-4-augustus-gene-96.65-mRNA-1	4	OsR498G0408970400.01.T01	100,00	0,00E+00	893	Os04g0536300	100,00	0,00E+00	893	0	NA	NA	
maker-4-augustus-gene-97.3-mRNA-1	4	OsR498G0408118000.01.T02	100,00	0,00E+00	1770	Os04g0326300	100,00	0,00E+00	1741	-29	NA	Indica	
maker-4-augustus-gene-99.32-mRNA-1	4	OsR498G0408135500.01.T01	100,00	1,51E-135	482	Os04g0328900	100,00	5,24E-136	482	0	NA	NA	
maker-4-augustus-gene-99.40-mRNA-1	4	OsR498G0408139600.01.T01	99,79	0,00E+00	2526	Os04g0329500	99,85	0,00E+00	2452	-74	NA	NA	
maker-4-augustus-gene-99.46-mRNA-1	4	OsR498G0408142400.01.T01	100,00	0,00E+00	1645	Os04g0330200	100,00	0,00E+00	1023	-622	Indica	NA	
maker-4-augustus-gene-99.48-mRNA-1	4	OsR498G0408144600.01.T01	100,00	0,00E+00	5627	Os04g0201000	97,16	0,00E+00	5278	-349	NA	NA	
augustus_masked-5-processed-gene-10.1-mRNA-1	5	OsR498G0509614300.01.T01	99,88	0,00E+00	1544	Os05g0119000	100,00	0,00E+00	1548	4	NA	Japonica	
augustus_masked-5-processed-gene-10.16-mRNA-1	5	OsR498G0509618900.01.T01	99,76	0,00E+00	3765	Os05g0120200	100,00	0,00E+00	3782	17	NA	Japonica	
augustus_masked-5-processed-gene-10.17-mRNA-1	5	OsR498G0509620300.01.T01	98,07	0,00E+00	1156	Os05g0120300	100,00	0,00E+00	1196	40	NA	Japonica	
augustus_masked-5-processed-gene-109.42-mRNA-1	5	OsR498G0510225500.01.T03	100,00	0,00E+00	682	Os05g0270400	100,00	0,00E+00	682	0	NA	NA	
augustus_masked-5-processed-gene-110.11-mRNA-1	5	OsR498G0510230500.01.T01	100,00	0,00E+00	682	Os01g0248000	76,27	2,10E-39	161	-521	Indica	NA	
augustus_masked-5-processed-gene-110.25-mRNA-1	5	OsR498G0510234100.01.T01	100,00	0,00E+00	2646	Os05g0271900	100,00	0,00E+00	2646	0	NA	NA	
augustus_masked-5-processed-gene-121.10-mRNA-1	5	OsR498G0510294300.01.T01	100,00	0,00E+00	985	Os05g0283200	100,00	0,00E+00	985	0	NA	NA	
augustus_masked-5-processed-gene-126.11-mRNA-1	5	OsR498G0510480600.01.T01	76,58	0,00E+00	1859	Os05g0317700	99,96	0,00E+00	4552	2693	Japonica	NA	Kinase-TM
augustus_masked-5-processed-gene-126.9-mRNA-1	5	OsR498G0510480600.01.T01	76,58	0,00E+00	1859	Os05g0317700	99,96	0,00E+00	4552	2693	Japonica	NA	Kinase-TM
augustus_masked-5-processed-gene-13.16-mRNA-1	5	OsR498G0509634700.01.T01	99,44	0,00E+00	2535	Os05g0123200	99,89	0,00E+00	1625	-910	Indica	Japonica	
augustus_masked-5-processed-gene-136.35-mRNA-1	5	OsR498G0510374400.01.T01	100,00	0,00E+00	1948	Os05g0319900	86,11	0,00E+00	1266	-682	Indica	NA	
augustus_masked-5-processed-gene-139.21-mRNA-1	5	OsR498G0510398900.01.T01	99,87	0,00E+00	1381	Os05g0302600	99,87	0,00E+00	1381	0	NA	Indica	
augustus_masked-5-processed-gene-14.15-mRNA-1	5	OsR498G0509646500.01.T01	99,53	0,00E+00	4128	Os05g0125200	99,83	0,00E+00	4159	31	NA	Japonica	Kinase-TM
augustus_masked-5-processed-gene-140.24-mRNA-1	5	OsR498G0510409700.01.T01	100,00	0,00E+00	1775	Os05g0304800	99,70	0,00E+00	1762	-13	NA	Indica	
augustus_masked-5-processed-gene-141.42-mRNA-1	5	OsR498G0510413500.01.T01	99,74	0,00E+00	3433	Os05g0305600	100,00	0,00E+00	6244	2811	Japonica	Japonica	LRR-NBS
augustus_masked-5-processed-gene-146.21-mRNA-1	5	OsR498G0510437500.01.T06	99,23	0,00E+00	2302	Os05g0311000	99,23	0,00E+00	2287	-15	NA	NA	
augustus_masked-5-processed-gene-15.0-mRNA-1	5	OsR498G0509648100.01.T01	99,68	0,00E+00	1675	Os05g0125600	99,92	0,00E+00	2177	502	Japonica	Japonica	
augustus_masked-5-processed-gene-151.19-mRNA-1	5	OsR498G0510480600.01.T01	76,58	0,00E+00	1859	Os05g0317700	99,96	0,00E+00	4552	2693	Japonica	NA	Kinase-TM
augustus_masked-5-processed-gene-151.20-mRNA-1	5	OsR498G0510480600.01.T01	77,20	0,00E+00	1911	Os05g0317900	100,00	0,00E+00	4583	2672	Japonica	Japonica	CC-Kinase-TM
augustus_masked-5-processed-gene-155.12-mRNA-1	5	OsR498G0510496700.01.T01	100,00	0,00E+00	3149	Os05g0324000	100,00	0,00E+00	3149	0	NA	NA	
augustus_masked-5-processed-gene-155.32-mRNA-1	5	OsR498G0510503700.01.T01	100,00	0,00E+00	1925	Os12g0468900	80,72	0,00E+00	776	-1149	Indica	Indica	
augustus_masked-5-processed-gene-155.34-mRNA-1	5	OsR498G0510504100.01.T02	100,00	0,00E+00	812	Os05g0326400	99,78	0,00E+00	808	-4	NA	Indica	
augustus_masked-5-processed-gene-159.0-mRNA-1	5	OsR498G0510518700.01.T01	100,00	0,00E+00	818	Os05g0329100	99,78	0,00E+00	813	-5	NA	NA	
augustus_masked-5-processed-gene-159.10-mRNA-1	5	OsR498G0510519500.01.T01	100,00	0,00E+00	807	Os05g0329100	98,68	0,00E+00	792	-15	NA	NA	
augustus_masked-5-processed-gene-159.11-mRNA-1	5	OsR498G0510519700.01.T01	100,00	0,00E+00	818	Os05g0329100	100,00	0,00E+00	818	0	NA	NA	
augustus_masked-5-processed-gene-159.12-mRNA-1	5	OsR498G0510519700.01.T01	99,56	0,00E+00	809	Os05g0329100	99,56	0,00E+00	809	0	NA	NA	
augustus_masked-5-processed-gene-159.13-mRNA-1	5	OsR498G0510519700.01.T01	99,56	0,00E+00	809	Os05g0329400	99,56	0,00E+00	809	0	NA	NA	
augustus_masked-5-processed-gene-159.8-mRNA-1	5	OsR498G0510519700.01.T01	100,00	0,00E+00	818	Os05g0329100	100,00	0,00E+00	818	0	NA	NA	
augustus_masked-5-processed-gene-160.0-mRNA-1	5	OsR498G0510521700.01.T01	99,78	0,00E+00	813	Os05g0329400	99,12	0,00E+00	800	-13	NA	NA	
augustus_masked-5-processed-gene-160.12-mRNA-1	5	OsR498G0510523500.01.T01	100,00	0,00E+00	818	Os05g0330600	99,78	0,00E+00	813	-5	NA	Indica	
augustus_masked-5-processed-gene-161.28-mRNA-1	5	OsR498G0510526000.01.T01	100,00	0,00E+00	834	Os05g0331550	100,00	0,00E+00	834	0	NA	Indica	
augustus_masked-5-processed-gene-161.9-mRNA-1	5	OsR498G0510529300.01.T01	100,00	0,00E+00	2479	Os05g0332300	99,93	0,00E+00	2474	-5	NA	Indica	Kinase-TM
augustus_masked-5-processed-gene-169.12-mRNA-1	5	OsR498G0510328900.01.T01	75,42	4,13E-46	186	Os07g0433200	95,64	4,67E-135	480	294	Japonica	Indica	
augustus_masked-5-processed-gene-17.20-mRNA-1	5	OsR498G0100416900.01.T01	96,80	1,68E-115	416	Os05g0129400	100,00	0,00E+00	666	250	Japonica	Japonica	
augustus_masked-5-processed-gene-170.3-mRNA-1	5	OsR498G0510586600.01.T01	99,83	0,00E+00	1040	Os05g0346300	100,00	0,00E+00	1045	5	NA	Japonica	
augustus_masked-5-processed-gene-176.24-mRNA-1	5	OsR498G0510623700.01.T02	99,92	0,00E+00	2198	Os05g0355200	99,95	0,00E+00	3421	1223	Japonica	NA	
augustus_masked-5-processed-gene-179.11-mRNA-1	5	OsR498G0510650500.01.T01	98,68	0,00E+00	1451	Os05g0360400	99,83	0,00E+00	1084	-367	Indica	Japonica	
augustus_masked-5-processed-gene-179.17-mRNA-1	5	OsR498G0510653900.01.T01	97,36	0,00E+00	2454	Os05g0361000	99,76	0,00E+00	2947	493	Japonica	Japonica	
augustus_masked-5-processed-gene-183.26-mRNA-1	5	OsR498G0510671900.01.T01	100,00	0,00E+00	2013	Os05g0365000	94,98	0,00E+00	1801	-212	Indica	Indica	
augustus_masked-5-processed-gene-183.36-mRNA-1	5	OsR498G0510674100.01.T01	100,00	0,00E+00	4919	Os05g0365500	97,81	0,00E+00	4663	-256	NA	Indica	
augustus_masked-5-processed-gene-185.33-mRNA-1	5	OsR498G0510682900.01.T01	100,00	0,00E+00	996	Os05g0368000	99,64	0,00E+00	987	-9	NA	NA	
augustus_masked-5-processed-gene-187.18-mRNA-1	5	OsR498G0510689700.01.T01	100,00	0,00E+00	682	Os05g0369300	71,46	4,63E-54	210	-472	Indica	NA	
augustus_masked-5-processed-gene-187.21-mRNA-1	5	OsR498G0510692500.01.T06	100,00	0,00E+00	4194	Os05g0370600	99,96	0,00E+00	4189	-5	NA	Indica	
augustus_masked-5-processed-gene-189.55-mRNA-1	5	OsR498G0510704700.01.T02	99,92	0,00E+00	2382	Os05g0373900	99,70	0,00E+00	2369	-13	NA	Indica	
augustus_masked-5-processed-gene-19.31-mRNA-1	5	OsR498G0509678900.01.T01	98,41	0,00E+00	3605	Os05g0133900	98,61	0,00E+00	3535	-70	NA	Japonica	
augustus_masked-5-processed-gene-193.19-mRNA-1	5	OsR498G0510731100.01.T01	91,86	0,00E+00	3920	Os05g0380800	100,00	0,00E+00	7808	3888	Japonica	Japonica	NBS-TM
augustus_masked-5-processed-gene-194.27-mRNA-1	5	OsR498G0510736300.01.T01	100,00	0,00E+00	5844	Os05g0381700	100,00	0,00E+00	3485	-2359	Indica	NA	
augustus_masked-5-processed-gene-194.37-mRNA-1	5	OsR498G0510739700.01.T01	99,68	0,00E+00	2829	Os01g0951500	77,23	0,00E+00	1167	-1662	Indica	Japonica	
augustus_masked-5-processed-gene-195.20-mRNA-1	5	OsR498G0510749100.01.T01	99,11	0,00E+00	1187	Os05g0385400	100,00	0,00E+00	1213	26	NA	Japonica	
augustus_masked-5-processed-gene-196.18-mRNA-1	5	OsR498G0510752300.01.T01	99,91	0,00E+00	1946	Os05g0386000	100,00	0,00E+00	1954	8	NA	Japonica	
augustus_masked-5-processed-gene-2.69-mRNA-1	5	OsR498G0509566700.01.T01	70,83	8,37E-125	448	Os05g0104600	100,00	0,00E+00	1840	1392	Japonica	NA	LRR-TM
augustus_masked-5-processed-gene-20.46-mRNA-1	5	OsR498G0509688100.01.T02	99,66	0,00E+00	2131	Os05g0135700	99,75	0,00E+00	2135	4	NA	NA	
augustus_masked-5-processed-gene-200.46-mRNA-1	5	OsR498G0510775900.01.T01	99,73	0,00E+00	1333	Os05g0393200	100,00	0,00E+00	1341	8	NA	Japonica	
augustus_masked-5-processed-gene-203.37-mRNA-1	5	OsR498G0510790300.01.T04	99,51	0,00E+00	722	Os05g0397800	100,00	2,11E-147	520	-202	Indica	Japonica	
augustus_masked-5-processed-gene-203.56-mRNA-1	5	OsR498G0510793300.01.T01	98,23	0,00E+00	1076	Os05g0399300	99,04	0,00E+00	1098	22	NA	Japonica	
augustus_masked-5-processed-gene-206.0-mRNA-1	5	OsR498G0510802100.01.T01	99,85	0,00E+00	2361	Os05g0402900	100,00	0,00E+00	2370	9	NA	Japonica	
augustus_masked-5-processed-gene-206.1-mRNA-1	5	OsR498G0510802300.01.T01	100,00	0,00E+00	2370	Os05g0403000	100,00	0,00E+00	2370	0	NA	Japonica	
augustus_masked-5-processed-gene-206.15-mRNA-1	5	OsR498G0510804300.01.T01	98,04	0,00E+00	2632	Os05g0404000	100,00	0,00E+00	2755	123	NA	Japonica	
augustus_masked-5-processed-gene-206.3-mRNA-1	5	OsR498G0510803300.01.T01	98,47	0,00E+00	2373	Os05g0403300	100,00	0,00E+00	2468	95	NA	Japonica	
augustus_masked-5-processed-gene-210.15-mRNA-1	5	OsR498G0510822100.01.T01	99,86	0,00E+00	1298	Os05g0409100	100,00	0,00E+00	1698	400	Japonica	Japonica	
augustus_masked-5-processed-gene-211.21-mRNA-1	5	OsR498G0510825100.01.T01	99,92	0,00E+00	2171	Os05g0409800	100,00	0,00E+00	2176	5	NA	Japonica	
augustus_masked-5-processed-gene-213.18-mRNA-1	5	OsR498G0510836900.01.T02	99,66	0,00E+00	2123	Os05g0413000	99,92	0,00E+00	2133	10	NA	Indica	
augustus_masked-5-processed-gene-215.17-mRNA-1	5	OsR498G0510852300.01.T01	100,00	0,00E+00	677	Os05g0417700	99,32	1,60E-148	524	-153	Indica	NA	
augustus_masked-5-processed-gene-218.0-mRNA-1	5	OsR498G0612271200.01.T01	92,59	0,00E+00	754	Os05g0422200	100,00	0,00E+00	1070	316	Japonica	Japonica	
augustus_masked-5-processed-gene-219.23-mRNA-1	5	OsR498G0510877500.01.T01	99,12	0,00E+00	803	Os06g0261300	79,83	3,34E-82	304	-499	Indica	Japonica	
augustus_masked-5-processed-gene-22.10-mRNA-1	5	OsR498G0509697700.01.T05	99,65	0,00E+00	1009	Os05g0137600	100,00	0,00E+00	1023	14	NA	Japonica	
augustus_masked-5-processed-gene-22.13-mRNA-1	5	OsR498G0100702400.01.T01	78,98	1,92E-176	620	Os05g0138200	99,89	0,00E+00	1598	978	Japonica	Japonica	
augustus_masked-5-processed-gene-220.31-mRNA-1	5	OsR498G0510884500.01.T01	99,48	0,00E+00	1021	Os05g0426900	100,00	0,00E+00	1034	13	NA	Japonica	
augustus_masked-5-processed-gene-220.34-mRNA-1	5	OsR498G0510884900.01.T02	99,82	0,00E+00	1977	Os05g0427100	100,00	0,00E+00	1986	9	NA	Japonica	
augustus_masked-5-processed-gene-222.18-mRNA-1	5	OsR498G0510899100.01.T01	100,00	0,00E+00	2933	Os05g0430800	100,00	0,00E+00	2933	0	NA	NA	
augustus_masked-5-processed-gene-224.3-mRNA-1	5	OsR498G0510908700.01.T01	99,69	8,65E-164	577	Os05g0434200	92,86	2,31E-159	560	-17	NA	NA	
augustus_masked-5-processed-gene-225.1-mRNA-1	5	OsR498G0510915300.01.T07	100,00	0,00E+00	1862	Os05g0436400	100,00	0,00E+00	1537	-325	Indica	NA	
augustus_masked-5-processed-gene-225.19-mRNA-1	5	OsR498G0510917300.01.T01	100,00	0,00E+00	1975	Os05g0437133	100,00	0,00E+00	1240	-735	Indica	NA	
augustus_masked-5-processed-gene-226.19-mRNA-1	5	OsR498G0510925700.01.T01	100,00	0,00E+00	2452	Os05g0439400	100,00	0,00E+00	2452	0	NA	NA	
augustus_masked-5-processed-gene-226.22-mRNA-1	5	OsR498G0510927500.01.T01	99,59	0,00E+00	1757	Os05g0440000	100,00	0,00E+00	1775	18	NA	Japonica	
augustus_masked-5-processed-gene-227.2-mRNA-1	5	OsR498G0510929500.01.T01	99,57	0,00E+00	1246	Os05g0440900	99,86	0,00E+00	1251	5	NA	Japonica	
augustus_masked-5-processed-gene-229.13-mRNA-1	5	OsR498G0510943100.01.T01	99,55	0,00E+00	2782	Os05g0445100	99,94	0,00E+00	2810	28	NA	Japonica	
augustus_masked-5-processed-gene-23.6-mRNA-1	5	OsR498G0509710100.01.T01	98,25	0,00E+00	991	Os05g0140500	100,00	0,00E+00	771	-220	Indica	Japonica	
augustus_masked-5-processed-gene-232.3-mRNA-1	5	OsR498G0510960100.01.T01	99,45	0,00E+00	3548	Os05g0450600	100,00	0,00E+00	2554	-994	Indica	Japonica	
augustus_masked-5-processed-gene-233.26-mRNA-1	5	OsR498G0510966100.01.T01	99,61	0,00E+00	917	Os05g0452800	100,00	0,00E+00	926	9	NA	Japonica	
augustus_masked-5-processed-gene-234.11-mRNA-1	5	OsR498G0510977300.01.T03	99,92	0,00E+00	4726	Os05g0455900	100,00	0,00E+00	4735	9	NA	Japonica	
augustus_masked-5-processed-gene-236.8-mRNA-1	5	OsR498G0510985100.01.T02	99,85	0,00E+00	1208	Os05g0459300	100,00	0,00E+00	1213	5	NA	Japonica	
augustus_masked-5-processed-gene-238.0-mRNA-1	5	OsR498G0510997100.01.T02	99,86	0,00E+00	2475	Os05g0462500	100,00	0,00E+00	2484	9	NA	Japonica	
augustus_masked-5-processed-gene-239.31-mRNA-1	5	OsR498G0510997800.01.T01	99,35	9,34E-156	549	Os05g0466600	100,00	6,27E-159	558	9	NA	Japonica	
augustus_masked-5-processed-gene-240.55-mRNA-1	5	OsR498G0511013500.01.T01	76,75	2,42E-170	600	Os05g0468200	100,00	0,00E+00	2129	1529	Japonica	Japonica	LRR-TM
augustus_masked-5-processed-gene-241.37-mRNA-1	5	OsR498G0511020500.01.T01	99,76	0,00E+00	3029	Os05g0470700	100,00	0,00E+00	3047	18	NA	Japonica	
augustus_masked-5-processed-gene-243.16-mRNA-1	5	OsR498G0511029900.01.T01	99,85	0,00E+00	1192	Os05g0474400	100,00	0,00E+00	1196	4	NA	Japonica	
augustus_masked-5-processed-gene-244.18-mRNA-1	5	OsR498G0511031100.01.T01	99,81	0,00E+00	927	Os05g0474700	100,00	0,00E+00	931	4	NA	Japonica	
augustus_masked-5-processed-gene-244.29-mRNA-1	5	OsR498G0511033100.01.T01	99,31	5,17E-146	517	Os04g0544200	94,92	4,84E-84	309	-208	Indica	Japonica	
augustus_masked-5-processed-gene-245.14-mRNA-1	5	OsR498G0511042100.01.T01	99,66	0,00E+00	3637	Os05g0476700	100,00	0,00E+00	3669	32	NA	Japonica	
augustus_masked-5-processed-gene-245.15-mRNA-1	5	OsR498G0511043500.01.T01	98,95	0,00E+00	2506	Os05g0477200	100,00	0,00E+00	2560	54	NA	Japonica	
augustus_masked-5-processed-gene-245.17-mRNA-1	5	OsR498G0511045900.01.T01	99,74	0,00E+00	700	Os05g0477900	99,74	0,00E+00	700	0	NA	Japonica	
augustus_masked-5-processed-gene-248.14-mRNA-1	5	OsR498G0511060710.01.T02	100,00	0,00E+00	2316	Os05g0482400	100,00	0,00E+00	2489	173	NA	Japonica	
augustus_masked-5-processed-gene-251.49-mRNA-1	5	OsR498G0511080900.01.T03	99,90	0,00E+00	1728	Os05g0489100	100,00	0,00E+00	1732	4	NA	Japonica	
augustus_masked-5-processed-gene-252.1-mRNA-1	5	OsR498G0511083100.01.T01	100,00	1,03E-163	576	Os05g0490100	100,00	3,60E-164	576	0	NA	Japonica	
augustus_masked-5-processed-gene-253.16-mRNA-1	5	OsR498G0511090900.01.T01	80,07	0,00E+00	1694	Os05g0492600	100,00	0,00E+00	3404	1710	Japonica	Japonica	CC-LRR-NBS-TM
augustus_masked-5-processed-gene-254.30-mRNA-1	5	OsR498G0511097100.01.T04	99,81	0,00E+00	1912	Os05g0494500	100,00	0,00E+00	1921	9	NA	Japonica	
augustus_masked-5-processed-gene-254.42-mRNA-1	5	OsR498G0511098700.01.T01	100,00	0,00E+00	1997	Os05g0495100	100,00	0,00E+00	1997	0	NA	Japonica	
augustus_masked-5-processed-gene-254.47-mRNA-1	5	OsR498G0511102100.01.T01	99,75	0,00E+00	716	Os05g0496000	100,00	0,00E+00	720	4	NA	Japonica	
augustus_masked-5-processed-gene-255.40-mRNA-1	5	OsR498G0511105800.01.T06	99,95	0,00E+00	3531	Os05g0497500	100,00	0,00E+00	2664	-867	Indica	Japonica	
augustus_masked-5-processed-gene-257.22-mRNA-1	5	OsR498G0511113900.01.T01	97,27	0,00E+00	2466	Os05g0500000	100,00	0,00E+00	2630	164	NA	Japonica	
augustus_masked-5-processed-gene-257.25-mRNA-1	5	OsR498G0511115500.01.T01	99,61	0,00E+00	2283	Os05g0500600	100,00	0,00E+00	2305	22	NA	Japonica	
augustus_masked-5-processed-gene-257.26-mRNA-1	5	OsR498G0511115700.01.T01	99,08	0,00E+00	1150	Os05g0500700	100,00	0,00E+00	1245	95	NA	Japonica	
augustus_masked-5-processed-gene-257.3-mRNA-1	5	OsR498G0511113100.01.T01	99,62	0,00E+00	2805	Os05g0499600	100,00	0,00E+00	2636	-169	NA	Japonica	
augustus_masked-5-processed-gene-257.5-mRNA-1	5	OsR498G0511113500.01.T01	99,59	0,00E+00	2598	Os05g0499800	100,00	0,00E+00	2625	27	NA	Japonica	
augustus_masked-5-processed-gene-259.3-mRNA-1	5	OsR498G0511123500.01.T01	98,53	8,78E-97	353	Os01g0778400	72,99	3,07E-21	100	-253	Indica	Japonica	
augustus_masked-5-processed-gene-261.38-mRNA-1	5	OsR498G0511140700.01.T01	99,78	0,00E+00	1615	Os05g0506000	100,00	0,00E+00	1624	9	NA	Japonica	
augustus_masked-5-processed-gene-261.52-mRNA-1	5	OsR498G0511143300.01.T01	94,81	0,00E+00	799	Os05g0507000	100,00	0,00E+00	904	105	Japonica	Japonica	
augustus_masked-5-processed-gene-261.53-mRNA-1	5	OsR498G0511143700.01.T02	99,74	0,00E+00	2045	Os05g0507300	100,00	0,00E+00	1734	-311	Indica	Japonica	
augustus_masked-5-processed-gene-263.13-mRNA-1	5	OsR498G0511160900.01.T02	99,86	0,00E+00	1284	Os05g0510100	100,00	0,00E+00	1288	4	NA	Japonica	
augustus_masked-5-processed-gene-264.42-mRNA-1	5	OsR498G0511161700.01.T02	99,76	0,00E+00	2222	Os05g0510400	100,00	0,00E+00	2235	13	NA	Japonica	
augustus_masked-5-processed-gene-265.1-mRNA-1	5	OsR498G0511168700.01.T02	99,61	9,42E-130	463	Os05g0512200	100,00	2,70E-131	466	3	NA	Japonica	
augustus_masked-5-processed-gene-265.3-mRNA-1	5	OsR498G0511170100.01.T01	95,94	0,00E+00	1048	Os05g0512600	100,00	0,00E+00	1122	74	NA	Japonica	
augustus_masked-5-processed-gene-266.19-mRNA-1	5	OsR498G0511175300.01.T02	99,27	0,00E+00	2681	Os05g0514200	100,00	0,00E+00	2755	74	NA	Japonica	Kinase-TM
augustus_masked-5-processed-gene-266.30-mRNA-1	5	OsR498G0511177100.01.T01	99,61	0,00E+00	1842	Os05g0514500	99,90	0,00E+00	1859	17	NA	Indica	
augustus_masked-5-processed-gene-267.47-mRNA-1	5	OsR498G0511181500.01.T01	99,60	0,00E+00	2252	Os05g0515700	99,60	0,00E+00	2252	0	NA	Japonica	
augustus_masked-5-processed-gene-267.60-mRNA-1	5	OsR498G0511182700.01.T04	99,58	0,00E+00	841	Os05g0516300	100,00	0,00E+00	839	-2	NA	Japonica	
augustus_masked-5-processed-gene-268.21-mRNA-1	5	OsR498G0511193300.01.T01	100,00	0,00E+00	1467	Os05g0519000	100,00	0,00E+00	1467	0	NA	Japonica	
augustus_masked-5-processed-gene-269.37-mRNA-1	5	OsR498G0511194100.01.T05	99,91	0,00E+00	1937	Os05g0519200	100,00	0,00E+00	1941	4	NA	Japonica	Kinase-TM
augustus_masked-5-processed-gene-269.52-mRNA-1	5	OsR498G0511196700.01.T01	98,16	0,00E+00	2051	Os02g0563800	80,55	0,00E+00	1127	-924	Indica	Japonica	
augustus_masked-5-processed-gene-273.24-mRNA-1	5	OsR498G0511217700.01.T01	99,20	0,00E+00	1102	Os05g0526600	100,00	0,00E+00	1126	24	NA	Japonica	
augustus_masked-5-processed-gene-273.26-mRNA-1	5	OsR498G0101954200.01.T01	77,17	0,00E+00	691	Os05g0526900	100,00	0,00E+00	2587	1896	Japonica	Japonica	
augustus_masked-5-processed-gene-277.2-mRNA-1	5	OsR498G0511248100.01.T07	100,00	0,00E+00	1404	Os05g0535500	100,00	0,00E+00	1404	0	NA	NA	
augustus_masked-5-processed-gene-279.0-mRNA-1	5	OsR498G0511260900.01.T01	100,00	0,00E+00	2322	Os05g0539300	76,32	0,00E+00	847	-1475	Indica	NA	
augustus_masked-5-processed-gene-279.1-mRNA-1	5	OsR498G0511261300.01.T01	100,00	0,00E+00	2159	Os05g0539300	78,08	0,00E+00	970	-1189	Indica	NA	
augustus_masked-5-processed-gene-279.2-mRNA-1	5	OsR498G0511262700.01.T01	100,00	0,00E+00	2284	Os05g0539300	100,00	0,00E+00	2111	-173	NA	NA	
augustus_masked-5-processed-gene-28.69-mRNA-1	5	OsR498G0509740900.01.T01	96,83	0,00E+00	2073	Os05g0150100	99,92	0,00E+00	2148	75	NA	Japonica	
augustus_masked-5-processed-gene-280.46-mRNA-1	5	OsR498G0511268300.01.T01	100,00	0,00E+00	2257	Os05g0541400	99,28	0,00E+00	2205	-52	NA	Indica	
augustus_masked-5-processed-gene-280.62-mRNA-1	5	OsR498G0511271700.01.T01	100,00	0,00E+00	1088	Os05g0542500	100,00	0,00E+00	1088	0	NA	NA	
augustus_masked-5-processed-gene-280.65-mRNA-1	5	OsR498G0511272500.01.T06	95,58	0,00E+00	2182	Os05g0542800	91,84	0,00E+00	1992	-190	NA	NA	
augustus_masked-5-processed-gene-282.0-mRNA-1	5	OsR498G0511283900.01.T02	99,52	0,00E+00	1485	Os05g0545500	98,57	0,00E+00	1461	-24	NA	NA	
augustus_masked-5-processed-gene-282.8-mRNA-1	5	OsR498G0511285700.01.T01	86,00	0,00E+00	659	Os05g0546300	99,85	0,00E+00	1224	565	Japonica	Indica	
augustus_masked-5-processed-gene-283.54-mRNA-1	5	OsR498G0511288100.01.T01	99,64	0,00E+00	1486	Os05g0548600	99,62	0,00E+00	2364	878	Japonica	Indica	
augustus_masked-5-processed-gene-283.56-mRNA-1	5	OsR498G0511290700.01.T01	98,90	0,00E+00	1758	Os05g0549300	98,70	0,00E+00	1749	-9	NA	NA	
augustus_masked-5-processed-gene-283.60-mRNA-1	5	OsR498G0511291300.01.T01	99,74	0,00E+00	2796	Os05g0549700	99,49	0,00E+00	2790	-6	NA	NA	
augustus_masked-5-processed-gene-284.0-mRNA-1	5	OsR498G0511292500.01.T01	98,65	0,00E+00	2072	Os05g0549800	99,33	0,00E+00	2102	30	NA	NA	
augustus_masked-5-processed-gene-285.8-mRNA-1	5	OsR498G0511305300.01.T01	98,51	0,00E+00	1402	Os05g0552600	86,72	0,00E+00	1342	-60	NA	NA	
augustus_masked-5-processed-gene-289.12-mRNA-1	5	OsR498G0511335100.01.T01	99,81	0,00E+00	1869	Os05g0560600	99,81	0,00E+00	1869	0	NA	NA	
augustus_masked-5-processed-gene-292.28-mRNA-1	5	OsR498G0511348300.01.T03	100,00	0,00E+00	1651	Os05g0564200	100,00	0,00E+00	1651	0	NA	Indica	
augustus_masked-5-processed-gene-294.14-mRNA-1	5	OsR498G0511364900.01.T01	100,00	0,00E+00	1245	Os05g0568800	99,57	0,00E+00	1232	-13	NA	Indica	
augustus_masked-5-processed-gene-295.30-mRNA-1	5	OsR498G0511372100.01.T01	100,00	0,00E+00	2760	Os05g0571100	99,41	0,00E+00	2719	-41	NA	Indica	
augustus_masked-5-processed-gene-296.0-mRNA-1	5	OsR498G0511373500.01.T01	99,41	0,00E+00	902	Os05g0571600	99,80	0,00E+00	911	9	NA	NA	
augustus_masked-5-processed-gene-299.37-mRNA-1	5	OsR498G0511397100.01.T02	99,83	0,00E+00	2048	Os05g0578500	99,91	0,00E+00	2052	4	NA	Indica	
augustus_masked-5-processed-gene-299.52-mRNA-1	5	OsR498G0511399700.01.T01	99,69	0,00E+00	3489	Os05g0579200	100,00	0,00E+00	3517	28	NA	Japonica	
augustus_masked-5-processed-gene-299.59-mRNA-1	5	OsR498G0511403100.01.T01	99,60	0,00E+00	7648	Os05g0579900	100,00	0,00E+00	3837	-3811	Indica	Japonica	
augustus_masked-5-processed-gene-3.56-mRNA-1	5	OsR498G0509568900.01.T03	100,00	0,00E+00	7661	Os01g0888500	96,05	0,00E+00	3864	-3797	Indica	NA	
augustus_masked-5-processed-gene-3.57-mRNA-1	5	OsR498G0509566700.01.T01	71,43	1,55E-128	461	Os05g0104700	100,00	0,00E+00	2176	1715	Japonica	NA	LRR-TM
augustus_masked-5-processed-gene-30.20-mRNA-1	5	OsR498G0509751100.01.T01	100,00	0,00E+00	1651	Os05g0152900	98,90	0,00E+00	2069	418	Japonica	Japonica	
augustus_masked-5-processed-gene-30.27-mRNA-1	5	OsR498G0509751900.01.T03	99,73	0,00E+00	3322	Os05g0153200	99,89	0,00E+00	3335	13	NA	Japonica	
augustus_masked-5-processed-gene-30.28-mRNA-1	5	OsR498G0509752700.01.T01	100,00	0,00E+00	2749	Os05g0153400	99,93	0,00E+00	2745	-4	NA	Japonica	
augustus_masked-5-processed-gene-30.29-mRNA-1	5	OsR498G0816083200.01.T02	95,41	0,00E+00	973	Os05g0128900	95,74	0,00E+00	982	9	NA	Japonica	
augustus_masked-5-processed-gene-300.17-mRNA-1	5	OsR498G0511407900.01.T03	99,13	0,00E+00	2620	Os05g0581800	97,59	0,00E+00	2551	-69	NA	Japonica	
augustus_masked-5-processed-gene-301.25-mRNA-1	5	OsR498G0511410700.01.T01	99,63	0,00E+00	2397	Os05g0582500	100,00	0,00E+00	2468	71	NA	Japonica	
augustus_masked-5-processed-gene-301.26-mRNA-1	5	OsR498G0511410900.01.T01	99,34	0,00E+00	2406	Os05g0582600	100,00	0,00E+00	2446	40	NA	Japonica	
augustus_masked-5-processed-gene-301.27-mRNA-1	5	OsR498G0511411500.01.T01	99,77	0,00E+00	2384	Os05g0582800	100,00	0,00E+00	2397	13	NA	Japonica	
augustus_masked-5-processed-gene-301.45-mRNA-1	5	OsR498G0511413700.01.T01	98,84	5,51E-126	451	Os05g0583400	100,00	2,51E-131	466	15	NA	Japonica	
augustus_masked-5-processed-gene-301.47-mRNA-1	5	OsR498G0511414500.01.T01	99,47	0,00E+00	1666	Os05g0583600	99,88	0,00E+00	1462	-204	Indica	Japonica	
augustus_masked-5-processed-gene-302.32-mRNA-1	5	OsR498G0101562800.01.T03	74,19	0,00E+00	1166	Os05g0585800	99,95	0,00E+00	3783	2617	Japonica	Japonica	Kinase-TM
augustus_masked-5-processed-gene-304.14-mRNA-1	5	OsR498G0511432300.01.T02	99,33	0,00E+00	2382	Os05g0588250	99,93	0,00E+00	2431	49	NA	Japonica	Kinase-LRR-TM
augustus_masked-5-processed-gene-304.15-mRNA-1	5	OsR498G0511432300.01.T01	99,69	0,00E+00	1144	Os05g0588300	100,00	0,00E+00	1104	-40	NA	Japonica	Kinase
augustus_masked-5-processed-gene-305.12-mRNA-1	5	OsR498G0511439500.01.T01	99,64	0,00E+00	1498	Os05g0591300	99,93	0,00E+00	2452	954	Japonica	Japonica	
augustus_masked-5-processed-gene-305.14-mRNA-1	5	OsR498G0511440100.01.T01	99,76	0,00E+00	1514	Os05g0591400	100,00	0,00E+00	2300	786	Japonica	Japonica	
augustus_masked-5-processed-gene-308.18-mRNA-1	5	OsR498G0511459500.01.T01	100,00	0,00E+00	2089	Os05g0596500	100,00	0,00E+00	2089	0	NA	NA	
augustus_masked-5-processed-gene-31.3-mRNA-1	5	OsR498G0509767100.01.T01	99,95	0,00E+00	3855	Os05g0154700	100,00	0,00E+00	2527	-1328	Indica	Japonica	
augustus_masked-5-processed-gene-33.40-mRNA-1	5	OsR498G0509774500.01.T02	98,49	0,00E+00	917	Os05g0157100	100,00	0,00E+00	953	36	NA	Japonica	
augustus_masked-5-processed-gene-37.37-mRNA-1	5	OsR498G0509802100.01.T01	98,98	0,00E+00	862	Os05g0164200	99,26	0,00E+00	951	89	NA	Japonica	
augustus_masked-5-processed-gene-38.0-mRNA-1	5	OsR498G0509806700.01.T01	99,81	0,00E+00	921	Os05g0165200	99,80	0,00E+00	882	-39	NA	NA	
augustus_masked-5-processed-gene-39.47-mRNA-1	5	OsR498G1019116500.01.T01	84,91	2,13E-107	389	Os05g0457000	85,37	3,15E-106	384	-5	NA	Japonica	

augustus_masked-5-processed-gene-4.1-mRNA-1	5	OsR498G0509574900.01.T01	99,96	0,00E+00	4963	Os05g0106700	99,95	0,00E+00	3468	-1495	Indica	NA	
augustus_masked-5-processed-gene-4.11-mRNA-1	5	OsR498G0509576500.01.T05	99,70	0,00E+00	3624	Os05g0107300	99,85	0,00E+00	3637	13	NA	Japonica	
augustus_masked-5-processed-gene-47.0-mRNA-1	5	OsR498G0509858900.01.T01	99,30	9,13E-143	506	Os05g0179100	99,65	2,61E-144	510	4	NA	Japonica	
augustus_masked-5-processed-gene-5.13-mRNA-1	5	OsR498G0509583500.01.T01	100,00	0,00E+00	2094	Os05g0110000	99,90	0,00E+00	1803	-291	Indica	Japonica	
augustus_masked-5-processed-gene-52.27-mRNA-1	5	OsR498G0509887900.01.T01	100,00	0,00E+00	3265	Os01g0187400	78,39	0,00E+00	1911	-1354	Indica	NA	
augustus_masked-5-processed-gene-53.36-mRNA-1	5	OsR498G0509898500.01.T01	99,87	0,00E+00	1367	Os05g0187500	100,00	0,00E+00	2695	1328	Japonica	NA	
augustus_masked-5-processed-gene-53.39-mRNA-1	5	OsR498G0509901500.01.T01	100,00	2,31E-137	488	Os05g0188100	100,00	8,07E-138	488	0	NA	Japonica	
augustus_masked-5-processed-gene-58.31-mRNA-1	5	OsR498G0202873200.01.T01	80,11	6,12E-90	332	Os05g0457000	82,02	1,74E-129	461	129	Japonica	NA	
augustus_masked-5-processed-gene-59.17-mRNA-1	5	OsR498G0509943900.01.T03	99,66	0,00E+00	1036	Os05g0198200	99,52	0,00E+00	744	-292	Indica	NA	
augustus_masked-5-processed-gene-6.59-mRNA-1	5	OsR498G0509588500.01.T01	99,77	0,00E+00	792	Os05g0111200	100,00	0,00E+00	796	4	NA	Japonica	
augustus_masked-5-processed-gene-62.19-mRNA-1	5	OsR498G0509966100.01.T01	97,60	0,00E+00	708	Os12g0117400	87,56	3,24E-69	260	-448	Indica	NA	
augustus_masked-5-processed-gene-63.9-mRNA-1	5	OsR498G0509974900.01.T01	100,00	0,00E+00	1965	Os05g0203500	99,44	0,00E+00	1895	-70	NA	NA	
augustus_masked-5-processed-gene-66.0-mRNA-1	5	OsR498G0509990300.01.T01	99,75	0,00E+00	2130	Os05g0207200	99,79	0,00E+00	2519	389	Japonica	NA	
augustus_masked-5-processed-gene-67.8-mRNA-1	5	OsR498G0509995200.01.T01	98,77	0,00E+00	848	Os05g0208900	100,00	0,00E+00	1040	192	Japonica	Japonica	
augustus_masked-5-processed-gene-69.1-mRNA-1	5	OsR498G0510003100.01.T01	97,90	0,00E+00	3661	Os05g0212100	100,00	0,00E+00	4453	792	Japonica	Japonica	
augustus_masked-5-processed-gene-7.3-mRNA-1	5	OsR498G0509596900.01.T01	99,85	0,00E+00	3569	Os05g0112900	99,95	0,00E+00	3578	9	NA	Japonica	
augustus_masked-5-processed-gene-71.9-mRNA-1	5	OsR498G0510025900.01.T02	100,00	0,00E+00	751	Os05g0215600	99,28	0,00E+00	741	-10	NA	Indica	
augustus_masked-5-processed-gene-76.11-mRNA-1	5	OsR498G0510060100.01.T01	99,46	0,00E+00	2318	Os05g0224700	99,17	0,00E+00	2122	-196	NA	NA	Kinase-TM
augustus_masked-5-processed-gene-87.12-mRNA-1	5	OsR498G0101343800.01.T01	74,47	0,00E+00	741	Os05g0242000	100,00	0,00E+00	2408	1667	Japonica	Japonica	
augustus_masked-5-processed-gene-9.20-mRNA-1	5	OsR498G0509612100.01.T01	99,54	0,00E+00	1535	Os05g0118000	100,00	0,00E+00	1553	18	NA	Japonica	
augustus_masked-5-processed-gene-90.19-mRNA-1	5	OsR498G0510139500.01.T01	99,89	0,00E+00	1608	Os05g0248200	100,00	0,00E+00	1613	5	NA	Japonica	
augustus-5-processed-gene-1.24-mRNA-1	5	OsR498G0509559600.01.T01	100,00	0,00E+00	2024	Os05g0102000	100,00	0,00E+00	2024	0	NA	NA	
augustus-5-processed-gene-10.31-mRNA-1	5	OsR498G0509618600.01.T01	99,67	0,00E+00	3263	Os05g0120000	100,00	0,00E+00	3685	422	Japonica	Japonica	
augustus-5-processed-gene-11.16-mRNA-1	5	OsR498G0509622800.01.T01	99,77	0,00E+00	1577	Os05g0120900	100,00	0,00E+00	1586	9	NA	Japonica	
augustus-5-processed-gene-113.6-mRNA-1	5	OsR498G0510247800.01.T05	100,00	0,00E+00	4296	Os05g0275000	100,00	0,00E+00	4296	0	NA	NA	
augustus-5-processed-gene-118.14-mRNA-1	5	OsR498G0510277400.01.T01	100,00	0,00E+00	4708	Os05g0280700	100,00	0,00E+00	4708	0	NA	NA	Kinase-TM
augustus-5-processed-gene-12.45-mRNA-1	5	OsR498G0509629600.01.T01	99,39	0,00E+00	2638	Os05g0122500	99,80	0,00E+00	2665	27	NA	Japonica	
augustus-5-processed-gene-126.6-mRNA-1	5	OsR498G0714239700.01.T01	71,73	1,13E-171	605	Os05g0317300	99,96	0,00E+00	4198	3593	Japonica	NA	
augustus-5-processed-gene-13.33-mRNA-1	5	OsR498G0509638000.01.T02	99,64	0,00E+00	1970	Os05g0124000	100,00	0,00E+00	1988	18	NA	Japonica	
augustus-5-processed-gene-135.28-mRNA-1	5	OsR498G0510369800.01.T01	100,00	0,00E+00	1613	Os09g0103700	73,92	4,51E-179	627	-986	Indica	NA	
augustus-5-processed-gene-136.18-mRNA-1	5	OsR498G0510373800.01.T02	100,00	0,00E+00	1705	Os05g0298500	99,86	0,00E+00	1295	-410	Indica	NA	
augustus-5-processed-gene-137.17-mRNA-1	5	OsR498G0510378400.01.T04	100,00	0,00E+00	1559	Os05g0299600	100,00	0,00E+00	1559	0	NA	NA	
augustus-5-processed-gene-137.23-mRNA-1	5	OsR498G0510380400.01.T04	100,00	0,00E+00	948	Os05g0299700	100,00	0,00E+00	948	0	NA	NA	
augustus-5-processed-gene-14.7-mRNA-1	5	OsR498G0509641800.01.T05	99,79	0,00E+00	1731	Os05g0124000	86,13	0,00E+00	1207	-524	Indica	Japonica	
augustus-5-processed-gene-141.37-mRNA-1	5	OsR498G0510413000.01.T01	99,24	0,00E+00	4418	Os05g0305300	99,77	0,00E+00	4589	171	NA	Japonica	
augustus-5-processed-gene-141.59-mRNA-1	5	OsR498G0510414000.01.T01	100,00	0,00E+00	946	Os05g0305700	100,00	0,00E+00	946	0	NA	Indica	
augustus-5-processed-gene-147.9-mRNA-1	5	OsR498G0510442200.01.T01	99,81	0,00E+00	1842	Os03g0356900	75,81	2,22E-101	369	-1473	Indica	Japonica	
augustus-5-processed-gene-148.47-mRNA-1	5	OsR498G0510453400.01.T01	99,84	0,00E+00	3277	Os05g0313700	100,00	0,00E+00	3290	13	NA	Japonica	
augustus-5-processed-gene-149.16-mRNA-1	5	OsR498G0510463400.01.T01	99,38	7,29E-164	576	Os05g0314700	100,00	6,37E-172	601	25	NA	Japonica	
augustus-5-processed-gene-15.32-mRNA-1	5	OsR498G0509652400.01.T01	97,37	1,68E-106	385	Os01g0881800	89,76	2,35E-42	171	-214	Indica	Japonica	
augustus-5-processed-gene-151.23-mRNA-1	5	OsR498G0510474100.01.T03	99,86	0,00E+00	3920	Os05g0317200	100,00	0,00E+00	3934	14	NA	Japonica	
augustus-5-processed-gene-151.34-mRNA-1	5	OsR498G0714239700.01.T01	71,79	2,65E-173	611	Os05g0317300	100,00	0,00E+00	4204	3593	Japonica	NA	
augustus-5-processed-gene-152.19-mRNA-1	5	OsR498G0510480600.01.T01	99,92	0,00E+00	4763	Os05g0318700	100,00	0,00E+00	4318	-445	NA	Japonica	Kinase-TM
augustus-5-processed-gene-153.24-mRNA-1	5	OsR498G0510483600.01.T01	97,79	0,00E+00	3883	Os05g0319800	98,36	0,00E+00	5088	1205	Japonica	Japonica	
augustus-5-processed-gene-153.25-mRNA-1	5	OsR498G0510483800.01.T01	99,66	0,00E+00	3122	Os05g0319900	100,00	0,00E+00	3149	27	NA	Japonica	
augustus-5-processed-gene-153.26-mRNA-1	5	OsR498G0510484400.01.T03	99,83	0,00E+00	2064	Os05g0320100	100,00	0,00E+00	2037	-27	NA	Japonica	
augustus-5-processed-gene-154.16-mRNA-1	5	OsR498G0510492300.01.T01	99,90	0,00E+00	1819	Os05g0323100	99,90	0,00E+00	1819	0	NA	NA	
augustus-5-processed-gene-155.22-mRNA-1	5	OsR498G0510499000.01.T01	100,00	0,00E+00	1413	Os05g0324600	99,75	0,00E+00	1404	-9	NA	NA	
augustus-5-processed-gene-16.30-mRNA-1	5	OsR498G0509662200.01.T03	99,92	0,00E+00	2263	Os05g0128400	100,00	0,00E+00	2268	5	NA	Japonica	
augustus-5-processed-gene-160.10-mRNA-1	5	OsR498G0510521900.01.T01	99,56	0,00E+00	809	Os05g0329400	99,12	0,00E+00	800	-9	NA	NA	
augustus-5-processed-gene-161.19-mRNA-1	5	OsR498G0510526000.01.T01	96,97	0,00E+00	771	Os05g0332000	99,13	0,00E+00	816	45	NA	Indica	
augustus-5-processed-gene-161.27-mRNA-1	5	OsR498G0510530600.01.T01	99,94	0,00E+00	3048	Os05g0332600	99,92	0,00E+00	2236	-812	Indica	Indica	
augustus-5-processed-gene-161.5-mRNA-1	5	OsR498G0510526000.01.T01	97,62	0,00E+00	784	Os05g0332000	97,62	0,00E+00	784	0	NA	Indica	
augustus-5-processed-gene-162.23-mRNA-1	5	OsR498G1019239600.01.T06	76,41	2,04E-35	149	Os05g0333000	99,06	2,82E-104	376	227	Japonica	Indica	
augustus-5-processed-gene-164.6-mRNA-1	5	OsR498G0510544200.01.T01	100,00	0,00E+00	2718	Os05g0335800	99,88	0,00E+00	1527	-1191	Indica	Indica	
augustus-5-processed-gene-17.8-mRNA-1	5	OsR498G0509662700.01.T04	99,73	0,00E+00	2650	Os05g0129000	100,00	0,00E+00	2668	18	NA	Japonica	
augustus-5-processed-gene-173.13-mRNA-1	5	OsR498G0510603600.01.T01	100,00	0,00E+00	899	Os05g0350900	99,89	0,00E+00	1641	742	Japonica	Indica	
augustus-5-processed-gene-173.16-mRNA-1	5	OsR498G0510607000.01.T03	100,00	6,86E-167	587	Os05g0351500	100,00	2,39E-167	587	0	NA	NA	
augustus-5-processed-gene-181.28-mRNA-1	5	OsR498G0510666200.01.T01	100,00	4,58E-128	457	Os05g0363600	99,61	6,78E-127	453	-4	NA	Indica	
augustus-5-processed-gene-185.27-mRNA-1	5	OsR498G0510679800.01.T01	100,00	1,83E-171	601	Os05g0367000	100,00	6,37E-172	601	0	NA	NA	
augustus-5-processed-gene-187.11-mRNA-1	5	OsR498G0510689400.01.T01	100,00	0,00E+00	3166	Os05g0370000	98,90	0,00E+00	3498	332	NA	Indica	
augustus-5-processed-gene-187.32-mRNA-1	5	OsR498G0510690200.01.T01	100,00	3,91E-121	434	Os05g0370000	97,08	8,09E-112	402	-32	NA	NA	
augustus-5-processed-gene-187.33-mRNA-1	5	OsR498G0510689400.01.T01	95,67	0,00E+00	2823	Os05g0370000	96,04	0,00E+00	3241	418	Japonica	NA	
augustus-5-processed-gene-187.34-mRNA-1	5	OsR498G0714930700.01.T01	70,14	2,12E-49	198	Os05g0370300	98,68	0,00E+00	1591	1393	Japonica	Indica	
augustus-5-processed-gene-190.32-mRNA-1	5	OsR498G0510710800.01.T02	100,00	0,00E+00	1813	Os05g0375400	99,80	0,00E+00	1804	-9	NA	Indica	
augustus-5-processed-gene-191.14-mRNA-1	5	OsR498G1221118000.01.T01	95,50	0,00E+00	994	Os05g0376000	100,00	0,00E+00	1801	807	Japonica	NA	
augustus-5-processed-gene-191.26-mRNA-1	5	OsR498G0510716000.01.T03	95,24	0,00E+00	1641	Os05g0376300	95,24	0,00E+00	1641	0	NA	NA	
augustus-5-processed-gene-194.45-mRNA-1	5	OsR498G0612562800.01.T01	85,56	2,45E-82	306	Os05g0382550	100,00	0,00E+00	737	431	Japonica	Japonica	
augustus-5-processed-gene-195.28-mRNA-1	5	OsR498G0510745600.01.T01	99,30	0,00E+00	2539	Os05g0384300	100,00	0,00E+00	2587	48	NA	Japonica	
augustus-5-processed-gene-195.29-mRNA-1	5	OsR498G0510747200.01.T01	99,84	0,00E+00	3378	Os05g0384600	100,00	0,00E+00	3391	13	NA	Japonica	NBS-TM
augustus-5-processed-gene-199.1-mRNA-1	5	OsR498G0510767800.01.T01	98,23	0,00E+00	874	Os05g0390800	100,00	0,00E+00	904	30	NA	Japonica	
augustus-5-processed-gene-2.80-mRNA-1	5	OsR498G0509565400.01.T01	100,00	0,00E+00	980	Os05g0103500	100,00	0,00E+00	980	0	NA	NA	
augustus-5-processed-gene-201.32-mRNA-1	5	OsR498G0510782000.01.T03	99,95	0,00E+00	3313	Os05g0395000	100,00	0,00E+00	3317	4	NA	Japonica	
augustus-5-processed-gene-202.25-mRNA-1	5	OsR498G0510787600.01.T01	99,83	0,00E+00	3244	Os05g0396900	100,00	0,00E+00	3258	14	NA	Japonica	
augustus-5-processed-gene-204.46-mRNA-1	5	OsR498G0510794200.01.T01	98,05	0,00E+00	1318	Os05g0399800	100,00	0,00E+00	1386	68	NA	Japonica	LRR-TM
augustus-5-processed-gene-206.10-mRNA-1	5	OsR498G0510803600.01.T01	98,31	0,00E+00	2046	Os05g0403400	99,75	0,00E+00	2122	76	NA	Japonica	
augustus-5-processed-gene-206.11-mRNA-1	5	OsR498G0510804000.01.T01	98,90	0,00E+00	2079	Os05g0403600	100,00	0,00E+00	2165	86	NA	Japonica	
augustus-5-processed-gene-208.36-mRNA-1	5	OsR498G0510805800.01.T01	99,82	0,00E+00	1003	Os05g0404600	100,00	0,00E+00	915	-88	NA	Japonica	
augustus-5-processed-gene-208.37-mRNA-1	5	OsR498G0510806000.01.T01	100,00	0,00E+00	935	Os05g0404700	100,00	0,00E+00	935	0	NA	Japonica	
augustus-5-processed-gene-209.36-mRNA-1	5	OsR498G0510812600.01.T01	98,18	0,00E+00	663	Os05g0406600	100,00	0,00E+00	682	19	NA	Japonica	
augustus-5-processed-gene-210.25-mRNA-1	5	OsR498G0510821800.01.T01	100,00	0,00E+00	3344	Os05g0409000	100,00	0,00E+00	3344	0	NA	Japonica	
augustus-5-processed-gene-210.5-mRNA-1	5	OsR498G0510818600.01.T04	99,87	0,00E+00	2691	Os05g0407900	100,00	0,00E+00	2679	-12	NA	Japonica	
augustus-5-processed-gene-217.56-mRNA-1	5	OsR498G0203891300.01.T01	83,44	0,00E+00	699	Os05g0420900	100,00	0,00E+00	1375	676	Japonica	Japonica	
augustus-5-processed-gene-219.17-mRNA-1	5	OsR498G0510875200.01.T01	99,63	0,00E+00	2911	Os05g0424300	100,00	0,00E+00	2814	-97	NA	Japonica	
augustus-5-processed-gene-22.36-mRNA-1	5	OsR498G0509702800.01.T01	99,33	0,00E+00	1595	Os05g0139200	99,83	0,00E+00	2156	561	Japonica	Japonica	
augustus-5-processed-gene-220.45-mRNA-1	5	OsR498G0204355400.01.T01	88,29	0,00E+00	2742	Os05g0427400	100,00	0,00E+00	3880	1138	Japonica	Japonica	
augustus-5-processed-gene-221.26-mRNA-1	5	OsR498G0510892400.01.T01	99,49	0,00E+00	699	Os05g0428800	100,00	0,00E+00	1012	313	Japonica	Japonica	
augustus-5-processed-gene-222.3-mRNA-1	5	OsR498G0510895200.01.T01	99,75	0,00E+00	4329	Os05g0429700	100,00	0,00E+00	4356	27	NA	Japonica	
augustus-5-processed-gene-224.33-mRNA-1	5	OsR498G0102409900.01.T01	76,81	0,00E+00	1691	Os05g0435800	100,00	0,00E+00	4123	2432	Japonica	NA	
augustus-5-processed-gene-226.36-mRNA-1	5	OsR498G0510924800.01.T01	99,94	0,00E+00	2983	Os05g0439300	100,00	0,00E+00	2987	4	NA	NA	
augustus-5-processed-gene-226.8-mRNA-1	5	OsR498G0611661000.01.T01	93,67	3,20E-177	621	Os05g0438700	100,00	0,00E+00	742	121	Japonica	NA	
augustus-5-processed-gene-227.10-mRNA-1	5	OsR498G0510928400.01.T02	99,67	2,44E-152	539	Os05g0440100	88,59	1,18E-106	385	-154	Indica	Japonica	
augustus-5-processed-gene-227.18-mRNA-1	5	OsR498G0509612600.01.T01	95,47	0,00E+00	775	Os10g0326400	94,51	7,35E-110	396	-379	Indica	Japonica	
augustus-5-processed-gene-227.20-mRNA-1	5	OsR498G0510930400.01.T01	98,18	0,00E+00	1617	Os05g0441400	97,92	0,00E+00	1324	-293	Indica	Japonica	
augustus-5-processed-gene-23.16-mRNA-1	5	OsR498G0509711600.01.T02	97,54	3,76E-115	414	Os05g0141200	83,90	1,16E-65	249	-165	Indica	Japonica	
augustus-5-processed-gene-23.18-mRNA-1	5	OsR498G0713871400.01.T01	73,42	0,00E+00	786	Os05g0141300	99,93	0,00E+00	2404	1618	Japonica	Japonica	
augustus-5-processed-gene-23.20-mRNA-1	5	OsR498G0509713200.01.T03	99,62	0,00E+00	4657	Os05g0141500	99,77	0,00E+00	4675	18	NA	Japonica	
augustus-5-processed-gene-230.35-mRNA-1	5	OsR498G0510949800.01.T01	99,80	0,00E+00	909	Os05g0447000	100,00	0,00E+00	937	28	NA	Japonica	
augustus-5-processed-gene-230.37-mRNA-1	5	OsR498G0510950000.01.T01	99,84	0,00E+00	2195	Os05g0447200	100,00	0,00E+00	2205	10	NA	Japonica	
augustus-5-processed-gene-231.43-mRNA-1	5	OsR498G0510954600.01.T01	99,72	0,00E+00	2585	Os05g0449200	100,00	0,00E+00	2603	18	NA	Japonica	
augustus-5-processed-gene-238.4-mRNA-1	5	OsR498G0510997800.01.T01	100,00	1,80E-158	558	Os05g0462700	100,00	6,27E-159	558	0	NA	Japonica	
augustus-5-processed-gene-242.44-mRNA-1	5	OsR498G0511022200.01.T01	99,74	0,00E+00	1388	Os05g0471100	100,00	0,00E+00	1397	9	NA	Japonica	
augustus-5-processed-gene-243.31-mRNA-1	5	OsR498G0511029000.01.T02	99,48	0,00E+00	3403	Os05g0473500	99,94	0,00E+00	3203	-200	NA	Japonica	
augustus-5-processed-gene-244.42-mRNA-1	5	OsR498G0511033800.01.T01	99,10	0,00E+00	2548	Os05g0475400	99,24	0,00E+00	2085	-463	Indica	Japonica	
augustus-5-processed-gene-248.2-mRNA-1	5	OsR498G0511058000.01.T02	99,90	0,00E+00	1847	Os05g0481900	100,00	0,00E+00	2370	523	Japonica	Japonica	
augustus-5-processed-gene-249.30-mRNA-1	5	OsR498G0511067600.01.T01	100,00	0,00E+00	1245	Os05g0484000	78,12	3,69E-147	520	-725	Indica	NA	
augustus-5-processed-gene-249.50-mRNA-1	5	OsR498G0511070200.01.T01	99,91	0,00E+00	4179	Os05g0485000	99,96	0,00E+00	4184	5	NA	NA	
augustus-5-processed-gene-25.28-mRNA-1	5	OsR498G0509725400.01.T01	97,82	0,00E+00	858	Os05g0145100	97,79	0,00E+00	924	66	NA	Indica	
augustus-5-processed-gene-250.30-mRNA-1	5	OsR498G0511076000.01.T01	99,73	0,00E+00	662	Os05g0486200	100,00	0,00E+00	666	4	NA	Japonica	
augustus-5-processed-gene-251.63-mRNA-1	5	OsR498G0511080200.01.T03	99,69	0,00E+00	1740	Os05g0488800	100,00	0,00E+00	1647	-93	NA	Japonica	
augustus-5-processed-gene-252.18-mRNA-1	5	OsR498G0511085200.01.T01	99,74	0,00E+00	2764	Os05g0490400	100,00	0,00E+00	2782	18	NA	Japonica	
augustus-5-processed-gene-252.21-mRNA-1	5	OsR498G0511085800.01.T02	99,95	0,00E+00	3637	Os05g0490700	100,00	0,00E+00	3642	5	NA	Japonica	
augustus-5-processed-gene-254.55-mRNA-1	5	OsR498G0511099200.01.T03	99,85	0,00E+00	1208	Os05g0495200	100,00	0,00E+00	1218	10	NA	Japonica	
augustus-5-processed-gene-255.34-mRNA-1	5	OsR498G0511105200.01.T01	100,00	0,00E+00	1283	Os05g0497300	100,00	0,00E+00	1283	0	NA	Japonica	
augustus-5-processed-gene-256.29-mRNA-1	5	OsR498G0511109000.01.T01	99,49	0,00E+00	1053	Os05g0498200	100,00	0,00E+00	1067	14	NA	Japonica	
augustus-5-processed-gene-257.40-mRNA-1	5	OsR498G0511115200.01.T01	99,19	0,00E+00	1311	Os05g0500400	100,00	0,00E+00	1846	535	Japonica	NA	
augustus-5-processed-gene-264.47-mRNA-1	5	OsR498G0511162200.01.T01	99,65	0,00E+00	3036	Os05g0510600	99,88	0,00E+00	3054	18	NA	Japonica	
augustus-5-processed-gene-264.67-mRNA-1	5	OsR498G0511165400.01.T02	95,78	0,00E+00	1867	Os05g0511300	95,95	0,00E+00	1876	9	NA	Japonica	
augustus-5-processed-gene-266.40-mRNA-1	5	OsR498G0511177800.01.T01	99,56	0,00E+00	1208	Os05g0514600	100,00	0,00E+00	717	-491	Indica	Japonica	
augustus-5-processed-gene-266.41-mRNA-1	5	OsR498G0511177800.01.T03	99,24	0,00E+00	701	Os05g0514600	98,29	6,35E-173	605	-96	Indica	Japonica	
augustus-5-processed-gene-267.54-mRNA-1	5	OsR498G0511181400.01.T01	98,83	0,00E+00	1949	Os05g0515600	100,00	0,00E+00	2008	59	NA	Japonica	
augustus-5-processed-gene-27.9-mRNA-1	5	OsR498G0509734200.01.T01	97,53	0,00E+00	1920	Os05g0147700	99,91	0,00E+00	2043	123	NA	Japonica	
augustus-5-processed-gene-271.36-mRNA-1	5	OsR498G0511207200.01.T01	99,43	0,00E+00	1243	Os05g0522700	100,00	0,00E+00	1261	18	NA	Japonica	
augustus-5-processed-gene-272.36-mRNA-1	5	OsR498G0511216000.01.T01	100,00	0,00E+00	1386	Os05g0525900	100,00	0,00E+00	1386	0	NA	Japonica	
augustus-5-processed-gene-273.35-mRNA-1	5	OsR498G0511218600.01.T01	96,55	0,00E+00	2339	Os05g0527000	100,00	0,00E+00	2560	221	NA	Japonica	
augustus-5-processed-gene-273.36-mRNA-1	5	OsR498G0511219800.01.T01	97,46	0,00E+00	2397	Os05g0527100	100,00	0,00E+00	2457	60	NA	Japonica	
augustus-5-processed-gene-273.52-mRNA-1	5	OsR498G0511221000.01.T01	84,76	0,00E+00	912	Os05g0527400	100,00	0,00E+00	1483	571	Japonica	Japonica	
augustus-5-processed-gene-273.55-mRNA-1	5	OsR498G0511221000.01.T01	99,19	0,00E+00	1089	Os05g0527700	100,00	0,00E+00	1283	194	Japonica	Japonica	
augustus-5-processed-gene-274.50-mRNA-1	5	OsR498G0511226400.01.T01	100,00	0,00E+00	1278	Os12g0265500	96,60	0,00E+00	1116	-162	Indica	NA	
augustus-5-processed-gene-274.72-mRNA-1	5	OsR498G0511233000.01.T01	100,00	0,00E+00	2554	Os05g0530701	100,00	0,00E+00	2554	0	NA	NA	LRR-TM
augustus-5-processed-gene-275.27-mRNA-1	5	OsR498G0511239400.01.T01	100,00	0,00E+00	2805	Os05g0532500	100,00	0,00E+00	3290	485	Japonica	NA	
augustus-5-processed-gene-277.28-mRNA-1	5	OsR498G0511251800.01.T01	100,00	0,00E+00	2993	Os05g0536800	100,00	0,00E+00	2830	-163	NA	NA	
augustus-5-processed-gene-279.26-mRNA-1	5	OsR498G0511263800.01.T02	98,56	0,00E+00	1937	Os05g0539700	98,56	0,00E+00	1937	0	NA	NA	
augustus-5-processed-gene-279.32-mRNA-1	5	OsR498G0511266200.01.T04	100,00	0,00E+00	3047	Os05g0540600	100,00	0,00E+00	3047	0	NA	NA	
augustus-5-processed-gene-28.60-mRNA-1	5	OsR498G0509738700.01.T01	99,37	0,00E+00	840	Os05g0149300	100,00	0,00E+00	856	16	NA	Japonica	
augustus-5-processed-gene-281.47-mRNA-1	5	OsR498G0511283000.01.T01	99,62	0,00E+00	2359	Os05g0545400	99,45	0,00E+00	2254	-105	NA	NA	Kinase-TM
augustus-5-processed-gene-285.12-mRNA-1	5	OsR498G0511302800.01.T01	100,00	3,25E-168	590	Os05g0552000	99,69	4,80E-167	586	-4	NA	NA	
augustus-5-processed-gene-285.14-mRNA-1	5	OsR498G0511303400.01.T01	99,63	0,00E+00	1920	Os05g0552200	99,63	0,00E+00	1920	0	NA	Japonica	
augustus-5-processed-gene-285.15-mRNA-1	5	OsR498G0511303600.01.T01	99,89	0,00E+00	1608	Os05g0552300	99,89	0,00E+00	1608	0	NA	NA	
augustus-5-processed-gene-285.19-mRNA-1	5	OsR498G0511306200.01.T01	97,30	0,00E+00	2498	Os05g0552700	95,94	0,00E+00	2566	68	NA	NA	
augustus-5-processed-gene-285.2-mRNA-1	5	OsR498G0101792300.01.T01	82,19	0,00E+00	832	Os05g0551700	99,82	0,00E+00	1945	1113	Japonica	Japonica	
augustus-5-processed-gene-286.59-mRNA-1	5	OsR498G0511312600.01.T01	99,83	0,00E+00	1073	Os05g0554100	99,83	0,00E+00	1073	0	NA	Indica	
augustus-5-processed-gene-287.26-mRNA-1	5	OsR498G0511321600.01.T01	99,91	0,00E+00	2074	Os05g0556800	99,66	0,00E+00	2120	46	NA	NA	
augustus-5-processed-gene-287.4-mRNA-1	5	OsR498G0511318100.01.T02	99,40	0,00E+00	6222	Os01g0681900	82,15	0,00E+00	3419	-2803	Indica	Indica	
augustus-5-processed-gene-288.20-mRNA-1	5	OsR498G0511325800.01.T01	100,00	0,00E+00	2251	Os05g0558000	100,00	0,00E+00	2251	0	NA	Indica	
augustus-5-processed-gene-288.25-mRNA-1	5	OsR498G0511327600.01.T01	100,00	3,31E-168	590	Os10g0549850	82,59	2,09E-51	202	-388	Indica	Indica	
augustus-5-processed-gene-289.23-mRNA-1	5	OsR498G0511333800.01.T01	99,85	0,00E+00	1208	Os05g0560200	99,55	0,00E+00	1199	-9	NA	NA	
augustus-5-processed-gene-292.54-mRNA-1	5	OsR498G0511352200.01.T01	100,00	0,00E+00	1687	Os05g0565500	99,89	0,00E+00	1682	-5	NA	Indica	
augustus-5-processed-gene-293.32-mRNA-1	5	OsR498G0511359200.01.T06	100,00	0,00E+00	1586	Os05g0567500	99,20	0,00E+00	1555	-31	NA	Indica	
augustus-5-processed-gene-293.4-mRNA-1	5	OsR498G0511355000.01.T05	100,00	0,00E+00	1599	Os05g0566400	99,77	0,00E+00	1590	-9	NA	Indica	Kinase-TM
augustus-5-processed-gene-293.5-mRNA-1	5	OsR498G0511356000.01.T01	100,00	0,00E+00	3171	Os05g0566500	99,77	0,00E+00	3153	-18	NA	Indica	
augustus-5-processed-gene-294.30-mRNA-1	5	OsR498G0511363600.01.T01	100,00	0,00E+00	975	Os05g0568300	99,82	0,00E+00	970	-5	NA	Indica	
augustus-5-processed-gene-295.46-mRNA-1	5	OsR498G0511370200.01.T01	100,00	0,00E+00	2186	Os05g0570300	99,67	0,00E+00	2168	-18	NA	Indica	
augustus-5-processed-gene-296.27-mRNA-1	5	OsR498G0511376600.01.T01	99,90	0,00E+00	3714	Os05g0572900	99,81	0,00E+00	3705	-9	NA	Indica	
augustus-5-processed-gene-298.25-mRNA-1	5	OsR498G0511394400.01.T01	100,00	0,00E+00	908	Os05g0577500	99,81	0,00E+00	938	30	NA	Indica	
augustus-5-processed-gene-298.6-mRNA-1	5	OsR498G0511391600.01.T01	99,81	0,00E+00	954	Os05g0576600	100,00	0,00E+00	958	4	NA	Indica	
augustus-5-processed-gene-299.42-mRNA-1	5	OsR498G0511396000.01.T01	100,00	0,00E+00	1656	Os05g0578100	99,67	0,00E+00	1646	-10	NA	NA	
augustus-5-processed-gene-299.66-mRNA-1	5	OsR498G0511400300.01.T01	99,10	0,00E+00	1366	Os05g0579300	100,00	0,00E+00	1386	20	NA	Japonica	
augustus-5-processed-gene-300.24-mRNA-1	5	OsR498G0511409400.01.T01	99,22	0,00E+00	1583	Os05g0582000	100,00	0,00E+00	1613	30	NA	Japonica	
augustus-5-processed-gene-300.9-mRNA-1	5	OsR498G0511404600.01.T01	98,57	0,00E+00	1454	Os08g0106900	97,61	0,00E+00	1418	-36	NA	Japonica	
augustus-5-processed-gene-305.4-mRNA-1	5	OsR498G0101471900.01.T01	81,66	0,00E+00	1250	Os05g0590000	99,93	0,00E+00	2604	1354	Japonica	Japonica	
augustus-5-processed-gene-306.77-mRNA-1	5	OsR498G0511448600.01.T01	99,17	0,00E+00	2563	Os05g0593900	100,00	0,00E+00	2571	8	NA	Japonica	
augustus-5-processed-gene-307.45-mRNA-1	5	OsR498G0511449400.01.T01	99,92	0,00E+00	2214	Os05g0594200	99,76	0,00E+00	2205	-9	NA	Indica	
augustus-5-processed-gene-307.57-mRNA-1	5	OsR498G0511453000.01.T03	100,00	0,00E+00	1342	Os05g0595200	100,00	0,00E+00	1315	-27	NA	NA	
augustus-5-processed-gene-31.10-mRNA-1	5	OsR498G0509766800.01.T01	99,85	0,00E+00	2372	Os05g0154600	100,00	0,00E+00	2365	-7	NA	Japonica	
augustus-5-processed-gene-32.51-mRNA-1	5	OsR498G0100884100.01.T02	70,82	3,29E-51	204	Os05g0155400	100,00	0,00E+00	785	581	Japonica	Japonica	
augustus-5-processed-gene-35.37-mRNA-1	5	OsR498G0509788400.01.T01	99,72	0,00E+00	635	Os05g0160300	100,00	0,00E+00	639	4	NA	Japonica	
augustus-5-processed-gene-36.27-mRNA-1	5	OsR498G0509798800.01.T02	100,00	0,00E+00	1494	Os05g0163300	100,00	0,00E+00	1494	0	NA	Japonica	
augustus-5-processed-gene-39.57-mRNA-1	5	OsR498G0509820600.01.T01	98,73	0,00E+00	3443	Os05g0168500	99,95	0,00E+00	3551	108	NA	Japonica	
augustus-5-processed-gene-39.59-mRNA-1	5	OsR498G0509822800.01.T01	99,95	0,00E+00	3378	Os05g0168800	99,73	0,00E+00	3360	-18	NA	NA	
augustus-5-processed-gene-40.60-mRNA-1	5	OsR498G0509829400.01.T02	100,00	2,45E-176	618	Os05g0170600	100,00	8,53E-177	618	0	NA	NA	
augustus-5-processed-gene-41.32-mRNA-1	5	OsR498G0509836200.01.T01	99,32	0,00E+00	2073	Os05g0172100	99,57	0,00E+00	2088	15	NA	NA	
augustus-5-processed-gene-46.28-mRNA-1	5	OsR498G0509853800.01.T01	100,00	0,00E+00	2001	Os05g0178400	100,00	0,00E+00	2001	0	NA	Indica	
augustus-5-processed-gene-46.7-mRNA-1	5	OsR498G0509850800.01.T01	99,69	0,00E+00	2927	Os05g0177500	99,88	0,00E+00	2940	13	NA	Japonica	
augustus-5-processed-gene-47.20-mRNA-1	5	OsR498G0509866000.01.T01	99,28	0,00E+00	2204	Os05g0180300	99,92	0,00E+00	2241	37	NA	Japonica	LRR-TM
augustus-5-processed-gene-5.23-mRNA-1	5	OsR498G0509581600.01.T01	99,22	0,00E+00	1362	Os05g0109100	99,78	0,00E+00	1593	231	Japonica	Japonica	
augustus-5-processed-gene-5.3-mRNA-1	5	OsR498G0509579900.01.T01	98,17	0,00E+00	3101	Os05g0108300	98,28	0,00E+00	3110	9	NA	Japonica	CC-Kinase
augustus-5-processed-gene-50.8-mRNA-1	5	OsR498G0509876800.01.T01	100,00	0,00E+00	3041	Os05g0183300	100,00	0,00E+00	1689	-1352	Indica	NA	
augustus-5-processed-gene-56.19-mRNA-1	5	OsR498G0509914200.01.T01	99,53	0,00E+00	1503	Os05g0192100	97,56	0,00E+00	1039	-464	Indica	Indica	
augustus-5-processed-gene-63.15-mRNA-1	5	OsR498G0509973600.01.T01	100,00	0,00E+00	747	Os05g0203000	100,00	0,00E+00	747	0	NA	NA	
augustus-5-processed-gene-63.17-mRNA-1	5	OsR498G0509974600.01.T01	100,00	3,67E-158	558	Os05g0203400	100,00	0,00E+00	1088	530	Japonica	NA	
augustus-5-processed-gene-65.26-mRNA-1	5	OsR498G0509988600.01.T01	99,39	0,00E+00	2601	Os05g0206600	99,39	0,00E+00	2601	0	NA	NA	
augustus-5-processed-gene-66.22-mRNA-1	5	OsR498G0816176500.01.T01	74,53	8,83E-145	516	Os05g0208100	99,85	0,00E+00	2369	1853	Japonica	Indica	Kinase-TM
augustus-5-processed-gene-67.20-mRNA-1	5	OsR498G0509996000.01.T01	99,62	0,00E+00	1399	Os05g0209300	100,00	0,00E+00	1413	14	NA	Japonica	
augustus-5-processed-gene-67.3-mRNA-1	5	OsR498G0509994400.01.T02	99,90	0,00E+00	1884	Os01g0209700	71,12	4,09E-136	485	-1399	Indica	Indica	
augustus-5-processed-gene-69.16-mRNA-1	5	OsR498G0510009000.01.T01	99,32	1,23E-147	522	Os05g0212900	100,00	8,26E-151	531	9	NA	Japonica	
augustus-5-processed-gene-7.13-mRNA-1	5	OsR498G0509596200.01.T01	100,00	0,00E+00	910	Os05g0112750	100,00	0,00E+00	910	0	NA	Japonica	
augustus-5-processed-gene-7.14-mRNA-1	5	OsR498G0509596400.01.T01	99,15	0,00E+00	1446	Os05g0112800	100,00	0,00E+00	1478	32	NA	Japonica	
augustus-5-processed-gene-70.12-mRNA-1	5	OsR498G0510014400.01.T01	100,00	0,00E+00	1050	Os05g0213900	100,00	0,00E+00	1050	0	NA	NA	
augustus-5-processed-gene-74.29-mRNA-1	5	OsR498G0510047800.01.T02	99,03	0,00E+00	909	Os03g0693400	88,83	0,00E+00	675	-234	Indica	NA	
augustus-5-processed-gene-75.27-mRNA-1	5	OsR498G0510053600.01.T01	100,00	0,00E+00	1029	Os05g0223000	99,30	0,00E+00	1007	-22	NA	Indica	
augustus-5-processed-gene-75.5-mRNA-1	5	OsR498G0510051200.01.T01	99,04	0,00E+00	1453	Os05g0217800	81,28	0,00E+00	796	-657	Indica	Indica	
augustus-5-processed-gene-77.27-mRNA-1	5	OsR498G0510064600.01.T01	99,08	0,00E+00	1146	Os05g0225800	99,21	0,00E+00	1348	202	Japonica	NA	
augustus-5-processed-gene-77.30-mRNA-1	5	OsR498G0510065600.01.T01	99,09	0,00E+00	1166	Os05g0226000	99,21	0,00E+00	1348	182	Japonica	NA	
augustus-5-processed-gene-79.10-mRNA-1	5	OsR498G0510076400.01.T06	100,00	2,31E-137	488	Os03g0772300	82,72	3,02E-48	190	-298	Indica	NA	
augustus-5-processed-gene-85.45-mRNA-1	5	OsR498G0510112800.01.T01	99,43	0,00E+00	1249	Os05g0238400	100,00	0,00E+00	1267	18	NA	Japonica	
augustus-5-processed-gene-87.38-mRNA-1	5	OsR498G0510126400.01.T01	97,97	0,00E+00	1180	Os05g0242600	100,00	0,00E+00	1240	60	NA	Japonica	
augustus-5-processed-gene-89.48-mRNA-1	5	OsR498G0510133000.01.T02	99,89	0,00E+00	1608	Os05g0247100	100,00	0,00E+00	1613	5	NA	Japonica	
augustus-5-processed-gene-9.33-mRNA-1	5	OsR498G0509612600.01.T01	97,73	0,00E+00	748	Os10g0326400	93,53	9,14E-96	350	-398	Indica	Japonica	
augustus-5-processed-gene-9.9-mRNA-1	5	OsR498G0509609000.01.T01	99,65	0,00E+00	2553	Os05g0116600	100,00	0,00E+00	2576	23	NA	Japonica	
augustus-5-processed-gene-92.27-mRNA-1	5	OsR498G0510143400.01.T01	75,49	1,40E-128	461	Os05g0251200	100,00	0,00E+00	1975	1514	Japonica	Japonica	
augustus-5-processed-gene-96.25-mRNA-1	5	OsR498G0510171400.01.T01	99,07	1,27E-160	566	Os05g0360900	77,03	1,19E-41	168	-398	Indica	Japonica	
maker-5-augustus-gene-0.29-mRNA-1	5	OsR498G0509554500.01.T03	100,00	0,00E+00	1272	Os05g0100100	100,00	0,00E+00	1272	0	NA	NA	
maker-5-augustus-gene-0.33-mRNA-1	5	OsR498G0509555200.01.T06	99,89	0,00E+00	4683	Os01g0109700	74,35	0,00E+00	2166	-2517	Indica	NA	
maker-5-augustus-gene-1.34-mRNA-1	5	OsR498G0509558300.01.T06	99,14	0,00E+00	2686	Os05g0101200	99,14	0,00E+00	2686	0	NA	NA	
maker-5-augustus-gene-1.41-mRNA-1	5	OsR498G0509557200.01.T07	99,95	0,00E+00	3410	Os01g0110500	80,50	0,00E+00	958	-2452	Indica	NA	Kinase-TM
maker-5-augustus-gene-1.45-mRNA-1	5	OsR498G0509557400.01.T02	100,00	0,00E+00	731	Os05g0100800	100,00	0,00E+00	731	0	NA	NA	
maker-5-augustus-gene-1.47-mRNA-1	5	OsR498G0509557800.01.T02	100,00	0,00E+00	3718	Os01g0108000	77,06	0,00E+00	1415	-2303	Indica	NA	
maker-5-augustus-gene-1.55-mRNA-1	5	OsR498G0509558800.01.T01	100,00	0,00E+00	747	Os05g0101400	100,00	0,00E+00	747	0	NA	NA	
maker-5-augustus-gene-1.57-mRNA-1	5	OsR498G0509559200.01.T02	100,00	0,00E+00	2825	Os05g0101600	99,43	0,00E+00	2788	-37	NA	NA	
maker-5-augustus-gene-1.59-mRNA-1	5	OsR498G0509562400.01.T04	100,00	0,00E+00	904	Os05g0102500	100,00	0,00E+00	904	0	NA	NA	
maker-5-augustus-gene-10.45-mRNA-1	5	OsR498G0509614800.01.T01	97,08	0,00E+00	810	Os05g0119100	97,19	0,00E+00	783	-27	NA	Japonica	

maker-5-augustus-gene-10.47-mRNA-1	5	OsR498G0509615200.01.T01	98,08	0,00E+00	901	Os05g0119200	100,00	0,00E+00	926	25	NA	Japonica	
maker-5-augustus-gene-10.49-mRNA-1	5	OsR498G0509615400.01.T02	99,50	0,00E+00	1058	Os05g0119300	100,00	0,00E+00	1072	14	NA	Japonica	
maker-5-augustus-gene-10.56-mRNA-1	5	OsR498G0509618800.01.T01	99,59	0,00E+00	1736	Os05g0120100	100,00	0,00E+00	1754	18	NA	Japonica	
maker-5-augustus-gene-101.5-mRNA-1	5	OsR498G0510192800.01.T06	100,00	0,00E+00	4818	Os04g0291900	88,33	0,00E+00	3399	-1419	Indica	NA	Kinase-LRR-TM
maker-5-augustus-gene-103.5-mRNA-1	5	OsR498G0510192800.01.T06	93,80	0,00E+00	3962	Os04g0291900	88,34	0,00E+00	3635	-327	NA	NA	Kinase-LRR-TM
maker-5-augustus-gene-104.30-mRNA-1	5	OsR498G0510199300.01.T01	99,89	0,00E+00	1608	Os10g0127700	97,20	0,00E+00	1500	-108	NA	NA	
maker-5-augustus-gene-104.35-mRNA-1	5	OsR498G0510203700.01.T01	93,75	0,00E+00	728	Os04g0291900	80,92	6,91E-113	407	-321	Indica	NA	LRR-TM
maker-5-augustus-gene-105.27-mRNA-1	5	OsR498G0510210000.01.T02	100,00	3,61E-155	547	Os05g0267001	100,00	2,43E-82	304	-243	Indica	NA	
maker-5-augustus-gene-107.37-mRNA-1	5	OsR498G0510213400.01.T01	100,00	0,00E+00	1344	Os05g0267732	100,00	0,00E+00	1088	-256	Indica	NA	
maker-5-augustus-gene-107.65-mRNA-1	5	OsR498G0510214400.01.T02	100,00	0,00E+00	657	Os05g0267800	100,00	0,00E+00	657	0	NA	NA	
maker-5-augustus-gene-108.31-mRNA-1	5	OsR498G0510217500.01.T01	100,00	0,00E+00	1485	Os01g0279400	75,89	2,03E-172	606	-879	Indica	NA	
maker-5-augustus-gene-108.34-mRNA-1	5	OsR498G0510217800.01.T07	98,67	0,00E+00	2098	Os05g0268500	98,74	0,00E+00	2094	-4	NA	NA	
maker-5-augustus-gene-108.37-mRNA-1	5	OsR498G0510219400.01.T01	100,00	0,00E+00	2338	Os05g0269100	100,00	0,00E+00	2338	0	NA	NA	
maker-5-augustus-gene-109.0-mRNA-1	5	OsR498G0510222700.01.T01	100,00	0,00E+00	1440	Os05g0270000	100,00	0,00E+00	1440	0	NA	NA	
maker-5-augustus-gene-109.10-mRNA-1	5	OsR498G0510221400.01.T02	97,15	0,00E+00	2233	Os05g0269500	97,15	0,00E+00	2233	0	NA	NA	
maker-5-augustus-gene-109.20-mRNA-1	5	OsR498G0510224000.01.T01	100,00	0,00E+00	2457	Os05g0270200	100,00	0,00E+00	2457	0	NA	NA	
maker-5-augustus-gene-109.22-mRNA-1	5	OsR498G0510225800.01.T01	100,00	0,00E+00	773	Os04g0295100	94,16	0,00E+00	679	-94	Indica	NA	
maker-5-augustus-gene-11.28-mRNA-1	5	OsR498G0509621700.01.T01	99,56	0,00E+00	5651	Os03g0281600	75,35	0,00E+00	1652	-3999	Indica	Japonica	
maker-5-augustus-gene-11.33-mRNA-1	5	OsR498G0509622300.01.T01	99,82	0,00E+00	970	Os05g0120800	100,00	0,00E+00	1056	86	NA	Japonica	
maker-5-augustus-gene-11.35-mRNA-1	5	OsR498G0509623500.01.T04	99,66	0,00E+00	1047	Os05g0121000	100,00	0,00E+00	1056	9	NA	Japonica	
maker-5-augustus-gene-11.38-mRNA-1	5	OsR498G0509623900.01.T03	99,88	0,00E+00	1435	Os12g0148600	71,06	3,33E-74	279	-1156	Indica	Japonica	
maker-5-augustus-gene-11.45-mRNA-1	5	OsR498G0509624200.01.T01	99,83	0,00E+00	2058	Os05g0121200	100,00	0,00E+00	2067	9	NA	Japonica	
maker-5-augustus-gene-110.0-mRNA-1	5	OsR498G0510230100.01.T04	100,00	0,00E+00	1721	Os05g0271300	100,00	0,00E+00	1012	-709	Indica	NA	
maker-5-augustus-gene-110.7-mRNA-1	5	OsR498G0510236300.01.T01	100,00	0,00E+00	2700	Os05g0272800	90,17	0,00E+00	2035	-665	Indica	NA	
maker-5-augustus-gene-111.0-mRNA-1	5	OsR498G0510237900.01.T02	100,00	0,00E+00	2852	Os05g0272800	98,67	0,00E+00	2772	-80	NA	NA	
maker-5-augustus-gene-111.4-mRNA-1	5	OsR498G0510241100.01.T01	100,00	0,00E+00	2747	Os05g0273500	100,00	0,00E+00	2746	-1	NA	NA	
maker-5-augustus-gene-111.6-mRNA-1	5	OsR498G0510238200.01.T01	100,00	0,00E+00	1202	Os05g0272900	100,00	0,00E+00	1202	0	NA	NA	
maker-5-augustus-gene-111.8-mRNA-1	5	OsR498G0510242400.01.T01	100,00	0,00E+00	1797	Os05g0273800	100,00	0,00E+00	1797	0	NA	NA	
maker-5-augustus-gene-112.4-mRNA-1	5	OsR498G0510244900.01.T02	100,00	0,00E+00	2073	Os05g0274300	100,00	0,00E+00	2073	0	NA	NA	
maker-5-augustus-gene-113.31-mRNA-1	5	OsR498G0510247500.01.T01	99,84	0,00E+00	1105	Os05g0274700	99,84	0,00E+00	1105	0	NA	NA	
maker-5-augustus-gene-113.40-mRNA-1	5	OsR498G0510250400.01.T01	100,00	0,00E+00	1856	Os05g0275700	100,00	0,00E+00	1856	0	NA	NA	
maker-5-augustus-gene-114.0-mRNA-1	5	OsR498G0510252100.01.T02	100,00	0,00E+00	4275	Os05g0276100	100,00	0,00E+00	4275	0	NA	NA	
maker-5-augustus-gene-114.2-mRNA-1	5	OsR498G0510255600.01.T01	100,00	0,00E+00	1386	Os05g0276500	100,00	0,00E+00	1386	0	NA	NA	
maker-5-augustus-gene-114.4-mRNA-1	5	OsR498G0510258600.01.T01	100,00	0,00E+00	1348	Os05g0277000	100,00	0,00E+00	1348	0	NA	NA	
maker-5-augustus-gene-115.0-mRNA-1	5	OsR498G0510262500.01.T01	100,00	0,00E+00	1202	Os05g0277500	100,00	0,00E+00	1202	0	NA	NA	
maker-5-augustus-gene-115.2-mRNA-1	5	OsR498G0510260000.01.T05	99,92	0,00E+00	2247	Os05g0277300	99,91	0,00E+00	1890	-357	Indica	NA	
maker-5-augustus-gene-116.22-mRNA-1	5	OsR498G0510266000.01.T01	100,00	0,00E+00	2343	Os05g0278500	100,00	0,00E+00	2343	0	NA	NA	
maker-5-augustus-gene-117.27-mRNA-1	5	OsR498G0510269100.01.T01	99,26	0,00E+00	4580	Os05g0279400	99,30	0,00E+00	4584	4	NA	NA	
maker-5-augustus-gene-117.36-mRNA-1	5	OsR498G0510271100.01.T01	100,00	0,00E+00	2630	Os05g0279900	100,00	0,00E+00	2630	0	NA	NA	
maker-5-augustus-gene-118.20-mRNA-1	5	OsR498G0510273200.01.T01	100,00	0,00E+00	1180	Os05g0280200	100,00	0,00E+00	1180	0	NA	NA	
maker-5-augustus-gene-118.22-mRNA-1	5	OsR498G0510274800.01.T01	100,00	0,00E+00	2911	Os05g0280500	100,00	0,00E+00	2911	0	NA	NA	
maker-5-augustus-gene-119.21-mRNA-1	5	OsR498G0510282100.01.T02	100,00	0,00E+00	699	Os05g0281400	100,00	0,00E+00	699	0	NA	NA	
maker-5-augustus-gene-12.0-mRNA-1	5	OsR498G0509627500.01.T02	99,57	0,00E+00	3288	Os05g0121800	99,78	0,00E+00	3308	20	NA	Japonica	
maker-5-augustus-gene-12.10-mRNA-1	5	OsR498G0509630100.01.T05	99,75	0,00E+00	2908	Os05g0122600	99,82	0,00E+00	2907	-1	NA	Japonica	
maker-5-augustus-gene-12.14-mRNA-1	5	OsR498G0509627400.01.T05	99,78	0,00E+00	1604	Os05g0121700	100,00	0,00E+00	1613	9	NA	Japonica	
maker-5-augustus-gene-12.17-mRNA-1	5	OsR498G0509627800.01.T01	96,51	0,00E+00	1772	Os05g0121900	96,60	0,00E+00	1776	4	NA	Japonica	
maker-5-augustus-gene-12.21-mRNA-1	5	OsR498G0509631400.01.T01	99,31	0,00E+00	766	Os05g0122900	99,59	6,07E-123	440	-326	Indica	Japonica	
maker-5-augustus-gene-125.2-mRNA-1	5	OsR498G0510322300.01.T04	100,00	0,00E+00	1700	Os05g0287800	100,00	0,00E+00	1700	0	NA	NA	
maker-5-augustus-gene-127.0-mRNA-1	5	OsR498G0510560100.01.T01	98,47	0,00E+00	677	Os05g0341100	99,81	0,00E+00	954	277	Japonica	NA	
maker-5-augustus-gene-129.19-mRNA-1	5	OsR498G0510328400.01.T01	100,00	0,00E+00	3918	Os05g0289400	100,00	0,00E+00	3918	0	NA	NA	
maker-5-augustus-gene-13.0-mRNA-1	5	OsR498G0509633500.01.T02	99,79	0,00E+00	1723	Os05g0123100	100,00	0,00E+00	1732	9	NA	Japonica	
maker-5-augustus-gene-131.43-mRNA-1	5	OsR498G0510346900.01.T01	100,00	0,00E+00	2096	Os05g0292200	100,00	0,00E+00	2096	0	NA	NA	
maker-5-augustus-gene-131.48-mRNA-1	5	OsR498G0510344200.01.T01	100,00	0,00E+00	2684	Os05g0291700	97,78	0,00E+00	2561	-123	NA	NA	
maker-5-augustus-gene-132.0-mRNA-1	5	OsR498G0510348500.01.T01	100,00	1,03E-174	612	Os05g0292800	100,00	3,59E-175	612	0	NA	NA	
maker-5-augustus-gene-132.2-mRNA-1	5	OsR498G0510352000.01.T01	100,00	0,00E+00	2024	Os05g0293500	100,00	0,00E+00	2024	0	NA	NA	
maker-5-augustus-gene-133.24-mRNA-1	5	OsR498G0510358500.01.T07	100,00	6,77E-171	600	Os05g0295100	100,00	2,35E-171	600	0	NA	NA	
maker-5-augustus-gene-133.25-mRNA-1	5	OsR498G0510358500.01.T07	100,00	0,00E+00	1597	Os05g0295100	100,00	0,00E+00	1597	0	NA	NA	
maker-5-augustus-gene-133.42-mRNA-1	5	OsR498G0510354800.01.T01	100,00	0,00E+00	4102	Os05g0294600	100,00	0,00E+00	4102	0	NA	NA	
maker-5-augustus-gene-134.6-mRNA-1	5	OsR498G0510362200.01.T02	97,44	0,00E+00	3853	Os05g0295900	97,44	0,00E+00	3853	0	NA	NA	
maker-5-augustus-gene-135.41-mRNA-1	5	OsR498G0510371300.01.T01	100,00	0,00E+00	839	Os05g0297900	100,00	0,00E+00	839	0	NA	NA	
maker-5-augustus-gene-136.0-mRNA-1	5	OsR498G0510373100.01.T04	100,00	0,00E+00	3631	Os05g0298200	100,00	0,00E+00	2794	-837	Indica	NA	
maker-5-augustus-gene-136.8-mRNA-1	5	OsR498G0510376800.01.T03	99,75	0,00E+00	718	Os05g0299300	99,75	0,00E+00	718	0	NA	NA	
maker-5-augustus-gene-137.0-mRNA-1	5	OsR498G0510378100.01.T04	100,00	0,00E+00	1240	Os05g0299500	100,00	0,00E+00	1240	0	NA	NA	
maker-5-augustus-gene-137.5-mRNA-1	5	OsR498G0510384700.01.T03	100,00	0,00E+00	2560	Os05g0300700	100,00	0,00E+00	2560	0	NA	NA	
maker-5-augustus-gene-138.0-mRNA-1	5	OsR498G0510392500.01.T05	97,37	0,00E+00	2077	Os05g0301500	97,37	0,00E+00	2077	0	NA	NA	
maker-5-augustus-gene-138.19-mRNA-1	5	OsR498G0510395600.01.T01	100,00	0,00E+00	5330	Os05g0302300	99,97	0,00E+00	5255	-75	NA	Indica	
maker-5-augustus-gene-138.6-mRNA-1	5	OsR498G0510392700.01.T02	100,00	0,00E+00	893	Os05g0301600	100,00	0,00E+00	893	0	NA	NA	
maker-5-augustus-gene-138.9-mRNA-1	5	OsR498G0510393200.01.T03	100,00	0,00E+00	1651	Os05g0301700	100,00	0,00E+00	1651	0	NA	NA	
maker-5-augustus-gene-139.0-mRNA-1	5	OsR498G0510400300.01.T01	100,00	0,00E+00	3734	Os05g0303000	100,00	0,00E+00	2489	-1245	Indica	NA	
maker-5-augustus-gene-139.8-mRNA-1	5	OsR498G0510399200.01.T02	100,00	0,00E+00	2062	Os05g0302700	100,00	0,00E+00	2062	0	NA	Indica	
maker-5-augustus-gene-14.34-mRNA-1	5	OsR498G0509643900.01.T01	99,84	0,00E+00	4414	Os05g0125000	99,96	0,00E+00	4427	13	NA	Japonica	
maker-5-augustus-gene-14.39-mRNA-1	5	OsR498G0509647100.01.T01	99,90	0,00E+00	1755	Os05g0125300	100,00	0,00E+00	2403	648	Japonica	Japonica	Kinase-TM
maker-5-augustus-gene-14.42-mRNA-1	5	OsR498G0509647700.01.T01	99,79	0,00E+00	1669	Os05g0125400	100,00	0,00E+00	2387	718	Japonica	Japonica	Kinase
maker-5-augustus-gene-14.50-mRNA-1	5	OsR498G0509648000.01.T05	99,85	0,00E+00	1167	Os05g0125500	100,00	0,00E+00	1171	4	NA	Japonica	
maker-5-augustus-gene-140.0-mRNA-1	5	OsR498G0510406900.01.T01	100,00	0,00E+00	2414	Os05g0304400	100,00	0,00E+00	2414	0	NA	Indica	
maker-5-augustus-gene-140.10-mRNA-1	5	OsR498G1120445500.01.T02	92,11	3,80E-57	221	Os05g0304200	99,47	2,39E-92	337	116	Japonica	Indica	
maker-5-augustus-gene-140.7-mRNA-1	5	OsR498G0510408500.01.T01	100,00	0,00E+00	4589	Os05g0304600	99,88	0,00E+00	4575	-14	NA	Indica	
maker-5-augustus-gene-140.9-mRNA-1	5	OsR498G0510483800.01.T01	92,59	0,00E+00	1348	Os05g0319900	93,14	0,00E+00	1370	22	NA	Indica	
maker-5-augustus-gene-141.0-mRNA-1	5	OsR498G0510411900.01.T02	99,91	0,00E+00	1966	Os05g0305100	99,82	0,00E+00	1961	-5	NA	Indica	
maker-5-augustus-gene-141.16-mRNA-1	5	OsR498G0510410400.01.T02	99,94	0,00E+00	3172	Os05g0304900	98,47	0,00E+00	2506	-666	Indica	Indica	
maker-5-augustus-gene-141.23-mRNA-1	5	OsR498G0510414800.01.T01	99,83	0,00E+00	2080	Os05g0306000	100,00	0,00E+00	2089	9	NA	Japonica	
maker-5-augustus-gene-141.6-mRNA-1	5	OsR498G0510412100.01.T01	99,90	0,00E+00	1809	Os05g0305200	99,90	0,00E+00	1809	0	NA	Japonica	
maker-5-augustus-gene-143.4-mRNA-1	5	OsR498G0510419200.01.T04	99,87	0,00E+00	2713	Os05g0307200	99,90	0,00E+00	3472	759	Japonica	Japonica	
maker-5-augustus-gene-145.39-mRNA-1	5	OsR498G0510436100.01.T03	99,86	0,00E+00	1268	Os05g0310500	100,00	0,00E+00	1272	4	NA	Japonica	
maker-5-augustus-gene-145.43-mRNA-1	5	OsR498G0510436600.01.T01	99,88	0,00E+00	1527	Os05g0310600	99,41	0,00E+00	1508	-19	NA	NA	
maker-5-augustus-gene-146.15-mRNA-1	5	OsR498G0510441500.01.T01	99,85	0,00E+00	1165	Os05g0311500	99,85	0,00E+00	1165	0	NA	NA	
maker-5-augustus-gene-146.4-mRNA-1	5	OsR498G0510437100.01.T01	100,00	0,00E+00	2837	Os05g0310800	100,00	0,00E+00	2837	0	NA	NA	
maker-5-augustus-gene-148.0-mRNA-1	5	OsR498G0510452500.01.T01	99,88	0,00E+00	1452	Os05g0313500	100,00	0,00E+00	830	-622	Indica	Japonica	
maker-5-augustus-gene-148.6-mRNA-1	5	OsR498G0510452900.01.T01	99,67	0,00E+00	2692	Os05g0313600	100,00	0,00E+00	3360	668	Japonica	Japonica	
maker-5-augustus-gene-148.8-mRNA-1	5	OsR498G0510448200.01.T03	99,87	0,00E+00	1360	Os05g0312500	100,00	0,00E+00	1364	4	NA	Japonica	
maker-5-augustus-gene-149.25-mRNA-1	5	OsR498G0510464300.01.T01	99,92	0,00E+00	2333	Os05g0315200	100,00	0,00E+00	2273	-60	NA	Japonica	
maker-5-augustus-gene-149.32-mRNA-1	5	OsR498G0510456400.01.T02	99,69	1,03E-161	570	Os05g0314100	99,69	3,58E-162	570	0	NA	Japonica	
maker-5-augustus-gene-149.41-mRNA-1	5	OsR498G0510465200.01.T01	97,99	3,28E-169	595	Os05g0315501	100,00	3,49E-138	489	-106	Indica	Japonica	
maker-5-augustus-gene-15.39-mRNA-1	5	OsR498G0509650100.01.T01	99,77	0,00E+00	1544	Os05g0126100	96,77	0,00E+00	1459	-85	NA	Japonica	
maker-5-augustus-gene-15.43-mRNA-1	5	OsR498G0509653300.01.T01	99,83	0,00E+00	3228	Os05g0127200	100,00	0,00E+00	3241	13	NA	Japonica	
maker-5-augustus-gene-15.52-mRNA-1	5	OsR498G0509655300.01.T01	99,69	0,00E+00	1735	Os05g0562300	71,27	1,51E-71	269	-1466	Indica	Japonica	
maker-5-augustus-gene-15.54-mRNA-1	5	OsR498G0509650600.01.T01	100,00	0,00E+00	1045	Os05g0126200	100,00	0,00E+00	1045	0	NA	Japonica	
maker-5-augustus-gene-15.59-mRNA-1	5	OsR498G0509652000.01.T01	99,77	0,00E+00	765	Os05g0126800	99,77	0,00E+00	765	0	NA	Japonica	
maker-5-augustus-gene-15.61-mRNA-1	5	OsR498G0509655800.01.T01	99,46	0,00E+00	1970	Os05g0127500	100,00	0,00E+00	1997	27	NA	Japonica	
maker-5-augustus-gene-150.15-mRNA-1	5	OsR498G0510468000.01.T02	100,00	0,00E+00	1878	Os05g0316100	100,00	0,00E+00	1878	0	NA	Japonica	
maker-5-augustus-gene-150.9-mRNA-1	5	OsR498G0510467600.01.T01	99,78	0,00E+00	2498	Os05g0316000	100,00	0,00E+00	2511	13	NA	Japonica	
maker-5-augustus-gene-152.30-mRNA-1	5	OsR498G0510478100.01.T01	99,80	0,00E+00	3526	Os05g0318100	99,82	0,00E+00	1947	-1579	Indica	Japonica	CC-Kinase-TM
maker-5-augustus-gene-152.33-mRNA-1	5	OsR498G1119671000.01.T02	90,96	0,00E+00	1067	Os05g0318300	100,00	0,00E+00	1342	275	Japonica	NA	
maker-5-augustus-gene-152.39-mRNA-1	5	OsR498G0510481500.01.T01	99,80	0,00E+00	2649	Os01g0663300	71,70	5,05E-118	425	-2224	Indica	Japonica	
maker-5-augustus-gene-152.41-mRNA-1	5	OsR498G0510482900.01.T01	94,63	0,00E+00	2357	Os05g0319700	97,49	0,00E+00	2513	156	NA	Japonica	Kinase-TM
maker-5-augustus-gene-152.45-mRNA-1	5	OsR498G0510482200.01.T01	99,70	0,00E+00	1787	Os01g0192900	84,62	0,00E+00	1040	-747	Indica	Japonica	
maker-5-augustus-gene-153.0-mRNA-1	5	OsR498G0510485300.01.T01	99,70	7,79E-170	597	Os05g0320300	100,00	1,83E-134	477	-120	Indica	Japonica	
maker-5-augustus-gene-153.15-mRNA-1	5	OsR498G0510486800.01.T01	99,87	0,00E+00	2702	Os05g0320700	100,00	0,00E+00	2711	9	NA	Japonica	
maker-5-augustus-gene-154.32-mRNA-1	5	OsR498G0510489900.01.T01	100,00	0,00E+00	2322	Os05g0321900	100,00	0,00E+00	2322	0	NA	NA	
maker-5-augustus-gene-154.34-mRNA-1	5	OsR498G0510491900.01.T01	100,00	0,00E+00	1220	Os05g0322900	100,00	0,00E+00	1220	0	NA	NA	WRKY45
maker-5-augustus-gene-154.40-mRNA-1	5	OsR498G0510496300.01.T04	98,77	0,00E+00	3865	Os05g0323800	98,39	0,00E+00	2269	-1596	Indica	NA	Kinase-TM
maker-5-augustus-gene-155.0-mRNA-1	5	OsR498G0510496500.01.T02	100,00	0,00E+00	740	Os05g0323900	100,00	0,00E+00	726	-14	NA	NA	
maker-5-augustus-gene-156.4-mRNA-1	5	OsR498G1221921500.01.T05	79,17	2,67E-163	577	Os05g0327100	99,72	0,00E+00	1299	722	Japonica	Indica	
maker-5-augustus-gene-16.0-mRNA-1	5	OsR498G0509661000.01.T01	98,63	0,00E+00	1021	Os05g0128300	100,00	0,00E+00	1975	954	Japonica	Japonica	
maker-5-augustus-gene-161.0-mRNA-1	5	OsR498G0510525000.01.T01	100,00	0,00E+00	3117	Os05g0331200	99,94	0,00E+00	3112	-5	NA	Indica	
maker-5-augustus-gene-162.19-mRNA-1	5	OsR498G0510534000.01.T01	100,00	0,00E+00	2425	Os05g0334400	99,93	0,00E+00	2420	-5	NA	Indica	
maker-5-augustus-gene-162.8-mRNA-1	5	OsR498G0510532800.01.T01	98,24	0,00E+00	2074	Os05g0333200	98,16	0,00E+00	2069	-5	NA	Indica	
maker-5-augustus-gene-163.1-mRNA-1	5	OsR498G0510534000.01.T01	100,00	0,00E+00	2425	Os05g0334400	99,93	0,00E+00	2420	-5	NA	Indica	
maker-5-augustus-gene-163.2-mRNA-1	5	OsR498G0510534000.01.T01	100,00	0,00E+00	2425	Os05g0334400	99,93	0,00E+00	2420	-5	NA	NA	
maker-5-augustus-gene-164.41-mRNA-1	5	OsR498G0510547400.01.T01	99,61	1,16E-129	463	Os05g0337200	99,61	4,03E-130	463	0	NA	Indica	
maker-5-augustus-gene-165.31-mRNA-1	5	OsR498G0510554500.01.T01	100,00	0,00E+00	3155	Os05g0338933	99,57	0,00E+00	1651	-1504	Indica	Indica	
maker-5-augustus-gene-165.35-mRNA-1	5	OsR498G0510555700.01.T02	100,00	0,00E+00	866	Os05g0339200	98,98	0,00E+00	1035	169	Japonica	Indica	
maker-5-augustus-gene-166.36-mRNA-1	5	OsR498G0510561200.01.T01	100,00	0,00E+00	1153	Os05g0341600	100,00	0,00E+00	1153	0	NA	Indica	
maker-5-augustus-gene-167.29-mRNA-1	5	OsR498G0510566900.01.T02	98,09	0,00E+00	808	Os05g0342900	100,00	2,42E-160	563	-245	Indica	Japonica	
maker-5-augustus-gene-168.0-mRNA-1	5	OsR498G0510567700.01.T02	98,84	0,00E+00	2573	Os05g0343400	98,84	0,00E+00	2573	0	NA	Japonica	
maker-5-augustus-gene-169.36-mRNA-1	5	OsR498G0510592600.01.T01	99,83	0,00E+00	1067	Os05g0348100	99,45	0,00E+00	646	-421	Indica	NA	
maker-5-augustus-gene-169.40-mRNA-1	5	OsR498G0510592600.01.T01	99,83	0,00E+00	1067	Os05g0348100	99,45	0,00E+00	646	-421	Indica	NA	
maker-5-augustus-gene-169.44-mRNA-1	5	OsR498G0510592600.01.T01	99,83	0,00E+00	1067	Os05g0348100	99,45	0,00E+00	646	-421	Indica	NA	
maker-5-augustus-gene-169.48-mRNA-1	5	OsR498G0510592600.01.T01	99,83	0,00E+00	1067	Os05g0348100	99,45	0,00E+00	646	-421	Indica	NA	
maker-5-augustus-gene-169.52-mRNA-1	5	OsR498G0510592600.01.T01	99,83	0,00E+00	1067	Os05g0348100	99,45	0,00E+00	646	-421	Indica	NA	
maker-5-augustus-gene-17.51-mRNA-1	5	OsR498G0509663100.01.T03	99,64	0,00E+00	2499	Os07g0461900	96,58	0,00E+00	1447	-1052	Indica	Japonica	
maker-5-augustus-gene-17.54-mRNA-1	5	OsR498G0509666100.01.T01	99,82	0,00E+00	970	Os05g0129800	99,73	0,00E+00	662	-308	Indica	Japonica	
maker-5-augustus-gene-17.62-mRNA-1	5	OsR498G0816083200.01.T02	97,21	0,00E+00	1024	Os05g0128900	100,00	0,00E+00	1242	218	Japonica	Japonica	
maker-5-augustus-gene-17.67-mRNA-1	5	OsR498G0509664800.01.T01	99,68	0,00E+00	1675	Os05g0129700	100,00	0,00E+00	1689	14	NA	Japonica	
maker-5-augustus-gene-17.69-mRNA-1	5	OsR498G0509666400.01.T02	99,88	0,00E+00	1475	Os05g0129900	100,00	0,00E+00	1462	-13	NA	Japonica	
maker-5-augustus-gene-170.31-mRNA-1	5	OsR498G0510588200.01.T01	99,50	0,00E+00	1075	Os05g0346800	100,00	0,00E+00	1088	13	NA	Japonica	
maker-5-augustus-gene-170.33-mRNA-1	5	OsR498G0510587200.01.T03	96,79	0,00E+00	2089	Os05g0346500	97,25	0,00E+00	2041	-48	NA	Japonica	
maker-5-augustus-gene-170.42-mRNA-1	5	OsR498G0510586200.01.T01	99,34	0,00E+00	1865	Os05g0346200	100,00	0,00E+00	1618	-247	Indica	Japonica	
maker-5-augustus-gene-170.53-mRNA-1	5	OsR498G0510584800.01.T02	97,58	0,00E+00	5174	Os05g0345400	97,68	0,00E+00	5187	13	NA	Japonica	
maker-5-augustus-gene-170.59-mRNA-1	5	OsR498G0510586700.01.T01	99,61	0,00E+00	917	Os05g0346400	100,00	0,00E+00	926	9	NA	Japonica	
maker-5-augustus-gene-170.61-mRNA-1	5	OsR498G0510585900.01.T02	99,86	0,00E+00	2590	Os05g0346100	100,00	0,00E+00	2599	9	NA	Japonica	
maker-5-augustus-gene-171.0-mRNA-1	5	OsR498G0510581000.01.T01	99,51	0,00E+00	1096	Os05g0344400	99,51	0,00E+00	1096	0	NA	Japonica	
maker-5-augustus-gene-172.0-mRNA-1	5	OsR498G0510598100.01.T06	99,12	0,00E+00	2018	Os05g0349700	99,12	0,00E+00	2018	0	NA	Indica	
maker-5-augustus-gene-172.19-mRNA-1	5	OsR498G0510597400.01.T02	100,00	0,00E+00	2549	Os05g0349500	99,86	0,00E+00	2540	-9	NA	Indica	
maker-5-augustus-gene-172.20-mRNA-1	5	OsR498G0510598400.01.T02	100,00	4,19E-147	520	Os05g0349800	100,00	1,46E-147	520	0	NA	Indica	
maker-5-augustus-gene-172.9-mRNA-1	5	OsR498G0510595800.01.T01	97,31	0,00E+00	3422	Os07g0693500	78,04	0,00E+00	1053	-2369	Indica	Indica	
maker-5-augustus-gene-173.21-mRNA-1	5	OsR498G0510601700.01.T01	100,00	0,00E+00	1957	Os05g0350500	99,91	0,00E+00	1953	-4	NA	Indica	
maker-5-augustus-gene-173.26-mRNA-1	5	OsR498G0510602300.01.T01	100,00	0,00E+00	1196	Os05g0350600	100,00	0,00E+00	1196	0	NA	Indica	
maker-5-augustus-gene-173.29-mRNA-1	5	OsR498G0510602900.01.T02	99,21	0,00E+00	6716	Os05g0350700	99,16	0,00E+00	6707	-9	NA	Indica	
maker-5-augustus-gene-174.10-mRNA-1	5	OsR498G0510613800.01.T01	100,00	0,00E+00	2890	Os05g0353500	100,00	0,00E+00	2544	-346	Indica	NA	Kinase-TM
maker-5-augustus-gene-174.4-mRNA-1	5	OsR498G0510612700.01.T01	100,00	0,00E+00	2646	Os05g0353300	100,00	0,00E+00	2646	0	NA	NA	
maker-5-augustus-gene-174.8-mRNA-1	5	OsR498G0510613500.01.T04	100,00	0,00E+00	4702	Os05g0353400	100,00	0,00E+00	4702	0	NA	NA	
maker-5-augustus-gene-175.24-mRNA-1	5	OsR498G0510618200.01.T02	100,00	0,00E+00	2879	Os05g0354400	99,94	0,00E+00	3089	210	NA	NA	
maker-5-augustus-gene-176.11-mRNA-1	5	OsR498G0510631900.01.T06	99,92	0,00E+00	2236	Os05g0357100	99,92	0,00E+00	2236	0	NA	NA	
maker-5-augustus-gene-176.16-mRNA-1	5	OsR498G0510624200.01.T01	99,86	0,00E+00	1289	Os05g0355300	97,59	0,00E+00	1066	-223	Indica	NA	
maker-5-augustus-gene-176.21-mRNA-1	5	OsR498G0510629600.01.T01	99,68	0,00E+00	2207	Os05g0356700	100,00	0,00E+00	2462	255	Japonica	NA	
maker-5-augustus-gene-176.5-mRNA-1	5	OsR498G0510625500.01.T01	100,00	0,00E+00	975	Os01g0303800	71,09	5,93E-61	233	-742	Indica	NA	
maker-5-augustus-gene-176.9-mRNA-1	5	OsR498G0510627700.01.T01	100,00	0,00E+00	978	Os05g0355900	100,00	0,00E+00	978	0	NA	NA	
maker-5-augustus-gene-177.40-mRNA-1	5	OsR498G0510632500.01.T01	100,00	0,00E+00	1519	Os05g0357200	100,00	0,00E+00	1519	0	NA	NA	
maker-5-augustus-gene-177.50-mRNA-1	5	OsR498G0510636300.01.T01	98,06	0,00E+00	1963	Os05g0357600	98,06	0,00E+00	1963	0	NA	NA	
maker-5-augustus-gene-177.63-mRNA-1	5	OsR498G0510639100.01.T01	100,00	0,00E+00	1673	Os05g0358400	100,00	0,00E+00	1673	0	NA	NA	
maker-5-augustus-gene-177.67-mRNA-1	5	OsR498G0510635800.01.T01	100,00	0,00E+00	737	Os01g0303800	96,37	4,34E-112	404	-333	Indica	NA	
maker-5-augustus-gene-177.72-mRNA-1	5	OsR498G0510637000.01.T04	100,00	0,00E+00	2192	Os05g0357800	100,00	0,00E+00	2192	0	NA	NA	
maker-5-augustus-gene-177.74-mRNA-1	5	OsR498G0510637400.01.T05	100,00	0,00E+00	756	Os05g0358000	100,00	1,89E-169	594	-162	Indica	NA	
maker-5-augustus-gene-177.84-mRNA-1	5	OsR498G0510639400.01.T04	100,00	0,00E+00	1916	Os05g0358500	100,00	0,00E+00	1916	0	NA	NA	TIR
maker-5-augustus-gene-179.35-mRNA-1	5	OsR498G0510654700.01.T05	99,78	0,00E+00	1615	Os05g0361200	99,89	0,00E+00	1619	4	NA	Japonica	
maker-5-augustus-gene-18.12-mRNA-1	5	OsR498G0509671200.01.T02	96,41	6,51E-101	367	Os05g0131100	92,83	1,63E-90	332	-35	NA	Japonica	
maker-5-augustus-gene-18.2-mRNA-1	5	OsR498G0509668800.01.T01	90,36	0,00E+00	1733	Os05g0130400	100,00	0,00E+00	2938	1205	Japonica	Japonica	
maker-5-augustus-gene-18.22-mRNA-1	5	OsR498G0509672600.01.T02	98,65	0,00E+00	3492	Os05g0132100	99,70	0,00E+00	3587	95	NA	Japonica	
maker-5-augustus-gene-18.6-mRNA-1	5	OsR498G0509670800.01.T01	99,53	0,00E+00	2636	Os05g0131000	100,00	0,00E+00	2857	221	NA	Japonica	
maker-5-augustus-gene-180.36-mRNA-1	5	OsR498G0510656300.01.T01	99,83	0,00E+00	3141	Os05g0361500	99,83	0,00E+00	3132	-9	NA	Indica	
maker-5-augustus-gene-180.38-mRNA-1	5	OsR498G0510657700.01.T01	98,33	0,00E+00	1037	Os05g0361700	98,33	0,00E+00	1037	0	NA	NA	
maker-5-augustus-gene-180.42-mRNA-1	5	OsR498G0510659700.01.T05	99,74	0,00E+00	2099	Os05g0362100	99,92	0,00E+00	2108	9	NA	Japonica	
maker-5-augustus-gene-180.49-mRNA-1	5	OsR498G0510660700.01.T01	99,26	0,00E+00	957	Os05g0362500	100,00	0,00E+00	971	14	NA	Japonica	
maker-5-augustus-gene-180.50-mRNA-1	5	OsR498G0510658800.01.T02	99,56	0,00E+00	816	Os05g0361900	100,00	0,00E+00	825	9	NA	Japonica	
maker-5-augustus-gene-180.54-mRNA-1	5	OsR498G0510661200.01.T01	99,66	4,24E-147	521	Os05g0362600	100,00	2,19E-126	450	-71	Indica	Japonica	
maker-5-augustus-gene-181.0-mRNA-1	5	OsR498G0510662300.01.T03	99,43	0,00E+00	1878	Os05g0363100	99,91	0,00E+00	1901	23	NA	Japonica	
maker-5-augustus-gene-181.3-mRNA-1	5	OsR498G0510662600.01.T01	98,51	0,00E+00	2339	Os05g0363200	98,74	0,00E+00	2352	13	NA	Japonica	
maker-5-augustus-gene-182.18-mRNA-1	5	OsR498G0510668900.01.T03	100,00	0,00E+00	1597	Os05g0364600	99,66	0,00E+00	1583	-14	NA	Indica	
maker-5-augustus-gene-182.23-mRNA-1	5	OsR498G0510669100.01.T01	90,07	0,00E+00	651	Os01g0586600	86,08	3,59E-139	493	-158	Indica	Indica	
maker-5-augustus-gene-183.21-mRNA-1	5	OsR498G0510676300.01.T03	100,00	0,00E+00	1622	Os05g0366000	97,37	0,00E+00	1555	-67	NA	Indica	
maker-5-augustus-gene-183.6-mRNA-1	5	OsR498G0510674700.01.T01	99,91	0,00E+00	2050	Os05g0366000	89,28	0,00E+00	1197	-853	Indica	Indica	
maker-5-augustus-gene-184.0-mRNA-1	5	OsR498G0510676700.01.T01	100,00	1,13E-118	426	Os05g0366000	83,48	6,23E-66	251	-175	Indica	Indica	
maker-5-augustus-gene-184.2-mRNA-1	5	OsR498G0510677300.01.T03	100,00	0,00E+00	1326	Os05g0366300	99,32	0,00E+00	1302	-24	NA	Indica	
maker-5-augustus-gene-184.6-mRNA-1	5	OsR498G0510677900.01.T01	99,88	0,00E+00	1493	Os05g0366600	99,40	0,00E+00	1475	-18	NA	Indica	
maker-5-augustus-gene-185.0-mRNA-1	5	OsR498G0510678900.01.T01	100,00	0,00E+00	1644	Os05g0366600	83,82	0,00E+00	1247	-397	Indica	NA	
maker-5-augustus-gene-185.12-mRNA-1	5	OsR498G0510680800.01.T01	99,63	0,00E+00	1441	Os05g0367400	99,26	0,00E+00	1427	-14	NA	Indica	
maker-5-augustus-gene-185.15-mRNA-1	5	OsR498G0510681800.01.T01	100,00	0,00E+00	3618	Os05g0367800	99,55	0,00E+00	3578	-40	NA	NA	
maker-5-augustus-gene-185.4-mRNA-1	5	OsR498G0510679400.01.T03	96,43	0,00E+00	1122	Os05g0366900	93,60	0,00E+00	1033	-89	NA	NA	
maker-5-augustus-gene-185.7-mRNA-1	5	OsR498G0510680000.01.T01	100,00	0,00E+00	1061	Os05g0367100	99,49	0,00E+00	1048	-13	NA	NA	
maker-5-augustus-gene-185.9-mRNA-1	5	OsR498G0510680400.01.T01	96,05	0,00E+00	1718	Os05g0367200	95,83	0,00E+00	1463	-255	Indica	NA	
maker-5-augustus-gene-186.0-mRNA-1	5	OsR498G0510685100.01.T01	100,00	0,00E+00	1425	Os05g0368300	99,37	0,00E+00	1403	-22	NA	Indica	
maker-5-augustus-gene-186.3-mRNA-1	5	OsR498G0510686600.01.T01	100,00	0,00E+00	1975	Os05g0368600	99,90	0,00E+00	1796	-179	NA	NA	
maker-5-augustus-gene-186.5-mRNA-1	5	OsR498G0510687800.01.T01	100,00	0,00E+00	3323	Os10g0568600	74,27	0,00E+00	1256	-2067	Indica	NA	CC-NBS-TM
maker-5-augustus-gene-188.24-mRNA-1	5	OsR498G0510696700.01.T01	100,00	0,00E+00	1667	Os05g0371200	99,89	0,00E+00	1663	-4	NA	Indica	
maker-5-augustus-gene-188.30-mRNA-1	5	OsR498G0510698900.01.T01	97,04	0,00E+00	2577	Os01g0936100	81,92	0,00E+00	1350	-1227	Indica	Indica	Kinase
maker-5-augustus-gene-188.34-mRNA-1	5	OsR498G0510694200.01.T01	100,00	0,00E+00	1618	Os05g0370700	99,67	0,00E+00	1605	-13	NA	Indica	
maker-5-augustus-gene-188.36-mRNA-1	5	OsR498G0510694600.01.T01	100,00	0,00E+00	760	Os05g0370700	87,41	1,75E-144	511	-249	Indica	Indica	
maker-5-augustus-gene-188.38-mRNA-1	5	OsR498G0510695800.01.T02	100,00	0,00E+00	4195	Os05g0371100	98,97	0,00E+00	6845	2650	Japonica	Indica	
maker-5-augustus-gene-188.47-mRNA-1	5	OsR498G0510698400.01.T01	100,00	0,00E+00	641	Os05g0372000	99,72	0,00E+00	636	-5	NA	Indica	
maker-5-augustus-gene-189.0-mRNA-1	5	OsR498G0510699300.01.T01	100,00	0,00E+00	1680	Os05g0372300	99,46	0,00E+00	1657	-23	NA	Indica	
maker-5-augustus-gene-189.12-mRNA-1	5	OsR498G0510703100.01.T01	100,00	0,00E+00	2423	Os05g0373400	99,85	0,00E+00	2414	-9	NA	Indica	
maker-5-augustus-gene-189.17-mRNA-1	5	OsR498G0510704100.01.T01	99,84	0,00E+00	1111	Os05g0373700	99,52	0,00E+00	1102	-9	NA	Indica	
maker-5-augustus-gene-189.2-mRNA-1	5	OsR498G0510700100.01.T01	98,43	0,00E+00	1327	Os05g0372800	98,29	0,00E+00	1323	-4	NA	Indica	
maker-5-augustus-gene-189.20-mRNA-1	5	OsR498G0510699600.01.T02	100,00	0,00E+00	1856	Os05g0372400	99,81	0,00E+00	1847	-9	NA	Indica	
maker-5-augustus-gene-189.27-mRNA-1	5	OsR498G0510701800.01.T01	100,00	0,00E+00	2210	Os05g0373000	99,59	0,00E+00	2187	-23	NA	Indica	
maker-5-augustus-gene-189.32-mRNA-1	5	OsR498G0510703600.01.T01	100,00	0,00E+00	2441	Os01g0938600	73,66	0,00E+00	738	-1703	Indica	Indica	
maker-5-augustus-gene-189.5-mRNA-1	5	OsR498G0510702700.01.T03	99,61	0,00E+00	1814	Os05g0373300	99,90	0,00E+00	1803	-11	NA	Indica	
maker-5-augustus-gene-19.0-mRNA-1	5	OsR498G0509676900.01.T01	99,82	0,00E+00	1972	Os05g0133401	100,00	0,00E+00	1981	9	NA	Japonica	
maker-5-augustus-gene-19.20-mRNA-1	5	OsR498G0509676800.01.T03	99,53	0,00E+00	1138	Os05g0133100	100,00	0,00E+00	1153	15	NA	Japonica	
maker-5-augustus-gene-190.0-mRNA-1	5	OsR498G0510705500.01.T01	100,00	0,00E+00	2825	Os05g0374200	99,68	0,00E+00	2802	-23	NA	Indica	
maker-5-augustus-gene-190.8-mRNA-1	5	OsR498G0510709000.01.T01	98,63	0,00E+00	2691	Os01g0940100	72,72	2,56E-172	605	-2086	Indica	Indica	

maker-5-augustus-gene-191.44-mRNA-1	5	OsR498G0510713900.01.T01	100,00	0,00E+00	2251	Os05g0375600	98,08	0,00E+00	2160	-91	NA	NA	
maker-5-augustus-gene-191.54-mRNA-1	5	OsR498G0408835700.01.T02	98,30	0,00E+00	1320	Os05g0376501	98,45	0,00E+00	2009	689	Japonica	Indica	
maker-5-augustus-gene-191.64-mRNA-1	5	OsR498G0510716400.01.T01	100,00	0,00E+00	3317	Os05g0376600	100,00	0,00E+00	3317	0	NA	NA	
maker-5-augustus-gene-191.72-mRNA-1	5	OsR498G0510716800.01.T01	100,00	0,00E+00	829	Os05g0376800	100,00	0,00E+00	829	0	NA	Japonica	
maker-5-augustus-gene-191.74-mRNA-1	5	OsR498G0510717000.01.T01	99,49	0,00E+00	695	Os05g0377000	100,00	0,00E+00	645	-50	NA	Japonica	
maker-5-augustus-gene-192.2-mRNA-1	5	OsR498G0510725000.01.T02	99,89	0,00E+00	3281	Os05g0378800	100,00	0,00E+00	3290	9	NA	Japonica	
maker-5-augustus-gene-192.6-mRNA-1	5	OsR498G0510725200.01.T01	99,96	0,00E+00	4433	Os05g0378900	100,00	0,00E+00	4437	4	NA	NA	
maker-5-augustus-gene-193.0-mRNA-1	5	OsR498G0510726700.01.T05	99,81	0,00E+00	3759	Os05g0379500	100,00	0,00E+00	3777	18	NA	Japonica	NBS-TM
maker-5-augustus-gene-193.3-mRNA-1	5	OsR498G0510726700.01.T05	97,28	0,00E+00	3520	Os05g0380300	100,00	0,00E+00	3777	257	NA	Japonica	NBS-TM
maker-5-augustus-gene-194.0-mRNA-1	5	OsR498G0510734900.01.T02	100,00	0,00E+00	2684	Os05g0381500	100,00	0,00E+00	1581	-1103	Indica	NA	
maker-5-augustus-gene-194.10-mRNA-1	5	OsR498G0510738600.01.T01	100,00	0,00E+00	2581	Os05g0382200	100,00	0,00E+00	2168	-413	Indica	Japonica	
maker-5-augustus-gene-194.17-mRNA-1	5	OsR498G0510740600.01.T01	100,00	0,00E+00	893	Os05g0383000	100,00	0,00E+00	893	0	NA	Japonica	
maker-5-augustus-gene-194.20-mRNA-1	5	OsR498G0510740800.01.T01	99,60	0,00E+00	2240	Os05g0383100	100,00	0,00E+00	2262	22	NA	Japonica	
maker-5-augustus-gene-194.7-mRNA-1	5	OsR498G0510740300.01.T01	99,64	0,00E+00	2004	Os05g0382900	100,00	0,00E+00	2019	15	NA	Japonica	
maker-5-augustus-gene-195.41-mRNA-1	5	OsR498G0510747400.01.T02	99,75	0,00E+00	5725	Os05g0384800	100,00	0,00E+00	5763	38	NA	Japonica	
maker-5-augustus-gene-196.5-mRNA-1	5	OsR498G0510753400.01.T01	98,89	0,00E+00	1746	Os05g0386201	100,00	0,00E+00	1792	46	NA	Japonica	
maker-5-augustus-gene-196.7-mRNA-1	5	OsR498G0510755200.01.T03	99,53	0,00E+00	1140	Os05g0386800	100,00	0,00E+00	1155	15	NA	Japonica	
maker-5-augustus-gene-197.43-mRNA-1	5	OsR498G0510755300.01.T03	99,62	0,00E+00	1896	Os05g0386900	100,00	0,00E+00	1911	15	NA	Japonica	
maker-5-augustus-gene-197.48-mRNA-1	5	OsR498G0510755500.01.T01	99,64	0,00E+00	2503	Os05g0387200	99,79	0,00E+00	2512	9	NA	Japonica	
maker-5-augustus-gene-197.50-mRNA-1	5	OsR498G0510758700.01.T01	99,89	0,00E+00	1567	Os01g0957000	80,03	0,00E+00	719	-848	Indica	Japonica	
maker-5-augustus-gene-197.55-mRNA-1	5	OsR498G0510759100.01.T01	96,51	0,00E+00	1865	Os05g0388500	96,77	0,00E+00	1879	14	NA	Japonica	
maker-5-augustus-gene-197.58-mRNA-1	5	OsR498G0510759300.01.T01	99,65	0,00E+00	2037	Os05g0388600	100,00	0,00E+00	2051	14	NA	Japonica	
maker-5-augustus-gene-197.62-mRNA-1	5	OsR498G0510759900.01.T01	99,14	0,00E+00	2260	Os05g0389000	100,00	0,00E+00	1629	-631	Indica	Japonica	
maker-5-augustus-gene-197.66-mRNA-1	5	OsR498G0510758000.01.T05	98,78	0,00E+00	1870	Os05g0387800	93,99	0,00E+00	1310	-560	Indica	Japonica	
maker-5-augustus-gene-197.72-mRNA-1	5	OsR498G0510758400.01.T03	99,67	0,00E+00	1068	Os05g0387900	100,00	0,00E+00	1077	9	NA	Japonica	
maker-5-augustus-gene-198.0-mRNA-1	5	OsR498G0510761300.01.T01	99,70	0,00E+00	2358	Os05g0389400	100,00	0,00E+00	2376	18	NA	Japonica	
maker-5-augustus-gene-198.11-mRNA-1	5	OsR498G0510763700.01.T05	99,73	0,00E+00	2617	Os05g0390100	99,86	0,00E+00	2626	9	NA	Japonica	
maker-5-augustus-gene-198.2-mRNA-1	5	OsR498G0510762500.01.T04	97,66	0,00E+00	1968	Os05g0389600	99,91	0,00E+00	2056	88	NA	Japonica	
maker-5-augustus-gene-198.34-mRNA-1	5	OsR498G0510763400.01.T01	98,56	0,00E+00	3022	Os05g0390000	100,00	0,00E+00	2590	-432	Indica	Japonica	
maker-5-augustus-gene-198.7-mRNA-1	5	OsR498G0510762700.01.T02	99,87	0,00E+00	2805	Os05g0389700	100,00	0,00E+00	2814	9	NA	Japonica	Kinase-TM
maker-5-augustus-gene-199.18-mRNA-1	5	OsR498G0510770300.01.T01	98,42	0,00E+00	5139	Os05g0391500	98,69	0,00E+00	5180	41	NA	Japonica	
maker-5-augustus-gene-199.24-mRNA-1	5	OsR498G0510770000.01.T01	99,66	0,00E+00	4217	Os05g0391200	100,00	0,00E+00	2397	-1820	Indica	Japonica	
maker-5-augustus-gene-199.26-mRNA-1	5	OsR498G0510770800.01.T01	99,60	0,00E+00	3087	Os05g0391600	100,00	0,00E+00	3115	28	NA	Japonica	
maker-5-augustus-gene-2.13-mRNA-1	5	OsR498G0509566500.01.T01	100,00	0,00E+00	4902	Os05g0104100	100,00	0,00E+00	4902	0	NA	NA	LRR-TM
maker-5-augustus-gene-2.18-mRNA-1	5	OsR498G0509563400.01.T06	100,00	0,00E+00	5173	Os05g0102800	100,00	0,00E+00	5173	0	NA	NA	
maker-5-augustus-gene-2.3-mRNA-1	5	OsR498G0509563700.01.T02	100,00	0,00E+00	1350	Os05g0102900	100,00	0,00E+00	1049	-301	Indica	NA	
maker-5-augustus-gene-2.30-mRNA-1	5	OsR498G0509564000.01.T04	100,00	0,00E+00	1462	Os05g0103000	100,00	0,00E+00	1462	0	NA	NA	
maker-5-augustus-gene-2.35-mRNA-1	5	OsR498G0509564200.01.T02	98,97	0,00E+00	1030	Os05g0103100	99,41	0,00E+00	1205	175	Japonica	Japonica	
maker-5-augustus-gene-2.38-mRNA-1	5	OsR498G0509565600.01.T01	98,21	0,00E+00	1744	Os05g0103600	98,21	0,00E+00	1744	0	NA	NA	
maker-5-augustus-gene-2.42-mRNA-1	5	OsR498G0100040000.01.T01	89,56	0,00E+00	1509	Os05g0103700	100,00	0,00E+00	1155	-354	Indica	NA	
maker-5-augustus-gene-2.50-mRNA-1	5	OsR498G0509566000.01.T01	100,00	1,35E-150	533	Os05g0103800	100,00	0,00E+00	1007	474	Japonica	NA	
maker-5-augustus-gene-2.7-mRNA-1	5	OsR498G0509564300.01.T01	100,00	0,00E+00	1359	Os05g0103200	100,00	0,00E+00	1359	0	NA	Japonica	
maker-5-augustus-gene-20.0-mRNA-1	5	OsR498G0509680700.01.T01	97,95	0,00E+00	2003	Os05g0134200	99,49	0,00E+00	2084	81	NA	Japonica	
maker-5-augustus-gene-20.11-mRNA-1	5	OsR498G0509684300.01.T01	98,71	0,00E+00	1760	Os05g0135000	99,90	0,00E+00	1814	54	NA	Japonica	
maker-5-augustus-gene-20.13-mRNA-1	5	OsR498G0509685100.01.T01	100,00	0,00E+00	1802	Os05g0135200	99,80	0,00E+00	1793	-9	NA	Japonica	
maker-5-augustus-gene-20.15-mRNA-1	5	OsR498G0509686100.01.T01	100,00	0,00E+00	1819	Os01g0327100	71,56	4,21E-123	442	-1377	Indica	NA	
maker-5-augustus-gene-20.17-mRNA-1	5	OsR498G0509686700.01.T01	99,71	0,00E+00	1854	Os05g0135500	99,61	0,00E+00	1849	-5	NA	NA	
maker-5-augustus-gene-20.20-mRNA-1	5	OsR498G0509679200.01.T01	99,76	0,00E+00	2216	Os05g0134000	100,00	0,00E+00	2230	14	NA	Japonica	Kinase-TM
maker-5-augustus-gene-20.28-mRNA-1	5	OsR498G0509684800.01.T02	99,84	0,00E+00	2226	Os05g0135100	99,68	0,00E+00	3902	1676	Japonica	NA	Kinase-TM
maker-5-augustus-gene-20.5-mRNA-1	5	OsR498G0509681700.01.T01	99,42	0,00E+00	1829	Os05g0134400	99,83	0,00E+00	1024	-805	Indica	NA	
maker-5-augustus-gene-20.7-mRNA-1	5	OsR498G0509682900.01.T01	97,56	0,00E+00	1806	Os05g0134700	97,90	0,00E+00	1797	-9	NA	Japonica	
maker-5-augustus-gene-20.9-mRNA-1	5	OsR498G0509684100.01.T01	97,42	0,00E+00	1767	Os05g0134800	97,14	0,00E+00	1754	-13	NA	Indica	
maker-5-augustus-gene-200.12-mRNA-1	5	OsR498G0510773500.01.T01	99,92	0,00E+00	2296	Os05g0392300	100,00	0,00E+00	2300	4	NA	Japonica	Kinase-TM
maker-5-augustus-gene-200.18-mRNA-1	5	OsR498G0510775500.01.T01	99,61	0,00E+00	2738	Os05g0393100	100,00	0,00E+00	2765	27	NA	Japonica	LRR-TM
maker-5-augustus-gene-200.29-mRNA-1	5	OsR498G0510775200.01.T01	99,80	0,00E+00	1783	Os05g0392700	100,00	0,00E+00	1792	9	NA	Japonica	
maker-5-augustus-gene-200.6-mRNA-1	5	OsR498G0510773300.01.T01	99,88	0,00E+00	1435	Os05g0392200	100,00	0,00E+00	915	-520	Indica	Japonica	
maker-5-augustus-gene-201.41-mRNA-1	5	OsR498G0510776500.01.T02	99,69	0,00E+00	1746	Os05g0393400	100,00	0,00E+00	1759	13	NA	Japonica	
maker-5-augustus-gene-201.46-mRNA-1	5	OsR498G0510777900.01.T01	99,92	0,00E+00	2243	Os05g0393800	100,00	0,00E+00	2248	5	NA	Japonica	
maker-5-augustus-gene-201.52-mRNA-1	5	OsR498G0713729200.01.T01	99,63	1,68E-135	483	Os05g0394100	100,00	1,38E-137	488	5	NA	Japonica	
maker-5-augustus-gene-201.54-mRNA-1	5	OsR498G0510782000.01.T02	99,28	0,00E+00	2200	Os05g0394900	100,00	0,00E+00	2024	-176	NA	Japonica	
maker-5-augustus-gene-202.30-mRNA-1	5	OsR498G0510783100.01.T01	99,87	0,00E+00	2854	Os05g0395300	99,93	0,00E+00	2665	-189	NA	Japonica	
maker-5-augustus-gene-203.10-mRNA-1	5	OsR498G0510792900.01.T01	98,34	0,00E+00	1251	Os05g0399100	100,00	0,00E+00	1543	292	Japonica	Japonica	
maker-5-augustus-gene-203.14-mRNA-1	5	OsR498G0510793100.01.T01	98,99	0,00E+00	1388	Os05g0399200	100,00	0,00E+00	1299	-89	NA	Japonica	
maker-5-augustus-gene-203.18-mRNA-1	5	OsR498G0510788800.01.T02	99,10	0,00E+00	783	Os05g0397300	100,00	0,00E+00	801	18	NA	Japonica	
maker-5-augustus-gene-203.20-mRNA-1	5	OsR498G0510790000.01.T03	98,55	0,00E+00	1686	Os05g0397650	100,00	0,00E+00	1721	35	NA	Japonica	
maker-5-augustus-gene-203.23-mRNA-1	5	OsR498G0510790200.01.T02	99,68	0,00E+00	2761	Os05g0397700	100,00	0,00E+00	2776	15	NA	Japonica	
maker-5-augustus-gene-203.3-mRNA-1	5	OsR498G0510790500.01.T01	99,76	0,00E+00	4524	Os05g0397900	100,00	0,00E+00	4551	27	NA	Japonica	
maker-5-augustus-gene-203.30-mRNA-1	5	OsR498G0510791000.01.T01	99,91	0,00E+00	4028	Os05g0398100	99,07	0,00E+00	3994	-34	NA	Japonica	
maker-5-augustus-gene-203.8-mRNA-1	5	OsR498G0510792100.01.T01	99,86	0,00E+00	2654	Os05g0398800	100,00	0,00E+00	2663	9	NA	Japonica	Kinase-TM
maker-5-augustus-gene-204.0-mRNA-1	5	OsR498G0510793500.01.T01	98,61	0,00E+00	1748	Os05g0399400	100,00	0,00E+00	1813	65	NA	Japonica	
maker-5-augustus-gene-204.11-mRNA-1	5	OsR498G0510795100.01.T04	98,73	0,00E+00	1242	Os05g0400600	99,58	0,00E+00	1269	27	NA	Japonica	
maker-5-augustus-gene-204.2-mRNA-1	5	OsR498G0510793900.01.T01	99,90	0,00E+00	1841	Os05g0399700	100,00	0,00E+00	1846	5	NA	Japonica	
maker-5-augustus-gene-204.28-mRNA-1	5	OsR498G0510795400.01.T01	99,25	0,00E+00	946	Os05g0400700	100,00	0,00E+00	964	18	NA	Japonica	
maker-5-augustus-gene-204.32-mRNA-1	5	OsR498G0510796000.01.T01	100,00	0,00E+00	1624	Os05g0401100	99,89	0,00E+00	1619	-5	NA	Indica	
maker-5-augustus-gene-204.4-mRNA-1	5	OsR498G0510794300.01.T01	95,66	0,00E+00	933	Os05g0400100	100,00	0,00E+00	1040	107	Japonica	Japonica	
maker-5-augustus-gene-204.6-mRNA-1	5	OsR498G0510794500.01.T01	98,04	0,00E+00	1225	Os05g0400100	86,27	0,00E+00	997	-228	Indica	Japonica	
maker-5-augustus-gene-204.8-mRNA-1	5	OsR498G0510794900.01.T01	99,22	0,00E+00	902	Os01g0247900	72,59	6,37E-54	210	-692	Indica	Japonica	
maker-5-augustus-gene-205.4-mRNA-1	5	OsR498G0510801400.01.T01	99,13	0,00E+00	1415	Os05g0402300	99,25	0,00E+00	1419	4	NA	Japonica	
maker-5-augustus-gene-206.34-mRNA-1	5	OsR498G0510804500.01.T01	98,99	0,00E+00	1214	Os05g0404200	100,00	0,00E+00	1245	31	NA	Japonica	
maker-5-augustus-gene-206.36-mRNA-1	5	OsR498G0510805200.01.T01	100,00	0,00E+00	1173	Os05g0404400	100,00	0,00E+00	1173	0	NA	Japonica	
maker-5-augustus-gene-208.1-mRNA-1	5	OsR498G0510809900.01.T01	99,90	0,00E+00	5430	Os05g0405900	100,00	0,00E+00	5443	13	NA	Japonica	
maker-5-augustus-gene-208.12-mRNA-1	5	OsR498G0510806800.01.T01	100,00	0,00E+00	4727	Os05g0405000	100,00	0,00E+00	4727	0	NA	Japonica	
maker-5-augustus-gene-208.13-mRNA-1	5	OsR498G0510806800.01.T05	100,00	5,28E-113	407	Os05g0405000	100,00	1,85E-113	407	0	NA	Japonica	
maker-5-augustus-gene-208.6-mRNA-1	5	OsR498G0510810100.01.T01	99,71	0,00E+00	1879	Os05g0406000	100,00	0,00E+00	948	-931	Indica	Japonica	
maker-5-augustus-gene-209.0-mRNA-1	5	OsR498G0510815700.01.T01	99,91	0,00E+00	1917	Os05g0407500	100,00	0,00E+00	1921	4	NA	Japonica	
maker-5-augustus-gene-209.14-mRNA-1	5	OsR498G0510814000.01.T02	99,86	0,00E+00	2502	Os05g0407200	100,00	0,00E+00	2511	9	NA	Japonica	
maker-5-augustus-gene-209.2-mRNA-1	5	OsR498G0510810400.01.T03	100,00	0,00E+00	2450	Os05g0406100	100,00	0,00E+00	2450	0	NA	Japonica	
maker-5-augustus-gene-209.8-mRNA-1	5	OsR498G0510811600.01.T01	100,00	0,00E+00	4435	Os05g0406200	100,00	0,00E+00	4551	116	NA	Japonica	
maker-5-augustus-gene-21.30-mRNA-1	5	OsR498G0509688700.01.T01	99,36	0,00E+00	1928	Os05g0135800	99,72	0,00E+00	1946	18	NA	Japonica	Kinase-TM
maker-5-augustus-gene-21.33-mRNA-1	5	OsR498G0509690700.01.T01	96,93	0,00E+00	1408	Os05g0136100	99,77	0,00E+00	1588	180	Japonica	Japonica	
maker-5-augustus-gene-21.35-mRNA-1	5	OsR498G0509694300.01.T01	98,55	0,00E+00	2294	Os05g0136900	99,77	0,00E+00	2355	61	NA	Japonica	
maker-5-augustus-gene-21.44-mRNA-1	5	OsR498G0509696300.01.T01	99,43	0,00E+00	2187	Os05g0137300	99,51	0,00E+00	2192	5	NA	NA	
maker-5-augustus-gene-21.48-mRNA-1	5	OsR498G0509689200.01.T02	96,27	0,00E+00	1780	Os05g0135900	96,36	0,00E+00	1784	4	NA	Japonica	
maker-5-augustus-gene-21.51-mRNA-1	5	OsR498G0509691000.01.T02	98,83	0,00E+00	2387	Os05g0136200	99,63	0,00E+00	2440	53	NA	Japonica	Kinase-TM
maker-5-augustus-gene-210.31-mRNA-1	5	OsR498G0510818900.01.T01	99,93	0,00E+00	2615	Os05g0408200	100,00	0,00E+00	2619	4	NA	Japonica	
maker-5-augustus-gene-210.35-mRNA-1	5	OsR498G0510819200.01.T01	99,86	0,00E+00	2545	Os05g0408300	100,00	0,00E+00	2554	9	NA	Japonica	
maker-5-augustus-gene-210.39-mRNA-1	5	OsR498G0510821200.01.T01	99,85	0,00E+00	2356	Os05g0408900	100,00	0,00E+00	2365	9	NA	Japonica	
maker-5-augustus-gene-211.0-mRNA-1	5	OsR498G0510822700.01.T04	99,07	0,00E+00	952	Os05g0409300	100,00	0,00E+00	975	23	NA	Japonica	
maker-5-augustus-gene-211.12-mRNA-1	5	OsR498G0510826800.01.T01	99,84	0,00E+00	3260	Os05g0410500	100,00	0,00E+00	3112	-148	NA	Japonica	
maker-5-augustus-gene-211.4-mRNA-1	5	OsR498G0510822900.01.T03	99,37	0,00E+00	2253	Os05g0409400	99,92	0,00E+00	2285	32	NA	Japonica	
maker-5-augustus-gene-211.9-mRNA-1	5	OsR498G0510823200.01.T01	99,60	0,00E+00	2254	Os05g0409500	100,00	0,00E+00	2278	24	NA	Japonica	
maker-5-augustus-gene-212.34-mRNA-1	5	OsR498G0510828700.01.T01	99,73	0,00E+00	3327	Os05g0410900	77,90	0,00E+00	1335	-1992	Indica	Indica	
maker-5-augustus-gene-212.39-mRNA-1	5	OsR498G0510828200.01.T01	99,89	0,00E+00	3232	Os05g0410900	100,00	0,00E+00	3301	69	NA	Japonica	
maker-5-augustus-gene-212.42-mRNA-1	5	OsR498G0510829000.01.T03	99,94	0,00E+00	3006	Os05g0411200	99,84	0,00E+00	2260	-746	Indica	Indica	
maker-5-augustus-gene-212.51-mRNA-1	5	OsR498G0510829200.01.T01	99,90	0,00E+00	3492	Os05g0411300	99,91	0,00E+00	1929	-1563	Indica	Indica	
maker-5-augustus-gene-213.0-mRNA-1	5	OsR498G0510837700.01.T02	99,93	0,00E+00	2404	Os05g0413200	99,93	0,00E+00	2404	0	NA	Indica	
maker-5-augustus-gene-213.3-mRNA-1	5	OsR498G0510838300.01.T01	100,00	0,00E+00	1591	Os05g0413400	99,66	0,00E+00	1578	-13	NA	Indica	
maker-5-augustus-gene-214.34-mRNA-1	5	OsR498G0510845100.01.T03	99,88	0,00E+00	2870	Os05g0415700	99,94	0,00E+00	2874	4	NA	Indica	
maker-5-augustus-gene-214.40-mRNA-1	5	OsR498G0510843600.01.T02	99,78	0,00E+00	3265	Os05g0414700	99,57	0,00E+00	3344	79	NA	Indica	Kinase-LRR-TM
maker-5-augustus-gene-214.43-mRNA-1	5	OsR498G0510844400.01.T01	99,89	0,00E+00	1673	Os05g0415400	99,57	0,00E+00	1663	-10	NA	NA	
maker-5-augustus-gene-215.30-mRNA-1	5	OsR498G0510849900.01.T01	99,89	0,00E+00	3319	Os05g0417100	99,78	0,00E+00	3310	-9	NA	Japonica	
maker-5-augustus-gene-215.35-mRNA-1	5	OsR498G0510850100.01.T01	99,60	0,00E+00	1768	Os05g0417200	99,60	0,00E+00	1768	0	NA	Japonica	
maker-5-augustus-gene-215.38-mRNA-1	5	OsR498G0510850300.01.T01	99,59	0,00E+00	1291	Os05g0417300	99,31	0,00E+00	1282	-9	NA	Indica	
maker-5-augustus-gene-215.41-mRNA-1	5	OsR498G0510848800.01.T01	99,88	0,00E+00	1517	Os05g0417000	99,29	0,00E+00	1492	-25	NA	Indica	
maker-5-augustus-gene-216.28-mRNA-1	5	OsR498G0510853700.01.T01	99,78	0,00E+00	2400	Os05g0418000	99,85	0,00E+00	2405	5	NA	Indica	
maker-5-augustus-gene-216.35-mRNA-1	5	OsR498G0510857700.01.T01	100,00	0,00E+00	1218	Os05g0419100	100,00	0,00E+00	1218	0	NA	NA	
maker-5-augustus-gene-216.40-mRNA-1	5	OsR498G0510854000.01.T02	99,80	0,00E+00	2633	Os05g0418100	96,03	0,00E+00	1542	-1091	Indica	Indica	
maker-5-augustus-gene-216.46-mRNA-1	5	OsR498G0510856200.01.T01	99,76	0,00E+00	2216	Os05g0418800	99,76	0,00E+00	2249	33	NA	NA	
maker-5-augustus-gene-216.48-mRNA-1	5	OsR498G0510857400.01.T01	96,65	0,00E+00	2106	Os05g0419000	96,65	0,00E+00	2106	0	NA	NA	
maker-5-augustus-gene-216.51-mRNA-1	5	OsR498G0510858000.01.T01	100,00	0,00E+00	2836	Os05g0419200	100,00	0,00E+00	2251	-585	Indica	NA	
maker-5-augustus-gene-216.56-mRNA-1	5	OsR498G0510859800.01.T02	100,00	0,00E+00	1586	Os05g0419500	99,66	0,00E+00	1571	-15	NA	Japonica	
maker-5-augustus-gene-217.0-mRNA-1	5	OsR498G0510860900.01.T02	99,92	0,00E+00	2128	Os05g0419800	100,00	0,00E+00	2132	4	NA	Japonica	
maker-5-augustus-gene-217.10-mRNA-1	5	OsR498G0510863900.01.T01	100,00	1,03E-174	612	Os05g0420600	100,00	3,59E-175	612	0	NA	Japonica	
maker-5-augustus-gene-217.23-mRNA-1	5	OsR498G0510866100.01.T02	99,89	0,00E+00	3249	Os05g0421300	100,00	0,00E+00	3258	9	NA	Japonica	
maker-5-augustus-gene-217.25-mRNA-1	5	OsR498G0510866900.01.T01	98,77	0,00E+00	1714	Os05g0421600	100,00	0,00E+00	1012	-702	Indica	Japonica	
maker-5-augustus-gene-217.3-mRNA-1	5	OsR498G0510861700.01.T01	99,32	0,00E+00	1043	Os05g0420200	100,00	0,00E+00	1911	868	Japonica	Japonica	
maker-5-augustus-gene-217.5-mRNA-1	5	OsR498G0510863700.01.T01	99,68	0,00E+00	3951	Os05g0420500	100,00	0,00E+00	3611	-340	NA	Japonica	
maker-5-augustus-gene-218.34-mRNA-1	5	OsR498G0510872100.01.T02	99,83	0,00E+00	2136	Os05g0423200	100,00	0,00E+00	2122	-14	NA	Japonica	
maker-5-augustus-gene-218.37-mRNA-1	5	OsR498G0510872700.01.T06	98,59	0,00E+00	868	Os05g0423400	95,92	0,00E+00	845	-23	NA	Japonica	
maker-5-augustus-gene-218.43-mRNA-1	5	OsR498G0510874300.01.T02	99,85	0,00E+00	2448	Os05g0424000	100,00	0,00E+00	2457	9	NA	Japonica	
maker-5-augustus-gene-218.46-mRNA-1	5	OsR498G0510868000.01.T01	99,52	0,00E+00	2244	Os01g0883800	81,32	0,00E+00	997	-1247	Indica	Japonica	
maker-5-augustus-gene-218.59-mRNA-1	5	OsR498G0510871200.01.T01	99,13	0,00E+00	2640	Os05g0422600	100,00	0,00E+00	3463	823	Japonica	Japonica	
maker-5-augustus-gene-219.3-mRNA-1	5	OsR498G0510879700.01.T01	99,65	0,00E+00	1025	Os05g0426000	100,00	0,00E+00	1034	9	NA	Japonica	
maker-5-augustus-gene-219.6-mRNA-1	5	OsR498G0510876200.01.T02	99,63	0,00E+00	1925	Os05g0424800	100,00	0,00E+00	1943	18	NA	Japonica	
maker-5-augustus-gene-22.4-mRNA-1	5	OsR498G0509702300.01.T01	100,00	0,00E+00	2901	Os05g0139100	100,00	0,00E+00	1867	-1034	Indica	Japonica	
maker-5-augustus-gene-22.8-mRNA-1	5	OsR498G0509698000.01.T01	99,66	0,00E+00	1047	Os05g0137700	100,00	0,00E+00	921	-126	Indica	Japonica	
maker-5-augustus-gene-220.0-mRNA-1	5	OsR498G0510880100.01.T01	99,61	0,00E+00	3237	Os05g0426100	99,95	0,00E+00	3583	346	NA	Japonica	
maker-5-augustus-gene-220.11-mRNA-1	5	OsR498G0510881400.01.T01	99,68	0,00E+00	3371	Os05g0426400	99,61	0,00E+00	1839	-1532	Indica	Japonica	
maker-5-augustus-gene-220.14-mRNA-1	5	OsR498G0510885600.01.T02	99,92	0,00E+00	2225	Os05g0427200	100,00	0,00E+00	2230	5	NA	Japonica	
maker-5-augustus-gene-220.18-mRNA-1	5	OsR498G0510885600.01.T06	99,65	0,00E+00	1009	Os05g0427300	100,00	0,00E+00	1018	9	NA	Japonica	
maker-5-augustus-gene-220.3-mRNA-1	5	OsR498G0510881100.01.T01	99,69	0,00E+00	2331	Os05g0426300	100,00	0,00E+00	2349	18	NA	Japonica	
maker-5-augustus-gene-220.6-mRNA-1	5	OsR498G0510884300.01.T01	99,65	0,00E+00	1025	Os05g0426800	100,00	0,00E+00	1034	9	NA	Japonica	
maker-5-augustus-gene-220.8-mRNA-1	5	OsR498G0510880400.01.T04	99,93	0,00E+00	2431	Os05g0426200	100,00	0,00E+00	2435	4	NA	Japonica	
maker-5-augustus-gene-221.38-mRNA-1	5	OsR498G0408675500.01.T01	89,90	7,45E-115	415	Os05g0429100	100,00	2,96E-165	580	165	Japonica	Japonica	
maker-5-augustus-gene-221.39-mRNA-1	5	OsR498G0510888800.01.T01	98,65	0,00E+00	1941	Os01g0875700	76,64	1,02E-118	426	-1515	Indica	Japonica	
maker-5-augustus-gene-221.41-mRNA-1	5	OsR498G0510889400.01.T01	97,87	0,00E+00	3876	Os05g0428100	98,23	0,00E+00	3912	36	NA	Japonica	
maker-5-augustus-gene-221.48-mRNA-1	5	OsR498G0510890400.01.T01	98,23	0,00E+00	1173	Os05g0428400	100,00	0,00E+00	1223	50	NA	Japonica	
maker-5-augustus-gene-221.51-mRNA-1	5	OsR498G0510891800.01.T01	99,77	0,00E+00	4605	Os05g0428700	100,00	0,00E+00	3068	-1537	Indica	Japonica	
maker-5-augustus-gene-221.56-mRNA-1	5	OsR498G0510892600.01.T01	97,59	0,00E+00	3202	Os05g0429000	100,00	0,00E+00	3171	-31	NA	Japonica	
maker-5-augustus-gene-221.60-mRNA-1	5	OsR498G0510893800.01.T01	97,65	0,00E+00	3059	Os05g0429400	100,00	0,00E+00	3171	112	NA	Japonica	
maker-5-augustus-gene-222.39-mRNA-1	5	OsR498G0510897900.01.T01	100,00	0,00E+00	2852	Os05g0430200	100,00	0,00E+00	1975	-877	Indica	NA	
maker-5-augustus-gene-222.51-mRNA-1	5	OsR498G0510897600.01.T01	100,00	0,00E+00	1413	Os05g0429900	100,00	0,00E+00	1413	0	NA	NA	
maker-5-augustus-gene-222.53-mRNA-1	5	OsR498G0510898600.01.T01	100,00	0,00E+00	2013	Os05g0430400	100,00	0,00E+00	2013	0	NA	NA	
maker-5-augustus-gene-222.58-mRNA-1	5	OsR498G0510898800.01.T01	99,72	0,00E+00	1917	Os05g0430700	99,72	0,00E+00	1917	0	NA	NA	
maker-5-augustus-gene-222.63-mRNA-1	5	OsR498G0510899400.01.T01	100,00	0,00E+00	2874	Os05g0430900	100,00	0,00E+00	2874	0	NA	NA	
maker-5-augustus-gene-223.48-mRNA-1	5	OsR498G0510902100.01.T01	100,00	4,30E-142	504	Os05g0432200	100,00	1,49E-142	504	0	NA	NA	
maker-5-augustus-gene-223.50-mRNA-1	5	OsR498G0510902300.01.T06	100,00	0,00E+00	2657	Os05g0432400	100,00	0,00E+00	2657	0	NA	NA	
maker-5-augustus-gene-223.53-mRNA-1	5	OsR498G0510902500.01.T01	100,00	0,00E+00	888	Os05g0432500	100,00	0,00E+00	888	0	NA	NA	
maker-5-augustus-gene-223.56-mRNA-1	5	OsR498G0510904500.01.T01	100,00	0,00E+00	1121	Os05g0433400	100,00	0,00E+00	1121	0	NA	NA	
maker-5-augustus-gene-223.57-mRNA-1	5	OsR498G0510905100.01.T01	100,00	0,00E+00	1391	Os05g0433100	76,82	1,36E-102	373	-1018	Indica	NA	
maker-5-augustus-gene-223.58-mRNA-1	5	OsR498G0510905700.01.T01	100,00	0,00E+00	1077	Os05g0433100	100,00	0,00E+00	1077	0	NA	NA	
maker-5-augustus-gene-223.68-mRNA-1	5	OsR498G0510901800.01.T04	100,00	0,00E+00	2459	Os05g0431700	100,00	0,00E+00	1478	-981	Indica	NA	
maker-5-augustus-gene-223.69-mRNA-1	5	OsR498G0510902800.01.T01	99,89	0,00E+00	1679	Os05g0432600	99,89	0,00E+00	1679	0	NA	NA	
maker-5-augustus-gene-223.74-mRNA-1	5	OsR498G0510903000.01.T01	100,00	0,00E+00	764	Os05g0432700	100,00	3,50E-170	596	-168	Indica	NA	
maker-5-augustus-gene-224.45-mRNA-1	5	OsR498G0510909100.01.T01	100,00	0,00E+00	1002	Os05g0434200	99,67	1,10E-152	539	-463	Indica	NA	
maker-5-augustus-gene-224.52-mRNA-1	5	OsR498G0510910900.01.T01	90,20	0,00E+00	937	Os05g0435300	100,00	0,00E+00	1196	259	Japonica	NA	
maker-5-augustus-gene-224.54-mRNA-1	5	OsR498G0510912100.01.T01	100,00	0,00E+00	1169	Os05g0435700	100,00	0,00E+00	1169	0	NA	NA	
maker-5-augustus-gene-224.57-mRNA-1	5	OsR498G0510914400.01.T01	100,00	0,00E+00	2863	Os05g0436100	98,83	0,00E+00	2844	-19	NA	NA	Kinase-TM
maker-5-augustus-gene-225.52-mRNA-1	5	OsR498G0510915900.01.T01	100,00	0,00E+00	2327	Os05g0436900	100,00	0,00E+00	2327	0	NA	NA	
maker-5-augustus-gene-225.56-mRNA-1	5	OsR498G0510917500.01.T03	100,00	0,00E+00	1413	Os05g0437200	100,00	0,00E+00	1413	0	NA	NA	
maker-5-augustus-gene-225.61-mRNA-1	5	OsR498G0510918900.01.T02	99,20	0,00E+00	1327	Os05g0437700	100,00	0,00E+00	834	-493	Indica	NA	
maker-5-augustus-gene-225.64-mRNA-1	5	OsR498G0510917800.01.T04	95,16	0,00E+00	1192	Os05g0437300	100,00	0,00E+00	1187	-5	NA	NA	
maker-5-augustus-gene-225.71-mRNA-1	5	OsR498G0510918800.01.T01	100,00	0,00E+00	1516	Os05g0437500	100,00	0,00E+00	1516	0	NA	NA	
maker-5-augustus-gene-225.73-mRNA-1	5	OsR498G0510920600.01.T01	100,00	0,00E+00	2414	Os05g0437900	100,00	0,00E+00	2414	0	NA	NA	
maker-5-augustus-gene-226.56-mRNA-1	5	OsR498G0510922900.01.T01	100,00	0,00E+00	1218	Os05g0438500	100,00	0,00E+00	1218	0	NA	NA	
maker-5-augustus-gene-226.59-mRNA-1	5	OsR498G0510924300.01.T02	100,00	0,00E+00	682	Os06g0669150	95,79	1,71E-162	572	-110	Indica	NA	
maker-5-augustus-gene-226.63-mRNA-1	5	OsR498G0510922000.01.T01	100,00	0,00E+00	791	Os09g0120033	97,03	0,00E+00	732	-59	NA	NA	
maker-5-augustus-gene-226.65-mRNA-1	5	OsR498G0510923200.01.T01	100,00	0,00E+00	1478	Os01g0866400	84,38	0,00E+00	880	-598	Indica	NA	
maker-5-augustus-gene-226.73-mRNA-1	5	OsR498G0510924000.01.T03	100,00	0,00E+00	2040	Os05g0438800	100,00	0,00E+00	2040	0	NA	NA	
maker-5-augustus-gene-226.75-mRNA-1	5	OsR498G0510927800.01.T01	99,88	0,00E+00	1484	Os05g0440050	100,00	0,00E+00	1489	5	NA	Japonica	
maker-5-augustus-gene-226.77-mRNA-1	5	OsR498G0510928200.01.T03	99,92	0,00E+00	2122	Os05g0440100	100,00	0,00E+00	2127	5	NA	Japonica	
maker-5-augustus-gene-227.41-mRNA-1	5	OsR498G0510929300.01.T06	99,65	0,00E+00	5063	Os05g0440800	100,00	0,00E+00	5108	45	NA	Japonica	Kinase
maker-5-augustus-gene-227.48-mRNA-1	5	OsR498G0510928400.01.T02	99,87	0,00E+00	1370	Os05g0440300	100,00	0,00E+00	1375	5	NA	Japonica	
maker-5-augustus-gene-227.51-mRNA-1	5	OsR498G0510935200.01.T01	99,45	0,00E+00	972	Os05g0442400	84,24	3,09E-172	603	-369	Indica	Japonica	
maker-5-augustus-gene-227.53-mRNA-1	5	OsR498G0510935600.01.T01	99,45	0,00E+00	977	Os05g0442400	100,00	0,00E+00	991	14	NA	Japonica	
maker-5-augustus-gene-228.0-mRNA-1	5	OsR498G0510938100.01.T08	99,86	0,00E+00	2507	Os05g0443900	99,66	0,00E+00	2107	-400	Indica	Japonica	
maker-5-augustus-gene-228.16-mRNA-1	5	OsR498G0510941100.01.T01	99,73	0,00E+00	1339	Os05g0444300	75,60	2,85E-137	488	-851	Indica	Japonica	
maker-5-augustus-gene-228.19-mRNA-1	5	OsR498G0510937000.01.T01	99,87	0,00E+00	4034	Os05g0443300	98,16	0,00E+00	3967	-67	NA	Japonica	
maker-5-augustus-gene-228.26-mRNA-1	5	OsR498G0510937400.01.T04	99,36	0,00E+00	2232	Os05g0443400	99,91	0,00E+00	1888	-344	Indica	Japonica	
maker-5-augustus-gene-228.31-mRNA-1	5	OsR498G0510938000.01.T04	99,79	0,00E+00	2546	Os05g0443800	100,00	0,00E+00	2560	14	NA	Japonica	
maker-5-augustus-gene-228.9-mRNA-1	5	OsR498G0510940500.01.T01	98,02	0,00E+00	1833	Os05g0444300	98,31	0,00E+00	1847	14	NA	Japonica	
maker-5-augustus-gene-229.0-mRNA-1	5	OsR498G0510944900.01.T01	100,00	5,94E-174	610	Os05g0445500	100,00	2,07E-174	610	0	NA	Japonica	
maker-5-augustus-gene-229.3-mRNA-1	5	OsR498G0510945100.01.T01	99,63	0,00E+00	3829	Os05g0445800	99,67	0,00E+00	3843	14	NA	Japonica	
maker-5-augustus-gene-23.30-mRNA-1	5	OsR498G0509709100.01.T03	99,77	0,00E+00	2306	Os05g0140100	99,69	0,00E+00	2302	-4	NA	Indica	
maker-5-augustus-gene-23.33-mRNA-1	5	OsR498G0509709900.01.T01	99,89	0,00E+00	1581	Os05g0140333	99,89	0,00E+00	1581	0	NA	Indica	
maker-5-augustus-gene-23.38-mRNA-1	5	OsR498G0509710600.01.T01	99,88	0,00E+00	1439	Os05g0140600	100,00	0,00E+00	1265	-174	Indica	Japonica	
maker-5-augustus-gene-23.41-mRNA-1	5	OsR498G0509711200.01.T01	98,69	0,00E+00	2003	Os05g0140800	99,82	0,00E+00	1972	-31	NA	Japonica	
maker-5-augustus-gene-23.43-mRNA-1	5	OsR498G0509711600.01.T01	99,62	0,00E+00	4677	Os05g0141000	98,38	0,00E+00	4617	-60	NA	Japonica	
maker-5-augustus-gene-23.46-mRNA-1	5	OsR498G0509712000.01.T01	99,70	0,00E+00	3582	Os05g0141200	99,97	0,00E+00	5179	1597	Japonica	Japonica	
maker-5-augustus-gene-23.49-mRNA-1	5	OsR498G0509712800.01.T01	99,70	0,00E+00	1198	Os05g0141400	99,56	0,00E+00	1206	8	NA	Japonica	
maker-5-augustus-gene-230.13-mRNA-1	5	OsR498G0510949400.01.T01	99,82	0,00E+00	2015	Os05g0446900	100,00	0,00E+00	2024	9	NA	Japonica	
maker-5-augustus-gene-230.2-mRNA-1	5	OsR498G0510948800.01.T01	99,90	0,00E+00	3481	Os05g0446800	100,00	0,00E+00	3490	9	NA	Japonica	
maker-5-augustus-gene-230.24-mRNA-1	5	OsR498G0510951600.01.T02	99,73	0,00E+00	2634	Os05g0447700	100,00	0,00E+00	2572	-62	NA	Japonica	
maker-5-augustus-gene-231.0-mRNA-1	5	OsR498G0510952700.01.T01	99,86	0,00E+00	2626	Os01g0855000	79,63	0,00E+00	1294	-1332	Indica	Japonica	
maker-5-augustus-gene-231.2-mRNA-1	5	OsR498G0510956300.01.T01	99,67	0,00E+00	3209	Os05g0449500	100,00	0,00E+00	3236	27	NA	Japonica	
maker-5-augustus-gene-231.4-mRNA-1	5	OsR498G0510956700.01.T01	99,89	0,00E+00	3276	Os05g0449600	99,90	0,00E+00	1798	-1478	Indica	Japonica	
maker-5-augustus-gene-231.8-mRNA-1	5	OsR498G0510957400.01.T01	99,68	0,00E+00	1691	Os05g0449900	100,00	0,00E+00	1710	19	NA	Japonica	
maker-5-augustus-gene-232.36-mRNA-1	5	OsR498G0510961900.01.T01	99,88	0,00E+00	2892	Os05g0451100	99,57	0,00E+00	2869	-23	NA	Japonica	
maker-5-augustus-gene-232.41-mRNA-1	5	OsR498G0510964300.01.T03	99,41	0,00E+00	3314	Os05g0451900	100,00	0,00E+00	2278	-1036	Indica	Japonica	
maker-5-augustus-gene-232.45-mRNA-1	5	OsR498G0510962600.01.T01	99,40	0,00E+00	891	Os05g0451300	100,00	0,00E+00	904	13	NA	Japonica	
maker-5-augustus-gene-233.0-mRNA-1	5	OsR498G0510970700.01.T01	100,00	0,00E+00	931	Os05g0453700	96,51	0,00E+00	862	-69	NA	Japonica	
maker-5-augustus-gene-233.10-mRNA-1	5	OsR498G0510972100.01.T01	99,54	0,00E+00	782	Os11g0620500	91,80	5,76E-155	546	-236	Indica	Japonica	Kinase

maker-5-augustus-gene-233.13-mRNA-1	5	OsR498G0510966400.01.T02	94,59	0,00E+00	864	Os05g0452900	94,77	0,00E+00	868	4	NA	Japonica		
maker-5-augustus-gene-233.20-mRNA-1	5	OsR498G0510968600.01.T01	99,82	0,00E+00	1988	Os05g0453300	100,00	0,00E+00	1997	9	NA	Japonica		
maker-5-augustus-gene-233.3-mRNA-1	5	OsR498G0510970900.01.T02	100,00	0,00E+00	1023	Os05g0453800	100,00	0,00E+00	1088	65	NA	Japonica		
maker-5-augustus-gene-233.7-mRNA-1	5	OsR498G0510971900.01.T01	99,77	0,00E+00	3061	Os05g0454200	100,00	0,00E+00	3079	18	NA	Japonica		
maker-5-augustus-gene-234.31-mRNA-1	5	OsR498G0510974500.01.T01	99,20	0,00E+00	3080	Os05g0455200	99,94	0,00E+00	3140	60	NA	Japonica		
maker-5-augustus-gene-234.42-mRNA-1	5	OsR498G0510977100.01.T02	99,75	0,00E+00	1445	Os05g0455800	100,00	0,00E+00	1454	9	NA	Japonica		
maker-5-augustus-gene-234.46-mRNA-1	5	OsR498G0510972600.01.T05	100,00	0,00E+00	785	Os05g0454500	100,00	0,00E+00	785	0	NA	Japonica		
maker-5-augustus-gene-234.50-mRNA-1	5	OsR498G0510976400.01.T01	97,66	0,00E+00	3738	Os05g0455500	97,80	0,00E+00	3752	14	NA	Japonica		
maker-5-augustus-gene-234.55-mRNA-1	5	OsR498G0510977000.01.T01	99,89	0,00E+00	1630	Os05g0455700	100,00	0,00E+00	1635	5	NA	Japonica		
maker-5-augustus-gene-235.0-mRNA-1	5	OsR498G0510978500.01.T01	100,00	0,00E+00	845	Os05g0456500	100,00	0,00E+00	845	0	NA	Japonica		
maker-5-augustus-gene-235.10-mRNA-1	5	OsR498G0510977600.01.T01	99,87	0,00E+00	1397	Os05g0456000	100,00	0,00E+00	1402	5	NA	Japonica		
maker-5-augustus-gene-235.12-mRNA-1	5	OsR498G0510978000.01.T01	99,86	0,00E+00	1311	Os05g0456200	100,00	0,00E+00	1122	-189	Indica	Japonica		
maker-5-augustus-gene-235.17-mRNA-1	5	OsR498G0510978200.01.T01	95,15	0,00E+00	1673	Os05g0456300	95,04	0,00E+00	1601	-72	NA	Japonica		
maker-5-augustus-gene-235.25-mRNA-1	5	OsR498G0510979800.01.T01	99,76	0,00E+00	2243	Os01g0846300	79,08	0,00E+00	851	-1392	Indica	Japonica		
maker-5-augustus-gene-235.29-mRNA-1	5	OsR498G0510980400.01.T02	99,79	0,00E+00	1746	Os05g0457700	100,00	0,00E+00	1755	9	NA	Japonica		
maker-5-augustus-gene-235.35-mRNA-1	5	OsR498G0510981600.01.T02	99,82	0,00E+00	1008	Os05g0458100	100,00	0,00E+00	1012	4	NA	Japonica		
maker-5-augustus-gene-235.37-mRNA-1	5	OsR498G0510982000.01.T01	99,43	0,00E+00	3121	Os05g0458300	100,00	0,00E+00	2690	-431	Indica	Japonica		
maker-5-augustus-gene-235.4-mRNA-1	5	OsR498G0510980700.01.T01	99,62	0,00E+00	2803	Os05g0457800	100,00	0,00E+00	2830	27	NA	Japonica		
maker-5-augustus-gene-235.6-mRNA-1	5	OsR498G0510981300.01.T01	99,48	0,00E+00	686	Os05g0458000	100,00	0,00E+00	695	9	NA	Japonica		
maker-5-augustus-gene-236.29-mRNA-1	5	OsR498G0510982500.01.T02	99,96	0,00E+00	4449	Os05g0458400	100,00	0,00E+00	4453	4	NA	Japonica		
maker-5-augustus-gene-236.32-mRNA-1	5	OsR498G0510983700.01.T01	99,60	0,00E+00	3086	Os05g0458600	99,77	0,00E+00	3100	14	NA	Japonica		
maker-5-augustus-gene-236.35-mRNA-1	5	OsR498G0510986100.01.T04	99,77	0,00E+00	3072	Os05g0459400	100,00	0,00E+00	3166	94	NA	Japonica		
maker-5-augustus-gene-236.41-mRNA-1	5	OsR498G0510986700.01.T01	95,31	0,00E+00	2858	Os05g0459600	100,00	0,00E+00	1842	-1016	Indica	Japonica		
maker-5-augustus-gene-236.46-mRNA-1	5	OsR498G0510987100.01.T01	99,92	0,00E+00	2377	Os05g0459700	100,00	0,00E+00	2560	183	NA	Japonica		
maker-5-augustus-gene-236.48-mRNA-1	5	OsR498G0510987900.01.T04	100,00	2,45E-176	618	Os05g0459900	100,00	8,53E-177	618	0	NA	Japonica		
maker-5-augustus-gene-236.50-mRNA-1	5	OsR498G0510983700.01.T01	99,60	0,00E+00	3086	Os05g0458600	99,77	0,00E+00	3100	14	NA	Japonica		
maker-5-augustus-gene-236.53-mRNA-1	5	OsR498G0510984600.01.T02	99,83	0,00E+00	3221	Os05g0459000	98,66	0,00E+00	3140	-81	NA	Japonica		
maker-5-augustus-gene-236.57-mRNA-1	5	OsR498G0510988400.01.T01	99,85	0,00E+00	3488	Os05g0460000	100,00	0,00E+00	3501	13	NA	Japonica		
maker-5-augustus-gene-237.0-mRNA-1	5	OsR498G0510989100.01.T01	96,67	0,00E+00	840	Os05g0460200	96,86	0,00E+00	845	5	NA	Japonica		
maker-5-augustus-gene-237.11-mRNA-1	5	OsR498G0510990000.01.T02	99,88	0,00E+00	2897	Os05g0460600	100,00	0,00E+00	2906	9	NA	Japonica		
maker-5-augustus-gene-237.16-mRNA-1	5	OsR498G0510990200.01.T01	99,79	0,00E+00	873	Os05g0460700	91,03	0,00E+00	866	-7	NA	Japonica		
maker-5-augustus-gene-237.20-mRNA-1	5	OsR498G0510990400.01.T05	84,33	5,41E-130	465	Os05g0460800	84,60	4,44E-132	470	5	NA	Japonica		
maker-5-augustus-gene-237.26-mRNA-1	5	OsR498G0510991000.01.T01	100,00	0,00E+00	964	Os05g0461000	100,00	0,00E+00	964	0	NA	Japonica		
maker-5-augustus-gene-237.29-mRNA-1	5	OsR498G0510991600.01.T02	99,69	0,00E+00	1166	Os05g0461300	100,00	0,00E+00	1175	9	NA	Japonica		
maker-5-augustus-gene-237.3-mRNA-1	5	OsR498G0305687300.01.T02	90,83	4,52E-91	334	Os05g0460500	96,75	9,25E-177	618	284	Japonica	Japonica		
maker-5-augustus-gene-237.32-mRNA-1	5	OsR498G0510992200.01.T02	100,00	0,00E+00	888	Os05g0461400	100,00	0,00E+00	888	0	NA	Japonica		
maker-5-augustus-gene-237.34-mRNA-1	5	OsR498G0510994000.01.T01	99,68	7,67E-157	554	Os05g0462000	100,00	6,27E-159	558	4	NA	Japonica		
maker-5-augustus-gene-237.5-mRNA-1	5	OsR498G0510993700.01.T03	93,19	0,00E+00	1077	Os05g0461900	95,76	0,00E+00	1003	-74	NA	Japonica		
maker-5-augustus-gene-238.42-mRNA-1	5	OsR498G0510999500.01.T03	99,72	0,00E+00	2587	Os05g0463400	100,00	0,00E+00	2605	18	NA	Japonica		
maker-5-augustus-gene-238.53-mRNA-1	5	OsR498G0510996600.01.T01	98,59	0,00E+00	5716	Os05g0462400	100,00	0,00E+00	4785	-931	Indica	Japonica		
maker-5-augustus-gene-238.58-mRNA-1	5	OsR498G0510997600.01.T01	99,87	0,00E+00	2711	Os05g0462600	99,80	0,00E+00	2705	-6	NA	Japonica		
maker-5-augustus-gene-238.66-mRNA-1	5	OsR498G0510998400.01.T01	99,92	0,00E+00	2258	Os05g0463000	100,00	0,00E+00	2262	4	NA	Japonica	Kinase-TM	
maker-5-augustus-gene-238.68-mRNA-1	5	OsR498G0510999400.01.T01	99,49	0,00E+00	2791	Os05g0463300	98,92	0,00E+00	2760	-31	NA	Japonica		
maker-5-augustus-gene-238.70-mRNA-1	5	OsR498G0511000000.01.T03	96,91	0,00E+00	807	Os05g0463800	95,82	5,90E-156	550	-257	Indica	Japonica		
maker-5-augustus-gene-239.0-mRNA-1	5	OsR498G0511004700.01.T05	100,00	0,00E+00	3162	Os05g0465800	100,00	0,00E+00	3162	0	NA	Japonica		
maker-5-augustus-gene-239.11-mRNA-1	5	OsR498G0511005600.01.T01	99,34	0,00E+00	1074	Os05g0466100	100,00	0,00E+00	1094	20	NA	Japonica		
maker-5-augustus-gene-239.13-mRNA-1	5	OsR498G0511006000.01.T01	99,81	0,00E+00	4685	Os05g0466200	100,00	0,00E+00	4708	23	NA	Japonica		
maker-5-augustus-gene-239.15-mRNA-1	5	OsR498G0511006800.01.T01	100,00	0,00E+00	829	Os05g0466700	100,00	0,00E+00	829	0	NA	Japonica		
maker-5-augustus-gene-239.5-mRNA-1	5	OsR498G0511004000.01.T04	99,72	0,00E+00	4465	Os05g0465100	100,00	0,00E+00	4497	32	NA	Japonica		
maker-5-augustus-gene-239.9-mRNA-1	5	OsR498G0511004400.01.T01	99,49	0,00E+00	1037	Os05g0465400	100,00	0,00E+00	1050	13	NA	Japonica		
maker-5-augustus-gene-24.0-mRNA-1	5	OsR498G0509713700.01.T03	99,19	0,00E+00	1735	Os05g0141700	100,00	0,00E+00	1602	-133	NA	Japonica		
maker-5-augustus-gene-24.12-mRNA-1	5	OsR498G0509720600.01.T01	99,71	0,00E+00	2443	Os05g0143300	99,78	0,00E+00	2447	4	NA	Japonica		
maker-5-augustus-gene-240.0-mRNA-1	5	OsR498G0511007500.01.T01	99,78	0,00E+00	831	Os01g0833100	76,76	1,31E-96	352	-479	Indica	Japonica		
maker-5-augustus-gene-240.11-mRNA-1	5	OsR498G0511011900.01.T01	99,77	0,00E+00	2368	Os05g0467700	100,00	0,00E+00	2381	13	NA	Japonica		
maker-5-augustus-gene-240.13-mRNA-1	5	OsR498G0511013500.01.T01	99,69	0,00E+00	2315	Os05g0468100	100,00	0,00E+00	2333	18	NA	Japonica		
maker-5-augustus-gene-240.15-mRNA-1	5	OsR498G0511013900.01.T01	99,67	0,00E+00	1079	Os05g0468200	73,50	2,57E-145	515	-564	Indica	Japonica		
maker-5-augustus-gene-240.17-mRNA-1	5	OsR498G0511014100.01.T01	99,62	0,00E+00	2326	Os05g0468400	100,00	0,00E+00	2349	23	NA	Japonica		
maker-5-augustus-gene-240.19-mRNA-1	5	OsR498G0511007800.01.T01	99,72	0,00E+00	3810	Os05g0466900	100,00	0,00E+00	3279	-531	Indica	Japonica	Kinase	
maker-5-augustus-gene-240.28-mRNA-1	5	OsR498G0511008400.01.T02	97,68	0,00E+00	2883	Os05g0467000	97,86	0,00E+00	2896	13	NA	Japonica		
maker-5-augustus-gene-240.3-mRNA-1	5	OsR498G0511009100.01.T01	86,49	0,00E+00	977	Os05g0467300	88,19	0,00E+00	1014	37	NA	Japonica		
maker-5-augustus-gene-240.31-mRNA-1	5	OsR498G0511011600.01.T01	99,57	0,00E+00	1250	Os05g0467600	100,00	0,00E+00	2262	1012	Japonica	Japonica		
maker-5-augustus-gene-240.34-mRNA-1	5	OsR498G0511014400.01.T01	99,35	0,00E+00	1922	Os05g0468500	100,00	0,00E+00	1954	32	NA	Japonica		
maker-5-augustus-gene-240.7-mRNA-1	5	OsR498G0511009500.01.T01	99,69	0,00E+00	2336	Os05g0467400	99,93	0,00E+00	2406	70	NA	Japonica		
maker-5-augustus-gene-241.0-mRNA-1	5	OsR498G0511014500.01.T02	99,81	0,00E+00	2833	Os05g0468600	100,00	0,00E+00	3631	798	Japonica	Japonica		
maker-5-augustus-gene-241.10-mRNA-1	5	OsR498G0511018700.01.T03	99,67	0,00E+00	3285	Os05g0469600	96,41	0,00E+00	3026	-259	NA	Japonica		
maker-5-augustus-gene-241.16-mRNA-1	5	OsR498G0511019800.01.T01	99,73	0,00E+00	2664	Os05g0470000	100,00	0,00E+00	2787	123	NA	Japonica		
maker-5-augustus-gene-241.5-mRNA-1	5	OsR498G0511014900.01.T01	99,66	0,00E+00	2613	Os05g0468700	100,00	0,00E+00	2636	23	NA	Japonica		
maker-5-augustus-gene-241.8-mRNA-1	5	OsR498G0511018100.01.T01	99,89	0,00E+00	3270	Os05g0469600	100,00	0,00E+00	3279	9	NA	Japonica		
maker-5-augustus-gene-242.17-mRNA-1	5	OsR498G0511025800.01.T01	99,90	0,00E+00	1711	Os05g0472300	100,00	0,00E+00	1716	5	NA	Japonica		
maker-5-augustus-gene-242.3-mRNA-1	5	OsR498G0511020800.01.T01	99,81	0,00E+00	2806	Os05g0470800	100,00	0,00E+00	2819	13	NA	Japonica		
maker-5-augustus-gene-242.5-mRNA-1	5	OsR498G0511021200.01.T03	99,66	0,00E+00	1587	Os05g0471000	100,00	0,00E+00	1600	13	NA	Japonica	Kinase-LRR-TM	
maker-5-augustus-gene-243.46-mRNA-1	5	OsR498G0511026300.01.T01	99,49	0,00E+00	1402	Os05g0472700	71,83	2,41E-95	349	-1053	Indica	Japonica		
maker-5-augustus-gene-243.54-mRNA-1	5	OsR498G0511026900.01.T01	100,00	0,00E+00	1916	Os05g0472700	100,00	0,00E+00	1916	0	NA	Japonica		
maker-5-augustus-gene-243.56-mRNA-1	5	OsR498G0511028500.01.T02	99,49	0,00E+00	1400	Os05g0473300	100,00	0,00E+00	1418	18	NA	Japonica		
maker-5-augustus-gene-243.58-mRNA-1	5	OsR498G0511028700.01.T01	99,04	0,00E+00	4422	Os05g0473400	99,28	0,00E+00	4449	27	NA	Japonica		
maker-5-augustus-gene-243.60-mRNA-1	5	OsR498G0511030100.01.T01	99,86	0,00E+00	2491	Os01g0827200	75,88	0,00E+00	817	-1674	Indica	Japonica		
maker-5-augustus-gene-243.62-mRNA-1	5	OsR498G0511030100.01.T06	99,96	0,00E+00	4021	Os01g0826900	79,92	0,00E+00	1710	-2311	Indica	Japonica		
maker-5-augustus-gene-244.0-mRNA-1	5	OsR498G0511033500.01.T01	99,68	0,00E+00	3360	Os05g0475300	100,00	0,00E+00	3388	28	NA	Japonica		
maker-5-augustus-gene-244.2-mRNA-1	5	OsR498G0511036300.01.T01	99,88	0,00E+00	3032	Os05g0475700	100,00	0,00E+00	3041	9	NA	Japonica		
maker-5-augustus-gene-244.4-mRNA-1	5	OsR498G0511031400.01.T01	99,54	0,00E+00	3065	Os05g0474800	100,00	0,00E+00	3101	36	NA	Japonica		
maker-5-augustus-gene-244.6-mRNA-1	5	OsR498G0511032000.01.T02	99,82	0,00E+00	2961	Os05g0474900	100,00	0,00E+00	2975	14	NA	Japonica		
maker-5-augustus-gene-245.40-mRNA-1	5	OsR498G0511037900.01.T01	99,59	0,00E+00	3035	Os05g0476100	99,94	0,00E+00	3064	29	NA	Japonica		
maker-5-augustus-gene-245.42-mRNA-1	5	OsR498G0511038100.01.T02	99,90	0,00E+00	3680	Os05g0476200	99,95	0,00E+00	3684	4	NA	Japonica		
maker-5-augustus-gene-245.46-mRNA-1	5	OsR498G0511039400.01.T01	99,03	0,00E+00	908	Os05g0476400	100,00	0,00E+00	807	-101	Indica	Japonica		
maker-5-augustus-gene-245.51-mRNA-1	5	OsR498G0511044800.01.T01	99,06	0,00E+00	1306	Os05g0477600	100,00	0,00E+00	1337	31	NA	Japonica		
maker-5-augustus-gene-245.53-mRNA-1	5	OsR498G0511045600.01.T01	94,94	0,00E+00	1046	Os05g0477800	95,81	0,00E+00	1217	171	Japonica	Japonica		
maker-5-augustus-gene-246.40-mRNA-1	5	OsR498G0511047500.01.T01	99,78	0,00E+00	4864	Os05g0478300	99,93	0,00E+00	4958	94	NA	Japonica	Kinase-LRR-TM	
maker-5-augustus-gene-246.48-mRNA-1	5	OsR498G0511050000.01.T01	96,34	0,00E+00	4900	Os05g0479700	100,00	0,00E+00	6130	1230	Japonica	Japonica	NBS-TM	
maker-5-augustus-gene-246.50-mRNA-1	5	OsR498G0511051000.01.T03	99,70	0,00E+00	1789	Os05g0479900	100,00	0,00E+00	1802	13	NA	Japonica		
maker-5-augustus-gene-247.14-mRNA-1	5	OsR498G0511055300.01.T09	100,00	0,00E+00	2405	Os05g0481100	99,93	0,00E+00	2400	-5	NA	Indica	Kinase-LRR-TM	
maker-5-augustus-gene-247.22-mRNA-1	5	OsR498G0511057300.01.T02	100,00	4,78E-100	363	Os05g0481600	100,00	1,66E-100	363	0	NA	NA		
maker-5-augustus-gene-247.24-mRNA-1	5	OsR498G0511057700.01.T01	99,91	0,00E+00	2099	Os05g0481800	100,00	0,00E+00	2104	5	NA	Japonica		
maker-5-augustus-gene-247.27-mRNA-1	5	OsR498G0511051600.01.T02	100,00	0,00E+00	737	Os05g0480200	100,00	0,00E+00	737	0	NA	NA		
maker-5-augustus-gene-247.29-mRNA-1	5	OsR498G0511051800.01.T01	99,90	0,00E+00	3442	Os05g0480400	100,00	0,00E+00	3451	9	NA	Indica	Kinase-LRR-TM	
maker-5-augustus-gene-247.3-mRNA-1	5	OsR498G0511053100.01.T01	100,00	0,00E+00	1256	Os05g0480700	100,00	0,00E+00	1256	0	NA	NA		
maker-5-augustus-gene-247.31-mRNA-1	5	OsR498G0511052800.01.T02	100,00	0,00E+00	3198	Os05g0480600	100,00	0,00E+00	3198	0	NA	NA		
maker-5-augustus-gene-247.33-mRNA-1	5	OsR498G0511055600.01.T01	99,89	0,00E+00	1637	Os05g0481400	100,00	0,00E+00	1508	-129	NA	Japonica		
maker-5-augustus-gene-247.37-mRNA-1	5	OsR498G0511056600.01.T03	99,74	0,00E+00	694	Os05g0481500	99,74	0,00E+00	694	0	NA	Japonica		
maker-5-augustus-gene-247.5-mRNA-1	5	OsR498G0511053500.01.T01	100,00	0,00E+00	1521	Os05g0480800	100,00	0,00E+00	1521	0	NA	NA		
maker-5-augustus-gene-247.7-mRNA-1	5	OsR498G0511054300.01.T01	100,00	0,00E+00	1263	Os05g0480800	72,21	3,19E-99	362	-901	Indica	NA		
maker-5-augustus-gene-247.9-mRNA-1	5	OsR498G0511054500.01.T01	100,00	0,00E+00	1380	Os05g0481000	100,00	0,00E+00	1380	0	NA	NA		
maker-5-augustus-gene-248.50-mRNA-1	5	OsR498G0511060700.01.T01	100,00	0,00E+00	641	Os05g0482400	100,00	0,00E+00	641	0	NA	Japonica		
maker-5-augustus-gene-248.52-mRNA-1	5	OsR498G0511061500.01.T02	96,96	0,00E+00	1725	Os05g0482600	96,96	0,00E+00	1725	0	NA	Japonica		
maker-5-augustus-gene-248.59-mRNA-1	5	OsR498G0511061800.01.T03	99,29	0,00E+00	2980	Os05g0482700	99,35	0,00E+00	2984	4	NA	Japonica		
maker-5-augustus-gene-249.15-mRNA-1	5	OsR498G0511074200.01.T01	99,82	0,00E+00	2992	Os01g0816700	82,30	0,00E+00	1644	-1348	Indica	Japonica		
maker-5-augustus-gene-249.2-mRNA-1	5	OsR498G0511072500.01.T01	99,75	0,00E+00	1420	Os05g0485300	100,00	0,00E+00	1429	9	NA	Japonica		
maker-5-augustus-gene-249.7-mRNA-1	5	OsR498G0511068200.01.T01	100,00	0,00E+00	1272	Os05g0484800	100,00	0,00E+00	1272	0	NA	NA		
maker-5-augustus-gene-25.37-mRNA-1	5	OsR498G0509721500.01.T06	100,00	0,00E+00	2938	Os11g0271100	85,16	0,00E+00	1702	-1236	Indica	Japonica	Kinase-TM	
maker-5-augustus-gene-25.45-mRNA-1	5	OsR498G0509722700.01.T01	99,58	0,00E+00	1682	Os05g0144100	100,00	0,00E+00	1700	18	NA	Japonica		
maker-5-augustus-gene-25.50-mRNA-1	5	OsR498G0509723500.01.T01	99,78	0,00E+00	4800	Os05g0144400	98,88	0,00E+00	4708	-92	NA	Japonica		
maker-5-augustus-gene-25.55-mRNA-1	5	OsR498G0509724700.01.T06	96,46	0,00E+00	2125	Os02g0143100	75,10	0,00E+00	874	-1251	Indica	NA		
maker-5-augustus-gene-25.61-mRNA-1	5	OsR498G0509722400.01.T01	99,78	0,00E+00	3245	Os05g0143800	100,00	0,00E+00	2327	-918	Indica	Japonica		
maker-5-augustus-gene-25.76-mRNA-1	5	OsR498G0509723200.01.T03	99,53	0,00E+00	3002	Os05g0144300	99,94	0,00E+00	3030	28	NA	Japonica		
maker-5-augustus-gene-25.80-mRNA-1	5	OsR498G0509724600.01.T02	99,78	0,00E+00	4085	Os05g0144800	99,74	0,00E+00	4080	-5	NA	Japonica		
maker-5-augustus-gene-25.84-mRNA-1	5	OsR498G0509725000.01.T01	98,78	0,00E+00	1147	Os05g0145000	98,63	0,00E+00	1141	-6	NA	Indica		
maker-5-augustus-gene-250.20-mRNA-1	5	OsR498G0511077000.01.T02	99,58	0,00E+00	854	Os05g0486700	99,79	0,00E+00	858	4	NA	Japonica		
maker-5-augustus-gene-250.6-mRNA-1	5	OsR498G0511075400.01.T01	99,14	0,00E+00	5156	Os05g0486100	99,90	0,00E+00	5235	79	NA	Japonica	Kinase-LRR-TM	
maker-5-augustus-gene-251.0-mRNA-1	5	OsR498G0511077900.01.T01	99,69	1,03E-161	570	Os05g0487300	100,00	1,17E-155	547	-23	NA	Japonica		
maker-5-augustus-gene-251.15-mRNA-1	5	OsR498G0511079200.01.T02	99,68	0,00E+00	2223	Os05g0488100	99,91	0,00E+00	2041	-182	NA	Japonica		
maker-5-augustus-gene-251.18-mRNA-1	5	OsR498G0511079800.01.T01	99,92	0,00E+00	2225	Os05g0488600	100,00	0,00E+00	2127	-98	NA	Japonica		
maker-5-augustus-gene-251.2-mRNA-1	5	OsR498G0511078900.01.T01	93,23	0,00E+00	866	Os05g0488000	99,82	0,00E+00	1008	142	Japonica	Japonica		
maker-5-augustus-gene-251.24-mRNA-1	5	OsR498G0511081600.01.T01	99,82	0,00E+00	972	Os05g0489600	100,00	0,00E+00	976	4	NA	Japonica		
maker-5-augustus-gene-251.4-mRNA-1	5	OsR498G0511080300.01.T01	97,69	0,00E+00	1486	Os05g0488900	97,92	0,00E+00	1495	9	NA	Japonica		
maker-5-augustus-gene-251.8-mRNA-1	5	OsR498G0511080700.01.T04	99,92	0,00E+00	2370	Os05g0489000	100,00	0,00E+00	2374	4	NA	Japonica		
maker-5-augustus-gene-252.40-mRNA-1	5	OsR498G0511082300.01.T04	98,06	0,00E+00	1592	Os05g0489700	100,00	0,00E+00	1673	81	NA	Japonica		
maker-5-augustus-gene-252.48-mRNA-1	5	OsR498G0511083300.01.T01	100,00	0,00E+00	2084	Os05g0490200	100,00	0,00E+00	2084	0	NA	Japonica		
maker-5-augustus-gene-252.54-mRNA-1	5	OsR498G0511082600.01.T01	89,29	0,00E+00	696	Os05g0489800	89,70	0,00E+00	705	9	NA	Japonica		
maker-5-augustus-gene-252.57-mRNA-1	5	OsR498G0511082800.01.T01	99,74	0,00E+00	2780	Os05g0489900	100,00	0,00E+00	2798	18	NA	Japonica	Kinase-TM	
maker-5-augustus-gene-252.59-mRNA-1	5	OsR498G0511083600.01.T04	99,53	0,00E+00	1881	Os05g0490300	99,12	0,00E+00	2208	327	Japonica	Japonica		
maker-5-augustus-gene-252.64-mRNA-1	5	OsR498G0511085600.01.T01	99,23	0,00E+00	2304	Os05g0490600	99,90	0,00E+00	1816	-488	Indica	Japonica		
maker-5-augustus-gene-252.67-mRNA-1	5	OsR498G0511086400.01.T01	100,00	0,00E+00	1166	Os01g0811100	85,43	0,00E+00	740	-426	Indica	Japonica		
maker-5-augustus-gene-252.72-mRNA-1	5	OsR498G0511086600.01.T01	99,81	0,00E+00	932	Os05g0490900	100,00	0,00E+00	937	5	NA	Japonica		
maker-5-augustus-gene-252.74-mRNA-1	5	OsR498G0511087000.01.T03	99,57	0,00E+00	836	Os05g0491000	100,00	0,00E+00	845	9	NA	Japonica		
maker-5-augustus-gene-252.76-mRNA-1	5	OsR498G0511087400.01.T01	99,78	0,00E+00	808	Os05g0491100	100,00	0,00E+00	812	4	NA	Japonica		
maker-5-augustus-gene-252.78-mRNA-1	5	OsR498G0511087600.01.T02	99,32	0,00E+00	788	Os05g0491200	100,00	0,00E+00	801	13	NA	Japonica		
maker-5-augustus-gene-252.82-mRNA-1	5	OsR498G0511088200.01.T01	99,69	0,00E+00	1166	Os05g0491400	100,00	0,00E+00	1175	9	NA	Japonica	LRR-TM	
maker-5-augustus-gene-252.84-mRNA-1	5	OsR498G0511088400.01.T03	97,60	0,00E+00	1355	Os05g0491500	97,73	0,00E+00	1360	5	NA	Japonica		
maker-5-augustus-gene-253.36-mRNA-1	5	OsR498G0511090500.01.T01	96,59	0,00E+00	1642	Os01g0279900	71,18	7,01E-95	347	-1295	Indica	Japonica		
maker-5-augustus-gene-253.38-mRNA-1	5	OsR498G0511092300.01.T01	97,04	0,00E+00	3543	Os05g0493100	97,43	0,00E+00	3579	36	NA	Japonica	Kinase-TM	
maker-5-augustus-gene-253.41-mRNA-1	5	OsR498G0511092900.01.T01	99,54	0,00E+00	2318	Os05g0493500	100,00	0,00E+00	2345	27	NA	Japonica		
maker-5-augustus-gene-253.45-mRNA-1	5	OsR498G0511094300.01.T01	99,84	0,00E+00	2183	Os05g0493800	100,00	0,00E+00	2192	9	NA	Japonica		
maker-5-augustus-gene-253.48-mRNA-1	5	OsR498G0511095500.01.T02	100,00	0,00E+00	2776	Os05g0494000	100,00	0,00E+00	2776	0	NA	Japonica		
maker-5-augustus-gene-253.51-mRNA-1	5	OsR498G0511090200.01.T01	97,72	0,00E+00	1576	Os05g0491900	99,89	0,00E+00	1652	76	NA	Japonica	Kinase-TM	
maker-5-augustus-gene-253.54-mRNA-1	5	OsR498G0511093400.01.T02	99,58	0,00E+00	1714	Os05g0493600	100,00	0,00E+00	2538	824	Japonica	Japonica		
maker-5-augustus-gene-253.56-mRNA-1	5	OsR498G0511094800.01.T01	99,14	3,87E-175	615	Os05g0493500	92,14	1,53E-168	590	-25	NA	Japonica		
maker-5-augustus-gene-254.0-mRNA-1	5	OsR498G0511095700.01.T06	100,00	0,00E+00	1575	Os05g0494100	100,00	0,00E+00	1575	0	NA	Japonica		
maker-5-augustus-gene-254.16-mRNA-1	5	OsR498G0511099700.01.T01	99,92	0,00E+00	4524	Os05g0495600	100,00	0,00E+00	4533	9	NA	Japonica		
maker-5-augustus-gene-254.21-mRNA-1	5	OsR498G0511100700.01.T01	99,79	0,00E+00	2508	Os05g0495700	100,00	0,00E+00	2993	485	Japonica	Japonica		
maker-5-augustus-gene-254.24-mRNA-1	5	OsR498G0511101500.01.T01	99,81	0,00E+00	1891	Os05g0495900	100,00	0,00E+00	1900	9	NA	Japonica		
maker-5-augustus-gene-254.26-mRNA-1	5	OsR498G0511096000.01.T01	99,12	0,00E+00	805	Os05g0494200	100,00	1,61E-168	590	-215	Indica	Japonica		
maker-5-augustus-gene-255.49-mRNA-1	5	OsR498G0511102000.01.T03	99,73	0,00E+00	2607	Os06g0668200	75,71	0,00E+00	850	-1757	Indica	Japonica		
maker-5-augustus-gene-255.55-mRNA-1	5	OsR498G0511102600.01.T02	99,84	0,00E+00	2210	Os05g0496400	100,00	0,00E+00	2219	9	NA	Japonica		
maker-5-augustus-gene-255.56-mRNA-1	5	OsR498G0511102600.01.T01	95,19	0,00E+00	1996	Os05g0496500	100,00	0,00E+00	2278	282	Japonica	Japonica		
maker-5-augustus-gene-256.48-mRNA-1	5	OsR498G0511106300.01.T01	99,84	0,00E+00	2250	Os05g0497650	99,90	0,00E+00	1801	-449	Indica	Japonica		
maker-5-augustus-gene-256.53-mRNA-1	5	OsR498G0511106500.01.T02	99,84	0,00E+00	2210	Os05g0497675	100,00	0,00E+00	2219	9	NA	Japonica		
maker-5-augustus-gene-256.57-mRNA-1	5	OsR498G0511109100.01.T03	98,90	0,00E+00	3207	Os05g0498300	99,05	0,00E+00	3353	146	NA	Japonica		
maker-5-augustus-gene-256.62-mRNA-1	5	OsR498G0511109500.01.T05	100,00	1,38E-179	628	Os05g0498400	100,00	4,80E-180	628	0	NA	Japonica		
maker-5-augustus-gene-256.73-mRNA-1	5	OsR498G0511106800.01.T03	99,81	0,00E+00	1925	Os05g0497700	100,00	0,00E+00	1934	9	NA	Japonica		
maker-5-augustus-gene-256.82-mRNA-1	5	OsR498G0511110800.01.T03	99,89	0,00E+00	1628	Os05g0498700	100,00	0,00E+00	1629	1	NA	Japonica		
maker-5-augustus-gene-256.86-mRNA-1	5	OsR498G0511111400.01.T01	100,00	0,00E+00	2625	Os05g0498900	100,00	0,00E+00	2625	0	NA	Japonica	Kinase-TM	
maker-5-augustus-gene-257.51-mRNA-1	5	OsR498G0511111700.01.T01	99,29	4,15E-141	500	Os09g0497500	90,03	7,02E-114	408	-92	Indica	Japonica		
maker-5-augustus-gene-257.53-mRNA-1	5	OsR498G0511112100.01.T01	99,69	0,00E+00	1756	Os05g0499300	100,00	0,00E+00	1770	14	NA	Japonica		
maker-5-augustus-gene-257.55-mRNA-1	5	OsR498G0511112500.01.T01	94,22	0,00E+00	1728	Os05g0499400	94,31	0,00E+00	1733	5	NA	Japonica		
maker-5-augustus-gene-257.57-mRNA-1	5	OsR498G0511115300.01.T01	99,69	9,37E-166	584	Os05g0500500	100,00	0,00E+00	1104	520	Japonica	Japonica		
maker-5-augustus-gene-257.59-mRNA-1	5	OsR498G0511117700.01.T02	98,78	0,00E+00	1003	Os05g0501200	100,00	0,00E+00	893	-110	Indica	Japonica		
maker-5-augustus-gene-257.60-mRNA-1	5	OsR498G0511117700.01.T04	99,52	5,93E-103	375	Os05g0501200	100,00	4,84E-105	379	4	NA	Japonica		
maker-5-augustus-gene-257.72-mRNA-1	5	OsR498G0511116600.01.T01	99,68	0,00E+00	3382	Os05g0500900	100,00	0,00E+00	3409	27	NA	Japonica		
maker-5-augustus-gene-258.0-mRNA-1	5	OsR498G0511118500.01.T04	99,53	0,00E+00	3012	Os05g0501400	99,94	0,00E+00	3044	32	NA	Japonica	Kinase-TM	
maker-5-augustus-gene-258.11-mRNA-1	5	OsR498G0511122100.01.T01	99,72	0,00E+00	1290	Os01g0780800	71,22	1,44E-95	349	-941	Indica	Japonica		
maker-5-augustus-gene-258.13-mRNA-1	5	OsR498G0511118000.01.T01	99,83	0,00E+00	1071	Os05g0501300	100,00	0,00E+00	1076	5	NA	Japonica		
maker-5-augustus-gene-258.15-mRNA-1	5	OsR498G0511118400.01.T01	99,53	0,00E+00	755	Os05g0493500	86,79	0,00E+00	700	-55	NA	Japonica		
maker-5-augustus-gene-258.21-mRNA-1	5	OsR498G0511122000.01.T03	99,20	0,00E+00	662	Os05g0502500	100,00	0,00E+00	672	10	NA	Japonica		
maker-5-augustus-gene-258.25-mRNA-1	5	OsR498G0511122400.01.T01	99,95	0,00E+00	3686	Os05g0502800	100,00	0,00E+00	3690	4	NA	Japonica		
maker-5-augustus-gene-258.28-mRNA-1	5	OsR498G0511123000.01.T01	99,88	0,00E+00	1527	Os05g0503000	100,00	0,00E+00	1532	5	NA	NA		
maker-5-augustus-gene-258.5-mRNA-1	5	OsR498G0511119100.01.T01	99,81	0,00E+00	3808	Os05g0501600	100,00	0,00E+00	3826	18	NA	Japonica	LRR-TM	
maker-5-augustus-gene-258.8-mRNA-1	5	OsR498G0511120900.01.T02	99,71	0,00E+00	3718	Os05g0502000	100,00	0,00E+00	3745	27	NA	Japonica		
maker-5-augustus-gene-259.27-mRNA-1	5	OsR498G0511123400.01.T03	99,94	0,00E+00	3223	Os05g0503300	100,00	0,00E+00	3227	4	NA	Japonica		
maker-5-augustus-gene-26.10-mRNA-1	5	OsR498G0509726600.01.T04	99,86	0,00E+00	1289	Os05g0145700	99,86	0,00E+00	1289	0	NA	Japonica		
maker-5-augustus-gene-26.14-mRNA-1	5	OsR498G0509727800.01.T02	98,98	0,00E+00	2921	Os05g0146100	98,92	0,00E+00	2910	-11	NA	NA		
maker-5-augustus-gene-26.2-mRNA-1	5	OsR498G0509726000.01.T02	99,88	0,00E+00	1524	Os05g0145400	99,88	0,00E+00	1524	0	NA	Japonica		
maker-5-augustus-gene-26.4-mRNA-1	5	OsR498G0509726400.01.T04	98,15	0,00E+00	1773	Os05g0145600	98,34	0,00E+00	1775	2	NA	NA		
maker-5-augustus-gene-261.14-mRNA-1	5	OsR498G0511141000.01.T01	99,83	0,00E+00	1058	Os05g0506100	100,00	0,00E+00	1786	728	Japonica	Japonica		
maker-5-augustus-gene-261.16-mRNA-1	5	OsR498G0511141600.01.T01	96,36	0,00E+00	1504	Os05g0506400	99,82	0,00E+00	1961	457	Japonica	Japonica		
maker-5-augustus-gene-261.19-mRNA-1	5	OsR498G0511141800.01.T01	99,59	0,00E+00	861	Os05g0506500	93,39	0,00E+00	1132	271	Japonica	Japonica		
maker-5-augustus-gene-261.22-mRNA-1	5	OsR498G0511142200.01.T01	98,21	0,00E+00	1923	Os05g0506600	100,00	0,00E+00	2013	90	NA	Japonica		
maker-5-augustus-gene-261.24-mRNA-1	5	OsR498G0511142400.01.T01	96,24	0,00E+00	781	Os05g0506700	100,00	0,00E+00	861	80	NA	Japonica		
maker-5-augustus-gene-261.25-mRNA-1	5	OsR498G0511142400.01.T01	94,24	0,00E+00	725	Os05g0506600	89,06	2,06E-167	588	-137	Indica	Japonica		
maker-5-augustus-gene-261.30-mRNA-1	5	OsR498G0511143000.01.T01	94,90	0,00E+00	1778	Os05g0506600	80,80	0,00E+00	1031	-747	Indica	Japonica		
maker-5-augustus-gene-261.32-mRNA-1	5	OsR498G0101111400.01.T01	92,55	0,00E+00	691	Os05g0506900	99,79	0,00E+00	862	171	Japonica	Japonica		
maker-5-augustus-gene-262.0-mRNA-1	5	OsR498G0511153900.01.T02	99,68	0,00E+00	1675	Os05g0508300	100,00	0,00E+00	1691	16	NA	Japonica		
maker-5-augustus-gene-262.3-mRNA-1	5	OsR498G0511154100.01.T02	99,79	0,00E+00	3411	Os05g0508400	100,00	0,00E+00	3270	-141	NA	Japonica		
maker-5-augustus-gene-262.6-mRNA-1	5	OsR498G0511154300.01.T02	99,17	1,12E-117	423	Os05g0508500	100,00	0,00E+00	661	238	Japonica	Japonica		
maker-5-augustus-gene-263.38-mRNA-1	5	OsR498G0511155300.01.T01	99,76	0,00E+00	6788	Os05g0508700	100,00	0,00E+00	4172	-2616	Indica	Japonica		
maker-5-augustus-gene-263.44-mRNA-1	5	OsR498G0511155500.01.T01	100,00	0,00E+00	2603	Os05g0508800	100,00	0,00E+00	2603	0	NA	Japonica		
maker-5-augustus-gene-263.50-mRNA-1	5	OsR498G0511158100.01.T01	100,00	0,00E+00	1306	Os05g0509200	100,00	0,00E+00	1306	0	NA	Japonica		
maker-5-augustus-gene-263.61-mRNA-1	5	OsR498G0511158600.01.T03	99,70	0,00E+00	1175	Os05g0509400	100,00	0,00E+00	1182	7	NA	Japonica		
maker-5-augustus-gene-263.65-mRNA-1	5	OsR498G0511158800.01.T01	99,93	0,00E+00	2615	Os05g0509500	100,00	0,00E+00	2619	4	NA	Japonica		
maker-5-augustus-gene-263.67-mRNA-1	5	OsR498G0511160200.01.T03	100,00	0,00E+00	1121	Os05g0509700	100,00	0,00E+00	1121	0	NA	Japonica		
maker-5-augustus-gene-263.70-mRNA-1	5	OsR498G0511160600.01.T03	99,83	0,00E+00	1051	Os05g0509900	100,00	0,00E+00	1056	5	NA	Japonica		
maker-5-augustus-gene-263.72-mRNA-1	5	OsR498G0511161200.01.T01	99,85	0,00E+00	1168	Os05g0510200	99,85	0,00E+00	1168	0	NA	Japonica		
maker-5-augustus-gene-264.0-mRNA-1	5	OsR498G0511161300.01.T02	99,73	0,00E+00	1966	Os05g0510300	100,00	0,00E+00	1979	13	NA	Japonica		
maker-5-augustus-gene-264.10-mRNA-1	5	OsR498G0511166900.01.T04	99,91	0,00E+00	1922	Os05g0511500	100,00	0,00E+00	1927	5	NA	Japonica		
maker-5-augustus-gene-264.14-mRNA-1	5	OsR498G0511167100.01.T04	96,90	0,00E+00	1361	Os05g0511700	97,03	0,00E+00	1365	4	NA	Japonica		
maker-5-augustus-gene-264.18-mRNA-1	5	OsR498G0511162600.01.T01	99,76	0,00E+00	3709	Os03g0770800	75,95	0,00E+00	1251	-2458	Indica	Japonica		
maker-5-augustus-gene-264.28-mRNA-1	5	OsR498G0511165600.01.T06	98,82	0,00E+00	2968	Os05g0511400	98,93	0,00E+00	2977	9	NA	Japonica	Kinase	
maker-5-augustus-gene-264.3-mRNA-1	5	OsR498G0511162300.01.T02	99,77	0,00E+00	2314	Os05g0510700	100,00	0,00E+00	1608	-706	Indica	Japonica		
maker-5-augustus-gene-264.37-mRNA-1	5	OsR498G0511167800.01.T02	99,77	0,00E+00	3944	Os05g0512000	99,96	0,00E+00	3962	18	NA	Japonica		
maker-5-augustus-gene-264.6-mRNA-1	5	OsR498G0511164300.01.T03	100,00	0,00E+00	1242	Os05g0511000	100,00	0,00E+00	1242	0	NA	Japonica		
maker-5-augustus-gene-265.41-mRNA-1	5	OsR498G0511168700.01.T02	99,68	0,00E+00	1654	Os05g0512200	99,89	0,00E+00	1661	7	NA	Japonica		
maker-5-augustus-gene-265.45-mRNA-1	5	OsR498G0511172100.01.T01	99,76	0,00E+00	1465	Os05g0513100	100,00	0,00E+00	1597	132	NA	Japonica		
maker-5-augustus-gene-265.46-mRNA-1	5	OsR498G0511173100.01.T01	99,72	0,00E+00	1892	Os05g0513200	100,00	0,00E+00	1905	13	NA	Japonica		
maker-5-augustus-gene-265.52-mRNA-1	5	OsR498G0511173300.01.T01	99,25	0,00E+00	1900	Os05g0513300	99,91	0,00E+00	1895	-5	NA	Japonica		

maker-5-augustus-gene-265.55-mRNA-1	5	OsR498G0511173500.01.T01	100,00	0,00E+00	1911	Os05g0513400	100,00	0,00E+00	1911	0	NA	Japonica	
maker-5-augustus-gene-265.60-mRNA-1	5	OsR498G0511174900.01.T02	99,85	0,00E+00	1159	Os05g0513800	100,00	0,00E+00	1164	5	NA	Japonica	
maker-5-augustus-gene-265.65-mRNA-1	5	OsR498G0511169600.01.T01	99,59	0,00E+00	2200	Os05g0512400	100,00	0,00E+00	1586	-614	Indica	Japonica	
maker-5-augustus-gene-265.67-mRNA-1	5	OsR498G0511169800.01.T01	99,73	0,00E+00	662	Os05g0512500	100,00	0,00E+00	666	4	NA	Japonica	
maker-5-augustus-gene-265.71-mRNA-1	5	OsR498G0511174200.01.T01	99,55	0,00E+00	2392	Os05g0513700	99,71	0,00E+00	3718	1326	Japonica	Japonica	
maker-5-augustus-gene-266.0-mRNA-1	5	OsR498G0511176500.01.T07	99,49	0,00E+00	1046	Os05g0514300	100,00	0,00E+00	1056	10	NA	Japonica	
maker-5-augustus-gene-266.16-mRNA-1	5	OsR498G0511176800.01.T01	98,64	0,00E+00	2697	Os05g0514400	98,48	0,00E+00	1599	-1098	Indica	NA	
maker-5-augustus-gene-266.6-mRNA-1	5	OsR498G0511177100.01.T01	100,00	3,14E-124	444	Os05g0514500	94,71	6,45E-96	350	-94	Indica	Indica	
maker-5-augustus-gene-266.8-mRNA-1	5	OsR498G0511179700.01.T02	99,81	0,00E+00	2817	Os05g0515200	100,00	0,00E+00	2830	13	NA	Japonica	
maker-5-augustus-gene-267.0-mRNA-1	5	OsR498G0511180700.01.T01	97,91	0,00E+00	3617	Os05g0515400	98,00	0,00E+00	3112	-505	Indica	Japonica	
maker-5-augustus-gene-267.14-mRNA-1	5	OsR498G0511182900.01.T01	99,16	0,00E+00	3976	Os05g0516400	99,96	0,00E+00	4698	722	Japonica	Japonica	Kinase-TM
maker-5-augustus-gene-267.16-mRNA-1	5	OsR498G0511183500.01.T01	99,84	0,00E+00	1116	Os05g0516600	100,00	0,00E+00	1121	5	NA	Japonica	
maker-5-augustus-gene-267.21-mRNA-1	5	OsR498G0511184300.01.T05	99,93	0,00E+00	2642	Os05g0516900	100,00	0,00E+00	2646	4	NA	Japonica	
maker-5-augustus-gene-267.25-mRNA-1	5	OsR498G0511185500.01.T01	99,78	0,00E+00	4843	Os05g0517200	100,00	0,00E+00	4870	27	NA	Japonica	
maker-5-augustus-gene-267.27-mRNA-1	5	OsR498G0511185700.01.T01	99,52	0,00E+00	738	Os05g0517400	100,00	0,00E+00	747	9	NA	Japonica	
maker-5-augustus-gene-267.29-mRNA-1	5	OsR498G0511186300.01.T01	99,34	1,25E-71	270	Os05g0517800	100,00	1,02E-73	275	5	NA	Japonica	
maker-5-augustus-gene-267.31-mRNA-1	5	OsR498G0511186500.01.T01	99,46	0,00E+00	994	Os05g0517900	100,00	3,16E-153	540	-454	Indica	Japonica	
maker-5-augustus-gene-267.35-mRNA-1	5	OsR498G0511184000.01.T01	99,85	0,00E+00	1192	Os05g0516800	100,00	0,00E+00	1196	4	NA	Japonica	
maker-5-augustus-gene-267.42-mRNA-1	5	OsR498G0511186200.01.T01	86,54	6,60E-166	584	Os05g0517600	100,00	0,00E+00	791	207	Japonica	Japonica	
maker-5-augustus-gene-267.5-mRNA-1	5	OsR498G0511180900.01.T01	97,65	0,00E+00	1874	Os05g0515500	100,00	0,00E+00	1992	118	NA	Japonica	
maker-5-augustus-gene-267.9-mRNA-1	5	OsR498G0511181900.01.T03	99,42	0,00E+00	923	Os01g0752800	77,80	2,76E-110	398	-525	Indica	Japonica	
maker-5-augustus-gene-268.0-mRNA-1	5	OsR498G0511187500.01.T01	99,50	0,00E+00	5642	Os05g0518200	99,97	0,00E+00	5709	67	NA	Japonica	
maker-5-augustus-gene-268.9-mRNA-1	5	OsR498G0511188200.01.T01	99,82	0,00E+00	1972	Os05g0518300	100,00	0,00E+00	1981	9	NA	Japonica	
maker-5-augustus-gene-269.10-mRNA-1	5	OsR498G0511197500.01.T01	100,00	0,00E+00	2094	Os05g0520300	100,00	0,00E+00	2094	0	NA	Japonica	
maker-5-augustus-gene-269.12-mRNA-1	5	OsR498G0511194400.01.T01	98,81	0,00E+00	1624	Os05g0519300	99,89	0,00E+00	1657	33	NA	Japonica	
maker-5-augustus-gene-269.14-mRNA-1	5	OsR498G0511195200.01.T01	98,34	0,00E+00	3868	Os05g0519400	98,43	0,00E+00	3877	9	NA	Japonica	
maker-5-augustus-gene-269.20-mRNA-1	5	OsR498G0511195400.01.T04	99,38	0,00E+00	2590	Os05g0519500	100,00	0,00E+00	2630	40	NA	Japonica	
maker-5-augustus-gene-269.23-mRNA-1	5	OsR498G0511196000.01.T01	99,71	0,00E+00	4908	Os05g0519700	100,00	0,00E+00	4940	32	NA	Japonica	
maker-5-augustus-gene-269.25-mRNA-1	5	OsR498G0511196600.01.T02	99,92	0,00E+00	4255	Os05g0519900	100,00	0,00E+00	4264	9	NA	Japonica	
maker-5-augustus-gene-269.29-mRNA-1	5	OsR498G0511197000.01.T01	99,80	0,00E+00	1788	Os05g0520100	100,00	0,00E+00	1139	-649	Indica	Japonica	
maker-5-augustus-gene-269.33-mRNA-1	5	OsR498G0511197200.01.T02	100,00	0,00E+00	1110	Os05g0520200	100,00	0,00E+00	1110	0	NA	Japonica	
maker-5-augustus-gene-27.54-mRNA-1	5	OsR498G0509736700.01.T01	99,78	0,00E+00	2465	Os05g0148700	99,93	0,00E+00	2474	9	NA	NA	
maker-5-augustus-gene-27.63-mRNA-1	5	OsR498G0509732400.01.T02	98,88	4,01E-85	315	Os05g0147100	99,44	1,15E-86	319	4	NA	NA	
maker-5-augustus-gene-27.71-mRNA-1	5	OsR498G0509733400.01.T01	99,88	0,00E+00	2892	Os05g0147400	99,88	0,00E+00	2892	0	NA	Japonica	
maker-5-augustus-gene-27.77-mRNA-1	5	OsR498G0509735400.01.T04	96,73	0,00E+00	1115	Os05g0148200	99,73	0,00E+00	1335	220	Japonica	NA	
maker-5-augustus-gene-27.85-mRNA-1	5	OsR498G0509736600.01.T01	99,50	0,00E+00	1438	Os05g0148600	99,38	0,00E+00	1434	-4	NA	NA	
maker-5-augustus-gene-270.35-mRNA-1	5	OsR498G0511203500.01.T02	97,35	0,00E+00	2372	Os05g0521500	97,63	0,00E+00	2390	18	NA	Japonica	
maker-5-augustus-gene-270.40-mRNA-1	5	OsR498G0511203700.01.T01	99,42	0,00E+00	2128	Os05g0521600	100,00	0,00E+00	2159	31	NA	Japonica	
maker-5-augustus-gene-270.54-mRNA-1	5	OsR498G0511204600.01.T03	99,46	0,00E+00	1965	Os05g0521900	100,00	0,00E+00	1970	5	NA	Japonica	
maker-5-augustus-gene-271.0-mRNA-1	5	OsR498G0511206700.01.T01	99,74	0,00E+00	2731	Os05g0522500	100,00	0,00E+00	2749	18	NA	Japonica	
maker-5-augustus-gene-271.11-mRNA-1	5	OsR498G0511208200.01.T01	99,69	0,00E+00	1718	Os05g0523300	100,00	0,00E+00	1732	14	NA	Japonica	
maker-5-augustus-gene-271.7-mRNA-1	5	OsR498G0511207000.01.T01	99,81	0,00E+00	5638	Os01g0742400	70,29	0,00E+00	1392	-4246	Indica	Japonica	Kinase-LRR-TM
maker-5-augustus-gene-272.40-mRNA-1	5	OsR498G0511211300.01.T01	99,81	0,00E+00	938	Os05g0524100	100,00	1,63E-137	488	-450	Indica	Japonica	
maker-5-augustus-gene-272.42-mRNA-1	5	OsR498G0101962600.01.T01	81,42	0,00E+00	1386	Os05g0524300	99,93	0,00E+00	2674	1288	Japonica	Japonica	
maker-5-augustus-gene-272.44-mRNA-1	5	OsR498G0511212100.01.T01	99,65	0,00E+00	3047	Os05g0524400	100,00	0,00E+00	3074	27	NA	Japonica	
maker-5-augustus-gene-272.49-mRNA-1	5	OsR498G0511212300.01.T01	98,37	0,00E+00	5002	Os05g0524500	98,82	0,00E+00	5061	59	NA	Japonica	Kinase-LRR-TM
maker-5-augustus-gene-272.52-mRNA-1	5	OsR498G0511212900.01.T03	99,54	0,00E+00	4653	Os05g0524600	100,00	0,00E+00	2558	-2095	Indica	Japonica	Kinase-LRR-TM
maker-5-augustus-gene-272.63-mRNA-1	5	OsR498G0511214700.01.T01	88,00	0,00E+00	2754	Os05g0525000	99,95	0,00E+00	3940	1186	Japonica	Japonica	Kinase-LRR-TM
maker-5-augustus-gene-272.65-mRNA-1	5	OsR498G0511214700.01.T01	98,99	0,00E+00	5075	Os05g0525550	98,59	0,00E+00	4845	-230	NA	Japonica	Kinase-LRR-TM
maker-5-augustus-gene-272.68-mRNA-1	5	OsR498G0511215100.01.T01	99,76	0,00E+00	3726	Os05g0525600	100,00	0,00E+00	3721	-5	NA	Japonica	Kinase-LRR-TM
maker-5-augustus-gene-272.71-mRNA-1	5	OsR498G0511215300.01.T01	97,96	0,00E+00	4809	Os05g0525550	77,39	0,00E+00	2088	-2721	Indica	Japonica	Kinase-LRR-TM
maker-5-augustus-gene-272.78-mRNA-1	5	OsR498G0511216500.01.T01	98,90	0,00E+00	1432	Os05g0526200	100,00	0,00E+00	1472	40	NA	Japonica	
maker-5-augustus-gene-272.80-mRNA-1	5	OsR498G0511211600.01.T01	94,16	0,00E+00	1215	Os05g0524200	94,94	0,00E+00	1244	29	NA	Japonica	
maker-5-augustus-gene-273.16-mRNA-1	5	OsR498G0511222400.01.T01	100,00	0,00E+00	661	Os05g0528200	100,00	0,00E+00	661	0	NA	Japonica	
maker-5-augustus-gene-273.3-mRNA-1	5	OsR498G0511217000.01.T02	98,15	0,00E+00	1301	Os05g0526400	100,00	0,00E+00	1364	63	NA	Japonica	
maker-5-augustus-gene-274.0-mRNA-1	5	OsR498G0511224500.01.T01	100,00	0,00E+00	1121	Os05g0528900	100,00	0,00E+00	1121	0	NA	NA	
maker-5-augustus-gene-274.10-mRNA-1	5	OsR498G0511231300.01.T01	100,00	0,00E+00	2489	Os05g0530400	100,00	0,00E+00	2489	0	NA	NA	
maker-5-augustus-gene-274.13-mRNA-1	5	OsR498G0511234900.01.T01	100,00	0,00E+00	1061	Os05g0531200	100,00	0,00E+00	1061	0	NA	Indica	
maker-5-augustus-gene-274.15-mRNA-1	5	OsR498G0511224800.01.T01	100,00	0,00E+00	1483	Os05g0529000	100,00	0,00E+00	1483	0	NA	NA	
maker-5-augustus-gene-274.19-mRNA-1	5	OsR498G0511225400.01.T02	100,00	0,00E+00	1200	Os05g0529200	100,00	0,00E+00	1200	0	NA	NA	
maker-5-augustus-gene-274.20-mRNA-1	5	OsR498G0511225600.01.T01	100,00	0,00E+00	1169	Os05g0529300	100,00	0,00E+00	1169	0	NA	NA	
maker-5-augustus-gene-274.24-mRNA-1	5	OsR498G0511228200.01.T06	100,00	0,00E+00	935	Os05g0529700	100,00	0,00E+00	935	0	NA	NA	
maker-5-augustus-gene-274.29-mRNA-1	5	OsR498G0511229400.01.T01	100,00	0,00E+00	1851	Os05g0529900	100,00	0,00E+00	1764	-87	NA	NA	
maker-5-augustus-gene-274.32-mRNA-1	5	OsR498G0511230600.01.T03	100,00	0,00E+00	1624	Os01g0729900	72,25	1,72E-142	507	-1117	Indica	NA	
maker-5-augustus-gene-274.36-mRNA-1	5	OsR498G0511231600.01.T04	98,64	0,00E+00	2696	Os05g0530500	98,25	0,00E+00	2670	-26	NA	NA	Kinase-TM
maker-5-augustus-gene-274.4-mRNA-1	5	OsR498G0511225700.01.T01	100,00	7,04E-159	560	Os05g0529400	100,00	2,44E-159	560	0	NA	NA	
maker-5-augustus-gene-274.7-mRNA-1	5	OsR498G0511227100.01.T01	100,00	0,00E+00	4174	Os05g0529600	100,00	0,00E+00	5434	1260	Japonica	NA	
maker-5-augustus-gene-275.44-mRNA-1	5	OsR498G0511235500.01.T02	100,00	0,00E+00	948	Os05g0531400	100,00	2,00E-155	547	-401	Indica	NA	
maker-5-augustus-gene-275.46-mRNA-1	5	OsR498G0511236100.01.T02	100,00	0,00E+00	2706	Os05g0531500	99,93	0,00E+00	2701	-5	NA	NA	
maker-5-augustus-gene-275.53-mRNA-1	5	OsR498G0511241500.01.T01	100,00	0,00E+00	2040	Os05g0533100	100,00	0,00E+00	1324	-716	Indica	NA	
maker-5-augustus-gene-275.59-mRNA-1	5	OsR498G0511239000.01.T01	100,00	0,00E+00	2197	Os05g0535200	82,73	0,00E+00	820	-1377	Indica	NA	
maker-5-augustus-gene-275.64-mRNA-1	5	OsR498G0511239800.01.T01	100,00	0,00E+00	2679	Os01g0722700	78,94	0,00E+00	722	-1957	Indica	NA	
maker-5-augustus-gene-276.0-mRNA-1	5	OsR498G0511243300.01.T02	99,96	0,00E+00	4725	Os05g0533600	100,00	0,00E+00	3901	-824	Indica	NA	
maker-5-augustus-gene-276.14-mRNA-1	5	OsR498G0511244300.01.T01	100,00	0,00E+00	1332	Os05g0534100	94,40	0,00E+00	1196	-136	Indica	NA	
maker-5-augustus-gene-276.18-mRNA-1	5	OsR498G0511244900.01.T01	100,00	0,00E+00	1142	Os05g0534400	100,00	0,00E+00	1142	0	NA	NA	
maker-5-augustus-gene-276.21-mRNA-1	5	OsR498G0511245900.01.T03	99,87	0,00E+00	2722	Os05g0534800	97,35	0,00E+00	2823	101	NA	NA	
maker-5-augustus-gene-276.28-mRNA-1	5	OsR498G0511245200.01.T02	100,00	0,00E+00	872	Os05g0534500	100,00	0,00E+00	872	0	NA	NA	
maker-5-augustus-gene-276.31-mRNA-1	5	OsR498G0511245800.01.T03	100,00	0,00E+00	3842	Os05g0534600	99,91	0,00E+00	3833	-9	NA	NA	
maker-5-augustus-gene-276.34-mRNA-1	5	OsR498G0511247200.01.T01	100,00	0,00E+00	2646	Os05g0535100	100,00	0,00E+00	2646	0	NA	NA	
maker-5-augustus-gene-276.5-mRNA-1	5	OsR498G0511243500.01.T01	100,00	0,00E+00	1110	Os05g0533700	100,00	0,00E+00	839	-271	Indica	NA	
maker-5-augustus-gene-276.7-mRNA-1	5	OsR498G0511243700.01.T03	100,00	0,00E+00	682	Os05g0533800	100,00	0,00E+00	682	0	NA	NA	
maker-5-augustus-gene-276.9-mRNA-1	5	OsR498G0511243900.01.T01	100,00	0,00E+00	2224	Os05g0533900	100,00	0,00E+00	1743	-481	Indica	NA	
maker-5-augustus-gene-277.39-mRNA-1	5	OsR498G0511247700.01.T03	100,00	0,00E+00	1535	Os05g0535400	100,00	0,00E+00	1535	0	NA	NA	
maker-5-augustus-gene-277.50-mRNA-1	5	OsR498G0511249100.01.T02	100,00	0,00E+00	2800	Os05g0535800	97,54	0,00E+00	2704	-96	NA	NA	
maker-5-augustus-gene-277.53-mRNA-1	5	OsR498G0511249700.01.T01	100,00	0,00E+00	2012	Os05g0535900	100,00	0,00E+00	2012	0	NA	NA	
maker-5-augustus-gene-277.56-mRNA-1	5	OsR498G0511250300.01.T01	97,98	0,00E+00	1453	Os05g0536200	97,98	0,00E+00	1453	0	NA	NA	
maker-5-augustus-gene-277.62-mRNA-1	5	OsR498G0511252100.01.T03	100,00	1,30E-169	596	Os05g0536900	100,00	4,53E-170	596	0	NA	NA	
maker-5-augustus-gene-277.67-mRNA-1	5	OsR498G0511248400.01.T03	99,73	0,00E+00	1951	Os05g0535600	96,03	0,00E+00	2005	54	NA	NA	
maker-5-augustus-gene-278.0-mRNA-1	5	OsR498G0511253300.01.T01	100,00	0,00E+00	1202	Os01g0714800	83,58	3,81E-96	351	-851	Indica	NA	
maker-5-augustus-gene-278.16-mRNA-1	5	OsR498G0101879400.01.T03	78,67	0,00E+00	884	Os05g0537300	100,00	0,00E+00	1948	1064	Japonica	NA	
maker-5-augustus-gene-278.18-mRNA-1	5	OsR498G0511254000.01.T02	100,00	0,00E+00	2100	Os05g0537400	100,00	0,00E+00	2100	0	NA	NA	
maker-5-augustus-gene-278.21-mRNA-1	5	OsR498G0511257000.01.T01	97,67	0,00E+00	884	Os09g0499600	89,68	1,97E-117	421	-463	Indica	NA	
maker-5-augustus-gene-278.23-mRNA-1	5	OsR498G0511257600.01.T01	100,00	0,00E+00	1819	Os09g0500100	81,02	0,00E+00	1080	-739	Indica	NA	
maker-5-augustus-gene-278.29-mRNA-1	5	OsR498G0917754400.01.T03	81,78	3,66E-159	563	Os05g0538900	100,00	0,00E+00	1310	747	Japonica	NA	
maker-5-augustus-gene-279.42-mRNA-1	5	OsR498G0511263100.01.T01	100,00	0,00E+00	1483	Os05g0539500	100,00	0,00E+00	1532	49	NA	NA	
maker-5-augustus-gene-279.49-mRNA-1	5	OsR498G0511264700.01.T04	100,00	0,00E+00	1418	Os05g0539900	100,00	0,00E+00	926	-492	Indica	NA	
maker-5-augustus-gene-279.53-mRNA-1	5	OsR498G0511264900.01.T01	100,00	0,00E+00	2013	Os05g0540000	100,00	0,00E+00	2013	0	NA	NA	
maker-5-augustus-gene-279.56-mRNA-1	5	OsR498G0511265300.01.T01	100,00	2,42E-163	574	Os05g0540200	100,00	8,42E-164	574	0	NA	NA	
maker-5-augustus-gene-279.58-mRNA-1	5	OsR498G0511266300.01.T07	98,99	0,00E+00	1572	Os05g0540800	98,99	0,00E+00	1572	0	NA	NA	
maker-5-augustus-gene-279.66-mRNA-1	5	OsR498G0511267100.01.T01	100,00	0,00E+00	2214	Os05g0541100	100,00	0,00E+00	2214	0	NA	NA	
maker-5-augustus-gene-279.69-mRNA-1	5	OsR498G0511267500.01.T02	100,00	0,00E+00	1213	Os05g0541200	100,00	0,00E+00	1213	0	NA	NA	
maker-5-augustus-gene-279.74-mRNA-1	5	OsR498G0511263000.01.T04	100,00	0,00E+00	2648	Os05g0539400	100,00	0,00E+00	2648	0	NA	NA	
maker-5-augustus-gene-279.87-mRNA-1	5	OsR498G0511264400.01.T04	100,00	0,00E+00	1267	Os05g0539800	100,00	0,00E+00	1267	0	NA	NA	
maker-5-augustus-gene-279.98-mRNA-1	5	OsR498G0511265600.01.T01	99,94	0,00E+00	3039	Os05g0540300	99,94	0,00E+00	3039	0	NA	NA	
maker-5-augustus-gene-28.0-mRNA-1	5	OsR498G0509736900.01.T05	100,00	7,17E-131	466	Os05g0148900	74,47	2,21E-24	111	-355	Indica	Japonica	
maker-5-augustus-gene-28.14-mRNA-1	5	OsR498G0509740100.01.T01	99,92	0,00E+00	2332	Os05g0149800	100,00	0,00E+00	2336	4	NA	Japonica	
maker-5-augustus-gene-28.2-mRNA-1	5	OsR498G0509736900.01.T01	99,39	0,00E+00	1162	Os05g0148900	99,65	0,00E+00	1034	-128	Indica	Japonica	
maker-5-augustus-gene-28.25-mRNA-1	5	OsR498G0509741900.01.T02	99,17	0,00E+00	6157	Os05g0150300	99,28	0,00E+00	6176	19	NA	Japonica	
maker-5-augustus-gene-28.31-mRNA-1	5	OsR498G0509738700.01.T01	97,20	7,57E-98	356	Os05g0149400	97,20	2,64E-98	356	0	NA	NA	
maker-5-augustus-gene-28.33-mRNA-1	5	OsR498G0509739800.01.T01	99,87	0,00E+00	4018	Os05g0149600	98,39	0,00E+00	3885	-133	NA	Japonica	
maker-5-augustus-gene-28.39-mRNA-1	5	OsR498G0509740600.01.T01	99,86	0,00E+00	1316	Os05g0150000	100,00	0,00E+00	1159	-157	Indica	Japonica	
maker-5-augustus-gene-28.44-mRNA-1	5	OsR498G0509742200.01.T03	99,66	0,00E+00	3187	Os05g0150400	99,94	0,00E+00	3210	23	NA	Japonica	
maker-5-augustus-gene-28.5-mRNA-1	5	OsR498G0509738100.01.T01	97,92	0,00E+00	3813	Os05g0149200	99,78	0,00E+00	5705	1892	Japonica	Japonica	
maker-5-augustus-gene-28.8-mRNA-1	5	OsR498G0509738700.01.T01	100,00	0,00E+00	1673	Os05g0149400	100,00	0,00E+00	1673	0	NA	Japonica	
maker-5-augustus-gene-280.15-mRNA-1	5	OsR498G0511272500.01.T01	100,00	0,00E+00	2414	Os05g0542900	100,00	0,00E+00	2414	0	NA	NA	
maker-5-augustus-gene-280.19-mRNA-1	5	OsR498G0511272700.01.T01	100,00	0,00E+00	1121	Os05g0543000	100,00	0,00E+00	1121	0	NA	NA	
maker-5-augustus-gene-280.2-mRNA-1	5	OsR498G0511270300.01.T02	100,00	0,00E+00	1099	Os05g0541800	100,00	0,00E+00	726	-373	Indica	Indica	
maker-5-augustus-gene-280.21-mRNA-1	5	OsR498G0511273300.01.T01	100,00	0,00E+00	1737	Os05g0543300	100,00	0,00E+00	1737	0	NA	NA	
maker-5-augustus-gene-280.25-mRNA-1	5	OsR498G0511270600.01.T01	100,00	0,00E+00	801	Os01g0706500	86,07	1,21E-144	512	-289	Indica	Indica	
maker-5-augustus-gene-280.29-mRNA-1	5	OsR498G0511270800.01.T05	100,00	0,00E+00	2170	Os05g0542200	100,00	0,00E+00	2170	0	NA	NA	
maker-5-augustus-gene-280.35-mRNA-1	5	OsR498G0511273000.01.T01	97,07	0,00E+00	2258	Os05g0543100	97,07	0,00E+00	2258	0	NA	NA	
maker-5-augustus-gene-280.40-mRNA-1	5	OsR498G0511273600.01.T01	99,72	0,00E+00	1917	Os05g0543400	99,63	0,00E+00	1912	-5	NA	NA	
maker-5-augustus-gene-280.6-mRNA-1	5	OsR498G0511272100.01.T03	96,14	0,00E+00	1332	Os05g0542600	96,02	0,00E+00	1327	-5	NA	NA	
maker-5-augustus-gene-281.0-mRNA-1	5	OsR498G0511275900.01.T01	99,62	0,00E+00	1425	Os05g0543600	98,93	0,00E+00	1481	56	NA	Indica	
maker-5-augustus-gene-281.14-mRNA-1	5	OsR498G0511276400.01.T01	99,88	0,00E+00	1482	Os05g0543800	99,64	0,00E+00	1473	-9	NA	NA	
maker-5-augustus-gene-281.2-mRNA-1	5	OsR498G0511280900.01.T06	98,33	0,00E+00	3028	Os05g0545000	98,45	0,00E+00	3037	9	NA	Indica	
maker-5-augustus-gene-281.21-mRNA-1	5	OsR498G0511281600.01.T02	99,93	0,00E+00	5422	Os01g0700200	74,76	0,00E+00	2021	-3401	Indica	Indica	
maker-5-augustus-gene-281.24-mRNA-1	5	OsR498G0511282400.01.T01	99,69	0,00E+00	2336	Os05g0545300	99,77	0,00E+00	2341	5	NA	NA	Kinase-TM
maker-5-augustus-gene-281.8-mRNA-1	5	OsR498G0511276200.01.T01	99,80	0,00E+00	1826	Os05g0543700	99,90	0,00E+00	1830	4	NA	Indica	
maker-5-augustus-gene-282.52-mRNA-1	5	OsR498G0511284300.01.T01	99,73	0,00E+00	2628	Os05g0545500	89,38	0,00E+00	1235	-1393	Indica	NA	
maker-5-augustus-gene-282.58-mRNA-1	5	OsR498G0511285100.01.T01	99,20	0,00E+00	1322	Os05g0545900	99,40	0,00E+00	1784	462	Japonica	Indica	
maker-5-augustus-gene-282.65-mRNA-1	5	OsR498G0511284300.01.T01	98,85	1,47E-127	456	Os05g0546600	99,62	9,84E-131	465	9	NA	Indica	
maker-5-augustus-gene-282.67-mRNA-1	5	OsR498G0511285100.01.T01	97,19	0,00E+00	1254	Os05g0546800	97,73	0,00E+00	2246	992	Japonica	Indica	
maker-5-augustus-gene-282.72-mRNA-1	5	OsR498G0511286400.01.T02	96,44	1,57E-100	365	Os05g0546500	100,00	4,23E-115	412	47	Japonica	Indica	
maker-5-augustus-gene-282.74-mRNA-1	5	OsR498G0511286400.01.T01	99,74	0,00E+00	689	Os05g0547850	98,70	0,00E+00	671	-18	NA	Indica	
maker-5-augustus-gene-283.12-mRNA-1	5	OsR498G0510529200.01.T01	86,93	0,00E+00	796	Os05g0548700	90,60	0,00E+00	1282	486	Japonica	NA	
maker-5-augustus-gene-283.20-mRNA-1	5	OsR498G0511289000.01.T03	99,75	0,00E+00	1456	Os01g0695100	83,25	0,00E+00	852	-604	Indica	NA	
maker-5-augustus-gene-283.30-mRNA-1	5	OsR498G0511289200.01.T02	99,90	0,00E+00	1746	Os05g0549000	99,79	0,00E+00	1741	-5	NA	Indica	
maker-5-augustus-gene-283.37-mRNA-1	5	OsR498G0511289600.01.T01	100,00	0,00E+00	2352	Os05g0549100	99,85	0,00E+00	2343	-9	NA	Indica	Kinase-TM
maker-5-augustus-gene-283.6-mRNA-1	5	OsR498G0511291100.01.T01	95,51	0,00E+00	1280	Os01g0693900	76,59	6,67E-132	471	-809	Indica	NA	
maker-5-augustus-gene-284.25-mRNA-1	5	OsR498G0511293700.01.T02	99,72	0,00E+00	1922	Os01g0692700	73,53	1,49E-97	357	-1565	Indica	NA	
maker-5-augustus-gene-284.39-mRNA-1	5	OsR498G0511293200.01.T01	94,68	0,00E+00	1485	Os05g0549900	97,71	0,00E+00	1347	-138	NA	NA	
maker-5-augustus-gene-284.45-mRNA-1	5	OsR498G0511294600.01.T01	99,85	0,00E+00	1192	Os01g0691400	71,86	7,32E-80	297	-895	Indica	NA	
maker-5-augustus-gene-284.49-mRNA-1	5	OsR498G0511297800.01.T01	99,85	0,00E+00	3529	Os05g0551100	99,87	0,00E+00	4027	498	Japonica	NA	
maker-5-augustus-gene-284.55-mRNA-1	5	OsR498G0511300200.01.T01	99,21	0,00E+00	1120	Os05g0551500	99,53	0,00E+00	1129	9	NA	Indica	
maker-5-augustus-gene-285.33-mRNA-1	5	OsR498G0511300500.01.T01	100,00	0,00E+00	1121	Os05g0551600	100,00	0,00E+00	1095	-26	NA	Indica	
maker-5-augustus-gene-285.36-mRNA-1	5	OsR498G0511303700.01.T02	86,40	2,80E-128	459	Os05g0552400	86,69	7,98E-130	463	4	NA	Indica	
maker-5-augustus-gene-285.46-mRNA-1	5	OsR498G0511303200.01.T01	99,77	0,00E+00	3139	Os05g0552100	99,94	0,00E+00	3152	13	NA	NA	
maker-5-augustus-gene-285.48-mRNA-1	5	OsR498G0511305200.01.T03	99,90	0,00E+00	1863	Os05g0552500	99,59	0,00E+00	1720	-143	NA	NA	
maker-5-augustus-gene-285.55-mRNA-1	5	OsR498G0511308800.01.T03	99,60	0,00E+00	3552	Os05g0552900	99,64	0,00E+00	3925	373	NA	NA	
maker-5-augustus-gene-285.59-mRNA-1	5	OsR498G0511309000.01.T01	99,70	0,00E+00	2970	Os05g0553000	99,64	0,00E+00	2966	-4	NA	NA	
maker-5-augustus-gene-286.17-mRNA-1	5	OsR498G0511313100.01.T02	99,64	0,00E+00	1995	Os05g0554500	99,91	0,00E+00	2009	14	NA	Indica	
maker-5-augustus-gene-286.20-mRNA-1	5	OsR498G0511316100.01.T01	99,87	0,00E+00	2756	Os05g0555100	99,73	0,00E+00	1989	-767	Indica	NA	
maker-5-augustus-gene-286.22-mRNA-1	5	OsR498G0511310400.01.T01	99,77	0,00E+00	1561	Os05g0553400	99,89	0,00E+00	1565	4	NA	NA	
maker-5-augustus-gene-286.26-mRNA-1	5	OsR498G0511315400.01.T01	99,85	0,00E+00	2399	Os05g0555000	99,43	0,00E+00	2515	116	NA	Indica	
maker-5-augustus-gene-286.31-mRNA-1	5	OsR498G0511316400.01.T03	96,28	0,00E+00	938	Os05g0555300	96,14	0,00E+00	902	-36	NA	NA	
maker-5-augustus-gene-286.7-mRNA-1	5	OsR498G0511311900.01.T01	100,00	0,00E+00	2711	Os01g0684900	75,81	0,00E+00	992	-1719	Indica	Indica	
maker-5-augustus-gene-287.35-mRNA-1	5	OsR498G0511318900.01.T02	99,70	1,85E-171	602	Os05g0556900	99,11	3,33E-169	593	-9	NA	NA	
maker-5-augustus-gene-287.38-mRNA-1	5	OsR498G0511319100.01.T01	96,35	0,00E+00	2102	Os05g0556000	96,19	0,00E+00	2093	-9	NA	Indica	
maker-5-augustus-gene-287.42-mRNA-1	5	OsR498G0511319700.01.T01	99,65	0,00E+00	3036	Os01g0681000	74,60	0,00E+00	965	-2071	Indica	NA	
maker-5-augustus-gene-287.46-mRNA-1	5	OsR498G0511319900.01.T01	99,91	0,00E+00	1998	Os05g0556400	99,91	0,00E+00	1998	0	NA	NA	
maker-5-augustus-gene-287.48-mRNA-1	5	OsR498G0511320500.01.T02	99,62	0,00E+00	1397	Os05g0556500	98,71	0,00E+00	2173	776	Japonica	Indica	
maker-5-augustus-gene-287.52-mRNA-1	5	OsR498G0511321100.01.T02	100,00	0,00E+00	2062	Os05g0556700	100,00	0,00E+00	1781	-281	Indica	NA	
maker-5-augustus-gene-287.58-mRNA-1	5	OsR498G0511321900.01.T04	99,70	1,85E-171	602	Os05g0556900	99,70	6,43E-172	602	0	NA	NA	
maker-5-augustus-gene-287.61-mRNA-1	5	OsR498G0511322100.01.T03	100,00	4,19E-147	520	Os05g0557000	100,00	1,37E-141	500	-20	NA	NA	
maker-5-augustus-gene-287.68-mRNA-1	5	OsR498G0511319400.01.T01	99,89	0,00E+00	3292	Os05g0556100	99,84	0,00E+00	3287	-5	NA	Indica	
maker-5-augustus-gene-287.77-mRNA-1	5	OsR498G0511324000.01.T01	99,93	0,00E+00	2427	Os05g0557400	99,70	0,00E+00	2414	-13	NA	NA	
maker-5-augustus-gene-288.38-mRNA-1	5	OsR498G0511327300.01.T04	96,15	0,00E+00	2107	Os05g0558400	96,15	0,00E+00	2107	0	NA	Indica	
maker-5-augustus-gene-288.67-mRNA-1	5	OsR498G0511328900.01.T02	99,85	0,00E+00	2437	Os05g0558800	99,85	0,00E+00	2437	0	NA	NA	
maker-5-augustus-gene-288.69-mRNA-1	5	OsR498G0511329100.01.T01	99,84	0,00E+00	2257	Os05g0558900	99,92	0,00E+00	2261	4	NA	Indica	
maker-5-augustus-gene-288.74-mRNA-1	5	OsR498G0511324400.01.T01	99,80	0,00E+00	2746	Os05g0557600	99,74	0,00E+00	2742	-4	NA	Indica	
maker-5-augustus-gene-288.81-mRNA-1	5	OsR498G0511325400.01.T01	100,00	0,00E+00	1359	Os05g0557800	100,00	0,00E+00	1359	0	NA	Indica	
maker-5-augustus-gene-288.85-mRNA-1	5	OsR498G0511325600.01.T03	98,74	0,00E+00	2083	Os05g0557900	98,74	0,00E+00	2083	0	NA	Indica	
maker-5-augustus-gene-289.31-mRNA-1	5	OsR498G0511331900.01.T02	99,85	0,00E+00	2425	Os05g0559600	99,78	0,00E+00	2420	-5	NA	Indica	
maker-5-augustus-gene-289.34-mRNA-1	5	OsR498G0511332700.01.T01	99,77	0,00E+00	766	Os05g0560000	99,53	0,00E+00	762	-4	NA	Indica	
maker-5-augustus-gene-289.36-mRNA-1	5	OsR498G0511334500.01.T03	99,34	0,00E+00	1891	Os05g0560400	100,00	0,00E+00	1865	-26	NA	NA	
maker-5-augustus-gene-289.46-mRNA-1	5	OsR498G0511331000.01.T04	99,47	0,00E+00	1335	Os05g0559400	99,20	0,00E+00	1326	-9	NA	Japonica	
maker-5-augustus-gene-289.50-mRNA-1	5	OsR498G0511332400.01.T05	100,00	0,00E+00	2661	Os05g0559900	99,93	0,00E+00	2613	-48	NA	Indica	
maker-5-augustus-gene-289.56-mRNA-1	5	OsR498G0511333400.01.T01	99,20	0,00E+00	2636	Os05g0560100	99,33	0,00E+00	2638	2	NA	Indica	
maker-5-augustus-gene-289.59-mRNA-1	5	OsR498G0511334000.01.T02	99,71	0,00E+00	2444	Os05g0560300	99,63	0,00E+00	2440	-4	NA	Indica	Kinase-TM
maker-5-augustus-gene-29.0-mRNA-1	5	OsR498G0509743900.01.T01	96,31	0,00E+00	1844	Os05g0150700	100,00	0,00E+00	1997	153	NA	Japonica	
maker-5-augustus-gene-29.11-mRNA-1	5	OsR498G0509750100.01.T02	98,83	0,00E+00	2260	Os05g0152400	100,00	0,00E+00	2163	-97	NA	Japonica	
maker-5-augustus-gene-29.18-mRNA-1	5	OsR498G0509745600.01.T01	99,59	0,00E+00	4745	Os05g0150900	99,31	0,00E+00	4080	-665	Indica	Japonica	
maker-5-augustus-gene-29.24-mRNA-1	5	OsR498G0509746000.01.T01	99,64	0,00E+00	9838	Os05g0151000	99,98	0,00E+00	9924	86	NA	Japonica	
maker-5-augustus-gene-29.30-mRNA-1	5	OsR498G0509747400.01.T01	99,08	0,00E+00	1343	Os05g0151300	99,61	0,00E+00	1361	18	NA	Japonica	
maker-5-augustus-gene-29.32-mRNA-1	5	OsR498G0509747600.01.T01	99,57	0,00E+00	7002	Os05g0151400	99,85	0,00E+00	7045	43	NA	Japonica	
maker-5-augustus-gene-29.4-mRNA-1	5	OsR498G0509745300.01.T01	99,89	0,00E+00	1581	Os05g0150800	99,90	0,00E+00	1778	197	Japonica	Japonica	
maker-5-augustus-gene-290.0-mRNA-1	5	OsR498G0511339900.01.T01	100,00	0,00E+00	2809	Os05g0561600	99,32	0,00E+00	2094	-715	Indica	Indica	
maker-5-augustus-gene-290.13-mRNA-1	5	OsR498G0511341400.01.T01	100,00	0,00E+00	1229	Os05g0562200	99,71	0,00E+00	1220	-9	NA	Indica	
maker-5-augustus-gene-290.17-mRNA-1	5	OsR498G0511341800.01.T01	100,00	0,00E+00	1965	Os05g0562300	99,82	0,00E+00	1956	-9	NA	Indica	
maker-5-augustus-gene-290.19-mRNA-1	5	OsR498G0511342000.01.T01	100,00	0,00E+00	10042	Os05g0562400	99,86	0,00E+00	7537	-2505	Indica	Indica	
maker-5-augustus-gene-290.2-mRNA-1	5	OsR498G0511337200.01.T01	99,91	0,00E+00	1911	Os05g0560900	99,81	0,00E+00	1907	-4	NA	Indica	
maker-5-augustus-gene-291.0-mRNA-1	5	OsR498G0511343100.01.T01	100,00	0,00E+00	1700	Os05g0563000	99,47	0,00E+00	1681	-19	NA	Indica	
maker-5-augustus-gene-291.2-mRNA-1	5	OsR498G0511345700.01.T09	99,03	0,00E+00	3817	Os05g0563400	98,39	0,00E+00	3777	-40	NA	Indica	
maker-5-augustus-gene-292.0-mRNA-1	5	OsR498G0511348700.01.T04	100,00	0,00E+00	2711	Os05g0564300	99,93	0,00E+00	2707	-4	NA	Indica	
maker-5-augustus-gene-292.10-mRNA-1	5	OsR498G0511350900.01.T01	100,00	0,00E+00	969	Os05g0565000	100,00	0,00E+00	969	0	NA	Indica	
maker-5-augustus-gene-292.13-mRNA-1	5	OsR498G0511351300.01.T01	100,00	0,00E+00	1413	Os05g0565100	100,00	0,00E+00	1413	0	NA	Indica	
maker-5-augustus-gene-292.15-mRNA-1	5	OsR498G0511351500.01.T01	100,00	0,00E+00	1543	Os05g0565200	99,53	0,00E+00	1525	-18	NA	Indica	
maker-5-augustus-gene-292.18-mRNA-1	5	OsR498G0511354100.01.T01	100,00	0,00E+00	1683	Os05g0565900	96,26	0,00E+00	2081	398	Japonica	Indica	
maker-5-augustus-gene-292.21-mRNA-1	5	OsR498G0511349200.01.T01	100,00	0,00E+00	1180	Os05g0564400	99,85	0,00E+00	1176	-4	NA	Indica	
maker-5-augustus-gene-292.4-mRNA-1	5	OsR498G0511349700.01.T01	100,00	0,00E+00	2787	Os05g0564500	99,72	0,00E+00	1890	-897	Indica	Indica	
maker-5-augustus-gene-293.47-mRNA-1	5	OsR498G0511357700.01.T03	100,00	0,00E+00	2922	Os05g0566900	99,82	0,00E+00	2909	-13	NA	Indica	
maker-5-augustus-gene-293.57-mRNA-1	5	OsR498G0511358900.01.T01	99,89	0,00E+00	3223	Os05g0567400	99,72	0,00E+00	3210	-13	NA	Indica	
maker-5-augustus-gene-293.67-mRNA-1	5	OsR498G0511360100.01.T04	100,00	0,00E+00	1472	Os05g0567800	99,88	0,00E+00	1468	-4	NA	Indica	
maker-5-augustus-gene-293.78-mRNA-1	5	OsR498G0511356200.01.T03	100,00	0,00E+00	764	Os05g0566600	99,07	7,83E-161	566	-198	Indica	Indica	
maker-5-augustus-gene-293.80-mRNA-1	5	OsR498G0511356600.01.T02	99,85	0,00E+00	1203	Os05g0566800	99,25	0,00E+00	1185	-18	NA	Indica	
maker-5-augustus-gene-294.44-mRNA-1	5	OsR498G0511362900.01.T01	100,00	0,00E+00	937	Os05g0568100	99,62	0,00E+00	928	-9	NA	Indica	
maker-5-augustus-gene-294.48-mRNA-1	5	OsR498G0511363300.01.T01	93,33	0,00E+00	1122	Os05g0568200	93,48	0,00E+00	1244	122	NA	Indica	
maker-5-augustus-gene-294.50-mRNA-1	5	OsR498G0511366100.01.T01	100,00	0,00E+00	850	Os05g0569200	99,58	0,00E+00	841	-9	NA	Indica	
maker-5-augustus-gene-294.52-mRNA-1	5	OsR498G0511366500.01.T01	100,00	0,00E+00	2062	Os05g0569300	99,48	0,00E+00	2035	-27	NA	Indica	
maker-5-augustus-gene-294.58-mRNA-1	5	OsR498G0511367100.01.T01	98,46	0,00E+00	1700	Os01g0658700	81,95	0,00E+00	957	-743	Indica	Indica	
maker-5-augustus-gene-294.62-mRNA-1	5	OsR498G0511367500.01.T01	100,00	0,00E+00	1294	Os05g0569500	99,86	0,00E+00	1289	-5	NA	Indica	

maker-5-augustus-gene-294.64-mRNA-1	5	OsR498G0511360600.01.T01	100,00	0,00E+00	1359	Os01g0662800	72,12	4,05E-109	394	-965	Indica	Indica	
maker-5-augustus-gene-294.66-mRNA-1	5	OsR498G0511365200.01.T02	100,00	0,00E+00	2370	Os05g0568900	99,70	0,00E+00	2352	-18	NA	Indica	
maker-5-augustus-gene-294.74-mRNA-1	5	OsR498G0511370300.01.T01	94,20	0,00E+00	1597	Os05g0569700	92,75	0,00E+00	1264	-333	Indica	Indica	
maker-5-augustus-gene-295.0-mRNA-1	5	OsR498G0204379800.01.T04	81,35	7,90E-180	630	Os05g0569900	100,00	0,00E+00	1175	545	Japonica	NA	
maker-5-augustus-gene-295.11-mRNA-1	5	OsR498G0511371400.01.T01	100,00	0,00E+00	985	Os05g0570900	99,45	0,00E+00	972	-13	NA	Indica	
maker-5-augustus-gene-295.13-mRNA-1	5	OsR498G0511371600.01.T02	95,61	0,00E+00	3995	Os05g0571000	95,37	0,00E+00	3968	-27	NA	Indica	
maker-5-augustus-gene-295.4-mRNA-1	5	OsR498G0511368600.01.T02	100,00	0,00E+00	1321	Os05g0569800	99,86	0,00E+00	1316	-5	NA	Indica	
maker-5-augustus-gene-295.6-mRNA-1	5	OsR498G0511370300.01.T01	99,89	0,00E+00	3281	Os05g0573300	71,91	0,00E+00	884	-2397	Indica	Indica	
maker-5-augustus-gene-295.9-mRNA-1	5	OsR498G0511368600.01.T02	100,00	0,00E+00	1321	Os05g0569800	99,86	0,00E+00	1316	-5	NA	NA	
maker-5-augustus-gene-296.37-mRNA-1	5	OsR498G0511377300.01.T04	99,92	0,00E+00	2231	Os05g0573200	99,76	0,00E+00	2222	-9	NA	Indica	
maker-5-augustus-gene-296.41-mRNA-1	5	OsR498G0511372800.01.T01	97,28	0,00E+00	2315	Os05g0571300	98,04	0,00E+00	3437	1122	Japonica	NA	
maker-5-augustus-gene-296.43-mRNA-1	5	OsR498G0511373200.01.T02	98,75	0,00E+00	976	Os05g0571300	88,87	0,00E+00	683	-293	Indica	NA	
maker-5-augustus-gene-296.46-mRNA-1	5	OsR498G0511373400.01.T02	99,80	0,00E+00	916	Os05g0571400	99,80	0,00E+00	916	0	NA	NA	
maker-5-augustus-gene-296.50-mRNA-1	5	OsR498G0511373800.01.T01	99,92	0,00E+00	2278	Os05g0571700	99,92	0,00E+00	2278	0	NA	NA	
maker-5-augustus-gene-296.53-mRNA-1	5	OsR498G0511375800.01.T01	99,83	0,00E+00	2058	Os05g0572700	99,91	0,00E+00	2063	5	NA	Indica	
maker-5-augustus-gene-296.56-mRNA-1	5	OsR498G0511376800.01.T01	99,92	0,00E+00	2171	Os05g0573000	99,85	0,00E+00	1167	-1004	Indica	Indica	
maker-5-augustus-gene-296.60-mRNA-1	5	OsR498G0511377600.01.T02	99,95	0,00E+00	3378	Os05g0573300	97,33	0,00E+00	3195	-183	NA	Indica	
maker-5-augustus-gene-297.46-mRNA-1	5	OsR498G0511379100.01.T02	97,25	0,00E+00	737	Os05g0573500	96,48	0,00E+00	706	-31	NA	Indica	
maker-5-augustus-gene-297.51-mRNA-1	5	OsR498G0511381100.01.T01	99,76	0,00E+00	2216	Os05g0574000	99,21	0,00E+00	1125	-1091	Indica	Indica	
maker-5-augustus-gene-297.53-mRNA-1	5	OsR498G0511385100.01.T01	99,95	0,00E+00	3789	Os05g0574800	99,84	0,00E+00	4571	782	Japonica	Indica	
maker-5-augustus-gene-297.54-mRNA-1	5	OsR498G0511387900.01.T01	99,69	0,00E+00	5133	Os05g0575300	99,53	0,00E+00	3006	-2127	Indica	Indica	
maker-5-augustus-gene-297.59-mRNA-1	5	OsR498G0511388300.01.T02	99,86	0,00E+00	2585	Os05g0575400	100,00	0,00E+00	1606	-979	Indica	Japonica	
maker-5-augustus-gene-297.65-mRNA-1	5	OsR498G0511380000.01.T01	99,93	0,00E+00	2662	Os05g0573700	99,93	0,00E+00	2662	0	NA	Indica	
maker-5-augustus-gene-297.74-mRNA-1	5	OsR498G0511381600.01.T01	99,92	0,00E+00	2204	Os05g0574100	99,76	0,00E+00	2195	-9	NA	Indica	
maker-5-augustus-gene-297.76-mRNA-1	5	OsR498G0511383800.01.T01	99,90	0,00E+00	1841	Os05g0574400	99,71	0,00E+00	1832	-9	NA	Indica	
maker-5-augustus-gene-297.79-mRNA-1	5	OsR498G0511384000.01.T01	100,00	0,00E+00	1202	Os05g0574500	99,85	0,00E+00	1197	-5	NA	Indica	
maker-5-augustus-gene-297.82-mRNA-1	5	OsR498G0511384800.01.T04	100,00	1,19E-169	596	Os05g0574700	100,00	4,16E-170	596	0	NA	Indica	
maker-5-augustus-gene-298.40-mRNA-1	5	OsR498G0511392300.01.T01	99,79	0,00E+00	1709	Os05g0576800	99,48	0,00E+00	1695	-14	NA	Indica	Kinase-TM
maker-5-augustus-gene-298.45-mRNA-1	5	OsR498G0511395300.01.T01	99,89	0,00E+00	1614	Os05g0578000	99,88	0,00E+00	1468	-146	NA	Indica	
maker-5-augustus-gene-298.53-mRNA-1	5	OsR498G0511393400.01.T01	99,94	0,00E+00	3199	Os01g0643300	76,08	0,00E+00	1262	-1937	Indica	Indica	
maker-5-augustus-gene-298.55-mRNA-1	5	OsR498G0511394200.01.T05	99,59	0,00E+00	2590	Os05g0577200	99,76	0,00E+00	2232	-358	Indica	Indica	
maker-5-augustus-gene-298.59-mRNA-1	5	OsR498G0511394600.01.T02	100,00	0,00E+00	2067	Os05g0577700	100,00	0,00E+00	2067	0	NA	Indica	Kinase-TM
maker-5-augustus-gene-298.63-mRNA-1	5	OsR498G0511395000.01.T03	99,85	0,00E+00	1196	Os05g0577800	99,85	0,00E+00	1196	0	NA	NA	
maker-5-augustus-gene-298.68-mRNA-1	5	OsR498G0511395200.01.T01	98,80	0,00E+00	1606	Os05g0577900	98,48	0,00E+00	1592	-14	NA	Indica	
maker-5-augustus-gene-299.16-mRNA-1	5	OsR498G0511397800.01.T02	100,00	0,00E+00	2895	Os05g0578600	99,79	0,00E+00	1685	-1210	Indica	Indica	
maker-5-augustus-gene-299.20-mRNA-1	5	OsR498G0511399000.01.T02	100,00	0,00E+00	1474	Os05g0579000	100,00	0,00E+00	1474	0	NA	Indica	
maker-5-augustus-gene-299.26-mRNA-1	5	OsR498G0511401000.01.T02	100,00	5,50E-139	493	Os05g0579600	100,00	1,92E-139	493	0	NA	Japonica	
maker-5-augustus-gene-299.28-mRNA-1	5	OsR498G0511401400.01.T01	99,70	1,85E-171	602	Os05g0579600	78,04	2,94E-43	175	-427	Indica	Japonica	
maker-5-augustus-gene-299.30-mRNA-1	5	OsR498G0511403400.01.T04	100,00	0,00E+00	1981	Os05g0580000	100,00	0,00E+00	1981	0	NA	Japonica	
maker-5-augustus-gene-299.6-mRNA-1	5	OsR498G0511400500.01.T01	88,40	1,90E-110	399	Os05g0579500	100,00	5,43E-150	529	130	Japonica	Japonica	
maker-5-augustus-gene-299.8-mRNA-1	5	OsR498G0511401700.01.T03	99,54	0,00E+00	780	Os05g0579800	99,56	0,00E+00	801	21	NA	Japonica	
maker-5-augustus-gene-3.10-mRNA-1	5	OsR498G0509571700.01.T05	100,00	0,00E+00	948	Os05g0105500	100,00	0,00E+00	677	-271	Indica	NA	
maker-5-augustus-gene-3.2-mRNA-1	5	OsR498G0509570500.01.T01	100,00	0,00E+00	1196	Os05g0105100	100,00	0,00E+00	1196	0	NA	NA	
maker-5-augustus-gene-3.24-mRNA-1	5	OsR498G0509573300.01.T01	100,00	0,00E+00	2084	Os05g0106200	100,00	0,00E+00	2084	0	NA	Japonica	
maker-5-augustus-gene-3.31-mRNA-1	5	OsR498G0509569200.01.T01	100,00	0,00E+00	1543	Os05g0104800	100,00	0,00E+00	1543	0	NA	NA	
maker-5-augustus-gene-3.38-mRNA-1	5	OsR498G0509569600.01.T01	100,00	0,00E+00	5871	Os05g0105000	100,00	0,00E+00	5871	0	NA	NA	
maker-5-augustus-gene-3.41-mRNA-1	5	OsR498G0509571000.01.T03	100,00	0,00E+00	1112	Os05g0105200	100,00	1,07E-178	625	-487	Indica	NA	
maker-5-augustus-gene-3.47-mRNA-1	5	OsR498G0509572200.01.T01	100,00	0,00E+00	1981	Os05g0105600	100,00	0,00E+00	1979	-2	NA	NA	
maker-5-augustus-gene-3.53-mRNA-1	5	OsR498G0509572800.01.T01	100,00	0,00E+00	904	Os05g0106100	100,00	0,00E+00	904	0	NA	NA	
maker-5-augustus-gene-30.0-mRNA-1	5	OsR498G0509751300.01.T07	94,24	0,00E+00	2325	Os05g0153000	94,58	0,00E+00	2348	23	NA	Japonica	
maker-5-augustus-gene-30.11-mRNA-1	5	OsR498G0509752400.01.T01	96,59	0,00E+00	2742	Os05g0153300	97,13	0,00E+00	2779	37	NA	Japonica	
maker-5-augustus-gene-30.9-mRNA-1	5	OsR498G0509696100.01.T04	94,14	6,13E-109	395	Os05g0153800	99,63	6,52E-135	480	85	Japonica	Japonica	
maker-5-augustus-gene-300.30-mRNA-1	5	OsR498G0511404300.01.T01	99,72	0,00E+00	3134	Os05g0580500	100,00	0,00E+00	3296	162	NA	Japonica	
maker-5-augustus-gene-300.37-mRNA-1	5	OsR498G0511373800.01.T01	89,56	0,00E+00	818	Os05g0581100	100,00	0,00E+00	1113	295	Japonica	Japonica	
maker-5-augustus-gene-300.50-mRNA-1	5	OsR498G0511408600.01.T03	99,07	0,00E+00	1333	Os05g0581900	96,18	0,00E+00	1306	-27	NA	Japonica	
maker-5-augustus-gene-300.52-mRNA-1	5	OsR498G0511409600.01.T02	99,87	0,00E+00	2684	Os05g0582100	100,00	0,00E+00	2634	-50	NA	Japonica	
maker-5-augustus-gene-301.0-mRNA-1	5	OsR498G0511410300.01.T01	99,84	0,00E+00	2177	Os12g0271700	84,41	0,00E+00	1210	-967	Indica	Japonica	
maker-5-augustus-gene-301.13-mRNA-1	5	OsR498G0511415500.01.T01	99,63	0,00E+00	953	Os05g0584000	99,89	0,00E+00	1673	720	Japonica	NA	
maker-5-augustus-gene-301.19-mRNA-1	5	OsR498G0511415200.01.T01	99,66	0,00E+00	2094	Os05g0583900	100,00	0,00E+00	2053	-41	NA	Japonica	
maker-5-augustus-gene-301.21-mRNA-1	5	OsR498G0511416200.01.T02	100,00	0,00E+00	823	Os05g0584200	100,00	0,00E+00	823	0	NA	NA	
maker-5-augustus-gene-301.4-mRNA-1	5	OsR498G0511410500.01.T01	99,85	0,00E+00	3681	Os01g0629900	78,42	0,00E+00	1490	-2191	Indica	Japonica	Kinase-TM
maker-5-augustus-gene-301.9-mRNA-1	5	OsR498G0511413900.01.T01	99,77	0,00E+00	6116	Os05g0583500	100,00	0,00E+00	6152	36	NA	Japonica	
maker-5-augustus-gene-302.0-mRNA-1	5	OsR498G0511420500.01.T02	99,81	0,00E+00	2892	Os05g0584900	99,92	0,00E+00	2177	-715	Indica	Japonica	
maker-5-augustus-gene-302.14-mRNA-1	5	OsR498G0511422000.01.T02	99,33	0,00E+00	2909	Os05g0585500	100,00	0,00E+00	2938	29	NA	Japonica	Kinase-TM
maker-5-augustus-gene-302.19-mRNA-1	5	OsR498G0511422400.01.T02	99,78	0,00E+00	824	Os05g0585700	100,00	0,00E+00	637	-187	Indica	Japonica	
maker-5-augustus-gene-302.2-mRNA-1	5	OsR498G0511421500.01.T05	100,00	0,00E+00	1361	Os05g0585400	100,00	0,00E+00	1361	0	NA	Japonica	
maker-5-augustus-gene-302.21-mRNA-1	5	OsR498G0511423000.01.T02	100,00	0,00E+00	1927	Os05g0585900	100,00	0,00E+00	1927	0	NA	Japonica	
maker-5-augustus-gene-302.5-mRNA-1	5	OsR498G0511417200.01.T01	98,96	0,00E+00	1525	Os05g0584400	97,48	0,00E+00	807	-718	Indica	NA	
maker-5-augustus-gene-302.7-mRNA-1	5	OsR498G0511418600.01.T02	99,84	0,00E+00	4614	Os05g0584600	100,00	0,00E+00	4632	18	NA	Japonica	
maker-5-augustus-gene-303.0-mRNA-1	5	OsR498G0511425100.01.T02	99,77	0,00E+00	3131	Os05g0586200	99,89	0,00E+00	3140	9	NA	Japonica	
maker-5-augustus-gene-303.19-mRNA-1	5	OsR498G0511427800.01.T01	99,63	0,00E+00	2407	Os05g0587000	99,93	0,00E+00	2425	18	NA	Japonica	
maker-5-augustus-gene-303.2-mRNA-1	5	OsR498G0511425700.01.T01	99,74	0,00E+00	4103	Os05g0586400	99,96	0,00E+00	4122	19	NA	Japonica	
maker-5-augustus-gene-303.23-mRNA-1	5	OsR498G0511428000.01.T01	99,93	0,00E+00	2658	Os05g0587100	100,00	0,00E+00	2663	5	NA	Japonica	
maker-5-augustus-gene-303.27-mRNA-1	5	OsR498G0511428600.01.T01	99,86	0,00E+00	3753	Os05g0587300	100,00	0,00E+00	2911	-842	Indica	Japonica	
maker-5-augustus-gene-303.32-mRNA-1	5	OsR498G0511430200.01.T03	99,61	0,00E+00	1828	Os05g0587400	100,00	0,00E+00	1846	18	NA	Japonica	
maker-5-augustus-gene-303.4-mRNA-1	5	OsR498G0511426100.01.T01	99,86	0,00E+00	2599	Os05g0586500	100,00	0,00E+00	2608	9	NA	Japonica	
maker-5-augustus-gene-303.6-mRNA-1	5	OsR498G0511426300.01.T02	99,80	0,00E+00	2698	Os05g0586600	99,93	0,00E+00	2707	9	NA	Japonica	
maker-5-augustus-gene-303.9-mRNA-1	5	OsR498G0511426500.01.T01	99,50	0,00E+00	1060	Os05g0586700	100,00	0,00E+00	1662	602	Japonica	Japonica	
maker-5-augustus-gene-304.45-mRNA-1	5	OsR498G0511434300.01.T01	99,81	0,00E+00	1896	Os05g0589000	100,00	0,00E+00	2051	155	NA	Japonica	
maker-5-augustus-gene-304.51-mRNA-1	5	OsR498G0511434500.01.T06	99,64	0,00E+00	2993	Os05g0589200	100,00	0,00E+00	2513	-480	Indica	Japonica	
maker-5-augustus-gene-304.58-mRNA-1	5	OsR498G0511435300.01.T01	99,48	0,00E+00	2051	Os05g0589600	100,00	0,00E+00	2078	27	NA	Japonica	
maker-5-augustus-gene-304.65-mRNA-1	5	OsR498G0511430600.01.T02	99,45	0,00E+00	2263	Os05g0587700	100,00	0,00E+00	1514	-749	Indica	Japonica	
maker-5-augustus-gene-304.69-mRNA-1	5	OsR498G0511431200.01.T01	99,74	0,00E+00	1371	Os01g0612500	81,36	0,00E+00	648	-723	Indica	Japonica	
maker-5-augustus-gene-304.75-mRNA-1	5	OsR498G0511432600.01.T01	99,44	0,00E+00	1270	Os05g0588500	100,00	0,00E+00	1288	18	NA	Japonica	
maker-5-augustus-gene-304.83-mRNA-1	5	OsR498G0511434000.01.T01	99,79	0,00E+00	2584	Os05g0588850	100,00	0,00E+00	2598	14	NA	Japonica	
maker-5-augustus-gene-304.85-mRNA-1	5	OsR498G0511434200.01.T01	96,50	0,00E+00	2431	Os01g0605100	74,70	7,18E-179	627	-1804	Indica	Japonica	
maker-5-augustus-gene-304.87-mRNA-1	5	OsR498G0511435200.01.T01	98,40	0,00E+00	1415	Os05g0589400	100,00	0,00E+00	1467	52	NA	NA	
maker-5-augustus-gene-305.39-mRNA-1	5	OsR498G0511436300.01.T01	99,77	0,00E+00	3130	Os05g0590100	99,94	0,00E+00	3172	42	NA	Japonica	
maker-5-augustus-gene-305.43-mRNA-1	5	OsR498G0511441300.01.T01	99,26	0,00E+00	1430	Os05g0591600	96,74	0,00E+00	1392	-38	NA	Japonica	
maker-5-augustus-gene-305.47-mRNA-1	5	OsR498G0511436800.01.T01	99,89	0,00E+00	1619	Os05g0590300	100,00	0,00E+00	1624	5	NA	Japonica	
maker-5-augustus-gene-306.13-mRNA-1	5	OsR498G0511445300.01.T07	97,32	0,00E+00	3928	Os05g0592500	97,49	0,00E+00	3946	18	NA	Japonica	
maker-5-augustus-gene-306.19-mRNA-1	5	OsR498G0511443400.01.T04	100,00	0,00E+00	1656	Os05g0591900	100,00	0,00E+00	1564	-92	NA	Japonica	
maker-5-augustus-gene-306.2-mRNA-1	5	OsR498G0511444700.01.T03	99,76	0,00E+00	1480	Os05g0592300	100,00	0,00E+00	1489	9	NA	Japonica	
maker-5-augustus-gene-306.22-mRNA-1	5	OsR498G0511444000.01.T01	99,91	0,00E+00	2090	Os05g0592100	100,00	0,00E+00	2094	4	NA	Japonica	
maker-5-augustus-gene-306.27-mRNA-1	5	OsR498G0511445600.01.T02	99,66	0,00E+00	6925	Os05g0592600	100,00	0,00E+00	6459	-466	NA	Japonica	
maker-5-augustus-gene-306.32-mRNA-1	5	OsR498G0511446600.01.T01	99,11	0,00E+00	2379	Os05g0592800	100,00	0,00E+00	2414	35	NA	Japonica	
maker-5-augustus-gene-306.36-mRNA-1	5	OsR498G0511447000.01.T01	100,00	0,00E+00	2046	Os05g0593100	100,00	0,00E+00	2046	0	NA	Japonica	
maker-5-augustus-gene-306.42-mRNA-1	5	OsR498G0511447200.01.T01	99,79	0,00E+00	856	Os05g0593200	100,00	0,00E+00	861	5	NA	Japonica	
maker-5-augustus-gene-306.8-mRNA-1	5	OsR498G0511445100.01.T02	98,11	0,00E+00	5628	Os05g0592400	98,29	0,00E+00	5655	27	NA	Japonica	
maker-5-augustus-gene-307.0-mRNA-1	5	OsR498G0511451100.01.T06	100,00	0,00E+00	2333	Os05g0594700	100,00	0,00E+00	2333	0	NA	NA	
maker-5-augustus-gene-307.15-mRNA-1	5	OsR498G0511456100.01.T04	100,00	0,00E+00	1673	Os05g0595300	100,00	0,00E+00	1673	0	NA	NA	
maker-5-augustus-gene-307.22-mRNA-1	5	OsR498G0511449600.01.T01	98,60	0,00E+00	3387	Os05g0594500	98,60	0,00E+00	3387	0	NA	NA	
maker-5-augustus-gene-307.27-mRNA-1	5	OsR498G0511451400.01.T03	100,00	0,00E+00	1620	Os05g0594800	100,00	0,00E+00	1620	0	NA	NA	
maker-5-augustus-gene-307.33-mRNA-1	5	OsR498G0511456600.01.T01	100,00	0,00E+00	1299	Os05g0595400	100,00	0,00E+00	1299	0	NA	NA	
maker-5-augustus-gene-307.38-mRNA-1	5	OsR498G0511456800.01.T02	100,00	0,00E+00	1314	Os05g0595500	100,00	0,00E+00	1314	0	NA	NA	
maker-5-augustus-gene-307.9-mRNA-1	5	OsR498G0511452700.01.T01	100,00	0,00E+00	1921	Os05g0595100	100,00	0,00E+00	1921	0	NA	NA	
maker-5-augustus-gene-308.55-mRNA-1	5	OsR498G0511462100.01.T01	99,94	0,00E+00	2856	Os05g0597400	100,00	0,00E+00	2861	5	NA	NA	
maker-5-augustus-gene-308.58-mRNA-1	5	OsR498G0511457400.01.T01	100,00	0,00E+00	5595	Os05g0595950	99,90	0,00E+00	5580	-15	NA	NA	Kinase-LRR-TM
maker-5-augustus-gene-308.61-mRNA-1	5	OsR498G0511458800.01.T02	100,00	0,00E+00	893	Os05g0596200	100,00	5,39E-156	549	-344	Indica	NA	
maker-5-augustus-gene-308.66-mRNA-1	5	OsR498G0511460400.01.T01	99,44	0,00E+00	5731	Os05g0596600	99,44	0,00E+00	5731	0	NA	NA	
maker-5-augustus-gene-308.73-mRNA-1	5	OsR498G0511462000.01.T01	100,00	0,00E+00	1591	Os05g0597100	99,89	0,00E+00	1587	-4	NA	NA	
maker-5-augustus-gene-31.23-mRNA-1	5	OsR498G0509767300.01.T01	99,61	0,00E+00	1361	Os05g0154800	100,00	0,00E+00	1375	14	NA	Japonica	
maker-5-augustus-gene-31.26-mRNA-1	5	OsR498G0509763000.01.T01	99,75	0,00E+00	709	Os05g0154300	99,77	0,00E+00	770	61	NA	Japonica	LRR-TM
maker-5-augustus-gene-31.28-mRNA-1	5	OsR498G0509766400.01.T01	97,18	0,00E+00	2711	Os05g0154500	97,37	0,00E+00	2725	14	NA	Japonica	
maker-5-augustus-gene-31.35-mRNA-1	5	OsR498G0509767600.01.T01	100,00	0,00E+00	796	Os05g0154900	100,00	0,00E+00	796	0	NA	Japonica	
maker-5-augustus-gene-312.40-mRNA-1	5	OsR498G0511415500.01.T01	99,63	0,00E+00	953	Os05g0584000	99,89	0,00E+00	1673	720	Japonica	NA	
maker-5-augustus-gene-312.43-mRNA-1	5	OsR498G0815920400.01.T01	99,49	0,00E+00	2801	Os05g0310800	85,80	0,00E+00	1824	-977	Indica	NA	
maker-5-augustus-gene-312.48-mRNA-1	5	OsR498G0511123000.01.T01	99,88	0,00E+00	1527	Os05g0503000	100,00	0,00E+00	1532	5	NA	NA	
maker-5-augustus-gene-312.53-mRNA-1	5	OsR498G0511416200.01.T02	100,00	0,00E+00	823	Os05g0584200	100,00	0,00E+00	823	0	NA	NA	
maker-5-augustus-gene-312.55-mRNA-1	5	OsR498G0511417200.01.T01	98,96	0,00E+00	1525	Os05g0584400	97,48	0,00E+00	807	-718	Indica	NA	
maker-5-augustus-gene-32.0-mRNA-1	5	OsR498G0509768100.01.T01	99,82	0,00E+00	1019	Os05g0155100	100,00	0,00E+00	1023	4	NA	Japonica	
maker-5-augustus-gene-32.11-mRNA-1	5	OsR498G0509772300.01.T01	99,65	0,00E+00	1551	Os05g0156200	100,00	0,00E+00	1564	13	NA	Japonica	
maker-5-augustus-gene-32.20-mRNA-1	5	OsR498G0509768000.01.T04	99,81	0,00E+00	1909	Os05g0155000	100,00	0,00E+00	1918	9	NA	Japonica	
maker-5-augustus-gene-32.26-mRNA-1	5	OsR498G0509768400.01.T02	99,72	0,00E+00	1939	Os05g0155200	100,00	0,00E+00	1952	13	NA	Japonica	
maker-5-augustus-gene-32.29-mRNA-1	5	OsR498G0509769800.01.T01	99,69	0,00E+00	2873	Os05g0155500	100,00	0,00E+00	2895	22	NA	Japonica	
maker-5-augustus-gene-32.3-mRNA-1	5	OsR498G0509768700.01.T01	99,76	0,00E+00	1469	Os05g0155300	100,00	0,00E+00	1478	9	NA	Japonica	
maker-5-augustus-gene-32.34-mRNA-1	5	OsR498G0509772600.01.T02	99,82	0,00E+00	1977	Os05g0156300	100,00	0,00E+00	1986	9	NA	Japonica	
maker-5-augustus-gene-32.40-mRNA-1	5	OsR498G0509773400.01.T02	100,00	0,00E+00	2544	Os05g0156600	100,00	0,00E+00	2544	0	NA	Japonica	
maker-5-augustus-gene-32.7-mRNA-1	5	OsR498G0509769900.01.T01	95,54	0,00E+00	1639	Os05g0155700	95,64	0,00E+00	1644	5	NA	Japonica	
maker-5-augustus-gene-33.0-mRNA-1	5	OsR498G0509774100.01.T01	99,47	0,00E+00	4335	Os01g0337600	72,41	0,00E+00	1439	-2896	Indica	Japonica	
maker-5-augustus-gene-33.21-mRNA-1	5	OsR498G0509773600.01.T02	99,83	0,00E+00	3185	Os01g0337900	86,21	0,00E+00	1819	-1366	Indica	Japonica	
maker-5-augustus-gene-33.26-mRNA-1	5	OsR498G0509774400.01.T01	98,44	0,00E+00	4013	Os05g0156900	100,00	0,00E+00	4172	159	NA	Japonica	
maker-5-augustus-gene-33.28-mRNA-1	5	OsR498G0509774800.01.T01	99,21	0,00E+00	892	Os05g0157200	100,00	0,00E+00	910	18	NA	Japonica	
maker-5-augustus-gene-33.31-mRNA-1	5	OsR498G0509775000.01.T02	99,64	0,00E+00	1495	Os05g0157300	99,64	0,00E+00	1495	0	NA	Japonica	
maker-5-augustus-gene-33.5-mRNA-1	5	OsR498G0509780300.01.T01	80,54	0,00E+00	663	Os05g0158200	80,69	0,00E+00	667	4	NA	Japonica	
maker-5-augustus-gene-33.8-mRNA-1	5	OsR498G0509776500.01.T01	99,93	0,00E+00	2517	Os05g0157600	99,62	0,00E+00	2810	293	Japonica	Japonica	
maker-5-augustus-gene-34.22-mRNA-1	5	OsR498G0509780300.01.T01	99,65	0,00E+00	2526	Os05g0158200	100,00	0,00E+00	2549	23	NA	Japonica	
maker-5-augustus-gene-34.28-mRNA-1	5	OsR498G0509781100.01.T01	99,93	0,00E+00	2494	Os05g0158400	99,93	0,00E+00	2494	0	NA	Japonica	
maker-5-augustus-gene-34.33-mRNA-1	5	OsR498G0509783500.01.T01	100,00	0,00E+00	2073	Os05g0158600	100,00	0,00E+00	2073	0	NA	Japonica	
maker-5-augustus-gene-34.35-mRNA-1	5	OsR498G0509784700.01.T01	99,91	0,00E+00	4159	Os05g0159000	100,00	0,00E+00	5579	1420	Japonica	Japonica	
maker-5-augustus-gene-34.41-mRNA-1	5	OsR498G0509784900.01.T02	100,00	0,00E+00	1040	Os05g0159100	100,00	0,00E+00	998	-42	NA	Japonica	
maker-5-augustus-gene-34.45-mRNA-1	5	OsR498G0509781400.01.T02	99,59	0,00E+00	2588	Os05g0158500	100,00	0,00E+00	2614	26	NA	Japonica	
maker-5-augustus-gene-34.50-mRNA-1	5	OsR498G0509785200.01.T01	100,00	0,00E+00	2338	Os05g0159200	100,00	0,00E+00	2338	0	NA	Japonica	
maker-5-augustus-gene-35.0-mRNA-1	5	OsR498G0509787700.01.T01	100,00	0,00E+00	2257	Os01g0328700	80,99	0,00E+00	1178	-1079	Indica	Japonica	
maker-5-augustus-gene-35.16-mRNA-1	5	OsR498G0509791800.01.T01	98,80	0,00E+00	1607	Os05g0160600	100,00	0,00E+00	1595	-12	NA	Japonica	
maker-5-augustus-gene-35.2-mRNA-1	5	OsR498G0509787900.01.T01	100,00	0,00E+00	2203	Os05g0160100	100,00	0,00E+00	2203	0	NA	NA	
maker-5-augustus-gene-35.8-mRNA-1	5	OsR498G0509792300.01.T01	99,58	0,00E+00	1701	Os05g0160800	99,69	0,00E+00	1706	5	NA	Japonica	
maker-5-augustus-gene-36.36-mRNA-1	5	OsR498G0509792700.01.T01	99,83	0,00E+00	3147	Os05g0161200	100,00	0,00E+00	3160	13	NA	Japonica	
maker-5-augustus-gene-36.39-mRNA-1	5	OsR498G0509792900.01.T06	99,81	0,00E+00	1921	Os05g0161400	100,00	0,00E+00	1930	9	NA	Japonica	
maker-5-augustus-gene-36.44-mRNA-1	5	OsR498G0509793900.01.T01	99,70	0,00E+00	3008	Os05g0161500	100,00	0,00E+00	3030	22	NA	Japonica	
maker-5-augustus-gene-36.46-mRNA-1	5	OsR498G0509794300.01.T01	99,19	0,00E+00	3074	Os05g0161800	99,25	0,00E+00	3078	4	NA	Indica	
maker-5-augustus-gene-36.55-mRNA-1	5	OsR498G0509795000.01.T01	99,35	0,00E+00	1917	Os05g0162000	99,07	0,00E+00	1903	-14	NA	NA	
maker-5-augustus-gene-36.57-mRNA-1	5	OsR498G0509795200.01.T01	99,91	0,00E+00	1969	Os01g0155400	77,55	0,00E+00	847	-1122	Indica	NA	
maker-5-augustus-gene-36.69-mRNA-1	5	OsR498G0509797800.01.T01	100,00	0,00E+00	1402	Os05g0163100	100,00	0,00E+00	1402	0	NA	Japonica	
maker-5-augustus-gene-37.0-mRNA-1	5	OsR498G0509801500.01.T02	99,88	0,00E+00	1468	Os05g0163900	99,63	0,00E+00	1459	-9	NA	NA	
maker-5-augustus-gene-37.12-mRNA-1	5	OsR498G0509800400.01.T02	99,85	0,00E+00	2324	Os05g0163700	99,92	0,00E+00	2328	4	NA	Japonica	
maker-5-augustus-gene-37.22-mRNA-1	5	OsR498G0509804000.01.T02	100,00	0,00E+00	2143	Os05g0164800	100,00	0,00E+00	2143	0	NA	Japonica	
maker-5-augustus-gene-37.24-mRNA-1	5	OsR498G0509804600.01.T01	99,87	0,00E+00	5725	Os05g0164900	99,97	0,00E+00	5677	-48	NA	Japonica	
maker-5-augustus-gene-37.3-mRNA-1	5	OsR498G0509801900.01.T06	99,87	0,00E+00	4169	Os05g0164100	99,79	0,00E+00	4164	-5	NA	Indica	
maker-5-augustus-gene-37.9-mRNA-1	5	OsR498G0509799000.01.T04	99,75	0,00E+00	2872	Os05g0163400	100,00	0,00E+00	2890	18	NA	Japonica	
maker-5-augustus-gene-38.32-mRNA-1	5	OsR498G0509807300.01.T05	99,71	8,68E-179	627	Os05g0165400	100,00	0,00E+00	1094	467	Japonica	Japonica	
maker-5-augustus-gene-38.35-mRNA-1	5	OsR498G0509807600.01.T01	96,01	0,00E+00	1075	Os05g0165450	96,75	0,00E+00	1241	166	Japonica	Japonica	
maker-5-augustus-gene-39.0-mRNA-1	5	OsR498G0509820300.01.T01	97,67	0,00E+00	1166	Os05g0168400	99,85	0,00E+00	1230	64	NA	Japonica	
maker-5-augustus-gene-39.10-mRNA-1	5	OsR498G0509819800.01.T01	94,21	0,00E+00	1209	Os05g0168200	98,07	0,00E+00	1335	126	NA	NA	
maker-5-augustus-gene-39.14-mRNA-1	5	OsR498G0509820000.01.T02	99,31	0,00E+00	1282	Os05g0168300	99,86	0,00E+00	1300	18	NA	Japonica	
maker-5-augustus-gene-39.20-mRNA-1	5	OsR498G0509822000.01.T03	100,00	0,00E+00	921	Os05g0168700	100,00	0,00E+00	921	0	NA	Japonica	
maker-5-augustus-gene-39.26-mRNA-1	5	OsR498G0509823000.01.T01	100,00	0,00E+00	720	Os05g0169000	100,00	0,00E+00	720	0	NA	Japonica	
maker-5-augustus-gene-39.29-mRNA-1	5	OsR498G0509825000.01.T01	100,00	0,00E+00	3020	Os05g0169400	100,00	0,00E+00	2718	-302	Indica	NA	
maker-5-augustus-gene-39.3-mRNA-1	5	OsR498G0509823300.01.T02	99,85	0,00E+00	1168	Os05g0169100	99,85	0,00E+00	1168	0	NA	Japonica	
maker-5-augustus-gene-39.6-mRNA-1	5	OsR498G0509823500.01.T03	100,00	0,00E+00	2073	Os05g0169200	100,00	0,00E+00	2073	0	NA	NA	
maker-5-augustus-gene-39.8-mRNA-1	5	OsR498G0509824100.01.T01	100,00	0,00E+00	2392	Os05g0169300	100,00	0,00E+00	2392	0	NA	NA	
maker-5-augustus-gene-4.28-mRNA-1	5	OsR498G0509574300.01.T01	99,86	0,00E+00	1246	Os05g0106500	99,86	0,00E+00	1246	0	NA	NA	
maker-5-augustus-gene-4.40-mRNA-1	5	OsR498G0102771100.01.T01	71,01	1,05E-46	187	Os05g0107700	84,11	1,67E-108	391	204	Japonica	Japonica	
maker-5-augustus-gene-4.44-mRNA-1	5	OsR498G0509577900.01.T03	97,24	0,00E+00	921	Os05g0107900	97,05	0,00E+00	917	-4	NA	Japonica	
maker-5-augustus-gene-4.48-mRNA-1	5	OsR498G0509574600.01.T04	100,00	0,00E+00	2046	Os05g0106600	100,00	0,00E+00	2046	0	NA	NA	
maker-5-augustus-gene-4.55-mRNA-1	5	OsR498G0509576000.01.T01	99,60	0,00E+00	1327	Os05g0106900	100,00	0,00E+00	937	-390	Indica	Japonica	
maker-5-augustus-gene-4.58-mRNA-1	5	OsR498G0509576200.01.T01	99,34	0,00E+00	2949	Os05g0107000	100,00	0,00E+00	1929	-1020	Indica	Japonica	
maker-5-augustus-gene-4.60-mRNA-1	5	OsR498G0509577000.01.T01	99,91	0,00E+00	6113	Os05g0107600	99,97	0,00E+00	6122	9	NA	Japonica	
maker-5-augustus-gene-40.0-mRNA-1	5	OsR498G0509825300.01.T01	100,00	0,00E+00	6141	Os01g0170300	85,41	0,00E+00	3211	-2930	Indica	Japonica	Kinase-LRR-TM
maker-5-augustus-gene-40.11-mRNA-1	5	OsR498G0509832300.01.T01	99,84	0,00E+00	2242	Os05g0171300	100,00	0,00E+00	2251	9	NA	Japonica	
maker-5-augustus-gene-40.14-mRNA-1	5	OsR498G0509825800.01.T02	97,81	0,00E+00	2355	Os01g0170500	80,82	0,00E+00	1095	-1260	Indica	NA	
maker-5-augustus-gene-40.17-mRNA-1	5	OsR498G0509826200.01.T01	100,00	0,00E+00	2165	Os05g0170000	100,00	0,00E+00	2165	0	NA	NA	
maker-5-augustus-gene-40.2-mRNA-1	5	OsR498G0509827700.01.T01	100,00	0,00E+00	5335	Os05g0170300	100,00	0,00E+00	5335	0	NA	NA	LRR-TM
maker-5-augustus-gene-40.23-mRNA-1	5	OsR498G0509830600.01.T01	100,00	0,00E+00	969	Os05g0170700	100,00	0,00E+00	969	0	NA	NA	LRR-TM
maker-5-augustus-gene-40.26-mRNA-1	5	OsR498G0509830800.01.T02	100,00	0,00E+00	1629	Os05g0170800	100,00	0,00E+00	1629	0	NA	NA	
maker-5-augustus-gene-40.29-mRNA-1	5	OsR498G0509831400.01.T02	100,00	0,00E+00	4464	Os05g0171000	100,00	0,00E+00	3676	-788	Indica	NA	
maker-5-augustus-gene-40.4-mRNA-1	5	OsR498G0509831100.01.T03	100,00	0,00E+00	1874	Os01g0172100	75,84	3,82E-143	508	-1366	Indica	NA	
maker-5-augustus-gene-40.9-mRNA-1	5	OsR498G0509831700.01.T01	100,00	0,00E+00	980	Os05g0171200	100,00	0,00E+00	980	0	NA	NA	
maker-5-augustus-gene-41.0-mRNA-1	5	OsR498G0509835300.01.T04	100,00	0,00E+00	1131	Os05g0171900	100,00	0,00E+00	1131	0	NA	NA	
maker-5-augustus-gene-45.11-mRNA-1	5	OsR498G0511038100.01.T02	80,77	6,25E-82	304	Os05g0173700	100,00	2,64E-157	553	249	Japonica	NA	
maker-5-augustus-gene-45.13-mRNA-1	5	OsR498G0509840800.01.T04	100,00	0,00E+00	2578	Os05g0173500	99,83	0,00E+00	2098	-480	Indica	Indica	
maker-5-augustus-gene-45.16-mRNA-1	5	OsR498G0509847200.01.T01	99,85	0,00E+00	5805	Os05g0176100	99,94	0,00E+00	5818	13	NA	Japonica	
maker-5-augustus-gene-45.22-mRNA-1	5	OsR498G0509848000.01.T01	99,39	0,00E+00	2633	Os05g0176400	99,66	0,00E+00	2651	18	NA	NA	
maker-5-augustus-gene-45.25-mRNA-1	5	OsR498G0509848400.01.T02	99,75	0,00E+00	1447	Os05g0176500	99,88	0,00E+00	1452	5	NA	Indica	
maker-5-augustus-gene-45.30-mRNA-1	5	OsR498G0509849600.01.T01	96,49	0,00E+00	3430	Os05g0176800	98,10	0,00E+00	3546	116	NA	NA	
maker-5-augustus-gene-45.9-mRNA-1	5	OsR498G0509849900.01.T01	99,89	0,00E+00	1645	Os05g0177000	99,73	0,00E+00	1991	346	Japonica	Japonica	
maker-5-augustus-gene-46.40-mRNA-1	5	OsR498G0509852900.01.T01	99,80	0,00E+00	1793	Os05g0178000	99,93	0,00E+00	2712	919	Japonica	Japonica	
maker-5-augustus-gene-46.45-mRNA-1	5	OsR498G0509856700.01.T03	100,00	0,00E+00	2208	Os05g0179000	100,00	0,00E+00	2208	0	NA	NA	
maker-5-augustus-gene-46.47-mRNA-1	5	OsR498G0509853400.01.T01	98,96	0,00E+00	2033	Os05g0178100	100,00	0,00E+00	2084	51	NA	Japonica	
maker-5-augustus-gene-46.49-mRNA-1	5	OsR498G0509854200.01.T01	100,00	0,00E+00	1159	Os05g0178600	99,69	0,00E+00	1144	-15	NA	Indica	
maker-5-augustus-gene-47.29-mRNA-1	5	OsR498G0509864300.01.T03	99,79	0,00E+00	1680	Os05g0179800	100,00	0,00E+00	1689	9	NA	Japonica	
maker-5-augustus-gene-47.32-mRNA-1	5	OsR498G0509860600.01.T01	99,92	0,00E+00	2377	Os05g0179300	100,00	0,00E+00	2381	4	NA	Japonica	
maker-5-augustus-gene-47.36-mRNA-1	5	OsR498G0509865400.01.T01	99,45	0,00E+00	2569	Os05g0179900	100,00	0,00E+00	4307	1738	Japonica	Japonica	
maker-5-augustus-gene-48.0-mRNA-1	5	OsR498G0509868500.01.T01	99,26	0,00E+00	4535	Os05g0181000	99,96	0,00E+00	4617	82	NA	Japonica	
maker-5-augustus-gene-48.15-mRNA-1	5	OsR498G0509869000.01.T01	100,00	4,00E-180	630	Os01g0183633	78,28	8,81E-63	240	-390	Indica	Japonica	
maker-5-augustus-gene-48.3-mRNA-1	5	OsR498G0509867000.01.T01	97,76	0,00E+00	3799	Os05g0180400	100,00	0,00E+00	3555	-244	NA	Japonica	
maker-5-augustus-gene-48.6-mRNA-1	5	OsR498G0509867200.01.T02	97,78	0,00E+00	1683	Os05g0180500	100,00	0,00E+00	1443	-240	Indica	Japonica	
maker-5-augustus-gene-48.9-mRNA-1	5	OsR498G0509867800.01.T06	99,73	0,00E+00	1982	Os05g0180600	100,00	0,00E+00	1997	15	NA	Japonica	
maker-5-augustus-gene-49.0-mRNA-1	5	OsR498G0509872900.01.T03	89,29	0,00E+00	678	Os05g0182500	89,50	0,00E+00	682	4	NA	Japonica	
maker-5-augustus-gene-49.13-mRNA-1	5	OsR498G0509872400.01.T02	98,23	0,00E+00	1362	Os05g0182100	100,00	0,00E+00	1402	40	NA	Japonica	
maker-5-augustus-gene-49.18-mRNA-1	5	OsR498G0509873600.01.T01	100,00	0,00E+00	4313	Os05g0182800	100,00	0,00E+00	4313	0	NA	NA	
maker-5-augustus-gene-49.25-mRNA-1	5	OsR498G0509873800.01.T01	100,00	0,00E+00	2003	Os05g0182900	100,00	0,00E+00	1878	-125	NA	NA	

maker-5-augustus-gene-49.28-mRNA-1	5	OsR498G0509875200.01.T01	100,00	0,00E+00	1056	Os05g0183100	100,00	0,00E+00	1056	0	NA	NA	WRKY67
maker-5-augustus-gene-49.3-mRNA-1	5	OsR498G0509873100.01.T01	100,00	0,00E+00	3469	Os05g0182600	100,00	0,00E+00	3469	0	NA	NA	
maker-5-augustus-gene-49.7-mRNA-1	5	OsR498G0509873300.01.T02	100,00	0,00E+00	1553	Os05g0182700	100,00	0,00E+00	1553	0	NA	NA	
maker-5-augustus-gene-5.49-mRNA-1	5	OsR498G0509580700.01.T01	99,75	0,00E+00	721	Os07g0573600	86,24	3,12E-69	261	-460	Indica	Japonica	
maker-5-augustus-gene-5.50-mRNA-1	5	OsR498G0509580900.01.T01	99,91	0,00E+00	1939	Os05g0108600	100,00	0,00E+00	1943	4	NA	Japonica	
maker-5-augustus-gene-5.52-mRNA-1	5	OsR498G0509581100.01.T01	99,76	0,00E+00	743	Os05g0108800	100,00	0,00E+00	747	4	NA	Japonica	
maker-5-augustus-gene-5.58-mRNA-1	5	OsR498G0509585100.01.T03	99,55	0,00E+00	1573	Os05g0110300	100,00	0,00E+00	1591	18	NA	Japonica	
maker-5-augustus-gene-5.61-mRNA-1	5	OsR498G0509578200.01.T02	99,78	0,00E+00	2436	Os05g0108000	100,00	0,00E+00	2450	14	NA	Japonica	
maker-5-augustus-gene-5.65-mRNA-1	5	OsR498G0509578400.01.T02	99,71	0,00E+00	1242	Os05g0108100	99,86	0,00E+00	1246	4	NA	Japonica	
maker-5-augustus-gene-5.74-mRNA-1	5	OsR498G0509582000.01.T02	99,78	0,00E+00	3234	Os01g0971900	75,84	0,00E+00	1279	-1955	Indica	Japonica	
maker-5-augustus-gene-52.18-mRNA-1	5	OsR498G0509890000.01.T06	100,00	0,00E+00	753	Os05g0186000	91,37	1,05E-176	618	-135	Indica	NA	
maker-5-augustus-gene-52.20-mRNA-1	5	OsR498G0509890200.01.T01	100,00	0,00E+00	717	Os05g0186100	99,25	0,00E+00	707	-10	NA	NA	
maker-5-augustus-gene-52.23-mRNA-1	5	OsR498G0509891000.01.T01	100,00	0,00E+00	3090	Os05g0186300	100,00	0,00E+00	3090	0	NA	NA	
maker-5-augustus-gene-52.6-mRNA-1	5	OsR498G0509889700.01.T04	100,00	0,00E+00	3344	Os05g0185800	100,00	0,00E+00	3344	0	NA	NA	
maker-5-augustus-gene-52.9-mRNA-1	5	OsR498G0509888600.01.T03	100,00	0,00E+00	1249	Os05g0185700	100,00	0,00E+00	1249	0	NA	NA	
maker-5-augustus-gene-53.0-mRNA-1	5	OsR498G0509894900.01.T02	100,00	0,00E+00	1240	Os01g0190300	73,66	9,87E-104	377	-863	Indica	NA	
maker-5-augustus-gene-53.15-mRNA-1	5	OsR498G0509895600.01.T01	97,07	0,00E+00	2431	Os05g0187100	96,20	0,00E+00	1836	-595	Indica	NA	
maker-5-augustus-gene-53.3-mRNA-1	5	OsR498G0509895300.01.T05	100,00	0,00E+00	1478	Os05g0187000	100,00	0,00E+00	1478	0	NA	NA	
maker-5-augustus-gene-54.37-mRNA-1	5	OsR498G0509902300.01.T01	99,82	0,00E+00	5992	Os05g0188500	100,00	0,00E+00	6019	27	NA	Japonica	
maker-5-augustus-gene-54.45-mRNA-1	5	OsR498G0509903100.01.T01	99,79	0,00E+00	2581	Os05g0188600	100,00	0,00E+00	2594	13	NA	Japonica	
maker-5-augustus-gene-54.53-mRNA-1	5	OsR498G0509903500.01.T02	99,90	0,00E+00	1782	Os05g0188800	100,00	0,00E+00	1786	4	NA	Japonica	
maker-5-augustus-gene-54.56-mRNA-1	5	OsR498G0509907200.01.T01	75,78	3,88E-69	262	Os05g0188900	99,69	1,10E-165	582	320	Japonica	Japonica	
maker-5-augustus-gene-54.58-mRNA-1	5	OsR498G0509906000.01.T01	99,74	0,00E+00	1355	Os05g0189300	99,34	0,00E+00	1342	-13	NA	NA	
maker-5-augustus-gene-54.60-mRNA-1	5	OsR498G0509907200.01.T01	98,94	0,00E+00	1321	Os05g0189900	99,07	0,00E+00	1325	4	NA	NA	
maker-5-augustus-gene-55.4-mRNA-1	5	OsR498G0509909600.01.T01	99,73	0,00E+00	1312	Os05g0190300	99,73	0,00E+00	1312	0	NA	NA	
maker-5-augustus-gene-55.6-mRNA-1	5	OsR498G0509910600.01.T01	99,62	0,00E+00	1426	Os05g0190500	99,25	0,00E+00	1413	-13	NA	NA	
maker-5-augustus-gene-57.0-mRNA-1	5	OsR498G0509918900.01.T01	99,94	0,00E+00	2934	Os05g0194900	99,89	0,00E+00	1560	-1374	Indica	Indica	
maker-5-augustus-gene-57.3-mRNA-1	5	OsR498G0509917000.01.T01	98,97	0,00E+00	1878	Os01g0191300	77,72	5,09E-135	481	-1397	Indica	NA	
maker-5-augustus-gene-57.8-mRNA-1	5	OsR498G0509922600.01.T02	99,54	0,00E+00	1541	Os05g0195700	99,42	0,00E+00	1536	-5	NA	Indica	
maker-5-augustus-gene-58.13-mRNA-1	5	OsR498G0509933500.01.T03	99,45	0,00E+00	2250	Os05g0196800	99,29	0,00E+00	2239	-11	NA	NA	
maker-5-augustus-gene-58.20-mRNA-1	5	OsR498G0509928200.01.T04	99,78	0,00E+00	1662	Os01g0192600	79,43	1,46E-167	589	-1073	Indica	NA	
maker-5-augustus-gene-58.6-mRNA-1	5	OsR498G0509930700.01.T01	99,84	0,00E+00	6677	Os05g0196500	99,87	0,00E+00	6681	4	NA	NA	
maker-5-augustus-gene-59.0-mRNA-1	5	OsR498G0509938500.01.T05	99,70	9,49E-169	593	Os05g0198050	99,66	9,46E-151	532	-61	Indica	NA	
maker-5-augustus-gene-59.2-mRNA-1	5	OsR498G0509935200.01.T04	99,04	0,00E+00	2942	Os05g0197300	99,04	0,00E+00	2942	0	NA	NA	
maker-5-augustus-gene-6.0-mRNA-1	5	OsR498G0509585700.01.T01	99,89	0,00E+00	3195	Os05g0110500	100,00	0,00E+00	3204	9	NA	Japonica	
maker-5-augustus-gene-6.20-mRNA-1	5	OsR498G0509591700.01.T03	99,46	0,00E+00	981	Os01g0974500	74,91	9,75E-157	553	-428	Indica	Japonica	
maker-5-augustus-gene-6.26-mRNA-1	5	OsR498G0509586000.01.T01	100,00	0,00E+00	693	Os05g0110600	100,00	0,00E+00	693	0	NA	Japonica	
maker-5-augustus-gene-6.28-mRNA-1	5	OsR498G0509586200.01.T01	99,23	0,00E+00	1382	Os05g0110700	99,74	0,00E+00	1400	18	NA	Japonica	
maker-5-augustus-gene-6.31-mRNA-1	5	OsR498G0509586800.01.T01	99,83	0,00E+00	2134	Os05g0110900	99,83	0,00E+00	2134	0	NA	NA	Kinase-TM
maker-5-augustus-gene-6.35-mRNA-1	5	OsR498G0509588400.01.T02	99,65	1,78E-145	516	Os05g0111100	100,00	1,46E-147	520	4	NA	Japonica	
maker-5-augustus-gene-6.37-mRNA-1	5	OsR498G0509592000.01.T01	99,63	0,00E+00	2418	Os05g0112000	99,70	0,00E+00	2423	5	NA	Japonica	
maker-5-augustus-gene-6.4-mRNA-1	5	OsR498G0509586500.01.T01	96,01	0,00E+00	1409	Os05g0110800	99,80	0,00E+00	884	-525	Indica	Japonica	
maker-5-augustus-gene-6.41-mRNA-1	5	OsR498G0509593800.01.T01	99,62	0,00E+00	1893	Os05g0112200	100,00	0,00E+00	1575	-318	Indica	Japonica	
maker-5-augustus-gene-6.8-mRNA-1	5	OsR498G0509588100.01.T01	99,79	0,00E+00	5124	Os05g0111000	99,90	0,00E+00	5138	14	NA	Japonica	
maker-5-augustus-gene-60.0-mRNA-1	5	OsR498G0509944500.01.T01	99,74	0,00E+00	2070	Os05g0198400	99,74	0,00E+00	2070	0	NA	NA	
maker-5-augustus-gene-60.3-mRNA-1	5	OsR498G0509949300.01.T02	99,02	0,00E+00	1077	Os05g0198700	99,69	1,69E-163	575	-502	Indica	NA	
maker-5-augustus-gene-61.22-mRNA-1	5	OsR498G0509955700.01.T01	96,44	0,00E+00	2250	Os05g0199800	96,15	0,00E+00	2067	-183	NA	NA	
maker-5-augustus-gene-61.24-mRNA-1	5	OsR498G0509955000.01.T02	100,00	0,00E+00	1481	Os05g0199500	100,00	0,00E+00	1481	0	NA	NA	
maker-5-augustus-gene-61.27-mRNA-1	5	OsR498G0509956600.01.T01	99,85	0,00E+00	1170	Os05g0200100	99,86	0,00E+00	1251	81	NA	NA	
maker-5-augustus-gene-62.0-mRNA-1	5	OsR498G0509961300.01.T02	99,22	0,00E+00	2027	Os01g0197100	74,02	0,00E+00	887	-1140	Indica	NA	
maker-5-augustus-gene-62.4-mRNA-1	5	OsR498G0509961600.01.T03	100,00	0,00E+00	2626	Os05g0200500	100,00	0,00E+00	2626	0	NA	NA	
maker-5-augustus-gene-63.35-mRNA-1	5	OsR498G0509975600.01.T01	100,00	0,00E+00	883	Os05g0203800	83,08	1,10E-145	516	-367	Indica	NA	
maker-5-augustus-gene-64.34-mRNA-1	5	OsR498G0509981100.01.T01	99,74	0,00E+00	2036	Os05g0204600	99,47	0,00E+00	2024	-12	NA	Indica	
maker-5-augustus-gene-64.36-mRNA-1	5	OsR498G0509982500.01.T03	98,31	0,00E+00	2567	Os05g0204900	98,31	0,00E+00	2567	0	NA	Indica	
maker-5-augustus-gene-64.43-mRNA-1	5	OsR498G0509978600.01.T05	100,00	0,00E+00	668	Os05g0203900	100,00	0,00E+00	666	-2	NA	NA	
maker-5-augustus-gene-64.46-mRNA-1	5	OsR498G0509982800.01.T01	99,88	0,00E+00	1549	Os02g0232900	77,83	9,57E-156	550	-999	Indica	Indica	
maker-5-augustus-gene-66.31-mRNA-1	5	OsR498G0509990500.01.T01	100,00	0,00E+00	985	Os05g0207300	100,00	0,00E+00	985	0	NA	NA	
maker-5-augustus-gene-66.35-mRNA-1	5	OsR498G0509992300.01.T03	99,79	0,00E+00	2590	Os05g0207900	99,24	0,00E+00	2553	-37	NA	Indica	
maker-5-augustus-gene-66.41-mRNA-1	5	OsR498G0509992900.01.T01	100,00	0,00E+00	1662	Os05g0208000	100,00	0,00E+00	1662	0	NA	Indica	
maker-5-augustus-gene-66.49-mRNA-1	5	OsR498G0509990800.01.T01	99,80	0,00E+00	911	Os05g0207400	99,61	0,00E+00	906	-5	NA	Indica	
maker-5-augustus-gene-66.51-mRNA-1	5	OsR498G0509991200.01.T01	100,00	0,00E+00	2181	Os05g0207500	99,67	0,00E+00	2163	-18	NA	Indica	Kinase-TM
maker-5-augustus-gene-66.56-mRNA-1	5	OsR498G0509991800.01.T03	99,89	0,00E+00	1645	Os05g0207700	99,67	0,00E+00	1636	-9	NA	Indica	Kinase-TM
maker-5-augustus-gene-67.23-mRNA-1	5	OsR498G0509996500.01.T01	99,49	0,00E+00	1384	Os05g0209400	100,00	0,00E+00	1402	18	NA	Japonica	
maker-5-augustus-gene-67.25-mRNA-1	5	OsR498G0509996700.01.T01	99,62	1,73E-132	472	Os05g0209500	100,00	1,42E-134	477	5	NA	Japonica	
maker-5-augustus-gene-67.27-mRNA-1	5	OsR498G0509997700.01.T01	99,54	0,00E+00	1937	Os05g0209600	100,00	0,00E+00	1992	55	NA	Japonica	
maker-5-augustus-gene-67.34-mRNA-1	5	OsR498G0509995400.01.T01	99,80	0,00E+00	2723	Os05g0209100	100,00	0,00E+00	2736	13	NA	Japonica	
maker-5-augustus-gene-68.1-mRNA-1	5	OsR498G0509999700.01.T02	99,54	0,00E+00	1564	Os05g0210100	99,43	0,00E+00	1556	-8	NA	Japonica	
maker-5-augustus-gene-68.10-mRNA-1	5	OsR498G0510001000.01.T02	98,47	0,00E+00	4085	Os05g0210500	97,61	0,00E+00	6279	2194	Japonica	Japonica	
maker-5-augustus-gene-68.14-mRNA-1	5	OsR498G0510002400.01.T01	96,69	0,00E+00	2502	Os05g0211100	99,93	0,00E+00	2599	97	NA	Japonica	
maker-5-augustus-gene-68.3-mRNA-1	5	OsR498G0510000700.01.T01	97,03	0,00E+00	1069	Os05g0210400	100,00	0,00E+00	1131	62	NA	Japonica	
maker-5-augustus-gene-68.5-mRNA-1	5	OsR498G0510001300.01.T02	98,11	0,00E+00	3363	Os05g0210600	100,00	0,00E+00	1979	-1384	Indica	Japonica	
maker-5-augustus-gene-69.29-mRNA-1	5	OsR498G0510003400.01.T04	94,92	0,00E+00	720	Os05g0212200	100,00	0,00E+00	827	107	Japonica	Japonica	
maker-5-augustus-gene-69.32-mRNA-1	5	OsR498G0510004600.01.T01	97,49	0,00E+00	3172	Os05g0212300	100,00	0,00E+00	3409	237	NA	Japonica	
maker-5-augustus-gene-69.34-mRNA-1	5	OsR498G0510005600.01.T05	99,49	0,00E+00	1053	Os05g0212400	100,00	0,00E+00	1067	14	NA	Japonica	
maker-5-augustus-gene-69.42-mRNA-1	5	OsR498G0510009000.01.T01	98,85	0,00E+00	1367	Os05g0212900	99,74	0,00E+00	1398	31	NA	Japonica	
maker-5-augustus-gene-69.44-mRNA-1	5	OsR498G0510010200.01.T01	97,29	0,00E+00	1983	Os05g0212900	92,88	0,00E+00	1748	-235	Indica	Japonica	
maker-5-augustus-gene-7.43-mRNA-1	5	OsR498G0509597100.01.T01	99,03	0,00E+00	2357	Os05g0113000	99,75	0,00E+00	2113	-244	Indica	Japonica	
maker-5-augustus-gene-7.46-mRNA-1	5	OsR498G0509597500.01.T01	99,92	0,00E+00	4563	Os05g0113300	100,00	0,00E+00	4572	9	NA	Japonica	
maker-5-augustus-gene-7.48-mRNA-1	5	OsR498G0509597700.01.T01	100,00	0,00E+00	758	Os05g0113400	100,00	0,00E+00	753	-5	NA	Japonica	
maker-5-augustus-gene-7.50-mRNA-1	5	OsR498G0509598000.01.T02	99,49	0,00E+00	1395	Os05g0113500	100,00	0,00E+00	1413	18	NA	Japonica	
maker-5-augustus-gene-7.52-mRNA-1	5	OsR498G0509597400.01.T02	100,00	0,00E+00	1175	Os05g0114000	100,00	0,00E+00	1175	0	NA	Japonica	
maker-5-augustus-gene-70.0-mRNA-1	5	OsR498G0510012300.01.T01	100,00	0,00E+00	1137	Os05g0213500	100,00	0,00E+00	1137	0	NA	Indica	
maker-5-augustus-gene-70.2-mRNA-1	5	OsR498G0510016900.01.T01	100,00	0,00E+00	1278	Os05g0214400	99,58	0,00E+00	1264	-14	NA	NA	
maker-5-augustus-gene-70.4-mRNA-1	5	OsR498G0510016900.01.T01	100,00	0,00E+00	1278	Os05g0214400	99,58	0,00E+00	1264	-14	NA	NA	
maker-5-augustus-gene-71.26-mRNA-1	5	OsR498G0510020300.01.T01	100,00	0,00E+00	2284	Os05g0214900	100,00	0,00E+00	1301	-983	Indica	Indica	
maker-5-augustus-gene-71.29-mRNA-1	5	OsR498G0510022000.01.T01	100,00	0,00E+00	1474	Os05g0215000	94,49	0,00E+00	1306	-168	Indica	Indica	
maker-5-augustus-gene-72.0-mRNA-1	5	OsR498G0510033900.01.T01	99,88	0,00E+00	1538	Os05g0217800	95,60	0,00E+00	1387	-151	NA	Indica	
maker-5-augustus-gene-72.2-mRNA-1	5	OsR498G0510026400.01.T02	100,00	0,00E+00	1819	Os05g0215800	100,00	0,00E+00	1819	0	NA	Indica	
maker-5-augustus-gene-72.7-mRNA-1	5	OsR498G0612586700.01.T02	77,55	1,15E-25	117	Os05g0217000	100,00	3,98E-102	369	252	Japonica	Indica	
maker-5-augustus-gene-73.26-mRNA-1	5	OsR498G0510036200.01.T03	100,00	0,00E+00	2791	Os01g0227200	77,93	0,00E+00	754	-2037	Indica	Indica	Kinase-TM
maker-5-augustus-gene-73.31-mRNA-1	5	OsR498G0510635800.01.T01	91,91	8,29E-171	600	Os01g0303800	90,32	1,01E-94	346	-254	Indica	Indica	
maker-5-augustus-gene-74.40-mRNA-1	5	OsR498G0510046700.01.T01	99,77	0,00E+00	1535	Os05g0220900	99,53	0,00E+00	1526	-9	NA	Indica	
maker-5-augustus-gene-74.48-mRNA-1	5	OsR498G0510043600.01.T01	99,84	0,00E+00	3358	Os05g0219900	99,63	0,00E+00	3340	-18	NA	Indica	
maker-5-augustus-gene-74.50-mRNA-1	5	OsR498G0510044200.01.T04	99,33	0,00E+00	1863	Os05g0220100	99,62	0,00E+00	1876	13	NA	Indica	
maker-5-augustus-gene-74.53-mRNA-1	5	OsR498G0510046200.01.T02	99,88	0,00E+00	1495	Os05g0220600	100,00	0,00E+00	1499	4	NA	NA	
maker-5-augustus-gene-74.58-mRNA-1	5	OsR498G0510048200.01.T01	99,85	0,00E+00	1165	Os01g0750500	75,15	9,83E-124	444	-721	Indica	Indica	
maker-5-augustus-gene-75.36-mRNA-1	5	OsR498G0510053900.01.T01	100,00	0,00E+00	645	Os05g0223200	99,68	2,30E-160	564	-81	Indica	Indica	
maker-5-augustus-gene-75.38-mRNA-1	5	OsR498G0510054500.01.T01	100,00	1,80E-158	558	Os05g0223300	100,00	6,27E-159	558	0	NA	NA	
maker-5-augustus-gene-75.40-mRNA-1	5	OsR498G0510054900.01.T01	100,00	1,80E-158	558	Os05g0223300	89,97	1,38E-116	418	-140	Indica	NA	
maker-5-augustus-gene-75.42-mRNA-1	5	OsR498G0510052200.01.T01	99,95	0,00E+00	3789	Os05g0222200	99,86	0,00E+00	3780	-9	NA	Indica	NBS-TM
maker-5-augustus-gene-76.31-mRNA-1	5	OsR498G0510059900.01.T01	99,43	0,00E+00	938	Os05g0224700	99,32	0,00E+00	786	-152	Indica	NA	
maker-5-augustus-gene-78.30-mRNA-1	5	OsR498G0510071700.01.T02	100,00	0,00E+00	2046	Os05g0227800	100,00	0,00E+00	2046	0	NA	NA	
maker-5-augustus-gene-78.32-mRNA-1	5	OsR498G0510071600.01.T01	100,00	0,00E+00	2868	Os05g0227700	100,00	0,00E+00	2868	0	NA	NA	
maker-5-augustus-gene-78.35-mRNA-1	5	OsR498G0510072400.01.T02	100,00	0,00E+00	1954	Os05g0228000	100,00	0,00E+00	1954	0	NA	NA	
maker-5-augustus-gene-79.39-mRNA-1	5	OsR498G0510076400.01.T05	100,00	1,56E-106	385	Os05g0229000	100,00	5,44E-107	385	0	NA	NA	
maker-5-augustus-gene-79.41-mRNA-1	5	OsR498G0815323400.01.T01	70,26	2,01E-26	120	Os05g0229300	100,00	0,00E+00	737	617	Japonica	Indica	
maker-5-augustus-gene-8.16-mRNA-1	5	OsR498G0509600600.01.T01	99,67	0,00E+00	2678	Os05g0114500	100,00	0,00E+00	2971	293	NA	Japonica	
maker-5-augustus-gene-8.5-mRNA-1	5	OsR498G0509604700.01.T01	97,81	0,00E+00	1409	Os05g0115100	100,00	0,00E+00	1467	58	NA	Japonica	
maker-5-augustus-gene-8.7-mRNA-1	5	OsR498G0509605500.01.T01	99,13	0,00E+00	1017	Os05g0115600	100,00	0,00E+00	1321	304	Japonica	Japonica	
maker-5-augustus-gene-8.9-mRNA-1	5	OsR498G0509605700.01.T01	100,00	0,00E+00	969	Os05g0115700	100,00	0,00E+00	969	0	NA	Japonica	
maker-5-augustus-gene-80.0-mRNA-1	5	OsR498G0510081500.01.T01	99,86	0,00E+00	5135	Os05g0230600	100,00	0,00E+00	5153	18	NA	Japonica	
maker-5-augustus-gene-80.11-mRNA-1	5	OsR498G0510087900.01.T04	99,27	0,00E+00	1691	Os05g0232200	100,00	0,00E+00	1721	30	NA	Japonica	
maker-5-augustus-gene-80.16-mRNA-1	5	OsR498G0510083800.01.T01	99,81	0,00E+00	1891	Os05g0230900	100,00	0,00E+00	1581	-310	Indica	Japonica	
maker-5-augustus-gene-80.23-mRNA-1	5	OsR498G0510087000.01.T01	99,14	0,00E+00	1637	Os05g0231900	100,00	0,00E+00	1673	36	NA	Japonica	
maker-5-augustus-gene-80.6-mRNA-1	5	OsR498G0510083100.01.T03	100,00	0,00E+00	1397	Os01g0231000	78,68	0,00E+00	693	-704	Indica	Japonica	
maker-5-augustus-gene-80.9-mRNA-1	5	OsR498G0510086700.01.T02	99,87	0,00E+00	1360	Os05g0231700	100,00	0,00E+00	1364	4	NA	Japonica	
maker-5-augustus-gene-81.0-mRNA-1	5	OsR498G0510088700.01.T01	99,36	0,00E+00	2496	Os05g0232500	100,00	0,00E+00	2533	37	NA	Japonica	
maker-5-augustus-gene-83.15-mRNA-1	5	OsR498G0510103900.01.T10	99,25	0,00E+00	7553	Os05g0235300	99,31	0,00E+00	7164	-389	NA	Japonica	
maker-5-augustus-gene-83.29-mRNA-1	5	OsR498G0510105500.01.T01	98,56	0,00E+00	4361	Os05g0235800	98,72	0,00E+00	4379	18	NA	Japonica	
maker-5-augustus-gene-84.38-mRNA-1	5	OsR498G0510107300.01.T04	99,72	0,00E+00	1919	Os05g0237100	100,00	0,00E+00	1110	-809	Indica	Japonica	
maker-5-augustus-gene-84.44-mRNA-1	5	OsR498G0510107900.01.T01	98,85	0,00E+00	3057	Os05g0237400	100,00	0,00E+00	3112	55	NA	Japonica	Kinase-TM
maker-5-augustus-gene-84.55-mRNA-1	5	OsR498G0612375300.01.T06	92,33	2,38E-139	495	Os05g0237700	100,00	4,28E-175	612	117	Japonica	Japonica	
maker-5-augustus-gene-85.0-mRNA-1	5	OsR498G0510111700.01.T02	99,36	0,00E+00	1928	Os05g0238200	100,00	0,00E+00	1959	31	NA	Japonica	
maker-5-augustus-gene-85.11-mRNA-1	5	OsR498G0510111400.01.T01	99,70	0,00E+00	2423	Os08g0127100	77,32	0,00E+00	1008	-1415	Indica	Japonica	
maker-5-augustus-gene-86.0-mRNA-1	5	OsR498G0510124100.01.T01	99,67	0,00E+00	2172	Os05g0241200	100,00	0,00E+00	2192	20	NA	Japonica	
maker-5-augustus-gene-86.11-mRNA-1	5	OsR498G0510123000.01.T02	99,90	0,00E+00	3617	Os05g0241000	100,00	0,00E+00	3626	9	NA	Japonica	
maker-5-augustus-gene-86.13-mRNA-1	5	OsR498G0510125200.01.T02	99,12	0,00E+00	800	Os05g0241400	100,00	0,00E+00	818	18	NA	Japonica	
maker-5-augustus-gene-86.6-mRNA-1	5	OsR498G0510120000.01.T03	99,90	0,00E+00	7506	Os05g0240200	100,00	0,00E+00	7120	-386	NA	Japonica	NBS-TM
maker-5-augustus-gene-87.2-mRNA-1	5	OsR498G0510127200.01.T04	98,25	4,54E-109	394	Os05g0243300	96,48	1,48E-103	374	-20	NA	Japonica	
maker-5-augustus-gene-87.7-mRNA-1	5	OsR498G0510127600.01.T02	99,43	0,00E+00	1243	Os05g0243300	100,00	0,00E+00	1036	-207	Indica	Japonica	
maker-5-augustus-gene-88.0-mRNA-1	5	OsR498G0510128200.01.T02	99,27	0,00E+00	2177	Os05g0244500	100,00	0,00E+00	2217	40	NA	Japonica	
maker-5-augustus-gene-88.1-mRNA-1	5	OsR498G0510128200.01.T04	98,29	5,40E-173	607	Os05g0244500	100,00	0,00E+00	634	27	NA	Japonica	
maker-5-augustus-gene-88.10-mRNA-1	5	OsR498G0510128800.01.T01	96,24	0,00E+00	1701	Os05g0244700	100,00	0,00E+00	1846	145	NA	Japonica	
maker-5-augustus-gene-88.8-mRNA-1	5	OsR498G0510128400.01.T01	99,54	0,00E+00	782	Os05g0244600	100,00	0,00E+00	953	171	Japonica	Japonica	
maker-5-augustus-gene-89.4-mRNA-1	5	OsR498G0510129800.01.T01	99,59	0,00E+00	874	Os05g0245300	100,00	0,00E+00	883	9	NA	Japonica	
maker-5-augustus-gene-89.6-mRNA-1	5	OsR498G0510131000.01.T01	99,87	0,00E+00	1430	Os05g0246300	100,00	0,00E+00	1434	4	NA	Japonica	
maker-5-augustus-gene-9.45-mRNA-1	5	OsR498G0509610300.01.T01	99,59	0,00E+00	881	Os05g0117500	99,59	0,00E+00	881	0	NA	Japonica	
maker-5-augustus-gene-9.51-mRNA-1	5	OsR498G0509610500.01.T01	99,88	0,00E+00	4386	Os05g0117600	98,03	0,00E+00	4222	-164	NA	Japonica	
maker-5-augustus-gene-9.57-mRNA-1	5	OsR498G0509611500.01.T02	99,81	0,00E+00	5692	Os05g0117798	100,00	0,00E+00	5719	27	NA	Japonica	
maker-5-augustus-gene-9.64-mRNA-1	5	OsR498G0509606200.01.T04	95,37	0,00E+00	1446	Os05g0115900	95,37	0,00E+00	1446	0	NA	Japonica	
maker-5-augustus-gene-9.70-mRNA-1	5	OsR498G0509606600.01.T01	99,72	0,00E+00	1935	Os05g0116000	100,00	0,00E+00	1948	13	NA	Japonica	
maker-5-augustus-gene-9.72-mRNA-1	5	OsR498G0509606800.01.T01	99,78	0,00E+00	1604	Os05g0116100	100,00	0,00E+00	1159	-445	Indica	Japonica	
maker-5-augustus-gene-9.76-mRNA-1	5	OsR498G0509608800.01.T01	90,39	0,00E+00	731	Os05g0116500	89,90	1,82E-168	590	-141	Indica	Japonica	
maker-5-augustus-gene-9.78-mRNA-1	5	OsR498G0509611800.01.T02	99,34	0,00E+00	6751	Os05g0117864	100,00	0,00E+00	6071	-680	Indica	Japonica	
maker-5-augustus-gene-90.0-mRNA-1	5	OsR498G0510138100.01.T01	99,77	0,00E+00	1575	Os05g0247800	100,00	0,00E+00	1584	9	NA	Japonica	
maker-5-augustus-gene-90.3-mRNA-1	5	OsR498G0510138800.01.T02	99,92	0,00E+00	2294	Os05g0247900	100,00	0,00E+00	2298	4	NA	Japonica	
maker-5-augustus-gene-91.1-mRNA-1	5	OsR498G0510143400.01.T01	99,92	0,00E+00	2229	Os05g0250101	76,73	0,00E+00	988	-1241	Indica	Japonica	
maker-5-augustus-gene-91.2-mRNA-1	5	OsR498G0510145600.01.T01	98,14	0,00E+00	647	Os05g0249100	100,00	0,00E+00	726	79	Japonica	Japonica	
maker-5-augustus-gene-92.40-mRNA-1	5	OsR498G0510151900.01.T01	99,65	0,00E+00	1538	Os05g0251400	100,00	0,00E+00	1553	15	NA	Japonica	
maker-5-augustus-gene-92.46-mRNA-1	5	OsR498G0510147800.01.T01	99,64	0,00E+00	2003	Os05g0251300	73,66	9,83E-177	619	-1384	Indica	Japonica	
maker-5-augustus-gene-92.48-mRNA-1	5	OsR498G0510151800.01.T01	78,95	0,00E+00	724	Os05g0251300	76,31	0,00E+00	803	79	NA	Japonica	
maker-5-augustus-gene-92.53-mRNA-1	5	OsR498G0510151800.01.T01	99,34	0,00E+00	1890	Os05g0251300	100,00	0,00E+00	2212	322	Japonica	Japonica	
maker-5-augustus-gene-93.2-mRNA-1	5	OsR498G0510154600.01.T01	99,91	0,00E+00	2030	Os05g0251900	100,00	0,00E+00	2035	5	NA	Japonica	
maker-5-augustus-gene-93.5-mRNA-1	5	OsR498G0510155000.01.T01	99,83	0,00E+00	2057	Os05g0252000	99,91	0,00E+00	2061	4	NA	Japonica	
maker-5-augustus-gene-93.7-mRNA-1	5	OsR498G0510155400.01.T01	97,96	0,00E+00	2531	Os05g0252100	100,00	0,00E+00	2646	115	NA	Japonica	
maker-5-augustus-gene-94.42-mRNA-1	5	OsR498G0510160500.01.T03	99,68	1,88E-158	559	Os05g0257100	80,83	4,43E-66	251	-308	Indica	Japonica	
maker-5-augustus-gene-94.45-mRNA-1	5	OsR498G0510160500.01.T05	98,17	0,00E+00	2462	Os04g0291900	80,31	0,00E+00	1314	-1148	Indica	Japonica	Kinase-LRR-TM
maker-5-augustus-gene-95.0-mRNA-1	5	OsR498G0510164300.01.T01	100,00	0,00E+00	1251	Os05g0255600	100,00	0,00E+00	1251	0	NA	Japonica	
maker-5-augustus-gene-95.16-mRNA-1	5	OsR498G0510174900.01.T02	82,37	0,00E+00	2298	Os04g0291900	80,76	0,00E+00	2134	-164	NA	Japonica	Kinase-LRR-TM
maker-5-augustus-gene-95.9-mRNA-1	5	OsR498G0510166100.01.T02	100,00	0,00E+00	1332	Os05g0256000	100,00	0,00E+00	1332	0	NA	Japonica	
maker-5-augustus-gene-96.10-mRNA-1	5	OsR498G0510172500.01.T03	99,96	0,00E+00	4196	Os05g0257100	100,00	0,00E+00	3003	-1193	Indica	Japonica	Kinase-LRR-TM
maker-5-augustus-gene-96.3-mRNA-1	5	OsR498G0510169700.01.T01	99,78	0,00E+00	1642	Os05g0257100	79,50	0,00E+00	740	-902	Indica	Japonica	LRR-TM
maker-5-augustus-gene-96.6-mRNA-1	5	OsR498G0510169900.01.T02	99,66	0,00E+00	2129	Os08g0203400	81,38	0,00E+00	1148	-981	Indica	Japonica	Kinase-TM
maker-5-augustus-gene-97.1-mRNA-1	5	OsR498G0510174900.01.T01	99,78	0,00E+00	4994	Os04g0291900	81,30	0,00E+00	2559	-2435	Indica	NA	Kinase-LRR-TM
maker-5-augustus-gene-97.14-mRNA-1	5	OsR498G0510177900.01.T01	99,77	0,00E+00	2294	Os04g0341500	80,13	0,00E+00	1150	-1144	Indica	NA	
augustus_masked-6-processed-gene-100.11-mRNA-1	6	OsR498G0612149700.01.T01	98,94	0,00E+00	1324	Os06g0285200	99,87	0,00E+00	1354	30	NA	Japonica	
augustus_masked-6-processed-gene-100.33-mRNA-1	6	OsR498G0612151000.01.T01	99,40	0,00E+00	885	Os06g0272601	72,86	3,43E-76	285	-600	Indica	NA	
augustus_masked-6-processed-gene-102.20-mRNA-1	6	OsR498G0612167300.01.T01	99,29	0,00E+00	2509	Os06g0288200	98,02	0,00E+00	2428	-81	NA	Indica	
augustus_masked-6-processed-gene-102.31-mRNA-1	6	OsR498G0612167200.01.T01	99,22	0,00E+00	2724	Os06g0288100	97,60	0,00E+00	2617	-107	NA	Indica	Kinase-TM
augustus_masked-6-processed-gene-103.24-mRNA-1	6	OsR498G0612168500.01.T01	99,03	0,00E+00	2535	Os06g0289900	90,82	0,00E+00	1992	-543	Indica	Indica	
augustus_masked-6-processed-gene-103.3-mRNA-1	6	OsR498G0612167500.01.T01	98,84	0,00E+00	2559	Os06g0289200	97,67	0,00E+00	2480	-79	NA	Indica	
augustus_masked-6-processed-gene-103.30-mRNA-1	6	OsR498G0612168000.01.T01	99,17	0,00E+00	2554	Os06g0289900	99,31	0,00E+00	2563	9	NA	NA	
augustus_masked-6-processed-gene-104.34-mRNA-1	6	OsR498G0612172400.01.T01	99,71	0,00E+00	5002	Os06g0292100	99,96	0,00E+00	4543	-459	NA	Japonica	
augustus_masked-6-processed-gene-104.35-mRNA-1	6	OsR498G0612172800.01.T02	99,95	0,00E+00	6587	Os06g0292400	100,00	0,00E+00	3956	-2631	Indica	Japonica	
augustus_masked-6-processed-gene-107.37-mRNA-1	6	OsR498G0612189500.01.T01	99,83	0,00E+00	5210	Os06g0297500	100,00	0,00E+00	5232	22	NA	Japonica	
augustus_masked-6-processed-gene-108.11-mRNA-1	6	OsR498G0612194000.01.T01	99,54	0,00E+00	1163	Os06g0298700	96,34	0,00E+00	1727	564	Japonica	Japonica	
augustus_masked-6-processed-gene-109.10-mRNA-1	6	OsR498G0612202300.01.T02	100,00	0,00E+00	1613	Os06g0300700	100,00	0,00E+00	1613	0	NA	Japonica	
augustus_masked-6-processed-gene-11.0-mRNA-1	6	OsR498G0611527900.01.T01	99,85	0,00E+00	1181	Os06g0119900	99,85	0,00E+00	1181	0	NA	NA	
augustus_masked-6-processed-gene-11.1-mRNA-1	6	OsR498G0611529100.01.T01	100,00	0,00E+00	4150	Os06g0120200	100,00	0,00E+00	4150	0	NA	NA	
augustus_masked-6-processed-gene-11.11-mRNA-1	6	OsR498G0611527800.01.T01	100,00	0,00E+00	1186	Os06g0119800	100,00	0,00E+00	1186	0	NA	NA	
augustus_masked-6-processed-gene-11.23-mRNA-1	6	OsR498G0611532700.01.T01	100,00	0,00E+00	4323	Os06g0121200	100,00	0,00E+00	4291	-32	NA	NA	
augustus_masked-6-processed-gene-11.28-mRNA-1	6	OsR498G0611529100.01.T01	71,15	0,00E+00	745	Os06g0122200	100,00	0,00E+00	4372	3627	Japonica	NA	
augustus_masked-6-processed-gene-11.41-mRNA-1	6	OsR498G0611534200.01.T01	100,00	0,00E+00	1229	Os06g0121300	100,00	0,00E+00	1229	0	NA	NA	
augustus_masked-6-processed-gene-110.8-mRNA-1	6	OsR498G0612208900.01.T01	99,18	0,00E+00	3011	Os06g0302000	93,32	0,00E+00	1513	-1498	Indica	Japonica	
augustus_masked-6-processed-gene-112.0-mRNA-1	6	OsR498G0612215700.01.T04	98,84	0,00E+00	1966	Os06g0303700	100,00	0,00E+00	1997	31	NA	Japonica	
augustus_masked-6-processed-gene-112.1-mRNA-1	6	OsR498G0612215700.01.T01	99,78	0,00E+00	822	Os06g0303700	100,00	0,00E+00	827	5	NA	Japonica	
augustus_masked-6-processed-gene-113.17-mRNA-1	6	OsR498G0612221700.01.T01	98,62	0,00E+00	1898	Os06g0306600	100,00	0,00E+00	1954	56	NA	Japonica	
augustus_masked-6-processed-gene-118.1-mRNA-1	6	OsR498G0612258000.01.T01	99,75	0,00E+00	4345	Os06g0314100	100,00	0,00E+00	5373	1028	Japonica	Japonica	
augustus_masked-6-processed-gene-118.3-mRNA-1	6	OsR498G0612260000.01.T01	98,51	0,00E+00	1745	Os06g0314501	100,00	0,00E+00	1532	-213	Indica	Japonica	
augustus_masked-6-processed-gene-120.19-mRNA-1	6	OsR498G0612267300.01.T01	98,56	1,62E-172	606	Os06g0317200	89,66	2,56E-139	494	-112	Indica	Japonica	
augustus_masked-6-processed-gene-120.45-mRNA-1	6	OsR498G0612271200.01.T01	96,22	0,00E+00	813	Os05g0422200	90,10	0,00E+00	1289	476	Japonica	Japonica	
augustus_masked-6-processed-gene-122.2-mRNA-1	6	OsR498G0612275800.01.T01	100,00	0,00E+00	791	Os06g0320300	100,00	0,00E+00	791	0	NA	Japonica	
augustus_masked-6-processed-gene-123.1-mRNA-1	6	OsR498G0612281500.01.T01	91,49	9,60E-131	466	Os06g0322300	99,68	1,62E-160	564	98	Japonica	Japonica	
augustus_masked-6-processed-gene-125.0-mRNA-1	6	OsR498G0815038200.01.T01	77,68	0,00E+00	1157	Os06g0325900	100,00	0,00E+00	2782	1625	Japonica	Japonica	
augustus_masked-6-processed-gene-125.10-mRNA-1	6	OsR498G0612296100.01.T02	99,39	0,00E+00	1733	Os06g0326900	100,00	0,00E+00	1748	15	NA	Japonica	
augustus_masked-6-processed-gene-128.25-mRNA-1	6	OsR498G0612561200.01.T01	99,75	0,00E+00	3598	Os06g0331900	100,00	0,00E+00	3620	22	NA	Japonica	
augustus_masked-6-processed-gene-129.38-mRNA-1	6	OsR498G0612558900.01.T01	99,75	0,00E+00	710	Os06g0332800	100,00	0,00E+00	715	5	NA	Japonica	
augustus_masked-6-processed-gene-13.0-mRNA-1	6	OsR498G0611544100.01.T01	100,00	0,00E+00	4275	Os06g0124300	100,00	0,00E+00	4275	0	NA	NA	
augustus_masked-6-processed-gene-13.13-mRNA-1	6	OsR498G0611547300.01.T02	100,00	0,00E+00	2901	Os06g0125300	100,00	0,00E+00	2901	0	NA	NA	
augustus_masked-6-processed-gene-13.26-mRNA-1	6	OsR498G0611548600.01.T02	100,00	0,00E+00	2244	Os06g0125500	100,00	0,00E+00	2244	0	NA	NA	
augustus_masked-6-processed-gene-133.14-mRNA-1	6	OsR498G0815054500.01.T01	89,59	1,06E-117	423	Os06g0339800	100,00	2,02E-178	623	200	Japonica	Japonica	
augustus_masked-6-processed-gene-134.16-mRNA-1	6	OsR498G0612524900.01.T01	99,32	0,00E+00	5772	Os06g0343100	100,00	0,00E+00	5871	99	NA	Japonica	
augustus_masked-6-processed-gene-134.17-mRNA-1	6	OsR498G0612523700.01.T01	99,79	0,00E+00	2573	Os06g0343600	100,00	0,00E+00	2587	14	NA	Japonica	
augustus_masked-6-processed-gene-134.3-mRNA-1	6	OsR498G0612527500.01.T01	99,30	0,00E+00	2789	Os06g0342000	99,30	0,00E+00	2791	2	NA	Japonica	
augustus_masked-6-processed-gene-139.6-mRNA-1	6	OsR498G0612504300.01.T01	99,79	0,00E+00	840	Os06g0349800	100,00	0,00E+00	845	5	NA	Japonica	
augustus_masked-6-processed-gene-14.45-mRNA-1	6	OsR498G0611551300.01.T03	100,00	0,00E+00	1775	Os06g0126500	96,47	0,00E+00	1706	-69	NA	NA	
augustus_masked-6-processed-gene-14.47-mRNA-1	6	OsR498G0611552100.01.T05	100,00	0,00E+00	1267	Os06g0127000	100,00	0,00E+00	1267	0	NA	NA	
augustus_masked-6-processed-gene-14.48-mRNA-1	6	OsR498G0611552700.01.T01	99,85	0,00E+00	1159	Os06g0127100	99,85	0,00E+00	1159	0	NA	NA	
augustus_masked-6-processed-gene-14.52-mRNA-1	6	OsR498G0611554900.01.T01	100,00	0,00E+00	1873	Os06g0127900	100,00	0,00E+00	1873	0	NA	NA	
augustus_masked-6-processed-gene-14.69-mRNA-1	6	OsR498G0611554400.01.T01	100,00	0,00E+00	3344	Os06g0127800	100,00	0,00E+00	3344	0	NA	NA	
augustus_masked-6-processed-gene-140.31-mRNA-1	6	OsR498G0612495900.01.T01	99,61	0,00E+00	1361	Os06g0352200	100,00	0,00E+00	1375	14	NA	Japonica	
augustus_masked-6-processed-gene-141.10-mRNA-1	6	OsR498G0612492500.01.T02	99,83	0,00E+00	4156	Os06g0352900	100,00	0,00E+00	4578	422	NA	Japonica	

augustus_masked-6-processed-gene-142.11-mRNA-1	6	OsR498G0612489400.01.T01	97,62	0,00E+00	713	Os06g0354601	100,00	0,00E+00	758	45	NA	Japonica	
augustus_masked-6-processed-gene-142.12-mRNA-1	6	OsR498G0612488000.01.T03	100,00	0,00E+00	2217	Os06g0354700	100,00	0,00E+00	2217	0	NA	Japonica	
augustus_masked-6-processed-gene-144.5-mRNA-1	6	OsR498G0612478700.01.T01	99,71	0,00E+00	1249	Os06g0356800	100,00	0,00E+00	1348	99	NA	NA	
augustus_masked-6-processed-gene-15.0-mRNA-1	6	OsR498G0611555700.01.T01	100,00	0,00E+00	3350	Os02g0675700	73,22	0,00E+00	987	-2363	Indica	NA	
augustus_masked-6-processed-gene-15.10-mRNA-1	6	OsR498G0611558300.01.T01	100,00	0,00E+00	2132	Os06g0129000	100,00	0,00E+00	2104	-28	NA	NA	
augustus_masked-6-processed-gene-15.12-mRNA-1	6	OsR498G0611560300.01.T01	100,00	0,00E+00	3687	Os04g0573000	70,51	0,00E+00	911	-2776	Indica	NA	
augustus_masked-6-processed-gene-154.8-mRNA-1	6	OsR498G0612438500.01.T06	99,57	0,00E+00	2052	Os06g0468300	100,00	0,00E+00	2078	26	NA	Japonica	
augustus_masked-6-processed-gene-156.17-mRNA-1	6	OsR498G0612396600.01.T01	99,91	0,00E+00	2058	Os06g0472700	99,74	0,00E+00	2047	-11	NA	Japonica	
augustus_masked-6-processed-gene-156.9-mRNA-1	6	OsR498G0612388200.01.T02	99,79	0,00E+00	3461	Os06g0472300	100,00	0,00E+00	2154	-1307	Indica	Japonica	
augustus_masked-6-processed-gene-157.11-mRNA-1	6	OsR498G0612402300.01.T01	99,81	0,00E+00	927	Os06g0473800	100,00	0,00E+00	931	4	NA	Japonica	
augustus_masked-6-processed-gene-158.0-mRNA-1	6	OsR498G0612405300.01.T01	99,86	0,00E+00	2594	Os06g0474300	100,00	0,00E+00	2603	9	NA	Japonica	
augustus_masked-6-processed-gene-159.19-mRNA-1	6	OsR498G0714121500.01.T06	79,31	4,97E-39	162	Os06g0476900	100,00	3,54E-162	569	407	Japonica	Japonica	
augustus_masked-6-processed-gene-16.54-mRNA-1	6	OsR498G0611565400.01.T02	99,70	0,00E+00	1783	Os06g0130600	99,70	0,00E+00	1783	0	NA	NA	
augustus_masked-6-processed-gene-161.13-mRNA-1	6	OsR498G0612420500.01.T01	99,77	0,00E+00	2341	Os06g0478600	100,00	0,00E+00	2354	13	NA	Japonica	LRR-TM
augustus_masked-6-processed-gene-166.12-mRNA-1	6	OsR498G0612361200.01.T01	97,79	0,00E+00	849	Os06g0114366	91,97	0,00E+00	719	-130	Indica	Japonica	
augustus_masked-6-processed-gene-168.7-mRNA-1	6	OsR498G0612352500.01.T02	99,35	9,34E-156	549	Os06g0490000	100,00	6,27E-159	558	9	NA	Japonica	
augustus_masked-6-processed-gene-17.16-mRNA-1	6	OsR498G0611574800.01.T05	100,00	0,00E+00	2462	Os06g0132100	100,00	0,00E+00	2462	0	NA	NA	
augustus_masked-6-processed-gene-17.17-mRNA-1	6	OsR498G0611575200.01.T02	100,00	0,00E+00	1040	Os06g0132300	100,00	0,00E+00	1040	0	NA	NA	
augustus_masked-6-processed-gene-170.19-mRNA-1	6	OsR498G0612334700.01.T04	99,85	0,00E+00	3623	Os06g0493800	100,00	0,00E+00	3609	-14	NA	Japonica	
augustus_masked-6-processed-gene-171.20-mRNA-1	6	OsR498G0612329400.01.T01	99,69	1,41E-166	586	Os06g0494700	100,00	1,15E-168	590	4	NA	Japonica	
augustus_masked-6-processed-gene-171.7-mRNA-1	6	OsR498G0612334000.01.T02	100,00	1,48E-166	585	Os06g0494000	100,00	4,80E-180	628	43	NA	Japonica	
augustus_masked-6-processed-gene-176.24-mRNA-1	6	OsR498G0612571700.01.T01	99,87	0,00E+00	2688	Os06g0501900	100,00	0,00E+00	2738	50	NA	Japonica	
augustus_masked-6-processed-gene-177.23-mRNA-1	6	OsR498G0612565500.01.T01	99,63	0,00E+00	3343	Os06g0503100	99,65	0,00E+00	2515	-828	Indica	NA	
augustus_masked-6-processed-gene-177.4-mRNA-1	6	OsR498G0612566000.01.T02	98,81	0,00E+00	2658	Os06g0502900	100,00	0,00E+00	2733	75	NA	Japonica	
augustus_masked-6-processed-gene-179.11-mRNA-1	6	OsR498G0612587700.01.T03	99,83	0,00E+00	4132	Os06g0506100	100,00	0,00E+00	4150	18	NA	Japonica	
augustus_masked-6-processed-gene-180.0-mRNA-1	6	OsR498G0612592000.01.T01	98,49	0,00E+00	691	Os06g0507101	100,00	0,00E+00	720	29	NA	Japonica	
augustus_masked-6-processed-gene-180.1-mRNA-1	6	OsR498G0612592400.01.T01	99,84	0,00E+00	1089	Os06g0507300	100,00	0,00E+00	1094	5	NA	Japonica	
augustus_masked-6-processed-gene-182.21-mRNA-1	6	OsR498G0612565500.01.T01	99,73	0,00E+00	3360	Os06g0503100	99,79	0,00E+00	2531	-829	Indica	NA	NBS-TM
augustus_masked-6-processed-gene-185.19-mRNA-1	6	OsR498G0612611200.01.T01	98,42	0,00E+00	875	Os06g0513754	99,58	0,00E+00	841	-34	NA	NA	
augustus_masked-6-processed-gene-185.4-mRNA-1	6	OsR498G0612611200.01.T01	98,42	0,00E+00	875	Os06g0513754	100,00	0,00E+00	850	-25	NA	NA	
augustus_masked-6-processed-gene-19.71-mRNA-1	6	OsR498G0611590800.01.T01	99,40	0,00E+00	1765	Os06g0136900	99,40	0,00E+00	1765	0	NA	NA	
augustus_masked-6-processed-gene-191.31-mRNA-1	6	OsR498G0612633600.01.T01	99,86	0,00E+00	1286	Os12g0119800	74,11	7,43E-93	340	-946	Indica	Japonica	
augustus_masked-6-processed-gene-192.18-mRNA-1	6	OsR498G0612638500.01.T03	99,79	0,00E+00	3386	Os06g0523100	100,00	0,00E+00	2511	-875	Indica	Japonica	
augustus_masked-6-processed-gene-193.7-mRNA-1	6	OsR498G0612640000.01.T01	100,00	2,37E-150	531	Os06g0523300	100,00	8,26E-151	531	0	NA	Japonica	
augustus_masked-6-processed-gene-193.8-mRNA-1	6	OsR498G0612640400.01.T06	100,00	9,24E-156	549	Os06g0523400	100,00	1,37E-154	544	-5	NA	Japonica	
augustus_masked-6-processed-gene-196.4-mRNA-1	6	OsR498G0100964300.01.T01	81,16	0,00E+00	1244	Os06g0528700	99,86	0,00E+00	2621	1377	Japonica	Japonica	
augustus_masked-6-processed-gene-199.12-mRNA-1	6	OsR498G0612688700.01.T01	98,49	0,00E+00	1150	Os06g0534900	100,00	0,00E+00	2235	1085	Japonica	Japonica	
augustus_masked-6-processed-gene-199.9-mRNA-1	6	OsR498G0612688300.01.T01	99,81	0,00E+00	1880	Os06g0534800	100,00	0,00E+00	1853	-27	NA	Japonica	
augustus_masked-6-processed-gene-2.14-mRNA-1	6	OsR498G1119695000.01.T01	82,01	0,00E+00	1382	Os06g0103200	99,93	0,00E+00	2496	1114	Japonica	Japonica	
augustus_masked-6-processed-gene-20.66-mRNA-1	6	OsR498G0611597900.01.T01	100,00	0,00E+00	1900	Os06g0138900	100,00	0,00E+00	1900	0	NA	NA	
augustus_masked-6-processed-gene-20.68-mRNA-1	6	OsR498G0611598700.01.T01	100,00	0,00E+00	1283	Os01g0881800	83,93	2,56E-149	528	-755	Indica	NA	
augustus_masked-6-processed-gene-20.71-mRNA-1	6	OsR498G0611599500.01.T01	100,00	0,00E+00	1591	Os06g0139800	100,00	0,00E+00	1591	0	NA	NA	
augustus_masked-6-processed-gene-20.86-mRNA-1	6	OsR498G0611596000.01.T01	100,00	0,00E+00	2078	Os06g0138500	100,00	0,00E+00	2078	0	NA	NA	
augustus_masked-6-processed-gene-201.14-mRNA-1	6	OsR498G0612697700.01.T01	100,00	0,00E+00	818	Os06g0537600	99,56	0,00E+00	809	-9	NA	NA	
augustus_masked-6-processed-gene-201.39-mRNA-1	6	OsR498G0612699600.01.T01	99,64	0,00E+00	987	Os06g0538400	99,84	0,00E+00	1122	135	Japonica	NA	
augustus_masked-6-processed-gene-206.15-mRNA-1	6	OsR498G0612721800.01.T01	99,63	0,00E+00	2877	Os06g0548100	99,57	0,00E+00	2873	-4	NA	Indica	
augustus_masked-6-processed-gene-206.16-mRNA-1	6	OsR498G0612722200.01.T01	99,44	0,00E+00	2858	Os06g0548200	99,88	0,00E+00	2886	28	NA	NA	
augustus_masked-6-processed-gene-208.2-mRNA-1	6	OsR498G0612734500.01.T01	98,91	0,00E+00	1443	Os02g0232900	75,18	3,64E-129	461	-982	Indica	Japonica	
augustus_masked-6-processed-gene-209.35-mRNA-1	6	OsR498G0612743600.01.T03	99,69	0,00E+00	2295	Os06g0554300	100,00	0,00E+00	2289	-6	NA	Japonica	
augustus_masked-6-processed-gene-209.36-mRNA-1	6	OsR498G0612745200.01.T01	99,88	0,00E+00	1527	Os06g0554600	100,00	0,00E+00	1445	-82	NA	Japonica	
augustus_masked-6-processed-gene-21.33-mRNA-1	6	OsR498G0611600200.01.T01	99,97	0,00E+00	5201	Os06g0140000	99,97	0,00E+00	5201	0	NA	NA	LRR-TM
augustus_masked-6-processed-gene-21.35-mRNA-1	6	OsR498G0611600600.01.T01	100,00	0,00E+00	3148	Os06g0140200	100,00	0,00E+00	3148	0	NA	NA	LRR-TM
augustus_masked-6-processed-gene-21.36-mRNA-1	6	OsR498G0611601000.01.T01	100,00	0,00E+00	4789	Os06g0140300	100,00	0,00E+00	4789	0	NA	NA	LRR-TM
augustus_masked-6-processed-gene-21.42-mRNA-1	6	OsR498G0611601900.01.T01	100,00	0,00E+00	921	Os06g0140700	100,00	0,00E+00	921	0	NA	NA	
augustus_masked-6-processed-gene-210.24-mRNA-1	6	OsR498G0612751900.01.T01	99,80	0,00E+00	1773	Os06g0556200	99,51	0,00E+00	1089	-684	Indica	Japonica	
augustus_masked-6-processed-gene-211.22-mRNA-1	6	OsR498G0612758000.01.T01	94,57	0,00E+00	2552	Os06g0557400	100,00	0,00E+00	2949	397	Japonica	Japonica	LRR-TM
augustus_masked-6-processed-gene-214.4-mRNA-1	6	OsR498G0612770700.01.T01	99,36	0,00E+00	1108	Os06g0562200	100,00	0,00E+00	1126	18	NA	Japonica	
augustus_masked-6-processed-gene-216.0-mRNA-1	6	OsR498G0612787500.01.T02	100,00	0,00E+00	2571	Os06g0565000	100,00	0,00E+00	3723	1152	Japonica	Japonica	
augustus_masked-6-processed-gene-217.12-mRNA-1	6	OsR498G0612796100.01.T01	99,13	1,49E-113	408	Os06g0567000	100,00	1,01E-116	417	9	NA	Japonica	
augustus_masked-6-processed-gene-218.16-mRNA-1	6	OsR498G0612797900.01.T01	99,11	0,00E+00	789	Os06g0568800	100,00	0,00E+00	948	159	Japonica	Japonica	
augustus_masked-6-processed-gene-218.17-mRNA-1	6	OsR498G0816542100.01.T01	86,18	3,77E-153	542	Os06g0569300	100,00	0,00E+00	1434	892	Japonica	Japonica	
augustus_masked-6-processed-gene-218.31-mRNA-1	6	OsR498G0612797400.01.T01	96,21	0,00E+00	2323	Os06g0568600	96,35	0,00E+00	2332	9	NA	Japonica	
augustus_masked-6-processed-gene-220.23-mRNA-1	6	OsR498G0916743100.01.T07	75,62	1,14E-39	163	Os06g0572000	100,00	2,51E-131	466	303	Japonica	Japonica	
augustus_masked-6-processed-gene-221.22-mRNA-1	6	OsR498G0612810500.01.T02	99,52	0,00E+00	2605	Os06g0573700	100,00	0,00E+00	2625	20	NA	Japonica	
augustus_masked-6-processed-gene-222.36-mRNA-1	6	OsR498G0612819600.01.T01	99,71	0,00E+00	1821	Os04g0205200	70,29	1,71E-97	356	-1465	Indica	Japonica	
augustus_masked-6-processed-gene-226.9-mRNA-1	6	OsR498G0612842700.01.T01	97,82	0,00E+00	3442	Os06g0585200	98,12	0,00E+00	2736	-706	Indica	NA	
augustus_masked-6-processed-gene-23.0-mRNA-1	6	OsR498G0611611000.01.T02	98,58	0,00E+00	3564	Os06g0143700	98,58	0,00E+00	3564	0	NA	NA	
augustus_masked-6-processed-gene-231.29-mRNA-1	6	OsR498G0612856100.01.T01	97,94	0,00E+00	5077	Os06g0587500	94,61	0,00E+00	3357	-1720	Indica	Japonica	Kinase-LRR-TM
augustus_masked-6-processed-gene-231.42-mRNA-1	6	OsR498G0612856600.01.T02	99,91	0,00E+00	2090	Os06g0588900	100,00	0,00E+00	2094	4	NA	Japonica	
augustus_masked-6-processed-gene-232.15-mRNA-1	6	OsR498G0612863700.01.T01	99,59	0,00E+00	2603	Os06g0590700	99,73	0,00E+00	2612	9	NA	NA	
augustus_masked-6-processed-gene-232.33-mRNA-1	6	OsR498G0612864400.01.T01	99,47	0,00E+00	2659	Os06g0590700	88,66	0,00E+00	1903	-756	Indica	NA	
augustus_masked-6-processed-gene-233.37-mRNA-1	6	OsR498G0612872400.01.T01	99,72	0,00E+00	2524	Os06g0593800	100,00	0,00E+00	2544	20	NA	Japonica	
augustus_masked-6-processed-gene-234.17-mRNA-1	6	OsR498G0612874800.01.T01	99,51	0,00E+00	3958	Os06g0594200	100,00	0,00E+00	7651	3693	Japonica	Japonica	CC-NBS-TM
augustus_masked-6-processed-gene-234.23-mRNA-1	6	OsR498G0612875900.01.T01	99,74	0,00E+00	2054	Os06g0594400	99,91	0,00E+00	2063	9	NA	NA	
augustus_masked-6-processed-gene-236.10-mRNA-1	6	OsR498G0612883400.01.T01	99,74	0,00E+00	2796	Os06g0597500	99,25	0,00E+00	2819	23	NA	Indica	
augustus_masked-6-processed-gene-236.14-mRNA-1	6	OsR498G0612885700.01.T02	99,48	0,00E+00	2760	Os06g0598800	99,29	0,00E+00	2746	-14	NA	Japonica	
augustus_masked-6-processed-gene-236.15-mRNA-1	6	OsR498G0204145300.01.T01	94,08	0,00E+00	2310	Os06g0599200	99,93	0,00E+00	2707	397	Japonica	Indica	
augustus_masked-6-processed-gene-237.8-mRNA-1	6	OsR498G0612888300.01.T01	99,69	0,00E+00	2894	Os06g0600400	100,00	0,00E+00	2917	23	NA	Japonica	
augustus_masked-6-processed-gene-238.11-mRNA-1	6	OsR498G0612892900.01.T01	97,14	0,00E+00	2126	Os06g0602400	100,00	0,00E+00	2273	147	NA	Japonica	
augustus_masked-6-processed-gene-238.19-mRNA-1	6	OsR498G0612893400.01.T01	99,67	0,00E+00	4369	Os06g0602500	100,00	0,00E+00	4405	36	NA	Japonica	Kinase-TM
augustus_masked-6-processed-gene-24.16-mRNA-1	6	OsR498G0611623700.01.T01	100,00	1,72E-178	625	Os06g0147250	100,00	5,96E-179	625	0	NA	NA	
augustus_masked-6-processed-gene-24.17-mRNA-1	6	OsR498G0611624700.01.T01	100,00	0,00E+00	829	Os06g0147300	100,00	0,00E+00	655	-174	Indica	NA	
augustus_masked-6-processed-gene-24.27-mRNA-1	6	OsR498G0611622000.01.T02	72,01	1,63E-39	164	Os06g0147000	100,00	0,00E+00	645	481	Japonica	NA	
augustus_masked-6-processed-gene-24.30-mRNA-1	6	OsR498G0203221200.01.T05	70,23	1,83E-14	81,5	Os06g0147600	100,00	0,00E+00	764	682,5	Japonica	NA	
augustus_masked-6-processed-gene-241.41-mRNA-1	6	OsR498G0612914100.01.T01	99,78	0,00E+00	1658	Os06g0607100	100,00	0,00E+00	1667	9	NA	Japonica	
augustus_masked-6-processed-gene-241.49-mRNA-1	6	OsR498G0612911400.01.T03	99,90	0,00E+00	1883	Os06g0606800	99,14	0,00E+00	1850	-33	NA	Japonica	
augustus_masked-6-processed-gene-244.40-mRNA-1	6	OsR498G0612925400.01.T01	99,80	0,00E+00	2676	Os06g0611200	100,00	0,00E+00	3333	657	Japonica	Japonica	
augustus_masked-6-processed-gene-245.16-mRNA-1	6	OsR498G0612933300.01.T05	99,81	0,00E+00	927	Os06g0612800	100,00	0,00E+00	931	4	NA	Japonica	
augustus_masked-6-processed-gene-246.2-mRNA-1	6	OsR498G1019138600.01.T01	70,59	9,89E-09	61,7	Os06g0614400	100,00	9,87E-124	441	379,3	Japonica	Japonica	
augustus_masked-6-processed-gene-247.13-mRNA-1	6	OsR498G0101162300.01.T01	75,00	2,84E-37	157	Os06g0616900	100,00	0,00E+00	747	590	Japonica	Japonica	
augustus_masked-6-processed-gene-248.39-mRNA-1	6	OsR498G0612961000.01.T01	98,59	0,00E+00	984	Os06g0618700	100,00	0,00E+00	1023	39	NA	Japonica	
augustus_masked-6-processed-gene-249.45-mRNA-1	6	OsR498G0613021000.01.T01	95,35	4,59E-134	478	Os06g0621300	100,00	1,23E-148	524	46	NA	NA	
augustus_masked-6-processed-gene-25.27-mRNA-1	6	OsR498G0611628500.01.T01	100,00	0,00E+00	2132	Os06g0148600	100,00	0,00E+00	2132	0	NA	NA	
augustus_masked-6-processed-gene-25.39-mRNA-1	6	OsR498G0611629300.01.T01	100,00	0,00E+00	2154	Os06g0148800	100,00	0,00E+00	2080	-74	NA	NA	
augustus_masked-6-processed-gene-251.1-mRNA-1	6	OsR498G0612972300.01.T01	99,33	0,00E+00	1577	Os07g0543600	71,46	2,97E-112	405	-1172	Indica	Japonica	
augustus_masked-6-processed-gene-251.7-mRNA-1	6	OsR498G0612972200.01.T01	99,15	0,00E+00	1667	Os07g0543600	72,11	6,43E-102	370	-1297	Indica	Japonica	
augustus_masked-6-processed-gene-252.18-mRNA-1	6	OsR498G0612977500.01.T03	99,74	0,00E+00	1373	Os06g0624900	100,00	0,00E+00	1499	126	NA	Japonica	
augustus_masked-6-processed-gene-252.19-mRNA-1	6	OsR498G0612978300.01.T01	99,50	0,00E+00	1075	Os06g0625000	100,00	0,00E+00	720	-355	Indica	Japonica	
augustus_masked-6-processed-gene-252.24-mRNA-1	6	OsR498G0612980700.01.T01	99,95	0,00E+00	3610	Os06g0625300	100,00	0,00E+00	3615	5	NA	NA	Kinase-TM
augustus_masked-6-processed-gene-253.26-mRNA-1	6	OsR498G0612988400.01.T01	99,73	0,00E+00	2000	Os06g0626600	100,00	0,00E+00	2013	13	NA	Japonica	
augustus_masked-6-processed-gene-254.48-mRNA-1	6	OsR498G0613102400.01.T05	99,83	0,00E+00	2114	Os06g0659300	100,00	0,00E+00	2123	9	NA	NA	
augustus_masked-6-processed-gene-254.49-mRNA-1	6	OsR498G0612992400.01.T01	99,41	1,59E-172	605	Os06g0627400	100,00	1,07E-175	614	9	NA	Japonica	
augustus_masked-6-processed-gene-255.13-mRNA-1	6	OsR498G0612993000.01.T01	99,55	0,00E+00	2376	Os06g0627500	100,00	0,00E+00	2403	27	NA	Japonica	LRR-TM
augustus_masked-6-processed-gene-258.14-mRNA-1	6	OsR498G0613018300.01.T02	98,82	0,00E+00	2076	Os06g0633900	98,99	0,00E+00	2085	9	NA	Japonica	
augustus_masked-6-processed-gene-259.35-mRNA-1	6	OsR498G0613019800.01.T03	99,79	0,00E+00	1703	Os06g0634300	100,00	0,00E+00	1645	-58	NA	Japonica	
augustus_masked-6-processed-gene-26.1-mRNA-1	6	OsR498G0612168000.01.T01	99,17	0,00E+00	2554	Os06g0289900	99,31	0,00E+00	2563	9	NA	NA	
augustus_masked-6-processed-gene-26.11-mRNA-1	6	OsR498G0611636200.01.T01	71,50	0,00E+00	697	Os06g0150550	100,00	0,00E+00	2571	1874	Japonica	NA	
augustus_masked-6-processed-gene-26.19-mRNA-1	6	OsR498G0612168000.01.T01	99,17	0,00E+00	2554	Os06g0289900	99,31	0,00E+00	2563	9	NA	NA	
augustus_masked-6-processed-gene-26.23-mRNA-1	6	OsR498G0611633400.01.T01	100,00	0,00E+00	1196	Os06g0150200	100,00	0,00E+00	1196	0	NA	NA	
augustus_masked-6-processed-gene-261.16-mRNA-1	6	OsR498G0613035300.01.T01	99,72	0,00E+00	1897	Os06g0639300	99,91	0,00E+00	1906	9	NA	Japonica	
augustus_masked-6-processed-gene-261.17-mRNA-1	6	OsR498G0613036700.01.T01	99,74	0,00E+00	3392	Os06g0639500	100,00	0,00E+00	3415	23	NA	Japonica	Kinase-TM
augustus_masked-6-processed-gene-262.33-mRNA-1	6	OsR498G0613042000.01.T01	98,49	0,00E+00	909	Os02g0186800	79,44	5,07E-106	383	-526	Indica	NA	
augustus_masked-6-processed-gene-262.7-mRNA-1	6	OsR498G0613038900.01.T01	97,71	0,00E+00	1556	Os06g0640500	100,00	0,00E+00	1651	95	NA	NA	
augustus_masked-6-processed-gene-262.8-mRNA-1	6	OsR498G0613038900.01.T01	99,54	0,00E+00	1154	Os06g0640500	99,54	0,00E+00	1154	0	NA	NA	
augustus_masked-6-processed-gene-263.24-mRNA-1	6	OsR498G0613042200.01.T01	98,73	0,00E+00	960	Os02g0186800	82,01	1,17E-120	432	-528	Indica	NA	
augustus_masked-6-processed-gene-263.25-mRNA-1	6	OsR498G0613042400.01.T01	98,02	0,00E+00	1211	Os06g0641900	100,00	0,00E+00	1674	463	Japonica	NA	
augustus_masked-6-processed-gene-263.34-mRNA-1	6	OsR498G0613043700.01.T01	99,26	0,00E+00	959	Os11g0635500	78,63	3,29E-121	434	-525	Indica	NA	
augustus_masked-6-processed-gene-263.35-mRNA-1	6	OsR498G0613043900.01.T01	99,34	0,00E+00	1081	Os11g0635500	72,71	1,10E-161	569	-512	Indica	NA	
augustus_masked-6-processed-gene-267.10-mRNA-1	6	OsR498G0613069100.01.T01	99,74	0,00E+00	4741	Os06g0649700	100,00	0,00E+00	4763	22	NA	NA	
augustus_masked-6-processed-gene-268.43-mRNA-1	6	OsR498G0613072900.01.T04	96,41	0,00E+00	3290	Os06g0650600	96,72	0,00E+00	3317	27	NA	NA	
augustus_masked-6-processed-gene-268.47-mRNA-1	6	OsR498G0613076300.01.T01	99,80	0,00E+00	2719	Os06g0652100	100,00	0,00E+00	3474	755	Japonica	NA	
augustus_masked-6-processed-gene-269.18-mRNA-1	6	OsR498G0613077300.01.T01	99,48	0,00E+00	1726	Os06g0652400	99,90	0,00E+00	1744	18	NA	NA	
augustus_masked-6-processed-gene-269.31-mRNA-1	6	OsR498G0613079500.01.T01	99,67	0,00E+00	3261	Os06g0653000	99,95	0,00E+00	3284	23	NA	NA	
augustus_masked-6-processed-gene-269.43-mRNA-1	6	OsR498G0613080000.01.T01	100,00	0,00E+00	931	Os06g0653200	100,00	0,00E+00	931	0	NA	NA	
augustus_masked-6-processed-gene-269.46-mRNA-1	6	OsR498G0613081000.01.T01	99,81	0,00E+00	1853	Os06g0653800	100,00	0,00E+00	1862	9	NA	NA	
augustus_masked-6-processed-gene-27.51-mRNA-1	6	OsR498G0611636200.01.T01	100,00	0,00E+00	2592	Os06g0151100	100,00	0,00E+00	2592	0	NA	NA	
augustus_masked-6-processed-gene-270.25-mRNA-1	6	OsR498G0613087200.01.T01	99,44	4,08E-180	630	Os06g0655200	100,00	0,00E+00	639	9	NA	NA	
augustus_masked-6-processed-gene-270.26-mRNA-1	6	OsR498G0613088000.01.T01	99,74	0,00E+00	2070	Os06g0655500	99,91	0,00E+00	2079	9	NA	NA	
augustus_masked-6-processed-gene-271.28-mRNA-1	6	OsR498G0613091300.01.T04	99,54	0,00E+00	1163	Os06g0656300	100,00	0,00E+00	724	-439	Indica	NA	
augustus_masked-6-processed-gene-271.47-mRNA-1	6	OsR498G0613093400.01.T01	98,28	0,00E+00	1511	Os06g0656800	91,61	0,00E+00	1272	-239	Indica	NA	
augustus_masked-6-processed-gene-273.23-mRNA-1	6	OsR498G0613104200.01.T02	98,77	0,00E+00	852	Os06g0659800	100,00	0,00E+00	877	25	NA	NA	
augustus_masked-6-processed-gene-274.61-mRNA-1	6	OsR498G0613111100.01.T02	100,00	2,22E-124	444	Os06g0661600	100,00	7,78E-125	444	0	NA	NA	
augustus_masked-6-processed-gene-275.22-mRNA-1	6	OsR498G0613113200.01.T01	98,32	2,57E-176	618	Os06g0655200	84,62	2,25E-108	390	-228	Indica	NA	
augustus_masked-6-processed-gene-275.23-mRNA-1	6	OsR498G0613113600.01.T01	99,72	0,00E+00	640	Os06g0662300	99,72	0,00E+00	640	0	NA	NA	
augustus_masked-6-processed-gene-275.24-mRNA-1	6	OsR498G0613114800.01.T01	100,00	0,00E+00	639	Os06g0662500	100,00	0,00E+00	639	0	NA	NA	
augustus_masked-6-processed-gene-275.25-mRNA-1	6	OsR498G0613115200.01.T01	98,87	2,09E-177	621	Os06g0662600	100,00	0,00E+00	639	18	NA	NA	
augustus_masked-6-processed-gene-275.45-mRNA-1	6	OsR498G0613116400.01.T01	99,44	4,03E-180	630	Os06g0662500	97,74	1,95E-172	603	-27	NA	NA	
augustus_masked-6-processed-gene-275.51-mRNA-1	6	OsR498G0613118400.01.T01	99,15	2,09E-178	626	Os06g0663300	100,00	0,00E+00	639	13	NA	NA	
augustus_masked-6-processed-gene-277.25-mRNA-1	6	OsR498G0613133500.01.T01	99,69	0,00E+00	1150	Os06g0666400	100,00	0,00E+00	1159	9	NA	NA	
augustus_masked-6-processed-gene-277.26-mRNA-1	6	OsR498G0613134900.01.T01	99,08	0,00E+00	963	Os06g0666500	100,00	0,00E+00	985	22	NA	NA	
augustus_masked-6-processed-gene-278.1-mRNA-1	6	OsR498G0613136300.01.T01	99,68	9,31E-156	550	Os06g0666800	99,68	3,24E-156	550	0	NA	NA	
augustus_masked-6-processed-gene-278.12-mRNA-1	6	OsR498G0613137700.01.T01	100,00	0,00E+00	1359	Os06g0667100	99,87	0,00E+00	1354	-5	NA	NA	
augustus_masked-6-processed-gene-278.2-mRNA-1	6	OsR498G0613137300.01.T01	99,97	0,00E+00	5742	Os06g0667000	99,97	0,00E+00	5742	0	NA	NA	Kinase-LRR-TM
augustus_masked-6-processed-gene-278.36-mRNA-1	6	OsR498G0613142000.01.T01	100,00	0,00E+00	1932	Os06g0668400	100,00	0,00E+00	1932	0	NA	NA	
augustus_masked-6-processed-gene-28.0-mRNA-1	6	OsR498G0611639300.01.T01	100,00	0,00E+00	3761	Os06g0152500	100,00	0,00E+00	3635	-126	NA	NA	
augustus_masked-6-processed-gene-28.13-mRNA-1	6	OsR498G0611643900.01.T05	99,95	0,00E+00	3897	Os06g0154200	100,00	0,00E+00	3901	4	NA	NA	
augustus_masked-6-processed-gene-281.0-mRNA-1	6	OsR498G0613158400.01.T03	99,64	0,00E+00	1489	Os06g0672400	99,64	0,00E+00	1489	0	NA	NA	
augustus_masked-6-processed-gene-282.29-mRNA-1	6	OsR498G0613165600.01.T01	99,84	0,00E+00	2226	Os06g0675200	100,00	0,00E+00	2235	9	NA	NA	
augustus_masked-6-processed-gene-282.43-mRNA-1	6	OsR498G0613167000.01.T02	99,56	1,87E-112	406	Os06g0675700	100,00	1,54E-114	410	4	NA	NA	
augustus_masked-6-processed-gene-283.10-mRNA-1	6	OsR498G0613174100.01.T01	99,84	0,00E+00	2188	Os06g0677700	100,00	0,00E+00	2197	9	NA	NA	
augustus_masked-6-processed-gene-283.16-mRNA-1	6	OsR498G0613174000.01.T02	99,43	3,49E-176	618	Os06g0677600	99,43	1,22E-176	618	0	NA	NA	
augustus_masked-6-processed-gene-286.37-mRNA-1	6	OsR498G0613198100.01.T01	98,19	0,00E+00	858	Os06g0114366	94,26	0,00E+00	836	-22	NA	NA	
augustus_masked-6-processed-gene-286.52-mRNA-1	6	OsR498G0613200700.01.T01	99,74	0,00E+00	678	Os06g0683400	100,00	0,00E+00	682	4	NA	NA	
augustus_masked-6-processed-gene-286.53-mRNA-1	6	OsR498G0613202700.01.T01	99,85	0,00E+00	2329	Os06g0683900	100,00	0,00E+00	2338	9	NA	NA	
augustus_masked-6-processed-gene-286.65-mRNA-1	6	OsR498G0613200200.01.T03	99,89	0,00E+00	1574	Os06g0683100	97,26	0,00E+00	1488	-86	NA	NA	
augustus_masked-6-processed-gene-286.69-mRNA-1	6	OsR498G0613203000.01.T01	99,83	0,00E+00	3233	Os06g0683950	100,00	0,00E+00	3247	14	NA	NA	
augustus_masked-6-processed-gene-287.43-mRNA-1	6	OsR498G0613204700.01.T01	99,90	0,00E+00	3692	Os06g0685100	100,00	0,00E+00	3701	9	NA	NA	
augustus_masked-6-processed-gene-287.45-mRNA-1	6	OsR498G0613206500.01.T01	98,19	0,00E+00	667	Os02g0633200	88,50	4,45E-67	253	-414	Indica	NA	
augustus_masked-6-processed-gene-288.54-mRNA-1	6	OsR498G0613210300.01.T01	99,56	0,00E+00	2012	Os06g0687200	100,00	0,00E+00	2035	23	NA	Japonica	
augustus_masked-6-processed-gene-289.7-mRNA-1	6	OsR498G0613213400.01.T01	97,93	0,00E+00	663	Os09g0472900	77,50	2,30E-64	244	-419	Indica	Japonica	
augustus_masked-6-processed-gene-289.8-mRNA-1	6	OsR498G0613213600.01.T01	95,70	4,21E-125	448	Os09g0472900	77,50	2,84E-52	205	-243	Indica	Japonica	
augustus_masked-6-processed-gene-29.59-mRNA-1	6	OsR498G0611653500.01.T01	99,92	0,00E+00	2187	Os06g0156700	99,92	0,00E+00	2187	0	NA	NA	
augustus_masked-6-processed-gene-290.36-mRNA-1	6	OsR498G0613224200.01.T01	99,72	0,00E+00	5717	Os06g0692600	87,62	0,00E+00	4001	-1716	Indica	Japonica	Kinase-LRR-TM
augustus_masked-6-processed-gene-290.37-mRNA-1	6	OsR498G0613225000.01.T01	99,07	0,00E+00	5666	Os06g0692600	99,97	0,00E+00	5769	103	NA	Japonica	Kinase-LRR-TM
augustus_masked-6-processed-gene-290.7-mRNA-1	6	OsR498G0613218100.01.T02	99,73	0,00E+00	4555	Os06g0690900	100,00	0,00E+00	5368	813	Japonica	Japonica	
augustus_masked-6-processed-gene-290.8-mRNA-1	6	OsR498G0613218700.01.T01	99,30	0,00E+00	1009	Os06g0691100	100,00	0,00E+00	1023	14	NA	Japonica	
augustus_masked-6-processed-gene-290.9-mRNA-1	6	OsR498G0613219300.01.T02	100,00	0,00E+00	1364	Os06g0691200	100,00	0,00E+00	1364	0	NA	Japonica	
augustus_masked-6-processed-gene-291.11-mRNA-1	6	OsR498G0613228500.01.T01	99,79	0,00E+00	1669	Os06g0693700	99,89	0,00E+00	1673	4	NA	Japonica	
augustus_masked-6-processed-gene-291.33-mRNA-1	6	OsR498G0613230800.01.T01	100,00	0,00E+00	1337	Os06g0694600	100,00	0,00E+00	2625	1288	Japonica	Japonica	
augustus_masked-6-processed-gene-292.26-mRNA-1	6	OsR498G0613235300.01.T01	99,72	0,00E+00	3241	Os06g0695700	100,00	0,00E+00	3263	22	NA	Japonica	
augustus_masked-6-processed-gene-292.34-mRNA-1	6	OsR498G0613236200.01.T01	99,50	0,00E+00	2136	Os06g0695900	100,00	0,00E+00	2159	23	NA	Japonica	
augustus_masked-6-processed-gene-293.18-mRNA-1	6	OsR498G0613242700.01.T01	100,00	0,00E+00	2273	Os06g0697500	95,89	0,00E+00	2479	206	NA	Indica	
augustus_masked-6-processed-gene-294.12-mRNA-1	6	OsR498G0613243200.01.T01	99,80	0,00E+00	2709	Os06g0697600	99,27	0,00E+00	2684	-25	NA	Indica	
augustus_masked-6-processed-gene-294.22-mRNA-1	6	OsR498G0613245900.01.T01	99,85	0,00E+00	3634	Os06g0698600	99,95	0,00E+00	3643	9	NA	Indica	
augustus_masked-6-processed-gene-294.33-mRNA-1	6	OsR498G0613246200.01.T02	99,70	0,00E+00	2423	Os06g0698674	99,63	0,00E+00	2418	-5	NA	NA	
augustus_masked-6-processed-gene-294.4-mRNA-1	6	OsR498G0613244900.01.T01	100,00	1,34E-153	542	Os06g0698400	100,00	4,67E-154	542	0	NA	NA	
augustus_masked-6-processed-gene-295.37-mRNA-1	6	OsR498G0613249300.01.T03	99,91	0,00E+00	1998	Os06g0699400	99,73	0,00E+00	1989	-9	NA	Indica	Kinase-TM
augustus_masked-6-processed-gene-295.52-mRNA-1	6	OsR498G0613251700.01.T01	99,72	0,00E+00	4411	Os06g0700000	99,72	0,00E+00	4411	0	NA	Indica	
augustus_masked-6-processed-gene-295.53-mRNA-1	6	OsR498G0613251900.01.T01	99,93	0,00E+00	2577	Os06g0700100	99,72	0,00E+00	2563	-14	NA	Indica	
augustus_masked-6-processed-gene-296.7-mRNA-1	6	OsR498G0613255900.01.T01	100,00	0,00E+00	661	Os01g0532700	81,85	4,18E-86	316	-345	Indica	Indica	
augustus_masked-6-processed-gene-297.23-mRNA-1	6	OsR498G0613262200.01.T01	93,24	0,00E+00	892	Os06g0702200	98,91	0,00E+00	1934	1042	Japonica	NA	
augustus_masked-6-processed-gene-297.26-mRNA-1	6	OsR498G0613262800.01.T01	99,70	0,00E+00	1816	Os06g0702400	99,41	0,00E+00	1802	-14	NA	Indica	
augustus_masked-6-processed-gene-297.9-mRNA-1	6	OsR498G0613261700.01.T03	99,72	0,00E+00	1285	Os06g0702100	99,70	0,00E+00	1200	-85	NA	Japonica	
augustus_masked-6-processed-gene-299.1-mRNA-1	6	OsR498G0613270100.01.T01	99,89	0,00E+00	1661	Os06g0704100	100,00	0,00E+00	1665	4	NA	Indica	
augustus_masked-6-processed-gene-299.12-mRNA-1	6	OsR498G0613272700.01.T01	95,23	0,00E+00	1731	Os06g0705000	99,38	0,00E+00	1421	-310	Indica	NA	
augustus_masked-6-processed-gene-299.26-mRNA-1	6	OsR498G0613274200.01.T01	99,76	0,00E+00	3783	Os02g0191200	90,39	0,00E+00	2905	-878	Indica	NA	
augustus_masked-6-processed-gene-3.1-mRNA-1	6	OsR498G0611474500.01.T01	99,89	0,00E+00	1636	Os06g0104100	100,00	0,00E+00	1640	4	NA	Japonica	
augustus_masked-6-processed-gene-3.22-mRNA-1	6	OsR498G0611476400.01.T01	99,91	0,00E+00	1949	Os06g0104900	100,00	0,00E+00	1954	5	NA	Japonica	
augustus_masked-6-processed-gene-300.24-mRNA-1	6	OsR498G0613277600.01.T01	99,59	0,00E+00	1309	Os02g0623600	72,53	8,98E-105	380	-929	Indica	Indica	Kinase
augustus_masked-6-processed-gene-300.26-mRNA-1	6	OsR498G0613278000.01.T01	94,58	4,27E-167	588	Os06g0706700	99,72	4,85E-180	629	41	NA	NA	
augustus_masked-6-processed-gene-300.30-mRNA-1	6	OsR498G0613279000.01.T01	99,65	0,00E+00	2535	Os06g0707300	99,72	0,00E+00	2540	5	NA	NA	
augustus_masked-6-processed-gene-301.13-mRNA-1	6	OsR498G0613284300.01.T01	99,91	0,00E+00	2079	Os06g0709000	99,91	0,00E+00	2079	0	NA	Indica	
augustus_masked-6-processed-gene-301.30-mRNA-1	6	OsR498G0613286600.01.T01	99,70	0,00E+00	2423	Os06g0710700	99,63	0,00E+00	2418	-5	NA	NA	
augustus_masked-6-processed-gene-302.14-mRNA-1	6	OsR498G0613291300.01.T01	98,50	0,00E+00	1280	Os06g0712200	100,00	0,00E+00	1310	30	NA	NA	
augustus_masked-6-processed-gene-302.26-mRNA-1	6	OsR498G0613289800.01.T02	99,47	0,00E+00	1015	Os06g0711800	97,60	0,00E+00	994	-21	NA	Indica	
augustus_masked-6-processed-gene-302.6-mRNA-1	6	OsR498G0613287200.01.T01	99,93	0,00E+00	2409	Os06g0710900	100,00	0,00E+00	1581	-828	Indica	Indica	
augustus_masked-6-processed-gene-302.7-mRNA-1	6	OsR498G0613287400.01.T01	98,99	0,00E+00	1040	Os06g0711000	99,68	0,00E+00	1122	82	NA	Indica	
augustus_masked-6-processed-gene-302.8-mRNA-1	6	OsR498G0613288400.01.T01	99,66	0,00E+00	2125	Os06g0711700	99,54	0,00E+00	2343	218	NA	Indica	
augustus_masked-6-processed-gene-303.39-mRNA-1	6	OsR498G0613296500.01.T01	99,31	0,00E+00	1801	Os06g0713000	99,67	0,00E+00	1095	-706	Indica	Indica	
augustus_masked-6-processed-gene-304.44-mRNA-1	6	OsR498G0613300200.01.T02	100,00	0,00E+00	742	Os06g0714300	96,01	0,00E+00	701	-41	NA	Indica	
augustus_masked-6-processed-gene-304.60-mRNA-1	6	OsR498G0917833300.01.T01	75,40	1,27E-126	454	Os06g0714600	100,00	0,00E+00	1223	769	Japonica	Indica	
augustus_masked-6-processed-gene-304.61-mRNA-1	6	OsR498G0613301800.01.T01	100,00	0,00E+00	937	Os06g0714700	100,00	0,00E+00	937	0	NA	Indica	
augustus_masked-6-processed-gene-305.1-mRNA-1	6	OsR498G0613306500.01.T01	100,00	0,00E+00	726	Os06g0716100	99,75	0,00E+00	721	-5	NA	Indica	
augustus_masked-6-processed-gene-305.23-mRNA-1	6	OsR498G0613307800.01.T01	100,00	0,00E+00	1970	Os06g0716800	99,76	0,00E+00	1469	-501	Indica	Indica	
augustus_masked-6-processed-gene-308.35-mRNA-1	6	OsR498G0613330300.01.T01	99,77	0,00E+00	1566	Os06g0725700	72,23	1,44E-96	353	-1213	Indica	Indica	
augustus_masked-6-processed-gene-309.47-mRNA-1	6	OsR498G0613335700.01.T03	93,90	0,00E+00	1783	Os06g0727700	72,94	2,29E-177	622	-1161	Indica	Indica	
augustus_masked-6-processed-gene-309.56-mRNA-1	6	OsR498G0613333800.01.T01	100,00	0,00E+00	1002	Os06g0727000	99,82	0,00E+00	997	-5	NA	Indica	
augustus_masked-6-processed-gene-31.37-mRNA-1	6	OsR498G0611659900.01.T01	100,00	0,00E+00	1673	Os06g0158800	100,00	0,00E+00	1673	0	NA	NA	
augustus_masked-6-processed-gene-310.34-mRNA-1	6	OsR498G0613336100.01.T01	99,92	0,00E+00	2290	Os06g0727900	99,94	0,00E+00	2923	633	Japonica	Indica	
augustus_masked-6-processed-gene-310.37-mRNA-1	6	OsR498G0613337700.01.T04	100,00	0,00E+00	2024	Os06g0728500	99,64	0,00E+00	2006	-18	NA	Indica	
augustus_masked-6-processed-gene-312.31-mRNA-1	6	OsR498G0613277600.01.T01	99,59	0,00E+00	1309	Os02g0623600	72,53	8,98E-105	380	-929	Indica	NA	Kinase
augustus_masked-6-processed-gene-312.33-mRNA-1	6	OsR498G0613278000.01.T01	94,58	4,27E-167	588	Os06g0706700	99,72	4,85E-180	629	41	NA	NA	

augustus_masked-6-processed-gene-32.72-mRNA-1	6	OsR498G0611670700.01.T01	100,00	0,00E+00	1058	Os06g0161900	71,94	5,31E-75	280	-778	Indica	NA	
augustus_masked-6-processed-gene-34.59-mRNA-1	6	OsR498G0611683900.01.T01	99,76	0,00E+00	737	Os05g0393900	70,13	1,66E-47	189	-548	Indica	NA	
augustus_masked-6-processed-gene-4.0-mRNA-1	6	OsR498G0611477700.01.T01	98,76	0,00E+00	2522	Os06g0105200	100,00	0,00E+00	1815	-707	Indica	Japonica	
augustus_masked-6-processed-gene-4.29-mRNA-1	6	OsR498G0611482600.01.T02	99,85	0,00E+00	1165	Os06g0106900	100,00	0,00E+00	1169	4	NA	Japonica	
augustus_masked-6-processed-gene-4.5-mRNA-1	6	OsR498G0611478000.01.T02	99,00	0,00E+00	3006	Os06g0105400	100,00	0,00E+00	2929	-77	NA	Japonica	
augustus_masked-6-processed-gene-40.2-mRNA-1	6	OsR498G0611718100.01.T01	100,00	0,00E+00	3177	Os06g0179500	100,00	0,00E+00	3177	0	NA	NA	
augustus_masked-6-processed-gene-42.44-mRNA-1	6	OsR498G0611733800.01.T03	99,81	0,00E+00	1891	Os06g0183900	100,00	0,00E+00	1900	9	NA	NA	
augustus_masked-6-processed-gene-43.0-mRNA-1	6	OsR498G0611736100.01.T01	99,71	0,00E+00	1839	Os06g0184400	99,81	0,00E+00	1844	5	NA	Japonica	
augustus_masked-6-processed-gene-43.10-mRNA-1	6	OsR498G0611739300.01.T01	98,79	0,00E+00	2313	Os06g0185500	91,87	0,00E+00	1897	-416	Indica	Indica	
augustus_masked-6-processed-gene-43.21-mRNA-1	6	OsR498G0611738000.01.T01	100,00	0,00E+00	2105	Os06g0184866	98,46	0,00E+00	2024	-81	NA	Indica	
augustus_masked-6-processed-gene-43.8-mRNA-1	6	OsR498G0611738500.01.T01	99,93	0,00E+00	2398	Os06g0184900	97,01	0,00E+00	2230	-168	NA	Indica	
augustus_masked-6-processed-gene-44.42-mRNA-1	6	OsR498G0611740100.01.T01	99,77	0,00E+00	3137	Os06g0185700	96,80	0,00E+00	3374	237	NA	Indica	
augustus_masked-6-processed-gene-44.48-mRNA-1	6	OsR498G0611740600.01.T01	100,00	0,00E+00	3328	Os06g0185800	97,97	0,00E+00	4048	720	Japonica	Indica	
augustus_masked-6-processed-gene-45.29-mRNA-1	6	OsR498G0611746400.01.T01	99,87	0,00E+00	2783	Os06g0187500	99,87	0,00E+00	2783	0	NA	NA	
augustus_masked-6-processed-gene-47.12-mRNA-1	6	OsR498G0204906600.01.T01	83,93	0,00E+00	1090	Os06g0191300	99,76	0,00E+00	1512	422	Japonica	Japonica	Kinase-TM
augustus_masked-6-processed-gene-47.21-mRNA-1	6	OsR498G0611761500.01.T01	99,90	0,00E+00	1738	Os06g0192800	100,00	0,00E+00	1754	16	NA	Japonica	
augustus_masked-6-processed-gene-48.3-mRNA-1	6	OsR498G0611762700.01.T02	95,63	0,00E+00	2864	Os06g0193200	99,94	0,00E+00	3167	303	NA	Japonica	
augustus_masked-6-processed-gene-49.31-mRNA-1	6	OsR498G0611778200.01.T02	99,95	0,00E+00	3659	Os07g0110200	97,01	0,00E+00	1728	-1931	Indica	Indica	
augustus_masked-6-processed-gene-49.32-mRNA-1	6	OsR498G0611779600.01.T01	99,84	0,00E+00	1122	Os06g0196200	99,84	0,00E+00	1122	0	NA	Indica	
augustus_masked-6-processed-gene-5.39-mRNA-1	6	OsR498G0611488200.01.T03	99,82	0,00E+00	1961	Os06g0109000	100,00	0,00E+00	1970	9	NA	Japonica	
augustus_masked-6-processed-gene-5.41-mRNA-1	6	OsR498G0611490800.01.T01	99,61	0,00E+00	1378	Os06g0109500	99,89	0,00E+00	1641	263	Japonica	Japonica	
augustus_masked-6-processed-gene-51.1-mRNA-1	6	OsR498G0611789100.01.T01	99,95	0,00E+00	3751	Os06g0198900	100,00	0,00E+00	3755	4	NA	NA	Kinase-LRR-TM
augustus_masked-6-processed-gene-51.2-mRNA-1	6	OsR498G0611840000.01.T01	75,59	8,16E-79	296	Os06g0199000	74,22	3,94E-71	268	-28	NA	NA	
augustus_masked-6-processed-gene-52.0-mRNA-1	6	OsR498G0611796500.01.T01	100,00	0,00E+00	1370	Os06g0201000	100,00	0,00E+00	1370	0	NA	NA	
augustus_masked-6-processed-gene-52.21-mRNA-1	6	OsR498G0611801700.01.T01	99,48	0,00E+00	1021	Os06g0202400	99,48	0,00E+00	1021	0	NA	NA	
augustus_masked-6-processed-gene-52.22-mRNA-1	6	OsR498G0611801500.01.T03	99,87	0,00E+00	1408	Os06g0202700	73,07	2,33E-93	342	-1066	Indica	NA	
augustus_masked-6-processed-gene-55.11-mRNA-1	6	OsR498G0611824500.01.T04	100,00	0,00E+00	4589	Os06g0207500	100,00	0,00E+00	3901	-688	Indica	NA	
augustus_masked-6-processed-gene-56.39-mRNA-1	6	OsR498G0611834100.01.T01	99,85	0,00E+00	2453	Os06g0209100	99,85	0,00E+00	2453	0	NA	NA	
augustus_masked-6-processed-gene-56.40-mRNA-1	6	OsR498G0611835500.01.T02	99,78	0,00E+00	1599	Os02g0768700	72,70	1,38E-90	333	-1266	Indica	Indica	
augustus_masked-6-processed-gene-57.31-mRNA-1	6	OsR498G0611837900.01.T01	99,91	0,00E+00	1993	Os06g0210500	99,82	0,00E+00	1988	-5	NA	Indica	
augustus_masked-6-processed-gene-57.46-mRNA-1	6	OsR498G1019094500.01.T01	76,94	0,00E+00	1381	Os06g0210400	97,80	0,00E+00	3629	2248	Japonica	Indica	Kinase-TM
augustus_masked-6-processed-gene-57.50-mRNA-1	6	OsR498G0611840000.01.T01	71,62	5,33E-63	242	Os06g0211100	71,62	1,85E-63	242	0	NA	NA	
augustus_masked-6-processed-gene-58.29-mRNA-1	6	OsR498G0204823700.01.T01	70,74	0,00E+00	662	Os06g0212600	99,82	0,00E+00	2957	2295	Japonica	Indica	
augustus_masked-6-processed-gene-59.23-mRNA-1	6	OsR498G0611848400.01.T01	100,00	1,34E-153	542	Os02g0762900	83,74	6,09E-83	306	-236	Indica	Indica	
augustus_masked-6-processed-gene-59.28-mRNA-1	6	OsR498G0611849700.01.T01	100,00	1,38E-179	628	Os06g0213900	100,00	4,80E-180	628	0	NA	Indica	
augustus_masked-6-processed-gene-6.12-mRNA-1	6	OsR498G0611495300.01.T01	99,92	0,00E+00	2122	Os06g0111200	100,00	0,00E+00	2127	5	NA	Japonica	
augustus_masked-6-processed-gene-6.13-mRNA-1	6	OsR498G0611496300.01.T02	99,86	0,00E+00	2594	Os06g0111500	100,00	0,00E+00	2603	9	NA	Japonica	
augustus_masked-6-processed-gene-6.27-mRNA-1	6	OsR498G0611495200.01.T01	99,84	0,00E+00	1109	Os06g0111100	100,00	0,00E+00	1113	4	NA	Japonica	
augustus_masked-6-processed-gene-6.8-mRNA-1	6	OsR498G0611494100.01.T01	99,67	1,15E-154	546	Os06g0110200	100,00	1,49E-160	563	17	NA	Japonica	
augustus_masked-6-processed-gene-60.36-mRNA-1	6	OsR498G0611853300.01.T01	100,00	0,00E+00	2251	Os06g0215100	99,52	0,00E+00	2224	-27	NA	Indica	
augustus_masked-6-processed-gene-60.49-mRNA-1	6	OsR498G0611856800.01.T01	99,79	0,00E+00	2590	Os10g0122000	89,68	0,00E+00	1959	-631	Indica	Indica	
augustus_masked-6-processed-gene-60.53-mRNA-1	6	OsR498G0611857800.01.T01	99,65	0,00E+00	2548	Os06g0216400	99,65	0,00E+00	2548	0	NA	Indica	
augustus_masked-6-processed-gene-61.17-mRNA-1	6	OsR498G0611860600.01.T05	100,00	9,82E-136	482	Os06g0217500	100,00	1,46E-96	351	-131	Indica	Indica	
augustus_masked-6-processed-gene-63.34-mRNA-1	6	OsR498G0611872700.01.T01	98,45	0,00E+00	1677	Os06g0220300	81,24	0,00E+00	802	-875	Indica	NA	
augustus_masked-6-processed-gene-63.35-mRNA-1	6	OsR498G0611872900.01.T01	98,61	0,00E+00	1628	Os06g0220300	99,78	0,00E+00	1647	19	NA	NA	
augustus_masked-6-processed-gene-63.45-mRNA-1	6	OsR498G0611870800.01.T02	100,00	0,00E+00	2132	Os06g0219800	99,83	0,00E+00	2123	-9	NA	Indica	
augustus_masked-6-processed-gene-63.46-mRNA-1	6	OsR498G0611871000.01.T01	99,89	0,00E+00	1700	Os06g0219900	99,68	0,00E+00	1691	-9	NA	Indica	
augustus_masked-6-processed-gene-64.15-mRNA-1	6	OsR498G0611875500.01.T01	100,00	2,31E-137	488	Os06g0220900	99,26	4,18E-135	479	-9	NA	Indica	
augustus_masked-6-processed-gene-65.17-mRNA-1	6	OsR498G0611880000.01.T01	99,82	0,00E+00	1961	Os02g0752800	76,42	2,69E-151	535	-1426	Indica	NA	
augustus_masked-6-processed-gene-65.20-mRNA-1	6	OsR498G0611882900.01.T01	99,09	0,00E+00	966	Os06g0223000	100,00	0,00E+00	1476	510	Japonica	Japonica	
augustus_masked-6-processed-gene-65.35-mRNA-1	6	OsR498G0611883600.01.T01	99,10	0,00E+00	1560	Os02g0751800	83,15	0,00E+00	862	-698	Indica	Japonica	
augustus_masked-6-processed-gene-66.35-mRNA-1	6	OsR498G0611907700.01.T02	100,00	3,17E-103	374	Os06g0224100	100,00	1,11E-103	374	0	NA	NA	
augustus_masked-6-processed-gene-66.37-mRNA-1	6	OsR498G0611910300.01.T01	100,00	0,00E+00	1056	Os06g0225000	100,00	0,00E+00	1056	0	NA	NA	
augustus_masked-6-processed-gene-66.38-mRNA-1	6	OsR498G0611911100.01.T01	100,00	0,00E+00	2132	Os06g0225200	100,00	0,00E+00	2132	0	NA	NA	
augustus_masked-6-processed-gene-66.47-mRNA-1	6	OsR498G0611909400.01.T01	100,00	0,00E+00	2408	Os06g0224600	100,00	0,00E+00	2111	-297	Indica	NA	
augustus_masked-6-processed-gene-66.48-mRNA-1	6	OsR498G0611909600.01.T01	100,00	0,00E+00	2203	Os06g0224650	100,00	0,00E+00	2203	0	NA	NA	
augustus_masked-6-processed-gene-66.49-mRNA-1	6	OsR498G0611909800.01.T04	100,00	0,00E+00	2132	Os06g0224700	100,00	0,00E+00	2132	0	NA	NA	
augustus_masked-6-processed-gene-66.50-mRNA-1	6	OsR498G0611910200.01.T03	100,00	0,00E+00	2051	Os06g0224900	100,00	0,00E+00	2051	0	NA	NA	
augustus_masked-6-processed-gene-66.51-mRNA-1	6	OsR498G0611910600.01.T01	100,00	0,00E+00	2192	Os06g0225100	100,00	0,00E+00	2192	0	NA	NA	
augustus_masked-6-processed-gene-67.33-mRNA-1	6	OsR498G0611914100.01.T01	100,00	0,00E+00	1548	Os06g0226100	100,00	0,00E+00	2078	530	Japonica	NA	
augustus_masked-6-processed-gene-67.35-mRNA-1	6	OsR498G0611917700.01.T01	100,00	0,00E+00	2100	Os06g0226700	100,00	0,00E+00	2100	0	NA	NA	
augustus_masked-6-processed-gene-7.27-mRNA-1	6	OsR498G0611498500.01.T01	99,86	0,00E+00	3964	Os06g0112000	100,00	0,00E+00	3977	13	NA	Japonica	
augustus_masked-6-processed-gene-71.33-mRNA-1	6	OsR498G0611947800.01.T01	100,00	0,00E+00	2365	Os06g0233200	100,00	0,00E+00	2365	0	NA	NA	
augustus_masked-6-processed-gene-74.22-mRNA-1	6	OsR498G0611967700.01.T01	99,81	0,00E+00	2795	Os06g0238000	100,00	0,00E+00	2809	14	NA	Japonica	
augustus_masked-6-processed-gene-74.39-mRNA-1	6	OsR498G0611970300.01.T01	99,91	0,00E+00	2009	Os06g0239100	100,00	0,00E+00	2013	4	NA	Japonica	
augustus_masked-6-processed-gene-74.55-mRNA-1	6	OsR498G0611970000.01.T02	99,68	0,00E+00	1664	Os06g0238900	100,00	0,00E+00	1678	14	NA	Japonica	
augustus_masked-6-processed-gene-75.5-mRNA-1	6	OsR498G0611972800.01.T01	95,63	0,00E+00	1796	Os06g0240800	99,84	0,00E+00	1156	-640	Indica	Japonica	
augustus_masked-6-processed-gene-78.10-mRNA-1	6	OsR498G0611991000.01.T01	98,98	1,11E-144	513	Os06g0246600	99,67	3,38E-152	537	24	NA	NA	
augustus_masked-6-processed-gene-78.15-mRNA-1	6	OsR498G0611993700.01.T02	99,91	0,00E+00	2009	Os02g0739400	86,67	0,00E+00	1160	-849	Indica	NA	
augustus_masked-6-processed-gene-78.17-mRNA-1	6	OsR498G0611996700.01.T05	100,00	0,00E+00	3746	Os06g0247800	100,00	0,00E+00	3746	0	NA	Indica	
augustus_masked-6-processed-gene-79.23-mRNA-1	6	OsR498G0612005800.01.T04	100,00	0,00E+00	2208	Os06g0249500	100,00	0,00E+00	3119	911	Japonica	NA	
augustus_masked-6-processed-gene-8.13-mRNA-1	6	OsR498G0611506700.01.T01	100,00	0,00E+00	1927	Os06g0114333	100,00	0,00E+00	1927	0	NA	NA	
augustus_masked-6-processed-gene-8.34-mRNA-1	6	OsR498G0611509600.01.T02	100,00	0,00E+00	3474	Os06g0115200	100,00	0,00E+00	3474	0	NA	NA	
augustus_masked-6-processed-gene-86.15-mRNA-1	6	OsR498G0612046000.01.T01	83,54	0,00E+00	1394	Os06g0262200	100,00	0,00E+00	2657	1263	Japonica	Japonica	
augustus_masked-6-processed-gene-87.24-mRNA-1	6	OsR498G0612054600.01.T01	96,80	0,00E+00	2539	Os06g0263400	99,93	0,00E+00	2728	189	NA	Japonica	
augustus_masked-6-processed-gene-87.3-mRNA-1	6	OsR498G0612046000.01.T01	84,42	0,00E+00	1444	Os06g0262800	99,73	0,00E+00	2661	1217	Japonica	Japonica	
augustus_masked-6-processed-gene-88.8-mRNA-1	6	OsR498G0612055300.01.T01	98,62	0,00E+00	1897	Os06g0264200	100,00	0,00E+00	2608	711	Japonica	Japonica	
augustus_masked-6-processed-gene-89.31-mRNA-1	6	OsR498G0612062300.01.T01	99,92	0,00E+00	2128	Os06g0265900	99,83	0,00E+00	2123	-5	NA	Indica	
augustus_masked-6-processed-gene-9.58-mRNA-1	6	OsR498G0611515500.01.T01	100,00	0,00E+00	980	Os06g0119100	79,31	5,57E-112	404	-576	Indica	NA	
augustus_masked-6-processed-gene-9.71-mRNA-1	6	OsR498G0611514600.01.T01	100,00	0,00E+00	1310	Os06g0116900	100,00	0,00E+00	1310	0	NA	NA	
augustus_masked-6-processed-gene-92.22-mRNA-1	6	OsR498G0612075700.01.T01	99,72	0,00E+00	2563	Os06g0271000	96,65	0,00E+00	2365	-198	NA	Indica	
augustus_masked-6-processed-gene-92.44-mRNA-1	6	OsR498G0612082400.01.T01	95,05	0,00E+00	2019	Os06g0272800	95,71	0,00E+00	4038	2019	Japonica	NA	
augustus_masked-6-processed-gene-94.23-mRNA-1	6	OsR498G0612092700.01.T01	99,63	0,00E+00	4809	Os06g0275500	99,63	0,00E+00	4809	0	NA	Indica	
augustus_masked-6-processed-gene-98.11-mRNA-1	6	OsR498G0612136200.01.T01	99,32	0,00E+00	2334	Os06g0282000	99,38	0,00E+00	2571	237	NA	NA	
augustus_masked-6-processed-gene-98.31-mRNA-1	6	OsR498G0612143000.01.T01	99,57	0,00E+00	3753	Os06g0283400	99,57	0,00E+00	3753	0	NA	Japonica	
augustus-6-processed-gene-111.20-mRNA-1	6	OsR498G0815345200.01.T02	79,12	0,00E+00	1185	Os06g0303400	100,00	0,00E+00	1808	623	Japonica	NA	
augustus-6-processed-gene-112.27-mRNA-1	6	OsR498G0612219700.01.T01	99,11	0,00E+00	7742	Os06g0305200	100,00	0,00E+00	7918	176	NA	Japonica	
augustus-6-processed-gene-120.40-mRNA-1	6	OsR498G0612270500.01.T03	99,94	0,00E+00	3167	Os06g0318200	100,00	0,00E+00	3171	4	NA	Japonica	
augustus-6-processed-gene-124.5-mRNA-1	6	OsR498G0612283100.01.T01	99,74	0,00E+00	2758	Os02g0716800	75,62	0,00E+00	1081	-1677	Indica	Japonica	
augustus-6-processed-gene-171.32-mRNA-1	6	OsR498G0612327800.01.T02	95,61	0,00E+00	1938	Os06g0494400	84,63	0,00E+00	1357	-581	Indica	Japonica	
augustus-6-processed-gene-26.33-mRNA-1	6	OsR498G0611633600.01.T01	100,00	0,00E+00	931	Os06g0150400	100,00	0,00E+00	931	0	NA	NA	
augustus-6-processed-gene-260.26-mRNA-1	6	OsR498G0613025400.01.T02	99,40	0,00E+00	1177	Os06g0636300	100,00	0,00E+00	1196	19	NA	Japonica	
augustus-6-processed-gene-266.15-mRNA-1	6	OsR498G0613060500.01.T01	99,66	0,00E+00	2660	Os06g0647200	100,00	0,00E+00	3332	672	Japonica	NA	
augustus-6-processed-gene-288.73-mRNA-1	6	OsR498G0613209600.01.T01	99,45	0,00E+00	3900	Os06g0687000	99,96	0,00E+00	4075	175	NA	Japonica	
augustus-6-processed-gene-291.18-mRNA-1	6	OsR498G0613227500.01.T01	99,83	0,00E+00	2118	Os06g0693200	100,00	0,00E+00	2127	9	NA	Japonica	Kinase-TM
augustus-6-processed-gene-4.32-mRNA-1	6	OsR498G0611480000.01.T01	99,86	0,00E+00	1246	Os06g0106100	100,00	0,00E+00	1251	5	NA	Japonica	
augustus-6-processed-gene-40.30-mRNA-1	6	OsR498G0611719200.01.T01	100,00	0,00E+00	2554	Os06g0179900	100,00	0,00E+00	2554	0	NA	NA	
augustus-6-processed-gene-41.7-mRNA-1	6	OsR498G0611725400.01.T02	100,00	0,00E+00	4708	Os06g0181500	100,00	0,00E+00	4648	-60	NA	NA	
augustus-6-processed-gene-44.67-mRNA-1	6	OsR498G0611742200.01.T02	99,93	0,00E+00	2636	Os06g0186400	98,36	0,00E+00	2527	-109	NA	Indica	
augustus-6-processed-gene-54.13-mRNA-1	6	OsR498G0611814400.01.T02	100,00	0,00E+00	850	Os06g0205250	99,79	0,00E+00	844	-6	NA	NA	
augustus-6-processed-gene-76.24-mRNA-1	6	OsR498G0611978800.01.T01	97,59	0,00E+00	1049	Os06g0244000	85,22	0,00E+00	690	-359	Indica	Japonica	
augustus-6-processed-gene-9.46-mRNA-1	6	OsR498G0611510900.01.T01	100,00	0,00E+00	1884	Os06g0115600	100,00	0,00E+00	1884	0	NA	NA	
augustus-6-processed-gene-91.47-mRNA-1	6	OsR498G0612071400.01.T01	99,04	0,00E+00	2734	Os06g0269300	99,10	0,00E+00	2738	4	NA	NA	
augustus-6-processed-gene-93.17-mRNA-1	6	OsR498G0612087700.01.T01	98,30	0,00E+00	3355	Os06g0274500	99,84	0,00E+00	3455	100	NA	Japonica	Kinase-LRR-TM
maker-6-augustus-gene-1.41-mRNA-1	6	OsR498G0611466900.01.T01	99,76	0,00E+00	1463	Os06g0101100	100,00	0,00E+00	1472	9	NA	Japonica	
maker-6-augustus-gene-1.43-mRNA-1	6	OsR498G0611468800.01.T01	99,90	0,00E+00	3660	Os06g0101800	100,00	0,00E+00	1921	-1739	Indica	Japonica	
maker-6-augustus-gene-10.0-mRNA-1	6	OsR498G0613108200.01.T01	98,69	0,00E+00	1061	Os06g0660600	94,61	0,00E+00	1717	656	Japonica	NA	
maker-6-augustus-gene-10.2-mRNA-1	6	OsR498G0611524600.01.T01	95,59	0,00E+00	2037	Os06g0117500	97,86	0,00E+00	3819	1782	Japonica	NA	
maker-6-augustus-gene-10.4-mRNA-1	6	OsR498G0611518800.01.T01	100,00	0,00E+00	2219	Os06g0119600	87,39	0,00E+00	1292	-927	Indica	NA	
maker-6-augustus-gene-10.6-mRNA-1	6	OsR498G0611521800.01.T01	100,00	0,00E+00	2379	Os06g0118700	100,00	0,00E+00	2379	0	NA	NA	
maker-6-augustus-gene-100.0-mRNA-1	6	OsR498G0612156900.01.T01	99,70	0,00E+00	1198	Os06g0286228	99,85	0,00E+00	1203	5	NA	NA	
maker-6-augustus-gene-100.2-mRNA-1	6	OsR498G0612153400.01.T01	99,29	0,00E+00	1244	Os06g0285900	99,40	0,00E+00	1184	-60	NA	NA	
maker-6-augustus-gene-100.6-mRNA-1	6	OsR498G0612154200.01.T01	99,37	0,00E+00	1124	Os06g0286146	72,42	3,39E-52	205	-919	Indica	Japonica	
maker-6-augustus-gene-100.8-mRNA-1	6	OsR498G0612156000.01.T01	93,83	0,00E+00	881	Os06g0286146	94,36	0,00E+00	894	13	NA	Indica	
maker-6-augustus-gene-101.19-mRNA-1	6	OsR498G0612158300.01.T01	99,61	0,00E+00	1367	Os06g0286310	100,00	0,00E+00	904	-463	Indica	Indica	
maker-6-augustus-gene-101.22-mRNA-1	6	OsR498G0612159700.01.T01	99,28	0,00E+00	5168	Os06g0286500	99,32	0,00E+00	5173	5	NA	NA	NBS-TM
maker-6-augustus-gene-101.24-mRNA-1	6	OsR498G0612158600.01.T01	99,09	0,00E+00	8367	Os06g0286351	99,09	0,00E+00	8367	0	NA	NA	
maker-6-augustus-gene-104.4-mRNA-1	6	OsR498G0612174700.01.T01	99,86	0,00E+00	5144	Os06g0293100	99,94	0,00E+00	3134	-2010	Indica	Japonica	
maker-6-augustus-gene-104.6-mRNA-1	6	OsR498G0612170400.01.T02	99,89	0,00E+00	3184	Os06g0291600	99,89	0,00E+00	3184	0	NA	Japonica	Kinase-TM
maker-6-augustus-gene-105.11-mRNA-1	6	OsR498G0612180200.01.T01	99,23	0,00E+00	2755	Os06g0294600	100,00	0,00E+00	2809	54	NA	Japonica	
maker-6-augustus-gene-105.5-mRNA-1	6	OsR498G0612176400.01.T01	99,91	0,00E+00	3979	Os06g0293500	99,81	0,00E+00	4615	636	Japonica	Indica	
maker-6-augustus-gene-105.7-mRNA-1	6	OsR498G0612178800.01.T02	99,77	0,00E+00	3836	Os06g0294000	99,89	0,00E+00	4959	1123	Japonica	Japonica	
maker-6-augustus-gene-105.9-mRNA-1	6	OsR498G0612179200.01.T01	99,61	0,00E+00	4527	Os06g0294300	100,00	0,00E+00	4572	45	NA	Japonica	*Pi2 R* gene
maker-6-augustus-gene-106.10-mRNA-1	6	OsR498G1222153000.01.T01	70,34	5,10E-80	298	Os06g0296500	91,02	0,00E+00	721	423	Japonica	Japonica	
maker-6-augustus-gene-106.13-mRNA-1	6	OsR498G0612180400.01.T01	97,50	0,00E+00	2096	Os06g0294200	83,49	0,00E+00	1694	-402	Indica	Japonica	
maker-6-augustus-gene-106.15-mRNA-1	6	OsR498G0612183000.01.T01	97,71	2,91E-80	298	Os06g0295500	100,00	3,30E-131	466	168	Japonica	Japonica	
maker-6-augustus-gene-106.17-mRNA-1	6	OsR498G0612185200.01.T02	99,63	4,45E-134	478	Os06g0296200	100,00	1,77E-108	390	-88	Indica	Japonica	
maker-6-augustus-gene-106.19-mRNA-1	6	OsR498G0612185600.01.T01	99,77	0,00E+00	3169	Os06g0296700	100,00	0,00E+00	4610	1441	Japonica	Japonica	
maker-6-augustus-gene-107.0-mRNA-1	6	OsR498G0612190700.01.T02	99,90	0,00E+00	3503	Os06g0297700	100,00	0,00E+00	3512	9	NA	Japonica	
maker-6-augustus-gene-107.2-mRNA-1	6	OsR498G0612190900.01.T05	97,13	0,00E+00	2725	Os06g0297800	97,13	0,00E+00	2725	0	NA	Japonica	
maker-6-augustus-gene-107.7-mRNA-1	6	OsR498G0612189800.01.T01	100,00	0,00E+00	2105	Os02g0724500	80,77	6,34E-166	583	-1522	Indica	Japonica	
maker-6-augustus-gene-108.40-mRNA-1	6	OsR498G0100204400.01.T01	78,89	1,13E-86	320	Os06g0298300	100,00	0,00E+00	747	427	Japonica	Japonica	
maker-6-augustus-gene-108.42-mRNA-1	6	OsR498G0612192700.01.T02	99,69	0,00E+00	5235	Os06g0298400	100,00	0,00E+00	4507	-728	Indica	Japonica	
maker-6-augustus-gene-108.48-mRNA-1	6	OsR498G0612192000.01.T01	100,00	0,00E+00	2214	Os06g0298200	99,76	0,00E+00	2198	-16	NA	Japonica	
maker-6-augustus-gene-108.54-mRNA-1	6	OsR498G0612193200.01.T02	99,83	0,00E+00	1062	Os06g0298500	100,00	0,00E+00	1063	1	NA	Japonica	
maker-6-augustus-gene-108.58-mRNA-1	6	OsR498G0612194600.01.T01	99,60	0,00E+00	1768	Os06g0299100	100,00	0,00E+00	1786	18	NA	Japonica	
maker-6-augustus-gene-108.60-mRNA-1	6	OsR498G0612197200.01.T01	91,71	0,00E+00	810	Os06g0299200	100,00	0,00E+00	1608	798	Japonica	Japonica	
maker-6-augustus-gene-108.62-mRNA-1	6	OsR498G0612197200.01.T01	99,80	0,00E+00	1783	Os06g0299300	100,00	0,00E+00	1792	9	NA	Japonica	
maker-6-augustus-gene-109.45-mRNA-1	6	OsR498G0612200000.01.T01	99,18	0,00E+00	1944	Os06g0300000	99,73	0,00E+00	1967	23	NA	Japonica	
maker-6-augustus-gene-109.46-mRNA-1	6	OsR498G0612200600.01.T01	99,59	0,00E+00	1746	Os06g0300000	77,62	1,62E-153	542	-1204	Indica	Japonica	
maker-6-augustus-gene-109.53-mRNA-1	6	OsR498G0612201000.01.T01	99,79	0,00E+00	846	Os06g0300300	100,00	0,00E+00	850	4	NA	Japonica	
maker-6-augustus-gene-109.55-mRNA-1	6	OsR498G0612202000.01.T01	99,77	0,00E+00	3175	Os06g0300500	100,00	0,00E+00	2587	-588	Indica	Japonica	
maker-6-augustus-gene-109.60-mRNA-1	6	OsR498G0612202200.01.T01	99,87	0,00E+00	1343	Os06g0300600	100,00	0,00E+00	1348	5	NA	Japonica	
maker-6-augustus-gene-109.63-mRNA-1	6	OsR498G0612204600.01.T01	99,87	0,00E+00	2702	Os06g0301000	100,00	0,00E+00	2711	9	NA	Japonica	
maker-6-augustus-gene-109.67-mRNA-1	6	OsR498G0612204800.01.T02	99,86	0,00E+00	1311	Os04g0470300	71,21	2,03E-49	196	-1115	Indica	Japonica	
maker-6-augustus-gene-11.47-mRNA-1	6	OsR498G0611535100.01.T01	100,00	0,00E+00	1517	Os06g0121800	96,65	0,00E+00	2459	942	Japonica	NA	
maker-6-augustus-gene-11.50-mRNA-1	6	OsR498G0203481100.01.T01	81,93	0,00E+00	1379	Os06g0119600	100,00	0,00E+00	2684	1305	Japonica	NA	
maker-6-augustus-gene-110.30-mRNA-1	6	OsR498G0612205500.01.T05	99,85	0,00E+00	8557	Os06g0301300	99,06	0,00E+00	7901	-656	NA	Indica	
maker-6-augustus-gene-110.40-mRNA-1	6	OsR498G0612210100.01.T01	98,99	0,00E+00	699	Os06g0303200	100,00	0,00E+00	899	200	Japonica	Japonica	
maker-6-augustus-gene-110.42-mRNA-1	6	OsR498G0612205800.01.T01	98,47	0,00E+00	2601	Os06g0301400	99,85	0,00E+00	2343	-258	NA	Japonica	
maker-6-augustus-gene-111.0-mRNA-1	6	OsR498G0815345200.01.T05	82,42	0,00E+00	645	Os06g0303400	100,00	0,00E+00	1151	506	Japonica	Japonica	
maker-6-augustus-gene-112.49-mRNA-1	6	OsR498G0612215900.01.T01	99,19	0,00E+00	870	Os06g0303800	100,00	0,00E+00	747	-123	Indica	Japonica	
maker-6-augustus-gene-112.53-mRNA-1	6	OsR498G0612217900.01.T05	99,81	0,00E+00	1880	Os06g0304500	100,00	0,00E+00	1889	9	NA	Japonica	
maker-6-augustus-gene-113.1-mRNA-1	6	OsR498G0612221100.01.T01	99,90	0,00E+00	1792	Os06g0306300	100,00	0,00E+00	1398	-394	Indica	Japonica	
maker-6-augustus-gene-114.35-mRNA-1	6	OsR498G0612226900.01.T01	99,91	0,00E+00	2000	Os06g0308000	100,00	0,00E+00	2004	4	NA	Japonica	
maker-6-augustus-gene-114.41-mRNA-1	6	OsR498G0612227900.01.T02	99,75	0,00E+00	1447	Os06g0308200	99,90	0,00E+00	1792	345	Japonica	Japonica	
maker-6-augustus-gene-114.45-mRNA-1	6	OsR498G0612229300.01.T01	99,93	0,00E+00	2642	Os06g0308800	100,00	0,00E+00	2646	4	NA	Japonica	
maker-6-augustus-gene-114.49-mRNA-1	6	OsR498G0612226600.01.T03	99,57	0,00E+00	2908	Os06g0307900	100,00	0,00E+00	2836	-72	NA	Japonica	
maker-6-augustus-gene-114.53-mRNA-1	6	OsR498G0612227400.01.T02	100,00	0,00E+00	760	Os06g0308100	98,58	0,00E+00	734	-26	NA	Japonica	
maker-6-augustus-gene-114.57-mRNA-1	6	OsR498G0612228200.01.T01	99,37	0,00E+00	3073	Os06g0308300	100,00	0,00E+00	3122	49	NA	Japonica	
maker-6-augustus-gene-114.63-mRNA-1	6	OsR498G0612230200.01.T03	100,00	0,00E+00	1727	Os06g0309000	99,90	0,00E+00	1713	-14	NA	Japonica	
maker-6-augustus-gene-114.70-mRNA-1	6	OsR498G0408570300.01.T06	99,67	0,00E+00	1601	Os06g0310200	99,89	0,00E+00	1610	9	NA	NA	
maker-6-augustus-gene-115.3-mRNA-1	6	OsR498G0408570300.01.T02	99,63	0,00E+00	3833	Os06g0310200	97,73	0,00E+00	4381	548	Japonica	NA	
maker-6-augustus-gene-116.0-mRNA-1	6	OsR498G0612244900.01.T02	99,85	0,00E+00	1163	Os06g0311300	100,00	0,00E+00	1168	5	NA	Japonica	
maker-6-augustus-gene-117.33-mRNA-1	6	OsR498G0612253000.01.T03	99,46	0,00E+00	654	Os06g0313440	99,46	0,00E+00	654	0	NA	Japonica	
maker-6-augustus-gene-117.34-mRNA-1	6	OsR498G0612253000.01.T03	99,60	0,00E+00	1352	Os06g0313440	100,00	0,00E+00	1187	-165	Indica	Japonica	
maker-6-augustus-gene-118.23-mRNA-1	6	OsR498G0612257800.01.T01	99,50	0,00E+00	4595	Os06g0313900	100,00	0,00E+00	2717	-1878	Indica	Japonica	
maker-6-augustus-gene-118.25-mRNA-1	6	OsR498G0612260200.01.T02	99,37	0,00E+00	1971	Os06g0314600	100,00	0,00E+00	1835	-136	NA	Japonica	
maker-6-augustus-gene-118.28-mRNA-1	6	OsR498G0612261200.01.T01	99,54	0,00E+00	1951	Os06g0315000	100,00	0,00E+00	1948	-3	NA	Japonica	
maker-6-augustus-gene-118.31-mRNA-1	6	OsR498G0612262400.01.T01	98,70	0,00E+00	1885	Os06g0315000	84,26	0,00E+00	1182	-703	Indica	Japonica	
maker-6-augustus-gene-119.5-mRNA-1	6	OsR498G0612263300.01.T01	99,24	0,00E+00	1618	Os06g0316100	100,00	0,00E+00	1651	33	NA	Japonica	
maker-6-augustus-gene-119.7-mRNA-1	6	OsR498G0612264100.01.T01	99,60	0,00E+00	901	Os06g0316300	100,00	0,00E+00	910	9	NA	Japonica	
maker-6-augustus-gene-12.45-mRNA-1	6	OsR498G0611538700.01.T02	100,00	0,00E+00	1411	Os06g0122600	100,00	0,00E+00	1773	362	Japonica	NA	
maker-6-augustus-gene-12.48-mRNA-1	6	OsR498G0611540200.01.T01	99,65	0,00E+00	1020	Os06g0123000	99,65	0,00E+00	1020	0	NA	NA	
maker-6-augustus-gene-120.0-mRNA-1	6	OsR498G0612266700.01.T01	98,22	2,90E-165	581	Os06g0316800	74,92	1,16E-63	243	-338	Indica	Japonica	
maker-6-augustus-gene-120.12-mRNA-1	6	OsR498G0612264800.01.T01	92,82	0,00E+00	653	Os06g0316800	100,00	0,00E+00	780	127	Japonica	Japonica	
maker-6-augustus-gene-120.14-mRNA-1	6	OsR498G0612270800.01.T01	99,63	0,00E+00	1453	Os06g0318300	100,00	0,00E+00	1467	14	NA	Japonica	
maker-6-augustus-gene-120.2-mRNA-1	6	OsR498G0612268100.01.T01	94,03	0,00E+00	764	Os06g0317400	95,06	0,00E+00	786	22	NA	Japonica	
maker-6-augustus-gene-120.9-mRNA-1	6	OsR498G0612271500.01.T01	99,38	0,00E+00	2874	Os06g0318500	97,48	0,00E+00	2026	-848	Indica	Japonica	
maker-6-augustus-gene-121.0-mRNA-1	6	OsR498G0612271900.01.T02	96,21	0,00E+00	2018	Os06g0318600	96,00	0,00E+00	1738	-280	Indica	Japonica	
maker-6-augustus-gene-121.14-mRNA-1	6	OsR498G0612274100.01.T01	99,47	0,00E+00	668	Os06g0319700	100,00	0,00E+00	677	9	NA	Japonica	
maker-6-augustus-gene-121.17-mRNA-1	6	OsR498G0612275100.01.T03	98,81	0,00E+00	2945	Os06g0320100	99,11	0,00E+00	2967	22	NA	Japonica	
maker-6-augustus-gene-121.19-mRNA-1	6	OsR498G0612275000.01.T01	100,00	0,00E+00	758	Os06g0320000	100,00	0,00E+00	758	0	NA	Japonica	
maker-6-augustus-gene-121.5-mRNA-1	6	OsR498G0612272100.01.T02	99,82	0,00E+00	1961	Os06g0318700	100,00	0,00E+00	1970	9	NA	Japonica	
maker-6-augustus-gene-121.8-mRNA-1	6	OsR498G0612273700.01.T05	97,09	0,00E+00	3160	Os06g0319600	97,48	0,00E+00	3347	187	NA	Japonica	
maker-6-augustus-gene-122.26-mRNA-1	6	OsR498G0612275900.01.T01	99,73	0,00E+00	1301	Os06g0320500	100,00	0,00E+00	1310	9	NA	Japonica	
maker-6-augustus-gene-122.29-mRNA-1	6	OsR498G0612276200.01.T01	99,66	0,00E+00	3191	Os06g0320700	100,00	0,00E+00	3218	27	NA	Japonica	
maker-6-augustus-gene-123.24-mRNA-1	6	OsR498G0612281500.01.T01	91,51	0,00E+00	909	Os06g0322500	100,00	0,00E+00	1148	239	Japonica	Japonica	
maker-6-augustus-gene-123.26-mRNA-1	6	OsR498G0409452300.01.T01	89,88	0,00E+00	1562	Os06g0323100	100,00	0,00E+00	1894	332	Japonica	Japonica	
maker-6-augustus-gene-124.34-mRNA-1	6	OsR498G0612284500.01.T01	99,72	0,00E+00	3136	Os06g0325200	93,10	0,00E+00	2391	-745	Indica	Japonica	
maker-6-augustus-gene-124.36-mRNA-1	6	OsR498G0612286700.01.T04	100,00	0,00E+00	926	Os06g0325500	100,00	0,00E+00	926	0	NA	Japonica	
maker-6-augustus-gene-124.42-mRNA-1	6	OsR498G0612283600.01.T01	99,68	8,84E-157	554	Os06g0324400	100,00	1,15E-124	444	-110	Indica	Japonica	
maker-6-augustus-gene-124.44-mRNA-1	6	OsR498G0612285800.01.T01	99,82	0,00E+00	2979	Os06g0325200	100,00	0,00E+00	2993	14	NA	Japonica	
maker-6-augustus-gene-125.24-mRNA-1	6	OsR498G0612293700.01.T01	97,43	0,00E+00	3129	Os06g0326400	97,54	0,00E+00	3138	9	NA	Japonica	
maker-6-augustus-gene-125.30-mRNA-1	6	OsR498G0612294100.01.T01	99,63	0,00E+00	1448	Os06g0326500	100,00	0,00E+00	1462	14	NA	Japonica	
maker-6-augustus-gene-125.37-mRNA-1	6	OsR498G0612291200.01.T01	99,82	0,00E+00	2042	Os06g0326000	100,00	0,00E+00	2051	9	NA	Japonica	
maker-6-augustus-gene-126.30-mRNA-1	6	OsR498G0612301900.01.T02	99,48	0,00E+00	4084	Os06g0328400	100,00	0,00E+00	3382	-702	Indica	Japonica	Kinase-TM
maker-6-augustus-gene-126.32-mRNA-1	6	OsR498G0612297400.01.T01	99,73	0,00E+00	1984	Os06g0327300	100,00	0,00E+00	1773	-211	Indica	Japonica	
maker-6-augustus-gene-127.0-mRNA-1	6	OsR498G0612303700.01.T02	99,55	0,00E+00	2793	Os06g0328800	100,00	0,00E+00	2825	32	NA	Japonica	
maker-6-augustus-gene-127.2-mRNA-1	6	OsR498G0612308100.01.T04	99,66	0,00E+00	2123	Os06g0329900	96,66	0,00E+00	1909	-214	Indica	Japonica	
maker-6-augustus-gene-128.2-mRNA-1	6	OsR498G0612310000.01.T01	94,05	0,00E+00	1261	Os06g0330400	100,00	0,00E+00	1483	222	Japonica	Japonica	
maker-6-augustus-gene-129.0-mRNA-1	6	OsR498G0612560000.01.T01	97,74	0,00E+00	3420	Os06g0332400	97,89	0,00E+00	3433	13	NA	Japonica	
maker-6-augustus-gene-129.11-mRNA-1	6	OsR498G0612559300.01.T02	99,80	0,00E+00	2698	Os06g0332700	100,00	0,00E+00	2430	-268	NA	Japonica	
maker-6-augustus-gene-129.17-mRNA-1	6	OsR498G0612558500.01.T03	98,96	0,00E+00	1693	Os06g0332900	100,00	0,00E+00	1727	34	NA	Japonica	
maker-6-augustus-gene-129.6-mRNA-1	6	OsR498G0612559600.01.T02	99,67	4,27E-155	548	Os06g0332600	100,00	3,49E-157	553	5	NA	Japonica	
maker-6-augustus-gene-129.9-mRNA-1	6	OsR498G0612559700.01.T02	99,39	5,49E-166	583	Os06g0332500	100,00	5,48E-129	459	-124	Indica	Japonica	
maker-6-augustus-gene-13.34-mRNA-1	6	OsR498G0611547100.01.T01	100,00	0,00E+00	785	Os06g0125200	100,00	0,00E+00	785	0	NA	NA	
maker-6-augustus-gene-13.36-mRNA-1	6	OsR498G0611548700.01.T03	100,00	0,00E+00	2765	Os06g0125600	99,89	0,00E+00	1700	-1065	Indica	NA	
maker-6-augustus-gene-13.38-mRNA-1	6	OsR498G0611546000.01.T05	100,00	0,00E+00	5454	Os06g0125000	100,00	0,00E+00	5454	0	NA	NA	LRR-NBS-TM
maker-6-augustus-gene-13.40-mRNA-1	6	OsR498G0611547600.01.T02	100,00	0,00E+00	4123	Os06g0125500	88,80	0,00E+00	2943	-1180	Indica	NA	
maker-6-augustus-gene-130.0-mRNA-1	6	OsR498G0612553000.01.T02	99,96	0,00E+00	4573	Os06g0334300	100,00	0,00E+00	4578	5	NA	Japonica	Kinase-TM
maker-6-augustus-gene-130.2-mRNA-1	6	OsR498G0612552700.01.T02	99,86	0,00E+00	2589	Os06g0334400	100,00	0,00E+00	2598	9	NA	Japonica	Kinase

maker-6-augustus-gene-131.0-mRNA-1	6	OsR498G0612550400.01.T01	99,13	0,00E+00	1425	Os06g0335500	100,00	0,00E+00	1445	20	NA	Japonica	
maker-6-augustus-gene-131.4-mRNA-1	6	OsR498G0612548600.01.T01	98,29	0,00E+00	1618	Os06g0335900	100,00	0,00E+00	1689	71	NA	Japonica	
maker-6-augustus-gene-131.6-mRNA-1	6	OsR498G0612546600.01.T01	99,73	0,00E+00	1339	Os06g0336200	100,00	0,00E+00	841	-498	Indica	Japonica	
maker-6-augustus-gene-132.0-mRNA-1	6	OsR498G0612542400.01.T01	99,68	0,00E+00	3377	Os06g0338200	100,00	0,00E+00	3404	27	NA	Japonica	
maker-6-augustus-gene-132.7-mRNA-1	6	OsR498G0612539300.01.T01	99,81	0,00E+00	1921	Os06g0338900	100,00	0,00E+00	1930	9	NA	Japonica	
maker-6-augustus-gene-133.0-mRNA-1	6	OsR498G0612530800.01.T01	99,76	0,00E+00	1496	Os06g0341300	100,00	0,00E+00	1505	9	NA	Japonica	
maker-6-augustus-gene-133.2-mRNA-1	6	OsR498G0612530600.01.T01	99,36	0,00E+00	1378	Os06g0341500	100,00	0,00E+00	1402	24	NA	Japonica	
maker-6-augustus-gene-133.4-mRNA-1	6	OsR498G0612534900.01.T02	99,80	0,00E+00	2681	Os06g0340200	100,00	0,00E+00	2695	14	NA	Japonica	
maker-6-augustus-gene-133.7-mRNA-1	6	OsR498G0612533500.01.T06	99,59	0,00E+00	3048	Os02g0290500	75,17	0,00E+00	1090	-1958	Indica	Japonica	
maker-6-augustus-gene-133.9-mRNA-1	6	OsR498G0612529700.01.T01	99,84	0,00E+00	4583	Os06g0341600	100,00	0,00E+00	5113	530	Japonica	Japonica	
maker-6-augustus-gene-134.34-mRNA-1	6	OsR498G0612527300.01.T02	96,48	0,00E+00	710	Os06g0342100	96,48	0,00E+00	710	0	NA	Japonica	
maker-6-augustus-gene-135.25-mRNA-1	6	OsR498G0612521200.01.T01	99,73	0,00E+00	2011	Os06g0344900	100,00	0,00E+00	2024	13	NA	Japonica	
maker-6-augustus-gene-135.27-mRNA-1	6	OsR498G0612517200.01.T03	99,57	0,00E+00	2054	Os06g0345200	100,00	0,00E+00	2071	17	NA	Japonica	
maker-6-augustus-gene-136.0-mRNA-1	6	OsR498G0612514800.01.T01	88,15	0,00E+00	1273	Os06g0347100	86,69	0,00E+00	898	-375	Indica	Japonica	
maker-6-augustus-gene-137.25-mRNA-1	6	OsR498G0612514800.01.T01	99,73	0,00E+00	1306	Os06g0347100	100,00	0,00E+00	1315	9	NA	Japonica	
maker-6-augustus-gene-138.29-mRNA-1	6	OsR498G0612507400.01.T01	99,71	0,00E+00	2450	Os06g0348800	99,90	0,00E+00	1708	-742	Indica	Japonica	
maker-6-augustus-gene-139.37-mRNA-1	6	OsR498G0612505100.01.T01	99,19	0,00E+00	1958	Os06g0349700	99,93	0,00E+00	2593	635	Japonica	Japonica	
maker-6-augustus-gene-14.13-mRNA-1	6	OsR498G0611553900.01.T01	100,00	0,00E+00	4118	Os06g0127700	100,00	0,00E+00	4118	0	NA	NA	
maker-6-augustus-gene-14.18-mRNA-1	6	OsR498G0611549600.01.T04	99,96	0,00E+00	4346	Os06g0126000	99,95	0,00E+00	3911	-435	Indica	NA	
maker-6-augustus-gene-14.24-mRNA-1	6	OsR498G0611550400.01.T01	99,80	0,00E+00	907	Os06g0126400	99,80	0,00E+00	907	0	NA	NA	
maker-6-augustus-gene-14.3-mRNA-1	6	OsR498G0611549100.01.T04	96,15	0,00E+00	1290	Os06g0125800	96,15	0,00E+00	1290	0	NA	NA	
maker-6-augustus-gene-140.0-mRNA-1	6	OsR498G0612499400.01.T01	99,38	0,00E+00	2020	Os06g0351500	100,00	0,00E+00	2051	31	NA	Japonica	
maker-6-augustus-gene-140.2-mRNA-1	6	OsR498G0612498400.01.T01	99,43	0,00E+00	1878	Os06g0351700	100,00	0,00E+00	1997	119	NA	Japonica	
maker-6-augustus-gene-142.42-mRNA-1	6	OsR498G0612489500.01.T01	93,62	0,00E+00	3108	Os06g0354500	93,81	0,00E+00	3126	18	NA	Japonica	
maker-6-augustus-gene-142.43-mRNA-1	6	OsR498G0612489500.01.T03	99,80	0,00E+00	887	Os06g0354500	100,00	0,00E+00	892	5	NA	Japonica	
maker-6-augustus-gene-143.2-mRNA-1	6	OsR498G0612482600.01.T01	99,81	0,00E+00	925	Os08g0109400	97,41	0,00E+00	718	-207	Indica	Japonica	
maker-6-augustus-gene-143.7-mRNA-1	6	OsR498G0612482400.01.T04	97,70	0,00E+00	2661	Os08g0109500	96,74	0,00E+00	2591	-70	NA	Japonica	
maker-6-augustus-gene-145.0-mRNA-1	6	OsR498G0612466400.01.T01	99,64	0,00E+00	1486	Os06g0358800	100,00	0,00E+00	1624	138	NA	Japonica	
maker-6-augustus-gene-145.2-mRNA-1	6	OsR498G0612464700.01.T04	99,76	0,00E+00	739	Os06g0359400	100,00	0,00E+00	742	3	NA	Japonica	
maker-6-augustus-gene-146.31-mRNA-1	6	OsR498G0612461900.01.T02	100,00	0,00E+00	823	Os06g0360300	100,00	0,00E+00	789	-34	NA	Japonica	
maker-6-augustus-gene-15.36-mRNA-1	6	OsR498G0611556500.01.T01	98,85	0,00E+00	3807	Os06g0128400	98,11	0,00E+00	2256	-1551	Indica	NA	
maker-6-augustus-gene-15.52-mRNA-1	6	OsR498G0611559300.01.T01	99,16	0,00E+00	1895	Os03g0115700	78,81	0,00E+00	794	-1101	Indica	NA	
maker-6-augustus-gene-15.63-mRNA-1	6	OsR498G0611560700.01.T01	99,92	0,00E+00	2153	Os06g0156700	80,40	0,00E+00	1077	-1076	Indica	NA	
maker-6-augustus-gene-15.68-mRNA-1	6	OsR498G0611556000.01.T02	100,00	0,00E+00	2765	Os06g0128200	100,00	0,00E+00	2765	0	NA	NA	
maker-6-augustus-gene-15.77-mRNA-1	6	OsR498G0611556400.01.T02	100,00	0,00E+00	2677	Os06g0128300	100,00	0,00E+00	2677	0	NA	NA	
maker-6-augustus-gene-15.83-mRNA-1	6	OsR498G0611559800.01.T03	100,00	2,16E-160	565	Os06g0129300	100,00	7,53E-161	565	0	NA	NA	
maker-6-augustus-gene-15.86-mRNA-1	6	OsR498G0611561000.01.T01	99,90	0,00E+00	1760	Os06g0129700	100,00	0,00E+00	1548	-212	Indica	NA	
maker-6-augustus-gene-151.0-mRNA-1	6	OsR498G0612442200.01.T07	97,07	0,00E+00	2721	Os06g0367100	97,20	0,00E+00	2730	9	NA	Japonica	
maker-6-augustus-gene-151.8-mRNA-1	6	OsR498G0612440300.01.T06	99,56	0,00E+00	1226	Os06g0367500	100,00	0,00E+00	1083	-143	Indica	Japonica	
maker-6-augustus-gene-152.10-mRNA-1	6	OsR498G0612439300.01.T06	99,78	0,00E+00	1629	Os06g0367900	100,00	0,00E+00	1638	9	NA	Japonica	Kinase-TM
maker-6-augustus-gene-155.35-mRNA-1	6	OsR498G0612426800.01.T01	99,87	0,00E+00	2832	Os06g0470150	100,00	0,00E+00	2841	9	NA	Japonica	
maker-6-augustus-gene-155.39-mRNA-1	6	OsR498G0612424200.01.T05	98,82	0,00E+00	741	Os06g0470800	100,00	0,00E+00	756	15	NA	Japonica	
maker-6-augustus-gene-156.22-mRNA-1	6	OsR498G0612423400.01.T03	99,81	0,00E+00	943	Os06g0471100	100,00	0,00E+00	962	19	NA	Japonica	
maker-6-augustus-gene-156.25-mRNA-1	6	OsR498G0612388400.01.T01	99,18	0,00E+00	1071	Os06g0472200	99,84	0,00E+00	1089	18	NA	Japonica	
maker-6-augustus-gene-156.28-mRNA-1	6	OsR498G0612388000.01.T02	99,78	0,00E+00	4003	Os06g0472400	100,00	0,00E+00	4026	23	NA	Japonica	
maker-6-augustus-gene-156.31-mRNA-1	6	OsR498G0612389500.01.T02	99,35	0,00E+00	1094	Os06g0472000	100,00	0,00E+00	1153	59	NA	Japonica	
maker-6-augustus-gene-156.34-mRNA-1	6	OsR498G0612396400.01.T02	96,38	0,00E+00	1012	Os06g0472600	94,66	0,00E+00	1233	221	Japonica	Japonica	
maker-6-augustus-gene-157.33-mRNA-1	6	OsR498G0612399500.01.T01	99,83	0,00E+00	2120	Os06g0472900	100,00	0,00E+00	2132	12	NA	Japonica	
maker-6-augustus-gene-157.45-mRNA-1	6	OsR498G0612401000.01.T06	99,74	0,00E+00	2027	Os06g0473200	98,06	0,00E+00	1423	-604	Indica	Japonica	Kinase-TM
maker-6-augustus-gene-158.29-mRNA-1	6	OsR498G0612406400.01.T01	99,77	0,00E+00	2341	Os06g0474500	100,00	0,00E+00	2354	13	NA	Japonica	
maker-6-augustus-gene-158.32-mRNA-1	6	OsR498G0612408600.01.T03	99,67	0,00E+00	1627	Os06g0474800	100,00	0,00E+00	1088	-539	Indica	Japonica	
maker-6-augustus-gene-159.0-mRNA-1	6	OsR498G0612412710.01.T04	96,32	0,00E+00	1900	Os06g0476200	97,72	0,00E+00	2939	1039	Japonica	Japonica	
maker-6-augustus-gene-159.2-mRNA-1	6	OsR498G0612409400.01.T01	99,77	0,00E+00	2335	Os06g0475400	100,00	0,00E+00	2349	14	NA	Japonica	
maker-6-augustus-gene-16.0-mRNA-1	6	OsR498G0611561500.01.T02	100,00	0,00E+00	1251	Os06g0129800	100,00	0,00E+00	1243	-8	NA	NA	
maker-6-augustus-gene-16.11-mRNA-1	6	OsR498G0611564900.01.T01	99,81	0,00E+00	2764	Os06g0130400	99,81	0,00E+00	2764	0	NA	NA	
maker-6-augustus-gene-16.13-mRNA-1	6	OsR498G0611565100.01.T01	100,00	0,00E+00	1142	Os06g0130500	100,00	0,00E+00	872	-270	Indica	NA	
maker-6-augustus-gene-16.15-mRNA-1	6	OsR498G0611566100.01.T03	99,66	0,00E+00	2109	Os06g0131100	99,66	0,00E+00	2109	0	NA	NA	
maker-6-augustus-gene-16.21-mRNA-1	6	OsR498G0611561800.01.T01	100,00	0,00E+00	2847	Os06g0129900	100,00	0,00E+00	2847	0	NA	NA	
maker-6-augustus-gene-16.31-mRNA-1	6	OsR498G0611565600.01.T02	99,59	0,00E+00	1291	Os06g0130800	99,59	0,00E+00	1291	0	NA	NA	
maker-6-augustus-gene-16.33-mRNA-1	6	OsR498G0611565400.01.T03	99,27	0,00E+00	728	Os06g0130900	99,27	0,00E+00	728	0	NA	NA	
maker-6-augustus-gene-16.4-mRNA-1	6	OsR498G0611562300.01.T06	100,00	0,00E+00	1559	Os06g0130000	100,00	0,00E+00	1559	0	NA	NA	
maker-6-augustus-gene-16.8-mRNA-1	6	OsR498G0611562900.01.T01	99,86	0,00E+00	5290	Os06g0130100	97,97	0,00E+00	5178	-112	NA	NA	Kinase-LRR-TM
maker-6-augustus-gene-160.0-mRNA-1	6	OsR498G0916869000.01.T01	86,53	4,01E-163	575	Os06g0357800	85,00	2,71E-109	394	-181	Indica	Japonica	
maker-6-augustus-gene-162.38-mRNA-1	6	OsR498G0612387100.01.T03	99,79	0,00E+00	3472	Os06g0480000	100,00	0,00E+00	3490	18	NA	Japonica	
maker-6-augustus-gene-164.29-mRNA-1	6	OsR498G0612369200.01.T01	98,04	0,00E+00	879	Os06g0484600	100,00	0,00E+00	996	117	Japonica	Japonica	
maker-6-augustus-gene-164.32-mRNA-1	6	OsR498G0612369900.01.T02	96,81	0,00E+00	1367	Os06g0483900	100,00	0,00E+00	1099	-268	Indica	Japonica	
maker-6-augustus-gene-165.0-mRNA-1	6	OsR498G0612366600.01.T01	97,47	0,00E+00	2771	Os06g0486300	100,00	0,00E+00	2863	92	NA	Japonica	
maker-6-augustus-gene-165.11-mRNA-1	6	OsR498G0612365700.01.T01	99,88	0,00E+00	3050	Os06g0486400	99,94	0,00E+00	3055	5	NA	Japonica	CC-Kinase-TM
maker-6-augustus-gene-165.4-mRNA-1	6	OsR498G0612365000.01.T01	99,82	0,00E+00	2031	Os06g0486800	100,00	0,00E+00	2040	9	NA	Japonica	
maker-6-augustus-gene-165.8-mRNA-1	6	OsR498G0612367500.01.T02	99,15	0,00E+00	3967	Os06g0486000	100,00	0,00E+00	4053	86	NA	Japonica	Kinase-TM
maker-6-augustus-gene-166.23-mRNA-1	6	OsR498G0612364400.01.T01	99,82	0,00E+00	2042	Os06g0486900	100,00	0,00E+00	2051	9	NA	Japonica	
maker-6-augustus-gene-167.0-mRNA-1	6	OsR498G0612359600.01.T01	99,86	0,00E+00	1295	Os03g0568800	76,62	9,06E-146	517	-778	Indica	Japonica	Kinase
maker-6-augustus-gene-167.19-mRNA-1	6	OsR498G0612355300.01.T02	99,81	0,00E+00	3741	Os06g0489200	100,00	0,00E+00	3759	18	NA	Japonica	
maker-6-augustus-gene-167.2-mRNA-1	6	OsR498G0612359200.01.T03	98,64	0,00E+00	3735	Os06g0488200	98,64	0,00E+00	3735	0	NA	Japonica	
maker-6-augustus-gene-167.7-mRNA-1	6	OsR498G0612354800.01.T02	99,52	0,00E+00	8508	Os06g0489500	100,00	0,00E+00	8608	100	NA	Japonica	
maker-6-augustus-gene-168.35-mRNA-1	6	OsR498G0612351800.01.T01	99,69	0,00E+00	1746	Os06g0490400	100,00	0,00E+00	1759	13	NA	Japonica	
maker-6-augustus-gene-168.37-mRNA-1	6	OsR498G0612350400.01.T04	99,85	0,00E+00	1221	Os06g0490700	100,00	0,00E+00	1027	-194	Indica	Japonica	
maker-6-augustus-gene-169.3-mRNA-1	6	OsR498G0612341000.01.T01	98,81	0,00E+00	734	Os06g0492700	100,00	0,00E+00	648	-86	Indica	Japonica	
maker-6-augustus-gene-169.7-mRNA-1	6	OsR498G0612339600.01.T02	99,75	0,00E+00	2812	Os06g0492900	100,00	0,00E+00	2922	110	NA	Japonica	
maker-6-augustus-gene-17.31-mRNA-1	6	OsR498G0611572100.01.T01	99,93	0,00E+00	2431	Os06g0131500	99,93	0,00E+00	2431	0	NA	NA	
maker-6-augustus-gene-17.33-mRNA-1	6	OsR498G0611577100.01.T01	99,14	0,00E+00	3276	Os06g0133000	98,81	0,00E+00	3250	-26	NA	NA	
maker-6-augustus-gene-17.36-mRNA-1	6	OsR498G0611573000.01.T03	99,92	0,00E+00	2139	Os06g0131700	99,83	0,00E+00	1062	-1077	Indica	NA	
maker-6-augustus-gene-17.38-mRNA-1	6	OsR498G0611575600.01.T01	96,91	0,00E+00	2620	Os06g0132500	100,00	0,00E+00	2706	86	NA	NA	
maker-6-augustus-gene-17.40-mRNA-1	6	OsR498G0611576400.01.T05	100,00	0,00E+00	1651	Os06g0132600	100,00	0,00E+00	1460	-191	Indica	NA	
maker-6-augustus-gene-170.2-mRNA-1	6	OsR498G0612335000.01.T02	99,91	0,00E+00	4035	Os06g0493600	100,00	0,00E+00	4044	9	NA	Japonica	
maker-6-augustus-gene-172.26-mRNA-1	6	OsR498G0612326000.01.T01	99,56	0,00E+00	1638	Os06g0495800	100,00	0,00E+00	1086	-552	Indica	Japonica	
maker-6-augustus-gene-172.29-mRNA-1	6	OsR498G0612324600.01.T01	98,37	0,00E+00	3623	Os06g0496000	98,61	0,00E+00	3660	37	NA	Japonica	
maker-6-augustus-gene-173.10-mRNA-1	6	OsR498G0612317500.01.T03	99,71	9,75E-178	624	Os06g0497600	100,00	0,00E+00	818	194	Japonica	Japonica	
maker-6-augustus-gene-173.5-mRNA-1	6	OsR498G0612318600.01.T03	99,49	0,00E+00	2783	Os06g0497200	100,00	0,00E+00	2755	-28	NA	Japonica	
maker-6-augustus-gene-173.7-mRNA-1	6	OsR498G0612318000.01.T01	99,23	0,00E+00	1379	Os06g0497400	100,00	0,00E+00	1353	-26	NA	Japonica	
maker-6-augustus-gene-174.28-mRNA-1	6	OsR498G0612315800.01.T01	99,49	0,00E+00	4544	Os06g0498500	99,65	0,00E+00	4562	18	NA	Japonica	
maker-6-augustus-gene-174.32-mRNA-1	6	OsR498G0612313400.01.T01	99,95	0,00E+00	3686	Os06g0498900	100,00	0,00E+00	3690	4	NA	Japonica	
maker-6-augustus-gene-174.34-mRNA-1	6	OsR498G0612314100.01.T01	100,00	0,00E+00	958	Os06g0498800	100,00	0,00E+00	958	0	NA	Japonica	
maker-6-augustus-gene-175.40-mRNA-1	6	OsR498G0612311300.01.T02	99,92	0,00E+00	2160	Os06g0499500	100,00	0,00E+00	2165	5	NA	Japonica	
maker-6-augustus-gene-175.42-mRNA-1	6	OsR498G0612310700.01.T01	90,43	0,00E+00	2114	Os06g0499900	90,02	0,00E+00	1993	-121	NA	Japonica	
maker-6-augustus-gene-175.49-mRNA-1	6	OsR498G0612310300.01.T01	99,49	0,00E+00	1406	Os06g0500300	100,00	0,00E+00	1424	18	NA	Japonica	
maker-6-augustus-gene-175.51-mRNA-1	6	OsR498G0612310100.01.T01	97,83	0,00E+00	2590	Os06g0497200	84,67	0,00E+00	1693	-897	Indica	Japonica	
maker-6-augustus-gene-177.33-mRNA-1	6	OsR498G0612564600.01.T01	99,50	0,00E+00	1422	Os06g0503400	100,00	0,00E+00	1440	18	NA	Japonica	
maker-6-augustus-gene-177.36-mRNA-1	6	OsR498G0612567100.01.T01	97,49	0,00E+00	1086	Os11g0481200	78,29	4,60E-146	517	-569	Indica	Japonica	
maker-6-augustus-gene-178.0-mRNA-1	6	OsR498G0612582000.01.T04	97,50	3,30E-72	271	Os06g0503600	100,00	4,58E-141	499	228	Japonica	Japonica	
maker-6-augustus-gene-179.30-mRNA-1	6	OsR498G0612586100.01.T01	99,80	0,00E+00	898	Os06g0505400	100,00	0,00E+00	902	4	NA	Japonica	
maker-6-augustus-gene-179.35-mRNA-1	6	OsR498G0612587500.01.T01	99,50	0,00E+00	1411	Os06g0506000	100,00	0,00E+00	1429	18	NA	Japonica	
maker-6-augustus-gene-179.47-mRNA-1	6	OsR498G0612588000.01.T03	99,80	0,00E+00	911	Os06g0506200	100,00	0,00E+00	915	4	NA	Japonica	
maker-6-augustus-gene-179.51-mRNA-1	6	OsR498G0612589000.01.T02	100,00	0,00E+00	1142	Os02g0261100	86,52	5,34E-151	533	-609	Indica	Japonica	
maker-6-augustus-gene-18.0-mRNA-1	6	OsR498G0611582700.01.T01	100,00	0,00E+00	2792	Os06g0133900	100,00	0,00E+00	2792	0	NA	NA	
maker-6-augustus-gene-18.10-mRNA-1	6	OsR498G1018250900.01.T02	82,18	1,12E-92	340	Os06g0134000	100,00	0,00E+00	699	359	Japonica	NA	
maker-6-augustus-gene-18.12-mRNA-1	6	OsR498G0611583000.01.T04	100,00	0,00E+00	1002	Os06g0134050	100,00	0,00E+00	993	-9	NA	NA	
maker-6-augustus-gene-18.16-mRNA-1	6	OsR498G0611584400.01.T01	100,00	0,00E+00	2257	Os06g0134300	100,00	0,00E+00	2257	0	NA	NA	
maker-6-augustus-gene-18.19-mRNA-1	6	OsR498G0611584800.01.T02	100,00	0,00E+00	1398	Os06g0134400	100,00	0,00E+00	1128	-270	Indica	NA	
maker-6-augustus-gene-18.22-mRNA-1	6	OsR498G0611585200.01.T03	100,00	0,00E+00	2132	Os06g0134500	85,56	0,00E+00	1053	-1079	Indica	NA	
maker-6-augustus-gene-18.26-mRNA-1	6	OsR498G0611585600.01.T01	100,00	0,00E+00	1723	Os06g0134500	100,00	0,00E+00	1723	0	NA	NA	
maker-6-augustus-gene-18.29-mRNA-1	6	OsR498G0611586600.01.T01	100,00	0,00E+00	2176	Os06g0134800	100,00	0,00E+00	2176	0	NA	NA	
maker-6-augustus-gene-18.32-mRNA-1	6	OsR498G0611586800.01.T02	100,00	0,00E+00	1629	Os06g0134900	100,00	0,00E+00	1629	0	NA	NA	
maker-6-augustus-gene-18.4-mRNA-1	6	OsR498G0611585900.01.T01	100,00	0,00E+00	3674	Os06g0134700	100,00	0,00E+00	3642	-32	NA	NA	Kinase-LRR-TM
maker-6-augustus-gene-18.6-mRNA-1	6	OsR498G0611582200.01.T01	100,00	0,00E+00	4026	Os06g0133800	100,00	0,00E+00	3674	-352	NA	NA	
maker-6-augustus-gene-180.30-mRNA-1	6	OsR498G0612592600.01.T02	99,63	0,00E+00	1952	Os06g0507400	100,00	0,00E+00	1970	18	NA	Japonica	
maker-6-augustus-gene-180.36-mRNA-1	6	OsR498G0612595200.01.T01	99,88	0,00E+00	4319	Os06g0508700	100,00	0,00E+00	3939	-380	NA	Japonica	
maker-6-augustus-gene-181.5-mRNA-1	6	OsR498G0612598800.01.T01	99,51	0,00E+00	728	Os06g0512700	85,05	1,80E-129	462	-266	Indica	Japonica	
maker-6-augustus-gene-184.24-mRNA-1	6	OsR498G0612611600.01.T01	97,79	0,00E+00	696	Os06g0512700	100,00	0,00E+00	737	41	NA	NA	
maker-6-augustus-gene-184.25-mRNA-1	6	OsR498G0612604400.01.T01	99,76	0,00E+00	732	Os06g0514800	100,00	0,00E+00	737	5	NA	NA	
maker-6-augustus-gene-185.36-mRNA-1	6	OsR498G0612611600.01.T01	99,51	0,00E+00	728	Os06g0513862	100,00	0,00E+00	737	9	NA	NA	
maker-6-augustus-gene-185.38-mRNA-1	6	OsR498G0612611600.01.T01	99,51	0,00E+00	728	Os06g0513862	100,00	0,00E+00	737	9	NA	NA	
maker-6-augustus-gene-185.40-mRNA-1	6	OsR498G0612611600.01.T01	99,76	0,00E+00	732	Os06g0513943	100,00	0,00E+00	737	5	NA	NA	
maker-6-augustus-gene-187.0-mRNA-1	6	OsR498G0612604400.01.T01	99,27	0,00E+00	723	Os06g0514100	100,00	0,00E+00	737	14	NA	NA	
maker-6-augustus-gene-187.2-mRNA-1	6	OsR498G0612604400.01.T01	99,76	0,00E+00	732	Os06g0513943	99,51	0,00E+00	728	-4	NA	Japonica	
maker-6-augustus-gene-19.12-mRNA-1	6	OsR498G0611589100.01.T01	100,00	0,00E+00	1613	Os06g0136800	100,00	0,00E+00	1613	0	NA	NA	
maker-6-augustus-gene-19.32-mRNA-1	6	OsR498G0611587800.01.T01	100,00	0,00E+00	1564	Os06g0136000	100,00	0,00E+00	1564	0	NA	NA	
maker-6-augustus-gene-19.35-mRNA-1	6	OsR498G0611588000.01.T02	100,00	0,00E+00	2214	Os06g0136100	100,00	0,00E+00	2214	0	NA	NA	
maker-6-augustus-gene-19.39-mRNA-1	6	OsR498G0611588800.01.T01	100,00	0,00E+00	2419	Os06g0136600	100,00	0,00E+00	2419	0	NA	NA	
maker-6-augustus-gene-19.7-mRNA-1	6	OsR498G0611588900.01.T07	99,65	0,00E+00	4104	Os06g0136700	99,65	0,00E+00	4104	0	NA	NA	
maker-6-augustus-gene-190.27-mRNA-1	6	OsR498G0612625900.01.T02	99,59	0,00E+00	3470	Os06g0520600	78,50	0,00E+00	1681	-1789	Indica	Japonica	
maker-6-augustus-gene-190.32-mRNA-1	6	OsR498G0612627900.01.T01	100,00	2,48E-176	618	Os03g0730500	85,99	1,05E-118	425	-193	Indica	NA	
maker-6-augustus-gene-191.0-mRNA-1	6	OsR498G0612633100.01.T01	100,00	0,00E+00	1748	Os06g0521200	100,00	0,00E+00	1748	0	NA	Japonica	
maker-6-augustus-gene-191.5-mRNA-1	6	OsR498G0612633800.01.T01	99,80	0,00E+00	1777	Os06g0521400	100,00	0,00E+00	1786	9	NA	Japonica	
maker-6-augustus-gene-191.7-mRNA-1	6	OsR498G0612634000.01.T01	99,80	0,00E+00	1804	Os06g0521500	100,00	0,00E+00	1813	9	NA	Japonica	
maker-6-augustus-gene-192.0-mRNA-1	6	OsR498G0612635600.01.T01	99,90	0,00E+00	1830	Os06g0521900	100,00	0,00E+00	1835	5	NA	Japonica	
maker-6-augustus-gene-192.2-mRNA-1	6	OsR498G0612636600.01.T01	98,92	0,00E+00	1785	Os06g0522300	100,00	0,00E+00	1835	50	NA	Japonica	
maker-6-augustus-gene-193.34-mRNA-1	6	OsR498G0510328400.01.T03	88,75	0,00E+00	2190	Os06g0523800	97,22	0,00E+00	2937	747	Japonica	Japonica	
maker-6-augustus-gene-193.37-mRNA-1	6	OsR498G0612644700.01.T02	99,71	0,00E+00	4270	Os06g0524300	100,00	0,00E+00	4295	25	NA	Japonica	
maker-6-augustus-gene-193.41-mRNA-1	6	OsR498G0612645500.01.T01	99,92	0,00E+00	2306	Os06g0524400	100,00	0,00E+00	2311	5	NA	Japonica	
maker-6-augustus-gene-194.41-mRNA-1	6	OsR498G0612650500.01.T01	99,73	0,00E+00	2016	Os06g0526100	99,29	0,00E+00	1994	-22	NA	Japonica	
maker-6-augustus-gene-194.43-mRNA-1	6	OsR498G0612647000.01.T02	99,42	6,57E-174	610	Os06g0525000	100,00	0,00E+00	875	265	Japonica	Japonica	
maker-6-augustus-gene-194.45-mRNA-1	6	OsR498G0612651400.01.T01	98,62	0,00E+00	884	Os06g0527800	83,99	6,73E-155	546	-338	Indica	NA	
maker-6-augustus-gene-194.47-mRNA-1	6	OsR498G0612651400.01.T01	86,10	2,88E-82	306	Os06g0526466	99,16	0,00E+00	843	537	Japonica	NA	
maker-6-augustus-gene-194.49-mRNA-1	6	OsR498G0612663800.01.T01	99,83	0,00E+00	4224	Os01g0618400	71,36	0,00E+00	692	-3532	Indica	NA	
maker-6-augustus-gene-194.52-mRNA-1	6	OsR498G0612664000.01.T02	95,81	0,00E+00	1727	Os06g0526700	99,65	0,00E+00	1031	-696	Indica	NA	
maker-6-augustus-gene-195.0-mRNA-1	6	OsR498G0612664900.01.T01	99,76	0,00E+00	3738	Os06g0527100	99,66	0,00E+00	3729	-9	NA	Japonica	
maker-6-augustus-gene-195.18-mRNA-1	6	OsR498G0612664400.01.T03	99,79	0,00E+00	1712	Os06g0526800	99,48	0,00E+00	1021	-691	Indica	NA	
maker-6-augustus-gene-195.5-mRNA-1	6	OsR498G0612666700.01.T02	99,48	0,00E+00	3075	Os06g0527100	95,65	0,00E+00	2809	-266	NA	Japonica	
maker-6-augustus-gene-195.9-mRNA-1	6	OsR498G0612669900.01.T06	100,00	0,00E+00	1393	Os06g0528600	100,00	0,00E+00	1393	0	NA	Japonica	
maker-6-augustus-gene-197.0-mRNA-1	6	OsR498G0612674500.01.T01	99,31	0,00E+00	1541	Os06g0530300	98,20	0,00E+00	1337	-204	Indica	Indica	
maker-6-augustus-gene-197.10-mRNA-1	6	OsR498G0612678800.01.T01	99,25	0,00E+00	2370	Os06g0531200	99,33	0,00E+00	2113	-257	Indica	Japonica	
maker-6-augustus-gene-197.4-mRNA-1	6	OsR498G0612677900.01.T01	99,67	0,00E+00	2190	Os06g0531000	99,91	0,00E+00	2085	-105	NA	Japonica	
maker-6-augustus-gene-197.7-mRNA-1	6	OsR498G0612675000.01.T02	99,56	0,00E+00	2018	Os06g0530400	99,73	0,00E+00	1346	-672	Indica	NA	
maker-6-augustus-gene-197.9-mRNA-1	6	OsR498G0612676800.01.T03	99,77	0,00E+00	1568	Os06g0530700	99,66	0,00E+00	1563	-5	NA	Japonica	
maker-6-augustus-gene-198.21-mRNA-1	6	OsR498G0612679600.01.T01	99,63	0,00E+00	1927	Os06g0531600	99,91	0,00E+00	1940	13	NA	Japonica	
maker-6-augustus-gene-198.23-mRNA-1	6	OsR498G0612680000.01.T05	99,40	1,08E-169	596	Os10g0128400	98,68	1,60E-149	527	-69	Indica	Japonica	
maker-6-augustus-gene-198.25-mRNA-1	6	OsR498G0612680600.01.T01	99,83	0,00E+00	2134	Os06g0531900	99,92	0,00E+00	2139	5	NA	Japonica	
maker-6-augustus-gene-2.36-mRNA-1	6	OsR498G0611469100.01.T01	99,74	0,00E+00	2791	Os06g0102100	99,94	0,00E+00	2804	13	NA	Japonica	
maker-6-augustus-gene-2.38-mRNA-1	6	OsR498G0611469300.01.T01	99,67	0,00E+00	2700	Os06g0102300	98,60	0,00E+00	1369	-1331	Indica	Japonica	
maker-6-augustus-gene-2.44-mRNA-1	6	OsR498G0611470700.01.T02	100,00	3,61E-125	448	Os06g0102600	79,52	7,42E-173	606	158	Japonica	Japonica	
maker-6-augustus-gene-2.46-mRNA-1	6	OsR498G0611470900.01.T02	99,34	0,00E+00	3223	Os06g0102700	100,00	0,00E+00	3274	51	NA	Japonica	
maker-6-augustus-gene-2.62-mRNA-1	6	OsR498G0611472700.01.T01	99,85	0,00E+00	3612	Os06g0103500	100,00	0,00E+00	3626	14	NA	Japonica	
maker-6-augustus-gene-2.67-mRNA-1	6	OsR498G0611472900.01.T09	99,74	0,00E+00	3410	Os06g0103600	100,00	0,00E+00	3433	23	NA	Japonica	
maker-6-augustus-gene-2.82-mRNA-1	6	OsR498G0611472200.01.T04	99,57	0,00E+00	1246	Os06g0103300	100,00	0,00E+00	1260	14	NA	Japonica	
maker-6-augustus-gene-2.89-mRNA-1	6	OsR498G0611473200.01.T01	99,22	0,00E+00	1821	Os06g0103700	100,00	0,00E+00	1856	35	NA	Japonica	
maker-6-augustus-gene-20.0-mRNA-1	6	OsR498G0611593100.01.T01	100,00	0,00E+00	1716	Os06g0137300	100,00	0,00E+00	1716	0	NA	NA	
maker-6-augustus-gene-20.13-mRNA-1	6	OsR498G0611595300.01.T01	96,92	0,00E+00	3092	Os06g0138200	96,92	0,00E+00	3092	0	NA	NA	
maker-6-augustus-gene-20.18-mRNA-1	6	OsR498G0611599100.01.T02	100,00	0,00E+00	1689	Os06g0139700	100,00	0,00E+00	1689	0	NA	NA	
maker-6-augustus-gene-20.36-mRNA-1	6	OsR498G0611595200.01.T01	100,00	0,00E+00	1380	Os06g0138100	100,00	0,00E+00	1380	0	NA	NA	
maker-6-augustus-gene-20.38-mRNA-1	6	OsR498G0611597400.01.T01	100,00	0,00E+00	2650	Os06g0139000	100,00	0,00E+00	2419	-231	NA	NA	
maker-6-augustus-gene-20.41-mRNA-1	6	OsR498G0611599800.01.T01	100,00	0,00E+00	1337	Os06g0139900	100,00	0,00E+00	1337	0	NA	NA	
maker-6-augustus-gene-20.5-mRNA-1	6	OsR498G0611594100.01.T05	100,00	0,00E+00	1137	Os06g0137600	100,00	0,00E+00	1137	0	NA	NA	
maker-6-augustus-gene-200.46-mRNA-1	6	OsR498G0612692700.01.T01	88,38	5,40E-143	508	Os06g0696900	89,18	0,00E+00	762	254	Japonica	Japonica	
maker-6-augustus-gene-201.0-mRNA-1	6	OsR498G0612698700.01.T01	100,00	0,00E+00	3025	Os06g0538000	100,00	0,00E+00	3025	0	NA	Japonica	
maker-6-augustus-gene-201.7-mRNA-1	6	OsR498G0612698000.01.T01	99,75	0,00E+00	714	Os06g0537700	99,75	0,00E+00	714	0	NA	NA	
maker-6-augustus-gene-202.48-mRNA-1	6	OsR498G0612706500.01.T01	96,90	0,00E+00	1847	Os02g0244600	76,16	0,00E+00	726	-1121	Indica	Indica	
maker-6-augustus-gene-202.53-mRNA-1	6	OsR498G0612707100.01.T02	99,29	0,00E+00	1507	Os06g0542200	99,53	0,00E+00	1516	9	NA	NA	
maker-6-augustus-gene-202.57-mRNA-1	6	OsR498G0612707400.01.T01	99,64	0,00E+00	987	Os06g0542300	99,46	0,00E+00	983	-4	NA	NA	
maker-6-augustus-gene-203.26-mRNA-1	6	OsR498G0612711400.01.T01	99,39	0,00E+00	2904	Os06g0543200	99,39	0,00E+00	2902	-2	NA	NA	
maker-6-augustus-gene-204.29-mRNA-1	6	OsR498G0612716800.01.T01	99,27	0,00E+00	1945	Os06g0545400	94,43	0,00E+00	1700	-245	Indica	NA	
maker-6-augustus-gene-204.31-mRNA-1	6	OsR498G0612717800.01.T01	99,44	0,00E+00	2207	Os06g0545400	99,60	0,00E+00	2213	6	NA	Indica	
maker-6-augustus-gene-205.44-mRNA-1	6	OsR498G0612718200.01.T02	99,62	0,00E+00	3249	Os06g0545900	99,89	0,00E+00	3259	10	NA	NA	
maker-6-augustus-gene-205.50-mRNA-1	6	OsR498G0612719200.01.T01	99,27	0,00E+00	1695	Os06g0546500	99,79	0,00E+00	1718	23	NA	Japonica	
maker-6-augustus-gene-205.52-mRNA-1	6	OsR498G0612720200.01.T01	99,80	0,00E+00	1817	Os06g0547100	99,59	0,00E+00	1741	-76	NA	Indica	
maker-6-augustus-gene-205.55-mRNA-1	6	OsR498G0612720400.01.T01	99,90	0,00E+00	1755	Os06g0547400	99,90	0,00E+00	1755	0	NA	NA	
maker-6-augustus-gene-205.57-mRNA-1	6	OsR498G0612720400.01.T01	99,90	0,00E+00	1755	Os06g0547400	99,90	0,00E+00	1755	0	NA	NA	
maker-6-augustus-gene-206.0-mRNA-1	6	OsR498G0612721100.01.T02	99,90	0,00E+00	1870	Os06g0547900	100,00	0,00E+00	1873	3	NA	Japonica	Kinase-TM
maker-6-augustus-gene-206.5-mRNA-1	6	OsR498G0612721600.01.T02	100,00	0,00E+00	2333	Os06g0548000	99,71	0,00E+00	2489	156	NA	NA	
maker-6-augustus-gene-207.44-mRNA-1	6	OsR498G0612730300.01.T02	97,88	1,57E-159	563	Os06g0551500	100,00	1,13E-168	590	27	NA	Japonica	
maker-6-augustus-gene-207.48-mRNA-1	6	OsR498G0612727000.01.T01	100,00	0,00E+00	991	Os06g0550000	100,00	0,00E+00	991	0	NA	Japonica	
maker-6-augustus-gene-207.54-mRNA-1	6	OsR498G0612730000.01.T01	100,00	0,00E+00	778	Os06g0551400	100,00	0,00E+00	774	-4	NA	NA	
maker-6-augustus-gene-208.19-mRNA-1	6	OsR498G0612733300.01.T01	99,53	0,00E+00	1902	Os06g0552300	100,00	0,00E+00	1927	25	NA	Japonica	
maker-6-augustus-gene-208.23-mRNA-1	6	OsR498G0612733600.01.T03	99,38	0,00E+00	2581	Os06g0552400	99,93	0,00E+00	2606	25	NA	Japonica	
maker-6-augustus-gene-208.27-mRNA-1	6	OsR498G0612736000.01.T01	100,00	0,00E+00	942	Os06g0552900	100,00	0,00E+00	942	0	NA	Japonica	
maker-6-augustus-gene-208.29-mRNA-1	6	OsR498G0612737600.01.T01	99,52	0,00E+00	1496	Os02g0232000	78,79	9,93E-143	507	-989	Indica	Japonica	
maker-6-augustus-gene-208.31-mRNA-1	6	OsR498G0612738400.01.T01	98,25	0,00E+00	987	Os06g0553200	100,00	0,00E+00	1012	25	NA	Japonica	
maker-6-augustus-gene-209.10-mRNA-1	6	OsR498G0612746800.01.T03	98,95	0,00E+00	8026	Os06g0554800	100,00	0,00E+00	6314	-1712	Indica	Japonica	
maker-6-augustus-gene-209.8-mRNA-1	6	OsR498G0612742200.01.T01	98,92	0,00E+00	1136	Os06g0553800	99,08	0,00E+00	767	-369	Indica	Japonica	
maker-6-augustus-gene-21.0-mRNA-1	6	OsR498G0611601500.01.T02	100,00	0,00E+00	1391	Os06g0140400	100,00	0,00E+00	1391	0	NA	NA	
maker-6-augustus-gene-21.14-mRNA-1	6	OsR498G0611603000.01.T01	100,00	0,00E+00	2819	Os06g0141000	100,00	0,00E+00	2560	-259	NA	NA	
maker-6-augustus-gene-21.16-mRNA-1	6	OsR498G0611603400.01.T01	100,00	0,00E+00	2197	Os06g0141100	100,00	0,00E+00	2197	0	NA	NA	
maker-6-augustus-gene-21.2-mRNA-1	6	OsR498G0611605700.01.T04	99,43	0,00E+00	2826	Os06g0142000	95,18	0,00E+00	2425	-401	Indica	NA	
maker-6-augustus-gene-21.20-mRNA-1	6	OsR498G0611603800.01.T01	99,77	0,00E+00	772	Os06g0141200	99,77	0,00E+00	772	0	NA	NA	
maker-6-augustus-gene-21.22-mRNA-1	6	OsR498G0611604200.01.T02	100,00	2,11E-145	515	Os06g0141400	100,00	1,85E-134	477	-38	NA	NA	
maker-6-augustus-gene-21.25-mRNA-1	6	OsR498G0611604400.01.T01	100,00	0,00E+00	634	Os06g0142350	96,87	1,81E-166	584	-50	NA	NA	
maker-6-augustus-gene-21.27-mRNA-1	6	OsR498G0611604600.01.T01	100,00	1,38E-179	628	Os06g0141700	100,00	4,80E-180	628	0	NA	NA	
maker-6-augustus-gene-21.4-mRNA-1	6	OsR498G0611600400.01.T03	100,00	0,00E+00	1846	Os06g0140100	99,90	0,00E+00	1841	-5	NA	NA	
maker-6-augustus-gene-21.9-mRNA-1	6	OsR498G0611602200.01.T02	100,00	0,00E+00	4367	Os06g0140800	100,00	0,00E+00	4367	0	NA	NA	CC-Kinase-TM
maker-6-augustus-gene-210.0-mRNA-1	6	OsR498G0612749100.01.T01	100,00	0,00E+00	753	Os02g0229000	95,80	0,00E+00	655	-98	Indica	Japonica	
maker-6-augustus-gene-210.3-mRNA-1	6	OsR498G0612751100.01.T01	99,86	0,00E+00	2632	Os06g0556000	100,00	0,00E+00	2641	9	NA	Japonica	
maker-6-augustus-gene-210.6-mRNA-1	6	OsR498G0612752700.01.T01	99,84	0,00E+00	1111	Os06g0556600	100,00	0,00E+00	953	-158	Indica	Japonica	

maker-6-augustus-gene-210.8-mRNA-1	6	OsR498G0612749400.01.T01	99,88	0,00E+00	2940	Os06g0555500	100,00	0,00E+00	2949	9	NA	Japonica	
maker-6-augustus-gene-211.0-mRNA-1	6	OsR498G0612756500.01.T04	99,94	0,00E+00	5629	Os06g0557100	100,00	0,00E+00	5638	9	NA	Japonica	Kinase-LRR-TM
maker-6-augustus-gene-211.2-mRNA-1	6	OsR498G0612758100.01.T01	99,57	0,00E+00	1663	Os06g0557700	100,00	0,00E+00	1682	19	NA	Japonica	Kinase-LRR-TM
maker-6-augustus-gene-212.0-mRNA-1	6	OsR498G0612763700.01.T01	97,99	0,00E+00	1189	Os06g0559400	99,57	0,00E+00	1235	46	NA	Japonica	
maker-6-augustus-gene-212.15-mRNA-1	6	OsR498G0612766200.01.T02	99,50	3,90E-98	359	Os06g0560400	100,00	3,18E-100	363	4	NA	Japonica	
maker-6-augustus-gene-212.2-mRNA-1	6	OsR498G0612764700.01.T01	99,66	0,00E+00	2602	Os06g0560000	100,00	0,00E+00	2625	23	NA	Japonica	
maker-6-augustus-gene-212.6-mRNA-1	6	OsR498G0612762400.01.T03	99,73	0,00E+00	3939	Os06g0558700	99,81	0,00E+00	3808	-131	NA	Japonica	
maker-6-augustus-gene-213.0-mRNA-1	6	OsR498G0612766900.01.T01	95,97	0,00E+00	2009	Os06g0560700	99,27	0,00E+00	2179	170	NA	Japonica	
maker-6-augustus-gene-213.2-mRNA-1	6	OsR498G0612767700.01.T05	99,67	0,00E+00	1621	Os06g0561000	99,78	0,00E+00	1626	5	NA	Japonica	
maker-6-augustus-gene-213.8-mRNA-1	6	OsR498G0612768900.01.T01	99,82	0,00E+00	2021	Os06g0561200	99,91	0,00E+00	2025	4	NA	NA	
maker-6-augustus-gene-214.32-mRNA-1	6	OsR498G0612773400.01.T01	99,50	0,00E+00	2865	Os06g0562600	100,00	0,00E+00	2901	36	NA	Japonica	
maker-6-augustus-gene-215.0-mRNA-1	6	OsR498G0612775700.01.T02	99,67	0,00E+00	2185	Os06g0563300	99,67	0,00E+00	2188	3	NA	Japonica	
maker-6-augustus-gene-215.14-mRNA-1	6	OsR498G0612777500.01.T02	98,14	0,00E+00	1847	Os06g0564500	100,00	0,00E+00	1938	91	NA	Japonica	
maker-6-augustus-gene-215.18-mRNA-1	6	OsR498G0612785600.01.T01	98,57	0,00E+00	976	Os06g0564500	72,40	1,76E-114	413	-563	Indica	Japonica	
maker-6-augustus-gene-215.20-mRNA-1	6	OsR498G0612786600.01.T01	100,00	0,00E+00	1799	Os06g0564700	100,00	0,00E+00	1799	0	NA	Japonica	
maker-6-augustus-gene-215.9-mRNA-1	6	OsR498G0612774000.01.T01	99,90	0,00E+00	1884	Os06g0563000	100,00	0,00E+00	1889	5	NA	Japonica	
maker-6-augustus-gene-216.30-mRNA-1	6	OsR498G0612788300.01.T01	99,70	0,00E+00	1782	Os06g0565200	100,00	0,00E+00	1797	15	NA	Japonica	
maker-6-augustus-gene-216.48-mRNA-1	6	OsR498G0612791500.01.T01	99,76	0,00E+00	2193	Os06g0566100	100,00	0,00E+00	1548	-645	Indica	Japonica	
maker-6-augustus-gene-216.59-mRNA-1	6	OsR498G0612787000.01.T03	98,54	0,00E+00	1425	Os03g0180700	91,77	0,00E+00	1188	-237	Indica	Japonica	
maker-6-augustus-gene-216.71-mRNA-1	6	OsR498G0612787800.01.T01	100,00	0,00E+00	2928	Os06g0565100	100,00	0,00E+00	2928	0	NA	Japonica	
maker-6-augustus-gene-216.73-mRNA-1	6	OsR498G0612788600.01.T01	99,83	0,00E+00	1084	Os06g0565300	100,00	0,00E+00	1948	864	Japonica	Japonica	
maker-6-augustus-gene-218.43-mRNA-1	6	OsR498G0612796500.01.T03	97,79	0,00E+00	3000	Os06g0567900	100,00	0,00E+00	2053	-947	Indica	Japonica	
maker-6-augustus-gene-218.49-mRNA-1	6	OsR498G0612796800.01.T01	99,17	0,00E+00	3408	Os06g0568000	100,00	0,00E+00	2062	-1346	Indica	Japonica	
maker-6-augustus-gene-218.51-mRNA-1	6	OsR498G0612797000.01.T01	99,11	0,00E+00	2588	Os06g0568200	100,00	0,00E+00	2646	58	NA	Japonica	
maker-6-augustus-gene-218.62-mRNA-1	6	OsR498G0612799200.01.T01	99,70	0,00E+00	1771	Os06g0569500	100,00	0,00E+00	1784	13	NA	Japonica	
maker-6-augustus-gene-219.28-mRNA-1	6	OsR498G0612803900.01.T01	98,43	0,00E+00	2094	Os06g0570950	100,00	0,00E+00	3052	958	Japonica	Japonica	
maker-6-augustus-gene-219.34-mRNA-1	6	OsR498G0612801600.01.T01	98,34	0,00E+00	2811	Os06g0570100	98,62	0,00E+00	1772	-1039	Indica	Japonica	
maker-6-augustus-gene-219.40-mRNA-1	6	OsR498G0612801000.01.T01	91,53	0,00E+00	2156	Os06g0570100	94,16	0,00E+00	2334	178	NA	Japonica	
maker-6-augustus-gene-219.44-mRNA-1	6	OsR498G0612804400.01.T03	100,00	0,00E+00	2273	Os06g0571100	100,00	0,00E+00	1911	-362	Indica	Japonica	
maker-6-augustus-gene-219.48-mRNA-1	6	OsR498G0612805400.01.T01	99,60	0,00E+00	2234	Os06g0571400	100,00	0,00E+00	2257	23	NA	Japonica	
maker-6-augustus-gene-22.1-mRNA-1	6	OsR498G0611605700.01.T03	99,81	0,00E+00	2766	Os06g0142100	99,81	0,00E+00	2766	0	NA	NA	
maker-6-augustus-gene-22.12-mRNA-1	6	OsR498G0611610100.01.T02	100,00	0,00E+00	2316	Os06g0143400	100,00	0,00E+00	2316	0	NA	NA	
maker-6-augustus-gene-22.16-mRNA-1	6	OsR498G0611606000.01.T02	100,00	1,38E-179	628	Os06g0142200	100,00	4,80E-180	628	0	NA	NA	
maker-6-augustus-gene-22.19-mRNA-1	6	OsR498G0611606400.01.T01	100,00	1,98E-179	628	Os06g0142300	100,00	6,91E-180	628	0	NA	NA	
maker-6-augustus-gene-22.22-mRNA-1	6	OsR498G0611606600.01.T01	100,00	0,00E+00	634	Os06g0142350	100,00	0,00E+00	839	205	Japonica	NA	
maker-6-augustus-gene-22.25-mRNA-1	6	OsR498G0611606800.01.T01	100,00	1,38E-179	628	Os06g0142400	100,00	4,80E-180	628	0	NA	NA	
maker-6-augustus-gene-22.27-mRNA-1	6	OsR498G0611607800.01.T01	99,95	0,00E+00	3956	Os06g0142600	100,00	0,00E+00	2250	-1706	Indica	NA	
maker-6-augustus-gene-22.31-mRNA-1	6	OsR498G0611608400.01.T01	100,00	0,00E+00	839	Os06g0142700	100,00	0,00E+00	839	0	NA	NA	
maker-6-augustus-gene-22.36-mRNA-1	6	OsR498G0611608800.01.T01	99,95	0,00E+00	3908	Os06g0142900	99,94	0,00E+00	3040	-868	Indica	NA	
maker-6-augustus-gene-22.5-mRNA-1	6	OsR498G0611608500.01.T01	100,00	0,00E+00	2062	Os06g0142800	100,00	0,00E+00	2062	0	NA	NA	
maker-6-augustus-gene-22.8-mRNA-1	6	OsR498G0611608900.01.T01	100,00	0,00E+00	1386	Os06g0143000	100,00	0,00E+00	1386	0	NA	NA	
maker-6-augustus-gene-220.0-mRNA-1	6	OsR498G0612810100.01.T01	99,63	0,00E+00	3850	Os06g0573600	100,00	0,00E+00	3874	24	NA	Japonica	
maker-6-augustus-gene-220.9-mRNA-1	6	OsR498G0612807400.01.T01	99,51	0,00E+00	1460	Os06g0572400	100,00	0,00E+00	1478	18	NA	Japonica	
maker-6-augustus-gene-221.12-mRNA-1	6	OsR498G0612812000.01.T01	99,96	0,00E+00	4362	Os06g0574200	100,00	0,00E+00	4367	5	NA	Japonica	Kinase-TM
maker-6-augustus-gene-221.19-mRNA-1	6	OsR498G0612815400.01.T01	99,24	0,00E+00	696	Os06g0574900	100,00	0,00E+00	710	14	NA	Japonica	
maker-6-augustus-gene-221.5-mRNA-1	6	OsR498G0612814100.01.T01	100,00	0,00E+00	1662	Os06g0574500	100,00	0,00E+00	1662	0	NA	Japonica	
maker-6-augustus-gene-223.32-mRNA-1	6	OsR498G0612827900.01.T01	98,69	0,00E+00	3060	Os06g0576900	99,94	0,00E+00	3150	90	NA	Japonica	
maker-6-augustus-gene-223.39-mRNA-1	6	OsR498G0612825400.01.T01	99,58	0,00E+00	3368	Os06g0578100	99,95	0,00E+00	3334	-34	NA	Japonica	
maker-6-augustus-gene-224.21-mRNA-1	6	OsR498G0612834500.01.T01	99,48	0,00E+00	1368	Os06g0581300	97,01	0,00E+00	1282	-86	NA	Japonica	
maker-6-augustus-gene-224.26-mRNA-1	6	OsR498G0612832000.01.T01	98,76	0,00E+00	3095	Os06g0581000	99,04	0,00E+00	3118	23	NA	Japonica	
maker-6-augustus-gene-225.34-mRNA-1	6	OsR498G0612836400.01.T01	99,69	0,00E+00	3472	Os12g0498650	70,80	0,00E+00	757	-2715	Indica	Japonica	Kinase-LRR-TM
maker-6-augustus-gene-225.37-mRNA-1	6	OsR498G0612837400.01.T02	93,74	0,00E+00	5202	Os06g0587900	87,58	0,00E+00	2976	-2226	Indica	Japonica	Kinase-LRR-TM
maker-6-augustus-gene-225.39-mRNA-1	6	OsR498G0612840200.01.T03	98,65	0,00E+00	2714	Os06g0583400	99,10	0,00E+00	2746	32	NA	NA	
maker-6-augustus-gene-227.0-mRNA-1	6	OsR498G0612844500.01.T01	99,92	0,00E+00	4323	Os06g0586150	73,40	0,00E+00	1700	-2623	Indica	Indica	Kinase-LRR-TM
maker-6-augustus-gene-227.3-mRNA-1	6	OsR498G0612845300.01.T01	99,79	0,00E+00	1705	Os06g0587500	75,74	0,00E+00	639	-1066	Indica	Indica	Kinase-LRR-TM
maker-6-augustus-gene-228.0-mRNA-1	6	OsR498G0612846900.01.T01	99,60	0,00E+00	3140	Os06g0586150	88,64	0,00E+00	3491	351	Japonica	Japonica	Kinase-LRR-TM
maker-6-augustus-gene-228.11-mRNA-1	6	OsR498G0612847800.01.T01	98,17	4,52E-103	374	Os06g0587100	75,00	1,30E-28	125	-249	Indica	Japonica	
maker-6-augustus-gene-228.6-mRNA-1	6	OsR498G0612847900.01.T01	89,41	0,00E+00	4020	Os06g0586150	75,46	0,00E+00	2128	-1892	Indica	NA	Kinase-LRR-TM
maker-6-augustus-gene-23.27-mRNA-1	6	OsR498G0611613700.01.T02	100,00	0,00E+00	1884	Os06g0144000	100,00	0,00E+00	1884	0	NA	NA	
maker-6-augustus-gene-23.31-mRNA-1	6	OsR498G0611616300.01.T01	100,00	0,00E+00	2430	Os06g0144900	100,00	0,00E+00	2430	0	NA	NA	
maker-6-augustus-gene-23.33-mRNA-1	6	OsR498G0611614200.01.T01	98,12	0,00E+00	2285	Os06g0144200	98,12	0,00E+00	2285	0	NA	NA	
maker-6-augustus-gene-23.37-mRNA-1	6	OsR498G0611617900.01.T01	100,00	0,00E+00	2587	Os06g0145600	100,00	0,00E+00	2587	0	NA	NA	
maker-6-augustus-gene-23.43-mRNA-1	6	OsR498G0611613600.01.T05	99,85	0,00E+00	4919	Os06g0143900	99,85	0,00E+00	4862	-57	NA	NA	
maker-6-augustus-gene-23.47-mRNA-1	6	OsR498G0611614200.01.T01	100,00	0,00E+00	2441	Os06g0144200	100,00	0,00E+00	2441	0	NA	NA	
maker-6-augustus-gene-23.56-mRNA-1	6	OsR498G0611616200.01.T03	98,69	0,00E+00	2138	Os06g0144800	98,77	0,00E+00	2137	-1	NA	NA	
maker-6-augustus-gene-230.2-mRNA-1	6	OsR498G0612852500.01.T01	99,73	0,00E+00	6057	Os06g0587500	82,20	0,00E+00	2293	-3764	Indica	NA	Kinase-LRR-TM
maker-6-augustus-gene-230.6-mRNA-1	6	OsR498G0612852800.01.T02	99,74	0,00E+00	683	Os06g0587300	79,41	2,95E-50	197	-486	Indica	NA	
maker-6-augustus-gene-231.4-mRNA-1	6	OsR498G0612857700.01.T01	99,71	0,00E+00	1838	Os06g0589500	98,56	0,00E+00	1819	-19	NA	Japonica	
maker-6-augustus-gene-231.7-mRNA-1	6	OsR498G0612839000.01.T02	99,97	0,00E+00	6191	Os06g0587900	99,19	0,00E+00	4347	-1844	Indica	Indica	Kinase-LRR-TM
maker-6-augustus-gene-231.9-mRNA-1	6	OsR498G0612857000.01.T01	99,54	0,00E+00	5082	Os06g0589300	100,00	0,00E+00	5140	58	NA	Japonica	
maker-6-augustus-gene-232.54-mRNA-1	6	OsR498G0612863300.01.T01	99,89	0,00E+00	1668	Os06g0590500	100,00	0,00E+00	1673	5	NA	Japonica	
maker-6-augustus-gene-232.58-mRNA-1	6	OsR498G0612866100.01.T01	89,01	5,78E-70	265	Os06g0591400	93,86	2,14E-152	537	272	Japonica	Japonica	
maker-6-augustus-gene-232.62-mRNA-1	6	OsR498G0612863600.01.T01	100,00	0,00E+00	2619	Os06g0590600	100,00	0,00E+00	2619	0	NA	NA	
maker-6-augustus-gene-233.50-mRNA-1	6	OsR498G0612870300.01.T01	96,78	0,00E+00	2717	Os01g0591000	74,57	0,00E+00	897	-1820	Indica	Japonica	
maker-6-augustus-gene-233.53-mRNA-1	6	OsR498G0612871100.01.T01	99,81	0,00E+00	1934	Os06g0593100	100,00	0,00E+00	1943	9	NA	Japonica	
maker-6-augustus-gene-233.56-mRNA-1	6	OsR498G0612867200.01.T01	99,74	0,00E+00	685	Os06g0591600	99,74	0,00E+00	685	0	NA	Japonica	
maker-6-augustus-gene-234.11-mRNA-1	6	OsR498G0612877000.01.T03	99,93	0,00E+00	2415	Os06g0594600	99,41	0,00E+00	1493	-922	Indica	Indica	
maker-6-augustus-gene-234.7-mRNA-1	6	OsR498G0612878300.01.T01	99,47	0,00E+00	2326	Os06g0595800	100,00	0,00E+00	1180	-1146	Indica	Japonica	
maker-6-augustus-gene-235.25-mRNA-1	6	OsR498G0612879900.01.T01	98,05	0,00E+00	2204	Os06g0596300	97,95	0,00E+00	1753	-451	Indica	NA	
maker-6-augustus-gene-235.30-mRNA-1	6	OsR498G0612881500.01.T01	99,49	0,00E+00	1042	Os06g0597000	99,15	0,00E+00	1031	-11	NA	Japonica	
maker-6-augustus-gene-235.32-mRNA-1	6	OsR498G0612881900.01.T02	100,00	0,00E+00	1786	Os06g0597200	99,90	0,00E+00	1782	-4	NA	Japonica	
maker-6-augustus-gene-235.34-mRNA-1	6	OsR498G0612878800.01.T01	98,95	0,00E+00	3692	Os06g0595900	98,77	0,00E+00	3260	-432	Indica	NA	
maker-6-augustus-gene-236.36-mRNA-1	6	OsR498G0612883100.01.T02	98,11	0,00E+00	5136	Os06g0597400	99,01	0,00E+00	2667	-2469	Indica	Indica	
maker-6-augustus-gene-236.43-mRNA-1	6	OsR498G0612883500.01.T01	99,29	0,00E+00	993	Os06g0597600	96,96	0,00E+00	933	-60	NA	NA	
maker-6-augustus-gene-236.44-mRNA-1	6	OsR498G0612883500.01.T01	99,86	0,00E+00	1313	Os06g0597600	95,57	0,00E+00	1200	-113	NA	Indica	
maker-6-augustus-gene-236.48-mRNA-1	6	OsR498G0612883900.01.T04	96,99	0,00E+00	2070	Os06g0597900	96,31	0,00E+00	1885	-185	NA	Indica	
maker-6-augustus-gene-236.51-mRNA-1	6	OsR498G0612886000.01.T01	96,91	0,00E+00	1801	Os06g0598900	96,26	0,00E+00	1773	-28	NA	Indica	
maker-6-augustus-gene-237.23-mRNA-1	6	OsR498G0612887100.01.T01	99,76	0,00E+00	2281	Os06g0600100	100,00	0,00E+00	2295	14	NA	Japonica	
maker-6-augustus-gene-237.25-mRNA-1	6	OsR498G0612887300.01.T01	100,00	0,00E+00	1204	Os06g0600301	100,00	0,00E+00	1229	25	NA	Japonica	
maker-6-augustus-gene-237.30-mRNA-1	6	OsR498G0612890300.01.T02	99,89	0,00E+00	1608	Os06g0601000	100,00	0,00E+00	1613	5	NA	Japonica	
maker-6-augustus-gene-237.33-mRNA-1	6	OsR498G0612889800.01.T03	99,43	0,00E+00	1238	Os06g0600700	99,86	0,00E+00	1251	13	NA	Japonica	
maker-6-augustus-gene-238.31-mRNA-1	6	OsR498G0612891900.01.T01	99,56	0,00E+00	1226	Os09g0422500	70,62	2,36E-67	255	-971	Indica	Japonica	
maker-6-augustus-gene-238.35-mRNA-1	6	OsR498G0306739200.01.T01	94,66	0,00E+00	749	Os06g0602200	100,00	1,38E-118	425	-324	Indica	Japonica	
maker-6-augustus-gene-238.42-mRNA-1	6	OsR498G0612893900.01.T01	100,00	0,00E+00	720	Os06g0602600	100,00	0,00E+00	720	0	NA	Japonica	
maker-6-augustus-gene-238.44-mRNA-1	6	OsR498G0612895100.01.T01	99,93	0,00E+00	2404	Os03g0692000	72,88	1,32E-124	446	-1958	Indica	Japonica	
maker-6-augustus-gene-238.47-mRNA-1	6	OsR498G0612894200.01.T03	99,76	0,00E+00	2281	Os05g0171300	74,62	6,47E-160	564	-1717	Indica	Japonica	
maker-6-augustus-gene-238.49-mRNA-1	6	OsR498G0612895400.01.T01	99,84	0,00E+00	1123	Os06g0602900	100,00	0,00E+00	1249	126	Japonica	Japonica	
maker-6-augustus-gene-238.52-mRNA-1	6	OsR498G0612895800.01.T01	100,00	0,00E+00	1570	Os06g0603000	100,00	0,00E+00	1570	0	NA	Japonica	
maker-6-augustus-gene-239.11-mRNA-1	6	OsR498G0612897800.01.T01	99,66	0,00E+00	1589	Os06g0603600	100,00	0,00E+00	1602	13	NA	Japonica	
maker-6-augustus-gene-239.15-mRNA-1	6	OsR498G0612899800.01.T01	99,45	0,00E+00	1303	Os02g0202000	78,71	3,44E-116	418	-885	Indica	Japonica	
maker-6-augustus-gene-239.17-mRNA-1	6	OsR498G0612900400.01.T01	99,80	0,00E+00	4485	Os06g0604200	100,00	0,00E+00	4507	22	NA	Japonica	
maker-6-augustus-gene-239.2-mRNA-1	6	OsR498G0612901100.01.T01	99,76	0,00E+00	4534	Os06g0604300	100,00	0,00E+00	4562	28	NA	Japonica	
maker-6-augustus-gene-239.4-mRNA-1	6	OsR498G0612901700.01.T01	99,84	0,00E+00	4408	Os06g0604400	100,00	0,00E+00	4426	18	NA	Japonica	
maker-6-augustus-gene-239.6-mRNA-1	6	OsR498G0612902300.01.T02	99,81	0,00E+00	2779	Os06g0604500	100,00	0,00E+00	2792	13	NA	Japonica	
maker-6-augustus-gene-239.9-mRNA-1	6	OsR498G0612903300.01.T02	100,00	0,00E+00	715	Os06g0604600	100,00	0,00E+00	715	0	NA	Japonica	
maker-6-augustus-gene-24.42-mRNA-1	6	OsR498G0611619500.01.T01	100,00	0,00E+00	1478	Os06g0145800	100,00	0,00E+00	978	-500	Indica	NA	
maker-6-augustus-gene-24.47-mRNA-1	6	OsR498G0611619900.01.T05	100,00	0,00E+00	2203	Os06g0146100	100,00	0,00E+00	2203	0	NA	NA	NBS-TM Pi9
maker-6-augustus-gene-24.51-mRNA-1	6	OsR498G0611621200.01.T02	100,00	0,00E+00	1846	Os06g0146300	100,00	0,00E+00	1213	-633	Indica	NA	
maker-6-augustus-gene-24.58-mRNA-1	6	OsR498G0611621400.01.T02	100,00	0,00E+00	1012	Os06g0146400	100,00	0,00E+00	1012	0	NA	NA	
maker-6-augustus-gene-240.33-mRNA-1	6	OsR498G0612908900.01.T02	99,85	0,00E+00	3585	Os02g0200900	78,15	0,00E+00	1122	-2463	Indica	Japonica	
maker-6-augustus-gene-240.40-mRNA-1	6	OsR498G0612909400.01.T01	99,63	0,00E+00	2434	Os06g0606000	100,00	0,00E+00	2457	23	NA	Japonica	Kinase-TM
maker-6-augustus-gene-241.12-mRNA-1	6	OsR498G0612912700.01.T01	99,75	0,00E+00	2184	Os06g0607000	100,00	0,00E+00	2197	13	NA	Japonica	
maker-6-augustus-gene-241.18-mRNA-1	6	OsR498G0612914400.01.T03	99,30	0,00E+00	1766	Os02g0200000	80,29	0,00E+00	890	-876	Indica	Japonica	
maker-6-augustus-gene-242.38-mRNA-1	6	OsR498G0612914900.01.T01	99,32	0,00E+00	3669	Os06g0607700	99,57	0,00E+00	3691	22	NA	Japonica	
maker-6-augustus-gene-242.40-mRNA-1	6	OsR498G0612915100.01.T01	99,59	0,00E+00	2153	Os06g0607800	100,00	0,00E+00	2176	23	NA	Japonica	
maker-6-augustus-gene-242.43-mRNA-1	6	OsR498G0612915300.01.T01	99,88	0,00E+00	5846	Os06g0607900	100,00	0,00E+00	5864	18	NA	Japonica	
maker-6-augustus-gene-242.45-mRNA-1	6	OsR498G0612916700.01.T01	97,74	0,00E+00	895	Os06g0608401	100,00	0,00E+00	807	-88	NA	Japonica	
maker-6-augustus-gene-242.47-mRNA-1	6	OsR498G0612916900.01.T02	100,00	0,00E+00	1196	Os06g0608500	100,00	0,00E+00	1196	0	NA	Japonica	
maker-6-augustus-gene-242.52-mRNA-1	6	OsR498G0612917100.01.T04	99,73	0,00E+00	651	Os06g0608600	100,00	0,00E+00	655	4	NA	Japonica	
maker-6-augustus-gene-242.55-mRNA-1	6	OsR498G0612917100.01.T05	99,91	0,00E+00	1895	Os06g0608700	100,00	0,00E+00	1900	5	NA	Japonica	
maker-6-augustus-gene-242.59-mRNA-1	6	OsR498G0510566200.01.T01	89,19	0,00E+00	1216	Os06g0607400	99,89	0,00E+00	1614	398	Japonica	Japonica	
maker-6-augustus-gene-242.62-mRNA-1	6	OsR498G0612916400.01.T01	99,74	0,00E+00	4822	Os06g0608100	100,00	0,00E+00	2814	-2008	Indica	Japonica	
maker-6-augustus-gene-242.67-mRNA-1	6	OsR498G0612916600.01.T01	99,91	0,00E+00	3878	Os06g0608300	100,00	0,00E+00	3887	9	NA	Japonica	
maker-6-augustus-gene-242.71-mRNA-1	6	OsR498G0612917400.01.T01	99,93	0,00E+00	2420	Os06g0608800	100,00	0,00E+00	2425	5	NA	Japonica	
maker-6-augustus-gene-243.26-mRNA-1	6	OsR498G0612919100.01.T01	99,54	0,00E+00	4234	Os02g0198700	72,89	0,00E+00	1220	-3014	Indica	Japonica	
maker-6-augustus-gene-243.28-mRNA-1	6	OsR498G0612919300.01.T02	99,90	0,00E+00	1711	Os02g0198300	77,58	0,00E+00	744	-967	Indica	Japonica	
maker-6-augustus-gene-243.33-mRNA-1	6	OsR498G0612919500.01.T05	95,36	0,00E+00	1687	Os06g0609500	95,65	0,00E+00	1700	13	NA	Japonica	
maker-6-augustus-gene-243.36-mRNA-1	6	OsR498G0612920500.01.T01	99,55	0,00E+00	3200	Os06g0609800	100,00	0,00E+00	2181	-1019	Indica	Japonica	
maker-6-augustus-gene-243.40-mRNA-1	6	OsR498G0612920700.01.T01	99,55	0,00E+00	1185	Os06g0609900	100,00	0,00E+00	812	-373	Indica	Japonica	
maker-6-augustus-gene-243.42-mRNA-1	6	OsR498G0612924300.01.T02	99,55	0,00E+00	2352	Os06g0610800	100,00	0,00E+00	2379	27	NA	Japonica	
maker-6-augustus-gene-243.45-mRNA-1	6	OsR498G0612919800.01.T02	99,85	0,00E+00	1214	Os06g0609600	100,00	0,00E+00	1218	4	NA	Japonica	
maker-6-augustus-gene-243.48-mRNA-1	6	OsR498G0612920200.01.T02	100,00	0,00E+00	1691	Os06g0609700	100,00	0,00E+00	1691	0	NA	Japonica	
maker-6-augustus-gene-243.52-mRNA-1	6	OsR498G0612921400.01.T01	99,29	0,00E+00	752	Os06g0610100	100,00	0,00E+00	1045	293	Japonica	Japonica	
maker-6-augustus-gene-244.0-mRNA-1	6	OsR498G0612924700.01.T01	99,49	0,00E+00	3109	Os06g0611000	100,00	0,00E+00	3149	40	NA	Japonica	
maker-6-augustus-gene-244.21-mRNA-1	6	OsR498G0612927600.01.T02	99,75	0,00E+00	723	Os01g0711800	96,28	0,00E+00	660	-63	NA	Japonica	
maker-6-augustus-gene-244.24-mRNA-1	6	OsR498G0612928200.01.T02	99,94	0,00E+00	5575	Os01g0711400	96,46	0,00E+00	5114	-461	NA	Japonica	
maker-6-augustus-gene-244.3-mRNA-1	6	OsR498G0612926500.01.T01	98,58	0,00E+00	2091	Os06g0611400	100,00	0,00E+00	2161	70	NA	Japonica	
maker-6-augustus-gene-244.5-mRNA-1	6	OsR498G0612926900.01.T01	99,69	0,00E+00	2287	Os06g0611500	100,00	0,00E+00	2305	18	NA	Japonica	
maker-6-augustus-gene-244.7-mRNA-1	6	OsR498G0612927300.01.T01	96,03	0,00E+00	901	Os01g0712700	85,94	0,00E+00	674	-227	Indica	Japonica	
maker-6-augustus-gene-245.38-mRNA-1	6	OsR498G0612934500.01.T01	99,53	0,00E+00	1895	Os06g0613400	100,00	0,00E+00	1918	23	NA	Japonica	
maker-6-augustus-gene-245.40-mRNA-1	6	OsR498G0612936500.01.T02	99,75	0,00E+00	719	Os06g0613800	99,75	0,00E+00	727	8	NA	Japonica	
maker-6-augustus-gene-245.45-mRNA-1	6	OsR498G0612933600.01.T02	99,68	0,00E+00	3397	Os06g0612900	100,00	0,00E+00	3885	488	Japonica	Japonica	
maker-6-augustus-gene-245.54-mRNA-1	6	OsR498G0612934200.01.T01	99,75	0,00E+00	2877	Os06g0613100	100,00	0,00E+00	3198	321	Japonica	Japonica	
maker-6-augustus-gene-245.63-mRNA-1	6	OsR498G0612935800.01.T01	99,75	0,00E+00	2843	Os06g0613600	100,00	0,00E+00	2863	20	NA	Japonica	
maker-6-augustus-gene-246.28-mRNA-1	6	OsR498G0612938100.01.T03	99,48	0,00E+00	2066	Os06g0614300	100,00	0,00E+00	2093	27	NA	Japonica	
maker-6-augustus-gene-246.34-mRNA-1	6	OsR498G0612937400.01.T01	99,93	0,00E+00	2490	Os06g0614100	99,92	0,00E+00	2335	-155	NA	Japonica	
maker-6-augustus-gene-248.0-mRNA-1	6	OsR498G0612959500.01.T01	98,19	0,00E+00	2105	Os06g0618000	100,00	0,00E+00	2165	60	NA	Japonica	
maker-6-augustus-gene-248.10-mRNA-1	6	OsR498G0612958800.01.T01	99,92	0,00E+00	2160	Os06g0617800	100,00	0,00E+00	1838	-322	Indica	Japonica	
maker-6-augustus-gene-248.15-mRNA-1	6	OsR498G0612959800.01.T01	99,87	0,00E+00	1369	Os06g0618100	100,00	0,00E+00	1373	4	NA	Japonica	
maker-6-augustus-gene-248.4-mRNA-1	6	OsR498G0612960500.01.T04	98,39	0,00E+00	2926	Os06g0618600	97,93	0,00E+00	2003	-923	Indica	Japonica	
maker-6-augustus-gene-249.57-mRNA-1	6	OsR498G0612961500.01.T01	99,81	0,00E+00	4742	Os06g0619000	100,00	0,00E+00	5337	595	Japonica	Japonica	NBS-TM
maker-6-augustus-gene-249.59-mRNA-1	6	OsR498G0612964900.01.T01	97,76	0,00E+00	1370	Os06g0620600	100,00	0,00E+00	1451	81	NA	Japonica	
maker-6-augustus-gene-249.63-mRNA-1	6	OsR498G0612965800.01.T01	84,65	0,00E+00	1530	Os06g0621500	100,00	0,00E+00	2699	1169	Japonica	Japonica	LRR-TM
maker-6-augustus-gene-25.0-mRNA-1	6	OsR498G0611627300.01.T03	97,78	0,00E+00	1857	Os06g0148200	97,78	0,00E+00	1857	0	NA	NA	
maker-6-augustus-gene-25.13-mRNA-1	6	OsR498G0611626600.01.T01	100,00	0,00E+00	1261	Os06g0148000	100,00	0,00E+00	787	-474	Indica	NA	
maker-6-augustus-gene-25.3-mRNA-1	6	OsR498G0611630700.01.T01	99,91	0,00E+00	1949	Os06g0149100	98,19	0,00E+00	1053	-896	Indica	NA	
maker-6-augustus-gene-25.8-mRNA-1	6	OsR498G0611631300.01.T03	99,74	0,00E+00	2036	Os06g0149400	100,00	0,00E+00	2051	15	NA	NA	
maker-6-augustus-gene-250.30-mRNA-1	6	OsR498G0612967500.01.T01	99,87	0,00E+00	1408	Os06g0621900	100,00	0,00E+00	1413	5	NA	Japonica	
maker-6-augustus-gene-250.32-mRNA-1	6	OsR498G0612968500.01.T02	99,71	0,00E+00	2482	Os06g0622300	100,00	0,00E+00	2500	18	NA	Japonica	
maker-6-augustus-gene-250.36-mRNA-1	6	OsR498G0612970100.01.T01	99,89	0,00E+00	1646	Os06g0622700	100,00	0,00E+00	1651	5	NA	Japonica	
maker-6-augustus-gene-251.30-mRNA-1	6	OsR498G0612975700.01.T01	98,44	0,00E+00	4024	Os06g0624100	98,48	0,00E+00	4124	100	NA	Japonica	
maker-6-augustus-gene-251.35-mRNA-1	6	OsR498G0612976300.01.T01	99,49	0,00E+00	2794	Os06g0624700	100,00	0,00E+00	2444	-350	Indica	Japonica	
maker-6-augustus-gene-251.37-mRNA-1	6	OsR498G0612970800.01.T06	99,44	0,00E+00	637	Os06g0622900	100,00	0,00E+00	646	9	NA	Japonica	
maker-6-augustus-gene-251.44-mRNA-1	6	OsR498G0612971600.01.T02	98,62	5,61E-180	631	Os06g0623200	100,00	0,00E+00	679	48	NA	Japonica	
maker-6-augustus-gene-251.46-mRNA-1	6	OsR498G0612971800.01.T01	99,79	0,00E+00	1734	Os06g0623300	100,00	0,00E+00	1743	9	NA	Japonica	
maker-6-augustus-gene-251.51-mRNA-1	6	OsR498G0612972800.01.T02	95,86	0,00E+00	1593	Os06g0623600	95,96	0,00E+00	1598	5	NA	Japonica	
maker-6-augustus-gene-252.13-mRNA-1	6	OsR498G0612983000.01.T01	98,08	0,00E+00	2788	Os06g0625900	98,88	0,00E+00	2840	52	NA	Japonica	
maker-6-augustus-gene-252.2-mRNA-1	6	OsR498G0612982500.01.T02	99,56	0,00E+00	1203	Os02g0192500	82,23	0,00E+00	688	-515	Indica	Japonica	
maker-6-augustus-gene-252.7-mRNA-1	6	OsR498G0612981000.01.T05	100,00	0,00E+00	5003	Os06g0625400	99,96	0,00E+00	4561	-442	NA	NA	
maker-6-augustus-gene-254.0-mRNA-1	6	OsR498G0612965100.01.T01	97,15	0,00E+00	2171	Os06g0620200	99,93	0,00E+00	2510	339	Japonica	NA	Kinase-TM
maker-6-augustus-gene-254.17-mRNA-1	6	OsR498G0612992800.01.T01	99,87	0,00E+00	1374	Os04g0483700	99,80	0,00E+00	878	-496	Indica	Japonica	
maker-6-augustus-gene-255.0-mRNA-1	6	OsR498G0612996700.01.T01	99,32	2,38E-147	522	Os03g0168000	77,54	1,71E-137	488	-34	NA	Japonica	
maker-6-augustus-gene-255.4-mRNA-1	6	OsR498G0612997600.01.T01	99,93	0,00E+00	2709	Os10g0330600	93,48	0,00E+00	2271	-438	Indica	Japonica	
maker-6-augustus-gene-256.2-mRNA-1	6	OsR498G0613010200.01.T01	100,00	0,00E+00	710	Os06g0630300	100,00	0,00E+00	975	265	Japonica	Japonica	
maker-6-augustus-gene-257.3-mRNA-1	6	OsR498G0613011200.01.T02	99,52	0,00E+00	2581	Os06g0632200	99,89	0,00E+00	1663	-918	Indica	Indica	
maker-6-augustus-gene-258.33-mRNA-1	6	OsR498G0613017700.01.T02	99,71	0,00E+00	2466	Os06g0633800	100,00	0,00E+00	2484	18	NA	Japonica	
maker-6-augustus-gene-259.17-mRNA-1	6	OsR498G0613020400.01.T02	99,78	0,00E+00	2433	Os06g0634600	100,00	0,00E+00	2446	13	NA	Japonica	
maker-6-augustus-gene-259.20-mRNA-1	6	OsR498G0613021200.01.T01	99,93	0,00E+00	2447	Os06g0635200	100,00	0,00E+00	2452	5	NA	Japonica	
maker-6-augustus-gene-259.27-mRNA-1	6	OsR498G0613022800.01.T01	97,54	0,00E+00	1733	Os06g0635500	100,00	0,00E+00	2040	307	Japonica	Japonica	
maker-6-augustus-gene-259.9-mRNA-1	6	OsR498G0613020200.01.T02	99,77	0,00E+00	3836	Os02g0190500	76,96	0,00E+00	1602	-2234	Indica	Japonica	Kinase-LRR-TM
maker-6-augustus-gene-26.39-mRNA-1	6	OsR498G0611631700.01.T02	100,00	0,00E+00	639	Os06g0149500	100,00	0,00E+00	639	0	NA	NA	
maker-6-augustus-gene-26.41-mRNA-1	6	OsR498G0611632100.01.T01	100,00	0,00E+00	1840	Os06g0149700	100,00	0,00E+00	1840	0	NA	NA	
maker-6-augustus-gene-26.45-mRNA-1	6	OsR498G0611632500.01.T02	100,00	0,00E+00	1884	Os06g0149900	100,00	0,00E+00	1884	0	NA	NA	
maker-6-augustus-gene-26.53-mRNA-1	6	OsR498G0611633000.01.T01	100,00	0,00E+00	1548	Os06g0150100	100,00	0,00E+00	1548	0	NA	NA	
maker-6-augustus-gene-260.32-mRNA-1	6	OsR498G0613026300.01.T03	100,00	0,00E+00	1283	Os06g0636600	100,00	0,00E+00	1283	0	NA	Japonica	Kinase
maker-6-augustus-gene-260.36-mRNA-1	6	OsR498G0613026600.01.T01	99,74	0,00E+00	2063	Os06g0636700	100,00	0,00E+00	2073	10	NA	Japonica	
maker-6-augustus-gene-261.44-mRNA-1	6	OsR498G0613032100.01.T01	99,14	0,00E+00	1028	Os06g0638200	100,00	4,03E-152	536	-492	Indica	Japonica	
maker-6-augustus-gene-261.60-mRNA-1	6	OsR498G0613031800.01.T01	99,77	0,00E+00	1550	Os06g0638100	100,00	0,00E+00	1559	9	NA	Japonica	
maker-6-augustus-gene-261.64-mRNA-1	6	OsR498G0613033400.01.T04	99,86	0,00E+00	1248	Os06g0638700	99,86	0,00E+00	1248	0	NA	Japonica	
maker-6-augustus-gene-261.82-mRNA-1	6	OsR498G0613037000.01.T02	98,74	0,00E+00	1112	Os06g0639600	100,00	0,00E+00	1148	36	NA	Japonica	
maker-6-augustus-gene-262.42-mRNA-1	6	OsR498G0613037900.01.T01	99,56	0,00E+00	2829	Os06g0639800	100,00	0,00E+00	2863	34	NA	Japonica	
maker-6-augustus-gene-262.43-mRNA-1	6	OsR498G0613038100.01.T01	99,56	0,00E+00	2820	Os06g0639800	90,64	0,00E+00	2183	-637	Indica	Japonica	
maker-6-augustus-gene-262.48-mRNA-1	6	OsR498G0613039900.01.T01	93,83	0,00E+00	2377	Os06g0640800	94,02	0,00E+00	2390	13	NA	NA	
maker-6-augustus-gene-262.49-mRNA-1	6	OsR498G0613041700.01.T02	97,54	0,00E+00	1448	Os06g0641500	100,00	0,00E+00	1543	95	NA	NA	
maker-6-augustus-gene-262.55-mRNA-1	6	OsR498G0613040900.01.T01	97,48	0,00E+00	2349	Os06g0640800	84,40	0,00E+00	1739	-610	Indica	NA	
maker-6-augustus-gene-262.59-mRNA-1	6	OsR498G0613042000.01.T01	98,94	0,00E+00	1656	Os11g0635500	72,39	3,92E-142	504	-1152	Indica	NA	
maker-6-augustus-gene-263.0-mRNA-1	6	OsR498G0613044300.01.T01	99,63	0,00E+00	2917	Os06g0671300	82,54	0,00E+00	1156	-1761	Indica	NA	
maker-6-augustus-gene-263.12-mRNA-1	6	OsR498G0613046400.01.T01	99,57	0,00E+00	2918	Os06g0643000	99,82	0,00E+00	2936	18	NA	NA	
maker-6-augustus-gene-263.4-mRNA-1	6	OsR498G0613044600.01.T01	99,55	0,00E+00	2360	Os02g0186800	71,72	0,00E+00	682	-1678	Indica	NA	
maker-6-augustus-gene-263.9-mRNA-1	6	OsR498G0613045600.01.T01	99,64	0,00E+00	1480	Os06g0642900	100,00	0,00E+00	1494	14	NA	NA	
maker-6-augustus-gene-264.26-mRNA-1	6	OsR498G0613050100.01.T01	99,84	0,00E+00	1111	Os06g0644100	100,00	0,00E+00	1115	4	NA	NA	
maker-6-augustus-gene-264.30-mRNA-1	6	OsR498G0613050500.01.T01	99,87	0,00E+00	4164	Os06g0644200	100,00	0,00E+00	4237	73	NA	NA	
maker-6-augustus-gene-264.50-mRNA-1	6	OsR498G0613046600.01.T01	94,47	0,00E+00	1040	Os06g0643100	94,47	0,00E+00	1040	0	NA	NA	
maker-6-augustus-gene-264.54-mRNA-1	6	OsR498G0613048200.01.T01	99,22	0,00E+00	2028	Os06g0643600	99,48	0,00E+00	2041	13	NA	NA	
maker-6-augustus-gene-264.58-mRNA-1	6	OsR498G0613048600.01.T01	99,91	0,00E+00	2036	Os06g0643700	100,00	0,00E+00	2040	4	NA	NA	
maker-6-augustus-gene-264.61-mRNA-1	6	OsR498G0613048800.01.T01	99,83	0,00E+00	5269	Os02g0184400	82,73	0,00E+00	2993	-2276	Indica	NA	
maker-6-augustus-gene-264.75-mRNA-1	6	OsR498G0613049800.01.T01	99,58	0,00E+00	2554	Os06g0643900	100,00	0,00E+00	2581	27	NA	NA	
maker-6-augustus-gene-265.39-mRNA-1	6	OsR498G0613053500.01.T02	98,44	0,00E+00	6125	Os02g0183700	79,72	0,00E+00	1721	-4404	Indica	NA	
maker-6-augustus-gene-265.47-mRNA-1	6	OsR498G0613052600.01.T01	99,67	0,00E+00	1603	Os06g0644800	100,00	0,00E+00	1618	15	NA	NA	
maker-6-augustus-gene-265.61-mRNA-1	6	OsR498G0613055600.01.T01	99,90	0,00E+00	1868	Os06g0646000	100,00	0,00E+00	1873	5	NA	Japonica	
maker-6-augustus-gene-266.44-mRNA-1	6	OsR498G0613057500.01.T01	100,00	0,00E+00	1223	Os06g0646500	99,63	0,00E+00	1462	239	Japonica	NA	
maker-6-augustus-gene-266.49-mRNA-1	6	OsR498G0613059700.01.T01	100,00	0,00E+00	796	Os06g0647100	100,00	0,00E+00	796	0	NA	NA	

maker-6-augustus-gene-266.55-mRNA-1	6	OsR498G0613057800.01.T04	99,59	0,00E+00	1746	Os06g0646600	100,00	0,00E+00	1682	-64	NA	NA	
maker-6-augustus-gene-266.70-mRNA-1	6	OsR498G0613061000.01.T01	98,95	0,00E+00	2852	Os06g0647400	99,26	0,00E+00	2874	22	NA	NA	
maker-6-augustus-gene-266.74-mRNA-1	6	OsR498G0613062800.01.T01	99,69	5,98E-161	568	Os06g0647900	100,00	2,08E-161	567	-1	NA	NA	
maker-6-augustus-gene-266.76-mRNA-1	6	OsR498G0613064000.01.T01	99,43	0,00E+00	1883	Os06g0648500	100,00	0,00E+00	1909	26	NA	NA	
maker-6-augustus-gene-267.24-mRNA-1	6	OsR498G0613067300.01.T02	98,08	0,00E+00	2605	Os06g0649500	98,41	0,00E+00	2627	22	NA	NA	
maker-6-augustus-gene-267.28-mRNA-1	6	OsR498G0613065200.01.T01	100,00	0,00E+00	1793	Os06g0649000	100,00	0,00E+00	1793	0	NA	NA	
maker-6-augustus-gene-267.30-mRNA-1	6	OsR498G0613069400.01.T01	98,83	0,00E+00	2260	Os06g0649800	95,74	0,00E+00	2079	-181	NA	NA	
maker-6-augustus-gene-267.34-mRNA-1	6	OsR498G0613070000.01.T03	99,10	0,00E+00	2741	Os06g0649900	100,00	0,00E+00	2776	35	NA	NA	
maker-6-augustus-gene-268.16-mRNA-1	6	OsR498G0613073200.01.T01	99,77	0,00E+00	2351	Os06g0650800	100,00	0,00E+00	2365	14	NA	NA	
maker-6-augustus-gene-268.20-mRNA-1	6	OsR498G0613074000.01.T01	92,64	0,00E+00	706	Os06g0650900	92,86	0,00E+00	710	4	NA	NA	
maker-6-augustus-gene-268.22-mRNA-1	6	OsR498G0613074200.01.T01	99,45	0,00E+00	1938	Os06g0651000	99,64	0,00E+00	976	-962	Indica	NA	
maker-6-augustus-gene-268.25-mRNA-1	6	OsR498G0613074600.01.T01	99,54	0,00E+00	1920	Os06g0651100	100,00	0,00E+00	1943	23	NA	NA	
maker-6-augustus-gene-268.28-mRNA-1	6	OsR498G0613075000.01.T01	99,86	0,00E+00	2643	Os06g0651300	100,00	0,00E+00	2448	-195	NA	NA	
maker-6-augustus-gene-268.31-mRNA-1	6	OsR498G0613075200.01.T03	99,64	0,00E+00	1979	Os06g0651600	100,00	0,00E+00	1997	18	NA	Japonica	
maker-6-augustus-gene-268.36-mRNA-1	6	OsR498G0613076600.01.T01	99,88	0,00E+00	1502	Os02g0179500	81,69	4,06E-175	615	-887	Indica	NA	
maker-6-augustus-gene-268.7-mRNA-1	6	OsR498G0613075900.01.T01	99,24	0,00E+00	700	Os06g0651900	100,00	9,93E-120	428	-272	Indica	NA	
maker-6-augustus-gene-268.9-mRNA-1	6	OsR498G0613076100.01.T01	96,74	0,00E+00	2634	Os06g0652000	96,94	0,00E+00	2647	13	NA	NA	
maker-6-augustus-gene-269.11-mRNA-1	6	OsR498G0613081600.01.T01	99,77	0,00E+00	3130	Os06g0654000	99,83	0,00E+00	3134	4	NA	NA	
maker-6-augustus-gene-269.2-mRNA-1	6	OsR498G0613077700.01.T01	99,42	0,00E+00	2780	Os06g0652600	100,00	0,00E+00	2809	29	NA	NA	
maker-6-augustus-gene-269.7-mRNA-1	6	OsR498G0613079700.01.T01	99,63	0,00E+00	1448	Os09g0425900	74,84	6,91E-132	470	-978	Indica	NA	
maker-6-augustus-gene-269.9-mRNA-1	6	OsR498G0613081200.01.T02	100,00	6,53E-105	379	Os06g0653900	100,00	2,28E-105	379	0	NA	NA	
maker-6-augustus-gene-27.0-mRNA-1	6	OsR498G0611636900.01.T02	100,00	0,00E+00	3355	Os06g0151600	100,00	0,00E+00	3355	0	NA	NA	
maker-6-augustus-gene-27.11-mRNA-1	6	OsR498G0611638100.01.T01	100,00	0,00E+00	715	Os06g0152100	100,00	0,00E+00	715	0	NA	NA	
maker-6-augustus-gene-27.14-mRNA-1	6	OsR498G0611638500.01.T01	100,00	0,00E+00	1954	Os06g0152200	100,00	0,00E+00	1954	0	NA	NA	
maker-6-augustus-gene-27.17-mRNA-1	6	OsR498G0611638700.01.T01	100,00	0,00E+00	1775	Os06g0152300	100,00	0,00E+00	1775	0	NA	NA	
maker-6-augustus-gene-27.23-mRNA-1	6	OsR498G0611639100.01.T01	94,44	0,00E+00	1208	Os06g0152400	100,00	0,00E+00	1063	-145	Indica	NA	
maker-6-augustus-gene-27.28-mRNA-1	6	OsR498G0611636400.01.T02	99,58	0,00E+00	1262	Os06g0151200	97,46	0,00E+00	1208	-54	NA	NA	
maker-6-augustus-gene-27.35-mRNA-1	6	OsR498G0611636600.01.T04	99,77	0,00E+00	775	Os06g0151300	99,77	0,00E+00	775	0	NA	NA	
maker-6-augustus-gene-27.39-mRNA-1	6	OsR498G0611637200.01.T01	100,00	0,00E+00	2884	Os06g0151700	100,00	0,00E+00	2884	0	NA	NA	Kinase-TM
maker-6-augustus-gene-27.41-mRNA-1	6	OsR498G0611637600.01.T02	96,96	0,00E+00	1838	Os05g0524400	73,09	3,49E-164	578	-1260	Indica	NA	
maker-6-augustus-gene-270.0-mRNA-1	6	OsR498G0613083100.01.T01	99,93	0,00E+00	5234	Os06g0654300	99,94	0,00E+00	5771	537	NA	NA	
maker-6-augustus-gene-270.4-mRNA-1	6	OsR498G0613085100.01.T01	98,37	0,00E+00	2130	Os06g0654500	100,00	0,00E+00	2176	46	NA	NA	Kinase-TM
maker-6-augustus-gene-270.8-mRNA-1	6	OsR498G0613087000.01.T01	99,84	0,00E+00	3309	Os06g0655100	97,46	0,00E+00	3232	-77	NA	NA	
maker-6-augustus-gene-271.0-mRNA-1	6	OsR498G0613090100.01.T01	99,94	0,00E+00	3204	Os06g0656000	100,00	0,00E+00	3209	5	NA	NA	
maker-6-augustus-gene-271.9-mRNA-1	6	OsR498G0613092000.01.T01	99,70	0,00E+00	3008	Os06g0656500	100,00	0,00E+00	3030	22	NA	NA	
maker-6-augustus-gene-272.30-mRNA-1	6	OsR498G0613096300.01.T01	98,58	0,00E+00	2458	Os06g0657500	99,36	0,00E+00	2507	49	NA	NA	
maker-6-augustus-gene-272.32-mRNA-1	6	OsR498G0613099100.01.T02	99,70	0,00E+00	1200	Os06g0658500	100,00	0,00E+00	1209	9	NA	NA	
maker-6-augustus-gene-273.27-mRNA-1	6	OsR498G0613101300.01.T01	98,52	0,00E+00	2235	Os06g0659100	100,00	0,00E+00	2311	76	NA	NA	
maker-6-augustus-gene-273.32-mRNA-1	6	OsR498G0613103300.01.T01	99,28	0,00E+00	732	Os06g0659500	100,00	4,46E-118	423	-309	Indica	NA	
maker-6-augustus-gene-273.35-mRNA-1	6	OsR498G0613102400.01.T03	99,30	0,00E+00	4804	Os06g0659300	99,45	0,00E+00	4822	18	NA	NA	
maker-6-augustus-gene-274.0-mRNA-1	6	OsR498G0613105900.01.T01	99,79	0,00E+00	3397	Os06g0660200	100,00	0,00E+00	3415	18	NA	NA	
maker-6-augustus-gene-274.13-mRNA-1	6	OsR498G0613110700.01.T05	99,38	0,00E+00	2005	Os06g0661400	99,81	0,00E+00	1848	-157	NA	NA	
maker-6-augustus-gene-274.18-mRNA-1	6	OsR498G0613111500.01.T06	99,75	0,00E+00	716	Os06g0661766	99,47	0,00E+00	665	-51	NA	NA	
maker-6-augustus-gene-274.2-mRNA-1	6	OsR498G0613108700.01.T03	96,58	0,00E+00	1553	Os06g0660700	96,07	0,00E+00	1292	-261	Indica	NA	
maker-6-augustus-gene-274.23-mRNA-1	6	OsR498G0613111000.01.T01	99,61	0,00E+00	917	Os06g0661500	100,00	0,00E+00	926	9	NA	NA	
maker-6-augustus-gene-274.25-mRNA-1	6	OsR498G0613111400.01.T03	99,87	0,00E+00	4023	Os06g0661700	100,00	0,00E+00	2681	-1342	Indica	NA	
maker-6-augustus-gene-274.30-mRNA-1	6	OsR498G0613111800.01.T01	99,37	0,00E+00	3091	Os06g0661800	99,45	0,00E+00	2911	-180	NA	NA	
maker-6-augustus-gene-274.33-mRNA-1	6	OsR498G0613112000.01.T04	100,00	0,00E+00	2051	Os06g0661900	100,00	0,00E+00	2073	22	NA	NA	
maker-6-augustus-gene-274.37-mRNA-1	6	OsR498G0613112200.01.T01	99,41	0,00E+00	3326	Os06g0662000	99,52	0,00E+00	3335	9	NA	NA	
maker-6-augustus-gene-275.0-mRNA-1	6	OsR498G0613118900.01.T01	99,85	0,00E+00	2313	Os06g0663400	100,00	0,00E+00	2322	9	NA	NA	Kinase-TM
maker-6-augustus-gene-275.10-mRNA-1	6	OsR498G0613117600.01.T02	99,87	0,00E+00	1405	Os06g0663200	100,00	0,00E+00	1409	4	NA	NA	Kinase
maker-6-augustus-gene-275.15-mRNA-1	6	OsR498G0613119800.01.T05	100,00	0,00E+00	939	Os06g0663600	100,00	0,00E+00	939	0	NA	NA	
maker-6-augustus-gene-275.2-mRNA-1	6	OsR498G0613112800.01.T01	99,89	0,00E+00	1598	Os06g0662200	100,00	0,00E+00	1602	4	NA	NA	
maker-6-augustus-gene-276.41-mRNA-1	6	OsR498G0613121100.01.T03	99,70	0,00E+00	2986	Os06g0663900	100,00	0,00E+00	2940	-46	NA	NA	Kinase
maker-6-augustus-gene-276.47-mRNA-1	6	OsR498G0613122300.01.T02	98,10	0,00E+00	3370	Os06g0664300	98,31	0,00E+00	3388	18	NA	NA	
maker-6-augustus-gene-276.53-mRNA-1	6	OsR498G0613123100.01.T01	99,91	0,00E+00	1969	Os06g0664800	100,00	0,00E+00	1898	-71	NA	Japonica	
maker-6-augustus-gene-276.55-mRNA-1	6	OsR498G0613124300.01.T02	99,58	0,00E+00	1698	Os06g0665000	100,00	0,00E+00	1716	18	NA	NA	
maker-6-augustus-gene-276.58-mRNA-1	6	OsR498G0613120200.01.T01	99,53	0,00E+00	1132	Os06g0663800	100,00	0,00E+00	1186	54	NA	NA	
maker-6-augustus-gene-276.63-mRNA-1	6	OsR498G0613121600.01.T01	99,94	0,00E+00	2797	Os11g0540100	91,59	0,00E+00	2255	-542	Indica	NA	
maker-6-augustus-gene-276.72-mRNA-1	6	OsR498G0613121800.01.T02	99,73	0,00E+00	1305	Os06g0664200	100,00	0,00E+00	1314	9	NA	NA	
maker-6-augustus-gene-276.74-mRNA-1	6	OsR498G0613123000.01.T01	99,54	0,00E+00	2342	Os06g0664400	100,00	0,00E+00	2365	23	NA	NA	
maker-6-augustus-gene-276.77-mRNA-1	6	OsR498G0613124600.01.T01	100,00	0,00E+00	1418	Os06g0665100	100,00	0,00E+00	1418	0	NA	NA	
maker-6-augustus-gene-276.81-mRNA-1	6	OsR498G0613126200.01.T02	100,00	0,00E+00	1801	Os06g0665500	100,00	0,00E+00	1801	0	NA	NA	
maker-6-augustus-gene-277.1-mRNA-1	6	OsR498G0613129900.01.T01	91,31	0,00E+00	833	Os06g0665900	91,32	0,00E+00	849	16	NA	NA	
maker-6-augustus-gene-277.4-mRNA-1	6	OsR498G0613130900.01.T02	99,75	0,00E+00	1406	Os06g0666100	99,87	0,00E+00	1410	4	NA	NA	
maker-6-augustus-gene-278.47-mRNA-1	6	OsR498G0613138500.01.T01	100,00	0,00E+00	926	Os06g0667200	99,81	0,00E+00	921	-5	NA	NA	
maker-6-augustus-gene-278.51-mRNA-1	6	OsR498G0613139300.01.T03	100,00	0,00E+00	975	Os03g0322500	72,01	1,43E-44	179	-796	Indica	NA	
maker-6-augustus-gene-278.56-mRNA-1	6	OsR498G0613135200.01.T02	100,00	0,00E+00	2251	Os06g0666600	100,00	0,00E+00	2251	0	NA	NA	
maker-6-augustus-gene-278.62-mRNA-1	6	OsR498G0613139600.01.T01	100,00	0,00E+00	872	Os06g0667600	100,00	0,00E+00	872	0	NA	NA	
maker-6-augustus-gene-278.65-mRNA-1	6	OsR498G0613141000.01.T01	100,00	0,00E+00	2176	Os06g0668200	100,00	0,00E+00	2176	0	NA	NA	
maker-6-augustus-gene-279.34-mRNA-1	6	OsR498G0613145900.01.T02	100,00	0,00E+00	1537	Os06g0669800	100,00	0,00E+00	1234	-303	Indica	NA	
maker-6-augustus-gene-279.45-mRNA-1	6	OsR498G0613148900.01.T01	100,00	0,00E+00	1391	Os06g0670300	100,00	0,00E+00	1391	0	NA	NA	
maker-6-augustus-gene-279.47-mRNA-1	6	OsR498G0613149500.01.T03	100,00	0,00E+00	2825	Os06g0670500	89,88	0,00E+00	2133	-692	Indica	NA	
maker-6-augustus-gene-279.53-mRNA-1	6	OsR498G0613149700.01.T05	100,00	0,00E+00	3057	Os06g0670500	100,00	0,00E+00	3057	0	NA	NA	
maker-6-augustus-gene-279.61-mRNA-1	6	OsR498G0613144600.01.T02	100,00	0,00E+00	758	Os06g0669150	100,00	0,00E+00	641	-117	Indica	NA	
maker-6-augustus-gene-279.71-mRNA-1	6	OsR498G0613145400.01.T05	100,00	0,00E+00	3663	Os06g0669400	100,00	0,00E+00	3204	-459	Indica	NA	
maker-6-augustus-gene-279.80-mRNA-1	6	OsR498G0613145800.01.T01	99,12	0,00E+00	1798	Os06g0669700	98,77	0,00E+00	1278	-520	Indica	NA	
maker-6-augustus-gene-279.86-mRNA-1	6	OsR498G0613146600.01.T01	100,00	0,00E+00	2241	Os06g0670100	100,00	0,00E+00	2241	0	NA	NA	
maker-6-augustus-gene-28.33-mRNA-1	6	OsR498G0611641900.01.T07	100,00	0,00E+00	2338	Os06g0153200	99,44	0,00E+00	2521	183	NA	NA	
maker-6-augustus-gene-28.38-mRNA-1	6	OsR498G0611639800.01.T01	100,00	0,00E+00	2966	Os06g0152700	100,00	0,00E+00	2966	0	NA	NA	
maker-6-augustus-gene-28.42-mRNA-1	6	OsR498G0611641600.01.T03	98,95	0,00E+00	671	Os06g0125200	89,39	4,50E-150	529	-142	Indica	NA	
maker-6-augustus-gene-28.48-mRNA-1	6	OsR498G0611643000.01.T04	100,00	0,00E+00	1829	Os06g0153600	100,00	0,00E+00	1829	0	NA	NA	
maker-6-augustus-gene-28.51-mRNA-1	6	OsR498G0611642800.01.T03	100,00	0,00E+00	1274	Os06g0153800	100,00	0,00E+00	1501	227	Japonica	NA	
maker-6-augustus-gene-280.26-mRNA-1	6	OsR498G0613151300.01.T04	98,46	0,00E+00	4081	Os06g0671000	98,46	0,00E+00	4081	0	NA	NA	
maker-6-augustus-gene-280.37-mRNA-1	6	OsR498G0613156100.01.T02	99,94	0,00E+00	2795	Os06g0671800	99,94	0,00E+00	2795	0	NA	NA	
maker-6-augustus-gene-280.40-mRNA-1	6	OsR498G0613153800.01.T01	100,00	0,00E+00	1820	Os06g0671300	100,00	0,00E+00	1471	-349	Indica	NA	
maker-6-augustus-gene-280.42-mRNA-1	6	OsR498G0613156400.01.T01	100,00	0,00E+00	2419	Os06g0671900	97,09	0,00E+00	2274	-145	NA	NA	
maker-6-augustus-gene-281.33-mRNA-1	6	OsR498G0613160200.01.T01	99,78	0,00E+00	819	Os06g0672801	100,00	0,00E+00	848	29	NA	NA	
maker-6-augustus-gene-281.37-mRNA-1	6	OsR498G0613163800.01.T02	99,59	0,00E+00	2619	Os06g0674000	100,00	0,00E+00	2646	27	NA	NA	
maker-6-augustus-gene-281.39-mRNA-1	6	OsR498G0613164400.01.T02	99,72	0,00E+00	640	Os06g0674100	100,00	0,00E+00	823	183	Japonica	NA	
maker-6-augustus-gene-282.18-mRNA-1	6	OsR498G0613170800.01.T03	99,74	0,00E+00	2059	Os06g0676600	100,00	0,00E+00	2073	14	NA	NA	Kinase-TM
maker-6-augustus-gene-282.5-mRNA-1	6	OsR498G0613166500.01.T01	98,91	0,00E+00	1609	Os06g0675600	100,00	0,00E+00	1651	42	NA	NA	
maker-6-augustus-gene-282.7-mRNA-1	6	OsR498G0613167000.01.T01	99,61	0,00E+00	3161	Os06g0675700	99,94	0,00E+00	3188	27	NA	NA	
maker-6-augustus-gene-283.22-mRNA-1	6	OsR498G0613171300.01.T01	99,66	0,00E+00	4759	Os06g0676700	100,00	0,00E+00	4800	41	NA	NA	
maker-6-augustus-gene-283.25-mRNA-1	6	OsR498G0613173300.01.T01	99,01	0,00E+00	2489	Os06g0677300	99,36	0,00E+00	2512	23	NA	NA	
maker-6-augustus-gene-283.29-mRNA-1	6	OsR498G0613173500.01.T04	99,90	0,00E+00	1852	Os06g0677400	100,00	0,00E+00	1856	4	NA	NA	
maker-6-augustus-gene-283.34-mRNA-1	6	OsR498G0613173700.01.T01	99,66	0,00E+00	3713	Os06g0677500	100,00	0,00E+00	3745	32	NA	NA	LRR
maker-6-augustus-gene-283.45-mRNA-1	6	OsR498G0613171800.01.T02	100,00	0,00E+00	1573	Os06g0677000	100,00	0,00E+00	1822	249	Japonica	NA	
maker-6-augustus-gene-283.47-mRNA-1	6	OsR498G0613174400.01.T03	99,74	0,00E+00	4060	Os06g0677800	99,96	0,00E+00	4083	23	NA	NA	
maker-6-augustus-gene-283.52-mRNA-1	6	OsR498G0613178200.01.T01	99,85	0,00E+00	2324	Os06g0678200	100,00	0,00E+00	2333	9	NA	NA	
maker-6-augustus-gene-284.34-mRNA-1	6	OsR498G0204447900.01.T01	98,17	0,00E+00	942	Os06g0679100	100,00	0,00E+00	987	45	NA	NA	
maker-6-augustus-gene-284.41-mRNA-1	6	OsR498G0613183300.01.T01	99,81	0,00E+00	1869	Os06g0679500	100,00	0,00E+00	1878	9	NA	NA	
maker-6-augustus-gene-284.46-mRNA-1	6	OsR498G0613183900.01.T01	98,62	0,00E+00	3942	Os06g0679800	100,00	0,00E+00	2141	-1801	Indica	NA	
maker-6-augustus-gene-284.56-mRNA-1	6	OsR498G0613181000.01.T01	99,75	0,00E+00	2920	Os06g0678800	100,00	0,00E+00	2938	18	NA	NA	
maker-6-augustus-gene-284.59-mRNA-1	6	OsR498G0613183000.01.T01	99,11	0,00E+00	2372	Os06g0679400	99,78	0,00E+00	2409	37	NA	NA	
maker-6-augustus-gene-285.0-mRNA-1	6	OsR498G0613191100.01.T03	99,67	0,00E+00	3200	Os06g0680500	100,00	0,00E+00	2926	-274	NA	NA	
maker-6-augustus-gene-285.12-mRNA-1	6	OsR498G0613193200.01.T01	98,77	0,00E+00	851	Os11g0599101	87,79	1,58E-151	535	-316	Indica	NA	
maker-6-augustus-gene-285.14-mRNA-1	6	OsR498G0613194000.01.T01	99,81	0,00E+00	1874	Os06g0681100	99,75	0,00E+00	2823	949	Japonica	NA	
maker-6-augustus-gene-285.16-mRNA-1	6	OsR498G0613194400.01.T01	99,64	0,00E+00	1502	Os06g0681200	99,88	0,00E+00	1511	9	NA	NA	
maker-6-augustus-gene-285.7-mRNA-1	6	OsR498G0613192000.01.T01	98,15	0,00E+00	2523	Os06g0680900	100,00	0,00E+00	2587	64	NA	NA	
maker-6-augustus-gene-286.0-mRNA-1	6	OsR498G0613197300.01.T01	99,80	0,00E+00	1820	Os06g0681600	100,00	0,00E+00	1829	9	NA	NA	
maker-6-augustus-gene-286.13-mRNA-1	6	OsR498G0613199200.01.T01	99,74	0,00E+00	3433	Os06g0682750	100,00	0,00E+00	4731	1298	Japonica	NA	
maker-6-augustus-gene-286.25-mRNA-1	6	OsR498G0613199600.01.T02	99,45	0,00E+00	1618	Os06g0682900	100,00	0,00E+00	1640	22	NA	NA	
maker-6-augustus-gene-286.3-mRNA-1	6	OsR498G0613197700.01.T01	99,70	0,00E+00	2421	Os06g0681700	99,93	0,00E+00	2436	15	NA	NA	
maker-6-augustus-gene-286.31-mRNA-1	6	OsR498G0613200400.01.T01	92,85	0,00E+00	880	Os06g0683200	93,19	0,00E+00	889	9	NA	NA	
maker-6-augustus-gene-286.5-mRNA-1	6	OsR498G0613199900.01.T01	99,64	0,00E+00	2499	Os06g0683000	100,00	0,00E+00	2522	23	NA	NA	
maker-6-augustus-gene-286.7-mRNA-1	6	OsR498G0613200500.01.T05	99,80	0,00E+00	2703	Os06g0683300	100,00	0,00E+00	1577	-1126	Indica	NA	
maker-6-augustus-gene-287.10-mRNA-1	6	OsR498G0613206900.01.T04	99,56	0,00E+00	3635	Os06g0685700	99,95	0,00E+00	3668	33	NA	NA	
maker-6-augustus-gene-287.13-mRNA-1	6	OsR498G0613203200.01.T03	99,94	0,00E+00	3183	Os06g0684000	100,00	0,00E+00	3187	4	NA	NA	
maker-6-augustus-gene-287.22-mRNA-1	6	OsR498G0613205200.01.T01	99,84	0,00E+00	2253	Os06g0685200	100,00	0,00E+00	2226	-27	NA	NA	
maker-6-augustus-gene-288.13-mRNA-1	6	OsR498G0613211900.01.T02	99,41	0,00E+00	1797	Os06g0687600	100,00	0,00E+00	1824	27	NA	Japonica	
maker-6-augustus-gene-288.15-mRNA-1	6	OsR498G0613212500.01.T03	99,27	0,00E+00	2895	Os06g0687800	100,00	0,00E+00	2949	54	NA	Japonica	
maker-6-augustus-gene-288.2-mRNA-1	6	OsR498G0613207700.01.T01	99,78	0,00E+00	813	Os06g0686400	99,70	5,59E-168	590	-223	Indica	Japonica	
maker-6-augustus-gene-288.24-mRNA-1	6	OsR498G0613210800.01.T02	99,91	0,00E+00	2068	Os02g0158800	82,28	0,00E+00	1146	-922	Indica	Japonica	
maker-6-augustus-gene-288.33-mRNA-1	6	OsR498G0613212200.01.T01	99,41	0,00E+00	2991	Os06g0687700	100,00	0,00E+00	1640	-1351	Indica	Japonica	
maker-6-augustus-gene-289.52-mRNA-1	6	OsR498G0613213100.01.T02	99,60	0,00E+00	2706	Os06g0688100	100,00	0,00E+00	2733	27	NA	Japonica	
maker-6-augustus-gene-289.68-mRNA-1	6	OsR498G0613215600.01.T01	99,40	0,00E+00	1772	Os06g0693000	88,36	0,00E+00	1437	-335	Indica	Japonica	Kinase-TM
maker-6-augustus-gene-289.74-mRNA-1	6	OsR498G0613217600.01.T01	99,24	0,00E+00	4404	Os06g0690700	100,00	0,00E+00	4453	49	NA	Japonica	
maker-6-augustus-gene-29.11-mRNA-1	6	OsR498G0611650500.01.T06	100,00	0,00E+00	1916	Os06g0155300	100,00	0,00E+00	1916	0	NA	NA	
maker-6-augustus-gene-29.13-mRNA-1	6	OsR498G0611650900.01.T01	100,00	0,00E+00	3485	Os06g0155600	100,00	0,00E+00	3485	0	NA	NA	
maker-6-augustus-gene-29.18-mRNA-1	6	OsR498G0611651500.01.T01	100,00	0,00E+00	1128	Os06g0155900	100,00	0,00E+00	1128	0	NA	NA	
maker-6-augustus-gene-29.22-mRNA-1	6	OsR498G0611653300.01.T01	100,00	0,00E+00	1954	Os06g0156600	100,00	0,00E+00	1954	0	NA	NA	
maker-6-augustus-gene-29.29-mRNA-1	6	OsR498G0611646000.01.T03	100,00	0,00E+00	926	Os06g0154500	100,00	0,00E+00	926	0	NA	NA	
maker-6-augustus-gene-29.33-mRNA-1	6	OsR498G0917948700.01.T07	91,70	1,64E-104	379	Os06g0154600	100,00	7,95E-135	479	100	Japonica	NA	
maker-6-augustus-gene-29.37-mRNA-1	6	OsR498G0611650800.01.T03	100,00	0,00E+00	2511	Os06g0155500	100,00	0,00E+00	2511	0	NA	NA	
maker-6-augustus-gene-29.42-mRNA-1	6	OsR498G0611652400.01.T01	100,00	0,00E+00	1299	Os06g0156400	100,00	0,00E+00	1029	-270	Indica	NA	
maker-6-augustus-gene-290.0-mRNA-1	6	OsR498G0613218600.01.T01	99,79	0,00E+00	6061	Os06g0691000	100,00	0,00E+00	6093	32	NA	Japonica	
maker-6-augustus-gene-290.5-mRNA-1	6	OsR498G0613219600.01.T01	99,09	0,00E+00	2703	Os06g0691400	100,00	0,00E+00	2765	62	NA	Japonica	
maker-6-augustus-gene-291.44-mRNA-1	6	OsR498G0613226300.01.T03	98,75	0,00E+00	1954	Os06g0693000	99,91	0,00E+00	2012	58	NA	Japonica	Kinase-TM
maker-6-augustus-gene-291.54-mRNA-1	6	OsR498G0613228100.01.T01	99,67	0,00E+00	2147	Os06g0693500	100,00	0,00E+00	2165	18	NA	Japonica	
maker-6-augustus-gene-291.63-mRNA-1	6	OsR498G0613229300.01.T02	99,92	0,00E+00	2269	Os06g0694100	100,00	0,00E+00	2273	4	NA	Japonica	
maker-6-augustus-gene-291.66-mRNA-1	6	OsR498G0613230500.01.T01	99,85	0,00E+00	1187	Os06g0694500	100,00	0,00E+00	1191	4	NA	Japonica	
maker-6-augustus-gene-291.69-mRNA-1	6	OsR498G0613225200.01.T03	99,95	0,00E+00	3542	Os06g0692700	100,00	0,00E+00	3546	4	NA	Japonica	LRR-TM
maker-6-augustus-gene-291.71-mRNA-1	6	OsR498G0613225600.01.T02	98,23	0,00E+00	2041	Os06g0692800	100,00	0,00E+00	2136	95	NA	Japonica	
maker-6-augustus-gene-291.81-mRNA-1	6	OsR498G0613229200.01.T02	99,89	0,00E+00	3406	Os06g0694000	100,00	0,00E+00	3415	9	NA	Japonica	
maker-6-augustus-gene-291.83-mRNA-1	6	OsR498G0613230200.01.T01	99,86	0,00E+00	1316	Os06g0694400	100,00	0,00E+00	1321	5	NA	Japonica	
maker-6-augustus-gene-291.85-mRNA-1	6	OsR498G0613231400.01.T01	100,00	0,00E+00	1029	Os06g0694800	100,00	0,00E+00	1029	0	NA	Japonica	
maker-6-augustus-gene-292.0-mRNA-1	6	OsR498G0613232300.01.T01	99,26	0,00E+00	1673	Os06g0695200	99,89	0,00E+00	1700	27	NA	Japonica	
maker-6-augustus-gene-292.2-mRNA-1	6	OsR498G0613232700.01.T01	99,48	0,00E+00	1709	Os06g0695400	100,00	0,00E+00	1732	23	NA	Japonica	
maker-6-augustus-gene-292.4-mRNA-1	6	OsR498G0613233900.01.T01	100,00	0,00E+00	1829	Os06g0695500	100,00	0,00E+00	1829	0	NA	Japonica	
maker-6-augustus-gene-292.6-mRNA-1	6	OsR498G0613232600.01.T01	99,15	0,00E+00	1669	Os06g0695200	97,99	0,00E+00	1619	-50	NA	Japonica	
maker-6-augustus-gene-292.8-mRNA-1	6	OsR498G0613234400.01.T01	99,65	0,00E+00	3103	Os06g0695600	100,00	0,00E+00	3130	27	NA	Japonica	
maker-6-augustus-gene-293.39-mRNA-1	6	OsR498G0613237300.01.T01	99,89	0,00E+00	1587	Os06g0696400	100,00	0,00E+00	1160	-427	Indica	Japonica	
maker-6-augustus-gene-293.43-mRNA-1	6	OsR498G0613237900.01.T01	99,56	0,00E+00	1606	Os04g0604900	73,05	2,88E-80	299	-1307	Indica	Japonica	
maker-6-augustus-gene-293.45-mRNA-1	6	OsR498G0613238700.01.T01	99,77	0,00E+00	1555	Os06g0696600	100,00	0,00E+00	1564	9	NA	Japonica	
maker-6-augustus-gene-293.47-mRNA-1	6	OsR498G0613241100.01.T01	99,73	0,00E+00	3338	Os02g0150100	78,43	0,00E+00	1150	-2188	Indica	Indica	
maker-6-augustus-gene-293.49-mRNA-1	6	OsR498G0613241500.01.T01	100,00	0,00E+00	807	Os06g0697300	99,11	0,00E+00	789	-18	NA	Indica	
maker-6-augustus-gene-293.51-mRNA-1	6	OsR498G0613240400.01.T01	100,00	0,00E+00	1575	Os06g0697000	100,00	0,00E+00	1575	0	NA	NA	
maker-6-augustus-gene-293.54-mRNA-1	6	OsR498G0613241800.01.T01	99,78	0,00E+00	802	Os03g0845700	82,53	8,65E-123	439	-363	Indica	Indica	
maker-6-augustus-gene-294.41-mRNA-1	6	OsR498G0613247300.01.T01	99,52	0,00E+00	1474	Os06g0698785	99,16	0,00E+00	1461	-13	NA	NA	
maker-6-augustus-gene-294.52-mRNA-1	6	OsR498G0613248000.01.T01	99,77	0,00E+00	3895	Os06g0698859	100,00	0,00E+00	3918	23	NA	NA	
maker-6-augustus-gene-294.56-mRNA-1	6	OsR498G0613248200.01.T01	98,93	0,00E+00	1808	Os02g0148500	75,87	4,89E-154	544	-1264	Indica	NA	
maker-6-augustus-gene-295.16-mRNA-1	6	OsR498G0613249000.01.T01	100,00	0,00E+00	2152	Os06g0699200	99,74	0,00E+00	2052	-100	NA	NA	
maker-6-augustus-gene-295.19-mRNA-1	6	OsR498G0613249800.01.T01	100,00	1,38E-179	628	Os06g0699500	99,71	2,04E-178	624	-4	NA	Indica	
maker-6-augustus-gene-295.22-mRNA-1	6	OsR498G0613251400.01.T02	99,67	0,00E+00	4852	Os09g0326800	96,19	0,00E+00	4434	-418	NA	NA	
maker-6-augustus-gene-295.32-mRNA-1	6	OsR498G0613253200.01.T05	92,92	0,00E+00	928	Os06g0700500	93,08	0,00E+00	932	4	NA	NA	
maker-6-augustus-gene-296.21-mRNA-1	6	OsR498G0613253600.01.T02	98,32	0,00E+00	5547	Os06g0700650	98,06	0,00E+00	3177	-2370	Indica	NA	
maker-6-augustus-gene-296.25-mRNA-1	6	OsR498G0613254600.01.T04	100,00	0,00E+00	2246	Os06g0701100	100,00	0,00E+00	2246	0	NA	NA	
maker-6-augustus-gene-296.27-mRNA-1	6	OsR498G0613254800.01.T01	99,89	0,00E+00	3330	Os06g0701200	99,78	0,00E+00	3321	-9	NA	NA	
maker-6-augustus-gene-296.33-mRNA-1	6	OsR498G0613255000.01.T01	99,54	0,00E+00	5076	Os06g0701300	99,77	0,00E+00	3135	-1941	Indica	Japonica	
maker-6-augustus-gene-296.41-mRNA-1	6	OsR498G0613255600.01.T01	98,89	4,07E-180	630	Os06g0701400	99,17	0,00E+00	635	5	NA	Indica	
maker-6-augustus-gene-296.43-mRNA-1	6	OsR498G0613256400.01.T02	99,67	0,00E+00	2737	Os06g0701600	99,48	0,00E+00	2724	-13	NA	NA	
maker-6-augustus-gene-296.46-mRNA-1	6	OsR498G0613256600.01.T01	99,81	0,00E+00	2860	Os06g0701700	99,69	0,00E+00	2851	-9	NA	Indica	
maker-6-augustus-gene-297.42-mRNA-1	6	OsR498G0613258400.01.T02	100,00	0,00E+00	2874	Os06g0701700	93,59	0,00E+00	2412	-462	Indica	Indica	
maker-6-augustus-gene-298.0-mRNA-1	6	OsR498G0613263100.01.T06	94,99	0,00E+00	2571	Os06g0702500	95,05	0,00E+00	2575	4	NA	Indica	
maker-6-augustus-gene-298.12-mRNA-1	6	OsR498G0613265100.01.T03	100,00	0,00E+00	1826	Os06g0702800	99,80	0,00E+00	1817	-9	NA	Indica	
maker-6-augustus-gene-298.14-mRNA-1	6	OsR498G0613266700.01.T02	98,45	0,00E+00	3349	Os06g0703300	98,77	0,00E+00	4274	925	Japonica	NA	
maker-6-augustus-gene-298.18-mRNA-1	6	OsR498G0613267900.01.T01	98,42	0,00E+00	1215	Os06g0703800	99,83	0,00E+00	1067	-148	Indica	Japonica	
maker-6-augustus-gene-298.22-mRNA-1	6	OsR498G0613265000.01.T01	99,83	0,00E+00	1030	Os06g0702700	100,00	0,00E+00	1034	4	NA	Indica	
maker-6-augustus-gene-298.25-mRNA-1	6	OsR498G0613265800.01.T01	99,92	0,00E+00	2177	Os06g0703000	99,59	0,00E+00	2159	-18	NA	Indica	Kinase-TM
maker-6-augustus-gene-298.32-mRNA-1	6	OsR498G0613267000.01.T02	99,86	0,00E+00	2567	Os06g0703500	99,65	0,00E+00	2553	-14	NA	Indica	
maker-6-augustus-gene-298.4-mRNA-1	6	OsR498G0613264700.01.T02	100,00	0,00E+00	6282	Os06g0702600	99,63	0,00E+00	3873	-2409	Indica	Indica	
maker-6-augustus-gene-299.36-mRNA-1	6	OsR498G0202933000.01.T03	73,60	0,00E+00	639	Os06g0703900	99,79	0,00E+00	2584	1945	Japonica	Indica	
maker-6-augustus-gene-299.43-mRNA-1	6	OsR498G0613270500.01.T03	99,88	0,00E+00	1497	Os06g0704300	99,11	0,00E+00	1386	-111	NA	NA	
maker-6-augustus-gene-299.48-mRNA-1	6	OsR498G0613272300.01.T01	99,50	0,00E+00	1073	Os06g0704900	99,34	0,00E+00	1068	-5	NA	NA	
maker-6-augustus-gene-299.56-mRNA-1	6	OsR498G0613272900.01.T01	100,00	0,00E+00	1299	Os06g0705100	99,86	0,00E+00	1295	-4	NA	NA	
maker-6-augustus-gene-299.60-mRNA-1	6	OsR498G0613270400.01.T01	99,60	2,25E-124	445	Os06g0704200	99,58	6,07E-120	429	-16	NA	NA	
maker-6-augustus-gene-299.65-mRNA-1	6	OsR498G0613271600.01.T06	99,85	0,00E+00	3498	Os06g0704700	99,80	0,00E+00	3494	-4	NA	NA	
maker-6-augustus-gene-299.73-mRNA-1	6	OsR498G0202925100.01.T04	74,39	9,84E-155	548	Os06g0705300	99,76	0,00E+00	1516	968	Japonica	NA	
maker-6-augustus-gene-299.75-mRNA-1	6	OsR498G0613274600.01.T01	99,64	0,00E+00	993	Os06g0705500	99,64	0,00E+00	993	0	NA	NA	
maker-6-augustus-gene-3.32-mRNA-1	6	OsR498G0611473500.01.T02	99,46	0,00E+00	4296	Os06g0103800	99,90	0,00E+00	3692	-604	Indica	Japonica	
maker-6-augustus-gene-3.37-mRNA-1	6	OsR498G0611474200.01.T03	99,95	0,00E+00	3415	Os06g0103900	100,00	0,00E+00	3420	5	NA	Japonica	
maker-6-augustus-gene-3.39-mRNA-1	6	OsR498G0611474400.01.T02	99,81	0,00E+00	3865	Os06g0104000	100,00	0,00E+00	3883	18	NA	Japonica	
maker-6-augustus-gene-3.42-mRNA-1	6	OsR498G0611474800.01.T01	100,00	0,00E+00	1975	Os06g0104200	100,00	0,00E+00	1975	0	NA	Japonica	
maker-6-augustus-gene-3.44-mRNA-1	6	OsR498G0611475400.01.T01	99,78	0,00E+00	3196	Os06g0104300	100,00	0,00E+00	3214	18	NA	Japonica	
maker-6-augustus-gene-3.47-mRNA-1	6	OsR498G0611475800.01.T01	99,92	0,00E+00	2285	Os06g0104400	100,00	0,00E+00	1714	-571	Indica	Japonica	
maker-6-augustus-gene-30.37-mRNA-1	6	OsR498G0611653900.01.T01	100,00	0,00E+00	1997	Os06g0156900	100,00	0,00E+00	1997	0	NA	NA	
maker-6-augustus-gene-30.41-mRNA-1	6	OsR498G0611656900.01.T01	90,37	0,00E+00	738	Os06g0157500	100,00	0,00E+00	969	231	Japonica	NA	
maker-6-augustus-gene-30.43-mRNA-1	6	OsR498G0611656900.01.T01	99,82	0,00E+00	970	Os06g0157700	100,00	0,00E+00	975	5	NA	NA	
maker-6-augustus-gene-30.47-mRNA-1	6	OsR498G0611658100.01.T03	100,00	0,00E+00	1938	Os06g0158000	100,00	0,00E+00	1938	0	NA	NA	
maker-6-augustus-gene-30.53-mRNA-1	6	OsR498G0611658300.01.T02	100,00	0,00E+00	2122	Os06g0158100	100,00	0,00E+00	2122	0	NA	NA	
maker-6-augustus-gene-30.60-mRNA-1	6	OsR498G0611654200.01.T02	100,00	0,00E+00	1997	Os06g0157000	100,00	0,00E+00	1584	-413	Indica	NA	
maker-6-augustus-gene-30.64-mRNA-1	6	OsR498G0611659000.01.T02	99,95	0,00E+00	3246	Os06g0158300	99,95	0,00E+00	3246	0	NA	NA	NBS-TM
maker-6-augustus-gene-30.68-mRNA-1	6	OsR498G0611659400.01.T01	100,00	0,00E+00	5167	Os06g0158500	100,00	0,00E+00	5167	0	NA	NA	CC-LRR-NBS-TM
maker-6-augustus-gene-30.70-mRNA-1	6	OsR498G0611659600.01.T01	100,00	0,00E+00	1083	Os06g0158600	100,00	0,00E+00	1083	0	NA	NA	
maker-6-augustus-gene-300.44-mRNA-1	6	OsR498G0613275100.01.T02	100,00	0,00E+00	791	Os06g0705600	100,00	0,00E+00	668	-123	Indica	Indica	
maker-6-augustus-gene-300.45-mRNA-1	6	OsR498G0613275100.01.T03	99,92	0,00E+00	2139	Os06g0705600	99,91	0,00E+00	2103	-36	NA	Indica	
maker-6-augustus-gene-300.52-mRNA-1	6	OsR498G0613275500.01.T01	99,65	0,00E+00	3057	Os06g0705700	99,65	0,00E+00	3057	0	NA	Indica	
maker-6-augustus-gene-300.54-mRNA-1	6	OsR498G0613276300.01.T01	99,94	0,00E+00	3096	Os06g0705900	99,65	0,00E+00	3072	-24	NA	Indica	
maker-6-augustus-gene-300.56-mRNA-1	6	OsR498G0613276500.01.T06	99,83	0,00E+00	3093	Os06g0705700	89,79	0,00E+00	2286	-807	Indica	Indica	
maker-6-augustus-gene-300.61-mRNA-1	6	OsR498G0613278300.01.T09	100,00	0,00E+00	1094	Os06g0707100	99,51	0,00E+00	1080	-14	NA	Indica	
maker-6-augustus-gene-300.67-mRNA-1	6	OsR498G0613278200.01.T01	99,79	0,00E+00	2508	Os06g0707000	94,71	0,00E+00	2199	-309	Indica	NA	

maker-6-augustus-gene-300.70-mRNA-1	6	OsR498G0613278600.01.T01	99,93	0,00E+00	2490	Os06g0707200	99,93	0,00E+00	2490	0	NA	NA	
maker-6-augustus-gene-301.39-mRNA-1	6	OsR498G0613281300.01.T01	100,00	0,00E+00	3144	Os06g0708000	99,89	0,00E+00	3135	-9	NA	Indica	Kinase
maker-6-augustus-gene-301.51-mRNA-1	6	OsR498G0613282300.01.T01	99,81	0,00E+00	959	Os01g0106200	83,08	1,32E-151	535	-424	Indica	Indica	
maker-6-augustus-gene-301.53-mRNA-1	6	OsR498G0613282500.01.T01	100,00	0,00E+00	1131	Os06g0708500	78,15	1,33E-139	495	-636	Indica	Indica	
maker-6-augustus-gene-301.55-mRNA-1	6	OsR498G0613282700.01.T01	100,00	0,00E+00	1196	Os06g0708500	99,85	0,00E+00	1192	-4	NA	Indica	
maker-6-augustus-gene-301.61-mRNA-1	6	OsR498G0613284500.01.T03	100,00	0,00E+00	1065	Os06g0709100	98,88	0,00E+00	938	-127	Indica	Indica	
maker-6-augustus-gene-301.65-mRNA-1	6	OsR498G0613286900.01.T05	100,00	0,00E+00	2331	Os06g0710800	99,85	0,00E+00	2320	-11	NA	NA	
maker-6-augustus-gene-301.71-mRNA-1	6	OsR498G0613283200.01.T02	99,85	0,00E+00	2361	Os06g0708700	99,56	0,00E+00	1635	-726	Indica	Indica	
maker-6-augustus-gene-301.81-mRNA-1	6	OsR498G0613285800.01.T02	100,00	0,00E+00	1402	Os06g0710300	99,36	0,00E+00	1378	-24	NA	Indica	
maker-6-augustus-gene-302.38-mRNA-1	6	OsR498G0613287700.01.T04	99,89	0,00E+00	1630	Os06g0711100	99,34	0,00E+00	1606	-24	NA	Indica	
maker-6-augustus-gene-302.41-mRNA-1	6	OsR498G0613287900.01.T01	99,81	0,00E+00	927	Os06g0711200	99,42	0,00E+00	918	-9	NA	Indica	
maker-6-augustus-gene-302.43-mRNA-1	6	OsR498G0613288100.01.T02	100,00	0,00E+00	1900	Os06g0711600	96,96	0,00E+00	1778	-122	NA	Indica	
maker-6-augustus-gene-302.46-mRNA-1	6	OsR498G0613290700.01.T01	99,82	0,00E+00	1013	Os06g0711900	99,50	0,00E+00	706	-307	Indica	NA	
maker-6-augustus-gene-302.48-mRNA-1	6	OsR498G0613291900.01.T02	99,88	0,00E+00	3005	Os06g0712300	99,94	0,00E+00	3010	5	NA	NA	
maker-6-augustus-gene-302.52-mRNA-1	6	OsR498G0613292600.01.T02	97,85	0,00E+00	3846	Os06g0712400	97,76	0,00E+00	3837	-9	NA	Indica	
maker-6-augustus-gene-303.0-mRNA-1	6	OsR498G0613293100.01.T01	100,00	0,00E+00	3187	Os09g0531900	80,78	0,00E+00	1399	-1788	Indica	Indica	
maker-6-augustus-gene-303.11-mRNA-1	6	OsR498G0613296000.01.T01	99,61	0,00E+00	2261	Os06g0712900	99,84	0,00E+00	2275	14	NA	NA	
maker-6-augustus-gene-303.13-mRNA-1	6	OsR498G0613297400.01.T01	100,00	0,00E+00	1213	Os06g0713100	99,85	0,00E+00	1208	-5	NA	Indica	
maker-6-augustus-gene-303.17-mRNA-1	6	OsR498G0613298200.01.T02	96,35	0,00E+00	1048	Os06g0713900	94,26	0,00E+00	635	-413	Indica	Indica	
maker-6-augustus-gene-303.21-mRNA-1	6	OsR498G0613298600.01.T04	97,49	0,00E+00	2247	Os06g0714000	97,41	0,00E+00	2242	-5	NA	Indica	
maker-6-augustus-gene-303.27-mRNA-1	6	OsR498G0613298800.01.T01	87,72	5,10E-129	462	Os06g0714100	87,72	1,77E-129	462	0	NA	NA	
maker-6-augustus-gene-303.3-mRNA-1	6	OsR498G0613294100.01.T01	99,72	0,00E+00	1939	Os06g0712600	99,28	0,00E+00	1230	-709	Indica	Indica	
maker-6-augustus-gene-303.5-mRNA-1	6	OsR498G0613295700.01.T02	99,90	0,00E+00	3508	Os11g0186300	74,83	0,00E+00	1053	-2455	Indica	NA	
maker-6-augustus-gene-303.8-mRNA-1	6	OsR498G0613297500.01.T02	99,25	0,00E+00	2378	Os06g0713800	99,48	0,00E+00	2383	5	NA	Indica	
maker-6-augustus-gene-304.0-mRNA-1	6	OsR498G0613299500.01.T02	100,00	0,00E+00	3128	Os10g0510700	74,20	0,00E+00	960	-2168	Indica	Indica	
maker-6-augustus-gene-304.13-mRNA-1	6	OsR498G0613303900.01.T05	100,00	0,00E+00	1840	Os06g0715400	99,80	0,00E+00	1831	-9	NA	Indica	
maker-6-augustus-gene-304.15-mRNA-1	6	OsR498G0613303900.01.T06	100,00	0,00E+00	1269	Os06g0715500	99,74	0,00E+00	1388	119	NA	Indica	
maker-6-augustus-gene-304.20-mRNA-1	6	OsR498G0613302400.01.T04	100,00	0,00E+00	1099	Os06g0714900	100,00	0,00E+00	1099	0	NA	Indica	Kinase
maker-6-augustus-gene-304.28-mRNA-1	6	OsR498G0613303600.01.T02	100,00	0,00E+00	2809	Os06g0715300	99,49	0,00E+00	2773	-36	NA	Indica	
maker-6-augustus-gene-304.30-mRNA-1	6	OsR498G0613304200.01.T01	100,00	0,00E+00	2105	Os06g0715600	99,91	0,00E+00	2101	-4	NA	Indica	
maker-6-augustus-gene-304.35-mRNA-1	6	OsR498G0613304400.01.T01	100,00	0,00E+00	1867	Os06g0715700	99,71	0,00E+00	1854	-13	NA	Indica	
maker-6-augustus-gene-304.4-mRNA-1	6	OsR498G0613301300.01.T01	100,00	0,00E+00	2132	Os06g0714500	99,83	0,00E+00	2123	-9	NA	Indica	TM
maker-6-augustus-gene-304.6-mRNA-1	6	OsR498G0613302100.01.T01	100,00	0,00E+00	726	Os06g0714800	97,57	0,00E+00	700	-26	NA	Indica	
maker-6-augustus-gene-304.8-mRNA-1	6	OsR498G0613303100.01.T02	100,00	0,00E+00	2089	Os06g0715000	99,40	0,00E+00	2069	-20	NA	Indica	
maker-6-augustus-gene-305.35-mRNA-1	6	OsR498G0613305900.01.T01	97,76	0,00E+00	1608	Os06g0716000	96,61	0,00E+00	1583	-25	NA	Indica	
maker-6-augustus-gene-305.40-mRNA-1	6	OsR498G0613306700.01.T01	100,00	0,00E+00	1727	Os06g0716200	99,90	0,00E+00	1711	-16	NA	Indica	
maker-6-augustus-gene-305.43-mRNA-1	6	OsR498G0613308900.01.T01	100,00	2,08E-140	499	Os06g0717300	100,00	1,14E-163	574	75	Japonica	Indica	
maker-6-augustus-gene-305.45-mRNA-1	6	OsR498G0613309900.01.T01	100,00	0,00E+00	1261	Os06g0717600	100,00	0,00E+00	1261	0	NA	Japonica	
maker-6-augustus-gene-305.50-mRNA-1	6	OsR498G0613311900.01.T01	100,00	0,00E+00	1429	Os06g0718100	99,75	0,00E+00	1420	-9	NA	Indica	
maker-6-augustus-gene-305.52-mRNA-1	6	OsR498G0613307600.01.T01	100,00	0,00E+00	4275	Os06g0716700	99,96	0,00E+00	4270	-5	NA	Indica	
maker-6-augustus-gene-305.58-mRNA-1	6	OsR498G0613308200.01.T01	100,00	0,00E+00	2273	Os06g0717100	99,60	0,00E+00	2250	-23	NA	Indica	
maker-6-augustus-gene-305.61-mRNA-1	6	OsR498G0613308600.01.T01	100,00	0,00E+00	5384	Os06g0717200	99,67	0,00E+00	5339	-45	NA	Indica	Kinase-LRR-TM
maker-6-augustus-gene-305.63-mRNA-1	6	OsR498G0613309200.01.T01	96,96	0,00E+00	2333	Os06g0717400	96,88	0,00E+00	2329	-4	NA	Indica	
maker-6-augustus-gene-305.67-mRNA-1	6	OsR498G0613310200.01.T01	99,92	0,00E+00	2119	Os06g0717800	99,83	0,00E+00	2118	-1	NA	Indica	
maker-6-augustus-gene-306.33-mRNA-1	6	OsR498G0613312700.01.T01	100,00	0,00E+00	996	Os06g0721800	99,40	0,00E+00	889	-107	Indica	Indica	
maker-6-augustus-gene-306.49-mRNA-1	6	OsR498G0613324000.01.T01	99,58	0,00E+00	1287	Os06g0724000	99,01	0,00E+00	885	-402	Indica	NA	
maker-6-augustus-gene-306.63-mRNA-1	6	OsR498G0613323500.01.T01	100,00	0,00E+00	2646	Os06g0722100	99,86	0,00E+00	2637	-9	NA	Indica	
maker-6-augustus-gene-307.40-mRNA-1	6	OsR498G0613323500.01.T01	100,00	0,00E+00	2646	Os06g0722100	99,86	0,00E+00	2637	-9	NA	NA	
maker-6-augustus-gene-307.42-mRNA-1	6	OsR498G0613324700.01.T01	100,00	0,00E+00	2646	Os06g0724100	99,94	0,00E+00	2842	196	NA	Indica	
maker-6-augustus-gene-307.48-mRNA-1	6	OsR498G0613324900.01.T02	100,00	0,00E+00	2105	Os06g0724200	99,66	0,00E+00	2107	2	NA	Indica	
maker-6-augustus-gene-307.51-mRNA-1	6	OsR498G0613325900.01.T01	100,00	0,00E+00	1440	Os06g0724600	100,00	0,00E+00	715	-725	Indica	NA	
maker-6-augustus-gene-307.57-mRNA-1	6	OsR498G0613327500.01.T01	100,00	0,00E+00	1305	Os06g0724800	100,00	0,00E+00	991	-314	Indica	Japonica	
maker-6-augustus-gene-307.59-mRNA-1	6	OsR498G0613323500.01.T01	100,00	0,00E+00	2646	Os06g0722100	99,86	0,00E+00	2637	-9	NA	Indica	
maker-6-augustus-gene-307.61-mRNA-1	6	OsR498G0613323500.01.T01	100,00	0,00E+00	2646	Os06g0722100	99,86	0,00E+00	2637	-9	NA	NA	
maker-6-augustus-gene-307.66-mRNA-1	6	OsR498G0613324000.01.T01	99,72	0,00E+00	1291	Os06g0724000	99,21	0,00E+00	890	-401	Indica	Indica	
maker-6-augustus-gene-307.73-mRNA-1	6	OsR498G0613325200.01.T02	100,00	0,00E+00	1005	Os06g0724300	99,82	0,00E+00	1001	-4	NA	Indica	
maker-6-augustus-gene-307.77-mRNA-1	6	OsR498G0613325600.01.T01	99,68	0,00E+00	1106	Os06g0724400	99,84	0,00E+00	1111	5	NA	NA	
maker-6-augustus-gene-307.80-mRNA-1	6	OsR498G0613325800.01.T01	99,58	0,00E+00	2576	Os06g0724500	99,72	0,00E+00	2585	9	NA	NA	
maker-6-augustus-gene-308.0-mRNA-1	6	OsR498G0613327700.01.T03	100,00	0,00E+00	1811	Os06g0724900	99,70	0,00E+00	1798	-13	NA	NA	Kinase-TM
maker-6-augustus-gene-308.16-mRNA-1	6	OsR498G0613328200.01.T01	99,91	0,00E+00	2063	Os06g0725100	100,00	0,00E+00	2067	4	NA	NA	
maker-6-augustus-gene-308.18-mRNA-1	6	OsR498G0613328600.01.T01	99,74	0,00E+00	2092	Os06g0725200	99,69	0,00E+00	1753	-339	Indica	NA	
maker-6-augustus-gene-308.21-mRNA-1	6	OsR498G0613331200.01.T01	100,00	0,00E+00	3718	Os06g0725900	99,76	0,00E+00	3695	-23	NA	Indica	
maker-6-augustus-gene-308.5-mRNA-1	6	OsR498G0613329300.01.T02	99,40	2,29E-79	296	Os06g0725400	99,51	2,29E-99	361	65	Japonica	NA	
maker-6-augustus-gene-309.0-mRNA-1	6	OsR498G0816324600.01.T01	76,86	0,00E+00	1416	Os06g0726900	99,89	0,00E+00	3357	1941	Japonica	Indica	
maker-6-augustus-gene-309.14-mRNA-1	6	OsR498G0613332800.01.T02	100,00	0,00E+00	4442	Os06g0726400	99,92	0,00E+00	4433	-9	NA	Indica	
maker-6-augustus-gene-309.19-mRNA-1	6	OsR498G0613333000.01.T01	100,00	0,00E+00	2598	Os06g0726600	99,86	0,00E+00	2589	-9	NA	Indica	
maker-6-augustus-gene-309.2-mRNA-1	6	OsR498G0613333900.01.T01	100,00	0,00E+00	2668	Os06g0727200	99,87	0,00E+00	2659	-9	NA	Indica	
maker-6-augustus-gene-309.24-mRNA-1	6	OsR498G0613333200.01.T03	100,00	0,00E+00	1175	Os06g0726800	100,00	0,00E+00	1175	0	NA	Indica	
maker-6-augustus-gene-309.29-mRNA-1	6	OsR498G0613334200.01.T02	100,00	0,00E+00	1851	Os06g0727300	100,00	0,00E+00	1981	130	NA	Indica	
maker-6-augustus-gene-309.34-mRNA-1	6	OsR498G0613334400.01.T01	100,00	0,00E+00	2246	Os06g0727400	99,60	0,00E+00	2223	-23	NA	Indica	Kinase
maker-6-augustus-gene-309.7-mRNA-1	6	OsR498G0613334900.01.T01	97,23	0,00E+00	1164	Os06g0727700	85,98	0,00E+00	755	-409	Indica	Indica	
maker-6-augustus-gene-309.9-mRNA-1	6	OsR498G0613335300.01.T01	96,75	0,00E+00	930	Os06g0727700	96,75	0,00E+00	930	0	NA	Indica	
maker-6-augustus-gene-31.0-mRNA-1	6	OsR498G0611659700.01.T01	100,00	1,14E-91	336	Os06g0158700	100,00	3,95E-92	336	0	NA	NA	
maker-6-augustus-gene-31.14-mRNA-1	6	OsR498G0611660200.01.T02	100,00	0,00E+00	8029	Os12g0562700	70,17	0,00E+00	1305	-6724	Indica	NA	
maker-6-augustus-gene-31.2-mRNA-1	6	OsR498G0611660500.01.T02	97,60	0,00E+00	2002	Os06g0159400	97,60	0,00E+00	2002	0	NA	NA	
maker-6-augustus-gene-31.22-mRNA-1	6	OsR498G0611661000.01.T01	93,80	4,40E-174	611	Os06g0159750	99,75	0,00E+00	727	116	Japonica	NA	
maker-6-augustus-gene-31.24-mRNA-1	6	OsR498G0611662600.01.T01	100,00	0,00E+00	2057	Os06g0160200	100,00	0,00E+00	2057	0	NA	NA	
maker-6-augustus-gene-31.26-mRNA-1	6	OsR498G0611662800.01.T01	100,00	0,00E+00	1122	Os06g0160400	100,00	0,00E+00	836	-286	Indica	NA	
maker-6-augustus-gene-31.28-mRNA-1	6	OsR498G0611664800.01.T02	96,80	0,00E+00	2367	Os06g0160600	96,80	0,00E+00	2367	0	NA	NA	
maker-6-augustus-gene-31.5-mRNA-1	6	OsR498G0611661100.01.T02	100,00	0,00E+00	1995	Os06g0159600	100,00	0,00E+00	1995	0	NA	NA	
maker-6-augustus-gene-31.8-mRNA-1	6	OsR498G0611662100.01.T05	100,00	0,00E+00	1021	Os06g0159900	100,00	0,00E+00	1018	-3	NA	NA	
maker-6-augustus-gene-310.0-mRNA-1	6	OsR498G0613336700.01.T02	100,00	0,00E+00	1202	Os06g0728000	100,00	0,00E+00	856	-346	Indica	Indica	
maker-6-augustus-gene-310.10-mRNA-1	6	OsR498G0613337900.01.T01	99,89	0,00E+00	3186	Os06g0728600	99,61	0,00E+00	3163	-23	NA	Indica	
maker-6-augustus-gene-310.21-mRNA-1	6	OsR498G0613338800.01.T01	100,00	0,00E+00	2446	Os06g0728700	99,48	0,00E+00	2415	-31	NA	Indica	
maker-6-augustus-gene-310.30-mRNA-1	6	OsR498G0613340000.01.T02	99,76	0,00E+00	2269	Os06g0729000	99,61	0,00E+00	2260	-9	NA	Indica	
maker-6-augustus-gene-310.7-mRNA-1	6	OsR498G0613337300.01.T01	100,00	0,00E+00	3584	Os06g0728200	99,04	0,00E+00	4928	1344	Japonica	Indica	
maker-6-augustus-gene-311.0-mRNA-1	6	OsR498G0613342300.01.T03	100,00	0,00E+00	699	Os06g0729400	96,02	0,00E+00	658	-41	NA	Indica	
maker-6-augustus-gene-311.10-mRNA-1	6	OsR498G0613347500.01.T01	100,00	0,00E+00	942	Os06g0730500	100,00	0,00E+00	942	0	NA	Indica	
maker-6-augustus-gene-311.14-mRNA-1	6	OsR498G0613347900.01.T01	99,46	0,00E+00	981	Os06g0730800	99,28	0,00E+00	976	-5	NA	Indica	
maker-6-augustus-gene-311.2-mRNA-1	6	OsR498G0613345100.01.T01	100,00	0,00E+00	2700	Os02g0634700	70,03	8,74E-96	352	-2348	Indica	Indica	
maker-6-augustus-gene-311.27-mRNA-1	6	OsR498G0613347800.01.T05	99,93	0,00E+00	2512	Os06g0730600	99,71	0,00E+00	2498	-14	NA	Indica	
maker-6-augustus-gene-311.4-mRNA-1	6	OsR498G0613346900.01.T02	100,00	0,00E+00	729	Os07g0172200	98,02	0,00E+00	690	-39	NA	Indica	
maker-6-augustus-gene-311.7-mRNA-1	6	OsR498G0613347100.01.T03	100,00	0,00E+00	2295	Os06g0730300	99,69	0,00E+00	2277	-18	NA	Indica	
maker-6-augustus-gene-312.51-mRNA-1	6	OsR498G0613349700.01.T01	100,00	0,00E+00	1315	Os06g0731400	99,86	0,00E+00	1311	-4	NA	Indica	
maker-6-augustus-gene-312.55-mRNA-1	6	OsR498G0613351300.01.T01	93,77	0,00E+00	1815	Os06g0731900	93,68	0,00E+00	1810	-5	NA	Indica	
maker-6-augustus-gene-312.68-mRNA-1	6	OsR498G0613349200.01.T01	100,00	0,00E+00	5844	Os06g0731200	99,80	0,00E+00	5362	-482	NA	Indica	
maker-6-augustus-gene-312.73-mRNA-1	6	OsR498G0613349400.01.T01	100,00	0,00E+00	899	Os06g0731300	99,75	0,00E+00	727	-172	Indica	Indica	
maker-6-augustus-gene-312.79-mRNA-1	6	OsR498G0613350600.01.T01	100,00	0,00E+00	3317	Os06g0731700	99,84	0,00E+00	3304	-13	NA	Indica	
maker-6-augustus-gene-312.83-mRNA-1	6	OsR498G0613351200.01.T01	99,89	0,00E+00	1576	Os06g0731800	99,66	0,00E+00	1567	-9	NA	Indica	
maker-6-augustus-gene-312.86-mRNA-1	6	OsR498G0613267900.01.T02	100,00	0,00E+00	1018	Os06g0703800	99,47	0,00E+00	668	-350	Indica	NA	
maker-6-augustus-gene-312.90-mRNA-1	6	OsR498G0613278200.01.T01	99,79	0,00E+00	2508	Os06g0707000	94,71	0,00E+00	2199	-309	Indica	NA	
maker-6-augustus-gene-32.10-mRNA-1	6	OsR498G0611670100.01.T01	100,00	0,00E+00	883	Os06g0161900	74,67	4,01E-97	354	-529	Indica	NA	
maker-6-augustus-gene-32.19-mRNA-1	6	OsR498G0611670900.01.T01	100,00	0,00E+00	1911	Os06g0162500	100,00	0,00E+00	1911	0	NA	NA	
maker-6-augustus-gene-32.23-mRNA-1	6	OsR498G0611673700.01.T01	100,00	0,00E+00	1274	Os06g0163200	100,00	0,00E+00	1274	0	NA	NA	
maker-6-augustus-gene-32.30-mRNA-1	6	OsR498G0611671800.01.T02	99,72	0,00E+00	644	Os06g0162600	99,72	0,00E+00	644	0	NA	NA	
maker-6-augustus-gene-32.36-mRNA-1	6	OsR498G0611672200.01.T01	93,81	0,00E+00	1067	Os06g0162800	93,81	0,00E+00	1067	0	NA	NA	
maker-6-augustus-gene-32.42-mRNA-1	6	OsR498G0611673200.01.T03	100,00	0,00E+00	4019	Os06g0163000	100,00	0,00E+00	4019	0	NA	NA	CC-Kinase-TM
maker-6-augustus-gene-32.5-mRNA-1	6	OsR498G0611668700.01.T01	100,00	0,00E+00	3388	Os06g0161400	100,00	0,00E+00	3388	0	NA	NA	
maker-6-augustus-gene-32.7-mRNA-1	6	OsR498G0611669500.01.T01	100,00	0,00E+00	910	Os06g0161800	100,00	0,00E+00	910	0	NA	Japonica	
maker-6-augustus-gene-32.9-mRNA-1	6	OsR498G0611669700.01.T01	99,81	0,00E+00	932	Os06g0161900	100,00	0,00E+00	937	5	NA	NA	
maker-6-augustus-gene-33.0-mRNA-1	6	OsR498G0611674100.01.T02	100,00	0,00E+00	899	Os06g0163300	100,00	0,00E+00	899	0	NA	NA	
maker-6-augustus-gene-33.2-mRNA-1	6	OsR498G0611674700.01.T01	100,00	0,00E+00	2890	Os06g0163400	100,00	0,00E+00	2890	0	NA	NA	
maker-6-augustus-gene-33.29-mRNA-1	6	OsR498G0611676000.01.T01	100,00	0,00E+00	1766	Os06g0163800	100,00	0,00E+00	1766	0	NA	NA	LRR-NBS-TM
maker-6-augustus-gene-33.31-mRNA-1	6	OsR498G0611676600.01.T02	99,93	0,00E+00	2662	Os06g0164000	100,00	0,00E+00	2666	4	NA	NA	
maker-6-augustus-gene-33.41-mRNA-1	6	OsR498G0611677000.01.T01	100,00	0,00E+00	1748	Os06g0164100	100,00	0,00E+00	1748	0	NA	NA	
maker-6-augustus-gene-33.8-mRNA-1	6	OsR498G0611674900.01.T01	98,04	0,00E+00	4765	Os06g0163500	97,43	0,00E+00	3570	-1195	Indica	NA	
maker-6-augustus-gene-34.11-mRNA-1	6	OsR498G0611684900.01.T05	100,00	0,00E+00	4713	Os06g0167100	100,00	0,00E+00	4713	0	NA	NA	
maker-6-augustus-gene-34.19-mRNA-1	6	OsR498G0611681600.01.T02	96,22	0,00E+00	1228	Os06g0166000	96,22	0,00E+00	1228	0	NA	NA	
maker-6-augustus-gene-34.2-mRNA-1	6	OsR498G0611680700.01.T03	99,34	0,00E+00	1618	Os06g0165800	99,49	0,00E+00	1395	-223	Indica	NA	
maker-6-augustus-gene-34.23-mRNA-1	6	OsR498G0611683600.01.T02	99,64	0,00E+00	987	Os06g0166500	99,64	0,00E+00	987	0	NA	NA	
maker-6-augustus-gene-34.7-mRNA-1	6	OsR498G0611684100.01.T01	99,91	0,00E+00	1987	Os06g0166900	99,91	0,00E+00	1987	0	NA	NA	Kinase-TM
maker-6-augustus-gene-35.35-mRNA-1	6	OsR498G0714733700.01.T03	95,55	0,00E+00	1619	Os06g0167400	99,89	0,00E+00	1619	0	NA	NA	
maker-6-augustus-gene-35.37-mRNA-1	6	OsR498G0611685900.01.T01	93,46	0,00E+00	3202	Os06g0167500	93,46	0,00E+00	3202	0	NA	NA	LRR-TM
maker-6-augustus-gene-35.46-mRNA-1	6	OsR498G0611686700.01.T01	99,52	0,00E+00	1487	Os06g0168000	99,52	0,00E+00	1487	0	NA	NA	
maker-6-augustus-gene-35.57-mRNA-1	6	OsR498G0611687600.01.T01	99,78	0,00E+00	1615	Os06g0168500	99,78	0,00E+00	1615	0	NA	NA	
maker-6-augustus-gene-35.62-mRNA-1	6	OsR498G0611687800.01.T02	99,96	0,00E+00	4406	Os06g0168600	99,96	0,00E+00	4406	0	NA	Japonica	
maker-6-augustus-gene-35.67-mRNA-1	6	OsR498G0611688600.01.T01	99,67	0,00E+00	2181	Os06g0168800	99,75	0,00E+00	2186	5	NA	Japonica	Kinase-TM
maker-6-augustus-gene-36.0-mRNA-1	6	OsR498G0611689300.01.T01	99,76	0,00E+00	748	Os06g0170200	100,00	0,00E+00	749	1	NA	NA	
maker-6-augustus-gene-36.15-mRNA-1	6	OsR498G0611692400.01.T01	99,86	0,00E+00	2605	Os06g0170500	97,79	0,00E+00	2470	-135	NA	NA	
maker-6-augustus-gene-36.2-mRNA-1	6	OsR498G0611689700.01.T01	99,52	0,00E+00	744	Os06g0169900	99,52	0,00E+00	744	0	NA	Indica	
maker-6-augustus-gene-36.5-mRNA-1	6	OsR498G0611693600.01.T02	100,00	0,00E+00	726	Os06g0161900	85,55	0,00E+00	724	-2	NA	Japonica	
maker-6-augustus-gene-37.0-mRNA-1	6	OsR498G0611692400.01.T01	99,86	0,00E+00	2605	Os06g0170500	97,79	0,00E+00	2470	-135	NA	NA	
maker-6-augustus-gene-37.14-mRNA-1	6	OsR498G0204888100.01.T01	99,26	0,00E+00	1440	Os06g0161900	74,43	1,55E-96	352	-1088	Indica	Japonica	
maker-6-augustus-gene-37.16-mRNA-1	6	OsR498G0611697700.01.T01	88,82	0,00E+00	634	Os06g0171700	88,82	0,00E+00	634	0	NA	Indica	
maker-6-augustus-gene-37.18-mRNA-1	6	OsR498G0611698300.01.T03	100,00	0,00E+00	1265	Os06g0171900	99,86	0,00E+00	1260	-5	NA	Japonica	
maker-6-augustus-gene-37.22-mRNA-1	6	OsR498G0611698500.01.T01	99,87	0,00E+00	2762	Os06g0172000	100,00	0,00E+00	2771	9	NA	Japonica	
maker-6-augustus-gene-37.26-mRNA-1	6	OsR498G0611699900.01.T01	100,00	0,00E+00	672	Os06g0172600	100,00	1,07E-93	342	-330	Indica	Japonica	
maker-6-augustus-gene-37.28-mRNA-1	6	OsR498G0611697600.01.T01	99,02	0,00E+00	3424	Os06g0171600	98,30	0,00E+00	1740	-1684	Indica	NA	
maker-6-augustus-gene-37.38-mRNA-1	6	OsR498G0611702200.01.T01	100,00	0,00E+00	4556	Os06g0172800	100,00	0,00E+00	2369	-2187	Indica	NA	
maker-6-augustus-gene-38.36-mRNA-1	6	OsR498G0611703100.01.T02	99,30	0,00E+00	2290	Os06g0173100	99,30	0,00E+00	2290	0	NA	NA	
maker-6-augustus-gene-38.41-mRNA-1	6	OsR498G0611707300.01.T02	100,00	0,00E+00	839	Os06g0174325	99,50	0,00E+00	709	-130	Indica	NA	
maker-6-augustus-gene-38.45-mRNA-1	6	OsR498G0611707500.01.T02	100,00	0,00E+00	3122	Os06g0174350	100,00	0,00E+00	1613	-1509	Indica	NA	
maker-6-augustus-gene-38.54-mRNA-1	6	OsR498G0611708000.01.T03	100,00	0,00E+00	1038	Os06g0174400	100,00	0,00E+00	1038	0	NA	NA	
maker-6-augustus-gene-39.46-mRNA-1	6	OsR498G0611712100.01.T02	99,91	0,00E+00	1888	Os06g0175800	99,62	0,00E+00	1873	-15	NA	NA	
maker-6-augustus-gene-39.53-mRNA-1	6	OsR498G0611713100.01.T04	100,00	0,00E+00	1296	Os06g0176200	100,00	0,00E+00	1296	0	NA	NA	
maker-6-augustus-gene-39.56-mRNA-1	6	OsR498G0611713300.01.T02	98,25	0,00E+00	1590	Os06g0177600	98,16	0,00E+00	1785	195	Japonica	NA	
maker-6-augustus-gene-39.58-mRNA-1	6	OsR498G0611713700.01.T02	100,00	0,00E+00	1916	Os06g0177700	100,00	0,00E+00	1786	-130	NA	NA	
maker-6-augustus-gene-39.59-mRNA-1	6	OsR498G0611713700.01.T03	100,00	0,00E+00	1900	Os06g0178100	100,00	0,00E+00	1900	0	NA	NA	
maker-6-augustus-gene-39.72-mRNA-1	6	OsR498G0611714100.01.T03	94,18	0,00E+00	1311	Os06g0178300	100,00	0,00E+00	989	-322	Indica	NA	
maker-6-augustus-gene-39.73-mRNA-1	6	OsR498G0611714100.01.T04	100,00	0,00E+00	1043	Os06g0178700	100,00	0,00E+00	1043	0	NA	NA	
maker-6-augustus-gene-39.82-mRNA-1	6	OsR498G0611710800.01.T01	100,00	0,00E+00	2116	Os06g0175400	100,00	0,00E+00	2116	0	NA	NA	
maker-6-augustus-gene-39.84-mRNA-1	6	OsR498G0611711000.01.T01	100,00	0,00E+00	3090	Os06g0175500	100,00	0,00E+00	3090	0	NA	NA	
maker-6-augustus-gene-4.38-mRNA-1	6	OsR498G0611478100.01.T01	99,80	0,00E+00	2743	Os06g0105500	100,00	0,00E+00	2756	13	NA	Japonica	
maker-6-augustus-gene-4.44-mRNA-1	6	OsR498G0611479500.01.T03	99,61	0,00E+00	5463	Os06g0105900	99,90	0,00E+00	5504	41	NA	Japonica	
maker-6-augustus-gene-4.49-mRNA-1	6	OsR498G0611479700.01.T01	100,00	0,00E+00	1662	Os06g0106000	100,00	0,00E+00	1662	0	NA	Japonica	
maker-6-augustus-gene-4.53-mRNA-1	6	OsR498G0611482700.01.T01	98,91	0,00E+00	1443	Os06g0107000	100,00	0,00E+00	1018	-425	Indica	Japonica	
maker-6-augustus-gene-4.56-mRNA-1	6	OsR498G0611482900.01.T01	99,80	0,00E+00	2627	Os06g0107100	99,12	0,00E+00	1602	-1025	Indica	Japonica	
maker-6-augustus-gene-4.71-mRNA-1	6	OsR498G0611478800.01.T02	100,00	0,00E+00	1386	Os06g0105700	100,00	0,00E+00	1386	0	NA	Japonica	
maker-6-augustus-gene-4.75-mRNA-1	6	OsR498G0611479000.01.T02	93,10	3,86E-158	559	Os06g0105800	93,37	3,15E-160	563	4	NA	Japonica	
maker-6-augustus-gene-4.85-mRNA-1	6	OsR498G0611482000.01.T01	99,86	0,00E+00	2515	Os06g0106800	100,00	0,00E+00	2524	9	NA	Japonica	
maker-6-augustus-gene-40.38-mRNA-1	6	OsR498G0611716100.01.T01	100,00	0,00E+00	4783	Os06g0178900	100,00	0,00E+00	4156	-627	Indica	NA	
maker-6-augustus-gene-40.49-mRNA-1	6	OsR498G0611724500.01.T01	100,00	0,00E+00	2311	Os06g0181300	100,00	0,00E+00	2311	0	NA	NA	
maker-6-augustus-gene-40.52-mRNA-1	6	OsR498G0611716600.01.T01	100,00	0,00E+00	2847	Os06g0179000	99,81	0,00E+00	2837	-10	NA	NA	
maker-6-augustus-gene-40.53-mRNA-1	6	OsR498G0611717600.01.T01	100,00	0,00E+00	3074	Os06g0179200	100,00	0,00E+00	3074	0	NA	NA	
maker-6-augustus-gene-40.68-mRNA-1	6	OsR498G0611718800.01.T01	100,00	0,00E+00	1797	Os06g0179700	100,00	0,00E+00	1797	0	NA	NA	
maker-6-augustus-gene-40.71-mRNA-1	6	OsR498G0611719000.01.T04	99,95	0,00E+00	3280	Os06g0179800	100,00	0,00E+00	3285	5	NA	NA	LRR-TM
maker-6-augustus-gene-40.76-mRNA-1	6	OsR498G0611719400.01.T02	100,00	0,00E+00	1975	Os06g0180000	100,00	0,00E+00	1537	-438	Indica	NA	
maker-6-augustus-gene-41.25-mRNA-1	6	OsR498G0611724700.01.T05	100,00	0,00E+00	1333	Os06g0181400	100,00	0,00E+00	1050	-283	Indica	NA	
maker-6-augustus-gene-41.31-mRNA-1	6	OsR498G0611726300.01.T01	100,00	0,00E+00	1110	Os06g0181700	100,00	0,00E+00	969	-141	Indica	NA	
maker-6-augustus-gene-41.33-mRNA-1	6	OsR498G0611729300.01.T06	99,88	0,00E+00	1533	Os06g0182400	99,88	0,00E+00	1533	0	NA	Japonica	
maker-6-augustus-gene-41.37-mRNA-1	6	OsR498G0611729500.01.T03	99,87	0,00E+00	1390	Os06g0182500	100,00	0,00E+00	1395	5	NA	Japonica	
maker-6-augustus-gene-41.44-mRNA-1	6	OsR498G0611728600.01.T01	94,08	0,00E+00	1288	Os06g0182100	98,90	0,00E+00	1435	147	Japonica	Japonica	
maker-6-augustus-gene-42.0-mRNA-1	6	OsR498G0611732100.01.T01	99,95	0,00E+00	3767	Os06g0183100	100,00	0,00E+00	3772	5	NA	NA	
maker-6-augustus-gene-42.20-mRNA-1	6	OsR498G0611735400.01.T01	98,49	0,00E+00	1494	Os06g0184200	97,53	0,00E+00	1516	22	NA	Indica	
maker-6-augustus-gene-42.22-mRNA-1	6	OsR498G0611735600.01.T01	99,82	0,00E+00	5069	Os06g0184300	99,89	0,00E+00	4856	-213	NA	Japonica	
maker-6-augustus-gene-43.44-mRNA-1	6	OsR498G0611736400.01.T01	99,64	0,00E+00	3448	Os06g0184500	99,33	0,00E+00	3433	-15	NA	NA	
maker-6-augustus-gene-43.46-mRNA-1	6	OsR498G0611737800.01.T01	100,00	0,00E+00	731	Os06g0184800	97,84	2,12E-109	395	-336	Indica	Indica	
maker-6-augustus-gene-44.0-mRNA-1	6	OsR498G0611740700.01.T01	99,86	0,00E+00	1257	Os02g0664000	72,34	1,60E-69	262	-995	Indica	Indica	
maker-6-augustus-gene-44.14-mRNA-1	6	OsR498G0611744100.01.T03	100,00	0,00E+00	2758	Os06g0187000	97,99	0,00E+00	2646	-112	NA	Indica	
maker-6-augustus-gene-44.21-mRNA-1	6	OsR498G0611741800.01.T01	99,53	0,00E+00	5717	Os06g0186300	74,23	0,00E+00	1524	-4193	Indica	Indica	Kinase-LRR-TM
maker-6-augustus-gene-44.23-mRNA-1	6	OsR498G0611742000.01.T01	99,61	0,00E+00	3660	Os06g0186300	98,34	0,00E+00	5302	1642	Japonica	Indica	Kinase-LRR-TM
maker-6-augustus-gene-44.31-mRNA-1	6	OsR498G0611743000.01.T01	99,56	0,00E+00	1217	Os06g0186600	99,42	0,00E+00	1213	-4	NA	Indica	
maker-6-augustus-gene-44.38-mRNA-1	6	OsR498G0611743800.01.T01	100,00	0,00E+00	2587	Os06g0186900	98,68	0,00E+00	2499	-88	NA	Indica	
maker-6-augustus-gene-44.40-mRNA-1	6	OsR498G0611744800.01.T01	99,93	0,00E+00	2452	Os06g0187200	99,79	0,00E+00	1669	-783	Indica	Indica	
maker-6-augustus-gene-45.0-mRNA-1	6	OsR498G0611747100.01.T08	99,45	0,00E+00	965	Os06g0187900	99,26	0,00E+00	958	-7	NA	Japonica	
maker-6-augustus-gene-45.11-mRNA-1	6	OsR498G0611747800.01.T02	99,79	0,00E+00	851	Os06g0188100	99,37	0,00E+00	842	-9	NA	Indica	
maker-6-augustus-gene-45.4-mRNA-1	6	OsR498G0611747500.01.T01	99,70	0,00E+00	1794	Os06g0188000	99,73	0,00E+00	1323	-471	Indica	Indica	
maker-6-augustus-gene-46.19-mRNA-1	6	OsR498G0611751000.01.T01	99,76	0,00E+00	752	Os06g0188800	93,08	1,23E-178	626	-126	Indica	NA	
maker-6-augustus-gene-46.23-mRNA-1	6	OsR498G0611753200.01.T01	85,14	0,00E+00	1599	Os06g0190700	82,41	0,00E+00	1452	-147	NA	Japonica	
maker-6-augustus-gene-46.5-mRNA-1	6	OsR498G0611753200.01.T01	85,27	0,00E+00	1675	Os06g0190700	82,67	0,00E+00	1536	-139	NA	Indica	
maker-6-augustus-gene-46.6-mRNA-1	6	OsR498G0611748600.01.T01	98,00	0,00E+00	2049	Os06g0190700	71,04	3,03E-140	499	-1550	Indica	NA	
maker-6-augustus-gene-47.42-mRNA-1	6	OsR498G0611754700.01.T01	98,68	0,00E+00	1061	Os06g0191200	100,00	0,00E+00	1357	296	Japonica	Japonica	
maker-6-augustus-gene-47.44-mRNA-1	6	OsR498G0611757300.01.T01	99,81	0,00E+00	2898	Os06g0191700	99,94	0,00E+00	2907	9	NA	Japonica	
maker-6-augustus-gene-47.46-mRNA-1	6	OsR498G0611758700.01.T01	99,78	0,00E+00	2449	Os06g0192100	100,00	0,00E+00	2462	13	NA	Japonica	
maker-6-augustus-gene-47.52-mRNA-1	6	OsR498G0611759900.01.T01	99,92	0,00E+00	2306	Os06g0192600	100,00	0,00E+00	2311	5	NA	Japonica	
maker-6-augustus-gene-47.57-mRNA-1	6	OsR498G0611753200.01.T01	98,24	0,00E+00	2551	Os06g0190700	95,74	0,00E+00	2827	276	NA	Japonica	
maker-6-augustus-gene-47.60-mRNA-1	6	OsR498G0611753600.01.T01	97,18	0,00E+00	1607	Os06g0190800	100,00	0,00E+00	1728	121	NA	Japonica	
maker-6-augustus-gene-47.62-mRNA-1	6	OsR498G0611757600.01.T01	98,73	0,00E+00	2620	Os06g0191800	100,00	0,00E+00	2717	97	NA	Japonica	
maker-6-augustus-gene-47.64-mRNA-1	6	OsR498G0611757800.01.T01	97,03	0,00E+00	4036	Os02g0787200	79,53	0,00E+00	1880	-2156	Indica	Japonica	CC-Kinase-TM
maker-6-augustus-gene-47.67-mRNA-1	6	OsR498G0611759200.01.T01	99,60	0,00E+00	1773	Os06g0192400	100,00	0,00E+00	1792	19	NA	Japonica	
maker-6-augustus-gene-48.32-mRNA-1	6	OsR498G0611762100.01.T02	99,91	0,00E+00	1939	Os06g0192900	99,81	0,00E+00	1934	-5	NA	Japonica	
maker-6-augustus-gene-48.36-mRNA-1	6	OsR498G0611765100.01.T01	99,63	0,00E+00	1947	Os06g0194000	99,91	0,00E+00	1960	13	NA	Japonica	
maker-6-augustus-gene-48.38-mRNA-1	6	OsR498G0611769500.01.T01	99,72	0,00E+00	1279	Os06g0194400	99,86	0,00E+00	1284	5	NA	Japonica	
maker-6-augustus-gene-48.42-mRNA-1	6	OsR498G0611762600.01.T01	100,00	0,00E+00	1305	Os06g0193000	100,00	0,00E+00	1305	0	NA	Japonica	
maker-6-augustus-gene-48.46-mRNA-1	6	OsR498G0611763000.01.T01	97,66	0,00E+00	651	Os06g0193300	100,00	0,00E+00	693	42	NA	Japonica	
maker-6-augustus-gene-48.49-mRNA-1	6	OsR498G0611763200.01.T03	98,75	0,00E+00	2511	Os06g0193400	99,93	0,00E+00	2588	77	NA	Japonica	
maker-6-augustus-gene-49.0-mRNA-1	6	OsR498G0611777900.01.T01	100,00	0,00E+00	1256	Os06g0195800	100,00	0,00E+00	1083	-173	Indica	Indica	
maker-6-augustus-gene-49.4-mRNA-1	6	OsR498G0611772600.01.T05	99,88	0,00E+00	4364	Os06g0194900	99,84	0,00E+00	4360	-4	NA	Japonica	
maker-6-augustus-gene-49.7-mRNA-1	6	OsR498G0611776200.01.T02	99,36	0,00E+00	4158	Os06g0195600	99,23	0,00E+00	4145	-13	NA	Indica	
maker-6-augustus-gene-5.52-mRNA-1	6	OsR498G0611484500.01.T02	99,73	0,00E+00	1957	Os06g0107600	100,00	0,00E+00	1608	-349	Indica	Japonica	
maker-6-augustus-gene-5.64-mRNA-1	6	OsR498G0611487900.01.T01	98,62	0,00E+00	2783	Os06g0108900	100,00	0,00E+00	2874	91	NA	Japonica	
maker-6-augustus-gene-5.69-mRNA-1	6	OsR498G0611490100.01.T01	100,00	0,00E+00	1943	Os06g0109200	100,00	0,00E+00	1943	0	NA	Japonica	

maker-6-augustus-gene-5.72-mRNA-1	6	OsR498G0611490300.01.T01	99,72	0,00E+00	1940	Os06g0109300	100,00	0,00E+00	1954	14	NA	Japonica	
maker-6-augustus-gene-5.75-mRNA-1	6	OsR498G0611490500.01.T01	99,74	0,00E+00	4145	Os06g0109400	100,00	0,00E+00	4172	27	NA	Japonica	NBS-TM
maker-6-augustus-gene-5.78-mRNA-1	6	OsR498G0611484800.01.T01	99,73	0,00E+00	1951	Os06g0107700	100,00	0,00E+00	1965	14	NA	Japonica	
maker-6-augustus-gene-5.82-mRNA-1	6	OsR498G0611486000.01.T01	99,70	1,30E-169	597	Os03g0596800	89,96	0,00E+00	725	128	Japonica	Japonica	
maker-6-augustus-gene-5.85-mRNA-1	6	OsR498G0611486400.01.T01	99,77	0,00E+00	1534	Os01g0274500	71,43	8,94E-55	214	-1320	Indica	Japonica	
maker-6-augustus-gene-5.87-mRNA-1	6	OsR498G0611488400.01.T02	99,79	0,00E+00	840	Os06g0109100	100,00	0,00E+00	1061	221	Japonica	Japonica	
maker-6-augustus-gene-50.58-mRNA-1	6	OsR498G0611782900.01.T05	99,83	0,00E+00	1057	Os06g0196900	99,83	0,00E+00	1057	0	NA	NA	
maker-6-augustus-gene-50.59-mRNA-1	6	OsR498G0611782900.01.T06	100,00	3,68E-85	315	Os02g0781400	79,31	9,23E-37	152	-163	Indica	Indica	
maker-6-augustus-gene-50.68-mRNA-1	6	OsR498G0611780000.01.T01	100,00	0,00E+00	1440	Os06g0196300	100,00	0,00E+00	810	-630	Indica	Indica	
maker-6-augustus-gene-50.71-mRNA-1	6	OsR498G0611780600.01.T01	100,00	0,00E+00	2837	Os06g0196500	100,00	0,00E+00	3276	439	Japonica	Indica	
maker-6-augustus-gene-50.75-mRNA-1	6	OsR498G0611780800.01.T03	100,00	0,00E+00	1582	Os06g0196600	100,00	0,00E+00	1582	0	NA	NA	
maker-6-augustus-gene-51.49-mRNA-1	6	OsR498G0611788200.01.T01	99,49	0,00E+00	1051	Os06g0198500	99,49	0,00E+00	1051	0	NA	NA	
maker-6-augustus-gene-51.51-mRNA-1	6	OsR498G0611788400.01.T01	99,40	0,00E+00	1190	Os06g0198500	92,72	0,00E+00	873	-317	Indica	NA	
maker-6-augustus-gene-51.59-mRNA-1	6	OsR498G0611789800.01.T07	99,61	0,00E+00	6916	Os06g0199100	99,53	0,00E+00	5740	-1176	Indica	NA	
maker-6-augustus-gene-51.62-mRNA-1	6	OsR498G0611790200.01.T06	100,00	0,00E+00	2150	Os06g0199200	100,00	0,00E+00	2150	0	NA	NA	
maker-6-augustus-gene-51.66-mRNA-1	6	OsR498G0611791800.01.T04	100,00	0,00E+00	1196	Os06g0199500	100,00	0,00E+00	1196	0	NA	NA	
maker-6-augustus-gene-52.42-mRNA-1	6	OsR498G0611802900.01.T01	98,84	0,00E+00	1360	Os06g0202700	100,00	0,00E+00	1110	-250	Indica	NA	
maker-6-augustus-gene-52.47-mRNA-1	6	OsR498G0611803600.01.T01	99,85	0,00E+00	1214	Os06g0202800	90,86	0,00E+00	814	-400	Indica	NA	
maker-6-augustus-gene-52.50-mRNA-1	6	OsR498G0611804800.01.T01	100,00	0,00E+00	2311	Os06g0202900	100,00	0,00E+00	2311	0	NA	Japonica	Kinase-TM
maker-6-augustus-gene-53.0-mRNA-1	6	OsR498G0611807100.01.T03	99,84	0,00E+00	1120	Os02g0778400	78,45	5,12E-158	556	-564	Indica	NA	
maker-6-augustus-gene-53.21-mRNA-1	6	OsR498G0611805800.01.T02	100,00	0,00E+00	2803	Os06g0203200	100,00	0,00E+00	2803	0	NA	NA	
maker-6-augustus-gene-53.26-mRNA-1	6	OsR498G0611808400.01.T02	100,00	0,00E+00	5297	Os06g0203800	100,00	0,00E+00	5297	0	NA	NA	Kinase-LRR-TM
maker-6-augustus-gene-53.31-mRNA-1	6	OsR498G0611811000.01.T04	99,90	0,00E+00	1823	Os06g0204400	99,74	0,00E+00	1379	-444	Indica	NA	
maker-6-augustus-gene-53.5-mRNA-1	6	OsR498G0611807100.01.T15	100,00	0,00E+00	2322	Os06g0203600	100,00	0,00E+00	2322	0	NA	NA	
maker-6-augustus-gene-54.47-mRNA-1	6	OsR498G0611814100.01.T02	98,26	0,00E+00	1094	Os06g0205100	99,51	0,00E+00	1458	364	Japonica	NA	
maker-6-augustus-gene-54.49-mRNA-1	6	OsR498G0611817500.01.T02	100,00	0,00E+00	1748	Os06g0206000	100,00	0,00E+00	1458	-290	Indica	NA	
maker-6-augustus-gene-54.55-mRNA-1	6	OsR498G0611817700.01.T04	97,34	0,00E+00	2174	Os06g0206100	96,47	0,00E+00	2131	-43	NA	NA	
maker-6-augustus-gene-54.59-mRNA-1	6	OsR498G0611818100.01.T01	100,00	0,00E+00	866	Os06g0206200	100,00	0,00E+00	866	0	NA	NA	
maker-6-augustus-gene-54.66-mRNA-1	6	OsR498G0611816600.01.T04	100,00	0,00E+00	1748	Os06g0205600	100,00	0,00E+00	2641	893	Japonica	NA	
maker-6-augustus-gene-55.29-mRNA-1	6	OsR498G0611820300.01.T05	99,40	0,00E+00	889	Os06g0206700	100,00	0,00E+00	906	17	NA	NA	
maker-6-augustus-gene-55.38-mRNA-1	6	OsR498G0611822700.01.T01	100,00	0,00E+00	3341	Os06g0207000	100,00	0,00E+00	3341	0	NA	Indica	
maker-6-augustus-gene-55.50-mRNA-1	6	OsR498G0611825200.01.T02	100,00	0,00E+00	3983	Os02g0773400	77,73	0,00E+00	1775	-2208	Indica	NA	
maker-6-augustus-gene-56.0-mRNA-1	6	OsR498G0611830100.01.T01	99,52	0,00E+00	744	Os06g0208200	99,49	0,00E+00	697	-47	NA	NA	
maker-6-augustus-gene-56.15-mRNA-1	6	OsR498G0611830400.01.T05	99,83	0,00E+00	3132	Os02g0772000	73,56	0,00E+00	1046	-2086	Indica	NA	
maker-6-augustus-gene-56.3-mRNA-1	6	OsR498G0611833100.01.T03	100,00	0,00E+00	733	Os06g0208700	100,00	0,00E+00	733	0	NA	NA	
maker-6-augustus-gene-56.7-mRNA-1	6	OsR498G0611833300.01.T02	99,76	0,00E+00	2205	Os02g0769200	82,83	0,00E+00	678	-1527	Indica	NA	
maker-6-augustus-gene-57.14-mRNA-1	6	OsR498G0611839300.01.T01	99,80	0,00E+00	2633	Os06g0210900	99,73	0,00E+00	2623	-10	NA	Indica	
maker-6-augustus-gene-57.2-mRNA-1	6	OsR498G0611836500.01.T01	99,92	0,00E+00	2106	Os02g0768300	82,42	0,00E+00	1015	-1091	Indica	Indica	
maker-6-augustus-gene-57.5-mRNA-1	6	OsR498G0611836900.01.T01	99,84	0,00E+00	1132	Os06g0210100	97,14	0,00E+00	1171	39	NA	Indica	
maker-6-augustus-gene-57.8-mRNA-1	6	OsR498G0611837100.01.T01	99,93	0,00E+00	2501	Os06g0210200	98,88	0,00E+00	2351	-150	NA	Indica	
maker-6-augustus-gene-58.40-mRNA-1	6	OsR498G0611841300.01.T05	99,79	0,00E+00	1743	Os06g0211200	99,49	0,00E+00	1729	-14	NA	Indica	
maker-6-augustus-gene-58.43-mRNA-1	6	OsR498G0611841700.01.T05	100,00	0,00E+00	666	Os06g0211300	100,00	0,00E+00	666	0	NA	NA	
maker-6-augustus-gene-58.45-mRNA-1	6	OsR498G0611842900.01.T01	99,88	0,00E+00	3054	Os06g0211600	99,76	0,00E+00	3045	-9	NA	Indica	
maker-6-augustus-gene-58.47-mRNA-1	6	OsR498G0611844900.01.T01	99,93	0,00E+00	2669	Os06g0212500	99,87	0,00E+00	2664	-5	NA	NA	
maker-6-augustus-gene-58.49-mRNA-1	6	OsR498G0611843200.01.T01	99,88	0,00E+00	3065	Os06g0211700	98,47	0,00E+00	2957	-108	NA	Indica	
maker-6-augustus-gene-58.51-mRNA-1	6	OsR498G0611844000.01.T02	99,82	0,00E+00	3001	Os06g0212100	99,88	0,00E+00	3005	4	NA	NA	
maker-6-augustus-gene-58.53-mRNA-1	6	OsR498G0611844600.01.T01	99,80	0,00E+00	2723	Os06g0212300	99,44	0,00E+00	3170	447	Japonica	NA	
maker-6-augustus-gene-59.0-mRNA-1	6	OsR498G0611849900.01.T01	100,00	0,00E+00	1808	Os06g0214100	99,90	0,00E+00	1803	-5	NA	Indica	
maker-6-augustus-gene-59.12-mRNA-1	6	OsR498G0611849000.01.T03	99,84	0,00E+00	1100	Os06g0213400	99,67	0,00E+00	1095	-5	NA	NA	
maker-6-augustus-gene-6.36-mRNA-1	6	OsR498G0611491500.01.T02	98,10	0,00E+00	1452	Os06g0109600	97,90	0,00E+00	1230	-222	Indica	Japonica	
maker-6-augustus-gene-6.42-mRNA-1	6	OsR498G0611493500.01.T01	99,80	0,00E+00	2730	Os06g0110000	100,00	0,00E+00	2744	14	NA	Japonica	
maker-6-augustus-gene-6.45-mRNA-1	6	OsR498G0611493700.01.T01	99,89	0,00E+00	1619	Os06g0110100	100,00	0,00E+00	1624	5	NA	Japonica	
maker-6-augustus-gene-6.58-mRNA-1	6	OsR498G0611495800.01.T01	99,87	0,00E+00	5469	Os06g0111300	100,00	0,00E+00	3757	-1712	Indica	Japonica	
maker-6-augustus-gene-6.65-mRNA-1	6	OsR498G0611496000.01.T01	99,81	0,00E+00	4674	Os06g0111400	100,00	0,00E+00	4697	23	NA	Japonica	
maker-6-augustus-gene-60.0-mRNA-1	6	OsR498G0611853000.01.T01	100,00	0,00E+00	3629	Os06g0214900	99,94	0,00E+00	2921	-708	Indica	Indica	
maker-6-augustus-gene-60.15-mRNA-1	6	OsR498G0611854400.01.T08	99,93	0,00E+00	2590	Os06g0215400	99,72	0,00E+00	2576	-14	NA	Indica	
maker-6-augustus-gene-60.21-mRNA-1	6	OsR498G0611854600.01.T01	99,92	0,00E+00	2117	Os06g0215500	99,83	0,00E+00	2113	-4	NA	NA	
maker-6-augustus-gene-60.23-mRNA-1	6	OsR498G0611854800.01.T01	99,73	0,00E+00	2016	Os06g0215600	99,56	0,00E+00	2007	-9	NA	NA	
maker-6-augustus-gene-60.25-mRNA-1	6	OsR498G0611856400.01.T01	98,87	0,00E+00	2026	Os06g0215900	97,73	0,00E+00	1955	-71	NA	Indica	
maker-6-augustus-gene-60.27-mRNA-1	6	OsR498G0611856600.01.T01	95,80	0,00E+00	1843	Os06g0216300	87,46	0,00E+00	1379	-464	Indica	Indica	
maker-6-augustus-gene-60.29-mRNA-1	6	OsR498G0611857200.01.T01	99,92	0,00E+00	2333	Os06g0216200	97,88	0,00E+00	1932	-401	Indica	Indica	
maker-6-augustus-gene-60.32-mRNA-1	6	OsR498G0611857600.01.T01	100,00	0,00E+00	2062	Os06g0216300	97,55	0,00E+00	1936	-126	NA	Indica	
maker-6-augustus-gene-60.34-mRNA-1	6	OsR498G0611858400.01.T01	100,00	0,00E+00	812	Os06g0216700	100,00	0,00E+00	812	0	NA	Indica	
maker-6-augustus-gene-60.7-mRNA-1	6	OsR498G0611854200.01.T01	100,00	0,00E+00	1308	Os06g0215200	99,45	0,00E+00	1290	-18	NA	Indica	
maker-6-augustus-gene-60.9-mRNA-1	6	OsR498G0611854400.01.T01	99,87	0,00E+00	4061	Os06g0215300	99,76	0,00E+00	3679	-382	NA	Indica	
maker-6-augustus-gene-61.27-mRNA-1	6	OsR498G0611858600.01.T02	94,52	0,00E+00	2015	Os06g0216800	94,44	0,00E+00	2011	-4	NA	Indica	
maker-6-augustus-gene-61.32-mRNA-1	6	OsR498G0611859800.01.T01	99,55	0,00E+00	1188	Os06g0217300	99,55	0,00E+00	1188	0	NA	Indica	
maker-6-augustus-gene-61.50-mRNA-1	6	OsR498G0611862200.01.T01	99,56	0,00E+00	1206	Os06g0217700	98,52	0,00E+00	1177	-29	NA	Indica	
maker-6-augustus-gene-62.16-mRNA-1	6	OsR498G0611867000.01.T01	98,92	0,00E+00	2436	Os06g0218500	98,77	0,00E+00	2427	-9	NA	Indica	
maker-6-augustus-gene-62.21-mRNA-1	6	OsR498G0611867800.01.T04	99,91	0,00E+00	3865	Os06g0218900	99,77	0,00E+00	3852	-13	NA	Indica	
maker-6-augustus-gene-62.7-mRNA-1	6	OsR498G0611867100.01.T01	99,83	0,00E+00	1073	Os06g0218600	99,33	0,00E+00	1059	-14	NA	Indica	
maker-6-augustus-gene-63.0-mRNA-1	6	OsR498G0611869700.01.T02	100,00	0,00E+00	2154	Os06g0219400	99,91	0,00E+00	1971	-183	NA	Indica	
maker-6-augustus-gene-63.11-mRNA-1	6	OsR498G0611873300.01.T01	99,93	0,00E+00	2701	Os06g0220500	99,34	0,00E+00	2681	-20	NA	NA	
maker-6-augustus-gene-63.13-mRNA-1	6	OsR498G0611874100.01.T02	89,27	0,00E+00	737	Os06g0220800	89,27	0,00E+00	737	0	NA	Indica	
maker-6-augustus-gene-63.16-mRNA-1	6	OsR498G0611870000.01.T01	99,87	0,00E+00	1343	Os06g0219500	100,00	0,00E+00	1348	5	NA	Indica	
maker-6-augustus-gene-63.18-mRNA-1	6	OsR498G0611870400.01.T01	99,90	0,00E+00	1717	Os06g0219700	99,79	0,00E+00	1712	-5	NA	NA	
maker-6-augustus-gene-63.7-mRNA-1	6	OsR498G0611870100.01.T02	100,00	0,00E+00	1110	Os06g0219600	100,00	0,00E+00	1110	0	NA	NA	
maker-6-augustus-gene-64.0-mRNA-1	6	OsR498G0611876100.01.T01	99,79	0,00E+00	1728	Os06g0221000	98,97	0,00E+00	1704	-24	NA	Indica	
maker-6-augustus-gene-64.11-mRNA-1	6	OsR498G0611878400.01.T02	100,00	0,00E+00	2046	Os06g0222100	99,41	0,00E+00	1795	-251	Indica	Indica	
maker-6-augustus-gene-64.3-mRNA-1	6	OsR498G0611877100.01.T01	100,00	0,00E+00	1721	Os06g0221200	99,90	0,00E+00	1717	-4	NA	Indica	
maker-6-augustus-gene-64.5-mRNA-1	6	OsR498G0611877700.01.T01	99,87	0,00E+00	1408	Os05g0477800	84,99	0,00E+00	781	-627	Indica	Indica	
maker-6-augustus-gene-64.7-mRNA-1	6	OsR498G0611876600.01.T01	100,00	0,00E+00	1997	Os06g0221100	99,73	0,00E+00	1984	-13	NA	Indica	
maker-6-augustus-gene-65.0-mRNA-1	6	OsR498G0611883700.01.T01	99,26	0,00E+00	1918	Os06g0223300	100,00	0,00E+00	1954	36	NA	Japonica	
maker-6-augustus-gene-65.3-mRNA-1	6	OsR498G0611882600.01.T01	99,92	0,00E+00	2187	Os06g0222800	99,35	0,00E+00	2168	-19	NA	Indica	
maker-6-augustus-gene-65.5-mRNA-1	6	OsR498G0611882800.01.T02	99,19	0,00E+00	2851	Os06g0222900	100,00	0,00E+00	2890	39	NA	Japonica	
maker-6-augustus-gene-66.15-mRNA-1	6	OsR498G0611908000.01.T03	99,95	0,00E+00	3848	Os06g0224200	99,95	0,00E+00	3848	0	NA	NA	
maker-6-augustus-gene-66.18-mRNA-1	6	OsR498G0611909200.01.T01	100,00	0,00E+00	870	Os06g0224500	100,00	0,00E+00	838	-32	NA	NA	
maker-6-augustus-gene-66.25-mRNA-1	6	OsR498G0611911800.01.T06	100,00	0,00E+00	940	Os06g0225400	100,00	0,00E+00	832	-108	Indica	NA	
maker-6-augustus-gene-66.6-mRNA-1	6	OsR498G0611885200.01.T01	99,77	0,00E+00	770	Os06g0223700	100,00	0,00E+00	774	4	NA	Japonica	
maker-6-augustus-gene-67.0-mRNA-1	6	OsR498G0611913300.01.T01	100,00	0,00E+00	791	Os06g0226050	100,00	0,00E+00	791	0	NA	NA	
maker-6-augustus-gene-67.17-mRNA-1	6	OsR498G0611916800.01.T02	100,00	0,00E+00	1050	Os06g0226600	99,83	0,00E+00	1046	-4	NA	NA	
maker-6-augustus-gene-67.2-mRNA-1	6	OsR498G0611916500.01.T01	100,00	4,07E-134	477	Os06g0226500	100,00	1,42E-134	477	0	NA	NA	
maker-6-augustus-gene-67.20-mRNA-1	6	OsR498G0611920000.01.T01	100,00	0,00E+00	1434	Os06g0226950	100,00	0,00E+00	1434	0	NA	NA	
maker-6-augustus-gene-67.4-mRNA-1	6	OsR498G0611912000.01.T04	100,00	0,00E+00	838	Os06g0225800	100,00	0,00E+00	838	0	NA	NA	
maker-6-augustus-gene-68.0-mRNA-1	6	OsR498G0611930900.01.T01	100,00	0,00E+00	2241	Os06g0228500	100,00	0,00E+00	2241	0	NA	NA	
maker-6-augustus-gene-68.2-mRNA-1	6	OsR498G0611920800.01.T01	99,91	0,00E+00	3806	Os06g0227200	99,95	0,00E+00	3810	4	NA	NA	
maker-6-augustus-gene-68.4-mRNA-1	6	OsR498G0611921600.01.T01	100,00	0,00E+00	2733	Os02g0745400	76,90	0,00E+00	1145	-1588	Indica	NA	
maker-6-augustus-gene-68.6-mRNA-1	6	OsR498G0611927800.01.T02	100,00	0,00E+00	1618	Os06g0228200	100,00	0,00E+00	1618	0	NA	NA	
maker-6-augustus-gene-68.9-mRNA-1	6	OsR498G0611931400.01.T01	100,00	0,00E+00	2625	Os06g0228600	100,00	0,00E+00	2625	0	NA	NA	
maker-6-augustus-gene-69.0-mRNA-1	6	OsR498G0611931900.01.T02	100,00	0,00E+00	3671	Os06g0228900	99,25	0,00E+00	3565	-106	NA	NA	
maker-6-augustus-gene-69.16-mRNA-1	6	OsR498G0611934500.01.T01	100,00	0,00E+00	1965	Os06g0229400	100,00	0,00E+00	1965	0	NA	NA	
maker-6-augustus-gene-69.18-mRNA-1	6	OsR498G0611935700.01.T01	99,06	0,00E+00	4299	Os06g0229800	99,10	0,00E+00	4304	5	NA	NA	
maker-6-augustus-gene-69.2-mRNA-1	6	OsR498G0611933700.01.T04	100,00	0,00E+00	2324	Os06g0229200	100,00	0,00E+00	2322	-2	NA	NA	
maker-6-augustus-gene-69.26-mRNA-1	6	OsR498G0611937500.01.T03	100,00	0,00E+00	1002	Os06g0231050	100,00	0,00E+00	1002	0	NA	NA	
maker-6-augustus-gene-69.28-mRNA-1	6	OsR498G0611931600.01.T01	100,00	0,00E+00	2749	Os06g0228800	100,00	0,00E+00	2749	0	NA	NA	
maker-6-augustus-gene-69.30-mRNA-1	6	OsR498G0611932200.01.T01	100,00	0,00E+00	3718	Os06g0229066	100,00	0,00E+00	2630	-1088	Indica	Indica	
maker-6-augustus-gene-69.8-mRNA-1	6	OsR498G0611934300.01.T01	100,00	0,00E+00	2847	Os06g0229300	100,00	0,00E+00	2847	0	NA	NA	
maker-6-augustus-gene-69.9-mRNA-1	6	OsR498G0611934300.01.T02	100,00	0,00E+00	1445	Os06g0229300	100,00	0,00E+00	1445	0	NA	NA	
maker-6-augustus-gene-7.0-mRNA-1	6	OsR498G0611500300.01.T01	99,87	0,00E+00	1403	Os06g0112200	100,00	0,00E+00	1407	4	NA	Japonica	
maker-6-augustus-gene-7.10-mRNA-1	6	OsR498G0611501300.01.T01	99,73	0,00E+00	1973	Os06g0112700	100,00	0,00E+00	1992	19	NA	Japonica	
maker-6-augustus-gene-7.13-mRNA-1	6	OsR498G0611497000.01.T02	99,94	0,00E+00	6327	Os06g0111800	100,00	0,00E+00	6336	9	NA	Japonica	
maker-6-augustus-gene-7.15-mRNA-1	6	OsR498G0611498800.01.T02	99,90	0,00E+00	1857	Os06g0112100	100,00	0,00E+00	1862	5	NA	Japonica	
maker-6-augustus-gene-7.4-mRNA-1	6	OsR498G0611500500.01.T01	95,48	0,00E+00	863	Os06g0112300	96,02	0,00E+00	873	10	NA	Japonica	
maker-6-augustus-gene-7.6-mRNA-1	6	OsR498G0611500700.01.T02	99,87	0,00E+00	1387	Os06g0112400	100,00	0,00E+00	1391	4	NA	Japonica	
maker-6-augustus-gene-70.23-mRNA-1	6	OsR498G0611937900.01.T02	100,00	0,00E+00	1673	Os06g0231300	100,00	0,00E+00	1673	0	NA	NA	
maker-6-augustus-gene-70.26-mRNA-1	6	OsR498G0611941700.01.T01	100,00	0,00E+00	2154	Os06g0232200	100,00	0,00E+00	2134	-20	NA	NA	
maker-6-augustus-gene-70.28-mRNA-1	6	OsR498G0611943700.01.T01	100,00	0,00E+00	3209	Os06g0232300	99,94	0,00E+00	3204	-5	NA	NA	
maker-6-augustus-gene-70.30-mRNA-1	6	OsR498G0611939200.01.T01	100,00	0,00E+00	1229	Os06g0231600	99,70	0,00E+00	1192	-37	NA	NA	
maker-6-augustus-gene-70.33-mRNA-1	6	OsR498G0611939800.01.T01	100,00	0,00E+00	2067	Os06g0232000	100,00	0,00E+00	2067	0	NA	NA	
maker-6-augustus-gene-70.35-mRNA-1	6	OsR498G0611940400.01.T04	98,99	0,00E+00	5563	Os06g0232100	100,00	0,00E+00	3034	-2529	Indica	NA	CC-Kinase-TM
maker-6-augustus-gene-71.16-mRNA-1	6	OsR498G0611948000.01.T01	100,00	0,00E+00	1359	Os06g0233400	100,00	0,00E+00	1359	0	NA	NA	
maker-6-augustus-gene-71.2-mRNA-1	6	OsR498G0611951500.01.T06	100,00	0,00E+00	3404	Os06g0234100	100,00	0,00E+00	3404	0	NA	NA	
maker-6-augustus-gene-71.21-mRNA-1	6	OsR498G0611949400.01.T01	100,00	0,00E+00	2013	Os06g0233800	100,00	0,00E+00	2013	0	NA	NA	
maker-6-augustus-gene-71.9-mRNA-1	6	OsR498G0611945200.01.T03	100,00	0,00E+00	854	Os06g0232600	100,00	0,00E+00	854	0	NA	NA	
maker-6-augustus-gene-72.41-mRNA-1	6	OsR498G0611952100.01.T04	100,00	0,00E+00	1169	Os06g0234200	100,00	0,00E+00	1169	0	NA	NA	
maker-6-augustus-gene-72.44-mRNA-1	6	OsR498G0611957100.01.T03	100,00	0,00E+00	5312	Os06g0235300	100,00	0,00E+00	5312	0	NA	NA	
maker-6-augustus-gene-72.48-mRNA-1	6	OsR498G0611953000.01.T01	99,71	0,00E+00	1218	Os06g0234600	75,19	3,02E-126	452	-766	Indica	NA	
maker-6-augustus-gene-72.49-mRNA-1	6	OsR498G0611953800.01.T01	98,98	0,00E+00	2753	Os06g0234600	99,64	0,00E+00	1988	-765	Indica	NA	
maker-6-augustus-gene-72.52-mRNA-1	6	OsR498G0611955400.01.T01	99,92	0,00E+00	2350	Os07g0256700	73,11	0,00E+00	712	-1638	Indica	NA	
maker-6-augustus-gene-72.54-mRNA-1	6	OsR498G0611957000.01.T01	99,79	0,00E+00	2528	Os06g0235200	100,00	0,00E+00	2010	-518	Indica	NA	
maker-6-augustus-gene-73.0-mRNA-1	6	OsR498G0611960900.01.T01	100,00	0,00E+00	1029	Os06g0236300	100,00	0,00E+00	1029	0	NA	Japonica	
maker-6-augustus-gene-73.10-mRNA-1	6	OsR498G0611962200.01.T01	99,82	0,00E+00	1961	Os06g0236600	100,00	0,00E+00	1858	-103	NA	Japonica	
maker-6-augustus-gene-73.15-mRNA-1	6	OsR498G0611964000.01.T03	99,19	0,00E+00	1962	Os06g0237200	100,00	0,00E+00	1999	37	NA	Japonica	
maker-6-augustus-gene-73.20-mRNA-1	6	OsR498G0611964200.01.T01	97,90	0,00E+00	5280	Os06g0237300	100,00	0,00E+00	7056	1776	Japonica	Japonica	
maker-6-augustus-gene-73.30-mRNA-1	6	OsR498G0611965600.01.T01	93,61	0,00E+00	1773	Os02g0741100	82,51	0,00E+00	1012	-761	Indica	Japonica	
maker-6-augustus-gene-73.36-mRNA-1	6	OsR498G0611966600.01.T02	99,83	0,00E+00	1071	Os06g0237900	100,00	0,00E+00	969	-102	NA	Japonica	
maker-6-augustus-gene-73.5-mRNA-1	6	OsR498G0611963700.01.T01	99,52	0,00E+00	1123	Os06g0237100	100,00	0,00E+00	1095	-28	NA	Japonica	
maker-6-augustus-gene-74.14-mRNA-1	6	OsR498G0611972500.01.T02	96,94	0,00E+00	1358	Os06g0239700	93,68	0,00E+00	1222	-136	Indica	Japonica	
maker-6-augustus-gene-74.17-mRNA-1	6	OsR498G0611968200.01.T02	98,80	0,00E+00	1461	Os06g0238300	98,48	0,00E+00	1147	-314	Indica	NA	
maker-6-augustus-gene-74.20-mRNA-1	6	OsR498G0611969400.01.T01	99,90	0,00E+00	1819	Os06g0238700	100,00	0,00E+00	2150	331	Japonica	Japonica	
maker-6-augustus-gene-74.5-mRNA-1	6	OsR498G0611971500.01.T01	96,19	0,00E+00	2663	Os06g0239500	100,00	0,00E+00	2901	238	NA	Japonica	
maker-6-augustus-gene-75.33-mRNA-1	6	OsR498G0611973800.01.T01	94,54	0,00E+00	1513	Os06g0240900	100,00	0,00E+00	1752	239	Japonica	Japonica	
maker-6-augustus-gene-75.37-mRNA-1	6	OsR498G0713492600.01.T02	99,40	0,00E+00	2372	Os06g0241100	100,00	0,00E+00	2408	36	NA	Japonica	Kinase-TM
maker-6-augustus-gene-76.28-mRNA-1	6	OsR498G0611977000.01.T01	99,47	0,00E+00	1997	Os06g0240900	87,28	0,00E+00	1333	-664	Indica	Japonica	
maker-6-augustus-gene-77.25-mRNA-1	6	OsR498G0611981300.01.T04	99,48	0,00E+00	2746	Os06g0244100	99,94	0,00E+00	2775	29	NA	Japonica	
maker-6-augustus-gene-77.30-mRNA-1	6	OsR498G0611983500.01.T03	99,95	0,00E+00	3329	Os06g0244700	99,89	0,00E+00	3324	-5	NA	NA	
maker-6-augustus-gene-77.34-mRNA-1	6	OsR498G0611980400.01.T02	99,52	0,00E+00	1856	Os06g0244000	100,00	0,00E+00	1878	22	NA	Japonica	
maker-6-augustus-gene-77.38-mRNA-1	6	OsR498G0611981600.01.T03	92,28	0,00E+00	944	Os06g0244200	95,86	0,00E+00	1673	729	Japonica	Japonica	
maker-6-augustus-gene-77.42-mRNA-1	6	OsR498G0611986800.01.T03	99,88	0,00E+00	1462	Os06g0245600	99,88	0,00E+00	1462	0	NA	NA	
maker-6-augustus-gene-78.41-mRNA-1	6	OsR498G0611988700.01.T01	99,92	0,00E+00	2301	Os06g0245900	99,92	0,00E+00	2301	0	NA	NA	
maker-6-augustus-gene-78.43-mRNA-1	6	OsR498G0611990500.01.T01	99,75	0,00E+00	2146	Os06g0246500	99,83	0,00E+00	2150	4	NA	NA	
maker-6-augustus-gene-78.50-mRNA-1	6	OsR498G0611994900.01.T01	99,75	0,00E+00	710	Os06g0247200	99,40	4,84E-168	589	-121	Indica	NA	
maker-6-augustus-gene-78.57-mRNA-1	6	OsR498G0611989000.01.T01	100,00	2,31E-137	488	Os06g0246000	100,00	8,07E-138	488	0	NA	NA	
maker-6-augustus-gene-78.63-mRNA-1	6	OsR498G0611995400.01.T01	99,88	0,00E+00	3065	Os06g0247500	99,88	0,00E+00	3065	0	NA	NA	
maker-6-augustus-gene-78.69-mRNA-1	6	OsR498G0611997000.01.T02	99,07	0,00E+00	2856	Os06g0247900	99,77	0,00E+00	2315	-541	Indica	NA	
maker-6-augustus-gene-79.35-mRNA-1	6	OsR498G0611999900.01.T02	100,00	0,00E+00	3097	Os06g0248400	100,00	0,00E+00	2305	-792	Indica	Japonica	
maker-6-augustus-gene-8.42-mRNA-1	6	OsR498G0611504100.01.T02	99,62	0,00E+00	1410	Os06g0113600	100,00	0,00E+00	2219	809	Japonica	Japonica	
maker-6-augustus-gene-8.45-mRNA-1	6	OsR498G0611506300.01.T02	100,00	0,00E+00	1029	Os02g0128600	71,78	2,50E-72	271	-758	Indica	NA	
maker-6-augustus-gene-8.48-mRNA-1	6	OsR498G0611507900.01.T02	100,00	0,00E+00	2235	Os06g0114600	100,00	0,00E+00	2235	0	NA	NA	NBS-TM
maker-6-augustus-gene-8.51-mRNA-1	6	OsR498G0611508100.01.T01	100,00	0,00E+00	1067	Os06g0114650	99,78	0,00E+00	793	-274	Indica	NA	
maker-6-augustus-gene-8.54-mRNA-1	6	OsR498G0611509300.01.T02	100,00	0,00E+00	742	Os06g0115100	100,00	4,43E-118	423	-319	Indica	NA	
maker-6-augustus-gene-8.57-mRNA-1	6	OsR498G0611504000.01.T01	99,48	0,00E+00	1375	Os06g0113500	100,00	0,00E+00	1393	18	NA	Japonica	
maker-6-augustus-gene-8.58-mRNA-1	6	OsR498G0611505200.01.T01	99,89	0,00E+00	3162	Os06g0113900	100,00	0,00E+00	3171	9	NA	Japonica	
maker-6-augustus-gene-8.60-mRNA-1	6	OsR498G0611505600.01.T01	89,08	1,45E-83	309	Os06g0113950	88,70	2,62E-62	238	-71	Indica	Japonica	
maker-6-augustus-gene-8.63-mRNA-1	6	OsR498G0611505800.01.T02	100,00	0,00E+00	3258	Os06g0114000	100,00	0,00E+00	3258	0	NA	Japonica	
maker-6-augustus-gene-8.69-mRNA-1	6	OsR498G0611507200.01.T01	100,00	0,00E+00	850	Os06g0114400	100,00	0,00E+00	764	-86	Indica	NA	
maker-6-augustus-gene-8.71-mRNA-1	6	OsR498G0611507600.01.T03	100,00	3,91E-121	434	Os06g0114500	100,00	1,37E-121	434	0	NA	NA	
maker-6-augustus-gene-8.73-mRNA-1	6	OsR498G0611508400.01.T01	100,00	0,00E+00	1115	Os06g0114700	100,00	0,00E+00	1115	0	NA	NA	
maker-6-augustus-gene-80.15-mRNA-1	6	OsR498G0611641600.01.T03	93,22	3,64E-149	528	Os06g0125200	90,15	5,76E-97	353	-175	Indica	Indica	
maker-6-augustus-gene-80.17-mRNA-1	6	OsR498G0612011400.01.T01	100,00	0,00E+00	3296	Os06g0251700	99,95	0,00E+00	3291	-5	NA	Indica	
maker-6-augustus-gene-80.8-mRNA-1	6	OsR498G0612010300.01.T01	98,26	0,00E+00	3302	Os06g0251100	100,00	0,00E+00	3366	64	NA	Indica	
maker-6-augustus-gene-81.47-mRNA-1	6	OsR498G0612016600.01.T01	97,65	0,00E+00	3398	Os10g0441900	86,77	0,00E+00	2597	-801	Indica	NA	Kinase-TM
maker-6-augustus-gene-81.48-mRNA-1	6	OsR498G0612017400.01.T01	97,52	0,00E+00	1642	Os06g0253600	97,42	0,00E+00	1637	-5	NA	NA	
maker-6-augustus-gene-82.26-mRNA-1	6	OsR498G0612021100.01.T01	97,20	0,00E+00	8798	Os06g0255300	99,94	0,00E+00	8651	-147	NA	Japonica	
maker-6-augustus-gene-82.29-mRNA-1	6	OsR498G0612023500.01.T03	99,90	0,00E+00	5179	Os06g0255700	99,93	0,00E+00	5112	-67	NA	NA	
maker-6-augustus-gene-82.33-mRNA-1	6	OsR498G0612019400.01.T04	99,86	0,00E+00	1311	Os06g0254700	100,00	0,00E+00	1112	-199	Indica	NA	
maker-6-augustus-gene-82.38-mRNA-1	6	OsR498G0612020800.01.T03	97,31	0,00E+00	1066	Os06g0255000	99,91	0,00E+00	1976	910	Japonica	Japonica	
maker-6-augustus-gene-82.40-mRNA-1	6	OsR498G0612021000.01.T01	93,84	0,00E+00	966	Os06g0255100	99,64	0,00E+00	1508	542	Japonica	Japonica	
maker-6-augustus-gene-83.0-mRNA-1	6	OsR498G0612024500.01.T01	99,72	0,00E+00	3197	Os06g0255900	99,84	0,00E+00	2226	-971	Indica	NA	
maker-6-augustus-gene-83.19-mRNA-1	6	OsR498G0612029500.01.T01	99,27	0,00E+00	1215	Os06g0256800	99,56	0,00E+00	1224	9	NA	Japonica	
maker-6-augustus-gene-83.21-mRNA-1	6	OsR498G0612025400.01.T02	99,85	0,00E+00	1208	Os06g0256200	99,85	0,00E+00	1168	-40	NA	NA	
maker-6-augustus-gene-83.26-mRNA-1	6	OsR498G0612029800.01.T02	99,80	0,00E+00	2681	Os06g0256900	99,93	0,00E+00	2691	10	NA	Japonica	
maker-6-augustus-gene-83.6-mRNA-1	6	OsR498G0612026500.01.T02	99,93	0,00E+00	2452	Os06g0256300	99,93	0,00E+00	2452	0	NA	NA	
maker-6-augustus-gene-83.9-mRNA-1	6	OsR498G0612027700.01.T07	97,20	0,00E+00	2970	Os06g0256500	97,26	0,00E+00	2975	5	NA	NA	
maker-6-augustus-gene-84.39-mRNA-1	6	OsR498G0612032300.01.T01	100,00	0,00E+00	2116	Os06g0257600	100,00	0,00E+00	2116	0	NA	NA	
maker-6-augustus-gene-84.41-mRNA-1	6	OsR498G0612033300.01.T01	99,88	0,00E+00	1506	Os06g0258000	96,06	0,00E+00	1387	-119	NA	Japonica	
maker-6-augustus-gene-84.43-mRNA-1	6	OsR498G0612038100.01.T01	99,83	0,00E+00	7419	Os06g0258900	100,00	0,00E+00	7451	32	NA	Japonica	
maker-6-augustus-gene-85.0-mRNA-1	6	OsR498G0612043200.01.T01	99,88	0,00E+00	1527	Os06g0260000	100,00	0,00E+00	1451	-76	NA	Japonica	
maker-6-augustus-gene-88.32-mRNA-1	6	OsR498G0612055700.01.T01	99,84	0,00E+00	2232	Os06g0264300	100,00	0,00E+00	2241	9	NA	Japonica	
maker-6-augustus-gene-88.38-mRNA-1	6	OsR498G0612057700.01.T01	99,93	0,00E+00	2701	Os06g0264500	99,93	0,00E+00	2701	0	NA	Japonica	
maker-6-augustus-gene-88.41-mRNA-1	6	OsR498G0612058800.01.T01	100,00	0,00E+00	850	Os06g0264800	100,00	0,00E+00	850	0	NA	Japonica	
maker-6-augustus-gene-88.44-mRNA-1	6	OsR498G0612059200.01.T03	95,06	0,00E+00	1082	Os06g0264900	100,00	0,00E+00	1142	60	NA	Japonica	
maker-6-augustus-gene-88.51-mRNA-1	6	OsR498G0612059400.01.T02	100,00	0,00E+00	1388	Os06g0265000	100,00	0,00E+00	1388	0	NA	NA	
maker-6-augustus-gene-89.0-mRNA-1	6	OsR498G0612082600.01.T01	92,28	0,00E+00	1873	Os06g0272800	70,09	1,26E-124	447	-1426	Indica	NA	
maker-6-augustus-gene-89.12-mRNA-1	6	OsR498G0612063600.01.T01	99,76	0,00E+00	745	Os02g0731400	70,19	5,58E-41	167	-578	Indica	Indica	
maker-6-augustus-gene-89.14-mRNA-1	6	OsR498G0612064200.01.T01	100,00	1,03E-174	612	Os06g0266800	96,55	6,95E-121	432	-180	Indica	Indica	
maker-6-augustus-gene-89.16-mRNA-1	6	OsR498G0612064600.01.T01	96,69	0,00E+00	1660	Os06g0267400	96,75	0,00E+00	1588	-72	NA	NA	
maker-6-augustus-gene-89.3-mRNA-1	6	OsR498G0612065100.01.T01	98,64	0,00E+00	2177	Os06g0267500	98,52	0,00E+00	1286	-891	Indica	NA	
maker-6-augustus-gene-89.8-mRNA-1	6	OsR498G0612061600.01.T03	99,71	0,00E+00	2502	Os06g0265400	99,91	0,00E+00	2085	-417	Indica	Indica	
maker-6-augustus-gene-9.13-mRNA-1	6	OsR498G0611513900.01.T05	100,00	0,00E+00	1234	Os06g0116600	100,00	0,00E+00	820	-414	Indica	NA	
maker-6-augustus-gene-9.17-mRNA-1	6	OsR498G0611510400.01.T01	100,00	1,00E-136	486	Os06g0115300	100,00	3,49E-137	486	0	NA	NA	
maker-6-augustus-gene-9.19-mRNA-1	6	OsR498G0611510600.01.T01	97,96	0,00E+00	2030	Os06g0115400	97,96	0,00E+00	2030	0	NA	NA	
maker-6-augustus-gene-9.24-mRNA-1	6	OsR498G0611510800.01.T01	100,00	0,00E+00	1131	Os06g0115500	100,00	0,00E+00	1131	0	NA	NA	
maker-6-augustus-gene-9.27-mRNA-1	6	OsR498G0611511400.01.T01	100,00	0,00E+00	2013	Os07g0159500	95,07	0,00E+00	1764	-249	Indica	NA	
maker-6-augustus-gene-9.3-mRNA-1	6	OsR498G0611512100.01.T03	100,00	0,00E+00	2614	Os06g0116100	100,00	0,00E+00	2614	0	NA	NA	Kinase-TM
maker-6-augustus-gene-9.30-mRNA-1	6	OsR498G0611512600.01.T03	98,88	0,00E+00	1884	Os06g0116200	100,00	0,00E+00	1880	-4	NA	NA	
maker-6-augustus-gene-9.33-mRNA-1	6	OsR498G0611513200.01.T02	100,00	0,00E+00	1452	Os06g0116300	100,00	0,00E+00	877	-575	Indica	NA	
maker-6-augustus-gene-9.38-mRNA-1	6	OsR498G0611513600.01.T01	100,00	0,00E+00	1992	Os06g0116400	100,00	0,00E+00	1992	0	NA	NA	
maker-6-augustus-gene-9.40-mRNA-1	6	OsR498G0611514400.01.T02	96,09	0,00E+00	2205	Os06g0116800	96,09	0,00E+00	2205	0	NA	NA	
maker-6-augustus-gene-9.8-mRNA-1	6	OsR498G0611513700.01.T01	97,49	0,00E+00	3467	Os02g0102800	76,06	0,00E+00	1383	-2084	Indica	NA	
maker-6-augustus-gene-91.6-mRNA-1	6	OsR498G0612074400.01.T02	99,91	0,00E+00	1957	Os06g0270200	99,91	0,00E+00	1957	0	NA	NA	
maker-6-augustus-gene-92.0-mRNA-1	6	OsR498G0612075300.01.T02	99,82	0,00E+00	2963	Os06g0270900	99,46	0,00E+00	2936	-27	NA	Indica	

maker-6-augustus-gene-92.10-mRNA-1	6	OsR498G0612083900.01.T01	98,29	0,00E+00	2128	Os06g0273500	99,92	0,00E+00	2198	70	NA	Japonica	
maker-6-augustus-gene-92.12-mRNA-1	6	OsR498G0612078200.01.T01	96,37	0,00E+00	5425	Os06g0272000	96,38	0,00E+00	5392	-33	NA	NA	Kinase-LRR-TM
maker-6-augustus-gene-92.18-mRNA-1	6	OsR498G0612082800.01.T01	96,65	0,00E+00	1626	Os06g0272900	97,29	0,00E+00	2229	603	Japonica	NA	
maker-6-augustus-gene-92.8-mRNA-1	6	OsR498G0612083700.01.T01	95,01	0,00E+00	2098	Os06g0273200	99,85	0,00E+00	2367	269	Japonica	Japonica	
maker-6-augustus-gene-93.28-mRNA-1	6	OsR498G0612084700.01.T01	89,29	4,84E-104	379	Os06g0274000	85,52	8,16E-96	350	-29	NA	NA	
maker-6-augustus-gene-93.31-mRNA-1	6	OsR498G0612085100.01.T01	99,11	0,00E+00	796	Os06g0274200	99,11	0,00E+00	796	0	NA	NA	
maker-6-augustus-gene-93.36-mRNA-1	6	OsR498G0612087300.01.T01	87,74	0,00E+00	954	Os06g0274500	79,70	0,00E+00	705	-249	Indica	NA	LRR-TM
maker-6-augustus-gene-93.48-mRNA-1	6	OsR498G0612084600.01.T03	90,62	0,00E+00	865	Os06g0273800	90,62	0,00E+00	865	0	NA	NA	
maker-6-augustus-gene-93.67-mRNA-1	6	OsR498G0612088200.01.T01	100,00	0,00E+00	1813	Os06g0274800	100,00	0,00E+00	1813	0	NA	NA	
maker-6-augustus-gene-94.12-mRNA-1	6	OsR498G0612095700.01.T02	97,65	0,00E+00	2324	Os06g0275800	97,94	0,00E+00	2340	16	NA	NA	
maker-6-augustus-gene-94.15-mRNA-1	6	OsR498G0612096100.01.T01	95,92	0,00E+00	3778	Os06g0275900	96,05	0,00E+00	3791	13	NA	NA	
maker-6-augustus-gene-94.21-mRNA-1	6	OsR498G0612093400.01.T03	97,83	0,00E+00	5283	Os06g0275700	97,79	0,00E+00	5021	-262	NA	NA	
maker-6-augustus-gene-94.6-mRNA-1	6	OsR498G0612093100.01.T03	95,81	0,00E+00	2164	Os06g0275600	95,81	0,00E+00	2164	0	NA	Indica	
maker-6-augustus-gene-95.0-mRNA-1	6	OsR498G0612116600.01.T01	98,05	0,00E+00	2111	Os01g0550800	90,90	0,00E+00	1729	-382	Indica	NA	
maker-6-augustus-gene-97.0-mRNA-1	6	OsR498G0613178300.01.T01	95,73	0,00E+00	766	Os03g0615700	71,70	3,22E-38	158	-608	Indica	NA	
maker-6-augustus-gene-97.2-mRNA-1	6	OsR498G0612134400.01.T01	99,29	0,00E+00	1988	Os06g0281300	98,67	0,00E+00	1840	-148	NA	Indica	
maker-6-augustus-gene-98.0-mRNA-1	6	OsR498G0612142500.01.T01	99,70	0,00E+00	4101	Os06g0283200	100,00	0,00E+00	4134	33	NA	Japonica	
maker-6-augustus-gene-98.2-mRNA-1	6	OsR498G0612142700.01.T02	99,77	0,00E+00	2341	Os06g0283300	99,77	0,00E+00	2341	0	NA	NA	Kinase-TM
maker-6-augustus-gene-99.26-mRNA-1	6	OsR498G0612148900.01.T01	99,37	0,00E+00	1687	Os06g0284800	100,00	0,00E+00	1710	23	NA	Japonica	
maker-6-augustus-gene-99.29-mRNA-1	6	OsR498G0612146400.01.T03	99,84	0,00E+00	2305	Os06g0284200	100,00	0,00E+00	2315	10	NA	Japonica	
augustus_masked-7-processed-gene-253.24-mRNA-1	7	OsR498G0714765300.01.T03	100,00	1,31E-140	499	Os07g0619200	100,00	4,58E-141	499	0	NA	Japonica	
augustus_masked-7-processed-gene-74.35-mRNA-1	7	OsR498G0713860700.01.T01	99,73	0,00E+00	2668	Os07g0240600	100,00	0,00E+00	2571	-97	NA	Japonica	
augustus_masked-7-processed-gene-89.22-mRNA-1	7	OsR498G0713942500.01.T04	99,51	4,86E-100	364	Os07g0264000	100,00	3,98E-102	369	5	NA	Japonica	
augustus-7-processed-gene-0.5-mRNA-1	7	OsR498G0713353000.01.T01	99,58	0,00E+00	1709	Os07g0100200	99,79	0,00E+00	1718	9	NA	NA	
augustus-7-processed-gene-0.7-mRNA-1	7	OsR498G0713355400.01.T02	99,78	0,00E+00	1647	Os07g0100900	99,67	0,00E+00	1643	-4	NA	NA	
augustus-7-processed-gene-1.0-mRNA-1	7	OsR498G0713357100.01.T04	99,78	0,00E+00	3980	Os07g0101200	99,96	0,00E+00	3994	14	NA	Indica	
augustus-7-processed-gene-1.24-mRNA-1	7	OsR498G0713361200.01.T01	100,00	0,00E+00	765	Os07g0102500	100,00	0,00E+00	765	0	NA	NA	
augustus-7-processed-gene-10.16-mRNA-1	7	OsR498G0713445900.01.T01	100,00	0,00E+00	937	Os07g0127500	100,00	0,00E+00	937	0	NA	Indica	
augustus-7-processed-gene-10.20-mRNA-1	7	OsR498G0713447700.01.T01	100,00	0,00E+00	931	Os01g0382400	70,35	8,97E-27	120	-811	Indica	Indica	
augustus-7-processed-gene-100.22-mRNA-1	7	OsR498G1221660800.01.T01	70,45	6,41E-81	303	Os07g0277400	99,87	0,00E+00	1374	1071	Japonica	Japonica	
augustus-7-processed-gene-103.25-mRNA-1	7	OsR498G0713993200.01.T01	99,72	1,39E-179	629	Os07g0281200	100,00	0,00E+00	634	5	NA	Japonica	
augustus-7-processed-gene-104.13-mRNA-1	7	OsR498G0713999000.01.T01	99,55	0,00E+00	3160	Os07g0283050	100,00	0,00E+00	3196	36	NA	Japonica	Kinase-TM
augustus-7-processed-gene-109.15-mRNA-1	7	OsR498G0714023700.01.T01	96,80	1,60E-146	518	Os07g0290200	77,91	8,29E-50	196	-322	Indica	Japonica	
augustus-7-processed-gene-109.21-mRNA-1	7	OsR498G0714025000.01.T01	98,99	0,00E+00	1572	Os01g0860500	70,83	3,72E-104	378	-1194	Indica	Japonica	
augustus-7-processed-gene-109.35-mRNA-1	7	OsR498G0714026000.01.T01	98,67	0,00E+00	921	Os07g0291100	100,00	0,00E+00	953	32	NA	Japonica	
augustus-7-processed-gene-11.19-mRNA-1	7	OsR498G0713452700.01.T01	100,00	0,00E+00	915	Os07g0129200	100,00	0,00E+00	915	0	NA	NA	
augustus-7-processed-gene-11.21-mRNA-1	7	OsR498G0713453500.01.T01	100,00	0,00E+00	899	Os07g0129300	100,00	0,00E+00	899	0	NA	NA	
augustus-7-processed-gene-11.23-mRNA-1	7	OsR498G0713453500.01.T01	100,00	0,00E+00	899	Os07g0129300	100,00	0,00E+00	899	0	NA	NA	
augustus-7-processed-gene-11.6-mRNA-1	7	OsR498G0713449300.01.T01	100,00	0,00E+00	937	Os07g0129200	90,79	1,39E-176	618	-319	Indica	Indica	
augustus-7-processed-gene-113.19-mRNA-1	7	OsR498G0714043200.01.T03	97,52	0,00E+00	1295	Os07g0297300	99,87	0,00E+00	1383	88	NA	Japonica	
augustus-7-processed-gene-116.5-mRNA-1	7	OsR498G0714058400.01.T01	98,57	0,00E+00	1098	Os07g0302100	100,00	0,00E+00	1137	39	NA	Japonica	
augustus-7-processed-gene-12.18-mRNA-1	7	OsR498G0713460400.01.T01	100,00	0,00E+00	3690	Os07g0130100	99,90	0,00E+00	3681	-9	NA	Indica	Kinase-TM
augustus-7-processed-gene-12.19-mRNA-1	7	OsR498G0713461200.01.T01	100,00	0,00E+00	3728	Os07g0130200	99,76	0,00E+00	3706	-22	NA	Indica	Kinase-TM
augustus-7-processed-gene-12.20-mRNA-1	7	OsR498G0713999000.01.T01	72,06	0,00E+00	966	Os07g0130300	100,00	0,00E+00	3636	2670	Japonica	Indica	Kinase-TM
augustus-7-processed-gene-12.22-mRNA-1	7	OsR498G0916878800.01.T01	70,78	0,00E+00	909	Os07g0130600	99,95	0,00E+00	3605	2696	Japonica	NA	Kinase-TM
augustus-7-processed-gene-12.23-mRNA-1	7	OsR498G0713461800.01.T01	100,00	0,00E+00	1948	Os07g0130700	99,95	0,00E+00	3497	1549	Japonica	NA	Kinase-TM
augustus-7-processed-gene-12.25-mRNA-1	7	OsR498G0713462000.01.T01	100,00	0,00E+00	3663	Os07g0130800	100,00	0,00E+00	3663	0	NA	Indica	Kinase-TM
augustus-7-processed-gene-12.31-mRNA-1	7	OsR498G0713464000.01.T01	100,00	0,00E+00	3382	Os07g0132150	98,52	0,00E+00	3400	18	NA	Indica	Kinase-TM
augustus-7-processed-gene-123.1-mRNA-1	7	OsR498G0714077500.01.T01	99,87	0,00E+00	1419	Os07g0407900	100,00	0,00E+00	1424	5	NA	Japonica	
augustus-7-processed-gene-124.34-mRNA-1	7	OsR498G0714085000.01.T05	99,83	0,00E+00	3103	Os07g0409400	100,00	0,00E+00	3117	14	NA	Japonica	
augustus-7-processed-gene-124.37-mRNA-1	7	OsR498G0714085700.01.T01	99,83	0,00E+00	1030	Os07g0409500	100,00	0,00E+00	1034	4	NA	Japonica	
augustus-7-processed-gene-125.1-mRNA-1	7	OsR498G0714091600.01.T01	100,00	0,00E+00	1299	Os07g0410700	100,00	0,00E+00	1299	0	NA	Japonica	
augustus-7-processed-gene-127.1-mRNA-1	7	OsR498G0714098100.01.T01	98,71	0,00E+00	2443	Os07g0414000	100,00	0,00E+00	2538	95	NA	Japonica	
augustus-7-processed-gene-127.20-mRNA-1	7	OsR498G0714100900.01.T01	100,00	0,00E+00	1067	Os07g0415200	100,00	0,00E+00	1067	0	NA	Japonica	
augustus-7-processed-gene-128.21-mRNA-1	7	OsR498G0714108300.01.T01	98,81	0,00E+00	1906	Os07g0417200	100,00	0,00E+00	2073	167	NA	Japonica	
augustus-7-processed-gene-128.31-mRNA-1	7	OsR498G0714107400.01.T02	98,30	0,00E+00	2026	Os07g0416900	100,00	0,00E+00	2116	90	NA	Japonica	
augustus-7-processed-gene-129.18-mRNA-1	7	OsR498G0714110200.01.T01	98,80	0,00E+00	1313	Os07g0417600	100,00	0,00E+00	1353	40	NA	Japonica	
augustus-7-processed-gene-13.27-mRNA-1	7	OsR498G0713465300.01.T01	100,00	0,00E+00	3718	Os07g0130200	70,59	0,00E+00	889	-2829	Indica	Indica	Kinase-TM
augustus-7-processed-gene-13.37-mRNA-1	7	OsR498G0713465800.01.T01	100,00	0,00E+00	2192	Os07g0133100	84,29	0,00E+00	883	-1309	Indica	Indica	Kinase-TM
augustus-7-processed-gene-130.12-mRNA-1	7	OsR498G0714114600.01.T01	97,98	0,00E+00	1269	Os07g0418600	100,00	0,00E+00	1342	73	NA	Japonica	
augustus-7-processed-gene-130.17-mRNA-1	7	OsR498G0714118700.01.T07	100,00	0,00E+00	3059	Os04g0131900	75,94	0,00E+00	1046	-2013	Indica	Japonica	
augustus-7-processed-gene-130.25-mRNA-1	7	OsR498G0714820100.01.T01	82,60	6,45E-135	481	Os07g0418500	98,35	0,00E+00	841	360	Japonica	Japonica	
augustus-7-processed-gene-130.28-mRNA-1	7	OsR498G0714121200.01.T03	99,92	0,00E+00	2182	Os07g0419800	99,87	0,00E+00	1363	-819	Indica	Japonica	
augustus-7-processed-gene-130.6-mRNA-1	7	OsR498G0714114600.01.T01	95,97	0,00E+00	1207	Os07g0418700	100,00	0,00E+00	1342	135	Japonica	Japonica	
augustus-7-processed-gene-131.27-mRNA-1	7	OsR498G0714126600.01.T02	99,50	0,00E+00	2132	Os07g0421000	99,82	0,00E+00	1984	-148	NA	Japonica	
augustus-7-processed-gene-131.8-mRNA-1	7	OsR498G0714121900.01.T01	98,97	0,00E+00	1361	Os11g0701800	70,03	3,36E-85	315	-1046	Indica	Japonica	
augustus-7-processed-gene-132.14-mRNA-1	7	OsR498G0714125300.01.T05	82,49	1,91E-44	180	Os07g0421300	100,00	2,03E-108	390	210	Japonica	Japonica	
augustus-7-processed-gene-133.14-mRNA-1	7	OsR498G0714131800.01.T03	99,67	0,00E+00	2195	Os07g0423000	100,00	0,00E+00	2214	19	NA	Japonica	
augustus-7-processed-gene-142.18-mRNA-1	7	OsR498G0714158500.01.T05	99,83	0,00E+00	2085	Os07g0436600	100,00	0,00E+00	2787	702	Japonica	Japonica	
augustus-7-processed-gene-142.27-mRNA-1	7	OsR498G0714159000.01.T02	99,83	0,00E+00	2145	Os07g0437000	99,75	0,00E+00	2144	-1	NA	Japonica	
augustus-7-processed-gene-142.9-mRNA-1	7	OsR498G0714156900.01.T02	99,90	0,00E+00	3698	Os07g0435900	100,00	0,00E+00	3707	9	NA	Japonica	
augustus-7-processed-gene-143.5-mRNA-1	7	OsR498G0714162100.01.T05	92,04	0,00E+00	1205	Os07g0437500	92,17	0,00E+00	1210	5	NA	Japonica	
augustus-7-processed-gene-144.41-mRNA-1	7	OsR498G0714170000.01.T03	70,35	4,38E-123	443	Os07g0440100	70,79	5,67E-129	461	18	NA	Japonica	
augustus-7-processed-gene-146.14-mRNA-1	7	OsR498G0714178700.01.T01	99,63	9,82E-136	483	Os07g0443500	100,00	5,10E-115	412	-71	Indica	Japonica	
augustus-7-processed-gene-149.7-mRNA-1	7	OsR498G0509895600.01.T01	71,04	0,00E+00	679	Os07g0446800	100,00	0,00E+00	2700	2021	Japonica	Japonica	
augustus-7-processed-gene-152.6-mRNA-1	7	OsR498G0714197900.01.T01	84,71	0,00E+00	853	Os07g0451300	100,00	0,00E+00	2765	1912	Japonica	Japonica	
augustus-7-processed-gene-155.23-mRNA-1	7	OsR498G0714218600.01.T01	99,57	0,00E+00	4159	Os07g0456900	99,96	0,00E+00	4200	41	NA	Japonica	
augustus-7-processed-gene-156.20-mRNA-1	7	OsR498G0714219900.01.T01	99,36	0,00E+00	1662	Os07g0457400	100,00	0,00E+00	1321	-341	Indica	Japonica	
augustus-7-processed-gene-158.9-mRNA-1	7	OsR498G0509914200.01.T01	84,00	6,94E-121	434	Os07g0460100	99,75	0,00E+00	727	293	Japonica	Indica	
augustus-7-processed-gene-159.35-mRNA-1	7	OsR498G0714240400.01.T04	99,87	0,00E+00	2681	Os05g0129000	97,79	0,00E+00	2543	-138	NA	Japonica	
augustus-7-processed-gene-16.18-mRNA-1	7	OsR498G0713482500.01.T01	100,00	0,00E+00	9414	Os07g0137900	100,00	0,00E+00	6087	-3327	Indica	NA	
augustus-7-processed-gene-165.32-mRNA-1	7	OsR498G0714265800.01.T02	100,00	9,98E-149	526	Os07g0470400	100,00	3,47E-149	526	0	NA	Japonica	
augustus-7-processed-gene-169.15-mRNA-1	7	OsR498G0714287500.01.T01	100,00	0,00E+00	655	Os07g0475700	100,00	0,00E+00	655	0	NA	Japonica	
augustus-7-processed-gene-17.0-mRNA-1	7	OsR498G0713487700.01.T06	99,76	0,00E+00	2270	Os07g0139400	99,76	0,00E+00	2270	0	NA	NA	
augustus-7-processed-gene-17.17-mRNA-1	7	OsR498G0713493700.01.T04	99,86	0,00E+00	2616	Os07g0141600	99,86	0,00E+00	2616	0	NA	NA	
augustus-7-processed-gene-17.28-mRNA-1	7	OsR498G0713488800.01.T01	96,33	0,00E+00	989	Os03g0233200	93,65	5,67E-163	573	-416	Indica	NA	
augustus-7-processed-gene-170.35-mRNA-1	7	OsR498G0714295500.01.T01	99,16	0,00E+00	4221	Os07g0477500	99,37	0,00E+00	4243	22	NA	Japonica	
augustus-7-processed-gene-174.27-mRNA-1	7	OsR498G0714312400.01.T04	99,90	0,00E+00	5462	Os07g0483500	100,00	0,00E+00	5476	14	NA	Japonica	
augustus-7-processed-gene-175.13-mRNA-1	7	OsR498G0714317500.01.T02	99,43	0,00E+00	1256	Os07g0485000	100,00	0,00E+00	1272	16	NA	Japonica	
augustus-7-processed-gene-175.9-mRNA-1	7	OsR498G0714315500.01.T02	93,64	0,00E+00	1292	Os07g0484500	94,00	0,00E+00	1306	14	NA	Japonica	
augustus-7-processed-gene-177.15-mRNA-1	7	OsR498G0714323900.01.T01	94,27	0,00E+00	2304	Os07g0487100	100,00	0,00E+00	2636	332	Japonica	Japonica	
augustus-7-processed-gene-178.20-mRNA-1	7	OsR498G0714328400.01.T01	99,28	0,00E+00	2466	Os07g0489200	99,35	0,00E+00	2470	4	NA	NA	
augustus-7-processed-gene-178.21-mRNA-1	7	OsR498G0714329200.01.T01	99,60	0,00E+00	2670	Os07g0489300	99,60	0,00E+00	2247	-423	Indica	NA	
augustus-7-processed-gene-179.47-mRNA-1	7	OsR498G0714332300.01.T01	99,19	0,00E+00	2603	Os07g0489950	99,46	0,00E+00	2621	18	NA	NA	
augustus-7-processed-gene-179.48-mRNA-1	7	OsR498G0714332300.01.T01	87,70	0,00E+00	1802	Os07g0490100	99,86	0,00E+00	2594	792	Japonica	NA	
augustus-7-processed-gene-179.66-mRNA-1	7	OsR498G0714340300.01.T01	100,00	0,00E+00	2419	Os07g0491500	99,75	0,00E+00	3522	1103	Japonica	NA	
augustus-7-processed-gene-18.0-mRNA-1	7	OsR498G0713494900.01.T01	100,00	0,00E+00	1337	Os07g0142000	100,00	0,00E+00	1337	0	NA	NA	
augustus-7-processed-gene-18.1-mRNA-1	7	OsR498G0713495500.01.T01	99,61	0,00E+00	911	Os07g0142200	99,61	0,00E+00	911	0	NA	NA	
augustus-7-processed-gene-18.13-mRNA-1	7	OsR498G0713496100.01.T01	96,80	0,00E+00	783	Os07g0142600	96,80	0,00E+00	783	0	NA	NA	
augustus-7-processed-gene-18.31-mRNA-1	7	OsR498G0713496600.01.T01	99,72	0,00E+00	2554	Os07g0142700	99,72	0,00E+00	2554	0	NA	NA	
augustus-7-processed-gene-181.23-mRNA-1	7	OsR498G0714347500.01.T01	83,90	0,00E+00	2092	Os07g0493800	83,80	0,00E+00	2083	-9	NA	Indica	Kinase-TM
augustus-7-processed-gene-184.26-mRNA-1	7	OsR498G0714367900.01.T01	99,75	0,00E+00	1458	Os02g0452800	70,80	5,71E-76	284	-1174	Indica	Indica	
augustus-7-processed-gene-185.14-mRNA-1	7	OsR498G0714373300.01.T01	99,80	0,00E+00	900	Os07g0501700	99,80	0,00E+00	900	0	NA	NA	
augustus-7-processed-gene-186.13-mRNA-1	7	OsR498G0714376200.01.T01	78,89	0,00E+00	1227	Os07g0503300	99,87	0,00E+00	2681	1454	Japonica	NA	
augustus-7-processed-gene-190.4-mRNA-1	7	OsR498G0714398700.01.T01	99,82	0,00E+00	1939	Os07g0508900	99,82	0,00E+00	1939	0	NA	NA	
augustus-7-processed-gene-191.7-mRNA-1	7	OsR498G0714405200.01.T01	99,17	0,00E+00	2560	Os07g0510400	99,17	0,00E+00	2560	0	NA	NA	
augustus-7-processed-gene-192.0-mRNA-1	7	OsR498G0714407900.01.T01	97,32	0,00E+00	1835	Os07g0511100	99,35	0,00E+00	1917	82	NA	NA	
augustus-7-processed-gene-193.51-mRNA-1	7	OsR498G0714418200.01.T02	99,91	0,00E+00	4062	Os07g0513200	99,82	0,00E+00	4053	-9	NA	Japonica	
augustus-7-processed-gene-193.58-mRNA-1	7	OsR498G0306608800.01.T06	70,00	1,12E-15	84,2	Os07g0514801	95,30	1,26E-142	505	420,8	Japonica	Indica	
augustus-7-processed-gene-194.16-mRNA-1	7	OsR498G0714424300.01.T03	96,30	0,00E+00	1843	Os07g0515700	99,65	0,00E+00	2039	196	NA	NA	
augustus-7-processed-gene-195.13-mRNA-1	7	OsR498G0714428500.01.T01	99,42	1,27E-173	609	Os07g0516400	99,42	4,42E-174	609	0	NA	NA	
augustus-7-processed-gene-196.1-mRNA-1	7	OsR498G0714434300.01.T01	99,33	0,00E+00	2379	Os07g0517700	99,31	0,00E+00	2314	-65	NA	NA	
augustus-7-processed-gene-197.81-mRNA-1	7	OsR498G0714438700.01.T01	97,80	0,00E+00	2493	Os07g0518500	97,80	0,00E+00	2493	0	NA	NA	
augustus-7-processed-gene-197.89-mRNA-1	7	OsR498G0714443500.01.T01	98,86	0,00E+00	1688	Os07g0520300	98,86	0,00E+00	1688	0	NA	NA	
augustus-7-processed-gene-198.23-mRNA-1	7	OsR498G0714451300.01.T01	100,00	0,00E+00	4807	Os07g0521800	100,00	0,00E+00	4807	0	NA	Indica	CC-NBS-TM
augustus-7-processed-gene-200.12-mRNA-1	7	OsR498G0714469300.01.T01	94,04	0,00E+00	2069	Os07g0527600	89,22	0,00E+00	1293	-776	Indica	NA	
augustus-7-processed-gene-200.18-mRNA-1	7	OsR498G0714472500.01.T01	99,86	0,00E+00	2491	Os07g0526600	99,35	0,00E+00	2458	-33	NA	Indica	
augustus-7-processed-gene-205.42-mRNA-1	7	OsR498G0714494400.01.T01	99,93	0,00E+00	2480	Os07g0533800	99,93	0,00E+00	2480	0	NA	Indica	
augustus-7-processed-gene-206.27-mRNA-1	7	OsR498G0714500100.01.T01	99,83	0,00E+00	2058	Os07g0535700	99,70	0,00E+00	1805	-253	Indica	NA	
augustus-7-processed-gene-21.26-mRNA-1	7	OsR498G1221267000.01.T01	73,71	8,73E-71	269	Os07g0148600	100,00	0,00E+00	969	700	Japonica	Japonica	
augustus-7-processed-gene-21.6-mRNA-1	7	OsR498G0713511000.01.T02	99,80	0,00E+00	4577	Os07g0147600	100,00	0,00E+00	4599	22	NA	Japonica	Kinase-TM
augustus-7-processed-gene-210.26-mRNA-1	7	OsR498G0714520000.01.T02	97,37	0,00E+00	1347	Os07g0542400	90,54	0,00E+00	1087	-260	Indica	Japonica	
augustus-7-processed-gene-211.28-mRNA-1	7	OsR498G0714523900.01.T01	98,12	0,00E+00	1281	Os07g0543600	99,28	0,00E+00	1728	447	Japonica	Japonica	
augustus-7-processed-gene-212.0-mRNA-1	7	OsR498G0714527300.01.T01	98,83	0,00E+00	3005	Os07g0545800	100,00	0,00E+00	3095	90	NA	Japonica	
augustus-7-processed-gene-212.12-mRNA-1	7	OsR498G0714529100.01.T04	99,53	0,00E+00	3041	Os07g0546400	100,00	0,00E+00	2565	-476	Indica	Japonica	
augustus-7-processed-gene-213.14-mRNA-1	7	OsR498G0714534100.01.T01	92,90	0,00E+00	1612	Os07g0548500	100,00	0,00E+00	1131	-481	Indica	Japonica	
augustus-7-processed-gene-213.24-mRNA-1	7	OsR498G0714534100.01.T01	71,41	1,26E-122	441	Os07g0548000	99,91	0,00E+00	1890	1449	Japonica	Japonica	
augustus-7-processed-gene-214.36-mRNA-1	7	OsR498G0714537700.01.T01	100,00	1,80E-158	558	Os07g0549900	100,00	6,27E-159	558	0	NA	Japonica	
augustus-7-processed-gene-216.33-mRNA-1	7	OsR498G0714550000.01.T01	99,64	0,00E+00	4424	Os07g0553633	100,00	0,00E+00	4464	40	NA	Japonica	Kinase-TM
augustus-7-processed-gene-216.35-mRNA-1	7	OsR498G0714554100.01.T01	84,64	0,00E+00	1359	Os07g0554200	99,84	0,00E+00	2210	851	Japonica	Indica	
augustus-7-processed-gene-217.17-mRNA-1	7	OsR498G0714554100.01.T01	99,92	0,00E+00	2241	Os07g0554500	99,76	0,00E+00	2232	-9	NA	NA	
augustus-7-processed-gene-217.19-mRNA-1	7	OsR498G0714555500.01.T02	99,49	0,00E+00	1388	Os07g0554900	99,07	0,00E+00	2266	878	Japonica	NA	
augustus-7-processed-gene-217.31-mRNA-1	7	OsR498G0714555900.01.T01	99,84	0,00E+00	2172	Os07g0555100	99,84	0,00E+00	2172	0	NA	NA	
augustus-7-processed-gene-217.38-mRNA-1	7	OsR498G0714559100.01.T01	100,00	0,00E+00	1223	Os07g0556200	100,00	0,00E+00	1223	0	NA	NA	
augustus-7-processed-gene-219.36-mRNA-1	7	OsR498G0714569500.01.T01	100,00	2,12E-111	401	Os07g0559100	100,00	1,18E-96	351	-50	Indica	Japonica	
augustus-7-processed-gene-22.35-mRNA-1	7	OsR498G0713519100.01.T01	100,00	0,00E+00	753	Os07g0150200	100,00	0,00E+00	931	178	Japonica	NA	
augustus-7-processed-gene-220.17-mRNA-1	7	OsR498G0714577900.01.T01	99,38	0,00E+00	2845	Os07g0562400	99,44	0,00E+00	2849	4	NA	Japonica	
augustus-7-processed-gene-220.35-mRNA-1	7	OsR498G0714577600.01.T01	99,51	0,00E+00	2911	Os07g0562300	96,82	0,00E+00	2725	-186	NA	NA	
augustus-7-processed-gene-221.2-mRNA-1	7	OsR498G0714629300.01.T01	99,25	0,00E+00	1172	Os07g0579000	99,69	0,00E+00	1171	-1	NA	NA	
augustus-7-processed-gene-222.32-mRNA-1	7	OsR498G0714585000.01.T01	95,16	6,89E-171	600	Os07g0564200	100,00	1,62E-116	417	-183	Indica	Japonica	
augustus-7-processed-gene-223.21-mRNA-1	7	OsR498G0714589800.01.T01	99,15	1,72E-178	626	Os07g0565800	100,00	0,00E+00	639	13	NA	Japonica	
augustus-7-processed-gene-223.6-mRNA-1	7	OsR498G0714587700.01.T03	100,00	0,00E+00	1276	Os04g0613400	99,86	0,00E+00	1271	-5	NA	Japonica	
augustus-7-processed-gene-224.10-mRNA-1	7	OsR498G0714593500.01.T01	99,69	0,00E+00	1704	Os07g0567000	100,00	0,00E+00	1718	14	NA	Japonica	
augustus-7-processed-gene-224.26-mRNA-1	7	OsR498G0714592800.01.T01	99,61	0,00E+00	2283	Os07g0566500	100,00	0,00E+00	2305	22	NA	Japonica	
augustus-7-processed-gene-225.2-mRNA-1	7	OsR498G0714596900.01.T03	97,77	0,00E+00	3467	Os07g0568300	100,00	0,00E+00	3561	94	NA	Japonica	
augustus-7-processed-gene-226.0-mRNA-1	7	OsR498G0714601500.01.T01	96,88	0,00E+00	1333	Os07g0569500	100,00	0,00E+00	1445	112	NA	Japonica	
augustus-7-processed-gene-226.3-mRNA-1	7	OsR498G0714602900.01.T01	94,44	0,00E+00	1480	Os07g0569800	100,00	0,00E+00	3187	1707	Japonica	Japonica	Kinase-TM
augustus-7-processed-gene-227.42-mRNA-1	7	OsR498G0714609700.01.T02	99,63	0,00E+00	1455	Os06g0338200	78,89	0,00E+00	666	-789	Indica	NA	
augustus-7-processed-gene-227.45-mRNA-1	7	OsR498G0714610500.01.T01	99,53	0,00E+00	1525	Os07g0572300	99,53	0,00E+00	1525	0	NA	NA	
augustus-7-processed-gene-229.3-mRNA-1	7	OsR498G0714613300.01.T01	100,00	0,00E+00	710	Os07g0573200	100,00	0,00E+00	666	-44	NA	Japonica	
augustus-7-processed-gene-23.2-mRNA-1	7	OsR498G0713525100.01.T01	99,31	0,00E+00	1027	Os04g0393500	73,70	1,71E-55	216	-811	Indica	Japonica	
augustus-7-processed-gene-230.27-mRNA-1	7	OsR498G0714620600.01.T01	99,81	0,00E+00	3759	Os07g0575600	99,90	0,00E+00	3768	9	NA	Japonica	Kinase-TM
augustus-7-processed-gene-230.37-mRNA-1	7	OsR498G0714622300.01.T01	99,78	0,00E+00	817	Os07g0576100	99,78	0,00E+00	819	2	NA	Japonica	
augustus-7-processed-gene-230.47-mRNA-1	7	OsR498G0714621000.01.T01	99,85	0,00E+00	3623	Os07g0575700	99,90	0,00E+00	3627	4	NA	Japonica	Kinase-TM
augustus-7-processed-gene-230.48-mRNA-1	7	OsR498G0714621200.01.T01	99,43	0,00E+00	3433	Os07g0575600	73,88	0,00E+00	1220	-2213	Indica	Japonica	Kinase-TM
augustus-7-processed-gene-231.4-mRNA-1	7	OsR498G0714622900.01.T06	97,02	0,00E+00	2608	Os07g0577300	96,95	0,00E+00	2603	-5	NA	Indica	
augustus-7-processed-gene-231.6-mRNA-1	7	OsR498G0714623100.01.T02	99,91	0,00E+00	1911	Os07g0577500	99,81	0,00E+00	1907	-4	NA	NA	
augustus-7-processed-gene-232.23-mRNA-1	7	OsR498G0714629300.01.T01	99,25	0,00E+00	1172	Os07g0579000	99,69	0,00E+00	1171	-1	NA	NA	
augustus-7-processed-gene-232.59-mRNA-1	7	OsR498G0714633000.01.T01	94,27	0,00E+00	4830	Os07g0580700	94,18	0,00E+00	4817	-13	NA	NA	
augustus-7-processed-gene-233.32-mRNA-1	7	OsR498G0714642400.01.T01	100,00	2,04E-101	369	Os07g0583000	100,00	7,09E-102	369	0	NA	Japonica	
augustus-7-processed-gene-234.40-mRNA-1	7	OsR498G0714645900.01.T01	99,88	0,00E+00	2940	Os07g0583600	100,00	0,00E+00	2949	9	NA	Japonica	
augustus-7-processed-gene-235.37-mRNA-1	7	OsR498G0714651700.01.T02	99,32	0,00E+00	1573	Os07g0585100	100,00	0,00E+00	1600	27	NA	Japonica	
augustus-7-processed-gene-235.43-mRNA-1	7	OsR498G0714655100.01.T01	98,74	0,00E+00	1801	Os07g0585700	100,00	0,00E+00	1862	61	NA	Japonica	
augustus-7-processed-gene-235.56-mRNA-1	7	OsR498G0714654200.01.T02	98,72	0,00E+00	2875	Os03g0431200	80,23	0,00E+00	1374	-1501	Indica	Japonica	
augustus-7-processed-gene-236.18-mRNA-1	7	OsR498G0714665300.01.T01	99,84	0,00E+00	2215	Os07g0587500	100,00	0,00E+00	1965	-250	Indica	Japonica	
augustus-7-processed-gene-236.26-mRNA-1	7	OsR498G0714664800.01.T03	100,00	0,00E+00	2370	Os07g0587400	100,00	0,00E+00	2370	0	NA	Japonica	
augustus-7-processed-gene-236.9-mRNA-1	7	OsR498G0714658200.01.T01	98,97	0,00E+00	1021	Os07g0586600	100,00	0,00E+00	1045	24	NA	Japonica	
augustus-7-processed-gene-237.10-mRNA-1	7	OsR498G0714669700.01.T01	71,80	4,35E-50	199	Os07g0588600	100,00	0,00E+00	1196	997	Japonica	Japonica	
augustus-7-processed-gene-237.12-mRNA-1	7	OsR498G0714669700.01.T01	98,25	0,00E+00	787	Os07g0588700	100,00	0,00E+00	1229	442	Japonica	Japonica	
augustus-7-processed-gene-237.13-mRNA-1	7	OsR498G0714670300.01.T04	99,76	0,00E+00	1485	Os07g0588800	100,00	0,00E+00	1494	9	NA	Japonica	
augustus-7-processed-gene-237.17-mRNA-1	7	OsR498G0714672500.01.T01	99,58	0,00E+00	2538	Os07g0589200	100,00	0,00E+00	2500	-38	NA	Japonica	
augustus-7-processed-gene-238.25-mRNA-1	7	OsR498G0714673400.01.T01	99,77	0,00E+00	1582	Os07g0589600	100,00	0,00E+00	1548	-34	NA	Japonica	
augustus-7-processed-gene-238.31-mRNA-1	7	OsR498G0714674100.01.T01	99,33	0,00E+00	1065	Os07g0590100	100,00	0,00E+00	1083	18	NA	Japonica	
augustus-7-processed-gene-238.32-mRNA-1	7	OsR498G0714675900.01.T01	99,77	0,00E+00	3164	Os07g0590600	100,00	0,00E+00	4248	1084	Japonica	Japonica	
augustus-7-processed-gene-238.49-mRNA-1	7	OsR498G0714678000.01.T01	100,00	0,00E+00	1186	Os07g0591100	100,00	0,00E+00	2062	876	Japonica	NA	
augustus-7-processed-gene-239.20-mRNA-1	7	OsR498G0714681300.01.T02	99,68	7,67E-157	554	Os07g0592000	99,68	2,67E-157	554	0	NA	NA	
augustus-7-processed-gene-239.21-mRNA-1	7	OsR498G0714681500.01.T02	99,67	0,00E+00	1616	Os07g0592100	99,78	0,00E+00	1620	4	NA	NA	
augustus-7-processed-gene-239.35-mRNA-1	7	OsR498G0714684100.01.T01	99,65	0,00E+00	1014	Os07g0593000	99,47	0,00E+00	1010	-4	NA	NA	
augustus-7-processed-gene-24.0-mRNA-1	7	OsR498G0713530300.01.T01	100,00	0,00E+00	2414	Os07g0152000	100,00	0,00E+00	2414	0	NA	NA	
augustus-7-processed-gene-241.42-mRNA-1	7	OsR498G0714697900.01.T01	100,00	1,38E-179	628	Os12g0230600	78,80	1,69E-46	185	-443	Indica	Indica	
augustus-7-processed-gene-241.46-mRNA-1	7	OsR498G0714699700.01.T01	99,33	0,00E+00	1325	Os07g0598200	99,47	0,00E+00	1330	5	NA	NA	
augustus-7-processed-gene-242.11-mRNA-1	7	OsR498G0714706300.01.T01	96,51	0,00E+00	2076	Os07g0600300	96,51	0,00E+00	2076	0	NA	NA	
augustus-7-processed-gene-243.15-mRNA-1	7	OsR498G0714710500.01.T01	98,22	0,00E+00	2119	Os07g0601600	100,00	0,00E+00	2224	105	NA	Japonica	
augustus-7-processed-gene-244.0-mRNA-1	7	OsR498G0714714100.01.T01	99,64	0,00E+00	5412	Os07g0602700	99,70	0,00E+00	5421	9	NA	NA	Kinase-LRR-TM
augustus-7-processed-gene-244.17-mRNA-1	7	OsR498G1018815700.01.T01	92,06	5,51E-149	528	Os07g0603500	99,52	0,00E+00	747	219	Japonica	NA	
augustus-7-processed-gene-244.2-mRNA-1	7	OsR498G0714714600.01.T01	99,82	0,00E+00	1999	Os07g0602800	98,14	0,00E+00	1577	-422	Indica	NA	
augustus-7-processed-gene-244.6-mRNA-1	7	OsR498G0714716900.01.T02	100,00	0,00E+00	3020	Os07g0603200	99,94	0,00E+00	3015	-5	NA	NA	
augustus-7-processed-gene-245.12-mRNA-1	7	OsR498G0714724300.01.T01	100,00	4,19E-147	520	Os07g0604700	100,00	1,46E-147	520	0	NA	Japonica	
augustus-7-processed-gene-245.24-mRNA-1	7	OsR498G0714723800.01.T02	99,85	0,00E+00	1203	Os07g0604500	100,00	0,00E+00	1207	4	NA	Japonica	
augustus-7-processed-gene-245.3-mRNA-1	7	OsR498G0714721300.01.T03	98,91	0,00E+00	2567	Os07g0603900	99,86	0,00E+00	2626	59	NA	Japonica	
augustus-7-processed-gene-246.22-mRNA-1	7	OsR498G0714728400.01.T01	99,81	0,00E+00	1853	Os07g0605800	100,00	0,00E+00	1862	9	NA	Japonica	
augustus-7-processed-gene-246.5-mRNA-1	7	OsR498G0714725500.01.T04	99,85	0,00E+00	1163	Os07g0605200	100,00	0,00E+00	1162	-1	NA	Japonica	
augustus-7-processed-gene-246.7-mRNA-1	7	OsR498G0714726500.01.T01	99,55	9,00E-110	397	Os07g0605900	83,85	4,69E-51	199	-198	Indica	Japonica	
augustus-7-processed-gene-247.10-mRNA-1	7	OsR498G0714730000.01.T02	99,70	0,00E+00	1209	Os07g0606600	100,00	0,00E+00	1218	9	NA	Japonica	
augustus-7-processed-gene-247.2-mRNA-1	7	OsR498G0714731100.01.T01	99,61	0,00E+00	1817	Os07g0606800	98,75	0,00E+00	1962	145	NA	Japonica	
augustus-7-processed-gene-247.23-mRNA-1	7	OsR498G0714734900.01.T01	99,86	0,00E+00	1327	Os07g0608100	100,00	0,00E+00	1332	5	NA	Japonica	
augustus-7-processed-gene-247.33-mRNA-1	7	OsR498G0714733000.01.T01	99,83	0,00E+00	1062	Os07g0607200	100,00	0,00E+00	1067	5	NA	Japonica	
augustus-7-processed-gene-247.6-mRNA-1	7	OsR498G0714729200.01.T01	99,52	0,00E+00	1113	Os10g0190100	72,27	5,64E-100	363	-750	Indica	Japonica	
augustus-7-processed-gene-248.17-mRNA-1	7	OsR498G0714737500.01.T05	91,88	0,00E+00	1180	Os07g0609000	100,00	0,00E+00	1418	238	Japonica	Japonica	
augustus-7-processed-gene-249.33-mRNA-1	7	OsR498G0714740700.01.T01	97,82	0,00E+00	1331	Os07g0611500	100,00	0,00E+00	1407	76	NA	Japonica	
augustus-7-processed-gene-25.1-mRNA-1	7	OsR498G0713538700.01.T01	100,00	0,00E+00	3155	Os07g0154100	99,95	0,00E+00	3361	206	NA	NA	
augustus-7-processed-gene-25.6-mRNA-1	7	OsR498G0713539500.01.T01	100,00	2,88E-116	417	Os07g0154300	100,00	1,01E-116	417	0	NA	NA	
augustus-7-processed-gene-250.37-mRNA-1	7	OsR498G0714741500.01.T02	80,43	2,05E-169	596	Os07g0611700	100,00	0,00E+00	1505	909	Japonica	Japonica	
augustus-7-processed-gene-252.0-mRNA-1	7	OsR498G0714756300.01.T02	99,81	0,00E+00	1864	Os07g0616500	100,00	0,00E+00	1050	-814	Indica	Japonica	
augustus-7-processed-gene-252.7-mRNA-1	7	OsR498G0714759700.01.T01	99,43	0,00E+00	939	Os07g0617100	100,00	0,00E+00	953	14	NA	Japonica	
augustus-7-processed-gene-253.19-mRNA-1	7	OsR498G0714766900.01.T01	99,50	0,00E+00	1762	Os07g0619500	100,00	0,00E+00	1786	24	NA	Japonica	
augustus-7-processed-gene-254.36-mRNA-1	7	OsR498G0714770300.01.T01	99,41	0,00E+00	2117	Os07g0620200	100,00	0,00E+00	2149	32	NA	Japonica	
augustus-7-processed-gene-254.46-mRNA-1	7	OsR498G0714772900.01.T01	99,55	0,00E+00	2799	Os07g0621100	100,00	0,00E+00	3346	547	Japonica	Japonica	
augustus-7-processed-gene-254.61-mRNA-1	7	OsR498G0714774200.01.T01	99,83	0,00E+00	2107	Os07g0621800	100,00	0,00E+00	2116	9	NA	Japonica	
augustus-7-processed-gene-255.1-mRNA-1	7	OsR498G0714775300.01.T01	100,00	0,00E+00	1359	Os07g0622100	100,00	0,00E+00	1359	0	NA	Japonica	
augustus-7-processed-gene-255.14-mRNA-1	7	OsR498G0714778400.01.T01	99,80	0,00E+00	1766	Os07g0622700	100,00	0,00E+00	1775	9	NA	Japonica	
augustus-7-processed-gene-255.15-mRNA-1	7	OsR498G0714778600.01.T01	100,00	4,47E-127	453	Os07g0622900	100,00	1,56E-127	453	0	NA	Japonica	

augustus-7-processed-gene-256.33-mRNA-1	7	OsR498G0714786700.01.T01	97,79	0,00E+00	1162	Os07g0625500	74,52	7,36E-61	233	-929	Indica	Japonica	
augustus-7-processed-gene-257.36-mRNA-1	7	OsR498G0714792000.01.T01	99,79	0,00E+00	2552	Os07g0626800	100,00	0,00E+00	2154	-398	Indica	Japonica	
augustus-7-processed-gene-259.23-mRNA-1	7	OsR498G0714808300.01.T02	99,84	0,00E+00	1154	Os07g0631700	100,00	0,00E+00	1159	5	NA	Japonica	
augustus-7-processed-gene-259.31-mRNA-1	7	OsR498G0714804800.01.T02	99,75	0,00E+00	2178	Os07g0630800	100,00	0,00E+00	2192	14	NA	Japonica	
augustus-7-processed-gene-260.3-mRNA-1	7	OsR498G0714810500.01.T03	99,71	0,00E+00	1836	Os07g0632600	100,00	0,00E+00	1849	13	NA	Japonica	
augustus-7-processed-gene-260.35-mRNA-1	7	OsR498G0714814400.01.T01	99,77	0,00E+00	3162	Os07g0633600	100,00	0,00E+00	3171	9	NA	Japonica	
augustus-7-processed-gene-261.41-mRNA-1	7	OsR498G0714820500.01.T01	99,04	0,00E+00	2757	Os07g0635300	100,00	0,00E+00	2663	-94	NA	Japonica	
augustus-7-processed-gene-261.57-mRNA-1	7	OsR498G0714822200.01.T01	99,67	0,00E+00	3223	Os07g0636000	100,00	0,00E+00	3252	29	NA	Japonica	
augustus-7-processed-gene-262.45-mRNA-1	7	OsR498G0714824300.01.T01	99,78	0,00E+00	799	Os07g0636400	100,00	0,00E+00	803	4	NA	Japonica	
augustus-7-processed-gene-263.0-mRNA-1	7	OsR498G0714825200.01.T01	88,56	0,00E+00	782	Os07g0637700	100,00	0,00E+00	1018	236	Japonica	Japonica	
augustus-7-processed-gene-263.10-mRNA-1	7	OsR498G0714830900.01.T01	99,85	0,00E+00	1192	Os07g0638300	100,00	0,00E+00	1196	4	NA	Japonica	
augustus-7-processed-gene-263.33-mRNA-1	7	OsR498G0714833300.01.T01	78,69	0,00E+00	742	Os07g0638600	100,00	0,00E+00	1829	1087	Japonica	Japonica	
augustus-7-processed-gene-263.8-mRNA-1	7	OsR498G0714830500.01.T01	99,65	0,00E+00	3540	Os07g0638100	100,00	0,00E+00	3571	31	NA	Japonica	
augustus-7-processed-gene-264.74-mRNA-1	7	OsR498G0714834500.01.T01	99,90	0,00E+00	1863	Os07g0639400	100,00	0,00E+00	1867	4	NA	Japonica	
augustus-7-processed-gene-264.97-mRNA-1	7	OsR498G0714840300.01.T01	100,00	5,64E-152	536	Os07g0641700	100,00	1,96E-152	536	0	NA	Japonica	
augustus-7-processed-gene-265.14-mRNA-1	7	OsR498G0714846000.01.T01	85,03	0,00E+00	1141	Os07g0643000	100,00	0,00E+00	1884	743	Japonica	Japonica	
augustus-7-processed-gene-265.15-mRNA-1	7	OsR498G0714825200.01.T01	87,18	0,00E+00	744	Os07g0643800	99,85	0,00E+00	1158	414	Japonica	Japonica	
augustus-7-processed-gene-265.23-mRNA-1	7	OsR498G0714846000.01.T01	97,76	0,00E+00	1746	Os07g0643100	100,00	0,00E+00	1873	127	NA	Japonica	
augustus-7-processed-gene-265.27-mRNA-1	7	OsR498G0714846800.01.T01	97,44	0,00E+00	1782	Os07g0643400	100,00	0,00E+00	1921	139	NA	Japonica	
augustus-7-processed-gene-267.21-mRNA-1	7	OsR498G0714855400.01.T01	99,92	0,00E+00	2187	Os07g0646300	82,86	0,00E+00	1009	-1178	Indica	Japonica	
augustus-7-processed-gene-267.5-mRNA-1	7	OsR498G0714853500.01.T01	99,85	0,00E+00	1214	Os07g0645300	100,00	0,00E+00	1023	-191	Indica	Japonica	
augustus-7-processed-gene-268.75-mRNA-1	7	OsR498G0714863900.01.T01	98,84	0,00E+00	2424	Os07g0647400	100,00	0,00E+00	2495	71	NA	Japonica	
augustus-7-processed-gene-269.14-mRNA-1	7	OsR498G0714870700.01.T01	94,68	0,00E+00	2589	Os07g0649300	100,00	0,00E+00	2980	391	Japonica	Japonica	
augustus-7-processed-gene-274.58-mRNA-1	7	OsR498G0714881300.01.T02	99,69	0,00E+00	2847	Os07g0656400	100,00	0,00E+00	2874	27	NA	Japonica	
augustus-7-processed-gene-275.51-mRNA-1	7	OsR498G0714885700.01.T01	99,76	0,00E+00	2991	Os07g0657400	100,00	0,00E+00	3009	18	NA	Japonica	
augustus-7-processed-gene-275.60-mRNA-1	7	OsR498G0714887700.01.T04	98,86	0,00E+00	4593	Os07g0658300	99,89	0,00E+00	4708	115	NA	Japonica	
augustus-7-processed-gene-275.71-mRNA-1	7	OsR498G0714888800.01.T01	97,95	0,00E+00	4100	Os07g0658700	100,00	0,00E+00	4286	186	NA	Japonica	
augustus-7-processed-gene-277.8-mRNA-1	7	OsR498G0714897400.01.T03	99,79	0,00E+00	835	Os07g0661400	100,00	0,00E+00	839	4	NA	Japonica	
augustus-7-processed-gene-278.114-mRNA-1	7	OsR498G0714906100.01.T01	100,00	0,00E+00	1427	Os07g0663800	100,00	0,00E+00	1427	0	NA	NA	
augustus-7-processed-gene-278.124-mRNA-1	7	OsR498G0714909900.01.T01	100,00	0,00E+00	1667	Os07g0664500	100,00	0,00E+00	1667	0	NA	NA	
augustus-7-processed-gene-28.12-mRNA-1	7	OsR498G0713564300.01.T01	99,71	0,00E+00	2448	Os07g0160600	100,00	0,00E+00	2466	18	NA	Japonica	
augustus-7-processed-gene-28.9-mRNA-1	7	OsR498G0713563100.01.T01	100,00	0,00E+00	1083	Os07g0160300	99,67	0,00E+00	1074	-9	NA	NA	NBS-TM
augustus-7-processed-gene-280.63-mRNA-1	7	OsR498G0714924900.01.T01	100,00	0,00E+00	2381	Os07g0668800	100,00	0,00E+00	2381	0	NA	NA	
augustus-7-processed-gene-280.81-mRNA-1	7	OsR498G0714924000.01.T01	100,00	0,00E+00	1521	Os07g0668600	100,00	0,00E+00	1521	0	NA	NA	
augustus-7-processed-gene-281.55-mRNA-1	7	OsR498G0714930300.01.T01	99,57	0,00E+00	1248	Os07g0670200	100,00	0,00E+00	1261	13	NA	Japonica	
augustus-7-processed-gene-281.56-mRNA-1	7	OsR498G0714930700.01.T01	97,75	0,00E+00	1598	Os07g0670300	100,00	0,00E+00	1683	85	NA	Japonica	
augustus-7-processed-gene-282.50-mRNA-1	7	OsR498G0714931700.01.T03	99,69	1,03E-161	570	Os07g0671000	100,00	8,42E-164	574	4	NA	Japonica	
augustus-7-processed-gene-282.56-mRNA-1	7	OsR498G0714932400.01.T01	99,51	0,00E+00	2544	Os07g0671200	100,00	0,00E+00	2987	443	Japonica	Japonica	
augustus-7-processed-gene-283.29-mRNA-1	7	OsR498G0714939900.01.T02	99,83	0,00E+00	2048	Os07g0673700	100,00	0,00E+00	2057	9	NA	Japonica	
augustus-7-processed-gene-283.41-mRNA-1	7	OsR498G0305924500.01.T01	75,13	1,61E-66	253	Os07g0673801	100,00	0,00E+00	872	619	Japonica	Japonica	
augustus-7-processed-gene-283.58-mRNA-1	7	OsR498G0714944100.01.T01	99,83	0,00E+00	1057	Os07g0674700	100,00	0,00E+00	1061	4	NA	Japonica	
augustus-7-processed-gene-283.66-mRNA-1	7	OsR498G0714942400.01.T01	99,59	0,00E+00	3953	Os07g0674300	100,00	0,00E+00	3993	40	NA	Japonica	
augustus-7-processed-gene-284.1-mRNA-1	7	OsR498G0714945700.01.T01	98,16	0,00E+00	1319	Os07g0675250	100,00	0,00E+00	1516	197	Japonica	Japonica	
augustus-7-processed-gene-285.36-mRNA-1	7	OsR498G0306613200.01.T01	70,24	5,93E-97	357	Os07g0678300	100,00	0,00E+00	2700	2343	Japonica	NA	Kinase-TM
augustus-7-processed-gene-285.47-mRNA-1	7	OsR498G0714955800.01.T01	100,00	0,00E+00	2403	Os07g0678600	100,00	0,00E+00	2403	0	NA	NA	Kinase-TM
augustus-7-processed-gene-286.0-mRNA-1	7	OsR498G0714958700.01.T01	99,49	0,00E+00	1737	Os07g0679200	100,00	0,00E+00	1792	55	NA	Japonica	
augustus-7-processed-gene-287.10-mRNA-1	7	OsR498G0714966300.01.T03	97,31	0,00E+00	1004	Os07g0681400	100,00	0,00E+00	1072	68	NA	Japonica	
augustus-7-processed-gene-289.31-mRNA-1	7	OsR498G0714985300.01.T01	99,57	0,00E+00	4186	Os07g0685900	100,00	0,00E+00	4231	45	NA	Japonica	
augustus-7-processed-gene-289.41-mRNA-1	7	OsR498G0714984400.01.T02	99,61	0,00E+00	3181	Os07g0685700	100,00	0,00E+00	3214	33	NA	Japonica	
augustus-7-processed-gene-29.24-mRNA-1	7	OsR498G0713566700.01.T01	99,62	0,00E+00	944	Os07g0161900	71,17	4,16E-88	324	-620	Indica	Japonica	
augustus-7-processed-gene-29.25-mRNA-1	7	OsR498G0713566700.01.T01	71,17	1,28E-87	324	Os07g0161900	100,00	0,00E+00	1018	694	Japonica	Japonica	
augustus-7-processed-gene-29.44-mRNA-1	7	OsR498G0713570600.01.T01	98,91	0,00E+00	1773	Os07g0162600	100,00	0,00E+00	1819	46	NA	Japonica	
augustus-7-processed-gene-29.45-mRNA-1	7	OsR498G0713571400.01.T01	99,72	0,00E+00	1892	Os07g0162700	100,00	0,00E+00	1905	13	NA	Japonica	
augustus-7-processed-gene-290.2-mRNA-1	7	OsR498G0714990300.01.T01	100,00	0,00E+00	1186	Os07g0686600	100,00	0,00E+00	1186	0	NA	Japonica	
augustus-7-processed-gene-290.20-mRNA-1	7	OsR498G0714994900.01.T04	99,79	0,00E+00	1734	Os07g0688000	100,00	0,00E+00	1743	9	NA	Japonica	
augustus-7-processed-gene-291.43-mRNA-1	7	OsR498G0714996600.01.T01	99,81	0,00E+00	959	Os03g0311600	74,10	9,91E-71	267	-692	Indica	Japonica	
augustus-7-processed-gene-292.9-mRNA-1	7	OsR498G0715005400.01.T01	99,31	0,00E+00	3599	Os07g0690900	100,00	0,00E+00	3658	59	NA	Japonica	
augustus-7-processed-gene-293.46-mRNA-1	7	OsR498G0715011300.01.T01	100,00	0,00E+00	2625	Os07g0692401	100,00	0,00E+00	2625	0	NA	Japonica	
augustus-7-processed-gene-293.48-mRNA-1	7	OsR498G0715011900.01.T01	99,79	0,00E+00	5225	Os07g0692900	99,97	0,00E+00	5248	23	NA	Japonica	
augustus-7-processed-gene-294.43-mRNA-1	7	OsR498G0715018100.01.T02	99,65	0,00E+00	3030	Os07g0694500	99,84	0,00E+00	2250	-780	Indica	Japonica	
augustus-7-processed-gene-294.52-mRNA-1	7	OsR498G0715018400.01.T01	96,93	0,00E+00	1862	Os07g0694600	97,11	0,00E+00	1871	9	NA	Japonica	
augustus-7-processed-gene-294.64-mRNA-1	7	OsR498G0715020300.01.T01	100,00	0,00E+00	1517	Os07g0695300	100,00	0,00E+00	1517	0	NA	Japonica	Kinase
augustus-7-processed-gene-3.14-mRNA-1	7	OsR498G0713392900.01.T01	100,00	0,00E+00	2581	Os07g0111900	100,00	0,00E+00	2581	0	NA	NA	
augustus-7-processed-gene-3.4-mRNA-1	7	OsR498G0713392100.01.T02	100,00	0,00E+00	2511	Os07g0111700	100,00	0,00E+00	2484	-27	NA	NA	
augustus-7-processed-gene-30.9-mRNA-1	7	OsR498G0713577500.01.T01	98,55	0,00E+00	1446	Os07g0164800	100,00	0,00E+00	1494	48	NA	Japonica	
augustus-7-processed-gene-31.25-mRNA-1	7	OsR498G0713579700.01.T01	97,44	0,00E+00	1646	Os12g0468900	80,71	0,00E+00	786	-860	Indica	Japonica	
augustus-7-processed-gene-31.28-mRNA-1	7	OsR498G0713578600.01.T01	99,74	0,00E+00	5579	Os07g0165100	100,00	0,00E+00	5616	37	NA	Japonica	
augustus-7-processed-gene-32.27-mRNA-1	7	OsR498G0713590600.01.T03	100,00	0,00E+00	980	Os07g0168800	100,00	0,00E+00	877	-103	Indica	NA	
augustus-7-processed-gene-34.14-mRNA-1	7	OsR498G0713602400.01.T01	99,96	0,00E+00	4259	Os07g0172600	100,00	0,00E+00	4724	465	NA	Indica	
augustus-7-processed-gene-35.1-mRNA-1	7	OsR498G0713607100.01.T02	100,00	0,00E+00	1846	Os07g0174700	96,96	0,00E+00	1534	-312	Indica	Indica	
augustus-7-processed-gene-35.23-mRNA-1	7	OsR498G0713615500.01.T02	99,94	0,00E+00	3002	Os07g0176200	99,94	0,00E+00	3002	0	NA	NA	
augustus-7-processed-gene-36.16-mRNA-1	7	OsR498G0713623600.01.T01	80,00	1,54E-117	423	Os07g0177400	100,00	0,00E+00	888	465	Japonica	NA	
augustus-7-processed-gene-36.9-mRNA-1	7	OsR498G0713620600.01.T01	100,00	0,00E+00	1521	Os07g0176900	100,00	0,00E+00	1521	0	NA	NA	
augustus-7-processed-gene-37.14-mRNA-1	7	OsR498G0713629900.01.T01	98,99	0,00E+00	2782	Os07g0180300	77,31	0,00E+00	996	-1786	Indica	Japonica	
augustus-7-processed-gene-37.4-mRNA-1	7	OsR498G0713626300.01.T05	99,59	0,00E+00	3902	Os07g0179000	100,00	0,00E+00	3939	37	NA	Japonica	
augustus-7-processed-gene-4.29-mRNA-1	7	OsR498G0713398700.01.T01	99,79	0,00E+00	3422	Os07g0113500	99,95	0,00E+00	3432	10	NA	NA	
augustus-7-processed-gene-4.34-mRNA-1	7	OsR498G0713400200.01.T01	100,00	0,00E+00	5476	Os07g0113700	100,00	0,00E+00	5476	0	NA	NA	
augustus-7-processed-gene-40.24-mRNA-1	7	OsR498G0713641500.01.T01	99,42	0,00E+00	2465	Os07g0184200	99,13	0,00E+00	2450	-15	NA	NA	
augustus-7-processed-gene-41.21-mRNA-1	7	OsR498G0713645400.01.T01	99,81	0,00E+00	948	Os07g0185300	100,00	0,00E+00	953	5	NA	Japonica	
augustus-7-processed-gene-41.40-mRNA-1	7	OsR498G0713651200.01.T01	99,41	0,00E+00	3615	Os07g0186200	99,92	0,00E+00	4525	910	Japonica	Japonica	Kinase-TM
augustus-7-processed-gene-42.1-mRNA-1	7	OsR498G0713652100.01.T01	99,89	0,00E+00	4696	Os07g0186500	100,00	0,00E+00	4709	13	NA	Japonica	LRR-NBS-TM
augustus-7-processed-gene-43.29-mRNA-1	7	OsR498G0306754100.01.T02	74,49	7,23E-64	244	Os07g0189400	100,00	0,00E+00	758	514	Japonica	Japonica	
augustus-7-processed-gene-44.7-mRNA-1	7	OsR498G0713665100.01.T01	100,00	0,00E+00	1299	Os07g0190400	100,00	0,00E+00	1299	0	NA	NA	
augustus-7-processed-gene-45.11-mRNA-1	7	OsR498G0713671300.01.T01	100,00	0,00E+00	2982	Os07g0191500	100,00	0,00E+00	2982	0	NA	NA	
augustus-7-processed-gene-45.16-mRNA-1	7	OsR498G0713673300.01.T01	100,00	0,00E+00	2774	Os07g0192000	100,00	0,00E+00	2798	24	NA	NA	
augustus-7-processed-gene-45.2-mRNA-1	7	OsR498G0713669500.01.T06	98,75	0,00E+00	5049	Os07g0191200	98,75	0,00E+00	5049	0	NA	NA	
augustus-7-processed-gene-46.24-mRNA-1	7	OsR498G0713676200.01.T01	99,90	0,00E+00	1816	Os07g0192800	100,00	0,00E+00	2700	884	Japonica	NA	
augustus-7-processed-gene-47.19-mRNA-1	7	OsR498G0713687000.01.T01	99,47	0,00E+00	2342	Os07g0195300	100,00	0,00E+00	2246	-96	NA	Japonica	
augustus-7-processed-gene-47.8-mRNA-1	7	OsR498G0713682000.01.T01	100,00	2,42E-163	574	Os07g0194300	90,44	9,02E-94	342	-232	Indica	NA	
augustus-7-processed-gene-48.12-mRNA-1	7	OsR498G0713690500.01.T01	98,19	0,00E+00	2090	Os07g0196300	100,00	0,00E+00	2192	102	NA	Japonica	
augustus-7-processed-gene-48.26-mRNA-1	7	OsR498G0713692900.01.T02	99,62	0,00E+00	1891	Os07g0242400	76,82	0,00E+00	788	-1103	Indica	Japonica	
augustus-7-processed-gene-48.37-mRNA-1	7	OsR498G0713691600.01.T01	98,03	0,00E+00	959	Os07g0196700	100,00	0,00E+00	1817	858	Japonica	Japonica	
augustus-7-processed-gene-5.27-mRNA-1	7	OsR498G0713412200.01.T02	99,54	0,00E+00	2734	Os07g0117000	83,33	0,00E+00	1588	-1146	Indica	Japonica	
augustus-7-processed-gene-50.35-mRNA-1	7	OsR498G0713703300.01.T01	99,81	0,00E+00	4793	Os07g0201100	100,00	0,00E+00	4816	23	NA	Japonica	
augustus-7-processed-gene-51.1-mRNA-1	7	OsR498G0713705500.01.T01	99,05	0,00E+00	2585	Os07g0201500	90,50	0,00E+00	2024	-561	Indica	Japonica	
augustus-7-processed-gene-51.3-mRNA-1	7	OsR498G0713706700.01.T01	99,86	0,00E+00	2524	Os07g0202000	100,00	0,00E+00	2706	182	NA	Japonica	
augustus-7-processed-gene-52.62-mRNA-1	7	OsR498G0713712600.01.T03	98,73	0,00E+00	1652	Os07g0204500	98,58	0,00E+00	1600	-52	NA	Japonica	
augustus-7-processed-gene-53.15-mRNA-1	7	OsR498G0713723000.01.T01	100,00	0,00E+00	1274	Os07g0206800	100,00	0,00E+00	1272	-2	NA	NA	
augustus-7-processed-gene-54.45-mRNA-1	7	OsR498G0713726400.01.T02	93,54	0,00E+00	1381	Os07g0208100	84,12	0,00E+00	665	-716	Indica	NA	
augustus-7-processed-gene-56.7-mRNA-1	7	OsR498G0713740600.01.T06	100,00	0,00E+00	1925	Os07g0210100	100,00	0,00E+00	1737	-188	NA	NA	
augustus-7-processed-gene-56.9-mRNA-1	7	OsR498G0713742400.01.T01	95,41	0,00E+00	1387	Os03g0643100	99,88	0,00E+00	1448	61	NA	NA	
augustus-7-processed-gene-58.16-mRNA-1	7	OsR498G0713761200.01.T01	100,00	9,98E-149	526	Os07g0213800	81,68	9,35E-68	255	-271	Indica	NA	
augustus-7-processed-gene-58.19-mRNA-1	7	OsR498G0713762000.01.T01	100,00	0,00E+00	888	Os07g0214100	100,00	0,00E+00	888	0	NA	NA	
augustus-7-processed-gene-58.20-mRNA-1	7	OsR498G0713762400.01.T01	100,00	0,00E+00	1023	Os07g0214300	100,00	0,00E+00	904	-119	Indica	NA	
augustus-7-processed-gene-59.26-mRNA-1	7	OsR498G0713769000.01.T01	100,00	0,00E+00	872	Os07g0215500	100,00	0,00E+00	872	0	NA	NA	
augustus-7-processed-gene-59.6-mRNA-1	7	OsR498G0713764600.01.T01	100,00	0,00E+00	856	Os07g0214600	100,00	0,00E+00	856	0	NA	NA	
augustus-7-processed-gene-6.32-mRNA-1	7	OsR498G0713415100.01.T01	95,54	0,00E+00	683	Os07g0118200	100,00	0,00E+00	767	84	Japonica	Japonica	
augustus-7-processed-gene-6.33-mRNA-1	7	OsR498G0713423300.01.T01	88,40	3,82E-156	551	Os07g0118250	100,00	0,00E+00	774	223	Japonica	Japonica	
augustus-7-processed-gene-60.16-mRNA-1	7	OsR498G0713776200.01.T01	100,00	0,00E+00	861	Os07g0216600	100,00	0,00E+00	861	0	NA	NA	
augustus-7-processed-gene-60.20-mRNA-1	7	OsR498G0713778000.01.T01	100,00	0,00E+00	866	Os07g0216700	100,00	0,00E+00	866	0	NA	NA	
augustus-7-processed-gene-61.0-mRNA-1	7	OsR498G0713782100.01.T01	100,00	0,00E+00	1680	Os07g0217600	100,00	0,00E+00	1680	0	NA	Japonica	
augustus-7-processed-gene-64.39-mRNA-1	7	OsR498G0713794900.01.T02	99,60	0,00E+00	1333	Os07g0223700	99,87	0,00E+00	1342	9	NA	Japonica	
augustus-7-processed-gene-64.42-mRNA-1	7	OsR498G0713795700.01.T01	99,38	0,00E+00	864	Os07g0224000	100,00	0,00E+00	877	13	NA	Japonica	
augustus-7-processed-gene-65.9-mRNA-1	7	OsR498G0713804500.01.T01	99,63	0,00E+00	2863	Os07g0224900	99,74	0,00E+00	2054	-809	Indica	Japonica	
augustus-7-processed-gene-67.20-mRNA-1	7	OsR498G0713819100.01.T01	99,28	6,78E-138	489	Os07g0227200	100,00	4,58E-141	499	10	NA	Japonica	
augustus-7-processed-gene-68.18-mRNA-1	7	OsR498G0713825000.01.T01	99,52	0,00E+00	735	Os07g0229900	100,00	0,00E+00	744	9	NA	Japonica	
augustus-7-processed-gene-68.3-mRNA-1	7	OsR498G0713822600.01.T01	99,24	0,00E+00	1156	Os07g0229200	74,83	1,19E-134	480	-676	Indica	Japonica	
augustus-7-processed-gene-69.27-mRNA-1	7	OsR498G0713829700.01.T01	98,66	0,00E+00	1297	Os07g0231500	100,00	0,00E+00	1342	45	NA	Japonica	
augustus-7-processed-gene-7.55-mRNA-1	7	OsR498G0713426300.01.T04	78,79	0,00E+00	1299	Os07g0121000	100,00	0,00E+00	2776	1477	Japonica	Japonica	
augustus-7-processed-gene-7.56-mRNA-1	7	OsR498G0713426300.01.T04	99,54	0,00E+00	2718	Os07g0120900	100,00	0,00E+00	2749	31	NA	Japonica	
augustus-7-processed-gene-7.57-mRNA-1	7	OsR498G0713426900.01.T02	99,67	0,00E+00	2743	Os07g0121100	100,00	0,00E+00	2765	22	NA	Japonica	
augustus-7-processed-gene-7.68-mRNA-1	7	OsR498G0713423300.01.T01	87,70	8,40E-152	537	Os07g0120000	100,00	0,00E+00	774	237	Japonica	Japonica	
augustus-7-processed-gene-7.71-mRNA-1	7	OsR498G0713415100.01.T01	93,01	0,00E+00	639	Os07g0120500	94,16	0,00E+00	660	21	NA	Japonica	
augustus-7-processed-gene-7.75-mRNA-1	7	OsR498G0713423300.01.T01	93,97	0,00E+00	659	Os07g0120600	100,00	0,00E+00	774	115	Japonica	Japonica	
augustus-7-processed-gene-72.24-mRNA-1	7	OsR498G0713848400.01.T02	98,80	0,00E+00	1752	Os07g0236700	100,00	0,00E+00	1802	50	NA	Japonica	
augustus-7-processed-gene-72.28-mRNA-1	7	OsR498G0613330300.01.T01	70,17	1,63E-13	77	Os07g0237900	100,00	3,54E-162	569	492	Japonica	Japonica	
augustus-7-processed-gene-73.34-mRNA-1	7	OsR498G0713856800.01.T03	99,26	0,00E+00	3838	Os07g0239600	100,00	0,00E+00	3901	63	NA	Japonica	
augustus-7-processed-gene-75.37-mRNA-1	7	OsR498G0713868400.01.T01	99,62	1,73E-132	472	Os07g0243000	99,61	1,33E-128	458	-14	NA	Japonica	
augustus-7-processed-gene-76.42-mRNA-1	7	OsR498G0713872200.01.T01	99,01	0,00E+00	2305	Os07g0244400	100,00	0,00E+00	2360	55	NA	Japonica	
augustus-7-processed-gene-77.14-mRNA-1	7	OsR498G0713877300.01.T02	99,69	0,00E+00	2282	Os07g0246200	100,00	0,00E+00	2300	18	NA	Japonica	
augustus-7-processed-gene-77.23-mRNA-1	7	OsR498G0713877600.01.T02	99,90	0,00E+00	1706	Os07g0246300	100,00	0,00E+00	1710	4	NA	Japonica	
augustus-7-processed-gene-78.2-mRNA-1	7	OsR498G0713879300.01.T01	99,63	0,00E+00	1941	Os07g0247000	100,00	0,00E+00	1959	18	NA	Japonica	
augustus-7-processed-gene-79.29-mRNA-1	7	OsR498G0714323900.01.T01	81,94	8,46E-102	371	Os07g0250133	100,00	0,00E+00	798	427	Japonica	Japonica	
augustus-7-processed-gene-8.0-mRNA-1	7	OsR498G0511374000.01.T01	83,85	3,49E-91	335	Os07g0121600	100,00	1,58E-134	477	142	Japonica	Japonica	
augustus-7-processed-gene-8.5-mRNA-1	7	OsR498G0713429500.01.T03	99,87	0,00E+00	2740	Os07g0122200	100,00	0,00E+00	2749	9	NA	Japonica	
augustus-7-processed-gene-8.6-mRNA-1	7	OsR498G0713429700.01.T01	99,72	0,00E+00	2598	Os07g0122300	100,00	0,00E+00	2616	18	NA	Japonica	
augustus-7-processed-gene-80.14-mRNA-1	7	OsR498G0713892000.01.T01	99,53	0,00E+00	1145	Os07g0251200	100,00	0,00E+00	1472	327	Japonica	Japonica	
augustus-7-processed-gene-83.15-mRNA-1	7	OsR498G0713577000.01.T01	97,07	0,00E+00	1084	Os07g0255200	100,00	0,00E+00	1169	85	NA	Japonica	
augustus-7-processed-gene-85.12-mRNA-1	7	OsR498G0713916200.01.T02	99,76	0,00E+00	1487	Os07g0257200	100,00	0,00E+00	1496	9	NA	Japonica	
augustus-7-processed-gene-9.32-mRNA-1	7	OsR498G0713435600.01.T01	98,97	0,00E+00	853	Os07g0123800	100,00	0,00E+00	877	24	NA	Japonica	
augustus-7-processed-gene-9.33-mRNA-1	7	OsR498G0713436000.01.T01	99,51	0,00E+00	728	Os07g0123900	100,00	0,00E+00	737	9	NA	Japonica	
augustus-7-processed-gene-93.17-mRNA-1	7	OsR498G0713959500.01.T02	99,00	0,00E+00	2644	Os07g0268800	100,00	0,00E+00	3961	1317	Japonica	Japonica	
augustus-7-processed-gene-94.12-mRNA-1	7	OsR498G0713961500.01.T01	99,35	0,00E+00	1086	Os07g0268800	77,27	6,29E-131	466	-620	Indica	Japonica	
augustus-7-processed-gene-97.10-mRNA-1	7	OsR498G0713971700.01.T01	99,87	0,00E+00	2686	Os07g0272400	100,00	0,00E+00	2695	9	NA	Japonica	
augustus-7-processed-gene-97.23-mRNA-1	7	OsR498G0713975100.01.T01	99,56	0,00E+00	3243	Os07g0272700	100,00	0,00E+00	3279	36	NA	Japonica	
maker-7-augustus-gene-0.22-mRNA-1	7	OsR498G0713353900.01.T02	99,74	0,00E+00	3403	Os07g0100500	99,67	0,00E+00	2705	-698	Indica	NA	
maker-7-augustus-gene-0.28-mRNA-1	7	OsR498G0713355200.01.T04	98,71	0,00E+00	2314	Os07g0100800	98,78	0,00E+00	2443	129	NA	NA	
maker-7-augustus-gene-0.30-mRNA-1	7	OsR498G0713356000.01.T01	98,97	0,00E+00	3085	Os07g0101000	99,66	0,00E+00	3133	48	NA	NA	
maker-7-augustus-gene-0.32-mRNA-1	7	OsR498G0713357000.01.T04	99,54	0,00E+00	782	Os07g0101101	99,60	3,41E-126	451	-331	Indica	NA	
maker-7-augustus-gene-1.36-mRNA-1	7	OsR498G0713358100.01.T04	97,46	0,00E+00	1414	Os07g0101500	97,46	0,00E+00	1414	0	NA	NA	
maker-7-augustus-gene-1.38-mRNA-1	7	OsR498G0713359500.01.T01	99,81	0,00E+00	4636	Os07g0102100	99,77	0,00E+00	3886	-750	Indica	NA	
maker-7-augustus-gene-1.40-mRNA-1	7	OsR498G0713357600.01.T01	99,92	0,00E+00	2139	Os07g0101400	99,92	0,00E+00	2139	0	NA	NA	
maker-7-augustus-gene-1.46-mRNA-1	7	OsR498G0713360000.01.T01	98,51	0,00E+00	1872	Os07g0102200	97,89	0,00E+00	2776	904	Japonica	NA	
maker-7-augustus-gene-1.48-mRNA-1	7	OsR498G0713360600.01.T05	100,00	0,00E+00	1362	Os07g0102300	100,00	0,00E+00	1563	201	Japonica	NA	
maker-7-augustus-gene-1.53-mRNA-1	7	OsR498G0713360800.01.T01	100,00	0,00E+00	866	Os07g0102400	100,00	0,00E+00	866	0	NA	NA	
maker-7-augustus-gene-1.56-mRNA-1	7	OsR498G0713361200.01.T01	99,84	0,00E+00	1105	Os07g0102500	99,84	0,00E+00	1105	0	NA	Indica	
maker-7-augustus-gene-102.37-mRNA-1	7	OsR498G0713992100.01.T01	99,76	0,00E+00	3007	Os07g0280200	100,00	0,00E+00	3025	18	NA	Japonica	
maker-7-augustus-gene-103.36-mRNA-1	7	OsR498G0713994900.01.T01	99,73	0,00E+00	7341	Os07g0282300	97,37	0,00E+00	4233	-3108	Indica	Japonica	
maker-7-augustus-gene-103.43-mRNA-1	7	OsR498G0713993000.01.T01	99,59	0,00E+00	2608	Os07g0281000	100,00	0,00E+00	2636	28	NA	Japonica	
maker-7-augustus-gene-104.23-mRNA-1	7	OsR498G0713994900.01.T01	97,94	0,00E+00	3906	Os07g0282300	100,00	0,00E+00	4118	212	NA	Japonica	
maker-7-augustus-gene-104.28-mRNA-1	7	OsR498G0713999800.01.T01	99,80	0,00E+00	3532	Os07g0283125	100,00	0,00E+00	3788	256	NA	Japonica	Kinase-TM
maker-7-augustus-gene-104.29-mRNA-1	7	OsR498G0713999800.01.T05	99,86	0,00E+00	1324	Os07g0283200	100,00	0,00E+00	1328	4	NA	Japonica	
maker-7-augustus-gene-106.28-mRNA-1	7	OsR498G0714008000.01.T01	99,65	0,00E+00	2532	Os07g0285200	100,00	0,00E+00	2554	22	NA	Japonica	
maker-7-augustus-gene-106.30-mRNA-1	7	OsR498G0714008200.01.T01	99,63	0,00E+00	1430	Os07g0285700	77,58	0,00E+00	733	-697	Indica	Japonica	
maker-7-augustus-gene-106.32-mRNA-1	7	OsR498G0714008400.01.T01	99,59	0,00E+00	2195	Os07g0285700	100,00	0,00E+00	2576	381	Japonica	Japonica	
maker-7-augustus-gene-107.27-mRNA-1	7	OsR498G0714014300.01.T02	99,48	0,00E+00	2064	Os07g0287000	99,91	0,00E+00	2081	17	NA	Japonica	
maker-7-augustus-gene-107.30-mRNA-1	7	OsR498G0714014500.01.T06	100,00	3,11E-118	425	Os07g0287100	100,00	1,08E-118	425	0	NA	Japonica	
maker-7-augustus-gene-109.0-mRNA-1	7	OsR498G0714024700.01.T01	99,20	0,00E+00	1554	Os07g0290800	100,00	0,00E+00	1586	32	NA	Japonica	
maker-7-augustus-gene-109.11-mRNA-1	7	OsR498G0714029400.01.T01	97,75	0,00E+00	910	Os07g0293000	82,29	4,59E-151	533	-377	Indica	Japonica	
maker-7-augustus-gene-109.9-mRNA-1	7	OsR498G0714026200.01.T01	99,23	0,00E+00	1141	Os03g0848400	80,34	3,44E-166	583	-558	Indica	Japonica	
maker-7-augustus-gene-11.2-mRNA-1	7	OsR498G0713450900.01.T01	100,00	0,00E+00	942	Os07g0128800	96,55	0,00E+00	859	-83	NA	Indica	
maker-7-augustus-gene-110.0-mRNA-1	7	OsR498G0714030900.01.T01	98,98	0,00E+00	866	Os07g0292800	100,00	0,00E+00	888	22	NA	Japonica	
maker-7-augustus-gene-110.3-mRNA-1	7	OsR498G0714031400.01.T01	97,39	0,00E+00	1877	Os07g0293000	100,00	0,00E+00	2214	337	Japonica	Japonica	
maker-7-augustus-gene-111.29-mRNA-1	7	OsR498G0714035300.01.T01	99,67	0,00E+00	2185	Os07g0294800	100,00	0,00E+00	2203	18	NA	Japonica	
maker-7-augustus-gene-111.31-mRNA-1	7	OsR498G0714037500.01.T01	96,59	0,00E+00	2053	Os07g0295400	100,00	0,00E+00	2186	133	NA	Japonica	
maker-7-augustus-gene-111.36-mRNA-1	7	OsR498G0714035600.01.T02	98,51	0,00E+00	1395	Os07g0295000	100,00	0,00E+00	1449	54	NA	Japonica	
maker-7-augustus-gene-111.40-mRNA-1	7	OsR498G0714035800.01.T02	99,79	0,00E+00	844	Os07g0295200	100,00	0,00E+00	848	4	NA	Japonica	
maker-7-augustus-gene-112.24-mRNA-1	7	OsR498G0714040700.01.T01	97,44	4,70E-148	524	Os07g0296800	100,00	3,36E-157	553	29	NA	Japonica	
maker-7-augustus-gene-112.26-mRNA-1	7	OsR498G0714041500.01.T01	99,35	0,00E+00	815	Os09g0272250	74,38	1,99E-53	209	-606	Indica	Japonica	
maker-7-augustus-gene-112.37-mRNA-1	7	OsR498G0714040200.01.T01	99,75	0,00E+00	2124	Os07g0296200	100,00	0,00E+00	2138	14	NA	Japonica	
maker-7-augustus-gene-113.0-mRNA-1	7	OsR498G0714042700.01.T01	99,20	0,00E+00	1102	Os07g0297100	100,00	0,00E+00	1124	22	NA	Japonica	
maker-7-augustus-gene-113.10-mRNA-1	7	OsR498G0714044000.01.T01	99,69	0,00E+00	1142	Os07g0298100	100,00	0,00E+00	1624	482	Japonica	Japonica	
maker-7-augustus-gene-114.26-mRNA-1	7	OsR498G0714051300.01.T03	100,00	7,71E-170	596	Os07g0299600	100,00	2,68E-170	596	0	NA	Japonica	
maker-7-augustus-gene-114.28-mRNA-1	7	OsR498G0714047000.01.T01	99,55	0,00E+00	2781	Os07g0298900	100,00	0,00E+00	2268	-513	Indica	Japonica	
maker-7-augustus-gene-114.30-mRNA-1	7	OsR498G0714051800.01.T02	99,80	0,00E+00	3629	Os07g0299800	100,00	0,00E+00	3647	18	NA	Japonica	
maker-7-augustus-gene-115.22-mRNA-1	7	OsR498G0714052900.01.T04	99,83	0,00E+00	5149	Os07g0300200	100,00	0,00E+00	5171	22	NA	Japonica	
maker-7-augustus-gene-115.29-mRNA-1	7	OsR498G0714055800.01.T01	99,81	0,00E+00	2763	Os07g0301200	100,00	0,00E+00	2776	13	NA	Japonica	
maker-7-augustus-gene-115.33-mRNA-1	7	OsR498G0714056600.01.T01	94,58	0,00E+00	1142	Os07g0301550	100,00	0,00E+00	1121	-21	NA	Japonica	Kinase-TM
maker-7-augustus-gene-119.0-mRNA-1	7	OsR498G0714065600.01.T01	99,79	0,00E+00	4171	Os07g0404300	100,00	0,00E+00	3507	-664	Indica	Japonica	
maker-7-augustus-gene-12.46-mRNA-1	7	OsR498G0713458300.01.T01	99,51	0,00E+00	1098	Os07g0129700	99,51	0,00E+00	1098	0	NA	Indica	
maker-7-augustus-gene-12.47-mRNA-1	7	OsR498G0713458300.01.T01	99,79	0,00E+00	835	Os07g0129700	99,79	0,00E+00	835	0	NA	Indica	
maker-7-augustus-gene-12.50-mRNA-1	7	OsR498G0713463100.01.T01	100,00	0,00E+00	2830	Os07g0131600	97,84	0,00E+00	2684	-146	NA	Indica	
maker-7-augustus-gene-12.52-mRNA-1	7	OsR498G0713458800.01.T01	100,00	0,00E+00	3647	Os07g0129800	99,85	0,00E+00	3666	19	NA	Indica	Kinase-TM
maker-7-augustus-gene-120.23-mRNA-1	7	OsR498G0714070300.01.T08	99,94	0,00E+00	3004	Os07g0405100	100,00	0,00E+00	3009	5	NA	Japonica	
maker-7-augustus-gene-122.29-mRNA-1	7	OsR498G0714075300.01.T10	99,14	0,00E+00	817	Os08g0110700	87,96	0,00E+00	669	-148	Indica	Japonica	
maker-7-augustus-gene-122.35-mRNA-1	7	OsR498G0714073200.01.T01	99,77	0,00E+00	3173	Os07g0406300	99,83	0,00E+00	3177	4	NA	Japonica	
maker-7-augustus-gene-122.40-mRNA-1	7	OsR498G0714075800.01.T04	99,75	0,00E+00	1409	Os07g0406800	100,00	0,00E+00	1418	9	NA	Japonica	
maker-7-augustus-gene-123.28-mRNA-1	7	OsR498G0714079000.01.T04	99,58	0,00E+00	1692	Os07g0408100	99,68	0,00E+00	1695	3	NA	Japonica	
maker-7-augustus-gene-123.29-mRNA-1	7	OsR498G0714079000.01.T06	99,75	0,00E+00	710	Os07g0408100	100,00	5,50E-168	589	-121	Indica	Japonica	
maker-7-augustus-gene-123.40-mRNA-1	7	OsR498G0714081800.01.T01	99,93	0,00E+00	2608	Os07g0408500	100,00	0,00E+00	2612	4	NA	Japonica	
maker-7-augustus-gene-123.43-mRNA-1	7	OsR498G0714083200.01.T01	99,90	0,00E+00	1749	Os07g0408700	100,00	0,00E+00	1754	5	NA	Japonica	
maker-7-augustus-gene-124.0-mRNA-1	7	OsR498G0714084300.01.T01	99,94	0,00E+00	2788	Os07g0409100	100,00	0,00E+00	2792	4	NA	Japonica	
maker-7-augustus-gene-124.10-mRNA-1	7	OsR498G0714086600.01.T03	100,00	0,00E+00	2576	Os07g0409700	100,00	0,00E+00	2576	0	NA	Japonica	
maker-7-augustus-gene-124.16-mRNA-1	7	OsR498G0714087400.01.T03	95,57	0,00E+00	2479	Os07g0409900	95,63	0,00E+00	2483	4	NA	Japonica	
maker-7-augustus-gene-124.24-mRNA-1	7	OsR498G0714088800.01.T02	99,75	0,00E+00	2915	Os07g0410100	100,00	0,00E+00	2933	18	NA	Japonica	
maker-7-augustus-gene-125.33-mRNA-1	7	OsR498G0714096700.01.T02	97,12	0,00E+00	936	Os07g0412400	97,84	0,00E+00	954	18	NA	Japonica	
maker-7-augustus-gene-125.35-mRNA-1	7	OsR498G0714094200.01.T01	99,92	0,00E+00	2193	Os07g0411300	100,00	0,00E+00	2197	4	NA	Japonica	
maker-7-augustus-gene-125.37-mRNA-1	7	OsR498G0714096400.01.T03	99,29	0,00E+00	3237	Os07g0412100	100,00	0,00E+00	3296	59	NA	Japonica	
maker-7-augustus-gene-126.24-mRNA-1	7	OsR498G0714097100.01.T06	98,34	0,00E+00	4185	Os07g0413700	98,76	0,00E+00	4230	45	NA	Japonica	
maker-7-augustus-gene-127.38-mRNA-1	7	OsR498G0714098600.01.T04	98,47	0,00E+00	1477	Os07g0414200	100,00	0,00E+00	1532	55	NA	Japonica	
maker-7-augustus-gene-127.47-mRNA-1	7	OsR498G0714099400.01.T02	99,88	0,00E+00	1455	Os07g0414800	100,00	0,00E+00	1169	-286	Indica	Japonica	
maker-7-augustus-gene-127.52-mRNA-1	7	OsR498G1018346900.01.T03	93,25	0,00E+00	1218	Os07g0415600	99,90	0,00E+00	1711	493	Japonica	Japonica	

maker-7-augustus-gene-129.0-mRNA-1	7	OsR498G0714111500.01.T02	98,80	0,00E+00	1307	Os07g0418200	100,00	0,00E+00	1348	41	NA	Japonica	
maker-7-augustus-gene-129.2-mRNA-1	7	OsR498G0714821100.01.T01	83,66	0,00E+00	1640	Os07g0418500	100,00	0,00E+00	2814	1174	Japonica	Japonica	
maker-7-augustus-gene-129.8-mRNA-1	7	OsR498G0714111400.01.T02	99,00	0,00E+00	1054	Os07g0418000	100,00	0,00E+00	998	-56	NA	Japonica	
maker-7-augustus-gene-13.0-mRNA-1	7	OsR498G0713464300.01.T05	100,00	0,00E+00	845	Os07g0132300	99,77	0,00E+00	792	-53	NA	Indica	
maker-7-augustus-gene-13.4-mRNA-1	7	OsR498G0713467300.01.T04	100,00	0,00E+00	1288	Os07g0133700	100,00	0,00E+00	648	-640	Indica	NA	
maker-7-augustus-gene-13.7-mRNA-1	7	OsR498G0713466600.01.T01	100,00	0,00E+00	2506	Os07g0133500	100,00	0,00E+00	2506	0	NA	Indica	
maker-7-augustus-gene-13.9-mRNA-1	7	OsR498G0713468000.01.T01	97,02	0,00E+00	2555	Os07g0134000	96,95	0,00E+00	2551	-4	NA	Indica	
maker-7-augustus-gene-130.33-mRNA-1	7	OsR498G0714117100.01.T01	99,79	0,00E+00	1685	Os07g0419300	100,00	0,00E+00	1499	-186	Indica	Japonica	
maker-7-augustus-gene-130.44-mRNA-1	7	OsR498G0714821100.01.T01	81,71	0,00E+00	643	Os07g0418500	95,90	0,00E+00	1066	423	Japonica	Japonica	
maker-7-augustus-gene-132.12-mRNA-1	7	OsR498G0714130100.01.T01	93,87	0,00E+00	820	Os07g0422100	100,00	0,00E+00	942	122	Japonica	Japonica	
maker-7-augustus-gene-132.4-mRNA-1	7	OsR498G0714128500.01.T01	100,00	0,00E+00	2544	Os10g0195600	70,04	1,22E-169	597	-1947	Indica	Japonica	
maker-7-augustus-gene-134.27-mRNA-1	7	OsR498G0714136000.01.T01	99,09	0,00E+00	5853	Os07g0424400	99,18	0,00E+00	5866	13	NA	Japonica	
maker-7-augustus-gene-134.35-mRNA-1	7	OsR498G0714138800.01.T01	99,87	0,00E+00	2789	Os07g0425000	100,00	0,00E+00	1764	-1025	Indica	Japonica	
maker-7-augustus-gene-137.0-mRNA-1	7	OsR498G0714151500.01.T02	99,65	1,78E-145	516	Os07g0429700	100,00	1,46E-147	520	4	NA	Japonica	
maker-7-augustus-gene-139.25-mRNA-1	7	OsR498G0714152700.01.T01	98,96	9,24E-143	507	Os07g0429700	98,61	3,92E-142	502	-5	NA	NA	
maker-7-augustus-gene-139.27-mRNA-1	7	OsR498G0714152700.01.T01	98,79	4,29E-121	434	Os07g0432201	100,00	6,82E-126	448	14	NA	NA	
maker-7-augustus-gene-14.40-mRNA-1	7	OsR498G0713472500.01.T01	97,20	0,00E+00	2366	Os07g0134700	97,87	0,00E+00	3150	784	Japonica	NA	
maker-7-augustus-gene-14.43-mRNA-1	7	OsR498G0713470200.01.T01	100,00	0,00E+00	5541	Os07g0134200	100,00	0,00E+00	4693	-848	Indica	NA	Kinase-LRR-TM
maker-7-augustus-gene-14.50-mRNA-1	7	OsR498G0713472800.01.T01	100,00	0,00E+00	3415	Os07g0134800	100,00	0,00E+00	3415	0	NA	NA	
maker-7-augustus-gene-141.42-mRNA-1	7	OsR498G0714154900.01.T01	99,33	0,00E+00	797	Os07g0435300	100,00	4,20E-138	489	-308	Indica	Japonica	
maker-7-augustus-gene-141.44-mRNA-1	7	OsR498G0714154400.01.T02	99,60	0,00E+00	4041	Os06g0255700	97,08	0,00E+00	3799	-242	NA	Japonica	
maker-7-augustus-gene-141.50-mRNA-1	7	OsR498G0714154600.01.T02	98,52	7,97E-167	588	Os07g0434700	100,00	2,95E-173	607	19	NA	Japonica	
maker-7-augustus-gene-141.55-mRNA-1	7	OsR498G0714154800.01.T01	99,50	0,00E+00	1433	Os07g0435100	100,00	0,00E+00	1451	18	NA	Japonica	
maker-7-augustus-gene-142.0-mRNA-1	7	OsR498G0714155200.01.T02	99,86	0,00E+00	2515	Os07g0435400	100,00	0,00E+00	2524	9	NA	Japonica	
maker-7-augustus-gene-142.5-mRNA-1	7	OsR498G0714157400.01.T01	99,80	0,00E+00	3537	Os07g0436000	100,00	0,00E+00	2953	-584	Indica	Japonica	
maker-7-augustus-gene-143.41-mRNA-1	7	OsR498G0714165300.01.T03	99,59	0,00E+00	6533	Os07g0438500	100,00	0,00E+00	4666	-1867	Indica	Japonica	Kinase-TM
maker-7-augustus-gene-143.47-mRNA-1	7	OsR498G0714165500.01.T01	99,15	1,72E-178	626	Os07g0438600	100,00	0,00E+00	639	13	NA	Japonica	
maker-7-augustus-gene-144.2-mRNA-1	7	OsR498G0714166700.01.T02	99,92	0,00E+00	2306	Os07g0438800	100,00	0,00E+00	2280	-26	NA	Japonica	
maker-7-augustus-gene-145.26-mRNA-1	7	OsR498G0714170700.01.T02	99,09	0,00E+00	2320	Os07g0442000	100,00	0,00E+00	2509	189	NA	Japonica	
maker-7-augustus-gene-146.25-mRNA-1	7	OsR498G0714174300.01.T01	97,78	0,00E+00	1075	Os07g0442900	100,00	0,00E+00	1126	51	NA	Japonica	
maker-7-augustus-gene-147.0-mRNA-1	7	OsR498G0714179800.01.T03	97,94	0,00E+00	2658	Os10g0473400	77,39	0,00E+00	910	-1748	Indica	Japonica	
maker-7-augustus-gene-148.2-mRNA-1	7	OsR498G0714182300.01.T02	100,00	3,99E-156	551	Os02g0622400	75,57	1,93E-53	209	-342	Indica	Japonica	
maker-7-augustus-gene-148.4-mRNA-1	7	OsR498G0714182900.01.T01	99,21	0,00E+00	1335	Os07g0446000	100,00	0,00E+00	1364	29	NA	Japonica	
maker-7-augustus-gene-148.6-mRNA-1	7	OsR498G0714181800.01.T01	99,20	0,00E+00	4656	Os07g0445600	100,00	0,00E+00	4221	-435	NA	Japonica	
maker-7-augustus-gene-149.22-mRNA-1	7	OsR498G0714183600.01.T01	97,52	0,00E+00	2686	Os07g0446100	97,78	0,00E+00	2704	18	NA	Japonica	
maker-7-augustus-gene-149.26-mRNA-1	7	OsR498G0714188400.01.T01	86,57	2,05E-67	255	Os07g0446900	100,00	4,23E-115	412	157	Japonica	Japonica	
maker-7-augustus-gene-149.28-mRNA-1	7	OsR498G0714190000.01.T01	98,31	0,00E+00	3478	Os07g0447000	99,95	0,00E+00	3902	424	Japonica	Japonica	
maker-7-augustus-gene-15.49-mRNA-1	7	OsR498G0713476700.01.T01	100,00	1,38E-179	628	Os07g0136300	100,00	4,80E-180	628	0	NA	NA	
maker-7-augustus-gene-15.58-mRNA-1	7	OsR498G0713479300.01.T02	96,91	8,93E-72	270	Os07g0139500	82,25	4,05E-39	159	-111	Indica	NA	
maker-7-augustus-gene-15.60-mRNA-1	7	OsR498G0713479900.01.T01	100,00	5,75E-165	580	Os07g0137200	99,69	8,51E-164	575	-5	NA	NA	
maker-7-augustus-gene-15.66-mRNA-1	7	OsR498G0713481800.01.T01	100,00	0,00E+00	1948	Os07g0137800	99,94	0,00E+00	2799	851	Japonica	NA	Kinase-TM
maker-7-augustus-gene-150.25-mRNA-1	7	OsR498G0714191700.01.T02	96,16	0,00E+00	2668	Os07g0447800	97,26	0,00E+00	2749	81	NA	Japonica	
maker-7-augustus-gene-150.31-mRNA-1	7	OsR498G0714192100.01.T01	100,00	0,00E+00	1553	Os07g0448100	98,37	0,00E+00	1490	-63	NA	NA	
maker-7-augustus-gene-150.32-mRNA-1	7	OsR498G0714192300.01.T01	99,77	0,00E+00	1544	Os07g0448100	94,08	0,00E+00	1324	-220	Indica	NA	
maker-7-augustus-gene-150.33-mRNA-1	7	OsR498G0714192500.01.T01	99,41	0,00E+00	1515	Os07g0448100	91,16	0,00E+00	1213	-302	Indica	Indica	
maker-7-augustus-gene-150.52-mRNA-1	7	OsR498G0714195500.01.T01	99,89	0,00E+00	1571	Os07g0448800	100,00	0,00E+00	1575	4	NA	Japonica	
maker-7-augustus-gene-151.0-mRNA-1	7	OsR498G0714197200.01.T01	92,99	0,00E+00	948	Os04g0524300	81,02	4,16E-89	328	-620	Indica	Japonica	
maker-7-augustus-gene-151.2-mRNA-1	7	OsR498G0714196800.01.T02	97,78	6,02E-152	537	Os07g0450000	99,68	1,51E-160	564	27	NA	Japonica	
maker-7-augustus-gene-151.4-mRNA-1	7	OsR498G0408173900.01.T01	85,90	8,87E-178	623	Os07g0450225	100,00	2,90E-134	477	-146	Indica	NA	
maker-7-augustus-gene-152.31-mRNA-1	7	OsR498G0714200500.01.T01	99,21	0,00E+00	2674	Os07g0452400	99,60	0,00E+00	2701	27	NA	Japonica	
maker-7-augustus-gene-152.36-mRNA-1	7	OsR498G0714198600.01.T01	92,65	0,00E+00	872	Os06g0279950	92,64	0,00E+00	745	-127	Indica	Japonica	
maker-7-augustus-gene-152.40-mRNA-1	7	OsR498G0714200800.01.T01	91,48	0,00E+00	803	Os07g0452500	91,67	0,00E+00	808	5	NA	Japonica	
maker-7-augustus-gene-154.28-mRNA-1	7	OsR498G0714212500.01.T02	99,77	0,00E+00	775	Os07g0454700	100,00	0,00E+00	780	5	NA	Japonica	
maker-7-augustus-gene-154.47-mRNA-1	7	OsR498G0714213200.01.T03	99,78	0,00E+00	4832	Os07g0455100	96,52	0,00E+00	2213	-2619	Indica	Japonica	
maker-7-augustus-gene-155.28-mRNA-1	7	OsR498G0714218100.01.T04	99,64	0,00E+00	2949	Os07g0456700	100,00	0,00E+00	2976	27	NA	Japonica	
maker-7-augustus-gene-155.32-mRNA-1	7	OsR498G0714217600.01.T04	99,81	0,00E+00	1880	Os07g0456400	100,00	0,00E+00	1889	9	NA	Japonica	
maker-7-augustus-gene-156.0-mRNA-1	7	OsR498G0714219700.01.T01	99,69	0,00E+00	3425	Os07g0457300	100,00	0,00E+00	3452	27	NA	Japonica	
maker-7-augustus-gene-156.11-mRNA-1	7	OsR498G0714228700.01.T01	99,58	0,00E+00	1269	Os07g0458800	100,00	0,00E+00	1283	14	NA	Japonica	
maker-7-augustus-gene-156.8-mRNA-1	7	OsR498G0714228300.01.T03	97,80	0,00E+00	853	Os07g0458500	94,25	0,00E+00	718	-135	Indica	Japonica	
maker-7-augustus-gene-157.28-mRNA-1	7	OsR498G0714230100.01.T03	100,00	0,00E+00	1478	Os07g0459400	100,00	0,00E+00	1478	0	NA	Japonica	
maker-7-augustus-gene-157.39-mRNA-1	7	OsR498G0714233600.01.T01	99,27	0,00E+00	1212	Os05g0168200	94,54	0,00E+00	1089	-123	Indica	Japonica	
maker-7-augustus-gene-158.4-mRNA-1	7	OsR498G0714239200.01.T01	99,73	0,00E+00	1957	Os07g0461500	100,00	0,00E+00	1970	13	NA	Japonica	
maker-7-augustus-gene-158.6-mRNA-1	7	OsR498G0714239400.01.T09	98,09	0,00E+00	4524	Os07g0461600	99,88	0,00E+00	2929	-1595	Indica	Japonica	
maker-7-augustus-gene-159.0-mRNA-1	7	OsR498G0714240500.01.T01	99,49	0,00E+00	1042	Os07g0461700	100,00	0,00E+00	1813	771	Japonica	Japonica	
maker-7-augustus-gene-159.10-mRNA-1	7	OsR498G0714245100.01.T05	98,73	0,00E+00	1924	Os07g0463400	100,00	0,00E+00	1959	35	NA	Japonica	
maker-7-augustus-gene-159.14-mRNA-1	7	OsR498G0509663100.01.T03	94,48	0,00E+00	2098	Os07g0461900	99,66	0,00E+00	1572	-526	Indica	Japonica	
maker-7-augustus-gene-159.26-mRNA-1	7	OsR498G0714244000.01.T01	100,00	0,00E+00	715	Os07g0463100	100,00	0,00E+00	715	0	NA	Japonica	
maker-7-augustus-gene-159.4-mRNA-1	7	OsR498G0714242700.01.T01	100,00	0,00E+00	915	Os07g0462700	100,00	0,00E+00	915	0	NA	Japonica	
maker-7-augustus-gene-159.6-mRNA-1	7	OsR498G0714248200.01.T01	85,03	0,00E+00	1187	Os07g0464200	84,84	0,00E+00	1181	-6	NA	Japonica	
maker-7-augustus-gene-16.12-mRNA-1	7	OsR498G0713483300.01.T01	100,00	0,00E+00	1894	Os03g0777000	78,84	9,63E-157	553	-1341	Indica	NA	NAC66
maker-7-augustus-gene-16.5-mRNA-1	7	OsR498G0713482700.01.T06	100,00	0,00E+00	2625	Os07g0138100	100,00	0,00E+00	2625	0	NA	NA	
maker-7-augustus-gene-160.40-mRNA-1	7	OsR498G0714249900.01.T01	96,16	0,00E+00	2334	Os07g0464600	96,37	0,00E+00	2348	14	NA	Japonica	
maker-7-augustus-gene-160.46-mRNA-1	7	OsR498G0714245400.01.T01	99,04	0,00E+00	918	Os07g0462700	89,30	0,00E+00	679	-239	Indica	Japonica	
maker-7-augustus-gene-160.49-mRNA-1	7	OsR498G0714245600.01.T01	99,11	0,00E+00	1785	Os07g0463600	100,00	0,00E+00	1954	169	NA	Japonica	
maker-7-augustus-gene-160.52-mRNA-1	7	OsR498G0714248200.01.T01	99,43	0,00E+00	1865	Os07g0464200	100,00	0,00E+00	1900	35	NA	Japonica	
maker-7-augustus-gene-161.26-mRNA-1	7	OsR498G0714254700.01.T01	98,66	0,00E+00	2085	Os07g0527900	79,26	3,36E-163	574	-1511	Indica	Japonica	
maker-7-augustus-gene-162.27-mRNA-1	7	OsR498G0714254900.01.T06	100,00	0,00E+00	2657	Os07g0466200	100,00	0,00E+00	2549	-108	NA	Japonica	
maker-7-augustus-gene-162.38-mRNA-1	7	OsR498G0714255400.01.T01	100,00	6,40E-155	547	Os07g0466300	100,00	2,23E-155	547	0	NA	Japonica	
maker-7-augustus-gene-162.40-mRNA-1	7	OsR498G0714258600.01.T01	99,39	5,46E-167	587	Os07g0467200	100,00	3,66E-170	596	9	NA	Japonica	
maker-7-augustus-gene-163.21-mRNA-1	7	OsR498G0714259700.01.T01	99,63	0,00E+00	1452	Os07g0467500	99,63	0,00E+00	1452	0	NA	Japonica	
maker-7-augustus-gene-164.26-mRNA-1	7	OsR498G0714162100.01.T05	99,89	0,00E+00	1605	Os07g0437500	100,00	0,00E+00	1609	4	NA	NA	
maker-7-augustus-gene-164.34-mRNA-1	7	OsR498G0714275200.01.T01	99,70	0,00E+00	3600	Os07g0472300	99,95	0,00E+00	3623	23	NA	NA	
maker-7-augustus-gene-165.37-mRNA-1	7	OsR498G0611686100.01.T01	93,60	0,00E+00	802	Os07g0468800	100,00	0,00E+00	820	18	NA	Japonica	
maker-7-augustus-gene-166.27-mRNA-1	7	OsR498G0714267700.01.T01	96,78	0,00E+00	1926	Os07g0470700	97,05	0,00E+00	1945	19	NA	Japonica	
maker-7-augustus-gene-166.33-mRNA-1	7	OsR498G0714268500.01.T01	100,00	0,00E+00	3595	Os07g0471000	100,00	0,00E+00	3595	0	NA	Japonica	Kinase-TM
maker-7-augustus-gene-166.40-mRNA-1	7	OsR498G0714269100.01.T01	99,89	0,00E+00	1562	Os07g0471100	99,89	0,00E+00	1562	0	NA	Japonica	
maker-7-augustus-gene-167.27-mRNA-1	7	OsR498G0714273300.01.T01	99,77	0,00E+00	2342	Os07g0471900	100,00	0,00E+00	2365	23	NA	Japonica	
maker-7-augustus-gene-167.38-mRNA-1	7	OsR498G0714275700.01.T01	97,46	0,00E+00	3958	Os07g0472400	100,00	0,00E+00	2944	-1014	Indica	Japonica	Kinase-TM
maker-7-augustus-gene-167.41-mRNA-1	7	OsR498G0714276100.01.T01	99,87	0,00E+00	4294	Os07g0472500	100,00	0,00E+00	4307	13	NA	Japonica	
maker-7-augustus-gene-167.49-mRNA-1	7	OsR498G0714275200.01.T01	99,75	0,00E+00	3608	Os07g0472300	100,00	0,00E+00	3631	23	NA	Japonica	
maker-7-augustus-gene-168.0-mRNA-1	7	OsR498G0714282100.01.T01	99,60	0,00E+00	1334	Os07g0474300	100,00	0,00E+00	1348	14	NA	Japonica	
maker-7-augustus-gene-168.2-mRNA-1	7	OsR498G0714282700.01.T03	99,76	0,00E+00	4448	Os12g0267200	70,68	0,00E+00	1161	-3287	Indica	Japonica	
maker-7-augustus-gene-168.8-mRNA-1	7	OsR498G0100612800.01.T01	89,35	0,00E+00	697	Os07g0513300	81,75	0,00E+00	984	287	Japonica	Japonica	
maker-7-augustus-gene-169.29-mRNA-1	7	OsR498G0714283900.01.T01	97,71	0,00E+00	893	Os07g0475000	100,00	0,00E+00	886	-7	NA	Japonica	
maker-7-augustus-gene-169.31-mRNA-1	7	OsR498G0714285400.01.T01	99,64	0,00E+00	1486	Os07g0475400	100,00	0,00E+00	1499	13	NA	Japonica	
maker-7-augustus-gene-169.35-mRNA-1	7	OsR498G0714289000.01.T01	99,75	0,00E+00	2854	Os07g0475900	100,00	0,00E+00	2376	-478	Indica	Japonica	Kinase-TM
maker-7-augustus-gene-17.59-mRNA-1	7	OsR498G0713488000.01.T01	99,55	0,00E+00	5185	Os07g0139500	99,52	0,00E+00	4464	-721	Indica	NA	
maker-7-augustus-gene-17.65-mRNA-1	7	OsR498G0713492400.01.T01	98,96	0,00E+00	2373	Os07g0141100	98,96	0,00E+00	2373	0	NA	NA	
maker-7-augustus-gene-17.67-mRNA-1	7	OsR498G0713492600.01.T02	99,70	0,00E+00	2390	Os06g0241100	99,40	0,00E+00	2372	-18	NA	NA	Kinase-TM
maker-7-augustus-gene-17.69-mRNA-1	7	OsR498G0713493200.01.T01	99,74	0,00E+00	1371	Os07g0141400	99,87	0,00E+00	1376	5	NA	NA	
maker-7-augustus-gene-170.11-mRNA-1	7	OsR498G0714294200.01.T03	100,00	0,00E+00	1278	Os07g0476500	100,00	0,00E+00	1278	0	NA	Japonica	
maker-7-augustus-gene-170.16-mRNA-1	7	OsR498G0714295000.01.T02	99,84	0,00E+00	1095	Os07g0477000	100,00	0,00E+00	1099	4	NA	Japonica	
maker-7-augustus-gene-171.26-mRNA-1	7	OsR498G0714302100.01.T07	97,72	0,00E+00	1051	Os07g0479100	99,84	0,00E+00	1091	40	NA	Japonica	
maker-7-augustus-gene-171.30-mRNA-1	7	OsR498G0714302300.01.T01	100,00	0,00E+00	1326	Os07g0479200	100,00	0,00E+00	1326	0	NA	Japonica	
maker-7-augustus-gene-171.36-mRNA-1	7	OsR498G0714302800.01.T01	99,62	0,00E+00	2814	Os07g0479300	100,00	0,00E+00	2841	27	NA	Japonica	
maker-7-augustus-gene-171.42-mRNA-1	7	OsR498G0714303800.01.T01	100,00	0,00E+00	1245	Os03g0108400	79,76	4,22E-102	370	-875	Indica	Japonica	
maker-7-augustus-gene-171.48-mRNA-1	7	OsR498G0714304000.01.T02	99,61	0,00E+00	922	Os07g0479600	100,00	0,00E+00	805	-117	Indica	Japonica	
maker-7-augustus-gene-172.0-mRNA-1	7	OsR498G0714307700.01.T01	99,23	0,00E+00	1603	Os10g0545500	79,78	0,00E+00	721	-882	Indica	Japonica	
maker-7-augustus-gene-172.14-mRNA-1	7	OsR498G0714308900.01.T01	99,36	0,00E+00	4975	Os07g0481300	100,00	0,00E+00	5196	221	NA	Japonica	LRR-NBS-TM
maker-7-augustus-gene-172.16-mRNA-1	7	OsR498G0714308600.01.T01	97,91	0,00E+00	2380	Os07g0481100	100,00	0,00E+00	2495	115	NA	Japonica	
maker-7-augustus-gene-172.3-mRNA-1	7	OsR498G0714308100.01.T01	96,09	0,00E+00	1831	Os07g0480900	100,00	0,00E+00	2040	209	Japonica	Japonica	
maker-7-augustus-gene-172.5-mRNA-1	7	OsR498G0714308300.01.T04	98,39	0,00E+00	1938	Os07g0481000	100,00	0,00E+00	2062	124	NA	Japonica	
maker-7-augustus-gene-173.0-mRNA-1	7	OsR498G0714309200.01.T01	98,52	0,00E+00	5633	Os07g0481400	100,00	0,00E+00	5849	216	NA	Japonica	CC-NBS-TM
maker-7-augustus-gene-174.0-mRNA-1	7	OsR498G0714311900.01.T01	97,54	0,00E+00	761	Os07g0483400	97,99	0,00E+00	770	9	NA	Japonica	
maker-7-augustus-gene-174.3-mRNA-1	7	OsR498G0714310200.01.T01	98,85	0,00E+00	1682	Os07g0482975	100,00	0,00E+00	1732	50	NA	Japonica	
maker-7-augustus-gene-174.4-mRNA-1	7	OsR498G0714310200.01.T01	97,80	0,00E+00	767	Os07g0483050	100,00	1,23E-163	574	-193	Indica	Japonica	
maker-7-augustus-gene-175.26-mRNA-1	7	OsR498G0714314300.01.T01	97,56	0,00E+00	694	Os07g0484200	98,53	0,00E+00	712	18	NA	Japonica	
maker-7-augustus-gene-175.35-mRNA-1	7	OsR498G0408431700.01.T02	95,78	0,00E+00	789	Os07g0484700	100,00	0,00E+00	886	97	Japonica	Japonica	
maker-7-augustus-gene-175.44-mRNA-1	7	OsR498G0714314610.01.T04	99,69	0,00E+00	2313	Os07g0484300	100,00	0,00E+00	3090	777	Japonica	Japonica	
maker-7-augustus-gene-175.50-mRNA-1	7	OsR498G0714316600.01.T01	99,47	0,00E+00	1004	Os07g0484800	100,00	0,00E+00	1018	14	NA	Japonica	
maker-7-augustus-gene-175.54-mRNA-1	7	OsR498G0714317800.01.T01	98,62	0,00E+00	1140	Os07g0485100	100,00	0,00E+00	1180	40	NA	Japonica	
maker-7-augustus-gene-176.12-mRNA-1	7	OsR498G0714323300.01.T01	98,56	0,00E+00	1930	Os07g0486500	100,00	0,00E+00	2003	73	NA	Japonica	
maker-7-augustus-gene-176.16-mRNA-1	7	OsR498G0714320000.01.T01	99,83	0,00E+00	2080	Os07g0485500	100,00	0,00E+00	1844	-236	Indica	Japonica	
maker-7-augustus-gene-176.19-mRNA-1	7	OsR498G0714320400.01.T02	100,00	0,00E+00	917	Os07g0485600	100,00	1,26E-158	558	-359	Indica	Japonica	
maker-7-augustus-gene-176.22-mRNA-1	7	OsR498G0714321600.01.T02	99,05	0,00E+00	1485	Os07g0486100	100,00	0,00E+00	1521	36	NA	Japonica	
maker-7-augustus-gene-177.0-mRNA-1	7	OsR498G0714323900.01.T01	85,33	0,00E+00	1594	Os07g0486700	100,00	0,00E+00	2668	1074	Japonica	Japonica	
maker-7-augustus-gene-178.2-mRNA-1	7	OsR498G0714331100.01.T02	99,68	0,00E+00	1133	Os07g0489800	99,53	0,00E+00	1129	-4	NA	NA	
maker-7-augustus-gene-178.6-mRNA-1	7	OsR498G0714327600.01.T01	99,42	0,00E+00	912	Os07g0489000	100,00	0,00E+00	926	14	NA	Japonica	
maker-7-augustus-gene-179.0-mRNA-1	7	OsR498G0714335100.01.T03	93,95	0,00E+00	1226	Os07g0490400	93,82	0,00E+00	1222	-4	NA	NA	
maker-7-augustus-gene-179.17-mRNA-1	7	OsR498G0714334200.01.T01	99,81	0,00E+00	3727	Os07g0490200	99,76	0,00E+00	3722	-5	NA	NA	
maker-7-augustus-gene-179.18-mRNA-1	7	OsR498G0714334200.01.T06	100,00	1,07E-111	403	Os07g0490200	100,00	5,91E-97	352	-51	Indica	NA	
maker-7-augustus-gene-179.34-mRNA-1	7	OsR498G0714336400.01.T01	97,24	0,00E+00	1416	Os07g0490600	99,76	0,00E+00	1496	80	NA	NA	
maker-7-augustus-gene-179.8-mRNA-1	7	OsR498G0714336900.01.T03	100,00	2,37E-150	531	Os07g0490700	100,00	8,26E-151	531	0	NA	NA	
maker-7-augustus-gene-18.47-mRNA-1	7	OsR498G0713496900.01.T02	99,66	0,00E+00	1061	Os07g0142900	99,83	0,00E+00	1066	5	NA	NA	
maker-7-augustus-gene-18.52-mRNA-1	7	OsR498G0713497100.01.T02	93,08	0,00E+00	1831	Os07g0143000	93,08	0,00E+00	1831	0	NA	NA	
maker-7-augustus-gene-18.56-mRNA-1	7	OsR498G0713498100.01.T03	99,50	0,00E+00	2116	Os07g0143200	99,48	0,00E+00	2053	-63	NA	NA	
maker-7-augustus-gene-18.61-mRNA-1	7	OsR498G0713499200.01.T01	97,64	0,00E+00	795	Os07g0143600	97,40	0,00E+00	654	-141	Indica	NA	
maker-7-augustus-gene-18.64-mRNA-1	7	OsR498G0713499600.01.T01	99,79	0,00E+00	1685	Os07g0143700	99,76	0,00E+00	1507	-178	Indica	NA	
maker-7-augustus-gene-18.69-mRNA-1	7	OsR498G0713499800.01.T01	100,00	0,00E+00	1932	Os07g0143900	100,00	0,00E+00	1932	0	NA	NA	
maker-7-augustus-gene-180.2-mRNA-1	7	OsR498G0714342200.01.T01	99,64	0,00E+00	2461	Os07g0491900	99,86	0,00E+00	2475	14	NA	NA	
maker-7-augustus-gene-180.4-mRNA-1	7	OsR498G0714342400.01.T02	100,00	0,00E+00	812	Os07g0492000	100,00	0,00E+00	812	0	NA	NA	
maker-7-augustus-gene-180.8-mRNA-1	7	OsR498G0714342800.01.T02	99,93	0,00E+00	4991	Os07g0492100	99,93	0,00E+00	4991	0	NA	NA	
maker-7-augustus-gene-181.0-mRNA-1	7	OsR498G0714350300.01.T06	100,00	0,00E+00	1678	Os07g0495000	100,00	0,00E+00	1678	0	NA	Indica	
maker-7-augustus-gene-181.16-mRNA-1	7	OsR498G0714351000.01.T01	100,00	0,00E+00	1115	Os07g0495200	99,84	0,00E+00	1111	-4	NA	Indica	
maker-7-augustus-gene-181.19-mRNA-1	7	OsR498G0714351200.01.T03	100,00	0,00E+00	2938	Os07g0495300	98,30	0,00E+00	2440	-498	Indica	Indica	
maker-7-augustus-gene-181.4-mRNA-1	7	OsR498G0714350500.01.T01	99,88	0,00E+00	1549	Os07g0495100	99,19	0,00E+00	1534	-15	NA	Indica	
maker-7-augustus-gene-182.0-mRNA-1	7	OsR498G0714352300.01.T01	99,90	0,00E+00	6897	Os07g0495900	99,91	0,00E+00	6057	-840	Indica	NA	
maker-7-augustus-gene-182.14-mRNA-1	7	OsR498G0714354100.01.T03	99,74	0,00E+00	1382	Os07g0496250	99,53	0,00E+00	760	-622	Indica	NA	
maker-7-augustus-gene-182.20-mRNA-1	7	OsR498G0714356900.01.T02	99,37	0,00E+00	2520	Os07g0496900	99,21	0,00E+00	2473	-47	NA	Indica	
maker-7-augustus-gene-182.24-mRNA-1	7	OsR498G0714353000.01.T01	100,00	0,00E+00	1131	Os07g0496000	100,00	0,00E+00	1131	0	NA	Indica	
maker-7-augustus-gene-182.8-mRNA-1	7	OsR498G0714353900.01.T01	99,88	0,00E+00	2906	Os07g0496200	99,88	0,00E+00	2906	0	NA	Indica	
maker-7-augustus-gene-183.12-mRNA-1	7	OsR498G0714363100.01.T01	99,92	0,00E+00	2236	Os07g0498900	99,76	0,00E+00	2227	-9	NA	Indica	
maker-7-augustus-gene-183.20-mRNA-1	7	OsR498G0714358000.01.T01	99,67	0,00E+00	2158	Os07g0497500	99,17	0,00E+00	2129	-29	NA	Indica	
maker-7-augustus-gene-183.22-mRNA-1	7	OsR498G0714359600.01.T01	99,95	0,00E+00	6809	Os07g0498400	99,77	0,00E+00	6862	53	NA	Indica	Kinase-LRR-TM
maker-7-augustus-gene-183.24-mRNA-1	7	OsR498G0714362600.01.T04	99,83	0,00E+00	5210	Os07g0498800	99,76	0,00E+00	5201	-9	NA	Indica	
maker-7-augustus-gene-184.0-mRNA-1	7	OsR498G0714366700.01.T02	99,60	0,00E+00	2243	Os07g0499800	99,29	0,00E+00	2242	-1	NA	Indica	
maker-7-augustus-gene-185.37-mRNA-1	7	OsR498G0714368300.01.T01	99,80	0,00E+00	914	Os07g0500300	98,81	0,00E+00	886	-28	NA	Indica	
maker-7-augustus-gene-185.41-mRNA-1	7	OsR498G0714371600.01.T01	99,84	0,00E+00	2188	Os07g0501100	99,92	0,00E+00	2193	5	NA	Indica	
maker-7-augustus-gene-185.43-mRNA-1	7	OsR498G0714373200.01.T01	100,00	3,43E-135	480	Os11g0270500	75,76	1,37E-33	141	-339	Indica	NA	
maker-7-augustus-gene-185.46-mRNA-1	7	OsR498G0714373600.01.T01	99,79	0,00E+00	3440	Os07g0501800	99,84	0,00E+00	3444	4	NA	NA	LRR-TM
maker-7-augustus-gene-186.34-mRNA-1	7	OsR498G0714374800.01.T01	99,88	0,00E+00	3059	Os07g0502200	99,72	0,00E+00	2596	-463	Indica	Indica	
maker-7-augustus-gene-187.39-mRNA-1	7	OsR498G0714378500.01.T01	98,54	0,00E+00	1556	Os07g0503700	98,88	0,00E+00	1570	14	NA	NA	
maker-7-augustus-gene-187.51-mRNA-1	7	OsR498G0714384000.01.T01	93,91	0,00E+00	1086	Os07g0505200	93,91	0,00E+00	1086	0	NA	NA	
maker-7-augustus-gene-188.30-mRNA-1	7	OsR498G0714390500.01.T04	99,71	0,00E+00	4286	Os07g0506600	99,62	0,00E+00	4277	-9	NA	NA	
maker-7-augustus-gene-188.32-mRNA-1	7	OsR498G0714387600.01.T01	100,00	0,00E+00	1635	Os07g0506000	99,89	0,00E+00	1630	-5	NA	Indica	
maker-7-augustus-gene-189.27-mRNA-1	7	OsR498G0714393300.01.T02	99,86	0,00E+00	2616	Os07g0507700	99,79	0,00E+00	2611	-5	NA	Indica	
maker-7-augustus-gene-189.51-mRNA-1	7	OsR498G0714392600.01.T04	99,81	0,00E+00	1865	Os07g0507300	99,81	0,00E+00	1864	-1	NA	Indica	Kinase-TM
maker-7-augustus-gene-189.60-mRNA-1	7	OsR498G0714393000.01.T01	99,66	0,00E+00	3166	Os07g0507500	99,66	0,00E+00	3166	0	NA	NA	
maker-7-augustus-gene-189.63-mRNA-1	7	OsR498G0714393200.01.T03	99,69	0,00E+00	1733	Os07g0507600	99,59	0,00E+00	1728	-5	NA	Indica	
maker-7-augustus-gene-189.66-mRNA-1	7	OsR498G0714394000.01.T03	98,27	0,00E+00	5681	Os07g0508000	98,24	0,00E+00	5677	-4	NA	NA	
maker-7-augustus-gene-189.71-mRNA-1	7	OsR498G0714394600.01.T01	96,58	0,00E+00	1815	Os07g0508300	96,58	0,00E+00	1815	0	NA	NA	
maker-7-augustus-gene-19.28-mRNA-1	7	OsR498G0713501900.01.T02	100,00	0,00E+00	2241	Os07g0144500	100,00	0,00E+00	2241	0	NA	NA	
maker-7-augustus-gene-19.33-mRNA-1	7	OsR498G0713506600.01.T01	100,00	0,00E+00	5768	Os07g0145400	100,00	0,00E+00	5768	0	NA	NA	Kinase-LRR-TM
maker-7-augustus-gene-190.24-mRNA-1	7	OsR498G0714394700.01.T03	99,94	0,00E+00	3031	Os07g0508500	99,72	0,00E+00	1902	-1129	Indica	NA	
maker-7-augustus-gene-190.32-mRNA-1	7	OsR498G0714401700.01.T01	99,63	0,00E+00	1925	Os07g0509600	99,62	0,00E+00	1865	-60	NA	Japonica	
maker-7-augustus-gene-190.34-mRNA-1	7	OsR498G0714403500.01.T01	99,72	0,00E+00	1924	Os07g0510100	99,81	0,00E+00	1929	5	NA	NA	
maker-7-augustus-gene-190.38-mRNA-1	7	OsR498G0714400000.01.T01	99,83	0,00E+00	3123	Os04g0610700	70,60	0,00E+00	634	-2489	Indica	NA	
maker-7-augustus-gene-190.45-mRNA-1	7	OsR498G0714402400.01.T01	99,79	0,00E+00	2562	Os07g0509800	99,79	0,00E+00	2562	0	NA	Japonica	
maker-7-augustus-gene-191.29-mRNA-1	7	OsR498G0714404300.01.T01	99,57	0,00E+00	2889	Os07g0510200	99,32	0,00E+00	2879	-10	NA	Japonica	
maker-7-augustus-gene-191.32-mRNA-1	7	OsR498G0714407100.01.T01	97,79	0,00E+00	2886	Os06g0678800	71,11	0,00E+00	829	-2057	Indica	NA	
maker-7-augustus-gene-191.35-mRNA-1	7	OsR498G0714407300.01.T01	95,97	0,00E+00	3296	Os07g0511000	96,02	0,00E+00	3301	5	NA	NA	
maker-7-augustus-gene-191.39-mRNA-1	7	OsR498G0714404600.01.T01	96,05	0,00E+00	1550	Os07g0510300	94,90	0,00E+00	1264	-286	Indica	NA	
maker-7-augustus-gene-192.21-mRNA-1	7	OsR498G0714410700.01.T03	99,73	0,00E+00	4638	Os07g0511500	99,89	0,00E+00	3377	-1261	Indica	NA	
maker-7-augustus-gene-192.34-mRNA-1	7	OsR498G0714413800.01.T01	99,68	0,00E+00	3299	Os07g0512100	99,60	0,00E+00	3150	-149	NA	NA	
maker-7-augustus-gene-193.6-mRNA-1	7	OsR498G0714414800.01.T01	100,00	0,00E+00	650	Os07g0512200	99,72	0,00E+00	645	-5	NA	NA	
maker-7-augustus-gene-193.8-mRNA-1	7	OsR498G0714419600.01.T04	99,68	0,00E+00	1106	Os07g0514400	99,52	0,00E+00	1102	-4	NA	NA	
maker-7-augustus-gene-194.31-mRNA-1	7	OsR498G0714421000.01.T03	99,88	0,00E+00	2881	Os07g0515100	99,81	0,00E+00	2876	-5	NA	Japonica	CC-Kinase-TM
maker-7-augustus-gene-195.37-mRNA-1	7	OsR498G0714424710.01.T03	100,00	0,00E+00	663	Os07g0515700	81,96	1,39E-124	444	-219	Indica	Japonica	
maker-7-augustus-gene-195.39-mRNA-1	7	OsR498G0714425300.01.T05	99,65	0,00E+00	1029	Os07g0516000	99,48	0,00E+00	1024	-5	NA	NA	
maker-7-augustus-gene-195.42-mRNA-1	7	OsR498G0714429900.01.T02	99,66	0,00E+00	2649	Os07g0516600	99,66	0,00E+00	2649	0	NA	NA	
maker-7-augustus-gene-195.46-mRNA-1	7	OsR498G0714430500.01.T04	99,72	0,00E+00	2558	Os03g0569900	70,91	1,02E-113	411	-2147	Indica	NA	
maker-7-augustus-gene-195.51-mRNA-1	7	OsR498G0714431500.01.T01	96,94	0,00E+00	876	Os07g0517100	96,94	0,00E+00	876	0	NA	NA	
maker-7-augustus-gene-195.53-mRNA-1	7	OsR498G0714432100.01.T01	98,49	0,00E+00	2997	Os07g0517300	98,56	0,00E+00	2774	-223	NA	NA	
maker-7-augustus-gene-195.58-mRNA-1	7	OsR498G0714430800.01.T01	99,70	0,00E+00	4151	Os07g0517000	99,75	0,00E+00	2823	-1328	Indica	NA	
maker-7-augustus-gene-196.37-mRNA-1	7	OsR498G0714433900.01.T01	96,54	0,00E+00	1859	Os07g0517600	96,98	0,00E+00	2432	573	Japonica	NA	
maker-7-augustus-gene-196.38-mRNA-1	7	OsR498G0714433900.01.T01	90,34	0,00E+00	1537	Os07g0517600	88,51	0,00E+00	1849	312	Japonica	NA	
maker-7-augustus-gene-196.43-mRNA-1	7	OsR498G0714435800.01.T05	99,09	0,00E+00	2130	Os07g0517900	99,34	0,00E+00	1602	-528	Indica	NA	
maker-7-augustus-gene-197.1-mRNA-1	7	OsR498G0714440300.01.T03	88,99	0,00E+00	1618	Os07g0519300	86,84	0,00E+00	1477	-141	NA	NA	
maker-7-augustus-gene-197.2-mRNA-1	7	OsR498G0714440300.01.T03	98,85	0,00E+00	1677	Os07g0519300	88,63	0,00E+00	1212	-465	Indica	NA	
maker-7-augustus-gene-197.4-mRNA-1	7	OsR498G0714440500.01.T05	98,53	0,00E+00	2491	Os07g0519300	98,51	0,00E+00	2099	-392	Indica	NA	
maker-7-augustus-gene-197.5-mRNA-1	7	OsR498G0714441900.01.T01	98,88	0,00E+00	2504	Os07g0519500	98,91	0,00E+00	2253	-251	Indica	NA	
maker-7-augustus-gene-197.68-mRNA-1	7	OsR498G0714447300.01.T03	99,49	0,00E+00	695	Os07g0521000	99,49	0,00E+00	695	0	NA	NA	
maker-7-augustus-gene-198.15-mRNA-1	7	OsR498G0714458300.01.T01	99,89	0,00E+00	1695	Os07g0522900	99,80	0,00E+00	911	-784	Indica	NA	
maker-7-augustus-gene-198.20-mRNA-1	7	OsR498G1222153700.01.T02	99,05	2,24E-157	555	Os07g0523700	99,68	1,51E-160	564	9	NA	NA	
maker-7-augustus-gene-198.9-mRNA-1	7	OsR498G0714455300.01.T04	99,86	0,00E+00	5030	Os07g0522600	99,79	0,00E+00	3433	-1597	Indica	NA	
maker-7-augustus-gene-199.32-mRNA-1	7	OsR498G1222153700.01.T02	99,37	5,28E-159	560	Os07g0523100	99,05	7,80E-158	555	-5	NA	NA	
maker-7-augustus-gene-199.37-mRNA-1	7	OsR498G1222153700.01.T02	100,00	3,80E-162	571	Os07g0523100	99,50	0,00E+00	717	146	Japonica	NA	
maker-7-augustus-gene-199.44-mRNA-1	7	OsR498G0714462100.01.T05	99,26	0,00E+00	1188	Os07g0524000	99,22	0,00E+00	1134	-54	NA	NA	
maker-7-augustus-gene-199.47-mRNA-1	7	OsR498G0714463500.01.T04	99,66	0,00E+00	1590	Os07g0524100	99,66	0,00E+00	1590	0	NA	NA	
maker-7-augustus-gene-199.58-mRNA-1	7	OsR498G0714459600.01.T01	99,53	0,00E+00	2271	Os07g0523600	99,76	0,00E+00	1485	-786	Indica	NA	
maker-7-augustus-gene-199.63-mRNA-1	7	OsR498G0714465200.01.T01	99,73	0,00E+00	1960	Os07g0524900	99,73	0,00E+00	1960	0	NA	NA	
maker-7-augustus-gene-2.0-mRNA-1	7	OsR498G0713362700.01.T01	99,30	0,00E+00	5097	Os07g0103100	99,76	0,00E+00	5147	50	NA	NA	

maker-7-augustus-gene-2.12-mRNA-1	7	OsR498G0713550700.01.T01	100,00	0,00E+00	1489	Os07g0158200	100,00	0,00E+00	1489	0	NA	NA	
maker-7-augustus-gene-2.29-mRNA-1	7	OsR498G0713551000.01.T01	100,00	0,00E+00	1889	Os07g0158300	100,00	0,00E+00	1889	0	NA	NA	
maker-7-augustus-gene-2.3-mRNA-1	7	OsR498G0713549900.01.T01	100,00	0,00E+00	1743	Os07g0157000	100,00	0,00E+00	1656	-87	NA	NA	
maker-7-augustus-gene-2.32-mRNA-1	7	OsR498G0713390000.01.T01	100,00	0,00E+00	1575	Os07g0111400	100,00	0,00E+00	1575	0	NA	NA	
maker-7-augustus-gene-200.40-mRNA-1	7	OsR498G0714467900.01.T01	99,74	0,00E+00	1393	Os07g0525400	99,74	0,00E+00	1393	0	NA	NA	
maker-7-augustus-gene-200.42-mRNA-1	7	OsR498G0714468400.01.T01	99,75	0,00E+00	2146	Os07g0525500	99,75	0,00E+00	2146	0	NA	NA	
maker-7-augustus-gene-200.44-mRNA-1	7	OsR498G0714469600.01.T01	99,67	0,00E+00	2141	Os07g0525900	99,00	0,00E+00	2105	-36	NA	Japonica	
maker-7-augustus-gene-200.46-mRNA-1	7	OsR498G0714472000.01.T01	97,87	0,00E+00	2002	Os07g0526400	96,52	0,00E+00	1990	-12	NA	NA	
maker-7-augustus-gene-201.0-mRNA-1	7	OsR498G0714474100.01.T01	99,86	0,00E+00	1320	Os07g0527000	97,77	0,00E+00	1224	-96	NA	Indica	
maker-7-augustus-gene-201.7-mRNA-1	7	OsR498G0714476000.01.T01	99,77	0,00E+00	2366	Os07g0527600	96,44	0,00E+00	3450	1084	Japonica	Indica	
maker-7-augustus-gene-202.35-mRNA-1	7	OsR498G0714477500.01.T01	99,15	0,00E+00	3553	Os07g0527800	99,39	0,00E+00	3490	-63	NA	NA	
maker-7-augustus-gene-202.43-mRNA-1	7	OsR498G0714480300.01.T01	99,57	0,00E+00	830	Os07g0528900	98,51	0,00E+00	1523	693	Japonica	Indica	
maker-7-augustus-gene-202.46-mRNA-1	7	OsR498G0714482300.01.T01	99,44	0,00E+00	1903	Os07g0529600	99,91	0,00E+00	1922	19	NA	Indica	DR8
maker-7-augustus-gene-203.0-mRNA-1	7	OsR498G0714482500.01.T01	99,79	0,00E+00	1669	Os07g0529700	99,81	0,00E+00	929	-740	Indica	NA	
maker-7-augustus-gene-203.10-mRNA-1	7	OsR498G0714487700.01.T01	99,74	0,00E+00	4084	Os07g0531700	99,75	0,00E+00	3585	-499	Indica	Japonica	
maker-7-augustus-gene-203.13-mRNA-1	7	OsR498G0714482800.01.T02	99,76	0,00E+00	732	Os07g0529800	99,71	2,42E-178	624	-108	Indica	NA	
maker-7-augustus-gene-203.16-mRNA-1	7	OsR498G0714483400.01.T01	99,85	0,00E+00	3623	Os07g0530100	99,70	0,00E+00	3609	-14	NA	Indica	
maker-7-augustus-gene-203.24-mRNA-1	7	OsR498G0714484800.01.T02	99,26	0,00E+00	957	Os07g0530700	99,82	0,00E+00	970	13	NA	NA	
maker-7-augustus-gene-203.27-mRNA-1	7	OsR498G0714487600.01.T01	99,91	0,00E+00	2003	Os07g0531600	99,91	0,00E+00	2003	0	NA	Japonica	
maker-7-augustus-gene-203.3-mRNA-1	7	OsR498G0714484500.01.T03	99,85	0,00E+00	2399	Os07g0530600	99,93	0,00E+00	2393	-6	NA	NA	
maker-7-augustus-gene-203.8-mRNA-1	7	OsR498G0714487100.01.T01	100,00	0,00E+00	1846	Os07g0531400	98,34	0,00E+00	1767	-79	NA	Indica	
maker-7-augustus-gene-204.2-mRNA-1	7	OsR498G0714492200.01.T02	88,87	0,00E+00	2138	Os07g0532500	98,64	0,00E+00	2180	42	NA	NA	
maker-7-augustus-gene-204.5-mRNA-1	7	OsR498G0714492200.01.T02	99,81	0,00E+00	2898	Os07g0532500	88,49	0,00E+00	1618	-1280	Indica	Japonica	
maker-7-augustus-gene-205.14-mRNA-1	7	OsR498G0714497100.01.T02	99,27	0,00E+00	3632	Os07g0534700	96,64	0,00E+00	3415	-217	NA	Indica	Kinase-TM
maker-7-augustus-gene-206.0-mRNA-1	7	OsR498G0714500300.01.T06	99,43	0,00E+00	3737	Os07g0535800	99,08	0,00E+00	3067	-670	Indica	NA	Kinase-TM
maker-7-augustus-gene-206.10-mRNA-1	7	OsR498G0714502100.01.T01	96,73	0,00E+00	1955	Os07g0628700	71,11	2,09E-149	530	-1425	Indica	Indica	Kinase-TM
maker-7-augustus-gene-206.4-mRNA-1	7	OsR498G0714501700.01.T01	92,75	0,00E+00	3063	Os07g0537000	95,08	0,00E+00	3180	117	NA	Japonica	Kinase-TM
maker-7-augustus-gene-207.52-mRNA-1	7	OsR498G0714503300.01.T01	99,74	0,00E+00	2783	Os07g0537900	99,68	0,00E+00	2779	-4	NA	NA	Kinase-TM
maker-7-augustus-gene-207.55-mRNA-1	7	OsR498G0714507100.01.T01	95,27	0,00E+00	2055	Os07g0538400	99,13	0,00E+00	1816	-239	Indica	Japonica	CC-Kinase-TM
maker-7-augustus-gene-207.58-mRNA-1	7	OsR498G0714507500.01.T01	99,51	0,00E+00	3645	Os07g0537900	83,00	0,00E+00	1994	-1651	Indica	Indica	Kinase-TM
maker-7-augustus-gene-207.62-mRNA-1	7	OsR498G0714502600.01.T01	98,27	0,00E+00	3480	Os07g0537500	97,37	0,00E+00	3421	-59	NA	Japonica	Kinase-TM
maker-7-augustus-gene-207.66-mRNA-1	7	OsR498G0714503600.01.T01	99,59	0,00E+00	3058	Os07g0538000	99,41	0,00E+00	2982	-76	NA	NA	
maker-7-augustus-gene-207.68-mRNA-1	7	OsR498G0714504000.01.T01	99,71	0,00E+00	3761	Os07g0538200	99,91	0,00E+00	3779	18	NA	NA	Kinase-TM
maker-7-augustus-gene-207.71-mRNA-1	7	OsR498G0714504400.01.T04	98,95	0,00E+00	2345	Os07g0537900	86,20	0,00E+00	1979	-366	Indica	Indica	Kinase-TM
maker-7-augustus-gene-207.76-mRNA-1	7	OsR498G0714508400.01.T02	99,72	0,00E+00	2581	Os07g0539100	99,52	0,00E+00	2593	12	NA	Indica	
maker-7-augustus-gene-208.31-mRNA-1	7	OsR498G0714510100.01.T01	97,22	0,00E+00	2905	Os07g0537900	84,40	0,00E+00	1872	-1033	Indica	Japonica	Kinase-TM
maker-7-augustus-gene-208.36-mRNA-1	7	OsR498G0714516300.01.T01	96,56	0,00E+00	3608	Os07g0541000	96,69	0,00E+00	3620	12	NA	Indica	Kinase-TM
maker-7-augustus-gene-208.38-mRNA-1	7	OsR498G0714510800.01.T02	91,13	0,00E+00	1939	Os07g0539900	99,70	0,00E+00	2370	431	Japonica	Indica	
maker-7-augustus-gene-208.46-mRNA-1	7	OsR498G0714513000.01.T03	98,41	0,00E+00	1523	Os07g0540200	98,86	0,00E+00	1541	18	NA	NA	
maker-7-augustus-gene-209.0-mRNA-1	7	OsR498G0714516900.01.T02	99,67	0,00E+00	3751	Os07g0541400	98,00	0,00E+00	3597	-154	NA	Indica	Kinase-TM
maker-7-augustus-gene-209.11-mRNA-1	7	OsR498G0714515800.01.T01	100,00	0,00E+00	1617	Os07g0540800	99,89	0,00E+00	1612	-5	NA	Indica	Kinase-TM
maker-7-augustus-gene-209.12-mRNA-1	7	OsR498G0714516800.01.T01	98,33	0,00E+00	2788	Os07g0541000	88,60	0,00E+00	2785	-3	NA	Indica	Kinase-TM
maker-7-augustus-gene-209.21-mRNA-1	7	OsR498G0714517200.01.T02	99,79	0,00E+00	1678	Os07g0541500	91,61	0,00E+00	1207	-471	Indica	Indica	Kinase-TM
maker-7-augustus-gene-209.24-mRNA-1	7	OsR498G0714517300.01.T03	83,67	0,00E+00	789	Os07g0541500	80,90	0,00E+00	758	-31	NA	Japonica	
maker-7-augustus-gene-209.26-mRNA-1	7	OsR498G0714517600.01.T05	99,55	0,00E+00	1994	Os07g0541800	99,55	0,00E+00	1994	0	NA	NA	Kinase-TM
maker-7-augustus-gene-209.30-mRNA-1	7	OsR498G0714517800.01.T05	99,18	0,00E+00	1940	Os07g0541900	97,73	0,00E+00	1876	-64	NA	NA	Kinase-TM
maker-7-augustus-gene-209.7-mRNA-1	7	OsR498G0714517300.01.T01	99,43	0,00E+00	3452	Os07g0541500	86,24	0,00E+00	2269	-1183	Indica	Japonica	Kinase-TM
maker-7-augustus-gene-21.37-mRNA-1	7	OsR498G0713510500.01.T02	90,36	2,32E-161	570	Os07g0147500	90,36	8,05E-162	570	0	NA	Japonica	
maker-7-augustus-gene-21.41-mRNA-1	7	OsR498G0713510700.01.T01	99,29	0,00E+00	745	Os07g0147500	77,40	1,39E-86	318	-427	Indica	Japonica	
maker-7-augustus-gene-21.44-mRNA-1	7	OsR498G0713511300.01.T04	99,81	0,00E+00	1902	Os09g0133700	73,22	1,44E-154	545	-1357	Indica	Japonica	
maker-7-augustus-gene-21.47-mRNA-1	7	OsR498G0713512500.01.T01	99,86	0,00E+00	2542	Os07g0148200	100,00	0,00E+00	2551	9	NA	Japonica	
maker-7-augustus-gene-21.56-mRNA-1	7	OsR498G0713515500.01.T01	99,75	0,00E+00	721	Os07g0148900	100,00	0,00E+00	726	5	NA	Japonica	
maker-7-augustus-gene-21.59-mRNA-1	7	OsR498G0713511600.01.T05	99,67	0,00E+00	3783	Os07g0147800	100,00	0,00E+00	3815	32	NA	Japonica	
maker-7-augustus-gene-21.62-mRNA-1	7	OsR498G0713512200.01.T01	99,74	0,00E+00	2041	Os07g0147900	100,00	0,00E+00	2051	10	NA	Japonica	
maker-7-augustus-gene-210.39-mRNA-1	7	OsR498G0714521300.01.T01	96,19	0,00E+00	981	Os07g0542900	96,89	0,00E+00	976	-5	NA	Japonica	
maker-7-augustus-gene-210.41-mRNA-1	7	OsR498G0714521700.01.T02	97,89	0,00E+00	3243	Os07g0543100	100,00	0,00E+00	3252	9	NA	Japonica	
maker-7-augustus-gene-210.44-mRNA-1	7	OsR498G0714520000.01.T01	86,98	0,00E+00	2414	Os07g0542400	79,61	0,00E+00	1644	-770	Indica	Japonica	Kinase-TM
maker-7-augustus-gene-210.47-mRNA-1	7	OsR498G0714519200.01.T05	99,40	0,00E+00	2067	Os07g0542400	98,84	0,00E+00	1962	-105	NA	Indica	Kinase-TM
maker-7-augustus-gene-210.56-mRNA-1	7	OsR498G0714521600.01.T02	99,11	0,00E+00	791	Os07g0543000	99,33	0,00E+00	795	4	NA	Japonica	
maker-7-augustus-gene-211.0-mRNA-1	7	OsR498G0714522100.01.T01	97,88	0,00E+00	2503	Os07g0543200	99,93	0,00E+00	2642	139	NA	Japonica	
maker-7-augustus-gene-211.11-mRNA-1	7	OsR498G0714525700.01.T01	100,00	0,00E+00	737	Os07g0545400	100,00	0,00E+00	737	0	NA	Japonica	
maker-7-augustus-gene-211.13-mRNA-1	7	OsR498G0714524800.01.T02	100,00	0,00E+00	1110	Os07g0544900	100,00	0,00E+00	1110	0	NA	Japonica	
maker-7-augustus-gene-211.15-mRNA-1	7	OsR498G0714526000.01.T01	99,11	0,00E+00	4956	Os07g0545500	100,00	0,00E+00	5032	76	NA	Japonica	
maker-7-augustus-gene-211.2-mRNA-1	7	OsR498G0714522500.01.T01	97,61	0,00E+00	3541	Os07g0543500	99,93	0,00E+00	5377	1836	Japonica	Japonica	
maker-7-augustus-gene-211.5-mRNA-1	7	OsR498G0714524300.01.T01	99,85	0,00E+00	1176	Os07g0544800	99,69	0,00E+00	1171	-5	NA	Japonica	
maker-7-augustus-gene-211.7-mRNA-1	7	OsR498G0714525100.01.T01	99,40	0,00E+00	891	Os07g0545100	100,00	0,00E+00	904	13	NA	Japonica	
maker-7-augustus-gene-211.9-mRNA-1	7	OsR498G0714525300.01.T01	99,51	0,00E+00	728	Os07g0545300	99,76	0,00E+00	732	4	NA	Japonica	
maker-7-augustus-gene-212.39-mRNA-1	7	OsR498G0714527900.01.T01	99,72	0,00E+00	1285	Os07g0546000	99,86	0,00E+00	1289	4	NA	Japonica	
maker-7-augustus-gene-212.43-mRNA-1	7	OsR498G0714528100.01.T03	99,88	0,00E+00	2962	Os07g0546100	96,84	0,00E+00	2773	-189	NA	Japonica	
maker-7-augustus-gene-212.52-mRNA-1	7	OsR498G0714530800.01.T02	97,71	0,00E+00	2091	Os07g0546600	98,94	0,00E+00	2159	68	NA	Japonica	
maker-7-augustus-gene-213.32-mRNA-1	7	OsR498G0714533900.01.T02	99,70	0,00E+00	3555	Os07g0548300	100,00	0,00E+00	3582	27	NA	Japonica	
maker-7-augustus-gene-213.37-mRNA-1	7	OsR498G0714534700.01.T01	99,84	0,00E+00	1095	Os07g0548800	100,00	0,00E+00	1099	4	NA	Japonica	
maker-7-augustus-gene-213.42-mRNA-1	7	OsR498G0714534100.01.T01	70,31	0,00E+00	648	Os07g0548100	83,44	0,00E+00	1686	1038	Japonica	Japonica	
maker-7-augustus-gene-213.44-mRNA-1	7	OsR498G0714533800.01.T01	99,09	0,00E+00	1746	Os07g0548100	100,00	0,00E+00	3371	1625	Japonica	Japonica	
maker-7-augustus-gene-213.47-mRNA-1	7	OsR498G0714535000.01.T01	99,55	0,00E+00	1568	Os07g0548900	94,30	0,00E+00	1006	-562	Indica	Japonica	
maker-7-augustus-gene-214.10-mRNA-1	7	OsR498G0714537100.01.T01	99,50	0,00E+00	717	Os07g0549800	100,00	0,00E+00	726	9	NA	Japonica	
maker-7-augustus-gene-214.12-mRNA-1	7	OsR498G0714539100.01.T01	99,48	0,00E+00	2375	Os07g0550600	100,00	0,00E+00	2403	28	NA	Japonica	
maker-7-augustus-gene-214.14-mRNA-1	7	OsR498G0714535600.01.T01	99,75	0,00E+00	1415	Os07g0549200	100,00	0,00E+00	1424	9	NA	Japonica	
maker-7-augustus-gene-214.16-mRNA-1	7	OsR498G0714536000.01.T02	93,87	0,00E+00	733	Os10g0388500	94,58	0,00E+00	820	87	Japonica	Japonica	
maker-7-augustus-gene-214.21-mRNA-1	7	OsR498G0714541400.01.T01	99,23	0,00E+00	4598	Os07g0550900	100,00	0,00E+00	4679	81	NA	Japonica	Kinase-TM
maker-7-augustus-gene-214.25-mRNA-1	7	OsR498G0714542600.01.T02	99,96	0,00E+00	4617	Os07g0551300	100,00	0,00E+00	4621	4	NA	Japonica	Kinase-TM
maker-7-augustus-gene-215.42-mRNA-1	7	OsR498G0714543800.01.T01	97,44	0,00E+00	4102	Os07g0551500	100,00	0,00E+00	3041	-1061	Indica	Japonica	
maker-7-augustus-gene-215.43-mRNA-1	7	OsR498G0714545000.01.T01	98,07	0,00E+00	4526	Os07g0551700	100,00	0,00E+00	4800	274	NA	Japonica	
maker-7-augustus-gene-216.0-mRNA-1	7	OsR498G0714550700.01.T02	99,70	3,25E-171	602	Os07g0449100	82,79	9,31E-97	353	-249	Indica	Japonica	
maker-7-augustus-gene-216.3-mRNA-1	7	OsR498G0714548600.01.T01	99,41	0,00E+00	3629	Os07g0553400	100,00	0,00E+00	3683	54	NA	Japonica	
maker-7-augustus-gene-217.13-mRNA-1	7	OsR498G0714558200.01.T01	99,68	0,00E+00	1691	Os07g0556000	99,90	0,00E+00	1729	38	NA	NA	
maker-7-augustus-gene-217.5-mRNA-1	7	OsR498G0714556700.01.T01	99,66	0,00E+00	2091	Os07g0555300	99,74	0,00E+00	1364	-727	Indica	NA	
maker-7-augustus-gene-218.0-mRNA-1	7	OsR498G0714563100.01.T02	99,65	0,00E+00	3034	Os07g0557500	100,00	0,00E+00	3061	27	NA	Japonica	
maker-7-augustus-gene-218.12-mRNA-1	7	OsR498G0714562400.01.T01	99,84	0,00E+00	2259	Os07g0557200	99,36	0,00E+00	2232	-27	NA	NA	
maker-7-augustus-gene-218.16-mRNA-1	7	OsR498G0714563000.01.T01	100,00	0,00E+00	1148	Os07g0557200	77,45	6,04E-140	498	-650	Indica	Indica	
maker-7-augustus-gene-218.17-mRNA-1	7	OsR498G0714563000.01.T01	99,12	0,00E+00	1204	Os07g0557400	99,12	0,00E+00	1204	0	NA	Indica	
maker-7-augustus-gene-218.26-mRNA-1	7	OsR498G0714563600.01.T01	99,70	0,00E+00	1187	Os07g0557700	100,00	0,00E+00	1819	632	Japonica	Japonica	
maker-7-augustus-gene-218.28-mRNA-1	7	OsR498G0714565000.01.T04	98,45	0,00E+00	3250	Os07g0558000	98,88	0,00E+00	3286	36	NA	Japonica	
maker-7-augustus-gene-218.7-mRNA-1	7	OsR498G0714561800.01.T02	96,98	0,00E+00	2124	Os07g0557100	96,90	0,00E+00	2120	-4	NA	Japonica	
maker-7-augustus-gene-219.0-mRNA-1	7	OsR498G0714569100.01.T01	100,00	0,00E+00	1543	Os07g0558800	100,00	0,00E+00	1543	0	NA	Japonica	
maker-7-augustus-gene-219.12-mRNA-1	7	OsR498G0714567400.01.T01	100,00	0,00E+00	1707	Os07g0558300	100,00	0,00E+00	1707	0	NA	Japonica	
maker-7-augustus-gene-219.15-mRNA-1	7	OsR498G0714568000.01.T01	99,79	0,00E+00	1685	Os07g0558400	100,00	0,00E+00	1575	-110	NA	Japonica	
maker-7-augustus-gene-219.17-mRNA-1	7	OsR498G0714568200.01.T02	99,88	0,00E+00	1554	Os07g0558500	100,00	0,00E+00	1559	5	NA	Japonica	
maker-7-augustus-gene-219.20-mRNA-1	7	OsR498G0714569800.01.T01	99,64	0,00E+00	1470	Os07g0559400	100,00	0,00E+00	1483	13	NA	Japonica	
maker-7-augustus-gene-219.22-mRNA-1	7	OsR498G0714570800.01.T01	100,00	0,00E+00	2874	Os07g0559700	100,00	0,00E+00	2874	0	NA	Japonica	
maker-7-augustus-gene-219.25-mRNA-1	7	OsR498G0714572400.01.T02	99,43	0,00E+00	3140	Os07g0560300	100,00	0,00E+00	3182	42	NA	Japonica	
maker-7-augustus-gene-219.6-mRNA-1	7	OsR498G0714565800.01.T01	99,64	0,00E+00	1979	Os07g0558100	100,00	0,00E+00	1997	18	NA	Japonica	
maker-7-augustus-gene-219.8-mRNA-1	7	OsR498G0714567400.01.T01	81,95	0,00E+00	1330	Os07g0558200	96,99	0,00E+00	2193	863	Japonica	Japonica	
maker-7-augustus-gene-22.12-mRNA-1	7	OsR498G0713521200.01.T02	99,64	0,00E+00	5010	Os07g0150500	99,64	0,00E+00	5010	0	NA	NA	
maker-7-augustus-gene-22.17-mRNA-1	7	OsR498G0713521400.01.T01	100,00	0,00E+00	2435	Os07g0150700	99,78	0,00E+00	2425	-10	NA	NA	Kinase-TM
maker-7-augustus-gene-22.7-mRNA-1	7	OsR498G0713518200.01.T01	99,40	0,00E+00	1182	Os07g0149900	100,00	0,00E+00	704	-478	Indica	Japonica	
maker-7-augustus-gene-22.9-mRNA-1	7	OsR498G0713518400.01.T01	99,73	0,00E+00	3934	Os07g0150100	100,00	0,00E+00	3961	27	NA	Japonica	
maker-7-augustus-gene-220.0-mRNA-1	7	OsR498G0714578700.01.T01	99,63	0,00E+00	1432	Os07g0562700	100,00	0,00E+00	1445	13	NA	NA	
maker-7-augustus-gene-220.2-mRNA-1	7	OsR498G0714575800.01.T01	93,19	0,00E+00	841	Os07g0561300	93,37	0,00E+00	846	5	NA	Japonica	
maker-7-augustus-gene-221.41-mRNA-1	7	OsR498G0714579000.01.T05	97,59	0,00E+00	1056	Os07g0562800	97,75	0,00E+00	1060	4	NA	NA	
maker-7-augustus-gene-221.42-mRNA-1	7	OsR498G0714579000.01.T01	97,67	0,00E+00	4696	Os07g0562800	97,40	0,00E+00	3794	-902	Indica	NA	
maker-7-augustus-gene-221.48-mRNA-1	7	OsR498G0714578700.01.T01	99,63	0,00E+00	1432	Os07g0562700	100,00	0,00E+00	1445	13	NA	NA	
maker-7-augustus-gene-221.58-mRNA-1	7	OsR498G0714578700.01.T01	99,63	0,00E+00	1432	Os07g0562700	100,00	0,00E+00	1445	13	NA	NA	
maker-7-augustus-gene-221.60-mRNA-1	7	OsR498G0714579000.01.T01	99,80	0,00E+00	4407	Os07g0562800	99,95	0,00E+00	3497	-910	Indica	Japonica	
maker-7-augustus-gene-222.47-mRNA-1	7	OsR498G0714583500.01.T01	99,70	0,00E+00	7218	Os07g0563700	100,00	0,00E+00	7272	54	NA	Japonica	
maker-7-augustus-gene-222.60-mRNA-1	7	OsR498G0714585900.01.T02	99,76	0,00E+00	3027	Os07g0564500	100,00	0,00E+00	3047	20	NA	Japonica	
maker-7-augustus-gene-222.62-mRNA-1	7	OsR498G0714582200.01.T01	100,00	0,00E+00	1808	Os07g0563400	100,00	0,00E+00	1808	0	NA	Japonica	
maker-7-augustus-gene-222.64-mRNA-1	7	OsR498G0714583800.01.T01	99,93	0,00E+00	2577	Os07g0563800	100,00	0,00E+00	2581	4	NA	Japonica	
maker-7-augustus-gene-223.43-mRNA-1	7	OsR498G0714589500.01.T01	99,77	0,00E+00	2330	Os07g0565600	100,00	0,00E+00	2343	13	NA	Japonica	
maker-7-augustus-gene-223.49-mRNA-1	7	OsR498G0714586200.01.T03	99,46	0,00E+00	1643	Os07g0564600	99,68	0,00E+00	1652	9	NA	Japonica	
maker-7-augustus-gene-223.63-mRNA-1	7	OsR498G0714587200.01.T02	99,92	0,00E+00	2296	Os07g0564800	100,00	0,00E+00	2116	-180	NA	Japonica	
maker-7-augustus-gene-223.66-mRNA-1	7	OsR498G0714587200.01.T05	100,00	1,44E-156	553	Os07g0565000	100,00	9,07E-154	542	-11	NA	Japonica	
maker-7-augustus-gene-223.68-mRNA-1	7	OsR498G0714587600.01.T01	94,74	0,00E+00	824	Os07g0565000	92,59	0,00E+00	774	-50	NA	NA	
maker-7-augustus-gene-223.71-mRNA-1	7	OsR498G0714588000.01.T02	99,66	0,00E+00	2658	Os07g0565300	100,00	0,00E+00	2679	21	NA	Japonica	
maker-7-augustus-gene-224.42-mRNA-1	7	OsR498G0714591800.01.T05	100,00	0,00E+00	1440	Os07g0566200	100,00	0,00E+00	1440	0	NA	Japonica	
maker-7-augustus-gene-224.48-mRNA-1	7	OsR498G0714593200.01.T01	99,71	0,00E+00	1236	Os07g0566800	100,00	0,00E+00	1059	-177	Indica	Japonica	TIR
maker-7-augustus-gene-224.50-mRNA-1	7	OsR498G0714594400.01.T01	99,66	0,00E+00	2624	Os07g0567300	100,00	0,00E+00	3162	538	Japonica	Japonica	
maker-7-augustus-gene-225.38-mRNA-1	7	OsR498G0714596100.01.T01	99,77	0,00E+00	3104	Os07g0568100	100,00	0,00E+00	3301	197	NA	Japonica	Kinase-LRR-TM
maker-7-augustus-gene-225.41-mRNA-1	7	OsR498G0714601100.01.T01	99,83	0,00E+00	3185	Os07g0569000	100,00	0,00E+00	5357	2172	Japonica	Japonica	
maker-7-augustus-gene-225.44-mRNA-1	7	OsR498G0714596000.01.T01	99,87	0,00E+00	4175	Os07g0568000	100,00	0,00E+00	4188	13	NA	Japonica	
maker-7-augustus-gene-225.49-mRNA-1	7	OsR498G0714596400.01.T01	99,66	0,00E+00	3655	Os07g0568200	100,00	0,00E+00	3781	126	NA	Japonica	
maker-7-augustus-gene-225.57-mRNA-1	7	OsR498G0714597800.01.T03	99,69	0,00E+00	1708	Os07g0568500	100,00	0,00E+00	1478	-230	Indica	Japonica	
maker-7-augustus-gene-225.59-mRNA-1	7	OsR498G0714598000.01.T01	99,70	0,00E+00	2959	Os07g0568600	100,00	0,00E+00	2982	23	NA	Japonica	
maker-7-augustus-gene-225.64-mRNA-1	7	OsR498G0714600800.01.T02	100,00	0,00E+00	910	Os07g0568900	100,00	0,00E+00	910	0	NA	Japonica	
maker-7-augustus-gene-225.67-mRNA-1	7	OsR498G0714601400.01.T02	97,11	0,00E+00	1614	Os07g0569100	100,00	0,00E+00	1716	102	NA	Japonica	
maker-7-augustus-gene-226.27-mRNA-1	7	OsR498G0714604900.01.T01	85,61	0,00E+00	723	Os07g0570700	85,79	0,00E+00	728	5	NA	Japonica	
maker-7-augustus-gene-226.32-mRNA-1	7	OsR498G0714602000.01.T04	100,00	0,00E+00	1050	Os07g0569600	100,00	0,00E+00	1050	0	NA	Japonica	
maker-7-augustus-gene-226.40-mRNA-1	7	OsR498G0714603600.01.T02	99,24	0,00E+00	1860	Os07g0570300	100,00	0,00E+00	1896	36	NA	Japonica	
maker-7-augustus-gene-226.49-mRNA-1	7	OsR498G0714603600.01.T02	98,85	0,00E+00	3355	Os07g0570300	99,95	0,00E+00	3461	106	NA	Japonica	
maker-7-augustus-gene-226.52-mRNA-1	7	OsR498G0714604000.01.T02	99,29	0,00E+00	5249	Os07g0570300	89,48	0,00E+00	3939	-1310	Indica	Japonica	
maker-7-augustus-gene-226.56-mRNA-1	7	OsR498G0714604600.01.T01	89,63	0,00E+00	874	Os07g0570550	87,34	3,77E-146	517	-357	Indica	Japonica	
maker-7-augustus-gene-227.11-mRNA-1	7	OsR498G0714610900.01.T01	99,79	0,00E+00	1696	Os07g0572500	100,00	0,00E+00	1553	-143	NA	Japonica	
maker-7-augustus-gene-227.12-mRNA-1	7	OsR498G0714606400.01.T05	99,67	0,00E+00	8196	Os07g0571100	99,60	0,00E+00	5835	-2361	Indica	Japonica	
maker-7-augustus-gene-227.20-mRNA-1	7	OsR498G0714608200.01.T01	100,00	0,00E+00	1489	Os07g0571600	96,00	0,00E+00	1365	-124	NA	NA	
maker-7-augustus-gene-227.25-mRNA-1	7	OsR498G0714608600.01.T01	95,32	0,00E+00	2491	Os07g0571700	95,07	0,00E+00	2477	-14	NA	NA	
maker-7-augustus-gene-227.27-mRNA-1	7	OsR498G0714609200.01.T01	99,92	0,00E+00	2371	Os07g0571900	99,92	0,00E+00	2371	0	NA	Japonica	
maker-7-augustus-gene-227.3-mRNA-1	7	OsR498G0714609300.01.T01	99,21	0,00E+00	1800	Os07g0572000	99,31	0,00E+00	1804	4	NA	NA	
maker-7-augustus-gene-227.5-mRNA-1	7	OsR498G0714609700.01.T01	99,77	0,00E+00	2337	Os07g0572100	99,78	0,00E+00	2406	69	NA	NA	
maker-7-augustus-gene-227.9-mRNA-1	7	OsR498G0714610700.01.T01	100,00	1,44E-128	459	Os07g0572400	99,61	2,13E-127	454	-5	NA	NA	
maker-7-augustus-gene-228.0-mRNA-1	7	OsR498G0714611100.01.T01	100,00	0,00E+00	3020	Os07g0572600	100,00	0,00E+00	3020	0	NA	Japonica	
maker-7-augustus-gene-228.2-mRNA-1	7	OsR498G0714612300.01.T06	99,76	0,00E+00	3792	Os07g0572800	99,67	0,00E+00	3783	-9	NA	Japonica	Kinase-TM
maker-7-augustus-gene-229.43-mRNA-1	7	OsR498G0714613100.01.T02	97,62	0,00E+00	1787	Os07g0573100	99,04	0,00E+00	1839	52	NA	Japonica	NBS
maker-7-augustus-gene-229.47-mRNA-1	7	OsR498G0714613700.01.T01	99,30	0,00E+00	2773	Os07g0573300	100,00	0,00E+00	1418	-1355	Indica	Indica	
maker-7-augustus-gene-229.52-mRNA-1	7	OsR498G0714613900.01.T02	99,92	0,00E+00	2328	Os07g0573400	100,00	0,00E+00	2333	5	NA	Japonica	
maker-7-augustus-gene-229.57-mRNA-1	7	OsR498G0714617700.01.T01	82,81	2,46E-68	260	Os07g0574633	100,00	1,19E-98	358	98	Japonica	Japonica	
maker-7-augustus-gene-229.59-mRNA-1	7	OsR498G0714619100.01.T01	99,78	0,00E+00	2427	Os07g0574800	100,00	0,00E+00	2441	14	NA	Japonica	
maker-7-augustus-gene-229.63-mRNA-1	7	OsR498G0714614400.01.T01	99,75	0,00E+00	710	Os07g0573600	99,75	0,00E+00	710	0	NA	Japonica	
maker-7-augustus-gene-229.65-mRNA-1	7	OsR498G0714614600.01.T01	99,72	0,00E+00	1297	Os07g0573700	100,00	0,00E+00	1306	9	NA	Japonica	
maker-7-augustus-gene-229.70-mRNA-1	7	OsR498G0714615200.01.T01	97,23	0,00E+00	1773	Os07g0573800	98,19	0,00E+00	1815	42	NA	Japonica	
maker-7-augustus-gene-229.75-mRNA-1	7	OsR498G0714615800.01.T01	99,89	0,00E+00	3243	Os07g0574100	99,78	0,00E+00	1663	-1580	Indica	Japonica	Kinase-LRR-TM
maker-7-augustus-gene-23.23-mRNA-1	7	OsR498G0713526100.01.T01	98,02	0,00E+00	1214	Os07g0151300	100,00	0,00E+00	1278	64	NA	Japonica	
maker-7-augustus-gene-23.25-mRNA-1	7	OsR498G0713527500.01.T01	100,00	0,00E+00	1321	Os07g0151700	100,00	0,00E+00	1959	638	Japonica	Japonica	
maker-7-augustus-gene-23.27-mRNA-1	7	OsR498G0713527200.01.T01	92,97	0,00E+00	679	Os07g0151800	70,08	1,91E-53	209	-470	Indica	Japonica	
maker-7-augustus-gene-23.29-mRNA-1	7	OsR498G0713527800.01.T01	100,00	0,00E+00	699	Os07g0151800	100,00	0,00E+00	699	0	NA	NA	
maker-7-augustus-gene-23.32-mRNA-1	7	OsR498G0713527800.01.T01	81,71	7,29E-42	170	Os07g0151901	100,00	7,78E-125	444	274	Japonica	Indica	
maker-7-augustus-gene-230.10-mRNA-1	7	OsR498G0714619800.01.T01	99,82	0,00E+00	5903	Os07g0575100	99,97	0,00E+00	5872	-31	NA	Japonica	
maker-7-augustus-gene-230.17-mRNA-1	7	OsR498G0714620200.01.T01	99,89	0,00E+00	3205	Os07g0575500	100,00	0,00E+00	3207	2	NA	Japonica	
maker-7-augustus-gene-230.5-mRNA-1	7	OsR498G0714621700.01.T01	99,64	0,00E+00	2955	Os07g0575900	99,94	0,00E+00	2782	-173	NA	Japonica	
maker-7-augustus-gene-230.7-mRNA-1	7	OsR498G0714621900.01.T01	99,74	0,00E+00	2043	Os07g0576000	99,91	0,00E+00	2052	9	NA	Japonica	
maker-7-augustus-gene-231.45-mRNA-1	7	OsR498G0714623100.01.T05	100,00	0,00E+00	655	Os07g0577400	99,51	0,00E+00	1086	431	Japonica	NA	
maker-7-augustus-gene-231.48-mRNA-1	7	OsR498G0714623900.01.T01	99,87	0,00E+00	1425	Os07g0577600	100,00	0,00E+00	1429	4	NA	Japonica	
maker-7-augustus-gene-231.56-mRNA-1	7	OsR498G0714626700.01.T03	99,88	0,00E+00	1542	Os07g0578200	100,00	0,00E+00	1546	4	NA	Japonica	
maker-7-augustus-gene-231.60-mRNA-1	7	OsR498G0714622800.01.T01	99,89	0,00E+00	3259	Os07g0576500	98,46	0,00E+00	2370	-889	Indica	Indica	
maker-7-augustus-gene-231.62-mRNA-1	7	OsR498G0714625600.01.T01	99,73	0,00E+00	656	Os07g0577900	100,00	0,00E+00	661	5	NA	Japonica	
maker-7-augustus-gene-232.0-mRNA-1	7	OsR498G0714628300.01.T01	99,76	0,00E+00	1523	Os07g0578600	100,00	0,00E+00	1532	9	NA	Japonica	
maker-7-augustus-gene-232.11-mRNA-1	7	OsR498G0714631800.01.T01	100,00	0,00E+00	893	Os07g0580200	85,81	2,98E-140	497	-396	Indica	NA	
maker-7-augustus-gene-232.14-mRNA-1	7	OsR498G0714632200.01.T01	99,89	0,00E+00	1636	Os07g0580500	98,71	0,00E+00	1625	-11	NA	NA	
maker-7-augustus-gene-232.16-mRNA-1	7	OsR498G0714634400.01.T03	99,91	0,00E+00	1982	Os07g0580900	99,91	0,00E+00	1982	0	NA	Japonica	
maker-7-augustus-gene-232.18-mRNA-1	7	OsR498G0714634800.01.T01	99,89	0,00E+00	1636	Os07g0581000	100,00	0,00E+00	1640	4	NA	Japonica	
maker-7-augustus-gene-232.4-mRNA-1	7	OsR498G0714628800.01.T03	99,71	0,00E+00	3123	Os07g0578800	99,76	0,00E+00	2960	-163	NA	Japonica	
maker-7-augustus-gene-232.9-mRNA-1	7	OsR498G0714631800.01.T01	85,81	7,65E-140	497	Os07g0580200	100,00	0,00E+00	801	304	Japonica	NA	
maker-7-augustus-gene-233.50-mRNA-1	7	OsR498G0714639300.01.T01	98,37	0,00E+00	1278	Os07g0581700	100,00	0,00E+00	1305	27	NA	Japonica	
maker-7-augustus-gene-233.52-mRNA-1	7	OsR498G0714641300.01.T01	99,89	0,00E+00	3119	Os07g0582400	100,00	0,00E+00	3128	9	NA	Japonica	
maker-7-augustus-gene-233.55-mRNA-1	7	OsR498G0714641600.01.T01	99,67	0,00E+00	2748	Os07g0582500	100,00	0,00E+00	2722	-26	NA	Japonica	
maker-7-augustus-gene-233.57-mRNA-1	7	OsR498G0714642200.01.T01	98,87	0,00E+00	2478	Os07g0582850	99,93	0,00E+00	2517	39	NA	Japonica	
maker-7-augustus-gene-234.0-mRNA-1	7	OsR498G0714643900.01.T01	99,67	0,00E+00	2705	Os07g0583200	100,00	0,00E+00	2727	22	NA	Japonica	
maker-7-augustus-gene-234.11-mRNA-1	7	OsR498G0714647700.01.T01	99,82	0,00E+00	3889	Os07g0584200	100,00	0,00E+00	2866	-1023	Indica	Japonica	
maker-7-augustus-gene-234.14-mRNA-1	7	OsR498G0714649100.01.T01	99,51	0,00E+00	2530	Os07g0584500	99,65	0,00E+00	2539	9	NA	Japonica	
maker-7-augustus-gene-234.20-mRNA-1	7	OsR498G0714645200.01.T04	99,00	0,00E+00	1752	Os07g0583500	99,92	0,00E+00	2187	435	Japonica	Japonica	
maker-7-augustus-gene-234.23-mRNA-1	7	OsR498G0714646200.01.T01	99,68	0,00E+00	3321	Os07g0583700	100,00	0,00E+00	3350	29	NA	Japonica	
maker-7-augustus-gene-234.9-mRNA-1	7	OsR498G0714647100.01.T01	99,90	0,00E+00	1771	Os07g0584100	100,00	0,00E+00	1775	4	NA	Japonica	Kinase
maker-7-augustus-gene-235.0-mRNA-1	7	OsR498G0714650500.01.T01	99,86	0,00E+00	2524	Os07g0584900	100,00	0,00E+00	2533	9	NA	Japonica	
maker-7-augustus-gene-235.11-mRNA-1	7	OsR498G0714652400.01.T01	99,35	0,00E+00	1092	Os07g0585250	100,00	0,00E+00	1110	18	NA	Japonica	
maker-7-augustus-gene-235.19-mRNA-1	7	OsR498G0714655400.01.T01	100,00	3,02E-159	562	Os07g0585800	100,00	1,05E-159	562	0	NA	Japonica	
maker-7-augustus-gene-235.23-mRNA-1	7	OsR498G0714656200.01.T02	99,80	0,00E+00	1828	Os01g0593100	93,56	0,00E+00	791	-1037	Indica	Japonica	
maker-7-augustus-gene-235.27-mRNA-1	7	OsR498G0714656600.01.T02	99,92	0,00E+00	2355	Os07g0586100	100,00	0,00E+00	1703	-652	Indica	Japonica	
maker-7-augustus-gene-236.32-mRNA-1	7	OsR498G0714657900.01.T03	97,40	0,00E+00	3016	Os07g0586500	95,83	0,00E+00	1780	-1236	Indica	Japonica	
maker-7-augustus-gene-236.41-mRNA-1	7	OsR498G0714656800.01.T02	100,00	0,00E+00	1142	Os07g0586200	100,00	0,00E+00	2122	980	Japonica	Japonica	
maker-7-augustus-gene-236.44-mRNA-1	7	OsR498G0714658400.01.T01	99,79	0,00E+00	3353	Os07g0586700	100,00	0,00E+00	3371	18	NA	Japonica	
maker-7-augustus-gene-236.46-mRNA-1	7	OsR498G0714663400.01.T01	99,77	0,00E+00	2297	Os07g0587100	100,00	0,00E+00	2311	14	NA	Japonica	
maker-7-augustus-gene-237.32-mRNA-1	7	OsR498G0714667100.01.T03	99,77	0,00E+00	2292	Os07g0588000	99,92	0,00E+00	2301	9	NA	Japonica	
maker-7-augustus-gene-237.38-mRNA-1	7	OsR498G0714668100.01.T01	99,66	0,00E+00	4158	Os07g0588200	100,00	0,00E+00	4194	36	NA	Japonica	
maker-7-augustus-gene-237.40-mRNA-1	7	OsR498G0714668300.01.T01	100,00	0,00E+00	1927	Os07g0588300	100,00	0,00E+00	3317	1390	Japonica	Japonica	
maker-7-augustus-gene-237.43-mRNA-1	7	OsR498G0714669100.01.T02	99,59	0,00E+00	1295	Os07g0588500	100,00	0,00E+00	1308	13	NA	Japonica	
maker-7-augustus-gene-238.0-mRNA-1	7	OsR498G0714672700.01.T01	99,90	0,00E+00	1744	Os07g0589400	100,00	0,00E+00	2284	540	Japonica	Japonica	
maker-7-augustus-gene-238.10-mRNA-1	7	OsR498G0714680900.01.T01	99,59	0,00E+00	863	Os07g0591700	100,00	0,00E+00	872	9	NA	NA	
maker-7-augustus-gene-239.12-mRNA-1	7	OsR498G0714682600.01.T01	99,89	0,00E+00	3270	Os07g0592600	99,89	0,00E+00	3270	0	NA	NA	
maker-7-augustus-gene-239.14-mRNA-1	7	OsR498G0714684600.01.T02	86,76	1,71E-154	546	Os07g0593200	83,08	5,25E-105	380	-166	Indica	Japonica	
maker-7-augustus-gene-239.9-mRNA-1	7	OsR498G0714682000.01.T03	99,56	0,00E+00	3259	Os07g0592300	99,67	0,00E+00	3268	9	NA	Japonica	
maker-7-augustus-gene-24.26-mRNA-1	7	OsR498G0713532700.01.T02	100,00	0,00E+00	888	Os07g0152700	99,83	0,00E+00	1048	160	Japonica	NA	
maker-7-augustus-gene-24.29-mRNA-1	7	OsR498G0713533300.01.T08	100,00	0,00E+00	1516	Os07g0152800	100,00	0,00E+00	1516	0	NA	NA	
maker-7-augustus-gene-24.32-mRNA-1	7	OsR498G0713533300.01.T09	100,00	0,00E+00	2003	Os07g0152900	100,00	0,00E+00	2003	0	NA	NA	

maker-7-augustus-gene-24.37-mRNA-1	7	OsR498G0713533800.01.T01	100,00	0,00E+00	1007	Os07g0153000	100,00	0,00E+00	1007	0	NA	NA	
maker-7-augustus-gene-24.43-mRNA-1	7	OsR498G0713534800.01.T02	100,00	0,00E+00	1759	Os07g0153300	99,00	0,00E+00	878	-881	Indica	NA	
maker-7-augustus-gene-240.35-mRNA-1	7	OsR498G0714690500.01.T02	99,78	0,00E+00	808	Os07g0595800	99,78	0,00E+00	808	0	NA	Japonica	
maker-7-augustus-gene-240.39-mRNA-1	7	OsR498G0714702700.01.T04	91,97	1,47E-157	557	Os07g0598900	91,96	1,37E-152	538	-19	NA	Japonica	
maker-7-augustus-gene-240.47-mRNA-1	7	OsR498G0714690400.01.T01	99,47	0,00E+00	1339	Os07g0595700	100,00	0,00E+00	1202	-137	Indica	Japonica	
maker-7-augustus-gene-240.62-mRNA-1	7	OsR498G0714693400.01.T01	99,58	0,00E+00	3778	Os07g0596600	99,86	0,00E+00	3805	27	NA	Japonica	Kinase
maker-7-augustus-gene-240.65-mRNA-1	7	OsR498G0714695200.01.T01	99,63	0,00E+00	1914	Os07g0596900	99,91	0,00E+00	1928	14	NA	Japonica	
maker-7-augustus-gene-241.17-mRNA-1	7	OsR498G0714695600.01.T02	99,59	0,00E+00	863	Os07g0597000	100,00	0,00E+00	872	9	NA	Japonica	
maker-7-augustus-gene-241.2-mRNA-1	7	OsR498G0714699100.01.T01	99,90	0,00E+00	1847	Os07g0598000	99,90	0,00E+00	1847	0	NA	Indica	
maker-7-augustus-gene-241.21-mRNA-1	7	OsR498G0714696400.01.T01	99,70	0,00E+00	5981	Os07g0597200	99,88	0,00E+00	5963	-18	NA	Japonica	Kinase-LRR-TM
maker-7-augustus-gene-241.24-mRNA-1	7	OsR498G0714700600.01.T01	99,75	0,00E+00	2850	Os07g0598400	99,87	0,00E+00	2859	9	NA	Japonica	
maker-7-augustus-gene-241.26-mRNA-1	7	OsR498G0714700800.01.T01	99,87	0,00E+00	2735	Os07g0598500	99,93	0,00E+00	2739	4	NA	Japonica	
maker-7-augustus-gene-241.8-mRNA-1	7	OsR498G0714700300.01.T01	97,85	0,00E+00	3599	Os07g0598300	97,85	0,00E+00	3599	0	NA	Japonica	
maker-7-augustus-gene-242.44-mRNA-1	7	OsR498G0714702900.01.T01	98,68	0,00E+00	3317	Os07g0599000	99,79	0,00E+00	3351	34	NA	Japonica	
maker-7-augustus-gene-242.47-mRNA-1	7	OsR498G0714708100.01.T01	98,47	0,00E+00	1021	Os07g0600700	99,49	0,00E+00	1042	21	NA	Japonica	
maker-7-augustus-gene-242.49-mRNA-1	7	OsR498G0714702000.01.T02	99,92	0,00E+00	2263	Os07g0598700	99,77	0,00E+00	2379	116	NA	Indica	
maker-7-augustus-gene-242.56-mRNA-1	7	OsR498G0714706600.01.T03	99,09	0,00E+00	2311	Os07g0600400	99,09	0,00E+00	2311	0	NA	NA	
maker-7-augustus-gene-243.39-mRNA-1	7	OsR498G0714712900.01.T04	100,00	0,00E+00	1676	Os07g0602200	99,89	0,00E+00	1672	-4	NA	Indica	
maker-7-augustus-gene-243.44-mRNA-1	7	OsR498G0714708800.01.T01	97,94	0,00E+00	1738	Os07g0601000	100,00	0,00E+00	1835	97	NA	Japonica	
maker-7-augustus-gene-243.47-mRNA-1	7	OsR498G0714709000.01.T01	95,27	0,00E+00	1178	Os07g0601100	100,00	0,00E+00	1324	146	Japonica	Japonica	
maker-7-augustus-gene-243.48-mRNA-1	7	OsR498G0714709000.01.T01	99,28	7,27E-138	489	Os07g0601100	100,00	4,88E-141	499	10	NA	Japonica	
maker-7-augustus-gene-243.51-mRNA-1	7	OsR498G0714710800.01.T01	97,91	0,00E+00	1795	Os07g0602000	85,63	0,00E+00	1218	-577	Indica	Japonica	
maker-7-augustus-gene-243.54-mRNA-1	7	OsR498G0714711200.01.T01	99,34	0,00E+00	1613	Os07g0602000	99,45	0,00E+00	1618	5	NA	Japonica	
maker-7-augustus-gene-243.59-mRNA-1	7	OsR498G0714713800.01.T01	99,49	0,00E+00	704	Os07g0602600	99,86	0,00E+00	1289	585	Japonica	NA	
maker-7-augustus-gene-244.33-mRNA-1	7	OsR498G0714715000.01.T01	98,14	0,00E+00	1669	Os07g0602900	99,81	0,00E+00	948	-721	Indica	Japonica	
maker-7-augustus-gene-244.35-mRNA-1	7	OsR498G0714716800.01.T01	99,71	0,00E+00	3062	Os07g0603100	99,71	0,00E+00	3062	0	NA	NA	
maker-7-augustus-gene-244.40-mRNA-1	7	OsR498G0714717400.01.T02	98,50	0,00E+00	4999	Os07g0603300	98,53	0,00E+00	5003	4	NA	Japonica	
maker-7-augustus-gene-245.31-mRNA-1	7	OsR498G0714720100.01.T01	99,83	0,00E+00	2075	Os07g0603600	99,76	0,00E+00	1507	-568	Indica	Japonica	
maker-7-augustus-gene-245.34-mRNA-1	7	OsR498G0714720500.01.T01	99,64	0,00E+00	2982	Os07g0603700	99,87	0,00E+00	2837	-145	NA	Japonica	
maker-7-augustus-gene-245.36-mRNA-1	7	OsR498G0714721100.01.T03	99,56	0,00E+00	1638	Os07g0603800	99,89	0,00E+00	1652	14	NA	Japonica	
maker-7-augustus-gene-245.40-mRNA-1	7	OsR498G0714722100.01.T05	99,76	0,00E+00	1512	Os07g0604000	99,84	0,00E+00	1149	-363	Indica	Japonica	
maker-7-augustus-gene-245.46-mRNA-1	7	OsR498G0714724100.01.T01	100,00	5,74E-147	520	Os07g0604600	100,00	0,00E+00	693	173	Japonica	Japonica	
maker-7-augustus-gene-245.50-mRNA-1	7	OsR498G0714724500.01.T01	99,73	0,00E+00	1973	Os07g0604800	99,82	0,00E+00	1977	4	NA	Japonica	
maker-7-augustus-gene-247.53-mRNA-1	7	OsR498G0714733700.01.T03	99,70	0,00E+00	1805	Os07g0607700	100,00	0,00E+00	1624	-181	Indica	Japonica	
maker-7-augustus-gene-247.60-mRNA-1	7	OsR498G0714731400.01.T01	96,75	0,00E+00	1599	Os07g0606900	100,00	0,00E+00	1986	387	Japonica	Japonica	
maker-7-augustus-gene-247.66-mRNA-1	7	OsR498G0714733400.01.T02	94,11	0,00E+00	1233	Os07g0607400	94,60	0,00E+00	1248	15	NA	Japonica	
maker-7-augustus-gene-247.69-mRNA-1	7	OsR498G0714733600.01.T01	99,60	0,00E+00	884	Os07g0607500	100,00	0,00E+00	893	9	NA	Japonica	
maker-7-augustus-gene-247.75-mRNA-1	7	OsR498G0714735400.01.T02	99,56	0,00E+00	1633	Os07g0608200	100,00	0,00E+00	1651	18	NA	Japonica	
maker-7-augustus-gene-248.38-mRNA-1	7	OsR498G0714736100.01.T03	99,89	0,00E+00	1563	Os07g0608300	100,00	0,00E+00	1281	-282	Indica	Japonica	
maker-7-augustus-gene-248.42-mRNA-1	7	OsR498G0714736300.01.T01	99,40	0,00E+00	1486	Os07g0608400	100,00	0,00E+00	1510	24	NA	Japonica	
maker-7-augustus-gene-248.46-mRNA-1	7	OsR498G0714737100.01.T01	99,87	0,00E+00	1372	Os07g0608800	99,87	0,00E+00	1372	0	NA	Japonica	
maker-7-augustus-gene-248.50-mRNA-1	7	OsR498G0714736800.01.T01	100,00	0,00E+00	1267	Os07g0608500	100,00	0,00E+00	1267	0	NA	Japonica	
maker-7-augustus-gene-248.55-mRNA-1	7	OsR498G0714737000.01.T01	99,59	3,96E-121	434	Os07g0608700	96,23	4,54E-96	350	-84	Indica	Japonica	
maker-7-augustus-gene-249.0-mRNA-1	7	OsR498G0714738100.01.T01	99,28	0,00E+00	2450	Os07g0609766	100,00	0,00E+00	2495	45	NA	Japonica	
maker-7-augustus-gene-249.4-mRNA-1	7	OsR498G0714741100.01.T01	95,19	0,00E+00	977	Os07g0611600	95,65	0,00E+00	987	10	NA	Japonica	
maker-7-augustus-gene-250.0-mRNA-1	7	OsR498G0714741500.01.T02	97,62	0,00E+00	1649	Os07g0611700	80,64	1,92E-171	601	-1048	Indica	Japonica	
maker-7-augustus-gene-250.12-mRNA-1	7	OsR498G0714747900.01.T01	99,11	0,00E+00	1195	Os07g0614000	98,08	0,00E+00	1161	-34	NA	Japonica	
maker-7-augustus-gene-250.13-mRNA-1	7	OsR498G0714748500.01.T01	99,56	0,00E+00	2018	Os07g0614000	94,84	0,00E+00	1710	-308	Indica	Japonica	
maker-7-augustus-gene-250.24-mRNA-1	7	OsR498G0714741800.01.T02	100,00	0,00E+00	1694	Os07g0612400	100,00	0,00E+00	1694	0	NA	Japonica	
maker-7-augustus-gene-250.25-mRNA-1	7	OsR498G0714742400.01.T01	78,62	1,54E-160	567	Os07g0612500	78,62	5,34E-161	567	0	NA	Japonica	
maker-7-augustus-gene-250.3-mRNA-1	7	OsR498G0714744100.01.T04	99,82	0,00E+00	3965	Os07g0613300	99,96	0,00E+00	3978	13	NA	Japonica	
maker-7-augustus-gene-250.32-mRNA-1	7	OsR498G0714748200.01.T02	100,00	0,00E+00	1164	Os07g0614100	99,85	0,00E+00	1159	-5	NA	Japonica	
maker-7-augustus-gene-250.8-mRNA-1	7	OsR498G0714745300.01.T02	99,93	0,00E+00	2544	Os07g0613500	99,58	0,00E+00	2526	-18	NA	Japonica	Kinase-TM
maker-7-augustus-gene-251.36-mRNA-1	7	OsR498G0714749700.01.T01	99,85	0,00E+00	1224	Os07g0614400	99,89	0,00E+00	1598	374	Japonica	Japonica	
maker-7-augustus-gene-251.42-mRNA-1	7	OsR498G0714752500.01.T05	99,32	0,00E+00	1304	Os07g0615200	99,86	0,00E+00	1322	18	NA	Japonica	
maker-7-augustus-gene-251.45-mRNA-1	7	OsR498G0714753100.01.T01	99,69	0,00E+00	2298	Os07g0615400	99,92	0,00E+00	2312	14	NA	Japonica	
maker-7-augustus-gene-251.54-mRNA-1	7	OsR498G0714754700.01.T03	99,85	0,00E+00	7364	Os07g0616000	100,00	0,00E+00	7391	27	NA	Japonica	
maker-7-augustus-gene-251.58-mRNA-1	7	OsR498G0714750200.01.T01	99,57	0,00E+00	1232	Os07g0614500	99,86	0,00E+00	1241	9	NA	Japonica	
maker-7-augustus-gene-251.61-mRNA-1	7	OsR498G0714751200.01.T04	97,96	0,00E+00	1265	Os03g0406100	71,19	1,11E-90	334	-931	Indica	Japonica	
maker-7-augustus-gene-251.63-mRNA-1	7	OsR498G0714753400.01.T01	97,36	0,00E+00	1023	Os07g0615500	98,51	0,00E+00	1051	28	NA	Japonica	
maker-7-augustus-gene-251.65-mRNA-1	7	OsR498G0714754000.01.T02	100,00	0,00E+00	634	Os07g0615800	100,00	1,30E-174	610	-24	NA	NA	
maker-7-augustus-gene-251.67-mRNA-1	7	OsR498G0714755000.01.T01	99,67	0,00E+00	2727	Os07g0616200	100,00	0,00E+00	2749	22	NA	Japonica	
maker-7-augustus-gene-252.31-mRNA-1	7	OsR498G0714758500.01.T02	99,76	0,00E+00	4394	Os07g0616800	100,00	0,00E+00	4421	27	NA	Japonica	
maker-7-augustus-gene-252.33-mRNA-1	7	OsR498G0714758700.01.T01	99,93	0,00E+00	2501	Os07g0616900	100,00	0,00E+00	2506	5	NA	Japonica	
maker-7-augustus-gene-252.36-mRNA-1	7	OsR498G0714756800.01.T01	99,67	0,00E+00	1643	Os07g0616600	99,89	0,00E+00	1652	9	NA	Japonica	
maker-7-augustus-gene-252.41-mRNA-1	7	OsR498G0714759000.01.T01	98,64	0,00E+00	1801	Os07g0617000	98,93	0,00E+00	1811	10	NA	Japonica	
maker-7-augustus-gene-252.43-mRNA-1	7	OsR498G0714762200.01.T01	99,76	0,00E+00	2942	Os07g0617800	100,00	0,00E+00	1786	-1156	Indica	Japonica	
maker-7-augustus-gene-253.31-mRNA-1	7	OsR498G0714763500.01.T01	99,91	0,00E+00	3957	Os07g0618400	100,00	0,00E+00	3966	9	NA	Japonica	Kinase-LRR-TM
maker-7-augustus-gene-253.38-mRNA-1	7	OsR498G0714763700.01.T06	99,75	0,00E+00	2866	Os07g0618500	100,00	0,00E+00	2884	18	NA	Japonica	
maker-7-augustus-gene-253.40-mRNA-1	7	OsR498G0714763900.01.T01	95,44	0,00E+00	1324	Os07g0618600	95,94	0,00E+00	1342	18	NA	Japonica	
maker-7-augustus-gene-253.44-mRNA-1	7	OsR498G0714764300.01.T01	99,80	0,00E+00	916	Os07g0618800	100,00	0,00E+00	921	5	NA	Japonica	
maker-7-augustus-gene-253.49-mRNA-1	7	OsR498G0714765100.01.T02	99,67	0,00E+00	4322	Os07g0619100	100,00	0,00E+00	4040	-282	NA	Japonica	
maker-7-augustus-gene-254.0-mRNA-1	7	OsR498G0714770500.01.T02	99,61	0,00E+00	1378	Os07g0620300	99,74	0,00E+00	1382	4	NA	Japonica	
maker-7-augustus-gene-254.14-mRNA-1	7	OsR498G0714768800.01.T02	99,66	0,00E+00	3180	Os07g0619800	100,00	0,00E+00	2417	-763	Indica	Japonica	
maker-7-augustus-gene-254.19-mRNA-1	7	OsR498G0714770800.01.T01	99,31	0,00E+00	1034	Os07g0620400	99,83	0,00E+00	1048	14	NA	Japonica	
maker-7-augustus-gene-254.22-mRNA-1	7	OsR498G0714772200.01.T02	99,91	0,00E+00	1928	Os07g0620800	100,00	0,00E+00	1932	4	NA	Japonica	
maker-7-augustus-gene-254.6-mRNA-1	7	OsR498G0714773300.01.T01	100,00	0,00E+00	1054	Os07g0621300	100,00	0,00E+00	812	-242	Indica	Japonica	
maker-7-augustus-gene-254.9-mRNA-1	7	OsR498G0714768600.01.T02	99,75	0,00E+00	2850	Os07g0619700	97,23	0,00E+00	1473	-1377	Indica	Japonica	
maker-7-augustus-gene-255.37-mRNA-1	7	OsR498G0306074200.01.T01	72,57	4,74E-42	173	Os07g0623200	99,80	0,00E+00	875	702	Japonica	Japonica	
maker-7-augustus-gene-255.40-mRNA-1	7	OsR498G0714779900.01.T06	99,67	9,91E-153	541	Os07g0623300	100,00	8,09E-155	545	4	NA	Japonica	
maker-7-augustus-gene-255.45-mRNA-1	7	OsR498G0714775600.01.T01	99,94	0,00E+00	2907	Os07g0622200	100,00	0,00E+00	2911	4	NA	Japonica	
maker-7-augustus-gene-255.57-mRNA-1	7	OsR498G0714780200.01.T03	99,72	0,00E+00	640	Os07g0623400	100,00	2,74E-145	513	-127	Indica	Japonica	
maker-7-augustus-gene-255.61-mRNA-1	7	OsR498G0714780600.01.T01	99,65	0,00E+00	1551	Os07g0623600	100,00	0,00E+00	1564	13	NA	Japonica	
maker-7-augustus-gene-256.0-mRNA-1	7	OsR498G0714783700.01.T01	99,82	0,00E+00	1977	Os07g0624600	100,00	0,00E+00	1986	9	NA	Japonica	
maker-7-augustus-gene-257.42-mRNA-1	7	OsR498G0714789300.01.T01	99,74	0,00E+00	4876	Os07g0626200	100,00	0,00E+00	3288	-1588	Indica	Japonica	
maker-7-augustus-gene-257.46-mRNA-1	7	OsR498G0714789500.01.T01	99,80	0,00E+00	2627	Os07g0626300	99,62	0,00E+00	2330	-297	Indica	Japonica	
maker-7-augustus-gene-257.52-mRNA-1	7	OsR498G0714789700.01.T01	93,75	0,00E+00	1343	Os07g0626400	93,75	0,00E+00	1343	0	NA	Japonica	
maker-7-augustus-gene-257.75-mRNA-1	7	OsR498G0714794700.01.T02	99,70	0,00E+00	2387	Os07g0627300	100,00	0,00E+00	2406	19	NA	Japonica	
maker-7-augustus-gene-257.79-mRNA-1	7	OsR498G0714795100.01.T01	99,86	0,00E+00	1284	Os07g0627400	100,00	0,00E+00	1288	4	NA	Japonica	
maker-7-augustus-gene-257.81-mRNA-1	7	OsR498G0714788600.01.T01	94,01	0,00E+00	842	Os07g0625800	94,38	0,00E+00	851	9	NA	Japonica	
maker-7-augustus-gene-257.83-mRNA-1	7	OsR498G0714790400.01.T01	99,87	0,00E+00	5717	Os07g0626500	100,00	0,00E+00	5735	18	NA	Japonica	Kinase-LRR-TM
maker-7-augustus-gene-257.86-mRNA-1	7	OsR498G0714791600.01.T01	99,78	0,00E+00	2427	Os07g0626600	100,00	0,00E+00	2441	14	NA	Japonica	
maker-7-augustus-gene-258.0-mRNA-1	7	OsR498G0714796200.01.T04	100,00	0,00E+00	1402	Os07g0627700	100,00	0,00E+00	1402	0	NA	Japonica	
maker-7-augustus-gene-258.11-mRNA-1	7	OsR498G0714798000.01.T02	99,25	0,00E+00	1413	Os07g0628500	100,00	0,00E+00	1440	27	NA	Japonica	
maker-7-augustus-gene-258.14-mRNA-1	7	OsR498G0714798400.01.T01	99,21	0,00E+00	1574	Os07g0628600	100,00	0,00E+00	1602	28	NA	Japonica	
maker-7-augustus-gene-258.17-mRNA-1	7	OsR498G0714798600.01.T01	99,85	0,00E+00	3655	Os07g0628700	100,00	0,00E+00	3669	14	NA	Japonica	Kinase-TM
maker-7-augustus-gene-258.21-mRNA-1	7	OsR498G0714798800.01.T02	99,81	0,00E+00	1847	Os07g0628900	100,00	0,00E+00	1856	9	NA	Japonica	Kinase-TM
maker-7-augustus-gene-258.26-mRNA-1	7	OsR498G0714799400.01.T01	99,71	0,00E+00	1838	Os08g0437200	81,27	1,94E-146	518	-1320	Indica	Japonica	
maker-7-augustus-gene-258.3-mRNA-1	7	OsR498G0714796600.01.T01	100,00	0,00E+00	2003	Os07g0627800	100,00	0,00E+00	2003	0	NA	Japonica	
maker-7-augustus-gene-259.47-mRNA-1	7	OsR498G0714803500.01.T01	99,87	0,00E+00	1387	Os07g0630400	100,00	0,00E+00	1391	4	NA	Japonica	
maker-7-augustus-gene-259.51-mRNA-1	7	OsR498G0714805300.01.T01	98,15	0,00E+00	3091	Os07g0630900	98,27	0,00E+00	1702	-1389	Indica	Japonica	
maker-7-augustus-gene-259.56-mRNA-1	7	OsR498G0714805700.01.T01	100,00	2,31E-137	488	Os07g0631100	100,00	8,07E-138	488	0	NA	Japonica	
maker-7-augustus-gene-259.59-mRNA-1	7	OsR498G0714805600.01.T01	99,67	0,00E+00	1621	Os07g0631000	100,00	0,00E+00	1635	14	NA	Japonica	
maker-7-augustus-gene-26.0-mRNA-1	7	OsR498G0713544300.01.T01	100,00	0,00E+00	2122	Os07g0155100	100,00	0,00E+00	2122	0	NA	NA	
maker-7-augustus-gene-26.11-mRNA-1	7	OsR498G0713549900.01.T01	100,00	0,00E+00	1743	Os07g0157000	100,00	0,00E+00	1656	-87	NA	NA	
maker-7-augustus-gene-26.20-mRNA-1	7	OsR498G0713550700.01.T01	99,88	0,00E+00	1484	Os07g0158200	99,88	0,00E+00	1484	0	NA	NA	
maker-7-augustus-gene-26.3-mRNA-1	7	OsR498G0713546500.01.T02	100,00	0,00E+00	6937	Os07g0155600	99,97	0,00E+00	6932	-5	NA	Indica	
maker-7-augustus-gene-26.31-mRNA-1	7	OsR498G0713551000.01.T01	100,00	0,00E+00	1889	Os07g0158300	100,00	0,00E+00	1889	0	NA	NA	
maker-7-augustus-gene-26.9-mRNA-1	7	OsR498G0713549700.01.T01	100,00	0,00E+00	1889	Os07g0156000	100,00	0,00E+00	1835	-54	NA	NA	
maker-7-augustus-gene-260.44-mRNA-1	7	OsR498G0714808700.01.T01	98,99	0,00E+00	1398	Os07g0631900	100,00	0,00E+00	1434	36	NA	Japonica	
maker-7-augustus-gene-260.51-mRNA-1	7	OsR498G0714810900.01.T01	100,00	0,00E+00	1251	Os07g0632700	100,00	0,00E+00	1251	0	NA	Japonica	
maker-7-augustus-gene-260.54-mRNA-1	7	OsR498G0714811700.01.T01	99,71	1,13E-177	624	Os07g0633000	100,00	0,00E+00	1052	428	Japonica	Japonica	
maker-7-augustus-gene-260.58-mRNA-1	7	OsR498G0714812300.01.T01	99,69	0,00E+00	1150	Os07g0633200	100,00	0,00E+00	1159	9	NA	Japonica	
maker-7-augustus-gene-260.62-mRNA-1	7	OsR498G0714813700.01.T01	97,88	0,00E+00	3093	Os07g0633400	97,99	0,00E+00	3102	9	NA	Japonica	
maker-7-augustus-gene-260.70-mRNA-1	7	OsR498G0714812000.01.T02	100,00	0,00E+00	1186	Os07g0633100	100,00	0,00E+00	1186	0	NA	Japonica	
maker-7-augustus-gene-260.72-mRNA-1	7	OsR498G0714814000.01.T01	99,80	0,00E+00	2703	Os07g0633500	100,00	0,00E+00	2717	14	NA	Japonica	
maker-7-augustus-gene-260.74-mRNA-1	7	OsR498G0714815000.01.T01	99,88	0,00E+00	1455	Os07g0634000	100,00	0,00E+00	2305	850	Japonica	Japonica	
maker-7-augustus-gene-261.0-mRNA-1	7	OsR498G0714816100.01.T02	100,00	0,00E+00	1196	Os07g0634200	100,00	0,00E+00	1196	0	NA	Japonica	
maker-7-augustus-gene-261.11-mRNA-1	7	OsR498G0714820100.01.T01	99,55	0,00E+00	2788	Os07g0635200	100,00	0,00E+00	2322	-466	Indica	Japonica	
maker-7-augustus-gene-261.13-mRNA-1	7	OsR498G0714821100.01.T01	99,23	0,00E+00	2749	Os07g0635500	100,00	0,00E+00	2803	54	NA	Japonica	
maker-7-augustus-gene-261.18-mRNA-1	7	OsR498G0714821700.01.T01	99,56	0,00E+00	2853	Os07g0635700	100,00	0,00E+00	2884	31	NA	Japonica	
maker-7-augustus-gene-261.2-mRNA-1	7	OsR498G0714817700.01.T01	95,31	0,00E+00	1999	Os07g0634600	95,47	0,00E+00	2008	9	NA	Japonica	
maker-7-augustus-gene-261.21-mRNA-1	7	OsR498G0714815200.01.T01	99,48	0,00E+00	2382	Os07g0634100	100,00	0,00E+00	2105	-277	Indica	Japonica	
maker-7-augustus-gene-261.24-mRNA-1	7	OsR498G0714816400.01.T01	99,74	0,00E+00	1393	Os07g0634300	100,00	0,00E+00	1402	9	NA	Japonica	
maker-7-augustus-gene-261.26-mRNA-1	7	OsR498G0714816600.01.T01	99,88	0,00E+00	1500	Os07g0634400	100,00	0,00E+00	1505	5	NA	Japonica	
maker-7-augustus-gene-262.0-mRNA-1	7	OsR498G0714823900.01.T01	99,69	0,00E+00	4016	Os07g0636200	100,00	0,00E+00	4048	32	NA	Japonica	
maker-7-augustus-gene-262.24-mRNA-1	7	OsR498G0714828500.01.T01	100,00	0,00E+00	1445	Os07g0637400	100,00	0,00E+00	1445	0	NA	Japonica	
maker-7-augustus-gene-262.30-mRNA-1	7	OsR498G0714827200.01.T01	99,24	0,00E+00	3470	Os07g0637200	100,00	0,00E+00	3534	64	NA	Japonica	
maker-7-augustus-gene-262.32-mRNA-1	7	OsR498G0714827600.01.T01	99,64	0,00E+00	2006	Os07g0637300	97,33	0,00E+00	1911	-95	NA	Japonica	
maker-7-augustus-gene-262.39-mRNA-1	7	OsR498G0714828800.01.T02	99,78	0,00E+00	3207	Os07g0637500	97,99	0,00E+00	3351	144	NA	Japonica	
maker-7-augustus-gene-263.44-mRNA-1	7	OsR498G0714831300.01.T01	99,85	0,00E+00	1192	Os07g0638400	100,00	0,00E+00	1196	4	NA	Japonica	
maker-7-augustus-gene-263.46-mRNA-1	7	OsR498G0714831500.01.T01	99,67	0,00E+00	1097	Os07g0638500	100,00	0,00E+00	1106	9	NA	Japonica	
maker-7-augustus-gene-264.0-mRNA-1	7	OsR498G0714835900.01.T01	99,86	0,00E+00	1306	Os07g0639800	100,00	0,00E+00	1310	4	NA	Japonica	
maker-7-augustus-gene-264.10-mRNA-1	7	OsR498G0714836700.01.T01	99,76	0,00E+00	3029	Os07g0640200	100,00	0,00E+00	3047	18	NA	Japonica	
maker-7-augustus-gene-264.15-mRNA-1	7	OsR498G0714838300.01.T02	97,58	0,00E+00	1613	Os07g0640900	100,00	0,00E+00	1683	70	NA	Japonica	
maker-7-augustus-gene-264.21-mRNA-1	7	OsR498G0714835000.01.T04	100,00	0,00E+00	1191	Os07g0639600	100,00	0,00E+00	1191	0	NA	Japonica	
maker-7-augustus-gene-264.31-mRNA-1	7	OsR498G0714837000.01.T04	99,12	6,25E-112	403	Os08g0522100	96,07	4,52E-102	370	-33	NA	Japonica	
maker-7-augustus-gene-264.4-mRNA-1	7	OsR498G0714836300.01.T01	99,30	0,00E+00	3306	Os07g0640000	100,00	0,00E+00	1911	-1395	Indica	Japonica	
maker-7-augustus-gene-264.57-mRNA-1	7	OsR498G0714839200.01.T01	99,22	0,00E+00	3163	Os03g0366200	85,16	0,00E+00	2023	-1140	Indica	Japonica	Kinase-TM
maker-7-augustus-gene-264.63-mRNA-1	7	OsR498G0714840200.01.T01	89,29	4,98E-168	591	Os07g0641600	89,29	1,74E-168	591	0	NA	Japonica	
maker-7-augustus-gene-264.67-mRNA-1	7	OsR498G0714840600.01.T01	99,69	1,03E-161	570	Os07g0641800	99,42	2,27E-82	305	-265	Indica	Japonica	
maker-7-augustus-gene-264.8-mRNA-1	7	OsR498G0714836500.01.T01	99,75	0,00E+00	710	Os07g0640100	100,00	0,00E+00	715	5	NA	Japonica	
maker-7-augustus-gene-265.41-mRNA-1	7	OsR498G0714842300.01.T03	99,82	0,00E+00	1013	Os07g0642300	100,00	0,00E+00	1018	5	NA	Japonica	
maker-7-augustus-gene-265.46-mRNA-1	7	OsR498G0714842500.01.T01	99,83	0,00E+00	3093	Os07g0642400	100,00	0,00E+00	4448	1355	Japonica	Japonica	
maker-7-augustus-gene-265.48-mRNA-1	7	OsR498G0714844300.01.T01	98,11	0,00E+00	2553	Os07g0642800	100,00	0,00E+00	2679	126	NA	Japonica	
maker-7-augustus-gene-265.53-mRNA-1	7	OsR498G0714841600.01.T01	99,22	0,00E+00	682	Os09g0557300	87,45	7,74E-90	330	-352	Indica	Japonica	
maker-7-augustus-gene-265.55-mRNA-1	7	OsR498G0714842200.01.T01	99,75	0,00E+00	2108	Os07g0642200	100,00	0,00E+00	2122	14	NA	Japonica	
maker-7-augustus-gene-265.57-mRNA-1	7	OsR498G0714844000.01.T01	98,67	0,00E+00	2363	Os07g0642600	100,00	0,00E+00	2441	78	NA	Japonica	
maker-7-augustus-gene-265.62-mRNA-1	7	OsR498G0714844600.01.T01	97,69	0,00E+00	1397	Os07g0642900	100,00	0,00E+00	1483	86	NA	Japonica	
maker-7-augustus-gene-265.67-mRNA-1	7	OsR498G0714848000.01.T02	100,00	0,00E+00	1012	Os07g0644000	100,00	0,00E+00	1012	0	NA	Japonica	
maker-7-augustus-gene-266.0-mRNA-1	7	OsR498G0714848100.01.T01	97,65	0,00E+00	2824	Os07g0644100	100,00	0,00E+00	2980	156	NA	Japonica	
maker-7-augustus-gene-266.10-mRNA-1	7	OsR498G0714848600.01.T01	98,71	0,00E+00	4881	Os07g0644300	100,00	0,00E+00	5043	162	NA	Japonica	
maker-7-augustus-gene-266.13-mRNA-1	7	OsR498G0714852800.01.T01	99,49	0,00E+00	1042	Os07g0645000	100,00	0,00E+00	1056	14	NA	NA	
maker-7-augustus-gene-266.2-mRNA-1	7	OsR498G0714848300.01.T04	97,40	0,00E+00	842	Os07g0644200	100,00	0,00E+00	901	59	NA	Japonica	
maker-7-augustus-gene-266.4-mRNA-1	7	OsR498G0714848700.01.T01	98,41	0,00E+00	2945	Os07g0644600	100,00	0,00E+00	3068	123	NA	Japonica	
maker-7-augustus-gene-266.8-mRNA-1	7	OsR498G0714852800.01.T01	99,49	0,00E+00	1042	Os07g0645000	100,00	0,00E+00	1056	14	NA	NA	
maker-7-augustus-gene-267.25-mRNA-1	7	OsR498G0714853900.01.T01	99,93	0,00E+00	2728	Os07g0645400	100,00	0,00E+00	2733	5	NA	Japonica	
maker-7-augustus-gene-267.44-mRNA-1	7	OsR498G0714852800.01.T01	99,49	0,00E+00	1042	Os07g0645000	100,00	0,00E+00	1056	14	NA	NA	
maker-7-augustus-gene-267.54-mRNA-1	7	OsR498G0714854200.01.T01	99,73	0,00E+00	1323	Os07g0645500	100,00	0,00E+00	1352	29	NA	Japonica	
maker-7-augustus-gene-268.11-mRNA-1	7	OsR498G0714864900.01.T01	99,63	0,00E+00	2901	Os07g0647900	100,00	0,00E+00	2928	27	NA	Japonica	
maker-7-augustus-gene-268.16-mRNA-1	7	OsR498G0714855800.01.T02	95,90	0,00E+00	2296	Os07g0646100	100,00	0,00E+00	1645	-651	Indica	Japonica	
maker-7-augustus-gene-268.19-mRNA-1	7	OsR498G0714860400.01.T02	100,00	0,00E+00	1095	Os07g0646600	100,00	0,00E+00	1095	0	NA	NA	
maker-7-augustus-gene-268.22-mRNA-1	7	OsR498G0714860400.01.T01	100,00	0,00E+00	1045	Os07g0646600	100,00	0,00E+00	1045	0	NA	NA	
maker-7-augustus-gene-268.23-mRNA-1	7	OsR498G0714860400.01.T01	100,00	0,00E+00	1647	Os07g0646600	100,00	0,00E+00	1528	-119	NA	NA	
maker-7-augustus-gene-268.3-mRNA-1	7	OsR498G0714862300.01.T01	95,34	0,00E+00	1643	Os07g0647100	95,54	0,00E+00	1652	9	NA	Japonica	
maker-7-augustus-gene-268.38-mRNA-1	7	OsR498G0714862600.01.T01	100,00	0,00E+00	2911	Os07g0647200	100,00	0,00E+00	2911	0	NA	Japonica	
maker-7-augustus-gene-268.49-mRNA-1	7	OsR498G0714864800.01.T03	99,88	0,00E+00	1545	Os07g0647800	100,00	0,00E+00	1274	-271	Indica	Japonica	
maker-7-augustus-gene-268.54-mRNA-1	7	OsR498G0714865200.01.T01	99,63	0,00E+00	4359	Os07g0648000	100,00	0,00E+00	4399	40	NA	Japonica	
maker-7-augustus-gene-269.44-mRNA-1	7	OsR498G0714872500.01.T01	85,72	0,00E+00	1876	Os07g0649500	100,00	0,00E+00	3584	1708	Japonica	Japonica	
maker-7-augustus-gene-27.31-mRNA-1	7	OsR498G0713555000.01.T04	100,00	0,00E+00	2690	Os07g0158900	99,67	0,00E+00	2667	-23	NA	Indica	
maker-7-augustus-gene-27.35-mRNA-1	7	OsR498G0713558200.01.T02	100,00	0,00E+00	2008	Os07g0159500	99,82	0,00E+00	1999	-9	NA	Indica	
maker-7-augustus-gene-270.4-mRNA-1	7	OsR498G0714874500.01.T01	97,97	0,00E+00	3037	Os07g0543500	85,13	0,00E+00	3297	260	NA	Japonica	
maker-7-augustus-gene-270.6-mRNA-1	7	OsR498G0714876300.01.T02	88,14	0,00E+00	1731	Os07g0650600	100,00	0,00E+00	2452	721	Japonica	Japonica	
maker-7-augustus-gene-272.31-mRNA-1	7	OsR498G0714875500.01.T01	88,12	0,00E+00	1261	Os07g0653150	100,00	0,00E+00	1974	713	Japonica	Japonica	
maker-7-augustus-gene-273.22-mRNA-1	7	OsR498G0714874300.01.T01	95,90	0,00E+00	2804	Os07g0543500	70,76	1,22E-152	540	-2264	Indica	Japonica	
maker-7-augustus-gene-273.24-mRNA-1	7	OsR498G0714874500.01.T01	95,66	0,00E+00	2264	Os07g0543500	81,15	0,00E+00	1302	-962	Indica	Japonica	
maker-7-augustus-gene-273.25-mRNA-1	7	OsR498G0714876300.01.T02	81,57	0,00E+00	1402	Os07g0654450	100,00	0,00E+00	2623	1221	Japonica	Japonica	
maker-7-augustus-gene-273.32-mRNA-1	7	OsR498G0714877700.01.T01	99,39	0,00E+00	2330	Os07g0655300	100,00	0,00E+00	2354	24	NA	Japonica	
maker-7-augustus-gene-273.34-mRNA-1	7	OsR498G0714878300.01.T01	98,84	0,00E+00	1666	Os07g0655400	100,00	0,00E+00	1716	50	NA	Japonica	
maker-7-augustus-gene-274.0-mRNA-1	7	OsR498G0714878700.01.T02	99,20	0,00E+00	4661	Os07g0655500	100,00	0,00E+00	4727	66	NA	Japonica	
maker-7-augustus-gene-274.12-mRNA-1	7	OsR498G0714880900.01.T05	99,74	0,00E+00	2747	Os07g0656200	100,00	0,00E+00	1793	-954	Indica	Japonica	
maker-7-augustus-gene-274.2-mRNA-1	7	OsR498G0714879300.01.T01	94,26	0,00E+00	1737	Os07g0655600	95,07	0,00E+00	1578	-159	NA	Japonica	
maker-7-augustus-gene-274.21-mRNA-1	7	OsR498G0714881900.01.T01	98,03	0,00E+00	1401	Os07g0656600	100,00	0,00E+00	1445	44	NA	Japonica	
maker-7-augustus-gene-274.23-mRNA-1	7	OsR498G0714882100.01.T01	100,00	0,00E+00	1553	Os07g0656700	100,00	0,00E+00	866	-687	Indica	Japonica	
maker-7-augustus-gene-274.31-mRNA-1	7	OsR498G0714880400.01.T01	99,67	0,00E+00	2136	Os07g0656100	100,00	0,00E+00	2744	608	Japonica	Japonica	
maker-7-augustus-gene-274.37-mRNA-1	7	OsR498G0305849500.01.T02	97,17	0,00E+00	3548	Os07g0656500	99,95	0,00E+00	3601	53	NA	Japonica	
maker-7-augustus-gene-274.40-mRNA-1	7	OsR498G0714882800.01.T01	99,71	0,00E+00	1875	Os07g0656900	100,00	0,00E+00	1889	14	NA	Japonica	
maker-7-augustus-gene-274.43-mRNA-1	7	OsR498G0714884200.01.T02	99,55	0,00E+00	1177	Os07g0657100	100,00	0,00E+00	1137	-40	NA	Japonica	
maker-7-augustus-gene-274.5-mRNA-1	7	OsR498G0714879900.01.T01	99,75	0,00E+00	1452	Os07g0655900	100,00	0,00E+00	1462	10	NA	Japonica	
maker-7-augustus-gene-275.0-mRNA-1	7	OsR498G0714884300.01.T04	99,82	0,00E+00	3930	Os07g0657200	100,00	0,00E+00	3948	18	NA	Japonica	
maker-7-augustus-gene-275.23-mRNA-1	7	OsR498G0714886200.01.T01	95,55	0,00E+00	1444	Os07g0657600	100,00	0,00E+00	2268	824	Japonica	Japonica	
maker-7-augustus-gene-275.32-mRNA-1	7	OsR498G0714887400.01.T05	98,56	0,00E+00	2169	Os07g0658200	100,00	0,00E+00	1137	-1032	Indica	Japonica	
maker-7-augustus-gene-275.6-mRNA-1	7	OsR498G0714885900.01.T01	99,20	0,00E+00	1098	Os07g0657500	100,00	0,00E+00	1021	-77	NA	Japonica	
maker-7-augustus-gene-275.9-mRNA-1	7	OsR498G0714886500.01.T01	98,84	0,00E+00	2709	Os07g0657900	100,00	0,00E+00	2580	-129	NA	Japonica	
maker-7-augustus-gene-276.19-mRNA-1	7	OsR498G0714891200.01.T01	99,76	0,00E+00	1469	Os07g0659400	100,00	0,00E+00	1651	182	Japonica	Japonica	
maker-7-augustus-gene-276.22-mRNA-1	7	OsR498G0714892200.01.T01	98,72	0,00E+00	3562	Os07g0659500	98,97	0,00E+00	3585	23	NA	Japonica	
maker-7-augustus-gene-276.3-mRNA-1	7	OsR498G0714892700.01.T02	100,00	0,00E+00	1640	Os07g0659800	100,00	0,00E+00	1640	0	NA	NA	
maker-7-augustus-gene-276.5-mRNA-1	7	OsR498G0714893500.01.T02	96,55	0,00E+00	2085	Os03g0669000	78,80	0,00E+00	1085	-1000	Indica	Japonica	
maker-7-augustus-gene-276.9-mRNA-1	7	OsR498G0714894900.01.T01	99,81	0,00E+00	1907	Os07g0660700	100,00	0,00E+00	1916	9	NA	Japonica	
maker-7-augustus-gene-277.36-mRNA-1	7	OsR498G0714895100.01.T01	99,37	0,00E+00	1966	Os07g0661000	100,00	0,00E+00	1997	31	NA	Japonica	
maker-7-augustus-gene-277.41-mRNA-1	7	OsR498G0714896900.01.T01	99,82	0,00E+00	3012	Os07g0661300	100,00	0,00E+00	2652	-360	Indica	Japonica	
maker-7-augustus-gene-277.45-mRNA-1	7	OsR498G0305954400.01.T03	84,62	0,00E+00	1011	Os07g0661800	100,00	0,00E+00	1642	631	Japonica	NA	
maker-7-augustus-gene-277.47-mRNA-1	7	OsR498G0714900500.01.T01	100,00	0,00E+00	1218	Os07g0662500	100,00	0,00E+00	1218	0	NA	NA	
maker-7-augustus-gene-277.51-mRNA-1	7	OsR498G0714900900.01.T01	100,00	0,00E+00	1353	Os07g0662600	100,00	0,00E+00	1353	0	NA	NA	
maker-7-augustus-gene-277.54-mRNA-1	7	OsR498G0714895400.01.T01	99,77	0,00E+00	2319	Os07g0661100	100,00	0,00E+00	2333	14	NA	Japonica	
maker-7-augustus-gene-277.59-mRNA-1	7	OsR498G0714898800.01.T02	99,96	0,00E+00	8799	Os07g0661500	100,00	0,00E+00	9149	350	NA	NA	
maker-7-augustus-gene-277.64-mRNA-1	7	OsR498G0714899400.01.T04	99,86	0,00E+00	1311	Os07g0661600	100,00	0,00E+00	1315	4	NA	Japonica	
maker-7-augustus-gene-277.68-mRNA-1	7	OsR498G0714899600.01.T03	100,00	3,19E-155	547	Os07g0661700	100,00	1,11E-155	547	0	NA	NA	
maker-7-augustus-gene-277.70-mRNA-1	7	OsR498G0714900000.01.T02	100,00	0,00E+00	2387	Os07g0661900	100,00	0,00E+00	2387	0	NA	NA	
maker-7-augustus-gene-278.0-mRNA-1	7	OsR498G0714902300.01.T05	97,94	0,00E+00	3519	Os07g0662900	98,08	0,00E+00	3518	-1	NA	NA	
maker-7-augustus-gene-278.10-mRNA-1	7	OsR498G0714906900.01.T01	100,00	0,00E+00	1635	Os07g0664000	100,00	0,00E+00	1635	0	NA	NA	
maker-7-augustus-gene-278.11-mRNA-1	7	OsR498G0714908300.01.T01	100,00	0,00E+00	1602	Os07g0664200	100,00	0,00E+00	1602	0	NA	Japonica	

maker-7-augustus-gene-278.12-mRNA-1	7	OsR498G0714909500.01.T01	100,00	0,00E+00	1618	Os07g0664300	100,00	0,00E+00	1618	0	NA	NA	
maker-7-augustus-gene-278.13-mRNA-1	7	OsR498G0714909700.01.T01	100,00	0,00E+00	1618	Os07g0664400	100,00	0,00E+00	1618	0	NA	NA	
maker-7-augustus-gene-278.7-mRNA-1	7	OsR498G0714904500.01.T03	100,00	0,00E+00	1608	Os07g0663500	99,89	0,00E+00	1664	56	NA	NA	
maker-7-augustus-gene-278.8-mRNA-1	7	OsR498G0714905300.01.T01	100,00	0,00E+00	1640	Os07g0663600	100,00	0,00E+00	1640	0	NA	NA	
maker-7-augustus-gene-278.9-mRNA-1	7	OsR498G0714905900.01.T01	100,00	0,00E+00	1629	Os07g0663700	100,00	0,00E+00	1629	0	NA	NA	
maker-7-augustus-gene-278.92-mRNA-1	7	OsR498G0714910700.01.T02	100,00	0,00E+00	1710	Os07g0664500	91,54	0,00E+00	1276	-434	Indica	NA	
maker-7-augustus-gene-278.95-mRNA-1	7	OsR498G0714904000.01.T01	100,00	0,00E+00	2316	Os07g0663300	100,00	0,00E+00	2316	0	NA	NA	
maker-7-augustus-gene-279.31-mRNA-1	7	OsR498G0714911100.01.T01	99,89	0,00E+00	1562	Os07g0665000	93,18	0,00E+00	1251	-311	Indica	NA	
maker-7-augustus-gene-279.33-mRNA-1	7	OsR498G0714911700.01.T01	100,00	0,00E+00	1662	Os07g0665000	100,00	0,00E+00	1662	0	NA	NA	
maker-7-augustus-gene-279.35-mRNA-1	7	OsR498G0714913300.01.T03	99,77	0,00E+00	788	Os07g0665900	100,00	0,00E+00	792	4	NA	Japonica	
maker-7-augustus-gene-279.40-mRNA-1	7	OsR498G0714912000.01.T02	100,00	0,00E+00	829	Os07g0665200	100,00	0,00E+00	829	0	NA	NA	SODCC2
maker-7-augustus-gene-279.44-mRNA-1	7	OsR498G0714913000.01.T04	91,49	0,00E+00	823	Os07g0665300	100,00	0,00E+00	1041	218	Japonica	NA	
maker-7-augustus-gene-279.50-mRNA-1	7	OsR498G0714913000.01.T03	100,00	0,00E+00	2535	Os07g0665700	100,00	0,00E+00	2057	-478	Indica	NA	
maker-7-augustus-gene-279.58-mRNA-1	7	OsR498G0714915400.01.T01	93,39	0,00E+00	749	Os07g0666400	100,00	0,00E+00	1434	685	Japonica	Japonica	
maker-7-augustus-gene-279.60-mRNA-1	7	OsR498G0714916000.01.T01	99,77	0,00E+00	1588	Os07g0666600	100,00	0,00E+00	1597	9	NA	Japonica	
maker-7-augustus-gene-28.29-mRNA-1	7	OsR498G0713560100.01.T01	100,00	0,00E+00	829	Os07g0159800	100,00	0,00E+00	829	0	NA	NA	
maker-7-augustus-gene-28.33-mRNA-1	7	OsR498G0713560700.01.T01	100,00	0,00E+00	4753	Os07g0160000	99,96	0,00E+00	4912	159	NA	NA	
maker-7-augustus-gene-28.45-mRNA-1	7	OsR498G0713558800.01.T02	100,00	0,00E+00	1640	Os07g0159700	99,82	0,00E+00	1008	-632	Indica	Indica	Kinase-TM
maker-7-augustus-gene-28.49-mRNA-1	7	OsR498G0713563400.01.T01	100,00	0,00E+00	1023	Os07g0160400	100,00	0,00E+00	1023	0	NA	Indica	
maker-7-augustus-gene-28.51-mRNA-1	7	OsR498G0713565400.01.T01	99,82	0,00E+00	2952	Os07g0161000	100,00	0,00E+00	2966	14	NA	Japonica	
maker-7-augustus-gene-280.0-mRNA-1	7	OsR498G0714918300.01.T01	100,00	0,00E+00	2205	Os07g0666900	100,00	0,00E+00	2205	0	NA	Japonica	
maker-7-augustus-gene-280.12-mRNA-1	7	OsR498G0714922100.01.T01	100,00	0,00E+00	3112	Os03g0349200	84,47	0,00E+00	1464	-1648	Indica	Japonica	Kinase-TM
maker-7-augustus-gene-280.19-mRNA-1	7	OsR498G0714924700.01.T02	100,00	0,00E+00	1885	Os07g0668700	100,00	0,00E+00	1878	-7	NA	NA	
maker-7-augustus-gene-280.23-mRNA-1	7	OsR498G0714919000.01.T01	99,92	0,00E+00	2285	Os07g0667100	100,00	0,00E+00	2836	551	Japonica	Japonica	
maker-7-augustus-gene-280.26-mRNA-1	7	OsR498G0714919400.01.T01	99,48	0,00E+00	2035	Os07g0667300	100,00	0,00E+00	2062	27	NA	Japonica	
maker-7-augustus-gene-280.28-mRNA-1	7	OsR498G0714919600.01.T02	100,00	0,00E+00	1317	Os07g0667400	100,00	0,00E+00	1317	0	NA	Japonica	
maker-7-augustus-gene-280.30-mRNA-1	7	OsR498G0714922400.01.T02	100,00	0,00E+00	3350	Os09g0442400	73,78	0,00E+00	939	-2411	Indica	NA	
maker-7-augustus-gene-280.35-mRNA-1	7	OsR498G0714922800.01.T02	100,00	0,00E+00	1380	Os07g0668200	100,00	0,00E+00	1380	0	NA	NA	
maker-7-augustus-gene-280.39-mRNA-1	7	OsR498G0714923000.01.T01	100,00	0,00E+00	2084	Os07g0668300	100,00	0,00E+00	1218	-866	Indica	NA	
maker-7-augustus-gene-280.42-mRNA-1	7	OsR498G0714925200.01.T01	100,00	0,00E+00	2598	Os07g0668900	100,00	0,00E+00	2598	0	NA	NA	Kinase-TM
maker-7-augustus-gene-280.6-mRNA-1	7	OsR498G0714918700.01.T01	99,84	0,00E+00	2194	Os07g0667000	100,00	0,00E+00	2203	9	NA	Japonica	
maker-7-augustus-gene-281.0-mRNA-1	7	OsR498G0714925300.01.T02	100,00	0,00E+00	796	Os07g0669000	100,00	0,00E+00	796	0	NA	NA	
maker-7-augustus-gene-281.10-mRNA-1	7	OsR498G0714925900.01.T01	100,00	0,00E+00	4075	Os07g0669200	100,00	0,00E+00	4075	0	NA	NA	
maker-7-augustus-gene-281.22-mRNA-1	7	OsR498G0714929900.01.T01	100,00	0,00E+00	666	Os07g0670100	85,21	1,47E-104	379	-287	Indica	Japonica	
maker-7-augustus-gene-281.27-mRNA-1	7	OsR498G0714925800.01.T02	100,00	0,00E+00	2598	Os07g0669100	100,00	0,00E+00	2598	0	NA	NA	
maker-7-augustus-gene-281.34-mRNA-1	7	OsR498G0714928400.01.T02	99,75	0,00E+00	705	Os07g0669600	100,00	0,00E+00	704	-1	NA	NA	
maker-7-augustus-gene-281.37-mRNA-1	7	OsR498G0714928600.01.T03	100,00	0,00E+00	4394	Os07g0669700	100,00	0,00E+00	4394	0	NA	NA	
maker-7-augustus-gene-282.0-mRNA-1	7	OsR498G0714931700.01.T02	99,61	6,47E-128	458	Os07g0671100	100,00	0,00E+00	774	316	Japonica	Japonica	
maker-7-augustus-gene-282.15-mRNA-1	7	OsR498G0714935100.01.T01	99,87	0,00E+00	1381	Os07g0671800	100,00	0,00E+00	1613	232	Japonica	NA	
maker-7-augustus-gene-282.19-mRNA-1	7	OsR498G0714936900.01.T05	99,77	0,00E+00	775	Os07g0672500	100,00	0,00E+00	780	5	NA	Japonica	
maker-7-augustus-gene-282.20-mRNA-1	7	OsR498G0714936900.01.T01	99,75	0,00E+00	3596	Os07g0672500	100,00	0,00E+00	3618	22	NA	Japonica	
maker-7-augustus-gene-282.33-mRNA-1	7	OsR498G0714938300.01.T02	99,60	0,00E+00	1763	Os07g0673000	100,00	0,00E+00	1442	-321	Indica	Japonica	
maker-7-augustus-gene-282.4-mRNA-1	7	OsR498G0714933500.01.T04	99,80	0,00E+00	1804	Os07g0671400	99,61	0,00E+00	1358	-446	Indica	Japonica	
maker-7-augustus-gene-282.43-mRNA-1	7	OsR498G0714938600.01.T01	99,87	0,00E+00	1403	Os07g0673100	100,00	0,00E+00	1407	4	NA	Japonica	
maker-7-augustus-gene-282.47-mRNA-1	7	OsR498G0714938800.01.T02	99,64	0,00E+00	994	Os07g0673200	100,00	0,00E+00	1003	9	NA	Japonica	
maker-7-augustus-gene-282.7-mRNA-1	7	OsR498G0714934900.01.T01	100,00	0,00E+00	2949	Os07g0671700	100,00	0,00E+00	2798	-151	NA	Japonica	
maker-7-augustus-gene-283.0-mRNA-1	7	OsR498G0714939500.01.T01	99,81	0,00E+00	927	Os07g0673400	100,00	0,00E+00	931	4	NA	Japonica	
maker-7-augustus-gene-283.11-mRNA-1	7	OsR498G0714943300.01.T01	99,53	0,00E+00	755	Os07g0674400	100,00	0,00E+00	764	9	NA	Japonica	
maker-7-augustus-gene-283.13-mRNA-1	7	OsR498G0714943500.01.T01	99,77	0,00E+00	786	Os07g0674500	100,00	0,00E+00	791	5	NA	Japonica	
maker-7-augustus-gene-283.2-mRNA-1	7	OsR498G0714940500.01.T04	100,00	9,95E-157	553	Os07g0673900	100,00	0,00E+00	710	157	Japonica	Japonica	
maker-7-augustus-gene-283.20-mRNA-1	7	OsR498G0714939400.01.T02	99,78	0,00E+00	1620	Os07g0673500	99,89	0,00E+00	1603	-17	NA	Japonica	
maker-7-augustus-gene-283.25-mRNA-1	7	OsR498G0714944400.01.T01	100,00	0,00E+00	1175	Os07g0674800	100,00	0,00E+00	1175	0	NA	Japonica	
maker-7-augustus-gene-283.5-mRNA-1	7	OsR498G0714941700.01.T01	99,93	0,00E+00	2409	Os07g0674100	100,00	0,00E+00	2414	5	NA	Japonica	
maker-7-augustus-gene-283.9-mRNA-1	7	OsR498G0714941900.01.T01	100,00	0,00E+00	710	Os07g0674200	100,00	0,00E+00	710	0	NA	Japonica	
maker-7-augustus-gene-284.33-mRNA-1	7	OsR498G0714949700.01.T05	100,00	0,00E+00	856	Os07g0676600	100,00	0,00E+00	688	-168	Indica	Japonica	
maker-7-augustus-gene-284.38-mRNA-1	7	OsR498G0714945200.01.T01	99,89	0,00E+00	3130	Os07g0675100	100,00	0,00E+00	3139	9	NA	Japonica	
maker-7-augustus-gene-285.0-mRNA-1	7	OsR498G0714950500.01.T04	98,95	0,00E+00	1666	Os07g0677100	99,37	0,00E+00	1684	18	NA	Japonica	
maker-7-augustus-gene-285.1-mRNA-1	7	OsR498G0714950500.01.T06	99,06	0,00E+00	1682	Os07g0677200	99,37	0,00E+00	1695	13	NA	Japonica	
maker-7-augustus-gene-285.4-mRNA-1	7	OsR498G0714952500.01.T01	98,79	0,00E+00	2764	Os07g0677900	99,05	0,00E+00	2782	18	NA	Japonica	
maker-7-augustus-gene-285.6-mRNA-1	7	OsR498G0714951400.01.T01	99,90	0,00E+00	1738	Os07g0677600	100,00	0,00E+00	1743	5	NA	Japonica	
maker-7-augustus-gene-285.9-mRNA-1	7	OsR498G0714957000.01.T03	99,87	0,00E+00	2767	Os07g0679000	99,87	0,00E+00	2767	0	NA	NA	
maker-7-augustus-gene-286.29-mRNA-1	7	OsR498G0714959300.01.T05	96,32	0,00E+00	1322	Os07g0679300	97,03	0,00E+00	1337	15	NA	Japonica	
maker-7-augustus-gene-286.35-mRNA-1	7	OsR498G0714960400.01.T01	99,04	0,00E+00	2012	Os07g0679500	100,00	0,00E+00	1775	-237	Indica	Japonica	
maker-7-augustus-gene-286.38-mRNA-1	7	OsR498G0714960800.01.T01	99,41	0,00E+00	2700	Os07g0679700	100,00	0,00E+00	2740	40	NA	Japonica	
maker-7-augustus-gene-286.42-mRNA-1	7	OsR498G0714961800.01.T02	99,87	0,00E+00	2857	Os07g0680000	100,00	0,00E+00	2866	9	NA	Japonica	
maker-7-augustus-gene-286.48-mRNA-1	7	OsR498G0714962600.01.T01	99,66	1,01E-148	526	Os07g0680300	100,00	3,52E-92	336	-190	Indica	Japonica	
maker-7-augustus-gene-286.59-mRNA-1	7	OsR498G0714963400.01.T02	99,90	0,00E+00	3470	Os07g0680600	100,00	0,00E+00	3479	9	NA	Japonica	
maker-7-augustus-gene-287.22-mRNA-1	7	OsR498G0714963900.01.T06	99,73	0,00E+00	2607	Os07g0680900	100,00	0,00E+00	2273	-334	Indica	Japonica	Kinase
maker-7-augustus-gene-287.25-mRNA-1	7	OsR498G0714965700.01.T01	99,86	0,00E+00	1322	Os07g0681200	100,00	0,00E+00	1326	4	NA	Japonica	
maker-7-augustus-gene-287.27-mRNA-1	7	OsR498G0714966100.01.T01	99,75	0,00E+00	2877	Os07g0681300	100,00	0,00E+00	2852	-25	NA	Japonica	
maker-7-augustus-gene-287.29-mRNA-1	7	OsR498G0714966500.01.T01	99,25	0,00E+00	7046	Os07g0681500	99,40	0,00E+00	7073	27	NA	Japonica	
maker-7-augustus-gene-287.36-mRNA-1	7	OsR498G0714966700.01.T03	99,56	0,00E+00	2419	Os07g0681600	100,00	0,00E+00	2450	31	NA	Japonica	
maker-7-augustus-gene-287.40-mRNA-1	7	OsR498G0714966900.01.T03	99,36	0,00E+00	3337	Os07g0681700	100,00	0,00E+00	3388	51	NA	Japonica	
maker-7-augustus-gene-287.43-mRNA-1	7	OsR498G0714967300.01.T03	99,18	0,00E+00	1504	Os07g0682000	99,65	0,00E+00	1522	18	NA	Japonica	
maker-7-augustus-gene-287.45-mRNA-1	7	OsR498G0714964200.01.T02	99,88	0,00E+00	1452	Os07g0681000	100,00	0,00E+00	1456	4	NA	Japonica	
maker-7-augustus-gene-287.50-mRNA-1	7	OsR498G0714964600.01.T02	99,79	0,00E+00	3451	Os07g0681100	100,00	0,00E+00	3469	18	NA	Japonica	Kinase-LRR-TM
maker-7-augustus-gene-287.53-mRNA-1	7	OsR498G0714968400.01.T01	99,56	0,00E+00	3656	Os07g0682400	99,71	0,00E+00	3670	14	NA	Japonica	
maker-7-augustus-gene-288.0-mRNA-1	7	OsR498G0714973300.01.T01	99,64	0,00E+00	2510	Os07g0682800	100,00	0,00E+00	2533	23	NA	Japonica	
maker-7-augustus-gene-288.10-mRNA-1	7	OsR498G0714976700.01.T01	100,00	0,00E+00	1110	Os07g0684000	100,00	0,00E+00	1110	0	NA	Japonica	
maker-7-augustus-gene-288.26-mRNA-1	7	OsR498G0714973000.01.T02	99,64	0,00E+00	2489	Os07g0682700	100,00	0,00E+00	2511	22	NA	Japonica	
maker-7-augustus-gene-288.32-mRNA-1	7	OsR498G0714977000.01.T01	99,64	0,00E+00	1502	Os07g0684100	100,00	0,00E+00	1516	14	NA	Japonica	
maker-7-augustus-gene-288.39-mRNA-1	7	OsR498G0714978600.01.T02	100,00	0,00E+00	1635	Os07g0684800	100,00	0,00E+00	1635	0	NA	Japonica	
maker-7-augustus-gene-288.9-mRNA-1	7	OsR498G0714976300.01.T03	99,81	0,00E+00	1880	Os07g0683900	100,00	0,00E+00	1858	-22	NA	Japonica	
maker-7-augustus-gene-289.0-mRNA-1	7	OsR498G0714981500.01.T01	100,00	0,00E+00	1424	Os07g0684900	100,00	0,00E+00	1424	0	NA	Japonica	
maker-7-augustus-gene-289.12-mRNA-1	7	OsR498G0714981800.01.T01	99,86	0,00E+00	2572	Os07g0685000	100,00	0,00E+00	2581	9	NA	Japonica	
maker-7-augustus-gene-289.13-mRNA-1	7	OsR498G0714982400.01.T01	99,71	0,00E+00	1859	Os07g0685300	100,00	0,00E+00	1873	14	NA	Japonica	
maker-7-augustus-gene-289.3-mRNA-1	7	OsR498G0714983100.01.T01	98,74	0,00E+00	2362	Os07g0685500	98,89	0,00E+00	2371	9	NA	Japonica	
maker-7-augustus-gene-289.5-mRNA-1	7	OsR498G0714984700.01.T01	99,90	0,00E+00	1728	Os07g0685800	100,00	0,00E+00	2024	296	Japonica	Japonica	
maker-7-augustus-gene-289.9-mRNA-1	7	OsR498G0714987100.01.T02	98,57	0,00E+00	1458	Os07g0686100	100,00	0,00E+00	1489	31	NA	Japonica	
maker-7-augustus-gene-29.13-mRNA-1	7	OsR498G0713568400.01.T01	97,96	0,00E+00	1434	Os07g0162100	100,00	0,00E+00	1505	71	NA	Japonica	
maker-7-augustus-gene-29.2-mRNA-1	7	OsR498G0713568900.01.T01	99,29	0,00E+00	1992	Os07g0162300	99,64	0,00E+00	2010	18	NA	Japonica	
maker-7-augustus-gene-29.9-mRNA-1	7	OsR498G0713566200.01.T08	97,87	0,00E+00	2997	Os07g0161600	98,10	0,00E+00	3015	18	NA	Japonica	
maker-7-augustus-gene-290.46-mRNA-1	7	OsR498G0714989100.01.T01	99,81	0,00E+00	1921	Os07g0686500	100,00	0,00E+00	1930	9	NA	Japonica	
maker-7-augustus-gene-290.48-mRNA-1	7	OsR498G0714990900.01.T01	99,64	0,00E+00	4407	Os07g0686700	100,00	0,00E+00	3620	-787	Indica	Japonica	
maker-7-augustus-gene-290.51-mRNA-1	7	OsR498G0714991100.01.T01	100,00	0,00E+00	2132	Os07g0686800	100,00	0,00E+00	1775	-357	Indica	Japonica	Kinase
maker-7-augustus-gene-290.60-mRNA-1	7	OsR498G0714991900.01.T01	99,85	0,00E+00	2405	Os03g0319400	79,37	0,00E+00	1140	-1265	Indica	Japonica	Kinase-TM
maker-7-augustus-gene-290.65-mRNA-1	7	OsR498G0714992900.01.T01	94,06	0,00E+00	1438	Os07g0687500	94,39	0,00E+00	1452	14	NA	Japonica	
maker-7-augustus-gene-290.71-mRNA-1	7	OsR498G0714993500.01.T06	99,77	0,00E+00	1543	Os07g0687700	100,00	0,00E+00	1552	9	NA	Japonica	
maker-7-augustus-gene-290.74-mRNA-1	7	OsR498G0714995900.01.T01	99,89	0,00E+00	1625	Os07g0688200	100,00	0,00E+00	1629	4	NA	Japonica	
maker-7-augustus-gene-290.76-mRNA-1	7	OsR498G0714988600.01.T01	99,91	0,00E+00	4024	Os07g0686400	100,00	0,00E+00	4033	9	NA	Japonica	
maker-7-augustus-gene-290.79-mRNA-1	7	OsR498G0714992200.01.T01	99,73	0,00E+00	1333	Os07g0687100	99,19	0,00E+00	1316	-17	NA	Japonica	
maker-7-augustus-gene-290.84-mRNA-1	7	OsR498G0714992400.01.T01	99,78	0,00E+00	808	Os07g0687200	100,00	0,00E+00	812	4	NA	Japonica	
maker-7-augustus-gene-290.86-mRNA-1	7	OsR498G0714992600.01.T01	100,00	0,00E+00	1716	Os07g0687300	100,00	0,00E+00	1716	0	NA	Japonica	
maker-7-augustus-gene-290.88-mRNA-1	7	OsR498G0714994000.01.T01	99,70	0,00E+00	1767	Os07g0687900	100,00	0,00E+00	1781	14	NA	Japonica	
maker-7-augustus-gene-290.90-mRNA-1	7	OsR498G0714996200.01.T07	99,71	0,00E+00	3073	Os07g0688300	100,00	0,00E+00	2506	-567	Indica	Japonica	
maker-7-augustus-gene-291.1-mRNA-1	7	OsR498G0714996300.01.T01	99,42	0,00E+00	1522	Os07g0688500	92,25	0,00E+00	1068	-454	Indica	Japonica	
maker-7-augustus-gene-291.11-mRNA-1	7	OsR498G0715003300.01.T02	99,61	0,00E+00	3237	Os07g0689800	100,00	0,00E+00	3268	31	NA	Japonica	
maker-7-augustus-gene-291.22-mRNA-1	7	OsR498G0714997800.01.T04	90,02	0,00E+00	685	Os07g0689150	100,00	0,00E+00	1104	419	Japonica	Japonica	
maker-7-augustus-gene-291.31-mRNA-1	7	OsR498G0714999400.01.T01	99,80	0,00E+00	5400	Os07g0689300	100,00	0,00E+00	4011	-1389	Indica	Japonica	
maker-7-augustus-gene-291.36-mRNA-1	7	OsR498G0715003800.01.T02	99,58	0,00E+00	5039	Os07g0689900	100,00	0,00E+00	5093	54	NA	Japonica	
maker-7-augustus-gene-291.4-mRNA-1	7	OsR498G0714999700.01.T03	99,32	0,00E+00	2856	Os07g0689400	99,63	0,00E+00	2878	22	NA	Japonica	
maker-7-augustus-gene-292.30-mRNA-1	7	OsR498G0715004500.01.T01	99,91	0,00E+00	1935	Os07g0690400	100,00	0,00E+00	1939	4	NA	Japonica	
maker-7-augustus-gene-292.42-mRNA-1	7	OsR498G0715009300.01.T02	97,92	0,00E+00	4378	Os07g0691700	98,74	0,00E+00	3351	-1027	Indica	Japonica	
maker-7-augustus-gene-292.47-mRNA-1	7	OsR498G0715004000.01.T02	99,58	0,00E+00	4210	Os07g0690300	99,77	0,00E+00	2375	-1835	Indica	Japonica	
maker-7-augustus-gene-292.49-mRNA-1	7	OsR498G0715009600.01.T01	99,63	0,00E+00	2407	Os07g0691800	100,00	0,00E+00	2430	23	NA	Japonica	
maker-7-augustus-gene-293.15-mRNA-1	7	OsR498G0715013300.01.T01	99,88	0,00E+00	4402	Os07g0693500	100,00	0,00E+00	4415	13	NA	Japonica	
maker-7-augustus-gene-293.26-mRNA-1	7	OsR498G0715015100.01.T01	99,92	0,00E+00	2236	Os07g0693800	100,00	0,00E+00	2241	5	NA	Japonica	
maker-7-augustus-gene-293.28-mRNA-1	7	OsR498G0715015300.01.T03	99,79	0,00E+00	5119	Os07g0693900	100,00	0,00E+00	5146	27	NA	Japonica	
maker-7-augustus-gene-293.35-mRNA-1	7	OsR498G0715011000.01.T01	99,34	0,00E+00	1068	Os07g0692200	100,00	0,00E+00	1083	15	NA	Japonica	
maker-7-augustus-gene-293.37-mRNA-1	7	OsR498G0715011800.01.T01	99,80	0,00E+00	1826	Os07g0692800	100,00	0,00E+00	1835	9	NA	Japonica	
maker-7-augustus-gene-293.39-mRNA-1	7	OsR498G0715013600.01.T01	99,28	0,00E+00	2709	Os07g0693600	100,00	0,00E+00	3279	570	Japonica	Japonica	
maker-7-augustus-gene-293.44-mRNA-1	7	OsR498G0715015800.01.T02	99,78	0,00E+00	3223	Os07g0694000	100,00	0,00E+00	3241	18	NA	Japonica	
maker-7-augustus-gene-293.6-mRNA-1	7	OsR498G0715012100.01.T01	99,87	0,00E+00	2702	Os07g0693000	100,00	0,00E+00	2711	9	NA	Japonica	Kinase-TM
maker-7-augustus-gene-294.0-mRNA-1	7	OsR498G0715020100.01.T04	99,55	0,00E+00	3970	Os07g0695100	100,00	0,00E+00	4015	45	NA	Japonica	
maker-7-augustus-gene-294.14-mRNA-1	7	OsR498G0715021700.01.T01	99,46	0,00E+00	1972	Os07g0695900	99,91	0,00E+00	1994	22	NA	Japonica	
maker-7-augustus-gene-294.23-mRNA-1	7	OsR498G0715016800.01.T01	99,67	0,00E+00	1607	Os07g0694400	100,00	0,00E+00	1620	13	NA	Japonica	
maker-7-augustus-gene-294.34-mRNA-1	7	OsR498G0715019000.01.T04	99,75	0,00E+00	2124	Os07g0694800	100,00	0,00E+00	2138	14	NA	Japonica	
maker-7-augustus-gene-294.38-mRNA-1	7	OsR498G0715020600.01.T01	98,51	0,00E+00	6217	Os07g0695400	98,82	0,00E+00	6267	50	NA	Japonica	
maker-7-augustus-gene-294.6-mRNA-1	7	OsR498G0715021300.01.T05	100,00	0,00E+00	931	Os07g0695700	99,42	0,00E+00	1228	297	Japonica	Japonica	
maker-7-augustus-gene-294.9-mRNA-1	7	OsR498G0715021500.01.T01	99,87	0,00E+00	5442	Os07g0695800	100,00	0,00E+00	5460	18	NA	Japonica	
maker-7-augustus-gene-3.50-mRNA-1	7	OsR498G0713391900.01.T01	100,00	0,00E+00	3539	Os07g0111600	100,00	0,00E+00	3663	124	NA	NA	
maker-7-augustus-gene-3.52-mRNA-1	7	OsR498G0713394500.01.T04	99,93	0,00E+00	2636	Os07g0112100	99,94	0,00E+00	2761	125	NA	NA	
maker-7-augustus-gene-3.54-mRNA-1	7	OsR498G0713396500.01.T06	100,00	0,00E+00	883	Os07g0112800	100,00	0,00E+00	883	0	NA	NA	
maker-7-augustus-gene-3.63-mRNA-1	7	OsR498G0713398300.01.T01	100,00	6,33E-122	437	Os07g0111001	94,56	2,68E-102	371	-66	Indica	NA	
maker-7-augustus-gene-3.67-mRNA-1	7	OsR498G0713396200.01.T02	99,57	0,00E+00	1235	Os07g0112700	99,44	0,00E+00	959	-276	Indica	NA	
maker-7-augustus-gene-3.69-mRNA-1	7	OsR498G0713397600.01.T02	100,00	0,00E+00	639	Os07g0113200	100,00	0,00E+00	639	0	NA	NA	
maker-7-augustus-gene-30.43-mRNA-1	7	OsR498G0713575100.01.T01	99,77	0,00E+00	765	Os07g0164200	100,00	0,00E+00	769	4	NA	Japonica	
maker-7-augustus-gene-30.49-mRNA-1	7	OsR498G0713576300.01.T01	100,00	0,00E+00	1178	Os07g0164600	100,00	0,00E+00	1083	-95	NA	Japonica	
maker-7-augustus-gene-30.52-mRNA-1	7	OsR498G0713578300.01.T01	99,88	0,00E+00	2946	Os07g0165000	100,00	0,00E+00	2046	-900	Indica	Japonica	
maker-7-augustus-gene-30.58-mRNA-1	7	OsR498G0713574400.01.T01	99,64	0,00E+00	2962	Os07g0163900	100,00	0,00E+00	3599	637	Japonica	Japonica	
maker-7-augustus-gene-30.63-mRNA-1	7	OsR498G0713574800.01.T01	99,73	0,00E+00	3354	Os07g0164100	100,00	0,00E+00	3377	23	NA	Japonica	
maker-7-augustus-gene-30.67-mRNA-1	7	OsR498G0713575400.01.T01	99,66	0,00E+00	1599	Os07g0164300	99,89	0,00E+00	1608	9	NA	Japonica	
maker-7-augustus-gene-31.11-mRNA-1	7	OsR498G0713581400.01.T02	99,27	0,00E+00	1686	Os07g0165700	100,00	0,00E+00	1718	32	NA	Japonica	
maker-7-augustus-gene-31.13-mRNA-1	7	OsR498G0713582600.01.T01	99,78	0,00E+00	3278	Os07g0166100	100,00	0,00E+00	3296	18	NA	Japonica	
maker-7-augustus-gene-31.2-mRNA-1	7	OsR498G0203722500.01.T01	91,60	1,89E-143	509	Os07g0165500	99,83	0,00E+00	1076	567	Japonica	Japonica	
maker-7-augustus-gene-31.7-mRNA-1	7	OsR498G0713578800.01.T01	99,78	0,00E+00	5780	Os07g0165200	100,00	0,00E+00	4082	-1698	Indica	Japonica	
maker-7-augustus-gene-32.32-mRNA-1	7	OsR498G0713584300.01.T02	99,79	0,00E+00	4296	Os07g0166700	99,62	0,00E+00	4272	-24	NA	Japonica	Kinase-LRR-TM
maker-7-augustus-gene-32.36-mRNA-1	7	OsR498G0713585100.01.T01	99,84	0,00E+00	2242	Os07g0167000	95,59	0,00E+00	2039	-203	NA	Japonica	
maker-7-augustus-gene-32.40-mRNA-1	7	OsR498G0713585300.01.T01	99,77	0,00E+00	3960	Os07g0167100	100,00	0,00E+00	3079	-881	Indica	Japonica	
maker-7-augustus-gene-32.46-mRNA-1	7	OsR498G0713585900.01.T02	99,43	7,16E-177	619	Os07g0167300	100,00	4,80E-180	628	9	NA	Japonica	
maker-7-augustus-gene-32.50-mRNA-1	7	OsR498G0713590100.01.T01	100,00	0,00E+00	2598	Os07g0168600	100,00	0,00E+00	3025	427	Japonica	NA	
maker-7-augustus-gene-32.52-mRNA-1	7	OsR498G0713584800.01.T01	99,78	0,00E+00	824	Os07g0166800	100,00	0,00E+00	829	5	NA	Japonica	
maker-7-augustus-gene-32.57-mRNA-1	7	OsR498G0713585800.01.T01	99,61	0,00E+00	2278	Os07g0167200	100,00	0,00E+00	2300	22	NA	Japonica	
maker-7-augustus-gene-32.60-mRNA-1	7	OsR498G0713586200.01.T04	99,11	1,19E-168	593	Os07g0167500	99,70	7,99E-172	602	9	NA	Japonica	
maker-7-augustus-gene-32.62-mRNA-1	7	OsR498G0713588000.01.T03	100,00	0,00E+00	2434	Os07g0168000	99,93	0,00E+00	2429	-5	NA	NA	
maker-7-augustus-gene-33.50-mRNA-1	7	OsR498G0713593500.01.T03	100,00	0,00E+00	1481	Os07g0169600	100,00	0,00E+00	1481	0	NA	NA	
maker-7-augustus-gene-33.54-mRNA-1	7	OsR498G0713593700.01.T01	100,00	0,00E+00	1992	Os07g0169700	100,00	0,00E+00	1992	0	NA	NA	
maker-7-augustus-gene-33.56-mRNA-1	7	OsR498G0713596500.01.T01	100,00	0,00E+00	1532	Os07g0170200	100,00	0,00E+00	1532	0	NA	NA	
maker-7-augustus-gene-33.63-mRNA-1	7	OsR498G0713599300.01.T02	100,00	0,00E+00	911	Os07g0170900	100,00	0,00E+00	915	4	NA	NA	
maker-7-augustus-gene-33.66-mRNA-1	7	OsR498G0713599500.01.T01	100,00	0,00E+00	1819	Os07g0171100	99,85	0,00E+00	1172	-647	Indica	NA	
maker-7-augustus-gene-33.70-mRNA-1	7	OsR498G0713600100.01.T02	100,00	0,00E+00	3680	Os07g0171300	100,00	0,00E+00	3680	0	NA	NA	
maker-7-augustus-gene-33.82-mRNA-1	7	OsR498G0713596800.01.T02	100,00	0,00E+00	3263	Os07g0170300	100,00	0,00E+00	3263	0	NA	NA	
maker-7-augustus-gene-33.86-mRNA-1	7	OsR498G0713599800.01.T01	100,00	0,00E+00	1905	Os07g0171200	100,00	0,00E+00	1040	-865	Indica	Indica	
maker-7-augustus-gene-34.54-mRNA-1	7	OsR498G0713603500.01.T05	100,00	0,00E+00	2809	Os07g0172900	100,00	0,00E+00	2590	-219	NA	Indica	
maker-7-augustus-gene-34.70-mRNA-1	7	OsR498G0713605700.01.T01	100,00	0,00E+00	829	Os07g0173700	99,13	0,00E+00	810	-19	NA	Indica	
maker-7-augustus-gene-34.73-mRNA-1	7	OsR498G0713606300.01.T01	100,00	0,00E+00	1018	Os07g0174400	94,89	3,42E-159	561	-457	Indica	Indica	
maker-7-augustus-gene-34.75-mRNA-1	7	OsR498G0713602200.01.T01	100,00	0,00E+00	655	Os07g0172500	100,00	0,00E+00	655	0	NA	Indica	
maker-7-augustus-gene-34.79-mRNA-1	7	OsR498G0713604200.01.T03	99,94	0,00E+00	2891	Os07g0173200	100,00	0,00E+00	2232	-659	Indica	Indica	
maker-7-augustus-gene-35.44-mRNA-1	7	OsR498G0713608300.01.T01	90,69	0,00E+00	758	Os07g0175000	99,82	0,00E+00	981	223	Japonica	Indica	
maker-7-augustus-gene-35.46-mRNA-1	7	OsR498G0713610100.01.T01	100,00	0,00E+00	3766	Os07g0175400	97,70	0,00E+00	3589	-177	NA	Indica	
maker-7-augustus-gene-35.49-mRNA-1	7	OsR498G0713610300.01.T01	100,00	0,00E+00	1088	Os07g0175500	98,37	0,00E+00	1063	-25	NA	Indica	
maker-7-augustus-gene-35.51-mRNA-1	7	OsR498G0713610900.01.T01	100,00	0,00E+00	926	Os07g0175600	100,00	0,00E+00	926	0	NA	Indica	
maker-7-augustus-gene-35.58-mRNA-1	7	OsR498G0713608000.01.T01	100,00	0,00E+00	931	Os07g0174900	99,81	0,00E+00	927	-4	NA	Indica	
maker-7-augustus-gene-35.62-mRNA-1	7	OsR498G0713609400.01.T01	100,00	0,00E+00	1294	Os07g0175300	100,00	0,00E+00	1108	-186	Indica	Indica	
maker-7-augustus-gene-35.63-mRNA-1	7	OsR498G0713613000.01.T03	99,93	0,00E+00	2425	Os07g0175900	99,93	0,00E+00	2425	0	NA	NA	
maker-7-augustus-gene-36.35-mRNA-1	7	OsR498G0713618700.01.T01	97,62	0,00E+00	2969	Os07g0176600	97,62	0,00E+00	2969	0	NA	NA	Kinase-TM
maker-7-augustus-gene-36.44-mRNA-1	7	OsR498G0713624300.01.T02	99,85	0,00E+00	4782	Os07g0178100	100,00	0,00E+00	4800	18	NA	Japonica	
maker-7-augustus-gene-36.47-mRNA-1	7	OsR498G0713621000.01.T02	99,49	0,00E+00	1046	Os07g0177000	99,39	0,00E+00	878	-168	Indica	NA	
maker-7-augustus-gene-36.52-mRNA-1	7	OsR498G0713621400.01.T01	100,00	0,00E+00	1213	Os07g0177200	100,00	0,00E+00	1342	129	NA	NA	
maker-7-augustus-gene-36.54-mRNA-1	7	OsR498G0713621600.01.T01	100,00	0,00E+00	1326	Os07g0177300	100,00	0,00E+00	1326	0	NA	NA	
maker-7-augustus-gene-37.32-mRNA-1	7	OsR498G0713625500.01.T01	99,82	0,00E+00	2010	Os07g0178600	99,91	0,00E+00	2014	4	NA	Japonica	
maker-7-augustus-gene-37.34-mRNA-1	7	OsR498G0713625700.01.T01	100,00	0,00E+00	1088	Os07g0178700	100,00	0,00E+00	1088	0	NA	Japonica	
maker-7-augustus-gene-37.36-mRNA-1	7	OsR498G0713625900.01.T01	99,48	0,00E+00	1031	Os07g0178800	99,83	0,00E+00	1040	9	NA	Japonica	
maker-7-augustus-gene-37.46-mRNA-1	7	OsR498G0713626200.01.T02	99,85	0,00E+00	2459	Os01g0837500	93,71	0,00E+00	2080	-379	Indica	Japonica	
maker-7-augustus-gene-37.54-mRNA-1	7	OsR498G0713627400.01.T01	99,80	0,00E+00	1766	Os12g0615400	81,24	0,00E+00	772	-994	Indica	Japonica	
maker-7-augustus-gene-37.56-mRNA-1	7	OsR498G0713627600.01.T01	99,83	0,00E+00	1078	Os07g0179400	100,00	0,00E+00	1003	-75	NA	Japonica	
maker-7-augustus-gene-37.60-mRNA-1	7	OsR498G0713628000.01.T05	99,73	0,00E+00	1951	Os07g0179500	100,00	0,00E+00	1608	-343	Indica	Japonica	
maker-7-augustus-gene-38.0-mRNA-1	7	OsR498G0713631300.01.T04	98,80	0,00E+00	1163	Os11g0135900	74,61	2,00E-102	372	-791	Indica	Indica	
maker-7-augustus-gene-38.10-mRNA-1	7	OsR498G0713631900.01.T01	99,03	0,00E+00	3069	Os07g0181000	99,80	0,00E+00	2709	-360	Indica	NA	
maker-7-augustus-gene-38.13-mRNA-1	7	OsR498G0510043600.01.T01	83,10	0,00E+00	1326	Os07g0181100	99,95	0,00E+00	3378	2052	Japonica	NA	
maker-7-augustus-gene-38.15-mRNA-1	7	OsR498G0713633300.01.T04	98,42	0,00E+00	2079	Os07g0181500	99,00	0,00E+00	1055	-1024	Indica	Japonica	
maker-7-augustus-gene-38.18-mRNA-1	7	OsR498G0713633700.01.T01	99,18	0,00E+00	3237	Os07g0181700	99,13	0,00E+00	3232	-5	NA	Japonica	
maker-7-augustus-gene-38.23-mRNA-1	7	OsR498G0713631600.01.T02	98,59	0,00E+00	2094	Os07g0180800	94,97	0,00E+00	3006	912	Japonica	Japonica	
maker-7-augustus-gene-38.8-mRNA-1	7	OsR498G0713631700.01.T01	99,02	0,00E+00	2143	Os07g0180900	99,45	0,00E+00	2275	132	NA	Japonica	
maker-7-augustus-gene-39.0-mRNA-1	7	OsR498G0713634900.01.T03	99,89	0,00E+00	1587	Os03g0797600	79,98	0,00E+00	793	-794	Indica	NA	
maker-7-augustus-gene-39.4-mRNA-1	7	OsR498G0713634400.01.T01	100,00	0,00E+00	1786	Os07g0182100	100,00	0,00E+00	1786	0	NA	Japonica	
maker-7-augustus-gene-39.9-mRNA-1	7	OsR498G0713635400.01.T01	97,85	0,00E+00	1109	Os07g0182400	99,39	0,00E+00	1153	44	NA	Japonica	
maker-7-augustus-gene-4.18-mRNA-1	7	OsR498G0713401800.01.T01	100,00	0,00E+00	2046	Os07g0114000	100,00	0,00E+00	2046	0	NA	NA	
maker-7-augustus-gene-4.2-mRNA-1	7	OsR498G0713402900.01.T03	100,00	6,87E-118	423	Os07g0114300	100,00	2,41E-118	423	0	NA	NA	
maker-7-augustus-gene-4.20-mRNA-1	7	OsR498G0713403200.01.T01	99,69	0,00E+00	1704	Os07g0114400	99,69	0,00E+00	1704	0	NA	NA	Kinase-TM
maker-7-augustus-gene-4.27-mRNA-1	7	OsR498G0713405600.01.T01	100,00	0,00E+00	1229	Os07g0115200	100,00	0,00E+00	1229	0	NA	NA	
maker-7-augustus-gene-4.5-mRNA-1	7	OsR498G0713403700.01.T01	100,00	0,00E+00	1045	Os07g0114500	99,48	0,00E+00	1031	-14	NA	NA	
maker-7-augustus-gene-40.47-mRNA-1	7	OsR498G0713638100.01.T01	99,67	0,00E+00	2190	Os07g0183200	99,92	0,00E+00	2204	14	NA	Japonica	
maker-7-augustus-gene-40.61-mRNA-1	7	OsR498G0713643500.01.T01	99,03	0,00E+00	1447	Os07g0184800	99,88	0,00E+00	1506	59	NA	Japonica	
maker-7-augustus-gene-40.64-mRNA-1	7	OsR498G0713643700.01.T04	99,85	0,00E+00	1212	Os07g0184900	100,00	0,00E+00	1216	4	NA	Japonica	
maker-7-augustus-gene-40.65-mRNA-1	7	OsR498G0713643700.01.T05	99,73	0,00E+00	1335	Os07g0184900	99,60	0,00E+00	1324	-11	NA	Japonica	
maker-7-augustus-gene-40.77-mRNA-1	7	OsR498G0713641800.01.T01	99,57	0,00E+00	830	Os03g0798600	92,69	0,00E+00	686	-144	Indica	Japonica	
maker-7-augustus-gene-40.80-mRNA-1	7	OsR498G0713644600.01.T01	99,45	0,00E+00	3214	Os07g0185000	100,00	0,00E+00	3258	44	NA	Japonica	Kinase
maker-7-augustus-gene-41.10-mRNA-1	7	OsR498G0713645200.01.T01	99,59	0,00E+00	2633	Os07g0185200	99,63	0,00E+00	1916	-717	Indica	Japonica	
maker-7-augustus-gene-41.14-mRNA-1	7	OsR498G0713649800.01.T01	100,00	0,00E+00	666	Os07g0186000	100,00	0,00E+00	666	0	NA	Indica	
maker-7-augustus-gene-41.2-mRNA-1	7	OsR498G0713648100.01.T01	99,78	0,00E+00	3245	Os07g0185700	100,00	0,00E+00	3263	18	NA	Japonica	
maker-7-augustus-gene-41.7-mRNA-1	7	OsR498G0713648500.01.T03	100,00	1,73E-58	224	Os07g0185800	100,00	6,11E-59	224	0	NA	Japonica	

maker-7-augustus-gene-42.24-mRNA-1	7	OsR498G0713651900.01.T05	95,70	0,00E+00	724	Os07g0186400	96,60	0,00E+00	885	161	Japonica	Japonica	
maker-7-augustus-gene-42.35-mRNA-1	7	OsR498G0713655300.01.T01	99,66	0,00E+00	2082	Os07g0187700	99,74	0,00E+00	2086	4	NA	Japonica	
maker-7-augustus-gene-42.40-mRNA-1	7	OsR498G0713654000.01.T01	100,00	0,00E+00	1673	Os07g0187300	100,00	0,00E+00	1673	0	NA	Japonica	
maker-7-augustus-gene-43.44-mRNA-1	7	OsR498G0713658300.01.T03	99,73	0,00E+00	669	Os07g0188233	100,00	0,00E+00	668	-1	NA	Japonica	
maker-7-augustus-gene-43.48-mRNA-1	7	OsR498G0713658300.01.T01	99,48	0,00E+00	1708	Os07g0188266	100,00	0,00E+00	1797	89	NA	Japonica	
maker-7-augustus-gene-43.51-mRNA-1	7	OsR498G0713658500.01.T01	98,19	0,00E+00	3140	Os07g0188700	100,00	0,00E+00	3285	145	NA	Japonica	
maker-7-augustus-gene-43.53-mRNA-1	7	OsR498G0713658700.01.T02	99,38	0,00E+00	2289	Os07g0188800	99,92	0,00E+00	2323	34	NA	Japonica	
maker-7-augustus-gene-43.59-mRNA-1	7	OsR498G0713661100.01.T02	98,83	0,00E+00	1191	Os07g0189700	100,00	0,00E+00	1223	32	NA	Japonica	
maker-7-augustus-gene-43.61-mRNA-1	7	OsR498G0713657800.01.T01	99,63	0,00E+00	2917	Os07g0188000	99,94	0,00E+00	2939	22	NA	Japonica	
maker-7-augustus-gene-43.66-mRNA-1	7	OsR498G0713661400.01.T01	99,27	0,00E+00	1206	Os07g0189800	100,00	0,00E+00	1229	23	NA	Japonica	
maker-7-augustus-gene-44.29-mRNA-1	7	OsR498G0713662100.01.T01	99,72	0,00E+00	3837	Os07g0190000	100,00	0,00E+00	3864	27	NA	Japonica	
maker-7-augustus-gene-44.36-mRNA-1	7	OsR498G0713666000.01.T03	100,00	0,00E+00	3350	Os03g0687000	71,68	0,00E+00	870	-2480	Indica	NA	
maker-7-augustus-gene-44.38-mRNA-1	7	OsR498G0713666200.01.T01	99,76	0,00E+00	748	Os07g0190800	100,00	0,00E+00	753	5	NA	NA	
maker-7-augustus-gene-44.40-mRNA-1	7	OsR498G0713666400.01.T01	100,00	0,00E+00	4686	Os07g0190900	100,00	0,00E+00	4686	0	NA	NA	
maker-7-augustus-gene-45.35-mRNA-1	7	OsR498G0713666500.01.T02	100,00	0,00E+00	1846	Os07g0191000	100,00	0,00E+00	1846	0	NA	NA	
maker-7-augustus-gene-45.47-mRNA-1	7	OsR498G0713672300.01.T01	100,00	0,00E+00	6293	Os07g0191700	100,00	0,00E+00	6293	0	NA	NA	
maker-7-augustus-gene-46.0-mRNA-1	7	OsR498G0713676500.01.T01	100,00	0,00E+00	2911	Os07g0192900	99,63	0,00E+00	2882	-29	NA	NA	
maker-7-augustus-gene-46.10-mRNA-1	7	OsR498G0713679800.01.T01	100,00	0,00E+00	2951	Os07g0193600	100,00	0,00E+00	2951	0	NA	NA	
maker-7-augustus-gene-47.29-mRNA-1	7	OsR498G0713681100.01.T01	94,44	0,00E+00	1122	Os07g0194000	94,44	0,00E+00	1122	0	NA	NA	
maker-7-augustus-gene-47.32-mRNA-1	7	OsR498G0713681500.01.T01	99,61	0,00E+00	2736	Os07g0194100	99,61	0,00E+00	2736	0	NA	NA	Kinase-TM
maker-7-augustus-gene-47.34-mRNA-1	7	OsR498G0713682400.01.T01	100,00	1,80E-158	558	Os07g0194350	100,00	3,25E-137	486	-72	Indica	NA	
maker-7-augustus-gene-47.36-mRNA-1	7	OsR498G0713682800.01.T01	99,90	0,00E+00	1728	Os07g0194500	100,00	0,00E+00	1732	4	NA	NA	
maker-7-augustus-gene-47.38-mRNA-1	7	OsR498G0713684800.01.T01	100,00	0,00E+00	3550	Os03g0803900	71,21	0,00E+00	731	-2819	Indica	NA	
maker-7-augustus-gene-47.40-mRNA-1	7	OsR498G0713686000.01.T01	93,10	0,00E+00	1078	Os07g0195100	93,25	0,00E+00	1082	4	NA	NA	
maker-7-augustus-gene-47.42-mRNA-1	7	OsR498G0713686200.01.T01	99,70	0,00E+00	3567	Os07g0195200	100,00	0,00E+00	3593	26	NA	NA	
maker-7-augustus-gene-47.46-mRNA-1	7	OsR498G0713687600.01.T01	99,65	0,00E+00	3020	Os07g0195400	100,00	0,00E+00	3047	27	NA	Japonica	
maker-7-augustus-gene-48.0-mRNA-1	7	OsR498G0713693700.01.T01	99,67	0,00E+00	2737	Os07g0197100	100,00	0,00E+00	2760	23	NA	Japonica	
maker-7-augustus-gene-48.6-mRNA-1	7	OsR498G0713693400.01.T01	99,85	0,00E+00	1181	Os07g0197000	100,00	0,00E+00	1068	-113	NA	Japonica	
maker-7-augustus-gene-49.0-mRNA-1	7	OsR498G0713697500.01.T01	99,52	0,00E+00	1113	Os07g0198300	100,00	0,00E+00	1126	13	NA	Japonica	
maker-7-augustus-gene-5.37-mRNA-1	7	OsR498G0713405700.01.T01	100,00	0,00E+00	1792	Os07g0115300	100,00	0,00E+00	1792	0	NA	NA	
maker-7-augustus-gene-5.41-mRNA-1	7	OsR498G0713407100.01.T01	100,00	0,00E+00	883	Os03g0762900	73,54	3,84E-69	261	-622	Indica	NA	
maker-7-augustus-gene-5.44-mRNA-1	7	OsR498G0713408500.01.T01	100,00	0,00E+00	1992	Os03g0643100	99,73	0,00E+00	2007	15	NA	NA	
maker-7-augustus-gene-5.49-mRNA-1	7	OsR498G0713410800.01.T02	99,92	0,00E+00	2150	Os03g0764000	78,83	0,00E+00	1003	-1147	Indica	Japonica	
maker-7-augustus-gene-50.18-mRNA-1	7	OsR498G0713703700.01.T01	99,74	0,00E+00	2758	Os07g0201200	100,00	0,00E+00	2727	-31	NA	Japonica	
maker-7-augustus-gene-50.24-mRNA-1	7	OsR498G0713699500.01.T01	99,62	0,00E+00	4230	Os07g0200000	100,00	0,00E+00	4269	39	NA	Japonica	
maker-7-augustus-gene-50.6-mRNA-1	7	OsR498G0713702900.01.T08	100,00	0,00E+00	1581	Os07g0200700	100,00	0,00E+00	1581	0	NA	Japonica	
maker-7-augustus-gene-51.42-mRNA-1	7	OsR498G0713704700.01.T01	99,02	0,00E+00	2685	Os07g0201500	87,35	0,00E+00	1902	-783	Indica	Japonica	
maker-7-augustus-gene-51.45-mRNA-1	7	OsR498G0713706100.01.T01	99,73	0,00E+00	1315	Os07g0201500	78,13	1,26E-169	597	-718	Indica	Japonica	
maker-7-augustus-gene-51.47-mRNA-1	7	OsR498G0713708100.01.T04	99,80	0,00E+00	1750	Os07g0202900	100,00	0,00E+00	2143	393	Japonica	Japonica	
maker-7-augustus-gene-51.56-mRNA-1	7	OsR498G0713707000.01.T01	99,79	0,00E+00	3443	Os07g0202100	100,00	0,00E+00	3458	15	NA	Japonica	
maker-7-augustus-gene-51.61-mRNA-1	7	OsR498G0713707600.01.T01	100,00	0,00E+00	774	Os07g0202450	100,00	0,00E+00	774	0	NA	Japonica	
maker-7-augustus-gene-52.0-mRNA-1	7	OsR498G0713710700.01.T01	99,92	0,00E+00	2108	Os07g0203900	100,00	0,00E+00	3288	1180	Japonica	Japonica	Kinase-TM
maker-7-augustus-gene-52.13-mRNA-1	7	OsR498G0713714100.01.T04	99,65	0,00E+00	1012	Os07g0205000	100,00	0,00E+00	953	-59	NA	Japonica	
maker-7-augustus-gene-52.16-mRNA-1	7	OsR498G0713714700.01.T05	98,20	0,00E+00	1831	Os06g0649500	80,92	0,00E+00	1038	-793	Indica	Japonica	
maker-7-augustus-gene-52.3-mRNA-1	7	OsR498G0713711700.01.T01	99,73	0,00E+00	674	Os07g0204100	100,00	0,00E+00	1029	355	Japonica	Japonica	
maker-7-augustus-gene-52.5-mRNA-1	7	OsR498G0713713900.01.T01	99,77	0,00E+00	3115	Os07g0204900	100,00	0,00E+00	3133	18	NA	Japonica	
maker-7-augustus-gene-53.20-mRNA-1	7	OsR498G0713716300.01.T01	99,92	0,00E+00	2209	Os07g0205500	100,00	0,00E+00	2214	5	NA	Japonica	
maker-7-augustus-gene-53.25-mRNA-1	7	OsR498G0713723100.01.T01	100,00	0,00E+00	2170	Os03g0807700	74,52	0,00E+00	736	-1434	Indica	NA	
maker-7-augustus-gene-53.27-mRNA-1	7	OsR498G0713716800.01.T01	98,93	0,00E+00	3783	Os07g0205700	100,00	0,00E+00	3891	108	NA	Japonica	
maker-7-augustus-gene-53.32-mRNA-1	7	OsR498G0713717000.01.T04	99,75	0,00E+00	5737	Os07g0205900	100,00	0,00E+00	5773	36	NA	Japonica	
maker-7-augustus-gene-53.36-mRNA-1	7	OsR498G0713721400.01.T01	99,48	0,00E+00	2756	Os07g0206600	100,00	0,00E+00	2792	36	NA	Japonica	
maker-7-augustus-gene-53.38-mRNA-1	7	OsR498G0713721600.01.T03	100,00	0,00E+00	2028	Os07g0206700	100,00	0,00E+00	1763	-265	Indica	Japonica	
maker-7-augustus-gene-54.0-mRNA-1	7	OsR498G0713724300.01.T01	100,00	0,00E+00	1499	Os03g0807800	87,53	0,00E+00	1012	-487	Indica	NA	
maker-7-augustus-gene-54.13-mRNA-1	7	OsR498G0713732500.01.T02	100,00	0,00E+00	1424	Os07g0208600	100,00	0,00E+00	1424	0	NA	NA	
maker-7-augustus-gene-54.15-mRNA-1	7	OsR498G0713723400.01.T01	99,11	0,00E+00	4784	Os07g0207100	99,16	0,00E+00	5073	289	NA	NA	Kinase-LRR-TM
maker-7-augustus-gene-54.2-mRNA-1	7	OsR498G0713726900.01.T04	100,00	0,00E+00	1537	Os07g0207700	99,64	0,00E+00	1489	-48	NA	NA	
maker-7-augustus-gene-54.20-mRNA-1	7	OsR498G0713727200.01.T01	100,00	0,00E+00	1705	Os07g0208100	95,21	0,00E+00	1024	-681	Indica	NA	
maker-7-augustus-gene-54.22-mRNA-1	7	OsR498G0713727400.01.T02	100,00	0,00E+00	710	Os07g0208000	100,00	0,00E+00	710	0	NA	NA	
maker-7-augustus-gene-54.24-mRNA-1	7	OsR498G0713729200.01.T01	100,00	3,96E-137	488	Os05g0394100	99,63	5,85E-136	483	-5	NA	NA	
maker-7-augustus-gene-54.26-mRNA-1	7	OsR498G0713731400.01.T02	100,00	0,00E+00	5463	Os07g0208500	100,00	0,00E+00	5463	0	NA	NA	
maker-7-augustus-gene-54.8-mRNA-1	7	OsR498G0713727500.01.T02	100,00	0,00E+00	1319	Os07g0208100	100,00	0,00E+00	1218	-101	NA	NA	
maker-7-augustus-gene-55.27-mRNA-1	7	OsR498G0713733900.01.T01	100,00	0,00E+00	3009	Os07g0208900	100,00	0,00E+00	1683	-1326	Indica	NA	
maker-7-augustus-gene-55.31-mRNA-1	7	OsR498G0713734100.01.T01	99,81	0,00E+00	1867	Os07g0209000	100,00	0,00E+00	1876	9	NA	NA	
maker-7-augustus-gene-55.38-mRNA-1	7	OsR498G0713736900.01.T10	100,00	0,00E+00	774	Os07g0209500	100,00	8,91E-167	585	-189	Indica	NA	
maker-7-augustus-gene-55.41-mRNA-1	7	OsR498G0713738700.01.T01	100,00	0,00E+00	2105	Os07g0209800	100,00	0,00E+00	1115	-990	Indica	NA	
maker-7-augustus-gene-55.47-mRNA-1	7	OsR498G0713740300.01.T01	100,00	0,00E+00	3793	Os07g0210000	100,00	0,00E+00	2727	-1066	Indica	NA	
maker-7-augustus-gene-55.50-mRNA-1	7	OsR498G0713734600.01.T01	100,00	0,00E+00	4134	Os07g0209100	100,00	0,00E+00	4134	0	NA	Japonica	
maker-7-augustus-gene-56.41-mRNA-1	7	OsR498G0713747300.01.T01	100,00	0,00E+00	3171	Os07g0211400	100,00	0,00E+00	3171	0	NA	NA	
maker-7-augustus-gene-56.50-mRNA-1	7	OsR498G0713744000.01.T05	100,00	0,00E+00	796	Os07g0211000	100,00	0,00E+00	796	0	NA	NA	
maker-7-augustus-gene-57.28-mRNA-1	7	OsR498G0713751900.01.T03	100,00	0,00E+00	668	Os07g0212200	100,00	0,00E+00	668	0	NA	NA	
maker-7-augustus-gene-57.29-mRNA-1	7	OsR498G0713751900.01.T04	100,00	0,00E+00	1052	Os07g0212200	100,00	0,00E+00	1052	0	NA	NA	
maker-7-augustus-gene-57.38-mRNA-1	7	OsR498G0713750000.01.T02	100,00	0,00E+00	969	Os07g0211800	100,00	0,00E+00	969	0	NA	NA	
maker-7-augustus-gene-57.46-mRNA-1	7	OsR498G0713752200.01.T01	100,00	0,00E+00	991	Os07g0212300	100,00	0,00E+00	991	0	NA	NA	
maker-7-augustus-gene-58.30-mRNA-1	7	OsR498G0713759900.01.T01	100,00	0,00E+00	1090	Os07g0213400	100,00	0,00E+00	1090	0	NA	NA	
maker-7-augustus-gene-58.36-mRNA-1	7	OsR498G0713759600.01.T07	100,00	0,00E+00	3254	Os07g0213300	100,00	0,00E+00	2415	-839	Indica	NA	
maker-7-augustus-gene-59.32-mRNA-1	7	OsR498G0713765800.01.T01	100,00	0,00E+00	2186	Os07g0214900	100,00	0,00E+00	2186	0	NA	NA	
maker-7-augustus-gene-59.34-mRNA-1	7	OsR498G0713768000.01.T06	100,00	0,00E+00	720	Os07g0215200	100,00	7,25E-166	581	-139	Indica	NA	
maker-7-augustus-gene-6.0-mRNA-1	7	OsR498G0713415700.01.T01	100,00	0,00E+00	715	Os07g0118300	100,00	0,00E+00	1283	568	Japonica	Japonica	
maker-7-augustus-gene-6.11-mRNA-1	7	OsR498G0713431000.01.T01	98,87	0,00E+00	2635	Os07g0117400	84,47	0,00E+00	1645	-990	Indica	Japonica	
maker-7-augustus-gene-6.14-mRNA-1	7	OsR498G0713419000.01.T01	99,69	0,00E+00	4048	Os07g0119000	100,00	0,00E+00	4080	32	NA	Japonica	Kinase-TM
maker-7-augustus-gene-6.5-mRNA-1	7	OsR498G0713418300.01.T01	99,16	0,00E+00	2716	Os07g0118800	99,91	0,00E+00	2009	-707	Indica	Japonica	
maker-7-augustus-gene-6.8-mRNA-1	7	OsR498G0713418700.01.T01	99,13	0,00E+00	1819	Os07g0118900	99,75	0,00E+00	2189	370	Japonica	Japonica	
maker-7-augustus-gene-6.9-mRNA-1	7	OsR498G0713430800.01.T01	99,31	0,00E+00	2578	Os07g0117000	99,94	0,00E+00	3078	500	Japonica	Japonica	
maker-7-augustus-gene-62.26-mRNA-1	7	OsR498G0408175300.01.T01	83,89	0,00E+00	1590	Os07g0218700	100,00	0,00E+00	2814	1224	Japonica	NA	
maker-7-augustus-gene-62.28-mRNA-1	7	OsR498G0408175300.01.T01	85,60	0,00E+00	1680	Os07g0218700	93,97	0,00E+00	2388	708	Japonica	NA	
maker-7-augustus-gene-63.21-mRNA-1	7	OsR498G0713790800.01.T01	99,69	0,00E+00	1715	Os07g0222300	100,00	0,00E+00	1728	13	NA	Japonica	
maker-7-augustus-gene-64.0-mRNA-1	7	OsR498G0713792500.01.T01	99,95	0,00E+00	3302	Os07g0222700	99,91	0,00E+00	2000	-1302	Indica	Japonica	
maker-7-augustus-gene-64.11-mRNA-1	7	OsR498G0713793300.01.T03	100,00	0,00E+00	1283	Os07g0223400	96,30	0,00E+00	886	-397	Indica	Japonica	
maker-7-augustus-gene-64.16-mRNA-1	7	OsR498G0713793700.01.T01	95,37	0,00E+00	1187	Os07g0223400	96,78	0,00E+00	939	-248	Indica	Japonica	
maker-7-augustus-gene-64.4-mRNA-1	7	OsR498G0713792700.01.T01	100,00	0,00E+00	715	Os07g0222800	100,00	0,00E+00	715	0	NA	Japonica	
maker-7-augustus-gene-67.0-mRNA-1	7	OsR498G0713819700.01.T01	95,35	0,00E+00	1473	Os07g0227400	95,35	0,00E+00	1473	0	NA	Japonica	
maker-7-augustus-gene-67.4-mRNA-1	7	OsR498G0713821100.01.T02	98,18	0,00E+00	3909	Os07g0227700	99,17	0,00E+00	4481	572	Japonica	Japonica	
maker-7-augustus-gene-68.28-mRNA-1	7	OsR498G0713823200.01.T01	76,79	2,71E-115	416	Os07g0228900	99,71	1,22E-177	622	206	Japonica	Japonica	
maker-7-augustus-gene-68.29-mRNA-1	7	OsR498G0713823200.01.T01	99,83	0,00E+00	3082	Os07g0229100	100,00	0,00E+00	3095	13	NA	Japonica	
maker-7-augustus-gene-68.31-mRNA-1	7	OsR498G0713823800.01.T01	100,00	0,00E+00	1889	Os07g0229200	100,00	0,00E+00	1889	0	NA	Japonica	
maker-7-augustus-gene-68.33-mRNA-1	7	OsR498G0713824600.01.T01	99,83	0,00E+00	2048	Os07g0229700	100,00	0,00E+00	2057	9	NA	Japonica	
maker-7-augustus-gene-68.35-mRNA-1	7	OsR498G0713824800.01.T03	97,09	0,00E+00	2387	Os07g0229800	94,83	0,00E+00	1274	-1113	Indica	Japonica	
maker-7-augustus-gene-68.42-mRNA-1	7	OsR498G0713825800.01.T01	94,73	0,00E+00	1541	Os07g0230500	100,00	0,00E+00	2392	851	Japonica	Japonica	
maker-7-augustus-gene-69.0-mRNA-1	7	OsR498G0713827300.01.T01	99,14	0,00E+00	2250	Os07g0231400	100,00	0,00E+00	1894	-356	Indica	Japonica	
maker-7-augustus-gene-69.3-mRNA-1	7	OsR498G0713830700.01.T03	99,80	0,00E+00	2671	Os07g0231800	99,93	0,00E+00	2680	9	NA	Japonica	
maker-7-augustus-gene-69.6-mRNA-1	7	OsR498G0713831300.01.T02	99,83	0,00E+00	1042	Os07g0232200	100,00	0,00E+00	1047	5	NA	Japonica	
maker-7-augustus-gene-69.9-mRNA-1	7	OsR498G0713826600.01.T01	99,54	0,00E+00	1150	Os07g0230700	100,00	0,00E+00	1164	14	NA	Japonica	
maker-7-augustus-gene-7.0-mRNA-1	7	OsR498G0713419300.01.T01	99,68	5,28E-156	552	Os07g0119100	100,00	0,00E+00	841	289	Japonica	Japonica	
maker-7-augustus-gene-7.11-mRNA-1	7	OsR498G0713425000.01.T01	99,64	0,00E+00	2012	Os07g0120400	78,44	0,00E+00	902	-1110	Indica	Japonica	
maker-7-augustus-gene-7.12-mRNA-1	7	OsR498G0713425200.01.T03	99,46	0,00E+00	1314	Os07g0120400	100,00	0,00E+00	1997	683	Japonica	Japonica	
maker-7-augustus-gene-7.3-mRNA-1	7	OsR498G0713421700.01.T01	99,81	0,00E+00	1918	Os07g0119300	100,00	0,00E+00	1927	9	NA	Japonica	
maker-7-augustus-gene-7.39-mRNA-1	7	OsR498G0713427200.01.T01	99,48	0,00E+00	5138	Os07g0121200	99,97	0,00E+00	5401	263	NA	Japonica	Kinase-LRR-TM
maker-7-augustus-gene-7.6-mRNA-1	7	OsR498G0713422500.01.T01	99,81	0,00E+00	2894	Os07g0119400	100,00	0,00E+00	2908	14	NA	Japonica	
maker-7-augustus-gene-70.33-mRNA-1	7	OsR498G0713832100.01.T01	98,51	0,00E+00	1168	Os07g0534500	89,14	0,00E+00	882	-286	Indica	Japonica	Kinase-TM
maker-7-augustus-gene-70.43-mRNA-1	7	OsR498G0713834000.01.T01	99,70	0,00E+00	5397	Os07g0232900	99,97	0,00E+00	5433	36	NA	Japonica	
maker-7-augustus-gene-70.46-mRNA-1	7	OsR498G0713834800.01.T02	95,51	0,00E+00	1203	Os07g0233300	95,51	0,00E+00	1203	0	NA	Japonica	
maker-7-augustus-gene-71.0-mRNA-1	7	OsR498G0713843700.01.T07	99,30	0,00E+00	1769	Os07g0235800	99,70	0,00E+00	1787	18	NA	Japonica	
maker-7-augustus-gene-72.37-mRNA-1	7	OsR498G0713849700.01.T03	100,00	0,00E+00	1056	Os07g0236800	100,00	0,00E+00	922	-134	Indica	Japonica	
maker-7-augustus-gene-72.42-mRNA-1	7	OsR498G0713850000.01.T03	98,35	0,00E+00	1052	Os07g0237100	98,54	0,00E+00	1073	21	NA	Japonica	
maker-7-augustus-gene-73.0-mRNA-1	7	OsR498G0713854700.01.T03	99,59	0,00E+00	881	Os07g0238700	100,00	0,00E+00	991	110	Japonica	Japonica	
maker-7-augustus-gene-73.3-mRNA-1	7	OsR498G0713855000.01.T01	99,40	0,00E+00	1182	Os12g0106900	70,02	3,56E-52	205	-977	Indica	Japonica	
maker-7-augustus-gene-73.5-mRNA-1	7	OsR498G0713855600.01.T01	99,09	0,00E+00	1347	Os07g0239200	100,00	0,00E+00	1375	28	NA	Japonica	
maker-7-augustus-gene-73.7-mRNA-1	7	OsR498G0713855800.01.T01	99,01	0,00E+00	708	Os07g0239400	100,00	0,00E+00	726	18	NA	Japonica	
maker-7-augustus-gene-74.0-mRNA-1	7	OsR498G0713858900.01.T01	99,71	0,00E+00	1877	Os07g0240200	100,00	0,00E+00	1891	14	NA	Japonica	
maker-7-augustus-gene-74.2-mRNA-1	7	OsR498G0713859300.01.T01	98,30	0,00E+00	2351	Os07g0240300	98,45	0,00E+00	2360	9	NA	Japonica	
maker-7-augustus-gene-74.8-mRNA-1	7	OsR498G0713861300.01.T01	99,65	0,00E+00	2575	Os07g0240700	100,00	0,00E+00	2556	-19	NA	Japonica	
maker-7-augustus-gene-75.44-mRNA-1	7	OsR498G0713863300.01.T01	99,65	0,00E+00	2586	Os07g0241500	100,00	0,00E+00	2608	22	NA	Japonica	
maker-7-augustus-gene-75.46-mRNA-1	7	OsR498G0713863500.01.T01	99,82	0,00E+00	981	Os07g0241600	100,00	0,00E+00	985	4	NA	Japonica	
maker-7-augustus-gene-75.47-mRNA-1	7	OsR498G0713864300.01.T01	99,43	0,00E+00	2480	Os07g0241700	100,00	0,00E+00	2516	36	NA	Japonica	
maker-7-augustus-gene-75.53-mRNA-1	7	OsR498G0713868400.01.T01	96,98	0,00E+00	1885	Os07g0243000	100,00	0,00E+00	1350	-535	Indica	Japonica	
maker-7-augustus-gene-76.52-mRNA-1	7	OsR498G0713871900.01.T01	100,00	0,00E+00	982	Os07g0244300	100,00	0,00E+00	982	0	NA	Japonica	
maker-7-augustus-gene-76.56-mRNA-1	7	OsR498G0713873700.01.T02	99,85	0,00E+00	1214	Os07g0244600	100,00	0,00E+00	2414	1200	Japonica	Japonica	
maker-7-augustus-gene-76.58-mRNA-1	7	OsR498G0713868800.01.T02	100,00	5,29E-75	280	Os07g0243100	100,00	1,85E-75	280	0	NA	Japonica	
maker-7-augustus-gene-76.61-mRNA-1	7	OsR498G0713869000.01.T03	98,78	0,00E+00	1874	Os07g0243200	100,00	0,00E+00	1927	53	NA	Japonica	
maker-7-augustus-gene-76.66-mRNA-1	7	OsR498G0713871400.01.T01	99,56	0,00E+00	2419	Os07g0244200	100,00	0,00E+00	2446	27	NA	Japonica	
maker-7-augustus-gene-76.68-mRNA-1	7	OsR498G0713874400.01.T01	99,42	0,00E+00	912	Os07g0244900	100,00	0,00E+00	926	14	NA	Japonica	
maker-7-augustus-gene-77.29-mRNA-1	7	OsR498G0713875100.01.T04	99,85	0,00E+00	1203	Os07g0245100	99,82	0,00E+00	985	-218	Indica	Japonica	
maker-7-augustus-gene-77.38-mRNA-1	7	OsR498G0713875600.01.T04	99,78	0,00E+00	2406	Os07g0245200	100,00	0,00E+00	2419	13	NA	Japonica	
maker-7-augustus-gene-78.38-mRNA-1	7	OsR498G0713879500.01.T02	94,88	0,00E+00	1731	Os07g0247100	95,25	0,00E+00	1749	18	NA	Japonica	
maker-7-augustus-gene-78.42-mRNA-1	7	OsR498G0713878200.01.T05	99,45	0,00E+00	1297	Os07g0246600	99,86	0,00E+00	1311	14	NA	Japonica	
maker-7-augustus-gene-79.40-mRNA-1	7	OsR498G0713886300.01.T01	100,00	0,00E+00	1348	Os07g0249200	100,00	0,00E+00	1348	0	NA	Japonica	
maker-7-augustus-gene-79.43-mRNA-1	7	OsR498G0713887500.01.T01	99,74	0,00E+00	2059	Os07g0249600	100,00	0,00E+00	2073	14	NA	Japonica	
maker-7-augustus-gene-79.49-mRNA-1	7	OsR498G0713889300.01.T01	99,69	5,81E-165	581	Os07g0250300	100,00	4,75E-167	585	4	NA	Japonica	
maker-7-augustus-gene-79.52-mRNA-1	7	OsR498G0713885000.01.T02	99,78	0,00E+00	2470	Os07g0248800	99,75	0,00E+00	2178	-292	Indica	Japonica	
maker-7-augustus-gene-79.56-mRNA-1	7	OsR498G0713885800.01.T01	99,80	0,00E+00	4465	Os07g0249100	100,00	0,00E+00	4488	23	NA	Japonica	
maker-7-augustus-gene-79.57-mRNA-1	7	OsR498G0713887800.01.T01	98,55	0,00E+00	2398	Os03g0836900	73,42	0,00E+00	723	-1675	Indica	Japonica	
maker-7-augustus-gene-79.59-mRNA-1	7	OsR498G0713888000.01.T01	99,48	0,00E+00	2377	Os07g0249800	100,00	0,00E+00	1537	-840	Indica	Japonica	
maker-7-augustus-gene-79.62-mRNA-1	7	OsR498G0713888200.01.T01	97,81	0,00E+00	2260	Os03g0836900	73,54	0,00E+00	719	-1541	Indica	Japonica	
maker-7-augustus-gene-8.43-mRNA-1	7	OsR498G0713430100.01.T01	99,30	0,00E+00	2514	Os07g0122400	99,58	0,00E+00	2528	14	NA	Japonica	
maker-7-augustus-gene-8.46-mRNA-1	7	OsR498G0713432100.01.T02	100,00	0,00E+00	2008	Os07g0122900	100,00	0,00E+00	1921	-87	NA	Japonica	
maker-7-augustus-gene-8.49-mRNA-1	7	OsR498G0101324300.01.T01	72,01	8,29E-164	578	Os07g0123000	100,00	0,00E+00	2246	1668	Japonica	Japonica	
maker-7-augustus-gene-8.52-mRNA-1	7	OsR498G0713433200.01.T03	99,78	0,00E+00	1604	Os07g0123300	100,00	0,00E+00	1328	-276	Indica	Japonica	
maker-7-augustus-gene-8.54-mRNA-1	7	OsR498G0713433400.01.T01	99,71	0,00E+00	3102	Os03g0769000	79,33	0,00E+00	1058	-2044	Indica	Japonica	
maker-7-augustus-gene-8.58-mRNA-1	7	OsR498G0713434400.01.T01	99,75	0,00E+00	2839	Os07g0123500	100,00	0,00E+00	2857	18	NA	Japonica	
maker-7-augustus-gene-81.0-mRNA-1	7	OsR498G0713893500.01.T01	99,31	0,00E+00	2562	Os07g0251900	100,00	0,00E+00	2599	37	NA	Japonica	Kinase-LRR-TM
maker-7-augustus-gene-81.1-mRNA-1	7	OsR498G0713894700.01.T03	100,00	0,00E+00	1384	Os07g0252000	100,00	0,00E+00	1384	0	NA	Japonica	
maker-7-augustus-gene-81.12-mRNA-1	7	OsR498G0713897300.01.T01	98,50	0,00E+00	814	Os07g0252800	100,00	0,00E+00	845	31	NA	Japonica	
maker-7-augustus-gene-81.14-mRNA-1	7	OsR498G0713898100.01.T02	100,00	0,00E+00	677	Os07g0253000	100,00	0,00E+00	677	0	NA	Japonica	
maker-7-augustus-gene-81.7-mRNA-1	7	OsR498G0713895900.01.T03	99,80	0,00E+00	3604	Os07g0252400	99,95	0,00E+00	3617	13	NA	Japonica	
maker-7-augustus-gene-84.0-mRNA-1	7	OsR498G0713912700.01.T01	99,81	0,00E+00	4626	Os07g0256200	100,00	0,00E+00	4648	22	NA	Japonica	
maker-7-augustus-gene-84.2-mRNA-1	7	OsR498G0713913700.01.T01	99,70	0,00E+00	2369	Os07g0256700	100,00	0,00E+00	2387	18	NA	Japonica	
maker-7-augustus-gene-84.4-mRNA-1	7	OsR498G0713911400.01.T01	99,42	0,00E+00	2432	Os07g0256050	99,83	0,00E+00	2051	-381	Indica	Japonica	
maker-7-augustus-gene-85.0-mRNA-1	7	OsR498G0102089900.01.T03	70,61	2,07E-95	352	Os07g0258000	100,00	0,00E+00	1692	1340	Japonica	Japonica	
maker-7-augustus-gene-85.6-mRNA-1	7	OsR498G0713921000.01.T02	99,62	0,00E+00	2333	Os07g0258100	100,00	0,00E+00	2013	-320	Indica	Japonica	
maker-7-augustus-gene-86.23-mRNA-1	7	OsR498G0713923500.01.T04	99,78	0,00E+00	2431	Os07g0258400	100,00	0,00E+00	2444	13	NA	Japonica	
maker-7-augustus-gene-86.29-mRNA-1	7	OsR498G0713924700.01.T02	99,87	0,00E+00	1333	Os07g0258700	100,00	0,00E+00	1337	4	NA	Japonica	
maker-7-augustus-gene-86.34-mRNA-1	7	OsR498G0713926400.01.T01	99,36	0,00E+00	4142	Os07g0259100	100,00	0,00E+00	4210	68	NA	Japonica	
maker-7-augustus-gene-86.36-mRNA-1	7	OsR498G0713929600.01.T02	99,76	0,00E+00	3039	Os07g0259700	100,00	0,00E+00	3057	18	NA	Japonica	
maker-7-augustus-gene-87.28-mRNA-1	7	OsR498G0713933300.01.T01	99,08	0,00E+00	1342	Os07g0260300	100,00	0,00E+00	1370	28	NA	Japonica	
maker-7-augustus-gene-87.36-mRNA-1	7	OsR498G0713933600.01.T02	99,90	0,00E+00	3647	Os07g0260400	100,00	0,00E+00	3653	6	NA	Japonica	
maker-7-augustus-gene-88.0-mRNA-1	7	OsR498G0713938000.01.T03	99,89	0,00E+00	1565	Os07g0262200	100,00	0,00E+00	1570	5	NA	Japonica	
maker-7-augustus-gene-89.0-mRNA-1	7	OsR498G0713942700.01.T04	93,35	0,00E+00	1244	Os07g0264100	93,35	0,00E+00	1244	0	NA	NA	
maker-7-augustus-gene-89.4-mRNA-1	7	OsR498G0713942700.01.T04	95,95	0,00E+00	1467	Os07g0264100	96,29	0,00E+00	1480	13	NA	Japonica	
maker-7-augustus-gene-9.0-mRNA-1	7	OsR498G0713437900.01.T03	99,50	0,00E+00	5007	Os07g0124500	99,96	0,00E+00	5066	59	NA	Japonica	
maker-7-augustus-gene-9.17-mRNA-1	7	OsR498G0713436800.01.T01	99,76	0,00E+00	743	Os07g0124100	100,00	9,40E-128	455	-288	Indica	Japonica	
maker-7-augustus-gene-9.19-mRNA-1	7	OsR498G0713440600.01.T01	100,00	0,00E+00	3718	Os03g0770800	80,27	0,00E+00	1625	-2093	Indica	Indica	
maker-7-augustus-gene-9.22-mRNA-1	7	OsR498G0713441000.01.T02	99,70	2,08E-167	590	Os07g0124800	99,40	8,82E-167	585	-5	NA	Indica	
maker-7-augustus-gene-9.3-mRNA-1	7	OsR498G0713438100.01.T02	100,00	0,00E+00	2046	Os07g0124600	100,00	0,00E+00	2046	0	NA	NA	
maker-7-augustus-gene-9.5-mRNA-1	7	OsR498G0713440300.01.T02	99,53	0,00E+00	3443	Os07g0124700	98,90	0,00E+00	1751	-1692	Indica	Indica	
maker-7-augustus-gene-90.30-mRNA-1	7	OsR498G0713943700.01.T02	98,65	0,00E+00	1544	Os07g0264800	100,00	0,00E+00	1597	53	NA	Japonica	
maker-7-augustus-gene-90.34-mRNA-1	7	OsR498G0713944100.01.T02	86,38	1,02E-149	529	Os07g0265100	86,86	6,87E-153	538	9	NA	Japonica	
maker-7-augustus-gene-91.0-mRNA-1	7	OsR498G0713955100.01.T01	100,00	0,00E+00	886	Os07g0265600	100,00	0,00E+00	886	0	NA	Japonica	
maker-7-augustus-gene-92.3-mRNA-1	7	OsR498G1221850900.01.T02	85,34	0,00E+00	2593	Os07g0267400	100,00	0,00E+00	4360	1767	Japonica	Japonica	
maker-7-augustus-gene-96.27-mRNA-1	7	OsR498G0713968500.01.T03	100,00	0,00E+00	1489	Os07g0270900	100,00	0,00E+00	1489	0	NA	Japonica	
maker-7-augustus-gene-96.32-mRNA-1	7	OsR498G0713970500.01.T01	97,04	0,00E+00	2039	Os07g0271500	100,00	0,00E+00	2181	142	NA	Japonica	
maker-7-augustus-gene-96.39-mRNA-1	7	OsR498G0713969200.01.T02	96,39	0,00E+00	2598	Os07g0271000	95,89	0,00E+00	2213	-385	Indica	Japonica	
maker-7-augustus-gene-97.4-mRNA-1	7	OsR498G0713971400.01.T01	99,05	0,00E+00	1663	Os03g0843300	71,53	4,92E-125	448	-1215	Indica	Japonica	
maker-7-augustus-gene-98.0-mRNA-1	7	OsR498G0713984800.01.T01	99,63	9,82E-136	483	Os07g0274400	100,00	8,07E-138	488	5	NA	Japonica	
maker-7-augustus-gene-98.10-mRNA-1	7	OsR498G0713985100.01.T04	99,42	0,00E+00	2160	Os07g0274100	100,00	0,00E+00	2122	-38	NA	Japonica	
maker-7-augustus-gene-98.2-mRNA-1	7	OsR498G0713984400.01.T01	97,11	0,00E+00	1172	Os07g0274700	99,49	0,00E+00	698	-474	Indica	Japonica	
maker-7-augustus-gene-98.5-mRNA-1	7	OsR498G0713986100.01.T01	99,45	0,00E+00	7111	Os07g0273700	100,00	0,00E+00	7126	15	NA	Japonica	LRR-NBS-TM
maker-7-augustus-gene-99.0-mRNA-1	7	OsR498G0713984000.01.T01	100,00	0,00E+00	2803	Os07g0274800	100,00	0,00E+00	2803	0	NA	Japonica	
maker-7-augustus-gene-99.2-mRNA-1	7	OsR498G0713983000.01.T01	100,00	0,00E+00	1353	Os12g0564400	92,33	0,00E+00	1084	-269	Indica	Japonica	
maker-7-augustus-gene-99.5-mRNA-1	7	OsR498G0713981800.01.T02	92,02	0,00E+00	994	Os07g0275300	91,67	0,00E+00	961	-33	NA	Japonica	
augustus_masked-8-processed-gene-1.7-mRNA-1	8	OsR498G0815025500.01.T01	99,26	0,00E+00	2400	Os08g0101500	99,12	0,00E+00	2420	20	NA	Japonica	
augustus_masked-8-processed-gene-100.8-mRNA-1	8	OsR498G0815571100.01.T01	97,95	0,00E+00	1003	Os08g0260400	99,48	0,00E+00	1031	28	NA	NA	
augustus_masked-8-processed-gene-101.5-mRNA-1	8	OsR498G0815572500.01.T03	99,44	0,00E+00	2540	Os08g0261000	99,53	0,00E+00	2653	113	NA	NA	CC-NBS-TM
augustus_masked-8-processed-gene-104.22-mRNA-1	8	OsR498G0815589400.01.T02	92,89	0,00E+00	1297	Os08g0267800	92,43	0,00E+00	1283	-14	NA	NA	
augustus_masked-8-processed-gene-105.3-mRNA-1	8	OsR498G0815592700.01.T01	99,46	0,00E+00	995	Os06g0159750	98,69	4,70E-151	533	-462	Indica	NA	
augustus_masked-8-processed-gene-109.9-mRNA-1	8	OsR498G0815613300.01.T01	99,79	0,00E+00	3424	Os08g0275200	99,79	0,00E+00	3424	0	NA	NA	Kinase-TM
augustus_masked-8-processed-gene-115.9-mRNA-1	8	OsR498G0815638900.01.T01	99,68	0,00E+00	1664	Os08g0282100	98,96	0,00E+00	2885	1221	Japonica	NA	
augustus_masked-8-processed-gene-121.16-mRNA-1	8	OsR498G0815665700.01.T01	97,78	0,00E+00	767	Os08g0290500	98,81	0,00E+00	734	-33	NA	NA	
augustus_masked-8-processed-gene-121.18-mRNA-1	8	OsR498G0815666900.01.T01	99,32	0,00E+00	777	Os06g0543400	87,50	1,19E-144	512	-265	Indica	NA	
augustus_masked-8-processed-gene-122.18-mRNA-1	8	OsR498G0815673500.01.T02	100,00	0,00E+00	1350	Os08g0292600	100,00	0,00E+00	1350	0	NA	NA	
augustus_masked-8-processed-gene-149.9-mRNA-1	8	OsR498G0815788300.01.T01	98,66	0,00E+00	3386	Os08g0327100	98,81	0,00E+00	3396	10	NA	NA	
augustus_masked-8-processed-gene-153.21-mRNA-1	8	OsR498G0815812500.01.T01	99,94	0,00E+00	3226	Os08g0334900	99,72	0,00E+00	3208	-18	NA	Indica	
augustus_masked-8-processed-gene-161.8-mRNA-1	8	OsR498G0815852300.01.T01	100,00	0,00E+00	1337	Os08g0346400	100,00	0,00E+00	1337	0	NA	Indica	
augustus_masked-8-processed-gene-164.10-mRNA-1	8	OsR498G0815862900.01.T01	96,12	0,00E+00	924	Os08g0349100	96,65	0,00E+00	938	14	NA	NA	
augustus_masked-8-processed-gene-165.0-mRNA-1	8	OsR498G0815866300.01.T03	99,71	0,00E+00	2428	Os08g0351200	94,07	0,00E+00	1137	-1291	Indica	Indica	
augustus_masked-8-processed-gene-17.55-mRNA-1	8	OsR498G0815106700.01.T03	99,69	0,00E+00	1166	Os08g0128000	99,85	0,00E+00	1170	4	NA	Japonica	
augustus_masked-8-processed-gene-17.59-mRNA-1	8	OsR498G0815108100.01.T01	99,64	0,00E+00	1990	Os08g0128700	99,91	0,00E+00	2003	13	NA	Japonica	
augustus_masked-8-processed-gene-18.47-mRNA-1	8	OsR498G0815118100.01.T01	100,00	0,00E+00	740	Os08g0131200	100,00	0,00E+00	637	-103	Indica	Japonica	
augustus_masked-8-processed-gene-190.21-mRNA-1	8	OsR498G0815950900.01.T01	99,84	0,00E+00	1122	Os08g0375400	99,68	0,00E+00	1117	-5	NA	NA	
augustus_masked-8-processed-gene-191.12-mRNA-1	8	OsR498G0815952700.01.T01	97,26	0,00E+00	857	Os08g0375700	96,86	0,00E+00	848	-9	NA	Indica	
augustus_masked-8-processed-gene-191.13-mRNA-1	8	OsR498G0815952700.01.T01	98,43	0,00E+00	884	Os08g0375700	99,02	0,00E+00	898	14	NA	NA	
augustus_masked-8-processed-gene-191.14-mRNA-1	8	OsR498G0815953100.01.T01	99,76	0,00E+00	3749	Os08g0375800	99,86	0,00E+00	3758	9	NA	NA	
augustus_masked-8-processed-gene-192.7-mRNA-1	8	OsR498G0815959900.01.T01	97,07	0,00E+00	975	Os08g0377300	95,93	0,00E+00	721	-254	Indica	NA	

augustus_masked-8-processed-gene-194.11-mRNA-1	8	OsR498G0815968100.01.T01	100,00	0,00E+00	991	Os08g0381600	100,00	0,00E+00	632	-359	Indica	Indica	
augustus_masked-8-processed-gene-195.42-mRNA-1	8	OsR498G0815976100.01.T01	98,39	0,00E+00	645	Os05g0510100	74,90	5,93E-40	164	-481	Indica	Indica	
augustus_masked-8-processed-gene-198.16-mRNA-1	8	OsR498G0815986100.01.T03	99,25	0,00E+00	1407	Os08g0388300	99,25	0,00E+00	1407	0	NA	NA	NBS-TM
augustus_masked-8-processed-gene-199.25-mRNA-1	8	OsR498G0815993500.01.T04	98,93	0,00E+00	3295	Os08g0390000	98,88	0,00E+00	3290	-5	NA	NA	
augustus_masked-8-processed-gene-2.8-mRNA-1	8	OsR498G0713661400.01.T01	77,91	4,51E-48	193	Os08g0102800	100,00	0,00E+00	850	657	Japonica	Japonica	
augustus_masked-8-processed-gene-204.31-mRNA-1	8	OsR498G0816031100.01.T01	99,41	0,00E+00	6890	Os08g0400200	99,64	0,00E+00	6929	39	NA	NA	
augustus_masked-8-processed-gene-205.21-mRNA-1	8	OsR498G0816036700.01.T01	99,41	0,00E+00	2415	Os08g0402100	100,00	0,00E+00	2311	-104	NA	Japonica	
augustus_masked-8-processed-gene-209.19-mRNA-1	8	OsR498G0816066900.01.T01	98,36	0,00E+00	2228	Os08g0409500	100,00	0,00E+00	2311	83	NA	Japonica	
augustus_masked-8-processed-gene-209.5-mRNA-1	8	OsR498G0816061900.01.T01	99,41	0,00E+00	1497	Os08g0408500	99,88	0,00E+00	1517	20	NA	Japonica	
augustus_masked-8-processed-gene-210.0-mRNA-1	8	OsR498G0816070500.01.T01	99,79	0,00E+00	2588	Os08g0410300	98,60	0,00E+00	1997	-591	Indica	Japonica	
augustus_masked-8-processed-gene-211.3-mRNA-1	8	OsR498G0816078500.01.T01	99,76	0,00E+00	2276	Os08g0413100	87,23	0,00E+00	1530	-746	Indica	Japonica	
augustus_masked-8-processed-gene-212.16-mRNA-1	8	OsR498G0816084700.01.T01	99,51	0,00E+00	3635	Os08g0415600	100,00	0,00E+00	3663	28	NA	Japonica	
augustus_masked-8-processed-gene-214.23-mRNA-1	8	OsR498G0816093100.01.T01	99,44	0,00E+00	961	Os08g0419100	99,72	0,00E+00	1283	322	Japonica	Japonica	
augustus_masked-8-processed-gene-215.10-mRNA-1	8	OsR498G0816100100.01.T01	99,46	0,00E+00	979	Os08g0421700	97,32	0,00E+00	1240	261	Japonica	Indica	
augustus_masked-8-processed-gene-22.53-mRNA-1	8	OsR498G0815137300.01.T01	83,60	3,22E-171	602	Os08g0137900	99,82	0,00E+00	1019	417	Japonica	Japonica	
augustus_masked-8-processed-gene-223.0-mRNA-1	8	OsR498G0816147700.01.T01	100,00	9,32E-123	439	Os08g0433000	100,00	4,54E-115	412	-27	NA	NA	
augustus_masked-8-processed-gene-223.1-mRNA-1	8	OsR498G0816147700.01.T01	100,00	6,87E-118	423	Os08g0433000	100,00	3,13E-104	376	-47	Indica	NA	
augustus_masked-8-processed-gene-223.4-mRNA-1	8	OsR498G0816148900.01.T01	99,39	0,00E+00	2027	Os08g0433300	99,13	0,00E+00	2021	-6	NA	Indica	
augustus_masked-8-processed-gene-227.32-mRNA-1	8	OsR498G0816169900.01.T01	99,92	0,00E+00	2177	Os08g0439600	98,94	0,00E+00	2146	-31	NA	Indica	
augustus_masked-8-processed-gene-227.33-mRNA-1	8	OsR498G0816170300.01.T01	100,00	0,00E+00	978	Os08g0439900	99,82	0,00E+00	974	-4	NA	Indica	
augustus_masked-8-processed-gene-228.11-mRNA-1	8	OsR498G0816176500.01.T01	99,85	0,00E+00	2437	Os08g0441100	99,89	0,00E+00	1646	-791	Indica	Indica	Kinase-TM
augustus_masked-8-processed-gene-229.14-mRNA-1	8	OsR498G0816189900.01.T01	99,52	0,00E+00	1487	Os08g0443800	100,00	0,00E+00	1505	18	NA	Japonica	
augustus_masked-8-processed-gene-23.0-mRNA-1	8	OsR498G0815138300.01.T01	99,30	0,00E+00	1016	Os08g0138200	100,00	0,00E+00	1034	18	NA	Japonica	
augustus_masked-8-processed-gene-230.11-mRNA-1	8	OsR498G0816195700.01.T01	98,77	1,06E-161	569	Os07g0597625	92,52	1,47E-135	480	-89	Indica	Indica	
augustus_masked-8-processed-gene-230.8-mRNA-1	8	OsR498G0816195500.01.T01	99,53	0,00E+00	2680	Os08g0445000	99,40	0,00E+00	2674	-6	NA	NA	
augustus_masked-8-processed-gene-232.17-mRNA-1	8	OsR498G0816215500.01.T01	99,47	0,00E+00	1688	Os08g0450100	98,74	0,00E+00	1656	-32	NA	Indica	
augustus_masked-8-processed-gene-232.18-mRNA-1	8	OsR498G0816215500.01.T01	100,00	0,00E+00	1332	Os08g0450100	98,24	0,00E+00	1277	-55	NA	Indica	
augustus_masked-8-processed-gene-233.2-mRNA-1	8	OsR498G0816216500.01.T01	99,34	0,00E+00	1070	Os05g0350900	75,53	9,23E-53	206	-864	Indica	Japonica	
augustus_masked-8-processed-gene-234.10-mRNA-1	8	OsR498G0816220900.01.T02	99,79	0,00E+00	4290	Os08g0452100	100,00	0,00E+00	4313	23	NA	Japonica	
augustus_masked-8-processed-gene-235.25-mRNA-1	8	OsR498G0816228700.01.T06	99,71	0,00E+00	1229	Os08g0453700	100,00	0,00E+00	1597	368	Japonica	Japonica	
augustus_masked-8-processed-gene-235.31-mRNA-1	8	OsR498G0816230500.01.T02	98,37	2,17E-118	425	Os08g0454400	100,00	1,05E-129	461	36	NA	Japonica	
augustus_masked-8-processed-gene-236.19-mRNA-1	8	OsR498G0816233900.01.T01	99,68	0,00E+00	3398	Os08g0455700	100,00	0,00E+00	3425	27	NA	Japonica	
augustus_masked-8-processed-gene-237.14-mRNA-1	8	OsR498G0816237900.01.T01	99,33	0,00E+00	799	Os08g0457100	78,52	5,22E-86	316	-483	Indica	Japonica	
augustus_masked-8-processed-gene-240.26-mRNA-1	8	OsR498G0816258900.01.T01	99,89	0,00E+00	1625	Os08g0463500	100,00	0,00E+00	1629	4	NA	Japonica	
augustus_masked-8-processed-gene-241.10-mRNA-1	8	OsR498G0816261100.01.T01	99,89	0,00E+00	1684	Os08g0464400	100,00	0,00E+00	1148	-536	Indica	Japonica	
augustus_masked-8-processed-gene-241.21-mRNA-1	8	OsR498G0816264700.01.T02	99,93	0,00E+00	2707	Os08g0465700	100,00	0,00E+00	2711	4	NA	Japonica	
augustus_masked-8-processed-gene-242.18-mRNA-1	8	OsR498G0816265900.01.T01	99,67	0,00E+00	3745	Os08g0466200	100,00	0,00E+00	3777	32	NA	Japonica	
augustus_masked-8-processed-gene-242.37-mRNA-1	8	OsR498G0816271500.01.T01	99,92	0,00E+00	2113	Os08g0467300	100,00	0,00E+00	2149	36	NA	Japonica	
augustus_masked-8-processed-gene-243.13-mRNA-1	8	OsR498G0816272300.01.T01	99,67	0,00E+00	1621	Os08g0467600	100,00	0,00E+00	2462	841	Japonica	Japonica	
augustus_masked-8-processed-gene-243.22-mRNA-1	8	OsR498G0816277700.01.T01	99,81	0,00E+00	1918	Os08g0469000	100,00	0,00E+00	2430	512	Japonica	Japonica	
augustus_masked-8-processed-gene-245.8-mRNA-1	8	OsR498G0816288900.01.T01	98,64	0,00E+00	1663	Os08g0472000	100,00	0,00E+00	1597	-66	NA	Japonica	
augustus_masked-8-processed-gene-246.32-mRNA-1	8	OsR498G0816296700.01.T01	99,76	0,00E+00	1523	Os08g0474000	100,00	0,00E+00	899	-624	Indica	Japonica	
augustus_masked-8-processed-gene-246.35-mRNA-1	8	OsR498G0816363600.01.T01	78,26	0,00E+00	940	Os08g0474400	100,00	0,00E+00	2949	2009	Japonica	Japonica	
augustus_masked-8-processed-gene-247.16-mRNA-1	8	OsR498G0611850100.01.T01	70,35	1,01E-72	276	Os08g0475300	100,00	0,00E+00	1737	1461	Japonica	Japonica	
augustus_masked-8-processed-gene-247.23-mRNA-1	8	OsR498G0816303700.01.T01	99,87	0,00E+00	1376	Os08g0476600	100,00	0,00E+00	1375	-1	NA	Japonica	
augustus_masked-8-processed-gene-248.19-mRNA-1	8	OsR498G0816307300.01.T01	95,99	0,00E+00	1967	Os08g0477700	99,75	0,00E+00	2139	172	NA	Japonica	
augustus_masked-8-processed-gene-25.15-mRNA-1	8	OsR498G0815150600.01.T01	99,47	0,00E+00	1010	Os08g0142500	100,00	0,00E+00	1061	51	NA	Japonica	
augustus_masked-8-processed-gene-25.22-mRNA-1	8	OsR498G0815152700.01.T01	99,83	0,00E+00	2161	Os08g0143600	100,00	0,00E+00	2608	447	Japonica	Japonica	
augustus_masked-8-processed-gene-250.9-mRNA-1	8	OsR498G0816322700.01.T01	100,00	0,00E+00	2307	Os08g0481100	100,00	0,00E+00	2307	0	NA	NA	
augustus_masked-8-processed-gene-251.25-mRNA-1	8	OsR498G0816325700.01.T05	100,00	1,56E-167	589	Os08g0481500	100,00	1,90E-110	398	-191	Indica	NA	
augustus_masked-8-processed-gene-251.42-mRNA-1	8	OsR498G0816327700.01.T01	100,00	0,00E+00	796	Os08g0482300	100,00	0,00E+00	796	0	NA	NA	
augustus_masked-8-processed-gene-252.40-mRNA-1	8	OsR498G0816335900.01.T01	99,92	0,00E+00	2236	Os08g0484200	99,92	0,00E+00	2236	0	NA	NA	
augustus_masked-8-processed-gene-252.44-mRNA-1	8	OsR498G0816337700.01.T01	100,00	0,00E+00	1710	Os08g0484700	100,00	0,00E+00	1710	0	NA	NA	
augustus_masked-8-processed-gene-253.55-mRNA-1	8	OsR498G0816347100.01.T02	100,00	0,00E+00	893	Os08g0486700	100,00	0,00E+00	893	0	NA	NA	
augustus_masked-8-processed-gene-255.13-mRNA-1	8	OsR498G0816360300.01.T01	100,00	0,00E+00	1499	Os08g0490100	100,00	0,00E+00	1499	0	NA	NA	
augustus_masked-8-processed-gene-255.16-mRNA-1	8	OsR498G0816362100.01.T02	99,92	0,00E+00	2274	Os08g0490700	99,92	0,00E+00	2274	0	NA	NA	
augustus_masked-8-processed-gene-256.1-mRNA-1	8	OsR498G0816363900.01.T01	100,00	0,00E+00	2365	Os08g0491400	100,00	0,00E+00	2365	0	NA	NA	
augustus_masked-8-processed-gene-257.1-mRNA-1	8	OsR498G0816372100.01.T01	100,00	0,00E+00	5979	Os08g0493800	100,00	0,00E+00	5104	-875	Indica	NA	Kinase-LRR-TM
augustus_masked-8-processed-gene-257.19-mRNA-1	8	OsR498G0816378100.01.T02	100,00	0,00E+00	1007	Os08g0495200	100,00	0,00E+00	1207	200	Japonica	NA	
augustus_masked-8-processed-gene-258.24-mRNA-1	8	OsR498G0816380100.01.T01	99,47	0,00E+00	4032	Os08g0495800	99,43	0,00E+00	3731	-301	NA	NA	
augustus_masked-8-processed-gene-258.25-mRNA-1	8	OsR498G0816380700.01.T02	100,00	0,00E+00	2614	Os08g0496000	100,00	0,00E+00	2614	0	NA	NA	
augustus_masked-8-processed-gene-258.27-mRNA-1	8	OsR498G0816383100.01.T01	100,00	0,00E+00	1543	Os09g0480400	79,65	0,00E+00	758	-785	Indica	NA	
augustus_masked-8-processed-gene-258.36-mRNA-1	8	OsR498G0816384200.01.T01	85,37	0,00E+00	948	Os08g0496400	99,60	0,00E+00	1342	394	Japonica	NA	
augustus_masked-8-processed-gene-259.16-mRNA-1	8	OsR498G0816396900.01.T01	100,00	0,00E+00	2094	Os08g0499400	100,00	0,00E+00	2094	0	NA	NA	
augustus_masked-8-processed-gene-260.36-mRNA-1	8	OsR498G0816397900.01.T02	100,00	0,00E+00	2403	Os08g0499800	100,00	0,00E+00	2403	0	NA	Indica	
augustus_masked-8-processed-gene-260.48-mRNA-1	8	OsR498G0816401500.01.T01	100,00	0,00E+00	3025	Os08g0500500	100,00	0,00E+00	3025	0	NA	Indica	
augustus_masked-8-processed-gene-260.51-mRNA-1	8	OsR498G0816402300.01.T01	100,00	0,00E+00	2666	Os08g0500800	100,00	0,00E+00	2666	0	NA	Japonica	
augustus_masked-8-processed-gene-261.38-mRNA-1	8	OsR498G0816413100.01.T01	100,00	0,00E+00	832	Os08g0503000	100,00	4,75E-150	529	-303	Indica	NA	
augustus_masked-8-processed-gene-262.23-mRNA-1	8	OsR498G0816418300.01.T02	99,31	0,00E+00	1286	Os08g0504500	100,00	0,00E+00	1321	35	NA	Japonica	
augustus_masked-8-processed-gene-262.27-mRNA-1	8	OsR498G0917718500.01.T01	73,72	0,00E+00	833	Os08g0504800	100,00	0,00E+00	2071	1238	Japonica	Japonica	Kinase-TM
augustus_masked-8-processed-gene-262.29-mRNA-1	8	OsR498G0816421500.01.T02	100,00	7,71E-170	596	Os08g0505001	100,00	1,59E-141	500	-96	Indica	Japonica	
augustus_masked-8-processed-gene-263.16-mRNA-1	8	OsR498G0816425900.01.T01	99,20	0,00E+00	3533	Os08g0506100	100,00	0,00E+00	4255	722	Japonica	Japonica	LRR-TM
augustus_masked-8-processed-gene-264.15-mRNA-1	8	OsR498G0816432100.01.T01	96,55	0,00E+00	2325	Os08g0507400	100,00	0,00E+00	2511	186	NA	Japonica	
augustus_masked-8-processed-gene-264.16-mRNA-1	8	OsR498G0816434700.01.T01	91,50	0,00E+00	1966	Os08g0508000	100,00	0,00E+00	2744	778	Japonica	Japonica	
augustus_masked-8-processed-gene-265.12-mRNA-1	8	OsR498G0816439300.01.T02	99,84	0,00E+00	3342	Os08g0508700	99,95	0,00E+00	3351	9	NA	Japonica	
augustus_masked-8-processed-gene-266.11-mRNA-1	8	OsR498G0816446900.01.T01	99,87	0,00E+00	1390	Os08g0511000	99,94	0,00E+00	2772	1382	Japonica	Japonica	
augustus_masked-8-processed-gene-266.12-mRNA-1	8	OsR498G0816449300.01.T03	100,00	0,00E+00	672	Os08g0511500	83,87	3,27E-119	427	-245	Indica	Japonica	
augustus_masked-8-processed-gene-269.21-mRNA-1	8	OsR498G0816465900.01.T01	98,68	4,87E-147	520	Os08g0517100	100,00	1,69E-147	520	0	NA	Japonica	
augustus_masked-8-processed-gene-270.47-mRNA-1	8	OsR498G0816472500.01.T01	99,90	0,00E+00	1706	Os08g0518900	99,90	0,00E+00	1706	0	NA	Japonica	
augustus_masked-8-processed-gene-271.11-mRNA-1	8	OsR498G0816477900.01.T01	100,00	0,00E+00	4340	Os08g0519800	99,50	0,00E+00	5390	1050	Japonica	Japonica	
augustus_masked-8-processed-gene-274.24-mRNA-1	8	OsR498G0816491700.01.T01	99,88	0,00E+00	1486	Os08g0524400	100,00	0,00E+00	1429	-57	NA	Japonica	
augustus_masked-8-processed-gene-277.11-mRNA-1	8	OsR498G0816515500.01.T01	99,84	0,00E+00	3271	Os08g0530300	99,78	0,00E+00	3267	-4	NA	Indica	
augustus_masked-8-processed-gene-282.14-mRNA-1	8	OsR498G0816537900.01.T03	72,73	2,86E-45	183	Os08g0537001	75,67	7,16E-54	211	28	Japonica	Japonica	
augustus_masked-8-processed-gene-283.51-mRNA-1	8	OsR498G0816542100.01.T01	99,49	0,00E+00	2443	Os08g0538100	100,00	0,00E+00	1447	-996	Indica	Japonica	
augustus_masked-8-processed-gene-285.26-mRNA-1	8	OsR498G0816556100.01.T02	99,89	0,00E+00	1661	Os08g0542600	100,00	0,00E+00	1665	4	NA	Japonica	
augustus_masked-8-processed-gene-286.27-mRNA-1	8	OsR498G0816559700.01.T02	100,00	0,00E+00	682	Os08g0543200	100,00	0,00E+00	1115	433	Japonica	Japonica	
augustus_masked-8-processed-gene-289.12-mRNA-1	8	OsR498G0816579100.01.T01	99,90	0,00E+00	3492	Os08g0548500	100,00	0,00E+00	3501	9	NA	Japonica	
augustus_masked-8-processed-gene-289.16-mRNA-1	8	OsR498G0816580100.01.T01	100,00	0,00E+00	1353	Os08g0548900	100,00	0,00E+00	1353	0	NA	Japonica	
augustus_masked-8-processed-gene-290.32-mRNA-1	8	OsR498G0816583500.01.T01	100,00	0,00E+00	652	Os08g0549900	100,00	0,00E+00	693	41	NA	Japonica	
augustus_masked-8-processed-gene-290.51-mRNA-1	8	OsR498G0816589700.01.T01	73,64	5,31E-81	302	Os08g0551300	100,00	0,00E+00	1278	976	Japonica	Japonica	
augustus_masked-8-processed-gene-290.53-mRNA-1	8	OsR498G0816590300.01.T01	73,57	4,33E-120	432	Os08g0551500	100,00	0,00E+00	1272	840	Japonica	Japonica	
augustus_masked-8-processed-gene-290.57-mRNA-1	8	OsR498G0816589300.01.T01	89,97	0,00E+00	957	Os08g0552300	100,00	0,00E+00	1278	321	Japonica	Japonica	
augustus_masked-8-processed-gene-291.32-mRNA-1	8	OsR498G0816589300.01.T01	89,41	0,00E+00	939	Os08g0553000	90,13	0,00E+00	921	-18	NA	Japonica	
augustus_masked-8-processed-gene-291.33-mRNA-1	8	OsR498G0816589300.01.T01	90,54	0,00E+00	974	Os08g0553000	90,12	0,00E+00	920	-54	NA	Japonica	
augustus_masked-8-processed-gene-291.36-mRNA-1	8	OsR498G0816588900.01.T01	99,86	0,00E+00	1309	Os08g0553050	100,00	0,00E+00	2533	1224	Japonica	Japonica	
augustus_masked-8-processed-gene-291.37-mRNA-1	8	OsR498G0816589300.01.T01	100,00	0,00E+00	1278	Os08g0553200	95,20	0,00E+00	1124	-154	Indica	Japonica	
augustus_masked-8-processed-gene-291.39-mRNA-1	8	OsR498G0816590300.01.T01	80,09	0,00E+00	640	Os08g0553400	100,00	0,00E+00	1272	632	Japonica	Japonica	
augustus_masked-8-processed-gene-291.55-mRNA-1	8	OsR498G0816589700.01.T01	88,84	0,00E+00	965	Os08g0553600	100,00	0,00E+00	1315	350	Japonica	Japonica	
augustus_masked-8-processed-gene-291.57-mRNA-1	8	OsR498G0816590300.01.T01	99,86	0,00E+00	1273	Os08g0552300	87,01	0,00E+00	863	-410	Indica	Japonica	
augustus_masked-8-processed-gene-291.62-mRNA-1	8	OsR498G0816591500.01.T01	99,59	0,00E+00	2589	Os08g0554300	99,95	0,00E+00	3280	691	Japonica	Japonica	
augustus_masked-8-processed-gene-292.19-mRNA-1	8	OsR498G0816598100.01.T01	99,93	0,00E+00	2519	Os08g0556400	100,00	0,00E+00	2524	5	NA	Japonica	
augustus_masked-8-processed-gene-293.69-mRNA-1	8	OsR498G0816603700.01.T01	99,14	0,00E+00	1022	Os08g0558300	100,00	0,00E+00	1045	23	NA	Japonica	
augustus_masked-8-processed-gene-295.10-mRNA-1	8	OsR498G0816616100.01.T01	99,67	0,00E+00	1095	Os08g0561700	100,00	0,00E+00	1104	9	NA	Japonica	
augustus_masked-8-processed-gene-295.11-mRNA-1	8	OsR498G0408675500.01.T01	89,55	5,82E-167	587	Os08g0561800	100,00	9,21E-172	601	14	NA	Japonica	
augustus_masked-8-processed-gene-296.10-mRNA-1	8	OsR498G0816621300.01.T01	99,51	0,00E+00	733	Os05g0437133	74,67	1,36E-29	130	-603	Indica	Japonica	
augustus_masked-8-processed-gene-296.23-mRNA-1	8	OsR498G0816622500.01.T01	99,81	0,00E+00	2882	Os08g0564000	100,00	0,00E+00	2895	13	NA	Japonica	
augustus_masked-8-processed-gene-296.25-mRNA-1	8	OsR498G0816623900.01.T03	99,70	0,00E+00	4205	Os08g0564300	99,93	0,00E+00	2525	-1680	Indica	Japonica	
augustus_masked-8-processed-gene-296.28-mRNA-1	8	OsR498G0816627100.01.T01	96,45	1,67E-172	606	Os08g0564750	100,00	1,46E-142	504	-102	Indica	Japonica	
augustus_masked-8-processed-gene-31.12-mRNA-1	8	OsR498G0815197900.01.T01	100,00	0,00E+00	2138	Os08g0150800	100,00	0,00E+00	2138	0	NA	Indica	
augustus_masked-8-processed-gene-36.9-mRNA-1	8	OsR498G0815236700.01.T01	100,00	0,00E+00	1764	Os08g0159000	99,59	0,00E+00	1746	-18	NA	Indica	
augustus_masked-8-processed-gene-39.34-mRNA-1	8	OsR498G0815251700.01.T02	99,63	0,00E+00	2874	Os08g0162200	99,88	0,00E+00	2892	18	NA	Japonica	
augustus_masked-8-processed-gene-39.39-mRNA-1	8	OsR498G0815253700.01.T01	96,95	0,00E+00	997	Os08g0162700	96,95	0,00E+00	997	0	NA	Japonica	
augustus_masked-8-processed-gene-4.18-mRNA-1	8	OsR498G0815041300.01.T01	98,63	0,00E+00	3559	Os08g0107000	100,00	0,00E+00	5086	1527	Japonica	Japonica	CC-NBS-TM
augustus_masked-8-processed-gene-40.0-mRNA-1	8	OsR498G0815258700.01.T01	89,82	0,00E+00	1130	Os08g0164300	89,82	0,00E+00	1130	0	NA	Japonica	
augustus_masked-8-processed-gene-40.19-mRNA-1	8	OsR498G0815259700.01.T01	90,09	0,00E+00	877	Os08g0165200	100,00	0,00E+00	1898	1021	Japonica	Japonica	
augustus_masked-8-processed-gene-42.1-mRNA-1	8	OsR498G0815272900.01.T01	99,81	0,00E+00	938	Os08g0168600	99,62	0,00E+00	944	6	NA	Indica	
augustus_masked-8-processed-gene-44.35-mRNA-1	8	OsR498G0815295300.01.T01	98,91	0,00E+00	962	Os08g0173300	100,00	0,00E+00	991	29	NA	Japonica	
augustus_masked-8-processed-gene-47.11-mRNA-1	8	OsR498G0815308500.01.T02	96,45	0,00E+00	2823	Os08g0178300	100,00	0,00E+00	2960	137	NA	Japonica	
augustus_masked-8-processed-gene-47.12-mRNA-1	8	OsR498G0815309100.01.T01	100,00	0,00E+00	931	Os08g0178500	100,00	3,79E-139	493	-438	Indica	Japonica	
augustus_masked-8-processed-gene-5.49-mRNA-1	8	OsR498G0815051500.01.T01	99,37	0,00E+00	4477	Os08g0109800	100,00	0,00E+00	4529	52	NA	Japonica	Kinase-TM
augustus_masked-8-processed-gene-53.1-mRNA-1	8	OsR498G0815338100.01.T01	99,70	0,00E+00	1209	Os08g0189300	99,85	0,00E+00	1214	5	NA	NA	
augustus_masked-8-processed-gene-53.22-mRNA-1	8	OsR498G0815344900.01.T01	97,88	0,00E+00	2337	Os08g0191000	97,95	0,00E+00	2342	5	NA	Japonica	
augustus_masked-8-processed-gene-54.50-mRNA-1	8	OsR498G0815347700.01.T01	99,51	0,00E+00	3259	Os08g0191900	99,29	0,00E+00	3244	-15	NA	NA	
augustus_masked-8-processed-gene-56.11-mRNA-1	8	OsR498G0815358900.01.T01	99,86	0,00E+00	3823	Os11g0638801	70,35	2,74E-167	590	-3233	Indica	Japonica	
augustus_masked-8-processed-gene-57.11-mRNA-1	8	OsR498G0815366500.01.T01	99,93	0,00E+00	2418	Os08g0197300	99,86	0,00E+00	1266	-1152	Indica	NA	
augustus_masked-8-processed-gene-62.5-mRNA-1	8	OsR498G0815388100.01.T01	99,43	0,00E+00	2500	Os08g0203800	99,29	0,00E+00	2491	-9	NA	NA	
augustus_masked-8-processed-gene-63.34-mRNA-1	8	OsR498G0815393500.01.T02	97,04	0,00E+00	1975	Os08g0205300	100,00	0,00E+00	1346	-629	Indica	Japonica	
augustus_masked-8-processed-gene-73.23-mRNA-1	8	OsR498G0815510700.01.T03	93,99	1,01E-96	352	Os08g0239300	100,00	1,50E-114	410	58	Japonica	NA	
augustus_masked-8-processed-gene-75.7-mRNA-1	8	OsR498G0815443700.01.T01	99,04	1,40E-155	550	Os08g0221000	96,41	0,00E+00	742	192	Japonica	Japonica	
augustus_masked-8-processed-gene-76.10-mRNA-1	8	OsR498G0815449300.01.T01	99,39	5,20E-166	583	Os08g0219100	100,00	0,00E+00	634	51	NA	Japonica	
augustus_masked-8-processed-gene-77.2-mRNA-1	8	OsR498G0815450300.01.T01	99,85	0,00E+00	1214	Os08g0218700	100,00	0,00E+00	1218	4	NA	Japonica	
augustus_masked-8-processed-gene-8.43-mRNA-1	8	OsR498G0204365600.01.T01	97,00	0,00E+00	659	Os08g0114000	100,00	0,00E+00	778	119	Japonica	NA	
augustus_masked-8-processed-gene-81.26-mRNA-1	8	OsR498G0815478400.01.T01	99,68	0,00E+00	1117	Os08g0227400	100,00	0,00E+00	1575	458	Japonica	Japonica	
augustus_masked-8-processed-gene-83.0-mRNA-1	8	OsR498G0815484900.01.T02	100,00	0,00E+00	2614	Os08g0230200	99,93	0,00E+00	2609	-5	NA	Japonica	
augustus_masked-8-processed-gene-84.13-mRNA-1	8	OsR498G0815493900.01.T01	99,94	0,00E+00	2847	Os08g0232700	99,94	0,00E+00	2847	0	NA	NA	
augustus_masked-8-processed-gene-87.33-mRNA-1	8	OsR498G1119809300.01.T02	98,22	0,00E+00	2609	Os08g0238100	100,00	0,00E+00	3232	623	Japonica	Japonica	
augustus_masked-8-processed-gene-88.1-mRNA-1	8	OsR498G0815510700.01.T03	93,99	1,01E-96	352	Os08g0239300	100,00	1,50E-114	410	58	Japonica	Japonica	
augustus_masked-8-processed-gene-88.13-mRNA-1	8	OsR498G0815512300.01.T01	99,77	0,00E+00	1566	Os08g0240533	74,67	7,22E-139	493	-1073	Indica	Japonica	
augustus_masked-8-processed-gene-93.26-mRNA-1	8	OsR498G0815530100.01.T01	99,63	0,00E+00	3421	Os08g0246300	100,00	0,00E+00	3452	31	NA	Japonica	LRR-NBS-TM
augustus_masked-8-processed-gene-94.1-mRNA-1	8	OsR498G0815537100.01.T01	97,82	0,00E+00	3668	Os10g0495200	83,07	0,00E+00	2399	-1269	Indica	Japonica	
augustus_masked-8-processed-gene-96.14-mRNA-1	8	OsR498G0815553900.01.T01	97,20	1,76E-152	539	Os06g0192100	85,00	9,73E-100	361	-178	Indica	NA	
augustus-8-processed-gene-1.19-mRNA-1	8	OsR498G0815026400.01.T01	99,86	0,00E+00	1289	Os08g0101700	100,00	0,00E+00	1294	5	NA	Japonica	
augustus-8-processed-gene-103.21-mRNA-1	8	OsR498G0815581600.01.T04	99,59	3,96E-121	434	Os08g0265350	99,58	2,06E-119	427	-7	NA	NA	
augustus-8-processed-gene-104.16-mRNA-1	8	OsR498G0815588200.01.T01	99,85	0,00E+00	2423	Os08g0267000	98,94	0,00E+00	2323	-100	NA	Indica	
augustus-8-processed-gene-11.47-mRNA-1	8	OsR498G0815087200.01.T06	99,35	0,00E+00	1095	Os08g0119800	100,00	0,00E+00	1175	80	NA	Japonica	
augustus-8-processed-gene-110.4-mRNA-1	8	OsR498G0815617800.01.T01	99,76	0,00E+00	4453	Os08g0276400	99,72	0,00E+00	4449	-4	NA	NA	Kinase-LRR-TM
augustus-8-processed-gene-111.14-mRNA-1	8	OsR498G0815626400.01.T02	100,00	7,41E-144	509	Os08g0278600	100,00	2,59E-144	509	0	NA	NA	
augustus-8-processed-gene-116.32-mRNA-1	8	OsR498G0815645000.01.T01	99,46	0,00E+00	2296	Os08g0285100	99,60	0,00E+00	2679	383	Japonica	NA	
augustus-8-processed-gene-121.30-mRNA-1	8	OsR498G0815664400.01.T06	99,83	0,00E+00	4338	Os08g0290000	99,55	0,00E+00	4318	-20	NA	NA	
augustus-8-processed-gene-14.24-mRNA-1	8	OsR498G0815098200.01.T02	96,63	0,00E+00	1914	Os08g0124100	100,00	0,00E+00	2630	716	Japonica	Japonica	Kinase-TM
augustus-8-processed-gene-141.27-mRNA-1	8	OsR498G0815757200.01.T04	99,55	0,00E+00	1183	Os08g0318500	99,70	0,00E+00	1187	4	NA	NA	
augustus-8-processed-gene-145.21-mRNA-1	8	OsR498G0815777800.01.T01	99,85	0,00E+00	1176	Os08g0323400	99,85	0,00E+00	1176	0	NA	NA	
augustus-8-processed-gene-151.41-mRNA-1	8	OsR498G0815802000.01.T01	93,69	0,00E+00	663	Os05g0225100	81,02	1,09E-81	303	-360	Indica	NA	
augustus-8-processed-gene-158.35-mRNA-1	8	OsR498G0815837200.01.T01	99,84	0,00E+00	6552	Os08g0342300	99,70	0,00E+00	6528	-24	NA	Japonica	Kinase-LRR-TM
augustus-8-processed-gene-159.31-mRNA-1	8	OsR498G0815843000.01.T01	97,87	0,00E+00	2020	Os08g0343600	97,55	0,00E+00	2713	693	Japonica	NA	
augustus-8-processed-gene-16.47-mRNA-1	8	OsR498G0815103200.01.T01	73,21	0,00E+00	720	Os08g0126800	100,00	0,00E+00	2010	1290	Japonica	Japonica	
augustus-8-processed-gene-160.19-mRNA-1	8	OsR498G0815848400.01.T01	99,86	0,00E+00	5254	Os08g0345500	99,88	0,00E+00	6020	766	Japonica	NA	
augustus-8-processed-gene-162.16-mRNA-1	8	OsR498G0815858000.01.T02	98,81	3,08E-123	442	Os10g0461100	74,60	2,72E-36	150	-292	Indica	Japonica	
augustus-8-processed-gene-17.50-mRNA-1	8	OsR498G0815105800.01.T01	99,87	0,00E+00	1392	Os08g0127800	100,00	0,00E+00	1397	5	NA	Japonica	
augustus-8-processed-gene-170.30-mRNA-1	8	OsR498G0917625500.01.T01	73,18	2,42E-138	494	Os08g0474800	100,00	0,00E+00	2008	1514	Japonica	NA	
augustus-8-processed-gene-174.21-mRNA-1	8	OsR498G0815875600.01.T01	100,00	0,00E+00	2960	Os08g0356700	100,00	0,00E+00	2960	0	NA	Japonica	
augustus-8-processed-gene-192.14-mRNA-1	8	OsR498G0815961000.01.T01	99,72	0,00E+00	1263	Os08g0378800	98,02	0,00E+00	1213	-50	NA	Indica	
augustus-8-processed-gene-192.16-mRNA-1	8	OsR498G0815961400.01.T05	98,59	0,00E+00	3093	Os08g0379000	97,37	0,00E+00	3018	-75	NA	Indica	
augustus-8-processed-gene-197.37-mRNA-1	8	OsR498G0815983300.01.T01	98,67	1,64E-148	526	Os08g0387100	86,89	1,03E-107	389	-137	Indica	Japonica	
augustus-8-processed-gene-199.43-mRNA-1	8	OsR498G0815994400.01.T01	99,83	0,00E+00	2140	Os08g0390200	99,92	0,00E+00	2144	4	NA	NA	
augustus-8-processed-gene-199.46-mRNA-1	8	OsR498G0815995400.01.T01	99,82	0,00E+00	2021	Os08g0392100	92,97	0,00E+00	1663	-358	Indica	NA	
augustus-8-processed-gene-199.51-mRNA-1	8	OsR498G0815999400.01.T01	99,12	0,00E+00	1995	Os08g0392100	99,03	0,00E+00	1991	-4	NA	NA	
augustus-8-processed-gene-207.40-mRNA-1	8	OsR498G0816055400.01.T01	99,32	0,00E+00	2091	Os08g0406500	100,00	0,00E+00	2127	36	NA	Japonica	
augustus-8-processed-gene-208.19-mRNA-1	8	OsR498G0816056200.01.T01	97,67	0,00E+00	2400	Os08g0406600	99,92	0,00E+00	2171	-229	NA	Japonica	
augustus-8-processed-gene-208.39-mRNA-1	8	OsR498G0816061400.01.T01	94,16	0,00E+00	727	Os08g0408300	100,00	0,00E+00	1299	572	Japonica	Japonica	
augustus-8-processed-gene-210.7-mRNA-1	8	OsR498G0816070200.01.T01	99,58	0,00E+00	2128	Os08g0410266	99,90	0,00E+00	3449	1321	Japonica	Japonica	
augustus-8-processed-gene-211.45-mRNA-1	8	OsR498G0816081800.01.T01	99,62	7,27E-131	467	Os08g0414500	100,00	1,01E-123	441	-26	NA	NA	
augustus-8-processed-gene-212.31-mRNA-1	8	OsR498G0816085600.01.T01	98,51	0,00E+00	930	Os08g0416000	99,44	0,00E+00	948	18	NA	Japonica	
augustus-8-processed-gene-213.7-mRNA-1	8	OsR498G0816090200.01.T01	99,89	0,00E+00	1700	Os08g0417100	94,78	0,00E+00	1599	-101	NA	Japonica	
augustus-8-processed-gene-215.20-mRNA-1	8	OsR498G0816100800.01.T01	99,75	0,00E+00	2828	Os08g0421850	98,41	0,00E+00	3050	222	NA	Indica	
augustus-8-processed-gene-223.31-mRNA-1	8	OsR498G0816151600.01.T02	99,83	0,00E+00	2145	Os08g0434300	99,35	0,00E+00	1901	-244	Indica	Indica	
augustus-8-processed-gene-226.16-mRNA-1	8	OsR498G0816166000.01.T06	99,63	0,00E+00	1947	Os08g0438400	98,90	0,00E+00	1910	-37	NA	NA	
augustus-8-processed-gene-228.28-mRNA-1	8	OsR498G0816183200.01.T02	99,63	0,00E+00	966	Os08g0441900	100,00	1,93E-168	590	-376	Indica	Japonica	
augustus-8-processed-gene-229.25-mRNA-1	8	OsR498G0816186400.01.T01	98,10	0,00E+00	1175	Os08g0442900	100,00	0,00E+00	1234	59	NA	Japonica	
augustus-8-processed-gene-230.5-mRNA-1	8	OsR498G0816191600.01.T01	100,00	7,41E-144	509	Os08g0444500	100,00	2,59E-144	509	0	NA	Japonica	
augustus-8-processed-gene-231.5-mRNA-1	8	OsR498G0816198200.01.T01	99,93	0,00E+00	2562	Os08g0446200	99,51	0,00E+00	2535	-27	NA	Indica	Kinase-LRR-TM
augustus-8-processed-gene-233.23-mRNA-1	8	OsR498G0816219600.01.T02	98,65	0,00E+00	1681	Os08g0451700	100,00	0,00E+00	2502	821	Japonica	Japonica	
augustus-8-processed-gene-236.27-mRNA-1	8	OsR498G0816234300.01.T01	100,00	0,00E+00	1056	Os08g0455800	100,00	0,00E+00	1056	0	NA	Japonica	
augustus-8-processed-gene-236.33-mRNA-1	8	OsR498G0816235600.01.T01	99,62	0,00E+00	2792	Os08g0456200	100,00	0,00E+00	2819	27	NA	Japonica	
augustus-8-processed-gene-238.17-mRNA-1	8	OsR498G0816246800.01.T01	99,85	0,00E+00	1224	Os08g0459300	100,00	0,00E+00	1229	5	NA	Japonica	
augustus-8-processed-gene-24.24-mRNA-1	8	OsR498G0815146000.01.T01	99,62	0,00E+00	2809	Os08g0140500	100,00	0,00E+00	2836	27	NA	Japonica	
augustus-8-processed-gene-24.34-mRNA-1	8	OsR498G0815146600.01.T01	78,89	0,00E+00	760	Os08g0141000	99,93	0,00E+00	2436	1676	Japonica	Japonica	
augustus-8-processed-gene-24.40-mRNA-1	8	OsR498G0815146800.01.T01	94,79	0,00E+00	1297	Os08g0140700	98,13	0,00E+00	2507	1210	Japonica	Japonica	
augustus-8-processed-gene-24.44-mRNA-1	8	OsR498G0815149600.01.T01	99,13	0,00E+00	1015	Os08g0141700	100,00	0,00E+00	1040	25	NA	Japonica	
augustus-8-processed-gene-240.18-mRNA-1	8	OsR498G0816257400.01.T01	99,47	0,00E+00	1688	Os08g0462800	100,00	0,00E+00	1710	22	NA	Japonica	
augustus-8-processed-gene-243.45-mRNA-1	8	OsR498G0816276200.01.T01	99,60	0,00E+00	4909	Os08g0468700	99,93	0,00E+00	4949	40	NA	Japonica	
augustus-8-processed-gene-244.22-mRNA-1	8	OsR498G0102262600.01.T01	97,60	0,00E+00	1715	Os08g0471100	99,90	0,00E+00	3499	1784	Japonica	Japonica	
augustus-8-processed-gene-245.21-mRNA-1	8	OsR498G0816290400.01.T01	99,92	0,00E+00	2155	Os08g0472400	100,00	0,00E+00	2122	-33	NA	Japonica	
augustus-8-processed-gene-246.49-mRNA-1	8	OsR498G0816298400.01.T01	97,60	0,00E+00	1198	Os08g0474500	100,00	0,00E+00	1342	144	Japonica	Japonica	
augustus-8-processed-gene-247.10-mRNA-1	8	OsR498G0816299200.01.T01	99,50	0,00E+00	1764	Os08g0475000	100,00	0,00E+00	1786	22	NA	Japonica	
augustus-8-processed-gene-249.26-mRNA-1	8	OsR498G0816316600.01.T02	100,00	0,00E+00	1570	Os08g0479400	99,89	0,00E+00	1565	-5	NA	NA	
augustus-8-processed-gene-249.4-mRNA-1	8	OsR498G0816310200.01.T01	99,39	0,00E+00	1730	Os08g0478466	99,90	0,00E+00	1755	25	NA	Japonica	
augustus-8-processed-gene-25.12-mRNA-1	8	OsR498G0815150800.01.T01	99,57	0,00E+00	836	Os08g0142900	100,00	0,00E+00	1034	198	Japonica	Japonica	
augustus-8-processed-gene-250.16-mRNA-1	8	OsR498G0816319800.01.T04	100,00	0,00E+00	3382	Os08g0480100	100,00	0,00E+00	3382	0	NA	NA	
augustus-8-processed-gene-254.33-mRNA-1	8	OsR498G0816351000.01.T01	99,88	0,00E+00	1468	Os08g0487500	99,88	0,00E+00	1468	0	NA	NA	
augustus-8-processed-gene-254.36-mRNA-1	8	OsR498G0816354000.01.T03	99,74	0,00E+00	1364	Os08g0488800	93,45	0,00E+00	1784	420	Japonica	NA	
augustus-8-processed-gene-255.27-mRNA-1	8	OsR498G0816360800.01.T03	100,00	0,00E+00	3268	Os08g0490300	100,00	0,00E+00	3268	0	NA	NA	
augustus-8-processed-gene-255.9-mRNA-1	8	OsR498G0816358000.01.T01	99,79	0,00E+00	3409	Os08g0489800	99,79	0,00E+00	3409	0	NA	NA	
augustus-8-processed-gene-256.40-mRNA-1	8	OsR498G0816368200.01.T01	100,00	0,00E+00	910	Os08g0492500	100,00	0,00E+00	910	0	NA	NA	
augustus-8-processed-gene-257.7-mRNA-1	8	OsR498G0816373000.01.T01	100,00	0,00E+00	1261	Os08g0494000	99,86	0,00E+00	1257	-4	NA	NA	
augustus-8-processed-gene-258.52-mRNA-1	8	OsR498G0816388500.01.T02	96,32	0,00E+00	3059	Os08g0497300	96,26	0,00E+00	3055	-4	NA	NA	
augustus-8-processed-gene-258.57-mRNA-1	8	OsR498G0816384400.01.T01	100,00	0,00E+00	1023	Os08g0499400	79,44	2,64E-135	480	-543	Indica	NA	
augustus-8-processed-gene-259.30-mRNA-1	8	OsR498G0816393800.01.T02	96,61	0,00E+00	1630	Os08g0499000	100,00	0,00E+00	1754	124	NA	NA	
augustus-8-processed-gene-261.46-mRNA-1	8	OsR498G0816408800.01.T01	100,00	0,00E+00	1489	Os08g0502400	100,00	0,00E+00	1489	0	NA	NA	
augustus-8-processed-gene-263.22-mRNA-1	8	OsR498G0816425400.01.T01	99,30	0,00E+00	2024	Os08g0505900	100,00	0,00E+00	2062	38	NA	Japonica	LRR-TM
augustus-8-processed-gene-264.24-mRNA-1	8	OsR498G0816430400.01.T01	99,41	0,00E+00	2701	Os08g0507100	100,00	0,00E+00	2738	37	NA	Japonica	
augustus-8-processed-gene-266.22-mRNA-1	8	OsR498G0816446400.01.T01	99,64	0,00E+00	1972	Os08g0510800	100,00	0,00E+00	2051	79	NA	Japonica	

augustus-8-processed-gene-269.28-mRNA-1	8	OsR498G0816464400.01.T01	100,00	0,00E+00	688	Os08g0516600	100,00	0,00E+00	688	0	NA	Japonica	
augustus-8-processed-gene-270.36-mRNA-1	8	OsR498G0816467200.01.T04	100,00	0,00E+00	812	Os08g0517600	91,58	0,00E+00	883	71	NA	Japonica	
augustus-8-processed-gene-270.56-mRNA-1	8	OsR498G0816469400.01.T01	99,67	0,00E+00	2186	Os08g0518200	100,00	0,00E+00	3447	1261	Japonica	Japonica	
augustus-8-processed-gene-270.61-mRNA-1	8	OsR498G0816473200.01.T01	99,94	0,00E+00	2905	Os08g0519100	99,85	0,00E+00	2399	-506	Indica	Japonica	
augustus-8-processed-gene-271.3-mRNA-1	8	OsR498G1120763500.01.T01	81,43	0,00E+00	870	Os08g0519300	100,00	0,00E+00	1537	667	Japonica	NA	
augustus-8-processed-gene-272.41-mRNA-1	8	OsR498G0816481000.01.T01	98,59	0,00E+00	1231	Os08g0520600	100,00	0,00E+00	1278	47	NA	Japonica	
augustus-8-processed-gene-274.53-mRNA-1	8	OsR498G0917763200.01.T01	86,67	0,00E+00	1673	Os08g0526100	99,86	0,00E+00	2583	910	Japonica	Japonica	
augustus-8-processed-gene-276.57-mRNA-1	8	OsR498G0816511900.01.T01	99,91	0,00E+00	2009	Os08g0529300	99,73	0,00E+00	2000	-9	NA	NA	
augustus-8-processed-gene-287.15-mRNA-1	8	OsR498G0816564400.01.T02	99,91	0,00E+00	2020	Os08g0544800	100,00	0,00E+00	2024	4	NA	Japonica	
augustus-8-processed-gene-287.17-mRNA-1	8	OsR498G0816566200.01.T01	99,16	0,00E+00	1256	Os08g0545400	100,00	0,00E+00	1283	27	NA	Japonica	
augustus-8-processed-gene-287.18-mRNA-1	8	OsR498G0917817800.01.T01	72,07	1,63E-81	305	Os08g0545500	100,00	0,00E+00	1364	1059	Japonica	Japonica	
augustus-8-processed-gene-287.21-mRNA-1	8	OsR498G0816568200.01.T01	99,63	0,00E+00	2879	Os08g0545900	100,00	0,00E+00	2625	-254	NA	Japonica	
augustus-8-processed-gene-288.48-mRNA-1	8	OsR498G0816572100.01.T01	99,92	0,00E+00	2297	Os08g0547200	100,00	0,00E+00	2302	5	NA	Japonica	
augustus-8-processed-gene-289.1-mRNA-1	8	OsR498G0816576400.01.T01	99,61	0,00E+00	2776	Os08g0547900	100,00	0,00E+00	2722	-54	NA	Japonica	
augustus-8-processed-gene-290.76-mRNA-1	8	OsR498G0816586000.01.T02	99,54	0,00E+00	771	Os08g0550700	100,00	0,00E+00	780	9	NA	Japonica	
augustus-8-processed-gene-293.57-mRNA-1	8	OsR498G0816600400.01.T01	99,80	0,00E+00	1813	Os08g0557200	100,00	0,00E+00	1840	27	NA	Japonica	
augustus-8-processed-gene-295.24-mRNA-1	8	OsR498G0816619400.01.T06	98,63	0,00E+00	1526	Os08g0562600	100,00	0,00E+00	1581	55	NA	Japonica	
augustus-8-processed-gene-295.4-mRNA-1	8	OsR498G0816613600.01.T02	100,00	0,00E+00	1104	Os08g0560900	100,00	0,00E+00	1104	0	NA	NA	
augustus-8-processed-gene-296.13-mRNA-1	8	OsR498G0816620000.01.T01	96,72	0,00E+00	1006	Os08g0563200	100,00	0,00E+00	1278	272	Japonica	Japonica	
augustus-8-processed-gene-296.37-mRNA-1	8	OsR498G0816624200.01.T01	100,00	0,00E+00	677	Os08g0564500	99,73	0,00E+00	653	-24	NA	Japonica	
augustus-8-processed-gene-296.38-mRNA-1	8	OsR498G0816627000.01.T01	99,95	0,00E+00	3583	Os08g0564700	100,00	0,00E+00	3588	5	NA	Japonica	Kinase-LRR-TM
augustus-8-processed-gene-297.53-mRNA-1	8	OsR498G0816629600.01.T01	99,31	0,00E+00	1291	Os08g0565200	100,00	0,00E+00	1251	-40	NA	Japonica	
augustus-8-processed-gene-297.80-mRNA-1	8	OsR498G0816630800.01.T05	99,87	0,00E+00	2767	Os08g0565700	100,00	0,00E+00	3220	453	Japonica	Japonica	
augustus-8-processed-gene-3.20-mRNA-1	8	OsR498G0815038200.01.T01	99,48	0,00E+00	2729	Os08g0105400	100,00	0,00E+00	2895	166	NA	Japonica	
augustus-8-processed-gene-3.21-mRNA-1	8	OsR498G0815038400.01.T01	98,71	0,00E+00	945	Os08g0105600	100,00	0,00E+00	1846	901	Japonica	Japonica	
augustus-8-processed-gene-3.23-mRNA-1	8	OsR498G0815038600.01.T01	99,32	0,00E+00	2874	Os08g0105400	76,82	0,00E+00	1129	-1745	Indica	Japonica	
augustus-8-processed-gene-3.3-mRNA-1	8	OsR498G0815034200.01.T01	92,38	4,53E-134	478	Os08g0104400	100,00	3,47E-149	526	48	NA	Japonica	
augustus-8-processed-gene-32.23-mRNA-1	8	OsR498G0815203800.01.T01	100,00	0,00E+00	1965	Os08g0152600	99,91	0,00E+00	1953	-12	NA	Indica	
augustus-8-processed-gene-33.7-mRNA-1	8	OsR498G0815207400.01.T04	100,00	0,00E+00	3323	Os08g0153600	99,63	0,00E+00	2926	-397	Indica	Indica	
augustus-8-processed-gene-33.8-mRNA-1	8	OsR498G0815207800.01.T01	100,00	0,00E+00	1342	Os08g0153800	100,00	0,00E+00	1278	-64	NA	Indica	
augustus-8-processed-gene-36.22-mRNA-1	8	OsR498G0815235000.01.T01	99,94	0,00E+00	2864	Os08g0158200	99,94	0,00E+00	2864	0	NA	Indica	
augustus-8-processed-gene-37.1-mRNA-1	8	OsR498G0815240000.01.T01	99,77	0,00E+00	1561	Os08g0159700	100,00	0,00E+00	1570	9	NA	NA	
augustus-8-processed-gene-37.9-mRNA-1	8	OsR498G0815240800.01.T04	99,81	0,00E+00	5654	Os08g0159900	100,00	0,00E+00	5681	27	NA	Japonica	
augustus-8-processed-gene-39.47-mRNA-1	8	OsR498G0815252800.01.T01	99,78	0,00E+00	2454	Os08g0162400	99,85	0,00E+00	2459	5	NA	Japonica	
augustus-8-processed-gene-39.73-mRNA-1	8	OsR498G0815255400.01.T02	99,12	0,00E+00	805	Os08g0163300	99,16	6,03E-117	419	-386	Indica	NA	
augustus-8-processed-gene-4.32-mRNA-1	8	OsR498G0815041800.01.T04	99,80	0,00E+00	1820	Os08g0107100	100,00	0,00E+00	1829	9	NA	Japonica	
augustus-8-processed-gene-4.36-mRNA-1	8	OsR498G0815044600.01.T01	97,08	0,00E+00	1141	Os08g0108100	100,00	0,00E+00	1234	93	NA	Japonica	
augustus-8-processed-gene-4.37-mRNA-1	8	OsR498G0815045600.01.T01	97,77	0,00E+00	1763	Os08g0108300	100,00	0,00E+00	1856	93	NA	Japonica	
augustus-8-processed-gene-43.37-mRNA-1	8	OsR498G0815282400.01.T01	100,00	9,98E-149	526	Os08g0170100	80,29	3,27E-48	191	-335	Indica	NA	
augustus-8-processed-gene-45.24-mRNA-1	8	OsR498G0815298200.01.T02	96,74	0,00E+00	867	Os08g0174500	99,89	0,00E+00	1608	741	Japonica	Japonica	
augustus-8-processed-gene-45.6-mRNA-1	8	OsR498G0815297000.01.T01	99,62	0,00E+00	944	Os08g0173700	100,00	0,00E+00	953	9	NA	Japonica	
augustus-8-processed-gene-47.15-mRNA-1	8	OsR498G0815307700.01.T01	100,00	0,00E+00	2679	Os08g0178100	100,00	0,00E+00	2679	0	NA	Japonica	
augustus-8-processed-gene-5.39-mRNA-1	8	OsR498G0815046800.01.T01	99,57	0,00E+00	1644	Os08g0108500	100,00	0,00E+00	1662	18	NA	Japonica	
augustus-8-processed-gene-59.34-mRNA-1	8	OsR498G0815382600.01.T01	100,00	0,00E+00	996	Os08g0201100	100,00	0,00E+00	996	0	NA	Japonica	
augustus-8-processed-gene-6.21-mRNA-1	8	OsR498G0815058200.01.T01	99,17	0,00E+00	2544	Os08g0111300	99,93	0,00E+00	2593	49	NA	Japonica	
augustus-8-processed-gene-6.6-mRNA-1	8	OsR498G0815055200.01.T01	99,88	0,00E+00	1455	Os08g0110300	100,00	0,00E+00	1460	5	NA	Japonica	
augustus-8-processed-gene-62.12-mRNA-1	8	OsR498G0815388600.01.T01	99,81	0,00E+00	1847	Os08g0203900	99,81	0,00E+00	1847	0	NA	Indica	
augustus-8-processed-gene-64.34-mRNA-1	8	OsR498G1018122200.01.T01	94,84	1,42E-154	546	Os08g0208300	100,00	0,00E+00	682	136	Japonica	Japonica	
augustus-8-processed-gene-69.15-mRNA-1	8	OsR498G0815441600.01.T01	93,86	0,00E+00	820	Os08g0214900	100,00	0,00E+00	1007	187	Japonica	NA	
augustus-8-processed-gene-7.22-mRNA-1	8	OsR498G0815060200.01.T01	97,99	0,00E+00	2399	Os08g0112200	100,00	0,00E+00	2542	143	NA	Japonica	
augustus-8-processed-gene-7.23-mRNA-1	8	OsR498G0815060400.01.T01	99,72	0,00E+00	2520	Os08g0112300	100,00	0,00E+00	2538	18	NA	Japonica	
augustus-8-processed-gene-7.24-mRNA-1	8	OsR498G0815060600.01.T01	99,45	0,00E+00	3565	Os08g0112400	100,00	0,00E+00	3615	50	NA	Japonica	
augustus-8-processed-gene-7.5-mRNA-1	8	OsR498G0815058600.01.T01	99,71	0,00E+00	2498	Os08g0111800	99,93	0,00E+00	2512	14	NA	Japonica	
augustus-8-processed-gene-75.19-mRNA-1	8	OsR498G0815445900.01.T01	98,83	0,00E+00	2094	Os08g0220400	100,00	0,00E+00	2138	44	NA	Japonica	
augustus-8-processed-gene-79.25-mRNA-1	8	OsR498G0815469000.01.T03	100,00	3,51E-111	401	Os08g0225100	100,00	0,00E+00	634	233	Japonica	Japonica	
augustus-8-processed-gene-79.5-mRNA-1	8	OsR498G0815462000.01.T01	99,92	0,00E+00	4506	Os08g0224000	100,00	0,00E+00	4515	9	NA	NA	CC-LRR-NBS-TM
augustus-8-processed-gene-8.33-mRNA-1	8	OsR498G0815064300.01.T01	98,88	0,00E+00	1254	Os08g0113500	99,88	0,00E+00	1477	223	Japonica	Japonica	
augustus-8-processed-gene-80.22-mRNA-1	8	OsR498G0815473500.01.T01	77,53	7,85E-55	214	Os08g0226800	94,43	7,34E-126	449	235	Japonica	Japonica	
augustus-8-processed-gene-80.23-mRNA-1	8	OsR498G0815471800.01.T01	99,30	0,00E+00	1254	Os08g0226800	94,52	0,00E+00	1105	-149	Indica	Japonica	
augustus-8-processed-gene-81.7-mRNA-1	8	OsR498G0815475400.01.T02	99,83	0,00E+00	2085	Os08g0227100	100,00	0,00E+00	2094	9	NA	Japonica	
augustus-8-processed-gene-81.8-mRNA-1	8	OsR498G0815475600.01.T01	99,25	0,00E+00	1891	Os08g0227200	100,00	0,00E+00	1927	36	NA	Japonica	
augustus-8-processed-gene-83.24-mRNA-1	8	OsR498G0815486600.01.T02	99,63	0,00E+00	2856	Os08g0230500	100,00	0,00E+00	2874	18	NA	Japonica	
augustus-8-processed-gene-86.13-mRNA-1	8	OsR498G0815502400.01.T01	99,46	0,00E+00	1643	Os08g0235800	100,00	0,00E+00	1667	24	NA	Japonica	
augustus-8-processed-gene-86.28-mRNA-1	8	OsR498G0815488100.01.T01	86,53	0,00E+00	1448	Os08g0230800	86,75	0,00E+00	2801	1353	Japonica	Japonica	CC-Kinase-TM
augustus-8-processed-gene-90.27-mRNA-1	8	OsR498G0815517200.01.T01	98,35	0,00E+00	4713	Os02g0797300	78,77	0,00E+00	2273	-2440	Indica	Japonica	
augustus-8-processed-gene-91.51-mRNA-1	8	OsR498G0815525200.01.T04	99,69	0,00E+00	3447	Os08g0243900	99,95	0,00E+00	3470	23	NA	Japonica	
augustus-8-processed-gene-93.30-mRNA-1	8	OsR498G0815530100.01.T01	99,15	0,00E+00	2080	Os08g0246300	99,75	0,00E+00	2108	28	NA	Japonica	
augustus-8-processed-gene-99.30-mRNA-1	8	OsR498G0815569000.01.T01	99,23	0,00E+00	2742	Os08g0258700	99,87	0,00E+00	2787	45	NA	Japonica	NBS-TM
maker-8-augustus-gene-0.26-mRNA-1	8	OsR498G0815037800.01.T01	99,76	0,00E+00	3034	Os08g0105000	99,94	0,00E+00	3048	14	NA	NA	
maker-8-augustus-gene-0.29-mRNA-1	8	OsR498G0815038000.01.T02	99,65	0,00E+00	3041	Os08g0105100	100,00	0,00E+00	3016	-25	NA	NA	
maker-8-augustus-gene-0.31-mRNA-1	8	OsR498G0815100800.01.T01	78,49	0,00E+00	1444	Os08g0124900	99,93	0,00E+00	2710	1266	Japonica	NA	Kinase-TM
maker-8-augustus-gene-1.26-mRNA-1	8	OsR498G0815023300.01.T06	99,60	0,00E+00	4457	Os08g0100500	100,00	0,00E+00	4502	45	NA	Japonica	
maker-8-augustus-gene-1.35-mRNA-1	8	OsR498G0815023900.01.T02	99,38	0,00E+00	4020	Os08g0100700	100,00	0,00E+00	2895	-1125	Indica	Japonica	
maker-8-augustus-gene-1.43-mRNA-1	8	OsR498G0815024100.01.T06	99,37	0,00E+00	1397	Os08g0100800	99,62	0,00E+00	1407	10	NA	Japonica	
maker-8-augustus-gene-1.47-mRNA-1	8	OsR498G0815025100.01.T01	99,79	0,00E+00	2525	Os10g0188900	96,17	0,00E+00	1278	-1247	Indica	Japonica	
maker-8-augustus-gene-1.56-mRNA-1	8	OsR498G0815026500.01.T01	98,97	0,00E+00	3067	Os08g0101800	99,83	0,00E+00	3129	62	NA	Japonica	
maker-8-augustus-gene-1.58-mRNA-1	8	OsR498G0815026700.01.T01	100,00	0,00E+00	1288	Os04g0460400	71,14	5,70E-50	197	-1091	Indica	Japonica	
maker-8-augustus-gene-1.61-mRNA-1	8	OsR498G0815022800.01.T03	99,63	0,00E+00	1435	Os08g0100200	100,00	0,00E+00	1133	-302	Indica	Japonica	
maker-8-augustus-gene-1.64-mRNA-1	8	OsR498G0815023200.01.T02	99,78	0,00E+00	4816	Os08g0100400	100,00	0,00E+00	3425	-1391	Indica	Japonica	
maker-8-augustus-gene-1.76-mRNA-1	8	OsR498G0815025000.01.T02	98,70	0,00E+00	1072	Os08g0101100	100,00	0,00E+00	1104	32	NA	Japonica	
maker-8-augustus-gene-1.78-mRNA-1	8	OsR498G0815025800.01.T01	99,79	0,00E+00	3460	Os08g0101600	100,00	0,00E+00	3474	14	NA	Japonica	
maker-8-augustus-gene-10.30-mRNA-1	8	OsR498G0815078900.01.T01	100,00	0,00E+00	1597	Os08g0117000	90,68	0,00E+00	1346	-251	Indica	NA	
maker-8-augustus-gene-10.38-mRNA-1	8	OsR498G0815079100.01.T01	100,00	0,00E+00	1732	Os08g0117100	100,00	0,00E+00	1732	0	NA	NA	
maker-8-augustus-gene-10.40-mRNA-1	8	OsR498G0815080100.01.T01	100,00	0,00E+00	3804	Os08g0117700	100,00	0,00E+00	3804	0	NA	NA	Kinase-LRR-TM
maker-8-augustus-gene-10.42-mRNA-1	8	OsR498G0815080500.01.T01	100,00	0,00E+00	4470	Os08g0117800	100,00	0,00E+00	4470	0	NA	NA	
maker-8-augustus-gene-10.53-mRNA-1	8	OsR498G0815078400.01.T01	100,00	0,00E+00	1424	Os08g0116800	100,00	0,00E+00	1424	0	NA	NA	
maker-8-augustus-gene-10.57-mRNA-1	8	OsR498G0815078600.01.T01	100,00	0,00E+00	2105	Os08g0116900	100,00	0,00E+00	2105	0	NA	NA	
maker-8-augustus-gene-10.63-mRNA-1	8	OsR498G0815079400.01.T01	100,00	0,00E+00	823	Os08g0117200	100,00	0,00E+00	823	0	NA	NA	
maker-8-augustus-gene-10.65-mRNA-1	8	OsR498G0815079600.01.T01	89,46	0,00E+00	685	Os08g0117300	89,46	0,00E+00	685	0	NA	NA	
maker-8-augustus-gene-10.69-mRNA-1	8	OsR498G0815080800.01.T02	100,00	0,00E+00	1781	Os08g0117900	100,00	0,00E+00	1781	0	NA	NA	
maker-8-augustus-gene-100.29-mRNA-1	8	OsR498G0815569500.01.T04	97,72	0,00E+00	1490	Os08g0260000	99,83	0,00E+00	1057	-433	Indica	Japonica	
maker-8-augustus-gene-100.32-mRNA-1	8	OsR498G0815571400.01.T01	99,27	0,00E+00	1456	Os08g0260600	99,03	0,00E+00	1447	-9	NA	Indica	
maker-8-augustus-gene-101.27-mRNA-1	8	OsR498G0815573000.01.T01	99,19	0,00E+00	872	Os08g0261100	99,19	0,00E+00	872	0	NA	NA	
maker-8-augustus-gene-103.12-mRNA-1	8	OsR498G0815585300.01.T04	98,96	0,00E+00	1179	Os08g0266600	99,51	0,00E+00	1091	-88	NA	NA	
maker-8-augustus-gene-103.14-mRNA-1	8	OsR498G0815585700.01.T01	100,00	0,00E+00	3945	Os08g0266700	99,91	0,00E+00	3936	-9	NA	Indica	
maker-8-augustus-gene-103.6-mRNA-1	8	OsR498G0815584300.01.T02	99,79	0,00E+00	835	Os08g0266300	99,66	0,00E+00	1596	761	Japonica	NA	
maker-8-augustus-gene-104.10-mRNA-1	8	OsR498G0815589400.01.T01	99,64	0,00E+00	2505	Os08g0267800	99,22	0,00E+00	2481	-24	NA	Indica	
maker-8-augustus-gene-104.8-mRNA-1	8	OsR498G0815588800.01.T01	100,00	0,00E+00	1575	Os08g0267800	82,91	0,00E+00	1051	-524	Indica	Indica	
maker-8-augustus-gene-105.29-mRNA-1	8	OsR498G0815595900.01.T01	98,41	0,00E+00	1088	Os08g0270200	98,41	0,00E+00	1088	0	NA	Indica	
maker-8-augustus-gene-105.31-mRNA-1	8	OsR498G0815593200.01.T01	99,75	0,00E+00	710	Os08g0269500	100,00	0,00E+00	715	5	NA	NA	
maker-8-augustus-gene-105.33-mRNA-1	8	OsR498G0815593400.01.T01	100,00	0,00E+00	850	Os08g0269600	96,25	3,23E-133	473	-377	Indica	Indica	
maker-8-augustus-gene-105.35-mRNA-1	8	OsR498G0815593600.01.T01	99,76	0,00E+00	737	Os08g0269700	99,76	0,00E+00	737	0	NA	Indica	
maker-8-augustus-gene-105.37-mRNA-1	8	OsR498G0815593800.01.T02	99,68	0,00E+00	1691	Os08g0269800	99,65	0,00E+00	1031	-660	Indica	Indica	
maker-8-augustus-gene-106.29-mRNA-1	8	OsR498G0815597900.01.T03	99,56	0,00E+00	1221	Os08g0270800	99,71	0,00E+00	1225	4	NA	NA	
maker-8-augustus-gene-106.31-mRNA-1	8	OsR498G0815597600.01.T01	99,53	0,00E+00	1913	Os08g0270466	99,42	0,00E+00	1531	-382	Indica	Indica	
maker-8-augustus-gene-107.37-mRNA-1	8	OsR498G0815603900.01.T01	99,72	0,00E+00	1940	Os08g0272200	99,56	0,00E+00	2831	891	Japonica	NA	
maker-8-augustus-gene-107.41-mRNA-1	8	OsR498G0815603000.01.T01	99,59	9,45E-123	440	Os08g0272000	99,18	1,71E-120	430	-10	NA	Japonica	
maker-8-augustus-gene-108.41-mRNA-1	8	OsR498G0815607300.01.T01	99,83	0,00E+00	1046	Os08g0273300	100,00	0,00E+00	1050	4	NA	NA	
maker-8-augustus-gene-108.43-mRNA-1	8	OsR498G0815612100.01.T06	100,00	2,06E-161	569	Os08g0274700	100,00	0,00E+00	1104	535	Japonica	Japonica	
maker-8-augustus-gene-109.0-mRNA-1	8	OsR498G0815615900.01.T02	99,65	0,00E+00	2521	Os08g0276100	99,65	0,00E+00	2521	0	NA	NA	
maker-8-augustus-gene-11.0-mRNA-1	8	OsR498G0815081500.01.T01	99,37	0,00E+00	1988	Os08g0118000	100,00	0,00E+00	2019	31	NA	Japonica	
maker-8-augustus-gene-11.14-mRNA-1	8	OsR498G0815082600.01.T01	99,70	0,00E+00	2374	Os08g0118100	100,00	0,00E+00	2392	18	NA	Japonica	
maker-8-augustus-gene-11.16-mRNA-1	8	OsR498G0815087000.01.T01	99,85	0,00E+00	1161	Os08g0119700	100,00	0,00E+00	1577	416	Japonica	Japonica	
maker-8-augustus-gene-11.19-mRNA-1	8	OsR498G0815087400.01.T04	99,76	0,00E+00	1517	Os08g0120000	100,00	0,00E+00	1526	9	NA	Japonica	
maker-8-augustus-gene-11.4-mRNA-1	8	OsR498G0815084900.01.T01	100,00	0,00E+00	1624	Os08g0118900	100,00	0,00E+00	1624	0	NA	Japonica	
maker-8-augustus-gene-11.7-mRNA-1	8	OsR498G0815085300.01.T02	99,45	0,00E+00	2279	Os08g0119000	100,00	0,00E+00	2311	32	NA	Japonica	
maker-8-augustus-gene-110.33-mRNA-1	8	OsR498G0815620900.01.T01	99,90	0,00E+00	1852	Os08g0277200	99,61	0,00E+00	1838	-14	NA	Indica	
maker-8-augustus-gene-110.35-mRNA-1	8	OsR498G0815621600.01.T01	99,76	0,00E+00	1498	Os08g0240000	70,85	1,02E-137	489	-1009	Indica	Indica	
maker-8-augustus-gene-111.30-mRNA-1	8	OsR498G0815624700.01.T01	99,86	0,00E+00	1278	Os08g0278100	99,72	0,00E+00	1274	-4	NA	NA	
maker-8-augustus-gene-111.33-mRNA-1	8	OsR498G0815628300.01.T01	99,24	2,91E-131	468	Os08g0278966	99,72	0,00E+00	635	167	Japonica	Japonica	
maker-8-augustus-gene-112.26-mRNA-1	8	OsR498G0815633100.01.T06	99,42	0,00E+00	2777	Os08g0280100	99,42	0,00E+00	2777	0	NA	NA	
maker-8-augustus-gene-113.38-mRNA-1	8	OsR498G0815634100.01.T01	96,55	0,00E+00	3490	Os02g0116400	71,10	0,00E+00	747	-2743	Indica	NA	
maker-8-augustus-gene-114.0-mRNA-1	8	OsR498G0815634100.01.T01	99,72	0,00E+00	3793	Os02g0116300	70,52	0,00E+00	839	-2954	Indica	Indica	
maker-8-augustus-gene-115.41-mRNA-1	8	OsR498G0815638800.01.T01	100,00	0,00E+00	769	Os08g0282000	99,77	0,00E+00	765	-4	NA	Indica	
maker-8-augustus-gene-116.43-mRNA-1	8	OsR498G0815641700.01.T01	100,00	0,00E+00	1570	Os08g0282400	100,00	0,00E+00	1570	0	NA	NA	
maker-8-augustus-gene-116.50-mRNA-1	8	OsR498G0815646200.01.T03	100,00	0,00E+00	2408	Os05g0319700	72,38	7,77E-115	414	-1994	Indica	NA	Kinase-TM
maker-8-augustus-gene-119.0-mRNA-1	8	OsR498G0815658200.01.T02	96,61	0,00E+00	2723	Os08g0288200	96,29	0,00E+00	2467	-256	NA	Indica	
maker-8-augustus-gene-119.5-mRNA-1	8	OsR498G0815659600.01.T01	99,82	0,00E+00	4002	Os08g0288500	99,87	0,00E+00	4007	5	NA	NA	
maker-8-augustus-gene-12.33-mRNA-1	8	OsR498G0815088900.01.T03	99,75	0,00E+00	4302	Os08g0120200	100,00	0,00E+00	4329	27	NA	Japonica	
maker-8-augustus-gene-12.36-mRNA-1	8	OsR498G0815089600.01.T01	99,91	0,00E+00	3871	Os08g0120500	100,00	0,00E+00	3880	9	NA	Japonica	
maker-8-augustus-gene-12.40-mRNA-1	8	OsR498G0815090000.01.T01	99,73	0,00E+00	1951	Os08g0120600	100,00	0,00E+00	1965	14	NA	Japonica	
maker-8-augustus-gene-120.0-mRNA-1	8	OsR498G0815661100.01.T01	99,38	0,00E+00	1423	Os08g0289000	99,63	0,00E+00	1432	9	NA	NA	
maker-8-augustus-gene-121.41-mRNA-1	8	OsR498G0815667700.01.T01	99,20	0,00E+00	1994	Os08g0290700	99,29	0,00E+00	1999	5	NA	NA	
maker-8-augustus-gene-121.43-mRNA-1	8	OsR498G0815665000.01.T03	99,45	0,00E+00	2254	Os08g0290100	99,83	0,00E+00	2168	-86	NA	Japonica	
maker-8-augustus-gene-121.47-mRNA-1	8	OsR498G0815665600.01.T01	100,00	0,00E+00	834	Os08g0290400	100,00	0,00E+00	834	0	NA	NA	
maker-8-augustus-gene-122.2-mRNA-1	8	OsR498G0815672300.01.T02	99,03	0,00E+00	4181	Os08g0292000	99,16	0,00E+00	4196	15	NA	NA	
maker-8-augustus-gene-123.0-mRNA-1	8	OsR498G0815676500.01.T01	98,94	0,00E+00	1995	Os08g0292900	99,83	0,00E+00	1048	-947	Indica	Japonica	
maker-8-augustus-gene-123.5-mRNA-1	8	OsR498G0815678600.01.T02	99,67	0,00E+00	4916	Os08g0293300	99,71	0,00E+00	4920	4	NA	Japonica	CC-NBS-TM
maker-8-augustus-gene-125.24-mRNA-1	8	OsR498G0815726600.01.T05	99,81	0,00E+00	1885	Os08g0307400	99,71	0,00E+00	1881	-4	NA	Indica	
maker-8-augustus-gene-125.31-mRNA-1	8	OsR498G0815686000.01.T01	100,00	0,00E+00	2599	Os08g0295300	99,93	0,00E+00	2595	-4	NA	Indica	
maker-8-augustus-gene-126.0-mRNA-1	8	OsR498G0815725100.01.T05	100,00	1,37E-166	585	Os08g0307000	99,69	2,02E-165	581	-4	NA	Indica	
maker-8-augustus-gene-127.40-mRNA-1	8	OsR498G0815720600.01.T05	99,02	0,00E+00	5391	Os08g0305300	99,08	0,00E+00	5400	9	NA	Japonica	
maker-8-augustus-gene-128.20-mRNA-1	8	OsR498G0815704000.01.T02	99,93	0,00E+00	2573	Os08g0300200	99,93	0,00E+00	2573	0	NA	Japonica	
maker-8-augustus-gene-128.28-mRNA-1	8	OsR498G0815705100.01.T01	99,80	0,00E+00	3640	Os08g0300300	99,88	0,00E+00	2908	-732	Indica	NA	
maker-8-augustus-gene-129.31-mRNA-1	8	OsR498G0815698400.01.T02	99,55	0,00E+00	1183	Os08g0299300	99,31	0,00E+00	1027	-156	Indica	NA	
maker-8-augustus-gene-129.37-mRNA-1	8	OsR498G0815699500.01.T02	100,00	0,00E+00	1115	Os07g0421000	84,77	0,00E+00	690	-425	Indica	NA	
maker-8-augustus-gene-129.39-mRNA-1	8	OsR498G0815699300.01.T01	99,79	0,00E+00	1671	Os08g0299400	99,89	0,00E+00	1675	4	NA	NA	
maker-8-augustus-gene-129.42-mRNA-1	8	OsR498G0815695900.01.T01	99,55	0,00E+00	1600	Os08g0299000	99,44	0,00E+00	1596	-4	NA	NA	
maker-8-augustus-gene-13.37-mRNA-1	8	OsR498G0815093500.01.T01	100,00	0,00E+00	872	Os08g0121800	99,59	0,00E+00	863	-9	NA	Indica	
maker-8-augustus-gene-13.42-mRNA-1	8	OsR498G0815094500.01.T01	99,57	0,00E+00	839	Os08g0395500	95,24	1,66E-150	532	-307	Indica	NA	
maker-8-augustus-gene-13.46-mRNA-1	8	OsR498G0815093800.01.T01	99,77	0,00E+00	3148	Os08g0121900	100,00	0,00E+00	3166	18	NA	Japonica	
maker-8-augustus-gene-13.48-mRNA-1	8	OsR498G0815094200.01.T01	99,87	0,00E+00	2789	Os08g0122000	99,74	0,00E+00	2780	-9	NA	Japonica	
maker-8-augustus-gene-130.28-mRNA-1	8	OsR498G0815691300.01.T01	99,80	0,00E+00	1810	Os08g0297800	99,70	0,00E+00	1805	-5	NA	NA	
maker-8-augustus-gene-131.0-mRNA-1	8	OsR498G0815689800.01.T03	99,77	0,00E+00	3888	Os08g0296900	99,77	0,00E+00	3888	0	NA	NA	
maker-8-augustus-gene-132.0-mRNA-1	8	OsR498G0815689000.01.T01	99,68	0,00E+00	4975	Os08g0296600	99,68	0,00E+00	4975	0	NA	NA	CC-LRR-NBS-TM
maker-8-augustus-gene-132.15-mRNA-1	8	OsR498G0815710000.01.T01	98,22	0,00E+00	966	Os08g0301600	97,98	2,00E-168	591	-375	Indica	NA	
maker-8-augustus-gene-132.2-mRNA-1	8	OsR498G0815709500.01.T03	99,91	0,00E+00	5760	Os08g0301500	99,94	0,00E+00	5764	4	NA	NA	
maker-8-augustus-gene-132.7-mRNA-1	8	OsR498G0815712500.01.T01	99,91	0,00E+00	1987	Os08g0302000	99,91	0,00E+00	1987	0	NA	NA	
maker-8-augustus-gene-132.9-mRNA-1	8	OsR498G0815689300.01.T01	99,80	0,00E+00	8216	Os08g0296700	99,75	0,00E+00	8473	257	NA	NA	CC-LRR-NBS-TM
maker-8-augustus-gene-133.30-mRNA-1	8	OsR498G0815719900.01.T05	99,29	1,65E-140	499	Os08g0304900	99,29	5,75E-141	499	0	NA	NA	
maker-8-augustus-gene-134.35-mRNA-1	8	OsR498G0815730600.01.T01	99,90	0,00E+00	1765	Os08g0308100	100,00	0,00E+00	1770	5	NA	NA	
maker-8-augustus-gene-134.45-mRNA-1	8	OsR498G0815733400.01.T02	99,83	0,00E+00	1040	Os08g0309225	100,00	9,42E-154	542	-498	Indica	NA	
maker-8-augustus-gene-134.46-mRNA-1	8	OsR498G0815736000.01.T04	99,76	0,00E+00	743	Os08g0309300	99,76	0,00E+00	741	-2	NA	NA	
maker-8-augustus-gene-135.37-mRNA-1	8	OsR498G0815738100.01.T02	99,62	0,00E+00	930	Os08g0310100	99,62	0,00E+00	930	0	NA	NA	
maker-8-augustus-gene-135.39-mRNA-1	8	OsR498G0815742700.01.T01	100,00	0,00E+00	1278	Os08g0313200	100,00	0,00E+00	1278	0	NA	NA	
maker-8-augustus-gene-136.0-mRNA-1	8	OsR498G0815746500.01.T01	99,80	0,00E+00	3559	Os08g0314800	99,80	0,00E+00	3559	0	NA	NA	
maker-8-augustus-gene-138.24-mRNA-1	8	OsR498G0815539600.01.T01	99,95	0,00E+00	3253	Os08g0249100	100,00	0,00E+00	3258	5	NA	NA	Kinase-TM
maker-8-augustus-gene-138.26-mRNA-1	8	OsR498G0815539600.01.T01	99,95	0,00E+00	3253	Os08g0249100	100,00	0,00E+00	3258	5	NA	NA	Kinase-TM
maker-8-augustus-gene-141.0-mRNA-1	8	OsR498G0815757200.01.T03	98,93	2,81E-138	492	Os08g0318500	99,29	2,29E-140	497	5	NA	NA	
maker-8-augustus-gene-143.0-mRNA-1	8	OsR498G0815763300.01.T01	99,91	0,00E+00	2058	Os08g0150800	92,68	0,00E+00	1697	-361	Indica	Japonica	
maker-8-augustus-gene-143.13-mRNA-1	8	OsR498G0815765100.01.T02	99,57	0,00E+00	832	Os08g0320300	95,28	0,00E+00	756	-76	NA	NA	
maker-8-augustus-gene-143.20-mRNA-1	8	OsR498G0815765900.01.T03	99,44	0,00E+00	1907	Os09g0255400	73,38	1,07E-176	619	-1288	Indica	NA	
maker-8-augustus-gene-143.29-mRNA-1	8	OsR498G0815763200.01.T02	99,03	0,00E+00	1453	Os08g0319900	97,70	0,00E+00	1410	-43	NA	Japonica	
maker-8-augustus-gene-143.33-mRNA-1	8	OsR498G0815765000.01.T02	98,12	0,00E+00	3875	Os08g0320200	98,17	0,00E+00	3880	5	NA	Japonica	
maker-8-augustus-gene-143.35-mRNA-1	8	OsR498G0815767600.01.T01	99,73	0,00E+00	2618	Os08g0321000	99,84	0,00E+00	2242	-376	Indica	NA	
maker-8-augustus-gene-145.29-mRNA-1	8	OsR498G0815772300.01.T01	99,75	0,00E+00	4286	Os08g0322500	99,91	0,00E+00	4060	-226	NA	NA	LRR-TM
maker-8-augustus-gene-145.31-mRNA-1	8	OsR498G0815773200.01.T01	99,68	0,00E+00	2250	Os08g0322600	99,76	0,00E+00	2254	4	NA	NA	
maker-8-augustus-gene-145.35-mRNA-1	8	OsR498G0815774200.01.T01	99,93	0,00E+00	2388	Os08g0323000	99,68	0,00E+00	2253	-135	NA	NA	
maker-8-augustus-gene-146.0-mRNA-1	8	OsR498G0815779000.01.T02	99,88	0,00E+00	5858	Os08g0323600	99,85	0,00E+00	5854	-4	NA	NA	
maker-8-augustus-gene-148.32-mRNA-1	8	OsR498G0815786100.01.T01	99,67	0,00E+00	2681	Os08g0326500	99,77	0,00E+00	3065	384	Japonica	Japonica	
maker-8-augustus-gene-148.34-mRNA-1	8	OsR498G0815784400.01.T01	99,84	0,00E+00	2275	Os08g0326100	99,76	0,00E+00	2270	-5	NA	Japonica	
maker-8-augustus-gene-148.36-mRNA-1	8	OsR498G0815785200.01.T01	99,87	0,00E+00	1394	Os08g0326400	99,87	0,00E+00	1394	0	NA	Japonica	
maker-8-augustus-gene-149.29-mRNA-1	8	OsR498G0815786500.01.T01	99,76	0,00E+00	2931	Os08g0326600	99,82	0,00E+00	2936	5	NA	NA	
maker-8-augustus-gene-149.39-mRNA-1	8	OsR498G0815789900.01.T05	100,00	0,00E+00	2035	Os08g0327400	99,91	0,00E+00	2030	-5	NA	Indica	
maker-8-augustus-gene-149.44-mRNA-1	8	OsR498G0815788600.01.T01	96,20	0,00E+00	1835	Os08g0327200	96,20	0,00E+00	1835	0	NA	Indica	
maker-8-augustus-gene-150.35-mRNA-1	8	OsR498G0815792700.01.T05	99,84	0,00E+00	2286	Os08g0328300	100,00	0,00E+00	2275	-11	NA	Japonica	
maker-8-augustus-gene-152.35-mRNA-1	8	OsR498G0815804700.01.T04	99,80	0,00E+00	9072	Os09g0281900	75,80	0,00E+00	1948	-7124	Indica	Indica	
maker-8-augustus-gene-152.46-mRNA-1	8	OsR498G0815804200.01.T04	96,96	0,00E+00	2830	Os08g0332700	97,02	0,00E+00	2835	5	NA	Japonica	
maker-8-augustus-gene-152.52-mRNA-1	8	OsR498G0815805400.01.T01	99,80	0,00E+00	1808	Os08g0333100	99,90	0,00E+00	1812	4	NA	NA	
maker-8-augustus-gene-152.53-mRNA-1	8	OsR498G0815805400.01.T01	99,77	0,00E+00	793	Os08g0333100	99,77	0,00E+00	793	0	NA	NA	
maker-8-augustus-gene-154.36-mRNA-1	8	OsR498G0815813300.01.T02	99,10	0,00E+00	1762	Os08g0334300	78,81	0,00E+00	646	-1116	Indica	NA	
maker-8-augustus-gene-154.43-mRNA-1	8	OsR498G0815817100.01.T01	99,82	0,00E+00	5048	Os08g0337100	99,87	0,00E+00	2718	-2330	Indica	NA	
maker-8-augustus-gene-155.35-mRNA-1	8	OsR498G0815818800.01.T01	99,76	0,00E+00	2260	Os08g0337700	99,84	0,00E+00	2264	4	NA	NA	
maker-8-augustus-gene-156.26-mRNA-1	8	OsR498G0815827700.01.T03	96,38	0,00E+00	1336	Os08g0339200	96,63	0,00E+00	1345	9	NA	Japonica	
maker-8-augustus-gene-156.35-mRNA-1	8	OsR498G0815825400.01.T01	99,90	0,00E+00	1814	Os08g0338700	99,83	0,00E+00	1076	-738	Indica	NA	
maker-8-augustus-gene-156.39-mRNA-1	8	OsR498G0815826400.01.T02	100,00	0,00E+00	2457	Os08g0338900	100,00	0,00E+00	2457	0	NA	NA	
maker-8-augustus-gene-158.0-mRNA-1	8	OsR498G0815835100.01.T02	99,85	0,00E+00	3549	Os08g0341700	99,89	0,00E+00	3397	-152	NA	NA	
maker-8-augustus-gene-158.16-mRNA-1	8	OsR498G0815837600.01.T01	99,86	0,00E+00	4971	Os08g0342400	99,74	0,00E+00	4107	-864	Indica	NA	
maker-8-augustus-gene-158.8-mRNA-1	8	OsR498G0815840500.01.T03	100,00	0,00E+00	1323	Os08g0343000	99,73	0,00E+00	1314	-9	NA	Japonica	Kinase-TM
maker-8-augustus-gene-159.0-mRNA-1	8	OsR498G0815842100.01.T01	99,93	0,00E+00	2501	Os08g0343300	99,86	0,00E+00	2497	-4	NA	NA	
maker-8-augustus-gene-159.13-mRNA-1	8	OsR498G0815843700.01.T03	99,70	1,85E-171	602	Os08g0344300	100,00	4,63E-142	502	-100	Indica	Indica	
maker-8-augustus-gene-159.16-mRNA-1	8	OsR498G0815845300.01.T01	99,76	0,00E+00	2195	Os08g0344600	99,92	0,00E+00	2204	9	NA	Indica	
maker-8-augustus-gene-159.8-mRNA-1	8	OsR498G0815843500.01.T02	99,78	0,00E+00	2427	Os08g0344100	99,71	0,00E+00	2423	-4	NA	Japonica	
maker-8-augustus-gene-16.0-mRNA-1	8	OsR498G0815102700.01.T04	98,16	0,00E+00	1788	Os08g0126300	98,26	0,00E+00	1792	4	NA	Japonica	
maker-8-augustus-gene-16.11-mRNA-1	8	OsR498G0815100800.01.T01	78,11	0,00E+00	1413	Os08g0125200	99,94	0,00E+00	2939	1526	Japonica	Japonica	Kinase-TM
maker-8-augustus-gene-16.13-mRNA-1	8	OsR498G0815100800.01.T01	99,63	0,00E+00	3909	Os08g0125800	100,00	0,00E+00	2576	-1333	Indica	Japonica	CC-Kinase-TM
maker-8-augustus-gene-16.6-mRNA-1	8	OsR498G0815103300.01.T02	99,19	0,00E+00	3487	Os08g0126700	100,00	0,00E+00	3274	-213	NA	Japonica	
maker-8-augustus-gene-160.28-mRNA-1	8	OsR498G0815848100.01.T02	99,44	0,00E+00	1599	Os08g0345400	99,82	0,00E+00	1003	-596	Indica	Indica	
maker-8-augustus-gene-160.37-mRNA-1	8	OsR498G0815846800.01.T01	99,77	0,00E+00	2308	Os08g0363000	96,35	0,00E+00	2106	-202	NA	NA	
maker-8-augustus-gene-160.39-mRNA-1	8	OsR498G0815850400.01.T01	100,00	0,00E+00	2787	Os08g0345800	99,77	0,00E+00	2332	-455	Indica	Indica	
maker-8-augustus-gene-161.1-mRNA-1	8	OsR498G0815853100.01.T03	99,35	0,00E+00	1640	Os09g0299200	73,95	6,93E-158	557	-1083	Indica	Japonica	
maker-8-augustus-gene-163.0-mRNA-1	8	OsR498G0815862900.01.T01	98,93	0,00E+00	989	Os08g0349100	99,47	0,00E+00	1003	14	NA	NA	

maker-8-augustus-gene-163.2-mRNA-1	8	OsR498G0815861800.01.T01	99,05	7,75E-157	554	Os08g0352100	98,40	1,70E-153	541	-13	NA	NA	
maker-8-augustus-gene-166.0-mRNA-1	8	OsR498G0815873600.01.T01	99,68	1,89E-158	559	Os08g0352100	99,36	7,99E-158	554	-5	NA	NA	
maker-8-augustus-gene-167.31-mRNA-1	8	OsR498G0815862900.01.T01	97,16	0,00E+00	944	Os08g0349100	97,69	0,00E+00	957	13	NA	Indica	
maker-8-augustus-gene-168.20-mRNA-1	8	OsR498G0815862900.01.T01	95,56	0,00E+00	903	Os08g0349100	96,09	0,00E+00	917	14	NA	Indica	
maker-8-augustus-gene-17.0-mRNA-1	8	OsR498G0815104300.01.T01	99,85	0,00E+00	2349	Os08g0127100	100,00	0,00E+00	2358	9	NA	Japonica	
maker-8-augustus-gene-17.10-mRNA-1	8	OsR498G0815106300.01.T01	99,75	0,00E+00	2861	Os08g0127900	99,94	0,00E+00	2874	13	NA	Japonica	
maker-8-augustus-gene-17.15-mRNA-1	8	OsR498G0815107100.01.T02	99,66	0,00E+00	2595	Os08g0128200	99,86	0,00E+00	2610	15	NA	Japonica	
maker-8-augustus-gene-17.17-mRNA-1	8	OsR498G0815107500.01.T01	99,65	9,30E-144	510	Os08g0128500	100,00	7,60E-146	515	5	NA	Japonica	
maker-8-augustus-gene-17.25-mRNA-1	8	OsR498G0815105400.01.T01	99,66	0,00E+00	1571	Os08g0127600	99,77	0,00E+00	1575	4	NA	Japonica	
maker-8-augustus-gene-17.3-mRNA-1	8	OsR498G0815105100.01.T01	100,00	0,00E+00	1002	Os08g0127500	100,00	0,00E+00	1002	0	NA	Japonica	
maker-8-augustus-gene-17.31-mRNA-1	8	OsR498G0815107400.01.T02	98,19	0,00E+00	2283	Os08g0128300	100,00	0,00E+00	1371	-912	Indica	Japonica	
maker-8-augustus-gene-17.36-mRNA-1	8	OsR498G0815110600.01.T01	94,36	0,00E+00	1672	Os08g0129500	94,36	0,00E+00	1672	0	NA	Indica	
maker-8-augustus-gene-17.39-mRNA-1	8	OsR498G0815110800.01.T02	99,92	0,00E+00	4314	Os08g0129600	99,92	0,00E+00	4314	0	NA	Indica	
maker-8-augustus-gene-17.7-mRNA-1	8	OsR498G0815105500.01.T01	99,81	0,00E+00	2892	Os08g0127700	99,88	0,00E+00	2897	5	NA	Japonica	
maker-8-augustus-gene-170.39-mRNA-1	8	OsR498G0815955500.01.T01	98,20	3,80E-131	469	Os08g0376500	98,20	1,33E-131	469	0	NA	NA	
maker-8-augustus-gene-170.40-mRNA-1	8	OsR498G0815955500.01.T01	99,72	0,00E+00	1290	Os08g0376500	99,86	0,00E+00	1295	5	NA	NA	
maker-8-augustus-gene-170.43-mRNA-1	8	OsR498G0816298700.01.T04	99,63	0,00E+00	1907	Os08g0474700	99,91	0,00E+00	1920	13	NA	NA	
maker-8-augustus-gene-170.53-mRNA-1	8	OsR498G0816298400.01.T01	97,60	0,00E+00	1198	Os08g0474500	100,00	0,00E+00	1301	103	NA	NA	
maker-8-augustus-gene-174.0-mRNA-1	8	OsR498G0815875000.01.T01	99,93	0,00E+00	2718	Os08g0356500	99,93	0,00E+00	2718	0	NA	Japonica	
maker-8-augustus-gene-175.17-mRNA-1	8	OsR498G0815877900.01.T01	98,90	0,00E+00	953	Os09g0306400	72,42	2,37E-53	208	-745	Indica	Japonica	
maker-8-augustus-gene-175.20-mRNA-1	8	OsR498G0815877000.01.T01	97,26	0,00E+00	2514	Os08g0356800	99,93	0,00E+00	2696	182	NA	Japonica	
maker-8-augustus-gene-176.0-mRNA-1	8	OsR498G0815882700.01.T01	99,28	2,44E-139	495	Os08g0358800	100,00	0,00E+00	737	242	Japonica	Japonica	
maker-8-augustus-gene-176.12-mRNA-1	8	OsR498G0815885000.01.T03	100,00	0,00E+00	1683	Os08g0359200	100,00	0,00E+00	1683	0	NA	Japonica	
maker-8-augustus-gene-176.15-mRNA-1	8	OsR498G0815885200.01.T02	100,00	0,00E+00	937	Os08g0359300	100,00	0,00E+00	937	0	NA	Japonica	
maker-8-augustus-gene-176.16-mRNA-1	8	OsR498G0815885200.01.T02	100,00	0,00E+00	657	Os08g0359300	100,00	3,49E-177	619	-38	NA	Japonica	
maker-8-augustus-gene-176.3-mRNA-1	8	OsR498G0815883700.01.T01	97,84	0,00E+00	1435	Os08g0359000	97,84	0,00E+00	1435	0	NA	Japonica	
maker-8-augustus-gene-176.8-mRNA-1	8	OsR498G0815884600.01.T02	99,68	0,00E+00	1670	Os08g0359100	100,00	0,00E+00	1683	13	NA	Japonica	
maker-8-augustus-gene-177.44-mRNA-1	8	OsR498G0815887100.01.T02	100,00	0,00E+00	720	Os08g0360000	100,00	0,00E+00	720	0	NA	NA	
maker-8-augustus-gene-177.48-mRNA-1	8	OsR498G0815890300.01.T01	97,39	0,00E+00	1678	Os08g0451700	91,66	0,00E+00	1984	306	Japonica	Japonica	
maker-8-augustus-gene-177.57-mRNA-1	8	OsR498G0815886000.01.T01	98,38	0,00E+00	968	Os08g0359500	100,00	0,00E+00	1005	37	NA	Japonica	
maker-8-augustus-gene-177.60-mRNA-1	8	OsR498G0815886400.01.T01	100,00	1,37E-166	585	Os08g0359600	99,69	2,02E-165	581	-4	NA	Japonica	
maker-8-augustus-gene-177.62-mRNA-1	8	OsR498G0815887400.01.T01	98,95	0,00E+00	2188	Os08g0360100	98,95	0,00E+00	2188	0	NA	Japonica	
maker-8-augustus-gene-178.30-mRNA-1	8	OsR498G0407773900.01.T01	93,10	0,00E+00	976	Os08g0361300	99,36	0,00E+00	1112	136	Japonica	Japonica	
maker-8-augustus-gene-18.0-mRNA-1	8	OsR498G0815111500.01.T01	99,75	0,00E+00	2119	Os08g0129700	99,83	0,00E+00	2123	4	NA	Japonica	
maker-8-augustus-gene-18.11-mRNA-1	8	OsR498G0815117900.01.T02	99,48	0,00E+00	2742	Os08g0131100	99,87	0,00E+00	2837	95	NA	Japonica	
maker-8-augustus-gene-18.13-mRNA-1	8	OsR498G0815112600.01.T02	99,86	0,00E+00	2535	Os08g0130000	100,00	0,00E+00	2533	-2	NA	Japonica	
maker-8-augustus-gene-18.15-mRNA-1	8	OsR498G0815112800.01.T01	100,00	0,00E+00	801	Os08g0130100	100,00	0,00E+00	801	0	NA	Japonica	
maker-8-augustus-gene-18.17-mRNA-1	8	OsR498G0815115800.01.T01	99,79	0,00E+00	3371	Os08g0130300	99,89	0,00E+00	3380	9	NA	Japonica	
maker-8-augustus-gene-18.22-mRNA-1	8	OsR498G0815116000.01.T03	99,58	0,00E+00	1271	Os08g0130400	100,00	0,00E+00	1285	14	NA	Japonica	
maker-8-augustus-gene-18.27-mRNA-1	8	OsR498G0815116200.01.T01	99,90	0,00E+00	1728	Os08g0130500	99,90	0,00E+00	1728	0	NA	Japonica	
maker-8-augustus-gene-18.33-mRNA-1	8	OsR498G0815116400.01.T02	99,48	0,00E+00	3429	Os08g0130600	99,95	0,00E+00	3470	41	NA	Japonica	
maker-8-augustus-gene-18.5-mRNA-1	8	OsR498G0815112300.01.T03	99,77	0,00E+00	781	Os08g0129900	100,00	0,00E+00	717	-64	NA	Japonica	
maker-8-augustus-gene-180.24-mRNA-1	8	OsR498G0815846800.01.T01	96,22	0,00E+00	1723	Os08g0363000	99,62	0,00E+00	1878	155	NA	Indica	
maker-8-augustus-gene-181.39-mRNA-1	8	OsR498G0815911100.01.T05	99,91	0,00E+00	1922	Os08g0364900	97,76	0,00E+00	1829	-93	NA	NA	
maker-8-augustus-gene-181.45-mRNA-1	8	OsR498G0815927700.01.T02	95,52	0,00E+00	783	Os08g0363800	99,41	0,00E+00	905	122	Japonica	NA	
maker-8-augustus-gene-182.25-mRNA-1	8	OsR498G0815912500.01.T01	99,53	0,00E+00	765	Os08g0366100	100,00	0,00E+00	774	9	NA	NA	
maker-8-augustus-gene-182.28-mRNA-1	8	OsR498G0815911800.01.T04	96,86	0,00E+00	2942	Os08g0365900	97,74	0,00E+00	3043	101	NA	NA	
maker-8-augustus-gene-182.31-mRNA-1	8	OsR498G0815912200.01.T01	98,45	0,00E+00	5019	Os08g0366000	99,86	0,00E+00	5204	185	NA	NA	
maker-8-augustus-gene-182.36-mRNA-1	8	OsR498G0815913800.01.T01	98,04	0,00E+00	788	Os08g0366200	78,40	1,19E-101	370	-418	Indica	NA	
maker-8-augustus-gene-183.19-mRNA-1	8	OsR498G0815920400.01.T01	99,49	0,00E+00	2801	Os05g0310800	85,80	0,00E+00	1824	-977	Indica	NA	
maker-8-augustus-gene-186.22-mRNA-1	8	OsR498G0815926300.01.T02	97,67	0,00E+00	3091	Os08g0369000	96,54	0,00E+00	1788	-1303	Indica	Japonica	
maker-8-augustus-gene-186.28-mRNA-1	8	OsR498G0815926700.01.T02	99,81	0,00E+00	2757	Os08g0369200	99,94	0,00E+00	2766	9	NA	Japonica	
maker-8-augustus-gene-186.31-mRNA-1	8	OsR498G0815927300.01.T01	97,12	9,87E-113	407	Os08g0369400	100,00	5,81E-123	439	32	NA	Japonica	
maker-8-augustus-gene-186.34-mRNA-1	8	OsR498G0815927700.01.T02	99,20	0,00E+00	886	Os08g0369700	99,80	0,00E+00	900	14	NA	Japonica	
maker-8-augustus-gene-186.38-mRNA-1	8	OsR498G0815927000.01.T02	96,71	0,00E+00	2245	Os08g0369300	94,89	0,00E+00	1924	-321	Indica	Japonica	
maker-8-augustus-gene-186.46-mRNA-1	8	OsR498G0815927600.01.T02	97,14	0,00E+00	1300	Os08g0369600	97,66	0,00E+00	1318	18	NA	Japonica	
maker-8-augustus-gene-187.0-mRNA-1	8	OsR498G0815932400.01.T03	99,19	0,00E+00	1095	Os08g0371476	83,07	4,85E-127	453	-642	Indica	NA	
maker-8-augustus-gene-187.1-mRNA-1	8	OsR498G0815932400.01.T03	98,75	0,00E+00	698	Os08g0371564	82,32	1,03E-100	365	-333	Indica	Japonica	
maker-8-augustus-gene-187.6-mRNA-1	8	OsR498G0815932400.01.T01	99,77	0,00E+00	790	Os08g0371200	99,74	0,00E+00	696	-94	Indica	Japonica	
maker-8-augustus-gene-188.33-mRNA-1	8	OsR498G0815939400.01.T01	99,01	0,00E+00	1948	Os08g0372900	100,00	0,00E+00	2105	157	NA	Japonica	
maker-8-augustus-gene-189.21-mRNA-1	8	OsR498G0815943600.01.T01	97,15	0,00E+00	765	Os08g0374000	100,00	0,00E+00	823	58	NA	Japonica	
maker-8-augustus-gene-189.23-mRNA-1	8	OsR498G0815943800.01.T01	100,00	0,00E+00	996	Os08g0374100	100,00	0,00E+00	996	0	NA	Indica	
maker-8-augustus-gene-189.26-mRNA-1	8	OsR498G0815944200.01.T01	99,83	0,00E+00	1062	Os08g0374200	100,00	5,31E-176	616	-446	Indica	Indica	
maker-8-augustus-gene-189.30-mRNA-1	8	OsR498G0815946000.01.T01	99,81	0,00E+00	1874	Os08g0374400	99,61	0,00E+00	1849	-25	NA	NA	
maker-8-augustus-gene-19.1-mRNA-1	8	OsR498G0815119900.01.T01	98,69	0,00E+00	1462	Os08g0132600	97,73	0,00E+00	1425	-37	NA	NA	
maker-8-augustus-gene-19.2-mRNA-1	8	OsR498G0815123300.01.T01	87,06	2,18E-119	429	Os08g0132100	98,05	1,28E-148	524	95	Japonica	NA	
maker-8-augustus-gene-19.6-mRNA-1	8	OsR498G0815123300.01.T01	100,00	0,00E+00	2435	Os08g0132600	100,00	0,00E+00	2435	0	NA	Japonica	
maker-8-augustus-gene-19.9-mRNA-1	8	OsR498G0815124300.01.T02	100,00	0,00E+00	1231	Os08g0133000	99,85	0,00E+00	1226	-5	NA	Indica	
maker-8-augustus-gene-190.0-mRNA-1	8	OsR498G0815946800.01.T03	99,32	0,00E+00	2600	Os08g0374600	99,40	0,00E+00	2061	-539	Indica	Indica	Kinase-TM
maker-8-augustus-gene-190.2-mRNA-1	8	OsR498G0815947600.01.T01	99,92	0,00E+00	2209	Os08g0374800	99,92	0,00E+00	2209	0	NA	Indica	
maker-8-augustus-gene-190.8-mRNA-1	8	OsR498G0815948000.01.T01	99,84	0,00E+00	1116	Os08g0374900	99,84	0,00E+00	1116	0	NA	NA	
maker-8-augustus-gene-191.0-mRNA-1	8	OsR498G0815953300.01.T02	99,87	0,00E+00	1354	Os08g0375900	100,00	0,00E+00	1359	5	NA	NA	
maker-8-augustus-gene-191.10-mRNA-1	8	OsR498G0815955000.01.T01	99,83	0,00E+00	5269	Os08g0376300	99,80	0,00E+00	5265	-4	NA	Indica	Kinase-LRR-TM
maker-8-augustus-gene-191.2-mRNA-1	8	OsR498G0815954100.01.T01	99,86	0,00E+00	1257	Os08g0376200	99,15	0,00E+00	827	-430	Indica	NA	
maker-8-augustus-gene-191.6-mRNA-1	8	OsR498G0815955500.01.T01	99,83	0,00E+00	2123	Os08g0376500	99,92	0,00E+00	2128	5	NA	NA	
maker-8-augustus-gene-191.8-mRNA-1	8	OsR498G0815956900.01.T01	97,81	2,16E-177	621	Os08g0358800	94,27	9,17E-120	428	-193	Indica	Japonica	
maker-8-augustus-gene-192.29-mRNA-1	8	OsR498G0815959500.01.T01	99,18	0,00E+00	647	Os08g0358800	93,91	3,21E-119	427	-220	Indica	NA	
maker-8-augustus-gene-192.31-mRNA-1	8	OsR498G0815960700.01.T01	99,75	0,00E+00	1415	Os08g0377500	99,37	0,00E+00	1401	-14	NA	NA	
maker-8-augustus-gene-192.33-mRNA-1	8	OsR498G0815960600.01.T05	99,89	0,00E+00	1571	Os08g0377400	97,14	0,00E+00	1464	-107	NA	Indica	
maker-8-augustus-gene-192.35-mRNA-1	8	OsR498G0815961200.01.T02	98,25	0,00E+00	984	Os08g0378900	98,25	0,00E+00	984	0	NA	Indica	
maker-8-augustus-gene-193.36-mRNA-1	8	OsR498G0815961900.01.T06	99,94	0,00E+00	3237	Os08g0379300	96,96	0,00E+00	3032	-205	NA	Indica	
maker-8-augustus-gene-193.41-mRNA-1	8	OsR498G0815964500.01.T03	99,95	0,00E+00	3305	Os08g0380100	99,89	0,00E+00	3301	-4	NA	Indica	
maker-8-augustus-gene-193.43-mRNA-1	8	OsR498G0815962200.01.T01	99,83	0,00E+00	2107	Os08g0379400	99,74	0,00E+00	2103	-4	NA	Indica	
maker-8-augustus-gene-194.3-mRNA-1	8	OsR498G0815970500.01.T05	100,00	0,00E+00	2316	Os08g0382400	99,77	0,00E+00	2301	-15	NA	NA	
maker-8-augustus-gene-195.13-mRNA-1	8	OsR498G0815974800.01.T01	100,00	0,00E+00	1683	Os08g0384100	99,26	0,00E+00	1663	-20	NA	Indica	
maker-8-augustus-gene-195.17-mRNA-1	8	OsR498G0815975800.01.T02	99,80	0,00E+00	5411	Os08g0384500	99,78	0,00E+00	4112	-1299	Indica	Indica	
maker-8-augustus-gene-195.4-mRNA-1	8	OsR498G0815974500.01.T01	96,43	0,00E+00	1211	Os08g0384000	94,33	0,00E+00	668	-543	Indica	Indica	
maker-8-augustus-gene-195.7-mRNA-1	8	OsR498G0815976700.01.T06	99,75	0,00E+00	1427	Os08g0384900	99,75	0,00E+00	1427	0	NA	Japonica	
maker-8-augustus-gene-196.26-mRNA-1	8	OsR498G0815979300.01.T01	99,90	0,00E+00	1728	Os08g0386200	100,00	0,00E+00	1732	4	NA	NA	
maker-8-augustus-gene-197.0-mRNA-1	8	OsR498G0815980500.01.T01	98,34	0,00E+00	1782	Os08g0386700	98,34	0,00E+00	1782	0	NA	NA	
maker-8-augustus-gene-197.20-mRNA-1	8	OsR498G0815983900.01.T01	99,72	0,00E+00	2526	Os08g0387200	99,72	0,00E+00	2526	0	NA	NA	
maker-8-augustus-gene-197.3-mRNA-1	8	OsR498G0815981100.01.T02	97,41	0,00E+00	2167	Os08g0386800	97,41	0,00E+00	2167	0	NA	NA	
maker-8-augustus-gene-198.39-mRNA-1	8	OsR498G0815984500.01.T01	100,00	0,00E+00	2798	Os08g0387400	100,00	0,00E+00	2798	0	NA	NA	
maker-8-augustus-gene-198.41-mRNA-1	8	OsR498G0815986100.01.T07	99,52	0,00E+00	1494	Os08g0388300	99,52	0,00E+00	1494	0	NA	NA	LRR-NBS-TM
maker-8-augustus-gene-198.47-mRNA-1	8	OsR498G0815985000.01.T02	99,85	0,00E+00	2378	Os08g0387500	99,86	0,00E+00	2626	248	NA	NA	
maker-8-augustus-gene-198.49-mRNA-1	8	OsR498G0815985400.01.T03	99,78	0,00E+00	4983	Os08g0387700	99,78	0,00E+00	4983	0	NA	NA	LRR-NBS-TM
maker-8-augustus-gene-199.0-mRNA-1	8	OsR498G0815992300.01.T02	97,86	0,00E+00	2390	Os08g0389700	97,86	0,00E+00	2390	0	NA	NA	
maker-8-augustus-gene-199.18-mRNA-1	8	OsR498G0815993800.01.T01	99,90	0,00E+00	5268	Os08g0390100	99,74	0,00E+00	2796	-2472	Indica	NA	
maker-8-augustus-gene-2.37-mRNA-1	8	OsR498G0815031300.01.T05	99,34	0,00E+00	2406	Os08g0103300	100,00	0,00E+00	2446	40	NA	Japonica	
maker-8-augustus-gene-2.40-mRNA-1	8	OsR498G0815027200.01.T01	99,82	0,00E+00	2995	Os08g0102100	100,00	0,00E+00	3009	14	NA	Japonica	
maker-8-augustus-gene-2.55-mRNA-1	8	OsR498G0815031600.01.T05	99,72	0,00E+00	1290	Os08g0103400	100,00	0,00E+00	1727	437	Japonica	Japonica	
maker-8-augustus-gene-2.59-mRNA-1	8	OsR498G0815031800.01.T02	99,27	0,00E+00	2188	Os08g0103500	100,00	0,00E+00	2230	42	NA	Japonica	
maker-8-augustus-gene-201.2-mRNA-1	8	OsR498G0816006600.01.T02	96,81	0,00E+00	1147	Os08g0393700	86,44	0,00E+00	851	-296	Indica	NA	
maker-8-augustus-gene-202.2-mRNA-1	8	OsR498G0816011100.01.T02	98,38	0,00E+00	748	Os10g0368400	92,13	2,11E-179	627	-121	Indica	NA	
maker-8-augustus-gene-203.30-mRNA-1	8	OsR498G0816026100.01.T01	100,00	0,00E+00	1043	Os08g0397900	99,48	0,00E+00	1030	-13	NA	Indica	
maker-8-augustus-gene-203.33-mRNA-1	8	OsR498G0816026500.01.T01	99,65	0,00E+00	2544	Os08g0398300	98,18	0,00E+00	2451	-93	NA	Indica	
maker-8-augustus-gene-203.36-mRNA-1	8	OsR498G0816026900.01.T01	99,86	0,00E+00	3899	Os08g0398350	97,22	0,00E+00	2470	-1429	Indica	Indica	
maker-8-augustus-gene-203.41-mRNA-1	8	OsR498G0816027300.01.T06	99,77	0,00E+00	1534	Os08g0398400	99,18	0,00E+00	1511	-23	NA	Indica	
maker-8-augustus-gene-203.45-mRNA-1	8	OsR498G0816027700.01.T01	100,00	0,00E+00	2940	Os08g0398700	99,94	0,00E+00	2936	-4	NA	Indica	
maker-8-augustus-gene-203.51-mRNA-1	8	OsR498G0816026400.01.T01	98,90	0,00E+00	5126	Os08g0398000	98,63	0,00E+00	5090	-36	NA	Indica	
maker-8-augustus-gene-203.55-mRNA-1	8	OsR498G0816027600.01.T01	100,00	0,00E+00	661	Os08g0398500	100,00	0,00E+00	661	0	NA	Indica	
maker-8-augustus-gene-203.57-mRNA-1	8	OsR498G0816028000.01.T01	100,00	0,00E+00	1229	Os08g0398800	99,71	0,00E+00	1220	-9	NA	NA	
maker-8-augustus-gene-204.12-mRNA-1	8	OsR498G0816030200.01.T03	99,82	0,00E+00	1968	Os08g0399600	99,82	0,00E+00	1968	0	NA	NA	
maker-8-augustus-gene-204.2-mRNA-1	8	OsR498G0816031900.01.T04	99,86	0,00E+00	2599	Os08g0400300	99,72	0,00E+00	2590	-9	NA	Indica	
maker-8-augustus-gene-204.6-mRNA-1	8	OsR498G0816032500.01.T02	100,00	1,16E-161	569	Os08g0400800	100,00	2,10E-102	370	-199	Indica	Indica	
maker-8-augustus-gene-204.9-mRNA-1	8	OsR498G0816033100.01.T01	97,72	0,00E+00	3612	Os08g0401100	99,33	0,00E+00	3672	60	NA	Indica	
maker-8-augustus-gene-205.0-mRNA-1	8	OsR498G1221669700.01.T01	86,99	2,42E-103	376	Os08g0402700	100,00	1,84E-175	614	238	Japonica	Japonica	
maker-8-augustus-gene-205.11-mRNA-1	8	OsR498G0816040000.01.T02	99,43	0,00E+00	1563	Os08g0402800	100,00	0,00E+00	1456	-107	NA	Japonica	
maker-8-augustus-gene-205.2-mRNA-1	8	OsR498G0816041700.01.T01	100,00	0,00E+00	818	Os08g0403300	100,00	0,00E+00	818	0	NA	Japonica	
maker-8-augustus-gene-205.4-mRNA-1	8	OsR498G0816035400.01.T02	100,00	0,00E+00	3166	Os08g0401500	100,00	0,00E+00	3166	0	NA	Indica	
maker-8-augustus-gene-205.7-mRNA-1	8	OsR498G0816036000.01.T01	97,97	0,00E+00	4425	Os08g0401800	98,44	0,00E+00	4447	22	NA	Indica	
maker-8-augustus-gene-206.0-mRNA-1	8	OsR498G0816044700.01.T01	99,67	0,00E+00	2676	Os08g0404000	100,00	0,00E+00	2695	19	NA	Japonica	
maker-8-augustus-gene-206.5-mRNA-1	8	OsR498G0816046500.01.T01	99,54	0,00E+00	782	Os08g0404300	100,00	0,00E+00	791	9	NA	NA	
maker-8-augustus-gene-206.7-mRNA-1	8	OsR498G0816046500.01.T01	99,54	0,00E+00	782	Os08g0404300	100,00	0,00E+00	791	9	NA	NA	
maker-8-augustus-gene-207.0-mRNA-1	8	OsR498G0816052700.01.T01	98,56	0,00E+00	1086	Os08g0405700	100,00	0,00E+00	1115	29	NA	Japonica	
maker-8-augustus-gene-207.3-mRNA-1	8	OsR498G0816054900.01.T01	99,90	0,00E+00	3546	Os03g0195800	71,01	0,00E+00	837	-2709	Indica	Japonica	
maker-8-augustus-gene-208.11-mRNA-1	8	OsR498G0816059800.01.T01	99,67	0,00E+00	1074	Os08g0407600	100,00	0,00E+00	1083	9	NA	Japonica	
maker-8-augustus-gene-208.3-mRNA-1	8	OsR498G0816057700.01.T04	99,83	0,00E+00	3185	Os08g0407200	100,00	0,00E+00	3198	13	NA	Japonica	
maker-8-augustus-gene-208.6-mRNA-1	8	OsR498G0816060900.01.T01	99,85	0,00E+00	2399	Os08g0408200	100,00	0,00E+00	2408	9	NA	Japonica	
maker-8-augustus-gene-209.0-mRNA-1	8	OsR498G0816064500.01.T01	99,46	0,00E+00	1981	Os02g0753000	79,38	0,00E+00	751	-1230	Indica	Japonica	
maker-8-augustus-gene-209.3-mRNA-1	8	OsR498G0816068700.01.T01	99,80	0,00E+00	2741	Os08g0409900	100,00	0,00E+00	2755	14	NA	Japonica	
maker-8-augustus-gene-21.34-mRNA-1	8	OsR498G0815131100.01.T01	100,00	0,00E+00	1317	Os08g0134900	100,00	0,00E+00	1317	0	NA	Japonica	
maker-8-augustus-gene-21.38-mRNA-1	8	OsR498G0815132500.01.T01	97,15	0,00E+00	1880	Os08g0135400	97,06	0,00E+00	1875	-5	NA	Indica	
maker-8-augustus-gene-21.42-mRNA-1	8	OsR498G0815133100.01.T02	99,72	0,00E+00	5164	Os08g0135800	98,47	0,00E+00	5002	-162	NA	Indica	
maker-8-augustus-gene-21.44-mRNA-1	8	OsR498G0815133300.01.T01	99,93	0,00E+00	2588	Os08g0135900	96,99	0,00E+00	2385	-203	NA	Indica	
maker-8-augustus-gene-21.47-mRNA-1	8	OsR498G0815131800.01.T02	94,18	0,00E+00	1548	Os08g0135100	95,00	0,00E+00	1584	36	NA	Japonica	
maker-8-augustus-gene-21.58-mRNA-1	8	OsR498G0815133600.01.T01	99,49	0,00E+00	4211	Os04g0627000	78,77	0,00E+00	1820	-2391	Indica	Indica	
maker-8-augustus-gene-210.52-mRNA-1	8	OsR498G0816076200.01.T01	99,61	0,00E+00	3172	Os08g0412100	99,27	0,00E+00	5109	1937	Japonica	Japonica	CC-LRR-NBS-TM
maker-8-augustus-gene-210.54-mRNA-1	8	OsR498G0816076400.01.T02	99,85	0,00E+00	1159	Os08g0412200	99,69	0,00E+00	1155	-4	NA	NA	
maker-8-augustus-gene-210.59-mRNA-1	8	OsR498G0816077400.01.T03	100,00	1,67E-74	279	Os08g0412500	99,34	1,05E-71	269	-10	NA	Indica	
maker-8-augustus-gene-211.53-mRNA-1	8	OsR498G0816080700.01.T01	99,80	0,00E+00	1761	Os08g0414200	97,15	0,00E+00	1644	-117	NA	Indica	
maker-8-augustus-gene-211.55-mRNA-1	8	OsR498G0816080900.01.T01	99,62	0,00E+00	939	Os08g0414300	96,57	0,00E+00	867	-72	NA	Indica	
maker-8-augustus-gene-211.59-mRNA-1	8	OsR498G0816078200.01.T01	99,59	0,00E+00	4365	Os08g0413000	98,12	0,00E+00	4393	28	NA	Indica	
maker-8-augustus-gene-212.49-mRNA-1	8	OsR498G0816083200.01.T01	99,61	0,00E+00	4623	Os08g0414700	99,34	0,00E+00	4591	-32	NA	Indica	
maker-8-augustus-gene-212.53-mRNA-1	8	OsR498G0816085600.01.T03	100,00	0,00E+00	964	Os08g0416000	100,00	0,00E+00	946	-18	NA	Japonica	
maker-8-augustus-gene-212.56-mRNA-1	8	OsR498G0816086800.01.T01	98,76	0,00E+00	4513	Os08g0416100	98,72	0,00E+00	4497	-16	NA	Indica	
maker-8-augustus-gene-213.36-mRNA-1	8	OsR498G0816088900.01.T01	99,47	0,00E+00	672	Os08g0416900	100,00	3,86E-178	623	-49	NA	Japonica	
maker-8-augustus-gene-213.41-mRNA-1	8	OsR498G0816092100.01.T01	92,57	0,00E+00	760	Os08g0418600	94,23	0,00E+00	992	232	Japonica	Japonica	
maker-8-augustus-gene-213.43-mRNA-1	8	OsR498G0816092100.01.T01	92,57	0,00E+00	760	Os08g0418600	94,23	0,00E+00	992	232	Japonica	Japonica	
maker-8-augustus-gene-213.45-mRNA-1	8	OsR498G0816092700.01.T02	99,55	0,00E+00	792	Os08g0419000	100,00	0,00E+00	1137	345	Japonica	Japonica	
maker-8-augustus-gene-213.47-mRNA-1	8	OsR498G0816089800.01.T01	99,37	0,00E+00	1683	Os08g0417000	98,73	0,00E+00	1663	-20	NA	Japonica	
maker-8-augustus-gene-214.11-mRNA-1	8	OsR498G0816094900.01.T02	99,32	0,00E+00	777	Os08g0419600	100,00	0,00E+00	767	-10	NA	Japonica	
maker-8-augustus-gene-214.19-mRNA-1	8	OsR498G0816096900.01.T03	97,98	0,00E+00	2818	Os08g0420600	97,92	0,00E+00	2813	-5	NA	NA	
maker-8-augustus-gene-215.36-mRNA-1	8	OsR498G0816098100.01.T01	98,45	0,00E+00	1008	Os08g0420700	98,27	0,00E+00	1003	-5	NA	NA	
maker-8-augustus-gene-215.38-mRNA-1	8	OsR498G0816101500.01.T04	99,55	0,00E+00	1181	Os08g0421900	99,37	0,00E+00	1119	-62	NA	NA	
maker-8-augustus-gene-215.41-mRNA-1	8	OsR498G0816101700.01.T01	100,00	0,00E+00	2212	Os08g0422000	100,00	0,00E+00	2212	0	NA	Japonica	
maker-8-augustus-gene-215.47-mRNA-1	8	OsR498G0816102300.01.T03	100,00	1,07E-156	553	Os08g0422300	100,00	1,39E-124	444	-109	Indica	NA	
maker-8-augustus-gene-215.52-mRNA-1	8	OsR498G0816100400.01.T01	98,22	0,00E+00	2243	Os08g0421800	97,37	0,00E+00	2194	-49	NA	Indica	CC-Kinase-TM Disease Resistance *Pi33 R* gene
maker-8-augustus-gene-216.32-mRNA-1	8	OsR498G0816105900.01.T03	99,88	0,00E+00	1495	Os08g0423500	99,76	0,00E+00	1490	-5	NA	Japonica	
maker-8-augustus-gene-216.34-mRNA-1	8	OsR498G0816107900.01.T01	99,28	0,00E+00	1472	Os08g0423500	95,28	0,00E+00	1313	-159	Indica	NA	
maker-8-augustus-gene-216.36-mRNA-1	8	OsR498G0816108500.01.T01	99,16	0,00E+00	1473	Os08g0424100	99,76	0,00E+00	1496	23	NA	Japonica	
maker-8-augustus-gene-216.38-mRNA-1	8	OsR498G0816109100.01.T03	96,81	0,00E+00	1581	Os08g0424500	96,81	0,00E+00	1581	0	NA	Japonica	
maker-8-augustus-gene-216.44-mRNA-1	8	OsR498G0816104600.01.T02	98,63	0,00E+00	1145	Os01g0259900	96,94	0,00E+00	1090	-55	NA	Japonica	
maker-8-augustus-gene-216.50-mRNA-1	8	OsR498G0816108800.01.T01	99,83	0,00E+00	3087	Os08g0424200	100,00	0,00E+00	1683	-1404	Indica	Japonica	
maker-8-augustus-gene-217.34-mRNA-1	8	OsR498G0816110100.01.T01	98,84	0,00E+00	2889	Os08g0425000	99,09	0,00E+00	2907	18	NA	Japonica	
maker-8-augustus-gene-217.38-mRNA-1	8	OsR498G0816110300.01.T01	99,25	0,00E+00	2839	Os08g0425100	99,44	0,00E+00	2853	14	NA	Japonica	
maker-8-augustus-gene-217.42-mRNA-1	8	OsR498G0816110700.01.T01	99,82	0,00E+00	1008	Os08g0425200	100,00	0,00E+00	1012	4	NA	Japonica	
maker-8-augustus-gene-217.44-mRNA-1	8	OsR498G0816112500.01.T01	98,45	0,00E+00	1682	Os08g0425700	100,00	0,00E+00	1743	61	NA	Japonica	
maker-8-augustus-gene-217.46-mRNA-1	8	OsR498G0816112000.01.T01	99,52	0,00E+00	2241	Os08g0425500	98,65	0,00E+00	2197	-44	NA	NA	
maker-8-augustus-gene-217.48-mRNA-1	8	OsR498G0816112800.01.T01	99,41	0,00E+00	1200	Os08g0425800	100,00	0,00E+00	1218	18	NA	Japonica	
maker-8-augustus-gene-218.38-mRNA-1	8	OsR498G0816115300.01.T02	99,67	0,00E+00	2183	Os08g0426866	100,00	0,00E+00	2201	18	NA	Japonica	
maker-8-augustus-gene-218.43-mRNA-1	8	OsR498G0816115700.01.T04	100,00	0,00E+00	2293	Os08g0427300	100,00	0,00E+00	2293	0	NA	Japonica	
maker-8-augustus-gene-218.48-mRNA-1	8	OsR498G0816115900.01.T04	99,03	0,00E+00	2724	Os08g0427500	100,00	0,00E+00	2764	40	NA	Japonica	
maker-8-augustus-gene-218.54-mRNA-1	8	OsR498G0816116900.01.T01	100,00	0,00E+00	731	Os08g0427700	99,51	0,00E+00	723	-8	NA	Japonica	
maker-8-augustus-gene-218.58-mRNA-1	8	OsR498G0816116200.01.T01	99,80	0,00E+00	1793	Os09g0400500	72,46	0,00E+00	1068	-725	Indica	Japonica	Kinase-LRR-TM
maker-8-augustus-gene-218.61-mRNA-1	8	OsR498G0816117600.01.T02	100,00	0,00E+00	1097	Os08g0427900	95,23	0,00E+00	977	-120	Indica	Japonica	
maker-8-augustus-gene-219.31-mRNA-1	8	OsR498G0816118900.01.T04	99,17	0,00E+00	1710	Os08g0428100	100,00	0,00E+00	931	-779	Indica	Japonica	
maker-8-augustus-gene-219.34-mRNA-1	8	OsR498G0816119300.01.T02	99,68	0,00E+00	1664	Os08g0428200	100,00	0,00E+00	1678	14	NA	Japonica	
maker-8-augustus-gene-219.36-mRNA-1	8	OsR498G0816120800.01.T06	95,43	1,79E-139	496	Os08g0428300	94,34	1,37E-116	417	-79	Indica	NA	
maker-8-augustus-gene-219.38-mRNA-1	8	OsR498G0816121400.01.T01	99,69	0,00E+00	2325	Os08g0428400	98,85	0,00E+00	2285	-40	NA	Japonica	
maker-8-augustus-gene-219.43-mRNA-1	8	OsR498G0816124800.01.T01	99,63	0,00E+00	960	Os08g0428800	100,00	0,00E+00	969	9	NA	Japonica	
maker-8-augustus-gene-219.45-mRNA-1	8	OsR498G0816126000.01.T01	99,76	0,00E+00	8774	Os08g0428900	99,98	0,00E+00	8820	46	NA	Japonica	
maker-8-augustus-gene-22.0-mRNA-1	8	OsR498G0815134800.01.T01	86,01	0,00E+00	969	Os08g0136800	100,00	0,00E+00	1499	530	Japonica	Japonica	
maker-8-augustus-gene-22.12-mRNA-1	8	OsR498G0815136100.01.T01	99,79	0,00E+00	2514	Os08g0137100	82,10	0,00E+00	1120	-1394	Indica	Japonica	
maker-8-augustus-gene-22.15-mRNA-1	8	OsR498G0815136700.01.T02	99,73	0,00E+00	2655	Os08g0137300	99,93	0,00E+00	2669	14	NA	Japonica	
maker-8-augustus-gene-22.17-mRNA-1	8	OsR498G0815136900.01.T01	95,58	0,00E+00	884	Os08g0137400	100,00	0,00E+00	948	64	NA	Japonica	
maker-8-augustus-gene-22.19-mRNA-1	8	OsR498G0815137300.01.T01	98,60	0,00E+00	998	Os08g0137800	100,00	0,00E+00	1034	36	NA	Japonica	
maker-8-augustus-gene-22.2-mRNA-1	8	OsR498G0815135500.01.T01	99,82	0,00E+00	2031	Os08g0137100	99,91	0,00E+00	2036	5	NA	Japonica	
maker-8-augustus-gene-22.21-mRNA-1	8	OsR498G0815134400.01.T01	99,88	0,00E+00	1489	Os08g0136300	100,00	0,00E+00	1494	5	NA	Japonica	
maker-8-augustus-gene-22.23-mRNA-1	8	OsR498G0815134600.01.T01	99,17	0,00E+00	1489	Os08g0136500	99,76	0,00E+00	1512	23	NA	Japonica	
maker-8-augustus-gene-22.25-mRNA-1	8	OsR498G0815134800.01.T01	99,27	0,00E+00	1462	Os08g0136700	100,00	0,00E+00	1489	27	NA	Japonica	
maker-8-augustus-gene-22.7-mRNA-1	8	OsR498G0815135700.01.T01	95,02	0,00E+00	2455	Os08g0137200	95,75	0,00E+00	2273	-182	NA	Japonica	
maker-8-augustus-gene-220.11-mRNA-1	8	OsR498G0816132000.01.T01	99,75	0,00E+00	2877	Os09g0403300	82,20	0,00E+00	1562	-1315	Indica	Japonica	
maker-8-augustus-gene-220.3-mRNA-1	8	OsR498G0816128800.01.T01	98,50	0,00E+00	1158	Os08g0429250	100,00	0,00E+00	1110	-48	NA	Japonica	
maker-8-augustus-gene-221.0-mRNA-1	8	OsR498G0816133300.01.T01	100,00	0,00E+00	1456	Os08g0430100	100,00	0,00E+00	1456	0	NA	Japonica	
maker-8-augustus-gene-221.11-mRNA-1	8	OsR498G0816137900.01.T02	99,47	0,00E+00	4727	Os08g0431200	100,00	0,00E+00	4778	51	NA	Japonica	
maker-8-augustus-gene-221.14-mRNA-1	8	OsR498G0816132600.01.T01	99,86	0,00E+00	4949	Os08g0430000	100,00	0,00E+00	7921	2972	Japonica	Japonica	
maker-8-augustus-gene-221.2-mRNA-1	8	OsR498G0816133900.01.T03	99,75	0,00E+00	2189	Os08g0430200	100,00	0,00E+00	2203	14	NA	Japonica	
maker-8-augustus-gene-221.25-mRNA-1	8	OsR498G0816135200.01.T01	100,00	0,00E+00	1391	Os08g0430500	99,87	0,00E+00	1387	-4	NA	Japonica	
maker-8-augustus-gene-221.28-mRNA-1	8	OsR498G0816135600.01.T04	99,58	0,00E+00	857	Os08g0430600	99,79	0,00E+00	862	5	NA	Japonica	
maker-8-augustus-gene-221.31-mRNA-1	8	OsR498G0816135800.01.T01	99,83	0,00E+00	2134	Os08g0430700	100,00	0,00E+00	2143	9	NA	Japonica	
maker-8-augustus-gene-221.36-mRNA-1	8	OsR498G0816138200.01.T02	99,86	0,00E+00	2567	Os08g0431300	99,93	0,00E+00	2571	4	NA	Japonica	
maker-8-augustus-gene-221.6-mRNA-1	8	OsR498G0816137300.01.T01	99,27	0,00E+00	3618	Os08g0431100	99,67	0,00E+00	3200	-418	Indica	NA	
maker-8-augustus-gene-221.9-mRNA-1	8	OsR498G0816137700.01.T01	100,00	4,56E-129	461	Os09g0408500	82,92	9,38E-63	239	-222	Indica	Japonica	
maker-8-augustus-gene-222.16-mRNA-1	8	OsR498G0816140400.01.T04	99,62	7,45E-131	467	Os08g0431900	99,23	3,18E-130	462	-5	NA	NA	
maker-8-augustus-gene-222.21-mRNA-1	8	OsR498G0816144000.01.T01	99,80	0,00E+00	878	Os08g0432500	100,00	0,00E+00	883	5	NA	Indica	
maker-8-augustus-gene-222.25-mRNA-1	8	OsR498G0816145600.01.T01	97,64	0,00E+00	1812	Os08g0432800	99,62	0,00E+00	1855	43	NA	Indica	
maker-8-augustus-gene-223.39-mRNA-1	8	OsR498G0816148700.01.T03	99,68	2,50E-159	563	Os08g0433200	100,00	3,70E-158	556	-7	NA	Indica	
maker-8-augustus-gene-223.41-mRNA-1	8	OsR498G0816150900.01.T05	99,88	0,00E+00	3070	Os08g0434000	99,65	0,00E+00	3052	-18	NA	Indica	
maker-8-augustus-gene-223.42-mRNA-1	8	OsR498G0816151100.01.T01	99,71	0,00E+00	1236	Os08g0434100	99,28	0,00E+00	1223	-13	NA	Indica	
maker-8-augustus-gene-223.44-mRNA-1	8	OsR498G0816148400.01.T03	99,71	0,00E+00	1252	Os08g0433100	99,82	0,00E+00	986	-266	Indica	NA	
maker-8-augustus-gene-223.50-mRNA-1	8	OsR498G0816149200.01.T05	100,00	0,00E+00	1938	Os08g0433400	100,00	0,00E+00	1938	0	NA	Indica	

maker-8-augustus-gene-223.52-mRNA-1	8	OsR498G0816149600.01.T01	95,86	0,00E+00	1666	Os08g0433500	99,21	0,00E+00	1781	115	NA	NA	
maker-8-augustus-gene-225.31-mRNA-1	8	OsR498G0816157500.01.T01	99,74	0,00E+00	1380	Os08g0435700	99,64	0,00E+00	1984	604	Japonica	Indica	
maker-8-augustus-gene-225.34-mRNA-1	8	OsR498G0816159500.01.T04	96,14	0,00E+00	1939	Os08g0436100	95,74	0,00E+00	1816	-123	NA	Indica	
maker-8-augustus-gene-225.43-mRNA-1	8	OsR498G0816161300.01.T02	100,00	0,00E+00	2089	Os08g0436700	99,91	0,00E+00	2085	-4	NA	NA	
maker-8-augustus-gene-225.45-mRNA-1	8	OsR498G0816158800.01.T02	99,75	0,00E+00	1458	Os08g0435800	99,88	0,00E+00	1462	4	NA	Indica	
maker-8-augustus-gene-225.48-mRNA-1	8	OsR498G0816159000.01.T01	99,73	0,00E+00	1317	Os08g0435900	98,91	0,00E+00	1290	-27	NA	Indica	
maker-8-augustus-gene-225.51-mRNA-1	8	OsR498G0816159200.01.T01	99,80	0,00E+00	1795	Os08g0436000	99,70	0,00E+00	1791	-4	NA	Indica	
maker-8-augustus-gene-225.57-mRNA-1	8	OsR498G0816162000.01.T01	99,44	0,00E+00	636	Os08g0436800	99,72	0,00E+00	640	4	NA	NA	
maker-8-augustus-gene-226.22-mRNA-1	8	OsR498G0816162900.01.T01	99,89	0,00E+00	1673	Os08g0437200	99,57	0,00E+00	1663	-10	NA	NA	
maker-8-augustus-gene-226.24-mRNA-1	8	OsR498G0816166700.01.T02	99,89	0,00E+00	3193	Os08g0438600	99,71	0,00E+00	3046	-147	NA	Indica	
maker-8-augustus-gene-227.0-mRNA-1	8	OsR498G0816168300.01.T02	99,84	0,00E+00	3397	Os08g0439100	99,89	0,00E+00	3402	5	NA	Japonica	
maker-8-augustus-gene-227.20-mRNA-1	8	OsR498G0816170600.01.T03	99,82	0,00E+00	977	Os08g0440000	100,00	0,00E+00	975	-2	NA	Indica	
maker-8-augustus-gene-228.34-mRNA-1	8	OsR498G0816174900.01.T01	100,00	0,00E+00	2706	Os08g0440800	99,93	0,00E+00	2701	-5	NA	Indica	
maker-8-augustus-gene-228.38-mRNA-1	8	OsR498G0816180100.01.T02	98,12	0,00E+00	641	Os08g0441400	100,00	0,00E+00	672	31	NA	Japonica	
maker-8-augustus-gene-228.40-mRNA-1	8	OsR498G0816182700.01.T01	99,77	0,00E+00	1534	Os08g0441600	100,00	0,00E+00	1543	9	NA	Japonica	
maker-8-augustus-gene-228.52-mRNA-1	8	OsR498G0816180400.01.T01	96,19	0,00E+00	1874	Os08g0441500	96,28	0,00E+00	1879	5	NA	Japonica	
maker-8-augustus-gene-228.59-mRNA-1	8	OsR498G0816183400.01.T03	99,46	0,00E+00	1312	Os08g0442000	100,00	0,00E+00	1330	18	NA	Japonica	
maker-8-augustus-gene-229.39-mRNA-1	8	OsR498G0816183900.01.T01	87,90	0,00E+00	1000	Os08g0442300	100,00	0,00E+00	953	-47	NA	Japonica	
maker-8-augustus-gene-229.43-mRNA-1	8	OsR498G0816183900.01.T05	99,00	0,00E+00	2633	Os08g0442350	100,00	0,00E+00	2700	67	NA	Japonica	
maker-8-augustus-gene-229.45-mRNA-1	8	OsR498G0816184900.01.T01	98,66	0,00E+00	2212	Os08g0442400	99,29	0,00E+00	2236	24	NA	Japonica	
maker-8-augustus-gene-229.51-mRNA-1	8	OsR498G0816183800.01.T01	97,27	0,00E+00	1789	Os08g0442200	100,00	0,00E+00	1900	111	NA	Japonica	
maker-8-augustus-gene-229.54-mRNA-1	8	OsR498G0816185600.01.T01	99,85	0,00E+00	3661	Os08g0442700	100,00	0,00E+00	3674	13	NA	Japonica	Kinase-LRR-TM
maker-8-augustus-gene-23.40-mRNA-1	8	OsR498G0815138700.01.T01	99,83	0,00E+00	1030	Os08g0138400	100,00	0,00E+00	1034	4	NA	Japonica	
maker-8-augustus-gene-23.42-mRNA-1	8	OsR498G0815139000.01.T01	97,38	0,00E+00	1297	Os08g0138500	100,00	0,00E+00	1375	78	NA	Japonica	
maker-8-augustus-gene-23.49-mRNA-1	8	OsR498G0815140000.01.T01	99,73	0,00E+00	667	Os08g0138900	100,00	0,00E+00	672	5	NA	Japonica	
maker-8-augustus-gene-23.51-mRNA-1	8	OsR498G0815140200.01.T02	99,67	1,14E-151	537	Os08g0139000	100,00	9,32E-154	542	5	NA	Japonica	
maker-8-augustus-gene-23.54-mRNA-1	8	OsR498G0815140400.01.T03	98,06	0,00E+00	890	Os08g0139100	100,00	0,00E+00	931	41	NA	Japonica	
maker-8-augustus-gene-23.57-mRNA-1	8	OsR498G0815140600.01.T01	100,00	0,00E+00	1104	Os08g0139200	100,00	0,00E+00	1104	0	NA	Japonica	
maker-8-augustus-gene-23.60-mRNA-1	8	OsR498G0815141800.01.T01	98,97	0,00E+00	681	Os06g0696900	95,14	0,00E+00	920	239	Japonica	Japonica	
maker-8-augustus-gene-230.0-mRNA-1	8	OsR498G0816196900.01.T01	99,88	0,00E+00	4451	Os08g0445700	99,76	0,00E+00	4437	-14	NA	Indica	
maker-8-augustus-gene-231.32-mRNA-1	8	OsR498G0816199700.01.T01	100,00	0,00E+00	1992	Os03g0643100	99,76	0,00E+00	2227	235	Japonica	NA	
maker-8-augustus-gene-231.39-mRNA-1	8	OsR498G0816202800.01.T01	99,79	0,00E+00	3348	Os06g0655100	72,03	0,00E+00	942	-2406	Indica	Indica	
maker-8-augustus-gene-231.42-mRNA-1	8	OsR498G0816204200.01.T01	99,95	0,00E+00	3578	Os08g0447200	99,60	0,00E+00	3546	-32	NA	Indica	
maker-8-augustus-gene-232.51-mRNA-1	8	OsR498G0816205500.01.T02	100,00	0,00E+00	2516	Os08g0447900	99,79	0,00E+00	2503	-13	NA	Indica	
maker-8-augustus-gene-232.53-mRNA-1	8	OsR498G0816204600.01.T04	95,84	0,00E+00	1227	Os08g0447300	96,25	0,00E+00	1550	323	Japonica	Indica	
maker-8-augustus-gene-232.62-mRNA-1	8	OsR498G0816205800.01.T02	99,32	0,00E+00	2873	Os08g0448000	99,88	0,00E+00	2913	40	NA	Indica	
maker-8-augustus-gene-233.35-mRNA-1	8	OsR498G0816217100.01.T03	99,91	0,00E+00	2025	Os08g0451000	100,00	0,00E+00	2030	5	NA	Japonica	
maker-8-augustus-gene-233.43-mRNA-1	8	OsR498G0816218600.01.T06	99,68	0,00E+00	2831	Os08g0451400	99,87	0,00E+00	2845	14	NA	Japonica	
maker-8-augustus-gene-234.0-mRNA-1	8	OsR498G0816220100.01.T01	99,33	0,00E+00	1063	Os08g0451800	100,00	0,00E+00	1710	647	Japonica	Japonica	
maker-8-augustus-gene-234.2-mRNA-1	8	OsR498G0816222500.01.T01	97,08	0,00E+00	691	Os08g0452500	100,00	0,00E+00	726	35	NA	Japonica	
maker-8-augustus-gene-235.0-mRNA-1	8	OsR498G0816226700.01.T01	98,31	0,00E+00	717	Os09g0437500	75,46	7,66E-79	294	-423	Indica	Japonica	
maker-8-augustus-gene-235.19-mRNA-1	8	OsR498G0816230800.01.T01	99,03	0,00E+00	722	Os08g0454500	100,00	0,00E+00	1359	637	Japonica	Japonica	
maker-8-augustus-gene-235.2-mRNA-1	8	OsR498G0816228700.01.T01	99,86	0,00E+00	2585	Os08g0453700	100,00	0,00E+00	2592	7	NA	Japonica	
maker-8-augustus-gene-237.0-mRNA-1	8	OsR498G0816240500.01.T01	99,81	0,00E+00	3847	Os08g0457600	100,00	0,00E+00	3865	18	NA	Japonica	
maker-8-augustus-gene-237.7-mRNA-1	8	OsR498G0816242700.01.T01	99,36	0,00E+00	1101	Os08g0458200	100,00	0,00E+00	1115	14	NA	Japonica	
maker-8-augustus-gene-237.9-mRNA-1	8	OsR498G0816238400.01.T01	99,86	0,00E+00	2583	Os08g0457400	100,00	0,00E+00	2592	9	NA	Japonica	Kinase
maker-8-augustus-gene-238.42-mRNA-1	8	OsR498G0816247500.01.T01	99,83	0,00E+00	2129	Os08g0459600	100,00	0,00E+00	2138	9	NA	Japonica	
maker-8-augustus-gene-238.46-mRNA-1	8	OsR498G0816244400.01.T01	99,13	0,00E+00	3256	Os08g0458600	100,00	0,00E+00	3328	72	NA	Japonica	
maker-8-augustus-gene-238.48-mRNA-1	8	OsR498G0816246400.01.T01	99,38	0,00E+00	3407	Os08g0459100	100,00	0,00E+00	3458	51	NA	Japonica	
maker-8-augustus-gene-239.12-mRNA-1	8	OsR498G0816254600.01.T02	82,64	0,00E+00	1898	Os08g0460900	97,09	0,00E+00	2973	1075	Japonica	Japonica	
maker-8-augustus-gene-239.14-mRNA-1	8	OsR498G0816253400.01.T03	99,19	0,00E+00	1084	Os08g0461000	100,00	0,00E+00	1487	403	Japonica	Japonica	
maker-8-augustus-gene-239.15-mRNA-1	8	OsR498G0816253600.01.T01	99,69	0,00E+00	2277	Os08g0461100	97,13	0,00E+00	3079	802	Japonica	Japonica	
maker-8-augustus-gene-239.21-mRNA-1	8	OsR498G0816254600.01.T02	99,67	0,00E+00	3267	Os08g0461300	100,00	0,00E+00	2336	-931	Indica	Japonica	
maker-8-augustus-gene-239.24-mRNA-1	8	OsR498G0816254800.01.T01	99,70	2,21E-167	590	Os08g0461400	100,00	0,00E+00	715	125	Japonica	Japonica	
maker-8-augustus-gene-239.26-mRNA-1	8	OsR498G0816255200.01.T04	99,57	0,00E+00	1241	Os08g0461700	100,00	0,00E+00	1218	-23	NA	Japonica	
maker-8-augustus-gene-239.3-mRNA-1	8	OsR498G0816252400.01.T01	99,28	0,00E+00	1957	Os08g0460700	100,00	0,00E+00	1462	-495	Indica	Japonica	
maker-8-augustus-gene-239.31-mRNA-1	8	OsR498G0816255600.01.T03	99,64	0,00E+00	2472	Os08g0461800	100,00	0,00E+00	1283	-1189	Indica	Japonica	
maker-8-augustus-gene-239.6-mRNA-1	8	OsR498G0816252800.01.T02	99,60	0,00E+00	3112	Os08g0461100	83,58	0,00E+00	1968	-1144	Indica	Japonica	
maker-8-augustus-gene-24.10-mRNA-1	8	OsR498G0815148400.01.T01	99,71	0,00E+00	3122	Os05g0331200	72,39	0,00E+00	870	-2252	Indica	Japonica	
maker-8-augustus-gene-24.5-mRNA-1	8	OsR498G0815146600.01.T01	98,51	0,00E+00	1167	Os08g0140700	83,33	0,00E+00	675	-492	Indica	Japonica	
maker-8-augustus-gene-240.0-mRNA-1	8	OsR498G0816257100.01.T01	99,09	0,00E+00	2124	Os08g0462700	100,00	0,00E+00	2154	30	NA	Japonica	
maker-8-augustus-gene-240.10-mRNA-1	8	OsR498G0816260000.01.T01	99,91	0,00E+00	1928	Os08g0464000	100,00	0,00E+00	1932	4	NA	Japonica	
maker-8-augustus-gene-240.3-mRNA-1	8	OsR498G0816259500.01.T02	100,00	0,00E+00	1018	Os08g0463900	100,00	0,00E+00	1018	0	NA	Japonica	
maker-8-augustus-gene-240.7-mRNA-1	8	OsR498G0816256800.01.T01	99,68	0,00E+00	2250	Os08g0462300	99,92	0,00E+00	2263	13	NA	Japonica	
maker-8-augustus-gene-240.9-mRNA-1	8	OsR498G0816257600.01.T01	99,89	0,00E+00	1668	Os08g0462900	100,00	0,00E+00	1673	5	NA	Japonica	
maker-8-augustus-gene-241.4-mRNA-1	8	OsR498G0816265000.01.T02	99,80	0,00E+00	2703	Os08g0465800	100,00	0,00E+00	2717	14	NA	Japonica	
maker-8-augustus-gene-242.13-mRNA-1	8	OsR498G0816272000.01.T05	94,52	0,00E+00	1801	Os08g0467400	94,05	0,00E+00	1801	0	NA	Japonica	
maker-8-augustus-gene-242.7-mRNA-1	8	OsR498G0816272100.01.T03	100,00	0,00E+00	726	Os08g0467500	100,00	1,09E-125	448	-278	Indica	Japonica	
maker-8-augustus-gene-243.0-mRNA-1	8	OsR498G0816274900.01.T01	99,93	0,00E+00	4953	Os08g0468100	100,00	0,00E+00	4962	9	NA	Japonica	
maker-8-augustus-gene-243.11-mRNA-1	8	OsR498G0816279200.01.T01	99,30	0,00E+00	2287	Os09g0454300	77,67	0,00E+00	1013	-1274	Indica	Japonica	
maker-8-augustus-gene-243.3-mRNA-1	8	OsR498G0816275500.01.T01	99,94	0,00E+00	3188	Os08g0468400	100,00	0,00E+00	2455	-733	Indica	Japonica	
maker-8-augustus-gene-243.6-mRNA-1	8	OsR498G0816279900.01.T03	99,88	0,00E+00	1549	Os08g0469700	100,00	0,00E+00	1420	-129	NA	Japonica	
maker-8-augustus-gene-244.32-mRNA-1	8	OsR498G0816282000.01.T01	99,88	0,00E+00	1489	Os08g0423500	73,00	5,44E-108	390	-1099	Indica	Japonica	
maker-8-augustus-gene-244.34-mRNA-1	8	OsR498G0816284600.01.T01	99,76	0,00E+00	1463	Os08g0470700	100,00	0,00E+00	921	-542	Indica	Japonica	
maker-8-augustus-gene-244.37-mRNA-1	8	OsR498G0816285600.01.T01	99,13	0,00E+00	2021	Os09g0455200	71,31	4,78E-142	504	-1517	Indica	Japonica	
maker-8-augustus-gene-245.25-mRNA-1	8	OsR498G0816288300.01.T04	95,98	0,00E+00	2579	Os08g0471800	97,31	0,00E+00	2663	84	NA	Japonica	
maker-8-augustus-gene-245.33-mRNA-1	8	OsR498G0816291500.01.T01	100,00	0,00E+00	1615	Os08g0472600	100,00	0,00E+00	1615	0	NA	Japonica	
maker-8-augustus-gene-245.39-mRNA-1	8	OsR498G0816288800.01.T01	96,41	0,00E+00	2491	Os08g0471900	96,75	0,00E+00	2516	25	NA	Japonica	
maker-8-augustus-gene-246.3-mRNA-1	8	OsR498G0816294500.01.T01	99,85	0,00E+00	2361	Os08g0473600	100,00	0,00E+00	2370	9	NA	Japonica	
maker-8-augustus-gene-246.5-mRNA-1	8	OsR498G0816295500.01.T01	99,65	0,00E+00	2559	Os08g0473900	100,00	0,00E+00	2581	22	NA	Japonica	
maker-8-augustus-gene-246.7-mRNA-1	8	OsR498G0816297500.01.T01	98,57	0,00E+00	852	Os08g0474100	100,00	0,00E+00	836	-16	NA	Japonica	
maker-8-augustus-gene-246.8-mRNA-1	8	OsR498G0816292400.01.T01	99,22	0,00E+00	2718	Os08g0472800	100,00	0,00E+00	2765	47	NA	Japonica	
maker-8-augustus-gene-247.42-mRNA-1	8	OsR498G0816298700.01.T04	99,63	0,00E+00	1907	Os08g0474700	99,91	0,00E+00	1920	13	NA	NA	
maker-8-augustus-gene-247.57-mRNA-1	8	OsR498G0917631500.01.T03	76,76	3,94E-75	283	Os08g0476100	100,00	0,00E+00	774	491	Japonica	Japonica	
maker-8-augustus-gene-247.59-mRNA-1	8	OsR498G0816303100.01.T02	99,41	0,00E+00	1210	Os08g0476300	100,00	0,00E+00	1148	-62	NA	Japonica	
maker-8-augustus-gene-248.43-mRNA-1	8	OsR498G0816307900.01.T02	99,38	0,00E+00	2581	Os08g0477800	99,61	0,00E+00	2276	-305	Indica	Japonica	
maker-8-augustus-gene-248.50-mRNA-1	8	OsR498G0816308300.01.T01	99,85	0,00E+00	1208	Os08g0477900	100,00	0,00E+00	1213	5	NA	Japonica	
maker-8-augustus-gene-248.52-mRNA-1	8	OsR498G0816305600.01.T01	98,14	0,00E+00	2224	Os08g0476900	100,00	0,00E+00	2333	109	NA	Japonica	
maker-8-augustus-gene-248.54-mRNA-1	8	OsR498G0816306000.01.T01	97,53	0,00E+00	2195	Os08g0477100	100,00	0,00E+00	2338	143	NA	Japonica	
maker-8-augustus-gene-248.56-mRNA-1	8	OsR498G0816307000.01.T03	99,84	0,00E+00	2188	Os03g0393900	70,38	1,12E-99	364	-1824	Indica	Japonica	
maker-8-augustus-gene-248.61-mRNA-1	8	OsR498G0816307200.01.T02	99,46	0,00E+00	1308	Os08g0477600	100,00	0,00E+00	1326	18	NA	Japonica	
maker-8-augustus-gene-248.65-mRNA-1	8	OsR498G0816309000.01.T04	99,87	0,00E+00	1331	Os08g0478100	100,00	0,00E+00	1335	4	NA	Japonica	
maker-8-augustus-gene-249.32-mRNA-1	8	OsR498G0816314700.01.T01	98,28	0,00E+00	2009	Os08g0479300	99,91	0,00E+00	2074	65	NA	Japonica	
maker-8-augustus-gene-249.36-mRNA-1	8	OsR498G0816310400.01.T01	99,90	0,00E+00	5284	Os08g0478500	100,00	0,00E+00	5297	13	NA	Japonica	
maker-8-augustus-gene-249.42-mRNA-1	8	OsR498G0816313600.01.T05	98,24	0,00E+00	1875	Os08g0478800	98,52	0,00E+00	1889	14	NA	Japonica	
maker-8-augustus-gene-25.38-mRNA-1	8	OsR498G0815151700.01.T01	99,55	0,00E+00	3168	Os08g0143300	100,00	0,00E+00	3204	36	NA	Japonica	
maker-8-augustus-gene-25.40-mRNA-1	8	OsR498G0815152100.01.T01	99,93	0,00E+00	2523	Os08g0143500	100,00	0,00E+00	2527	4	NA	Japonica	
maker-8-augustus-gene-25.45-mRNA-1	8	OsR498G0815154900.01.T01	100,00	0,00E+00	2078	Os08g0144000	100,00	0,00E+00	2078	0	NA	NA	
maker-8-augustus-gene-25.47-mRNA-1	8	OsR498G0815152000.01.T01	99,35	0,00E+00	4066	Os08g0143400	99,96	0,00E+00	4130	64	NA	Japonica	
maker-8-augustus-gene-25.49-mRNA-1	8	OsR498G0815153200.01.T01	99,72	0,00E+00	1886	Os08g0143700	100,00	0,00E+00	1900	14	NA	Japonica	
maker-8-augustus-gene-250.27-mRNA-1	8	OsR498G0816319500.01.T04	100,00	0,00E+00	2195	Os08g0480000	100,00	0,00E+00	2195	0	NA	NA	
maker-8-augustus-gene-250.32-mRNA-1	8	OsR498G0816321900.01.T01	100,00	0,00E+00	1434	Os08g0480800	100,00	0,00E+00	1434	0	NA	NA	
maker-8-augustus-gene-250.44-mRNA-1	8	OsR498G0816320000.01.T02	100,00	0,00E+00	1829	Os08g0480200	99,90	0,00E+00	1825	-4	NA	NA	
maker-8-augustus-gene-250.46-mRNA-1	8	OsR498G0816320800.01.T01	100,00	0,00E+00	1445	Os09g0471000	80,56	1,50E-165	581	-864	Indica	NA	
maker-8-augustus-gene-250.49-mRNA-1	8	OsR498G0816321400.01.T04	100,00	0,00E+00	807	Os08g0480500	100,00	0,00E+00	969	162	Japonica	NA	
maker-8-augustus-gene-250.52-mRNA-1	8	OsR498G0816322600.01.T01	100,00	0,00E+00	3052	Os08g0481000	99,94	0,00E+00	3048	-4	NA	NA	
maker-8-augustus-gene-251.0-mRNA-1	8	OsR498G0816327100.01.T02	100,00	0,00E+00	2960	Os02g0572400	78,84	0,00E+00	1027	-1933	Indica	NA	
maker-8-augustus-gene-251.10-mRNA-1	8	OsR498G0816324600.01.T01	100,00	0,00E+00	3479	Os08g0481200	100,00	0,00E+00	3479	0	NA	NA	
maker-8-augustus-gene-251.12-mRNA-1	8	OsR498G0816325400.01.T01	100,00	0,00E+00	1462	Os08g0481400	100,00	0,00E+00	1462	0	NA	NA	
maker-8-augustus-gene-251.17-mRNA-1	8	OsR498G0816328200.01.T01	100,00	0,00E+00	937	Os08g0482600	100,00	0,00E+00	937	0	NA	NA	
maker-8-augustus-gene-251.19-mRNA-1	8	OsR498G0816328800.01.T01	100,00	0,00E+00	985	Os08g0482700	100,00	0,00E+00	985	0	NA	NA	
maker-8-augustus-gene-251.21-mRNA-1	8	OsR498G0816331200.01.T01	100,00	0,00E+00	1169	Os08g0483100	100,00	0,00E+00	1169	0	NA	NA	
maker-8-augustus-gene-251.23-mRNA-1	8	OsR498G0816331600.01.T03	100,00	0,00E+00	1462	Os08g0483200	100,00	0,00E+00	1462	0	NA	NA	
maker-8-augustus-gene-251.3-mRNA-1	8	OsR498G0816327300.01.T06	100,00	0,00E+00	4785	Os08g0482100	100,00	0,00E+00	4596	-189	NA	NA	
maker-8-augustus-gene-252.11-mRNA-1	8	OsR498G0816337900.01.T01	96,43	0,00E+00	1544	Os08g0484800	100,00	0,00E+00	1911	367	Japonica	NA	
maker-8-augustus-gene-252.16-mRNA-1	8	OsR498G0816333400.01.T02	100,00	0,00E+00	1970	Os08g0483900	100,00	0,00E+00	1970	0	NA	NA	
maker-8-augustus-gene-252.25-mRNA-1	8	OsR498G0816336600.01.T01	100,00	0,00E+00	2738	Os08g0484500	100,00	0,00E+00	2738	0	NA	NA	
maker-8-augustus-gene-252.4-mRNA-1	8	OsR498G0816337100.01.T01	100,00	0,00E+00	2760	Os08g0484600	100,00	0,00E+00	2760	0	NA	NA	Kinase-TM
maker-8-augustus-gene-253.0-mRNA-1	8	OsR498G0816340500.01.T03	99,88	0,00E+00	1468	Os08g0485400	99,30	0,00E+00	1266	-202	Indica	NA	
maker-8-augustus-gene-253.10-mRNA-1	8	OsR498G0816342700.01.T04	100,00	0,00E+00	3934	Os08g0485700	100,00	0,00E+00	2172	-1762	Indica	NA	
maker-8-augustus-gene-253.28-mRNA-1	8	OsR498G0816347700.01.T01	100,00	2,41E-146	518	Os08g0487000	100,00	8,40E-147	518	0	NA	Japonica	
maker-8-augustus-gene-253.31-mRNA-1	8	OsR498G0816343400.01.T02	100,00	0,00E+00	863	Os08g0485900	100,00	0,00E+00	863	0	NA	NA	
maker-8-augustus-gene-253.36-mRNA-1	8	OsR498G0816344600.01.T06	100,00	0,00E+00	1615	Os08g0486200	100,00	0,00E+00	1561	-54	NA	NA	
maker-8-augustus-gene-253.38-mRNA-1	8	OsR498G0816345400.01.T01	100,00	0,00E+00	1938	Os08g0486300	100,00	0,00E+00	1938	0	NA	NA	
maker-8-augustus-gene-253.4-mRNA-1	8	OsR498G0816341300.01.T01	99,66	0,00E+00	1050	Os08g0485500	100,00	0,00E+00	634	-416	Indica	NA	
maker-8-augustus-gene-253.6-mRNA-1	8	OsR498G0816342100.01.T01	100,00	0,00E+00	3703	Os08g0485600	100,00	0,00E+00	3703	0	NA	NA	
maker-8-augustus-gene-254.0-mRNA-1	8	OsR498G0816351900.01.T01	100,00	0,00E+00	1981	Os08g0487700	100,00	0,00E+00	1981	0	NA	NA	
maker-8-augustus-gene-254.10-mRNA-1	8	OsR498G0816352400.01.T01	100,00	0,00E+00	2246	Os08g0487900	100,00	0,00E+00	2246	0	NA	NA	
maker-8-augustus-gene-254.2-mRNA-1	8	OsR498G0816353700.01.T01	93,94	0,00E+00	833	Os08g0488700	100,00	0,00E+00	841	8	NA	NA	
maker-8-augustus-gene-254.5-mRNA-1	8	OsR498G0816352200.01.T02	99,96	0,00E+00	4249	Os08g0487800	99,96	0,00E+00	4249	0	NA	NA	
maker-8-augustus-gene-255.31-mRNA-1	8	OsR498G0816355900.01.T01	96,18	0,00E+00	1695	Os08g0489300	96,18	0,00E+00	1695	0	NA	NA	
maker-8-augustus-gene-255.37-mRNA-1	8	OsR498G0816359300.01.T01	100,00	0,00E+00	2755	Os08g0490000	100,00	0,00E+00	2755	0	NA	NA	
maker-8-augustus-gene-256.55-mRNA-1	8	OsR498G0917686300.01.T03	81,94	0,00E+00	1651	Os08g0491200	100,00	0,00E+00	3220	1569	Japonica	NA	Kinase-TM
maker-8-augustus-gene-256.60-mRNA-1	8	OsR498G0816366500.01.T02	100,00	0,00E+00	4075	Os08g0492000	100,00	0,00E+00	4075	0	NA	NA	
maker-8-augustus-gene-256.66-mRNA-1	8	OsR498G0816370100.01.T01	100,00	0,00E+00	1550	Os08g0493100	100,00	0,00E+00	1550	0	NA	NA	
maker-8-augustus-gene-257.41-mRNA-1	8	OsR498G0816372500.01.T01	100,00	0,00E+00	2408	Os08g0493900	100,00	0,00E+00	2408	0	NA	NA	
maker-8-augustus-gene-257.55-mRNA-1	8	OsR498G0816378300.01.T01	100,00	0,00E+00	1867	Os08g0494400	91,05	0,00E+00	892	-975	Indica	NA	
maker-8-augustus-gene-257.66-mRNA-1	8	OsR498G0816373600.01.T02	100,00	0,00E+00	2149	Os08g0494300	100,00	0,00E+00	2149	0	NA	NA	
maker-8-augustus-gene-258.15-mRNA-1	8	OsR498G0816385500.01.T02	100,00	0,00E+00	2241	Os08g0496800	99,76	0,00E+00	2225	-16	NA	NA	
maker-8-augustus-gene-258.23-mRNA-1	8	OsR498G0816386000.01.T01	100,00	0,00E+00	3402	Os08g0496900	100,00	0,00E+00	3402	0	NA	NA	
maker-8-augustus-gene-259.41-mRNA-1	8	OsR498G0816389500.01.T01	97,69	0,00E+00	2231	Os08g0497600	97,61	0,00E+00	2226	-5	NA	Japonica	
maker-8-augustus-gene-259.46-mRNA-1	8	OsR498G0816394900.01.T01	100,00	0,00E+00	5321	Os08g0499100	100,00	0,00E+00	5321	0	NA	NA	
maker-8-augustus-gene-259.48-mRNA-1	8	OsR498G0816395300.01.T04	100,00	0,00E+00	2502	Os08g0499200	100,00	0,00E+00	2502	0	NA	NA	
maker-8-augustus-gene-259.59-mRNA-1	8	OsR498G0816389800.01.T01	100,00	0,00E+00	2506	Os08g0497900	100,00	0,00E+00	2506	0	NA	NA	
maker-8-augustus-gene-259.61-mRNA-1	8	OsR498G0816390800.01.T01	100,00	0,00E+00	1370	Os08g0498100	100,00	0,00E+00	1370	0	NA	NA	
maker-8-augustus-gene-259.63-mRNA-1	8	OsR498G0816391800.01.T03	99,76	0,00E+00	1501	Os08g0498400	99,76	0,00E+00	1501	0	NA	NA	
maker-8-augustus-gene-259.66-mRNA-1	8	OsR498G0816392000.01.T01	100,00	0,00E+00	1272	Os08g0498600	100,00	0,00E+00	1272	0	NA	NA	
maker-8-augustus-gene-259.68-mRNA-1	8	OsR498G0816395800.01.T07	100,00	0,00E+00	3653	Os08g0499300	100,00	0,00E+00	3653	0	NA	NA	
maker-8-augustus-gene-26.1-mRNA-1	8	OsR498G0815163200.01.T07	99,93	0,00E+00	2681	Os08g0144400	99,93	0,00E+00	2681	0	NA	NA	
maker-8-augustus-gene-260.0-mRNA-1	8	OsR498G0816398100.01.T01	100,00	0,00E+00	3355	Os08g0499900	100,00	0,00E+00	2396	-959	Indica	Indica	LRR-TM
maker-8-augustus-gene-260.16-mRNA-1	8	OsR498G0816402500.01.T04	100,00	0,00E+00	1575	Os08g0500900	100,00	0,00E+00	1575	0	NA	NA	
maker-8-augustus-gene-260.19-mRNA-1	8	OsR498G0816398400.01.T02	100,00	0,00E+00	928	Os08g0500000	100,00	0,00E+00	1095	167	Japonica	NA	
maker-8-augustus-gene-260.2-mRNA-1	8	OsR498G0816398500.01.T04	98,64	0,00E+00	3870	Os08g0500100	98,60	0,00E+00	3865	-5	NA	NA	
maker-8-augustus-gene-260.23-mRNA-1	8	OsR498G0816399000.01.T01	100,00	0,00E+00	2782	Os08g0500200	100,00	0,00E+00	2782	0	NA	NA	
maker-8-augustus-gene-260.26-mRNA-1	8	OsR498G0816399200.01.T02	100,00	0,00E+00	2879	Os08g0500300	100,00	0,00E+00	2879	0	NA	Indica	
maker-8-augustus-gene-260.29-mRNA-1	8	OsR498G0816402000.01.T01	100,00	0,00E+00	3788	Os08g0500700	100,00	0,00E+00	3561	-227	NA	NA	
maker-8-augustus-gene-260.33-mRNA-1	8	OsR498G0816405000.01.T01	99,67	0,00E+00	2149	Os08g0501500	100,00	0,00E+00	2170	21	NA	Japonica	Kinase-TM
maker-8-augustus-gene-261.0-mRNA-1	8	OsR498G0816405300.01.T01	100,00	0,00E+00	2181	Os08g0501600	98,56	0,00E+00	4016	1835	Japonica	Japonica	Kinase-TM
maker-8-augustus-gene-261.13-mRNA-1	8	OsR498G0816407600.01.T02	100,00	0,00E+00	2296	Os08g0502000	100,00	0,00E+00	2296	0	NA	NA	
maker-8-augustus-gene-261.19-mRNA-1	8	OsR498G0816409800.01.T02	100,00	0,00E+00	2181	Os08g0502700	100,00	0,00E+00	2181	0	NA	NA	
maker-8-augustus-gene-261.3-mRNA-1	8	OsR498G0816405900.01.T01	99,86	0,00E+00	3866	Os08g0501700	100,00	0,00E+00	2679	-1187	Indica	NA	Kinase-TM
maker-8-augustus-gene-261.6-mRNA-1	8	OsR498G0816410700.01.T01	98,62	0,00E+00	2141	Os08g0502800	99,92	0,00E+00	2198	57	NA	Japonica	
maker-8-augustus-gene-261.9-mRNA-1	8	OsR498G0816414100.01.T01	100,00	0,00E+00	2235	Os08g0503200	100,00	0,00E+00	2235	0	NA	NA	
maker-8-augustus-gene-262.0-mRNA-1	8	OsR498G0816415100.01.T01	99,88	0,00E+00	2962	Os08g0503700	99,88	0,00E+00	2962	0	NA	NA	
maker-8-augustus-gene-262.2-mRNA-1	8	OsR498G0816415500.01.T04	100,00	0,00E+00	2861	Os08g0503800	100,00	0,00E+00	2861	0	NA	NA	
maker-8-augustus-gene-262.5-mRNA-1	8	OsR498G0816418500.01.T02	99,58	0,00E+00	2544	Os08g0504600	100,00	0,00E+00	2571	27	NA	Japonica	
maker-8-augustus-gene-262.9-mRNA-1	8	OsR498G0816418200.01.T03	99,50	0,00E+00	1418	Os08g0504400	99,87	0,00E+00	1430	12	NA	Japonica	
maker-8-augustus-gene-263.34-mRNA-1	8	OsR498G0816422700.01.T05	99,71	0,00E+00	1845	Os08g0505200	100,00	0,00E+00	1858	13	NA	Japonica	
maker-8-augustus-gene-263.37-mRNA-1	8	OsR498G0816425700.01.T03	99,70	0,00E+00	1794	Os08g0506000	100,00	0,00E+00	1736	-58	NA	Japonica	
maker-8-augustus-gene-263.40-mRNA-1	8	OsR498G0816427500.01.T03	99,80	0,00E+00	3543	Os08g0506400	99,78	0,00E+00	2438	-1105	Indica	Japonica	Kinase-LRR-TM
maker-8-augustus-gene-263.42-mRNA-1	8	OsR498G0816423000.01.T01	100,00	0,00E+00	666	Os06g0319700	85,67	8,14E-108	389	-277	Indica	Japonica	
maker-8-augustus-gene-263.44-mRNA-1	8	OsR498G0816427800.01.T03	99,75	0,00E+00	709	Os08g0506500	100,00	0,00E+00	1038	329	Japonica	Japonica	
maker-8-augustus-gene-264.35-mRNA-1	8	OsR498G0816428500.01.T01	98,77	0,00E+00	2124	Os08g0506700	100,00	0,00E+00	2165	41	NA	Japonica	
maker-8-augustus-gene-265.32-mRNA-1	8	OsR498G0816438900.01.T01	99,17	0,00E+00	1058	Os08g0508600	100,00	0,00E+00	1077	19	NA	Japonica	
maker-8-augustus-gene-265.35-mRNA-1	8	OsR498G0816440900.01.T02	99,66	0,00E+00	4167	Os08g0509100	100,00	0,00E+00	5097	930	Japonica	Japonica	
maker-8-augustus-gene-265.37-mRNA-1	8	OsR498G0816442300.01.T03	97,49	0,00E+00	846	Os08g0509500	100,00	0,00E+00	1234	388	Japonica	Japonica	
maker-8-augustus-gene-265.39-mRNA-1	8	OsR498G0917724200.01.T05	80,91	0,00E+00	645	Os08g0508500	99,86	0,00E+00	1262	617	Japonica	Japonica	
maker-8-augustus-gene-265.41-mRNA-1	8	OsR498G0816440900.01.T02	89,90	0,00E+00	3141	Os08g0508800	100,00	0,00E+00	5005	1864	Japonica	Japonica	
maker-8-augustus-gene-265.43-mRNA-1	8	OsR498G0816441200.01.T01	99,68	0,00E+00	1684	Os08g0509200	100,00	0,00E+00	2706	1022	Japonica	Japonica	
maker-8-augustus-gene-265.46-mRNA-1	8	OsR498G0917725800.01.T01	71,61	4,93E-162	572	Os08g0509400	95,89	0,00E+00	1807	1235	Japonica	Japonica	
maker-8-augustus-gene-265.48-mRNA-1	8	OsR498G0816442600.01.T01	99,92	0,00E+00	2359	Os08g0509600	99,76	0,00E+00	2252	-107	NA	Japonica	
maker-8-augustus-gene-266.41-mRNA-1	8	OsR498G0816445100.01.T01	99,68	0,00E+00	2235	Os08g0510300	99,76	0,00E+00	2240	5	NA	Japonica	
maker-8-augustus-gene-266.47-mRNA-1	8	OsR498G0816446700.01.T01	99,90	0,00E+00	1765	Os08g0510900	100,00	0,00E+00	2154	389	Japonica	Japonica	
maker-8-augustus-gene-266.50-mRNA-1	8	OsR498G0816445400.01.T03	99,79	0,00E+00	844	Os08g0510400	100,00	0,00E+00	1543	699	Japonica	Japonica	
maker-8-augustus-gene-266.55-mRNA-1	8	OsR498G0816445800.01.T04	99,82	0,00E+00	1992	Os08g0510700	99,95	0,00E+00	3572	1580	Japonica	Japonica	
maker-8-augustus-gene-266.57-mRNA-1	8	OsR498G0816447200.01.T01	99,90	0,00E+00	1879	Os08g0511200	100,00	0,00E+00	1846	-33	NA	Japonica	
maker-8-augustus-gene-267.13-mRNA-1	8	OsR498G0816453300.01.T01	100,00	0,00E+00	1640	Os08g0512600	100,00	0,00E+00	1770	130	NA	Japonica	Kinase-TM
maker-8-augustus-gene-267.16-mRNA-1	8	OsR498G0816454300.01.T01	99,73	0,00E+00	1962	Os08g0512700	100,00	0,00E+00	2273	311	Japonica	Japonica	
maker-8-augustus-gene-267.19-mRNA-1	8	OsR498G0816454500.01.T01	99,62	2,99E-130	465	Os08g0512900	100,00	1,11E-155	547	82	Japonica	Japonica	
maker-8-augustus-gene-267.32-mRNA-1	8	OsR498G0816450400.01.T01	100,00	0,00E+00	639	Os08g0511800	100,00	0,00E+00	639	0	NA	Japonica	
maker-8-augustus-gene-267.38-mRNA-1	8	OsR498G0816451600.01.T03	97,00	0,00E+00	1857	Os08g0512400	95,92	0,00E+00	1410	-447	Indica	NA	
maker-8-augustus-gene-267.4-mRNA-1	8	OsR498G0816451300.01.T01	99,52	0,00E+00	1102	Os08g0512300	100,00	0,00E+00	1732	630	Japonica	Japonica	
maker-8-augustus-gene-267.9-mRNA-1	8	OsR498G0816453100.01.T01	100,00	0,00E+00	1315	Os08g0512500	100,00	0,00E+00	1315	0	NA	Japonica	
maker-8-augustus-gene-268.36-mRNA-1	8	OsR498G0816459900.01.T01	98,43	0,00E+00	3429	Os08g0514000	99,19	0,00E+00	3935	506	Japonica	Japonica	Kinase-TM
maker-8-augustus-gene-268.38-mRNA-1	8	OsR498G0816460300.01.T01	99,06	0,00E+00	4097	Os06g0328400	72,84	0,00E+00	1058	-3039	Indica	Japonica	Kinase-TM
maker-8-augustus-gene-269.35-mRNA-1	8	OsR498G0917737700.01.T01	72,36	0,00E+00	827	Os08g0515700	99,87	0,00E+00	2720	1893	Japonica	Japonica	
maker-8-augustus-gene-269.39-mRNA-1	8	OsR498G0816465500.01.T01	98,78	0,00E+00	979	Os08g0516900	100,00	0,00E+00	1337	358	Japonica	Japonica	
maker-8-augustus-gene-27.0-mRNA-1	8	OsR498G0815168200.01.T04	100,00	0,00E+00	1692	Os08g0144400	84,23	0,00E+00	1038	-654	Indica	NA	
maker-8-augustus-gene-270.0-mRNA-1	8	OsR498G0816468300.01.T03	100,00	0,00E+00	1126	Os08g0517700	100,00	0,00E+00	2102	976	Japonica	Japonica	
maker-8-augustus-gene-270.11-mRNA-1	8	OsR498G0816466800.01.T04	100,00	0,00E+00	1851	Os08g0517300	99,85	0,00E+00	3497	1646	Japonica	Japonica	
maker-8-augustus-gene-270.15-mRNA-1	8	OsR498G0202973500.01.T01	78,53	0,00E+00	646	Os08g0517500	100,00	0,00E+00	2013	1367	Japonica	Japonica	
maker-8-augustus-gene-270.23-mRNA-1	8	OsR498G0816468600.01.T01	99,64	0,00E+00	1497	Os10g0146200	70,34	1,48E-91	336	-1161	Indica	Japonica	
maker-8-augustus-gene-270.25-mRNA-1	8	OsR498G0816468800.01.T01	100,00	0,00E+00	747	Os08g0517900	100,00	0,00E+00	747	0	NA	Japonica	
maker-8-augustus-gene-271.28-mRNA-1	8	OsR498G0816476700.01.T02	100,00	0,00E+00	2425	Os08g0519600	100,00	0,00E+00	2425	0	NA	NA	
maker-8-augustus-gene-271.38-mRNA-1	8	OsR498G0816475800.01.T07	100,00	0,00E+00	1370	Os08g0519400	100,00	0,00E+00	1370	0	NA	NA	
maker-8-augustus-gene-271.45-mRNA-1	8	OsR498G0917743900.01.T01	79,93	0,00E+00	1006	Os08g0520000	100,00	0,00E+00	2089	1083	Japonica	Japonica	
maker-8-augustus-gene-271.49-mRNA-1	8	OsR498G0816479800.01.T03	100,00	0,00E+00	5021	Os08g0520500	100,00	0,00E+00	5021	0	NA	NA	
maker-8-augustus-gene-272.0-mRNA-1	8	OsR498G0816482100.01.T01	99,15	0,00E+00	1030	Os08g0521000	99,87	0,00E+00	1354	324	Japonica	Japonica	
maker-8-augustus-gene-272.13-mRNA-1	8	OsR498G0816482400.01.T01	99,62	0,00E+00	3264	Os08g0521200	100,00	0,00E+00	3885	621	Japonica	Japonica	Kinase-LRR-TM
maker-8-augustus-gene-272.15-mRNA-1	8	OsR498G0816482800.01.T01	99,32	0,00E+00	2335	Os08g0521300	100,00	0,00E+00	1709	-626	Indica	Japonica	
maker-8-augustus-gene-272.18-mRNA-1	8	OsR498G0816483800.01.T01	100,00	0,00E+00	1413	Os08g0521800	100,00	0,00E+00	1413	0	NA	Japonica	
maker-8-augustus-gene-272.2-mRNA-1	8	OsR498G0816482900.01.T05	99,59	0,00E+00	1318	Os08g0521400	99,89	0,00E+00	1565	247	Japonica	Japonica	

maker-8-augustus-gene-272.36-mRNA-1	8	OsR498G0816484800.01.T01	99,90	0,00E+00	1711	Os08g0522500	100,00	0,00E+00	1716	5	NA	Japonica	
maker-8-augustus-gene-273.40-mRNA-1	8	OsR498G0816484900.01.T01	98,13	0,00E+00	1384	Os08g0522600	98,26	0,00E+00	1388	4	NA	Japonica	
maker-8-augustus-gene-273.46-mRNA-1	8	OsR498G0816487100.01.T01	99,46	0,00E+00	986	Os08g0564800	94,18	3,73E-165	580	-406	Indica	Japonica	
maker-8-augustus-gene-273.50-mRNA-1	8	OsR498G0917754500.01.T01	80,99	0,00E+00	1652	Os08g0524100	98,03	0,00E+00	5341	3689	Japonica	Japonica	
maker-8-augustus-gene-273.53-mRNA-1	8	OsR498G0816489400.01.T02	100,00	0,00E+00	1391	Os08g0524000	100,00	0,00E+00	1391	0	NA	Japonica	
maker-8-augustus-gene-273.56-mRNA-1	8	OsR498G0816490600.01.T01	99,22	0,00E+00	1122	Os08g0524200	100,00	0,00E+00	2116	994	Japonica	Japonica	
maker-8-augustus-gene-274.0-mRNA-1	8	OsR498G0816492300.01.T01	99,50	0,00E+00	715	Os08g0524600	100,00	0,00E+00	823	108	Japonica	Japonica	
maker-8-augustus-gene-274.13-mRNA-1	8	OsR498G0816495600.01.T01	99,06	0,00E+00	1113	Os08g0525000	100,00	0,00E+00	1148	35	NA	Japonica	
maker-8-augustus-gene-274.17-mRNA-1	8	OsR498G0816497000.01.T01	99,19	0,00E+00	5691	Os08g0525700	99,97	0,00E+00	5774	83	NA	Japonica	
maker-8-augustus-gene-274.20-mRNA-1	8	OsR498G0917759000.01.T01	77,17	0,00E+00	1006	Os08g0525800	100,00	0,00E+00	2531	1525	Japonica	Japonica	
maker-8-augustus-gene-274.3-mRNA-1	8	OsR498G0917757000.01.T01	72,66	1,62E-109	398	Os08g0524800	99,51	0,00E+00	2177	1779	Japonica	Japonica	
maker-8-augustus-gene-274.6-mRNA-1	8	OsR498G0816496700.01.T01	98,99	0,00E+00	1913	Os08g0525600	99,49	0,00E+00	3123	1210	Japonica	Japonica	
maker-8-augustus-gene-275.32-mRNA-1	8	OsR498G0816500100.01.T02	95,86	0,00E+00	828	Os08g0526500	95,27	0,00E+00	814	-14	NA	Indica	
maker-8-augustus-gene-275.42-mRNA-1	8	OsR498G0816499600.01.T02	99,77	0,00E+00	1568	Os08g0526400	99,74	0,00E+00	1361	-207	Indica	Indica	
maker-8-augustus-gene-275.44-mRNA-1	8	OsR498G0816502800.01.T01	99,79	0,00E+00	5167	Os08g0527100	99,72	0,00E+00	5158	-9	NA	Indica	
maker-8-augustus-gene-275.48-mRNA-1	8	OsR498G0816503600.01.T01	99,79	0,00E+00	1718	Os08g0527400	77,87	0,00E+00	983	-735	Indica	NA	
maker-8-augustus-gene-276.0-mRNA-1	8	OsR498G0816508900.01.T02	100,00	0,00E+00	1793	Os08g0528900	99,50	0,00E+00	1769	-24	NA	Indica	
maker-8-augustus-gene-276.11-mRNA-1	8	OsR498G0816500700.01.T02	94,91	0,00E+00	1932	Os08g0527900	99,96	0,00E+00	4059	2127	Japonica	Japonica	
maker-8-augustus-gene-276.12-mRNA-1	8	OsR498G0816506000.01.T01	99,74	0,00E+00	2700	Os08g0528000	99,49	0,00E+00	4210	1510	Japonica	NA	
maker-8-augustus-gene-276.2-mRNA-1	8	OsR498G0816510300.01.T01	100,00	0,00E+00	1191	Os08g0529100	99,70	0,00E+00	1182	-9	NA	Indica	
maker-8-augustus-gene-276.22-mRNA-1	8	OsR498G0816507800.01.T02	98,94	0,00E+00	1489	Os08g0528500	98,47	0,00E+00	1475	-14	NA	Indica	
maker-8-augustus-gene-276.26-mRNA-1	8	OsR498G0816508200.01.T02	100,00	2,39E-114	412	Os08g0528601	100,00	1,23E-112	405	-7	NA	Indica	
maker-8-augustus-gene-276.30-mRNA-1	8	OsR498G0816508600.01.T01	99,94	0,00E+00	2912	Os08g0528800	99,81	0,00E+00	2903	-9	NA	Indica	
maker-8-augustus-gene-276.32-mRNA-1	8	OsR498G0816509400.01.T01	99,81	0,00E+00	1860	Os08g0529000	99,81	0,00E+00	1874	14	NA	Indica	
maker-8-augustus-gene-276.34-mRNA-1	8	OsR498G0816510600.01.T01	100,00	0,00E+00	1142	Os08g0529200	99,92	0,00E+00	2263	1121	Japonica	NA	
maker-8-augustus-gene-277.31-mRNA-1	8	OsR498G0816513700.01.T01	99,86	0,00E+00	2654	Os08g0530000	99,80	0,00E+00	2649	-5	NA	NA	
maker-8-augustus-gene-277.37-mRNA-1	8	OsR498G0816513900.01.T01	100,00	0,00E+00	1862	Os07g0294800	70,54	1,23E-80	301	-1561	Indica	Indica	
maker-8-augustus-gene-277.39-mRNA-1	8	OsR498G0816515900.01.T01	100,00	0,00E+00	1261	Os08g0530400	99,86	0,00E+00	1257	-4	NA	Indica	
maker-8-augustus-gene-277.45-mRNA-1	8	OsR498G0816517100.01.T01	98,53	2,60E-97	355	Os08g0531000	92,86	4,12E-83	306	-49	Indica	NA	
maker-8-augustus-gene-277.46-mRNA-1	8	OsR498G0816517100.01.T02	99,89	0,00E+00	3368	Os08g0531000	100,00	0,00E+00	3377	9	NA	Indica	
maker-8-augustus-gene-277.54-mRNA-1	8	OsR498G0816517500.01.T06	99,93	0,00E+00	2627	Os08g0531100	99,66	0,00E+00	2608	-19	NA	Indica	
maker-8-augustus-gene-277.60-mRNA-1	8	OsR498G0816512800.01.T01	99,62	0,00E+00	2348	Os08g0529800	99,24	0,00E+00	2327	-21	NA	Indica	
maker-8-augustus-gene-277.63-mRNA-1	8	OsR498G0816514600.01.T01	100,00	0,00E+00	931	Os08g0530200	99,81	0,00E+00	927	-4	NA	Indica	
maker-8-augustus-gene-277.67-mRNA-1	8	OsR498G0816516200.01.T01	99,84	0,00E+00	1116	Os08g0530500	97,03	0,00E+00	1075	-41	NA	Indica	
maker-8-augustus-gene-278.0-mRNA-1	8	OsR498G0816518100.01.T01	92,71	0,00E+00	1529	Os08g0531300	92,41	0,00E+00	1514	-15	NA	Indica	
maker-8-augustus-gene-278.18-mRNA-1	8	OsR498G0816520500.01.T01	99,89	0,00E+00	3216	Os08g0532400	100,00	0,00E+00	3225	9	NA	Japonica	
maker-8-augustus-gene-278.20-mRNA-1	8	OsR498G0816520200.01.T01	99,58	0,00E+00	2565	Os08g0532200	100,00	0,00E+00	2592	27	NA	Japonica	
maker-8-augustus-gene-278.23-mRNA-1	8	OsR498G0816520400.01.T01	100,00	0,00E+00	666	Os08g0532300	100,00	0,00E+00	666	0	NA	Japonica	
maker-8-augustus-gene-278.26-mRNA-1	8	OsR498G0816521200.01.T01	99,90	0,00E+00	1836	Os08g0532700	100,00	0,00E+00	1840	4	NA	Japonica	
maker-8-augustus-gene-278.28-mRNA-1	8	OsR498G0816521800.01.T01	89,95	0,00E+00	845	Os08g0532800	90,91	0,00E+00	868	23	NA	Japonica	
maker-8-augustus-gene-278.30-mRNA-1	8	OsR498G0816522000.01.T01	99,37	0,00E+00	1135	Os08g0532900	100,00	0,00E+00	1153	18	NA	Japonica	
maker-8-augustus-gene-278.5-mRNA-1	8	OsR498G0816518900.01.T01	99,05	0,00E+00	2409	Os08g0531600	100,00	0,00E+00	2468	59	NA	Japonica	
maker-8-augustus-gene-278.8-mRNA-1	8	OsR498G0816519300.01.T05	99,53	0,00E+00	755	Os08g0531700	100,00	0,00E+00	753	-2	NA	Japonica	
maker-8-augustus-gene-279.30-mRNA-1	8	OsR498G0816523500.01.T01	97,23	0,00E+00	2446	Os08g0533300	96,06	0,00E+00	2468	22	NA	Japonica	
maker-8-augustus-gene-279.32-mRNA-1	8	OsR498G0816524700.01.T02	99,71	0,00E+00	2493	Os08g0533600	100,00	0,00E+00	2511	18	NA	Japonica	
maker-8-augustus-gene-279.36-mRNA-1	8	OsR498G0816524900.01.T03	100,00	0,00E+00	1274	Os08g0533700	100,00	0,00E+00	1274	0	NA	Japonica	
maker-8-augustus-gene-279.45-mRNA-1	8	OsR498G0816525300.01.T04	99,81	0,00E+00	1869	Os08g0534900	100,00	0,00E+00	1878	9	NA	Japonica	
maker-8-augustus-gene-280.103-mRNA-1	8	OsR498G0816528200.01.T01	99,83	0,00E+00	2075	Os08g0535100	100,00	0,00E+00	2084	9	NA	Japonica	
maker-8-augustus-gene-280.105-mRNA-1	8	OsR498G0816529000.01.T01	99,89	0,00E+00	1663	Os08g0535200	100,00	0,00E+00	1667	4	NA	Japonica	
maker-8-augustus-gene-280.34-mRNA-1	8	OsR498G0816525900.01.T01	99,67	3,23E-155	548	Os02g0305801	100,00	7,57E-139	491	-57	Indica	NA	
maker-8-augustus-gene-280.35-mRNA-1	8	OsR498G0816525300.01.T04	99,81	0,00E+00	1869	Os08g0534900	100,00	0,00E+00	1878	9	NA	NA	
maker-8-augustus-gene-280.37-mRNA-1	8	OsR498G0816525900.01.T01	99,67	3,23E-155	548	Os02g0305801	100,00	7,57E-139	491	-57	Indica	NA	
maker-8-augustus-gene-281.20-mRNA-1	8	OsR498G0816530900.01.T01	99,73	0,00E+00	1305	Os08g0535700	100,00	0,00E+00	2100	795	Japonica	Japonica	
maker-8-augustus-gene-281.24-mRNA-1	8	OsR498G0816531500.01.T01	98,87	0,00E+00	1237	Os08g0535800	100,00	0,00E+00	1261	24	NA	Japonica	
maker-8-augustus-gene-281.27-mRNA-1	8	OsR498G0816532500.01.T01	99,73	0,00E+00	2016	Os08g0536000	99,91	0,00E+00	2025	9	NA	Japonica	
maker-8-augustus-gene-281.31-mRNA-1	8	OsR498G0816532700.01.T01	99,86	0,00E+00	3877	Os08g0536100	100,00	0,00E+00	2899	-978	Indica	Japonica	
maker-8-augustus-gene-281.36-mRNA-1	8	OsR498G0816532900.01.T01	99,01	0,00E+00	891	Os08g0536200	100,00	0,00E+00	915	24	NA	Japonica	
maker-8-augustus-gene-281.38-mRNA-1	8	OsR498G0816530400.01.T02	99,85	0,00E+00	1217	Os08g0535400	100,00	0,00E+00	1231	14	NA	Japonica	
maker-8-augustus-gene-281.41-mRNA-1	8	OsR498G1220874000.01.T02	70,80	6,95E-10	64,4	Os08g0535600	100,00	7,41E-112	401	336,6	Japonica	Japonica	
maker-8-augustus-gene-281.43-mRNA-1	8	OsR498G0816533200.01.T01	97,23	0,00E+00	2509	Os08g0536300	98,16	0,00E+00	2544	35	NA	Japonica	
maker-8-augustus-gene-282.2-mRNA-1	8	OsR498G0816537200.01.T02	99,89	0,00E+00	1576	Os09g0510500	76,84	1,45E-153	543	-1033	Indica	Japonica	
maker-8-augustus-gene-282.7-mRNA-1	8	OsR498G0816540800.01.T02	99,39	0,00E+00	4359	Os08g0537600	100,00	0,00E+00	4423	64	NA	Japonica	
maker-8-augustus-gene-283.16-mRNA-1	8	OsR498G0816542900.01.T03	99,45	0,00E+00	2269	Os08g0538600	100,00	0,00E+00	2300	31	NA	Japonica	
maker-8-augustus-gene-283.19-mRNA-1	8	OsR498G0816544900.01.T01	100,00	0,00E+00	2300	Os08g0539300	100,00	0,00E+00	2300	0	NA	Japonica	
maker-8-augustus-gene-283.37-mRNA-1	8	OsR498G0816543400.01.T03	99,41	0,00E+00	1205	Os08g0538800	99,71	0,00E+00	1214	9	NA	Japonica	
maker-8-augustus-gene-283.4-mRNA-1	8	OsR498G0816541900.01.T01	99,91	0,00E+00	4001	Os08g0538000	100,00	0,00E+00	3782	-219	NA	Japonica	
maker-8-augustus-gene-283.9-mRNA-1	8	OsR498G0816542500.01.T02	99,83	0,00E+00	2145	Os08g0538300	100,00	0,00E+00	2127	-18	NA	Japonica	Kinase-TM
maker-8-augustus-gene-284.0-mRNA-1	8	OsR498G0816548500.01.T01	98,93	0,00E+00	1475	Os09g0513000	75,23	8,84E-164	577	-898	Indica	Japonica	
maker-8-augustus-gene-284.12-mRNA-1	8	OsR498G0816551500.01.T01	99,78	0,00E+00	3272	Os08g0540300	100,00	0,00E+00	3290	18	NA	Japonica	
maker-8-augustus-gene-284.17-mRNA-1	8	OsR498G0816548000.01.T01	91,37	0,00E+00	3188	Os08g0539400	100,00	0,00E+00	3981	793	Japonica	Japonica	CC-NBS-TM
maker-8-augustus-gene-284.25-mRNA-1	8	OsR498G0816552000.01.T01	97,24	0,00E+00	2951	Os08g0540400	97,92	0,00E+00	2993	42	NA	Japonica	
maker-8-augustus-gene-284.4-mRNA-1	8	OsR498G0816548900.01.T01	99,76	0,00E+00	6598	Os08g0539700	100,00	0,00E+00	6639	41	NA	Japonica	CC-LRR-NBS-TM PIBH8
maker-8-augustus-gene-284.6-mRNA-1	8	OsR498G0816550100.01.T01	99,81	0,00E+00	1920	Os08g0539700	78,66	0,00E+00	899	-1021	Indica	Japonica	PIBH8
maker-8-augustus-gene-284.7-mRNA-1	8	OsR498G0816550500.01.T01	88,94	0,00E+00	2536	Os08g0539700	79,70	0,00E+00	1952	-584	Indica	Japonica	LRR-NBS-TM PIBH8
maker-8-augustus-gene-284.9-mRNA-1	8	OsR498G0816550900.01.T01	99,91	0,00E+00	2063	Os08g0540100	100,00	0,00E+00	2067	4	NA	Japonica	
maker-8-augustus-gene-285.44-mRNA-1	8	OsR498G0816552100.01.T04	96,02	0,00E+00	1154	Os08g0540500	99,86	0,00E+00	1264	110	NA	Japonica	
maker-8-augustus-gene-285.49-mRNA-1	8	OsR498G0816552900.01.T01	99,66	0,00E+00	1583	Os08g0541400	100,00	0,00E+00	1418	-165	Indica	Japonica	
maker-8-augustus-gene-285.52-mRNA-1	8	OsR498G0816554900.01.T01	99,87	0,00E+00	1343	Os08g0542100	100,00	0,00E+00	1348	5	NA	Japonica	
maker-8-augustus-gene-285.62-mRNA-1	8	OsR498G0816557100.01.T01	99,94	0,00E+00	3015	Os08g0542700	100,00	0,00E+00	3020	5	NA	Japonica	
maker-8-augustus-gene-285.64-mRNA-1	8	OsR498G0816554000.01.T01	99,82	0,00E+00	3909	Os08g0541500	100,00	0,00E+00	3972	63	NA	Japonica	
maker-8-augustus-gene-285.66-mRNA-1	8	OsR498G0816554800.01.T02	97,91	0,00E+00	2395	Os12g0628500	85,13	0,00E+00	1589	-806	Indica	Japonica	
maker-8-augustus-gene-286.0-mRNA-1	8	OsR498G0816557500.01.T01	99,77	0,00E+00	2303	Os08g0542900	99,92	0,00E+00	2312	9	NA	Japonica	
maker-8-augustus-gene-286.5-mRNA-1	8	OsR498G0816559900.01.T04	99,75	0,00E+00	2130	Os08g0543400	100,00	0,00E+00	2397	267	Japonica	Japonica	
maker-8-augustus-gene-286.8-mRNA-1	8	OsR498G0816562300.01.T06	99,57	0,00E+00	2489	Os08g0543900	100,00	0,00E+00	2516	27	NA	Japonica	
maker-8-augustus-gene-287.35-mRNA-1	8	OsR498G0816564900.01.T03	100,00	0,00E+00	1889	Os08g0544900	100,00	0,00E+00	1889	0	NA	Japonica	
maker-8-augustus-gene-287.42-mRNA-1	8	OsR498G0816564000.01.T06	98,70	0,00E+00	3764	Os08g0544500	98,79	0,00E+00	3773	9	NA	Japonica	
maker-8-augustus-gene-287.47-mRNA-1	8	OsR498G0816567400.01.T01	98,73	0,00E+00	821	Os08g0545800	100,00	0,00E+00	850	29	NA	Japonica	
maker-8-augustus-gene-287.49-mRNA-1	8	OsR498G0816568800.01.T01	99,90	0,00E+00	1711	Os08g0546100	99,93	0,00E+00	2712	1001	Japonica	Japonica	
maker-8-augustus-gene-288.14-mRNA-1	8	OsR498G0816570400.01.T01	96,92	0,00E+00	1204	Os08g0546700	97,20	0,00E+00	1213	9	NA	Japonica	
maker-8-augustus-gene-288.18-mRNA-1	8	OsR498G0816570600.01.T01	99,46	0,00E+00	2312	Os08g0546800	100,00	0,00E+00	2859	547	Japonica	Japonica	
maker-8-augustus-gene-288.2-mRNA-1	8	OsR498G0816571500.01.T01	99,70	0,00E+00	1762	Os08g0547100	100,00	0,00E+00	1775	13	NA	Japonica	
maker-8-augustus-gene-288.22-mRNA-1	8	OsR498G0816571000.01.T01	100,00	0,00E+00	1553	Os08g0546900	100,00	0,00E+00	1553	0	NA	Japonica	
maker-8-augustus-gene-288.24-mRNA-1	8	OsR498G0816572600.01.T01	99,40	0,00E+00	2942	Os08g0547300	99,94	0,00E+00	2983	41	NA	Japonica	
maker-8-augustus-gene-288.26-mRNA-1	8	OsR498G0816573200.01.T01	99,23	0,00E+00	5286	Os08g0547500	99,30	0,00E+00	5294	8	NA	Japonica	
maker-8-augustus-gene-288.31-mRNA-1	8	OsR498G0816574600.01.T02	99,56	0,00E+00	1622	Os08g0547600	100,00	0,00E+00	1640	18	NA	Japonica	
maker-8-augustus-gene-288.8-mRNA-1	8	OsR498G0816569600.01.T01	99,77	0,00E+00	3140	Os08g0546400	99,83	0,00E+00	3145	5	NA	Japonica	
maker-8-augustus-gene-289.33-mRNA-1	8	OsR498G0816577700.01.T01	99,88	0,00E+00	1473	Os08g0548200	100,00	0,00E+00	1478	5	NA	Japonica	
maker-8-augustus-gene-289.38-mRNA-1	8	OsR498G0816577900.01.T01	97,93	0,00E+00	1580	Os08g0548300	100,00	0,00E+00	1656	76	NA	Japonica	
maker-8-augustus-gene-289.44-mRNA-1	8	OsR498G0816579700.01.T02	99,87	0,00E+00	1379	Os09g0536400	78,11	6,55E-159	561	-818	Indica	Japonica	
maker-8-augustus-gene-289.48-mRNA-1	8	OsR498G0816577200.01.T03	99,75	0,00E+00	1415	Os08g0548000	100,00	0,00E+00	953	-462	Indica	Japonica	
maker-8-augustus-gene-289.50-mRNA-1	8	OsR498G0816581200.01.T01	99,59	0,00E+00	1307	Os08g0549000	100,00	0,00E+00	1321	14	NA	Japonica	
maker-8-augustus-gene-289.52-mRNA-1	8	OsR498G0816581800.01.T02	100,00	0,00E+00	1573	Os08g0549100	100,00	0,00E+00	1573	0	NA	Japonica	
maker-8-augustus-gene-29.4-mRNA-1	8	OsR498G0815184400.01.T01	100,00	0,00E+00	948	Os08g0148233	99,35	2,44E-154	544	-404	Indica	NA	
maker-8-augustus-gene-290.0-mRNA-1	8	OsR498G0816581900.01.T01	99,70	0,00E+00	2950	Os08g0549200	100,00	0,00E+00	2973	23	NA	Japonica	
maker-8-augustus-gene-290.11-mRNA-1	8	OsR498G0816586500.01.T01	99,76	0,00E+00	2942	Os08g0551200	100,00	0,00E+00	2960	18	NA	Japonica	
maker-8-augustus-gene-290.15-mRNA-1	8	OsR498G0816582400.01.T01	100,00	0,00E+00	753	Os08g0549300	100,00	0,00E+00	753	0	NA	Japonica	
maker-8-augustus-gene-290.19-mRNA-1	8	OsR498G0816584000.01.T01	99,80	0,00E+00	2622	Os08g0550100	100,00	0,00E+00	2636	14	NA	Japonica	
maker-8-augustus-gene-290.23-mRNA-1	8	OsR498G0816584200.01.T01	99,81	0,00E+00	2817	Os08g0550200	100,00	0,00E+00	2830	13	NA	Japonica	
maker-8-augustus-gene-290.27-mRNA-1	8	OsR498G0816584800.01.T01	98,72	0,00E+00	1371	Os08g0550400	100,00	0,00E+00	1413	42	NA	Japonica	
maker-8-augustus-gene-290.3-mRNA-1	8	OsR498G0816583100.01.T01	98,19	0,00E+00	761	Os08g0549600	100,00	0,00E+00	796	35	NA	Japonica	
maker-8-augustus-gene-290.9-mRNA-1	8	OsR498G0816585700.01.T01	99,96	0,00E+00	4422	Os08g0550600	100,00	0,00E+00	4426	4	NA	Japonica	
maker-8-augustus-gene-291.0-mRNA-1	8	OsR498G0816589300.01.T01	89,55	0,00E+00	942	Os08g0553000	89,47	0,00E+00	1829	887	Japonica	Japonica	
maker-8-augustus-gene-291.1-mRNA-1	8	OsR498G0816589700.01.T01	100,00	0,00E+00	1294	Os08g0553500	100,00	0,00E+00	1294	0	NA	Japonica	
maker-8-augustus-gene-291.11-mRNA-1	8	OsR498G0816591100.01.T03	100,00	0,00E+00	2616	Os08g0554300	96,87	0,00E+00	3035	419	Japonica	Japonica	
maker-8-augustus-gene-291.2-mRNA-1	8	OsR498G0816590700.01.T06	96,85	0,00E+00	1765	Os08g0554000	100,00	0,00E+00	1727	-38	NA	Japonica	
maker-8-augustus-gene-291.27-mRNA-1	8	OsR498G0816590200.01.T02	99,83	0,00E+00	2085	Os08g0553800	99,91	0,00E+00	2090	5	NA	Japonica	
maker-8-augustus-gene-292.40-mRNA-1	8	OsR498G0816593500.01.T01	99,66	0,00E+00	2094	Os08g0554900	99,74	0,00E+00	2099	5	NA	Japonica	
maker-8-augustus-gene-292.41-mRNA-1	8	OsR498G0816593500.01.T01	99,74	0,00E+00	2099	Os08g0554900	99,83	0,00E+00	2104	5	NA	Japonica	
maker-8-augustus-gene-292.42-mRNA-1	8	OsR498G0816594700.01.T01	99,06	0,00E+00	2063	Os08g0555200	100,00	0,00E+00	2089	26	NA	Japonica	
maker-8-augustus-gene-292.61-mRNA-1	8	OsR498G0816594900.01.T02	99,56	0,00E+00	1626	Os09g0557800	73,05	3,67E-152	539	-1087	Indica	Japonica	
maker-8-augustus-gene-292.63-mRNA-1	8	OsR498G0816595900.01.T05	93,38	0,00E+00	1524	Os08g0555800	100,00	0,00E+00	1663	139	NA	Japonica	
maker-8-augustus-gene-292.68-mRNA-1	8	OsR498G0816597300.01.T02	100,00	0,00E+00	737	Os08g0556200	100,00	0,00E+00	737	0	NA	Japonica	
maker-8-augustus-gene-292.79-mRNA-1	8	OsR498G0816598500.01.T01	99,63	2,34E-137	489	Os08g0556600	100,00	1,92E-139	493	4	NA	Japonica	
maker-8-augustus-gene-292.83-mRNA-1	8	OsR498G0816597000.01.T02	99,68	0,00E+00	3353	Os08g0556000	100,00	0,00E+00	3382	29	NA	Japonica	
maker-8-augustus-gene-292.88-mRNA-1	8	OsR498G0816597800.01.T01	99,56	0,00E+00	1205	Os08g0556300	100,00	0,00E+00	769	-436	Indica	Japonica	
maker-8-augustus-gene-293.0-mRNA-1	8	OsR498G0816599700.01.T01	99,71	0,00E+00	1245	Os08g0557000	99,86	0,00E+00	1250	5	NA	Japonica	
maker-8-augustus-gene-293.11-mRNA-1	8	OsR498G0816602100.01.T01	100,00	0,00E+00	2760	Os08g0557800	100,00	0,00E+00	2760	0	NA	Japonica	
maker-8-augustus-gene-293.15-mRNA-1	8	OsR498G0816603500.01.T01	100,00	0,00E+00	1851	Os08g0558200	100,00	0,00E+00	1851	0	NA	Japonica	
maker-8-augustus-gene-293.26-mRNA-1	8	OsR498G0816600000.01.T01	99,46	0,00E+00	1637	Os08g0557100	100,00	0,00E+00	975	-662	Indica	Japonica	
maker-8-augustus-gene-293.29-mRNA-1	8	OsR498G0816600800.01.T01	99,75	0,00E+00	1447	Os08g0557400	100,00	0,00E+00	1456	9	NA	Japonica	
maker-8-augustus-gene-293.32-mRNA-1	8	OsR498G0816602400.01.T03	99,89	0,00E+00	1648	Os08g0557900	100,00	0,00E+00	1651	3	NA	Japonica	
maker-8-augustus-gene-293.35-mRNA-1	8	OsR498G0816602800.01.T03	99,24	0,00E+00	1168	Os08g0558100	100,00	0,00E+00	1186	18	NA	Japonica	
maker-8-augustus-gene-293.4-mRNA-1	8	OsR498G0917967100.01.T03	81,73	0,00E+00	1282	Os08g0557600	100,00	0,00E+00	2360	1078	Japonica	Japonica	
maker-8-augustus-gene-293.45-mRNA-1	8	OsR498G0816605000.01.T02	100,00	0,00E+00	1310	Os08g0558600	100,00	0,00E+00	1310	0	NA	Japonica	
maker-8-augustus-gene-293.6-mRNA-1	8	OsR498G0816601700.01.T01	99,32	0,00E+00	788	Os08g0557700	99,78	0,00E+00	797	9	NA	Japonica	
maker-8-augustus-gene-294.46-mRNA-1	8	OsR498G0816605700.01.T01	99,59	1,28E-122	440	Os08g0558900	100,00	1,05E-124	444	4	NA	Japonica	
maker-8-augustus-gene-294.48-mRNA-1	8	OsR498G0816606100.01.T01	100,00	3,25E-168	590	Os08g0559200	99,69	4,80E-167	586	-4	NA	Japonica	
maker-8-augustus-gene-294.51-mRNA-1	8	OsR498G0816606900.01.T01	98,14	0,00E+00	2974	Os08g0559300	98,14	0,00E+00	2974	0	NA	Japonica	
maker-8-augustus-gene-294.57-mRNA-1	8	OsR498G0816607900.01.T01	99,89	0,00E+00	3211	Os08g0559600	99,89	0,00E+00	3211	0	NA	Japonica	
maker-8-augustus-gene-294.62-mRNA-1	8	OsR498G0816608100.01.T04	100,00	0,00E+00	785	Os08g0559700	100,00	0,00E+00	785	0	NA	Japonica	
maker-8-augustus-gene-294.65-mRNA-1	8	OsR498G0816608500.01.T02	99,74	0,00E+00	2765	Os08g0559900	99,55	0,00E+00	2755	-10	NA	Japonica	
maker-8-augustus-gene-294.69-mRNA-1	8	OsR498G0816609100.01.T01	99,65	0,00E+00	2060	Os08g0560000	99,83	0,00E+00	2069	9	NA	NA	
maker-8-augustus-gene-294.71-mRNA-1	8	OsR498G0816612500.01.T01	99,79	0,00E+00	2525	Os08g0560500	100,00	0,00E+00	2538	13	NA	Japonica	
maker-8-augustus-gene-294.76-mRNA-1	8	OsR498G0816605200.01.T01	99,72	0,00E+00	1263	Os08g0558700	100,00	0,00E+00	1272	9	NA	Japonica	
maker-8-augustus-gene-294.79-mRNA-1	8	OsR498G0816606000.01.T02	100,00	6,53E-105	379	Os08g0559000	98,32	4,12E-83	306	-73	Indica	Japonica	
maker-8-augustus-gene-294.81-mRNA-1	8	OsR498G0816607600.01.T02	100,00	0,00E+00	958	Os08g0559400	93,22	0,00E+00	824	-134	Indica	Japonica	
maker-8-augustus-gene-294.88-mRNA-1	8	OsR498G0816608400.01.T01	99,92	0,00E+00	2220	Os08g0559800	100,00	0,00E+00	1135	-1085	Indica	Japonica	
maker-8-augustus-gene-294.91-mRNA-1	8	OsR498G0816611600.01.T01	99,50	0,00E+00	1075	Os08g0560300	100,00	0,00E+00	1085	10	NA	NA	
maker-8-augustus-gene-294.93-mRNA-1	8	OsR498G0816612800.01.T01	99,40	0,00E+00	1461	Os08g0560600	99,85	0,00E+00	1165	-296	Indica	Japonica	
maker-8-augustus-gene-295.41-mRNA-1	8	OsR498G0816615300.01.T01	99,06	0,00E+00	2070	Os08g0561500	99,29	0,00E+00	2250	180	NA	Japonica	
maker-8-augustus-gene-295.47-mRNA-1	8	OsR498G0816616300.01.T01	99,75	0,00E+00	1452	Os08g0561900	100,00	0,00E+00	1462	10	NA	Japonica	
maker-8-augustus-gene-295.49-mRNA-1	8	OsR498G0816616900.01.T01	99,92	0,00E+00	2344	Os08g0562100	100,00	0,00E+00	2349	5	NA	Japonica	
maker-8-augustus-gene-295.52-mRNA-1	8	OsR498G0816615600.01.T01	99,86	0,00E+00	2533	Os08g0561600	99,57	0,00E+00	2516	-17	NA	Japonica	
maker-8-augustus-gene-295.55-mRNA-1	8	OsR498G0816617200.01.T01	99,80	0,00E+00	3537	Os08g0562200	100,00	0,00E+00	3555	18	NA	Japonica	
maker-8-augustus-gene-295.59-mRNA-1	8	OsR498G0816619200.01.T01	99,85	0,00E+00	2405	Os08g0562500	100,00	0,00E+00	2414	9	NA	Japonica	
maker-8-augustus-gene-296.2-mRNA-1	8	OsR498G0816620200.01.T01	99,16	1,65E-179	629	Os08g0563300	99,72	0,00E+00	638	9	NA	Japonica	
maker-8-augustus-gene-296.4-mRNA-1	8	OsR498G0816623400.01.T01	100,00	0,00E+00	3209	Os08g0564100	100,00	0,00E+00	3209	0	NA	Japonica	
maker-8-augustus-gene-297.15-mRNA-1	8	OsR498G0816632900.01.T01	100,00	0,00E+00	682	Os08g0566600	100,00	0,00E+00	682	0	NA	Japonica	
maker-8-augustus-gene-297.17-mRNA-1	8	OsR498G0816634100.01.T04	100,00	4,70E-149	527	Os08g0566900	100,00	1,64E-149	527	0	NA	Japonica	
maker-8-augustus-gene-297.19-mRNA-1	8	OsR498G0816634700.01.T01	99,91	0,00E+00	2003	Os08g0567000	100,00	0,00E+00	2008	5	NA	Japonica	
maker-8-augustus-gene-297.2-mRNA-1	8	OsR498G0816631300.01.T01	99,60	0,00E+00	884	Os08g0565800	100,00	0,00E+00	893	9	NA	Japonica	
maker-8-augustus-gene-297.24-mRNA-1	8	OsR498G0816627600.01.T02	99,77	0,00E+00	7107	Os08g0564800	100,00	0,00E+00	7148	41	NA	Japonica	
maker-8-augustus-gene-297.32-mRNA-1	8	OsR498G0816631800.01.T01	99,64	0,00E+00	2481	Os08g0566000	99,93	0,00E+00	2499	18	NA	Japonica	
maker-8-augustus-gene-297.35-mRNA-1	8	OsR498G0816632600.01.T01	99,92	0,00E+00	2344	Os08g0566100	100,00	0,00E+00	2349	5	NA	Japonica	
maker-8-augustus-gene-297.40-mRNA-1	8	OsR498G0816633400.01.T01	99,24	0,00E+00	2566	Os08g0566700	100,00	0,00E+00	3842	1276	Japonica	Japonica	
maker-8-augustus-gene-297.42-mRNA-1	8	OsR498G0816635000.01.T03	100,00	0,00E+00	1182	Os08g0567200	100,00	0,00E+00	1182	0	NA	NA	
maker-8-augustus-gene-297.7-mRNA-1	8	OsR498G0816631500.01.T01	99,49	0,00E+00	2074	Os08g0565900	100,00	0,00E+00	2100	26	NA	Japonica	
maker-8-augustus-gene-297.9-mRNA-1	8	OsR498G0816632700.01.T01	93,91	0,00E+00	1324	Os08g0566400	93,91	0,00E+00	1324	0	NA	Japonica	
maker-8-augustus-gene-3.34-mRNA-1	8	OsR498G0815035700.01.T02	99,83	0,00E+00	6462	Os08g0104700	100,00	0,00E+00	6489	27	NA	Japonica	
maker-8-augustus-gene-3.37-mRNA-1	8	OsR498G0815039700.01.T01	99,30	0,00E+00	1772	Os08g0106300	79,56	0,00E+00	1336	-436	Indica	Japonica	
maker-8-augustus-gene-3.39-mRNA-1	8	OsR498G0815033400.01.T01	99,63	0,00E+00	971	Os08g0104100	100,00	0,00E+00	980	9	NA	Japonica	
maker-8-augustus-gene-3.42-mRNA-1	8	OsR498G0815037400.01.T01	99,70	0,00E+00	1816	Os08g0104900	100,00	0,00E+00	1829	13	NA	NA	
maker-8-augustus-gene-3.44-mRNA-1	8	OsR498G0815037800.01.T01	99,76	0,00E+00	3034	Os08g0105000	99,94	0,00E+00	3048	14	NA	NA	
maker-8-augustus-gene-3.47-mRNA-1	8	OsR498G0815038000.01.T02	99,65	0,00E+00	3041	Os08g0105100	100,00	0,00E+00	3016	-25	NA	Japonica	
maker-8-augustus-gene-3.51-mRNA-1	8	OsR498G0815039000.01.T01	99,64	0,00E+00	2949	Os08g0105800	99,88	0,00E+00	2967	18	NA	Japonica	
maker-8-augustus-gene-30.34-mRNA-1	8	OsR498G0815189100.01.T01	100,00	0,00E+00	1160	Os08g0148500	98,91	0,00E+00	1130	-30	NA	Indica	
maker-8-augustus-gene-30.44-mRNA-1	8	OsR498G0815188200.01.T01	100,00	2,42E-120	432	Os08g0149000	81,15	5,32E-60	230	-202	Indica	NA	
maker-8-augustus-gene-30.46-mRNA-1	8	OsR498G0815188200.01.T03	99,88	0,00E+00	1491	Os08g0144400	70,66	4,89E-58	225	-1266	Indica	NA	
maker-8-augustus-gene-30.48-mRNA-1	8	OsR498G0815188200.01.T02	100,00	0,00E+00	949	Os08g0149000	81,78	1,28E-146	518	-431	Indica	NA	
maker-8-augustus-gene-31.29-mRNA-1	8	OsR498G0815197100.01.T01	100,00	0,00E+00	1624	Os08g0150700	99,89	0,00E+00	1619	-5	NA	Indica	
maker-8-augustus-gene-31.44-mRNA-1	8	OsR498G0815199900.01.T01	89,89	7,55E-105	380	Os08g0151400	100,00	3,68E-97	352	-28	NA	NA	
maker-8-augustus-gene-31.49-mRNA-1	8	OsR498G0815200500.01.T01	100,00	0,00E+00	1348	Os08g0151600	100,00	0,00E+00	1348	0	NA	NA	
maker-8-augustus-gene-31.51-mRNA-1	8	OsR498G0815200500.01.T03	97,67	0,00E+00	3096	Os08g0151700	97,67	0,00E+00	3096	0	NA	NA	
maker-8-augustus-gene-31.54-mRNA-1	8	OsR498G0815200700.01.T03	100,00	0,00E+00	1829	Os08g0151800	100,00	0,00E+00	1631	-198	Indica	NA	
maker-8-augustus-gene-31.65-mRNA-1	8	OsR498G0815199200.01.T01	100,00	0,00E+00	1948	Os08g0151300	99,91	0,00E+00	1944	-4	NA	Indica	
maker-8-augustus-gene-32.38-mRNA-1	8	OsR498G0815204100.01.T01	100,00	0,00E+00	2408	Os08g0152700	99,70	0,00E+00	2390	-18	NA	Indica	
maker-8-augustus-gene-32.46-mRNA-1	8	OsR498G0815203600.01.T04	100,00	0,00E+00	1456	Os08g0152500	100,00	0,00E+00	1456	0	NA	Indica	
maker-8-augustus-gene-32.50-mRNA-1	8	OsR498G0815204400.01.T02	99,91	0,00E+00	1949	Os02g0106800	71,15	9,95E-138	490	-1459	Indica	Indica	
maker-8-augustus-gene-33.40-mRNA-1	8	OsR498G0815208700.01.T02	99,67	0,00E+00	1081	Os08g0154000	99,67	0,00E+00	1081	0	NA	Indica	
maker-8-augustus-gene-33.46-mRNA-1	8	OsR498G0815210500.01.T01	100,00	0,00E+00	888	Os08g0154700	99,29	2,66E-141	500	-388	Indica	Indica	
maker-8-augustus-gene-33.51-mRNA-1	8	OsR498G0815211900.01.T02	100,00	0,00E+00	1662	Os08g0154900	100,00	0,00E+00	1662	0	NA	NA	
maker-8-augustus-gene-33.57-mRNA-1	8	OsR498G0815212500.01.T01	93,70	0,00E+00	1122	Os08g0155100	93,70	0,00E+00	1122	0	NA	NA	
maker-8-augustus-gene-33.61-mRNA-1	8	OsR498G0815209400.01.T01	98,27	0,00E+00	4633	Os08g0159900	85,36	0,00E+00	2029	-2604	Indica	Indica	
maker-8-augustus-gene-33.64-mRNA-1	8	OsR498G0815209800.01.T01	100,00	0,00E+00	3252	Os08g0154250	100,00	0,00E+00	1611	-1641	Indica	Indica	
maker-8-augustus-gene-33.66-mRNA-1	8	OsR498G0815210200.01.T01	100,00	0,00E+00	2165	Os08g0154300	99,75	0,00E+00	2151	-14	NA	Indica	
maker-8-augustus-gene-33.69-mRNA-1	8	OsR498G0815212200.01.T01	100,00	0,00E+00	1591	Os08g0155000	100,00	0,00E+00	1175	-416	Indica	NA	
maker-8-augustus-gene-34.30-mRNA-1	8	OsR498G0815213400.01.T01	100,00	0,00E+00	3268	Os08g0155400	100,00	0,00E+00	2847	-421	Indica	NA	
maker-8-augustus-gene-34.33-mRNA-1	8	OsR498G0815217400.01.T03	100,00	0,00E+00	8267	Os05g0151000	93,92	0,00E+00	7019	-1248	Indica	NA	
maker-8-augustus-gene-34.34-mRNA-1	8	OsR498G0815219000.01.T02	99,96	0,00E+00	4516	Os05g0151000	93,18	0,00E+00	3694	-822	Indica	NA	
maker-8-augustus-gene-35.24-mRNA-1	8	OsR498G0815224500.01.T01	100,00	0,00E+00	2706	Os08g0156600	100,00	0,00E+00	2706	0	NA	NA	
maker-8-augustus-gene-35.26-mRNA-1	8	OsR498G0815224900.01.T01	100,00	0,00E+00	645	Os08g0156800	100,00	0,00E+00	645	0	NA	NA	
maker-8-augustus-gene-35.28-mRNA-1	8	OsR498G0815225900.01.T01	100,00	0,00E+00	2138	Os08g0157000	100,00	0,00E+00	2138	0	NA	NA	Kinase-TM
maker-8-augustus-gene-35.30-mRNA-1	8	OsR498G0815224800.01.T01	100,00	0,00E+00	1070	Os08g0156700	100,00	0,00E+00	1070	0	NA	Indica	
maker-8-augustus-gene-35.35-mRNA-1	8	OsR498G0815225400.01.T01	98,72	0,00E+00	2058	Os08g0156900	98,72	0,00E+00	2058	0	NA	NA	
maker-8-augustus-gene-35.43-mRNA-1	8	OsR498G0815228600.01.T01	100,00	0,00E+00	1997	Os08g0157500	100,00	0,00E+00	1997	0	NA	Indica	
maker-8-augustus-gene-36.34-mRNA-1	8	OsR498G0815231500.01.T07	100,00	0,00E+00	4091	Os08g0157600	98,86	0,00E+00	3233	-858	Indica	Indica	
maker-8-augustus-gene-36.39-mRNA-1	8	OsR498G0815235900.01.T03	100,00	0,00E+00	2046	Os08g0158500	99,91	0,00E+00	2041	-5	NA	Indica	
maker-8-augustus-gene-36.46-mRNA-1	8	OsR498G0815236300.01.T01	100,00	0,00E+00	731	Os08g0158600	99,72	0,00E+00	645	-86	Indica	NA	
maker-8-augustus-gene-36.48-mRNA-1	8	OsR498G0815236900.01.T04	100,00	0,00E+00	706	Os08g0159100	100,00	0,00E+00	706	0	NA	Indica	
maker-8-augustus-gene-36.51-mRNA-1	8	OsR498G0815232800.01.T01	100,00	0,00E+00	3891	Os08g0157800	100,00	0,00E+00	3891	0	NA	Indica	
maker-8-augustus-gene-36.55-mRNA-1	8	OsR498G0815233600.01.T02	100,00	0,00E+00	3950	Os08g0157900	99,95	0,00E+00	3946	-4	NA	Indica	
maker-8-augustus-gene-36.58-mRNA-1	8	OsR498G0815236600.01.T02	100,00	0,00E+00	2511	Os08g0158900	100,00	0,00E+00	2511	0	NA	Indica	

maker-8-augustus-gene-37.16-mRNA-1	8	OsR498G0815241900.01.T02	94,93	0,00E+00	1613	Os08g0160300	98,35	0,00E+00	1371	-242	Indica	Japonica	
maker-8-augustus-gene-37.19-mRNA-1	8	OsR498G0815239000.01.T04	100,00	0,00E+00	973	Os08g0159500	99,81	0,00E+00	968	-5	NA	Indica	
maker-8-augustus-gene-37.21-mRNA-1	8	OsR498G0815240600.01.T01	98,01	0,00E+00	1903	Os08g0159800	100,00	0,00E+00	1992	89	NA	Japonica	
maker-8-augustus-gene-37.23-mRNA-1	8	OsR498G0815241200.01.T01	100,00	0,00E+00	1315	Os08g0160000	100,00	0,00E+00	1315	0	NA	Japonica	
maker-8-augustus-gene-37.25-mRNA-1	8	OsR498G0815243400.01.T01	99,72	0,00E+00	5121	Os08g0160500	99,90	0,00E+00	5143	22	NA	Japonica	
maker-8-augustus-gene-38.10-mRNA-1	8	OsR498G0815249300.01.T01	99,84	0,00E+00	1095	Os05g0243300	89,37	0,00E+00	759	-336	Indica	Japonica	
maker-8-augustus-gene-38.17-mRNA-1	8	OsR498G0815247400.01.T02	99,66	0,00E+00	1043	Os08g0161500	99,83	0,00E+00	1048	5	NA	Japonica	
maker-8-augustus-gene-38.2-mRNA-1	8	OsR498G0815247900.01.T01	100,00	0,00E+00	639	Os08g0161700	100,00	0,00E+00	639	0	NA	Japonica	
maker-8-augustus-gene-38.21-mRNA-1	8	OsR498G0815248400.01.T01	100,00	0,00E+00	1020	Os08g0161800	100,00	0,00E+00	1020	0	NA	Japonica	
maker-8-augustus-gene-38.23-mRNA-1	8	OsR498G0815248600.01.T01	99,71	0,00E+00	1843	Os08g0161900	100,00	0,00E+00	1038	-805	Indica	Japonica	
maker-8-augustus-gene-38.6-mRNA-1	8	OsR498G0815249100.01.T01	99,78	0,00E+00	3191	Os03g0237000	75,02	0,00E+00	1137	-2054	Indica	Japonica	
maker-8-augustus-gene-39.0-mRNA-1	8	OsR498G0815251300.01.T01	99,91	0,00E+00	6122	Os08g0162100	100,00	0,00E+00	6136	14	NA	Japonica	
maker-8-augustus-gene-39.13-mRNA-1	8	OsR498G0815253500.01.T01	99,87	0,00E+00	1408	Os08g0162600	100,00	0,00E+00	1413	5	NA	Japonica	
maker-8-augustus-gene-39.19-mRNA-1	8	OsR498G0815255900.01.T01	96,84	0,00E+00	2723	Os08g0163400	96,78	0,00E+00	2718	-5	NA	Japonica	
maker-8-augustus-gene-39.23-mRNA-1	8	OsR498G0815257100.01.T01	100,00	0,00E+00	2381	Os08g0163500	100,00	0,00E+00	2381	0	NA	NA	
maker-8-augustus-gene-39.25-mRNA-1	8	OsR498G0815254000.01.T01	100,00	9,15E-137	486	Os08g0162800	100,00	3,18E-137	486	0	NA	NA	
maker-8-augustus-gene-39.28-mRNA-1	8	OsR498G0306434400.01.T02	96,48	0,00E+00	2384	Os08g0163100	99,86	0,00E+00	2540	156	NA	Japonica	
maker-8-augustus-gene-39.8-mRNA-1	8	OsR498G0815253300.01.T01	99,81	0,00E+00	921	Os08g0162500	100,00	0,00E+00	926	5	NA	Japonica	
maker-8-augustus-gene-4.44-mRNA-1	8	OsR498G0815040500.01.T01	99,37	0,00E+00	2794	Os08g0106300	100,00	0,00E+00	2841	47	NA	Japonica	
maker-8-augustus-gene-4.46-mRNA-1	8	OsR498G0815042100.01.T01	99,86	0,00E+00	1311	Os08g0107300	99,86	0,00E+00	1311	0	NA	Japonica	
maker-8-augustus-gene-4.49-mRNA-1	8	OsR498G0815042500.01.T02	100,00	0,00E+00	1938	Os08g0107500	100,00	0,00E+00	1938	0	NA	Japonica	
maker-8-augustus-gene-4.51-mRNA-1	8	OsR498G0815042700.01.T01	99,80	0,00E+00	3575	Os08g0107600	100,00	0,00E+00	3593	18	NA	Japonica	
maker-8-augustus-gene-4.54-mRNA-1	8	OsR498G0815044300.01.T02	98,09	0,00E+00	810	Os08g0107900	100,00	0,00E+00	868	58	NA	Japonica	
maker-8-augustus-gene-4.56-mRNA-1	8	OsR498G0815045300.01.T01	99,93	0,00E+00	2734	Os08g0108200	100,00	0,00E+00	2738	4	NA	Japonica	
maker-8-augustus-gene-40.53-mRNA-1	8	OsR498G0815258700.01.T01	100,00	0,00E+00	1463	Os08g0164300	100,00	0,00E+00	1745	282	Japonica	Japonica	
maker-8-augustus-gene-40.61-mRNA-1	8	OsR498G0815262100.01.T01	100,00	0,00E+00	1088	Os08g0163900	91,68	0,00E+00	1518	430	Japonica	Indica	
maker-8-augustus-gene-40.64-mRNA-1	8	OsR498G0815257600.01.T01	99,83	0,00E+00	1084	Os08g0163900	96,64	0,00E+00	1749	665	Japonica	Japonica	
maker-8-augustus-gene-40.69-mRNA-1	8	OsR498G0815258200.01.T01	99,46	0,00E+00	2306	Os08g0164100	99,92	0,00E+00	2333	27	NA	Japonica	
maker-8-augustus-gene-41.11-mRNA-1	8	OsR498G0815269800.01.T01	100,00	0,00E+00	2446	Os08g0167400	99,63	0,00E+00	2398	-48	NA	Indica	
maker-8-augustus-gene-41.14-mRNA-1	8	OsR498G0815270400.01.T01	100,00	2,57E-92	338	Os08g0167600	95,46	6,47E-120	429	91	Japonica	Indica	
maker-8-augustus-gene-41.4-mRNA-1	8	OsR498G0815270100.01.T02	100,00	0,00E+00	1375	Os08g0167500	100,00	0,00E+00	1375	0	NA	Indica	
maker-8-augustus-gene-41.7-mRNA-1	8	OsR498G0815271100.01.T02	91,91	0,00E+00	941	Os08g0168000	99,54	0,00E+00	1159	218	Japonica	Indica	
maker-8-augustus-gene-41.9-mRNA-1	8	OsR498G0917743600.01.T01	85,17	0,00E+00	707	Os08g0168150	93,46	0,00E+00	935	228	Japonica	Indica	
maker-8-augustus-gene-42.33-mRNA-1	8	OsR498G0815272900.01.T02	100,00	0,00E+00	2587	Os08g0168700	100,00	0,00E+00	2587	0	NA	Indica	
maker-8-augustus-gene-42.39-mRNA-1	8	OsR498G0815275500.01.T01	88,89	0,00E+00	707	Os08g0169300	100,00	0,00E+00	1142	435	Japonica	Japonica	
maker-8-augustus-gene-42.48-mRNA-1	8	OsR498G0815277100.01.T03	99,74	0,00E+00	698	Os08g0169800	100,00	0,00E+00	702	4	NA	Japonica	
maker-8-augustus-gene-42.55-mRNA-1	8	OsR498G0815277600.01.T01	100,00	0,00E+00	5104	Os08g0170700	73,56	0,00E+00	1699	-3405	Indica	Japonica	CC-LRR-NBS-TM
maker-8-augustus-gene-43.0-mRNA-1	8	OsR498G0815279900.01.T01	100,00	0,00E+00	1894	Os08g0170200	100,00	0,00E+00	1894	0	NA	NA	NBS-TM
maker-8-augustus-gene-43.13-mRNA-1	8	OsR498G0815285000.01.T01	100,00	0,00E+00	5609	Os05g0240200	72,51	0,00E+00	1249	-4360	Indica	NA	CC-NBS-TM
maker-8-augustus-gene-43.17-mRNA-1	8	OsR498G0815285200.01.T01	100,00	0,00E+00	3961	Os08g0170900	100,00	0,00E+00	3961	0	NA	NA	
maker-8-augustus-gene-43.19-mRNA-1	8	OsR498G0815286000.01.T03	100,00	0,00E+00	655	Os08g0171100	100,00	8,00E-89	325	-330	Indica	NA	
maker-8-augustus-gene-43.3-mRNA-1	8	OsR498G0815285700.01.T01	100,00	0,00E+00	1754	Os08g0171000	100,00	0,00E+00	1754	0	NA	NA	
maker-8-augustus-gene-44.11-mRNA-1	8	OsR498G0815288000.01.T01	100,00	0,00E+00	6736	Os04g0641000	71,49	0,00E+00	1012	-5724	Indica	NA	
maker-8-augustus-gene-44.2-mRNA-1	8	OsR498G0815291700.01.T04	94,80	0,00E+00	1421	Os08g0172200	100,00	0,00E+00	1162	-259	Indica	NA	
maker-8-augustus-gene-45.30-mRNA-1	8	OsR498G0815296500.01.T01	98,89	1,18E-180	632	Os08g0173600	99,60	3,37E-125	447	-185	Indica	Japonica	
maker-8-augustus-gene-45.32-mRNA-1	8	OsR498G0815297700.01.T01	95,90	0,00E+00	2306	Os08g0174300	100,00	0,00E+00	2544	238	NA	Japonica	
maker-8-augustus-gene-45.34-mRNA-1	8	OsR498G0815299300.01.T01	99,31	0,00E+00	2067	Os08g0174700	100,00	0,00E+00	2104	37	NA	Japonica	Kinase-LRR-TM
maker-8-augustus-gene-45.40-mRNA-1	8	OsR498G0815299300.01.T01	95,13	0,00E+00	1001	Os08g0174800	100,00	0,00E+00	1198	197	Japonica	Japonica	Kinase-TM
maker-8-augustus-gene-45.42-mRNA-1	8	OsR498G0815300300.01.T01	99,77	0,00E+00	2308	Os08g0174900	100,00	0,00E+00	2322	14	NA	Japonica	
maker-8-augustus-gene-45.44-mRNA-1	8	OsR498G0816183800.01.T01	81,61	0,00E+00	949	Os08g0442200	80,73	0,00E+00	906	-43	NA	Japonica	
maker-8-augustus-gene-45.45-mRNA-1	8	OsR498G0815297600.01.T01	96,89	0,00E+00	2410	Os08g0174100	100,00	0,00E+00	1377	-1033	Indica	Japonica	
maker-8-augustus-gene-45.47-mRNA-1	8	OsR498G0815301600.01.T01	99,05	0,00E+00	2973	Os08g0175200	99,82	0,00E+00	3031	58	NA	Japonica	
maker-8-augustus-gene-46.37-mRNA-1	8	OsR498G0815302500.01.T01	96,08	0,00E+00	1425	Os08g0176100	97,92	0,00E+00	1491	66	NA	Japonica	
maker-8-augustus-gene-46.39-mRNA-1	8	OsR498G0815299300.01.T01	89,50	0,00E+00	1378	Os08g0176200	100,00	0,00E+00	1667	289	Japonica	NA	Kinase-TM
maker-8-augustus-gene-46.46-mRNA-1	8	OsR498G0815301800.01.T04	98,52	0,00E+00	3752	Os08g0175300	100,00	0,00E+00	3896	144	NA	Japonica	
maker-8-augustus-gene-46.47-mRNA-1	8	OsR498G0815302200.01.T03	98,22	0,00E+00	3687	Os08g0175300	95,41	0,00E+00	3491	-196	NA	Japonica	
maker-8-augustus-gene-47.28-mRNA-1	8	OsR498G0815307300.01.T01	90,77	6,34E-133	474	Os08g0177700	90,77	2,20E-133	474	0	NA	Japonica	
maker-8-augustus-gene-47.47-mRNA-1	8	OsR498G0815305000.01.T05	96,66	0,00E+00	5522	Os08g0177300	97,09	0,00E+00	5584	62	NA	Japonica	
maker-8-augustus-gene-47.56-mRNA-1	8	OsR498G0815306800.01.T01	100,00	2,90E-66	251	Os04g0635900	73,19	1,09E-15	83,3	-167,7	Indica	Japonica	
maker-8-augustus-gene-47.58-mRNA-1	8	OsR498G0815307000.01.T01	100,00	0,00E+00	857	Os04g0635900	73,30	2,37E-91	335	-522	Indica	Japonica	
maker-8-augustus-gene-48.37-mRNA-1	8	OsR498G0815313300.01.T02	99,78	0,00E+00	1620	Os08g0179900	100,00	0,00E+00	1629	9	NA	Japonica	
maker-8-augustus-gene-48.47-mRNA-1	8	OsR498G0815309600.01.T01	99,67	0,00E+00	2700	Os08g0178700	100,00	0,00E+00	2722	22	NA	Japonica	
maker-8-augustus-gene-48.53-mRNA-1	8	OsR498G0408688000.01.T01	99,57	0,00E+00	2088	Os08g0178900	100,00	0,00E+00	2111	23	NA	Japonica	
maker-8-augustus-gene-48.59-mRNA-1	8	OsR498G0815313600.01.T01	92,06	0,00E+00	989	Os08g0180000	92,21	0,00E+00	994	5	NA	Japonica	
maker-8-augustus-gene-49.0-mRNA-1	8	OsR498G0815314100.01.T02	98,94	0,00E+00	4164	Os08g0180300	99,07	0,00E+00	4177	13	NA	Japonica	
maker-8-augustus-gene-5.0-mRNA-1	8	OsR498G0815048900.01.T01	99,83	0,00E+00	2167	Os08g0109000	100,00	0,00E+00	2176	9	NA	Japonica	
maker-8-augustus-gene-5.13-mRNA-1	8	OsR498G0612486200.01.T01	99,51	0,00E+00	1454	Os08g0109200	100,00	0,00E+00	2289	835	Japonica	Japonica	
maker-8-augustus-gene-5.17-mRNA-1	8	OsR498G0815049700.01.T06	99,87	0,00E+00	1333	Os08g0109400	100,00	0,00E+00	1337	4	NA	Japonica	
maker-8-augustus-gene-5.21-mRNA-1	8	OsR498G0815049900.01.T01	99,74	0,00E+00	2798	Os08g0109500	99,94	0,00E+00	2811	13	NA	Japonica	
maker-8-augustus-gene-5.25-mRNA-1	8	OsR498G0815048200.01.T01	96,05	0,00E+00	1171	Os08g0108700	100,00	0,00E+00	1278	107	NA	Japonica	
maker-8-augustus-gene-5.28-mRNA-1	8	OsR498G0815049400.01.T01	99,89	0,00E+00	1571	Os08g0109300	100,00	0,00E+00	1575	4	NA	Japonica	
maker-8-augustus-gene-50.33-mRNA-1	8	OsR498G0815323400.01.T01	99,83	0,00E+00	1073	Os08g0182400	100,00	0,00E+00	1077	4	NA	Japonica	
maker-8-augustus-gene-51.36-mRNA-1	8	OsR498G0815328600.01.T03	99,84	0,00E+00	1132	Os08g0183900	99,05	0,00E+00	920	-212	Indica	NA	
maker-8-augustus-gene-52.0-mRNA-1	8	OsR498G0815332900.01.T01	97,60	0,00E+00	3210	Os04g0627000	81,16	0,00E+00	1838	-1372	Indica	Japonica	
maker-8-augustus-gene-52.10-mRNA-1	8	OsR498G0815336300.01.T01	98,79	0,00E+00	1159	Os08g0188900	98,79	0,00E+00	1159	0	NA	NA	
maker-8-augustus-gene-52.12-mRNA-1	8	OsR498G0815336500.01.T01	99,39	0,00E+00	1171	Os08g0189100	99,55	0,00E+00	1188	17	NA	NA	
maker-8-augustus-gene-52.14-mRNA-1	8	OsR498G0815333200.01.T01	99,68	0,00E+00	3872	Os08g0187700	99,63	0,00E+00	3867	-5	NA	Indica	
maker-8-augustus-gene-52.17-mRNA-1	8	OsR498G0815334200.01.T01	99,55	0,00E+00	1962	Os08g0188000	99,82	0,00E+00	1941	-21	NA	NA	
maker-8-augustus-gene-52.4-mRNA-1	8	OsR498G0815333700.01.T03	99,58	0,00E+00	2110	Os08g0187800	99,57	0,00E+00	2065	-45	NA	NA	
maker-8-augustus-gene-53.35-mRNA-1	8	OsR498G0815337700.01.T01	99,05	0,00E+00	1110	Os08g0189200	99,21	0,00E+00	1114	4	NA	Indica	
maker-8-augustus-gene-53.37-mRNA-1	8	OsR498G0815338500.01.T01	99,12	0,00E+00	1196	Os08g0189400	99,84	0,00E+00	1105	-91	NA	NA	
maker-8-augustus-gene-53.39-mRNA-1	8	OsR498G0815338900.01.T01	100,00	0,00E+00	1223	Os08g0189500	99,85	0,00E+00	1219	-4	NA	Japonica	
maker-8-augustus-gene-53.41-mRNA-1	8	OsR498G0815339300.01.T01	99,12	0,00E+00	1196	Os08g0189600	99,26	0,00E+00	1201	5	NA	NA	
maker-8-augustus-gene-53.43-mRNA-1	8	OsR498G0815340100.01.T01	98,37	0,00E+00	1168	Os08g0189700	99,70	0,00E+00	1209	41	NA	NA	
maker-8-augustus-gene-53.45-mRNA-1	8	OsR498G0815340500.01.T01	99,70	0,00E+00	1209	Os08g0189850	99,70	0,00E+00	1209	0	NA	NA	
maker-8-augustus-gene-53.47-mRNA-1	8	OsR498G0815341300.01.T01	99,41	0,00E+00	1200	Os08g0189900	99,70	0,00E+00	1209	9	NA	NA	
maker-8-augustus-gene-53.49-mRNA-1	8	OsR498G0815344500.01.T02	99,36	0,00E+00	2215	Os08g0190700	99,90	0,00E+00	3649	1434	Japonica	Indica	
maker-8-augustus-gene-53.51-mRNA-1	8	OsR498G0815342400.01.T04	96,65	0,00E+00	2187	Os04g0625800	79,29	0,00E+00	1122	-1065	Indica	NA	
maker-8-augustus-gene-53.54-mRNA-1	8	OsR498G0815343400.01.T01	95,37	0,00E+00	4291	Os08g0190300	95,84	0,00E+00	5036	745	Japonica	Indica	CC-LRR-NBS-TM
maker-8-augustus-gene-53.56-mRNA-1	8	OsR498G0815344800.01.T02	99,62	2,70E-132	472	Os08g0190800	98,26	5,93E-110	396	-76	Indica	NA	
maker-8-augustus-gene-54.0-mRNA-1	8	OsR498G0815345500.01.T01	98,30	0,00E+00	2736	Os08g0191200	98,23	0,00E+00	2731	-5	NA	NA	
maker-8-augustus-gene-54.13-mRNA-1	8	OsR498G0815348700.01.T05	99,76	0,00E+00	743	Os08g0192200	99,52	0,00E+00	738	-5	NA	NA	
maker-8-augustus-gene-54.16-mRNA-1	8	OsR498G0815345200.01.T01	98,20	0,00E+00	5166	Os03g0136900	75,88	0,00E+00	1863	-3303	Indica	Japonica	
maker-8-augustus-gene-54.2-mRNA-1	8	OsR498G0815345700.01.T01	99,74	0,00E+00	6092	Os04g0624600	77,84	0,00E+00	2260	-3832	Indica	Indica	
maker-8-augustus-gene-54.22-mRNA-1	8	OsR498G0815347200.01.T01	100,00	0,00E+00	1581	Os08g0191700	100,00	0,00E+00	1581	0	NA	NA	
maker-8-augustus-gene-54.27-mRNA-1	8	OsR498G0815347600.01.T01	99,72	0,00E+00	1910	Os08g0191800	99,81	0,00E+00	1914	4	NA	NA	
maker-8-augustus-gene-54.31-mRNA-1	8	OsR498G0815349000.01.T01	97,16	0,00E+00	1903	Os08g0192400	100,00	0,00E+00	1306	-597	Indica	Japonica	
maker-8-augustus-gene-54.6-mRNA-1	8	OsR498G0815346100.01.T04	99,71	1,35E-174	613	Os08g0191600	100,00	1,53E-168	590	-23	NA	Indica	
maker-8-augustus-gene-55.0-mRNA-1	8	OsR498G0815350700.01.T01	98,36	0,00E+00	2536	Os08g0192800	98,43	0,00E+00	2541	5	NA	NA	
maker-8-augustus-gene-55.14-mRNA-1	8	OsR498G0815351000.01.T01	99,07	0,00E+00	3040	Os08g0192900	99,30	0,00E+00	3058	18	NA	NA	
maker-8-augustus-gene-55.20-mRNA-1	8	OsR498G0815353200.01.T01	85,56	0,00E+00	756	Os08g0193200	95,85	0,00E+00	1589	833	Japonica	NA	
maker-8-augustus-gene-55.22-mRNA-1	8	OsR498G0815353200.01.T01	98,27	0,00E+00	1199	Os08g0193200	85,31	0,00E+00	798	-401	Indica	Japonica	
maker-8-augustus-gene-55.24-mRNA-1	8	OsR498G0815353600.01.T01	99,11	0,00E+00	2369	Os08g0193500	99,55	0,00E+00	2397	28	NA	Japonica	
maker-8-augustus-gene-55.26-mRNA-1	8	OsR498G0815356200.01.T01	99,50	0,00E+00	2120	Os08g0193900	100,00	0,00E+00	2149	29	NA	Japonica	
maker-8-augustus-gene-56.0-mRNA-1	8	OsR498G0815361000.01.T03	97,96	0,00E+00	1336	Os08g0195400	99,76	0,00E+00	734	-602	Indica	Japonica	
maker-8-augustus-gene-56.4-mRNA-1	8	OsR498G0815362200.01.T01	99,81	0,00E+00	930	Os08g0196200	100,00	0,00E+00	1440	510	Japonica	Japonica	
maker-8-augustus-gene-57.41-mRNA-1	8	OsR498G0815364700.01.T02	97,60	0,00E+00	985	Os08g0196700	99,83	0,00E+00	1046	61	NA	Japonica	
maker-8-augustus-gene-57.45-mRNA-1	8	OsR498G0815364900.01.T03	99,91	0,00E+00	2074	Os08g0196900	95,57	0,00E+00	1890	-184	NA	Japonica	
maker-8-augustus-gene-57.49-mRNA-1	8	OsR498G0815365300.01.T01	100,00	0,00E+00	2798	Os08g0197000	99,94	0,00E+00	2793	-5	NA	NA	
maker-8-augustus-gene-57.52-mRNA-1	8	OsR498G0815365500.01.T01	100,00	0,00E+00	2911	Os08g0197050	100,00	0,00E+00	2131	-780	Indica	NA	
maker-8-augustus-gene-57.56-mRNA-1	8	OsR498G0815365700.01.T01	100,00	0,00E+00	2938	Os08g0197100	100,00	0,00E+00	2116	-822	Indica	Japonica	
maker-8-augustus-gene-57.59-mRNA-1	8	OsR498G0815366100.01.T01	99,93	0,00E+00	2620	Os08g0197200	100,00	0,00E+00	2625	5	NA	NA	
maker-8-augustus-gene-57.61-mRNA-1	8	OsR498G0815366900.01.T01	100,00	0,00E+00	2987	Os08g0197500	86,02	0,00E+00	1933	-1054	Indica	Indica	
maker-8-augustus-gene-57.63-mRNA-1	8	OsR498G0815367100.01.T01	100,00	0,00E+00	2944	Os08g0197500	99,94	0,00E+00	2939	-5	NA	Japonica	
maker-8-augustus-gene-57.65-mRNA-1	8	OsR498G0815367900.01.T04	100,00	0,00E+00	1128	Os08g0197800	100,00	0,00E+00	1128	0	NA	Japonica	
maker-8-augustus-gene-58.10-mRNA-1	8	OsR498G0815375700.01.T05	100,00	0,00E+00	1449	Os08g0199300	100,00	0,00E+00	1449	0	NA	Japonica	
maker-8-augustus-gene-58.18-mRNA-1	8	OsR498G0815376000.01.T01	99,84	0,00E+00	2280	Os08g0199400	99,29	0,00E+00	2252	-28	NA	Japonica	
maker-8-augustus-gene-59.0-mRNA-1	8	OsR498G0815381500.01.T04	99,73	0,00E+00	1989	Os08g0200500	100,00	0,00E+00	2001	12	NA	NA	Kinase
maker-8-augustus-gene-59.13-mRNA-1	8	OsR498G0815382000.01.T01	100,00	0,00E+00	1591	Os08g0200600	100,00	0,00E+00	1591	0	NA	Japonica	
maker-8-augustus-gene-59.2-mRNA-1	8	OsR498G0815379800.01.T01	99,84	0,00E+00	2183	Os08g0200100	100,00	0,00E+00	2192	9	NA	Japonica	
maker-8-augustus-gene-59.6-mRNA-1	8	OsR498G0815380800.01.T01	89,73	7,52E-178	624	Os08g0200300	89,73	2,61E-178	624	0	NA	Japonica	
maker-8-augustus-gene-59.8-mRNA-1	8	OsR498G0815381000.01.T03	97,84	0,00E+00	3348	Os08g0200400	97,14	0,00E+00	2317	-1031	Indica	Japonica	
maker-8-augustus-gene-6.30-mRNA-1	8	OsR498G0815055900.01.T01	99,56	0,00E+00	4415	Os08g0110500	100,00	0,00E+00	4464	49	NA	Japonica	
maker-8-augustus-gene-6.32-mRNA-1	8	OsR498G0815056700.01.T01	99,95	0,00E+00	3268	Os08g0110800	100,00	0,00E+00	2105	-1163	Indica	Japonica	
maker-8-augustus-gene-6.36-mRNA-1	8	OsR498G0815053400.01.T03	99,89	0,00E+00	1608	Os08g0109900	98,35	0,00E+00	1581	-27	NA	Japonica	
maker-8-augustus-gene-6.42-mRNA-1	8	OsR498G0815053800.01.T01	100,00	0,00E+00	2159	Os08g0110000	100,00	0,00E+00	2159	0	NA	Japonica	
maker-8-augustus-gene-6.46-mRNA-1	8	OsR498G0815055800.01.T02	99,84	0,00E+00	2199	Os08g0110400	100,00	0,00E+00	2208	9	NA	Japonica	
maker-8-augustus-gene-6.48-mRNA-1	8	OsR498G0815056400.01.T01	99,87	0,00E+00	1425	Os08g0110600	99,87	0,00E+00	1349	-76	NA	Japonica	
maker-8-augustus-gene-6.50-mRNA-1	8	OsR498G0815056600.01.T04	99,89	0,00E+00	4894	Os08g0110700	99,96	0,00E+00	4903	9	NA	Japonica	
maker-8-augustus-gene-6.55-mRNA-1	8	OsR498G0815057200.01.T01	99,87	0,00E+00	4279	Os08g0111200	98,36	0,00E+00	4148	-131	NA	Japonica	
maker-8-augustus-gene-60.1-mRNA-1	8	OsR498G0815383300.01.T05	98,84	0,00E+00	3494	Os08g0201700	96,13	0,00E+00	3978	484	Japonica	Japonica	Kinase-LRR-TM
maker-8-augustus-gene-60.12-mRNA-1	8	OsR498G0815386000.01.T02	99,85	0,00E+00	2329	Os08g0202400	99,38	0,00E+00	2302	-27	NA	Indica	NBS-TM
maker-8-augustus-gene-60.8-mRNA-1	8	OsR498G0815385700.01.T13	85,47	0,00E+00	2250	Os08g0201700	78,25	0,00E+00	1609	-641	Indica	Indica	CC-Kinase-LRR-TM
maker-8-augustus-gene-61.30-mRNA-1	8	OsR498G0815386700.01.T02	98,61	0,00E+00	3267	Os08g0203300	99,95	0,00E+00	3379	112	NA	Japonica	Kinase-LRR-TM
maker-8-augustus-gene-61.45-mRNA-1	8	OsR498G0815387700.01.T03	99,78	0,00E+00	3330	Os08g0203400	90,79	0,00E+00	2715	-615	Indica	Japonica	Kinase-LRR-TM
maker-8-augustus-gene-61.50-mRNA-1	8	OsR498G0815387900.01.T03	99,23	0,00E+00	4823	Os08g0203700	99,78	0,00E+00	4852	29	NA	Japonica	Kinase-LRR-TM
maker-8-augustus-gene-62.0-mRNA-1	8	OsR498G0306965200.01.T01	97,57	8,55E-137	487	Os08g0204632	100,00	6,13E-146	515	28	NA	Japonica	
maker-8-augustus-gene-62.2-mRNA-1	8	OsR498G0815389800.01.T01	99,86	0,00E+00	1259	Os08g0204800	99,86	0,00E+00	1259	0	NA	Japonica	
maker-8-augustus-gene-63.0-mRNA-1	8	OsR498G0815390700.01.T01	76,72	0,00E+00	691	Os08g0205100	100,00	0,00E+00	1689	998	Japonica	Japonica	CC-NBS-TM
maker-8-augustus-gene-63.1-mRNA-1	8	OsR498G0815390700.01.T02	96,93	0,00E+00	2558	Os08g0205150	100,00	0,00E+00	2771	213	NA	Japonica	CC-NBS-TM
maker-8-augustus-gene-63.12-mRNA-1	8	OsR498G0815397300.01.T01	99,12	0,00E+00	4211	Os04g0613900	83,31	0,00E+00	2496	-1715	Indica	Japonica	
maker-8-augustus-gene-63.17-mRNA-1	8	OsR498G0815398700.01.T02	99,33	0,00E+00	3199	Os08g0206600	100,00	0,00E+00	3252	53	NA	Japonica	
maker-8-augustus-gene-63.22-mRNA-1	8	OsR498G0815393200.01.T01	97,73	0,00E+00	2168	Os08g0205200	100,00	0,00E+00	2295	127	NA	Japonica	
maker-8-augustus-gene-63.24-mRNA-1	8	OsR498G0815394600.01.T02	99,22	0,00E+00	2711	Os08g0205900	100,00	0,00E+00	2765	54	NA	Japonica	
maker-8-augustus-gene-63.29-mRNA-1	8	OsR498G0815397600.01.T01	99,73	0,00E+00	1344	Os08g0206500	99,00	0,00E+00	1590	246	Japonica	Japonica	
maker-8-augustus-gene-63.7-mRNA-1	8	OsR498G0815393700.01.T01	100,00	0,00E+00	785	Os08g0205400	100,00	1,45E-124	444	-341	Indica	Japonica	
maker-8-augustus-gene-64.47-mRNA-1	8	OsR498G0815399300.01.T02	95,60	0,00E+00	2493	Os08g0206900	95,67	0,00E+00	2498	5	NA	Japonica	
maker-8-augustus-gene-64.53-mRNA-1	8	OsR498G0815402500.01.T01	99,92	0,00E+00	2144	Os08g0207500	100,00	0,00E+00	2149	5	NA	Japonica	
maker-8-augustus-gene-64.64-mRNA-1	8	OsR498G0815400000.01.T01	99,77	0,00E+00	768	Os04g0613600	86,50	1,95E-148	524	-244	Indica	Japonica	
maker-8-augustus-gene-67.33-mRNA-1	8	OsR498G0815426000.01.T02	98,81	0,00E+00	734	Os08g0216900	99,22	0,00E+00	680	-54	NA	NA	
maker-8-augustus-gene-69.0-mRNA-1	8	OsR498G0815381500.01.T04	99,73	0,00E+00	1989	Os08g0200500	100,00	0,00E+00	2001	12	NA	NA	Kinase
maker-8-augustus-gene-69.5-mRNA-1	8	OsR498G0815382000.01.T02	100,00	0,00E+00	1242	Os08g0200600	100,00	0,00E+00	1242	0	NA	NA	
maker-8-augustus-gene-7.33-mRNA-1	8	OsR498G0815059100.01.T01	98,01	0,00E+00	1466	Os08g0112000	100,00	0,00E+00	1631	165	Japonica	Japonica	
maker-8-augustus-gene-7.41-mRNA-1	8	OsR498G0815061500.01.T01	99,54	0,00E+00	771	Os08g0112600	100,00	0,00E+00	780	9	NA	Japonica	
maker-8-augustus-gene-7.44-mRNA-1	8	OsR498G0815061700.01.T01	93,57	0,00E+00	1047	Os08g0112700	93,72	0,00E+00	1051	4	NA	Japonica	
maker-8-augustus-gene-7.47-mRNA-1	8	OsR498G0815062300.01.T01	99,91	0,00E+00	2063	Os08g0112900	100,00	0,00E+00	2067	4	NA	Japonica	
maker-8-augustus-gene-7.50-mRNA-1	8	OsR498G0815062900.01.T01	99,88	0,00E+00	1446	Os08g0113100	99,88	0,00E+00	1446	0	NA	Japonica	
maker-8-augustus-gene-7.53-mRNA-1	8	OsR498G0815061000.01.T02	99,73	0,00E+00	4026	Os08g0112500	100,00	0,00E+00	4053	27	NA	Japonica	CC-Kinase-TM
maker-8-augustus-gene-7.57-mRNA-1	8	OsR498G0815062200.01.T01	99,76	0,00E+00	741	Os08g0112800	100,00	0,00E+00	746	5	NA	Japonica	
maker-8-augustus-gene-72.29-mRNA-1	8	OsR498G0815426000.01.T02	99,13	0,00E+00	1012	Os08g0216900	100,00	0,00E+00	991	-21	NA	Japonica	
maker-8-augustus-gene-73.1-mRNA-1	8	OsR498G0815510700.01.T01	97,74	0,00E+00	829	Os08g0239300	99,80	0,00E+00	875	46	NA	NA	
maker-8-augustus-gene-74.2-mRNA-1	8	OsR498G0815443000.01.T01	99,85	0,00E+00	1224	Os08g0222100	100,00	0,00E+00	1229	5	NA	Japonica	
maker-8-augustus-gene-75.2-mRNA-1	8	OsR498G0815444300.01.T03	99,79	0,00E+00	3397	Os08g0220600	100,00	0,00E+00	3415	18	NA	Japonica	
maker-8-augustus-gene-78.0-mRNA-1	8	OsR498G0815457900.01.T06	98,73	0,00E+00	2337	Os08g0223700	99,72	0,00E+00	1910	-427	Indica	Japonica	
maker-8-augustus-gene-79.30-mRNA-1	8	OsR498G0815461300.01.T05	100,00	0,00E+00	1472	Os08g0223900	100,00	0,00E+00	1472	0	NA	Japonica	
maker-8-augustus-gene-79.35-mRNA-1	8	OsR498G0815462500.01.T01	100,00	0,00E+00	2262	Os08g0224100	100,00	0,00E+00	2262	0	NA	Japonica	Kinase-TM
maker-8-augustus-gene-79.37-mRNA-1	8	OsR498G0815464100.01.T01	98,92	0,00E+00	2607	Os08g0224500	99,93	0,00E+00	2674	67	NA	Japonica	
maker-8-augustus-gene-79.39-mRNA-1	8	OsR498G0815464500.01.T01	99,93	0,00E+00	5337	Os08g0224700	100,00	0,00E+00	5346	9	NA	Japonica	
maker-8-augustus-gene-79.45-mRNA-1	8	OsR498G0815465100.01.T01	99,85	0,00E+00	2324	Os10g0387000	97,37	0,00E+00	2184	-140	NA	Japonica	
maker-8-augustus-gene-79.51-mRNA-1	8	OsR498G0815462800.01.T01	99,94	0,00E+00	3123	Os08g0224200	100,00	0,00E+00	3128	5	NA	Japonica	
maker-8-augustus-gene-79.61-mRNA-1	8	OsR498G0815465600.01.T01	97,51	0,00E+00	1090	Os08g0224900	95,13	0,00E+00	1100	10	NA	Japonica	
maker-8-augustus-gene-8.12-mRNA-1	8	OsR498G0815068300.01.T01	100,00	0,00E+00	2836	Os08g0114200	100,00	0,00E+00	2836	0	NA	NA	
maker-8-augustus-gene-8.14-mRNA-1	8	OsR498G0815063200.01.T03	97,82	0,00E+00	4328	Os08g0113200	98,21	0,00E+00	4373	45	NA	Japonica	
maker-8-augustus-gene-8.26-mRNA-1	8	OsR498G0815068800.01.T01	100,00	0,00E+00	1792	Os08g0114300	100,00	0,00E+00	3193	1401	Japonica	NA	
maker-8-augustus-gene-8.28-mRNA-1	8	OsR498G0815070600.01.T01	100,00	0,00E+00	2944	Os08g0115200	100,00	0,00E+00	2944	0	NA	NA	
maker-8-augustus-gene-8.9-mRNA-1	8	OsR498G0815066500.01.T02	100,00	0,00E+00	1418	Os08g0114100	100,00	0,00E+00	1418	0	NA	NA	
maker-8-augustus-gene-80.0-mRNA-1	8	OsR498G0815470300.01.T01	99,90	0,00E+00	1803	Os08g0225400	100,00	0,00E+00	1808	5	NA	Japonica	
maker-8-augustus-gene-80.2-mRNA-1	8	OsR498G0815473200.01.T01	99,52	0,00E+00	1839	Os08g0226300	100,00	0,00E+00	2123	284	Japonica	Japonica	
maker-8-augustus-gene-81.32-mRNA-1	8	OsR498G0815471800.01.T01	94,34	0,00E+00	1251	Os08g0226800	100,00	0,00E+00	1975	724	Japonica	Japonica	
maker-8-augustus-gene-82.29-mRNA-1	8	OsR498G0815481500.01.T01	99,16	0,00E+00	1055	Os08g0228200	99,91	0,00E+00	2012	957	Japonica	Japonica	
maker-8-augustus-gene-83.42-mRNA-1	8	OsR498G0815485200.01.T03	99,90	0,00E+00	1751	Os08g0230300	99,35	0,00E+00	2463	712	Japonica	Japonica	
maker-8-augustus-gene-83.46-mRNA-1	8	OsR498G0815489200.01.T01	99,41	0,00E+00	1196	Os08g0231400	99,85	0,00E+00	1214	18	NA	Japonica	
maker-8-augustus-gene-85.10-mRNA-1	8	OsR498G0815501900.01.T01	99,73	0,00E+00	4649	Os08g0235600	99,93	0,00E+00	2709	-1940	Indica	Japonica	NBS-TM
maker-8-augustus-gene-85.14-mRNA-1	8	OsR498G0815500400.01.T02	95,73	9,58E-105	379	Os08g0236900	73,91	3,81E-22	104	-275	Indica	Indica	
maker-8-augustus-gene-85.4-mRNA-1	8	OsR498G0815500900.01.T01	99,19	0,00E+00	875	Os08g0235400	99,89	0,00E+00	1576	701	Japonica	Japonica	
maker-8-augustus-gene-85.5-mRNA-1	8	OsR498G0815500900.01.T01	99,07	2,49E-104	378	Os08g0235400	100,00	4,23E-96	349	-29	NA	Japonica	
maker-8-augustus-gene-86.0-mRNA-1	8	OsR498G0815503500.01.T01	98,32	0,00E+00	2065	Os08g0236700	99,91	0,00E+00	1920	-145	NA	Japonica	
maker-8-augustus-gene-86.3-mRNA-1	8	OsR498G0815506900.01.T01	99,66	0,00E+00	1562	Os08g0237000	99,54	0,00E+00	1557	-5	NA	NA	
maker-8-augustus-gene-87.0-mRNA-1	8	OsR498G1119807900.01.T01	97,39	0,00E+00	1419	Os08g0238200	99,76	0,00E+00	1490	71	NA	Japonica	
maker-8-augustus-gene-87.24-mRNA-1	8	OsR498G0815508800.01.T01	99,87	0,00E+00	1425	Os08g0237800	99,67	0,00E+00	1619	194	Japonica	Japonica	
maker-8-augustus-gene-87.6-mRNA-1	8	OsR498G0815509300.01.T01	96,43	0,00E+00	1397	Os11g0275000	99,39	0,00E+00	1453	56	NA	NA	
maker-8-augustus-gene-88.38-mRNA-1	8	OsR498G0815510300.01.T01	97,56	0,00E+00	1183	Os08g0239100	94,96	3,48E-160	563	-620	Indica	Indica	
maker-8-augustus-gene-88.44-mRNA-1	8	OsR498G0815510700.01.T01	97,75	0,00E+00	831	Os08g0239300	99,80	0,00E+00	876	45	NA	NA	
maker-8-augustus-gene-88.51-mRNA-1	8	OsR498G0815510900.01.T01	97,38	0,00E+00	707	Os08g0239900	99,77	0,00E+00	790	83	Japonica	Japonica	
maker-8-augustus-gene-88.52-mRNA-1	8	OsR498G0815510900.01.T01	93,78	0,00E+00	744	Os08g0239900	100,00	0,00E+00	874	130	Japonica	Japonica	
maker-8-augustus-gene-88.57-mRNA-1	8	OsR498G0815511900.01.T02	99,88	0,00E+00	1549	Os08g0240533	100,00	0,00E+00	1434	-115	NA	Japonica	
maker-8-augustus-gene-88.61-mRNA-1	8	OsR498G0815510900.01.T01	82,46	0,00E+00	890	Os08g0240000	100,00	0,00E+00	1889	999	Japonica	Japonica	
maker-8-augustus-gene-88.63-mRNA-1	8	OsR498G0815512600.01.T01	97,59	0,00E+00	1062	Os08g0240600	97,59	0,00E+00	1062	0	NA	Japonica	
maker-8-augustus-gene-89.0-mRNA-1	8	OsR498G0815512900.01.T05	97,84	0,00E+00	3022	Os08g0240800	97,95	0,00E+00	3031	9	NA	Japonica	
maker-8-augustus-gene-89.12-mRNA-1	8	OsR498G0815516200.01.T02	97,26	0,00E+00	1737	Os04g0658000	74,36	8,04E-166	584	-1153	Indica	Japonica	
maker-8-augustus-gene-89.7-mRNA-1	8	OsR498G0409229200.01.T06	73,78	3,53E-40	165	Os08g0241300	100,00	4,58E-141	499	334	Japonica	Japonica	
maker-8-augustus-gene-89.9-mRNA-1	8	OsR498G0815515100.01.T04	99,62	0,00E+00	1898	Os08g0241400	100,00	0,00E+00	1894	-4	NA	Japonica	
maker-8-augustus-gene-9.15-mRNA-1	8	OsR498G0815077300.01.T03	98,81	4,43E-167	587	Os08g0116500	98,81	1,54E-167	587	0	NA	NA	
maker-8-augustus-gene-9.18-mRNA-1	8	OsR498G0815076800.01.T01	100,00	0,00E+00	1640	Os08g0116400	99,59	0,00E+00	1306	-334	Indica	NA	
maker-8-augustus-gene-90.0-mRNA-1	8	OsR498G0815521100.01.T01	97,06	0,00E+00	2992	Os08g0242800	97,28	0,00E+00	3010	18	NA	Japonica	
maker-8-augustus-gene-90.12-mRNA-1	8	OsR498G0815519200.01.T01	99,77	0,00E+00	1534	Os08g0242400	100,00	0,00E+00	1543	9	NA	Japonica	
maker-8-augustus-gene-90.18-mRNA-1	8	OsR498G0815521400.01.T03	92,58	0,00E+00	1735	Os08g0242900	93,41	0,00E+00	1775	40	NA	Japonica	
maker-8-augustus-gene-90.4-mRNA-1	8	OsR498G0815521900.01.T04	99,48	0,00E+00	1704	Os08g0243100	99,90	0,00E+00	1722	18	NA	Japonica	
maker-8-augustus-gene-91.0-mRNA-1	8	OsR498G0815523900.01.T01	99,77	0,00E+00	3874	Os08g0243500	99,80	0,00E+00	3510	-364	NA	Japonica	
maker-8-augustus-gene-91.13-mRNA-1	8	OsR498G0815524400.01.T01	99,86	0,00E+00	1300	Os08g0243600	100,00	0,00E+00	1305	5	NA	NA	
maker-8-augustus-gene-91.16-mRNA-1	8	OsR498G0815525200.01.T02	99,67	0,00E+00	3800	Os06g0255700	77,29	0,00E+00	1479	-2321	Indica	Japonica	
maker-8-augustus-gene-91.18-mRNA-1	8	OsR498G0815525400.01.T01	94,86	0,00E+00	1503	Os08g0244100	95,18	0,00E+00	1517	14	NA	Japonica	

maker-8-augustus-gene-91.5-mRNA-1	8	OsR498G0714509400.01.T03	90,42	0,00E+00	757	Os08g0244500	98,73	0,00E+00	961	204	Japonica	Indica	
maker-8-augustus-gene-91.6-mRNA-1	8	OsR498G0714509400.01.T01	96,88	9,07E-119	426	Os08g0244500	100,00	4,38E-130	462	36	NA	Indica	
maker-8-augustus-gene-92.0-mRNA-1	8	OsR498G0815527100.01.T01	99,47	0,00E+00	3017	Os08g0245200	100,00	0,00E+00	3057	40	NA	Japonica	
maker-8-augustus-gene-92.2-mRNA-1	8	OsR498G0815527400.01.T01	99,80	0,00E+00	4425	Os08g0245400	100,00	0,00E+00	4448	23	NA	Japonica	
maker-8-augustus-gene-92.7-mRNA-1	8	OsR498G0815527800.01.T03	99,16	6,27E-179	627	Os08g0245800	100,00	5,13E-124	443	-184	Indica	NA	
maker-8-augustus-gene-93.0-mRNA-1	8	OsR498G0815530900.01.T02	98,96	0,00E+00	675	Os08g0246400	100,00	0,00E+00	693	18	NA	Japonica	
maker-8-augustus-gene-93.6-mRNA-1	8	OsR498G0815535500.01.T02	84,00	0,00E+00	2014	Os08g0248100	83,17	0,00E+00	1945	-69	NA	Japonica	Kinase-LRR-TM
maker-8-augustus-gene-93.7-mRNA-1	8	OsR498G0815535500.01.T02	90,23	0,00E+00	3865	Os08g0248100	88,30	0,00E+00	3585	-280	NA	Japonica	Kinase-LRR-TM
maker-8-augustus-gene-93.8-mRNA-1	8	OsR498G0815535700.01.T01	93,79	0,00E+00	4370	Os08g0248100	100,00	0,00E+00	5476	1106	Japonica	Japonica	Kinase-LRR-TM
maker-8-augustus-gene-94.23-mRNA-1	8	OsR498G0815537500.01.T01	98,21	0,00E+00	1648	Os08g0248700	100,00	0,00E+00	1716	68	NA	Japonica	
maker-8-augustus-gene-94.26-mRNA-1	8	OsR498G0815538100.01.T05	99,75	0,00E+00	719	Os08g0248900	99,73	0,00E+00	674	-45	NA	Japonica	
maker-8-augustus-gene-94.34-mRNA-1	8	OsR498G0815537800.01.T01	99,73	0,00E+00	1957	Os08g0248800	99,73	0,00E+00	1957	0	NA	Japonica	
maker-8-augustus-gene-94.36-mRNA-1	8	OsR498G0815539600.01.T01	99,95	0,00E+00	3253	Os08g0249100	100,00	0,00E+00	3258	5	NA	NA	Kinase-TM
maker-8-augustus-gene-94.38-mRNA-1	8	OsR498G0204909000.01.T03	74,09	4,73E-53	209	Os08g0249500	100,00	0,00E+00	693	484	Japonica	NA	
maker-8-augustus-gene-94.40-mRNA-1	8	OsR498G0815542000.01.T06	98,68	0,00E+00	2908	Os08g0249600	99,02	0,00E+00	3419	511	Japonica	NA	
maker-8-augustus-gene-95.0-mRNA-1	8	OsR498G0815547500.01.T02	99,84	0,00E+00	1138	Os08g0250700	99,84	0,00E+00	1138	0	NA	NA	
maker-8-augustus-gene-95.10-mRNA-1	8	OsR498G0815549800.01.T01	99,62	0,00E+00	5588	Os08g0250900	100,00	0,00E+00	5265	-323	NA	Japonica	
maker-8-augustus-gene-95.8-mRNA-1	8	OsR498G0100355100.01.T03	97,83	1,50E-38	158	Os08g0250000	98,96	2,91E-42	169	11	NA	NA	
maker-8-augustus-gene-96.0-mRNA-1	8	OsR498G0815556900.01.T02	99,46	0,00E+00	3981	Os08g0253800	98,90	0,00E+00	2548	-1433	Indica	NA	
maker-8-augustus-gene-96.10-mRNA-1	8	OsR498G0815559000.01.T01	99,71	0,00E+00	1231	Os08g0254600	97,68	0,00E+00	1173	-58	NA	Indica	
maker-8-augustus-gene-96.5-mRNA-1	8	OsR498G0815558700.01.T05	99,82	0,00E+00	3012	Os08g0254500	97,91	0,00E+00	2864	-148	NA	Indica	
maker-8-augustus-gene-96.9-mRNA-1	8	OsR498G0815557200.01.T01	99,21	9,34E-125	446	Os03g0663800	89,16	1,38E-123	442	-4	NA	NA	
maker-8-augustus-gene-97.0-mRNA-1	8	OsR498G0815559700.01.T01	99,58	0,00E+00	1703	Os08g0254900	98,53	0,00E+00	1658	-45	NA	Indica	
maker-8-augustus-gene-99.0-mRNA-1	8	OsR498G0815565000.01.T02	99,13	0,00E+00	2626	Os08g0258200	99,80	0,00E+00	2671	45	NA	Japonica	
augustus_masked-9-processed-gene-102.1-mRNA-1	9	OsR498G0917174700.01.T01	100,00	0,00E+00	872	Os09g0332100	97,93	0,00E+00	827	-45	NA	Indica	
augustus_masked-9-processed-gene-106.11-mRNA-1	9	OsR498G0917216400.01.T01	95,62	0,00E+00	700	Os04g0461800	89,05	2,91E-178	624	-76	Indica	Japonica	
augustus_masked-9-processed-gene-110.29-mRNA-1	9	OsR498G0917198400.01.T01	99,85	0,00E+00	2426	Os09g0342000	97,12	0,00E+00	2265	-161	NA	Indica	
augustus_masked-9-processed-gene-110.30-mRNA-1	9	OsR498G0917198200.01.T01	99,77	0,00E+00	2308	Os09g0341600	99,85	0,00E+00	2313	5	NA	Japonica	
augustus_masked-9-processed-gene-111.0-mRNA-1	9	OsR498G0917198000.01.T01	95,86	0,00E+00	2301	Os09g0341500	99,89	0,00E+00	1646	-655	Indica	Japonica	
augustus_masked-9-processed-gene-114.24-mRNA-1	9	OsR498G1220975100.01.T01	88,85	4,34E-96	351	Os09g0336500	100,00	1,09E-142	504	153	Japonica	Japonica	
augustus_masked-9-processed-gene-118.25-mRNA-1	9	OsR498G0917242100.01.T03	95,82	0,00E+00	4127	Os09g0352000	74,48	0,00E+00	950	-3177	Indica	Japonica	Kinase-LRR-TM
augustus_masked-9-processed-gene-119.44-mRNA-1	9	OsR498G0917249500.01.T02	97,35	0,00E+00	2506	Os09g0362500	97,45	0,00E+00	2471	-35	NA	Japonica	
augustus_masked-9-processed-gene-120.13-mRNA-1	9	OsR498G0917253500.01.T01	99,73	0,00E+00	3300	Os09g0364000	100,00	0,00E+00	3323	23	NA	Japonica	
augustus_masked-9-processed-gene-121.10-mRNA-1	9	OsR498G0917256500.01.T01	98,97	0,00E+00	853	Os09g0364800	99,33	2,09E-148	524	-329	Indica	Japonica	
augustus_masked-9-processed-gene-121.19-mRNA-1	9	OsR498G0917259200.01.T01	89,20	0,00E+00	2889	Os09g0365300	100,00	0,00E+00	3988	1099	Japonica	Japonica	LRR-NBS-TM
augustus_masked-9-processed-gene-123.14-mRNA-1	9	OsR498G0917276700.01.T01	99,54	0,00E+00	1563	Os09g0369000	100,00	0,00E+00	1581	18	NA	Japonica	
augustus_masked-9-processed-gene-124.0-mRNA-1	9	OsR498G0917279900.01.T04	99,86	0,00E+00	1280	Os09g0369400	99,92	0,00E+00	2243	963	Japonica	Japonica	
augustus_masked-9-processed-gene-126.1-mRNA-1	9	OsR498G0917293100.01.T01	97,12	0,00E+00	1278	Os09g0373200	95,17	0,00E+00	1249	-29	NA	Japonica	
augustus_masked-9-processed-gene-129.0-mRNA-1	9	OsR498G0917310300.01.T01	99,49	0,00E+00	3809	Os09g0378700	100,00	0,00E+00	3858	49	NA	Japonica	
augustus_masked-9-processed-gene-146.33-mRNA-1	9	OsR498G0917433500.01.T02	99,26	0,00E+00	1438	Os09g0410500	100,00	0,00E+00	1290	-148	Indica	Japonica	
augustus_masked-9-processed-gene-147.54-mRNA-1	9	OsR498G0917439900.01.T01	99,34	0,00E+00	2682	Os09g0412900	100,00	0,00E+00	2727	45	NA	Japonica	
augustus_masked-9-processed-gene-150.22-mRNA-1	9	OsR498G0917456300.01.T01	99,93	0,00E+00	2404	Os09g0416600	100,00	0,00E+00	1288	-1116	Indica	Japonica	
augustus_masked-9-processed-gene-150.23-mRNA-1	9	OsR498G0917457300.01.T01	99,79	0,00E+00	853	Os09g0416800	100,00	0,00E+00	1521	668	Japonica	Japonica	
augustus_masked-9-processed-gene-150.28-mRNA-1	9	OsR498G0917462100.01.T01	99,62	0,00E+00	3318	Os09g0417500	99,84	0,00E+00	3336	18	NA	Japonica	
augustus_masked-9-processed-gene-151.10-mRNA-1	9	OsR498G0917465900.01.T01	99,84	0,00E+00	2183	Os09g0418500	99,01	0,00E+00	2138	-45	NA	NA	Kinase-TM
augustus_masked-9-processed-gene-154.8-mRNA-1	9	OsR498G0917480900.01.T01	99,93	0,00E+00	5202	Os09g0423300	99,86	0,00E+00	5193	-9	NA	NA	
augustus_masked-9-processed-gene-160.21-mRNA-1	9	OsR498G0917518300.01.T01	99,68	7,67E-157	554	Os09g0433600	100,00	6,27E-159	558	4	NA	Japonica	
augustus_masked-9-processed-gene-160.38-mRNA-1	9	OsR498G0917520500.01.T01	99,00	0,00E+00	2102	Os09g0434500	99,92	0,00E+00	2144	42	NA	Japonica	
augustus_masked-9-processed-gene-163.13-mRNA-1	9	OsR498G0917538300.01.T01	99,79	0,00E+00	3440	Os09g0439600	100,00	0,00E+00	3458	18	NA	Japonica	
augustus_masked-9-processed-gene-165.9-mRNA-1	9	OsR498G0917550500.01.T02	99,53	0,00E+00	3423	Os09g0442400	100,00	0,00E+00	3463	40	NA	Japonica	
augustus_masked-9-processed-gene-167.14-mRNA-1	9	OsR498G0917561300.01.T01	99,48	0,00E+00	1708	Os09g0445500	99,72	0,00E+00	1294	-414	Indica	Japonica	
augustus_masked-9-processed-gene-167.17-mRNA-1	9	OsR498G0917563300.01.T01	99,60	0,00E+00	2679	Os09g0445700	100,00	0,00E+00	3555	876	Japonica	Japonica	
augustus_masked-9-processed-gene-170.23-mRNA-1	9	OsR498G0917589100.01.T01	95,78	0,00E+00	2179	Os09g0451500	95,78	0,00E+00	2179	0	NA	Japonica	
augustus_masked-9-processed-gene-170.35-mRNA-1	9	OsR498G0917591700.01.T01	99,93	0,00E+00	2388	Os09g0452400	100,00	0,00E+00	2392	4	NA	Japonica	
augustus_masked-9-processed-gene-172.11-mRNA-1	9	OsR498G0917602900.01.T02	99,86	0,00E+00	1278	Os09g0455300	100,00	0,00E+00	1283	5	NA	Japonica	
augustus_masked-9-processed-gene-173.0-mRNA-1	9	OsR498G0917608700.01.T01	99,07	0,00E+00	1131	Os09g0456800	100,00	0,00E+00	1159	28	NA	Japonica	
augustus_masked-9-processed-gene-174.20-mRNA-1	9	OsR498G0917615900.01.T02	83,04	2,28E-62	240	Os09g0458800	100,00	8,42E-164	574	334	Japonica	Japonica	
augustus_masked-9-processed-gene-174.25-mRNA-1	9	OsR498G0917620100.01.T03	99,63	0,00E+00	971	Os09g0460000	100,00	0,00E+00	942	-29	NA	Japonica	
augustus_masked-9-processed-gene-175.15-mRNA-1	9	OsR498G0917605400.01.T01	73,03	2,89E-73	278	Os09g0461700	100,00	0,00E+00	1732	1454	Japonica	Japonica	
augustus_masked-9-processed-gene-175.17-mRNA-1	9	OsR498G0917625500.01.T01	99,83	0,00E+00	2053	Os09g0462100	100,00	0,00E+00	2062	9	NA	Japonica	
augustus_masked-9-processed-gene-175.18-mRNA-1	9	OsR498G0816299500.01.T01	73,86	1,55E-89	332	Os09g0462300	100,00	0,00E+00	1786	1454	Japonica	Japonica	
augustus_masked-9-processed-gene-175.2-mRNA-1	9	OsR498G0917621300.01.T01	99,91	0,00E+00	2095	Os09g0460300	100,00	0,00E+00	2100	5	NA	Japonica	
augustus_masked-9-processed-gene-175.3-mRNA-1	9	OsR498G0917621300.01.T01	85,65	0,00E+00	1055	Os09g0460400	100,00	0,00E+00	1710	655	Japonica	Japonica	
augustus_masked-9-processed-gene-175.5-mRNA-1	9	OsR498G0917622300.01.T01	99,57	0,00E+00	1676	Os09g0460700	100,00	0,00E+00	1694	18	NA	Japonica	
augustus_masked-9-processed-gene-175.6-mRNA-1	9	OsR498G0917622500.01.T01	99,89	0,00E+00	1684	Os09g0460800	100,00	0,00E+00	1689	5	NA	Japonica	
augustus_masked-9-processed-gene-176.18-mRNA-1	9	OsR498G0917633900.01.T01	100,00	0,00E+00	1326	Os09g0463600	100,00	0,00E+00	1326	0	NA	Japonica	
augustus_masked-9-processed-gene-176.19-mRNA-1	9	OsR498G0917634300.01.T02	98,24	0,00E+00	1973	Os09g0463700	100,00	0,00E+00	2019	46	NA	Japonica	
augustus_masked-9-processed-gene-181.10-mRNA-1	9	OsR498G0917668500.01.T01	99,92	0,00E+00	2121	Os09g0473400	100,00	0,00E+00	2165	44	NA	Japonica	
augustus_masked-9-processed-gene-181.6-mRNA-1	9	OsR498G0917666300.01.T01	100,00	0,00E+00	731	Os09g0472700	100,00	0,00E+00	710	-21	NA	Japonica	
augustus_masked-9-processed-gene-181.7-mRNA-1	9	OsR498G0917667700.01.T01	99,75	0,00E+00	727	Os09g0472900	100,00	0,00E+00	731	4	NA	Japonica	
augustus_masked-9-processed-gene-182.22-mRNA-1	9	OsR498G0917669300.01.T01	99,59	0,00E+00	863	Os09g0473600	99,77	0,00E+00	772	-91	Indica	Japonica	
augustus_masked-9-processed-gene-183.24-mRNA-1	9	OsR498G0917678300.01.T01	98,14	0,00E+00	1202	Os09g0475800	99,57	0,00E+00	1235	33	NA	Japonica	
augustus_masked-9-processed-gene-183.29-mRNA-1	9	OsR498G0917681700.01.T01	100,00	2,22E-124	444	Os09g0477100	100,00	7,78E-125	444	0	NA	NA	
augustus_masked-9-processed-gene-184.10-mRNA-1	9	OsR498G0917689100.01.T06	99,90	0,00E+00	1841	Os09g0517800	85,15	0,00E+00	1161	-680	Indica	Japonica	
augustus_masked-9-processed-gene-185.10-mRNA-1	9	OsR498G0917693300.01.T01	100,00	0,00E+00	883	Os09g0480200	100,00	0,00E+00	1153	270	Japonica	Japonica	
augustus_masked-9-processed-gene-186.43-mRNA-1	9	OsR498G0917698300.01.T01	99,83	0,00E+00	5367	Os09g0481600	100,00	0,00E+00	2744	-2623	Indica	Japonica	
augustus_masked-9-processed-gene-187.20-mRNA-1	9	OsR498G0917709300.01.T01	99,53	0,00E+00	755	Os09g0483300	100,00	0,00E+00	764	9	NA	Japonica	
augustus_masked-9-processed-gene-188.10-mRNA-1	9	OsR498G0917718500.01.T01	99,85	0,00E+00	2459	Os09g0486700	100,00	0,00E+00	2468	9	NA	Japonica	Kinase-TM
augustus_masked-9-processed-gene-190.13-mRNA-1	9	OsR498G0917732900.01.T04	99,66	0,00E+00	1562	Os09g0491756	100,00	0,00E+00	1575	13	NA	Japonica	
augustus_masked-9-processed-gene-193.57-mRNA-1	9	OsR498G0917746500.01.T10	98,63	0,00E+00	887	Os09g0498100	100,00	0,00E+00	742	-145	Indica	Japonica	
augustus_masked-9-processed-gene-194.40-mRNA-1	9	OsR498G0917753100.01.T01	99,77	0,00E+00	3819	Os02g0179600	72,68	0,00E+00	673	-3146	Indica	Japonica	
augustus_masked-9-processed-gene-195.0-mRNA-1	9	OsR498G0917755900.01.T01	99,50	0,00E+00	1411	Os08g0524400	73,88	7,70E-125	446	-965	Indica	Japonica	
augustus_masked-9-processed-gene-195.19-mRNA-1	9	OsR498G0917762300.01.T01	99,85	0,00E+00	5908	Os09g0503400	100,00	0,00E+00	5930	22	NA	Japonica	
augustus_masked-9-processed-gene-197.76-mRNA-1	9	OsR498G0917772100.01.T04	97,18	0,00E+00	2453	Os09g0507100	100,00	0,00E+00	2560	107	NA	Japonica	
augustus_masked-9-processed-gene-203.27-mRNA-1	9	OsR498G0917804100.01.T01	99,11	0,00E+00	2782	Os09g0517800	100,00	0,00E+00	2214	-568	Indica	Japonica	
augustus_masked-9-processed-gene-203.37-mRNA-1	9	OsR498G0917804500.01.T01	99,87	0,00E+00	1358	Os09g0518000	100,00	0,00E+00	2711	1353	Japonica	Japonica	
augustus_masked-9-processed-gene-205.19-mRNA-1	9	OsR498G0917819100.01.T01	97,82	0,00E+00	2522	Os09g0522800	100,00	0,00E+00	2643	121	NA	Japonica	
augustus_masked-9-processed-gene-208.23-mRNA-1	9	OsR498G0917834700.01.T01	99,60	2,25E-124	445	Os09g0528100	100,00	1,86E-126	450	5	NA	NA	
augustus_masked-9-processed-gene-209.0-mRNA-1	9	OsR498G0917840500.01.T01	99,42	0,00E+00	1828	Os09g0529900	100,00	0,00E+00	1874	46	NA	Japonica	
augustus_masked-9-processed-gene-210.47-mRNA-1	9	OsR498G0917851900.01.T01	100,00	0,00E+00	2203	Os09g0532800	98,05	0,00E+00	2113	-90	NA	NA	
augustus_masked-9-processed-gene-210.49-mRNA-1	9	OsR498G0917852300.01.T08	100,00	0,00E+00	2181	Os09g0532800	100,00	0,00E+00	2221	40	NA	NA	
augustus_masked-9-processed-gene-211.43-mRNA-1	9	OsR498G0917856700.01.T01	99,66	0,00E+00	1562	Os11g0138300	89,44	0,00E+00	1088	-474	Indica	Japonica	
augustus_masked-9-processed-gene-212.1-mRNA-1	9	OsR498G0917860300.01.T01	99,08	0,00E+00	2293	Os09g0535400	100,00	0,00E+00	2327	34	NA	Japonica	
augustus_masked-9-processed-gene-212.2-mRNA-1	9	OsR498G0917860900.01.T02	97,96	3,30E-142	504	Os07g0593400	87,57	3,53E-54	210	-294	Indica	Japonica	
augustus_masked-9-processed-gene-212.23-mRNA-1	9	OsR498G0917864300.01.T01	100,00	0,00E+00	1792	Os09g0536300	100,00	0,00E+00	1792	0	NA	NA	
augustus_masked-9-processed-gene-212.24-mRNA-1	9	OsR498G0917866300.01.T01	100,00	0,00E+00	1694	Os09g0536400	100,00	0,00E+00	1694	0	NA	NA	
augustus_masked-9-processed-gene-212.26-mRNA-1	9	OsR498G0917868900.01.T01	100,00	0,00E+00	1889	Os09g0537200	100,00	0,00E+00	3177	1288	Japonica	NA	
augustus_masked-9-processed-gene-212.30-mRNA-1	9	OsR498G0917870300.01.T01	100,00	0,00E+00	1326	Os09g0537533	100,00	0,00E+00	1326	0	NA	NA	
augustus_masked-9-processed-gene-212.5-mRNA-1	9	OsR498G0917863900.01.T01	99,79	0,00E+00	3445	Os09g0536200	100,00	0,00E+00	3427	-18	NA	Japonica	
augustus_masked-9-processed-gene-213.15-mRNA-1	9	OsR498G0917877500.01.T01	99,70	0,00E+00	1803	Os09g0538750	100,00	0,00E+00	1817	14	NA	Japonica	
augustus_masked-9-processed-gene-215.26-mRNA-1	9	OsR498G0917890300.01.T01	100,00	0,00E+00	1380	Os09g0542900	100,00	0,00E+00	1380	0	NA	NA	
augustus_masked-9-processed-gene-215.29-mRNA-1	9	OsR498G0917894100.01.T01	99,92	0,00E+00	2366	Os09g0543900	100,00	0,00E+00	2370	4	NA	Japonica	
augustus_masked-9-processed-gene-217.11-mRNA-1	9	OsR498G0917901900.01.T06	100,00	0,00E+00	710	Os09g0545360	100,00	0,00E+00	710	0	NA	NA	
augustus_masked-9-processed-gene-217.12-mRNA-1	9	OsR498G0917903100.01.T01	100,00	0,00E+00	769	Os09g0545700	100,00	0,00E+00	769	0	NA	NA	
augustus_masked-9-processed-gene-217.30-mRNA-1	9	OsR498G0917903500.01.T04	100,00	0,00E+00	866	Os09g0546000	100,00	0,00E+00	785	-81	NA	NA	
augustus_masked-9-processed-gene-217.31-mRNA-1	9	OsR498G0917903700.01.T01	100,00	0,00E+00	769	Os09g0546100	100,00	0,00E+00	769	0	NA	NA	
augustus_masked-9-processed-gene-217.34-mRNA-1	9	OsR498G0917903700.01.T01	76,74	1,27E-73	277	Os09g0546500	100,00	0,00E+00	785	508	Japonica	NA	
augustus_masked-9-processed-gene-217.35-mRNA-1	9	OsR498G0917905100.01.T01	100,00	1,76E-145	515	Os09g0545400	82,00	3,41E-35	148	-367	Indica	NA	
augustus_masked-9-processed-gene-217.36-mRNA-1	9	OsR498G0917905300.01.T01	100,00	0,00E+00	785	Os09g0545700	84,06	1,88E-72	271	-514	Indica	NA	
augustus_masked-9-processed-gene-217.38-mRNA-1	9	OsR498G0917903100.01.T01	85,38	7,31E-77	288	Os09g0547100	100,00	0,00E+00	818	530	Japonica	NA	
augustus_masked-9-processed-gene-22.21-mRNA-1	9	OsR498G0916735500.01.T01	99,74	0,00E+00	683	Os09g0126600	99,73	0,00E+00	1328	645	Japonica	Japonica	
augustus_masked-9-processed-gene-220.58-mRNA-1	9	OsR498G0917928900.01.T01	99,75	0,00E+00	705	Os09g0553800	100,00	1,38E-111	401	-304	Indica	Japonica	
augustus_masked-9-processed-gene-221.53-mRNA-1	9	OsR498G0917933500.01.T01	99,74	0,00E+00	2050	Os09g0555150	100,00	0,00E+00	2084	34	NA	Japonica	
augustus_masked-9-processed-gene-221.55-mRNA-1	9	OsR498G0917934300.01.T01	99,66	0,00E+00	3133	Os09g0555300	100,00	0,00E+00	3166	33	NA	Japonica	
augustus_masked-9-processed-gene-223.14-mRNA-1	9	OsR498G0917943500.01.T01	98,52	0,00E+00	1760	Os09g0558200	100,00	0,00E+00	1808	48	NA	Japonica	
augustus_masked-9-processed-gene-223.18-mRNA-1	9	OsR498G0917946500.01.T02	99,75	0,00E+00	716	Os09g0559600	99,34	2,22E-153	540	-176	Indica	Japonica	
augustus_masked-9-processed-gene-225.0-mRNA-1	9	OsR498G0917953300.01.T02	99,59	0,00E+00	4312	Os09g0562600	100,00	0,00E+00	4345	33	NA	Japonica	Kinase-TM WAK92
augustus_masked-9-processed-gene-227.15-mRNA-1	9	OsR498G0917961300.01.T03	98,84	0,00E+00	1655	Os09g0565700	99,92	0,00E+00	2171	516	Japonica	Japonica	
augustus_masked-9-processed-gene-227.17-mRNA-1	9	OsR498G0917961300.01.T03	98,89	0,00E+00	1582	Os09g0566000	100,00	0,00E+00	3063	1481	Japonica	Japonica	
augustus_masked-9-processed-gene-228.76-mRNA-1	9	OsR498G0917971500.01.T01	99,57	0,00E+00	2056	Os09g0569100	100,00	0,00E+00	2078	22	NA	Japonica	
augustus_masked-9-processed-gene-229.63-mRNA-1	9	OsR498G0816609100.01.T01	72,15	2,47E-157	556	Os09g0570800	100,00	0,00E+00	2030	1474	Japonica	Japonica	
augustus_masked-9-processed-gene-229.64-mRNA-1	9	OsR498G0917977900.01.T01	99,76	0,00E+00	2200	Os09g0571100	100,00	0,00E+00	2214	14	NA	Japonica	
augustus_masked-9-processed-gene-229.68-mRNA-1	9	OsR498G0917980300.01.T01	98,47	0,00E+00	1248	Os09g0572000	100,00	0,00E+00	1294	46	NA	Japonica	
augustus_masked-9-processed-gene-230.0-mRNA-1	9	OsR498G0917980900.01.T03	99,28	0,00E+00	1710	Os09g0572200	100,00	0,00E+00	1730	20	NA	Japonica	
augustus_masked-9-processed-gene-230.10-mRNA-1	9	OsR498G0917982900.01.T01	99,42	0,00E+00	923	Os09g0572700	100,00	0,00E+00	937	14	NA	Japonica	
augustus_masked-9-processed-gene-230.16-mRNA-1	9	OsR498G0917864300.01.T01	100,00	0,00E+00	1792	Os09g0536300	100,00	0,00E+00	1792	0	NA	NA	
augustus_masked-9-processed-gene-28.17-mRNA-1	9	OsR498G0916754500.01.T01	99,21	0,00E+00	674	Os09g0132200	99,73	0,00E+00	667	-7	NA	Japonica	
augustus_masked-9-processed-gene-30.9-mRNA-1	9	OsR498G0916767700.01.T01	99,70	0,00E+00	4232	Os09g0135400	99,94	0,00E+00	2945	-1287	Indica	Japonica	
augustus_masked-9-processed-gene-35.25-mRNA-1	9	OsR498G0916787100.01.T01	99,53	0,00E+00	2273	Os09g0241550	99,77	0,00E+00	2287	14	NA	Japonica	
augustus_masked-9-processed-gene-37.10-mRNA-1	9	OsR498G1019219100.01.T02	88,24	2,32E-48	192	Os09g0244600	99,52	1,47E-102	371	179	Japonica	Japonica	
augustus_masked-9-processed-gene-46.10-mRNA-1	9	OsR498G0916851500.01.T01	90,93	0,00E+00	1119	Os09g0258700	90,54	0,00E+00	711	-408	Indica	Japonica	
augustus_masked-9-processed-gene-47.13-mRNA-1	9	OsR498G0916844700.01.T01	98,34	0,00E+00	2919	Os09g0255900	99,88	0,00E+00	3016	97	NA	Japonica	
augustus_masked-9-processed-gene-48.0-mRNA-1	9	OsR498G0916845900.01.T01	97,02	0,00E+00	1678	Os09g0256100	100,00	0,00E+00	1521	-157	NA	Japonica	
augustus_masked-9-processed-gene-5.28-mRNA-1	9	OsR498G0916647700.01.T01	99,74	0,00E+00	2032	Os09g0103700	100,00	0,00E+00	2046	14	NA	Japonica	
augustus_masked-9-processed-gene-50.22-mRNA-1	9	OsR498G0916854500.01.T01	99,84	0,00E+00	2264	Os09g0260000	99,76	0,00E+00	2260	-4	NA	NA	
augustus_masked-9-processed-gene-53.22-mRNA-1	9	OsR498G0916869900.01.T01	95,13	0,00E+00	2507	Os05g0525550	74,01	0,00E+00	981	-1526	Indica	NA	Kinase-LRR-TM
augustus_masked-9-processed-gene-55.15-mRNA-1	9	OsR498G0916877500.01.T05	98,64	0,00E+00	1156	Os09g0267600	98,64	0,00E+00	1156	0	NA	NA	
augustus_masked-9-processed-gene-55.25-mRNA-1	9	OsR498G0916878900.01.T01	98,22	0,00E+00	3479	Os07g0283125	70,58	0,00E+00	870	-2609	Indica	Japonica	Kinase-TM
augustus_masked-9-processed-gene-55.39-mRNA-1	9	OsR498G0916878800.01.T01	98,61	0,00E+00	3494	Os07g0130600	70,84	0,00E+00	913	-2581	Indica	NA	Kinase-TM
augustus_masked-9-processed-gene-73.12-mRNA-1	9	OsR498G0916991700.01.T01	100,00	0,00E+00	2138	Os09g0292900	99,75	0,00E+00	2124	-14	NA	Indica	
augustus_masked-9-processed-gene-77.12-mRNA-1	9	OsR498G0917012900.01.T01	99,56	0,00E+00	2804	Os07g0559700	78,57	0,00E+00	1205	-1599	Indica	Japonica	
augustus_masked-9-processed-gene-82.11-mRNA-1	9	OsR498G0917038900.01.T01	99,73	0,00E+00	2661	Os09g0304500	100,00	0,00E+00	2679	18	NA	Japonica	
augustus_masked-9-processed-gene-83.12-mRNA-1	9	OsR498G0917041300.01.T01	98,22	0,00E+00	2137	Os09g0305300	98,55	0,00E+00	2155	18	NA	NA	
augustus_masked-9-processed-gene-87.7-mRNA-1	9	OsR498G0917061900.01.T01	100,00	0,00E+00	1245	Os09g0309500	100,00	0,00E+00	1245	0	NA	NA	
augustus_masked-9-processed-gene-87.8-mRNA-1	9	OsR498G0917062300.01.T01	98,26	0,00E+00	695	Os09g0309600	98,54	0,00E+00	836	141	Japonica	NA	
augustus_masked-9-processed-gene-96.18-mRNA-1	9	OsR498G0917155300.01.T01	99,36	0,00E+00	2517	Os09g0324300	100,00	0,00E+00	2538	21	NA	Japonica	
augustus_masked-9-processed-gene-96.19-mRNA-1	9	OsR498G0917155900.01.T01	99,85	0,00E+00	2318	Os09g0324400	100,00	0,00E+00	2327	9	NA	Japonica	
augustus_masked-9-processed-gene-96.2-mRNA-1	9	OsR498G0917154900.01.T01	99,55	0,00E+00	2365	Os09g0324200	100,00	0,00E+00	2392	27	NA	Japonica	
augustus_masked-9-processed-gene-96.23-mRNA-1	9	OsR498G0917159900.01.T01	99,63	0,00E+00	1925	Os09g0325700	100,00	0,00E+00	1943	18	NA	Japonica	
augustus_masked-9-processed-gene-97.17-mRNA-1	9	OsR498G0917161300.01.T01	98,45	0,00E+00	4722	Os09g0326800	98,63	0,00E+00	4745	23	NA	Japonica	
augustus_masked-9-processed-gene-98.4-mRNA-1	9	OsR498G0917161700.01.T06	99,70	0,00E+00	2423	Os09g0326900	100,00	0,00E+00	2441	18	NA	Japonica	
augustus_masked-9-processed-gene-99.19-mRNA-1	9	OsR498G0917165300.01.T01	97,10	0,00E+00	3468	Os09g0329000	99,71	0,00E+00	3713	245	NA	Japonica	
augustus-9-processed-gene-100.30-mRNA-1	9	OsR498G0917168200.01.T01	100,00	0,00E+00	2170	Os09g0329700	99,92	0,00E+00	2166	-4	NA	NA	
augustus-9-processed-gene-103.24-mRNA-1	9	OsR498G0917178000.01.T05	85,31	2,25E-74	279	Os09g0333600	100,00	4,63E-141	499	220	Japonica	Indica	
augustus-9-processed-gene-104.52-mRNA-1	9	OsR498G0917228700.01.T01	99,32	0,00E+00	788	Os09g0353400	99,74	0,00E+00	700	-88	Indica	Japonica	
augustus-9-processed-gene-108.28-mRNA-1	9	OsR498G0917209900.01.T01	98,87	0,00E+00	2935	Os09g0347300	99,33	0,00E+00	3188	253	NA	Japonica	
augustus-9-processed-gene-112.18-mRNA-1	9	OsR498G0917192300.01.T01	99,64	0,00E+00	2461	Os09g0338400	100,00	0,00E+00	2480	19	NA	Japonica	
augustus-9-processed-gene-116.37-mRNA-1	9	OsR498G0815285000.01.T01	75,61	1,12E-42	174	Os09g0357500	100,00	0,00E+00	758	584	Japonica	NA	
augustus-9-processed-gene-119.47-mRNA-1	9	OsR498G0917246900.01.T01	98,91	0,00E+00	1448	Os09g0361400	100,00	0,00E+00	1489	41	NA	Japonica	
augustus-9-processed-gene-121.26-mRNA-1	9	OsR498G0917259000.01.T01	99,51	0,00E+00	3982	Os09g0365000	100,00	0,00E+00	6157	2175	Japonica	Japonica	LRR-NBS-TM
augustus-9-processed-gene-125.71-mRNA-1	9	OsR498G0917292200.01.T02	99,91	0,00E+00	2052	Os09g0372900	100,00	0,00E+00	2048	-4	NA	Japonica	
augustus-9-processed-gene-126.24-mRNA-1	9	OsR498G0917660800.01.T02	74,73	4,40E-46	186	Os09g0373800	100,00	1,52E-160	563	377	Japonica	NA	Kinase
augustus-9-processed-gene-127.29-mRNA-1	9	OsR498G0917299200.01.T01	98,93	6,86E-138	490	Os09g0376400	100,00	1,97E-101	367	-123	Indica	Japonica	
augustus-9-processed-gene-129.12-mRNA-1	9	OsR498G0917311200.01.T01	95,44	0,00E+00	2177	Os09g0379300	100,00	0,00E+00	2598	421	Japonica	Japonica	
augustus-9-processed-gene-129.34-mRNA-1	9	OsR498G0917313200.01.T01	99,73	0,00E+00	3983	Os09g0379900	100,00	0,00E+00	3750	-233	NA	Japonica	
augustus-9-processed-gene-132.23-mRNA-1	9	OsR498G0917335000.01.T01	96,49	0,00E+00	2014	Os09g0384900	96,41	0,00E+00	2010	-4	NA	Indica	
augustus-9-processed-gene-133.28-mRNA-1	9	OsR498G0917343200.01.T03	99,83	0,00E+00	1024	Os09g0387150	99,80	0,00E+00	916	-108	Indica	Japonica	
augustus-9-processed-gene-133.3-mRNA-1	9	OsR498G0917340000.01.T01	99,70	0,00E+00	2943	Os09g0386200	99,88	0,00E+00	2957	14	NA	Japonica	
augustus-9-processed-gene-138.2-mRNA-1	9	OsR498G0917376000.01.T02	99,86	0,00E+00	1287	Os09g0394300	100,00	0,00E+00	1292	5	NA	Japonica	
augustus-9-processed-gene-141.32-mRNA-1	9	OsR498G0917406000.01.T01	100,00	0,00E+00	3512	Os09g0400500	99,85	0,00E+00	3498	-14	NA	Indica	Kinase-LRR-TM
augustus-9-processed-gene-144.27-mRNA-1	9	OsR498G0917419800.01.T01	99,75	0,00E+00	727	Os09g0404900	100,00	0,00E+00	731	4	NA	NA	
augustus-9-processed-gene-144.32-mRNA-1	9	OsR498G0917422800.01.T01	99,08	0,00E+00	3241	Os09g0407800	99,18	0,00E+00	3250	9	NA	Indica	
augustus-9-processed-gene-147.41-mRNA-1	9	OsR498G0917435800.01.T03	99,70	0,00E+00	2948	Os09g0411600	100,00	0,00E+00	2971	23	NA	Japonica	
augustus-9-processed-gene-147.71-mRNA-1	9	OsR498G0917441800.01.T03	99,76	0,00E+00	3776	Os09g0413300	100,00	0,00E+00	4388	612	Japonica	Japonica	
augustus-9-processed-gene-149.17-mRNA-1	9	OsR498G0917452000.01.T01	98,01	0,00E+00	1031	Os09g0415300	100,00	0,00E+00	1081	50	NA	Japonica	
augustus-9-processed-gene-154.23-mRNA-1	9	OsR498G0917484800.01.T01	99,83	0,00E+00	2118	Os09g0424300	100,00	0,00E+00	2127	9	NA	Japonica	
augustus-9-processed-gene-155.13-mRNA-1	9	OsR498G0917486200.01.T01	97,28	0,00E+00	1482	Os09g0425200	100,00	0,00E+00	1440	-42	NA	Japonica	
augustus-9-processed-gene-159.1-mRNA-1	9	OsR498G0917513200.01.T01	100,00	0,00E+00	926	Os09g0431900	100,00	0,00E+00	704	-222	Indica	Japonica	
augustus-9-processed-gene-159.21-mRNA-1	9	OsR498G0917516800.01.T01	99,89	0,00E+00	1695	Os09g0433000	100,00	0,00E+00	1700	5	NA	Japonica	
augustus-9-processed-gene-160.31-mRNA-1	9	OsR498G0917518800.01.T01	100,00	0,00E+00	1267	Os09g0433700	100,00	0,00E+00	1267	0	NA	Japonica	
augustus-9-processed-gene-160.32-mRNA-1	9	OsR498G0917518800.01.T01	99,91	0,00E+00	2076	Os09g0433700	100,00	0,00E+00	2080	4	NA	Japonica	
augustus-9-processed-gene-160.54-mRNA-1	9	OsR498G0917523000.01.T01	98,77	0,00E+00	1281	Os09g0434900	100,00	0,00E+00	1319	38	NA	Japonica	
augustus-9-processed-gene-162.28-mRNA-1	9	OsR498G0917532800.01.T01	98,99	0,00E+00	1223	Os09g0438100	100,00	0,00E+00	1256	33	NA	Japonica	
augustus-9-processed-gene-165.3-mRNA-1	9	OsR498G0917547600.01.T01	98,45	0,00E+00	1679	Os08g0499400	79,86	0,00E+00	893	-786	Indica	Japonica	
augustus-9-processed-gene-166.41-mRNA-1	9	OsR498G0917558800.01.T01	98,53	0,00E+00	829	Os05g0567400	92,51	1,81E-174	611	-218	Indica	Japonica	
augustus-9-processed-gene-168.25-mRNA-1	9	OsR498G0917576300.01.T01	99,27	0,00E+00	3640	Os09g0449000	100,00	0,00E+00	3680	40	NA	Japonica	
augustus-9-processed-gene-168.30-mRNA-1	9	OsR498G0917580500.01.T06	79,39	0,00E+00	1050	Os09g0448100	100,00	0,00E+00	2343	1293	Japonica	Japonica	
augustus-9-processed-gene-168.7-mRNA-1	9	OsR498G0917572600.01.T01	99,55	0,00E+00	791	Os01g0587300	81,37	7,63E-141	499	-292	Indica	Japonica	
augustus-9-processed-gene-17.15-mRNA-1	9	OsR498G0916710400.01.T01	98,29	0,00E+00	2634	Os09g0119600	99,54	0,00E+00	2701	67	NA	NA	
augustus-9-processed-gene-172.34-mRNA-1	9	OsR498G0917604800.01.T01	99,59	0,00E+00	1719	Os09g0455500	100,00	0,00E+00	1737	18	NA	Japonica	
augustus-9-processed-gene-172.41-mRNA-1	9	OsR498G0917605400.01.T01	99,79	0,00E+00	1723	Os09g0455900	100,00	0,00E+00	1732	9	NA	Japonica	
augustus-9-processed-gene-173.1-mRNA-1	9	OsR498G0917608700.01.T01	99,63	1,33E-134	480	Os09g0456800	100,00	1,09E-136	484	4	NA	Japonica	
augustus-9-processed-gene-173.22-mRNA-1	9	OsR498G1220975100.01.T01	89,19	2,99E-93	342	Os09g0336500	90,00	5,76E-97	352	10	NA	Japonica	
augustus-9-processed-gene-173.3-mRNA-1	9	OsR498G0917608000.01.T01	99,58	0,00E+00	2126	Os09g0456700	100,00	0,00E+00	2149	23	NA	Japonica	
augustus-9-processed-gene-174.37-mRNA-1	9	OsR498G0917618800.01.T01	99,21	0,00E+00	2255	Os09g0459600	100,00	0,00E+00	2280	25	NA	Japonica	
augustus-9-processed-gene-175.34-mRNA-1	9	OsR498G0917625800.01.T01	99,69	0,00E+00	1746	Os09g0462200	100,00	0,00E+00	1759	13	NA	Japonica	
augustus-9-processed-gene-176.35-mRNA-1	9	OsR498G0917637200.01.T01	99,80	0,00E+00	1766	Os08g0478466	74,93	3,43E-143	508	-1258	Indica	Japonica	
augustus-9-processed-gene-178.20-mRNA-1	9	OsR498G0917649000.01.T01	93,72	0,00E+00	1159	Os09g0467500	100,00	0,00E+00	1366	207	Japonica	Japonica	
augustus-9-processed-gene-178.23-mRNA-1	9	OsR498G0917650800.01.T01	100,00	0,00E+00	1808	Os09g0468150	100,00	0,00E+00	1808	0	NA	Japonica	
augustus-9-processed-gene-178.25-mRNA-1	9	OsR498G0917651200.01.T01	98,20	0,00E+00	2116	Os09g0468300	100,00	0,00E+00	2127	11	NA	Japonica	
augustus-9-processed-gene-178.6-mRNA-1	9	OsR498G0917645800.01.T01	99,40	0,00E+00	1488	Os09g0466400	100,00	0,00E+00	1510	22	NA	Japonica	
augustus-9-processed-gene-182.56-mRNA-1	9	OsR498G0917674800.01.T01	99,85	0,00E+00	3563	Os09g0474800	100,00	0,00E+00	3577	14	NA	Japonica	
augustus-9-processed-gene-183.44-mRNA-1	9	OsR498G0917679000.01.T03	100,00	0,00E+00	3274	Os09g0476100	100,00	0,00E+00	3274	0	NA	NA	
augustus-9-processed-gene-184.19-mRNA-1	9	OsR498G0917690900.01.T01	99,93	0,00E+00	2544	Os09g0479300	100,00	0,00E+00	2549	5	NA	NA	
augustus-9-processed-gene-185.20-mRNA-1	9	OsR498G0917694500.01.T01	100,00	0,00E+00	3831	Os09g0480400	100,00	0,00E+00	3831	0	NA	NA	
augustus-9-processed-gene-187.13-mRNA-1	9	OsR498G0917706600.01.T02	100,00	0,00E+00	1451	Os09g0482780	99,68	0,00E+00	1117	-334	Indica	Japonica	
augustus-9-processed-gene-187.38-mRNA-1	9	OsR498G0917707800.01.T01	99,50	0,00E+00	1058	Os09g0482860	100,00	0,00E+00	1072	14	NA	Japonica	
augustus-9-processed-gene-187.40-mRNA-1	9	OsR498G0917708600.01.T01	99,65	0,00E+00	2564	Os09g0482900	100,00	0,00E+00	2587	23	NA	Japonica	
augustus-9-processed-gene-187.42-mRNA-1	9	OsR498G0917709600.01.T03	99,90	0,00E+00	1755	Os09g0483600	100,00	0,00E+00	1948	193	NA	Japonica	
augustus-9-processed-gene-189.20-mRNA-1	9	OsR498G0917725400.01.T02	99,62	0,00E+00	3278	Os09g0490200	99,94	0,00E+00	3055	-223	NA	Japonica	
augustus-9-processed-gene-190.20-mRNA-1	9	OsR498G0917731000.01.T01	99,64	0,00E+00	1974	Os09g0491676	100,00	0,00E+00	1992	18	NA	Japonica	
augustus-9-processed-gene-192.7-mRNA-1	9	OsR498G0917738000.01.T04	99,90	0,00E+00	1787	Os04g0577000	70,08	2,14E-120	433	-1354	Indica	Japonica	

augustus-9-processed-gene-196.50-mRNA-1	9	OsR498G0917763200.01.T01	99,60	0,00E+00	2673	Os09g0504000	100,00	0,00E+00	2700	27	NA	Japonica	
augustus-9-processed-gene-201.109-mRNA-1	9	OsR498G0917793800.01.T02	98,74	0,00E+00	3065	Os09g0514200	99,14	0,00E+00	3096	31	NA	Japonica	
augustus-9-processed-gene-205.9-mRNA-1	9	OsR498G0917817400.01.T01	100,00	0,00E+00	1337	Os09g0522100	100,00	0,00E+00	1337	0	NA	Japonica	
augustus-9-processed-gene-207.21-mRNA-1	9	OsR498G0917831800.01.T01	95,39	0,00E+00	1381	Os09g0526800	95,39	0,00E+00	1381	0	NA	NA	
augustus-9-processed-gene-212.14-mRNA-1	9	OsR498G0917860600.01.T01	99,54	0,00E+00	1152	Os09g0535500	99,85	0,00E+00	1176	24	NA	Japonica	
augustus-9-processed-gene-214.39-mRNA-1	9	OsR498G0917882200.01.T01	99,68	0,00E+00	2228	Os09g0540500	100,00	0,00E+00	2246	18	NA	Japonica	
augustus-9-processed-gene-214.52-mRNA-1	9	OsR498G0917882700.01.T01	98,82	0,00E+00	892	Os09g0540800	100,00	0,00E+00	1274	382	Japonica	Japonica	
augustus-9-processed-gene-214.59-mRNA-1	9	OsR498G0917884600.01.T01	99,49	0,00E+00	691	Os09g0541100	100,00	0,00E+00	1213	522	Japonica	Japonica	
augustus-9-processed-gene-215.21-mRNA-1	9	OsR498G0917890000.01.T03	99,94	0,00E+00	2939	Os09g0542800	100,00	0,00E+00	2944	5	NA	NA	
augustus-9-processed-gene-215.37-mRNA-1	9	OsR498G0917892000.01.T01	100,00	0,00E+00	2246	Os09g0543400	100,00	0,00E+00	2214	-32	NA	Japonica	
augustus-9-processed-gene-217.23-mRNA-1	9	OsR498G0917902400.01.T01	100,00	0,00E+00	753	Os09g0546800	80,08	1,92E-59	228	-525	Indica	NA	
augustus-9-processed-gene-217.54-mRNA-1	9	OsR498G0917905600.01.T01	100,00	0,00E+00	769	Os09g0546800	100,00	0,00E+00	769	0	NA	NA	
augustus-9-processed-gene-217.56-mRNA-1	9	OsR498G0917906000.01.T01	100,00	0,00E+00	769	Os09g0547000	100,00	0,00E+00	769	0	NA	NA	
augustus-9-processed-gene-217.57-mRNA-1	9	OsR498G0917906200.01.T01	100,00	0,00E+00	753	Os09g0545700	83,10	4,80E-124	444	-309	Indica	NA	
augustus-9-processed-gene-218.78-mRNA-1	9	OsR498G0917917200.01.T02	99,76	0,00E+00	1490	Os09g0549500	100,00	0,00E+00	1489	-1	NA	Japonica	
augustus-9-processed-gene-218.79-mRNA-1	9	OsR498G0917917600.01.T02	100,00	0,00E+00	666	Os09g0549700	100,00	0,00E+00	964	298	Japonica	Japonica	
augustus-9-processed-gene-220.73-mRNA-1	9	OsR498G0917927000.01.T01	100,00	1,80E-158	558	Os09g0553100	100,00	6,27E-159	558	0	NA	Japonica	
augustus-9-processed-gene-221.46-mRNA-1	9	OsR498G0917930200.01.T01	99,84	0,00E+00	1089	Os09g0554200	100,00	0,00E+00	1094	5	NA	Japonica	
augustus-9-processed-gene-222.40-mRNA-1	9	OsR498G0917939700.01.T01	97,47	0,00E+00	875	Os02g0184100	70,67	1,14E-31	136	-739	Indica	Japonica	
augustus-9-processed-gene-223.32-mRNA-1	9	OsR498G0917945400.01.T01	98,60	0,00E+00	2615	Os09g0559000	100,00	0,00E+00	2847	232	NA	Japonica	
augustus-9-processed-gene-223.33-mRNA-1	9	OsR498G0917945800.01.T01	98,47	0,00E+00	1928	Os09g0559100	100,00	0,00E+00	2004	76	NA	Japonica	
augustus-9-processed-gene-223.35-mRNA-1	9	OsR498G0917946400.01.T01	99,26	0,00E+00	4063	Os09g0559400	100,00	0,00E+00	2051	-2012	Indica	Japonica	
augustus-9-processed-gene-224.58-mRNA-1	9	OsR498G0917948400.01.T01	98,34	7,83E-85	314	Os09g0560300	99,45	5,27E-88	323	9	NA	Japonica	
augustus-9-processed-gene-226.9-mRNA-1	9	OsR498G0917959000.01.T01	99,91	0,00E+00	1944	Os09g0564000	76,51	3,61E-175	614	-1330	Indica	Japonica	
augustus-9-processed-gene-227.41-mRNA-1	9	OsR498G0917963800.01.T01	97,41	0,00E+00	2148	Os09g0566400	100,00	0,00E+00	2296	148	NA	Japonica	
augustus-9-processed-gene-227.42-mRNA-1	9	OsR498G0917963800.01.T01	81,46	0,00E+00	795	Os09g0566400	82,06	0,00E+00	818	23	NA	Japonica	
augustus-9-processed-gene-227.49-mRNA-1	9	OsR498G0917966400.01.T01	99,72	0,00E+00	1917	Os08g0557300	73,52	1,90E-121	436	-1481	Indica	Japonica	
augustus-9-processed-gene-228.93-mRNA-1	9	OsR498G0917970600.01.T01	99,85	0,00E+00	1159	Os09g0568700	86,83	0,00E+00	759	-400	Indica	Japonica	
augustus-9-processed-gene-228.94-mRNA-1	9	OsR498G0917971000.01.T01	100,00	0,00E+00	1164	Os09g0568700	100,00	0,00E+00	1164	0	NA	Japonica	
augustus-9-processed-gene-229.83-mRNA-1	9	OsR498G0816619400.01.T06	79,37	0,00E+00	746	Os09g0571200	100,00	0,00E+00	1575	829	Japonica	Japonica	
augustus-9-processed-gene-23.22-mRNA-1	9	OsR498G0916739000.01.T01	100,00	0,00E+00	661	Os09g0127700	100,00	0,00E+00	661	0	NA	Japonica	
augustus-9-processed-gene-30.29-mRNA-1	9	OsR498G0916770000.01.T01	99,77	0,00E+00	3142	Os09g0135800	99,94	0,00E+00	3156	14	NA	Japonica	
augustus-9-processed-gene-4.39-mRNA-1	9	OsR498G0916647000.01.T01	99,61	0,00E+00	922	Os09g0103400	100,00	0,00E+00	1797	875	Japonica	Japonica	
augustus-9-processed-gene-44.19-mRNA-1	9	OsR498G0916836300.01.T01	99,56	0,00E+00	2430	Os09g0251250	99,90	0,00E+00	3525	1095	Japonica	Japonica	Kinase-TM
augustus-9-processed-gene-44.26-mRNA-1	9	OsR498G0100917100.01.T04	97,00	0,00E+00	4395	Os09g0251500	99,96	0,00E+00	4746	351	NA	Japonica	
augustus-9-processed-gene-57.20-mRNA-1	9	OsR498G0916883200.01.T02	95,73	0,00E+00	1775	Os09g0270900	100,00	0,00E+00	1986	211	Japonica	Japonica	
augustus-9-processed-gene-57.22-mRNA-1	9	OsR498G0916884000.01.T01	98,76	0,00E+00	707	Os09g0271100	100,00	6,03E-167	585	-122	Indica	Japonica	
augustus-9-processed-gene-59.32-mRNA-1	9	OsR498G0916892000.01.T01	99,01	0,00E+00	891	Os09g0273800	100,00	0,00E+00	910	19	NA	Japonica	
augustus-9-processed-gene-59.9-mRNA-1	9	OsR498G0916894000.01.T01	72,83	2,32E-65	251	Os09g0272900	100,00	0,00E+00	1007	756	Japonica	Japonica	
augustus-9-processed-gene-60.6-mRNA-1	9	OsR498G0916892000.01.T01	95,64	0,00E+00	814	Os09g0273800	96,61	0,00E+00	829	15	NA	Japonica	
augustus-9-processed-gene-7.4-mRNA-1	9	OsR498G0916657400.01.T01	99,58	0,00E+00	1709	Os09g0106700	99,90	0,00E+00	1728	19	NA	Japonica	
augustus-9-processed-gene-71.13-mRNA-1	9	OsR498G1220975100.01.T01	90,61	3,20E-105	381	Os09g0336500	90,71	2,62E-107	387	6	NA	NA	
augustus-9-processed-gene-74.22-mRNA-1	9	OsR498G0917002400.01.T01	99,92	0,00E+00	6561	Os09g0293500	100,00	0,00E+00	6574	13	NA	Japonica	Kinase-LRR-TM
augustus-9-processed-gene-76.0-mRNA-1	9	OsR498G0917006600.01.T01	99,46	0,00E+00	2632	Os09g0295000	100,00	0,00E+00	2684	52	NA	Japonica	
augustus-9-processed-gene-76.3-mRNA-1	9	OsR498G0917007400.01.T01	98,76	0,00E+00	1128	Os09g0295700	100,00	0,00E+00	1386	258	Japonica	Japonica	
augustus-9-processed-gene-87.17-mRNA-1	9	OsR498G0917062600.01.T03	99,69	0,00E+00	1713	Os09g0309700	99,69	0,00E+00	1713	0	NA	NA	
augustus-9-processed-gene-88.4-mRNA-1	9	OsR498G0917083400.01.T01	100,00	0,00E+00	1862	Os09g0314333	100,00	0,00E+00	1862	0	NA	NA	
augustus-9-processed-gene-88.5-mRNA-1	9	OsR498G0917084400.01.T01	99,91	0,00E+00	1987	Os09g0314400	99,91	0,00E+00	1987	0	NA	NA	
augustus-9-processed-gene-89.34-mRNA-1	9	OsR498G0917110200.01.T01	99,60	0,00E+00	2657	Os09g0317700	99,60	0,00E+00	2657	0	NA	NA	
augustus-9-processed-gene-99.31-mRNA-1	9	OsR498G0917166200.01.T01	99,47	0,00E+00	2681	Os09g0329200	99,93	0,00E+00	2712	31	NA	Japonica	
maker-9-augustus-gene-10.0-mRNA-1	9	OsR498G0916674500.01.T03	99,70	0,00E+00	1760	Os09g0110400	100,00	0,00E+00	1773	13	NA	Japonica	
maker-9-augustus-gene-10.4-mRNA-1	9	OsR498G0916678000.01.T01	99,64	0,00E+00	1957	Os09g0111100	100,00	0,00E+00	1975	18	NA	Japonica	
maker-9-augustus-gene-10.6-mRNA-1	9	OsR498G0916679400.01.T02	99,69	0,00E+00	1155	Os09g0111800	100,00	0,00E+00	1164	9	NA	Japonica	
maker-9-augustus-gene-10.9-mRNA-1	9	OsR498G0916681000.01.T01	100,00	0,00E+00	866	Os09g0112400	100,00	0,00E+00	866	0	NA	Japonica	
maker-9-augustus-gene-100.40-mRNA-1	9	OsR498G0917172500.01.T01	100,00	0,00E+00	2890	Os09g0330700	100,00	0,00E+00	3837	947	Japonica	Indica	
maker-9-augustus-gene-102.26-mRNA-1	9	OsR498G0917174600.01.T01	100,00	0,00E+00	673	Os09g0331900	98,97	0,00E+00	686	13	NA	Indica	
maker-9-augustus-gene-102.30-mRNA-1	9	OsR498G0917178000.01.T01	99,50	0,00E+00	7778	Os01g0609066	70,38	0,00E+00	1948	-5830	Indica	Indica	
maker-9-augustus-gene-103.30-mRNA-1	9	OsR498G0917179000.01.T01	89,54	0,00E+00	752	Os09g0333600	100,00	0,00E+00	807	55	NA	Indica	
maker-9-augustus-gene-104.2-mRNA-1	9	OsR498G0917230700.01.T01	81,56	0,00E+00	1088	Os09g0353700	79,84	0,00E+00	1023	-65	NA	Indica	
maker-9-augustus-gene-104.4-mRNA-1	9	OsR498G0917230700.01.T01	99,29	0,00E+00	994	Os09g0354501	99,47	0,00E+00	1015	21	NA	Japonica	
maker-9-augustus-gene-104.5-mRNA-1	9	OsR498G0917230300.01.T01	97,83	0,00E+00	1805	Os09g0353700	100,00	0,00E+00	1894	89	NA	Japonica	
maker-9-augustus-gene-105.27-mRNA-1	9	OsR498G0917227800.01.T02	98,43	0,00E+00	4668	Os09g0353200	97,99	0,00E+00	2833	-1835	Indica	Japonica	Kinase-LRR-TM
maker-9-augustus-gene-105.30-mRNA-1	9	OsR498G0917225800.01.T02	99,83	0,00E+00	2085	Os09g0352400	100,00	0,00E+00	2093	8	NA	Japonica	
maker-9-augustus-gene-105.36-mRNA-1	9	OsR498G0917223000.01.T02	99,82	0,00E+00	4082	Os09g0352000	100,00	0,00E+00	4616	534	Japonica	Japonica	Kinase-LRR-TM
maker-9-augustus-gene-106.28-mRNA-1	9	OsR498G0917219200.01.T02	92,15	0,00E+00	2067	Os09g0350900	92,57	0,00E+00	2094	27	NA	Japonica	Kinase-LRR-TM
maker-9-augustus-gene-107.12-mRNA-1	9	OsR498G0917214100.01.T01	82,38	0,00E+00	1063	Os09g0349100	100,00	0,00E+00	1231	168	Japonica	NA	Kinase-TM
maker-9-augustus-gene-107.2-mRNA-1	9	OsR498G0917216200.01.T01	97,25	0,00E+00	4337	Os09g0352000	81,19	0,00E+00	2428	-1909	Indica	Japonica	Kinase-LRR-TM
maker-9-augustus-gene-107.4-mRNA-1	9	OsR498G0917216200.01.T01	92,53	0,00E+00	3115	Os09g0349800	100,00	0,00E+00	3503	388	Japonica	Japonica	Kinase-LRR-TM
maker-9-augustus-gene-108.43-mRNA-1	9	OsR498G0917211800.01.T02	98,84	1,03E-171	603	Os02g0814900	93,08	1,17E-146	518	-85	Indica	Japonica	
maker-9-augustus-gene-108.51-mRNA-1	9	OsR498G0917211200.01.T06	99,65	0,00E+00	2553	Os09g0347700	100,00	0,00E+00	2576	23	NA	Japonica	
maker-9-augustus-gene-108.52-mRNA-1	9	OsR498G0917211200.01.T02	99,93	0,00E+00	2571	Os09g0347700	97,90	0,00E+00	2441	-130	NA	Japonica	
maker-9-augustus-gene-108.67-mRNA-1	9	OsR498G0917213300.01.T01	96,56	0,00E+00	672	Os09g0348300	78,45	1,47E-92	339	-333	Indica	Japonica	Kinase-TM
maker-9-augustus-gene-108.69-mRNA-1	9	OsR498G0917213100.01.T01	98,21	0,00E+00	667	Os09g0348300	83,29	4,99E-112	403	-264	Indica	Japonica	Kinase-TM
maker-9-augustus-gene-108.77-mRNA-1	9	OsR498G0917211500.01.T01	96,36	0,00E+00	2314	Os09g0347800	96,43	0,00E+00	2318	4	NA	Japonica	
maker-9-augustus-gene-109.13-mRNA-1	9	OsR498G0917205400.01.T02	100,00	0,00E+00	1764	Os09g0345700	99,90	0,00E+00	1760	-4	NA	Japonica	
maker-9-augustus-gene-109.16-mRNA-1	9	OsR498G0917206100.01.T01	99,79	0,00E+00	3380	Os09g0346400	99,84	0,00E+00	3385	5	NA	NA	
maker-9-augustus-gene-109.20-mRNA-1	9	OsR498G0917204700.01.T01	97,86	0,00E+00	1031	Os09g0345500	99,83	0,00E+00	1084	53	NA	Japonica	
maker-9-augustus-gene-109.5-mRNA-1	9	OsR498G0917208000.01.T02	99,93	0,00E+00	5326	Os09g0346700	99,93	0,00E+00	5326	0	NA	Japonica	
maker-9-augustus-gene-109.7-mRNA-1	9	OsR498G0917207600.01.T03	99,82	0,00E+00	1994	Os09g0346600	99,82	0,00E+00	1994	0	NA	Indica	
maker-9-augustus-gene-110.0-mRNA-1	9	OsR498G0917203400.01.T02	100,00	0,00E+00	634	Os09g0345000	100,00	0,00E+00	634	0	NA	Japonica	
maker-9-augustus-gene-110.10-mRNA-1	9	OsR498G0917202900.01.T05	96,54	0,00E+00	2422	Os09g0344800	100,00	0,00E+00	2608	186	NA	Japonica	
maker-9-augustus-gene-110.15-mRNA-1	9	OsR498G0917200700.01.T01	97,51	0,00E+00	2177	Os09g0344400	97,31	0,00E+00	2065	-112	NA	NA	
maker-9-augustus-gene-110.2-mRNA-1	9	OsR498G0917201600.01.T01	98,78	0,00E+00	1289	Os09g0344500	99,54	0,00E+00	1953	664	Japonica	Japonica	
maker-9-augustus-gene-110.8-mRNA-1	9	OsR498G0917203100.01.T03	100,00	0,00E+00	1234	Os09g0344900	100,00	0,00E+00	1234	0	NA	Japonica	
maker-9-augustus-gene-111.31-mRNA-1	9	OsR498G0917196300.01.T01	97,15	0,00E+00	1352	Os09g0341100	99,62	0,00E+00	2332	980	Japonica	Japonica	Kinase
maker-9-augustus-gene-112.27-mRNA-1	9	OsR498G0917194500.01.T01	99,04	0,00E+00	2579	Os09g0339000	98,43	0,00E+00	2541	-38	NA	NA	Kinase-TM
maker-9-augustus-gene-114.0-mRNA-1	9	OsR498G0917183000.01.T01	99,51	0,00E+00	3277	Os09g0334900	100,00	0,00E+00	3723	446	Japonica	Japonica	
maker-9-augustus-gene-114.3-mRNA-1	9	OsR498G0917182700.01.T01	99,59	0,00E+00	3877	Os09g0334800	99,96	0,00E+00	3967	90	NA	Japonica	Kinase-TM
maker-9-augustus-gene-115.4-mRNA-1	9	OsR498G0917182100.01.T01	99,36	0,00E+00	1928	Os09g0334500	100,00	0,00E+00	1959	31	NA	Japonica	
maker-9-augustus-gene-115.7-mRNA-1	9	OsR498G0917236000.01.T01	98,39	0,00E+00	3011	Os09g0352000	76,23	0,00E+00	1166	-1845	Indica	Japonica	Kinase-LRR-TM
maker-9-augustus-gene-116.0-mRNA-1	9	OsR498G0917236300.01.T05	91,56	0,00E+00	1030	Os09g0356200	100,00	0,00E+00	1306	276	Japonica	Japonica	Kinase-TM
maker-9-augustus-gene-116.11-mRNA-1	9	OsR498G0305320000.01.T01	98,27	0,00E+00	1395	Os09g0357250	100,00	0,00E+00	1451	56	NA	NA	
maker-9-augustus-gene-116.5-mRNA-1	9	OsR498G0917239000.01.T01	94,26	0,00E+00	1588	Os09g0356800	100,00	0,00E+00	1842	254	Japonica	Japonica	Kinase-LRR-TM
maker-9-augustus-gene-116.6-mRNA-1	9	OsR498G0917239000.01.T02	94,49	0,00E+00	958	Os09g0356800	100,00	0,00E+00	1108	150	Japonica	Japonica	
maker-9-augustus-gene-117.0-mRNA-1	9	OsR498G0917239000.01.T02	90,69	0,00E+00	1916	Os09g0356800	89,87	0,00E+00	1860	-56	NA	Japonica	Kinase-LRR-TM
maker-9-augustus-gene-117.3-mRNA-1	9	OsR498G0917240400.01.T05	97,98	0,00E+00	1523	Os09g0356800	71,99	8,31E-122	438	-1085	Indica	Indica	Kinase-TM
maker-9-augustus-gene-118.12-mRNA-1	9	OsR498G0917243500.01.T04	99,83	0,00E+00	1062	Os09g0360400	100,00	0,00E+00	1067	5	NA	Japonica	
maker-9-augustus-gene-118.15-mRNA-1	9	OsR498G0917242800.01.T02	99,83	0,00E+00	2091	Os09g0359800	99,91	0,00E+00	2095	4	NA	Japonica	
maker-9-augustus-gene-118.20-mRNA-1	9	OsR498G0917243800.01.T01	99,17	0,00E+00	2560	Os09g0360500	99,93	0,00E+00	2609	49	NA	Japonica	
maker-9-augustus-gene-118.6-mRNA-1	9	OsR498G0917242500.01.T01	99,77	0,00E+00	766	Os09g0359700	100,00	0,00E+00	1131	365	Japonica	Japonica	
maker-9-augustus-gene-118.9-mRNA-1	9	OsR498G0917242900.01.T01	99,78	0,00E+00	1644	Os09g0359900	100,00	0,00E+00	1653	9	NA	Japonica	
maker-9-augustus-gene-119.0-mRNA-1	9	OsR498G0917245500.01.T05	99,21	0,00E+00	2700	Os08g0398300	77,85	0,00E+00	1207	-1493	Indica	Japonica	
maker-9-augustus-gene-119.10-mRNA-1	9	OsR498G0917246300.01.T01	98,38	0,00E+00	1489	Os09g0361200	100,00	0,00E+00	1508	19	NA	Japonica	
maker-9-augustus-gene-119.28-mRNA-1	9	OsR498G0917246600.01.T01	96,67	0,00E+00	693	Os09g0361300	99,76	0,00E+00	759	66	NA	Japonica	
maker-9-augustus-gene-119.5-mRNA-1	9	OsR498G0917245900.01.T01	99,09	0,00E+00	2713	Os09g0361100	100,00	0,00E+00	2084	-629	Indica	Japonica	Kinase
maker-9-augustus-gene-12.0-mRNA-1	9	OsR498G0916689300.01.T01	99,84	0,00E+00	5587	Os09g0114500	99,94	0,00E+00	5597	10	NA	Japonica	
maker-9-augustus-gene-120.40-mRNA-1	9	OsR498G0917250300.01.T01	99,71	0,00E+00	3660	Os09g0362900	100,00	0,00E+00	3411	-249	NA	Japonica	
maker-9-augustus-gene-120.42-mRNA-1	9	OsR498G0917251300.01.T02	99,72	0,00E+00	5095	Os09g0363100	100,00	0,00E+00	5130	35	NA	Japonica	
maker-9-augustus-gene-120.50-mRNA-1	9	OsR498G0917251900.01.T03	99,71	0,00E+00	2493	Os09g0363500	100,00	0,00E+00	2511	18	NA	Japonica	
maker-9-augustus-gene-120.52-mRNA-1	9	OsR498G0917252700.01.T01	100,00	0,00E+00	2057	Os09g0363800	96,24	0,00E+00	1459	-598	Indica	Japonica	
maker-9-augustus-gene-120.62-mRNA-1	9	OsR498G0917252200.01.T03	99,74	0,00E+00	2049	Os09g0363600	100,00	0,00E+00	1480	-569	Indica	Japonica	
maker-9-augustus-gene-120.68-mRNA-1	9	OsR498G0917252400.01.T01	99,24	0,00E+00	696	Os09g0363700	100,00	0,00E+00	710	14	NA	Japonica	
maker-9-augustus-gene-120.70-mRNA-1	9	OsR498G0917253000.01.T01	99,83	0,00E+00	3158	Os09g0363900	100,00	0,00E+00	3171	13	NA	Japonica	
maker-9-augustus-gene-121.0-mRNA-1	9	OsR498G0917255300.01.T01	99,84	0,00E+00	3260	Os09g0364500	100,00	0,00E+00	2268	-992	Indica	Japonica	
maker-9-augustus-gene-121.6-mRNA-1	9	OsR498G0917260400.01.T01	100,00	0,00E+00	3128	Os09g0507300	74,44	0,00E+00	1084	-2044	Indica	Japonica	
maker-9-augustus-gene-122.0-mRNA-1	9	OsR498G0917268500.01.T06	100,00	0,00E+00	1040	Os08g0407600	74,96	1,99E-94	345	-695	Indica	NA	
maker-9-augustus-gene-123.42-mRNA-1	9	OsR498G0917271000.01.T02	100,00	0,00E+00	2690	Os09g0368200	99,93	0,00E+00	2467	-223	NA	NA	
maker-9-augustus-gene-123.46-mRNA-1	9	OsR498G0917272200.01.T01	100,00	0,00E+00	1718	Os09g0368500	99,11	0,00E+00	1577	-141	NA	Japonica	
maker-9-augustus-gene-123.47-mRNA-1	9	OsR498G0917272200.01.T02	99,74	0,00E+00	692	Os09g0368500	100,00	0,00E+00	675	-17	NA	Japonica	
maker-9-augustus-gene-124.39-mRNA-1	9	OsR498G0917282000.01.T01	99,53	0,00E+00	1525	Os09g0370200	100,00	0,00E+00	1543	18	NA	Japonica	
maker-9-augustus-gene-125.0-mRNA-1	9	OsR498G0917285100.01.T01	99,62	0,00E+00	2791	Os09g0371000	99,94	0,00E+00	2815	24	NA	Japonica	
maker-9-augustus-gene-125.17-mRNA-1	9	OsR498G0917289300.01.T01	99,69	0,00E+00	2298	Os09g0372400	100,00	0,00E+00	1164	-1134	Indica	Japonica	
maker-9-augustus-gene-125.23-mRNA-1	9	OsR498G0917291500.01.T02	99,68	0,00E+00	1686	Os02g0581300	77,74	0,00E+00	716	-970	Indica	Japonica	
maker-9-augustus-gene-125.28-mRNA-1	9	OsR498G0917291800.01.T05	100,00	0,00E+00	863	Os09g0372800	100,00	0,00E+00	861	-2	NA	Japonica	
maker-9-augustus-gene-125.33-mRNA-1	9	OsR498G0917292600.01.T01	99,38	7,08E-164	576	Os09g0373000	100,00	4,75E-167	585	9	NA	Japonica	
maker-9-augustus-gene-125.7-mRNA-1	9	OsR498G0917286300.01.T01	98,00	0,00E+00	1289	Os09g0371300	100,00	0,00E+00	1243	-46	NA	Japonica	
maker-9-augustus-gene-125.8-mRNA-1	9	OsR498G0917286300.01.T01	96,75	0,00E+00	1425	Os09g0371300	100,00	0,00E+00	1508	83	NA	Japonica	
maker-9-augustus-gene-126.39-mRNA-1	9	OsR498G0917292900.01.T01	99,00	0,00E+00	1926	Os09g0373200	100,00	0,00E+00	1975	49	NA	Japonica	
maker-9-augustus-gene-126.43-mRNA-1	9	OsR498G0917293900.01.T01	99,12	0,00E+00	1809	Os09g0374900	100,00	0,00E+00	1965	156	NA	Japonica	
maker-9-augustus-gene-127.33-mRNA-1	9	OsR498G0917296300.01.T01	100,00	7,59E-157	553	Os09g0375900	100,00	2,64E-157	553	0	NA	Japonica	
maker-9-augustus-gene-127.35-mRNA-1	9	OsR498G0917298500.01.T01	99,63	0,00E+00	966	Os09g0376300	99,80	0,00E+00	902	-64	NA	Japonica	
maker-9-augustus-gene-127.39-mRNA-1	9	OsR498G0917296000.01.T01	99,63	0,00E+00	1936	Os09g0375600	100,00	0,00E+00	1045	-891	Indica	Japonica	Kinase-TM
maker-9-augustus-gene-128.2-mRNA-1	9	OsR498G0917302000.01.T05	99,73	0,00E+00	4644	Os09g0376800	100,00	0,00E+00	2792	-1852	Indica	Japonica	
maker-9-augustus-gene-128.5-mRNA-1	9	OsR498G0917303400.01.T02	99,94	0,00E+00	2750	Os09g0376900	100,00	0,00E+00	2755	5	NA	Japonica	
maker-9-augustus-gene-129.49-mRNA-1	9	OsR498G0917314300.01.T01	93,84	0,00E+00	1356	Os09g0380200	100,00	0,00E+00	791	-565	Indica	Japonica	
maker-9-augustus-gene-129.52-mRNA-1	9	OsR498G0917312000.01.T01	99,70	0,00E+00	1794	Os09g0379600	100,00	0,00E+00	1553	-241	Indica	Japonica	
maker-9-augustus-gene-13.31-mRNA-1	9	OsR498G0916693700.01.T01	99,00	0,00E+00	1764	Os09g0115900	98,73	0,00E+00	1510	-254	Indica	Indica	
maker-9-augustus-gene-13.39-mRNA-1	9	OsR498G0916691400.01.T02	99,85	0,00E+00	3574	Os09g0115400	99,90	0,00E+00	3579	5	NA	Japonica	
maker-9-augustus-gene-13.46-mRNA-1	9	OsR498G0916693000.01.T04	99,73	0,00E+00	1330	Os09g0115600	99,86	0,00E+00	1318	-12	NA	NA	
maker-9-augustus-gene-130.0-mRNA-1	9	OsR498G0917320100.01.T01	99,94	0,00E+00	2869	Os09g0381100	100,00	0,00E+00	2874	5	NA	Japonica	
maker-9-augustus-gene-130.13-mRNA-1	9	OsR498G0917322700.01.T01	94,85	0,00E+00	1063	Os09g0381600	94,85	0,00E+00	1063	0	NA	Japonica	
maker-9-augustus-gene-130.17-mRNA-1	9	OsR498G0917321000.01.T01	99,73	0,00E+00	1951	Os09g0381400	100,00	0,00E+00	1965	14	NA	Japonica	
maker-9-augustus-gene-131.0-mRNA-1	9	OsR498G0917329300.01.T03	99,81	0,00E+00	2902	Os09g0383300	100,00	0,00E+00	2917	15	NA	Japonica	CC-Kinase-TM
maker-9-augustus-gene-131.17-mRNA-1	9	OsR498G0917327400.01.T01	98,90	1,21E-134	480	Os09g0382500	100,00	1,92E-139	493	13	NA	Japonica	
maker-9-augustus-gene-131.6-mRNA-1	9	OsR498G0917329900.01.T01	97,35	0,00E+00	2954	Os09g0383400	100,00	0,00E+00	2691	-263	NA	Japonica	
maker-9-augustus-gene-133.40-mRNA-1	9	OsR498G0917341700.01.T02	99,78	0,00E+00	801	Os09g0386500	100,00	0,00E+00	756	-45	NA	Japonica	
maker-9-augustus-gene-133.43-mRNA-1	9	OsR498G0917341200.01.T02	99,92	0,00E+00	2214	Os09g0386400	99,92	0,00E+00	2214	0	NA	Japonica	
maker-9-augustus-gene-135.31-mRNA-1	9	OsR498G0917355600.01.T01	97,11	0,00E+00	1818	Os09g0389000	99,42	0,00E+00	2145	327	Japonica	Japonica	
maker-9-augustus-gene-137.1-mRNA-1	9	OsR498G0917367500.01.T01	100,00	0,00E+00	2960	Os09g0392000	99,94	0,00E+00	2956	-4	NA	Japonica	
maker-9-augustus-gene-137.19-mRNA-1	9	OsR498G0917375400.01.T03	99,42	0,00E+00	2755	Os09g0394100	100,00	0,00E+00	2776	21	NA	Japonica	
maker-9-augustus-gene-137.5-mRNA-1	9	OsR498G0917367700.01.T01	99,89	0,00E+00	3357	Os09g0392100	100,00	0,00E+00	3366	9	NA	Japonica	
maker-9-augustus-gene-138.32-mRNA-1	9	OsR498G0917379500.01.T05	100,00	0,00E+00	1310	Os09g0394900	100,00	0,00E+00	1310	0	NA	Japonica	
maker-9-augustus-gene-138.36-mRNA-1	9	OsR498G0917376000.01.T01	99,79	0,00E+00	1718	Os09g0394300	100,00	0,00E+00	1104	-614	Indica	Japonica	
maker-9-augustus-gene-138.39-mRNA-1	9	OsR498G0917379000.01.T02	99,90	0,00E+00	1859	Os09g0394600	100,00	0,00E+00	1864	5	NA	Japonica	
maker-9-augustus-gene-138.41-mRNA-1	9	OsR498G0917379000.01.T05	99,00	0,00E+00	2120	Os09g0394700	100,00	0,00E+00	1732	-388	Indica	Japonica	
maker-9-augustus-gene-139.0-mRNA-1	9	OsR498G0917383500.01.T01	100,00	0,00E+00	2185	Os09g0395300	100,00	0,00E+00	2185	0	NA	Indica	
maker-9-augustus-gene-139.12-mRNA-1	9	OsR498G0917385200.01.T01	99,90	0,00E+00	1798	Os09g0395600	99,81	0,00E+00	963	-835	Indica	Indica	
maker-9-augustus-gene-139.4-mRNA-1	9	OsR498G0917388100.01.T05	100,00	0,00E+00	1160	Os09g0396300	100,00	0,00E+00	1160	0	NA	NA	
maker-9-augustus-gene-139.8-mRNA-1	9	OsR498G0917389500.01.T01	100,00	0,00E+00	1337	Os09g0396900	100,00	0,00E+00	749	-588	Indica	NA	
maker-9-augustus-gene-14.36-mRNA-1	9	OsR498G0916695200.01.T01	99,33	0,00E+00	788	Os09g0116100	98,95	0,00E+00	670	-118	Indica	NA	
maker-9-augustus-gene-140.0-mRNA-1	9	OsR498G0917391500.01.T01	100,00	0,00E+00	893	Os09g0397100	100,00	0,00E+00	893	0	NA	NA	
maker-9-augustus-gene-140.14-mRNA-1	9	OsR498G0917394200.01.T02	100,00	0,00E+00	2251	Os09g0397400	100,00	0,00E+00	2251	0	NA	NA	
maker-9-augustus-gene-140.2-mRNA-1	9	OsR498G0917391700.01.T01	99,40	0,00E+00	884	Os09g0397200	99,40	0,00E+00	884	0	NA	NA	
maker-9-augustus-gene-140.5-mRNA-1	9	OsR498G0917393900.01.T03	99,93	0,00E+00	4785	Os09g0397300	99,96	0,00E+00	4640	-145	NA	NA	
maker-9-augustus-gene-140.8-mRNA-1	9	OsR498G0917394900.01.T04	97,06	0,00E+00	2739	Os09g0397900	97,06	0,00E+00	2739	0	NA	NA	
maker-9-augustus-gene-141.42-mRNA-1	9	OsR498G0917404400.01.T01	96,67	0,00E+00	2186	Os09g0400000	95,98	0,00E+00	1781	-405	Indica	Indica	
maker-9-augustus-gene-141.44-mRNA-1	9	OsR498G0917405100.01.T01	100,00	0,00E+00	1965	Os09g0400400	100,00	0,00E+00	1965	0	NA	Indica	
maker-9-augustus-gene-141.46-mRNA-1	9	OsR498G0917407900.01.T01	100,00	0,00E+00	1516	Os09g0401000	99,88	0,00E+00	1511	-5	NA	Indica	
maker-9-augustus-gene-141.48-mRNA-1	9	OsR498G0917404000.01.T01	100,00	0,00E+00	1948	Os09g0399800	100,00	0,00E+00	1948	0	NA	Indica	
maker-9-augustus-gene-141.50-mRNA-1	9	OsR498G0917404400.01.T01	100,00	0,00E+00	2199	Os09g0400000	100,00	0,00E+00	1959	-240	Indica	Indica	
maker-9-augustus-gene-142.33-mRNA-1	9	OsR498G0917409300.01.T01	100,00	0,00E+00	4735	Os09g0401100	99,81	0,00E+00	2763	-1972	Indica	Indica	
maker-9-augustus-gene-142.35-mRNA-1	9	OsR498G0917411900.01.T02	99,77	0,00E+00	1548	Os09g0401900	99,77	0,00E+00	1548	0	NA	NA	
maker-9-augustus-gene-142.44-mRNA-1	9	OsR498G0917409600.01.T06	94,54	0,00E+00	1610	Os09g0401200	94,54	0,00E+00	1610	0	NA	Indica	
maker-9-augustus-gene-142.50-mRNA-1	9	OsR498G0917410000.01.T01	99,34	0,00E+00	1884	Os09g0401300	99,91	0,00E+00	1899	15	NA	Indica	
maker-9-augustus-gene-142.55-mRNA-1	9	OsR498G0917411600.01.T01	98,77	0,00E+00	2281	Os09g0401600	98,67	0,00E+00	2232	-49	NA	Japonica	
maker-9-augustus-gene-143.25-mRNA-1	9	OsR498G0917417400.01.T01	99,44	0,00E+00	2861	Os09g0403300	99,94	0,00E+00	2896	35	NA	Japonica	
maker-9-augustus-gene-144.43-mRNA-1	9	OsR498G0917421300.01.T06	98,58	0,00E+00	1107	Os09g0407200	98,58	0,00E+00	1107	0	NA	Japonica	
maker-9-augustus-gene-144.53-mRNA-1	9	OsR498G0917419000.01.T01	98,72	0,00E+00	685	Os09g0406100	100,00	0,00E+00	708	23	NA	NA	
maker-9-augustus-gene-144.54-mRNA-1	9	OsR498G0917419000.01.T01	98,72	0,00E+00	685	Os09g0406100	100,00	0,00E+00	708	23	NA	Indica	
maker-9-augustus-gene-144.55-mRNA-1	9	OsR498G0917422000.01.T01	99,30	0,00E+00	3029	Os09g0407300	99,88	0,00E+00	3074	45	NA	Japonica	
maker-9-augustus-gene-144.59-mRNA-1	9	OsR498G0917422600.01.T02	99,34	0,00E+00	1347	Os09g0407700	100,00	0,00E+00	1020	-327	Indica	Japonica	
maker-9-augustus-gene-144.63-mRNA-1	9	OsR498G0917423000.01.T01	98,56	0,00E+00	2905	Os09g0407900	98,74	0,00E+00	2911	6	NA	NA	
maker-9-augustus-gene-145.39-mRNA-1	9	OsR498G0917427900.01.T01	99,93	0,00E+00	2458	Os09g0408800	99,56	0,00E+00	2434	-24	NA	Indica	Kinase-TM
maker-9-augustus-gene-145.41-mRNA-1	9	OsR498G0917427400.01.T01	98,34	0,00E+00	2602	Os09g0408600	98,67	0,00E+00	2625	23	NA	NA	
maker-9-augustus-gene-146.12-mRNA-1	9	OsR498G0917434200.01.T01	99,63	0,00E+00	1952	Os09g0410700	100,00	0,00E+00	1232	-720	Indica	Japonica	
maker-9-augustus-gene-146.6-mRNA-1	9	OsR498G0917431700.01.T04	99,57	2,29E-114	413	Os03g0706900	73,22	6,13E-34	144	-269	Indica	Japonica	
maker-9-augustus-gene-147.18-mRNA-1	9	OsR498G0917436200.01.T01	95,50	0,00E+00	762	Os09g0411700	95,72	0,00E+00	766	4	NA	Japonica	
maker-9-augustus-gene-147.2-mRNA-1	9	OsR498G0917439300.01.T01	99,35	3,93E-154	544	Os09g0412700	100,00	2,64E-157	553	9	NA	Japonica	
maker-9-augustus-gene-147.25-mRNA-1	9	OsR498G0917438600.01.T01	97,52	0,00E+00	824	Os09g0412400	100,00	8,89E-128	455	-369	Indica	Japonica	
maker-9-augustus-gene-147.30-mRNA-1	9	OsR498G0917442200.01.T01	99,17	0,00E+00	636	Os08g0436800	89,17	8,45E-133	472	-164	Indica	Japonica	
maker-9-augustus-gene-147.4-mRNA-1	9	OsR498G0917441100.01.T03	99,80	0,00E+00	914	Os08g0436200	81,16	4,37E-134	477	-437	Indica	Japonica	
maker-9-augustus-gene-148.0-mRNA-1	9	OsR498G0917444100.01.T03	99,64	0,00E+00	2001	Os09g0413700	100,00	0,00E+00	1487	-514	Indica	Japonica	
maker-9-augustus-gene-148.3-mRNA-1	9	OsR498G0917445700.01.T01	98,97	0,00E+00	1712	Os09g0414300	99,90	0,00E+00	1728	16	NA	Japonica	
maker-9-augustus-gene-148.5-mRNA-1	9	OsR498G0917442600.01.T01	99,21	0,00E+00	6066	Os09g0413600	100,00	0,00E+00	6152	86	NA	Japonica	
maker-9-augustus-gene-149.27-mRNA-1	9	OsR498G0917450500.01.T01	100,00	1,03E-174	612	Os09g0414900	100,00	3,59E-175	612	0	NA	Japonica	
maker-9-augustus-gene-149.33-mRNA-1	9	OsR498G0917452300.01.T01	99,57	0,00E+00	3303	Os09g0415700	100,00	0,00E+00	3339	36	NA	Japonica	
maker-9-augustus-gene-149.38-mRNA-1	9	OsR498G0917452800.01.T03	99,70	0,00E+00	2372	Os09g0415800	100,00	0,00E+00	2390	18	NA	Japonica	
maker-9-augustus-gene-15.0-mRNA-1	9	OsR498G0916701600.01.T01	98,88	2,19E-131	469	Os02g0826600	97,74	3,72E-104	377	-92	Indica	NA	LRR
maker-9-augustus-gene-150.0-mRNA-1	9	OsR498G0917453500.01.T01	99,74	0,00E+00	2753	Os09g0416200	100,00	0,00E+00	2771	18	NA	Japonica	
maker-9-augustus-gene-150.11-mRNA-1	9	OsR498G0917460800.01.T02	96,30	0,00E+00	1691	Os09g0417400	97,00	0,00E+00	2535	844	Japonica	Japonica	
maker-9-augustus-gene-150.13-mRNA-1	9	OsR498G0917462400.01.T01	99,90	0,00E+00	1771	Os09g0417600	99,49	0,00E+00	1753	-18	NA	NA	
maker-9-augustus-gene-150.6-mRNA-1	9	OsR498G0917458500.01.T01	99,39	0,00E+00	2607	Os09g0416900	100,00	0,00E+00	2646	39	NA	Japonica	
maker-9-augustus-gene-150.8-mRNA-1	9	OsR498G0917456800.01.T01	99,75	0,00E+00	705	Os09g0416700	99,75	0,00E+00	705	0	NA	Japonica	
maker-9-augustus-gene-151.29-mRNA-1	9	OsR498G0917463600.01.T02	99,79	0,00E+00	1718	Os09g0417800	99,90	0,00E+00	1722	4	NA	NA	
maker-9-augustus-gene-151.32-mRNA-1	9	OsR498G0917467800.01.T01	95,53	0,00E+00	1752	Os09g0419200	95,25	0,00E+00	1738	-14	NA	Indica	
maker-9-augustus-gene-152.0-mRNA-1	9	OsR498G0917469500.01.T01	99,67	0,00E+00	2141	Os09g0419500	99,69	0,00E+00	1157	-984	Indica	NA	
maker-9-augustus-gene-152.10-mRNA-1	9	OsR498G0917472200.01.T01	99,68	0,00E+00	2212	Os09g0420300	99,76	0,00E+00	2216	4	NA	NA	
maker-9-augustus-gene-152.15-mRNA-1	9	OsR498G0917472800.01.T01	92,40	0,00E+00	1022	Os09g0420600	92,40	0,00E+00	1022	0	NA	Japonica	
maker-9-augustus-gene-152.17-mRNA-1	9	OsR498G0917473600.01.T01	99,89	0,00E+00	3341	Os09g0420900	99,89	0,00E+00	3341	0	NA	Japonica	
maker-9-augustus-gene-152.2-mRNA-1	9	OsR498G0917469700.01.T01	99,55	0,00E+00	1188	Os09g0419600	99,70	0,00E+00	1193	5	NA	NA	
maker-9-augustus-gene-152.4-mRNA-1	9	OsR498G0917471500.01.T01	99,52	0,00E+00	1113	Os09g0420000	99,48	0,00E+00	1724	611	Japonica	NA	
maker-9-augustus-gene-152.8-mRNA-1	9	OsR498G0917471700.01.T01	99,60	0,00E+00	1784	Os09g0420100	99,77	0,00E+00	1577	-207	Indica	Japonica	

maker-9-augustus-gene-153.0-mRNA-1	9	OsR498G0917475900.01.T01	97,26	0,00E+00	797	Os11g0130200	90,89	1,17E-164	578	-219	Indica	Japonica	
maker-9-augustus-gene-153.2-mRNA-1	9	OsR498G0917476100.01.T01	99,67	0,00E+00	1068	Os09g0421500	100,00	0,00E+00	1077	9	NA	Japonica	
maker-9-augustus-gene-153.26-mRNA-1	9	OsR498G0917475600.01.T01	99,72	0,00E+00	4395	Os09g0421300	99,96	0,00E+00	4422	27	NA	Japonica	
maker-9-augustus-gene-153.31-mRNA-1	9	OsR498G0917476600.01.T01	99,19	0,00E+00	1745	Os09g0421700	99,90	0,00E+00	1776	31	NA	Japonica	
maker-9-augustus-gene-153.33-mRNA-1	9	OsR498G0917477000.01.T01	99,45	0,00E+00	1612	Os09g0421800	100,00	0,00E+00	2679	1067	Japonica	Japonica	
maker-9-augustus-gene-153.36-mRNA-1	9	OsR498G0917477200.01.T01	99,25	0,00E+00	2353	Os09g0422000	100,00	0,00E+00	2387	34	NA	Japonica	
maker-9-augustus-gene-153.40-mRNA-1	9	OsR498G0917480000.01.T04	97,74	0,00E+00	2109	Os09g0422600	99,76	0,00E+00	2223	114	NA	Japonica	
maker-9-augustus-gene-153.5-mRNA-1	9	OsR498G0917479700.01.T01	99,50	0,00E+00	5642	Os09g0422500	99,87	0,00E+00	5696	54	NA	Japonica	
maker-9-augustus-gene-153.8-mRNA-1	9	OsR498G0917475200.01.T02	99,84	0,00E+00	1138	Os09g0421100	100,00	0,00E+00	1572	434	Japonica	Japonica	
maker-9-augustus-gene-154.29-mRNA-1	9	OsR498G0917480300.01.T01	99,08	0,00E+00	2860	Os09g0423000	98,44	0,00E+00	4892	2032	Japonica	Japonica	CC-Kinase-LRR-TM
maker-9-augustus-gene-154.31-mRNA-1	9	OsR498G0917481100.01.T01	99,87	0,00E+00	2724	Os09g0423400	99,87	0,00E+00	2724	0	NA	NA	
maker-9-augustus-gene-154.33-mRNA-1	9	OsR498G0917482100.01.T01	99,65	0,00E+00	2541	Os09g0423500	100,00	0,00E+00	2565	24	NA	NA	
maker-9-augustus-gene-154.35-mRNA-1	9	OsR498G0917482700.01.T01	100,00	7,04E-113	407	Os09g0423700	100,00	2,45E-113	407	0	NA	Japonica	
maker-9-augustus-gene-154.41-mRNA-1	9	OsR498G0917483900.01.T01	98,91	0,00E+00	1441	Os09g0424200	100,00	0,00E+00	1478	37	NA	Japonica	
maker-9-augustus-gene-155.50-mRNA-1	9	OsR498G0917485100.01.T04	99,87	0,00E+00	1426	Os09g0424600	100,00	0,00E+00	1429	3	NA	Japonica	
maker-9-augustus-gene-155.55-mRNA-1	9	OsR498G0917486500.01.T01	99,69	6,55E-166	584	Os09g0425400	100,00	0,00E+00	785	201	Japonica	Japonica	
maker-9-augustus-gene-155.57-mRNA-1	9	OsR498G0917487700.01.T03	97,16	0,00E+00	716	Os10g0508300	90,64	1,31E-99	361	-355	Indica	Japonica	
maker-9-augustus-gene-155.59-mRNA-1	9	OsR498G0917488500.01.T01	99,76	0,00E+00	1490	Os09g0425900	100,00	0,00E+00	1499	9	NA	Japonica	
maker-9-augustus-gene-155.65-mRNA-1	9	OsR498G0917489000.01.T01	99,65	0,00E+00	1522	Os09g0426000	99,88	0,00E+00	1489	-33	NA	Japonica	
maker-9-augustus-gene-155.74-mRNA-1	9	OsR498G0917489400.01.T01	99,84	0,00E+00	1154	Os09g0426100	84,83	3,00E-173	608	-546	Indica	Japonica	
maker-9-augustus-gene-155.76-mRNA-1	9	OsR498G0917490000.01.T01	99,82	0,00E+00	2021	Os09g0426100	79,22	1,64E-160	565	-1456	Indica	Japonica	
maker-9-augustus-gene-156.0-mRNA-1	9	OsR498G0917491900.01.T01	99,73	0,00E+00	3333	Os09g0426800	100,00	0,00E+00	3355	22	NA	Japonica	
maker-9-augustus-gene-156.11-mRNA-1	9	OsR498G0917494200.01.T01	98,34	0,00E+00	723	Os09g0427300	100,00	0,00E+00	704	-19	NA	Japonica	
maker-9-augustus-gene-156.13-mRNA-1	9	OsR498G0917495200.01.T02	99,00	0,00E+00	3691	Os09g0428000	100,00	0,00E+00	2630	-1061	Indica	Japonica	
maker-9-augustus-gene-156.6-mRNA-1	9	OsR498G0917494500.01.T01	99,55	0,00E+00	4745	Os09g0427800	100,00	0,00E+00	3589	-1156	Indica	Japonica	
maker-9-augustus-gene-156.8-mRNA-1	9	OsR498G0917497500.01.T04	94,10	0,00E+00	1925	Os09g0428900	100,00	0,00E+00	2098	173	NA	Japonica	
maker-9-augustus-gene-157.0-mRNA-1	9	OsR498G0917500700.01.T07	90,86	6,53E-69	260	Os09g0429100	100,00	6,49E-146	515	255	Japonica	Japonica	
maker-9-augustus-gene-157.2-mRNA-1	9	OsR498G0917501300.01.T01	98,71	0,00E+00	810	Os09g0429350	100,00	0,00E+00	715	-95	Indica	Japonica	
maker-9-augustus-gene-157.6-mRNA-1	9	OsR498G0917501600.01.T03	96,64	0,00E+00	4377	Os09g0429400	99,96	0,00E+00	5055	678	Japonica	Japonica	
maker-9-augustus-gene-158.30-mRNA-1	9	OsR498G0917508100.01.T01	99,62	0,00E+00	5130	Os09g0431100	100,00	0,00E+00	5173	43	NA	Japonica	
maker-9-augustus-gene-158.32-mRNA-1	9	OsR498G0917508700.01.T01	99,72	0,00E+00	4548	Os09g0431200	100,00	0,00E+00	4580	32	NA	Japonica	
maker-9-augustus-gene-158.43-mRNA-1	9	OsR498G0917509600.01.T04	95,58	0,00E+00	811	Os09g0431500	100,00	0,00E+00	899	88	NA	Japonica	
maker-9-augustus-gene-16.0-mRNA-1	9	OsR498G0916695200.01.T01	88,18	3,61E-138	491	Os09g0116100	90,00	5,34E-137	486	-5	NA	NA	
maker-9-augustus-gene-16.2-mRNA-1	9	OsR498G0916707400.01.T01	99,88	0,00E+00	1457	Os09g0119100	99,75	0,00E+00	1452	-5	NA	NA	
maker-9-augustus-gene-160.0-mRNA-1	9	OsR498G0917519700.01.T01	99,62	0,00E+00	939	Os09g0433800	100,00	0,00E+00	948	9	NA	Japonica	
maker-9-augustus-gene-160.16-mRNA-1	9	OsR498G0917520400.01.T01	100,00	0,00E+00	1737	Os09g0434200	100,00	0,00E+00	1737	0	NA	Japonica	
maker-9-augustus-gene-160.18-mRNA-1	9	OsR498G0204973200.01.T01	92,37	6,28E-94	343	Os09g0434600	100,00	1,92E-139	493	150	Japonica	Japonica	
maker-9-augustus-gene-160.2-mRNA-1	9	OsR498G0917519900.01.T01	95,69	0,00E+00	2393	Os09g0433900	96,51	0,00E+00	2447	54	NA	Japonica	
maker-9-augustus-gene-160.8-mRNA-1	9	OsR498G0917518600.01.T01	98,33	0,00E+00	1659	Os09g0433650	100,00	0,00E+00	1635	-24	NA	Japonica	
maker-9-augustus-gene-160.9-mRNA-1	9	OsR498G0917520200.01.T02	99,65	0,00E+00	4109	Os08g0451400	77,31	0,00E+00	1761	-2348	Indica	Japonica	
maker-9-augustus-gene-161.56-mRNA-1	9	OsR498G0917526600.01.T02	99,75	0,00E+00	2124	Os09g0436300	100,00	0,00E+00	2138	14	NA	Japonica	
maker-9-augustus-gene-161.60-mRNA-1	9	OsR498G0917527400.01.T01	99,73	0,00E+00	2634	Os09g0436700	100,00	0,00E+00	2652	18	NA	Japonica	
maker-9-augustus-gene-162.0-mRNA-1	9	OsR498G0917531100.01.T04	100,00	0,00E+00	746	Os09g0437500	100,00	0,00E+00	746	0	NA	Japonica	
maker-9-augustus-gene-162.2-mRNA-1	9	OsR498G0917531500.01.T07	100,00	0,00E+00	765	Os09g0437900	100,00	0,00E+00	765	0	NA	Japonica	
maker-9-augustus-gene-162.7-mRNA-1	9	OsR498G0917532300.01.T01	99,80	0,00E+00	5431	Os09g0438000	100,00	0,00E+00	5454	23	NA	Japonica	
maker-9-augustus-gene-163.36-mRNA-1	9	OsR498G0917535900.01.T04	91,30	0,00E+00	919	Os03g0169400	80,77	0,00E+00	643	-276	Indica	Japonica	
maker-9-augustus-gene-163.42-mRNA-1	9	OsR498G0917537500.01.T01	99,75	0,00E+00	2834	Os09g0439400	100,00	0,00E+00	2852	18	NA	Japonica	
maker-9-augustus-gene-163.47-mRNA-1	9	OsR498G0917537700.01.T01	99,36	0,00E+00	1381	Os09g0439500	100,00	0,00E+00	1404	23	NA	Japonica	
maker-9-augustus-gene-163.49-mRNA-1	9	OsR498G0917535200.01.T01	99,59	0,00E+00	1318	Os09g0438700	100,00	0,00E+00	1332	14	NA	Japonica	
maker-9-augustus-gene-163.58-mRNA-1	9	OsR498G0917537200.01.T01	99,86	0,00E+00	1257	Os09g0439200	100,00	0,00E+00	1261	4	NA	Japonica	
maker-9-augustus-gene-163.60-mRNA-1	9	OsR498G0917538800.01.T03	99,74	0,00E+00	2072	Os09g0439700	100,00	0,00E+00	2085	13	NA	Japonica	
maker-9-augustus-gene-164.12-mRNA-1	9	OsR498G0917539200.01.T01	99,74	0,00E+00	1355	Os09g0439800	100,00	0,00E+00	1364	9	NA	Japonica	
maker-9-augustus-gene-164.14-mRNA-1	9	OsR498G0917542200.01.T01	99,86	0,00E+00	2502	Os09g0440300	100,00	0,00E+00	2511	9	NA	Japonica	
maker-9-augustus-gene-164.19-mRNA-1	9	OsR498G0917544800.01.T02	99,75	0,00E+00	2828	Os09g0441100	100,00	0,00E+00	2847	19	NA	Japonica	
maker-9-augustus-gene-164.4-mRNA-1	9	OsR498G0917541700.01.T04	99,74	0,00E+00	2104	Os09g0440200	99,87	0,00E+00	1388	-716	Indica	Japonica	
maker-9-augustus-gene-164.7-mRNA-1	9	OsR498G0917544100.01.T01	99,87	0,00E+00	4158	Os09g0441000	100,00	0,00E+00	2953	-1205	Indica	Japonica	
maker-9-augustus-gene-164.9-mRNA-1	9	OsR498G0917545900.01.T02	99,94	0,00E+00	2923	Os09g0441400	100,00	0,00E+00	2787	-136	NA	Japonica	
maker-9-augustus-gene-165.30-mRNA-1	9	OsR498G0917547300.01.T01	95,05	0,00E+00	948	Os09g0441900	95,22	0,00E+00	952	4	NA	Japonica	
maker-9-augustus-gene-165.33-mRNA-1	9	OsR498G0917550300.01.T01	94,79	0,00E+00	1298	Os09g0442300	94,79	0,00E+00	1298	0	NA	Japonica	
maker-9-augustus-gene-165.41-mRNA-1	9	OsR498G0917550700.01.T01	99,67	0,00E+00	3805	Os09g0442600	100,00	0,00E+00	2612	-1193	Indica	Japonica	
maker-9-augustus-gene-165.45-mRNA-1	9	OsR498G0917551100.01.T02	99,61	0,00E+00	917	Os09g0442800	100,00	0,00E+00	1207	290	Japonica	Japonica	
maker-9-augustus-gene-165.54-mRNA-1	9	OsR498G0917551500.01.T01	98,64	3,05E-144	511	Os01g0177400	88,03	1,29E-86	319	-192	Indica	Japonica	
maker-9-augustus-gene-165.55-mRNA-1	9	OsR498G0917546600.01.T01	99,75	0,00E+00	2812	Os09g0441400	77,32	0,00E+00	1177	-1635	Indica	Japonica	
maker-9-augustus-gene-165.57-mRNA-1	9	OsR498G0917547800.01.T01	100,00	0,00E+00	2484	Os09g0442100	100,00	0,00E+00	2484	0	NA	Japonica	CC-Kinase-TM
maker-9-augustus-gene-165.66-mRNA-1	9	OsR498G0917551800.01.T01	98,54	0,00E+00	1195	Os09g0443000	100,00	0,00E+00	2264	1069	Japonica	Japonica	
maker-9-augustus-gene-165.69-mRNA-1	9	OsR498G1220899000.01.T02	99,76	0,00E+00	1472	Os09g0443400	100,00	0,00E+00	1449	-23	NA	Japonica	
maker-9-augustus-gene-166.10-mRNA-1	9	OsR498G0917554200.01.T01	99,76	0,00E+00	3722	Os09g0444200	100,00	0,00E+00	3745	23	NA	Japonica	
maker-9-augustus-gene-166.2-mRNA-1	9	OsR498G0917555100.01.T01	97,27	0,00E+00	2043	Os08g0462700	73,44	0,00E+00	794	-1249	Indica	Japonica	
maker-9-augustus-gene-167.2-mRNA-1	9	OsR498G0917566900.01.T04	99,86	0,00E+00	3818	Os09g0446800	100,00	0,00E+00	3831	13	NA	Japonica	
maker-9-augustus-gene-168.42-mRNA-1	9	OsR498G0917573900.01.T02	99,78	0,00E+00	2494	Os09g0448200	100,00	0,00E+00	2507	13	NA	Japonica	
maker-9-augustus-gene-168.45-mRNA-1	9	OsR498G0917575300.01.T01	99,33	0,00E+00	1845	Os09g0448500	100,00	0,00E+00	1438	-407	Indica	Japonica	
maker-9-augustus-gene-168.58-mRNA-1	9	OsR498G0917570600.01.T01	99,74	0,00E+00	2720	Os09g0447500	100,00	0,00E+00	2738	18	NA	Japonica	
maker-9-augustus-gene-169.11-mRNA-1	9	OsR498G0917585300.01.T01	99,48	0,00E+00	688	Os09g0450600	100,00	4,98E-150	529	-159	Indica	Japonica	
maker-9-augustus-gene-169.14-mRNA-1	9	OsR498G0917582600.01.T02	99,67	0,00E+00	1601	Os09g0450300	99,78	0,00E+00	1606	5	NA	Japonica	
maker-9-augustus-gene-169.19-mRNA-1	9	OsR498G0917586800.01.T01	99,36	0,00E+00	1920	Os09g0451000	100,00	0,00E+00	1748	-172	NA	Japonica	
maker-9-augustus-gene-169.2-mRNA-1	9	OsR498G0917580500.01.T06	99,87	0,00E+00	2659	Os09g0448100	79,39	0,00E+00	1050	-1609	Indica	Japonica	
maker-9-augustus-gene-169.7-mRNA-1	9	OsR498G0917582300.01.T04	99,72	0,00E+00	1279	Os09g0450200	100,00	0,00E+00	801	-478	Indica	Japonica	
maker-9-augustus-gene-170.0-mRNA-1	9	OsR498G0917588300.01.T01	99,28	0,00E+00	1717	Os09g0451400	100,00	0,00E+00	1748	31	NA	Japonica	
maker-9-augustus-gene-170.12-mRNA-1	9	OsR498G0917592600.01.T02	99,92	0,00E+00	2128	Os09g0452900	100,00	0,00E+00	2132	4	NA	Japonica	
maker-9-augustus-gene-170.15-mRNA-1	9	OsR498G0917592800.01.T01	100,00	0,00E+00	769	Os09g0453000	100,00	0,00E+00	769	0	NA	Japonica	
maker-9-augustus-gene-170.18-mRNA-1	9	OsR498G0917593400.01.T02	99,90	0,00E+00	1728	Os09g0453300	100,00	0,00E+00	1732	4	NA	Japonica	
maker-9-augustus-gene-170.6-mRNA-1	9	OsR498G0917590900.01.T01	96,68	0,00E+00	2023	Os09g0452200	97,02	0,00E+00	2041	18	NA	Japonica	
maker-9-augustus-gene-170.9-mRNA-1	9	OsR498G0917593100.01.T01	99,84	0,00E+00	1149	Os09g0453200	100,00	0,00E+00	787	-362	Indica	Japonica	
maker-9-augustus-gene-171.1-mRNA-1	9	OsR498G0917594700.01.T01	99,79	0,00E+00	2514	Os09g0453800	100,00	0,00E+00	2527	13	NA	Japonica	
maker-9-augustus-gene-171.11-mRNA-1	9	OsR498G0917593800.01.T01	99,84	0,00E+00	2221	Os09g0453400	100,00	0,00E+00	2230	9	NA	Japonica	
maker-9-augustus-gene-171.16-mRNA-1	9	OsR498G0917594200.01.T03	98,78	0,00E+00	1580	Os09g0453500	100,00	0,00E+00	1609	29	NA	Japonica	
maker-9-augustus-gene-171.26-mRNA-1	9	OsR498G0917595200.01.T01	98,89	0,00E+00	2224	Os09g0453900	100,00	0,00E+00	2268	44	NA	Japonica	
maker-9-augustus-gene-171.29-mRNA-1	9	OsR498G0917597200.01.T01	99,86	0,00E+00	2617	Os09g0454200	100,00	0,00E+00	2626	9	NA	Japonica	
maker-9-augustus-gene-171.3-mRNA-1	9	OsR498G0917596900.01.T01	100,00	0,00E+00	1894	Os09g0454100	100,00	0,00E+00	1894	0	NA	Japonica	
maker-9-augustus-gene-171.31-mRNA-1	9	OsR498G0917598200.01.T02	99,78	0,00E+00	1653	Os09g0454500	100,00	0,00E+00	1040	-613	Indica	Japonica	
maker-9-augustus-gene-171.35-mRNA-1	9	OsR498G0917599400.01.T02	99,37	0,00E+00	4797	Os09g0454900	100,00	0,00E+00	4864	67	NA	Japonica	Kinase-TM
maker-9-augustus-gene-171.6-mRNA-1	9	OsR498G0917597900.01.T01	100,00	0,00E+00	2322	Os09g0454300	100,00	0,00E+00	2322	0	NA	Japonica	
maker-9-augustus-gene-171.8-mRNA-1	9	OsR498G0917598500.01.T01	99,91	0,00E+00	1890	Os09g0454600	100,00	0,00E+00	1894	4	NA	Japonica	
maker-9-augustus-gene-172.0-mRNA-1	9	OsR498G0917606100.01.T01	97,44	0,00E+00	2137	Os09g0456100	97,60	0,00E+00	2146	9	NA	Japonica	
maker-9-augustus-gene-172.5-mRNA-1	9	OsR498G0917606700.01.T02	99,72	0,00E+00	1892	Os09g0456200	100,00	0,00E+00	2035	143	NA	Japonica	
maker-9-augustus-gene-172.8-mRNA-1	9	OsR498G0917600600.01.T01	99,92	0,00E+00	2133	Os09g0455200	100,00	0,00E+00	2138	5	NA	Japonica	
maker-9-augustus-gene-173.27-mRNA-1	9	OsR498G0917610600.01.T01	99,73	0,00E+00	2693	Os09g0457100	100,00	0,00E+00	2711	18	NA	Japonica	
maker-9-augustus-gene-174.46-mRNA-1	9	OsR498G0917612100.01.T01	99,85	0,00E+00	2367	Os09g0457500	100,00	0,00E+00	2376	9	NA	Japonica	
maker-9-augustus-gene-174.49-mRNA-1	9	OsR498G0917612900.01.T01	99,93	0,00E+00	2458	Os09g0457800	100,00	0,00E+00	2370	-88	NA	Japonica	
maker-9-augustus-gene-174.56-mRNA-1	9	OsR498G0917614100.01.T01	99,49	0,00E+00	3152	Os09g0458000	100,00	0,00E+00	4524	1372	Japonica	Japonica	
maker-9-augustus-gene-174.58-mRNA-1	9	OsR498G0917614700.01.T01	99,67	0,00E+00	2743	Os09g0458300	100,00	0,00E+00	2765	22	NA	Japonica	LRR-TM
maker-9-augustus-gene-174.60-mRNA-1	9	OsR498G0917614900.01.T01	99,84	0,00E+00	1111	Os09g0458400	100,00	0,00E+00	1115	4	NA	Japonica	
maker-9-augustus-gene-174.66-mRNA-1	9	OsR498G0917619300.01.T01	99,74	0,00E+00	3378	Os09g0459800	99,95	0,00E+00	3396	18	NA	Japonica	
maker-9-augustus-gene-174.73-mRNA-1	9	OsR498G0917620300.01.T01	99,31	0,00E+00	1286	Os09g0460100	100,00	0,00E+00	1305	19	NA	Japonica	
maker-9-augustus-gene-174.75-mRNA-1	9	OsR498G0917614400.01.T01	99,58	0,00E+00	2961	Os09g0458100	100,00	0,00E+00	2993	32	NA	Japonica	
maker-9-augustus-gene-174.80-mRNA-1	9	OsR498G0917620000.01.T01	100,00	1,27E-174	612	Os09g0459900	100,00	9,77E-133	471	-141	Indica	Japonica	
maker-9-augustus-gene-175.41-mRNA-1	9	OsR498G0917621100.01.T01	99,58	0,00E+00	2545	Os08g0474700	83,94	0,00E+00	1540	-1005	Indica	Japonica	
maker-9-augustus-gene-175.47-mRNA-1	9	OsR498G0917626300.01.T05	99,77	0,00E+00	2306	Os09g0462400	100,00	0,00E+00	2093	-213	NA	Japonica	
maker-9-augustus-gene-176.42-mRNA-1	9	OsR498G0917629100.01.T01	99,77	0,00E+00	2351	Os09g0462700	100,00	0,00E+00	1817	-534	Indica	Japonica	
maker-9-augustus-gene-176.46-mRNA-1	9	OsR498G0917631500.01.T02	99,60	0,00E+00	3125	Os09g0463300	100,00	0,00E+00	3144	19	NA	Japonica	
maker-9-augustus-gene-176.50-mRNA-1	9	OsR498G0917635100.01.T01	98,47	0,00E+00	1136	Os08g0477900	75,00	2,07E-99	362	-774	Indica	Japonica	
maker-9-augustus-gene-176.52-mRNA-1	9	OsR498G0917629800.01.T01	100,00	0,00E+00	1460	Os09g0462901	98,73	0,00E+00	1655	195	Japonica	Japonica	
maker-9-augustus-gene-176.58-mRNA-1	9	OsR498G0917635400.01.T02	93,92	0,00E+00	1444	Os09g0464000	94,14	0,00E+00	1453	9	NA	Japonica	
maker-9-augustus-gene-177.10-mRNA-1	9	OsR498G0917644900.01.T01	100,00	0,00E+00	1092	Os09g0466300	100,00	0,00E+00	1045	-47	NA	Japonica	
maker-9-augustus-gene-177.14-mRNA-1	9	OsR498G0917638000.01.T01	99,84	0,00E+00	4473	Os09g0464400	98,53	0,00E+00	4645	172	NA	Japonica	
maker-9-augustus-gene-177.19-mRNA-1	9	OsR498G0917641000.01.T01	97,81	0,00E+00	3299	Os09g0465600	97,97	0,00E+00	3313	14	NA	Japonica	
maker-9-augustus-gene-177.24-mRNA-1	9	OsR498G0917641400.01.T03	100,00	0,00E+00	852	Os09g0465800	100,00	0,00E+00	852	0	NA	Japonica	
maker-9-augustus-gene-177.3-mRNA-1	9	OsR498G0917640700.01.T01	94,19	0,00E+00	1227	Os09g0465500	100,00	0,00E+00	1397	170	Japonica	Japonica	
maker-9-augustus-gene-177.7-mRNA-1	9	OsR498G0917643500.01.T01	99,63	0,00E+00	1914	Os09g0466100	100,00	0,00E+00	1932	18	NA	Japonica	
maker-9-augustus-gene-178.38-mRNA-1	9	OsR498G0917648500.01.T03	97,34	0,00E+00	2114	Os09g0467100	97,59	0,00E+00	2128	14	NA	Japonica	
maker-9-augustus-gene-178.44-mRNA-1	9	OsR498G0510836400.01.T01	71,27	3,39E-64	246	Os09g0467200	100,00	0,00E+00	1213	967	Japonica	Japonica	
maker-9-augustus-gene-178.46-mRNA-1	9	OsR498G0917650500.01.T01	99,72	0,00E+00	2596	Os09g0468000	100,00	0,00E+00	2614	18	NA	Japonica	
maker-9-augustus-gene-178.50-mRNA-1	9	OsR498G0917648000.01.T01	99,85	0,00E+00	2448	Os09g0466900	100,00	0,00E+00	2819	371	Japonica	Japonica	Kinase-TM
maker-9-augustus-gene-178.54-mRNA-1	9	OsR498G0917648800.01.T01	99,49	0,00E+00	2090	Os09g0467300	100,00	0,00E+00	2116	26	NA	Japonica	
maker-9-augustus-gene-178.56-mRNA-1	9	OsR498G0917649000.01.T01	99,72	0,00E+00	1946	Os09g0467400	96,32	0,00E+00	1779	-167	NA	Japonica	
maker-9-augustus-gene-178.57-mRNA-1	9	OsR498G0917649000.01.T01	94,60	0,00E+00	1185	Os09g0467400	100,00	0,00E+00	1370	185	Japonica	Japonica	
maker-9-augustus-gene-179.41-mRNA-1	9	OsR498G0917653900.01.T02	97,29	0,00E+00	1817	Os09g0468700	100,00	0,00E+00	1905	88	NA	Japonica	
maker-9-augustus-gene-179.43-mRNA-1	9	OsR498G0917654300.01.T01	99,83	0,00E+00	1024	Os09g0468900	100,00	0,00E+00	1029	5	NA	Japonica	
maker-9-augustus-gene-179.47-mRNA-1	9	OsR498G0917654500.01.T01	99,83	0,00E+00	1024	Os09g0469300	100,00	0,00E+00	829	-195	Indica	Japonica	
maker-9-augustus-gene-179.49-mRNA-1	9	OsR498G0917656500.01.T01	95,72	0,00E+00	2036	Os09g0469900	95,88	0,00E+00	2045	9	NA	Japonica	
maker-9-augustus-gene-18.5-mRNA-1	9	OsR498G0916718700.01.T01	99,50	0,00E+00	711	Os01g0214200	98,08	7,74E-153	538	-173	Indica	NA	
maker-9-augustus-gene-180.34-mRNA-1	9	OsR498G0917658500.01.T01	99,64	0,00E+00	1491	Os09g0470500	100,00	0,00E+00	1505	14	NA	Japonica	
maker-9-augustus-gene-180.38-mRNA-1	9	OsR498G0917659300.01.T01	98,19	0,00E+00	3153	Os09g0470900	98,35	0,00E+00	3167	14	NA	Japonica	
maker-9-augustus-gene-180.41-mRNA-1	9	OsR498G0917659900.01.T01	100,00	0,00E+00	1368	Os09g0471000	100,00	0,00E+00	1169	-199	Indica	Japonica	
maker-9-augustus-gene-180.45-mRNA-1	9	OsR498G0917660200.01.T01	99,45	0,00E+00	1930	Os09g0471100	100,00	0,00E+00	1954	24	NA	Japonica	
maker-9-augustus-gene-180.47-mRNA-1	9	OsR498G0917660800.01.T02	99,41	0,00E+00	2097	Os09g0471200	99,92	0,00E+00	2124	27	NA	Japonica	Kinase
maker-9-augustus-gene-181.33-mRNA-1	9	OsR498G0917664100.01.T01	99,77	0,00E+00	4740	Os09g0471900	100,00	0,00E+00	4767	27	NA	Japonica	
maker-9-augustus-gene-181.39-mRNA-1	9	OsR498G0917665300.01.T01	99,91	0,00E+00	3898	Os09g0472100	100,00	0,00E+00	3907	9	NA	Japonica	
maker-9-augustus-gene-181.48-mRNA-1	9	OsR498G0917668100.01.T01	99,74	0,00E+00	700	Os09g0472700	86,32	9,91E-127	452	-248	Indica	Japonica	
maker-9-augustus-gene-182.0-mRNA-1	9	OsR498G0917671300.01.T07	99,77	0,00E+00	2379	Os09g0474100	100,00	0,00E+00	2322	-57	NA	Japonica	
maker-9-augustus-gene-182.15-mRNA-1	9	OsR498G0917672200.01.T01	99,50	0,00E+00	4251	Os09g0474300	100,00	0,00E+00	3077	-1174	Indica	Japonica	
maker-9-augustus-gene-183.13-mRNA-1	9	OsR498G0917678200.01.T06	99,70	0,00E+00	1789	Os09g0475700	100,00	0,00E+00	1802	13	NA	Japonica	
maker-9-augustus-gene-183.4-mRNA-1	9	OsR498G0917678700.01.T01	100,00	0,00E+00	1440	Os09g0476000	100,00	0,00E+00	1440	0	NA	NA	
maker-9-augustus-gene-183.9-mRNA-1	9	OsR498G0917677600.01.T01	99,90	0,00E+00	1856	Os06g0284200	77,38	3,77E-157	554	-1302	Indica	Japonica	
maker-9-augustus-gene-184.26-mRNA-1	9	OsR498G0917683500.01.T06	98,32	0,00E+00	2480	Os09g0477900	98,32	0,00E+00	2480	0	NA	NA	
maker-9-augustus-gene-184.31-mRNA-1	9	OsR498G0917686300.01.T03	99,94	0,00E+00	3156	Os09g0478500	100,00	0,00E+00	3160	4	NA	NA	Kinase
maker-9-augustus-gene-184.33-mRNA-1	9	OsR498G0917689300.01.T04	99,80	0,00E+00	2714	Os09g0479100	100,00	0,00E+00	2727	13	NA	Japonica	
maker-9-augustus-gene-184.37-mRNA-1	9	OsR498G0917690700.01.T01	99,94	0,00E+00	6029	Os08g0493800	82,19	0,00E+00	2791	-3238	Indica	NA	Kinase-LRR-TM
maker-9-augustus-gene-184.41-mRNA-1	9	OsR498G0917684000.01.T01	100,00	0,00E+00	3993	Os09g0478100	100,00	0,00E+00	3993	0	NA	NA	
maker-9-augustus-gene-184.46-mRNA-1	9	OsR498G0917684200.01.T01	100,00	0,00E+00	3945	Os09g0478100	90,62	0,00E+00	3035	-910	Indica	NA	
maker-9-augustus-gene-184.49-mRNA-1	9	OsR498G0917685000.01.T04	100,00	8,17E-140	497	Os09g0478400	100,00	2,85E-140	497	0	NA	NA	
maker-9-augustus-gene-185.38-mRNA-1	9	OsR498G0917691100.01.T01	100,00	0,00E+00	3117	Os09g0479400	100,00	0,00E+00	3117	0	NA	NA	
maker-9-augustus-gene-185.41-mRNA-1	9	OsR498G0917692300.01.T01	99,96	0,00E+00	4169	Os02g0198700	72,98	0,00E+00	1034	-3135	Indica	Japonica	
maker-9-augustus-gene-185.48-mRNA-1	9	OsR498G0917697100.01.T05	99,80	0,00E+00	1772	Os09g0481100	100,00	0,00E+00	1781	9	NA	Japonica	
maker-9-augustus-gene-185.54-mRNA-1	9	OsR498G0917691400.01.T01	100,00	0,00E+00	2026	Os09g0479500	100,00	0,00E+00	2026	0	NA	Japonica	CC-NBS-TM
maker-9-augustus-gene-185.58-mRNA-1	9	OsR498G0917697800.01.T01	99,74	0,00E+00	1393	Os09g0481400	100,00	0,00E+00	1402	9	NA	Japonica	
maker-9-augustus-gene-186.0-mRNA-1	9	OsR498G0612924300.01.T02	70,30	1,88E-128	461	Os09g0482200	100,00	0,00E+00	2630	2169	Japonica	Japonica	
maker-9-augustus-gene-186.10-mRNA-1	9	OsR498G0917698800.01.T03	99,74	0,00E+00	3408	Os09g0481700	100,00	0,00E+00	3431	23	NA	Japonica	
maker-9-augustus-gene-186.13-mRNA-1	9	OsR498G0917700200.01.T01	99,58	0,00E+00	2126	Os09g0482000	100,00	0,00E+00	2149	23	NA	Japonica	
maker-9-augustus-gene-186.16-mRNA-1	9	OsR498G0917700400.01.T01	99,71	0,00E+00	3761	Os09g0482100	100,00	0,00E+00	3788	27	NA	Japonica	
maker-9-augustus-gene-186.17-mRNA-1	9	OsR498G0917701400.01.T01	99,81	0,00E+00	3770	Os09g0482610	100,00	0,00E+00	3788	18	NA	Japonica	
maker-9-augustus-gene-186.2-mRNA-1	9	OsR498G0917701900.01.T01	98,64	0,00E+00	2049	Os09g0482640	100,00	0,00E+00	2109	60	NA	Japonica	Kinase-TM
maker-9-augustus-gene-186.30-mRNA-1	9	OsR498G0917703200.01.T01	99,63	0,00E+00	4348	Os09g0482660	100,00	0,00E+00	2836	-1512	Indica	Japonica	
maker-9-augustus-gene-186.34-mRNA-1	9	OsR498G0917705000.01.T01	99,55	0,00E+00	1958	Os09g0482680	100,00	0,00E+00	1981	23	NA	Japonica	
maker-9-augustus-gene-186.4-mRNA-1	9	OsR498G0917705500.01.T01	99,89	0,00E+00	1630	Os09g0482740	100,00	0,00E+00	1635	5	NA	Japonica	
maker-9-augustus-gene-187.0-mRNA-1	9	OsR498G0917711700.01.T01	99,56	0,00E+00	809	Os09g0484200	100,00	0,00E+00	818	9	NA	Japonica	
maker-9-augustus-gene-187.3-mRNA-1	9	OsR498G0917712000.01.T01	99,48	0,00E+00	3753	Os09g0484300	100,00	0,00E+00	3799	46	NA	Japonica	
maker-9-augustus-gene-188.26-mRNA-1	9	OsR498G0917713300.01.T01	99,78	0,00E+00	1604	Os09g0484800	100,00	0,00E+00	1613	9	NA	Japonica	
maker-9-augustus-gene-188.28-mRNA-1	9	OsR498G0917714500.01.T02	99,82	0,00E+00	2930	Os09g0484900	100,00	0,00E+00	2944	14	NA	Japonica	
maker-9-augustus-gene-188.31-mRNA-1	9	OsR498G0917716700.01.T01	99,69	1,84E-161	570	Os09g0485800	100,00	0,00E+00	969	399	Japonica	Japonica	
maker-9-augustus-gene-188.35-mRNA-1	9	OsR498G0917716600.01.T01	100,00	0,00E+00	1034	Os09g0485900	100,00	0,00E+00	1034	0	NA	Japonica	
maker-9-augustus-gene-188.41-mRNA-1	9	OsR498G0917720000.01.T04	99,66	0,00E+00	4190	Os09g0487200	100,00	0,00E+00	4226	36	NA	Japonica	
maker-9-augustus-gene-189.29-mRNA-1	9	OsR498G0917721300.01.T04	99,71	0,00E+00	1242	Os09g0487700	100,00	0,00E+00	1251	9	NA	Japonica	
maker-9-augustus-gene-189.32-mRNA-1	9	OsR498G0917721500.01.T01	97,87	0,00E+00	2083	Os09g0487900	99,10	0,00E+00	2146	63	NA	Japonica	
maker-9-augustus-gene-189.36-mRNA-1	9	OsR498G0917722500.01.T01	99,56	0,00E+00	2047	Os09g0488600	99,91	0,00E+00	2065	18	NA	Japonica	
maker-9-augustus-gene-189.39-mRNA-1	9	OsR498G0917722900.01.T02	99,34	0,00E+00	2140	Os09g0488700	100,00	0,00E+00	1586	-554	Indica	Japonica	
maker-9-augustus-gene-189.42-mRNA-1	9	OsR498G0917721000.01.T01	99,93	0,00E+00	2474	Os09g0487600	98,91	0,00E+00	2420	-54	NA	Japonica	
maker-9-augustus-gene-189.44-mRNA-1	9	OsR498G0917723200.01.T01	99,46	0,00E+00	3272	Os09g0488800	100,00	0,00E+00	3306	34	NA	Japonica	
maker-9-augustus-gene-189.46-mRNA-1	9	OsR498G0917724200.01.T02	100,00	0,00E+00	2235	Os09g0489200	100,00	0,00E+00	2100	-135	NA	Japonica	
maker-9-augustus-gene-189.53-mRNA-1	9	OsR498G0917724400.01.T02	99,81	0,00E+00	2822	Os09g0489500	99,62	0,00E+00	2810	-12	NA	Japonica	
maker-9-augustus-gene-189.57-mRNA-1	9	OsR498G0917725800.01.T01	94,76	0,00E+00	2390	Os09g0491100	77,86	0,00E+00	1232	-1158	Indica	Japonica	
maker-9-augustus-gene-19.26-mRNA-1	9	OsR498G0916721100.01.T01	99,78	0,00E+00	4126	Os09g0123100	99,74	0,00E+00	4118	-8	NA	NA	
maker-9-augustus-gene-19.30-mRNA-1	9	OsR498G0916721700.01.T03	99,82	0,00E+00	3979	Os09g0123200	99,53	0,00E+00	3405	-574	Indica	NA	
maker-9-augustus-gene-190.36-mRNA-1	9	OsR498G0917727000.01.T01	98,74	0,00E+00	2626	Os09g0491100	100,00	0,00E+00	2711	85	NA	Japonica	
maker-9-augustus-gene-190.39-mRNA-1	9	OsR498G0917728000.01.T01	99,83	0,00E+00	2123	Os09g0491532	99,89	0,00E+00	1645	-478	Indica	Japonica	
maker-9-augustus-gene-190.45-mRNA-1	9	OsR498G0917731600.01.T01	100,00	0,00E+00	639	Os09g0491692	100,00	0,00E+00	639	0	NA	Japonica	
maker-9-augustus-gene-190.47-mRNA-1	9	OsR498G0917731800.01.T01	99,72	0,00E+00	1935	Os09g0491708	100,00	0,00E+00	1948	13	NA	Japonica	
maker-9-augustus-gene-190.55-mRNA-1	9	OsR498G0917733200.01.T01	97,52	0,00E+00	3581	Os09g0491772	98,00	0,00E+00	3626	45	NA	Japonica	
maker-9-augustus-gene-191.0-mRNA-1	9	OsR498G0917736100.01.T01	99,60	0,00E+00	3091	Os09g0492700	99,94	0,00E+00	3118	27	NA	Japonica	
maker-9-augustus-gene-191.14-mRNA-1	9	OsR498G0917735200.01.T03	98,23	0,00E+00	1570	Os09g0491852	98,34	0,00E+00	1574	4	NA	Japonica	
maker-9-augustus-gene-191.18-mRNA-1	9	OsR498G0917735400.01.T02	100,00	1,10E-138	493	Os09g0491868	100,00	0,00E+00	829	336	Japonica	Japonica	
maker-9-augustus-gene-191.3-mRNA-1	9	OsR498G0917733800.01.T01	99,73	0,00E+00	1978	Os09g0491788	100,00	0,00E+00	1862	-116	NA	Japonica	
maker-9-augustus-gene-191.5-mRNA-1	9	OsR498G0917734800.01.T01	99,12	0,00E+00	1003	Os09g0491820	99,30	0,00E+00	1007	4	NA	Japonica	
maker-9-augustus-gene-191.8-mRNA-1	9	OsR498G0917735000.01.T02	100,00	7,56E-86	316	Os09g0491852	90,54	4,79E-83	306	-10	NA	Japonica	
maker-9-augustus-gene-192.40-mRNA-1	9	OsR498G0917737700.01.T01	100,00	0,00E+00	1932	Os09g0493400	100,00	0,00E+00	1932	0	NA	Japonica	
maker-9-augustus-gene-192.46-mRNA-1	9	OsR498G0917739300.01.T01	99,80	0,00E+00	1761	Os09g0494200	100,00	0,00E+00	1770	9	NA	Japonica	
maker-9-augustus-gene-192.49-mRNA-1	9	OsR498G0917740300.01.T01	100,00	0,00E+00	1445	Os09g0494600	100,00	0,00E+00	1445	0	NA	Japonica	
maker-9-augustus-gene-192.53-mRNA-1	9	OsR498G0917742100.01.T01	99,23	0,00E+00	1384	Os09g0495200	100,00	0,00E+00	1411	27	NA	Japonica	
maker-9-augustus-gene-192.59-mRNA-1	9	OsR498G0917738000.01.T03	100,00	0,00E+00	1261	Os09g0493500	100,00	0,00E+00	1261	0	NA	Japonica	
maker-9-augustus-gene-192.61-mRNA-1	9	OsR498G0917738200.01.T01	99,85	0,00E+00	1219	Os09g0493600	100,00	0,00E+00	1223	4	NA	Japonica	
maker-9-augustus-gene-192.66-mRNA-1	9	OsR498G0917738400.01.T02	99,44	0,00E+00	3202	Os09g0493700	100,00	0,00E+00	3173	-29	NA	Japonica	
maker-9-augustus-gene-192.70-mRNA-1	9	OsR498G0917738600.01.T01	93,31	0,00E+00	1852	Os09g0493800	93,31	0,00E+00	1852	0	NA	Japonica	
maker-9-augustus-gene-192.73-mRNA-1	9	OsR498G0917739600.01.T01	100,00	0,00E+00	1710	Os09g0494300	100,00	0,00E+00	1710	0	NA	Japonica	
maker-9-augustus-gene-192.80-mRNA-1	9	OsR498G0917742800.01.T01	99,42	0,00E+00	918	Os09g0495400	100,00	6,41E-168	589	-329	Indica	Japonica	
maker-9-augustus-gene-193.0-mRNA-1	9	OsR498G0917743900.01.T05	99,82	0,00E+00	999	Os09g0497000	100,00	0,00E+00	1003	4	NA	Japonica	
maker-9-augustus-gene-193.11-mRNA-1	9	OsR498G0917745500.01.T02	99,76	0,00E+00	2258	Os09g0497700	99,92	0,00E+00	2267	9	NA	Japonica	
maker-9-augustus-gene-193.17-mRNA-1	9	OsR498G0917746700.01.T01	99,20	0,00E+00	1762	Os09g0536300	74,53	1,55E-109	396	-1366	Indica	Japonica	
maker-9-augustus-gene-193.19-mRNA-1	9	OsR498G0917748700.01.T02	99,68	0,00E+00	3315	Os09g0498600	100,00	0,00E+00	2159	-1156	Indica	Japonica	
maker-9-augustus-gene-193.22-mRNA-1	9	OsR498G0917743600.01.T01	98,11	0,00E+00	1361	Os08g0168150	87,97	0,00E+00	774	-587	Indica	Japonica	
maker-9-augustus-gene-193.31-mRNA-1	9	OsR498G0917744200.01.T02	99,76	0,00E+00	3805	Os09g0497100	100,00	0,00E+00	3826	21	NA	Japonica	
maker-9-augustus-gene-193.34-mRNA-1	9	OsR498G0917745200.01.T01	99,71	0,00E+00	1881	Os09g0497500	100,00	0,00E+00	1894	13	NA	Japonica	
maker-9-augustus-gene-193.41-mRNA-1	9	OsR498G0917746200.01.T01	99,86	0,00E+00	1248	Os09g0497900	100,00	0,00E+00	1252	4	NA	Japonica	

maker-9-augustus-gene-193.44-mRNA-1	9	OsR498G0917747400.01.T01	99,92	0,00E+00	2252	Os12g0543600	87,09	0,00E+00	1544	-708	Indica	Japonica	
maker-9-augustus-gene-193.5-mRNA-1	9	OsR498G0917744900.01.T04	99,87	0,00E+00	1360	Os09g0497400	100,00	0,00E+00	1364	4	NA	Japonica	
maker-9-augustus-gene-194.0-mRNA-1	9	OsR498G0917748900.01.T01	99,67	0,00E+00	1639	Os09g0498700	99,89	0,00E+00	1655	16	NA	Japonica	
maker-9-augustus-gene-194.11-mRNA-1	9	OsR498G0917749800.01.T01	99,78	0,00E+00	1622	Os09g0498800	99,89	0,00E+00	1627	5	NA	Japonica	
maker-9-augustus-gene-194.14-mRNA-1	9	OsR498G0917750600.01.T01	99,62	0,00E+00	1392	Os09g0499000	100,00	0,00E+00	1407	15	NA	Japonica	
maker-9-augustus-gene-194.19-mRNA-1	9	OsR498G0917753800.01.T01	99,81	0,00E+00	963	Os09g0500000	100,00	0,00E+00	967	4	NA	Japonica	
maker-9-augustus-gene-194.2-mRNA-1	9	OsR498G0917754500.01.T01	99,88	0,00E+00	4503	Os09g0500300	100,00	0,00E+00	4078	-425	NA	Japonica	
maker-9-augustus-gene-194.24-mRNA-1	9	OsR498G0917754200.01.T02	99,26	0,00E+00	1199	Os09g0500100	100,00	0,00E+00	1223	24	NA	Japonica	
maker-9-augustus-gene-194.27-mRNA-1	9	OsR498G0917754400.01.T02	95,76	0,00E+00	1355	Os09g0500200	95,50	0,00E+00	1269	-86	NA	Japonica	
maker-9-augustus-gene-194.8-mRNA-1	9	OsR498G0917754700.01.T01	99,68	0,00E+00	4515	Os09g0500600	100,00	0,00E+00	4551	36	NA	Japonica	
maker-9-augustus-gene-195.49-mRNA-1	9	OsR498G0917756100.01.T01	99,75	0,00E+00	721	Os09g0501100	100,00	0,00E+00	726	5	NA	Japonica	
maker-9-augustus-gene-195.52-mRNA-1	9	OsR498G0917759700.01.T01	99,71	0,00E+00	1832	Os09g0502500	99,90	0,00E+00	1841	9	NA	Japonica	
maker-9-augustus-gene-195.56-mRNA-1	9	OsR498G0917761900.01.T01	99,81	0,00E+00	1869	Os09g0503100	100,00	0,00E+00	1878	9	NA	Japonica	
maker-9-augustus-gene-195.60-mRNA-1	9	OsR498G0917755000.01.T01	99,33	0,00E+00	2100	Os08g0524200	74,22	0,00E+00	710	-1390	Indica	Japonica	
maker-9-augustus-gene-195.62-mRNA-1	9	OsR498G0917757000.01.T02	99,62	0,00E+00	1394	Os09g0501600	100,00	0,00E+00	1407	13	NA	Japonica	
maker-9-augustus-gene-195.66-mRNA-1	9	OsR498G0917757400.01.T01	99,61	0,00E+00	917	Os09g0501700	99,73	0,00E+00	653	-264	Indica	Japonica	
maker-9-augustus-gene-195.70-mRNA-1	9	OsR498G0917757600.01.T01	99,57	0,00E+00	830	Os09g0501850	100,00	0,00E+00	839	9	NA	Japonica	
maker-9-augustus-gene-195.72-mRNA-1	9	OsR498G0917757800.01.T01	99,75	0,00E+00	721	Os09g0502000	100,00	0,00E+00	726	5	NA	Japonica	
maker-9-augustus-gene-195.78-mRNA-1	9	OsR498G0917759000.01.T01	99,03	0,00E+00	2547	Os09g0502200	100,00	0,00E+00	2603	56	NA	Japonica	
maker-9-augustus-gene-195.82-mRNA-1	9	OsR498G0917760000.01.T02	96,70	0,00E+00	2638	Os09g0502600	100,00	0,00E+00	1429	-1209	Indica	Japonica	
maker-9-augustus-gene-196.0-mRNA-1	9	OsR498G0917764300.01.T01	95,15	0,00E+00	1238	Os09g0504400	95,40	0,00E+00	1247	9	NA	Japonica	
maker-9-augustus-gene-196.10-mRNA-1	9	OsR498G0917766700.01.T05	100,00	0,00E+00	1296	Os09g0505200	100,00	0,00E+00	2340	1044	Japonica	Japonica	
maker-9-augustus-gene-196.13-mRNA-1	9	OsR498G0917767700.01.T01	99,64	0,00E+00	1486	Os09g0505600	100,00	0,00E+00	1202	-284	Indica	Japonica	
maker-9-augustus-gene-196.16-mRNA-1	9	OsR498G0917767900.01.T04	100,00	0,00E+00	1240	Os09g0505700	99,42	0,00E+00	1222	-18	NA	Japonica	
maker-9-augustus-gene-196.21-mRNA-1	9	OsR498G0917768300.01.T01	99,67	0,00E+00	2671	Os09g0505800	99,80	0,00E+00	2680	9	NA	Japonica	
maker-9-augustus-gene-196.30-mRNA-1	9	OsR498G0917764600.01.T02	99,17	0,00E+00	642	Os09g0504700	99,73	0,00E+00	651	9	NA	Japonica	
maker-9-augustus-gene-196.32-mRNA-1	9	OsR498G0917765000.01.T02	100,00	0,00E+00	1438	Os09g0504900	100,00	0,00E+00	1438	0	NA	Japonica	
maker-9-augustus-gene-196.37-mRNA-1	9	OsR498G0917766200.01.T01	99,88	0,00E+00	5772	Os09g0505100	100,00	0,00E+00	5714	-58	NA	Japonica	
maker-9-augustus-gene-196.39-mRNA-1	9	OsR498G0917767000.01.T02	99,87	0,00E+00	1361	Os09g0505300	100,00	0,00E+00	1366	5	NA	Japonica	
maker-9-augustus-gene-196.43-mRNA-1	9	OsR498G0917767400.01.T01	98,97	0,00E+00	1705	Os09g0505400	100,00	0,00E+00	2159	454	Japonica	Japonica	
maker-9-augustus-gene-196.7-mRNA-1	9	OsR498G0917764700.01.T01	94,37	0,00E+00	1413	Os09g0504800	94,93	0,00E+00	1435	22	NA	Japonica	
maker-9-augustus-gene-197.0-mRNA-1	9	OsR498G0917768900.01.T07	99,63	0,00E+00	964	Os03g0202500	80,08	1,63E-134	480	-484	Indica	Japonica	
maker-9-augustus-gene-197.20-mRNA-1	9	OsR498G0917770300.01.T01	99,53	0,00E+00	1902	Os09g0506700	100,00	0,00E+00	2733	831	Japonica	Japonica	
maker-9-augustus-gene-197.24-mRNA-1	9	OsR498G0917770500.01.T06	99,69	0,00E+00	2287	Os09g0506800	99,92	0,00E+00	2301	14	NA	Japonica	
maker-9-augustus-gene-197.38-mRNA-1	9	OsR498G0917772700.01.T01	99,87	0,00E+00	1343	Os09g0507200	100,00	0,00E+00	1348	5	NA	Japonica	
maker-9-augustus-gene-197.42-mRNA-1	9	OsR498G0917773100.01.T01	99,94	0,00E+00	3107	Os09g0507300	100,00	0,00E+00	3112	5	NA	Japonica	
maker-9-augustus-gene-197.44-mRNA-1	9	OsR498G0917774100.01.T01	99,46	0,00E+00	654	Os09g0507400	100,00	0,00E+00	663	9	NA	Japonica	
maker-9-augustus-gene-197.48-mRNA-1	9	OsR498G0917768800.01.T01	99,79	0,00E+00	3397	Os09g0506000	100,00	0,00E+00	3415	18	NA	Japonica	
maker-9-augustus-gene-197.52-mRNA-1	9	OsR498G0917774400.01.T01	99,40	0,00E+00	1770	Os09g0507500	100,00	0,00E+00	1797	27	NA	Japonica	
maker-9-augustus-gene-197.54-mRNA-1	9	OsR498G0917774600.01.T04	99,84	0,00E+00	1143	Os08g0532900	83,15	0,00E+00	650	-493	Indica	Japonica	
maker-9-augustus-gene-198.35-mRNA-1	9	OsR498G0917777500.01.T02	99,44	0,00E+00	1911	Os09g0508500	100,00	0,00E+00	1310	-601	Indica	Japonica	
maker-9-augustus-gene-198.38-mRNA-1	9	OsR498G0917779100.01.T01	98,66	0,00E+00	1298	Os09g0509100	99,87	0,00E+00	1338	40	NA	Japonica	
maker-9-augustus-gene-198.40-mRNA-1	9	OsR498G0917779300.01.T01	99,65	0,00E+00	2022	Os09g0509200	100,00	0,00E+00	2040	18	NA	Japonica	
maker-9-augustus-gene-198.46-mRNA-1	9	OsR498G0917779700.01.T05	97,66	0,00E+00	3300	Os09g0509300	97,82	0,00E+00	3314	14	NA	Japonica	
maker-9-augustus-gene-198.50-mRNA-1	9	OsR498G0917780100.01.T02	99,71	0,00E+00	3105	Os09g0509400	100,00	0,00E+00	3128	23	NA	Japonica	
maker-9-augustus-gene-198.55-mRNA-1	9	OsR498G0917775000.01.T01	99,74	0,00E+00	1393	Os09g0507800	100,00	0,00E+00	1402	9	NA	Japonica	
maker-9-augustus-gene-198.60-mRNA-1	9	OsR498G0917776400.01.T02	99,75	0,00E+00	1415	Os09g0508200	100,00	0,00E+00	1424	9	NA	Japonica	
maker-9-augustus-gene-198.62-mRNA-1	9	OsR498G0917776800.01.T02	99,77	0,00E+00	1582	Os08g0535200	83,81	0,00E+00	644	-938	Indica	Japonica	
maker-9-augustus-gene-198.64-mRNA-1	9	OsR498G0917778000.01.T01	99,73	0,00E+00	1957	Os09g0508900	100,00	0,00E+00	1986	29	NA	Japonica	
maker-9-augustus-gene-198.73-mRNA-1	9	OsR498G0917778400.01.T01	99,75	0,00E+00	2135	Os09g0509000	100,00	0,00E+00	2149	14	NA	Japonica	
maker-9-augustus-gene-198.78-mRNA-1	9	OsR498G0917780400.01.T01	98,20	0,00E+00	3449	Os09g0509500	98,35	0,00E+00	3462	13	NA	Japonica	
maker-9-augustus-gene-199.34-mRNA-1	9	OsR498G0917782300.01.T01	99,48	0,00E+00	1021	Os09g0510200	100,00	0,00E+00	937	-84	NA	Japonica	
maker-9-augustus-gene-199.43-mRNA-1	9	OsR498G0917787500.01.T03	99,59	0,00E+00	872	Os09g0511500	99,80	0,00E+00	876	4	NA	Japonica	
maker-9-augustus-gene-199.47-mRNA-1	9	OsR498G0917780800.01.T01	98,02	0,00E+00	2448	Os08g0536300	71,44	5,18E-143	508	-1940	Indica	Japonica	
maker-9-augustus-gene-199.49-mRNA-1	9	OsR498G0917783800.01.T03	95,82	0,00E+00	1458	Os09g0510500	95,93	0,00E+00	1462	4	NA	Japonica	
maker-9-augustus-gene-199.53-mRNA-1	9	OsR498G0917784600.01.T01	99,80	0,00E+00	2694	Os09g0510700	100,00	0,00E+00	2708	14	NA	Japonica	
maker-9-augustus-gene-199.61-mRNA-1	9	OsR498G0917786400.01.T02	99,30	0,00E+00	2775	Os09g0325220	96,54	0,00E+00	2617	-158	NA	Japonica	
maker-9-augustus-gene-20.40-mRNA-1	9	OsR498G0916722500.01.T02	99,90	0,00E+00	1856	Os09g0123300	99,81	0,00E+00	1851	-5	NA	NA	Kinase-TM
maker-9-augustus-gene-20.49-mRNA-1	9	OsR498G0916723200.01.T01	99,92	0,00E+00	2317	Os09g0123500	99,77	0,00E+00	2308	-9	NA	NA	
maker-9-augustus-gene-200.49-mRNA-1	9	OsR498G0917788900.01.T01	99,53	0,00E+00	1893	Os09g0512000	100,00	0,00E+00	2527	634	Japonica	Japonica	
maker-9-augustus-gene-200.58-mRNA-1	9	OsR498G0917790700.01.T01	99,82	0,00E+00	1008	Os03g0851500	71,16	1,36E-56	219	-789	Indica	Japonica	
maker-9-augustus-gene-200.61-mRNA-1	9	OsR498G0917791100.01.T01	99,80	0,00E+00	918	Os09g0512900	100,00	0,00E+00	922	4	NA	Japonica	
maker-9-augustus-gene-200.64-mRNA-1	9	OsR498G0917791500.01.T02	99,80	0,00E+00	1777	Os09g0513000	100,00	0,00E+00	1911	134	NA	Japonica	
maker-9-augustus-gene-200.74-mRNA-1	9	OsR498G0917787800.01.T01	97,84	0,00E+00	2705	Os09g0511600	98,09	0,00E+00	2718	13	NA	Japonica	
maker-9-augustus-gene-200.77-mRNA-1	9	OsR498G0917788200.01.T04	96,21	0,00E+00	2752	Os09g0511900	88,47	0,00E+00	1966	-786	Indica	Japonica	
maker-9-augustus-gene-200.84-mRNA-1	9	OsR498G0917788200.01.T02	88,22	0,00E+00	1936	Os09g0511900	99,74	0,00E+00	2735	799	Japonica	Japonica	
maker-9-augustus-gene-200.88-mRNA-1	9	OsR498G0917792000.01.T05	99,82	0,00E+00	981	Os09g0513200	100,00	0,00E+00	985	4	NA	Japonica	
maker-9-augustus-gene-201.0-mRNA-1	9	OsR498G0917792700.01.T02	99,63	0,00E+00	1941	Os01g0899000	77,49	0,00E+00	696	-1245	Indica	Japonica	Kinase-TM
maker-9-augustus-gene-201.12-mRNA-1	9	OsR498G0917793300.01.T01	100,00	0,00E+00	2621	Os09g0514100	100,00	0,00E+00	2621	0	NA	Japonica	
maker-9-augustus-gene-201.17-mRNA-1	9	OsR498G0917794300.01.T03	99,91	0,00E+00	1926	Os09g0514300	100,00	0,00E+00	1537	-389	Indica	Japonica	
maker-9-augustus-gene-201.22-mRNA-1	9	OsR498G0917794500.01.T03	100,00	0,00E+00	1840	Os09g0514400	100,00	0,00E+00	1840	0	NA	Japonica	
maker-9-augustus-gene-201.30-mRNA-1	9	OsR498G0917795700.01.T04	99,56	0,00E+00	2432	Os09g0514700	100,00	0,00E+00	2868	436	Japonica	Japonica	
maker-9-augustus-gene-201.33-mRNA-1	9	OsR498G0917796100.01.T01	99,74	0,00E+00	3412	Os09g0514900	100,00	0,00E+00	3431	19	NA	Japonica	
maker-9-augustus-gene-201.35-mRNA-1	9	OsR498G0917796500.01.T01	97,86	0,00E+00	6139	Os09g0515100	98,25	0,00E+00	6199	60	NA	Japonica	
maker-9-augustus-gene-201.47-mRNA-1	9	OsR498G0917797500.01.T01	99,91	0,00E+00	3822	Os09g0515500	100,00	0,00E+00	3831	9	NA	Japonica	TM
maker-9-augustus-gene-201.51-mRNA-1	9	OsR498G0917745200.01.T01	89,24	8,21E-178	623	Os09g0513300	100,00	0,00E+00	646	23	NA	Japonica	
maker-9-augustus-gene-201.66-mRNA-1	9	OsR498G0917796800.01.T01	98,83	0,00E+00	1354	Os09g0515200	100,00	0,00E+00	1391	37	NA	Japonica	
maker-9-augustus-gene-201.7-mRNA-1	9	OsR498G0917792900.01.T06	99,89	0,00E+00	3178	Os09g0513800	99,69	0,00E+00	2927	-251	NA	Japonica	
maker-9-augustus-gene-202.0-mRNA-1	9	OsR498G0917797700.01.T04	100,00	0,00E+00	650	Os09g0515700	100,00	1,60E-129	461	-189	Indica	Japonica	
maker-9-augustus-gene-202.12-mRNA-1	9	OsR498G0917799000.01.T03	100,00	0,00E+00	2062	Os09g0516200	96,85	0,00E+00	1928	-134	NA	Japonica	
maker-9-augustus-gene-202.15-mRNA-1	9	OsR498G0917800000.01.T01	99,63	0,00E+00	4830	Os09g0516300	100,00	0,00E+00	4875	45	NA	Japonica	
maker-9-augustus-gene-202.25-mRNA-1	9	OsR498G0917800800.01.T01	94,52	0,00E+00	1168	Os09g0516600	94,66	0,00E+00	1172	4	NA	Japonica	
maker-9-augustus-gene-202.28-mRNA-1	9	OsR498G0917801000.01.T02	99,83	0,00E+00	4094	Os09g0516700	100,00	0,00E+00	2428	-1666	Indica	Japonica	
maker-9-augustus-gene-202.31-mRNA-1	9	OsR498G0917802400.01.T01	99,78	0,00E+00	3303	Os09g0516900	100,00	0,00E+00	3317	14	NA	Japonica	
maker-9-augustus-gene-202.41-mRNA-1	9	OsR498G0917803000.01.T01	99,75	0,00E+00	5033	Os09g0517200	100,00	0,00E+00	5103	70	NA	Japonica	CC-NBS-TM
maker-9-augustus-gene-202.5-mRNA-1	9	OsR498G0917803700.01.T01	99,83	0,00E+00	4251	Os09g0517600	100,00	0,00E+00	4269	18	NA	Japonica	
maker-9-augustus-gene-202.7-mRNA-1	9	OsR498G0917798400.01.T02	99,85	0,00E+00	2399	Os09g0515800	100,00	0,00E+00	2408	9	NA	Japonica	
maker-9-augustus-gene-203.0-mRNA-1	9	OsR498G0917804300.01.T01	99,86	0,00E+00	2605	Os09g0517900	100,00	0,00E+00	2614	9	NA	Japonica	
maker-9-augustus-gene-203.11-mRNA-1	9	OsR498G0917807300.01.T02	97,14	0,00E+00	768	Os09g0518800	100,00	8,92E-115	412	-356	Indica	Japonica	
maker-9-augustus-gene-203.13-mRNA-1	9	OsR498G0917804000.01.T02	99,79	0,00E+00	1692	Os09g0517700	99,89	0,00E+00	1697	5	NA	Japonica	
maker-9-augustus-gene-203.16-mRNA-1	9	OsR498G0917806800.01.T02	99,80	0,00E+00	1804	Os09g0518600	100,00	0,00E+00	1764	-40	NA	Japonica	
maker-9-augustus-gene-203.19-mRNA-1	9	OsR498G0917807000.01.T03	100,00	0,00E+00	1101	Os09g0518700	100,00	0,00E+00	1101	0	NA	Japonica	
maker-9-augustus-gene-203.3-mRNA-1	9	OsR498G0917805300.01.T01	99,01	0,00E+00	2492	Os09g0518200	100,00	0,00E+00	2544	52	NA	Japonica	
maker-9-augustus-gene-203.5-mRNA-1	9	OsR498G0917805700.01.T01	97,12	0,00E+00	2347	Os09g0518400	97,08	0,00E+00	2787	440	Japonica	Japonica	
maker-9-augustus-gene-203.8-mRNA-1	9	OsR498G0917806500.01.T05	99,82	0,00E+00	3033	Os09g0518500	100,00	0,00E+00	3047	14	NA	Japonica	
maker-9-augustus-gene-204.28-mRNA-1	9	OsR498G0917811300.01.T03	99,87	0,00E+00	1407	Os09g0520200	100,00	0,00E+00	1324	-83	NA	Japonica	
maker-9-augustus-gene-204.37-mRNA-1	9	OsR498G0917813300.01.T01	99,76	0,00E+00	3749	Os09g0520700	100,00	0,00E+00	3772	23	NA	Japonica	
maker-9-augustus-gene-204.42-mRNA-1	9	OsR498G0917815700.01.T01	99,74	0,00E+00	2025	Os09g0521400	100,00	0,00E+00	2039	14	NA	Japonica	
maker-9-augustus-gene-204.48-mRNA-1	9	OsR498G0917811200.01.T03	100,00	0,00E+00	715	Os09g0520100	100,00	0,00E+00	715	0	NA	Japonica	
maker-9-augustus-gene-204.50-mRNA-1	9	OsR498G0917812400.01.T01	99,66	6,19E-148	525	Os09g0520500	100,00	1,45E-150	531	6	NA	Japonica	
maker-9-augustus-gene-204.56-mRNA-1	9	OsR498G0917813000.01.T02	99,92	0,00E+00	2171	Os09g0520600	100,00	0,00E+00	2176	5	NA	Japonica	
maker-9-augustus-gene-204.61-mRNA-1	9	OsR498G0917813800.01.T01	99,87	0,00E+00	2670	Os09g0520800	100,00	0,00E+00	2679	9	NA	Japonica	
maker-9-augustus-gene-205.38-mRNA-1	9	OsR498G0917816000.01.T01	99,82	0,00E+00	1950	Os09g0521500	100,00	0,00E+00	1959	9	NA	Japonica	
maker-9-augustus-gene-206.0-mRNA-1	9	OsR498G0917825500.01.T01	99,88	0,00E+00	1462	Os09g0525200	100,00	0,00E+00	1467	5	NA	Japonica	
maker-9-augustus-gene-206.10-mRNA-1	9	OsR498G0917826400.01.T04	99,74	0,00E+00	691	Os09g0525400	100,00	0,00E+00	686	-5	NA	Japonica	
maker-9-augustus-gene-206.15-mRNA-1	9	OsR498G0917826800.01.T01	99,82	0,00E+00	3044	Os09g0525600	99,20	0,00E+00	2892	-152	NA	Japonica	
maker-9-augustus-gene-206.26-mRNA-1	9	OsR498G0917827400.01.T02	100,00	0,00E+00	1106	Os09g0525900	100,00	0,00E+00	1106	0	NA	Japonica	
maker-9-augustus-gene-206.5-mRNA-1	9	OsR498G0917824800.01.T01	98,68	0,00E+00	1328	Os09g0524800	100,00	0,00E+00	1353	25	NA	Japonica	
maker-9-augustus-gene-206.7-mRNA-1	9	OsR498G0917825800.01.T02	99,54	0,00E+00	1541	Os09g0525300	97,92	0,00E+00	1489	-52	NA	Japonica	
maker-9-augustus-gene-207.35-mRNA-1	9	OsR498G0101550000.01.T03	80,27	1,99E-78	294	Os09g0526000	91,91	0,00E+00	781	487	Japonica	Japonica	
maker-9-augustus-gene-207.37-mRNA-1	9	OsR498G0917832300.01.T01	91,80	1,96E-160	566	Os09g0527100	91,80	6,84E-161	566	0	NA	NA	
maker-9-augustus-gene-207.40-mRNA-1	9	OsR498G0917832900.01.T01	100,00	0,00E+00	1278	Os09g0527500	100,00	0,00E+00	1278	0	NA	NA	
maker-9-augustus-gene-207.42-mRNA-1	9	OsR498G0917833300.01.T01	100,00	0,00E+00	1299	Os09g0527600	100,00	0,00E+00	1299	0	NA	NA	
maker-9-augustus-gene-207.49-mRNA-1	9	OsR498G0917829800.01.T04	100,00	0,00E+00	2246	Os09g0526600	100,00	0,00E+00	2239	-7	NA	Japonica	
maker-9-augustus-gene-207.53-mRNA-1	9	OsR498G0917831400.01.T01	100,00	0,00E+00	2024	Os09g0526700	100,00	0,00E+00	2024	0	NA	NA	
maker-9-augustus-gene-207.61-mRNA-1	9	OsR498G0917833800.01.T01	100,00	0,00E+00	764	Os09g0527700	100,00	0,00E+00	764	0	NA	NA	
maker-9-augustus-gene-207.64-mRNA-1	9	OsR498G0917834200.01.T01	99,37	0,00E+00	1128	Os09g0527900	99,37	0,00E+00	1128	0	NA	NA	
maker-9-augustus-gene-208.0-mRNA-1	9	OsR498G0917834500.01.T01	99,08	0,00E+00	4622	Os09g0528000	99,08	0,00E+00	4622	0	NA	NA	
maker-9-augustus-gene-208.10-mRNA-1	9	OsR498G0917838600.01.T03	99,83	0,00E+00	3125	Os09g0528800	100,00	0,00E+00	3139	14	NA	Japonica	
maker-9-augustus-gene-208.13-mRNA-1	9	OsR498G0917839400.01.T01	99,90	0,00E+00	1755	Os09g0529100	100,00	0,00E+00	1462	-293	Indica	Japonica	
maker-9-augustus-gene-208.15-mRNA-1	9	OsR498G0917840000.01.T01	99,49	0,00E+00	1393	Os09g0529300	99,62	0,00E+00	1397	4	NA	Japonica	
maker-9-augustus-gene-208.5-mRNA-1	9	OsR498G0917836900.01.T01	98,96	0,00E+00	3036	Os09g0528700	100,00	0,00E+00	2998	-38	NA	Japonica	
maker-9-augustus-gene-208.8-mRNA-1	9	OsR498G0917836200.01.T01	100,00	0,00E+00	1034	Os09g0528300	100,00	0,00E+00	1034	0	NA	NA	
maker-9-augustus-gene-209.35-mRNA-1	9	OsR498G0917841700.01.T02	99,82	0,00E+00	1966	Os09g0530200	99,91	0,00E+00	1971	5	NA	Japonica	
maker-9-augustus-gene-209.39-mRNA-1	9	OsR498G0917842900.01.T01	99,80	0,00E+00	2736	Os09g0530300	100,00	0,00E+00	2749	13	NA	Japonica	
maker-9-augustus-gene-209.46-mRNA-1	9	OsR498G0917843700.01.T03	100,00	0,00E+00	776	Os09g0530700	100,00	0,00E+00	776	0	NA	Japonica	
maker-9-augustus-gene-209.51-mRNA-1	9	OsR498G0917844300.01.T01	99,74	0,00E+00	4140	Os09g0530800	100,00	0,00E+00	4167	27	NA	Japonica	
maker-9-augustus-gene-209.55-mRNA-1	9	OsR498G0917844800.01.T04	100,00	0,00E+00	1144	Os09g0531100	100,00	0,00E+00	1142	-2	NA	Japonica	
maker-9-augustus-gene-209.60-mRNA-1	9	OsR498G0917845200.01.T01	99,38	0,00E+00	1727	Os09g0531200	100,00	0,00E+00	1754	27	NA	Japonica	
maker-9-augustus-gene-209.62-mRNA-1	9	OsR498G0917846800.01.T01	99,79	0,00E+00	1701	Os09g0531600	100,00	0,00E+00	989	-712	Indica	Japonica	
maker-9-augustus-gene-21.36-mRNA-1	9	OsR498G0916727400.01.T01	99,54	0,00E+00	771	Os09g0125100	99,54	0,00E+00	771	0	NA	Japonica	
maker-9-augustus-gene-210.11-mRNA-1	9	OsR498G0917853100.01.T02	100,00	0,00E+00	2170	Os09g0533300	100,00	0,00E+00	2093	-77	NA	NA	
maker-9-augustus-gene-210.13-mRNA-1	9	OsR498G0917848400.01.T02	100,00	0,00E+00	2105	Os06g0654500	74,09	1,01E-150	533	-1572	Indica	NA	Kinase-TM
maker-9-augustus-gene-210.15-mRNA-1	9	OsR498G0917848800.01.T02	100,00	0,00E+00	2419	Os09g0531800	100,00	0,00E+00	2513	94	NA	NA	
maker-9-augustus-gene-210.19-mRNA-1	9	OsR498G0917849000.01.T01	99,86	0,00E+00	3751	Os09g0531900	99,95	0,00E+00	3762	11	NA	Japonica	
maker-9-augustus-gene-210.24-mRNA-1	9	OsR498G0917849600.01.T01	99,39	0,00E+00	1466	Os09g0532000	100,00	0,00E+00	1489	23	NA	Japonica	
maker-9-augustus-gene-210.26-mRNA-1	9	OsR498G0917850800.01.T01	98,61	0,00E+00	3274	Os09g0532400	98,77	0,00E+00	3287	13	NA	Japonica	
maker-9-augustus-gene-210.31-mRNA-1	9	OsR498G0917852800.01.T03	100,00	0,00E+00	1548	Os09g0532900	100,00	0,00E+00	1548	0	NA	NA	
maker-9-augustus-gene-210.34-mRNA-1	9	OsR498G0917853400.01.T01	99,55	0,00E+00	4741	Os09g0533400	99,55	0,00E+00	4741	0	NA	NA	CC-NBS-TM
maker-9-augustus-gene-210.4-mRNA-1	9	OsR498G0917852900.01.T03	97,01	0,00E+00	3312	Os09g0533100	96,14	0,00E+00	2506	-806	Indica	NA	
maker-9-augustus-gene-211.0-mRNA-1	9	OsR498G0917856500.01.T01	100,00	0,00E+00	1478	Os08g0548200	87,18	0,00E+00	1004	-474	Indica	Japonica	
maker-9-augustus-gene-211.12-mRNA-1	9	OsR498G0917858700.01.T01	95,60	0,00E+00	1604	Os09g0535100	95,71	0,00E+00	1608	4	NA	Japonica	
maker-9-augustus-gene-211.19-mRNA-1	9	OsR498G0917854200.01.T01	100,00	0,00E+00	2203	Os09g0533600	100,00	0,00E+00	2203	0	NA	NA	Kinase
maker-9-augustus-gene-211.25-mRNA-1	9	OsR498G0917855800.01.T01	99,71	0,00E+00	2448	Os09g0533900	100,00	0,00E+00	2466	18	NA	Japonica	
maker-9-augustus-gene-211.9-mRNA-1	9	OsR498G0917858300.01.T01	99,57	0,00E+00	1676	Os09g0534800	100,00	0,00E+00	1694	18	NA	Japonica	
maker-9-augustus-gene-212.47-mRNA-1	9	OsR498G0917860100.01.T02	100,00	0,00E+00	1581	Os09g0535300	100,00	0,00E+00	1581	0	NA	Japonica	
maker-9-augustus-gene-212.63-mRNA-1	9	OsR498G0917867600.01.T01	100,00	0,00E+00	2993	Os09g0536700	100,00	0,00E+00	2993	0	NA	NA	
maker-9-augustus-gene-213.38-mRNA-1	9	OsR498G0917870900.01.T01	100,00	0,00E+00	1169	Os09g0537600	100,00	0,00E+00	1169	0	NA	Japonica	
maker-9-augustus-gene-213.44-mRNA-1	9	OsR498G0917872700.01.T01	100,00	0,00E+00	1353	Os09g0538200	100,00	0,00E+00	1353	0	NA	Japonica	
maker-9-augustus-gene-213.46-mRNA-1	9	OsR498G0917875900.01.T01	99,42	0,00E+00	2753	Os09g0538500	100,00	0,00E+00	2792	39	NA	Japonica	
maker-9-augustus-gene-213.50-mRNA-1	9	OsR498G0917878700.01.T04	99,92	0,00E+00	4531	Os09g0539200	99,77	0,00E+00	2321	-2210	Indica	Japonica	
maker-9-augustus-gene-213.57-mRNA-1	9	OsR498G0917879100.01.T01	99,85	0,00E+00	1179	Os09g0539400	99,88	0,00E+00	1477	298	Japonica	Japonica	
maker-9-augustus-gene-213.59-mRNA-1	9	OsR498G0917871200.01.T01	99,87	0,00E+00	1365	Os09g0537700	100,00	0,00E+00	1370	5	NA	Japonica	
maker-9-augustus-gene-213.61-mRNA-1	9	OsR498G0917872200.01.T01	99,74	0,00E+00	1398	Os09g0538000	96,80	0,00E+00	1312	-86	NA	Japonica	
maker-9-augustus-gene-213.64-mRNA-1	9	OsR498G0917875600.01.T01	100,00	0,00E+00	1364	Os09g0538400	100,00	0,00E+00	1364	0	NA	Japonica	
maker-9-augustus-gene-213.72-mRNA-1	9	OsR498G0917877800.01.T04	99,88	0,00E+00	2913	Os09g0538800	99,94	0,00E+00	2918	5	NA	Japonica	
maker-9-augustus-gene-213.82-mRNA-1	9	OsR498G0917878400.01.T01	99,93	0,00E+00	2398	Os09g0539100	99,93	0,00E+00	2398	0	NA	Japonica	
maker-9-augustus-gene-213.85-mRNA-1	9	OsR498G0917879400.01.T03	100,00	0,00E+00	953	Os11g0456300	83,14	3,64E-152	537	-416	Indica	Japonica	
maker-9-augustus-gene-214.0-mRNA-1	9	OsR498G0917882300.01.T01	99,34	0,00E+00	2161	Os09g0540600	99,27	0,00E+00	2187	26	NA	Japonica	
maker-9-augustus-gene-214.10-mRNA-1	9	OsR498G0917882000.01.T01	95,07	0,00E+00	1862	Os09g0540400	95,33	0,00E+00	1875	13	NA	Japonica	
maker-9-augustus-gene-214.12-mRNA-1	9	OsR498G0917883600.01.T04	99,21	0,00E+00	2461	Os09g0540900	100,00	0,00E+00	3198	737	Japonica	Japonica	
maker-9-augustus-gene-214.2-mRNA-1	9	OsR498G0917884300.01.T01	99,89	0,00E+00	1571	Os09g0541000	100,00	0,00E+00	785	-786	Indica	Japonica	
maker-9-augustus-gene-214.21-mRNA-1	9	OsR498G0917887000.01.T01	99,66	0,00E+00	2618	Os09g0541900	100,00	0,00E+00	2641	23	NA	Japonica	
maker-9-augustus-gene-214.24-mRNA-1	9	OsR498G0917887200.01.T01	93,71	0,00E+00	1317	Os09g0542000	94,64	0,00E+00	1493	176	Japonica	Japonica	
maker-9-augustus-gene-214.4-mRNA-1	9	OsR498G0917886700.01.T01	100,00	0,00E+00	1056	Os09g0541700	100,00	0,00E+00	1056	0	NA	Japonica	
maker-9-augustus-gene-214.7-mRNA-1	9	OsR498G0917880200.01.T01	99,76	0,00E+00	732	Os08g0549300	78,11	1,40E-81	303	-429	Indica	Japonica	
maker-9-augustus-gene-215.2-mRNA-1	9	OsR498G0917891100.01.T02	98,75	0,00E+00	4348	Os09g0543100	98,51	0,00E+00	4322	-26	NA	Japonica	
maker-9-augustus-gene-215.7-mRNA-1	9	OsR498G0917888000.01.T01	94,90	0,00E+00	1232	Os09g0542200	95,17	0,00E+00	1212	-20	NA	Japonica	
maker-9-augustus-gene-216.0-mRNA-1	9	OsR498G0917897100.01.T01	99,95	0,00E+00	3264	Os09g0544300	100,00	0,00E+00	3268	4	NA	NA	Kinase-TM
maker-9-augustus-gene-216.15-mRNA-1	9	OsR498G0917899200.01.T02	100,00	0,00E+00	3041	Os09g0544800	100,00	0,00E+00	3041	0	NA	Japonica	
maker-9-augustus-gene-216.18-mRNA-1	9	OsR498G0917900200.01.T04	99,82	0,00E+00	983	Os09g0544900	100,00	0,00E+00	987	4	NA	Japonica	
maker-9-augustus-gene-216.6-mRNA-1	9	OsR498G0917897500.01.T01	99,99	0,00E+00	13935	Os09g0544400	100,00	0,00E+00	15542	1607	Japonica	NA	
maker-9-augustus-gene-217.0-mRNA-1	9	OsR498G0917909500.01.T07	99,42	0,00E+00	4908	Os09g0547300	100,00	0,00E+00	4978	70	NA	Japonica	
maker-9-augustus-gene-217.4-mRNA-1	9	OsR498G0917910100.01.T01	97,37	0,00E+00	1357	Os09g0547500	97,51	0,00E+00	1237	-120	NA	Japonica	
maker-9-augustus-gene-217.8-mRNA-1	9	OsR498G0917907000.01.T01	100,00	0,00E+00	2581	Os09g0547200	100,00	0,00E+00	2581	0	NA	NA	
maker-9-augustus-gene-218.0-mRNA-1	9	OsR498G0917911300.01.T02	99,92	0,00E+00	2177	Os09g0547800	100,00	0,00E+00	2181	4	NA	Japonica	
maker-9-augustus-gene-218.12-mRNA-1	9	OsR498G0917912500.01.T01	99,91	0,00E+00	2020	Os09g0548200	100,00	0,00E+00	2024	4	NA	Japonica	LRR-TM
maker-9-augustus-gene-218.18-mRNA-1	9	OsR498G0917916100.01.T01	99,49	0,00E+00	2424	Os09g0549400	100,00	0,00E+00	2305	-119	NA	Japonica	
maker-9-augustus-gene-218.24-mRNA-1	9	OsR498G0917917300.01.T02	99,84	0,00E+00	1127	Os09g0549600	100,00	0,00E+00	1131	4	NA	Japonica	
maker-9-augustus-gene-218.26-mRNA-1	9	OsR498G0917913000.01.T02	99,87	0,00E+00	1365	Os09g0548400	100,00	0,00E+00	1231	-134	NA	Japonica	
maker-9-augustus-gene-218.29-mRNA-1	9	OsR498G0917913400.01.T01	97,54	0,00E+00	2783	Os09g0548700	100,00	0,00E+00	2046	-737	Indica	Japonica	
maker-9-augustus-gene-218.32-mRNA-1	9	OsR498G0917915800.01.T01	99,62	0,00E+00	2830	Os09g0549300	100,00	0,00E+00	2857	27	NA	Japonica	
maker-9-augustus-gene-219.29-mRNA-1	9	OsR498G0917922900.01.T01	99,86	0,00E+00	3774	Os09g0551300	100,00	0,00E+00	3788	14	NA	Japonica	
maker-9-augustus-gene-219.38-mRNA-1	9	OsR498G0917921800.01.T02	83,95	0,00E+00	2235	Os09g0551500	86,83	0,00E+00	1584	-651	Indica	Japonica	Kinase-TM
maker-9-augustus-gene-219.40-mRNA-1	9	OsR498G0917917800.01.T02	99,24	0,00E+00	1388	Os09g0550000	100,00	0,00E+00	1416	28	NA	Japonica	
maker-9-augustus-gene-219.54-mRNA-1	9	OsR498G0917921800.01.T01	82,62	1,14E-131	471	Os09g0551500	80,23	1,70E-111	402	-69	Indica	Japonica	Kinase-TM
maker-9-augustus-gene-22.4-mRNA-1	9	OsR498G0916734200.01.T01	99,67	0,00E+00	3810	Os09g0250800	87,39	0,00E+00	1948	-1862	Indica	Japonica	
maker-9-augustus-gene-22.8-mRNA-1	9	OsR498G0916736800.01.T01	99,52	0,00E+00	1861	Os09g0127300	99,81	0,00E+00	1874	13	NA	Japonica	
maker-9-augustus-gene-220.0-mRNA-1	9	OsR498G0917924100.01.T05	99,50	0,00E+00	719	Os09g0551600	100,00	0,00E+00	673	-46	NA	Japonica	
maker-9-augustus-gene-220.13-mRNA-1	9	OsR498G0917928300.01.T02	100,00	0,00E+00	1555	Os09g0553600	100,00	0,00E+00	1555	0	NA	Japonica	
maker-9-augustus-gene-220.19-mRNA-1	9	OsR498G0917924400.01.T05	97,65	0,00E+00	6888	Os09g0551800	98,37	0,00E+00	3536	-3352	Indica	Japonica	
maker-9-augustus-gene-220.26-mRNA-1	9	OsR498G0917924800.01.T01	99,84	0,00E+00	2275	Os09g0552300	100,00	0,00E+00	2284	9	NA	Japonica	Kinase-TM
maker-9-augustus-gene-220.29-mRNA-1	9	OsR498G0917925800.01.T01	91,12	0,00E+00	972	Os09g0552400	100,00	0,00E+00	1764	792	Japonica	Japonica	
maker-9-augustus-gene-220.31-mRNA-1	9	OsR498G0917925800.01.T01	94,25	0,00E+00	1608	Os09g0552500	99,91	0,00E+00	1895	287	Japonica	Japonica	
maker-9-augustus-gene-220.33-mRNA-1	9	OsR498G0917925800.01.T01	99,91	0,00E+00	1917	Os09g0552600	100,00	0,00E+00	1921	4	NA	Japonica	
maker-9-augustus-gene-220.35-mRNA-1	9	OsR498G0917928000.01.T01	100,00	0,00E+00	1467	Os09g0553300	100,00	0,00E+00	996	-471	Indica	Japonica	
maker-9-augustus-gene-220.5-mRNA-1	9	OsR498G0917926500.01.T03	99,55	0,00E+00	1176	Os09g0552900	99,09	0,00E+00	1159	-17	NA	Japonica	
maker-9-augustus-gene-220.7-mRNA-1	9	OsR498G0917927700.01.T01	98,12	0,00E+00	2482	Os09g0553200	98,33	0,00E+00	2496	14	NA	Japonica	
maker-9-augustus-gene-221.0-mRNA-1	9	OsR498G0917931100.01.T01	99,92	0,00E+00	2317	Os09g0554300	100,00	0,00E+00	2322	5	NA	Japonica	
maker-9-augustus-gene-221.30-mRNA-1	9	OsR498G0917929400.01.T01	99,59	0,00E+00	1736	Os09g0554000	100,00	0,00E+00	987	-749	Indica	Japonica	
maker-9-augustus-gene-221.35-mRNA-1	9	OsR498G0917935000.01.T01	97,57	0,00E+00	2320	Os09g0555500	97,65	0,00E+00	2111	-209	NA	Japonica	
maker-9-augustus-gene-221.5-mRNA-1	9	OsR498G0917931100.01.T02	85,49	0,00E+00	1338	Os09g0555100	100,00	0,00E+00	2069	731	Japonica	Japonica	
maker-9-augustus-gene-222.0-mRNA-1	9	OsR498G0917939500.01.T02	99,82	0,00E+00	3048	Os09g0556200	99,94	0,00E+00	3156	108	NA	Japonica	
maker-9-augustus-gene-222.13-mRNA-1	9	OsR498G0917938400.01.T01	99,83	0,00E+00	3114	Os09g0555800	100,00	0,00E+00	3128	14	NA	Japonica	
maker-9-augustus-gene-222.22-mRNA-1	9	OsR498G0917940000.01.T06	99,83	0,00E+00	3071	Os09g0556500	100,00	0,00E+00	3085	14	NA	Japonica	
maker-9-augustus-gene-222.25-mRNA-1	9	OsR498G0917940400.01.T01	99,75	0,00E+00	2113	Os09g0556800	100,00	0,00E+00	2127	14	NA	Japonica	
maker-9-augustus-gene-222.9-mRNA-1	9	OsR498G0917935800.01.T03	97,75	0,00E+00	3901	Os09g0555600	97,89	0,00E+00	3914	13	NA	Japonica	
maker-9-augustus-gene-223.48-mRNA-1	9	OsR498G0917942100.01.T01	99,85	0,00E+00	3488	Os09g0557800	100,00	0,00E+00	3501	13	NA	Japonica	
maker-9-augustus-gene-223.52-mRNA-1	9	OsR498G0917942300.01.T01	100,00	0,00E+00	915	Os09g0557900	100,00	0,00E+00	915	0	NA	Japonica	
maker-9-augustus-gene-223.54-mRNA-1	9	OsR498G0917942900.01.T01	100,00	8,91E-110	396	Os09g0558100	100,00	3,10E-110	396	0	NA	Japonica	
maker-9-augustus-gene-223.65-mRNA-1	9	OsR498G0917947500.01.T02	99,90	0,00E+00	1737	Os09g0559800	100,00	0,00E+00	1745	8	NA	Japonica	
maker-9-augustus-gene-223.67-mRNA-1	9	OsR498G0917947700.01.T02	99,14	0,00E+00	3710	Os09g0559900	97,86	0,00E+00	3626	-84	NA	Japonica	Kinase-LRR-TM

maker-9-augustus-gene-223.70-mRNA-1	9	OsR498G0917941200.01.T01	99,70	0,00E+00	1209	Os09g0557400	100,00	0,00E+00	1218	9	NA	Japonica	
maker-9-augustus-gene-223.73-mRNA-1	9	OsR498G0917941600.01.T02	99,74	0,00E+00	2092	Os09g0557500	100,00	0,00E+00	1846	-246	Indica	Japonica	
maker-9-augustus-gene-223.75-mRNA-1	9	OsR498G0917942600.01.T01	100,00	0,00E+00	1905	Os09g0558000	100,00	0,00E+00	1898	-7	NA	Japonica	
maker-9-augustus-gene-223.80-mRNA-1	9	OsR498G0917945200.01.T01	99,58	0,00E+00	3814	Os09g0558900	100,00	0,00E+00	3869	55	NA	Japonica	
maker-9-augustus-gene-224.12-mRNA-1	9	OsR498G0917948700.01.T08	99,81	0,00E+00	1896	Os09g0560600	100,00	0,00E+00	1905	9	NA	Japonica	
maker-9-augustus-gene-224.14-mRNA-1	9	OsR498G0917948900.01.T02	97,25	0,00E+00	1649	Os09g0560700	100,00	0,00E+00	1759	110	NA	Japonica	
maker-9-augustus-gene-224.19-mRNA-1	9	OsR498G0917949100.01.T01	97,09	0,00E+00	1609	Os09g0560800	100,00	0,00E+00	1732	123	NA	Japonica	
maker-9-augustus-gene-224.22-mRNA-1	9	OsR498G0917950700.01.T01	84,83	0,00E+00	2419	Os09g0561500	100,00	0,00E+00	4231	1812	Japonica	Japonica	Kinase-TM
maker-9-augustus-gene-224.23-mRNA-1	9	OsR498G0917952300.01.T01	96,62	0,00E+00	3855	Os09g0561600	100,00	0,00E+00	3901	46	NA	Japonica	CC-Kinase-TM
maker-9-augustus-gene-224.31-mRNA-1	9	OsR498G1019198700.01.T01	82,58	1,31E-99	363	Os09g0562300	100,00	0,00E+00	810	447	Japonica	Japonica	
maker-9-augustus-gene-224.41-mRNA-1	9	OsR498G0917948000.01.T01	99,16	0,00E+00	4418	Os09g0560000	100,00	0,00E+00	4513	95	NA	Japonica	
maker-9-augustus-gene-224.45-mRNA-1	9	OsR498G0917948600.01.T01	93,57	0,00E+00	921	Os09g0560400	90,11	1,98E-143	508	-413	Indica	Japonica	
maker-9-augustus-gene-224.9-mRNA-1	9	OsR498G0917948700.01.T07	99,52	0,00E+00	1118	Os09g0560500	100,00	0,00E+00	1131	13	NA	Japonica	
maker-9-augustus-gene-225.30-mRNA-1	9	OsR498G0917955300.01.T01	98,86	0,00E+00	3685	Os09g0563250	100,00	0,00E+00	2565	-1120	Indica	Japonica	
maker-9-augustus-gene-225.41-mRNA-1	9	OsR498G0917955900.01.T01	99,72	0,00E+00	3820	Os09g0563700	100,00	0,00E+00	3847	27	NA	Japonica	
maker-9-augustus-gene-225.46-mRNA-1	9	OsR498G0917955200.01.T01	98,89	0,00E+00	3789	Os09g0563200	99,01	0,00E+00	3201	-588	Indica	Japonica	
maker-9-augustus-gene-225.55-mRNA-1	9	OsR498G0917956200.01.T02	95,64	0,00E+00	1801	Os09g0563800	100,00	0,00E+00	1734	-67	NA	Japonica	
maker-9-augustus-gene-225.64-mRNA-1	9	OsR498G0917957000.01.T01	99,88	0,00E+00	1533	Os09g0564000	100,00	0,00E+00	1537	4	NA	Japonica	
maker-9-augustus-gene-226.52-mRNA-1	9	OsR498G0917957400.01.T01	99,82	0,00E+00	1994	Os09g0564200	100,00	0,00E+00	1159	-835	Indica	Japonica	
maker-9-augustus-gene-226.53-mRNA-1	9	OsR498G0917958800.01.T01	78,60	0,00E+00	902	Os09g0564600	78,41	0,00E+00	893	-9	NA	Japonica	
maker-9-augustus-gene-226.54-mRNA-1	9	OsR498G0917958800.01.T01	99,64	0,00E+00	2012	Os09g0564600	100,00	0,00E+00	2003	-9	NA	Japonica	
maker-9-augustus-gene-226.84-mRNA-1	9	OsR498G0917960200.01.T01	99,81	0,00E+00	1912	Os09g0565200	100,00	0,00E+00	1748	-164	NA	Japonica	
maker-9-augustus-gene-226.91-mRNA-1	9	OsR498G0917960600.01.T01	99,72	0,00E+00	3203	Os09g0565400	100,00	0,00E+00	3225	22	NA	Japonica	
maker-9-augustus-gene-227.6-mRNA-1	9	OsR498G0917963000.01.T01	99,84	0,00E+00	4563	Os09g0566100	100,00	0,00E+00	4581	18	NA	Japonica	
maker-9-augustus-gene-227.8-mRNA-1	9	OsR498G0917963800.01.T01	78,19	0,00E+00	989	Os09g0566400	77,34	0,00E+00	849	-140	Indica	Japonica	
maker-9-augustus-gene-228.0-mRNA-1	9	OsR498G0917967100.01.T03	99,92	0,00E+00	2133	Os09g0567300	99,92	0,00E+00	2133	0	NA	NA	
maker-9-augustus-gene-228.12-mRNA-1	9	OsR498G0917968500.01.T01	99,80	0,00E+00	5254	Os09g0567700	100,00	0,00E+00	5281	27	NA	Japonica	
maker-9-augustus-gene-228.18-mRNA-1	9	OsR498G0917971300.01.T01	100,00	0,00E+00	672	Os09g0568900	100,00	0,00E+00	672	0	NA	Japonica	
maker-9-augustus-gene-228.2-mRNA-1	9	OsR498G0917967100.01.T06	99,91	0,00E+00	2085	Os09g0567350	100,00	0,00E+00	2089	4	NA	Japonica	
maker-9-augustus-gene-228.28-mRNA-1	9	OsR498G0917973100.01.T01	99,56	0,00E+00	2846	Os09g0569800	100,00	0,00E+00	2877	31	NA	Japonica	CC-Kinase-TM
maker-9-augustus-gene-228.32-mRNA-1	9	OsR498G0917968800.01.T02	99,57	0,00E+00	2050	Os09g0567800	100,00	0,00E+00	2073	23	NA	Japonica	
maker-9-augustus-gene-228.36-mRNA-1	9	OsR498G0917969200.01.T01	99,75	0,00E+00	1443	Os09g0567900	100,00	0,00E+00	1452	9	NA	Japonica	
maker-9-augustus-gene-228.40-mRNA-1	9	OsR498G0917969800.01.T01	99,41	0,00E+00	896	Os09g0568200	100,00	0,00E+00	636	-260	Indica	Japonica	
maker-9-augustus-gene-228.43-mRNA-1	9	OsR498G0917971200.01.T01	100,00	3,25E-168	590	Os09g0568800	100,00	1,13E-168	590	0	NA	Japonica	
maker-9-augustus-gene-228.49-mRNA-1	9	OsR498G0917972400.01.T02	99,37	0,00E+00	3964	Os09g0569400	99,42	0,00E+00	3968	4	NA	Japonica	
maker-9-augustus-gene-228.5-mRNA-1	9	OsR498G0917967300.01.T01	100,00	0,00E+00	812	Os09g0567400	100,00	0,00E+00	812	0	NA	Japonica	
maker-9-augustus-gene-228.9-mRNA-1	9	OsR498G0917968100.01.T01	100,00	0,00E+00	2738	Os09g0567500	100,00	0,00E+00	2738	0	NA	Japonica	
maker-9-augustus-gene-229.0-mRNA-1	9	OsR498G0917973500.01.T05	99,75	0,00E+00	2818	Os09g0570000	100,00	0,00E+00	1954	-864	Indica	Japonica	CC-Kinase-TM
maker-9-augustus-gene-229.13-mRNA-1	9	OsR498G0917974100.01.T01	99,90	0,00E+00	1706	Os09g0570300	100,00	0,00E+00	1716	10	NA	Japonica	
maker-9-augustus-gene-229.17-mRNA-1	9	OsR498G0917974300.01.T01	99,81	0,00E+00	1936	Os09g0570400	100,00	0,00E+00	1945	9	NA	Japonica	
maker-9-augustus-gene-229.21-mRNA-1	9	OsR498G0917974900.01.T02	99,90	0,00E+00	3557	Os09g0570500	100,00	0,00E+00	2446	-1111	Indica	Japonica	
maker-9-augustus-gene-229.26-mRNA-1	9	OsR498G0917975100.01.T02	97,91	0,00E+00	2690	Os08g0559900	74,37	0,00E+00	725	-1965	Indica	Japonica	
maker-9-augustus-gene-229.33-mRNA-1	9	OsR498G0917976200.01.T01	99,87	0,00E+00	2702	Os09g0570900	100,00	0,00E+00	1384	-1318	Indica	Japonica	
maker-9-augustus-gene-229.4-mRNA-1	9	OsR498G0917973700.01.T02	97,23	0,00E+00	3149	Os09g0570100	99,91	0,00E+00	1928	-1221	Indica	Japonica	CC-Kinase-TM
maker-9-augustus-gene-229.43-mRNA-1	9	OsR498G0917977400.01.T01	97,64	0,00E+00	2173	Os09g0571000	99,86	0,00E+00	2563	390	Japonica	Japonica	
maker-9-augustus-gene-229.47-mRNA-1	9	OsR498G0917978400.01.T01	99,75	0,00E+00	1452	Os09g0571500	99,75	0,00E+00	1452	0	NA	Japonica	
maker-9-augustus-gene-229.8-mRNA-1	9	OsR498G0917973900.01.T05	99,91	0,00E+00	1901	Os09g0570200	100,00	0,00E+00	1905	4	NA	Japonica	
maker-9-augustus-gene-23.0-mRNA-1	9	OsR498G0916739400.01.T02	99,95	0,00E+00	6587	Os09g0127800	99,95	0,00E+00	6587	0	NA	Japonica	
maker-9-augustus-gene-230.44-mRNA-1	9	OsR498G0917982100.01.T01	99,75	0,00E+00	2861	Os09g0572500	99,39	0,00E+00	2911	50	NA	Japonica	
maker-9-augustus-gene-230.46-mRNA-1	9	OsR498G0917982500.01.T01	99,76	0,00E+00	2260	Os09g0572600	100,00	0,00E+00	2273	13	NA	Japonica	Kinase-TM
maker-9-augustus-gene-230.63-mRNA-1	9	OsR498G0917866300.01.T01	100,00	0,00E+00	1375	Os09g0536400	100,00	0,00E+00	1375	0	NA	NA	
maker-9-augustus-gene-230.65-mRNA-1	9	OsR498G0917981400.01.T03	99,71	0,00E+00	3094	Os09g0572400	99,88	0,00E+00	3108	14	NA	Japonica	
maker-9-augustus-gene-230.75-mRNA-1	9	OsR498G0917984400.01.T01	95,16	0,00E+00	1139	Os09g0573100	95,30	0,00E+00	1143	4	NA	Japonica	
maker-9-augustus-gene-24.5-mRNA-1	9	OsR498G0916742000.01.T04	96,64	0,00E+00	2145	Os09g0128600	96,71	0,00E+00	2150	5	NA	Japonica	
maker-9-augustus-gene-25.33-mRNA-1	9	OsR498G0916742300.01.T01	100,00	4,37E-91	334	Os09g0128700	98,02	8,39E-95	346	12	NA	Japonica	
maker-9-augustus-gene-25.38-mRNA-1	9	OsR498G0916743100.01.T06	92,24	0,00E+00	800	Os09g0129400	100,00	0,00E+00	749	-51	NA	NA	
maker-9-augustus-gene-26.0-mRNA-1	9	OsR498G0916748200.01.T02	99,79	0,00E+00	3355	Os09g0130800	99,95	0,00E+00	3369	14	NA	Japonica	
maker-9-augustus-gene-28.11-mRNA-1	9	OsR498G0916757600.01.T01	99,68	1,00E-156	554	Os09g0133000	100,00	8,20E-159	558	4	NA	Japonica	
maker-9-augustus-gene-28.13-mRNA-1	9	OsR498G0916758200.01.T02	99,74	0,00E+00	1366	Os09g0133200	99,87	0,00E+00	1370	4	NA	Japonica	
maker-9-augustus-gene-28.2-mRNA-1	9	OsR498G0916757300.01.T01	100,00	0,00E+00	1564	Os09g0132900	97,23	0,00E+00	1473	-91	NA	Japonica	
maker-9-augustus-gene-28.4-mRNA-1	9	OsR498G0916760900.01.T01	95,10	0,00E+00	1556	Os09g0133600	92,89	0,00E+00	976	-580	Indica	Japonica	
maker-9-augustus-gene-28.6-mRNA-1	9	OsR498G0916756400.01.T01	99,75	0,00E+00	2146	Os09g0132600	100,00	0,00E+00	2159	13	NA	Japonica	
maker-9-augustus-gene-29.31-mRNA-1	9	OsR498G0916761500.01.T01	99,90	0,00E+00	1879	Os09g0133700	100,00	0,00E+00	1884	5	NA	Japonica	
maker-9-augustus-gene-29.37-mRNA-1	9	OsR498G0916762900.01.T03	99,72	0,00E+00	1287	Os09g0134400	100,00	0,00E+00	1296	9	NA	Japonica	
maker-9-augustus-gene-29.40-mRNA-1	9	OsR498G0916761800.01.T02	99,85	0,00E+00	3569	Os09g0133800	99,90	0,00E+00	3573	4	NA	Japonica	
maker-9-augustus-gene-29.42-mRNA-1	9	OsR498G0916763200.01.T02	100,00	0,00E+00	2663	Os09g0134500	100,00	0,00E+00	2663	0	NA	Japonica	
maker-9-augustus-gene-3.0-mRNA-1	9	OsR498G0916642200.01.T02	98,03	0,00E+00	2345	Os09g0101100	100,00	0,00E+00	2300	-45	NA	Japonica	
maker-9-augustus-gene-3.5-mRNA-1	9	OsR498G0916644200.01.T04	99,15	0,00E+00	3540	Os09g0101800	100,00	0,00E+00	3617	77	NA	Japonica	
maker-9-augustus-gene-30.0-mRNA-1	9	OsR498G0916769500.01.T05	100,00	0,00E+00	780	Os09g0135700	100,00	0,00E+00	706	-74	NA	Japonica	
maker-9-augustus-gene-30.5-mRNA-1	9	OsR498G0916768000.01.T06	99,80	0,00E+00	900	Os05g0340000	87,40	3,06E-168	591	-309	Indica	Japonica	
maker-9-augustus-gene-31.0-mRNA-1	9	OsR498G0916849000.01.T03	99,05	0,00E+00	1481	Os09g0258000	99,64	0,00E+00	1504	23	NA	NA	
maker-9-augustus-gene-33.27-mRNA-1	9	OsR498G0916776000.01.T05	99,92	0,00E+00	2258	Os09g0237600	100,00	0,00E+00	1943	-315	Indica	Japonica	
maker-9-augustus-gene-34.22-mRNA-1	9	OsR498G0916781010.01.T01	99,68	0,00E+00	1117	Os09g0240200	99,84	0,00E+00	1122	5	NA	Japonica	
maker-9-augustus-gene-35.14-mRNA-1	9	OsR498G0916788900.01.T01	99,58	0,00E+00	2565	Os09g0242300	99,93	0,00E+00	2514	-51	NA	Japonica	
maker-9-augustus-gene-35.7-mRNA-1	9	OsR498G0916784100.01.T01	99,79	0,00E+00	6065	Os09g0241100	99,96	0,00E+00	5058	-1007	Indica	Japonica	
maker-9-augustus-gene-39.3-mRNA-1	9	OsR498G0916804200.01.T01	99,17	0,00E+00	4678	Os09g0246200	99,41	0,00E+00	5471	793	Japonica	Japonica	
maker-9-augustus-gene-4.6-mRNA-1	9	OsR498G0916646300.01.T02	97,49	0,00E+00	3136	Os09g0103200	100,00	0,00E+00	3014	-122	NA	Japonica	
maker-9-augustus-gene-40.26-mRNA-1	9	OsR498G0916815400.01.T01	99,81	0,00E+00	1923	Os09g0247600	100,00	0,00E+00	1932	9	NA	Japonica	
maker-9-augustus-gene-41.0-mRNA-1	9	OsR498G0916816700.01.T07	99,27	0,00E+00	973	Os08g0339200	73,93	1,93E-89	330	-643	Indica	Japonica	
maker-9-augustus-gene-41.13-mRNA-1	9	OsR498G0916818400.01.T03	99,77	0,00E+00	3126	Os09g0248200	99,94	0,00E+00	2871	-255	NA	Japonica	
maker-9-augustus-gene-41.17-mRNA-1	9	OsR498G0916818800.01.T01	99,94	0,00E+00	2934	Os09g0248300	100,00	0,00E+00	2938	4	NA	Japonica	
maker-9-augustus-gene-41.6-mRNA-1	9	OsR498G0916818100.01.T01	99,01	0,00E+00	2128	Os09g0248100	99,01	0,00E+00	2128	0	NA	Indica	
maker-9-augustus-gene-42.0-mRNA-1	9	OsR498G0916826900.01.T02	96,09	0,00E+00	835	Os09g0249900	99,62	0,00E+00	929	94	Japonica	Japonica	
maker-9-augustus-gene-42.14-mRNA-1	9	OsR498G0916823600.01.T01	99,60	0,00E+00	1788	Os09g0249000	99,70	0,00E+00	1792	4	NA	Japonica	
maker-9-augustus-gene-42.15-mRNA-1	9	OsR498G0916825600.01.T02	99,72	0,00E+00	4443	Os09g0249600	100,00	0,00E+00	2538	-1905	Indica	Japonica	
maker-9-augustus-gene-42.19-mRNA-1	9	OsR498G0916826000.01.T03	99,89	0,00E+00	3173	Os09g0249700	100,00	0,00E+00	3182	9	NA	Japonica	
maker-9-augustus-gene-42.9-mRNA-1	9	OsR498G0916823200.01.T01	100,00	7,84E-154	544	Os09g0248900	100,00	2,73E-154	544	0	NA	Japonica	
maker-9-augustus-gene-43.0-mRNA-1	9	OsR498G0916833700.01.T06	99,79	0,00E+00	2521	Os09g0250700	99,86	0,00E+00	2529	8	NA	Japonica	
maker-9-augustus-gene-44.37-mRNA-1	9	OsR498G0916837100.01.T01	99,81	0,00E+00	927	Os09g0251800	100,00	0,00E+00	931	4	NA	Japonica	
maker-9-augustus-gene-44.47-mRNA-1	9	OsR498G0916834400.01.T01	99,57	0,00E+00	1658	Os09g0250800	99,57	0,00E+00	1658	0	NA	Japonica	
maker-9-augustus-gene-45.17-mRNA-1	9	OsR498G0916841100.01.T01	100,00	3,91E-121	434	Os09g0253000	100,00	1,37E-121	434	0	NA	Japonica	
maker-9-augustus-gene-45.5-mRNA-1	9	OsR498G0916838900.01.T05	100,00	0,00E+00	2176	Os09g0252100	100,00	0,00E+00	2349	173	NA	Japonica	
maker-9-augustus-gene-46.37-mRNA-1	9	OsR498G0916851500.01.T01	88,57	0,00E+00	662	Os09g0254600	100,00	0,00E+00	1121	459	Japonica	Japonica	
maker-9-augustus-gene-47.40-mRNA-1	9	OsR498G0916842100.01.T02	98,88	0,00E+00	3144	Os09g0255000	99,50	0,00E+00	3194	50	NA	Japonica	
maker-9-augustus-gene-47.47-mRNA-1	9	OsR498G0916843600.01.T01	99,90	0,00E+00	1798	Os09g0255400	100,00	0,00E+00	1802	4	NA	Japonica	
maker-9-augustus-gene-49.0-mRNA-1	9	OsR498G0916851500.01.T01	98,64	0,00E+00	893	Os09g0258700	99,16	0,00E+00	1048	155	Japonica	NA	
maker-9-augustus-gene-49.11-mRNA-1	9	OsR498G0916851000.01.T01	99,61	0,00E+00	919	Os09g0258600	100,00	0,00E+00	928	9	NA	Japonica	
maker-9-augustus-gene-49.4-mRNA-1	9	OsR498G0916849000.01.T03	99,05	0,00E+00	1481	Os09g0258000	99,64	0,00E+00	1504	23	NA	Japonica	
maker-9-augustus-gene-49.9-mRNA-1	9	OsR498G0916850600.01.T01	99,82	0,00E+00	3060	Os09g0258500	99,94	0,00E+00	3069	9	NA	Japonica	Kinase
maker-9-augustus-gene-5.0-mRNA-1	9	OsR498G0916647100.01.T01	99,71	0,00E+00	2504	Os09g0103500	98,61	0,00E+00	2391	-113	NA	Japonica	
maker-9-augustus-gene-5.13-mRNA-1	9	OsR498G0916649300.01.T03	99,62	0,00E+00	944	Os09g0104300	100,00	0,00E+00	953	9	NA	Japonica	
maker-9-augustus-gene-5.15-mRNA-1	9	OsR498G0916647400.01.T01	99,75	0,00E+00	2113	Os09g0103600	100,00	0,00E+00	2127	14	NA	Japonica	
maker-9-augustus-gene-5.4-mRNA-1	9	OsR498G0916648300.01.T02	99,57	0,00E+00	2927	Os09g0103800	97,26	0,00E+00	2793	-134	NA	Japonica	
maker-9-augustus-gene-51.11-mRNA-1	9	OsR498G0916863300.01.T01	99,57	0,00E+00	1667	Os09g0262000	99,39	0,00E+00	2030	363	Japonica	NA	
maker-9-augustus-gene-51.16-mRNA-1	9	OsR498G0916858000.01.T01	99,92	0,00E+00	2189	Os09g0261100	99,92	0,00E+00	2189	0	NA	NA	
maker-9-augustus-gene-51.17-mRNA-1	9	OsR498G0916858600.01.T01	99,81	0,00E+00	929	Os09g0261300	100,00	0,00E+00	933	4	NA	Japonica	
maker-9-augustus-gene-52.39-mRNA-1	9	OsR498G0916863800.01.T01	99,17	0,00E+00	2537	Os09g0264400	92,04	0,00E+00	2275	-262	Indica	Indica	
maker-9-augustus-gene-53.27-mRNA-1	9	OsR498G0916870200.01.T01	96,76	0,00E+00	1330	Os09g0262000	78,71	0,00E+00	649	-681	Indica	NA	
maker-9-augustus-gene-53.31-mRNA-1	9	OsR498G0916863800.01.T01	91,27	0,00E+00	2003	Os09g0264400	97,56	0,00E+00	2636	633	Japonica	NA	
maker-9-augustus-gene-53.34-mRNA-1	9	OsR498G0916869900.01.T01	94,30	0,00E+00	1493	Os05g0524500	73,77	2,92E-169	595	-898	Indica	Indica	
maker-9-augustus-gene-54.0-mRNA-1	9	OsR498G0916872700.01.T01	100,00	0,00E+00	4044	Os09g0266400	99,91	0,00E+00	4035	-9	NA	Indica	
maker-9-augustus-gene-54.12-mRNA-1	9	OsR498G0916873200.01.T02	98,69	0,00E+00	3068	Os09g0266600	99,68	0,00E+00	2232	-836	Indica	NA	
maker-9-augustus-gene-55.0-mRNA-1	9	OsR498G0916877300.01.T02	99,61	0,00E+00	4153	Os09g0267500	99,61	0,00E+00	4153	0	NA	NA	
maker-9-augustus-gene-55.10-mRNA-1	9	OsR498G0916879300.01.T01	99,02	0,00E+00	2703	Os09g0268300	99,41	0,00E+00	2730	27	NA	Japonica	
maker-9-augustus-gene-57.0-mRNA-1	9	OsR498G0916882900.01.T01	99,01	0,00E+00	3198	Os09g0270700	100,00	0,00E+00	3279	81	NA	Japonica	CC-NBS-TM
maker-9-augustus-gene-58.34-mRNA-1	9	OsR498G0916887300.01.T02	98,84	0,00E+00	2570	Os09g0272300	100,00	0,00E+00	2646	76	NA	Japonica	
maker-9-augustus-gene-58.36-mRNA-1	9	OsR498G0916886400.01.T03	92,86	0,00E+00	820	Os09g0272000	100,00	0,00E+00	939	119	Japonica	Japonica	
maker-9-augustus-gene-58.38-mRNA-1	9	OsR498G0916887200.01.T01	97,20	1,80E-152	539	Os09g0272250	100,00	5,85E-166	581	42	NA	Japonica	
maker-9-augustus-gene-58.42-mRNA-1	9	OsR498G0916888000.01.T02	97,61	0,00E+00	848	Os09g0272500	99,86	0,00E+00	1284	436	Japonica	Japonica	
maker-9-augustus-gene-59.39-mRNA-1	9	OsR498G0916888300.01.T01	99,51	4,86E-100	364	Os09g0272600	100,00	3,98E-102	369	5	NA	Japonica	
maker-9-augustus-gene-60.30-mRNA-1	9	OsR498G0916887200.01.T01	81,93	1,04E-93	343	Os09g0275300	100,00	0,00E+00	785	442	Japonica	Japonica	
maker-9-augustus-gene-60.32-mRNA-1	9	OsR498G0916897000.01.T01	99,74	0,00E+00	2742	Os09g0275400	100,00	0,00E+00	2760	18	NA	Japonica	
maker-9-augustus-gene-62.32-mRNA-1	9	OsR498G0916899900.01.T05	96,15	0,00E+00	1884	Os09g0277800	97,64	0,00E+00	1960	76	NA	Japonica	
maker-9-augustus-gene-62.38-mRNA-1	9	OsR498G0916901800.01.T02	99,54	0,00E+00	1156	Os09g0278300	100,00	0,00E+00	1169	13	NA	Japonica	
maker-9-augustus-gene-62.46-mRNA-1	9	OsR498G0916903600.01.T02	99,76	0,00E+00	2209	Os09g0279000	99,68	0,00E+00	2205	-4	NA	Japonica	
maker-9-augustus-gene-63.0-mRNA-1	9	OsR498G0916906700.01.T01	99,78	0,00E+00	1636	Os09g0279400	99,86	0,00E+00	1295	-341	Indica	Japonica	
maker-9-augustus-gene-63.10-mRNA-1	9	OsR498G0916908100.01.T01	97,90	0,00E+00	1641	Os09g0280300	98,11	0,00E+00	1650	9	NA	Japonica	
maker-9-augustus-gene-63.15-mRNA-1	9	OsR498G0916909900.01.T02	99,72	0,00E+00	1299	Os09g0280500	100,00	0,00E+00	1308	9	NA	Japonica	
maker-9-augustus-gene-63.19-mRNA-1	9	OsR498G0916907000.01.T01	99,73	0,00E+00	1323	Os09g0279500	100,00	0,00E+00	1332	9	NA	Japonica	
maker-9-augustus-gene-63.2-mRNA-1	9	OsR498G0916907100.01.T05	99,92	0,00E+00	2171	Os09g0279600	100,00	0,00E+00	2176	5	NA	Japonica	
maker-9-augustus-gene-63.22-mRNA-1	9	OsR498G0916910400.01.T01	86,46	0,00E+00	827	Os09g0280600	86,46	0,00E+00	827	0	NA	Japonica	
maker-9-augustus-gene-64.28-mRNA-1	9	OsR498G0916915500.01.T05	99,69	0,00E+00	2320	Os09g0281600	100,00	0,00E+00	1570	-750	Indica	Japonica	
maker-9-augustus-gene-64.33-mRNA-1	9	OsR498G0916915900.01.T01	99,84	0,00E+00	1143	Os10g0377400	78,26	1,85E-119	429	-714	Indica	Japonica	
maker-9-augustus-gene-64.35-mRNA-1	9	OsR498G0916917100.01.T01	99,40	0,00E+00	2362	Os09g0281800	99,55	0,00E+00	2371	9	NA	Japonica	
maker-9-augustus-gene-64.37-mRNA-1	9	OsR498G0916914400.01.T02	99,57	0,00E+00	839	Os09g0281300	100,00	0,00E+00	848	9	NA	Japonica	
maker-9-augustus-gene-64.39-mRNA-1	9	OsR498G0916917400.01.T01	99,81	0,00E+00	9375	Os09g0281900	100,00	0,00E+00	9420	45	NA	Japonica	
maker-9-augustus-gene-66.41-mRNA-1	9	OsR498G0916926100.01.T01	98,59	0,00E+00	1734	Os09g0283600	100,00	0,00E+00	1797	63	NA	Japonica	
maker-9-augustus-gene-66.43-mRNA-1	9	OsR498G0916928500.01.T03	99,79	0,00E+00	853	Os09g0284200	100,00	0,00E+00	810	-43	NA	Japonica	
maker-9-augustus-gene-66.45-mRNA-1	9	OsR498G0916928900.01.T01	98,89	8,09E-85	313	Os09g0284300	99,44	2,32E-86	317	4	NA	Japonica	
maker-9-augustus-gene-66.49-mRNA-1	9	OsR498G0916929200.01.T01	95,38	0,00E+00	1482	Os09g0284400	95,60	0,00E+00	1491	9	NA	Japonica	
maker-9-augustus-gene-66.51-mRNA-1	9	OsR498G0916929800.01.T01	99,86	0,00E+00	2529	Os08g0338900	78,23	0,00E+00	1142	-1387	Indica	Japonica	
maker-9-augustus-gene-68.0-mRNA-1	9	OsR498G0916940800.01.T01	100,00	0,00E+00	2684	Os09g0286300	99,58	0,00E+00	2557	-127	NA	NA	
maker-9-augustus-gene-68.11-mRNA-1	9	OsR498G0916944400.01.T02	100,00	0,00E+00	1332	Os09g0287000	100,00	0,00E+00	1332	0	NA	NA	
maker-9-augustus-gene-68.13-mRNA-1	9	OsR498G0916948000.01.T03	100,00	1,74E-155	549	Os09g0287300	100,00	6,07E-156	549	0	NA	NA	
maker-9-augustus-gene-68.9-mRNA-1	9	OsR498G0916941200.01.T03	96,40	0,00E+00	2039	Os09g0286400	96,40	0,00E+00	2039	0	NA	NA	
maker-9-augustus-gene-74.27-mRNA-1	9	OsR498G0917001700.01.T02	100,00	0,00E+00	807	Os09g0293400	100,00	0,00E+00	807	0	NA	Japonica	
maker-9-augustus-gene-75.0-mRNA-1	9	OsR498G0917003300.01.T01	97,02	0,00E+00	2151	Os09g0293900	94,25	0,00E+00	1988	-163	NA	Japonica	
maker-9-augustus-gene-75.14-mRNA-1	9	OsR498G0917004800.01.T02	99,82	0,00E+00	999	Os09g0294300	100,00	0,00E+00	1003	4	NA	Japonica	
maker-9-augustus-gene-75.9-mRNA-1	9	OsR498G0917003600.01.T01	99,96	0,00E+00	4952	Os09g0294000	100,00	0,00E+00	4956	4	NA	Japonica	
maker-9-augustus-gene-77.41-mRNA-1	9	OsR498G0917011700.01.T02	99,59	0,00E+00	1725	Os09g0296800	100,00	0,00E+00	1743	18	NA	Japonica	
maker-9-augustus-gene-77.44-mRNA-1	9	OsR498G0917011900.01.T01	99,84	0,00E+00	2304	Os09g0296900	100,00	0,00E+00	2313	9	NA	Japonica	
maker-9-augustus-gene-77.48-mRNA-1	9	OsR498G0917012300.01.T06	99,70	0,00E+00	1173	Os09g0297000	99,70	0,00E+00	1173	0	NA	Japonica	
maker-9-augustus-gene-77.53-mRNA-1	9	OsR498G0917014900.01.T04	99,70	0,00E+00	2397	Os08g0345700	77,39	0,00E+00	1038	-1359	Indica	Japonica	
maker-9-augustus-gene-77.57-mRNA-1	9	OsR498G0917016700.01.T01	99,90	0,00E+00	1798	Os09g0298400	100,00	0,00E+00	1802	4	NA	Japonica	
maker-9-augustus-gene-77.63-mRNA-1	9	OsR498G0917012600.01.T01	100,00	0,00E+00	807	Os09g0297100	100,00	0,00E+00	807	0	NA	Japonica	
maker-9-augustus-gene-77.66-mRNA-1	9	OsR498G0917013200.01.T02	99,92	0,00E+00	2209	Os09g0297400	100,00	0,00E+00	2214	5	NA	Japonica	
maker-9-augustus-gene-77.71-mRNA-1	9	OsR498G0917014000.01.T01	99,62	0,00E+00	939	Os09g0297800	100,00	0,00E+00	948	9	NA	Japonica	
maker-9-augustus-gene-77.73-mRNA-1	9	OsR498G0917015400.01.T02	99,27	0,00E+00	2673	Os09g0298200	100,00	0,00E+00	2711	38	NA	Japonica	
maker-9-augustus-gene-78.26-mRNA-1	9	OsR498G0917018900.01.T02	100,00	0,00E+00	1052	Os08g0346400	79,57	1,44E-126	452	-600	Indica	Japonica	
maker-9-augustus-gene-78.30-mRNA-1	9	OsR498G0917019700.01.T04	99,77	0,00E+00	1555	Os09g0299200	100,00	0,00E+00	1564	9	NA	Japonica	
maker-9-augustus-gene-78.32-mRNA-1	9	OsR498G0917017000.01.T01	99,94	0,00E+00	2880	Os09g0298500	100,00	0,00E+00	2884	4	NA	Japonica	
maker-9-augustus-gene-78.34-mRNA-1	9	OsR498G0917018000.01.T02	98,50	0,00E+00	5337	Os09g0298700	98,72	0,00E+00	5369	32	NA	Japonica	
maker-9-augustus-gene-78.36-mRNA-1	9	OsR498G0917020200.01.T01	99,20	0,00E+00	2427	Os09g0299400	99,92	0,00E+00	2135	-292	Indica	Japonica	
maker-9-augustus-gene-78.39-mRNA-1	9	OsR498G0917021600.01.T03	96,40	0,00E+00	2040	Os07g0296200	89,42	0,00E+00	1686	-354	Indica	Japonica	
maker-9-augustus-gene-8.26-mRNA-1	9	OsR498G0916666100.01.T02	99,81	0,00E+00	1858	Os09g0108400	100,00	0,00E+00	1867	9	NA	Japonica	
maker-9-augustus-gene-8.29-mRNA-1	9	OsR498G1018169000.01.T01	71,96	3,51E-137	490	Os09g0108600	100,00	0,00E+00	1546	1056	Japonica	Japonica	
maker-9-augustus-gene-82.25-mRNA-1	9	OsR498G0917038800.01.T01	89,31	0,00E+00	1104	Os09g0304400	100,00	0,00E+00	989	-115	Indica	Japonica	
maker-9-augustus-gene-83.31-mRNA-1	9	OsR498G0917044700.01.T01	100,00	0,00E+00	834	Os09g0306400	100,00	0,00E+00	834	0	NA	Japonica	
maker-9-augustus-gene-84.13-mRNA-1	9	OsR498G0917046000.01.T05	100,00	0,00E+00	953	Os09g0306650	99,62	0,00E+00	937	-16	NA	NA	
maker-9-augustus-gene-84.26-mRNA-1	9	OsR498G0917046800.01.T01	96,36	0,00E+00	2993	Os09g0306800	96,38	0,00E+00	3004	11	NA	Japonica	
maker-9-augustus-gene-84.7-mRNA-1	9	OsR498G0917047700.01.T03	100,00	6,60E-140	497	Os09g0307300	99,64	9,76E-139	492	-5	NA	NA	
maker-9-augustus-gene-85.22-mRNA-1	9	OsR498G0917051400.01.T03	99,27	0,00E+00	1219	Os09g0307800	99,27	0,00E+00	1219	0	NA	NA	
maker-9-augustus-gene-86.10-mRNA-1	9	OsR498G0917060600.01.T01	99,91	0,00E+00	3929	Os09g0309100	99,91	0,00E+00	3929	0	NA	NA	
maker-9-augustus-gene-86.3-mRNA-1	9	OsR498G0917061100.01.T02	99,70	0,00E+00	2347	Os09g0309200	99,70	0,00E+00	2347	0	NA	NA	
maker-9-augustus-gene-86.6-mRNA-1	9	OsR498G0917060400.01.T03	91,90	0,00E+00	1447	Os09g0308900	91,90	0,00E+00	1447	0	NA	NA	
maker-9-augustus-gene-87.4-mRNA-1	9	OsR498G0917079600.01.T01	97,78	0,00E+00	5598	Os09g0313500	97,81	0,00E+00	5603	5	NA	NA	CC-NBS-TM
maker-9-augustus-gene-88.29-mRNA-1	9	OsR498G0917083100.01.T01	100,00	0,00E+00	2062	Os09g0314300	100,00	0,00E+00	2062	0	NA	NA	
maker-9-augustus-gene-88.44-mRNA-1	9	OsR498G0917090600.01.T01	98,94	0,00E+00	5130	Os08g0366000	85,57	0,00E+00	3333	-1797	Indica	NA	
maker-9-augustus-gene-88.47-mRNA-1	9	OsR498G0917093200.01.T03	99,93	0,00E+00	2539	Os09g0315800	99,93	0,00E+00	2539	0	NA	NA	
maker-9-augustus-gene-88.51-mRNA-1	9	OsR498G0917094200.01.T02	99,72	0,00E+00	644	Os04g0151900	70,38	5,23E-36	151	-493	Indica	NA	
maker-9-augustus-gene-89.42-mRNA-1	9	OsR498G0917109300.01.T01	99,72	0,00E+00	2538	Os09g0317600	99,63	0,00E+00	2929	391	Japonica	NA	
maker-9-augustus-gene-9.0-mRNA-1	9	OsR498G0916670300.01.T01	99,75	0,00E+00	707	Os09g0109600	100,00	7,67E-115	412	-295	Indica	Japonica	
maker-9-augustus-gene-9.10-mRNA-1	9	OsR498G0916670000.01.T05	86,92	1,29E-149	530	Os09g0109500	86,83	1,91E-148	525	-5	NA	Japonica	
maker-9-augustus-gene-9.16-mRNA-1	9	OsR498G0916673800.01.T01	99,71	0,00E+00	3696	Os09g0110200	99,95	0,00E+00	3718	22	NA	Japonica	
maker-9-augustus-gene-9.18-mRNA-1	9	OsR498G0916674400.01.T01	99,50	0,00E+00	1433	Os09g0110300	100,00	0,00E+00	1451	18	NA	Japonica	
maker-9-augustus-gene-9.8-mRNA-1	9	OsR498G0916673100.01.T01	99,83	0,00E+00	4284	Os09g0110100	100,00	0,00E+00	4302	18	NA	Japonica	Kinase-LRR-TM
maker-9-augustus-gene-90.13-mRNA-1	9	OsR498G0917118200.01.T02	100,00	0,00E+00	2857	Os09g0318600	100,00	0,00E+00	2857	0	NA	Indica	
maker-9-augustus-gene-92.0-mRNA-1	9	OsR498G0407643700.01.T01	72,53	6,36E-61	235	Os09g0319800	100,00	0,00E+00	1251	1016	Japonica	NA	
maker-9-augustus-gene-92.2-mRNA-1	9	OsR498G0917128900.01.T01	88,89	4,94E-56	218	Os02g0571100	71,94	7,83E-23	106	-112	Indica	Japonica	
maker-9-augustus-gene-93.0-mRNA-1	9	OsR498G0917137100.01.T01	100,00	0,00E+00	2847	Os08g0369000	81,46	0,00E+00	997	-1850	Indica	Japonica	
maker-9-augustus-gene-93.5-mRNA-1	9	OsR498G0917140600.01.T01	99,88	0,00E+00	2922	Os09g0321200	97,65	0,00E+00	3069	147	NA	Japonica	
maker-9-augustus-gene-94.22-mRNA-1	9	OsR498G0917147300.01.T02	99,82	0,00E+00	992	Os09g0321900	100,00	0,00E+00	996	4	NA	Japonica	
maker-9-augustus-gene-94.27-mRNA-1	9	OsR498G0917148400.01.T02	99,24	0,00E+00	3256	Os09g0322000	100,00	0,00E+00	2607	-649	Indica	Japonica	
maker-9-augustus-gene-95.0-mRNA-1	9	OsR498G0917149700.01.T01	99,88	0,00E+00	2892	Os09g0322800	100,00	0,00E+00	4787	1895	Japonica	Japonica	CC-LRR-NBS-TM
maker-9-augustus-gene-95.15-mRNA-1	9	OsR498G0917151200.01.T05	97,26	0,00E+00	1300	Os09g0323000	97,26	0,00E+00	1300	0	NA	Japonica	
maker-9-augustus-gene-95.4-mRNA-1	9	OsR498G0917150300.01.T01	84,07	1,81E-120	432	Os09g0322900	100,00	6,27E-159	558	126	Japonica	Japonica	
maker-9-augustus-gene-96.59-mRNA-1	9	OsR498G0917153500.01.T01	99,70	0,00E+00	1767	Os09g0323700	100,00	0,00E+00	1781	14	NA	Japonica	
maker-9-augustus-gene-96.61-mRNA-1	9	OsR498G0917156900.01.T02	99,93	0,00E+00	2647	Os09g0325220	100,00	0,00E+00	1456	-1191	Indica	Japonica	
maker-9-augustus-gene-96.67-mRNA-1	9	OsR498G0917152800.01.T01	100,00	0,00E+00	1445	Os09g0323500	100,00	0,00E+00	1445	0	NA	Japonica	
maker-9-augustus-gene-96.72-mRNA-1	9	OsR498G0917154200.01.T01	99,90	0,00E+00	1776	Os09g0323900	100,00	0,00E+00	1797	21	NA	Japonica	
maker-9-augustus-gene-96.74-mRNA-1	9	OsR498G0917156400.01.T04	100,00	0,00E+00	883	Os09g0325140	100,00	0,00E+00	1061	178	Japonica	Japonica	Kinase-TM
maker-9-augustus-gene-97.48-mRNA-1	9	OsR498G0917161200.01.T01	98,76	0,00E+00	5075	Os09g0326100	100,00	0,00E+00	5238	163	NA	Japonica	Kinase-LRR-TM
maker-9-augustus-gene-98.25-mRNA-1	9	OsR498G0917161900.01.T01	100,00	0,00E+00	1456	Os09g0327000	100,00	0,00E+00	1431	-25	NA	Japonica	
maker-9-augustus-gene-98.28-mRNA-1	9	OsR498G0917162500.01.T01	99,24	0,00E+00	2101	Os09g0327300	100,00	0,00E+00	2143	42	NA	Japonica	
maker-9-augustus-gene-98.33-mRNA-1	9	OsR498G0917162200.01.T01	100,00	0,00E+00	1175	Os09g0327100	100,00	0,00E+00	1175	0	NA	Japonica	
maker-9-augustus-gene-98.35-mRNA-1	9	OsR498G0917162400.01.T02	98,80	0,00E+00	3793	Os09g0327200	100,00	0,00E+00	4004	211	NA	Japonica	
maker-9-augustus-gene-98.39-mRNA-1	9	OsR498G0917162800.01.T03	99,69	0,00E+00	1718	Os09g0327400	100,00	0,00E+00	1732	14	NA	Japonica	
maker-9-augustus-gene-99.4-mRNA-1	9	OsR498G0917164800.01.T01	75,94	0,00E+00	996	Os09g0328600	100,00	0,00E+00	3415	2419	Japonica	NA	
augustus_masked-10-processed-gene-1.26-mRNA-1	10	OsR498G0713353000.01.T01	90,72	0,00E+00	1331	Os10g0100700	100,00	0,00E+00	1694	363	Japonica	Indica	
augustus_masked-10-processed-gene-104.39-mRNA-1	10	OsR498G1120366000.01.T05	72,00	1,22E-34	148	Os10g0427900	100,00	4,80E-180	628	480	Japonica	NA	
augustus_masked-10-processed-gene-107.8-mRNA-1	10	OsR498G1018774700.01.T01	70,66	1,19E-116	422	Os10g0434200	99,90	0,00E+00	1706	1284	Japonica	Japonica	
augustus_masked-10-processed-gene-112.18-mRNA-1	10	OsR498G1018804000.01.T01	99,69	0,00E+00	2905	Os10g0444700	99,14	0,00E+00	2868	-37	NA	Indica	

augustus_masked-10-processed-gene-13.18-mRNA-1	10	OsR498G1018044000.01.T01	98,83	0,00E+00	1797	Os10g0114500	100,00	0,00E+00	1847	50	NA	Japonica	
augustus_masked-10-processed-gene-13.19-mRNA-1	10	OsR498G1018044000.01.T01	98,83	0,00E+00	1797	Os10g0114500	100,00	0,00E+00	1847	50	NA	Japonica	
augustus_masked-10-processed-gene-14.23-mRNA-1	10	OsR498G1018044000.01.T01	89,63	0,00E+00	840	Os10g0115500	100,00	0,00E+00	1862	1022	Japonica	Japonica	
augustus_masked-10-processed-gene-154.16-mRNA-1	10	OsR498G1018825800.01.T06	90,12	4,61E-155	547	Os10g0450900	99,76	0,00E+00	759	212	Japonica	NA	
augustus_masked-10-processed-gene-155.25-mRNA-1	10	OsR498G1018827600.01.T01	73,96	1,59E-68	260	Os10g0451700	73,96	5,52E-69	260	0	NA	Indica	
augustus_masked-10-processed-gene-155.26-mRNA-1	10	OsR498G1018828000.01.T01	71,43	1,33E-29	132	Os10g0451900	87,12	2,09E-110	398	266	Japonica	NA	
augustus_masked-10-processed-gene-155.27-mRNA-1	10	OsR498G1018828000.01.T01	72,77	4,00E-27	123	Os10g0451700	73,84	3,06E-42	171	48	Japonica	NA	
augustus_masked-10-processed-gene-155.29-mRNA-1	10	OsR498G1018828200.01.T01	72,54	4,62E-42	173	Os10g0452300	72,89	1,96E-41	168	-5	NA	Japonica	
augustus_masked-10-processed-gene-155.30-mRNA-1	10	OsR498G1018828400.01.T01	72,60	6,69E-40	166	Os10g0452201	72,89	3,46E-38	158	-8	NA	Japonica	
augustus_masked-10-processed-gene-155.31-mRNA-1	10	OsR498G1018828200.01.T01	72,89	5,70E-41	168	Os10g0452300	76,06	2,76E-52	205	37	Japonica	NA	
augustus_masked-10-processed-gene-155.32-mRNA-1	10	OsR498G1018828400.01.T01	71,08	2,27E-33	143	Os10g0452400	71,67	6,49E-35	148	5	NA	NA	
augustus_masked-10-processed-gene-155.33-mRNA-1	10	OsR498G1018829200.01.T01	97,19	0,00E+00	1375	Os10g0452500	97,44	0,00E+00	1384	9	NA	NA	
augustus_masked-10-processed-gene-156.31-mRNA-1	10	OsR498G1018832400.01.T01	99,66	0,00E+00	1036	Os10g0453900	99,66	0,00E+00	1036	0	NA	Japonica	
augustus_masked-10-processed-gene-156.34-mRNA-1	10	OsR498G1018834200.01.T01	99,66	0,00E+00	1041	Os10g0454300	99,83	0,00E+00	1046	5	NA	NA	
augustus_masked-10-processed-gene-156.35-mRNA-1	10	OsR498G1018834800.01.T01	98,96	0,00E+00	1012	Os10g0454500	99,83	0,00E+00	1035	23	NA	Japonica	
augustus_masked-10-processed-gene-156.36-mRNA-1	10	OsR498G1018835400.01.T01	99,31	0,00E+00	1027	Os10g0454700	99,83	0,00E+00	1040	13	NA	NA	
augustus_masked-10-processed-gene-156.5-mRNA-1	10	OsR498G1018829600.01.T01	99,83	0,00E+00	1040	Os10g0452700	99,66	0,00E+00	1036	-4	NA	Japonica	
augustus_masked-10-processed-gene-156.8-mRNA-1	10	OsR498G1018830200.01.T01	99,47	0,00E+00	1015	Os10g0452900	99,65	0,00E+00	1020	5	NA	Indica	
augustus_masked-10-processed-gene-156.9-mRNA-1	10	OsR498G1018830800.01.T01	99,66	0,00E+00	1041	Os10g0453000	100,00	0,00E+00	1050	9	NA	NA	
augustus_masked-10-processed-gene-157.36-mRNA-1	10	OsR498G1018835400.01.T01	93,58	0,00E+00	824	Os10g0455000	99,82	0,00E+00	974	150	Japonica	Japonica	
augustus_masked-10-processed-gene-157.37-mRNA-1	10	OsR498G1018836000.01.T01	96,17	0,00E+00	937	Os10g0455000	88,72	0,00E+00	746	-191	Indica	Japonica	
augustus_masked-10-processed-gene-157.38-mRNA-1	10	OsR498G1018836200.01.T01	97,91	0,00E+00	980	Os10g0455100	100,00	0,00E+00	1034	54	NA	Japonica	
augustus_masked-10-processed-gene-159.40-mRNA-1	10	OsR498G1018848000.01.T01	99,84	0,00E+00	3331	Os10g0459700	99,03	0,00E+00	3283	-48	NA	Indica	
augustus_masked-10-processed-gene-160.49-mRNA-1	10	OsR498G1018854400.01.T03	100,00	0,00E+00	740	Os10g0462300	99,42	1,04E-176	618	-122	Indica	NA	
augustus_masked-10-processed-gene-163.36-mRNA-1	10	OsR498G1018866600.01.T02	100,00	0,00E+00	845	Os10g0466200	99,79	0,00E+00	840	-5	NA	NA	
augustus_masked-10-processed-gene-168.21-mRNA-1	10	OsR498G1018896800.01.T01	99,66	0,00E+00	3162	Os10g0474966	90,43	0,00E+00	2248	-914	Indica	Japonica	
augustus_masked-10-processed-gene-169.20-mRNA-1	10	OsR498G1018901200.01.T01	99,83	0,00E+00	3233	Os10g0476900	100,00	0,00E+00	3247	14	NA	Japonica	
augustus_masked-10-processed-gene-169.21-mRNA-1	10	OsR498G1018901800.01.T01	99,64	0,00E+00	4494	Os10g0477200	100,00	0,00E+00	3788	-706	Indica	Japonica	
augustus_masked-10-processed-gene-17.31-mRNA-1	10	OsR498G1018069800.01.T01	97,72	0,00E+00	1266	Os10g0123300	99,78	0,00E+00	1647	381	Japonica	NA	Kinase-TM
augustus_masked-10-processed-gene-170.36-mRNA-1	10	OsR498G1018908200.01.T01	99,42	0,00E+00	2443	Os10g0478400	100,00	0,00E+00	2479	36	NA	Japonica	
augustus_masked-10-processed-gene-173.8-mRNA-1	10	OsR498G1018923400.01.T01	97,36	7,72E-164	577	Os10g0481300	100,00	0,00E+00	634	57	NA	Japonica	
augustus_masked-10-processed-gene-174.59-mRNA-1	10	OsR498G1018936200.01.T02	99,66	0,00E+00	3106	Os10g0484300	100,00	0,00E+00	3133	27	NA	Japonica	
augustus_masked-10-processed-gene-176.10-mRNA-1	10	OsR498G1018946600.01.T01	99,73	0,00E+00	1994	Os10g0487400	100,00	0,00E+00	1651	-343	Indica	Japonica	
augustus_masked-10-processed-gene-177.58-mRNA-1	10	OsR498G1018955800.01.T02	99,60	0,00E+00	1801	Os10g0489800	100,00	0,00E+00	948	-853	Indica	Japonica	
augustus_masked-10-processed-gene-178.7-mRNA-1	10	OsR498G1018957600.01.T01	99,28	0,00E+00	1224	Os10g0490900	100,00	0,00E+00	975	-249	Indica	Japonica	
augustus_masked-10-processed-gene-179.66-mRNA-1	10	OsR498G1018971000.01.T01	99,62	0,00E+00	1403	Os10g0494100	100,00	0,00E+00	1416	13	NA	Japonica	
augustus_masked-10-processed-gene-18.40-mRNA-1	10	OsR498G1018071200.01.T02	94,19	0,00E+00	1298	Os10g0124300	100,00	0,00E+00	1521	223	Japonica	Japonica	CC-NBS
augustus_masked-10-processed-gene-180.3-mRNA-1	10	OsR498G1018978200.01.T01	97,40	0,00E+00	3962	Os10g0495200	99,96	0,00E+00	4232	270	NA	Japonica	
augustus_masked-10-processed-gene-185.4-mRNA-1	10	OsR498G1019007500.01.T01	78,11	5,07E-70	264	Os10g0505500	100,00	6,13E-146	515	251	Japonica	Japonica	
augustus_masked-10-processed-gene-187.32-mRNA-1	10	OsR498G1019016200.01.T01	99,69	0,00E+00	1155	Os10g0508700	100,00	0,00E+00	1164	9	NA	Japonica	
augustus_masked-10-processed-gene-187.33-mRNA-1	10	OsR498G1019017000.01.T01	98,41	2,42E-121	435	Os10g0508900	100,00	0,00E+00	883	448	Japonica	Japonica	
augustus_masked-10-processed-gene-187.59-mRNA-1	10	OsR498G1019022800.01.T01	99,65	0,00E+00	2017	Os10g0510300	100,00	0,00E+00	2035	18	NA	Japonica	
augustus_masked-10-processed-gene-188.32-mRNA-1	10	OsR498G1019024000.01.T01	96,83	0,00E+00	635	Os10g0510500	100,00	0,00E+00	682	47	NA	Japonica	
augustus_masked-10-processed-gene-189.16-mRNA-1	10	OsR498G1019030200.01.T01	99,11	0,00E+00	792	Os10g0512500	100,00	0,00E+00	812	20	NA	Japonica	
augustus_masked-10-processed-gene-190.20-mRNA-1	10	OsR498G1019034400.01.T01	96,45	0,00E+00	2754	Os10g0514600	100,00	0,00E+00	2933	179	NA	Japonica	
augustus_masked-10-processed-gene-195.52-mRNA-1	10	OsR498G1019068000.01.T01	99,59	0,00E+00	2191	Os10g0523700	100,00	0,00E+00	2214	23	NA	Japonica	
augustus_masked-10-processed-gene-195.55-mRNA-1	10	OsR498G1019071400.01.T01	99,69	0,00E+00	4081	Os10g0524600	100,00	0,00E+00	4112	31	NA	Japonica	
augustus_masked-10-processed-gene-25.18-mRNA-1	10	OsR498G1018108800.01.T01	95,89	0,00E+00	1783	Os10g0134500	85,71	0,00E+00	1414	-369	Indica	Japonica	
augustus_masked-10-processed-gene-25.20-mRNA-1	10	OsR498G1018109000.01.T02	97,62	0,00E+00	1352	Os10g0136400	99,88	0,00E+00	1434	82	NA	Japonica	
augustus_masked-10-processed-gene-26.16-mRNA-1	10	OsR498G1018109800.01.T01	95,57	0,00E+00	1447	Os10g0136900	98,67	0,00E+00	1579	132	NA	Japonica	
augustus_masked-10-processed-gene-26.17-mRNA-1	10	OsR498G1018110000.01.T01	98,47	0,00E+00	1933	Os10g0137000	99,82	0,00E+00	2001	68	NA	Japonica	
augustus_masked-10-processed-gene-27.36-mRNA-1	10	OsR498G1018115600.01.T01	99,44	0,00E+00	2231	Os10g0139300	99,36	0,00E+00	2226	-5	NA	NA	
augustus_masked-10-processed-gene-27.39-mRNA-1	10	OsR498G1018117600.01.T01	99,69	0,00E+00	2849	Os10g0139700	99,81	0,00E+00	2860	11	NA	Indica	
augustus_masked-10-processed-gene-28.25-mRNA-1	10	OsR498G1018118600.01.T01	100,00	0,00E+00	1040	Os10g0140700	98,80	0,00E+00	1020	-20	NA	Indica	
augustus_masked-10-processed-gene-28.27-mRNA-1	10	OsR498G1018119400.01.T01	99,81	0,00E+00	1851	Os10g0140300	99,90	0,00E+00	1856	5	NA	Indica	
augustus_masked-10-processed-gene-30.28-mRNA-1	10	OsR498G1018134000.01.T02	99,73	0,00E+00	2012	Os10g0145100	99,82	0,00E+00	2017	5	NA	Japonica	
augustus_masked-10-processed-gene-30.29-mRNA-1	10	OsR498G1018134400.01.T02	99,74	0,00E+00	2030	Os10g0145200	99,74	0,00E+00	2030	0	NA	NA	
augustus_masked-10-processed-gene-31.37-mRNA-1	10	OsR498G1018144800.01.T02	99,95	0,00E+00	3572	Os10g0147900	99,60	0,00E+00	3541	-31	NA	Indica	
augustus_masked-10-processed-gene-36.28-mRNA-1	10	OsR498G1018162400.01.T01	95,30	0,00E+00	1557	Os10g0154500	98,16	0,00E+00	1683	126	NA	NA	Kinase-TM
augustus_masked-10-processed-gene-36.6-mRNA-1	10	OsR498G1018157400.01.T01	99,72	0,00E+00	1924	Os10g0152000	99,67	0,00E+00	1607	-317	Indica	Japonica	Kinase-TM
augustus_masked-10-processed-gene-36.7-mRNA-1	10	OsR498G1018157400.01.T01	99,10	0,00E+00	1559	Os10g0152000	99,55	0,00E+00	1577	18	NA	NA	
augustus_masked-10-processed-gene-38.31-mRNA-1	10	OsR498G1018162400.01.T01	84,14	0,00E+00	974	Os10g0157400	95,45	0,00E+00	1551	577	Japonica	Indica	Kinase
augustus_masked-10-processed-gene-39.19-mRNA-1	10	OsR498G1018179200.01.T01	98,36	0,00E+00	2874	Os10g0158100	98,05	0,00E+00	2752	-122	NA	Indica	
augustus_masked-10-processed-gene-41.11-mRNA-1	10	OsR498G1018192000.01.T01	100,00	0,00E+00	2145	Os10g0160100	99,75	0,00E+00	2131	-14	NA	Indica	
augustus_masked-10-processed-gene-45.15-mRNA-1	10	OsR498G1018221800.01.T01	99,63	0,00E+00	3878	Os02g0156400	84,69	0,00E+00	2331	-1547	Indica	Japonica	LRR-TM
augustus_masked-10-processed-gene-46.19-mRNA-1	10	OsR498G1018230000.01.T01	99,31	2,17E-144	511	Os10g0163201	88,82	3,45E-54	211	-300	Indica	Japonica	
augustus_masked-10-processed-gene-5.20-mRNA-1	10	OsR498G1018012400.01.T01	100,00	0,00E+00	3112	Os10g0104800	98,90	0,00E+00	3038	-74	NA	Indica	Kinase-TM
augustus_masked-10-processed-gene-51.21-mRNA-1	10	OsR498G1018251600.01.T01	100,00	0,00E+00	818	Os11g0708500	94,91	2,72E-121	434	-384	Indica	Indica	
augustus_masked-10-processed-gene-54.31-mRNA-1	10	OsR498G1018205800.01.T02	75,22	4,56E-80	299	Os10g0174800	98,42	0,00E+00	879	580	Japonica	Indica	Kinase
augustus_masked-10-processed-gene-57.18-mRNA-1	10	OsR498G1018274000.01.T03	97,87	4,43E-134	477	Os03g0658800	73,77	2,99E-23	107	-370	Indica	NA	
augustus_masked-10-processed-gene-63.36-mRNA-1	10	OsR498G1018312800.01.T01	99,20	0,00E+00	879	Os10g0188800	100,00	0,00E+00	926	47	NA	Japonica	
augustus_masked-10-processed-gene-75.28-mRNA-1	10	OsR498G1018354200.01.T01	99,90	0,00E+00	1711	Os10g0200700	99,90	0,00E+00	1711	0	NA	Japonica	
augustus_masked-10-processed-gene-87.19-mRNA-1	10	OsR498G1018399800.01.T01	99,70	0,00E+00	1198	Os10g0213700	99,80	0,00E+00	884	-314	Indica	Indica	
augustus-10-processed-gene-1.21-mRNA-1	10	OsR498G1017996700.01.T01	99,96	0,00E+00	4427	Os10g0101000	98,74	0,00E+00	4294	-133	NA	Indica	Kinase-TM
augustus-10-processed-gene-104.0-mRNA-1	10	OsR498G1018759900.01.T02	94,67	0,00E+00	1732	Os10g0427800	80,76	0,00E+00	979	-753	Indica	NA	
augustus-10-processed-gene-104.20-mRNA-1	10	OsR498G1018762300.01.T01	98,90	0,00E+00	1916	Os10g0428500	89,69	0,00E+00	1437	-479	Indica	Japonica	
augustus-10-processed-gene-104.22-mRNA-1	10	OsR498G1018762100.01.T01	79,42	0,00E+00	988	Os10g0428900	100,00	0,00E+00	1948	960	Japonica	NA	
augustus-10-processed-gene-105.68-mRNA-1	10	OsR498G1018764700.01.T01	99,22	0,00E+00	901	Os10g0430950	100,00	0,00E+00	915	14	NA	Japonica	
augustus-10-processed-gene-107.3-mRNA-1	10	OsR498G1018774700.01.T01	99,33	0,00E+00	2107	Os10g0435400	70,92	1,63E-116	419	-1688	Indica	Japonica	
augustus-10-processed-gene-108.15-mRNA-1	10	OsR498G1018781100.01.T02	97,28	0,00E+00	1308	Os03g0748500	70,90	2,73E-92	339	-969	Indica	Japonica	
augustus-10-processed-gene-108.17-mRNA-1	10	OsR498G1018781700.01.T01	99,17	0,00E+00	3173	Os10g0436900	99,94	0,00E+00	3232	59	NA	Japonica	
augustus-10-processed-gene-109.50-mRNA-1	10	OsR498G1018789500.01.T01	99,22	0,00E+00	678	Os10g0438800	99,75	0,00E+00	721	43	NA	Japonica	
augustus-10-processed-gene-111.13-mRNA-1	10	OsR498G1018800100.01.T02	99,93	0,00E+00	2669	Os10g0442700	98,18	0,00E+00	2552	-117	NA	Indica	
augustus-10-processed-gene-111.2-mRNA-1	10	OsR498G1018798100.01.T01	99,62	0,00E+00	3795	Os10g0442000	99,77	0,00E+00	3809	14	NA	Japonica	Kinase-TM
augustus-10-processed-gene-12.15-mRNA-1	10	OsR498G1018038100.01.T01	100,00	0,00E+00	1743	Os10g0113000	99,69	0,00E+00	1729	-14	NA	Indica	
augustus-10-processed-gene-157.47-mRNA-1	10	OsR498G1018837500.01.T01	99,79	0,00E+00	3445	Os10g0456400	100,00	0,00E+00	3463	18	NA	Japonica	
augustus-10-processed-gene-159.29-mRNA-1	10	OsR498G1018847300.01.T01	99,78	0,00E+00	3186	Os10g0459600	98,93	0,00E+00	3118	-68	NA	Indica	
augustus-10-processed-gene-162.0-mRNA-1	10	OsR498G1018861500.01.T01	100,00	0,00E+00	682	Os10g0463900	100,00	0,00E+00	682	0	NA	NA	
augustus-10-processed-gene-162.20-mRNA-1	10	OsR498G1018865300.01.T01	99,44	0,00E+00	2860	Os10g0465700	99,50	0,00E+00	2865	5	NA	NA	
augustus-10-processed-gene-168.11-mRNA-1	10	OsR498G1018898300.01.T01	99,60	0,00E+00	1763	Os10g0475900	100,00	0,00E+00	1781	18	NA	Japonica	
augustus-10-processed-gene-168.12-mRNA-1	10	OsR498G1018898500.01.T03	98,84	0,00E+00	1517	Os10g0476000	100,00	0,00E+00	1559	42	NA	Japonica	
augustus-10-processed-gene-17.15-mRNA-1	10	OsR498G1018066900.01.T01	97,79	0,00E+00	2163	Os10g0122500	97,86	0,00E+00	2786	623	Japonica	NA	
augustus-10-processed-gene-17.2-mRNA-1	10	OsR498G1018063500.01.T01	99,66	0,00E+00	2645	Os10g0122000	99,53	0,00E+00	2636	-9	NA	NA	
augustus-10-processed-gene-172.0-mRNA-1	10	OsR498G1018914700.01.T01	99,43	0,00E+00	3728	Os10g0479900	100,00	0,00E+00	2012	-1716	Indica	Japonica	
augustus-10-processed-gene-172.5-mRNA-1	10	OsR498G1018918700.01.T01	100,00	0,00E+00	942	Os07g0693100	83,62	7,43E-85	313	-629	Indica	Japonica	
augustus-10-processed-gene-177.26-mRNA-1	10	OsR498G1018950500.01.T02	99,84	0,00E+00	3280	Os10g0488900	99,90	0,00E+00	1839	-1441	Indica	Japonica	
augustus-10-processed-gene-179.42-mRNA-1	10	OsR498G1018966500.01.T04	100,00	0,00E+00	1736	Os10g0493600	100,00	0,00E+00	1736	0	NA	Japonica	
augustus-10-processed-gene-181.75-mRNA-1	10	OsR498G1018975500.01.T02	94,62	0,00E+00	755	Os10g0495000	94,41	0,00E+00	751	-4	NA	Japonica	
augustus-10-processed-gene-182.45-mRNA-1	10	OsR498G1018987100.01.T01	99,80	0,00E+00	885	Os10g0498500	100,00	0,00E+00	890	5	NA	Japonica	
augustus-10-processed-gene-182.59-mRNA-1	10	OsR498G1018991500.01.T01	99,46	0,00E+00	2319	Os10g0499500	100,00	0,00E+00	2351	32	NA	Japonica	
augustus-10-processed-gene-182.61-mRNA-1	10	OsR498G1018978200.01.T01	93,57	5,93E-166	584	Os10g0499800	100,00	0,00E+00	715	131	Japonica	Japonica	
augustus-10-processed-gene-183.9-mRNA-1	10	OsR498G1018999100.01.T01	100,00	3,43E-135	480	Os10g0502100	100,00	8,07E-138	488	8	NA	Japonica	
augustus-10-processed-gene-184.39-mRNA-1	10	OsR498G1019001800.01.T04	99,71	0,00E+00	1841	Os10g0503200	99,90	0,00E+00	1850	9	NA	Japonica	
augustus-10-processed-gene-185.13-mRNA-1	10	OsR498G1019008700.01.T01	99,73	0,00E+00	2021	Os10g0506000	100,00	0,00E+00	2749	728	Japonica	Japonica	
augustus-10-processed-gene-186.1-mRNA-1	10	OsR498G1019011500.01.T02	93,29	0,00E+00	1126	Os10g0507500	99,87	0,00E+00	1343	217	Japonica	Japonica	
augustus-10-processed-gene-186.15-mRNA-1	10	OsR498G1019014300.01.T02	98,94	0,00E+00	2481	Os10g0508100	100,00	0,00E+00	2549	68	NA	Japonica	
augustus-10-processed-gene-188.30-mRNA-1	10	OsR498G1019023100.01.T04	99,79	0,00E+00	3418	Os10g0510400	99,95	0,00E+00	3432	14	NA	Japonica	
augustus-10-processed-gene-188.39-mRNA-1	10	OsR498G1019027100.01.T02	99,81	0,00E+00	1838	Os10g0511400	100,00	0,00E+00	1842	4	NA	Japonica	
augustus-10-processed-gene-189.23-mRNA-1	10	OsR498G1019032900.01.T03	97,25	0,00E+00	2324	Os10g0513300	100,00	0,00E+00	2452	128	NA	Japonica	
augustus-10-processed-gene-189.24-mRNA-1	10	OsR498G1019033100.01.T01	97,63	0,00E+00	2722	Os10g0513550	100,00	0,00E+00	1867	-855	Indica	Japonica	
augustus-10-processed-gene-190.0-mRNA-1	10	OsR498G1019033100.01.T01	85,10	0,00E+00	1799	Os10g0513900	100,00	0,00E+00	2819	1020	Japonica	Japonica	
augustus-10-processed-gene-190.2-mRNA-1	10	OsR498G1019033500.01.T01	99,03	0,00E+00	1806	Os10g0513900	81,06	0,00E+00	977	-829	Indica	Japonica	
augustus-10-processed-gene-190.27-mRNA-1	10	OsR498G1019034900.01.T01	98,41	0,00E+00	2721	Os10g0515200	100,00	0,00E+00	2836	115	NA	Japonica	
augustus-10-processed-gene-190.3-mRNA-1	10	OsR498G1019033500.01.T01	98,46	1,56E-159	563	Os10g0513900	87,65	9,20E-113	405	-158	Indica	Japonica	
augustus-10-processed-gene-190.30-mRNA-1	10	OsR498G1019035500.01.T01	98,78	0,00E+00	2706	Os10g0515900	100,00	0,00E+00	2798	92	NA	Japonica	
augustus-10-processed-gene-192.22-mRNA-1	10	OsR498G1019053500.01.T01	99,09	0,00E+00	2331	Os10g0519800	100,00	0,00E+00	2381	50	NA	Japonica	
augustus-10-processed-gene-193.32-mRNA-1	10	OsR498G1019053700.01.T01	99,52	0,00E+00	1850	Os10g0520100	100,00	0,00E+00	2197	347	Japonica	Japonica	
augustus-10-processed-gene-193.33-mRNA-1	10	OsR498G1019054500.01.T01	94,80	0,00E+00	1764	Os10g0520400	100,00	0,00E+00	2013	249	Japonica	Japonica	
augustus-10-processed-gene-195.38-mRNA-1	10	OsR498G1019069700.01.T01	100,00	5,87E-165	580	Os10g0524300	100,00	1,13E-168	590	10	NA	Japonica	
augustus-10-processed-gene-20.32-mRNA-1	10	OsR498G1018085500.01.T04	100,00	4,35E-173	607	Os10g0128400	100,00	1,24E-155	547	-60	NA	Japonica	
augustus-10-processed-gene-21.31-mRNA-1	10	OsR498G0203391400.01.T01	78,45	0,00E+00	656	Os10g0130200	100,00	0,00E+00	1948	1292	Japonica	Japonica	
augustus-10-processed-gene-22.0-mRNA-1	10	OsR498G1018101100.01.T01	88,94	0,00E+00	848	Os10g0131000	100,00	0,00E+00	1137	289	Japonica	Japonica	
augustus-10-processed-gene-23.14-mRNA-1	10	OsR498G1018102300.01.T01	97,45	0,00E+00	2451	Os10g0133500	86,83	0,00E+00	1781	-670	Indica	Japonica	
augustus-10-processed-gene-24.12-mRNA-1	10	OsR498G1018104100.01.T01	99,58	0,00E+00	2531	Os10g0134400	88,22	0,00E+00	1251	-1280	Indica	Japonica	
augustus-10-processed-gene-24.14-mRNA-1	10	OsR498G1018104900.01.T01	90,45	0,00E+00	1997	Os10g0134700	99,93	0,00E+00	2615	618	Japonica	Japonica	
augustus-10-processed-gene-24.15-mRNA-1	10	OsR498G1018105500.01.T01	98,36	0,00E+00	844	Os10g0135100	90,39	0,00E+00	668	-176	Indica	Japonica	
augustus-10-processed-gene-24.16-mRNA-1	10	OsR498G1018105900.01.T01	99,52	0,00E+00	2575	Os10g0135100	88,29	0,00E+00	1828	-747	Indica	Japonica	
augustus-10-processed-gene-24.19-mRNA-1	10	OsR498G1018107100.01.T03	99,68	0,00E+00	1699	Os10g0135300	100,00	0,00E+00	2251	552	Japonica	Japonica	
augustus-10-processed-gene-25.0-mRNA-1	10	OsR498G1018107700.01.T05	99,83	0,00E+00	2085	Os10g0135400	100,00	0,00E+00	2094	9	NA	Japonica	
augustus-10-processed-gene-25.2-mRNA-1	10	OsR498G1018108500.01.T01	99,20	0,00E+00	1324	Os10g0135600	99,60	0,00E+00	1338	14	NA	Japonica	
augustus-10-processed-gene-27.20-mRNA-1	10	OsR498G1018116300.01.T01	99,00	0,00E+00	1406	Os10g0137700	72,59	2,89E-130	464	-942	Indica	NA	
augustus-10-processed-gene-28.44-mRNA-1	10	OsR498G1018122500.01.T01	99,35	0,00E+00	1086	Os10g0123200	72,82	3,49E-39	161	-925	Indica	NA	
augustus-10-processed-gene-29.2-mRNA-1	10	OsR498G1018125100.01.T01	97,90	0,00E+00	2116	Os10g0142500	97,72	0,00E+00	2014	-102	NA	NA	
augustus-10-processed-gene-29.3-mRNA-1	10	OsR498G1018125300.01.T01	93,06	0,00E+00	2917	Os10g0142600	92,80	0,00E+00	2896	-21	NA	Indica	Kinase
augustus-10-processed-gene-3.2-mRNA-1	10	OsR498G1018003900.01.T01	100,00	0,00E+00	921	Os01g0524500	85,10	4,28E-132	471	-450	Indica	Indica	
augustus-10-processed-gene-31.30-mRNA-1	10	OsR498G1018144900.01.T01	100,00	1,38E-179	628	Os10g0148000	99,71	2,04E-178	624	-4	NA	Indica	
augustus-10-processed-gene-34.19-mRNA-1	10	OsR498G1018151700.01.T01	92,13	3,23E-105	381	Os10g0150200	92,52	9,22E-107	386	5	NA	Indica	
augustus-10-processed-gene-35.7-mRNA-1	10	OsR498G1018156300.01.T01	99,45	0,00E+00	3207	Os10g0151100	94,52	0,00E+00	2810	-397	Indica	Japonica	Kinase-TM
augustus-10-processed-gene-37.10-mRNA-1	10	OsR498G1018168100.01.T01	100,00	0,00E+00	2057	Os10g0155300	99,83	0,00E+00	2048	-9	NA	Indica	
augustus-10-processed-gene-38.5-mRNA-1	10	OsR498G1018161300.01.T01	83,10	0,00E+00	948	Os10g0157400	96,04	0,00E+00	1599	651	Japonica	NA	Kinase-TM
augustus-10-processed-gene-39.13-mRNA-1	10	OsR498G1018182700.01.T01	99,67	0,00E+00	2163	Os10g0158700	99,84	0,00E+00	2177	14	NA	NA	
augustus-10-processed-gene-46.22-mRNA-1	10	OsR498G1018228800.01.T01	100,00	0,00E+00	1321	Os10g0163201	70,33	1,57E-63	243	-1078	Indica	Japonica	
augustus-10-processed-gene-47.0-mRNA-1	10	OsR498G1018230300.01.T01	99,67	0,00E+00	1601	Os10g0164600	98,84	0,00E+00	1655	54	NA	Indica	
augustus-10-processed-gene-47.2-mRNA-1	10	OsR498G1018231300.01.T01	100,00	0,00E+00	2192	Os10g0165000	99,84	0,00E+00	2183	-9	NA	Indica	
augustus-10-processed-gene-49.26-mRNA-1	10	OsR498G1018238900.01.T02	99,21	0,00E+00	1574	Os10g0166600	88,83	0,00E+00	1148	-426	Indica	Indica	
augustus-10-processed-gene-50.23-mRNA-1	10	OsR498G1018246900.01.T01	100,00	5,75E-165	580	Os10g0169200	100,00	5,03E-154	542	-38	NA	Indica	
augustus-10-processed-gene-51.30-mRNA-1	10	OsR498G1018251900.01.T01	99,75	0,00E+00	1402	Os12g0435200	77,17	3,46E-167	588	-814	Indica	Indica	
augustus-10-processed-gene-51.42-mRNA-1	10	OsR498G1018303700.01.T01	72,00	9,53E-44	178	Os10g0171400	96,03	0,00E+00	737	559	Japonica	Indica	
augustus-10-processed-gene-53.2-mRNA-1	10	OsR498G1018256500.01.T02	100,00	0,00E+00	715	Os10g0173000	93,00	1,93E-179	627	-88	Indica	Indica	
augustus-10-processed-gene-61.1-mRNA-1	10	OsR498G1018303700.01.T01	99,56	0,00E+00	1618	Os10g0351300	72,05	3,07E-152	539	-1079	Indica	NA	
augustus-10-processed-gene-64.22-mRNA-1	10	OsR498G1018319700.01.T01	100,00	0,00E+00	996	Os10g0190000	99,78	0,00E+00	801	-195	Indica	NA	
augustus-10-processed-gene-65.7-mRNA-1	10	OsR498G1018325900.01.T01	99,80	0,00E+00	1810	Os10g0191900	99,90	0,00E+00	1814	4	NA	NA	
augustus-10-processed-gene-7.10-mRNA-1	10	OsR498G1018024500.01.T01	100,00	0,00E+00	2425	Os10g0108700	81,20	0,00E+00	1272	-1153	Indica	Indica	
augustus-10-processed-gene-77.12-mRNA-1	10	OsR498G1018361500.01.T01	99,64	0,00E+00	982	Os03g0845100	83,06	0,00E+00	726	-256	Indica	Japonica	
augustus-10-processed-gene-80.22-mRNA-1	10	OsR498G1018382300.01.T01	99,69	0,00E+00	5826	Os11g0692100	80,81	0,00E+00	3018	-2808	Indica	NA	Kinase-LRR-TM
augustus-10-processed-gene-9.12-mRNA-1	10	OsR498G1018033700.01.T01	100,00	0,00E+00	2035	Os10g0152000	79,70	0,00E+00	1801	-234	Indica	NA	Kinase-TM
augustus-10-processed-gene-94.21-mRNA-1	10	OsR498G1018411500.01.T01	99,66	3,88E-148	525	Os05g0267732	87,32	2,14E-95	347	-178	Indica	Japonica	
augustus-10-processed-gene-98.1-mRNA-1	10	OsR498G1018420100.01.T01	99,57	0,00E+00	2869	Os10g0317900	99,63	0,00E+00	2874	5	NA	NA	
maker-10-augustus-gene-1.0-mRNA-1	10	OsR498G1017994900.01.T04	100,00	0,00E+00	3984	Os10g0100500	99,78	0,00E+00	2447	-1537	Indica	Indica	CC-Kinase-TM
maker-10-augustus-gene-105.10-mRNA-1	10	OsR498G1018763700.01.T01	100,00	0,00E+00	1061	Os10g0430700	100,00	0,00E+00	1061	0	NA	Japonica	
maker-10-augustus-gene-105.30-mRNA-1	10	OsR498G1018764400.01.T03	99,46	0,00E+00	4974	Os10g0430900	99,96	0,00E+00	5026	52	NA	Japonica	Kinase-TM
maker-10-augustus-gene-105.4-mRNA-1	10	OsR498G1018763500.01.T01	99,49	0,00E+00	1400	Os10g0430600	100,00	0,00E+00	1418	18	NA	Japonica	
maker-10-augustus-gene-105.9-mRNA-1	10	OsR498G1018763700.01.T03	99,55	3,77E-109	395	Os10g0430700	100,00	3,10E-111	399	4	NA	Japonica	
maker-10-augustus-gene-106.0-mRNA-1	10	OsR498G1018765300.01.T01	99,49	0,00E+00	4902	Os10g0431000	100,00	0,00E+00	2562	-2340	Indica	Japonica	
maker-10-augustus-gene-106.13-mRNA-1	10	OsR498G1018768400.01.T02	99,83	0,00E+00	2053	Os10g0431900	99,91	0,00E+00	2058	5	NA	Japonica	Kinase
maker-10-augustus-gene-106.16-mRNA-1	10	OsR498G1018769200.01.T03	96,54	0,00E+00	1155	Os10g0432300	97,38	0,00E+00	1170	15	NA	Japonica	
maker-10-augustus-gene-106.7-mRNA-1	10	OsR498G1018765500.01.T01	98,73	0,00E+00	829	Os03g0159600	72,79	1,93E-66	251	-578	Indica	Japonica	
maker-10-augustus-gene-108.44-mRNA-1	10	OsR498G1018782300.01.T01	99,84	0,00E+00	2269	Os10g0437000	100,00	0,00E+00	2278	9	NA	Japonica	
maker-10-augustus-gene-108.48-mRNA-1	10	OsR498G1018782800.01.T02	99,73	0,00E+00	3349	Os10g0437100	100,00	0,00E+00	3371	22	NA	Japonica	
maker-10-augustus-gene-109.0-mRNA-1	10	OsR498G1018785700.01.T01	99,63	0,00E+00	976	Os10g0437500	100,00	0,00E+00	985	9	NA	Japonica	
maker-10-augustus-gene-109.10-mRNA-1	10	OsR498G1018788500.01.T01	99,75	0,00E+00	1415	Os10g0438500	99,62	0,00E+00	1410	-5	NA	NA	
maker-10-augustus-gene-109.12-mRNA-1	10	OsR498G1018789100.01.T02	100,00	9,98E-149	526	Os10g0438700	100,00	3,47E-149	526	0	NA	NA	
maker-10-augustus-gene-109.14-mRNA-1	10	OsR498G1018790700.01.T01	99,74	0,00E+00	1377	Os03g0428700	85,91	0,00E+00	839	-538	Indica	NA	
maker-10-augustus-gene-109.16-mRNA-1	10	OsR498G1018787000.01.T01	98,46	0,00E+00	1583	Os10g0438000	98,02	0,00E+00	1565	-18	NA	Japonica	
maker-10-augustus-gene-109.19-mRNA-1	10	OsR498G1018787600.01.T01	98,17	0,00E+00	2259	Os10g0438300	99,37	0,00E+00	2819	560	Japonica	Japonica	
maker-10-augustus-gene-109.2-mRNA-1	10	OsR498G1018786300.01.T05	97,75	0,00E+00	3842	Os10g0437600	97,70	0,00E+00	3837	-5	NA	Japonica	
maker-10-augustus-gene-109.22-mRNA-1	10	OsR498G1018789000.01.T01	100,00	0,00E+00	1943	Os10g0438600	100,00	0,00E+00	1943	0	NA	NA	
maker-10-augustus-gene-109.24-mRNA-1	10	OsR498G1018791000.01.T01	99,87	0,00E+00	1397	Os10g0439200	98,71	0,00E+00	1361	-36	NA	NA	
maker-10-augustus-gene-109.6-mRNA-1	10	OsR498G1018786700.01.T01	99,28	0,00E+00	984	Os10g0437900	97,66	0,00E+00	941	-43	NA	NA	
maker-10-augustus-gene-109.8-mRNA-1	10	OsR498G1018787300.01.T01	99,70	0,00E+00	1187	Os10g0438100	100,00	0,00E+00	1196	9	NA	Japonica	
maker-10-augustus-gene-110.49-mRNA-1	10	OsR498G1018793600.01.T01	99,10	0,00E+00	2755	Os10g0440000	99,23	0,00E+00	2764	9	NA	NA	
maker-10-augustus-gene-110.50-mRNA-1	10	OsR498G1018793000.01.T01	99,37	0,00E+00	2794	Os10g0439800	99,73	0,00E+00	2691	-103	NA	NA	
maker-10-augustus-gene-110.60-mRNA-1	10	OsR498G1018794000.01.T02	99,86	0,00E+00	1237	Os10g0440100	99,71	0,00E+00	1232	-5	NA	NA	
maker-10-augustus-gene-111.36-mRNA-1	10	OsR498G1018797700.01.T01	96,89	0,00E+00	1293	Os10g0441800	97,67	0,00E+00	1322	29	NA	Japonica	
maker-10-augustus-gene-111.39-mRNA-1	10	OsR498G1018799900.01.T01	99,88	0,00E+00	4364	Os03g0151800	87,53	0,00E+00	3015	-1349	Indica	Indica	
maker-10-augustus-gene-111.45-mRNA-1	10	OsR498G1018800500.01.T01	99,86	0,00E+00	3910	Os10g0442900	97,13	0,00E+00	3830	-80	NA	Indica	
maker-10-augustus-gene-111.57-mRNA-1	10	OsR498G1018798800.01.T01	99,54	0,00E+00	2705	Os10g0442300	99,13	0,00E+00	1629	-1076	Indica	Indica	
maker-10-augustus-gene-112.34-mRNA-1	10	OsR498G1018801300.01.T01	99,85	0,00E+00	1197	Os10g0443200	97,74	0,00E+00	1510	313	Japonica	Indica	
maker-10-augustus-gene-112.37-mRNA-1	10	OsR498G1018801700.01.T01	99,13	5,85E-174	611	Os10g0443800	97,12	9,89E-166	581	-30	NA	Indica	
maker-10-augustus-gene-112.38-mRNA-1	10	OsR498G1018802500.01.T02	99,90	0,00E+00	1722	Os10g0444400	99,58	0,00E+00	1709	-13	NA	Indica	
maker-10-augustus-gene-113.37-mRNA-1	10	OsR498G1018810100.01.T02	100,00	0,00E+00	738	Os10g0445500	100,00	0,00E+00	738	0	NA	NA	
maker-10-augustus-gene-113.49-mRNA-1	10	OsR498G1018812900.01.T01	99,86	0,00E+00	1246	Os10g0446400	99,57	0,00E+00	1239	-7	NA	NA	
maker-10-augustus-gene-113.51-mRNA-1	10	OsR498G1018809400.01.T02	99,86	0,00E+00	1278	Os10g0445400	99,85	0,00E+00	1161	-117	NA	Japonica	
maker-10-augustus-gene-113.55-mRNA-1	10	OsR498G1018811400.01.T01	99,16	0,00E+00	1683	Os10g0446100	99,69	0,00E+00	2329	646	Japonica	Japonica	
maker-10-augustus-gene-12.38-mRNA-1	10	OsR498G1018036700.01.T02	100,00	0,00E+00	3009	Os10g0370700	70,05	1,88E-136	487	-2522	Indica	Indica	
maker-10-augustus-gene-12.42-mRNA-1	10	OsR498G1018038500.01.T01	100,00	0,00E+00	1748	Os10g0113100	99,90	0,00E+00	1744	-4	NA	Japonica	
maker-10-augustus-gene-12.44-mRNA-1	10	OsR498G1018036600.01.T02	100,00	0,00E+00	913	Os10g0112100	93,28	0,00E+00	777	-136	Indica	Indica	
maker-10-augustus-gene-12.48-mRNA-1	10	OsR498G1018037200.01.T01	99,89	0,00E+00	3178	Os10g0112600	98,76	0,00E+00	3090	-88	NA	Indica	
maker-10-augustus-gene-13.28-mRNA-1	10	OsR498G1018041500.01.T01	99,52	0,00E+00	1866	Os10g0113900	100,00	0,00E+00	1792	-74	NA	Japonica	
maker-10-augustus-gene-13.31-mRNA-1	10	OsR498G1018042100.01.T03	99,81	0,00E+00	1847	Os10g0114300	100,00	0,00E+00	1856	9	NA	Japonica	
maker-10-augustus-gene-14.0-mRNA-1	10	OsR498G1018044900.01.T01	99,37	5,28E-159	560	Os10g0115550	100,00	9,51E-157	551	-9	NA	Japonica	
maker-10-augustus-gene-14.7-mRNA-1	10	OsR498G1018045600.01.T03	97,82	0,00E+00	2435	Os10g0115600	100,00	0,00E+00	2511	76	NA	Japonica	
maker-10-augustus-gene-15.10-mRNA-1	10	OsR498G1018051200.01.T01	99,72	0,00E+00	3201	Os10g0116900	99,89	0,00E+00	3216	15	NA	Japonica	
maker-10-augustus-gene-15.14-mRNA-1	10	OsR498G1018051400.01.T01	99,51	0,00E+00	1818	Os03g0239000	77,88	0,00E+00	666	-1152	Indica	NA	
maker-10-augustus-gene-15.19-mRNA-1	10	OsR498G1018056400.01.T01	99,18	0,00E+00	1946	Os10g0118200	99,27	0,00E+00	1950	4	NA	Japonica	
maker-10-augustus-gene-15.3-mRNA-1	10	OsR498G1018050700.01.T01	99,50	0,00E+00	1796	Os10g0116800	99,01	0,00E+00	1773	-23	NA	NA	
maker-10-augustus-gene-15.5-mRNA-1	10	OsR498G1018054500.01.T02	98,92	0,00E+00	2111	Os10g0117800	97,50	0,00E+00	2050	-61	NA	Japonica	
maker-10-augustus-gene-156.0-mRNA-1	10	OsR498G1018829400.01.T03	99,69	0,00E+00	1737	Os10g0452800	99,90	0,00E+00	1746	9	NA	NA	
maker-10-augustus-gene-157.12-mRNA-1	10	OsR498G1018837200.01.T01	92,19	0,00E+00	815	Os10g0456200	92,19	0,00E+00	815	0	NA	Japonica	LRR
maker-10-augustus-gene-157.24-mRNA-1	10	OsR498G1018838600.01.T01	99,70	0,00E+00	1198	Os10g0456600	100,00	0,00E+00	1207	9	NA	Japonica	
maker-10-augustus-gene-158.24-mRNA-1	10	OsR498G1018839900.01.T02	100,00	0,00E+00	1927	Os10g0457000	100,00	0,00E+00	1683	-244	Indica	Japonica	
maker-10-augustus-gene-158.29-mRNA-1	10	OsR498G1018841300.01.T01	97,05	0,00E+00	1829	Os10g0457400	97,32	0,00E+00	1843	14	NA	Japonica	

maker-10-augustus-gene-158.32-mRNA-1	10	OsR498G1018841900.01.T01	100,00	0,00E+00	2500	Os10g0457600	99,86	0,00E+00	2491	-9	NA	NA	
maker-10-augustus-gene-158.39-mRNA-1	10	OsR498G1018842700.01.T02	99,86	0,00E+00	6326	Os10g0457700	99,63	0,00E+00	6288	-38	NA	Indica	
maker-10-augustus-gene-158.46-mRNA-1	10	OsR498G1018839000.01.T03	94,11	0,00E+00	1341	Os10g0456800	94,35	0,00E+00	1350	9	NA	Japonica	
maker-10-augustus-gene-158.65-mRNA-1	10	OsR498G1018840200.01.T01	99,53	0,00E+00	2631	Os10g0457200	99,29	0,00E+00	2232	-399	Indica	NA	
maker-10-augustus-gene-158.69-mRNA-1	10	OsR498G1018841600.01.T01	92,68	0,00E+00	920	Os10g0457500	93,04	0,00E+00	955	35	NA	Japonica	
maker-10-augustus-gene-159.0-mRNA-1	10	OsR498G1018845900.01.T03	99,77	0,00E+00	1573	Os10g0458700	99,77	0,00E+00	1573	0	NA	NA	
maker-10-augustus-gene-16.50-mRNA-1	10	OsR498G1018061000.01.T01	99,03	0,00E+00	5819	Os10g0119200	99,25	0,00E+00	5695	-124	NA	Japonica	Kinase-LRR-TM
maker-10-augustus-gene-160.0-mRNA-1	10	OsR498G1018849700.01.T01	99,16	0,00E+00	1057	Os10g0460500	99,24	0,00E+00	1632	575	Japonica	NA	
maker-10-augustus-gene-160.14-mRNA-1	10	OsR498G1018854400.01.T02	98,96	0,00E+00	1686	Os10g0462300	99,37	0,00E+00	1696	10	NA	NA	
maker-10-augustus-gene-160.6-mRNA-1	10	OsR498G1018850800.01.T01	95,98	0,00E+00	1874	Os10g0461100	95,14	0,00E+00	1396	-478	Indica	Japonica	
maker-10-augustus-gene-160.9-mRNA-1	10	OsR498G1018853400.01.T01	99,54	0,00E+00	771	Os10g0461100	99,77	0,00E+00	783	12	NA	Japonica	
maker-10-augustus-gene-161.40-mRNA-1	10	OsR498G1018856700.01.T01	98,29	0,00E+00	3045	Os10g0462900	98,17	0,00E+00	3036	-9	NA	Indica	
maker-10-augustus-gene-161.46-mRNA-1	10	OsR498G0305217400.01.T06	81,87	0,00E+00	958	Os10g0463100	99,90	0,00E+00	1838	880	Japonica	NA	
maker-10-augustus-gene-161.48-mRNA-1	10	OsR498G1018857700.01.T01	100,00	0,00E+00	2116	Os10g0463200	99,83	0,00E+00	2107	-9	NA	NA	
maker-10-augustus-gene-161.50-mRNA-1	10	OsR498G1018857900.01.T01	99,20	0,00E+00	1098	Os10g0463300	99,52	0,00E+00	1107	9	NA	NA	
maker-10-augustus-gene-162.49-mRNA-1	10	OsR498G1018864100.01.T01	99,36	2,22E-157	554	Os05g0112000	97,07	3,75E-111	400	-154	Indica	NA	
maker-10-augustus-gene-162.53-mRNA-1	10	OsR498G1018865700.01.T02	97,00	0,00E+00	2078	Os10g0465900	93,75	0,00E+00	1101	-977	Indica	Japonica	
maker-10-augustus-gene-162.59-mRNA-1	10	OsR498G1018861400.01.T04	99,54	0,00E+00	1953	Os10g0463800	99,73	0,00E+00	1962	9	NA	NA	
maker-10-augustus-gene-162.63-mRNA-1	10	OsR498G1018861800.01.T01	100,00	0,00E+00	1586	Os10g0464000	99,89	0,00E+00	1581	-5	NA	NA	
maker-10-augustus-gene-162.65-mRNA-1	10	OsR498G1018862000.01.T04	99,38	0,00E+00	3448	Os10g0464100	98,87	0,00E+00	3425	-23	NA	Japonica	
maker-10-augustus-gene-162.69-mRNA-1	10	OsR498G1018862400.01.T05	99,46	0,00E+00	1657	Os10g0464400	99,89	0,00E+00	1675	18	NA	Japonica	
maker-10-augustus-gene-162.76-mRNA-1	10	OsR498G1018862600.01.T02	98,99	0,00E+00	3482	Os10g0464500	99,17	0,00E+00	3837	355	NA	NA	
maker-10-augustus-gene-162.78-mRNA-1	10	OsR498G1018863400.01.T04	99,81	0,00E+00	2876	Os10g0465000	99,68	0,00E+00	1686	-1190	Indica	Indica	
maker-10-augustus-gene-162.84-mRNA-1	10	OsR498G1018865600.01.T01	99,80	0,00E+00	889	Os10g0465800	100,00	0,00E+00	893	4	NA	NA	
maker-10-augustus-gene-162.86-mRNA-1	10	OsR498G1018866000.01.T03	99,64	0,00E+00	3486	Os10g0466000	99,88	0,00E+00	3011	-475	Indica	Japonica	
maker-10-augustus-gene-163.13-mRNA-1	10	OsR498G1018869700.01.T01	99,84	0,00E+00	1116	Os10g0467600	100,00	0,00E+00	1121	5	NA	Indica	
maker-10-augustus-gene-163.16-mRNA-1	10	OsR498G1018869900.01.T01	99,88	0,00E+00	5739	Os10g0467800	99,75	0,00E+00	5721	-18	NA	Indica	
maker-10-augustus-gene-163.19-mRNA-1	10	OsR498G1018866800.01.T01	99,87	0,00E+00	2825	Os10g0466300	99,87	0,00E+00	2825	0	NA	NA	
maker-10-augustus-gene-163.24-mRNA-1	10	OsR498G1018867600.01.T01	100,00	0,00E+00	764	Os10g0466700	100,00	0,00E+00	764	0	NA	Japonica	
maker-10-augustus-gene-163.28-mRNA-1	10	OsR498G1018870200.01.T01	99,28	0,00E+00	5891	Os10g0467900	99,51	0,00E+00	5069	-822	Indica	Indica	Kinase-LRR-TM
maker-10-augustus-gene-164.25-mRNA-1	10	OsR498G1018872900.01.T01	96,79	0,00E+00	6041	Os10g0468500	96,93	0,00E+00	6064	23	NA	Japonica	Kinase-LRR-TM
maker-10-augustus-gene-164.27-mRNA-1	10	OsR498G1018875100.01.T01	100,00	0,00E+00	3290	Os10g0469900	98,90	0,00E+00	3200	-90	NA	Indica	
maker-10-augustus-gene-164.29-mRNA-1	10	OsR498G1018872900.01.T01	88,25	0,00E+00	1865	Os10g0469600	89,20	0,00E+00	2647	782	Japonica	Indica	LRR-TM
maker-10-augustus-gene-165.0-mRNA-1	10	OsR498G1018878300.01.T02	99,62	0,00E+00	3264	Os10g0470700	99,29	0,00E+00	3249	-15	NA	Japonica	
maker-10-augustus-gene-165.7-mRNA-1	10	OsR498G1018879700.01.T01	99,86	0,00E+00	2507	Os10g0470900	99,86	0,00E+00	2507	0	NA	NA	
maker-10-augustus-gene-165.9-mRNA-1	10	OsR498G1018880100.01.T01	99,49	0,00E+00	5255	Os03g0138600	73,14	0,00E+00	987	-4268	Indica	NA	
maker-10-augustus-gene-166.15-mRNA-1	10	OsR498G1018883200.01.T03	99,77	0,00E+00	3917	Os10g0471400	100,00	0,00E+00	3939	22	NA	Japonica	
maker-10-augustus-gene-166.17-mRNA-1	10	OsR498G1018884800.01.T01	94,18	0,00E+00	978	Os10g0471700	94,66	0,00E+00	992	14	NA	Japonica	
maker-10-augustus-gene-166.4-mRNA-1	10	OsR498G1018881800.01.T01	99,27	0,00E+00	2175	Os10g0471100	100,00	0,00E+00	2215	40	NA	Japonica	
maker-10-augustus-gene-167.0-mRNA-1	10	OsR498G1018887500.01.T01	99,44	0,00E+00	1574	Os10g0472400	99,90	0,00E+00	1728	154	NA	Japonica	
maker-10-augustus-gene-167.13-mRNA-1	10	OsR498G1018890800.01.T03	96,57	0,00E+00	4666	Os10g0473400	96,68	0,00E+00	4680	14	NA	Japonica	
maker-10-augustus-gene-167.19-mRNA-1	10	OsR498G1018893600.01.T01	99,51	0,00E+00	3277	Os10g0474800	100,00	0,00E+00	3328	51	NA	Japonica	
maker-10-augustus-gene-167.20-mRNA-1	10	OsR498G1018893600.01.T01	92,79	0,00E+00	636	Os10g0474900	99,80	0,00E+00	889	253	Japonica	Japonica	
maker-10-augustus-gene-167.3-mRNA-1	10	OsR498G1018889900.01.T01	99,93	0,00E+00	2517	Os10g0472900	100,00	0,00E+00	2522	5	NA	Japonica	
maker-10-augustus-gene-167.5-mRNA-1	10	OsR498G1018892300.01.T01	99,73	0,00E+00	8770	Os10g0474500	99,94	0,00E+00	8815	45	NA	Japonica	CC-LRR-NBS-TM
maker-10-augustus-gene-167.6-mRNA-1	10	OsR498G1018892700.01.T01	99,57	0,00E+00	821	Os10g0474600	100,00	0,00E+00	1564	743	Japonica	Japonica	
maker-10-augustus-gene-168.35-mRNA-1	10	OsR498G1018894000.01.T01	87,36	0,00E+00	788	Os10g0474933	92,11	0,00E+00	1049	261	Japonica	Japonica	
maker-10-augustus-gene-168.44-mRNA-1	10	OsR498G1018898700.01.T01	99,58	0,00E+00	2957	Os10g0476100	98,14	0,00E+00	2979	22	NA	Japonica	Kinase-TM
maker-10-augustus-gene-168.50-mRNA-1	10	OsR498G1018894000.01.T01	98,89	0,00E+00	3144	Os10g0474300	100,00	0,00E+00	3489	345	NA	Japonica	
maker-10-augustus-gene-168.53-mRNA-1	10	OsR498G1018896600.01.T01	99,74	0,00E+00	4091	Os10g0474966	100,00	0,00E+00	4118	27	NA	Japonica	
maker-10-augustus-gene-169.27-mRNA-1	10	OsR498G1018899100.01.T01	99,73	0,00E+00	1957	Os10g0476300	99,91	0,00E+00	1966	9	NA	Japonica	Kinase
maker-10-augustus-gene-169.30-mRNA-1	10	OsR498G1018900500.01.T03	97,25	0,00E+00	2843	Os10g0476600	97,31	0,00E+00	2847	4	NA	Japonica	
maker-10-augustus-gene-169.35-mRNA-1	10	OsR498G1018901300.01.T01	99,02	0,00E+00	4303	Os10g0477000	99,22	0,00E+00	4325	22	NA	Japonica	
maker-10-augustus-gene-169.38-mRNA-1	10	OsR498G1018901500.01.T05	99,95	0,00E+00	3340	Os10g0477100	100,00	0,00E+00	3344	4	NA	Japonica	
maker-10-augustus-gene-169.43-mRNA-1	10	OsR498G1018899600.01.T02	100,00	0,00E+00	1153	Os10g0476400	100,00	0,00E+00	1153	0	NA	Japonica	
maker-10-augustus-gene-17.37-mRNA-1	10	OsR498G1018069300.01.T01	96,33	0,00E+00	801	Os10g0123200	100,00	0,00E+00	879	78	NA	Japonica	
maker-10-augustus-gene-17.38-mRNA-1	10	OsR498G1018067200.01.T01	99,28	0,00E+00	3183	Os10g0122600	99,22	0,00E+00	3178	-5	NA	NA	
maker-10-augustus-gene-170.0-mRNA-1	10	OsR498G1018903500.01.T01	99,18	0,00E+00	1723	Os10g0477600	100,00	0,00E+00	1759	36	NA	Japonica	
maker-10-augustus-gene-170.10-mRNA-1	10	OsR498G1018906500.01.T01	99,90	0,00E+00	1722	Os10g0478200	100,00	0,00E+00	1727	5	NA	Japonica	
maker-10-augustus-gene-170.15-mRNA-1	10	OsR498G1018904200.01.T01	99,47	0,00E+00	1677	Os04g0630100	70,08	7,57E-107	387	-1290	Indica	Japonica	
maker-10-augustus-gene-170.17-mRNA-1	10	OsR498G1018905000.01.T01	97,77	0,00E+00	1075	Os10g0478000	100,00	0,00E+00	1110	35	NA	Japonica	
maker-10-augustus-gene-170.18-mRNA-1	10	OsR498G1018906000.01.T05	99,82	0,00E+00	972	Os10g0478100	100,00	0,00E+00	975	3	NA	Japonica	
maker-10-augustus-gene-170.24-mRNA-1	10	OsR498G1018907600.01.T01	99,76	0,00E+00	1490	Os10g0478300	100,00	0,00E+00	1499	9	NA	Japonica	
maker-10-augustus-gene-171.0-mRNA-1	10	OsR498G1018910500.01.T01	99,45	0,00E+00	977	Os10g0478900	100,00	1,24E-145	515	-462	Indica	Japonica	
maker-10-augustus-gene-171.10-mRNA-1	10	OsR498G1018913100.01.T03	94,23	0,00E+00	1840	Os10g0479700	94,57	0,00E+00	1858	18	NA	Japonica	
maker-10-augustus-gene-171.22-mRNA-1	10	OsR498G1018910000.01.T05	97,99	0,00E+00	766	Os10g0478600	100,00	0,00E+00	807	41	NA	Japonica	
maker-10-augustus-gene-171.3-mRNA-1	10	OsR498G1018911300.01.T01	99,86	0,00E+00	3753	Os10g0479200	100,00	0,00E+00	4295	542	Japonica	Japonica	
maker-10-augustus-gene-171.7-mRNA-1	10	OsR498G1018912700.01.T01	99,84	0,00E+00	1105	Os10g0479500	100,00	0,00E+00	1110	5	NA	Japonica	
maker-10-augustus-gene-172.21-mRNA-1	10	OsR498G1018916700.01.T06	99,70	0,00E+00	4738	Os10g0480200	100,00	0,00E+00	4551	-187	NA	Japonica	
maker-10-augustus-gene-172.27-mRNA-1	10	OsR498G1018917600.01.T02	99,81	0,00E+00	2773	Os10g0480500	100,00	0,00E+00	2787	14	NA	Japonica	
maker-10-augustus-gene-173.42-mRNA-1	10	OsR498G1018923100.01.T01	97,42	0,00E+00	857	Os10g0481100	100,00	0,00E+00	888	31	NA	Japonica	
maker-10-augustus-gene-173.44-mRNA-1	10	OsR498G1018924900.01.T01	98,49	0,00E+00	2535	Os10g0481500	99,93	0,00E+00	2626	91	NA	Japonica	
maker-10-augustus-gene-173.46-mRNA-1	10	OsR498G1018923600.01.T02	100,00	0,00E+00	654	Os10g0481400	100,00	1,63E-129	461	-193	Indica	Japonica	
maker-10-augustus-gene-173.54-mRNA-1	10	OsR498G1018930000.01.T01	99,80	0,00E+00	2676	Os10g0481500	74,89	0,00E+00	723	-1953	Indica	Japonica	
maker-10-augustus-gene-173.56-mRNA-1	10	OsR498G1018931000.01.T01	99,73	0,00E+00	2688	Os10g0481500	78,19	0,00E+00	1221	-1467	Indica	Japonica	
maker-10-augustus-gene-173.58-mRNA-1	10	OsR498G1018931200.01.T01	99,43	0,00E+00	3764	Os10g0482900	100,00	0,00E+00	3815	51	NA	Japonica	
maker-10-augustus-gene-174.0-mRNA-1	10	OsR498G1120115000.01.T01	89,42	0,00E+00	2448	Os10g0483400	100,00	0,00E+00	2100	-348	Indica	Japonica	Kinase-TM
maker-10-augustus-gene-174.14-mRNA-1	10	OsR498G1018932000.01.T01	100,00	0,00E+00	1308	Os10g0483100	100,00	0,00E+00	1308	0	NA	Japonica	
maker-10-augustus-gene-174.16-mRNA-1	10	OsR498G1018936400.01.T01	99,79	0,00E+00	1745	Os10g0484400	100,00	0,00E+00	1754	9	NA	Japonica	
maker-10-augustus-gene-174.18-mRNA-1	10	OsR498G1018937800.01.T01	99,84	0,00E+00	2226	Os10g0484700	100,00	0,00E+00	2235	9	NA	Japonica	
maker-10-augustus-gene-174.23-mRNA-1	10	OsR498G1018938800.01.T03	98,80	0,00E+00	5692	Os10g0485000	98,98	0,00E+00	5719	27	NA	Japonica	
maker-10-augustus-gene-174.27-mRNA-1	10	OsR498G1018939200.01.T03	99,76	0,00E+00	6739	Os10g0485100	100,00	0,00E+00	4632	-2107	Indica	Japonica	
maker-10-augustus-gene-174.3-mRNA-1	10	OsR498G1018934100.01.T02	99,94	0,00E+00	2853	Os10g0483500	100,00	0,00E+00	2857	4	NA	Japonica	
maker-10-augustus-gene-174.6-mRNA-1	10	OsR498G1018938300.01.T01	99,93	0,00E+00	2409	Os10g0484800	100,00	0,00E+00	2111	-298	Indica	Japonica	
maker-10-augustus-gene-174.9-mRNA-1	10	OsR498G1018931800.01.T03	100,00	0,00E+00	893	Os10g0483000	100,00	0,00E+00	893	0	NA	Japonica	
maker-10-augustus-gene-175.14-mRNA-1	10	OsR498G1018941500.01.T02	94,94	0,00E+00	1149	Os10g0486000	94,94	0,00E+00	1149	0	NA	Japonica	
maker-10-augustus-gene-175.19-mRNA-1	10	OsR498G1018945500.01.T01	100,00	0,00E+00	971	Os10g0486900	100,00	0,00E+00	971	0	NA	Japonica	
maker-10-augustus-gene-175.22-mRNA-1	10	OsR498G1018945900.01.T01	96,42	0,00E+00	1242	Os10g0487200	100,00	0,00E+00	791	-451	Indica	Japonica	
maker-10-augustus-gene-175.24-mRNA-1	10	OsR498G1018946300.01.T01	99,58	0,00E+00	3004	Os10g0487300	100,00	0,00E+00	3036	32	NA	Japonica	
maker-10-augustus-gene-175.30-mRNA-1	10	OsR498G1018941400.01.T01	97,93	0,00E+00	1408	Os10g0485900	100,00	0,00E+00	1472	64	NA	Japonica	
maker-10-augustus-gene-175.32-mRNA-1	10	OsR498G1018941800.01.T01	99,76	0,00E+00	3018	Os10g0486100	100,00	0,00E+00	3014	-4	NA	Japonica	
maker-10-augustus-gene-175.34-mRNA-1	10	OsR498G1018943600.01.T02	99,47	0,00E+00	1003	Os10g0486300	100,00	0,00E+00	1510	507	Japonica	Japonica	
maker-10-augustus-gene-175.6-mRNA-1	10	OsR498G1018940100.01.T01	100,00	0,00E+00	1997	Os10g0485500	100,00	0,00E+00	1997	0	NA	Japonica	
maker-10-augustus-gene-176.30-mRNA-1	10	OsR498G1018946700.01.T02	99,83	0,00E+00	1028	Os10g0487500	100,00	0,00E+00	1032	4	NA	Japonica	
maker-10-augustus-gene-176.33-mRNA-1	10	OsR498G1018946900.01.T04	99,32	0,00E+00	1829	Os10g0487600	99,90	0,00E+00	1852	23	NA	Japonica	
maker-10-augustus-gene-176.44-mRNA-1	10	OsR498G1018947300.01.T04	96,52	0,00E+00	1729	Os10g0488300	96,52	0,00E+00	1729	0	NA	Japonica	
maker-10-augustus-gene-177.0-mRNA-1	10	OsR498G1018951100.01.T01	99,88	0,00E+00	1556	Os10g0489100	100,00	0,00E+00	1561	5	NA	Japonica	
maker-10-augustus-gene-177.11-mRNA-1	10	OsR498G1018951400.01.T02	99,75	0,00E+00	4930	Os10g0489200	100,00	0,00E+00	4448	-482	NA	Japonica	
maker-10-augustus-gene-177.18-mRNA-1	10	OsR498G1018952600.01.T01	99,08	0,00E+00	2681	Os10g0489500	100,00	0,00E+00	2516	-165	NA	Japonica	
maker-10-augustus-gene-177.8-mRNA-1	10	OsR498G1018950800.01.T03	100,00	0,00E+00	1164	Os10g0489000	100,00	0,00E+00	1128	-36	NA	Japonica	
maker-10-augustus-gene-178.40-mRNA-1	10	OsR498G1018957900.01.T01	97,25	0,00E+00	1159	Os10g0491000	100,00	0,00E+00	1245	86	NA	Japonica	
maker-10-augustus-gene-178.42-mRNA-1	10	OsR498G1018960700.01.T01	99,67	1,44E-155	550	Os10g0491900	100,00	1,18E-157	554	4	NA	Japonica	
maker-10-augustus-gene-178.46-mRNA-1	10	OsR498G1018963100.01.T01	99,75	0,00E+00	1425	Os10g0492600	100,00	0,00E+00	1434	9	NA	Japonica	
maker-10-augustus-gene-178.64-mRNA-1	10	OsR498G1018962000.01.T03	98,61	0,00E+00	2640	Os10g0492200	100,00	0,00E+00	2711	71	NA	Japonica	
maker-10-augustus-gene-178.66-mRNA-1	10	OsR498G1018962600.01.T01	99,93	0,00E+00	2393	Os10g0492300	96,54	0,00E+00	2226	-167	NA	Japonica	
maker-10-augustus-gene-178.72-mRNA-1	10	OsR498G1018963400.01.T02	99,82	0,00E+00	1972	Os10g0492800	100,00	0,00E+00	1094	-878	Indica	Japonica	
maker-10-augustus-gene-179.15-mRNA-1	10	OsR498G0306568200.01.T03	89,67	0,00E+00	918	Os10g0494200	99,86	0,00E+00	1237	319	Japonica	Japonica	
maker-10-augustus-gene-179.18-mRNA-1	10	OsR498G1018972100.01.T02	99,54	0,00E+00	3874	Os10g0494300	100,00	0,00E+00	3918	44	NA	Japonica	
maker-10-augustus-gene-179.21-mRNA-1	10	OsR498G1018972500.01.T01	99,80	0,00E+00	2709	Os10g0494500	100,00	0,00E+00	2446	-263	NA	Japonica	
maker-10-augustus-gene-179.24-mRNA-1	10	OsR498G1018975500.01.T02	94,95	0,00E+00	1367	Os10g0495000	94,83	0,00E+00	1362	-5	NA	Japonica	
maker-10-augustus-gene-179.27-mRNA-1	10	OsR498G1018969000.01.T01	100,00	0,00E+00	1727	Os10g0494000	100,00	0,00E+00	1727	0	NA	Japonica	
maker-10-augustus-gene-179.33-mRNA-1	10	OsR498G1018972800.01.T04	99,61	0,00E+00	3613	Os10g0494800	100,00	0,00E+00	3649	36	NA	Japonica	
maker-10-augustus-gene-179.7-mRNA-1	10	OsR498G1018968500.01.T04	99,65	0,00E+00	1524	Os10g0493800	100,00	0,00E+00	1537	13	NA	Japonica	
maker-10-augustus-gene-18.0-mRNA-1	10	OsR498G1018070300.01.T01	97,48	2,12E-152	539	Os10g0124000	100,00	0,00E+00	688	149	Japonica	Japonica	
maker-10-augustus-gene-18.3-mRNA-1	10	OsR498G1018070700.01.T01	93,95	0,00E+00	1209	Os10g0124100	99,94	0,00E+00	2815	1606	Japonica	Japonica	
maker-10-augustus-gene-18.8-mRNA-1	10	OsR498G1018071800.01.T01	99,34	0,00E+00	2154	Os10g0124700	100,00	0,00E+00	2127	-27	NA	Japonica	
maker-10-augustus-gene-180.43-mRNA-1	10	OsR498G1018972900.01.T01	99,44	0,00E+00	948	Os10g0494950	100,00	0,00E+00	962	14	NA	Japonica	
maker-10-augustus-gene-180.46-mRNA-1	10	OsR498G1018975500.01.T01	99,91	0,00E+00	4120	Os10g0495000	100,00	0,00E+00	4129	9	NA	Japonica	
maker-10-augustus-gene-180.51-mRNA-1	10	OsR498G1018979500.01.T01	99,82	0,00E+00	992	Os04g0678200	71,89	1,63E-55	216	-776	Indica	Japonica	
maker-10-augustus-gene-180.53-mRNA-1	10	OsR498G1018982300.01.T01	99,34	0,00E+00	2143	Os10g0496900	99,89	0,00E+00	1558	-585	Indica	Japonica	
maker-10-augustus-gene-180.57-mRNA-1	10	OsR498G1018978600.01.T01	99,70	0,00E+00	4747	Os10g0495300	100,00	0,00E+00	5000	253	NA	Japonica	
maker-10-augustus-gene-180.58-mRNA-1	10	OsR498G1018978800.01.T02	98,47	0,00E+00	1934	Os10g0495400	100,00	0,00E+00	2004	70	NA	Japonica	
maker-10-augustus-gene-180.84-mRNA-1	10	OsR498G1018981000.01.T01	100,00	6,42E-89	327	Os10g0493600	81,87	2,24E-51	201	-126	Indica	Japonica	
maker-10-augustus-gene-181.0-mRNA-1	10	OsR498G1018982700.01.T05	98,13	0,00E+00	833	Os10g0497000	100,00	0,00E+00	870	37	NA	Japonica	
maker-10-augustus-gene-181.1-mRNA-1	10	OsR498G1018982900.01.T01	96,52	0,00E+00	1733	Os10g0497100	96,52	0,00E+00	1733	0	NA	Japonica	
maker-10-augustus-gene-181.2-mRNA-1	10	OsR498G1018983100.01.T03	98,45	0,00E+00	1902	Os10g0497300	100,00	0,00E+00	1979	77	NA	Japonica	
maker-10-augustus-gene-181.28-mRNA-1	10	OsR498G1018975500.01.T01	97,31	4,06E-141	500	Os10g0495000	97,31	1,41E-141	500	0	NA	Japonica	
maker-10-augustus-gene-181.34-mRNA-1	10	OsR498G1018985300.01.T01	99,61	0,00E+00	2736	Os10g0497600	99,80	0,00E+00	2750	14	NA	Japonica	Kinase-TM
maker-10-augustus-gene-181.39-mRNA-1	10	OsR498G1018985500.01.T04	99,63	0,00E+00	966	Os10g0497700	100,00	0,00E+00	975	9	NA	Japonica	
maker-10-augustus-gene-181.43-mRNA-1	10	OsR498G1018985700.01.T02	99,90	0,00E+00	1738	Os10g0497800	100,00	0,00E+00	1743	5	NA	Japonica	
maker-10-augustus-gene-181.47-mRNA-1	10	OsR498G1018985900.01.T02	99,34	0,00E+00	2140	Os10g0497900	99,55	0,00E+00	2398	258	Japonica	Japonica	
maker-10-augustus-gene-181.50-mRNA-1	10	OsR498G1018986700.01.T01	99,37	0,00E+00	1700	Os10g0498200	100,00	0,00E+00	1727	27	NA	Japonica	
maker-10-augustus-gene-181.53-mRNA-1	10	OsR498G1018986900.01.T01	99,17	0,00E+00	1692	Os10g0498300	99,50	0,00E+00	1780	88	NA	Japonica	
maker-10-augustus-gene-181.56-mRNA-1	10	OsR498G1018986200.01.T01	99,79	0,00E+00	1680	Os10g0498000	100,00	0,00E+00	1689	9	NA	Japonica	
maker-10-augustus-gene-182.16-mRNA-1	10	OsR498G1018991100.01.T01	99,66	0,00E+00	2071	Os10g0499400	100,00	0,00E+00	1496	-575	Indica	Japonica	
maker-10-augustus-gene-182.22-mRNA-1	10	OsR498G1018993300.01.T01	99,45	0,00E+00	1297	Os10g0500000	100,00	0,00E+00	1315	18	NA	Japonica	
maker-10-augustus-gene-182.27-mRNA-1	10	OsR498G1018993700.01.T01	99,76	0,00E+00	1480	Os10g0500500	100,00	0,00E+00	1489	9	NA	Japonica	
maker-10-augustus-gene-182.29-mRNA-1	10	OsR498G1018994500.01.T03	99,91	0,00E+00	2063	Os10g0500600	100,00	0,00E+00	2067	4	NA	Japonica	
maker-10-augustus-gene-182.3-mRNA-1	10	OsR498G1018988100.01.T04	99,68	0,00E+00	5034	Os10g0498700	100,00	0,00E+00	5074	40	NA	Japonica	
maker-10-augustus-gene-182.33-mRNA-1	10	OsR498G1018994500.01.T06	99,73	0,00E+00	2645	Os10g0500700	98,86	0,00E+00	1993	-652	Indica	Japonica	
maker-10-augustus-gene-182.35-mRNA-1	10	OsR498G1018995300.01.T01	84,72	0,00E+00	1025	Os10g0501000	100,00	0,00E+00	2446	1421	Japonica	Japonica	
maker-10-augustus-gene-182.37-mRNA-1	10	OsR498G1018988800.01.T01	97,80	0,00E+00	1718	Os10g0498900	97,90	0,00E+00	1722	4	NA	Japonica	
maker-10-augustus-gene-182.41-mRNA-1	10	OsR498G1018992400.01.T01	99,13	1,28E-173	609	Os08g0248700	70,53	1,91E-20	99,6	-509,4	Indica	Japonica	
maker-10-augustus-gene-182.43-mRNA-1	10	OsR498G1018993600.01.T01	99,43	0,00E+00	930	Os10g0500400	100,00	0,00E+00	964	34	NA	Japonica	
maker-10-augustus-gene-183.40-mRNA-1	10	OsR498G1018995300.01.T01	99,70	0,00E+00	2936	Os10g0501300	87,70	0,00E+00	1845	-1091	Indica	Japonica	
maker-10-augustus-gene-183.42-mRNA-1	10	OsR498G1018995500.01.T01	99,29	0,00E+00	2732	Os10g0501300	100,00	0,00E+00	2782	50	NA	Japonica	
maker-10-augustus-gene-183.44-mRNA-1	10	OsR498G1018997100.01.T01	99,11	0,00E+00	1784	Os10g0501500	100,00	0,00E+00	1824	40	NA	Japonica	
maker-10-augustus-gene-183.47-mRNA-1	10	OsR498G1018998500.01.T01	99,54	0,00E+00	1946	Os10g0501900	99,82	0,00E+00	1959	13	NA	Japonica	
maker-10-augustus-gene-183.49-mRNA-1	10	OsR498G1018998800.01.T01	99,86	0,00E+00	1278	Os10g0502000	100,00	0,00E+00	1283	5	NA	Japonica	
maker-10-augustus-gene-183.51-mRNA-1	10	OsR498G1019000200.01.T01	99,88	0,00E+00	2902	Os10g0502400	100,00	0,00E+00	2911	9	NA	Japonica	
maker-10-augustus-gene-183.53-mRNA-1	10	OsR498G1019000800.01.T01	99,68	0,00E+00	1117	Os10g0502500	100,00	0,00E+00	1126	9	NA	Japonica	
maker-10-augustus-gene-183.56-mRNA-1	10	OsR498G1019001200.01.T04	99,57	0,00E+00	1246	Os10g0502600	100,00	0,00E+00	1261	15	NA	Japonica	
maker-10-augustus-gene-183.58-mRNA-1	10	OsR498G1019001400.01.T02	99,69	0,00E+00	1753	Os10g0502900	100,00	0,00E+00	1743	-10	NA	Japonica	
maker-10-augustus-gene-183.61-mRNA-1	10	OsR498G0204401000.01.T01	76,95	2,97E-109	396	Os10g0503100	99,56	0,00E+00	1203	807	Japonica	Japonica	
maker-10-augustus-gene-184.0-mRNA-1	10	OsR498G1019003300.01.T01	99,79	0,00E+00	3342	Os10g0503500	100,00	0,00E+00	3360	18	NA	Japonica	
maker-10-augustus-gene-184.15-mRNA-1	10	OsR498G1019004000.01.T01	91,34	2,12E-149	530	Os10g0503600	91,34	7,35E-150	530	0	NA	Japonica	
maker-10-augustus-gene-184.19-mRNA-1	10	OsR498G1019004200.01.T01	99,20	0,00E+00	2645	Os10g0503700	99,87	0,00E+00	2670	25	NA	Japonica	
maker-10-augustus-gene-184.23-mRNA-1	10	OsR498G1019004800.01.T01	99,61	0,00E+00	922	Os02g0824500	71,03	3,34E-32	138	-784	Indica	Japonica	
maker-10-augustus-gene-184.25-mRNA-1	10	OsR498G1019005800.01.T01	99,86	0,00E+00	1246	Os10g0504200	100,00	0,00E+00	1251	5	NA	Japonica	
maker-10-augustus-gene-184.7-mRNA-1	10	OsR498G1019006300.01.T01	99,81	0,00E+00	4800	Os10g0504600	99,93	0,00E+00	4814	14	NA	Japonica	
maker-10-augustus-gene-185.37-mRNA-1	10	OsR498G1019008300.01.T01	99,48	0,00E+00	2388	Os10g0505900	100,00	0,00E+00	2419	31	NA	Japonica	
maker-10-augustus-gene-185.52-mRNA-1	10	OsR498G1019009000.01.T02	99,76	0,00E+00	732	Os10g0506100	100,00	0,00E+00	737	5	NA	Japonica	
maker-10-augustus-gene-185.54-mRNA-1	10	OsR498G1019010000.01.T02	98,90	0,00E+00	4125	Os10g0506800	99,96	0,00E+00	4238	113	NA	Japonica	
maker-10-augustus-gene-186.25-mRNA-1	10	OsR498G1019013500.01.T01	97,92	0,00E+00	3152	Os01g0100400	73,12	0,00E+00	885	-2267	Indica	Japonica	
maker-10-augustus-gene-186.28-mRNA-1	10	OsR498G1019015500.01.T01	100,00	0,00E+00	1211	Os10g0508400	100,00	0,00E+00	1211	0	NA	Japonica	
maker-10-augustus-gene-186.33-mRNA-1	10	OsR498G1019011800.01.T01	99,04	0,00E+00	1462	Os10g0507600	99,59	0,00E+00	1289	-173	Indica	Japonica	
maker-10-augustus-gene-186.37-mRNA-1	10	OsR498G1019013400.01.T01	96,61	0,00E+00	2710	Os10g0507800	96,86	0,00E+00	2888	178	NA	Japonica	
maker-10-augustus-gene-186.43-mRNA-1	10	OsR498G1019015000.01.T01	97,65	0,00E+00	1234	Os10g0508300	100,00	0,00E+00	1889	655	Japonica	Japonica	
maker-10-augustus-gene-187.0-mRNA-1	10	OsR498G1019015900.01.T01	92,25	0,00E+00	838	Os10g0508600	93,69	0,00E+00	970	132	Japonica	Japonica	
maker-10-augustus-gene-187.14-mRNA-1	10	OsR498G1019021500.01.T02	99,91	0,00E+00	2041	Os10g0510000	100,00	0,00E+00	2046	5	NA	Japonica	
maker-10-augustus-gene-187.16-mRNA-1	10	OsR498G1019015800.01.T02	99,64	0,00E+00	3933	Os10g0508500	98,18	0,00E+00	4588	655	Japonica	Japonica	
maker-10-augustus-gene-187.3-mRNA-1	10	OsR498G1019017300.01.T07	98,44	0,00E+00	4360	Os10g0509000	100,00	0,00E+00	2863	-1497	Indica	Japonica	
maker-10-augustus-gene-187.9-mRNA-1	10	OsR498G1019017900.01.T05	99,26	0,00E+00	719	Os10g0509100	100,00	0,00E+00	731	12	NA	Japonica	
maker-10-augustus-gene-188.19-mRNA-1	10	OsR498G1019025000.01.T01	99,94	0,00E+00	3168	Os10g0510700	100,00	0,00E+00	3604	436	Japonica	Japonica	
maker-10-augustus-gene-188.25-mRNA-1	10	OsR498G1019028800.01.T02	99,83	0,00E+00	2156	Os10g0512100	100,00	0,00E+00	3447	1291	Japonica	Japonica	
maker-10-augustus-gene-188.8-mRNA-1	10	OsR498G1019027700.01.T03	96,50	0,00E+00	2526	Os10g0511400	73,81	0,00E+00	867	-1659	Indica	Japonica	
maker-10-augustus-gene-189.0-mRNA-1	10	OsR498G1019030000.01.T01	99,94	0,00E+00	2869	Os10g0512400	100,00	0,00E+00	2874	5	NA	Japonica	
maker-10-augustus-gene-189.2-mRNA-1	10	OsR498G1019030400.01.T01	100,00	0,00E+00	2008	Os10g0512700	100,00	0,00E+00	2008	0	NA	Japonica	
maker-10-augustus-gene-19.36-mRNA-1	10	OsR498G1018080600.01.T01	99,85	0,00E+00	1192	Os10g0126500	100,00	0,00E+00	1380	188	Japonica	Japonica	
maker-10-augustus-gene-19.38-mRNA-1	10	OsR498G1018081000.01.T01	99,62	0,00E+00	1873	Os10g0126600	100,00	0,00E+00	1891	18	NA	Japonica	
maker-10-augustus-gene-19.40-mRNA-1	10	OsR498G1018081200.01.T01	99,70	0,00E+00	2347	Os10g0126700	100,00	0,00E+00	2500	153	NA	Japonica	
maker-10-augustus-gene-190.57-mRNA-1	10	OsR498G1019036300.01.T01	100,00	0,00E+00	893	Os10g0516100	100,00	0,00E+00	893	0	NA	Japonica	
maker-10-augustus-gene-190.59-mRNA-1	10	OsR498G1019038500.01.T07	98,63	0,00E+00	3061	Os10g0516300	99,65	0,00E+00	2029	-1032	Indica	Japonica	
maker-10-augustus-gene-190.66-mRNA-1	10	OsR498G1019036800.01.T01	99,82	0,00E+00	1972	Os03g0113000	83,15	0,00E+00	920	-1052	Indica	Japonica	Kinase
maker-10-augustus-gene-191.0-mRNA-1	10	OsR498G1019043100.01.T01	99,73	0,00E+00	2672	Os10g0516800	100,00	0,00E+00	2690	18	NA	Japonica	
maker-10-augustus-gene-191.13-mRNA-1	10	OsR498G1019042200.01.T01	99,11	0,00E+00	1578	Os10g0516600	100,00	0,00E+00	1613	35	NA	Japonica	
maker-10-augustus-gene-191.16-mRNA-1	10	OsR498G1019043400.01.T01	96,80	1,27E-147	522	Os10g0516900	100,00	1,11E-155	547	25	NA	Japonica	
maker-10-augustus-gene-191.20-mRNA-1	10	OsR498G1019047400.01.T01	99,93	0,00E+00	2512	Os10g0517500	100,00	0,00E+00	2516	4	NA	Japonica	
maker-10-augustus-gene-191.6-mRNA-1	10	OsR498G1019046900.01.T05	99,91	0,00E+00	2030	Os10g0517400	100,00	0,00E+00	2035	5	NA	Japonica	
maker-10-augustus-gene-192.39-mRNA-1	10	OsR498G1019048900.01.T02	99,83	0,00E+00	3163	Os10g0518100	100,00	0,00E+00	3177	14	NA	Japonica	
maker-10-augustus-gene-192.42-mRNA-1	10	OsR498G1019049300.01.T03	98,49	0,00E+00	2541	Os10g0518300	97,33	0,00E+00	2480	-61	NA	Japonica	
maker-10-augustus-gene-192.47-mRNA-1	10	OsR498G1019051100.01.T01	99,80	0,00E+00	3645	Os10g0518800	99,95	0,00E+00	3659	14	NA	Japonica	Kinase-TM
maker-10-augustus-gene-192.53-mRNA-1	10	OsR498G1019053100.01.T01	99,93	0,00E+00	2469	Os10g0519600	100,00	0,00E+00	2473	4	NA	Japonica	
maker-10-augustus-gene-192.62-mRNA-1	10	OsR498G1019051400.01.T01	99,74	0,00E+00	2086	Os10g0518900	100,00	0,00E+00	2100	14	NA	Japonica	
maker-10-augustus-gene-193.16-mRNA-1	10	OsR498G1019058200.01.T01	100,00	0,00E+00	834	Os10g0521100	100,00	0,00E+00	776	-58	NA	Japonica	
maker-10-augustus-gene-193.26-mRNA-1	10	OsR498G1019060000.01.T01	100,00	0,00E+00	2544	Os10g0521700	100,00	0,00E+00	2544	0	NA	Japonica	Kinase-TM
maker-10-augustus-gene-193.5-mRNA-1	10	OsR498G1019058900.01.T01	99,62	0,00E+00	1898	Os10g0521500	100,00	0,00E+00	1916	18	NA	Japonica	
maker-10-augustus-gene-193.7-mRNA-1	10	OsR498G1019056600.01.T01	99,71	0,00E+00	2507	Os10g0520600	100,00	0,00E+00	1465	-1042	Indica	Japonica	
maker-10-augustus-gene-193.9-mRNA-1	10	OsR498G1019056800.01.T01	99,53	0,00E+00	2250	Os10g0520700	100,00	0,00E+00	2278	28	NA	Japonica	
maker-10-augustus-gene-194.0-mRNA-1	10	OsR498G1019060300.01.T01	99,80	0,00E+00	1793	Os10g0521900	99,80	0,00E+00	1827	34	NA	Japonica	
maker-10-augustus-gene-194.34-mRNA-1	10	OsR498G1019066700.01.T02	98,03	0,00E+00	1391	Os10g0523100	100,00	0,00E+00	1463	72	NA	Japonica	
maker-10-augustus-gene-194.5-mRNA-1	10	OsR498G1019063700.01.T01	99,68	0,00E+00	1670	Os10g0522400	100,00	0,00E+00	892	-778	Indica	Japonica	
maker-10-augustus-gene-195.0-mRNA-1	10	OsR498G1019068700.01.T01	99,62	0,00E+00	2348	Os10g0524000	100,00	0,00E+00	2176	-172	NA	Japonica	
maker-10-augustus-gene-195.15-mRNA-1	10	OsR498G1019071800.01.T01	99,61	0,00E+00	2744	Os10g0525000	89,42	0,00E+00	2067	-677	Indica	Japonica	
maker-10-augustus-gene-195.18-mRNA-1	10	OsR498G1019073200.01.T01	99,92	0,00E+00	2166	Os10g0525000	100,00	0,00E+00	2792	626	Japonica	Japonica	
maker-10-augustus-gene-195.2-mRNA-1	10	OsR498G1019070300.01.T01	99,54	0,00E+00	3080	Os10g0524500	100,00	0,00E+00	3104	24	NA	Japonica	
maker-10-augustus-gene-195.21-mRNA-1	10	OsR498G1019073800.01.T01	99,38	0,00E+00	2839	Os10g0525200	100,00	0,00E+00	2798	-41	NA	Japonica	
maker-10-augustus-gene-195.23-mRNA-1	10	OsR498G1019074000.01.T01	98,10	0,00E+00	1263	Os10g0525400	100,00	0,00E+00	1326	63	NA	Japonica	
maker-10-augustus-gene-195.25-mRNA-1	10	OsR498G1019074400.01.T01	91,36	0,00E+00	1128	Os10g0525800	82,81	0,00E+00	751	-377	Indica	Japonica	
maker-10-augustus-gene-195.27-mRNA-1	10	OsR498G1019075200.01.T01	96,54	7,83E-177	620	Os10g0525800	100,00	5,26E-161	565	-55	NA	Japonica	
maker-10-augustus-gene-195.4-mRNA-1	10	OsR498G1019074700.01.T01	98,43	0,00E+00	1212	Os10g0525600	100,00	0,00E+00	1261	49	NA	Japonica	
maker-10-augustus-gene-195.6-mRNA-1	10	OsR498G1019069000.01.T01	99,62	0,00E+00	1865	Os10g0524100	100,00	0,00E+00	1884	19	NA	Japonica	

maker-10-augustus-gene-195.9-mRNA-1	10	OsR498G1019070200.01.T02	99,62	0,00E+00	5621	Os10g0524400	100,00	0,00E+00	5665	44	NA	Japonica	
maker-10-augustus-gene-2.0-mRNA-1	10	OsR498G1017997000.01.T05	100,00	0,00E+00	1397	Os10g0101100	98,84	0,00E+00	1352	-45	NA	Indica	
maker-10-augustus-gene-2.4-mRNA-1	10	OsR498G1017997800.01.T02	100,00	0,00E+00	2560	Os10g0101200	99,93	0,00E+00	2555	-5	NA	Indica	
maker-10-augustus-gene-20.0-mRNA-1	10	OsR498G1018082400.01.T02	99,46	0,00E+00	2632	Os10g0126600	93,16	0,00E+00	1574	-1058	Indica	Japonica	
maker-10-augustus-gene-20.3-mRNA-1	10	OsR498G1018082800.01.T01	99,86	0,00E+00	3764	Os10g0127700	100,00	0,00E+00	3777	13	NA	Japonica	
maker-10-augustus-gene-21.0-mRNA-1	10	OsR498G1018089500.01.T01	97,99	0,00E+00	674	Os10g0130500	99,59	0,00E+00	871	197	Japonica	Japonica	
maker-10-augustus-gene-21.12-mRNA-1	10	OsR498G1018091000.01.T01	99,82	0,00E+00	1957	Os10g0130600	83,68	0,00E+00	1206	-751	Indica	Japonica	NBS-TM
maker-10-augustus-gene-22.26-mRNA-1	10	OsR498G1018095600.01.T01	100,00	0,00E+00	3800	Os11g0689100	73,56	0,00E+00	973	-2827	Indica	Japonica	NBS-TM
maker-10-augustus-gene-22.27-mRNA-1	10	OsR498G1018095600.01.T01	99,90	0,00E+00	1742	Os10g0131901	100,00	0,00E+00	1746	4	NA	Japonica	CC-NBS-TM
maker-10-augustus-gene-23.27-mRNA-1	10	OsR498G1018099300.01.T02	99,75	0,00E+00	4231	Os10g0132500	99,87	0,00E+00	4245	14	NA	Japonica	LRR-NBS-TM
maker-10-augustus-gene-23.34-mRNA-1	10	OsR498G1018101100.01.T01	98,22	0,00E+00	1452	Os10g0131000	88,41	0,00E+00	806	-646	Indica	Japonica	CC-NBS-TM
maker-10-augustus-gene-23.37-mRNA-1	10	OsR498G1018102900.01.T01	99,17	0,00E+00	2560	Os10g0133500	99,93	0,00E+00	2609	49	NA	Japonica	
maker-10-augustus-gene-23.39-mRNA-1	10	OsR498G1018098400.01.T01	99,35	0,00E+00	820	Os10g0132300	99,35	0,00E+00	820	0	NA	NA	
maker-10-augustus-gene-24.0-mRNA-1	10	OsR498G1018104900.01.T01	96,85	0,00E+00	2581	Os10g0134500	100,00	0,00E+00	2361	-220	NA	Japonica	
maker-10-augustus-gene-24.2-mRNA-1	10	OsR498G1018106600.01.T02	99,71	0,00E+00	1231	Os10g0135200	99,85	0,00E+00	1235	4	NA	Japonica	
maker-10-augustus-gene-25.33-mRNA-1	10	OsR498G1018109100.01.T04	94,62	0,00E+00	1852	Os10g0136500	99,58	0,00E+00	2101	249	Japonica	Japonica	Kinase-TM
maker-10-augustus-gene-25.38-mRNA-1	10	OsR498G1018108800.01.T03	92,69	0,00E+00	1801	Os10g0136200	99,67	0,00E+00	2163	362	Japonica	Japonica	
maker-10-augustus-gene-26.0-mRNA-1	10	OsR498G1018112500.01.T01	99,17	0,00E+00	7260	Os10g0138100	99,43	0,00E+00	7462	202	NA	Japonica	
maker-10-augustus-gene-26.3-mRNA-1	10	OsR498G1018113300.01.T01	99,76	0,00E+00	1485	Os10g0138300	99,52	0,00E+00	1476	-9	NA	Indica	
maker-10-augustus-gene-27.0-mRNA-1	10	OsR498G1018113700.01.T01	99,61	0,00E+00	2731	Os10g0138600	99,58	0,00E+00	2535	-196	NA	Indica	
maker-10-augustus-gene-28.0-mRNA-1	10	OsR498G1018123300.01.T02	99,92	0,00E+00	2144	Os10g0141900	99,75	0,00E+00	2135	-9	NA	Indica	
maker-10-augustus-gene-28.6-mRNA-1	10	OsR498G1018123500.01.T03	100,00	0,00E+00	850	Os10g0142100	100,00	0,00E+00	850	0	NA	NA	
maker-10-augustus-gene-3.24-mRNA-1	10	OsR498G1018004100.01.T01	100,00	0,00E+00	2415	Os07g0248800	98,04	0,00E+00	2095	-320	Indica	Indica	
maker-10-augustus-gene-30.0-mRNA-1	10	OsR498G1018131800.01.T01	100,00	0,00E+00	1685	Os10g0144700	99,61	0,00E+00	2765	1080	Japonica	Indica	
maker-10-augustus-gene-30.11-mRNA-1	10	OsR498G1018137200.01.T02	99,75	0,00E+00	1440	Os10g0146200	99,46	0,00E+00	1314	-126	NA	Indica	
maker-10-augustus-gene-30.6-mRNA-1	10	OsR498G1018135400.01.T02	100,00	0,00E+00	1104	Os12g0210400	90,70	0,00E+00	1012	-92	NA	NA	LRR-TM
maker-10-augustus-gene-31.0-mRNA-1	10	OsR498G1018140100.01.T01	100,00	0,00E+00	1781	Os03g0244200	77,23	0,00E+00	647	-1134	Indica	Indica	
maker-10-augustus-gene-31.2-mRNA-1	10	OsR498G1018142600.01.T01	99,76	0,00E+00	2947	Os10g0147400	99,82	0,00E+00	2952	5	NA	NA	
maker-10-augustus-gene-31.4-mRNA-1	10	OsR498G1018145800.01.T01	99,88	0,00E+00	1468	Os10g0148100	99,76	0,00E+00	1463	-5	NA	Indica	
maker-10-augustus-gene-32.0-mRNA-1	10	OsR498G1018147200.01.T01	99,87	0,00E+00	2686	Os10g0148400	95,58	0,00E+00	2691	5	NA	Indica	
maker-10-augustus-gene-32.10-mRNA-1	10	OsR498G1018149800.01.T01	100,00	0,00E+00	1223	Os10g0149200	95,71	0,00E+00	1087	-136	Indica	Indica	
maker-10-augustus-gene-32.2-mRNA-1	10	OsR498G1018147400.01.T01	99,76	0,00E+00	1480	Os10g0148700	98,07	0,00E+00	1418	-62	NA	Indica	
maker-10-augustus-gene-32.8-mRNA-1	10	OsR498G1018148800.01.T01	99,70	0,00E+00	1182	Os10g0149000	99,39	0,00E+00	1173	-9	NA	NA	
maker-10-augustus-gene-32.9-mRNA-1	10	OsR498G1018149000.01.T01	100,00	0,00E+00	1364	Os10g0149200	96,59	0,00E+00	1114	-250	Indica	Indica	
maker-10-augustus-gene-33.23-mRNA-1	10	OsR498G1018150600.01.T01	99,35	0,00E+00	1097	Os10g0149400	99,00	0,00E+00	1230	133	Japonica	Indica	
maker-10-augustus-gene-33.24-mRNA-1	10	OsR498G1018151400.01.T01	99,84	0,00E+00	1116	Os10g0149800	99,52	0,00E+00	1107	-9	NA	NA	
maker-10-augustus-gene-34.0-mRNA-1	10	OsR498G1018151500.01.T01	99,84	0,00E+00	1143	Os10g0149900	98,74	0,00E+00	1112	-31	NA	Japonica	
maker-10-augustus-gene-34.11-mRNA-1	10	OsR498G1018157400.01.T01	93,38	0,00E+00	3218	Os10g0151100	99,42	0,00E+00	3701	483	Japonica	Japonica	Kinase-TM
maker-10-augustus-gene-34.14-mRNA-1	10	OsR498G1018152000.01.T01	99,83	0,00E+00	1062	Os10g0150300	99,49	0,00E+00	1053	-9	NA	Indica	
maker-10-augustus-gene-34.16-mRNA-1	10	OsR498G1018152200.01.T01	92,87	0,00E+00	1025	Os10g0150400	99,56	0,00E+00	1205	180	Japonica	Japonica	
maker-10-augustus-gene-34.5-mRNA-1	10	OsR498G1018152300.01.T01	93,46	0,00E+00	1039	Os10g0150600	100,00	0,00E+00	1245	206	Japonica	Japonica	
maker-10-augustus-gene-34.7-mRNA-1	10	OsR498G1018152700.01.T01	97,83	0,00E+00	1177	Os10g0150700	100,00	0,00E+00	1245	68	NA	Japonica	
maker-10-augustus-gene-34.9-mRNA-1	10	OsR498G1018152900.01.T01	94,78	0,00E+00	1083	Os10g0150800	99,86	0,00E+00	1241	158	Japonica	Japonica	
maker-10-augustus-gene-36.47-mRNA-1	10	OsR498G1018157200.01.T02	98,13	0,00E+00	2496	Os10g0151800	97,50	0,00E+00	2455	-41	NA	Indica	
maker-10-augustus-gene-37.38-mRNA-1	10	OsR498G1018168500.01.T04	98,74	0,00E+00	1673	Os10g0155400	99,49	0,00E+00	2782	1109	Japonica	Indica	
maker-10-augustus-gene-37.42-mRNA-1	10	OsR498G1018170700.01.T01	99,20	0,00E+00	5499	Os10g0155733	99,42	0,00E+00	6168	669	Japonica	Indica	Kinase-LRR-TM
maker-10-augustus-gene-37.44-mRNA-1	10	OsR498G1018169000.01.T01	99,73	0,00E+00	1951	Os10g0155500	99,73	0,00E+00	1951	0	NA	Indica	
maker-10-augustus-gene-38.0-mRNA-1	10	OsR498G1018171900.01.T01	99,89	0,00E+00	4932	Os10g0155800	99,87	0,00E+00	4088	-844	Indica	NA	Kinase-LRR-TM
maker-10-augustus-gene-39.25-mRNA-1	10	OsR498G1018181100.01.T01	99,25	0,00E+00	2119	Os10g0158400	99,00	0,00E+00	2105	-14	NA	NA	
maker-10-augustus-gene-4.11-mRNA-1	10	OsR498G1018011400.01.T01	100,00	9,94E-176	616	Os10g0104500	100,00	3,46E-176	616	0	NA	Indica	
maker-10-augustus-gene-4.12-mRNA-1	10	OsR498G1018011400.01.T01	100,00	0,00E+00	1992	Os10g0104500	99,55	0,00E+00	1969	-23	NA	Indica	
maker-10-augustus-gene-4.3-mRNA-1	10	OsR498G1018006400.01.T01	100,00	0,00E+00	1348	Os10g0561800	77,32	3,52E-78	291	-1057	Indica	Indica	
maker-10-augustus-gene-4.6-mRNA-1	10	OsR498G1018008200.01.T01	100,00	0,00E+00	1921	Os10g0103800	99,91	0,00E+00	1917	-4	NA	Indica	
maker-10-augustus-gene-40.0-mRNA-1	10	OsR498G1018187400.01.T01	99,91	0,00E+00	2052	Os10g0159600	96,45	0,00E+00	1709	-343	Indica	Indica	
maker-10-augustus-gene-40.3-mRNA-1	10	OsR498G1018188000.01.T01	99,66	0,00E+00	1063	Os10g0159700	98,32	0,00E+00	1027	-36	NA	Indica	
maker-10-augustus-gene-40.8-mRNA-1	10	OsR498G1018188600.01.T01	100,00	0,00E+00	2186	Os10g0159800	99,59	0,00E+00	2164	-22	NA	Indica	
maker-10-augustus-gene-41.20-mRNA-1	10	OsR498G0305555000.01.T04	70,71	7,94E-26	118	Os10g0141400	83,26	4,38E-68	258	140	Japonica	Indica	
maker-10-augustus-gene-41.28-mRNA-1	10	OsR498G1018196400.01.T01	100,00	0,00E+00	648	Os10g0160700	98,18	0,00E+00	663	15	NA	Indica	
maker-10-augustus-gene-42.42-mRNA-1	10	OsR498G1018198900.01.T02	100,00	0,00E+00	1714	Os10g0161700	100,00	0,00E+00	1714	0	NA	Indica	
maker-10-augustus-gene-42.43-mRNA-1	10	OsR498G1018201700.01.T05	99,87	0,00E+00	5642	Os10g0162400	99,63	0,00E+00	4852	-790	Indica	Indica	
maker-10-augustus-gene-43.32-mRNA-1	10	OsR498G1018202600.01.T01	100,00	0,00E+00	1462	Os10g0162600	99,51	0,00E+00	1443	-19	NA	Indica	
maker-10-augustus-gene-43.39-mRNA-1	10	OsR498G1018204400.01.T01	99,80	0,00E+00	1788	Os10g0174800	74,79	9,65E-176	617	-1171	Indica	Indica	Kinase-TM
maker-10-augustus-gene-44.23-mRNA-1	10	OsR498G1018221300.01.T01	99,41	0,00E+00	1515	Os01g0946300	71,41	6,86E-128	457	-1058	Indica	Japonica	
maker-10-augustus-gene-45.0-mRNA-1	10	OsR498G1018222500.01.T01	99,70	0,00E+00	1816	Os10g0158400	92,40	0,00E+00	1749	-67	NA	Japonica	
maker-10-augustus-gene-45.2-mRNA-1	10	OsR498G1018222000.01.T01	99,17	0,00E+00	2114	Os10g0158400	94,65	0,00E+00	1871	-243	Indica	Japonica	
maker-10-augustus-gene-47.26-mRNA-1	10	OsR498G1018232300.01.T01	99,80	0,00E+00	2703	Os10g0166600	83,40	0,00E+00	1599	-1104	Indica	Indica	
maker-10-augustus-gene-48.36-mRNA-1	10	OsR498G1018238100.01.T01	99,66	0,00E+00	1578	Os10g0166600	99,66	0,00E+00	1578	0	NA	Indica	
maker-10-augustus-gene-49.0-mRNA-1	10	OsR498G1018238900.01.T02	95,76	0,00E+00	2053	Os10g0166600	86,32	0,00E+00	1513	-540	Indica	NA	
maker-10-augustus-gene-49.10-mRNA-1	10	OsR498G1018243700.01.T01	100,00	0,00E+00	2159	Os10g0167900	85,75	0,00E+00	1360	-799	Indica	Indica	
maker-10-augustus-gene-49.12-mRNA-1	10	OsR498G1018239200.01.T05	99,62	0,00E+00	2368	Os10g0167300	99,57	0,00E+00	2058	-310	Indica	NA	
maker-10-augustus-gene-49.2-mRNA-1	10	OsR498G1018239500.01.T02	99,70	0,00E+00	2981	Os10g0167500	99,76	0,00E+00	3697	716	Japonica	Indica	
maker-10-augustus-gene-49.7-mRNA-1	10	OsR498G1018241300.01.T01	100,00	0,00E+00	2165	Os10g0167900	99,16	0,00E+00	2094	-71	NA	Indica	
maker-10-augustus-gene-5.14-mRNA-1	10	OsR498G1018015000.01.T01	100,00	0,00E+00	2576	Os10g0105900	98,97	0,00E+00	2403	-173	NA	Indica	
maker-10-augustus-gene-5.9-mRNA-1	10	OsR498G1018013200.01.T01	100,00	0,00E+00	6368	Os10g0105400	99,07	0,00E+00	6237	-131	NA	Indica	
maker-10-augustus-gene-50.2-mRNA-1	10	OsR498G1018248100.01.T06	100,00	0,00E+00	2100	Os10g0169800	99,74	0,00E+00	2086	-14	NA	Indica	
maker-10-augustus-gene-50.6-mRNA-1	10	OsR498G1018249900.01.T01	98,69	0,00E+00	1203	Os10g0170000	98,69	0,00E+00	1203	0	NA	NA	
maker-10-augustus-gene-51.0-mRNA-1	10	OsR498G1018250900.01.T02	100,00	0,00E+00	710	Os10g0170200	100,00	0,00E+00	710	0	NA	Indica	
maker-10-augustus-gene-51.3-mRNA-1	10	OsR498G1018251300.01.T01	97,41	0,00E+00	2571	Os10g0170300	97,41	0,00E+00	2571	0	NA	Indica	
maker-10-augustus-gene-52.4-mRNA-1	10	OsR498G1018255800.01.T02	100,00	0,00E+00	1732	Os06g0725100	71,17	2,88E-93	343	-1389	Indica	NA	
maker-10-augustus-gene-55.1-mRNA-1	10	OsR498G1018265600.01.T01	99,75	0,00E+00	2106	Os10g0177200	98,81	0,00E+00	2058	-48	NA	Indica	
maker-10-augustus-gene-56.23-mRNA-1	10	OsR498G1018269500.01.T01	99,87	0,00E+00	1416	Os10g0178200	100,00	0,00E+00	1083	-333	Indica	NA	
maker-10-augustus-gene-56.25-mRNA-1	10	OsR498G1018222500.01.T01	85,04	0,00E+00	982	Os10g0177300	98,60	0,00E+00	1496	514	Japonica	Indica	
maker-10-augustus-gene-56.31-mRNA-1	10	OsR498G1018272600.01.T01	99,86	0,00E+00	5241	Os10g0180800	77,91	0,00E+00	2366	-2875	Indica	NA	Kinase-TM WAK112
maker-10-augustus-gene-57.30-mRNA-1	10	OsR498G1018274200.01.T03	99,13	0,00E+00	2218	Os10g0180000	98,89	0,00E+00	2203	-15	NA	NA	
maker-10-augustus-gene-58.36-mRNA-1	10	OsR498G1018281900.01.T02	99,85	0,00E+00	1187	Os10g0181600	99,69	0,00E+00	1166	-21	NA	NA	
maker-10-augustus-gene-58.41-mRNA-1	10	OsR498G1018282900.01.T01	100,00	0,00E+00	2926	Os10g0182000	99,94	0,00E+00	2921	-5	NA	Indica	
maker-10-augustus-gene-58.47-mRNA-1	10	OsR498G1018281600.01.T07	99,86	0,00E+00	3917	Os10g0181200	99,77	0,00E+00	5374	1457	Japonica	Japonica	
maker-10-augustus-gene-58.52-mRNA-1	10	OsR498G1018282200.01.T01	98,81	0,00E+00	3254	Os10g0181700	98,70	0,00E+00	3245	-9	NA	Indica	
maker-10-augustus-gene-59.35-mRNA-1	10	OsR498G1018292300.01.T01	100,00	0,00E+00	1975	Os10g0183900	100,00	0,00E+00	1975	0	NA	Japonica	
maker-10-augustus-gene-59.37-mRNA-1	10	OsR498G1018288800.01.T04	99,95	0,00E+00	3502	Os10g0183000	99,95	0,00E+00	3502	0	NA	Japonica	NBS-TM
maker-10-augustus-gene-6.1-mRNA-1	10	OsR498G1018015700.01.T01	93,69	0,00E+00	1759	Os10g0106701	99,48	0,00E+00	2384	625	Japonica	Indica	
maker-10-augustus-gene-60.0-mRNA-1	10	OsR498G1018295100.01.T01	99,90	0,00E+00	3490	Os03g0251500	82,51	0,00E+00	1961	-1529	Indica	Japonica	
maker-10-augustus-gene-62.11-mRNA-1	10	OsR498G1018308400.01.T02	99,94	0,00E+00	2880	Os10g0188100	99,25	0,00E+00	2834	-46	NA	Indica	
maker-10-augustus-gene-63.22-mRNA-1	10	OsR498G1018314600.01.T01	99,81	0,00E+00	2759	Os10g0189100	100,00	0,00E+00	2773	14	NA	Japonica	
maker-10-augustus-gene-63.27-mRNA-1	10	OsR498G1018314600.01.T02	99,76	0,00E+00	732	Os10g0189100	100,00	0,00E+00	693	-39	NA	NA	
maker-10-augustus-gene-64.16-mRNA-1	10	OsR498G1018320200.01.T01	99,46	0,00E+00	1965	Os10g0190100	99,30	0,00E+00	1782	-183	NA	NA	
maker-10-augustus-gene-64.18-mRNA-1	10	OsR498G1018321600.01.T03	99,81	0,00E+00	3775	Os10g0190500	99,81	0,00E+00	3775	0	NA	Indica	
maker-10-augustus-gene-64.6-mRNA-1	10	OsR498G0408361700.01.T01	79,73	0,00E+00	1249	Os10g0190900	99,88	0,00E+00	1434	185	Japonica	Japonica	
maker-10-augustus-gene-64.9-mRNA-1	10	OsR498G1018317800.01.T02	99,93	0,00E+00	2609	Os10g0189600	99,93	0,00E+00	2609	0	NA	Japonica	
maker-10-augustus-gene-66.0-mRNA-1	10	OsR498G1018327500.01.T01	98,42	0,00E+00	875	Os10g0193100	99,64	0,00E+00	1003	128	Japonica	Japonica	
maker-10-augustus-gene-66.2-mRNA-1	10	OsR498G1018329300.01.T01	99,76	0,00E+00	750	Os10g0193500	100,00	0,00E+00	755	5	NA	Japonica	
maker-10-augustus-gene-66.3-mRNA-1	10	OsR498G1018331900.01.T06	100,00	0,00E+00	1921	Os10g0194200	99,91	0,00E+00	1928	7	NA	NA	
maker-10-augustus-gene-7.33-mRNA-1	10	OsR498G1018023900.01.T01	100,00	0,00E+00	1891	Os10g0108700	99,33	0,00E+00	1859	-32	NA	Indica	
maker-10-augustus-gene-70.0-mRNA-1	10	OsR498G1018336100.01.T01	99,04	0,00E+00	2567	Os10g0195600	99,11	0,00E+00	2571	4	NA	NA	
maker-10-augustus-gene-71.21-mRNA-1	10	OsR498G1018339300.01.T01	91,76	0,00E+00	2209	Os05g0494000	75,51	0,00E+00	1074	-1135	Indica	NA	
maker-10-augustus-gene-72.39-mRNA-1	10	OsR498G1018339300.01.T01	99,18	0,00E+00	1944	Os10g0196100	99,50	0,00E+00	1078	-866	Indica	Japonica	
maker-10-augustus-gene-73.33-mRNA-1	10	OsR498G1018343700.01.T03	100,00	0,00E+00	3057	Os10g0198600	99,88	0,00E+00	3048	-9	NA	Japonica	
maker-10-augustus-gene-73.49-mRNA-1	10	OsR498G1018341600.01.T01	100,00	0,00E+00	1045	Os10g0198100	99,66	0,00E+00	1036	-9	NA	Japonica	
maker-10-augustus-gene-74.5-mRNA-1	10	OsR498G1018348300.01.T03	99,90	0,00E+00	1787	Os10g0199500	99,67	0,00E+00	1092	-695	Indica	Japonica	
maker-10-augustus-gene-75.3-mRNA-1	10	OsR498G1018354600.01.T02	88,95	1,59E-149	530	Os03g0129000	87,37	2,68E-141	501	-29	NA	Japonica	
maker-10-augustus-gene-77.25-mRNA-1	10	OsR498G1018362000.01.T01	99,66	1,57E-147	523	Os10g0202900	100,00	0,00E+00	646	123	Japonica	Japonica	
maker-10-augustus-gene-77.27-mRNA-1	10	OsR498G1018362600.01.T01	99,85	0,00E+00	2468	Os10g0203000	99,93	0,00E+00	2472	4	NA	Japonica	
maker-10-augustus-gene-78.5-mRNA-1	10	OsR498G1018368400.01.T01	99,22	0,00E+00	2260	Os10g0204100	99,84	0,00E+00	2296	36	NA	Japonica	
maker-10-augustus-gene-78.7-mRNA-1	10	OsR498G1018371000.01.T02	99,75	0,00E+00	2126	Os10g0204400	99,66	0,00E+00	2122	-4	NA	Indica	
maker-10-augustus-gene-79.25-mRNA-1	10	OsR498G1018374100.01.T02	99,82	0,00E+00	1003	Os10g0205200	99,82	0,00E+00	1003	0	NA	NA	
maker-10-augustus-gene-79.36-mRNA-1	10	OsR498G1018374400.01.T03	99,81	0,00E+00	1896	Os10g0205300	99,81	0,00E+00	1896	0	NA	NA	
maker-10-augustus-gene-79.39-mRNA-1	10	OsR498G1018376400.01.T02	99,88	0,00E+00	1495	Os10g0205700	99,88	0,00E+00	1495	0	NA	NA	
maker-10-augustus-gene-8.0-mRNA-1	10	OsR498G1018027200.01.T02	100,00	0,00E+00	1067	Os10g0109300	99,83	0,00E+00	1062	-5	NA	Indica	
maker-10-augustus-gene-8.5-mRNA-1	10	OsR498G1018031200.01.T01	100,00	0,00E+00	3057	Os10g0110600	99,54	0,00E+00	3077	20	NA	Indica	
maker-10-augustus-gene-80.0-mRNA-1	10	OsR498G1018378900.01.T01	99,47	0,00E+00	1010	Os10g0206500	99,65	0,00E+00	1014	4	NA	NA	
maker-10-augustus-gene-80.3-mRNA-1	10	OsR498G1018380100.01.T08	99,77	0,00E+00	2368	Os10g0206800	99,70	0,00E+00	2363	-5	NA	Indica	
maker-10-augustus-gene-81.0-mRNA-1	10	OsR498G1018384500.01.T01	99,80	0,00E+00	916	Os10g0207500	99,63	2,88E-134	478	-438	Indica	Indica	
maker-10-augustus-gene-81.2-mRNA-1	10	OsR498G1018383500.01.T01	99,60	0,00E+00	899	Os10g0207700	91,98	0,00E+00	920	21	NA	Indica	
maker-10-augustus-gene-81.5-mRNA-1	10	OsR498G1018385400.01.T01	99,41	2,98E-172	605	Os02g0586400	79,01	1,26E-152	538	-67	Indica	Indica	
maker-10-augustus-gene-82.2-mRNA-1	10	OsR498G1018387600.01.T01	99,70	5,02E-169	595	Os10g0208600	99,33	0,00E+00	1054	459	Japonica	Indica	
maker-10-augustus-gene-83.3-mRNA-1	10	OsR498G1018862400.01.T01	95,65	0,00E+00	1917	Os10g0464400	96,07	0,00E+00	1939	22	NA	Indica	
maker-10-augustus-gene-84.0-mRNA-1	10	OsR498G1018391500.01.T01	99,62	0,00E+00	1410	Os10g0209700	98,49	0,00E+00	1371	-39	NA	Japonica	
maker-10-augustus-gene-84.4-mRNA-1	10	OsR498G1018393600.01.T03	99,59	0,00E+00	868	Os10g0210500	99,38	0,00E+00	864	-4	NA	NA	
maker-10-augustus-gene-87.32-mRNA-1	10	OsR498G1018396800.01.T03	99,69	5,99E-165	581	Os10g0214400	98,88	4,60E-85	313	-268	Indica	NA	
maker-10-augustus-gene-87.34-mRNA-1	10	OsR498G1018399600.01.T05	99,36	1,48E-156	553	Os10g0213800	99,81	0,00E+00	930	377	Japonica	NA	
maker-10-augustus-gene-9.27-mRNA-1	10	OsR498G1018031600.01.T06	100,00	0,00E+00	3068	Os10g0370700	84,45	0,00E+00	1884	-1184	Indica	Indica	
maker-10-augustus-gene-9.32-mRNA-1	10	OsR498G1018032200.01.T01	100,00	0,00E+00	3144	Os10g0370700	70,49	0,00E+00	775	-2369	Indica	Indica	
maker-10-augustus-gene-9.40-mRNA-1	10	OsR498G1018034200.01.T01	100,00	0,00E+00	3122	Os10g0111700	99,30	0,00E+00	2764	-358	Indica	Indica	
maker-10-augustus-gene-90.0-mRNA-1	10	OsR498G1018408000.01.T01	99,58	0,00E+00	1280	Os10g0254720	99,58	0,00E+00	1286	6	NA	NA	
maker-10-augustus-gene-92.20-mRNA-1	10	OsR498G1018408000.01.T01	99,16	0,00E+00	1267	Os10g0254720	99,44	0,00E+00	1281	14	NA	NA	
maker-10-augustus-gene-94.0-mRNA-1	10	OsR498G1018411100.01.T04	99,94	0,00E+00	3188	Os10g0315400	99,94	0,00E+00	3188	0	NA	NA	
maker-10-augustus-gene-96.27-mRNA-1	10	OsR498G1018414400.01.T01	99,78	0,00E+00	8136	Os10g0316400	99,78	0,00E+00	8136	0	NA	NA	
maker-10-augustus-gene-99.36-mRNA-1	10	OsR498G1018427100.01.T02	100,00	0,00E+00	2852	Os10g0320100	99,87	0,00E+00	2843	-9	NA	NA	
maker-10-augustus-gene-99.38-mRNA-1	10	OsR498G1018427700.01.T01	100,00	0,00E+00	1759	Os10g0320400	99,90	0,00E+00	1755	-4	NA	NA	
augustus_masked-11-processed-gene-0.37-mRNA-1	11	OsR498G1119278000.01.T04	100,00	0,00E+00	1305	Os11g0102700	100,00	0,00E+00	1305	0	NA	NA	
augustus_masked-11-processed-gene-10.82-mRNA-1	11	OsR498G1119380200.01.T01	98,10	0,00E+00	2279	Os11g0135900	98,10	0,00E+00	2279	0	NA	Japonica	
augustus_masked-11-processed-gene-11.10-mRNA-1	11	OsR498G1119390400.01.T01	99,80	0,00E+00	2741	Os11g0138300	92,36	0,00E+00	2231	-510	Indica	Japonica	
augustus_masked-11-processed-gene-111.22-mRNA-1	11	OsR498G1120005800.01.T01	99,87	0,00E+00	2659	Os11g0429100	100,00	0,00E+00	3025	366	Japonica	Japonica	NBS-TM
augustus_masked-11-processed-gene-12.26-mRNA-1	11	OsR498G1119397800.01.T01	99,41	0,00E+00	902	Os12g0137900	94,60	0,00E+00	1034	132	Japonica	Japonica	
augustus_masked-11-processed-gene-125.1-mRNA-1	11	OsR498G1120065200.01.T01	99,49	0,00E+00	1744	Os11g0450300	99,80	0,00E+00	1752	8	NA	Japonica	
augustus_masked-11-processed-gene-128.18-mRNA-1	11	OsR498G1120082000.01.T03	100,00	1,02E-161	569	Os11g0456000	100,00	3,54E-162	569	0	NA	Japonica	
augustus_masked-11-processed-gene-129.4-mRNA-1	11	OsR498G1120085200.01.T01	99,58	0,00E+00	2571	Os11g0457300	100,00	0,00E+00	2598	27	NA	Japonica	
augustus_masked-11-processed-gene-14.10-mRNA-1	11	OsR498G1119406400.01.T01	99,59	0,00E+00	2587	Os11g0145200	100,00	0,00E+00	2614	27	NA	Japonica	
augustus_masked-11-processed-gene-14.9-mRNA-1	11	OsR498G1119406200.01.T03	99,87	0,00E+00	1381	Os11g0145100	100,00	0,00E+00	1386	5	NA	Japonica	
augustus_masked-11-processed-gene-151.6-mRNA-1	11	OsR498G0612724200.01.T01	72,50	0,00E+00	807	Os11g0495950	100,00	0,00E+00	2928	2121	Japonica	Japonica	
augustus_masked-11-processed-gene-159.51-mRNA-1	11	OsR498G1120215400.01.T01	99,69	0,00E+00	5177	Os11g0514100	99,97	0,00E+00	5213	36	NA	Japonica	
augustus_masked-11-processed-gene-159.56-mRNA-1	11	OsR498G1120218800.01.T01	99,70	0,00E+00	2948	Os11g0515000	100,00	0,00E+00	1519	-1429	Indica	Japonica	
augustus_masked-11-processed-gene-162.15-mRNA-1	11	OsR498G1120235000.01.T02	100,00	0,00E+00	807	Os11g0521500	100,00	0,00E+00	807	0	NA	NA	
augustus_masked-11-processed-gene-167.45-mRNA-1	11	OsR498G1120265000.01.T01	99,57	1,49E-113	409	Os07g0501100	80,30	5,60E-44	177	-232	Indica	Japonica	
augustus_masked-11-processed-gene-168.20-mRNA-1	11	OsR498G1120275200.01.T01	99,43	0,00E+00	1243	Os11g0531900	100,00	0,00E+00	1261	18	NA	NA	
augustus_masked-11-processed-gene-169.20-mRNA-1	11	OsR498G1120278000.01.T02	100,00	0,00E+00	1135	Os11g0533100	100,00	0,00E+00	1056	-79	NA	Japonica	
augustus_masked-11-processed-gene-169.21-mRNA-1	11	OsR498G1120278400.01.T01	99,80	0,00E+00	905	Os11g0533400	100,00	0,00E+00	910	5	NA	Japonica	
augustus_masked-11-processed-gene-17.31-mRNA-1	11	OsR498G1119430200.01.T01	99,50	0,00E+00	715	Os10g0326400	93,97	2,13E-97	354	-361	Indica	Japonica	
augustus_masked-11-processed-gene-17.6-mRNA-1	11	OsR498G1119422800.01.T02	99,35	0,00E+00	820	Os11g0151300	99,80	0,00E+00	882	62	NA	Japonica	
augustus_masked-11-processed-gene-17.7-mRNA-1	11	OsR498G1119425200.01.T01	99,74	0,00E+00	2801	Os11g0151400	100,00	0,00E+00	2819	18	NA	Japonica	
augustus_masked-11-processed-gene-171.47-mRNA-1	11	OsR498G0407604900.01.T01	77,72	6,87E-38	159	Os11g0537800	100,00	0,00E+00	639	480	Japonica	Japonica	
augustus_masked-11-processed-gene-175.7-mRNA-1	11	OsR498G1120313000.01.T03	99,43	0,00E+00	2497	Os11g0544300	100,00	0,00E+00	2533	36	NA	Japonica	
augustus_masked-11-processed-gene-176.62-mRNA-1	11	OsR498G1120322800.01.T01	99,65	0,00E+00	2537	Os11g0546500	100,00	0,00E+00	2619	82	NA	Japonica	
augustus_masked-11-processed-gene-177.15-mRNA-1	11	OsR498G1120324400.01.T01	99,68	0,00E+00	3391	Os11g0547000	100,00	0,00E+00	3415	24	NA	Japonica	
augustus_masked-11-processed-gene-18.46-mRNA-1	11	OsR498G1119432200.01.T01	100,00	0,00E+00	661	Os11g0152900	100,00	0,00E+00	661	0	NA	Japonica	
augustus_masked-11-processed-gene-180.64-mRNA-1	11	OsR498G1120340000.01.T01	92,70	0,00E+00	1557	Os11g0552500	100,00	0,00E+00	1873	316	Japonica	Japonica	
augustus_masked-11-processed-gene-181.6-mRNA-1	11	OsR498G1120341200.01.T01	100,00	0,00E+00	975	Os10g0445600	74,32	1,70E-80	299	-676	Indica	Japonica	
augustus_masked-11-processed-gene-181.7-mRNA-1	11	OsR498G1120341600.01.T01	99,95	0,00E+00	3675	Os11g0553500	100,00	0,00E+00	3680	5	NA	Japonica	Kinase-TM
augustus_masked-11-processed-gene-182.53-mRNA-1	11	OsR498G1120348600.01.T01	99,53	0,00E+00	1143	Os11g0557300	100,00	0,00E+00	1157	14	NA	Japonica	
augustus_masked-11-processed-gene-182.55-mRNA-1	11	OsR498G1120348600.01.T02	100,00	0,00E+00	1180	Os11g0557300	100,00	0,00E+00	1180	0	NA	Japonica	
augustus_masked-11-processed-gene-182.58-mRNA-1	11	OsR498G1120351600.01.T01	99,68	0,00E+00	4466	Os11g0558400	100,00	0,00E+00	5557	1091	Japonica	Japonica	LRR-TM
augustus_masked-11-processed-gene-183.19-mRNA-1	11	OsR498G1120357400.01.T01	99,47	0,00E+00	5019	Os11g0560000	100,00	0,00E+00	5254	235	NA	Japonica	LRR-NBS-TM
augustus_masked-11-processed-gene-183.4-mRNA-1	11	OsR498G1120352700.01.T01	99,54	0,00E+00	5076	Os11g0558900	100,00	0,00E+00	5135	59	NA	Japonica	LRR-TM
augustus_masked-11-processed-gene-19.28-mRNA-1	11	OsR498G0611661000.01.T01	93,66	1,11E-176	619	Os11g0155900	100,00	0,00E+00	742	123	Japonica	Japonica	
augustus_masked-11-processed-gene-192.34-mRNA-1	11	OsR498G0815097600.01.T01	76,86	7,42E-16	84,2	Os11g0576700	100,00	2,59E-111	399	314,8	Japonica	Japonica	
augustus_masked-11-processed-gene-193.38-mRNA-1	11	OsR498G1120404900.01.T02	98,78	0,00E+00	4732	Os11g0578500	100,00	0,00E+00	3382	-1350	Indica	Japonica	
augustus_masked-11-processed-gene-197.24-mRNA-1	11	OsR498G1120421800.01.T05	99,61	0,00E+00	3633	Os11g0587100	100,00	0,00E+00	3669	36	NA	Japonica	
augustus_masked-11-processed-gene-2.45-mRNA-1	11	OsR498G0612633600.01.T01	74,05	4,71E-89	329	Os11g0120000	99,54	0,00E+00	1551	1222	Japonica	Japonica	
augustus_masked-11-processed-gene-2.47-mRNA-1	11	OsR498G1119335000.01.T01	99,55	0,00E+00	1183	Os12g0120100	98,95	0,00E+00	1167	-16	NA	Japonica	
augustus_masked-11-processed-gene-20.42-mRNA-1	11	OsR498G1119449800.01.T01	99,71	0,00E+00	1843	Os11g0157300	100,00	0,00E+00	1856	13	NA	Japonica	
augustus_masked-11-processed-gene-201.35-mRNA-1	11	OsR498G1120437600.01.T01	99,57	0,00E+00	836	Os11g0592900	100,00	0,00E+00	845	9	NA	Japonica	
augustus_masked-11-processed-gene-201.67-mRNA-1	11	OsR498G1120441400.01.T01	98,81	0,00E+00	2495	Os11g0594400	100,00	0,00E+00	2668	173	NA	Japonica	
augustus_masked-11-processed-gene-203.21-mRNA-1	11	OsR498G1120448400.01.T01	99,79	0,00E+00	7659	Os11g0598300	100,00	0,00E+00	7699	40	NA	Japonica	CC-NBS-TM
augustus_masked-11-processed-gene-204.54-mRNA-1	11	OsR498G1120454800.01.T01	99,86	0,00E+00	2648	Os11g0599200	100,00	0,00E+00	2657	9	NA	Japonica	
augustus_masked-11-processed-gene-206.12-mRNA-1	11	OsR498G1120466600.01.T01	97,40	0,00E+00	972	Os11g0600500	100,00	0,00E+00	1040	68	NA	Japonica	
augustus_masked-11-processed-gene-207.40-mRNA-1	11	OsR498G1120475600.01.T01	99,62	0,00E+00	2321	Os11g0602800	100,00	0,00E+00	2343	22	NA	Japonica	
augustus_masked-11-processed-gene-208.22-mRNA-1	11	OsR498G1019153500.01.T01	72,52	4,77E-42	172	Os11g0604500	100,00	0,00E+00	953	781	Japonica	Japonica	
augustus_masked-11-processed-gene-211.3-mRNA-1	11	OsR498G1120489600.01.T01	99,90	0,00E+00	3476	Os11g0609700	100,00	0,00E+00	3388	-88	NA	Japonica	CC-NBS-TM
augustus_masked-11-processed-gene-217.29-mRNA-1	11	OsR498G1120525600.01.T03	99,57	0,00E+00	1660	Os11g0621000	100,00	0,00E+00	1678	18	NA	Japonica	
augustus_masked-11-processed-gene-218.4-mRNA-1	11	OsR498G1120530800.01.T01	99,73	0,00E+00	1994	Os11g0622600	100,00	0,00E+00	2008	14	NA	Japonica	
augustus_masked-11-processed-gene-219.12-mRNA-1	11	OsR498G1120539400.01.T03	99,93	0,00E+00	5353	Os11g0623800	100,00	0,00E+00	5708	355	NA	Japonica	CC-LRR-NBS-TM
augustus_masked-11-processed-gene-219.32-mRNA-1	11	OsR498G1120543200.01.T03	94,97	0,00E+00	856	Os11g0624600	100,00	0,00E+00	942	86	NA	Japonica	Kinase-LRR-TM
augustus_masked-11-processed-gene-227.3-mRNA-1	11	OsR498G1120603000.01.T01	99,86	0,00E+00	2572	Os11g0637200	100,00	0,00E+00	2581	9	NA	Japonica	
augustus_masked-11-processed-gene-228.26-mRNA-1	11	OsR498G1120610200.01.T02	99,26	0,00E+00	4998	Os11g0639600	100,00	0,00E+00	5065	67	NA	Japonica	LRR-NBS-TM
augustus_masked-11-processed-gene-228.29-mRNA-1	11	OsR498G1120611600.01.T01	99,74	0,00E+00	3489	Os11g0640000	100,00	0,00E+00	3512	23	NA	Japonica	CC-LRR-NBS-TM
augustus_masked-11-processed-gene-23.6-mRNA-1	11	OsR498G0611509300.01.T02	91,78	3,38E-90	332	Os11g0161033	100,00	5,74E-120	428	96	Japonica	Japonica	
augustus_masked-11-processed-gene-230.2-mRNA-1	11	OsR498G1120621600.01.T01	100,00	0,00E+00	2170	Os11g0642100	100,00	0,00E+00	2170	0	NA	NA	
augustus_masked-11-processed-gene-230.34-mRNA-1	11	OsR498G1120673600.01.T01	97,36	2,43E-144	511	Os08g0364700	85,51	3,61E-67	253	-258	Indica	NA	
augustus_masked-11-processed-gene-230.4-mRNA-1	11	OsR498G1120622600.01.T01	100,00	0,00E+00	2414	Os11g0642400	100,00	0,00E+00	2414	0	NA	NA	
augustus_masked-11-processed-gene-234.34-mRNA-1	11	OsR498G1120645800.01.T01	100,00	0,00E+00	1012	Os11g0649500	100,00	0,00E+00	1012	0	NA	Japonica	
augustus_masked-11-processed-gene-237.27-mRNA-1	11	OsR498G1120666400.01.T01	96,86	0,00E+00	4062	Os11g0654800	100,00	0,00E+00	4399	337	NA	Japonica	CC-NBS-TM
augustus_masked-11-processed-gene-240.39-mRNA-1	11	OsR498G1120730000.01.T01	72,92	3,17E-38	160	Os11g0659600	100,00	0,00E+00	931	771	Japonica	Japonica	
augustus_masked-11-processed-gene-244.4-mRNA-1	11	OsR498G1120691000.01.T01	86,34	0,00E+00	1287	Os11g0664000	86,46	0,00E+00	1306	19	NA	Japonica	CC-Kinase-TM
augustus_masked-11-processed-gene-245.11-mRNA-1	11	OsR498G1120693800.01.T01	99,84	0,00E+00	4516	Os11g0668000	99,92	0,00E+00	4430	-86	NA	Japonica	CC-NBS
augustus_masked-11-processed-gene-245.12-mRNA-1	11	OsR498G1120693800.01.T01	99,72	0,00E+00	640	Os11g0668000	100,00	0,00E+00	634	-6	NA	Japonica	
augustus_masked-11-processed-gene-247.40-mRNA-1	11	OsR498G1120707000.01.T03	99,74	0,00E+00	2774	Os11g0673000	99,87	0,00E+00	2783	9	NA	Japonica	
augustus_masked-11-processed-gene-249.38-mRNA-1	11	OsR498G1120716000.01.T01	98,98	0,00E+00	2405	Os11g0677400	99,20	0,00E+00	2418	13	NA	NA	
augustus_masked-11-processed-gene-25.9-mRNA-1	11	OsR498G1220787900.01.T02	93,45	6,85E-122	438	Os11g0166201	100,00	0,00E+00	866	428	Japonica	Japonica	
augustus_masked-11-processed-gene-252.23-mRNA-1	11	OsR498G1120730000.01.T01	99,83	0,00E+00	2149	Os11g0683700	99,92	0,00E+00	2153	4	NA	Japonica	
augustus_masked-11-processed-gene-252.24-mRNA-1	11	OsR498G1120730400.01.T01	99,22	0,00E+00	2262	Os11g0683800	99,90	0,00E+00	1872	-390	Indica	Indica	
augustus_masked-11-processed-gene-253.37-mRNA-1	11	OsR498G1120736800.01.T01	99,44	0,00E+00	1585	Os11g0686900	99,88	0,00E+00	1484	-101	NA	Indica	NBS-TM
augustus_masked-11-processed-gene-26.21-mRNA-1	11	OsR498G1119549700.01.T01	97,54	0,00E+00	1167	Os11g0191800	100,00	0,00E+00	1245	78	NA	NA	

augustus_masked-11-processed-gene-26.47-mRNA-1	11	OsR498G1119545900.01.T01	97,75	0,00E+00	910	Os11g0194600	100,00	0,00E+00	1012	102	Japonica	NA	
augustus_masked-11-processed-gene-26.49-mRNA-1	11	OsR498G1119545900.01.T01	97,74	0,00E+00	902	Os11g0194600	99,64	0,00E+00	996	94	NA	NA	
augustus_masked-11-processed-gene-260.33-mRNA-1	11	OsR498G1120761400.01.T01	94,71	0,00E+00	1036	Os11g0701000	88,15	0,00E+00	1194	158	Japonica	Japonica	
augustus_masked-11-processed-gene-261.41-mRNA-1	11	OsR498G1120763800.01.T02	100,00	0,00E+00	1609	Os11g0701900	100,00	0,00E+00	1629	20	NA	Japonica	
augustus_masked-11-processed-gene-261.42-mRNA-1	11	OsR498G1120764000.01.T01	98,12	0,00E+00	1558	Os11g0702100	99,78	0,00E+00	1626	68	NA	Japonica	
augustus_masked-11-processed-gene-261.43-mRNA-1	11	OsR498G1120764200.01.T01	99,56	0,00E+00	1622	Os11g0702200	99,56	0,00E+00	1622	0	NA	Japonica	
augustus_masked-11-processed-gene-261.9-mRNA-1	11	OsR498G1120761400.01.T01	88,92	0,00E+00	882	Os11g0701000	99,89	0,00E+00	1690	808	Japonica	Japonica	
augustus_masked-11-processed-gene-263.100-mRNA-1	11	OsR498G1120777600.01.T01	99,74	0,00E+00	2038	Os11g0707900	99,52	0,00E+00	2241	203	NA	Japonica	
augustus_masked-11-processed-gene-263.91-mRNA-1	11	OsR498G1120775000.01.T01	99,54	3,79E-108	391	Os11g0707100	99,51	5,26E-101	366	-25	NA	Japonica	
augustus_masked-11-processed-gene-27.58-mRNA-1	11	OsR498G1119478400.01.T01	99,77	0,00E+00	781	Os11g0168000	100,00	0,00E+00	785	4	NA	Japonica	
augustus_masked-11-processed-gene-28.25-mRNA-1	11	OsR498G1119484000.01.T03	99,71	0,00E+00	3105	Os11g0169700	100,00	0,00E+00	3128	23	NA	Japonica	
augustus_masked-11-processed-gene-29.18-mRNA-1	11	OsR498G0407767500.01.T01	80,03	2,25E-177	623	Os11g0170900	99,25	0,00E+00	2135	1512	Japonica	NA	
augustus_masked-11-processed-gene-29.21-mRNA-1	11	OsR498G1119486600.01.T01	99,80	0,00E+00	2633	Os11g0171400	100,00	0,00E+00	1315	-1318	Indica	Japonica	
augustus_masked-11-processed-gene-3.21-mRNA-1	11	OsR498G1119343800.01.T03	99,68	0,00E+00	1106	Os11g0123033	100,00	0,00E+00	1115	9	NA	Japonica	
augustus_masked-11-processed-gene-32.50-mRNA-1	11	OsR498G1119504600.01.T02	99,92	0,00E+00	2371	Os11g0175700	100,00	0,00E+00	2273	-98	NA	Japonica	
augustus_masked-11-processed-gene-34.21-mRNA-1	11	OsR498G1119516000.01.T01	97,02	0,00E+00	897	Os11g0179100	80,15	2,05E-136	485	-412	Indica	Japonica	
augustus_masked-11-processed-gene-34.5-mRNA-1	11	OsR498G1119514600.01.T02	100,00	0,00E+00	958	Os11g0178800	100,00	0,00E+00	958	0	NA	Japonica	
augustus_masked-11-processed-gene-34.6-mRNA-1	11	OsR498G1119515000.01.T01	98,88	0,00E+00	942	Os11g0179000	100,00	0,00E+00	969	27	NA	Japonica	
augustus_masked-11-processed-gene-36.6-mRNA-1	11	OsR498G1119524400.01.T01	99,71	0,00E+00	1236	Os11g0182100	100,00	0,00E+00	1391	155	Japonica	Japonica	
augustus_masked-11-processed-gene-39.32-mRNA-1	11	OsR498G1119540600.01.T01	85,39	1,01E-144	514	Os11g0187600	85,62	8,24E-147	518	4	NA	NA	
augustus_masked-11-processed-gene-41.32-mRNA-1	11	OsR498G1119549700.01.T01	97,54	0,00E+00	1167	Os11g0191800	100,00	0,00E+00	1245	78	NA	NA	
augustus_masked-11-processed-gene-41.37-mRNA-1	11	OsR498G1119549700.01.T01	99,27	0,00E+00	1217	Os11g0193800	100,00	0,00E+00	1240	23	NA	NA	
augustus_masked-11-processed-gene-49.6-mRNA-1	11	OsR498G1119787000.01.T01	99,33	0,00E+00	1325	Os11g0270000	100,00	0,00E+00	1359	34	NA	Japonica	
augustus_masked-11-processed-gene-54.29-mRNA-1	11	OsR498G1119726400.01.T03	99,80	0,00E+00	2665	Os11g0256100	100,00	0,00E+00	3155	490	Japonica	Japonica	
augustus_masked-11-processed-gene-57.11-mRNA-1	11	OsR498G1119695000.01.T01	99,79	0,00E+00	2579	Os06g0103200	81,94	0,00E+00	1378	-1201	Indica	Japonica	
augustus_masked-11-processed-gene-58.3-mRNA-1	11	OsR498G1119697000.01.T01	98,86	0,00E+00	2145	Os11g0244600	99,92	0,00E+00	2139	-6	NA	Japonica	
augustus_masked-11-processed-gene-59.18-mRNA-1	11	OsR498G1119707600.01.T01	100,00	2,42E-163	574	Os11g0247000	100,00	8,42E-164	574	0	NA	Japonica	
augustus_masked-11-processed-gene-59.6-mRNA-1	11	OsR498G1119702200.01.T01	99,05	0,00E+00	2029	Os11g0246200	100,00	0,00E+00	2078	49	NA	Japonica	
augustus_masked-11-processed-gene-6.97-mRNA-1	11	OsR498G1119354800.01.T01	99,56	0,00E+00	803	Os11g0127900	100,00	0,00E+00	812	9	NA	Japonica	
augustus_masked-11-processed-gene-62.37-mRNA-1	11	OsR498G1119545900.01.T01	97,75	0,00E+00	910	Os11g0194600	100,00	0,00E+00	1012	102	Japonica	NA	
augustus_masked-11-processed-gene-63.31-mRNA-1	11	OsR498G1119554600.01.T01	98,41	1,16E-154	546	Os11g0195700	99,68	1,51E-160	564	18	NA	Japonica	
augustus_masked-11-processed-gene-65.23-mRNA-1	11	OsR498G1119568600.01.T01	99,03	0,00E+00	1445	Os11g0199600	100,00	0,00E+00	1483	38	NA	Japonica	
augustus_masked-11-processed-gene-69.27-mRNA-1	11	OsR498G1119583000.01.T04	100,00	0,00E+00	2208	Os11g0206200	99,43	0,00E+00	2175	-33	NA	NA	
augustus_masked-11-processed-gene-69.28-mRNA-1	11	OsR498G1119583600.01.T01	99,93	0,00E+00	2480	Os11g0206300	99,93	0,00E+00	2480	0	NA	NA	
augustus_masked-11-processed-gene-7.32-mRNA-1	11	OsR498G1119369000.01.T01	99,19	0,00E+00	875	Os11g0132100	100,00	0,00E+00	893	18	NA	Japonica	
augustus_masked-11-processed-gene-7.7-mRNA-1	11	OsR498G1119364000.01.T01	96,99	0,00E+00	1401	Os11g0130400	100,00	0,00E+00	1499	98	NA	Japonica	
augustus_masked-11-processed-gene-70.20-mRNA-1	11	OsR498G1119592200.01.T01	99,62	0,00E+00	1410	Os11g0207300	100,00	0,00E+00	2333	923	Japonica	Japonica	
augustus_masked-11-processed-gene-71.36-mRNA-1	11	OsR498G1119598200.01.T01	99,51	0,00E+00	4387	Os04g0103700	75,86	0,00E+00	1723	-2664	Indica	Japonica	Kinase-TM
augustus_masked-11-processed-gene-73.23-mRNA-1	11	OsR498G1119605600.01.T01	100,00	7,59E-157	553	Os11g0210201	100,00	2,64E-157	553	0	NA	Japonica	
augustus_masked-11-processed-gene-74.21-mRNA-1	11	OsR498G1119611200.01.T01	100,00	0,00E+00	2219	Os11g0213800	71,82	5,23E-180	630	-1589	Indica	NA	CC-NBS-TM
augustus_masked-11-processed-gene-76.32-mRNA-1	11	OsR498G1119618400.01.T01	95,12	0,00E+00	2039	Os11g0215400	100,00	0,00E+00	2322	283	Japonica	Japonica	
augustus_masked-11-processed-gene-77.16-mRNA-1	11	OsR498G1119621400.01.T01	95,08	0,00E+00	1905	Os11g0216400	100,00	0,00E+00	2030	125	NA	Japonica	
augustus_masked-11-processed-gene-79.20-mRNA-1	11	OsR498G1119634100.01.T01	99,85	0,00E+00	1214	Os11g0220800	100,00	0,00E+00	1218	4	NA	NA	
augustus_masked-11-processed-gene-8.106-mRNA-1	11	OsR498G1119372000.01.T01	99,13	0,00E+00	4457	Os11g0133100	100,00	0,00E+00	4556	99	NA	Japonica	Kinase-TM
augustus_masked-11-processed-gene-8.107-mRNA-1	11	OsR498G0306499400.01.T02	90,31	0,00E+00	1141	Os11g0133200	99,87	0,00E+00	1381	240	Japonica	Japonica	
augustus_masked-11-processed-gene-8.108-mRNA-1	11	OsR498G1220884400.01.T01	98,07	0,00E+00	4269	Os11g0133500	100,00	0,00E+00	4486	217	NA	Japonica	Kinase-TM
augustus_masked-11-processed-gene-8.82-mRNA-1	11	OsR498G1119369400.01.T01	98,93	0,00E+00	980	Os11g0132100	81,29	4,28E-133	474	-506	Indica	Japonica	
augustus_masked-11-processed-gene-80.5-mRNA-1	11	OsR498G1119637400.01.T01	99,66	0,00E+00	4770	Os11g0222000	100,00	0,00E+00	4394	-376	NA	Japonica	Kinase-TM
augustus_masked-11-processed-gene-81.41-mRNA-1	11	OsR498G1119640600.01.T01	98,76	0,00E+00	1553	Os11g0224400	100,00	0,00E+00	1602	49	NA	Japonica	
augustus_masked-11-processed-gene-81.43-mRNA-1	11	OsR498G1119641200.01.T01	99,19	0,00E+00	2614	Os11g0224600	100,00	0,00E+00	2668	54	NA	Japonica	
augustus_masked-11-processed-gene-81.45-mRNA-1	11	OsR498G1119642200.01.T01	88,44	0,00E+00	1377	Os11g0224900	99,35	0,00E+00	1910	533	Japonica	Japonica	LRR-TM
augustus_masked-11-processed-gene-82.26-mRNA-1	11	OsR498G1119644200.01.T01	93,99	0,00E+00	4185	Os11g0226933	99,95	0,00E+00	3491	-694	Indica	Japonica	CC-NBS-TM
augustus_masked-11-processed-gene-84.45-mRNA-1	11	OsR498G1119654600.01.T01	96,79	0,00E+00	2016	Os11g0231400	92,21	0,00E+00	1642	-374	Indica	Indica	
augustus_masked-11-processed-gene-85.7-mRNA-1	11	OsR498G1119658200.01.T01	99,67	0,00E+00	2678	Os11g0232100	80,45	0,00E+00	1361	-1317	Indica	Japonica	LRR-TM
augustus_masked-11-processed-gene-88.46-mRNA-1	11	OsR498G1119681000.01.T01	92,47	0,00E+00	1533	Os11g0240000	99,74	0,00E+00	2038	505	Japonica	Japonica	
augustus_masked-11-processed-gene-88.49-mRNA-1	11	OsR498G1119681000.01.T01	99,72	0,00E+00	1892	Os11g0240600	100,00	0,00E+00	1905	13	NA	Japonica	
augustus-11-processed-gene-0.48-mRNA-1	11	OsR498G1119298400.01.T01	99,42	0,00E+00	921	Os12g0118800	92,68	0,00E+00	795	-126	Indica	NA	
augustus-11-processed-gene-0.51-mRNA-1	11	OsR498G1119298400.01.T01	100,00	0,00E+00	1391	Os12g0118800	93,65	0,00E+00	1167	-224	Indica	NA	
augustus-11-processed-gene-1.13-mRNA-1	11	OsR498G1220832400.01.T01	93,83	0,00E+00	2026	Os11g0119200	100,00	0,00E+00	2414	388	Japonica	Japonica	
augustus-11-processed-gene-11.17-mRNA-1	11	OsR498G1119390700.01.T01	99,92	0,00E+00	2247	Os11g0138700	100,00	0,00E+00	2251	4	NA	Japonica	
augustus-11-processed-gene-11.19-mRNA-1	11	OsR498G1119391100.01.T01	99,72	0,00E+00	1919	Os11g0138900	100,00	0,00E+00	1932	13	NA	Japonica	
augustus-11-processed-gene-11.20-mRNA-1	11	OsR498G1119391300.01.T01	99,14	0,00E+00	1441	Os11g0139000	100,00	0,00E+00	1472	31	NA	Japonica	
augustus-11-processed-gene-11.22-mRNA-1	11	OsR498G1119391900.01.T01	99,49	0,00E+00	2078	Os11g0139400	100,00	0,00E+00	2105	27	NA	Japonica	
augustus-11-processed-gene-11.3-mRNA-1	11	OsR498G1119389500.01.T01	99,51	0,00E+00	2902	Os11g0138300	99,94	0,00E+00	2934	32	NA	Japonica	
augustus-11-processed-gene-110.13-mRNA-1	11	OsR498G1119995500.01.T01	99,39	0,00E+00	2915	Os11g0427700	100,00	0,00E+00	2960	45	NA	Japonica	
augustus-11-processed-gene-110.14-mRNA-1	11	OsR498G1119996500.01.T01	99,71	3,39E-176	618	Os11g0427800	100,00	2,78E-178	623	5	NA	Japonica	
augustus-11-processed-gene-113.24-mRNA-1	11	OsR498G1120014500.01.T02	99,79	0,00E+00	1669	Os11g0432400	100,00	0,00E+00	1678	9	NA	Japonica	
augustus-11-processed-gene-113.27-mRNA-1	11	OsR498G1120016500.01.T01	99,95	0,00E+00	3367	Os11g0433100	100,00	0,00E+00	3371	4	NA	Japonica	
augustus-11-processed-gene-114.4-mRNA-1	11	OsR498G1120018500.01.T01	97,33	0,00E+00	3350	Os11g0433600	99,93	0,00E+00	2636	-714	Indica	Japonica	
augustus-11-processed-gene-119.2-mRNA-1	11	OsR498G1120029400.01.T01	98,59	0,00E+00	2231	Os11g0441900	100,00	0,00E+00	2057	-174	NA	Japonica	Kinase-TM
augustus-11-processed-gene-121.11-mRNA-1	11	OsR498G1120050700.01.T01	99,77	0,00E+00	4659	Os11g0445300	100,00	0,00E+00	4686	27	NA	Japonica	Kinase-TM
augustus-11-processed-gene-13.26-mRNA-1	11	OsR498G1220908700.01.T01	92,70	0,00E+00	680	Os11g0142300	100,00	2,78E-146	517	-163	Indica	Japonica	
augustus-11-processed-gene-13.53-mRNA-1	11	OsR498G1119405700.01.T01	99,89	0,00E+00	4786	Os11g0144900	100,00	0,00E+00	4800	14	NA	Japonica	
augustus-11-processed-gene-130.0-mRNA-1	11	OsR498G1120090900.01.T01	98,82	0,00E+00	2370	Os11g0459300	99,93	0,00E+00	2436	66	NA	Japonica	
augustus-11-processed-gene-131.15-mRNA-1	11	OsR498G1120094300.01.T01	98,16	0,00E+00	656	Os12g0575000	90,83	4,97E-136	482	-174	Indica	Japonica	
augustus-11-processed-gene-131.26-mRNA-1	11	OsR498G1120095300.01.T01	99,36	0,00E+00	2508	Os11g0457300	85,53	0,00E+00	1568	-940	Indica	Japonica	
augustus-11-processed-gene-131.27-mRNA-1	11	OsR498G1120097100.01.T01	100,00	0,00E+00	2075	Os11g0462100	100,00	0,00E+00	2075	0	NA	Japonica	
augustus-11-processed-gene-133.6-mRNA-1	11	OsR498G1120104500.01.T01	99,69	2,45E-163	575	Os11g0180000	84,11	6,14E-96	350	-225	Indica	Japonica	
augustus-11-processed-gene-137.11-mRNA-1	11	OsR498G1120125900.01.T01	100,00	1,34E-149	529	Os11g0473000	100,00	4,66E-150	529	0	NA	Japonica	
augustus-11-processed-gene-14.0-mRNA-1	11	OsR498G1119406500.01.T02	99,74	0,00E+00	676	Os12g0143150	99,55	5,61E-110	397	-279	Indica	Japonica	
augustus-11-processed-gene-14.24-mRNA-1	11	OsR498G1119407500.01.T01	99,39	0,00E+00	869	Os12g0143750	82,17	6,94E-85	314	-555	Indica	Japonica	
augustus-11-processed-gene-144.6-mRNA-1	11	OsR498G1120148300.01.T01	99,57	0,00E+00	4127	Os11g0482400	100,00	0,00E+00	4172	45	NA	Japonica	
augustus-11-processed-gene-145.40-mRNA-1	11	OsR498G1120152900.01.T01	97,97	0,00E+00	2615	Os11g0484500	99,94	0,00E+00	2750	135	NA	Japonica	
augustus-11-processed-gene-146.34-mRNA-1	11	OsR498G0409297100.01.T04	74,42	0,00E+00	649	Os11g0486000	98,07	0,00E+00	1685	1036	Japonica	Indica	
augustus-11-processed-gene-147.18-mRNA-1	11	OsR498G1120164300.01.T01	98,79	0,00E+00	1590	Os11g0490100	98,90	0,00E+00	1595	5	NA	NA	
augustus-11-processed-gene-154.17-mRNA-1	11	OsR498G1120198900.01.T01	99,08	0,00E+00	963	Os11g0505200	73,56	1,01E-70	266	-697	Indica	Japonica	
augustus-11-processed-gene-155.4-mRNA-1	11	OsR498G1120199500.01.T01	99,24	0,00E+00	2104	Os11g0505200	100,00	0,00E+00	2040	-64	NA	Japonica	
augustus-11-processed-gene-158.17-mRNA-1	11	OsR498G1120212500.01.T01	97,64	0,00E+00	1441	Os11g0512200	99,76	0,00E+00	759	-682	Indica	Japonica	
augustus-11-processed-gene-16.34-mRNA-1	11	OsR498G1119419500.01.T01	100,00	0,00E+00	2749	Os11g0149900	100,00	0,00E+00	2749	0	NA	Japonica	
augustus-11-processed-gene-165.25-mRNA-1	11	OsR498G1120251900.01.T01	99,73	0,00E+00	2639	Os11g0525200	100,00	0,00E+00	2657	18	NA	Japonica	
augustus-11-processed-gene-169.13-mRNA-1	11	OsR498G1120275200.01.T01	99,43	0,00E+00	1243	Os11g0531900	100,00	0,00E+00	1261	18	NA	Japonica	
augustus-11-processed-gene-169.14-mRNA-1	11	OsR498G1120278500.01.T06	99,71	0,00E+00	2434	Os11g0533500	100,00	0,00E+00	2452	18	NA	Japonica	
augustus-11-processed-gene-17.15-mRNA-1	11	OsR498G1119427900.01.T01	99,77	0,00E+00	1534	Os12g0151000	91,20	0,00E+00	1199	-335	Indica	Japonica	
augustus-11-processed-gene-170.12-mRNA-1	11	OsR498G1120283900.01.T04	99,62	0,00E+00	1864	Os11g0535600	100,00	0,00E+00	1884	20	NA	Japonica	
augustus-11-processed-gene-171.26-mRNA-1	11	OsR498G1120288100.01.T01	99,70	0,00E+00	1198	Os11g0537300	100,00	0,00E+00	1207	9	NA	Japonica	
augustus-11-processed-gene-171.37-mRNA-1	11	OsR498G1120290900.01.T01	82,18	0,00E+00	1067	Os11g0538600	100,00	0,00E+00	3252	2185	Japonica	Japonica	
augustus-11-processed-gene-173.1-mRNA-1	11	OsR498G1120300300.01.T01	100,00	4,35E-173	607	Os11g0540900	100,00	1,51E-173	607	0	NA	Japonica	
augustus-11-processed-gene-173.2-mRNA-1	11	OsR498G1120301100.01.T01	99,15	0,00E+00	1037	Os11g0541600	72,75	3,58E-45	181	-856	Indica	Japonica	
augustus-11-processed-gene-175.0-mRNA-1	11	OsR498G1120311700.01.T06	95,81	0,00E+00	2281	Os11g0544000	100,00	0,00E+00	1285	-996	Indica	Japonica	
augustus-11-processed-gene-178.46-mRNA-1	11	OsR498G1120330300.01.T02	100,00	2,51E-119	428	Os11g0549635	100,00	8,71E-120	428	0	NA	Japonica	
augustus-11-processed-gene-180.30-mRNA-1	11	OsR498G1120333300.01.T05	99,89	0,00E+00	4928	Os11g0550500	100,00	0,00E+00	4940	12	NA	NA	LRR-NBS-TM
augustus-11-processed-gene-180.33-mRNA-1	11	OsR498G1120334900.01.T01	99,89	0,00E+00	1652	Os11g0551300	100,00	0,00E+00	1927	275	Japonica	Japonica	CC-LRR-NBS-TM
augustus-11-processed-gene-182.37-mRNA-1	11	OsR498G1120348700.01.T01	99,51	0,00E+00	3660	Os11g0557500	100,00	0,00E+00	3707	47	NA	Japonica	Kinase-TM
augustus-11-processed-gene-182.41-mRNA-1	11	OsR498G1120351100.01.T01	97,63	0,00E+00	3475	Os11g0558300	97,93	0,00E+00	3502	27	NA	Japonica	
augustus-11-processed-gene-184.34-mRNA-1	11	OsR498G1120361100.01.T02	100,00	0,00E+00	1366	Os11g0562100	100,00	0,00E+00	1249	-117	NA	Japonica	
augustus-11-processed-gene-185.22-mRNA-1	11	OsR498G1120370300.01.T01	99,13	0,00E+00	5080	Os11g0565000	100,00	0,00E+00	5140	60	NA	Japonica	LRR-TM
augustus-11-processed-gene-187.24-mRNA-1	11	OsR498G1120379500.01.T02	99,78	0,00E+00	797	Os11g0568500	100,00	0,00E+00	801	4	NA	Japonica	
augustus-11-processed-gene-189.16-mRNA-1	11	OsR498G1120390900.01.T01	99,46	0,00E+00	2299	Os11g0571900	100,00	0,00E+00	2333	34	NA	Japonica	
augustus-11-processed-gene-19.24-mRNA-1	11	OsR498G1119444100.01.T01	99,71	0,00E+00	1838	Os11g0156300	100,00	0,00E+00	1851	13	NA	Japonica	
augustus-11-processed-gene-193.13-mRNA-1	11	OsR498G0307190300.01.T02	77,69	0,00E+00	992	Os11g0577800	100,00	0,00E+00	2668	1676	Japonica	Japonica	
augustus-11-processed-gene-193.14-mRNA-1	11	OsR498G1120402700.01.T01	99,52	2,10E-101	370	Os03g0804800	91,82	4,04E-86	316	-54	Indica	Japonica	
augustus-11-processed-gene-193.22-mRNA-1	11	OsR498G1120403100.01.T01	99,43	0,00E+00	5620	Os11g0578100	100,00	0,00E+00	5698	78	NA	Japonica	
augustus-11-processed-gene-193.30-mRNA-1	11	OsR498G1120405100.01.T01	96,88	0,00E+00	1439	Os11g0578700	100,00	0,00E+00	1559	120	NA	Japonica	
augustus-11-processed-gene-194.0-mRNA-1	11	OsR498G1120448400.01.T01	99,18	0,00E+00	7538	Os11g0598300	99,20	0,00E+00	7542	4	NA	NA	CC-NBS-TM
augustus-11-processed-gene-194.1-mRNA-1	11	OsR498G1120405500.01.T01	98,24	0,00E+00	1764	Os11g0579600	100,00	0,00E+00	2192	428	Japonica	Japonica	
augustus-11-processed-gene-196.0-mRNA-1	11	OsR498G1120415500.01.T01	99,67	0,00E+00	2665	Os11g0584100	100,00	0,00E+00	2690	25	NA	Japonica	
augustus-11-processed-gene-197.15-mRNA-1	11	OsR498G1120421300.01.T02	98,35	7,42E-180	630	Os11g0586900	100,00	0,00E+00	657	27	NA	Japonica	
augustus-11-processed-gene-2.36-mRNA-1	11	OsR498G1220832200.01.T01	98,29	4,56E-173	607	Os11g0119700	100,00	0,00E+00	634	27	NA	Japonica	
augustus-11-processed-gene-200.40-mRNA-1	11	OsR498G1120433100.01.T01	99,58	0,00E+00	2126	Os11g0591600	100,00	0,00E+00	2149	23	NA	Japonica	
augustus-11-processed-gene-200.53-mRNA-1	11	OsR498G1120434500.01.T01	98,64	0,00E+00	769	Os11g0592200	100,00	0,00E+00	796	27	NA	Japonica	
augustus-11-processed-gene-201.32-mRNA-1	11	OsR498G1120437900.01.T01	96,34	0,00E+00	2301	Os11g0593100	100,00	0,00E+00	2511	210	NA	Japonica	
augustus-11-processed-gene-201.44-mRNA-1	11	OsR498G1120438300.01.T01	97,16	0,00E+00	2411	Os11g0593500	81,33	0,00E+00	1397	-1014	Indica	Japonica	
augustus-11-processed-gene-201.45-mRNA-1	11	OsR498G1120439100.01.T01	95,87	0,00E+00	1621	Os11g0593500	100,00	0,00E+00	2641	1020	Japonica	Japonica	
augustus-11-processed-gene-201.48-mRNA-1	11	OsR498G1120444100.01.T01	96,50	0,00E+00	707	Os11g0594700	100,00	0,00E+00	774	67	NA	Japonica	
augustus-11-processed-gene-201.49-mRNA-1	11	OsR498G1120444500.01.T01	96,50	0,00E+00	705	Os11g0594800	100,00	0,00E+00	774	69	NA	Japonica	
augustus-11-processed-gene-201.50-mRNA-1	11	OsR498G1120445100.01.T01	99,30	0,00E+00	759	Os11g0595000	100,00	0,00E+00	688	-71	NA	Japonica	
augustus-11-processed-gene-204.32-mRNA-1	11	OsR498G1120448400.01.T01	83,45	0,00E+00	4199	Os11g0598500	100,00	0,00E+00	6985	2786	Japonica	Japonica	CC-NBS
augustus-11-processed-gene-205.0-mRNA-1	11	OsR498G1120456900.01.T01	99,67	0,00E+00	1079	Os09g0434900	76,05	2,82E-97	354	-725	Indica	Japonica	
augustus-11-processed-gene-206.1-mRNA-1	11	OsR498G1120466900.01.T02	98,55	0,00E+00	1198	Os11g0600700	98,98	0,00E+00	1212	14	NA	Japonica	
augustus-11-processed-gene-206.17-mRNA-1	11	OsR498G1120470500.01.T01	98,37	0,00E+00	1379	Os11g0601500	100,00	0,00E+00	2556	1177	Japonica	Japonica	Kinase-TM
augustus-11-processed-gene-206.20-mRNA-1	11	OsR498G1120470700.01.T01	99,64	0,00E+00	3506	Os11g0601600	99,85	0,00E+00	3524	18	NA	Japonica	
augustus-11-processed-gene-206.5-mRNA-1	11	OsR498G1119745100.01.T01	97,31	0,00E+00	2065	Os07g0298700	97,69	0,00E+00	1397	-668	Indica	NA	
augustus-11-processed-gene-207.20-mRNA-1	11	OsR498G1120473300.01.T05	99,84	0,00E+00	4385	Os11g0602200	100,00	0,00E+00	4405	20	NA	Japonica	
augustus-11-processed-gene-207.23-mRNA-1	11	OsR498G0612676800.01.T03	88,40	0,00E+00	1124	Os11g0602400	100,00	0,00E+00	1581	457	Japonica	Japonica	
augustus-11-processed-gene-207.24-mRNA-1	11	OsR498G1120474500.01.T03	99,83	0,00E+00	1057	Os11g0602600	98,47	0,00E+00	1021	-36	NA	Japonica	
augustus-11-processed-gene-209.35-mRNA-1	11	OsR498G1120485700.01.T01	82,99	0,00E+00	3110	Os11g0606500	100,00	0,00E+00	5416	2306	Japonica	Japonica	NBS-TM
augustus-11-processed-gene-209.38-mRNA-1	11	OsR498G1120486300.01.T02	98,91	0,00E+00	3538	Os11g0607100	100,00	0,00E+00	3636	98	NA	Japonica	
augustus-11-processed-gene-211.0-mRNA-1	11	OsR498G0204795800.01.T02	89,53	1,77E-143	509	Os11g0609900	100,00	0,00E+00	702	193	Japonica	NA	
augustus-11-processed-gene-211.12-mRNA-1	11	OsR498G0204795800.01.T02	89,53	1,77E-143	509	Os11g0609900	100,00	0,00E+00	702	193	Japonica	NA	
augustus-11-processed-gene-211.13-mRNA-1	11	OsR498G1120490700.01.T02	99,26	0,00E+00	2160	Os11g0610600	100,00	0,00E+00	2197	37	NA	Japonica	
augustus-11-processed-gene-214.19-mRNA-1	11	OsR498G1120508300.01.T01	97,95	0,00E+00	1002	Os11g0615600	100,00	0,00E+00	1056	54	NA	Japonica	
augustus-11-processed-gene-215.12-mRNA-1	11	OsR498G1120511100.01.T01	99,68	0,00E+00	1112	Os11g0616200	100,00	0,00E+00	1121	9	NA	Japonica	
augustus-11-processed-gene-218.12-mRNA-1	11	OsR498G1120532700.01.T01	96,83	0,00E+00	1810	Os11g0622800	100,00	0,00E+00	1862	52	NA	Japonica	
augustus-11-processed-gene-219.23-mRNA-1	11	OsR498G1120542900.01.T01	98,41	0,00E+00	1848	Os11g0624500	100,00	0,00E+00	1326	-522	Indica	Japonica	
augustus-11-processed-gene-219.3-mRNA-1	11	OsR498G1120539100.01.T01	100,00	0,00E+00	948	Os11g0624000	100,00	0,00E+00	948	0	NA	NA	
augustus-11-processed-gene-22.12-mRNA-1	11	OsR498G1119464700.01.T01	99,39	0,00E+00	2633	Os11g0160800	100,00	0,00E+00	2673	40	NA	Japonica	
augustus-11-processed-gene-22.6-mRNA-1	11	OsR498G1119460700.01.T01	99,59	0,00E+00	1291	Os11g0160000	78,71	4,71E-165	581	-710	Indica	Japonica	
augustus-11-processed-gene-22.7-mRNA-1	11	OsR498G1119461100.01.T01	99,59	0,00E+00	1302	Os11g0160000	78,75	1,36E-165	582	-720	Indica	Japonica	
augustus-11-processed-gene-22.9-mRNA-1	11	OsR498G1119463100.01.T01	95,02	0,00E+00	1535	Os11g0160500	92,54	0,00E+00	868	-667	Indica	Japonica	
augustus-11-processed-gene-223.15-mRNA-1	11	OsR498G1120574300.01.T01	99,55	0,00E+00	1199	Os11g0631600	100,00	0,00E+00	1213	14	NA	Japonica	
augustus-11-processed-gene-228.19-mRNA-1	11	OsR498G1120612100.01.T01	98,24	0,00E+00	5203	Os11g0640300	100,00	0,00E+00	6055	852	Japonica	Japonica	LRR-NBS-TM
augustus-11-processed-gene-228.9-mRNA-1	11	OsR498G1120609300.01.T01	99,50	0,00E+00	3883	Os11g0639300	100,00	0,00E+00	3934	51	NA	Japonica	
augustus-11-processed-gene-229.22-mRNA-1	11	OsR498G1120617100.01.T01	100,00	0,00E+00	1283	Os11g0641300	100,00	0,00E+00	1283	0	NA	NA	
augustus-11-processed-gene-229.9-mRNA-1	11	OsR498G1120614900.01.T02	100,00	0,00E+00	3360	Os11g0640700	100,00	0,00E+00	3360	0	NA	NA	CC-LRR-NBS
augustus-11-processed-gene-23.0-mRNA-1	11	OsR498G1119465100.01.T03	98,91	0,00E+00	2563	Os11g0161000	100,00	0,00E+00	2636	73	NA	Japonica	
augustus-11-processed-gene-23.11-mRNA-1	11	OsR498G1119466100.01.T01	90,37	0,00E+00	934	Os04g0538400	70,92	1,69E-43	177	-757	Indica	Japonica	
augustus-11-processed-gene-230.17-mRNA-1	11	OsR498G1120623900.01.T01	100,00	0,00E+00	1294	Os11g0515000	87,28	0,00E+00	649	-645	Indica	NA	
augustus-11-processed-gene-230.18-mRNA-1	11	OsR498G1120624500.01.T01	100,00	0,00E+00	2430	Os11g0643100	100,00	0,00E+00	2430	0	NA	NA	
augustus-11-processed-gene-230.23-mRNA-1	11	OsR498G1120628100.01.T01	100,00	0,00E+00	2100	Os11g0644100	100,00	0,00E+00	2154	54	NA	NA	LRR-TM
augustus-11-processed-gene-231.21-mRNA-1	11	OsR498G1120631700.01.T01	99,73	0,00E+00	1301	Os11g0644700	100,00	0,00E+00	872	-429	Indica	Japonica	NBS-LRR (*Pi1*)
augustus-11-processed-gene-235.0-mRNA-1	11	OsR498G0306323800.01.T01	84,71	0,00E+00	1429	Os11g0650100	100,00	0,00E+00	3139	1710	Japonica	Japonica	
augustus-11-processed-gene-235.26-mRNA-1	11	OsR498G1120654000.01.T01	99,87	0,00E+00	7118	Os11g0652100	100,00	0,00E+00	7140	22	NA	Japonica	CC-NBS-TM
augustus-11-processed-gene-237.18-mRNA-1	11	OsR498G1120667300.01.T01	99,86	0,00E+00	5247	Os11g0655300	100,00	0,00E+00	5265	18	NA	Japonica	CC-NBS-TM
augustus-11-processed-gene-24.1-mRNA-1	11	OsR498G1119469700.01.T03	99,69	0,00E+00	2342	Os11g0163500	100,00	0,00E+00	2619	277	Japonica	Japonica	
augustus-11-processed-gene-24.10-mRNA-1	11	OsR498G1119470500.01.T01	99,60	0,00E+00	2249	Os11g0163800	100,00	0,00E+00	2273	24	NA	Japonica	
augustus-11-processed-gene-243.9-mRNA-1	11	OsR498G1120691200.01.T01	95,52	2,75E-97	354	Os11g0664133	100,00	7,41E-112	401	47	Japonica	NA	
augustus-11-processed-gene-247.35-mRNA-1	11	OsR498G1120706700.01.T01	100,00	0,00E+00	1846	Os11g0672900	100,00	0,00E+00	1846	0	NA	Japonica	
augustus-11-processed-gene-25.0-mRNA-1	11	OsR498G1119471900.01.T01	99,28	0,00E+00	1966	Os11g0166100	100,00	0,00E+00	2003	37	NA	Japonica	
augustus-11-processed-gene-250.11-mRNA-1	11	OsR498G1018527700.01.T01	82,58	0,00E+00	1894	Os11g0560000	81,07	0,00E+00	1798	-96	NA	Indica	
augustus-11-processed-gene-252.13-mRNA-1	11	OsR498G1120731300.01.T01	99,53	0,00E+00	3011	Os11g0684200	99,70	0,00E+00	3024	13	NA	Indica	CC-NBS-TM
augustus-11-processed-gene-252.35-mRNA-1	11	OsR498G1120735400.01.T01	90,93	0,00E+00	4888	Os11g0684100	92,04	0,00E+00	4726	-162	NA	NA	NBS-TM
augustus-11-processed-gene-253.11-mRNA-1	11	OsR498G1120733900.01.T01	98,90	0,00E+00	2232	Os05g0479700	84,87	0,00E+00	1414	-818	Indica	NA	
augustus-11-processed-gene-256.22-mRNA-1	11	OsR498G1120759300.01.T01	99,15	0,00E+00	1875	Os11g0691240	99,91	0,00E+00	1910	35	NA	NA	Kinase-TM
augustus-11-processed-gene-26.15-mRNA-1	11	OsR498G1119549500.01.T01	99,13	0,00E+00	2033	Os11g0192400	99,91	0,00E+00	2074	41	NA	NA	
augustus-11-processed-gene-26.27-mRNA-1	11	OsR498G1119548300.01.T01	99,13	0,00E+00	1218	Os11g0192500	100,00	0,00E+00	1245	27	NA	NA	
augustus-11-processed-gene-26.29-mRNA-1	11	OsR498G1119549700.01.T01	99,27	0,00E+00	1217	Os11g0193800	100,00	0,00E+00	1240	23	NA	NA	
augustus-11-processed-gene-26.34-mRNA-1	11	OsR498G1119549700.01.T01	97,54	0,00E+00	1167	Os11g0191800	100,00	0,00E+00	1245	78	NA	NA	
augustus-11-processed-gene-26.56-mRNA-1	11	OsR498G1119549500.01.T01	99,13	0,00E+00	2033	Os11g0192400	99,91	0,00E+00	2074	41	NA	NA	
augustus-11-processed-gene-261.0-mRNA-1	11	OsR498G1120762300.01.T01	96,69	0,00E+00	1446	Os11g0701100	99,89	0,00E+00	1571	125	NA	Japonica	
augustus-11-processed-gene-261.1-mRNA-1	11	OsR498G1120762500.01.T01	96,47	0,00E+00	1445	Os11g0701200	99,77	0,00E+00	1577	132	NA	Japonica	
augustus-11-processed-gene-261.13-mRNA-1	11	OsR498G1120763100.01.T01	99,42	0,00E+00	916	Os11g0701600	100,00	0,00E+00	675	-241	Indica	NA	
augustus-11-processed-gene-261.14-mRNA-1	11	OsR498G1120763100.01.T01	100,00	5,05E-113	407	Os11g0701500	90,19	3,42E-78	290	-117	Indica	NA	
augustus-11-processed-gene-261.18-mRNA-1	11	OsR498G1120763500.01.T01	99,78	0,00E+00	1642	Os11g0701800	100,00	0,00E+00	1651	9	NA	Japonica	
augustus-11-processed-gene-261.22-mRNA-1	11	OsR498G1221905100.01.T01	71,14	2,16E-79	297	Os11g0703000	96,00	0,00E+00	1053	756	Japonica	Indica	
augustus-11-processed-gene-261.26-mRNA-1	11	OsR498G1120766900.01.T02	97,72	0,00E+00	1275	Os11g0703600	97,72	0,00E+00	1275	0	NA	Indica	
augustus-11-processed-gene-261.3-mRNA-1	11	OsR498G1120762700.01.T01	98,39	0,00E+00	1507	Os11g0701400	100,00	0,00E+00	1570	63	NA	Indica	
augustus-11-processed-gene-262.22-mRNA-1	11	OsR498G1120771100.01.T01	99,33	0,00E+00	3688	Os11g0705200	99,95	0,00E+00	3745	57	NA	Japonica	
augustus-11-processed-gene-263.49-mRNA-1	11	OsR498G1120772300.01.T01	99,79	0,00E+00	3440	Os11g0705200	87,45	0,00E+00	1976	-1464	Indica	Japonica	
augustus-11-processed-gene-263.50-mRNA-1	11	OsR498G1120772300.01.T01	73,79	0,00E+00	833	Os11g0705900	100,00	0,00E+00	3479	2646	Japonica	Japonica	
augustus-11-processed-gene-263.65-mRNA-1	11	OsR498G1120778700.01.T01	99,89	0,00E+00	1686	Os11g0708500	98,90	0,00E+00	1922	236	Japonica	Japonica	
augustus-11-processed-gene-27.39-mRNA-1	11	OsR498G1119475900.01.T01	99,51	0,00E+00	3275	Os11g0167600	100,00	0,00E+00	3750	475	Japonica	Japonica	
augustus-11-processed-gene-29.29-mRNA-1	11	OsR498G1119486400.01.T02	99,83	0,00E+00	2096	Os11g0171300	100,00	0,00E+00	2105	9	NA	Japonica	
augustus-11-processed-gene-30.12-mRNA-1	11	OsR498G1119491700.01.T01	91,68	0,00E+00	1728	Os11g0172800	100,00	0,00E+00	2190	462	Japonica	Japonica	Kinase-LRR-TM
augustus-11-processed-gene-31.0-mRNA-1	11	OsR498G1119492700.01.T01	93,76	0,00E+00	4609	Os11g0171800	73,29	0,00E+00	1655	-2954	Indica	Japonica	Kinase-LRR-TM
augustus-11-processed-gene-34.15-mRNA-1	11	OsR498G1119517500.01.T01	99,28	0,00E+00	984	Os11g0179700	99,45	0,00E+00	648	-336	Indica	Japonica	
augustus-11-processed-gene-34.18-mRNA-1	11	OsR498G1119518700.01.T02	99,82	0,00E+00	1003	Os11g0180000	100,00	0,00E+00	1007	4	NA	Japonica	
augustus-11-processed-gene-36.0-mRNA-1	11	OsR498G1119522900.01.T07	99,90	0,00E+00	1782	Os11g0181800	100,00	0,00E+00	1786	4	NA	Japonica	
augustus-11-processed-gene-37.29-mRNA-1	11	OsR498G1119530100.01.T04	96,70	0,00E+00	1356	Os11g0184100	100,00	0,00E+00	1478	122	NA	Japonica	
augustus-11-processed-gene-39.2-mRNA-1	11	OsR498G1119536300.01.T02	99,92	0,00E+00	2317	Os11g0186800	100,00	0,00E+00	2322	5	NA	Japonica	
augustus-11-processed-gene-40.28-mRNA-1	11	OsR498G1119541700.01.T01	99,34	2,12E-151	535	Os11g0562100	76,53	6,09E-39	159	-376	Indica	NA	
augustus-11-processed-gene-41.20-mRNA-1	11	OsR498G1119545900.01.T01	99,81	0,00E+00	939	Os11g0194600	96,44	0,00E+00	919	-20	NA	Japonica	
augustus-11-processed-gene-41.22-mRNA-1	11	OsR498G1119549500.01.T01	99,13	0,00E+00	2033	Os11g0192400	99,91	0,00E+00	2074	41	NA	NA	
augustus-11-processed-gene-41.23-mRNA-1	11	OsR498G1119545900.01.T01	97,75	0,00E+00	910	Os11g0194600	100,00	0,00E+00	1012	102	Japonica	NA	
augustus-11-processed-gene-43.12-mRNA-1	11	OsR498G1119742100.01.T04	99,05	0,00E+00	2214	Os11g0261900	100,00	0,00E+00	2268	54	NA	Japonica	
augustus-11-processed-gene-43.45-mRNA-1	11	OsR498G1119746200.01.T02	99,24	0,00E+00	4652	Os11g0263000	100,00	0,00E+00	6536	1884	Japonica	Japonica	NBS-TM
augustus-11-processed-gene-46.39-mRNA-1	11	OsR498G1119770300.01.T01	99,51	0,00E+00	1465	Os11g0267000	100,00	0,00E+00	1483	18	NA	Japonica	
augustus-11-processed-gene-49.13-mRNA-1	11	OsR498G1119789300.01.T01	100,00	0,00E+00	4239	Os11g0270500	100,00	0,00E+00	4239	0	NA	Japonica	CC-LRR-NBS-TM
augustus-11-processed-gene-6.102-mRNA-1	11	OsR498G1119357300.01.T01	99,34	0,00E+00	3220	Os11g0128400	99,50	0,00E+00	3233	13	NA	Japonica	
augustus-11-processed-gene-61.7-mRNA-1	11	OsR498G1119716500.01.T01	99,34	0,00E+00	3231	Os11g0249900	100,00	0,00E+00	2717	-514	Indica	Japonica	Kinase-LRR-TM
augustus-11-processed-gene-62.24-mRNA-1	11	OsR498G1119549700.01.T01	97,54	0,00E+00	1167	Os11g0191800	100,00	0,00E+00	1245	78	NA	NA	
augustus-11-processed-gene-69.17-mRNA-1	11	OsR498G1119583900.01.T05	99,92	0,00E+00	2122	Os11g0206600	99,83	0,00E+00	2118	-4	NA	NA	
augustus-11-processed-gene-7.15-mRNA-1	11	OsR498G1119366100.01.T01	98,38	0,00E+00	965	Os11g0131100	100,00	0,00E+00	985	20	NA	Japonica	

augustus-11-processed-gene-70.2-mRNA-1	11	OsR498G1119587900.01.T03	99,78	0,00E+00	3247	Os11g0207100	99,95	0,00E+00	3260	13	NA	Japonica	
augustus-11-processed-gene-73.31-mRNA-1	11	OsR498G1119608900.01.T02	99,63	4,12E-134	478	Os11g0211800	97,75	4,71E-128	455	-23	NA	Indica	
augustus-11-processed-gene-75.39-mRNA-1	11	OsR498G1119614900.01.T01	99,79	0,00E+00	2535	Os11g0213500	100,00	0,00E+00	2549	14	NA	Japonica	LRR-TM
augustus-11-processed-gene-75.40-mRNA-1	11	OsR498G1119617700.01.T01	77,18	1,16E-72	274	Os11g0214100	100,00	6,37E-172	601	327	Japonica	NA	
augustus-11-processed-gene-75.43-mRNA-1	11	OsR498G1119617700.01.T01	86,38	0,00E+00	655	Os11g0214400	100,00	0,00E+00	1045	390	Japonica	Japonica	
augustus-11-processed-gene-76.18-mRNA-1	11	OsR498G1119617900.01.T01	96,18	0,00E+00	893	Os11g0215100	100,00	0,00E+00	985	92	NA	Japonica	
augustus-11-processed-gene-76.4-mRNA-1	11	OsR498G1119617700.01.T01	95,10	4,26E-88	324	Os11g0214700	100,00	3,73E-96	349	25	NA	Japonica	
augustus-11-processed-gene-79.18-mRNA-1	11	OsR498G1119635700.01.T01	99,81	0,00E+00	927	Os11g0221300	100,00	0,00E+00	931	4	NA	Japonica	
augustus-11-processed-gene-8.94-mRNA-1	11	OsR498G1119371300.01.T01	100,00	0,00E+00	1975	Os11g0132900	100,00	0,00E+00	1975	0	NA	Japonica	CC-Kinase-TM
augustus-11-processed-gene-8.96-mRNA-1	11	OsR498G1119373100.01.T01	99,76	0,00E+00	4551	Os12g0130300	85,94	0,00E+00	2926	-1625	Indica	Japonica	Kinase-TM
augustus-11-processed-gene-8.97-mRNA-1	11	OsR498G1119374500.01.T01	99,66	0,00E+00	1580	Os11g0133600	100,00	0,00E+00	1590	10	NA	Japonica	
augustus-11-processed-gene-81.11-mRNA-1	11	OsR498G1120733700.01.T01	87,88	3,68E-104	378	Os07g0543700	87,61	1,20E-117	422	44	Japonica	Japonica	
augustus-11-processed-gene-83.15-mRNA-1	11	OsR498G1119649900.01.T03	98,93	0,00E+00	4772	Os11g0229400	98,90	0,00E+00	4767	-5	NA	Japonica	LRR-NBS-TM
augustus-11-processed-gene-83.16-mRNA-1	11	OsR498G1119650700.01.T02	93,34	0,00E+00	3200	Os11g0227800	95,71	0,00E+00	4384	1184	Japonica	NA	LRR-NBS-TM
augustus-11-processed-gene-89.15-mRNA-1	11	OsR498G1119684100.01.T01	99,43	0,00E+00	1867	Os11g0241000	100,00	0,00E+00	1894	27	NA	Japonica	
maker-11-augustus-gene-0.11-mRNA-1	11	OsR498G1119278000.01.T03	100,00	9,66E-146	517	Os12g0102300	99,66	2,76E-147	521	4	NA	NA	
maker-11-augustus-gene-0.17-mRNA-1	11	OsR498G1119325200.01.T01	100,00	0,00E+00	1218	Os11g0116900	100,00	0,00E+00	1364	146	Japonica	Japonica	
maker-11-augustus-gene-0.8-mRNA-1	11	OsR498G1119298500.01.T01	100,00	0,00E+00	3074	Os12g0108000	98,53	0,00E+00	2952	-122	NA	NA	
maker-11-augustus-gene-1.39-mRNA-1	11	OsR498G1119329500.01.T03	99,75	0,00E+00	1447	Os12g0117100	98,39	0,00E+00	1397	-50	NA	Japonica	
maker-11-augustus-gene-1.47-mRNA-1	11	OsR498G1119332100.01.T03	98,40	0,00E+00	1517	Os11g0119100	100,00	0,00E+00	1566	49	NA	Japonica	
maker-11-augustus-gene-1.51-mRNA-1	11	OsR498G1119332500.01.T01	99,94	0,00E+00	2766	Os12g0118900	95,98	0,00E+00	1551	-1215	Indica	Japonica	
maker-11-augustus-gene-1.53-mRNA-1	11	OsR498G1119327000.01.T01	100,00	0,00E+00	1521	Os11g0117400	100,00	0,00E+00	1429	-92	NA	NA	
maker-11-augustus-gene-1.55-mRNA-1	11	OsR498G1119327000.01.T01	100,00	0,00E+00	1521	Os11g0117400	100,00	0,00E+00	1429	-92	NA	Japonica	
maker-11-augustus-gene-1.57-mRNA-1	11	OsR498G1119327400.01.T01	99,17	0,00E+00	1701	Os11g0117500	100,00	0,00E+00	1737	36	NA	Japonica	
maker-11-augustus-gene-1.60-mRNA-1	11	OsR498G1119327800.01.T01	98,40	0,00E+00	1734	Os11g0117600	99,90	0,00E+00	1782	48	NA	Japonica	
maker-11-augustus-gene-1.62-mRNA-1	11	OsR498G1119328600.01.T01	99,38	0,00E+00	4614	Os11g0117700	100,00	0,00E+00	4686	72	NA	Japonica	
maker-11-augustus-gene-1.64-mRNA-1	11	OsR498G1119330000.01.T01	99,56	0,00E+00	2034	Os12g0117250	97,02	0,00E+00	1793	-241	Indica	Japonica	
maker-11-augustus-gene-1.67-mRNA-1	11	OsR498G1119330200.01.T02	99,59	0,00E+00	3069	Os11g0118300	99,83	0,00E+00	3085	16	NA	Japonica	
maker-11-augustus-gene-1.70-mRNA-1	11	OsR498G1119330600.01.T01	99,77	0,00E+00	2346	Os12g0117600	98,17	0,00E+00	2251	-95	NA	Japonica	
maker-11-augustus-gene-1.72-mRNA-1	11	OsR498G1119331000.01.T02	99,54	0,00E+00	1541	Os12g0117800	99,88	0,00E+00	1554	13	NA	Japonica	
maker-11-augustus-gene-1.73-mRNA-1	11	OsR498G1119331600.01.T01	99,68	0,00E+00	2217	Os11g0118800	99,91	0,00E+00	2014	-203	NA	Japonica	
maker-11-augustus-gene-10.3-mRNA-1	11	OsR498G1119382100.01.T03	99,88	0,00E+00	1533	Os11g0136500	100,00	0,00E+00	1537	4	NA	Japonica	
maker-11-augustus-gene-10.40-mRNA-1	11	OsR498G1119382000.01.T03	99,73	0,00E+00	1958	Os11g0136400	100,00	0,00E+00	1784	-174	NA	Japonica	
maker-11-augustus-gene-10.49-mRNA-1	11	OsR498G1119382600.01.T02	100,00	1,96E-161	569	Os11g0136701	100,00	4,03E-152	536	-33	NA	Japonica	
maker-11-augustus-gene-10.53-mRNA-1	11	OsR498G1119382800.01.T02	99,91	0,00E+00	1928	Os11g0136800	99,91	0,00E+00	1928	0	NA	Japonica	
maker-11-augustus-gene-10.58-mRNA-1	11	OsR498G1119383800.01.T01	99,88	0,00E+00	2994	Os11g0137000	100,00	0,00E+00	3003	9	NA	Japonica	
maker-11-augustus-gene-10.6-mRNA-1	11	OsR498G1119382300.01.T01	100,00	0,00E+00	2933	Os11g0136600	100,00	0,00E+00	2933	0	NA	Japonica	Kinase-TM
maker-11-augustus-gene-10.60-mRNA-1	11	OsR498G1119384000.01.T01	99,42	0,00E+00	2764	Os12g0133900	96,22	0,00E+00	2610	-154	NA	Japonica	
maker-11-augustus-gene-10.65-mRNA-1	11	OsR498G1119384600.01.T01	99,91	0,00E+00	9803	Os11g0137300	100,00	0,00E+00	6010	-3793	Indica	Japonica	
maker-11-augustus-gene-10.9-mRNA-1	11	OsR498G1119384300.01.T04	100,00	0,00E+00	2100	Os12g0134000	97,15	0,00E+00	1528	-572	Indica	Japonica	
maker-11-augustus-gene-101.39-mRNA-1	11	OsR498G1119927600.01.T02	99,89	0,00E+00	3130	Os11g0416900	99,94	0,00E+00	3134	4	NA	Japonica	
maker-11-augustus-gene-102.0-mRNA-1	11	OsR498G1119930100.01.T01	100,00	0,00E+00	2170	Os11g0417400	100,00	0,00E+00	2170	0	NA	Japonica	
maker-11-augustus-gene-105.33-mRNA-1	11	OsR498G1119966200.01.T01	99,62	0,00E+00	944	Os11g0421800	100,00	0,00E+00	953	9	NA	Japonica	
maker-11-augustus-gene-106.0-mRNA-1	11	OsR498G1119975500.01.T07	95,83	0,00E+00	1582	Os11g0423800	91,25	0,00E+00	740	-842	Indica	Japonica	
maker-11-augustus-gene-107.0-mRNA-1	11	OsR498G1119977800.01.T01	99,80	0,00E+00	1755	Os11g0424400	100,00	0,00E+00	1764	9	NA	Japonica	
maker-11-augustus-gene-108.35-mRNA-1	11	OsR498G1119982900.01.T01	99,60	0,00E+00	895	Os11g0425100	100,00	0,00E+00	1081	186	Japonica	Japonica	
maker-11-augustus-gene-108.38-mRNA-1	11	OsR498G1119984600.01.T03	99,48	0,00E+00	2046	Os11g0425300	99,36	0,00E+00	1953	-93	NA	Japonica	
maker-11-augustus-gene-109.0-mRNA-1	11	OsR498G1119987500.01.T02	100,00	2,00E-106	385	Os11g0425800	100,00	7,00E-107	385	0	NA	Japonica	
maker-11-augustus-gene-109.2-mRNA-1	11	OsR498G1119990700.01.T01	96,35	0,00E+00	638	Os11g0426400	100,00	3,46E-144	509	-129	Indica	Japonica	
maker-11-augustus-gene-11.49-mRNA-1	11	OsR498G1119392100.01.T01	99,80	0,00E+00	2694	Os12g0136200	98,25	0,00E+00	2649	-45	NA	Japonica	
maker-11-augustus-gene-11.55-mRNA-1	11	OsR498G1119394100.01.T05	99,78	0,00E+00	5596	Os12g0136900	97,89	0,00E+00	5330	-266	NA	Japonica	
maker-11-augustus-gene-11.66-mRNA-1	11	OsR498G1119390600.01.T03	100,00	0,00E+00	1218	Os11g0138600	100,00	0,00E+00	1218	0	NA	Japonica	
maker-11-augustus-gene-11.71-mRNA-1	11	OsR498G1119391800.01.T01	99,58	0,00E+00	857	Os11g0139100	100,00	0,00E+00	866	9	NA	Japonica	
maker-11-augustus-gene-11.76-mRNA-1	11	OsR498G1119393400.01.T04	99,83	0,00E+00	1035	Os11g0139850	100,00	0,00E+00	1040	5	NA	Japonica	
maker-11-augustus-gene-11.79-mRNA-1	11	OsR498G1119393800.01.T04	99,36	0,00E+00	837	Os11g0140100	100,00	0,00E+00	843	6	NA	Japonica	
maker-11-augustus-gene-111.32-mRNA-1	11	OsR498G1120003500.01.T01	99,73	0,00E+00	673	Os11g0428800	100,00	0,00E+00	677	4	NA	Japonica	
maker-11-augustus-gene-111.33-mRNA-1	11	OsR498G1120003100.01.T01	98,05	0,00E+00	966	Os08g0560300	89,55	3,83E-159	560	-406	Indica	Japonica	
maker-11-augustus-gene-111.40-mRNA-1	11	OsR498G1120007200.01.T01	99,83	0,00E+00	1057	Os11g0429600	100,00	0,00E+00	911	-146	Indica	Japonica	
maker-11-augustus-gene-111.43-mRNA-1	11	OsR498G1120008800.01.T02	99,52	0,00E+00	1481	Os11g0430000	100,00	0,00E+00	1629	148	NA	Japonica	
maker-11-augustus-gene-112.27-mRNA-1	11	OsR498G1120010000.01.T02	99,52	0,00E+00	1123	Os11g0432900	80,87	4,66E-146	517	-606	Indica	Japonica	
maker-11-augustus-gene-113.3-mRNA-1	11	OsR498G1120015100.01.T01	99,60	0,00E+00	1351	Os11g0432600	100,00	0,00E+00	1364	13	NA	Japonica	
maker-11-augustus-gene-113.5-mRNA-1	11	OsR498G1120011800.01.T03	99,53	0,00E+00	1525	Os11g0432900	82,14	0,00E+00	728	-797	Indica	Japonica	
maker-11-augustus-gene-113.7-mRNA-1	11	OsR498G1120015800.01.T02	99,67	0,00E+00	1076	Os11g0432800	100,00	0,00E+00	1085	9	NA	Japonica	
maker-11-augustus-gene-114.31-mRNA-1	11	OsR498G1120016900.01.T02	99,94	0,00E+00	2891	Os11g0433200	100,00	0,00E+00	1920	-971	Indica	Japonica	
maker-11-augustus-gene-114.42-mRNA-1	11	OsR498G1120018900.01.T01	88,66	1,60E-148	526	Os07g0546700	86,10	4,00E-100	364	-162	Indica	Japonica	
maker-11-augustus-gene-114.46-mRNA-1	11	OsR498G1120019200.01.T01	99,21	0,00E+00	674	Os11g0434000	100,00	0,00E+00	688	14	NA	Japonica	
maker-11-augustus-gene-117.0-mRNA-1	11	OsR498G1120043200.01.T01	99,53	0,00E+00	762	Os11g0438700	75,40	1,90E-80	298	-464	Indica	Japonica	
maker-11-augustus-gene-117.3-mRNA-1	11	OsR498G1120041800.01.T03	99,62	0,00E+00	1421	Os11g0438700	100,00	0,00E+00	1434	13	NA	Japonica	
maker-11-augustus-gene-117.8-mRNA-1	11	OsR498G1120039600.01.T01	99,76	0,00E+00	759	Os11g0439100	100,00	0,00E+00	816	57	NA	Japonica	
maker-11-augustus-gene-118.30-mRNA-1	11	OsR498G1120037500.01.T01	99,82	0,00E+00	1988	Os11g0440200	100,00	0,00E+00	1997	9	NA	Japonica	
maker-11-augustus-gene-118.36-mRNA-1	11	OsR498G1120037500.01.T01	99,82	0,00E+00	1988	Os11g0440200	100,00	0,00E+00	1997	9	NA	Japonica	
maker-11-augustus-gene-119.31-mRNA-1	11	OsR498G1120030500.01.T01	99,72	0,00E+00	2574	Os11g0441500	100,00	0,00E+00	2538	-36	NA	Japonica	
maker-11-augustus-gene-119.34-mRNA-1	11	OsR498G1120026900.01.T01	96,04	0,00E+00	1893	Os11g0442650	99,91	0,00E+00	1890	-3	NA	Japonica	
maker-11-augustus-gene-12.50-mRNA-1	11	OsR498G1119395900.01.T01	100,00	0,00E+00	1324	Os11g0140900	100,00	0,00E+00	1324	0	NA	Japonica	
maker-11-augustus-gene-12.63-mRNA-1	11	OsR498G1119400100.01.T02	99,73	0,00E+00	2661	Os11g0141900	99,93	0,00E+00	2674	13	NA	Japonica	
maker-11-augustus-gene-12.66-mRNA-1	11	OsR498G1119394800.01.T02	99,89	0,00E+00	1668	Os11g0140600	100,00	0,00E+00	1673	5	NA	Japonica	
maker-11-augustus-gene-120.33-mRNA-1	11	OsR498G1120046000.01.T01	99,62	0,00E+00	1399	Os11g0443700	99,87	0,00E+00	1408	9	NA	Japonica	
maker-11-augustus-gene-120.35-mRNA-1	11	OsR498G1120044700.01.T01	99,93	0,00E+00	2458	Os11g0444000	100,00	0,00E+00	2462	4	NA	Japonica	
maker-11-augustus-gene-121.22-mRNA-1	11	OsR498G1120048900.01.T01	99,56	0,00E+00	1205	Os11g0444700	100,00	0,00E+00	1218	13	NA	Japonica	
maker-11-augustus-gene-121.24-mRNA-1	11	OsR498G1120049700.01.T01	99,08	0,00E+00	1148	Os11g0444700	90,02	0,00E+00	932	-216	Indica	Japonica	
maker-11-augustus-gene-121.26-mRNA-1	11	OsR498G1120054300.01.T01	98,94	0,00E+00	2482	Os11g0446000	97,28	0,00E+00	1437	-1045	Indica	Japonica	
maker-11-augustus-gene-122.25-mRNA-1	11	OsR498G1120055700.01.T02	99,91	0,00E+00	5971	Os11g0446500	99,83	0,00E+00	3080	-2891	Indica	Indica	
maker-11-augustus-gene-123.41-mRNA-1	11	OsR498G1120059500.01.T01	99,92	0,00E+00	4336	Os11g0448000	99,85	0,00E+00	4922	586	Japonica	Indica	Kinase-LRR-TM
maker-11-augustus-gene-123.45-mRNA-1	11	OsR498G1120060300.01.T02	99,58	0,00E+00	2986	Os11g0448400	100,00	0,00E+00	3020	34	NA	Japonica	
maker-11-augustus-gene-126.0-mRNA-1	11	OsR498G1120068200.01.T02	99,59	0,00E+00	1752	Os11g0451700	99,90	0,00E+00	1765	13	NA	Japonica	
maker-11-augustus-gene-127.31-mRNA-1	11	OsR498G1120073200.01.T01	99,78	0,00E+00	819	Os11g0453900	100,00	0,00E+00	823	4	NA	Japonica	
maker-11-augustus-gene-127.33-mRNA-1	11	OsR498G1120074000.01.T01	99,80	0,00E+00	889	Os11g0454000	100,00	0,00E+00	893	4	NA	Japonica	
maker-11-augustus-gene-127.35-mRNA-1	11	OsR498G1120074800.01.T01	98,99	0,00E+00	871	Os11g0454200	100,00	0,00E+00	893	22	NA	Japonica	
maker-11-augustus-gene-127.37-mRNA-1	11	OsR498G1120075200.01.T01	86,03	0,00E+00	682	Os11g0454300	100,00	0,00E+00	937	255	Japonica	Japonica	
maker-11-augustus-gene-128.30-mRNA-1	11	OsR498G1120081300.01.T01	99,55	0,00E+00	2781	Os11g0455800	99,93	0,00E+00	2689	-92	NA	Japonica	
maker-11-augustus-gene-128.33-mRNA-1	11	OsR498G1120082900.01.T01	97,70	0,00E+00	894	Os11g0456300	97,51	0,00E+00	890	-4	NA	Japonica	
maker-11-augustus-gene-128.38-mRNA-1	11	OsR498G1120079200.01.T01	99,59	0,00E+00	2603	Os11g0455500	99,61	0,00E+00	2758	155	NA	Japonica	
maker-11-augustus-gene-128.40-mRNA-1	11	OsR498G1120082200.01.T01	99,74	0,00E+00	2092	Os11g0456100	100,00	0,00E+00	1905	-187	NA	Japonica	
maker-11-augustus-gene-128.43-mRNA-1	11	OsR498G1120082600.01.T01	99,65	0,00E+00	1025	Os11g0456200	100,00	0,00E+00	1034	9	NA	Japonica	
maker-11-augustus-gene-128.45-mRNA-1	11	OsR498G1120085200.01.T01	85,93	0,00E+00	703	Os11g0457000	94,23	0,00E+00	1158	455	Japonica	Japonica	
maker-11-augustus-gene-13.13-mRNA-1	11	OsR498G1119403200.01.T02	99,47	0,00E+00	2681	Os11g0143200	100,00	0,00E+00	2717	36	NA	Japonica	
maker-11-augustus-gene-13.19-mRNA-1	11	OsR498G1119403800.01.T01	99,72	0,00E+00	3138	Os11g0143500	100,00	0,00E+00	3236	98	NA	Japonica	
maker-11-augustus-gene-13.9-mRNA-1	11	OsR498G1220910900.01.T01	98,85	1,83E-173	609	Os11g0143300	99,71	1,01E-177	622	13	NA	Japonica	
maker-11-augustus-gene-130.32-mRNA-1	11	OsR498G1120091900.01.T04	99,52	0,00E+00	747	Os11g0460800	81,44	6,53E-124	444	-303	Indica	Japonica	
maker-11-augustus-gene-131.7-mRNA-1	11	OsR498G1120091900.01.T04	93,23	0,00E+00	1018	Os11g0460800	82,97	0,00E+00	706	-312	Indica	Japonica	
maker-11-augustus-gene-132.30-mRNA-1	11	OsR498G1120101100.01.T02	99,32	0,00E+00	1559	Os11g0463600	100,00	0,00E+00	1023	-536	Indica	Japonica	
maker-11-augustus-gene-132.35-mRNA-1	11	OsR498G1120101600.01.T03	100,00	0,00E+00	677	Os11g0463700	100,00	0,00E+00	677	0	NA	Japonica	
maker-11-augustus-gene-133.28-mRNA-1	11	OsR498G1119949600.01.T01	85,10	1,05E-120	434	Os08g0381000	73,02	9,81E-78	290	-144	Indica	Japonica	
maker-11-augustus-gene-133.30-mRNA-1	11	OsR498G1120113400.01.T01	75,93	0,00E+00	917	Os11g0465200	100,00	0,00E+00	2782	1865	Japonica	Indica	
maker-11-augustus-gene-135.29-mRNA-1	11	OsR498G1120113400.01.T01	98,41	0,00E+00	1414	Os11g0469900	99,70	0,00E+00	1175	-239	Indica	Japonica	
maker-11-augustus-gene-135.30-mRNA-1	11	OsR498G1120113400.01.T01	97,32	8,77E-179	627	Os11g0465200	74,69	5,52E-100	363	-264	Indica	Japonica	
maker-11-augustus-gene-135.35-mRNA-1	11	OsR498G1120115000.01.T01	99,53	0,00E+00	3444	Os11g0470200	100,00	0,00E+00	2094	-1350	Indica	Japonica	Kinase-TM
maker-11-augustus-gene-136.26-mRNA-1	11	OsR498G1120119800.01.T02	99,83	0,00E+00	1067	Os11g0472000	100,00	0,00E+00	1072	5	NA	Japonica	
maker-11-augustus-gene-137.33-mRNA-1	11	OsR498G1120121900.01.T06	100,00	0,00E+00	841	Os11g0472500	90,34	0,00E+00	674	-167	Indica	Japonica	
maker-11-augustus-gene-137.50-mRNA-1	11	OsR498G1120126200.01.T01	99,66	0,00E+00	1052	Os11g0473100	100,00	0,00E+00	1061	9	NA	Japonica	
maker-11-augustus-gene-138.0-mRNA-1	11	OsR498G1120128800.01.T01	99,78	0,00E+00	1647	Os11g0474800	86,21	0,00E+00	857	-790	Indica	Indica	
maker-11-augustus-gene-138.2-mRNA-1	11	OsR498G0204151800.01.T05	73,18	0,00E+00	1366	Os11g0474800	92,31	0,00E+00	3586	2220	Japonica	Japonica	
maker-11-augustus-gene-14.50-mRNA-1	11	OsR498G1119406700.01.T01	95,78	0,00E+00	2081	Os11g0146500	100,00	0,00E+00	2278	197	NA	Japonica	
maker-11-augustus-gene-14.62-mRNA-1	11	OsR498G1119408900.01.T01	100,00	0,00E+00	1076	Os11g0147100	100,00	0,00E+00	1072	-4	NA	Japonica	
maker-11-augustus-gene-14.66-mRNA-1	11	OsR498G1119411100.01.T02	99,68	0,00E+00	2768	Os11g0147500	99,94	0,00E+00	2788	20	NA	Japonica	
maker-11-augustus-gene-14.68-mRNA-1	11	OsR498G1119406000.01.T01	98,99	0,00E+00	3498	Os12g0141100	97,63	0,00E+00	3374	-124	NA	Japonica	
maker-11-augustus-gene-14.71-mRNA-1	11	OsR498G1119406000.01.T01	96,45	0,00E+00	2175	Os11g0146100	100,00	0,00E+00	2428	253	Japonica	Indica	
maker-11-augustus-gene-14.74-mRNA-1	11	OsR498G1119407400.01.T02	99,77	0,00E+00	3056	Os11g0146700	100,00	0,00E+00	3074	18	NA	Japonica	
maker-11-augustus-gene-14.81-mRNA-1	11	OsR498G1119407800.01.T03	100,00	0,00E+00	2122	Os11g0146800	100,00	0,00E+00	1786	-336	Indica	Japonica	
maker-11-augustus-gene-14.86-mRNA-1	11	OsR498G1119408600.01.T02	99,37	0,00E+00	2834	Os11g0147000	99,75	0,00E+00	2861	27	NA	Japonica	
maker-11-augustus-gene-142.34-mRNA-1	11	OsR498G1120142300.01.T01	97,74	0,00E+00	678	Os11g0479000	100,00	0,00E+00	715	37	NA	Japonica	
maker-11-augustus-gene-142.36-mRNA-1	11	OsR498G1120143900.01.T01	96,49	0,00E+00	2912	Os11g0479232	99,75	0,00E+00	2142	-770	Indica	Japonica	
maker-11-augustus-gene-142.46-mRNA-1	11	OsR498G1120143800.01.T02	97,49	0,00E+00	3446	Os11g0479100	100,00	0,00E+00	3820	374	NA	Japonica	CC-NBS-TM
maker-11-augustus-gene-143.25-mRNA-1	11	OsR498G1120145600.01.T01	95,55	0,00E+00	2673	Os11g0481200	95,31	0,00E+00	2662	-11	NA	Indica	
maker-11-augustus-gene-144.30-mRNA-1	11	OsR498G1120147500.01.T01	99,67	0,00E+00	3202	Os11g0482100	100,00	0,00E+00	3128	-74	NA	Japonica	
maker-11-augustus-gene-144.36-mRNA-1	11	OsR498G1120146800.01.T01	98,83	0,00E+00	1051	Os11g0482000	100,00	0,00E+00	1083	32	NA	Japonica	
maker-11-augustus-gene-144.38-mRNA-1	11	OsR498G1120147800.01.T01	100,00	0,00E+00	890	Os11g0482200	100,00	2,05E-168	590	-300	Indica	Japonica	
maker-11-augustus-gene-144.41-mRNA-1	11	OsR498G1120148800.01.T01	99,82	0,00E+00	4988	Os11g0482500	100,00	0,00E+00	3853	-1135	Indica	Japonica	
maker-11-augustus-gene-145.0-mRNA-1	11	OsR498G1120151100.01.T04	99,48	4,47E-94	344	Os11g0483600	100,00	3,67E-96	349	5	NA	Japonica	
maker-11-augustus-gene-145.11-mRNA-1	11	OsR498G1120152300.01.T01	99,79	0,00E+00	5178	Os11g0484300	100,00	0,00E+00	5205	27	NA	Japonica	
maker-11-augustus-gene-145.18-mRNA-1	11	OsR498G1120153700.01.T02	99,07	0,00E+00	2849	Os11g0487600	99,87	0,00E+00	2697	-152	NA	Japonica	
maker-11-augustus-gene-145.21-mRNA-1	11	OsR498G1120151800.01.T02	99,81	0,00E+00	2795	Os11g0484000	99,81	0,00E+00	2795	0	NA	Japonica	
maker-11-augustus-gene-145.5-mRNA-1	11	OsR498G1120151500.01.T04	99,79	0,00E+00	846	Os11g0483900	93,27	0,00E+00	1109	263	Japonica	Japonica	
maker-11-augustus-gene-146.0-mRNA-1	11	OsR498G1120154700.01.T02	99,84	0,00E+00	1138	Os11g0488600	100,00	0,00E+00	1040	-98	NA	Japonica	
maker-11-augustus-gene-146.10-mRNA-1	11	OsR498G1120155000.01.T01	99,46	0,00E+00	988	Os11g0488500	100,00	0,00E+00	996	8	NA	Japonica	
maker-11-augustus-gene-146.7-mRNA-1	11	OsR498G1120156700.01.T02	99,64	0,00E+00	2012	Os11g0487100	97,52	0,00E+00	1921	-91	NA	NA	
maker-11-augustus-gene-147.32-mRNA-1	11	OsR498G1120163700.01.T02	97,13	0,00E+00	3798	Os11g0485200	94,06	0,00E+00	3489	-309	NA	NA	
maker-11-augustus-gene-147.38-mRNA-1	11	OsR498G1120160600.01.T05	99,47	0,00E+00	2693	Os11g0485200	99,54	0,00E+00	2698	5	NA	Indica	
maker-11-augustus-gene-148.29-mRNA-1	11	OsR498G1120166100.01.T01	99,41	0,00E+00	2095	Os11g0490600	99,32	0,00E+00	2089	-6	NA	Japonica	
maker-11-augustus-gene-148.34-mRNA-1	11	OsR498G1120167900.01.T01	99,45	0,00E+00	1297	Os11g0490900	99,59	0,00E+00	1302	5	NA	Japonica	
maker-11-augustus-gene-148.36-mRNA-1	11	OsR498G1120168100.01.T03	99,37	0,00E+00	2531	Os11g0491400	100,00	0,00E+00	2316	-215	NA	Japonica	
maker-11-augustus-gene-149.35-mRNA-1	11	OsR498G1120174100.01.T01	93,88	0,00E+00	1363	Os11g0492700	100,00	0,00E+00	930	-433	Indica	Japonica	
maker-11-augustus-gene-149.41-mRNA-1	11	OsR498G1120172400.01.T01	99,43	0,00E+00	939	Os11g0492100	80,00	2,45E-85	315	-624	Indica	Japonica	
maker-11-augustus-gene-15.28-mRNA-1	11	OsR498G1119414500.01.T01	99,86	0,00E+00	2529	Os12g0145400	84,54	0,00E+00	1384	-1145	Indica	Japonica	
maker-11-augustus-gene-15.30-mRNA-1	11	OsR498G1119414700.01.T01	99,65	0,00E+00	2017	Os12g0145500	89,09	0,00E+00	1430	-587	Indica	Japonica	
maker-11-augustus-gene-15.35-mRNA-1	11	OsR498G1119414900.01.T02	98,94	0,00E+00	3323	Os11g0148400	96,62	0,00E+00	3166	-157	NA	Japonica	
maker-11-augustus-gene-15.41-mRNA-1	11	OsR498G1119413400.01.T03	100,00	0,00E+00	1288	Os11g0147800	100,00	0,00E+00	1288	0	NA	Japonica	
maker-11-augustus-gene-15.47-mRNA-1	11	OsR498G1119415200.01.T01	99,94	0,00E+00	2853	Os11g0148500	100,00	0,00E+00	2857	4	NA	Japonica	
maker-11-augustus-gene-15.56-mRNA-1	11	OsR498G1119415400.01.T01	99,29	0,00E+00	994	Os11g0148600	100,00	0,00E+00	1012	18	NA	Japonica	
maker-11-augustus-gene-15.58-mRNA-1	11	OsR498G1119415600.01.T01	99,79	0,00E+00	9240	Os11g0148700	100,00	0,00E+00	5400	-3840	Indica	Japonica	
maker-11-augustus-gene-15.62-mRNA-1	11	OsR498G1119415800.01.T04	99,91	0,00E+00	1939	Os11g0148800	100,00	0,00E+00	1267	-672	Indica	Japonica	
maker-11-augustus-gene-151.31-mRNA-1	11	OsR498G1120184700.01.T01	99,80	0,00E+00	1772	Os11g0497000	100,00	0,00E+00	1781	9	NA	Japonica	
maker-11-augustus-gene-152.33-mRNA-1	11	OsR498G1120186500.01.T01	99,89	0,00E+00	1657	Os11g0498400	100,00	0,00E+00	1662	5	NA	Japonica	
maker-11-augustus-gene-153.0-mRNA-1	11	OsR498G1120188500.01.T01	99,62	0,00E+00	1903	Os11g0499600	100,00	0,00E+00	1921	18	NA	Japonica	
maker-11-augustus-gene-153.10-mRNA-1	11	OsR498G1120193400.01.T01	99,49	0,00E+00	2436	Os11g0501500	99,91	0,00E+00	2095	-341	Indica	Japonica	
maker-11-augustus-gene-154.41-mRNA-1	11	OsR498G1120196500.01.T01	98,52	0,00E+00	702	Os11g0502700	100,00	2,93E-177	619	-83	Indica	Japonica	
maker-11-augustus-gene-154.43-mRNA-1	11	OsR498G1120197300.01.T02	99,40	0,00E+00	2681	Os11g0503400	100,00	0,00E+00	2722	41	NA	Japonica	
maker-11-augustus-gene-154.45-mRNA-1	11	OsR498G1120197700.01.T01	99,52	0,00E+00	1839	Os11g0503900	95,64	0,00E+00	1695	-144	NA	Japonica	
maker-11-augustus-gene-156.31-mRNA-1	11	OsR498G1120205300.01.T01	97,70	0,00E+00	2370	Os11g0507200	100,00	0,00E+00	2500	130	NA	Japonica	
maker-11-augustus-gene-156.35-mRNA-1	11	OsR498G1120206200.01.T01	96,08	0,00E+00	2434	Os11g0507400	96,77	0,00E+00	2951	517	Japonica	Japonica	
maker-11-augustus-gene-156.38-mRNA-1	11	OsR498G1120210200.01.T01	99,45	0,00E+00	1621	Os11g0508600	100,00	0,00E+00	1645	24	NA	Japonica	
maker-11-augustus-gene-158.0-mRNA-1	11	OsR498G1120212100.01.T01	98,34	0,00E+00	1562	Os11g0512000	100,00	0,00E+00	1629	67	NA	Japonica	
maker-11-augustus-gene-159.18-mRNA-1	11	OsR498G1120215800.01.T01	99,67	0,00E+00	1079	Os11g0514400	91,96	0,00E+00	989	-90	NA	Japonica	LRR-TM
maker-11-augustus-gene-159.21-mRNA-1	11	OsR498G1120216000.01.T01	99,87	0,00E+00	1354	Os11g0514500	100,00	0,00E+00	785	-569	Indica	Japonica	LRR-TM
maker-11-augustus-gene-159.26-mRNA-1	11	OsR498G1120216600.01.T01	100,00	0,00E+00	1290	Os11g0514600	93,59	0,00E+00	972	-318	Indica	Japonica	LRR-TM
maker-11-augustus-gene-159.29-mRNA-1	11	OsR498G1120218000.01.T01	85,22	0,00E+00	855	Os11g0514700	100,00	0,00E+00	1364	509	Japonica	Japonica	LRR-TM
maker-11-augustus-gene-159.32-mRNA-1	11	OsR498G1120218600.01.T02	98,91	0,00E+00	2241	Os11g0514800	99,77	0,00E+00	2290	49	NA	Japonica	
maker-11-augustus-gene-159.4-mRNA-1	11	OsR498G1120216800.01.T02	98,89	0,00E+00	5054	Os11g0514000	99,61	0,00E+00	5511	457	NA	Japonica	
maker-11-augustus-gene-16.0-mRNA-1	11	OsR498G1119416900.01.T01	99,91	0,00E+00	1971	Os11g0149100	100,00	0,00E+00	1986	15	NA	Japonica	
maker-11-augustus-gene-16.12-mRNA-1	11	OsR498G1119421100.01.T02	99,33	0,00E+00	1055	Os03g0793100	98,34	0,00E+00	938	-117	Indica	Japonica	
maker-11-augustus-gene-16.16-mRNA-1	11	OsR498G1119421300.01.T01	96,94	0,00E+00	1849	Os11g0150700	100,00	0,00E+00	2484	635	Japonica	Japonica	Kinase-TM
maker-11-augustus-gene-16.18-mRNA-1	11	OsR498G1119420600.01.T01	99,71	1,06E-174	613	Os11g0150400	100,00	8,69E-177	618	5	NA	Japonica	
maker-11-augustus-gene-16.20-mRNA-1	11	OsR498G1119420800.01.T02	99,61	0,00E+00	1361	Os11g0150450	100,00	0,00E+00	798	-563	Indica	Japonica	
maker-11-augustus-gene-16.3-mRNA-1	11	OsR498G1119418100.01.T01	99,72	0,00E+00	645	Os11g0149400	100,00	0,00E+00	650	5	NA	Japonica	
maker-11-augustus-gene-16.6-mRNA-1	11	OsR498G1119419700.01.T04	100,00	0,00E+00	2307	Os11g0150100	100,00	0,00E+00	2307	0	NA	Japonica	
maker-11-augustus-gene-16.9-mRNA-1	11	OsR498G1119420100.01.T04	99,90	0,00E+00	1830	Os11g0150200	100,00	0,00E+00	1835	5	NA	Japonica	
maker-11-augustus-gene-160.0-mRNA-1	11	OsR498G1120222300.01.T01	100,00	0,00E+00	2646	Os11g0516000	100,00	0,00E+00	2646	0	NA	NA	
maker-11-augustus-gene-160.10-mRNA-1	11	OsR498G1120221800.01.T06	100,00	0,00E+00	688	Os11g0515900	99,72	0,00E+00	638	-50	NA	NA	
maker-11-augustus-gene-160.15-mRNA-1	11	OsR498G1120224000.01.T02	100,00	0,00E+00	1126	Os11g0517200	100,00	0,00E+00	1279	153	Japonica	NA	
maker-11-augustus-gene-160.20-mRNA-1	11	OsR498G1120224200.01.T01	100,00	5,82E-178	623	Os11g0517400	100,00	2,02E-178	623	0	NA	NA	
maker-11-augustus-gene-160.8-mRNA-1	11	OsR498G1120221400.01.T02	99,82	0,00E+00	3066	Os11g0515500	100,00	0,00E+00	3079	13	NA	Japonica	
maker-11-augustus-gene-161.19-mRNA-1	11	OsR498G1120227300.01.T02	100,00	0,00E+00	1261	Os11g0518600	100,00	0,00E+00	1261	0	NA	NA	
maker-11-augustus-gene-162.26-mRNA-1	11	OsR498G1120233300.01.T03	100,00	0,00E+00	1220	Os11g0522000	99,86	0,00E+00	1289	69	NA	NA	
maker-11-augustus-gene-162.33-mRNA-1	11	OsR498G1120234600.01.T01	100,00	0,00E+00	6904	Os11g0521900	100,00	0,00E+00	3599	-3305	Indica	NA	
maker-11-augustus-gene-163.0-mRNA-1	11	OsR498G1120235500.01.T01	93,32	0,00E+00	1745	Os11g0521000	93,32	0,00E+00	1745	0	NA	NA	
maker-11-augustus-gene-163.3-mRNA-1	11	OsR498G1120235900.01.T02	99,85	0,00E+00	2432	Os11g0520500	99,94	0,00E+00	2772	340	Japonica	Japonica	
maker-11-augustus-gene-163.8-mRNA-1	11	OsR498G1120238700.01.T01	99,65	0,00E+00	1533	Os11g0522900	100,00	0,00E+00	1546	13	NA	Japonica	
maker-11-augustus-gene-164.0-mRNA-1	11	OsR498G1120240900.01.T01	99,78	0,00E+00	1642	Os11g0523110	100,00	0,00E+00	1651	9	NA	Japonica	
maker-11-augustus-gene-164.4-mRNA-1	11	OsR498G1120244700.01.T01	99,49	0,00E+00	2805	Os11g0523700	100,00	0,00E+00	2841	36	NA	Japonica	
maker-11-augustus-gene-164.6-mRNA-1	11	OsR498G1120243200.01.T01	98,21	0,00E+00	673	Os03g0193700	94,25	2,63E-135	480	-193	Indica	Japonica	
maker-11-augustus-gene-164.9-mRNA-1	11	OsR498G1120245800.01.T02	99,61	0,00E+00	4576	Os11g0523800	99,88	0,00E+00	4606	30	NA	Japonica	
maker-11-augustus-gene-165.0-mRNA-1	11	OsR498G1120247700.01.T01	100,00	0,00E+00	1494	Os11g0524300	100,00	0,00E+00	1494	0	NA	Japonica	
maker-11-augustus-gene-165.6-mRNA-1	11	OsR498G1120252900.01.T01	97,68	0,00E+00	3626	Os11g0525600	97,73	0,00E+00	3630	4	NA	Japonica	
maker-11-augustus-gene-166.0-mRNA-1	11	OsR498G1120253100.01.T01	99,35	0,00E+00	3534	Os11g0525700	97,43	0,00E+00	3394	-140	NA	Japonica	
maker-11-augustus-gene-166.11-mRNA-1	11	OsR498G1120253400.01.T01	95,49	0,00E+00	1014	Os11g0525800	95,65	0,00E+00	1019	5	NA	Japonica	
maker-11-augustus-gene-166.4-mRNA-1	11	OsR498G1120254900.01.T01	100,00	0,00E+00	843	Os11g0526200	99,71	1,62E-175	615	-228	Indica	Japonica	
maker-11-augustus-gene-166.9-mRNA-1	11	OsR498G1120261100.01.T02	99,64	1,90E-139	496	Os07g0278866	95,52	1,05E-86	319	-177	Indica	Japonica	
maker-11-augustus-gene-167.13-mRNA-1	11	OsR498G1120263000.01.T01	99,56	0,00E+00	814	Os11g0528500	100,00	0,00E+00	823	9	NA	Japonica	
maker-11-augustus-gene-167.16-mRNA-1	11	OsR498G1120263200.01.T01	99,83	0,00E+00	3195	Os11g0528700	100,00	0,00E+00	2390	-805	Indica	Japonica	
maker-11-augustus-gene-167.2-mRNA-1	11	OsR498G1120262500.01.T01	100,00	0,00E+00	1012	Os11g0528300	100,00	0,00E+00	1012	0	NA	Japonica	
maker-11-augustus-gene-167.20-mRNA-1	11	OsR498G1120265600.01.T01	99,36	0,00E+00	2205	Os11g0529500	100,00	0,00E+00	2241	36	NA	Japonica	
maker-11-augustus-gene-167.22-mRNA-1	11	OsR498G1120266400.01.T01	98,93	0,00E+00	1794	Os11g0529900	100,00	0,00E+00	2208	414	Japonica	Japonica	
maker-11-augustus-gene-167.7-mRNA-1	11	OsR498G1120266100.01.T01	99,78	0,00E+00	1636	Os11g0529800	100,00	0,00E+00	2176	540	Japonica	Japonica	
maker-11-augustus-gene-167.9-mRNA-1	11	OsR498G1120262800.01.T02	99,62	0,00E+00	1397	Os11g0528400	100,00	0,00E+00	1411	14	NA	Japonica	
maker-11-augustus-gene-168.27-mRNA-1	11	OsR498G1120272700.01.T01	99,22	0,00E+00	904	Os11g0531300	100,00	0,00E+00	922	18	NA	Japonica	
maker-11-augustus-gene-168.29-mRNA-1	11	OsR498G1120274700.01.T02	99,46	0,00E+00	1319	Os11g0531700	100,00	0,00E+00	1337	18	NA	Japonica	
maker-11-augustus-gene-168.32-mRNA-1	11	OsR498G1120269200.01.T01	99,83	0,00E+00	2150	Os11g0530600	100,00	0,00E+00	2159	9	NA	Japonica	
maker-11-augustus-gene-169.30-mRNA-1	11	OsR498G1120276200.01.T01	97,99	0,00E+00	1806	Os11g0532600	100,00	0,00E+00	2376	570	Japonica	Japonica	
maker-11-augustus-gene-169.45-mRNA-1	11	OsR498G1120278000.01.T01	99,94	0,00E+00	3194	Os11g0533100	100,00	0,00E+00	3198	4	NA	Japonica	
maker-11-augustus-gene-169.54-mRNA-1	11	OsR498G1120279400.01.T07	99,73	0,00E+00	1967	Os11g0533800	100,00	0,00E+00	1981	14	NA	Japonica	
maker-11-augustus-gene-17.42-mRNA-1	11	OsR498G1119422500.01.T01	100,00	0,00E+00	1434	Os11g0151100	100,00	0,00E+00	1085	-349	Indica	Japonica	
maker-11-augustus-gene-17.45-mRNA-1	11	OsR498G1119427900.01.T01	99,16	0,00E+00	838	Os11g0151800	100,00	0,00E+00	751	-87	Indica	Japonica	

maker-11-augustus-gene-17.50-mRNA-1	11	OsR498G1119421800.01.T01	99,04	0,00E+00	2560	Os12g0149900	85,89	0,00E+00	1618	-942	Indica	Japonica	
maker-11-augustus-gene-17.54-mRNA-1	11	OsR498G1119426800.01.T02	99,78	0,00E+00	2433	Os11g0151500	100,00	0,00E+00	2425	-8	NA	Japonica	
maker-11-augustus-gene-17.65-mRNA-1	11	OsR498G1119427200.01.T01	99,54	0,00E+00	3065	Os11g0151700	100,00	0,00E+00	2425	-640	Indica	Japonica	
maker-11-augustus-gene-17.67-mRNA-1	11	OsR498G1119431200.01.T01	99,81	0,00E+00	938	Os11g0152500	100,00	0,00E+00	942	4	NA	Japonica	
maker-11-augustus-gene-17.70-mRNA-1	11	OsR498G1119432000.01.T01	99,86	0,00E+00	2616	Os11g0152700	100,00	0,00E+00	2625	9	NA	Japonica	
maker-11-augustus-gene-170.27-mRNA-1	11	OsR498G1120281000.01.T01	99,91	0,00E+00	1955	Os11g0534300	100,00	0,00E+00	1959	4	NA	Japonica	
maker-11-augustus-gene-171.12-mRNA-1	11	OsR498G1120287000.01.T02	97,28	0,00E+00	2675	Os11g0536800	97,41	0,00E+00	2684	9	NA	Japonica	
maker-11-augustus-gene-171.2-mRNA-1	11	OsR498G1119336000.01.T01	91,26	1,02E-77	290	Os11g0120700	91,26	3,58E-78	290	0	NA	Japonica	
maker-11-augustus-gene-171.4-mRNA-1	11	OsR498G1120292700.01.T01	100,00	0,00E+00	2560	Os11g0538900	100,00	0,00E+00	2560	0	NA	Japonica	
maker-11-augustus-gene-172.0-mRNA-1	11	OsR498G1120293500.01.T01	99,78	0,00E+00	1593	Os11g0539200	100,00	0,00E+00	1602	9	NA	Japonica	
maker-11-augustus-gene-172.18-mRNA-1	11	OsR498G1120296000.01.T02	99,58	0,00E+00	2982	Os11g0540100	90,89	0,00E+00	2202	-780	Indica	Japonica	
maker-11-augustus-gene-172.2-mRNA-1	11	OsR498G1120295700.01.T01	97,09	0,00E+00	2470	Os11g0539700	100,00	0,00E+00	1835	-635	Indica	Japonica	
maker-11-augustus-gene-172.5-mRNA-1	11	OsR498G1120297100.01.T01	99,19	0,00E+00	2413	Os11g0540600	100,00	0,00E+00	2462	49	NA	Japonica	
maker-11-augustus-gene-172.7-mRNA-1	11	OsR498G1120294800.01.T01	96,30	0,00E+00	1773	Os11g0539500	100,00	0,00E+00	2035	262	Japonica	Japonica	
maker-11-augustus-gene-172.9-mRNA-1	11	OsR498G1120295400.01.T01	98,15	0,00E+00	2512	Os11g0539600	100,00	0,00E+00	1754	-758	Indica	Japonica	
maker-11-augustus-gene-173.49-mRNA-1	11	OsR498G1120304700.01.T02	98,77	0,00E+00	1282	Os11g0540900	71,05	2,34E-80	299	-983	Indica	Japonica	
maker-11-augustus-gene-173.50-mRNA-1	11	OsR498G1120304700.01.T01	97,82	0,00E+00	1172	Os11g0541600	100,00	0,00E+00	1305	133	Japonica	Japonica	
maker-11-augustus-gene-174.3-mRNA-1	11	OsR498G1120311100.01.T01	94,97	0,00E+00	2056	Os11g0543300	100,00	0,00E+00	2722	666	Japonica	Japonica	
maker-11-augustus-gene-174.5-mRNA-1	11	OsR498G1120311400.01.T01	97,20	0,00E+00	1867	Os11g0543500	100,00	0,00E+00	1959	92	NA	Japonica	
maker-11-augustus-gene-175.42-mRNA-1	11	OsR498G1120314100.01.T01	100,00	0,00E+00	2554	Os11g0544600	100,00	0,00E+00	2554	0	NA	NA	
maker-11-augustus-gene-175.46-mRNA-1	11	OsR498G1120314500.01.T03	100,00	0,00E+00	1344	Os11g0544700	100,00	0,00E+00	1344	0	NA	NA	
maker-11-augustus-gene-175.54-mRNA-1	11	OsR498G1120312000.01.T01	99,77	0,00E+00	2287	Os11g0544100	100,00	0,00E+00	2652	365	Japonica	Japonica	
maker-11-augustus-gene-175.56-mRNA-1	11	OsR498G1120312200.01.T02	100,00	0,00E+00	884	Os11g0544200	100,00	0,00E+00	883	-1	NA	Japonica	
maker-11-augustus-gene-175.66-mRNA-1	11	OsR498G1120315000.01.T02	99,76	0,00E+00	2931	Os11g0544800	100,00	0,00E+00	2949	18	NA	Japonica	
maker-11-augustus-gene-175.71-mRNA-1	11	OsR498G1120315800.01.T02	99,36	0,00E+00	1394	Os11g0545000	100,00	0,00E+00	1644	250	Japonica	Japonica	
maker-11-augustus-gene-176.2-mRNA-1	11	OsR498G1120321500.01.T01	99,71	0,00E+00	3046	Os11g0546000	99,88	0,00E+00	3059	13	NA	Japonica	
maker-11-augustus-gene-176.25-mRNA-1	11	OsR498G1120321000.01.T01	99,92	0,00E+00	2301	Os11g0545800	99,92	0,00E+00	2164	-137	NA	Japonica	
maker-11-augustus-gene-176.32-mRNA-1	11	OsR498G1120322200.01.T03	96,65	0,00E+00	1883	Os11g0546300	100,00	0,00E+00	1992	109	NA	Japonica	
maker-11-augustus-gene-176.6-mRNA-1	11	OsR498G1120321700.01.T01	99,61	0,00E+00	2756	Os11g0546100	100,00	0,00E+00	2787	31	NA	Japonica	
maker-11-augustus-gene-177.0-mRNA-1	11	OsR498G1120324100.01.T01	99,88	0,00E+00	1435	Os11g0546900	100,00	0,00E+00	1440	5	NA	Japonica	
maker-11-augustus-gene-177.4-mRNA-1	11	OsR498G1120323400.01.T01	98,39	0,00E+00	861	Os03g0708000	73,45	1,77E-54	212	-649	Indica	Japonica	
maker-11-augustus-gene-178.27-mRNA-1	11	OsR498G0305541500.01.T01	75,32	8,34E-118	425	Os11g0549615	100,00	0,00E+00	1261	836	Japonica	Japonica	
maker-11-augustus-gene-178.29-mRNA-1	11	OsR498G1120328800.01.T01	98,39	0,00E+00	1719	Os11g0549620	100,00	0,00E+00	1792	73	NA	Japonica	
maker-11-augustus-gene-179.38-mRNA-1	11	OsR498G1120331100.01.T01	99,74	0,00E+00	2039	Os11g0549700	99,89	0,00E+00	1659	-380	Indica	Japonica	
maker-11-augustus-gene-179.45-mRNA-1	11	OsR498G1120331000.01.T01	89,93	0,00E+00	1042	Os11g0549600	90,94	0,00E+00	976	-66	NA	NA	
maker-11-augustus-gene-179.54-mRNA-1	11	OsR498G1120331000.01.T01	95,09	0,00E+00	1551	Os11g0549600	93,39	0,00E+00	1062	-489	Indica	Japonica	
maker-11-augustus-gene-18.0-mRNA-1	11	OsR498G1119433900.01.T02	99,45	0,00E+00	2576	Os11g0153400	99,38	0,00E+00	2577	1	NA	Japonica	
maker-11-augustus-gene-18.15-mRNA-1	11	OsR498G1119435300.01.T01	100,00	5,14E-108	390	Os11g0153800	100,00	7,60E-107	385	-5	NA	Japonica	
maker-11-augustus-gene-18.23-mRNA-1	11	OsR498G1119435900.01.T01	100,00	0,00E+00	1618	Os11g0153900	100,00	0,00E+00	1618	0	NA	Japonica	
maker-11-augustus-gene-18.28-mRNA-1	11	OsR498G1119437300.01.T01	99,21	0,00E+00	1565	Os11g0154500	100,00	0,00E+00	1597	32	NA	Japonica	NAC17
maker-11-augustus-gene-18.3-mRNA-1	11	OsR498G1119434700.01.T02	99,30	0,00E+00	1761	Os11g0153700	81,77	0,00E+00	948	-813	Indica	Japonica	
maker-11-augustus-gene-18.34-mRNA-1	11	OsR498G1119438100.01.T01	99,74	0,00E+00	2034	Os12g0156400	82,68	0,00E+00	1131	-903	Indica	Japonica	
maker-11-augustus-gene-18.9-mRNA-1	11	OsR498G1119435100.01.T02	96,22	0,00E+00	1848	Os11g0153700	96,22	0,00E+00	1848	0	NA	Japonica	
maker-11-augustus-gene-180.11-mRNA-1	11	OsR498G1120334400.01.T01	99,02	0,00E+00	1443	Os11g0550900	100,00	0,00E+00	2035	592	Japonica	Japonica	
maker-11-augustus-gene-180.15-mRNA-1	11	OsR498G1120335400.01.T03	99,72	0,00E+00	645	Os11g0551350	100,00	5,34E-91	333	-312	Indica	Japonica	
maker-11-augustus-gene-180.19-mRNA-1	11	OsR498G1120336600.01.T05	100,00	1,12E-162	572	Os11g0551800	100,00	3,90E-163	572	0	NA	Japonica	
maker-11-augustus-gene-180.26-mRNA-1	11	OsR498G1120337800.01.T01	99,96	0,00E+00	4389	Os11g0552100	100,00	0,00E+00	4394	5	NA	Japonica	
maker-11-augustus-gene-180.4-mRNA-1	11	OsR498G1120336900.01.T01	99,76	0,00E+00	2205	Os11g0551900	100,00	0,00E+00	2219	14	NA	Japonica	
maker-11-augustus-gene-181.32-mRNA-1	11	OsR498G1120340200.01.T01	99,53	0,00E+00	3439	Os11g0552600	100,00	0,00E+00	1997	-1442	Indica	Japonica	
maker-11-augustus-gene-182.0-mRNA-1	11	OsR498G1120348100.01.T01	98,95	1,36E-140	499	Os11g0557000	100,00	6,13E-146	515	16	NA	Japonica	
maker-11-augustus-gene-182.2-mRNA-1	11	OsR498G1120349100.01.T01	98,80	0,00E+00	1314	Os11g0557700	99,20	0,00E+00	1327	13	NA	Japonica	
maker-11-augustus-gene-182.21-mRNA-1	11	OsR498G1120350400.01.T01	99,60	0,00E+00	1773	Os11g0558200	100,00	0,00E+00	1792	19	NA	Japonica	
maker-11-augustus-gene-182.6-mRNA-1	11	OsR498G1120349900.01.T01	100,00	6,16E-159	560	Os11g0557900	100,00	2,14E-159	560	0	NA	Japonica	
maker-11-augustus-gene-183.28-mRNA-1	11	OsR498G1120353500.01.T01	99,70	0,00E+00	1794	Os11g0559200	100,00	0,00E+00	1808	14	NA	Japonica	Kinase-LRR-TM
maker-11-augustus-gene-185.0-mRNA-1	11	OsR498G1120370600.01.T01	96,32	0,00E+00	1553	Os11g0565300	99,79	0,00E+00	1683	130	NA	Japonica	Kinase-TM
maker-11-augustus-gene-186.38-mRNA-1	11	OsR498G1120376700.01.T01	94,31	0,00E+00	1849	Os11g0566800	100,00	0,00E+00	2165	316	Japonica	Japonica	
maker-11-augustus-gene-187.0-mRNA-1	11	OsR498G1120379700.01.T04	99,90	0,00E+00	1868	Os11g0568600	100,00	0,00E+00	1873	5	NA	Japonica	
maker-11-augustus-gene-187.12-mRNA-1	11	OsR498G1120381200.01.T01	99,74	0,00E+00	700	Os11g0568800	100,00	0,00E+00	1171	471	Japonica	Japonica	Kinase
maker-11-augustus-gene-187.15-mRNA-1	11	OsR498G1120383600.01.T02	98,50	0,00E+00	4730	Os11g0569300	100,00	0,00E+00	5800	1070	Japonica	Japonica	Kinase-LRR-TM
maker-11-augustus-gene-187.20-mRNA-1	11	OsR498G1120381200.01.T01	70,77	2,29E-108	395	Os11g0569500	100,00	0,00E+00	1629	1234	Japonica	Japonica	Kinase-LRR-TM
maker-11-augustus-gene-187.5-mRNA-1	11	OsR498G1120380300.01.T05	97,63	0,00E+00	2897	Os11g0568700	97,69	0,00E+00	2902	5	NA	Japonica	
maker-11-augustus-gene-187.9-mRNA-1	11	OsR498G1120379000.01.T01	99,18	0,00E+00	2156	Os11g0568300	99,26	0,00E+00	2160	4	NA	Japonica	
maker-11-augustus-gene-188.40-mRNA-1	11	OsR498G1120386200.01.T01	99,13	0,00E+00	2240	Os11g0569600	82,07	0,00E+00	1249	-991	Indica	Japonica	Kinase-LRR-TM
maker-11-augustus-gene-188.43-mRNA-1	11	OsR498G1120381200.01.T01	75,40	0,00E+00	1700	Os11g0569800	100,00	0,00E+00	4453	2753	Japonica	NA	Kinase-LRR-TM
maker-11-augustus-gene-188.45-mRNA-1	11	OsR498G1120387200.01.T01	100,00	0,00E+00	1081	Os11g0570400	100,00	0,00E+00	1081	0	NA	Japonica	
maker-11-augustus-gene-189.4-mRNA-1	11	OsR498G1120391500.01.T01	99,67	0,00E+00	5493	Os11g0572100	100,00	0,00E+00	5526	33	NA	Japonica	NBS-TM
maker-11-augustus-gene-19.0-mRNA-1	11	OsR498G1119440500.01.T01	99,40	0,00E+00	3257	Os11g0155500	99,95	0,00E+00	3296	39	NA	Japonica	
maker-11-augustus-gene-19.11-mRNA-1	11	OsR498G1119443000.01.T01	99,76	0,00E+00	1507	Os11g0156000	100,00	0,00E+00	1516	9	NA	Japonica	
maker-11-augustus-gene-19.14-mRNA-1	11	OsR498G1119443600.01.T01	99,68	0,00E+00	2260	Os11g0156200	100,00	0,00E+00	2280	20	NA	Japonica	
maker-11-augustus-gene-190.0-mRNA-1	11	OsR498G1120393700.01.T01	98,06	0,00E+00	2471	Os11g0572400	100,00	0,00E+00	2603	132	NA	Japonica	
maker-11-augustus-gene-190.22-mRNA-1	11	OsR498G1120394400.01.T01	97,53	0,00E+00	2681	Os11g0572800	100,00	0,00E+00	2825	144	NA	Japonica	
maker-11-augustus-gene-190.5-mRNA-1	11	OsR498G1120394100.01.T01	98,88	0,00E+00	2667	Os11g0572700	100,00	0,00E+00	1893	-774	Indica	Japonica	
maker-11-augustus-gene-191.36-mRNA-1	11	OsR498G1120403500.01.T01	98,57	0,00E+00	731	Os11g0574200	100,00	0,00E+00	645	-86	Indica	Japonica	
maker-11-augustus-gene-192.0-mRNA-1	11	OsR498G0307013100.01.T01	70,23	0,00E+00	1132	Os11g0575600	100,00	0,00E+00	4702	3570	Japonica	NA	
maker-11-augustus-gene-194.34-mRNA-1	11	OsR498G1120405900.01.T02	98,48	5,40E-162	572	Os11g0579800	100,00	2,79E-160	563	-9	NA	Japonica	
maker-11-augustus-gene-194.36-mRNA-1	11	OsR498G1120406500.01.T01	99,89	0,00E+00	3362	Os11g0580000	100,00	0,00E+00	3371	9	NA	Japonica	
maker-11-augustus-gene-194.38-mRNA-1	11	OsR498G1120410300.01.T01	99,53	0,00E+00	760	Os11g0581700	100,00	6,38E-167	585	-175	Indica	Japonica	
maker-11-augustus-gene-194.42-mRNA-1	11	OsR498G1120406200.01.T01	98,44	0,00E+00	3885	Os11g0579900	100,00	0,00E+00	4042	157	NA	Japonica	
maker-11-augustus-gene-194.46-mRNA-1	11	OsR498G1120407000.01.T01	99,28	0,00E+00	1223	Os11g0545000	82,13	0,00E+00	698	-525	Indica	Japonica	
maker-11-augustus-gene-194.48-mRNA-1	11	OsR498G1120407400.01.T01	99,73	0,00E+00	1333	Os11g0580800	100,00	0,00E+00	1342	9	NA	Japonica	
maker-11-augustus-gene-195.0-mRNA-1	11	OsR498G1120411100.01.T01	99,61	0,00E+00	1388	Os11g0581900	100,00	0,00E+00	1402	14	NA	Japonica	
maker-11-augustus-gene-195.12-mRNA-1	11	OsR498G1120413700.01.T03	99,90	0,00E+00	3752	Os11g0583200	100,00	0,00E+00	3600	-152	NA	Japonica	
maker-11-augustus-gene-195.2-mRNA-1	11	OsR498G1120411700.01.T01	96,42	0,00E+00	3195	Os11g0582300	96,68	0,00E+00	3217	22	NA	Japonica	NBS-TM
maker-11-augustus-gene-195.20-mRNA-1	11	OsR498G1120411600.01.T01	99,52	0,00E+00	1102	Os11g0582100	100,00	0,00E+00	1115	13	NA	Japonica	
maker-11-augustus-gene-195.8-mRNA-1	11	OsR498G1120412300.01.T01	100,00	0,00E+00	720	Os11g0582500	100,00	0,00E+00	720	0	NA	Japonica	
maker-11-augustus-gene-196.42-mRNA-1	11	OsR498G1120418500.01.T02	99,70	0,00E+00	4763	Os11g0585900	100,00	0,00E+00	4643	-120	NA	Japonica	
maker-11-augustus-gene-197.48-mRNA-1	11	OsR498G1120419800.01.T02	99,88	0,00E+00	3059	Os11g0586300	100,00	0,00E+00	2526	-533	Indica	Japonica	
maker-11-augustus-gene-197.58-mRNA-1	11	OsR498G1120422600.01.T01	99,60	0,00E+00	3962	Os11g0587300	100,00	0,00E+00	3983	21	NA	NA	
maker-11-augustus-gene-198.0-mRNA-1	11	OsR498G1120426500.01.T01	99,61	0,00E+00	1838	Os07g0485500	73,36	1,13E-156	553	-1285	Indica	Japonica	
maker-11-augustus-gene-198.10-mRNA-1	11	OsR498G1120424800.01.T01	99,72	0,00E+00	1901	Os07g0525400	77,41	0,00E+00	826	-1075	Indica	Japonica	
maker-11-augustus-gene-198.2-mRNA-1	11	OsR498G1120426900.01.T01	100,00	0,00E+00	724	Os11g0588300	100,00	0,00E+00	724	0	NA	Japonica	
maker-11-augustus-gene-198.7-mRNA-1	11	OsR498G1120426900.01.T01	92,73	1,13E-168	593	Os11g0588300	92,73	3,91E-169	593	0	NA	Japonica	
maker-11-augustus-gene-199.32-mRNA-1	11	OsR498G1120429100.01.T01	98,45	0,00E+00	3246	Os11g0590400	100,00	0,00E+00	4778	1532	Japonica	Japonica	NBS-TM
maker-11-augustus-gene-199.35-mRNA-1	11	OsR498G1120430700.01.T02	75,01	0,00E+00	1686	Os11g0590700	100,00	0,00E+00	4605	2919	Japonica	Japonica	CC-NBS-TM
maker-11-augustus-gene-199.37-mRNA-1	11	OsR498G1120430300.01.T01	99,71	0,00E+00	1870	Os11g0590900	100,00	0,00E+00	1884	14	NA	Japonica	CC-NBS-TM
maker-11-augustus-gene-199.40-mRNA-1	11	OsR498G1120430700.01.T02	99,27	0,00E+00	5057	Os11g0590900	75,61	0,00E+00	1896	-3161	Indica	Japonica	CC-LRR-NBS-TM
maker-11-augustus-gene-199.42-mRNA-1	11	OsR498G1120430700.01.T02	98,81	0,00E+00	1022	Os11g0591000	100,00	0,00E+00	953	-69	NA	NA	
maker-11-augustus-gene-2.11-mRNA-1	11	OsR498G1119336000.01.T01	100,00	0,00E+00	1445	Os11g0120700	100,00	0,00E+00	1409	-36	NA	Japonica	
maker-11-augustus-gene-2.14-mRNA-1	11	OsR498G1119336800.01.T03	98,99	0,00E+00	1212	Os12g0120500	96,67	0,00E+00	1140	-72	NA	Japonica	
maker-11-augustus-gene-2.17-mRNA-1	11	OsR498G1119338600.01.T02	100,00	0,00E+00	666	Os11g0121300	100,00	0,00E+00	666	0	NA	Japonica	
maker-11-augustus-gene-2.19-mRNA-1	11	OsR498G1119338800.01.T01	100,00	0,00E+00	2241	Os11g0121400	100,00	0,00E+00	2241	0	NA	Japonica	Kinase
maker-11-augustus-gene-2.2-mRNA-1	11	OsR498G1119333300.01.T02	98,77	0,00E+00	1984	Os12g0119700	94,33	0,00E+00	1064	-920	Indica	Japonica	
maker-11-augustus-gene-2.4-mRNA-1	11	OsR498G1119335700.01.T01	97,81	0,00E+00	1096	Os11g0120600	100,00	0,00E+00	803	-293	Indica	Japonica	
maker-11-augustus-gene-2.9-mRNA-1	11	OsR498G1119333800.01.T01	99,81	0,00E+00	3819	Os11g0120200	100,00	0,00E+00	3837	18	NA	Japonica	
maker-11-augustus-gene-20.0-mRNA-1	11	OsR498G1119445300.01.T01	99,69	0,00E+00	1166	Os11g0156600	100,00	0,00E+00	1180	14	NA	Japonica	
maker-11-augustus-gene-20.11-mRNA-1	11	OsR498G1119447400.01.T01	100,00	0,00E+00	1909	Os11g0157000	96,78	0,00E+00	2269	360	Japonica	Japonica	
maker-11-augustus-gene-20.13-mRNA-1	11	OsR498G1119448000.01.T01	98,87	0,00E+00	774	Os11g0157000	100,00	0,00E+00	675	-99	Indica	Japonica	
maker-11-augustus-gene-20.17-mRNA-1	11	OsR498G1119450600.01.T03	99,80	0,00E+00	1768	Os11g0157400	100,00	0,00E+00	1777	9	NA	Japonica	
maker-11-augustus-gene-20.3-mRNA-1	11	OsR498G1119446100.01.T01	99,69	0,00E+00	4542	Os11g0156800	100,00	0,00E+00	2533	-2009	Indica	Japonica	
maker-11-augustus-gene-200.14-mRNA-1	11	OsR498G1120436500.01.T01	97,55	0,00E+00	2094	Os11g0592600	97,63	0,00E+00	2099	5	NA	Japonica	
maker-11-augustus-gene-200.19-mRNA-1	11	OsR498G1120431000.01.T02	99,73	0,00E+00	2000	Os11g0591100	100,00	0,00E+00	2013	13	NA	Japonica	
maker-11-augustus-gene-200.2-mRNA-1	11	OsR498G1120433700.01.T01	98,95	0,00E+00	838	Os11g0591800	100,00	0,00E+00	861	23	NA	Japonica	
maker-11-augustus-gene-200.24-mRNA-1	11	OsR498G1120432000.01.T01	99,67	0,00E+00	2745	Os11g0591200	100,00	0,00E+00	2343	-402	Indica	Japonica	
maker-11-augustus-gene-200.28-mRNA-1	11	OsR498G1120434800.01.T05	99,89	0,00E+00	1614	Os11g0592300	100,00	0,00E+00	1618	4	NA	Japonica	
maker-11-augustus-gene-200.4-mRNA-1	11	OsR498G1120433900.01.T01	98,90	0,00E+00	795	Os11g0592000	100,00	0,00E+00	818	23	NA	Japonica	
maker-11-augustus-gene-200.6-mRNA-1	11	OsR498G1120434100.01.T01	99,78	0,00E+00	819	Os11g0592100	100,00	0,00E+00	829	10	NA	Japonica	
maker-11-augustus-gene-201.0-mRNA-1	11	OsR498G1120440500.01.T01	99,87	0,00E+00	2821	Os06g0141000	77,78	0,00E+00	1096	-1725	Indica	Japonica	
maker-11-augustus-gene-201.10-mRNA-1	11	OsR498G1120445500.01.T02	98,89	0,00E+00	1426	Os11g0595200	100,00	0,00E+00	1467	41	NA	Japonica	
maker-11-augustus-gene-201.12-mRNA-1	11	OsR498G1120437200.01.T01	99,78	0,00E+00	3234	Os11g0592800	100,00	0,00E+00	3252	18	NA	Japonica	
maker-11-augustus-gene-201.14-mRNA-1	11	OsR498G1120437800.01.T01	99,50	0,00E+00	2865	Os11g0593000	100,00	0,00E+00	1781	-1084	Indica	Japonica	
maker-11-augustus-gene-201.2-mRNA-1	11	OsR498G1120441100.01.T01	99,93	0,00E+00	2599	Os11g0594200	100,00	0,00E+00	2603	4	NA	Japonica	
maker-11-augustus-gene-201.30-mRNA-1	11	OsR498G1120445400.01.T01	99,19	0,00E+00	2168	Os11g0595100	100,00	0,00E+00	2214	46	NA	Japonica	
maker-11-augustus-gene-202.8-mRNA-1	11	OsR498G1120452900.01.T05	95,72	0,00E+00	2554	Os11g0596400	100,00	0,00E+00	2933	379	Japonica	Japonica	
maker-11-augustus-gene-203.30-mRNA-1	11	OsR498G1120452300.01.T01	91,18	0,00E+00	1626	Os11g0597700	100,00	0,00E+00	2215	589	Japonica	Japonica	
maker-11-augustus-gene-203.34-mRNA-1	11	OsR498G1120448000.01.T01	99,75	0,00E+00	1436	Os11g0579600	98,15	0,00E+00	1389	-47	NA	Japonica	
maker-11-augustus-gene-204.0-mRNA-1	11	OsR498G1120452900.01.T01	98,56	0,00E+00	4962	Os11g0598800	99,27	0,00E+00	5110	148	NA	Japonica	
maker-11-augustus-gene-204.24-mRNA-1	11	OsR498G1120454300.01.T02	99,38	0,00E+00	4892	Os11g0599101	100,00	0,00E+00	3324	-1568	Indica	Japonica	
maker-11-augustus-gene-204.28-mRNA-1	11	OsR498G1120456100.01.T01	99,73	0,00E+00	3354	Os11g0599500	100,00	0,00E+00	3377	23	NA	Japonica	
maker-11-augustus-gene-206.49-mRNA-1	11	OsR498G1120467700.01.T01	99,64	0,00E+00	2472	Os01g0127900	75,70	0,00E+00	870	-1602	Indica	Japonica	
maker-11-augustus-gene-206.57-mRNA-1	11	OsR498G1120471000.01.T01	99,28	0,00E+00	1480	Os11g0601700	99,52	0,00E+00	1489	9	NA	Japonica	
maker-11-augustus-gene-207.0-mRNA-1	11	OsR498G1120474100.01.T02	99,58	0,00E+00	1687	Os11g0602300	100,00	0,00E+00	1705	18	NA	Japonica	
maker-11-augustus-gene-207.11-mRNA-1	11	OsR498G1120477300.01.T01	99,02	5,77E-152	537	Os11g0603000	99,02	2,01E-152	537	0	NA	Japonica	
maker-11-augustus-gene-207.13-mRNA-1	11	OsR498G1120478100.01.T02	99,66	0,00E+00	2093	Os11g0603200	100,00	0,00E+00	2111	18	NA	Japonica	
maker-11-augustus-gene-207.18-mRNA-1	11	OsR498G1120477800.01.T01	98,52	0,00E+00	705	Os11g0603100	100,00	0,00E+00	731	26	NA	Japonica	
maker-11-augustus-gene-207.5-mRNA-1	11	OsR498G1120475100.01.T01	99,32	0,00E+00	1043	Os11g0602600	100,00	0,00E+00	1061	18	NA	Japonica	
maker-11-augustus-gene-207.8-mRNA-1	11	OsR498G1120476500.01.T01	99,29	0,00E+00	1253	Os11g0602900	100,00	0,00E+00	1278	25	NA	NA	
maker-11-augustus-gene-208.0-mRNA-1	11	OsR498G1120482500.01.T01	99,18	0,00E+00	1727	Os11g0604600	100,00	0,00E+00	1764	37	NA	Japonica	
maker-11-augustus-gene-209.1-mRNA-1	11	OsR498G1120485700.01.T01	96,66	0,00E+00	4755	Os11g0606900	100,00	0,00E+00	5189	434	NA	Japonica	NBS-TM
maker-11-augustus-gene-209.14-mRNA-1	11	OsR498G1120487000.01.T01	99,82	0,00E+00	2921	Os11g0607200	99,94	0,00E+00	2930	9	NA	Japonica	Kinase-LRR-TM
maker-11-augustus-gene-209.8-mRNA-1	11	OsR498G1120484800.01.T01	97,38	0,00E+00	3086	Os11g0606400	100,00	0,00E+00	4944	1858	Japonica	Japonica	LRR-NBS-TM
maker-11-augustus-gene-209.9-mRNA-1	11	OsR498G1120485600.01.T01	99,18	0,00E+00	3885	Os11g0606400	85,69	0,00E+00	1998	-1887	Indica	Japonica	LRR-NBS-TM
maker-11-augustus-gene-21.37-mRNA-1	11	OsR498G1119451500.01.T01	99,52	0,00E+00	3746	Os11g0157600	100,00	0,00E+00	3377	-369	NA	Japonica	
maker-11-augustus-gene-21.43-mRNA-1	11	OsR498G1119453100.01.T01	99,91	0,00E+00	2058	Os11g0158200	100,00	0,00E+00	2062	4	NA	Japonica	
maker-11-augustus-gene-21.45-mRNA-1	11	OsR498G1119453700.01.T02	98,00	0,00E+00	3894	Os11g0158400	98,14	0,00E+00	3908	14	NA	Japonica	
maker-11-augustus-gene-21.48-mRNA-1	11	OsR498G1119454100.01.T02	97,87	0,00E+00	4761	Os12g0160400	72,79	0,00E+00	1179	-3582	Indica	NA	
maker-11-augustus-gene-21.56-mRNA-1	11	OsR498G1119454400.01.T03	100,00	0,00E+00	3674	Os11g0158600	100,00	0,00E+00	3674	0	NA	NA	
maker-11-augustus-gene-211.54-mRNA-1	11	OsR498G1120491500.01.T01	100,00	0,00E+00	2158	Os11g0610900	100,00	0,00E+00	2158	0	NA	Japonica	
maker-11-augustus-gene-211.55-mRNA-1	11	OsR498G1120491500.01.T03	100,00	1,33E-173	609	Os11g0610900	100,00	4,63E-174	609	0	NA	Japonica	
maker-11-augustus-gene-211.69-mRNA-1	11	OsR498G1120491200.01.T01	97,31	0,00E+00	1897	Os11g0610700	97,31	0,00E+00	1897	0	NA	Japonica	
maker-11-augustus-gene-214.40-mRNA-1	11	OsR498G1120503300.01.T01	99,20	0,00E+00	2217	Os11g0614400	100,00	0,00E+00	2262	45	NA	Japonica	
maker-11-augustus-gene-214.42-mRNA-1	11	OsR498G1120504700.01.T01	99,37	0,00E+00	1128	Os11g0614800	100,00	0,00E+00	1164	36	NA	Japonica	
maker-11-augustus-gene-214.45-mRNA-1	11	OsR498G1120505100.01.T05	99,92	0,00E+00	2160	Os11g0614900	100,00	0,00E+00	1278	-882	Indica	Japonica	
maker-11-augustus-gene-214.55-mRNA-1	11	OsR498G1120506100.01.T01	99,33	0,00E+00	2651	Os11g0615100	97,78	0,00E+00	2561	-90	NA	Japonica	
maker-11-augustus-gene-214.58-mRNA-1	11	OsR498G1120508900.01.T01	99,24	0,00E+00	1397	Os11g0615800	100,00	0,00E+00	1424	27	NA	Japonica	
maker-11-augustus-gene-214.62-mRNA-1	11	OsR498G1120506400.01.T01	100,00	0,00E+00	1115	Os11g0615200	100,00	0,00E+00	1115	0	NA	Japonica	
maker-11-augustus-gene-214.64-mRNA-1	11	OsR498G1120508800.01.T01	96,50	0,00E+00	1176	Os11g0615700	100,00	0,00E+00	1288	112	NA	Japonica	
maker-11-augustus-gene-215.0-mRNA-1	11	OsR498G1120510900.01.T01	99,14	0,00E+00	1430	Os11g0616100	100,00	0,00E+00	1465	35	NA	Japonica	Kinase-TM
maker-11-augustus-gene-215.5-mRNA-1	11	OsR498G1120512200.01.T01	97,40	0,00E+00	2532	Os11g0616300	100,00	0,00E+00	2673	141	NA	Japonica	
maker-11-augustus-gene-216.38-mRNA-1	11	OsR498G1120518800.01.T01	97,99	0,00E+00	2043	Os11g0618600	100,00	0,00E+00	1629	-414	Indica	Japonica	
maker-11-augustus-gene-217.42-mRNA-1	11	OsR498G1120524900.01.T01	98,03	0,00E+00	3582	Os11g0620500	100,00	0,00E+00	3777	195	NA	Japonica	Kinase-LRR-TM
maker-11-augustus-gene-217.44-mRNA-1	11	OsR498G1120523200.01.T03	99,55	0,00E+00	798	Os11g0620000	99,71	7,50E-175	613	-185	Indica	Japonica	
maker-11-augustus-gene-217.48-mRNA-1	11	OsR498G1120523800.01.T02	97,13	0,00E+00	2216	Os11g0620100	97,29	0,00E+00	2225	9	NA	Japonica	
maker-11-augustus-gene-217.57-mRNA-1	11	OsR498G1120526200.01.T02	100,00	0,00E+00	715	Os11g0621400	100,00	0,00E+00	834	119	Japonica	Japonica	
maker-11-augustus-gene-218.23-mRNA-1	11	OsR498G1120530800.01.T01	91,76	8,05E-180	630	Os11g0622150	100,00	0,00E+00	838	208	Japonica	Japonica	
maker-11-augustus-gene-22.29-mRNA-1	11	OsR498G1119460300.01.T01	99,82	0,00E+00	1008	Os11g0160000	100,00	0,00E+00	946	-62	NA	Japonica	
maker-11-augustus-gene-22.35-mRNA-1	11	OsR498G1119461400.01.T01	99,54	0,00E+00	3837	Os11g0160100	100,00	0,00E+00	2435	-1402	Indica	Japonica	
maker-11-augustus-gene-22.37-mRNA-1	11	OsR498G1119462200.01.T01	99,77	0,00E+00	2368	Os11g0160300	100,00	0,00E+00	2381	13	NA	Japonica	CC-Kinase-TM
maker-11-augustus-gene-22.40-mRNA-1	11	OsR498G1119464000.01.T02	99,93	0,00E+00	5142	Os11g0160700	100,00	0,00E+00	5151	9	NA	Japonica	
maker-11-augustus-gene-220.23-mRNA-1	11	OsR498G1120550600.01.T01	99,79	0,00E+00	4344	Os11g0625900	100,00	0,00E+00	5294	950	Japonica	Japonica	Kinase-LRR-TM
maker-11-augustus-gene-221.0-mRNA-1	11	OsR498G1120550600.01.T01	84,04	0,00E+00	2620	Os11g0628000	100,00	0,00E+00	5984	3364	Japonica	Japonica	Kinase-LRR-TM
maker-11-augustus-gene-221.12-mRNA-1	11	OsR498G1120561500.01.T01	100,00	0,00E+00	1148	Os11g0629300	100,00	0,00E+00	1148	0	NA	Japonica	
maker-11-augustus-gene-221.4-mRNA-1	11	OsR498G1120561100.01.T02	99,89	0,00E+00	1639	Os11g0629100	100,00	0,00E+00	1644	5	NA	Japonica	
maker-11-augustus-gene-221.6-mRNA-1	11	OsR498G1120561300.01.T01	99,89	0,00E+00	1646	Os11g0629200	100,00	0,00E+00	1651	5	NA	Japonica	
maker-11-augustus-gene-222.46-mRNA-1	11	OsR498G1120562000.01.T01	100,00	1,38E-179	628	Os11g0629400	100,00	4,80E-180	628	0	NA	Japonica	
maker-11-augustus-gene-222.48-mRNA-1	11	OsR498G1120562200.01.T03	99,66	0,00E+00	1594	Os11g0629500	100,00	0,00E+00	1608	14	NA	Japonica	
maker-11-augustus-gene-223.2-mRNA-1	11	OsR498G1120576600.01.T02	99,11	4,22E-168	591	Os02g0701833	79,55	4,81E-67	253	-338	Indica	Japonica	
maker-11-augustus-gene-224.0-mRNA-1	11	OsR498G1120581500.01.T03	99,78	0,00E+00	1615	Os11g0633500	100,00	0,00E+00	1602	-13	NA	Japonica	LRR-NBS-TM
maker-11-augustus-gene-224.3-mRNA-1	11	OsR498G1120584100.01.T01	98,86	0,00E+00	2761	Os11g0633800	100,00	0,00E+00	2836	75	NA	Japonica	
maker-11-augustus-gene-224.5-mRNA-1	11	OsR498G1120584800.01.T01	99,52	0,00E+00	1123	Os11g0634200	100,00	0,00E+00	1137	14	NA	Japonica	
maker-11-augustus-gene-225.0-mRNA-1	11	OsR498G1120587100.01.T02	99,54	3,79E-108	391	Os11g0634500	100,00	2,73E-79	293	-98	Indica	Japonica	
maker-11-augustus-gene-225.3-mRNA-1	11	OsR498G1120588100.01.T01	99,78	0,00E+00	1642	Os07g0573100	72,38	5,65E-83	308	-1334	Indica	Japonica	NBS-TM
maker-11-augustus-gene-225.6-mRNA-1	11	OsR498G1120590900.01.T01	99,26	0,00E+00	2863	Os11g0635500	96,56	0,00E+00	2537	-326	Indica	Japonica	
maker-11-augustus-gene-225.8-mRNA-1	11	OsR498G1120591500.01.T01	99,22	0,00E+00	2722	Os11g0635500	100,00	0,00E+00	2776	54	NA	Japonica	
maker-11-augustus-gene-226.2-mRNA-1	11	OsR498G1120596000.01.T03	99,16	0,00E+00	2306	Os11g0636200	100,00	0,00E+00	2917	611	Japonica	Japonica	
maker-11-augustus-gene-226.7-mRNA-1	11	OsR498G1120601800.01.T01	99,42	0,00E+00	3078	Os11g0636900	99,65	0,00E+00	3096	18	NA	Japonica	
maker-11-augustus-gene-227.36-mRNA-1	11	OsR498G1120603300.01.T01	99,46	0,00E+00	994	Os11g0637300	100,00	0,00E+00	1370	376	Japonica	Japonica	
maker-11-augustus-gene-227.38-mRNA-1	11	OsR498G1120604700.01.T02	98,65	0,00E+00	2451	Os11g0637700	100,00	0,00E+00	2533	82	NA	Japonica	
maker-11-augustus-gene-227.48-mRNA-1	11	OsR498G1120602400.01.T01	99,72	0,00E+00	2574	Os11g0637200	90,23	0,00E+00	1957	-617	Indica	Japonica	
maker-11-augustus-gene-227.53-mRNA-1	11	OsR498G1120604000.01.T02	99,82	0,00E+00	3002	Os11g0637200	84,42	0,00E+00	1564	-1438	Indica	Japonica	
maker-11-augustus-gene-227.55-mRNA-1	11	OsR498G1120604200.01.T01	94,59	0,00E+00	738	Os11g0637400	99,66	1,55E-149	528	-210	Indica	Japonica	
maker-11-augustus-gene-228.6-mRNA-1	11	OsR498G1120613000.01.T01	99,73	0,00E+00	5894	Os11g0640600	100,00	0,00E+00	5963	69	NA	Japonica	CC-LRR-NBS
maker-11-augustus-gene-229.5-mRNA-1	11	OsR498G1120617700.01.T01	100,00	0,00E+00	3198	Os11g0641500	100,00	0,00E+00	3198	0	NA	NA	
maker-11-augustus-gene-229.7-mRNA-1	11	OsR498G1120619100.01.T01	100,00	0,00E+00	3144	Os11g0641800	100,00	0,00E+00	3182	38	NA	NA	
maker-11-augustus-gene-23.40-mRNA-1	11	OsR498G1119465100.01.T02	97,43	0,00E+00	2562	Os11g0161000	97,26	0,00E+00	2455	-107	NA	Japonica	
maker-11-augustus-gene-23.42-mRNA-1	11	OsR498G1119465700.01.T05	96,66	0,00E+00	2227	Os11g0161650	100,00	0,00E+00	2616	389	Japonica	Japonica	
maker-11-augustus-gene-23.48-mRNA-1	11	OsR498G1119466700.01.T01	100,00	1,44E-103	376	Os11g0162200	100,00	5,00E-104	376	0	NA	Japonica	
maker-11-augustus-gene-230.46-mRNA-1	11	OsR498G1120623700.01.T08	98,20	0,00E+00	2880	Os11g0642800	98,17	0,00E+00	2842	-38	NA	NA	
maker-11-augustus-gene-230.58-mRNA-1	11	OsR498G1120627100.01.T01	100,00	0,00E+00	1880	Os06g0693200	70,43	3,85E-73	275	-1605	Indica	NA	Kinase-TM
maker-11-augustus-gene-231.7-mRNA-1	11	OsR498G1120634500.01.T01	74,81	1,44E-41	171	Os11g0645700	100,00	0,00E+00	720	549	Japonica	Japonica	
maker-11-augustus-gene-233.29-mRNA-1	11	OsR498G1120635000.01.T01	98,78	0,00E+00	2865	Os11g0648000	100,00	0,00E+00	2955	90	NA	Japonica	
maker-11-augustus-gene-233.38-mRNA-1	11	OsR498G1120641000.01.T02	98,01	0,00E+00	1912	Os11g0648400	97,71	0,00E+00	1576	-336	Indica	Japonica	
maker-11-augustus-gene-234.11-mRNA-1	11	OsR498G1120646000.01.T01	100,00	0,00E+00	1110	Os11g0649600	100,00	0,00E+00	1110	0	NA	Japonica	
maker-11-augustus-gene-234.13-mRNA-1	11	OsR498G1120646200.01.T01	99,64	0,00E+00	993	Os11g0649700	100,00	0,00E+00	1002	9	NA	Japonica	
maker-11-augustus-gene-236.36-mRNA-1	11	OsR498G1120655200.01.T01	99,59	0,00E+00	3031	Os11g0652600	100,00	0,00E+00	1894	-1137	Indica	Japonica	
maker-11-augustus-gene-236.39-mRNA-1	11	OsR498G1120655800.01.T02	99,75	0,00E+00	1425	Os11g0652900	100,00	0,00E+00	1434	9	NA	Japonica	
maker-11-augustus-gene-237.33-mRNA-1	11	OsR498G1120661900.01.T01	100,00	2,96E-152	538	Os11g0655000	100,00	0,00E+00	682	144	Japonica	Japonica	
maker-11-augustus-gene-237.37-mRNA-1	11	OsR498G1120665000.01.T01	100,00	0,00E+00	1456	Os11g0654400	100,00	0,00E+00	1456	0	NA	Japonica	
maker-11-augustus-gene-238.30-mRNA-1	11	OsR498G1120669300.01.T05	100,00	0,00E+00	1489	Os11g0655800	100,00	0,00E+00	1489	0	NA	Japonica	
maker-11-augustus-gene-238.37-mRNA-1	11	OsR498G1120670900.01.T02	98,79	0,00E+00	3614	Os11g0657000	98,68	0,00E+00	2793	-821	Indica	Japonica	
maker-11-augustus-gene-238.40-mRNA-1	11	OsR498G1120671100.01.T01	100,00	0,00E+00	1142	Os11g0657100	100,00	0,00E+00	1142	0	NA	Japonica	
maker-11-augustus-gene-238.46-mRNA-1	11	OsR498G1120671500.01.T02	100,00	8,85E-130	462	Os11g0657300	100,00	3,10E-130	462	0	NA	Japonica	
maker-11-augustus-gene-238.50-mRNA-1	11	OsR498G1120671400.01.T01	100,00	0,00E+00	1407	Os11g0657200	100,00	0,00E+00	1407	0	NA	Japonica	

maker-11-augustus-gene-239.0-mRNA-1	11	OsR498G1120679700.01.T02	97,56	0,00E+00	1895	Os11g0658700	100,00	0,00E+00	1997	102	NA	Japonica	
maker-11-augustus-gene-239.13-mRNA-1	11	OsR498G1120680000.01.T02	99,93	0,00E+00	2431	Os11g0658800	100,00	0,00E+00	2435	4	NA	Japonica	
maker-11-augustus-gene-24.35-mRNA-1	11	OsR498G1119468300.01.T04	99,82	0,00E+00	2037	Os11g0163100	100,00	0,00E+00	2046	9	NA	Japonica	
maker-11-augustus-gene-24.40-mRNA-1	11	OsR498G1119469900.01.T05	99,47	0,00E+00	2355	Os11g0163600	100,00	0,00E+00	1721	-634	Indica	Japonica	
maker-11-augustus-gene-24.44-mRNA-1	11	OsR498G1119471500.01.T01	99,79	0,00E+00	856	Os11g0165700	100,00	0,00E+00	861	5	NA	Japonica	
maker-11-augustus-gene-240.0-mRNA-1	11	OsR498G1120681700.01.T01	99,56	0,00E+00	809	Os11g0659200	100,00	0,00E+00	818	9	NA	Japonica	
maker-11-augustus-gene-240.11-mRNA-1	11	OsR498G1120683400.01.T02	99,87	0,00E+00	2762	Os11g0660000	100,00	0,00E+00	2771	9	NA	Japonica	
maker-11-augustus-gene-240.18-mRNA-1	11	OsR498G1120685800.01.T01	100,00	0,00E+00	915	Os11g0660500	100,00	0,00E+00	915	0	NA	Japonica	
maker-11-augustus-gene-240.2-mRNA-1	11	OsR498G1120683700.01.T01	99,69	0,00E+00	1740	Os11g0660300	100,00	0,00E+00	1754	14	NA	Japonica	
maker-11-augustus-gene-240.4-mRNA-1	11	OsR498G1120682400.01.T03	99,58	0,00E+00	1701	Os11g0659500	100,00	0,00E+00	1721	20	NA	Japonica	
maker-11-augustus-gene-241.10-mRNA-1	11	OsR498G1120687800.01.T02	98,63	0,00E+00	5772	Os07g0672500	70,16	0,00E+00	840	-4932	Indica	Japonica	
maker-11-augustus-gene-241.6-mRNA-1	11	OsR498G1120687500.01.T01	99,90	0,00E+00	1782	Os11g0661300	100,00	0,00E+00	1786	4	NA	NA	
maker-11-augustus-gene-241.8-mRNA-1	11	OsR498G1120688300.01.T01	97,52	0,00E+00	1706	Os11g0661600	100,00	0,00E+00	1819	113	NA	Japonica	
maker-11-augustus-gene-243.0-mRNA-1	11	OsR498G1120691000.01.T01	89,68	0,00E+00	2157	Os11g0664000	100,00	0,00E+00	2040	-117	NA	NA	CC-Kinase-TM
maker-11-augustus-gene-243.1-mRNA-1	11	OsR498G1120691200.01.T01	82,88	0,00E+00	843	Os11g0664000	80,45	0,00E+00	743	-100	Indica	NA	CC-Kinase-TM
maker-11-augustus-gene-243.4-mRNA-1	11	OsR498G1120688900.01.T01	79,09	0,00E+00	1355	Os11g0664800	100,00	0,00E+00	2080	725	Japonica	Japonica	
maker-11-augustus-gene-243.6-mRNA-1	11	OsR498G1120688800.01.T01	93,66	0,00E+00	1127	Os11g0665600	74,10	2,80E-60	233	-894	Indica	Japonica	
maker-11-augustus-gene-244.53-mRNA-1	11	OsR498G1120691200.01.T01	88,63	0,00E+00	2135	Os11g0666200	100,00	0,00E+00	2911	776	Japonica	Japonica	CC-Kinase-TM
maker-11-augustus-gene-245.50-mRNA-1	11	OsR498G1120697100.01.T01	99,84	0,00E+00	3336	Os11g0669100	100,00	0,00E+00	3350	14	NA	Japonica	
maker-11-augustus-gene-246.17-mRNA-1	11	OsR498G1120700800.01.T01	100,00	0,00E+00	682	Os11g0671000	97,88	0,00E+00	646	-36	NA	Indica	
maker-11-augustus-gene-246.7-mRNA-1	11	OsR498G1120699900.01.T01	98,77	4,41E-76	284	Os09g0529700	94,44	9,12E-67	252	-32	Indica	Japonica	
maker-11-augustus-gene-247.10-mRNA-1	11	OsR498G1120707400.01.T01	99,84	0,00E+00	1143	Os11g0673100	99,69	0,00E+00	1139	-4	NA	NA	
maker-11-augustus-gene-247.3-mRNA-1	11	OsR498G1120704100.01.T05	99,77	0,00E+00	788	Os11g0672300	99,77	0,00E+00	788	0	NA	Japonica	
maker-11-augustus-gene-247.8-mRNA-1	11	OsR498G1120706200.01.T01	99,59	0,00E+00	3061	Os11g0672700	99,65	0,00E+00	3066	5	NA	NA	
maker-11-augustus-gene-248.27-mRNA-1	11	OsR498G1120709900.01.T01	93,00	0,00E+00	4598	Os11g0677101	92,16	0,00E+00	4478	-120	NA	Indica	CC-LRR-NBS-TM
maker-11-augustus-gene-248.32-mRNA-1	11	OsR498G1120709100.01.T03	74,25	0,00E+00	1497	Os11g0673900	99,53	0,00E+00	4974	3477	Japonica	Japonica	LRR-NBS
maker-11-augustus-gene-248.45-mRNA-1	11	OsR498G1120707800.01.T01	98,74	0,00E+00	4145	Os11g0673200	99,95	0,00E+00	3556	-589	Indica	Indica	
maker-11-augustus-gene-249.0-mRNA-1	11	OsR498G1120716200.01.T01	99,65	0,00E+00	1020	Os11g0677500	94,89	0,00E+00	984	-36	NA	NA	
maker-11-augustus-gene-25.39-mRNA-1	11	OsR498G1119474600.01.T04	98,99	0,00E+00	873	Os12g0165700	76,08	6,81E-101	368	-505	Indica	NA	
maker-11-augustus-gene-25.43-mRNA-1	11	OsR498G0611509300.01.T02	91,78	3,38E-90	332	Os11g0161033	100,00	5,74E-120	428	96	Japonica	NA	
maker-11-augustus-gene-250.32-mRNA-1	11	OsR498G1120719100.01.T01	99,75	0,00E+00	3634	Os12g0562500	70,28	6,93E-169	594	-3040	Indica	Japonica	CC-Kinase-TM
maker-11-augustus-gene-250.37-mRNA-1	11	OsR498G1120723300.01.T01	98,30	0,00E+00	2837	Os11g0679700	100,00	0,00E+00	2944	107	NA	Japonica	
maker-11-augustus-gene-252.38-mRNA-1	11	OsR498G1120728700.01.T06	99,82	0,00E+00	2035	Os11g0682300	99,65	0,00E+00	2028	-7	NA	Japonica	
maker-11-augustus-gene-252.48-mRNA-1	11	OsR498G1120729800.01.T02	100,00	0,00E+00	3182	Os11g0683500	100,00	0,00E+00	3182	0	NA	Japonica	
maker-11-augustus-gene-252.53-mRNA-1	11	OsR498G1120730600.01.T01	98,37	0,00E+00	1485	Os11g0684000	99,88	0,00E+00	1544	59	NA	Japonica	
maker-11-augustus-gene-252.57-mRNA-1	11	OsR498G1120731800.01.T01	99,56	0,00E+00	818	Os11g0684600	99,86	0,00E+00	1322	504	Japonica	Japonica	NBS
maker-11-augustus-gene-253.1-mRNA-1	11	OsR498G1120735400.01.T01	99,42	0,00E+00	6780	Os11g0684100	91,70	0,00E+00	4668	-2112	Indica	Japonica	NBS-TM
maker-11-augustus-gene-254.0-mRNA-1	11	OsR498G1120737300.01.T01	99,46	0,00E+00	3321	Os11g0687100	99,41	0,00E+00	3316	-5	NA	Indica	
maker-11-augustus-gene-254.2-mRNA-1	11	OsR498G1120737500.01.T01	96,57	0,00E+00	2978	Os11g0687200	94,16	0,00E+00	2808	-170	NA	Indica	
maker-11-augustus-gene-254.4-mRNA-1	11	OsR498G1120737300.01.T01	95,79	0,00E+00	3066	Os11g0687100	95,64	0,00E+00	3052	-14	NA	Japonica	
maker-11-augustus-gene-254.7-mRNA-1	11	OsR498G1120737500.01.T01	99,44	0,00E+00	3197	Os11g0687200	93,61	0,00E+00	2762	-435	Indica	Japonica	
maker-11-augustus-gene-255.0-mRNA-1	11	OsR498G1120737900.01.T02	99,70	0,00E+00	1782	Os11g0689100	85,05	0,00E+00	917	-865	Indica	Japonica	NBS-TM PIK, PIKM
maker-11-augustus-gene-255.12-mRNA-1	11	OsR498G1120738400.01.T01	99,76	0,00E+00	3754	Os11g0689300	98,42	0,00E+00	3628	-126	NA	Indica	
maker-11-augustus-gene-255.2-mRNA-1	11	OsR498G1120738500.01.T02	99,96	0,00E+00	4348	Os11g0689400	98,88	0,00E+00	4231	-117	NA	Indica	
maker-11-augustus-gene-256.34-mRNA-1	11	OsR498G1120743300.01.T02	98,54	0,00E+00	1783	Os11g0695850	98,59	0,00E+00	1727	-56	NA	Indica	Kinase-TM
maker-11-augustus-gene-256.48-mRNA-1	11	OsR498G1120759300.01.T01	99,44	0,00E+00	1914	Os11g0691240	99,53	0,00E+00	1892	-22	NA	NA	Kinase-TM
maker-11-augustus-gene-257.26-mRNA-1	11	OsR498G1120747200.01.T01	99,78	0,00E+00	5823	Os11g0695700	75,65	0,00E+00	1605	-4218	Indica	Japonica	Kinase-LRR-TM
maker-11-augustus-gene-258.22-mRNA-1	11	OsR498G1120747600.01.T01	85,15	0,00E+00	1047	Os11g0695600	83,80	0,00E+00	992	-55	NA	Japonica	Kinase-LRR-TM
maker-11-augustus-gene-259.37-mRNA-1	11	OsR498G1120756900.01.T01	99,69	0,00E+00	2277	Os12g0566300	84,43	0,00E+00	1402	-875	Indica	Indica	
maker-11-augustus-gene-259.44-mRNA-1	11	OsR498G1120757100.01.T01	98,36	0,00E+00	3911	Os11g0696400	98,74	0,00E+00	4279	368	NA	Indica	
maker-11-augustus-gene-259.46-mRNA-1	11	OsR498G1120756600.01.T02	99,79	0,00E+00	4207	Os11g0695900	95,86	0,00E+00	3823	-384	NA	Indica	
maker-11-augustus-gene-260.0-mRNA-1	11	OsR498G1120757300.01.T01	93,86	0,00E+00	1616	Os11g0699100	99,72	0,00E+00	1897	281	Japonica	Japonica	CC-NBS-TM
maker-11-augustus-gene-260.5-mRNA-1	11	OsR498G1120760800.01.T01	98,88	0,00E+00	1252	Os11g0700500	100,00	0,00E+00	1288	36	NA	Japonica	
maker-11-augustus-gene-261.63-mRNA-1	11	OsR498G1120766700.01.T01	98,27	1,01E-169	597	Os11g0703500	100,00	2,53E-121	434	-163	Indica	NA	
maker-11-augustus-gene-261.65-mRNA-1	11	OsR498G1120766200.01.T01	100,00	0,00E+00	1218	Os11g0703400	99,70	0,00E+00	1209	-9	NA	Indica	
maker-11-augustus-gene-262.39-mRNA-1	11	OsR498G1120767700.01.T01	98,77	0,00E+00	3409	Os11g0703900	98,77	0,00E+00	3409	0	NA	Indica	
maker-11-augustus-gene-262.42-mRNA-1	11	OsR498G1120768100.01.T01	97,78	0,00E+00	844	Os11g0704000	97,58	0,00E+00	839	-5	NA	Indica	
maker-11-augustus-gene-262.44-mRNA-1	11	OsR498G1120769100.01.T01	99,81	0,00E+00	2849	Os11g0704300	99,81	0,00E+00	2849	0	NA	Indica	
maker-11-augustus-gene-262.46-mRNA-1	11	OsR498G1120769500.01.T01	100,00	5,05E-113	407	Os11g0704500	99,56	7,53E-112	402	-5	NA	NA	
maker-11-augustus-gene-262.53-mRNA-1	11	OsR498G1120770400.01.T02	83,78	0,00E+00	838	Os11g0704700	83,90	0,00E+00	843	5	NA	Japonica	
maker-11-augustus-gene-262.58-mRNA-1	11	OsR498G1120770600.01.T01	99,77	0,00E+00	1575	Os11g0704800	99,77	0,00E+00	1575	0	NA	Japonica	
maker-11-augustus-gene-263.16-mRNA-1	11	OsR498G0203365400.01.T04	73,22	5,37E-71	269	Os11g0706100	78,64	4,68E-98	357	88	Japonica	Japonica	
maker-11-augustus-gene-263.21-mRNA-1	11	OsR498G1120773800.01.T02	100,00	0,00E+00	1532	Os11g0706600	100,00	0,00E+00	1270	-262	Indica	Japonica	
maker-11-augustus-gene-263.25-mRNA-1	11	OsR498G1120774600.01.T03	100,00	0,00E+00	2484	Os11g0707000	96,46	0,00E+00	1792	-692	Indica	Japonica	
maker-11-augustus-gene-263.30-mRNA-1	11	OsR498G1120774600.01.T02	71,16	4,00E-179	629	Os11g0707100	99,89	0,00E+00	1630	1001	Japonica	Japonica	
maker-11-augustus-gene-263.32-mRNA-1	11	OsR498G1120776600.01.T01	100,00	0,00E+00	1445	Os11g0707600	100,00	0,00E+00	1445	0	NA	Japonica	
maker-11-augustus-gene-263.37-mRNA-1	11	OsR498G1120776800.01.T01	100,00	0,00E+00	677	Os11g0707700	100,00	0,00E+00	677	0	NA	Japonica	
maker-11-augustus-gene-263.39-mRNA-1	11	OsR498G1120779000.01.T02	99,70	0,00E+00	2401	Os11g0708600	99,55	0,00E+00	1958	-443	Indica	Japonica	
maker-11-augustus-gene-263.46-mRNA-1	11	OsR498G1120422600.01.T01	99,60	0,00E+00	3962	Os11g0587300	100,00	0,00E+00	3983	21	NA	NA	
maker-11-augustus-gene-263.6-mRNA-1	11	OsR498G1120778500.01.T01	100,00	0,00E+00	1413	Os11g0708400	100,00	0,00E+00	1413	0	NA	Japonica	
maker-11-augustus-gene-263.9-mRNA-1	11	OsR498G0407560600.01.T01	92,29	0,00E+00	3067	Os11g0708900	100,00	0,00E+00	3793	726	Japonica	Japonica	
maker-11-augustus-gene-27.12-mRNA-1	11	OsR498G1119478900.01.T01	100,00	0,00E+00	2111	Os11g0168200	100,00	0,00E+00	2111	0	NA	Japonica	
maker-11-augustus-gene-27.16-mRNA-1	11	OsR498G1119474600.01.T02	99,51	0,00E+00	1816	Os11g0166800	100,00	0,00E+00	1294	-522	Indica	Japonica	
maker-11-augustus-gene-27.20-mRNA-1	11	OsR498G1119475600.01.T01	99,68	0,00E+00	4970	Os11g0167300	100,00	0,00E+00	5011	41	NA	Japonica	
maker-11-augustus-gene-27.22-mRNA-1	11	OsR498G1119476600.01.T01	100,00	0,00E+00	753	Os11g0167800	100,00	0,00E+00	753	0	NA	Japonica	
maker-11-augustus-gene-27.8-mRNA-1	11	OsR498G1119478500.01.T02	99,63	0,00E+00	958	Os01g0963300	72,58	2,31E-73	276	-682	Indica	Japonica	
maker-11-augustus-gene-28.36-mRNA-1	11	OsR498G0408582600.01.T02	73,52	5,01E-80	298	Os11g0168300	100,00	0,00E+00	879	581	Japonica	Japonica	
maker-11-augustus-gene-28.42-mRNA-1	11	OsR498G1119480300.01.T01	99,40	0,00E+00	2074	Os02g0194400	71,50	1,31E-79	297	-1777	Indica	Japonica	Kinase-TM
maker-11-augustus-gene-28.48-mRNA-1	11	OsR498G1119481900.01.T01	99,96	0,00E+00	4433	Os11g0169100	100,00	0,00E+00	4437	4	NA	Japonica	
maker-11-augustus-gene-28.50-mRNA-1	11	OsR498G1119483300.01.T01	99,15	0,00E+00	2290	Os11g0169400	100,00	0,00E+00	2257	-33	NA	Japonica	
maker-11-augustus-gene-28.52-mRNA-1	11	OsR498G1119484100.01.T01	99,43	0,00E+00	2788	Os11g0169800	99,91	0,00E+00	1966	-822	Indica	Japonica	
maker-11-augustus-gene-28.57-mRNA-1	11	OsR498G1119479600.01.T01	99,27	0,00E+00	1695	Os11g0168500	100,00	0,00E+00	1727	32	NA	Japonica	
maker-11-augustus-gene-28.59-mRNA-1	11	OsR498G1119482600.01.T01	99,86	0,00E+00	2594	Os11g0169200	100,00	0,00E+00	2603	9	NA	Japonica	
maker-11-augustus-gene-28.63-mRNA-1	11	OsR498G1119484400.01.T01	100,00	0,00E+00	899	Os11g0169900	100,00	0,00E+00	899	0	NA	Japonica	
maker-11-augustus-gene-28.66-mRNA-1	11	OsR498G1221019600.01.T03	78,43	0,00E+00	1547	Os11g0170000	98,31	0,00E+00	3388	1841	Japonica	Japonica	
maker-11-augustus-gene-28.68-mRNA-1	11	OsR498G1119484800.01.T07	99,52	0,00E+00	4822	Os11g0170200	100,00	0,00E+00	4881	59	NA	Japonica	
maker-11-augustus-gene-29.32-mRNA-1	11	OsR498G1119485200.01.T02	98,04	0,00E+00	2902	Os12g0169400	79,06	0,00E+00	1277	-1625	Indica	Japonica	
maker-11-augustus-gene-29.41-mRNA-1	11	OsR498G1119487000.01.T02	99,87	0,00E+00	2773	Os11g0171500	100,00	0,00E+00	2782	9	NA	Japonica	
maker-11-augustus-gene-30.26-mRNA-1	11	OsR498G1119488300.01.T02	99,67	0,00E+00	5443	Os11g0171800	99,97	0,00E+00	5484	41	NA	Japonica	Kinase-LRR-TM
maker-11-augustus-gene-30.27-mRNA-1	11	OsR498G1119488700.01.T01	98,68	0,00E+00	1709	Os01g0788400	71,32	3,31E-120	432	-1277	Indica	Japonica	
maker-11-augustus-gene-30.28-mRNA-1	11	OsR498G1119496900.01.T01	71,91	0,00E+00	1566	Os11g0172133	99,93	0,00E+00	5155	3589	Japonica	NA	Kinase-LRR-TM
maker-11-augustus-gene-30.62-mRNA-1	11	OsR498G1119488300.01.T02	71,82	0,00E+00	791	Os11g0172700	100,00	0,00E+00	2670	1879	Japonica	Japonica	Kinase-LRR-TM
maker-11-augustus-gene-30.66-mRNA-1	11	OsR498G1119488200.01.T01	99,79	0,00E+00	5991	Os11g0171700	99,97	0,00E+00	6018	27	NA	Japonica	
maker-11-augustus-gene-31.34-mRNA-1	11	OsR498G1119493500.01.T01	99,62	0,00E+00	5129	Os11g0173700	83,26	0,00E+00	3039	-2090	Indica	Japonica	Kinase-LRR-TM
maker-11-augustus-gene-31.36-mRNA-1	11	OsR498G1119494900.01.T07	99,77	0,00E+00	5450	Os11g0173500	100,00	0,00E+00	4776	-674	Indica	Japonica	Kinase-LRR-TM
maker-11-augustus-gene-31.38-mRNA-1	11	OsR498G1119493500.01.T01	86,65	0,00E+00	1804	Os11g0173700	97,79	0,00E+00	3039	1235	Japonica	Japonica	Kinase-LRR-TM
maker-11-augustus-gene-31.40-mRNA-1	11	OsR498G1119496700.01.T02	98,88	0,00E+00	5333	Os11g0173800	100,00	0,00E+00	4823	-510	NA	Japonica	Kinase-LRR-TM
maker-11-augustus-gene-31.44-mRNA-1	11	OsR498G1119496900.01.T01	99,80	0,00E+00	5460	Os11g0173900	99,97	0,00E+00	5332	-128	NA	Japonica	Kinase-LRR-TM
maker-11-augustus-gene-31.54-mRNA-1	11	OsR498G1119497200.01.T02	99,41	0,00E+00	5089	Os11g0174000	100,00	0,00E+00	5162	73	NA	Japonica	
maker-11-augustus-gene-32.0-mRNA-1	11	OsR498G1119504300.01.T02	95,14	0,00E+00	1403	Os11g0175500	94,47	0,00E+00	1215	-188	Indica	NA	
maker-11-augustus-gene-32.16-mRNA-1	11	OsR498G1119506400.01.T02	100,00	0,00E+00	6244	Os02g0793100	89,00	0,00E+00	4525	-1719	Indica	NA	
maker-11-augustus-gene-32.5-mRNA-1	11	OsR498G1119506700.01.T01	99,62	0,00E+00	2815	Os11g0176000	99,62	0,00E+00	2815	0	NA	NA	
maker-11-augustus-gene-32.7-mRNA-1	11	OsR498G1119502200.01.T01	100,00	0,00E+00	1196	Os11g0175000	100,00	0,00E+00	1198	2	NA	NA	
maker-11-augustus-gene-32.9-mRNA-1	11	OsR498G1119503600.01.T03	100,00	0,00E+00	2971	Os11g0175400	100,00	0,00E+00	2971	0	NA	NA	
maker-11-augustus-gene-33.0-mRNA-1	11	OsR498G1119507100.01.T01	96,64	0,00E+00	1997	Os11g0176100	96,56	0,00E+00	1993	-4	NA	NA	
maker-11-augustus-gene-33.14-mRNA-1	11	OsR498G1119507600.01.T02	100,00	0,00E+00	5433	Os11g0176200	100,00	0,00E+00	5433	0	NA	NA	
maker-11-augustus-gene-33.20-mRNA-1	11	OsR498G1119510400.01.T02	99,49	0,00E+00	3489	Os11g0177400	100,00	0,00E+00	2598	-891	Indica	Japonica	
maker-11-augustus-gene-33.6-mRNA-1	11	OsR498G1119507700.01.T03	100,00	0,00E+00	2452	Os11g0176300	100,00	0,00E+00	2048	-404	Indica	NA	
maker-11-augustus-gene-34.38-mRNA-1	11	OsR498G1119519000.01.T01	99,55	0,00E+00	2392	Os11g0180100	100,00	0,00E+00	1559	-833	Indica	Japonica	
maker-11-augustus-gene-35.33-mRNA-1	11	OsR498G1119521900.01.T01	98,57	0,00E+00	2324	Os11g0181100	98,65	0,00E+00	2328	4	NA	Japonica	
maker-11-augustus-gene-35.42-mRNA-1	11	OsR498G1119522700.01.T01	99,85	0,00E+00	1235	Os11g0181500	100,00	0,00E+00	1240	5	NA	Japonica	
maker-11-augustus-gene-35.44-mRNA-1	11	OsR498G1119519800.01.T04	99,86	0,00E+00	1284	Os12g0174800	71,05	1,37E-98	360	-924	Indica	Japonica	
maker-11-augustus-gene-36.43-mRNA-1	11	OsR498G1119523700.01.T02	99,74	0,00E+00	1368	Os11g0181900	100,00	0,00E+00	1377	9	NA	Japonica	
maker-11-augustus-gene-36.46-mRNA-1	11	OsR498G0816559700.01.T01	71,66	0,00E+00	683	Os11g0182200	100,00	0,00E+00	2425	1742	Japonica	Japonica	
maker-11-augustus-gene-36.48-mRNA-1	11	OsR498G1119525400.01.T01	99,10	0,00E+00	2342	Os11g0182400	99,32	0,00E+00	2355	13	NA	Japonica	
maker-11-augustus-gene-36.54-mRNA-1	11	OsR498G1119526200.01.T01	94,74	0,00E+00	834	Os11g0182500	100,00	0,00E+00	1040	206	Japonica	Japonica	
maker-11-augustus-gene-36.56-mRNA-1	11	OsR498G1119527200.01.T02	97,89	0,00E+00	3495	Os11g0182900	100,00	0,00E+00	3205	-290	NA	Japonica	
maker-11-augustus-gene-37.0-mRNA-1	11	OsR498G1119529300.01.T01	97,33	0,00E+00	3974	Os11g0183700	99,96	0,00E+00	4243	269	NA	Japonica	
maker-11-augustus-gene-37.12-mRNA-1	11	OsR498G0917727000.01.T01	84,20	0,00E+00	793	Os11g0184200	100,00	0,00E+00	2076	1283	Japonica	Japonica	
maker-11-augustus-gene-37.17-mRNA-1	11	OsR498G1119531400.01.T01	98,29	0,00E+00	1719	Os11g0184900	100,00	0,00E+00	1786	67	NA	Japonica	NBS-TM
maker-11-augustus-gene-38.28-mRNA-1	11	OsR498G1119534500.01.T01	99,79	0,00E+00	2600	Os11g0186200	99,86	0,00E+00	2605	5	NA	Indica	
maker-11-augustus-gene-38.32-mRNA-1	11	OsR498G1119534700.01.T01	98,96	0,00E+00	3387	Os11g0186300	97,26	0,00E+00	3253	-134	NA	Indica	
maker-11-augustus-gene-38.36-mRNA-1	11	OsR498G1119535300.01.T01	99,62	0,00E+00	3291	Os01g0221100	77,82	0,00E+00	997	-2294	Indica	Japonica	
maker-11-augustus-gene-38.38-mRNA-1	11	OsR498G1119535000.01.T01	97,45	0,00E+00	5693	Os11g0186400	97,57	0,00E+00	5711	18	NA	NA	
maker-11-augustus-gene-39.43-mRNA-1	11	OsR498G1119538700.01.T01	96,95	0,00E+00	2901	Os11g0187500	96,72	0,00E+00	2883	-18	NA	NA	
maker-11-augustus-gene-39.45-mRNA-1	11	OsR498G1119536600.01.T01	97,07	0,00E+00	1612	Os11g0186900	97,38	0,00E+00	1626	14	NA	Japonica	
maker-11-augustus-gene-39.51-mRNA-1	11	OsR498G1119540600.01.T01	94,55	0,00E+00	1857	Os11g0187600	94,76	0,00E+00	1498	-359	Indica	NA	
maker-11-augustus-gene-39.53-mRNA-1	11	OsR498G1119541000.01.T01	94,44	0,00E+00	2770	Os11g0187500	91,15	0,00E+00	2516	-254	NA	Japonica	
maker-11-augustus-gene-4.52-mRNA-1	11	OsR498G1119346300.01.T01	99,56	0,00E+00	4496	Os12g0121600	97,26	0,00E+00	4234	-262	NA	Japonica	
maker-11-augustus-gene-4.54-mRNA-1	11	OsR498G1119347500.01.T01	99,75	0,00E+00	3549	Os11g0124300	98,79	0,00E+00	3480	-69	NA	Japonica	
maker-11-augustus-gene-4.56-mRNA-1	11	OsR498G1119348100.01.T01	100,00	0,00E+00	1537	Os11g0124500	100,00	0,00E+00	823	-714	Indica	Japonica	
maker-11-augustus-gene-4.58-mRNA-1	11	OsR498G1220855500.01.T04	85,17	4,17E-53	209	Os11g0125600	100,00	8,53E-177	618	409	Japonica	Japonica	
maker-11-augustus-gene-4.65-mRNA-1	11	OsR498G1119346000.01.T02	99,69	0,00E+00	1166	Os11g0123500	100,00	0,00E+00	1175	9	NA	Japonica	
maker-11-augustus-gene-4.70-mRNA-1	11	OsR498G1119348600.01.T01	98,77	0,00E+00	3415	Os11g0124900	99,08	0,00E+00	3443	28	NA	Japonica	
maker-11-augustus-gene-41.9-mRNA-1	11	OsR498G1119545300.01.T02	99,69	0,00E+00	2334	Os11g0191400	100,00	0,00E+00	2352	18	NA	Japonica	
maker-11-augustus-gene-44.36-mRNA-1	11	OsR498G1119754700.01.T02	99,60	0,00E+00	1784	Os11g0264200	100,00	0,00E+00	1593	-191	Indica	Japonica	
maker-11-augustus-gene-44.38-mRNA-1	11	OsR498G1119753600.01.T01	98,66	0,00E+00	3117	Os11g0641500	84,12	0,00E+00	1940	-1177	Indica	Japonica	
maker-11-augustus-gene-45.0-mRNA-1	11	OsR498G1119761300.01.T01	99,67	0,00E+00	2159	Os11g0264500	99,92	0,00E+00	2173	14	NA	Japonica	
maker-11-augustus-gene-45.10-mRNA-1	11	OsR498G1119763500.01.T01	99,86	0,00E+00	2637	Os11g0265000	100,00	0,00E+00	2646	9	NA	Japonica	
maker-11-augustus-gene-45.17-mRNA-1	11	OsR498G1119764700.01.T01	100,00	0,00E+00	1781	Os11g0265400	100,00	0,00E+00	1781	0	NA	Japonica	
maker-11-augustus-gene-45.24-mRNA-1	11	OsR498G1119762000.01.T01	99,77	0,00E+00	2384	Os11g0264700	99,89	0,00E+00	1679	-705	Indica	Japonica	
maker-11-augustus-gene-45.28-mRNA-1	11	OsR498G1119764400.01.T02	99,85	0,00E+00	2329	Os11g0265200	100,00	0,00E+00	2338	9	NA	Japonica	
maker-11-augustus-gene-46.18-mRNA-1	11	OsR498G1119767600.01.T01	99,90	0,00E+00	1717	Os11g0266000	100,00	0,00E+00	1683	-34	NA	Japonica	
maker-11-augustus-gene-46.22-mRNA-1	11	OsR498G1119768400.01.T01	100,00	0,00E+00	771	Os11g0265900	92,38	0,00E+00	650	-121	Indica	Japonica	NBS-TM
maker-11-augustus-gene-46.6-mRNA-1	11	OsR498G1119766900.01.T01	99,79	0,00E+00	1667	Os11g0265800	100,00	0,00E+00	1954	287	Japonica	Japonica	
maker-11-augustus-gene-46.8-mRNA-1	11	OsR498G1119768700.01.T02	99,65	0,00E+00	3014	Os11g0266500	98,41	0,00E+00	3942	928	Japonica	Japonica	NBS-TM
maker-11-augustus-gene-47.33-mRNA-1	11	OsR498G1119770200.01.T04	99,80	0,00E+00	916	Os11g0267100	100,00	0,00E+00	921	5	NA	Japonica	
maker-11-augustus-gene-47.42-mRNA-1	11	OsR498G1119771200.01.T01	99,86	0,00E+00	5030	Os11g0267400	100,00	0,00E+00	5048	18	NA	Japonica	
maker-11-augustus-gene-48.25-mRNA-1	11	OsR498G1119779800.01.T01	99,42	0,00E+00	4901	Os11g0267400	77,46	0,00E+00	1562	-3339	Indica	Japonica	
maker-11-augustus-gene-49.33-mRNA-1	11	OsR498G1119793700.01.T06	99,47	0,00E+00	2664	Os11g0271100	100,00	0,00E+00	2717	53	NA	Japonica	Kinase-TM
maker-11-augustus-gene-49.37-mRNA-1	11	OsR498G1119786800.01.T01	99,75	0,00E+00	1404	Os11g0269900	100,00	0,00E+00	1297	-107	NA	Japonica	
maker-11-augustus-gene-5.40-mRNA-1	11	OsR498G1119360200.01.T01	97,33	0,00E+00	2535	Os11g0126100	99,87	0,00E+00	2686	151	NA	NA	
maker-11-augustus-gene-5.47-mRNA-1	11	OsR498G1119350900.01.T01	99,26	0,00E+00	1670	Os11g0126400	95,09	0,00E+00	1892	222	Japonica	Japonica	
maker-11-augustus-gene-5.50-mRNA-1	11	OsR498G1119351300.01.T01	99,49	0,00E+00	2443	Os11g0126400	86,43	0,00E+00	1283	-1160	Indica	Japonica	
maker-11-augustus-gene-5.57-mRNA-1	11	OsR498G1119352900.01.T02	99,81	0,00E+00	1896	Os11g0127000	100,00	0,00E+00	1905	9	NA	Japonica	
maker-11-augustus-gene-5.62-mRNA-1	11	OsR498G1119353500.01.T01	99,82	0,00E+00	1939	Os11g0127600	100,00	0,00E+00	1948	9	NA	Japonica	
maker-11-augustus-gene-52.38-mRNA-1	11	OsR498G1119717500.01.T01	94,04	6,87E-129	461	Os11g0252900	100,00	0,00E+00	922	461	Japonica	Japonica	
maker-11-augustus-gene-52.53-mRNA-1	11	OsR498G1119797800.01.T03	99,25	0,00E+00	1417	Os11g0274300	99,50	0,00E+00	1426	9	NA	Japonica	CC-Kinase-TM
maker-11-augustus-gene-52.59-mRNA-1	11	OsR498G1119719010.01.T01	92,88	0,00E+00	1970	Os11g0251200	70,76	2,69E-133	475	-1495	Indica	Japonica	
maker-11-augustus-gene-54.41-mRNA-1	11	OsR498G1119727500.01.T01	99,38	0,00E+00	1146	Os11g0257200	75,74	1,01E-135	482	-664	Indica	Japonica	
maker-11-augustus-gene-54.43-mRNA-1	11	OsR498G1119728500.01.T01	99,38	0,00E+00	1144	Os11g0257200	90,84	0,00E+00	896	-248	Indica	Japonica	
maker-11-augustus-gene-54.47-mRNA-1	11	OsR498G1119729900.01.T01	99,56	0,00E+00	2012	Os11g0257200	100,00	0,00E+00	2035	23	NA	Japonica	
maker-11-augustus-gene-54.49-mRNA-1	11	OsR498G1119723500.01.T01	99,47	0,00E+00	2024	Os11g0255300	100,00	0,00E+00	2051	27	NA	Japonica	
maker-11-augustus-gene-54.52-mRNA-1	11	OsR498G1119723100.01.T01	99,35	0,00E+00	1917	Os11g0255500	100,00	0,00E+00	1948	31	NA	Japonica	
maker-11-augustus-gene-54.54-mRNA-1	11	OsR498G1119725800.01.T01	100,00	0,00E+00	2581	Os02g0608600	75,05	0,00E+00	682	-1899	Indica	Japonica	
maker-11-augustus-gene-54.58-mRNA-1	11	OsR498G1119726600.01.T01	100,00	0,00E+00	807	Os11g0256200	100,00	0,00E+00	807	0	NA	Japonica	
maker-11-augustus-gene-55.0-mRNA-1	11	OsR498G1119733300.01.T01	90,41	0,00E+00	1001	Os11g0257200	80,03	0,00E+00	651	-350	Indica	Japonica	
maker-11-augustus-gene-55.6-mRNA-1	11	OsR498G1119733300.01.T01	99,22	0,00E+00	1129	Os11g0257200	83,39	0,00E+00	673	-456	Indica	Japonica	
maker-11-augustus-gene-55.8-mRNA-1	11	OsR498G1119734000.01.T01	99,56	0,00E+00	2858	Os11g0258500	98,45	0,00E+00	5066	2208	Japonica	Japonica	CC-LRR-NBS-TM
maker-11-augustus-gene-56.31-mRNA-1	11	OsR498G1119728500.01.T01	71,61	1,01E-168	595	Os11g0259700	98,48	0,00E+00	2068	1473	Japonica	NA	
maker-11-augustus-gene-56.33-mRNA-1	11	OsR498G1119728500.01.T01	77,23	1,96E-100	367	Os11g0260100	100,00	0,00E+00	1629	1262	Japonica	NA	
maker-11-augustus-gene-56.36-mRNA-1	11	OsR498G1119735000.01.T01	98,39	0,00E+00	646	Os04g0557800	94,10	1,03E-163	574	-72	Indica	Japonica	
maker-11-augustus-gene-56.37-mRNA-1	11	OsR498G1119735000.01.T01	98,58	0,00E+00	2341	Os11g0258700	95,12	0,00E+00	2225	-116	NA	Japonica	
maker-11-augustus-gene-58.29-mRNA-1	11	OsR498G1119698900.01.T01	99,96	0,00E+00	4451	Os12g0210400	71,14	0,00E+00	1124	-3327	Indica	Japonica	Kinase-LRR-TM
maker-11-augustus-gene-58.33-mRNA-1	11	OsR498G1119695800.01.T01	99,81	0,00E+00	4708	Os12g0209300	75,46	0,00E+00	1811	-2897	Indica	Japonica	
maker-11-augustus-gene-58.36-mRNA-1	11	OsR498G1119697600.01.T01	99,76	0,00E+00	750	Os11g0244800	100,00	0,00E+00	1380	630	Japonica	Japonica	
maker-11-augustus-gene-58.42-mRNA-1	11	OsR498G1119700200.01.T01	100,00	0,00E+00	1467	Os11g0245500	100,00	0,00E+00	1467	0	NA	Japonica	
maker-11-augustus-gene-59.25-mRNA-1	11	OsR498G1119701700.01.T01	99,75	0,00E+00	2155	Os11g0246100	99,92	0,00E+00	2164	9	NA	Japonica	
maker-11-augustus-gene-59.32-mRNA-1	11	OsR498G1119707300.01.T01	99,73	0,00E+00	3278	Os11g0246900	99,94	0,00E+00	3042	-236	NA	Japonica	
maker-11-augustus-gene-6.0-mRNA-1	11	OsR498G1119354300.01.T01	97,69	0,00E+00	1259	Os11g0127800	94,29	0,00E+00	1170	-89	NA	Japonica	
maker-11-augustus-gene-6.11-mRNA-1	11	OsR498G1119354000.01.T01	99,79	0,00E+00	1685	Os11g0127700	100,00	0,00E+00	1694	9	NA	Japonica	
maker-11-augustus-gene-6.17-mRNA-1	11	OsR498G1119355200.01.T01	98,58	0,00E+00	1596	Os12g0124400	98,26	0,00E+00	1582	-14	NA	NA	
maker-11-augustus-gene-6.19-mRNA-1	11	OsR498G1119358600.01.T01	99,89	0,00E+00	1668	Os11g0128500	100,00	0,00E+00	1673	5	NA	Japonica	
maker-11-augustus-gene-6.24-mRNA-1	11	OsR498G1119359200.01.T02	97,05	0,00E+00	2011	Os11g0128800	99,91	0,00E+00	2079	68	NA	Japonica	
maker-11-augustus-gene-6.31-mRNA-1	11	OsR498G1119360200.01.T01	99,51	0,00E+00	2557	Os11g0126100	97,11	0,00E+00	2391	-166	NA	Japonica	
maker-11-augustus-gene-6.35-mRNA-1	11	OsR498G1119360400.01.T03	99,86	0,00E+00	1262	Os11g0128900	99,86	0,00E+00	1264	2	NA	Japonica	
maker-11-augustus-gene-6.4-mRNA-1	11	OsR498G1119361700.01.T02	91,54	0,00E+00	1058	Os12g0126200	91,26	0,00E+00	1049	-9	NA	Japonica	
maker-11-augustus-gene-6.84-mRNA-1	11	OsR498G1119362000.01.T01	98,21	0,00E+00	2025	Os12g0126300	98,36	0,00E+00	2324	299	Japonica	Japonica	
maker-11-augustus-gene-60.0-mRNA-1	11	OsR498G1119708900.01.T01	99,93	0,00E+00	2442	Os11g0247300	100,00	0,00E+00	2446	4	NA	Japonica	
maker-11-augustus-gene-60.13-mRNA-1	11	OsR498G1119712400.01.T01	99,27	0,00E+00	2174	Os11g0248200	100,00	0,00E+00	1921	-253	Indica	Japonica	
maker-11-augustus-gene-60.4-mRNA-1	11	OsR498G1119717400.01.T04	71,72	5,38E-136	486	Os11g0252400	71,82	1,53E-137	490	4	NA	Japonica	
maker-11-augustus-gene-60.8-mRNA-1	11	OsR498G1119709200.01.T02	99,89	0,00E+00	1598	Os11g0247400	100,00	0,00E+00	1602	4	NA	Japonica	
maker-11-augustus-gene-61.35-mRNA-1	11	OsR498G1119714500.01.T01	99,53	0,00E+00	2262	Os11g0249000	100,00	0,00E+00	2801	539	Japonica	Japonica	CC-NBS-TM
maker-11-augustus-gene-61.49-mRNA-1	11	OsR498G1119717000.01.T01	97,73	0,00E+00	2397	Os10g0151800	88,29	0,00E+00	1854	-543	Indica	Japonica	
maker-11-augustus-gene-61.54-mRNA-1	11	OsR498G1119717400.01.T03	72,62	0,00E+00	936	Os11g0251200	99,94	0,00E+00	3051	2115	Japonica	NA	
maker-11-augustus-gene-62.0-mRNA-1	11	OsR498G1119550500.01.T01	100,00	6,43E-94	343	Os11g0194800	100,00	2,25E-94	343	0	NA	Japonica	
maker-11-augustus-gene-62.2-mRNA-1	11	OsR498G1119717200.01.T01	98,80	0,00E+00	3071	Os11g0251400	100,00	0,00E+00	3166	95	NA	Japonica	
maker-11-augustus-gene-62.4-mRNA-1	11	OsR498G1119717400.01.T03	99,59	0,00E+00	3037	Os11g0252400	100,00	0,00E+00	3068	31	NA	Japonica	
maker-11-augustus-gene-62.7-mRNA-1	11	OsR498G1119551000.01.T02	99,73	0,00E+00	4006	Os11g0194900	100,00	0,00E+00	3615	-391	NA	Japonica	Kinase-TM
maker-11-augustus-gene-63.0-mRNA-1	11	OsR498G1119553300.01.T05	98,67	0,00E+00	3555	Os11g0195100	99,06	0,00E+00	4519	964	Japonica	Japonica	
maker-11-augustus-gene-63.4-mRNA-1	11	OsR498G1119553700.01.T03	98,88	0,00E+00	3449	Os11g0195500	100,00	0,00E+00	3003	-446	Indica	Japonica	
maker-11-augustus-gene-63.8-mRNA-1	11	OsR498G1119554100.01.T01	95,25	0,00E+00	2290	Os11g0195600	96,16	0,00E+00	2349	59	NA	Japonica	
maker-11-augustus-gene-64.3-mRNA-1	11	OsR498G1119559800.01.T02	99,45	0,00E+00	1621	Os01g0882300	72,47	6,22E-153	541	-1080	Indica	Japonica	
maker-11-augustus-gene-64.9-mRNA-1	11	OsR498G1119561000.01.T01	97,63	0,00E+00	2078	Os11g0197600	97,54	0,00E+00	2074	-4	NA	NA	
maker-11-augustus-gene-65.33-mRNA-1	11	OsR498G1119567600.01.T02	98,18	0,00E+00	2644	Os11g0199200	98,18	0,00E+00	2644	0	NA	NA	
maker-11-augustus-gene-66.39-mRNA-1	11	OsR498G1119570700.01.T03	100,00	0,00E+00	2489	Os11g0200600	97,68	0,00E+00	2340	-149	NA	Indica	
maker-11-augustus-gene-66.50-mRNA-1	11	OsR498G1119571800.01.T02	99,34	0,00E+00	2140	Os11g0201332	99,61	0,00E+00	2283	143	NA	Japonica	
maker-11-augustus-gene-67.51-mRNA-1	11	OsR498G1119574200.01.T01	99,89	0,00E+00	1693	Os11g0201800	99,92	0,00E+00	2370	677	Japonica	Japonica	
maker-11-augustus-gene-67.55-mRNA-1	11	OsR498G1119574400.01.T01	99,89	0,00E+00	1673	Os11g0201800	77,95	0,00E+00	803	-870	Indica	Japonica	
maker-11-augustus-gene-67.57-mRNA-1	11	OsR498G1119574800.01.T01	100,00	0,00E+00	2457	Os11g0202000	100,00	0,00E+00	2457	0	NA	Japonica	
maker-11-augustus-gene-69.35-mRNA-1	11	OsR498G1119581200.01.T01	97,68	0,00E+00	3809	Os11g0205500	99,87	0,00E+00	4229	420	NA	Indica	
maker-11-augustus-gene-69.39-mRNA-1	11	OsR498G1119582800.01.T01	99,75	0,00E+00	2159	Os11g0205900	97,97	0,00E+00	2547	388	Japonica	Indica	

maker-11-augustus-gene-69.44-mRNA-1	11	OsR498G1119575200.01.T01	92,12	1,56E-173	609	Os11g0208400	76,39	1,20E-36	153	-456	Indica	NA	
maker-11-augustus-gene-69.50-mRNA-1	11	OsR498G1119585800.01.T01	99,81	0,00E+00	4615	Os11g0206700	99,96	0,00E+00	4633	18	NA	Japonica	
maker-11-augustus-gene-7.42-mRNA-1	11	OsR498G1119363500.01.T01	99,71	0,00E+00	1821	Os11g0130300	100,00	0,00E+00	1835	14	NA	Japonica	
maker-11-augustus-gene-7.46-mRNA-1	11	OsR498G1119364700.01.T01	99,88	0,00E+00	1482	Os11g0130600	100,00	0,00E+00	1487	5	NA	Japonica	
maker-11-augustus-gene-7.53-mRNA-1	11	OsR498G1119368100.01.T04	99,85	0,00E+00	3553	Os11g0131900	100,00	0,00E+00	3566	13	NA	Japonica	
maker-11-augustus-gene-7.54-mRNA-1	11	OsR498G1119362400.01.T01	99,67	0,00E+00	2743	Os11g0129800	100,00	0,00E+00	1703	-1040	Indica	Japonica	
maker-11-augustus-gene-7.65-mRNA-1	11	OsR498G1119363200.01.T01	99,64	0,00E+00	1480	Os11g0130200	99,88	0,00E+00	1489	9	NA	Japonica	
maker-11-augustus-gene-7.73-mRNA-1	11	OsR498G1119365800.01.T01	99,69	0,00E+00	5176	Os08g0170200	71,35	0,00E+00	1084	-4092	Indica	Japonica	NBS-TM
maker-11-augustus-gene-7.75-mRNA-1	11	OsR498G1119366400.01.T02	92,84	0,00E+00	1196	Os11g0131200	98,72	0,00E+00	684	-512	Indica	Japonica	
maker-11-augustus-gene-7.80-mRNA-1	11	OsR498G1220874000.01.T02	98,31	4,40E-114	410	Os11g0130900	99,16	2,97E-117	420	10	NA	Japonica	
maker-11-augustus-gene-70.39-mRNA-1	11	OsR498G1119591700.01.T01	99,90	0,00E+00	3530	Os11g0207200	100,00	0,00E+00	2993	-537	Indica	Japonica	Kinase-TM
maker-11-augustus-gene-70.45-mRNA-1	11	OsR498G1119593300.01.T01	99,88	0,00E+00	1446	Os11g0207600	100,00	0,00E+00	1451	5	NA	Japonica	
maker-11-augustus-gene-71.0-mRNA-1	11	OsR498G1119594500.01.T03	99,92	0,00E+00	2301	Os11g0208000	100,00	0,00E+00	2305	4	NA	Japonica	
maker-11-augustus-gene-71.10-mRNA-1	11	OsR498G1119599000.01.T01	98,29	0,00E+00	4943	Os11g0208900	100,00	0,00E+00	5817	874	Japonica	Japonica	Kinase-LRR-TM
maker-11-augustus-gene-71.3-mRNA-1	11	OsR498G1119595800.01.T02	99,61	0,00E+00	2278	Os11g0208100	100,00	0,00E+00	2300	22	NA	Japonica	
maker-11-augustus-gene-71.9-mRNA-1	11	OsR498G1119598200.01.T01	96,03	0,00E+00	1105	Os07g0186200	83,53	0,00E+00	722	-383	Indica	Japonica	Kinase-TM
maker-11-augustus-gene-72.24-mRNA-1	11	OsR498G1119599800.01.T01	99,60	0,00E+00	2207	Os11g0209100	100,00	0,00E+00	2230	23	NA	Japonica	
maker-11-augustus-gene-72.26-mRNA-1	11	OsR498G1119600000.01.T01	99,85	0,00E+00	2322	Os11g0209200	100,00	0,00E+00	2331	9	NA	Japonica	
maker-11-augustus-gene-72.31-mRNA-1	11	OsR498G1119601400.01.T02	99,20	0,00E+00	1980	Os11g0209500	100,00	0,00E+00	1586	-394	Indica	Japonica	
maker-11-augustus-gene-72.41-mRNA-1	11	OsR498G1119604000.01.T01	99,87	0,00E+00	2762	Os11g0209900	100,00	0,00E+00	3571	809	Japonica	Japonica	
maker-11-augustus-gene-72.43-mRNA-1	11	OsR498G1119605000.01.T01	98,01	0,00E+00	1724	Os12g0191500	80,33	0,00E+00	837	-887	Indica	Japonica	
maker-11-augustus-gene-73.0-mRNA-1	11	OsR498G1119608300.01.T06	99,64	0,00E+00	989	Os06g0614300	83,49	1,73E-166	585	-404	Indica	Indica	
maker-11-augustus-gene-73.12-mRNA-1	11	OsR498G1119607600.01.T02	100,00	3,52E-142	504	Os11g0210600	100,00	1,22E-142	504	0	NA	Indica	
maker-11-augustus-gene-73.13-mRNA-1	11	OsR498G1119607600.01.T03	99,88	0,00E+00	1556	Os11g0210600	99,84	0,00E+00	1107	-449	Indica	Indica	
maker-11-augustus-gene-73.3-mRNA-1	11	OsR498G1119605800.01.T01	99,83	0,00E+00	2048	Os11g0210300	99,83	0,00E+00	2048	0	NA	Japonica	
maker-11-augustus-gene-73.9-mRNA-1	11	OsR498G1119606600.01.T01	99,91	0,00E+00	2052	Os11g0210500	99,12	0,00E+00	2011	-41	NA	Japonica	
maker-11-augustus-gene-75.13-mRNA-1	11	OsR498G1119615200.01.T01	97,83	0,00E+00	2451	Os11g0213600	98,11	0,00E+00	2469	18	NA	Japonica	
maker-11-augustus-gene-75.17-mRNA-1	11	OsR498G1119615600.01.T02	99,30	0,00E+00	4587	Os11g0213700	100,00	0,00E+00	4037	-550	Indica	Japonica	LRR-TM
maker-11-augustus-gene-75.20-mRNA-1	11	OsR498G1119615600.01.T05	99,51	0,00E+00	2181	Os11g0213800	100,00	0,00E+00	2208	27	NA	Japonica	CC-NBS-TM
maker-11-augustus-gene-76.0-mRNA-1	11	OsR498G1119618200.01.T01	83,77	5,22E-148	525	Os11g0215300	100,00	0,00E+00	764	239	Japonica	Japonica	
maker-11-augustus-gene-77.35-mRNA-1	11	OsR498G1119619100.01.T03	99,94	0,00E+00	2853	Os11g0216000	100,00	0,00E+00	2857	4	NA	Japonica	
maker-11-augustus-gene-77.39-mRNA-1	11	OsR498G1119622100.01.T02	99,64	0,00E+00	2012	Os11g0216900	100,00	0,00E+00	2030	18	NA	Japonica	
maker-11-augustus-gene-77.44-mRNA-1	11	OsR498G1119622300.01.T03	99,72	0,00E+00	1902	Os11g0217300	100,00	0,00E+00	1916	14	NA	Japonica	
maker-11-augustus-gene-77.48-mRNA-1	11	OsR498G1119624300.01.T01	100,00	0,00E+00	1975	Os11g0218000	100,00	0,00E+00	1975	0	NA	Japonica	
maker-11-augustus-gene-77.51-mRNA-1	11	OsR498G1119619600.01.T01	99,80	0,00E+00	3640	Os11g0216300	100,00	0,00E+00	3699	59	NA	Japonica	
maker-11-augustus-gene-77.60-mRNA-1	11	OsR498G1119622600.01.T01	99,80	0,00E+00	3638	Os12g0193200	78,30	0,00E+00	1671	-1967	Indica	Japonica	
maker-11-augustus-gene-79.40-mRNA-1	11	OsR498G1119634900.01.T01	98,11	0,00E+00	1819	Os11g0221000	100,00	0,00E+00	1480	-339	Indica	Japonica	
maker-11-augustus-gene-8.2-mRNA-1	11	OsR498G1119370100.01.T06	96,57	0,00E+00	1755	Os11g0132600	96,76	0,00E+00	1764	9	NA	Japonica	
maker-11-augustus-gene-8.45-mRNA-1	11	OsR498G1119375500.01.T01	99,64	0,00E+00	2447	Os11g0133800	96,69	0,00E+00	2442	-5	NA	Japonica	
maker-11-augustus-gene-8.49-mRNA-1	11	OsR498G1119369600.01.T02	98,66	0,00E+00	778	Os12g0129000	96,14	1,57E-168	590	-188	Indica	Japonica	
maker-11-augustus-gene-8.55-mRNA-1	11	OsR498G1119370400.01.T05	100,00	0,00E+00	1061	Os11g0132700	100,00	0,00E+00	1061	0	NA	Japonica	
maker-11-augustus-gene-8.76-mRNA-1	11	OsR498G1119375800.01.T02	99,37	7,00E-159	560	Os11g0133900	100,00	0,00E+00	738	178	Japonica	Japonica	
maker-11-augustus-gene-80.32-mRNA-1	11	OsR498G1119638500.01.T01	99,01	0,00E+00	1594	Os07g0179600	78,81	1,50E-172	606	-988	Indica	Japonica	
maker-11-augustus-gene-80.34-mRNA-1	11	OsR498G1119638200.01.T01	99,24	0,00E+00	2330	Os11g0222225	100,00	0,00E+00	2138	-192	NA	Japonica	
maker-11-augustus-gene-81.0-mRNA-1	11	OsR498G1119638500.01.T01	99,08	0,00E+00	963	Os07g0179600	77,47	1,96E-118	425	-538	Indica	Japonica	
maker-11-augustus-gene-82.0-mRNA-1	11	OsR498G1119642700.01.T03	73,57	0,00E+00	1687	Os11g0225300	99,44	0,00E+00	6950	5263	Japonica	Indica	CC-NBS-TM RGA5, Pia
maker-11-augustus-gene-82.6-mRNA-1	11	OsR498G1119646100.01.T02	97,66	0,00E+00	4990	Os11g0226700	100,00	0,00E+00	3810	-1180	Indica	Japonica	CC-NBS-TM
maker-11-augustus-gene-83.3-mRNA-1	11	OsR498G1119648000.01.T02	98,49	0,00E+00	4707	Os11g0227100	99,50	0,00E+00	7861	3154	Japonica	Indica	CC-LRR-NBS-TM
maker-11-augustus-gene-84.0-mRNA-1	11	OsR498G1119652500.01.T01	95,75	0,00E+00	2462	Os11g0230400	96,32	0,00E+00	1333	-1129	Indica	Japonica	
maker-11-augustus-gene-84.1-mRNA-1	11	OsR498G1119652700.01.T01	94,71	0,00E+00	1564	Os11g0230700	95,36	0,00E+00	1305	-259	Indica	NA	
maker-11-augustus-gene-84.4-mRNA-1	11	OsR498G1119653900.01.T01	97,06	0,00E+00	2100	Os11g0230700	81,92	0,00E+00	785	-1315	Indica	Japonica	
maker-11-augustus-gene-84.6-mRNA-1	11	OsR498G1119654700.01.T01	99,42	0,00E+00	2150	Os11g0231600	99,09	0,00E+00	2131	-19	NA	Indica	
maker-11-augustus-gene-84.8-mRNA-1	11	OsR498G1119656400.01.T01	98,31	0,00E+00	5121	Os11g0232100	98,45	0,00E+00	5139	18	NA	NA	Kinase-LRR-TM
maker-11-augustus-gene-86.10-mRNA-1	11	OsR498G1119664600.01.T01	99,43	6,56E-179	627	Os04g0631200	88,59	1,76E-117	421	-206	Indica	NA	
maker-11-augustus-gene-86.3-mRNA-1	11	OsR498G1119661800.01.T01	98,54	0,00E+00	3212	Os11g0234200	99,29	0,00E+00	3255	43	NA	Indica	
maker-11-augustus-gene-86.6-mRNA-1	11	OsR498G1119662400.01.T01	99,84	0,00E+00	3325	Os11g0235200	97,83	0,00E+00	3232	-93	NA	Indica	
maker-11-augustus-gene-87.36-mRNA-1	11	OsR498G1119671300.01.T01	99,81	0,00E+00	1855	Os11g0237900	100,00	0,00E+00	1864	9	NA	Japonica	CC-LRR-NBS-TM
maker-11-augustus-gene-87.38-mRNA-1	11	OsR498G1119671000.01.T02	98,90	0,00E+00	1272	Os05g0318300	93,45	0,00E+00	1085	-187	Indica	Japonica	
maker-11-augustus-gene-89.0-mRNA-1	11	OsR498G1119683100.01.T04	99,83	0,00E+00	2118	Os11g0240900	100,00	0,00E+00	2127	9	NA	Japonica	
maker-11-augustus-gene-89.7-mRNA-1	11	OsR498G1119686000.01.T01	99,93	0,00E+00	2452	Os11g0241900	100,00	0,00E+00	2457	5	NA	Japonica	
maker-11-augustus-gene-9.0-mRNA-1	11	OsR498G1119377100.01.T07	99,92	0,00E+00	2371	Os12g0132200	98,79	0,00E+00	2304	-67	NA	Japonica	Kinase-TM
maker-11-augustus-gene-9.17-mRNA-1	11	OsR498G1119377400.01.T01	99,28	0,00E+00	978	Os12g0132300	98,73	0,00E+00	965	-13	NA	Japonica	
maker-11-augustus-gene-9.18-mRNA-1	11	OsR498G1119377600.01.T01	95,99	0,00E+00	2311	Os11g0134500	96,35	0,00E+00	2333	22	NA	Japonica	
maker-11-augustus-gene-9.40-mRNA-1	11	OsR498G1119378200.01.T02	96,65	0,00E+00	2449	Os11g0135000	96,72	0,00E+00	2453	4	NA	Japonica	
maker-11-augustus-gene-9.50-mRNA-1	11	OsR498G1119380000.01.T01	99,90	0,00E+00	1733	Os08g0130500	95,02	0,00E+00	1519	-214	Indica	Japonica	
maker-11-augustus-gene-9.6-mRNA-1	11	OsR498G1119376200.01.T01	99,84	0,00E+00	3396	Os12g0131900	92,36	0,00E+00	3028	-368	Indica	Japonica	
maker-11-augustus-gene-90.0-mRNA-1	11	OsR498G1119686500.01.T06	99,83	0,00E+00	5123	Os11g0242100	100,00	0,00E+00	5146	23	NA	Japonica	
maker-11-augustus-gene-90.12-mRNA-1	11	OsR498G1119689500.01.T01	89,97	5,18E-149	527	Os11g0242700	91,47	2,51E-179	627	100	Japonica	Japonica	
maker-11-augustus-gene-90.15-mRNA-1	11	OsR498G1119689900.01.T02	99,53	0,00E+00	1519	Os11g0242800	100,00	0,00E+00	1537	18	NA	Japonica	
maker-11-augustus-gene-90.20-mRNA-1	11	OsR498G1119688800.01.T03	99,85	0,00E+00	1161	Os11g0242400	99,85	0,00E+00	1161	0	NA	Japonica	
maker-11-augustus-gene-90.7-mRNA-1	11	OsR498G1119687300.01.T01	98,67	0,00E+00	2110	Os11g0242200	99,92	0,00E+00	2150	40	NA	Japonica	
maker-11-augustus-gene-90.9-mRNA-1	11	OsR498G1119688900.01.T03	99,65	0,00E+00	3068	Os11g0242500	100,00	0,00E+00	3095	27	NA	Japonica	Kinase-TM
maker-11-augustus-gene-99.5-mRNA-1	11	OsR498G1119939800.01.T02	97,65	0,00E+00	2251	Os11g0414000	100,00	0,00E+00	2228	-23	NA	Japonica	
augustus_masked-12-processed-gene-1.54-mRNA-1	12	OsR498G1220784400.01.T01	98,19	0,00E+00	2573	Os11g0100800	99,73	0,00E+00	2625	52	NA	Japonica	
augustus_masked-12-processed-gene-1.78-mRNA-1	12	OsR498G1220788600.01.T03	98,89	0,00E+00	1262	Os12g0102300	100,00	0,00E+00	1287	25	NA	Japonica	
augustus_masked-12-processed-gene-10.8-mRNA-1	12	OsR498G1119335000.01.T01	98,35	0,00E+00	1154	Os12g0120100	100,00	0,00E+00	1207	53	NA	Japonica	
augustus_masked-12-processed-gene-102.18-mRNA-1	12	OsR498G1221277200.01.T02	96,45	0,00E+00	1394	Os12g0273000	100,00	0,00E+00	1526	132	NA	Japonica	CC-NBS-TM
augustus_masked-12-processed-gene-108.24-mRNA-1	12	OsR498G1221311800.01.T01	99,87	0,00E+00	5577	Os12g0281300	100,00	0,00E+00	5595	18	NA	Japonica	NBS-TM Pi-ta
augustus_masked-12-processed-gene-109.7-mRNA-1	12	OsR498G1221316000.01.T02	99,30	0,00E+00	757	Os12g0282000	100,00	0,00E+00	893	136	Japonica	Japonica	
augustus_masked-12-processed-gene-111.17-mRNA-1	12	OsR498G1221327800.01.T01	97,31	0,00E+00	813	Os12g0286200	100,00	0,00E+00	872	59	NA	Japonica	
augustus_masked-12-processed-gene-113.3-mRNA-1	12	OsR498G1221338800.01.T02	99,87	0,00E+00	2713	Os12g0288400	99,87	0,00E+00	2702	-11	NA	Indica	
augustus_masked-12-processed-gene-113.30-mRNA-1	12	OsR498G1221344000.01.T05	99,51	0,00E+00	3602	Os12g0289800	99,85	0,00E+00	3634	32	NA	NA	
augustus_masked-12-processed-gene-12.54-mRNA-1	12	OsR498G1119355200.01.T02	98,16	0,00E+00	1683	Os12g0124400	98,58	0,00E+00	1594	-89	NA	NA	
augustus_masked-12-processed-gene-126.16-mRNA-1	12	OsR498G1221371000.01.T01	98,56	0,00E+00	1202	Os12g0407400	78,23	8,16E-124	444	-758	Indica	Japonica	
augustus_masked-12-processed-gene-126.35-mRNA-1	12	OsR498G1221371000.01.T01	80,84	7,53E-81	300	Os12g0407400	100,00	4,72E-154	542	242	Japonica	Japonica	
augustus_masked-12-processed-gene-127.26-mRNA-1	12	OsR498G1221377600.01.T02	99,69	2,01E-166	586	Os12g0409901	98,97	0,00E+00	677	91	Japonica	NA	NBS
augustus_masked-12-processed-gene-13.28-mRNA-1	12	OsR498G1220870600.01.T01	100,00	0,00E+00	1494	Os12g0127200	100,00	0,00E+00	1494	0	NA	NA	
augustus_masked-12-processed-gene-132.11-mRNA-1	12	OsR498G1221397600.01.T01	94,65	0,00E+00	643	Os12g0415400	99,76	0,00E+00	737	94	Japonica	NA	
augustus_masked-12-processed-gene-132.22-mRNA-1	12	OsR498G1221397600.01.T01	99,27	0,00E+00	728	Os12g0415800	99,27	0,00E+00	728	0	NA	NA	
augustus_masked-12-processed-gene-14.78-mRNA-1	12	OsR498G1220875000.01.T01	99,48	0,00E+00	1026	Os11g0132100	76,20	1,07E-89	330	-696	Indica	Japonica	
augustus_masked-12-processed-gene-140.24-mRNA-1	12	OsR498G1221431000.01.T01	99,73	0,00E+00	3945	Os12g0427600	99,77	0,00E+00	3949	4	NA	NA	
augustus_masked-12-processed-gene-145.10-mRNA-1	12	OsR498G1221528600.01.T01	89,41	0,00E+00	848	Os12g0453500	100,00	0,00E+00	1148	300	Japonica	NA	
augustus_masked-12-processed-gene-149.15-mRNA-1	12	OsR498G1221463200.01.T01	99,47	0,00E+00	3010	Os12g0435200	100,00	0,00E+00	3050	40	NA	Japonica	
augustus_masked-12-processed-gene-15.24-mRNA-1	12	OsR498G1119372000.01.T01	96,32	0,00E+00	4146	Os12g0130300	100,00	0,00E+00	4524	378	NA	Japonica	Kinase-TM
augustus_masked-12-processed-gene-15.29-mRNA-1	12	OsR498G1220884400.01.T01	99,72	0,00E+00	4454	Os11g0133500	98,03	0,00E+00	4265	-189	NA	Japonica	Kinase-TM
augustus_masked-12-processed-gene-152.21-mRNA-1	12	OsR498G1221472800.01.T01	100,00	3,72E-135	480	Os12g0438900	100,00	3,47E-149	526	46	NA	Japonica	
augustus_masked-12-processed-gene-155.9-mRNA-1	12	OsR498G1221505800.01.T01	99,06	6,65E-159	561	Os12g0442700	100,00	1,05E-163	574	13	NA	Japonica	
augustus_masked-12-processed-gene-162.11-mRNA-1	12	OsR498G1221528600.01.T01	99,38	0,00E+00	1141	Os12g0453500	90,03	0,00E+00	866	-275	Indica	Japonica	
augustus_masked-12-processed-gene-178.3-mRNA-1	12	OsR498G1221574600.01.T01	94,54	0,00E+00	2375	Os12g0470700	100,00	0,00E+00	2565	190	NA	Japonica	
augustus_masked-12-processed-gene-19.38-mRNA-1	12	OsR498G1220896200.01.T01	99,89	0,00E+00	1571	Os12g0134600	100,00	0,00E+00	2419	848	Japonica	Japonica	
augustus_masked-12-processed-gene-192.21-mRNA-1	12	OsR498G1221649100.01.T01	99,92	0,00E+00	2299	Os12g0489800	100,00	0,00E+00	2305	6	NA	Japonica	NBS-TM
augustus_masked-12-processed-gene-192.6-mRNA-1	12	OsR498G1221645800.01.T01	99,57	0,00E+00	4125	Os12g0488900	100,00	0,00E+00	4167	42	NA	Japonica	
augustus_masked-12-processed-gene-2.34-mRNA-1	12	OsR498G1119284600.01.T05	98,62	2,35E-104	378	Os12g0104400	99,52	4,25E-102	370	-8	NA	NA	
augustus_masked-12-processed-gene-20.28-mRNA-1	12	OsR498G1220904600.01.T01	99,53	0,00E+00	1508	Os12g0137600	100,00	0,00E+00	1321	-187	Indica	Japonica	
augustus_masked-12-processed-gene-20.29-mRNA-1	12	OsR498G1220904600.01.T02	99,80	0,00E+00	1820	Os12g0137700	100,00	0,00E+00	1829	9	NA	Japonica	
augustus_masked-12-processed-gene-203.27-mRNA-1	12	OsR498G1221682400.01.T01	99,09	0,00E+00	773	Os12g0506100	100,00	0,00E+00	1391	618	Japonica	Japonica	
augustus_masked-12-processed-gene-203.49-mRNA-1	12	OsR498G1221689000.01.T01	99,80	0,00E+00	1772	Os12g0507400	100,00	0,00E+00	1781	9	NA	Japonica	
augustus_masked-12-processed-gene-205.48-mRNA-1	12	OsR498G1221701800.01.T01	99,04	0,00E+00	1104	Os12g0510750	100,00	0,00E+00	969	-135	Indica	Japonica	
augustus_masked-12-processed-gene-207.17-mRNA-1	12	OsR498G1221706000.01.T05	99,84	0,00E+00	2275	Os12g0512700	99,86	0,00E+00	1239	-1036	Indica	Japonica	
augustus_masked-12-processed-gene-21.42-mRNA-1	12	OsR498G1220912600.01.T01	96,33	0,00E+00	985	Os12g0140700	100,00	0,00E+00	1072	87	NA	Japonica	
augustus_masked-12-processed-gene-23.34-mRNA-1	12	OsR498G1220919200.01.T02	99,87	0,00E+00	1403	Os12g0142600	100,00	0,00E+00	1407	4	NA	Japonica	
augustus_masked-12-processed-gene-23.36-mRNA-1	12	OsR498G1220919400.01.T01	100,00	0,00E+00	969	Os02g0711400	84,81	3,71E-114	410	-559	Indica	Japonica	
augustus_masked-12-processed-gene-23.54-mRNA-1	12	OsR498G1220920800.01.T05	99,55	7,61E-111	400	Os12g0143300	100,00	6,27E-113	405	5	NA	Japonica	
augustus_masked-12-processed-gene-230.38-mRNA-1	12	OsR498G1221868800.01.T03	99,76	0,00E+00	3749	Os12g0557800	97,99	0,00E+00	3616	-133	NA	Japonica	
augustus_masked-12-processed-gene-233.33-mRNA-1	12	OsR498G1221881400.01.T01	99,72	0,00E+00	2587	Os10g0122000	74,14	0,00E+00	951	-1636	Indica	Japonica	
augustus_masked-12-processed-gene-234.9-mRNA-1	12	OsR498G1221887800.01.T01	100,00	5,50E-139	493	Os03g0158000	97,44	1,14E-129	462	-31	NA	NA	
augustus_masked-12-processed-gene-238.53-mRNA-1	12	OsR498G1221911600.01.T01	100,00	0,00E+00	1370	Os12g0571300	100,00	0,00E+00	1370	0	NA	Japonica	
augustus_masked-12-processed-gene-240.16-mRNA-1	12	OsR498G1221924200.01.T03	99,18	0,00E+00	1947	Os12g0574800	100,00	0,00E+00	2652	705	Japonica	Japonica	
augustus_masked-12-processed-gene-242.24-mRNA-1	12	OsR498G1221937000.01.T02	100,00	0,00E+00	2089	Os12g0578700	100,00	0,00E+00	2089	0	NA	NA	
augustus_masked-12-processed-gene-244.21-mRNA-1	12	OsR498G1221955800.01.T01	100,00	0,00E+00	2598	Os12g0582666	100,00	0,00E+00	3090	492	Japonica	NA	
augustus_masked-12-processed-gene-246.3-mRNA-1	12	OsR498G1221976200.01.T03	100,00	0,00E+00	2268	Os12g0585200	100,00	0,00E+00	2268	0	NA	NA	
augustus_masked-12-processed-gene-250.25-mRNA-1	12	OsR498G1222005000.01.T01	99,59	0,00E+00	861	Os12g0590400	100,00	0,00E+00	872	11	NA	Japonica	
augustus_masked-12-processed-gene-257.18-mRNA-1	12	OsR498G0306613200.01.T01	81,69	0,00E+00	1256	Os12g0603700	98,53	0,00E+00	2373	1117	Japonica	NA	Kinase-TM
augustus_masked-12-processed-gene-26.21-mRNA-1	12	OsR498G1220943800.01.T01	99,50	0,00E+00	2480	Os12g0149300	100,00	0,00E+00	2511	31	NA	Japonica	
augustus_masked-12-processed-gene-26.24-mRNA-1	12	OsR498G1220945800.01.T01	100,00	0,00E+00	818	Os12g0150100	100,00	0,00E+00	818	0	NA	Japonica	
augustus_masked-12-processed-gene-26.25-mRNA-1	12	OsR498G1220946600.01.T01	99,80	0,00E+00	2723	Os12g0150200	100,00	0,00E+00	2760	37	NA	Japonica	
augustus_masked-12-processed-gene-260.34-mRNA-1	12	OsR498G1120733700.01.T01	88,89	6,18E-134	478	Os12g0166700	88,80	1,76E-135	480	2	NA	Japonica	
augustus_masked-12-processed-gene-260.37-mRNA-1	12	OsR498G1222080600.01.T01	97,47	0,00E+00	3479	Os12g0608900	99,90	0,00E+00	3687	208	NA	Japonica	Kinase-TM
augustus_masked-12-processed-gene-260.42-mRNA-1	12	OsR498G1222082800.01.T01	97,18	0,00E+00	890	Os12g0609600	99,81	0,00E+00	938	48	NA	Japonica	
augustus_masked-12-processed-gene-260.9-mRNA-1	12	OsR498G1222075300.01.T01	100,00	0,00E+00	2722	Os12g0608500	84,80	0,00E+00	1579	-1143	Indica	NA	Kinase-TM
augustus_masked-12-processed-gene-261.35-mRNA-1	12	OsR498G1222083400.01.T01	97,90	0,00E+00	1471	Os12g0609900	99,89	0,00E+00	1688	217	Japonica	Japonica	
augustus_masked-12-processed-gene-264.61-mRNA-1	12	OsR498G1222106200.01.T03	97,87	0,00E+00	4331	Os12g0615800	100,00	0,00E+00	4572	241	NA	Japonica	
augustus_masked-12-processed-gene-265.12-mRNA-1	12	OsR498G1222111000.01.T01	99,75	0,00E+00	1442	Os12g0616800	99,88	0,00E+00	1446	4	NA	Japonica	
augustus_masked-12-processed-gene-267.18-mRNA-1	12	OsR498G1222122000.01.T01	100,00	0,00E+00	1981	Os12g0618800	100,00	0,00E+00	1981	0	NA	NA	
augustus_masked-12-processed-gene-27.26-mRNA-1	12	OsR498G1220948000.01.T01	99,41	0,00E+00	1211	Os12g0150750	100,00	0,00E+00	1575	364	Japonica	Japonica	
augustus_masked-12-processed-gene-272.42-mRNA-1	12	OsR498G1222150400.01.T01	93,03	0,00E+00	790	Os12g0628600	99,62	0,00E+00	949	159	Japonica	Japonica	
augustus_masked-12-processed-gene-272.44-mRNA-1	12	OsR498G1222152600.01.T01	99,64	0,00E+00	982	Os12g0630100	99,82	0,00E+00	986	4	NA	NA	
augustus_masked-12-processed-gene-28.55-mRNA-1	12	OsR498G1119432200.01.T01	71,30	5,87E-33	141	Os12g0153200	100,00	0,00E+00	704	563	Japonica	NA	
augustus_masked-12-processed-gene-29.31-mRNA-1	12	OsR498G1220962000.01.T01	93,22	0,00E+00	862	Os12g0155000	100,00	0,00E+00	1032	170	Japonica	NA	
augustus_masked-12-processed-gene-3.8-mRNA-1	12	OsR498G1220796000.01.T01	100,00	0,00E+00	733	Os12g0104900	100,00	0,00E+00	812	79	NA	Japonica	
augustus_masked-12-processed-gene-31.22-mRNA-1	12	OsR498G1220979400.01.T02	99,61	0,00E+00	1370	Os12g0158400	100,00	0,00E+00	1380	10	NA	NA	
augustus_masked-12-processed-gene-31.25-mRNA-1	12	OsR498G1119445600.01.T01	79,81	1,03E-94	347	Os12g0158700	100,00	0,00E+00	942	595	Japonica	NA	
augustus_masked-12-processed-gene-31.28-mRNA-1	12	OsR498G1220983000.01.T01	100,00	0,00E+00	1045	Os12g0159000	100,00	0,00E+00	1045	0	NA	NA	
augustus_masked-12-processed-gene-34.46-mRNA-1	12	OsR498G1221003600.01.T02	99,85	0,00E+00	2334	Os12g0163500	100,00	0,00E+00	2343	9	NA	Japonica	
augustus_masked-12-processed-gene-34.47-mRNA-1	12	OsR498G1221004000.01.T01	99,67	0,00E+00	3247	Os12g0163600	100,00	0,00E+00	3274	27	NA	Japonica	
augustus_masked-12-processed-gene-5.58-mRNA-1	12	OsR498G1119303000.01.T01	97,35	0,00E+00	2602	Os12g0109300	99,38	0,00E+00	3442	840	Japonica	NA	
augustus_masked-12-processed-gene-51.11-mRNA-1	12	OsR498G1221047500.01.T01	82,20	0,00E+00	1377	Os12g0195100	100,00	0,00E+00	2538	1161	Japonica	Japonica	
augustus_masked-12-processed-gene-54.11-mRNA-1	12	OsR498G0816429700.01.T01	70,84	2,16E-178	627	Os12g0199800	100,00	0,00E+00	2646	2019	Japonica	NA	
augustus_masked-12-processed-gene-57.32-mRNA-1	12	OsR498G1221100000.01.T02	97,59	0,00E+00	2632	Os12g0205500	100,00	0,00E+00	5086	2454	Japonica	Japonica	NBS-TM
augustus_masked-12-processed-gene-60.19-mRNA-1	12	OsR498G1221115800.01.T01	99,63	0,00E+00	4348	Os12g0211500	100,00	0,00E+00	5442	1094	Japonica	Japonica	LRR-TM
augustus_masked-12-processed-gene-60.2-mRNA-1	12	OsR498G1221110600.01.T01	98,34	1,76E-178	625	Os12g0210500	100,00	0,00E+00	632	7	NA	Japonica	
augustus_masked-12-processed-gene-63.37-mRNA-1	12	OsR498G1221115800.01.T01	86,96	0,00E+00	2914	Os12g0215950	100,00	0,00E+00	5324	2410	Japonica	NA	LRR-TM
augustus_masked-12-processed-gene-7.59-mRNA-1	12	OsR498G1220822800.01.T01	99,55	0,00E+00	2354	Os12g0113500	100,00	0,00E+00	2527	173	NA	Japonica	Kinase-TM
augustus_masked-12-processed-gene-73.22-mRNA-1	12	OsR498G1221156200.01.T01	97,31	0,00E+00	2131	Os12g0230133	100,00	0,00E+00	2284	153	NA	Japonica	
augustus_masked-12-processed-gene-75.47-mRNA-1	12	OsR498G1221165600.01.T05	99,80	0,00E+00	3575	Os12g0233200	100,00	0,00E+00	3557	-18	NA	Japonica	
augustus_masked-12-processed-gene-78.39-mRNA-1	12	OsR498G1221184800.01.T01	95,58	1,95E-132	472	Os09g0552200	87,16	2,53E-100	363	-109	Indica	Japonica	
augustus_masked-12-processed-gene-81.3-mRNA-1	12	OsR498G1221196400.01.T01	74,36	7,77E-53	208	Os12g0242100	73,48	2,07E-48	193	-15	NA	Japonica	
augustus_masked-12-processed-gene-9.51-mRNA-1	12	OsR498G1220835700.01.T01	99,53	0,00E+00	3054	Os12g0117400	100,00	0,00E+00	3204	150	NA	Japonica	
augustus-12-processed-gene-0.4-mRNA-1	12	OsR498G1220782900.01.T03	99,80	0,00E+00	914	Os11g0100200	100,00	0,00E+00	921	7	NA	Japonica	
augustus-12-processed-gene-10.19-mRNA-1	12	OsR498G1220825200.01.T01	99,68	4,43E-157	555	Os12g0120900	100,00	0,00E+00	1077	522	Japonica	Japonica	
augustus-12-processed-gene-10.21-mRNA-1	12	OsR498G1220848300.01.T01	99,76	0,00E+00	4518	Os12g0121600	100,00	0,00E+00	4545	27	NA	Japonica	
augustus-12-processed-gene-110.0-mRNA-1	12	OsR498G1221320300.01.T01	100,00	0,00E+00	1240	Os12g0283400	100,00	0,00E+00	1240	0	NA	Japonica	
augustus-12-processed-gene-113.8-mRNA-1	12	OsR498G1221339400.01.T01	99,56	0,00E+00	3672	Os12g0288600	99,88	0,00E+00	3032	-640	Indica	NA	
augustus-12-processed-gene-12.45-mRNA-1	12	OsR498G1220863300.01.T01	99,45	0,00E+00	3229	Os12g0124700	99,45	0,00E+00	3229	0	NA	NA	
augustus-12-processed-gene-123.17-mRNA-1	12	OsR498G1221322300.01.T01	98,71	5,59E-113	407	Os12g0285100	85,77	8,33E-74	275	-132	Indica	NA	Ptr
augustus-12-processed-gene-124.16-mRNA-1	12	OsR498G1221366300.01.T01	99,71	0,00E+00	2444	Os12g0405100	100,00	0,00E+00	2462	18	NA	Japonica	
augustus-12-processed-gene-126.23-mRNA-1	12	OsR498G1221372700.01.T03	98,48	0,00E+00	2395	Os12g0407800	99,93	0,00E+00	2485	90	NA	Indica	
augustus-12-processed-gene-137.1-mRNA-1	12	OsR498G1221417500.01.T01	98,75	0,00E+00	1115	Os01g0389200	73,52	1,54E-75	282	-833	Indica	Japonica	
augustus-12-processed-gene-137.11-mRNA-1	12	OsR498G1221421300.01.T01	100,00	0,00E+00	2154	Os12g0422200	98,91	0,00E+00	2095	-59	NA	Indica	
augustus-12-processed-gene-137.12-mRNA-1	12	OsR498G1221421500.01.T01	99,58	0,00E+00	2142	Os12g0424600	97,50	0,00E+00	2030	-112	NA	Indica	
augustus-12-processed-gene-14.53-mRNA-1	12	OsR498G1220872300.01.T01	100,00	0,00E+00	1040	Os12g0127800	100,00	0,00E+00	1005	-35	NA	NA	
augustus-12-processed-gene-14.55-mRNA-1	12	OsR498G1220873500.01.T01	99,67	0,00E+00	3252	Os12g0128300	96,76	0,00E+00	3026	-226	NA	Japonica	
augustus-12-processed-gene-142.18-mRNA-1	12	OsR498G1221439900.01.T01	99,75	0,00E+00	2814	Os12g0431100	99,64	0,00E+00	2501	-313	Indica	NA	
augustus-12-processed-gene-143.10-mRNA-1	12	OsR498G1221448500.01.T01	99,82	0,00E+00	1003	Os12g0432600	99,82	0,00E+00	1003	0	NA	Indica	
augustus-12-processed-gene-146.12-mRNA-1	12	OsR498G1221448500.01.T01	99,46	0,00E+00	986	Os12g0432600	99,46	0,00E+00	986	0	NA	NA	
augustus-12-processed-gene-15.14-mRNA-1	12	OsR498G1220884900.01.T01	99,66	0,00E+00	1581	Os12g0131000	100,00	0,00E+00	1586	5	NA	Japonica	
augustus-12-processed-gene-15.2-mRNA-1	12	OsR498G1220879500.01.T01	75,31	0,00E+00	714	Os12g0129700	94,52	0,00E+00	1487	773	Japonica	Japonica	
augustus-12-processed-gene-151.7-mRNA-1	12	OsR498G1221468900.01.T01	99,22	0,00E+00	2722	Os12g0438300	100,00	0,00E+00	2776	54	NA	Japonica	NBS-TM
augustus-12-processed-gene-156.16-mRNA-1	12	OsR498G1221501200.01.T01	99,79	0,00E+00	2590	Os12g0443500	99,93	0,00E+00	2599	9	NA	Japonica	
augustus-12-processed-gene-156.17-mRNA-1	12	OsR498G1221500400.01.T03	99,58	0,00E+00	2576	Os12g0443600	100,00	0,00E+00	2603	27	NA	Japonica	
augustus-12-processed-gene-165.3-mRNA-1	12	OsR498G1221528600.01.T01	89,41	0,00E+00	848	Os12g0453500	100,00	0,00E+00	1148	300	Japonica	NA	
augustus-12-processed-gene-167.10-mRNA-1	12	OsR498G1221537900.01.T01	100,00	1,31E-140	499	Os12g0455000	100,00	4,58E-141	499	0	NA	Japonica	
augustus-12-processed-gene-167.22-mRNA-1	12	OsR498G1221540900.01.T05	99,95	0,00E+00	3760	Os12g0456100	100,00	0,00E+00	6049	2289	Japonica	Japonica	
augustus-12-processed-gene-167.8-mRNA-1	12	OsR498G1221536100.01.T01	97,79	0,00E+00	3187	Os12g0454800	100,00	0,00E+00	5194	2007	Japonica	Japonica	Kinase-TM
augustus-12-processed-gene-170.13-mRNA-1	12	OsR498G1221549300.01.T01	96,84	0,00E+00	1155	Os12g0460000	100,00	0,00E+00	1256	101	NA	Japonica	
augustus-12-processed-gene-173.17-mRNA-1	12	OsR498G1221564100.01.T01	100,00	1,78E-165	581	Os12g0465100	77,39	5,83E-65	247	-334	Indica	Japonica	
augustus-12-processed-gene-173.6-mRNA-1	12	OsR498G1221563300.01.T01	99,05	0,00E+00	2409	Os12g0464200	100,00	0,00E+00	2511	102	NA	Japonica	
augustus-12-processed-gene-175.1-mRNA-1	12	OsR498G1221571300.01.T01	98,91	0,00E+00	1611	Os12g0467200	89,67	0,00E+00	1275	-336	Indica	Japonica	
augustus-12-processed-gene-175.12-mRNA-1	12	OsR498G1221572700.01.T01	94,23	0,00E+00	2351	Os12g0467700	100,00	0,00E+00	2765	414	Japonica	NA	
augustus-12-processed-gene-175.16-mRNA-1	12	OsR498G1221572700.01.T01	94,23	0,00E+00	2351	Os12g0467700	100,00	0,00E+00	2765	414	Japonica	NA	
augustus-12-processed-gene-175.2-mRNA-1	12	OsR498G1221571300.01.T01	89,35	0,00E+00	1261	Os12g0467200	100,00	0,00E+00	2214	953	Japonica	NA	
augustus-12-processed-gene-178.12-mRNA-1	12	OsR498G1221576100.01.T01	99,81	0,00E+00	2849	Os12g0471100	100,00	0,00E+00	2863	14	NA	Japonica	
augustus-12-processed-gene-179.10-mRNA-1	12	OsR498G1221579700.01.T01	97,54	0,00E+00	1173	Os12g0472500	100,00	0,00E+00	1229	56	NA	Japonica	
augustus-12-processed-gene-18.10-mRNA-1	12	OsR498G1221077900.01.T02	99,58	0,00E+00	1264	Os12g0194400	100,00	0,00E+00	1402	138	NA	NA	
augustus-12-processed-gene-182.13-mRNA-1	12	OsR498G1221588100.01.T01	99,91	0,00E+00	4114	Os12g0477100	88,66	0,00E+00	2969	-1145	Indica	Japonica	CC-LRR-NBS-TM
augustus-12-processed-gene-187.0-mRNA-1	12	OsR498G1221614700.01.T01	97,02	0,00E+00	901	Os12g0482600	90,06	0,00E+00	762	-139	Indica	Japonica	
augustus-12-processed-gene-188.25-mRNA-1	12	OsR498G1221621100.01.T02	100,00	0,00E+00	2145	Os12g0484900	100,00	0,00E+00	1180	-965	Indica	Japonica	
augustus-12-processed-gene-19.11-mRNA-1	12	OsR498G1119389500.01.T01	97,09	0,00E+00	2646	Os12g0134900	100,00	0,00E+00	2798	152	NA	Japonica	
augustus-12-processed-gene-19.12-mRNA-1	12	OsR498G1119390700.01.T01	97,12	0,00E+00	2089	Os12g0135200	100,00	0,00E+00	2251	162	NA	Japonica	
augustus-12-processed-gene-19.18-mRNA-1	12	OsR498G1220899500.01.T01	99,65	0,00E+00	2033	Os12g0136100	100,00	0,00E+00	2291	258	Japonica	Japonica	
augustus-12-processed-gene-19.20-mRNA-1	12	OsR498G1220899900.01.T02	99,85	0,00E+00	1224	Os05g0495200	71,30	6,19E-43	174	-1050	Indica	Japonica	
augustus-12-processed-gene-198.43-mRNA-1	12	OsR498G1221664100.01.T01	99,83	0,00E+00	3213	Os12g0498650	100,00	0,00E+00	3804	591	Japonica	Japonica	Kinase-LRR-TM
augustus-12-processed-gene-198.45-mRNA-1	12	OsR498G1221666100.01.T01	99,54	0,00E+00	1152	Os12g0498900	100,00	0,00E+00	1169	17	NA	Japonica	
augustus-12-processed-gene-200.10-mRNA-1	12	OsR498G1221670300.01.T04	98,97	0,00E+00	1364	Os12g0502200	100,00	0,00E+00	1402	38	NA	Japonica	
augustus-12-processed-gene-204.10-mRNA-1	12	OsR498G1221692700.01.T02	98,37	8,66E-150	530	Os12g0508400	100,00	2,64E-157	553	23	NA	Japonica	
augustus-12-processed-gene-207.6-mRNA-1	12	OsR498G1221705000.01.T01	99,74	0,00E+00	2764	Os12g0512100	100,00	0,00E+00	2782	18	NA	Japonica	
augustus-12-processed-gene-209.6-mRNA-1	12	OsR498G1221715900.01.T02	99,57	0,00E+00	3308	Os12g0515500	99,25	0,00E+00	3293	-15	NA	Indica	
augustus-12-processed-gene-209.8-mRNA-1	12	OsR498G1221718100.01.T02	100,00	0,00E+00	2614	Os12g0516900	99,32	0,00E+00	2080	-534	Indica	Indica	
augustus-12-processed-gene-21.14-mRNA-1	12	OsR498G1220910900.01.T01	100,00	0,00E+00	1094	Os12g0139400	100,00	0,00E+00	1094	0	NA	Japonica	
augustus-12-processed-gene-21.18-mRNA-1	12	OsR498G1119404100.01.T01	93,20	8,76E-131	467	Os12g0140200	100,00	3,25E-156	549	82	Japonica	Japonica	
augustus-12-processed-gene-21.24-mRNA-1	12	OsR498G1220914900.01.T04	99,81	0,00E+00	927	Os12g0140600	100,00	0,00E+00	931	4	NA	Japonica	
augustus-12-processed-gene-210.14-mRNA-1	12	OsR498G1221720100.01.T01	100,00	0,00E+00	2608	Os12g0517000	99,10	0,00E+00	2550	-58	NA	Indica	

augustus-12-processed-gene-210.15-mRNA-1	12	OsR498G1221720500.01.T01	100,00	0,00E+00	2657	Os12g0517100	100,00	0,00E+00	2657	0	NA	Indica	
augustus-12-processed-gene-210.16-mRNA-1	12	OsR498G1221720900.01.T02	100,00	1,03E-174	612	Os12g0517200	99,71	1,53E-173	608	-4	NA	Indica	
augustus-12-processed-gene-212.13-mRNA-1	12	OsR498G1221728500.01.T01	99,86	0,00E+00	2556	Os12g0529900	96,67	0,00E+00	1340	-1216	Indica	Indica	
augustus-12-processed-gene-217.2-mRNA-1	12	OsR498G1221764900.01.T01	100,00	0,00E+00	3690	Os12g0535900	100,00	0,00E+00	3247	-443	Indica	NA	
augustus-12-processed-gene-218.22-mRNA-1	12	OsR498G1221773900.01.T01	100,00	4,29E-160	563	Os12g0538600	100,00	1,49E-160	563	0	NA	NA	
augustus-12-processed-gene-219.15-mRNA-1	12	OsR498G1221781100.01.T01	100,00	0,00E+00	1144	Os12g0540000	100,00	0,00E+00	1144	0	NA	Japonica	
augustus-12-processed-gene-22.0-mRNA-1	12	OsR498G1220915700.01.T01	99,42	0,00E+00	1216	Os11g0144900	96,24	0,00E+00	1084	-132	Indica	Japonica	
augustus-12-processed-gene-22.1-mRNA-1	12	OsR498G1119405700.01.T01	97,16	0,00E+00	3314	Os12g0141000	100,00	0,00E+00	3561	247	NA	Japonica	
augustus-12-processed-gene-224.15-mRNA-1	12	OsR498G1221827700.01.T03	97,86	1,92E-158	559	Os12g0545900	97,73	1,38E-148	525	-34	NA	NA	
augustus-12-processed-gene-226.12-mRNA-1	12	OsR498G1221839300.01.T03	94,69	2,50E-98	358	Os12g0548300	95,58	1,69E-101	367	9	NA	NA	
augustus-12-processed-gene-227.9-mRNA-1	12	OsR498G1221851600.01.T01	98,95	0,00E+00	2166	Os12g0261300	70,86	0,00E+00	1118	-1048	Indica	Japonica	
augustus-12-processed-gene-229.35-mRNA-1	12	OsR498G1221864300.01.T01	98,02	0,00E+00	1133	Os12g0556100	97,83	0,00E+00	952	-181	Indica	Japonica	
augustus-12-processed-gene-229.43-mRNA-1	12	OsR498G1221864900.01.T02	99,71	0,00E+00	3100	Os12g0556200	100,00	0,00E+00	3122	22	NA	Japonica	
augustus-12-processed-gene-229.48-mRNA-1	12	OsR498G1221867300.01.T01	97,83	0,00E+00	784	Os12g0556900	100,00	0,00E+00	1629	845	Japonica	Japonica	
augustus-12-processed-gene-234.3-mRNA-1	12	OsR498G1221888700.01.T01	100,00	0,00E+00	1353	Os12g0563600	100,00	0,00E+00	1104	-249	Indica	Japonica	
augustus-12-processed-gene-237.22-mRNA-1	12	OsR498G1221905900.01.T03	98,57	0,00E+00	728	Os11g0703900	81,82	9,28E-121	432	-296	Indica	Japonica	
augustus-12-processed-gene-237.23-mRNA-1	12	OsR498G1221906500.01.T01	98,62	0,00E+00	1767	Os12g0569900	100,00	0,00E+00	1802	35	NA	Japonica	
augustus-12-processed-gene-238.40-mRNA-1	12	OsR498G1221912500.01.T01	95,73	5,91E-172	603	Os12g0571700	100,00	0,00E+00	672	69	Japonica	Japonica	
augustus-12-processed-gene-239.1-mRNA-1	12	OsR498G1018848000.01.T01	70,00	7,83E-134	480	Os12g0572700	100,00	0,00E+00	2852	2372	Japonica	Japonica	
augustus-12-processed-gene-239.13-mRNA-1	12	OsR498G1221918700.01.T01	99,73	0,00E+00	3972	Os12g0573200	100,00	0,00E+00	3999	27	NA	Japonica	
augustus-12-processed-gene-243.22-mRNA-1	12	OsR498G1221944300.01.T01	100,00	0,00E+00	2690	Os12g0580400	99,93	0,00E+00	2416	-274	Indica	Japonica	
augustus-12-processed-gene-243.28-mRNA-1	12	OsR498G1221945900.01.T01	99,75	0,00E+00	2112	Os12g0580900	100,00	0,00E+00	2127	15	NA	Japonica	
augustus-12-processed-gene-245.3-mRNA-1	12	OsR498G1221957900.01.T01	100,00	0,00E+00	2419	Os12g0583300	100,00	0,00E+00	2419	0	NA	NA	
augustus-12-processed-gene-248.16-mRNA-1	12	OsR498G1221992910.01.T02	99,09	0,00E+00	2323	Os12g0587200	100,00	0,00E+00	2376	53	NA	Japonica	
augustus-12-processed-gene-25.23-mRNA-1	12	OsR498G1220936500.01.T01	99,77	0,00E+00	792	Os12g0147200	100,00	0,00E+00	796	4	NA	Japonica	
augustus-12-processed-gene-250.31-mRNA-1	12	OsR498G1222008300.01.T01	99,82	0,00E+00	4896	Os12g0590900	100,00	0,00E+00	4919	23	NA	Japonica	
augustus-12-processed-gene-250.39-mRNA-1	12	OsR498G1222010700.01.T01	99,72	0,00E+00	1905	Os12g0592300	83,60	0,00E+00	1222	-683	Indica	Japonica	
augustus-12-processed-gene-251.7-mRNA-1	12	OsR498G1222011000.01.T01	98,12	0,00E+00	2029	Os12g0591600	100,00	0,00E+00	1034	-995	Indica	Japonica	
augustus-12-processed-gene-253.57-mRNA-1	12	OsR498G1222032500.01.T01	99,78	0,00E+00	1627	Os12g0595800	74,42	2,99E-175	614	-1013	Indica	Japonica	
augustus-12-processed-gene-253.59-mRNA-1	12	OsR498G1222033300.01.T01	99,76	0,00E+00	1480	Os12g0596300	99,89	0,00E+00	1675	195	Japonica	Japonica	
augustus-12-processed-gene-254.0-mRNA-1	12	OsR498G1222036300.01.T03	100,00	0,00E+00	2446	Os12g0597600	100,00	0,00E+00	1575	-871	Indica	Japonica	
augustus-12-processed-gene-255.23-mRNA-1	12	OsR498G1222046500.01.T01	100,00	0,00E+00	1137	Os12g0599800	100,00	0,00E+00	1137	0	NA	Japonica	
augustus-12-processed-gene-255.25-mRNA-1	12	OsR498G1222046700.01.T08	100,00	0,00E+00	688	Os12g0600000	100,00	0,00E+00	688	0	NA	NA	
augustus-12-processed-gene-26.12-mRNA-1	12	OsR498G1220942300.01.T03	99,70	0,00E+00	1767	Os12g0149000	99,91	0,00E+00	1940	173	NA	Japonica	
augustus-12-processed-gene-260.18-mRNA-1	12	OsR498G1222080600.01.T01	88,15	0,00E+00	2583	Os12g0608500	100,00	0,00E+00	3723	1140	Japonica	Japonica	Kinase-TM
augustus-12-processed-gene-260.19-mRNA-1	12	OsR498G0816121400.01.T01	85,18	1,44E-138	494	Os12g0609200	100,00	0,00E+00	1182	688	Japonica	Japonica	
augustus-12-processed-gene-260.4-mRNA-1	12	OsR498G1222075700.01.T01	99,77	0,00E+00	1528	Os12g0607800	91,67	0,00E+00	1041	-487	Indica	Japonica	
augustus-12-processed-gene-260.7-mRNA-1	12	OsR498G1222076300.01.T01	99,79	0,00E+00	2530	Os12g0607800	99,72	0,00E+00	2526	-4	NA	NA	
augustus-12-processed-gene-261.31-mRNA-1	12	OsR498G1222083900.01.T01	93,13	0,00E+00	2214	Os12g0610200	100,00	0,00E+00	2414	200	NA	Japonica	
augustus-12-processed-gene-262.8-mRNA-1	12	OsR498G1222092500.01.T02	98,81	0,00E+00	1917	Os12g0611900	100,00	0,00E+00	1975	58	NA	Japonica	
augustus-12-processed-gene-264.32-mRNA-1	12	OsR498G1222101900.01.T01	99,79	0,00E+00	2611	Os12g0614600	100,00	0,00E+00	2625	14	NA	Japonica	Kinase-TM
augustus-12-processed-gene-264.34-mRNA-1	12	OsR498G1222102900.01.T01	98,10	0,00E+00	3518	Os12g0615300	95,65	0,00E+00	2808	-710	Indica	Japonica	Kinase-TM
augustus-12-processed-gene-264.46-mRNA-1	12	OsR498G1222104500.01.T04	99,83	0,00E+00	4111	Os12g0615300	100,00	0,00E+00	3961	-150	NA	Japonica	Kinase-TM
augustus-12-processed-gene-264.49-mRNA-1	12	OsR498G1222106300.01.T01	96,34	0,00E+00	1618	Os12g0615900	99,60	0,00E+00	1343	-275	Indica	Japonica	
augustus-12-processed-gene-266.21-mRNA-1	12	OsR498G1222114300.01.T01	99,78	0,00E+00	3305	Os12g0617400	100,00	0,00E+00	3323	18	NA	Japonica	
augustus-12-processed-gene-268.23-mRNA-1	12	OsR498G1222128900.01.T02	99,71	0,00E+00	1843	Os12g0621000	100,00	0,00E+00	1853	10	NA	Japonica	
augustus-12-processed-gene-269.45-mRNA-1	12	OsR498G1222134700.01.T01	99,79	0,00E+00	1734	Os12g0622500	99,45	0,00E+00	1939	205	Japonica	Japonica	
augustus-12-processed-gene-27.0-mRNA-1	12	OsR498G0815138700.01.T01	72,76	5,36E-74	279	Os12g0150500	100,00	0,00E+00	1034	755	Japonica	Japonica	
augustus-12-processed-gene-27.15-mRNA-1	12	OsR498G1220949700.01.T01	99,83	0,00E+00	2140	Os12g0151500	100,00	0,00E+00	2149	9	NA	Japonica	
augustus-12-processed-gene-270.2-mRNA-1	12	OsR498G1222139900.01.T01	99,43	0,00E+00	3135	Os12g0624200	100,00	0,00E+00	3182	47	NA	Japonica	
augustus-12-processed-gene-272.25-mRNA-1	12	OsR498G1222149500.01.T01	92,50	0,00E+00	1468	Os12g0628200	100,00	0,00E+00	1781	313	Japonica	Japonica	
augustus-12-processed-gene-272.27-mRNA-1	12	OsR498G1222152700.01.T01	99,03	0,00E+00	1274	Os12g0630200	99,86	0,00E+00	1289	15	NA	Japonica	
augustus-12-processed-gene-274.19-mRNA-1	12	OsR498G1222161300.01.T01	98,05	0,00E+00	5179	Os12g0632800	97,75	0,00E+00	5147	-32	NA	NA	Kinase-LRR-TM
augustus-12-processed-gene-28.43-mRNA-1	12	OsR498G1220958300.01.T01	100,00	1,95E-145	515	Os03g0672700	97,89	8,83E-113	406	-109	Indica	NA	
augustus-12-processed-gene-28.46-mRNA-1	12	OsR498G1220958900.01.T01	100,00	0,00E+00	2506	Os12g0153600	100,00	0,00E+00	2506	0	NA	NA	
augustus-12-processed-gene-29.25-mRNA-1	12	OsR498G1220961100.01.T01	100,00	0,00E+00	939	Os12g0154600	100,00	0,00E+00	939	0	NA	NA	
augustus-12-processed-gene-29.41-mRNA-1	12	OsR498G1220967900.01.T01	100,00	0,00E+00	899	Os12g0156000	100,00	0,00E+00	899	0	NA	NA	
augustus-12-processed-gene-29.43-mRNA-1	12	OsR498G1220969300.01.T01	100,00	0,00E+00	1667	Os12g0156100	100,00	0,00E+00	1667	0	NA	NA	
augustus-12-processed-gene-31.18-mRNA-1	12	OsR498G1220984300.01.T03	100,00	7,04E-159	560	Os12g0159400	100,00	2,44E-159	560	0	NA	NA	
augustus-12-processed-gene-32.0-mRNA-1	12	OsR498G1220985700.01.T01	100,00	0,00E+00	742	Os12g0160000	100,00	0,00E+00	742	0	NA	NA	
augustus-12-processed-gene-35.38-mRNA-1	12	OsR498G1119470500.01.T01	78,77	0,00E+00	1149	Os12g0164600	100,00	0,00E+00	2246	1097	Japonica	Japonica	
augustus-12-processed-gene-37.39-mRNA-1	12	OsR498G1221019100.01.T03	99,60	0,00E+00	2209	Os12g0168800	100,00	0,00E+00	2246	37	NA	Japonica	
augustus-12-processed-gene-39.32-mRNA-1	12	OsR498G1221028300.01.T01	99,36	0,00E+00	2223	Os12g0173333	100,00	0,00E+00	2268	45	NA	Japonica	
augustus-12-processed-gene-4.20-mRNA-1	12	OsR498G1119296700.01.T01	98,64	0,00E+00	1156	Os12g0107600	98,79	0,00E+00	1160	4	NA	Japonica	
augustus-12-processed-gene-40.23-mRNA-1	12	OsR498G1221031900.01.T01	99,45	0,00E+00	1301	Os12g0174300	99,86	0,00E+00	1318	17	NA	Japonica	
augustus-12-processed-gene-41.14-mRNA-1	12	OsR498G1221041300.01.T01	99,24	0,00E+00	2579	Os11g0186200	72,61	0,00E+00	789	-1790	Indica	Japonica	
augustus-12-processed-gene-43.43-mRNA-1	12	OsR498G1221049100.01.T04	98,69	0,00E+00	2392	Os12g0181900	100,00	0,00E+00	3166	774	Japonica	Japonica	
augustus-12-processed-gene-44.36-mRNA-1	12	OsR498G1221050900.01.T01	98,26	0,00E+00	990	Os12g0182500	100,00	0,00E+00	1023	33	NA	Japonica	
augustus-12-processed-gene-5.47-mRNA-1	12	OsR498G1220812100.01.T01	99,49	0,00E+00	2084	Os12g0109600	100,00	0,00E+00	2111	27	NA	Japonica	
augustus-12-processed-gene-5.48-mRNA-1	12	OsR498G1220813700.01.T01	99,42	0,00E+00	3984	Os12g0109800	99,96	0,00E+00	4003	19	NA	Japonica	
augustus-12-processed-gene-50.15-mRNA-1	12	OsR498G1221077900.01.T02	99,70	0,00E+00	1180	Os12g0194400	99,85	0,00E+00	1179	-1	NA	NA	
augustus-12-processed-gene-53.29-mRNA-1	12	OsR498G1221091800.01.T01	99,78	0,00E+00	1658	Os12g0198700	100,00	0,00E+00	1667	9	NA	Japonica	
augustus-12-processed-gene-53.30-mRNA-1	12	OsR498G1221093800.01.T01	96,40	3,19E-113	409	Os12g0199000	100,00	1,04E-126	452	43	NA	Japonica	
augustus-12-processed-gene-57.3-mRNA-1	12	OsR498G1221097900.01.T01	99,43	0,00E+00	3404	Os12g0204700	100,00	0,00E+00	3528	124	NA	Japonica	CC-NBS-TM
augustus-12-processed-gene-58.0-mRNA-1	12	OsR498G1221102500.01.T03	99,32	0,00E+00	777	Os12g0206700	100,00	0,00E+00	791	14	NA	Japonica	
augustus-12-processed-gene-6.95-mRNA-1	12	OsR498G1220818300.01.T05	99,59	0,00E+00	1318	Os12g0112250	99,73	0,00E+00	1323	5	NA	Japonica	
augustus-12-processed-gene-66.22-mRNA-1	12	OsR498G1221134900.01.T01	98,91	0,00E+00	4674	Os12g0218500	86,27	0,00E+00	3546	-1128	Indica	Japonica	LRR-TM
augustus-12-processed-gene-68.16-mRNA-1	12	OsR498G1221130500.01.T01	99,71	0,00E+00	4270	Os12g0222800	99,97	0,00E+00	5212	942	Japonica	NA	LRR-TM
augustus-12-processed-gene-68.17-mRNA-1	12	OsR498G1221134900.01.T01	84,30	0,00E+00	2913	Os12g0222900	99,90	0,00E+00	5139	2226	Japonica	NA	CC-LRR-TM
augustus-12-processed-gene-7.39-mRNA-1	12	OsR498G1119317100.01.T03	96,15	0,00E+00	2072	Os12g0113900	100,00	0,00E+00	2295	223	NA	Japonica	
augustus-12-processed-gene-7.42-mRNA-1	12	OsR498G1220847000.01.T01	99,91	0,00E+00	2095	Os12g0114400	100,00	0,00E+00	2733	638	Japonica	NA	
augustus-12-processed-gene-7.44-mRNA-1	12	OsR498G1220845600.01.T01	98,85	4,52E-173	607	Os11g0115466	98,85	1,57E-173	607	0	NA	Japonica	
augustus-12-processed-gene-7.46-mRNA-1	12	OsR498G1220845200.01.T01	100,00	2,45E-176	618	Os12g0115100	100,00	8,53E-177	618	0	NA	Japonica	
augustus-12-processed-gene-71.43-mRNA-1	12	OsR498G1221147500.01.T01	96,92	0,00E+00	1734	Os12g0226900	85,35	0,00E+00	1194	-540	Indica	Japonica	
augustus-12-processed-gene-75.37-mRNA-1	12	OsR498G1221166700.01.T01	99,58	0,00E+00	1268	Os12g0233900	100,00	0,00E+00	1281	13	NA	Japonica	
augustus-12-processed-gene-8.0-mRNA-1	12	OsR498G1220844800.01.T01	100,00	1,38E-179	628	Os12g0115300	100,00	4,80E-180	628	0	NA	Japonica	
augustus-12-processed-gene-81.16-mRNA-1	12	OsR498G1221200500.01.T01	97,66	0,00E+00	651	Os12g0243100	100,00	6,10E-173	605	-46	NA	Japonica	
augustus-12-processed-gene-87.36-mRNA-1	12	OsR498G0100846800.01.T01	91,52	0,00E+00	1306	Os12g0468900	84,76	0,00E+00	1024	-282	Indica	NA	
augustus-12-processed-gene-9.82-mRNA-1	12	OsR498G1220833300.01.T06	97,20	0,00E+00	2668	Os12g0118800	97,52	0,00E+00	2691	23	NA	Japonica	
augustus-12-processed-gene-91.12-mRNA-1	12	OsR498G1221223100.01.T02	99,28	0,00E+00	1722	Os12g0257500	100,00	0,00E+00	1754	32	NA	Japonica	
augustus-12-processed-gene-96.25-mRNA-1	12	OsR498G1221246300.01.T06	100,00	0,00E+00	998	Os12g0264500	100,00	0,00E+00	996	-2	NA	Japonica	
augustus-12-processed-gene-97.14-mRNA-1	12	OsR498G0306920300.01.T01	99,69	0,00E+00	3496	Os12g0265500	100,00	0,00E+00	3548	52	NA	Japonica	
maker-12-augustus-gene-0.43-mRNA-1	12	OsR498G1220783200.01.T01	98,65	0,00E+00	3863	Os11g0100300	99,96	0,00E+00	4573	710	Japonica	Japonica	
maker-12-augustus-gene-0.45-mRNA-1	12	OsR498G1220783400.01.T01	98,79	0,00E+00	1737	Os12g0100500	99,70	0,00E+00	1789	52	NA	Japonica	
maker-12-augustus-gene-1.0-mRNA-1	12	OsR498G1220784700.01.T03	99,04	0,00E+00	2922	Os12g0101000	100,00	0,00E+00	3784	862	Japonica	Japonica	
maker-12-augustus-gene-1.22-mRNA-1	12	OsR498G1220788900.01.T02	99,88	0,00E+00	1509	Os12g0102400	99,88	0,00E+00	1509	0	NA	Japonica	
maker-12-augustus-gene-1.23-mRNA-1	12	OsR498G1220788900.01.T03	99,68	0,00E+00	2226	Os12g0102400	99,92	0,00E+00	2240	14	NA	Japonica	
maker-12-augustus-gene-1.30-mRNA-1	12	OsR498G1119278000.01.T03	99,83	0,00E+00	1040	Os12g0102300	99,83	0,00E+00	1049	9	NA	NA	
maker-12-augustus-gene-10.42-mRNA-1	12	OsR498G1119333300.01.T02	94,62	0,00E+00	1073	Os12g0119700	100,00	0,00E+00	1242	169	Japonica	Japonica	
maker-12-augustus-gene-10.52-mRNA-1	12	OsR498G1220829700.01.T01	99,91	0,00E+00	3828	Os12g0120000	100,00	0,00E+00	3837	9	NA	Japonica	
maker-12-augustus-gene-10.54-mRNA-1	12	OsR498G1220827300.01.T01	99,50	0,00E+00	1422	Os11g0120700	97,57	0,00E+00	1322	-100	NA	Japonica	
maker-12-augustus-gene-10.57-mRNA-1	12	OsR498G1119336800.01.T03	96,09	0,00E+00	1120	Os12g0120500	100,00	0,00E+00	1240	120	NA	Japonica	
maker-12-augustus-gene-10.60-mRNA-1	12	OsR498G1220824900.01.T02	100,00	0,00E+00	699	Os12g0121000	100,00	0,00E+00	699	0	NA	Japonica	
maker-12-augustus-gene-10.62-mRNA-1	12	OsR498G1220824700.01.T01	99,84	0,00E+00	2232	Os11g0121400	98,63	0,00E+00	2164	-68	NA	Japonica	Kinase
maker-12-augustus-gene-10.73-mRNA-1	12	OsR498G1220823700.01.T03	100,00	0,00E+00	1175	Os12g0121400	100,00	0,00E+00	1175	0	NA	Japonica	
maker-12-augustus-gene-100.37-mRNA-1	12	OsR498G1221264000.01.T03	99,86	0,00E+00	2524	Os12g0270100	100,00	0,00E+00	2533	9	NA	Japonica	
maker-12-augustus-gene-100.40-mRNA-1	12	OsR498G1221267000.01.T01	99,71	0,00E+00	1821	Os12g0270900	99,86	0,00E+00	1239	-582	Indica	Japonica	
maker-12-augustus-gene-101.37-mRNA-1	12	OsR498G1221270500.01.T02	99,61	0,00E+00	3179	Os12g0271600	99,94	0,00E+00	3206	27	NA	Japonica	
maker-12-augustus-gene-101.39-mRNA-1	12	OsR498G1221270800.01.T01	99,92	0,00E+00	2241	Os12g0271700	100,00	0,00E+00	2019	-222	NA	Japonica	
maker-12-augustus-gene-102.0-mRNA-1	12	OsR498G1221272600.01.T01	93,80	0,00E+00	3638	Os12g0273266	100,00	0,00E+00	4881	1243	Japonica	Japonica	CC-NBS-TM
maker-12-augustus-gene-103.40-mRNA-1	12	OsR498G1221278700.01.T03	99,91	0,00E+00	2047	Os12g0273700	100,00	0,00E+00	1359	-688	Indica	Japonica	
maker-12-augustus-gene-103.44-mRNA-1	12	OsR498G1221278900.01.T01	99,81	0,00E+00	1891	Os12g0273900	100,00	0,00E+00	1002	-889	Indica	Japonica	
maker-12-augustus-gene-103.49-mRNA-1	12	OsR498G1221280200.01.T01	99,50	0,00E+00	2140	Os12g0274200	99,75	0,00E+00	1449	-691	Indica	Japonica	
maker-12-augustus-gene-103.51-mRNA-1	12	OsR498G1221281600.01.T01	98,67	0,00E+00	921	Os12g0274700	100,00	0,00E+00	953	32	NA	Japonica	
maker-12-augustus-gene-104.35-mRNA-1	12	OsR498G1221283500.01.T01	96,07	0,00E+00	712	Os12g0275500	96,88	0,00E+00	808	96	Japonica	Japonica	
maker-12-augustus-gene-104.42-mRNA-1	12	OsR498G1221290200.01.T02	100,00	0,00E+00	888	Os12g0276100	100,00	0,00E+00	888	0	NA	Japonica	
maker-12-augustus-gene-105.23-mRNA-1	12	OsR498G1221297100.01.T01	99,89	0,00E+00	3124	Os12g0277500	100,00	0,00E+00	3133	9	NA	Japonica	
maker-12-augustus-gene-105.29-mRNA-1	12	OsR498G1221295000.01.T01	99,25	0,00E+00	4492	Os12g0277000	99,33	0,00E+00	4501	9	NA	Japonica	
maker-12-augustus-gene-105.31-mRNA-1	12	OsR498G1221297000.01.T01	100,00	0,00E+00	4367	Os12g0277400	100,00	0,00E+00	4367	0	NA	Japonica	
maker-12-augustus-gene-106.36-mRNA-1	12	OsR498G1221303800.01.T03	100,00	0,00E+00	1489	Os12g0278700	100,00	0,00E+00	1355	-134	NA	Japonica	
maker-12-augustus-gene-107.19-mRNA-1	12	OsR498G1221304600.01.T03	97,28	0,00E+00	1681	Os12g0278800	97,38	0,00E+00	1685	4	NA	Japonica	
maker-12-augustus-gene-107.23-mRNA-1	12	OsR498G1221305600.01.T01	98,06	3,41E-149	528	Os12g0278900	100,00	7,01E-140	495	-33	NA	Japonica	
maker-12-augustus-gene-107.27-mRNA-1	12	OsR498G1221307200.01.T02	99,91	0,00E+00	2052	Os12g0279600	100,00	0,00E+00	2057	5	NA	Japonica	
maker-12-augustus-gene-109.40-mRNA-1	12	OsR498G1221312700.01.T02	99,88	0,00E+00	5909	Os12g0281600	100,00	0,00E+00	6044	135	NA	Japonica	CC-NBS-TM Pi42
maker-12-augustus-gene-109.48-mRNA-1	12	OsR498G1221319200.01.T01	99,93	0,00E+00	5348	Os12g0283100	100,00	0,00E+00	5357	9	NA	Japonica	
maker-12-augustus-gene-11.0-mRNA-1	12	OsR498G1220851500.01.T01	99,80	0,00E+00	3559	Os12g0122000	100,00	0,00E+00	3577	18	NA	Japonica	
maker-12-augustus-gene-11.2-mRNA-1	12	OsR498G1220852100.01.T01	99,76	0,00E+00	1523	Os12g0122100	100,00	0,00E+00	823	-700	Indica	Japonica	
maker-12-augustus-gene-11.32-mRNA-1	12	OsR498G1220854200.01.T01	97,53	0,00E+00	3387	Os11g0124900	95,73	0,00E+00	3208	-179	NA	Japonica	
maker-12-augustus-gene-11.36-mRNA-1	12	OsR498G1119349400.01.T02	95,20	0,00E+00	1058	Os12g0123100	100,00	0,00E+00	1227	169	Japonica	NA	
maker-12-augustus-gene-11.7-mRNA-1	12	OsR498G1220856100.01.T01	99,93	0,00E+00	2442	Os12g0123500	100,00	0,00E+00	2446	4	NA	NA	
maker-12-augustus-gene-11.8-mRNA-1	12	OsR498G1119351300.01.T01	96,53	0,00E+00	852	Os12g0123600	100,00	0,00E+00	648	-204	Indica	NA	
maker-12-augustus-gene-110.39-mRNA-1	12	OsR498G1221321500.01.T01	97,83	0,00E+00	3467	Os12g0283900	100,00	0,00E+00	3658	191	NA	Japonica	
maker-12-augustus-gene-110.41-mRNA-1	12	OsR498G1221322100.01.T01	99,47	0,00E+00	2350	Os12g0284000	98,13	0,00E+00	2761	411	Japonica	Japonica	
maker-12-augustus-gene-110.45-mRNA-1	12	OsR498G1221322300.01.T01	98,71	5,59E-113	407	Os12g0285100	85,77	8,33E-74	275	-132	Indica	Japonica	Ptr
maker-12-augustus-gene-111.26-mRNA-1	12	OsR498G1221325200.01.T02	95,25	0,00E+00	4180	Os12g0285100	100,00	0,00E+00	4702	522	Japonica	Japonica	Ptr
maker-12-augustus-gene-111.37-mRNA-1	12	OsR498G1221326600.01.T03	98,82	0,00E+00	3993	Os12g0285600	100,00	0,00E+00	4096	103	NA	Japonica	
maker-12-augustus-gene-112.28-mRNA-1	12	OsR498G1221331700.01.T03	99,80	0,00E+00	911	Os12g0287200	100,00	0,00E+00	755	-156	Indica	Japonica	
maker-12-augustus-gene-112.30-mRNA-1	12	OsR498G1221330000.01.T01	99,88	0,00E+00	1498	Os12g0286600	100,00	0,00E+00	1503	5	NA	Japonica	
maker-12-augustus-gene-112.36-mRNA-1	12	OsR498G1221332000.01.T03	100,00	0,00E+00	2235	Os12g0287300	100,00	0,00E+00	1462	-773	Indica	Japonica	
maker-12-augustus-gene-112.42-mRNA-1	12	OsR498G1221337600.01.T03	99,33	0,00E+00	1318	Os12g0288000	99,46	0,00E+00	1323	5	NA	NA	
maker-12-augustus-gene-113.58-mRNA-1	12	OsR498G1221340400.01.T01	99,35	0,00E+00	1092	Os12g0288900	100,00	0,00E+00	1110	18	NA	Japonica	
maker-12-augustus-gene-114.0-mRNA-1	12	OsR498G1221346100.01.T01	99,92	0,00E+00	2353	Os05g0511400	76,47	0,00E+00	1032	-1321	Indica	Japonica	Kinase
maker-12-augustus-gene-114.10-mRNA-1	12	OsR498G1221348500.01.T01	98,86	0,00E+00	926	Os12g0291100	99,24	0,00E+00	935	9	NA	NA	
maker-12-augustus-gene-115.19-mRNA-1	12	OsR498G1221348500.01.T01	87,90	2,70E-62	240	Os12g0291200	99,19	0,00E+00	658	418	Japonica	Japonica	
maker-12-augustus-gene-115.21-mRNA-1	12	OsR498G1221349500.01.T01	98,30	0,00E+00	912	Os12g0291400	99,81	0,00E+00	948	36	NA	Japonica	
maker-12-augustus-gene-115.23-mRNA-1	12	OsR498G1221350300.01.T01	99,24	0,00E+00	935	Os12g0292400	99,81	0,00E+00	948	13	NA	Japonica	
maker-12-augustus-gene-116.24-mRNA-1	12	OsR498G1221351800.01.T01	98,84	0,00E+00	6639	Os12g0293100	99,45	0,00E+00	6764	125	NA	Japonica	
maker-12-augustus-gene-118.2-mRNA-1	12	OsR498G1221360600.01.T01	99,54	0,00E+00	4646	Os12g0297500	99,89	0,00E+00	6516	1870	Japonica	Japonica	
maker-12-augustus-gene-12.0-mRNA-1	12	OsR498G1220859100.01.T01	100,00	0,00E+00	2149	Os12g0123800	100,00	0,00E+00	2149	0	NA	NA	
maker-12-augustus-gene-12.14-mRNA-1	12	OsR498G1220863800.01.T01	100,00	0,00E+00	1689	Os12g0125000	100,00	0,00E+00	1689	0	NA	NA	
maker-12-augustus-gene-12.16-mRNA-1	12	OsR498G1220865000.01.T01	98,89	0,00E+00	3324	Os12g0125300	98,89	0,00E+00	3324	0	NA	NA	
maker-12-augustus-gene-12.17-mRNA-1	12	OsR498G1220865000.01.T01	100,00	2,09E-164	578	Os12g0125300	100,00	7,27E-165	578	0	NA	NA	
maker-12-augustus-gene-12.2-mRNA-1	12	OsR498G1220859700.01.T02	100,00	0,00E+00	1505	Os12g0124000	100,00	0,00E+00	1240	-265	Indica	NA	
maker-12-augustus-gene-12.20-mRNA-1	12	OsR498G1220865200.01.T01	100,00	0,00E+00	2084	Os12g0125400	100,00	0,00E+00	2084	0	NA	NA	
maker-12-augustus-gene-12.6-mRNA-1	12	OsR498G1220859400.01.T03	100,00	0,00E+00	1694	Os12g0123900	100,00	0,00E+00	1694	0	NA	NA	
maker-12-augustus-gene-122.10-mRNA-1	12	OsR498G1221362900.01.T03	98,50	0,00E+00	928	Os12g0403800	100,00	0,00E+00	870	-58	NA	Japonica	
maker-12-augustus-gene-125.29-mRNA-1	12	OsR498G1221366900.01.T02	100,00	0,00E+00	1101	Os12g0405200	100,00	0,00E+00	1101	0	NA	Japonica	
maker-12-augustus-gene-125.34-mRNA-1	12	OsR498G1221368800.01.T03	96,07	0,00E+00	2396	Os12g0406100	96,69	0,00E+00	2436	40	NA	Japonica	
maker-12-augustus-gene-125.38-mRNA-1	12	OsR498G1221369000.01.T01	99,52	0,00E+00	1107	Os12g0406200	99,68	0,00E+00	1112	5	NA	Japonica	
maker-12-augustus-gene-126.4-mRNA-1	12	OsR498G1221372600.01.T03	99,70	0,00E+00	3539	Os12g0407500	100,00	0,00E+00	3566	27	NA	Japonica	
maker-12-augustus-gene-127.39-mRNA-1	12	OsR498G1221374800.01.T01	98,94	0,00E+00	2802	Os12g0409000	99,93	0,00E+00	2498	-304	Indica	Japonica	
maker-12-augustus-gene-127.42-mRNA-1	12	OsR498G1221377600.01.T01	99,81	0,00E+00	2831	Os12g0409700	99,87	0,00E+00	2800	-31	NA	Japonica	
maker-12-augustus-gene-129.27-mRNA-1	12	OsR498G1221382800.01.T01	99,91	0,00E+00	3855	Os12g0411700	99,91	0,00E+00	3855	0	NA	Indica	
maker-12-augustus-gene-13.37-mRNA-1	12	OsR498G1220868300.01.T03	100,00	2,32E-153	542	Os12g0126200	100,00	8,09E-154	542	0	NA	NA	
maker-12-augustus-gene-13.44-mRNA-1	12	OsR498G1220869900.01.T02	100,00	0,00E+00	717	Os12g0127100	100,00	0,00E+00	717	0	NA	NA	
maker-12-augustus-gene-13.49-mRNA-1	12	OsR498G1220870900.01.T01	100,00	0,00E+00	1975	Os12g0127500	100,00	0,00E+00	1975	0	NA	NA	
maker-12-augustus-gene-13.64-mRNA-1	12	OsR498G1220867600.01.T01	99,93	0,00E+00	2745	Os12g0126000	100,00	0,00E+00	2749	4	NA	NA	
maker-12-augustus-gene-13.73-mRNA-1	12	OsR498G1220868600.01.T01	99,06	0,00E+00	2056	Os12g0126300	100,00	0,00E+00	2403	347	Japonica	NA	
maker-12-augustus-gene-13.76-mRNA-1	12	OsR498G1220869400.01.T02	100,00	0,00E+00	3171	Os12g0126800	100,00	0,00E+00	3171	0	NA	NA	
maker-12-augustus-gene-13.82-mRNA-1	12	OsR498G1220869600.01.T01	100,00	0,00E+00	1521	Os11g0130200	94,04	0,00E+00	1276	-245	Indica	NA	
maker-12-augustus-gene-13.84-mRNA-1	12	OsR498G1220870800.01.T03	100,00	0,00E+00	1943	Os12g0127400	100,00	0,00E+00	1943	0	NA	NA	
maker-12-augustus-gene-13.88-mRNA-1	12	OsR498G1220871400.01.T02	100,00	0,00E+00	3676	Os12g0127600	100,00	0,00E+00	3434	-242	NA	NA	
maker-12-augustus-gene-131.20-mRNA-1	12	OsR498G1221394200.01.T01	98,03	0,00E+00	3251	Os12g0414500	98,03	0,00E+00	3251	0	NA	Indica	
maker-12-augustus-gene-132.40-mRNA-1	12	OsR498G1221400300.01.T05	99,39	6,46E-165	581	Os12g0416500	100,00	2,74E-145	513	-68	Indica	Japonica	
maker-12-augustus-gene-135.0-mRNA-1	12	OsR498G1221412500.01.T01	99,46	0,00E+00	1317	Os12g0420100	98,31	0,00E+00	1328	11	NA	Japonica	
maker-12-augustus-gene-135.11-mRNA-1	12	OsR498G1221413300.01.T03	100,00	0,00E+00	1131	Os12g0420400	99,84	0,00E+00	1127	-4	NA	Indica	
maker-12-augustus-gene-135.13-mRNA-1	12	OsR498G0408810600.01.T03	90,15	0,00E+00	655	Os12g0420000	92,76	0,00E+00	863	208	Japonica	Indica	
maker-12-augustus-gene-135.2-mRNA-1	12	OsR498G1221412700.01.T01	99,91	0,00E+00	2036	Os12g0420200	99,91	0,00E+00	2036	0	NA	Indica	
maker-12-augustus-gene-139.0-mRNA-1	12	OsR498G1221425300.01.T01	99,92	0,00E+00	2263	Os12g0425600	99,28	0,00E+00	2227	-36	NA	Indica	
maker-12-augustus-gene-14.0-mRNA-1	12	OsR498G1220874100.01.T06	99,37	0,00E+00	3653	Os12g0128700	99,70	0,00E+00	3551	-102	NA	Japonica	
maker-12-augustus-gene-14.26-mRNA-1	12	OsR498G1220874000.01.T02	100,00	1,64E-119	428	Os12g0128600	100,00	5,74E-120	428	0	NA	Japonica	
maker-12-augustus-gene-14.28-mRNA-1	12	OsR498G1220874600.01.T01	99,72	0,00E+00	635	Os12g0129000	100,00	0,00E+00	1142	507	Japonica	Japonica	
maker-12-augustus-gene-14.6-mRNA-1	12	OsR498G1220874300.01.T01	99,60	0,00E+00	2247	Os12g0128800	99,92	0,00E+00	2265	18	NA	Japonica	
maker-12-augustus-gene-140.0-mRNA-1	12	OsR498G1221432700.01.T01	98,31	0,00E+00	714	Os12g0428000	97,96	0,00E+00	834	120	Japonica	NA	
maker-12-augustus-gene-140.21-mRNA-1	12	OsR498G1221433900.01.T01	86,92	5,34E-104	378	Os12g0428700	96,33	1,63E-111	402	24	NA	NA	
maker-12-augustus-gene-141.27-mRNA-1	12	OsR498G1221435100.01.T01	99,24	0,00E+00	696	Os12g0430500	99,03	0,00E+00	726	30	NA	NA	
maker-12-augustus-gene-141.31-mRNA-1	12	OsR498G1221434400.01.T01	99,71	0,00E+00	1247	Os12g0429200	100,00	0,00E+00	1256	9	NA	NA	
maker-12-augustus-gene-141.34-mRNA-1	12	OsR498G1221434400.01.T01	99,27	0,00E+00	3654	Os12g0429200	99,37	0,00E+00	3663	9	NA	NA	
maker-12-augustus-gene-142.37-mRNA-1	12	OsR498G1221441100.01.T01	99,81	0,00E+00	2828	Os12g0431700	99,94	0,00E+00	2837	9	NA	NA	
maker-12-augustus-gene-142.51-mRNA-1	12	OsR498G1221440200.01.T01	99,87	0,00E+00	4113	Os12g0431300	99,74	0,00E+00	4100	-13	NA	NA	
maker-12-augustus-gene-147.0-mRNA-1	12	OsR498G0305782200.01.T01	86,47	1,33E-64	246	Os12g0433700	99,62	6,44E-133	472	226	Japonica	NA	
maker-12-augustus-gene-147.6-mRNA-1	12	OsR498G1221451200.01.T03	99,76	0,00E+00	3767	Os12g0433500	99,67	0,00E+00	3758	-9	NA	Indica	Kinase-TM
maker-12-augustus-gene-148.23-mRNA-1	12	OsR498G1221453900.01.T01	99,94	0,00E+00	2907	Os12g0434400	96,75	0,00E+00	2741	-166	NA	Indica	
maker-12-augustus-gene-15.46-mRNA-1	12	OsR498G1220880500.01.T01	99,46	0,00E+00	994	Os12g0130200	100,00	0,00E+00	984	-10	NA	Japonica	Kinase-TM
maker-12-augustus-gene-15.49-mRNA-1	12	OsR498G1220885500.01.T01	99,79	0,00E+00	2566	Os12g0131200	100,00	0,00E+00	2437	-129	NA	Japonica	
maker-12-augustus-gene-15.54-mRNA-1	12	OsR498G1119370400.01.T05	93,91	6,19E-120	431	Os12g0131300	100,00	9,15E-138	488	57	Japonica	Japonica	
maker-12-augustus-gene-15.61-mRNA-1	12	OsR498G1220884800.01.T01	99,87	0,00E+00	1360	Os12g0130900	100,00	0,00E+00	1395	35	NA	Japonica	
maker-12-augustus-gene-15.71-mRNA-1	12	OsR498G1220885800.01.T01	99,06	1,09E-157	557	Os12g0131400	100,00	0,00E+00	917	360	Japonica	Japonica	
maker-12-augustus-gene-15.73-mRNA-1	12	OsR498G1220879500.01.T01	89,43	0,00E+00	1212	Os12g0131600	100,00	0,00E+00	841	-371	Indica	Japonica	
maker-12-augustus-gene-151.29-mRNA-1	12	OsR498G1221468200.01.T01	100,00	0,00E+00	737	Os12g0438000	100,00	0,00E+00	737	0	NA	Japonica	
maker-12-augustus-gene-152.31-mRNA-1	12	OsR498G1221471600.01.T01	99,85	0,00E+00	3542	Os12g0438600	100,00	0,00E+00	3498	-44	NA	Japonica	
maker-12-augustus-gene-152.32-mRNA-1	12	OsR498G1221471600.01.T02	99,36	7,75E-157	553	Os12g0438600	100,00	1,31E-91	334	-219	Indica	Japonica	
maker-12-augustus-gene-154.0-mRNA-1	12	OsR498G1221486600.01.T01	99,54	0,00E+00	1926	Os12g0445200	86,55	0,00E+00	1199	-727	Indica	Japonica	
maker-12-augustus-gene-154.2-mRNA-1	12	OsR498G1221488200.01.T01	96,07	0,00E+00	1812	Os12g0444800	87,39	0,00E+00	1319	-493	Indica	Japonica	
maker-12-augustus-gene-155.18-mRNA-1	12	OsR498G1221505500.01.T01	99,83	0,00E+00	2161	Os12g0442800	100,00	0,00E+00	2170	9	NA	Japonica	
maker-12-augustus-gene-155.22-mRNA-1	12	OsR498G1221505100.01.T03	99,81	0,00E+00	965	Os12g0442900	100,00	0,00E+00	969	4	NA	Japonica	
maker-12-augustus-gene-156.2-mRNA-1	12	OsR498G1221500100.01.T01	100,00	0,00E+00	2765	Os12g0443700	100,00	0,00E+00	2765	0	NA	Japonica	
maker-12-augustus-gene-157.0-mRNA-1	12	OsR498G1221494600.01.T01	97,22	0,00E+00	1459	Os12g0444800	100,00	0,00E+00	1970	511	Japonica	Japonica	
maker-12-augustus-gene-157.2-mRNA-1	12	OsR498G1221486600.01.T01	86,85	0,00E+00	1213	Os12g0445200	100,00	0,00E+00	1819	606	Japonica	Japonica	
maker-12-augustus-gene-158.33-mRNA-1	12	OsR498G1221506800.01.T02	99,68	0,00E+00	1122	Os12g0446700	100,00	0,00E+00	1021	-101	NA	Japonica	
maker-12-augustus-gene-16.0-mRNA-1	12	OsR498G1119377100.01.T07	98,71	0,00E+00	2299	Os12g0132200	100,00	0,00E+00	2376	77	NA	Japonica	Kinase-TM
maker-12-augustus-gene-16.11-mRNA-1	12	OsR498G1220890000.01.T01	99,46	0,00E+00	983	Os12g0132300	100,00	0,00E+00	996	13	NA	Japonica	

maker-12-augustus-gene-16.12-mRNA-1	12	OsR498G1220890200.01.T02	96,28	0,00E+00	2329	Os11g0134500	94,78	0,00E+00	2234	-95	NA	Japonica	
maker-12-augustus-gene-16.28-mRNA-1	12	OsR498G1220890400.01.T05	97,85	0,00E+00	2486	Os12g0132800	97,92	0,00E+00	2490	4	NA	Japonica	
maker-12-augustus-gene-16.42-mRNA-1	12	OsR498G1220891200.01.T01	99,92	0,00E+00	2204	Os12g0133000	95,96	0,00E+00	2241	37	NA	Japonica	
maker-12-augustus-gene-16.47-mRNA-1	12	OsR498G1220891400.01.T01	99,79	0,00E+00	1728	Os08g0130500	94,19	0,00E+00	1483	-245	Indica	Japonica	
maker-12-augustus-gene-16.53-mRNA-1	12	OsR498G1220891600.01.T02	96,69	0,00E+00	2286	Os11g0135900	95,36	0,00E+00	2199	-87	NA	Japonica	
maker-12-augustus-gene-16.6-mRNA-1	12	OsR498G1220889000.01.T01	99,58	0,00E+00	3389	Os12g0131900	98,10	0,00E+00	3560	171	NA	Japonica	
maker-12-augustus-gene-160.0-mRNA-1	12	OsR498G1221518200.01.T03	99,42	0,00E+00	1531	Os12g0448900	100,00	0,00E+00	1553	22	NA	Japonica	
maker-12-augustus-gene-166.0-mRNA-1	12	OsR498G1221535900.01.T01	99,86	0,00E+00	1268	Os12g0454600	100,00	0,00E+00	1164	-104	NA	Japonica	
maker-12-augustus-gene-167.3-mRNA-1	12	OsR498G1221541200.01.T01	99,75	0,00E+00	1404	Os12g0456200	100,00	0,00E+00	1413	9	NA	Japonica	
maker-12-augustus-gene-168.0-mRNA-1	12	OsR498G0816414100.01.T04	76,08	0,00E+00	813	Os12g0456700	96,84	0,00E+00	2295	1482	Japonica	NA	
maker-12-augustus-gene-169.0-mRNA-1	12	OsR498G1221544200.01.T01	99,85	0,00E+00	2394	Os12g0458100	88,67	0,00E+00	1740	-654	Indica	Japonica	
maker-12-augustus-gene-169.2-mRNA-1	12	OsR498G1221546000.01.T01	99,55	0,00E+00	787	Os12g0458100	100,00	0,00E+00	796	9	NA	Japonica	
maker-12-augustus-gene-169.4-mRNA-1	12	OsR498G1221546000.01.T01	91,56	0,00E+00	1281	Os12g0458100	91,78	0,00E+00	1290	9	NA	Japonica	
maker-12-augustus-gene-17.27-mRNA-1	12	OsR498G1220892100.01.T01	92,76	0,00E+00	1095	Os12g0518200	91,24	0,00E+00	1320	225	Japonica	Japonica	
maker-12-augustus-gene-17.30-mRNA-1	12	OsR498G1220892700.01.T02	95,72	0,00E+00	1459	Os12g0133400	95,61	0,00E+00	1452	-7	NA	Japonica	
maker-12-augustus-gene-17.34-mRNA-1	12	OsR498G1119382300.01.T01	99,46	0,00E+00	2303	Os12g0133500	100,00	0,00E+00	3101	798	Japonica	Japonica	Kinase-TM
maker-12-augustus-gene-17.36-mRNA-1	12	OsR498G1220895100.01.T03	99,83	0,00E+00	2091	Os12g0134000	100,00	0,00E+00	1645	-446	Indica	NA	
maker-12-augustus-gene-17.42-mRNA-1	12	OsR498G1220895400.01.T01	99,83	0,00E+00	4134	Os12g0134200	99,87	0,00E+00	4139	5	NA	NA	
maker-12-augustus-gene-17.48-mRNA-1	12	OsR498G1220894600.01.T03	99,51	0,00E+00	2167	Os12g0133900	99,84	0,00E+00	2167	0	NA	NA	
maker-12-augustus-gene-17.62-mRNA-1	12	OsR498G1220893400.01.T01	99,53	0,00E+00	1910	Os12g0133700	100,00	0,00E+00	1932	22	NA	Japonica	
maker-12-augustus-gene-17.67-mRNA-1	12	OsR498G1220894000.01.T01	99,46	0,00E+00	2984	Os11g0137000	97,26	0,00E+00	2819	-165	NA	Japonica	
maker-12-augustus-gene-17.69-mRNA-1	12	OsR498G1220894600.01.T01	99,75	0,00E+00	2919	Os12g0133900	100,00	0,00E+00	2938	19	NA	Japonica	
maker-12-augustus-gene-17.73-mRNA-1	12	OsR498G1220895400.01.T01	99,96	0,00E+00	4719	Os12g0134200	100,00	0,00E+00	4724	5	NA	NA	
maker-12-augustus-gene-17.76-mRNA-1	12	OsR498G1220895100.01.T03	99,73	0,00E+00	1315	Os12g0134000	100,00	0,00E+00	1324	9	NA	NA	
maker-12-augustus-gene-171.0-mRNA-1	12	OsR498G1221553400.01.T03	99,66	0,00E+00	2120	Os01g0837900	73,19	5,07E-168	591	-1529	Indica	Japonica	
maker-12-augustus-gene-173.40-mRNA-1	12	OsR498G1221563700.01.T01	96,56	0,00E+00	1788	Os12g0464400	98,01	0,00E+00	2085	297	Japonica	Japonica	
maker-12-augustus-gene-174.0-mRNA-1	12	OsR498G1221566500.01.T01	97,42	0,00E+00	652	Os12g0465100	73,94	1,11E-55	215	-437	Indica	Japonica	
maker-12-augustus-gene-174.2-mRNA-1	12	OsR498G1221566900.01.T01	98,16	0,00E+00	656	Os12g0465100	100,00	0,00E+00	688	32	NA	Japonica	
maker-12-augustus-gene-176.28-mRNA-1	12	OsR498G1221573200.01.T01	98,83	0,00E+00	3891	Os03g0646300	78,52	0,00E+00	1436	-2455	Indica	Japonica	
maker-12-augustus-gene-176.31-mRNA-1	12	OsR498G1221572900.01.T01	97,80	0,00E+00	2400	Os12g0468600	100,00	0,00E+00	2554	154	NA	Japonica	
maker-12-augustus-gene-179.0-mRNA-1	12	OsR498G1221576100.01.T01	87,11	0,00E+00	1976	Os12g0471100	87,11	0,00E+00	1976	0	NA	Japonica	
maker-12-augustus-gene-179.3-mRNA-1	12	OsR498G1221580600.01.T01	97,23	0,00E+00	1957	Os12g0472800	100,00	0,00E+00	2051	94	NA	Japonica	
maker-12-augustus-gene-18.35-mRNA-1	12	OsR498G1220859700.01.T02	100,00	0,00E+00	1505	Os12g0124000	100,00	0,00E+00	1240	-265	Indica	NA	
maker-12-augustus-gene-18.42-mRNA-1	12	OsR498G1220895100.01.T03	99,69	0,00E+00	1162	Os12g0134000	99,85	0,00E+00	1167	5	NA	NA	
maker-12-augustus-gene-18.56-mRNA-1	12	OsR498G1220894600.01.T03	99,30	0,00E+00	1767	Os12g0133900	99,70	0,00E+00	1787	20	NA	NA	
maker-12-augustus-gene-182.24-mRNA-1	12	OsR498G1221587200.01.T01	96,98	0,00E+00	1490	Os11g0508600	83,28	0,00E+00	698	-792	Indica	Japonica	
maker-12-augustus-gene-183.27-mRNA-1	12	OsR498G1221589500.01.T01	99,11	0,00E+00	1381	Os12g0477400	100,00	0,00E+00	1413	32	NA	Japonica	
maker-12-augustus-gene-184.25-mRNA-1	12	OsR498G1221591800.01.T01	98,89	0,00E+00	1572	Os09g0466300	70,86	4,55E-46	185	-1387	Indica	Japonica	
maker-12-augustus-gene-184.27-mRNA-1	12	OsR498G1221596400.01.T01	91,44	0,00E+00	887	Os12g0479100	92,09	0,00E+00	976	89	NA	Japonica	
maker-12-augustus-gene-185.18-mRNA-1	12	OsR498G1221601700.01.T01	99,09	0,00E+00	2730	Os12g0479900	100,00	0,00E+00	2765	35	NA	Japonica	
maker-12-augustus-gene-185.29-mRNA-1	12	OsR498G1221602100.01.T02	99,71	1,04E-174	613	Os12g0480100	100,00	8,53E-177	618	5	NA	Japonica	
maker-12-augustus-gene-185.37-mRNA-1	12	OsR498G1221602400.01.T01	99,24	0,00E+00	4211	Os12g0480200	100,00	0,00E+00	2192	-2019	Indica	Japonica	Kinase
maker-12-augustus-gene-186.37-mRNA-1	12	OsR498G1221605700.01.T01	99,76	0,00E+00	743	Os12g0481200	100,00	1,66E-142	504	-239	Indica	Japonica	
maker-12-augustus-gene-186.42-mRNA-1	12	OsR498G1221605200.01.T01	98,34	0,00E+00	1977	Os11g0543300	71,65	0,00E+00	808	-1169	Indica	Japonica	
maker-12-augustus-gene-186.44-mRNA-1	12	OsR498G1221605600.01.T01	99,96	0,00E+00	4341	Os12g0481100	100,00	0,00E+00	4507	166	NA	Japonica	
maker-12-augustus-gene-186.49-mRNA-1	12	OsR498G1221614600.01.T01	91,59	0,00E+00	1827	Os12g0482500	100,00	0,00E+00	2300	473	Japonica	Japonica	
maker-12-augustus-gene-187.37-mRNA-1	12	OsR498G1221615000.01.T01	99,69	0,00E+00	1702	Os12g0482700	100,00	0,00E+00	1716	14	NA	Japonica	
maker-12-augustus-gene-188.13-mRNA-1	12	OsR498G1221621600.01.T02	94,74	0,00E+00	1617	Os12g0485000	94,94	0,00E+00	1626	9	NA	Japonica	
maker-12-augustus-gene-188.18-mRNA-1	12	OsR498G1221623000.01.T01	100,00	0,00E+00	726	Os12g0485500	100,00	0,00E+00	726	0	NA	Japonica	
maker-12-augustus-gene-188.2-mRNA-1	12	OsR498G1221622500.01.T02	99,82	0,00E+00	2930	Os12g0485400	100,00	0,00E+00	2944	14	NA	Japonica	
maker-12-augustus-gene-188.20-mRNA-1	12	OsR498G1221624200.01.T01	99,78	0,00E+00	1627	Os12g0485600	100,00	0,00E+00	1636	9	NA	Japonica	
maker-12-augustus-gene-188.7-mRNA-1	12	OsR498G1221620800.01.T01	99,70	2,97E-171	602	Os12g0484700	100,00	4,39E-170	596	-6	NA	Japonica	
maker-12-augustus-gene-189.12-mRNA-1	12	OsR498G1221625000.01.T01	93,40	0,00E+00	2783	Os12g0485900	100,00	0,00E+00	5525	2742	Japonica	Japonica	CC-NBS-TM
maker-12-augustus-gene-19.56-mRNA-1	12	OsR498G1220899700.01.T01	99,82	0,00E+00	2952	Os12g0136200	99,81	0,00E+00	2773	-179	NA	Japonica	
maker-12-augustus-gene-19.62-mRNA-1	12	OsR498G1220895400.01.T01	99,84	0,00E+00	9807	Os11g0137300	99,01	0,00E+00	5889	-3918	Indica	Japonica	
maker-12-augustus-gene-19.78-mRNA-1	12	OsR498G1220897400.01.T02	100,00	0,00E+00	1218	Os11g0138600	99,70	0,00E+00	1209	-9	NA	Japonica	
maker-12-augustus-gene-19.83-mRNA-1	12	OsR498G1220899400.01.T01	99,29	0,00E+00	752	Os11g0139100	98,35	0,00E+00	734	-18	NA	Japonica	
maker-12-augustus-gene-190.28-mRNA-1	12	OsR498G1221633300.01.T01	99,53	0,00E+00	1884	Os12g0486600	100,00	0,00E+00	1907	23	NA	Japonica	
maker-12-augustus-gene-190.29-mRNA-1	12	OsR498G1221633300.01.T01	99,13	0,00E+00	1411	Os12g0486600	99,38	0,00E+00	1420	9	NA	Japonica	
maker-12-augustus-gene-190.35-mRNA-1	12	OsR498G1221633900.01.T01	99,46	0,00E+00	1645	Os12g0486900	100,00	0,00E+00	2435	790	Japonica	Japonica	Kinase-TM
maker-12-augustus-gene-192.30-mRNA-1	12	OsR498G1120417100.01.T01	78,40	0,00E+00	646	Os12g0488600	100,00	0,00E+00	1640	994	Japonica	Japonica	
maker-12-augustus-gene-192.32-mRNA-1	12	OsR498G1221645100.01.T01	99,73	0,00E+00	2623	Os12g0488700	100,00	0,00E+00	2641	18	NA	Japonica	
maker-12-augustus-gene-192.50-mRNA-1	12	OsR498G1221647600.01.T01	99,22	0,00E+00	902	Os12g0489300	100,00	0,00E+00	921	19	NA	Japonica	
maker-12-augustus-gene-194.22-mRNA-1	12	OsR498G1221651000.01.T01	96,68	3,04E-140	499	Os12g0491800	86,56	1,57E-100	366	-133	Indica	Japonica	
maker-12-augustus-gene-194.24-mRNA-1	12	OsR498G1221651400.01.T02	97,15	0,00E+00	2470	Os12g0491800	100,00	0,00E+00	2659	189	NA	Japonica	
maker-12-augustus-gene-195.41-mRNA-1	12	OsR498G1221652500.01.T01	96,23	0,00E+00	2613	Os12g0493900	100,00	0,00E+00	2819	206	NA	Japonica	
maker-12-augustus-gene-196.0-mRNA-1	12	OsR498G1221658200.01.T01	98,83	0,00E+00	3156	Os12g0495600	100,00	0,00E+00	3247	91	NA	Japonica	CC-NBS-TM
maker-12-augustus-gene-197.5-mRNA-1	12	OsR498G1221661000.01.T01	98,76	0,00E+00	848	Os12g0496900	100,00	0,00E+00	875	27	NA	Japonica	
maker-12-augustus-gene-197.8-mRNA-1	12	OsR498G1221661600.01.T01	98,69	0,00E+00	2001	Os12g0497400	99,88	0,00E+00	1488	-513	Indica	Japonica	
maker-12-augustus-gene-198.11-mRNA-1	12	OsR498G1221664300.01.T01	99,72	0,00E+00	1256	Os12g0498700	100,00	0,00E+00	1265	9	NA	Japonica	
maker-12-augustus-gene-198.18-mRNA-1	12	OsR498G1221663000.01.T02	98,59	0,00E+00	1238	Os12g0498400	100,00	0,00E+00	1283	45	NA	Japonica	
maker-12-augustus-gene-198.2-mRNA-1	12	OsR498G1221662700.01.T01	99,80	0,00E+00	2633	Os05g0482600	70,02	2,61E-159	562	-2071	Indica	Japonica	
maker-12-augustus-gene-198.24-mRNA-1	12	OsR498G1221665000.01.T01	99,90	0,00E+00	1787	Os12g0498800	100,00	0,00E+00	1792	5	NA	Japonica	
maker-12-augustus-gene-199.0-mRNA-1	12	OsR498G1221667300.01.T05	94,78	0,00E+00	1478	Os12g0500700	95,10	0,00E+00	1491	13	NA	Japonica	
maker-12-augustus-gene-2.46-mRNA-1	12	OsR498G1119279500.01.T02	97,23	0,00E+00	2070	Os12g0102900	99,09	0,00E+00	2720	650	Japonica	NA	
maker-12-augustus-gene-2.57-mRNA-1	12	OsR498G1220792700.01.T01	96,34	0,00E+00	1877	Os12g0104200	95,47	0,00E+00	1482	-395	Indica	NA	
maker-12-augustus-gene-2.66-mRNA-1	12	OsR498G1220794300.01.T01	97,37	0,00E+00	3568	Os12g0104300	97,61	0,00E+00	3590	22	NA	Japonica	
maker-12-augustus-gene-2.69-mRNA-1	12	OsR498G1220790000.01.T01	97,29	0,00E+00	2790	Os12g0102700	95,51	0,00E+00	3938	1148	Japonica	NA	
maker-12-augustus-gene-20.40-mRNA-1	12	OsR498G1220901700.01.T01	99,74	0,00E+00	5591	Os12g0136900	100,00	0,00E+00	5627	36	NA	Japonica	
maker-12-augustus-gene-20.55-mRNA-1	12	OsR498G1220903700.01.T01	99,47	0,00E+00	1350	Os11g0140900	95,43	0,00E+00	1201	-149	Indica	Japonica	
maker-12-augustus-gene-20.57-mRNA-1	12	OsR498G1220904300.01.T01	99,76	0,00E+00	2254	Os12g0137500	100,00	0,00E+00	2268	14	NA	Japonica	
maker-12-augustus-gene-20.65-mRNA-1	12	OsR498G1220907100.01.T01	99,53	0,00E+00	2645	Os11g0141900	98,52	0,00E+00	2581	-64	NA	Japonica	
maker-12-augustus-gene-20.68-mRNA-1	12	OsR498G1220901400.01.T01	99,20	0,00E+00	1102	Os11g0140100	93,73	0,00E+00	946	-156	Indica	Japonica	
maker-12-augustus-gene-20.71-mRNA-1	12	OsR498G1220902400.01.T01	99,84	0,00E+00	2307	Os12g0137100	100,00	0,00E+00	1586	-721	Indica	Japonica	
maker-12-augustus-gene-200.32-mRNA-1	12	OsR498G1221671900.01.T01	95,37	0,00E+00	1580	Os12g0502500	95,57	0,00E+00	1589	9	NA	Japonica	
maker-12-augustus-gene-200.35-mRNA-1	12	OsR498G1221672300.01.T02	99,29	0,00E+00	3011	Os12g0502700	100,00	0,00E+00	3061	50	NA	Japonica	
maker-12-augustus-gene-200.38-mRNA-1	12	OsR498G1221669000.01.T02	94,35	0,00E+00	1072	Os12g0501400	100,00	0,00E+00	1223	151	Japonica	Japonica	
maker-12-augustus-gene-200.41-mRNA-1	12	OsR498G1221669400.01.T01	89,77	0,00E+00	966	Os12g0501700	100,00	0,00E+00	1267	301	Japonica	Japonica	
maker-12-augustus-gene-200.45-mRNA-1	12	OsR498G1221670000.01.T02	100,00	7,59E-157	553	Os12g0502000	100,00	2,64E-157	553	0	NA	Japonica	
maker-12-augustus-gene-200.50-mRNA-1	12	OsR498G1221671000.01.T01	99,80	0,00E+00	2644	Os12g0502300	100,00	0,00E+00	2657	13	NA	Japonica	
maker-12-augustus-gene-200.54-mRNA-1	12	OsR498G1221671600.01.T01	99,44	0,00E+00	1580	Os12g0502400	100,00	0,00E+00	1602	22	NA	Japonica	
maker-12-augustus-gene-200.62-mRNA-1	12	OsR498G1221672600.01.T01	98,58	0,00E+00	2340	Os12g0502800	98,54	0,00E+00	2155	-185	NA	Japonica	
maker-12-augustus-gene-201.31-mRNA-1	12	OsR498G1221672800.01.T02	97,85	0,00E+00	2146	Os12g0503000	100,00	0,00E+00	2268	122	NA	Japonica	
maker-12-augustus-gene-203.0-mRNA-1	12	OsR498G1221685500.01.T01	99,10	0,00E+00	2538	Os12g0506700	100,00	0,00E+00	2587	49	NA	Japonica	
maker-12-augustus-gene-203.12-mRNA-1	12	OsR498G1221687500.01.T01	100,00	0,00E+00	872	Os12g0507200	100,00	0,00E+00	872	0	NA	Japonica	
maker-12-augustus-gene-203.16-mRNA-1	12	OsR498G1221688500.01.T01	99,54	0,00E+00	2705	Os12g0507300	100,00	0,00E+00	2691	-14	NA	Japonica	
maker-12-augustus-gene-203.9-mRNA-1	12	OsR498G1221686500.01.T07	100,00	0,00E+00	1552	Os12g0507000	100,00	0,00E+00	1543	-9	NA	Japonica	
maker-12-augustus-gene-204.39-mRNA-1	12	OsR498G1221690700.01.T01	100,00	0,00E+00	818	Os12g0508100	100,00	0,00E+00	1283	465	Japonica	Japonica	
maker-12-augustus-gene-204.51-mRNA-1	12	OsR498G1221691000.01.T01	99,79	0,00E+00	840	Os12g0508200	100,00	0,00E+00	845	5	NA	Japonica	
maker-12-augustus-gene-204.53-mRNA-1	12	OsR498G1221691800.01.T01	99,22	1,36E-127	455	Os12g0508266	100,00	9,21E-131	464	9	NA	Japonica	
maker-12-augustus-gene-205.13-mRNA-1	12	OsR498G1221697300.01.T01	99,79	0,00E+00	866	Os12g0509300	100,00	0,00E+00	1153	287	Japonica	Japonica	
maker-12-augustus-gene-206.21-mRNA-1	12	OsR498G1221702900.01.T01	95,42	0,00E+00	2030	Os12g0511200	100,00	0,00E+00	2268	238	Japonica	Japonica	
maker-12-augustus-gene-206.23-mRNA-1	12	OsR498G1221703100.01.T02	99,42	0,00E+00	1835	Os12g0511300	100,00	0,00E+00	1862	27	NA	Japonica	
maker-12-augustus-gene-206.28-mRNA-1	12	OsR498G1221703400.01.T01	98,88	0,00E+00	6717	Os12g0511400	100,00	0,00E+00	6882	165	NA	Japonica	NBS-TM
maker-12-augustus-gene-206.34-mRNA-1	12	OsR498G1221704000.01.T02	92,90	0,00E+00	1463	Os12g0511400	84,02	0,00E+00	1068	-395	Indica	Japonica	CC-NBS-TM
maker-12-augustus-gene-207.32-mRNA-1	12	OsR498G1221704700.01.T01	99,60	0,00E+00	1329	Os12g0512000	100,00	0,00E+00	2430	1101	Japonica	Japonica	
maker-12-augustus-gene-207.55-mRNA-1	12	OsR498G1221706400.01.T01	99,75	0,00E+00	2812	Os03g0850200	73,13	0,00E+00	826	-1986	Indica	Japonica	
maker-12-augustus-gene-208.41-mRNA-1	12	OsR498G1221708200.01.T01	99,59	0,00E+00	2614	Os12g0514000	100,00	0,00E+00	2641	27	NA	Japonica	
maker-12-augustus-gene-208.48-mRNA-1	12	OsR498G1221710800.01.T01	99,92	0,00E+00	2195	Os12g0514500	100,00	0,00E+00	2199	4	NA	Japonica	
maker-12-augustus-gene-208.54-mRNA-1	12	OsR498G1221711000.01.T01	99,74	0,00E+00	678	Os12g0514600	100,00	0,00E+00	682	4	NA	Japonica	
maker-12-augustus-gene-209.21-mRNA-1	12	OsR498G1221715500.01.T01	99,94	0,00E+00	2966	Os12g0515400	99,94	0,00E+00	2966	0	NA	Indica	
maker-12-augustus-gene-209.28-mRNA-1	12	OsR498G1221715000.01.T01	97,40	0,00E+00	2094	Os12g0515300	99,92	0,00E+00	2214	120	NA	Indica	
maker-12-augustus-gene-209.30-mRNA-1	12	OsR498G1221716600.01.T01	99,82	0,00E+00	1984	Os12g0515600	99,19	0,00E+00	1951	-33	NA	Indica	
maker-12-augustus-gene-21.50-mRNA-1	12	OsR498G1220908500.01.T01	99,88	0,00E+00	1517	Os12g0138800	100,00	0,00E+00	1602	85	NA	Japonica	
maker-12-augustus-gene-21.52-mRNA-1	12	OsR498G1220908900.01.T01	99,64	0,00E+00	1995	Os11g0142400	97,99	0,00E+00	1446	-549	Indica	Japonica	
maker-12-augustus-gene-21.55-mRNA-1	12	OsR498G1220909100.01.T02	100,00	0,00E+00	1838	Os12g0138900	100,00	0,00E+00	1838	0	NA	Japonica	
maker-12-augustus-gene-21.67-mRNA-1	12	OsR498G1220910200.01.T01	99,73	0,00E+00	2699	Os12g0139300	100,00	0,00E+00	2717	18	NA	Japonica	
maker-12-augustus-gene-21.70-mRNA-1	12	OsR498G1220911200.01.T01	99,71	0,00E+00	1225	Os12g0139500	100,00	0,00E+00	1234	9	NA	Japonica	
maker-12-augustus-gene-21.73-mRNA-1	12	OsR498G1220911800.01.T01	99,77	0,00E+00	3142	Os11g0143500	98,16	0,00E+00	3087	-55	NA	Japonica	
maker-12-augustus-gene-21.84-mRNA-1	12	OsR498G1220913400.01.T01	99,58	0,00E+00	2549	Os12g0140300	100,00	0,00E+00	3463	914	Japonica	Japonica	
maker-12-augustus-gene-211.37-mRNA-1	12	OsR498G1221723300.01.T01	99,94	0,00E+00	3172	Os12g0527500	99,38	0,00E+00	3127	-45	NA	Indica	
maker-12-augustus-gene-211.42-mRNA-1	12	OsR498G1221725100.01.T01	99,94	0,00E+00	2772	Os12g0527850	99,76	0,00E+00	1496	-1276	Indica	Indica	
maker-12-augustus-gene-211.49-mRNA-1	12	OsR498G1221725400.01.T02	99,93	0,00E+00	2528	Os12g0528000	99,61	0,00E+00	2261	-267	Indica	Indica	
maker-12-augustus-gene-212.40-mRNA-1	12	OsR498G1221726300.01.T01	100,00	0,00E+00	3355	Os12g0528300	99,36	0,00E+00	2233	-1122	Indica	Indica	
maker-12-augustus-gene-212.49-mRNA-1	12	OsR498G1221728300.01.T02	100,00	0,00E+00	1131	Os12g0529400	96,33	0,00E+00	1029	-102	NA	Indica	
maker-12-augustus-gene-212.54-mRNA-1	12	OsR498G1221729000.01.T01	100,00	0,00E+00	753	Os12g0530000	99,28	0,00E+00	739	-14	NA	Indica	
maker-12-augustus-gene-212.56-mRNA-1	12	OsR498G1221729200.01.T02	99,81	0,00E+00	1874	Os12g0530100	99,52	0,00E+00	1861	-13	NA	Indica	
maker-12-augustus-gene-216.39-mRNA-1	12	OsR498G1221756300.01.T01	100,00	0,00E+00	2358	Os12g0533400	100,00	0,00E+00	1975	-383	Indica	NA	
maker-12-augustus-gene-216.41-mRNA-1	12	OsR498G1221757300.01.T01	99,63	0,00E+00	2407	Os12g0533700	99,61	0,00E+00	2305	-102	NA	NA	
maker-12-augustus-gene-216.50-mRNA-1	12	OsR498G1221757200.01.T02	99,73	0,00E+00	667	Os12g0533600	99,73	0,00E+00	669	2	NA	NA	
maker-12-augustus-gene-216.53-mRNA-1	12	OsR498G1221757600.01.T04	99,61	0,00E+00	922	Os12g0533800	99,60	0,00E+00	890	-32	NA	NA	
maker-12-augustus-gene-216.57-mRNA-1	12	OsR498G1221758000.01.T05	99,91	0,00E+00	1973	Os01g0375200	70,37	3,77E-132	471	-1502	Indica	Japonica	
maker-12-augustus-gene-216.63-mRNA-1	12	OsR498G1221758800.01.T01	100,00	0,00E+00	937	Os12g0534100	100,00	0,00E+00	937	0	NA	NA	
maker-12-augustus-gene-216.65-mRNA-1	12	OsR498G1221759000.01.T01	100,00	4,78E-128	457	Os12g0534200	100,00	1,66E-128	457	0	NA	Indica	
maker-12-augustus-gene-217.38-mRNA-1	12	OsR498G1221765500.01.T02	99,12	0,00E+00	1803	Os12g0536200	98,74	0,00E+00	1251	-552	Indica	NA	
maker-12-augustus-gene-217.47-mRNA-1	12	OsR498G0305726100.01.T03	96,16	0,00E+00	681	Os12g0535400	99,37	0,00E+00	851	170	Japonica	NA	
maker-12-augustus-gene-217.50-mRNA-1	12	OsR498G1221764600.01.T01	100,00	0,00E+00	1434	Os12g0535600	95,29	0,00E+00	1961	527	Japonica	Indica	
maker-12-augustus-gene-218.55-mRNA-1	12	OsR498G1221772300.01.T02	100,00	0,00E+00	1480	Os12g0538300	100,00	0,00E+00	1480	0	NA	NA	
maker-12-augustus-gene-218.61-mRNA-1	12	OsR498G1221773300.01.T01	95,44	0,00E+00	1506	Os12g0538500	95,44	0,00E+00	1506	0	NA	NA	
maker-12-augustus-gene-218.66-mRNA-1	12	OsR498G1221771400.01.T01	100,00	0,00E+00	1072	Os12g0538100	100,00	0,00E+00	1072	0	NA	NA	
maker-12-augustus-gene-219.41-mRNA-1	12	OsR498G1221780100.01.T02	100,00	0,00E+00	686	Os01g0630700	84,29	8,53E-117	419	-267	Indica	NA	
maker-12-augustus-gene-219.47-mRNA-1	12	OsR498G1221780300.01.T02	100,00	0,00E+00	756	Os03g0855700	82,50	6,68E-131	467	-289	Indica	NA	
maker-12-augustus-gene-219.54-mRNA-1	12	OsR498G1221775200.01.T01	99,94	0,00E+00	3103	Os12g0538900	100,00	0,00E+00	3108	5	NA	NA	
maker-12-augustus-gene-219.59-mRNA-1	12	OsR498G1221780600.01.T01	100,00	0,00E+00	1299	Os12g0539800	93,06	0,00E+00	1026	-273	Indica	NA	
maker-12-augustus-gene-22.37-mRNA-1	12	OsR498G1220916400.01.T02	100,00	0,00E+00	3573	Os12g0141100	100,00	0,00E+00	3573	0	NA	Japonica	
maker-12-augustus-gene-22.39-mRNA-1	12	OsR498G1220917600.01.T01	99,89	0,00E+00	3205	Os01g0776500	71,17	4,15E-126	452	-2753	Indica	Japonica	
maker-12-augustus-gene-220.24-mRNA-1	12	OsR498G1221784900.01.T02	100,00	0,00E+00	1018	Os12g0540700	100,00	8,03E-180	628	-390	Indica	Japonica	
maker-12-augustus-gene-220.27-mRNA-1	12	OsR498G1221784900.01.T04	99,80	0,00E+00	1766	Os12g0540800	100,00	0,00E+00	1775	9	NA	Japonica	
maker-12-augustus-gene-220.36-mRNA-1	12	OsR498G1221787700.01.T01	99,67	0,00E+00	1068	Os12g0541400	98,78	0,00E+00	1150	82	NA	NA	
maker-12-augustus-gene-220.41-mRNA-1	12	OsR498G1221787900.01.T03	99,85	0,00E+00	6059	Os12g0541500	99,88	0,00E+00	6064	5	NA	Japonica	
maker-12-augustus-gene-220.43-mRNA-1	12	OsR498G1221785200.01.T01	100,00	0,00E+00	2214	Os12g0540900	100,00	0,00E+00	2214	0	NA	Japonica	
maker-12-augustus-gene-223.27-mRNA-1	12	OsR498G1221807500.01.T01	98,97	0,00E+00	2220	Os12g0543600	99,12	0,00E+00	2207	-13	NA	NA	
maker-12-augustus-gene-225.0-mRNA-1	12	OsR498G1221833900.01.T01	96,32	0,00E+00	1445	Os12g0546800	95,99	0,00E+00	1435	-10	NA	NA	
maker-12-augustus-gene-225.11-mRNA-1	12	OsR498G1221838400.01.T02	99,96	0,00E+00	4891	Os12g0548100	99,16	0,00E+00	4815	-76	NA	Indica	
maker-12-augustus-gene-226.0-mRNA-1	12	OsR498G1221839300.01.T01	92,34	0,00E+00	730	Os12g0548300	92,34	0,00E+00	730	0	NA	NA	
maker-12-augustus-gene-226.10-mRNA-1	12	OsR498G1221848400.01.T01	97,97	0,00E+00	3746	Os12g0551200	95,77	0,00E+00	2443	-1303	Indica	Indica	
maker-12-augustus-gene-226.4-mRNA-1	12	OsR498G1221839000.01.T01	99,75	0,00E+00	4912	Os12g0548200	99,67	0,00E+00	4903	-9	NA	Indica	
maker-12-augustus-gene-227.29-mRNA-1	12	OsR498G1221850700.01.T01	98,64	0,00E+00	1279	Os03g0393900	76,64	0,00E+00	862	-417	Indica	NA	
maker-12-augustus-gene-227.31-mRNA-1	12	OsR498G1221850900.01.T02	98,55	0,00E+00	3117	Os07g0267400	87,43	0,00E+00	2084	-1033	Indica	NA	
maker-12-augustus-gene-227.32-mRNA-1	12	OsR498G1221850900.01.T02	98,87	0,00E+00	779	Os12g0551900	98,65	0,00E+00	773	-6	NA	NA	
maker-12-augustus-gene-227.45-mRNA-1	12	OsR498G1221852400.01.T03	74,56	4,33E-80	299	Os12g0552400	98,97	0,00E+00	849	550	Japonica	NA	
maker-12-augustus-gene-227.47-mRNA-1	12	OsR498G1221852400.01.T03	97,18	0,00E+00	900	Os12g0552500	96,71	0,00E+00	851	-49	NA	NA	
maker-12-augustus-gene-228.29-mRNA-1	12	OsR498G1221853700.01.T01	99,95	0,00E+00	3551	Os12g0552700	98,35	0,00E+00	1884	-1667	Indica	Indica	
maker-12-augustus-gene-228.32-mRNA-1	12	OsR498G1221854300.01.T01	98,12	0,00E+00	1733	Os12g0552800	90,97	0,00E+00	2182	449	Japonica	NA	
maker-12-augustus-gene-228.36-mRNA-1	12	OsR498G1221857500.01.T01	82,92	0,00E+00	732	Os12g0553900	82,92	0,00E+00	732	0	NA	Japonica	
maker-12-augustus-gene-228.39-mRNA-1	12	OsR498G1221861300.01.T01	99,06	0,00E+00	2443	Os12g0554800	99,84	0,00E+00	2286	-157	NA	NA	
maker-12-augustus-gene-228.43-mRNA-1	12	OsR498G1221861500.01.T01	100,00	0,00E+00	872	Os12g0555000	99,79	0,00E+00	867	-5	NA	NA	
maker-12-augustus-gene-228.45-mRNA-1	12	OsR498G1221862100.01.T01	99,79	0,00E+00	856	Os12g0555100	99,79	0,00E+00	856	0	NA	NA	
maker-12-augustus-gene-228.48-mRNA-1	12	OsR498G1221855600.01.T01	96,91	0,00E+00	4085	Os12g0516300	95,74	0,00E+00	4521	436	NA	NA	CC-LRR-NBS-TM
maker-12-augustus-gene-228.50-mRNA-1	12	OsR498G1221857800.01.T03	96,86	0,00E+00	2278	Os12g0516800	95,62	0,00E+00	2222	-56	NA	Japonica	
maker-12-augustus-gene-228.55-mRNA-1	12	OsR498G1221858600.01.T01	99,75	0,00E+00	4340	Os12g0554400	99,75	0,00E+00	4340	0	NA	NA	
maker-12-augustus-gene-228.61-mRNA-1	12	OsR498G1221858800.01.T02	99,80	0,00E+00	2698	Os12g0554500	99,78	0,00E+00	3292	594	Japonica	Indica	
maker-12-augustus-gene-229.0-mRNA-1	12	OsR498G1221862700.01.T01	99,79	0,00E+00	856	Os12g0555200	99,37	0,00E+00	847	-9	NA	NA	
maker-12-augustus-gene-229.17-mRNA-1	12	OsR498G1221865700.01.T02	99,72	0,00E+00	2533	Os12g0556300	100,00	0,00E+00	2551	18	NA	Japonica	
maker-12-augustus-gene-229.19-mRNA-1	12	OsR498G1221865900.01.T02	99,60	3,14E-124	445	Os12g0556400	100,00	1,51E-173	607	162	Japonica	Japonica	
maker-12-augustus-gene-229.2-mRNA-1	12	OsR498G1221863100.01.T01	97,54	0,00E+00	762	Os12g0555300	97,19	0,00E+00	663	-99	Indica	NA	
maker-12-augustus-gene-229.21-mRNA-1	12	OsR498G1221866100.01.T02	99,85	0,00E+00	3623	Os12g0556500	100,00	0,00E+00	3636	13	NA	Japonica	
maker-12-augustus-gene-229.25-mRNA-1	12	OsR498G1221863800.01.T01	99,40	0,00E+00	6487	Os06g0336500	72,76	0,00E+00	1562	-4925	Indica	Japonica	
maker-12-augustus-gene-229.27-mRNA-1	12	OsR498G1221866400.01.T01	98,31	0,00E+00	3083	Os12g0556600	98,38	0,00E+00	2903	-180	NA	Japonica	
maker-12-augustus-gene-229.4-mRNA-1	12	OsR498G1221863300.01.T01	95,64	0,00E+00	825	Os12g0555400	96,67	0,00E+00	1002	177	Japonica	Japonica	
maker-12-augustus-gene-229.6-mRNA-1	12	OsR498G1221863500.01.T01	99,16	0,00E+00	843	Os12g0555500	100,00	0,00E+00	861	18	NA	Japonica	
maker-12-augustus-gene-23.0-mRNA-1	12	OsR498G1220920500.01.T01	99,84	0,00E+00	2237	Os12g0143200	100,00	0,00E+00	2246	9	NA	Japonica	
maker-12-augustus-gene-23.14-mRNA-1	12	OsR498G1220924800.01.T03	99,71	0,00E+00	1875	Os12g0143800	100,00	0,00E+00	1786	-89	NA	Japonica	
maker-12-augustus-gene-23.19-mRNA-1	12	OsR498G1220925600.01.T03	99,79	0,00E+00	1707	Os12g0143900	100,00	0,00E+00	1709	2	NA	Japonica	
maker-12-augustus-gene-23.2-mRNA-1	12	OsR498G1220925900.01.T01	99,86	0,00E+00	1327	Os11g0147100	91,97	0,00E+00	1041	-286	Indica	Japonica	
maker-12-augustus-gene-23.7-mRNA-1	12	OsR498G1119407400.01.T02	94,74	0,00E+00	1903	Os12g0143400	100,00	0,00E+00	2906	1003	Japonica	Japonica	
maker-12-augustus-gene-230.0-mRNA-1	12	OsR498G1221868900.01.T02	99,55	0,00E+00	1600	Os12g0557900	99,89	0,00E+00	1632	32	NA	Japonica	
maker-12-augustus-gene-230.3-mRNA-1	12	OsR498G1221867600.01.T01	99,69	0,00E+00	1155	Os12g0557400	100,00	0,00E+00	1164	9	NA	Japonica	
maker-12-augustus-gene-230.5-mRNA-1	12	OsR498G1221868900.01.T02	87,71	0,00E+00	900	Os12g0558400	100,00	0,00E+00	1283	383	Japonica	NA	
maker-12-augustus-gene-231.30-mRNA-1	12	OsR498G1221874400.01.T02	98,99	0,00E+00	4874	Os12g0559200	99,57	0,00E+00	4947	73	NA	Japonica	
maker-12-augustus-gene-231.39-mRNA-1	12	OsR498G1221875800.01.T01	94,76	0,00E+00	2732	Os12g0559500	99,35	0,00E+00	2995	263	NA	Japonica	CC-NBS-TM
maker-12-augustus-gene-232.31-mRNA-1	12	OsR498G1221876500.01.T01	99,45	0,00E+00	4558	Os12g0559200	90,56	0,00E+00	3498	-1060	Indica	Japonica	
maker-12-augustus-gene-232.38-mRNA-1	12	OsR498G1221877300.01.T01	99,71	2,48E-176	618	Os12g0560400	100,00	3,91E-162	569	-49	NA	Japonica	
maker-12-augustus-gene-232.41-mRNA-1	12	OsR498G1221877900.01.T01	100,00	0,00E+00	839	Os12g0560600	100,00	0,00E+00	839	0	NA	Japonica	
maker-12-augustus-gene-232.44-mRNA-1	12	OsR498G1221877200.01.T01	99,66	0,00E+00	1041	Os12g0560300	100,00	0,00E+00	1050	9	NA	Japonica	
maker-12-augustus-gene-232.47-mRNA-1	12	OsR498G1221877800.01.T01	99,90	0,00E+00	1868	Os12g0560500	100,00	0,00E+00	1873	5	NA	Japonica	
maker-12-augustus-gene-232.49-mRNA-1	12	OsR498G1221878200.01.T02	100,00	0,00E+00	2675	Os12g0560700	100,00	0,00E+00	2675	0	NA	Japonica	
maker-12-augustus-gene-232.53-mRNA-1	12	OsR498G1221879000.01.T01	100,00	1,09E-108	392	Os12g0561000	100,00	1,41E-95	347	-45	Indica	Japonica	
maker-12-augustus-gene-232.55-mRNA-1	12	OsR498G1221879200.01.T02	99,15	0,00E+00	2714	Os12g0561200	99,90	0,00E+00	1785	-929	Indica	Japonica	
maker-12-augustus-gene-233.0-mRNA-1	12	OsR498G1221881500.01.T02	98,86	0,00E+00	768	Os12g0562000	100,00	3,18E-177	619	-149	Indica	Japonica	
maker-12-augustus-gene-233.14-mRNA-1	12	OsR498G1221885500.01.T01	100,00	0,00E+00	5220	Os12g0562700	99,34	0,00E+00	6440	1220	Japonica	NA	
maker-12-augustus-gene-233.19-mRNA-1	12	OsR498G1221885700.01.T01	100,00	0,00E+00	1067	Os12g0562900	100,00	0,00E+00	1067	0	NA	NA	
maker-12-augustus-gene-233.24-mRNA-1	12	OsR498G1221882000.01.T04	99,66	0,00E+00	3177	Os12g0562100	99,94	0,00E+00	3199	22	NA	Japonica	
maker-12-augustus-gene-233.9-mRNA-1	12	OsR498G1221884500.01.T01	100,00	0,00E+00	2464	Os12g0562500	100,00	0,00E+00	2475	11	NA	NA	CC-Kinase
maker-12-augustus-gene-234.53-mRNA-1	12	OsR498G1221886900.01.T01	99,80	0,00E+00	2736	Os12g0563000	100,00	0,00E+00	3025	289	NA	Japonica	
maker-12-augustus-gene-234.57-mRNA-1	12	OsR498G1221887900.01.T04	99,84	0,00E+00	3372	Os12g0563400	99,84	0,00E+00	3372	0	NA	NA	
maker-12-augustus-gene-234.62-mRNA-1	12	OsR498G1221888100.01.T01	100,00	0,00E+00	1797	Os12g0563500	100,00	0,00E+00	1797	0	NA	NA	
maker-12-augustus-gene-234.70-mRNA-1	12	OsR498G1221891300.01.T01	100,00	0,00E+00	1353	Os12g0564400	100,00	0,00E+00	1353	0	NA	NA	
maker-12-augustus-gene-234.75-mRNA-1	12	OsR498G1221893300.01.T01	99,30	0,00E+00	2799	Os12g0565100	100,00	0,00E+00	3559	760	Japonica	Japonica	CC-NBS-TM
maker-12-augustus-gene-234.79-mRNA-1	12	OsR498G1221889200.01.T01	100,00	0,00E+00	2246	Os12g0563700	100,00	0,00E+00	2246	0	NA	Japonica	
maker-12-augustus-gene-234.86-mRNA-1	12	OsR498G1221891600.01.T01	100,00	0,00E+00	6277	Os12g0564600	100,00	0,00E+00	6277	0	NA	NA	
maker-12-augustus-gene-234.92-mRNA-1	12	OsR498G1221892000.01.T02	100,00	0,00E+00	1644	Os12g0564800	99,34	0,00E+00	1618	-26	NA	Japonica	NBS-TM

maker-12-augustus-gene-235.0-mRNA-1	12	OsR498G1221893900.01.T01	99,82	0,00E+00	3006	Os12g0565200	100,00	0,00E+00	3020	14	NA	Japonica	CC-LRR-NBS-TM
maker-12-augustus-gene-235.13-mRNA-1	12	OsR498G1221895900.01.T03	98,41	0,00E+00	2078	Os12g0566300	98,49	0,00E+00	2081	3	NA	Japonica	
maker-12-augustus-gene-235.17-mRNA-1	12	OsR498G1221896100.01.T02	96,45	0,00E+00	794	Os12g0566400	100,00	3,77E-165	580	-214	Indica	Japonica	
maker-12-augustus-gene-235.20-mRNA-1	12	OsR498G1221894200.01.T01	99,91	0,00E+00	3925	Os12g0565300	100,00	0,00E+00	3934	9	NA	Japonica	
maker-12-augustus-gene-235.25-mRNA-1	12	OsR498G1221895200.01.T01	99,63	1,70E-135	483	Os12g0566100	100,00	1,59E-111	401	-82	Indica	Japonica	
maker-12-augustus-gene-235.3-mRNA-1	12	OsR498G1221894900.01.T01	99,86	0,00E+00	3839	Os12g0566000	100,00	0,00E+00	3853	14	NA	Japonica	
maker-12-augustus-gene-236.56-mRNA-1	12	OsR498G1221900900.01.T01	99,87	0,00E+00	1414	Os12g0567700	100,00	0,00E+00	1418	4	NA	Japonica	
maker-12-augustus-gene-236.58-mRNA-1	12	OsR498G1221896800.01.T01	99,50	0,00E+00	2154	Os12g0566700	100,00	0,00E+00	1272	-882	Indica	Japonica	
maker-12-augustus-gene-236.61-mRNA-1	12	OsR498G1221897000.01.T03	99,71	0,00E+00	1242	Os12g0566900	95,82	0,00E+00	1120	-122	NA	Japonica	
maker-12-augustus-gene-236.63-mRNA-1	12	OsR498G1221897000.01.T02	99,04	0,00E+00	1100	Os12g0566800	100,00	0,00E+00	646	-454	Indica	Japonica	
maker-12-augustus-gene-236.67-mRNA-1	12	OsR498G1221897200.01.T01	99,78	0,00E+00	2389	Os12g0566900	93,36	0,00E+00	1958	-431	Indica	Japonica	
maker-12-augustus-gene-236.68-mRNA-1	12	OsR498G1221897200.01.T06	99,23	0,00E+00	685	Os12g0566900	100,00	0,00E+00	697	12	NA	Japonica	
maker-12-augustus-gene-236.77-mRNA-1	12	OsR498G1221897800.01.T01	99,73	0,00E+00	1306	Os12g0567300	100,00	0,00E+00	1315	9	NA	Japonica	
maker-12-augustus-gene-236.80-mRNA-1	12	OsR498G1221899800.01.T01	99,52	0,00E+00	4453	Os12g0567500	100,00	0,00E+00	5254	801	Japonica	Japonica	Kinase-LRR-TM
maker-12-augustus-gene-236.82-mRNA-1	12	OsR498G1221901200.01.T01	99,16	8,49E-117	419	Os12g0567800	100,00	5,74E-120	428	9	NA	Japonica	
maker-12-augustus-gene-236.86-mRNA-1	12	OsR498G1221901600.01.T01	100,00	3,91E-121	434	Os12g0571000	91,80	8,11E-74	275	-159	Indica	NA	
maker-12-augustus-gene-236.88-mRNA-1	12	OsR498G1221902200.01.T01	99,16	2,47E-117	421	Os12g0571100	95,83	1,78E-107	387	-34	NA	NA	
maker-12-augustus-gene-237.33-mRNA-1	12	OsR498G1221903300.01.T01	99,45	0,00E+00	977	Os12g0568700	100,00	0,00E+00	991	14	NA	Japonica	
maker-12-augustus-gene-237.35-mRNA-1	12	OsR498G1221903700.01.T01	99,92	0,00E+00	4715	Os12g0568800	100,00	0,00E+00	4724	9	NA	Japonica	
maker-12-augustus-gene-237.37-mRNA-1	12	OsR498G0306484600.01.T01	87,55	3,98E-171	602	Os12g0569700	100,00	0,00E+00	850	248	Japonica	Japonica	
maker-12-augustus-gene-237.38-mRNA-1	12	OsR498G1221902600.01.T01	100,00	3,91E-121	434	Os12g0571100	93,33	2,48E-99	360	-74	Indica	Indica	
maker-12-augustus-gene-237.41-mRNA-1	12	OsR498G1221907400.01.T01	98,14	0,00E+00	3611	Os12g0570000	99,90	0,00E+00	3438	-173	NA	Japonica	
maker-12-augustus-gene-238.0-mRNA-1	12	OsR498G1221909300.01.T02	99,44	0,00E+00	961	Os12g0570400	100,00	0,00E+00	975	14	NA	Japonica	
maker-12-augustus-gene-238.10-mRNA-1	12	OsR498G1221910900.01.T01	100,00	1,64E-119	428	Os12g0571100	100,00	5,74E-120	428	0	NA	Japonica	
maker-12-augustus-gene-238.13-mRNA-1	12	OsR498G1221911300.01.T01	99,65	3,15E-142	505	Os12g0571200	100,00	2,59E-144	509	4	NA	Japonica	
maker-12-augustus-gene-238.15-mRNA-1	12	OsR498G1221912900.01.T06	99,78	0,00E+00	2476	Os12g0571900	100,00	0,00E+00	2489	13	NA	Japonica	
maker-12-augustus-gene-238.2-mRNA-1	12	OsR498G1221909500.01.T03	99,30	0,00E+00	4573	Os12g0570500	99,77	0,00E+00	4633	60	NA	Japonica	
maker-12-augustus-gene-238.22-mRNA-1	12	OsR498G1221913200.01.T01	99,03	0,00E+00	1804	Os12g0572000	99,81	0,00E+00	1842	38	NA	Japonica	
maker-12-augustus-gene-238.7-mRNA-1	12	OsR498G1221910700.01.T01	98,70	6,34E-112	404	Os12g0571000	99,50	4,29E-96	350	-54	Indica	Japonica	
maker-12-augustus-gene-239.25-mRNA-1	12	OsR498G1221916500.01.T04	99,47	0,00E+00	2008	Os12g0572500	100,00	0,00E+00	2037	29	NA	Japonica	
maker-12-augustus-gene-239.30-mRNA-1	12	OsR498G1221918300.01.T02	99,46	0,00E+00	1314	Os12g0573000	99,26	0,00E+00	957	-357	Indica	Japonica	
maker-12-augustus-gene-239.34-mRNA-1	12	OsR498G1221920100.01.T01	93,88	0,00E+00	749	Os12g0573700	99,40	0,00E+00	894	145	Japonica	NA	
maker-12-augustus-gene-239.36-mRNA-1	12	OsR498G1221920100.01.T01	89,03	0,00E+00	856	Os12g0573800	100,00	0,00E+00	1088	232	Japonica	NA	
maker-12-augustus-gene-239.40-mRNA-1	12	OsR498G1221916200.01.T01	99,87	0,00E+00	1425	Os12g0572400	100,00	0,00E+00	1429	4	NA	Japonica	
maker-12-augustus-gene-239.45-mRNA-1	12	OsR498G1221917200.01.T01	99,92	0,00E+00	4646	Os12g0572800	100,00	0,00E+00	3956	-690	Indica	Japonica	
maker-12-augustus-gene-24.12-mRNA-1	12	OsR498G1220932300.01.T01	99,68	1,75E-160	566	Os11g0148400	83,81	1,54E-92	339	-227	Indica	Japonica	
maker-12-augustus-gene-24.15-mRNA-1	12	OsR498G1220930000.01.T01	100,00	0,00E+00	1294	Os12g0145100	100,00	0,00E+00	1294	0	NA	Japonica	
maker-12-augustus-gene-24.19-mRNA-1	12	OsR498G1220932600.01.T01	99,94	0,00E+00	2853	Os12g0145700	100,00	0,00E+00	2857	4	NA	Japonica	
maker-12-augustus-gene-24.3-mRNA-1	12	OsR498G1220928100.01.T02	99,56	0,00E+00	2810	Os12g0144600	100,00	0,00E+00	2841	31	NA	Japonica	
maker-12-augustus-gene-24.5-mRNA-1	12	OsR498G1220931300.01.T01	98,55	0,00E+00	2406	Os12g0145400	98,65	0,00E+00	2328	-78	NA	Japonica	
maker-12-augustus-gene-24.8-mRNA-1	12	OsR498G1220931500.01.T01	99,91	0,00E+00	2003	Os12g0145500	100,00	0,00E+00	2008	5	NA	Japonica	
maker-12-augustus-gene-240.36-mRNA-1	12	OsR498G1221921500.01.T05	99,72	0,00E+00	5023	Os12g0574400	100,00	0,00E+00	5059	36	NA	Japonica	
maker-12-augustus-gene-240.39-mRNA-1	12	OsR498G1221920800.01.T01	99,94	0,00E+00	3215	Os12g0574000	100,00	0,00E+00	3220	5	NA	Japonica	
maker-12-augustus-gene-240.41-mRNA-1	12	OsR498G1221924400.01.T04	100,00	0,00E+00	2311	Os12g0574900	100,00	0,00E+00	2311	0	NA	Japonica	
maker-12-augustus-gene-240.46-mRNA-1	12	OsR498G1221924600.01.T01	99,83	0,00E+00	1030	Os12g0575000	100,00	0,00E+00	1034	4	NA	Japonica	
maker-12-augustus-gene-241.38-mRNA-1	12	OsR498G1221929100.01.T03	99,90	0,00E+00	1814	Os12g0577100	100,00	0,00E+00	1819	5	NA	Japonica	
maker-12-augustus-gene-241.51-mRNA-1	12	OsR498G1221932100.01.T04	100,00	0,00E+00	3063	Os12g0577600	100,00	0,00E+00	3063	0	NA	NA	
maker-12-augustus-gene-241.56-mRNA-1	12	OsR498G1221927000.01.T02	99,74	0,00E+00	3419	Os12g0576300	100,00	0,00E+00	2230	-1189	Indica	Japonica	
maker-12-augustus-gene-241.59-mRNA-1	12	OsR498G1221927800.01.T02	97,28	0,00E+00	3186	Os12g0576600	100,00	0,00E+00	3306	120	NA	Japonica	
maker-12-augustus-gene-241.64-mRNA-1	12	OsR498G1221928000.01.T03	99,90	0,00E+00	1717	Os12g0576700	100,00	0,00E+00	1721	4	NA	Japonica	
maker-12-augustus-gene-241.69-mRNA-1	12	OsR498G1221928600.01.T01	98,35	0,00E+00	3265	Os12g0576600	73,64	0,00E+00	1100	-2165	Indica	Japonica	
maker-12-augustus-gene-241.71-mRNA-1	12	OsR498G1221928800.01.T01	100,00	0,00E+00	2663	Os12g0576900	97,76	0,00E+00	2539	-124	NA	Japonica	
maker-12-augustus-gene-241.74-mRNA-1	12	OsR498G1221932600.01.T02	100,00	0,00E+00	3090	Os02g0559300	73,96	1,11E-158	560	-2530	Indica	NA	Kinase-TM
maker-12-augustus-gene-242.34-mRNA-1	12	OsR498G1221935500.01.T01	100,00	0,00E+00	1146	Os12g0578200	100,00	0,00E+00	1146	0	NA	NA	
maker-12-augustus-gene-242.36-mRNA-1	12	OsR498G1221935700.01.T01	100,00	0,00E+00	3350	Os12g0578300	100,00	0,00E+00	3350	0	NA	NA	
maker-12-augustus-gene-242.38-mRNA-1	12	OsR498G1221936100.01.T01	100,00	0,00E+00	2033	Os12g0578400	100,00	0,00E+00	2033	0	NA	NA	
maker-12-augustus-gene-242.43-mRNA-1	12	OsR498G1221937900.01.T01	100,00	0,00E+00	3593	Os12g0579000	100,00	0,00E+00	3593	0	NA	NA	
maker-12-augustus-gene-242.53-mRNA-1	12	OsR498G1221932800.01.T01	100,00	0,00E+00	4259	Os12g0577800	100,00	0,00E+00	4259	0	NA	NA	
maker-12-augustus-gene-242.55-mRNA-1	12	OsR498G1221936400.01.T01	100,00	0,00E+00	3003	Os12g0578500	100,00	0,00E+00	3003	0	NA	NA	
maker-12-augustus-gene-243.0-mRNA-1	12	OsR498G1221943900.01.T04	100,00	0,00E+00	1131	Os12g0580300	100,00	0,00E+00	1131	0	NA	NA	
maker-12-augustus-gene-243.4-mRNA-1	12	OsR498G1221944900.01.T02	99,80	0,00E+00	1755	Os12g0580500	99,90	0,00E+00	1760	5	NA	Japonica	
maker-12-augustus-gene-243.5-mRNA-1	12	OsR498G1221945300.01.T04	99,33	0,00E+00	1065	Os12g0580600	100,00	0,00E+00	1099	34	NA	Japonica	
maker-12-augustus-gene-243.8-mRNA-1	12	OsR498G1221945700.01.T01	100,00	0,00E+00	1018	Os12g0580700	100,00	0,00E+00	1018	0	NA	Japonica	
maker-12-augustus-gene-244.29-mRNA-1	12	OsR498G1221950700.01.T01	100,00	0,00E+00	2132	Os12g0581700	100,00	0,00E+00	2132	0	NA	NA	
maker-12-augustus-gene-244.32-mRNA-1	12	OsR498G1221951100.01.T01	100,00	0,00E+00	2089	Os12g0581800	100,00	0,00E+00	2089	0	NA	NA	
maker-12-augustus-gene-244.34-mRNA-1	12	OsR498G1221947400.01.T02	99,58	0,00E+00	2527	Os03g0606100	80,06	0,00E+00	1234	-1293	Indica	Japonica	
maker-12-augustus-gene-244.37-mRNA-1	12	OsR498G1221949000.01.T02	100,00	0,00E+00	1581	Os11g0432900	76,83	2,73E-158	559	-1022	Indica	NA	
maker-12-augustus-gene-244.40-mRNA-1	12	OsR498G1221949800.01.T01	99,94	0,00E+00	2929	Os12g0581600	100,00	0,00E+00	2933	4	NA	NA	
maker-12-augustus-gene-244.42-mRNA-1	12	OsR498G1221951400.01.T01	100,00	0,00E+00	1061	Os12g0581900	100,00	0,00E+00	1061	0	NA	NA	
maker-12-augustus-gene-244.43-mRNA-1	12	OsR498G1221952800.01.T01	100,00	0,00E+00	1770	Os12g0582000	100,00	0,00E+00	2906	1136	Japonica	NA	
maker-12-augustus-gene-244.48-mRNA-1	12	OsR498G1221956000.01.T01	99,94	0,00E+00	2885	Os12g0582700	99,94	0,00E+00	2873	-12	NA	NA	
maker-12-augustus-gene-245.26-mRNA-1	12	OsR498G1221956500.01.T01	100,00	0,00E+00	1849	Os12g0582800	100,00	0,00E+00	1849	0	NA	NA	
maker-12-augustus-gene-245.32-mRNA-1	12	OsR498G1221958100.01.T02	100,00	0,00E+00	827	Os12g0583400	98,90	0,00E+00	801	-26	NA	NA	
maker-12-augustus-gene-245.37-mRNA-1	12	OsR498G1221960500.01.T11	100,00	0,00E+00	3014	Os12g0583900	100,00	0,00E+00	3014	0	NA	NA	
maker-12-augustus-gene-245.42-mRNA-1	12	OsR498G1221957400.01.T01	100,00	0,00E+00	1104	Os12g0583000	100,00	0,00E+00	1104	0	NA	NA	
maker-12-augustus-gene-245.47-mRNA-1	12	OsR498G1221958400.01.T02	100,00	0,00E+00	3458	Os12g0583500	100,00	0,00E+00	3458	0	NA	NA	
maker-12-augustus-gene-245.50-mRNA-1	12	OsR498G1221963400.01.T05	100,00	0,00E+00	872	Os12g0584100	99,75	0,00E+00	725	-147	Indica	Japonica	
maker-12-augustus-gene-247.0-mRNA-1	12	OsR498G1221987100.01.T02	100,00	0,00E+00	4653	Os12g0586000	100,00	0,00E+00	3240	-1413	Indica	NA	CC-LRR-NBS-TM
maker-12-augustus-gene-247.13-mRNA-1	12	OsR498G1221987400.01.T01	91,88	0,00E+00	1773	Os12g0586100	91,88	0,00E+00	1773	0	NA	NA	Kinase-TM
maker-12-augustus-gene-247.3-mRNA-1	12	OsR498G1221989900.01.T01	98,91	0,00E+00	2086	Os12g0586300	100,00	0,00E+00	2143	57	NA	Japonica	
maker-12-augustus-gene-247.6-mRNA-1	12	OsR498G1221990100.01.T01	99,39	0,00E+00	1448	Os03g0240900	95,71	0,00E+00	1313	-135	NA	Japonica	
maker-12-augustus-gene-248.31-mRNA-1	12	OsR498G1221990700.01.T02	99,87	0,00E+00	5506	Os12g0586600	100,00	0,00E+00	5525	19	NA	Japonica	
maker-12-augustus-gene-248.45-mRNA-1	12	OsR498G1222005100.01.T04	96,21	0,00E+00	1434	Os12g0572800	91,46	0,00E+00	1341	-93	NA	Japonica	
maker-12-augustus-gene-249.33-mRNA-1	12	OsR498G1221996700.01.T01	98,27	1,81E-80	299	Os12g0588400	100,00	1,30E-108	390	91	Japonica	Japonica	
maker-12-augustus-gene-249.35-mRNA-1	12	OsR498G1222005000.01.T01	90,69	0,00E+00	2053	Os12g0589400	100,00	0,00E+00	2488	435	Japonica	Japonica	
maker-12-augustus-gene-249.36-mRNA-1	12	OsR498G1221999600.01.T01	99,00	0,00E+00	1929	Os12g0588800	100,00	0,00E+00	1981	52	NA	Japonica	
maker-12-augustus-gene-249.40-mRNA-1	12	OsR498G1222000200.01.T02	99,54	0,00E+00	1555	Os12g0588900	100,00	0,00E+00	1573	18	NA	Japonica	
maker-12-augustus-gene-249.42-mRNA-1	12	OsR498G1222000400.01.T01	100,00	0,00E+00	1083	Os03g0617800	76,18	3,24E-92	339	-744	Indica	Japonica	
maker-12-augustus-gene-249.44-mRNA-1	12	OsR498G1222001000.01.T01	99,45	0,00E+00	1287	Os12g0589100	100,00	0,00E+00	1305	18	NA	Japonica	
maker-12-augustus-gene-25.0-mRNA-1	12	OsR498G1220936100.01.T01	98,97	0,00E+00	1696	Os12g0147000	100,00	0,00E+00	2024	328	Japonica	Japonica	
maker-12-augustus-gene-25.3-mRNA-1	12	OsR498G1220937300.01.T01	98,43	0,00E+00	1548	Os12g0147500	100,00	0,00E+00	1609	61	NA	Japonica	
maker-12-augustus-gene-25.6-mRNA-1	12	OsR498G1220938900.01.T01	99,68	7,67E-157	554	Os12g0147800	100,00	6,27E-159	558	4	NA	Japonica	
maker-12-augustus-gene-25.9-mRNA-1	12	OsR498G1220935000.01.T02	99,28	0,00E+00	976	Os11g0148600	85,41	0,00E+00	644	-332	Indica	Japonica	
maker-12-augustus-gene-250.12-mRNA-1	12	OsR498G1222005000.01.T01	99,80	0,00E+00	1817	Os12g0589400	90,49	0,00E+00	1130	-687	Indica	Japonica	
maker-12-augustus-gene-251.28-mRNA-1	12	OsR498G1222012100.01.T01	100,00	5,17E-140	497	Os12g0592200	100,00	6,28E-140	495	-2	NA	Japonica	
maker-12-augustus-gene-251.30-mRNA-1	12	OsR498G1222012900.01.T01	100,00	0,00E+00	1023	Os12g0589100	79,21	9,19E-129	459	-564	Indica	Japonica	
maker-12-augustus-gene-251.35-mRNA-1	12	OsR498G1222012400.01.T02	99,91	0,00E+00	1982	Os12g0592300	100,00	0,00E+00	1986	4	NA	Japonica	
maker-12-augustus-gene-251.37-mRNA-1	12	OsR498G1222022200.01.T02	99,89	0,00E+00	4840	Os12g0593000	100,00	0,00E+00	3366	-1474	Indica	Japonica	
maker-12-augustus-gene-252.13-mRNA-1	12	OsR498G1222027900.01.T01	100,00	0,00E+00	856	Os12g0594600	100,00	0,00E+00	856	0	NA	Japonica	
maker-12-augustus-gene-252.19-mRNA-1	12	OsR498G1222029400.01.T01	100,00	0,00E+00	1548	Os12g0595000	100,00	0,00E+00	1548	0	NA	Japonica	
maker-12-augustus-gene-252.9-mRNA-1	12	OsR498G1222027100.01.T01	99,97	0,00E+00	5769	Os12g0594300	99,94	0,00E+00	5993	224	NA	Japonica	Kinase-TM
maker-12-augustus-gene-253.0-mRNA-1	12	OsR498G1222032500.01.T02	99,82	0,00E+00	3910	Os12g0595800	100,00	0,00E+00	3929	19	NA	Japonica	Kinase-TM
maker-12-augustus-gene-253.10-mRNA-1	12	OsR498G1222034300.01.T02	99,84	0,00E+00	1111	Os12g0596900	100,00	0,00E+00	1115	4	NA	Japonica	
maker-12-augustus-gene-253.13-mRNA-1	12	OsR498G1222034900.01.T05	100,00	0,00E+00	1223	Os12g0597000	100,00	0,00E+00	1223	0	NA	Japonica	
maker-12-augustus-gene-253.23-mRNA-1	12	OsR498G1222028700.01.T01	85,63	0,00E+00	1866	Os12g0595200	100,00	0,00E+00	2870	1004	Japonica	Japonica	
maker-12-augustus-gene-253.25-mRNA-1	12	OsR498G1222030800.01.T03	99,87	0,00E+00	2800	Os12g0595300	100,00	0,00E+00	3020	220	NA	Japonica	
maker-12-augustus-gene-253.27-mRNA-1	12	OsR498G1222028700.01.T01	75,03	0,00E+00	666	Os12g0594700	75,03	0,00E+00	666	0	NA	Japonica	
maker-12-augustus-gene-253.29-mRNA-1	12	OsR498G1222031600.01.T01	100,00	0,00E+00	785	Os12g0595500	100,00	0,00E+00	785	0	NA	NA	
maker-12-augustus-gene-253.31-mRNA-1	12	OsR498G1222031800.01.T01	100,00	0,00E+00	834	Os12g0595600	100,00	1,82E-162	571	-263	Indica	Japonica	
maker-12-augustus-gene-253.34-mRNA-1	12	OsR498G1222032800.01.T02	100,00	0,00E+00	834	Os12g0596000	100,00	0,00E+00	834	0	NA	Japonica	
maker-12-augustus-gene-253.36-mRNA-1	12	OsR498G1222036000.01.T01	99,87	0,00E+00	2686	Os12g0597400	100,00	0,00E+00	2695	9	NA	Japonica	
maker-12-augustus-gene-253.6-mRNA-1	12	OsR498G1222034100.01.T01	100,00	0,00E+00	2556	Os12g0596800	100,00	0,00E+00	2556	0	NA	Japonica	
maker-12-augustus-gene-254.44-mRNA-1	12	OsR498G1222036200.01.T01	98,49	0,00E+00	2540	Os12g0597500	98,21	0,00E+00	2030	-510	Indica	Japonica	
maker-12-augustus-gene-254.48-mRNA-1	12	OsR498G1222037200.01.T02	100,00	0,00E+00	1981	Os12g0597700	100,00	0,00E+00	1885	-96	NA	Japonica	
maker-12-augustus-gene-255.48-mRNA-1	12	OsR498G1222046700.01.T05	100,00	0,00E+00	1261	Os12g0599900	100,00	0,00E+00	1261	0	NA	Japonica	
maker-12-augustus-gene-255.52-mRNA-1	12	OsR498G1222046700.01.T05	82,17	0,00E+00	1239	Os12g0599900	82,17	0,00E+00	1239	0	NA	NA	
maker-12-augustus-gene-255.60-mRNA-1	12	OsR498G1222047000.01.T01	100,00	0,00E+00	2549	Os12g0600200	100,00	0,00E+00	2044	-505	Indica	NA	
maker-12-augustus-gene-256.0-mRNA-1	12	OsR498G1222049900.01.T02	100,00	0,00E+00	2484	Os12g0601000	99,88	0,00E+00	3048	564	Japonica	Indica	
maker-12-augustus-gene-256.18-mRNA-1	12	OsR498G1222050600.01.T01	100,00	0,00E+00	1505	Os12g0601300	100,00	0,00E+00	1505	0	NA	Indica	
maker-12-augustus-gene-256.3-mRNA-1	12	OsR498G1222050300.01.T04	100,00	0,00E+00	928	Os12g0601200	100,00	0,00E+00	928	0	NA	Indica	NBS-TM
maker-12-augustus-gene-256.9-mRNA-1	12	OsR498G1222051500.01.T01	100,00	0,00E+00	1072	Os12g0601400	100,00	0,00E+00	1072	0	NA	Japonica	
maker-12-augustus-gene-257.24-mRNA-1	12	OsR498G1222057600.01.T01	99,65	0,00E+00	1511	Os12g0603300	100,00	0,00E+00	1198	-313	Indica	NA	
maker-12-augustus-gene-258.0-mRNA-1	12	OsR498G1222064900.01.T01	100,00	0,00E+00	1745	Os12g0604500	100,00	0,00E+00	2527	782	Japonica	NA	
maker-12-augustus-gene-258.13-mRNA-1	12	OsR498G1222064000.01.T01	99,93	0,00E+00	2599	Os03g0635800	70,91	2,11E-160	565	-2034	Indica	NA	
maker-12-augustus-gene-258.16-mRNA-1	12	OsR498G1222065800.01.T02	99,94	0,00E+00	3199	Os12g0604700	100,00	0,00E+00	3204	5	NA	NA	Kinase
maker-12-augustus-gene-258.21-mRNA-1	12	OsR498G1222066600.01.T01	100,00	0,00E+00	1759	Os12g0604800	100,00	0,00E+00	1759	0	NA	NA	
maker-12-augustus-gene-258.26-mRNA-1	12	OsR498G1222068600.01.T01	99,27	0,00E+00	2187	Os12g0605300	99,27	0,00E+00	2187	0	NA	NA	
maker-12-augustus-gene-258.29-mRNA-1	12	OsR498G1222069000.01.T01	96,29	0,00E+00	761	Os12g0605400	96,29	0,00E+00	761	0	NA	NA	
maker-12-augustus-gene-258.32-mRNA-1	12	OsR498G1222069200.01.T03	100,00	0,00E+00	2773	Os12g0605500	100,00	0,00E+00	2773	0	NA	NA	
maker-12-augustus-gene-258.7-mRNA-1	12	OsR498G1222069300.01.T01	98,36	0,00E+00	3915	Os12g0605800	98,80	0,00E+00	3940	25	NA	NA	
maker-12-augustus-gene-259.0-mRNA-1	12	OsR498G1222069500.01.T01	99,67	0,00E+00	2152	Os12g0605900	100,00	0,00E+00	2170	18	NA	Japonica	Kinase
maker-12-augustus-gene-259.14-mRNA-1	12	OsR498G1222072300.01.T02	100,00	0,00E+00	2462	Os12g0606600	100,00	0,00E+00	2462	0	NA	Indica	
maker-12-augustus-gene-259.20-mRNA-1	12	OsR498G1222073500.01.T01	100,00	0,00E+00	1856	Os12g0607000	100,00	0,00E+00	1856	0	NA	NA	
maker-12-augustus-gene-259.21-mRNA-1	12	OsR498G1222073700.01.T01	100,00	0,00E+00	2516	Os12g0607100	100,00	0,00E+00	2516	0	NA	NA	
maker-12-augustus-gene-259.58-mRNA-1	12	OsR498G1222074600.01.T04	100,00	0,00E+00	872	Os05g0497700	92,01	1,08E-126	452	-420	Indica	NA	
maker-12-augustus-gene-26.32-mRNA-1	12	OsR498G0509623900.01.T03	71,06	9,44E-74	279	Os12g0148600	100,00	0,00E+00	1131	852	Japonica	Japonica	
maker-12-augustus-gene-26.35-mRNA-1	12	OsR498G1119421300.01.T01	90,21	0,00E+00	1527	Os12g0149700	100,00	0,00E+00	1835	308	Japonica	Japonica	Kinase-TM
maker-12-augustus-gene-26.37-mRNA-1	12	OsR498G1220944900.01.T03	99,77	0,00E+00	786	Os12g0149800	100,00	0,00E+00	791	5	NA	Japonica	
maker-12-augustus-gene-26.39-mRNA-1	12	OsR498G1220940400.01.T02	99,71	0,00E+00	3100	Os11g0137300	85,71	0,00E+00	2401	-699	Indica	Japonica	
maker-12-augustus-gene-26.44-mRNA-1	12	OsR498G1220941200.01.T01	99,84	0,00E+00	2210	Os12g0148700	100,00	0,00E+00	2219	9	NA	Japonica	
maker-12-augustus-gene-26.49-mRNA-1	12	OsR498G1220945200.01.T01	99,93	0,00E+00	2582	Os12g0149900	100,00	0,00E+00	2587	5	NA	Japonica	
maker-12-augustus-gene-260.56-mRNA-1	12	OsR498G1222073900.01.T01	98,59	0,00E+00	1857	Os12g0608500	74,63	0,00E+00	800	-1057	Indica	NA	Kinase-TM
maker-12-augustus-gene-260.58-mRNA-1	12	OsR498G1222079400.01.T01	99,62	0,00E+00	1403	Os12g0608600	100,00	0,00E+00	1416	13	NA	Japonica	
maker-12-augustus-gene-260.61-mRNA-1	12	OsR498G1222080200.01.T01	87,13	0,00E+00	1661	Os12g0608700	100,00	0,00E+00	2410	749	Japonica	Japonica	Kinase-TM
maker-12-augustus-gene-260.63-mRNA-1	12	OsR498G1222081400.01.T02	95,92	0,00E+00	1946	Os12g0609000	100,00	0,00E+00	2170	224	Japonica	Japonica	Kinase-TM
maker-12-augustus-gene-260.64-mRNA-1	12	OsR498G1222082600.01.T01	93,88	0,00E+00	1319	Os12g0609500	97,50	0,00E+00	1435	116	NA	Japonica	
maker-12-augustus-gene-261.0-mRNA-1	12	OsR498G1222082900.01.T01	99,42	0,00E+00	4255	Os12g0609800	100,00	0,00E+00	4318	63	NA	Japonica	
maker-12-augustus-gene-261.11-mRNA-1	12	OsR498G1222086200.01.T01	99,15	0,00E+00	1039	Os12g0610800	100,00	0,00E+00	1061	22	NA	Japonica	
maker-12-augustus-gene-261.13-mRNA-1	12	OsR498G1222086600.01.T01	97,96	4,67E-142	504	Os12g0611000	99,66	4,97E-149	526	22	NA	Japonica	
maker-12-augustus-gene-261.18-mRNA-1	12	OsR498G1222086800.01.T03	98,22	0,00E+00	1646	Os12g0611100	100,00	0,00E+00	1342	-304	Indica	Japonica	Kinase-TM
maker-12-augustus-gene-261.27-mRNA-1	12	OsR498G1222088000.01.T01	99,58	0,00E+00	2571	Os12g0611300	99,40	0,00E+00	1779	-792	Indica	Japonica	
maker-12-augustus-gene-261.9-mRNA-1	12	OsR498G1222084900.01.T05	98,46	0,00E+00	1352	Os12g0610500	100,00	0,00E+00	1406	54	NA	Japonica	
maker-12-augustus-gene-262.29-mRNA-1	12	OsR498G1222093000.01.T01	99,28	0,00E+00	1961	Os12g0612100	100,00	0,00E+00	1997	36	NA	Japonica	
maker-12-augustus-gene-262.32-mRNA-1	12	OsR498G1222093200.01.T02	99,25	0,00E+00	3048	Os12g0612300	100,00	0,00E+00	3106	58	NA	Japonica	
maker-12-augustus-gene-262.35-mRNA-1	12	OsR498G1222093800.01.T02	99,51	0,00E+00	2913	Os12g0612400	100,00	0,00E+00	2949	36	NA	Japonica	
maker-12-augustus-gene-262.39-mRNA-1	12	OsR498G1222094400.01.T06	99,68	0,00E+00	1655	Os12g0612500	100,00	0,00E+00	1678	23	NA	Japonica	
maker-12-augustus-gene-262.42-mRNA-1	12	OsR498G1222095000.01.T01	99,82	0,00E+00	2986	Os12g0612700	99,94	0,00E+00	2995	9	NA	Japonica	
maker-12-augustus-gene-263.0-mRNA-1	12	OsR498G1222097900.01.T03	99,41	0,00E+00	2397	Os12g0613500	100,00	0,00E+00	2430	33	NA	Japonica	
maker-12-augustus-gene-263.10-mRNA-1	12	OsR498G1222099300.01.T05	99,55	0,00E+00	1183	Os12g0614000	100,00	0,00E+00	1256	73	NA	Japonica	
maker-12-augustus-gene-263.13-mRNA-1	12	OsR498G1222095800.01.T01	99,85	0,00E+00	1181	Os12g0613000	99,54	0,00E+00	1170	-11	NA	Japonica	
maker-12-augustus-gene-263.17-mRNA-1	12	OsR498G1222096200.01.T01	99,50	0,00E+00	3233	Os03g0641200	85,87	0,00E+00	2043	-1190	Indica	Japonica	
maker-12-augustus-gene-263.22-mRNA-1	12	OsR498G1222097400.01.T01	99,74	0,00E+00	3486	Os12g0613250	100,00	0,00E+00	2392	-1094	Indica	Japonica	
maker-12-augustus-gene-263.25-mRNA-1	12	OsR498G1222097800.01.T03	99,66	0,00E+00	1599	Os12g0613300	100,00	0,00E+00	1613	14	NA	Japonica	
maker-12-augustus-gene-263.28-mRNA-1	12	OsR498G1222099000.01.T02	99,70	0,00E+00	4834	Os12g0613700	100,00	0,00E+00	4870	36	NA	Japonica	
maker-12-augustus-gene-263.5-mRNA-1	12	OsR498G1222098700.01.T01	98,70	0,00E+00	1888	Os12g0613600	100,00	0,00E+00	1945	57	NA	Japonica	
maker-12-augustus-gene-264.0-mRNA-1	12	OsR498G1222103900.01.T01	99,92	0,00E+00	2323	Os12g0615000	100,00	0,00E+00	4053	1730	Japonica	Japonica	Kinase-TM
maker-12-augustus-gene-264.11-mRNA-1	12	OsR498G1222105000.01.T02	100,00	0,00E+00	1889	Os12g0615400	100,00	0,00E+00	1889	0	NA	Japonica	
maker-12-augustus-gene-264.17-mRNA-1	12	OsR498G1222105600.01.T01	98,98	0,00E+00	858	Os12g0615500	100,00	0,00E+00	881	23	NA	Japonica	
maker-12-augustus-gene-264.29-mRNA-1	12	OsR498G1222107000.01.T01	100,00	0,00E+00	681	Os12g0616200	100,00	0,00E+00	681	0	NA	Japonica	
maker-12-augustus-gene-265.21-mRNA-1	12	OsR498G1222108900.01.T01	99,54	0,00E+00	4296	Os12g0616500	100,00	0,00E+00	4345	49	NA	Japonica	
maker-12-augustus-gene-265.30-mRNA-1	12	OsR498G1222111900.01.T01	98,82	0,00E+00	2086	Os12g0616900	98,99	0,00E+00	2095	9	NA	Japonica	
maker-12-augustus-gene-265.35-mRNA-1	12	OsR498G1222112800.01.T05	99,72	0,00E+00	3203	Os12g0617100	100,00	0,00E+00	3177	-26	NA	Japonica	
maker-12-augustus-gene-266.13-mRNA-1	12	OsR498G1222119800.01.T01	100,00	0,00E+00	3241	Os12g0618300	100,00	0,00E+00	3241	0	NA	NA	
maker-12-augustus-gene-266.17-mRNA-1	12	OsR498G1222120200.01.T01	100,00	0,00E+00	1099	Os12g0618400	100,00	0,00E+00	1023	-76	NA	NA	
maker-12-augustus-gene-266.2-mRNA-1	12	OsR498G1222118000.01.T02	100,00	0,00E+00	4286	Os12g0617800	100,00	0,00E+00	4286	0	NA	NA	
maker-12-augustus-gene-266.8-mRNA-1	12	OsR498G1222118200.01.T01	98,71	0,00E+00	5320	Os12g0617900	98,71	0,00E+00	5320	0	NA	NA	
maker-12-augustus-gene-267.12-mRNA-1	12	OsR498G1222127200.01.T02	100,00	0,00E+00	4080	Os12g0620600	100,00	0,00E+00	4080	0	NA	NA	
maker-12-augustus-gene-267.2-mRNA-1	12	OsR498G1222120400.01.T02	100,00	0,00E+00	1689	Os12g0618600	100,00	0,00E+00	1689	0	NA	NA	
maker-12-augustus-gene-267.7-mRNA-1	12	OsR498G1222122200.01.T01	100,00	0,00E+00	2397	Os12g0619000	100,00	0,00E+00	2397	0	NA	NA	
maker-12-augustus-gene-268.14-mRNA-1	12	OsR498G1222131900.01.T03	98,50	0,00E+00	3030	Os12g0621500	98,62	0,00E+00	3039	9	NA	Japonica	Kinase-TM
maker-12-augustus-gene-268.17-mRNA-1	12	OsR498G1222132700.01.T01	99,95	0,00E+00	3356	Os12g0621700	100,00	0,00E+00	3360	4	NA	Japonica	
maker-12-augustus-gene-268.21-mRNA-1	12	OsR498G1222132200.01.T01	99,30	0,00E+00	759	Os12g0621600	100,00	0,00E+00	769	10	NA	Japonica	
maker-12-augustus-gene-268.7-mRNA-1	12	OsR498G1222129900.01.T01	99,76	0,00E+00	746	Os12g0621100	100,00	0,00E+00	751	5	NA	Japonica	
maker-12-augustus-gene-269.18-mRNA-1	12	OsR498G1222137200.01.T02	99,04	0,00E+00	3285	Os12g0623500	100,00	0,00E+00	3366	81	NA	Japonica	
maker-12-augustus-gene-269.25-mRNA-1	12	OsR498G1222137600.01.T01	99,78	0,00E+00	1609	Os12g0623600	100,00	0,00E+00	1618	9	NA	Japonica	
maker-12-augustus-gene-269.5-mRNA-1	12	OsR498G1222136100.01.T01	99,27	0,00E+00	1686	Os12g0622900	100,00	0,00E+00	1718	32	NA	Japonica	
maker-12-augustus-gene-27.37-mRNA-1	12	OsR498G1220949100.01.T01	99,85	0,00E+00	2405	Os12g0151000	100,00	0,00E+00	2414	9	NA	Japonica	
maker-12-augustus-gene-27.39-mRNA-1	12	OsR498G1220950900.01.T03	100,00	0,00E+00	1009	Os12g0151900	100,00	0,00E+00	1009	0	NA	Japonica	
maker-12-augustus-gene-27.41-mRNA-1	12	OsR498G1220951300.01.T01	99,90	0,00E+00	1857	Os12g0152100	100,00	0,00E+00	1862	5	NA	Japonica	
maker-12-augustus-gene-27.43-mRNA-1	12	OsR498G1220948000.01.T01	99,72	0,00E+00	640	Os11g0151700	84,91	4,07E-105	380	-260	Indica	Japonica	
maker-12-augustus-gene-27.45-mRNA-1	12	OsR498G1220951200.01.T01	100,00	0,00E+00	942	Os12g0152000	100,00	0,00E+00	942	0	NA	Japonica	
maker-12-augustus-gene-270.31-mRNA-1	12	OsR498G1222138500.01.T02	99,52	0,00E+00	4101	Os12g0623900	100,00	0,00E+00	4150	49	NA	Japonica	
maker-12-augustus-gene-270.36-mRNA-1	12	OsR498G1222139100.01.T03	99,61	0,00E+00	4110	Os12g0624000	100,00	0,00E+00	4150	40	NA	Japonica	
maker-12-augustus-gene-270.42-mRNA-1	12	OsR498G1222141300.01.T01	99,79	0,00E+00	1669	Os12g0624900	99,89	0,00E+00	1673	4	NA	Japonica	
maker-12-augustus-gene-270.47-mRNA-1	12	OsR498G1222141000.01.T02	99,69	0,00E+00	2289	Os12g0624800	100,00	0,00E+00	3182	893	Japonica	Japonica	
maker-12-augustus-gene-270.49-mRNA-1	12	OsR498G1222141600.01.T01	99,76	0,00E+00	2200	Os12g0625000	100,00	0,00E+00	1743	-457	Indica	Japonica	
maker-12-augustus-gene-271.34-mRNA-1	12	OsR498G1222146600.01.T01	92,90	0,00E+00	1035	Os12g0626000	100,00	0,00E+00	1337	302	Japonica	Japonica	
maker-12-augustus-gene-271.36-mRNA-1	12	OsR498G1222146100.01.T01	99,48	0,00E+00	682	Os12g0626100	99,74	0,00E+00	687	5	NA	Japonica	
maker-12-augustus-gene-271.46-mRNA-1	12	OsR498G1222144900.01.T01	100,00	0,00E+00	2159	Os12g0626400	99,51	0,00E+00	1454	-705	Indica	Japonica	
maker-12-augustus-gene-272.10-mRNA-1	12	OsR498G1222149200.01.T02	98,08	0,00E+00	717	Os12g0628100	100,00	0,00E+00	753	36	NA	Japonica	
maker-12-augustus-gene-272.2-mRNA-1	12	OsR498G1222149900.01.T04	97,66	0,00E+00	1237	Os12g0628400	98,62	0,00E+00	1269	32	NA	Japonica	
maker-12-augustus-gene-272.5-mRNA-1	12	OsR498G1222150100.01.T01	97,67	0,00E+00	2349	Os12g0628500	98,47	0,00E+00	2398	49	NA	Japonica	
maker-12-augustus-gene-273.36-mRNA-1	12	OsR498G1222155700.01.T01	99,67	0,00E+00	1095	Os12g0631100	99,18	0,00E+00	1082	-13	NA	Indica	
maker-12-augustus-gene-273.45-mRNA-1	12	OsR498G1222159300.01.T01	97,07	0,00E+00	2192	Os12g0631600	98,69	0,00E+00	1996	-196	NA	Indica	
maker-12-augustus-gene-273.47-mRNA-1	12	OsR498G1222154600.01.T04	100,00	0,00E+00	874	Os12g0630700	98,76	0,00E+00	847	-27	NA	Indica	
maker-12-augustus-gene-273.56-mRNA-1	12	OsR498G1222159600.01.T01	99,70	0,00E+00	1773	Os12g0631800	98,37	0,00E+00	3058	1285	Japonica	Indica	
maker-12-augustus-gene-274.0-mRNA-1	12	OsR498G1222159700.01.T01	99,38	0,00E+00	864	Os12g0632000	97,14	0,00E+00	818	-46	NA	Indica	
maker-12-augustus-gene-274.2-mRNA-1	12	OsR498G1222160800.01.T01	97,48	0,00E+00	1811	Os12g0632700	97,29	0,00E+00	1802	-9	NA	NA	
maker-12-augustus-gene-278.33-mRNA-1	12	OsR498G0713382800.01.T01	99,95	0,00E+00	3610	Os12g0579000	95,47	0,00E+00	3219	-391	Indica	NA	
maker-12-augustus-gene-28.0-mRNA-1	12	OsR498G1220951300.01.T01	88,31	2,15E-135	482	Os12g0152500	100,00	0,00E+00	1012	530	Japonica	NA	
maker-12-augustus-gene-28.12-mRNA-1	12	OsR498G1220953200.01.T03	99,72	0,00E+00	1904	Os12g0152600	99,72	0,00E+00	1904	0	NA	NA	
maker-12-augustus-gene-28.14-mRNA-1	12	OsR498G1220953400.01.T02	100,00	0,00E+00	1128	Os12g0152700	100,00	0,00E+00	989	-139	Indica	NA	

maker-12-augustus-gene-28.19-mRNA-1	12	OsR498G1220954200.01.T01	99,92	0,00E+00	2223	Os12g0152900	99,92	0,00E+00	2223	0	NA	NA	
maker-12-augustus-gene-28.25-mRNA-1	12	OsR498G1220960000.01.T03	100,00	0,00E+00	1808	Os12g0154600	96,71	0,00E+00	1659	-149	NA	NA	
maker-12-augustus-gene-28.4-mRNA-1	12	OsR498G1220958500.01.T01	99,88	0,00E+00	1522	Os12g0153500	100,00	0,00E+00	1526	4	NA	NA	
maker-12-augustus-gene-285.1-mRNA-1	12	OsR498G1222106000.01.T03	97,13	2,32E-113	409	Os04g0564300	90,46	1,36E-104	379	-30	NA	NA	
maker-12-augustus-gene-288.88-mRNA-1	12	OsR498G1221807500.01.T01	98,97	0,00E+00	2220	Os12g0543600	99,12	0,00E+00	2207	-13	NA	NA	
maker-12-augustus-gene-29.0-mRNA-1	12	OsR498G1220960500.01.T01	100,00	0,00E+00	1716	Os04g0530400	98,84	0,00E+00	1666	-50	NA	Japonica	
maker-12-augustus-gene-29.14-mRNA-1	12	OsR498G1220961400.01.T01	99,86	0,00E+00	1241	Os12g0154700	100,00	0,00E+00	1245	4	NA	NA	
maker-12-augustus-gene-29.16-mRNA-1	12	OsR498G1220962000.01.T01	100,00	0,00E+00	1245	Os12g0154800	100,00	0,00E+00	1245	0	NA	NA	
maker-12-augustus-gene-29.18-mRNA-1	12	OsR498G1220962200.01.T01	100,00	0,00E+00	1245	Os12g0154900	100,00	0,00E+00	1137	-108	NA	NA	
maker-12-augustus-gene-29.20-mRNA-1	12	OsR498G1220963800.01.T02	100,00	0,00E+00	2684	Os11g0153400	81,00	0,00E+00	1433	-1251	Indica	NA	
maker-12-augustus-gene-3.107-mRNA-1	12	OsR498G1220799800.01.T01	99,74	0,00E+00	1377	Os12g0106000	100,00	0,00E+00	1386	9	NA	Japonica	
maker-12-augustus-gene-3.35-mRNA-1	12	OsR498G1220795300.01.T01	100,00	0,00E+00	1364	Os12g0104700	100,00	0,00E+00	1364	0	NA	Japonica	
maker-12-augustus-gene-3.60-mRNA-1	12	OsR498G1220796300.01.T01	97,27	0,00E+00	1116	Os11g0205500	73,73	1,27E-180	632	-484	Indica	Japonica	
maker-12-augustus-gene-3.62-mRNA-1	12	OsR498G1220799900.01.T01	100,00	0,00E+00	1016	Os12g0106100	100,00	0,00E+00	1223	207	Japonica	Japonica	
maker-12-augustus-gene-3.69-mRNA-1	12	OsR498G1220800900.01.T01	99,60	0,00E+00	2695	Os12g0106300	99,74	0,00E+00	2704	9	NA	Japonica	
maker-12-augustus-gene-3.74-mRNA-1	12	OsR498G1220797200.01.T01	99,01	0,00E+00	1244	Os11g0105400	98,02	0,00E+00	1213	-31	NA	Japonica	
maker-12-augustus-gene-3.78-mRNA-1	12	OsR498G1220797600.01.T03	99,73	0,00E+00	4633	Os11g0105750	98,37	0,00E+00	4766	133	NA	Japonica	
maker-12-augustus-gene-3.85-mRNA-1	12	OsR498G1220798000.01.T01	99,76	0,00E+00	1463	Os12g0105500	100,00	0,00E+00	1521	58	NA	Japonica	
maker-12-augustus-gene-3.90-mRNA-1	12	OsR498G1220798200.01.T01	99,71	0,00E+00	1252	Os12g0105600	100,00	0,00E+00	1261	9	NA	Japonica	
maker-12-augustus-gene-3.93-mRNA-1	12	OsR498G1119292200.01.T01	98,92	0,00E+00	2753	Os12g0105700	100,00	0,00E+00	2825	72	NA	Japonica	
maker-12-augustus-gene-30.0-mRNA-1	12	OsR498G1220970700.01.T01	100,00	0,00E+00	2030	Os12g0156400	100,00	0,00E+00	2030	0	NA	NA	
maker-12-augustus-gene-30.14-mRNA-1	12	OsR498G1220973000.01.T01	100,00	0,00E+00	1462	Os12g0157000	100,00	0,00E+00	890	-572	Indica	NA	
maker-12-augustus-gene-30.16-mRNA-1	12	OsR498G1220974400.01.T03	100,00	0,00E+00	2338	Os12g0157200	98,61	0,00E+00	2269	-69	NA	NA	
maker-12-augustus-gene-30.6-mRNA-1	12	OsR498G1220972100.01.T01	100,00	0,00E+00	3252	Os11g0155500	90,28	0,00E+00	2383	-869	Indica	NA	
maker-12-augustus-gene-30.9-mRNA-1	12	OsR498G1220975700.01.T01	100,00	0,00E+00	672	Os12g0157700	100,00	0,00E+00	1321	649	Japonica	NA	
maker-12-augustus-gene-31.0-mRNA-1	12	OsR498G1220981300.01.T01	99,53	0,00E+00	1132	Os12g0158600	100,00	0,00E+00	807	-325	Indica	Japonica	
maker-12-augustus-gene-31.5-mRNA-1	12	OsR498G1220982600.01.T01	100,00	0,00E+00	2419	Os12g0158800	100,00	0,00E+00	2419	0	NA	NA	
maker-12-augustus-gene-32.40-mRNA-1	12	OsR498G1220988300.01.T02	99,92	0,00E+00	4758	Os12g0160400	100,00	0,00E+00	4767	9	NA	Japonica	
maker-12-augustus-gene-32.47-mRNA-1	12	OsR498G1220985400.01.T01	99,91	0,00E+00	2070	Os12g0159700	100,00	0,00E+00	2978	908	Japonica	NA	
maker-12-augustus-gene-32.51-mRNA-1	12	OsR498G1220986800.01.T01	100,00	0,00E+00	1310	Os12g0160200	100,00	0,00E+00	1310	0	NA	NA	
maker-12-augustus-gene-32.53-mRNA-1	12	OsR498G1220988600.01.T02	99,85	0,00E+00	3531	Os12g0160500	99,92	0,00E+00	2254	-1277	Indica	Japonica	
maker-12-augustus-gene-33.0-mRNA-1	12	OsR498G1221000100.01.T01	98,80	0,00E+00	1750	Os12g0162500	100,00	0,00E+00	1781	31	NA	Japonica	
maker-12-augustus-gene-33.16-mRNA-1	12	OsR498G1220999600.01.T01	99,71	0,00E+00	1220	Os12g0162400	100,00	0,00E+00	1229	9	NA	Japonica	
maker-12-augustus-gene-33.3-mRNA-1	12	OsR498G1220994200.01.T01	99,79	0,00E+00	3478	Os12g0161500	100,00	0,00E+00	2874	-604	Indica	Japonica	
maker-12-augustus-gene-33.5-mRNA-1	12	OsR498G1220995600.01.T01	93,23	0,00E+00	1256	Os12g0161700	92,61	0,00E+00	1111	-145	Indica	NA	
maker-12-augustus-gene-34.12-mRNA-1	12	OsR498G1221003000.01.T01	99,70	0,00E+00	2396	Os12g0163400	100,00	0,00E+00	2414	18	NA	Japonica	
maker-12-augustus-gene-34.15-mRNA-1	12	OsR498G1221004400.01.T01	99,91	0,00E+00	2058	Os12g0163700	95,54	0,00E+00	1874	-184	NA	Japonica	
maker-12-augustus-gene-34.2-mRNA-1	12	OsR498G1221002500.01.T01	99,79	0,00E+00	3407	Os12g0163200	100,00	0,00E+00	3425	18	NA	Japonica	
maker-12-augustus-gene-34.7-mRNA-1	12	OsR498G1221002000.01.T01	99,80	0,00E+00	3516	Os12g0163000	99,29	0,00E+00	3468	-48	NA	Japonica	
maker-12-augustus-gene-34.9-mRNA-1	12	OsR498G1221002800.01.T02	99,69	0,00E+00	1718	Os12g0163300	100,00	0,00E+00	1732	14	NA	Japonica	
maker-12-augustus-gene-35.0-mRNA-1	12	OsR498G1221005700.01.T02	99,76	0,00E+00	759	Os12g0164300	100,00	2,99E-152	536	-223	Indica	Japonica	
maker-12-augustus-gene-35.23-mRNA-1	12	OsR498G1221007600.01.T01	98,00	0,00E+00	3984	Os12g0165000	98,05	0,00E+00	3989	5	NA	Japonica	
maker-12-augustus-gene-35.30-mRNA-1	12	OsR498G1221007800.01.T01	99,79	0,00E+00	2546	Os12g0165200	100,00	0,00E+00	2560	14	NA	Japonica	
maker-12-augustus-gene-35.32-mRNA-1	12	OsR498G1221008600.01.T01	99,80	0,00E+00	1837	Os12g0165700	100,00	0,00E+00	1846	9	NA	Japonica	
maker-12-augustus-gene-36.19-mRNA-1	12	OsR498G1221014600.01.T01	97,39	0,00E+00	1291	Os12g0168100	99,61	0,00E+00	1818	527	Japonica	Japonica	
maker-12-augustus-gene-36.2-mRNA-1	12	OsR498G1221014100.01.T01	99,83	0,00E+00	2102	Os12g0167900	100,00	0,00E+00	2111	9	NA	Japonica	
maker-12-augustus-gene-36.6-mRNA-1	12	OsR498G1221014300.01.T03	100,00	0,00E+00	908	Os12g0168000	100,00	0,00E+00	908	0	NA	Japonica	
maker-12-augustus-gene-36.9-mRNA-1	12	OsR498G1221016100.01.T01	99,68	0,00E+00	2255	Os12g0168400	100,00	0,00E+00	2273	18	NA	Japonica	
maker-12-augustus-gene-37.0-mRNA-1	12	OsR498G1221017900.01.T04	99,91	0,00E+00	3773	Os12g0168700	100,00	0,00E+00	3782	9	NA	Japonica	
maker-12-augustus-gene-37.11-mRNA-1	12	OsR498G1221019600.01.T03	99,89	0,00E+00	1650	Os11g0170000	82,64	0,00E+00	930	-720	Indica	Japonica	
maker-12-augustus-gene-37.18-mRNA-1	12	OsR498G1221020200.01.T02	99,59	0,00E+00	5661	Os12g0169100	100,00	0,00E+00	5719	58	NA	Japonica	
maker-12-augustus-gene-37.23-mRNA-1	12	OsR498G1221020400.01.T01	99,57	0,00E+00	2911	Os12g0169400	99,82	0,00E+00	2929	18	NA	Japonica	
maker-12-augustus-gene-37.27-mRNA-1	12	OsR498G1221021400.01.T02	99,87	0,00E+00	2843	Os12g0169800	100,00	0,00E+00	2852	9	NA	Japonica	
maker-12-augustus-gene-37.3-mRNA-1	12	OsR498G1221021100.01.T01	100,00	0,00E+00	1483	Os12g0169700	100,00	0,00E+00	1483	0	NA	Japonica	
maker-12-augustus-gene-37.34-mRNA-1	12	OsR498G1221022200.01.T02	99,67	0,00E+00	3285	Os12g0170100	100,00	0,00E+00	3312	27	NA	Japonica	
maker-12-augustus-gene-37.36-mRNA-1	12	OsR498G1221022400.01.T01	99,88	0,00E+00	1549	Os12g0170200	100,00	0,00E+00	1553	4	NA	Japonica	
maker-12-augustus-gene-37.8-mRNA-1	12	OsR498G1221019400.01.T01	99,60	0,00E+00	890	Os12g0168900	100,00	0,00E+00	899	9	NA	Japonica	
maker-12-augustus-gene-38.30-mRNA-1	12	OsR498G1221022900.01.T02	96,41	0,00E+00	2171	Os12g0170600	94,38	0,00E+00	2071	-100	NA	Japonica	
maker-12-augustus-gene-38.40-mRNA-1	12	OsR498G1221023500.01.T01	99,21	0,00E+00	1120	Os12g0170800	100,00	0,00E+00	1142	22	NA	Japonica	
maker-12-augustus-gene-38.42-mRNA-1	12	OsR498G1221024300.01.T02	99,49	0,00E+00	3161	Os12g0171200	100,00	0,00E+00	2911	-250	NA	Japonica	
maker-12-augustus-gene-38.48-mRNA-1	12	OsR498G1221024900.01.T02	98,41	0,00E+00	763	Os12g0171600	100,00	0,00E+00	991	228	Japonica	Japonica	
maker-12-augustus-gene-39.1-mRNA-1	12	OsR498G1221030100.01.T02	98,15	0,00E+00	4189	Os12g0174000	100,00	0,00E+00	4121	-68	NA	Japonica	
maker-12-augustus-gene-39.3-mRNA-1	12	OsR498G1221030300.01.T01	98,98	0,00E+00	2595	Os12g0174100	100,00	0,00E+00	2663	68	NA	Japonica	
maker-12-augustus-gene-39.8-mRNA-1	12	OsR498G1221026000.01.T02	100,00	0,00E+00	1783	Os12g0172500	100,00	0,00E+00	1783	0	NA	Japonica	
maker-12-augustus-gene-39.9-mRNA-1	12	OsR498G1221026000.01.T05	99,72	0,00E+00	1261	Os12g0172500	100,00	0,00E+00	1270	9	NA	Japonica	
maker-12-augustus-gene-4.46-mRNA-1	12	OsR498G1220801500.01.T01	99,94	0,00E+00	3094	Os12g0106500	100,00	0,00E+00	3070	-24	NA	Japonica	
maker-12-augustus-gene-4.60-mRNA-1	12	OsR498G1220804500.01.T01	99,59	0,00E+00	4298	Os11g0107700	96,64	0,00E+00	3495	-803	Indica	Japonica	Kinase-LRR-TM
maker-12-augustus-gene-4.63-mRNA-1	12	OsR498G1220805900.01.T01	99,94	0,00E+00	3069	Os12g0108000	100,00	0,00E+00	3074	5	NA	Japonica	
maker-12-augustus-gene-4.66-mRNA-1	12	OsR498G1220802000.01.T01	100,00	6,53E-105	379	Os12g0106550	100,00	2,28E-105	379	0	NA	NA	
maker-12-augustus-gene-4.68-mRNA-1	12	OsR498G1220802400.01.T03	99,56	0,00E+00	1627	Os12g0106700	100,00	0,00E+00	1916	289	Japonica	Japonica	
maker-12-augustus-gene-4.71-mRNA-1	12	OsR498G1220802600.01.T03	99,88	0,00E+00	1511	Os12g0106900	100,00	0,00E+00	1359	-152	Indica	Japonica	
maker-12-augustus-gene-40.0-mRNA-1	12	OsR498G1221036900.01.T01	99,63	0,00E+00	1435	Os12g0175400	100,00	0,00E+00	1445	10	NA	Japonica	
maker-12-augustus-gene-40.18-mRNA-1	12	OsR498G1221038000.01.T01	100,00	0,00E+00	1548	Os12g0175500	100,00	0,00E+00	1548	0	NA	Japonica	
maker-12-augustus-gene-40.20-mRNA-1	12	OsR498G1221038600.01.T03	99,79	0,00E+00	1674	Os12g0175900	100,00	0,00E+00	1683	9	NA	Japonica	
maker-12-augustus-gene-40.3-mRNA-1	12	OsR498G1221038300.01.T03	98,76	0,00E+00	2697	Os12g0175700	98,76	0,00E+00	2697	0	NA	Japonica	
maker-12-augustus-gene-40.7-mRNA-1	12	OsR498G1221038900.01.T01	98,70	0,00E+00	1205	Os12g0176200	99,56	0,00E+00	1219	14	NA	Japonica	
maker-12-augustus-gene-40.9-mRNA-1	12	OsR498G1221033800.01.T02	99,60	0,00E+00	1763	Os12g0174800	100,00	0,00E+00	1781	18	NA	Japonica	
maker-12-augustus-gene-41.34-mRNA-1	12	OsR498G1221039500.01.T01	99,80	0,00E+00	1831	Os12g0176500	100,00	0,00E+00	1840	9	NA	Japonica	
maker-12-augustus-gene-41.43-mRNA-1	12	OsR498G1221039900.01.T04	96,21	0,00E+00	4075	Os12g0176600	98,76	0,00E+00	3828	-247	NA	Japonica	
maker-12-augustus-gene-41.52-mRNA-1	12	OsR498G1221040200.01.T01	98,93	0,00E+00	2449	Os12g0176800	100,00	0,00E+00	2516	67	NA	Japonica	
maker-12-augustus-gene-41.56-mRNA-1	12	OsR498G1221041600.01.T01	99,89	0,00E+00	1673	Os12g0178100	100,00	0,00E+00	1678	5	NA	Japonica	
maker-12-augustus-gene-41.61-mRNA-1	12	OsR498G1221042000.01.T01	99,05	0,00E+00	1664	Os12g0178200	100,00	0,00E+00	1707	43	NA	Japonica	
maker-12-augustus-gene-42.0-mRNA-1	12	OsR498G1221042700.01.T01	99,66	0,00E+00	1049	Os09g0254600	87,72	0,00E+00	641	-408	Indica	Japonica	
maker-12-augustus-gene-42.14-mRNA-1	12	OsR498G1221045200.01.T03	99,36	0,00E+00	1663	Os12g0179800	100,00	0,00E+00	1691	28	NA	Japonica	
maker-12-augustus-gene-42.2-mRNA-1	12	OsR498G1221045900.01.T01	93,90	1,94E-165	581	Os12g0180400	100,00	5,56E-110	396	-185	Indica	Japonica	
maker-12-augustus-gene-42.6-mRNA-1	12	OsR498G1221044000.01.T01	98,87	0,00E+00	3103	Os12g0178633	98,67	0,00E+00	2376	-727	Indica	Japonica	
maker-12-augustus-gene-42.9-mRNA-1	12	OsR498G1221044200.01.T02	99,32	0,00E+00	3122	Os12g0178700	99,65	0,00E+00	3076	-46	NA	Japonica	
maker-12-augustus-gene-43.14-mRNA-1	12	OsR498G1221046400.01.T01	97,82	0,00E+00	1719	Os12g0181300	100,00	0,00E+00	1819	100	NA	Japonica	
maker-12-augustus-gene-43.17-mRNA-1	12	OsR498G1221047500.01.T01	99,01	0,00E+00	2493	Os12g0181600	100,00	0,00E+00	2576	83	NA	Japonica	
maker-12-augustus-gene-43.2-mRNA-1	12	OsR498G1221047500.01.T01	99,44	0,00E+00	2520	Os12g0181500	100,00	0,00E+00	2576	56	NA	Japonica	
maker-12-augustus-gene-43.4-mRNA-1	12	OsR498G0408793100.01.T02	84,13	1,97E-32	138	Os12g0180800	100,00	3,16E-56	215	77	Japonica	Japonica	
maker-12-augustus-gene-43.6-mRNA-1	12	OsR498G0409083600.01.T03	95,39	2,52E-53	208	Os12g0180833	100,00	1,22E-64	244	36	Japonica	NA	
maker-12-augustus-gene-44.0-mRNA-1	12	OsR498G1221051500.01.T01	99,85	0,00E+00	2394	Os12g0182700	100,00	0,00E+00	2403	9	NA	Japonica	
maker-12-augustus-gene-44.10-mRNA-1	12	OsR498G1221052100.01.T03	99,66	0,00E+00	2618	Os12g0183100	99,80	0,00E+00	2626	8	NA	Japonica	
maker-12-augustus-gene-44.16-mRNA-1	12	OsR498G1221050000.01.T01	98,93	0,00E+00	2464	Os12g0182200	100,00	0,00E+00	2533	69	NA	Japonica	
maker-12-augustus-gene-44.21-mRNA-1	12	OsR498G1221050600.01.T01	99,93	0,00E+00	5261	Os12g0182300	100,00	0,00E+00	5270	9	NA	Japonica	Kinase-LRR-TM
maker-12-augustus-gene-44.26-mRNA-1	12	OsR498G1221052400.01.T03	99,54	0,00E+00	1931	Os12g0183200	100,00	0,00E+00	1954	23	NA	Japonica	
maker-12-augustus-gene-44.30-mRNA-1	12	OsR498G1221052800.01.T01	99,54	0,00E+00	1920	Os12g0183300	100,00	0,00E+00	1943	23	NA	Japonica	
maker-12-augustus-gene-46.35-mRNA-1	12	OsR498G1221057100.01.T01	99,12	0,00E+00	2798	Os12g0188500	100,00	0,00E+00	2758	-40	NA	Japonica	
maker-12-augustus-gene-46.38-mRNA-1	12	OsR498G1221056000.01.T01	96,21	0,00E+00	645	Os12g0187800	100,00	0,00E+00	715	70	NA	Japonica	
maker-12-augustus-gene-46.40-mRNA-1	12	OsR498G1221058200.01.T02	99,04	0,00E+00	914	Os12g0188700	100,00	0,00E+00	937	23	NA	Japonica	
maker-12-augustus-gene-47.12-mRNA-1	12	OsR498G1221061300.01.T01	99,76	0,00E+00	2238	Os12g0189900	100,00	0,00E+00	2251	13	NA	Japonica	
maker-12-augustus-gene-47.17-mRNA-1	12	OsR498G1221061900.01.T03	99,87	0,00E+00	1365	Os12g0190100	100,00	0,00E+00	1370	5	NA	Japonica	
maker-12-augustus-gene-47.20-mRNA-1	12	OsR498G1221059800.01.T01	99,22	0,00E+00	2043	Os12g0189500	100,00	0,00E+00	2019	-24	NA	Japonica	
maker-12-augustus-gene-47.23-mRNA-1	12	OsR498G1221061600.01.T01	99,47	0,00E+00	2344	Os12g0190000	100,00	0,00E+00	2376	32	NA	Japonica	
maker-12-augustus-gene-47.26-mRNA-1	12	OsR498G1221062200.01.T01	99,76	0,00E+00	1474	Os12g0190200	100,00	0,00E+00	1483	9	NA	Japonica	
maker-12-augustus-gene-47.9-mRNA-1	12	OsR498G1221059300.01.T01	93,46	0,00E+00	689	Os12g0189400	100,00	0,00E+00	812	123	Japonica	Japonica	
maker-12-augustus-gene-48.0-mRNA-1	12	OsR498G1221064700.01.T01	97,89	0,00E+00	1627	Os01g0729600	72,72	2,36E-115	416	-1211	Indica	Japonica	
maker-12-augustus-gene-48.2-mRNA-1	12	OsR498G1221062400.01.T01	99,52	0,00E+00	3342	Os01g0729600	71,53	8,44E-129	462	-2880	Indica	Japonica	
maker-12-augustus-gene-48.4-mRNA-1	12	OsR498G1221064200.01.T06	99,32	0,00E+00	1041	Os05g0281400	83,98	2,60E-110	398	-643	Indica	Japonica	
maker-12-augustus-gene-48.6-mRNA-1	12	OsR498G1221065400.01.T01	99,59	0,00E+00	1757	Os12g0191500	100,00	0,00E+00	1775	18	NA	Japonica	
maker-12-augustus-gene-49.0-mRNA-1	12	OsR498G1221069500.01.T01	99,56	0,00E+00	2824	Os12g0192500	100,00	0,00E+00	2856	32	NA	Japonica	
maker-12-augustus-gene-5.0-mRNA-1	12	OsR498G1220808300.01.T01	99,27	0,00E+00	3637	Os12g0108500	99,27	0,00E+00	3637	0	NA	Japonica	
maker-12-augustus-gene-5.16-mRNA-1	12	OsR498G1119298800.01.T04	88,01	1,67E-128	459	Os12g0109800	100,00	2,82E-158	556	97	Japonica	Japonica	Kinase
maker-12-augustus-gene-5.18-mRNA-1	12	OsR498G1220806200.01.T01	97,72	0,00E+00	2712	Os12g0108100	97,72	0,00E+00	2712	0	NA	Japonica	Kinase-TM
maker-12-augustus-gene-5.7-mRNA-1	12	OsR498G1220808500.01.T01	99,61	0,00E+00	2260	Os12g0108600	100,00	0,00E+00	2581	321	Japonica	Japonica	
maker-12-augustus-gene-50.40-mRNA-1	12	OsR498G1221072900.01.T02	99,78	0,00E+00	1658	Os12g0193800	100,00	0,00E+00	3236	1578	Japonica	Japonica	
maker-12-augustus-gene-50.46-mRNA-1	12	OsR498G1221077900.01.T03	99,76	0,00E+00	734	Os12g0194600	100,00	0,00E+00	818	84	Japonica	Japonica	
maker-12-augustus-gene-50.58-mRNA-1	12	OsR498G1221078200.01.T01	100,00	3,25E-168	590	Os12g0194700	100,00	1,13E-168	590	0	NA	Japonica	
maker-12-augustus-gene-51.37-mRNA-1	12	OsR498G1221079100.01.T01	99,86	0,00E+00	2529	Os12g0194900	100,00	0,00E+00	2538	9	NA	Japonica	
maker-12-augustus-gene-52.0-mRNA-1	12	OsR498G1221087100.01.T02	98,77	0,00E+00	4122	Os12g0197400	100,00	0,00E+00	4253	131	NA	Japonica	
maker-12-augustus-gene-52.2-mRNA-1	12	OsR498G1221085600.01.T01	99,53	0,00E+00	1503	Os12g0196700	100,00	0,00E+00	2771	1268	Japonica	Japonica	
maker-12-augustus-gene-52.4-mRNA-1	12	OsR498G1221086600.01.T01	99,81	0,00E+00	2795	Os12g0197100	100,00	0,00E+00	2809	14	NA	Japonica	
maker-12-augustus-gene-52.6-mRNA-1	12	OsR498G1221087400.01.T01	99,04	0,00E+00	3118	Os12g0197500	100,00	0,00E+00	3195	77	NA	Japonica	
maker-12-augustus-gene-52.8-mRNA-1	12	OsR498G1221087800.01.T01	94,93	0,00E+00	2615	Os12g0197700	100,00	0,00E+00	3696	1081	Japonica	Japonica	
maker-12-augustus-gene-53.34-mRNA-1	12	OsR498G1221087900.01.T02	99,77	0,00E+00	2368	Os11g0240900	75,71	0,00E+00	910	-1458	Indica	Japonica	
maker-12-augustus-gene-53.41-mRNA-1	12	OsR498G1221092300.01.T02	99,54	0,00E+00	1926	Os09g0517200	71,22	1,30E-161	570	-1356	Indica	Japonica	CC-NBS-TM
maker-12-augustus-gene-53.42-mRNA-1	12	OsR498G1221088800.01.T01	98,12	0,00E+00	2204	Os12g0198200	100,00	0,00E+00	2284	80	NA	Japonica	
maker-12-augustus-gene-56.0-mRNA-1	12	OsR498G1221094300.01.T02	85,31	0,00E+00	728	Os12g0202700	100,00	0,00E+00	1312	584	Japonica	NA	
maker-12-augustus-gene-56.2-mRNA-1	12	OsR498G1221094300.01.T02	93,23	1,15E-156	554	Os12g0202800	99,74	0,00E+00	682	128	Japonica	Japonica	
maker-12-augustus-gene-58.16-mRNA-1	12	OsR498G1221103500.01.T01	99,52	0,00E+00	1476	Os12g0207000	98,19	0,00E+00	1435	-41	NA	Japonica	
maker-12-augustus-gene-58.18-mRNA-1	12	OsR498G1221103700.01.T01	99,32	0,00E+00	1575	Os12g0207200	100,00	0,00E+00	1602	27	NA	Japonica	
maker-12-augustus-gene-58.20-mRNA-1	12	OsR498G1221104100.01.T02	100,00	4,03E-77	288	Os12g0207300	100,00	3,11E-73	273	-15	NA	Japonica	
maker-12-augustus-gene-59.26-mRNA-1	12	OsR498G1221107500.01.T01	97,98	0,00E+00	1112	Os12g0209200	100,00	0,00E+00	1142	30	NA	Japonica	
maker-12-augustus-gene-59.28-mRNA-1	12	OsR498G1221108500.01.T01	98,34	0,00E+00	6160	Os12g0209700	99,47	0,00E+00	5731	-429	NA	Japonica	
maker-12-augustus-gene-59.35-mRNA-1	12	OsR498G1221106400.01.T02	97,06	0,00E+00	2071	Os12g0209000	100,00	0,00E+00	2013	-58	NA	Japonica	
maker-12-augustus-gene-59.41-mRNA-1	12	OsR498G1221107800.01.T02	99,63	0,00E+00	6299	Os12g0209300	99,01	0,00E+00	6227	-72	NA	Japonica	
maker-12-augustus-gene-6.0-mRNA-1	12	OsR498G1220815500.01.T04	99,44	0,00E+00	1279	Os12g0110400	100,00	0,00E+00	937	-342	Indica	Japonica	
maker-12-augustus-gene-6.10-mRNA-1	12	OsR498G1220816700.01.T02	99,73	0,00E+00	656	Os11g0112150	96,67	0,00E+00	1134	478	Japonica	Japonica	
maker-12-augustus-gene-6.14-mRNA-1	12	OsR498G1220817300.01.T01	100,00	0,00E+00	1342	Os11g0112200	95,99	0,00E+00	1297	-45	NA	NA	
maker-12-augustus-gene-6.16-mRNA-1	12	OsR498G1220818100.01.T01	99,19	0,00E+00	1751	Os12g0112000	100,00	0,00E+00	1775	24	NA	Japonica	
maker-12-augustus-gene-6.25-mRNA-1	12	OsR498G1220818700.01.T05	99,20	0,00E+00	1982	Os12g0112300	99,82	0,00E+00	2013	31	NA	Japonica	
maker-12-augustus-gene-6.39-mRNA-1	12	OsR498G1220814600.01.T02	99,63	0,00E+00	4778	Os12g0110000	100,00	0,00E+00	4819	41	NA	Japonica	
maker-12-augustus-gene-6.5-mRNA-1	12	OsR498G1220815900.01.T01	97,93	0,00E+00	827	Os11g0111800	96,14	1,72E-130	465	-362	Indica	Japonica	
maker-12-augustus-gene-6.53-mRNA-1	12	OsR498G1220815000.01.T04	96,98	0,00E+00	2510	Os12g0110100	97,18	0,00E+00	2524	14	NA	Japonica	
maker-12-augustus-gene-6.57-mRNA-1	12	OsR498G1220815400.01.T01	98,36	0,00E+00	1700	Os12g0110300	98,47	0,00E+00	1704	4	NA	Japonica	
maker-12-augustus-gene-6.63-mRNA-1	12	OsR498G1119309400.01.T01	97,59	0,00E+00	1404	Os12g0111500	100,00	0,00E+00	1494	90	NA	Japonica	
maker-12-augustus-gene-6.67-mRNA-1	12	OsR498G1119310800.01.T01	94,57	6,17E-172	604	Os12g0111900	100,00	0,00E+00	699	95	Japonica	Japonica	
maker-12-augustus-gene-60.24-mRNA-1	12	OsR498G1221109700.01.T07	100,00	0,00E+00	760	Os12g0210200	100,00	0,00E+00	760	0	NA	Japonica	
maker-12-augustus-gene-60.27-mRNA-1	12	OsR498G1221109700.01.T07	77,76	3,33E-140	499	Os12g0210300	100,00	0,00E+00	1153	654	Japonica	Japonica	
maker-12-augustus-gene-60.29-mRNA-1	12	OsR498G1221109900.01.T01	99,97	0,00E+00	5233	Os12g0210400	100,00	0,00E+00	5238	5	NA	Japonica	Kinase-LRR-TM
maker-12-augustus-gene-60.44-mRNA-1	12	OsR498G1221112500.01.T01	96,76	0,00E+00	1275	Os12g0211000	100,00	0,00E+00	1543	268	Japonica	Japonica	
maker-12-augustus-gene-60.46-mRNA-1	12	OsR498G1221113500.01.T01	96,73	0,00E+00	1214	Os12g0211100	100,00	0,00E+00	1341	127	NA	Japonica	
maker-12-augustus-gene-60.57-mRNA-1	12	OsR498G1221115300.01.T01	98,24	0,00E+00	2468	Os12g0211400	100,00	0,00E+00	2571	103	NA	Japonica	
maker-12-augustus-gene-64.30-mRNA-1	12	OsR498G1221121400.01.T01	100,00	5,82E-178	623	Os12g0217300	99,70	4,18E-168	590	-33	NA	Japonica	
maker-12-augustus-gene-67.22-mRNA-1	12	OsR498G1221121400.01.T01	99,71	2,48E-176	618	Os12g0217300	99,39	5,09E-167	585	-33	NA	NA	
maker-12-augustus-gene-69.0-mRNA-1	12	OsR498G1221135300.01.T01	99,12	0,00E+00	800	Os12g0223300	100,00	0,00E+00	818	18	NA	Japonica	
maker-12-augustus-gene-69.8-mRNA-1	12	OsR498G1221138400.01.T01	99,68	0,00E+00	2835	Os12g0224400	100,00	0,00E+00	2028	-807	Indica	Japonica	
maker-12-augustus-gene-7.0-mRNA-1	12	OsR498G1220819500.01.T01	99,87	0,00E+00	4104	Os12g0112600	100,00	0,00E+00	4118	14	NA	Japonica	
maker-12-augustus-gene-7.13-mRNA-1	12	OsR498G1220847100.01.T02	96,08	0,00E+00	2839	Os11g0114700	94,97	0,00E+00	2742	-97	NA	Japonica	
maker-12-augustus-gene-7.2-mRNA-1	12	OsR498G1220819900.01.T01	95,70	2,02E-125	449	Os11g0113300	97,78	1,12E-129	461	12	NA	Japonica	
maker-12-augustus-gene-7.4-mRNA-1	12	OsR498G1220846600.01.T01	99,78	0,00E+00	813	Os12g0114500	100,00	0,00E+00	650	-163	Indica	Japonica	
maker-12-augustus-gene-7.9-mRNA-1	12	OsR498G1220847300.01.T01	99,73	0,00E+00	3333	Os12g0114100	100,00	0,00E+00	3355	22	NA	Japonica	Kinase
maker-12-augustus-gene-70.0-mRNA-1	12	OsR498G1221142300.01.T01	99,58	0,00E+00	2104	Os12g0225200	100,00	0,00E+00	1104	-1000	Indica	Japonica	
maker-12-augustus-gene-70.11-mRNA-1	12	OsR498G1221142200.01.T01	95,41	0,00E+00	2308	Os11g0248200	71,42	6,23E-148	524	-1784	Indica	Japonica	
maker-12-augustus-gene-70.6-mRNA-1	12	OsR498G1221138800.01.T02	99,75	0,00E+00	714	Os12g0224500	100,00	0,00E+00	719	5	NA	Japonica	
maker-12-augustus-gene-71.0-mRNA-1	12	OsR498G1221145100.01.T04	99,23	0,00E+00	1840	Os12g0226900	79,69	0,00E+00	922	-918	Indica	Japonica	
maker-12-augustus-gene-71.10-mRNA-1	12	OsR498G1221146500.01.T01	99,19	9,54E-123	439	Os12g0226300	83,82	3,58E-34	143	-296	Indica	Japonica	
maker-12-augustus-gene-71.12-mRNA-1	12	OsR498G1221146700.01.T01	96,54	0,00E+00	1716	Os12g0226700	100,00	0,00E+00	1878	162	NA	Japonica	
maker-12-augustus-gene-71.16-mRNA-1	12	OsR498G1221146900.01.T01	96,99	0,00E+00	1053	Os12g0226900	100,00	0,00E+00	1139	86	NA	Japonica	
maker-12-augustus-gene-71.22-mRNA-1	12	OsR498G1221147900.01.T01	100,00	0,00E+00	839	Os12g0227500	100,00	0,00E+00	839	0	NA	Japonica	
maker-12-augustus-gene-73.29-mRNA-1	12	OsR498G1221156900.01.T01	98,23	0,00E+00	2934	Os12g0230200	98,58	0,00E+00	2961	27	NA	Japonica	
maker-12-augustus-gene-73.31-mRNA-1	12	OsR498G1221157700.01.T01	95,05	0,00E+00	1054	Os12g0230600	100,00	0,00E+00	1169	115	NA	Japonica	
maker-12-augustus-gene-73.34-mRNA-1	12	OsR498G1221155800.01.T02	99,96	0,00E+00	4974	Os12g0230100	100,00	0,00E+00	4978	4	NA	Japonica	
maker-12-augustus-gene-74.1-mRNA-1	12	OsR498G1221158500.01.T01	99,54	0,00E+00	3124	Os12g0231000	100,00	0,00E+00	3160	36	NA	Japonica	
maker-12-augustus-gene-74.9-mRNA-1	12	OsR498G1221161500.01.T01	99,06	0,00E+00	941	Os01g0218500	70,40	1,06E-57	224	-717	Indica	Japonica	
maker-12-augustus-gene-75.0-mRNA-1	12	OsR498G1221165700.01.T01	98,97	0,00E+00	1193	Os12g0233100	99,76	0,00E+00	737	-456	Indica	Japonica	
maker-12-augustus-gene-75.16-mRNA-1	12	OsR498G1221167000.01.T02	99,49	0,00E+00	2089	Os12g0233800	100,00	0,00E+00	2116	27	NA	Japonica	
maker-12-augustus-gene-75.2-mRNA-1	12	OsR498G1221165900.01.T03	93,28	0,00E+00	1029	Os12g0233300	94,10	0,00E+00	1044	15	NA	Japonica	
maker-12-augustus-gene-75.7-mRNA-1	12	OsR498G1221166100.01.T06	99,81	0,00E+00	966	Os12g0233400	100,00	0,00E+00	971	5	NA	Japonica	
maker-12-augustus-gene-76.35-mRNA-1	12	OsR498G1221171100.01.T01	99,66	0,00E+00	4217	Os11g0300600	76,19	0,00E+00	1119	-3098	Indica	Japonica	Kinase-LRR-TM
maker-12-augustus-gene-76.37-mRNA-1	12	OsR498G1221171700.01.T01	99,86	0,00E+00	2599	Os12g0235800	100,00	0,00E+00	2608	9	NA	Japonica	
maker-12-augustus-gene-77.0-mRNA-1	12	OsR498G1221174100.01.T01	99,59	0,00E+00	1297	Os12g0236400	100,00	0,00E+00	1310	13	NA	Japonica	
maker-12-augustus-gene-77.13-mRNA-1	12	OsR498G1221175000.01.T02	100,00	0,00E+00	1635	Os12g0236500	100,00	0,00E+00	1635	0	NA	Japonica	
maker-12-augustus-gene-77.8-mRNA-1	12	OsR498G1221179700.01.T01	96,04	0,00E+00	1377	Os12g0236900	97,36	0,00E+00	1420	43	NA	Japonica	
maker-12-augustus-gene-78.2-mRNA-1	12	OsR498G1221183200.01.T01	99,90	0,00E+00	5397	Os12g0238000	100,00	0,00E+00	5411	14	NA	Japonica	
maker-12-augustus-gene-78.9-mRNA-1	12	OsR498G1221184600.01.T01	98,13	0,00E+00	4268	Os12g0238100	98,30	0,00E+00	4286	18	NA	Japonica	
maker-12-augustus-gene-79.16-mRNA-1	12	OsR498G1221185500.01.T02	99,91	0,00E+00	2056	Os12g0238900	97,18	0,00E+00	2716	660	Japonica	Japonica	
maker-12-augustus-gene-79.18-mRNA-1	12	OsR498G1221186200.01.T01	99,86	0,00E+00	6335	Os12g0239000	100,00	0,00E+00	6358	23	NA	Japonica	
maker-12-augustus-gene-79.23-mRNA-1	12	OsR498G1221186800.01.T03	99,70	0,00E+00	5437	Os12g0239200	100,00	0,00E+00	3487	-1950	Indica	Japonica	
maker-12-augustus-gene-8.47-mRNA-1	12	OsR498G1220842800.01.T03	100,00	0,00E+00	684	Os12g0115900	100,00	0,00E+00	684	0	NA	Japonica	
maker-12-augustus-gene-8.56-mRNA-1	12	OsR498G1220843100.01.T03	99,37	0,00E+00	1133	Os12g0115700	99,53	0,00E+00	1138	5	NA	Japonica	
maker-12-augustus-gene-8.63-mRNA-1	12	OsR498G1220842300.01.T01	99,78	0,00E+00	3323	Os12g0116000	100,00	0,00E+00	3341	18	NA	Japonica	
maker-12-augustus-gene-8.67-mRNA-1	12	OsR498G1220841100.01.T01	100,00	0,00E+00	1218	Os11g0116900	98,02	0,00E+00	1293	75	NA	Japonica	
maker-12-augustus-gene-8.72-mRNA-1	12	OsR498G1220839300.01.T01	99,59	0,00E+00	1725	Os11g0117500	94,36	0,00E+00	1508	-217	Indica	Japonica	
maker-12-augustus-gene-8.75-mRNA-1	12	OsR498G1220838500.01.T02	100,00	0,00E+00	1819	Os11g0117600	88,32	0,00E+00	1326	-493	Indica	Japonica	
maker-12-augustus-gene-80.25-mRNA-1	12	OsR498G1221191800.01.T01	99,82	0,00E+00	2042	Os12g0240900	100,00	0,00E+00	2035	-7	NA	Japonica	
maker-12-augustus-gene-81.28-mRNA-1	12	OsR498G1221198900.01.T04	96,40	0,00E+00	1531	Os12g0242700	95,33	0,00E+00	1150	-381	Indica	Japonica	
maker-12-augustus-gene-81.31-mRNA-1	12	OsR498G1221199400.01.T01	99,75	0,00E+00	1415	Os12g0242900	100,00	0,00E+00	1424	9	NA	Japonica	
maker-12-augustus-gene-82.10-mRNA-1	12	OsR498G1221201400.01.T01	99,01	0,00E+00	891	Os12g0244500	100,00	0,00E+00	910	19	NA	Japonica	
maker-12-augustus-gene-82.2-mRNA-1	12	OsR498G1221200700.01.T01	99,24	0,00E+00	3722	Os12g0244100	100,00	0,00E+00	3782	60	NA	Japonica	
maker-12-augustus-gene-82.6-mRNA-1	12	OsR498G1221201100.01.T01	98,21	0,00E+00	2312	Os12g0244400	100,00	0,00E+00	2419	107	NA	Japonica	
maker-12-augustus-gene-82.8-mRNA-1	12	OsR498G1221203300.01.T01	93,87	0,00E+00	2230	Os12g0245100	100,00	0,00E+00	2587	357	Japonica	Japonica	
maker-12-augustus-gene-84.0-mRNA-1	12	OsR498G0407486900.01.T01	85,78	0,00E+00	2070	Os12g0246700	100,00	0,00E+00	5081	3011	Japonica	NA	CC-NBS-TM
maker-12-augustus-gene-85.2-mRNA-1	12	OsR498G1221215500.01.T01	99,11	0,00E+00	794	Os03g0853200	70,82	7,79E-113	407	-387	Indica	Japonica	
maker-12-augustus-gene-85.4-mRNA-1	12	OsR498G1221217600.01.T01	90,07	0,00E+00	833	Os12g0248000	100,00	0,00E+00	1975	1142	Japonica	Japonica	Kinase-TM
maker-12-augustus-gene-86.0-mRNA-1	12	OsR498G1221216600.01.T01	99,70	0,00E+00	1767	Os12g0249900	81,03	0,00E+00	864	-903	Indica	Japonica	Kinase-TM
maker-12-augustus-gene-87.0-mRNA-1	12	OsR498G0305726100.01.T03	91,28	1,85E-63	243	Os12g0535400	90,39	1,24E-85	315	72	Japonica	NA	
maker-12-augustus-gene-87.1-mRNA-1	12	OsR498G1221763400.01.T07	96,51	2,51E-118	425	Os12g0535400	96,51	8,73E-119	425	0	NA	NA	
maker-12-augustus-gene-9.0-mRNA-1	12	OsR498G1220836800.01.T01	99,38	0,00E+00	1434	Os12g0117100	100,00	0,00E+00	1456	22	NA	Japonica	
maker-12-augustus-gene-9.18-mRNA-1	12	OsR498G1220832200.01.T02	98,05	0,00E+00	2653	Os12g0118900	100,00	0,00E+00	1662	-991	Indica	Japonica	
maker-12-augustus-gene-9.21-mRNA-1	12	OsR498G1220832200.01.T01	99,68	0,00E+00	2811	Os12g0119000	100,00	0,00E+00	2830	19	NA	Japonica	

maker-12-augustus-gene-9.23-mRNA-1	12	OsR498G1119333300.01.T02	95,77	0,00E+00	1526	Os12g0119700	94,49	0,00E+00	1070	-456	Indica	Japonica	
maker-12-augustus-gene-9.25-mRNA-1	12	OsR498G1220831200.01.T01	100,00	5,82E-178	623	Os12g0119500	100,00	2,02E-178	623	0	NA	Japonica	
maker-12-augustus-gene-9.27-mRNA-1	12	OsR498G1220837700.01.T01	99,92	0,00E+00	4677	Os11g0117700	97,84	0,00E+00	4430	-247	NA	Japonica	
maker-12-augustus-gene-9.29-mRNA-1	12	OsR498G1220836100.01.T01	99,91	0,00E+00	2068	Os12g0117250	100,00	0,00E+00	1934	-134	NA	Japonica	
maker-12-augustus-gene-9.32-mRNA-1	12	OsR498G1220834900.01.T01	99,90	0,00E+00	3519	Os12g0117600	100,00	0,00E+00	3528	9	NA	Japonica	
maker-12-augustus-gene-9.34-mRNA-1	12	OsR498G1119331000.01.T02	99,65	0,00E+00	1547	Os12g0117800	100,00	0,00E+00	1561	14	NA	Japonica	
maker-12-augustus-gene-9.35-mRNA-1	12	OsR498G1220834100.01.T01	94,76	0,00E+00	1893	Os11g0118800	93,43	0,00E+00	1804	-89	NA	Japonica	
maker-12-augustus-gene-9.4-mRNA-1	12	OsR498G1220836400.01.T03	100,00	3,00E-113	408	Os12g0117200	100,00	1,04E-113	408	0	NA	Japonica	
maker-12-augustus-gene-9.8-mRNA-1	12	OsR498G0713610100.01.T02	95,26	2,46E-81	303	Os12g0118400	97,48	3,40E-131	467	164	Japonica	NA	
maker-12-augustus-gene-9.9-mRNA-1	12	OsR498G1119332100.01.T03	94,96	0,00E+00	2186	Os12g0118400	100,00	0,00E+00	2549	363	Japonica	Japonica	
maker-12-augustus-gene-90.37-mRNA-1	12	OsR498G1221220100.01.T01	98,34	0,00E+00	2291	Os12g0256000	100,00	0,00E+00	2392	101	NA	Japonica	
maker-12-augustus-gene-90.39-mRNA-1	12	OsR498G1221220300.01.T01	94,40	0,00E+00	2734	Os12g0256300	100,00	0,00E+00	3113	379	Japonica	Japonica	
maker-12-augustus-gene-90.50-mRNA-1	12	OsR498G1221222200.01.T03	99,64	0,00E+00	1990	Os12g0257000	99,73	0,00E+00	1994	4	NA	Japonica	
maker-12-augustus-gene-91.0-mRNA-1	12	OsR498G1221223500.01.T01	98,03	0,00E+00	3145	Os12g0259800	81,48	0,00E+00	1684	-1461	Indica	Japonica	
maker-12-augustus-gene-91.3-mRNA-1	12	OsR498G1221224100.01.T01	94,61	0,00E+00	2422	Os12g0257900	100,00	0,00E+00	2399	-23	NA	Japonica	Kinase-TM
maker-12-augustus-gene-91.5-mRNA-1	12	OsR498G1221224400.01.T03	99,73	0,00E+00	1330	Os12g0258200	100,00	0,00E+00	1339	9	NA	Japonica	
maker-12-augustus-gene-92.32-mRNA-1	12	OsR498G1221227900.01.T01	99,61	0,00E+00	3210	Os12g0258700	100,00	0,00E+00	3139	-71	NA	Japonica	
maker-12-augustus-gene-93.0-mRNA-1	12	OsR498G1221230900.01.T01	99,71	0,00E+00	3132	Os12g0259800	100,00	0,00E+00	3128	-4	NA	Japonica	
maker-12-augustus-gene-93.6-mRNA-1	12	OsR498G1221233200.01.T01	99,67	0,00E+00	1643	Os12g0260500	100,00	0,00E+00	1656	13	NA	Japonica	
maker-12-augustus-gene-94.20-mRNA-1	12	OsR498G1221234700.01.T01	98,23	4,96E-166	583	Os12g0551200	77,20	1,17E-35	149	-434	Indica	Japonica	
maker-12-augustus-gene-94.23-mRNA-1	12	OsR498G1221234900.01.T01	98,73	0,00E+00	2750	Os12g0261300	100,00	0,00E+00	2812	62	NA	Japonica	
maker-12-augustus-gene-95.32-mRNA-1	12	OsR498G1221238700.01.T01	99,89	0,00E+00	1576	Os12g0262700	100,00	0,00E+00	1581	5	NA	Japonica	
maker-12-augustus-gene-95.36-mRNA-1	12	OsR498G1221239800.01.T02	99,88	0,00E+00	2919	Os12g0263000	100,00	0,00E+00	2262	-657	Indica	Japonica	
maker-12-augustus-gene-95.46-mRNA-1	12	OsR498G1221240800.01.T01	99,94	0,00E+00	2853	Os12g0263100	100,00	0,00E+00	2857	4	NA	Japonica	
maker-12-augustus-gene-95.48-mRNA-1	12	OsR498G1221241000.01.T01	94,71	0,00E+00	816	Os12g0265100	86,10	1,02E-120	433	-383	Indica	Japonica	
maker-12-augustus-gene-95.50-mRNA-1	12	OsR498G1221241000.01.T03	98,27	0,00E+00	1096	Os12g0263200	100,00	0,00E+00	1148	52	NA	Japonica	
maker-12-augustus-gene-95.52-mRNA-1	12	OsR498G1221242000.01.T03	90,97	0,00E+00	914	Os12g0263200	84,79	0,00E+00	738	-176	Indica	Japonica	
maker-12-augustus-gene-96.18-mRNA-1	12	OsR498G1221249000.01.T01	100,00	0,00E+00	1705	Os12g0265100	100,00	0,00E+00	1705	0	NA	Japonica	
maker-12-augustus-gene-97.1-mRNA-1	12	OsR498G1221250200.01.T04	95,61	0,00E+00	1865	Os12g0265600	100,00	0,00E+00	1339	-526	Indica	Japonica	
maker-12-augustus-gene-97.10-mRNA-1	12	OsR498G1221252200.01.T03	99,46	0,00E+00	1979	Os12g0266200	99,91	0,00E+00	2000	21	NA	Japonica	
maker-12-augustus-gene-97.8-mRNA-1	12	OsR498G1221251800.01.T01	99,71	0,00E+00	1838	Os12g0265900	100,00	0,00E+00	2760	922	Japonica	Japonica	CC-Kinase-TM
maker-12-augustus-gene-98.0-mRNA-1	12	OsR498G0714282700.01.T02	75,00	6,20E-32	139	Os12g0267100	100,00	1,65E-103	374	235	Japonica	Japonica	
maker-12-augustus-gene-98.2-mRNA-1	12	OsR498G1221257500.01.T01	99,31	0,00E+00	2567	Os12g0267200	100,00	0,00E+00	4257	1690	Japonica	Japonica	
maker-12-augustus-gene-98.6-mRNA-1	12	OsR498G1221260700.01.T01	98,90	0,00E+00	959	Os12g0267900	100,00	0,00E+00	985	26	NA	Japonica	
