# Supplementary material for: Development and Genome-Wide Analysis of a Blast-Resistant japonica Rice Variety
Source: Plants (Basel). 2023 Oct 11;12(20):3536. doi: 10.3390/plants12203536 (PMC10667994; doi:10.3390/plants12203536)
Supplement: Supplementary file 1 [file plants-12-03536-s001.zip › Supplementary Material Escol_Suppl Table S4 R_defense-related genes.pdf]

Supplemental Table S4. List of Resistance (R) genes and defense-related genes identified in the genome of COPSEMAR9. The DRAGO2 pipeline was used on the predicted proteome to identify and classify Resistance (R) and defense regulator genes. In red, blast resistance (*Pi*) genes. CC-NBS-LRR, Coiled-Coil-Nucleotide-Binding Site-Leucine-Rich Repeat; TM, transmembrane.

Locus	Assignment Variants	Assignment Similarity	Resistance Gene Class	Japonica Locus	Indica Locus	Description
maker-8-augustus-gene-60.8-mRNA-1	Indica	Indica	CC-Kinase-LRR-TM	Os08g0201700	OsR498G0815385700.01.T13	Non-specific serine/threonine protein kinase
augustus_masked-3-processed-gene-134.57-mRNA-1	Japonica	NA	CC-Kinase-LRR-TM	Os10g0531700	OsR498G1019085800.01.T01	LRR receptor-like serine/threonine-protein kinase
maker-3-augustus-gene-102.3-mRNA-1	Japonica	Indica	CC-Kinase-LRR-TM	Os03g0297800	OsR498G0305757700.01.T01	Probably inactive leucine-rich repeat receptor-like protein kinase IMK2
maker-4-augustus-gene-70.0-mRNA-1	Japonica	Japonica	CC-Kinase-LRR-TM	Os04g0291900	OsR498G0510192800.01.T08	Non-specific serine/threonine protein kinase
maker-9-augustus-gene-154.29-mRNA-1	Japonica	Japonica	CC-Kinase-LRR-TM	Os09g0423000	OsR498G0917480300.01.T01	putative leucine-rich repeat receptor-like serine/threonine-protein kinase At2g24130
maker-8-augustus-gene-60.8-mRNA-1	NA	Indica	CC-Kinase-LRR-TM	Os08g0201700	OsR498G0815385700.01.T13	Non-specific serine/threonine protein kinase
maker-9-augustus-gene-154.29-mRNA-1	NA	Japonica	CC-Kinase-LRR-TM	Os09g0423000	OsR498G0917480300.01.T01	putative leucine-rich repeat receptor-like serine/threonine-protein kinase At2g24130
augustus-1-processed-gene-8.0-mRNA-1	Indica	Indica	CC-Kinase-TM	Os01g0113350	OsR498G0100044500.01.T01	Putative receptor serine/threonine kinase PR5K
maker-10-augustus-gene-1.0-mRNA-1	Indica	Indica	CC-Kinase-TM	Os10g0100500	OsR498G1017994900.01.T04	E3 ubiquitin ligase
maker-10-augustus-gene-61.19-mRNA-1	Indica	NA	CC-Kinase-TM	Os03g0251700	OsR498G0305588100.01.T01	Receptor-like serine/threonine-protein kinase
maker-11-augustus-gene-247.0-mRNA-1	Indica	NA	CC-Kinase-TM			S-receptor kinase-like protein 1-like
maker-4-augustus-gene-125.39-mRNA-1	Indica	NA	CC-Kinase-TM		OsR498G0408339000.01.T01	Jacalin-like lectin domain containing protein
augustus-1-processed-gene-151.21-mRNA-1	Japonica	Indica	CC-Kinase-TM	Os01g0364400	OsR498G0100957400.01.T01	Wall-associated receptor kinase 3
augustus-11-processed-gene-8.94-mRNA-1	Japonica	NA	CC-Kinase-TM	Os11g0132900	OsR498G1119371300.01.T01	Non-specific serine/threonine protein kinase
augustus-2-processed-gene-257.1-mRNA-1	Japonica	Japonica	CC-Kinase-TM	Os02g0625300	OsR498G0204350900.01.T01	Phosphoenolpyruvate carboxylase kinase
augustus-8-processed-gene-86.28-mRNA-1	Japonica	Japonica	CC-Kinase-TM	Os08g0230800	OsR498G0815488100.01.T01	Non-specific serine/threonine protein kinase
augustus_masked-11-processed-gene-244.4-mRNA-1	Japonica	NA	CC-Kinase-TM	Os11g0664000	OsR498G1120691000.01.T01	L-type lectin-domain containing receptor kinase IX.1
augustus_masked-3-processed-gene-142.65-mRNA-1	Japonica	NA	CC-Kinase-TM	Os10g0548300	OsR498G1019140600.01.T06	E3 ubiquitin ligase
augustus_masked-3-processed-gene-405.19-mRNA-1	Japonica	NA	CC-Kinase-TM	Os03g0678100	OsR498G0306787500.01.T01	Serinethreonine-protein kinase blus1
augustus_masked-5-processed-gene-151.20-mRNA-1	Japonica	Japonica	CC-Kinase-TM	Os05g0317900	OsR498G0510480600.01.T01	Receptor-like protein kinase FERONIA
augustus_masked-5-processed-gene-168.18-mRNA-1	Japonica	NA	CC-Kinase-TM		OsR498G0510577100.01.T01	Receptor-like protein kinase FERONIA
augustus_masked-5-processed-gene-197.3-mRNA-1	Japonica	NA	CC-Kinase-TM	Os05g0387600		serine/threonine-protein kinase-like protein At3g51990
maker-1-augustus-gene-232.7-mRNA-1	Japonica	NA	CC-Kinase-TM	Os01g0581400	OsR498G0101403700.01.T01	Lycopene epsilon cyclase, chloroplastic
maker-1-augustus-gene-309.56-mRNA-1	Japonica	Indica	CC-Kinase-TM	Os01g0725800	OsR498G0101926500.01.T05	Protein kinase-like domain, Coatomer beta' subunit (COPB2), WD40 repeat, conserved site
maker-11-augustus-gene-22.37-mRNA-1	Japonica	NA	CC-Kinase-TM	Os11g0160300	OsR498G1119462200.01.T01	Probable serine/threonine-protein kinase WNK6
maker-11-augustus-gene-244.53-mRNA-1	Japonica	Japonica	CC-Kinase-TM	Os11g0666200	OsR498G1120691200.01.T01	Protein kinase domain-containing protein
maker-11-augustus-gene-250.32-mRNA-1	Japonica	Indica	CC-Kinase-TM	Os12g0562500	OsR498G1120719100.01.T01	Serine/threonine-protein kinase CTR1
maker-11-augustus-gene-52.53-mRNA-1	Japonica	NA	CC-Kinase-TM	Os11g0274300	OsR498G1119797800.01.T03	Protein kinase domain containing protein, expressed
maker-12-augustus-gene-97.8-mRNA-1	Japonica	Japonica	CC-Kinase-TM	Os12g0265900	OsR498G1221251800.01.T01	Wall-associated receptor kinase 2
maker-2-augustus-gene-216.2-mRNA-1	Japonica	NA	CC-Kinase-TM	Os02g0559300	OsR498G0204120100.01.T02	Putative serine/threonine-protein kinase
maker-2-augustus-gene-278.3-mRNA-1	Japonica	NA	CC-Kinase-TM		OsR498G0204478300.01.T02	E3 ubiquitin ligase
maker-2-augustus-gene-278.7-mRNA-1	Japonica	NA	CC-Kinase-TM		OsR498G0204478700.01.T01	E3 ubiquitin ligase
maker-2-augustus-gene-319.34-mRNA-1	Japonica	Indica	CC-Kinase-TM	Os02g0743500	OsR498G0204756500.01.T01	Serine/threonine-protein kinase CTR1
maker-2-augustus-gene-371.0-mRNA-1	Japonica	NA	CC-Kinase-TM	Os02g0787200	OsR498G0204905900.01.T01	Serine threonine kinase
maker-2-augustus-gene-373.1-mRNA-1	Japonica	Indica	CC-Kinase-TM	Os02g0791700	OsR498G0204919500.01.T02	Serine/threonine-protein kinase BLUS1
maker-2-augustus-gene-67.47-mRNA-1	Japonica	Indica	CC-Kinase-TM	Os02g0218600	OsR498G0203227300.01.T01	E3 ubiquitin ligase
maker-2-augustus-gene-67.58-mRNA-1	Japonica	NA	CC-Kinase-TM	Os02g0218400	OsR498G0203227000.01.T01	U-box domain-containing protein 52
maker-3-augustus-gene-143.72-mRNA-1	Japonica	NA	CC-Kinase-TM	Os10g0548700	OsR498G1019141000.01.T03	E3 ubiquitin ligase
maker-3-augustus-gene-214.43-mRNA-1	Japonica	Japonica	CC-Kinase-TM	Os03g0424000	OsR498G0306240800.01.T02	E3 ubiquitin ligase
maker-3-augustus-gene-446.22-mRNA-1	Japonica	NA	CC-Kinase-TM	Os03g0749800	OsR498G0307052200.01.T02	Serine/threonine-protein kinase TOUSLED
maker-3-augustus-gene-492.41-mRNA-1	Japonica	NA	CC-Kinase-TM	Os03g0841100	OsR498G0307348200.01.T01	Wall-associated receptor kinase 3
maker-3-augustus-gene-97.64-mRNA-1	Japonica	NA	CC-Kinase-TM	Os03g0289100	OsR498G0305728500.01.T03	Serine/threonine protein kinase OSK4
maker-4-augustus-gene-125.39-mRNA-1	Japonica	NA	CC-Kinase-TM		OsR498G0408339000.01.T01	Jacalin-like lectin domain containing protein
maker-4-augustus-gene-277.10-mRNA-1	Japonica	Japonica	CC-Kinase-TM	Os04g0651500	OsR498G0409395900.01.T01	Wall-associated receptor kinase 3
maker-5-augustus-gene-152.30-mRNA-1	Japonica	Indica	CC-Kinase-TM	Os05g0318100	OsR498G0510478100.01.T01	Wall-associated receptor kinase-like protein 20
maker-6-augustus-gene-165.11-mRNA-1	Japonica	NA	CC-Kinase-TM	Os06g0486400	OsR498G0612365700.01.T01	Kinase superfamily protein isoform 1
maker-6-augustus-gene-47.64-mRNA-1	Japonica	Indica	CC-Kinase-TM	Os02g0787200	OsR498G0611757800.01.T01	U-box domain-containing protein 35-like isoform X2
maker-7-augustus-gene-194.31-mRNA-1	Japonica	NA	CC-Kinase-TM	Os07g0515100	OsR498G0714421000.01.T03	Calcium-dependent protein kinase
maker-7-augustus-gene-207.55-mRNA-1	Japonica	Indica	CC-Kinase-TM	Os07g0538400	OsR498G0714507100.01.T01	Cysteine-rich receptor-like protein kinase 6
maker-8-augustus-gene-16.13-mRNA-1	Japonica	Indica	CC-Kinase-TM	Os08g0125800	OsR498G0815100800.01.T01	Putative receptor-type protein kinase LRK1
maker-8-augustus-gene-7.53-mRNA-1	Japonica	NA	CC-Kinase-TM	Os08g0112500	OsR498G0815061000.01.T02	Putative serine/threonine-protein kinase
maker-8-augustus-gene-83.40-mRNA-1	Japonica	NA	CC-Kinase-TM	Os08g0230800		Non-specific serine/threonine protein kinase
maker-9-augustus-gene-131.0-mRNA-1	Japonica	NA	CC-Kinase-TM	Os09g0383300	OsR498G0917329300.01.T03	Mitogen-activated protein kinase kinase kinase NPK1
maker-9-augustus-gene-165.57-mRNA-1	Japonica	NA	CC-Kinase-TM	Os09g0442100	OsR498G0917547800.01.T01	Serine/threonine-protein kinase RIPK
maker-9-augustus-gene-166.8-mRNA-1	Japonica	NA	CC-Kinase-TM			Serine-threonine protein kinase
maker-9-augustus-gene-224.23-mRNA-1	Japonica	NA	CC-Kinase-TM	Os09g0561600	OsR498G0917952300.01.T01	Wall-associated receptor kinase 3
maker-9-augustus-gene-228.28-mRNA-1	Japonica	NA	CC-Kinase-TM	Os09g0569800	OsR498G0917973100.01.T01	E3 ubiquitin ligase
maker-9-augustus-gene-229.0-mRNA-1	Japonica	Indica	CC-Kinase-TM	Os09g0570000	OsR498G0917973500.01.T05	E3 ubiquitin ligase
maker-9-augustus-gene-229.4-mRNA-1	Japonica	Indica	CC-Kinase-TM	Os09g0570100	OsR498G0917973700.01.T02	U-box domain-containing protein 33
augustus-8-processed-gene-86.28-mRNA-1	NA	Japonica	CC-Kinase-TM	Os08g0230800	OsR498G0815488100.01.T01	Non-specific serine/threonine protein kinase
augustus_masked-1-processed-gene-502.62-mRNA-1	NA	NA	CC-Kinase-TM	0	OsR498G0102752000.01.T01	Non-specific serine/threonine protein kinase
augustus_masked-11-processed-gene-244.4-mRNA-1	NA	NA	CC-Kinase-TM	Os11g0664000	OsR498G1120691000.01.T01	L-type lectin-domain containing receptor kinase IX.1
augustus_masked-4-processed-gene-127.3-mRNA-1	NA	Indica	CC-Kinase-TM	Os04g0371700	OsR498G0408350000.01.T01	Wall-associated receptor kinase 3
augustus_masked-5-processed-gene-168.18-mRNA-1	NA	NA	CC-Kinase-TM	0	OsR498G0510577100.01.T01	Receptor-like protein kinase FERONIA
maker-1-augustus-gene-500.6-mRNA-1	NA	NA	CC-Kinase-TM	Os01g0958000	OsR498G0102734300.01.T01	Cyclin-dependent kinase
maker-10-augustus-gene-61.19-mRNA-1	NA	NA	CC-Kinase-TM	Os03g0251700	OsR498G0305588100.01.T01	Receptor-like serine/threonine-protein kinase
maker-11-augustus-gene-243.0-mRNA-1	NA	NA	CC-Kinase-TM	Os11g0664000	OsR498G1120691000.01.T01	Serine/threonine protein kinase (Fragment)
maker-11-augustus-gene-243.1-mRNA-1	NA	Indica	CC-Kinase-TM	Os11g0664000	OsR498G1120691200.01.T01	Putative receptor-like protein kinase FERONIA (Fragment)
maker-11-augustus-gene-247.0-mRNA-1	NA	NA	CC-Kinase-TM			S-receptor kinase-like protein 1-like
maker-11-augustus-gene-250.32-mRNA-1	NA	Indica	CC-Kinase-TM	Os12g0562500	OsR498G1120719100.01.T01	Serine/threonine-protein kinase CTR1
maker-2-augustus-gene-233.0-mRNA-1	NA	Indica	CC-Kinase-TM	Os02g0591900	OsR498G0204217300.01.T05	Serine/threonine protein kinase
maker-2-augustus-gene-29.8-mRNA-1	NA	NA	CC-Kinase-TM	Os02g0152300	OsR498G0202986300.01.T01	Cyclophilin-like peptidyl-prolyl cis-trans isomerase family protein isoform 1
maker-3-augustus-gene-117.71-mRNA-1	NA	NA	CC-Kinase-TM	Os03g0101200	OsR498G0305069200.01.T01	E3 ubiquitin ligase
maker-3-augustus-gene-121.13-mRNA-1	NA	NA	CC-Kinase-TM	Os10g0561500	OsR498G1019196900.01.T01	E3 ubiquitin ligase
maker-3-augustus-gene-16.62-mRNA-1	NA	NA	CC-Kinase-TM	Os03g0132800	OsR498G0305179900.01.T01	serine/threonine-protein kinase BSK1
maker-6-augustus-gene-21.9-mRNA-1	NA	NA	CC-Kinase-TM	Os06g0140800	OsR498G0611602200.01.T02	E3 ubiquitin ligase
maker-6-augustus-gene-32.42-mRNA-1	NA	NA	CC-Kinase-TM	Os06g0163000	OsR498G0611673200.01.T03	E3 ubiquitin ligase
maker-6-augustus-gene-70.35-mRNA-1	NA	Indica	CC-Kinase-TM	Os06g0232100	OsR498G0611940400.01.T04	Serine/threonine-protein kinase CTR1
maker-7-augustus-gene-194.31-mRNA-1	NA	NA	CC-Kinase-TM	Os07g0515100	OsR498G0714421000.01.T03	Calcium-dependent protein kinase
maker-8-augustus-gene-215.52-mRNA-1	Indica	NA	CC-Kinase-TM Pi33	Os08g0421800	OsR498G0816100400.01.T01	Pyricularia oryzae resistance-33(t) Pi33 R gene
maker-3-augustus-gene-127.37-mRNA-1	Japonica	NA	CC-LRR	Os10g0572300	OsR498G1019235500.01.T03	Plant intracellular Ras-group-related LRR protein 5
maker-4-augustus-gene-185.12-mRNA-1	Japonica	Indica	CC-LRR	Os04g0476700	OsR498G0408763900.01.T01	Plant intracellular Ras-group-related LRR protein 3
maker-11-augustus-gene-228.6-mRNA-1	Japonica	NA	CC-LRR-NBS	Os11g0640600	OsR498G1120613000.01.T01	Blast resistance protein Pi54 variant (Fragment)
maker-9-augustus-gene-202.37-mRNA-1	Japonica	NA	CC-LRR-NBS		OsR498G0917802800.01.T01	Pib variant protein (Fragment)
augustus-11-processed-gene-229.9-mRNA-1	NA	NA	CC-LRR-NBS	Os11g0640700	OsR498G1120614900.01.T02	Leucine Rich Repeat family protein, expressed
augustus_masked-11-processed-gene-83.18-mRNA-1	Indica	NA	CC-LRR-NBS-TM			NBS-LRR type protein (Fragment)
maker-10-augustus-gene-144.32-mRNA-1	Indica	NA	CC-LRR-NBS-TM	Os04g0111900	OsR498G0407452000.01.T01	Putative NB-ARC domain-containing protein (Fragment)
maker-11-augustus-gene-248.27-mRNA-1	Indica	NA	CC-LRR-NBS-TM	Os11g0677101	OsR498G1120709900.01.T01	Putative nucleotide-binding leucine-rich-repeat protein 1
maker-11-augustus-gene-83.3-mRNA-1	Indica	Japonica	CC-LRR-NBS-TM	Os11g0227100	OsR498G1119648000.01.T02	NBS-LRR type resistance protein (Fragment)
maker-8-augustus-gene-210.50-mRNA-1	Indica	NA	CC-LRR-NBS-TM		OsR498G0816075400.01.T01	Disease resistance protein RPM1
maker-8-augustus-gene-53.54-mRNA-1	Indica	Japonica	CC-LRR-NBS-TM	Os08g0190300	OsR498G0815343400.01.T01	
augustus-11-processed-gene-180.33-mRNA-1	Japonica	Japonica	CC-LRR-NBS-TM	Os11g0551300	OsR498G1120334900.01.T01	Disease resistance protein RPM1
augustus-11-processed-gene-49.13-mRNA-1	Japonica	NA	CC-LRR-NBS-TM	Os11g0270500	OsR498G1119789300.01.T01	NB-ARC domain, putative
augustus-12-processed-gene-182.13-mRNA-1	Japonica	Indica	CC-LRR-NBS-TM	Os12g0477100	OsR498G1221588100.01.T01	NB-ARC domain containing protein, expressed
augustus-2-processed-gene-154.19-mRNA-1	Japonica	NA	CC-LRR-NBS-TM	Os02g0456800	OsR498G0203724300.01.T01	Putative NB-ARC domain-containing protein (Fragment)
augustus-2-processed-gene-51.36-mRNA-1	Japonica	Japonica	CC-LRR-NBS-TM	Os02g0191000	OsR498G0203022300.01.T01	Disease resistance protein RPM1
augustus-4-processed-gene-192.20-mRNA-1	Japonica	NA	CC-LRR-NBS-TM	Os04g0491100	OsR498G0408811900.01.T02	Disease resistance RPP13-like protein 4
augustus_masked-11-processed-gene-219.12-mRNA-1	Japonica	NA	CC-LRR-NBS-TM	Os11g0623800	OsR498G1120539400.01.T03	Putative nucleotide-binding leucine-rich-repeat protein 1
augustus_masked-11-processed-gene-228.29-mRNA-1	Japonica	NA	CC-LRR-NBS-TM	Os11g0640000	OsR498G1120611600.01.T01	Leucine Rich Repeat family protein, expressed
augustus_masked-11-processed-gene-57.10-mRNA-1	Japonica	NA	CC-LRR-NBS-TM		OsR498G1119693000.01.T02	Putative nucleotide-binding leucine-rich-repeat protein 1
augustus_masked-11-processed-gene-83.18-mRNA-1	Japonica	NA	CC-LRR-NBS-TM			NBS-LRR type protein (Fragment)
augustus_masked-3-processed-gene-148.50-mRNA-1	Japonica	NA	CC-LRR-NBS-TM	Os03g0324600	OsR498G0305854800.01.T01	NB-ARC domain containing protein, expressed
augustus_masked-5-processed-gene-253.1-mRNA-1	Japonica	NA	CC-LRR-NBS-TM		OsR498G0511090900.01.T01	NBS-LRR type resistance protein (Fragment)
augustus_masked-5-processed-gene-253.16-mRNA-1	Japonica	Japonica	CC-LRR-NBS-TM	Os05g0492600	OsR498G0511090900.01.T01	NBS-LRR type resistance protein (Fragment)
maker-1-augustus-gene-133.42-mRNA-1	Japonica	NA	CC-LRR-NBS-TM	Os01g0335700	OsR498G0100857600.01.T01	Putative NB-ARC domain-containing protein (Fragment)
maker-10-augustus-gene-167.5-mRNA-1	Japonica	NA	CC-LRR-NBS-TM	Os10g0474500	OsR498G1018892300.01.T01	Putative disease resistance protein RGA3
maker-11-augustus-gene-199.40-mRNA-1	Japonica	Indica	CC-LRR-NBS-TM	Os11g0590900	OsR498G1120430700.01.T02	Putative CC-NBS-LRR resistance protein MLA13
maker-11-augustus-gene-248.27-mRNA-1	Japonica	NA	CC-LRR-NBS-TM	Os11g0677101	OsR498G1120709900.01.T01	Putative nucleotide-binding leucine-rich-repeat protein 1
maker-11-augustus-gene-55.8-mRNA-1	Japonica	Japonica	CC-LRR-NBS-TM	Os11g0258500	OsR498G1119734000.01.T01	NB-ARC domain, putative
maker-11-augustus-gene-74.5-mRNA-1	Japonica	NA	CC-LRR-NBS-TM		OsR498G1119611800.01.T01	NBS-LRR disease resistance protein family-4
maker-11-augustus-gene-83.3-mRNA-1	Japonica	Japonica	CC-LRR-NBS-TM	Os11g0227100	OsR498G1119648000.01.T02	NBS-LRR type resistance protein (Fragment)
maker-11-augustus-gene-87.36-mRNA-1	Japonica	NA	CC-LRR-NBS-TM	Os11g0237900	OsR498G1119671300.01.T01	Putative disease resistance RPP13-like protein 3
maker-12-augustus-gene-235.0-mRNA-1	Japonica	NA	CC-LRR-NBS-TM	Os12g0565200	OsR498G1221893900.01.T01	NB-ARC domain containing protein, expressed
maker-2-augustus-gene-100.35-mRNA-1	Japonica	Indica	CC-LRR-NBS-TM	Os02g0272900	OsR498G0203396700.01.T01	AAA domain-containing protein
maker-2-augustus-gene-115.6-mRNA-1	Japonica	NA	CC-LRR-NBS-TM		OsR498G0203491300.01.T01	Putative stripe rust resistance protein Yr10
maker-3-augustus-gene-412.14-mRNA-1	Japonica	NA	CC-LRR-NBS-TM	Os03g0689800	OsR498G0306832100.01.T01	NB-ARC domain containing protein
maker-3-augustus-gene-412.19-mRNA-1	Japonica	NA	CC-LRR-NBS-TM	Os03g0689400		Disease resistance protein RGA2
maker-3-augustus-gene-79.50-mRNA-1	Japonica	Indica	CC-LRR-NBS-TM	Os03g0254000	OsR498G0305596400.01.T01	Disease resistance protein RPS2
maker-4-augustus-gene-263.42-mRNA-1	Japonica	NA	CC-LRR-NBS-TM	Os04g0622600	OsR498G0409301900.01.T01	BED-type domain-containing protein
maker-8-augustus-gene-210.52-mRNA-1	Japonica	Japonica	CC-LRR-NBS-TM	Os08g0412100	OsR498G0816076200.01.T01	Disease resistance protein RPM1
maker-8-augustus-gene-42.55-mRNA-1	Japonica	Indica	CC-LRR-NBS-TM	Os08g0170700	OsR498G0815277600.01.T01	NAC domain-containing protein
maker-9-augustus-gene-95.0-mRNA-1	Japonica	Japonica	CC-LRR-NBS-TM	Os09g0322800	OsR498G0917149700.01.T01	NBS-LRR disease resistance protein NBS39
augustus-7-processed-gene-197.118-mRNA-1	NA	NA	CC-LRR-NBS-TM	0	OsR498G0714447800.01.T03	Putative resistance complex protein I2C-2
augustus-8-processed-gene-79.5-mRNA-1	NA	NA	CC-LRR-NBS-TM	Os08g0224000	OsR498G0815462000.01.T01	Putative P-loop containing nucleoside triphosphate hydrolase, leucine-rich repeat domain, L
augustus_masked-11-processed-gene-219.12-mRNA-1	NA	NA	CC-LRR-NBS-TM	Os11g0623800	OsR498G1120539400.01.T03	Putative nucleotide-binding leucine-rich-repeat protein 1
augustus_masked-11-processed-gene-83.18-mRNA-1	NA	NA	CC-LRR-NBS-TM			NBS-LRR type protein (Fragment)
augustus_masked-6-processed-gene-264.11-mRNA-1	NA	NA	CC-LRR-NBS-TM	Os06g0644300		Formate dehydrogenase
maker-10-augustus-gene-144.32-mRNA-1	NA	NA	CC-LRR-NBS-TM	Os04g0111900	OsR498G0407452000.01.T01	Putative NB-ARC domain-containing protein (Fragment)
maker-11-augustus-gene-198.9-mRNA-1	NA	NA	CC-LRR-NBS-TM			NB-ARC domain containing protein, expressed
maker-11-augustus-gene-199.40-mRNA-1	NA	Indica	CC-LRR-NBS-TM	Os11g0590900	OsR498G1120430700.01.T02	Putative CC-NBS-LRR resistance protein MLA13
maker-11-augustus-gene-83.3-mRNA-1	NA	Japonica	CC-LRR-NBS-TM	Os11g0227100	OsR498G1119648000.01.T02	NBS-LRR type resistance protein (Fragment)
maker-12-augustus-gene-228.48-mRNA-1	NA	NA	CC-LRR-NBS-TM	Os12g0516300	OsR498G1221855600.01.T01	NB-ARC domain containing protein, expressed
maker-12-augustus-gene-247.0-mRNA-1	NA	Indica	CC-LRR-NBS-TM	Os12g0586000	OsR498G1221987100.01.T02	RPW8 domain-containing protein
maker-3-augustus-gene-240.31-mRNA-1	NA	NA	CC-LRR-NBS-TM	0	OsR498G0306443900.01.T02	Disease resistance protein RGA3
maker-3-augustus-gene-301.29-mRNA-1	NA	NA	CC-LRR-NBS-TM	Os10g0370400	OsR498G1018560100.01.T01	Disease resistance protein RPP13
maker-3-augustus-gene-349.3-mRNA-1	NA	NA	CC-LRR-NBS-TM	0	OsR498G0306443900.01.T02	Disease resistance protein RGA3
maker-3-augustus-gene-400.3-mRNA-1	NA	NA	CC-LRR-NBS-TM	0	OsR498G0306756300.01.T01	TTF-type domain-containing protein
maker-4-augustus-gene-203.16-mRNA-1	NA	NA	CC-LRR-NBS-TM	Os04g0512900	OsR498G0408883400.01.T01	Disease resistance gene analog (Fragment)
maker-6-augustus-gene-30.68-mRNA-1	NA	NA	CC-LRR-NBS-TM	Os06g0158500	OsR498G0611659400.01.T01	Putative NB-ARC domain-containing protein (Fragment)
maker-8-augustus-gene-132.0-mRNA-1	NA	NA	CC-LRR-NBS-TM	Os08g0296600	OsR498G0815689000.01.T01	NBS resistance protein (Fragment)
maker-8-augustus-gene-132.9-mRNA-1	NA	NA	CC-LRR-NBS-TM	Os08g0296700	OsR498G0815689300.01.T01	Putative bacterial blight-resistance protein Xa1
maker-8-augustus-gene-210.50-mRNA-1	NA	NA	CC-LRR-NBS-TM	0	OsR498G0816075400.01.T01	Disease resistance protein RPM1
maker-8-augustus-gene-210.52-mRNA-1	NA	Japonica	CC-LRR-NBS-TM	Os08g0412100	OsR498G0816076200.01.T01	Disease resistance protein RPM1
maker-8-augustus-gene-42.55-mRNA-1	NA	Indica	CC-LRR-NBS-TM	Os08g0170700	OsR498G0815277600.01.T01	NAC domain-containing protein

maker-8-augustus-gene-53.54-mRNA-1	NA	Japonica	CC-LRR-NBS-TM	Os08g0190300	OsR498G0815343400.01.T01	
maker-8-augustus-gene-284.4-mRNA-1	Japonica	NA	CC-LRR-NBS-TM	Os08g0539700	OsR498G0816548900.01.T01	Brown planthopper resistance protein BPH1/9 (PIBH8)
maker-2-augustus-gene-391.32-mRNA-1	Japonica	NA	CC-LRR-TM	Os02g0826600	OsR498G0205039000.01.T01	Plant intracellular Ras-group-related LRR protein 9
augustus-12-processed-gene-68.17-mRNA-1	NA	Japonica	CC-LRR-TM	Os12g0222900	OsR498G1221134900.01.T01	Leucine Rich Repeat family protein, expressed
augustus-11-processed-gene-204.32-mRNA-1	Japonica	Japonica	CC-NBS	Os11g0598500	OsR498G1120448400.01.T01	Leucine Rich Repeat family protein, expressed
augustus_masked-10-processed-gene-18.40-mRNA-1	Japonica	Japonica	CC-NBS	Os10g0124300	OsR498G1018071200.01.T02	Disease resistance protein RPM1
augustus_masked-11-processed-gene-245.11-mRNA-1	Japonica	NA	CC-NBS	Os11g0668000	OsR498G1120693800.01.T01	Putative NBS-LRR class RGA
maker-2-augustus-gene-211.4-mRNA-1	Japonica	NA	CC-NBS		OsR498G0204086500.01.T01	
maker-8-augustus-gene-92.8-mRNA-1	Japonica	NA	CC-NBS		OsR498G0815527800.01.T03	Disease resistance protein RPP13
maker-4-augustus-gene-85.2-mRNA-1	NA	NA	CC-NBS	0	OsR498G0408112900.01.T01	Putative NBS-LRR class RGA
maker-8-augustus-gene-92.8-mRNA-1	NA	NA	CC-NBS	0	OsR498G0815527800.01.T03	Disease resistance protein RPP13
augustus-11-processed-gene-252.13-mRNA-1	Indica	NA	CC-NBS-TM	Os11g0684200	OsR498G1120731300.01.T01	NB-ARC domain containing protein, expressed
augustus-7-processed-gene-198.23-mRNA-1	Indica	NA	CC-NBS-TM	Os07g0521800	OsR498G0714451300.01.T01	Putative resistance complex protein I2C-2
augustus_masked-4-processed-gene-29.12-mRNA-1	Indica	Japonica	CC-NBS-TM	Os04g0219600	OsR498G1119645400.01.T01	NBS-LRR type protein (Fragment)
augustus_masked-5-processed-gene-213.32-mRNA-1	Indica	NA	CC-NBS-TM		OsR498G0510842500.01.T01	Putative disease resistance RPP13-like protein 1
augustus_masked-8-processed-gene-33.2-mRNA-1	Indica	NA	CC-NBS-TM		OsR498G0815208900.01.T01	NB-ARC domain containing protein, expressed
maker-1-augustus-gene-94.39-mRNA-1	Indica	NA	CC-NBS-TM	Os01g0269500	OsR498G0100629900.01.T03	Putative NBS type R protein
maker-11-augustus-gene-149.39-mRNA-1	Indica	NA	CC-NBS-TM			Putative NBS-LRR type disease resistance protein
maker-4-augustus-gene-86.32-mRNA-1	Indica	NA	CC-NBS-TM		OsR498G0408112900.01.T01	Putative NBS-LRR class RGA
maker-6-augustus-gene-102.41-mRNA-1	Indica	NA	CC-NBS-TM			NBS-LRR
augustus-11-processed-gene-235.26-mRNA-1	Japonica	NA	CC-NBS-TM	Os11g0652100	OsR498G1120654000.01.T01	Disease resistance protein RPM1
augustus-11-processed-gene-237.18-mRNA-1	Japonica	NA	CC-NBS-TM	Os11g0655300	OsR498G1120667300.01.T01	NB-ARC domain containing protein, expressed
augustus-12-processed-gene-57.3-mRNA-1	Japonica	NA	CC-NBS-TM	Os12g0204700	OsR498G1221097900.01.T01	NB-ARC domain containing protein, expressed
augustus-4-processed-gene-182.0-mRNA-1	Japonica	Japonica	CC-NBS-TM	Os04g0470500	OsR498G0408741100.01.T01	Disease resistance protein RPS2
augustus-4-processed-gene-262.14-mRNA-1	Japonica	Japonica	CC-NBS-TM	Os04g0621750	OsR498G0409301900.01.T01	Disease resistance protein RGA2
augustus-8-processed-gene-286.21-mRNA-1	Japonica	NA	CC-NBS-TM	Os08g0543100		Putative NBS-LRR resistance protein RGH1
augustus_masked-1-processed-gene-341.24-mRNA-1	Japonica	NA	CC-NBS-TM	Os01g0788500	OsR498G0102125800.01.T03	Disease resistance protein RPS2
augustus_masked-1-processed-gene-488.23-mRNA-1	Japonica	Japonica	CC-NBS-TM	Os01g0937300	OsR498G0102662000.01.T03	Disease resistance protein RGA2
augustus_masked-10-processed-gene-144.26-mRNA-1	Japonica	NA	CC-NBS-TM			NBS-LRR resistant protein (Fragment)
augustus_masked-11-processed-gene-203.21-mRNA-1	Japonica	NA	CC-NBS-TM	Os11g0598300	OsR498G1120448400.01.T01	Leucine Rich Repeat family protein, expressed
augustus_masked-11-processed-gene-211.3-mRNA-1	Japonica	NA	CC-NBS-TM	Os11g0609700	OsR498G1120489600.01.T01	Disease resistance protein RPM1
augustus_masked-11-processed-gene-236.22-mRNA-1	Japonica	NA	CC-NBS-TM	Os11g0653300		NBS-LRR type resistance protein-like
augustus_masked-11-processed-gene-237.27-mRNA-1	Japonica	NA	CC-NBS-TM	Os11g0654800	OsR498G1120666400.01.T01	Disease resistance protein RPM1
augustus_masked-11-processed-gene-239.33-mRNA-1	Japonica	NA	CC-NBS-TM		OsR498G1120676400.01.T02	Putative disease resistance RPP8-like protein 2
augustus_masked-11-processed-gene-82.26-mRNA-1	Japonica	Indica	CC-NBS-TM	Os11g0226933	OsR498G1119644200.01.T01	NBS-LRR type protein (Fragment)
augustus_masked-12-processed-gene-100.23-mRNA-1	Japonica	NA	CC-NBS-TM			NBS-LRR disease resistance protein, putative
augustus_masked-12-processed-gene-102.18-mRNA-1	Japonica	NA	CC-NBS-TM	Os12g0273000	OsR498G1221277200.01.T02	Disease resistance protein RPM1
augustus_masked-12-processed-gene-50.21-mRNA-1	Japonica	NA	CC-NBS-TM		OsR498G1221076200.01.T01	Disease resistance protein RPM1
augustus_masked-12-processed-gene-59.18-mRNA-1	Japonica	NA	CC-NBS-TM			Disease resistance protein RGA2
augustus_masked-6-processed-gene-234.17-mRNA-1	Japonica	Japonica	CC-NBS-TM	Os06g0594200	OsR498G0612874800.01.T01	Putative disease resistance RPP13-like protein 1
augustus_masked-8-processed-gene-4.18-mRNA-1	Japonica	Japonica	CC-NBS-TM	Os08g0107000	OsR498G0815041300.01.T01	Disease resistance protein RPP13
maker-1-augustus-gene-147.35-mRNA-1	Japonica	NA	CC-NBS-TM		OsR498G0100940800.01.T01	Powdery mildew resistance protein PM3 variant
maker-1-augustus-gene-192.32-mRNA-1	Japonica	NA	CC-NBS-TM		OsR498G0101131600.01.T02	Blight resistance protein RGA1-like protein
maker-1-augustus-gene-482.51-mRNA-1	Japonica	NA	CC-NBS-TM		OsR498G0102617400.01.T01	Disease resistance protein RPP13
maker-1-augustus-gene-488.70-mRNA-1	Japonica	NA	CC-NBS-TM	Os01g0937400	OsR498G0102663200.01.T06	Disease resistance protein RGA2
maker-10-augustus-gene-139.30-mRNA-1	Japonica	NA	CC-NBS-TM	Os12g0246700	OsR498G0407486900.01.T01	NB-ARC domain containing protein, expressed
maker-10-augustus-gene-22.27-mRNA-1	Japonica	NA	CC-NBS-TM	Os10g0131901	OsR498G1018095600.01.T01	Disease resistance protein Pikm2-TS
maker-10-augustus-gene-23.34-mRNA-1	Japonica	Indica	CC-NBS-TM	Os10g0131000	OsR498G1018101100.01.T01	Disease resistance protein RPM1
maker-11-augustus-gene-142.46-mRNA-1	Japonica	NA	CC-NBS-TM	Os11g0479100	OsR498G1120143800.01.T02	NB-ARC domain, putative
maker-11-augustus-gene-149.40-mRNA-1	Japonica	NA	CC-NBS-TM	Os11g0493700		NB-ARC domain containing protein, expressed
maker-11-augustus-gene-150.0-mRNA-1	Japonica	NA	CC-NBS-TM		OsR498G1120176700.01.T01	NBS-LRR type resistance protein (Fragment)
maker-11-augustus-gene-150.2-mRNA-1	Japonica	NA	CC-NBS-TM	Os11g0495032		NB-ARC domain containing protein, expressed
maker-11-augustus-gene-165.14-mRNA-1	Japonica	NA	CC-NBS-TM		OsR498G1120249800.01.T02	NBS-LRR-like resistance protein (Fragment)
maker-11-augustus-gene-180.2-mRNA-1	Japonica	NA	CC-NBS-TM	Os11g0551700		Disease resistance protein RPM1
maker-11-augustus-gene-181.30-mRNA-1	Japonica	NA	CC-NBS-TM			Rust resistance protein RP1-D homolog
maker-11-augustus-gene-199.35-mRNA-1	Japonica	Japonica	CC-NBS-TM	Os11g0590700	OsR498G1120430700.01.T02	Putative CC-NBS-LRR resistance protein MLA13
maker-11-augustus-gene-199.37-mRNA-1	Japonica	NA	CC-NBS-TM	Os11g0590900	OsR498G1120430300.01.T01	NB-ARC domain containing protein, expressed
maker-11-augustus-gene-222.39-mRNA-1	Japonica	NA	CC-NBS-TM		OsR498G1120562500.01.T05	Disease resistance protein RPM1
maker-11-augustus-gene-260.0-mRNA-1	Japonica	Japonica	CC-NBS-TM	Os11g0699100	OsR498G1120757300.01.T01	Disease resistance protein RPP13
maker-11-augustus-gene-42.0-mRNA-1	Japonica	NA	CC-NBS-TM			NB-ARC domain, putative
maker-11-augustus-gene-61.35-mRNA-1	Japonica	Japonica	CC-NBS-TM	Os11g0249000	OsR498G1119714500.01.T01	Disease resistance protein RPM1
maker-11-augustus-gene-75.20-mRNA-1	Japonica	NA	CC-NBS-TM	Os11g0213800	OsR498G1119615600.01.T05	NBS-LRR type protein (Fragment)
maker-11-augustus-gene-82.6-mRNA-1	Japonica	Indica	CC-NBS-TM	Os11g0226700	OsR498G1119646100.01.T02	NBS-LRR type protein (Fragment)
maker-11-augustus-gene-82.8-mRNA-1	Japonica	NA	CC-NBS-TM			Protein kinase domain-containing protein
maker-12-augustus-gene-102.0-mRNA-1	Japonica	Japonica	CC-NBS-TM	Os12g0273266	OsR498G1221272600.01.T01	Disease resistance protein RPM1, putative, expressed
maker-12-augustus-gene-102.3-mRNA-1	Japonica	NA	CC-NBS-TM			NB-ARC domain containing protein, expressed
maker-12-augustus-gene-189.12-mRNA-1	Japonica	Japonica	CC-NBS-TM	Os12g0485900	OsR498G1221625000.01.T01	NB-ARC domain, putative
maker-12-augustus-gene-196.0-mRNA-1	Japonica	NA	CC-NBS-TM	Os12g0495600	OsR498G1221658200.01.T01	Putative CC-NBS-LRR resistance protein MLA13
maker-12-augustus-gene-206.31-mRNA-1	Japonica	NA	CC-NBS-TM		OsR498G1221703800.01.T01	NB-ARC domain containing protein, expressed
maker-12-augustus-gene-206.34-mRNA-1	Japonica	Indica	CC-NBS-TM	Os12g0511400	OsR498G1221704000.01.T02	NB-ARC domain containing protein, expressed
maker-12-augustus-gene-231.39-mRNA-1	Japonica	NA	CC-NBS-TM	Os12g0559500	OsR498G1221875800.01.T01	Disease resistance protein RPP13
maker-12-augustus-gene-234.75-mRNA-1	Japonica	Japonica	CC-NBS-TM	Os12g0565100	OsR498G1221893300.01.T01	NB-ARC domain containing protein, expressed
maker-12-augustus-gene-53.41-mRNA-1	Japonica	Indica	CC-NBS-TM	Os09g0517200	OsR498G1221092300.01.T02	Disease resistance protein RPM1
maker-2-augustus-gene-105.32-mRNA-1	Japonica	Japonica	CC-NBS-TM	Os02g0281900	OsR498G0203420100.01.T02	Disease resistance protein RGA2
maker-2-augustus-gene-117.50-mRNA-1	Japonica	NA	CC-NBS-TM		OsR498G0203501200.01.T03	LZ-NBS-LRR class RGA
maker-2-augustus-gene-130.6-mRNA-1	Japonica	NA	CC-NBS-TM	Os02g0325100	OsR498G0203583700.01.T01	26S proteasome regulatory subunit 6B homolog
maker-2-augustus-gene-182.28-mRNA-1	Japonica	NA	CC-NBS-TM		OsR498G0203882300.01.T01	CC-NBS-LRR type disease resistance protein NLR2-V
maker-2-augustus-gene-216.0-mRNA-1	Japonica	NA	CC-NBS-TM		OsR498G0204117300.01.T01	Disease resistance protein RPM1
maker-2-augustus-gene-58.42-mRNA-1	Japonica	NA	CC-NBS-TM	Os02g0203500	OsR498G0203179000.01.T01	NB-ARC domain-containing protein (Fragment)
maker-3-augustus-gene-106.29-mRNA-1	Japonica	NA	CC-NBS-TM	Os03g0306400	OsR498G0305788600.01.T02	Lon protease, mitochondrial
maker-3-augustus-gene-285.44-mRNA-1	Japonica	NA	CC-NBS-TM		OsR498G1018641100.01.T02	Putative disease resistance protein RGA3
maker-3-augustus-gene-495.19-mRNA-1	Japonica	Japonica	CC-NBS-TM	Os03g0848700	OsR498G0307368000.01.T01	Powdery mildew resistance protein PM3 variant
maker-3-augustus-gene-85.63-mRNA-1	Japonica	Indica	CC-NBS-TM	Os03g0264800	OsR498G0305631400.01.T02	AAA domain-containing protein (Fragment)
maker-4-augustus-gene-244.7-mRNA-1	Japonica	NA	CC-NBS-TM		OsR498G0409181900.01.T01	Multidrug resistance-associated protein/mitoxantrone resistance protein, ABC superfamily
maker-4-augustus-gene-262.49-mRNA-1	Japonica	NA	CC-NBS-TM			Disease Resistance RGA protein (Pi69)
maker-6-augustus-gene-102.41-mRNA-1	Japonica	NA	CC-NBS-TM			NBS-LRR
maker-7-augustus-gene-173.0-mRNA-1	Japonica	NA	CC-NBS-TM	Os07g0481400	OsR498G0714309200.01.T01	Putative NB-ARC domain-containing protein (Fragment)
maker-8-augustus-gene-123.5-mRNA-1	Japonica	NA	CC-NBS-TM	Os08g0293300	OsR498G0815678600.01.T02	Disease resistance protein RPM1
maker-8-augustus-gene-284.17-mRNA-1	Japonica	Japonica	CC-NBS-TM	Os08g0539400	OsR498G0816548000.01.T01	Resistance protein RGA2
maker-8-augustus-gene-45.38-mRNA-1	Japonica	NA	CC-NBS-TM		OsR498G0815299900.01.T02	Putative CC-NBS-LRR resistance protein MLA13
maker-8-augustus-gene-63.0-mRNA-1	Japonica	Japonica	CC-NBS-TM	Os08g0205100	OsR498G0815390700.01.T01	CC-NBS-LRR type disease resistance protein NLR1-V
maker-8-augustus-gene-63.1-mRNA-1	Japonica	NA	CC-NBS-TM	Os08g0205150	OsR498G0815390700.01.T02	CC-NBS-LRR type disease resistance protein NLR1-V
maker-9-augustus-gene-185.54-mRNA-1	Japonica	NA	CC-NBS-TM	Os09g0479500	OsR498G0917691400.01.T01	Disease resistance protein RPM1
maker-9-augustus-gene-202.41-mRNA-1	Japonica	NA	CC-NBS-TM	Os09g0517200	OsR498G0917803000.01.T01	NB-ARC domain-containing protein (Fragment)
maker-9-augustus-gene-57.0-mRNA-1	Japonica	NA	CC-NBS-TM	Os09g0270700	OsR498G0916882900.01.T01	Disease resistance protein RPM1
augustus-11-processed-gene-194.0-mRNA-1	NA	NA	CC-NBS-TM	Os11g0598300	OsR498G1120448400.01.T01	Leucine Rich Repeat family protein, expressed
augustus-12-processed-gene-53.32-mRNA-1	NA	NA	CC-NBS-TM	Os12g0199100		Putative NB-ARC domain-containing protein (Fragment)
augustus-2-processed-gene-10.1-mRNA-1	NA	NA	CC-NBS-TM			Truncated Sr21
augustus-2-processed-gene-56.51-mRNA-1	NA	NA	CC-NBS-TM	0	OsR498G0203162900.01.T01	NB-ARC domain-containing protein
augustus-4-processed-gene-182.0-mRNA-1	NA	Japonica	CC-NBS-TM	Os04g0470500	OsR498G0408741100.01.T01	Disease resistance protein RPS2
augustus-4-processed-gene-84.19-mRNA-1	NA	Japonica	CC-NBS-TM	Os04g0312000	OsR498G0612066600.01.T01	Protein kinase domain-containing protein
augustus_masked-10-processed-gene-144.26-mRNA-1	NA	NA	CC-NBS-TM			NBS-LRR resistant protein (Fragment)
augustus_masked-11-processed-gene-203.21-mRNA-1	NA	NA	CC-NBS-TM	Os11g0598300	OsR498G1120448400.01.T01	Leucine Rich Repeat family protein, expressed
augustus_masked-11-processed-gene-211.3-mRNA-1	NA	NA	CC-NBS-TM	Os11g0609700	OsR498G1120489600.01.T01	Disease resistance protein RPM1
augustus_masked-11-processed-gene-74.21-mRNA-1	NA	Indica	CC-NBS-TM	Os11g0213800	OsR498G1119611200.01.T01	NBS-LRR type protein (Fragment)
augustus-11-processed-gene-231.21-mRNA-1	Japonica	Indica	NBS-LRR	Os11g0644700	OsR498G1120631700.01.T01	Disease Resistance protein Pi1 (allele at the Pik locus)
maker-6-augustus-gene-105.9-mRNA-1	Japonica	NA		Os06g0294300	OsR498G0612179200.01.T01	Disease Resistance protein Pi2
augustus_masked-12-processed-gene-276.25-mRNA-1	NA	NA	CC-NBS-TM	Os07g0105900		NB-ARC domain-containing protein
augustus_masked-8-processed-gene-101.5-mRNA-1	NA	NA	CC-NBS-TM	Os08g0261000	OsR498G0815572500.01.T03	NBS-type RGA protein (Fragment)
augustus_masked-9-processed-gene-86.16-mRNA-1	NA	NA	CC-NBS-TM	Os09g0308500		Putative nucleotide-binding leucine-rich-repeat protein 1
maker-1-augustus-gene-247.25-mRNA-1	NA	NA	CC-NBS-TM	0	OsR498G1120729200.01.T01	AAA domain-containing protein
maker-1-augustus-gene-411.31-mRNA-1	NA	NA	CC-NBS-TM	0	OsR498G1120729200.01.T01	AAA domain-containing protein
maker-1-augustus-gene-462.16-mRNA-1	NA	NA	CC-NBS-TM	0	OsR498G1120729200.01.T01	AAA domain-containing protein
maker-1-augustus-gene-94.39-mRNA-1	NA	NA	CC-NBS-TM	Os01g0269500	OsR498G0100629900.01.T03	Putative NBS type R protein
maker-1-augustus-gene-94.41-mRNA-1	NA	NA	CC-NBS-TM	Os01g0269800	OsR498G0100631100.01.T01	NBS-LRR disease resistance protein-like
maker-1-augustus-gene-94.49-mRNA-1	NA	Indica	CC-NBS-TM	Os01g0269800	OsR498G0100630400.01.T01	Putative NB-ARC domain-containing protein (Fragment)
maker-10-augustus-gene-139.30-mRNA-1	NA	NA	CC-NBS-TM	Os12g0246700	OsR498G0407486900.01.T01	NB-ARC domain containing protein, expressed
maker-11-augustus-gene-149.39-mRNA-1	NA	NA	CC-NBS-TM			Putative NBS-LRR type disease resistance protein
maker-11-augustus-gene-165.14-mRNA-1	NA	NA	CC-NBS-TM	0	OsR498G1120249800.01.T02	NBS-LRR-like resistance protein (Fragment)
maker-11-augustus-gene-232.0-mRNA-1	NA	NA	CC-NBS-TM	Os11g0646300		Leucine Rich Repeat family protein, expressed
maker-11-augustus-gene-260.0-mRNA-1	NA	Japonica	CC-NBS-TM	Os11g0699100	OsR498G1120757300.01.T01	Disease resistance protein RPP13
maker-11-augustus-gene-80.36-mRNA-1	NA	NA	CC-NBS-TM	Os11g0222900		Putative NB-ARC domain-containing protein (Fragment)
maker-12-augustus-gene-175.32-mRNA-1	NA	NA	CC-NBS-TM	Os12g0467300		NB-ARC domain containing protein, expressed
maker-12-augustus-gene-206.34-mRNA-1	NA	Indica	CC-NBS-TM	Os12g0511400	OsR498G1221704000.01.T02	NB-ARC domain containing protein, expressed
maker-12-augustus-gene-84.0-mRNA-1	NA	Japonica	CC-NBS-TM	Os12g0246700	OsR498G0407486900.01.T01	NB-ARC domain containing protein, expressed
maker-4-augustus-gene-96.56-mRNA-1	NA	Japonica	CC-NBS-TM	Os04g0470500	OsR498G0408741100.01.T01	Disease resistance protein RPS2
maker-5-augustus-gene-186.5-mRNA-1	NA	Indica	CC-NBS-TM	Os10g0568600	OsR498G0510687800.01.T01	Putative stripe rust resistance protein Yr10
maker-6-augustus-gene-102.41-mRNA-1	NA	NA	CC-NBS-TM			NBS-LRR
maker-7-augustus-gene-148.0-mRNA-1	NA	NA	CC-NBS-TM			Mitogen-activated protein kinase kinase 1
maker-8-augustus-gene-123.5-mRNA-1	NA	NA	CC-NBS-TM	Os08g0293300	OsR498G0815678600.01.T02	Disease resistance protein RPM1
maker-8-augustus-gene-31.27-mRNA-1	NA	NA	CC-NBS-TM	0	OsR498G0815193900.01.T01	ADP-ribosyl cyclase/cyclic ADP-ribose hydrolase
maker-8-augustus-gene-43.13-mRNA-1	NA	Indica	CC-NBS-TM	Os05g0240200	OsR498G0815285000.01.T01	AAA domain-containing protein
maker-9-augustus-gene-185.54-mRNA-1	NA	NA	CC-NBS-TM	Os09g0479500	OsR498G0917691400.01.T01	Disease resistance protein RPM1
maker-9-augustus-gene-210.34-mRNA-1	NA	NA	CC-NBS-TM	Os09g0533400	OsR498G0917853400.01.T01	Lon protease homolog 2, peroxisomal
maker-9-augustus-gene-87.4-mRNA-1	NA	NA	CC-NBS-TM	Os09g0313500	OsR498G0917079600.01.T01	Putative nucleotide-binding leucine-rich-repeat protein 1
maker-12-augustus-gene-109.40-mRNA-1	Japonica	NA	CC-NBS-TM Pi42	Os12g0281600	OsR498G1221312700.01.T02	NB-ARC domain containing protein, expressed
maker-11-augustus-gene-82.0-mRNA-1	Indica	Japonica	CC-NBS-TM RGA5, *Pia*	Os11g0225300	OsR498G1119642700.01.T03	NBS-LRR type protein (Fragment)
augustus-10-processed-gene-24.15-mRNA-1	Japonica	Indica		Os10g0135100	OsR498G1018105500.01.T01	Putative disease resistance protein RGA4
augustus-4-processed-gene-262.8-mRNA-1	Japonica	Japonica		Os04g0621300	OsR498G0409297100.01.T04	Disease Resistance RGA protein fragment (Pi63)
augustus-9-processed-gene-65.24-mRNA-1	Japonica	NA			OsR498G0916922600.01.T01	Putative disease resistance protein RGA3
augustus_masked-11-processed-gene-245.12-mRNA-1	Japonica	NA		Os11g0668000	OsR498G1120693800.01.T01	Putative NBS-LRR class RGA
augustus_masked-5-processed-gene-141.41-mRNA-1	Japonica	NA			OsR498G0510413500.01.T01	Putative disease resistance protein RGA3
maker-2-augustus-gene-10.48-mRNA-1	NA	NA				Disease resistance protein RGA2
augustus-2-processed-gene-30.39-mRNA-1	Indica	NA	Kinase-LRR-TM	Os02g0153100	OsR498G0202989000.01.T01	Putative Phytosulfokine receptor
augustus-2-processed-gene-30.40-mRNA-1	Indica	NA	Kinase-LRR-TM	Os02g0153200	OsR498G0202989400.01.T01	Putative Phytosulfokine receptor
augustus-2-processed-gene-30.41-mRNA-1	Indica	NA	Kinase-LRR-TM			Putative leucine-rich repeat receptor-like kinase
augustus-2-processed-gene-30.42-mRNA-1	Indica	Japonica	Kinase-LRR-TM	Os02g0153700	OsR498G0202991600.01.T01	Putative leucine-rich repeat receptor-like kinase
augustus-2-processed-gene-30.44-mRNA-1	Indica	NA	Kinase-LRR-TM	Os02g0153900	OsR498G0202990800.01.T01	Putative leucine-rich repeat receptor-like kinase

augustus-8-processed-gene-231.5-mRNA-1	Indica	NA	Kinase-LRR-TM	Os08g0446200	OsR498G0816198200.01.T01	Leucine-rich repeat receptor-like protein kinase PEPR1
augustus-9-processed-gene-141.32-mRNA-1	Indica	NA	Kinase-LRR-TM	Os09g0400500	OsR498G0917406000.01.T01	Atypical receptor-like kinase MARK
maker-1-augustus-gene-18.17-mRNA-1	Indica	NA	Kinase-LRR-TM	Os01g0133900	OsR498G0100117900.01.T02	probable leucine-rich repeat receptor-like protein kinase At1g68400
maker-1-augustus-gene-27.62-mRNA-1	Indica	Indica	Kinase-LRR-TM	Os01g0149700	OsR498G0100178400.01.T01	Putative LRR receptor-like serine/threonine-protein kinase
maker-1-augustus-gene-351.20-mRNA-1	Indica	Indica	Kinase-LRR-TM	Os01g0810533	OsR498G0102188400.01.T04	Receptor-like protein kinase
maker-1-augustus-gene-352.53-mRNA-1	Indica	NA	Kinase-LRR-TM		OsR498G0102191000.01.T01	Putative light repressible receptor protein kinase
maker-1-augustus-gene-352.57-mRNA-1	Indica	NA	Kinase-LRR-TM	Os01g0810900	OsR498G0102192000.01.T04	Putative light repressible receptor protein kinase
maker-1-augustus-gene-355.0-mRNA-1	Indica	NA	Kinase-LRR-TM		OsR498G0102210700.01.T02	Leucine-rich repeat protein kinase family protein
maker-1-augustus-gene-356.16-mRNA-1	Indica	Indica	Kinase-LRR-TM	Os05g0480400	OsR498G0102218900.01.T03	Inactive receptor kinase
maker-1-augustus-gene-356.23-mRNA-1	Indica	NA	Kinase-LRR-TM		OsR498G0102217000.01.T01	Non-specific serine/threonine protein kinase (Fragment)
maker-1-augustus-gene-358.18-mRNA-1	Indica	Indica	Kinase-LRR-TM	Os01g0821900	OsR498G0102230200.01.T01	Serine/threonine kinase
maker-1-augustus-gene-36.36-mRNA-1	Indica	Indica	Kinase-LRR-TM	Os01g0170300	OsR498G0100236300.01.T02	Mitogen-activated protein kinase kinase kinase
maker-10-augustus-gene-163.28-mRNA-1	Indica	Indica	Kinase-LRR-TM	Os10g0467900	OsR498G1018870200.01.T01	Receptor protein kinase
maker-10-augustus-gene-37.42-mRNA-1	Indica	Japonica	Kinase-LRR-TM	Os10g0155733	OsR498G1018170700.01.T01	Leucine-rich repeat receptor-like protein kinase PEPR1
maker-11-augustus-gene-123.41-mRNA-1	Indica	Japonica	Kinase-LRR-TM	Os11g0448000	OsR498G1120059500.01.T01	Receptor-like kinase TMK4 isoform A
maker-2-augustus-gene-30.20-mRNA-1	Indica	Indica	Kinase-LRR-TM	Os02g0154000	OsR498G0202991600.01.T01	Putative leucine-rich repeat receptor-like kinase
maker-2-augustus-gene-41.14-mRNA-1	Indica	NA	Kinase-LRR-TM		OsR498G0203068900.01.T01	Pollen receptor-like kinase
maker-3-augustus-gene-197.33-mRNA-1	Indica	Indica	Kinase-LRR-TM	Os07g0618400	OsR498G0306114400.01.T02	Protein STRUBBELIG-RECEPTOR FAMILY 6
maker-3-augustus-gene-298.46-mRNA-1	Indica	NA	Kinase-LRR-TM	Os10g0375000	OsR498G1018574700.01.T03	Putative receptor like protein kinase
maker-3-augustus-gene-299.43-mRNA-1	Indica	NA	Kinase-LRR-TM	Os10g0374666	OsR498G1018573900.01.T01	LRR receptor-like serine/threonine-protein kinase EFR isoform B
maker-3-augustus-gene-307.8-mRNA-1	Indica	NA	Kinase-LRR-TM	Os10g0360933	OsR498G1018534900.01.T01	Putative LRR receptor-like serine/threonine-protein kinase
maker-5-augustus-gene-214.40-mRNA-1	Indica	NA	Kinase-LRR-TM	Os05g0414700	OsR498G0510843600.01.T02	Serine/threonine protein kinase
maker-5-augustus-gene-247.14-mRNA-1	Indica	NA	Kinase-LRR-TM	Os05g0481100	OsR498G0511055300.01.T09	Putative LRR receptor-like serine/threonine-protein kinase-like
maker-5-augustus-gene-247.29-mRNA-1	Indica	NA	Kinase-LRR-TM	Os05g0480400	OsR498G0511051800.01.T01	Putative inactive receptor kinase
maker-6-augustus-gene-227.0-mRNA-1	Indica	Indica	Kinase-LRR-TM	Os06g0586150	OsR498G0612844500.01.T01	Putative LRR receptor-like serine/threonine-protein kinase
maker-6-augustus-gene-227.3-mRNA-1	Indica	Indica	Kinase-LRR-TM	Os06g0587500	OsR498G0612845300.01.T01	Putative LRR receptor-like serine/threonine-protein kinase
maker-6-augustus-gene-228.1-mRNA-1	Indica	NA	Kinase-LRR-TM		OsR498G0612847900.01.T01	Putative LRR receptor-like serine/threonine-protein kinase
maker-6-augustus-gene-231.7-mRNA-1	Indica	Indica	Kinase-LRR-TM	Os06g0587900	OsR498G0612839000.01.T02	Putative LRR receptor-like serine/threonine-protein kinase
maker-6-augustus-gene-305.61-mRNA-1	Indica	NA	Kinase-LRR-TM	Os06g0717200	OsR498G0613308600.01.T01	Leucine-rich repeat receptor-like kinase protein THICK TASSEL DWARF1
maker-6-augustus-gene-44.21-mRNA-1	Indica	Indica	Kinase-LRR-TM	Os06g0186300	OsR498G0611741800.01.T01	Serine/threonine protein kinase
maker-6-augustus-gene-44.23-mRNA-1	Indica	Japonica	Kinase-LRR-TM	Os06g0186300	OsR498G0611742000.01.T01	Serine/threonine protein kinase
maker-6-augustus-gene-44.5-mRNA-1	Indica	NA	Kinase-LRR-TM			LRR receptor-like serine/threonine-protein kinase EFR
maker-7-augustus-gene-183.22-mRNA-1	Indica	NA	Kinase-LRR-TM	Os07g0498400	OsR498G0714359600.01.T01	LRR receptor-like serine/threonine-protein kinase
maker-8-augustus-gene-191.10-mRNA-1	Indica	NA	Kinase-LRR-TM	Os08g0376300	OsR498G0815955000.01.T01	Receptor-like protein kinase 5
maker-8-augustus-gene-231.37-mRNA-1	Indica	NA	Kinase-LRR-TM		OsR498G0816198400.01.T04	Receptor-like protein kinase
augustus-1-processed-gene-78.1-mRNA-1	Japonica	NA	Kinase-LRR-TM	Os01g0239700	OsR498G0100512300.01.T01	Receptor-like protein kinase HSL1
augustus-10-processed-gene-178.18-mRNA-1	Japonica	NA	Kinase-LRR-TM		OsR498G1018962700.01.T01	Tyrosine-protein kinase
augustus-11-processed-gene-30.12-mRNA-1	Japonica	Japonica	Kinase-LRR-TM	Os11g0172800	OsR498G1119491700.01.T01	Protein kinase domain-containing protein
augustus-11-processed-gene-31.0-mRNA-1	Japonica	Indica	Kinase-LRR-TM	Os11g0171800	OsR498G1119492700.01.T01	Leucine-rich repeat receptor kinase (LRR-RK)
augustus-11-processed-gene-61.7-mRNA-1	Japonica	Indica	Kinase-LRR-TM	Os11g0249900	OsR498G1119716500.01.T01	Probably inactive leucine-rich repeat receptor-like protein kinase
augustus-12-processed-gene-198.43-mRNA-1	Japonica	Japonica	Kinase-LRR-TM	Os12g0498650	OsR498G1221664100.01.T01	Protein kinase domain-containing protein (Fragment)
augustus-2-processed-gene-213.6-mRNA-1	Japonica	Indica	Kinase-LRR-TM	Os01g0296000	OsR498G0204100400.01.T01	Putative LRR receptor-like serine/threonine-protein kinase
augustus-2-processed-gene-259.25-mRNA-1	Japonica	NA	Kinase-LRR-TM	Os02g0629400	OsR498G0204364800.01.T01	Phytosulfokine receptor 1
augustus-2-processed-gene-4.37-mRNA-1	Japonica	NA	Kinase-LRR-TM	Os02g0211200		Putative LRR receptor-like serine/threonine-protein kinase
augustus-2-processed-gene-6.27-mRNA-1	Japonica	NA	Kinase-LRR-TM	Os02g0111800	OsR498G0202835600.01.T01	Leucine-rich repeat receptor-like protein kinase PXL2
augustus-4-processed-gene-237.18-mRNA-1	Japonica	Indica	Kinase-LRR-TM	Os04g0576900	OsR498G0409129100.01.T01	Probable inactive leucine-rich repeat receptor kinase XIAO
augustus-4-processed-gene-35.36-mRNA-1	Japonica	NA	Kinase-LRR-TM			Leucine-rich repeat protein kinase family protein
augustus-6-processed-gene-93.17-mRNA-1	Japonica	NA	Kinase-LRR-TM	Os06g0274500	OsR498G0612087700.01.T01	Putative somatic embryogenesis protein kinase 1
augustus-8-processed-gene-158.35-mRNA-1	Japonica	NA	Kinase-LRR-TM	Os08g0342300	OsR498G0815837200.01.T01	Brassinosteroid LRR receptor kinase BRL3
augustus-8-processed-gene-296.38-mRNA-1	Japonica	NA	Kinase-LRR-TM	Os08g0564700	OsR498G0816627000.01.T01	Serine/threonine protein kinase
augustus-9-processed-gene-74.22-mRNA-1	Japonica	NA	Kinase-LRR-TM	Os09g0293500	OsR498G0917002400.01.T01	Brassinosteroid LRR receptor kinase BRL1
augustus_masked-1-processed-gene-306.22-mRNA-1	Japonica	NA	Kinase-LRR-TM	Os01g0718300	OsR498G0101902000.01.T01	Brassinosteroid-insensitive 1
augustus_masked-10-processed-gene-13.17-mRNA-1	Japonica	NA	Kinase-LRR-TM	Os10g0114400		Mitogen-activated protein kinase kinase kinase
augustus_masked-11-processed-gene-219.32-mRNA-1	Japonica	NA	Kinase-LRR-TM	Os11g0624600	OsR498G1120543200.01.T03	GPCR kinase
augustus_masked-11-processed-gene-74.40-mRNA-1	Japonica	NA	Kinase-LRR-TM			Putative LRR receptor-like serine/threonine-protein kinase (Fragment)
augustus_masked-3-processed-gene-153.48-mRNA-1	Japonica	NA	Kinase-LRR-TM	Os03g0332900	OsR498G0305887500.01.T03	Putative inactive receptor kinase
augustus_masked-3-processed-gene-22.39-mRNA-1	Japonica	NA	Kinase-LRR-TM	Os03g0145000	OsR498G0305221600.01.T01	Leucine-rich repeat receptor-like protein kinase PXL2
augustus_masked-3-processed-gene-41.18-mRNA-1	Japonica	NA	Kinase-LRR-TM	Os03g0183800	OsR498G0305342500.01.T01	Strubbelig-receptor family 6
augustus_masked-3-processed-gene-450.56-mRNA-1	Japonica	NA	Kinase-LRR-TM	Os03g0756200	OsR498G0307070000.01.T03	Lrr receptor-like serinethreonine-protein kinase rpk2
augustus_masked-4-processed-gene-199.39-mRNA-1	Japonica	NA	Kinase-LRR-TM			probable leucine-rich repeat receptor-like protein kinase At2g33170
augustus_masked-4-processed-gene-288.15-mRNA-1	Japonica	NA	Kinase-LRR-TM	Os04g0672100	OsR498G0409468600.01.T01	Phytosulfokine receptor
augustus_masked-5-processed-gene-304.14-mRNA-1	Japonica	NA	Kinase-LRR-TM	Os05g0588250	OsR498G0511432300.01.T02	Putative inactive receptor kinase
augustus_masked-6-processed-gene-226.0-mRNA-1	Japonica	NA	Kinase-LRR-TM			U-box domain-containing protein 33-like isoform X1
augustus_masked-6-processed-gene-231.29-mRNA-1	Japonica	Indica	Kinase-LRR-TM	Os06g0587500	OsR498G0612856100.01.T01	Leucine-rich repeat protein kinase family protein
augustus_masked-6-processed-gene-290.36-mRNA-1	Japonica	Indica	Kinase-LRR-TM	Os06g0692600	OsR498G0613224200.01.T01	Tyrosine-sulfated glycopeptide receptor 1
augustus_masked-6-processed-gene-290.37-mRNA-1	Japonica	NA	Kinase-LRR-TM	Os06g0692600	OsR498G0613225000.01.T01	Tyrosine-sulfated glycopeptide receptor 1
augustus_masked-8-processed-gene-151.29-mRNA-1	Japonica	NA	Kinase-LRR-TM		OsR498G0815800700.01.T01	LRR receptor-like serine/threonine-protein kinase EFR
augustus_masked-8-processed-gene-270.46-mRNA-1	Japonica	NA	Kinase-LRR-TM	Os08g0518400		Serine/threonine-protein kinase
augustus_masked-9-processed-gene-118.25-mRNA-1	Japonica	Indica	Kinase-LRR-TM	Os09g0352000	OsR498G0917242100.01.T03	Putative leucine-rich repeat receptor-like serine/threonine-protein kinase
maker-1-augustus-gene-189.11-mRNA-1	Japonica	NA	Kinase-LRR-TM	Os01g0514700	OsR498G0101114500.01.T01	Receptor protein kinase-like protein ZAR1
maker-1-augustus-gene-189.13-mRNA-1	Japonica	NA	Kinase-LRR-TM			Protein kinase domain-containing protein
maker-1-augustus-gene-230.10-mRNA-1	Japonica	NA	Kinase-LRR-TM	Os01g0577600	OsR498G0101394600.01.T01	Leucine-rich repeat receptor protein kinase EXS
maker-1-augustus-gene-28.30-mRNA-1	Japonica	NA	Kinase-LRR-TM	Os01g0152000	OsR498G0100185300.01.T01	LRR receptor-like serine/threonine-protein kinase EFR
maker-1-augustus-gene-284.39-mRNA-1	Japonica	NA	Kinase-LRR-TM		OsR498G0101747700.01.T01	Protein MALE DISCOVERER 1
maker-1-augustus-gene-29.59-mRNA-1	Japonica	Japonica	Kinase-LRR-TM	Os01g0152600	OsR498G0100187100.01.T01	Putative LRR receptor-like serine/threonine-protein kinase
maker-1-augustus-gene-29.60-mRNA-1	Japonica	Japonica	Kinase-LRR-TM	Os01g0152800	OsR498G0100189500.01.T01	LRR receptor-like serine/threonine-protein kinase EFR
maker-1-augustus-gene-293.3-mRNA-1	Japonica	NA	Kinase-LRR-TM			Receptor kinase MRKa
maker-1-augustus-gene-293.5-mRNA-1	Japonica	NA	Kinase-LRR-TM			Leucine-rich repeat receptor kinase-like protein
maker-1-augustus-gene-302.17-mRNA-1	Japonica	NA	Kinase-LRR-TM	Os01g0711200	OsR498G0101870300.01.T01	Leucine-rich repeat transmembrane protein kinase
maker-1-augustus-gene-317.35-mRNA-1	Japonica	NA	Kinase-LRR-TM	Os01g0742400	OsR498G0101973900.01.T01	Protein kinase family protein with leucine-rich repeat domain
maker-1-augustus-gene-477.5-mRNA-1	Japonica	NA	Kinase-LRR-TM	Os01g0917500	OsR498G0102576500.01.T05	Leucine-rich repeat receptor protein kinase MSP1
maker-1-augustus-gene-499.14-mRNA-1	Japonica	NA	Kinase-LRR-TM	Os01g0957100	OsR498G0102731100.01.T01	Inactive leucine-rich repeat receptor-like protein kinase-like
maker-1-augustus-gene-501.23-mRNA-1	Japonica	NA	Kinase-LRR-TM	Os01g0960400	OsR498G0102740800.01.T02	Putative leucine-rich repeat receptor-like serine/threonine-protein kinase
maker-1-augustus-gene-509.16-mRNA-1	Japonica	Indica	Kinase-LRR-TM	Os01g0976900	OsR498G0102790800.01.T02	Mitogen-activated protein kinase kinase kinase
maker-1-augustus-gene-71.50-mRNA-1	Japonica	Japonica	Kinase-LRR-TM	Os01g0228200	OsR498G0100470100.01.T01	Putative receptor kinase TRKc
maker-10-augustus-gene-16.50-mRNA-1	Japonica	NA	Kinase-LRR-TM	Os10g0119200	OsR498G1018061000.01.T01	Putative LRR receptor-like serine/threonine-protein kinase
maker-10-augustus-gene-164.25-mRNA-1	Japonica	NA	Kinase-LRR-TM	Os10g0468500	OsR498G1018872900.01.T01	MDIS1-interacting receptor like kinase 2
maker-11-augustus-gene-183.28-mRNA-1	Japonica	NA	Kinase-LRR-TM	Os11g0559200	OsR498G1120353500.01.T01	Receptor kinase, putative, expressed
maker-11-augustus-gene-187.15-mRNA-1	Japonica	Japonica	Kinase-LRR-TM	Os11g0569300	OsR498G1120383600.01.T02	Leucin-rich receptor-like kinase
maker-11-augustus-gene-187.20-mRNA-1	Japonica	Japonica	Kinase-LRR-TM	Os11g0569500	OsR498G1120381200.01.T01	Putative LRR receptor-like serine/threonine-protein kinase
maker-11-augustus-gene-188.40-mRNA-1	Japonica	Indica	Kinase-LRR-TM	Os11g0569600	OsR498G1120386200.01.T01	Receptor kinase-like protein (Fragment)
maker-11-augustus-gene-209.14-mRNA-1	Japonica	NA	Kinase-LRR-TM	Os11g0607200	OsR498G1120487000.01.T01	BRASSINOSTEROID INSENSITIVE 1-associated receptor kinase 1
maker-11-augustus-gene-217.42-mRNA-1	Japonica	NA	Kinase-LRR-TM	Os11g0620500	OsR498G1120524900.01.T01	Leucine-rich repeat receptor protein kinase EXS, putative
maker-11-augustus-gene-220.23-mRNA-1	Japonica	Japonica	Kinase-LRR-TM	Os11g0625900	OsR498G1120550600.01.T01	Putative LRR receptor-like serine/threonine-protein kinase
maker-11-augustus-gene-221.0-mRNA-1	Japonica	Japonica	Kinase-LRR-TM	Os11g0628000	OsR498G1120550600.01.T01	Putative LRR receptor-like serine/threonine-protein kinase
maker-11-augustus-gene-257.26-mRNA-1	Japonica	Indica	Kinase-LRR-TM	Os11g0695700	OsR498G1120747200.01.T01	Putative receptor kinase TRKc
maker-11-augustus-gene-257.28-mRNA-1	Japonica	NA	Kinase-LRR-TM		OsR498G1120751000.01.T01	Protein kinase domain containing protein, expressed
maker-11-augustus-gene-258.22-mRNA-1	Japonica	NA	Kinase-LRR-TM	Os11g0695600	OsR498G1120747600.01.T01	Serine-threonine protein kinase plant-type
maker-11-augustus-gene-30.26-mRNA-1	Japonica	NA	Kinase-LRR-TM	Os11g0171800	OsR498G1119488300.01.T02	Putative LRR receptor-like serine/threonine-protein kinase
maker-11-augustus-gene-30.62-mRNA-1	Japonica	Japonica	Kinase-LRR-TM	Os11g0172700	OsR498G1119488300.01.T02	Putative LRR receptor-like serine/threonine-protein kinase
maker-11-augustus-gene-31.34-mRNA-1	Japonica	Indica	Kinase-LRR-TM	Os11g0173700	OsR498G1119493500.01.T01	Putative LRR receptor-like serine/threonine-protein kinase
maker-11-augustus-gene-31.36-mRNA-1	Japonica	Indica	Kinase-LRR-TM	Os11g0173500	OsR498G1119494900.01.T07	Putative LRR receptor-like serine/threonine-protein kinase
maker-11-augustus-gene-31.38-mRNA-1	Japonica	Japonica	Kinase-LRR-TM	Os11g0173700	OsR498G1119493500.01.T01	Putative LRR receptor-like serine/threonine-protein kinase
maker-11-augustus-gene-31.40-mRNA-1	Japonica	NA	Kinase-LRR-TM	Os11g0173800	OsR498G1119496700.01.T02	Leucine Rich Repeat family protein, expressed
maker-11-augustus-gene-31.44-mRNA-1	Japonica	NA	Kinase-LRR-TM	Os11g0173900	OsR498G1119496900.01.T01	Leucine-rich repeat receptor kinase (LRR-RK)
maker-11-augustus-gene-58.29-mRNA-1	Japonica	Indica	Kinase-LRR-TM	Os12g0210400	OsR498G1119698900.01.T01	probable leucine-rich repeat receptor-like protein kinase At5g49770
maker-11-augustus-gene-71.10-mRNA-1	Japonica	Japonica	Kinase-LRR-TM	Os11g0208900	OsR498G1119599000.01.T01	Serine-threonine protein kinase plant-type
maker-11-augustus-gene-85.28-mRNA-1	Japonica	NA	Kinase-LRR-TM		OsR498G1119660500.01.T01	Leucine-rich repeat receptor-like serine/threonine-protein kinase BAM3
maker-12-augustus-gene-1.34-mRNA-1	Japonica	NA	Kinase-LRR-TM		OsR498G1220789200.01.T06	Leucine-rich repeat protein kinase family protein
maker-12-augustus-gene-236.80-mRNA-1	Japonica	Japonica	Kinase-LRR-TM	Os12g0567500	OsR498G1221899800.01.T01	Expressed protein kinase
maker-12-augustus-gene-24.29-mRNA-1	Japonica	NA	Kinase-LRR-TM		OsR498G1220933000.01.T02	Leucine-rich repeat receptor-like protein kinase PXC1
maker-12-augustus-gene-4.60-mRNA-1	Japonica	Indica	Kinase-LRR-TM	Os11g0107700	OsR498G1220804500.01.T01	probable leucine-rich repeat receptor-like protein kinase IMK3
maker-12-augustus-gene-44.21-mRNA-1	Japonica	NA	Kinase-LRR-TM	Os12g0182300	OsR498G1221050600.01.T01	Tyrosine-protein kinase
maker-12-augustus-gene-60.29-mRNA-1	Japonica	NA	Kinase-LRR-TM	Os12g0210400	OsR498G1221109900.01.T01	Leucine-rich repeat receptor-like protein kinase
maker-12-augustus-gene-76.35-mRNA-1	Japonica	Indica	Kinase-LRR-TM	Os11g0300600	OsR498G1221171100.01.T01	Putative inactive leucine-rich repeat receptor-like protein kinase
maker-2-augustus-gene-102.24-mRNA-1	Japonica	NA	Kinase-LRR-TM		OsR498G0203410200.01.T02	Putative leucine-rich repeat receptor-like protein kinase
maker-2-augustus-gene-263.37-mRNA-1	Japonica	Indica	Kinase-LRR-TM	Os11g0569800	OsR498G0204384300.01.T02	Putative LRR receptor-like serine/threonine-protein kinase
maker-2-augustus-gene-311.70-mRNA-1	Japonica	Indica	Kinase-LRR-TM	Os06g0274500	OsR498G0204694200.01.T01	Putative somatic embryogenesis protein kinase 1
maker-2-augustus-gene-365.0-mRNA-1	Japonica	Indica	Kinase-LRR-TM	Os06g0203800	OsR498G0204869500.01.T02	LRR receptor-like serine/threonine-protein kinase ERECTA
maker-2-augustus-gene-392.80-mRNA-1	Japonica	NA	Kinase-LRR-TM		OsR498G0205050200.01.T01	Inactive leucine-rich repeat receptor-like serine/threonine-protein kinase
maker-2-augustus-gene-51.0-mRNA-1	Japonica	NA	Kinase-LRR-TM	Os02g0190500	OsR498G0203132100.01.T01	Protein STRUBBELIG-RECEPTOR FAMILY 8 isoform X1
maker-2-augustus-gene-53.60-mRNA-1	Japonica	Indica	Kinase-LRR-TM	Os01g0917500	OsR498G0203145000.01.T01	Leucine-rich repeat receptor protein kinase EXS
maker-2-augustus-gene-62.5-mRNA-1	Japonica	NA	Kinase-LRR-TM	Os02g0210700	OsR498G0203202700.01.T01	Putative LRR receptor-like serine/threonine-protein kinase
maker-2-augustus-gene-63.14-mRNA-1	Japonica	NA	Kinase-LRR-TM	Os02g0211200		Putative LRR receptor-like serine/threonine-protein kinase
maker-2-augustus-gene-63.16-mRNA-1	Japonica	NA	Kinase-LRR-TM	Os02g0211200		Putative LRR receptor-like serine/threonine-protein kinase
maker-2-augustus-gene-65.48-mRNA-1	Japonica	Japonica	Kinase-LRR-TM	Os02g0215500	OsR498G0203219100.01.T01	Putative LRR receptor-like serine/threonine-protein kinase
maker-2-augustus-gene-65.50-mRNA-1	Japonica	NA	Kinase-LRR-TM		OsR498G0203220300.01.T01	Putative LRR receptor-like serine/threonine-protein kinase
maker-2-augustus-gene-65.52-mRNA-1	Japonica	NA	Kinase-LRR-TM	Os02g0215700		Putative LRR receptor-like serine/threonine-protein kinase
maker-2-augustus-gene-66.58-mRNA-1	Japonica	Japonica	Kinase-LRR-TM	Os02g0215700	OsR498G0203219100.01.T01	Putative LRR receptor-like serine/threonine-protein kinase
maker-2-augustus-gene-70.0-mRNA-1	Japonica	NA	Kinase-LRR-TM		OsR498G0203239100.01.T01	receptor-like protein kinase 5 isoform X1
maker-2-augustus-gene-70.2-mRNA-1	Japonica	NA	Kinase-LRR-TM		OsR498G0203240500.01.T01	Serine/threonine protein kinase
maker-2-augustus-gene-76.10-mRNA-1	Japonica	NA	Kinase-LRR-TM			Mitogen-activated protein kinase kinase kinase
maker-2-augustus-gene-76.20-mRNA-1	Japonica	NA	Kinase-LRR-TM	Os02g0231700	OsR498G0203282600.01.T01	Leucine-rich repeat protein kinase family protein
maker-2-augustus-gene-78.31-mRNA-1	Japonica	NA	Kinase-LRR-TM	Os02g0236100	OsR498G0203300000.01.T02	LRR receptor-like serine/threonine-protein kinase
maker-2-augustus-gene-81.15-mRNA-1	Japonica	NA	Kinase-LRR-TM	Os02g0241100	OsR498G0203310000.01.T01	Serine/threonine protein kinase
maker-3-augustus-gene-113.36-mRNA-1	Japonica	NA	Kinase-LRR-TM		OsR498G0305838900.01.T01	Putative inactive receptor kinase
maker-3-augustus-gene-13.9-mRNA-1	Japonica	NA	Kinase-LRR-TM	Os03g0127700	OsR498G0305161700.01.T01	Leucine-rich repeat transmembrane protein kinase, putative, expressed
maker-3-augustus-gene-152.0-mRNA-1	Japonica	NA	Kinase-LRR-TM		OsR498G0305877700.01.T01	probable leucine-rich repeat receptor-like protein kinase At5g49770
maker-3-augustus-gene-155.37-mRNA-1	Japonica	NA	Kinase-LRR-TM	Os03g0335500	OsR498G0305895900.01.T01	Mitogen-activated protein kinase kinase kinase
maker-3-augustus-gene-288.2-mRNA-1	Japonica	NA	Kinase-LRR-TM	Os10g0389800	OsR498G1018625400.01.T01	Strubbelig-receptor family 6
maker-3-augustus-gene-419.41-mRNA-1	Japonica	Indica	Kinase-LRR-TM	Os03g0703200	OsR498G0306885000.01.T01	LRR receptor-like serine/threonine-protein kinase
maker-3-augustus-gene-425.67-mRNA-1	Japonica	NA	Kinase-LRR-TM		OsR498G0306918600.01.T01	Atypical receptor-like kinase MARK
maker-3-augustus-gene-427.53-mRNA-1	Japonica	Indica	Kinase-LRR-TM	Os03g0717000	OsR498G0306934600.01.T01	Receptor-like kinase TMK2
maker-3-augustus-gene-431.40-mRNA-1	Japonica	NA	Kinase-LRR-TM		OsR498G0306963400.01.T02	Lrr receptor protein kinase
maker-3-augustus-gene-443.8-mRNA-1	Japonica	NA	Kinase-LRR-TM		OsR498G0307031400.01.T02	Serine/threonine protein kinase
maker-3-augustus-gene-458.28-mRNA-1	Japonica	NA	Kinase-LRR-TM	Os03g0773300	OsR498G0307128800.01.T01	Putative LRR receptor-like serine/threonine-protein kinase
maker-3-augustus-gene-459.39-mRNA-1	Japonica	Indica	Kinase-LRR-TM	Os03g0773700	OsR498G0307131300.01.T03	Leucine-rich repeat receptor-like serine/threonine-protein kinase BAM1
maker-3-augustus-gene-467.11-mRNA-1	Japonica	NA	Kinase-LRR-TM	Os03g0791700	OsR498G0307187900.01.T02	Leucine-rich repeat transmembrane protein kinase, putative, expressed

maker-3-augustus-gene-62.58-mRNA-1	Japonica	NA	Kinase-LRR-TM	Os03g0223000	OsR498G0305477500.01.T01	Atypical receptor-like kinase MARK
maker-3-augustus-gene-66.8-mRNA-1	Japonica	NA	Kinase-LRR-TM	Os03g0228800	OsR498G0305501000.01.T01	Leucine-rich repeat receptor-like serine/threonine-protein kinase BAM1
maker-3-augustus-gene-86.1-mRNA-1	Japonica	NA	Kinase-LRR-TM	Os03g0266800	OsR498G0305638100.01.T01	LRR receptor-like serine/threonine-protein kinase FEI 1
maker-4-augustus-gene-175.77-mRNA-1	Japonica	NA	Kinase-LRR-TM	Os04g0457800	OsR498G0408690200.01.T01	Non-specific serine/threonine protein kinase
maker-4-augustus-gene-183.9-mRNA-1	Japonica	NA	Kinase-LRR-TM	Os04g0472500	OsR498G0408749300.01.T01	Inactive leucine-rich repeat receptor-like protein kinase
maker-4-augustus-gene-259.21-mRNA-1	Japonica	Indica	Kinase-LRR-TM	Os08g0203700	OsR498G0409280800.01.T01	Non-specific serine/threonine protein kinase
maker-4-augustus-gene-260.19-mRNA-1	Japonica	Japonica	Kinase-LRR-TM	Os04g0616700	OsR498G0409282800.01.T02	Leucine-rich repeat transmembrane protein kinase
maker-4-augustus-gene-261.7-mRNA-1	Japonica	NA	Kinase-LRR-TM	Os04g0618700	OsR498G0409289200.01.T01	LRR receptor-like protein serine/threonine-protein kinase FLS2
maker-4-augustus-gene-276.47-mRNA-1	Japonica	NA	Kinase-LRR-TM	Os04g0649700	OsR498G0409389000.01.T01	Putative inactive receptor kinase
maker-4-augustus-gene-294.22-mRNA-1	Japonica	NA	Kinase-LRR-TM		OsR498G0409512300.01.T01	Serine/threonine protein kinase
maker-4-augustus-gene-296.8-mRNA-1	Japonica	NA	Kinase-LRR-TM	Os04g0685900	OsR498G0409521200.01.T01	Receptor-like kinase TMK3
maker-4-augustus-gene-35.2-mRNA-1	Japonica	NA	Kinase-LRR-TM		OsR498G0407776100.01.T01	Putative LRR receptor-like serine/threonine-protein kinase
maker-4-augustus-gene-35.3-mRNA-1	Japonica	NA	Kinase-LRR-TM			Putative LRR receptor-like serine/threonine-protein kinase
maker-5-augustus-gene-141.9-mRNA-1	Japonica	NA	Kinase-LRR-TM		OsR498G0510414500.01.T01	Serine/threonine protein kinase
maker-5-augustus-gene-242.5-mRNA-1	Japonica	NA	Kinase-LRR-TM	Os05g0471000	OsR498G0511021200.01.T03	Inactive receptor-like serine/threonine-protein kinase (Fragment)
maker-5-augustus-gene-246.40-mRNA-1	Japonica	NA	Kinase-LRR-TM	Os05g0478300	OsR498G0511047500.01.T01	Protein kinase domain-containing protein (Fragment)
maker-5-augustus-gene-250.6-mRNA-1	Japonica	NA	Kinase-LRR-TM	Os05g0486100	OsR498G0511075400.01.T01	Putative leucine-rich repeat receptor-like protein kinase
maker-5-augustus-gene-271.7-mRNA-1	Japonica	Indica	Kinase-LRR-TM	Os01g0742400	OsR498G0511207000.01.T01	Protein kinase family protein with leucine-rich repeat domain
maker-5-augustus-gene-272.49-mRNA-1	Japonica	NA	Kinase-LRR-TM	Os05g0524500	OsR498G0511212300.01.T01	Putative receptor like protein kinase
maker-5-augustus-gene-272.52-mRNA-1	Japonica	Indica	Kinase-LRR-TM	Os05g0524600	OsR498G0511212900.01.T03	Putative serine/threonine-specific receptor protein kinase
maker-5-augustus-gene-272.63-mRNA-1	Japonica	Japonica	Kinase-LRR-TM	Os05g0525000	OsR498G0511214700.01.T01	Putative serine/threonine-specific receptor protein kinase
maker-5-augustus-gene-272.65-mRNA-1	Japonica	NA	Kinase-LRR-TM	Os05g0525550	OsR498G0511214700.01.T01	Putative receptor like protein kinase
maker-5-augustus-gene-272.68-mRNA-1	Japonica	NA	Kinase-LRR-TM	Os05g0525600	OsR498G0511215100.01.T01	Putative leucine-rich repeat receptor-like protein kinase
maker-5-augustus-gene-272.71-mRNA-1	Japonica	Indica	Kinase-LRR-TM	Os05g0525550	OsR498G0511215300.01.T01	Putative serine/threonine-specific receptor protein kinase
maker-5-augustus-gene-40.0-mRNA-1	Japonica	Indica	Kinase-LRR-TM	Os01g0170300	OsR498G0509825300.01.T01	Mitogen-activated protein kinase kinase kinase
maker-5-augustus-gene-89.0-mRNA-1	Japonica	NA	Kinase-LRR-TM			Putative leucine-rich repeat receptor-like protein kinase
maker-5-augustus-gene-94.45-mRNA-1	Japonica	Indica	Kinase-LRR-TM	Os04g0291900	OsR498G0510160500.01.T05	Non-specific serine/threonine protein kinase
maker-5-augustus-gene-95.16-mRNA-1	Japonica	NA	Kinase-LRR-TM	Os04g0291900	OsR498G0510174900.01.T02	Non-specific serine/threonine protein kinase
maker-5-augustus-gene-96.10-mRNA-1	Japonica	Indica	Kinase-LRR-TM	Os05g0257100	OsR498G0510172500.01.T03	Non-specific serine/threonine protein kinase
maker-6-augustus-gene-132.5-mRNA-1	Japonica	NA	Kinase-LRR-TM		OsR498G0612537000.01.T03	GPCR kinase
maker-6-augustus-gene-211.0-mRNA-1	Japonica	NA	Kinase-LRR-TM	Os06g0557100	OsR498G0612756500.01.T04	Protein kinase family protein with leucine-rich repeat domain
maker-6-augustus-gene-211.2-mRNA-1	Japonica	NA	Kinase-LRR-TM	Os06g0557700	OsR498G0612758100.01.T01	Serine/threonine protein kinase
maker-6-augustus-gene-224.30-mRNA-1	Japonica	NA	Kinase-LRR-TM			LRR receptor-like serine/threonine-protein kinase EFR
maker-6-augustus-gene-225.34-mRNA-1	Japonica	Indica	Kinase-LRR-TM	Os12g0498650	OsR498G0612836400.01.T01	Putative LRR receptor-like serine/threonine-protein kinase
maker-6-augustus-gene-225.37-mRNA-1	Japonica	Indica	Kinase-LRR-TM	Os06g0587900	OsR498G0612837400.01.T02	Putative LRR receptor-like serine/threonine-protein kinase
maker-6-augustus-gene-228.0-mRNA-1	Japonica	Japonica	Kinase-LRR-TM	Os06g0586150	OsR498G0612846900.01.T01	Putative LRR receptor-like serine/threonine-protein kinase
maker-6-augustus-gene-228.1-mRNA-1	Japonica	NA	Kinase-LRR-TM		OsR498G0612847900.01.T01	Putative LRR receptor-like serine/threonine-protein kinase
maker-6-augustus-gene-229.0-mRNA-1	Japonica	NA	Kinase-LRR-TM		OsR498G0612851100.01.T02	Leucine-rich repeat protein kinase family protein
maker-6-augustus-gene-230.0-mRNA-1	Japonica	NA	Kinase-LRR-TM			Protein kinase domain-containing protein (Fragment)
maker-6-augustus-gene-232.45-mRNA-1	Japonica	NA	Kinase-LRR-TM		OsR498G0612858300.01.T01	Leucine-rich repeat protein kinase family protein
maker-6-augustus-gene-259.9-mRNA-1	Japonica	Indica	Kinase-LRR-TM	Os02g0190500	OsR498G0613020200.01.T02	Putative STRUBBELIG family receptor protein kinase
maker-6-augustus-gene-261.62-mRNA-1	Japonica	NA	Kinase-LRR-TM		OsR498G0613032800.01.T01	Leucine-rich repeat protein kinase family protein isoform 2
maker-7-augustus-gene-223.74-mRNA-1	Japonica	NA	Kinase-LRR-TM		OsR498G0714588200.01.T01	Protein kinase domain-containing protein
maker-7-augustus-gene-225.38-mRNA-1	Japonica	NA	Kinase-LRR-TM	Os07g0568100	OsR498G0714596100.01.T01	Nodulation receptor kinase A
maker-7-augustus-gene-229.75-mRNA-1	Japonica	Indica	Kinase-LRR-TM	Os07g0574100	OsR498G0714615800.01.T01	Receptor-kinase isolog
maker-7-augustus-gene-241.21-mRNA-1	Japonica	NA	Kinase-LRR-TM	Os07g0597200	OsR498G0714696400.01.T01	Leucine-rich repeat transmembrane protein kinase
maker-7-augustus-gene-253.31-mRNA-1	Japonica	NA	Kinase-LRR-TM	Os07g0618400	OsR498G0714763500.01.T01	Protein STRUBBELIG-RECEPTOR FAMILY 7
maker-7-augustus-gene-257.83-mRNA-1	Japonica	NA	Kinase-LRR-TM	Os07g0626500	OsR498G0714790400.01.T01	Inactive receptor kinase
maker-7-augustus-gene-287.50-mRNA-1	Japonica	NA	Kinase-LRR-TM	Os07g0681100	OsR498G0714964600.01.T02	Putative inactive receptor kinase isoform A
maker-7-augustus-gene-32.32-mRNA-1	Japonica	NA	Kinase-LRR-TM	Os07g0166700	OsR498G0713584300.01.T02	Leucine-rich repeat transmembrane protein kinase, putative, expressed
maker-7-augustus-gene-7.39-mRNA-1	Japonica	NA	Kinase-LRR-TM	Os07g0121200	OsR498G0713427200.01.T01	Putative LRR receptor-like serine/threonine-protein kinase
maker-7-augustus-gene-81.0-mRNA-1	Japonica	NA	Kinase-LRR-TM	Os07g0251900	OsR498G0713893500.01.T01	Putative LRR receptor-like serine/threonine-protein kinase
maker-8-augustus-gene-218.58-mRNA-1	Japonica	Indica	Kinase-LRR-TM	Os09g0400500	OsR498G0816116200.01.T01	Mitogen-activated protein kinase kinase kinase
maker-8-augustus-gene-229.54-mRNA-1	Japonica	NA	Kinase-LRR-TM	Os08g0442700	OsR498G0816185600.01.T01	Serine/threonine protein kinase
maker-8-augustus-gene-263.40-mRNA-1	Japonica	Indica	Kinase-LRR-TM	Os08g0506400	OsR498G0816427500.01.T03	Receptor protein kinase-like protein ZAR1
maker-8-augustus-gene-272.13-mRNA-1	Japonica	Japonica	Kinase-LRR-TM	Os08g0521200	OsR498G0816482400.01.T01	Putative transferase, protein kinase RLK-Pelle-LRR-III family
maker-8-augustus-gene-45.34-mRNA-1	Japonica	NA	Kinase-LRR-TM	Os08g0174700	OsR498G0815299300.01.T01	Non-specific serine/threonine protein kinase
maker-8-augustus-gene-60.1-mRNA-1	Japonica	Japonica	Kinase-LRR-TM	Os08g0201700	OsR498G0815383300.01.T05	Non-specific serine/threonine protein kinase
maker-8-augustus-gene-61.24-mRNA-1	Japonica	NA	Kinase-LRR-TM			Non-specific serine/threonine protein kinase
maker-8-augustus-gene-61.30-mRNA-1	Japonica	NA	Kinase-LRR-TM	Os08g0203300	OsR498G0815386700.01.T02	Non-specific serine/threonine protein kinase
maker-8-augustus-gene-61.36-mRNA-1	Japonica	NA	Kinase-LRR-TM			Putative Receptor-like serine/threonine kinase(RFK1)
maker-8-augustus-gene-61.45-mRNA-1	Japonica	Indica	Kinase-LRR-TM	Os08g0203400	OsR498G0815387700.01.T03	Non-specific serine/threonine protein kinase
maker-8-augustus-gene-61.50-mRNA-1	Japonica	NA	Kinase-LRR-TM	Os08g0203700	OsR498G0815387900.01.T03	Non-specific serine/threonine protein kinase
maker-8-augustus-gene-93.6-mRNA-1	Japonica	NA	Kinase-LRR-TM	Os08g0248100	OsR498G0815535500.01.T02	Putative LRR receptor-like serine/threonine-protein kinase
maker-8-augustus-gene-93.7-mRNA-1	Japonica	NA	Kinase-LRR-TM	Os08g0248100	OsR498G0815535500.01.T02	Putative LRR receptor-like serine/threonine-protein kinase
maker-8-augustus-gene-93.8-mRNA-1	Japonica	Japonica	Kinase-LRR-TM	Os08g0248100	OsR498G0815535700.01.T01	Receptor kinase
maker-9-augustus-gene-105.27-mRNA-1	Japonica	Indica	Kinase-LRR-TM	Os09g0353200	OsR498G0917227800.01.T02	Putative LRR receptor-like serine/threonine-protein kinase
maker-9-augustus-gene-105.36-mRNA-1	Japonica	Japonica	Kinase-LRR-TM	Os09g0352000	OsR498G0917223000.01.T02	Putative leucine-rich repeat receptor-like serine/threonine-protein kinase
maker-9-augustus-gene-106.25-mRNA-1	Japonica	NA	Kinase-LRR-TM	Os09g0351700		Serine/threonine-specific receptor protein kinase-like
maker-9-augustus-gene-106.28-mRNA-1	Japonica	NA	Kinase-LRR-TM	Os09g0350900	OsR498G0917219200.01.T02	Serine/threonine-specific receptor protein kinase-like
maker-9-augustus-gene-107.2-mRNA-1	Japonica	Indica	Kinase-LRR-TM	Os09g0352000	OsR498G0917216200.01.T01	Serine/threonine-specific receptor protein kinase-like
maker-9-augustus-gene-107.4-mRNA-1	Japonica	Japonica	Kinase-LRR-TM	Os09g0349800	OsR498G0917216200.01.T01	Putative LRR receptor-like serine/threonine-protein kinase
maker-9-augustus-gene-109.22-mRNA-1	Japonica	NA	Kinase-LRR-TM			Leucine rich repeat malectin kinase 1
maker-9-augustus-gene-115.0-mRNA-1	Japonica	NA	Kinase-LRR-TM	Os09g0355400		Senescence-induced receptor-like serine/threonine-protein kinase
maker-9-augustus-gene-115.7-mRNA-1	Japonica	Indica	Kinase-LRR-TM	Os09g0352000	OsR498G0917236000.01.T01	Putative leucine-rich repeat receptor-like serine/threonine-protein kinase
maker-9-augustus-gene-116.5-mRNA-1	Japonica	Japonica	Kinase-LRR-TM	Os09g0356800	OsR498G0917239000.01.T01	Leucine-rich repeat protein kinase-like
maker-9-augustus-gene-117.0-mRNA-1	Japonica	NA	Kinase-LRR-TM	Os09g0356800	OsR498G0917239000.01.T02	Putative LRR receptor-like serine/threonine-protein kinase
maker-9-augustus-gene-127.37-mRNA-1	Japonica	NA	Kinase-LRR-TM	Os09g0376600		Nodulation receptor kinase
maker-9-augustus-gene-223.67-mRNA-1	Japonica	NA	Kinase-LRR-TM	Os09g0559900	OsR498G0917947700.01.T02	STRUBBELIG-receptor family 6
maker-9-augustus-gene-9.8-mRNA-1	Japonica	NA	Kinase-LRR-TM	Os09g0110100	OsR498G0916673100.01.T01	LRR-RLK
maker-9-augustus-gene-97.48-mRNA-1	Japonica	NA	Kinase-LRR-TM	Os09g0326100	OsR498G0917161200.01.T01	Receptor-like protein kinase HSL1
augustus-1-processed-gene-388.2-mRNA-1	NA	NA	Kinase-LRR-TM	Os01g0878300	OsR498G0102446100.01.T01	receptor-like protein kinase HAIKU2
augustus-1-processed-gene-412.37-mRNA-1	NA	Indica	Kinase-LRR-TM	Os05g0588250	OsR498G0101507200.01.T01	Serine/threonine protein kinase
augustus-1-processed-gene-439.40-mRNA-1	NA	NA	Kinase-LRR-TM	Os01g0878300	OsR498G0102446100.01.T01	receptor-like protein kinase HAIKU2
augustus-1-processed-gene-463.44-mRNA-1	NA	Indica	Kinase-LRR-TM	Os05g0588250	OsR498G0101507200.01.T01	Serine/threonine protein kinase
augustus-10-processed-gene-80.22-mRNA-1	NA	Indica	Kinase-LRR-TM	Os11g0692100	OsR498G1018382300.01.T01	Leucine-rich repeat receptor kinase-type protein
augustus-12-processed-gene-274.19-mRNA-1	NA	NA	Kinase-LRR-TM	Os12g0632800	OsR498G1222161300.01.T01	Receptor-like protein kinase HAIKU2
augustus-2-processed-gene-259.25-mRNA-1	NA	NA	Kinase-LRR-TM	Os02g0629400	OsR498G0204364800.01.T01	Phytosulfokine receptor 1
augustus-2-processed-gene-331.30-mRNA-1	NA	NA	Kinase-LRR-TM	Os06g0667000	OsR498G0613137300.01.T01	Protein kinase domain-containing protein (Fragment)
augustus-2-processed-gene-334.32-mRNA-1	NA	NA	Kinase-LRR-TM	0	OsR498G0613116800.01.T01	Pollen receptor-like kinase 4
augustus-2-processed-gene-9.69-mRNA-1	NA	NA	Kinase-LRR-TM	Os02g0116700	OsR498G0202850700.01.T01	Phytosulfokine receptor 1
augustus-4-processed-gene-190.60-mRNA-1	NA	NA	Kinase-LRR-TM	Os04g0487200	OsR498G0408795300.01.T01	Inactive LRR receptor-like serine/threonine-protein kinase
augustus-7-processed-gene-244.0-mRNA-1	NA	NA	Kinase-LRR-TM	Os07g0602700	OsR498G0714714100.01.T01	LRR receptor-like serine/threonine-protein kinase
augustus-8-processed-gene-110.4-mRNA-1	NA	NA	Kinase-LRR-TM	Os08g0276400	OsR498G0815617800.01.T01	Tyrosine-protein kinase
augustus-8-processed-gene-158.35-mRNA-1	NA	NA	Kinase-LRR-TM	Os08g0342300	OsR498G0815837200.01.T01	Brassinosteroid LRR receptor kinase BRL3
augustus_masked-4-processed-gene-199.39-mRNA-1	NA	NA	Kinase-LRR-TM			probable leucine-rich repeat receptor-like protein kinase At2g33170
augustus_masked-6-processed-gene-226.0-mRNA-1	NA	NA	Kinase-LRR-TM			U-box domain-containing protein 33-like isoform X1
augustus_masked-6-processed-gene-278.2-mRNA-1	NA	NA	Kinase-LRR-TM	Os06g0667000	OsR498G0613137300.01.T01	Protein kinase domain-containing protein (Fragment)
augustus_masked-6-processed-gene-51.1-mRNA-1	NA	NA	Kinase-LRR-TM	Os06g0198900	OsR498G0611789100.01.T01	Pollen receptor-like protein kinase 3
augustus_masked-8-processed-gene-257.1-mRNA-1	NA	Indica	Kinase-LRR-TM	Os08g0493800	OsR498G0816372100.01.T01	LRR receptor-like serine/threonine-protein kinase
augustus_masked-8-processed-gene-270.46-mRNA-1	NA	NA	Kinase-LRR-TM	Os08g0518400		Serine/threonine-protein kinase
augustus_masked-9-processed-gene-53.22-mRNA-1	NA	Indica	Kinase-LRR-TM	Os05g0525550	OsR498G0916869900.01.T01	Putative serine/threonine-specific protein kinase
maker-1-augustus-gene-13.58-mRNA-1	NA	Indica	Kinase-LRR-TM	Os05g0524500	OsR498G0100082200.01.T01	Putative serine/threonine-specific receptor protein kinase
maker-1-augustus-gene-246.65-mRNA-1	NA	Indica	Kinase-LRR-TM	Os05g0588250	OsR498G0101507200.01.T01	Serine/threonine protein kinase
maker-1-augustus-gene-29.61-mRNA-1	NA	NA	Kinase-LRR-TM	Os01g0153000	OsR498G0100190500.01.T02	LRR receptor-like serine/threonine-protein kinase EFR
maker-1-augustus-gene-351.29-mRNA-1	NA	NA	Kinase-LRR-TM	0	OsR498G0102189200.01.T01	Putative light repressible receptor protein kinase
maker-1-augustus-gene-352.48-mRNA-1	NA	NA	Kinase-LRR-TM	0	OsR498G0102192000.01.T01	Putative light repressible receptor protein kinase
maker-1-augustus-gene-37.14-mRNA-1	NA	NA	Kinase-LRR-TM	0	OsR498G0100238400.01.T01	Leucine-rich repeat receptor-like kinase
maker-1-augustus-gene-58.40-mRNA-1	NA	NA	Kinase-LRR-TM	Os01g0206800	OsR498G0100378000.01.T01	Transmembrane kinase
maker-1-augustus-gene-68.41-mRNA-1	NA	Indica	Kinase-LRR-TM	Os01g0223600	OsR498G0100446600.01.T01	Inactive leucine-rich repeat receptor-like serine/threonine-protein kinase
maker-10-augustus-gene-133.6-mRNA-1	NA	NA	Kinase-LRR-TM	Os04g0132500	OsR498G0407514500.01.T01	Lrr receptor-like serinethreonine-protein kinase rch1
maker-10-augustus-gene-163.28-mRNA-1	NA	Indica	Kinase-LRR-TM	Os10g0467900	OsR498G1018870200.01.T01	Receptor protein kinase
maker-10-augustus-gene-164.25-mRNA-1	NA	NA	Kinase-LRR-TM	Os10g0468500	OsR498G1018872900.01.T01	MDIS1-interacting receptor like kinase 2
maker-10-augustus-gene-38.0-mRNA-1	NA	Indica	Kinase-LRR-TM	Os10g0155800	OsR498G1018171900.01.T01	Leucine-rich repeat receptor-like protein kinase PEPR1
maker-11-augustus-gene-123.41-mRNA-1	NA	Japonica	Kinase-LRR-TM	Os11g0448000	OsR498G1120059500.01.T01	Receptor-like kinase TMK4 isoform A
maker-11-augustus-gene-148.27-mRNA-1	NA	NA	Kinase-LRR-TM	Os11g0490200		Putative LRR receptor-like serine/threonine-protein kinase
maker-11-augustus-gene-188.43-mRNA-1	NA	Japonica	Kinase-LRR-TM	Os11g0569800	OsR498G1120381200.01.T01	Putative LRR receptor-like serine/threonine-protein kinase
maker-11-augustus-gene-221.0-mRNA-1	NA	Japonica	Kinase-LRR-TM	Os11g0628000	OsR498G1120550600.01.T01	Putative LRR receptor-like serine/threonine-protein kinase
maker-11-augustus-gene-30.28-mRNA-1	NA	Japonica	Kinase-LRR-TM	Os11g0172133	OsR498G1119496900.01.T01	Protein kinase domain containing protein, expressed
maker-11-augustus-gene-58.29-mRNA-1	NA	Indica	Kinase-LRR-TM	Os12g0210400	OsR498G1119698900.01.T01	probable leucine-rich repeat receptor-like protein kinase At5g49770
maker-11-augustus-gene-84.8-mRNA-1	NA	NA	Kinase-LRR-TM	Os11g0232100	OsR498G1119656400.01.T01	Receptor-like protein kinase HSL1
maker-11-augustus-gene-85.28-mRNA-1	NA	NA	Kinase-LRR-TM	0	OsR498G1119660500.01.T01	Leucine-rich repeat receptor-like serine/threonine-protein kinase BAM3
maker-12-augustus-gene-1.34-mRNA-1	NA	NA	Kinase-LRR-TM	0	OsR498G1220789200.01.T06	Leucine-rich repeat protein kinase family protein
maker-12-augustus-gene-267.0-mRNA-1	NA	NA	Kinase-LRR-TM	0	OsR498G1222125700.01.T01	EF-TU receptor
maker-2-augustus-gene-102.24-mRNA-1	NA	NA	Kinase-LRR-TM	0	OsR498G0203410200.01.T02	Putative leucine-rich repeat receptor-like protein kinase
maker-2-augustus-gene-107.34-mRNA-1	NA	NA	Kinase-LRR-TM	Os02g0283800	OsR498G0203424500.01.T01	BRASSINOSTEROID INSENSITIVE 1-associated receptor kinase 1, putative, expressed
maker-2-augustus-gene-19.12-mRNA-1	NA	Indica	Kinase-LRR-TM	Os02g0136900	OsR498G0202924100.01.T01	Serine/threonine protein kinase
maker-2-augustus-gene-245.12-mRNA-1	NA	NA	Kinase-LRR-TM			Protein kinase domain-containing protein (Fragment)
maker-2-augustus-gene-245.3-mRNA-1	NA	NA	Kinase-LRR-TM			Protein kinase domain-containing protein (Fragment)
maker-2-augustus-gene-245.8-mRNA-1	NA	Indica	Kinase-LRR-TM	Os02g0615500	OsR498G0204301900.01.T02	LRR receptor-like serine/threonine-protein kinase EFR
maker-2-augustus-gene-249.0-mRNA-1	NA	Indica	Kinase-LRR-TM	Os02g0615300	OsR498G0204315700.01.T02	LRR receptor-like serine/threonine-protein kinase EFR
maker-2-augustus-gene-29.19-mRNA-1	NA	NA	Kinase-LRR-TM	0	OsR498G0202983400.01.T02	Putative leucine-rich repeat receptor-like protein kinase
maker-2-augustus-gene-335.60-mRNA-1	NA	NA	Kinase-LRR-TM	0	OsR498G0613109100.01.T01	Protein STRUBBELIG-RECEPTOR FAMILY 1
maker-2-augustus-gene-42.26-mRNA-1	NA	NA	Kinase-LRR-TM	Os02g0176100	OsR498G0203075000.01.T09	Protein STRUBBELIG-RECEPTOR FAMILY 1
maker-2-augustus-gene-74.43-mRNA-1	NA	NA	Kinase-LRR-TM	Os02g0228300	OsR498G0203268600.01.T02	Receptor-like protein kinase HSL1
maker-3-augustus-gene-321.44-mRNA-1	NA	NA	Kinase-LRR-TM	Os10g0337400	OsR498G1018480300.01.T01	Receptor protein kinase
maker-4-augustus-gene-178.10-mRNA-1	NA	NA	Kinase-LRR-TM	Os04g0463000	OsR498G0408711000.01.T01	Putative inactive receptor kinase
maker-4-augustus-gene-214.6-mRNA-1	NA	NA	Kinase-LRR-TM	0	OsR498G0408964900.01.T01	Putative LRR receptor-like serine/threonine-protein kinase
maker-5-augustus-gene-101.5-mRNA-1	NA	Indica	Kinase-LRR-TM	Os04g0291900	OsR498G0510192800.01.T06	Non-specific serine/threonine protein kinase
maker-5-augustus-gene-103.5-mRNA-1	NA	NA	Kinase-LRR-TM	Os04g0291900	OsR498G0510192800.01.T06	Non-specific serine/threonine protein kinase
maker-5-augustus-gene-141.9-mRNA-1	NA	NA	Kinase-LRR-TM	0	OsR498G0510414500.01.T01	Serine/threonine protein kinase
maker-5-augustus-gene-247.14-mRNA-1	NA	NA	Kinase-LRR-TM	Os05g0481100	OsR498G0511055300.01.T09	Putative LRR receptor-like serine/threonine-protein kinase-like
maker-5-augustus-gene-247.29-mRNA-1	NA	NA	Kinase-LRR-TM	Os05g0480400	OsR498G0511051800.01.T01	Putative inactive receptor kinase
maker-5-augustus-gene-250.6-mRNA-1	NA	NA	Kinase-LRR-TM	Os05g0486100	OsR498G0511075400.01.T01	Putative leucine-rich repeat receptor-like protein kinase
maker-5-augustus-gene-308.58-mRNA-1	NA	NA	Kinase-LRR-TM	Os05g0595950	OsR498G0511457400.01.T01	Leucine-rich repeat receptor-like serine/threonine-protein kinase BAM3
maker-5-augustus-gene-40.0-mRNA-1	NA	Indica	Kinase-LRR-TM	Os01g0170300	OsR498G0509825300.01.T01	Mitogen-activated protein kinase kinase kinase
maker-5-augustus-gene-96.10-mRNA-1	NA	Indica	Kinase-LRR-TM	Os05g0257100	OsR498G0510172500.01.T03	Non-specific serine/threonine protein kinase
maker-5-augustus-gene-97.1-mRNA-1	NA	Indica	Kinase-LRR-TM	Os04g0291900	OsR498G0510174900.01.T01	Non-specific serine/threonine protein kinase

maker-6-augustus-gene-16.8-mRNA-1	NA	NA	Kinase-LRR-TM	Os06g0130100	OsR498G0611562900.01.T01	LRR receptor-like serine/threonine-protein kinase ERECTA
maker-6-augustus-gene-18.4-mRNA-1	NA	NA	Kinase-LRR-TM	Os06g0134700	OsR498G0611585900.01.T01	Somatic embryogenesis receptor kinase 4
maker-6-augustus-gene-203.24-mRNA-1	NA	NA	Kinase-LRR-TM	0	OsR498G0612714500.01.T01	Calmodulin-binding receptor kinase CaMRLK
maker-6-augustus-gene-225.34-mRNA-1	NA	Indica	Kinase-LRR-TM	Os12g0498650	OsR498G0612836400.01.T01	Putative LRR receptor-like serine/threonine-protein kinase
maker-6-augustus-gene-228.0-mRNA-1	NA	Japonica	Kinase-LRR-TM	Os06g0586150	OsR498G0612846900.01.T01	Putative LRR receptor-like serine/threonine-protein kinase
maker-6-augustus-gene-228.6-mRNA-1	NA	Indica	Kinase-LRR-TM	Os06g0586150	OsR498G0612847900.01.T01	Leucine-rich repeat protein kinase family protein
maker-6-augustus-gene-229.0-mRNA-1	NA	NA	Kinase-LRR-TM	0	OsR498G0612851100.01.T02	Leucine-rich repeat protein kinase family protein
maker-6-augustus-gene-230.0-mRNA-1	NA	NA	Kinase-LRR-TM			Protein kinase domain-containing protein (Fragment)
maker-6-augustus-gene-230.2-mRNA-1	NA	Indica	Kinase-LRR-TM	Os06g0587500	OsR498G0612852500.01.T01	Putative LRR receptor-like serine/threonine-protein kinase
maker-6-augustus-gene-231.7-mRNA-1	NA	Indica	Kinase-LRR-TM	Os06g0587900	OsR498G0612839000.01.T02	Putative LRR receptor-like serine/threonine-protein kinase
maker-6-augustus-gene-274.6-mRNA-1	NA	NA	Kinase-LRR-TM	0	OsR498G0613109100.01.T01	Protein STRUBBELIG-RECEPTOR FAMILY 1
maker-6-augustus-gene-275.4-mRNA-1	NA	NA	Kinase-LRR-TM	0	OsR498G0613116800.01.T01	Pollen receptor-like kinase 4
maker-6-augustus-gene-53.26-mRNA-1	NA	NA	Kinase-LRR-TM	Os06g0203800	OsR498G0611808400.01.T02	LRR receptor-like serine/threonine-protein kinase ERECTA
maker-6-augustus-gene-66.20-mRNA-1	NA	NA	Kinase-LRR-TM			Non-specific serine/threonine protein kinase
maker-6-augustus-gene-92.12-mRNA-1	NA	NA	Kinase-LRR-TM	Os06g0272000	OsR498G0612078200.01.T01	Receptor kinase MRKa
maker-7-augustus-gene-14.37-mRNA-1	NA	NA	Kinase-LRR-TM	0	OsR498G0713472300.01.T02	Putative LRR receptor-like serine/threonine-protein kinase
maker-7-augustus-gene-14.43-mRNA-1	NA	Indica	Kinase-LRR-TM	Os07g0134200	OsR498G0713470200.01.T01	Leucine-rich repeat receptor-like serine/threonine-protein kinase BAM1
maker-7-augustus-gene-19.33-mRNA-1	NA	NA	Kinase-LRR-TM	Os07g0145400	OsR498G0713506600.01.T01	Tyrosine-protein kinase
maker-7-augustus-gene-24.35-mRNA-1	NA	NA	Kinase-LRR-TM	0	OsR498G0713530800.01.T02	Leucine-rich repeat receptor-like serine/threonine-protein kinase At1g17230
maker-7-augustus-gene-54.15-mRNA-1	NA	NA	Kinase-LRR-TM	Os07g0207100	OsR498G0713723400.01.T01	Leucine-rich repeat receptor-like tyrosine-protein kinase PXC3
maker-8-augustus-gene-10.40-mRNA-1	NA	NA	Kinase-LRR-TM	Os08g0117700	OsR498G0815080100.01.T01	Somatic embryogenesis receptor kinase 5-like
maker-8-augustus-gene-191.10-mRNA-1	NA	NA	Kinase-LRR-TM	Os08g0376300	OsR498G0815955000.01.T01	Receptor-like protein kinase 5
maker-8-augustus-gene-29.0-mRNA-1	NA	NA	Kinase-LRR-TM	0	OsR498G0815185900.01.T01	Leucine-rich repeat receptor-like protein kinase TDR
maker-9-augustus-gene-106.25-mRNA-1	NA	NA	Kinase-LRR-TM	Os09g0351700		Serine/threonine-specific receptor protein kinase-like
maker-9-augustus-gene-107.2-mRNA-1	NA	Indica	Kinase-LRR-TM	Os09g0352000	OsR498G0917216200.01.T01	Serine/threonine-specific receptor protein kinase-like
maker-9-augustus-gene-107.4-mRNA-1	NA	Japonica	Kinase-LRR-TM	Os09g0349800	OsR498G0917216200.01.T01	Putative LRR receptor-like serine/threonine-protein kinase
maker-9-augustus-gene-115.0-mRNA-1	NA	NA	Kinase-LRR-TM	Os09g0355400		Senescence-induced receptor-like serine/threonine-protein kinase
maker-9-augustus-gene-117.0-mRNA-1	NA	NA	Kinase-LRR-TM	Os09g0356800	OsR498G0917239000.01.T02	Putative LRR receptor-like serine/threonine-protein kinase
maker-9-augustus-gene-184.37-mRNA-1	NA	Indica	Kinase-LRR-TM	Os08g0493800	OsR498G0917690700.01.T01	Serine/threonine protein kinase
augustus-2-processed-gene-28.42-mRNA-1	Japonica	NA	Kinase-LRR-NBS			Disease resistance protein RGA2
augustus_masked-11-processed-gene-211.2-mRNA-1	Japonica	NA	LRR	Os11g0609700		Disease resistance protein RPM1
maker-10-augustus-gene-157.12-mRNA-1	Japonica	NA	LRR	Os10g0456200	OsR498G1018837200.01.T01	LRR repeat and ubiquitin-like domain-containing protein isoform X2
maker-11-augustus-gene-30.29-mRNA-1	Japonica	NA	LRR	Os11g0172300		Protein kinase domain-containing protein
maker-2-augustus-gene-283.29-mRNA-1	Japonica	NA	LRR	Os02g0675800	OsR498G0204514700.01.T03	RING/U-box superfamily protein
maker-3-augustus-gene-56.49-mRNA-1	Japonica	NA	LRR		OsR498G0305445900.01.T02	Plant intracellular Ras-group-related LRR protein 7
maker-6-augustus-gene-73.27-mRNA-1	Japonica	NA	LRR		OsR498G0611964800.01.T03	NLR family CARD domain-containing protein 3
maker-8-augustus-gene-285.55-mRNA-1	Japonica	NA	LRR		OsR498G0816555300.01.T02	NB-ARC domain-containing protein
maker-12-augustus-gene-276.42-mRNA-1	NA	NA	LRR	Os07g0106100	OsR498G0713370900.01.T01	Dynein assembly factor 1, axonemal
maker-2-augustus-gene-326.42-mRNA-1	NA	NA	LRR	Os06g0677500	OsR498G0613173700.01.T01	Protein geranylgeranyltransferase type II
maker-4-augustus-gene-252.6-mRNA-1	NA	NA	LRR	Os04g0605300	OsR498G0409234800.01.T02	Plant intracellular Ras-group-related LRR protein 1
maker-6-augustus-gene-283.34-mRNA-1	NA	NA	LRR	Os06g0677500	OsR498G0613173700.01.T01	Protein geranylgeranyltransferase type II
maker-6-augustus-gene-75.28-mRNA-1	NA	NA	LRR			LRR receptor-like serine/threonine-protein kinase ERECTA
maker-9-augustus-gene-15.0-mRNA-1	NA	Indica	LRR	Os02g0826600	OsR498G0916701600.01.T01	Plant intracellular Ras-group-related LRR protein 6
augustus_masked-5-processed-gene-141.42-mRNA-1	Japonica	Japonica	LRR-NBS	Os05g0305600	OsR498G0510413500.01.T01	NB-ARC domain containing protein, expressed
maker-11-augustus-gene-248.32-mRNA-1	Japonica	Japonica	LRR-NBS	Os11g0673900	OsR498G1120709100.01.T03	Putative nucleotide-binding leucine-rich-repeat protein 1
maker-11-augustus-gene-248.32-mRNA-1	NA	Japonica	LRR-NBS	Os11g0673900	OsR498G1120709100.01.T03	Putative nucleotide-binding leucine-rich-repeat protein 1
maker-7-augustus-gene-17.71-mRNA-1	NA	NA	LRR-NBS	Os07g0141700		Disease resistance protein RPM1
augustus_masked-10-processed-gene-25.16-mRNA-1	Indica	NA	LRR-NBS-TM	Os10g0136100		NB-ARC domain containing protein, expressed
maker-9-augustus-gene-99.0-mRNA-1	Indica	NA	LRR-NBS-TM	Os09g0327800		NBS-LRR disease resistance protein-like
augustus-10-processed-gene-44.0-mRNA-1	Japonica	NA	LRR-NBS-TM		OsR498G1018206100.01.T04	NB-ARC domain containing protein, expressed
augustus-11-processed-gene-228.19-mRNA-1	Japonica	Japonica	LRR-NBS-TM	Os11g0640300	OsR498G1120612100.01.T01	Leucine Rich Repeat family protein, expressed
augustus-11-processed-gene-83.15-mRNA-1	Japonica	NA	LRR-NBS-TM	Os11g0229400	OsR498G1119649900.01.T03	NBS-LRR type protein (Fragment)
augustus-7-processed-gene-42.1-mRNA-1	Japonica	NA	LRR-NBS-TM	Os07g0186500	OsR498G0713652100.01.T01	Disease resistance protein RPM1
augustus-9-processed-gene-121.26-mRNA-1	Japonica	Japonica	LRR-NBS-TM	Os09g0365000	OsR498G0917259000.01.T01	PPC domain-containing protein
augustus_masked-1-processed-gene-147.22-mRNA-1	Japonica	Japonica	LRR-NBS-TM	Os01g0359800	OsR498G0100941400.01.T02	Powdery mildew resistance protein PM3 variant
augustus_masked-11-processed-gene-183.19-mRNA-1	Japonica	NA	LRR-NBS-TM	Os11g0560000	OsR498G1120357400.01.T01	Disease resistance protein RPM1
augustus_masked-11-processed-gene-228.26-mRNA-1	Japonica	NA	LRR-NBS-TM	Os11g0639600	OsR498G1120610200.01.T02	NB-ARC domain containing protein, expressed
augustus_masked-3-processed-gene-341.23-mRNA-1	Japonica	NA	LRR-NBS-TM	Os03g0566700		NBS-LRR protein (Fragment)
augustus_masked-4-processed-gene-221.11-mRNA-1	Japonica	Indica	LRR-NBS-TM	Os04g0548100	OsR498G0409024200.01.T01	Disease resistance protein RPM1
augustus_masked-8-processed-gene-93.26-mRNA-1	Japonica	NA	LRR-NBS-TM	Os08g0246300	OsR498G0815530100.01.T01	Disease resistance protein RPM1
augustus_masked-9-processed-gene-121.19-mRNA-1	Japonica	Japonica	LRR-NBS-TM	Os09g0365300	OsR498G0917259200.01.T01	Protein kinase domain-containing protein
maker-1-augustus-gene-116.31-mRNA-1	Japonica	NA	LRR-NBS-TM	Os01g0308300	OsR498G0100770300.01.T01	Putative NB-ARC domain-containing protein (Fragment)
maker-1-augustus-gene-497.55-mRNA-1	Japonica	Japonica	LRR-NBS-TM	Os01g0953400	OsR498G0102712500.01.T01	Leucine Rich Repeat family protein, expressed
maker-10-augustus-gene-23.27-mRNA-1	Japonica	NA	LRR-NBS-TM	Os10g0132500	OsR498G1018099300.01.T02	Putative retrotransposable elements TNP2
maker-11-augustus-gene-209.8-mRNA-1	Japonica	Japonica	LRR-NBS-TM	Os11g0606400	OsR498G1120484800.01.T01	NB-ARC domain containing protein, expressed
maker-11-augustus-gene-209.9-mRNA-1	Japonica	Indica	LRR-NBS-TM	Os11g0606400	OsR498G1120485600.01.T01	NB-ARC domain containing protein, expressed
maker-11-augustus-gene-224.0-mRNA-1	Japonica	NA	LRR-NBS-TM	Os11g0633500	OsR498G1120581500.01.T03	Disease resistance protein RPP8
maker-11-augustus-gene-254.11-mRNA-1	Japonica	NA	LRR-NBS-TM		OsR498G1120737800.01.T01	Disease resistance protein Piks-1
maker-3-augustus-gene-125.41-mRNA-1	Japonica	NA	LRR-NBS-TM	Os10g0568600	OsR498G1019224400.01.T01	NB-ARC domain-containing protein (Fragment)
maker-7-augustus-gene-156.17-mRNA-1	Japonica	NA	LRR-NBS-TM		OsR498G0714220800.01.T01	NB-ARC domain, putative
maker-7-augustus-gene-172.14-mRNA-1	Japonica	NA	LRR-NBS-TM	Os07g0481300	OsR498G0714308900.01.T01	Putative disease resistance protein RGA3
maker-7-augustus-gene-98.5-mRNA-1	Japonica	NA	LRR-NBS-TM	Os07g0273700	OsR498G0713986100.01.T01	WRKY domain-containing protein
augustus-11-processed-gene-180.30-mRNA-1	NA	NA	LRR-NBS-TM	Os11g0550500	OsR498G1120333300.01.T05	Disease resistance protein RPM1
augustus-11-processed-gene-83.15-mRNA-1	NA	NA	LRR-NBS-TM	Os11g0229400	OsR498G1119649900.01.T03	NBS-LRR type protein (Fragment)
augustus-11-processed-gene-83.16-mRNA-1	NA	Japonica	LRR-NBS-TM	Os11g0227800	OsR498G1119650700.01.T02	NBS-LRR type protein (Fragment)
augustus-6-processed-gene-33.72-mRNA-1	NA	NA	LRR-NBS-TM			Leaf rust resistance protein Lr10
augustus_masked-10-processed-gene-25.16-mRNA-1	NA	NA	LRR-NBS-TM	Os10g0136100		NB-ARC domain containing protein, expressed
augustus_masked-3-processed-gene-251.52-mRNA-1	NA	NA	LRR-NBS-TM	Os03g0599400		Leucine Rich Repeat family protein, expressed
augustus_masked-3-processed-gene-345.38-mRNA-1	NA	NA	LRR-NBS-TM	Os03g0573500	OsR498G0306422200.01.T01	Putative disease resistance protein RGA3
augustus_masked-3-processed-gene-360.14-mRNA-1	NA	NA	LRR-NBS-TM	Os03g0599400		Leucine Rich Repeat family protein, expressed
maker-1-augustus-gene-494.48-mRNA-1	NA	NA	LRR-NBS-TM	Os01g0947700		Beta 1,3-glucanase
maker-2-augustus-gene-108.14-mRNA-1	NA	NA	LRR-NBS-TM	Os02g0286700		Putative stripe rust resistance protein Yr10
maker-3-augustus-gene-236.44-mRNA-1	NA	NA	LRR-NBS-TM	Os03g0573500	OsR498G0306422200.01.T01	Putative disease resistance protein RGA3
maker-3-augustus-gene-239.29-mRNA-1	NA	NA	LRR-NBS-TM			Putative NBS-LRR type disease resistance protein
maker-3-augustus-gene-349.0-mRNA-1	NA	NA	LRR-NBS-TM	0	OsR498G0306441900.01.T01	Putative NBS-LRR type disease resistance protein
maker-3-augustus-gene-360.49-mRNA-1	NA	Japonica	LRR-NBS-TM	Os03g0599000	OsR498G0306507000.01.T01	NB-ARC domain-containing protein
maker-3-augustus-gene-495.20-mRNA-1	NA	Japonica	LRR-NBS-TM	Os03g0849100	OsR498G0307368000.01.T01	Powdery mildew resistance protein PM3 variant
maker-6-augustus-gene-13.38-mRNA-1	NA	NA	LRR-NBS-TM	Os06g0125000	OsR498G0611546000.01.T05	NBS-LRR type R protein Pigm-R8
maker-6-augustus-gene-33.29-mRNA-1	NA	NA	LRR-NBS-TM	Os06g0163800	OsR498G0611676000.01.T01	Disease resistance protein RPP13
maker-8-augustus-gene-198.41-mRNA-1	NA	NA	LRR-NBS-TM	Os08g0388300	OsR498G0815986100.01.T07	
maker-8-augustus-gene-198.49-mRNA-1	NA	NA	LRR-NBS-TM	Os08g0387700	OsR498G0815985400.01.T03	Disease resistance protein RPM1
maker-9-augustus-gene-55.8-mRNA-1	NA	NA	LRR-NBS-TM	0	OsR498G0916877900.01.T01	Disease resistance protein RPM1
maker-9-augustus-gene-99.0-mRNA-1	NA	NA	LRR-NBS-TM	Os09g0327800		NBS-LRR disease resistance protein-like
maker-8-augustus-gene-284.7-mRNA-1	Japonica	Indica	LRR-NBS-TM PIBH8	Os08g0539700	OsR498G0816550500.01.T01	Pib variant protein (Fragment)
augustus-2-processed-gene-166.21-mRNA-1	Indica	NA	LRR-TM		OsR498G0203803900.01.T01	NB-ARC domain-containing protein
augustus-2-processed-gene-247.15-mRNA-1	Indica	Indica	LRR-TM	Os02g0615300	OsR498G0204309700.01.T01	Putative LRR receptor-like serine/threonine-protein kinase (Fragment)
augustus-2-processed-gene-251.11-mRNA-1	Indica	NA	LRR-TM		OsR498G0204320700.01.T01	Leucine-rich repeat-like protein
augustus-2-processed-gene-31.11-mRNA-1	Indica	NA	LRR-TM	Os02g0155700		Tyrosine-sulfated glycopeptide receptor 1
augustus-2-processed-gene-34.2-mRNA-1	Indica	Japonica	LRR-TM	Os02g0161700	OsR498G0203018700.01.T01	LRR receptor-like serine/threonine-protein kinase FLS2
augustus-8-processed-gene-103.47-mRNA-1	Indica	NA	LRR-TM	Os08g0266400		Putative somatic embryogenesis protein kinase 1
maker-1-augustus-gene-350.22-mRNA-1	Indica	NA	LRR-TM		OsR498G0102182300.01.T02	BRASSINOSTEROID INSENSITIVE 1-associated receptor kinase 1
maker-10-augustus-gene-164.29-mRNA-1	Indica	Japonica	LRR-TM	Os10g0469600	OsR498G1018872900.01.T01	MDIS1-interacting receptor like kinase 2
maker-11-augustus-gene-74.2-mRNA-1	Indica	NA	LRR-TM		OsR498G1119610600.01.T03	NBS-LRR disease resistance protein family-4
maker-2-augustus-gene-247.3-mRNA-1	Indica	NA	LRR-TM		OsR498G0204309100.01.T01	LRR receptor-like serine/threonine-protein kinase EFR
maker-3-augustus-gene-199.10-mRNA-1	Indica	NA	LRR-TM	Os03g0400850	OsR498G0306129200.01.T01	Putative LRR receptor-like serine/threonine-protein kinase
maker-3-augustus-gene-298.34-mRNA-1	Indica	NA	LRR-TM	Os10g0376200	OsR498G1018578500.01.T02	Dynein assembly factor 1, axonemal
maker-4-augustus-gene-112.45-mRNA-1	Indica	Indica	LRR-TM	Os04g0349700	OsR498G0408227200.01.T02	Putative verticillium wilt disease resistance protein
maker-8-augustus-gene-260.0-mRNA-1	Indica	Indica	LRR-TM	Os08g0499900	OsR498G0816398100.01.T01	F-box protein At-B
augustus-1-processed-gene-311.1-mRNA-1	Japonica	NA	LRR-TM	Os01g0729400	OsR498G0101933900.01.T01	Receptor like protein 29
augustus-11-processed-gene-185.22-mRNA-1	Japonica	NA	LRR-TM	Os11g0565000	OsR498G1120370300.01.T01	Leucine Rich Repeat family protein, expressed
augustus-11-processed-gene-75.39-mRNA-1	Japonica	NA	LRR-TM	Os11g0213500	OsR498G1119614900.01.T01	Pentatricopeptide repeat-containing protein
augustus-12-processed-gene-66.22-mRNA-1	Japonica	Indica	LRR-TM	Os12g0218500	OsR498G1221134900.01.T01	Leucine Rich Repeat family protein, expressed
augustus-2-processed-gene-20.27-mRNA-1	Japonica	NA	LRR-TM	Os02g0138000	OsR498G0202928200.01.T01	Leucine-rich repeat extensin-like protein 1
augustus-2-processed-gene-238.27-mRNA-1	Japonica	NA	LRR-TM	Os02g0603100	OsR498G0204256200.01.T01	Leucine-rich repeat receptor-like protein CLAVATA2
augustus-2-processed-gene-241.48-mRNA-1	Japonica	NA	LRR-TM	Os02g0609900		Receptor-like protein kinase
augustus-2-processed-gene-242.0-mRNA-1	Japonica	NA	LRR-TM		OsR498G0204282100.01.T01	Protein kinase domain-containing protein (Fragment)
augustus-2-processed-gene-31.36-mRNA-1	Japonica	NA	LRR-TM	Os02g0156400	OsR498G0202998000.01.T01	Putative Phytosulfokine receptor
augustus-2-processed-gene-34.2-mRNA-1	Japonica	Japonica	LRR-TM	Os02g0161700	OsR498G0203018700.01.T01	LRR receptor-like serine/threonine-protein kinase FLS2
augustus-4-processed-gene-275.33-mRNA-1	Japonica	NA	LRR-TM			Phytosulfokine receptor 1
augustus-5-processed-gene-204.46-mRNA-1	Japonica	NA	LRR-TM	Os05g0399800	OsR498G0510794200.01.T01	Inactive LRR receptor-like serine/threonine-protein kinase BIR2
augustus-5-processed-gene-47.20-mRNA-1	Japonica	NA	LRR-TM	Os05g0180300	OsR498G0509866000.01.T01	Leucine-rich repeat extensin-like protein 3
augustus-8-processed-gene-263.22-mRNA-1	Japonica	NA	LRR-TM	Os08g0505900	OsR498G0816425400.01.T01	DNA-damage-repair/toleration protein DRT100
augustus_masked-1-processed-gene-42.29-mRNA-1	Japonica	NA	LRR-TM	Os01g0180000	OsR498G0100270600.01.T01	Leucine-rich repeat extensin-like protein 3
augustus_masked-10-processed-gene-45.15-mRNA-1	Japonica	Indica	LRR-TM	Os02g0156400	OsR498G1018221800.01.T01	Putative Phytosulfokine receptor
augustus_masked-11-processed-gene-182.58-mRNA-1	Japonica	Japonica	LRR-TM	Os11g0558400	OsR498G1120351600.01.T01	Leucine Rich Repeat family protein, expressed
augustus_masked-11-processed-gene-183.18-mRNA-1	Japonica	NA	LRR-TM			Leucine-rich repeat receptor protein kinase MSL1
augustus_masked-11-processed-gene-183.4-mRNA-1	Japonica	NA	LRR-TM	Os11g0558900	OsR498G1120352700.01.T01	Leucine Rich Repeat family protein, expressed
augustus_masked-11-processed-gene-81.45-mRNA-1	Japonica	Japonica	LRR-TM	Os11g0224900	OsR498G1119642200.01.T01	NBS-LRR type protein (Fragment)
augustus_masked-11-processed-gene-85.7-mRNA-1	Japonica	Indica	LRR-TM	Os11g0232100	OsR498G1119658200.01.T01	Receptor protein-tyrosine kinase CEPR2
augustus_masked-12-processed-gene-60.19-mRNA-1	Japonica	Japonica	LRR-TM	Os12g0211500	OsR498G1221115800.01.T01	Leucine Rich Repeat family protein, expressed
augustus_masked-3-processed-gene-381.22-mRNA-1	Japonica	NA	LRR-TM	Os03g0637600	OsR498G0306623400.01.T01	Leucine-rich repeat extensin-like protein 6
augustus_masked-3-processed-gene-415.31-mRNA-1	Japonica	NA	LRR-TM		OsR498G0306857800.01.T01	Putative LRR receptor-like protein kinase
augustus_masked-4-processed-gene-289.5-mRNA-1	Japonica	Japonica	LRR-TM	Os04g0672600	OsR498G0409468600.01.T01	Phytosulfokine receptor 2
augustus_masked-5-processed-gene-240.55-mRNA-1	Japonica	Japonica	LRR-TM	Os05g0468200	OsR498G0511013500.01.T01	DUF1618 domain-containing protein (Fragment)
augustus_masked-6-processed-gene-161.13-mRNA-1	Japonica	NA	LRR-TM	Os06g0478600	OsR498G0612420500.01.T01	Leucine-rich repeat receptor-like serine/threonine-protein kinase BAM2
augustus_masked-6-processed-gene-211.22-mRNA-1	Japonica	Japonica	LRR-TM	Os06g0557400	OsR498G0612758000.01.T01	LRR receptor-like serine/threonine-protein kinase HSL2
augustus_masked-6-processed-gene-255.13-mRNA-1	Japonica	NA	LRR-TM	Os06g0627500	OsR498G0612993000.01.T01	piriformospora indica-insensitive protein 2
augustus_masked-8-processed-gene-263.16-mRNA-1	Japonica	Japonica	LRR-TM	Os08g0506100	OsR498G0816425900.01.T01	RNase H domain-containing protein (Fragment)
augustus_masked-9-processed-gene-26.17-mRNA-1	Japonica	NA	LRR-TM	Os09g0131200		Receptor like protein 44 isoform 2
maker-1-augustus-gene-100.20-mRNA-1	Japonica	NA	LRR-TM	Os01g0279800	OsR498G0100670400.01.T01	Somatic embryoproteinsis receptor kinase 5
maker-1-augustus-gene-503.45-mRNA-1	Japonica	NA	LRR-TM	Os01g0966400	OsR498G0102757400.01.T01	Protein phosphatase 1 regulatory subunit like
maker-1-augustus-gene-6.37-mRNA-1	Japonica	Indica	LRR-TM	Os01g0110800	OsR498G0100035200.01.T01	Piriformospora indica-insensitive protein 2
maker-10-augustus-gene-139.21-mRNA-1	Japonica	NA	LRR-TM		OsR498G0407494400.01.T01	Protein kinase domain-containing protein
maker-10-augustus-gene-164.29-mRNA-1	Japonica	Japonica	LRR-TM	Os10g0469600	OsR498G1018872900.01.T01	MDIS1-interacting receptor like kinase 2
maker-11-augustus-gene-159.18-mRNA-1	Japonica	NA	LRR-TM	Os11g0514400	OsR498G1120215800.01.T01	Somatic embryogenesis receptor kinase 1
maker-11-augustus-gene-159.21-mRNA-1	Japonica	Indica	LRR-TM	Os11g0514500	OsR498G1120216000.01.T01	BRASSINOSTEROID INSENSITIVE 1-associated receptor kinase 1, putative, expressed
maker-11-augustus-gene-159.26-mRNA-1	Japonica	Indica	LRR-TM	Os11g0514600	OsR498G1120216600.01.T01	Somatic embryogenesis receptor kinase 1
maker-11-augustus-gene-159.29-mRNA-1	Japonica	Japonica	LRR-TM	Os11g0514700	OsR498G1120218000.01.T01	Somatic embryogenesis receptor kinase 1

maker-11-augustus-gene-184.0-mRNA-1	Japonica	NA	LRR-TM			
maker-11-augustus-gene-74.2-mRNA-1	Japonica	NA	LRR-TM		OsR498G1119610600.01.T03	NBS-LRR disease resistance protein family-4
maker-11-augustus-gene-75.17-mRNA-1	Japonica	Indica	LRR-TM	Os11g0213700	OsR498G1119615600.01.T02	Disease resistance protein RGA2
maker-2-augustus-gene-100.57-mRNA-1	Japonica	NA	LRR-TM	Os02g0274200		Receptor-like protein 12
maker-2-augustus-gene-190.3-mRNA-1	Japonica	NA	LRR-TM			
maker-2-augustus-gene-244.45-mRNA-1	Japonica	NA	LRR-TM	Os02g0613200	OsR498G0204293400.01.T01	Regulatory subunit
maker-2-augustus-gene-31.64-mRNA-1	Japonica	NA	LRR-TM	Os02g0156800	OsR498G0203001200.01.T02	Tyrosine-sulfated glycopeptide receptor 1
maker-2-augustus-gene-74.39-mRNA-1	Japonica	NA	LRR-TM	Os02g0230100	OsR498G0203274700.01.T03	Protein phosphatase 1 regulatory subunit pprA
maker-3-augustus-gene-224.0-mRNA-1	Japonica	NA	LRR-TM	Os03g0440900	OsR498G0306297500.01.T01	Somatic embryogenesis receptor kinase 1
maker-3-augustus-gene-42.49-mRNA-1	Japonica	NA	LRR-TM	Os03g0184400	OsR498G0305345100.01.T01	Leucine-rich repeat receptor-like serine/threonine-protein kinase
maker-3-augustus-gene-44.53-mRNA-1	Japonica	Indica	LRR-TM	Os03g0190300	OsR498G0305366800.01.T02	Dynein assembly factor 1, axonemal
maker-3-augustus-gene-469.25-mRNA-1	Japonica	NA	LRR-TM	Os03g0795300	OsR498G0307198000.01.T01	Leucine-rich repeat extensin-like protein 6
maker-3-augustus-gene-56.75-mRNA-1	Japonica	NA	LRR-TM	Os03g0211900	OsR498G0305445400.01.T01	probable leucine-rich repeat receptor-like protein kinase At1g35710
maker-4-augustus-gene-187.12-mRNA-1	Japonica	NA	LRR-TM	Os04g0480500	OsR498G0408770900.01.T01	Serine/threonine-protein kinase BRI1-like protein 2
maker-4-augustus-gene-198.30-mRNA-1	Japonica	NA	LRR-TM	Os04g0503500	OsR498G0408848000.01.T07	F-box/LRR-repeat protein 14-like isoform X2
maker-4-augustus-gene-259.29-mRNA-1	Japonica	Indica	LRR-TM	Os04g0616700	OsR498G0409282000.01.T05	Non-specific serine/threonine protein kinase
maker-4-augustus-gene-259.33-mRNA-1	Japonica	Indica	LRR-TM	Os04g0616700	OsR498G0409282200.01.T05	Non-specific serine/threonine protein kinase
maker-4-augustus-gene-259.37-mRNA-1	Japonica	Indica	LRR-TM	Os04g0616700	OsR498G0409282400.01.T05	Non-specific serine/threonine protein kinase
maker-4-augustus-gene-275.3-mRNA-1	Japonica	NA	LRR-TM		OsR498G0409378300.01.T01	LRR binding protein
maker-5-augustus-gene-200.18-mRNA-1	Japonica	NA	LRR-TM	Os05g0393100	OsR498G0510775500.01.T01	Serine-threonine protein kinase, plant-type, putative isoform 2
maker-5-augustus-gene-252.82-mRNA-1	Japonica	NA	LRR-TM	Os05g0491400	OsR498G0511088200.01.T01	Somatic embryoproteinsis receptor kinase 5
maker-5-augustus-gene-258.5-mRNA-1	Japonica	NA	LRR-TM	Os05g0501600	OsR498G0511119100.01.T01	Dynein light chain 1, axonemal
maker-5-augustus-gene-31.26-mRNA-1	Japonica	NA	LRR-TM	Os05g0154300	OsR498G0509763000.01.T01	Putative receptor protein kinase TMK1
maker-5-augustus-gene-96.3-mRNA-1	Japonica	Indica	LRR-TM	Os05g0257100	OsR498G0510169700.01.T01	Non-specific serine/threonine protein kinase
maker-6-augustus-gene-249.63-mRNA-1	Japonica	Japonica	LRR-TM	Os06g0621500	OsR498G0612965800.01.T01	NAF domain-containing protein
maker-6-augustus-gene-250.44-mRNA-1	Japonica	NA	LRR-TM	Os06g0621500		Putative disease resistance RPP13-like protein 1
maker-6-augustus-gene-291.69-mRNA-1	Japonica	NA	LRR-TM	Os06g0692700	OsR498G0613225200.01.T03	Tyrosine-sulfated glycopeptide receptor 1
maker-8-augustus-gene-267.30-mRNA-1	Japonica	NA	LRR-TM		OsR498G0816450000.01.T02	Plant intracellular Ras-group-related LRR protein 4
maker-9-augustus-gene-174.58-mRNA-1	Japonica	NA	LRR-TM	Os09g0458300	OsR498G0917614700.01.T01	Putative leucine-rich repeat receptor-like serine/threonine-protein kinase
maker-9-augustus-gene-218.12-mRNA-1	Japonica	NA	LRR-TM	Os09g0548200	OsR498G0917912500.01.T01	Chitin elicitor-binding protein
augustus-1-processed-gene-240.28-mRNA-1	NA	Indica	LRR-TM	Os01g0594300	OsR498G0101457300.01.T01	Leucine-rich repeat extensin-like protein 3
augustus-1-processed-gene-29.2-mRNA-1	NA	Japonica	LRR-TM	Os01g0153000	OsR498G0100190500.01.T02	Serine/threonine kinase
augustus-1-processed-gene-394.52-mRNA-1	NA	NA	LRR-TM	Os01g0891700	OsR498G0102493700.01.T01	RNA polymerase Rpb7-like, N-terminal domain
augustus-1-processed-gene-395.39-mRNA-1	NA	NA	LRR-TM	Os01g0892300	OsR498G0102496500.01.T01	Putative systemin receptor SR160
augustus-1-processed-gene-403.32-mRNA-1	NA	NA	LRR-TM	0	OsR498G0101567200.01.T01	Protein TOO MANY MOUTHS like
augustus-1-processed-gene-446.16-mRNA-1	NA	NA	LRR-TM	Os01g0892300	OsR498G0102496500.01.T01	Putative systemin receptor SR160
augustus-1-processed-gene-446.3-mRNA-1	NA	NA	LRR-TM	Os01g0891700	OsR498G0102493700.01.T01	RNA polymerase Rpb7-like, N-terminal domain
augustus-1-processed-gene-454.40-mRNA-1	NA	NA	LRR-TM	0	OsR498G0101567200.01.T01	Protein TOO MANY MOUTHS like
augustus-1-processed-gene-466.5-mRNA-1	NA	NA	LRR-TM	Os01g0601700		LRR receptor-like serine/threonine-protein kinase GSO1
augustus-11-processed-gene-230.23-mRNA-1	NA	NA	LRR-TM	Os11g0644100	OsR498G1120628100.01.T01	Leucine-rich repeat receptor-like kinase protein THICK TASSEL DWARF1
augustus-12-processed-gene-68.16-mRNA-1	NA	Japonica	LRR-TM	Os12g0222800	OsR498G1221130500.01.T01	Leucine Rich Repeat family protein, expressed
augustus-2-processed-gene-166.21-mRNA-1	NA	NA	LRR-TM	0	OsR498G0203803900.01.T01	NB-ARC domain-containing protein
augustus-2-processed-gene-246.15-mRNA-1	NA	NA	LRR-TM	0	OsR498G0204306700.01.T01	Leucine-rich repeat-like protein
augustus-2-processed-gene-247.15-mRNA-1	NA	Indica	LRR-TM	Os02g0615300	OsR498G0204309700.01.T01	Putative LRR receptor-like serine/threonine-protein kinase (Fragment)
augustus-4-processed-gene-146.12-mRNA-1	NA	NA	LRR-TM	0	OsR498G0408499300.01.T01	Protein kinase domain containing protein, expressed
augustus-5-processed-gene-274.72-mRNA-1	NA	NA	LRR-TM	Os05g0530701	OsR498G0511233000.01.T01	Too many mouths protein
augustus-8-processed-gene-103.47-mRNA-1	NA	NA	LRR-TM	Os08g0266400		Putative somatic embryogenesis protein kinase 1
augustus_masked-1-processed-gene-255.17-mRNA-1	NA	NA	LRR-TM	0	OsR498G0101567200.01.T01	Protein TOO MANY MOUTHS like
augustus_masked-1-processed-gene-418.57-mRNA-1	NA	Indica	LRR-TM	Os01g0594300	OsR498G0101457300.01.T01	Leucine-rich repeat extensin-like protein 3
augustus_masked-1-processed-gene-470.9-mRNA-1	NA	Indica	LRR-TM	Os01g0594300	OsR498G0101457300.01.T01	Leucine-rich repeat extensin-like protein 3
augustus_masked-12-processed-gene-63.37-mRNA-1	NA	Japonica	LRR-TM	Os12g0215950	OsR498G1221115800.01.T01	Leucine Rich Repeat family protein, expressed
augustus_masked-3-processed-gene-16.27-mRNA-1	NA	NA	LRR-TM	Os03g0133400	OsR498G0305181500.01.T01	Chitin elicitor-binding protein (Fragment)
augustus_masked-5-processed-gene-2.69-mRNA-1	NA	Japonica	LRR-TM	Os05g0104600	OsR498G0509566700.01.T01	Polygalacturonase inhibitor
augustus_masked-5-processed-gene-3.57-mRNA-1	NA	Japonica	LRR-TM	Os05g0104700	OsR498G0509566700.01.T01	Polygalacturonase inhibitor
augustus_masked-6-processed-gene-21.33-mRNA-1	NA	NA	LRR-TM	Os06g0140000	OsR498G0611600200.01.T01	Serine/threonine-protein kinase bri1, putative
augustus_masked-6-processed-gene-21.35-mRNA-1	NA	NA	LRR-TM	Os06g0140200	OsR498G0611600600.01.T01	Leucine-rich repeat domain, L domain-like protein
augustus_masked-6-processed-gene-21.36-mRNA-1	NA	NA	LRR-TM	Os06g0140300	OsR498G0611601000.01.T01	[Phosphatase 2A protein]-leucine-carboxy methyltransferase 1
maker-1-augustus-gene-22.25-mRNA-1	NA	NA	LRR-TM	Os01g0140400	OsR498G0100141500.01.T06	Di-glucose binding protein with Leucine-rich repeat domain
maker-1-augustus-gene-32.49-mRNA-1	NA	NA	LRR-TM			LRR receptor-like serine/threonine-protein kinase FLS2
maker-10-augustus-gene-139.21-mRNA-1	NA	NA	LRR-TM	0	OsR498G0407494400.01.T01	Protein kinase domain-containing protein
maker-10-augustus-gene-164.29-mRNA-1	NA	Japonica	LRR-TM	Os10g0469600	OsR498G1018872900.01.T01	MDIS1-interacting receptor like kinase 2
maker-10-augustus-gene-30.6-mRNA-1	NA	NA	LRR-TM	Os12g0210400	OsR498G1018135400.01.T02	probable leucine-rich repeat receptor-like protein kinase At5g49770
maker-11-augustus-gene-74.2-mRNA-1	NA	NA	LRR-TM	0	OsR498G1119610600.01.T03	NBS-LRR disease resistance protein family-4
maker-12-augustus-gene-12.12-mRNA-1	NA	NA	LRR-TM			NB-ARC domain-containing protein
maker-12-augustus-gene-18.48-mRNA-1	NA	NA	LRR-TM			NB-ARC domain-containing protein
maker-12-augustus-gene-281.10-mRNA-1	NA	NA	LRR-TM	Os07g0115400		LRR receptor-like serine/threonine-protein kinase FLS2
maker-2-augustus-gene-190.3-mRNA-1	NA	NA	LRR-TM			
maker-2-augustus-gene-234.0-mRNA-1	NA	NA	LRR-TM	Os02g0593600	OsR498G0204224100.01.T01	Plant intracellular Ras-group-related LRR protein 2
maker-2-augustus-gene-247.5-mRNA-1	NA	Indica	LRR-TM	Os02g0615800	OsR498G0204310500.01.T04	LRR receptor-like serine/threonine-protein kinase EFR
maker-2-augustus-gene-248.0-mRNA-1	NA	Indica	LRR-TM	Os02g0615300	OsR498G0204314300.01.T03	Putative receptor like protein 12
maker-2-augustus-gene-31.58-mRNA-1	NA	NA	LRR-TM	Os02g0157150		Protein kinase domain-containing protein (Fragment)
maker-2-augustus-gene-31.66-mRNA-1	NA	NA	LRR-TM			Tyrosine-sulfated glycopeptide receptor 1
maker-2-augustus-gene-31.67-mRNA-1	NA	Japonica	LRR-TM	Os02g0157200	OsR498G0202990800.01.T01	Tyrosine-sulfated glycopeptide receptor 1
maker-4-augustus-gene-275.10-mRNA-1	NA	NA	LRR-TM			Phytosulfokine receptor 1
maker-5-augustus-gene-104.35-mRNA-1	NA	Indica	LRR-TM	Os04g0291900	OsR498G0510203700.01.T01	Non-specific serine/threonine protein kinase
maker-5-augustus-gene-2.13-mRNA-1	NA	NA	LRR-TM	Os05g0104100	OsR498G0509566500.01.T01	Vacuolar sorting protein 39/Transforming growth factor beta receptor-associated domain
maker-5-augustus-gene-40.2-mRNA-1	NA	NA	LRR-TM	Os05g0170300	OsR498G0509827700.01.T01	LRR receptor-like serine/threonine-protein kinase
maker-5-augustus-gene-40.23-mRNA-1	NA	NA	LRR-TM	Os05g0170700	OsR498G0509830600.01.T01	Protein NSP-INTERACTING KINASE 2
maker-5-augustus-gene-98.23-mRNA-1	NA	NA	LRR-TM	Os04g0291900	OsR498G0407958200.01.T02	Non-specific serine/threonine protein kinase
maker-6-augustus-gene-249.63-mRNA-1	NA	Japonica	LRR-TM	Os06g0621500	OsR498G0612965800.01.T01	NAF domain-containing protein
maker-6-augustus-gene-35.37-mRNA-1	NA	NA	LRR-TM	Os06g0167500	OsR498G0611685900.01.T01	Malectin domain-containing protein (Fragment)
maker-6-augustus-gene-40.71-mRNA-1	NA	NA	LRR-TM	Os06g0179800	OsR498G0611719000.01.T04	Putative leucine-rich repeat domain, L domain-containing protein
maker-6-augustus-gene-93.36-mRNA-1	NA	Indica	LRR-TM	Os06g0274500	OsR498G0612087300.01.T01	Somatic embryogenesis receptor kinase 1-like
maker-7-augustus-gene-185.46-mRNA-1	NA	NA	LRR-TM	Os07g0501800	OsR498G0714373600.01.T01	Receptor like protein 4
maker-7-augustus-gene-197.75-mRNA-1	NA	NA	LRR-TM	0	OsR498G0714446800.01.T02	Patatin
maker-7-augustus-gene-5.39-mRNA-1	NA	NA	LRR-TM	Os07g0115400		LRR receptor-like serine/threonine-protein kinase FLS2
maker-8-augustus-gene-145.29-mRNA-1	NA	NA	LRR-TM	Os08g0322500	OsR498G0815772300.01.T01	Putative LRR receptor-like serine/threonine-protein kinase
maker-8-augustus-gene-260.0-mRNA-1	NA	Indica	LRR-TM	Os08g0499900	OsR498G0816398100.01.T01	F-box protein At-B
maker-11-augustus-gene-87.44-mRNA-1	Indica	NA	NBS	Os12g0263800		Cyprosin
maker-1-augustus-gene-259.42-mRNA-1	Japonica	Indica	NBS	Os12g0539800	OsR498G0101591800.01.T01	
maker-1-augustus-gene-325.2-mRNA-1	Japonica	NA	NBS	Os01g0757400	OsR498G0102022500.01.T02	AAA domain-containing protein (Fragment)
maker-11-augustus-gene-252.57-mRNA-1	Japonica	Japonica	NBS	Os11g0684600	OsR498G1120731800.01.T01	NBS-LRR disease resistance protein homologue-like
maker-4-augustus-gene-271.50-mRNA-1	Japonica	NA	NBS	Os04g0640600	OsR498G0409354200.01.T01	Shikimate kinase
maker-7-augustus-gene-229.43-mRNA-1	Japonica	NA	NBS	Os07g0573100	OsR498G0714613100.01.T02	Adenylyl-sulfate kinase
maker-8-augustus-gene-92.16-mRNA-1	Japonica	NA	NBS			Resistance protein PSH-RGH6
augustus_masked-12-processed-gene-127.26-mRNA-1	NA	Japonica	NBS	Os12g0409901	OsR498G1221377600.01.T02	ABC transporter domain-containing protein
maker-11-augustus-gene-252.57-mRNA-1	NA	Japonica	NBS	Os11g0684600	OsR498G1120731800.01.T01	NBS-LRR disease resistance protein homologue-like
maker-3-augustus-gene-401.28-mRNA-1	NA	NA	NBS	Os03g0670100	OsR498G0306764000.01.T02	Protein TRIGALACTOSYLDIACYLGLYCEROL 3 chloroplastic
maker-8-augustus-gene-46.41-mRNA-1	NA	NA	NBS	Os08g0176633		Disease resistance RPP13-like protein 4
augustus-8-processed-gene-189.5-mRNA-1	Indica	NA	NBS-TM	Os08g0373400		CC-NBS-LRR type disease resistance protein NLR1-V
augustus_masked-11-processed-gene-253.37-mRNA-1	Indica	NA	NBS-TM	Os11g0686900	OsR498G1120736800.01.T01	NBS-LRR disease resistance protein homologue-like
augustus_masked-6-processed-gene-102.3-mRNA-1	Indica	NA	NBS-TM		OsR498G0612165700.01.T01	Disease resistance protein RPM1
maker-1-augustus-gene-473.15-mRNA-1	Indica	NA	NBS-TM	Os01g0911300	OsR498G0102556000.01.T01	ABC transporter B family member 26, chloroplastic
maker-11-augustus-gene-142.50-mRNA-1	Indica	NA	NBS-TM			Pollen signalling protein with adenylyl cyclase activity (Fragment)
maker-12-augustus-gene-256.3-mRNA-1	Indica	NA	NBS-TM	Os12g0601200	OsR498G1222050300.01.T04	PRK domain-containing protein (Fragment)
maker-2-augustus-gene-182.49-mRNA-1	Indica	NA	NBS-TM			Disease resistance protein RGA2
maker-3-augustus-gene-301.25-mRNA-1	Indica	NA	NBS-TM	Os10g0370100		calmodulin-binding transcription activator 3
maker-3-augustus-gene-304.14-mRNA-1	Indica	NA	NBS-TM		OsR498G1018551100.01.T01	NB-ARC domain containing protein, expressed
maker-5-augustus-gene-75.42-mRNA-1	Indica	NA	NBS-TM	Os05g0222200	OsR498G0510052200.01.T01	Eceriferum 5 (Fragment)
maker-6-augustus-gene-102.42-mRNA-1	Indica	NA	NBS-TM			Disease resistance protein RPM1
maker-8-augustus-gene-60.12-mRNA-1	Indica	NA	NBS-TM	Os08g0202400	OsR498G0815386000.01.T02	NB-ARC domain-containing protein
maker-9-augustus-gene-88.25-mRNA-1	Indica	NA	NBS-TM			NBS-LRR protein (Fragment)
augustus-1-processed-gene-120.2-mRNA-1	Japonica	NA	NBS-TM		OsR498G0100788500.01.T01	NB-ARC domain-containing protein
augustus-1-processed-gene-345.2-mRNA-1	Japonica	NA	NBS-TM	Os01g0799100	OsR498G0102144900.01.T02	NB-ARC domain-containing protein
augustus-11-processed-gene-209.35-mRNA-1	Japonica	Japonica	NBS-TM	Os11g0606500	OsR498G1120485700.01.T01	Nucleotide binding-leucine rich repeat protein
augustus-11-processed-gene-236.18-mRNA-1	Japonica	NA	NBS-TM	Os11g0654100		NB-ARC domain containing protein, expressed
augustus-11-processed-gene-43.45-mRNA-1	Japonica	Japonica	NBS-TM	Os11g0263000	OsR498G1119746200.01.T02	Similar to NBS-LRR disease resistance protein homologue
augustus-12-processed-gene-151.7-mRNA-1	Japonica	NA	NBS-TM	Os12g0438300	OsR498G1221468900.01.T01	Disease resistance protein RGA2
augustus-12-processed-gene-205.35-mRNA-1	Japonica	NA	NBS-TM		OsR498G1221700900.01.T01	NB-ARC domain containing protein, expressed
augustus-2-processed-gene-93.14-mRNA-1	Japonica	Japonica	NBS-TM	Os02g0262800	OsR498G0203369300.01.T01	Powdery mildew resistance protein
augustus-5-processed-gene-195.29-mRNA-1	Japonica	NA	NBS-TM	Os05g0384600	OsR498G0510747200.01.T01	ATPase, coupled to transmembrane movement of substances
augustus-8-processed-gene-99.30-mRNA-1	Japonica	NA	NBS-TM	Os08g0258700	OsR498G0815569000.01.T01	Nucleotide-binding leucine-rich-repeat protein 1
augustus_masked-1-processed-gene-147.20-mRNA-1	Japonica	NA	NBS-TM	Os01g0359400	OsR498G0100940200.01.T05	Truncated powdery mildew resistance protein Pm3
augustus_masked-10-processed-gene-21.43-mRNA-1	Japonica	NA	NBS-TM			Powdery mildew resistance protein PM17
augustus_masked-11-processed-gene-111.22-mRNA-1	Japonica	Japonica	NBS-TM	Os11g0429100	OsR498G1120005800.01.T01	NBS-LRR type resistance protein (Fragment)
augustus_masked-11-processed-gene-253.37-mRNA-1	Japonica	NA	NBS-TM	Os11g0686900	OsR498G1120736800.01.T01	NBS-LRR disease resistance protein homologue-like
augustus_masked-11-processed-gene-70.21-mRNA-1	Japonica	NA	NBS-TM	Os11g0207400		NB-ARC domain-containing protein
augustus_masked-12-processed-gene-192.21-mRNA-1	Japonica	NA	NBS-TM	Os12g0489800	OsR498G1221649100.01.T01	NB-ARC domain-containing protein
augustus_masked-12-processed-gene-57.32-mRNA-1	Japonica	Japonica	NBS-TM	Os12g0205500	OsR498G1221100000.01.T02	NB-ARC domain containing protein, expressed
augustus_masked-3-processed-gene-309.6-mRNA-1	Japonica	NA	NBS-TM			Disease resistance protein RPM1
augustus_masked-4-processed-gene-300.96-mRNA-1	Japonica	NA	NBS-TM		OsR498G0409545600.01.T01	White-brown-complex ABC transporter family
augustus_masked-5-processed-gene-193.19-mRNA-1	Japonica	Japonica	NBS-TM	Os05g0380800	OsR498G0510731100.01.T01	Disease resistance protein RPM1
augustus_masked-8-processed-gene-217.0-mRNA-1	Japonica	NA	NBS-TM	Os08g0424700		NB-ARC domain-containing protein
maker-1-augustus-gene-141.37-mRNA-1	Japonica	NA	NBS-TM		OsR498G0100907000.01.T01	NB-ARC domain-containing protein
maker-1-augustus-gene-148.26-mRNA-1	Japonica	Indica	NBS-TM	Os01g0359800	OsR498G0100942600.01.T01	Powdery mildew resistance protein PM3 variant
maker-1-augustus-gene-228.33-mRNA-1	Japonica	NA	NBS-TM	Os01g0574400	OsR498G0101378500.01.T03	ATP-dependent zinc metalloprotease FTSH 4 mitochondrial
maker-1-augustus-gene-228.36-mRNA-1	Japonica	NA	NBS-TM	Os01g0574500	OsR498G0101378700.01.T01	ATP-dependent zinc metalloprotease FTSH 4 mitochondrial
maker-1-augustus-gene-283.43-mRNA-1	Japonica	NA	NBS-TM		OsR498G0101739800.01.T04	Katanin p60 ATPase-containing subunit
maker-10-augustus-gene-21.12-mRNA-1	Japonica	Indica	NBS-TM	Os10g0130600	OsR498G1018091000.01.T01	Disease resistance protein RGA4
maker-10-augustus-gene-59.37-mRNA-1	Japonica	NA	NBS-TM	Os10g0183000	OsR498G1018288800.01.T04	NB-ARC domain containing protein, expressed
maker-11-augustus-gene-132.28-mRNA-1	Japonica	NA	NBS-TM			F-box protein FBX14
maker-11-augustus-gene-176.28-mRNA-1	Japonica	NA	NBS-TM		OsR498G1120322000.01.T02	Armadillo repeat-containing protein 7
maker-11-augustus-gene-189.4-mRNA-1	Japonica	NA	NBS-TM	Os11g0572100	OsR498G1120391500.01.T01	Replication factor C subunit 1
maker-11-augustus-gene-195.2-mRNA-1	Japonica	NA	NBS-TM	Os11g0582300	OsR498G1120411700.01.T01	Protein ROOT HAIR DEFECTIVE 3 homolog
maker-11-augustus-gene-199.32-mRNA-1	Japonica	Japonica	NBS-TM	Os11g0590400	OsR498G1120429100.01.T01	Putative CC-NBS-LRR resistance protein MLA13
maker-11-augustus-gene-200.10-mRNA-1	Japonica	NA	NBS-TM			Protein-serine/threonine phosphatase
maker-11-augustus-gene-209.1-mRNA-1	Japonica	NA	NBS-TM	Os11g0606900	OsR498G1120485700.01.T01	Disease resistance protein RPM1
maker-11-augustus-gene-222.42-mRNA-1	Japonica	NA	NBS-TM		OsR498G1120563300.01.T01	NB-ARC domain containing protein, expressed

maker-11-augustus-gene-225.3-mRNA-1	Japonica	Indica	NBS-TM	Os07g0573100	OsR498G1120588100.01.T01	Adenylyl-sulfate kinase
maker-11-augustus-gene-253.1-mRNA-1	Japonica	Indica	NBS-TM	Os11g0684100	OsR498G1120735400.01.T01	NBS-LRR disease resistance protein homologue-like
maker-11-augustus-gene-37.17-mRNA-1	Japonica	NA	NBS-TM	Os11g0184900	OsR498G1119531400.01.T01	NAC domain transcription factor
maker-11-augustus-gene-46.22-mRNA-1	Japonica	Indica	NBS-TM	Os11g0265900	OsR498G1119768400.01.T01	NB-ARC domain, putative
maker-11-augustus-gene-46.8-mRNA-1	Japonica	Japonica	NBS-TM	Os11g0266500	OsR498G1119768700.01.T02	Disease resistance protein RPM1
maker-11-augustus-gene-59.22-mRNA-1	Japonica	NA	NBS-TM		OsR498G1119700900.01.T04	Leucine Rich Repeat family protein, expressed
maker-11-augustus-gene-7.73-mRNA-1	Japonica	Indica	NBS-TM	Os08g0170200	OsR498G1119365800.01.T01	Disease resistance protein RPM1
maker-12-augustus-gene-197.0-mRNA-1	Japonica	NA	NBS-TM		OsR498G1221658300.01.T01	NB-ARC domain containing protein, expressed
maker-12-augustus-gene-206.28-mRNA-1	Japonica	NA	NBS-TM	Os12g0511400	OsR498G1221703400.01.T01	NB-ARC domain containing protein, expressed
maker-12-augustus-gene-206.36-mRNA-1	Japonica	NA	NBS-TM			NB-ARC domain containing protein, expressed
maker-12-augustus-gene-207.37-mRNA-1	Japonica	NA	NBS-TM		OsR498G1221704200.01.T01	NB-ARC domain containing protein, expressed
maker-12-augustus-gene-234.92-mRNA-1	Japonica	NA	NBS-TM	Os12g0564800	OsR498G1221892000.01.T02	NB-ARC domain containing protein expressed
maker-12-augustus-gene-36.0-mRNA-1	Japonica	NA	NBS-TM		OsR498G1221011500.01.T01	Disease resistance protein RPM1
maker-2-augustus-gene-127.48-mRNA-1	Japonica	Indica	NBS-TM	Os02g0318500	OsR498G0203559000.01.T05	PDR-type ABC transporter
maker-3-augustus-gene-450.0-mRNA-1	Japonica	NA	NBS-TM	Os03g0755100	OsR498G0307065300.01.T01	Transporter associated with antigen processing protein 2
maker-3-augustus-gene-51.40-mRNA-1	Japonica	Indica	NBS-TM	Os07g0573100	OsR498G0305414000.01.T04	Adenylyl-sulfate kinase
maker-3-augustus-gene-99.37-mRNA-1	Japonica	NA	NBS-TM		OsR498G0305742500.01.T01	Macro domain-containing protein
maker-4-augustus-gene-262.52-mRNA-1	Japonica	Japonica	NBS-TM	Os04g0621500	OsR498G0409298500.01.T01	NBS-type RGA protein (Fragment)
maker-5-augustus-gene-193.0-mRNA-1	Japonica	NA	NBS-TM	Os05g0379500	OsR498G0510726700.01.T05	Powdery mildew resistance protein PM3 variant
maker-5-augustus-gene-193.3-mRNA-1	Japonica	NA	NBS-TM	Os05g0380300	OsR498G0510726700.01.T05	Powdery mildew resistance protein PM3 variant
maker-5-augustus-gene-246.48-mRNA-1	Japonica	Japonica	NBS-TM	Os05g0479700	OsR498G0511050000.01.T01	NBS-LRR disease resistance protein homologue-like
maker-5-augustus-gene-86.6-mRNA-1	Japonica	NA	NBS-TM	Os05g0240200	OsR498G0510120000.01.T03	TOPLESS-related 1 isoform 2
maker-6-augustus-gene-102.42-mRNA-1	Japonica	NA	NBS-TM			Disease resistance protein RPM1
maker-6-augustus-gene-102.49-mRNA-1	Japonica	NA	NBS-TM		OsR498G0612165900.01.T03	Disease resistance protein RPM1
maker-6-augustus-gene-194.39-mRNA-1	Japonica	NA	NBS-TM			Disease resistance protein RPP13
maker-6-augustus-gene-249.57-mRNA-1	Japonica	Japonica	NBS-TM	Os06g0619000	OsR498G0612961500.01.T01	Putative disease resistance protein RGA3
maker-6-augustus-gene-5.75-mRNA-1	Japonica	NA	NBS-TM	Os06g0109400	OsR498G0611490500.01.T01	Cell division control protein C
maker-7-augustus-gene-42.45-mRNA-1	Japonica	NA	NBS-TM		OsR498G0713654200.01.T01	AAA domain-containing protein
maker-7-augustus-gene-97.8-mRNA-1	Japonica	NA	NBS-TM		OsR498G0713986700.01.T02	Putative disease resistance RPP13-like protein 1
maker-8-augustus-gene-46.43-mRNA-1	Japonica	NA	NBS-TM			Disease resistance RPP13-like protein 4
maker-8-augustus-gene-60.12-mRNA-1	Japonica	NA	NBS-TM	Os08g0202400	OsR498G0815386000.01.T02	NB-ARC domain-containing protein
maker-8-augustus-gene-85.10-mRNA-1	Japonica	Indica	NBS-TM	Os08g0235600	OsR498G0815501900.01.T01	NB-ARC domain-containing protein
maker-9-augustus-gene-87.0-mRNA-1	Japonica	NA	NBS-TM			NB-ARC domain-containing protein
augustus-1-processed-gene-244.17-mRNA-1	NA	NA	NBS-TM	0	OsR498G0101487100.01.T01	Disease resistance protein RPP13
augustus-1-processed-gene-407.14-mRNA-1	NA	NA	NBS-TM	Os01g0615500	OsR498G0101542000.01.T01	ATPase, coupled to transmembrane movement of substances
augustus-1-processed-gene-459.1-mRNA-1	NA	NA	NBS-TM	Os01g0615500	OsR498G0101542000.01.T01	ATPase, coupled to transmembrane movement of substances
augustus-11-processed-gene-208.33-mRNA-1	NA	NA	NBS-TM	Os11g0605100		NB-ARC domain containing protein, expressed
augustus-11-processed-gene-252.35-mRNA-1	NA	NA	NBS-TM	Os11g0684100	OsR498G1120735400.01.T01	NBS-LRR disease resistance protein homologue-like
augustus-11-processed-gene-43.45-mRNA-1	NA	Japonica	NBS-TM	Os11g0263000	OsR498G1119746200.01.T02	Similar to NBS-LRR disease resistance protein homologue
augustus-4-processed-gene-129.53-mRNA-1	NA	NA	NBS-TM	Os04g0375300	OsR498G0408366900.01.T01	NB-ARC domain-containing protein
augustus-4-processed-gene-130.56-mRNA-1	NA	NA	NBS-TM	0	OsR498G0408382700.01.T01	Disease resistance protein RGA2
augustus-7-processed-gene-28.9-mRNA-1	NA	NA	NBS-TM	Os07g0160300	OsR498G0713563100.01.T01	P-loop containing nucleoside triphosphate hydrolase
augustus-8-processed-gene-100.23-mRNA-1	NA	NA	NBS-TM	0	OsR498G0815571800.01.T01	Disease resistance protein RPM1
augustus-8-processed-gene-189.5-mRNA-1	NA	NA	NBS-TM	Os08g0373400		CC-NBS-LRR type disease resistance protein NLR1-V
augustus-8-processed-gene-43.39-mRNA-1	NA	NA	NBS-TM			Disease resistance protein RPP13
augustus-8-processed-gene-99.30-mRNA-1	NA	NA	NBS-TM	Os08g0258700	OsR498G0815569000.01.T01	Nucleotide-binding leucine-rich-repeat protein 1
augustus_masked-1-processed-gene-251.5-mRNA-1	NA	NA	NBS-TM	Os01g0615500	OsR498G0101542000.01.T01	ATPase, coupled to transmembrane movement of substances
augustus_masked-11-processed-gene-262.11-mRNA-1	NA	NA	NBS-TM	Os05g0250700	OsR498G0510149400.01.T01	NB-ARC domain, putative
augustus_masked-3-processed-gene-309.6-mRNA-1	NA	NA	NBS-TM			Disease resistance protein RPM1
augustus_masked-6-processed-gene-102.3-mRNA-1	NA	NA	NBS-TM	0	OsR498G0612165700.01.T01	Disease resistance protein RPM1
augustus_masked-6-processed-gene-182.21-mRNA-1	NA	Indica	NBS-TM	Os06g0503100	OsR498G0612565500.01.T01	ATPase, coupled to transmembrane movement of substances
augustus_masked-8-processed-gene-198.16-mRNA-1	NA	NA	NBS-TM	Os08g0388300	OsR498G0815986100.01.T03	NB-ARC domain containing protein, expressed
maker-1-augustus-gene-414.45-mRNA-1	NA	NA	NBS-TM	0	OsR498G0101487100.01.T01	Mitochondrial import inner membrane translocase subunit
maker-1-augustus-gene-465.15-mRNA-1	NA	NA	NBS-TM	0	OsR498G0101487100.01.T01	Mitochondrial import inner membrane translocase subunit
maker-11-augustus-gene-253.1-mRNA-1	NA	Indica	NBS-TM	Os11g0684100	OsR498G1120735400.01.T01	NBS-LRR disease resistance protein homologue-like
maker-12-augustus-gene-176.30-mRNA-1	NA	NA	NBS-TM			Putative NB-ARC domain-containing protein (Fragment)
maker-12-augustus-gene-197.0-mRNA-1	NA	NA	NBS-TM	0	OsR498G1221658300.01.T01	NB-ARC domain containing protein, expressed
maker-12-augustus-gene-234.92-mRNA-1	NA	NA	NBS-TM	Os12g0564800	OsR498G1221892000.01.T02	NB-ARC domain containing protein expressed
maker-2-augustus-gene-10.50-mRNA-1	NA	NA	NBS-TM			Putative disease resistance protein RGA4
maker-3-augustus-gene-185.7-mRNA-1	NA	Indica	NBS-TM	Os07g0501500	OsR498G0306049500.01.T01	Disease resistance protein RPM1
maker-3-augustus-gene-251.24-mRNA-1	NA	Japonica	NBS-TM	Os03g0599000	OsR498G0306507000.01.T01	Putative NBS-LRR type resistance protein
maker-3-augustus-gene-301.25-mRNA-1	NA	NA	NBS-TM	Os10g0370100		calmodulin-binding transcription activator 3
maker-3-augustus-gene-304.14-mRNA-1	NA	NA	NBS-TM	0	OsR498G1018551100.01.T01	NB-ARC domain containing protein, expressed
maker-4-augustus-gene-129.11-mRNA-1	NA	Indica	NBS-TM	Os04g0374175	OsR498G0408364900.01.T02	NB-ARC domain-containing protein
maker-4-augustus-gene-150.52-mRNA-1	NA	NA	NBS-TM	Os04g0413000		ABC transporter B family member 29 chloroplastic
maker-5-augustus-gene-193.0-mRNA-1	NA	NA	NBS-TM	Os05g0379500	OsR498G0510726700.01.T05	Powdery mildew resistance protein PM3 variant
maker-5-augustus-gene-193.3-mRNA-1	NA	NA	NBS-TM	Os05g0380300	OsR498G0510726700.01.T05	Powdery mildew resistance protein PM3 variant
maker-5-augustus-gene-75.42-mRNA-1	NA	NA	NBS-TM	Os05g0222200	OsR498G0510052200.01.T01	Eceriferum 5 (Fragment)
maker-5-augustus-gene-86.6-mRNA-1	NA	NA	NBS-TM	Os05g0240200	OsR498G0510120000.01.T03	TOPLESS-related 1 isoform 2
maker-6-augustus-gene-101.22-mRNA-1	NA	NA	NBS-TM	Os06g0286500	OsR498G0612159700.01.T01	NBS-LRR disease resistance protein homologue
maker-6-augustus-gene-102.42-mRNA-1	NA	NA	NBS-TM			Disease resistance protein RPM1
maker-6-augustus-gene-102.49-mRNA-1	NA	NA	NBS-TM	0	OsR498G0612165900.01.T03	Disease resistance protein RPM1
maker-6-augustus-gene-24.47-mRNA-1	NA	NA	NBS-TM	Os06g0146100	OsR498G0611619900.01.T05	NBS-LRR type R protein Pizh-1
maker-6-augustus-gene-30.64-mRNA-1	NA	NA	NBS-TM	Os06g0158300	OsR498G0611659000.01.T02	Putative NB-ARC domain-containing protein (Fragment)
maker-6-augustus-gene-8.48-mRNA-1	NA	NA	NBS-TM	Os06g0114600	OsR498G0611507900.01.T02	Gluconate kinase
maker-7-augustus-gene-242.53-mRNA-1	NA	NA	NBS-TM	Os07g0599100		Pentatricopeptide repeat
maker-8-augustus-gene-152.44-mRNA-1	NA	NA	NBS-TM			NBS-LRR disease resistance protein homologue (Fragment)
maker-8-augustus-gene-43.0-mRNA-1	NA	NA	NBS-TM	Os08g0170200	OsR498G0815279900.01.T01	Disease resistance protein RPP13
maker-9-augustus-gene-87.0-mRNA-1	NA	NA	NBS-TM			NB-ARC domain-containing protein
maker-9-augustus-gene-88.25-mRNA-1	NA	NA	NBS-TM			NBS-LRR protein (Fragment)
maker-10-augustus-gene-22.26-mRNA-1	Japonica	Indica	NBS-TM PIK, PIKM	Os11g0689100	OsR498G1018095600.01.T01	Disease resistance protein Piks-2
maker-11-augustus-gene-255.0-mRNA-1	Japonica	Indica	NBS-TM PIK, PIKM	Os11g0689100	OsR498G1120737900.01.T02	Disease resistance protein Piks-2
maker-6-augustus-gene-128.0-mRNA-1	Japonica	NA	NBS-TM PID3	Os06g0330100		Resistance protein (Fragment) PiD3
augustus_masked-12-processed-gene-108.24-mRNA-1	Japonica	NA	NBS-TM PITA	Os12g0281300	OsR498G1221311800.01.T01	Blast resistance protein Pi-ta
maker-8-augustus-gene-284.6-mRNA-1	Japonica	Indica	PIBH8	Os08g0539700	OsR498G0816550100.01.T01	Brown planthopper resistance protein BPH1/9
maker-12-augustus-gene-110.45-mRNA-1	Japonica	Indica	PTR	Os12g0285100	OsR498G1221322300.01.T01	Disease resistance protein
maker-12-augustus-gene-111.26-mRNA-1	Japonica	Japonica	PTR	Os12g0285100	OsR498G1221325200.01.T02	Disease resistance protein (Fragment)
augustus-1-processed-gene-30.34-mRNA-1	Indica	NA	Kinase-TM	Os01g0155500	OsR498G0100197900.01.T01	Receptor-like protein kinase HERK 1
augustus-1-processed-gene-8.3-mRNA-1	Indica	Japonica	Kinase-TM	Os01g0113650	OsR498G0100044500.01.T01	Putative receptor serine/threonine kinase PR5K
augustus-10-processed-gene-1.21-mRNA-1	Indica	NA	Kinase-TM	Os10g0101000	OsR498G1017996700.01.T01	Non-specific serine/threonine protein kinase
augustus-2-processed-gene-33.34-mRNA-1	Indica	NA	Kinase-TM	Os02g0161000	OsR498G0203015000.01.T02	Non-specific serine/threonine protein kinase
augustus-4-processed-gene-154.22-mRNA-1	Indica	NA	Kinase-TM	Os04g0420600	OsR498G0408563300.01.T01	Non-specific serine/threonine protein kinase (Fragment)
augustus-5-processed-gene-293.4-mRNA-1	Indica	NA	Kinase-TM	Os05g0566400	OsR498G0511355000.01.T05	Mitogen-activated protein kinase
augustus-5-processed-gene-66.22-mRNA-1	Indica	Japonica	Kinase-TM	Os05g0208100	OsR498G0816176500.01.T01	Non-specific serine/threonine protein kinase
augustus-7-processed-gene-12.18-mRNA-1	Indica	NA	Kinase-TM	Os07g0130100	OsR498G0713460400.01.T01	L-type lectin-domain containing receptor kinase IV.2
augustus-7-processed-gene-12.19-mRNA-1	Indica	NA	Kinase-TM	Os07g0130200	OsR498G0713461200.01.T01	Putative receptor kinase Lecrk
augustus-7-processed-gene-12.20-mRNA-1	Indica	Japonica	Kinase-TM	Os07g0130300	OsR498G0713999000.01.T01	L-type lectin-domain containing receptor kinase IV.1
augustus-7-processed-gene-12.25-mRNA-1	Indica	NA	Kinase-TM	Os07g0130800	OsR498G0713462000.01.T01	Putative receptor kinase Lecrk
augustus-7-processed-gene-12.31-mRNA-1	Indica	NA	Kinase-TM	Os07g0132150	OsR498G0713464000.01.T01	L-type lectin-domain containing receptor kinase IV.2
augustus-7-processed-gene-13.14-mRNA-1	Indica	NA	Kinase-TM		OsR498G0713464300.01.T06	Putative receptor-type protein kinase LRK1
augustus-7-processed-gene-13.27-mRNA-1	Indica	Indica	Kinase-TM	Os07g0130200	OsR498G0713465300.01.T01	Putative receptor-type protein kinase LRK1
augustus-7-processed-gene-13.37-mRNA-1	Indica	Indica	Kinase-TM	Os07g0133100	OsR498G0713465800.01.T01	Non-specific serine/threonine protein kinase
augustus-7-processed-gene-181.23-mRNA-1	Indica	NA	Kinase-TM	Os07g0493800	OsR498G0714347500.01.T01	Wall-associated protein kinase
augustus-7-processed-gene-208.7-mRNA-1	Indica	NA	Kinase-TM			Receptor-like serine/threonine-protein kinase
augustus_masked-1-processed-gene-8.12-mRNA-1	Indica	NA	Kinase-TM	Os01g0113400	OsR498G0100045000.01.T04	Cysteine-rich receptor-like protein kinase 37
augustus_masked-1-processed-gene-9.31-mRNA-1	Indica	Indica	Kinase-TM	Os01g0117400	OsR498G0100056000.01.T03	Receptor-like kinase
augustus_masked-1-processed-gene-9.4-mRNA-1	Indica	NA	Kinase-TM	Os01g0115700	OsR498G0100051200.01.T01	Receptor-like kinase
augustus_masked-10-processed-gene-148.16-mRNA-1	Indica	NA	Kinase-TM	Os04g0103500	OsR498G0407421200.01.T01	G-type lectin S-receptor-like serine/threonine-protein kinase
augustus_masked-10-processed-gene-5.20-mRNA-1	Indica	NA	Kinase-TM	Os10g0104800	OsR498G1018012400.01.T01	Proline-rich receptor-like protein kinase PERK4
augustus_masked-4-processed-gene-154.33-mRNA-1	Indica	Japonica	Kinase-TM	Os04g0420900	OsR498G0408563300.01.T01	Non-specific serine/threonine protein kinase (Fragment)
augustus_masked-4-processed-gene-22.27-mRNA-1	Indica	Indica	Kinase-TM	Os04g0202350	OsR498G0407718200.01.T01	Non-specific serine/threonine protein kinase
augustus_masked-5-processed-gene-161.9-mRNA-1	Indica	NA	Kinase-TM	Os05g0332300	OsR498G0510529300.01.T01	Non-specific serine/threonine protein kinase
augustus_masked-6-processed-gene-102.31-mRNA-1	Indica	NA	Kinase-TM	Os06g0288100	OsR498G0612167200.01.T01	Leucine-rich repeat receptor-like serine/threonine/tyrosine-protein kinase SOBIR1
augustus_masked-6-processed-gene-295.37-mRNA-1	Indica	NA	Kinase-TM	Os06g0699400	OsR498G0613249300.01.T03	Mitogen-activated protein kinase
augustus_masked-6-processed-gene-57.46-mRNA-1	Indica	Japonica	Kinase-TM	Os06g0210400	OsR498G1019094500.01.T01	Mitogen-activated protein kinase kinase kinase
augustus_masked-8-processed-gene-228.11-mRNA-1	Indica	Indica	Kinase-TM	Os08g0441100	OsR498G0816176500.01.T01	Non-specific serine/threonine protein kinase
maker-1-augustus-gene-19.21-mRNA-1	Indica	NA	Kinase-TM	Os01g0136800	OsR498G0100126800.01.T01	WAK2-OsWAK receptor-like cytoplasmic kinase (OsWAK-RLCK)
maker-1-augustus-gene-19.9-mRNA-1	Indica	NA	Kinase-TM		OsR498G0100125200.01.T02	Putative serine/threonine-protein kinase
maker-1-augustus-gene-20.0-mRNA-1	Indica	NA	Kinase-TM			Protein kinase domain-containing protein (Fragment)
maker-1-augustus-gene-20.11-mRNA-1	Indica	NA	Kinase-TM			Putative receptor serine/threonine kinase PR5K
maker-1-augustus-gene-20.13-mRNA-1	Indica	NA	Kinase-TM		OsR498G0100130500.01.T01	Putative receptor serine/threonine kinase PR5K
maker-1-augustus-gene-20.18-mRNA-1	Indica	NA	Kinase-TM			Protein kinase domain-containing protein (Fragment)
maker-1-augustus-gene-20.9-mRNA-1	Indica	NA	Kinase-TM			Protein kinase domain-containing protein (Fragment)
maker-1-augustus-gene-21.2-mRNA-1	Indica	NA	Kinase-TM	Os01g0138400	OsR498G0100134300.01.T01	Stress-induced receptor-like kinase 2
maker-1-augustus-gene-351.26-mRNA-1	Indica	NA	Kinase-TM	Os01g0810600		Putative light repressible receptor protein kinase
maker-1-augustus-gene-358.20-mRNA-1	Indica	NA	Kinase-TM	Os01g0822200	OsR498G0102231000.01.T04	Serine/threonine-protein kinase NAK
maker-1-augustus-gene-360.3-mRNA-1	Indica	NA	Kinase-TM	Os01g0824600	OsR498G0102242100.01.T06	Non-specific serine/threonine protein kinase
maker-1-augustus-gene-445.9-mRNA-1	Indica	Indica	Kinase-TM	Os01g0890600	OsR498G0102491100.01.T02	Receptor-like serine/threonine-protein kinase
maker-1-augustus-gene-47.42-mRNA-1	Indica	NA	Kinase-TM	Os01g0186700	OsR498G0100292200.01.T03	AGC (cAMP-dependent cGMP-dependent and protein kinase C) kinase family protein
maker-1-augustus-gene-7.25-mRNA-1	Indica	NA	Kinase-TM	Os01g0113200	OsR498G0100043400.01.T02	Receptor serine/threonine kinase
maker-1-augustus-gene-7.9-mRNA-1	Indica	NA	Kinase-TM	Os01g0113300	OsR498G0100043700.01.T01	Iron-deficiency induced receptor-like kinase
maker-1-augustus-gene-8.133-mRNA-1	Indica	Indica	Kinase-TM	Os01g0113400	OsR498G0100045600.01.T01	Serine/threonine protein kinase
maker-1-augustus-gene-8.134-mRNA-1	Indica	Japonica	Kinase-TM	Os01g0114100	OsR498G0100046400.01.T01	Putative rust resistance kinase Lr10
maker-1-augustus-gene-8.139-mRNA-1	Indica	Indica	Kinase-TM	Os01g0116200	OsR498G0100046400.01.T07	Putative rust resistance kinase Lr10
maker-1-augustus-gene-8.141-mRNA-1	Indica	Japonica	Kinase-TM	Os01g0114600	OsR498G0100047600.01.T01	Receptor-like kinase
maker-1-augustus-gene-8.47-mRNA-1	Indica	Indica	Kinase-TM	Os01g0113800	OsR498G0100045100.01.T01	Putative rust resistance kinase Lr10
maker-1-augustus-gene-8.48-mRNA-1	Indica	NA	Kinase-TM	Os01g0113800	OsR498G0100045700.01.T01	Resistance-related receptor-like kinase
maker-1-augustus-gene-8.49-mRNA-1	Indica	NA	Kinase-TM	Os01g0115900	OsR498G0100046700.01.T01	Receptor-like protein kinase (Fragment)
maker-1-augustus-gene-8.50-mRNA-1	Indica	Japonica	Kinase-TM	Os01g0114500	OsR498G0100047300.01.T01	Receptor serine/threonine kinase
maker-1-augustus-gene-8.51-mRNA-1	Indica	NA	Kinase-TM	Os01g0114700	OsR498G0100047900.01.T01	Resistance-related receptor-like kinase
maker-1-augustus-gene-9.101-mRNA-1	Indica	NA	Kinase-TM	Os01g0116400	OsR498G0100053600.01.T01	Receptor-like kinase
maker-1-augustus-gene-9.105-mRNA-1	Indica	Indica	Kinase-TM	Os01g0117400	OsR498G0100056600.01.T01	Putative rust resistance kinase Lr10
maker-1-augustus-gene-9.51-mRNA-1	Indica	NA	Kinase-TM	Os01g0115900	OsR498G0100052100.01.T01	Receptor-like protein kinase (Fragment)
maker-1-augustus-gene-9.52-mRNA-1	Indica	Japonica	Kinase-TM	Os01g0114500	OsR498G0100045700.01.T01	Protein kinase domain-containing protein
maker-1-augustus-gene-9.75-mRNA-1	Indica	Indica	Kinase-TM	Os01g0117500	OsR498G0100056100.01.T02	Putative rust resistance kinase Lr10
maker-1-augustus-gene-9.81-mRNA-1	Indica	Japonica	Kinase-TM	Os01g0117100	OsR498G0100056700.01.T02	Receptor kinase
maker-1-augustus-gene-9.86-mRNA-1	Indica	NA	Kinase-TM	Os01g0115750	OsR498G0100051800.01.T01	Putative rust resistance kinase Lr10

maker-1-augustus-gene-9.99-mRNA-1	Indica	Indica	Kinase-TM	Os01g0116000	OsR498G0100052000.01.T01	Serine/threonine protein kinase
maker-10-augustus-gene-127.46-mRNA-1	Indica	NA	Kinase-TM	Os04g0141400	OsR498G0407537100.01.T01	Putative receptor-type protein kinase LRK1
maker-10-augustus-gene-127.48-mRNA-1	Indica	NA	Kinase-TM	Os04g0141200	OsR498G0407536900.01.T01	Receptor-like serine/threonine-protein kinase
maker-10-augustus-gene-131.28-mRNA-1	Indica	NA	Kinase-TM	Os04g0125700	OsR498G0407519500.01.T01	Lectin-domain containing receptor kinase A4.3
maker-10-augustus-gene-142.3-mRNA-1	Indica	NA	Kinase-TM	Os01g0136400	OsR498G0100125200.01.T04	Putative serine/threonine-protein kinase
maker-10-augustus-gene-145.10-mRNA-1	Indica	NA	Kinase-TM	Os04g0109100	OsR498G0407442600.01.T01	Non-specific serine/threonine protein kinase
maker-10-augustus-gene-35.0-mRNA-1	Indica	NA	Kinase-TM			Wall-associated receptor kinase 4
maker-10-augustus-gene-43.39-mRNA-1	Indica	Indica	Kinase-TM	Os10g0174800	OsR498G1018204400.01.T01	Wall associated kinase
maker-11-augustus-gene-256.34-mRNA-1	Indica	NA	Kinase-TM	Os11g0695850	OsR498G1120743300.01.T02	Wall-associated receptor kinase
maker-11-augustus-gene-256.35-mRNA-1	Indica	NA	Kinase-TM			Putative inactive receptor kinase RLK902
maker-11-augustus-gene-74.11-mRNA-1	Indica	NA	Kinase-TM		OsR498G1119612600.01.T01	Protein kinase domain containing protein, expressed
maker-12-augustus-gene-147.6-mRNA-1	Indica	NA	Kinase-TM	Os12g0433500	OsR498G1221451200.01.T03	serine/threonine-protein kinase TIO
maker-2-augustus-gene-189.0-mRNA-1	Indica	NA	Kinase-TM	Os02g0513000	OsR498G0203912300.01.T01	Serine/threonine-protein kinase PBS1
maker-2-augustus-gene-260.6-mRNA-1	Indica	Japonica	Kinase-TM	Os02g0632100	OsR498G0509684800.01.T02	Wall-associated receptor kinase 3
maker-2-augustus-gene-262.30-mRNA-1	Indica	NA	Kinase-TM			Wall-associated receptor kinase 4
maker-2-augustus-gene-31.54-mRNA-1	Indica	NA	Kinase-TM		OsR498G0202996100.01.T01	L-type lectin-domain containing receptor kinase IX.2
maker-2-augustus-gene-41.37-mRNA-1	Indica	NA	Kinase-TM	Os02g0174200	OsR498G0203068200.01.T01	serine/threonine-protein kinase STY13
maker-2-augustus-gene-46.50-mRNA-1	Indica	NA	Kinase-TM		OsR498G0203101600.01.T02	Phytosulfokine receptor
maker-4-augustus-gene-126.0-mRNA-1	Indica	NA	Kinase-TM			Protein kinase domain-containing protein (Fragment)
maker-4-augustus-gene-205.9-mRNA-1	Indica	NA	Kinase-TM	Os04g0517500	OsR498G0408901300.01.T01	Phosphoenolpyruvate carboxylase kinase
maker-4-augustus-gene-255.7-mRNA-1	Indica	NA	Kinase-TM	Os04g0608900	OsR498G0409252600.01.T01	serine/threonine-protein kinase STY13
maker-4-augustus-gene-26.27-mRNA-1	Indica	Indica	Kinase-TM	Os11g0692100	OsR498G0407758000.01.T01	Bacterial blight resistance protein XA26
maker-4-augustus-gene-268.3-mRNA-1	Indica	NA	Kinase-TM	Os04g0631800	OsR498G0409332600.01.T02	Serine/threonine-protein kinase
maker-4-augustus-gene-268.39-mRNA-1	Indica	Japonica	Kinase-TM	Os04g0634000	OsR498G0409338400.01.T01	G-type lectin S-receptor-like serine/threonine-protein kinase
maker-4-augustus-gene-268.7-mRNA-1	Indica	NA	Kinase-TM	Os04g0632100	OsR498G0409332800.01.T04	Non-specific serine/threonine protein kinase
maker-4-augustus-gene-31.39-mRNA-1	Indica	Indica	Kinase-TM	Os11g0692100	OsR498G0407758000.01.T01	Receptor kinase MRKa
maker-5-augustus-gene-188.44-mRNA-1	Indica	NA	Kinase-TM		OsR498G0510697600.01.T02	Receptor-like protein kinase ANXUR1
maker-5-augustus-gene-283.37-mRNA-1	Indica	NA	Kinase-TM	Os05g0549100	OsR498G0511289600.01.T01	Serine/threonine-protein kinase SNT7
maker-5-augustus-gene-284.29-mRNA-1	Indica	NA	Kinase-TM		OsR498G0511296500.01.T02	Wall-associated kinase
maker-5-augustus-gene-289.59-mRNA-1	Indica	NA	Kinase-TM	Os05g0560300	OsR498G0511334000.01.T02	Protein MALE DISCOVERER 1
maker-5-augustus-gene-298.40-mRNA-1	Indica	NA	Kinase-TM	Os05g0576800	OsR498G0511392300.01.T01	Mitogen-activated protein kinase
maker-5-augustus-gene-298.59-mRNA-1	Indica	NA	Kinase-TM	Os05g0577700	OsR498G0511394600.01.T02	MAP kinase kinase kinase55
maker-5-augustus-gene-66.51-mRNA-1	Indica	NA	Kinase-TM	Os05g0207500	OsR498G0509991200.01.T01	Non-specific serine/threonine protein kinase
maker-5-augustus-gene-66.56-mRNA-1	Indica	NA	Kinase-TM	Os05g0207700	OsR498G0509991800.01.T03	Serine/threonine-protein kinase CDL1
maker-5-augustus-gene-73.26-mRNA-1	Indica	Indica	Kinase-TM	Os01g0227200	OsR498G0510036200.01.T03	Receptor protein kinase PERK1
maker-6-augustus-gene-298.25-mRNA-1	Indica	NA	Kinase-TM	Os06g0703000	OsR498G0613265800.01.T01	Putative serine/threonine-protein kinase PBL17 isoform B
maker-7-augustus-gene-12.52-mRNA-1	Indica	NA	Kinase-TM	Os07g0129800	OsR498G0713458800.01.T01	Putative receptor kinase Lecrk
maker-7-augustus-gene-12.54-mRNA-1	Indica	NA	Kinase-TM	Os07g0131000		L-type lectin-domain containing receptor kinase IV.1
maker-7-augustus-gene-189.51-mRNA-1	Indica	NA	Kinase-TM	Os07g0507300	OsR498G0714392600.01.T04	germinal center kinase 1 isoform X3
maker-7-augustus-gene-205.14-mRNA-1	Indica	NA	Kinase-TM	Os07g0534700	OsR498G0714497100.01.T02	Cysteine-rich receptor-like protein kinase 25
maker-7-augustus-gene-205.9-mRNA-1	Indica	NA	Kinase-TM		OsR498G0714496300.01.T01	Receptor-like serine/threonine-protein kinase
maker-7-augustus-gene-206.10-mRNA-1	Indica	Indica	Kinase-TM	Os07g0628700	OsR498G0714502100.01.T01	Cysteine-rich receptor-like protein kinase 8
maker-7-augustus-gene-207.46-mRNA-1	Indica	NA	Kinase-TM		OsR498G0714502300.01.T01	Cysteine-rich receptor-like protein kinase 8
maker-7-augustus-gene-207.58-mRNA-1	Indica	Indica	Kinase-TM	Os07g0537900	OsR498G0714507500.01.T01	Cysteine-rich receptor-like protein kinase 8
maker-7-augustus-gene-207.71-mRNA-1	Indica	Indica	Kinase-TM	Os07g0537900	OsR498G0714504400.01.T04	Cysteine-rich receptor-like protein kinase 8
maker-7-augustus-gene-208.36-mRNA-1	Indica	NA	Kinase-TM	Os07g0541000	OsR498G0714516300.01.T01	Receptor-like serine-threonine protein kinase
maker-7-augustus-gene-209.0-mRNA-1	Indica	NA	Kinase-TM	Os07g0541400	OsR498G0714516900.01.T02	Cysteine-rich receptor kinase
maker-7-augustus-gene-209.11-mRNA-1	Indica	NA	Kinase-TM	Os07g0540800	OsR498G0714515800.01.T01	Receptor-like serine-threonine protein kinase
maker-7-augustus-gene-209.12-mRNA-1	Indica	NA	Kinase-TM	Os07g0541000	OsR498G0714516800.01.T01	Cysteine-rich receptor-like protein kinase 10
maker-7-augustus-gene-209.21-mRNA-1	Indica	Indica	Kinase-TM	Os07g0541500	OsR498G0714517200.01.T02	Cysteine-rich receptor kinase
maker-7-augustus-gene-210.47-mRNA-1	Indica	NA	Kinase-TM	Os07g0542400	OsR498G0714519200.01.T05	Putative Zea mays retrotransposon Opie-2
maker-7-augustus-gene-28.45-mRNA-1	Indica	Indica	Kinase-TM	Os07g0159700	OsR498G0713558800.01.T02	Protein kinase domain-containing protein (Fragment)
maker-8-augustus-gene-190.0-mRNA-1	Indica	Indica	Kinase-TM	Os08g0374600	OsR498G0815946800.01.T03	Cytokinin-regulated kinase-like
maker-9-augustus-gene-115.2-mRNA-1	Indica	NA	Kinase-TM			Serine/threonine-specific receptor protein kinase-like
maker-9-augustus-gene-117.3-mRNA-1	Indica	Indica	Kinase-TM	Os09g0356800	OsR498G0917240400.01.T05	Protein kinase domain-containing protein
maker-9-augustus-gene-145.39-mRNA-1	Indica	NA	Kinase-TM	Os09g0408800	OsR498G0917427900.01.T01	Cysteine-rich receptor-like protein kinase 10
augustus-1-processed-gene-162.25-mRNA-1	Japonica	Indica	Kinase-TM	Os01g0384300	OsR498G0101005100.01.T01	LRR receptor-like serine/threonine-protein kinase RKF3
augustus-1-processed-gene-296.25-mRNA-1	Japonica	Japonica	Kinase-TM	Os01g0699400	OsR498G0101831600.01.T01	Mitogen-activated protein kinase kinase kinase A
augustus-1-processed-gene-331.52-mRNA-1	Japonica	NA	Kinase-TM	Os01g0769700	OsR498G0102064500.01.T01	Receptor-like protein kinase FERONIA
augustus-1-processed-gene-337.1-mRNA-1	Japonica	NA	Kinase-TM	Os01g0779300	OsR498G0102097100.01.T01	Non-specific serine/threonine protein kinase
augustus-1-processed-gene-339.2-mRNA-1	Japonica	Japonica	Kinase-TM	Os01g0784200	OsR498G0102109300.01.T01	Receptor-like serine/threonine-protein kinase
augustus-1-processed-gene-339.4-mRNA-1	Japonica	NA	Kinase-TM	Os01g0784200		Non-specific serine/threonine protein kinase
augustus-1-processed-gene-484.34-mRNA-1	Japonica	NA	Kinase-TM	Os01g0927500	OsR498G0102625300.01.T03	Inactive leucine-rich repeat receptor-like protein kinase CORYNE
augustus-1-processed-gene-68.14-mRNA-1	Japonica	NA	Kinase-TM	Os01g0223900	OsR498G0100448100.01.T01	Non-specific serine/threonine protein kinase (Fragment)
augustus-10-processed-gene-111.2-mRNA-1	Japonica	NA	Kinase-TM	Os10g0442000	OsR498G1018798100.01.T01	Putative receptor-type protein kinase LRK1
augustus-10-processed-gene-35.7-mRNA-1	Japonica	Indica	Kinase-TM	Os10g0151100	OsR498G1018156300.01.T01	Wall associated kinase
augustus-11-processed-gene-119.2-mRNA-1	Japonica	NA	Kinase-TM	Os11g0441900	OsR498G1120029400.01.T01	G-type lectin S-receptor-like serine/threonine-protein kinase
augustus-11-processed-gene-121.11-mRNA-1	Japonica	NA	Kinase-TM	Os11g0445300	OsR498G1120050700.01.T01	Brassinosteroid insensitive 1-associated receptor kinase 1
augustus-11-processed-gene-182.37-mRNA-1	Japonica	NA	Kinase-TM	Os11g0557500	OsR498G1120348700.01.T01	Proline-rich receptor-like protein kinase PERK14
augustus-11-processed-gene-206.17-mRNA-1	Japonica	Japonica	Kinase-TM	Os11g0601500	OsR498G1120470500.01.T01	Putative serine/threonine-specific protein kinase
augustus-11-processed-gene-8.96-mRNA-1	Japonica	Indica	Kinase-TM	Os12g0130300	OsR498G1119373100.01.T01	Receptor-like serine/threonine-protein kinase
augustus-12-processed-gene-167.8-mRNA-1	Japonica	Japonica	Kinase-TM	Os12g0454800	OsR498G1221536100.01.T01	Histidine kinase2
augustus-12-processed-gene-260.18-mRNA-1	Japonica	Japonica	Kinase-TM	Os12g0608500	OsR498G1222080600.01.T01	Lectin protein kinase, putative, expressed
augustus-12-processed-gene-264.32-mRNA-1	Japonica	NA	Kinase-TM	Os12g0614600	OsR498G1222101900.01.T01	Protein kinase PINOID
augustus-12-processed-gene-264.34-mRNA-1	Japonica	Indica	Kinase-TM	Os12g0615300	OsR498G1222102900.01.T01	Wall-associated receptor kinase 5
augustus-12-processed-gene-264.46-mRNA-1	Japonica	NA	Kinase-TM	Os12g0615300	OsR498G1222104500.01.T04	Wall-associated receptor kinase 5
augustus-12-processed-gene-41.13-mRNA-1	Japonica	NA	Kinase-TM		OsR498G1221041100.01.T01	Non-specific serine/threonine protein kinase
augustus-2-processed-gene-114.14-mRNA-1	Japonica	Indica	Kinase-TM	Os02g0298200	OsR498G0203485500.01.T01	Putative receptor-type protein kinase LRK1
augustus-2-processed-gene-115.18-mRNA-1	Japonica	Indica	Kinase-TM	Os02g0298200	OsR498G0203485500.01.T01	Putative receptor-type protein kinase LRK1
augustus-2-processed-gene-156.10-mRNA-1	Japonica	NA	Kinase-TM	Os02g0459600	OsR498G0203737300.01.T03	Non-specific serine/threonine protein kinase
augustus-2-processed-gene-218.61-mRNA-1	Japonica	NA	Kinase-TM	Os02g0565500	OsR498G0204133200.01.T01	Tyrosine-protein kinase
augustus-2-processed-gene-238.26-mRNA-1	Japonica	NA	Kinase-TM	Os02g0603000	OsR498G0204255800.01.T01	Serine/threonine-protein kinase OXI1
augustus-2-processed-gene-265.58-mRNA-1	Japonica	NA	Kinase-TM	Os02g0640500	OsR498G0204399600.01.T01	L-type lectin-domain containing receptor kinase IV.2
augustus-2-processed-gene-359.68-mRNA-1	Japonica	NA	Kinase-TM	Os02g0767400	OsR498G0204832400.01.T01	Non-specific serine/threonine protein kinase
augustus-2-processed-gene-52.12-mRNA-1	Japonica	NA	Kinase-TM		OsR498G0203139900.01.T01	Proline-rich receptor-like protein kinase PERK9 (Fragment)
augustus-3-processed-gene-491.56-mRNA-1	Japonica	NA	Kinase-TM			probable receptor-like serine/threonine-protein kinase At5g57670
augustus-4-processed-gene-268.79-mRNA-1	Japonica	Japonica	Kinase-TM	Os04g0633800	OsR498G0409334600.01.T02	Non-specific serine/threonine protein kinase
augustus-4-processed-gene-279.43-mRNA-1	Japonica	Japonica	Kinase-TM	Os04g0655000	OsR498G0409408100.01.T01	Serine/threonine-protein kinase
augustus-4-processed-gene-295.1-mRNA-1	Japonica	Indica	Kinase-TM	Os04g0684200	OsR498G0409514300.01.T06	CASP-like protein 1
augustus-4-processed-gene-82.4-mRNA-1	Japonica	Japonica	Kinase-TM	Os04g0307900	OsR498G0408016900.01.T01	Wall-associated receptor kinase 5
augustus-4-processed-gene-82.5-mRNA-1	Japonica	NA	Kinase-TM			Wall-associated receptor kinase 3
augustus-5-processed-gene-152.17-mRNA-1	Japonica	NA	Kinase-TM		OsR498G0510479400.01.T04	Receptor-like protein kinase FERONIA
augustus-5-processed-gene-152.19-mRNA-1	Japonica	NA	Kinase-TM	Os05g0318700	OsR498G0510480600.01.T01	Receptor-like protein kinase FERONIA
augustus-6-processed-gene-291.18-mRNA-1	Japonica	NA	Kinase-TM	Os06g0693200	OsR498G0613227500.01.T01	probable receptor-like serine/threonine-protein kinase At5g57670
augustus-7-processed-gene-104.13-mRNA-1	Japonica	NA	Kinase-TM	Os07g0283050	OsR498G0713999000.01.T01	Putative receptor-type protein kinase LRK1
augustus-7-processed-gene-21.6-mRNA-1	Japonica	NA	Kinase-TM	Os07g0147600	OsR498G0713511000.01.T02	Mitogen-activated protein kinase kinase kinase
augustus-7-processed-gene-216.33-mRNA-1	Japonica	NA	Kinase-TM	Os07g0553633	OsR498G0714550000.01.T01	Non-specific serine/threonine protein kinase (Fragment)
augustus-7-processed-gene-226.3-mRNA-1	Japonica	Japonica	Kinase-TM	Os07g0569800	OsR498G0714602900.01.T01	L-type lectin-domain containing receptor kinase IX.1
augustus-7-processed-gene-230.27-mRNA-1	Japonica	NA	Kinase-TM	Os07g0575600	OsR498G0714620600.01.T01	Putative receptor-type protein kinase LRK1
augustus-7-processed-gene-230.47-mRNA-1	Japonica	NA	Kinase-TM	Os07g0575700	OsR498G0714621000.01.T01	Lectin receptor-type kinase
augustus-7-processed-gene-230.48-mRNA-1	Japonica	Indica	Kinase-TM	Os07g0575600	OsR498G0714621200.01.T01	Lectin receptor-type kinase
augustus-7-processed-gene-41.40-mRNA-1	Japonica	Japonica	Kinase-TM	Os07g0186200	OsR498G0713651200.01.T01	Non-specific serine/threonine protein kinase (Fragment)
augustus-8-processed-gene-14.24-mRNA-1	Japonica	Japonica	Kinase-TM	Os08g0124100	OsR498G0815098200.01.T02	Putative receptor-type protein kinase LRK1
augustus-8-processed-gene-153.14-mRNA-1	Japonica	NA	Kinase-TM		OsR498G0815808800.01.T01	Putative glycerophosphodiester phosphodiesterase, protein kinase RLK-Pelle-LRK10L-2 family
augustus-9-processed-gene-230.3-mRNA-1	Japonica	NA	Kinase-TM	Os09g0572100		lysM domain receptor-like kinase 3
augustus-9-processed-gene-44.19-mRNA-1	Japonica	Japonica	Kinase-TM	Os09g0251250	OsR498G0916836300.01.T01	Serine/threonine protein kinase
augustus_masked-1-processed-gene-280.18-mRNA-1	Japonica	Japonica	Kinase-TM	Os01g0668600	OsR498G0101726500.01.T01	Serine/threonine-protein kinase
augustus_masked-1-processed-gene-280.20-mRNA-1	Japonica	NA	Kinase-TM	Os01g0669100	OsR498G0101727800.01.T01	Non-specific serine/threonine protein kinase
augustus_masked-1-processed-gene-281.23-mRNA-1	Japonica	NA	Kinase-TM	Os01g0670100	OsR498G0101730000.01.T01	Non-specific serine/threonine protein kinase
augustus_masked-1-processed-gene-326.20-mRNA-1	Japonica	NA	Kinase-TM	Os01g0759200	OsR498G0102036200.01.T01	Non-specific serine/threonine protein kinase
augustus_masked-1-processed-gene-326.21-mRNA-1	Japonica	NA	Kinase-TM	Os01g0759400	OsR498G0102036600.01.T01	Non-specific serine/threonine protein kinase
augustus_masked-1-processed-gene-68.24-mRNA-1	Japonica	Indica	Kinase-TM	Os01g0223700	OsR498G0100448000.01.T02	Non-specific serine/threonine protein kinase (Fragment)
augustus_masked-1-processed-gene-68.25-mRNA-1	Japonica	Japonica	Kinase-TM	Os01g0224000	OsR498G0100448100.01.T01	Non-specific serine/threonine protein kinase (Fragment)
augustus_masked-10-processed-gene-36.6-mRNA-1	Japonica	Indica	Kinase-TM	Os10g0152000	OsR498G1018157400.01.T01	Wall-associated receptor kinase 2
augustus_masked-11-processed-gene-181.7-mRNA-1	Japonica	NA	Kinase-TM	Os11g0553500	OsR498G1120341600.01.T01	Wall-associated receptor kinase 3
augustus_masked-11-processed-gene-182.29-mRNA-1	Japonica	NA	Kinase-TM	Os11g0556400		Wall-associated receptor kinase 1
augustus_masked-11-processed-gene-71.36-mRNA-1	Japonica	Indica	Kinase-TM	Os04g0103700	OsR498G1119598200.01.T01	Non-specific serine/threonine protein kinase (Fragment)
augustus_masked-11-processed-gene-8.106-mRNA-1	Japonica	NA	Kinase-TM	Os11g0133100	OsR498G1119372000.01.T01	Receptor-like serine/threonine-protein kinase
augustus_masked-11-processed-gene-8.108-mRNA-1	Japonica	NA	Kinase-TM	Os11g0133500	OsR498G1220884400.01.T01	Receptor-like serine/threonine-protein kinase
augustus_masked-11-processed-gene-80.5-mRNA-1	Japonica	NA	Kinase-TM	Os11g0222000	OsR498G1119637400.01.T01	Serine/threonine-protein kinase-like protein CCR4
augustus_masked-12-processed-gene-15.24-mRNA-1	Japonica	NA	Kinase-TM	Os12g0130300	OsR498G1119372000.01.T01	Receptor-like serine/threonine-protein kinase
augustus_masked-12-processed-gene-15.26-mRNA-1	Japonica	NA	Kinase-TM	Os11g0133100	OsR498G1119373100.01.T01	Receptor-like serine/threonine-protein kinase
augustus_masked-12-processed-gene-15.29-mRNA-1	Japonica	NA	Kinase-TM	Os11g0133500	OsR498G1220884400.01.T01	Receptor-like serine/threonine-protein kinase
augustus_masked-12-processed-gene-240.15-mRNA-1	Japonica	NA	Kinase-TM		OsR498G1221922000.01.T01	Mitogen-activated protein kinase kinase kinase
augustus_masked-12-processed-gene-260.37-mRNA-1	Japonica	NA	Kinase-TM	Os12g0608900	OsR498G1222080600.01.T01	Lectin protein kinase, putative, expressed
augustus_masked-12-processed-gene-7.59-mRNA-1	Japonica	NA	Kinase-TM	Os12g0113500	OsR498G1220822800.01.T01	Non-specific serine/threonine protein kinase
augustus_masked-3-processed-gene-12.55-mRNA-1	Japonica	NA	Kinase-TM	Os03g0124200	OsR498G0305151700.01.T01	Receptor-like protein kinase HERK 1 isoform A
augustus_masked-3-processed-gene-135.13-mRNA-1	Japonica	NA	Kinase-TM	Os10g0533800	OsR498G1019094500.01.T01	L-type lectin-domain containing receptor kinase IV.1
augustus_masked-3-processed-gene-136.7-mRNA-1	Japonica	NA	Kinase-TM	Os10g0534500	OsR498G1019095800.01.T03	Receptor-like protein kinase THESEUS 1
augustus_masked-3-processed-gene-15.54-mRNA-1	Japonica	NA	Kinase-TM			Serine/threonine-protein kinase Cx32, chloroplast, putative, expressed
augustus_masked-3-processed-gene-154.30-mRNA-1	Japonica	Japonica	Kinase-TM	Os03g0333200	OsR498G0305888100.01.T01	Receptor-like protein kinase FERONIA
augustus_masked-3-processed-gene-215.25-mRNA-1	Japonica	Indica	Kinase-TM	Os03g0426300	OsR498G0306245800.01.T01	G-type lectin S-receptor-like serine/threonine-protein kinase
augustus_masked-3-processed-gene-29.19-mRNA-1	Japonica	NA	Kinase-TM	Os03g0159100	OsR498G0305257900.01.T01	probable serine/threonine-protein kinase PBL16
augustus_masked-3-processed-gene-334.0-mRNA-1	Japonica	NA	Kinase-TM	Os03g0556600	OsR498G0306350500.01.T01	Non-specific serine/threonine protein kinase
augustus_masked-3-processed-gene-35.26-mRNA-1	Japonica	Indica	Kinase-TM	Os10g0405100	OsR498G0305298200.01.T01	probable serine/threonine-protein kinase PBL3
augustus_masked-3-processed-gene-381.27-mRNA-1	Japonica	NA	Kinase-TM	Os03g0637800	OsR498G0306624300.01.T01	Receptor protein kinase CRINKLY4
augustus_masked-3-processed-gene-426.1-mRNA-1	Japonica	Indica	Kinase-TM	Os03g0225100	OsR498G0306923500.01.T01	Mitogen-activated protein kinase kinase 7
augustus_masked-3-processed-gene-458.45-mRNA-1	Japonica	Indica	Kinase-TM	Os03g0772600	OsR498G0307124500.01.T01	Lectin protein kinase, putative, expressed
augustus_masked-3-processed-gene-483.47-mRNA-1	Japonica	NA	Kinase-TM	Os03g0821900	OsR498G0307292500.01.T01	Protein kinase APK1A chloroplastic
augustus_masked-3-processed-gene-486.14-mRNA-1	Japonica	NA	Kinase-TM	Os03g0828800	OsR498G0307310600.01.T02	Dual specificity testis-specific protein kinase 2
augustus_masked-3-processed-gene-490.33-mRNA-1	Japonica	NA	Kinase-TM	Os03g0838100	OsR498G0307339000.01.T02	Non-specific serine/threonine protein kinase (Fragment)
augustus_masked-3-processed-gene-61.13-mRNA-1	Japonica	NA	Kinase-TM	Os03g0221700		G-type lectin S-receptor-like serine/threonine-protein kinase
augustus_masked-3-processed-gene-78.2-mRNA-1	Japonica	NA	Kinase-TM	Os03g0251700	OsR498G0305588100.01.T01	Serine/threonine-protein kinase PBS1
augustus_masked-3-processed-gene-79.1-mRNA-1	Japonica	NA	Kinase-TM	Os03g0253200	OsR498G0305593900.01.T01	Serine/threonine-protein kinase WAG2
augustus_masked-3-processed-gene-99.11-mRNA-1	Japonica	NA	Kinase-TM		OsR498G0305741500.01.T01	Mitogen-activated protein kinase kinase kinase 2
augustus_masked-4-processed-gene-165.41-mRNA-1	Japonica	NA	Kinase-TM	Os04g0439600	OsR498G0408622600.01.T01	Serine/threonine-protein kinase-like protein CCR4
augustus_masked-4-processed-gene-261.39-mRNA-1	Japonica	NA	Kinase-TM	Os04g0619600	OsR498G0409294000.01.T01	Mitogen-activated protein kinase kinase kinase
augustus_masked-4-processed-gene-60.12-mRNA-1	Japonica	NA	Kinase-TM	Os04g0275100	OsR498G0407914000.01.T01	Wall-associated receptor kinase 3

augustus_masked-4-processed-gene-78.23-mRNA-1	Japonica	Japonica	Kinase-TM	Os04g0303300	OsR498G0407999200.01.T01	Receptor-like serine/threonine-protein kinase
augustus_masked-4-processed-gene-81.46-mRNA-1	Japonica	NA	Kinase-TM	Os04g0307500	OsR498G0408014500.01.T02	Wall-associated receptor kinase 1
augustus_masked-5-processed-gene-14.15-mRNA-1	Japonica	NA	Kinase-TM	Os05g0125200	OsR498G0509646500.01.T01	Receptor-like kinase LIP2
augustus_masked-5-processed-gene-15.21-mRNA-1	Japonica	NA	Kinase-TM		OsR498G0509654700.01.T01	Serine/threonine-protein kinase-like protein CCR3
augustus_masked-5-processed-gene-266.19-mRNA-1	Japonica	NA	Kinase-TM	Os05g0514200	OsR498G0511175300.01.T02	Non-specific serine/threonine protein kinase
augustus_masked-5-processed-gene-269.37-mRNA-1	Japonica	NA	Kinase-TM	Os05g0519200	OsR498G0511194100.01.T05	Serine/threonine protein kinase
augustus_masked-5-processed-gene-302.32-mRNA-1	Japonica	Japonica	Kinase-TM	Os05g0585800	OsR498G0101562800.01.T03	mitogen-activated protein kinase kinase kinase 7
augustus_masked-5-processed-gene-36.15-mRNA-1	Japonica	NA	Kinase-TM		OsR498G0509796100.01.T01	Receptor-like protein kinase HERK 1
augustus_masked-6-processed-gene-238.19-mRNA-1	Japonica	NA	Kinase-TM	Os06g0602500	OsR498G0612893400.01.T01	Non-specific serine/threonine protein kinase
augustus_masked-6-processed-gene-261.17-mRNA-1	Japonica	NA	Kinase-TM	Os06g0639500	OsR498G0613036700.01.T01	E3 ubiquitin-protein ligase KEG
augustus_masked-6-processed-gene-4.4-mRNA-1	Japonica	NA	Kinase-TM		OsR498G0611477400.01.T01	Receptor-like serine/threonine-protein kinase
augustus_masked-6-processed-gene-47.12-mRNA-1	Japonica	Japonica	Kinase-TM	Os06g0191300	OsR498G0204906600.01.T01	MAP kinase kinase
augustus_masked-8-processed-gene-262.27-mRNA-1	Japonica	Japonica	Kinase-TM	Os08g0504800	OsR498G0917718500.01.T01	Serine/threonine-protein kinase UCNL
augustus_masked-8-processed-gene-5.49-mRNA-1	Japonica	NA	Kinase-TM	Os08g0109800	OsR498G0815051500.01.T01	Serine/threonine-protein kinase-like protein CCR4
augustus_masked-9-processed-gene-188.10-mRNA-1	Japonica	NA	Kinase-TM	Os09g0486700	OsR498G0917718500.01.T01	Serine/threonine-protein kinase UCNL
augustus_masked-9-processed-gene-55.25-mRNA-1	Japonica	Indica	Kinase-TM	Os07g0283125	OsR498G0916878900.01.T01	Lectin-domain containing receptor kinase A4.2
maker-1-augustus-gene-107.29-mRNA-1	Japonica	NA	Kinase-TM	Os01g0292200	OsR498G0100720500.01.T01	Non-specific serine/threonine protein kinase
maker-1-augustus-gene-109.26-mRNA-1	Japonica	NA	Kinase-TM	Os01g0296000	OsR498G0100728600.01.T01	Putative LRR receptor-like serine/threonine-protein kinase
maker-1-augustus-gene-109.5-mRNA-1	Japonica	NA	Kinase-TM	Os01g0296100	OsR498G0100728700.01.T04	Non-specific serine/threonine protein kinase
maker-1-augustus-gene-118.0-mRNA-1	Japonica	Japonica	Kinase-TM	Os01g0310400	OsR498G0100776900.01.T03	Wall-associated receptor kinase 5
maker-1-augustus-gene-118.6-mRNA-1	Japonica	Indica	Kinase-TM	Os01g0310400	OsR498G0100778100.01.T02	PTI1-like tyrosine-protein kinase 3
maker-1-augustus-gene-124.2-mRNA-1	Japonica	NA	Kinase-TM	Os01g0323000	OsR498G0100818500.01.T02	Serine/threonine protein kinase
maker-1-augustus-gene-125.7-mRNA-1	Japonica	NA	Kinase-TM	Os01g0323100	OsR498G0100818800.01.T01	Pto-interacting protein 1
maker-1-augustus-gene-150.40-mRNA-1	Japonica	NA	Kinase-TM	Os01g0364800	OsR498G0100956900.01.T01	Wall-associated receptor kinase
maker-1-augustus-gene-225.4-mRNA-1	Japonica	NA	Kinase-TM	Os01g0568400		receptor-like kinase TMK3
maker-1-augustus-gene-236.9-mRNA-1	Japonica	NA	Kinase-TM	Os01g0587400	OsR498G0101426800.01.T01	Receptor-like serine/threonine-protein kinase
maker-1-augustus-gene-258.10-mRNA-1	Japonica	NA	Kinase-TM	Os01g0629900	OsR498G0101587800.01.T02	Mitogen-activated protein kinase
maker-1-augustus-gene-259.33-mRNA-1	Japonica	NA	Kinase-TM	Os01g0631700	OsR498G0101594500.01.T03	Receptor-like protein kinase
maker-1-augustus-gene-264.49-mRNA-1	Japonica	NA	Kinase-TM	Os01g0641000	OsR498G0101624900.01.T01	Serine/threonine-protein kinase STY8
maker-1-augustus-gene-277.80-mRNA-1	Japonica	NA	Kinase-TM	Os01g0664200	OsR498G0101706400.01.T01	Putative serine/threonine-protein kinase
maker-1-augustus-gene-278.40-mRNA-1	Japonica	NA	Kinase-TM	Os01g0665200	OsR498G0101709700.01.T01	Mitogen-activated protein kinase
maker-1-augustus-gene-291.26-mRNA-1	Japonica	Indica	Kinase-TM	Os01g0689900	OsR498G0101799400.01.T01	Wall-associated receptor-like kinase 2
maker-1-augustus-gene-292.1-mRNA-1	Japonica	NA	Kinase-TM	Os01g0690800	OsR498G0101800500.01.T01	Protein kinase domain-containing protein (Fragment)
maker-1-augustus-gene-292.4-mRNA-1	Japonica	NA	Kinase-TM		OsR498G0101800900.01.T01	Acidic endochitinase
maker-1-augustus-gene-3.26-mRNA-1	Japonica	NA	Kinase-TM	Os01g0104000	OsR498G0100015400.01.T01	C-type lectin receptor-like tyrosine-protein kinase
maker-1-augustus-gene-301.40-mRNA-1	Japonica	NA	Kinase-TM	Os01g0709500	OsR498G0101865600.01.T04	Receptor-like serine/threonine-protein kinase ALE2
maker-1-augustus-gene-315.36-mRNA-1	Japonica	NA	Kinase-TM	Os01g0738300	OsR498G0101960100.01.T01	Proline-rich receptor-like protein kinase PERK10 isoform A
maker-1-augustus-gene-316.24-mRNA-1	Japonica	NA	Kinase-TM	Os01g0741200	OsR498G0101969600.01.T01	Putative serine/threonine-protein kinase RLCKVII
maker-1-augustus-gene-319.46-mRNA-1	Japonica	NA	Kinase-TM	Os01g0747400	OsR498G0101987200.01.T01	Serine/threonine-protein kinase HT1
maker-1-augustus-gene-321.19-mRNA-1	Japonica	NA	Kinase-TM	Os01g0750600	OsR498G0102002200.01.T01	proline-rich receptor-like protein kinase PERK5
maker-1-augustus-gene-339.39-mRNA-1	Japonica	Japonica	Kinase-TM	Os01g0783800	OsR498G0102108700.01.T06	G-type lectin S-receptor-like serine/threonine-protein kinase
maker-1-augustus-gene-339.42-mRNA-1	Japonica	NA	Kinase-TM			Serine/threonine-protein kinase
maker-1-augustus-gene-339.61-mRNA-1	Japonica	NA	Kinase-TM	Os01g0784700	OsR498G0102110200.01.T02	Serine/threonine-protein kinase
maker-1-augustus-gene-483.51-mRNA-1	Japonica	Indica	Kinase-TM	Os03g0349200	OsR498G0102619200.01.T01	probable serine/threonine-protein kinase At1g54610
maker-1-augustus-gene-485.26-mRNA-1	Japonica	NA	Kinase-TM		OsR498G0102633200.01.T02	proline-rich receptor-like protein kinase PERK3 isoform X2
maker-1-augustus-gene-488.53-mRNA-1	Japonica	Indica	Kinase-TM	Os01g0935800	OsR498G0102655400.01.T01	Receptor-like protein kinase ANXUR1
maker-1-augustus-gene-5.48-mRNA-1	Japonica	NA	Kinase-TM		OsR498G0100026400.01.T01	Serine/threonine protein kinase
maker-1-augustus-gene-503.3-mRNA-1	Japonica	NA	Kinase-TM		OsR498G0102753900.01.T01	Putative serine-threonine/tyrosine-protein kinase catalytic domain-containing protein
maker-1-augustus-gene-507.39-mRNA-1	Japonica	NA	Kinase-TM	Os01g0973500	OsR498G0102780200.01.T01	receptor-like cytoplasmic kinase 176
maker-1-augustus-gene-55.18-mRNA-1	Japonica	NA	Kinase-TM	Os01g0201200	OsR498G0100357200.01.T04	Serine/threonine-protein kinase
maker-1-augustus-gene-6.31-mRNA-1	Japonica	NA	Kinase-TM	Os01g0110500	OsR498G0100034200.01.T01	Proline-rich receptor-like protein kinase PERK8
maker-1-augustus-gene-71.33-mRNA-1	Japonica	NA	Kinase-TM	Os01g0227200	OsR498G0100467900.01.T01	Receptor protein kinase PERK1
maker-1-augustus-gene-74.47-mRNA-1	Japonica	NA	Kinase-TM	Os01g0233800	OsR498G0100488900.01.T01	Serine/threonine-protein kinase
maker-10-augustus-gene-105.30-mRNA-1	Japonica	NA	Kinase-TM	Os10g0430900	OsR498G1018764400.01.T03	Serine/threonine-protein kinase EDR1
maker-10-augustus-gene-127.41-mRNA-1	Japonica	NA	Kinase-TM	Os04g0125700	OsR498G0407534800.01.T01	Non-specific serine/threonine protein kinase
maker-10-augustus-gene-142.3-mRNA-1	Japonica	NA	Kinase-TM	Os01g0136400	OsR498G0100125200.01.T04	Putative serine/threonine-protein kinase
maker-10-augustus-gene-168.44-mRNA-1	Japonica	NA	Kinase-TM	Os10g0476100	OsR498G1018898700.01.T01	serine/threonine-protein kinase tricorner isoform X1
maker-10-augustus-gene-174.0-mRNA-1	Japonica	Indica	Kinase-TM	Os10g0483400	OsR498G1120115000.01.T01	Protein kinase domain containing protein, expressed
maker-10-augustus-gene-181.34-mRNA-1	Japonica	NA	Kinase-TM	Os10g0497600	OsR498G1018985300.01.T01	Putative serine/threonine-protein kinase
maker-10-augustus-gene-192.47-mRNA-1	Japonica	NA	Kinase-TM	Os10g0518800	OsR498G1019051100.01.T01	Protein kinase domain containing protein, expressed
maker-10-augustus-gene-193.26-mRNA-1	Japonica	NA	Kinase-TM	Os10g0521700	OsR498G1019060000.01.T01	Non-specific serine/threonine protein kinase
maker-10-augustus-gene-25.33-mRNA-1	Japonica	Japonica	Kinase-TM	Os10g0136500	OsR498G1018109100.01.T04	Protein kinase domain containing protein, expressed
maker-10-augustus-gene-34.11-mRNA-1	Japonica	Japonica	Kinase-TM	Os10g0151100	OsR498G1018157400.01.T01	Wall-associated receptor kinase 2
maker-10-augustus-gene-35.0-mRNA-1	Japonica	NA	Kinase-TM			Wall-associated receptor kinase 4
maker-10-augustus-gene-43.39-mRNA-1	Japonica	Indica	Kinase-TM	Os10g0174800	OsR498G1018204400.01.T01	Wall associated kinase
maker-10-augustus-gene-43.42-mRNA-1	Japonica	NA	Kinase-TM		OsR498G1018205800.01.T01	Wall associated kinase
maker-11-augustus-gene-10.6-mRNA-1	Japonica	NA	Kinase-TM	Os11g0136600	OsR498G1119382300.01.T01	Calcium-dependent protein kinase
maker-11-augustus-gene-123.43-mRNA-1	Japonica	NA	Kinase-TM		OsR498G1120059900.01.T01	probable serine/threonine-protein kinase PBL28
maker-11-augustus-gene-123.48-mRNA-1	Japonica	NA	Kinase-TM			Putative serine/threonine-protein kinase WNK9 (Fragment)
maker-11-augustus-gene-135.35-mRNA-1	Japonica	Indica	Kinase-TM	Os11g0470200	OsR498G1120115000.01.T01	Protein kinase domain containing protein, expressed
maker-11-augustus-gene-16.16-mRNA-1	Japonica	Japonica	Kinase-TM	Os11g0150700	OsR498G1119421300.01.T01	Serine/threonine-protein kinase D6PKL2
maker-11-augustus-gene-185.0-mRNA-1	Japonica	NA	Kinase-TM	Os11g0565300	OsR498G1120370600.01.T01	Wall-associated receptor kinase 2
maker-11-augustus-gene-215.0-mRNA-1	Japonica	NA	Kinase-TM	Os11g0616100	OsR498G1120510900.01.T01	Leucine-rich repeat receptor protein kinase EXS
maker-11-augustus-gene-216.34-mRNA-1	Japonica	NA	Kinase-TM		OsR498G1120517600.01.T01	Protein kinase domain-containing protein
maker-11-augustus-gene-216.36-mRNA-1	Japonica	NA	Kinase-TM		OsR498G1120517800.01.T01	Protein kinase domain containing protein, expressed
maker-11-augustus-gene-244.49-mRNA-1	Japonica	NA	Kinase-TM		OsR498G1120691300.01.T01	Cytokinin-regulated kinase-like
maker-11-augustus-gene-245.48-mRNA-1	Japonica	NA	Kinase-TM		OsR498G1120696900.01.T05	Protein kinase domain containing protein, expressed
maker-11-augustus-gene-245.56-mRNA-1	Japonica	NA	Kinase-TM		OsR498G1120696900.01.T05	G-type lectin S-receptor-like serine/threonine-protein kinase
maker-11-augustus-gene-251.40-mRNA-1	Japonica	NA	Kinase-TM		OsR498G1120726800.01.T02	Receptor protein kinase-like
maker-11-augustus-gene-256.34-mRNA-1	Japonica	NA	Kinase-TM	Os11g0695850	OsR498G1120743300.01.T02	Wall-associated receptor kinase
maker-11-augustus-gene-256.35-mRNA-1	Japonica	NA	Kinase-TM			Putative inactive receptor kinase RLK902
maker-11-augustus-gene-28.42-mRNA-1	Japonica	Indica	Kinase-TM	Os02g0194400	OsR498G1119480300.01.T01	Leucine-rich repeat receptor protein kinase MSL1
maker-11-augustus-gene-49.33-mRNA-1	Japonica	NA	Kinase-TM	Os11g0271100	OsR498G1119793700.01.T06	Mitogen-activated protein kinase
maker-11-augustus-gene-62.7-mRNA-1	Japonica	NA	Kinase-TM	Os11g0194900	OsR498G1119551000.01.T02	Protein kinase domain-containing protein
maker-11-augustus-gene-70.39-mRNA-1	Japonica	Indica	Kinase-TM	Os11g0207200	OsR498G1119591700.01.T01	Mitogen-activated protein kinase kinase kinase 3
maker-11-augustus-gene-71.9-mRNA-1	Japonica	Indica	Kinase-TM	Os07g0186200	OsR498G1119598200.01.T01	Serine/threonine-protein kinase
maker-11-augustus-gene-75.2-mRNA-1	Japonica	NA	Kinase-TM		OsR498G1119613600.01.T01	Coatomer beta subunit, putative
maker-11-augustus-gene-75.8-mRNA-1	Japonica	NA	Kinase-TM		OsR498G1119614000.01.T02	Putative mitogen-activated protein (MAP) kinase kinase kinase Ste11, Cryptococcus
maker-11-augustus-gene-9.0-mRNA-1	Japonica	NA	Kinase-TM	Os12g0132200	OsR498G1119377100.01.T07	Non-specific serine/threonine protein kinase
maker-11-augustus-gene-90.9-mRNA-1	Japonica	NA	Kinase-TM	Os11g0242500	OsR498G1119688900.01.T03	Cyclin-dependent kinase C
maker-12-augustus-gene-1.5-mRNA-1	Japonica	NA	Kinase-TM			Nonphototropic hypocotyl 1
maker-12-augustus-gene-15.46-mRNA-1	Japonica	NA	Kinase-TM	Os12g0130200	OsR498G1220880500.01.T01	Receptor-like serine/threonine-protein kinase
maker-12-augustus-gene-16.0-mRNA-1	Japonica	NA	Kinase-TM	Os12g0132200	OsR498G1119377100.01.T07	Non-specific serine/threonine protein kinase
maker-12-augustus-gene-17.34-mRNA-1	Japonica	Japonica	Kinase-TM	Os12g0133500	OsR498G1119382300.01.T01	Calcium-dependent protein kinase
maker-12-augustus-gene-190.35-mRNA-1	Japonica	Japonica	Kinase-TM	Os12g0486900	OsR498G1221633900.01.T01	receptor-like serine/threonine-protein kinase ALE2 isoform X2
maker-12-augustus-gene-252.9-mRNA-1	Japonica	NA	Kinase-TM	Os12g0594300	OsR498G1222027100.01.T01	Serine/threonine-protein kinase CTR1
maker-12-augustus-gene-253.0-mRNA-1	Japonica	NA	Kinase-TM	Os12g0595800	OsR498G1222032500.01.T02	Wall-associated receptor kinase-like 14
maker-12-augustus-gene-26.35-mRNA-1	Japonica	Japonica	Kinase-TM	Os12g0149700	OsR498G1119421300.01.T01	Serine/threonine-protein kinase D6PKL2
maker-12-augustus-gene-260.61-mRNA-1	Japonica	Japonica	Kinase-TM	Os12g0608700	OsR498G1222080200.01.T01	L-type lectin-domain containing receptor kinase IX.1
maker-12-augustus-gene-260.63-mRNA-1	Japonica	Japonica	Kinase-TM	Os12g0609000	OsR498G1222081400.01.T02	Wall-associated receptor kinase-like 21
maker-12-augustus-gene-261.18-mRNA-1	Japonica	Indica	Kinase-TM	Os12g0611100	OsR498G1222086800.01.T03	Serine/threonine-protein kinase receptor
maker-12-augustus-gene-264.0-mRNA-1	Japonica	Japonica	Kinase-TM	Os12g0615000	OsR498G1222103900.01.T01	Wall-associated receptor kinase 3
maker-12-augustus-gene-264.3-mRNA-1	Japonica	NA	Kinase-TM			Wall-associated receptor kinase 5
maker-12-augustus-gene-268.14-mRNA-1	Japonica	NA	Kinase-TM	Os12g0621500	OsR498G1222131900.01.T03	Probable serine/threonine protein kinase IREH1 isoform X1
maker-12-augustus-gene-3.97-mRNA-1	Japonica	NA	Kinase-TM		OsR498G1220798800.01.T02	Putative LRR receptor-like serine/threonine-protein kinase MRH1-like
maker-12-augustus-gene-34.19-mRNA-1	Japonica	NA	Kinase-TM		OsR498G1221004600.01.T03	Serine/threonine-protein kinase STY46
maker-12-augustus-gene-42.18-mRNA-1	Japonica	NA	Kinase-TM		OsR498G1221046200.01.T01	Protein kinase protein with adenine nucleotide alpha hydrolase-like domain
maker-12-augustus-gene-43.8-mRNA-1	Japonica	NA	Kinase-TM		OsR498G1221046200.01.T01	Protein kinase protein with adenine nucleotide alpha hydrolase-like domain
maker-12-augustus-gene-5.18-mRNA-1	Japonica	NA	Kinase-TM	Os12g0108100	OsR498G1220806200.01.T01	Putative serine/threonine-protein kinase
maker-12-augustus-gene-56.4-mRNA-1	Japonica	NA	Kinase-TM	Os11g0242500	OsR498G1119688900.01.T03	Serine/threonine-protein kinase
maker-12-augustus-gene-85.4-mRNA-1	Japonica	Japonica	Kinase-TM	Os12g0248000	OsR498G1221217600.01.T01	Interleukin-1 receptor-associated kinase 4
maker-12-augustus-gene-86.0-mRNA-1	Japonica	Indica	Kinase-TM	Os12g0249900	OsR498G1221216600.01.T01	Protein kinase domain-containing protein
maker-12-augustus-gene-86.2-mRNA-1	Japonica	NA	Kinase-TM	Os12g0249900		Proline-rich receptor-like protein kinase PERK2 isoform X2
maker-12-augustus-gene-91.3-mRNA-1	Japonica	NA	Kinase-TM	Os12g0257900	OsR498G1221224100.01.T01	Non-specific serine/threonine protein kinase
maker-2-augustus-gene-119.6-mRNA-1	Japonica	NA	Kinase-TM			probable serine/threonine-protein kinase At1g54610
maker-2-augustus-gene-129.0-mRNA-1	Japonica	NA	Kinase-TM	Os02g0322400	OsR498G0203569900.01.T01	Putative MAPKKK family protein kinase
maker-2-augustus-gene-163.26-mRNA-1	Japonica	NA	Kinase-TM	Os02g0472700	OsR498G0203785700.01.T05	Putative pathogenesis-related protein 1
maker-2-augustus-gene-210.34-mRNA-1	Japonica	NA	Kinase-TM	Os02g0549200	OsR498G0204084500.01.T02	probable serine/threonine-protein kinase At1g01540
maker-2-augustus-gene-212.12-mRNA-1	Japonica	NA	Kinase-TM	Os02g0551100	OsR498G0204092600.01.T02	Serine/threonine-protein kinase SAPK7
maker-2-augustus-gene-214.54-mRNA-1	Japonica	NA	Kinase-TM	Os02g0555900	OsR498G0204108200.01.T03	Mitogen-activated protein kinase kinase kinase YODA
maker-2-augustus-gene-241.17-mRNA-1	Japonica	NA	Kinase-TM	Os02g0608500	OsR498G0204275600.01.T03	Dual specificity protein kinase shkC
maker-2-augustus-gene-256.18-mRNA-1	Japonica	Indica	Kinase-TM	Os02g0623600	OsR498G0204344200.01.T02	Wall-associated receptor kinase-like 8
maker-2-augustus-gene-256.20-mRNA-1	Japonica	NA	Kinase-TM		OsR498G0204345600.01.T01	Putative wall-associated receptor kinase-like 16
maker-2-augustus-gene-260.6-mRNA-1	Japonica	Japonica	Kinase-TM	Os02g0632100	OsR498G0509684800.01.T02	Wall-associated receptor kinase 3
maker-2-augustus-gene-262.27-mRNA-1	Japonica	Indica	Kinase-TM	Os02g0633066	OsR498G0204379000.01.T01	Wall-associated receptor kinase 2-like
maker-2-augustus-gene-264.73-mRNA-1	Japonica	Japonica	Kinase-TM	Os02g0639100	OsR498G0204395600.01.T01	cold-responsive protein kinase 1
maker-2-augustus-gene-269.36-mRNA-1	Japonica	NA	Kinase-TM	Os02g0648100	OsR498G0204421800.01.T01	Putative LRR receptor-like serine/threonine-protein kinase
maker-2-augustus-gene-27.43-mRNA-1	Japonica	NA	Kinase-TM	Os02g0148100	OsR498G0202972400.01.T02	Mitogen-activated protein kinase
maker-2-augustus-gene-270.11-mRNA-1	Japonica	NA	Kinase-TM	Os02g0650500	OsR498G0204428800.01.T03	probable serine/threonine-protein kinase PBL19
maker-2-augustus-gene-273.8-mRNA-1	Japonica	NA	Kinase-TM	Os02g0655800	OsR498G0204447000.01.T01	Nodulation receptor kinase
maker-2-augustus-gene-278.15-mRNA-1	Japonica	NA	Kinase-TM	Os02g0666300	OsR498G0204480400.01.T02	Putative MAPKKK family protein kinase isoform 1
maker-2-augustus-gene-281.24-mRNA-1	Japonica	NA	Kinase-TM	Os02g0672800	OsR498G0204501800.01.T01	Serine/threonine-protein kinase WNK8
maker-2-augustus-gene-286.7-mRNA-1	Japonica	Indica	Kinase-TM	Os02g0681700	OsR498G0204533500.01.T01	Serine/threonine receptor-like protein kinase NFP
maker-2-augustus-gene-288.6-mRNA-1	Japonica	NA	Kinase-TM	Os02g0685900	OsR498G0204551100.01.T01	Calcium-dependent protein kinase
maker-2-augustus-gene-302.32-mRNA-1	Japonica	NA	Kinase-TM	Os02g0710500	OsR498G0204635700.01.T01	Liguleless narrow
maker-2-augustus-gene-303.16-mRNA-1	Japonica	NA	Kinase-TM	Os02g0712600	OsR498G0204641600.01.T01	Lectin receptor kinase
maker-2-augustus-gene-303.18-mRNA-1	Japonica	NA	Kinase-TM	Os02g0712700	OsR498G0204641800.01.T01	Lectin receptor kinase 1
maker-2-augustus-gene-309.15-mRNA-1	Japonica	NA	Kinase-TM	Os02g0725000	OsR498G0204682900.01.T03	serine/threonine-protein kinase D6PK
maker-2-augustus-gene-344.16-mRNA-1	Japonica	NA	Kinase-TM	Os06g0654500	OsR498G0613085100.01.T01	Concanavalin A-like lectin/glucanase domain, Serine/threonine-protein kinase, SIK1/2
maker-2-augustus-gene-361.35-mRNA-1	Japonica	NA	Kinase-TM	Os02g0769700	OsR498G0204841300.01.T04	Mitogen-activated protein kinase kinase kinase 1
maker-2-augustus-gene-365.16-mRNA-1	Japonica	Indica	Kinase-TM	Os06g0202900	OsR498G0204870400.01.T01	Mitogen-activated protein kinase kinase kinase
maker-2-augustus-gene-370.21-mRNA-1	Japonica	Indica	Kinase-TM	Os02g0786200	OsR498G0204902300.01.T02	Serine/threonine-protein kinase STY8 (Fragment)
maker-2-augustus-gene-380.40-mRNA-1	Japonica	NA	Kinase-TM	Os02g0807000	OsR498G0204976400.01.T01	Calcium-dependent protein kinase, isoform AK1
maker-2-augustus-gene-381.46-mRNA-1	Japonica	NA	Kinase-TM	Os02g0811200	OsR498G0204979300.01.T01	Wall-associated receptor kinase 3
maker-2-augustus-gene-381.48-mRNA-1	Japonica	NA	Kinase-TM	Os02g0807200		Wall-associated receptor kinase 3
maker-2-augustus-gene-381.49-mRNA-1	Japonica	Indica	Kinase-TM	Os02g0807800	OsR498G0204977600.01.T01	Wall-associated receptor kinase 3
maker-2-augustus-gene-381.50-mRNA-1	Japonica	Indica	Kinase-TM	Os02g0811200	OsR498G0204978800.01.T01	Wall-associated receptor kinase 3
maker-2-augustus-gene-385.38-mRNA-1	Japonica	NA	Kinase-TM	Os02g0815900	OsR498G0205003400.01.T01	Receptor-like serine/threonine-protein kinase ALE2 isoform A

maker-2-augustus-gene-385.47-mRNA-1	Japonica	NA	Kinase-TM		OsR498G0205006800.01.T01	AarF domain-containing kinase
maker-2-augustus-gene-43.67-mRNA-1	Japonica	NA	Kinase-TM	Os02g0178000	OsR498G0203083200.01.T03	Non-specific serine/threonine protein kinase
maker-2-augustus-gene-53.43-mRNA-1	Japonica	NA	Kinase-TM	Os02g0194600	OsR498G0203145700.01.T02	Putative STRUBBELIG family receptor protein kinase
maker-2-augustus-gene-6.44-mRNA-1	Japonica	NA	Kinase-TM		OsR498G0202833400.01.T05	Receptor-like serine/threonine-protein kinase NCRK
maker-2-augustus-gene-6.52-mRNA-1	Japonica	Japonica	Kinase-TM	Os02g0111600	OsR498G0202835200.01.T02	Wall-associated receptor kinase 5
maker-2-augustus-gene-68.16-mRNA-1	Japonica	NA	Kinase-TM		OsR498G0203233800.01.T02	(Rice Genome Annotation Project) MAP3K delta-1 protein kinase
maker-2-augustus-gene-79.10-mRNA-1	Japonica	NA	Kinase-TM	Os02g0236200	OsR498G0203300400.01.T01	Non-specific serine/threonine protein kinase
maker-2-augustus-gene-81.8-mRNA-1	Japonica	NA	Kinase-TM	Os02g0241600	OsR498G0203312300.01.T01	Serine/threonine-protein kinase EDR1
maker-2-augustus-gene-96.25-mRNA-1	Japonica	NA	Kinase-TM	Os01g0711200	OsR498G0101870300.01.T01	Putative leucine-rich repeat receptor-like protein kinase
maker-3-augustus-gene-10.20-mRNA-1	Japonica	NA	Kinase-TM	Os03g0122000	OsR498G0305141900.01.T02	Serine/threonine-protein kinase ATG1a
maker-3-augustus-gene-100.19-mRNA-1	Japonica	NA	Kinase-TM	Os03g0294800	OsR498G0305747600.01.T01	Putative LRR receptor-like serine/threonine-protein kinase MRH1
maker-3-augustus-gene-101.30-mRNA-1	Japonica	NA	Kinase-TM	Os03g0295600	OsR498G0305749600.01.T01	Serine/threonine-protein kinase STY46
maker-3-augustus-gene-113.44-mRNA-1	Japonica	NA	Kinase-TM	Os03g0319400	OsR498G0305833000.01.T03	CBL-interacting protein kinase
maker-3-augustus-gene-12.18-mRNA-1	Japonica	Indica	Kinase-TM	Os10g0533150	OsR498G0305155100.01.T01	probable serine/threonine-protein kinase At1g01540
maker-3-augustus-gene-126.57-mRNA-1	Japonica	NA	Kinase-TM	Os10g0571300	OsR498G1019232500.01.T01	probable serine/threonine-protein kinase At4g35230
maker-3-augustus-gene-13.17-mRNA-1	Japonica	Indica	Kinase-TM	Os11g0134300	OsR498G0305158600.01.T04	Non-specific serine/threonine protein kinase
maker-3-augustus-gene-131.90-mRNA-1	Japonica	NA	Kinase-TM			Cyclin-dependent kinase E
maker-3-augustus-gene-135.46-mRNA-1	Japonica	Japonica	Kinase-TM	Os10g0533150	OsR498G0305155100.01.T01	Protein kinase domain containing protein, expressed
maker-3-augustus-gene-138.0-mRNA-1	Japonica	NA	Kinase-TM	Os10g0539600	OsR498G1019112100.01.T04	Calcium-dependent protein kinase isoform 1
maker-3-augustus-gene-14.0-mRNA-1	Japonica	NA	Kinase-TM	Os03g0128700	OsR498G0305164700.01.T02	Calcium-dependent protein kinase
maker-3-augustus-gene-140.5-mRNA-1	Japonica	Indica	Kinase-TM	Os03g0132800	OsR498G1019125100.01.T01	Serine/threonine-protein kinase BSK1-2
maker-3-augustus-gene-15.13-mRNA-1	Japonica	NA	Kinase-TM	Os03g0130900	OsR498G0305172900.01.T01	Putative serine/threonine-protein kinase PBL21
maker-3-augustus-gene-177.10-mRNA-1	Japonica	NA	Kinase-TM	Os03g0364400	OsR498G0306001300.01.T01	probable serine/threonine-protein kinase PBL15
maker-3-augustus-gene-178.12-mRNA-1	Japonica	NA	Kinase-TM	Os03g0366200	OsR498G0306008700.01.T02	Calcium/calmodulin-dependent serine/threonine-protein kinase 1
maker-3-augustus-gene-191.31-mRNA-1	Japonica	NA	Kinase-TM	Os03g0390200	OsR498G0306085400.01.T01	Serine/threonine-protein kinase SRK2C
maker-3-augustus-gene-214.34-mRNA-1	Japonica	Indica	Kinase-TM	Os03g0424200	OsR498G0306238100.01.T01	U-box domain-containing protein 57
maker-3-augustus-gene-214.48-mRNA-1	Japonica	NA	Kinase-TM	Os03g0424200	OsR498G0306240800.01.T02	U-box domain-containing protein 32
maker-3-augustus-gene-278.24-mRNA-1	Japonica	NA	Kinase-TM	Os10g0405100	OsR498G1018676700.01.T05	probable serine/threonine-protein kinase PBL3
maker-3-augustus-gene-29.65-mRNA-1	Japonica	NA	Kinase-TM	Os03g0160100	OsR498G0305260500.01.T01	Serine/threonine-protein kinase EDR1
maker-3-augustus-gene-3.4-mRNA-1	Japonica	Indica	Kinase-TM	Os03g0118400	OsR498G0305096900.01.T04	Cell division control protein A
maker-3-augustus-gene-325.29-mRNA-1	Japonica	NA	Kinase-TM	Os10g0329700	OsR498G1018459800.01.T01	Receptor-like serine/threonine-protein kinase
maker-3-augustus-gene-327.30-mRNA-1	Japonica	NA	Kinase-TM	Os10g0327000	OsR498G1018449400.01.T02	Receptor-like serine/threonine-protein kinase
maker-3-augustus-gene-342.3-mRNA-1	Japonica	NA	Kinase-TM	Os03g0568800	OsR498G0306402700.01.T01	Proline-rich receptor-like protein kinase PERK13
maker-3-augustus-gene-380.27-mRNA-1	Japonica	Indica	Kinase-TM	Os12g0604700	OsR498G0306621200.01.T01	Serine/threonine-protein kinase Nek2
maker-3-augustus-gene-384.43-mRNA-1	Japonica	NA	Kinase-TM		OsR498G0306653000.01.T01	Wall-associated receptor kinase 5
maker-3-augustus-gene-385.45-mRNA-1	Japonica	NA	Kinase-TM	Os03g0643250		Phosphonate metabolism protein, putative
maker-3-augustus-gene-39.11-mRNA-1	Japonica	NA	Kinase-TM	Os03g0179400	OsR498G0305326300.01.T01	Serine/threonine-protein kinase RIPK
maker-3-augustus-gene-420.19-mRNA-1	Japonica	NA	Kinase-TM	Os03g0703400	OsR498G0306885800.01.T03	Mitogen-activated protein kinase kinase kinase 1
maker-3-augustus-gene-420.9-mRNA-1	Japonica	Indica	Kinase-TM	Os03g0704400	OsR498G0306887900.01.T01	Serine/threonine-protein kinase mph1
maker-3-augustus-gene-424.23-mRNA-1	Japonica	NA	Kinase-TM	Os03g0711300	OsR498G0306914200.01.T02	Serinethreonine-protein kinase grik2
maker-3-augustus-gene-425.62-mRNA-1	Japonica	NA	Kinase-TM	Os03g0711800	OsR498G0306916400.01.T04	probable serine/threonine protein kinase IREH1
maker-3-augustus-gene-429.46-mRNA-1	Japonica	NA	Kinase-TM	Os03g0719850	OsR498G0306945400.01.T01	Protein kinase domain containing protein
maker-3-augustus-gene-449.11-mRNA-1	Japonica	NA	Kinase-TM	Os03g0755000	OsR498G0307065100.01.T02	Serine/threonine-protein kinase dst1
maker-3-augustus-gene-453.37-mRNA-1	Japonica	Indica	Kinase-TM	Os07g0114400	OsR498G0307088600.01.T01	Casein kinase II alpha subunit
maker-3-augustus-gene-454.22-mRNA-1	Japonica	NA	Kinase-TM	Os03g0764300	OsR498G0307097600.01.T02	Mitogen-activated protein kinase kinase kinase 5
maker-3-augustus-gene-454.4-mRNA-1	Japonica	NA	Kinase-TM	Os03g0763000	OsR498G0307094500.01.T01	Casein kinase II subunit alpha
maker-3-augustus-gene-455.0-mRNA-1	Japonica	NA	Kinase-TM	Os03g0764800	OsR498G0307099900.01.T01	Serine/threonine-protein kinase SAPK8
maker-3-augustus-gene-460.80-mRNA-1	Japonica	NA	Kinase-TM	Os03g0776100	OsR498G0307138400.01.T01	proline-rich receptor-like protein kinase PERK4
maker-3-augustus-gene-480.25-mRNA-1	Japonica	Indica	Kinase-TM	Os03g0816100	OsR498G0307269400.01.T01	Non-specific lipid-transfer protein-like isoform 1
maker-3-augustus-gene-485.0-mRNA-1	Japonica	Indica	Kinase-TM	Os03g0825300	OsR498G0307301100.01.T01	Putative serine/threonine-protein kinase BSK3
maker-3-augustus-gene-491.22-mRNA-1	Japonica	NA	Kinase-TM	Os03g0839900	OsR498G0307344800.01.T01	Protein kinase protein with adenine nucleotide alpha hydrolase-like domain
maker-3-augustus-gene-494.32-mRNA-1	Japonica	NA	Kinase-TM	Os03g0847600	OsR498G0307365500.01.T05	Serine/threonine protein kinase
maker-3-augustus-gene-5.37-mRNA-1	Japonica	NA	Kinase-TM	Os03g0113000	OsR498G0305110000.01.T01	Receptor protein kinase CRINKLY4
maker-3-augustus-gene-54.69-mRNA-1	Japonica	NA	Kinase-TM	Os03g0207300	OsR498G0305432000.01.T01	Protein kinase CK2 alpha chain
maker-3-augustus-gene-64.25-mRNA-1	Japonica	NA	Kinase-TM		OsR498G0305491900.01.T01	Proline-rich receptor-like protein kinase PERK4 isoform A
maker-3-augustus-gene-64.4-mRNA-1	Japonica	NA	Kinase-TM	Os03g0225700	OsR498G0305488100.01.T01	Wall-associated receptor kinase
maker-3-augustus-gene-64.8-mRNA-1	Japonica	NA	Kinase-TM	Os03g0226300	OsR498G0305489900.01.T01	Pto kinase interactor
maker-3-augustus-gene-65.20-mRNA-1	Japonica	NA	Kinase-TM	Os03g0227900	OsR498G0305497400.01.T02	probable serine/threonine-protein kinase At1g01540
maker-3-augustus-gene-73.26-mRNA-1	Japonica	NA	Kinase-TM		OsR498G0305548400.01.T03	serine/threonine-protein kinase RIPK-like isoform X1
maker-3-augustus-gene-8.0-mRNA-1	Japonica	NA	Kinase-TM	Os03g0118400	OsR498G0305126900.01.T01	Cell division control protein A
maker-3-augustus-gene-83.13-mRNA-1	Japonica	NA	Kinase-TM		OsR498G0305621000.01.T02	MAP kinase kinase kinase10
maker-3-augustus-gene-86.49-mRNA-1	Japonica	NA	Kinase-TM		OsR498G0305644000.01.T04	Serine/threonine-protein kinase ATG1c isoform C
maker-3-augustus-gene-87.18-mRNA-1	Japonica	NA	Kinase-TM	Os03g0269300	OsR498G0305649000.01.T01	Serine/threonine protein kinase
maker-3-augustus-gene-96.19-mRNA-1	Japonica	NA	Kinase-TM	Os03g0285800	OsR498G0305716200.01.T01	Mitogen-activated protein kinase
maker-4-augustus-gene-123.1-mRNA-1	Japonica	Indica	Kinase-TM	Os04g0366000	OsR498G0408332800.01.T02	Calcium binding EGF domain containing protein, expressed
maker-4-augustus-gene-124.35-mRNA-1	Japonica	NA	Kinase-TM	Os04g0368000	OsR498G0408337400.01.T01	Wall-associated receptor kinase 2
maker-4-augustus-gene-139.33-mRNA-1	Japonica	NA	Kinase-TM	Os04g0393900	OsR498G0408448500.01.T01	Serine/threonine-protein kinase NAK
maker-4-augustus-gene-159.60-mRNA-1	Japonica	Indica	Kinase-TM	Os04g0430000	OsR498G0408585300.01.T02	probable serine/threonine-protein kinase At1g01540
maker-4-augustus-gene-159.68-mRNA-1	Japonica	Indica	Kinase-TM	Os04g0430400	OsR498G0408586700.01.T01	Protein kinase domain-containing protein
maker-4-augustus-gene-160.11-mRNA-1	Japonica	NA	Kinase-TM	Os04g0432000	OsR498G0408595100.01.T04	Serine/threonine-protein kinase SAPK7
maker-4-augustus-gene-19.43-mRNA-1	Japonica	NA	Kinase-TM	Os04g0197200	OsR498G0407706500.01.T02	Cysteine-rich receptor-like protein kinase 6
maker-4-augustus-gene-19.50-mRNA-1	Japonica	Indica	Kinase-TM	Os04g0197200	OsR498G0407709800.01.T01	Cysteine-rich receptor-like protein kinase 6
maker-4-augustus-gene-191.39-mRNA-1	Japonica	NA	Kinase-TM	Os04g0488700	OsR498G0408802200.01.T01	Serine/threonine-protein kinase OXI1
maker-4-augustus-gene-201.39-mRNA-1	Japonica	NA	Kinase-TM		OsR498G0408871500.01.T01	Receptor-like serinethreonine-protein kinase ale2
maker-4-augustus-gene-220.0-mRNA-1	Japonica	NA	Kinase-TM	Os04g0546300	OsR498G0409018300.01.T01	Serine/threonine-protein kinase D6PKL1
maker-4-augustus-gene-221.65-mRNA-1	Japonica	Indica	Kinase-TM	Os02g0655800	OsR498G0409024600.01.T01	Putative leucine-rich repeat receptor-like protein kinase family protein
maker-4-augustus-gene-242.17-mRNA-1	Japonica	Indica	Kinase-TM	Os04g0584050	OsR498G0409164800.01.T01	Clade XII lectin receptor kinase
maker-4-augustus-gene-242.4-mRNA-1	Japonica	Indica	Kinase-TM	Os04g0584600	OsR498G0409166900.01.T02	Calcium-dependent protein kinase
maker-4-augustus-gene-248.40-mRNA-1	Japonica	NA	Kinase-TM	Os04g0598600		Wall-associated kinase
maker-4-augustus-gene-248.42-mRNA-1	Japonica	NA	Kinase-TM	Os04g0598800	OsR498G0409215700.01.T01	Wall-associated receptor kinase-like 22 (Fragment)
maker-4-augustus-gene-248.50-mRNA-1	Japonica	NA	Kinase-TM	Os04g0599000	OsR498G0409216600.01.T02	Wall-associated kinase
maker-4-augustus-gene-256.39-mRNA-1	Japonica	NA	Kinase-TM	Os04g0610900	OsR498G0409261700.01.T03	Serine/threonine-protein kinase CTR1
maker-4-augustus-gene-259.35-mRNA-1	Japonica	NA	Kinase-TM	Os04g0616600	OsR498G0409282400.01.T01	Leucine-rich repeat transmembrane protein kinase
maker-4-augustus-gene-261.12-mRNA-1	Japonica	NA	Kinase-TM	Os04g0619400	OsR498G0409292800.01.T02	Serine/threonine-protein kinase receptor
maker-4-augustus-gene-268.0-mRNA-1	Japonica	Indica	Kinase-TM	Os04g0633800	OsR498G0409337500.01.T02	Serine/threonine-protein kinase
maker-4-augustus-gene-268.22-mRNA-1	Japonica	Indica	Kinase-TM	Os04g0632700	OsR498G0409334800.01.T01	Serine/threonine-protein kinase
maker-4-augustus-gene-268.25-mRNA-1	Japonica	Japonica	Kinase-TM	Os04g0633200	OsR498G0409335600.01.T01	Non-specific serine/threonine protein kinase
maker-4-augustus-gene-268.31-mRNA-1	Japonica	NA	Kinase-TM	Os04g0633300	OsR498G0409336200.01.T01	Non-specific serine/threonine protein kinase
maker-4-augustus-gene-269.71-mRNA-1	Japonica	Indica	Kinase-TM	Os01g0783800	OsR498G0409339200.01.T03	Cysteine rich receptor like kinase
maker-4-augustus-gene-279.14-mRNA-1	Japonica	NA	Kinase-TM	Os04g0654600	OsR498G0409406500.01.T01	probable receptor-like serine/threonine-protein kinase At5g57670
maker-4-augustus-gene-279.21-mRNA-1	Japonica	NA	Kinase-TM	Os04g0655300	OsR498G0409409300.01.T01	G-type lectin S-receptor-like serine/threonine-protein kinase
maker-4-augustus-gene-279.23-mRNA-1	Japonica	Indica	Kinase-TM	Os04g0655400	OsR498G0409409600.01.T01	G-type lectin S-receptor-like serine/threonine-protein kinase
maker-4-augustus-gene-280.0-mRNA-1	Japonica	Indica	Kinase-TM	Os04g0655300	OsR498G0409410100.01.T01	Mitogen-activated protein kinase kinase kinase
maker-4-augustus-gene-281.48-mRNA-1	Japonica	Japonica	Kinase-TM	Os04g0658700	OsR498G0409421500.01.T01	Similar to putative receptor protein kinase from A. thaliana
maker-4-augustus-gene-292.61-mRNA-1	Japonica	NA	Kinase-TM	Os04g0679200	OsR498G0409497000.01.T02	Serine/threonine-protein kinase
maker-4-augustus-gene-296.20-mRNA-1	Japonica	NA	Kinase-TM		OsR498G0409524800.01.T04	Serine/threonine-protein kinase STY46
maker-4-augustus-gene-298.34-mRNA-1	Japonica	NA	Kinase-TM	Os04g0689400	OsR498G0409533700.01.T01	Serine/threonine protein kinase
maker-4-augustus-gene-299.42-mRNA-1	Japonica	NA	Kinase-TM	Os04g0691100	OsR498G0409538500.01.T02	Serine/threonine-protein kinase SAPK5
maker-4-augustus-gene-301.0-mRNA-1	Japonica	Indica	Kinase-TM	Os08g0112500	OsR498G0409548500.01.T02	probable serine/threonine-protein kinase At1g54610
maker-4-augustus-gene-31.39-mRNA-1	Japonica	Indica	Kinase-TM	Os11g0692100	OsR498G0407758000.01.T01	Receptor kinase MRKa
maker-4-augustus-gene-35.20-mRNA-1	Japonica	NA	Kinase-TM	Os04g0226600	OsR498G0407773100.01.T02	Non-specific serine/threonine protein kinase (Fragment)
maker-4-augustus-gene-66.41-mRNA-1	Japonica	NA	Kinase-TM	Os04g0286000		Calcium binding EGF domain containing protein, expressed
maker-4-augustus-gene-68.42-mRNA-1	Japonica	NA	Kinase-TM	Os04g0288500	OsR498G0407951200.01.T01	Putative receptor-type protein kinase LRK1
maker-4-augustus-gene-78.3-mRNA-1	Japonica	NA	Kinase-TM	Os04g0303500	OsR498G0408000400.01.T01	Receptor-like serine/threonine-protein kinase
maker-4-augustus-gene-79.14-mRNA-1	Japonica	NA	Kinase-TM			Putative LOV domain-containing protein
maker-5-augustus-gene-14.39-mRNA-1	Japonica	Japonica	Kinase-TM	Os05g0125300	OsR498G0509647100.01.T01	Receptor-like kinase LIP2
maker-5-augustus-gene-152.41-mRNA-1	Japonica	NA	Kinase-TM	Os05g0319700	OsR498G0510482900.01.T01	Phytosulfokine receptor
maker-5-augustus-gene-17.57-mRNA-1	Japonica	NA	Kinase-TM		OsR498G0509666500.01.T01	Cysteine-rich receptor-like protein kinase 3
maker-5-augustus-gene-198.7-mRNA-1	Japonica	NA	Kinase-TM	Os05g0389700	OsR498G0510762700.01.T02	Cyclin-dependent kinase
maker-5-augustus-gene-20.20-mRNA-1	Japonica	NA	Kinase-TM	Os05g0134000	OsR498G0509679200.01.T01	Non-specific serine/threonine protein kinase
maker-5-augustus-gene-200.12-mRNA-1	Japonica	NA	Kinase-TM	Os05g0392300	OsR498G0510773500.01.T01	Cdk activating kinase (CAK)/RNA polymerase II transcription initiation
maker-5-augustus-gene-200.22-mRNA-1	Japonica	NA	Kinase-TM		OsR498G0510772400.01.T05	RING-type E3 ubiquitin transferase
maker-5-augustus-gene-203.8-mRNA-1	Japonica	NA	Kinase-TM	Os05g0398800	OsR498G0510792100.01.T01	Putative serine/threonine-protein kinase RLCKVII (Fragment)
maker-5-augustus-gene-21.30-mRNA-1	Japonica	NA	Kinase-TM	Os05g0135800	OsR498G0509688700.01.T01	Pto-interacting protein 1
maker-5-augustus-gene-21.51-mRNA-1	Japonica	NA	Kinase-TM	Os05g0136200	OsR498G0509691000.01.T02	Non-specific serine/threonine protein kinase
maker-5-augustus-gene-238.66-mRNA-1	Japonica	NA	Kinase-TM	Os05g0463000	OsR498G0510998400.01.T01	Putative serine/threonine-protein kinase PBL26
maker-5-augustus-gene-247.0-mRNA-1	Japonica	NA	Kinase-TM		OsR498G0511051300.01.T01	Serine/threonine-protein kinase STN8 chloroplastic
maker-5-augustus-gene-25.37-mRNA-1	Japonica	Indica	Kinase-TM	Os11g0271100	OsR498G0509721500.01.T06	Mitogen-activated protein kinase
maker-5-augustus-gene-252.57-mRNA-1	Japonica	NA	Kinase-TM	Os05g0489900	OsR498G0511082800.01.T01	Calcium and calmodulin-dependent kinase
maker-5-augustus-gene-253.38-mRNA-1	Japonica	NA	Kinase-TM	Os05g0493100	OsR498G0511092300.01.T01	Cysteine-rich receptor-like protein kinase 25
maker-5-augustus-gene-253.51-mRNA-1	Japonica	NA	Kinase-TM	Os05g0491900	OsR498G0511090200.01.T01	Calcium-dependent protein kinase 34
maker-5-augustus-gene-256.86-mRNA-1	Japonica	NA	Kinase-TM	Os05g0498900	OsR498G0511111400.01.T01	probable serine/threonine-protein kinase PBL23
maker-5-augustus-gene-258.0-mRNA-1	Japonica	NA	Kinase-TM	Os05g0501400	OsR498G0511118500.01.T04	Serine/threonine-protein kinase
maker-5-augustus-gene-267.14-mRNA-1	Japonica	Japonica	Kinase-TM	Os05g0516400	OsR498G0511182900.01.T01	Proline-rich receptor-like protein kinase PERK1
maker-5-augustus-gene-301.4-mRNA-1	Japonica	Indica	Kinase-TM	Os01g0629900	OsR498G0511410500.01.T01	Mitogen-activated protein kinase
maker-5-augustus-gene-302.14-mRNA-1	Japonica	NA	Kinase-TM	Os05g0585500	OsR498G0511422000.01.T02	Calcium-dependent protein kinase 1 isoform A
maker-5-augustus-gene-304.89-mRNA-1	Japonica	NA	Kinase-TM		OsR498G0511435600.01.T05	Protein kinase protein with adenine nucleotide alpha hydrolase-like domain
maker-5-augustus-gene-80.21-mRNA-1	Japonica	NA	Kinase-TM		OsR498G0510084400.01.T01	Glyoxalase I
maker-5-augustus-gene-84.44-mRNA-1	Japonica	NA	Kinase-TM	Os05g0237400	OsR498G0510107900.01.T01	Serine/threonine-protein kinase D6PKL1
maker-5-augustus-gene-96.6-mRNA-1	Japonica	Indica	Kinase-TM	Os08g0203400	OsR498G0510169900.01.T02	Non-specific serine/threonine protein kinase
maker-6-augustus-gene-104.6-mRNA-1	Japonica	NA	Kinase-TM	Os06g0291600	OsR498G0612170400.01.T02	serine/threonine-protein kinase D6PK
maker-6-augustus-gene-126.30-mRNA-1	Japonica	Indica	Kinase-TM	Os06g0328400	OsR498G0612301900.01.T02	Lectin receptor kinase 1
maker-6-augustus-gene-130.0-mRNA-1	Japonica	NA	Kinase-TM	Os06g0334300	OsR498G0612553000.01.T02	Receptor-like protein kinase HERK 1 isoform A
maker-6-augustus-gene-152.10-mRNA-1	Japonica	NA	Kinase-TM	Os06g0367900	OsR498G0612439300.01.T06	Mitogen-activated protein kinase
maker-6-augustus-gene-157.45-mRNA-1	Japonica	Indica	Kinase-TM	Os06g0473200	OsR498G0612401000.01.T06	Mitogen-activated protein kinase kinase 3
maker-6-augustus-gene-165.8-mRNA-1	Japonica	NA	Kinase-TM	Os06g0486000	OsR498G0612367500.01.T02	Proline-rich receptor-like protein kinase PERK12
maker-6-augustus-gene-173.0-mRNA-1	Japonica	NA	Kinase-TM			Liguleless narrow
maker-6-augustus-gene-206.0-mRNA-1	Japonica	NA	Kinase-TM	Os06g0547900	OsR498G0612721100.01.T02	Non-specific serine/threonine protein kinase
maker-6-augustus-gene-221.12-mRNA-1	Japonica	NA	Kinase-TM	Os06g0574200	OsR498G0612812000.01.T01	E3 ubiquitin ligase
maker-6-augustus-gene-240.40-mRNA-1	Japonica	NA	Kinase-TM	Os06g0606000	OsR498G0612909400.01.T01	Non-specific serine/threonine protein kinase
maker-6-augustus-gene-289.68-mRNA-1	Japonica	Indica	Kinase-TM	Os06g0693000	OsR498G0613215600.01.T01	Cysteine-rich receptor-like protein kinase 19
maker-6-augustus-gene-291.44-mRNA-1	Japonica	NA	Kinase-TM	Os06g0693000	OsR498G0613226300.01.T03	Cysteine-rich receptor-like protein kinase 19
maker-6-augustus-gene-35.67-mRNA-1	Japonica	NA	Kinase-TM	Os06g0168800	OsR498G0611688600.01.T01	Mitogen-activated protein kinase kinase kinase
maker-6-augustus-gene-52.50-mRNA-1	Japonica	NA	Kinase-TM	Os06g0202900	OsR498G0611804800.01.T01	Serinethreonine-protein kinase
maker-6-augustus-gene-75.37-mRNA-1	Japonica	NA	Kinase-TM	Os06g0241100	OsR498G0713492600.01.T02	G-type lectin S-receptor-like serine/threonine-protein kinase
maker-7-augustus-gene-104.28-mRNA-1	Japonica	NA	Kinase-TM	Os07g0283125	OsR498G0713999800.01.T01	Putative receptor-type protein kinase LRK1
maker-7-augustus-gene-115.33-mRNA-1	Japonica	NA	Kinase-TM	Os07g0301550	OsR498G0714056600.01.T01	Receptor-like serine/threonine-protein kinase
maker-7-augustus-gene-127.36-mRNA-1	Japonica	NA	Kinase-TM			Non-specific serine/threonine protein kinase (Fragment)

maker-7-augustus-gene-143.41-mRNA-1	Japonica	Indica	Kinase-TM	Os07g0438500	OsR498G0714165300.01.T03	Putative serine/threonine-protein kinase SIS8 isoform A
maker-7-augustus-gene-157.34-mRNA-1	Japonica	NA	Kinase-TM		OsR498G0714229400.01.T02	ABC1 domain-containing protein (Fragment)
maker-7-augustus-gene-166.33-mRNA-1	Japonica	NA	Kinase-TM	Os07g0471000	OsR498G0714268500.01.T01	Serine/threonine-protein kinase/endoribonuclease IRE1a
maker-7-augustus-gene-167.38-mRNA-1	Japonica	Indica	Kinase-TM	Os07g0472400	OsR498G0714275700.01.T01	probable serine/threonine-protein kinase sky1
maker-7-augustus-gene-169.35-mRNA-1	Japonica	Indica	Kinase-TM	Os07g0475900	OsR498G0714289000.01.T01	serine/threonine-protein kinase STY46 isoform X2
maker-7-augustus-gene-177.3-mRNA-1	Japonica	NA	Kinase-TM	Os07g0487400		Receptor protein kinase-like protein
maker-7-augustus-gene-206.4-mRNA-1	Japonica	NA	Kinase-TM	Os07g0537000	OsR498G0714501700.01.T01	Receptor-like serine-threonine protein kinase
maker-7-augustus-gene-206.5-mRNA-1	Japonica	NA	Kinase-TM			G-type lectin S-receptor-like serine/threonine-protein kinase
maker-7-augustus-gene-207.62-mRNA-1	Japonica	NA	Kinase-TM	Os07g0537500	OsR498G0714502600.01.T01	Cysteine-rich receptor-like protein kinase 6
maker-7-augustus-gene-208.31-mRNA-1	Japonica	Indica	Kinase-TM	Os07g0537900	OsR498G0714510100.01.T01	Cysteine-rich receptor-like protein kinase 8
maker-7-augustus-gene-209.33-mRNA-1	Japonica	NA	Kinase-TM			Cysteine-rich receptor-like protein kinase 25 isoform C
maker-7-augustus-gene-209.7-mRNA-1	Japonica	Indica	Kinase-TM	Os07g0541500	OsR498G0714517300.01.T01	Cysteine-rich receptor kinase
maker-7-augustus-gene-210.35-mRNA-1	Japonica	NA	Kinase-TM			Pkinase domain-containing protein/Stress-antifung domain-containing protein
maker-7-augustus-gene-210.44-mRNA-1	Japonica	Indica	Kinase-TM	Os07g0542400	OsR498G0714520000.01.T01	Cysteine-rich receptor-like protein kinase 15
maker-7-augustus-gene-214.18-mRNA-1	Japonica	NA	Kinase-TM		OsR498G0714538800.01.T02	Non-specific serine/threonine protein kinase (Fragment)
maker-7-augustus-gene-214.21-mRNA-1	Japonica	NA	Kinase-TM	Os07g0550900	OsR498G0714541400.01.T01	Serine/threonine-protein kinase
maker-7-augustus-gene-214.25-mRNA-1	Japonica	NA	Kinase-TM	Os07g0551300	OsR498G0714542600.01.T02	Serine/threonine-protein kinase
maker-7-augustus-gene-228.2-mRNA-1	Japonica	NA	Kinase-TM	Os07g0572800	OsR498G0714612300.01.T06	Serine/threonine-protein kinase WNK1
maker-7-augustus-gene-250.8-mRNA-1	Japonica	NA	Kinase-TM	Os07g0613500	OsR498G0714745300.01.T02	Serine/threonine-protein kinase RIPK
maker-7-augustus-gene-255.26-mRNA-1	Japonica	NA	Kinase-TM			Serine/threonine-protein kinase SRK2C
maker-7-augustus-gene-258.17-mRNA-1	Japonica	NA	Kinase-TM	Os07g0628700	OsR498G0714798600.01.T01	Cysteine-rich receptor-like protein kinase 8
maker-7-augustus-gene-258.21-mRNA-1	Japonica	NA	Kinase-TM	Os07g0628900	OsR498G0714798800.01.T02	Non-specific serine/threonine protein kinase
maker-7-augustus-gene-260.64-mRNA-1	Japonica	NA	Kinase-TM		OsR498G0714811200.01.T01	serine/threonine-protein kinase STY8-like isoform X1
maker-7-augustus-gene-262.14-mRNA-1	Japonica	NA	Kinase-TM		OsR498G0714826500.01.T04	Non-specific serine/threonine protein kinase
maker-7-augustus-gene-264.57-mRNA-1	Japonica	Indica	Kinase-TM	Os03g0366200	OsR498G0714839200.01.T01	Calcium/calmodulin-dependent serine/threonine-protein kinase 1
maker-7-augustus-gene-280.12-mRNA-1	Japonica	Indica	Kinase-TM	Os03g0349200	OsR498G0714922100.01.T01	probable serine/threonine-protein kinase At1g54610
maker-7-augustus-gene-284.30-mRNA-1	Japonica	NA	Kinase-TM		OsR498G0714949300.01.T02	PEK protein kinase
maker-7-augustus-gene-290.60-mRNA-1	Japonica	Indica	Kinase-TM	Os03g0319400	OsR498G0714991900.01.T01	Non-specific serine/threonine protein kinase
maker-7-augustus-gene-293.6-mRNA-1	Japonica	NA	Kinase-TM	Os07g0693000	OsR498G0715012100.01.T01	Putative inactive receptor-like protein kinase
maker-7-augustus-gene-52.0-mRNA-1	Japonica	Japonica	Kinase-TM	Os07g0203900	OsR498G0713710700.01.T01	Proto-oncogene tyrosine-protein kinase YRK
maker-7-augustus-gene-6.14-mRNA-1	Japonica	NA	Kinase-TM	Os07g0119000	OsR498G0713419000.01.T01	Mitogen-activated protein kinase kinase kinase YODA
maker-7-augustus-gene-70.33-mRNA-1	Japonica	Indica	Kinase-TM	Os07g0534500	OsR498G0713832100.01.T01	Cysteine-rich receptor-like protein kinase 29
maker-7-augustus-gene-89.7-mRNA-1	Japonica	NA	Kinase-TM			Clade IV lectin receptor kinase
maker-8-augustus-gene-158.8-mRNA-1	Japonica	NA	Kinase-TM	Os08g0343000	OsR498G0815840500.01.T03	Proline-rich receptor-like protein kinase PERK14 isoform X5
maker-8-augustus-gene-16.11-mRNA-1	Japonica	Japonica	Kinase-TM	Os08g0125200	OsR498G0815100800.01.T01	Putative receptor-type protein kinase LRK1
maker-8-augustus-gene-2.45-mRNA-1	Japonica	NA	Kinase-TM		OsR498G0815030200.01.T02	Serine/threonine-protein kinase STY46
maker-8-augustus-gene-23.45-mRNA-1	Japonica	NA	Kinase-TM		OsR498G0815139400.01.T02	Calcium/calmodulin-regulated receptor-like protein kinase 1
maker-8-augustus-gene-260.33-mRNA-1	Japonica	NA	Kinase-TM	Os08g0501500	OsR498G0816405000.01.T01	Wall-associated receptor kinase 1
maker-8-augustus-gene-261.0-mRNA-1	Japonica	Japonica	Kinase-TM	Os08g0501600	OsR498G0816405300.01.T01	Wall associated kinase
maker-8-augustus-gene-267.13-mRNA-1	Japonica	NA	Kinase-TM	Os08g0512600	OsR498G0816453300.01.T01	Cyclin-dependent kinase B
maker-8-augustus-gene-268.36-mRNA-1	Japonica	Japonica	Kinase-TM	Os08g0514000	OsR498G0816459900.01.T01	L-type lectin-domain containing receptor kinase IV.2
maker-8-augustus-gene-268.38-mRNA-1	Japonica	Indica	Kinase-TM	Os06g0328400	OsR498G0816460300.01.T01	Mitogen-activated protein kinase kinase kinase
maker-8-augustus-gene-283.9-mRNA-1	Japonica	NA	Kinase-TM	Os08g0538300	OsR498G0816542500.01.T02	Chitin elicitor receptor kinase 1
maker-8-augustus-gene-45.40-mRNA-1	Japonica	Japonica	Kinase-TM	Os08g0174800	OsR498G0815299300.01.T01	LRR receptor kinase BAK1
maker-8-augustus-gene-48.33-mRNA-1	Japonica	NA	Kinase-TM		OsR498G0815310900.01.T05	Non-specific serine/threonine protein kinase
maker-8-augustus-gene-79.35-mRNA-1	Japonica	NA	Kinase-TM	Os08g0224100	OsR498G0815462500.01.T01	serine/threonine-protein kinase STY13
maker-9-augustus-gene-104.0-mRNA-1	Japonica	NA	Kinase-TM			Senescence-induced receptor-like serine/threonine-protein kinase
maker-9-augustus-gene-107.14-mRNA-1	Japonica	NA	Kinase-TM	Os09g0349600		Putative LRR receptor-like serine/threonine-protein kinase
maker-9-augustus-gene-108.67-mRNA-1	Japonica	Indica	Kinase-TM	Os09g0348300	OsR498G0917213300.01.T01	Serine/threonine-specific receptor protein kinase-like
maker-9-augustus-gene-108.69-mRNA-1	Japonica	Indica	Kinase-TM	Os09g0348300	OsR498G0917213100.01.T01	Serine/threonine-specific receptor protein kinase-like
maker-9-augustus-gene-110.4-mRNA-1	Japonica	NA	Kinase-TM			L-type lectin-domain containing receptor kinase IX.1
maker-9-augustus-gene-114.2-mRNA-1	Japonica	NA	Kinase-TM		OsR498G0917185900.01.T01	Clade XIV lectin receptor kinase
maker-9-augustus-gene-114.3-mRNA-1	Japonica	NA	Kinase-TM	Os09g0334800	OsR498G0917182700.01.T01	L-type lectin-domain containing receptor kinase IX.1
maker-9-augustus-gene-115.2-mRNA-1	Japonica	NA	Kinase-TM			Serine/threonine-specific receptor protein kinase-like
maker-9-augustus-gene-116.0-mRNA-1	Japonica	Japonica	Kinase-TM	Os09g0356200	OsR498G0917236300.01.T05	Putative LRR receptor-like serine/threonine-protein kinase
maker-9-augustus-gene-117.3-mRNA-1	Japonica	Indica	Kinase-TM	Os09g0356800	OsR498G0917240400.01.T05	Protein kinase domain-containing protein
maker-9-augustus-gene-127.39-mRNA-1	Japonica	Indica	Kinase-TM	Os09g0375600	OsR498G0917296000.01.T01	Serine/threonine-protein kinase CDL1
maker-9-augustus-gene-171.35-mRNA-1	Japonica	NA	Kinase-TM	Os09g0454900	OsR498G0917599400.01.T02	Non-specific serine/threonine protein kinase
maker-9-augustus-gene-178.50-mRNA-1	Japonica	Japonica	Kinase-TM	Os09g0466900	OsR498G0917648000.01.T01	Serine/threonine-protein kinase PEPKR2
maker-9-augustus-gene-180.51-mRNA-1	Japonica	NA	Kinase-TM		OsR498G0917661800.01.T01	Wall-associated receptor kinase-like 8
maker-9-augustus-gene-181.50-mRNA-1	Japonica	NA	Kinase-TM			Wall-associated receptor kinase
maker-9-augustus-gene-186.2-mRNA-1	Japonica	NA	Kinase-TM	Os09g0482640	OsR498G0917701900.01.T01	Wall associated kinase
maker-9-augustus-gene-199.37-mRNA-1	Japonica	NA	Kinase-TM		OsR498G0917786500.01.T01	Chitin elicitor receptor kinase 1
maker-9-augustus-gene-201.0-mRNA-1	Japonica	Indica	Kinase-TM	Os01g0899000	OsR498G0917792700.01.T02	Putative Pto kinase interactor 1
maker-9-augustus-gene-219.38-mRNA-1	Japonica	Indica	Kinase-TM	Os09g0551500	OsR498G0917921800.01.T02	Non-specific serine/threonine protein kinase (Fragment)
maker-9-augustus-gene-219.45-mRNA-1	Japonica	NA	Kinase-TM			Non-specific serine/threonine protein kinase (Fragment)
maker-9-augustus-gene-219.47-mRNA-1	Japonica	NA	Kinase-TM	Os09g0550500		Non-specific serine/threonine protein kinase (Fragment)
maker-9-augustus-gene-219.50-mRNA-1	Japonica	NA	Kinase-TM			Non-specific serine/threonine protein kinase
maker-9-augustus-gene-219.52-mRNA-1	Japonica	NA	Kinase-TM		OsR498G0917920000.01.T01	Serine/threonine-protein kinase
maker-9-augustus-gene-219.54-mRNA-1	Japonica	Indica	Kinase-TM	Os09g0551500	OsR498G0917921800.01.T01	Non-specific serine/threonine protein kinase
maker-9-augustus-gene-220.26-mRNA-1	Japonica	NA	Kinase-TM	Os09g0552300	OsR498G0917924800.01.T01	Serinethreonine-protein kinase afc3
maker-9-augustus-gene-224.22-mRNA-1	Japonica	Japonica	Kinase-TM	Os09g0561500	OsR498G0917950700.01.T01	Wall-associated receptor kinase 3
maker-9-augustus-gene-227.10-mRNA-1	Japonica	NA	Kinase-TM		OsR498G0917964200.01.T02	Tyrosine kinase
maker-9-augustus-gene-230.46-mRNA-1	Japonica	NA	Kinase-TM	Os09g0572600	OsR498G0917982500.01.T01	probable serine/threonine-protein kinase PBL23
maker-9-augustus-gene-96.74-mRNA-1	Japonica	Japonica	Kinase-TM	Os09g0325140	OsR498G0917156400.01.T04	LysM domain receptor-like kinase 10
augustus-1-processed-gene-151.26-mRNA-1	NA	NA	Kinase-TM	Os01g0366300		Cysteine-rich receptor-like protein kinase 10
augustus-1-processed-gene-383.64-mRNA-1	NA	NA	Kinase-TM	Os01g0869900	OsR498G0102413500.01.T01	Serine/threonine-protein kinase SAPK4
augustus-1-processed-gene-384.53-mRNA-1	NA	Indica	Kinase-TM	Os01g0889900	OsR498G0102416100.01.T01	Receptor-like serine/threonine-protein kinase
augustus-1-processed-gene-390.53-mRNA-1	NA	NA	Kinase-TM	Os01g0883000		Receptor-like protein kinase
augustus-1-processed-gene-390.54-mRNA-1	NA	Indica	Kinase-TM	Os10g0492400	OsR498G0102463700.01.T01	Tyrosine-protein kinase
augustus-1-processed-gene-392.0-mRNA-1	NA	NA	Kinase-TM	Os01g0885700	OsR498G0102473900.01.T01	Serine/threonine-protein kinase
augustus-1-processed-gene-395.54-mRNA-1	NA	NA	Kinase-TM	Os01g0892800	OsR498G0102498100.01.T02	Dual specificity protein kinase shkC
augustus-1-processed-gene-435.41-mRNA-1	NA	NA	Kinase-TM	Os01g0869900	OsR498G0102413500.01.T01	Serine/threonine-protein kinase SAPK4
augustus-1-processed-gene-435.54-mRNA-1	NA	Indica	Kinase-TM	Os01g0889900	OsR498G0102416100.01.T01	Receptor-like serine/threonine-protein kinase
augustus-1-processed-gene-441.20-mRNA-1	NA	NA	Kinase-TM	Os01g0883000		Receptor-like protein kinase
augustus-1-processed-gene-441.21-mRNA-1	NA	Indica	Kinase-TM	Os10g0492400	OsR498G0102463700.01.T01	Tyrosine-protein kinase
augustus-1-processed-gene-443.42-mRNA-1	NA	NA	Kinase-TM	Os01g0885700	OsR498G0102473900.01.T01	Serine/threonine-protein kinase
augustus-1-processed-gene-446.17-mRNA-1	NA	NA	Kinase-TM	Os01g0892800	OsR498G0102498100.01.T02	Dual specificity protein kinase shkC
augustus-1-processed-gene-58.69-mRNA-1	NA	Indica	Kinase-TM	Os01g0206300	OsR498G0100377100.01.T01	Non-specific serine/threonine protein kinase
augustus-1-processed-gene-67.44-mRNA-1	NA	Indica	Kinase-TM	Os01g0222800	OsR498G0100443500.01.T01	Serine/threonine-protein kinase
augustus-1-processed-gene-67.45-mRNA-1	NA	NA	Kinase-TM	0	OsR498G0100443900.01.T01	Serine/threonine-protein kinase
augustus-1-processed-gene-93.35-mRNA-1	NA	NA	Kinase-TM	Os01g0267800	OsR498G0100624700.01.T01	serine/threonine-protein kinase-like protein At5g23170
augustus-10-processed-gene-38.5-mRNA-1	NA	Japonica	Kinase-TM	Os10g0157400	OsR498G1018161300.01.T01	Putative cyclin-dependent kinase F-2
augustus-10-processed-gene-9.12-mRNA-1	NA	Indica	Kinase-TM	Os10g0152000	OsR498G1018033700.01.T01	Wall-associated receptor kinase 2
augustus-11-processed-gene-256.22-mRNA-1	NA	NA	Kinase-TM	Os11g0691240	OsR498G1120759300.01.T01	Wall associated kinase-like
augustus-2-processed-gene-261.6-mRNA-1	NA	NA	Kinase-TM	Os02g0632800	OsR498G0204378000.01.T02	Wall-associated receptor kinase-like protein 9
augustus-4-processed-gene-121.14-mRNA-1	NA	NA	Kinase-TM	0	OsR498G0408317100.01.T03	Wall-associated receptor kinase 3
augustus-4-processed-gene-212.65-mRNA-1	NA	NA	Kinase-TM	Os04g0531500	OsR498G0408956500.01.T01	Lectin receptor-type kinase
augustus-4-processed-gene-268.79-mRNA-1	NA	Japonica	Kinase-TM	Os04g0633800	OsR498G0409334600.01.T02	Non-specific serine/threonine protein kinase
augustus-5-processed-gene-118.14-mRNA-1	NA	NA	Kinase-TM	Os05g0280700	OsR498G0510277400.01.T01	Receptor-like protein kinase ANXUR1
augustus-5-processed-gene-281.47-mRNA-1	NA	NA	Kinase-TM	Os05g0545400	OsR498G0511283000.01.T01	Mitogen-activated protein kinase kinase kinase ANP1-like
augustus-7-processed-gene-12.20-mRNA-1	NA	Japonica	Kinase-TM	Os07g0130300	OsR498G0713999000.01.T01	L-type lectin-domain containing receptor kinase IV.1
augustus-7-processed-gene-12.22-mRNA-1	NA	Japonica	Kinase-TM	Os07g0130600	OsR498G0916878800.01.T01	Putative receptor kinase Lecrk
augustus-7-processed-gene-12.23-mRNA-1	NA	Japonica	Kinase-TM	Os07g0130700	OsR498G0713461800.01.T01	L-type lectin-domain containing receptor kinase IV.1
augustus-7-processed-gene-12.25-mRNA-1	NA	NA	Kinase-TM	Os07g0130800	OsR498G0713462000.01.T01	Putative receptor kinase Lecrk
augustus-7-processed-gene-181.23-mRNA-1	NA	NA	Kinase-TM	Os07g0493800	OsR498G0714347500.01.T01	Wall-associated protein kinase
augustus-7-processed-gene-208.7-mRNA-1	NA	NA	Kinase-TM			Receptor-like serine/threonine-protein kinase
augustus-7-processed-gene-285.36-mRNA-1	NA	Japonica	Kinase-TM	Os07g0678300	OsR498G0306613200.01.T01	Non-specific serine/threonine protein kinase
augustus-7-processed-gene-285.47-mRNA-1	NA	NA	Kinase-TM	Os07g0678600	OsR498G0714955800.01.T01	CBL-interacting protein kinase
augustus-9-processed-gene-126.25-mRNA-1	NA	NA	Kinase-TM			Wall-associated receptor kinase 2
augustus_masked-1-processed-gene-58.86-mRNA-1	NA	NA	Kinase-TM	Os01g0206700	OsR498G0100377600.01.T01	Non-specific serine/threonine protein kinase
augustus_masked-10-processed-gene-148.16-mRNA-1	NA	NA	Kinase-TM	Os04g0103500	OsR498G0407421200.01.T01	G-type lectin S-receptor-like serine/threonine-protein kinase
augustus_masked-10-processed-gene-17.31-mRNA-1	NA	Japonica	Kinase-TM	Os10g0123300	OsR498G1018069800.01.T01	Mitogen-activated protein kinase
augustus_masked-10-processed-gene-36.28-mRNA-1	NA	NA	Kinase-TM	Os10g0154500	OsR498G1018162400.01.T01	Cyclin-dependent kinase-like 2
augustus_masked-12-processed-gene-139.29-mRNA-1	NA	NA	Kinase-TM	Os12g0427000		Cyclin-dependent kinase
augustus_masked-12-processed-gene-199.30-mRNA-1	NA	NA	Kinase-TM	Os12g0500400		Lectin receptor kinase-like protein
augustus_masked-12-processed-gene-257.18-mRNA-1	NA	Japonica	Kinase-TM	Os12g0603700	OsR498G0306613200.01.T01	Non-specific serine/threonine protein kinase
augustus_masked-12-processed-gene-260.9-mRNA-1	NA	Indica	Kinase-TM	Os12g0608500	OsR498G1222075300.01.T01	L-type lectin-domain containing receptor kinase IX.1
augustus_masked-3-processed-gene-242.7-mRNA-1	NA	NA	Kinase-TM	Os03g0269300	OsR498G0305649000.01.T01	Protein kinase domain-containing protein (Fragment)
augustus_masked-3-processed-gene-318.0-mRNA-1	NA	NA	Kinase-TM	Os10g0342300	OsR498G0408563300.01.T01	Non-specific serine/threonine protein kinase (Fragment)
augustus_masked-3-processed-gene-502.39-mRNA-1	NA	NA	Kinase-TM	Os12g0427000		Cyclin-dependent kinase
augustus_masked-3-processed-gene-63.45-mRNA-1	NA	NA	Kinase-TM	Os03g0225100	OsR498G0305485800.01.T01	Mitogen-activated protein kinase kinase
augustus_masked-3-processed-gene-94.36-mRNA-1	NA	Japonica	Kinase-TM	Os03g0281500	OsR498G0305699500.01.T01	Receptor-like protein kinase HERK 1
augustus_masked-4-processed-gene-154.33-mRNA-1	NA	Japonica	Kinase-TM	Os04g0420900	OsR498G0408563300.01.T01	Non-specific serine/threonine protein kinase (Fragment)
augustus_masked-4-processed-gene-261.39-mRNA-1	NA	NA	Kinase-TM	Os04g0619600	OsR498G0409294000.01.T01	Mitogen-activated protein kinase kinase kinase
augustus_masked-5-processed-gene-126.11-mRNA-1	NA	Japonica	Kinase-TM	Os05g0317700	OsR498G0510480600.01.T01	Receptor-like protein kinase FERONIA
augustus_masked-5-processed-gene-126.9-mRNA-1	NA	Japonica	Kinase-TM	Os05g0317700	OsR498G0510480600.01.T01	Receptor-like protein kinase FERONIA
augustus_masked-5-processed-gene-151.19-mRNA-1	NA	Japonica	Kinase-TM	Os05g0317700	OsR498G0510480600.01.T01	Receptor-like protein kinase FERONIA
augustus_masked-5-processed-gene-76.11-mRNA-1	NA	NA	Kinase-TM	Os05g0224700	OsR498G0510060100.01.T01	Non-specific serine/threonine protein kinase
augustus_masked-6-processed-gene-100.26-mRNA-1	NA	NA	Kinase-TM	Os06g0285400		Clade XIV lectin receptor kinase
augustus_masked-6-processed-gene-252.22-mRNA-1	NA	NA	Kinase-TM	Os06g0625200		Proline-rich receptor-like protein kinase PERK14
augustus_masked-6-processed-gene-252.24-mRNA-1	NA	NA	Kinase-TM	Os06g0625300	OsR498G0612980700.01.T01	Proline-rich receptor-like protein kinase PERK9 (Fragment)
augustus_masked-6-processed-gene-295.37-mRNA-1	NA	NA	Kinase-TM	Os06g0699400	OsR498G0613249300.01.T03	Mitogen-activated protein kinase
augustus_masked-6-processed-gene-299.24-mRNA-1	NA	NA	Kinase-TM	Os02g0623600	OsR498G0204344200.01.T02	Wall-associated receptor kinase 3
augustus_masked-6-processed-gene-34.43-mRNA-1	NA	NA	Kinase-TM	Os06g0164900		Non-specific serine/threonine protein kinase (Fragment)
augustus_masked-8-processed-gene-109.9-mRNA-1	NA	NA	Kinase-TM	Os08g0275200	OsR498G0815613300.01.T01	Receptor-like protein kinase
augustus_masked-9-processed-gene-151.10-mRNA-1	NA	NA	Kinase-TM	Os09g0418500	OsR498G0917465900.01.T01	Non-specific serine/threonine protein kinase
augustus_masked-9-processed-gene-180.27-mRNA-1	NA	NA	Kinase-TM			Wall-associated receptor kinase
augustus_masked-9-processed-gene-55.25-mRNA-1	NA	Indica	Kinase-TM	Os07g0283125	OsR498G0916878900.01.T01	Lectin-domain containing receptor kinase A4.2
augustus_masked-9-processed-gene-55.39-mRNA-1	NA	Indica	Kinase-TM	Os07g0130600	OsR498G0916878800.01.T01	L-type lectin-domain containing receptor kinase IV.2
maker-1-augustus-gene-0.28-mRNA-1	NA	NA	Kinase-TM	Os01g0136800	OsR498G0100126800.01.T01	WAK2-OsWAK receptor-like cytoplasmic kinase (OsWAK-RLCK)
maker-1-augustus-gene-19.21-mRNA-1	NA	NA	Kinase-TM	Os01g0136800	OsR498G0100126800.01.T01	WAK2-OsWAK receptor-like cytoplasmic kinase (OsWAK-RLCK)
maker-1-augustus-gene-20.9-mRNA-1	NA	NA	Kinase-TM			Protein kinase domain-containing protein (Fragment)
maker-1-augustus-gene-201.9-mRNA-1	NA	NA	Kinase-TM	0	OsR498G0101184200.01.T02	Cysteine-rich receptor-like protein kinase 10
maker-1-augustus-gene-202.40-mRNA-1	NA	NA	Kinase-TM			Non-specific serine/threonine protein kinase
maker-1-augustus-gene-210.0-mRNA-1	NA	NA	Kinase-TM	0	OsR498G0101254500.01.T02	Proline-rich receptor-like protein kinase PERK8
maker-1-augustus-gene-211.43-mRNA-1	NA	NA	Kinase-TM	0	OsR498G0101271400.01.T01	Cysteine-rich receptor-like protein kinase
maker-1-augustus-gene-244.0-mRNA-1	NA	NA	Kinase-TM	0	OsR498G0101485500.01.T05	Kinase protein with adenine nucleotide alpha hydrolases-like domain isoform 1

maker-1-augustus-gene-254.15-mRNA-1	NA	NA	Kinase-TM	Os01g0621600	OsR498G0101562800.01.T02	Serine/threonine-protein kinase MARK2
maker-1-augustus-gene-255.29-mRNA-1	NA	Indica	Kinase-TM	Os05g0585500	OsR498G0101565900.01.T01	Calcium-dependent protein kinase 1 isoform A
maker-1-augustus-gene-264.49-mRNA-1	NA	NA	Kinase-TM	Os01g0641000	OsR498G0101624900.01.T01	Serine/threonine-protein kinase STY8
maker-1-augustus-gene-265.37-mRNA-1	NA	NA	Kinase-TM			Non-specific serine/threonine protein kinase
maker-1-augustus-gene-266.5-mRNA-1	NA	NA	Kinase-TM			Mitogen-activated protein kinase
maker-1-augustus-gene-278.40-mRNA-1	NA	NA	Kinase-TM	Os01g0665200	OsR498G0101709700.01.T01	Mitogen-activated protein kinase
maker-1-augustus-gene-292.1-mRNA-1	NA	NA	Kinase-TM	Os01g0690800	OsR498G0101800500.01.T01	Protein kinase domain-containing protein (Fragment)
maker-1-augustus-gene-374.48-mRNA-1	NA	Indica	Kinase-TM	Os03g0825800	OsR498G0102345600.01.T02	Serine/threonine protein kinase
maker-1-augustus-gene-384.34-mRNA-1	NA	Indica	Kinase-TM	Os01g0889900	OsR498G0102414800.01.T01	Receptor-like serine/threonine-protein kinase
maker-1-augustus-gene-384.35-mRNA-1	NA	NA	Kinase-TM	0	OsR498G0102415600.01.T01	Non-specific serine/threonine protein kinase (Fragment)
maker-1-augustus-gene-390.20-mRNA-1	NA	NA	Kinase-TM	0	OsR498G0102460400.01.T03	probable serine/threonine-protein kinase PBL16
maker-1-augustus-gene-394.0-mRNA-1	NA	Indica	Kinase-TM	Os01g0890600	OsR498G0102489700.01.T01	Non-specific serine/threonine protein kinase (Fragment)
maker-1-augustus-gene-394.19-mRNA-1	NA	NA	Kinase-TM	Os01g0889900	OsR498G0102488800.01.T01	Receptor-like serine/threonine-protein kinase
maker-1-augustus-gene-394.3-mRNA-1	NA	Indica	Kinase-TM	Os01g0890600	OsR498G0102491100.01.T02	Receptor-like serine/threonine-protein kinase
maker-1-augustus-gene-398.16-mRNA-1	NA	NA	Kinase-TM	Os01g0899000	OsR498G0102515200.01.T03	Tyrosine-protein kinase
maker-1-augustus-gene-404.31-mRNA-1	NA	NA	Kinase-TM	Os01g0621600	OsR498G0101562800.01.T02	Serine/threonine-protein kinase MARK2
maker-1-augustus-gene-414.47-mRNA-1	NA	NA	Kinase-TM	0	OsR498G0101485500.01.T05	Kinase protein with adenine nucleotide alpha hydrolases-like domain isoform 1
maker-1-augustus-gene-415.56-mRNA-1	NA	Indica	Kinase-TM	Os01g0601200	OsR498G0101477700.01.T01	Putative serine/threonine-protein kinase
maker-1-augustus-gene-425.19-mRNA-1	NA	Indica	Kinase-TM	Os03g0825800	OsR498G0102345600.01.T02	Serine/threonine protein kinase
maker-1-augustus-gene-435.37-mRNA-1	NA	Indica	Kinase-TM	Os01g0889900	OsR498G0102414800.01.T01	Receptor-like serine/threonine-protein kinase
maker-1-augustus-gene-435.39-mRNA-1	NA	NA	Kinase-TM	0	OsR498G0102415600.01.T01	Non-specific serine/threonine protein kinase (Fragment)
maker-1-augustus-gene-441.74-mRNA-1	NA	NA	Kinase-TM	0	OsR498G0102460400.01.T03	probable serine/threonine-protein kinase PBL16
maker-1-augustus-gene-445.24-mRNA-1	NA	NA	Kinase-TM	Os01g0889900	OsR498G0102488800.01.T01	Receptor-like serine/threonine-protein kinase
maker-1-augustus-gene-445.6-mRNA-1	NA	Indica	Kinase-TM	Os01g0890600	OsR498G0102489700.01.T01	Non-specific serine/threonine protein kinase (Fragment)
maker-1-augustus-gene-445.9-mRNA-1	NA	Indica	Kinase-TM	Os01g0890600	OsR498G0102491100.01.T02	Receptor-like serine/threonine-protein kinase
maker-1-augustus-gene-449.48-mRNA-1	NA	NA	Kinase-TM	Os01g0899000	OsR498G0102515200.01.T03	Tyrosine-protein kinase
maker-1-augustus-gene-455.37-mRNA-1	NA	NA	Kinase-TM	Os01g0621600	OsR498G0101562800.01.T02	Serine/threonine-protein kinase MARK2
maker-1-augustus-gene-465.17-mRNA-1	NA	NA	Kinase-TM	0	OsR498G0101485500.01.T05	Kinase protein with adenine nucleotide alpha hydrolases-like domain isoform 1
maker-1-augustus-gene-466.47-mRNA-1	NA	Indica	Kinase-TM	Os01g0601200	OsR498G0101477700.01.T01	Putative serine/threonine-protein kinase
maker-1-augustus-gene-47.42-mRNA-1	NA	NA	Kinase-TM	Os01g0186700	OsR498G0100292200.01.T03	AGC (cAMP-dependent cGMP-dependent and protein kinase C) kinase family protein
maker-1-augustus-gene-51.18-mRNA-1	NA	NA	Kinase-TM	0	OsR498G0100334000.01.T02	Protein kinase domain-containing protein
maker-1-augustus-gene-55.3-mRNA-1	NA	Indica	Kinase-TM	Os08g0112500	OsR498G0100356100.01.T01	probable serine/threonine-protein kinase At1g54610
maker-1-augustus-gene-58.31-mRNA-1	NA	Indica	Kinase-TM	Os05g0207500	OsR498G0100374600.01.T04	Non-specific serine/threonine protein kinase
maker-1-augustus-gene-82.9-mRNA-1	NA	NA	Kinase-TM	Os01g0247500	OsR498G0100545400.01.T01	receptor-like serine/threonine-protein kinase At1g78530
maker-1-augustus-gene-84.39-mRNA-1	NA	NA	Kinase-TM	Os01g0252100	OsR498G0100559700.01.T07	Non-specific serine/threonine protein kinase
maker-1-augustus-gene-84.72-mRNA-1	NA	NA	Kinase-TM	Os01g0253100	OsR498G0100562800.01.T03	proline-rich receptor-like protein kinase PERK3
maker-1-augustus-gene-87.33-mRNA-1	NA	NA	Kinase-TM	Os01g0259200	OsR498G0100587300.01.T01	Serine/threonine-protein kinase PBL27
maker-1-augustus-gene-87.52-mRNA-1	NA	NA	Kinase-TM	Os01g0259400	OsR498G0100587800.01.T01	Serine/threonine-protein kinase RUNKEL
maker-10-augustus-gene-127.40-mRNA-1	NA	NA	Kinase-TM	Os04g0140400	OsR498G0407534800.01.T01	Serine/threonine-protein kinase
maker-10-augustus-gene-127.41-mRNA-1	NA	NA	Kinase-TM	Os04g0125700	OsR498G0407534800.01.T01	Non-specific serine/threonine protein kinase
maker-10-augustus-gene-127.48-mRNA-1	NA	NA	Kinase-TM	Os04g0141200	OsR498G0407536900.01.T01	Receptor-like serine/threonine-protein kinase
maker-10-augustus-gene-132.28-mRNA-1	NA	NA	Kinase-TM	Os04g0125700	OsR498G0407520700.01.T01	Agglutinin-2
maker-10-augustus-gene-145.10-mRNA-1	NA	NA	Kinase-TM	Os04g0109100	OsR498G0407442600.01.T01	Non-specific serine/threonine protein kinase
maker-10-augustus-gene-56.31-mRNA-1	NA	Indica	Kinase-TM	Os10g0180800	OsR498G1018272600.01.T01	Calcium binding EGF domain containing protein, expressed
maker-11-augustus-gene-123.43-mRNA-1	NA	NA	Kinase-TM	0	OsR498G1120059900.01.T01	probable serine/threonine-protein kinase PBL28
maker-11-augustus-gene-230.58-mRNA-1	NA	Indica	Kinase-TM	Os06g0693200	OsR498G1120627100.01.T01	Protein kinase domain containing protein, expressed
maker-11-augustus-gene-256.34-mRNA-1	NA	NA	Kinase-TM	Os11g0695850	OsR498G1120743300.01.T02	Wall-associated receptor kinase
maker-11-augustus-gene-256.48-mRNA-1	NA	NA	Kinase-TM	Os11g0691240	OsR498G1120759300.01.T01	Wall associated kinase-like
maker-11-augustus-gene-75.2-mRNA-1	NA	NA	Kinase-TM	0	OsR498G1119613600.01.T01	Coatomer beta subunit, putative
maker-12-augustus-gene-1.5-mRNA-1	NA	NA	Kinase-TM			Nonphototropic hypocotyl 1
maker-12-augustus-gene-147.6-mRNA-1	NA	NA	Kinase-TM	Os12g0433500	OsR498G1221451200.01.T03	serine/threonine-protein kinase TIO
maker-12-augustus-gene-241.74-mRNA-1	NA	Indica	Kinase-TM	Os02g0559300	OsR498G1221932600.01.T02	Protein kinase domain containing protein, expressed
maker-12-augustus-gene-247.13-mRNA-1	NA	NA	Kinase-TM	Os12g0586100	OsR498G1221987400.01.T01	Serine/threonine-protein kinase SAPK10
maker-12-augustus-gene-252.9-mRNA-1	NA	NA	Kinase-TM	Os12g0594300	OsR498G1222027100.01.T01	Serine/threonine-protein kinase CTR1
maker-12-augustus-gene-259.2-mRNA-1	NA	NA	Kinase-TM	Os12g0606000		Lectin protein kinase, putative, expressed
maker-12-augustus-gene-260.56-mRNA-1	NA	Indica	Kinase-TM	Os12g0608500	OsR498G1222073900.01.T01	Protein kinase-like resistance protein (Fragment)
maker-12-augustus-gene-281.15-mRNA-1	NA	NA	Kinase-TM	Os07g0114400	OsR498G0713403200.01.T01	CK2 protein kinase alpha 4
maker-12-augustus-gene-283.35-mRNA-1	NA	NA	Kinase-TM	Os04g0560600	OsR498G0409068000.01.T01	Calcium-dependent protein kinase
maker-12-augustus-gene-284.41-mRNA-1	NA	NA	Kinase-TM			Mitogen-activated protein kinase pmk-2 (inferred by orthology to a C. elegans protein)
maker-12-augustus-gene-285.20-mRNA-1	NA	NA	Kinase-TM	Os04g0563900	OsR498G0409078000.01.T02	probable serine/threonine-protein kinase PIX13
maker-12-augustus-gene-288.123-mRNA-1	NA	NA	Kinase-TM	Os12g0606000		Lectin protein kinase, putative, expressed
maker-12-augustus-gene-288.90-mRNA-1	NA	NA	Kinase-TM	Os12g0606000		Lectin protein kinase, putative, expressed
maker-12-augustus-gene-43.8-mRNA-1	NA	NA	Kinase-TM	0	OsR498G1221046200.01.T01	Protein kinase protein with adenine nucleotide alpha hydrolase-like domain
maker-12-augustus-gene-43.9-mRNA-1	NA	NA	Kinase-TM	0	OsR498G1221046200.01.T01	Proline-rich receptor-like protein kinase PERK13
maker-12-augustus-gene-5.18-mRNA-1	NA	NA	Kinase-TM	Os12g0108100	OsR498G1220806200.01.T01	Putative serine/threonine-protein kinase
maker-2-augustus-gene-10.86-mRNA-1	NA	NA	Kinase-TM	Os02g0120100	OsR498G0202861000.01.T04	Serine/threonine-protein kinase STY46
maker-2-augustus-gene-107.44-mRNA-1	NA	NA	Kinase-TM	0	OsR498G0203430400.01.T02	serine/threonine-protein kinase RHS3
maker-2-augustus-gene-197.16-mRNA-1	NA	NA	Kinase-TM	0	OsR498G0203979600.01.T05	Mitogen activated kinase kinase kinase CTR1
maker-2-augustus-gene-234.7-mRNA-1	NA	NA	Kinase-TM	Os02g0594100	OsR498G0204225300.01.T02	serine/threonine-protein kinase STY13
maker-2-augustus-gene-247.8-mRNA-1	NA	Indica	Kinase-TM	Os02g0615500	OsR498G0204301900.01.T02	Putative LRR receptor-like serine/threonine-protein kinase
maker-2-augustus-gene-262.27-mRNA-1	NA	Indica	Kinase-TM	Os02g0633066	OsR498G0204379000.01.T01	Wall-associated receptor kinase 2-like
maker-2-augustus-gene-262.30-mRNA-1	NA	NA	Kinase-TM			Wall-associated receptor kinase 4
maker-2-augustus-gene-296.6-mRNA-1	NA	NA	Kinase-TM	Os02g0700600	OsR498G0204600900.01.T03	Serine/threonine-protein kinase MHK
maker-2-augustus-gene-326.33-mRNA-1	NA	NA	Kinase-TM	Os06g0676600	OsR498G0613170800.01.T03	Serine/threonine protein kinase Stpk-D
maker-2-augustus-gene-333.46-mRNA-1	NA	NA	Kinase-TM	Os06g0663400	OsR498G0613118900.01.T01	serine/threonine-protein kinase STY13
maker-2-augustus-gene-344.16-mRNA-1	NA	NA	Kinase-TM	Os06g0654500	OsR498G0613085100.01.T01	Concanavalin A-like lectin/glucanase domain, Serine/threonine-protein kinase, SIK1/2
maker-2-augustus-gene-348.34-mRNA-1	NA	NA	Kinase-TM	0	OsR498G0613057100.01.T01	Trithorax group protein osa-like
maker-2-augustus-gene-35.20-mRNA-1	NA	NA	Kinase-TM	Os02g0165100	OsR498G0203030300.01.T07	Serine/threonine protein kinase Stpk-B
maker-2-augustus-gene-383.0-mRNA-1	NA	Japonica	Kinase-TM	Os02g0811200	OsR498G0204979300.01.T01	Wall-associated receptor kinase 5
maker-2-augustus-gene-385.38-mRNA-1	NA	NA	Kinase-TM	Os02g0815900	OsR498G0205003400.01.T01	Receptor-like serine/threonine-protein kinase ALE2 isoform A
maker-2-augustus-gene-4.19-mRNA-1	NA	NA	Kinase-TM	Os02g0106900	OsR498G0202819600.01.T01	Senescence-induced receptor-like serine/threonine-protein kinase
maker-2-augustus-gene-46.50-mRNA-1	NA	NA	Kinase-TM	0	OsR498G0203101600.01.T02	Phytosulfokine receptor
maker-2-augustus-gene-73.17-mRNA-1	NA	NA	Kinase-TM	0	OsR498G0203266600.01.T01	Receptor-like protein kinase HSL1
maker-3-augustus-gene-122.0-mRNA-1	NA	NA	Kinase-TM	Os10g0562500	OsR498G1019201900.01.T03	Serine/threonine-protein kinase D6PKL2
maker-3-augustus-gene-122.11-mRNA-1	NA	NA	Kinase-TM	Os10g0563500	OsR498G1019206100.01.T02	Serine/threonine-protein kinase cdc7
maker-3-augustus-gene-123.59-mRNA-1	NA	NA	Kinase-TM	Os10g0564500	OsR498G1019209600.01.T02	Serinethreonine-protein kinase sapk3
maker-3-augustus-gene-157.53-mRNA-1	NA	NA	Kinase-TM	Os03g0339900	OsR498G0305910600.01.T02	Non-specific serine/threonine protein kinase
maker-3-augustus-gene-205.34-mRNA-1	NA	Japonica	Kinase-TM	Os03g0407900	OsR498G0306167000.01.T01	Serine/threonine-protein kinase RIPK
maker-3-augustus-gene-258.11-mRNA-1	NA	Indica	Kinase-TM	Os03g0610900	OsR498G0306549800.01.T02	Serine/threonine-protein kinase SAPK10
maker-3-augustus-gene-301.27-mRNA-1	NA	NA	Kinase-TM			Serinethreonine-protein kinase edr1
maker-3-augustus-gene-311.6-mRNA-1	NA	NA	Kinase-TM	Os10g0351500	OsR498G1018522700.01.T01	Tyrosine-protein kinase
maker-3-augustus-gene-327.30-mRNA-1	NA	NA	Kinase-TM	Os10g0327000	OsR498G1018449400.01.T02	Receptor-like serine/threonine-protein kinase
maker-3-augustus-gene-351.20-mRNA-1	NA	NA	Kinase-TM	Os03g0269300	OsR498G0305649000.01.T01	Proline-rich receptor-like protein kinase PERK3
maker-3-augustus-gene-367.9-mRNA-1	NA	Indica	Kinase-TM	Os03g0610900	OsR498G0306549800.01.T02	Serine/threonine-protein kinase SAPK10
maker-3-augustus-gene-444.40-mRNA-1	NA	Indica	Kinase-TM	Os03g0745700	OsR498G0307037500.01.T01	Serinethreonine-protein kinase sty17
maker-3-augustus-gene-5.25-mRNA-1	NA	NA	Kinase-TM	Os03g0114300	OsR498G0305114700.01.T01	Serine/threonine-protein kinase fray2
maker-3-augustus-gene-81.19-mRNA-1	NA	NA	Kinase-TM	0	OsR498G0305607600.01.T01	Putative receptor-type protein kinase LRK1
maker-3-augustus-gene-95.56-mRNA-1	NA	NA	Kinase-TM	Os03g0283900	OsR498G0305709600.01.T01	Serine/threonine-protein kinase RLCKVII
maker-4-augustus-gene-122.31-mRNA-1	NA	NA	Kinase-TM	Os04g0366000	OsR498G0408325600.01.T01	Wall-associated receptor kinase 1
maker-4-augustus-gene-124.35-mRNA-1	NA	NA	Kinase-TM	Os04g0368000	OsR498G0408337400.01.T01	Wall-associated receptor kinase 2
maker-4-augustus-gene-126.0-mRNA-1	NA	NA	Kinase-TM			Protein kinase domain-containing protein (Fragment)
maker-4-augustus-gene-179.56-mRNA-1	NA	Japonica	Kinase-TM	Os04g0465900	OsR498G0408725800.01.T01	Serine/threonine protein kinase
maker-4-augustus-gene-190.22-mRNA-1	NA	NA	Kinase-TM	Os04g0488000	OsR498G0408798300.01.T05	Cyclin-dependent kinase G-2
maker-4-augustus-gene-201.39-mRNA-1	NA	NA	Kinase-TM	0	OsR498G0408871500.01.T01	Receptor-like serinethreonine-protein kinase ale2
maker-4-augustus-gene-205.22-mRNA-1	NA	Indica	Kinase-TM	Os01g0364800	OsR498G0408902600.01.T01	Wall-associated receptor kinase-like 8
maker-4-augustus-gene-217.46-mRNA-1	NA	NA	Kinase-TM	Os04g0540900	OsR498G0408995400.01.T01	Leucine-rich repeat receptor protein kinase EXS
maker-4-augustus-gene-22.52-mRNA-1	NA	Japonica	Kinase-TM	Os04g0201900	OsR498G0407717600.01.T01	Non-specific serine/threonine protein kinase
maker-4-augustus-gene-227.38-mRNA-1	NA	NA	Kinase-TM	Os04g0560600	OsR498G0409068000.01.T01	Calcium-dependent protein kinase
maker-4-augustus-gene-229.42-mRNA-1	NA	NA	Kinase-TM			Protein kinase domain-containing protein
maker-4-augustus-gene-229.58-mRNA-1	NA	NA	Kinase-TM	Os04g0563900	OsR498G0409078000.01.T01	probable serine/threonine-protein kinase PIX13
maker-4-augustus-gene-250.43-mRNA-1	NA	NA	Kinase-TM	Os04g0602800	OsR498G0409227400.01.T04	Serine/threonine-protein kinase minibrain-like
maker-4-augustus-gene-255.7-mRNA-1	NA	NA	Kinase-TM	Os04g0608900	OsR498G0409252600.01.T01	serine/threonine-protein kinase STY13
maker-4-augustus-gene-268.10-mRNA-1	NA	NA	Kinase-TM	Os04g0632300		Non-specific serine/threonine protein kinase
maker-4-augustus-gene-268.13-mRNA-1	NA	NA	Kinase-TM	Os04g0632500	OsR498G0409334400.01.T03	Serine/threonine-protein kinase
maker-4-augustus-gene-268.16-mRNA-1	NA	Indica	Kinase-TM	Os04g0632600	OsR498G0409334600.01.T02	Non-specific serine/threonine protein kinase
maker-4-augustus-gene-268.22-mRNA-1	NA	Indica	Kinase-TM	Os04g0632700	OsR498G0409334800.01.T01	Serine/threonine-protein kinase
maker-4-augustus-gene-268.31-mRNA-1	NA	NA	Kinase-TM	Os04g0633300	OsR498G0409336200.01.T01	Non-specific serine/threonine protein kinase
maker-4-augustus-gene-268.36-mRNA-1	NA	NA	Kinase-TM	Os04g0633900	OsR498G0409338200.01.T01	Serine/threonine-protein kinase
maker-4-augustus-gene-268.39-mRNA-1	NA	Japonica	Kinase-TM	Os04g0634000	OsR498G0409338400.01.T01	G-type lectin S-receptor-like serine/threonine-protein kinase
maker-4-augustus-gene-268.42-mRNA-1	NA	NA	Kinase-TM	Os04g0634400	OsR498G0409339000.01.T02	Non-specific serine/threonine protein kinase
maker-4-augustus-gene-268.7-mRNA-1	NA	NA	Kinase-TM	Os04g0632100	OsR498G0409332800.01.T04	Non-specific serine/threonine protein kinase
maker-4-augustus-gene-296.20-mRNA-1	NA	NA	Kinase-TM	0	OsR498G0409524800.01.T04	Serine/threonine-protein kinase STY46
maker-4-augustus-gene-298.34-mRNA-1	NA	NA	Kinase-TM	Os04g0689400	OsR498G0409533700.01.T01	Serine/threonine protein kinase
maker-4-augustus-gene-301.31-mRNA-1	NA	NA	Kinase-TM	Os04g0689400	OsR498G0409533700.01.T02	Receptor-like protein kinase
maker-4-augustus-gene-66.41-mRNA-1	NA	NA	Kinase-TM	Os04g0286000		Calcium binding EGF domain containing protein, expressed
maker-5-augustus-gene-1.41-mRNA-1	NA	Indica	Kinase-TM	Os01g0110500	OsR498G0509557200.01.T07	Proline-rich receptor-like protein kinase PERK8
maker-5-augustus-gene-154.40-mRNA-1	NA	Indica	Kinase-TM	Os05g0323800	OsR498G0510496300.01.T04	protein ACTIVITY OF BC1 COMPLEX KINASE 3, chloroplastic
maker-5-augustus-gene-174.10-mRNA-1	NA	Indica	Kinase-TM	Os05g0353500	OsR498G0510613800.01.T01	Protein MALE DISCOVERER 2
maker-5-augustus-gene-20.28-mRNA-1	NA	Japonica	Kinase-TM	Os05g0135100	OsR498G0509684800.01.T02	Wall-associated receptor kinase 3
maker-5-augustus-gene-224.57-mRNA-1	NA	NA	Kinase-TM	Os05g0436100	OsR498G0510914400.01.T01	probable serine/threonine-protein kinase At1g01540
maker-5-augustus-gene-247.0-mRNA-1	NA	NA	Kinase-TM	0	OsR498G0511051300.01.T01	Serine/threonine-protein kinase STN8 chloroplastic
maker-5-augustus-gene-274.36-mRNA-1	NA	NA	Kinase-TM	Os05g0530500	OsR498G0511231600.01.T04	Non-specific serine/threonine protein kinase
maker-5-augustus-gene-281.24-mRNA-1	NA	NA	Kinase-TM	Os05g0545300	OsR498G0511282400.01.T01	Mitogen-activated protein kinase kinase kinase YODA
maker-5-augustus-gene-283.37-mRNA-1	NA	NA	Kinase-TM	Os05g0549100	OsR498G0511289600.01.T01	Serine/threonine-protein kinase SNT7
maker-5-augustus-gene-284.29-mRNA-1	NA	NA	Kinase-TM	0	OsR498G0511296500.01.T02	Wall-associated kinase
maker-5-augustus-gene-284.47-mRNA-1	NA	NA	Kinase-TM			Receptor serine/threonine kinase PR5K-like
maker-5-augustus-gene-289.59-mRNA-1	NA	NA	Kinase-TM	Os05g0560300	OsR498G0511334000.01.T02	Protein MALE DISCOVERER 1
maker-5-augustus-gene-310.11-mRNA-1	NA	NA	Kinase-TM	Os08g0365800		Putative WAK family receptor-like protein kinase
maker-5-augustus-gene-6.31-mRNA-1	NA	NA	Kinase-TM	Os05g0110900	OsR498G0509586800.01.T01	Receptor-like cytoplasmic kinase 176
maker-6-augustus-gene-104.6-mRNA-1	NA	NA	Kinase-TM	Os06g0291600	OsR498G0612170400.01.T02	serine/threonine-protein kinase D6PK
maker-6-augustus-gene-14.31-mRNA-1	NA	NA	Kinase-TM	0	OsR498G0611553400.01.T01	Calcium-dependent protein kinase, putative
maker-6-augustus-gene-206.0-mRNA-1	NA	NA	Kinase-TM	Os06g0547900	OsR498G0612721100.01.T02	Non-specific serine/threonine protein kinase
maker-6-augustus-gene-22.3-mRNA-1	NA	NA	Kinase-TM	Os06g0142500		Wall-associated receptor kinase 3
maker-6-augustus-gene-24.61-mRNA-1	NA	NA	Kinase-TM	0	OsR498G0611626200.01.T05	Mitogen-activated protein kinase kinase kinase
maker-6-augustus-gene-254.0-mRNA-1	NA	Japonica	Kinase-TM	Os06g0620200	OsR498G0612965100.01.T01	Non-specific serine/threonine protein kinase
maker-6-augustus-gene-266.37-mRNA-1	NA	NA	Kinase-TM	0	OsR498G0613057100.01.T01	Trithorax group protein osa-like
maker-6-augustus-gene-27.39-mRNA-1	NA	NA	Kinase-TM	Os06g0151700	OsR498G0611637200.01.T01	Protein kinase domain containing protein, expressed
maker-6-augustus-gene-270.4-mRNA-1	NA	NA	Kinase-TM	Os06g0654500	OsR498G0613085100.01.T01	Concanavalin A-like lectin/glucanase domain, Serine/threonine-protein kinase, SIK1/2

maker-6-augustus-gene-275.0-mRNA-1	NA	NA	Kinase-TM	Os06g0663400	OsR498G0613118900.01.T01	serine/threonine-protein kinase STY13
maker-6-augustus-gene-282.18-mRNA-1	NA	NA	Kinase-TM	Os06g0676600	OsR498G0613170800.01.T03	Putative leucine-rich repeat receptor-like serine/threonine-protein kinase
maker-6-augustus-gene-308.0-mRNA-1	NA	NA	Kinase-TM	Os06g0724900	OsR498G0613327700.01.T03	Serinethreonine-protein kinase sty46
maker-6-augustus-gene-34.7-mRNA-1	NA	NA	Kinase-TM	Os06g0166900	OsR498G0611684100.01.T01	Mitogen-activated protein kinase kinase kinase
maker-6-augustus-gene-35.67-mRNA-1	NA	NA	Kinase-TM	Os06g0168800	OsR498G0611688600.01.T01	Mitogen-activated protein kinase kinase kinase
maker-6-augustus-gene-36.12-mRNA-1	NA	NA	Kinase-TM	Os06g0170250		Putative WAK family receptor-like protein kinase
maker-6-augustus-gene-37.1-mRNA-1	NA	NA	Kinase-TM	Os06g0170250		Putative WAK family receptor-like protein kinase
maker-6-augustus-gene-40.43-mRNA-1	NA	NA	Kinase-TM	0	OsR498G0611724300.01.T05	Serine/threonine-protein kinase
maker-6-augustus-gene-81.47-mRNA-1	NA	Indica	Kinase-TM	Os10g0441900	OsR498G0612016600.01.T01	Putative receptor kinase Lecrk
maker-6-augustus-gene-9.3-mRNA-1	NA	NA	Kinase-TM	Os06g0116100	OsR498G0611512100.01.T03	GAMYB-binding protein
maker-6-augustus-gene-98.2-mRNA-1	NA	NA	Kinase-TM	Os06g0283300	OsR498G0612142700.01.T02	probable leucine-rich repeat receptor-like serine/threonine-protein kinase At5g15730 isoform X1
maker-7-augustus-gene-12.54-mRNA-1	NA	NA	Kinase-TM	Os07g0131000		L-type lectin-domain containing receptor kinase IV.1
maker-7-augustus-gene-15.66-mRNA-1	NA	Japonica	Kinase-TM	Os07g0137800	OsR498G0713481800.01.T01	Proline-rich receptor-like protein kinase PERK4
maker-7-augustus-gene-164.31-mRNA-1	NA	NA	Kinase-TM			Serine/threonine-protein kinase SRPK
maker-7-augustus-gene-167.38-mRNA-1	NA	Indica	Kinase-TM	Os07g0472400	OsR498G0714275700.01.T01	probable serine/threonine-protein kinase sky1
maker-7-augustus-gene-17.67-mRNA-1	NA	NA	Kinase-TM	Os06g0241100	OsR498G0713492600.01.T02	G-type lectin S-receptor-like serine/threonine-protein kinase
maker-7-augustus-gene-189.51-mRNA-1	NA	NA	Kinase-TM	Os07g0507300	OsR498G0714392600.01.T04	germinal center kinase 1 isoform X3
maker-7-augustus-gene-206.0-mRNA-1	NA	Indica	Kinase-TM	Os07g0535800	OsR498G0714500300.01.T06	Putative serine/threonine-specific protein kinase
maker-7-augustus-gene-206.10-mRNA-1	NA	Indica	Kinase-TM	Os07g0628700	OsR498G0714502100.01.T01	Cysteine-rich receptor-like protein kinase 8
maker-7-augustus-gene-206.4-mRNA-1	NA	NA	Kinase-TM	Os07g0537000	OsR498G0714501700.01.T01	Receptor-like serine-threonine protein kinase
maker-7-augustus-gene-206.5-mRNA-1	NA	NA	Kinase-TM			G-type lectin S-receptor-like serine/threonine-protein kinase
maker-7-augustus-gene-207.50-mRNA-1	NA	NA	Kinase-TM	0	OsR498G0714502900.01.T01	Cysteine-rich receptor-like protein kinase 8
maker-7-augustus-gene-207.52-mRNA-1	NA	NA	Kinase-TM	Os07g0537900	OsR498G0714503300.01.T01	Cysteine-rich receptor-like protein kinase 8
maker-7-augustus-gene-207.62-mRNA-1	NA	NA	Kinase-TM	Os07g0537500	OsR498G0714502600.01.T01	Cysteine-rich receptor-like protein kinase 6
maker-7-augustus-gene-207.68-mRNA-1	NA	NA	Kinase-TM	Os07g0538200	OsR498G0714504000.01.T01	Cysteine-rich receptor-like protein kinase 6
maker-7-augustus-gene-208.31-mRNA-1	NA	Indica	Kinase-TM	Os07g0537900	OsR498G0714510100.01.T01	Cysteine-rich receptor-like protein kinase 8
maker-7-augustus-gene-208.36-mRNA-1	NA	NA	Kinase-TM	Os07g0541000	OsR498G0714516300.01.T01	Receptor-like serine-threonine protein kinase
maker-7-augustus-gene-209.11-mRNA-1	NA	NA	Kinase-TM	Os07g0540800	OsR498G0714515800.01.T01	Receptor-like serine-threonine protein kinase
maker-7-augustus-gene-209.12-mRNA-1	NA	NA	Kinase-TM	Os07g0541000	OsR498G0714516800.01.T01	Cysteine-rich receptor-like protein kinase 10
maker-7-augustus-gene-209.26-mRNA-1	NA	NA	Kinase-TM	Os07g0541800	OsR498G0714517600.01.T05	Membrane binding protein
maker-7-augustus-gene-209.30-mRNA-1	NA	NA	Kinase-TM	Os07g0541900	OsR498G0714517800.01.T05	Cysteine-rich receptor-like protein kinase 10
maker-7-augustus-gene-210.47-mRNA-1	NA	NA	Kinase-TM	Os07g0542400	OsR498G0714519200.01.T05	Putative Zea mays retrotransposon Opie-2
maker-7-augustus-gene-22.17-mRNA-1	NA	NA	Kinase-TM	Os07g0150700	OsR498G0713521400.01.T01	Non-specific serine/threonine protein kinase
maker-7-augustus-gene-228.2-mRNA-1	NA	NA	Kinase-TM	Os07g0572800	OsR498G0714612300.01.T06	Serine/threonine-protein kinase WNK1
maker-7-augustus-gene-250.8-mRNA-1	NA	NA	Kinase-TM	Os07g0613500	OsR498G0714745300.01.T02	Serine/threonine-protein kinase RIPK
maker-7-augustus-gene-280.12-mRNA-1	NA	Indica	Kinase-TM	Os03g0349200	OsR498G0714922100.01.T01	probable serine/threonine-protein kinase At1g54610
maker-7-augustus-gene-280.16-mRNA-1	NA	NA	Kinase-TM	0	OsR498G0714923700.01.T02	Receptor-like serine/threonine-protein kinase
maker-7-augustus-gene-280.42-mRNA-1	NA	NA	Kinase-TM	Os07g0668900	OsR498G0714925200.01.T01	Serine/threonine-protein kinase PBL27
maker-7-augustus-gene-36.35-mRNA-1	NA	NA	Kinase-TM	Os07g0176600	OsR498G0713618700.01.T01	Serine/threonine-protein kinase Nek2
maker-7-augustus-gene-4.20-mRNA-1	NA	NA	Kinase-TM	Os07g0114400	OsR498G0713403200.01.T01	CK2 protein kinase alpha 4
maker-7-augustus-gene-47.32-mRNA-1	NA	NA	Kinase-TM	Os07g0194100	OsR498G0713681500.01.T01	Non-specific serine/threonine protein kinase
maker-8-augustus-gene-0.31-mRNA-1	NA	Japonica	Kinase-TM	Os08g0124900	OsR498G0815100800.01.T01	Putative receptor-type protein kinase LRK1
maker-8-augustus-gene-116.50-mRNA-1	NA	Indica	Kinase-TM	Os05g0319700	OsR498G0815646200.01.T03	Phytosulfokine receptor
maker-8-augustus-gene-138.24-mRNA-1	NA	NA	Kinase-TM	Os08g0249100	OsR498G0815539600.01.T01	probable receptor-like serine/threonine-protein kinase At5g57670
maker-8-augustus-gene-138.26-mRNA-1	NA	NA	Kinase-TM	Os08g0249100	OsR498G0815539600.01.T01	probable receptor-like serine/threonine-protein kinase At5g57670
maker-8-augustus-gene-158.8-mRNA-1	NA	NA	Kinase-TM	Os08g0343000	OsR498G0815840500.01.T03	Proline-rich receptor-like protein kinase PERK14 isoform X5
maker-8-augustus-gene-181.48-mRNA-1	NA	NA	Kinase-TM	Os08g0365800		Putative WAK family receptor-like protein kinase
maker-8-augustus-gene-190.0-mRNA-1	NA	Indica	Kinase-TM	Os08g0374600	OsR498G0815946800.01.T03	Cytokinin-regulated kinase-like
maker-8-augustus-gene-252.4-mRNA-1	NA	NA	Kinase-TM	Os08g0484600	OsR498G0816337100.01.T01	Serine/threonine protein kinase OSK4
maker-8-augustus-gene-256.55-mRNA-1	NA	Japonica	Kinase-TM	Os08g0491200	OsR498G0917686300.01.T03	Serine/threonine-protein kinase D6PKL1
maker-8-augustus-gene-261.3-mRNA-1	NA	Indica	Kinase-TM	Os08g0501700	OsR498G0816405900.01.T01	Wall-associated kinase-like
maker-8-augustus-gene-35.28-mRNA-1	NA	NA	Kinase-TM	Os08g0157000	OsR498G0815225900.01.T01	Mitogen-activated protein kinase
maker-8-augustus-gene-46.39-mRNA-1	NA	Japonica	Kinase-TM	Os08g0176200	OsR498G0815299300.01.T01	LRR receptor kinase SERK2
maker-8-augustus-gene-79.35-mRNA-1	NA	NA	Kinase-TM	Os08g0224100	OsR498G0815462500.01.T01	serine/threonine-protein kinase STY13
maker-8-augustus-gene-94.36-mRNA-1	NA	NA	Kinase-TM	Os08g0249100	OsR498G0815539600.01.T01	probable receptor-like serine/threonine-protein kinase At5g57670
maker-9-augustus-gene-104.0-mRNA-1	NA	NA	Kinase-TM			Senescence-induced receptor-like serine/threonine-protein kinase
maker-9-augustus-gene-107.12-mRNA-1	NA	Japonica	Kinase-TM	Os09g0349100	OsR498G0917214100.01.T01	Serine/threonine-specific receptor protein kinase-like
maker-9-augustus-gene-110.4-mRNA-1	NA	NA	Kinase-TM			L-type lectin-domain containing receptor kinase IX.1
maker-9-augustus-gene-112.27-mRNA-1	NA	NA	Kinase-TM	Os09g0339000	OsR498G0917194500.01.T01	L-type lectin-domain containing receptor kinase IX.1
maker-9-augustus-gene-115.2-mRNA-1	NA	NA	Kinase-TM			Serine/threonine-specific receptor protein kinase-like
maker-9-augustus-gene-116.0-mRNA-1	NA	Japonica	Kinase-TM	Os09g0356200	OsR498G0917236300.01.T05	Putative LRR receptor-like serine/threonine-protein kinase
maker-9-augustus-gene-145.39-mRNA-1	NA	NA	Kinase-TM	Os09g0408800	OsR498G0917427900.01.T01	Cysteine-rich receptor-like protein kinase 10
maker-9-augustus-gene-20.40-mRNA-1	NA	NA	Kinase-TM	Os09g0123300	OsR498G0916722500.01.T02	Putative leucine-rich repeat receptor-like protein kinase At4g00330
maker-9-augustus-gene-210.13-mRNA-1	NA	Indica	Kinase-TM	Os06g0654500	OsR498G0917848400.01.T02	Serine/threonine protein kinase
maker-9-augustus-gene-216.0-mRNA-1	NA	NA	Kinase-TM	Os09g0544300	OsR498G0917897100.01.T01	Serine/threonine-protein kinase STY46
maker-9-augustus-gene-88.42-mRNA-1	NA	NA	Kinase-TM	Os09g0315600		L-type lectin-domain containing receptor kinase IV.2
augustus_masked-9-processed-gene-225.0-mRNA-1	Japonica	NA	Kinase-TM WAK92	Os09g0562600	OsR498G0917953300.01.T02	Wall-associated receptor kinase 3
maker-4-augustus-gene-283.19-mRNA-1	Japonica	NA	Kinase-TM OsMAPKKK8	Os04g0660500	OsR498G0409429000.01.T02	MAP3K epsilon protein kinase 1
maker-2-augustus-gene-44.2-mRNA-1	Indica	NA	CC-Kinase		OsR498G0203087100.01.T01	*Mitogen-activated protein kinase kinase kinase ANP1*
augustus-1-processed-gene-152.17-mRNA-1	Japonica	NA	CC-Kinase	Os01g0367700	OsR498G0100962700.01.T05	*probable serine/threonine-protein kinase At1g54610*
augustus-5-processed-gene-5.3-mRNA-1	Japonica	NA	CC-Kinase	Os05g0108300	OsR498G0509579900.01.T01	*Non-specific serine/threonine protein kinase*
maker-2-augustus-gene-308.34-mRNA-1	Japonica	NA	CC-Kinase		OsR498G0204671700.01.T01	*Non-specific serine/threonine protein kinase*
maker-2-augustus-gene-389.0-mRNA-1	Japonica	NA	CC-Kinase		OsR498G0205028700.01.T03	*U-box domain-containing protein 33*
maker-3-augustus-gene-423.29-mRNA-1	Japonica	NA	CC-Kinase		OsR498G0306910800.01.T02	*Homologous-pairing protein 2 homolog*
maker-3-augustus-gene-429.41-mRNA-1	Japonica	NA	CC-Kinase		OsR498G0306944200.01.T01	*Putative serine/threonine-protein kinase dyrk2*
maker-9-augustus-gene-62.50-mRNA-1	Japonica	NA	CC-Kinase		OsR498G0916904200.01.T03	*Serine/threonine protein kinase*
maker-12-augustus-gene-233.9-mRNA-1	NA	NA	CC-Kinase	Os12g0562500	OsR498G1221884500.01.T01	*Putative serine/threonine-protein kinase roco5*
maker-2-augustus-gene-9.40-mRNA-1	NA	NA	CC-Kinase	Os02g0118200	OsR498G0202854300.01.T01	*probable serine/threonine-protein kinase PIX13*
maker-3-augustus-gene-162.14-mRNA-1	NA	Indica	CC-Kinase	Os03g0349200	OsR498G0305945000.01.T02	*probable serine/threonine-protein kinase At1g54610*
maker-9-augustus-gene-62.50-mRNA-1	NA	NA	CC-Kinase	0	OsR498G0916904200.01.T03	*Serine/threonine protein kinase*
augustus-1-processed-gene-15.3-mRNA-1	Indica	NA	Kinase		OsR498G0100091500.01.T01	G-type lectin S-receptor-like serine/threonine-protein kinase
augustus-10-processed-gene-29.3-mRNA-1	Indica	NA	Kinase	Os10g0142600	OsR498G1018125300.01.T01	Receptor-like protein kinase FERONIA
augustus_masked-10-processed-gene-38.31-mRNA-1	Indica	Japonica	Kinase	Os10g0157400	OsR498G1018162400.01.T01	Putative cyclin-dependent kinase F-2
augustus_masked-10-processed-gene-54.31-mRNA-1	Indica	Japonica	Kinase	Os10g0174800	OsR498G1018205800.01.T02	Wall-associated receptor kinase 2
augustus_masked-6-processed-gene-300.24-mRNA-1	Indica	Indica	Kinase	Os02g0623600	OsR498G0613277600.01.T01	Putative tyrosine-protein kinase, insulin-like receptor
maker-1-augustus-gene-448.23-mRNA-1	Indica	NA	Kinase		OsR498G0102507600.01.T01	Cell division control protein 2
maker-10-augustus-gene-111.62-mRNA-1	Indica	NA	Kinase		OsR498G1018800400.01.T01	Putative serine/threonine-protein kinase NAK
maker-11-augustus-gene-82.2-mRNA-1	Indica	NA	Kinase			Retrotransposon protein, putative, LINE subclass
maker-3-augustus-gene-199.0-mRNA-1	Indica	NA	Kinase		OsR498G0306131300.01.T02	Serine/threonine-protein kinase CTR1
maker-5-augustus-gene-188.30-mRNA-1	Indica	Indica	Kinase	Os01g0936100	OsR498G0510698900.01.T01	Serine/threonine-protein kinase PBL27
maker-6-augustus-gene-301.39-mRNA-1	Indica	NA	Kinase	Os06g0708000	OsR498G0613281300.01.T01	Mitogen-activated protein kinase
maker-6-augustus-gene-304.20-mRNA-1	Indica	NA	Kinase	Os06g0714900	OsR498G0613302400.01.T04	probable serine/threonine-protein kinase PBL19
maker-6-augustus-gene-309.34-mRNA-1	Indica	NA	Kinase	Os06g0727400	OsR498G0613334400.01.T01	probable serine/threonine-protein kinase PIX13
maker-7-augustus-gene-181.8-mRNA-1	Indica	NA	Kinase			Receptor-like serine/threonine-protein kinase
augustus-1-processed-gene-73.17-mRNA-1	Japonica	NA	Kinase	Os01g0231500	OsR498G0100481100.01.T06	Casein kinase (Serine/threonine/tyrosine protein kinase)
augustus-2-processed-gene-371.37-mRNA-1	Japonica	NA	Kinase	Os02g0787300	OsR498G0204906600.01.T01	MAP kinase kinase
augustus-3-processed-gene-493.97-mRNA-1	Japonica	NA	Kinase	Os03g0844100	OsR498G0307355200.01.T01	Tyrosine-protein kinase
augustus-7-processed-gene-294.64-mRNA-1	Japonica	NA	Kinase	Os07g0695300	OsR498G0715020300.01.T01	Putative serine/threonine-protein kinase PBL7
augustus_masked-11-processed-gene-2.65-mRNA-1	Japonica	NA	Kinase	Os11g0121800		Serine/threonine-protein kinase At5g01020
augustus_masked-3-processed-gene-289.48-mRNA-1	Japonica	NA	Kinase	Os09g0376600	OsR498G1018618100.01.T01	Putative leucine-rich repeat receptor-like protein kinase
augustus_masked-3-processed-gene-458.51-mRNA-1	Japonica	Indica	Kinase	Os04g0531500	OsR498G0307125400.01.T01	Non-specific serine/threonine protein kinase
augustus_masked-5-processed-gene-304.15-mRNA-1	Japonica	NA	Kinase	Os05g0588300	OsR498G0511432300.01.T01	Receptor-like protein kinase 1-like
maker-1-augustus-gene-186.38-mRNA-1	Japonica	NA	Kinase			MAPKK
maker-1-augustus-gene-236.19-mRNA-1	Japonica	NA	Kinase	Os01g0588500	OsR498G0101429400.01.T01	Putative serine/threonine-protein kinase PIX7
maker-1-augustus-gene-296.20-mRNA-1	Japonica	NA	Kinase	Os01g0699500	OsR498G0101831600.01.T01	Putative MAPKKK family protein kinase
maker-1-augustus-gene-301.12-mRNA-1	Japonica	Indica	Kinase	Os05g0593800	OsR498G0101862300.01.T01	Serine/threonine protein kinase
maker-1-augustus-gene-341.44-mRNA-1	Japonica	NA	Kinase			probable serine/threonine-protein kinase PBL23
maker-1-augustus-gene-488.40-mRNA-1	Japonica	NA	Kinase	Os01g0936100	OsR498G0102657500.01.T01	Serine/threonine-protein kinase PBL27
maker-10-augustus-gene-106.13-mRNA-1	Japonica	NA	Kinase	Os10g0431900	OsR498G1018768400.01.T02	Probable serine/threonine-protein kinase PBL16
maker-10-augustus-gene-169.27-mRNA-1	Japonica	NA	Kinase	Os10g0476300	OsR498G1018899100.01.T01	Casein kinase I isoform epsilon
maker-10-augustus-gene-190.66-mRNA-1	Japonica	Indica	Kinase	Os03g0113000	OsR498G1019036800.01.T01	Putative receptor protein kinase
maker-10-augustus-gene-75.0-mRNA-1	Japonica	NA	Kinase		OsR498G1018352600.01.T02	Putative receptor-like protein kinase
maker-11-augustus-gene-187.12-mRNA-1	Japonica	Japonica	Kinase	Os11g0568800	OsR498G1120381200.01.T01	Protein kinase domain-containing protein
maker-11-augustus-gene-2.19-mRNA-1	Japonica	NA	Kinase	Os11g0121400	OsR498G1119338800.01.T01	Serine/threonine-protein kinase NAK
maker-11-augustus-gene-2.20-mRNA-1	Japonica	NA	Kinase			Putative serine/threonine-protein kinase PBL8
maker-11-augustus-gene-256.36-mRNA-1	Japonica	NA	Kinase			Major disease resistance protein Xa4
maker-11-augustus-gene-82.2-mRNA-1	Japonica	NA	Kinase			Retrotransposon protein, putative, LINE subclass
maker-12-augustus-gene-10.62-mRNA-1	Japonica	NA	Kinase	Os11g0121400	OsR498G1220824700.01.T01	Serine-threonine kinase STK11
maker-12-augustus-gene-114.0-mRNA-1	Japonica	Indica	Kinase	Os05g0511400	OsR498G1221346100.01.T01	Serine/threonine-protein kinase CBK1
maker-12-augustus-gene-185.37-mRNA-1	Japonica	Indica	Kinase	Os12g0480200	OsR498G1221602400.01.T01	Serine/threonine-protein kinase KIPK
maker-12-augustus-gene-259.0-mRNA-1	Japonica	NA	Kinase	Os12g0605900	OsR498G1222069500.01.T01	CXC domain-containing TSO1-like protein 1
maker-12-augustus-gene-5.16-mRNA-1	Japonica	Japonica	Kinase	Os12g0109800	OsR498G1119298800.01.T04	Putative serine/threonine-protein kinase
maker-12-augustus-gene-7.9-mRNA-1	Japonica	NA	Kinase	Os12g0114100	OsR498G1220847300.01.T01	probable serine/threonine-protein kinase WNK5
maker-2-augustus-gene-232.7-mRNA-1	Japonica	NA	Kinase		OsR498G0204210800.01.T01	Serine/threonine-protein kinase Nek6
maker-2-augustus-gene-238.40-mRNA-1	Japonica	NA	Kinase	Os02g0602100	OsR498G0204253300.01.T07	Cyclin-dependent kinase G-1
maker-2-augustus-gene-272.35-mRNA-1	Japonica	NA	Kinase	Os02g0654300	OsR498G0204441700.01.T01	Serine/threonine-protein kinase
maker-2-augustus-gene-280.10-mRNA-1	Japonica	NA	Kinase	Os02g0670100	OsR498G0204492800.01.T01	probable serine/threonine-protein kinase PIX13
maker-2-augustus-gene-361.43-mRNA-1	Japonica	NA	Kinase		OsR498G0204841500.01.T02	Mitogen-activated protein kinase kinase kinase
maker-2-augustus-gene-382.45-mRNA-1	Japonica	NA	Kinase	Os02g0810400	OsR498G0204985500.01.T01	Diphthamide synthase
maker-2-augustus-gene-388.45-mRNA-1	Japonica	NA	Kinase	Os02g0821400	OsR498G0205024600.01.T01	Serine/threonine protein kinase
maker-2-augustus-gene-64.39-mRNA-1	Japonica	NA	Kinase		OsR498G1120698300.01.T01	Receptor-like serine/threonine-protein kinase
maker-3-augustus-gene-135.40-mRNA-1	Japonica	NA	Kinase	Os10g0533600	OsR498G1019094300.01.T01	Mitogen-activated protein kinase
maker-3-augustus-gene-154.23-mRNA-1	Japonica	NA	Kinase	Os03g0334000	OsR498G0305890800.01.T02	Non-specific serine/threonine protein kinase
maker-3-augustus-gene-25.38-mRNA-1	Japonica	NA	Kinase	Os03g0148700	OsR498G0305233300.01.T01	Serine/threonine protein kinase
maker-3-augustus-gene-285.37-mRNA-1	Japonica	NA	Kinase	Os10g0395000	OsR498G1018643000.01.T01	Serine/threonine-protein kinase RIPK
maker-3-augustus-gene-381.5-mRNA-1	Japonica	Indica	Kinase	Os12g0605900	OsR498G0306628100.01.T01	Serinethreonine-protein kinase sty17
maker-3-augustus-gene-429.53-mRNA-1	Japonica	NA	Kinase	Os11g0620500	OsR498G0510972100.01.T01	Pollen receptor-like kinase 6
maker-3-augustus-gene-445.69-mRNA-1	Japonica	NA	Kinase	Os03g0748400	OsR498G0307046900.01.T02	Serine/threonine-protein kinase AFC3
maker-3-augustus-gene-485.21-mRNA-1	Japonica	NA	Kinase	Os03g0825800	OsR498G0307302800.01.T02	Receptor-like protein kinase ANXUR2
maker-3-augustus-gene-492.48-mRNA-1	Japonica	NA	Kinase	Os03g0841800	OsR498G0307348800.01.T01	Non-specific serine/threonine protein kinase
maker-3-augustus-gene-84.64-mRNA-1	Japonica	NA	Kinase		OsR498G0305629300.01.T03	Putative LRR receptor-like serine/threonine-protein kinase
maker-3-augustus-gene-90.50-mRNA-1	Japonica	NA	Kinase	Os03g0274800	OsR498G0305669400.01.T01	receptor-like cytoplasmic kinase 176
maker-4-augustus-gene-192.44-mRNA-1	Japonica	Indica	Kinase	Os04g0490500	OsR498G0408809700.01.T02	Tyrosine-protein kinase
maker-4-augustus-gene-198.9-mRNA-1	Japonica	Indica	Kinase	Os09g0123300	OsR498G0408848300.01.T03	Mitogen-activated protein kinase kinase kinase
maker-4-augustus-gene-259.31-mRNA-1	Japonica	Japonica	Kinase	Os04g0616400	OsR498G0409282400.01.T01	Leucine-rich repeat transmembrane protein kinase
maker-4-augustus-gene-296.16-mRNA-1	Japonica	NA	Kinase		OsR498G0409524600.01.T05	Serinethreonine-protein kinase atg1t
maker-5-augustus-gene-14.42-mRNA-1	Japonica	Japonica	Kinase	Os05g0125400	OsR498G0509647700.01.T01	Receptor-like kinase LIP2
maker-5-augustus-gene-227.41-mRNA-1	Japonica	NA	Kinase	Os05g0440800	OsR498G0510929300.01.T06	Serine/threonine-protein kinase Nek5

maker-5-augustus-gene-233.10-mRNA-1	Japonica	Indica	Kinase	Os11g0620500	OsR498G0510972100.01.T01	Pollen receptor-like kinase 6
maker-5-augustus-gene-240.19-mRNA-1	Japonica	Indica	Kinase	Os05g0466900	OsR498G0511007800.01.T01	Putative serine/threonine-protein kinase dyrk2
maker-5-augustus-gene-264.28-mRNA-1	Japonica	NA	Kinase	Os05g0511400	OsR498G0511165600.01.T06	AGC (cAMP-dependent cGMP-dependent and protein kinase C) kinase family protein
maker-6-augustus-gene-130.2-mRNA-1	Japonica	NA	Kinase	Os06g0334400	OsR498G0612552700.01.T02	Cyclin-dependent kinase
maker-6-augustus-gene-167.0-mRNA-1	Japonica	Indica	Kinase	Os03g0568800	OsR498G0612359600.01.T01	Proline-rich receptor-like protein kinase PERK8
maker-6-augustus-gene-260.32-mRNA-1	Japonica	NA	Kinase	Os06g0636600	OsR498G0613026300.01.T03	Serine/threonine-protein kinase
maker-6-augustus-gene-291.61-mRNA-1	Japonica	NA	Kinase	Os06g0693900		Cyclin dependent protein kinase
maker-7-augustus-gene-205.7-mRNA-1	Japonica	NA	Kinase		OsR498G0714495300.01.T01	G-type lectin S-receptor-like serine/threonine-protein kinase
maker-7-augustus-gene-234.9-mRNA-1	Japonica	NA	Kinase	Os07g0584100	OsR498G0714647100.01.T01	probable serine/threonine-protein kinase WNK11
maker-7-augustus-gene-240.62-mRNA-1	Japonica	NA	Kinase	Os07g0596600	OsR498G0714693400.01.T01	Putative serine/threonine-protein kinase
maker-7-augustus-gene-287.22-mRNA-1	Japonica	Indica	Kinase	Os07g0680900	OsR498G0714963900.01.T06	Non-specific serine/threonine protein kinase
maker-7-augustus-gene-290.51-mRNA-1	Japonica	Indica	Kinase	Os07g0686800	OsR498G0714991100.01.T01	Putative serine/threonine-protein kinase PBL18
maker-7-augustus-gene-40.80-mRNA-1	Japonica	NA	Kinase	Os07g0185000	OsR498G0713644600.01.T01	Putative serine/threonine-protein kinase WNK9 (Fragment)
maker-8-augustus-gene-235.11-mRNA-1	Japonica	NA	Kinase		OsR498G0816229000.01.T01	Cyclin-dependent kinase C-3
maker-8-augustus-gene-237.9-mRNA-1	Japonica	NA	Kinase	Os08g0457400	OsR498G0816238400.01.T01	Serine/threonine-protein kinase RIPK
maker-9-augustus-gene-111.31-mRNA-1	Japonica	Japonica	Kinase	Os09g0341100	OsR498G0917196300.01.T01	L-type lectin-domain containing receptor kinase IX.1
maker-9-augustus-gene-119.5-mRNA-1	Japonica	Indica	Kinase	Os09g0361100	OsR498G0917245900.01.T01	Putative serine/threonine-protein kinase PIX7
maker-9-augustus-gene-180.47-mRNA-1	Japonica	NA	Kinase	Os09g0471200	OsR498G0917660800.01.T02	Wall-associated receptor kinase 3
maker-9-augustus-gene-49.9-mRNA-1	Japonica	NA	Kinase	Os09g0258500	OsR498G0916850600.01.T01	Serine/threonine-protein kinase D6PKL2
augustus-10-processed-gene-29.3-mRNA-1	NA	NA	Kinase	Os10g0142600	OsR498G1018125300.01.T01	Receptor-like protein kinase FERONIA
augustus-12-processed-gene-209.10-mRNA-1	NA	NA	Kinase	0	OsR498G1221718300.01.T01	probable serine/threonine-protein kinase PIX13 isoform X2
augustus-9-processed-gene-126.24-mRNA-1	NA	Japonica	Kinase	Os09g0373800	OsR498G0917660800.01.T02	Putative wall-associated kinase 4
augustus_masked-10-processed-gene-132.3-mRNA-1	NA	NA	Kinase	Os04g0125700	OsR498G0407520900.01.T01	Non-specific serine/threonine protein kinase
augustus_masked-6-processed-gene-300.24-mRNA-1	NA	Indica	Kinase	Os02g0623600	OsR498G0613277600.01.T01	Putative tyrosine-protein kinase, insulin-like receptor
augustus_masked-6-processed-gene-312.31-mRNA-1	NA	Indica	Kinase	Os02g0623600	OsR498G0613277600.01.T01	Putative tyrosine-protein kinase, insulin-like receptor
augustus_masked-6-processed-gene-35.26-mRNA-1	NA	NA	Kinase	0	OsR498G0611689200.01.T01	C2H2-type domain-containing protein
maker-1-augustus-gene-207.34-mRNA-1	NA	NA	Kinase	Os01g0541900	OsR498G0101225400.01.T04	Kinase family protein / protein phosphatase 2C (PP2C) family protein isoform 1
maker-1-augustus-gene-243.42-mRNA-1	NA	Indica	Kinase	Os01g0601200	OsR498G0101477700.01.T01	Serine/threonine protein kinase
maker-1-augustus-gene-381.17-mRNA-1	NA	NA	Kinase	0	OsR498G0102391800.01.T03	Serine/threonine-protein kinase Nek5
maker-1-augustus-gene-385.27-mRNA-1	NA	NA	Kinase	Os01g0872800	OsR498G0102422600.01.T01	Non-specific serine/threonine protein kinase
maker-1-augustus-gene-397.48-mRNA-1	NA	Indica	Kinase	Os08g0512600	OsR498G0102507600.01.T01	Cell division control protein 2
maker-1-augustus-gene-432.6-mRNA-1	NA	NA	Kinase	0	OsR498G0102391800.01.T03	Serine/threonine-protein kinase Nek5
maker-1-augustus-gene-436.78-mRNA-1	NA	NA	Kinase	Os01g0872800	OsR498G0102422600.01.T01	Non-specific serine/threonine protein kinase
maker-1-augustus-gene-448.23-mRNA-1	NA	NA	Kinase	0	OsR498G0102507600.01.T01	Cell division control protein 2
maker-11-augustus-gene-256.36-mRNA-1	NA	NA	Kinase			Major disease resistance protein Xa4
maker-11-augustus-gene-82.2-mRNA-1	NA	NA	Kinase			Retrotransposon protein, putative, LINE subclass
maker-12-augustus-gene-114.0-mRNA-1	NA	Indica	Kinase	Os05g0511400	OsR498G1221346100.01.T01	Serine/threonine-protein kinase CBK1
maker-12-augustus-gene-258.16-mRNA-1	NA	NA	Kinase	Os12g0604700	OsR498G1222065800.01.T02	Serine/threonine-protein kinase Nek2
maker-12-augustus-gene-282.64-mRNA-1	NA	NA	Kinase	Os04g0559800	OsR498G0409066400.01.T05	Putative MAPKKK family protein kinase isoform 1
maker-2-augustus-gene-12.47-mRNA-1	NA	Indica	Kinase	Os02g0123100	OsR498G0202873300.01.T01	Cell division control protein 2 like A
maker-2-augustus-gene-18.33-mRNA-1	NA	NA	Kinase	Os02g0135200	OsR498G0202916400.01.T05	Mitogen-activated protein kinase
maker-2-augustus-gene-19.16-mRNA-1	NA	NA	Kinase	0	OsR498G0202916600.01.T01	Putative cell cycle regulatory protein kinase Wee1 (Fragment)
maker-2-augustus-gene-238.40-mRNA-1	NA	NA	Kinase	Os02g0602100	OsR498G0204253300.01.T07	Cyclin-dependent kinase G-1
maker-2-augustus-gene-333.40-mRNA-1	NA	NA	Kinase	Os06g0663900	OsR498G0613121100.01.T03	Receptor-like cytosolic serine/threonine-protein kinase RBK1
maker-2-augustus-gene-334.0-mRNA-1	NA	NA	Kinase	Os06g0663200	OsR498G0613117600.01.T02	Putative serine/threonine-protein kinase PBL17
maker-2-augustus-gene-48.69-mRNA-1	NA	NA	Kinase	Os02g0186500	OsR498G0203116200.01.T02	Mitogen-activated protein kinase kinase kinase
maker-3-augustus-gene-199.0-mRNA-1	NA	NA	Kinase	0	OsR498G0306131300.01.T02	Serine/threonine-protein kinase CTR1
maker-4-augustus-gene-164.0-mRNA-1	NA	NA	Kinase	Os04g0437600	OsR498G0408615000.01.T01	Mitogen-activated protein kinase kinase kinase YODA
maker-4-augustus-gene-227.29-mRNA-1	NA	NA	Kinase	Os04g0559800	OsR498G0409066400.01.T05	Putative MAPKKK family protein kinase isoform 1
maker-4-augustus-gene-259.31-mRNA-1	NA	Japonica	Kinase	Os04g0616400	OsR498G0409282400.01.T01	Leucine-rich repeat transmembrane protein kinase
maker-5-augustus-gene-98.24-mRNA-1	NA	NA	Kinase	Os04g0291900	OsR498G0407958200.01.T01	Non-specific serine/threonine protein kinase
maker-6-augustus-gene-275.10-mRNA-1	NA	NA	Kinase	Os06g0663200	OsR498G0613117600.01.T02	Putative serine/threonine-protein kinase PBL17
maker-6-augustus-gene-276.41-mRNA-1	NA	NA	Kinase	Os06g0663900	OsR498G0613121100.01.T03	Receptor-like cytosolic serine/threonine-protein kinase RBK1
maker-7-augustus-gene-205.7-mRNA-1	NA	NA	Kinase	0	OsR498G0714495300.01.T01	G-type lectin S-receptor-like serine/threonine-protein kinase
maker-8-augustus-gene-59.0-mRNA-1	NA	NA	Kinase	Os08g0200500	OsR498G0815381500.01.T04	Serine/threonine-protein kinase receptor
maker-8-augustus-gene-69.0-mRNA-1	NA	NA	Kinase	Os08g0200500	OsR498G0815381500.01.T04	Serine/threonine-protein kinase receptor
maker-9-augustus-gene-184.31-mRNA-1	NA	NA	Kinase	Os09g0478500	OsR498G0917686300.01.T03	Serine/threonine-protein kinase D6PKL1
maker-9-augustus-gene-211.19-mRNA-1	NA	NA	Kinase	Os09g0533600	OsR498G0917854200.01.T01	probable serine/threonine-protein kinase PBL19
maker-1-augustus-gene-181.10-mRNA-1	Japonica	NA	NRAMP6	Os01g0503400	OsR498G0101068400.01.T03	Metal transporter
maker-3-augustus-gene-460.78-mRNA-1	Japonica	NA	ONAC066	Os03g0777000	OsR498G0307142500.01.T01	Transcription factor JUNGBRUNNEN 1
maker-11-augustus-gene-18.28-mRNA-1	Japonica	NA	NAC17	Os11g0154500	OsR498G1119437300.01.T01	NAC domain-containing protein 90
maker-2-augustus-gene-142.5-mRNA-1	Japonica	Indica	OsSNAP32	Os02g0437200	OsR498G0203643200.01.T06	SNAPous protein SNAP33
augustus_masked-3-processed-gene-439.14-mRNA-1	Japonica	Indica	OsSYP121	Os03g0787000	OsR498G0307007300.01.T01	Syntaxin-related protein KNOLLE
maker-1-augustus-gene-487.26-mRNA-1	Japonica	NA	PIR7A	Os01g0934900	OsR498G0102650800.01.T03	AB hydrolase-1 domain-containing protein (Fragment)
maker-1-augustus-gene-487.25-mRNA-1	Japonica	NA	PIR7B	Os01g0934800	OsR498G0102650800.01.T01	AB hydrolase-1 domain-containing protein
maker-3-augustus-gene-17.61-mRNA-1	Japonica	Japonica	RAI1	Os03g0135700	OsR498G0305187800.01.T01	BHLH transcription factor
maker-10-augustus-gene-175.19-mRNA-1	Japonica	NA	TPR1	Os10g0486900	OsR498G1018945500.01.T01	Tetratricopeptide repeat protein 1
maker-4-augustus-gene-235.33-mRNA-1	Japonica	NA	CCSD	Os04g0573200	OsR498G0409117900.01.T03	Putative superoxide dismutase, copper/zinc binding domain, heavy metal-associated domain, HMA
augustus-2-processed-gene-15.68-mRNA-1	NA	NA	CC-TM	Os02g0129800		Response to low sulfur 3
maker-3-augustus-gene-162.5-mRNA-1	Japonica	NA	CSD1	Os03g0351500	OsR498G0305951300.01.T01	Superoxide dismutase
maker-6-augustus-gene-304.4-mRNA-1	Indica	NA	TM	Os06g0714500	OsR498G0613301300.01.T01	ATPase family AAA domain-containing protein 1-A
maker-3-augustus-gene-457.9-mRNA-1	Japonica	NA	TM	Os03g0769600	OsR498G0307113800.01.T02	C-type cytochrome synthesis protein1
maker-9-augustus-gene-201.47-mRNA-1	Japonica	NA	TM	Os09g0515500	OsR498G0917797500.01.T01	Tr-type G domain-containing protein (Fragment)
maker-7-augustus-gene-224.48-mRNA-1	Japonica	Indica	TIR	Os07g0566800	OsR498G0714593200.01.T01	TIR domain-containing protein (Fragment)
maker-3-augustus-gene-263.0-mRNA-1	NA	NA	TIR	Os03g0620400	OsR498G0306573300.01.T02	B3 domain-containing protein (Fragment)
maker-3-augustus-gene-372.3-mRNA-1	NA	NA	TIR	Os03g0620400	OsR498G0306573300.01.T02	B3 domain-containing protein (Fragment)
maker-5-augustus-gene-177.84-mRNA-1	NA	NA	TIR	Os05g0358500	OsR498G0510639400.01.T04	PPM-type phosphatase domain-containing protein
augustus_masked-1-processed-gene-327.6-mRNA-1	Japonica	NA	TIR-TM	Os01g0760400	OsR498G0102039800.01.T01	ADP-ribosyl cyclase/cyclic ADP-ribose hydrolase
