# Supplementary material for: Development and Genome-Wide Analysis of a Blast-Resistant japonica Rice Variety
Source: Plants (Basel). 2023 Oct 11;12(20):3536. doi: 10.3390/plants12203536 (PMC10667994; doi:10.3390/plants12203536)
Supplement: Supplementary file 1 [file plants-12-03536-s001.zip › Supplementary Material Escol_Suppl Table S5 Pi1 Pi2 Pi33.pdf]

Supplemental Table S5. Chromosomal coordinates and nucleotide sequences of *Pi1*, *Pi2* and *Pi33*.

Locus	Name	Chromosome	Start	End	Region	Sequence
Os06g0294300	*Pi2*	6	10510800	10512346	Japonica	CACCAATGCGAAGACAAAGATGGATGTACGCATATGCTGTGAGCTCCCAGCGGCGTAGGCACCCATGAGACCAAACATGCCAGCCACCATGCAAACGGAGAGCGCATAGCTCT<br>GAATGGCTGGCCTGTACAAATTTGGGTTCACGAGAAGGATGATGAGGGCGATAGATGACATAAAGCTGGTGGTGTTGCAGTAGAAAAAGGCTTTAAAACGGTAAGGGTTGTT<br>GCTGAAGAGGACTGGCTCGCCTGCACGGTGACCATCACTATCATCTTGCCAGAAGCCACTTGGTGGGGTTAGCCCGGCTTGGTAGGTGATGGTTGCGGACAAGATGGCGAAA<br>AGGAGCAACCTCTTGCGCCTCTTCTCCAGCAATACATCTTCGTCGTGTCCTCTCTGGTCTAGTGTGAAGAACACGACATGGATCACAACATACACCAGCACGGCACCGGCTATGG<br>CCATAACATAAATGGAGGTACTCGCATCCCGACAGCTTCCCGCTGCATATGCACCCATGAGCCCAAACAAATCCAGAATCATGGCTGCCTCTAGCGCGTGGCGCTTGAGCAGAG<br>ACCTGCTCTGCACTAGGATGATGGCAAGTAAGGATGACACGAAGGCGGTCGAGTTGCAGTAGAAGAATGCCTTGTACCTTCTAGGGTTAGTTGTTAGGAGAATCGGGTTACCG<br>GCCTTGTGGTCGCCATTGTCATCCTGCCAATACCCACCCGGCGGATCTAGCGCAGCTTGGAAAGTGACGGTCGCTGCTAGAGTGGCGAGCAGTAGAACGAGAGAGCGAGCCTT<br>CTCCATAGCTCGGTTATGTTCCGCCTCCCTAATAATTGCAGCAAGAGTAGAAAAAAATTGATTATTCTCTTTCAACAAATAGTTAGAGAGTGAAACAAATGTGATTATTCTCAAAA<br>ACTATCAAACTCTCTTTGCCTCTCTAATCAAATTTGCTGCACAATGAGAGAGGGGACATGTAAATTCTTTTGTTTGTAAAAGAAAAAAAACAAATAGGAGTGCTTTACAAACCTGT<br>CAATAGTGGCCTGATCTACTTCTTGGTCTTGCTTGGGACAATATCGTCGTTTTGATATCGAAGAGTAGATCCGTGCGAGCTTCGTGTTGCCAAGCGCCTGCTTGACGCACGGCAC<br>GACGGTGAGGTGGAAGGCGATGTACACGAGCACGGCGCCGACGAGCGCCAGCACGTACACGGTGGTGTGCTCCGCCCTCGAGCTGCCGGCGGCGTACGCTCCGACGAGCCC<br>GACCAGCGCGACGACGATGCACCCGTACAGCTCCCAGGACCGCACCGTGCCGTCGCGCAGCTTCCGGTCGAGGAGCAGCACGATGATGAGCAGCGACGCGACGAACGCGGT<br>GGTGTTGCAGAGGAGGAACGCCGTCAGCCGCGCCGGGTGCCGCTCCCCCATGACCGCCTCGCCGGCGCGGTGGCCGGCGACGCCGTCCGCCCAGAACCCTCCCGGCGTGCTC<br>AGCCCGGCCACGTACGTGAGGCTCGTGGCGAACGTGGCGAGCAGCAGGAGCACCTTGCGCGACCGCTCCTTGGT
Os11g0644700	*Pi1*	11	23108014	23108779	Japonica	CAATCCATTCCTCCATCTAATCCCGATTGAAATGGCGCCCAAGGTGTTCGTCCCGCTCTGCGTCGTCGTCCTCGCCGCGGCGCTCGCCGCGGGGCCGCCGGTGGCGGACGCGGC<br>GTCGGCGCACCTCCACTTCTACATGCACGACGTGCTGGGCGCGTCGGCGGTGCAGGTGGTGCGGGGCCCGCGCGGCATGTTCGGGAACACGGTGGTGATGGACGACGTGCT<br>GACGGAGGGACCCGCGGCGACGTCGTCGGTGCTGGGGCGCGCCCAGGGGCAGTACATCGTGGCGTCGACGGGGAGCATGGACCTGATGGTCACCATGAACGTCGTTCTCAG<br>CTCCGGCCCGTTCGCCGGCAGCTCGGTGACCGTCGTCGGGCGCGACGACACCGGCGCGGCGGTGCGGGAGCTGACGGTGGTGGGGGGCACGGGGCAGTTCAGGATGGCGA<br>GAGGGTACGTGCTGTGGAGGACCATCCGCCCTGACCTGCTCGAGCTCGACGTCTACGTTAACCCGTAGAAAAGATAAGAGAGACGATGACGATGAGGATGATGATCTGTCGA<br>AAACGGACACGTCATTCGCCCAGTCAGCATCTGTGTTCGTGTGTATGTGTGTGACGTGGCGTGTTCTTGCACAGGTGTCGTACCGGTAAAGGCTTATCCTTGTGGTGTGTGTGT<br>GTCCGGTGAAAAAATAAGCTTGACATGGGCGAACCTGATGGGTATAATAATAATTCTTATATACTATATATTTAATTTACTGAACATTATTATC
Os08g0421800	*Pi33*	8	21513309	21518636	Indica	CGCATCCAAACCCTCGCCCTCCTCCTCGCCGCCGCCGCCGACGACGACCATCCCACCGACCGCGCGCGCGCGAGGGGGCTCTACTACCACCGGCGATGCGGCGGACGACGCC<br>GCTGTCGGAGGCGGCGGGGGGTTCCAGGAGCTGTTCGACTCGGTGCGCCGCTCCATCTCCTTCCGCCCCGGGGGCAGCGCCGCGGCCGCCACCCTCGACGAGCCCGCCTCGG<br>CTTCCGGCGGTGGGGGGTTCAGGGGGCGGATCAGCACGTGCCTCCGCAAGTCCAGGGGGATGGGGCTGCTCGGGATGGTGGCCTCCAAGAGCCCCTCGCCCCCGCGCCGCC<br>GCCTCCTTCCCCCGCCGCCTTCGCCGCCCCCCGCGCCAACGAAGTCGCTGTCGCCACAGCCTGCGGCGGCGGCTGCGGTGGTGTCCCAAGGGGGAGACGGTGCGGGTGGAAT<br>CGGAGGAGAGGAGGAGAACCCGCCGATCCGGTGGCGGAAGGGCGATCTCCTCGGGAGCGGCGCGTTCGGGTCGGTCTTCCTCGGCATGGATCTTGATTCCGGCGAGCTCCTC<br>GCCGTAAAGCAGGTCAGGAGGCCTCTTCCAAGATGCTGATGCTTCAAGTTTCCAGCGACGGCCGATTTGGTCTGGTTTCATCACCCTTCTGCTCTTTCTGGTTGCAGGTTCTGAT<br>CGGGAGCAGCAACGCAACGAGGGAGAAGGCCCAAGTGAGTGGCTTTGCTCAATTACTTGTTTACTATCTTTATTTTCCTGACCAATTGTGCCATTGAAATAGTCTTCTTTAACCTC<br>CTCTAAGCTCTAAGTGTTTGTAATTGGATTTTTTTTTGTTAGGGACACATAAGGGAGCTTGAGGACGAAGTGAAGCTACTCAAGAACCTCTCACACCCCAACATTGTTGTGAGTA<br>ATGCTAAATAACCACATATGTTTTGTTCTTCAATTCGGTGGTGGTGTTTGATCTATCTGGTGTGCTTTGAGCAGCGATATATTGGAACGGTGCGGGAGGAGAACTCGTTAAATAT<br>TCTGCTGGAGTTTGTGCCAGGAGGATCGATCCAGTCACTCCTTGGGAGGCTGGGATCATTCCCTGAGGCTGTAAGTACTAACTGTTATGTTCATCCTGTCTTCGGTAGTTTGGTT<br>AATCACAAAAGAATCGTGTTGATTGAATGCATTTATTTATCAGGTCATCAGGAAATACACTAAACAGATTTTGCATGGCCTGGAGTATTTACATCGCAATGGAATCATACACAGG<br>GACATAAAGGCATGAAGAGTATTCGAATTTCGAATTTTGTTGCCTTGAACCCTTTTGTTGTGTGGGTTATATATTTCTAATGTGGCATTACTGTTTTTTTTTCAATATTCAGGGAG<br>CAAATATACTTGTTGATAATAAAGGATGCATTAAGTTGGCAGACTTTGGGGCATCTAAGCAAGTGGAGAAGTTGGTGAGTAGATGAACTAATTGTTTTAATTTCGTTTGATCATT<br>TGTTCAACTATGTATTAGCTTAGCTCTAAAGTTAACTTTATAGATGCCAGGTTTATCTTGCATTTCCTTTTATCCATAGAATGCTAGTGTTTCACTTAAAAGTGAAAAGGTAGAAGA<br>CTGGAACTTACTTCATCACTGCATAGGACAGGGTTCTGTTGCTTCTTGTGTGTTTTAAATGCGGGATCATCCCTACTGTTACGTTCCACGTGTTTATATTTTTCTTTCACACGCTATT<br>ACCTTTTCAGGCGACCACTGCCAAGACAATGAAAGGGACACCATATTGGATGGCCCCAGAAGTAATTGTTGGGAGTGGGCATGACTTGTAAGTGGACAAGCTCTTTATCTCATC<br>GTTATAGTTAACTGCTCGTTGCTCAATCCTTAGTGATGCTTGTGGTCCTCTGGCTCTCTTATTTCTTTAAGAATAAGAATATCTGTGAACTAATGTTTTGGCTAATCATGCCACCAA<br>ATGACTTATTTTGTGAAAAATATCTTAAAGGACAAAAAAAAATCCTATGAAAAAAAAGGGATGGTACAATTAGCTTATTATCTTTTCTGGCCCCAATGCCCCATTTATTGTTGCCT<br>TTTCATTGGTGAAAAAATTTCACCAAGCTTGCTGTTCAATACCATATCCAGAAACTGTATGATGGTTTTGTTTCTTGAATTAATCCTTGTCATCTAACTATCTTTGCAGTTCAGCTGA<br>TATCTGGAGTGTCGGATGCACTGTAATTGAAATGGCAACTGGGAAAACTCCATGGAATCAGGAAATTCAAGAGGTAAATTTTGGCATCAGCATTTACCAAGAAACTTATAAGCA<br>TTGCTTATTCTTGACAATCACTATTTCAGGTTTCTCTTCTGTATTATGTTGGGACAACAAAGTCACATCCACCGATACCAGAGCATCTTTCCCCAGAAGCCAAGGATTTTCTGTTAA<br>AATGCTTACAGAAGTATGACTACAATCACACTTTTGAATAGATGTTGATTATTGCAGCAAAATTGACAATGGGACCTATAAAAGTTTAGAATTTTCACCACAAAGAAGTGGAAAA<br>ATCGATTCAGACAATCTAGTAAGAGAAACTGAGAAATAGTTTGGTTGTGCTAACTCAGTTTTTTTGCATAGCTGTTGTTAAACATTGAGCGCTCTTATGCGCTGTGAGTCATACCT<br>AGAATTACATAGGTGGAAATAAATCGCTCTCTCTGTATTTGAAGTTTTGAAAATTGAAGGTGGCATCCATCTGATAATGAACTGCATTACATGCATCCTAACAGCGCGATAGCTT<br>TATATATTCTTGTGGTAGCCTACTTCCAGTGAGCCATGTTTATGTACAACTTTGTGCTCCATGTATTTCAGTATGAATAGTTTCATTTCTTTCCATTGTTATTACCTGATTACATAATA<br>AGAGTAACGAATTCTAGATTTGTATGTGTAACTTGTGCTGTTTGCCTGATTCCTATCTTCACACTGCGTGATTTTTGTCTGTGTAGTTTTGTGTATGCGTTTCCAATTAACAGCTGC<br>AATTCAGTGGCATTTGTTGTGCAATTATCATATGTAAAGTTTATCTAGAAATGATGAGAATCAAGCTTCAAATGGCACATTTTCCTGCATTTGGATATTATAATTAGTAAATGTTAC<br>TTCATTGCCTTCAGCATTTTGTTACTCCTTTCAGGGAACCAGAACTGAGATCTACTGCATCAGACTTATTGCTGGTATAATAAACTCTCTCTTTCTCTCTCTCGGCATTTTAGCTGTT<br>GGGGAAGTCCTCTTGCTGTAAGGATATGTTATGTACTTTGTGAAGAGTAATACCTTAAAACATTTTTTTTCCAGCACCCATTTGTTACAGGAGGATTGGAAGATCTTCACCAGGTT<br>AATCATGCAGCACACAAGGTTGGATTACAATAAATCCTTTTACTTGTCTATTTATCCCAAAATGTTACTCTTACAAAATGATGTACTCACAGGAAACCTCTGAGCTTCCGGCATAC<br>GATATGCTAACTGGTGACCTGTAAGTACCTTGCAATCTTGAGCCAGGCAGATCGTTCATCTTCAGTTTGATTGAACAAATTCTTCAACTTGTAGGGGCTTGAATCATTCTGGCAAC<br>CCAAGTAACTTGAATTCCTATAAGTCATCAGACGTGAGACCAATATGGGATGGGCACAGCAGTGATGACATGTGTCAGTTTGCTGACAATGACGTGGTAATGATTGGATCGGTA<br>AGTATCCGTACCTAGTCCTGACTACTTTAGCCTTATGTTTTAGTTCGTACACAAGTCACATAAGAAATGCGTATTTTGTATTCCTACATGGTATACTTATAGTTACGTAATGTCATTT<br>CCAGAGCTTCAACCCTATGTCTGAACCATTTGATGACTGGGAAAGCCCAGAACAAAGATCTAGTCAATCAAGGGAATTCGGAGGATTAGCCAAACATGTTGAAAATAACATGTC<br>TGAAAATGATTTTACTTTCCCATGTGAGGGAGGCTGTGAAGAGGATGATGAACTCACAGAATCAAAAATAAAGGAATTTCTGGATGAAAAGGTATTTTTCATGCTTTGAGGCAG<br>CAATCCCTTCTGCTGCATGTCCAGTTGTTCTTAATCTACTATTGTTTACTTTTGCACTTAACAGGCCATAGATCTAAAGAAACTACAGACACCTTTGTATGAGTTCTACAACACAGT<br>AAATGCTGGACTTTCTCAAGGAGTTGGTGAAACTTGCCAAGTAGACAACATTACTGATTTGCAATTGCCCCTTCAAGGAAGATCACCTCCAATTCAGATGGAGGGAGTTGCAGC<br>AGTAGGACCAACCAGCGATGTTCTGAAGAGTGCCAGTCCAAAGAGCTGCACTAGGCGTTTCTCAAGAAGCGGTGTGGAGAATGGTCGAATTTTAAGAGAAATTGCTTCGCCTC<br>AACTCAATAAGTTTGAGGACAAGGTCCACGATATTCAAGAAAATCCCAGGTTTGTCTGGAGTAGATTGCTTTGGATATGAAATTATACATGAGACAGCTATCACTTTACAGTTAT<br>GCTACAAAAGACTGAATTCACTTATTCTCTTGCAAAAGTGCTCACCTTTTGGTCAATTTAATTATGGTGTTTTCACTCATGTGAGTTATTTACCCGTTATTTTTTTTTCAGCATTAGCT<br>TTTCTGAAAGACAGAAGAAGTGGAAAGAAGAGTTAGACCAAGAACTTGAGATCGGGAGAGGTAAATGAAAAGCATTTTCCTCTCAATATTAACATGAATTATCAGTGACTTACA<br>GATGCTTATCTTGCTCATTCCATTTGTGAACTCCAATAGTGATGAGGTTGGGTAGCTTCGGTAAAGCGCCATCACCAAAGAGCCGAGGCATGACCAGGAAACGGGATCGCGTCT<br>ACTGAATGGAAACGAGAATTGTCATCCGGCAGGACTAATGGTGACTTGACTACAACTGTACAATGTCAGCGATTGGTGGCCATACCAGTACAGTGCACTGCCTCGCCACTCGTG<br>TGACCTAACAAAAATTTTCATAGCTGCCAAATCCTTTTGGTGCTCATTTGAGTCTGATATGAAGGCTATTCCAGGTGGGCATTTGGATTTGAAGTCAGGCTGTCACGATTTGCCCC<br>CAGGGAAACAGTTTGGTGGCTGTTCCGCCTACAATATGCAGCTTTATGTTAACGATGCCTTGGCGATTTTGGGAGCCCTCCAAGTGTGGCAGGATGGCTTATGCGCAGAAGGA<br>ATTGTTGTACTTTTATTTTTTTTTCCTTTCTTTTCGTTGTTTTTGTAAGAAAGATACTCCAAGAGCTTGCCGTTTGCGAGCATGTAATAAGCAATGTAATGCTGAGTGTGTTATCATC<br>GAGAATGTAGTATGCGTTTTGC
